# Plasma proteins and osteosarcoma: Mendelian randomization analysis and targeted therapeutic discovery

**DOI:** 10.1097/MD.0000000000045704

**Published:** 2026-05-12

**Authors:** Liang Zhao, Juncheng Shen, Xuzhou Zheng, Qiao Yuan, Junwu Ye

**Affiliations:** a Department of Bone and Joint Surgery, Affiliated Hospital of Southwest Medical University, Luzhou Sichuan, China.

**Keywords:** drug targets, Mendelian randomization, osteosarcoma, plasma proteins, pQTLs

## Abstract

Prior research has hinted at a possible link between plasma protein metabolites and osteosarcoma (OS). However, no conclusive evidence has been presented to firmly establish a direct connection between particular plasma proteins and OS. To fill this research void, our study utilizes Mendelian randomization (MR) analysis to meticulously assess the causal impact of plasma proteins on the development of OS. Additionally, we aim to discover new plasma protein biomarkers that could serve as potential targets for therapeutic strategies in the treatment of OS. In this study, we leveraged the plasma protein quantitative trait locis data from Ferkingstad et al genome-wide analysis as the exposure variables. Meanwhile, the summary statistics of OS were sourced from the FinnGen database. We constructed a robust analytical framework that integrated enrichment analysis, protein–protein interaction network analysis, drug target prediction, and molecular docking. This comprehensive approach enabled us to thoroughly investigate the biological mechanisms and therapeutic prospects of the identified protein targets. In the MR analysis, a total of 81 genetic loci exhibited significant associations with the development of OS (*P* < .05). However, the reverse MR analysis demonstrated that the occurrence of OS did not significantly influence the levels of these 81 plasma proteins (*P* > .05). The protein–protein interaction network analysis pinpointed core genes, such as EGFR, IGFBP3, PLG, and TAGLN. These genes play crucial roles in the key pathological processes of OS, such as cell proliferation, extracellular matrix remodeling, and immune regulation. The drug prediction analysis identified potential therapeutic agents, including agarose, clofibrate, ascorbic acid (vitamin C), 6-aminohexanoic acid, and tetracycline. Molecular docking studies further revealed strong binding affinities between these drugs and the target proteins. This study systematically identified 81 potential therapeutic targets for OS, of which 10 key targets exhibited particularly high potential for clinical validation. These findings offer valuable insights into the molecular pathogenesis of OS and highlight novel opportunities for the development of targeted therapeutic strategies. Nevertheless, further functional investigations, large-scale cohort studies, and clinical trials are warranted to substantiate the causal relationships, assess therapeutic efficacy, and establish safety profiles, thereby advancing the prospects for clinical translation.

## 1. Introduction

Osteosarcoma (OS) is the most common primary malignant bone tumor, originating from mesenchymal stem cells and predominantly affecting children and adolescents.^[1]^ It typically arises in the metaphyses of long bones, particularly the proximal femur, proximal humerus, proximal tibia, and distal femur.^[2]^ OS exhibits high malignancy, aggressive invasiveness, rapid progression, and a generally poor prognosis.^[3]^ Clinically, patients commonly present with severe localized bone pain and swelling, which may be severe enough to disrupt sleep. Some cases may even involve pathological fractures and associated symptoms.^[4]^ The current standard treatment for OS combines surgery and chemotherapy, achieving long-term survival in approximately 60% of non-metastatic patients.^[4]^ However, due to its highly invasive and metastatic nature, 15% to 20% of patients already exhibit metastasis at initial diagnosis.^[5]^ The lungs are the most frequent metastatic site, followed by bone. Once pulmonary metastasis occurs, the prognosis worsens significantly, with the 5-year survival rate declining to around 20%.^[2,3,6]^ This poor survival outcome underscores the urgent need for more effective therapeutic strategies. Although research has identified multiple factors associated with OS development (including genetic susceptibility, gene mutations, and environmental exposures^[7,8]^) their interactions remain complex and incompletely understood.^[9]^ Consequently, further investigation into the molecular mechanisms and etiology of OS is essential to enable early diagnosis and precision therapy, representing a critical challenge in current research.

Proteins function as central regulatory molecules in all biological processes (BPs) and are closely linked to disease pathogenesis.^[10]^ The human plasma proteome consists of circulatory proteins that not only maintain essential blood functions but also facilitate critical inter-tissue communication.^[11]^ Given their fundamental roles in both physiological homeostasis and pathological mechanisms, plasma proteins represent prime targets for numerous approved therapeutics.^[12]^ Dysregulation of plasma proteins is a hallmark of various diseases, including OS.^[13]^ Emerging evidence suggests a potential causal relationship between plasma protein metabolites and OS,^[3]^ implicating plasma proteins in OS pathogenesis. However, no study has yet definitively established direct associations between specific plasma proteins and OS. Investigating this relationship could advance our understanding of OS molecular mechanisms and identify novel biomarkers or therapeutic targets.

Recent large-scale proteomic studies have identified over 18,000 plasma protein quantitative trait locis (pQTLs), each defined by its most strongly associated single nucleotide polymorphism (SNP) that significantly correlates with specific protein expression levels.^[14]^ The discovery of pQTLs offers critical insights into how genetic variation regulates protein expression and establishes a robust foundation for Mendelian randomization (MR)-based causal inference studies. MR is an important application of instrumental variable (IV) analysis, aiming to infer the causal relationship between exposure and outcome using genetic variation. In MR analysis, genetic variants (typically SNPs) are employed as IVs for the presumed exposure factors. The core principle of MR is based on Mendel second law, which states that genetic alleles are independently segregated from parents to offspring during gamete formation. This process mimics the random allocation in randomized controlled trials, effectively simulating random assignment and thereby reducing the impact of confounding and reverse causation, enhancing the reliability of causal inference.^[15]^

This study employs an integrative MR approach to systematically identify plasma proteins and potential drug targets with causal associations to OS. We validate the pharmacological activity of identified targets through drug prediction and molecular docking analyses. Furthermore, enrichment analysis and protein–protein interaction (PPI) network construction elucidate the biological significance of these targets and their roles in OS pathogenesis. These findings are anticipated to provide both theoretical foundations and practical guidance for OS precision medicine and personalized treatment strategies.

## 2. Materials and methods

### 2.1. Study design

**Figure 1.**
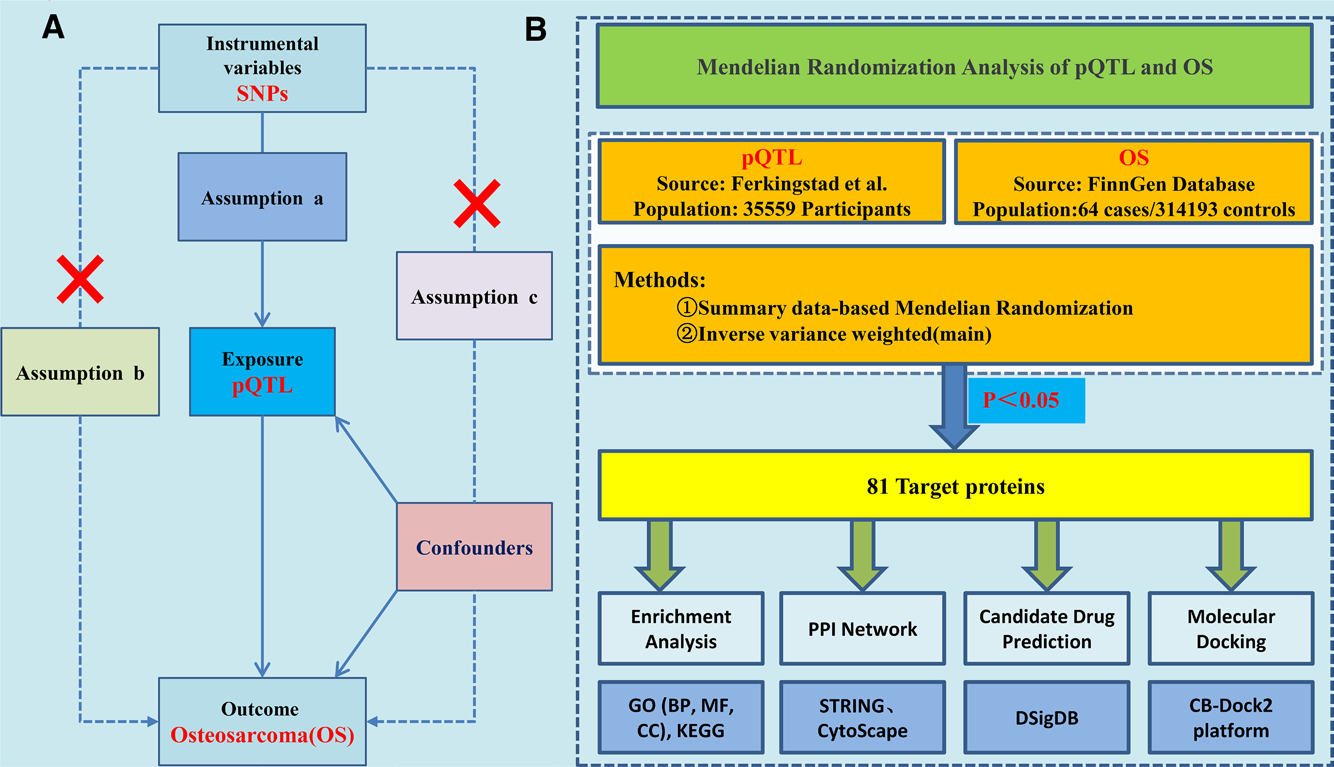
Schematic representation of the study design. (A) The 3 fundamental assumptions of MR analysis. (B) Overall analysis process. MR = Mendelian randomization.

This study utilized SNPs as IVs to evaluate causal relationships between plasma proteins (exposure) and OS (outcome). The analysis was predicated on 3 core MR assumptions (Fig. 1A): relevance – IVs must show significant association with the exposure; independence – IVs should affect the outcome exclusively through the exposure; and exclusion restriction – IVs must not correlate with confounding factors.^[16]^ Figure 1B illustrates the analytical framework developed based on these assumptions. This study only used publicly available summary-level genetic data, without involving individually identifiable information or direct contact with research subjects; therefore, neither ethical approval nor informed consent was required.

### 2.2. Data sources

#### 2.2.1. Source of exposure data

The genetic summary statistics for 4907 plasma proteins were obtained from a large-scale study by Ferkingstad et al,^[17]^ which analyzed pQTLs in 35,559 Icelandic individuals. These data are publicly available through the deCODE genetics database (https://www.decode.com/summarydata/).

#### 2.2.2. Source of outcome data

The OS genome-wide association study (GWAS) data were acquired from the FinnGen consortium (release December 10, 2023; https://www.finngen.fi/en/access_results).^[18]^ The dataset comprised 64 OS cases and 314,193 healthy controls.

#### 2.2.3. Selection of IVs

IVs were selected based on 3 key assumptions: significant association with exposure (*P* < 5 × 10^−8^); independence (*R*^2^ < 0.001) to minimize linkage disequilibrium; and adequate allele frequency (EAF > 0.01) to ensure statistical power. We calculated the *F* statistic (*F* = *R*^2^ × (N − 2)/(1 − *R*^2^)), retaining only SNPs with *F* > 10 to exclude weak instruments.^[19]^ Exposure and outcome variables were harmonized through standardized procedures including chromosome position matching, allele orientation adjustment, and exclusion of reverse causality SNPs. For reverse MR analysis of OS, we employed a relaxed threshold (*P* < 5 × 10^−6^) to obtain sufficient IVs while maintaining strong association with OS.^[20]^ Conversely, in forward MR analysis of plasma proteins, we maintained the conventional genome-wide significance threshold (*P* < 5 × 10^−8^) to ensure result robustness.

### 2.3. MR analysis

#### 2.3.1. Forward MR analysis

This study implemented a 2-sample MR framework to investigate potential causal relationships between circulating plasma proteins and OS risk.^[21]^ A total of 5 methods, including inverse-variance weighted (IVW) method, MR-Egger regression, weighted median, simple mode, and weighted mode analysis, were utilized to evaluate the causal effects of plasma proteins on the risk of OS. Among these methods, the IVW method was the primary analytical approach.

#### 2.3.2. Sensitivity analysis

To ensure the robustness and accuracy of the results, this study conducted a comprehensive sensitivity analysis using multiple methodological approaches. Heterogeneity was assessed using Cochrane *Q* test, with a *P*-value threshold of > .05 indicating no significant heterogeneity. Furthermore, potential pleiotropy was evaluated via the MR-Egger intercept test; similarly, a *P*-value > .05 suggested no directional pleiotropy. Importantly, directional pleiotropy was considered a violation of assumptions, and any protein exhibiting such pleiotropy (*P* ≤ .05) was excluded from subsequent analyses.

For variables showing statistically significant associations in the IVW analysis, we generated 4 diagnostic plots: scatter plots, forest plots, funnel plots, and leave-one-out plots (see Figures S1–S4, Supplemental Digital Content, https://links.lww.com/MD/Q853). The final selection required: consistent odds ratio (OR) directions across all 5 analytical methods (either all > 1.0 or all < 1.0), non-significant MR-Egger intercept test results (*P* ≥ .05), and retention of the most statistically significant measurement when repeated protein assessments were available.

#### 2.3.3. Reverse MR analysis

In this study, we performed reverse MR and sensitivity analyses by considering OS as the exposure and plasma proteins previously identified in forward MR analysis as outcomes. This analytical approach serves 2 key purposes: clarifying the causal direction between exposure and outcome, and detecting potential reverse causation, thereby minimizing confounding effects and enhancing result reliability.^[20]^ All statistical analyses, including forward MR, sensitivity analyses, and reverse MR, were conducted using the TwoSampleMR package (version 0.5.7) in R 4.3.3 (R Foundation for Statistical Computing, Vienna, Austria).

#### 2.3.4. Power analysis

To evaluate the statistical power of our study given the small number of OS cases in the FinnGen dataset (n = 64), we performed power analyses for all plasma proteins with putative causal associations. Power calculations were conducted using an online tool (https://sb452.shinyapps.io/power/). For each protein, we entered the sample size, case-to-control ratio, OR values, and other relevant parameters to calculate the power under a 2-sided significance level of α = 0.05.

### 2.4. Enrichment analysis

In this study, we systematically explored the biological functions of potential therapeutic targets using Gene Ontology (GO) and Kyoto Encyclopedia of Genes and Genomes (KEGG) enrichment analysis methods. We employed the R package ClusterProfiler^[22]^ to perform GO enrichment analysis (including BPs, molecular functions [MFs], and cellular components [CCs]) and KEGG pathway enrichment analysis on the key genes identified by MR. The screening criteria were set as the raw *P*-value < .05, and the Benjamini–Hochberg method was applied for multiple testing correction. The results were ultimately presented in an intuitive manner through bubble plots.

### 2.5. Construction of PPI network and identification of core genes

To gain a comprehensive understanding of the interactions between proteins within cells, this study utilized the STRING database (https://cn.string-db.org/) to construct a PPI network. During the construction process, the minimum interaction score was set at 0.15, and all unconnected nodes were removed. The remaining parameters were maintained at the default settings of the STRING database. Subsequently, the generated PPI network file was imported into the Cytoscape software (V3.10.3; University of California, San Diego) for visualization analysis. With the aid of the cytoHubba plugin in Cytoscape, the top 10 genes were screened out as core genes based on the node degree value.^[23]^ In the visualization display, nodes were color-coded according to their degree values, with red representing nodes of higher degree values, thereby intuitively presenting the distribution of key genes in the network.

### 2.6. Drug prediction

In drug target research, evaluating the interactions between proteins and drugs is essential for assessing the viability of potential drug targets. In this study, we employed the Drug Signatures Database (DSigDB) (http://dsigdb.tanlab.org/DSigDBv1.0/) to systematically analyze the associations between core genes and candidate drugs.^[24]^ Specifically, the screened core protein genes were uploaded to the DSigDB database, and its robust data analysis tools were utilized to predict potential drugs that may interact with these target genes. Subsequently, drug enrichment analysis was conducted using the ClusterProfiler package in R. To ensure the identification of drugs strongly associated with the core genes, we applied stringent significance thresholds, requiring both the *P*-value and adjusted *P*-value (*P*.adjust) to be below .05. The enrichment analysis was carried out using the hypergeometric test to determine whether core genes were significantly overrepresented in the target gene sets of specific drugs. Finally, significant results (*P* < .05) were selected, and the findings were visualized. Two types of charts were generated: a bar chart displaying the ranking of enriched drugs and their associated gene proportions, and a gene–drug interaction network diagram to visually represent the complex relationships between core genes and potential drugs.

### 2.7. Molecular docking

In this study, molecular docking was employed to assess the binding affinity and interaction patterns between candidate drugs and their targets. Based on the results from the previous drug enrichment analysis, the top 5 candidate drugs were selected for molecular docking. Prior to docking, the structural data for both the drugs and proteins had to be prepared. The drug structures were retrieved from the PubChem Compound Database (https://pubchem.ncbi.nlm.nih.gov/). Protein structures were identified through the UniProt database to obtain the corresponding UniProt ID. The target protein was then located and downloaded from the Protein Data Bank (https://www.rcsb.org/) using this ID for the docking analysis. Molecular docking was performed using the CB-Dock2 platform (https://cadd.labshare.cn/cb-dock2/index.php).^[25]^

## 3. Results

### 3.1. Forward MR Analysis

**Figure 2.**
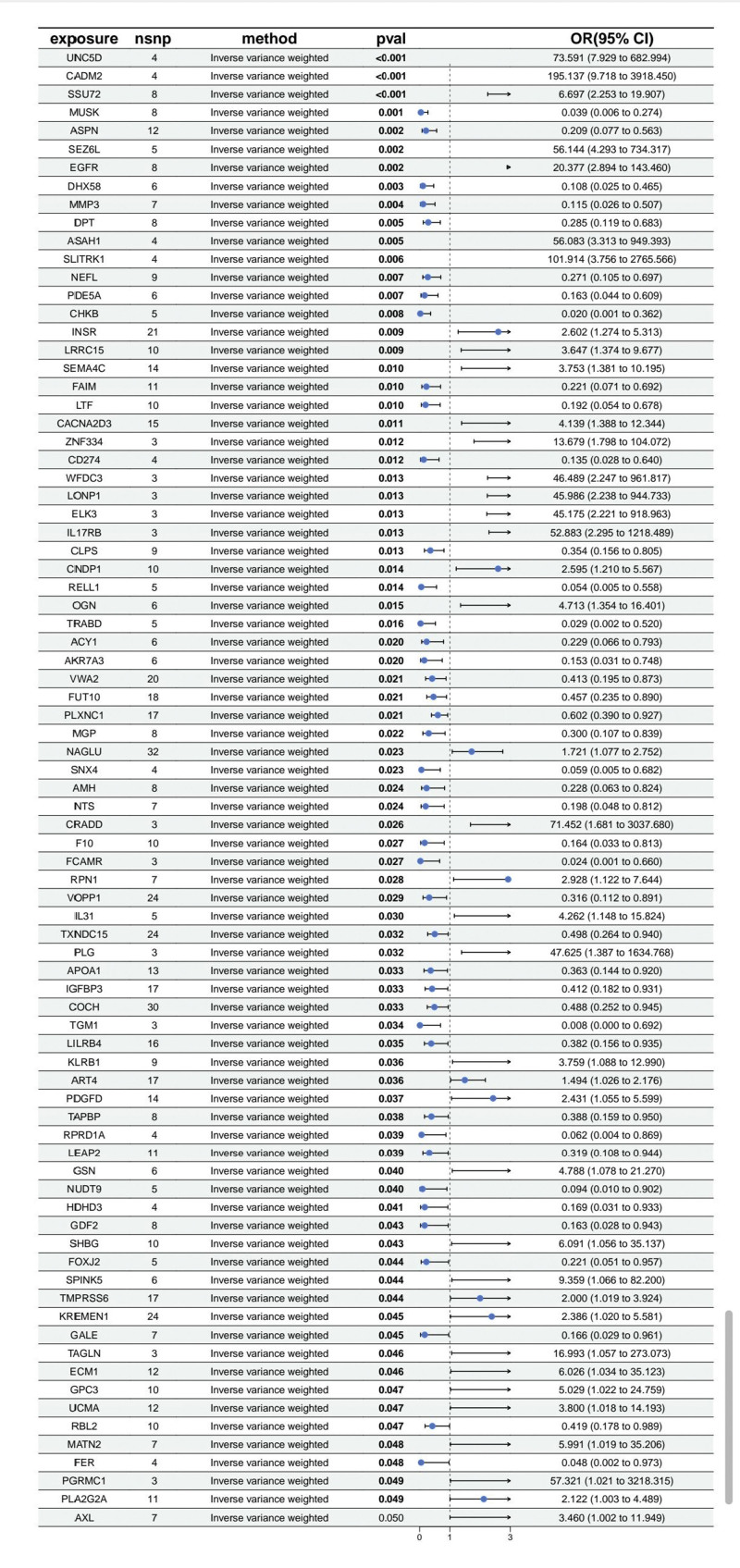
A forest plot summarizing the Mendelian randomization analysis results of plasma proteins associated with OS is presented. *P*val is determined from the IVW MR method. The blue dots represent odds ratios (OR), with horizontal lines indicating 95% confidence intervals (CI), and the vertical black dashed line represents OR = 1. OR = odds ratio, with values >1 indicating a positive association between the exposure and outcome, and values <1 indicating a negative association. CI = confidence interval, IVW = inverse variance weighted, MR = Mendelian randomization, OS = osteosarcoma.

After filtering, a total of 31,315 SNPs were retained for the final MR analysis. The results of the MR analysis, as depicted in Figure 2, unveiled 81 gene loci significantly associated with OS. For instance, the INSR locus (*P* = .009, OR = 2.602, 95% CI [1.274–5.313]) and the CNDP1 locus (*P* = .014, OR = 2.595, 95% CI [1.210–5.567]) exhibited positive correlations with OS, suggesting that increased expression of these proteins might augment the risk of OS occurrence. Conversely, the MUSK locus (*P* = .001, OR = 0.039, 95% CI [0.006–0.274]) and the ASPN locus (*P* = .002, OR = 0.209, 95% CI [0.077–0.563]) demonstrated negative correlations with OS, indicating that these proteins may exert protective effects by inhibiting or reversing OS. These findings suggest that alterations in the expression levels of specific proteins may serve as potential biomarkers influencing the phenotypic variation of OS. MR diagnostic plots for the 81 plasma proteins potentially causally associated with OS are provided in the Supplementary Figures: scatter plots in Figure S5, forest plots in Figure S6, and leave-one-out analyses in Figure S7, Supplemental Digital Content, https://links.lww.com/MD/Q853.

### 3.2. Sensitivity analysis

Heterogeneity and pleiotropy were assessed using Cochran *Q* test and Egger intercept test, respectively. The results indicated that for the 81 plasma proteins associated with OS, the *P*-values from Cochran *Q* test were all >.05, suggesting no significant heterogeneity. Similarly, the *P*-values from Egger intercept test for these analyses were also >.05, indicating no substantial horizontal pleiotropy. The sensitivity analysis thus confirmed the robustness of our findings (Table S1, Supplemental Digital Content, https://links.lww.com/MD/Q853).

### 3.3. Reverse MR analysis

Plasma protein levels served as exposures while OS was designated as the outcome variable in the forward MR. The results demonstrated statistically significant causal associations between 81 plasma proteins and OS, suggesting that dysregulation of these proteins may potentially influence both the incidence and progression of OS. To further investigate whether OS influences the expression of these 81 plasma proteins, we conducted an inverse MR analysis. For this purpose, we set a threshold of *P* < 5e-6 for the selection of IVs. After erforming association analysis and linkage disequilibrium adjustment on the GWAS data for OS, we identified 11 IVs. The IVW analysis revealed *P*-values exceeding .05 across the board, indicating that the occurrence of OS does not impact the dysregulated expression of these proteins (Table S2, Supplemental Digital Content, https://links.lww.com/MD/Q853).

### 3.4. Power analysis

we performed post hoc power analyses for all 81 plasma proteins with putative causal associations using the online tool (https://sb452.shinyapps.io/power/). The results showed that 28 proteins (34.6%) achieved ≥ 80% power to detect their corresponding ORs at a significance level of α = 0.05, whereas 53 proteins (65.4%) were underpowered (<80%). This finding highlights a substantial risk of type II error, suggesting that some true associations may have been missed. Detailed power estimates for each protein are provided in Table S3, Supplemental Digital Content, https://links.lww.com/MD/Q853.

### 3.5. Enrichment analysis

**Figure 3.**
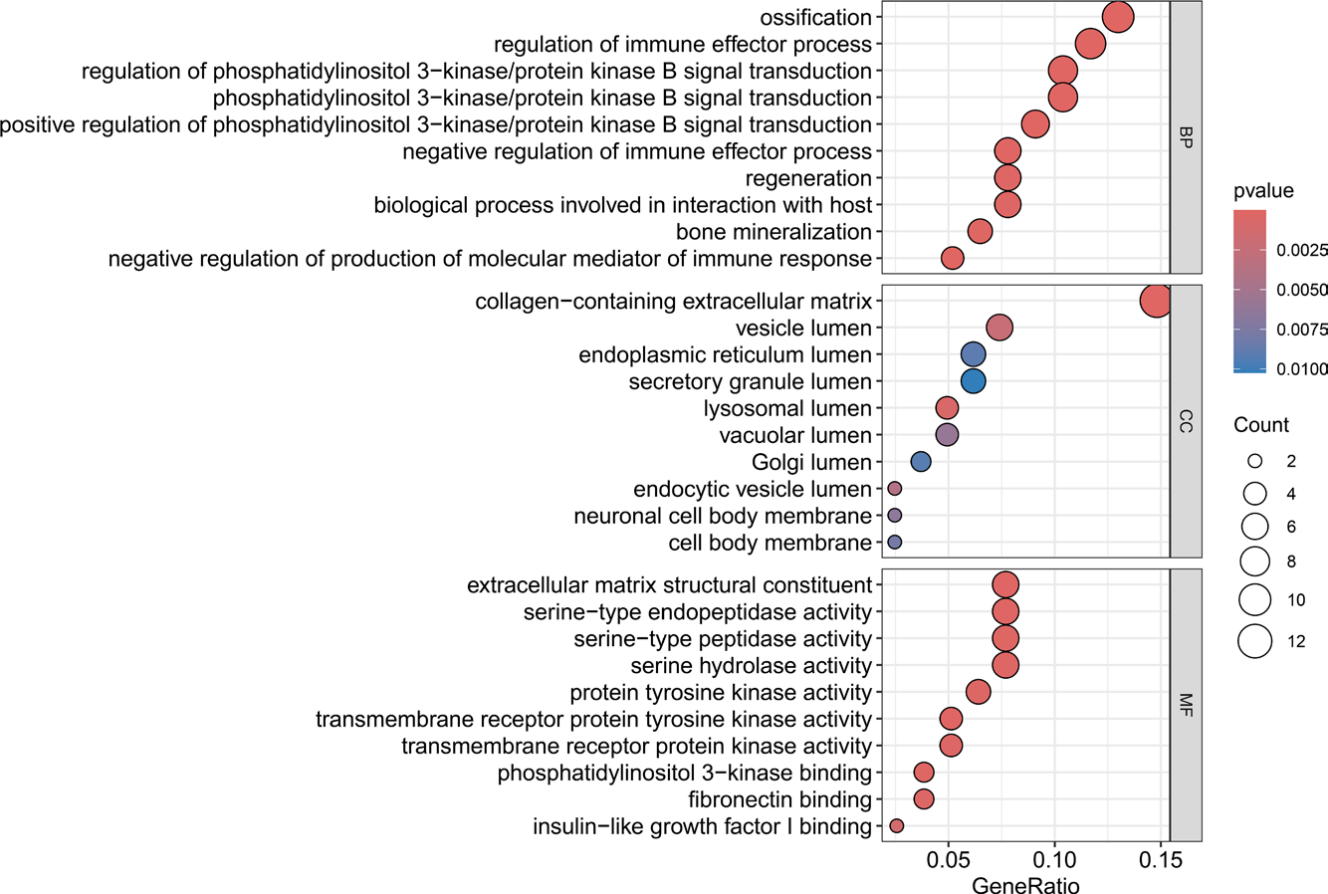
GO enrichment results for 3 terms. The color of the bubbles indicates the *P*-value, and the size of the bubbles represents the number of genes enriched in the term. GO = Gene Ontology.

**Figure 4.**
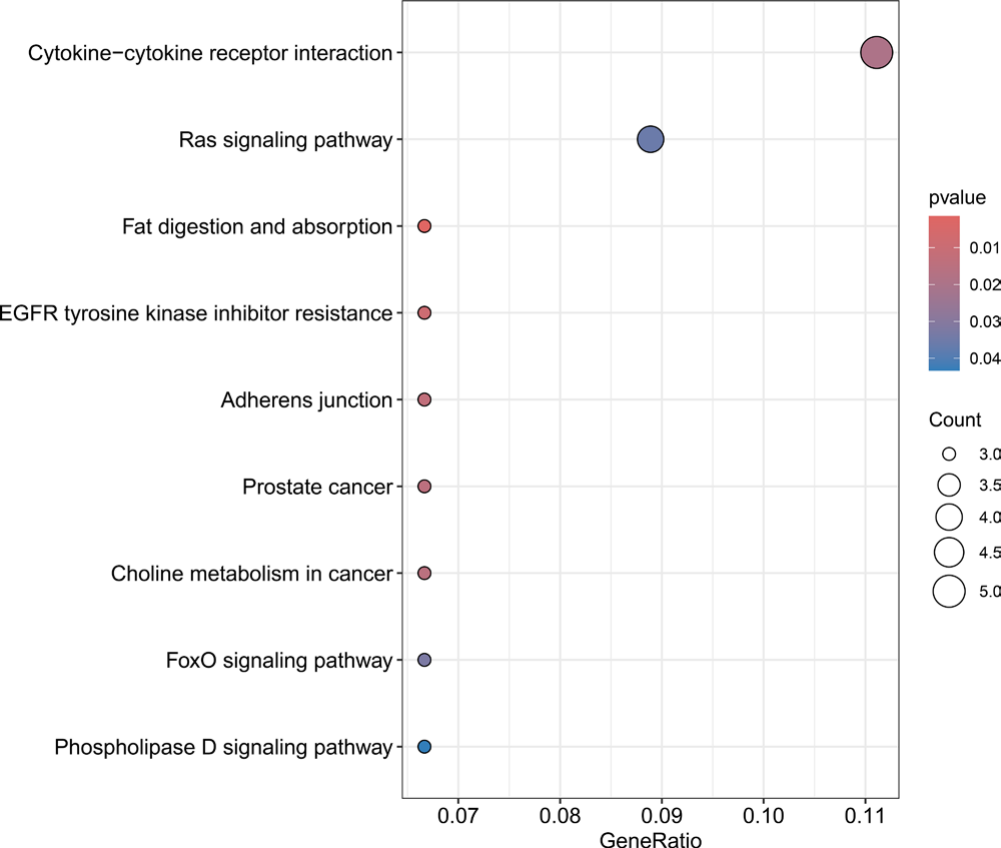
KEGG enrichment results. The color of the bubbles represents the *P*-value, and the size of the bubbles indicates the number of genes enriched in the pathway. KEGG = Kyoto Encyclopedia of Genes and Genomes.

To identify and comprehend the functional characteristics of genes and the biological processes in which they are involved, we conducted GO enrichment analysis, which encompasses 3 categories: BP, CC, and MF. Additionally, we used KEGG enrichment analysis to elucidate the roles of genes in specific biological pathways.^[26]^ As depicted in Figure 3, the significantly enriched terms within the BP category are associated with a variety of biological processes, such as the regulation of immune effector processes, the regulation of phosphatidylinositol 3-kinase/protein kinase B signaling, ossification, regeneration, and BPs interacting with the host, among others. In the CC category, the enrichment of terms such as extracellular matrix (ECM) components, vesicle lumen, endoplasmic reticulum lumen, and secretory granule lumen highlights active cellular interactions of ECM components, membrane microdomains, and synaptic vesicles, as well as cellular membrane structures and neurobiological functions. Moreover, in the MF category, the enriched terms, including ECM structural constituent, serine-type endopeptidase activity, and protein tyrosine kinase activity, emphasize the significance of gene products in biochemical functions such as cell signaling, membrane fusion, and protein binding. According to Figure 4, the top 5 pathways revealed by KEGG enrichment analysis are cytokine–cytokine receptor interaction, Ras signaling pathway, fat digestion and absorption, EGFR tyrosine kinase inhibitor resistance, and adherens junction.

### 3.6. PPI network construction and identification of hub genes

**Figure 5.**
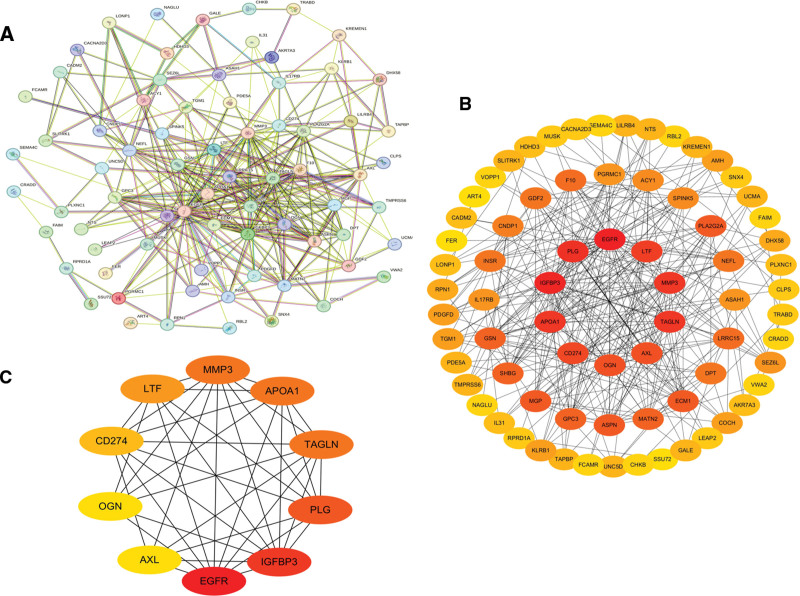
PPI network construction diagram. (A) PPI network built with STRING. (B) Full PPI network of selected genes. Key clusters with hub genes highlighted in red. (C) Core sub-network showing interactions among top hub genes. PPI = protein–protein interaction.

A total of 81 gene proteins were submitted to the STRING database for network construction. The resultant file was imported into Cytoscape software (version 3.10.3) for visualization, and the cytoHubba plugin was employed to identify hub genes. As depicted in Figure 5, the PPI network comprises 73 nodes and 656 edges. The identified hub genes include EGFR, IGFBP3, PLG, MMP3, APOA1, TAGLN, LTF, CD274, OGN, and AXL. Network analysis reveals that these genes are implicated in critical biological processes, such as cell proliferation, ECM remodeling, immune regulation, lipid metabolism, and signal transduction. These processes are inextricably linked to the occurrence and progression of OS.

### 3.7. Candidate drug prediction

**Figure 6.**
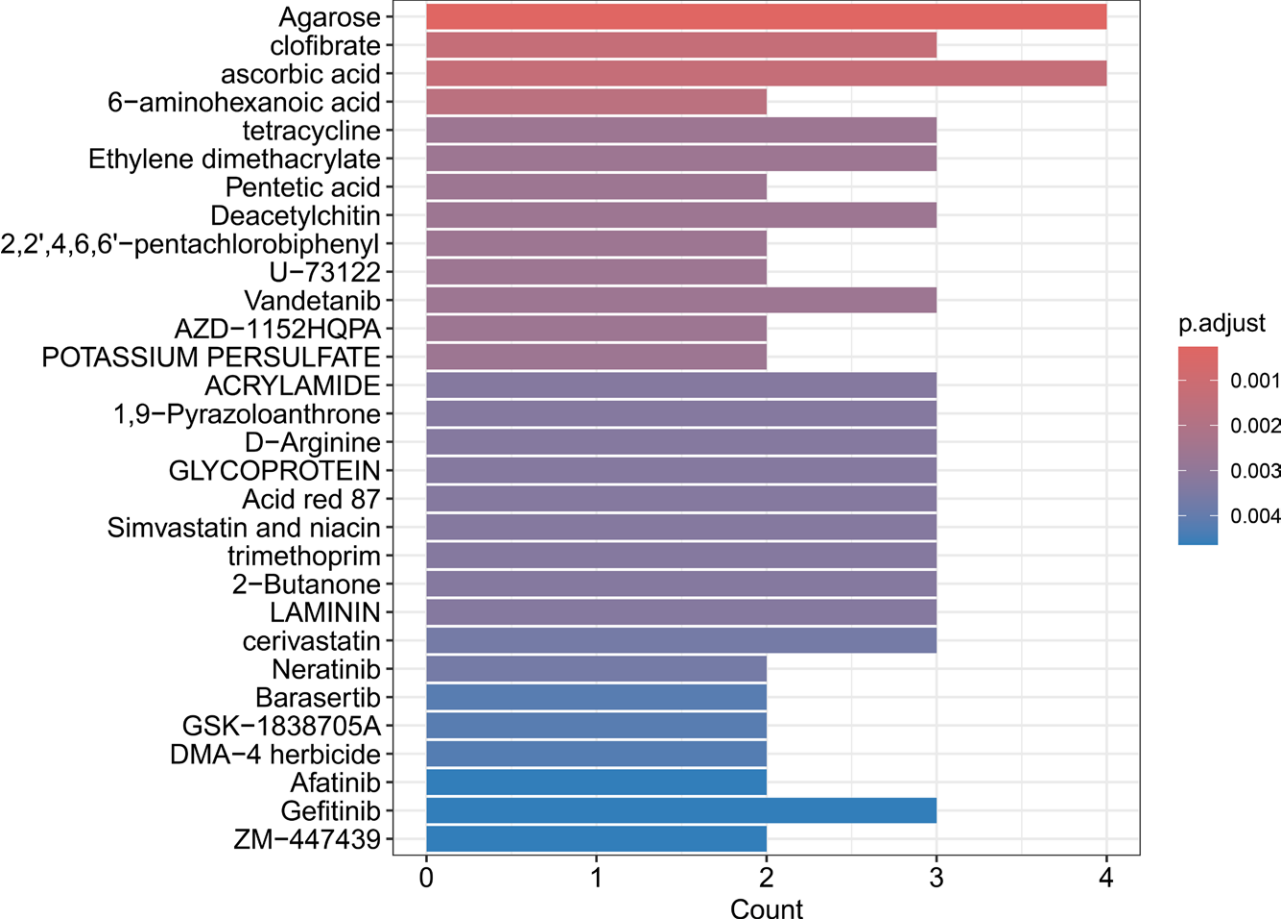
Bar chart of drug prediction results. The color of the bars represents the *P*-value.

**Figure 7.**
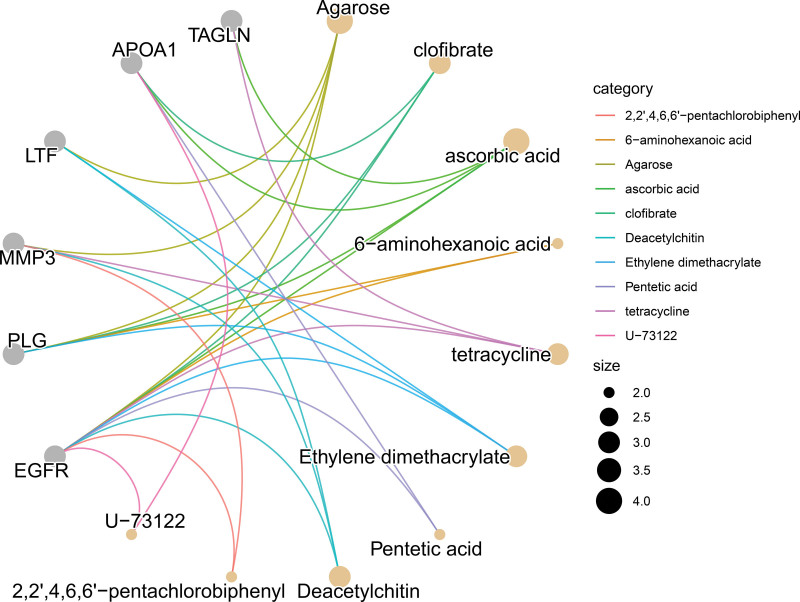
Gene–drug interaction network diagram.

Initially, the present study employed DSigDB to predict potential therapeutic interventions. Subsequently, drug enrichment analysis was conducted utilizing the R package clusterProfiler. The results indicated that 5’-adenylic acid (Agarose), clofibrate, ascorbic acid (vitamin C), and 6-aminohexanoic acid are key drugs associated with the hub genes (Figs. 6 and 7).

### 3.8. Molecular docking

**Figure 8.**
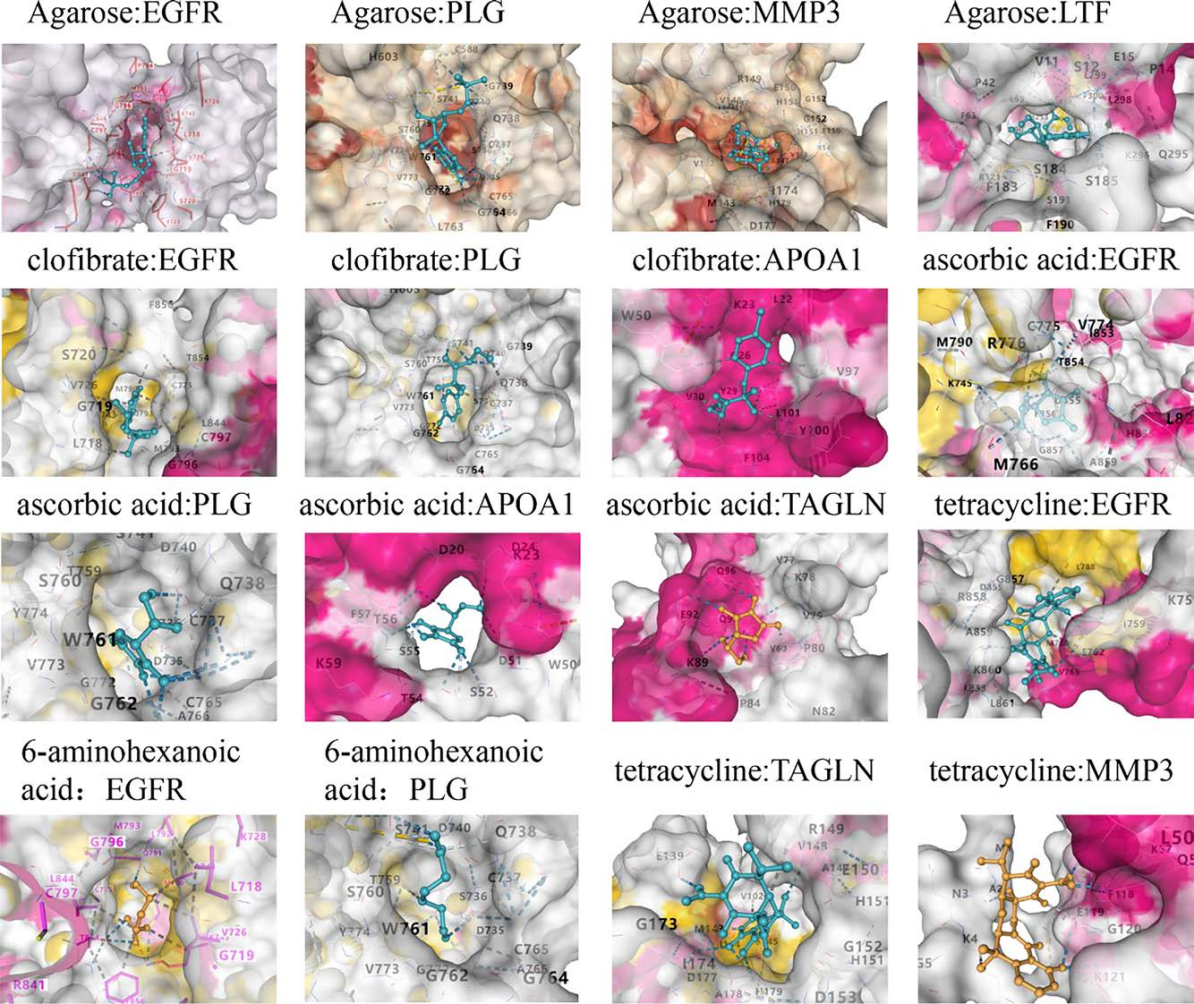
Docking results of available proteins small molecules. (*Note*: the images were generated using the online platform https://cadd.labshare.cn/cb-dock2/. Color coding: receptor proteins are shown as cyan or yellow stick representation, ligands are displayed in color, and hydrogen bonds are indicated by yellow dashed lines.)

In this study, molecular docking analysis was conducted to assess the binding affinity of candidate drugs to their respective targets and to evaluate the druggability of these targets. The CB-Dock2 platform was utilized to explore the binding interactions between 5 candidate drugs and their corresponding target proteins (Fig. 8). The analysis revealed that sorafenib had the most stable binding interaction with MAPK8, with a binding energy of −10.6 kcal/mol. The binding energy values for all drugs ranged from −4.4 to −10.6 kcal/mol (Table 1), indicating that each drug had a favorable binding affinity to its corresponding target protein. Detailed information on the interaction residues between the candidate drugs and the target proteins is shown in Figure S5, Supplemental Digital Content, https://links.lww.com/MD/Q853.

**Table 1:** Docking results of available proteins with small molecules.

Drug	PubChem ID	Target	UniProt ID	PDB ID	Binding energy (kcal/mol)
Agarose	6083	EGFR	P00533	5ug8	-7.2
Agarose	6083	PLG	P00747	5ugd	-7.4
Agarose	6083	MMP3	P08254	1c3i	-8.9
Agarose	6083	LTF	P02788	1h45	-8.2
Clofibrate	2796	EGFR	P00533	5ug8	-7.3
Clofibrate	2796	PLG	P00747	5ugd	-6
Clofibrate	2796	APOA1	P02647	3r2p	-5.4
Ascorbic acid	54670067	EGFR	P00533	5ug8	-5.2
Ascorbic acid	54670067	PLG	P00747	5ugd	-6.2
Ascorbic acid	54670067	APOA1	P02647	3r2p	-5.2
Ascorbic acid	54670067	TAGLN	Q01995	AF-Q01995-F1-model_v4	-5
6-Aminohexanoic acid	564	EGFR	P00533	5ug8	-4.7
6-Aminohexanoic acid	564	PLG	P00747	5ugd	-4.7
Tetracycline	54675776	EGFR	P00533	5ug8	-6.7
Tetracycline	54675776	MMP3	P08254	1c3i	-7.3
Tetracycline	54675776	TAGLN	Q01995	AF-Q01995-F1-model_v4	-6.8

## 4. Discussion

In this study, we performed MR analysis using GWAS data from 4907 plasma proteins, identifying 81 potential therapeutic targets within the plasma proteome associated with OS. To explore the biological significance of these targets, we conducted enrichment and PPI network analyses to identify key genes. Furthermore, we carried out drug prediction and molecular docking analyses, which revealed several drugs with potential anti-OS effects. These findings not only broaden the clinical applications of existing drugs but also provide a theoretical basis for their future use in OS treatment.

The core genes identified in this study include EGFR, IGFBP3, PLG, TAGLN, APOA1, MMP3, LTF, CD274, OGN, and AXL. EGFR plays a critical role in cell differentiation, apoptosis, proliferation, and migration.^[27,28]^ Abnormal EGFR expression has been observed in various cancers.^[29,30]^ In the context of OS, numerous studies have demonstrated a strong association between EGFR and disease progression. For instance, Zhao et al^[31]^ identified epi-EGFR as a hub gene through bioinformatics analysis, showing a significant correlation with the prognosis of OS patients, and confirming it as an independent prognostic factor for OS. Moreover, diallyl trisulfide has been reported to induce autophagy, inhibit cell migration, and promote apoptosis by suppressing EGFR and its downstream signaling pathways, achieving therapeutic effects in OS.^[32]^ IGFBP3 is a pleiotropic protein that inhibits cell growth and induces apoptosis.^[33]^ As a carrier of IGF-1, IGFBP3 inhibits cell proliferation by prolonging the half-life of IGF-1 and restricting its binding to the IGF-1 receptor (IGF-1R).^[34]^ Notably, even in the absence of IGF-1, IGFBP3 can regulate tumor development, metastasis, and recurrence by participating in various cell signaling pathways. Tan et al^[35]^ showed that miR-384 inhibits the malignant behavior of OS by regulating IGFBP3. The TAGLN gene encodes transgelin, a protein that stabilizes the cytoskeleton by binding to actin, regulating cell growth, differentiation, migration, invasion, and matrix remodeling.^[36–38]^ Zhao et al^[39]^ reported that TAGLN is a direct target of miR-144 in OS, and miR-144 suppresses TAGLN expression to exert its anti-metastatic effects in OS. APOA1 is the principal protein component of high-density lipoprotein cholesterol and a key mediator of cholesterol homeostasis. It exerts anti-tumor effects by modulating the immune system and inhibiting angiogenesis.^[40,41]^ Ma et al^[42]^ revealed through a retrospective study that OS patients with higher preoperative platelet-to-albumin ratio and apolipoprotein B-to-A1 ratio had significantly shorter overall survival, indicating that APOA1 is a protective factor in OS, consistent with the findings of this study. Matrix metalloproteinases (MMPs) are crucial enzymes involved in the degradation of the ECM, and their altered expression is closely linked to the invasion and metastasis of OS.^[43,44]^ Elevated MMP3 levels are often associated with an increased risk of metastasis in OS.^[45–47]^ However, our current study found that MMP3 may act as a protective factor in OS, a result that warrants further experimental validation. Lactoferrin (LTF), a principal iron-binding protein found in milk and various bodily secretions, exhibits a broad range of properties, including regulating iron homeostasis, providing anti-inflammatory effects, and preventing cancer development and metastasis. Bioinformatics studies have identified downregulated LTF expression in OS, suggesting a suppressive role in the pathogenesis and progression of this malignancy.^[48]^ Similarly, Liu et al^[49]^ conducted a systematic bioinformatics analysis and experimental validation, suggesting that LTF may serve as a prognostic biomarker and therapeutic target for OS. CD274, also known as programmed death-ligand 1, is a type I transmembrane protein with a molecular weight of 40 kDa. Studies using genomic analysis, transcriptomics, immunohistochemistry, and in vitro experiments have explored CD274’s role in OS. These studies have revealed copy number gains and overexpression of CD274 in OS, with elevated expression being significantly associated with poor prognosis. These findings suggest that CD274 may serve as an immunotherapeutic target in OS, particularly in patients with CD274 copy number amplification.^[50]^ Additionally, Wu et al^[51]^ identified CD274 overexpression in OS through bioinformatics and qRT-PCR analyses, which correlated with adverse prognosis, and suggested that CD274 may promote immune evasion by inhibiting the anti-tumor activity of T cells. These results indicate that CD274 could represent a potential immunotherapeutic target in OS, especially in patients with high expression of ZYX, CAV1, and ITGA5. AXL, a receptor tyrosine kinase, has been implicated in the aggressiveness, metastasis, and chemoresistance of various cancers. In OS, multifaceted approaches including in vitro model experiments, gene expression analysis, immunohistochemistry, and animal studies have demonstrated that AXL plays a pivotal role in OS metastasis. The high expression of AXL is closely associated with cellular dedifferentiation, proliferation, and migration. Inhibition of AXL has been shown to significantly reduce pulmonary metastasis of OS cells, making AXL a novel therapeutic target for OS.^[52]^ Currently, there is limited research on the relationship between PLG, OGN, and OS, underscoring the need for further investigation to clarify their roles in OS pathogenesis.

Immune factors within cytokine–cytokine receptor interactions, such as interleukin-6, can activate signal transducer and activator of transcription 3, thereby promoting the growth and metastasis of OS both in vivo and in vitro, facilitating the continuous progression of OS.^[53,54]^ The Ras signaling pathway exerts a significant influence on the oncogenesis, progression, and metastasis of OS by activating downstream molecules, such as mitogen-activated protein kinase, Rac1, and extracellular signal-regulated kinase 1/2.^[55–57]^ Although there is currently no definitive evidence explicitly linking the EGFR tyrosine kinase inhibitor resistance pathway to OS, the fact that EGFR can activate the downstream PI3K/AKT/mTOR pathway, which in turn promotes tumor proliferation, survival, invasion, and drug resistance,^[58]^ suggests that the EGFR tyrosine kinase inhibitor resistance pathway may potentially facilitate the progression of OS. Zhang et al^[59]^ employed machine learning to elucidate that the adherens junction pathway may influence the progression of OS by modulating cell adhesion and metastasis regulation. Although there is currently no direct evidence linking the Fat digestion and absorption pathway to OS, several studies have demonstrated a close relationship between lipid metabolism and OS.^[60,61]^ Therefore, it is plausible that the fat digestion and absorption pathway may indirectly contribute to OS progression by influencing metabolic processes. Collectively, these pathways are likely to synergistically promote the occurrence, development, and metastasis of OS, thereby providing potential therapeutic targets for its treatment.

The present study identified several potential therapeutic agents for OS treatment, among which ascorbic acid has been previously reported to possess potential anti-OS effects. For instance, Valenti et al^[62]^ demonstrated via PCR that ascorbic acid can induce OS cell death at relatively high concentrations. Furthermore, it has been shown that ascorbic acid exerts a certain cytotoxic effect on OS stem cells,^[63]^ which further corroborates the potential therapeutic efficacy of this agent against OS. Other compounds, such as agarose, clofibrate, 6-aminohexanoic acid, and tetracycline, currently lack direct evidence supporting their application in OS treatment. Therefore, future studies should employ cell-based and animal-based experiments to specifically evaluate whether agarose, clofibrate, 6-aminohexanoic acid, and tetracycline themselves possess anti-OS activity.

Despite the significant advancements made in this study, several limitations must be acknowledged. First, the study population primarily consisted of individuals of European descent, which may limit the generalizability of the findings. Future research involving diverse ethnic groups is essential to validate the broader applicability of these results. Second, the GWAS data for OS lacked stratification by specific subtypes, preventing us from conducting subtype-specific analyses. This limitation highlights the need for more detailed genetic data to explore potential differences in genetic predispositions across OS subtypes. Third, although we assessed the robustness of our findings through various sensitivity analyses, including MR-Egger regression, weighted median and mode methods, Cochran *Q* test, and the MR-PRESSO pleiotropy test, MR analysis remains vulnerable to the influence of unmeasured confounding factors and pleiotropy. These factors could introduce bias, affecting the validity of the causal inferences drawn from our analyses. Lastly, although we identified several potential therapeutic targets, the relatively small number of cases included in the analysis resulted in limited statistical power and a higher risk of type II error, leaving the clinical efficacy of these targets uncertain. Further experimental studies and clinical trials are needed to validate their therapeutic potential and translate these findings into effective treatment strategies for OS.

## 5. Conclusion

This study systematically examined the potential causal relationships between plasma proteins and OS using the MR approach. A total of 81 putative therapeutic targets were identified, and several core genes were further pinpointed. These key genes were mainly involved in critical biological processes, including cell proliferation, ECM remodeling, immune regulation, lipid metabolism, and signal transduction. Through drug enrichment analysis and molecular docking, several candidate therapeutic agents (such as agarose, clofibrate, and ascorbic acid) were identified that may hold therapeutic promise for OS. Importantly, these findings should be regarded as preliminary and hypothesis-generating due to the relatively small number of OS cases and the lack of experimental or clinical validation. They provide novel insights into the molecular mechanisms of OS and a basis for precision treatment strategies, but further functional studies, large-scale cohorts, and clinical trials are warranted to confirm the causal relationships, evaluate drug efficacy, and assess safety before potential clinical translation.

Supplemental digital content “Tables S4 and S5” are available for this article (https://links.lww.com/MD/Q853).

## Acknowledgments

We are grateful to the deCODE database for supplying us with the GWAS summary statistics used in our analysis. Our appreciation also goes to the participants and researchers involved in the FinnGen study. The FinnGen study represents a major genomics effort, having examined over 500,000 biobank samples from Finland and explored the links between genetic variations and health data in order to shed light on disease mechanisms and genetic predispositions. This project is a collaborative endeavor involving research institutions, biobanks in Finland, and international industry partners.

## Author contributions

**Conceptualization**: Liang Zhao, Junwu Ye.

**Data curation**: Liang Zhao.

**Formal analysis**: Liang Zhao, Juncheng Shen, Xuzhou Zheng, Qiao Yuan.

**Funding acquisition**: Junwu Ye.

**Methodology**: Liang Zhao.

**Project administration**: Junwu Ye.

**Software**: Liang Zhao.

**Supervision**: Junwu Ye.

**Visualization**: Liang Zhao, Juncheng Shen.

**Writing – original draft**: Liang Zhao.

**Writing – review & editing**: Liang Zhao, Juncheng Shen, Xuzhou Zheng, Qiao Yuan, Junwu Ye.

## Supplementary Material
