# Supplementary material for: Plasma proteins and osteosarcoma: Mendelian randomization analysis and targeted therapeutic discovery
Source: Medicine (Baltimore). 2026 May 12;104(49):e45704. doi: 10.1097/MD.0000000000045704 (PMC12688887; doi:10.1097/MD.0000000000045704)
Supplement: Supplementary file 1 [file medi-104-e45704-s001.pdf]

Table S1. Results of a sensitivity analysis of a positive mendelian randomization analysis for OS as an outcome.

Exposure	Heterogeneity analysis						Pleiotropy analysis		
	Inverse variance weighted			MR Egger			MR Egger		
	Q	Q_df	Q_pval	Q	Q_df	Q_pval	Egger_intercept	se	pval
UNC5D	1.37348223	3	0.71176225	1.3426694	2	0.5110261	0.052674148	0.30008	0.8768
CADM2	1.24667604	3	0.74183259	1.2358409	2	0.5390643	-0.037044086	0.35588	0.9266
SSU72	4.09444808	7	0.76883686	4.0853417	6	0.6651284	0.04197064	0.43982	0.9271
MUSK	5.92653831	7	0.54835289	5.4934323	6	0.4822509	-0.144180786	0.21908	0.5349
ASPN	12.7378503	11	0.31080417	12.732256	10	0.2390293	0.009190189	0.13865	0.9485
SEZ6L	3.3048689	4	0.50816119	3.0678468	3	0.3812824	-0.13709556	0.28476	0.6631
EGFR	7.34593577	7	0.39377108	4.6678004	6	0.5870695	-0.346098963	0.21149	0.1529
DHX58	4.37412959	5	0.49689896	4.3674246	4	0.3585582	0.015815128	0.20182	0.9413
MMP3	5.94927327	6	0.42889649	4.4971533	5	0.4802644	-0.276442473	0.22941	0.2821
DPT	1.7145005	7	0.9739427	1.3264926	6	0.9701768	-0.103425303	0.16604	0.5563
ASAH1	0.60079522	3	0.8962503	0.3077314	2	0.8573872	0.153430064	0.28342	0.6425
SLITRK1	2.04197662	3	0.56374017	2.0221711	2	0.3638238	0.084826125	0.60608	0.9015
NEFL	6.81214068	8	0.55703045	6.0764744	7	0.5308488	-0.103931133	0.12117	0.4194
PDE5A	0.92471771	5	0.96840644	0.8313136	4	0.9342014	0.082451126	0.26978	0.7751
CHKB	2.33771657	4	0.67391089	1.8825913	3	0.5971291	-0.283070508	0.41959	0.5482
INSR	20.5345188	20	0.42497036	20.007869	19	0.3941048	0.057084424	0.08072	0.488
LRRC15	9.78222363	9	0.36840156	9.7709574	8	0.2814712	0.01185574	0.12344	0.9258
SEMA4C	7.6772237	13	0.86397434	6.0470725	12	0.9136906	-0.265709572	0.20811	0.2258
FAIM	9.21779249	10	0.51156668	8.4984547	9	0.4847958	-0.134855557	0.159	0.4183
LTF	5.35013103	9	0.80278111	1.9384196	8	0.9828419	0.234910043	0.12718	0.1019
CACNA2D3	12.3640808	14	0.57708938	11.744946	13	0.548661	-0.083546401	0.10618	0.4455
ZNF334	1.6864519	2	0.43032009	0.5303663	1	0.4664529	-0.274526086	0.25532	0.4769
CD274	5.05828949	3	0.16757832	3.0353253	2	0.2192237	-0.250238797	0.21674	0.3676
WFDC3	0.44291723	2	0.80134909	0.2838825	1	0.5941677	0.159509848	0.39998	0.7584
LONP1	0.44924375	2	0.79881822	0.2789772	1	0.5973724	0.161225404	0.39072	0.7509
ELK3	0.4662982	2	0.79203547	0.3042039	1	0.5812589	0.165234481	0.41041	0.7563
IL17RB	0.4664984	2	0.79195619	0.3022719	1	0.5824616	0.165271426	0.40783	0.7549
CLPS	2.44853143	4	0.65387514	0.1994507	3	0.9776779	-0.419529892	0.27974	0.2307
CNDP1	2.75161371	9	0.97333429	2.737556	8	0.9497329	-0.027492194	0.23187	0.9085
RELL1	0.65381178	4	0.95690031	0.2770144	3	0.9642919	-0.156444486	0.25486	0.5827
OGN	1.77138666	5	0.87978635	1.5629786	4	0.8154301	-0.079115142	0.1733	0.6717
TRABD	1.45647899	4	0.83432168	1.4418837	3	0.695747	-0.094406105	0.78144	0.9115
ACY1	5.70071504	5	0.33643815	5.6579979	4	0.2261865	-0.03292355	0.18946	0.8705
AKR7A3	6.08162556	5	0.29835725	5.9940831	4	0.1995906	0.067101094	0.27762	0.8209
VWA2	16.107774	19	0.65007549	15.625195	18	0.6186869	0.087506806	0.12597	0.4961
FUT10	12.3849496	17	0.77628758	11.93885	16	0.7481784	0.061275632	0.09174	0.5137
PLXNC1	19.0660492	16	0.26525296	18.740489	15	0.2257354	0.053975986	0.10574	0.6172

MGP	7.12457425	7	0.41602562	6.0272454	6	0.420145	-0.183799666	0.17586	0.3362
NAGLU	24.0151401	31	0.80992626	21.579178	30	0.8689084	-0.153915328	0.09862	0.1291
SNX4	2.79849131	3	0.42374833	0.038244	2	0.9810597	0.6263804	0.37702	0.2385
AMH	4.68107694	7	0.69881702	4.6493803	6	0.5895022	0.023837451	0.13389	0.8646
NTS	6.95011788	6	0.32548494	6.8452968	5	0.2324024	0.035896399	0.12973	0.7931
CRADD	1.61874779	2	0.44513668	1.2620517	1	0.2612631	-0.212041024	0.39885	0.6889
F10	5.81438997	9	0.75834164	5.7557222	8	0.674573	0.068113432	0.34395	0.8527
FCAMR	1.63815009	2	0.44083922	0.0903775	1	0.7636978	1.695094049	1.36251	0.431
RPN1	4.38862554	6	0.62423914	4.3880481	5	0.4950005	-0.003325541	0.13839	0.9818
VOPP1	23.6827632	23	0.42154524	21.436111	22	0.4939437	0.182858154	0.122	0.1481
IL31	1.47183478	4	0.8316186	0.9907167	3	0.8034982	-0.131390431	0.18943	0.5378
TXNDC15	15.0691643	23	0.89213754	14.200297	22	0.894218	0.076951494	0.08255	0.3614
PLG	15.3966543	10	0.1182558	8.0045832	9	0.5336844	-0.394671288	0.14516	0.0237
APOA1	15.84745	12	0.19832347	13.44235	11	0.2653989	-0.207442214	0.14787	0.1882
IGFBP3	16.6814763	16	0.40649233	15.470889	15	0.4180597	0.111907894	0.10329	0.2957
COCH	24.6349257	29	0.69704548	21.894875	28	0.7861347	-0.101244594	0.06116	0.109
TGM1	2.71285275	2	0.25757963	1.4224088	1	0.2330073	1.48677104	1.56094	0.5155
LILRB4	10.9919656	15	0.75316371	10.694457	14	0.7098545	0.055790174	0.10228	0.594
KLRB1	4.33224318	8	0.82597257	4.2473303	7	0.7508898	-0.040313561	0.13835	0.7792
ART4	16.3037448	16	0.43197013	16.223752	15	0.3673388	-0.040180505	0.14775	0.7894
PDGFD	1.58412493	4	0.81164251	1.4424564	3	0.6956136	-0.103187228	0.27415	0.7317
TAPBP	5.52146627	7	0.59659591	3.8022559	6	0.7034158	-0.214716389	0.16376	0.2377
RPRD1A	2.44345019	3	0.48559764	1.9637587	2	0.3746064	-0.184901041	0.26697	0.5602
LEAP2	7.3006398	10	0.69679046	7.2702867	9	0.6090012	0.020082154	0.11527	0.8655
GSN	0.44048675	5	0.99414075	0.3436944	4	0.9868217	-0.079898149	0.25681	0.7713
NUDT9	3.02860305	4	0.55305021	2.6566631	3	0.4476423	-0.175975643	0.28855	0.585
HDHD3	1.52070415	3	0.67750013	0.710508	2	0.7009954	0.202903793	0.22542	0.4631
GDF2	6.20626041	7	0.51588262	6.1893677	6	0.402315	0.029473777	0.23032	0.9024
SHBG	13.0585406	9	0.15997521	11.694905	8	0.1653444	-0.173014077	0.17914	0.3624
FOXJ2	3.48171311	4	0.48066435	3.4120686	3	0.3323469	-0.038932276	0.15733	0.8205
SPINK5	1.76732141	5	0.88031163	1.680753	4	0.7942108	0.076089228	0.25861	0.7832
TMPRSS6	8.69243335	16	0.92553715	7.9518153	15	0.9256999	-0.128843987	0.14972	0.403
KREMEN1	18.2780818	23	0.74223569	17.873854	22	0.7134407	-0.05820019	0.09154	0.5315
GALE	7.35948208	6	0.28887756	7.3569162	5	0.1954202	-0.010649837	0.25503	0.9683
TAGLN	1.42479979	2	0.49046572	1.4199107	1	0.2334181	-0.025112365	0.42796	0.9627
ECM1	7.07546219	11	0.79293989	6.482375	10	0.7732402	-0.128880663	0.16735	0.459
GPC3	10.1717114	9	0.33676399	10.165691	8	0.2535887	-0.011488538	0.1669	0.9468
UCMA	10.7996121	11	0.46020078	10.792974	10	0.3738732	0.010677458	0.13615	0.939
RBL2	7.29848694	9	0.60607326	6.0536516	8	0.641222	0.140531913	0.12596	0.2969
MATN2	5.78346642	6	0.44787836	5.6757177	5	0.3390622	0.078624051	0.2552	0.7704
FER	2.24512606	3	0.52311472	2.00058	2	0.3677728	0.241015549	0.48745	0.67
PGRMC1	1.6428019	2	0.43981506	0.459918	1	0.4976623	-1.372291093	1.26176	0.4733
PLA2G2A	9.62461274	10	0.47402095	9.4041863	9	0.400831	-0.080223744	0.17467	0.6569

AXL	7.73966308	6	0.25780343	6.1466341	5	0.2922112	0.241921795	0.21252	0.3066

Table S2. Results of the reverse Mendelian randomization analysis.

exposure	outcome	method	nsnp	b	se	pval	lo_ci	up_ci	or	or_lci95	or_uci95
OS	UNC5D	IVW	2	0.002	0.015111	0.896967	-0.02766	0.031575	1.001959	0.972717	1.032079
OS	CADM2	IVW	2	-0.01	0.015106	0.505845	-0.03966	0.019557	0.99	0.961119	1.01975
OS	SSU72	IVW	2	-0.022	0.014862	0.135413	-0.05132	0.006939	0.978053	0.949973	1.006964
OS	MUSK	IVW	2	-0.012	0.014854	0.408122	-0.0414	0.016827	0.987788	0.959443	1.016969
OS	ASPN	IVW	2	0.0083	0.015008	0.578953	-0.02109	0.037744	1.008363	0.979133	1.038465
OS	SEZ6L	IVW	2	0.0032	0.01543	0.836132	-0.02705	0.033434	1.003197	0.973311	1.034
OS	EGFR	IVW	2	0.015	0.015439	0.330222	-0.01523	0.045293	1.015146	0.984887	1.046334
OS	DHX58	IVW	2	-0.014	0.014813	0.360002	-0.04259	0.015474	0.986532	0.958301	1.015595
OS	MMP3	IVW	2	-0.009	0.015056	0.570336	-0.03806	0.020965	0.991491	0.962659	1.021187
OS	DPT	IVW	2	-0.019	0.015435	0.211814	-0.04953	0.010981	0.980912	0.951681	1.011041
OS	ASAH1	IVW	2	-0.021	0.015097	0.163882	-0.05061	0.008573	0.979202	0.950651	1.00861
OS	SLITRK1	IVW	2	0.0176	0.01494	0.238913	-0.01169	0.046877	1.017751	0.988381	1.047994
OS	NEFL	IVW	2	-1E-04	0.025124	0.99671	-0.04935	0.04914	0.999896	0.951851	1.050367
OS	PDE5A	IVW	2	-0.014	0.017335	0.428849	-0.04769	0.020262	0.986379	0.953429	1.020468
OS	CHKB	IVW	2	-0.012	0.015159	0.426373	-0.04177	0.017654	0.988015	0.95909	1.017811
OS	INSR	IVW	2	0.0018	0.01563	0.910687	-0.02888	0.032388	1.001755	0.971532	1.032918
OS	LRRC15	IVW	2	0.0092	0.015551	0.555862	-0.02132	0.03964	1.009202	0.978905	1.040436
OS	SEMA4C	IVW	2	-0.019	0.015007	0.196622	-0.04879	0.010036	0.98081	0.952381	1.010086
OS	FAIM	IVW	2	-0.015	0.015297	0.32063	-0.04518	0.01479	0.984922	0.95583	1.0149
OS	LTF	IVW	2	-0.01	0.015327	0.526229	-0.03976	0.020328	0.990333	0.961024	1.020536
OS	CACNA2D3	IVW	2	0.0009	0.015399	0.952654	-0.02927	0.031096	1.000915	0.971156	1.031585
OS	ZNF334	IVW	2	-0.024	0.014794	0.105493	-0.05294	0.005048	0.976336	0.948433	1.005061
OS	CD274	IVW	2	0.0027	0.014616	0.854186	-0.02596	0.031334	1.00269	0.974373	1.03183
OS	WFDC3	IVW	2	-0.022	0.015009	0.140839	-0.05152	0.007314	0.978139	0.949782	1.007341
OS	LONP1	IVW	2	-0.016	0.015093	0.294113	-0.04542	0.013748	0.98429	0.955598	1.013843
OS	ELK3	IVW	2	-0.017	0.015111	0.261119	-0.0466	0.012636	0.983163	0.954472	1.012716
OS	IL17RB	IVW	2	-0.023	0.015119	0.130247	-0.05251	0.006756	0.977383	0.948845	1.006779
OS	CLPS	IVW	2	-0.014	0.017163	0.431094	-0.04715	0.020127	0.986578	0.953941	1.020331
OS	CNDP1	IVW	2	-0.023	0.015553	0.140797	-0.05339	0.007577	0.977353	0.948008	1.007606
OS	RELL1	IVW	2	-0.003	0.028925	0.913699	-0.05983	0.053559	0.99687	0.941926	1.055019
OS	OGN	IVW	2	0.0017	0.014295	0.907365	-0.02635	0.029681	1.001665	0.97399	1.030126
OS	TRABD	IVW	2	-0.015	0.01481	0.302757	-0.04429	0.013766	0.984853	0.956676	1.013861
OS	ACY1	IVW	2	-0.005	0.015516	0.737713	-0.03561	0.025215	0.994817	0.96502	1.025536
OS	AKR7A3	IVW	2	-0.004	0.015194	0.789914	-0.03383	0.025732	0.99596	0.966738	1.026066
OS	VWA2	IVW	2	0.0041	0.01549	0.792284	-0.02628	0.034439	1.004087	0.974062	1.035039
OS	FUT10	IVW	2	-6E-04	0.012958	0.961959	-0.02602	0.02478	0.999382	0.974319	1.02509
OS	PLXNC1	IVW	2	-0.006	0.015512	0.677934	-0.03684	0.023961	0.993579	0.963826	1.02425
OS	MGP	IVW	2	-0.016	0.015136	0.288187	-0.04574	0.01359	0.984053	0.955288	1.013683
OS	MGP	IVW	2	-0.016	0.015136	0.288187	-0.04574	0.01359	0.984053	0.955288	1.013683
OS	SNX4	IVW	2	-0.02	0.015305	0.189468	-0.05008	0.009916	0.980117	0.951152	1.009965

OS	AMH	IVW	2	-0.017	0.015301	0.26791	-0.04694	0.013038	0.983191	0.954144	1.013123
OS	NTS	IVW	2	-0.022	0.015229	0.152269	-0.05165	0.008047	0.978435	0.949662	1.00808
OS	CRADD	IVW	2	-0.011	0.0149	0.444974	-0.04059	0.017824	0.988683	0.960226	1.017983
OS	F10	IVW	2	0.0105	0.015608	0.499385	-0.02005	0.041134	1.010598	0.980151	1.041992
OS	FCAMR	IVW	2	-0.015	0.014717	0.31183	-0.04373	0.013961	0.985226	0.957213	1.014058
OS	RPN1	IVW	2	-0.004	0.014963	0.814349	-0.03284	0.025813	0.996493	0.967693	1.026149
OS	VOPP1	IVW	2	0.0051	0.015428	0.740789	-0.02514	0.035343	1.005117	0.975178	1.035975
OS	IL31	IVW	2	0.0008	0.014891	0.956258	-0.02837	0.030003	1.000817	0.972029	1.030457
OS	TXNDC15	IVW	2	-0.007	0.015572	0.654896	-0.03748	0.023561	0.993064	0.963212	1.023841
OS	PLG	IVW	2	-2E-04	0.015287	0.987132	-0.03021	0.029715	0.999753	0.970243	1.030161
OS	APOA1	IVW	2	-7E-04	0.015301	0.964439	-0.03067	0.029307	0.999318	0.969794	1.029741
OS	IGFBP3	IVW	2	0.0077	0.015203	0.6128	-0.0221	0.037492	1.007724	0.978139	1.038203
OS	COCH	IVW	2	0.0013	0.016077	0.934079	-0.03018	0.03284	1.001331	0.97027	1.033385
OS	TGM1	IVW	2	-0.013	0.014643	0.368428	-0.04187	0.01553	0.986916	0.958993	1.015652
OS	LILRB4	IVW	2	-0.025	0.018061	0.16545	-0.06045	0.010349	0.975261	0.941341	1.010403
OS	KLRB1	IVW	2	-0.013	0.014741	0.364007	-0.04227	0.015511	0.986708	0.958607	1.015632
OS	ART4	IVW	2	-0.019	0.01324	0.141825	-0.0454	0.006501	0.980738	0.955614	1.006522
OS	PDGFD	IVW	2	-0.013	0.015326	0.403011	-0.04286	0.017223	0.987265	0.958049	1.017372
OS	TAPBP	IVW	2	0.0006	0.015341	0.970192	-0.0295	0.030642	1.000573	0.970935	1.031117
OS	RPRD1A	IVW	2	-0.02	0.014668	0.172109	-0.04878	0.008721	0.980171	0.952393	1.008759
OS	LEAP2	IVW	2	-0.02	0.020125	0.325015	-0.05925	0.019637	0.980388	0.94247	1.019831
OS	GSN	IVW	2	0.0174	0.01498	0.245352	-0.01196	0.046764	1.017555	0.988113	1.047875
OS	NUDT9	IVW	2	-0.003	0.015086	0.83201	-0.03277	0.026369	0.996805	0.967761	1.02672
OS	HDHD3	IVW	2	0.0029	0.01509	0.845319	-0.02663	0.03252	1.002948	0.973719	1.033054
OS	GDF2	IVW	2	-0.007	0.015067	0.638371	-0.03661	0.02245	0.992944	0.96405	1.022704
OS	SHBG	IVW	2	-9E-04	0.015649	0.953931	-0.03158	0.029769	0.999096	0.968917	1.030216
OS	FOXJ2	IVW	2	-0.025	0.014877	0.089089	-0.05445	0.003865	0.975022	0.947002	1.003872
OS	SPINK5	IVW	2	-5E-04	0.015463	0.972142	-0.03085	0.029768	0.99946	0.969623	1.030216
OS	TMPRSS6	IVW	2	0.0077	0.015548	0.62166	-0.0228	0.038146	1.007702	0.977457	1.038883
OS	KREMEN1	IVW	2	-9E-04	0.018241	0.959916	-0.03667	0.034836	0.999084	0.963994	1.03545
OS	GALE	IVW	2	-0.013	0.015154	0.406564	-0.04228	0.017125	0.987501	0.958601	1.017273
OS	TAGLN	IVW	2	0.0168	0.015292	0.27228	-0.01318	0.046759	1.016929	0.986903	1.047869
OS	ECM1	IVW	2	-0.003	0.013962	0.835831	-0.03026	0.024472	0.997111	0.970195	1.024773
OS	GPC3	IVW	2	0.0152	0.015379	0.322361	-0.01492	0.045364	1.015336	0.985187	1.046408
OS	UCMA	IVW	2	-0.017	0.015237	0.276375	-0.04645	0.013279	0.983552	0.954614	1.013367
OS	RBL2	IVW	2	-0.014	0.014616	0.341135	-0.04256	0.014734	0.986183	0.958334	1.014843
OS	RBL2	IVW	2	-0.014	0.014616	0.341135	-0.04256	0.014734	0.986183	0.958334	1.014843
OS	FER	IVW	2	-0.009	0.015325	0.558545	-0.039	0.021071	0.991075	0.96175	1.021295
OS	PGRMC1	IVW	2	-0.001	0.014591	0.919218	-0.03008	0.027118	0.998521	0.97037	1.027489
OS	PLA2G2A	IVW	2	0.0025	0.015585	0.874402	-0.02808	0.03301	1.002467	0.972308	1.033561
OS	AXL	IVW	2	-0.021	0.014983	0.154926	-0.05068	0.008056	0.978914	0.950584	1.008088

Table S3: Power analysis of plasma proteins with putative causal relationships.

exposure	Sample size	Ratio of cases to controls	R^2	OR	power（%）
PLG	314257	0.0002	0.124	47.625	100
ECM1	314257	0.0002	0.141	6.026	100
TAPBP	314257	0.0002	0.442	0.388	99.9
EGFR	314257	0.0002	0.041	20.377	99.8
F10	314257	0.0002	0.097	0.164	99.4
CHKB	314257	0.0002	0.02	0.020	99.2
PDE5A	314257	0.0002	0.066	0.163	95.9
NEFL	314257	0.0002	0.123	0.271	95.2
ASAH1	314257	0.0002	0.012	56.083	93.8
FAIM	314257	0.0002	0.084	0.221	93.4
AMH	314257	0.0002	0.086	0.228	93
UNC5D	314257	0.0002	0.01	73.591	92.6
SEZ6L	314257	0.0002	0.011	56.144	91.8
MUSK	314257	0.0002	0.016	0.039	90.2
CADM2	314257	0.0002	0.006	195.137	89.9
SSU72	314257	0.0002	0.046	6.697	89.8
WFDC3	314257	0.0002	0.011	46.489	89.1
LONP1	314257	0.0002	0.011	45.986	88.9
ELK3	314257	0.0002	0.011	45.175	88.6
IL17RB	314257	0.0002	0.01	52.883	88.2
RELL1	314257	0.0002	0.018	0.054	87.4
NAGLU	314257	0.0002	0.517	1.721	87.2
ASPN	314257	0.0002	0.06	0.209	86
CACNA2D3	314257	0.0002	0.073	4.139	86
SEMA4C	314257	0.0002	0.082	3.753	85.1
DHX58	314257	0.0002	0.028	0.108	83.9
GSN	314257	0.0002	0.053	4.788	81.5
AXL	314257	0.0002	0.081	3.460	80
AKR7A3	314257	0.0002	0.035	0.153	79.5
LTF	314257	0.0002	0.044	0.192	78.3
FUT10	314257	0.0002	0.195	0.457	78.3
TRABD	314257	0.0002	0.009	0.029	75.9
MMP3	314257	0.0002	0.024	0.115	75.7
SHBG	314257	0.0002	0.034	6.091	75.2
CLPS	314257	0.0002	0.101	0.354	74.4
OGN	314257	0.0002	0.045	4.713	74.1
APOA1	314257	0.0002	0.105	0.363	74
SLITRK1	314257	0.0002	0.005	101.914	73.6
PDGFD	314257	0.0002	0.134	2.431	73.2
CD274	314257	0.0002	0.026	0.135	72.6
INSR	314257	0.0002	0.113	2.602	72.2

UCMA	314257	0.0002	0.057	3.800	71.4
ART4	314257	0.0002	0.615	1.494	70.4
FCAMR	314257	0.0002	0.007	0.024	69.6
SNX4	314257	0.0002	0.012	0.059	69.1
MGP	314257	0.0002	0.065	0.300	68.2
TGM1	314257	0.0002	0.004	0.008	67.7
DPT	314257	0.0002	0.057	0.285	66.1
ZNF334	314257	0.0002	0.013	13.679	65.7
RPN1	314257	0.0002	0.074	2.928	63.9
CNDP1	314257	0.0002	0.093	2.595	63.5
TXNDC15	314257	0.0002	0.172	0.498	63
VWA2	314257	0.0002	0.099	0.413	59.7
RPRD1A	314257	0.0002	0.01	0.062	59.6
VOPP1	314257	0.0002	0.056	0.316	58
GALE	314257	0.0002	0.023	0.166	57.9
LRRC15	314257	0.0002	0.044	3.647	57.6
KLRB1	314257	0.0002	0.04	3.759	55.5
ACY1	314257	0.0002	0.032	0.229	55.2
GPC3	314257	0.0002	0.026	5.029	54.2
IL31	314257	0.0002	0.032	4.262	53.8
FOXJ2	314257	0.0002	0.028	0.221	51.7
PLXNC1	314257	0.0002	0.244	0.602	51.1
GDF2	314257	0.0002	0.019	0.163	50.9
LILRB4	314257	0.0002	0.067	0.382	50.6
NUDT9	314257	0.0002	0.011	0.094	50.2
LEAP2	314257	0.0002	0.046	0.319	49.3
SPINK5	314257	0.0002	0.012	9.359	49.3
TMPRSS6	314257	0.0002	0.125	2.000	49.3
HDHD3	314257	0.0002	0.018	0.169	47.2
IGFBP3	314257	0.0002	0.071	0.412	46.5
FER	314257	0.0002	0.006	0.048	46.2
CRADD	314257	0.0002	0.003	71.452	45.8
RBL2	314257	0.0002	0.071	0.419	45.1
COCH	314257	0.0002	0.103	0.488	44.6
NTS	314257	0.0002	0.019	0.198	42.4
KREMEN1	314257	0.0002	0.065	2.386	42
PGRMC1	314257	0.0002	0.003	57.321	42
PLA2G2A	314257	0.0002	0.087	2.122	42
MATN2	314257	0.0002	0.015	5.991	41.2
TAGLN	314257	0.0002	0.005	16.993	35.5

Table S4: Details of the SNPs included in the analysis

exposure	SNP	F
CRYBB2	rs203688	100.3197064
CRYBB2	rs7160425	160.5832424
CRYBB2	rs2337918	29.82985706
CRYBB2	rs2932738	45.64727381
CRYBB2	rs697356	32.99347666
CRYBB2	rs6457457	33.61719662
RAF1	rs11601665	33.87918329
RAF1	rs9971407	31.76017262
RAF1	rs79041741	29.83554402
RAF1	rs1354034	51.04227786
ZNF41	rs368465	162.8856119
ZNF41	rs1706435	92.90794825
ZNF41	rs9501393	44.30641599
ZNF41	rs117125720	90.68282414
ZNF41	rs1042133	35.40447925
GUCA1A	rs184815320	30.39309201
GUCA1A	rs115977231	62.50198109
GUCA1A	rs61806985	984.5875424
GUCA1A	rs10800448	43.96317226
GUCA1A	rs2239853	92.3120076
GUCA1A	rs12765810	70.39109316
GUCA1A	rs892145	44.27808996
GUCA1A	rs62140549	37.95199523
GUCA1A	rs3020644	33.82498332
OCRL	rs145439832	30.01841904
OCRL	rs10922098	543.223079
OCRL	rs704	579.7980498
OCRL	rs1706435	68.8687809
OCRL	rs555007	88.44787691
OCRL	rs444921	179.7738971
OCRL	rs9269234	40.674203
OCRL	rs9272526	41.40728536
SPDEF	rs10922098	698.8412787
SPDEF	rs35351085	36.04238873
SPDEF	rs11569393	37.01734075
SPDEF	rs1707652	132.5082014
SPDEF	rs1265941	77.28098918
SPDEF	rs550671	189.9055611
SPDEF	rs9276606	33.16199255
SNAI2	rs10922098	158.3815519
SNAI2	rs1706435	43.781252
SNAI2	rs444921	43.95929765
SNAI2	rs34927613	35.02790344
KCNAB2	rs806109	69.78902688
KCNAB2	rs1295106	50.67426078
KCNAB2	rs10922098	31.30922384
KCNAB2	rs557011	33.13302757
VDR	rs10922098	355.0214725
VDR	rs13469	30.14108602
VDR	rs2686395	111.003762
VDR	rs9267139	33.03279862
VDR	rs9501393	109.3868446
VDR	rs7774739	74.14618642
VDR	rs9271766	33.60127345
HOGA1	rs10922098	251.2710908
HOGA1	rs1707652	44.89393953

HOGA1	rs9267139	30.89697274
HOGA1	rs9501393	87.36222068
HOGA1	rs7774739	59.91594603
HOGA1	rs9271683	33.23909457
DUSP4	rs10922094	187.0616462
DUSP4	rs6542680	53.29296705
DUSP4	rs1707652	54.67416522
DUSP4	rs6457457	38.72973009
DUSP4	rs9501393	52.89954254
DUSP4	rs11794541	33.07272751
ZHX3	rs10922094	179.3123927
ZHX3	rs62068364	45.73638589
ZHX3	rs6457457	102.5201203
ZHX3	rs6458	193.4598826
ZHX3	rs9269187	42.18769216
ZHX3	rs10982054	49.46456811
SIGLEC12	rs190502932	190.4655059
SIGLEC12	rs140987580	921.2482215
SIGLEC12	rs79506121	938.633059
SIGLEC12	rs61998206	81.76755023
SIGLEC12	rs112735728	762.8251314
SIGLEC12	rs73052899	204.8046622
SIGLEC12	rs111972944	77.3924196
SIGLEC12	rs117391293	55.75082712
SIGLEC12	rs368465	115.4922244
SIGLEC12	rs12971607	44.0015279
SIGLEC12	rs139978289	33.75054038
SIGLEC12	rs1042663	42.99522577
HNF4A	rs61747728	48.46576866
HNF4A	rs34825987	44.16782196
HNF4A	rs9501342	30.02977973
HNF4A	rs28366149	150.431308
HNF4A	rs148766553	112.4801782
HNF4A	rs6458	286.1937015
HNF4A	rs9273325	62.87884255
HNF4A	rs28616789	99.58961511
HNF4A	rs56294298	33.50376693
WNT10A	rs1697422	31.16657127
WNT10A	rs10922098	494.1688816
WNT10A	rs12478841	37.37715505
WNT10A	rs1707652	102.695806
WNT10A	rs1265941	63.42292303
WNT10A	rs550671	143.7882048
WNT10A	rs78897911	30.54072152
BIRC2	rs4251945	71.03025635
BIRC2	rs201842361	154.5299133
BIRC2	rs118147862	2626.242564
BIRC2	rs7254892	1182.358859
BIRC2	rs118004808	47.97440564
BIRC2	rs116490500	33.89632382
BIRC2	rs147910350	60.34228571
BIRC2	rs143675675	59.32820729
BIRC2	rs117909986	36.36462744
BIRC2	rs1619994	53.12157006
NCF2	rs10801548	73.62275539
NCF2	rs7546015	714.2358511
NCF2	rs118020353	31.48455232
NCF2	rs201028957	66.46065816

NCF2	rs76492422	34.52401615
NCF2	rs141947817	31.88924548
NCF2	rs145496844	2096.044807
NCF2	rs112963922	209.176184
NCF2	rs55698602	164.5689418
NCF2	rs72704666	115.2681212
NCF2	rs7153367	47.57896688
NCF2	rs148317773	59.28019069
CBFB	rs7535409	56.39016731
CBFB	rs10922094	121.9915656
CBFB	rs16919431	31.78540185
CBFB	rs1355538	63.27121105
CBFB	rs28573154	54.14482867
CBFB	rs34927613	40.33579806
ILK	rs802171	536.462198
ILK	rs4980320	33.49970532
ILK	rs342200	31.63369906
FOXM1	rs10876550	80.45011512
FOXM1	rs117828578	60.59357357
FOXM1	rs1354034	54.10427922
FOXM1	rs342292	77.25470763
FOXM1	rs10820606	104.9582339
FANCL	rs572515	176.6902352
FANCL	rs1706420	56.15988952
FANCL	rs28573154	57.768886
FANCL	rs7774739	46.85094537
RBBP9	rs10922094	36.97143387
RBBP9	rs5167	45.59804422
RBBP9	rs1355538	91.79990245
RBBP9	rs6457457	33.06913958
RBBP9	rs6458	62.65541784
RBBP9	rs6993770	34.50681335
BRD2	rs28573154	35.05405537
BRD2	rs10922098	150.7109344
BRD2	rs9864801	29.92154235
BRD2	rs6457457	42.40496123
ACBD6	rs80261041	36.5647057
ACBD6	rs4980320	31.21738403
ACBD6	rs7595829	31.5506573
ACBD6	rs1354034	90.97849631
BAG3	rs10922094	297.470476
BAG3	rs1707652	89.71983134
BAG3	rs9267139	40.93897054
BAG3	rs28573154	86.99751875
BAG3	rs7774739	60.30353503
BAG3	rs9271766	35.57534193
SATB2	rs1451915	34.13652876
SATB2	rs10922098	684.1235521
SATB2	rs6542680	59.7060074
SATB2	rs1707652	101.1095085
SATB2	rs555007	69.45916474
SATB2	rs550671	171.3099995
NEFL	rs1535	42.12450169
NEFL	rs76594250	44.5544182
NEFL	rs2722685	120.4547753
NEFL	rs58258919	82.9020621
NEFL	rs8103315	921.1461689
NEFL	rs416116	305.2702887

NEFL	rs3745150	2771.607688
NEFL	rs148352823	69.91264333
NEFL	rs149857247	103.4683133
NEFL	rs79471478	30.15063608
NEFL	rs780093	30.92439355
NEFL	rs1707652	41.33521829
STAR	rs11627993	30.64261338
STAR	rs6445035	65.85744377
STAR	rs3863087	30.50183343
STAR	rs28366149	49.9406448
STAR	rs6458	98.08982149
APRT	rs33992624	51.30020762
APRT	rs112783573	307.4848103
APRT	rs2272435	354.8641218
APRT	rs11640986	80.02466865
APRT	rs78016521	187.1662427
APRT	rs117132636	52.49466545
ASMTL	rs4980320	32.06147111
ASMTL	rs35495893	37.87678431
ASMTL	rs1354034	39.70255704
ENO2	rs2239165	299.3991905
ENO2	rs185781160	54.90242535
ENO2	rs10761739	94.35931697
ENO2	rs12313283	31.01662933
ENO2	rs6580981	38.75691083
ENO2	rs4734879	222.1712404
ENO2	rs60757417	32.59150854
PIAS4	rs10922094	239.567685
PIAS4	rs2686395	50.27632598
PIAS4	rs6457457	44.03626553
PIAS4	rs28573154	65.17251383
IL10RA	rs10733084	31.96160775
IL10RA	rs1329424	1106.110219
IL10RA	rs3900909	50.27059088
IL10RA	rs10801660	35.81696665
IL10RA	rs704	167.7280814
IL10RA	rs1047286	31.11457872
IL10RA	rs111696008	35.50428048
IL10RA	rs1707652	222.7075147
IL10RA	rs1265941	120.3629978
IL10RA	rs550671	314.2032275
IL10RA	rs9269234	52.28831894
STAT3	rs4796791	349.9794854
STAT3	rs12150483	31.1695395
STAT3	rs4980320	51.5710977
STAT3	rs7304860	35.87124774
STAT3	rs185009887	30.02326594
STAT3	rs1354034	66.83868387
STAT3	rs342200	30.98339125
JUN	rs10922098	604.800872
JUN	rs11569415	50.69878387
JUN	rs10208529	33.81280244
JUN	rs1707652	121.5355471
JUN	rs555007	62.75757618
JUN	rs444921	156.5404009
JUN	rs9272526	33.67953982
SMAD3	rs4980320	34.06758875
SMAD3	rs35979828	40.07946862

SMAD3	rs1354034	32.28895523
SMAD3	rs342200	29.73586667
SMAD2	rs4980320	40.71023805
SMAD2	rs35979828	38.91708176
SMAD2	rs342200	30.80987771
PDGFRA	rs2307050	112.7960397
PDGFRA	rs79285284	98.67180485
PDGFRA	rs56199187	43.04343048
PDGFRA	rs6703327	32.90292933
PDGFRA	rs112171832	206.595621
PDGFRA	rs144408988	84.75911802
PDGFRA	rs80012936	151.8109713
PDGFRA	rs150992058	643.2140151
PDGFRA	rs116688712	4246.049474
PDGFRA	rs138353023	2150.438888
PDGFRA	rs199692762	465.2996018
PDGFRA	rs9658810	60.50092217
PDGFRA	rs72747173	31.58545751
PDGFRA	rs145336675	57.4205818
PDGFRA	rs143737500	36.47674328
PDGFRA	rs201862321	44.85613041
PDGFRA	rs35495893	31.37942065
PDGFRA	rs697357	36.32175107
PDGFRA	rs189772645	34.89875491
STAT1	rs11417460	31.87564133
STAT1	rs4980320	40.20267119
STAT1	rs653178	42.62654239
STAT1	rs181209	38.98738379
STAT1	rs1354034	100.0669281
STAT6	rs774050	127.9659187
STAT6	rs150937490	170.3626261
STAT6	rs4980320	46.68080755
STAT6	rs78409925	29.77093511
STAT6	rs118188674	90.14171919
STAT6	rs374627156	54.29505759
STAT6	rs78062104	107.3057666
STAT6	rs12423621	171.9251977
STAT6	rs12810354	124.4083345
STAT6	rs185009887	31.23214397
STAT6	rs1354034	76.16787478
STAT6	rs1398617	55.35946478
ZNRF3	rs56312618	67.63264716
ZNRF3	rs4900222	73.79521636
ZNRF3	rs10512472	56.63627764
ZNRF3	rs1354034	309.1660592
ZNRF3	rs114315741	43.82841658
ZNRF3	rs1619994	75.92253267
ANGPTL3	rs17123220	410.6527341
ANGPTL3	rs10889352	662.0855095
ANGPTL3	rs144996885	48.66571936
ANGPTL3	rs113939762	61.44420092
ANGPTL3	rs144695160	77.79024328
ANGPTL3	rs138617180	171.5988614
ANGPTL3	rs17122460	42.94941419
ANGPTL3	rs115365379	284.8740634
ANGPTL3	rs138384996	196.9942058
ANGPTL3	rs12130644	34.29866754
ANGPTL3	rs964184	87.19646187

ANGPTL3	rs116843064	32.57529617
ANGPTL3	rs5112	42.97811314
ANGPTL3	rs2119690	35.72642153
STX12	rs12292693	121.6028741
STX12	rs185009887	31.46790544
STX12	rs1354034	35.32209839
SCARA5	rs60007820	157.6744788
SCARA5	rs2085765	147.0397922
SCARA5	rs2726951	913.9714528
SCARA5	rs66913624	34.13955649
SCARA5	rs352428	43.49905202
SCARA5	rs112771035	43.25433535
SCARA5	rs11872195	38.18586709
SCARA5	rs594793	52.44900419
SCARA5	rs111561142	31.06811765
SCARA5	rs780093	44.55452608
SCARA5	rs117955789	29.85811539
SCARA5	rs9344214	44.81112192
SCARA5	rs2952556	46.57328513
SCARA5	rs62492162	50.30565422
SCARA5	rs146247846	101.124418
NPDC1	rs9314872	45.44167945
NPDC1	rs368465	60.25213415
NPDC1	rs13208583	31.08534773
B4GALT5	rs10922094	157.7543768
B4GALT5	rs1355538	55.15218159
B4GALT5	rs9501393	52.1590624
B4GALT5	rs7774739	39.87732202
GOSR2	rs1451915	29.7261633
GOSR2	rs10922098	612.8763105
GOSR2	rs2686395	131.3420353
GOSR2	rs1265941	71.51388082
GOSR2	rs550671	153.2231575
SLC3A2	rs10922098	143.8372699
SLC3A2	rs72637119	162.2592765
SLC3A2	rs1355538	42.13393898
SLC3A2	rs4713498	29.74307825
AGA	rs35617250	132.5509401
AGA	rs411729	66.23287281
AGA	rs71655744	44.19949607
AGA	rs6829551	128.8240674
AGA	rs116430705	40.60650811
AGA	rs9501393	46.37488338
AGA	rs17205128	39.79210881
AGA	rs4735839	30.20613514
CSF2RA	rs660240	99.45704607
CSF2RA	rs3184504	39.43157967
CSF2RA	rs55714927	35.20076317
CSF2RA	rs9469051	47.9447268
CSF2RA	rs9469077	32.06693668
CLMP	rs7131073	71.76112696
CLMP	rs7946718	644.6426018
CLMP	rs56134443	43.57458476
CLMP	rs10922094	58.7311409
CLMP	rs77924615	40.41115333
CLMP	rs7206790	29.75281425
CLMP	rs147390813	33.48306261
CLMP	rs66962766	32.27527141

CLMP	rs9276171	45.96585971
TMEM190	rs12985351	33.23296156
TMEM190	rs62123425	63.21437513
TMEM190	rs12982674	168.5335037
TMEM190	rs8111781	102.0207168
TMEM190	rs79012440	1175.854089
TMEM190	rs45583840	920.4865256
TMEM190	rs75602785	496.4385264
TMEM190	rs7250117	485.7341279
TMEM190	rs73615245	152.0637207
TMEM190	rs150450992	36.13676393
TMEM190	rs77542162	47.05000589
APOM	rs12660700	30.05610251
APOM	rs805295	533.2760465
APOM	rs41285751	92.02537742
APOM	rs9272165	69.76077845
APOM	rs10922094	299.5153534
APOM	rs3782735	32.5933486
APOM	rs72786786	73.89684232
APOM	rs372434515	42.93417481
APOM	rs780093	44.6786657
APOM	rs1800961	118.7141032
APOM	rs1707652	41.02027472
APOM	rs139072234	46.13166697
APOM	rs1362076	111.6437676
APOM	rs364950	52.67153696
APOM	rs369560730	36.53032179
PABPC3	rs10761739	35.74795196
PABPC3	rs4980320	50.85399309
PABPC3	rs1354034	146.7209438
SHISA2	rs72748420	32.95188334
SHISA2	rs1329424	999.2891302
SHISA2	rs3900909	47.30712577
SHISA2	rs1047286	50.12121675
SHISA2	rs2686395	190.6997314
SHISA2	rs1265941	105.6950204
SHISA2	rs550671	254.9111084
SHISA2	rs112164276	32.50590727
SHISA2	rs41548618	39.31328692
NUCB1	rs12462111	31.30898773
NUCB1	rs3826817	251.2777799
NUCB1	rs646776	69.01384467
NUCB1	rs56199187	40.318527
NUCB1	rs860532	32.21500621
NUCB1	rs201862321	74.87295807
NUCB1	rs10169065	37.63495404
NUCB1	rs9270094	40.48846856
CEACAM20	rs28853072	277.2728188
CEACAM20	rs1355538	46.30583121
CEACAM20	rs550671	77.47467567
CEACAM20	rs28645249	30.68200492
IL31	rs2289567	56.11677129
IL31	rs241771	974.9103398
IL31	rs77263366	51.98490443
IL31	rs78814626	44.44192984
IL31	rs223032	31.50354738
ANTXR1	rs7424376	30.71826815
ANTXR1	rs10922098	40.81310318

ANTXR1	rs13329672	44.60149755
ANTXR1	rs3764261	243.6898991
ANTXR1	rs2686395	117.5923309
ANTXR1	rs13234157	37.92043013
ANTXR1	rs17410962	43.27027264
TMEM106A	rs79391435	45.29178889
TMEM106A	rs10922098	35.54215644
TMEM106A	rs10822155	62.71779299
TMEM106A	rs7157038	48.53148627
TMEM106A	rs72701845	92.98585812
TMEM106A	rs2071040	110.552782
TMEM106A	rs62258069	38.16179641
TMEM106A	rs1293303	47.59515106
PGRMC2	rs10922098	31.85469893
PGRMC2	rs7311982	115.4144715
PGRMC2	rs11050145	43.51227918
PGRMC2	rs1355538	42.11063722
ACRV1	rs10922098	124.2170919
ACRV1	rs13379369	166.0822132
ACRV1	rs11846842	43.98320326
ACRV1	rs139354903	32.31216144
ACRV1	rs1355538	39.46873386
ACRV1	rs451652	31.44642501
ACRV1	rs4713498	39.69248831
COL6A3	rs139126374	42.11936711
COL6A3	rs2646260	241.4746677
COL6A3	rs10922098	81.45501331
CSF2RB	rs78469	33.74686144
CSF2RB	rs77818863	78.53659325
CSF2RB	rs2284031	2112.861773
CSF2RB	rs2858236	120.5799611
CSF2RB	rs3218340	56.35581322
CSF2RB	rs145143750	30.43701786
CSF2RB	rs10922098	52.48714744
CSF2RB	rs113908050	43.04528466
KCNIP3	rs7546015	85.38597778
KCNIP3	rs11628176	34.88306166
KCNIP3	rs429608	42.30343463
KCNIP3	rs28455809	62.76335207
PTGDS	rs7040970	298.3417211
PTGDS	rs72704449	29.90728776
PTGDS	rs10892301	37.20154774
PTGDS	rs77924615	60.49040392
PTGDS	rs28611065	34.06695865
MXRA8	rs181929025	94.53985112
MXRA8	rs2313135	42.82706219
MXRA8	rs78705678	74.63849763
MXRA8	rs2477642	83.1992721
MXRA8	rs174536	31.37681691
MXRA8	rs5104	67.89895395
MXRA8	rs13329672	81.7471111
MXRA8	rs1800588	80.57947255
MXRA8	rs3764261	100.2149597
MXRA8	rs1065853	57.4119139
MXRA8	rs1260326	41.11829372
MXRA8	rs2542941	54.30234195
MXRA8	rs6805055	29.97773065
MXRA8	rs199980439	30.61694977

MXRA8	rs12353096	37.39805527
DHX9	rs10922098	455.9609296
DHX9	rs11569415	39.98456183
DHX9	rs1707652	143.6127164
DHX9	rs550671	167.5682655
DHX9	rs9469195	65.57237151
DHX9	rs9272526	36.57212346
PSMB6	rs10922098	169.7008142
PSMB6	rs5754116	39.69054285
PSMB6	rs1355538	30.96965122
PSMB6	rs9501600	45.85698379
COX6A2	rs10922098	123.0169691
COX6A2	rs3794468	38.06718017
COX6A2	rs11845744	62.62345821
COX6A2	rs62402680	37.52108828
COX6A2	rs77311729	62.69992498
COX6A2	rs9271732	117.5785281
CD99L2	rs10922098	305.2914147
CD99L2	rs704	65.42334155
CD99L2	rs1355538	55.90900438
CD99L2	rs28573154	104.3285151
CD99L2	rs7774739	66.63212635
CD99L2	rs9271766	30.72055886
TSPEAR	rs1451915	33.22604916
TSPEAR	rs10922098	577.6653316
TSPEAR	rs2686395	105.6327649
TSPEAR	rs389512	139.122904
TSPEAR	rs28645249	73.10448128
BMPR1B	rs10922098	439.6461191
BMPR1B	rs1707652	96.11143657
BMPR1B	rs555007	49.94628807
BMPR1B	rs550671	133.4672499
LAT	rs761972	36.58191152
LAT	rs2093214	32.04073131
LAT	rs113556713	241.4157663
LAT	rs12586321	63.44901945
LAT	rs79327907	32.72942368
LAT	rs9273429	75.25204344
KLRC4	rs572515	60.5282265
KLRC4	rs1707652	50.71227231
KLRC4	rs6993770	68.16752032
TEX29	rs41452950	203.5578398
TEX29	rs617283	203.4909455
TEX29	rs12529130	31.10031517
TEX29	rs6458	60.72011326
TEX29	rs9801685	30.095279
TEX29	rs76342676	52.30476367
TEX29	rs854547	1030.058034
TEX29	rs374185817	154.1055461
TEX29	rs2922965	42.85741461
TEX29	rs57971788	33.06267194
TEX29	rs72760268	36.51347893
PCDH9	rs192652452	98.59851154
PCDH9	rs4396423	121.9473858
PCDH9	rs9571710	419.0836526
PCDH9	rs5804316	482.077291
PCDH9	rs7087507	44.3261573
PCDH9	rs7310615	38.24585755

PCDH9	rs9738226	75.85994702
PCDH9	rs7327146	42.89624163
PCDH9	rs147278277	34.51052054
PCDH9	rs950941	38.15541594
PCDH9	rs2786946	195.3341628
PCDH9	rs55823018	36.3456273
PCDH9	rs55714927	56.48979654
PCDH9	rs736289	37.31504653
PCDH9	rs142546236	47.07209135
PCDH9	rs11720167	30.15061702
PCDH9	rs3887925	47.91494137
PCDH9	rs7660227	31.04943038
PCDH9	rs700589	35.94716387
PCDH9	rs77341267	29.82271097
PCDH9	rs7840212	56.72130472
PCDH9	rs10984499	52.78087226
ITM2C	rs10922098	97.03391932
ITM2C	rs893522	32.79461074
ITM2C	rs28573154	43.08958393
ITM2C	rs148919153	44.60329087
PGLYRP3	rs141738509	41.58656876
PGLYRP3	rs10922098	384.7582512
PGLYRP3	rs1047286	32.97090046
PGLYRP3	rs12478841	34.19092989
PGLYRP3	rs9864801	85.63559299
PGLYRP3	rs138073993	29.89635943
PGLYRP3	rs2075798	52.12192092
PGLYRP3	rs550671	119.2822285
SLITRK3	rs80336735	78.67828307
SLITRK3	rs62282368	2782.077991
SLITRK3	rs79598313	45.62962429
SLITRK3	rs7540799	31.8131644
SLITRK3	rs10761727	48.37123413
SLITRK3	rs1519125	30.71357368
SLITRK3	rs2259820	51.33538723
SLITRK3	rs74397112	31.64454194
SLITRK3	rs57526361	34.90271259
SLITRK3	rs56292801	51.77388319
SLITRK3	rs12601665	69.77966313
SLITRK3	rs28432811	40.15583255
SLITRK3	rs273492	33.21437835
SLITRK3	rs41278228	66.49897081
SLITRK3	rs1154988	96.95408443
SLITRK3	rs2036291	29.99385526
SLITRK3	rs11924462	56.47301351
SLITRK3	rs114464925	52.59419008
SLITRK3	rs147150049	72.90070238
SLITRK3	rs75357795	38.34379544
SLITRK3	rs2111557	40.03610793
SLITRK3	rs9462852	55.16182482
SLITRK3	rs2749005	44.45214842
SLITRK3	rs7740107	34.39970011
SLITRK3	rs36170987	70.20597111
SLITRK3	rs186735625	47.25906524
SLITRK3	rs148190310	33.21988374
SLITRK3	rs2976842	29.90975532
SLITRK3	rs722740	33.91526975
SCGB1A1	rs187347153	30.50450466

SCGB1A1	rs57585017	133.9756027
SCGB1A1	rs12217103	958.0767772
SCGB1A1	rs35496797	84.28739271
SCGB1A1	rs61767275	54.42983515
SCGB1A1	rs203688	32.95525139
SCGB1A1	rs1126605	264.8300298
SCGB1A1	rs1355538	89.41480515
SCGB1A1	rs7614709	96.83726432
SCGB1A1	rs143819005	35.44772797
MGAT1	rs634501	48.82758039
MGAT1	rs10922098	69.33652104
MGAT1	rs4448834	122.2827956
MGAT1	rs28873836	38.61617565
MGAT1	rs41285751	35.70803781
MGAT1	rs41556615	67.10829823
CST8	rs10922094	359.5782299
CST8	rs11535840	51.49271546
CST8	rs200957441	129.5368402
CST8	rs117199639	32.74713663
CST8	rs3748338	56.61869395
CST8	rs41324748	190.5082705
CST8	rs117517474	34.99319301
CST8	rs4251784	161.3826833
CST8	rs112930619	918.5167329
CST8	rs146038760	925.1368362
CST8	rs150951362	1191.070795
CST8	rs139210698	64.17020762
CST8	rs1707652	52.07650142
CST8	rs72548024	71.89884819
CST8	rs41285751	215.7044356
CST8	rs451652	189.3251605
CST8	rs67322126	40.43279342
AXL	rs143728110	95.2120563
AXL	rs141462616	31.82161189
AXL	rs146211397	128.5781073
AXL	rs11080372	143.9803474
AXL	rs4796158	46.24172165
AXL	rs4603604	1091.026352
AXL	rs9330240	663.5294548
AXL	rs75240299	37.16296994
AXL	rs61080193	32.55880635
AXL	rs113341849	663.6302154
PUF60	rs10922094	54.19097098
PUF60	rs4795734	30.79623051
PUF60	rs28873836	131.3291476
PUF60	rs4801902	37.45261857
PUF60	rs3129754	33.29056784
SFTA2	rs10922098	244.9665101
SFTA2	rs6445035	106.347432
SFTA2	rs3863087	40.57266531
SFTA2	rs28573154	90.11154963
SFTA2	rs7774739	72.70065358
POMK	rs10922098	443.6553611
POMK	rs141575023	34.92112937
POMK	rs1047286	32.66530027
POMK	rs2686395	101.3789765
POMK	rs3099847	29.8439627
POMK	rs555007	53.06158631

POMK	rs550671	140.4877752
NDUFS4	rs7535761	45.36481528
NDUFS4	rs6660584	51.62789527
NDUFS4	rs6664577	90.93874875
NDUFS4	rs77333802	47.0054087
NDUFS4	rs12035228	125.829942
NDUFS4	rs11588705	924.3479291
NDUFS4	rs148789508	142.8013425
NDUFS4	rs151334895	1180.110033
NDUFS4	rs116119767	184.9617064
NDUFS4	rs72732236	445.5505487
NDUFS4	rs79707161	330.0705691
NDUFS4	rs114990940	295.8091599
NDUFS4	rs10801548	3378.027816
NDUFS4	rs139784897	600.850809
NDUFS4	rs35788722	797.9870329
NDUFS4	rs112454880	337.3780091
NDUFS4	rs191086600	465.3834723
NDUFS4	rs384006	648.740049
NDUFS4	rs462382	14264.00621
NDUFS4	rs187739503	731.1941249
NDUFS4	rs148056042	576.7330731
NDUFS4	rs147814709	478.4076908
NDUFS4	rs142565493	331.927349
NDUFS4	rs191076788	373.4822013
NDUFS4	rs149681097	115.7027207
NDUFS4	rs185873389	36.27849191
NDUFS4	rs2635536	37.73043498
NDUFS4	rs76645026	30.25940671
NDUFS4	rs144743753	29.87073204
NDUFS4	rs7580569	50.03800739
NDUFS4	rs138259153	35.5251298
NDUFS4	rs1250258	316.1407853
NDUFS4	rs4674006	63.08958171
NDUFS4	rs74576202	32.53873162
CLGN	rs2270000	44.87031845
CLGN	rs144558125	42.19228804
CLGN	rs79906664	104.3609208
CLGN	rs6696924	1384.14266
CLGN	rs41286846	224.6416895
CLGN	rs4912425	243.0245046
CLGN	rs155298	35.56146331
CLGN	rs139128791	32.96778589
CLGN	rs28399664	35.74873227
CLGN	rs118170342	31.0786832
CLGN	rs1065853	275.9611334
CLGN	rs1707652	30.87645153
CLGN	rs138772443	30.46146917
CLGN	rs908830	327.3873979
HTN3	rs115000417	356.1608523
HTN3	rs376023333	34.81683813
HTN3	rs74801295	39.80113961
HTN3	rs10863962	62.65803799
HTN3	rs10832961	55.92324065
HTN3	rs7398041	627.7766533
HTN3	rs61930005	54.2295355
HTN3	rs4423274	69.15730014
HTN3	rs848097	31.26110093

HTN3	rs227727	30.3677611
HTN3	rs4793442	48.28717389
HTN3	rs681343	228.207571
HTN3	rs61762319	50.13473011
HTN3	rs150022487	73.09912338
HTN3	rs13357659	110.0165867
HTN3	rs12517853	39.11918139
HTN3	rs3749751	126.3509244
APMAP	rs6049886	72.63504173
APMAP	rs6036977	167.7177714
APMAP	rs10922098	62.34391159
APMAP	rs11417460	33.28741907
APMAP	rs1355538	78.63106819
TOR1AIP1	rs1926446	47.34866149
TOR1AIP1	rs2848407	56.77609628
TOR1AIP1	rs4089292	236.457521
TOR1AIP1	rs140489194	272.8817942
TOR1AIP1	rs56125452	30.12813133
TOR1AIP1	rs451637	122.4306317
TOR1AIP1	rs137893789	161.6015504
TOR1AIP1	rs35580488	41.62453818
TOR1AIP1	rs9281658	89.39885978
TOR1AIP1	rs370779031	59.33236633
HTN1	rs2511241	72.99754956
HTN1	rs11699344	33.09008775
HTN1	rs149110519	158.15914
HTN1	rs13312419	113.5343878
HTN1	rs11771936	752.0672757
HTN1	rs13245946	32.29816911
PLOD3	rs142309161	132.853345
PLOD3	rs62465594	30.03622375
PLOD3	rs2477664	58.85314374
PLOD3	rs692719	53.60972786
PLOD3	rs7964859	42.63838848
PLOD3	rs9862378	30.13059079
PLOD3	rs149062332	31.58543857
PLOD3	rs80028711	49.00987532
PLOD3	rs72816176	32.39086707
PLOD3	rs17691833	33.14246671
PLOD3	rs77339260	106.4831154
PLOD3	rs144769653	61.77707786
PLOD3	rs117207573	30.77093632
MPZ	rs2802788	60.6810456
MPZ	rs28417369	94.83777553
MPZ	rs7529009	765.678977
MPZ	rs6541311	266.4715321
MPZ	rs79242340	33.91248272
MPZ	rs78931415	35.82315923
MPZ	rs114459700	79.1505087
MPZ	rs139349382	38.59983756
MPZ	rs147712570	50.83636
MPZ	rs75327292	91.45025819
MPZ	rs117801489	562.9213268
MPZ	rs12581309	82.70344065
MPZ	rs370212152	50.50208945
MPZ	rs8035692	36.17755804
MPZ	rs6759217	32.02013624
MPZ	rs6746339	30.79177403

MPZ	rs1042445	132.5164265
MPZ	rs13107325	73.62176445
MPZ	rs10755398	30.52376997
MPZ	rs117350387	44.72140712
MPZ	rs3217964	135.4547189
MPZ	rs4724390	34.23915236
MPZ	rs13269700	34.536727
LRP2	rs10922098	189.2607956
LRP2	rs1706435	54.87999374
LRP2	rs3129957	38.78785291
MSMB	rs12412427	84.75723243
MSMB	rs144584548	445.7048941
MSMB	rs79786386	1050.988415
MSMB	rs61850100	4783.265628
MSMB	rs200779162	1209.688352
MSMB	rs77567868	455.9529966
MSMB	rs28710245	35.63806665
MSMB	rs61833942	29.91074626
MSMB	rs2256455	35.45981017
MSMB	rs17505094	90.83663783
MSMB	rs11009654	42.82069899
MSMB	rs138342429	50.21014664
MSMB	rs2486502	51.65105303
MSMB	rs1451189	35.86448082
MSMB	rs141362236	91.35335633
MSMB	rs36126966	31.53678627
MSMB	rs61859378	154.9772688
MSMB	rs4948583	332.404013
MSMB	rs75935865	196.4604668
MSMB	rs3013912	92.20874835
MSMB	rs4623821	36.11715016
MSMB	rs7912117	29.88106009
MSMB	rs28415845	60.50988917
MSMB	rs525202	34.60011933
MSMB	rs12985355	36.64281945
MSMB	rs7254067	65.27841231
MSMB	rs77487490	47.74477243
PRR16	rs13127052	41.18657588
PRR16	rs318261	34.48820775
PRR16	rs116869929	50.71428568
PRR16	rs3736639	160.4841756
SPINK13	rs10922098	90.12921138
SPINK13	rs71615484	32.22995162
SPINK13	rs13217434	31.39925009
APLP2	rs73583419	282.0652631
APLP2	rs1326273	37.02837351
APLP2	rs10822155	52.73501491
APLP2	rs741736	35.26317127
APLP2	rs775824	30.83997824
APLP2	rs1071960	31.02804492
APLP2	rs367768	39.15601369
APLP2	rs199772122	34.33033259
APLP2	rs6993770	150.7586101
HTATIP2	rs79225249	65.35503206
HTATIP2	rs1356981	107.2412096
HTATIP2	rs11025480	1035.797244
HTATIP2	rs3758807	209.563496
HTATIP2	rs1715284	152.9848285

HTATIP2	rs2003174	57.72640373
HTATIP2	rs368465	78.12071333
HTATIP2	rs34931250	87.40379733
HTATIP2	rs9864801	49.420904
HTATIP2	rs342294	36.91963082
OLFM3	rs10922098	297.1592853
OLFM3	rs72749892	31.49340134
OLFM3	rs704	72.05917094
OLFM3	rs2384656	29.98145342
OLFM3	rs1707652	68.26689158
OLFM3	rs9501393	81.48187499
OLFM3	rs7774739	62.55831159
OLFM3	rs77460382	37.1956317
ASCC1	rs74746093	34.88204967
ASCC1	rs118137024	95.43147565
ASCC1	rs62269916	32.32046509
ASCC1	rs1619994	50.06542517
ASCC1	rs2731672	29.78882829
GNPTG	rs58894215	43.56874558
GNPTG	rs4984644	1200.422468
GNPTG	rs6600145	69.49513427
GNPTG	rs1132368	31.97779688
GNPTG	rs72641004	65.64111596
GNPTG	rs660240	37.21344239
GNPTG	rs1008764	32.05634999
GNPTG	rs4764823	46.03644043
GNPTG	rs9928413	33.03508823
GNPTG	rs12945299	84.94584078
GNPTG	rs143501783	33.21034278
GNPTG	rs1861877	33.60062378
GNPTG	rs9272050	37.94672328
SFTPB	rs114068223	92.24742102
SFTPB	rs145297992	71.48668822
SFTPB	rs1130866	3498.534187
SFTPB	rs72925063	162.8644645
SFTPB	rs800245	40.03923825
SFTPB	rs35705950	48.7135323
SFTPB	rs11569415	177.6940211
SFTPB	rs12468241	31.02474866
SFTPB	rs71390144	63.31772388
SFTPB	rs199969223	37.39746598
SFTPB	rs11678909	33.62644787
TMX2	rs114009540	35.36691811
TMX2	rs10922098	686.412837
TMX2	rs3900909	33.9777608
TMX2	rs1047286	47.5699871
TMX2	rs2686395	163.7492886
TMX2	rs555007	80.77144792
TMX2	rs550671	198.7631092
TMX2	rs9272526	34.48787576
NDUFB4	rs145439832	35.77496619
NDUFB4	rs72748420	47.48857617
NDUFB4	rs1329424	1337.188804
NDUFB4	rs3900909	60.20649947
NDUFB4	rs10801660	47.52196814
NDUFB4	rs9282801	61.5004732
NDUFB4	rs117340335	41.49853177
NDUFB4	rs11272957	918.1083587

NDUFB4	rs1160610	39.74491142
NDUFB4	rs1047286	42.58804845
NDUFB4	rs1707652	202.0501768
NDUFB4	rs73169117	31.2900786
NDUFB4	rs1265941	134.7154825
NDUFB4	rs550671	371.3162134
NDUFB4	rs112164276	50.09350041
NDUFB4	rs41548618	53.0025307
KLRG2	rs79697519	37.46593625
KLRG2	rs111360378	60.74008165
KLRG2	rs10444770	30.3948323
KLRG2	rs142678764	38.06708035
KLRG2	rs34894886	1154.516836
KLRG2	rs9268560	50.04089247
WFDC10B	rs10922098	43.43612468
WFDC10B	rs11085197	45.66314143
WFDC10B	rs41547217	30.21371291
IGSF11	rs3761959	46.3986904
IGSF11	rs61804164	55.59939272
IGSF11	rs77333309	49.33017798
IGSF11	rs9273429	53.02697974
COL28A1	rs767298	32.750052
COL28A1	rs12126578	29.75520165
COL28A1	rs2129775	30.99476608
COL28A1	rs12945299	41.12021244
COL28A1	rs1050785	61.06128451
COL28A1	rs2646260	33.88561185
COL28A1	rs611462	30.2721486
COL28A1	rs3088026	44.53609091
COL28A1	rs10254101	31.99393088
SUDS3	rs10922098	224.8574817
SUDS3	rs11569415	36.44229823
SUDS3	rs9864801	53.13700376
SUDS3	rs2634465	30.09617803
SUDS3	rs1042663	70.54285796
SUDS3	rs9501602	36.57498225
GNRH2	rs2296236	379.2108314
GNRH2	rs6115777	51.18298812
GNRH2	rs10922098	76.7433713
GNRH2	rs11845659	38.30010309
GNRH2	rs6445180	33.92441768
ZPBP2	rs10922098	40.58576304
ZPBP2	rs67399078	48.13243567
ZPBP2	rs7749543	30.19571633
ZPBP2	rs10869516	35.19282873
ACE	rs188635580	70.05737419
ACE	rs4968748	1388.021658
ACE	rs4277404	214.3524708
ACE	rs142377191	113.8881637
ACE	rs111725844	384.894148
ACE	rs150241958	110.4827018
ACE	rs2584622	146.8333159
ACE	rs3744287	32.51037271
ACE	rs75163181	145.5075229
ACE	rs79593721	67.51242877
ACE	rs144762318	60.12539184
ACE	rs611593	50.3971368
ACE	rs35166255	170.0667019

ACE	rs12945299	31.23537154
ACE	rs143501783	33.21849403
ACE	rs189634497	75.94849568
ACE	rs74905978	46.41927048
ACE	rs8103531	30.61400039
ACE	rs3205136	281.734374
ACE	rs600038	954.15616
ACE	rs117608058	51.29511262
PSMD5	rs10922098	52.7439665
PSMD5	rs34160075	218.1794874
PSMD5	rs2284350	42.47394315
PSMD5	rs3917550	39.24847891
HS3ST5	rs12132412	33.6184895
HS3ST5	rs7535409	48.12638943
HS3ST5	rs2420378	36.90002077
HS3ST5	rs1914886	41.81488321
HS3ST5	rs704	1036.062874
HS3ST5	rs376533982	40.09111789
HS3ST5	rs36020655	49.6984849
HS3ST5	rs429358	87.89667305
HS3ST5	rs35136575	49.77911187
GAL3ST2	rs72748420	35.64878689
GAL3ST2	rs1329424	1103.72129
GAL3ST2	rs3900909	50.21974564
GAL3ST2	rs10801660	42.45860312
GAL3ST2	rs35136575	54.41912989
GAL3ST2	rs111696008	36.38533715
GAL3ST2	rs144202860	29.72935514
GAL3ST2	rs2686395	281.3601347
GAL3ST2	rs1265941	112.2893654
GAL3ST2	rs550671	267.8049748
GAL3ST2	rs9269234	47.7703564
SERPINB1	rs2293772	92.64825073
SERPINB1	rs10922094	119.8134258
SERPINB1	rs9971407	54.80193971
SERPINB1	rs6445180	35.94785329
SERPINB1	rs4713498	30.47718768
PCDHB2	rs10922098	576.6643446
PCDHB2	rs7084817	61.47179642
PCDHB2	rs11064498	47.31518844
PCDHB2	rs56317765	32.04001902
PCDHB2	rs11720167	99.43504256
PCDHB2	rs3863087	37.44362776
PCDHB2	rs550671	175.4598589
PCDHB2	rs9469195	74.43707233
PCDHB2	rs9272526	34.52683496
CDYL2	rs10922098	59.09652417
CDYL2	rs4452009	31.86492277
CDYL2	rs704	482.8262461
CDYL2	rs62083752	58.79475384
CDYL2	rs9501393	34.01417636
CDYL2	rs2299257	31.26282424
ADAM7	rs71427346	58.32396776
ADAM7	rs113954333	90.2897874
ADAM7	rs241777	937.504386
ADAM7	rs36020655	33.41595973
PROK2	rs2686274	88.47034979
PROK2	rs6549404	253.7047913

PROK2	rs4677042	4417.475292
PROK2	rs17009077	165.6046169
PROK2	rs9809964	42.6045661
PROK2	rs56318703	69.78468531
PROK2	rs10896415	44.89726426
PROK2	rs2511241	419.6745048
PROK2	rs3809627	32.12404744
PROK2	rs9898547	142.758234
PROK2	rs62135367	34.46752272
PROK2	rs116198731	30.79553451
PROK2	rs138866676	46.53114129
PROK2	rs4264815	38.46497299
PROK2	rs1071649	56.07913215
PROK2	rs9266228	88.37564659
KERA	rs10922094	190.7666169
KERA	rs4950781	31.5873477
KERA	rs142759151	37.85710918
KERA	rs10920636	919.5162141
KERA	rs117861214	30.81199659
KERA	rs186033146	67.30291634
KERA	rs1362245	32.44881408
KERA	rs2686395	54.96493834
KERA	rs1265941	41.72311756
KERA	rs550671	75.98629096
GRIA4	rs9280648	37.93931686
GRIA4	rs56194989	31.26786095
GRIA4	rs72548024	139.3976047
GRIA4	rs115884658	152.7845032
GRIA4	rs62395855	39.59268905
GRIA4	rs181063261	549.626274
GRIA4	rs2284179	228.0943803
GRIA4	rs451652	314.8189772
GRIA4	rs7774739	273.5398525
GRIA4	rs13196283	32.40637936
GRIA4	rs9501265	36.01737756
TMED2	rs10922094	215.4980889
TMED2	rs1355538	73.3982521
TMED2	rs454212	60.13041198
TMED2	rs34927613	37.53758865
KLRG2	rs10922098	82.11525237
KLRG2	rs142860569	36.38977177
KLRG2	rs2808466	518.650294
KLRG2	rs2491393	84.15145113
KLRG2	rs1107080	36.27433807
CSGALNACT2	rs2435382	188.5136203
CSGALNACT2	rs10922098	347.8802649
CSGALNACT2	rs6542680	147.7519708
CSGALNACT2	rs1355538	74.14228138
CSGALNACT2	rs550671	92.90468292
CSGALNACT2	rs62404084	37.73986126
CLEC4G	rs117133715	166.6904644
CLEC4G	rs62122042	31.56467875
CLEC4G	rs1801701	951.4613854
CLEC4G	rs115256181	33.02272735
CLEC4G	rs6445035	170.3848237
CLEC4G	rs3863087	61.01615553
CLEC4G	rs79428469	36.70415145
CLEC4G	rs78810414	39.81305845

LRRC52	rs61801825	34.36314044
LRRC52	rs111653192	31.2094126
LRRC52	rs12589177	619.6487446
LRRC52	rs61997798	58.9725718
LRRC52	rs41547217	52.45365171
CD300E	rs10922094	201.5366449
CD300E	rs1555405	35.22175064
CD300E	rs1706435	73.1350817
CD300E	rs9501600	70.20872638
SERPINH1	rs514887	233.3972919
SERPINH1	rs594708	85.1958248
SERPINH1	rs2588512	53.84718208
SERPINH1	rs9865425	61.95647116
SERPINH1	rs1354034	35.14020935
SERPINH1	rs9656588	60.77493486
SERPINH1	rs7782210	35.71169671
SERPINH1	rs72673731	40.06011911
SERPINH1	rs10098139	42.21488354
KLRB1	rs139517772	32.60169463
KLRB1	rs10691349	46.97413776
KLRB1	rs1135816	804.6236821
KLRB1	rs118021813	34.02174481
KLRB1	rs713452	66.02495261
KLRB1	rs10922094	149.5061769
KLRB1	rs7310615	36.57134478
KLRB1	rs8117436	29.88529854
KLRB1	rs697356	30.03738857
KLRB1	rs28455809	38.71515259
KLRB1	rs7862602	167.1768803
CD1A	rs114009540	31.26629772
CD1A	rs10922098	772.628736
CD1A	rs3900909	44.08158966
CD1A	rs1047286	31.57477963
CD1A	rs72848922	51.45543714
CD1A	rs111696008	31.45664354
CD1A	rs1707652	200.9001982
CD1A	rs550671	227.7365949
CD1A	rs28471056	71.23638496
CD1A	rs9272526	38.0656381
HABP4	rs473902	38.23907749
HABP4	rs58088508	1376.105492
HABP4	rs147602960	128.9347645
HABP4	rs28825490	40.28616934
HABP4	rs6695321	37.64316709
HABP4	rs116836559	38.2527284
PILRA	rs45454594	1167.982432
PILRA	rs199974872	259.0522399
PILRA	rs76265464	168.5609123
PILRA	rs75126895	453.6243561
PILRA	rs12705068	1415.87173
PILRA	rs371165331	1043.948067
PILRA	rs62482204	841.7533624
PILRA	rs149744179	213.6397434
PILRA	rs78690606	502.6170648
PILRA	rs73405382	1025.405713
PILRA	rs140711669	247.7324456
PILRA	rs55867908	581.6587506
PILRA	rs143364347	498.739316

PILRA	rs146216542	34.59001165
PILRA	rs5745757	32.07995501
PILRA	rs148292738	50.05209916
PILRA	rs117466579	31.28145419
PILRA	rs79858296	44.65146678
PILRA	rs62467851	39.61844397
PILRA	rs12670513	148.6287539
PILRA	rs117850863	1307.12642
PILRA	rs712846	262.94053
PILRA	rs143036628	111.9173884
PILRA	rs138702212	31.88227984
PILRA	rs73203019	39.52807388
PILRA	rs118045511	30.68829828
PILRA	rs1206383	31.62539992
TOMM20L	rs72748420	30.98369315
TOMM20L	rs1329424	1045.414394
TOMM20L	rs3900909	47.86088161
TOMM20L	rs10801660	36.54699007
TOMM20L	rs1047286	41.27520471
TOMM20L	rs2686395	197.600263
TOMM20L	rs1265941	104.8506182
TOMM20L	rs550671	280.2314036
TOMM20L	rs112164276	38.50564713
TOMM20L	rs41548618	39.66978529
SMPD1	rs57528411	46.9000696
SMPD1	rs142303644	58.0512954
SMPD1	rs1050228	1181.625676
SMPD1	rs117210486	133.109554
SMPD1	rs77690773	43.57287443
SMPD1	rs113199701	33.84624844
SMPD1	rs12743641	34.71079698
SMPD1	rs78444298	68.2480954
SMPD1	rs7897379	102.4361149
SMPD1	rs139736720	33.52794448
SMPD1	rs3923092	44.90472543
SMPD1	rs60167606	76.28031347
SMPD1	rs2251298	941.5134114
SMPD1	rs7980436	345.5639532
SMPD1	rs2946833	43.51842505
SMPD1	rs67057049	31.94354449
SMPD1	rs17532371	32.16756494
SMPD1	rs17092065	37.74805479
SMPD1	rs112963922	33.08277584
SMPD1	rs55707100	55.50606077
SMPD1	rs339969	46.19220075
SMPD1	rs62061733	37.09924055
SMPD1	rs2532362	33.71437151
SMPD1	rs56255430	81.80020719
SMPD1	rs429358	46.99774717
SMPD1	rs8736	78.05461112
SMPD1	rs738409	37.49503497
SMPD1	rs4681749	31.95401687
SMPD1	rs1154988	40.93280819
SMPD1	rs1707652	92.48370548
SMPD1	rs6858114	34.32978972
SMPD1	rs56055826	40.19575014
SMPD1	rs41280463	45.35923295
SMPD1	rs9639523	34.87897433

SMPD1	rs7036351	48.19114192
VSIG10	rs10922098	39.18653208
VSIG10	rs4448834	51.13136035
VSIG10	rs697356	39.27093309
B4GALT6	rs1816633	108.4425559
B4GALT6	rs78796387	440.3998185
B4GALT6	rs143674791	456.7289887
B4GALT6	rs142098862	100.9991124
B4GALT6	rs59477943	2788.367203
B4GALT6	rs113633457	326.731485
B4GALT6	rs17804730	80.47101777
B4GALT6	rs148295641	288.1004665
B4GALT6	rs78705678	33.27685111
B4GALT6	rs140118846	35.46802968
B4GALT6	rs181707610	48.79854073
B4GALT6	rs189321311	36.08717389
B4GALT6	rs147694814	70.16333415
B4GALT6	rs12608350	84.95892959
B4GALT6	rs138902810	104.3976723
B4GALT6	rs145766357	30.31556691
B4GALT6	rs117474269	39.42375928
B4GALT6	rs141745012	31.91426568
B4GALT6	rs8102873	33.1015462
B4GALT6	rs1260326	31.63389243
B4GALT6	rs4684847	43.79777596
B4GALT6	rs62621812	32.00861226
B4GALT6	rs2980858	44.08092978
HHIP	rs7438126	45.24413404
HHIP	rs140950906	33.35220072
HHIP	rs11727676	312.9017918
HHIP	rs78689694	35.06856223
HHIP	rs181707610	33.12881289
HHIP	rs718530	37.62441916
HHIP	rs118146573	42.15880799
HHIP	rs1065853	391.606441
HHIP	rs77945941	70.53963679
HHIP	rs9274536	56.96711097
A4GNT	rs2251550	58.28340009
A4GNT	rs61801011	34.09424387
A4GNT	rs203687	38.18482285
A4GNT	rs4974539	183.3091265
A4GNT	rs2073044	36.08711658
GCNT4	rs10922094	411.0093241
GCNT4	rs1555405	30.33942917
GCNT4	rs11569393	30.63307727
GCNT4	rs1355538	85.55594699
GCNT4	rs9267139	37.97953708
GCNT4	rs28573154	109.2057493
GCNT4	rs7774739	80.84968623
GCNT4	rs9271766	30.59765612
SIGLEC15	rs10305748	33.1188482
SIGLEC15	rs10922098	512.4825199
SIGLEC15	rs1047286	36.95010222
SIGLEC15	rs1707652	125.4391507
SIGLEC15	rs3130955	30.21883453
SIGLEC15	rs9501600	132.951794
SIGLEC15	rs35209896	43.55347577
BTNL3	rs10922098	253.3256077

BTNL3	rs6445035	111.4888961
BTNL3	rs3863087	46.0263962
BTNL3	rs9501600	128.5382194
BTNL3	rs35209896	44.53998705
EBI3	rs115770048	80.88089242
EBI3	rs34328206	74.82724396
EBI3	rs8108746	3970.811396
EBI3	rs9807813	5632.302667
EBI3	rs4807574	613.6418992
EBI3	rs7255715	305.1989391
EBI3	rs60103502	71.03529621
EBI3	rs143771088	35.70208813
EBI3	rs61872201	113.4911571
EBI3	rs61751507	473.5134117
EBI3	rs11594344	32.89294194
EBI3	rs3184504	41.56061662
EBI3	rs56115403	58.86554984
EBI3	rs704	215.4505525
EBI3	rs11666444	39.59423682
EBI3	rs4807876	104.5250394
EBI3	rs62165726	72.94872779
EBI3	rs10937241	62.59979463
EBI3	rs11711157	165.6229444
EBI3	rs5743618	109.9271398
HNRNPDL	rs4980320	34.7040611
HNRNPDL	rs1354034	123.058402
HNRNPDL	rs6897795	47.69669582
HNRNPDL	rs342200	30.31134696
BRAT1	rs10922094	82.1454705
BRAT1	rs1355538	30.81626616
BRAT1	rs9501393	36.10986718
BRAT1	rs7774739	31.69017956
FAM163B	rs1797077	883.8883553
FAM163B	rs10904861	50.38588543
FAM163B	rs61872201	49.17556835
FAM163B	rs61751507	274.9959866
FAM163B	rs704	47.90086728
FAM163B	rs7634194	31.94175804
DSC1	rs10922098	282.0062898
DSC1	rs1555405	30.86952386
DSC1	rs704	74.45320414
DSC1	rs1706435	69.92878804
DSC1	rs872914	118.7712534
DSC1	rs453821	88.21687937
DSC1	rs35426875	42.77219215
C6orf89	rs10922098	87.19855061
C6orf89	rs812426	79.86024687
C6orf89	rs1059216	373.2275408
C6orf89	rs2857383	32.8317244
C6orf89	rs117051919	40.11956961
C6orf89	rs1355538	53.85111638
MRAP2	rs17580	40.92416733
MRAP2	rs185009887	31.74707
MRAP2	rs8099840	40.7795425
MRAP2	rs6993770	36.08501265
LCTL	rs10922098	250.0506752
LCTL	rs9864801	44.78406398
LCTL	rs2242572	75.21780045

LCTL	rs9272068	37.37015287
OSMR	rs2921713	33.62905395
OSMR	rs77326070	189.911084
OSMR	rs10922098	167.7340614
OSMR	rs2106009	62.36888665
OSMR	rs697356	39.22102055
OSMR	rs28573154	56.08829031
OSMR	rs7774739	37.60594865
OSMR	rs9271766	30.55677169
TMPRSS11B	rs28853072	210.0863104
TMPRSS11B	rs2668202	41.12047012
TMPRSS11B	rs9501600	91.94451849
TMPRSS11B	rs72508422	33.27968787
STMN2	rs72882960	36.20759584
STMN2	rs4759076	107.8271838
STMN2	rs6436128	56.7945625
STMN2	rs1354034	174.012357
ASPRV1	rs3796097	64.08324173
ASPRV1	rs7545538	31.64754795
ASPRV1	rs224313	42.50422286
ASPRV1	rs17658212	125.0283401
ASPRV1	rs117562643	33.33056538
ASPRV1	rs113125305	107.7802533
ASPRV1	rs72802919	120.3044729
ASPRV1	rs74446154	44.00747763
ASPRV1	rs140510457	38.52314125
ASPRV1	rs117749902	41.69419007
ASPRV1	rs12610606	249.5058737
ASPRV1	rs145684392	87.43752952
ASPRV1	rs9845881	52.61133577
ASPRV1	rs188486759	30.9013138
ASPRV1	rs2702945	48.89305086
STX8	rs11078801	49.20510047
STX8	rs741735	29.81686368
STX8	rs12292693	93.34163276
STX8	rs1354034	37.7864123
NTM	rs4592429	34.42688238
NTM	rs12800878	507.4867074
NTM	rs2511504	2953.022003
NTM	rs12289924	124.4602686
NTM	rs7118465	216.3802272
NTM	rs2477669	54.1987272
NTM	rs78705678	202.9435345
NTM	rs2477642	194.3117427
NTM	rs112256242	32.19896294
NTM	rs138580446	43.67879356
NTM	rs540	33.9341662
NTM	rs1260326	126.9157869
NTM	rs2686401	77.71761279
GALNT13	rs16834610	45.8745652
GALNT13	rs10922098	55.09306529
GALNT13	rs2477642	35.78937717
GALNT13	rs12975366	55.38997958
CEACAM4	rs10922098	218.9217988
CEACAM4	rs1707652	51.5334813
CEACAM4	rs9501600	92.99388429
CEACAM4	rs35209896	30.78487408
PLA2R1	rs12471260	1015.193276

PLA2R1	rs113098364	957.5424537
PLA2R1	rs80339456	386.0474994
PLA2R1	rs6432620	4954.278179
PLA2R1	rs13001774	406.5720929
PLA2R1	rs757344	32.04736117
PLA2R1	rs6496748	32.37451063
PLA2R1	rs6430066	33.43628359
PLA2R1	rs12998722	33.82311396
PLA2R1	rs2091151	30.88655716
PLA2R1	rs12471193	74.22535846
PLA2R1	rs114994001	35.04346412
PLA2R1	rs116241488	62.02011788
PLA2R1	rs112083943	136.8444022
PLA2R1	rs62194171	76.20434515
PLA2R1	rs78489768	111.3233731
PLA2R1	rs115087160	71.65434149
PLA2R1	rs6747805	56.67253538
PLA2R1	rs80024373	86.58826123
PLA2R1	rs77298446	31.84953633
PLA2R1	rs61656332	51.97145829
PLA2R1	rs1377057	43.98422167
PLA2R1	rs11751347	63.41554908
PLA2R1	rs3798938	38.14936124
PLA2R1	rs2000242	32.37013027
GNGT2	rs182801617	45.22270344
GNGT2	rs10922094	35.22816653
GNGT2	rs28720282	30.56059736
CYB5D2	rs149208504	717.8270645
CYB5D2	rs17176065	359.0542914
CYB5D2	rs181444731	33.73565931
CYB5D2	rs7224464	36.74144785
CYB5D2	rs10922098	39.47770523
CYB5D2	rs1042445	46.42141594
CYB5D2	rs2523610	177.0150529
CYB5D2	rs2854001	177.3635785
CYB5D2	rs2523483	32.90282375
CYB5D2	rs41285751	51.74415273
CALN1	rs10922098	384.1324432
CALN1	rs11569415	31.30491875
CALN1	rs1355538	74.19840695
CALN1	rs453821	117.592644
CALN1	rs28471056	52.19896885
CD58	rs61789227	588.0044875
CD58	rs116614403	50.46919661
CD58	rs190942314	32.50388529
CD58	rs10922094	256.0012496
CD58	rs1706435	81.52050512
CD58	rs9501600	63.90219558
PLEKHA4	rs10922098	144.4779762
PLEKHA4	rs113747960	29.82815665
PLEKHA4	rs17580	156.8857737
PLEKHA4	rs2686395	66.72471105
PLEKHA4	rs9501600	76.44035696
SRL	rs11640587	202.3588137
SRL	rs7915908	56.19663275
SRL	rs932274	446.8658949
SRL	rs2736921	117.8988148
SRL	rs1355538	30.64001894

SRL	rs13190721	33.5095135
SRL	rs13208583	35.43945173
STX6	rs572515	199.789704
STX6	rs1706435	63.36309751
STX6	rs28573154	51.77190209
STX6	rs7774739	33.52158382
PLD3	rs7254808	47.49681498
PLD3	rs10922098	46.53459324
PLD3	rs11738994	52.48414558
PLD3	rs4631423	32.57196426
PLD3	rs6993770	48.39045723
CLEC2A	rs10922098	45.6063444
CLEC2A	rs7080536	1045.929223
CLEC2A	rs77774564	104.2339232
CLEC2A	rs11593575	52.83869634
CLEC2A	rs485305	54.72012444
CLEC2A	rs11196979	35.12508908
CLEC2A	rs9501393	47.65333828
CLEC2A	rs7774739	40.20688029
CLEC10A	rs10922098	151.5315035
CLEC10A	rs1706435	53.74973068
CLEC10A	rs9469017	33.03632664
CLEC10A	rs2844452	70.54336307
CLEC10A	rs550671	130.3526396
BET1L	rs2739240	184.3830577
BET1L	rs10922098	122.1349696
BET1L	rs1619994	30.58213889
BET1L	rs444921	54.91000301
BET1L	rs4734879	29.99295044
SPINK7	rs9784645	195.3205268
SPINK7	rs61816766	32.0810292
SPINK7	rs10922094	120.2122017
SPINK7	rs2727270	31.07404752
SPINK7	rs34529371	32.42754213
SPINK7	rs77924615	47.31820497
SPINK7	rs34155263	30.31554605
SPINK7	rs1355538	40.86630885
SPINK7	rs322372	32.66789579
SPINK7	rs453821	32.63471298
SPINK7	rs10971512	135.7550948
MUCL1	rs10922098	466.723605
MUCL1	rs2686395	74.40318408
MUCL1	rs9501600	116.887546
UCMA	rs185705950	166.6339586
UCMA	rs142421037	32.67453767
UCMA	rs138834887	336.0162072
UCMA	rs2399954	998.5997102
UCMA	rs112582999	35.31483422
UCMA	rs78560997	72.02624193
UCMA	rs117415981	35.22242087
UCMA	rs10906474	112.3861754
UCMA	rs75025577	94.97490401
UCMA	rs4755234	37.01243111
UCMA	rs6485513	32.68659506
UCMA	rs7952436	48.04196794
UCMA	rs7971039	62.93239086
NRG3	rs572515	44.66469462
NRG3	rs143959838	35.9366954

NRG3	rs143501783	32.55835424
LRRK2	rs10922094	207.5733648
LRRK2	rs964184	69.65767076
LRRK2	rs704	143.8963045
LRRK2	rs71352239	46.20264851
LRRK2	rs12478841	44.83776615
LRRK2	rs1291921	31.13005855
LRRK2	rs6445035	126.0022331
LRRK2	rs3863087	51.62166459
LRRK2	rs550671	96.49147769
LRRK2	rs34927613	60.51410618
BGLAP	rs12406047	33.71521417
BGLAP	rs3132487	41.2184164
BGLAP	rs77311729	251.1014451
BGLAP	rs6926251	334.697005
BGLAP	rs9272802	111.5051051
IL5	rs572515	52.54672963
IL5	rs1846190	43.52088419
IL5	rs11755689	31.09652675
CLC	rs10922098	169.8832084
CLC	rs9864801	49.00599249
CLC	rs9501600	60.12382528
CLC	rs9268744	31.50712551
N6AMT1	rs4980320	32.67956266
N6AMT1	rs17784482	38.70859877
N6AMT1	rs144176526	56.06479137
N6AMT1	rs146527756	48.69305586
N6AMT1	rs4541868	48.45889972
PDXK	rs4818855	248.2494387
PDXK	rs55704290	29.99749316
PDXK	rs75136590	37.3278565
PDXK	rs79039769	83.40246704
PDXK	rs144059351	41.20557315
PDXK	rs10057588	53.83404989
TLR4	rs10922094	39.98077834
TLR4	rs35802700	31.33665824
TLR4	rs67399078	33.24870382
REG4	rs12125319	35.03957903
REG4	rs80117153	32.8755552
REG4	rs3009182	204.3152898
REG4	rs72697218	70.5987507
REG4	rs191615076	75.50057259
REG4	rs7480143	52.91985427
REG4	rs10500661	30.53725747
REG4	rs3217810	47.72355933
REG4	rs731541	33.52298816
REG4	rs2979153	31.70065414
HSPB1	rs34179550	33.79603782
HSPB1	rs113088829	52.58907944
HSPB1	rs13240755	1180.156817
HSPB1	rs10231308	167.9292909
HSPB1	rs3972522	137.7581441
HSPB1	rs117296956	143.7118702
HSPB1	rs7926172	39.08487765
HSPB1	rs6926219	104.5363995
HSPB1	rs6460050	34.40065983
HSPB1	rs342200	48.93614435
CHI3L1	rs11584227	288.9267466

CHI3L1	rs7516546	141.2534653
CHI3L1	rs75228438	160.7898929
CHI3L1	rs143359688	1156.791856
CHI3L1	rs80241012	647.4310183
CHI3L1	rs6670714	42.57727377
CHI3L1	rs114072431	48.99837762
CHI3L1	rs1932023	123.7681057
CHI3L1	rs6679311	82.47241235
CHI3L1	rs1800894	32.04493022
CHI3L1	rs28601761	77.57700638
TENM3	rs10922094	91.31144074
TENM3	rs704	160.4181175
TENM3	rs9501393	39.71851896
TENM3	rs7774739	33.55683683
SVEP1	rs145419292	298.9942325
SVEP1	rs75412647	56.25431068
SVEP1	rs1327537	161.6942807
SVEP1	rs76038906	546.0906082
SVEP1	rs78742138	1018.704179
SVEP1	rs116853336	239.7914729
SVEP1	rs185970880	379.68634
SVEP1	rs146370544	58.73813584
SVEP1	rs72759341	155.1829455
SVEP1	rs115770375	44.34738642
SVEP1	rs41264285	262.0635139
SVEP1	rs200367989	47.72515387
SVEP1	rs147639000	565.56113
SVEP1	rs7935242	35.79190109
SVEP1	rs78689694	38.19468384
SVEP1	rs879620	33.6825426
SVEP1	rs12945299	32.18652621
SVEP1	rs34914463	29.85396595
SVEP1	rs12666169	45.82431524
SVEP1	rs117223414	100.7388078
SVEP1	rs576123	50.80569998
TMEM119	rs10922098	99.53086637
TMEM119	rs802171	62.32334393
TMEM119	rs1355538	34.60011728
C11orf87	rs1591734	97.79619154
C11orf87	rs9332701	544.0314846
C11orf87	rs6427202	306.5244299
C11orf87	rs78334957	97.37912144
C11orf87	rs927826	326.3426011
C11orf87	rs184924341	79.18121452
C11orf87	rs594793	31.15783916
C11orf87	rs62140549	60.02579879
C11orf87	rs34568880	39.61944628
C11orf87	rs34977123	190.6574847
C11orf87	rs9271669	68.80578013
SPATA20	rs147604653	34.60011883
SPATA20	rs77405995	43.41743519
SPATA20	rs17714829	67.74762643
SPATA20	rs9890200	2027.162512
SPATA20	rs10922094	62.2481762
SPATA20	rs4666000	30.6008394
SPATA20	rs3020644	69.1924786
SPATA20	rs6458	49.06323662
RAB17	rs72748420	43.70967466

RAB17	rs1329424	1279.945726
RAB17	rs3900909	57.67808216
RAB17	rs10801660	40.03504046
RAB17	rs1047286	37.0106746
RAB17	rs111696008	32.8480492
RAB17	rs1707652	232.9470942
RAB17	rs3099847	37.22688525
RAB17	rs1265941	128.7380914
RAB17	rs550671	336.0550627
RAB17	rs9276606	49.34752579
NAT14	rs10922098	213.8555061
NAT14	rs704	157.4627901
NAT14	rs1707652	63.41483201
NAT14	rs9501600	64.35845953
NAT14	rs35209896	29.95087072
TMEM132C	rs10922098	56.07845411
TMEM132C	rs117511908	269.1509978
TMEM132C	rs34735009	44.42607018
TMEM132C	rs141618758	59.59230751
TMEM132C	rs145639768	44.08836307
TMEM132C	rs146039342	33.99483247
BMP15	rs10922098	323.2185829
BMP15	rs58068569	29.91609802
BMP15	rs1707652	91.86709199
BMP15	rs9267139	32.95390487
BMP15	rs9501393	109.1821416
BMP15	rs7774739	83.77391947
BMP15	rs9271683	46.04497459
CACNB4	rs11502187	45.02976004
CACNB4	rs7975385	187.6697724
CACNB4	rs79977579	30.95266489
CACNB4	rs1354034	149.3840368
CACNB4	rs114694170	37.72886139
MTRF1L	rs2038332	404.3140525
MTRF1L	rs10922098	64.08377923
MTRF1L	rs6542680	30.71755606
MRPL55	rs6809081	31.76576798
MRPL55	rs116706170	184.6254087
MRPL55	rs9260109	157.4615
CSF2RB	rs10922098	414.1578935
CSF2RB	rs1707652	112.2743729
CSF2RB	rs550671	124.8065228
CSF2RB	rs34927613	57.10587702
RUNX3	rs10922094	46.26517122
RUNX3	rs1265564	32.44049135
RUNX3	rs4639017	47.86798445
COL1A1	rs138103546	60.42466521
COL1A1	rs116585561	202.2513928
COL1A1	rs118081843	61.49330225
COL1A1	rs6679677	36.63641689
COL1A1	rs10842700	31.07586384
COL1A1	rs1056692	30.51074608
COL1A1	rs9594738	144.712369
COL1A1	rs2663343	44.26498717
COL1A1	rs139072136	46.68307714
COL1A1	rs369441062	51.54775415
COL1A1	rs74485878	54.2846001
COL1A1	rs62071464	36.03631885

COL1A1	rs3928387	35.9371984
COL1A1	rs145495890	80.29535442
COL1A1	rs148047098	65.33426079
COL1A1	rs146385050	562.4249522
COL1A1	rs149631508	45.76210837
COL1A1	rs11726170	35.28003596
COL1A1	rs10955915	42.52046051
ANGPTL1	rs4457535	915.6545732
ANGPTL1	rs12725691	329.1929181
ANGPTL1	rs140477729	30.04344312
ANGPTL1	rs333947	31.31793946
ANGPTL1	rs6678237	38.39560907
ANGPTL1	rs12130811	53.62169889
ANGPTL1	rs78811502	80.69442584
ANGPTL1	rs115584967	32.94853277
ANGPTL1	rs2477664	32.0039191
ANGPTL1	rs4681671	30.84718223
ANGPTL1	rs10213052	32.11095745
OLFM3	rs33995407	30.48424939
OLFM3	rs572515	159.1607688
OLFM3	rs1355538	40.92004495
OLFM3	rs2075798	29.97839652
OLFM3	rs550671	50.59201459
KIAA1549L	rs11388755	102.3090912
KIAA1549L	rs4756649	923.1669264
KIAA1549L	rs762044	240.1890145
KIAA1549L	rs143077049	47.73589644
KIAA1549L	rs10922098	90.88516537
KIAA1549L	rs55714927	33.2061826
KIAA1549L	rs9864801	31.23426088
KIAA1549L	rs635634	80.96690154
TBX22	rs10922098	95.5596885
TBX22	rs1355538	41.1114865
TBX22	rs4713498	32.48298538
TLR1	rs10922098	95.45534717
TLR1	rs1555405	33.27177905
TLR1	rs56210800	32.56021082
TLR1	rs149468457	32.67827148
TLR1	rs9271642	40.9318999
KLK13	rs11669237	3632.109088
KLK13	rs2691230	104.7479404
KLK13	rs73051038	133.183443
KLK13	rs1039405	31.16401528
KLK13	rs116960573	193.8824229
KLK13	rs12368376	38.3166327
KLK13	rs5511	130.7508188
KLK13	rs55714927	54.55217461
KLK13	rs4072059	30.80961535
KLK13	rs17855739	60.44442043
KLK13	rs2548459	86.05410064
KLK13	rs1992880	55.28340111
KLK13	rs12712266	33.65699374
KLK13	rs2479016	37.99019447
NFE2L1	rs11451044	70.24440218
NFE2L1	rs1354034	38.08489687
NFE2L1	rs342292	62.08146402
COL6A5	rs10922098	60.8822089
COL6A5	rs2072634	30.25784686

COL6A5	rs9271642	43.7366525
RNF122	rs10922094	120.5476405
RNF122	rs964184	39.54542814
RNF122	rs71352239	81.56128445
RNF122	rs10208529	33.98013371
RNF122	rs111696008	31.63570873
RNF122	rs1707652	199.243485
RNF122	rs28573154	33.30371875
RNF122	rs34927613	30.26448112
EQTN	rs10922094	75.54294338
EQTN	rs704	110.6680591
EQTN	rs71549272	43.96255604
FAM162B	rs10922094	169.6710286
FAM162B	rs6542680	33.70342092
FAM162B	rs6445035	41.77493864
FAM162B	rs147906938	48.17478221
TENM2	rs10922098	371.4361105
TENM2	rs1355538	86.18015481
TENM2	rs9501600	114.5492513
TENM2	rs35209896	40.39201556
MTMR1	rs144704969	31.3169243
MTMR1	rs10922098	575.09365
MTMR1	rs1047286	31.78039276
MTMR1	rs12478841	33.66555432
MTMR1	rs1707652	120.0942624
MTMR1	rs1265941	67.44853749
MTMR1	rs550671	149.0809199
MTMR1	rs9276606	30.09700096
MEGF10	rs10922098	358.251867
MEGF10	rs1355538	65.43074087
MEGF10	rs9267139	31.1942673
MEGF10	rs9501393	97.510356
MEGF10	rs7774739	62.75407974
MEGF10	rs9271683	56.67668044
MILR1	rs10922098	70.02541765
MILR1	rs1926446	55.22170288
MILR1	rs1555405	33.05663967
MILR1	rs697356	33.05012426
MILR1	rs2057681	76.62781291
THSD7A	rs2808466	286.147368
THSD7A	rs45574833	74.00286797
THSD7A	rs28593765	36.81161936
THSD7A	rs1260326	33.93557611
THSD7A	rs71552082	36.94922164
THSD7A	rs79327907	40.70204185
THSD7A	rs9273429	93.09396835
KRT5	rs10506306	29.96232777
KRT5	rs641621	273.6484368
KRT5	rs113543611	108.0595213
KRT5	rs139214344	33.80664018
KRT5	rs10922094	60.18624021
KRT5	rs17580	108.042255
KRT5	rs118132312	41.95223988
KRT5	rs58539907	54.7713447
KRT5	rs186618212	71.84785604
KRT5	rs117484558	155.5425763
KRT5	rs138065021	61.57360607
KRT5	rs111494201	61.34242484

KRT5	rs7240884	86.98923543
KRT5	rs511515	34.63906763
SVEP1	rs145419292	228.9801253
SVEP1	rs7020788	525.817472
SVEP1	rs78742138	1188.864633
SVEP1	rs191541154	115.5681741
SVEP1	rs117584091	78.7956894
SVEP1	rs115770375	36.77753923
SVEP1	rs45444697	298.3030767
SVEP1	rs200367989	52.06034474
SVEP1	rs147639000	650.0300128
SVEP1	rs2923099	37.91542245
SVEP1	rs7952602	30.11277548
SVEP1	rs879620	31.69365254
SVEP1	rs74617664	37.4739389
SVEP1	rs117223414	74.42131119
SVEP1	rs151231214	54.38882404
SVEP1	rs112639900	203.5335528
SVEP1	rs75733144	92.74531695
SVEP1	rs11244049	55.21695283
TSSK1B	rs61804164	38.0925996
TSSK1B	rs13379369	58.36753295
TSSK1B	rs1830104	33.23780185
TSSK1B	rs3129880	37.46427333
SMARCC1	rs114009540	32.8471141
SMARCC1	rs10922098	495.3501292
SMARCC1	rs2230203	38.65034363
SMARCC1	rs157592	35.20721881
SMARCC1	rs2686395	106.1242445
SMARCC1	rs9267139	32.00389019
SMARCC1	rs9501393	90.37811206
SMARCC1	rs7774739	62.18279306
SMARCC1	rs9271683	39.70620528
GCH1	rs10922098	419.3015565
GCH1	rs2686395	81.55793253
GCH1	rs9501600	116.7292709
GCH1	rs35209896	40.1894667
CLEC12A	rs34735582	1050.736913
CLEC12A	rs486890	5254.370074
CLEC12A	rs80338457	695.6392763
CLEC12A	rs7132536	660.6016063
CLEC12A	rs113247892	422.7173946
CLEC12A	rs2244387	75.32692909
CLEC12A	rs138092566	122.2549879
CLEC12A	rs3759253	108.4680573
CLEC12A	rs71453409	321.3349152
CLEC12A	rs141864201	41.83334578
CLEC12A	rs145271031	87.1497265
CLEC12A	rs7300976	31.43727708
CLEC12A	rs12826805	103.2244643
CLEC12A	rs11888817	59.50161552
CLEC12A	rs2470750	135.8850274
CLEC12A	rs11920688	50.41040493
CLEC12A	rs509227	38.70427822
CLEC12A	rs4644106	45.99599144
CLEC12A	rs4455675	30.07436037
TINAGL1	rs112284033	46.89458455
TINAGL1	rs945214	163.8478038

TINAGL1	rs2128699	38.53775102
TINAGL1	rs897471	155.0079706
TINAGL1	rs2477664	36.47988088
TINAGL1	rs7310615	45.59601598
TINAGL1	rs544012	60.1058044
TINAGL1	rs11842143	56.67172053
TINAGL1	rs144600577	72.33281761
TINAGL1	rs28529205	92.56882012
TINAGL1	rs895394	60.97715931
TINAGL1	rs3032928	35.03085548
TINAGL1	rs2277998	34.30799423
TINAGL1	rs1260326	73.23779061
TINAGL1	rs6061230	86.99141226
TINAGL1	rs13088856	39.53486669
TINAGL1	rs2523535	30.7914337
COL6A3	rs11677932	59.10243933
COL6A3	rs200150873	31.95960771
COL6A3	rs10922094	85.88882756
COL6A3	rs12945299	35.58566995
COL6A3	rs3088026	32.66268662
CREB3L1	rs10922098	168.0409223
CREB3L1	rs1355538	50.94088822
CREB3L1	rs9501393	41.98664664
CREB3L1	rs7774739	38.08644708
CREB3L1	rs67399078	31.31124527
PUS1	rs10922098	80.51162021
PUS1	rs9501393	32.28000872
PUS1	rs7774739	30.27389168
TBX5	rs10922094	154.3005308
TBX5	rs7137828	30.58933422
TBX5	rs697357	39.16707043
TBX5	rs6457457	33.8222285
TBX5	rs9501393	42.16004442
ITGB7	rs12878578	58.62040035
ITGB7	rs78912960	31.82774305
ITGB7	rs2275132	29.78387032
MSR1	rs572515	198.8265279
MSR1	rs2304200	416.8806683
MSR1	rs1707652	46.18919997
MSR1	rs415620	42.55571363
MSR1	rs4713498	54.8397708
NAGPA	rs72766568	41.66815673
NAGPA	rs117959699	292.9594375
NAGPA	rs35416418	1100.145979
NAGPA	rs112366035	57.46053572
NAGPA	rs7196133	79.79660075
NAGPA	rs77294552	132.3309754
NAGPA	rs75520381	45.30143426
NAGPA	rs118096022	51.89348131
NAGPA	rs547187	33.02601799
NAGPA	rs12280645	32.37258226
NAGPA	rs55714927	45.1214211
NAGPA	rs635634	94.1019297
TBCE	rs34099250	42.35763728
TBCE	rs903347	145.3177657
TBCE	rs4980320	41.98955071
TBCE	rs10876550	38.11661251
TBCE	rs1109867	43.14664479

TBCE	rs1354034	70.53995206
TXNDC5	rs1043784	222.1855041
TXNDC5	rs149742777	270.7746231
TXNDC5	rs149679123	106.6225138
TXNDC5	rs13209436	54.19058342
TXNDC5	rs7903703	31.47417938
TXNDC5	rs8078222	40.65585371
TXNDC5	rs56238200	227.1905748
TXNDC5	rs112483799	68.04003784
TXNDC5	rs115447463	32.56325527
TXNDC5	rs6796	33.83121127
TXNDC5	rs78740585	64.47871435
DNAJB9	rs147800199	52.71671316
DNAJB9	rs661126	29.7314536
DNAJB9	rs17580	36.56359511
DNAJB9	rs112963922	87.47619566
DNAJB9	rs34095861	31.90150442
DNAJB9	rs28487964	35.23245425
DNAJB9	rs4788808	63.67999729
DNAJB9	rs9302635	136.5164392
DNAJB9	rs8178824	240.8713523
DNAJB9	rs111481700	31.09355748
DNAJB9	rs72613567	45.44455354
DNAJB9	rs3130955	50.47555899
DNAJB9	rs73394251	36.72349624
DNAJB9	rs188302747	49.83235527
CDH15	rs2281721	96.27592043
CDH15	rs7302925	32.7449975
CDH15	rs16959006	107.070771
CDH15	rs73069835	40.57948221
CDH15	rs115881877	70.0637556
CDH15	rs114704096	82.66064052
CDH15	rs113567935	107.2310421
CDH15	rs75097760	244.9044901
CDH15	rs11716402	33.7297722
CDH15	rs116347702	170.0478568
CDH15	rs79020595	51.89592715
CDH15	rs147408433	38.30907187
CDH15	rs116749582	72.08648525
CDH15	rs71640035	36.7331262
CDH15	rs75077631	134.4731394
TPMT	rs9332701	539.0722059
TPMT	rs10822155	65.78105224
TPMT	rs964184	93.31353426
TPMT	rs261290	31.59764392
TPMT	rs3816117	69.56159101
TPMT	rs775824	45.19928705
TPMT	rs12995461	39.09259208
TPMT	rs1355538	99.4087218
TPMT	rs28374650	32.57641567
TPMT	rs10096633	40.95998267
TPMT	rs6993770	65.00848028
FGFBP3	rs11186737	1035.108318
FGFBP3	rs2209972	63.39525238
FGFBP3	rs599839	56.99973142
FGFBP3	rs35769183	40.80028724
FGFBP3	rs3758538	30.1257788
FGFBP3	rs11190377	141.9542193

FGFBP3	rs61269268	37.4521334
FGFBP3	rs77542162	43.36382703
FGFBP3	rs13077145	38.01504351
FGFBP3	rs12640189	32.96388363
TMEM154	rs1591734	45.98643679
TMEM154	rs9332701	124.8198293
TMEM154	rs2239853	108.4083788
TMEM154	rs78334957	32.50686165
TMEM154	rs927826	111.5150996
TMEM154	rs184924341	31.68710558
TMEM154	rs6544941	30.57684194
TMEM154	rs2276815	46.29410859
TMEM154	rs3130511	35.67842705
UBE3A	rs10922094	35.74230792
UBE3A	rs4980320	40.63950659
UBE3A	rs1354034	47.81216209
UBE3A	rs1355538	30.33930405
RIC3	rs10922094	396.9182673
RIC3	rs12403425	29.85420898
RIC3	rs704	40.8972385
RIC3	rs1355538	93.708746
RIC3	rs941758	30.32073709
RIC3	rs453821	119.2822292
RIC3	rs28471056	61.8871942
ERN1	rs10922094	58.06828595
ERN1	rs507855	30.86999021
ERN1	rs9501393	35.79994273
ERN1	rs11244079	35.31816781
PCOLCE	rs7385804	352.5571858
PCOLCE	rs150731032	56.85346669
PCOLCE	rs13379042	40.03172579
PCOLCE	rs142931061	29.82111661
PCOLCE	rs150844304	97.04887913
PCOLCE	rs56139860	33.33367232
PCOLCE	rs339969	45.30978392
PCOLCE	rs704	31.45881346
PCOLCE	rs7671124	30.89122735
PCOLCE	rs55649657	52.5007367
TMPRSS6	rs10922094	46.23666693
TMPRSS6	rs111265231	46.10472148
TMPRSS6	rs147804221	63.79584637
TMPRSS6	rs190324353	40.28503893
TMPRSS6	rs201234732	65.18536513
TMPRSS6	rs11655538	148.9703182
TMPRSS6	rs140390732	89.39365049
TMPRSS6	rs2004584	293.8361953
TMPRSS6	rs4239214	1246.73045
TMPRSS6	rs144924964	931.7348241
TMPRSS6	rs117451747	322.0342188
TMPRSS6	rs117348742	161.1723342
TMPRSS6	rs16964261	697.623561
TMPRSS6	rs142216432	65.8675426
TMPRSS6	rs78119120	94.60674968
TMPRSS6	rs137940962	57.27813321
TMPRSS6	rs12478841	45.86909231
TMPRSS6	rs56210800	31.58694992
TMPRSS6	rs140431012	31.76565932
TMPRSS6	rs9501393	30.23507349

ASL	rs111439465	117.4667757
ASL	rs10950053	174.6083588
ASL	rs4753123	38.1392011
ASL	rs3747207	53.90579228
ASL	rs137996846	54.77848013
ASL	rs139112016	83.16729684
ASL	rs143906171	69.7348896
ASL	rs73134721	107.3113919
ASL	rs28601761	32.59640744
TGM1	rs572515	40.45157813
TGM1	rs974472	58.47528706
TGM1	rs601020	36.95206449
ADGRF1	rs704	335.7026957
ADGRF1	rs142820745	29.87233927
ADGRF1	rs429358	94.86847936
ADGRF1	rs35136575	64.82784521
ADGRF1	rs62269916	33.56243894
ADGRF1	rs1355538	57.59980332
NAGS	rs10922098	210.1162734
NAGS	rs6445035	51.40274774
NAGS	rs9501393	68.28206375
NAGS	rs7774739	41.46697814
UROS	rs12765966	53.26369503
UROS	rs11244609	29.88053556
UROS	rs1935451	516.5314847
UROS	rs6597746	40.15947839
UROS	rs3811444	33.88089662
UROS	rs57467915	133.6433759
UROS	rs7634917	30.27960315
LRCH4	rs1329424	211.8134105
LRCH4	rs1707652	66.13315569
LRCH4	rs9501600	74.3638896
PAPLN	rs10922098	137.8866382
PAPLN	rs6542680	31.18265728
PAPLN	rs1355538	50.81532033
PAPLN	rs1056429	30.71147737
PAPLN	rs9501600	53.53031278
QDPR	rs56320400	36.21829463
QDPR	rs75218392	106.1605459
QDPR	rs3822233	2744.669253
QDPR	rs1354034	30.55840774
QDPR	rs28363792	56.80286086
QDPR	rs79442348	34.06369073
MADCAM1	rs62269916	31.5782248
MADCAM1	rs1706435	63.87318864
MADCAM1	rs6993770	36.20205159
SUN5	rs368465	169.9439396
SUN5	rs7073753	67.55936535
SUN5	rs115378853	42.09630264
SUN5	rs183484749	32.84570479
SUN5	rs113375294	196.0025755
SUN5	rs148316141	70.71892778
SUN5	rs73189766	71.3098468
SUN5	rs116115132	62.6901116
SUN5	rs1398015	34.47625002
SUN5	rs11574452	39.37710752
TRABD	rs10922094	58.59795494
TRABD	rs964184	35.80168675

TRABD	rs71352239	73.39143909
TRABD	rs12478841	37.08778984
TRABD	rs1707652	67.00292059
TRABD	rs4713488	30.43378117
CASQ1	rs10922094	32.01245939
CASQ1	rs964184	40.68085274
CASQ1	rs371267418	40.95079752
CASQ1	rs113448098	68.92376111
CASQ1	rs11838267	245.7805668
CASQ1	rs117827303	38.27362159
CASQ1	rs12100587	37.01795437
CASQ1	rs72713385	72.20292403
CASQ1	rs1355538	74.812861
CASQ1	rs73725598	53.32261164
CASQ1	rs13217434	39.40209688
XDH	rs10915471	34.71544431
XDH	rs10922098	30.02836861
XDH	rs10138174	87.80115202
XDH	rs2753516	31.0320153
ALDH1A1	rs348452	118.4640106
ALDH1A1	rs738409	33.16793847
ALDH1A1	rs1331309	48.50638164
SELPLG	rs10658530	33.37257248
SELPLG	rs73191242	759.5985918
SELPLG	rs7298509	96.51085642
SELPLG	rs1251489	316.7098521
SELPLG	rs11162091	32.92158428
SELPLG	rs11066272	38.08600058
SELPLG	rs76428106	38.68871401
SELPLG	rs12945886	32.14705855
SELPLG	rs7221894	45.13202111
SELPLG	rs12110	103.1344232
SELPLG	rs1260326	37.59238513
SELPLG	rs112694524	35.63974302
SELPLG	rs2595507	49.45524109
SELPLG	rs66817580	46.58962256
SELPLG	rs1264708	34.150581
SELPLG	rs2256974	36.41935549
SELPLG	rs13205436	35.37162524
SELPLG	rs6796	67.91718724
SELPLG	rs5028373	86.67003281
SELPLG	rs139803760	40.22644028
SELPLG	rs7814163	30.37732043
SELPLG	rs117549241	35.60901012
SELPLG	rs2142306	482.0706616
SELPLG	rs62520288	357.2112451
SELPLG	rs13282872	36.55490141
SELPLG	rs60988380	35.72666067
SELPLG	rs871744	105.8833594
SELPLG	rs375021953	140.66986
SELPLG	rs1137654	156.5934339
CHCHD10	rs10922094	227.9113357
CHCHD10	rs1355538	67.00509726
CHCHD10	rs204995	84.79102178
CHCHD10	rs71789649	62.63258035
GSTT2B	rs4822435	39.53559032
GSTT2B	rs140977331	37.36561282
GSTT2B	rs5751764	997.8360878

GSTT2B	rs143467905	61.82927234
GSTT2B	rs1355538	30.09227473
LRP1B	rs10801548	104.8310573
LRP1B	rs1329424	1237.169683
LRP1B	rs35351085	46.18750175
LRP1B	rs1354034	98.97848567
ATF6	rs10918270	34.56711498
ATF6	rs149072800	34.86572582
ATF6	rs11164870	129.5451841
ATF6	rs2273967	99.20165026
ATF6	rs79688957	88.09068462
ATF6	rs61738953	1085.247396
ATF6	rs147456212	34.23814835
ATF6	rs9333273	47.32014179
ATF6	rs193096844	39.87145911
ATF6	rs2583223	85.28001053
ATF6	rs72706230	30.6490482
ATF6	rs6500548	30.727356
ATF6	rs58192144	32.25448337
ATF6	rs4796383	33.62287049
ATF6	rs8066387	31.0271106
ATF6	rs77262773	50.75835535
ATF6	rs737908	60.93068805
ATF6	rs9825233	49.06008469
ATF6	rs572553	56.90881735
ATF6	rs13107325	37.028657
ATF6	rs163279	38.68104432
ATF6	rs8111	623.286011
ATF6	rs9272803	185.7871746
ATF6	rs1794514	63.63184326
ATF6	rs8176743	45.08368312
COL11A2	rs146374908	35.12109239
COL11A2	rs3918140	49.54465871
COL11A2	rs2855437	773.0257896
COL11A2	rs12194518	73.07057711
COL11A2	rs61729512	32.1138638
COL11A2	rs7305932	35.43023271
COL11A2	rs2147161	34.33309825
COL11A2	rs8051661	61.95077018
COL11A2	rs9963868	46.42002795
COL11A2	rs12975366	69.99492981
COL11A2	rs10174098	30.53043569
COL11A2	rs1050414	49.39048044
COL11A2	rs148658859	38.74291497
COL11A2	rs271173	35.78697224
COL11A2	rs2450083	31.43049493
GAD1	rs10922098	157.673398
GAD1	rs4977155	43.3707585
GAD1	rs415620	39.62253088
GAD1	rs7774739	32.09018812
GAD1	rs147168394	31.64560869
GRB7	rs144704969	30.61415214
GRB7	rs10922098	821.1145398
GRB7	rs3900909	44.4019176
GRB7	rs704	106.208837
GRB7	rs1047286	46.69399576
GRB7	rs111696008	29.7347103
GRB7	rs1707652	158.4605391

GRB7	rs555007	101.4363525
GRB7	rs444921	225.1958867
GRB7	rs9269234	33.56705626
GRB7	rs9272526	49.36912808
DCAF5	rs10922098	413.0946038
DCAF5	rs11569415	31.10498504
DCAF5	rs1707652	81.48146002
DCAF5	rs2075798	45.58924719
DCAF5	rs550671	104.8964128
CYB5A	rs17806420	101.3893667
CYB5A	rs10922094	33.52293757
CYB5A	rs11645522	34.25185685
CYB5A	rs1065853	33.71883417
CYB5A	rs7669911	32.50722426
NT5C2	rs4919682	180.2966331
NT5C2	rs2073340	36.38294678
NT5C2	rs72815685	32.79174021
NT5C2	rs1354034	37.58830737
NT5C2	rs342200	31.66927867
SCARA3	rs1451915	36.76362272
SCARA3	rs10922098	751.5281751
SCARA3	rs7535557	39.89281169
SCARA3	rs6542680	91.41752881
SCARA3	rs1707652	131.0592602
SCARA3	rs3891366	244.4175575
SCARA3	rs7774739	158.7699946
SCARA3	rs28645249	63.03906574
SCARA3	rs116334170	41.59549457
LRRN1	rs10922098	76.63517915
LRRN1	rs144063062	54.04443312
LRRN1	rs140776740	77.77120121
LRRN1	rs10411127	73.94177841
LRRN1	rs55923289	950.1356651
LRRN1	rs6859	922.6859469
LRRN1	rs405509	656.767246
LRRN1	rs12459074	43.2867618
LRRN1	rs147442832	89.39882473
LRRN1	rs12971662	34.57824436
LRRN1	rs11720167	149.6098706
LRRN1	rs3863087	52.00799464
SORT1	rs10922094	137.7736722
SORT1	rs4977155	52.71153995
SORT1	rs1706435	46.06676925
SORT1	rs9501600	66.69719718
SORT1	rs74671299	32.15818356
SORT1	rs73403125	30.52803615
TNR	rs2235256	264.6560152
TNR	rs10922098	365.4569228
TNR	rs35166255	37.75769074
TNR	rs10151220	33.48982935
TNR	rs3817428	44.41231648
TNR	rs704	163.0678059
TNR	rs11720167	97.34866925
TNR	rs3863087	35.20126397
TNR	rs3099847	33.23695822
TNR	rs1265941	70.07547761
TNR	rs550671	134.2994395
TNR	rs2636882	38.40248389

SAMHD1	rs528298	332.0850608
SAMHD1	rs704	54.15311273
SAMHD1	rs1706435	94.11540756
SAMHD1	rs9501600	78.71722487
WWP1	rs7073753	31.11267062
WWP1	rs4980320	54.79890567
WWP1	rs10876550	39.70332684
WWP1	rs1354034	114.6790751
WWP1	rs342292	45.92433214
CREB3L4	rs28538178	30.13698096
CREB3L4	rs34074094	64.8674836
CREB3L4	rs6671362	617.5024478
CREB3L4	rs7937770	33.55893736
CREB3L4	rs2117168	32.93575458
CREB3L4	rs11925342	116.1296619
CREB3L4	rs146479329	82.13052475
CREB3L4	rs9468618	167.8156548
CREB3L4	rs138678612	1147.680502
CREB3L4	rs185289795	344.4287385
CREB3L4	rs111319575	43.25784299
CREB3L4	rs36229731	317.0934955
CREB3L4	rs111876947	142.694663
CREB3L4	rs79507667	287.6247246
CREB3L4	rs115100599	68.05558124
CREB3L4	rs73743304	38.96103891
CREB3L4	rs35385182	57.03978217
CREB3L4	rs3752617	59.66013609
CREB3L4	rs4841132	77.38820564
RAG1	rs10922098	65.97343904
RAG1	rs919791	740.3213151
RAG1	rs6457457	32.91128573
RAG1	rs28573154	44.99288823
PMS2	rs10922098	284.9530057
PMS2	rs1707652	74.74683378
PMS2	rs1042663	67.76009013
PMS2	rs9501602	30.17809596
PCBD1	rs118079346	44.46066492
PCBD1	rs72809253	43.06595899
PCBD1	rs149422142	64.52775832
PCBD1	rs2541232	43.93490926
PCBD1	rs844124	220.7557438
PCBD1	rs11596669	65.91346849
PCBD1	rs72805136	32.08995603
PCBD1	rs7489145	87.6928887
PCBD1	rs11611373	87.62946182
PCBD1	rs3747207	35.81553477
PCBD1	rs28374650	29.78443571
PPM1D	rs10922098	191.3508506
PPM1D	rs75327431	31.92709218
PPM1D	rs4639017	35.45520875
PPM1D	rs2695234	43.69094835
PPM1D	rs6457457	34.90832441
PPM1D	rs9501393	48.23718938
CHFR	rs10922094	117.5943784
CHFR	rs113448098	62.36336704
CHFR	rs12146727	217.2998031
CHFR	rs17580	54.49152553
CHFR	rs61984785	32.91278494

CHFR	rs1355538	70.05187201
CHFR	rs147906938	47.54126758
CHFR	rs9501602	30.09286873
BTG2	rs10801548	40.55037845
BTG2	rs1329424	949.4283632
BTG2	rs35351085	42.18872002
BTG2	rs6445035	182.6406079
BTG2	rs3863087	58.38457113
BTG2	rs1265941	94.70734161
BTG2	rs550671	248.6429912
BTG2	rs41548618	42.02254264
BTG2	rs9276577	34.71933072
CLK2	rs72748420	37.02618725
CLK2	rs1329424	1143.673965
CLK2	rs3900909	49.81749584
CLK2	rs10801660	38.42263132
CLK2	rs704	520.2403389
CLK2	rs1047286	37.30961506
CLK2	rs111696008	30.83014791
CLK2	rs1707652	203.082247
CLK2	rs3099847	50.52405392
CLK2	rs1265941	124.5377618
CLK2	rs550671	328.0413099
CLK2	rs78897911	30.83876449
CLK2	rs9276606	47.8204509
CSNK2B	rs78912960	37.15865727
CSNK2B	rs61269268	40.84031346
CSNK2B	rs6542680	219.3473811
CSNK2B	rs80220851	33.47779289
ARHGAP25	rs141732432	32.14509832
ARHGAP25	rs13413887	339.0281217
ARHGAP25	rs1354034	39.126739
ARHGAP25	rs6993770	51.03874621
FANCF	rs10922094	30.64587241
FANCF	rs2078687	38.44594062
FANCF	rs11845244	81.94355699
FANCF	rs6542680	33.75458041
TALDO1	rs146899805	105.8982162
TALDO1	rs143912793	113.7473608
TALDO1	rs72849433	37.95231383
TALDO1	rs35979828	36.94656624
TALDO1	rs1354034	38.33854941
TALDO1	rs2343596	30.81352171
P4HA2	rs10922094	34.44152584
P4HA2	rs113448098	70.63138744
P4HA2	rs12146727	193.4725306
P4HA2	rs1355538	35.96894895
P4HA2	rs114925146	33.95690501
P4HA2	rs56783668	31.12493062
P4HA2	rs1042303	56.72770599
STUB1	rs11417460	33.04001779
STUB1	rs11601665	33.59150001
STUB1	rs1354034	85.67905056
NHEJ1	rs72949337	74.08314064
NHEJ1	rs115279228	133.8913889
NHEJ1	rs142913615	78.99821778
NHEJ1	rs6993770	50.48426011
PLCB1	rs4980320	43.97380775

PLCB1	rs1354034	56.31415856
PLCB1	rs342293	43.29074589
GRB10	rs4980320	49.65768671
GRB10	rs1354034	204.0700853
GRB10	rs342293	47.97530311
GRB10	rs6993770	53.47534761
GRB10	rs11792848	79.26130689
RRM1	rs11031142	45.95553923
RRM1	rs4460801	33.18061038
RRM1	rs28594470	41.76426343
RRM1	rs3811444	89.8506431
RRM1	rs150158762	30.547129
RRM1	rs68149176	48.32578294
RRM1	rs377655768	35.24746434
RRM1	rs543798	59.72084163
RRM1	rs74717647	38.37944306
RRM1	rs11666771	31.54826239
RRM1	rs8887	67.53193403
RRM1	rs74459970	38.6882785
RRM1	rs11900746	57.7718474
RRM1	rs2425031	35.97766301
RRM1	rs146623855	38.14565127
RRM1	rs6017001	338.3680449
RRM1	rs6014993	43.06853397
RRM1	rs182172833	42.63208781
RRM1	rs9609519	35.84396188
RRM1	rs8141337	3086.245182
RRM1	rs17692518	352.3314462
RRM1	rs78038115	60.90920125
RRM1	rs113356676	67.49553789
RRM1	rs138116184	155.2744441
RRM1	rs76966048	34.26450854
RRM1	rs230508	241.4847353
RRM1	rs372691294	50.02799779
RRM1	rs9400888	55.30121847
RRM1	rs10281146	76.66186844
RRM1	rs58141407	39.44268335
RRM1	rs13301443	51.32439147
RRM1	rs73660574	65.67778953
AQP4	rs10922094	191.6464707
AQP4	rs114115200	48.80910214
AQP4	rs6457457	43.18525801
AQP4	rs9501393	61.48791681
ADH5	rs13125919	79.29587587
ADH5	rs28894371	347.578292
ADH5	rs59859017	36.32988428
ADH5	rs144873436	56.93551622
ADH5	rs72678667	53.97999744
ADH5	rs151006272	58.05751606
ADH5	rs138191461	66.44814593
ADH5	rs143884562	29.85334917
INPP5E	rs10822155	62.44083994
INPP5E	rs6542680	44.43342764
INPP5E	rs1398015	39.26395783
INPP5E	rs16850360	38.30122484
INPP5E	rs6993770	31.99186418
GPR101	rs10922094	116.8961309
GPR101	rs113747960	57.41536039

GPR101	rs28624101	31.47566712
ZNF18	rs10922098	562.7461996
ZNF18	rs1707652	209.4401156
ZNF18	rs3926704	31.18990185
ZNF18	rs444921	143.6819462
ZNF18	rs9270570	39.49774969
ZNF18	rs9469195	73.59098325
ADH7	rs4147541	87.86077289
ADH7	rs80121626	41.42552199
ADH7	rs1573496	769.6206872
ADH7	rs716556	48.17004698
ADH7	rs10922098	93.37902786
ADH7	rs78705678	48.20865364
ADH7	rs2461161	52.4702248
ADH7	rs72848256	47.33573892
ADH7	rs2275848	44.70646455
SYK	rs71509656	55.27638173
SYK	rs72729008	110.4105987
SYK	rs1354034	49.47292594
SYK	rs342200	30.47489336
RBM24	rs114009540	34.51693236
RBM24	rs10922098	829.7518176
RBM24	rs3900909	37.9155604
RBM24	rs1047286	59.77895044
RBM24	rs2686395	133.4384835
RBM24	rs1265941	76.25746564
RBM24	rs453821	184.3776526
RBM24	rs9276606	36.36134847
KRT7	rs10922098	530.5089399
KRT7	rs192777257	139.531938
KRT7	rs112189508	98.76904263
KRT7	rs16914574	278.0610689
KRT7	rs143666677	702.9089364
KRT7	rs117627469	88.73321084
KRT7	rs4977155	62.54281194
KRT7	rs1047286	30.57920595
KRT7	rs2686395	144.4148951
KRT7	rs550671	129.9403547
KRT7	rs28471056	56.60606861
ATF6B	rs62395789	39.52681003
ATF6B	rs449186	102.6786473
ATF6B	rs8111	471.5751898
ATF6B	rs41547217	109.428405
ATF6B	rs35352142	86.76417549
ATF6B	rs4846923	61.86855752
ATF6B	rs150344164	35.35366518
ATF6B	rs17580	105.2674744
ATF6B	rs112963922	38.00009789
ATF6B	rs613426	31.12627767
ATF6B	rs8178824	70.63236598
ATF6B	rs77542162	111.6274598
ATF6B	rs2967893	446.9021066
ATF6B	rs28578510	34.15852356
ATF6B	rs1800759	32.81238712
ATF6B	rs16869652	38.7814294
ATF6B	rs983309	69.79916401
WFDC2	rs2272953	50.48463536
WFDC2	rs12592898	36.56592089

WFDC2	rs77924615	49.62746345
WFDC2	rs681343	42.76362662
CA8	rs74985355	92.58828484
CA8	rs56227154	2935.520706
CA8	rs74701322	244.7992644
CA8	rs140403163	52.88956948
CA8	rs76601506	41.16805598
CA8	rs7819622	165.7041169
CA8	rs60757417	37.34445892
MVK	rs572515	73.52566893
MVK	rs1914886	39.42664343
MVK	rs113954333	144.1180603
MVK	rs614877	967.3578488
MVK	rs36020655	55.68734591
MVK	rs7412	41.5167255
MVK	rs1619994	42.45038912
DNAI1	rs10922098	418.5473269
DNAI1	rs1047286	33.05725056
DNAI1	rs1707652	91.60389019
DNAI1	rs1265941	51.68293832
DNAI1	rs550671	104.2334349
RNF146	rs10922098	155.0885126
RNF146	rs7084817	97.01357051
RNF146	rs111862412	34.88890993
RNF146	rs2284350	66.20175821
RNF146	rs1355538	48.82829105
RNF146	rs9501393	49.32860648
RNF146	rs28471056	40.15240149
KMT2C	rs10922094	165.0574309
KMT2C	rs11846959	226.6666263
KMT2C	rs1065853	101.0128314
KMT2C	rs1706435	75.64745688
KMT2C	rs9501393	36.40400207
CARD9	rs10922098	335.8125593
CARD9	rs697356	89.09422608
CARD9	rs6457457	63.45149441
CARD9	rs9501393	84.23456798
ACAD8	rs10922094	117.3164327
ACAD8	rs17580	71.48420946
ACAD8	rs1707652	30.09312136
ACAD8	rs9501600	37.47572847
PLSCR3	rs10922094	133.8726172
PLSCR3	rs1355538	47.22752198
PLSCR3	rs2242572	47.52486673
PLSCR3	rs28455809	58.56055761
FBXL4	rs73262897	34.67815824
FBXL4	rs1126605	116.405754
FBXL4	rs7412	58.67409736
FBXL4	rs111696008	57.49584134
FBXL4	rs144202860	34.3178176
FBXL4	rs111602331	260.8307717
FBXL4	rs6445035	323.6469453
FBXL4	rs3863087	117.4662031
FBXL4	rs4132620	34.26860303
FBXL4	rs11755689	29.95786766
EHD4	rs655029	366.8134156
EHD4	rs4420743	31.25864756
EHD4	rs11792848	42.28634558

FAH	rs11853785	32.53721567
FAH	rs145712044	77.47428221
FAH	rs11555096	4544.637379
FAH	rs77218477	162.235375
FAH	rs143477230	60.35155654
FAH	rs146479592	38.13343491
FAH	rs143855649	56.64585459
FAH	rs738409	31.23383794
RUFY1	rs1697422	34.32227753
RUFY1	rs10922098	164.2421842
RUFY1	rs12995461	43.92067703
RUFY1	rs56210800	42.76409782
RUFY1	rs140431012	42.84268607
RUFY1	rs1706435	60.03921146
RUFY1	rs6457457	44.13720775
RUFY1	rs9501393	59.15709002
HNRNPC	rs10922098	457.1684826
HNRNPC	rs2232613	111.7387195
HNRNPC	rs1707652	152.8560602
HNRNPC	rs9266722	30.39828899
HNRNPC	rs9267139	37.19911256
HNRNPC	rs9501393	132.3177046
HNRNPC	rs7774739	84.0668133
HNRNPC	rs9271766	42.84146235
DTX1	rs1591734	51.95263772
DTX1	rs9332701	223.478246
DTX1	rs2239853	132.0813924
DTX1	rs10922098	353.1020146
DTX1	rs75519859	48.40387448
DTX1	rs10159712	127.5369061
DTX1	rs11569415	35.25743103
DTX1	rs6544941	44.3180931
DTX1	rs2686395	74.00973599
DTX1	rs2075798	50.13598888
DTX1	rs3891366	139.941695
DTX1	rs28383417	99.25560276
RECQL	rs11613698	463.8666993
RECQL	rs112755939	763.9721488
RECQL	rs118171527	570.9617356
RECQL	rs76728072	590.9302262
RECQL	rs11602951	42.34459311
RECQL	rs146780586	61.57043855
RECQL	rs17363755	187.5291168
RECQL	rs16926592	467.9318376
RECQL	rs73064470	318.1834435
RECQL	rs11047515	85.23271661
RECQL	rs145627956	80.3292312
RECQL	rs1354034	231.5637427
PQBP1	rs10922098	160.7404805
PQBP1	rs11720167	48.94369036
PQBP1	rs6444156	31.78392693
PQBP1	rs4713498	35.4737417
TCTN2	rs617283	258.0989109
TCTN2	rs12067507	258.0863487
TCTN2	rs6542680	578.4474499
TCTN2	rs80220851	45.46377872
RHPN2	rs1451915	31.64065624
RHPN2	rs10922098	599.1862448

RHPN2	rs704	37.23581645
RHPN2	rs1047286	30.30958494
RHPN2	rs1707652	137.340487
RHPN2	rs550671	153.8215072
RHPN2	rs9270570	33.98341745
RHPN2	rs9469195	60.74958667
SOCS3	rs12134934	63.65299293
SOCS3	rs144744138	39.28424885
SOCS3	rs4666000	29.89570965
SOCS3	rs78623688	54.90706361
SOCS3	rs7609518	66.53601874
SOCS3	rs77418648	49.73081167
SOCS3	rs10171729	954.0298452
SOCS3	rs2097976	61.84798457
SOCS3	rs4675969	57.42470757
SOCS3	rs183812412	88.07032785
PYGL	rs17720606	52.783723
PYGL	rs73284790	185.2265975
PYGL	rs1001652	37.00018053
PYGL	rs11611373	30.59301445
PYGL	rs4759076	35.17248528
PYGL	rs117296551	31.92917076
PYGL	rs79419800	35.52020486
PYGL	rs143575738	37.11538981
PYGL	rs11380594	84.96064208
PYGL	rs3748136	180.9683592
POLR3F	rs10922098	56.33128088
POLR3F	rs6542680	41.21089618
POLR3F	rs557011	31.18866997
GALK1	rs17580	620.5064322
GALK1	rs1354034	30.15603793
GALK1	rs1619994	36.5295464
EIF3G	rs4980320	42.68693173
EIF3G	rs10876550	40.17808541
EIF3G	rs1354034	72.77728139
EIF3G	rs342200	42.31335187
EIF3G	rs3748136	134.7181483
MAGEB10	rs10922098	429.144762
MAGEB10	rs1047286	45.69037383
MAGEB10	rs2686395	87.70872835
MAGEB10	rs9267139	44.79173302
MAGEB10	rs28573154	104.7438536
MAGEB10	rs7774739	68.10014875
GALE	rs10922094	37.18354322
GALE	rs964184	91.51541429
GALE	rs704	35.99796328
GALE	rs59007384	69.07764836
GALE	rs8106813	348.6693728
GALE	rs148023052	47.93515822
GALE	rs1260326	66.46038168
GALE	rs1706435	72.83432796
GALE	rs147390813	31.96507463
ELL	rs144704969	29.83690859
ELL	rs10922098	643.7501624
ELL	rs3900909	33.8917284
ELL	rs704	33.66333957
ELL	rs1707652	169.2535132
ELL	rs550671	189.8817269

ELL	rs28471056	79.35588993
ELL	rs9272526	35.18714201
RELB	rs114009540	31.16979224
RELB	rs10922098	734.6572016
RELB	rs3900909	40.26579873
RELB	rs704	411.0454034
RELB	rs114115200	206.7260616
RELB	rs3863087	69.7516464
RELB	rs3099847	35.42010416
RELB	rs1265941	90.08730715
RELB	rs444921	211.5628876
RELB	rs9276606	34.95750354
GPR135	rs10922098	320.0347828
GPR135	rs1965605	31.64631342
GPR135	rs704	191.9428065
GPR135	rs6445035	70.46893876
GPR135	rs3863087	37.16579893
GPR135	rs9501393	90.20892636
GPR135	rs7774739	60.84563173
GPR135	rs9271683	32.16685932
RBM19	rs146335091	40.61659594
RBM19	rs148900432	83.3940121
RBM19	rs117320146	1215.176451
RBM19	rs2753517	33.54744208
RBM19	rs2093214	44.64472261
RBM19	rs144640687	29.74569468
RBM19	rs6139182	81.6079573
RBM19	rs76106586	32.3567849
RBM19	rs9272371	39.10241982
RBM19	rs11155297	49.64798597
RBM19	rs3778754	30.64215477
ALDH3A1	rs4646787	234.3056438
ALDH3A1	rs10922094	50.94075378
ALDH3A1	rs557011	37.48540719
HAVCR2	rs11845234	41.48050539
HAVCR2	rs8104156	29.80894671
HAVCR2	rs9271710	50.53509555
UBL4A	rs4980320	35.52077886
UBL4A	rs1354034	45.94722028
UBL4A	rs342292	42.22649339
APOL1	rs58899157	33.69953027
APOL1	rs377486200	48.55664896
APOL1	rs136148	2501.992333
APOL1	rs3886200	114.8548972
APOL1	rs738280	65.43534634
APOL1	rs2555366	34.27869742
APOL1	rs77589373	39.35328594
APOL1	rs16958649	107.3617002
APOL1	rs1463436	139.0080146
APOL1	rs34596355	1222.598447
APOL1	rs3794693	1247.17536
APOL1	rs61452478	978.6952914
APOL1	rs199742317	40.99015714
APOL1	rs11745587	45.45181622
APOL1	rs150611042	32.69228538
CD38	rs10922098	346.4544065
CD38	rs1707652	113.5728697
CD38	rs9501600	101.8727957

CD38	rs35209896	36.35266458
CD59	rs831630	455.2713082
CD59	rs4756046	37.81201677
CD59	rs10836121	77.12853357
CD59	rs10922098	114.7894197
CD59	rs77924615	51.67651362
CD59	rs9992101	31.06471684
CD59	rs368611	34.53299093
CD59	rs13230509	33.22198125
FABP1	rs2241883	162.0334894
FABP1	rs10922098	189.3365325
FABP1	rs1707652	179.0196315
FABP1	rs9501393	61.86558113
FABP1	rs28471056	40.11671244
HMBS	rs45510602	42.6360635
HMBS	rs10753508	31.83518855
HMBS	rs5754100	37.13225707
HMBS	rs2072860	49.60267925
HMBS	rs115740542	36.43944631
HMBS	rs34164109	42.03109057
LRIT3	rs9332701	120.0191192
LRIT3	rs7070003	38.58184253
LRIT3	rs6542680	34.11864732
LRIT3	rs211161	31.69141962
MYSM1	rs572515	34.43888082
MYSM1	rs704	58.44376271
MYSM1	rs697356	39.46797868
TFR2	rs10922098	233.6043304
TFR2	rs1355538	48.33601077
TFR2	rs113099710	34.3133011
TFR2	rs60805730	32.09403937
TFR2	rs369216897	49.47099441
TFR2	rs9501600	62.30141641
TFR2	rs74671299	30.73436177
TFR2	rs73403125	33.94644651
FOXO3	rs3813498	37.35469533
FOXO3	rs10876550	72.98968226
FOXO3	rs1354034	59.92934055
FOXO3	rs342293	47.30571292
FOXO3	rs10820606	80.89510901
TMEM230	rs10922094	122.7834069
TMEM230	rs1355538	61.11875941
TMEM230	rs2731702	72.15231315
TMEM230	rs9501393	44.29229962
TMEM230	rs7774739	41.24422503
LIMA1	rs7967954	55.27181577
LIMA1	rs10922098	241.6115075
LIMA1	rs1707652	74.12468497
LIMA1	rs555007	39.12371321
LIMA1	rs444921	81.81864781
CYGB	rs374896	29.72253224
CYGB	rs17580	31.88309963
CYGB	rs6445035	55.06115534
MUSK	rs4978949	65.10658191
MUSK	rs12134934	78.82001499
MUSK	rs7103589	55.37312181
MUSK	rs79940707	59.76666226
MUSK	rs1260326	35.6681787

MUSK	rs1250258	141.4471271
MUSK	rs4674006	36.38414017
MUSK	rs112875651	54.92708988
MUSK	rs9643300	38.27832288
ISL1	rs10922098	86.29591571
ISL1	rs77938199	47.30568093
ISL1	rs2300149	53.64792773
HMG20B	rs11676298	31.12940976
HMG20B	rs1354034	66.13671126
HMG20B	rs67399078	30.23266452
ARFGAP1	rs10922094	232.8085475
ARFGAP1	rs964184	72.0093434
ARFGAP1	rs1126605	71.38658563
ARFGAP1	rs71352239	167.0899347
ARFGAP1	rs12478841	53.13617924
ARFGAP1	rs1707652	133.6617457
ARFGAP1	rs2284179	43.02375502
ARFGAP1	rs11751024	67.4242704
SMURF1	rs10922098	52.56399403
SMURF1	rs4419136	201.7002602
SMURF1	rs1355538	35.83227752
NFATC4	rs10922098	245.5342217
NFATC4	rs11720167	46.51578807
NFATC4	rs9501600	51.35654378
SYTL4	rs10922098	243.5870655
SYTL4	rs704	84.49241194
SYTL4	rs71352239	55.32096061
SYTL4	rs4665985	38.41406109
SYTL4	rs1707652	103.0402727
SYTL4	rs9501393	83.08915554
SYTL4	rs7774739	60.10750597
SYTL4	rs9271766	32.50196958
ZNF23	rs10801548	34.23935814
ZNF23	rs28664709	203.5532506
ZNF23	rs4977155	39.53157626
ZNF23	rs28455809	33.3242408
ZNF10	rs146457388	29.73077457
ZNF10	rs6542680	402.8637167
ZNF10	rs80220851	30.77672603
FKBP1B	rs72782112	81.66509339
FKBP1B	rs55788848	674.334249
FKBP1B	rs11417460	29.91250938
FKBP1B	rs73396521	36.09765451
FKBP1B	rs62130490	32.86102628
FKBP1B	rs1354034	71.43045228
FKBP1B	rs342200	33.38587835
BCAP29	rs10922098	384.1596484
BCAP29	rs704	60.24673765
BCAP29	rs1047286	30.06808498
BCAP29	rs1707652	62.78129537
BCAP29	rs6457457	73.78049253
BCAP29	rs9501393	105.3468477
BCAP29	rs6451	30.7018847
KLRC3	rs72673550	49.50460304
KLRC3	rs12085435	739.9898341
KLRC3	rs617283	62.22873605
KLRC3	rs704	337.3496887
KLRC3	rs438811	102.022511

KLRC3	rs1355538	39.3468285
DNM2	rs2038480	42.42965719
DNM2	rs4980320	30.55718628
DNM2	rs342200	32.83697251
SRSF6	rs10828722	41.66076394
SRSF6	rs7310615	44.4741525
SRSF6	rs3129754	39.57101
MGA	rs9332701	30.94754741
MGA	rs10922098	101.6798856
MGA	rs704	189.6157163
MGA	rs6542680	216.9775242
MGA	rs9864801	34.85051352
MGA	rs9501393	49.74757073
MGA	rs9272068	36.40208391
PRC1	rs10922098	103.4428186
PRC1	rs1355538	40.0814296
PRC1	rs9271642	31.38530696
ELAVL1	rs572515	79.76188803
ELAVL1	rs4980320	40.5094919
ELAVL1	rs1354034	125.7808513
ELAVL1	rs1355538	31.49621451
CXCL9	rs10922098	109.6971992
CXCL9	rs1355538	42.39663534
CXCL9	rs415620	38.57451808
CXCL9	rs4713498	43.42416329
ZNF75D	rs10922094	46.28823461
ZNF75D	rs6542680	34.23423166
ZNF75D	rs67399078	31.40166181
DHX8	rs10922098	53.18644185
DHX8	rs12602457	32.05237192
DHX8	rs148966640	33.5091928
DHX8	rs60508832	36.59757675
DHX8	rs8071339	1103.343084
DHX8	rs77584326	56.35431362
DHX8	rs4791070	381.3554019
DHX8	rs16960087	40.02777546
DHX8	rs28672722	31.94758155
DHX8	rs3127580	36.94783745
DHX8	rs112839059	64.9248025
MAP1LC3B	rs3811444	48.49025504
MAP1LC3B	rs10761739	44.54947178
MAP1LC3B	rs7926172	57.31106611
MAP1LC3B	rs364220	41.12785443
MAP1LC3B	rs16975847	106.2545711
MAP1LC3B	rs185009887	33.58682764
MAP1LC3B	rs6437284	30.06960297
MAP1LC3B	rs1354034	450.6160832
MAP1LC3B	rs55665228	29.86206067
DAPP1	rs145432257	35.51416498
DAPP1	rs2282588	101.5429966
DAPP1	rs181207021	46.61327147
DAPP1	rs77265585	30.27870293
ITGAL	rs10922094	71.05565785
ITGAL	rs34160075	151.8040751
ITGAL	rs3820897	39.99703616
ITGAL	rs9864801	30.40009806
ITGAL	rs9501393	46.55549109
ITGAL	rs7774739	37.03644932

MYB	rs10922094	164.0025381
MYB	rs9864801	43.21677142
MYB	rs6457457	44.05025548
MYB	rs9501393	65.71680883
AHSA1	rs1329424	86.63272041
AHSA1	rs4980320	31.1744792
AHSA1	rs1354034	42.52387712
AHSA1	rs1398617	46.17608574
AHSA1	rs7742632	32.63571842
B3GALT2	rs10922098	88.70029436
B3GALT2	rs201488097	124.9451334
B3GALT2	rs72850959	152.5897452
B3GALT2	rs9635249	45.98922977
B3GALT2	rs148840367	37.94685664
B3GALT2	rs8178824	125.9838126
B3GALT2	rs182233516	34.41068536
B3GALT2	rs10071135	49.09415563
B3GALT2	rs28636516	32.33278237
B3GALT2	rs210142	31.60655938
B3GALT2	rs6993770	40.7822235
DTX3L	rs9917729	42.64382677
DTX3L	rs7113761	49.56467042
DTX3L	rs1354034	35.08352604
DTX3L	rs1355538	31.65008693
P4HA1	rs10922094	64.8943935
P4HA1	rs6542680	73.94406142
P4HA1	rs415620	32.16690486
P4HA1	rs13217434	32.7626555
CHST9	rs35072345	38.16782088
CHST9	rs1485803	117.4935291
CHST9	rs9961915	916.2526999
CHST9	rs9957937	38.12143868
CHST9	rs368465	45.20838716
CHST9	rs7964859	104.8822427
CHST9	rs9738226	100.9421516
CHST9	rs190543502	125.4589155
CHST9	rs79391862	37.28833155
CHST9	rs200210321	50.16846002
CHST9	rs1260326	62.79390034
CHST9	rs1800961	32.67467498
CHST9	rs1706435	29.87596826
CHST9	rs41268617	32.01179349
CHST9	rs28601761	31.40627311
FZD10	rs10922098	644.0798978
FZD10	rs35351085	30.71404115
FZD10	rs11569415	53.99208523
FZD10	rs2686395	105.9415649
FZD10	rs4293988	33.81248508
FZD10	rs555007	64.65379453
FZD10	rs444921	141.9442223
SMAP1	rs78936674	46.11544966
SMAP1	rs2691477	1104.430614
SMAP1	rs147236212	105.1335815
SMAP1	rs12203753	81.00901386
SMAP1	rs10822155	40.6532352
SMAP1	rs4980320	57.25270131
SMAP1	rs1354034	127.8071419
SMAP1	rs138174879	38.58915713

SMAP1	rs10944798	31.44909605
SMAP1	rs6454314	33.71641059
NRSN1	rs10922098	65.3120509
NRSN1	rs704	61.63269797
NRSN1	rs28615351	30.52164094
EVL	rs117136557	60.64434944
EVL	rs17099126	49.61540571
EVL	rs76401008	517.3200664
EVL	rs2767355	89.12277904
EVL	rs144704969	31.71297924
EVL	rs10922098	522.2587702
EVL	rs1707652	132.5523336
EVL	rs453821	139.2678268
EVL	rs28471056	64.9916849
EVL	rs9272526	31.09706065
SOCS7	rs10922094	99.79440045
SOCS7	rs6542680	32.08436462
SOCS7	rs1355538	32.79016258
SOCS7	rs7742632	33.75038207
SOCS7	rs3129773	31.4665211
CLINT1	rs62389053	44.93723204
CLINT1	rs12292693	116.0025141
CLINT1	rs1354034	38.19585979
CLINT1	rs210142	32.17977551
RGS8	rs72748420	30.63005611
RGS8	rs114009540	34.57802213
RGS8	rs10922098	915.1750377
RGS8	rs3900909	41.6524742
RGS8	rs1047286	71.38260801
RGS8	rs111696008	31.5772941
RGS8	rs1707652	193.742632
RGS8	rs555007	102.1875594
RGS8	rs550671	240.7157557
RGS8	rs41548618	32.48225978
RGS8	rs9272526	45.42210425
TNS2	rs10922098	40.8080479
TNS2	rs9852529	141.012905
TNS2	rs557011	30.53078993
BRPF1	rs10922094	443.768124
BRPF1	rs1706435	81.59133232
BRPF1	rs550671	111.6784676
BRPF1	rs28471056	57.66443939
KIF16B	rs1451915	30.70789164
KIF16B	rs10922098	614.0551253
KIF16B	rs1707652	128.0925503
KIF16B	rs1265941	74.13174852
KIF16B	rs444921	162.3499062
GJD2	rs10922098	89.93385622
GJD2	rs34793466	29.74852101
GJD2	rs8077564	56.58846402
GJD2	rs6445035	41.71657338
GJD2	rs9271642	33.25155524
AGFG1	rs55722757	61.93140141
AGFG1	rs11679534	135.4289356
AGFG1	rs72882962	31.20685672
AGFG1	rs185009887	38.11891435
AGFG1	rs1354034	69.15250356
AGFG1	rs511515	30.83453453

AGFG1	rs342200	34.36745522
TXNIP	rs114009540	37.10069875
TXNIP	rs10922098	755.6487239
TXNIP	rs3900909	32.2527629
TXNIP	rs1047286	42.30046956
TXNIP	rs1707652	128.2463299
TXNIP	rs1265941	73.98800988
TXNIP	rs550671	204.9601981
GGA3	rs4980320	45.91025557
GGA3	rs1354034	56.78895213
GGA3	rs342200	31.25724364
ANAPC7	rs1706435	31.16479926
ANAPC7	rs9501393	53.04937855
ANAPC7	rs7774739	45.69090431
SHC4	rs10922094	109.682241
SHC4	rs1555405	29.82896623
SHC4	rs6542680	34.72255902
SHC4	rs1355538	31.41851929
SHC4	rs147906938	43.59437559
CRABP2	rs10494305	33.7411528
CRABP2	rs4457570	1179.429783
CRABP2	rs703156	37.17553347
CRABP2	rs6687516	39.33950067
CRABP2	rs4795734	32.22945942
LCN1	rs56307603	69.05090658
LCN1	rs10922094	107.6713802
LCN1	rs281377	47.66535952
LCN1	rs9864801	82.99000948
LCN1	rs138073993	34.49671342
LCN1	rs4713498	39.87637949
LCN1	rs621089	32.76747519
CPT1B	rs115041442	30.2063209
CPT1B	rs76418562	30.14477259
CPT1B	rs10922098	505.6964514
CPT1B	rs12026141	73.21197542
CPT1B	rs117192879	30.66347632
CPT1B	rs77368797	49.52414792
CPT1B	rs74683816	38.77728376
CPT1B	rs77998935	29.84157358
CPT1B	rs73015723	38.1626528
CPT1B	rs375402329	59.97852076
CPT1B	rs142437389	31.74638937
CPT1B	rs2230203	65.57626931
CPT1B	rs55881728	75.59157228
CPT1B	rs111696008	149.6976384
CPT1B	rs144202860	77.96411146
CPT1B	rs483465	37.57006468
CPT1B	rs12485327	48.9936303
CPT1B	rs79330559	40.46474265
CPT1B	rs185693927	40.07648726
CPT1B	rs697356	961.1180881
CPT1B	rs186485515	72.68438327
CPT1B	rs555007	34.85653153
CPT1B	rs453821	106.6005946
CPT1B	rs72939784	32.95242959
PPARGC1A	rs10922098	86.474439
PPARGC1A	rs112731419	30.2694441
PPARGC1A	rs6542680	32.04435545

PPARGC1A	rs9501393	32.82489442
PIEZO1	rs572515	70.88579135
PIEZO1	rs1355538	41.0972772
PIEZO1	rs28573154	44.70247223
PIEZO1	rs7774739	31.1056104
PIEZO1	rs41547217	49.54068289
PCDHGB1	rs10922094	273.6440662
PCDHGB1	rs2232613	169.2278372
PCDHGB1	rs76730118	33.81067511
PCDHGB1	rs182482308	41.55352731
PCDHGB1	rs6445035	133.8002984
PCDHGB1	rs3863087	46.55298678
PCDHGB1	rs6457457	39.17261091
PCDHGB1	rs9501393	51.55810748
DLX4	rs7529925	33.21574144
DLX4	rs4759076	86.54440362
DLX4	rs1354034	52.42619859
DLX4	rs342293	98.7606173
DLX4	rs75129807	164.848017
DLX4	rs116870395	51.00248867
AAGAB	rs1574186	368.3366475
AAGAB	rs34930529	52.26523474
AAGAB	rs11251694	538.268005
AAGAB	rs2388595	799.1273616
AAGAB	rs12217524	35.0838983
AAGAB	rs61751507	660.853182
AAGAB	rs603424	40.18695804
AAGAB	rs4980320	30.85484892
AAGAB	rs35979828	30.24076627
AAGAB	rs1354034	35.83117957
AAGAB	rs1707654	30.47256408
INTS3	rs10922098	83.95027122
INTS3	rs13379369	56.36513371
INTS3	rs11846842	29.74329437
INTS3	rs41556615	31.55192272
IGDCC3	rs10922098	135.2005664
IGDCC3	rs6542680	30.74826158
IGDCC3	rs1355538	29.73566849
IGDCC3	rs9501600	41.19792807
IGDCC3	rs557011	30.49668125
ARHGAP1	rs2968478	57.89921991
ARHGAP1	rs1354034	35.03980256
ARHGAP1	rs7836609	31.64598781
ARHGAP1	rs6993770	100.6426031
CRABP1	rs10922098	721.3027416
CRABP1	rs3900909	35.27343959
CRABP1	rs11078072	30.87704887
CRABP1	rs704	38.2527646
CRABP1	rs1047286	62.39826196
CRABP1	rs111696008	34.64659794
CRABP1	rs1707652	174.7334438
CRABP1	rs1144709	41.00354564
CRABP1	rs1265941	124.8700113
CRABP1	rs444921	259.0924977
CRABP1	rs41291806	57.41398941
S100A2	rs61265945	113.8285502
S100A2	rs75282796	116.8792262
S100A2	rs10922098	97.09432601

S100A2	rs9864801	32.53313634
S100A2	rs9501600	49.13787689
PTPRH	rs10922098	220.5577312
PTPRH	rs1355538	51.6071316
PTPRH	rs9501600	75.46652057
EMC1	rs368465	68.79731392
EMC1	rs113747960	85.68745486
EMC1	rs1355538	32.32861557
EMC1	rs872914	48.19851209
EMC1	rs9267655	30.76853637
EMC1	rs4713498	39.0177496
TJP1	rs10922098	708.2224109
TJP1	rs11569393	30.44294869
TJP1	rs111696008	30.12060093
TJP1	rs1707652	165.2668778
TJP1	rs550671	177.6125209
TJP1	rs9270570	36.4730621
TJP1	rs9469195	64.98847367
CD7	rs118126964	53.48284499
CD7	rs140403190	39.77683425
CD7	rs3176831	527.590924
CD7	rs117850386	30.57728954
CD7	rs572515	43.88710328
CD7	rs150344164	84.31151432
CD7	rs7310615	55.68577964
CD7	rs56193570	34.67936402
PTS	rs10922098	125.7941228
PTS	rs1707652	49.13893419
PTS	rs3132135	34.82291555
PTS	rs3130217	36.10197045
TPK1	rs10922098	63.38265729
TPK1	rs9864801	32.20270964
TPK1	rs28573154	30.90146
BPGM	rs6467547	35.80575741
BPGM	rs3811444	66.75215929
BPGM	rs61751507	42.53209143
SMAD4	rs7073753	33.74879257
SMAD4	rs4980320	36.72886728
SMAD4	rs35979828	30.23662979
SMAD4	rs1354034	46.16557888
DES	rs10922098	849.7549503
DES	rs3900909	35.76484493
DES	rs10801660	32.40471627
DES	rs17580	31.42815958
DES	rs1047286	51.96433717
DES	rs111696008	37.43563291
DES	rs2686395	168.4046885
DES	rs1265941	88.07851374
DES	rs550671	248.675588
DES	rs9276606	44.12548304
BUB1	rs203688	48.63201888
BUB1	rs11147139	68.58609374
BUB1	rs1354034	46.85513402
BUB1	rs1355538	31.61004327
HSPA1L	rs3099847	33.42231326
HSPA1L	rs1265941	93.2624481
HSPA1L	rs550671	255.4894802
HSPA1L	rs9276606	49.23220804

HSPA1L	rs144704969	30.62687203
HSPA1L	rs10922094	911.5408249
HSPA1L	rs3900909	40.3415003
HSPA1L	rs704	339.4959602
HSPA1L	rs35136575	39.70655378
HSPA1L	rs111696008	39.51464489
HSPA1L	rs11720167	301.6752324
HSPA1L	rs3863087	105.221836
HSPA1L	rs4955716	38.7434085
TARDBP	rs10922098	239.295387
TARDBP	rs1707652	67.61384392
TARDBP	rs9267139	29.99208438
TARDBP	rs28573154	78.75260989
TARDBP	rs7774739	61.6687691
TARDBP	rs9271766	35.6168886
RPS6KA1	rs11589734	156.774794
RPS6KA1	rs74573933	34.14373822
RPS6KA1	rs6664577	71.67011459
RPS6KA1	rs145439832	125.4659826
RPS6KA1	rs76563384	155.8542727
RPS6KA1	rs144546772	107.8807
RPS6KA1	rs12035228	98.94644998
RPS6KA1	rs71631848	1217.610168
RPS6KA1	rs115656947	52.25522878
RPS6KA1	rs10801548	984.6649428
RPS6KA1	rs77881733	469.684288
RPS6KA1	rs76780329	1006.800639
RPS6KA1	rs115325629	142.0493658
RPS6KA1	rs142413464	265.2069724
RPS6KA1	rs17514403	215.5104187
RPS6KA1	rs17620514	139.9528772
RPS6KA1	rs200441686	115.0713838
EEF2K	rs12664075	33.47095677
EEF2K	rs9266557	182.7882309
EEF2K	rs3130921	239.2809781
EEF2K	rs707932	138.6001694
EEF2K	rs553108	960.5382793
EEF2K	rs72508422	367.839968
EEF2K	rs3846855	48.02652043
RPIA	rs144706079	260.7187044
RPIA	rs371291303	151.592101
RPIA	rs3811444	31.74163804
RPIA	rs11611373	79.62867975
RPIA	rs7305170	108.2672317
RPIA	rs148704808	34.58576447
RPIA	rs2221932	29.78829503
SHMT1	rs79791160	50.91803053
SHMT1	rs854784	2890.459062
SHMT1	rs604956	948.6902737
SHMT1	rs182947055	107.3961874
SHMT1	rs140644844	196.648819
SHMT1	rs4980320	29.95570116
SHMT1	rs74419753	41.32084993
SHMT1	rs117430595	58.11458176
SHMT1	rs141343208	95.06319279
SHMT1	rs201234732	91.64017296
SHMT1	rs150068036	53.52578505
SHMT1	rs35999898	187.4459829

SHMT1	rs150279116	73.76463082
SHMT1	rs144290802	36.07916463
SHMT1	rs138651100	41.60556168
SHMT1	rs77439074	33.62118867
SHMT1	rs1354034	48.20098212
SHMT1	rs72673751	46.65851364
EGFLAM	rs368353866	33.36605371
EGFLAM	rs35650653	59.63812117
EGFLAM	rs2589775	267.9599214
EGFLAM	rs113400125	679.0190231
EGFLAM	rs148393709	30.0495307
EGFLAM	rs10838529	38.2204899
EGFLAM	rs10734844	35.86351491
EGFLAM	rs17329885	31.61691017
EGFLAM	rs2304849	46.26986872
EGFLAM	rs620982	77.37618124
EGFLAM	rs113763631	67.60886727
EGFLAM	rs12975366	907.5809415
EGFLAM	rs780094	30.30997772
EGFLAM	rs2427312	29.71528844
EGFLAM	rs78804971	48.87537092
EGFLAM	rs2740594	54.0494015
DUSP6	rs10922098	58.30259389
DUSP6	rs17580	115.7432185
DUSP6	rs111490052	46.04040749
DUSP6	rs1355538	59.06675241
DUSP6	rs28573154	31.69243604
ANAPC10	rs10922098	77.95910729
ANAPC10	rs1355538	33.52363174
ANAPC10	rs391165	46.03394836
ANAPC10	rs28471056	34.61387894
CCM2	rs6958168	46.24245784
CCM2	rs10922094	267.2509111
CCM2	rs12289048	42.72356821
CCM2	rs1707652	110.6560518
CCM2	rs6457457	51.30727473
CCM2	rs28573154	90.00105853
ETS2	rs10922098	30.62578727
ETS2	rs143973988	34.35134755
ETS2	rs74625263	118.9426396
ETS2	rs16997771	170.1322945
ETS2	rs117511908	405.6099198
ETS2	rs141618758	103.1385301
ETS2	rs6542680	531.4992366
ETS2	rs80220851	32.80855749
ETS2	rs28383314	32.05071323
MRRF	rs10922094	174.0403415
MRRF	rs1706435	55.97210643
MRRF	rs9501393	56.0699203
MRRF	rs7774739	38.04797603
SNAP29	rs192565874	135.1260868
SNAP29	rs10922098	30.84714722
SNAP29	rs1354034	41.68752491
TRIB2	rs10922098	73.1503304
TRIB2	rs697357	44.37616167
TRIB2	rs9501600	41.33860026
CRYGD	rs34570682	193.7208477
CRYGD	rs61731517	77.45891732

CRYGD	rs4658046	104.9725577
CRYGD	rs117265578	43.46535358
APOF	rs141749430	73.11903905
APOF	rs2020854	460.7571115
APOF	rs111794954	36.90006197
APOF	rs4759252	95.32085438
APOF	rs3024961	42.06926665
APOF	rs150844304	66.36489192
APOF	rs77924615	41.83313492
APOF	rs374087027	61.8758329
APOF	rs56325564	77.16878701
APOF	rs201819916	29.88806725
APOF	rs483082	30.26751202
APOF	rs2668202	93.0815722
APOF	rs112875651	65.98238901
CDKN2D	rs2576570	32.55990703
CDKN2D	rs61851769	32.65461274
CDKN2D	rs4980320	50.70642032
CDKN2D	rs17106229	32.61124344
CDKN2D	rs1354034	208.7220826
CDKN2D	rs4541868	51.44221299
CDKN2D	rs55665228	33.37390579
TAPBP	rs73727907	33.31423334
TAPBP	rs369701142	141.1950366
TAPBP	rs114840990	2449.375433
TAPBP	rs11455464	1349.489996
TAPBP	rs145044106	170.7319542
TAPBP	rs9469680	68.07247928
TAPBP	rs3184504	45.6467893
TAPBP	rs123698	61.44331694
TAPBP	rs77470907	42.41084202
TAPBP	rs7775482	58.06473356
TAPBP	rs140117173	151.3190436
TAPBP	rs7739385	63.23838866
TAPBP	rs73410227	39.52112519
CBR1	rs16993715	82.86907852
CBR1	rs867224	1207.276722
CBR1	rs62232377	56.98565129
COPS7B	rs10922094	134.5964878
COPS7B	rs4950805	35.91972795
COPS7B	rs964184	59.72929767
COPS7B	rs2571068	81.25623932
COPS7B	rs62119281	111.9382156
COPS7B	rs394353	71.27532333
COPS7B	rs283813	217.6561604
COPS7B	rs73045691	36974.4424
COPS7B	rs77660932	605.5800969
COPS7B	rs182320411	50.32140222
COPS7B	rs1049739	292.8486484
COPS7B	rs9304661	55.36559246
COPS7B	rs78980904	36.02785629
COPS7B	rs111696008	68.92898488
COPS7B	rs144202860	38.87537761
COPS7B	rs887829	35.78500105
COPS7B	rs3755636	63.92817104
COPS7B	rs62290472	31.91056668
COPS7B	rs11720167	652.8069017
COPS7B	rs3863087	238.3528342

COPS7B	rs148077724	65.75371384
COPS7B	rs73169103	47.39203929
COPS7B	rs4704826	40.22975948
COPS7B	rs28573154	50.78858698
COPS7B	rs112692477	40.77088614
CAPN3	rs203688	46.60215985
CAPN3	rs710446	85.08930439
CAPN3	rs67036916	37.14492587
RNPEP	rs2678208	280.6033595
RNPEP	rs10920287	1070.999989
RNPEP	rs112482827	35.2131077
RNPEP	rs116853128	51.53625669
RNPEP	rs112451455	45.70439247
RNPEP	rs144457182	147.1424801
RNPEP	rs78705678	49.70928151
RNPEP	rs2477642	56.60571973
RNPEP	rs1042445	84.32334084
PDZD7	rs10922098	312.0465933
PDZD7	rs1706435	56.56367918
PDZD7	rs2075798	49.5649261
PDZD7	rs444921	90.94512941
ARL1	rs10822155	55.06256151
ARL1	rs1126605	38.0213142
ARL1	rs429358	285.6414501
ARL1	rs35136575	33.67026768
ARL1	rs1706435	42.03599712
ARL1	rs6993770	54.1497655
HIBCH	rs75949659	52.71086575
HIBCH	rs785223	34.00159006
HIBCH	rs62184386	941.9494943
HIBCH	rs11396180	245.3794105
HIBCH	rs12464143	52.59755989
HIBCH	rs200650699	31.49061497
CCDC50	rs884298	34.70748996
CCDC50	rs1867981	91.32204687
CCDC50	rs1800738	82.82500424
CCDC50	rs9568693	41.96158244
CCDC50	rs1354034	37.73191835
CCDC50	rs1293303	105.2010021
CCDC50	rs4375078	35.25596545
UBE2T	rs10922098	231.899486
UBE2T	rs114115200	44.29218125
UBE2T	rs9501393	72.17157299
UBE2T	rs7774739	51.89636669
STAMBPL1	rs303510	56.89479846
STAMBPL1	rs1147604	170.0379554
STAMBPL1	rs368465	101.8037121
STAMBPL1	rs1355538	39.79631216
STAMBPL1	rs4713498	35.21634204
RAB39B	rs10922098	317.0246111
RAB39B	rs1355538	92.20955725
RAB39B	rs9501600	107.1824014
RAB39B	rs35209896	32.61532109
RAB22A	rs35933296	60.1927913
RAB22A	rs329031	30.28575479
RAB22A	rs4570933	118.9323579
RAB22A	rs12607866	239.8297761
RAB22A	rs1354034	47.68017152

MAX	rs372555148	62.19551201
MAX	rs8181938	480.8476755
MAX	rs7073753	34.42085413
MAX	rs11082304	31.95620607
EDF1	rs10922098	421.5522739
EDF1	rs11569415	34.11060596
EDF1	rs1707652	121.5133639
EDF1	rs2075798	54.78943076
EDF1	rs550671	128.7028848
TPRKB	rs583104	34.48131901
TPRKB	rs10922094	362.3464105
TPRKB	rs10208529	33.39041891
TPRKB	rs111696008	35.15354448
TPRKB	rs6445035	200.9481358
TPRKB	rs3863087	76.8778931
TPRKB	rs73169117	31.18953927
TPRKB	rs9267139	33.36033612
TPRKB	rs9501393	115.0252887
TPRKB	rs7774739	69.57732209
TPRKB	rs9271766	39.38784303
GPD1L	rs145439832	33.3995745
GPD1L	rs72748420	47.65890621
GPD1L	rs1329424	1339.24531
GPD1L	rs3900909	60.51661732
GPD1L	rs10801660	40.65378389
GPD1L	rs61821527	29.73417337
GPD1L	rs11078072	33.05108842
GPD1L	rs704	140.6462167
GPD1L	rs1047286	38.48877537
GPD1L	rs111696008	40.27394734
GPD1L	rs1707652	274.7542262
GPD1L	rs73169117	33.6888838
GPD1L	rs3099847	45.5776688
GPD1L	rs1265941	129.6863836
GPD1L	rs444921	348.9542234
GPD1L	rs9276606	50.99475996
A1CF	rs10922094	88.11738489
A1CF	rs117023267	34.45013757
A1CF	rs114115200	39.24577458
A1CF	rs186023627	48.70257809
A1CF	rs79828486	41.67525466
A1CF	rs144274836	997.0875495
ARF6	rs10922098	180.7428539
ARF6	rs704	37.41624187
ARF6	rs6445035	137.2213732
ARF6	rs3863087	52.87221057
ARF6	rs9501600	72.80218877
CDC25B	rs10922098	314.2384081
CDC25B	rs704	72.68968463
CDC25B	rs429358	33.02635347
CDC25B	rs1706435	131.1588477
CDC25B	rs9501393	78.83813098
CDC25B	rs7774739	54.82724551
PAIP1	rs10922098	78.48665674
PAIP1	rs9864801	57.49947482
PAIP1	rs1042663	48.6325609
PELO	rs1499280	32.68430356
PELO	rs572515	30.22518971

PELO	rs1354034	37.96321248
CACYBP	rs79904921	220.0246646
CACYBP	rs35340377	34.12613866
CACYBP	rs4980320	46.55831866
CACYBP	rs35979828	37.28013851
CACYBP	rs1354034	121.386738
ARL11	rs111960598	834.9070065
ARL11	rs11254299	55.34161395
ARL11	rs117192879	66.09973248
ARL11	rs79879928	85.48542467
ARL11	rs77998935	75.41511984
ARL11	rs371267418	136.4654198
ARL11	rs117620934	39.64630457
ARL11	rs6445035	120.0966878
ARL11	rs3863087	35.73992846
GSTO1	rs142707907	55.82951649
GSTO1	rs4917404	192.2510231
GSTO1	rs805670	917.7435317
GSTO1	rs10883974	4066.389734
GSTO1	rs1490171	145.4322375
GSTO1	rs113474709	112.6203781
GSTO1	rs146191686	62.07319968
GSTO1	rs78837000	50.77281068
GSTO1	rs75950986	77.4612729
GSTO1	rs61870857	53.64080223
GSTO1	rs11817165	70.2464183
GSTO1	rs140911275	227.747008
GSTO1	rs4466753	39.54324435
GSTO1	rs185795781	67.6739555
GSTO1	rs61871328	43.36237771
GSTO1	rs1881451	35.82193467
GSTO1	rs3788375	50.80008647
ULK3	rs936228	92.85269652
ULK3	rs10922098	63.32966986
ULK3	rs1619994	32.30623943
ULK3	rs2731672	45.91830602
ULK3	rs3129773	33.66748807
IRF9	rs572515	203.511088
IRF9	rs34762726	77.30144517
IRF9	rs3778754	30.55729071
RHOD	rs493006	72.92251247
RHOD	rs4762802	41.80847328
RHOD	rs1926446	166.25591
ANKRD27	rs17693106	41.32419314
ANKRD27	rs10922098	224.8023542
ANKRD27	rs9971407	33.28454707
ANKRD27	rs1707652	103.7629375
ANKRD27	rs555007	32.2882157
ANKRD27	rs550671	67.50768899
PPIH	rs55716016	39.04249859
PPIH	rs61479385	31.65280787
PPIH	rs2229826	31.05811469
PPIH	rs192592903	41.84775719
PPIH	rs74856158	36.30808393
PPIH	rs2015255	46.92760379
PMVK	rs76977753	103.2355264
PMVK	rs144886967	90.71518791
PMVK	rs55935614	38.71625224

PMVK	rs11417460	33.25427445
PMVK	rs4980320	33.28214018
PMVK	rs35979828	41.01280839
PMVK	rs1354034	36.72886752
PMVK	rs1398617	35.58611982
PMVK	rs342200	40.39753361
PMVK	rs77265585	31.04679157
BACH1	rs72748418	29.76699301
BACH1	rs10922098	718.7114414
BACH1	rs3900909	35.88621716
BACH1	rs704	77.54757937
BACH1	rs1047286	36.1950059
BACH1	rs1707652	147.0003025
BACH1	rs555007	84.90838394
BACH1	rs550671	211.4298406
BACH1	rs9272526	38.50240271
MYNN	rs10922094	384.1517913
MYNN	rs704	53.05879749
MYNN	rs1047286	42.32090704
MYNN	rs697356	63.42446602
MYNN	rs444921	114.8027827
MYNN	rs28471056	52.59653068
PFKFB3	rs10922098	391.7080562
PFKFB3	rs1564879	52.24780966
PFKFB3	rs704	57.98161882
PFKFB3	rs74175068	31.31098172
PFKFB3	rs1706435	96.55639679
PFKFB3	rs2242572	104.7211644
PFKFB3	rs9501602	46.35668488
PFKFB3	rs13257521	35.35107819
ADI1	rs10179340	92.23875251
ADI1	rs10922094	31.88546744
ADI1	rs2297937	31.21625596
ADI1	rs704	92.63507585
ADI1	rs9864801	38.7937169
CHP1	rs10922094	31.22701775
CHP1	rs7297496	256.9935857
CHP1	rs9864801	59.75262219
PLEKHA1	rs2420998	37.63142259
PLEKHA1	rs10749451	919.0015015
PLEKHA1	rs10082476	75.66563899
PLEKHA1	rs142677258	99.04329376
PSMA7	rs113525195	98.77522816
PSMA7	rs2542582	33.06558838
PSMA7	rs5754116	30.94729711
PSMA7	rs9392465	114.2665388
PSMA7	rs371821058	56.035684
PSMA7	rs3211066	224.1709047
PSMA7	rs2284190	44.08193745
PSMA7	rs3780198	60.44356538
SIRT5	rs7076920	35.48028219
SIRT5	rs17580	62.57046669
SIRT5	rs1619994	30.77055052
SIRT5	rs2057681	57.02847791
SETMAR	rs10922094	59.34597478
SETMAR	rs4977155	35.16756595
SETMAR	rs16881504	30.05690365
SETMAR	rs67399078	30.3415793

KLHL13	rs10305748	30.35585475
KLHL13	rs10922098	506.2770047
KLHL13	rs704	99.54250466
KLHL13	rs1047286	33.20570748
KLHL13	rs1707652	142.4378199
KLHL13	rs555007	55.57278549
KLHL13	rs550671	153.4323458
MAPRE1	rs20654	57.17887593
MAPRE1	rs7073753	30.30122512
MAPRE1	rs3786314	41.62095239
MAPRE1	rs1354034	35.31393183
STAU1	rs10922098	35.13611653
STAU1	rs1976848	64.16714903
STAU1	rs10876550	39.79817718
STAU1	rs11553699	39.55315985
STAU1	rs9894057	80.83867255
STAU1	rs1354034	168.4847624
STAU1	rs1355538	49.02297907
KCTD5	rs10922098	370.71792
KCTD5	rs1047286	33.97550058
KCTD5	rs12478841	31.10188305
KCTD5	rs2686395	79.04484858
KCTD5	rs555007	65.16858979
KCTD5	rs550671	123.0162079
CLIC5	rs62401911	31.93584806
CLIC5	rs76801943	326.3474663
CLIC5	rs35822882	2548.274845
CLIC5	rs17462184	87.1292923
CLIC5	rs35181147	157.1520965
CLIC5	rs6600274	34.60731216
CLIC5	rs4813636	94.66106128
CLIC5	rs74557139	31.28394179
CLIC5	rs28471056	50.90157912
CLIC5	rs80209537	40.86175177
CLIC5	rs149957527	37.10146358
CLIC5	rs115172976	34.96280677
CLIC5	rs576123	185.3502664
RPL30	rs7080536	106.2805695
RPL30	rs1354034	104.8668834
RPL30	rs78937964	31.05474375
RPL30	rs3129755	63.61649362
PRKAR1B	rs10796982	32.66368395
PRKAR1B	rs10922098	56.22342577
PRKAR1B	rs12897483	37.81068446
PRKAR1B	rs9469195	42.36593138
PRKAR1B	rs28383461	79.80882643
OTUD5	rs1451915	37.83848523
OTUD5	rs10922098	786.7565843
OTUD5	rs1047286	63.39828666
OTUD5	rs1707652	153.655139
OTUD5	rs1265941	81.51076775
OTUD5	rs550671	200.0921303
OTUD5	rs9276606	29.76147403
BPTF	rs10922098	174.4849087
BPTF	rs429358	92.39786562
BPTF	rs1355538	84.16115051
BPTF	rs2075798	36.03858623
BPTF	rs453821	64.50749157

PITPNB	rs72748420	35.19695454
PITPNB	rs1329424	965.8239604
PITPNB	rs3900909	43.86115034
PITPNB	rs11569415	45.38430627
PITPNB	rs111696008	31.53144177
PITPNB	rs1707652	224.7264838
PITPNB	rs3099847	30.95993673
PITPNB	rs1265941	102.2009349
PITPNB	rs550671	258.9363671
PITPNB	rs9276606	40.4119003
GLRX2	rs148212596	1150.751041
GLRX2	rs1489261	41.4590889
GLRX2	rs16832407	44.75610277
GLRX2	rs201388663	124.3637341
GLRX2	rs6683772	140.6240747
GLRX2	rs12022395	100.2757269
GLRX2	rs1354034	44.78998068
MCTS1	rs17713196	58.82709024
MCTS1	rs3863087	42.07252652
MCTS1	rs67554133	30.42930026
MCTS1	rs74632039	74.51352822
MCTS1	rs6458	137.3420258
MCTS1	rs34115112	81.38652762
MCTS1	rs3129724	47.71367801
MCTS1	rs12378445	40.53275386
LAMTOR3	rs10922094	31.95673826
LAMTOR3	rs17580	385.6128911
LAMTOR3	rs7620024	544.0140953
LAMTOR3	rs71080520	178.3120963
LAMTOR3	rs71080562	63.84394317
LAMTOR3	rs2237582	146.2354779
LAMTOR3	rs854572	36.74704521
OTUB2	rs10922098	82.66028541
OTUB2	rs775824	30.05764021
OTUB2	rs2379887	32.93545681
OTUB2	rs72673751	36.78417402
GABARAPL2	rs364220	46.45356971
GABARAPL2	rs16975847	112.3570823
GABARAPL2	rs3811444	54.2638445
GABARAPL2	rs7073753	41.83295738
GABARAPL2	rs7926172	51.70206491
GABARAPL2	rs185009887	30.612217
GABARAPL2	rs78909033	32.16811935
GABARAPL2	rs6437284	31.46461893
GABARAPL2	rs1354034	422.3548604
NUDT16L1	rs2277413	256.1300095
NUDT16L1	rs140349498	39.3235694
NUDT16L1	rs1354034	45.80442556
NUDT16L1	rs342293	35.20761421
TAX1BP3	rs222773	119.8953135
TAX1BP3	rs826404	33.09540234
TAX1BP3	rs35340377	30.60830979
TAX1BP3	rs4980320	31.87833675
TAX1BP3	rs75092273	78.36976518
TAX1BP3	rs1354034	44.11279033
TAX1BP3	rs35733856	30.84761256
TAX1BP3	rs342200	30.67448375
SH3GL2	rs4980320	48.03681217

SH3GL2	rs4759076	63.4742971
SH3GL2	rs1354034	33.79049986
UBA2	rs12289048	33.02043216
UBA2	rs12146727	42.64606039
UBA2	rs185009887	31.4019009
UBA2	rs141622900	406.6381999
UBA2	rs5167	501.6684164
UBA2	rs186012201	34.83722822
UBA2	rs1354034	34.84531353
TBCA	rs174536	75.42850618
TBCA	rs112298726	38.48283495
TBCA	rs12978968	50.2866635
TBCA	rs2722757	103.5464158
TBCA	rs55923289	931.85066
TBCA	rs11668861	1096.181698
TBCA	rs2075649	3718.089054
TBCA	rs10408267	84.3492413
TBCA	rs111420738	126.7315847
TBCA	rs117884013	84.61069804
TBCA	rs60819212	32.86468364
GPR68	rs10800453	142.2383939
GPR68	rs704	46.55470941
GPR68	rs1858668	37.93080397
GPR68	rs11751024	30.96870734
LMOD1	rs10922098	225.9955448
LMOD1	rs2856876	77.88148874
LMOD1	rs2686395	63.1948149
LMOD1	rs415620	65.28996216
LMOD1	rs4713498	57.26182655
LMOD1	rs67399078	37.72713053
ITPKC	rs10922098	96.10193172
ITPKC	rs9864801	37.80394729
ITPKC	rs9501600	34.47364187
ITPKC	rs557011	34.55722395
COMMD1	rs232235	137.683089
COMMD1	rs1849787	98.22344495
COMMD1	rs3132124	50.87364237
COMMD1	rs9468873	62.86714871
COMMD1	rs28891435	42.10247052
COMMD1	rs113745074	51.91110612
STAP1	rs10922098	61.50755658
STAP1	rs76495028	30.18623086
STAP1	rs2232613	261.8873504
STAP1	rs1355538	79.55088057
STAP1	rs16849384	58.37233678
STAP1	rs147906938	30.41224323
SLC41A2	rs10922098	504.1674091
SLC41A2	rs11569415	31.54477309
SLC41A2	rs12478841	30.46858408
SLC41A2	rs9864801	65.40990898
SLC41A2	rs550671	97.30255841
SLC41A2	rs28645249	38.75468105
GLTP	rs572515	66.0831004
GLTP	rs77774564	73.31882369
GLTP	rs9864801	40.24260863
METTL1	rs10922094	164.6679679
METTL1	rs964184	95.92247965
METTL1	rs704	31.13800761

METTL1	rs429358	166.6775872
METTL1	rs35136575	77.35224955
METTL1	rs780093	34.86054493
METTL1	rs1707652	199.9124054
METTL1	rs453821	50.94735285
METTL1	rs28471056	39.63518234
METTL1	rs10096633	34.88974498
UCK2	rs10922098	73.53298532
UCK2	rs704	43.040849
UCK2	rs6445035	76.56699687
UCK2	rs9501393	37.17672763
TEAD4	rs10922098	223.6293153
TEAD4	rs1707652	52.6847681
TEAD4	rs28573154	69.21340924
TEAD4	rs7774739	47.5122403
TEAD4	rs9271766	35.06214453
PDCD5	rs146064596	30.6602279
PDCD5	rs75428591	69.73292548
PDCD5	rs117432447	40.03105556
PDCD5	rs6510263	1155.198173
PDCD5	rs141714582	533.0172224
PDCD5	rs200410659	87.20384124
PDCD5	rs11667586	240.5946102
PDCD5	rs150985471	32.46126018
PDCD5	rs1354034	45.59614744
CDKN2C	rs144704969	30.66642215
CDKN2C	rs1329424	802.4313775
CDKN2C	rs3900909	43.24593435
CDKN2C	rs11569393	30.18193369
CDKN2C	rs111696008	37.10455162
CDKN2C	rs1707652	216.6813969
CDKN2C	rs12629202	31.1621887
CDKN2C	rs550671	238.858803
CDKN2C	rs9469195	81.28539559
CDKN2C	rs9272526	48.92985789
RAD23B	rs35827173	41.68088095
RAD23B	rs1329424	37.78143167
RAD23B	rs66489920	30.25368019
SAT2	rs140474227	43.40998813
SAT2	rs858523	604.622226
SAT2	rs2642157	151.9374119
SAT2	rs10922098	94.20851697
SAT2	rs17580	38.85764369
SAT2	rs1706435	97.61360686
THRA	rs10922098	179.7965559
THRA	rs704	46.88443788
THRA	rs10208529	31.80118035
THRA	rs1706435	47.93147373
THRA	rs453821	46.47142026
THRA	rs35426875	31.625796
WRNIP1	rs10922098	231.2149895
WRNIP1	rs1706435	58.89639133
WRNIP1	rs9501393	64.19398548
WRNIP1	rs7774739	45.04208983
FKBP6	rs3741298	32.54059335
FKBP6	rs56136229	31.13508854
FKBP6	rs10775412	111.973916
FKBP6	rs7222245	207.1737693

FKBP6	rs1242299	1051.888793
FKBP6	rs16964949	80.17892247
FKBP6	rs9913470	68.13122928
FKBP6	rs2188951	47.32552012
FKBP6	rs919791	29.96094737
FKBP6	rs190712692	55.86853996
FKBP6	rs73045691	1309.044102
FKBP6	rs77660932	52.71880517
FKBP6	rs73036568	43.91555051
FKBP6	rs4002927	30.62247035
FKBP6	rs62294359	141.0318568
FKBP6	rs662	83.7495152
CKS1B	rs1354034	39.07922121
CKS1B	rs200482118	39.93029097
CKS1B	rs117383410	918.1633867
CKS1B	rs12533191	97.69114165
CKS1B	rs144407735	52.05387317
CYTH2	rs10922098	343.0342114
CYTH2	rs1707652	97.70777391
CYTH2	rs1265941	57.17955146
CYTH2	rs444921	110.9995928
CALCOCO2	rs318100	112.1253225
CALCOCO2	rs10922094	150.1005327
CALCOCO2	rs1355538	56.27894423
CALCOCO2	rs147906938	44.91577103
PURA	rs4980320	40.89450023
PURA	rs35979828	31.29184499
PURA	rs1354034	40.14037785
PURA	rs77265585	29.95611539
RGS7	rs429358	66.02951334
RGS7	rs35136575	30.17249193
RGS7	rs9864801	55.98037882
RHOG	rs1355538	37.81225578
RHOG	rs11755689	36.10041125
RHOG	rs6993770	44.84829666
SMTN	rs9971407	29.8124823
SMTN	rs4075583	36.37757843
SMTN	rs7281510	37.2812418
SMTN	rs1354034	30.0372595
SMTN	rs114694170	38.72223405
HPGDS	rs62310110	103.1258344
HPGDS	rs181514001	39.76451099
HPGDS	rs78621898	62.01792511
HPGDS	rs74354031	1002.701365
HPGDS	rs116232009	228.7424417
HPGDS	rs56028059	98.44874344
HPGDS	rs11729397	390.3334933
HPGDS	rs282451	69.92127347
HPGDS	rs181557011	113.2255667
HPGDS	rs28649197	161.38723
HPGDS	rs17022003	89.48229576
HPGDS	rs4657825	67.84397071
HPGDS	rs1047891	41.15001884
HPGDS	rs10000798	32.38140575
HPGDS	rs115074597	32.34384628
HPGDS	rs138042382	41.68559561
HPGDS	rs78736235	30.73944532
HPGDS	rs2797335	33.92333966

HPGDS	rs12342201	71.57854207
ITCH	rs2210913	33.99666496
ITCH	rs2093214	97.37536324
ITCH	rs3794468	379.613915
ITCH	rs7151816	652.4126348
ITCH	rs1106906	71.1332846
ITCH	rs3094689	37.44013851
ITCH	rs73403125	111.4349091
ITCH	rs77311729	98.86648258
ITCH	rs9271775	144.730903
VRK1	rs10922094	84.93123791
VRK1	rs6542680	37.42602261
VRK1	rs1846190	29.79138921
UBE2C	rs2200587	81.78266596
UBE2C	rs36051332	284.8997193
UBE2C	rs10922094	71.20962744
UBE2C	rs1619994	37.24408853
UBE2C	rs41556615	38.40901792
UBASH3B	rs11218734	217.3066805
UBASH3B	rs4936732	49.03121274
UBASH3B	rs4980320	39.88208596
UBASH3B	rs1354034	30.68697207
UBASH3B	rs67036916	33.32819538
NT5C	rs2706538	37.265527
NT5C	rs80155227	981.1969317
NT5C	rs1005714	88.21328114
NT5C	rs56158987	267.981799
NT5C	rs112529405	81.72108355
PKN1	rs117192879	61.82764641
PKN1	rs116985420	92.05961867
PKN1	rs371267418	130.8132042
PKN1	rs117620934	34.98555294
PKN1	rs113448098	66.80558094
PKN1	rs11064501	218.3422156
PKN1	rs12478841	36.71741669
PKN1	rs1619994	117.5985744
TNFAIP8	rs1035376	414.8032055
TNFAIP8	rs12234017	49.77745418
TNFAIP8	rs342200	30.29371197
ARL3	rs8354	322.0896725
ARL3	rs11191401	92.09945663
ARL3	rs1354034	35.41707463
EFS	rs10922094	39.55789689
EFS	rs964184	53.12489694
EFS	rs71352239	44.1436634
EFS	rs12478841	41.01902659
EFS	rs150687841	30.1627069
EFS	rs1706435	122.9250419
EFS	rs6457457	58.54154001
EFS	rs6458	109.3005513
EFS	rs78810414	37.70476936
TRIM3	rs67992843	38.21014539
TRIM3	rs35717222	171.7927687
TRIM3	rs12770589	32.84364916
TRIM3	rs7073753	44.38781436
TRIM3	rs9971407	56.25873649
TRIM3	rs202208968	30.57005236
MTHFD1	rs117510947	34.36848628

MTHFD1	rs11158538	144.963482
MTHFD1	rs72726297	58.06386609
MTHFD1	rs7297265	32.02495134
MTHFD1	rs112324047	48.72667555
MTHFD1	rs2039595	29.97765443
MTHFD1	rs72716450	43.56624853
MTHFD1	rs1354034	32.37410452
MTHFD1	rs77265585	30.39412022
MAGEA3	rs10922098	105.4983996
MAGEA3	rs17580	710.7996764
MAGEA3	rs56361564	38.75978359
MAGEA3	rs77241309	30.5364867
MAGEA3	rs5167	838.2984015
MAGEA3	rs1355538	81.38354877
MAGEA3	rs451652	30.14665054
MAGEA3	rs4713498	40.66075539
FEN1	rs10922094	174.3608826
FEN1	rs1619994	52.13275234
FEN1	rs41285751	64.12648189
FEN1	rs451652	48.58715513
PSMB5	rs10922094	396.2300546
PSMB5	rs11569393	38.10368937
PSMB5	rs1707652	100.2512287
PSMB5	rs3099847	32.16968628
PSMB5	rs9501600	132.9358601
PSMB5	rs35209896	43.22156097
IMPA2	rs10922094	207.7180155
IMPA2	rs1707652	52.92090511
IMPA2	rs147906938	46.31966925
IMPA2	rs78912960	35.97266323
ARAF	rs11605332	32.6696213
ARAF	rs1065853	87.07621931
ARAF	rs1354034	65.58065364
ARAF	rs854561	220.7122787
ARAF	rs342293	45.60019905
ERCC1	rs10922098	227.7416513
ERCC1	rs3136202	35.46436204
ERCC1	rs1707652	52.4824655
ERCC1	rs2242572	57.65889285
ERCC1	rs28624101	49.88125765
ARL2	rs10922098	293.7244412
ARL2	rs174536	43.83697153
ARL2	rs78264306	40.78532656
ARL2	rs55923289	308.9622258
ARL2	rs406315	326.3186298
ARL2	rs3745150	1171.080264
ARL2	rs10408267	82.32225304
ARL2	rs113280663	63.96446057
ARL2	rs111696008	53.13564717
ARL2	rs144202860	51.8707862
ARL2	rs2686395	330.8872747
ARL2	rs73169117	34.12105133
ARL2	rs550671	68.55005099
OPHN1	rs7113761	38.75277914
OPHN1	rs1354034	33.22101496
OPHN1	rs342292	34.04021376
PDRG1	rs4980320	34.15127507
PDRG1	rs10876550	32.71200815

PDRG1	rs5742938	40.87006785
PDRG1	rs1354034	116.1477827
GCA	rs377413955	256.5242636
GCA	rs17783344	1015.236837
GCA	rs13018302	31.45244948
GCA	rs184075645	31.49425871
GCA	rs1451831	36.92031506
PABPC4	rs7073753	37.72303513
PABPC4	rs4980320	51.06808346
PABPC4	rs1354034	166.8360398
PABPC4	rs342200	32.02226116
SCMH1	rs10801548	44.07866691
SCMH1	rs10922098	688.8444326
SCMH1	rs35351085	34.99362002
SCMH1	rs1706435	51.90297525
SCMH1	rs9267139	31.52803935
SCMH1	rs28573154	173.4525706
SCMH1	rs7774739	99.16550161
SCMH1	rs9271766	68.43242293
EXOSC3	rs203688	35.00026621
EXOSC3	rs12889189	105.9320128
EXOSC3	rs4977155	866.0134514
EXOSC3	rs376083359	40.42925601
EXOSC3	rs114607072	39.37865157
EXOSC3	rs9272742	32.9289025
PSMB1	rs138757877	52.50476778
PSMB1	rs10946271	921.2573181
PSMB1	rs6591561	54.81612951
PSMB1	rs117577063	30.75003331
NRBP1	rs12478841	30.52552474
NRBP1	rs10922094	180.5141305
NRBP1	rs9971407	55.96904135
NRBP1	rs429358	60.8721291
NRBP1	rs35136575	32.03086446
NRBP1	rs1707652	121.4612282
NRBP1	rs2242572	55.26023355
NRBP1	rs10107388	220.1034667
AKR1C1	rs10922094	220.1344304
AKR1C1	rs12478841	40.44987551
AKR1C1	rs1619994	46.3301509
AKR1C1	rs9501393	59.44813159
AKR1C1	rs7774739	38.7068418
PPP2R1A	rs111696008	39.57358918
PPP2R1A	rs6445035	383.1184879
PPP2R1A	rs3863087	135.7733464
PPP2R1A	rs73169103	34.41222583
JARID2	rs10922098	326.2134476
JARID2	rs111696008	37.58054757
JARID2	rs1707652	241.8084028
JARID2	rs438999	155.405758
JARID2	rs9501602	65.96258177
JARID2	rs9271529	38.29065283
ARFIP2	rs2275355	30.48941328
ARFIP2	rs3219110	71.83543066
ARFIP2	rs61953412	32.72353356
ARFIP2	rs112963922	30.97070287
ARFIP2	rs77542162	65.64899673
ARFIP2	rs1047891	44.34620107

ARFIP2	rs738409	68.45540565
ARFIP2	rs62294359	31.8101865
ARFIP2	rs36205397	43.73133174
ARFIP2	rs72649156	56.73029083
ARFIP2	rs116009877	47.39241951
ARFIP2	rs73224404	41.32230705
NAT1	rs10922094	82.9671148
NAT1	rs12257143	170.3345257
NAT1	rs10208529	34.4985782
NAT1	rs143827255	35.33936818
NAT1	rs62259725	66.99517423
NAT1	rs2878628	962.0363185
NAT1	rs34352856	37.9595381
NAT1	rs11920905	37.44270236
NAT1	rs9501393	46.97939561
PTPN9	rs111647176	63.47449456
PTPN9	rs60411925	34.08177177
PTPN9	rs10922098	62.49851546
PTPN9	rs1355538	54.90063258
BCAR3	rs203688	48.62052338
BCAR3	rs11594179	34.0454167
BCAR3	rs8050367	59.06051239
BCAR3	rs1355538	39.2524669
BCAR3	rs6980715	39.73409123
BCAR3	rs2417054	168.2814831
TRDMT1	rs10904881	914.472186
TRDMT1	rs398636	103.4808086
TRDMT1	rs2461164	33.31155618
TRDMT1	rs4311960	39.28283413
TRDMT1	rs1354034	51.78552955
SMYD2	rs10922094	157.573696
SMYD2	rs4566588	34.31873945
SMYD2	rs9501393	44.37419527
SMYD2	rs7774739	33.29634985
SMYD2	rs67399078	32.09138887
CRAT	rs6478861	109.6310154
CRAT	rs9409310	94.38358082
CRAT	rs11242109	44.54103787
CRAT	rs342200	29.97436977
INPP5B	rs12742776	35.95333702
INPP5B	rs139477416	608.8735508
INPP5B	rs4980320	46.40919636
INPP5B	rs35979828	40.71243381
INPP5B	rs1354034	46.13114
GNAI3	rs10922098	30.71622855
GNAI3	rs185009887	36.31163742
GNAI3	rs7412	210.2512369
GNAI3	rs1706435	42.0367619
PDK2	rs10922098	89.43154153
PDK2	rs1355538	32.04875969
PDK2	rs4713498	37.73985364
CSNK1G2	rs10922094	30.70197733
CSNK1G2	rs12289048	69.95776193
CSNK1G2	rs1354034	48.67619117
CSNK1G2	rs1355538	32.44488645
NAPB	rs10922098	166.9192658
NAPB	rs1355538	66.47737737
NAPB	rs872914	59.85449048

NAPB	rs9501600	47.60561879
KLC1	rs12884809	104.7872976
KLC1	rs4980320	38.78520121
KLC1	rs9971407	58.59645959
KLC1	rs450269	35.76206902
PANK3	rs17580	81.01781126
PANK3	rs4665383	35.74432669
PANK3	rs1619994	70.59822632
PANK3	rs11755689	37.61084937
PANK3	rs6993770	31.76104428
OLA1	rs139509080	278.7991499
OLA1	rs146824750	32.06865873
OLA1	rs1354034	39.06028977
ECH1	rs4802890	1053.928114
ECH1	rs10853721	44.67626323
ECH1	rs73544031	32.64935416
TST	rs3992625	35.0191024
TST	rs34877649	83.82608592
TST	rs11804953	514.8806263
TST	rs12737414	45.09940727
TST	rs3820201	616.2932055
TST	rs1383599	78.0442201
TST	rs149087463	47.71655519
TST	rs11805728	42.97978154
TST	rs16824380	31.8903685
TST	rs114505698	77.84345019
TST	rs116638028	81.47999058
TST	rs144691905	85.05283489
TST	rs71644803	55.07781618
TST	rs190119408	150.9958778
TST	rs12035228	112.4076865
TST	rs71631848	1382.150685
TST	rs116815955	1230.044468
TST	rs74550177	348.1129796
TST	rs142565493	225.8579632
TST	rs187717871	573.3320851
TST	rs3790370	631.1911856
TST	rs12120957	32.49453318
TST	rs34414349	333.9490721
TST	rs141323461	148.8747045
TST	rs79641904	102.8782545
TST	rs4084030	38.52538301
TST	rs12318001	60.18461841
TST	rs760715	31.06028739
TST	rs7624084	35.73172604
TST	rs6858276	35.47383827
TST	rs9272528	36.46320324
TST	rs35548455	75.30273657
TST	rs150325896	30.74287338
PTPN13	rs10922098	61.6642757
PTPN13	rs2284179	33.40793559
PTPN13	rs116751091	38.98859118
PTPN13	rs67399078	52.55142
GDA	rs146457388	97.63521469
GDA	rs188023612	46.32285975
GDA	rs17441745	52.076274
GDA	rs709275	44.23105197
GDA	rs6542680	1870.101191

GDA	rs80220851	71.75389477
GDA	rs3105782	34.33991425
GDA	rs11755689	31.70401918
VPS4B	rs1329424	347.8831031
VPS4B	rs11569393	31.04574749
VPS4B	rs1706435	95.76844703
VPS4B	rs555007	40.06615493
VPS4B	rs444921	99.7346861
HECW1	rs10922094	81.5785114
HECW1	rs1355538	53.68264922
HECW1	rs9501393	46.16093623
HECW1	rs7774739	37.27655839
SULT1B1	rs199817936	244.9300822
SULT1B1	rs12127364	1005.350816
SULT1B1	rs6029	503.5210407
SULT1B1	rs150192271	59.81052074
SULT1B1	rs114585353	35.16663933
SULT1B1	rs114862025	38.85352378
SULT1B1	rs78334957	42.94514196
SULT1B1	rs10159712	128.1234562
SULT1B1	rs1265067	36.98008976
SULT1B1	rs73407207	41.72857635
SULT1B1	rs6938008	65.67314654
PACSIN1	rs7080536	117.238833
PACSIN1	rs4759076	56.60746942
PACSIN1	rs185009887	35.45922879
PACSIN1	rs2304200	502.4671749
PACSIN1	rs183440743	32.85784722
PACSIN1	rs2143922	30.0984944
PACSIN1	rs2010366	218.0196603
PACSIN1	rs342200	30.23763107
PACSIN1	rs6993770	87.57667817
FLII	rs138865936	91.66291949
FLII	rs12292693	125.6221468
FLII	rs1354034	38.26222993
USP21	rs10922098	134.4971395
USP21	rs5167	238.4137566
USP21	rs1354034	39.38783794
USP21	rs1706435	91.84485341
USP21	rs28573154	48.70760338
USP21	rs34927613	35.2872982
DPYSL5	rs10922094	211.7691966
DPYSL5	rs1707652	55.88539843
DPYSL5	rs453821	65.5448027
DPYSL5	rs28471056	36.64792718
SCIN	rs6460966	124.4892511
SCIN	rs113448098	92.39593191
SCIN	rs113747960	296.2518119
SCIN	rs2232710	30.34658936
SCIN	rs10873559	54.21303232
SCIN	rs13469	31.76730326
HOMER2	rs1256430	66.55477149
HOMER2	rs1124025	36.75151437
HOMER2	rs3811444	36.81671623
HOMER2	rs116052829	44.59122071
HOMER2	rs2739240	43.64018244
HOMER2	rs4759076	32.79703584
HOMER2	rs139948926	37.67206444

HOMER2	rs5771045	42.825717
HOMER2	rs3213442	167.0047521
HOMER2	rs9616896	1131.163547
HOMER2	rs28689705	295.6189752
HOMER2	rs9616960	31.88401744
HOMER2	rs11705273	87.22074283
HOMER2	rs1354034	159.7167761
DECR1	rs9649958	66.77850745
DECR1	rs142973309	34.33970193
DECR1	rs74785202	56.99747268
RPS6KA6	rs3811463	45.60332442
RPS6KA6	rs4980320	36.51409909
RPS6KA6	rs11451044	47.31293221
RPS6KA6	rs1619994	38.41388113
ARPC1B	rs10633389	100.1305294
ARPC1B	rs35824732	40.80271406
ARPC1B	rs1354034	31.58143457
DOT1L	rs10922094	38.76722768
DOT1L	rs6445035	31.08649643
DOT1L	rs9348630	113.8268138
DOT1L	rs111437814	444.837774
DOT1L	rs148550521	111.9721243
DOT1L	rs2894038	110.3771631
SH3BGRL	rs10922098	206.9968448
SH3BGRL	rs1707652	56.539476
SH3BGRL	rs555007	32.06217154
SH3BGRL	rs444921	65.83171259
KPNA4	rs572515	40.50215426
KPNA4	rs7114094	63.25149309
KPNA4	rs1355538	30.70959044
ACLY	rs10922094	100.8673756
ACLY	rs6542680	32.93692821
ACLY	rs1355538	74.19355708
ACLY	rs147906938	41.54045783
EIF1B	rs6580980	34.10718092
EIF1B	rs1354034	67.5832638
EIF1B	rs16873418	32.54742209
PELI2	rs9332600	456.2443023
PELI2	rs572515	233.1170328
PELI2	rs1619994	31.66371677
SH2D3C	rs10922094	62.41933435
SH2D3C	rs10822155	29.972851
SH2D3C	rs704	34.49729502
SH2D3C	rs438811	127.1464399
SH2D3C	rs5112	31.95375581
SH2D3C	rs1706435	101.9707975
SH2D3C	rs6993770	49.01457695
HERC1	rs10922094	105.8088913
HERC1	rs117265578	32.139046
HERC1	rs9864801	43.04244439
HERC1	rs3828879	32.6010092
HERC1	rs9501393	37.11496775
CDC42BPA	rs17580	1068.898543
CDC42BPA	rs12437339	46.27890489
CDC42BPA	rs147863506	39.09606814
CDC42BPA	rs704	34.12818505
CDC42BPA	rs1354034	40.52927089
CDC42BPA	rs1355538	30.62494457

DPYSL3	rs342293	51.5739801
DPYSL3	rs35648025	42.9520013
DPYSL3	rs189016543	97.60078081
DPYSL3	rs114213881	109.5680305
L3MBTL2	rs4980320	42.66356629
L3MBTL2	rs35979828	31.9883534
L3MBTL2	rs1354034	41.26687259
HMG20A	rs572515	64.17990186
HMG20A	rs1354034	41.78605486
HMG20A	rs9864801	30.22131059
RGS19	rs10733084	31.81143467
RGS19	rs10922098	600.358233
RGS19	rs704	30.81605912
RGS19	rs1047286	31.15467067
RGS19	rs2384656	34.65782815
RGS19	rs1707652	148.9759664
RGS19	rs1265941	80.03796801
RGS19	rs550671	186.6022327
RGS19	rs9276606	37.33283561
RGS19	rs1916675	31.24141349
AP1G2	rs7147244	39.73281905
AP1G2	rs34015250	281.9138232
AP1G2	rs140983547	202.1645672
AP1G2	rs4980320	39.94805046
DCP1B	rs112848587	41.63867627
DCP1B	rs78111684	51.59398725
DCP1B	rs2073333	960.4225419
DCP1B	rs116936902	30.86245197
ZNF175	rs10922098	463.7569552
ZNF175	rs1047286	35.49404602
ZNF175	rs1707652	81.7028771
ZNF175	rs1265941	54.43194576
ZNF175	rs444921	117.3341151
TOX3	rs4980320	30.30627921
TOX3	rs59146411	31.17512036
TOX3	rs371314787	35.7709628
UBQLN4	rs4980320	39.0311853
UBQLN4	rs10876550	36.44882411
UBQLN4	rs185009887	30.31272766
UBQLN4	rs1354034	62.44911428
TAX1BP1	rs10922098	247.8258735
TAX1BP1	rs9864801	50.04941921
TAX1BP1	rs9501600	73.89073272
CIRBP	rs4980320	42.1632405
CIRBP	rs10876550	53.17278079
CIRBP	rs1354034	197.9686681
CIRBP	rs342200	30.48740973
PTGFRN	rs114576333	314.8217252
PTGFRN	rs139704902	220.6433223
PTGFRN	rs12037202	970.1319885
PTGFRN	rs12024571	636.7853674
PTGFRN	rs75567925	893.5261221
PTGFRN	rs113594400	188.3971631
PTGFRN	rs78896342	101.7019022
PTGFRN	rs11102890	68.59417622
PTGFRN	rs114911729	36.40034302
PTGFRN	rs149160089	65.16205354
PTGFRN	rs77421845	95.27316735

PTGFRN	rs143103033	53.56054668
PTGFRN	rs143737455	100.8106194
PTGFRN	rs140458621	64.04370493
PTGFRN	rs565847	41.42759293
PTGFRN	rs138866201	33.75657102
PTGFRN	rs429358	30.79443537
NDC80	rs10922094	312.9002205
NDC80	rs964184	37.09862522
NDC80	rs8106813	32.71174487
NDC80	rs10208529	33.9666068
NDC80	rs111696008	33.32121703
NDC80	rs1707652	212.170153
NDC80	rs444921	92.3513551
NDC80	rs9270570	33.12360716
NDC80	rs9469195	62.27307248
PLEKHA7	rs142056137	35.70486466
PLEKHA7	rs433373	1070.860122
PLEKHA7	rs12421951	121.2395759
PLEKHA7	rs148969064	59.58011773
PLEKHA7	rs199786165	55.03477077
PLEKHA7	rs117300835	77.54294978
PLEKHA7	rs75476113	77.43259443
PLEKHA7	rs55665228	31.89834504
MPP7	rs572515	99.77385782
MPP7	rs12995461	31.7940964
MPP7	rs541862	36.88516258
MPP7	rs9272068	34.06840114
NISCH	rs10922098	258.2339837
NISCH	rs1355538	59.45875069
NISCH	rs444921	82.85523668
NISCH	rs28471056	35.65120605
FEV	rs4980320	32.74062981
FEV	rs1354034	65.6049127
FEV	rs6993770	39.49902962
FEV	rs55665228	44.59634687
BAG5	rs12371	161.5925159
BAG5	rs10922094	54.43877043
BAG5	rs1355538	31.3938546
RBM3	rs4980320	43.22246142
RBM3	rs10876550	68.76272211
RBM3	rs72799293	75.2484951
RBM3	rs1354034	183.1763504
RBM3	rs114694170	34.59540129
RBM3	rs7749454	34.03302129
BRDT	rs1329424	158.4772735
BRDT	rs1355538	37.44227432
BRDT	rs550671	44.41861574
ITGB2	rs10922098	245.3374484
ITGB2	rs704	44.50921525
ITGB2	rs1355538	55.41068383
ITGB2	rs147906938	56.91443716
ITGB2	rs9501602	33.09917327
CSDC2	rs10922098	282.4290872
CSDC2	rs964184	35.93043892
CSDC2	rs1555405	31.67599584
CSDC2	rs7412	81.68215701
CSDC2	rs1707652	123.5646651
CSDC2	rs6457457	55.63060371

CSDC2	rs9501393	84.50712223
BACH2	rs10922098	240.3565625
BACH2	rs2686395	54.54932827
BACH2	rs28573154	115.6455232
BACH2	rs7774739	70.42047451
BACH2	rs3917538	84.42803562
GRID2	rs6679677	41.44077291
GRID2	rs2295616	30.3343771
GRID2	rs10922098	37.8460307
GRID2	rs17578693	30.61066091
GRID2	rs12798553	37.34356622
GRID2	rs1958991	34.54827485
GRID2	rs142364821	30.91857897
GRID2	rs146335091	54.6584644
GRID2	rs147878777	34.38141609
GRID2	rs117320146	3007.052493
GRID2	rs2857383	140.7013717
GRID2	rs11846842	91.69391481
GRID2	rs187963233	63.89593193
GRID2	rs11862962	33.14852873
GRID2	rs9302635	40.10685035
GRID2	rs4608411	29.82467921
GRID2	rs142279279	53.45879571
GRID2	rs3810560	82.2969322
GRID2	rs567189	828.7838392
GRID2	rs79395339	34.26917564
GRID2	rs147424791	32.10595315
GRID2	rs117894413	32.42523319
GRID2	rs6891054	34.44543382
GRID2	rs148813665	61.52398596
GRID2	rs76106586	71.66859594
GRID2	rs1264457	90.11362495
GRID2	rs9271378	59.06538968
GRID2	rs7785014	45.8565408
GRID2	rs2511737	71.95850095
ILF3	rs368465	99.45069115
ILF3	rs653178	30.11102035
ILF3	rs1354034	61.01074947
ZNF774	rs10922098	247.0580679
ZNF774	rs9864801	47.05775588
ZNF774	rs9266642	38.40977979
ZNF774	rs2075798	33.65113627
ZNF774	rs453821	65.34719745
APBB2	rs11606706	40.80418084
APBB2	rs113448098	142.2827707
APBB2	rs12146727	477.5999006
APBB2	rs1047286	46.22860684
ZNF334	rs10922098	33.78342335
ZNF334	rs112727502	61.37212414
ZNF334	rs77938199	138.3745917
ZNF334	rs2284351	231.4003301
ELMO1	rs56358455	36.7643391
ELMO1	rs7782999	107.3814689
ELMO1	rs28628397	69.39374632
ELMO1	rs78909033	38.177677
ELMO1	rs114115200	62.38195742
ELMO1	rs3863087	30.88529359
PPP1R3B	rs10131226	71.96640185

PPP1R3B	rs1801690	302.0006507
PPP1R3B	rs3094689	39.60315598
PPP1R3B	rs77311729	55.0995647
PPP1R3B	rs9271636	122.1836774
HMGB3	rs10922094	406.5473076
HMGB3	rs1706420	83.34467894
HMGB3	rs9501600	91.60850255
HMGB3	rs35209896	31.33774745
TARBP2	rs1354034	44.78681545
TARBP2	rs1355538	30.65951966
TARBP2	rs342200	36.25903204
HNRNPM	rs2583264	382.9263219
HNRNPM	rs7312381	50.97933481
HNRNPM	rs1354034	46.82690726
APBB3	rs10922098	105.5786294
APBB3	rs704	100.199775
APBB3	rs6441996	37.80303077
APBB3	rs3129773	32.14138246
GPCPD1	rs6133228	71.50761137
GPCPD1	rs2024886	68.17039217
GPCPD1	rs4980320	41.9392772
GPCPD1	rs1354034	32.8279452
CCDC51	rs572515	34.67753687
CCDC51	rs79740491	31.53429424
CCDC51	rs6542680	39.86213668
PIWIL1	rs10922098	30.90873817
PIWIL1	rs62265615	30.82950552
PIWIL1	rs56210800	30.60248046
NLRP4	rs10922098	151.7650569
NLRP4	rs6542680	48.28454818
NLRP4	rs9864801	42.6471451
NLRP4	rs6457457	47.98657602
NLRP4	rs9501393	39.93815138
ZNF566	rs10922098	109.7862718
ZNF566	rs1355538	37.18918342
ZNF566	rs9501600	43.55754114
SLC4A8	rs10922098	326.753158
SLC4A8	rs1619994	31.62511739
SLC4A8	rs9501393	59.53041893
SLC4A8	rs7774739	34.70163585
TPPP2	rs140084787	37.85434426
TPPP2	rs140481478	36.38334786
TPPP2	rs368391158	281.07753
TPPP2	rs7203074	667.990061
TPPP2	rs138498474	42.1080644
TPPP2	rs146973537	40.9401435
TPPP2	rs12210303	30.23192307
ARHGAP30	rs371267418	32.90611632
ARHGAP30	rs17713196	96.95661635
ARHGAP30	rs3863087	32.86212974
ARHGAP30	rs72673751	39.10806841
ZNF415	rs10922098	185.6309649
ZNF415	rs697356	33.81507155
ZNF415	rs415620	44.99140711
ZNF415	rs4713498	42.51492495
ACYP2	rs138650079	42.38565384
ACYP2	rs7559380	753.1373895
ACYP2	rs74399555	31.92735453

ACYP2	rs11417460	38.96850146
ACYP2	rs1354034	54.53387443
EHBP1	rs13389363	32.57339518
EHBP1	rs9332701	240.5235158
EHBP1	rs2142760	31.64414346
EHBP1	rs10922094	33.34002506
RNPC3	rs10922098	142.6468325
RNPC3	rs704	47.33702224
RNPC3	rs9864801	36.33863727
RNPC3	rs41285751	42.27026499
RNPC3	rs415620	38.53404968
GEM	rs147092838	40.91193552
GEM	rs140870715	43.72531833
GEM	rs147804221	33.06244463
GEM	rs201234732	37.06978001
GEM	rs9899944	81.11684064
GEM	rs140390732	75.99343569
GEM	rs182655273	92.39343304
GEM	rs28999383	40.36358122
GEM	rs9902833	1101.629303
GEM	rs12936368	307.6598079
GEM	rs10454088	175.2746232
GEM	rs36020655	200.0871067
GEM	rs76608048	47.72748788
GEM	rs140915766	54.43941129
GEM	rs12478841	35.26279522
GEM	rs56210800	47.44634622
GEM	rs140431012	47.43220107
GEM	rs9864801	36.0278545
SLC14A2	rs72702354	1137.643166
SLC14A2	rs12437339	72.16975936
SLC14A2	rs56252683	114.522975
SLC14A2	rs116947628	36.84453517
SLC14A2	rs140279936	34.89088973
SLC14A2	rs146527756	34.58656649
SLC14A2	rs16873418	35.88924348
GRAP	rs75693337	36.71643508
GRAP	rs10922098	122.6398538
GRAP	rs148328786	72.46974574
GRAP	rs1354034	36.02695275
GRAP	rs1355538	35.28722512
GRAP	rs1042445	65.43100207
GRAP	rs34192428	112.7094536
GRAP	rs550671	39.75939237
SLC5A5	rs138555008	35.8334427
SLC5A5	rs111874634	38.61118206
SLC5A5	rs55725193	37.86324938
SLC5A5	rs2923009	42.08383206
SLC5A5	rs77311729	80.57417948
SLC5A5	rs9271640	147.8985785
RGS3	rs10922098	337.0901293
RGS3	rs2230203	32.03805984
RGS3	rs10208529	30.8366365
RGS3	rs6445035	79.31735018
RGS3	rs1265941	44.22091872
RGS3	rs550671	118.5807427
TESC	rs11614628	106.8564009
TESC	rs143724565	33.82975546

TESC	rs4767461	950.333525
TESC	rs57576794	630.4377093
TESC	rs3862136	136.724546
TESC	rs71638510	43.11428014
TESC	rs78635633	48.1424817
TESC	rs897877	35.47419277
TESC	rs1354034	64.23716354
TESC	rs6993770	34.53548258
STX10	rs10876550	32.77431049
STX10	rs2057681	247.4497507
STX10	rs854572	30.28483959
SNX17	rs10922098	186.1809559
SNX17	rs1354034	31.07088007
SNX17	rs1706435	47.4742838
SNX17	rs28573154	60.332126
SNX17	rs7774739	45.3225415
ARHGEF2	rs10922098	133.4357605
ARHGEF2	rs12995461	32.1947742
ARHGEF2	rs9501393	49.06711661
ARHGEF2	rs7774739	30.08105367
POLM	rs139763375	74.68348541
POLM	rs141574506	121.4181878
POLM	rs142059440	273.2900348
CASS4	rs2426628	49.98533973
CASS4	rs185009887	31.44120425
CASS4	rs5167	144.1947561
CASS4	rs1355538	62.54396848
CASS4	rs342200	35.55623787
TMEM237	rs12038333	60.99223915
TMEM237	rs9864801	31.25834265
TMEM237	rs557011	30.73186945
ECI2	rs77482745	52.68535052
ECI2	rs72845845	63.58523209
ECI2	rs675073	1132.266482
ECI2	rs4544959	125.5015297
ECI2	rs115538711	34.27004082
ECI2	rs10876550	31.03191985
ECI2	rs74770429	33.36250647
ECI2	rs1354034	49.23711564
ARPP21	rs10922098	62.74708003
ARPP21	rs9501393	41.23219174
ARPP21	rs7774739	34.19290019
NEDD9	rs10922098	177.0013564
NEDD9	rs1355538	53.87609949
NEDD9	rs453821	52.27252511
NEDD9	rs28471056	30.24064984
NIPAL4	rs10922098	431.1507174
NIPAL4	rs11720167	105.7048707
NIPAL4	rs3863087	32.79888551
NIPAL4	rs9267139	36.28081368
NIPAL4	rs9501393	132.9448559
NIPAL4	rs7774739	103.4376301
NIPAL4	rs67965605	30.09163652
DYNLT3	rs368857	43.78241122
DYNLT3	rs7519758	83.60811468
DYNLT3	rs76080039	30.57457652
DDX6	rs4980320	34.17469337
DDX6	rs4759076	56.97060106

DDX6	rs185009887	31.20229756
DDX6	rs1354034	89.27333598
DDX6	rs342200	33.15422916
SPN	rs10922094	112.454447
SPN	rs113448098	49.72832391
SPN	rs12146727	166.5304472
SPN	rs71424600	30.65742147
SPN	rs1706435	100.8471654
SPN	rs28573154	40.82583336
RDH16	rs10922094	54.41628957
RDH16	rs704	50.83898279
RDH16	rs429358	226.4747885
RDH16	rs35136575	54.74250457
RDH16	rs1355538	50.34221267
RDH16	rs61751211	38.61376082
RDH16	rs28573154	33.4219395
MICALL2	rs1451915	30.0860674
MICALL2	rs10922098	560.420409
MICALL2	rs704	257.3305311
MICALL2	rs1047286	39.80607515
MICALL2	rs12478841	31.53111907
MICALL2	rs1707652	139.1069207
MICALL2	rs550671	154.262005
MICALL2	rs9270570	31.20553698
MICALL2	rs9469195	53.67516581
SYTL1	rs10922094	289.3948164
SYTL1	rs59228823	29.8812637
SYTL1	rs1706435	40.97136318
SYTL1	rs1265941	43.91540115
SYTL1	rs550671	88.10620166
PAXIP1	rs10922098	404.7248199
PAXIP1	rs1555405	31.0613811
PAXIP1	rs1291921	30.65566841
PAXIP1	rs1706435	89.67791304
PAXIP1	rs550671	156.4324261
PAXIP1	rs9270570	41.97183392
PAXIP1	rs9469195	60.39991533
DGKB	rs10922094	132.773914
DGKB	rs113448098	35.89238852
DGKB	rs12146727	102.1915254
DGKB	rs57516848	31.07952038
DGKB	rs11611373	31.12559344
DGKB	rs17580	214.4885711
DGKB	rs41285751	49.4073109
DGKB	rs147390813	71.20093044
DGKB	rs2057681	165.1331881
DGKB	rs485609	42.49799217
CUBN	rs10922098	124.0637492
CUBN	rs415620	39.35280196
CUBN	rs4713498	46.77898788
KRT17	rs10922098	295.3867487
KRT17	rs2686395	96.37863012
KRT17	rs2075798	45.12205112
KRT17	rs550671	113.1589943
AXIN2	rs114009540	33.55000266
AXIN2	rs10922098	781.7945883
AXIN2	rs3900909	40.02354113
AXIN2	rs1047286	32.25207203

AXIN2	rs1707652	134.4453458
AXIN2	rs550671	203.2488702
AXIN2	rs9469195	68.89907994
AXIN2	rs9272526	37.04391488
NR3C2	rs79874727	127.4083945
NR3C2	rs111736413	47.18896855
NR3C2	rs146958887	40.20170612
ENOX1	rs72748420	33.42579843
ENOX1	rs114009540	31.22027276
ENOX1	rs10922098	829.5398845
ENOX1	rs1047286	70.25645362
ENOX1	rs111696008	38.49519831
ENOX1	rs2686395	203.9758428
ENOX1	rs1144709	34.69430731
ENOX1	rs1265941	89.72112753
ENOX1	rs550671	236.2833183
ENOX1	rs112164276	31.36285985
ENOX1	rs3917538	33.36443665
HERC5	rs424535	76.80413572
HERC5	rs12478841	52.67894536
HERC5	rs1706435	53.65700009
HERC5	rs73425709	43.64765291
HERC5	rs4081560	65.22502387
HERC5	rs11965547	277.9563031
HERC5	rs444921	980.5502834
HERC5	rs970902	56.87911972
HERC5	rs12205398	34.81971409
HERC5	rs662	138.9131374
RLBP1	rs10922098	68.56267202
RLBP1	rs704	404.1615787
RLBP1	rs872914	30.58513277
RLBP1	rs9501393	39.00109033
POLR1C	rs72748420	30.49815301
POLR1C	rs1329424	1004.617079
POLR1C	rs3900909	45.73506532
POLR1C	rs10801660	34.78186814
POLR1C	rs1047286	59.26882392
POLR1C	rs111696008	43.17744729
POLR1C	rs1707652	214.2940153
POLR1C	rs1265941	102.9289084
POLR1C	rs550671	265.0389781
POLR1C	rs112164276	30.93800349
POLR1C	rs41548618	36.79667685
ALDH3B1	rs475325	206.9948476
ALDH3B1	rs10922098	110.4958052
ALDH3B1	rs1355538	34.28388245
ALDH3B1	rs9501600	34.48565778
RAB18	rs10922098	266.8205304
RAB18	rs697356	59.93295791
RAB18	rs6457457	95.48752863
RAB18	rs9501393	136.6603573
RAB18	rs6451	44.6590926
RAB18	rs41291802	42.29147093
PPARA	rs11553699	110.7002524
PPARA	rs704	183.793129
PPARA	rs34162626	43.42866692
UQCRB	rs10922094	499.5083516
UQCRB	rs11569393	32.99796867

UQCRB	rs1707652	156.1681023
UQCRB	rs550671	160.0501177
UQCRB	rs28471056	68.8483424
UQCRB	rs9272526	33.12349993
GCK	rs10922094	218.5257543
GCK	rs113747960	30.02756526
GCK	rs17580	67.48109508
GCK	rs704	33.10513768
GCK	rs12995461	46.15448104
GCK	rs62265615	37.40196858
GCK	rs56210800	36.0405083
GCK	rs114115200	53.54706091
GCK	rs9501393	95.89265269
GCK	rs9469195	67.34893671
GCK	rs17732416	29.83261575
CSRP2	rs1329424	147.2266661
CSRP2	rs927901	104.5858587
CSRP2	rs17437465	32.27208879
CSRP2	rs1707652	32.36719303
CSRP2	rs391165	42.60415482
STAU2	rs10922098	150.6127066
STAU2	rs7080536	32.98635514
STAU2	rs11601665	33.34485501
STAU2	rs71366466	36.55251565
STAU2	rs1354034	103.1514685
STAU2	rs1355538	33.89180757
STAU2	rs686250	40.89910317
KRT20	rs11650915	32.06718034
KRT20	rs4796667	49.80817153
KRT20	rs10922098	141.0678742
KRT20	rs368878614	35.48975394
KRT20	rs143746166	31.69996287
KRT20	rs632111	41.62095533
KRT20	rs1706435	31.19309025
KRT20	rs9501393	32.34902782
KRT20	rs9269314	41.52962895
KRT20	rs10235272	32.88901089
SORBS3	rs10922098	68.79718666
SORBS3	rs1355538	38.44498335
SORBS3	rs9501393	37.25787143
SORBS3	rs28471056	35.41595295
LIN7C	rs114009540	34.9359378
LIN7C	rs10922098	721.5260236
LIN7C	rs3900909	31.59455046
LIN7C	rs11569415	80.76505033
LIN7C	rs2686395	151.4926806
LIN7C	rs3099847	31.19410146
LIN7C	rs1265941	71.73411985
LIN7C	rs550671	192.8836231
LIN7C	rs9276606	29.80388244
CUL9	rs10922098	434.3905083
CUL9	rs12403425	31.2221978
CUL9	rs11569415	34.96970743
CUL9	rs1355538	79.84120579
CUL9	rs9267139	30.53637287
CUL9	rs28573154	106.300235
CUL9	rs7774739	71.43834681
CUL9	rs9271766	33.25872794

NPLOC4	rs1797081	75.03273756
NPLOC4	rs4980320	30.09290053
NPLOC4	rs74812261	52.75475715
LCN10	rs11208686	31.97652949
LCN10	rs10922018	35.71200429
LCN10	rs10801548	43.82525037
LCN10	rs1329424	1208.240378
LCN10	rs7513100	52.89424789
LCN10	rs1047286	41.79401952
LCN10	rs111696008	38.10407394
LCN10	rs1707652	251.3912918
LCN10	rs1265941	111.3709981
LCN10	rs550671	309.1172511
LCN10	rs112164276	40.56311439
LCN10	rs41548618	43.84815452
RPS10	rs72748420	45.74240085
RPS10	rs1329424	1277.24578
RPS10	rs3900909	65.80979588
RPS10	rs10801660	45.29044301
RPS10	rs1047286	32.15816487
RPS10	rs111696008	34.41478168
RPS10	rs1707652	253.4174146
RPS10	rs1265941	129.2512568
RPS10	rs550671	335.2854511
RPS10	rs112164276	50.00585107
RPS10	rs41548618	47.24811641
TP53I11	rs10889353	32.33264917
TP53I11	rs10922094	38.98683261
TP53I11	rs964184	178.9978742
TP53I11	rs141469619	39.8100626
TP53I11	rs1532085	45.57348985
TP53I11	rs261332	31.82797441
TP53I11	rs2289494	89.34329561
TP53I11	rs62120574	36.87469244
TP53I11	rs283815	930.3778545
TP53I11	rs143539524	124.2428395
TP53I11	rs8102957	48.39448445
TP53I11	rs66777718	56.50726981
TP53I11	rs2678379	36.78086347
TP53I11	rs780093	73.63790527
TP53I11	rs1707652	128.6190455
TP53I11	rs78810414	34.07360307
TP53I11	rs2954038	42.8261754
FRS2	rs10922098	267.9004629
FRS2	rs704	40.34817797
FRS2	rs1355538	78.05533527
FRS2	rs2075798	53.02901112
FRS2	rs453821	95.74234211
CBX7	rs28664709	77.72178764
CBX7	rs7080536	33.7365005
CBX7	rs11755689	33.88392465
BECN1	rs10922098	280.6607797
BECN1	rs1355538	91.81429156
BECN1	rs4293988	37.81271022
BECN1	rs9267139	32.81440582
BECN1	rs28573154	95.85024475
BECN1	rs7774739	83.20989169
BECN1	rs9271689	37.76276

PPP1R8	rs10922098	216.728547
PPP1R8	rs111696008	38.58529029
PPP1R8	rs483465	33.35112041
PPP1R8	rs11720167	289.5574345
PPP1R8	rs3863087	137.5694925
PPP1R8	rs9501600	55.98499779
TRAF4	rs10922098	98.50929511
TRAF4	rs114115200	61.8926699
TRAF4	rs9501600	40.00917702
KCNA10	rs10922098	355.2948605
KCNA10	rs79326407	31.69066653
KCNA10	rs2583264	519.1046259
KCNA10	rs7312381	46.8824214
KCNA10	rs111696008	33.69320853
KCNA10	rs6445035	186.0889069
KCNA10	rs3863087	69.73716878
KCNA10	rs9501393	110.5131566
KCNA10	rs7774739	67.01840423
KCNA10	rs9269467	34.76204621
ATAD2	rs10922094	250.6440123
ATAD2	rs550671	67.2562491
ATAD2	rs77089552	32.07430706
TSG101	rs1395319	296.2350037
TSG101	rs4980320	38.24957169
TSG101	rs10876550	36.64288166
TSG101	rs185009887	31.96999894
SLC6A14	rs10922094	126.1590561
SLC6A14	rs704	191.1676006
SLC6A14	rs1047286	33.17769696
SLC6A14	rs12478841	43.48858387
SLC6A14	rs7610437	49.66317609
SLC6A14	rs9856702	477.9027143
SLC6A14	rs9501393	59.25662605
PSD	rs1451915	34.12716828
PSD	rs10922098	885.3083161
PSD	rs2073333	321.7160259
PSD	rs704	99.38069344
PSD	rs1047286	34.49095337
PSD	rs199956232	33.59284537
PSD	rs429358	201.0982801
PSD	rs111696008	40.00025847
PSD	rs1707652	170.8101233
PSD	rs1265941	73.65343207
PSD	rs550671	215.3238876
SLC6A16	rs10922098	109.1416745
SLC6A16	rs4370937	30.39236308
SLC6A16	rs411337	52.27661416
SLC6A16	rs7756647	34.45099011
RFK	rs11144910	52.82508907
RFK	rs591411	194.3523157
RFK	rs10922098	464.9268227
RFK	rs11720167	61.99291152
RFK	rs3891366	151.6241897
RFK	rs7774739	95.38351575
RFK	rs28645249	59.08633522
RFK	rs116334170	34.47995378
EDC4	rs10922094	32.73075284
EDC4	rs964184	57.58341659

EDC4	rs75327431	30.23239117
EDC4	rs62087820	30.41891053
EDC4	rs5167	265.9362924
EDC4	rs62122484	149.0294625
EDC4	rs55881728	29.84215306
EDC4	rs111696008	63.84325484
EDC4	rs144202860	44.12455055
EDC4	rs2232613	31.32984592
EDC4	rs12485327	33.02335133
EDC4	rs6445035	521.9706867
EDC4	rs3863087	191.9258227
EDC4	rs148077724	44.21856255
EDC4	rs111266377	53.4234883
EDC4	rs139315015	34.31363816
PRKG1	rs10922094	119.5054012
PRKG1	rs78606665	83.10623932
PRKG1	rs4666000	40.68892145
PRKG1	rs1355538	70.54698181
PRKG1	rs147390813	37.38702174
PRKG1	rs4713498	44.49565822
PRKG1	rs2057681	124.5031482
CCS	rs498045	197.2355714
CCS	rs10922098	517.4660312
CCS	rs3900909	35.53627461
CCS	rs1047286	35.91582375
CCS	rs2686395	107.0342524
CCS	rs550671	162.9604703
CCS	rs9270570	32.93988945
CCS	rs9469195	59.83217219
ANP32A	rs10922094	302.0133203
ANP32A	rs2232613	70.5397108
ANP32A	rs1707652	163.0613561
ANP32A	rs6457457	61.74715812
ANP32A	rs28573154	88.00668397
FXR1	rs10922098	129.684822
FXR1	rs3847647	30.31647798
FXR1	rs10876550	33.17051806
FXR1	rs1354034	52.53238068
FXR1	rs1355538	42.91825774
FXR1	rs541862	53.73562926
CETN2	rs10922094	121.7622369
CETN2	rs4665383	39.07651694
CETN2	rs1619994	98.87521708
CETN2	rs9501393	50.70376506
CETN2	rs9272068	46.31796203
UNC45A	rs11417460	30.98400559
UNC45A	rs4980320	34.0843051
UNC45A	rs1354034	48.73030403
UNC45A	rs1355538	35.81900438
UNC45A	rs77265585	30.21081056
GLP1R	rs10922098	155.700595
GLP1R	rs9864801	35.87851641
GLP1R	rs6457457	40.58956519
GLP1R	rs9501393	46.792085
BTC	rs62316276	79.54874263
BTC	rs28549760	232.2967984
BTC	rs572515	44.36521466
HIF1A	rs10922094	81.35310858

HIF1A	rs9864801	32.25216197
HIF1A	rs2242572	33.58491806
S100A6	rs75742937	74.81038435
S100A6	rs76418562	73.30121187
S100A6	rs113724571	46.32286376
S100A6	rs77543908	127.2027425
S100A6	rs985242	114.485631
S100A6	rs79011663	29.80723019
S100A6	rs80322391	35.81387591
S100A6	rs6445035	121.10148
S100A6	rs3863087	30.8318165
S100A6	rs7776054	48.10065576
S100A6	rs4734879	32.18484458
SECTM1	rs4789763	518.18046
SECTM1	rs4073961	64.29577877
SECTM1	rs180677689	30.29006429
SECTM1	rs111912737	34.12561008
SECTM1	rs10922098	59.13967799
SECTM1	rs112070424	41.94730968
SECTM1	rs34953075	31.51079062
SECTM1	rs601338	413.8919875
SECTM1	rs4639017	38.6931518
SECTM1	rs9501393	38.51470671
SECTM1	rs67322126	29.74827379
SECTM1	rs600038	53.0588471
RSPO3	rs853974	84.74557946
RSPO3	rs9285458	709.9567795
RSPO3	rs1126605	99.41952004
RSPO3	rs14142	32.22967114
RSPO3	rs142707166	31.44201497
RSPO3	rs6932834	33.77334244
REG1A	rs146599142	141.4701807
REG1A	rs12990312	261.6561398
REG1A	rs76841471	1034.815227
REG1A	rs12999504	136.9420323
REG1A	rs116553900	118.2488621
REG1A	rs11598094	41.70654673
REG1A	rs7478186	441.4004059
REG1A	rs3013182	112.1250989
REG1A	rs80158569	33.81484114
REG1A	rs708686	134.5247914
REG1A	rs34953075	39.53236603
REG1A	rs492602	535.4750209
REG1A	rs114131585	34.85464352
REG1A	rs576123	212.2017705
BCL2L2	rs572515	241.5050072
BCL2L2	rs1707652	66.95557699
BCL2L2	rs2304456	72.26679819
BCL2L2	rs4861708	42.56239474
BCL2L2	rs6457457	48.75230441
BCL2L2	rs28573154	73.30286468
SOST	rs10922098	83.01687013
SOST	rs7294354	56.60746389
SOST	rs1051412	32.79398691
SOST	rs2669572	29.79399338
SOST	rs17138656	77.87592689
SOST	rs244730	37.54386822
SOST	rs28471056	30.74925872

SOST	rs10955898	30.49573624
SOST	rs1485307	113.6495256
FAM3D	rs1444188	88.3294751
FAM3D	rs12493131	898.1237487
FAM3D	rs12638242	34.39792043
FAM3D	rs708686	151.9757252
FAM3D	rs75940592	30.48855195
FAM3D	rs76321971	80.17993666
FAM3D	rs55941736	73.8781693
FAM3D	rs411482	1342.46092
FAM3D	rs80206894	191.4280912
FAM3D	rs9676447	52.12773798
FAM3D	rs146346242	45.05019691
FAM3D	rs4556017	69.49841976
FAM3D	rs375994438	1097.388447
FAM3D	rs176694	491.2150864
FAM3D	rs28446901	1014.932269
FAM3D	rs129902	54.98599185
FAM3D	rs117541350	66.802236
FAM3D	rs618439	42.0914221
FAM3D	rs139562936	49.64470734
SNAP25	rs10922098	209.3361877
SNAP25	rs145730801	974.7324619
SNAP25	rs1243160	80.61205697
SNAP25	rs61978278	32.99076804
SNAP25	rs111397271	39.9525492
SNAP25	rs12437339	35.14337593
SNAP25	rs56252683	64.15616299
SNAP25	rs11629238	34.28012765
SNAP25	rs1047286	31.56392414
SNAP25	rs1706435	67.05305333
SNAP25	rs9501393	61.6686384
SNAP25	rs7774739	39.19277933
LYPD3	rs35681564	197.4282166
LYPD3	rs10922098	348.5508796
LYPD3	rs11220477	32.32399796
LYPD3	rs8059597	38.07438725
LYPD3	rs62061425	30.41066893
LYPD3	rs276938	31.7729906
LYPD3	rs681343	68.11787124
LYPD3	rs1707652	66.38855983
LYPD3	rs9501600	87.66695625
LYPD3	rs635634	52.59726104
NEGR1	rs140320901	55.79702419
NEGR1	rs1026566	250.1690619
NEGR1	rs72704449	44.33503407
NEGR1	rs10922098	126.0329399
NEGR1	rs12280645	44.94752339
NEGR1	rs77924615	32.84572644
NEGR1	rs12945299	30.34613555
NEGR1	rs780094	47.66189626
NEGR1	rs2686395	58.26577784
NEGR1	rs35379448	33.40908197
NEGR1	rs17145750	30.38354236
BCL6	rs10922098	394.0845839
BCL6	rs704	40.92051744
BCL6	rs6445035	86.16983778
BCL6	rs3863087	30.87141737

BCL6	rs9267139	73.36126796
BCL6	rs444921	112.6194231
BCL6	rs75140942	43.63871175
FSTL1	rs9851825	85.67167845
FSTL1	rs1147707	315.203617
FSTL1	rs763665	102.0829932
FSTL1	rs12945299	37.00040308
FSTL1	rs77542162	34.49029241
FSTL1	rs574937	54.0319732
FSTL1	rs780094	38.36063736
FSTL1	rs10820967	36.50173017
SPP1	rs116237853	63.46723324
SPP1	rs9996125	56.32688567
SPP1	rs12132412	36.96293406
SPP1	rs10922094	140.2388736
SPP1	rs113497810	204.2887725
SPP1	rs1056523	554.4534604
SPP1	rs149063279	33.16124241
SPP1	rs181830063	88.20299141
SPP1	rs147390813	38.25127019
LUM	rs12309747	92.60133578
LUM	rs142086573	87.99606209
LUM	rs73210679	36.79544875
LUM	rs76912709	66.99379931
LUM	rs77751442	150.4433197
LUM	rs146369172	86.07028881
LUM	rs1803343	451.2556493
LUM	rs184515881	38.74158757
LUM	rs117789382	40.51065843
LUM	rs117801489	301.7204057
LUM	rs77262773	40.00883465
LUM	rs13081361	30.94218454
CD177	rs34137659	946.1511221
CD177	rs62113069	414.1268888
CD177	rs138605473	4564.424453
CD177	rs61995939	81.77089885
CD177	rs77043841	1251.315718
CD177	rs34577669	590.7487511
CD177	rs11668974	130.4738049
CD177	rs12973081	34.78589078
CD177	rs55734981	32.9966832
CD177	rs80142449	483.3370802
CD177	rs148595630	343.1286146
CD177	rs12974604	64.03565995
CD177	rs181069377	245.1329268
SMOC1	rs74277751	101.197373
SMOC1	rs1958078	418.2254764
SMOC1	rs227418	65.02272857
SMOC1	rs77542162	131.3859643
SERPINA10	rs10144067	37.50322751
SERPINA10	rs11621100	106.3777115
SERPINA10	rs10139196	36.41292243
SERPINA10	rs117955282	42.72706189
SERPINA10	rs34258981	971.9399435
SERPINA10	rs45505795	281.0116156
SERPINA10	rs150331426	1025.925433
SERPINA10	rs138179301	229.9425923
SERPINA10	rs76483535	33.52001166

SERPINA10	rs76605570	67.53752781
SERPINA10	rs116094872	182.9147228
SERPINA10	rs144634898	119.4366283
SERPINA10	rs12436634	58.57603624
SERPINA10	rs2255531	38.45586634
SERPINA10	rs746367	34.27771655
SERPINA10	rs72661955	198.9266306
SERPINA10	rs528218	419.2823898
SERPINA10	rs559054	5931.328578
SERPINA10	rs9604093	133.3027488
SERPINA10	rs4150746	55.72012196
SERPINA10	rs123698	31.66677794
SERPINA10	rs1260326	34.30459152
FLRT2	rs1667515	86.62709123
FLRT2	rs36101989	907.9859283
FLRT2	rs2747001	222.6001528
FLRT2	rs10689689	35.99218651
FLRT2	rs12881315	54.54816687
FLRT2	rs924204	35.46294314
FLRT2	rs3748770	213.6290052
FLRT2	rs150816167	47.76926223
FLRT2	rs17117780	41.02208878
FLRT2	rs77542162	64.83803337
FLRT2	rs780094	53.98979757
FLRT2	rs11473050	47.46949255
FLRT2	rs11908351	146.544507
FLRT2	rs13108218	33.14821226
FLRT3	rs6042044	66.30791371
FLRT3	rs56265150	954.0238785
FLRT3	rs117932517	324.2829271
FLRT3	rs4302276	62.59243201
FLRT3	rs11698358	4294.899668
FLRT3	rs142322639	514.8810896
FLRT3	rs144808273	418.3842482
FLRT3	rs149148521	518.4773195
FLRT3	rs145650062	181.1239531
FLRT3	rs138337674	1013.484157
FLRT3	rs117333363	48.21686995
FLRT3	rs12480498	131.5677054
FLRT3	rs2878349	47.90980944
FLRT3	rs1169288	204.8631574
FLRT3	rs1233749	73.64732833
FLRT3	rs201214	30.65683903
FLRT3	rs80173297	112.6003721
FLRT3	rs78473194	42.69806025
FLRT3	rs1800961	56.73546625
FLRT3	rs1279840	58.1959274
FLRT3	rs10818799	36.05340885
ISLR2	rs78619960	31.48533479
ISLR2	rs923118	499.8452114
ISLR2	rs874712	113.4387697
ISLR2	rs10922098	54.46696632
ISLR2	rs60843925	183.6599157
ISLR2	rs35257264	87.07164189
ISLR2	rs41280048	44.31512146
ISLR2	rs11552322	33.28534168
ISLR2	rs117633773	46.20939951
ISLR2	rs12945299	122.5159813

ISLR2	rs143501783	82.8130337
ISLR2	rs284663	46.34967967
ISLR2	rs143794981	35.03152506
ISLR2	rs117241334	53.21143222
ISLR2	rs111903631	32.21280272
ISLR2	rs2073926	104.8676859
ISLR2	rs12378537	1006.776984
ISLR2	rs75212266	54.58310793
ISLR2	rs590819	64.78402064
ISLR2	rs3124761	742.4342124
ISLR2	rs62572818	77.86091829
ISLR2	rs150730585	48.1111482
VTN	rs1382046	188.2925518
VTN	rs28999409	246.6512556
VTN	rs8082174	1034.813578
VTN	rs72847293	339.3334912
VTN	rs7219736	927.6717267
VTN	rs116963549	851.2004777
VTN	rs117581744	327.5741359
VTN	rs112266449	6965.391452
VTN	rs117974225	446.8471153
VTN	rs12132412	121.0799718
VTN	rs78247247	53.09667246
VTN	rs74628379	53.92845065
VTN	rs144361853	64.28780998
VTN	rs111649396	96.17118908
VTN	rs113963443	100.4710371
VTN	rs4247138	165.2477625
VTN	rs35596889	128.2970956
VTN	rs4392119	67.18434466
VTN	rs150882303	101.7093169
VTN	rs62079535	34.60360425
VTN	rs56210800	40.92516887
VTN	rs140431012	41.01157311
DSC2	rs1658125	359.3901347
DSC2	rs11664471	52.46749645
DSC2	rs117031963	31.33800396
DSC2	rs77924615	68.29748081
DSC2	rs9992101	38.45705111
DSC2	rs600038	31.61851657
HK1	rs144704969	30.41387642
HK1	rs10922098	908.5031207
HK1	rs3900909	40.82232176
HK1	rs1047286	38.22622787
HK1	rs2686395	150.8673035
HK1	rs1265941	87.92007602
HK1	rs444921	228.8354678
HK1	rs112164276	34.33003902
SEMA5A	rs11740334	44.83555542
SEMA5A	rs57582942	31.54293992
SEMA5A	rs75782831	62.23756549
SEMA5A	rs7734982	44.01150347
SEMA5A	rs148516956	106.4014644
SEMA5A	rs1805936	351.7990554
SEMA5A	rs116346391	1187.456227
SEMA5A	rs3798064	43.34894616
SEMA5A	rs72729191	20769.51085
SEMA5A	rs194089	148.8018631

SEMA5A	rs34534206	48.43416608
SEMA5A	rs2393986	34.63501034
SEMA5A	rs77262773	79.06053754
SEMA5A	rs113568066	40.13956349
SEMA5A	rs6745500	56.81724144
LTBP4	rs112009052	59.22900724
LTBP4	rs4657078	40.57677851
LTBP4	rs184924341	46.2228017
LTBP4	rs7952602	49.8910175
LTBP4	rs150179248	44.34615034
LTBP4	rs939629	32.134109
LTBP4	rs112725054	45.29856976
LTBP4	rs831428	37.36675338
LTBP4	rs201862321	62.74938175
LTBP4	rs4965561	31.1157906
LTBP4	rs55714927	96.40231551
MDM2	rs10922098	96.79512494
MDM2	rs550671	63.02264193
MDM2	rs34927613	38.67695052
MDM4	rs10922094	59.29891826
MDM4	rs148198842	31.12363551
MDM4	rs6457457	172.6757666
MDM4	rs148766553	189.6269006
MDM4	rs6458	345.9060183
MDM4	rs191795965	92.42074194
IGHG4	rs2472297	59.82675239
IGHG4	rs2867105	43.06511605
IGHG4	rs2106727	52.64997977
WNT3A	rs12132412	64.96246398
WNT3A	rs1591734	105.6276847
WNT3A	rs61808983	338.915441
WNT3A	rs2187955	257.0567824
WNT3A	rs10764161	96.43311952
WNT3A	rs10159712	257.8861716
WNT3A	rs10437425	44.84623701
WNT3A	rs11012153	36.36832905
WNT3A	rs429358	31.22507923
WNT3A	rs2117168	47.01146894
WNT3A	rs2274933	34.47939891
WNT3A	rs1265764	33.47520083
WNT3A	rs28366220	37.98334824
WNT3A	rs34958241	97.47285578
WNT3A	rs9271669	67.01945819
SHANK3	rs139948926	59.96056013
SHANK3	rs5771045	102.2196203
SHANK3	rs3213442	187.9407472
SHANK3	rs9616896	2130.590736
SHANK3	rs28689705	442.295497
SHANK3	rs11705273	95.17604247
SHANK3	rs7073753	51.96296085
SHANK3	rs7114094	48.05203216
SHANK3	rs11451044	36.81771438
SHANK3	rs74035509	36.24573239
SHANK3	rs2905426	31.55543489
SHANK3	rs1354034	34.53442446
SHANK3	rs2071805	32.24458351
SHANK3	rs576123	120.7747478
WNT5A	rs17123220	30.51774401

WNT5A	rs1168093	66.65378836
WNT5A	rs115365379	35.12361296
WNT5A	rs964184	422.2294842
WNT5A	rs141469619	54.10963566
WNT5A	rs150844304	46.59568346
WNT5A	rs261290	191.9523565
WNT5A	rs473224	68.3527905
WNT5A	rs3816117	310.5166186
WNT5A	rs34250990	33.07631824
WNT5A	rs116843064	57.95106638
WNT5A	rs5112	126.7837668
WNT5A	rs673548	83.72970003
WNT5A	rs780093	108.1948744
WNT5A	rs78058190	32.39688101
WNT5A	rs1154988	40.60646827
WNT5A	rs6445035	145.3535081
WNT5A	rs3863087	64.45713618
WNT5A	rs28731358	34.83896025
WNT5A	rs144503444	33.07105224
WNT5A	rs10096633	161.0369262
WNT5A	rs28601761	93.91641122
WNT5A	rs199650842	45.71713797
WNT5A	rs77391147	44.92741629
DEFB113	rs181774817	42.76727643
DEFB113	rs9501600	500.5814462
DEFB113	rs449186	81.29900826
DEFB113	rs3828785	68.29447742
DEFB113	rs9484759	110.9420775
DEFB113	rs150677950	31.10111236
DEFB113	rs9458012	33.73813329
CYB561D1	rs10922098	333.8315477
CYB561D1	rs2686395	87.62274611
CYB561D1	rs2242572	99.35233871
CYB561D1	rs9501602	51.07191061
CYB561D1	rs116881261	50.44286075
CYB561D1	rs75179845	111.779371
C5orf63	rs10922098	389.9697331
C5orf63	rs12478841	29.961687
C5orf63	rs139160896	40.75065759
C5orf63	rs1707652	78.75961575
C5orf63	rs550671	133.0819372
C5orf63	rs28645249	46.85463944
B4GALT1	rs78104081	34.47071459
B4GALT1	rs7019909	445.922661
B4GALT1	rs34007720	31.03075281
B4GALT1	rs61747728	85.57924767
B4GALT1	rs77924615	35.06890379
B4GALT1	rs12972279	42.96791635
B4GALT1	rs2191225	30.80371729
B4GALT1	rs28873836	600.331921
B4GALT1	rs4801902	71.16190947
B4GALT1	rs77042931	29.84598586
B4GALT1	rs116719962	46.96530378
B4GALT1	rs148451580	33.22477947
B4GALT1	rs73158188	35.39345748
B4GALT1	rs140678530	36.79881673
B4GALT1	rs635634	144.4878292
RNF34	rs10922098	293.6549109

RNF34	rs1706435	55.82709889
RNF34	rs9267139	30.07729423
RNF34	rs28573154	79.79282525
RNF34	rs7774739	64.18169907
EHF	rs10922098	55.57695178
EHF	rs58559635	32.43513275
EHF	rs116751091	39.72513846
EHF	rs67399078	57.0958214
PCSK1	rs10476544	68.19267916
PCSK1	rs144329796	134.8352506
PCSK1	rs76239650	2554.724891
PCSK1	rs6232	3343.252114
PCSK1	rs3811952	64.23292939
PCSK1	rs72772040	190.7884367
PCSK1	rs27373	200.5084035
PCSK1	rs6872684	31.23516377
PCSK1	rs309580	44.50393369
PCSK1	rs62364577	106.5069119
PCSK1	rs143213504	126.7228984
PCSK1	rs464906	243.2351478
PCSK1	rs10039569	49.95051697
PCSK1	rs152557	41.7264564
PCSK1	rs770184	42.33767226
ATP1B1	rs3956912	96.95142104
ATP1B1	rs79391862	83.45682563
ATP1B1	rs112779928	41.5040604
ATP1B1	rs1707652	61.99109894
ATP1B1	rs13144491	30.74946319
ATP1B1	rs148399718	152.177561
ATP1B1	rs4712818	215.2839535
ATP1B1	rs2744567	442.3585916
ATP1B1	rs2760180	295.9958665
ATP1B1	rs148550521	80.21220595
ATP1B1	rs1611593	34.81898107
ATP1B1	rs3132685	45.94838318
ATP1B1	rs2954021	33.18693607
DEFB4A	rs377283103	116.2425597
DEFB4A	rs6985641	222.567685
DEFB4A	rs112001035	59.1682513
DEFB4A	rs1541470	68.18614376
DEFB4A	rs12188300	69.9787518
DEFB4A	rs35789108	58.2159538
DEFB4A	rs9274130	45.29522566
DEFB4A	rs2269829	39.87819372
DEFB4A	rs74393288	151.2675306
DEFB4A	rs76923010	79.7619923
DEFB4A	rs35759534	40.35940551
DEFB4A	rs2684060	873.6191332
DEFB4A	rs185156723	39.62246286
DEFB4A	rs17122808	182.870651
RELL1	rs147653240	32.99799804
RELL1	rs16931960	61.79210566
RELL1	rs7487683	166.0107133
RELL1	rs12433460	36.04707897
RELL1	rs34562254	35.54543977
RELL1	rs232230	305.7192416
SPINK2	rs35535963	351.9703589
SPINK2	rs56215858	65.38009818

SPINK2	rs34393987	1018.551162
SPINK2	rs137942837	1000.325988
SPINK2	rs72637160	35.49730661
SPINK2	rs77262773	40.35461181
SPINK2	rs183229159	122.7871701
SPINK2	rs78740585	87.7121847
SPINK2	rs10982156	112.6238392
TMEM132D	rs117605016	34.38798002
TMEM132D	rs7309864	56.91922087
TMEM132D	rs74322448	105.3221801
TMEM132D	rs189863837	199.7041179
TMEM132D	rs55655444	1326.196874
TMEM132D	rs78705678	92.4205525
TMEM132D	rs2477642	90.02054722
TMEM132D	rs137927952	35.22041923
TMEM132D	rs35846994	38.67018914
TMEM132D	rs55974014	31.56551565
TMEM132D	rs2844572	31.00008051
TMEM132D	rs28600710	37.76622597
TMEM132D	rs635634	64.50414247
ENOX2	rs10922094	266.5900465
ENOX2	rs4784323	30.12237198
ENOX2	rs10208529	33.67845107
ENOX2	rs1706435	32.70299124
ENOX2	rs9501600	65.64610193
MAN1C1	rs34136392	36.58440882
MAN1C1	rs11247595	530.0713898
MAN1C1	rs333947	45.79381498
MAN1C1	rs61804164	65.43051203
MAN1C1	rs10752084	46.43613584
MAN1C1	rs34775765	68.63274743
MAN1C1	rs78705678	240.6209556
MAN1C1	rs2477642	290.0220299
MAN1C1	rs6589598	80.69565321
MAN1C1	rs143794981	40.48507724
MAN1C1	rs111903631	38.41852238
MAN1C1	rs28873836	500.8530413
MAN1C1	rs4801902	60.17748563
MAN1C1	rs12189420	30.73270387
MAN1C1	rs4704043	36.94416427
MAN1C1	rs9268485	32.41475384
MAN1C1	rs1126504	40.32104823
MAN1C1	rs10813948	57.38885513
WFDC10A	rs10922098	244.2961571
WFDC10A	rs2668202	43.84087887
WFDC10A	rs9267139	31.93670923
WFDC10A	rs453821	84.9757542
WFDC10A	rs6926251	52.2582443
SLC14A1	rs10922098	165.3286803
SLC14A1	rs10208529	30.59688636
SLC14A1	rs9864801	61.21520929
SLC14A1	rs550671	70.54771741
SLC14A1	rs28471056	36.32251597
PARVA	rs11022131	41.4412919
PARVA	rs4757383	104.1232244
PARVA	rs812426	37.85843474
PARVA	rs10822155	50.0942576
PARVA	rs342293	140.2655742

IL20RB	rs2270552	38.41442133
IL20RB	rs13379369	145.9514599
IL20RB	rs3761068	43.51401152
IL20RB	rs75492971	97.99160243
IL20RB	rs9271860	50.02692029
IL20RB	rs28414125	215.2958323
IL20RB	rs3129204	38.60402514
MAZ	rs469758	41.65637467
MAZ	rs9260042	83.69735441
MAZ	rs13203830	164.979249
MAZ	rs28374650	41.93838683
CHRD	rs34095724	115.2013651
CHRD	rs3761068	30.14516589
CHRD	rs373004254	118.5216376
CHRD	rs10947290	204.5092098
CHRD	rs17612845	149.4394299
CHRD	rs369900193	36.5185959
IQCF3	rs10922098	226.8382412
IQCF3	rs1355538	42.40859195
IQCF3	rs555007	42.86206368
IQCF3	rs550671	91.13485862
IQCF3	rs9272526	31.37615652
DIXDC1	rs10922094	147.6226401
DIXDC1	rs8106813	31.96263172
DIXDC1	rs11709525	32.6819473
DIXDC1	rs6445035	73.65194462
DIXDC1	rs3828879	30.96372673
DIXDC1	rs9501600	57.88607566
SPRN	rs10922094	30.58503661
SPRN	rs35837081	122.1875472
SPRN	rs8018815	30.11728739
SPRN	rs78111684	41.20418099
SPRN	rs2736894	38.27612898
SPRN	rs2073333	778.552541
SPRN	rs17090888	32.14215196
SPRN	rs2248747	47.42299948
SPRN	rs1355538	67.00737334
SPRN	rs4556105	59.4414087
USP8	rs62019074	86.95335648
USP8	rs4980320	39.64457912
USP8	rs10876550	45.98954483
USP8	rs185009887	31.86534513
USP8	rs78909033	325.4609604
USP8	rs1354034	80.71017826
USP8	rs342200	41.73929415
FNDC4	rs10922098	168.0109637
FNDC4	rs1706435	40.11246831
FNDC4	rs76359966	33.27051032
FNDC4	rs9271642	59.92916049
SMIM13	rs10922094	164.0623365
SMIM13	rs1355538	50.2851905
SMIM13	rs415620	60.57489881
SMIM13	rs7774739	52.63295634
SMIM13	rs188302747	38.48836211
MRPL33	rs10922094	55.893489
MRPL33	rs78114433	84.30302735
MRPL33	rs145115766	34.92118274
MRPL33	rs150331426	32.4524776

MRPL33	rs79247904	66.77770218
MRPL33	rs12590834	1112.128274
MRPL33	rs116922373	156.3561948
MRPL33	rs72706301	140.8880441
MRPL33	rs75285293	134.4131349
MRPL33	rs112963922	286.6238168
MRPL33	rs139421381	59.83022735
MRPL33	rs118168154	37.54353225
MRPL33	rs1355538	32.48298019
CHAD	rs28590506	34.40335995
CHAD	rs6679677	33.10364986
CHAD	rs61729512	35.26795336
CHAD	rs7305932	32.5076763
CHAD	rs9594738	132.0309081
CHAD	rs78667121	31.9647166
CHAD	rs56225285	35.71884027
CHAD	rs884205	38.64630003
CHAD	rs2361797	65.57528997
CHAD	rs219768	29.96290856
CHAD	rs11726170	44.35405711
CHAD	rs2055101	60.87209338
CHAD	rs635634	32.10280001
PXDN	rs77946397	33.35242697
PXDN	rs111613941	68.03737966
PXDN	rs73182757	260.2682773
PXDN	rs12621293	44.08496439
PXDN	rs77924615	39.27114128
PXDN	rs12945299	56.8007132
PXDN	rs368611	31.22415874
PXDN	rs11787310	30.86994028
RCAN1	rs41313005	52.29363734
RCAN1	rs10922094	106.7775413
RCAN1	rs72847562	59.62475352
RCAN1	rs1707652	104.3263924
RCAN1	rs57165260	65.62451197
RCAN1	rs451876	160.5774091
RCAN1	rs148766553	53.61181861
RCAN1	rs7774739	154.4832061
RCAN1	rs12378445	32.49391079
NEURL4	rs10922094	148.6315198
NEURL4	rs438811	91.57265699
NEURL4	rs5112	35.65186777
NEURL4	rs1706435	115.9333226
NEURL4	rs9501393	58.5446765
NEURL4	rs7774739	38.85530083
PTH1R	rs10922098	205.8885177
PTH1R	rs6542680	30.30662363
PTH1R	rs1706435	54.51949166
PTH1R	rs1056429	30.52844866
PTH1R	rs9501600	58.47108117
ITPKA	rs316617	75.19798396
ITPKA	rs572515	136.7023206
ITPKA	rs1355538	48.17256465
ITPKA	rs444921	43.98487283
LTB4R	rs12280510	43.41218104
LTB4R	rs11656427	38.35701694
LTB4R	rs75792246	48.78771417
LTB4R	rs7636726	36.65852622

LTB4R	rs12191772	139.9445373
LTB4R	rs342293	55.62132223
LTB4R	rs6993770	35.93260204
FAM171A2	rs10922098	85.11911263
FAM171A2	rs704	87.50655312
FAM171A2	rs12478841	29.71756582
FAM171A2	rs1706435	55.90869344
FAM171A2	rs9267139	32.57467692
FAM171A2	rs28573154	56.50888323
FAM171A2	rs7774739	49.67740319
TCEA2	rs6011275	287.2371883
TCEA2	rs10922094	80.04740739
TCEA2	rs1619994	33.87309831
TCEA2	rs9501393	30.3742922
CNOT1	rs4980320	32.56527054
CNOT1	rs7113761	42.45680534
CNOT1	rs11451044	32.14970472
COL1A1	rs10922098	110.8109211
COL1A1	rs9501393	40.61066496
COL1A1	rs7774739	30.34970995
TMEM87B	rs704	269.2686319
TMEM87B	rs13412535	61.90461363
TMEM87B	rs115329695	42.47115644
TMEM87B	rs139276089	90.61862658
ARFIP1	rs77450465	41.61435851
ARFIP1	rs56730233	36.23836287
ARFIP1	rs4619875	697.7771925
ARFIP1	rs4980320	44.56177053
ARFIP1	rs10876550	35.03037586
ARFIP1	rs1354034	77.95557627
PDE6D	rs1329424	111.0807399
PDE6D	rs4977155	55.43596098
PDE6D	rs4639017	36.60352757
PDE6D	rs453821	37.44340062
GRID1	rs10922098	155.5376313
GRID1	rs6445035	66.66193256
GRID1	rs3863087	32.05228626
GRID1	rs453821	52.50328767
GRID1	rs601020	32.28712934
GRID1	rs211161	30.57173855
HCAR2	rs424535	50.96904749
HCAR2	rs704	84.15691786
HCAR2	rs2230199	136.3709222
EIF3J	rs4980320	31.63123054
EIF3J	rs1354034	70.24050542
EIF3J	rs3748136	104.3765884
MIOX	rs10922098	62.72748387
MIOX	rs17580	1143.293728
MIOX	rs12437339	54.88467571
MIOX	rs147863506	41.42405295
MIOX	rs2232613	35.19689613
MIOX	rs9501393	32.19436648
F8	rs11603123	79.94514237
F8	rs2362482	122.339874
F8	rs2283335	237.4131899
F8	rs11111630	48.24084643
F8	rs78004438	34.79864202
F8	rs4981022	70.12634413

F8	rs188547348	38.43675864
F8	rs2277998	38.58513576
F8	rs9751083	32.95465875
F8	rs4703589	35.42186397
F8	rs9271610	39.90279374
F8	rs6928919	34.18743876
F8	rs11243569	33.68713382
F8	rs306535	37.93361996
F8	rs146437644	47.48212352
F8	rs10901252	961.005178
F8	rs3124761	455.7111475
F8	rs139562936	36.7352258
SLC35G2	rs1697422	44.19880781
SLC35G2	rs112613420	32.32229743
SLC35G2	rs10775412	102.6341126
SLC35G2	rs7222245	216.8499621
SLC35G2	rs8068072	919.8724736
SLC35G2	rs12941530	47.96158606
SLC35G2	rs56147019	49.52543556
SLC35G2	rs3760457	92.90010405
SLC35G2	rs11869913	38.5866827
SLC35G2	rs12478841	41.31086644
SLC35G2	rs2274933	34.70917994
SLC35G2	rs56210800	93.90978499
SLC35G2	rs140431012	93.80840872
SLC35G2	rs2228243	322.1127108
SLC35G2	rs941757	53.15157312
SLC26A11	rs10922098	392.6362345
SLC26A11	rs1555405	29.79806905
SLC26A11	rs704	94.7187998
SLC26A11	rs1707652	131.1058492
SLC26A11	rs1265941	43.31504999
SLC26A11	rs453821	113.1197968
DUS2	rs10922098	621.7206653
DUS2	rs3900909	31.46386268
DUS2	rs1047286	57.28510004
DUS2	rs2686395	98.78483554
DUS2	rs555007	77.16492941
DUS2	rs550671	160.0420614
DUS2	rs73726194	35.76187635
TRADD	rs1451915	31.2808835
TRADD	rs10922098	492.7169077
TRADD	rs704	55.37729709
TRADD	rs1047286	36.41402622
TRADD	rs71352239	43.8582342
TRADD	rs111696008	36.36405115
TRADD	rs1707652	215.9420217
TRADD	rs1265941	59.51224757
TRADD	rs453821	137.0328778
SCAMP5	rs10922094	218.179165
SCAMP5	rs1706435	52.15359246
SCAMP5	rs9267139	30.35686408
SCAMP5	rs9501393	76.66438862
SCAMP5	rs7774739	53.00061531
SCAMP5	rs9271766	32.18738115
ATP2A3	rs10922098	146.364486
ATP2A3	rs2232710	109.7403359
ATP2A3	rs147892697	34.14836531

ATP2A3	rs704	38.33904272
ATP2A3	rs6065906	151.6913995
ATP2A3	rs1355538	50.17877018
ATP2A3	rs550671	50.13827687
ATP2A3	rs28675927	35.89000945
HNRNPR	rs10922094	182.7593198
HNRNPR	rs1707652	78.79408104
HNRNPR	rs9501600	56.11876195
HNRNPR	rs557011	30.91634681
PIAS3	rs10922094	105.2337813
PIAS3	rs1355538	30.32764894
PIAS3	rs9501393	41.67226686
RAB35	rs144704969	31.69067416
RAB35	rs10922094	898.3230655
RAB35	rs3900909	49.76370214
RAB35	rs704	360.546887
RAB35	rs111696008	31.20449243
RAB35	rs1707652	171.4140942
RAB35	rs3099847	35.52817358
RAB35	rs1265941	101.3770805
RAB35	rs444921	252.9153372
RAB35	rs116334170	43.11272476
RPRD1A	rs10922094	69.09426375
RPRD1A	rs3751343	33.12623593
RPRD1A	rs9534312	177.8961427
RPRD1A	rs4665985	30.09909365
RPRD1A	rs557011	37.00523632
EMC4	rs411530	40.07691671
EMC4	rs147233090	108.3547944
EMC4	rs61690552	41.65961761
EMC4	rs374067329	61.68378366
EMC4	rs348356	74.36522455
EMC4	rs58668331	53.24271997
EMC4	rs11569538	2550.958793
EMC4	rs402756	645.7272379
EMC4	rs163915	151.547816
EMC4	rs1065853	239.4029811
EMC4	rs144585235	233.8443202
EMC4	rs114879139	30.08668247
EMC4	rs114382848	212.7112104
EMC4	rs112668737	32.4353428
PTCHD3	rs10922098	131.0929414
PTCHD3	rs697356	34.27037295
PTCHD3	rs9469077	30.35771975
ASAP2	rs1914886	56.34344339
ASAP2	rs117292610	36.8416847
ASAP2	rs2289567	79.19802703
ASAP2	rs11272957	1234.312466
ASAP2	rs80237247	52.05535981
ASAP2	rs1160610	39.86210855
ASAP2	rs78912960	41.83748057
ARHGEF25	rs10437954	214.5511415
ARHGEF25	rs4237845	35.19795016
ARHGEF25	rs10922098	131.5376679
ARHGEF25	rs1706435	40.44830602
ARHGEF25	rs9501393	35.35531112
ARHGEF25	rs7774739	30.952009
HS6ST2	rs146326139	47.64893399

HS6ST2	rs1860018	30.50343362
HS6ST2	rs704	44.66469641
HS6ST2	rs1260326	53.58727765
HS6ST2	rs7605890	40.27503402
HS6ST2	rs28365897	57.37580105
HS6ST2	rs17145750	50.72505324
HS6ST2	rs28601761	35.40955202
KCNG4	rs10922098	651.2793432
KCNG4	rs7084817	49.99400776
KCNG4	rs1047286	37.49298126
KCNG4	rs1707652	113.5302003
KCNG4	rs4293988	31.17797361
KCNG4	rs9267139	40.31014585
KCNG4	rs28573154	144.5973337
KCNG4	rs7774739	88.37560673
KCNG4	rs9271766	41.91115945
NAP1L2	rs964184	58.69457908
NAP1L2	rs56156922	32.47746781
NAP1L2	rs704	113.4022536
NAP1L2	rs1065853	35.851882
NAP1L2	rs12478841	36.86862618
NAP1L2	rs111696008	33.15170196
NAP1L2	rs1706435	154.6725917
UTS2R	rs10922098	94.78984
UTS2R	rs1974469	51.07331212
UTS2R	rs9501393	33.27580888
UTS2R	rs7774739	31.17916526
CNEP1R1	rs10922098	77.33333801
CNEP1R1	rs1355538	37.96106395
CNEP1R1	rs9501393	31.33890919
MYOM2	rs73184720	35.32979796
MYOM2	rs79012673	30.18198969
MYOM2	rs74455617	73.23455973
MYOM2	rs17617337	37.76846033
MYOM2	rs10833712	132.5644032
MYOM2	rs7318906	76.42239989
MYOM2	rs7211380	40.77144193
MYOM2	rs1799938	51.31552362
MYOM2	rs12463674	41.65763702
MYOM2	rs12473286	30.20115714
POLI	rs117050326	73.38691387
POLI	rs572515	36.82464831
POLI	rs1355538	61.45775028
POLI	rs9268581	37.39197967
KCNN1	rs10922098	638.4602103
KCNN1	rs3900909	36.76870805
KCNN1	rs1047286	55.50455968
KCNN1	rs1707652	125.0349696
KCNN1	rs555007	69.19751363
KCNN1	rs550671	178.5771219
KCNN1	rs9272526	37.45852092
GPR26	rs10922098	521.8590204
GPR26	rs1047286	37.72018856
GPR26	rs1707652	112.1995732
GPR26	rs1265941	55.43744172
GPR26	rs444921	137.0010972
MYCT1	rs572515	290.4445914
MYCT1	rs6542680	39.51218077

MYCT1	rs1706435	60.09795853
MYCT1	rs3891366	68.25636515
MYCT1	rs7774739	55.94767627
MYCT1	rs28645249	35.40269715
EIF1AD	rs72930984	534.3926821
EIF1AD	rs112856902	37.37322204
EIF1AD	rs607969	34.89994827
EIF1AD	rs1354034	60.17312004
EIF1AD	rs342200	30.91646403
ADAM29	rs10922098	162.7525419
ADAM29	rs1706435	44.51771734
ADAM29	rs415620	44.8046932
ADAM29	rs4713498	49.28987365
SWAP70	rs149150635	169.6676209
SWAP70	rs148133668	74.06233046
SWAP70	rs74736715	921.3516367
SWAP70	rs7948799	947.5854525
SWAP70	rs148650635	44.92525478
SWAP70	rs2252446	1365.936261
SWAP70	rs117295257	55.0566352
SWAP70	rs4910079	544.2831231
SWAP70	rs11607830	78.88693171
SWAP70	rs75964416	143.3951428
SWAP70	rs117697737	48.01089926
SWAP70	rs78548950	99.98068694
SWAP70	rs12377604	41.31633656
REPIN1	rs1329424	312.7969509
REPIN1	rs111696008	33.17804483
REPIN1	rs1355538	56.54600058
REPIN1	rs7774739	45.33481069
REPIN1	rs7742632	54.02573269
HTR6	rs7514261	50.84022225
HTR6	rs704	223.2140362
HTR6	rs28455809	31.43467441
ACBD7	rs4748140	32.88725392
ACBD7	rs572515	80.09529956
ACBD7	rs3917538	30.57758826
RBL2	rs73089364	38.33770562
RBL2	rs6780601	46.62159203
RBL2	rs62260770	160.5846017
RBL2	rs6802320	943.448304
RBL2	rs115063752	90.86759857
RBL2	rs6764116	80.50968742
RBL2	rs2087597	34.25337436
RBL2	rs4679256	968.1536034
RBL2	rs5852582	35.13512169
RBL2	rs62332415	36.0377736
RBL2	rs71595446	33.75097571
RBL2	rs1495741	58.53581666
RBL2	rs112875651	36.1119906
SSU72	rs13102801	51.95265902
SSU72	rs3775114	1185.071068
SSU72	rs73188365	50.84662614
SSU72	rs190152535	134.8316575
SSU72	rs115642734	119.1416179
SSU72	rs114959044	54.84206388
SSU72	rs3135900	47.42675167
SSU72	rs4646968	34.50467088

PSMD4	rs572515	39.7459736
PSMD4	rs3847647	31.48862984
PSMD4	rs1706435	39.86398745
HDLBP	rs4980320	41.31736825
HDLBP	rs10876550	30.31117952
HDLBP	rs1354034	85.98593457
PSMD11	rs4980320	37.65389109
PSMD11	rs7114094	49.34876017
PSMD11	rs185009887	34.51857951
PSMD11	rs7009435	37.93970781
ARPC3	rs10922094	184.9468452
ARPC3	rs1706435	58.80867299
ARPC3	rs7774739	49.89162088
ARPC3	rs7742632	66.01764085
RND1	rs10922098	193.6649743
RND1	rs111696008	36.46780229
RND1	rs11720167	87.15626286
RND1	rs9267139	40.59006815
RND1	rs550671	120.585984
RND1	rs6926251	38.23554279
DVL2	rs12058824	55.35115726
DVL2	rs10896631	161.3960445
DVL2	rs7948571	65.84277691
DVL2	rs113448098	373.1821104
DVL2	rs7962629	1111.979888
DVL2	rs145271606	33.8307652
DVL2	rs8010223	42.9340538
DVL2	rs11849582	378.6188834
DVL2	rs140391681	71.00327745
DVL2	rs3130955	32.65121814
UAP1	rs28384482	37.51714861
UAP1	rs10922094	34.07447738
UAP1	rs9567611	91.38275704
RACGAP1	rs28362944	57.02274033
RACGAP1	rs12146727	45.87660814
RACGAP1	rs10138174	39.28146504
RACGAP1	rs9348623	64.77850493
RACGAP1	rs187193008	37.98032894
RACGAP1	rs114916085	90.75604684
RACGAP1	rs9468618	46.24933791
RACGAP1	rs1264341	1034.892455
RACGAP1	rs3132444	969.2881738
RACGAP1	rs41316148	184.6399036
RACGAP1	rs182953657	861.1638299
RACGAP1	rs115490327	72.55083574
RACGAP1	rs9276980	135.2684744
RACGAP1	rs112393700	50.68145099
RACGAP1	rs35681945	343.3717008
RACGAP1	rs148263928	38.75353494
ANXA9	rs1329424	120.5566704
ANXA9	rs697356	31.30845078
ANXA9	rs28573154	44.40852402
ANXA9	rs41547217	34.15419235
APBB3	rs10922098	38.35707833
APBB3	rs28479572	32.61393041
APBB3	rs704	32.51952284
APBB3	rs1707654	66.8663441
REXO2	rs28679554	162.947041

REXO2	rs7073753	31.66927852
REXO2	rs1354034	101.0888298
PRIM1	rs10922098	160.4610094
PRIM1	rs9864801	45.15886009
PRIM1	rs9501393	31.87569734
THTPA	rs10922098	125.7341457
THTPA	rs6445035	206.0770804
THTPA	rs3863087	51.41601954
THTPA	rs12529130	37.2721343
THTPA	rs3891366	88.42360545
THTPA	rs7774739	97.24301331
RAB27B	rs10922098	408.8985114
RAB27B	rs1555405	31.00434866
RAB27B	rs1706435	85.94137647
RAB27B	rs453821	115.5573822
RAB27B	rs28471056	55.11670968
RAB31	rs4570933	96.42630268
RAB31	rs12607866	206.7612983
RAB31	rs17580	61.32618769
RAB31	rs11699178	67.33617198
RAB31	rs4811994	51.69233464
RABEPK	rs114009540	30.37954407
RABEPK	rs10922098	743.2093974
RABEPK	rs35351085	37.22566242
RABEPK	rs12146727	84.39925988
RABEPK	rs704	180.1348046
RABEPK	rs1047286	37.21482821
RABEPK	rs1707652	159.5261268
RABEPK	rs1265941	91.31925745
RABEPK	rs550671	226.9169073
RABEPK	rs9269234	35.51466717
RFESD	rs75044039	57.00874537
RFESD	rs35837081	38.39701147
RFESD	rs17580	38.40698306
RFESD	rs1005364	356.2543654
RFESD	rs1698237	42.82808875
NEURL1	rs10922098	553.7039239
NEURL1	rs1047286	31.34000714
NEURL1	rs1707652	149.3616637
NEURL1	rs9266729	31.85420116
NEURL1	rs555007	92.17748681
NEURL1	rs550671	183.9689797
NEURL1	rs9276606	42.14619917
ANXA10	rs10922098	247.8792882
ANXA10	rs1555405	29.95182485
ANXA10	rs1706435	53.44106003
ANXA10	rs550671	82.98813454
ANXA10	rs28675927	47.03404155
MAGEA10	rs10922094	126.7649458
MAGEA10	rs1574186	299.6262171
MAGEA10	rs11251695	521.1702893
MAGEA10	rs2388595	656.7279749
MAGEA10	rs1707652	84.49060455
MAGEA10	rs550671	77.90339892
MAGEA10	rs9469195	30.55504049
AK5	rs572515	358.6480133
AK5	rs58068569	31.30450584
AK5	rs1355538	47.01382454

AK5	rs9267139	35.12795863
AK5	rs28573154	81.0729361
AK5	rs7774739	55.34906108
CREBBP	rs10922098	42.55089026
CREBBP	rs56094641	33.06717422
CREBBP	rs704	72.71267379
CREBBP	rs829502	60.79262286
CREBBP	rs6776319	31.68414989
CREBBP	rs752721	58.59798402
CREBBP	rs6864541	156.1006078
CREBBP	rs62358178	308.8951373
CREBBP	rs62358228	39.36375283
CREBBP	rs190444444	79.61889853
CREBBP	rs62359169	33.72592038
CREBBP	rs16876270	55.38736107
CREBBP	rs3129773	36.33441664
MAD1L1	rs10922098	355.6497134
MAD1L1	rs113747960	30.76079804
MAD1L1	rs704	34.58257963
MAD1L1	rs1707652	75.38755361
MAD1L1	rs2242572	92.63170079
MAD1L1	rs9501602	44.21791775
AP2A2	rs7482118	55.32243723
AP2A2	rs3811444	61.09599036
AP2A2	rs185009887	31.61927728
AP2A2	rs1354034	51.33312566
PPP2R5A	rs11119862	49.06304563
PPP2R5A	rs4980320	29.95008631
PPP2R5A	rs1354034	38.44891685
NADK	rs4648629	233.7060564
NADK	rs142834130	33.01005816
NADK	rs7488360	36.16356273
NADK	rs11611373	38.10329667
NADK	rs61748920	68.99121253
NADK	rs35940802	37.40729183
GRB14	rs10922098	76.09572051
GRB14	rs964184	33.22419703
GRB14	rs75327431	30.62981024
GRB14	rs704	37.85600828
GRB14	rs71352239	45.01702937
GRB14	rs10208529	31.18006334
GRB14	rs1707652	111.3058794
GRB14	rs11755689	32.97969732
ATG4B	rs10138174	80.327976
ATG4B	rs190627972	46.33045437
ATG4B	rs41556615	36.14149361
ATG4B	rs34665982	56.61807067
ATG4B	rs4713555	64.74498811
RBBP5	rs10922098	219.2764607
RBBP5	rs1355538	46.4620139
RBBP5	rs9501393	56.77720294
RBBP5	rs7774739	40.72786031
RBBP5	rs9271766	31.3616669
ZYX	rs12539742	78.39286978
ZYX	rs35340377	30.86244169
ZYX	rs4980320	34.67672867
ZYX	rs342200	41.34664869
PIR	rs9590923	54.23309592

PIR	rs7331865	104.9648542
PIR	rs2806920	3130.911857
PIR	rs74799068	31.45091717
PIR	rs146091138	39.73146798
PIR	rs4734879	35.99551953
USO1	rs4980320	38.97341975
USO1	rs1354034	66.20943818
USO1	rs6993770	46.84075601
IFIT3	rs10509571	101.4027757
IFIT3	rs4639017	30.22093256
IFIT3	rs13153461	79.03144828
DHX38	rs10922098	584.08403
DHX38	rs17095637	33.2368676
DHX38	rs1047286	39.57266213
DHX38	rs1707652	93.0826146
DHX38	rs9501600	133.3820827
DHX38	rs72508422	41.85877438
DHX38	rs587495	38.86416077
KCNIP1	rs75215871	30.63576529
KCNIP1	rs10922094	734.4209652
KCNIP1	rs3900909	40.48112628
KCNIP1	rs12366520	47.0044624
KCNIP1	rs704	54.70981456
KCNIP1	rs1047286	53.47187377
KCNIP1	rs2686395	184.2964668
KCNIP1	rs555007	75.150902
KCNIP1	rs550671	218.7810555
KCNIP1	rs9272526	39.80798563
TOM1L1	rs10822186	37.19283547
TOM1L1	rs964184	49.38440609
TOM1L1	rs1926447	82.09911211
TOM1L1	rs438811	145.6387477
TOM1L1	rs5112	53.84457944
TOM1L1	rs10208529	35.40258081
TOM1L1	rs1355538	56.16031268
TOM1L1	rs6993770	41.30433323
MACROD1	rs10922098	699.5009433
MACROD1	rs704	53.84932255
MACROD1	rs1047286	39.46331833
MACROD1	rs1707652	197.528218
MACROD1	rs3099847	30.81570886
MACROD1	rs1265941	75.75795369
MACROD1	rs550671	208.1539244
MACROD1	rs9276606	39.21001066
ROCK2	rs4980320	57.35510277
ROCK2	rs1354034	44.76108141
ROCK2	rs342292	29.86471624
PNKP	rs2290774	153.8637436
PNKP	rs1354034	35.7601222
PNKP	rs16873418	33.17121064
PDGFC	rs10922098	534.4523343
PDGFC	rs11720167	94.38511956
PDGFC	rs3863087	30.97963554
PDGFC	rs2075798	47.49864292
PDGFC	rs444921	165.6830013
ATP13A1	rs17315646	91.80685343
ATP13A1	rs115881877	59.73223469
ATP13A1	rs80246156	63.03931909

ATP13A1	rs138142760	44.00164571
ATP13A1	rs113567935	109.7867682
ATP13A1	rs75097760	255.9414314
ATP13A1	rs74887469	61.48722075
ATP13A1	rs75453795	46.86018567
ATP13A1	rs147408433	33.55145915
ATP13A1	rs12509937	38.42368654
ATP13A1	rs1801020	105.3703229
PPP2R3A	rs114009540	40.88475488
PPP2R3A	rs10801548	69.42337151
PPP2R3A	rs1329424	938.6328269
PPP2R3A	rs190548044	30.75007225
PPP2R3A	rs35351085	40.38011153
PPP2R3A	rs1047286	41.89889108
PPP2R3A	rs550671	80.94187872
PPP2R3A	rs34927613	43.43209906
CA10	rs2106331	97.58666017
CA10	rs1263963	48.11206762
CA10	rs2938140	287.3738892
CA10	rs117399000	477.7558198
CA10	rs6672420	51.41607259
CA10	rs10157710	37.0082731
CA10	rs4246520	46.10514739
CA10	rs7528419	1022.936465
CA10	rs7917848	71.86676083
CA10	rs1250565	62.21720147
CA10	rs669	31.80487222
CA10	rs12945886	36.501717
CA10	rs79811137	50.85606158
CA10	rs10420053	37.77800099
CA10	rs57880821	35.2940588
CA10	rs10060626	77.66973719
CA10	rs7756288	40.21843546
CA10	rs17879496	133.9084355
CA10	rs9281300	38.01455195
FGFR3	rs112869746	31.02190102
FGFR3	rs2403274	345.3976023
FGFR3	rs3135835	152.1586501
FGFR3	rs114889926	48.59298252
FGFR3	rs72704449	47.27590409
FGFR3	rs17329885	46.03871042
FGFR3	rs600038	31.72097113
ELANE	rs351970	216.8413055
ELANE	rs10409474	1330.855679
ELANE	rs17216649	874.5088309
ELANE	rs78914045	150.5665203
ELANE	rs186642113	37.96398579
ELANE	rs141548740	47.60589038
ELANE	rs76428106	30.61366256
ELANE	rs12903325	57.80516903
ELANE	rs3830847	101.4173127
ELANE	rs7502514	59.02149103
ELANE	rs145870849	40.52944739
ELANE	rs13382133	34.13123927
ELANE	rs56185965	55.81366583
ELANE	rs6803805	40.95125948
ELANE	rs144317085	29.7475362
ELANE	rs9264681	49.26516058

ELANE	rs6605578	46.96166068
ELANE	rs2380606	44.90046799
ELANE	rs116855556	59.16118878
HSPA6	rs10922098	504.5160251
HSPA6	rs704	120.1887229
HSPA6	rs6445035	115.8879693
HSPA6	rs3863087	45.82775891
HSPA6	rs9267139	44.19055646
HSPA6	rs9501393	146.2065792
HSPA6	rs7774739	98.23931784
HSPA6	rs9271766	56.78532707
CCT7	rs2077612	33.61924813
CCT7	rs111696008	48.4818853
CCT7	rs6445035	293.8897241
CCT7	rs3863087	110.4863793
INHBB	rs17608972	37.69547112
INHBB	rs1880544	100.4129921
INHBB	rs28416518	41.18104161
INHBB	rs2311597	1046.881241
INHBB	rs117325986	52.08038448
INHBB	rs3741414	534.8141461
INHBB	rs374087027	46.46308606
INHBB	rs11868058	62.63994765
INHBB	rs148663274	66.20525476
INHBB	rs4738679	61.49746183
INHBB	rs10102041	31.4754344
CSNK2A2	rs1329424	521.7108929
CSNK2A2	rs1047286	31.355338
CSNK2A2	rs1707652	107.3668541
CSNK2A2	rs1265941	66.70046313
CSNK2A2	rs550671	167.3819261
CSF1R	rs2237084	169.8534135
CSF1R	rs7736526	66.80711954
CSF1R	rs79412885	92.6118836
CSF1R	rs34856868	41.82279784
CSF1R	rs73021423	99.903401
CSF1R	rs7945585	41.06890644
CSF1R	rs58628777	33.19989008
CSF1R	rs3184504	101.9697272
CSF1R	rs2461475	45.60291962
CSF1R	rs117902156	49.73426049
CSF1R	rs55714927	303.0473844
CSF1R	rs112723993	53.58297143
CSF1R	rs143501783	91.40200458
CSF1R	rs12453125	36.02353868
CSF1R	rs9892550	50.53203084
CSF1R	rs681343	68.01013414
CSF1R	rs62165726	233.3742014
CSF1R	rs35217204	37.91852656
CSF1R	rs3747207	55.84017842
CSF1R	rs1841542	109.9942825
CSF1R	rs13071075	103.7025454
CSF1R	rs1383910	288.7880949
CSF1R	rs11920688	127.4214372
CSF1R	rs7650880	33.81075043
CSF1R	rs3097671	41.43691542
CSF1R	rs13209376	42.80154278
CSF1R	rs7865362	124.0776117

CSF1R	rs635634	58.97627194
IL5RA	rs60095187	47.85336468
IL5RA	rs1391927	80.93033715
IL5RA	rs340829	482.5870842
IL5RA	rs7635810	1045.789073
IL5RA	rs6766682	82.61367444
IL5RA	rs9831674	610.4310935
IL5RA	rs16830188	60.14073441
IL5RA	rs12887521	42.31358603
IL5RA	rs885107	47.55973611
IL5RA	rs9901675	36.2904443
IL5RA	rs34562254	155.6852143
IL5RA	rs2872507	90.61193226
IL5RA	rs1005980	86.0262035
IL5RA	rs62127753	32.02424761
IL5RA	rs139015452	38.00708885
IL5RA	rs9858020	53.59088585
IL5RA	rs980455	33.76675194
IL5RA	rs62328135	48.60534991
IL5RA	rs12521868	41.7805001
IL5RA	rs3129015	38.30001633
IL5RA	rs12526858	45.41027183
IL5RA	rs13195237	108.4642609
IL5RA	rs72492324	70.95883007
IL5RA	rs7766960	35.11358196
IL5RA	rs4133115	33.92128055
IL5RA	rs7758383	45.06835087
IL5RA	rs6421315	61.34239094
IL5RA	rs113745074	271.4963443
IL5RA	rs56006671	56.41777342
IL5RA	rs2511737	64.57514478
IL5RA	rs1999148	34.94521495
ODC1	rs10922098	215.896372
ODC1	rs11036181	30.5793029
ODC1	rs12791308	43.29833909
ODC1	rs17242778	31.68433998
ODC1	rs16914574	85.37750988
ODC1	rs117983753	51.86508015
ODC1	rs143666677	167.5544482
ODC1	rs8078686	32.35159136
ODC1	rs13019325	47.74638056
ODC1	rs1707652	123.5061536
ODC1	rs1265941	55.44371969
ODC1	rs550671	141.8854335
SLC5A8	rs11591147	52.08306684
SLC5A8	rs7534572	32.29283435
SLC5A8	rs660240	105.9889103
SLC5A8	rs12026141	35.57450799
SLC5A8	rs964184	175.3713248
SLC5A8	rs261290	74.74785441
SLC5A8	rs473224	36.85268747
SLC5A8	rs3764261	140.7830482
SLC5A8	rs73013176	30.16351927
SLC5A8	rs8106503	83.31282585
SLC5A8	rs7412	295.5629007
SLC5A8	rs2288912	44.12785506
SLC5A8	rs11897611	38.91380883
SLC5A8	rs673548	459.21902

SLC5A8	rs74717300	40.71614869
SLC5A8	rs780093	33.37910354
SLC5A8	rs111696008	77.65635189
SLC5A8	rs144202860	40.53172298
SLC5A8	rs78058190	41.73599383
SLC5A8	rs483465	53.98359883
SLC5A8	rs62290472	48.04779222
SLC5A8	rs6445035	500.0200974
SLC5A8	rs3863087	202.8660078
SLC5A8	rs148077724	70.65881577
SLC5A8	rs73169103	39.34237514
SLC5A8	rs1794493	30.21526508
SLC5A8	rs10096633	94.17063559
SLC5A8	rs112875651	51.40766771
KIAA0319L	rs10922098	305.7265022
KIAA0319L	rs1707652	74.19103367
KIAA0319L	rs9267139	31.6393925
KIAA0319L	rs9501393	93.54186971
KIAA0319L	rs7774739	65.63528706
KIAA0319L	rs9271766	43.44564428
ANXA2	rs77538017	206.9831085
ANXA2	rs1912261	109.2889142
ANXA2	rs3743268	2467.600299
ANXA2	rs79120050	294.5580871
ANXA2	rs103947	111.2235612
ANXA2	rs16977653	30.26868331
ANXA2	rs114694170	45.21067374
ANXA2	rs342293	53.45195717
ANXA2	rs72759453	34.27226256
HMGCS2	rs10923900	45.20862892
HMGCS2	rs7519043	34.84633854
HMGCS2	rs76794363	47.2470689
HMGCS2	rs4503880	39.83502173
HMGCS2	rs67613802	43.3583696
CLTC	rs10922098	287.4724325
CLTC	rs1706435	86.83139835
CLTC	rs453821	70.17728991
CLTC	rs28471056	34.34409607
SEC22A	rs7114094	77.94767225
SEC22A	rs1354034	53.57201963
SEC22A	rs1355538	29.72801215
FCN2	rs35027349	50.1513782
FCN2	rs11103428	74.90477744
FCN2	rs117676361	78.81729606
FCN2	rs11103545	1020.9501
FCN2	rs183486037	5018.497124
FCN2	rs113909851	233.467615
FCN2	rs143506788	251.9650227
FCN2	rs7028584	121.6650506
FCN2	rs146595262	36.07396054
FCN2	rs72550870	264.1080686
FCN2	rs6684216	40.31374556
FCN2	rs7951844	973.6411964
FCN2	rs10891719	117.9453315
FCN2	rs117546606	68.42183669
FCN2	rs139801914	54.98325201
FCN2	rs6538879	34.87509089
FCN2	rs4969182	36.46915434

FCN2	rs35677470	47.56272537
FCN2	rs3093989	32.46400267
PAK4	rs2319153	30.12753101
PAK4	rs4980320	33.59830696
PAK4	rs4759076	60.80443265
PRTN3	rs116184613	64.88314967
PRTN3	rs59653410	171.8628944
PRTN3	rs10411119	1232.767487
PRTN3	rs1060442	249.9615509
PRTN3	rs4807455	80.62776166
PRTN3	rs62128522	37.64681627
PRTN3	rs34354538	57.98984277
PRTN3	rs2606725	31.21111727
PRTN3	rs76428106	48.95685904
PRTN3	rs2071408	32.46834157
PRTN3	rs28678897	92.97460176
PRTN3	rs3859194	33.41205333
PRTN3	rs12453341	52.85999577
PRTN3	rs8178414	137.5857103
PRTN3	rs28730837	77.40634278
PRTN3	rs186905046	55.42222458
PRTN3	rs55771110	40.05007261
PRTN3	rs2236947	38.72788215
PRTN3	rs6775011	47.79742657
PRTN3	rs79408316	29.73743884
PRTN3	rs74899816	128.6939935
PRTN3	rs79350467	144.2225371
PRTN3	rs9271369	350.5871419
PRTN3	rs439205	60.71912947
PRTN3	rs915125	65.52020899
PRTN3	rs2380606	34.17683119
PRTN3	rs13277237	78.92252132
FGF19	rs708686	96.45127149
FGF19	rs492602	845.7082711
FGF19	rs3795054	36.93162555
FGF19	rs1260326	37.02747651
FGF19	rs6854452	118.9739758
FGF19	rs374539739	34.1506796
FGF19	rs10107182	110.317543
FGF19	rs10102041	39.82650178
FGF19	rs11244049	65.52557445
SPOP	rs10922098	172.7007564
SPOP	rs35866131	45.85652248
SPOP	rs1707652	45.08895586
SPOP	rs9501393	51.10417469
SPOP	rs7774739	30.81217056
SFXN5	rs10922098	144.5623968
SFXN5	rs238238	372.3285597
SFXN5	rs8064438	103.8552178
SFXN5	rs697356	45.32275336
SFXN5	rs6457457	41.70670825
SFXN5	rs3891366	70.83021927
IL12B	rs111546564	351.2275408
IL12B	rs77343625	75.541292
IL12B	rs3213120	1229.352036
IL12B	rs12657636	1275.776175
IL12B	rs35595526	30.96049675
IL12B	rs72810115	97.00834597

IL12B	rs12358180	31.73649016
IL12B	rs2271194	31.78991437
IL12B	rs3184504	151.4212991
IL12B	rs76428106	44.7253312
IL12B	rs2877455	31.22154901
IL12B	rs12588969	146.8560785
IL12B	rs373013841	47.74652881
IL12B	rs11650773	32.71671152
IL12B	rs9815073	662.123298
IL12B	rs1145623	49.41102001
IL12B	rs6597255	31.79401118
IL12B	rs9265986	86.10700149
IL12B	rs2076536	59.65919167
IL12B	rs7788220	37.51293955
INHBA	rs73100943	104.0949098
INHBA	rs7132006	33.59656686
INHBA	rs7971133	135.9061822
INHBA	rs2244608	45.78276512
INHBA	rs273510	36.14238838
INHBA	rs1260326	143.5446788
INHBA	rs6957745	65.39710544
INHBA	rs28601761	58.52235407
FRZB	rs188423082	234.8423398
FRZB	rs114983291	66.7440567
FRZB	rs1561369	1267.286392
FRZB	rs72890325	1050.093795
FRZB	rs75781425	38.27546583
FRZB	rs113069888	46.40620429
FRZB	rs3184504	34.20874635
FRZB	rs77262773	30.34896368
FRZB	rs140418617	50.7716983
FRZB	rs77764200	156.5868541
FRZB	rs77194274	99.02140348
FRZB	rs3771074	33.90459123
FRZB	rs150442575	37.7540813
FRZB	rs6018653	40.99576158
FRZB	rs299628	32.48802371
FRZB	rs9827708	32.00403007
FRZB	rs13220896	30.70877906
FRZB	rs3117582	86.85749792
FRZB	rs147906938	105.778775
FRZB	rs41316148	52.17755584
FRZB	rs34115112	89.55460314
IL3RA	rs2622935	172.0234376
IL3RA	rs112564808	32.1306336
IL3RA	rs11603123	136.4596419
IL3RA	rs10850034	30.1287065
IL3RA	rs11066301	29.98852879
IL3RA	rs2940174	37.13717124
IL3RA	rs12945299	131.2469143
IL3RA	rs61764072	70.13406645
IL3RA	rs4802117	84.00212947
IL3RA	rs117877056	94.45481203
IL3RA	rs117241334	117.4455716
IL3RA	rs9842051	32.72652255
IL3RA	rs28367690	41.05869378
IL3RA	rs517076	38.64411037
IL3RA	rs35434910	981.2878557

IL3RA	rs9411492	1235.463531
IL3RA	rs11507716	409.3615783
IL3RA	rs3793628	84.14507377
IL3RA	rs34250484	137.774109
IL3RA	rs115676813	37.49260105
IL3RA	rs117369137	66.74980709
PKD2	rs10922094	136.8764467
PKD2	rs114115200	38.23241182
PKD2	rs9501600	47.6595434
CCL8	rs12943905	32.35483861
CCL8	rs317356	66.85187852
CCL8	rs10512462	1313.486555
CCL8	rs10853162	2811.032261
CCL8	rs2215230	304.7583305
CCL8	rs12451648	61.25882668
CCL8	rs3135980	151.062854
CCL8	rs12075	117.2572013
CCL8	rs10437655	38.87392854
CCL8	rs75015026	40.11657558
CCL8	rs149062779	31.28903941
CCL8	rs73988096	38.38753022
CCL8	rs319778	76.78489128
CCL8	rs79051484	118.9725481
CCL8	rs145626091	54.12212084
CCL8	rs7507204	29.78553814
RMI1	rs10922094	441.336719
RMI1	rs1047286	35.09771628
RMI1	rs1707652	102.8693747
RMI1	rs9501600	126.5189152
RMI1	rs35209896	41.99318684
CROT	rs7812191	30.61477435
CROT	rs809066	2498.918484
CROT	rs7810499	41.06422569
CROT	rs6972856	356.6066395
CROT	rs193163017	109.8556192
CROT	rs7128819	29.91230817
CROT	rs10876550	48.56764414
CROT	rs190125718	30.31011451
CROT	rs75967349	46.99046675
CROT	rs114694170	39.20062907
CROT	rs2371656	40.78737265
CROT	rs2722997	33.98479592
CROT	rs10264865	58.95928866
CROT	rs10253461	89.66834978
CROT	rs10087163	68.15783348
APOBEC3G	rs6519166	191.6357572
APOBEC3G	rs41281251	56.75751227
APOBEC3G	rs4980320	52.07125478
APOBEC3G	rs10876550	44.92281466
APOBEC3G	rs1037590	34.04787118
APOBEC3G	rs1354034	73.7771857
ARHGEF7	rs1451915	36.8840713
ARHGEF7	rs10922098	693.3339189
ARHGEF7	rs1047286	44.04386115
ARHGEF7	rs2686395	171.7577216
ARHGEF7	rs1265941	80.98255556
ARHGEF7	rs550671	185.2169748
ARHGEF7	rs9269234	31.44980526

NAB1	rs6542680	33.47878401
NAB1	rs1260326	43.59994836
NAB1	rs1354034	31.62164716
NAB1	rs62265615	54.26833805
NAB1	rs56210800	53.6721435
MCF2L	rs10492684	45.35691594
MCF2L	rs3011487	675.2240989
MCF2L	rs7114094	36.54734545
MCF2L	rs1963140	29.80303359
MCF2L	rs1354034	48.99892364
MCF2L	rs1355538	41.12922688
PGK2	rs10922098	50.20893475
PGK2	rs62205397	103.3782852
PGK2	rs9864801	36.24218452
UGP2	rs11046893	94.07799681
UGP2	rs12230154	34.33375205
UGP2	rs11611373	85.40169038
UGP2	rs10845139	61.04138944
UGP2	rs71309976	30.81211423
FARS2	rs10922098	298.5328074
FARS2	rs704	96.61238219
FARS2	rs1707652	140.5016754
FARS2	rs9267139	38.37028494
FARS2	rs9501393	114.7460701
FARS2	rs7774739	81.74398236
FARS2	rs9271766	45.27578179
SPSB1	rs10922098	128.8169022
SPSB1	rs6542680	46.77083602
SPSB1	rs12478841	30.42221334
SPSB1	rs9864801	35.00904256
SPSB1	rs6457457	42.52890308
SPSB1	rs9501393	37.44203832
DPY30	rs221205	68.36079744
DPY30	rs10876550	49.0897752
DPY30	rs1355538	54.58224415
DPY30	rs72806804	31.76182418
DPY30	rs342200	35.80336017
DPY30	rs6993770	32.34753956
DPY30	rs4610723	31.90575046
SULT1A3	rs143154922	49.68587382
SULT1A3	rs146344226	34.3708539
SULT1A3	rs148788997	267.0136848
SULT1A3	rs144319982	51.64200935
SULT1A3	rs4980320	32.89457128
SULT1A3	rs72790561	53.86091393
SULT1A3	rs142458994	35.71674719
SULT1A3	rs4785321	34.08653349
SULT1A3	rs59053293	36.19040226
DDX23	rs1451915	29.78997985
DDX23	rs10922094	455.3454643
DDX23	rs704	189.6656457
DDX23	rs6445035	86.99544117
DDX23	rs9266722	33.28214534
DDX23	rs9267139	34.80757078
DDX23	rs3891366	121.563716
DDX23	rs7774739	98.28930081
DDX23	rs9271766	50.32722368
NUDT12	rs73190041	2850.927238

NUDT12	rs140216035	59.18706125
NUDT12	rs113870602	59.8589124
NUDT12	rs10922098	39.10829546
NUDT12	rs13155553	58.4986712
NUDT12	rs369896358	32.4098776
NUDT12	rs75170310	30.08047755
NUDT12	rs1477619	75.03690491
GABBR2	rs4448834	348.971707
GABBR2	rs546575	42.56929741
GABBR2	rs3917545	147.4502189
GABBR2	rs4246129	31.8329947
DAPK1	rs2235547	38.70500321
DAPK1	rs7113761	32.30764904
DAPK1	rs10506328	66.82529229
DAPK1	rs34772505	796.1969925
DAPK1	rs79227106	66.00470407
DAPK1	rs74982409	29.81987716
DAPK1	rs113252264	31.26539246
DAPK1	rs1354034	52.24793884
LAP3	rs10939687	45.86099204
LAP3	rs114838597	84.43678285
LAP3	rs141974152	139.1500713
LAP3	rs61851769	52.34786485
LAP3	rs76551904	46.64824262
LAP3	rs115375955	50.12076483
LAP3	rs73117935	44.39450372
AGAP3	rs10922098	228.0756964
AGAP3	rs12147435	66.96291363
AGAP3	rs1355538	56.03146408
AGAP3	rs9267139	32.0278205
AGAP3	rs9501393	69.34792487
AGAP3	rs7774739	46.97050363
KIF3A	rs10922094	521.0248571
KIF3A	rs964184	38.61501877
KIF3A	rs12478841	42.30155343
KIF3A	rs2232613	191.9606057
KIF3A	rs11906988	30.55305634
KIF3A	rs6445035	187.0509131
KIF3A	rs3863087	74.42047658
KIF3A	rs9501393	96.70108246
KIF3A	rs35426875	72.72286534
KIF3A	rs3917538	41.71458469
KIF3A	rs17732416	34.40676436
MYOM3	rs72748420	33.5498284
MYOM3	rs144704969	31.67382324
MYOM3	rs1329424	761.1873903
MYOM3	rs1047286	38.96926584
MYOM3	rs2686395	149.2869467
MYOM3	rs444921	201.480109
MYOM3	rs9469195	72.4138006
MYOM3	rs9272526	38.77509134
KPNA6	rs140904849	55.62377128
KPNA6	rs4980320	30.01521999
KPNA6	rs342200	31.48487201
HSD17B14	rs35299026	80.42316687
HSD17B14	rs664914	44.90688363
HSD17B14	rs10922098	71.10349144
HSD17B14	rs62295961	33.12087887

HSD17B14	rs3020644	67.03606375
HSD17B14	rs6458	106.2213598
TTL	rs10194022	52.133736
TTL	rs11417460	30.09793094
TTL	rs4980320	44.06911511
TTL	rs11451044	30.84731829
TTL	rs1354034	65.99111405
PPFIA1	rs11605332	35.83998654
PPFIA1	rs11451044	32.22980891
PPFIA1	rs1354034	58.05726979
PPFIA1	rs450269	33.47176643
ARHGEF1	rs4980320	35.4658456
ARHGEF1	rs10876550	39.64110567
ARHGEF1	rs1354034	30.37834224
BARD1	rs10922098	188.2247484
BARD1	rs41446546	29.73995903
BARD1	rs1707652	62.53314002
BARD1	rs6457457	57.77728171
BARD1	rs28573154	76.0162254
RGS18	rs4495675	46.76772613
RGS18	rs7073753	31.84676381
RGS18	rs4980320	37.32911594
CRYZ	rs71655423	100.9863146
CRYZ	rs144889249	31.1997019
CRYZ	rs76296629	1008.221208
CRYZ	rs13375787	3466.430074
CRYZ	rs277392	1150.322712
CRYZ	rs75209031	176.869188
CRYZ	rs7529852	379.1672426
CRYZ	rs12141037	31.07278064
CRYZ	rs113846452	50.904086
CRYZ	rs115793338	167.0646814
CRYZ	rs111993682	79.65532075
CRYZ	rs147322213	123.4573425
CRYZ	rs79841811	84.507303
CRYZ	rs74957782	135.1990409
CRYZ	rs12023669	30.81608476
CRYZ	rs1537906	30.34328927
CRYZ	rs871895	33.8504494
DDX25	rs10922098	329.8663138
DDX25	rs1707652	76.5734605
DDX25	rs3099847	31.99967081
DDX25	rs555007	40.90169412
DDX25	rs550671	75.82220808
SMURF2	rs10922094	247.5831692
SMURF2	rs1355538	59.68297662
SMURF2	rs9501393	79.68477089
SMURF2	rs7774739	54.41997514
SMURF2	rs9271766	30.90409932
NMRAL1	rs116875306	116.923987
NMRAL1	rs143297875	232.7943028
NMRAL1	rs146827390	361.3449363
NMRAL1	rs117658699	2699.875584
NMRAL1	rs17880377	551.3514545
NMRAL1	rs147766888	232.0577484
NMRAL1	rs141319000	72.80461198
NMRAL1	rs148542202	49.01536031
NMRAL1	rs147380181	175.8491757

NMRAL1	rs141785524	170.2830636
NMRAL1	rs201454237	49.93508322
NMRAL1	rs79052177	65.55032922
NMRAL1	rs35979828	39.44188233
NMRAL1	rs117705141	71.10425668
NMRAL1	rs1354034	163.2640566
NMRAL1	rs342293	50.7564521
EIF4G3	rs10922094	202.5987637
EIF4G3	rs704	94.12281558
EIF4G3	rs1355538	45.49903972
EIF4G3	rs453821	45.1641747
NSF	rs138109415	113.712428
NSF	rs72430786	36.72007165
NSF	rs75104997	230.1314034
NSF	rs75327431	30.80894091
NSF	rs1355538	54.05730345
EPB41L1	rs204074	32.17874322
EPB41L1	rs12403568	31.99752873
EPB41L1	rs10443899	67.6006907
EPB41L1	rs17478374	33.83681117
EPB41L1	rs3896594	30.01528747
FBL	rs10922098	675.5235621
FBL	rs3900909	35.55747859
FBL	rs1047286	56.25894364
FBL	rs2686395	133.8027982
FBL	rs1265941	83.07342785
FBL	rs444921	172.2616419
FBL	rs9276606	30.80665914
DOCK9	rs10922094	115.7450301
DOCK9	rs34762726	31.12848986
DOCK9	rs1706435	39.63203511
DOCK9	rs6457457	48.11464636
DOCK9	rs9501393	46.33359153
GNMT	rs6940814	734.3015621
GNMT	rs9472021	88.84365685
GNMT	rs10922098	387.2593566
GNMT	rs1355538	65.26537998
GNMT	rs9501393	41.56605378
GNMT	rs72848261	32.11423593
PAPSS1	rs12355080	40.09691753
PAPSS1	rs2255683	247.2048315
PAPSS1	rs17173698	179.7855501
PAPSS1	rs1354034	69.49007227
PAPSS1	rs114694170	42.69718545
PAPSS1	rs77265585	29.86296811
DCP1A	rs72704449	41.88861284
DCP1A	rs12945299	77.39589857
DCP1A	rs143101359	41.6489704
DCP1A	rs8128660	32.33326518
DCP1A	rs8129972	191.370133
DCP1A	rs2839108	523.981641
DCP1A	rs66514606	75.58489252
DCP1A	rs1556341	38.3286145
DCP1A	rs2686395	122.2388971
DCP1A	rs113477191	222.6931472
TNFAIP3	rs59214750	226.2652456
TNFAIP3	rs5029937	245.6204429
TNFAIP3	rs342293	35.25237014

TNFAIP3	rs6993770	45.43532439
TNFAIP3	rs4962034	47.20121044
S100A11	rs10922094	68.41977476
S100A11	rs653178	34.10501337
S100A11	rs3827295	63.58466115
S100A11	rs2231495	1244.383951
S100A11	rs191655261	69.89837743
S100A11	rs9880223	365.2975912
S100A11	rs115028961	51.37014785
S100A11	rs78094070	39.45963581
DHX58	rs112616358	92.56882384
DHX58	rs4796771	474.3396306
DHX58	rs12150483	31.35719074
DHX58	rs1451915	30.06990296
DHX58	rs6695321	310.4677911
DHX58	rs73073557	39.30433909
DHX58	rs11078929	38.84886974
MED4	rs10922094	74.35225341
MED4	rs964184	29.86380805
MED4	rs73045691	988.8464341
MED4	rs17642665	59.37080555
MED4	rs1049739	43.79797259
MED4	rs6445035	126.7357687
MED4	rs3863087	46.88596512
EDA	rs1451915	37.24997235
EDA	rs10922094	640.7682421
EDA	rs1047286	46.39223673
EDA	rs1707652	107.5064252
EDA	rs555007	61.31732448
EDA	rs453821	146.0170747
TNFRSF9	rs10922098	59.08011333
TNFRSF9	rs1355538	59.03590601
TNFRSF9	rs6457457	31.34364364
TNFRSF9	rs28573154	34.25454846
IL17F	rs572515	223.8312399
IL17F	rs12895651	50.73379717
IL17F	rs11569415	30.53907597
IL17F	rs1707652	43.92031476
IL17F	rs415620	48.25355332
IL17F	rs7774739	36.31544288
PIK3C2A	rs10922098	724.2040238
PIK3C2A	rs35351085	34.51847902
PIK3C2A	rs2686395	146.1643614
PIK3C2A	rs9267139	60.19383773
PIK3C2A	rs3891366	198.7542633
PIK3C2A	rs7774739	127.9017012
PIK3C2A	rs9271766	50.01095961
COPS2	rs964184	39.09861422
COPS2	rs55870921	36.84042129
COPS2	rs704	905.0343881
COPS2	rs429358	78.1010096
COPS2	rs35136575	73.91434305
COPS2	rs12478841	53.57317891
COPS2	rs62265637	30.84786526
COPS2	rs1619994	56.8110051
TACSTD2	rs6445035	45.4507341
TACSTD2	rs9501600	54.81891732
TACSTD2	rs557011	31.46015047

KLK5	rs10922098	30.89350728
KLK5	rs117265578	30.88036656
KLK5	rs41547217	30.8702685
EEA1	rs1451915	34.13201335
EEA1	rs10922098	684.4192974
EEA1	rs1047286	60.05456054
EEA1	rs1707652	137.139353
EEA1	rs3099847	30.84132495
EEA1	rs1265941	86.21446343
EEA1	rs550671	187.2345363
NCOA2	rs11208704	33.13589527
NCOA2	rs10922098	258.7166816
NCOA2	rs12886125	97.46095821
NCOA2	rs241786	31.61869287
NCOA2	rs114115200	80.39312215
NCOA2	rs72681226	36.13327174
NCOA2	rs9266722	30.18088318
NCOA2	rs9267139	31.53397172
NCOA2	rs28573154	84.21810274
NCOA2	rs7774739	67.59584045
NCOA2	rs9271683	30.53548469
BDNF	rs116911082	30.18183668
BDNF	rs144178847	35.71721542
BDNF	rs10501089	101.8030308
BDNF	rs10822155	109.1043521
BDNF	rs12882815	35.64276578
BDNF	rs775824	40.28107444
BDNF	rs12468189	32.84911814
BDNF	rs75967349	42.06965072
BDNF	rs1071960	32.82479526
BDNF	rs1354034	37.860972
BDNF	rs5008180	29.86229816
BDNF	rs6993770	51.4317925
IL1RAP	rs893051	102.4729862
IL1RAP	rs9811674	3459.702334
IL1RAP	rs9856858	6833.462732
IL1RAP	rs34879831	3471.354822
IL1RAP	rs4624605	105.297471
IL1RAP	rs4308324	105.2571949
IL1RAP	rs9868783	224.0771422
IL1RAP	rs6805405	69.87337811
IL1RAP	rs7970695	34.66201176
IL1RAP	rs141866277	75.3637686
IL1RAP	rs200406618	103.0148293
IL1RAP	rs35491981	34.22361913
IL15RA	rs148235865	137.0869572
IL15RA	rs74116316	46.35654816
IL15RA	rs3136630	1003.833059
IL15RA	rs7914655	577.8381126
IL15RA	rs12722558	84.84453706
IL15RA	rs77347828	246.1049834
IL15RA	rs61747728	34.02017803
IL15RA	rs7310615	63.95188511
IL15RA	rs117753190	43.72930424
IL15RA	rs1260326	31.52851676
IL15RA	rs6873866	181.5312522
IL15RA	rs9269273	73.10365669
IL15RA	rs116334170	49.34152753

IL15RA	rs635634	89.29311142
CGA	rs10922094	290.4201751
CGA	rs1707652	48.03017589
CGA	rs9501600	68.85821754
NCF4	rs10922098	352.5263025
NCF4	rs1047286	64.51975426
NCF4	rs12478841	31.60071607
NCF4	rs9864801	35.95931824
NCF4	rs9501600	134.1236722
NCF4	rs35209896	42.45365053
TNFSF11	rs10922098	205.7809325
TNFSF11	rs34295723	163.7061095
TNFSF11	rs1707652	40.74958657
TNFSF11	rs415620	42.5356789
TNFSF11	rs4713498	55.65189675
TNFSF11	rs67399078	30.03558548
OSM	rs10922094	46.87083417
OSM	rs2511241	62.22777093
OSM	rs115497630	29.80678619
OSM	rs1042663	31.86405817
MAGI2	rs10499842	1123.574427
MAGI2	rs28412320	93.68904413
MAGI2	rs117656916	130.3134425
MAGI2	rs78438715	368.1068149
MAGI2	rs112043211	58.03759866
MAGI2	rs111348213	46.05983573
MAGI2	rs2667740	120.676573
MAGI2	rs62460682	41.21378672
MAGI2	rs147184500	101.8010022
MAGI2	rs140903980	167.6017999
MAGI2	rs77494173	90.72748437
MAGI2	rs342293	84.19906521
PKP2	rs9919823	158.3683572
PKP2	rs56969496	34.52770493
PKP2	rs12292693	121.37046
PKP2	rs1354034	52.92526397
PKP2	rs67036916	32.34788397
CA4	rs10922098	67.55383976
CA4	rs78878517	48.50278103
CA4	rs113120025	57.68442813
CA4	rs75357403	33.90695825
CA4	rs35524242	489.7996894
CA4	rs927914	75.04877313
CA4	rs61841389	29.74200877
CA4	rs2284350	58.32714367
ITSN1	rs10922094	247.1732084
ITSN1	rs704	52.6882216
ITSN1	rs1355538	74.03141036
ITSN1	rs444921	82.15232935
ITSN1	rs28471056	39.17606961
SHC2	rs10922094	126.919828
SHC2	rs697356	32.34512233
SHC2	rs147906938	31.64341815
SHC2	rs41547217	40.40444341
CST4	rs1998082	37.36766878
CST4	rs111257030	915.6633272
CST4	rs6049102	90.13387961
CST4	rs6138249	73.42474509

CST4	rs1970947	52.00334379
CST4	rs6427943	160.2255365
CST4	rs61880808	37.37422698
CST4	rs10836317	73.82639023
CST4	rs61912249	56.44257438
CST4	rs1998764	105.7153071
CST4	rs34230357	50.88282297
CST4	rs67020211	82.03089108
CST4	rs6113759	41.77509803
CST4	rs62216860	77.98108148
CST4	rs145979311	33.5325016
CST4	rs34223888	37.41002428
CST4	rs3749751	360.1479912
IL18R1	rs10186962	102.5540586
IL18R1	rs115232861	226.855523
IL18R1	rs183775293	43.07273697
IL18R1	rs55715763	133.1081382
IL18R1	rs55871806	3399.96299
IL18R1	rs1030025	1120.331027
IL18R1	rs183611009	227.5279289
IL18R1	rs80339564	116.824868
IL18R1	rs76161381	34.6499463
IL18R1	rs61801013	58.87189056
IL18R1	rs200250001	50.0800071
IL18R1	rs78705678	116.088532
IL18R1	rs2477642	127.4456111
IL18R1	rs61418148	33.91564453
IL18R1	rs117278560	35.40073451
IL18R1	rs8178824	58.71447042
IL18R1	rs78060698	36.94568801
IL18R1	rs139899816	31.43403479
IL18R1	rs114432729	36.64735846
IL18R1	rs11413708	72.01150251
IL18R1	rs141702610	96.49461127
IL18R1	rs72833729	32.83480267
IL18R1	rs13209376	31.17206876
TLN2	rs7113761	58.77168646
TLN2	rs1355538	34.54058339
TLN2	rs148772994	44.64608414
IGFBP6	rs10876406	62.45840957
IGFBP6	rs77924615	68.31319983
IGFBP6	rs9992101	34.97164993
IGFBP6	rs466059	31.07434173
IGFBP6	rs62435145	31.51647972
IGFBP6	rs7805747	43.70455421
DEF6	rs73407610	360.7232798
DEF6	rs369825175	233.1018947
DEF6	rs7747190	35.46840883
DEF6	rs9394333	87.14217864
DEF6	rs1354034	38.22520164
CBR3	rs138308942	36.35461589
CBR3	rs145416409	1335.980523
CBR3	rs117789622	60.99083862
CBR3	rs9980393	224.7290844
CBR3	rs73202262	99.63125093
CBR3	rs17814116	60.28257151
CBR3	rs35979828	68.02788209
CBR3	rs117203477	51.10357878

CBR3	rs117029697	36.20384897
CBR3	rs60757417	49.84666263
FLT3LG	rs10922094	138.3315039
FLT3LG	rs76428106	47.64316409
FLT3LG	rs5167	165.9998072
FLT3LG	rs1355538	74.00892823
FLT3LG	rs9501393	36.99773702
HBEGF	rs269767	35.57717149
HBEGF	rs3811444	68.2665421
HBEGF	rs10822155	290.4523816
HBEGF	rs80137017	52.70687806
HBEGF	rs77906229	34.54041494
HBEGF	rs11553699	201.4346381
HBEGF	rs150253538	58.06747029
HBEGF	rs2073775	37.38318978
HBEGF	rs1354034	88.39277047
HBEGF	rs2087609	44.37912634
HBEGF	rs116574109	32.83690096
HBEGF	rs13207171	49.79118535
HBEGF	rs199772122	41.19915252
HBEGF	rs77265585	30.85724168
HBEGF	rs10820606	81.15765062
HBEGF	rs6479631	59.07885891
C1QC	rs41266007	61.35958752
C1QC	rs145809479	238.2405758
C1QC	rs2744700	108.5313967
C1QC	rs144444914	126.2529991
C1QC	rs199812128	109.6704586
C1QC	rs209739	1158.220009
C1QC	rs77482787	99.5732193
C1QC	rs72651398	40.42211917
C1QC	rs292004	249.5751133
C1QC	rs142315752	195.0714542
C1QC	rs41312012	32.95047656
C1QC	rs184316561	34.93874435
C1QC	rs4934470	215.0905384
C1QC	rs117204878	49.40205093
C1QC	rs10849546	49.88226071
C1QC	rs2987530	33.92101103
C1QC	rs28671514	76.84576802
C1QC	rs74497536	43.71624877
C1QC	rs6532770	74.36497986
CNTFR	rs2183012	80.08313945
CNTFR	rs72735302	161.3840816
CNTFR	rs10972159	564.6045552
CNTFR	rs147165534	44.72569933
CNTFR	rs12035995	30.80267851
CNTFR	rs72704449	57.66802642
CNTFR	rs2298475	30.26938714
CNTFR	rs77924615	34.54033451
CNTFR	rs12945299	36.6487186
CNTFR	rs2725405	31.67688555
CNTFR	rs2008174	38.81890533
CNTFR	rs4690013	32.56514697
CNTFR	rs7027331	45.49342486
RAP1GDS1	rs10516428	32.69271446
RAP1GDS1	rs4980320	31.43161611
RAP1GDS1	rs1354034	41.22431571

RAP1GDS1	rs705016	33.63915983
MTHFS	rs1698221	190.6216514
MTHFS	rs1380642	993.46368
MTHFS	rs62026657	447.64225
MTHFS	rs72736560	90.51126276
MTHFS	rs71455308	79.78989223
MTHFS	rs80102939	35.56573732
MTHFS	rs28757801	30.55043345
MTHFS	rs1354034	89.9613108
MTHFS	rs10984361	41.60099649
THBS2	rs73041853	100.8253976
THBS2	rs10922098	30.18485269
THBS2	rs9272780	43.97430542
RELT	rs12293998	46.0285677
RELT	rs10922098	497.334392
RELT	rs2686395	106.5124927
RELT	rs4293988	30.42695852
RELT	rs2075798	66.11849936
RELT	rs444921	117.8201427
PIANP	rs71450153	29.77686891
PIANP	rs11064321	444.767415
PIANP	rs77924615	33.03540917
PIANP	rs485940	30.68181192
ADM	rs10849546	57.62822868
ADM	rs2304456	215.5742807
ADM	rs3856931	40.11134448
ADM	rs2731672	54.70155128
S100A4	rs41265162	116.589254
S100A4	rs11054061	64.40514433
S100A4	rs7975716	32.6193452
S100A4	rs5112	209.9157168
S100A4	rs12986064	145.1371849
S100A4	rs62217177	31.32549819
S100A4	rs1619994	52.34595841
S100A4	rs1863622	89.10604019
RNF43	rs10922098	413.4310192
RNF43	rs11078072	29.71970018
RNF43	rs241771	43.16214901
RNF43	rs1047286	32.9796643
RNF43	rs1707652	137.5206486
RNF43	rs555007	49.03382742
RNF43	rs550671	118.8360818
TNFRSF10D	rs10922098	253.6556633
TNFRSF10D	rs1047286	33.94855912
TNFRSF10D	rs7638018	70.32613923
TNFRSF10D	rs1706435	59.71589991
TNFRSF10D	rs9501600	83.35875669
TNFRSF10D	rs72508422	32.34829061
ZNRF3	rs378961	45.10664954
ZNRF3	rs1965605	50.82671102
ZNRF3	rs1706435	58.92142738
ZNRF3	rs415620	36.69283754
ZNRF3	rs4713498	44.14058954
EFNA2	rs78705678	49.69852079
EFNA2	rs2477642	61.98452737
EFNA2	rs2410942	43.91985554
EFNA2	rs12514540	153.0991755
IFNA10	rs10922098	118.3419879

IFNA10	rs1355538	67.52286172
IFNA10	rs411337	36.78436683
EFNB2	rs72632701	56.11752589
EFNB2	rs288667	45.3411345
EFNB2	rs72704449	51.43188136
EFNB2	rs143616286	33.93534334
EFNB2	rs77924615	61.88296848
EFNB2	rs11568581	31.19757529
EFNB2	rs1260326	34.88007099
EFNB2	rs13108218	32.7857155
EFNB2	rs10051765	34.91283133
EFNB2	rs10253736	34.42739156
EFNB2	rs635634	235.8366218
HHLA2	rs10922094	64.95212892
HHLA2	rs1706435	89.82367716
HHLA2	rs4541868	31.21150619
IL1R2	rs200669380	2415.09991
IL1R2	rs78423067	875.296502
IL1R2	rs76261424	123.7677101
IL1R2	rs469802	32.7983058
IL1R2	rs141094656	58.65544421
IL1R2	rs1870140	34.38686816
IL1R2	rs7120907	42.44145146
IL1R2	rs78689694	56.01978947
IL1R2	rs1169288	45.0116935
IL1R2	rs1892548	39.77387296
IL1R2	rs10521222	30.82261695
IL1R2	rs55714927	64.13115421
IL1R2	rs143501783	37.94795195
IL1R2	rs12940122	33.30104456
IL1R2	rs77542162	56.35559554
IL1R2	rs113506881	53.53303281
IL1R2	rs111645402	61.09309925
IL1R2	rs367794814	31.07551211
IL1R2	rs79204775	70.7035961
IL1R2	rs17030637	44.56585404
IL1R2	rs73405151	47.50790272
IL1R2	rs7779671	51.30928535
IL1R2	rs28601761	197.0145876
AMIGO2	rs79609374	127.340753
AMIGO2	rs832736	62.21121097
AMIGO2	rs215397	38.29622481
AMIGO2	rs10922098	45.94197351
AMIGO2	rs1993484	32.76979074
AMIGO2	rs7310615	88.83510794
AMIGO2	rs2127868	30.52744028
AMIGO2	rs55714927	54.63848053
AMIGO2	rs1354034	312.2893054
AMIGO2	rs12493830	49.89036648
AMIGO2	rs9275640	39.06640685
AMIGO2	rs241442	70.66702227
AMIGO2	rs9494145	36.4641158
AMIGO2	rs635634	124.8114713
RXFP1	rs10922098	212.9609925
RXFP1	rs6542680	55.26132005
RXFP1	rs1706435	51.43940766
RXFP1	rs6457457	46.03970936
RXFP1	rs3891366	57.45745969

CD93	rs10158500	32.32806044
CD93	rs12133018	33.08854678
CD93	rs72704449	85.58925223
CD93	rs33950747	32.04378401
CD93	rs1260326	41.5875893
CD93	rs13108218	35.58648534
CD93	rs9274934	32.32220247
CD93	rs60373308	30.74909234
CD93	rs2035141	33.06582893
CD93	rs11244049	73.11436794
IL36B	rs10922098	171.9186148
IL36B	rs1355538	48.71989908
IL36B	rs9501600	52.40750425
IL36A	rs198812	42.22306534
IL36A	rs114055010	47.14502157
IL36A	rs78852381	939.415387
IL36A	rs451637	466.9349174
IL36A	rs6450	47.89634262
IL36A	rs72508422	264.0603335
IL36A	rs9391753	61.84238699
ISG15	rs116030099	34.41322357
ISG15	rs3128110	1139.944257
ISG15	rs2465125	239.3166586
ISG15	rs147326918	62.27292955
ISG15	rs835608	39.60849899
ISG15	rs17849502	30.36327662
ISG15	rs9975738	125.4286386
ISG15	rs8134026	41.03159215
ISG15	rs1354034	33.25830122
ISG15	rs4917014	40.34621138
ISG15	rs4418446	30.15331439
EFNA3	rs67333363	40.78187367
EFNA3	rs10922098	47.23572685
EFNA3	rs78705678	121.7711378
EFNA3	rs2461161	142.1106388
EFNA3	rs1010163	35.94566013
EFNA3	rs9271377	29.96211309
YWHAB	rs6031847	391.7961437
YWHAB	rs11569649	30.29482396
YWHAB	rs1354034	39.07914865
YWHAE	rs10922098	90.03524677
YWHAE	rs1355538	128.6816207
YWHAE	rs28573154	52.42353276
YWHAE	rs7774739	33.01663252
ANXA5	rs7666178	102.3838292
ANXA5	rs9995591	285.8494424
ANXA5	rs10922098	40.47625795
ANXA5	rs9864801	77.19831032
ANXA5	rs73169064	34.36363769
SCP2D1	rs9970075	36.54293858
SCP2D1	rs450993	53.5176803
SCP2D1	rs187178091	36.69959695
SCP2D1	rs79379298	1358.244208
SCP2D1	rs16987300	112.6094866
SCP2D1	rs13057849	309.6503318
SCP2D1	rs165741	90.58882432
SCP2D1	rs9607986	257.0501072
SCP2D1	rs2856876	494.068951

SCP2D1	rs117164661	78.18649788
SCP2D1	rs111235858	208.5813934
SCP2D1	rs200834629	31.41228016
SCP2D1	rs1707652	57.06532128
SCP2D1	rs2524279	64.72579521
SCP2D1	rs3104405	30.55100318
SCP2D1	rs1042131	35.23829227
SCP2D1	rs113810201	37.40655171
PDCL2	rs10922094	170.7109198
PDCL2	rs1355538	57.5163146
PDCL2	rs9501393	47.05163308
PDCL2	rs28675927	33.7735969
PRDM1	rs10922098	730.4727153
PRDM1	rs35351085	30.49370942
PRDM1	rs1707652	174.285172
PRDM1	rs1265941	82.44698055
PRDM1	rs550671	202.757828
ANXA7	rs79554558	37.01370005
ANXA7	rs79083576	109.7380933
ANXA7	rs1354034	35.98929093
FOXJ2	rs12818933	69.55911171
FOXJ2	rs5751508	213.9141729
FOXJ2	rs2854096	597.4422277
FOXJ2	rs469412	75.33763781
FOXJ2	rs2923009	30.23817716
FOXJ2	rs2073044	31.94581164
APBB1	rs1329424	584.7185788
APBB1	rs1706435	107.8138341
APBB1	rs453821	84.37256257
APBB1	rs28471056	45.59891934
RBP7	rs1220424	178.1159986
RBP7	rs10922098	455.531024
RBP7	rs7513100	35.2740201
RBP7	rs62143198	30.52371617
RBP7	rs1707652	100.6891457
RBP7	rs9501600	92.46948959
RBP7	rs116334170	31.13437026
DDX46	rs114009540	29.83737493
DDX46	rs10922098	897.8031128
DDX46	rs3900909	42.33047142
DDX46	rs11064498	35.39234277
DDX46	rs704	197.2867922
DDX46	rs1047286	58.12121439
DDX46	rs71336056	32.97794441
DDX46	rs1707652	191.4776975
DDX46	rs73169117	30.5802709
DDX46	rs3099847	39.98387271
DDX46	rs1265941	102.4685124
DDX46	rs550671	260.203668
DDX46	rs9276606	51.75423443
BHMT2	rs10922098	482.4635611
BHMT2	rs17580	350.7771853
BHMT2	rs1047286	31.34186197
BHMT2	rs1706435	115.3105987
BHMT2	rs9267139	44.34403882
BHMT2	rs28573154	162.3629652
BHMT2	rs7774739	114.4382595
BHMT2	rs9271766	55.7247495

BHMT2	rs13321	64.29595345
MYL6B	rs10922098	38.42227455
MYL6B	rs115975731	84.38039768
MYL6B	rs1355538	63.28651273
MOCS3	rs4512628	30.63704096
MOCS3	rs114009540	31.95656606
MOCS3	rs1329424	964.6372398
MOCS3	rs35351085	45.35724052
MOCS3	rs1047286	55.91483311
MOCS3	rs111696008	31.86262412
MOCS3	rs2686395	208.2511539
MOCS3	rs12629202	30.61340399
MOCS3	rs542418	42.05715933
MOCS3	rs550671	261.2370911
MOCS3	rs9270570	49.37962244
MOCS3	rs9469195	97.53764572
SNX7	rs4512628	33.92083388
SNX7	rs114009540	30.01374937
SNX7	rs10922098	752.3415021
SNX7	rs1047286	60.24258012
SNX7	rs1707652	163.6372541
SNX7	rs555007	62.92914508
SNX7	rs550671	183.3712758
SNX7	rs9272526	30.65934693
DOK2	rs4980320	31.56143112
DOK2	rs342200	40.43299646
DOK2	rs6993770	42.70747325
DOK2	rs77265585	30.53342519
MID2	rs10922094	241.3363906
MID2	rs1707652	93.94364915
MID2	rs454212	87.28310536
BLMH	rs10922098	49.9328191
BLMH	rs2304200	97.2129115
BLMH	rs6542680	39.88562049
BLMH	rs28361039	35.98225379
BLMH	rs1846190	32.88924588
PTPN4	rs112864756	34.7610702
PTPN4	rs113448098	421.5416582
PTPN4	rs3919533	1017.298363
PTPN4	rs117827303	415.0905377
PTPN4	rs117392682	44.94679736
PTPN4	rs61984231	31.28192558
PTPN4	rs12433460	281.791354
PTPN4	rs2093214	31.44445472
NET1	rs73024307	35.99653303
NET1	rs7571427	32.66689925
NET1	rs149247216	34.05013332
NET1	rs147157073	29.89082724
SRXN1	rs1801701	39.19385359
SRXN1	rs62294359	33.60815921
SRXN1	rs73216598	111.3903863
SRXN1	rs2237582	951.2137318
SRXN1	rs854572	110.8800128
SRXN1	rs73243140	29.89816801
SRXN1	rs2299269	43.55182931
RAB6B	rs10212397	610.2691012
RAB6B	rs7114094	42.52458364
RAB6B	rs1354034	68.75394385

RAB6B	rs342200	29.9689231
RAB6B	rs10984373	34.49259381
RAB6B	rs60757417	33.04971154
PREP	rs221632	46.9328553
PREP	rs9377697	41.63516569
PREP	rs12192369	344.0075226
PREP	rs140157947	39.94026946
PREP	rs6800964	40.24514979
RAB14	rs10922098	68.73871029
RAB14	rs438811	264.7447105
RAB14	rs5112	35.13992185
RAB14	rs9864801	55.2642363
RAB14	rs28374650	31.56282722
NFATC1	rs1451915	33.32235813
NFATC1	rs10922098	578.0028285
NFATC1	rs12478841	36.40536046
NFATC1	rs1707652	114.5228105
NFATC1	rs1265941	70.46633505
NFATC1	rs550671	173.6647795
NFATC1	rs9276606	31.47036174
RAB5C	rs10922094	94.53609832
RAB5C	rs4980320	37.65352272
RAB5C	rs185009887	33.34623838
RAB5C	rs1354034	64.27655321
RAB5C	rs1355538	36.08333375
RAB5C	rs9501600	31.38062719
MBD1	rs1697422	44.21832476
MBD1	rs10922094	35.61860255
MBD1	rs4792597	30.34987175
MBD1	rs8080168	97.36915691
MBD1	rs62058594	45.44699516
MBD1	rs2945405	146.2435057
MBD1	rs12952423	90.94444634
MBD1	rs9902833	1050.321592
MBD1	rs3764866	384.959205
MBD1	rs111987450	110.4030086
MBD1	rs184713097	76.54776892
MBD1	rs887469	85.24923503
MBD1	rs28548111	30.8869995
MBD1	rs10208529	50.23835624
MBD1	rs6001394	34.90465926
MBD1	rs56210800	75.77430375
MBD1	rs17282078	75.75741829
MBD1	rs1355538	44.80695363
MBD1	rs6535457	31.09493651
HNRNPH1	rs10922094	161.5770646
HNRNPH1	rs78385612	42.67532548
HNRNPH1	rs9290835	70.45329631
HNRNPH1	rs55636108	825.3520194
HNRNPH1	rs4686435	30.25238666
HNRNPH1	rs4861708	109.3245099
HNRNPH1	rs6870182	29.90923009
HNRNPH1	rs9501600	56.48029542
PPIL2	rs1451915	39.68039091
PPIL2	rs10922098	854.6548948
PPIL2	rs10922341	31.03094782
PPIL2	rs1047286	52.6752725
PPIL2	rs1707652	182.5117096

PPIL2	rs542418	33.60448869
PPIL2	rs550671	219.3613842
PPIL2	rs9270570	42.95012528
PPIL2	rs9469195	77.36059515
VPS29	rs10922094	66.2203925
VPS29	rs964184	33.25329948
VPS29	rs4251945	104.8241288
VPS29	rs189902748	187.7523878
VPS29	rs111278137	125.5804382
VPS29	rs62117160	2584.602592
VPS29	rs7254892	3502.134017
VPS29	rs118170342	151.3023249
VPS29	rs12691088	367.3562691
VPS29	rs118004808	93.58597858
VPS29	rs2116891	62.63358373
VPS29	rs1706435	61.00984836
VPS29	rs11755689	32.73989419
UBE2E1	rs4980320	77.63815391
UBE2E1	rs10876550	34.52360377
UBE2E1	rs1354034	408.3556754
DIDO1	rs10922098	103.0082629
DIDO1	rs9501600	34.97482944
DIDO1	rs557011	30.30628386
DYNLT1	rs10922098	351.3407202
DYNLT1	rs61923082	30.07349033
DYNLT1	rs1047286	33.22748443
DYNLT1	rs1355538	65.23757705
DYNLT1	rs9501393	91.42124719
DYNLT1	rs7774739	61.7574223
RCVRN	rs10922098	391.5145542
RCVRN	rs111696008	31.94749724
RCVRN	rs2232613	49.25816564
RCVRN	rs1707652	130.5515067
RCVRN	rs9267139	30.08424899
RCVRN	rs28573154	111.4886897
RCVRN	rs7774739	77.02849641
RCVRN	rs9271766	35.16593763
TRAPPC3	rs61747728	42.97673499
TRAPPC3	rs77924615	31.2225326
TRAPPC3	rs409632	43.87872953
TRAPPC3	rs9501342	47.60507751
TRAPPC3	rs28474889	130.1843854
TRAPPC3	rs148766553	104.1434827
TRAPPC3	rs6458	237.7481542
TRAPPC3	rs9273325	65.1994271
TRAPPC3	rs28616789	87.84778459
TRAPPC3	rs56294298	31.55591365
MSTN	rs10922098	132.2654919
MSTN	rs6445035	53.82668553
MSTN	rs41285751	44.17465444
MSTN	rs147390813	46.76642192
FAM210A	rs704	145.1399108
FAM210A	rs429358	50.7321641
FAM210A	rs10208529	34.40743134
FAM210A	rs115063752	35.62671686
FAM210A	rs872914	30.73686661
STAB1	rs10922098	301.2273229
STAB1	rs12403425	31.36722221

STAB1	rs151301820	150.2139153
STAB1	rs11234734	79.10396212
STAB1	rs76745121	369.6492526
STAB1	rs2365384	48.12779551
STAB1	rs1707652	70.97218433
STAB1	rs9501600	107.1955542
STAB1	rs35209896	34.0322945
KIAA0040	rs61808983	77.25446879
KIAA0040	rs6427202	42.60516483
KIAA0040	rs12766994	32.04996224
KIAA0040	rs1354034	32.81058528
NCMAP	rs10922098	48.94245068
NCMAP	rs61937764	33.19089506
NCMAP	rs372462064	30.90805879
NCMAP	rs7139079	65.83903721
NCMAP	rs116853338	31.29117325
NCMAP	rs72713764	32.1563833
NCMAP	rs150844304	109.2381393
NCMAP	rs17282057	54.56902023
NCMAP	rs56210800	54.0050864
NCMAP	rs147390813	36.75614508
NCMAP	rs4921915	51.54283554
NCMAP	rs34971098	106.8828611
NCMAP	rs117475080	122.1203891
NCMAP	rs3858097	1051.243705
NCMAP	rs112095134	495.0782243
NCMAP	rs150392262	59.72696825
NCMAP	rs113979662	100.944833
NCMAP	rs140989966	108.8825644
ZNF382	rs10922098	53.97294554
ZNF382	rs67399078	33.51151267
ZNF382	rs877653	31.68079756
VOPP1	rs10229613	35.12274052
VOPP1	rs117042408	96.54185823
VOPP1	rs12117692	30.18457721
VOPP1	rs10922098	98.29995058
VOPP1	rs10751648	39.98075982
VOPP1	rs1373064	77.23413361
VOPP1	rs375402329	31.00101668
VOPP1	rs75203136	78.93764122
VOPP1	rs34579002	77.54609434
VOPP1	rs17580	49.46208363
VOPP1	rs4775041	45.03946276
VOPP1	rs1800588	61.8455993
VOPP1	rs3764261	269.2107778
VOPP1	rs34216592	34.50920869
VOPP1	rs2304262	150.581128
VOPP1	rs3752241	30.83326125
VOPP1	rs8106813	68.02019955
VOPP1	rs1260326	31.48506306
VOPP1	rs111696008	36.40649392
VOPP1	rs4810479	94.31306504
VOPP1	rs2686395	119.415366
VOPP1	rs1264701	30.90646668
VOPP1	rs9268494	91.35983675
VOPP1	rs77315897	36.69724412
VOPP1	rs111344982	53.38455885
VOPP1	rs3917538	33.45332806

VOPP1	rs705379	108.5008053
VOPP1	rs2066714	38.95112374
VOPP1	rs2740488	44.67800973
ZNF526	rs572515	62.9625919
ZNF526	rs1354034	56.00971068
ZNF526	rs6445035	54.87759489
SUMO3	rs4818737	48.51536844
SUMO3	rs2401949	31.356241
SUMO3	rs1354034	136.7848382
CTCF	rs10922098	95.44696612
CTCF	rs9501393	52.59817157
CTCF	rs7774739	45.46132474
FBXO3	rs13379369	455.74205
FBXO3	rs2753516	154.404602
FBXO3	rs139354903	41.05343542
FBXO3	rs12891460	49.41817755
DUSP16	rs1329424	266.3928956
DUSP16	rs1707652	64.98426205
DUSP16	rs550671	121.1703352
DUSP16	rs28645249	54.15926522
TMEM185A	rs1451915	35.50919175
TMEM185A	rs10922098	813.3765777
TMEM185A	rs7513100	35.88304522
TMEM185A	rs704	138.8144869
TMEM185A	rs1047286	43.50916446
TMEM185A	rs1707652	184.9948343
TMEM185A	rs12629202	30.62097889
TMEM185A	rs1265941	91.94792444
TMEM185A	rs550671	211.691564
TMEM185A	rs9276606	38.14785549
LHCGR	rs1329424	47.83973291
LHCGR	rs1008369	63.09599804
LHCGR	rs145663802	382.7309055
LHCGR	rs187979386	298.9750413
LHCGR	rs73074378	1240.781007
LHCGR	rs147367607	221.7542227
LHCGR	rs138404620	80.6377646
LHCGR	rs10644120	132.530712
LHCGR	rs34537989	53.05010057
LHCGR	rs113685194	163.812654
LHCGR	rs9271642	30.27194905
GSTA4	rs72748420	31.19647706
GSTA4	rs114009540	30.8342928
GSTA4	rs1329424	981.1821317
GSTA4	rs3900909	42.75197186
GSTA4	rs11078072	31.83166358
GSTA4	rs1047286	42.73520834
GSTA4	rs111696008	33.95557382
GSTA4	rs1707652	202.9928887
GSTA4	rs12629202	30.31379665
GSTA4	rs3099847	31.3344678
GSTA4	rs1265941	93.28570498
GSTA4	rs550671	236.9796511
GSTA4	rs9276606	48.11740414
DNAJC17	rs79709714	221.4477882
DNAJC17	rs185808193	83.33826121
DNAJC17	rs189936214	60.40481434
DNAJC17	rs11925835	30.4092647

MZF1	rs10922098	229.7656874
MZF1	rs11607339	40.42895019
MZF1	rs2035675	207.9922868
MZF1	rs117983753	46.80322297
MZF1	rs372434515	32.3648366
MZF1	rs13019325	49.72661958
MZF1	rs1707652	188.8206004
MZF1	rs41285751	74.40448808
MZF1	rs451652	68.18830301
RNF8	rs10922094	187.4264924
RNF8	rs11064498	30.34466164
RNF8	rs704	39.36174437
RNF8	rs10208529	34.77215236
RNF8	rs1706435	84.24030006
RNF8	rs2284179	36.17906423
RNF8	rs11751024	66.58216168
RNF8	rs2057681	169.6526332
RNF8	rs854572	33.17866572
SKIL	rs10922098	79.70328849
SKIL	rs34524896	80.68080037
SKIL	rs1707652	69.75956054
SKIL	rs541862	34.73308237
EIF4B	rs4980320	37.41463112
EIF4B	rs7297265	33.40230378
EIF4B	rs1354034	68.80841029
CAPN2	rs150394890	433.5128381
CAPN2	rs17599	106.0737914
CAPN2	rs187029480	36.38708576
CAPN2	rs317180	34.47734299
CAPN2	rs11064498	43.366892
CAPN2	rs12433324	221.3905998
CAPN2	rs144881474	43.20810356
CAPN2	rs2668202	43.47215592
CAPN2	rs144930535	41.56408309
CAPN2	rs5030087	45.75978406
AKT2	rs10922094	33.74289883
AKT2	rs438811	153.3475739
AKT2	rs5112	72.28455836
AKT2	rs6445035	103.8220007
AKT2	rs3863087	46.76694431
AKT2	rs146385031	30.33562408
AKT2	rs114925146	923.3154632
AKT2	rs56783668	942.7025892
AKT2	rs1042303	83.36398247
AKT2	rs927985	59.57823528
AKT2	rs9272050	33.12169394
PLIN3	rs10922098	307.8617798
PLIN3	rs1355538	46.88004338
PLIN3	rs550671	107.0410178
PLIN3	rs9469195	46.77620472
SEC13	rs10799692	33.39229281
SEC13	rs72702154	32.12580057
SEC13	rs7535409	241.4154674
SEC13	rs2420378	118.5325621
SEC13	rs76980224	69.98470642
SEC13	rs10159712	201.2531701
SEC13	rs10437425	30.14629149
SEC13	rs148108365	33.81059236

SEC13	rs594793	32.25589407
SEC13	rs679899	277.8310257
SEC13	rs74717300	36.58508807
SEC13	rs62140549	30.78450285
SEC13	rs1265109	30.56146525
SEC13	rs34977123	111.3938181
SEC13	rs3104374	52.93859042
ZNF276	rs72748420	44.21732245
ZNF276	rs1329424	1365.628557
ZNF276	rs3900909	64.09319308
ZNF276	rs10801660	40.43488731
ZNF276	rs1047286	34.16833137
ZNF276	rs111696008	32.65365016
ZNF276	rs1707652	229.6716643
ZNF276	rs1265941	127.6354905
ZNF276	rs550671	355.0371389
ZNF276	rs112164276	49.23522502
ZNF276	rs41548618	47.86133301
C8G	rs2151205	94.39103961
C8G	rs7032104	1122.170022
C8G	rs12555781	825.0188296
C8G	rs7861020	210.7691453
C8G	rs11523300	42.96131161
C8G	rs6696924	1143.209177
C8G	rs191533645	35.26789384
C8G	rs861361	129.0976825
C8G	rs114114065	78.2537395
C8G	rs12752978	48.1760233
C8G	rs76168185	37.73516907
C8G	rs3753395	63.54169819
C8G	rs73887969	44.90542185
C8G	rs16860992	133.2505624
CST6	rs76584642	55.85142244
CST6	rs72928860	350.0558195
CST6	rs80293268	423.4407983
CST6	rs28635651	49.85800564
CST6	rs7905367	65.2001919
CST6	rs7913336	44.63369652
CST6	rs34560261	123.3819892
CST6	rs77924615	43.24396552
CST6	rs148242830	33.17698068
CST6	rs35280626	37.56634218
CST6	rs181067358	29.95243128
CST6	rs12053317	35.88796256
CST6	rs310762	35.19866868
CST6	rs4694690	33.1117939
CST6	rs5965373	42.91485758
AZU1	rs10922098	57.48876685
AZU1	rs62294359	49.27819525
AZU1	rs16881504	32.36529961
AZU1	rs3129773	30.57603076
CRLF1	rs1985976	36.01894953
CRLF1	rs7246833	744.5285167
CRLF1	rs77050841	29.7552676
CRLF1	rs34931250	43.25650567
CRLF1	rs62294359	34.60011783
ARHGAP5	rs4980320	48.01841914
ARHGAP5	rs185009887	30.01761005

ARHGAP5	rs1354034	48.8842893
ARHGAP5	rs342292	34.59167497
ARHGAP5	rs4541868	30.98082417
IGF2	rs646776	36.3444521
IGF2	rs190545575	31.59113682
IGF2	rs35391516	85.3142841
IGF2	rs147400256	69.1357682
IGF2	rs139482112	124.7055804
IGF2	rs184995649	48.97499673
IGF2	rs77589580	57.36688844
IGF2	rs12484809	29.92423528
IGF2	rs4234797	47.5537412
IGF2	rs2854746	305.0966151
S100A3	rs76418562	30.22893951
S100A3	rs10922098	181.7773882
S100A3	rs6542680	33.00813314
S100A3	rs1355538	64.86096759
S100A3	rs7774739	43.83605447
S100A3	rs7742632	58.98469366
CCDC134	rs5996067	162.121948
CCDC134	rs73169175	32.17367923
CCDC134	rs3804749	58.73450392
CCDC134	rs34115112	81.11761474
CCDC134	rs9272068	52.65057072
CCDC134	rs9273369	96.87027377
REG3A	rs72913277	186.3082902
REG3A	rs4459759	425.9650849
REG3A	rs112787300	119.9860715
REG3A	rs191783020	95.78660297
REG3A	rs139564670	40.10544924
REG3A	rs4533495	70.52315758
REG3A	rs10910535	48.58205674
REG3A	rs3217810	31.86529183
REG3A	rs12368026	48.52539062
REG3A	rs17338413	373.579737
REG3A	rs117900576	54.81555299
REG3A	rs10853981	32.0774926
REG3A	rs8133137	41.68392433
REG3A	rs13073114	44.55698966
REG3A	rs17616434	33.63402058
REG3A	rs13215052	78.12057501
REG3A	rs10457139	86.95356824
REG3A	rs13253974	52.66595254
REG3A	rs673854	38.92116126
SCGN	rs9467475	32.26417751
SCGN	rs2064125	74.63557278
SCGN	rs11240503	56.78848377
SCGN	rs7480143	44.49152908
SCGN	rs601338	195.9061334
SCGN	rs6445035	32.40471643
SCGN	rs6859236	74.41678753
SCGN	rs10107182	30.20906884
VWC2	rs957814	65.64327107
VWC2	rs76473891	131.1986848
VWC2	rs79523632	244.6960402
VWC2	rs73347621	1047.769307
VWC2	rs72704449	44.28876799
VWC2	rs77924615	36.56789177

VWC2	rs143243910	142.2552224
VWC2	rs71301894	79.69359756
VWC2	rs71308053	34.90158345
VWC2	rs191798511	64.18509258
VWC2	rs149136248	33.07073808
VWC2	rs28645249	37.70533737
VWC2	rs74798443	79.19748975
VWC2	rs143603772	30.7040381
PRC1	rs10922098	205.4835506
PRC1	rs1706435	40.73020697
PRC1	rs9501600	64.53164062
PRC1	rs1846190	35.59539552
AGO1	rs4980320	32.93575474
AGO1	rs342200	31.98533062
AGO1	rs7009635	39.45997632
MAD2L1	rs10922098	269.5325281
MAD2L1	rs6445035	65.45590033
MAD2L1	rs2242572	78.62548304
MAD2L1	rs28455809	38.14579717
CUTC	rs10922098	274.9899975
CUTC	rs11720167	54.16464515
CUTC	rs1042663	54.26896318
TXNL4B	rs3826248	30.87474718
TXNL4B	rs116891509	268.2758866
TXNL4B	rs149721298	34.56795364
TXNL4B	rs35979828	62.1257049
TXNL4B	rs62057937	44.7039443
TXNL4B	rs1354034	44.09293205
TXNL4B	rs66489920	44.99076661
S100A10	rs10922094	252.4459707
S100A10	rs10135681	45.11258026
S100A10	rs10147337	280.3153732
S100A10	rs2284179	31.90899393
S100A10	rs11751024	69.16780877
CCNA1	rs10922094	39.03118505
CCNA1	rs6542680	47.54573938
CCNA1	rs1846190	35.88678681
CPLX2	rs76735111	42.9989756
CPLX2	rs77924615	43.32951382
CPLX2	rs4976689	30.385704
CRADD	rs52807869	32.99736603
CRADD	rs60169972	41.84232204
CRADD	rs3740888	32.90897606
FTL	rs45520632	34.1930843
FTL	rs199138	66.62840538
FTL	rs8263	38.01085228
FTL	rs10439301	30.62925468
FTL	rs12693541	34.89044292
FTL	rs115740542	39.87808683
GBP1	rs75391630	85.12240784
GBP1	rs4656073	198.318465
GBP1	rs17130751	312.9501472
GBP1	rs12410795	328.0345525
GBP1	rs115621185	91.46937918
GBP1	rs139795227	128.4782533
GBP1	rs114564514	84.76845126
GBP1	rs12821887	39.50448021
GBP1	rs10748098	31.66870482

GBP1	rs7310615	170.000175
GBP1	rs181209	37.13146884
GBP1	rs56373884	33.94498434
GBP1	rs9264764	33.45402943
GBP1	rs28414666	58.2881477
RBBP4	rs10922094	162.6511916
RBBP4	rs7310615	38.96491756
RBBP4	rs1354034	63.14250359
RBBP4	rs4639017	30.45894975
RBBP4	rs28414666	46.67081944
SDF2	rs704	882.0828615
SDF2	rs10922094	85.72058603
SDF2	rs55870921	42.32929948
SDF2	rs1706435	32.21221697
SDF2	rs1863622	41.30448968
SDF2	rs2237193	32.23994654
SDF2	rs111305232	46.67765866
SDF2	rs72834371	33.20340356
SDF2	rs115000510	131.2534123
SDF2	rs114173559	102.256597
SDF2	rs113969967	324.4475425
SDF2	rs115103442	1023.123841
SDF2	rs141440850	227.4499177
SDF2	rs9260280	160.0023478
SDF2	rs116633440	4348.061159
SDF2	rs6937167	1086.40803
SDF2	rs73741546	443.1240725
SDF2	rs114746521	202.9102367
SDF2	rs143425782	62.56007891
SDF2	rs148513395	330.753398
SDF2	rs17360657	275.5412829
SDF2	rs9462399	36.65769651
KNG1	rs7610464	86.85743938
KNG1	rs144274836	949.9579367
KNG1	rs115329695	1190.26403
KNG1	rs138437490	105.0678443
KNG1	rs139797297	64.27198397
KNG1	rs11680187	37.90029296
KNG1	rs4241822	56.17431504
IFNG	rs10922098	336.4322913
IFNG	rs1707652	70.99093334
IFNG	rs6457457	61.94648876
IFNG	rs28573154	91.20711272
SCG5	rs145439832	29.79835805
SCG5	rs72748420	47.40918217
SCG5	rs1329424	1375.71357
SCG5	rs3900909	61.46501034
SCG5	rs10801660	43.4922983
SCG5	rs1047286	53.13009894
SCG5	rs111696008	32.5736119
SCG5	rs1707652	209.2007606
SCG5	rs3099847	40.67020003
SCG5	rs1265941	135.5446278
SCG5	rs550671	358.5048373
SCG5	rs78897911	33.31035828
SCG5	rs9276606	55.39735985
ANKRD1	rs10922098	88.84495114
ANKRD1	rs1355538	42.01912636

ANKRD1	rs9501600	44.53503276
APOA5	rs17461214	44.05860376
APOA5	rs1145183	165.5592805
APOA5	rs61906076	2344.511756
APOA5	rs111683933	78.37215847
APOA5	rs7123666	442.7726203
APOA5	rs78751065	140.1463957
APOA5	rs71480353	210.6642523
APOA5	rs74076477	34.94771588
APOA5	rs146589617	51.93326161
APOA5	rs56228609	146.5837369
APOA5	rs35909200	49.39372815
APOA5	rs6507939	41.03528442
APOA5	rs11668738	56.12105763
APOA5	rs429358	98.39728407
APOA5	rs4665972	80.02213165
APOA5	rs111696008	35.60336553
APOA5	rs697356	38.48238572
APOA5	rs2575876	56.17887209
APOC1	rs62116266	83.07093195
APOC1	rs3852860	106.9755222
APOC1	rs5112	1367.362547
APOC1	rs148023052	71.84859103
APOC1	rs247616	111.7743296
APOC1	rs2156552	49.7581224
APOC1	rs116843064	37.289294
APOC1	rs58542926	31.31918135
APOC1	rs6874202	44.77647167
BPIFB1	rs1998112	78.10493269
BPIFB1	rs221982	1026.179808
BPIFB1	rs117258330	920.2944073
BPIFB1	rs293735	270.5562508
BPIFB1	rs56156922	89.23089276
BPIFB1	rs9922594	30.96243484
BPIFB1	rs62035712	48.52204314
BPIFB1	rs116843064	34.42665956
BPIFB1	rs492602	190.4884483
BPIFB1	rs116955791	79.6031965
BPIFB1	rs73129828	73.29906412
BPIFB1	rs74850097	30.60472912
BPIFB1	rs57532134	152.0067634
BPIFB1	rs6060309	74.84360808
BPIFB1	rs138836591	373.5445934
BPIFB1	rs62208272	126.5947529
BPIFB1	rs78156627	40.98701975
BPIFB1	rs112283936	207.2666521
BPIFB1	rs150077408	85.50487061
BPIFB1	rs66960403	47.49683209
BPIFB1	rs6791309	35.68627637
BPIFB1	rs12523203	30.08882355
BPIFB1	rs9503212	80.77606438
BPIFB1	rs7776054	114.0251084
BPIFB1	rs7841189	36.23915598
BPIFB1	rs1590743	56.73772377
BMPER	rs144850853	37.73437709
BMPER	rs60539122	124.9426333
BMPER	rs1861365	157.8988771
BMPER	rs138631784	32.36764819

BMPER	rs374896	33.85293682
BMPER	rs34220697	48.90655173
BOLA1	rs185180388	33.49415754
BOLA1	rs2664765	1158.111268
BOLA1	rs1321232	4476.839461
BOLA1	rs2089695	2455.11396
BOLA1	rs149171534	31.55778363
BOLA1	rs199941359	40.26664961
BOLA1	rs148013105	55.62797089
BOLA1	rs61803983	142.5866193
BOLA1	rs143154825	74.94378375
BOLA1	rs7526706	161.2902722
BOLA1	rs116812574	176.5308685
BOLA1	rs28780420	110.137297
BOLA1	rs148652381	32.7219556
BOLA1	rs4570974	30.20331848
BOLA1	rs1354034	62.15269096
BOLA1	rs7814758	31.20125908
BOLA1	rs138772443	30.52566108
BOLA1	rs2811739	48.20739801
BOLA1	rs113809617	3361.010198
BOLA1	rs41314459	90.67687097
BOLA1	rs146572882	32.27823042
BOLA1	rs376261370	51.48922273
BRD2	rs35940802	37.84616176
BRD2	rs203688	96.37714922
BRD2	rs1355538	35.44980066
BRD2	rs453821	30.58267209
GKN1	rs10922098	324.8997415
GKN1	rs2686395	90.28486644
GKN1	rs3099847	33.63826223
GKN1	rs2242572	102.1683215
GKN1	rs9501602	43.43236648
CPB1	rs2291671	600.7262297
CPB1	rs76876937	44.67598903
CPB1	rs11166273	30.18289524
CPB1	rs1164032	69.40996285
CPB1	rs78705678	71.39682701
CPB1	rs2477642	54.30873273
CPB1	rs28456	51.40082818
CPB1	rs507695	52.4340659
CPB1	rs7302145	42.78912274
CPB1	rs72802342	537.6398839
CPB1	rs77396851	34.80348667
CPB1	rs533406	302.0707653
CPB1	rs889573	68.26497061
CPB1	rs56850260	114.4316894
CPB1	rs34920409	29.72640997
CPB1	rs838133	47.04370126
CPB1	rs145185835	38.34496297
CPB1	rs17032925	64.06714136
CPB1	rs78058190	31.83965542
CPB1	rs9983358	31.87349456
CPB1	rs6842549	42.92167472
CPB1	rs7679386	43.22290304
CPB1	rs35901985	32.99569786
CPB1	rs2534795	45.47600295
CPB1	rs1591805	70.41233389

CPB1	rs13438309	51.79529554
CPB1	rs748208	41.52168104
CPB1	rs11244061	31.0778964
CTSE	rs58493320	2475.788412
CTSE	rs41297620	60.43352483
CTSE	rs2285170	39.53080838
CTSE	rs56160741	46.15474907
CTSE	rs78705678	61.62302109
CTSE	rs2477642	66.24606635
CTSE	rs62108438	44.40073927
CTSE	rs4671393	156.3167381
CTSE	rs34115112	31.3385637
CTSE	rs28672722	60.59899507
CTSE	rs9280312	32.53190325
CTSE	rs635634	164.727655
CTSE	rs41307428	33.82659996
EDN3	rs10922094	31.51756353
EDN3	rs704	186.9475275
EDN3	rs3917538	118.8016755
EDN3	rs705379	182.5598576
EDN3	rs4735839	31.01767899
KL	rs10467359	234.2791463
KL	rs7984515	71.5040159
KL	rs9536282	166.2177105
KL	rs79282194	31.6311892
KL	rs2974937	38.83142665
KL	rs4846806	60.44693878
KL	rs10752124	66.79354126
KL	rs2477639	50.63848816
KL	rs692440	30.78268942
KL	rs11605349	86.41332658
KL	rs7298766	589.4917704
KL	rs36078145	125.4807684
KL	rs79122344	33.20089897
KL	rs1169311	51.97110073
KL	rs117633773	34.2658695
KL	rs55714927	118.4524117
KL	rs143501783	50.56852653
KL	rs162000	32.67435659
KL	rs9961915	662.2977892
KL	rs17855739	37.10269695
KL	rs117877056	45.01001588
KL	rs117241334	55.26769676
KL	rs2823253	31.20472102
KL	rs71322420	47.31701327
KL	rs1264373	40.0532923
KL	rs66497154	41.15672801
KL	rs62505473	67.85968887
KL	rs7849977	70.28416832
KL	rs8176746	715.6352774
KL	rs739468	170.5926732
FABP2	rs298978	96.29323644
FABP2	rs11726993	1124.972713
FABP2	rs13111751	930.2654683
FABP2	rs75135627	2944.166027
FABP2	rs12502424	833.7046877
FABP2	rs424535	43.14005224
FABP2	rs281379	34.87618368

FABP2	rs2048493	55.70550552
FABP2	rs58289215	31.41415032
FABP2	rs10026650	190.8440187
FABP2	rs76350290	301.9714825
FABP2	rs77657103	130.061335
FABP2	rs13122213	48.27290863
FABP2	rs79551293	73.40898987
FABP2	rs116089146	30.45765457
FABP2	rs147390813	48.67221239
FABP2	rs28688825	32.47577564
FABP4	rs16909138	75.57808928
FABP4	rs1421085	61.41963711
FABP4	rs34674929	29.82769101
FABP4	rs2197423	81.47272929
FABP4	rs72961007	30.19449388
NRP2	rs16837000	207.3262126
NRP2	rs113213551	83.34009268
NRP2	rs141893000	67.08218446
NRP2	rs333947	39.23310812
NRP2	rs56199187	41.09978789
NRP2	rs7949696	30.65043941
NRP2	rs139404143	31.50704949
NRP2	rs11111630	41.91968914
NRP2	rs78004438	32.23914453
NRP2	rs4981022	64.70291968
NRP2	rs77262773	30.13103261
NRP2	rs145645281	30.50845085
NRP2	rs72915925	30.74747518
NRP2	rs115214655	47.80572932
NRP2	rs17629251	36.10223265
NRP2	rs75145867	118.161524
NRP2	rs72958127	32.20546924
NRP2	rs113341849	39.92508925
NRP2	rs115832719	44.894311
NRP2	rs143840518	31.43945856
NRP2	rs1364076	29.98435906
NRP2	rs2844572	74.12301331
NRP2	rs3096688	53.66111424
NRP2	rs56287412	41.6359048
FCGR3A	rs1466933	89.83123394
FCGR3A	rs143426473	377.0851733
FCGR3A	rs7531561	1419.163073
FCGR3A	rs4657041	8075.913837
FCGR3A	rs116787511	4887.160544
FCGR3A	rs10918341	620.163564
FCGR3A	rs12095544	167.8369583
FCGR3A	rs1935992	39.59114599
FCGR3A	rs35118879	32.72613598
FCGR3A	rs75609405	59.52727319
FCGR3A	rs4657260	92.37080112
FCGR3A	rs16849374	57.75397432
FCGR3A	rs653178	61.8145133
FCGR3A	rs7147722	29.7503834
FCGR3A	rs56115403	35.58924341
FCGR3A	rs278373	89.25107805
FCGR3A	rs11920688	45.34881815
FCGR3A	rs12665091	31.79490873
FCGR3A	rs7047770	41.69644575

GAL	rs10922098	637.364417
GAL	rs35351085	34.85215798
GAL	rs1707652	128.6446822
GAL	rs555007	77.07300854
GAL	rs550671	183.9412479
GAL	rs9269234	31.24897179
GAL	rs9272526	36.9665317
GAS6	rs111762974	45.97275233
GAS6	rs6602909	889.3814138
GAS6	rs6602900	93.56046741
GAS6	rs9525363	32.98946025
GAS6	rs9590402	43.05847669
GAS6	rs55714927	110.0824853
GAS6	rs143501783	62.4136735
GAS6	rs77262773	32.93271263
GAS6	rs338583	50.642809
GAS6	rs2304234	494.1515143
GAS6	rs13074787	47.75040483
GAS6	rs13108218	42.42210431
GAS6	rs1800759	43.25789438
GAS6	rs9268199	58.09960248
GAS6	rs6962006	32.52978745
GAS6	rs79624003	31.7454528
UNC5B	rs4517438	43.14820416
UNC5B	rs10823655	33.92788409
UNC5B	rs10999762	130.6513099
UNC5B	rs12262480	180.8227547
UNC5B	rs12133018	30.80964705
UNC5B	rs72704449	78.49364988
UNC5B	rs77924615	32.26127723
UNC5B	rs780094	40.04061848
UNC5B	rs2837995	32.10137491
UNC5B	rs8176743	225.0593771
GSTM1	rs116680878	35.78604574
GSTM1	rs12034332	57.49730636
GSTM1	rs150896402	72.03643349
GSTM1	rs115232080	118.7212009
GSTM1	rs115181845	1145.063555
GSTM1	rs2269340	3402.163851
GSTM1	rs115929572	4504.706105
GSTM1	rs115814431	313.182877
GSTM1	rs10776700	60.25626769
GSTM1	rs6541375	32.61961347
GSTM1	rs115614917	36.84331798
GSTM1	rs114240822	144.2174345
GSTM1	rs146795662	37.94317944
GSTM1	rs112298818	48.83735493
GSTM1	rs7141205	41.8518391
GSTM1	rs2073333	644.0366856
ID1	rs10922094	68.59173266
ID1	rs1706435	34.73677578
ID1	rs454212	34.03876929
IFNA4	rs10922098	129.3231365
IFNA4	rs391165	46.56698562
IFNA4	rs28675927	30.65545746
LCAT	rs704	146.705225
LCAT	rs56210800	39.58518451
LCAT	rs149468457	39.63767121

LCAT	rs1619994	49.28718542
LCAT	rs6444156	47.81783373
LDHA	rs111294128	99.51603231
LDHA	rs115655239	45.54980599
LDHA	rs11611373	44.00750779
LDHA	rs74626198	135.7486959
LDHA	rs12975366	49.75528288
LDHA	rs1354034	29.85311268
LDHA	rs3824496	37.4614745
LYG2	rs10922098	76.91996739
LYG2	rs10822155	58.05196135
LYG2	rs17580	51.39136762
LYG2	rs7172696	33.52114752
LYG2	rs13412535	34.62048132
LYG2	rs6993770	37.65513044
MAPK10	rs35340377	38.14355548
MAPK10	rs4980320	42.34705096
MAPK10	rs35979828	30.60707202
MAPK10	rs17784482	46.33317542
MAPK10	rs144176526	82.02522891
MAPK10	rs146527756	54.45072078
MAPK10	rs6993770	50.1978942
MMP20	rs10922098	550.763212
MMP20	rs11078072	34.21138009
MMP20	rs1047286	46.98318278
MMP20	rs1707652	113.658564
MMP20	rs9266329	33.63320506
MMP20	rs9267139	59.14025958
MMP20	rs550671	166.3451936
MMP20	rs9271645	41.3541972
NEU1	rs2073044	36.45368396
NEU1	rs174564	47.68614059
NEU1	rs112829508	33.03379578
NEU1	rs7964859	482.5707548
NEU1	rs10778161	44.97960463
NEU1	rs73001065	53.00367518
NEU1	rs9346805	49.43734733
NEU1	rs576123	33.97663992
LOXL3	rs17010021	160.9655092
LOXL3	rs5174	71.64484236
LOXL3	rs113520471	54.46131795
LOXL3	rs10127790	33.47013872
LOXL3	rs10822155	67.27556102
LOXL3	rs2576976	142.215472
LOXL3	rs1678388	224.228025
LOXL3	rs145897705	198.9755226
LOXL3	rs72636534	43.42549077
LOXL3	rs17268589	926.6658667
LOXL3	rs146205183	132.6420303
LOXL3	rs9562041	107.1779696
LOXL3	rs61966959	48.44550094
LOXL3	rs113906094	42.55506858
LOXL3	rs76373179	405.3604475
LOXL3	rs115745987	56.93385314
LOXL3	rs1654449	31.26786651
LOXL3	rs6897795	33.32217991
OPTC	rs10922098	578.1678471
OPTC	rs35351085	29.76542857

OPTC	rs10208529	31.45508047
OPTC	rs1707652	99.62330434
OPTC	rs1265941	66.32101014
OPTC	rs550671	145.2925876
OTC	rs7519043	81.3554177
OTC	rs12137164	46.06524755
OTC	rs151068477	34.32834756
OTC	rs9645500	35.38328213
OTC	rs4149067	33.67165356
OTC	rs147350387	36.78380889
OTC	rs28650012	35.73758264
OTC	rs4940689	71.89445183
OTC	rs4835265	118.7065579
OTC	rs10075805	32.11248545
OTOR	rs75098569	56.93613318
OTOR	rs143584132	45.50959191
OTOR	rs1329423	1057.902086
OTOR	rs2019727	1273.85008
OTOR	rs1747818	95.09059993
OTOR	rs141856814	49.6149063
OTOR	rs2230694	40.72970378
OTOR	rs147906938	31.91561198
PNP	rs1049564	247.0959662
PNP	rs1760928	71.30927118
PNP	rs4541868	36.33483664
RAMP3	rs10922094	123.0174589
RAMP3	rs6542680	76.85430596
RAMP3	rs1355538	45.84335478
RAMP3	rs9501600	38.92959595
PCSK2	rs6044699	104.8529012
PCSK2	rs368465	175.2727619
PCSK2	rs2509919	33.25815294
PCSK2	rs11720167	57.83860275
PCSK2	rs451652	43.96436553
PCSK2	rs4713498	61.2020932
GM2A	rs72794132	837.6007596
GM2A	rs375396	246.2573646
GM2A	rs66495175	60.55264112
GM2A	rs732975	29.83193622
GM2A	rs77924615	55.60203883
GM2A	rs11740355	49.79469138
SERPINB4	rs75064813	294.9831512
SERPINB4	rs12969816	55.49920789
SERPINB4	rs213129	32.93808899
SERPINB4	rs2516993	36.72427441
SERPINB4	rs78111136	49.9549853
SERPINB4	rs79922210	32.90183793
NRG1	rs9561963	114.9119019
NRG1	rs143185594	32.45217045
NRG1	rs1801690	378.3669976
NRG1	rs59357103	64.1704787
NRG1	rs149062332	65.71542678
NRG1	rs4713460	40.85159488
NRG1	rs12614	82.27745908
NRG1	rs2557773	141.708286
NRG1	rs33997473	32.90872446
SORD	rs142462144	39.61941637
SORD	rs56822449	60.18816167

SORD	rs72722045	693.3707983
SORD	rs56194896	66.37127892
SORD	rs2733251	31.13921161
SORD	rs11611373	91.59040548
SORD	rs75036712	92.11098147
SORD	rs690507	42.05772463
TIMD4	rs4704826	766.3714843
TIMD4	rs12513886	96.12329846
TIMD4	rs28638168	45.67430368
TIMD4	rs6555971	88.8575301
TIMD4	rs2282718	29.85956159
TIMD4	rs661126	38.48951248
TIMD4	rs2068888	37.30170707
TIMD4	rs1377416	107.5542425
TIMD4	rs11230633	33.40819767
TIMD4	rs73026960	49.29004955
TIMD4	rs112771035	705.9442201
TIMD4	rs6590207	180.3079296
TIMD4	rs10843390	79.91387629
TIMD4	rs10778118	30.18293222
TIMD4	rs10849819	31.7225011
TIMD4	rs894491	32.10929492
TIMD4	rs1864163	35.81648002
TIMD4	rs34318854	35.22095725
TIMD4	rs12945299	97.38653961
TIMD4	rs143501783	65.13960168
TIMD4	rs77262773	56.67220611
TIMD4	rs7214944	33.75087129
TIMD4	rs601338	38.13187388
TIMD4	rs780094	34.12750425
TIMD4	rs10208580	106.8979169
TIMD4	rs59870886	44.33980642
TIMD4	rs116678101	81.50077524
TIMD4	rs56206139	31.52267826
TIMD4	rs35068491	31.92383144
TIMD4	rs66817580	127.005149
TIMD4	rs7626629	39.85713514
TIMD4	rs6810489	42.04509488
TIMD4	rs115216147	55.23989471
TIMD4	rs27289	47.6776965
TIMD4	rs9281828	119.0296527
TIMD4	rs9273060	60.17276243
TIMD4	rs10456852	54.87118471
TIMD4	rs2737245	31.1944607
TIMD4	rs56097301	32.56219668
TIMD4	rs576123	33.44185961
NT5E	rs9294301	54.16220157
NT5E	rs9353298	37.94433901
NT5E	rs632350	2752.522199
NT5E	rs4296866	225.2625667
NT5E	rs4707234	583.2453434
NT5E	rs10944174	582.6254957
NT5E	rs77562907	47.78617008
NT5E	rs78705678	55.66989678
NT5E	rs2477642	39.72669576
NT5E	rs2393986	30.75509046
NT5E	rs174529	43.2525414
NT5E	rs35887873	32.99841207

NT5E	rs10144543	48.58801917
NT5E	rs2414577	33.74525357
NT5E	rs12945299	43.29950147
NT5E	rs8736	57.03314105
NT5E	rs416745	73.85660517
NT5E	rs111795396	236.9549049
NT5E	rs9450555	75.33711262
NT5E	rs77705726	88.80389794
NT5E	rs77448558	30.22240454
AMBP	rs28616789	144.4086191
AMBP	rs56294298	67.60458822
AMBP	rs61747728	33.75477565
AMBP	rs77924615	32.77679555
AMBP	rs143487297	32.52817892
AMBP	rs17190106	42.87260559
AMBP	rs2260051	106.36272
AMBP	rs6477	164.0459548
AMBP	rs8283	225.6879243
AMBP	rs421446	49.12745554
ADAM15	rs10922094	37.77057205
ADAM15	rs16914574	33.13556899
ADAM15	rs143666677	36.6082671
ADAM15	rs17580	74.15421207
ADAM15	rs112963922	54.82175084
ADAM15	rs5816953	30.20809541
ADAM15	rs1355538	44.08630614
ANPEP	rs10922094	51.98105098
ANPEP	rs34843303	46.89720125
ANPEP	rs9271573	30.93192661
DPP4	rs10922094	382.1002696
DPP4	rs1707652	119.5460461
DPP4	rs9267139	35.98277612
DPP4	rs3891366	133.2454084
DPP4	rs7774739	89.1687472
DPP4	rs9271766	42.65623255
CD8A	rs80170993	95.46557512
CD8A	rs34933869	1235.841425
CD8A	rs7587224	98.81900944
CD8A	rs111591143	54.75931228
CD8A	rs2478539	61.29924672
CD8A	rs28999107	30.3638516
CD8A	rs3184504	115.635483
CD8A	rs12978752	37.02431765
CD8A	rs7609387	55.1744776
CD8A	rs11682671	39.06763397
CD8A	rs71251947	54.32064793
CD8A	rs469783	56.01281222
CD8A	rs74976306	31.93610153
CD8A	rs55813680	70.69605032
CD8A	rs147107592	95.85661892
CD8A	rs7749652	105.9471395
CD8A	rs114464272	137.5189289
CD8A	rs13211972	917.0091535
CD8A	rs9266196	612.3847973
CD8A	rs2717539	37.12348668
CD8A	rs2075533	60.75889785
CD8A	rs635634	60.44815735
COL9A1	rs6455357	31.23690675

COL9A1	rs3806095	70.6955832
COL9A1	rs10922098	53.01623804
COL9A1	rs12190836	31.12955592
CTHRC1	rs72704449	32.99357299
CTHRC1	rs2638314	30.03695083
CTHRC1	rs13379042	37.42711957
CTHRC1	rs116853338	30.06163854
CTHRC1	rs150844304	90.47093176
CTHRC1	rs340005	56.54356673
CTHRC1	rs704	46.51539104
CTHRC1	rs6119254	37.27589397
CTHRC1	rs55649657	46.54329752
CTHRC1	rs9801017	406.9923415
CTHRC1	rs150731032	66.03036928
CFHR1	rs17696917	427.7355804
CFHR1	rs191753228	409.3174331
CFHR1	rs71524417	927.8612937
CFHR1	rs35449482	1105.702373
CFHR1	rs34231058	1022.212842
CFHR1	rs76831210	3343.517623
CFHR1	rs145456171	266.4520902
CFHR1	rs78805188	47.9285976
CFHR1	rs192413466	83.02033242
CFHR1	rs11554178	36.60923185
CFHR1	rs79473878	40.998776
CFHR1	rs115353164	72.4804643
CFHR1	rs1769325	49.26255637
CFHR1	rs79060385	75.3160125
CFHR1	rs11190375	1164.061794
CFHR1	rs11190387	145.7621682
CFHR1	rs147343606	83.12940262
CFHR1	rs1421085	31.62959738
CFHR1	rs9933029	47.91399781
CFHR1	rs704	109.0165043
CFHR1	rs715	33.36377426
CFHR1	rs62288099	128.6959082
HEXB	rs1696980	138.4689833
HEXB	rs13164140	895.8588695
HEXB	rs7442914	43.37364184
HEXB	rs9425328	34.84125989
HEXB	rs7897379	46.79384265
HEXB	rs75506960	32.95234649
HEXB	rs3813731	47.75847828
HEXB	rs12230154	47.33727642
HEXB	rs115655239	131.8989886
HEXB	rs11611373	131.5040553
HEXB	rs187229204	30.2830275
HEXB	rs76391683	44.68820695
HEXB	rs11110781	60.56757203
HEXB	rs78386135	36.27261365
HEXB	rs2251298	1251.835909
HEXB	rs150043343	107.8321605
HEXB	rs7980436	494.4586376
HEXB	rs9530288	38.50151536
HEXB	rs7147869	34.17872125
HEXB	rs147233090	47.66758519
HEXB	rs339969	33.98907394
HEXB	rs74949863	30.36883985

HEXB	rs62072124	32.37299015
HEXB	rs58542926	180.5816774
HEXB	rs429358	76.49488792
HEXB	rs8736	52.30739075
HEXB	rs738409	42.50101037
HEXB	rs71309976	40.75676216
HEXB	rs1154988	132.1350713
HEXB	rs4274960	58.58442577
HEXB	rs7763108	42.28966856
HEXB	rs28601761	48.26283967
PNLIPRP2	rs116329237	42.14291819
PNLIPRP2	rs7072869	265.4368837
PNLIPRP2	rs149271803	65.47139708
PNLIPRP2	rs11197747	5746.196737
PNLIPRP2	rs17735613	4655.008086
PNLIPRP2	rs1638410	91.65176916
PNLIPRP2	rs11197895	421.4304657
PNLIPRP2	rs242982	51.85616217
PNLIPRP2	rs11196736	60.2143294
PNLIPRP2	rs149069870	124.1994385
PNLIPRP2	rs112062107	45.52284583
PNLIPRP2	rs55993634	68.74251571
PNLIPRP2	rs516316	262.9072074
PNLIPRP2	rs8176693	93.35806746
LRP11	rs76976204	43.37713414
LRP11	rs17729786	126.9393869
LRP11	rs564961	924.3880943
LRP11	rs117937568	169.2000201
LRP11	rs7762285	329.6388826
LRP11	rs138604219	43.56625048
LRP11	rs647932	30.65347121
LRP11	rs12133018	33.12589035
LRP11	rs61747728	82.20130635
LRP11	rs77924615	42.11195303
LRP11	rs13108218	34.47192653
LRP11	rs6853156	39.4729854
LRP11	rs203134	31.25474576
LRP11	rs62431889	34.2506134
LRP11	rs76455993	73.90239081
LRP11	rs148653156	139.7286686
LRP11	rs145136225	109.8363635
LRP11	rs11203609	31.94839157
PLTP	rs76849991	207.9283402
PLTP	rs2200587	142.9222515
PLTP	rs190148453	134.2583337
PLTP	rs73620988	50.0239318
PLTP	rs79833227	1119.681843
PLTP	rs117479286	351.351227
PLTP	rs75053191	70.76456063
PLTP	rs76720462	102.9461003
PLTP	rs75591819	110.203609
PLTP	rs78689694	56.71896333
PLTP	rs117633773	32.47931992
PLTP	rs55714927	65.78568585
PLTP	rs143501783	80.97260944
PLTP	rs77262773	44.28451047
PLTP	rs492602	31.39570331
PLTP	rs143128743	46.63168436

PLTP	rs10935473	152.5705855
PLTP	rs7889	45.00544698
PLTP	rs635634	42.84251727
REG3G	rs115154697	43.2939984
REG3G	rs112834521	138.6891087
REG3G	rs4270377	2727.155369
REG3G	rs13030971	1059.499299
REG3G	rs3806561	123.0680398
REG3G	rs13417018	145.3977703
REG3G	rs2228347	30.43231911
REG3G	rs2793831	51.75422807
REG3G	rs7938011	29.96639826
REG3G	rs499241	44.1533472
REG3G	rs7970943	39.44379705
REG3G	rs28462048	29.78732472
REG3G	rs111869668	56.71014883
REG3G	rs1126464	40.51567126
REG3G	rs11658433	32.44335619
REG3G	rs1882682	40.7509157
REG3G	rs10182495	100.8278733
REG3G	rs35995534	58.96249215
REG3G	rs6137646	39.92152398
REG3G	rs9983358	35.68430967
REG3G	rs718768	32.00232061
REG3G	rs10013413	43.37264693
REG3G	rs71624710	38.24185863
REG3G	rs31863	32.03806009
REG3G	rs2523442	43.26080866
REG3G	rs9278208	39.09602467
REG3G	rs7766106	48.70026544
REG3G	rs13438309	63.20034052
REG3G	rs13256317	36.98549956
REG3G	rs576123	90.54025514
VNN2	rs72985829	66.18960607
VNN2	rs76988041	103.9120674
VNN2	rs12205095	8082.301571
VNN2	rs12195746	358.158018
VNN2	rs79709397	218.8587852
VNN2	rs117161286	93.6612018
VNN2	rs3917932	29.89923684
VNN2	rs2477639	43.24751858
VNN2	rs2511241	188.1841728
VNN2	rs34656952	66.16445332
VNN2	rs3862627	109.8410106
VNN2	rs921651	140.7312129
VNN2	rs8066582	192.957503
VNN2	rs4760	55.92418333
VNN2	rs6723921	81.85580374
VNN2	rs58719755	56.60919232
VNN2	rs6414311	31.86328058
VNN2	rs9266059	46.87988389
VNN2	rs375239297	29.95008736
VNN2	rs2627233	109.8419147
VNN2	rs77170649	145.4421389
VNN2	rs2347784	30.27210716
VNN2	rs149007767	37.77484013
VNN2	rs4512391	41.85802679
CAMP	rs55771110	89.07168262

CAMP	rs7518497	52.63749478
CAMP	rs4657179	37.84043992
CAMP	rs12762973	35.53650881
CAMP	rs61863078	37.70307918
CAMP	rs2074038	303.1191033
CAMP	rs964184	146.2132053
CAMP	rs12180	74.463026
CAMP	rs9554263	36.68468312
CAMP	rs1532085	42.05875315
CAMP	rs12444012	30.70716973
CAMP	rs7502514	62.70391769
CAMP	rs35362691	45.7684052
CAMP	rs60642791	126.0906512
CAMP	rs34216592	3565.399
CAMP	rs4147974	391.2750126
CAMP	rs17703558	365.9543609
CAMP	rs2521350	34.33924069
CAMP	rs149524607	71.39691859
CAMP	rs2041037	41.96999293
CAMP	rs6507939	35.40559437
CAMP	rs5117	43.58472285
CAMP	rs5112	56.3459443
CAMP	rs17019087	45.78626544
CAMP	rs243597	109.1871291
CAMP	rs2734031	57.39782222
CAMP	rs9840812	32.36479439
CAMP	rs1707652	220.1662801
CAMP	rs1471251	36.49791026
CAMP	rs4619875	35.71559052
CAMP	rs13165628	33.8270464
CAMP	rs1144700	34.33369822
CAMP	rs199842489	35.25104798
CAMP	rs73747624	69.95658943
CAMP	rs2734934	47.32344903
CAMP	rs41556417	73.19501532
CAMP	rs141874749	94.16926638
CAMP	rs11769630	162.2667635
CAMP	rs10103048	42.53402045
A2ML1	rs1558526	641.4465529
A2ML1	rs71453682	45.28646252
A2ML1	rs10922098	49.9116509
A2ML1	rs11220477	34.89252702
A2ML1	rs516316	79.96725054
A2ML1	rs9864801	35.50229948
AGRN	rs6657048	132.783676
AGRN	rs4970394	810.4566293
AGRN	rs11260546	88.34596102
AGRN	rs146525333	46.95113757
AGRN	rs144974594	41.2649325
AGRN	rs79203343	49.37276463
AGRN	rs3740777	106.0825208
AGRN	rs2526538	47.3136908
AGRN	rs77262773	40.46379197
AGRN	rs429358	29.95494557
AGRN	rs35136575	34.742994
AGRN	rs907866	31.17385536
AGRN	rs6061230	591.0892871
AGRN	rs62262670	31.34811876

AGRN	rs7628146	81.50773936
AGRN	rs13107325	36.90136798
AGRN	rs3130955	46.40182241
AGRN	rs6458	34.72647632
AGRN	rs10251094	30.63463136
AGRN	rs10758693	48.9491975
AOC1	rs10253089	58.11103672
AOC1	rs4725943	972.822901
AOC1	rs73163602	4326.440616
AOC1	rs10156191	8851.514583
AOC1	rs77855615	608.9311791
AOC1	rs2240629	355.4378042
AOC1	rs62479791	587.1483312
AOC1	rs2511241	31.02594913
AOC1	rs1260326	32.22611609
AOC1	rs3130484	34.46194307
AOC1	rs10952717	33.59423441
AOC1	rs56066958	222.7198071
AOC1	rs10237223	70.92140077
AOC1	rs36036364	52.49028732
AOC1	rs4454213	33.64530295
CES1	rs3859101	31.32543298
CES1	rs10922094	526.1229173
CES1	rs73001065	33.50173772
CES1	rs157592	30.83835975
CES1	rs1260326	78.09955057
CES1	rs1707652	117.1158014
CES1	rs1265941	69.54814357
CES1	rs550671	156.5656145
CES1	rs4240624	40.73124386
CD248	rs116884770	36.15766174
CD248	rs145928297	30.18167732
CD248	rs333947	38.93539696
CD248	rs61801021	57.3134963
CD248	rs11039216	33.60047721
CD248	rs150179248	42.74347022
CD248	rs147174770	35.40037833
CD248	rs10861032	50.08523827
CD248	rs201862321	73.74795392
CD248	rs150844304	30.63542527
CD248	rs1260326	60.58765045
CD248	rs74814272	31.74936558
CD248	rs186189054	55.16376334
CD248	rs3199918	55.18955
CD248	rs141359357	66.28520605
CD248	rs143840518	55.95723249
CD248	rs62292950	39.06029046
CD248	rs62446919	48.50029121
CD248	rs11768171	59.83198714
CD248	rs2142306	33.29265835
CD248	rs12544863	30.28827663
FGFBP1	rs2314019	78.62043528
FGFBP1	rs333947	39.11429651
FGFBP1	rs3184504	33.36081353
FGFBP1	rs77542162	41.98120004
FGFBP1	rs4665972	35.20874347
FGFBP1	rs2074735	46.27887837
FGFBP1	rs9269037	30.83459522

FGFBP1	rs3129761	47.70119144
FOLR3	rs78672796	989.984091
FOLR3	rs12798050	498.1822565
FOLR3	rs71464396	1057.563983
FOLR3	rs76848014	4376.814116
FOLR3	rs61895676	315.061964
FOLR3	rs77803271	43.5222252
FOLR3	rs75926779	658.478855
FOLR3	rs10922098	114.8932797
FOLR3	rs11230803	54.22345374
FOLR3	rs111490736	41.46841278
FOLR3	rs117976804	41.87834403
FOLR3	rs1623476	353.9190124
FOLR3	rs72983223	36.95878298
FOLR3	rs112240808	229.03122
FOLR3	rs17748438	33.44986355
FOLR3	rs186284494	77.1253178
FOLR3	rs34300064	44.524131
FOLR3	rs73504617	58.22059294
FOLR3	rs4482056	29.74988037
FOLR3	rs138312560	43.47250742
FOLR3	rs704	39.55973606
HS3ST1	rs10922098	377.4507702
HS3ST1	rs1707652	67.50902305
HS3ST1	rs3130955	34.69743996
HS3ST1	rs9501600	111.2310112
ATRN	rs144235722	38.83336034
ATRN	rs141486209	114.3476762
ATRN	rs74401648	61.6193973
ATRN	rs76034548	1143.421209
ATRN	rs182203670	67.85065319
ATRN	rs145194579	394.5219087
ATRN	rs6052017	96.55350444
ATRN	rs144469244	580.136648
ATRN	rs73610112	205.4548955
ATRN	rs11264230	44.59883073
ATRN	rs10624859	34.27735624
LEFTY2	rs115491302	42.40868008
LEFTY2	rs360058	458.5405398
LEFTY2	rs41304002	51.16201973
LEFTY2	rs597429	33.72657122
LEFTY2	rs72704449	41.32559197
LEFTY2	rs77924615	36.06695816
LEFTY2	rs1707652	76.95970697
LEFTY2	rs9501340	53.4542304
LEFTY2	rs28474889	142.325425
LEFTY2	rs148766553	130.8512597
LEFTY2	rs6458	289.4239805
LEFTY2	rs9273325	79.86395821
LEFTY2	rs12378445	58.22422765
LEFTY2	rs2615068	412.2195703
LEFTY2	rs2740168	7147.628497
LEFTY2	rs56301120	915.2021936
LEFTY2	rs145643169	479.2077488
LEFTY2	rs141415111	158.684077
LEFTY2	rs186015966	76.9208474
LEFTY2	rs144135113	32.05891445
LEFTY2	rs116224866	110.3897914

LEFTY2	rs114468433	62.94849873
LEFTY2	rs58012510	86.28874593
LEFTY2	rs10915730	132.995136
LEFTY2	rs77722489	51.82901645
LEFTY2	rs61824945	53.94360516
LEFTY2	rs144548279	39.49005773
LEFTY2	rs7532346	39.54989218
LEFTY2	rs61835449	41.67081001
LEFTY2	rs2241170	71.00855125
LEFTY2	rs10013413	33.0905897
LEFTY2	rs635634	59.37934251
BIRC7	rs10922098	31.86046863
BIRC7	rs146457388	35.08248191
BIRC7	rs6542680	639.8835138
BIRC7	rs80220851	41.01711218
LRP12	rs2942570	35.39371331
LRP12	rs203688	38.2732487
LRP12	rs6993770	38.62681356
NAGLU	rs1076188	161.7193844
NAGLU	rs190597324	273.4767465
NAGLU	rs58947745	1006.622998
NAGLU	rs12150483	162.5226293
NAGLU	rs148215732	3582.212856
NAGLU	rs139325057	47.82185152
NAGLU	rs77386381	340.9542452
NAGLU	rs56097721	362.378664
NAGLU	rs147677460	272.9610725
NAGLU	rs10797949	37.76197875
NAGLU	rs4651228	32.52821447
NAGLU	rs146409195	37.44762262
NAGLU	rs999763	48.52058067
NAGLU	rs10489705	36.01435527
NAGLU	rs150161290	45.7396986
NAGLU	rs140629687	86.357561
NAGLU	rs16838346	284.0160025
NAGLU	rs147319422	132.2296368
NAGLU	rs78379279	1212.275183
NAGLU	rs34251987	1295.154722
NAGLU	rs35449482	1238.483712
NAGLU	rs142413464	943.5899198
NAGLU	rs191076788	539.023275
NAGLU	rs7537588	5933.557397
NAGLU	rs4335403	445.604358
NAGLU	rs76505423	196.5710985
NAGLU	rs116514830	104.7128774
NAGLU	rs137905573	158.3267142
NAGLU	rs116394272	160.1664114
NAGLU	rs12565263	33.22513633
NAGLU	rs12083166	34.06637299
NAGLU	rs4597119	33.06346811
NAGLU	rs62076601	93.67183076
NAGLU	rs74939104	132.4512012
NAGLU	rs116930878	74.13181376
NAGLU	rs139322023	58.24332427
NAGLU	rs138488005	37.94728033
NPTXR	rs145515994	250.5583818
NPTXR	rs117773903	2525.053639
NPTXR	rs55935155	91.17838535

NPTXR	rs6001369	152.0613339
NPTXR	rs148578448	223.454209
NPTXR	rs78705678	58.95712366
NPTXR	rs2461161	56.02861356
NPTXR	rs151233481	73.62316897
NPTXR	rs117616798	82.28574591
NPTXR	rs62237039	47.13446648
NPTXR	rs181446844	156.6755814
NPTXR	rs112595670	120.7515352
NPTXR	rs2240046	32.30790248
PRSS8	rs55667375	43.60046929
PRSS8	rs10922094	83.71512503
PRSS8	rs149155722	81.77634379
PRSS8	rs147398533	30.20314123
PRSS8	rs76045563	41.45200692
PRSS8	rs1260326	52.85284043
PRSS8	rs1706435	52.36779194
PRSS8	rs62402680	31.9566922
PRSS8	rs28601761	31.72643146
BCHE	rs697356	3343.602579
BCHE	rs11924273	70.15284911
BCHE	rs77230198	133.3459119
BCHE	rs12059695	134.8808377
BCHE	rs4746203	41.93713702
BCHE	rs174564	36.6970245
BCHE	rs780093	37.60983093
BCHE	rs34788166	34.89311666
BCHE	rs3814365	234.2718841
BCHE	rs6435932	32.92278992
BCHE	rs111696008	385.2036103
BCHE	rs144202860	215.9357609
BCHE	rs887829	71.00532555
BCHE	rs687339	39.31726555
BCHE	rs73021373	49.07502727
BCHE	rs73173009	86.70525206
BCHE	rs9825572	39.43418737
BCHE	rs147033786	73.07308944
BCHE	rs114893996	30.28912759
BCHE	rs28732107	34.0460871
BCHE	rs10502	32.24547286
BCHE	rs11968875	59.31340568
BCHE	rs13244614	51.9879503
BCHE	rs4240624	54.63299993
BCHE	rs59844459	38.31982129
SAA1	rs4148620	37.63437416
SAA1	rs2299629	106.4898427
SAA1	rs79669707	1033.041904
SAA1	rs4609582	950.9577675
SAA1	rs4537730	75.15614014
SAA1	rs7131023	41.5642838
SAA1	rs11208691	256.7892853
SAA1	rs4071540	41.47853818
SAA1	rs12133641	56.16539019
SAA1	rs1864163	66.3561374
SAA1	rs429358	30.19683614
SAA1	rs6509571	46.56721351
SAA1	rs6445035	191.7507316
SAA1	rs3863087	108.4325258

SAA1	rs34434863	33.7868286
SAA4	rs117147437	4423.423598
SAA4	rs1356979	3133.451209
SAA4	rs34068567	355.9674243
SAA4	rs10766492	76.79564791
SAA4	rs73438377	47.01039026
SAA4	rs1648233	189.8414383
SAA4	rs12418493	142.3442289
SAA4	rs2702652	54.59074832
SAA4	rs58810621	31.94978146
SAA4	rs12720922	74.63176659
SAA4	rs28384491	111.0666351
CLSTN1	rs114116433	1308.819314
CLSTN1	rs114513597	297.9629756
CLSTN1	rs35960446	99.95258125
CLSTN1	rs58109538	169.8811985
CLSTN1	rs1188530	39.31559545
CLSTN1	rs143298504	30.78786119
CLSTN1	rs77542162	93.56437438
CLSTN1	rs5839310	38.66588688
CLSTN1	rs1354034	42.90702862
CLSTN1	rs600038	44.99424482
GLIPR2	rs72729437	84.35345927
GLIPR2	rs3739613	929.741732
GLIPR2	rs10973026	39.63066607
GLIPR2	rs423135	40.10732078
GLIPR2	rs117882248	57.22043305
GLIPR2	rs12342201	213.5430789
ALAD	rs146715110	101.8396874
ALAD	rs55917620	68.74357049
ALAD	rs1800435	816.9515942
ALAD	rs62555332	36.28938454
ALAD	rs149095400	30.44568925
ALAD	rs150823572	41.02492292
ADH1C	rs283415	245.4804552
ADH1C	rs4753123	31.46622881
ADH1C	rs738409	53.05887466
ADH1C	rs28601761	54.6142762
GSS	rs2424999	433.047455
GSS	rs11046893	34.06479506
GSS	rs6113872	30.87653993
GSS	rs73102628	43.14563676
GSS	rs62213218	31.28847231
GSS	rs4811529	36.76422764
EPHB4	rs35061193	42.05414555
EPHB4	rs314366	183.771521
EPHB4	rs72704449	46.79358022
EPHB4	rs724444	42.95845675
EPHB4	rs9558661	86.09275919
EPHB4	rs7329478	35.86112289
EPHB4	rs143616286	29.97536454
EPHB4	rs77924615	37.67362344
EPHB4	rs1260326	30.24383251
EPHB4	rs13108218	36.38713116
EPHB4	rs368611	29.86077435
EPHB4	rs146798134	69.27598554
EPHB4	rs635634	1016.298278
EPHB4	rs41307428	143.7225848

MSR1	rs78993928	50.30169959
MSR1	rs1993978	84.75568956
MSR1	rs59251421	511.7831401
MSR1	rs145734953	77.61224348
MSR1	rs13251172	156.9110083
MSR1	rs41271951	79.34039615
MSR1	rs6688272	30.92065435
MSR1	rs1613078	93.21065568
MSR1	rs683486	201.869636
MSR1	rs7933515	38.49163997
MSR1	rs12945299	172.6701474
MSR1	rs143501783	50.56939964
MSR1	rs62165726	35.92071837
MSR1	rs3747207	32.88026988
MSR1	rs13071075	44.74170463
MSR1	rs2062059	96.62305347
MSR1	rs11920688	50.20545636
MSR1	rs9998898	98.65399647
MSR1	rs9269492	47.23590918
MSR1	rs139299944	83.8948892
MSR1	rs75525829	36.30746468
MSR1	rs7865362	36.91117091
PRSS27	rs71386687	486.5269011
PRSS27	rs916947	32.71625742
PRSS27	rs113422568	35.53546857
PRSS27	rs7123489	32.11526571
PRSS27	rs35156054	140.0891248
PRSS27	rs11220477	301.8552585
PRSS27	rs35527562	38.90204984
PRSS27	rs150158762	30.29876666
PRSS27	rs708686	188.2596794
PRSS27	rs1504519	81.76226238
PRSS27	rs10418222	86.18496987
PRSS27	rs411482	1369.947418
PRSS27	rs80206894	129.406868
PRSS27	rs518388	103.708081
PRSS27	rs6763927	67.61986931
PRSS27	rs2479016	190.747107
PRSS27	rs10971420	52.45302805
PRSS27	rs10972823	237.8556799
PRSS27	rs3824496	40.64450038
SLITRK1	rs2876803	55.94658102
SLITRK1	rs4652418	44.61171802
SLITRK1	rs77924615	38.83685793
SLITRK1	rs1260326	33.89281055
VIM	rs796667	89.5429081
VIM	rs3902042	30.14478908
VIM	rs10904915	40.01191531
VIM	rs438811	100.7720582
VIM	rs5112	38.12266555
VIM	rs4639017	40.76341399
VIM	rs114694170	34.53332713
KLK14	rs2569441	217.5661941
KLK14	rs3745540	62.97031626
KLK14	rs17658926	376.388288
KLK14	rs34093024	521.4319586
KLK14	rs76195063	58.16848266
KLK14	rs2455070	36.78246237

KLK14	rs11190363	29.80813464
KLK14	rs61729512	131.9654926
KLK14	rs1849188	37.58049068
KLK14	rs11674978	40.97076887
KLK14	rs268794	35.30326744
KLK14	rs28724138	33.08012954
KLK14	rs7823296	45.17372867
KLK14	rs576123	49.08186625
PPP3R1	rs10822155	71.78958172
PPP3R1	rs4980320	32.88212845
PPP3R1	rs113661219	34.58030425
PPP3R1	rs68066031	44.40688911
PPP3R1	rs1355538	30.03366575
PPP3R1	rs13129744	44.07329452
PPP3R1	rs6993770	38.77796229
PHGDH	rs10922103	96.1290946
PHGDH	rs1535	37.75489566
PHGDH	rs189902748	147.7645378
PHGDH	rs75746043	227.0884005
PHGDH	rs111278137	116.7190903
PHGDH	rs118147862	2858.826488
PHGDH	rs28399664	998.9721916
PHGDH	rs7254892	1184.936545
PHGDH	rs118170342	377.7714191
PHGDH	rs112293672	30.8484604
PHGDH	rs370881	95.54655813
PHGDH	rs117565628	52.51778427
PHGDH	rs35609178	44.29351317
PHGDH	rs149857247	67.17657131
PHGDH	rs6445035	108.4045936
PHGDH	rs3863087	38.50536876
PHGDH	rs11755689	29.91435705
ACHE	rs572515	101.4652839
ACHE	rs1706435	78.09891147
ACHE	rs389512	57.16349337
AMY2B	rs75782947	106.7551174
AMY2B	rs200048932	291.1352873
AMY2B	rs111980575	1089.905076
AMY2B	rs114500706	917.1941184
AMY2B	rs4847148	178.4417395
AMY2B	rs76527254	95.77375072
AMY2B	rs11578575	113.7209455
AMY2B	rs76836317	50.7338291
AMY2B	rs114279212	53.43157707
AMY2B	rs138026918	201.7048239
AMY2B	rs6678423	64.38662693
AMY2B	rs114118243	53.06053592
AMY2B	rs1713235	41.74864962
AMY2B	rs78705678	49.50199271
AMY2B	rs2477642	44.02324052
AMY2B	rs2227296	104.2550798
AMY2B	rs34858945	42.3039979
AMY2B	rs4423274	36.22153355
AMY2B	rs533406	54.41177633
AMY2B	rs56850260	40.8486784
AMY2B	rs679574	87.84971923
AMY2B	rs10472392	60.44057318
AMY2B	rs9968918	29.81166714

AMY2B	rs635634	202.1734611
ENPEP	rs80008037	89.9709972
ENPEP	rs35895172	717.0021052
ENPEP	rs6833731	999.8854546
ENPEP	rs13120170	228.2761109
ENPEP	rs76716443	71.55297895
ENPEP	rs10761784	40.83398356
ENPEP	rs144496825	143.7466283
ENPEP	rs79797272	87.70767952
ENPEP	rs114702284	62.01515519
ENPEP	rs10029917	34.14732505
ANTXR2	rs6829364	152.7140756
ANTXR2	rs7674623	453.3582102
ANTXR2	rs2125236	41.26173165
ANTXR2	rs75178643	30.06619658
ANTXR2	rs13329672	55.06719551
ANTXR2	rs2070895	63.7858417
ANTXR2	rs12149545	116.4555636
ANTXR2	rs112001035	113.3569273
ANTXR2	rs55698825	30.74197981
ANTXR2	rs8106813	46.84890465
ANTXR2	rs1260326	49.68556021
ANTXR2	rs6073958	57.98450643
ANTXR2	rs2686395	125.1326966
ANTXR2	rs199980439	31.94790482
ANTXR2	rs145391587	34.36001791
ANTXR2	rs10988676	38.86263743
TCN2	rs140099	925.6560162
TCN2	rs5997658	1271.04275
TCN2	rs116997758	383.201312
TCN2	rs112812772	1118.707522
TCN2	rs11089474	1299.097922
TCN2	rs2413004	1160.085285
TCN2	rs4820944	410.1792894
TCN2	rs2927715	42.50256643
TCN2	rs8104777	235.9684763
TCN2	rs73004560	43.61032511
TCN2	rs74277563	76.10707554
TCN2	rs140910498	222.6943412
TCN2	rs112100051	97.5472191
TCN2	rs117528365	31.02339317
TCN2	rs117885227	30.7998575
GUSB	rs145851867	195.8938452
GUSB	rs34356500	288.2878659
GUSB	rs1800450	73.0869587
GUSB	rs174574	67.04034813
GUSB	rs111849297	46.94031715
GUSB	rs10778141	105.6208741
GUSB	rs4764822	916.8355588
GUSB	rs7955091	39.72050058
GUSB	rs56272941	31.86506563
GUSB	rs7970695	36.95162304
GUSB	rs17532301	30.98446772
GUSB	rs147233090	108.8966625
GUSB	rs11640695	37.25704815
GUSB	rs2696549	32.86209509
GUSB	rs1801690	302.9221226
GUSB	rs1788606	108.3639439

GUSB	rs2900660	62.15663582
GUSB	rs200210321	103.5336932
GUSB	rs4420638	30.63470788
GUSB	rs1047891	39.14446271
GUSB	rs1154988	114.8521606
GUSB	rs72613567	39.19116791
GUSB	rs116587722	100.5241714
GUSB	rs3837027	55.47333845
GUSB	rs149742777	74.99702038
GUSB	rs114406793	34.46978549
GUSB	rs62440901	52.59765094
GUSB	rs4574742	41.23464059
GUSB	rs117981302	45.09030391
GUSB	rs76476993	52.79191126
GUSB	rs142443403	108.2569664
GUSB	rs10949891	50.85736573
GUSB	rs190670827	34.67150423
GUSB	rs142333581	129.5075026
GUSB	rs62455836	36.14973129
MUC16	rs562108	78.49400578
MUC16	rs200974959	70.71174775
MUC16	rs61742668	339.2248258
MUC16	rs368465	34.0538741
MUC16	rs722414	46.61899394
MUC16	rs1760957	29.83597516
MUC16	rs34786260	60.00578186
MUC16	rs7185523	491.5474169
MUC16	rs7185367	44.65927083
MUC16	rs516316	211.3498381
MUC16	rs190367401	34.04395424
MUC16	rs62193222	35.5405828
MUC16	rs62193070	436.4154794
MUC16	rs62193113	61.53091392
MUC16	rs141711339	130.4224917
MUC16	rs1355538	29.71459233
MUC16	rs34434863	30.32075565
MUC16	rs71562420	42.31448627
MUC16	rs635634	76.37955912
CNN1	rs585790	155.9966575
CNN1	rs10922098	51.60609858
CNN1	rs1769758	81.59656801
CNN1	rs12767683	132.1298666
CNN1	rs73000929	69.66941033
CNN1	rs11553699	123.927029
CNN1	rs72716646	32.57367771
CNN1	rs141229230	32.41320676
CNN1	rs9745114	32.00309134
CNN1	rs146916271	62.03908811
CNN1	rs77554484	39.49748639
CNN1	rs4783187	30.3250295
CNN1	rs12445547	38.61867352
CNN1	rs680478	31.57559921
CNN1	rs1047891	96.03043641
CNN1	rs62191340	130.9244616
CNN1	rs5750957	46.57697855
CNN1	rs4693545	62.58747234
CNN1	rs114694170	76.74498924
CNN1	rs10268683	32.80176574

CNN1	rs342296	201.7227886
CNN1	rs6993770	357.3420363
CD247	rs10922098	113.7151758
CD247	rs4639017	31.84580825
CD247	rs9501393	42.99878981
CD247	rs17205128	37.27451258
COL2A1	rs8001611	88.26104406
COL2A1	rs138103546	33.91901986
COL2A1	rs7218591	31.08776192
COL2A1	rs116585561	104.6807756
COL2A1	rs118081843	38.30364603
COL2A1	rs147377769	35.56379089
COL2A1	rs145495890	51.07541527
COL2A1	rs146385050	347.7111212
COL2A1	rs184378334	33.91092095
CR2	rs3813948	49.90295215
CR2	rs61240730	424.127864
CR2	rs4844599	106.2269615
CR2	rs861212	33.53754592
CR2	rs11161815	46.63214226
CR2	rs1660367	79.34587775
CR2	rs1550494	46.07616564
CR2	rs10849448	29.96665745
CR2	rs76368427	39.34235297
CR2	rs7310615	57.3809775
CR2	rs76428106	31.15388765
CR2	rs111727450	38.12976292
CR2	rs7196849	30.60419064
CR2	rs56115403	79.03842134
CR2	rs41556717	41.15780074
CR2	rs116852327	50.23069364
CR2	rs12950377	66.67970418
CR2	rs1047286	43.76022055
CR2	rs6006310	31.13777214
CR2	rs73165110	42.40700348
CR2	rs113286101	29.98717256
CR2	rs10212190	82.63995403
CR2	rs13434265	36.76286698
CR2	rs67838388	48.68345678
CR2	rs11241688	54.3721791
CR2	rs4478317	37.2470149
CR2	rs9257052	34.13615852
CR2	rs601945	289.785383
CR2	rs9271669	183.4228718
CR2	rs2273215	65.31290608
CR2	rs1528151	97.96466801
CR2	rs2058269	44.46273003
CR2	rs227953	41.63753586
CR2	rs12535578	59.04559476
CR2	rs76324903	32.65269629
CR2	rs77328788	30.56641048
CR2	rs187099314	49.04498608
NCAN	rs2228603	422.6138971
NCAN	rs111406374	47.88086602
NCAN	rs4657078	40.52636419
NCAN	rs11588675	29.95241497
NCAN	rs4746103	62.74936037
NCAN	rs11039297	36.20384065

NCAN	rs7937987	34.50764062
NCAN	rs1704886	38.98102048
NCAN	rs60111444	54.75627165
NCAN	rs12148990	57.44099137
NCAN	rs17817497	34.61615094
NCAN	rs1126642	59.79793597
NCAN	rs1724394	29.95892466
NCAN	rs6504649	53.53245166
NCAN	rs12458752	37.6365792
NCAN	rs1877223	257.017796
NCAN	rs58078456	29.78986412
NCAN	rs112369494	61.47774216
CCNA2	rs10922098	46.38123051
CCNA2	rs6542680	93.59824219
CCNA2	rs557011	33.27938666
RNASE3	rs148300946	34.32644522
RNASE3	rs72659967	83.4790022
RNASE3	rs2006079	47.8415183
RNASE3	rs11156734	926.9045722
RNASE3	rs113022713	126.8050141
RNASE3	rs147599917	40.93752567
RNASE3	rs143057962	37.20979796
RNASE3	rs174599	29.82712161
RNASE3	rs7310615	52.60694965
RNASE3	rs76428106	36.51739023
RNASE3	rs4580194	37.539889
RNASE3	rs112058117	35.46979368
RNASE3	rs13390874	36.58965602
RNASE3	rs2593620	58.22534493
RNASE3	rs7576634	45.1479297
RNASE3	rs17354348	31.41596448
RNASE3	rs56185965	46.29703861
RNASE3	rs13089722	81.29445142
RNASE3	rs7612124	39.18753006
RNASE3	rs3774298	54.56017217
RNASE3	rs10068466	32.62526531
RNASE3	rs9348865	40.76707895
ENPP6	rs78293932	48.26378946
ENPP6	rs10922098	151.9197621
ENPP6	rs1707652	59.8268121
ENPP6	rs317900	40.24483527
ENPP6	rs2596540	112.9880215
ENPP6	rs395976	48.43108668
EPHA7	rs10922098	168.4644807
EPHA7	rs6602268	140.9536802
EPHA7	rs1355538	40.42033826
EPHA7	rs147906938	65.21824887
EPHA7	rs9501602	34.82632901
FCN1	rs74467699	36.85064009
FCN1	rs7037264	791.6544492
FCN1	rs10776914	1091.172316
FCN1	rs111481598	63.45932584
FCN1	rs75446386	29.89610798
FCN1	rs149892510	41.00987463
FCN1	rs11103734	44.00081977
FCN1	rs142369618	68.80653073
FCN1	rs73632737	39.49637491
FCN1	rs2344873	41.67622016

FCN1	rs705016	36.72065443
FCN1	rs5030049	47.67330252
FCN1	rs3129880	32.85869191
FCRLB	rs12121649	176.3253948
FCRLB	rs1801274	112.3209562
FCRLB	rs1954172	1972.087585
FCRLB	rs1063178	303.9709828
FCRLB	rs33975669	59.62276521
FCRLB	rs73021687	68.42734212
FCRLB	rs113810201	52.51075227
CFHR2	rs74320729	314.1292604
CFHR2	rs12035228	242.708499
CFHR2	rs71631848	1269.302752
CFHR2	rs116815955	956.6183647
CFHR2	rs34181066	5612.060528
CFHR2	rs114645367	6947.869461
CFHR2	rs72736416	1100.632328
CFHR2	rs137858566	34.54738644
CFHR2	rs12070395	63.21010953
CFHR2	rs149231156	33.69705998
CFHR2	rs150090318	36.20769726
CFHR2	rs1231768	80.82480022
CFHR2	rs114505698	72.26085108
CFHR2	rs143736195	64.54939421
CFHR2	rs190119408	284.9980937
CFHR2	rs141323461	455.3644426
CFHR2	rs144377305	123.6754373
CFHR2	rs143814257	449.0502871
CFHR2	rs112169695	142.4243191
CFHR2	rs7558604	31.14133233
FBLN5	rs2430349	83.28846126
FBLN5	rs368482801	67.98304556
FBLN5	rs3814834	32.63462548
FBLN5	rs203688	211.271275
FBLN5	rs143443947	32.48225505
FBLN5	rs1707652	40.50809365
FBLN5	rs41285751	49.3013985
FBLN5	rs451652	35.53756106
FOLR2	rs74603010	77.27961817
FOLR2	rs1801133	58.36404084
FOLR2	rs603424	38.93697507
FOLR2	rs1151102	30.05758088
FOLR2	rs950803	102.1771289
FOLR2	rs61729512	240.98596
FOLR2	rs7305932	52.20312134
FOLR2	rs4760	94.05389013
FOLR2	rs12986064	142.5294252
FOLR2	rs4665972	51.46427008
FOLR2	rs58936783	40.31275906
FOLR2	rs13071075	42.12129665
FOLR2	rs2470854	94.37273653
FOLR2	rs11920688	42.56761371
FOLR2	rs73403125	37.78442468
FOLR2	rs77311729	31.91669905
FOLR2	rs9270159	54.56725112
FOLR2	rs9271775	106.9240174
FOLR2	rs7865362	111.9478497
GC	rs7697091	204.1179265

GC	rs9994658	61.58288498
GC	rs10922098	82.4007037
GC	rs447115	38.06393786
GC	rs72729623	30.28962501
GC	rs2954021	35.65811397
HTRA1	rs61871680	110.6374682
HTRA1	rs6585831	36.10411773
HTRA1	rs12133641	30.26863998
HTRA1	rs150844304	61.49778037
HTRA1	rs157592	34.62001695
HTRA1	rs7606574	36.07310694
HTRA1	rs4698935	37.22038825
HTRA1	rs2269425	35.00206933
IL6R	rs111671159	157.5531274
IL6R	rs55953243	137.7524558
IL6R	rs148882162	422.3122077
IL6R	rs12121180	1361.674232
IL6R	rs57569414	1409.914195
IL6R	rs185104823	362.2106482
IL6R	rs142768042	491.4059272
IL6R	rs34569656	188.8022066
IL6R	rs1051614	106.7635481
IL6R	rs151256463	105.1409335
IL6R	rs61808553	36.26064715
IL6R	rs10802089	50.78322869
IL6R	rs116103231	79.91248885
IL6R	rs145134231	51.27385772
IL6R	rs72695200	59.83607811
IL6R	rs114192875	40.9967647
IL6R	rs3184504	36.31187442
IL6R	rs12945299	33.25359832
ITGA2	rs181769	122.8900453
ITGA2	rs34124994	30.7050303
ITGA2	rs4666078	30.36351402
MAPK9	rs145843394	68.99091644
MAPK9	rs3763131	338.852081
MAPK9	rs4980320	37.51635044
MAPK9	rs10876550	44.44193161
MAPK9	rs1354034	33.29824838
KRT19	rs10922094	35.64490395
KRT19	rs964184	94.36647581
KRT19	rs62119326	36.37915707
KRT19	rs438811	794.0809663
KRT19	rs5112	470.5982451
KRT19	rs875255	47.77314664
KRT19	rs73940367	38.76043642
KRT19	rs1260326	57.12325126
KRT19	rs114115200	105.1527502
KRT19	rs3863087	49.97396736
KRT19	rs11755689	30.65743863
PKLR	rs77350683	245.7061543
PKLR	rs61799715	33.20325066
PKLR	rs74979599	32.12997332
PKLR	rs6014993	33.08178102
PKLR	rs6976036	48.32667539
LAP3	rs62296952	32.64415676
LAP3	rs114838597	93.45372874
LAP3	rs141974152	133.3045729

LAP3	rs61851769	57.50529378
LAP3	rs7662287	37.70916552
LAP3	rs140947655	62.32204714
LAP3	rs79313834	61.81075192
LAP3	rs114697781	45.25742079
PNLIP	rs78206436	45.05701469
PNLIP	rs147414236	65.81826428
PNLIP	rs2934381	36.69184399
PNLIP	rs2816950	33.74887867
PNLIP	rs78705678	60.92067064
PNLIP	rs2477642	58.94062527
PNLIP	rs57255883	32.36222252
PNLIP	rs117358628	36.43497615
PNLIP	rs28456	49.53045264
PNLIP	rs507695	41.28329808
PNLIP	rs9517774	34.11344933
PNLIP	rs8022656	33.90043897
PNLIP	rs72802342	283.2652001
PNLIP	rs8058234	186.0433585
PNLIP	rs889573	46.81427223
PNLIP	rs56850260	90.83384191
PNLIP	rs679574	268.7297678
PNLIP	rs145185835	30.99055535
PNLIP	rs17032925	103.9894072
PNLIP	rs66473811	54.37934434
PNLIP	rs35901985	37.62638043
PNLIP	rs611735	35.81898937
PNLIP	rs11965209	369.0353462
PNLIP	rs112509700	40.09652019
PNLIP	rs12192273	78.07606073
PNLIP	rs67824503	35.25424008
PNLIP	rs8176743	795.9960721
PNLIP	rs9286383	38.37969235
LILRA2	rs149260334	57.67980201
LILRA2	rs75873152	2273.277002
LILRA2	rs11672845	266.5456812
LILRA2	rs2241524	2429.284888
LILRA2	rs2098953	102.6223255
LILRA2	rs11574588	398.8156986
LILRA2	rs181099569	148.4783035
LILRA2	rs75177735	63.70005744
LILRA2	rs4233366	36.28472603
LILRA2	rs78689694	34.62583239
LILRA2	rs3184504	45.75865817
LILRA2	rs12589177	660.9788372
LILRA2	rs61997798	31.11121932
LILRA2	rs12945886	41.98738278
LILRA2	rs10179986	30.01823635
LILRA2	rs74342641	40.47575086
LILRB3	rs117480526	46.50122065
LILRB3	rs147636352	54.10751391
LILRB3	rs112421468	428.7271304
LILRB3	rs2288334	1014.653697
LILRB3	rs372255675	4703.734604
LILRB3	rs11670991	816.8411886
LILRB3	rs4806729	8819.282501
LILRB3	rs10425360	111.0500325
LILRB3	rs62132207	114.4877467

LILRB3	rs7259673	74.51335552
LILRB3	rs272413	175.9794845
NLGN1	rs557382	217.9497716
NLGN1	rs67200533	41.33958011
NLGN1	rs78399800	45.85604513
NLGN1	rs61801011	66.49694102
NLGN1	rs150179248	40.43127721
NLGN1	rs138472902	30.19306739
NLGN1	rs112725054	39.8455666
NLGN1	rs831428	46.07416232
NLGN1	rs201862321	94.0192201
NLGN1	rs2271541	75.74706151
NLGN1	rs62257591	33.14289571
NLGN1	rs12699916	32.37526002
OPCML	rs7118465	138.461711
OPCML	rs1544300	34.33674533
OPCML	rs78705678	119.7589208
OPCML	rs2477642	123.7495136
OPCML	rs12800878	266.4931658
OPCML	rs2511504	953.5228677
OPCML	rs11222592	59.42996774
OPCML	rs763144	29.86029784
OPCML	rs780093	38.31982873
PDCD1	rs10922098	251.1347881
PDCD1	rs1707652	102.3106888
PDCD1	rs3130955	41.50703524
PDCD1	rs9501600	82.43551833
PDCD1	rs72508422	35.66030789
HSPG2	rs212548	31.28087541
HSPG2	rs113855594	82.67534885
HSPG2	rs2229475	870.6955572
HSPG2	rs76305492	76.87270818
HSPG2	rs144654395	53.19696852
HSPG2	rs72877151	36.8118036
HSPG2	rs333947	37.3857786
HSPG2	rs2477664	49.96831515
HSPG2	rs2461161	42.0444984
HSPG2	rs181707610	30.53873908
HSPG2	rs55714927	42.13542719
HSPG2	rs78804971	67.14092736
HSPG2	rs6870546	36.23209233
HSPG2	rs3135344	32.47115869
HSPG2	rs7778938	73.59396908
HSPG2	rs11244049	33.75018455
RBP4	rs10882283	99.57464235
RBP4	rs4243177	29.83059667
RBP4	rs1667244	77.94950978
RBP4	rs780093	38.62098708
RBP4	rs1883711	49.59697981
RBP4	rs2126263	34.85451547
SLIT1	rs10922098	67.87187195
SLIT1	rs6542680	42.076957
SLIT1	rs4639017	32.92878199
SLIT1	rs9501600	35.61717933
SMOC2	rs6936697	45.63540169
SMOC2	rs56348400	32.27493783
SMOC2	rs2763257	976.4123537
SMOC2	rs59291571	352.8426176

SMOC2	rs58602032	70.28906518
SMOC2	rs9455725	42.76532192
SMOC2	rs181707610	30.20396888
SMOC2	rs77924615	35.73006403
SMOC2	rs2215319	117.7930081
SMOC2	rs150522134	37.9652665
SORCS1	rs2210969	61.2741878
SORCS1	rs9749205	36.36261619
SORCS1	rs111405904	40.32497072
SORCS1	rs74580987	30.0094338
SORCS1	rs41274017	30.87090964
SORCS1	rs635634	52.54201247
TAGLN	rs144037495	59.37217138
TAGLN	rs80351364	52.64334977
TAGLN	rs77924615	47.56432284
TMEFF1	rs3983768	42.91285987
TMEFF1	rs10922098	61.44457523
TMEFF1	rs7073753	150.7069157
TMEFF1	rs1555405	31.61823999
BTD	rs13100619	4077.619682
BTD	rs17485446	222.9367119
BTD	rs78816655	42.84905279
BTD	rs114000715	31.83943935
BTD	rs62234692	118.8007826
BTD	rs7964859	41.99680402
BTD	rs1801689	34.95143207
BTD	rs11127048	35.37463496
BTD	rs56289618	37.54251437
BTD	rs3731143	36.18085215
BTD	rs55836238	57.3600043
BTD	rs12495284	174.0650298
BTD	rs73153390	129.1853556
BTD	rs74848493	53.54047306
BTD	rs74569567	29.85752717
COL10A1	rs11968601	98.0240595
COL10A1	rs9488842	819.0728007
COL10A1	rs980214	67.25570096
COL10A1	rs1084887	31.10628079
COL10A1	rs10922098	36.48989101
COL10A1	rs143875230	33.10913434
COL10A1	rs8121146	39.59270844
COL10A1	rs9271642	35.01168717
COL10A1	rs147319042	31.69794636
COL10A1	rs117724066	40.71862648
BMP2	rs572515	84.72379254
BMP2	rs4639017	32.35986665
BMP2	rs67399078	39.3031317
BMP4	rs74917046	39.02616878
BMP4	rs117029746	30.5361556
BMP4	rs6592590	164.6621762
BMP4	rs61389091	707.1220074
BMP4	rs35763136	621.8709451
BMP4	rs35887873	77.66873306
BMP4	rs10861032	36.53482672
BMP4	rs201862321	44.59311637
BMP4	rs138903858	38.04381152
BMP4	rs55714927	118.691383
BMP4	rs143501783	49.36445242

BMP4	rs6123685	61.79830924
BMP4	rs1355538	34.08677607
BMP4	rs7467847	57.44147539
BMP8B	rs1329424	1183.830066
BMP8B	rs3900909	54.64216847
BMP8B	rs10801660	37.00907618
BMP8B	rs11569393	31.53802837
BMP8B	rs111696008	30.73240697
BMP8B	rs2686395	175.606427
BMP8B	rs3099847	30.57061375
BMP8B	rs1265941	115.0336173
BMP8B	rs550671	324.0697208
BMP8B	rs78897911	36.37941279
BMP8B	rs9276606	54.36207017
BRAF	rs10922094	117.7457393
BRAF	rs4980320	32.12631277
BRAF	rs7332311	33.75132272
BRAF	rs704	54.77729169
BRAF	rs1354034	52.06759792
BRAF	rs1619994	33.01271067
C1QL2	rs10922098	689.120149
C1QL2	rs12740041	41.21789149
C1QL2	rs704	134.2923427
C1QL2	rs114115200	155.2608228
C1QL2	rs3863087	53.40015548
C1QL2	rs3099847	31.31651202
C1QL2	rs1265941	70.76187968
C1QL2	rs453821	185.5534173
C1QL2	rs9276606	38.2333696
CD79A	rs572515	36.41292121
CD79A	rs10822155	33.39300709
CD79A	rs4734879	35.34360449
CEBPB	rs10305748	30.09703431
CEBPB	rs72748420	37.07824776
CEBPB	rs1329424	1167.683391
CEBPB	rs3900909	55.79724251
CEBPB	rs10801660	38.91579073
CEBPB	rs1047286	39.44773336
CEBPB	rs111696008	34.72061743
CEBPB	rs2686395	258.2895867
CEBPB	rs1265941	112.0897473
CEBPB	rs550671	287.1920834
CEBPB	rs145704749	40.82533619
DKK2	rs4648825	30.6429688
DKK2	rs10888208	169.0665847
DKK2	rs141099634	325.449232
DKK2	rs9415731	129.5402022
DKK2	rs72795135	140.6073325
DKK2	rs7101062	155.1185851
DKK2	rs75580312	472.362955
DKK2	rs150513777	378.2417509
DKK2	rs117565303	51.42363886
DKK2	rs61850494	42.60004274
DKK2	rs145591269	30.16501751
DKK2	rs10822155	175.1584377
DKK2	rs34416903	32.83418299
DKK2	rs1801690	50.2752201
DKK2	rs6993770	202.5055527

INHBC	rs117325986	1106.661529
INHBC	rs183310037	235.1288469
INHBC	rs76545764	1010.77126
INHBC	rs113953523	1041.635986
INHBC	rs151337033	1094.224311
INHBC	rs79245439	117.6608781
INHBC	rs34633617	362.7641761
INHBC	rs182612562	186.5777143
INHBC	rs117176626	678.6851495
INHBC	rs12231914	31.24284449
INHBC	rs10876614	29.88705501
INHBC	rs149717212	131.8758945
INHBC	rs117385498	75.27739722
INHBC	rs78188443	37.94985375
INHBC	rs190135880	98.49562193
INHBC	rs150291370	91.88377348
INHBC	rs12816210	82.19741898
INHBC	rs9668323	33.67403966
INHBC	rs10878824	40.66704948
INHBC	rs76182087	59.62990021
INHBC	rs147618567	33.89219637
INHBC	rs9268557	29.90948793
LMAN1	rs12133641	85.77110381
LMAN1	rs10922098	261.5523138
LMAN1	rs116040950	29.83760799
LMAN1	rs1706435	80.29511102
LMAN1	rs28573154	72.25126888
LMAN1	rs7774739	51.53155593
NODAL	rs10922098	101.0118353
NODAL	rs1355538	36.14388095
NODAL	rs28573154	40.94003301
NODAL	rs7774739	42.63345531
NODAL	rs601020	32.30166573
NODAL	rs211161	32.46449688
TXLNA	rs10922098	31.06471639
TXLNA	rs4980320	37.53166088
TXLNA	rs10876550	32.71924049
TXLNA	rs1047286	59.30622441
TXLNA	rs1354034	70.49701309
TXLNA	rs1355538	31.75335769
TXLNA	rs342293	50.45091002
GPT	rs1055167	36.94750155
GPT	rs1451915	42.05047508
GPT	rs10922098	878.8623876
GPT	rs7513100	35.21045181
GPT	rs704	45.4650847
GPT	rs2686395	152.185216
GPT	rs1265941	94.09675847
GPT	rs550671	243.1100063
GPT	rs9276606	39.20893822
CDH8	rs72748420	43.2394909
CDH8	rs1329424	1321.021362
CDH8	rs3900909	65.4892144
CDH8	rs10801660	38.18801449
CDH8	rs429358	32.10788471
CDH8	rs111696008	43.44940058
CDH8	rs144202860	30.80207669
CDH8	rs2686395	318.4468747

CDH8	rs73169117	31.40747325
CDH8	rs7746061	31.69745922
CDH8	rs542418	49.02855791
CDH8	rs550671	351.0138258
CDH8	rs9270570	57.11369822
CDH8	rs9469195	118.9296982
FLT4	rs34221241	258.5424813
FLT4	rs11740644	36.04905812
FLT4	rs202046353	101.0618547
FLT4	rs148313519	38.46559653
FLT4	rs2003428	41.49002833
FLT4	rs61479154	32.63534137
FLT4	rs2837982	48.60960071
FLT4	rs116021462	36.48852322
FLT4	rs67562454	41.8513286
FLT4	rs7628076	61.14951891
FLT4	rs6782460	60.42441037
FLT4	rs62256583	42.34376433
FLT4	rs3749260	273.5014593
FLT4	rs3804622	2853.377849
FLT4	rs13071075	163.6711219
FLT4	rs73138077	85.35669909
FLT4	rs189392979	129.6842114
FLT4	rs56162907	33.71989888
FLT4	rs115022773	35.55070235
FLT4	rs76436027	127.2826125
FLT4	rs2394969	34.08688431
FLT4	rs3116990	30.8201638
FLT4	rs12535558	60.9687781
FLT4	rs3205136	159.021638
FLT4	rs600038	1335.086482
FLT4	rs11655	40.44076603
FLT4	rs117608058	67.77525928
FLT4	rs117541350	30.98977392
SHC1	rs4980320	51.10559686
SHC1	rs185009887	31.06028824
SHC1	rs1354034	142.9097319
SHC1	rs342292	36.60199801
OLR1	rs7296791	152.0980353
OLR1	rs146801898	30.49104394
OLR1	rs10922098	179.1128078
OLR1	rs2511241	55.04748402
OLR1	rs1355538	44.62906435
OLR1	rs2853923	30.02837389
OLR1	rs147906938	32.02964046
CFHR5	rs12035228	73.13728379
CFHR5	rs72732232	409.5484181
CFHR5	rs116688712	2543.30124
CFHR5	rs138353023	677.7482558
CFHR5	rs12746361	146.3093208
CFHR5	rs476325	958.8679574
CFHR5	rs45512792	191.1760108
CFHR5	rs78776940	72.88403275
CFHR5	rs192624559	40.48086321
CFHR5	rs111554873	36.60968386
CFHR5	rs139192950	59.39743486
CFHR5	rs150992058	334.4983696
CFHR5	rs7916868	42.69568227

CFHR5	rs1169294	60.88855527
IGF2R	rs6902339	197.4577541
IGF2R	rs13192111	1324.338657
IGF2R	rs117534432	110.853517
IGF2R	rs148234538	51.94019366
IGF2R	rs10922098	45.98526313
IGF2R	rs1805248	64.93575517
IGF2R	rs76889468	92.05607177
IGF2R	rs7978801	31.75269721
IGF2R	rs12984330	37.39087138
IGF2R	rs3132487	45.90753222
IGF2R	rs3117106	77.45263206
IGF2R	rs2065168	33.05250168
IGF2R	rs73012202	42.87427755
IGF2R	rs78207873	51.39415239
NID2	rs34598814	146.5215117
NID2	rs10148939	1114.376476
NID2	rs2749880	997.726204
NID2	rs754423	506.7706821
NID2	rs4901205	124.5932743
NID2	rs35579510	54.31502078
NID2	rs7073753	67.65478087
NID2	rs150394939	50.6481409
NID2	rs1671176	30.58609469
NID2	rs11757455	30.29751151
WIF1	rs466104	51.96654822
WIF1	rs10922098	195.15873
WIF1	rs6489548	77.94615404
WIF1	rs2230203	34.04936736
WIF1	rs6519245	70.97582859
WIF1	rs2686395	214.0466664
WIF1	rs9501600	39.5203817
WIF1	rs576123	37.3703034
GRAP2	rs148328786	64.15409587
GRAP2	rs10922094	121.6312111
GRAP2	rs9971407	88.91479557
GRAP2	rs704	69.38910773
GRAP2	rs1355538	55.6615187
GRAP2	rs415620	31.4656865
GRAP2	rs4713498	47.33159667
TEC	rs58762758	180.1352963
TEC	rs6447637	41.1703614
TEC	rs11417460	31.5001401
TEC	rs4980320	34.23682088
TEC	rs1354034	34.16399582
TEC	rs1398617	30.56016947
AKR1B10	rs72748418	33.18421683
AKR1B10	rs114056970	42.27550876
AKR1B10	rs10922098	886.3948195
AKR1B10	rs7513100	37.44706469
AKR1B10	rs704	311.4782125
AKR1B10	rs1047286	99.54999758
AKR1B10	rs4665988	32.18231663
AKR1B10	rs111696008	31.34487765
AKR1B10	rs1707652	150.5895389
AKR1B10	rs542418	38.27187535
AKR1B10	rs550671	233.7819625
AKR1B10	rs9270570	46.09504458

AKR1B10	rs9469195	92.81549426
EPHA4	rs190696118	48.91297249
EPHA4	rs4674589	115.0128621
EPHA4	rs114022440	72.37955157
EPHA4	rs71050338	218.7225005
EPHA4	rs4661359	65.98508771
EPHA4	rs72704449	49.74072264
EPHA4	rs77924615	34.82322476
EPHA4	rs12945299	36.51059197
EPHA4	rs1260326	44.76200947
EPHA4	rs112155714	68.74122155
EPHA4	rs13108218	33.44869596
EPHA4	rs62576557	29.7479364
EPHA4	rs146798134	127.9515457
EPHA4	rs75212266	72.09133488
EPHA4	rs7470777	939.5765749
EPHA4	rs739468	915.3539001
EPHA4	rs28477937	40.9064457
EPHA4	rs118167234	55.65085171
EPHA4	rs62572818	66.36099652
GHRH	rs7920986	61.69301448
GHRH	rs17580	125.523155
GHRH	rs1706435	40.37913446
GHRH	rs6884904	34.43286807
LGR5	rs7305333	50.34137406
LGR5	rs10922098	186.8833973
LGR5	rs1355538	34.74443511
LGR5	rs6457457	54.66326574
LGR5	rs9501393	76.58065331
PLA2G2D	rs10922098	67.94193506
PLA2G2D	rs4977155	31.14392526
PLA2G2D	rs557011	34.3497896
TIGIT	rs10922098	335.2948896
TIGIT	rs964184	66.61871882
TIGIT	rs7412	79.51258146
TIGIT	rs111696008	30.233489
TIGIT	rs1707652	215.2434244
TIGIT	rs35735140	31.65621418
TIGIT	rs9501600	103.2116849
TREM2	rs77705101	411.8615581
TREM2	rs188904277	944.6309029
TREM2	rs77790943	86.1840856
TREM2	rs146936090	398.2508735
TREM2	rs114431690	83.53524868
TREM2	rs10922094	54.32145953
TREM2	rs10838389	38.29002921
TREM2	rs11038540	73.86261045
TREM2	rs4752854	49.46577909
TREM2	rs67703379	82.83104078
TREM2	rs74883075	31.17983212
TREM2	rs112474179	53.72419908
TREM2	rs72912764	201.4811132
TREM2	rs1286289	2746.702978
TREM2	rs138508553	132.927174
TREM2	rs150271427	280.9463237
TREM2	rs117327140	73.74731627
TREM2	rs55756397	36.06328394
TREM2	rs141393619	32.39501731

TREM2	rs149069407	72.76153735
TREM2	rs147931202	72.76174363
TREM2	rs78053849	46.01590126
TREM2	rs4143898	49.86404162
TREM2	rs261290	44.57245003
TREM2	rs9939224	41.96084098
TREM2	rs432157	37.61453437
TREM2	rs738409	36.05696563
TREM2	rs114115200	179.2292771
TREM2	rs3863087	73.11275749
TREM2	rs111266377	33.80187282
TREM2	rs55640410	52.02775191
TREM2	rs144782411	119.4553196
TREM2	rs140378210	158.1340597
TREM2	rs116319373	181.6288222
TREM2	rs62414730	221.512216
TREM2	rs74730442	72.94234946
TREM2	rs143329063	54.75608933
TREM2	rs79289342	46.14842389
TREM2	rs913914	49.62901962
BPIFA2	rs3818222	87.86420226
BPIFA2	rs10922094	48.78111278
BPIFA2	rs964184	69.0369909
BPIFA2	rs2227296	106.5835494
BPIFA2	rs7217335	32.24154378
BPIFA2	rs704	911.710835
BPIFA2	rs16964821	36.71925056
BPIFA2	rs71352239	158.1279912
BPIFA2	rs679574	1033.407354
BPIFA2	rs67086575	38.055226
BPIFA2	rs1707652	124.6574036
BPIFA2	rs28676336	34.76062037
LGR4	rs2472632	157.579073
LGR4	rs137906310	60.43123168
LGR4	rs61808983	73.80027025
LGR4	rs10922098	75.28006698
LGR4	rs7625980	86.57074743
CDH11	rs140978623	42.36343131
CDH11	rs12094497	81.57508964
CDH11	rs12065184	38.44978831
CDH11	rs61804164	755.2406306
CDH11	rs7532925	47.5038118
CDH11	rs7154507	35.32324796
CDH11	rs186344496	35.79312526
CDH11	rs117572554	51.97416223
CDH11	rs73357588	53.15619662
CDH11	rs79697519	77.2571967
CDH11	rs143931049	80.4597527
CDH11	rs61983272	1118.946646
CDH11	rs10139374	288.9113198
CDH11	rs2516960	167.8961922
CDH11	rs12148488	35.38097442
CDH11	rs73292853	52.86274413
CDH11	rs56260553	70.87441731
CDH11	rs12152337	47.7735746
CDH11	rs390288	31.89000915
CDH11	rs1155043	36.19169098
CDH11	rs1841010	65.56904354

CDH11	rs66962766	39.54779661
CDH11	rs9271369	158.2214274
CDH11	rs241422	40.40865334
CDH11	rs3822960	261.6042059
UNC5D	rs2405810	148.0348272
UNC5D	rs61747728	78.77482369
UNC5D	rs77924615	58.99557205
UNC5D	rs1260326	42.11617877
UNC5D	rs117018654	36.2271589
BTLA	rs10922098	234.0212176
BTLA	rs697356	52.55292282
BTLA	rs28573154	54.65382634
BTLA	rs7774739	41.42608444
DKKL1	rs55689129	41.5067055
DKKL1	rs10733084	38.62017672
DKKL1	rs1329424	959.6942624
DKKL1	rs3900909	41.54004364
DKKL1	rs1555405	30.96900357
DKKL1	rs704	51.75032944
DKKL1	rs111696008	33.87893678
DKKL1	rs606725	41.36744711
DKKL1	rs2686395	263.2988007
DKKL1	rs550671	275.5258185
DKKL1	rs41548618	41.02953343
DKKL1	rs28471056	82.93504786
DKKL1	rs9272526	46.30523667
CDH6	rs10922094	467.5264262
CDH6	rs12478841	34.75327387
CDH6	rs2686395	81.6511305
CDH6	rs1265941	49.99867875
CDH6	rs550671	109.917306
FLT1	rs10922098	65.76943455
FLT1	rs10513546	31.53426671
FLT1	rs9501393	33.67240459
FLT1	rs6921438	596.7049577
FLT1	rs10757632	40.415967
DSG3	rs10822155	149.244632
DSG3	rs715440	34.02166884
DSG3	rs892090	64.5598623
DSG3	rs633323	33.82893855
DSG3	rs73191660	34.26338067
DSG3	rs6780053	29.91569512
DSG3	rs6993770	118.8507717
ACVRL1	rs34546728	41.18300237
ACVRL1	rs2277382	519.796148
ACVRL1	rs3741737	45.38918506
ACVRL1	rs78035300	45.0917479
CD320	rs10922098	80.21513507
CD320	rs73726194	29.79426242
CD320	rs71549285	69.44052446
NRXN3	rs34121288	351.2429462
NRXN3	rs72704449	48.35939505
NRXN3	rs11220464	40.06742174
NRXN3	rs17598373	94.98459186
NRXN3	rs4540997	131.5349767
NRXN3	rs2940174	34.43061793
NRXN3	rs12945299	129.9467291
NRXN3	rs17880604	68.17032494

NRXN3	rs1260326	64.04812703
NRXN3	rs4234797	53.05664902
NRXN3	rs3129781	67.05948518
TLR1	rs5743618	273.0691655
TLR1	rs10922098	533.1734132
TLR1	rs2686395	114.0403898
TLR1	rs144558021	32.00276566
TLR1	rs74716816	32.67899067
TLR1	rs1265941	62.22267197
TLR1	rs550671	136.8621503
HMGA1	rs10922094	62.76475209
HMGA1	rs34475883	30.99499949
HMGA1	rs1355538	30.0433165
HMGA1	rs3129773	39.69070009
STAT5B	rs10922098	637.453589
STAT5B	rs35351085	34.25351343
STAT5B	rs1291921	29.91598495
STAT5B	rs11720167	150.3122454
STAT5B	rs3863087	57.0676691
STAT5B	rs60946795	31.86071225
STAT5B	rs542418	35.76675608
STAT5B	rs550671	238.6950772
STAT5B	rs9270570	60.00573206
STAT5B	rs9469195	114.2890869
MYOC	rs7535044	120.2637263
MYOC	rs57118325	1051.993039
MYOC	rs114099765	153.7653948
MYOC	rs192038293	48.8380227
MYOC	rs180932522	76.6984595
MYOC	rs80089053	52.29151777
MYOC	rs859462	35.15434008
MYOC	rs61821073	48.00609317
MYOC	rs78705678	73.52245848
MYOC	rs2461161	60.5996008
MYOC	rs564708	31.30476377
MYOC	rs34262842	50.16240108
A1BG	rs79541297	98.95067816
A1BG	rs73058298	35.01125144
A1BG	rs58509147	2184.2094
A1BG	rs62114673	164.9132975
A1BG	rs373579	35.35337762
A1BG	rs35885418	43.03379658
A1BG	rs80079566	59.8948149
A1BG	rs117374109	116.579593
A1BG	rs117903814	100.0863256
A1BG	rs1260326	123.1938227
A1BG	rs13244614	58.3840013
NUDT2	rs111577519	40.02205272
NUDT2	rs71506168	395.2553803
NUDT2	rs7025269	941.0354278
NUDT2	rs10814111	149.7411326
NUDT2	rs1354034	33.8513936
B3GNT4	rs10922098	324.3980625
B3GNT4	rs704	43.31329275
B3GNT4	rs1047286	43.19480623
B3GNT4	rs1706435	53.45707904
B3GNT4	rs9266766	30.83163184
B3GNT4	rs9267139	30.09388066

B3GNT4	rs28573154	88.54983709
B3GNT4	rs7774739	58.10793591
CSN2	rs114009540	39.43000113
CSN2	rs10922098	484.60269
CSN2	rs1047286	38.43187207
CSN2	rs2686395	76.27368454
CSN2	rs9501600	106.4627557
HSPA5	rs4149000	32.10533441
HSPA5	rs201862321	41.95591358
HSPA5	rs9302635	50.15405503
CHIA	rs58468211	30.03535391
CHIA	rs10922098	276.7323557
CHIA	rs1706435	71.65701543
CHIA	rs9267139	41.78558022
CHIA	rs9501393	100.4465635
CHIA	rs7774739	66.31666372
CHIA	rs9271689	30.75796127
FADD	rs75327431	34.92488945
FADD	rs7999532	70.7532357
FADD	rs9595243	54.75027454
FADD	rs7989315	51.69565228
FADD	rs7987805	201.566428
FADD	rs2404726	1037.683167
FADD	rs11618144	201.8443064
FADD	rs4476072	66.39507612
FADD	rs3816117	34.44441915
FADD	rs5112	35.70280853
FADD	rs4666000	41.13581359
FAIM	rs111554649	328.5155239
FAIM	rs116831965	43.99918939
FAIM	rs641320	1905.597085
FAIM	rs141080172	77.98527914
FAIM	rs192325010	159.4671279
FAIM	rs34935748	104.9914071
FAIM	rs199799920	55.89230903
FAIM	rs142442169	31.1723049
FAIM	rs1354034	39.67783476
FAIM	rs138421205	91.02898403
FAIM	rs151053585	112.0850368
FAIM	rs189028619	75.66500952
FAIM	rs5030049	54.48011444
GLRX3	rs12248841	91.14720647
GLRX3	rs4980320	34.11445011
GLRX3	rs1354034	43.19813022
GLRX5	rs10922094	61.40458968
GLRX5	rs697356	36.5095038
GLRX5	rs4713498	32.40107607
GPN1	rs10208529	66.03709505
GPN1	rs3856240	34.12117164
GPN1	rs36004172	42.50918261
GPN1	rs3170660	602.6762864
GPN1	rs4980320	33.08986195
GPN1	rs2297380	53.36957348
GPN1	rs1354034	88.7880407
GPN1	rs342292	32.06989839
AKR1B1	rs2229542	41.81255685
AKR1B1	rs75627053	66.38313091
AKR1B1	rs16873418	33.05018586

AKR1B1	rs11792848	32.29998253
GSN	rs2159994	35.07772319
GSN	rs10985149	336.1099393
GSN	rs12338713	56.64929558
GSN	rs61288910	75.94706485
GSN	rs79727415	76.44005771
GSN	rs375109452	228.0223583
GSN	rs147338114	70.24339045
GSN	rs142791210	119.0583711
KIRREL2	rs117731902	719.5607814
KIRREL2	rs117704954	430.3203323
KIRREL2	rs150313799	56.80325369
KIRREL2	rs143750646	976.7558327
KIRREL2	rs443338	32.38329615
KIRREL2	rs421195	203.3578504
KIRREL2	rs8051363	61.43001998
KIRREL2	rs7405380	43.03677925
KIRREL2	rs116962404	30.04800967
KIRREL2	rs78958855	340.3241647
KIRREL2	rs190707972	177.7457051
KIRREL2	rs117240178	646.6250808
KIRREL2	rs73047940	513.5611101
KIRREL2	rs11665751	188.144128
KIRREL2	rs73048936	142.4744649
KIRREL2	rs148075933	61.69499072
KIRREL2	rs4960289	37.97502502
KIRREL2	rs1326757	33.93022309
KIRREL2	rs11965209	151.4663004
KIRREL2	rs15906	30.81825245
KIRREL2	rs8176743	569.6244876
KIRREL2	rs3094379	45.29614148
LRP10	rs117221168	103.369043
LRP10	rs117411993	43.10183012
LRP10	rs599839	46.07987686
LRP10	rs1805248	31.84395021
LRP10	rs77924615	29.74846161
LRP10	rs10901252	65.42087292
SDC3	rs10922094	455.0580844
SDC3	rs4665985	45.19082281
SDC3	rs1250259	92.18301272
SDC3	rs4674006	33.67842434
SDC3	rs1707652	85.24084448
SDC3	rs542418	34.60012341
SDC3	rs550671	153.5944204
SDC3	rs9469195	81.60471619
SDC3	rs28601761	33.64088949
CDH17	rs7839227	80.76874856
CDH17	rs3115920	75.47002152
CDH17	rs2446871	318.9066052
CDH17	rs7833351	966.6424614
CDH17	rs11120001	43.28476227
CDH17	rs78705678	62.58961283
CDH17	rs2461161	39.2567207
CDH17	rs11220467	58.84650625
CDH17	rs12791948	32.00540984
CDH17	rs55714927	34.03775395
CDH17	rs708686	201.7445241
CDH17	rs79775625	39.09009633

CDH17	rs61743291	33.29935949
CDH17	rs190161436	30.74695935
CDH17	rs76321971	68.85772169
CDH17	rs55941736	72.89471026
CDH17	rs411482	3150.938491
CDH17	rs80206894	212.5710068
CDH17	rs9676447	37.22879275
CDH17	rs146346242	42.19305857
CDH17	rs117543080	36.40541646
CDH17	rs1012143	203.9766065
CDH17	rs9815073	41.39482202
CDH17	rs2071805	80.50941733
CDH17	rs116232911	56.69352187
CDH17	rs2855422	39.62667233
CDH17	rs4556017	83.43576473
CDH17	rs145175118	40.03524741
CDH17	rs7037570	43.35084638
CDH17	rs62578578	63.58343051
CDH17	rs146798134	88.69633823
CDH17	rs76324624	35.93057499
CDH17	rs11244038	1032.460338
CDH17	rs590819	169.7903729
CDH17	rs3124761	601.1825194
RSPO1	rs10625758	307.7984703
RSPO1	rs36043533	462.2144614
RSPO1	rs114120637	29.91435428
RSPO1	rs11573156	29.81891861
RSPO1	rs569586	30.40184664
RSPO1	rs7310615	36.15366847
RSPO1	rs35287116	38.54840383
RSPO1	rs9458012	64.82745027
ENO3	rs2586528	1089.878583
ENO3	rs11653414	73.95396098
ENO3	rs887524	33.72699901
ENO3	rs4883279	92.23023009
ENO3	rs201847610	48.54105839
ENO3	rs12975366	104.9951569
ENO3	rs28672722	30.15768939
CD69	rs10922098	46.83210871
CD69	rs10761739	41.46041064
CD69	rs10850001	31.2917985
CD69	rs9271348	31.9903609
CD69	rs819441	75.4481263
CD69	rs6993770	43.09779481
LY75	rs13004043	1419.775446
LY75	rs139721273	177.2051642
LY75	rs60244750	5960.879789
LY75	rs115200150	3298.399479
LY75	rs7419274	691.4781326
LY75	rs55993371	45.6199095
LY75	rs78705678	41.77264788
LY75	rs2477642	50.34990589
LY75	rs142029184	33.2789558
LY75	rs2298475	54.43855395
LY75	rs116417234	32.38573471
LY75	rs7310615	97.49952934
LY75	rs78110020	45.24892796
LY75	rs117633773	30.6691126

LY75	rs62061425	97.22256331
LY75	rs143501783	52.63753665
LY75	rs681343	45.47423032
LY75	rs2890714	36.76410292
LY75	rs1838381	50.07405804
LY75	rs77080489	38.13926047
LY75	rs843203	58.92544492
LY75	rs180681025	130.332475
LY75	rs150948745	82.23120638
LY75	rs150685660	122.5473788
LY75	rs62194171	274.5967059
LY75	rs17705404	357.39667
LY75	rs2909447	97.99309893
LY75	rs62176383	33.52553439
LY75	rs6730459	55.4230119
LY75	rs10930409	33.47798068
LY75	rs79443047	54.88603225
LY75	rs10165086	30.26656415
LY75	rs28732085	36.01906824
LY75	rs17886232	56.21336022
LY75	rs78897208	32.01552244
LY75	rs635634	54.64535772
INHBB	rs10922098	237.2557179
INHBB	rs1706435	56.15137321
INHBB	rs9501600	67.54132618
BMP3	rs10922098	522.0999372
BMP3	rs1047286	54.85490522
BMP3	rs6445035	112.2020496
BMP3	rs3863087	31.17412322
BMP3	rs555007	47.05597298
BMP3	rs444921	113.8918262
GDF10	rs10922098	566.2175214
GDF10	rs11569415	35.25432097
GDF10	rs114115200	87.1406036
GDF10	rs555007	69.04796938
GDF10	rs550671	144.689241
COL6A2	rs149071379	70.06112195
COL6A2	rs73230562	42.88439748
COL6A2	rs35881321	131.7518933
COL6A2	rs35548026	783.1615798
COL6A2	rs148337125	342.8517177
COL6A2	rs13047780	84.57686565
COL6A2	rs368465	49.3809322
COL6A2	rs4639017	38.36136458
CASK	rs10922094	168.0349292
CASK	rs2753491	35.21137651
CASK	rs1355538	48.53787758
CASK	rs9295986	41.08906832
CASK	rs609061	62.68805333
GDF3	rs10922098	424.7026923
GDF3	rs1047286	41.41105921
GDF3	rs1707652	96.76521015
GDF3	rs9267139	36.96324267
GDF3	rs28573154	117.9491923
GDF3	rs7774739	90.22525405
IL26	rs10922094	46.33504227
IL26	rs964184	85.5041028
IL26	rs704	44.00242615

IL26	rs11549936	45.79309602
IL26	rs2571068	104.2424976
IL26	rs62119281	142.5812968
IL26	rs394353	86.58907169
IL26	rs283813	223.294531
IL26	rs73045691	44241.86522
IL26	rs77660932	601.1459661
IL26	rs182320411	61.53215395
IL26	rs1049739	291.6897304
IL26	rs8101216	58.71230214
IL26	rs78980904	32.59298711
IL26	rs111696008	36.05267988
IL26	rs483465	30.28870621
IL26	rs11720167	392.4062645
IL26	rs3863087	143.9030323
IL26	rs148077724	57.08162108
IL26	rs73169103	30.83920485
IL26	rs11134475	44.25539533
IL26	rs188302747	32.00504442
LECT2	rs4976285	185.6256332
LECT2	rs801576	2940.974164
LECT2	rs115645824	222.376051
LECT2	rs1989973	87.14281622
LECT2	rs149658926	635.7499037
LECT2	rs142363998	94.09511793
LECT2	rs346661	180.4465353
LECT2	rs10922098	95.25965308
LECT2	rs780093	34.99634128
LECT2	rs246342	38.71363996
LECT2	rs13360974	141.2108714
LECT2	rs184790893	36.12251469
LECT2	rs41285751	43.48993676
LECT2	rs451652	35.9428857
PPBP	rs7073753	124.3238904
PPBP	rs13412535	45.19528077
PPBP	rs6993770	54.1290006
REG1B	rs114506174	31.81330954
REG1B	rs146599142	146.9694498
REG1B	rs76841471	1045.758163
REG1B	rs72913277	337.4592627
REG1B	rs56170896	108.8629539
REG1B	rs141639277	38.55064082
REG1B	rs11598094	40.41955304
REG1B	rs7478186	393.0490027
REG1B	rs3013182	94.34465824
REG1B	rs55993634	36.73784588
REG1B	rs708686	91.24652808
REG1B	rs34953075	62.05089427
REG1B	rs679574	1107.062475
REG1B	rs35320397	57.48742172
REG1B	rs142996976	30.16894752
REG1B	rs77305423	44.88808801
REG1B	rs79672111	36.06196497
REG1B	rs139922251	55.78160186
REG1B	rs2870238	30.47255095
REG1B	rs576123	207.2250919
SCUBE3	rs144022875	77.34584808
SCUBE3	rs3800403	552.2227976

SCUBE3	rs6908638	57.98032054
SCUBE3	rs10922094	46.03440899
SCUBE3	rs11130032	39.60957958
SCUBE3	rs35959442	61.55621064
SCUBE3	rs7468080	71.75326835
ENGASE	rs117549034	208.3833132
ENGASE	rs142841419	252.4898637
ENGASE	rs12450779	914.8553131
ENGASE	rs117570724	65.05455818
ENGASE	rs3803780	327.3650461
ENGASE	rs140871359	384.1114587
ENGASE	rs117502073	3143.466523
ENGASE	rs117014247	655.5356929
ENGASE	rs150745719	130.2899825
ENGASE	rs4903104	132.9766039
DEFA5	rs188963550	40.09389593
DEFA5	rs16837903	33.94672837
DEFA5	rs10799448	54.1036115
DEFA5	rs10495440	58.69323389
DEFA5	rs12419620	41.20397743
DEFA5	rs2472632	178.6255168
DEFA5	rs11603094	62.30468495
DEFA5	rs55748041	37.09980014
DEFA5	rs80158569	53.13307283
DEFA5	rs12599863	33.10882929
DEFA5	rs72856733	69.40842414
DEFA5	rs7207133	34.09685184
DEFA5	rs2287133	485.5017552
DEFA5	rs4800364	63.67255204
DEFA5	rs485073	161.4165832
DEFA5	rs2881260	30.20910639
DEFA5	rs13029745	40.21519829
DEFA5	rs4444693	59.90960261
DEFA5	rs13073114	436.7326515
DEFA5	rs1131853	32.59693433
DEFA5	rs1064839	117.4595166
DEFA5	rs7693632	45.40571533
DEFA5	rs2522545	34.00544642
DEFA5	rs4703084	43.59022162
DEFA5	rs10248095	93.69912691
DEFA5	rs610891	39.87271059
DEFA5	rs28702953	67.66605607
DEFA5	rs635634	88.42192949
SIGLEC5	rs4802782	49.58002965
SIGLEC5	rs115494840	1048.431309
SIGLEC5	rs10401234	953.1749319
SIGLEC5	rs35692207	1117.036162
SIGLEC5	rs3829649	927.7942348
SIGLEC5	rs12610918	318.5347939
SIGLEC5	rs1241462	340.351572
SIGLEC5	rs653178	38.2295112
SIGLEC5	rs2708751	71.76875993
OCIAD1	rs114009540	30.15371719
OCIAD1	rs10922098	808.0388877
OCIAD1	rs3900909	38.49517269
OCIAD1	rs704	170.6703637
OCIAD1	rs1047286	47.67060411
OCIAD1	rs111696008	38.05695148

OCIAD1	rs2686395	183.5122515
OCIAD1	rs550671	250.4570721
OCIAD1	rs9469195	123.0939316
OCIAD1	rs9272526	53.14263079
PDE5A	rs75135627	281.6552267
PDE5A	rs9307476	229.2982012
PDE5A	rs2389874	462.4273466
PDE5A	rs28646193	36.5574132
PDE5A	rs7073753	49.86476059
PDE5A	rs190088137	49.34493044
TNNT3	rs10922098	728.5014926
TNNT3	rs3900909	35.33987244
TNNT3	rs61679841	30.55932381
TNNT3	rs1047286	48.80033981
TNNT3	rs1707652	162.3705447
TNNT3	rs542418	36.19488092
TNNT3	rs550671	202.2656815
TNNT3	rs9270570	44.99355387
TNNT3	rs9469195	82.80025489
NME4	rs3848364	53.03637595
NME4	rs6600214	409.7013638
NME4	rs117799316	31.27184234
NME4	rs9501602	35.47607122
NME4	rs7774739	35.62610099
FHL1	rs10922098	374.3879123
FHL1	rs11569415	33.63181132
FHL1	rs111696008	31.75778873
FHL1	rs11536949	85.29185083
FHL1	rs6445035	125.6391756
FHL1	rs3863087	46.97889926
FHL1	rs9267139	44.23156049
FHL1	rs28573154	102.799418
FHL1	rs7774739	79.44730945
FHL1	rs9271689	35.07006872
CDCP1	rs4682944	37.8550377
CDCP1	rs17077267	198.8190375
CDCP1	rs2276862	754.3805753
CDCP1	rs73064389	44.3160048
CDCP1	rs2436695	32.83784528
CDCP1	rs1613078	86.20011915
CDCP1	rs683486	215.1783135
CDCP1	rs11057841	39.15269481
CDCP1	rs12945299	51.90971687
CDCP1	rs3747207	35.08572944
CDCP1	rs7640056	37.94261728
CDCP1	rs9270747	55.52028643
CDCP1	rs75525829	38.83848423
APOL3	rs2307340	83.80283037
APOL3	rs75387341	306.9588408
APOL3	rs13053898	1186.583408
APOL3	rs143633479	880.7476454
APOL3	rs11704345	133.2483212
APOL3	rs5756164	32.75853221
APOL3	rs56380119	50.58059957
APOL3	rs646776	52.66494625
APOL3	rs7310615	78.39728587
APOL3	rs56156922	42.56307927
APOL3	rs34931250	165.2747089

APOL3	rs145113125	36.19653928
ATXN3	rs11621105	42.23398533
ATXN3	rs2273185	874.5951151
ATXN3	rs2093291	64.18295846
ATXN3	rs56365547	41.38759405
ATXN3	rs75924474	62.26245742
ATXN3	rs11925835	30.57490312
COL6A1	rs145942527	146.4664005
COL6A1	rs9984235	211.5849455
COL6A1	rs181341736	37.7322389
COL6A1	rs2838952	1137.530755
COL6A1	rs34627227	4930.184276
COL6A1	rs56221159	4761.435276
COL6A1	rs36119246	7523.991174
COL6A1	rs111443291	267.8606939
COL6A1	rs78705678	171.9870448
COL6A1	rs2461161	167.133342
COL6A1	rs142196190	34.18169101
COL6A1	rs113876738	45.45818985
COL6A1	rs78378324	72.32448133
COL6A1	rs199525389	108.686936
COL6A1	rs147321259	32.45635425
COL6A1	rs75191173	68.47332694
MPZL1	rs10922098	40.22794659
MPZL1	rs77303550	61.15715726
MPZL1	rs6542680	30.80828204
MPZL1	rs9272780	31.91118037
RGS5	rs10922098	359.8957991
RGS5	rs6542680	50.31882672
RGS5	rs11720167	75.07500684
RGS5	rs9501600	98.20386031
RGS5	rs35209896	38.96305593
PDZK1	rs10922098	374.8349894
PDZK1	rs1047286	32.93253807
PDZK1	rs1707652	76.19200647
PDZK1	rs9267139	37.73657286
PDZK1	rs28573154	100.1567071
PDZK1	rs7774739	80.4082521
PDZK1	rs9271766	29.87941458
LSM4	rs72748420	30.83825907
LSM4	rs114009540	31.05526964
LSM4	rs1329424	943.6204234
LSM4	rs3900909	43.28397301
LSM4	rs704	43.02881891
LSM4	rs1047286	44.01699114
LSM4	rs1707652	175.3115427
LSM4	rs550671	270.8578763
LSM4	rs9469195	100.1152321
LSM4	rs9272526	50.089043
LSM4	rs3917545	61.43661399
MAPRE2	rs3786314	329.5516585
MAPRE2	rs138948503	50.29317616
MAPRE2	rs35340377	30.8041832
MAPRE2	rs7073753	32.9763955
MAPRE2	rs34524896	39.71661076
RCN3	rs10922098	215.3585873
RCN3	rs3842763	47.84909254
RCN3	rs223046	201.9662902

RCN3	rs1707652	76.00559439
RCN3	rs3891366	74.40350014
RCN3	rs7774739	51.5044352
SH3GLB1	rs10922098	58.93403522
SH3GLB1	rs11679004	35.10300466
SH3GLB1	rs1619994	37.76665727
SH3GLB1	rs415620	40.87045143
SH3GLB1	rs4713498	52.58958706
NANP	rs7412	59.40144056
NANP	rs6542680	30.16319321
NANP	rs872914	162.18715
GSTZ1	rs75784865	140.1620715
GSTZ1	rs186433582	1055.082732
GSTZ1	rs79607813	55.41286744
GSTZ1	rs4467006	1197.442228
GSTZ1	rs45608147	1017.799923
GSTZ1	rs755072	1218.057982
GSTZ1	rs149721633	296.3417174
GSTZ1	rs56198483	485.6825442
GSTZ1	rs78371502	602.580698
GSTZ1	rs12881757	4577.940217
GSTZ1	rs9707306	59.9559031
GSTZ1	rs143101173	93.31828921
GSTZ1	rs114538974	67.73589879
GSTZ1	rs57979367	83.15402256
GSTZ1	rs257206	82.39144231
GSTZ1	rs71418988	101.0060403
GSTZ1	rs148041878	67.67479526
GSTZ1	rs72699510	50.68861624
MSRB2	rs10922098	184.7188129
MSRB2	rs9864801	64.93895685
MSRB2	rs9267139	36.10226213
MSRB2	rs454212	47.901163
MSRB2	rs3917538	100.9354602
PHPT1	rs4880150	522.4050255
PHPT1	rs62580411	32.08952839
PHPT1	rs1354034	30.7947337
RAB4A	rs10922094	135.5869414
RAB4A	rs1354034	43.43872228
RAB4A	rs9864801	34.93535085
RAB4A	rs28573154	37.60387399
RAB4A	rs6993770	31.16592537
PTRH2	rs10922094	119.9232827
PTRH2	rs1706435	97.80341719
PTRH2	rs11755689	38.53930425
ADAMTSL1	rs7847635	44.42805682
ADAMTSL1	rs35264941	570.4859733
ADAMTSL1	rs702208	788.7988409
ADAMTSL1	rs74413064	35.11299938
ADAMTSL1	rs116922118	55.40600278
ADAMTSL1	rs12337179	126.6219129
ADAMTSL1	rs72704449	50.21388757
ADAMTSL1	rs77542162	34.24556543
ADAMTSL1	rs7572682	29.92599869
ADAMTSL1	rs72713815	39.46565551
ADAMTSL1	rs192820461	66.16147327
ENPP2	rs147305264	35.95714427
ENPP2	rs7829390	362.126493

ENPP2	rs10109973	81.56770737
ENPP2	rs2264782	69.77282474
ENPP2	rs740516	35.57963755
ENPP2	rs11780884	31.59577046
ENPP2	rs73311781	32.393227
OSBPL9	rs572515	115.3550249
OSBPL9	rs1707652	104.4285645
OSBPL9	rs147906938	31.11475919
MDGA1	rs913499	206.1430829
MDGA1	rs1776441	618.4743638
MDGA1	rs1619327	1058.648492
MDGA1	rs112099259	384.4505142
MDGA1	rs12203552	368.8534402
MDGA1	rs11757527	756.4018945
MDGA1	rs56344867	46.9104645
MDGA1	rs55714927	43.43236163
MDGA1	rs117753190	40.629211
MDGA1	rs511868	37.01024462
MDGA1	rs114892943	30.42020199
MDGA1	rs75185110	39.79321292
MDGA1	rs9468618	37.86892397
MDGA1	rs3129891	75.33661525
MDGA1	rs112612978	79.23983766
MDGA1	rs9470960	201.4535847
MDGA1	rs9462551	149.7323863
MDGA1	rs3004074	104.8499972
MDGA1	rs72854204	46.21668271
CADM2	rs12133018	29.75284767
CADM2	rs72704449	49.14557683
CADM2	rs77924615	50.46833311
CADM2	rs1260326	35.58846982
CADM2	rs635634	33.13237847
OMG	rs12449302	116.8723154
OMG	rs141114572	86.66168704
OMG	rs79412885	31.06057843
OMG	rs1544300	40.61238585
OMG	rs78705678	200.6555423
OMG	rs2477642	159.3751564
OMG	rs181707610	32.3290841
OMG	rs72749499	37.12970328
OMG	rs4624605	34.57566013
OMG	rs4308324	34.5024368
OMG	rs187620717	35.58209896
OMG	rs113992041	41.09566884
OMG	rs3129960	38.18233361
OMG	rs2240046	53.21794851
OMG	rs35475338	42.32609834
OMG	rs183867209	31.36456275
RNASET2	rs13216122	2143.930703
RNASET2	rs12203820	165.1452632
RNASET2	rs2247315	917.2245763
RNASET2	rs66822994	37.14237761
RNASET2	rs71571499	78.62467366
RNASET2	rs3184504	38.69804333
RNASET2	rs13314114	166.4159229
RNASET2	rs79327907	35.17445319
RNASET2	rs9269890	34.6072533
RNASET2	rs2858320	77.21606205

RNASET2	rs2737246	45.87379932
CD14	rs78656209	36.09456957
CD14	rs192900523	48.04824403
CD14	rs114928388	146.739963
CD14	rs5744454	731.7273302
CD14	rs9425316	97.04531857
CD14	rs2948711	32.72278707
CD14	rs78705678	122.5270355
CD14	rs2461161	117.4577132
CD14	rs10769263	42.69715662
CD14	rs4760	32.2945572
CD14	rs1250258	61.53954484
CD14	rs9878990	55.95657601
CD14	rs13209376	31.00175322
CD14	rs56365312	35.01045109
CD14	rs635634	50.42587585
SEMA4A	rs7695	367.0356915
SEMA4A	rs150649461	109.4972936
SEMA4A	rs905938	38.02118793
SEMA4A	rs2477664	87.05034405
SEMA4A	rs2461161	70.86591937
SEMA4A	rs7899547	91.73837808
SEMA4A	rs11215043	188.0789288
SEMA4A	rs78689694	46.44436794
SEMA4A	rs2859660	32.06464867
SEMA4A	rs3184504	48.76198758
SEMA4A	rs76428106	120.5855471
SEMA4A	rs62019249	50.79561945
SEMA4A	rs55714927	48.59391445
SEMA4A	rs143501783	40.36868458
SEMA4A	rs9898547	43.32708587
SEMA4A	rs55974215	56.25563586
SEMA4A	rs58560372	50.1185541
SEMA4A	rs150918492	52.85243956
SEMA4A	rs3821801	39.09537793
SEMA4A	rs148925417	34.85052479
SEMA4A	rs2253491	70.24760305
SEMA4A	rs185319710	72.75096782
SEMA4A	rs11775560	32.88531061
SEMA4A	rs10107630	36.57068868
SEMA4A	rs11103564	87.70625236
SEMA4A	rs10858304	854.4272722
SEMA4A	rs4842221	38.23966256
SLITRK6	rs143781069	37.47472525
SLITRK6	rs9547175	444.281497
SLITRK6	rs368076061	441.5275921
SLITRK6	rs72631040	61.08783638
SLITRK6	rs72704449	71.49337285
SLITRK6	rs78705678	54.08067132
SLITRK6	rs2477642	50.74266346
SLITRK6	rs7137546	44.40914681
SLITRK6	rs118114074	33.7507143
SLITRK6	rs681343	44.9254307
SLITRK6	rs1260326	57.46407615
SLITRK6	rs6873324	92.3833374
SLITRK6	rs7859904	43.23343
TLR3	rs34928277	476.9014922
TLR3	rs34609719	980.82032

TLR3	rs62350493	151.6996415
TLR3	rs9312345	113.4532509
TLR3	rs111286548	255.6603107
TLR3	rs116591520	133.7296505
TLR3	rs77575931	74.15173327
TLR3	rs77994684	51.74216128
TLR3	rs114051073	30.72610788
DBI	rs78115599	718.6448219
DBI	rs7580437	45.73095466
DBI	rs2091255	47.25598342
PLXNB3	rs10922098	83.35815413
PLXNB3	rs9635249	136.1480108
PLXNB3	rs374229803	49.59052952
PLXNB3	rs41446546	29.82936092
PLXNB3	rs415620	34.66765657
PLXNB3	rs7774739	31.8949417
PLXNB3	rs147168394	31.50130672
PFN2	rs10922094	105.1159368
PFN2	rs9501393	48.36177343
PFN2	rs7774739	43.6924867
ALPL	rs61808983	173.5726625
ALPL	rs72840032	45.25929755
ALPL	rs6542680	838.9384004
ALPL	rs138936322	38.67634706
ALPL	rs1355538	85.16806384
ALPL	rs13080283	30.25433993
ALPL	rs77734842	32.97158826
ALPL	rs72682450	232.1087667
ALPL	rs9693053	56.48224745
SULT1E1	rs10922098	181.5078524
SULT1E1	rs1706435	51.80151868
SULT1E1	rs9501393	56.91848789
SULT1E1	rs7774739	41.61333248
HBB	rs10922094	186.6398548
HBB	rs7113917	35.36616408
HBB	rs2461286	76.57847527
HBB	rs34924625	111.0903672
HBB	rs73579049	52.62942134
HBB	rs12924886	137.5205844
HBB	rs117051919	197.1219949
HBB	rs118107049	45.2186188
HBB	rs35495893	62.25176444
HBB	rs111696008	32.04449131
HBB	rs11422232	32.65528059
HBB	rs2668202	229.2359265
HBB	rs5030049	85.33036659
HBB	rs41285751	44.8642724
HBB	rs147390813	67.15919824
GSTA1	rs4712001	62.14954096
GSTA1	rs11754704	92.50881204
GSTA1	rs2144693	923.203875
GSTA1	rs7752387	74.60659409
GSTA1	rs112692814	43.11674331
GSTA1	rs10922094	68.40022163
GSTA1	rs3747207	46.08726515
GSTA1	rs1706435	33.08051603
GSTA1	rs11755689	48.4627634
GSTA1	rs1553331	48.0088783

GSTA1	rs28601761	58.5544318
PDGFD	rs146014868	58.52271713
PDGFD	rs11225768	124.4066003
PDGFD	rs12223045	136.399188
PDGFD	rs2515082	311.6949067
PDGFD	rs11226172	1276.919415
PDGFD	rs1926231	32.26872012
PDGFD	rs561267	48.55742166
PDGFD	rs17662785	31.15437174
PDGFD	rs1542541	33.11811005
PDGFD	rs667695	32.75494173
PDGFD	rs113661219	37.87124517
PDGFD	rs1071960	30.94491728
PDGFD	rs342200	197.3740738
PDGFD	rs1460580	31.03845637
PDGFD	rs6993770	368.8953796
PDGFD	rs1475695	36.26894171
BLVRB	rs141109067	75.66148333
BLVRB	rs268664	54.51635986
BLVRB	rs35340377	56.63153082
BLVRB	rs3896477	37.42909349
BLVRB	rs4895441	43.99415668
KIR2DS4	rs10922094	228.1345503
KIR2DS4	rs113747960	34.75200486
KIR2DS4	rs9902706	153.1836986
KIR2DS4	rs1355538	42.74349719
KIR2DS4	rs555007	37.48899189
KIR2DS4	rs453821	80.11479589
KIR2DL3	rs8104813	49.37899768
KIR2DL3	rs140109311	295.3578997
KIR2DL3	rs648029	15970.01738
KIR2DL3	rs13344925	8255.647711
KIR2DL3	rs62122550	396.7499978
KIR2DL3	rs199881873	1020.410078
KIR2DL3	rs375664612	6081.693994
KIR2DL3	rs182610324	234.7310623
KIR2DL3	rs775901	40.76590525
KIR2DL3	rs7310615	45.54230891
KIR2DL3	rs142467654	48.73279459
KIR2DL3	rs76926657	29.72499805
KIR2DL3	rs2328848	77.94069484
KIR2DL3	rs116104346	87.65177996
KIR2DL3	rs9468618	171.5029586
KIR2DL3	rs3130648	1007.31826
KIR2DL3	rs28397298	1060.155383
KIR2DL3	rs116302614	1091.056641
KIR2DL3	rs147390813	441.6057515
KIR2DL3	rs7760260	104.4103139
KIR2DL3	rs17214852	135.1100098
KIR2DL3	rs4314495	38.11666472
PTGES3	rs72748420	38.68429635
PTGES3	rs1329424	1192.088199
PTGES3	rs3900909	51.12245022
PTGES3	rs10801660	35.67757579
PTGES3	rs11078072	38.55184912
PTGES3	rs704	69.19931414
PTGES3	rs1047286	48.76205618
PTGES3	rs111696008	32.29767065

PTGES3	rs1707652	194.5689365
PTGES3	rs3099847	33.68944839
PTGES3	rs1265941	109.0829672
PTGES3	rs550671	316.1413087
PTGES3	rs9276606	52.03825545
VPS28	rs72748420	45.1817481
VPS28	rs1329424	1216.884191
VPS28	rs3900909	59.55222649
VPS28	rs10801660	44.16109658
VPS28	rs61821527	30.53371181
VPS28	rs111696008	44.47828589
VPS28	rs12242	45.38229069
VPS28	rs1707652	334.2500986
VPS28	rs73169117	42.89135146
VPS28	rs1265941	109.3499645
VPS28	rs550671	316.6104995
VPS28	rs41291802	40.99641863
VPS28	rs41548618	46.5170107
DCLK1	rs36001943	44.3333372
DCLK1	rs1926318	486.8078265
DCLK1	rs9531602	51.73182076
DCLK1	rs12561036	55.45844064
DCLK1	rs9575912	55.36587493
DCLK1	rs9566079	55.35432052
DCLK1	rs904323	31.57669311
DCLK1	rs188530181	512.8340388
DCLK1	rs1539707	102.4798762
DCLK1	rs16849875	30.62615306
DCLK1	rs704	42.35161033
DCLK1	rs697976	485.1772766
DCLK1	rs26499	97.69654114
DCLK1	rs115269242	37.76562059
ANXA3	rs10922098	163.7412564
ANXA3	rs697356	45.70496704
ANXA3	rs9271642	39.4347834
ANXA4	rs17037076	911.6397065
ANXA4	rs148712348	33.89009857
ANXA4	rs10822155	38.89481144
ANXA4	rs373771501	36.05403191
PPBP	rs7080386	118.192491
PPBP	rs4541868	55.36695799
PPBP	rs77265585	31.02715823
CALCB	rs10832218	38.65497407
CALCB	rs10766205	813.2937533
CALCB	rs138183704	29.99091128
CALCB	rs10922098	71.52257321
STATH	rs1557550	176.9931587
STATH	rs9273560	73.14776028
STATH	rs28746806	34.14874471
STATH	rs9280312	107.6180483
STATH	rs3130160	49.48836436
MAP2K6	rs520820	47.02841503
MAP2K6	rs572515	101.7209694
MAP2K6	rs146472986	73.67260069
MAP2K6	rs1398617	52.94945402
MAP2K6	rs453821	39.42348885
HIF1AN	rs10922098	92.89360341
HIF1AN	rs6542680	33.21952136

HIF1AN	rs9864801	35.22282962
HIF1AN	rs9501393	33.75313546
KIR2DL1	rs1451915	30.32500536
KIR2DL1	rs10922098	597.0001141
KIR2DL1	rs1047286	56.54979134
KIR2DL1	rs111696008	31.43211071
KIR2DL1	rs1707652	123.5649078
KIR2DL1	rs1265941	89.73389359
KIR2DL1	rs550671	201.6813705
KIR2DL1	rs9276606	32.87270709
RAB5A	rs4980320	42.88934983
RAB5A	rs185009887	30.7719084
RAB5A	rs1354034	45.57555191
SULT2B1	rs10922098	554.112391
SULT2B1	rs11569415	35.94631749
SULT2B1	rs2232613	72.65478842
SULT2B1	rs2686395	194.289824
SULT2B1	rs1265941	68.05705019
SULT2B1	rs550671	169.5797926
TCL1A	rs370019376	88.05234332
TCL1A	rs78986913	483.6864439
TCL1A	rs11585450	34.1101974
TCL1A	rs7087507	76.95948931
TCL1A	rs76428106	30.85899544
TCL1A	rs7173724	41.01234928
TCL1A	rs66777340	38.84187797
TCL1A	rs8067378	87.55391291
TCL1A	rs752347	41.91070337
TCL1A	rs160044	45.56376998
TCL1A	rs111298234	40.66227463
TCL1A	rs4714797	32.29184613
TCL1A	rs2395903	44.62565377
OGN	rs7023804	40.52319105
OGN	rs62565677	182.7316421
OGN	rs7022820	1025.440863
OGN	rs144655897	127.5765466
OGN	rs705379	35.60493103
OGN	rs62554281	49.40345293
OGN	rs4744276	88.7605681
OGN	rs12378270	61.53412752
LCP1	rs76101695	65.0818647
LCP1	rs9562623	30.29756002
LCP1	rs17843980	141.3966534
LCP1	rs7990889	167.924007
LCP1	rs333947	38.9532021
LCP1	rs12041767	43.84635784
LCP1	rs115655239	108.3901732
LCP1	rs11611373	107.6262071
LCP1	rs16959006	109.0309785
LCP1	rs28374650	57.54027381
LCP1	rs12342201	80.11036001
AASDHPPT	rs11211830	31.16204269
AASDHPPT	rs4980320	33.1926343
AASDHPPT	rs185009887	30.39936812
AASDHPPT	rs1354034	81.26428231
AASDHPPT	rs77265585	33.75819826
GUK1	rs146563008	62.71761058
GUK1	rs148467842	122.7058166

GUK1	rs61825157	488.7798555
GUK1	rs115715830	43.84353392
GUK1	rs78142369	66.53575443
GUK1	rs72748172	53.3375072
GUK1	rs1354034	40.53806629
CNPY3	rs9471969	241.2442615
CNPY3	rs12801747	30.76846002
CNPY3	rs729940	569.5960432
CNPY3	rs137906439	34.11306373
BDH2	rs28444377	84.71683633
BDH2	rs2711897	625.2756781
BDH2	rs415143	36.6727416
BDH2	rs7695494	31.06410746
BDH2	rs139720552	29.82410094
KREMEN1	rs5762962	43.99581625
KREMEN1	rs2205771	410.6001097
KREMEN1	rs35755679	116.5078682
KREMEN1	rs333947	46.9405129
KREMEN1	rs1984769	41.4761857
KREMEN1	rs6695321	34.67321236
KREMEN1	rs2477669	39.6061105
KREMEN1	rs78705678	259.9404927
KREMEN1	rs2461161	259.4253735
KREMEN1	rs137927952	52.64288807
KREMEN1	rs1929423	41.538895
KREMEN1	rs56344867	33.80014404
KREMEN1	rs876556	31.20479461
KREMEN1	rs181707610	34.89937773
KREMEN1	rs12945299	69.39997441
KREMEN1	rs143501783	38.04351066
KREMEN1	rs34931250	50.85183376
KREMEN1	rs77542162	38.55704276
KREMEN1	rs11704754	68.17581934
KREMEN1	rs723183	110.8058314
KREMEN1	rs138013760	104.0656489
KREMEN1	rs147756739	48.40400151
KREMEN1	rs9862378	41.51730021
KREMEN1	rs10513807	34.48481324
KREMEN1	rs4704044	36.35344011
KREMEN1	rs7716761	43.6205375
KREMEN1	rs35139284	32.33732429
KREMEN1	rs635634	92.04771487
KREMEN1	rs117210485	45.59594807
VAMP2	rs6675393	53.10685051
VAMP2	rs45525531	91.74270008
VAMP2	rs3761068	31.98409558
VAMP2	rs2395199	129.0137085
VAMP2	rs9280312	42.95161876
ACAT2	rs2842970	1048.269568
ACAT2	rs151149709	41.2154983
ACAT2	rs622217	32.16611659
ACAT2	rs41272114	63.240944
AGR3	rs2239013	39.96253852
AGR3	rs35477610	30.43336158
AGR3	rs2020854	31.81572698
AGR3	rs8020861	41.69975449
AGR3	rs112001035	30.12364981
AGR3	rs438811	80.09605215

AGR3	rs12983058	86.34247806
AGR3	rs2235229	31.42573208
AGR3	rs709166	32.14253758
AGR3	rs6874202	143.6093899
AGR3	rs35062987	38.0330524
AGR3	rs41270871	36.31594367
AGR3	rs28601761	39.03213789
AGR3	rs34024143	45.00392165
SNPH	rs17779421	55.75241346
SNPH	rs1048621	53.25531685
SNPH	rs72681067	31.28558297
SNPH	rs7073753	36.42865506
SNPH	rs9268560	30.33806571
SNPH	rs342295	54.7621625
STOML1	rs11229075	37.35003986
STOML1	rs2302255	57.09254081
STOML1	rs73266929	40.1205904
STOML1	rs113448098	119.9974933
STOML1	rs12366520	448.7565669
STOML1	rs34985304	144.0038241
STOML1	rs11611373	108.0139998
STOML1	rs117006594	30.57829454
STOML1	rs11624581	98.36801629
FDCSP	rs77542162	108.1276102
FDCSP	rs7247412	32.08102652
FDCSP	rs62226440	34.03580472
FDCSP	rs1355538	33.00664682
SYNJ2BP	rs10922098	159.2806846
SYNJ2BP	rs1706435	39.84835912
SYNJ2BP	rs9501600	46.99475332
CHMP2B	rs76984117	59.2779955
CHMP2B	rs300996	835.6103436
CHMP2B	rs1354034	36.82160931
CHMP2B	rs13325641	34.26830541
IL1F10	rs10922094	102.423822
IL1F10	rs62269916	36.48090079
IL1F10	rs114115200	53.81871875
IL1F10	rs395976	32.48827196
IL1F10	rs28455809	38.21743905
SEPSECS	rs6448333	56.86273833
SEPSECS	rs10922098	73.09762537
SEPSECS	rs61923082	29.73835328
DCUN1D1	rs75863201	35.17952179
DCUN1D1	rs117943989	30.3130446
DCUN1D1	rs117881774	63.18154086
DCUN1D1	rs11172134	1023.136479
DCUN1D1	rs55852335	73.80340942
DCUN1D1	rs112887724	48.36229826
DCUN1D1	rs429358	44.0205294
DCUN1D1	rs1260326	47.05911117
DCUN1D1	rs1707652	155.67711
DCUN1D1	rs62277509	38.37577604
STMN1	rs10922094	117.4473469
STMN1	rs11601665	30.71271652
STMN1	rs4759076	80.69519253
STMN1	rs6436128	39.4238209
STMN1	rs1354034	116.9948863
STMN1	rs9501600	36.29644742

AKR1C3	rs76267270	102.8598248
AKR1C3	rs56375548	178.8855999
AKR1C3	rs7099757	55.59556717
AKR1C3	rs7903755	269.0101883
AKR1C3	rs117980121	76.73634616
AKR1C3	rs62626310	46.27235923
AKR1C3	rs35301881	60.61336844
AKR1C3	rs11208704	30.35409668
AKR1C3	rs10922098	104.2082969
AKR1C3	rs1707652	117.8210559
AKR1C3	rs2245801	50.56956707
AKR1C3	rs6592965	60.24787318
AKR1C3	rs11557154	364.8032474
AKR1C3	rs41279069	43.0410481
STARD5	rs12910527	43.15432004
STARD5	rs11634869	526.0202435
STARD5	rs4392019	484.6511929
STARD5	rs10405535	78.57586175
STARD5	rs141714582	37.20736341
STARD5	rs1354034	34.53348399
PFKM	rs4760682	404.3174215
PFKM	rs36090264	52.46004909
PFKM	rs704	96.54652379
PFKM	rs71077749	57.1727462
NFKBIB	rs528298	148.091692
NFKBIB	rs1707652	53.32473844
NFKBIB	rs2242572	57.05747248
NFKBIB	rs28455809	30.67086307
RBM17	rs140764661	537.4040705
RBM17	rs11256846	81.12155818
RBM17	rs7294894	46.77664208
RBM17	rs1354034	41.18930517
RBM17	rs342200	51.56730289
RBM17	rs11380594	31.76826797
RBM17	rs3748136	83.62900031
RBM17	rs6993770	56.07026206
ACADS	rs1408868	30.89388684
ACADS	rs10922098	663.9986701
ACADS	rs704	34.36843678
ACADS	rs111696008	53.94120125
ACADS	rs11720167	326.8071486
ACADS	rs3863087	119.952369
ACADS	rs73169103	34.89432788
ACADS	rs1265941	76.21855023
ACADS	rs550671	162.3481143
ACADS	rs9276606	31.09095026
ADH1A	rs189407772	149.2607825
ADH1A	rs3747207	45.7340491
ADH1A	rs66711128	39.76565767
ADH1A	rs139808768	38.22033012
ADH1A	rs13213821	32.28586039
ADH1A	rs28601761	33.7002633
HSD17B11	rs10922103	37.28593257
HSD17B11	rs6542680	51.08796196
HSD17B11	rs1846190	41.56995709
HMOX1	rs73168076	64.67097421
HMOX1	rs3761439	33.3517329
HMOX1	rs11913874	36.20240393

HMOX1	rs7080536	256.4607017
HMOX1	rs2727784	75.37237297
HMOX1	rs11216334	30.01037188
HMOX1	rs11057841	54.05423078
HMOX1	rs2414577	46.71232527
HMOX1	rs261334	31.29188875
HMOX1	rs247617	47.18187246
HMOX1	rs34931250	34.57713336
HMOX1	rs1065853	157.9027158
HMOX1	rs41555716	34.33362734
HMOX1	rs28394079	208.7132645
HMOX1	rs385076	327.0863586
HMOX1	rs34120451	36.81145078
HMOX1	rs2164162	86.10862013
HMOX1	rs4114200	58.21636401
HMOX1	rs145559505	116.3812189
HMOX1	rs6059696	275.6062599
HMOX1	rs1153284	31.9993461
HMOX1	rs73884366	52.72732031
HMOX1	rs6786226	43.74505775
HMOX1	rs371339882	53.23402282
HMOX1	rs251149	72.55896157
HMOX1	rs11134525	47.22792373
HMOX1	rs7889	35.16966315
HMOX1	rs2637681	65.69137389
HMOX1	rs1084887	30.82832486
HMOX1	rs7039090	31.94466545
HMOX1	rs576123	45.51547259
ACOT8	rs10399785	75.45607745
ACOT8	rs10922098	39.20600924
ACOT8	rs10771511	39.75152268
ACOT8	rs12897483	29.72912167
ACOT8	rs7161336	146.6295983
ACOT8	rs704	41.27426822
ACOT8	rs11704359	51.40989426
ACOT8	rs74770429	82.05553417
ACADSB	rs7913063	157.3351029
ACADSB	rs7894710	83.9515298
ACADSB	rs1706435	54.36374859
ACADSB	rs75865130	33.09067533
ACADSB	rs79254850	31.0869018
ARL5B	rs10922094	32.34372854
ARL5B	rs7206790	32.35159146
ARL5B	rs5167	42.13289063
ARL5B	rs853484	35.1508873
ARL5B	rs752721	57.94394884
ARL5B	rs6864541	150.5572748
ARL5B	rs62358178	292.4001895
ARL5B	rs62358228	40.46537119
ARL5B	rs190444444	82.58558527
ARL5B	rs62359169	30.45592536
ARL5B	rs16876270	53.33425952
ARL5B	rs3129773	39.8690157
PMM1	rs8139993	32.22300694
PMM1	rs56068340	71.00640218
PMM1	rs12891460	32.95444566
PMM1	rs148166993	51.27512029
PMM1	rs3171723	31.22914638

PMM1	rs33964890	29.95336182
CETN3	rs4873	81.09139034
CETN3	rs572515	61.07948556
CETN3	rs8138525	92.02024779
CETN3	rs1354034	46.9948909
CETN3	rs4699154	30.79411882
CETN3	rs6594371	71.54619803
CETN3	rs12523131	36.19072306
TES	rs143664050	63.13186421
TES	rs10922098	52.69808967
TES	rs1355538	30.13736707
AK4	rs10922098	260.309861
AK4	rs113654728	211.8121299
AK4	rs1555405	34.75078795
AK4	rs11569415	41.86368218
AK4	rs1707652	158.8288829
AK4	rs542418	29.92963964
AK4	rs550671	175.9111757
AK4	rs9270570	39.50406196
AK4	rs9469195	73.41756003
ETFA	rs78185702	46.92677389
ETFA	rs10922098	476.2208477
ETFA	rs704	258.6861778
ETFA	rs11569415	39.83101847
ETFA	rs10208529	36.62201038
ETFA	rs1707652	180.5054359
ETFA	rs13107461	46.05715275
ETFA	rs185528097	37.577219
ETFA	rs71655744	49.86426861
ETFA	rs115181106	86.99383338
ETFA	rs13117588	56.8197491
ETFA	rs1265941	76.4784936
ETFA	rs453821	180.3890699
ETFA	rs9269234	30.8739469
CSNK1G2	rs7113761	48.73544459
CSNK1G2	rs185009887	30.41120105
CSNK1G2	rs1354034	70.78282333
ALPI	rs10922098	77.34184109
ALPI	rs11878193	31.6854151
ALPI	rs34953075	36.35864321
ALPI	rs679574	931.429286
ALPI	rs697356	29.73145332
ALPI	rs9501393	37.0861308
ALPI	rs576123	788.3393836
ALPI	rs10901256	82.60327499
ALPI	rs579232	47.73342923
SFRP4	rs1052981	166.6137209
SFRP4	rs2598105	559.0838623
SFRP4	rs35887873	252.0618101
SFRP4	rs188003872	39.64125886
SFRP4	rs215223	137.2586193
SFRP4	rs4779525	32.20615123
SFRP4	rs763665	37.73305349
SFRP4	rs780094	32.10587082
SFRP4	rs10512861	30.05645692
SFRP4	rs10040716	34.55346666
CD9	rs10922098	604.2658295
CD9	rs12403425	33.77176648

CD9	rs1707652	166.6091144
CD9	rs12529130	35.31857972
CD9	rs444921	183.8289011
CD9	rs28471056	76.93332103
CD9	rs9272526	36.16153324
BCL2L11	rs10801548	59.84552684
BCL2L11	rs7546015	361.3293122
BCL2L11	rs1706435	58.29006808
BCL2L11	rs1265906	78.85392215
BCL2L11	rs9501602	33.60564692
CP	rs13089226	29.83336776
CP	rs11208686	30.21806278
CP	rs72748420	39.84689933
CP	rs114009540	34.64468293
CP	rs1329424	1310.356902
CP	rs3900909	53.85419594
CP	rs111696008	41.0872082
CP	rs2686395	276.538508
CP	rs542418	58.41144356
CP	rs550671	338.1841751
CP	rs9270570	63.10238673
CP	rs9469195	127.1946328
EGFL6	rs12022510	37.60795142
EGFL6	rs10761739	61.67637153
EGFL6	rs10432210	81.82316492
EGFL6	rs2032903	352.8070184
EGFL6	rs11700925	31.74814014
EGFL6	rs7280028	34.38510849
EGFL6	rs77323797	34.13141591
EGFL6	rs1460580	36.27552455
EGFL6	rs6993770	315.4464195
EGFL6	rs3780420	58.55521694
FOLR1	rs10922094	45.15247196
FOLR1	rs72548024	107.7032204
FOLR1	rs181063261	336.1380761
FOLR1	rs115277907	106.2496732
FOLR1	rs2284179	200.8303542
FOLR1	rs451652	288.0334377
FOLR1	rs7774739	238.0118201
FOLR1	rs71538535	33.56551923
GOLM1	rs113968521	222.0789932
GOLM1	rs7868110	654.8919262
GOLM1	rs138221898	46.49792788
GOLM1	rs2622935	126.1497899
GOLM1	rs184457882	30.35590204
GOLM1	rs3184504	31.21872592
GOLM1	rs12945299	49.66286372
GOLM1	rs75016991	46.13014894
GOLM1	rs34953075	46.48154488
GOLM1	rs679574	1124.019198
GOLM1	rs35320397	56.27402052
GOLM1	rs2518028	49.68379495
GOLM1	rs34115112	42.16895145
GOLM1	rs76518888	49.65815246
GOLM1	rs146798134	76.89676317
GOLM1	rs192129994	61.3581149
GOLM1	rs118070731	50.47597521
GOLM1	rs576123	5483.38586

GOLM1	rs55710199	79.18815848
GOLM1	rs117630211	55.81727898
GOLM1	rs11793922	58.1576175
MX1	rs2837969	48.12549037
MX1	rs457274	2147.738305
MX1	rs60687893	72.41768744
MX1	rs2838064	53.16454752
MX1	rs17849502	35.18053603
MX1	rs572515	106.649057
MX1	rs1904604	36.83168418
MX1	rs55805232	32.32084377
MX1	rs78456138	55.31381607
MX1	rs371314787	39.01618095
MX1	rs1207778	33.76943421
MX1	rs9272226	110.4477443
MX1	rs62447171	86.54549202
MX1	rs7042630	49.06746917
MX1	rs3923776	64.88154948
IRF1	rs10922098	145.9874394
IRF1	rs9864801	48.73311728
IRF1	rs28573154	59.32960428
IRF1	rs7774739	48.88431067
IRF1	rs112692477	36.26757504
IRF1	rs11794541	35.00351597
DHRS9	rs62116888	30.59916826
DHRS9	rs28399664	55.05949412
DHRS9	rs7412	945.4927175
DHRS9	rs140480140	35.4189272
DHRS9	rs2234197	214.764363
DHRS9	rs1354034	43.16995547
SUFU	rs10922098	268.8871561
SUFU	rs2686395	101.8268264
SUFU	rs9267139	34.91915387
SUFU	rs9501393	105.7801191
SUFU	rs7774739	78.36471271
SUFU	rs9271766	33.20660411
YEATS4	rs11615712	36.12004267
YEATS4	rs572515	49.47778873
YEATS4	rs1706435	36.92221795
PBK	rs10922098	601.4447397
PBK	rs704	41.62612912
PBK	rs11569393	29.72347872
PBK	rs111696008	31.85341984
PBK	rs1707652	177.0082236
PBK	rs555007	70.53218696
PBK	rs453821	175.6655925
PBK	rs9272526	37.71600944
SH3BGRL3	rs146701335	58.89355475
SH3BGRL3	rs6676630	1035.031172
SH3BGRL3	rs111486799	85.51797943
SH3BGRL3	rs114289390	94.35066028
SH3BGRL3	rs3117999	43.91812677
SH3BGRL3	rs4980320	35.17417127
SH3BGRL3	rs6580980	34.35498441
SH3BGRL3	rs72799293	43.52313755
SH3BGRL3	rs1354034	52.1285789
SH3BGRL3	rs342200	32.14036086
SIRT3	rs6578690	45.43707142

SIRT3	rs192777257	131.030525
SIRT3	rs112189508	111.3415159
SIRT3	rs7950251	39.16492369
SIRT3	rs16914574	153.6174149
SIRT3	rs113199701	47.80923177
SIRT3	rs143666677	499.3589245
SIRT3	rs117627469	110.5618216
SIRT3	rs1555405	31.87746913
SIRT3	rs13019325	79.18939267
SIRT3	rs142405255	32.04279137
SIRT3	rs1619994	81.05589402
NAALADL1	rs10922094	649.8104898
NAALADL1	rs76004643	32.45905339
NAALADL1	rs704	91.89913728
NAALADL1	rs429358	86.70082102
NAALADL1	rs12478841	42.77226552
NAALADL1	rs1707652	186.0109334
NAALADL1	rs112516307	38.97385846
NAALADL1	rs542418	39.01689283
NAALADL1	rs550671	196.075646
NAALADL1	rs9270570	54.30222948
NAALADL1	rs9469195	113.7270044
HPD	rs1619994	34.72450867
HPD	rs1157745	134.7321439
HPD	rs854572	49.71553292
SRP14	rs10922098	43.95495853
SRP14	rs79740491	29.96834989
SRP14	rs185009887	33.55827573
SRP14	rs62269916	30.65030899
SRP14	rs28573154	38.07947387
RPP30	rs10922098	467.6841084
RPP30	rs4665987	31.87263454
RPP30	rs1706435	87.37493629
RPP30	rs9267139	31.44695894
RPP30	rs9501393	138.2188913
RPP30	rs7774739	82.07642687
RPP30	rs9271683	35.53072877
ANXA11	rs2152546	342.6570867
ANXA11	rs1538821	38.69602274
ANXA11	rs149464173	34.74403648
ANXA11	rs1354034	44.95453932
HSPA13	rs2822638	124.2203869
HSPA13	rs629301	87.71498557
HSPA13	rs825038	73.81475844
HSPA13	rs7964859	837.7509324
HSPA13	rs140624338	109.0580053
HSPA13	rs79940707	49.45568237
HSPA13	rs55714539	30.60812653
HSPA13	rs58542926	52.66728652
HSPA13	rs12995479	31.41392701
HSPA13	rs483465	34.44549829
HSPA13	rs3116995	54.85097959
RAB3A	rs10922098	625.7399695
RAB3A	rs3900909	31.1377185
RAB3A	rs59228823	30.16312239
RAB3A	rs1047286	46.79934404
RAB3A	rs1707652	80.66360976
RAB3A	rs555007	54.07147087

RAB3A	rs550671	158.0861822
RAB3A	rs73726194	30.45724846
ING4	rs16840639	50.90075004
ING4	rs74428318	57.47658492
ING4	rs184844840	31.79335829
ING4	rs35681564	103.2634962
ING4	rs189902748	138.2278469
ING4	rs62117160	1268.634806
ING4	rs7254892	2438.24741
ING4	rs118004808	65.27974808
ING4	rs190086576	88.22925257
ING4	rs2116891	57.74382374
ING4	rs73048731	92.15250976
ING4	rs11705829	30.87499267
CA5B	rs10922098	600.5041374
CA5B	rs3900909	34.50505814
CA5B	rs1707652	113.3057597
CA5B	rs9267139	54.37830606
CA5B	rs28573154	184.2228649
CA5B	rs7774739	135.9758223
CA5B	rs9271683	44.5937446
ACOT13	rs112965562	47.53496874
ACOT13	rs9356943	34.69706527
ACOT13	rs1354034	69.03912672
CD160	rs3753436	274.6851694
CD160	rs10922098	183.5939582
CD160	rs7310615	56.3893123
CD160	rs438811	48.80625444
CD160	rs875063	44.03745547
CD160	rs2686395	40.94445838
CD160	rs41285751	72.65908691
CD160	rs451652	52.7296689
EPHB1	rs57512309	62.53147043
EPHB1	rs114727697	35.27246456
EPHB1	rs73229138	127.7507585
EPHB1	rs36187	1080.30847
EPHB1	rs7373413	88.12591299
CD46	rs60467252	45.44015148
CD46	rs10746399	860.8169338
CD46	rs12133018	29.84436407
CD46	rs72704449	81.90797846
CD46	rs972570	122.6363027
CD46	rs77924615	37.46540183
CD46	rs4601112	41.43930189
CD46	rs11244049	65.19102654
APOA4	rs1729410	46.10013179
APOA4	rs187929675	112.5890119
APOA4	rs72704449	40.99191822
APOA4	rs77924615	32.39883848
APOA4	rs731541	40.02066249
APOA4	rs35058673	52.4090381
TBCB	rs2231569	49.56499805
TBCB	rs7073753	40.40345958
TBCB	rs1354034	57.22189905
TPP1	rs325652	31.0917705
TPP1	rs3758978	263.3507514
TPP1	rs4758455	36.86563486
TPP1	rs150234237	111.8642347

TPP1	rs72846817	97.37459354
TPP1	rs2393986	41.77816257
TPP1	rs4883201	130.9785571
TPP1	rs7964859	188.4192478
TPP1	rs140624338	46.60313827
TPP1	rs17580	40.30228152
TPP1	rs15951	73.89220047
TPP1	rs9346805	30.2240241
TPP1	rs6993770	71.70067843
BTN3A3	rs201814970	65.61249516
BTN3A3	rs12195411	344.9219039
BTN3A3	rs140862681	1390.110654
BTN3A3	rs9379861	691.0573097
BTN3A3	rs143104579	247.7690396
BTN3A3	rs9379919	10252.00502
BTN3A3	rs193056830	176.8432442
BTN3A3	rs147207784	150.2238837
BTN3A3	rs112482034	102.2811814
BTN3A3	rs7310615	42.55193893
BTN3A3	rs1968905	29.9741869
BTN3A3	rs75996569	51.97749971
BTN3A3	rs148149244	961.0769153
BTN3A3	rs9461445	5724.910584
BTN3A3	rs9468618	35.58178093
BTN3A3	rs3129015	3085.978341
BTN3A3	rs12525100	72.50124138
BTN3A3	rs186239568	46.95780031
BTN3A3	rs11037	81.6556243
BTN3A3	rs145924676	78.36369922
BTN3A3	rs6934840	33.84886615
BTN3A3	rs148389811	50.70630788
PSME2	rs180953787	166.7565814
PSME2	rs4575	96.46312142
PSME2	rs117849039	78.58534363
OVCA2	rs147835681	59.2810243
OVCA2	rs7073753	30.84731716
OVCA2	rs1354034	41.24705954
WBP2	rs11870078	41.5553117
WBP2	rs368465	71.49326126
WBP2	rs1355538	34.44540374
UGT1A1	rs13001875	59.20667933
UGT1A1	rs6742078	690.6832849
UGT1A1	rs10922098	120.9904329
UGT1A1	rs72690530	55.6722116
UGT1A1	rs704	231.8314006
UGT1A1	rs1355538	70.50947717
UGT1A1	rs28573154	31.81257658
GMNN	rs10922098	201.245617
GMNN	rs1355538	48.28711266
GMNN	rs454212	54.41990503
GMNN	rs28471056	32.60243063
PPP1R1A	rs10876568	39.12819274
PPP1R1A	rs10922094	187.7280878
PPP1R1A	rs1707652	67.43779352
PPP1R1A	rs9501393	69.46610915
PPP1R1A	rs28471056	45.56419605
TPD52L1	rs10922098	518.9391875
TPD52L1	rs3900909	34.51514191

TPD52L1	rs11569393	30.59329881
TPD52L1	rs2686395	83.241143
TPD52L1	rs555007	60.59710925
TPD52L1	rs550671	126.6617114
VAMP1	rs61802335	34.29120573
VAMP1	rs10922098	34.59138423
VAMP1	rs2523451	31.41583281
VAMP1	rs41556615	95.59401786
VAMP1	rs9273165	43.68490586
IDI1	rs4880758	42.77723992
IDI1	rs7926172	45.93039051
IDI1	rs1354034	146.5397238
GAS7	rs10922098	309.5222472
GAS7	rs28636516	31.22093454
GAS7	rs375109452	74.60212735
GAS7	rs117074475	30.9239286
GAS7	rs10985149	79.58503259
GAS7	rs6478516	41.55218912
FKBP4	rs34728283	92.52115633
FKBP4	rs145074222	50.28139142
FKBP4	rs1354034	54.85995126
USH1C	rs1451915	30.3241701
USH1C	rs10922094	463.2157226
USH1C	rs2686395	130.1633763
USH1C	rs9267139	47.7435326
USH1C	rs28573154	132.0006323
USH1C	rs7774739	94.64048719
USH1C	rs9271766	38.34579287
SAR1A	rs870801	326.4684538
SAR1A	rs118085384	33.90839098
SAR1A	rs4980320	30.8094134
SAR1A	rs1354034	70.27462898
GABARAP	rs16975847	39.06029077
GABARAP	rs7601000	37.74368722
GABARAP	rs2606731	114.1892896
APTX	rs34331968	107.7277638
APTX	rs1412635	235.778709
APTX	rs1355538	34.20083181
IVD	rs1129264	85.80170008
IVD	rs12902310	180.787217
IVD	rs10922094	60.49254068
IVD	rs2393778	70.47615595
IVD	rs13394512	30.1584173
IVD	rs1706435	34.38067624
CRNN	rs10494275	262.3796654
CRNN	rs79343884	50.51677243
CRNN	rs704	42.34709277
RRAS	rs10922094	538.9651497
RRAS	rs1291921	31.38438498
RRAS	rs11720167	194.9337874
RRAS	rs3863087	76.14060515
RRAS	rs4955716	31.80566228
RRAS	rs28573154	156.1280255
RRAS	rs7774739	107.1707347
RRAS	rs112692477	51.03033957
UBE2A	rs10922098	434.932059
UBE2A	rs111696008	31.15693352
UBE2A	rs2686395	147.9870203

UBE2A	rs1265941	62.2277112
UBE2A	rs550671	137.1099397
RAB11A	rs72882962	32.13071121
RAB11A	rs7114094	38.1776958
RAB11A	rs185009887	33.6428559
RAB11A	rs1354034	57.41967326
RAB11A	rs78906361	31.18096382
FIS1	rs75487681	363.6006606
FIS1	rs1354034	38.75691059
FIS1	rs1706435	101.3541314
TRAF1	rs58468211	31.94475019
TRAF1	rs10922098	684.8152921
TRAF1	rs704	198.7582978
TRAF1	rs11569415	37.19571479
TRAF1	rs1707652	132.1746224
TRAF1	rs1265941	66.83417336
TRAF1	rs444921	189.7527541
TP53I3	rs370119287	68.93551971
TP53I3	rs12713147	2238.848441
TP53I3	rs4665681	257.6081397
TP53I3	rs4665722	101.1229684
TP53I3	rs3847327	30.77120983
TP53I3	rs62122092	47.80085368
TP53I3	rs4665806	38.6433546
TP53I3	rs935172	53.94776352
TP53I3	rs74942955	106.2764467
TP53I3	rs1354034	32.35361992
TP53I3	rs6993770	46.74412347
RRM2	rs72702154	66.43366131
RRM2	rs7535409	288.6575317
RRM2	rs3917751	153.5445693
RRM2	rs10922098	127.2886354
RRM2	rs10764161	77.29057726
RRM2	rs10159712	202.578635
RRM2	rs2270190	32.91109889
RRM2	rs550671	59.90243741
RRM2	rs9270423	46.25402709
RRM2	rs9272422	108.920977
CRYBB1	rs5752349	44.10203323
CRYBB1	rs4822754	420.0536344
CRYBB1	rs11594179	45.48293449
CRYBB1	rs79977579	44.35644296
CRYBB1	rs4783183	46.42530189
CRYBB1	rs9494145	41.152626
CRYBB1	rs342293	49.72106887
CRYBB1	rs1460580	42.00722615
CRYBB1	rs6993770	369.6511071
CRYBB1	rs10984405	75.89366998
ARG2	rs10922098	36.93457292
ARG2	rs9558305	30.45761725
ARG2	rs1008910	366.0327636
ARG2	rs9272780	58.58156624
ARG2	rs3763354	34.84888538
UGDH	rs7682824	76.374798
UGDH	rs7667751	1048.437517
UGDH	rs10023376	114.4244989
UGDH	rs1354034	92.59085339
UGDH	rs10517405	39.92533703

UGDH	rs4734879	44.07610024
DCTD	rs66892705	30.43766553
DCTD	rs2603350	99.80550103
DCTD	rs1354034	39.80783875
WDR5	rs10922094	162.8115653
WDR5	rs10208529	33.98172074
WDR5	rs1619994	49.14759616
WDR5	rs415620	35.68199204
WDR5	rs28414666	57.62625849
RHOC	rs10745330	148.9527448
RHOC	rs3811444	39.48398548
RHOC	rs11082304	36.87232224
RHOC	rs112595944	30.66260336
RHOC	rs7412	29.91147612
RHOC	rs1354034	70.7201924
RHOC	rs73165061	134.7781526
RHOC	rs3863087	39.18222264
RHOC	rs6993770	93.53253754
SNUPN	rs8028182	196.2420482
SNUPN	rs185490651	30.33173494
SNUPN	rs4980320	54.03320174
SNUPN	rs1354034	173.5810774
SNUPN	rs342200	39.74762602
NCF1	rs141302758	99.36680484
NCF1	rs118073108	36.75797571
NCF1	rs148380322	177.5448566
NCF1	rs71539253	298.9514766
NCF1	rs12154789	1273.080267
NCF1	rs34474108	72.08338901
NCF1	rs7916803	29.89724764
NCF1	rs117995283	55.73366655
EMC8	rs6686805	108.8297668
EMC8	rs11624734	36.0975445
EMC8	rs2856876	179.1281442
EMC8	rs80236486	30.85029541
CRCP	rs10922094	462.3008418
CRCP	rs704	71.84680228
CRCP	rs11569415	65.92737114
CRCP	rs1707652	104.3746878
CRCP	rs9267139	36.4088348
CRCP	rs3891366	124.443751
CRCP	rs7774739	83.83470696
CRCP	rs9271766	34.63980608
UBD	rs10922098	577.0570139
UBD	rs3900909	32.31566697
UBD	rs1047286	41.51838329
UBD	rs2686395	126.1925672
UBD	rs4293988	34.07790773
UBD	rs555007	70.25705185
UBD	rs550671	161.4486364
UBD	rs73726194	30.01761317
NAPG	rs9959985	53.36034235
NAPG	rs7241196	79.04169914
NAPG	rs10922098	32.61188622
NAPG	rs4980320	30.35704371
NAPG	rs1354034	44.99631633
SNRPD3	rs10922094	244.2726496
SNRPD3	rs10208529	32.13039382

SNRPD3	rs6445035	49.38312939
SNRPD3	rs9501393	57.95321087
SNRPD3	rs7774739	45.60472432
PECR	rs73072196	140.0603715
PECR	rs10922094	76.11405988
PECR	rs6542680	36.68246138
SDSL	rs116092857	56.09031286
SDSL	rs77607740	352.2264801
SDSL	rs71467906	60.39477671
SDSL	rs113152874	667.073634
SDSL	rs7294372	34.67757709
SDSL	rs61943032	158.3738137
SDSL	rs111286706	59.90963279
SDSL	rs162391	36.16881534
SDSL	rs1619994	43.46565111
RAB32	rs185009887	33.65587785
RAB32	rs342200	39.18678562
RAB32	rs6993770	56.03560749
MAP1LC3A	rs6120675	36.91808788
MAP1LC3A	rs826404	38.73993279
MAP1LC3A	rs35340377	32.41243876
MAP1LC3A	rs10761739	71.86657366
MAP1LC3A	rs12767683	43.93968634
MAP1LC3A	rs7926172	58.56121345
MAP1LC3A	rs11171911	33.62271764
MAP1LC3A	rs10878645	35.19149127
MAP1LC3A	rs11553699	31.82725989
MAP1LC3A	rs11071720	30.82067544
MAP1LC3A	rs72628299	84.57601766
MAP1LC3A	rs185009887	42.20317515
MAP1LC3A	rs1037590	37.51069038
MAP1LC3A	rs7610953	31.7868868
MAP1LC3A	rs142822219	36.40709209
MAP1LC3A	rs1354034	679.0340846
MAP1LC3A	rs7648740	34.47395635
MAP1LC3A	rs3112401	55.94779714
MAP1LC3A	rs114694170	32.91687023
MAP1LC3A	rs740290	31.2659502
MAP1LC3A	rs342293	286.3679798
MAP1LC3A	rs56225072	40.17765079
MAP1LC3A	rs10820606	97.84297762
ACAA1	rs112208526	44.42268523
ACAA1	rs2685096	91.63392153
ACAA1	rs2229528	2178.714663
ACAA1	rs7625290	83.58389195
ACAA1	rs1242331	41.72779397
ACAA1	rs143276016	37.58235973
ACAA1	rs72719670	83.03347547
ACAA1	rs9953884	47.1103592
ACAA1	rs74770429	44.72545177
ACAA1	rs114653015	55.26573109
ACAA1	rs28362641	55.66602784
ACAA1	rs138983321	48.8696582
ACAA1	rs4835265	45.70919938
MMAB	rs16939972	132.4184631
MMAB	rs4766598	1052.511905
MMAB	rs1344544	255.7447669
MMAB	rs190825201	120.0674866

MMAB	rs78925260	59.71539691
MMAB	rs117415952	33.07729137
MMAB	rs1355538	31.75781441
ALDH5A1	rs4418728	31.04192231
ALDH5A1	rs1008764	38.24979609
ALDH5A1	rs180756495	54.75967222
ALDH5A1	rs3803195	174.9012978
ALDH5A1	rs9534213	147.6128099
ALDH5A1	rs145430629	460.7962879
ALDH5A1	rs9316176	6896.292526
ALDH5A1	rs73186684	1039.80843
ALDH5A1	rs4942469	415.691888
ALDH5A1	rs144761544	88.63818695
ALDH5A1	rs9534515	155.5864289
ALDH5A1	rs3012123	29.99552377
ALDH5A1	rs112702177	47.06880424
ALDH5A1	rs17530689	67.55106823
ALDH5A1	rs189693782	33.13884671
ALDH5A1	rs9317599	45.19756314
ALDH5A1	rs7328400	30.79629414
ALDH5A1	rs780093	47.67866317
ALDH5A1	rs3127580	91.20710545
ALDH5A1	rs12212507	33.21373681
ALDH5A1	rs150683617	90.45823052
ALDH5A1	rs112839059	126.7768174
ALDH5A1	rs112049074	47.01002018
PMM2	rs79460322	30.33890623
PMM2	rs1641002	49.2409199
PMM2	rs1273428	138.8252439
PMM2	rs3826198	288.6137903
PMM2	rs138018010	40.76817275
IMPA1	rs6099277	33.84267331
IMPA1	rs2232613	471.3820396
IMPA1	rs11906988	76.98435849
IMPA1	rs117247004	31.82000292
IMPA1	rs4554007	79.68307597
IMPA1	rs3863087	38.15995828
CUL1	rs10922098	285.5643735
CUL1	rs1706435	91.14355813
CUL1	rs28573154	86.60332066
CUL1	rs7774739	55.07637378
CUL1	rs9271766	39.12073684
PGLS	rs11665799	119.4815678
PGLS	rs145226688	91.59028547
PGLS	rs117004311	149.0219245
PGLS	rs4380127	35.48107172
PGLS	rs12974746	40.39912841
PGLS	rs151242358	54.11839859
PGLS	rs1354034	35.39999878
PNPO	rs216311	188.3193977
PNPO	rs704	376.3464375
PNPO	rs1065853	276.7477725
PNPO	rs1355538	44.07455266
PNPO	rs8176693	43.48734961
SDCBP	rs10914357	34.23238661
SDCBP	rs10751648	48.0905531
SDCBP	rs755240	46.56714016
SDCBP	rs13043452	92.9648422

SDCBP	rs1354034	43.38193436
SDCBP	rs7759300	37.1977906
SDCBP	rs2245961	30.3731131
SDCBP	rs11557154	102.0589927
NIT2	rs62274147	113.8144267
NIT2	rs115103390	45.79039729
NIT2	rs115361890	45.9197934
SULT1C2	rs10922098	447.5508997
SULT1C2	rs10208529	32.45246746
SULT1C2	rs1706435	84.48863942
SULT1C2	rs9267139	35.91810598
SULT1C2	rs9501393	121.6364604
SULT1C2	rs7774739	83.12047133
SULT1C2	rs9271766	41.44890238
PTP4A1	rs3213017	33.79515704
PTP4A1	rs2706762	35.09922577
PTP4A1	rs1706435	43.06746458
BPHL	rs9503407	184.5250792
BPHL	rs200978408	160.7911883
BPHL	rs10922094	173.2750787
BPHL	rs1706435	74.75259139
BPHL	rs9501393	53.09221176
BPHL	rs28471056	41.16947329
DDIT4	rs10922098	286.4064854
DDIT4	rs1707652	50.51857482
DDIT4	rs1265941	36.15849431
DDIT4	rs550671	91.50280607
FAHD1	rs113189356	80.57690926
FAHD1	rs112311350	382.0841951
FAHD1	rs367911720	38.55118093
FAHD1	rs113304135	39.77104401
FAHD1	rs5757204	84.70220802
MLF1	rs10922094	99.24072392
MLF1	rs113448098	44.16271939
MLF1	rs12146727	133.269061
MLF1	rs117827303	31.29938543
MLF1	rs7412	31.04065302
MLF1	rs1801701	84.50809609
MLF1	rs111696008	39.55878399
MLF1	rs62290472	36.70891119
MLF1	rs6445035	241.1697458
MLF1	rs3863087	91.56122446
MLF1	rs3891366	42.30047476
MLF1	rs35426875	36.52354947
MLF1	rs17164545	45.78470648
MLF1	rs9801685	34.72187737
MLF1	rs76342676	78.30807385
MLF1	rs854547	1014.414258
MLF1	rs374185817	149.644281
MLF1	rs57971788	43.22189974
NMT2	rs4237446	55.02821521
NMT2	rs35340377	29.9119272
NMT2	rs1354034	75.42727979
NMT2	rs342293	32.3679078
RERG	rs10922098	425.6626277
RERG	rs1047286	40.46623139
RERG	rs10208529	29.96168743
RERG	rs1707652	79.88961799

RERG	rs9267139	33.79269302
RERG	rs28573154	101.2070017
RERG	rs7774739	70.97563215
RERG	rs9271766	40.77653021
EEF1D	rs3811444	145.2179761
EEF1D	rs4980320	43.97466684
EEF1D	rs10876550	32.92968898
EEF1D	rs1354034	57.09282603
EEF1D	rs342292	30.05592054
DYNLRB2	rs10922098	62.98459333
DYNLRB2	rs4977155	43.08260292
DYNLRB2	rs557011	31.41317921
S100A14	rs9330298	119.9981077
S100A14	rs10922098	41.72816128
S100A14	rs2020934	46.90579876
S100A14	rs1355538	37.63571846
S100A14	rs28471056	36.31211011
IDI2	rs61830965	313.0897871
IDI2	rs6560713	34.13235114
IDI2	rs572515	106.6723219
IDI2	rs1706435	42.27317668
IDI2	rs4713498	36.72433986
ANXA13	rs10922098	109.9909857
ANXA13	rs697356	36.45219101
ANXA13	rs3891366	35.39565247
S100A16	rs12044086	40.11927861
S100A16	rs141973811	114.4219956
S100A16	rs9726753	506.6967087
S100A16	rs137931238	46.66629037
S100A16	rs10922094	46.24127698
S100A16	rs12986064	33.67711663
S100A16	rs9469079	30.26580321
S100A16	rs9271323	47.8395439
S100A16	rs28471056	96.53781182
S100A16	rs576123	309.7153726
CLIC2	rs3811444	35.16270179
CLIC2	rs6730558	37.39673414
CLIC2	rs6084653	47.28757818
CLIC2	rs28817896	30.01211236
CLIC2	rs2637678	36.63423201
PPCS	rs2152315	147.7967865
PPCS	rs2762687	45.70487148
PPCS	rs1354034	47.83521034
DRG1	rs35340377	37.48224905
DRG1	rs4980320	32.64320773
DRG1	rs1354034	63.95813293
DRG1	rs342292	38.80156591
KLF4	rs10922098	92.92539704
KLF4	rs1706435	31.44201467
KLF4	rs7774739	30.90747742
KLF4	rs557011	38.40901847
RGS4	rs10922098	292.203445
RGS4	rs1355538	68.85430262
RGS4	rs9501600	80.39547357
RGS4	rs28826696	33.53630157
ACADL	rs10922098	123.6042406
ACADL	rs9902706	58.1959497
ACADL	rs9501393	52.32900519

ACADL	rs35426875	38.77680352
CASP8	rs10922094	329.5833811
CASP8	rs1047286	31.44763564
CASP8	rs1706435	70.3354767
CASP8	rs9501393	100.6511323
CASP8	rs7774739	75.45396067
PCMT1	rs572515	37.87000497
PCMT1	rs10822155	40.7841658
PCMT1	rs11574452	34.8712414
RHOB	rs704	69.50945728
RHOB	rs77262773	62.2984797
RHOB	rs2706762	139.143564
RHOB	rs1354034	58.10262566
RHOB	rs115959678	71.94556781
RHOB	rs2428492	70.5704011
RHOB	rs28414666	32.52571504
RHOB	rs799169	30.09893514
PYCR1	rs10922098	49.61282659
PYCR1	rs1126477	38.77805692
PYCR1	rs9501393	35.71532857
PYCR1	rs7774739	33.08561446
ASF1A	rs147886588	151.1416457
ASF1A	rs10922094	68.39714677
ASF1A	rs6489859	30.73869303
ASF1A	rs185009887	29.7241844
ASF1A	rs1706435	34.49059053
ASF1A	rs141789230	51.27468224
ASF1A	rs1393562	31.15737642
AKR7A3	rs189614440	48.01437415
AKR7A3	rs9793028	61.16921242
AKR7A3	rs72645633	133.6553908
AKR7A3	rs12094043	604.5859825
AKR7A3	rs35169864	52.1095069
AKR7A3	rs183015613	179.7806514
AKR7A3	rs3747207	82.43613668
AKR7A3	rs141748393	30.09097971
AKR7A3	rs28601761	49.37998553
VAT1	rs2356310	55.14180921
VAT1	rs77420750	167.3591488
VAT1	rs12342201	36.13647672
NAE1	rs10922098	288.9668796
NAE1	rs11720167	125.8800765
NAE1	rs3863087	55.4184205
NAE1	rs550671	104.9394904
NAE1	rs34927613	56.87120734
SMS	rs10922094	88.98578177
SMS	rs1355538	53.46136119
SMS	rs550671	37.23841539
SMS	rs28471056	30.87477231
SMS	rs62467349	36.26659153
TSSC4	rs11555995	42.14258551
TSSC4	rs1354034	39.91094322
TSSC4	rs3748136	97.10892898
PCK1	rs4512628	42.61904941
PCK1	rs1329424	1034.036299
PCK1	rs7513100	48.04640551
PCK1	rs1047286	39.08529925
PCK1	rs2686395	228.7373528

PCK1	rs555007	91.94100216
PCK1	rs550671	264.2951399
PCK1	rs73726194	40.2195413
LDLRAP1	rs11248997	33.00436439
LDLRAP1	rs75446219	125.1398529
LDLRAP1	rs35340377	31.58229793
LDLRAP1	rs10876550	63.96555104
LDLRAP1	rs72673751	42.95988185
ALDOB	rs76682371	83.80520082
ALDOB	rs4408267	43.58138752
ALDOB	rs12230154	30.16451589
ALDOB	rs11047129	49.85394173
ALDOB	rs1260326	97.36077149
ALDOB	rs3747207	64.68097053
ALDOB	rs13213821	42.48365044
ALDOB	rs28601761	41.78646106
ALDOB	rs77412152	34.3578179
ALDOB	rs5741694	35.59641767
ASPA	rs147403462	34.53363141
ASPA	rs572515	641.1265005
ASPA	rs1706435	104.0356142
ASPA	rs9267139	48.5742701
ASPA	rs28573154	211.4281416
ASPA	rs7774739	156.3834194
ASPA	rs9271683	53.4108538
GATM	rs2453541	385.5386534
GATM	rs28629977	32.53566094
GATM	rs4528202	30.71094242
GATM	rs342292	42.57984154
VPS25	rs33996599	48.20611611
VPS25	rs140835565	41.18617756
VPS25	rs56239087	56.38699655
CARD18	rs10922098	466.5335944
CARD18	rs4900225	37.38255038
CARD18	rs4448834	32.40780428
CARD18	rs111696008	45.17878948
CARD18	rs6445035	146.7416037
CARD18	rs3863087	55.32095875
CARD18	rs3099847	30.76059944
CARD18	rs1265941	66.312376
CARD18	rs550671	151.5864153
CARD18	rs3917538	44.7739979
FABP9	rs72748420	37.82565955
FABP9	rs114009540	37.53265859
FABP9	rs1329424	1032.907738
FABP9	rs3900909	61.47278722
FABP9	rs1047286	49.60321301
FABP9	rs2686395	215.3520849
FABP9	rs3926704	33.62405172
FABP9	rs550671	283.0436913
FABP9	rs9469195	102.1085235
FABP9	rs9272526	51.53796489
CKMT2	rs142913020	30.45263839
CKMT2	rs572515	401.4684221
CKMT2	rs75743765	39.21844568
CKMT2	rs1707652	111.2786234
CKMT2	rs4293988	35.28182594
CKMT2	rs9267139	42.30383007

CKMT2	rs28573154	123.6742568
CKMT2	rs7774739	91.54706629
CKMT2	rs9271766	31.07677758
HBQ1	rs1005364	137.462867
HBQ1	rs10922094	90.11426169
HBQ1	rs11227136	52.72774049
HBQ1	rs34924625	39.79483916
HBQ1	rs2287997	40.45550563
HBQ1	rs138620872	70.95167183
HBQ1	rs6445035	164.7886596
HBQ1	rs3863087	55.66410066
HBQ1	rs3891366	51.19035564
HBQ1	rs7774739	34.3560535
CD37	rs10922098	92.5452223
CD37	rs11536949	49.39307475
CD37	rs1707652	102.9987202
CD37	rs4293988	29.87548245
CD37	rs9267139	36.68009333
CD37	rs9501393	106.5980229
CD37	rs7774739	77.91056793
TCEA1	rs7219736	32.16589037
TCEA1	rs1354034	86.96308824
TCEA1	rs6976036	34.41933742
MAGEA6	rs10922094	377.8389921
MAGEA6	rs704	62.34934455
MAGEA6	rs6445035	116.751825
MAGEA6	rs3863087	41.26561925
MAGEA6	rs9267139	35.81724459
MAGEA6	rs3891366	114.1056623
MAGEA6	rs7774739	78.39184282
MAGEA6	rs9271766	43.75832156
ADH6	rs28730617	354.136738
ADH6	rs13125919	74.70626653
ADH6	rs151006272	56.10353777
ADH6	rs59859017	39.32345114
ADH6	rs144873436	61.52174786
ADH6	rs72678667	55.82293315
ADH6	rs138191461	73.09991666
CCNB2	rs72748420	34.60463102
CCNB2	rs1329424	1003.289966
CCNB2	rs3900909	44.06132275
CCNB2	rs10801660	30.19269068
CCNB2	rs1047286	30.72469574
CCNB2	rs111696008	34.00476496
CCNB2	rs2686395	254.9668412
CCNB2	rs12629202	31.04034297
CCNB2	rs1265941	116.1841386
CCNB2	rs550671	275.0223496
CCNB2	rs9269234	44.68849295
SPIN1	rs10922098	571.5704482
SPIN1	rs111696008	33.89356205
SPIN1	rs2232613	86.34766171
SPIN1	rs1707652	191.0365823
SPIN1	rs555007	65.0695392
SPIN1	rs550671	168.4025262
SPIN1	rs9272526	32.17825379
UBE2F	rs59412705	70.15771175
UBE2F	rs10822155	31.36380133

UBE2F	rs61751507	150.8114481
UBE2F	rs4980320	31.73199241
UBE2F	rs2816622	85.5009396
UBE2F	rs704	31.39746081
UBE2F	rs1354034	61.95432098
UBE2F	rs1619994	37.61454452
GCLM	rs12563495	109.8720504
GCLM	rs945067	62.9062557
GCLM	rs1354034	30.28538866
THG1L	rs62387542	106.5975681
THG1L	rs7704907	1009.876224
THG1L	rs115777975	66.68161727
THG1L	rs6580149	47.90086743
THG1L	rs149687752	89.06417706
THG1L	rs6556411	43.9227719
THG1L	rs4921371	30.09728167
IL11RA	rs148778217	1343.585448
IL11RA	rs7023421	1362.594209
IL11RA	rs78705678	120.4982295
IL11RA	rs2477642	156.4311305
IL11RA	rs12720926	31.67996528
IL11RA	rs12150028	32.9853544
IL11RA	rs112001035	339.5700511
IL11RA	rs77542162	53.68531935
IL11RA	rs7236292	33.75468767
IL11RA	rs7232942	40.78027073
IL11RA	rs4805762	54.68476212
IL11RA	rs1065853	31.56282354
IL11RA	rs1260326	37.7842796
IL11RA	rs55683663	50.2756929
IL11RA	rs139849225	31.27364049
IL11RA	rs72729437	54.29055721
IL11RA	rs61729933	32.52493546
CNRIP1	rs10199432	69.50195467
CNRIP1	rs72830075	32.46188895
CNRIP1	rs7592976	2487.549103
CNRIP1	rs140590049	150.1796875
CNRIP1	rs184832464	311.5223287
CNRIP1	rs72835044	100.3789642
CNRIP1	rs4280488	45.58763106
CNRIP1	rs78857843	32.14482652
CNRIP1	rs183550357	50.03848963
CNRIP1	rs145971222	53.80361232
CNRIP1	rs6014993	30.67997055
CNRIP1	rs9399136	65.1613721
SRA1	rs111745822	33.29003217
SRA1	rs2563253	202.7650091
SRA1	rs1354034	78.25739226
SH3GLB2	rs10739734	119.6098711
SH3GLB2	rs57294583	1286.998322
SH3GLB2	rs9409310	151.5047481
SH3GLB2	rs10733695	46.36741222
SH3GLB2	rs7606173	35.07815135
HEBP1	rs73068707	186.7781566
HEBP1	rs11055175	4353.815563
HEBP1	rs35465601	579.7888492
HEBP1	rs12368093	52.12057341
HEBP1	rs1379509	118.4841955

HEBP1	rs2723842	29.7796441
HEBP1	rs71459131	30.59955863
HEBP1	rs139174956	45.94646806
HEBP1	rs2016598	37.51458416
PRND	rs10801548	32.03544747
PRND	rs11806293	216.2239207
PRND	rs2230199	320.19147
PRND	rs73613564	153.1738326
PRND	rs2686395	75.10686022
PRND	rs7439493	32.06363468
PRND	rs9332739	102.1676682
PRND	rs114382848	85.86717598
PGP	rs116986044	53.99168723
PGP	rs77634115	34.04850667
PGP	rs62038850	75.44043432
PGP	rs116977380	491.345312
PGP	rs117145260	36.87665355
PGP	rs77818036	107.5914949
PGP	rs118033454	37.62803023
PGP	rs77769444	30.22048156
NRIP3	rs10922098	184.5582972
NRIP3	rs9864801	38.72906497
NRIP3	rs28573154	72.28221369
NRIP3	rs7774739	56.01234702
NRIP3	rs112692477	30.58315861
OMP	rs10922098	261.2353835
OMP	rs116040950	30.94927541
OMP	rs9864801	54.70328587
OMP	rs444921	63.48415206
OMP	rs34927613	39.47568355
CPOX	rs147816533	36.86750363
CPOX	rs1675513	257.6154449
CPOX	rs1841542	71.70732302
CPOX	rs192384676	37.08462463
CPOX	rs549893	29.73586833
CPOX	rs56086968	32.28542461
CPOX	rs543798	151.9136815
CPOX	rs4313850	87.75183479
CPOX	rs56387760	40.70842582
CPOX	rs5167	90.88453606
CPOX	rs855791	162.5303516
CPOX	rs7614298	80.55561248
CPOX	rs75191663	33.5605048
CPOX	rs202240117	68.49345955
CPOX	rs115740542	247.6998597
CPOX	rs200418405	42.89248768
CPOX	rs10947058	30.82429854
CPOX	rs9399136	40.13353685
CPOX	rs4878129	50.87787604
CPOX	rs147707926	78.12180373
CPOX	rs71491081	38.71790925
SPA17	rs62140549	66.07621997
SPA17	rs149062332	40.26424335
SPA17	rs1800789	86.07930719
CMPK1	rs35687416	109.2883617
CMPK1	rs203687	36.93627671
CMPK1	rs10822155	35.40393283
ANXA7	rs79554558	40.41720243

ANXA7	rs79083576	111.6492567
ANXA7	rs1354034	34.11019707
CYB5R1	rs10922098	313.7278344
CYB5R1	rs6542680	36.81144978
CYB5R1	rs371314787	45.04255911
CYB5R1	rs1706435	79.97254329
CYB5R1	rs9267139	42.10834211
CYB5R1	rs28573154	98.38266615
CYB5R1	rs7774739	73.98610397
CYB5R1	rs9271766	40.56496212
SRP19	rs12147435	37.51671815
SRP19	rs9897552	52.02434652
SRP19	rs1354034	85.43917697
MEOX2	rs10922098	303.4666292
MEOX2	rs28362944	35.23904408
MEOX2	rs113448098	37.45148927
MEOX2	rs12146727	132.4418951
MEOX2	rs11624581	43.28643381
MEOX2	rs1355538	67.31824515
MEOX2	rs9267139	51.71840491
MEOX2	rs28573154	137.1926043
MEOX2	rs7774739	120.2692844
CCDC25	rs411729	77.23117924
CCDC25	rs67908756	207.4812052
CCDC25	rs3891366	32.38195649
CCDC25	rs3757387	29.79791044
TAF10	rs72748420	46.68647603
TAF10	rs1329424	2086.123982
TAF10	rs3900909	74.9218488
TAF10	rs10801660	44.47717994
TAF10	rs11080290	29.88931129
TAF10	rs111696008	44.13706938
TAF10	rs1707652	327.1767787
TAF10	rs73169117	40.10058023
TAF10	rs1265941	146.7922985
TAF10	rs550671	366.2871696
TAF10	rs112164276	50.93868655
TAF10	rs41548618	61.55392081
OXSM	rs74564945	30.14311464
OXSM	rs73076560	63.32031953
OXSM	rs142824563	37.55761416
OXSM	rs7651161	144.1009294
OXSM	rs2304442	953.9454678
OXSM	rs115680081	59.08615317
OXSM	rs56052360	86.36247548
OXSM	rs184430520	46.89874566
SERF2	rs10922098	240.1749632
SERF2	rs9864801	70.18704108
SERF2	rs9501393	59.94077678
SERF2	rs7774739	43.45743636
CRIP1	rs11591147	65.16171272
CRIP1	rs660240	64.12785541
CRIP1	rs684818	31.61932448
CRIP1	rs964184	125.202545
CRIP1	rs3764261	39.84612094
CRIP1	rs6511720	77.03396496
CRIP1	rs147711004	30.81853548
CRIP1	rs7412	201.2853029

CRIP1	rs639463	35.74647958
CRIP1	rs10172650	950.7769991
CRIP1	rs754524	553.3305169
CRIP1	rs116609675	93.14802772
CRIP1	rs115092560	37.83301874
CRIP1	rs112709686	31.91375782
CRIP1	rs6740545	31.30131022
CRIP1	rs9272068	30.78685037
CRIP1	rs74869266	38.20675443
CRIP1	rs112875651	59.95546595
TCL1B	rs10922098	161.3351158
TCL1B	rs6542680	45.60853951
TCL1B	rs1355538	34.5062407
TCL1B	rs9501600	52.35733522
TRIAP1	rs10922098	150.318045
TRIAP1	rs1355538	37.23879433
TRIAP1	rs28573154	40.31924945
TRIAP1	rs72848261	29.95515978
TRIAP1	rs2057681	215.4273391
TRIAP1	rs854572	30.96219933
GNG11	rs10922098	761.8971103
GNG11	rs7513100	36.68616025
GNG11	rs11080290	29.87274094
GNG11	rs1047286	53.90936056
GNG11	rs2686395	168.1463809
GNG11	rs1265941	87.3435041
GNG11	rs550671	205.1428496
GNG11	rs9276606	38.46356189
SVIP	rs10922098	352.0918091
SVIP	rs1706435	82.94128132
SVIP	rs75380382	33.40411258
SVIP	rs147527033	79.57944267
SVIP	rs9281012	55.39724632
SVIP	rs147906938	184.3502042
SVIP	rs9501602	86.73812942
SVIP	rs115501522	35.61122229
ZWINT	rs10922094	187.7746743
ZWINT	rs1126605	446.1194284
ZWINT	rs9864801	47.05821854
ZWINT	rs6457457	46.94506851
ZWINT	rs28573154	67.11771015
CCL15	rs4796109	611.6050015
CCL15	rs62078063	1098.327991
CCL15	rs4795104	1058.759566
CCL15	rs12452733	121.8828718
CCL15	rs72826131	91.96676498
CCL15	rs17580	104.6035108
CCL15	rs112963922	45.02089629
CCL15	rs80066936	47.18169781
CCL15	rs35230108	34.66868614
CCL15	rs147124687	58.4332364
CCL15	rs75372608	38.13113775
CCL15	rs117954971	55.58571553
CCL15	rs73295531	67.79648403
CCL15	rs117974358	30.44748079
CCL15	rs34953075	42.53700983
CCL15	rs492602	624.5094016
CCL15	rs35320397	38.20849065

CCL15	rs635634	70.88788591
ANXA8	rs10922098	511.8385915
ANXA8	rs1707652	100.1808536
ANXA8	rs3891366	159.2072097
ANXA8	rs7774739	105.2923746
ANXA8	rs28645249	52.30019841
GRHPR	rs72737776	63.70841767
GRHPR	rs10973326	931.6601208
GRHPR	rs7874199	44.79365085
MRAS	rs10922098	402.551148
MRAS	rs1707652	96.89321255
MRAS	rs9501600	118.8603034
MRAS	rs72508422	38.90842497
MRAS	rs587495	36.21630275
CAPNS1	rs149003598	37.39431235
CAPNS1	rs11418026	72.74071817
CAPNS1	rs12292693	894.5913855
CAPNS1	rs182588186	49.93944715
CAPNS1	rs144930535	170.1227042
CAPNS1	rs5030088	203.1947157
PURB	rs4980320	41.40271837
PURB	rs35979828	34.97187051
PURB	rs1354034	48.57191858
NAIF1	rs4950771	136.6443278
NAIF1	rs72773851	48.4453548
NAIF1	rs113808467	971.4041901
NAIF1	rs141439100	29.83377621
NAIF1	rs2941223	47.60773554
NAIF1	rs1354034	42.54106574
NAIF1	rs9260109	87.17001102
NAIF1	rs74885417	90.58194435
ZWILCH	rs72748420	37.63916114
ZWILCH	rs1329424	1096.101255
ZWILCH	rs3900909	51.8154095
ZWILCH	rs10801660	31.44117899
ZWILCH	rs1047286	30.046669
ZWILCH	rs111696008	31.68625208
ZWILCH	rs2686395	208.0518442
ZWILCH	rs1265941	105.2152641
ZWILCH	rs550671	269.8603138
ZWILCH	rs9276606	43.43327352
BCCIP	rs10922098	171.3106759
BCCIP	rs704	198.6260915
BCCIP	rs1706435	57.43061488
BCCIP	rs9501393	58.27383203
BCCIP	rs28471056	41.54269994
PALM	rs10922098	633.4209072
PALM	rs1555405	29.81598971
PALM	rs1047286	36.40848678
PALM	rs2686395	124.0209326
PALM	rs9266766	30.63775716
PALM	rs555007	83.92798466
PALM	rs453821	169.8872196
PPA2	rs4699179	134.8849156
PPA2	rs7920986	45.16734978
PPA2	rs1354034	40.4822959
PPA2	rs1619994	40.32829752
STXBP6	rs10922094	75.5634792

STXBP6	rs964184	40.25819997
STXBP6	rs71352239	146.483539
STXBP6	rs75916142	74.56802402
STXBP6	rs6736362	941.9728346
STXBP6	rs71403033	112.3748805
STXBP6	rs6445035	139.51452
STXBP6	rs3863087	64.14103665
DCTN6	rs4980320	36.27136271
DCTN6	rs35633	62.60020322
DCTN6	rs1354034	45.76253723
DCTN6	rs10964532	34.59165321
NDRG3	rs62210514	81.08520746
NDRG3	rs111279459	120.115103
NDRG3	rs199678162	41.48415675
NDRG3	rs1354034	30.13736794
NDRG3	rs1619994	40.62909055
ASRGL1	rs113174841	51.71762503
ASRGL1	rs12275541	597.7737861
ASRGL1	rs572515	46.704589
ASRGL1	rs7073753	45.81885994
ASRGL1	rs149089405	38.18746339
ASRGL1	rs11671654	409.6722333
ASRGL1	rs1047891	93.71560776
ASRGL1	rs1354034	63.26023856
RTP4	rs79918843	40.06774652
RTP4	rs9865045	1096.123916
RTP4	rs7637064	122.7559469
RTP4	rs9830399	36.63384816
RTP4	rs9850403	38.19774223
RTP4	rs1452200	49.98832642
RTP4	rs78240249	37.30441019
RTP4	rs11502187	37.56679237
RTP4	rs12292693	30.14951538
RTP4	rs1354034	55.99935713
RTP4	rs10820606	34.51115277
SH3GL3	rs4980320	48.03512638
SH3GL3	rs4759076	67.41145372
SH3GL3	rs1354034	30.47610528
MTHFD2	rs72818091	70.19518618
MTHFD2	rs114388033	73.83953758
MTHFD2	rs34416903	45.40414856
MTHFD2	rs447673	39.73741267
MTHFD2	rs3771529	45.81862157
MTHFD2	rs10820606	63.26535194
JMJD6	rs704	36.91276519
JMJD6	rs1355538	86.41682564
JMJD6	rs6884904	30.16257816
SUOX	rs10922098	193.8066021
SUOX	rs55648536	197.8951273
SUOX	rs4774168	69.56288807
SUOX	rs1355538	84.57176897
SUOX	rs9501600	47.0308298
SUOX	rs9268744	34.60012336
SUOX	rs3778754	44.55522995
MAGEA8	rs535088	175.1366413
MAGEA8	rs2518694	33.96409216
MAGEA8	rs10922098	156.7461313
MAGEA8	rs11720167	52.78156751

MAGEA8	rs550671	59.17869876
MAGEA8	rs2909668	30.91683077
SNX5	rs4980320	30.7085626
SNX5	rs1354034	49.95558649
SNX5	rs1706435	56.72917044
SNX5	rs2438150	625.5910183
SNX5	rs80312727	60.74236039
PYCR2	rs10922098	704.1524139
PYCR2	rs35351085	30.79590841
PYCR2	rs1047286	32.10406529
PYCR2	rs111696008	35.63532043
PYCR2	rs1707652	230.3101591
PYCR2	rs3130955	39.16902279
PYCR2	rs9501600	187.5126144
PYCR2	rs35209896	50.04099778
PGM2	rs28434334	165.9227515
PGM2	rs77864400	33.50336173
PGM2	rs77566893	33.01376489
PGM2	rs1995963	34.63240155
PGM2	rs113793196	58.23872506
PGM2	rs10517374	37.34249612
PGM2	rs16995688	56.40168584
PGM2	rs150214082	52.52430586
CPLX1	rs7687101	50.26880779
CPLX1	rs10922098	96.09250098
CPLX1	rs1355538	42.91987255
CPLX1	rs6884904	30.45063331
CPLX1	rs4713498	33.64348438
IDH1	rs116643358	53.94160085
IDH1	rs73070958	438.6471559
IDH1	rs116165405	175.3399937
IDH1	rs142404086	37.19120305
PSMB3	rs10922094	105.7598192
PSMB3	rs1706435	57.90689834
PSMB3	rs72548024	50.28245628
PSMB3	rs7774739	137.1432656
PSMB3	rs7742632	139.4314475
PSMB3	rs3917538	39.77193272
PSMB4	rs3738478	120.7274644
PSMB4	rs2769265	978.7542604
PSMB4	rs41263712	31.115496
PSMB4	rs12134551	35.03502276
PSMB4	rs1611755	50.79854966
PSMB4	rs192355405	80.85814295
PSMB4	rs8013143	48.12145097
PSAT1	rs4745686	31.25206857
PSAT1	rs2277148	232.0888209
PSAT1	rs3747207	34.92090195
PSAT1	rs28601761	81.42650623
DECR2	rs9788833	222.9512962
DECR2	rs436075	1187.268879
DECR2	rs3826231	1188.957043
DECR2	rs12924790	70.04842594
DECR2	rs35582942	96.01659956
DECR2	rs10399785	153.9929786
DECR2	rs10822155	46.54121981
DECR2	rs74770429	60.05425442
DECR2	rs1354034	49.12659814

DECR2	rs342292	32.07709442
HNRNPD	rs3751343	30.296652
HNRNPD	rs1242105	30.61079334
HNRNPD	rs1354034	93.70782209
MYL3	rs113785232	40.69301245
MYL3	rs17617337	45.07769707
MYL3	rs56678255	31.39136162
MYL3	rs10833719	188.9012737
MYL3	rs7318906	138.2013141
MYL3	rs2289292	33.1731349
MYL3	rs144579769	36.36066186
MYL3	rs6431221	30.40638125
MYL3	rs12463674	50.26921692
MYL3	rs150115323	44.9584779
ALB	rs12026141	79.81810253
ALB	rs7603427	100.466866
ALB	rs111696008	169.4685449
ALB	rs144202860	106.4877754
ALB	rs1105880	42.18316145
ALB	rs9872765	44.46619127
ALB	rs79330559	44.8862514
ALB	rs697356	1110.779028
ALB	rs13095777	67.16588087
ALB	rs147033786	94.12027495
ALB	rs114595100	53.78807543
ALB	rs10502	47.3819751
ALDH2	rs10849939	99.68838548
ALDH2	rs7519043	48.49066206
ALDH2	rs116859940	37.61263163
ALDH2	rs58118359	34.06234168
ALDH2	rs4940691	29.73009698
ALDH2	rs13030345	33.69103484
ALDH2	rs4835265	48.45661743
COMT	rs165722	98.71724434
COMT	rs75406264	119.6330134
COMT	rs75506960	59.8814702
COMT	rs36136293	777.8562116
COMT	rs117813943	32.48702428
COMT	rs12433460	92.95278538
PSMD10	rs10922098	89.26055843
PSMD10	rs10822155	63.54493494
PSMD10	rs1355538	46.50014981
PSMD10	rs2367289	31.02639495
GLRX	rs6556885	86.59985171
GLRX	rs10922094	167.7068501
GLRX	rs1355538	68.42648399
GLRX	rs28573154	57.05756173
GLRX	rs7774739	41.34922423
IFNA1	rs10922098	124.965554
IFNA1	rs1355538	42.49520376
IFNA1	rs3891366	55.36175956
IFNA1	rs7774739	47.42499588
MAT2B	rs10799536	34.22101541
MAT2B	rs214085	48.26000718
MAT2B	rs76428106	36.51910625
MAT2B	rs113753171	35.0091892
MAT2B	rs62083752	179.5940449
MAT2B	rs190383363	125.5058985

MAT2B	rs200132449	101.2904819
MAT2B	rs145433937	119.9137446
MAT2B	rs111398308	68.0145245
MAT2B	rs6131985	35.25554765
MAT2B	rs2305637	48.02685411
MAT2B	rs744233	38.11565737
MAT2B	rs10103048	49.58815897
ADPRH	rs709275	31.58945529
ADPRH	rs6542680	611.064194
ADPRH	rs80220851	54.82550803
ADPRH	rs9864801	30.3087394
ADPRH	rs188302747	30.32658592
AKR1C4	rs75056746	114.4244643
AKR1C4	rs1892403	1273.720899
AKR1C4	rs111849400	109.189749
AKR1C4	rs2050359	1006.150167
AKR1C4	rs10904475	130.7898254
AKR1C4	rs72779812	59.84747624
AKR1C4	rs4747987	123.5482409
AKR1C4	rs1033779	37.52780212
AKR1C4	rs4746822	46.14749095
AKR1C4	rs1476029	37.95165739
AKR1C4	rs3747207	64.7924441
AKR1C4	rs2954038	37.10642676
AKAP7	rs1451915	35.50575521
AKAP7	rs10922098	703.0876174
AKAP7	rs7513100	30.09761203
AKAP7	rs1047286	38.85175884
AKAP7	rs1707652	157.4758296
AKAP7	rs1265941	69.21503286
AKAP7	rs550671	169.6684579
ALKBH2	rs10922098	40.56525041
ALKBH2	rs113493922	53.13106543
ALKBH2	rs76359966	38.61362801
ALKBH2	rs41547217	61.28409853
ALKBH2	rs9270599	33.99969878
ALKBH3	rs11037575	75.23219966
ALKBH3	rs2434484	783.7471792
ALKBH3	rs11037809	83.40769394
ALKBH3	rs35979828	37.718482
ALKBH3	rs1354034	44.22133609
ALKBH3	rs114694170	41.85848785
AMPD2	rs4980320	42.76320118
AMPD2	rs450269	36.73784546
AMPD2	rs72673751	32.91126999
ANKRD54	rs10922098	335.8450981
ANKRD54	rs1355538	65.57161436
ANKRD54	rs28573154	65.68669363
ANKRD54	rs7774739	48.68380887
AP1S2	rs10922094	48.98680358
AP1S2	rs1354034	30.71817266
AP1S2	rs1619994	73.43179089
ARF4	rs1354034	103.5707199
ARF4	rs62622492	43.0214133
ARF4	rs11417460	39.78586662
ARF4	rs4980320	31.45369476
ARF4	rs17580	57.26628563
ARF4	rs7412	31.80136107

ARF4	rs111696008	36.67670921
ARF4	rs6445035	348.219859
ARF4	rs3863087	121.7868409
ARF5	rs1329424	253.138614
ARF5	rs4980320	33.32970556
ARF5	rs704	35.76217981
ARF5	rs72952553	32.91939561
ARF5	rs6445035	233.8716686
ARF5	rs3863087	77.36448478
ARF5	rs454212	73.92622253
ARL14	rs10922098	263.0497477
ARL14	rs2686395	86.74381906
ARL14	rs9501600	84.95946681
ARL15	rs8178824	43.99991374
ARL15	rs7580569	50.66688713
ARL15	rs138259153	65.63152897
ARL15	rs1250259	293.6878891
ARL15	rs4674006	52.11082419
ARL15	rs28374650	30.07968666
ARL5A	rs10922098	130.1688441
ARL5A	rs1706435	64.41139114
ARL5A	rs9501393	47.1390392
ARL5A	rs7774739	37.97070309
AS3MT	rs4917993	35.98012078
AS3MT	rs10922098	394.1459292
AS3MT	rs2686395	110.8359829
AS3MT	rs2242572	116.357885
AS3MT	rs9501602	65.03275017
AS3MT	rs9271529	37.04535607
ARPC5	rs2254707	55.67297265
ARPC5	rs112057769	35.19953622
ARPC5	rs1354034	48.09321095
ARPC5	rs78912960	31.05020486
CDH1	rs374896	34.66241627
CDH1	rs601338	365.2074659
CDH1	rs635634	105.6332139
RALB	rs4849845	769.6747294
RALB	rs1354034	53.93654286
RALB	rs6445035	95.45306508
RALB	rs3863087	31.56446128
TBPL1	rs7073753	30.11676345
TBPL1	rs4980320	40.51216803
TBPL1	rs35095923	32.49724849
TBPL1	rs6151565	34.25312919
TBPL1	rs1354034	68.09194625
TBPL1	rs342292	47.08462458
TBPL1	rs11380594	53.33522449
TBPL1	rs3748136	254.3163314
TBPL1	rs140770261	34.99465373
TBPL1	rs6993770	37.71953953
UBXN2B	rs7000685	53.53220771
UBXN2B	rs61808983	36.36894142
UBXN2B	rs4448834	42.1158191
UBXN2B	rs1354034	54.69319504
PPIG	rs145439832	30.76421359
PPIG	rs72748420	35.99327265
PPIG	rs1329424	1257.602121
PPIG	rs3900909	63.20183275

PPIG	rs10801660	40.61056245
PPIG	rs111696008	35.35996652
PPIG	rs2686395	223.9845194
PPIG	rs555007	117.9768846
PPIG	rs550671	308.4744332
PPIG	rs41548618	40.16833398
PPIG	rs9272526	51.79806609
PTGES2	rs13283456	88.13304399
PTGES2	rs572515	114.4373778
PTGES2	rs4980320	34.71132474
PTGES2	rs1354034	37.26593975
PTGES2	rs28573154	43.35860179
PTGES2	rs7774739	34.20816112
RABIF	rs572515	282.0603198
RABIF	rs114115200	46.72925943
RABIF	rs9501600	79.21566545
CBX1	rs1974470	114.9546643
CBX1	rs11625314	45.42575739
CBX1	rs35567863	32.00043594
PPIC	rs111336721	64.30981646
PPIC	rs141771168	125.0440252
PPIC	rs1031707	1118.933248
PPIC	rs61372352	90.01246475
PPIC	rs113638297	52.6882294
PPIC	rs757081	406.4026206
PPIC	rs1801261	83.96316041
PPIC	rs491567	30.08756855
PPIC	rs77924615	35.98349633
PPIC	rs139578440	59.17431597
PPIC	rs146895994	38.30925428
PPIC	rs141357345	205.4736848
PPIC	rs80089406	156.4107168
PPIC	rs464531	81.31714689
PPIC	rs57792329	149.364595
ITLN1	rs141410828	41.17894782
ITLN1	rs74625706	99.10383334
ITLN1	rs10922098	364.7267364
ITLN1	rs10832616	39.52233063
ITLN1	rs62193065	42.04333831
ITLN1	rs1707652	84.79676707
ITLN1	rs6768262	31.65714515
ITLN1	rs12658587	35.05854079
ITLN1	rs9501600	103.3728977
ITLN1	rs35209896	31.73461969
ITLN1	rs4722168	31.51794591
LRIG1	rs17073215	64.92442069
LRIG1	rs73125752	77.91102409
LRIG1	rs3772197	1044.287572
LRIG1	rs77048548	123.6875726
LRIG1	rs74578384	5765.450126
LRIG1	rs77982189	55.26289976
LRIG1	rs4537545	29.85076121
LRIG1	rs11122456	73.64268396
LRIG1	rs78705678	67.20185066
LRIG1	rs2477642	64.76165045
LRIG1	rs1519125	31.2306811
LRIG1	rs7960688	54.34112382
LRIG1	rs75342935	43.73006373

LRIG1	rs6734238	34.93088543
LRIG1	rs149592170	44.48280544
LRIG1	rs145061772	46.1120011
LRIG1	rs74797254	37.33558846
LRIG1	rs78935718	101.8031532
LRIG1	rs7740612	43.47869031
SAA2	rs1818737	75.39005318
SAA2	rs11024589	2052.149482
SAA2	rs76204461	212.4673154
SAA2	rs12282271	135.043013
SAA2	rs11208691	287.5359351
SAA2	rs4071540	42.71619513
SAA2	rs4129267	63.14645309
SAA2	rs34927905	31.21551316
SAA2	rs8192300	40.03137167
SAA2	rs1371135	80.52677074
SAA2	rs9939224	59.05974693
SAA2	rs111696008	37.28027998
SAA2	rs6445035	265.0596302
SAA2	rs3863087	149.3917953
SAA2	rs3129773	48.21041193
SGTB	rs1329424	235.0649185
SGTB	rs1355538	60.59971664
SGTB	rs9267139	36.94165406
SGTB	rs9501393	88.38411756
SGTB	rs7774739	71.62190321
TG	rs180223	37.65177998
TG	rs2979029	87.69529286
TG	rs116909374	45.90822144
TG	rs13061952	82.15691653
TG	rs3872724	286.8044931
TG	rs2268533	133.0647832
TG	rs10032216	31.37562979
TG	rs9295128	49.37480674
SH2D1B	rs10922098	243.9217274
SH2D1B	rs56210800	39.69037978
SH2D1B	rs140431012	39.77317277
SH2D1B	rs11720167	92.18674714
SH2D1B	rs3863087	36.9217957
SH2D1B	rs9358339	29.93678128
SH2D1B	rs9267139	29.88195301
SH2D1B	rs28573154	93.31459366
SH2D1B	rs7774739	63.06420468
SERPINB13	rs17756365	30.90417222
SERPINB13	rs118015107	63.91623289
SERPINB13	rs77157727	497.2973018
SERPINB13	rs72704449	31.80393435
SERPINB13	rs7953031	76.20503751
SERPINB13	rs77924615	30.10977332
SERPINB13	rs492602	258.6186964
SERPINB13	rs10405357	99.63128025
SERPINB13	rs1260326	39.06029139
UBE2D2	rs10922094	261.0111256
UBE2D2	rs1065853	213.154512
UBE2D2	rs6445035	149.9192181
UBE2D2	rs3863087	57.5895946
UBE2D2	rs550671	81.25222403
UBE2D2	rs9469195	54.47495761

UBE2D2	rs73216598	65.5323165
UBE2D2	rs2057681	749.1442367
UBE2D2	rs854572	34.58312839
EIF4EBP3	rs10922098	69.28272473
EIF4EBP3	rs41285751	31.36331174
EIF4EBP3	rs28645249	41.43016743
CHST2	rs10922094	122.0556889
CHST2	rs2232613	34.08558722
CHST2	rs1619994	59.08644942
CHST2	rs9501600	35.3887049
PRSS3	rs10813910	70.63607051
PRSS3	rs12237401	87.9371904
PRSS3	rs138062707	193.5271264
PRSS3	rs72709664	930.6132264
PRSS3	rs74178897	729.7140022
PRSS3	rs61639723	118.5896742
PRSS3	rs10971925	512.457076
PRSS3	rs117173455	77.36994763
PRSS3	rs2742676	88.97179087
PRSS3	rs76544063	49.24147104
PRSS3	rs111969858	59.74220815
PRSS3	rs76335653	68.00203327
PRSS3	rs33948761	48.9426941
PRSS3	rs2579674	41.87710623
PRSS3	rs10994434	100.3496973
PRSS3	rs7068821	44.21714332
PRSS3	rs11622435	34.80547099
PRSS3	rs12927671	43.46358009
PRSS3	rs9652674	97.11826347
PRSS3	rs1673931	159.5213073
PRSS3	rs6501460	262.9407116
PRSS3	rs12604466	37.03483802
PRSS3	rs4804181	345.1787146
PRSS3	rs2161098	47.99046413
PRSS3	rs281377	38.10358203
PRSS3	rs3771138	35.88937948
PRSS3	rs4953699	78.47761588
PRSS3	rs2743283	38.73353179
PRSS3	rs6445387	86.93183147
PRSS3	rs1816308	61.30251797
PRSS3	rs7687718	79.47313576
PRSS3	rs11759956	64.79074928
PRSS3	rs2535253	30.08372246
PRSS3	rs2766594	36.94369006
PRSS3	rs9482770	32.28123163
PRSS3	rs1018941	46.89210723
PRSS3	rs157931	31.21872512
PRSS3	rs12666392	31.46295326
PRSS3	rs111726637	32.64784051
PRSS3	rs2583503	51.83706787
PRSS3	rs581752	120.8271112
PRSS3	rs183429905	45.09206495
PRSS3	rs9411464	69.01863392
PPP3R2	rs72748420	32.32072024
PPP3R2	rs114009540	35.490321
PPP3R2	rs1329424	930.1973513
PPP3R2	rs3900909	48.92116681
PPP3R2	rs704	140.4823684

PPP3R2	rs1047286	64.99135833
PPP3R2	rs111696008	35.08680051
PPP3R2	rs1707652	221.2103628
PPP3R2	rs3099847	32.45814438
PPP3R2	rs1265941	100.5622854
PPP3R2	rs550671	266.2181636
PPP3R2	rs9276606	39.35630465
CD3E	rs72748418	30.08934229
CD3E	rs10801548	34.8799191
CD3E	rs1329424	990.8704402
CD3E	rs7513100	47.37477519
CD3E	rs704	288.0874368
CD3E	rs2686395	152.0522409
CD3E	rs3099847	45.62749132
CD3E	rs1265941	101.473908
CD3E	rs550671	286.6996625
CD3E	rs78897911	33.77246177
CD3E	rs9276606	39.21742688
AIF1L	rs2789759	52.16753788
AIF1L	rs7853134	2577.252002
AIF1L	rs35509603	2397.538994
AIF1L	rs352954	111.5683961
AIF1L	rs12351921	203.1237921
AIF1L	rs7024748	32.27118466
AIF1L	rs28672722	30.24889661
CEACAM8	rs12459454	70.34434138
CEACAM8	rs377305051	46.39757916
CEACAM8	rs79757359	30.7285266
CEACAM8	rs2511241	67.29667781
CEACAM8	rs2606725	30.10709959
CEACAM8	rs184234067	36.85319799
CEACAM8	rs34656952	212.799652
CEACAM8	rs73017394	443.337641
CEACAM8	rs188003872	76.96182051
CEACAM8	rs486119	37.41042252
CEACAM8	rs7310615	33.02448951
CEACAM8	rs143959838	38.5432472
CEACAM8	rs9898547	113.9182346
CEACAM8	rs114641762	87.44861031
CEACAM8	rs28730837	77.40763515
CEACAM8	rs56378716	339.7449332
CEACAM8	rs186905046	78.90078668
CEACAM8	rs117513377	38.88346084
CEACAM8	rs4760	31.53991851
CEACAM8	rs149290349	37.86768065
CEACAM8	rs35202975	31.99839868
CEACAM8	rs2212930	30.75306956
CEACAM8	rs138150079	45.54937584
CEACAM8	rs112083412	38.1608222
CEACAM8	rs11155297	31.05859
CEACAM8	rs6796	55.72775724
CEACAM8	rs34950278	30.31361222
CEACAM8	rs77649030	66.30298669
CEACAM8	rs659118	41.64447803
CEACAM8	rs35440906	382.5335942
COL2A1	rs73110507	60.81254911
COL2A1	rs113542637	57.49547841
COL2A1	rs1541408	3412.910958

COL2A1	rs12423250	3313.177195
COL2A1	rs2731109	509.9461943
COL2A1	rs11168712	318.2408967
COL2A1	rs74102412	34.23660312
COL2A1	rs17547360	34.24142228
COL2A1	rs35073	41.16582551
COL2A1	rs139355963	50.73573172
COL2A1	rs77009487	137.0313616
COL2A1	rs74719879	179.0307729
COL2A1	rs12146732	75.73717634
COL2A1	rs61936282	34.24390221
COL2A1	rs61751612	73.97126008
COL2A1	rs61269268	207.9264469
COL2A1	rs3783383	71.26137252
COL2A1	rs12603344	50.29643727
COL2A1	rs9807368	38.68479566
COL2A1	rs8098085	34.88420963
COL2A1	rs3761188	41.14265483
COL2A1	rs3212591	47.22267042
COL2A1	rs2438150	90.2144827
COL2A1	rs2874705	37.37536578
COL2A1	rs3735528	43.39234679
COL2A1	rs878504	41.64056348
COL2A1	rs2905070	37.89163388
CHST4	rs58134724	83.48916097
CHST4	rs10922098	54.90670823
CHST4	rs147350387	62.68724897
CHST4	rs686250	30.59350313
GREM1	rs74525987	37.88006164
GREM1	rs11071897	147.5274632
GREM1	rs4779584	5077.738342
GREM1	rs2037841	54.18123923
GREM1	rs17816447	187.2250809
GREM1	rs144674978	180.8954295
GREM1	rs117880751	47.40760578
GREM1	rs141051032	87.2743223
GREM1	rs7553916	65.37019966
GREM1	rs10922098	62.89972274
GREM1	rs56111084	30.49570146
GREM1	rs79404501	30.76299622
GREM1	rs56031155	147.2851269
GREM1	rs114922365	47.2906862
GREM1	rs149062332	264.61929
GREM1	rs2090626	57.70761596
COL3A1	rs6739315	40.20892333
COL3A1	rs72914144	50.54874373
COL3A1	rs201862321	34.06444224
COL3A1	rs35700401	64.10904985
COL3A1	rs67080913	35.29491324
COL3A1	rs146385050	1018.567721
COL3A1	rs35688424	123.2763632
COL3A1	rs2251391	81.44589116
COL3A1	rs8071111	37.4128181
COL3A1	rs9895620	36.6469953
COL3A1	rs7650294	39.53682364
COL3A1	rs55818129	31.46379389
COL3A1	rs7444792	31.47931749
COL3A1	rs1364076	31.41657929

COL3A1	rs35139284	35.14633553
COL3A1	rs12544261	42.28006684
CLSTN2	rs2071386	52.05408274
CLSTN2	rs4073898	194.8876843
CLSTN2	rs35220837	728.3603832
CLSTN2	rs76824284	36.1319175
CLSTN2	rs113351462	34.4125973
CLSTN2	rs2220837	138.5530606
CLSTN2	rs77542162	76.33862969
CLSTN2	rs10413980	112.0125689
CLSTN2	rs5839310	34.19082661
CLSTN2	rs7569795	30.42381713
CLSTN2	rs12484809	31.9452636
CLSTN2	rs116412553	51.58406466
CLSTN2	rs13071095	32.06989869
CLSTN2	rs693293	68.5958402
CLSTN2	rs148302872	69.96841252
DNAJB4	rs187495986	74.57214584
DNAJB4	rs3767026	44.12564909
DNAJB4	rs6699769	347.741626
DNAJB4	rs3811444	82.65916859
DNAJB4	rs56375235	31.90462564
DNAJB4	rs4759076	61.05585466
DNAJB4	rs4795456	34.95187548
DNAJB4	rs78909033	69.49107311
DNAJB4	rs9608008	32.40518056
DNAJB4	rs1354034	86.51270855
DNAJB4	rs114694170	35.25089722
DNAJB4	rs342293	41.46646928
ENTPD2	rs10922098	108.8397059
ENTPD2	rs12147435	48.18155262
ENTPD2	rs201699945	33.29908993
FABP12	rs10922098	295.2991966
FABP12	rs1555405	31.5731616
FABP12	rs1355538	44.05318552
FABP12	rs9501393	62.1847616
FABP12	rs7774739	46.28963804
FGB	rs10922098	557.5406014
FGB	rs76004643	37.18789781
FGB	rs7920986	33.76713497
FGB	rs964184	31.30263455
FGB	rs704	46.76465129
FGB	rs71352239	43.51155014
FGB	rs111696008	45.10212674
FGB	rs144202860	33.47160929
FGB	rs62290472	37.76429335
FGB	rs6445035	305.0667248
FGB	rs3863087	112.4500059
FGB	rs73169117	38.97519818
FGB	rs542418	37.87822246
FGB	rs444921	191.5553114
FGB	rs9270570	59.92129547
FGB	rs9469195	104.6330669
GBP2	rs1886910	87.76371477
GBP2	rs5167	66.15140838
GBP2	rs4566588	48.69663109
GBP2	rs2395490	41.43976898
GBP2	rs7887	152.2588003

GBP2	rs9269041	34.38687195
GBP2	rs41317867	30.84907798
GBP2	rs662	172.5228098
GBP2	rs854572	55.82824221
GPX2	rs10922098	117.6928123
GPX2	rs9864801	41.23405134
GPX2	rs9501393	39.98688985
GSTM4	rs116680878	53.70233534
GSTM4	rs150896402	159.2204484
GSTM4	rs115181845	1123.967691
GSTM4	rs2269340	3293.947561
GSTM4	rs115929572	3227.611228
GSTM4	rs115814431	460.0589598
GSTM4	rs10776700	82.70092889
GSTM4	rs114799872	102.6135232
GSTM4	rs1760216	30.61896207
GSTM4	rs115614917	34.92620314
GSTM4	rs116219465	58.7628904
GSTM4	rs114240822	85.0978436
GSTM4	rs144619840	65.08874053
GSTM4	rs7515124	30.53107111
GSTM4	rs1354034	67.83520469
GSTM4	rs4541868	42.31585621
HS6ST3	rs9562027	133.032009
HS6ST3	rs2477664	32.51608635
HS6ST3	rs2461161	32.07734803
HS6ST3	rs73021415	65.06593543
HS6ST3	rs55714927	33.04602447
HS6ST3	rs77262773	57.8094091
HS6ST3	rs907866	34.5662229
HS6ST3	rs6761320	35.19502357
HDAC2	rs72652155	34.68339976
HDAC2	rs4980320	61.72012745
HDAC2	rs10405535	30.70652775
HDAC2	rs34524896	40.00993184
HDAC2	rs1354034	198.3143679
HDAC2	rs114694170	30.15169566
HDAC2	rs342200	36.92713424
HDAC2	rs6993770	38.96394489
HDGFL1	rs368465	73.55618022
HDGFL1	rs6457457	34.35863272
HDGFL1	rs9501393	42.19982878
HRAS	rs62294359	76.61918726
HRAS	rs369290435	66.04816384
HRAS	rs17178938	67.43239333
HSPA1B	rs9271323	39.77280129
HSPA1B	rs72848261	49.20813773
HSPA1B	rs79940707	39.97609375
HSPA1B	rs3747207	38.32917212
HSPA1B	rs1706435	54.6665918
HSPA1B	rs72801474	83.77253155
HSPA1B	rs2179919	33.97396044
KRT16	rs145730801	1030.292723
KRT16	rs12437339	58.64840415
KRT16	rs56252683	77.80781137
KRT16	rs140824580	39.85612676
KIR3DL1	rs2384656	36.84309342
KIR3DL1	rs1706435	35.16965753

KIR3DL1	rs13217434	36.32786479
KIR3DL1	rs9801017	140.4335811
EXOSC8	rs200071202	91.42492471
EXOSC8	rs11670725	33.44657692
EXOSC8	rs2056321	204.018679
LIN7A	rs7080536	123.7532222
LIN7A	rs2304200	293.993081
LIN7A	rs1355538	33.54355702
PAFAH2	rs4980320	33.12374382
PAFAH2	rs1354034	105.6777562
PAFAH2	rs9864801	42.42148796
ITPA	rs6115655	139.9278714
ITPA	rs77353335	57.12435088
ITPA	rs151029951	282.2207887
ITPA	rs144819505	3124.830493
ITPA	rs148006599	3541.189644
ITPA	rs73573863	1376.881983
ITPA	rs41281876	779.3669516
ITPA	rs192818776	235.8709032
ITPA	rs371455236	193.3893796
ITPA	rs57299529	65.77621352
ITPA	rs141648665	100.1865893
ITPA	rs11699626	47.96651248
AMY2A	rs200048932	245.2668577
AMY2A	rs111980575	999.6401501
AMY2A	rs114500706	2815.914226
AMY2A	rs4847148	278.6668367
AMY2A	rs76836317	58.14225887
AMY2A	rs115614347	31.68305917
AMY2A	rs76527254	65.78848912
AMY2A	rs11578575	63.63651199
AMY2A	rs75235839	34.98554561
AMY2A	rs116131946	87.16233793
AMY2A	rs138026918	361.4753111
AMY2A	rs114118243	36.65708558
AMY2A	rs78705678	44.26065437
AMY2A	rs2477642	39.73067391
AMY2A	rs61839967	40.6023595
AMY2A	rs10845225	31.84003725
AMY2A	rs28365941	42.20672023
AMY2A	rs8051363	43.42114934
AMY2A	rs533406	101.9516852
AMY2A	rs56850260	53.74558974
AMY2A	rs6501460	30.61797054
AMY2A	rs679574	57.2488684
AMY2A	rs10472392	33.71925275
AMY2A	rs9482770	49.61282794
AMY2A	rs67824503	55.49446929
AMY2A	rs635634	280.6278794
PDE4A	rs138570417	43.82431789
PDE4A	rs79803848	34.00431735
PDE4A	rs11667876	34.69436917
PDE4A	rs370630099	660.4282456
PDE4A	rs10922098	42.05913344
PDE4A	rs77897491	43.13224643
PDE4A	rs146704942	41.44870813
PDE4A	rs62269916	32.34543321
PDE4A	rs697356	31.08830033

PDE4A	rs9501600	32.75084077
PHEX	rs9332701	30.14783197
PHEX	rs10822155	41.14209773
PHEX	rs7187856	109.7235986
CD68	rs147621831	51.03023053
CD68	rs184469798	61.98732772
CD68	rs112379600	33.54102163
CD68	rs9568693	41.37791344
CD68	rs9635249	37.46829361
CD68	rs12468189	33.76436868
CD68	rs62258069	48.44850022
CD68	rs9494145	37.35290147
CD68	rs1293303	290.3089153
CD68	rs6993770	53.67946457
PSMA5	rs12132412	56.90690846
PSMA5	rs1591734	145.9921019
PSMA5	rs9332701	636.3954696
PSMA5	rs6670678	394.8003617
PSMA5	rs112616165	32.30638277
PSMA5	rs10764161	102.2062935
PSMA5	rs7070003	358.7939296
PSMA5	rs10437425	46.13536859
PSMA5	rs594793	30.51866708
PSMA5	rs6544941	55.15894785
PSMA5	rs2270190	57.51509035
PSMA5	rs9266557	65.32614783
PSMA5	rs60378094	66.37821855
PSMA5	rs9272422	148.85742
RAET1G	rs10922098	705.7001098
RAET1G	rs17580	105.641707
RAET1G	rs704	240.2964816
RAET1G	rs1047286	46.56788152
RAET1G	rs11720167	202.2253002
RAET1G	rs3863087	67.69440593
RAET1G	rs4955716	31.54060672
RAET1G	rs1265941	74.15762144
RAET1G	rs550671	193.0517968
RAET1G	rs9276606	35.01316239
SLIT2	rs116783529	52.12164437
SLIT2	rs677198	138.0393514
SLIT2	rs2477664	38.87172988
SLIT2	rs35887873	89.87702909
SLIT2	rs215226	39.3130891
SLIT2	rs181707610	38.27157174
SLIT2	rs62061425	31.02875171
SLIT2	rs3127580	37.24890803
SLIT2	rs11751347	88.60262207
SLIT2	rs1247562	37.51554373
SLIT3	rs192284873	53.95025509
SLIT3	rs17355830	34.73823088
SLIT3	rs187104477	125.8005148
SLIT3	rs113543611	53.08171847
SLIT3	rs201944701	332.1430365
SLIT3	rs143927812	38.89483948
SLIT3	rs56201169	487.9141053
SLIT3	rs62129666	33.5866372
NMNAT1	rs10922098	45.17070287
NMNAT1	rs2330106	114.6825762

NMNAT1	rs28752490	43.41314655
NMNAT1	rs2760994	51.01752883
TGM4	rs1995640	40.17275702
TGM4	rs10922098	99.84544697
TGM4	rs6001394	37.15204882
TGM4	rs6457457	42.53778046
TGM4	rs9501393	36.45823461
TGM2	rs742413	477.4116657
TGM2	rs11906988	29.71945356
TGM2	rs75644699	52.09845
TGM2	rs543798	70.10384255
TGM2	rs56387760	50.89547103
TGM2	rs12718598	43.83784429
TLR5	rs72697623	33.46683997
TLR5	rs4149056	61.64725778
TLR5	rs7970695	73.3338639
TLR5	rs17580	35.33304726
TLR5	rs28427460	45.84158831
TLR5	rs78118112	105.3242232
TLR5	rs2670298	914.6791191
TLR5	rs1525836	223.8371817
TLR5	rs4676673	36.01027407
TLR5	rs75465217	116.5076252
TLR5	rs34569113	274.0022994
TLR5	rs139880090	84.71491166
TLR5	rs56210800	33.56507979
TLR5	rs1394094	32.27977341
VLDLR	rs72748420	30.71337153
VLDLR	rs114009540	31.3574026
VLDLR	rs1329424	967.8973627
VLDLR	rs3900909	42.39102739
VLDLR	rs1047286	55.13618191
VLDLR	rs111696008	31.38780023
VLDLR	rs1707652	186.1262558
VLDLR	rs542418	37.10478251
VLDLR	rs550671	264.2700126
VLDLR	rs9270570	49.38454809
VLDLR	rs9469195	92.40569294
PSMB9	rs9501393	33.28214534
PSMB9	rs10922098	127.0459117
PSMB9	rs1706435	56.17360235
PSMB9	rs872914	53.66364935
PSMB9	rs73216598	135.4416863
PSMB9	rs76342676	46.94566683
PSMB9	rs2237582	1288.5741
PSMB9	rs854572	88.85356065
PSMB9	rs2706869	37.1506831
PSMB9	rs2299269	56.01799056
PTGR2	rs10922098	358.5463669
PTGR2	rs10208529	32.47857846
PTGR2	rs143243910	34.66106766
PTGR2	rs1707652	74.17758118
PTGR2	rs77444140	33.38914294
PTGR2	rs9266642	38.16997172
PTGR2	rs438999	167.1620736
PTGR2	rs9501602	73.98537121
PTGR2	rs9269386	55.05206412
RGS1	rs10922094	69.7852172

RGS1	rs6542680	207.9644981
RGS1	rs1706435	39.85609676
RGS1	rs11755689	43.11938246
LRRC3B	rs10922098	201.1782065
LRRC3B	rs71352239	36.24712797
LRRC3B	rs1707652	53.72174797
LRRC3B	rs9267139	35.36560192
LRRC3B	rs28573154	60.93145441
LRRC3B	rs7774739	50.65506069
RAC2	rs4980320	31.07693294
RAC2	rs185009887	30.64762646
RAC2	rs1619994	48.59610767
MECP2	rs10922098	104.1951707
MECP2	rs144744495	31.58908538
MECP2	rs9864801	33.58125351
MECP2	rs28573154	31.49081266
TCEAL8	rs10922098	216.8789466
TCEAL8	rs1706435	57.17420103
TCEAL8	rs9267139	32.94421726
TCEAL8	rs9501393	76.90431764
TCEAL8	rs7774739	56.30510422
MEMO1	rs4660100	160.0803935
MEMO1	rs12130136	40.92672179
MEMO1	rs4980320	43.75100551
MEMO1	rs1354034	42.64712726
MEMO1	rs342200	32.41024663
UBE2M	rs885928	33.47274277
UBE2M	rs3115673	56.11486813
UBE2M	rs148766553	108.2225376
UBE2M	rs406658	194.0403245
UBE2M	rs7774739	202.0479018
UBE2M	rs80163833	49.21609604
SH3BGRL2	rs2812679	151.5210394
SH3BGRL2	rs7073753	79.81114578
SH3BGRL2	rs1354034	79.5226603
SH3BGRL2	rs114694170	56.90695273
TK2	rs73216598	31.10530538
TK2	rs2057681	218.4984157
TK2	rs854572	83.4309598
PPP1R14A	rs77856404	40.86015607
PPP1R14A	rs71354995	469.8394301
PPP1R14A	rs62340386	29.72694395
UBQLN2	rs4980320	41.6106284
UBQLN2	rs10876550	36.51671397
UBQLN2	rs1354034	59.67000815
NFKBID	rs10922094	155.2133201
NFKBID	rs2239165	42.41255241
NFKBID	rs1126605	726.2833671
NFKBID	rs71067161	39.3031285
NFKBID	rs4883430	42.62014965
NFKBID	rs1706435	55.28990021
NFKBID	rs9501393	43.60389307
NFKBID	rs28471056	39.81521517
UBLCP1	rs1422670	229.3758619
UBLCP1	rs13158921	450.670047
UBLCP1	rs116554406	101.7835153
UBLCP1	rs11594179	42.09226045
UBLCP1	rs4759076	42.86014375

UBLCP1	rs1354034	64.89374214
UBLCP1	rs17629516	37.36732364
UBLCP1	rs145607345	41.06171113
UBLCP1	rs140084589	57.76730204
UBLCP1	rs6993770	230.7352533
RPRD1B	rs10922094	248.3463481
RPRD1B	rs1706435	72.00741596
RPRD1B	rs9501600	73.78164377
HSPB6	rs7095308	32.54800415
HSPB6	rs9318186	31.64815278
HSPB6	rs77303334	30.98512391
HSPB6	rs77924615	39.06774559
HSPB6	rs3734537	32.579661
HSPB6	rs9271738	44.65006007
HSPB6	rs12342201	61.8466856
MTHFSD	rs16940800	46.4031312
MTHFSD	rs75079463	124.3231382
MTHFSD	rs12920306	1000.664947
MTHFSD	rs12149377	116.4948049
MTHFSD	rs71390875	30.7748431
MTHFSD	rs7186698	53.01027825
MTHFSD	rs1354034	34.89868025
SERPINB8	rs9807661	115.1742206
SERPINB8	rs56321661	309.7055225
SERPINB8	rs1944270	126.5619269
SERPINB8	rs3826616	488.9435484
TIAL1	rs4980320	41.52780201
TIAL1	rs10876550	40.59937082
TIAL1	rs185009887	35.12913323
TIAL1	rs1354034	81.71996079
ALDH6A1	rs8013751	107.1886569
ALDH6A1	rs17782641	53.5121322
ALDH6A1	rs10922098	38.40333835
ALDH6A1	rs1355538	38.69257393
DAP	rs10922098	33.11605571
DAP	rs78311875	78.72217077
DAP	rs4686780	195.5271876
DAP	rs9858594	1003.606885
DAP	rs6797321	15988.22495
DAP	rs9852488	492.7825139
DAP	rs140915195	326.7903288
DAP	rs28412033	56.97142191
DAP	rs7611640	48.71083334
DAP	rs9830399	31.5071681
DAP	rs58624989	86.19519919
CYB5R2	rs11041116	57.45776879
CYB5R2	rs10500692	56.48439876
CYB5R2	rs7122082	255.9398549
CYB5R2	rs185375375	623.1305807
CYB5R2	rs75667685	31.44401211
CYB5R2	rs12787991	37.63871413
CYB5R2	rs4676872	50.73009909
CYB5R2	rs342293	39.70998938
MREG	rs74430756	48.57757511
MREG	rs17303078	38.80583458
MREG	rs149431443	189.1857365
MREG	rs2806920	3173.254728
MREG	rs11147999	62.44386701

MREG	rs11618078	38.67904466
MREG	rs17069137	41.94653868
MREG	rs112362282	51.35304244
MREG	rs1260326	39.45010298
MREG	rs3129773	30.41492364
MREG	rs3127580	63.62518584
MREG	rs3120137	72.54836193
MREG	rs116881261	58.67367519
MREG	rs150683617	41.51765455
TIMM8A	rs11121845	34.58287862
TIMM8A	rs342200	31.44590799
TIMM8A	rs72673751	30.81359975
MPPED2	rs61884729	30.58497601
MPPED2	rs11031140	236.240405
MPPED2	rs10922098	103.4592352
MPPED2	rs9864801	47.00804918
MPPED2	rs28573154	29.91628362
MPPED2	rs6993770	31.80911811
SERPINE2	rs6715495	66.90480161
SERPINE2	rs4674836	2637.916737
SERPINE2	rs4674862	485.7474527
SERPINE2	rs10498158	88.78975839
SERPINE2	rs373375672	123.8606491
SERPINE2	rs7172696	196.616522
SERPINE2	rs7561846	48.574368
SERPINE2	rs114264570	108.3670831
SERPINE2	rs6993770	55.07875548
SERPINE2	rs4631576	65.54272122
SERPINE2	rs60757417	41.37696386
USP15	rs348675	66.17247353
USP15	rs73136831	215.3431789
USP15	rs4980320	30.39873253
USP15	rs1354034	36.42420289
RPS19	rs203687	42.81876677
RPS19	rs72865316	71.00670942
RPS19	rs11553699	34.47345088
RPS19	rs1037590	93.64155078
RPS19	rs1354034	421.3794226
RPS19	rs9469195	30.22893789
FIBP	rs2077612	30.62208759
FIBP	rs185009887	31.35891379
FIBP	rs11082304	135.8773352
FIBP	rs1354034	43.83193455
FIBP	rs511515	35.08218265
POLE3	rs10922098	70.8947263
POLE3	rs6445035	123.1054524
POLE3	rs3863087	37.6820502
POLE3	rs9501393	31.96943598
POLE3	rs34481430	79.88433613
ZFAND1	rs117843941	57.58978246
ZFAND1	rs2130263	327.1493847
ZFAND1	rs1354034	41.79791835
ZFAND1	rs140810200	95.80220152
ZFAND1	rs148933704	54.79439301
MARCKSL1	rs10437655	35.80655117
MARCKSL1	rs78606665	51.94959196
MARCKSL1	rs2191225	41.67695498
MARCKSL1	rs28873836	542.0020962

MARCKSL1	rs4801903	73.97353432
MARCKSL1	rs77042931	31.22160283
MARCKSL1	rs1047891	47.9620169
MARCKSL1	rs373004254	62.65066254
MARCKSL1	rs2071805	101.4600874
MARCKSL1	rs6924874	41.40324868
MARCKSL1	rs2637678	157.7021381
MARCKSL1	rs1084887	72.01740712
MARCKSL1	rs147859186	42.58918571
MARCKSL1	rs12342201	703.2827274
MARCKSL1	rs80345890	66.74603066
MARCKSL1	rs10117697	30.94879239
GULP1	rs72748418	36.42900697
GULP1	rs114009540	37.00275382
GULP1	rs10801548	50.65567323
GULP1	rs1329424	1094.990664
GULP1	rs35351085	46.42656659
GULP1	rs12478841	30.44217018
GULP1	rs1707652	181.5325186
GULP1	rs1265941	100.4616802
GULP1	rs550671	249.9442213
GULP1	rs9276606	40.39378302
RPL12	rs113520074	51.47303414
RPL12	rs7926172	32.4585818
RPL12	rs6580980	38.90605551
RPL12	rs1354034	201.8421304
STAMBP	rs114808657	33.44423894
STAMBP	rs4980320	35.81203062
STAMBP	rs114507848	60.11597616
STAMBP	rs188400754	29.91833703
NAP1L4	rs737872	297.6695475
NAP1L4	rs3811444	31.25998646
NAP1L4	rs3847647	35.46193787
UBE2L6	rs5167	86.7449275
UBE2L6	rs1706435	47.55386686
UBE2L6	rs11755689	30.16528114
VBP1	rs7546015	55.7832645
VBP1	rs12975366	46.37515947
VBP1	rs13174778	39.55472994
VBP1	rs199967197	113.0871651
VBP1	rs151157472	119.5505917
VBP1	rs77444140	615.7275526
VBP1	rs143370251	518.1140594
VBP1	rs33997360	36.84129418
VBP1	rs138080110	30.67303949
VBP1	rs11955942	47.45569622
VBP1	rs139598601	60.40168524
DTD1	rs6081231	581.5461201
DTD1	rs6075377	263.9282243
DTD1	rs4759076	53.13121573
DTD1	rs1354034	82.23751906
RPS5	rs203687	43.15740557
RPS5	rs11606393	60.88244133
RPS5	rs1037590	69.32780497
RPS5	rs1354034	276.06664
HOPX	rs1697422	31.99752893
HOPX	rs10922098	171.199554
HOPX	rs9864801	36.87648354

HOPX	rs56270003	101.4318039
HOPX	rs9501393	49.09830658
HOPX	rs67322126	30.77454298
ACAT1	rs7105163	40.90882206
ACAT1	rs112087419	127.2258314
ACAT1	rs28629977	35.03446366
ACAT1	rs6489859	33.4029145
NUDT1	rs10922094	102.2290563
NUDT1	rs1706435	46.52972323
NUDT1	rs4693545	30.82858331
NUDT1	rs7774739	42.30047456
NUDT1	rs7742632	49.84941305
ADK	rs4746172	36.91607272
ADK	rs11000927	33.20317615
ADK	rs586689	36.67013849
ADK	rs75792246	33.45208384
ADK	rs1354034	55.5167866
ADK	rs71506168	62.66513852
ADK	rs10814104	96.99017759
ACVR2A	rs10922098	665.0325407
ACVR2A	rs3900909	32.02265068
ACVR2A	rs1047286	30.67099641
ACVR2A	rs111696008	35.23415306
ACVR2A	rs11720167	137.8144768
ACVR2A	rs3863087	40.24011127
ACVR2A	rs444921	178.8366034
ACVR2A	rs28471056	62.46299401
ACVR2A	rs9272526	34.40989596
SPINK4	rs1630171	629.3588404
SPINK4	rs10758227	38.14579175
SPINK4	rs144704969	29.74105826
SPINK4	rs10922098	735.5599057
SPINK4	rs3900909	38.44884875
SPINK4	rs1047286	44.17638062
SPINK4	rs1707652	117.6325886
SPINK4	rs1265941	81.20825629
SPINK4	rs550671	184.7773941
SPINK4	rs9276606	39.41139746
NDRG1	rs10922098	220.8296739
NDRG1	rs1355538	82.63995072
NDRG1	rs28573154	45.76787009
NDRG1	rs7774739	32.85681862
EIF1AY	rs4980320	33.67061824
EIF1AY	rs6580980	37.53107119
EIF1AY	rs1354034	125.413547
BATF	rs10922098	153.1793888
BATF	rs6542680	36.8444002
BATF	rs6445035	62.58157367
BATF	rs115341303	30.00316243
BATF	rs9501600	70.64102744
BATF	rs35209896	29.94684891
BAG2	rs113823023	51.29681713
BAG2	rs61430220	34.61903384
BAG2	rs117761837	178.7098067
HOMER1	rs2360962	38.30817566
HOMER1	rs35340377	41.62094113
HOMER1	rs116052829	48.56834132
HOMER1	rs7926172	40.06030826

HOMER1	rs4759076	35.87683214
HOMER1	rs2654210	31.61969449
HOMER1	rs1256430	70.28446933
HOMER1	rs139948926	39.83125926
HOMER1	rs5771045	50.36345642
HOMER1	rs3213442	183.7931852
HOMER1	rs9616896	1214.696832
HOMER1	rs28689705	312.8198116
HOMER1	rs9616960	29.91320853
HOMER1	rs11705273	97.70098396
HOMER1	rs1354034	172.1231861
HOMER1	rs68116612	32.17786309
DDT	rs5760055	46.85339537
DDT	rs41277305	232.0936624
DDT	rs56057548	48.00324542
DDT	rs10922098	345.9132357
DDT	rs181566122	86.56072733
DDT	rs148117344	44.95087857
DDT	rs58596209	141.590552
DDT	rs118130999	74.942373
DDT	rs12929353	121.4252623
DDT	rs117689831	124.3939627
DDT	rs115569302	957.7141942
DDT	rs40433	76.08495212
DDT	rs7187649	31.58329301
DDT	rs425921	100.0569072
DDT	rs4089292	86.18267993
DDT	rs1707652	84.93778922
DDT	rs9501600	90.65896728
GLUL	rs4652700	41.18537513
GLUL	rs10876550	81.04620431
GLUL	rs12464674	42.35784564
GLUL	rs114694170	37.95826432
GLUL	rs450269	32.67545857
ARF1	rs4980320	31.98427477
ARF1	rs2035675	36.69200204
ARF1	rs1354034	50.61777809
ARF1	rs6445035	102.9195793
ARF1	rs3863087	30.49829521
RBP5	rs2290239	372.3393905
RBP5	rs7137828	37.9011209
RBP5	rs28471056	30.3375484
RBP5	rs2637678	106.5637856
RBP5	rs1084887	40.95088105
RBP5	rs2954038	37.7884117
RBP5	rs12342201	36.2470077
PFDN1	rs10922098	90.00627606
PFDN1	rs9501600	44.48826617
PFDN1	rs557011	47.442397
PFDN2	rs572515	85.13599124
PFDN2	rs1354034	44.2144816
PFDN2	rs1355538	50.9798438
PFDN2	rs12668458	30.58627777
UBE2D1	rs1451915	38.45787734
UBE2D1	rs10922098	869.0537259
UBE2D1	rs7513100	39.96855676
UBE2D1	rs1047286	53.20920915
UBE2D1	rs1707652	142.3727936

UBE2D1	rs3099847	32.86453276
UBE2D1	rs1265941	84.88886234
UBE2D1	rs550671	198.2481589
UBE2D1	rs78897911	29.88575761
UBE2D1	rs9276606	30.9386464
MYCBP	rs4980320	39.33133961
MYCBP	rs1354034	36.69790961
MYCBP	rs1355538	49.39294183
MYCBP	rs342200	34.54045979
SRGN	rs35898435	62.27077312
SRGN	rs2229498	425.2974283
SRGN	rs143964554	51.99697132
SRGN	rs826404	35.00386618
SRGN	rs7907594	32.10695276
SRGN	rs147184402	78.25200954
SRGN	rs143941257	53.67969165
SRGN	rs4980320	31.85470025
SRGN	rs11439719	41.70641134
SRGN	rs9813363	30.25193363
SRGN	rs11734099	40.79285876
SRGN	rs450269	34.40219523
SRGN	rs6993770	49.52250812
GMPR	rs35504297	1285.082213
GMPR	rs114648232	4664.906021
GMPR	rs140659976	435.0361583
GMPR	rs138143229	351.7453914
GMPR	rs114787025	282.6895019
GMPR	rs141485758	1020.544434
GMPR	rs113594805	863.9519057
GMPR	rs116359566	177.0371321
GMPR	rs146753713	152.4825808
GMPR	rs59543599	383.245054
GMPR	rs12192318	43.72121101
GMPR	rs80228340	119.8717453
GMPR	rs71551019	124.9176321
GMPR	rs115007786	49.8274528
GMPR	rs172521	76.94427333
GMPR	rs115578457	35.98346524
CLTA	rs10922094	113.9550059
CLTA	rs3811444	125.875513
CLTA	rs9501393	35.35653168
CLTA	rs72848261	36.71135168
EIF2S1	rs4980320	40.00810963
EIF2S1	rs13426600	63.54316334
EIF2S1	rs1354034	52.07485194
EIF2S1	rs2544220	31.39448753
SDCBP2	rs6109526	31.34508081
SDCBP2	rs13043452	35.07147251
SDCBP2	rs1354034	40.64289255
SDCBP2	rs3129754	32.05387323
SDCBP2	rs62560860	35.3490988
SDCBP2	rs8176693	47.09866829
RPA2	rs10922098	232.4535783
RPA2	rs6542680	64.01518079
RPA2	rs1706435	74.64029325
RPA2	rs3128984	31.65042338
RPA2	rs9501600	73.7826135
TCP1	rs4980320	30.78553569

TCP1	rs2077612	29.98869569
TCP1	rs12386575	30.08813818
G3BP1	rs140252175	61.24417203
G3BP1	rs4980320	41.43906175
G3BP1	rs10876550	43.49930059
G3BP1	rs185009887	32.09747948
G3BP1	rs1354034	87.7073537
PEF1	rs583104	136.7201328
PEF1	rs10922094	41.34285211
PEF1	rs12026141	59.07506697
PEF1	rs964184	87.4733651
PEF1	rs1532085	132.925058
PEF1	rs2070895	96.05521239
PEF1	rs247617	51.32206971
PEF1	rs147711004	54.40187616
PEF1	rs7412	388.6284657
PEF1	rs10412986	40.5950385
PEF1	rs192571720	55.80276915
PEF1	rs11680700	44.38849971
PEF1	rs72784378	125.2202713
PEF1	rs78980904	2819.426068
PEF1	rs116498710	480.7879739
PEF1	rs10746529	182.7027401
PEF1	rs13390335	31.25072381
PEF1	rs76693471	121.0951829
PEF1	rs113840968	112.833103
PEF1	rs61754186	49.35745288
PEF1	rs55915628	38.48416807
PEF1	rs111696008	131.0975503
PEF1	rs144202860	74.3296028
PEF1	rs696516	37.77228965
PEF1	rs114833464	33.12945649
PEF1	rs697356	939.9578421
PEF1	rs77230198	64.19469165
PEF1	rs145242450	30.94777169
PEF1	rs147033786	76.83318134
PEF1	rs368220407	49.30009026
PEF1	rs9275596	33.10851837
PEF1	rs139315015	67.21841031
GSR	rs370802180	59.49325499
GSR	rs4733505	173.5242743
GSR	rs12230154	43.72905876
GSR	rs8815	73.16877675
GSR	rs181707610	30.69638438
GSR	rs28672722	46.44295946
GSR	rs12680844	36.60759893
GSR	rs10821074	37.42988045
SNTA1	rs35340377	31.36577636
SNTA1	rs4980320	47.23458078
SNTA1	rs12819689	45.33297596
SNTA1	rs1354034	90.97920139
SNTA1	rs114694170	44.43592036
SNTA1	rs342200	44.24722339
TSTD1	rs114395538	55.10616299
TSTD1	rs78714351	52.52517542
TSTD1	rs2990693	33.38751836
TSTD1	rs11580071	1032.154825
TSTD1	rs115503614	37.03961891

TSTD1	rs7073753	36.95429451
RBP1	rs2071387	85.07730377
RBP1	rs10922098	227.9746159
RBP1	rs1706435	85.41072418
RBP1	rs9501393	59.50393239
RBP1	rs7774739	45.92790115
UBE2D3	rs10922094	49.34847569
UBE2D3	rs964184	55.55637892
UBE2D3	rs73262897	38.67038829
UBE2D3	rs1126605	309.9770551
UBE2D3	rs1706435	95.06710821
UBE2D3	rs11755689	32.0641351
MAFG	rs11594179	36.75007797
MAFG	rs10876550	50.41032807
MAFG	rs342290	50.53170463
RAB13	rs4511431	33.02757305
RAB13	rs13412535	33.83760567
RAB13	rs697356	84.04332121
RAB13	rs342200	34.77215147
RAB13	rs77265585	29.90403321
UROD	rs68002039	63.33274096
UROD	rs7525733	976.9611994
UROD	rs10700834	38.24686712
UROD	rs4665483	30.22387374
UROD	rs7598665	34.66108474
VPS26A	rs10998638	378.6246881
VPS26A	rs3811444	38.62667952
VPS26A	rs7073753	36.00969665
VPS26A	rs2894058	30.14245471
VPS26A	rs4980320	30.60498148
VPS26A	rs2276265	195.5493124
VPS26A	rs1354034	196.2042408
VPS26A	rs342200	56.9286474
VPS26A	rs6993770	43.5613303
CRIPT	rs1451915	44.78381846
CRIPT	rs1329424	1084.265321
CRIPT	rs7513100	55.10935718
CRIPT	rs11720167	152.9854148
CRIPT	rs3863087	64.48068954
CRIPT	rs3130955	40.75291536
CRIPT	rs9501600	227.5967459
CRIPT	rs35209896	55.63740862
MYL7	rs954439	48.42341812
MYL7	rs73182279	475.5405971
MYL7	rs66965282	4283.498841
MYL7	rs3888001	1279.538985
MYL7	rs710446	277.3361707
MYL7	rs1501299	63.56448894
MYL7	rs9851238	118.1904108
MYL7	rs79154403	40.00610597
MYL7	rs142443324	48.30682632
MYL7	rs73191744	54.06779978
MYL7	rs74809616	42.4648584
G6PD	rs4980320	43.26297783
G6PD	rs4759076	42.55169735
G6PD	rs6993770	54.75354773
GIMAP6	rs10922098	141.2159972
GIMAP6	rs9864801	45.5734487

GIMAP6	rs9501393	60.10166577
GIMAP6	rs7774739	40.47886133
PET117	rs10922098	608.2245849
PET117	rs1047286	43.20083448
PET117	rs1707652	141.64984
PET117	rs9267139	40.95688828
PET117	rs28573154	137.9301019
PET117	rs7774739	98.80125491
PET117	rs9271683	43.94731641
COPE	rs10922098	639.1334157
COPE	rs3900909	30.00363909
COPE	rs2808466	108.0674781
COPE	rs11847600	50.41820893
COPE	rs7598430	30.86245221
COPE	rs1707652	152.1831186
COPE	rs12629202	29.84981876
COPE	rs3099847	31.56687873
COPE	rs2075798	42.21338175
COPE	rs453821	120.4899664
CRYGS	rs10922098	51.09352434
CRYGS	rs116040950	31.42029787
CRYGS	rs28573154	31.23945842
HSCB	rs72748420	37.02827766
HSCB	rs114009540	35.2161503
HSCB	rs1329424	1097.497332
HSCB	rs3900909	52.83533416
HSCB	rs704	34.66018491
HSCB	rs1047286	50.86602376
HSCB	rs111696008	34.5949505
HSCB	rs2686395	243.5086589
HSCB	rs3099847	37.10522938
HSCB	rs1265941	110.0188865
HSCB	rs550671	291.2460047
HSCB	rs9276606	46.53681109
EBAG9	rs28664709	77.61216645
EBAG9	rs744865	58.89166869
EBAG9	rs114531342	57.26303478
EBAG9	rs112430397	61.82373243
EBAG9	rs9280648	162.118768
EBAG9	rs143654430	916.1184964
EBAG9	rs9468883	563.8751965
EBAG9	rs146431191	90.42705145
EBAG9	rs3210148	331.5716346
EBAG9	rs181369017	332.2764766
EBAG9	rs3176349	66.97991927
EBAG9	rs150581397	39.15121654
HAT1	rs72748418	35.55827368
HAT1	rs1329424	1182.093979
HAT1	rs3900909	55.31282436
HAT1	rs10801660	38.04193945
HAT1	rs964184	35.76821391
HAT1	rs1367117	32.85354879
HAT1	rs111696008	30.58242876
HAT1	rs1707652	195.8675689
HAT1	rs3099847	47.34821549
HAT1	rs610265	110.7265065
HAT1	rs550671	304.9594796
HAT1	rs116334170	60.02555093

HIPK3	rs10922094	74.74775414
HIPK3	rs56210800	30.02046806
HIPK3	rs17282078	30.06062772
TXNDC12	rs111332301	36.53606668
TXNDC12	rs6686632	121.1310667
TXNDC12	rs2588511	35.0212024
LZIC	rs4265433	39.31376458
LZIC	rs10922094	82.05108152
LZIC	rs1354034	30.83232722
LZIC	rs1619994	42.34222055
LZIC	rs28573154	35.170024
ACBD6	rs2083385	61.58920943
ACBD6	rs12612942	972.8401234
ACBD6	rs1995916	106.9925387
CA12	rs7183733	33.58258423
CA12	rs11121817	29.90041302
CA12	rs10922094	37.00073042
OSTM1	rs10922098	215.1571817
OSTM1	rs1706435	64.32542776
OSTM1	rs2075798	31.20316111
OSTM1	rs454212	56.26329493
TOM1L2	rs72748420	35.50732585
TOM1L2	rs1329424	1167.846309
TOM1L2	rs3900909	54.58038607
TOM1L2	rs10801660	36.81947474
TOM1L2	rs704	93.3295627
TOM1L2	rs1047286	50.83337407
TOM1L2	rs111696008	37.40378497
TOM1L2	rs1707652	246.4640979
TOM1L2	rs73169117	31.6608792
TOM1L2	rs3099847	39.02829848
TOM1L2	rs1265941	109.6902322
TOM1L2	rs550671	298.7550835
TOM1L2	rs78897911	34.23779716
TOM1L2	rs9276606	45.27903462
LRRTM1	rs72748418	32.12218093
LRRTM1	rs10801548	126.1765236
LRRTM1	rs1329424	2483.974972
LRRTM1	rs190548044	30.93691958
LRRTM1	rs35351085	59.06450217
LRRTM1	rs704	33.97032339
LRRTM1	rs1706435	53.7999641
LRRTM1	rs9501600	63.12846085
MATN3	rs9306885	210.6570907
MATN3	rs11886092	3770.073004
MATN3	rs71391748	5324.126529
MATN3	rs3771248	377.2283512
MATN3	rs61635185	102.5259845
MATN3	rs896738	73.95502259
MATN3	rs11685815	68.98184132
MATN3	rs78879955	75.6518976
MATN3	rs11694634	35.51419582
MATN3	rs72871120	30.29351236
MATN3	rs6873866	93.51665057
MATN3	rs635634	32.91359419
PCNA	rs12102137	55.21500601
PCNA	rs1354034	38.53390959
PCNA	rs115910976	31.16636274

PEX26	rs10922098	457.1684892
PEX26	rs76004643	31.19099076
PEX26	rs11569415	38.26593634
PEX26	rs1707652	101.4130946
PEX26	rs550671	127.8734995
PEX26	rs9469195	43.88292372
HS3ST4	rs10922098	122.3597438
HS3ST4	rs17669311	95.07089283
HS3ST4	rs704	76.80759139
HS3ST4	rs1619994	39.17470001
HS3ST4	rs9501600	44.51889201
MDGA2	rs8011466	78.13521327
MDGA2	rs75705866	3085.501829
MDGA2	rs111629268	89.74714629
MDGA2	rs149817759	121.4521996
MDGA2	rs77544995	118.254138
MDGA2	rs10149770	38.91023531
MDGA2	rs148244051	40.89596296
MDGA2	rs139953520	290.8703075
MDGA2	rs147722382	30.53385583
MDGA2	rs12147146	311.3519057
MDGA2	rs4900811	3587.307949
MDGA2	rs79827742	97.21244942
MDGA2	rs116174082	2803.403506
MDGA2	rs112351914	84.35163988
MDGA2	rs75694823	143.0298895
MDGA2	rs146048337	33.05112325
MDGA2	rs34489253	115.6194525
MDGA2	rs57844210	54.22727679
MDGA2	rs4788706	30.00915382
MDGA2	rs4665972	35.32470995
MDGA2	rs191390916	53.57909051
MDGA2	rs34231016	30.18896084
MDGA2	rs849133	30.94235527
MDGA2	rs62621812	35.44283964
MTCP1	rs10922098	415.0946917
MTCP1	rs1047286	47.33711668
MTCP1	rs1706420	87.3305369
MTCP1	rs3130955	31.61687938
MTCP1	rs9501600	110.978186
MTCP1	rs35209896	31.71254659
OLFM2	rs73008030	44.51380093
OLFM2	rs111340603	166.5025714
OLFM2	rs182239486	58.17010282
OLFM2	rs74178184	671.0378467
OLFM2	rs8099922	2763.920323
OLFM2	rs7247115	206.4472165
OLFM2	rs2298475	45.67065517
OLFM2	rs148657904	64.36985073
OLFM2	rs62129144	44.67600245
OLFM2	rs6852182	142.8440784
OLFM2	rs899843	58.92415109
RAB3D	rs9971407	39.27857461
RAB3D	rs185009887	31.78235327
RAB3D	rs1354034	35.17933069
RAB3D	rs35408476	51.53129701
RGS21	rs6675393	82.53614178
RGS21	rs61804160	30.20814236

RGS21	rs2006166	74.25939787
RGS21	rs376549	77.80132269
RGS21	rs1557549	205.0401442
RGS21	rs17613292	57.03678574
RGS21	rs10627680	30.40189194
CINP	rs3759562	39.17490906
CINP	rs111454107	96.69054351
CINP	rs6017442	40.69453026
CINP	rs1354034	35.97422408
CDH12	rs144426992	33.95640942
CDH12	rs411729	35.17695035
CDH12	rs17020993	51.47980731
CDH12	rs141575023	51.99154885
CDH12	rs9269109	59.31535147
DDAH1	rs1976848	37.3092784
DDAH1	rs66678883	34.86377396
DDAH1	rs56690752	116.8267072
DDAH1	rs75184624	47.7304466
DDAH1	rs4240383	1317.578462
DDAH1	rs147187715	379.7099022
DDAH1	rs114721060	110.2092885
DDAH1	rs17391294	566.1727093
DDAH1	rs6877458	235.5484526
DDAH1	rs13161894	73.07006955
DDAH1	rs13154292	39.95916173
DDAH1	rs450269	57.5688551
DDAH1	rs372964615	127.3382555
DDAH1	rs7043132	461.4850696
VEGFA	rs2841642	81.15403417
VEGFA	rs2759270	61.49807149
VEGFA	rs78076125	52.82970046
VEGFA	rs4426996	218.6659115
VEGFA	rs7739450	4937.223091
VEGFA	rs9296424	114.8411737
VEGFA	rs7080386	146.090684
VEGFA	rs61050482	48.20991363
VEGFA	rs114694170	78.2217124
VEGFA	rs77737652	33.54564311
VEGFA	rs1460580	32.31782829
VEGFA	rs6993770	286.5741207
VEGFA	rs7852409	71.00781403
VEGFA	rs11789392	268.6668428
GMPR2	rs117627441	32.19775798
GMPR2	rs34354104	842.5397424
GMPR2	rs12878578	40.97724056
GMPR2	rs77075600	37.161146
GMPR2	rs4980320	32.59085118
GMPR2	rs6459465	951.9755462
GMPR2	rs9477098	74.60558517
GMPR2	rs6939612	33.72921822
UPP1	rs6950051	105.5784996
UPP1	rs7310615	31.91828206
UPP1	rs4639017	34.22532857
RBKS	rs10922098	269.2393056
RBKS	rs1706435	52.44257539
RBKS	rs9501600	73.08314188
RBKS	rs587495	32.05440754
HDHD3	rs1043836	449.3294245

HDHD3	rs203688	91.39683172
HDHD3	rs1355538	53.14499125
HDHD3	rs444921	32.36390387
KDSR	rs1329424	985.6747972
KDSR	rs3900909	49.85019852
KDSR	rs6445035	134.5232969
KDSR	rs3863087	50.8927251
KDSR	rs6910297	36.37165908
KDSR	rs9501600	278.5276818
KDSR	rs74671299	42.90438508
KDSR	rs73403125	46.48497696
KDSR	rs35209896	65.94177705
PDCD6	rs34511054	108.290145
PDCD6	rs62346174	48.63273049
PDCD6	rs3817059	30.40186987
PDCD6	rs72705167	95.11410788
PDCD6	rs607268	127.8853956
PDCD6	rs10922094	125.5523167
PDCD6	rs10842789	37.79844913
PDCD6	rs1707652	42.22522505
PDCD6	rs28573154	35.34714474
HSD17B8	rs10922094	33.90254424
HSD17B8	rs6857	56.86419036
HSD17B8	rs8106813	235.8529544
HSD17B8	rs780093	37.32034872
HSD17B8	rs1707652	44.33923262
KAAG1	rs2781083	65.35162432
KAAG1	rs75381154	122.537184
KAAG1	rs114159428	86.56864072
KAAG1	rs77639220	88.33344531
KAAG1	rs11579159	31.29198799
KAAG1	rs41274494	147.2509981
KAAG1	rs148414479	65.69625048
KAAG1	rs115102542	5049.62002
KAAG1	rs56410663	51.9025163
KAAG1	rs138200736	593.950855
KAAG1	rs12748809	56.98400093
KAAG1	rs113855303	84.67446077
KAAG1	rs57626622	95.50669427
KAAG1	rs115388123	52.27596241
KAAG1	rs5745984	44.04194275
KAAG1	rs150814911	123.6360528
KAAG1	rs10623052	33.27650294
KAAG1	rs4379693	31.65842351
KAAG1	rs1800450	255.2096116
KAAG1	rs215225	76.52136999
KAAG1	rs903107	36.43415014
KAAG1	rs6542680	35.39692273
KAAG1	rs1260326	36.61110794
KAAG1	rs1486236	31.72879909
KAAG1	rs73167083	58.78410098
KAAG1	rs698088	176.3962099
KAAG1	rs3774282	35.18574605
KAAG1	rs62467349	32.13140457
KAAG1	rs10102164	51.1886857
KAAG1	rs1038193	468.9609523
NPL	rs12138972	37.45781284
NPL	rs74646646	268.2262417

NPL	rs10922094	45.15767343
NPL	rs111696008	36.43739485
NPL	rs114115200	226.4858549
NPL	rs3863087	72.63763683
NPL	rs6993770	29.8323546
DTYMK	rs189110269	31.11751714
DTYMK	rs144303868	84.9520953
DTYMK	rs142623301	38.6769933
DTYMK	rs116616655	46.18184303
DTYMK	rs4980320	32.7645861
DTYMK	rs35979828	44.66244124
DTYMK	rs1354034	47.35325323
GLYAT	rs10922094	116.6333988
GLYAT	rs12896956	38.29968548
GLYAT	rs5752773	29.95786542
GLYAT	rs1707652	34.5921843
GLYAT	rs185107166	36.81005534
GLYAT	rs28672722	46.74493684
GLYAT	rs9280312	32.97976911
DCUN1D2	rs35617250	144.0562773
DCUN1D2	rs474300	51.1398891
DCUN1D2	rs9271642	33.62595361
HK3	rs10922098	723.4204732
HK3	rs3900909	39.96074378
HK3	rs2686395	115.1030108
HK3	rs555007	71.7579005
HK3	rs444921	166.2648551
HK3	rs9272526	29.75038701
PARK7	rs114009540	30.62990269
PARK7	rs10922098	817.9679355
PARK7	rs3900909	40.91996195
PARK7	rs1707652	105.7549343
PARK7	rs2075798	97.66339729
PARK7	rs550671	242.6582923
PARK7	rs9269234	33.51052351
PARK7	rs9272526	39.1917992
STX1A	rs576274	55.68853341
STX1A	rs232712	35.5434869
STX1A	rs4690014	275.4583673
STX1A	rs2498323	76.24405606
STX1A	rs73796110	92.86834794
CR1	rs6663887	224.5410497
CR1	rs7536931	925.2285446
CR1	rs2864861	30.86245251
CR1	rs145796852	123.7018108
CR1	rs10544723	112.4924153
CR1	rs35439639	106.4800026
CR1	rs75157360	36.99922677
CR1	rs2800932	43.4184861
CR1	rs4951510	64.19392489
CR1	rs78689694	103.7039506
CR1	rs76060115	33.24607339
CR1	rs7310615	66.7805831
CR1	rs117902156	63.96701002
CR1	rs55714927	316.6390173
CR1	rs112723993	32.03298114
CR1	rs143501783	95.5525846
CR1	rs117753190	54.25527231

CR1	rs681343	57.37253256
CR1	rs62165726	120.3048562
CR1	rs9677256	63.26530053
CR1	rs13064814	175.6845272
CR1	rs3936289	83.61961099
CR1	rs6908045	62.25769284
CR1	rs7390710	34.47801356
KLB	rs138208148	117.6117023
KLB	rs28712821	1073.173675
KLB	rs36101900	5168.844249
KLB	rs76857490	405.4801432
KLB	rs144510601	251.9898596
KLB	rs141390674	34.88499208
KLB	rs12643250	102.7793186
KLB	rs6701606	29.98026293
KLB	rs71391424	32.98111589
KLB	rs34931250	193.6464418
KLB	rs77262773	119.0061243
KLB	rs141773472	43.50453035
KLB	rs2925956	46.56467404
KLB	rs141560185	58.73693659
KLB	rs28773216	33.8809072
KLB	rs9489	37.9098303
KLB	rs112875651	31.28683242
KLB	rs7870697	49.0712964
LRP4	rs142929944	61.3560526
LRP4	rs4752926	1198.932772
LRP4	rs77682558	208.3441441
LRP4	rs411729	33.90962339
LRP4	rs16840639	180.9740065
LRP4	rs17181389	47.55514913
LRP4	rs17788257	30.27774196
LRP4	rs12792178	91.40929917
LRP4	rs117326158	38.09694361
LRP4	rs75361611	92.93626822
LRP4	rs654635	91.09841131
LRP4	rs117354805	43.33059446
LRP4	rs36093728	87.31409792
LRP4	rs11229778	31.68354422
LRP4	rs78320275	54.34980439
LRP4	rs62061425	45.75785701
LRP4	rs1800961	32.78130927
LRP4	rs6840113	50.65141306
LRP4	rs7850844	45.53070119
PLXNA4	rs6467443	94.14604648
PLXNA4	rs199886647	39.42520292
PLXNA4	rs2461161	31.11052841
PLXNA4	rs6993770	45.46049024
PLXND1	rs62266917	96.09498522
PLXND1	rs2255703	659.6635407
PLXND1	rs28850104	117.6790797
PLXND1	rs78705678	35.35303751
PLXND1	rs2477642	34.47299797
PLXND1	rs2936613	36.72886922
PLXND1	rs11603123	104.0030663
PLXND1	rs117633773	34.76672491
PLXND1	rs55714927	141.726723
PLXND1	rs143501783	66.2220551

PLXND1	rs117877056	48.76317462
PLXND1	rs117241334	57.81255043
PLXND1	rs111903631	39.52859028
PLXND1	rs2837988	46.45447741
PLXND1	rs62259780	32.52379498
PLXND1	rs4857414	79.21936795
PLXND1	rs75876642	44.85669475
PLXND1	rs3135387	68.09596017
PLXND1	rs73403125	35.10564661
PLXND1	rs77311729	33.49652168
PLXND1	rs2073926	79.37418777
PLXND1	rs12378537	1110.90777
PLXND1	rs11244084	1203.37224
PLXND1	rs3094377	377.4041807
PLXND1	rs62572818	134.171915
PLXND1	rs150730585	94.24725815
PLXND1	rs117369137	51.85573569
SEZ6L	rs117966464	91.65662314
SEZ6L	rs73158619	110.4020625
SEZ6L	rs6005031	78.30081353
SEZ6L	rs1260326	42.75136177
SEZ6L	rs73165969	49.87183621
EGFR	rs10922098	190.2627487
EGFR	rs13379369	57.77146289
EGFR	rs11846842	35.49094391
EGFR	rs2753537	121.9375471
EGFR	rs704	264.3070323
EGFR	rs1801690	64.47035924
EGFR	rs1619994	31.70337041
EGFR	rs2923009	31.13642207
EGFR	rs77311729	30.50292719
EGFR	rs9271775	48.19175771
IL15	rs17580	1205.386185
IL15	rs56361564	59.942068
IL15	rs1354034	43.68777793
IL15	rs1049296	31.94572514
CEBPG	rs10922098	156.7095288
CEBPG	rs11846452	30.88071287
CEBPG	rs62294359	38.39369992
CEBPG	rs9501600	41.04863201
CEBPG	rs557011	30.69286092
DOK2	rs117330214	32.22112107
DOK2	rs4980320	31.49412265
DOK2	rs67036916	37.57635188
DOK2	rs6993770	60.86876576
DEFA1	rs10922094	211.2072535
DEFA1	rs2686395	53.86447095
DEFA1	rs144040165	31.6065424
DEFA1	rs9468618	182.3314897
DEFA1	rs9258540	270.5831248
DEFA1	rs62389359	273.1140463
DEFA1	rs7765011	42.00655488
DEFA1	rs609061	66.39291989
IGFBP5	rs7598172	40.02726448
IGFBP5	rs13889	45.67693048
IGFBP5	rs139482112	39.19587056
IGFBP5	rs2949837	79.35843642
KPNA1	rs10733084	29.74698586

KPNA1	rs28853072	667.1480169
KPNA1	rs12403425	34.00317931
KPNA1	rs11227136	33.80161
KPNA1	rs72952553	31.48952358
KPNA1	rs1355538	171.0879295
KPNA1	rs73169117	34.5125096
KPNA1	rs1265941	60.93455241
KPNA1	rs454212	191.3933238
KPNA1	rs6451	36.87579696
SFTPD	rs71479646	64.37626426
SFTPD	rs4253536	930.8775788
SFTPD	rs191706102	596.0620007
SFTPD	rs2181205	3371.011996
SFTPD	rs2789693	107.8126725
SFTPD	rs148111397	52.21298267
SFTPD	rs7067934	122.0669749
SFTPD	rs9426886	59.94964516
SFTPD	rs56218657	82.04586113
SFTPD	rs192731179	35.16043553
SFTPD	rs12793244	41.82132036
SFTPD	rs117654599	29.95796901
SFTPD	rs12905199	30.68077469
SFTPD	rs9927461	671.2795161
SFTPD	rs13336938	57.54728152
SFTPD	rs1528074	55.72830883
SFTPD	rs1718859	60.17900097
SFTPD	rs2446301	93.89007465
SFTPD	rs70959640	46.53621025
SFTPD	rs2521994	40.57365651
SFTPD	rs72080416	32.95248065
SFTPD	rs140393761	41.04304205
SFTPD	rs9468618	71.18617705
SFTPD	rs1002045	157.3651444
SFTPD	rs34847854	925.3594485
SFTPD	rs1071649	1165.061067
SFTPD	rs9391844	128.6845762
SFTPD	rs79118723	595.1975012
SFTPD	rs373004254	364.1773365
SFTPD	rs76198540	30.41624348
SFTPD	rs2451951	279.2861397
SFTPD	rs74950512	31.74998006
BRF1	rs10305748	31.25849867
BRF1	rs10922098	512.1564429
BRF1	rs1047286	47.88173258
BRF1	rs111696008	42.69879241
BRF1	rs2232613	420.4232173
BRF1	rs11906988	41.05347595
BRF1	rs2145943	35.08300668
BRF1	rs6445035	226.6349481
BRF1	rs3863087	95.05181551
BRF1	rs73169117	30.32829913
BRF1	rs2242572	133.4106471
BRF1	rs9501602	63.74266923
BRF1	rs9271529	42.47653317
ASB9	rs333947	30.5862786
ASB9	rs12883070	281.9217105
ASB9	rs62006268	376.1932852
ASB9	rs74580987	41.10635442

ASB9	rs76983194	30.60441827
ASB9	rs635634	85.62300765
IHH	rs10164896	36.30212539
IHH	rs114006326	44.15972924
IHH	rs10922098	77.85026499
IHH	rs118146573	42.01555898
IHH	rs1065853	315.7634043
IHH	rs1707652	79.45701519
IHH	rs9501600	32.64848692
CD81	rs10922098	107.4983425
CD81	rs117790450	32.77456286
CD81	rs1355538	36.78835989
CD81	rs2242572	52.11182816
CD81	rs9272068	41.14659582
TCN1	rs11229862	50.23212905
TCN1	rs34324219	1363.159265
TCN1	rs17860470	124.2063407
TCN1	rs1801222	35.86559218
TCN1	rs10769106	48.99430881
TCN1	rs16938342	49.15960092
TCN1	rs113453659	79.28507838
TCN1	rs139128886	59.92978299
TCN1	rs61885288	51.19000609
TCN1	rs12799025	35.88855152
TCN1	rs72929778	99.2350387
TCN1	rs72916973	55.6763542
TCN1	rs80089870	64.04167811
TCN1	rs147981159	97.23689162
TCN1	rs117419973	32.7526945
TCN1	rs2511241	184.9789154
TCN1	rs143959838	33.65297064
TCN1	rs3826331	134.7547914
TCN1	rs681343	120.8889223
TCN1	rs1131603	103.5998482
TCN1	rs2347784	41.57373542
TCN1	rs1870028	41.23106358
NT5C3A	rs4316067	91.76249814
NT5C3A	rs10922098	139.1412011
NT5C3A	rs1619994	44.04817058
NT5C3A	rs28573154	55.60339726
NT5C3A	rs7774739	38.50161642
EFHD1	rs10922094	111.2724632
EFHD1	rs964184	50.29805489
EFHD1	rs113448098	62.27383443
EFHD1	rs12146727	213.0096895
EFHD1	rs117827303	51.30216788
EFHD1	rs141622900	48.56413789
EFHD1	rs111696008	33.74477207
EFHD1	rs144202860	30.5327163
EFHD1	rs1707652	215.9871857
EFHD1	rs13217434	43.42639094
EFHD1	rs74615535	36.5162045
EFHD1	rs6993770	38.32102091
PTGR1	rs78776206	32.01344983
PTGR1	rs144349540	212.7266892
PTGR1	rs74602773	918.9654371
PTGR1	rs200636047	948.4028328
PTGR1	rs74761683	66.70142058

PTGR1	rs117961590	496.81875
PTGR1	rs148199353	85.07815925
PTGR1	rs79384552	89.13412348
PTGR1	rs73535452	3072.069964
PTGR1	rs72748038	747.3911893
PTGR1	rs149486949	90.01246706
DLG2	rs11601665	31.47906859
DLG2	rs10876550	44.51884452
DLG2	rs185009887	33.21644064
DLG2	rs78909033	113.2790383
DLG2	rs1354034	62.4846468
DLG2	rs6782581	50.33987662
DLG2	rs342293	46.70941694
INHBA	rs73100943	102.5612637
INHBA	rs7311982	53.8188884
INHBA	rs7132006	39.98739566
INHBA	rs3741414	65.34464978
INHBA	rs2244608	61.63105319
INHBA	rs273510	40.66142567
INHBA	rs1260326	87.51017116
INHBA	rs12614487	35.34405906
INHBA	rs1047891	30.31628312
INHBA	rs6957745	73.80863379
INHBA	rs28601761	50.57119943
RPSA	rs424535	88.1908153
RPSA	rs114115200	57.44871035
RPSA	rs3891366	41.2980893
KNG1	rs5030062	808.3819241
KNG1	rs1846899	97.33569722
KNG1	rs1260326	36.26094986
KNG1	rs10935742	35.81804101
KNG1	rs1391867	39.6612379
KNG1	rs1970871	53.79798984
KNG1	rs76484380	58.56810632
KNG1	rs10013653	1017.58649
KNG1	rs75061705	111.7753955
KNG1	rs10010668	239.2414469
KNG1	rs139819017	43.9866485
KNG1	rs6875461	101.2826926
KNG1	rs4976691	916.5043572
KNG1	rs185903163	74.09865191
KNG1	rs11740757	33.52581677
KNG1	rs112260799	38.78772518
FGL2	rs66471312	42.70918756
FGL2	rs10922094	462.5610347
FGL2	rs2511241	54.51206961
FGL2	rs1707652	101.2964406
FGL2	rs2242572	127.9532539
FGL2	rs9272068	65.26450941
CRH	rs34556813	33.45678028
CRH	rs2224606	39.69833014
CRH	rs61803028	30.55745817
CRH	rs4906263	32.52291975
CRH	rs1801690	124.5216963
CRH	rs9258801	31.76107732
CRH	rs9273429	42.40356383
CRH	rs112647257	30.07785687
PDYN	rs2021652	38.1727178

PDYN	rs7073753	124.8679768
PDYN	rs4239252	390.0829869
PDYN	rs111887239	30.57733834
PDYN	rs1654447	38.37796775
PDYN	rs6993770	34.13517334
IAPP	rs6667019	47.970553
IAPP	rs10822143	40.18371239
IAPP	rs174560	35.42380485
IAPP	rs480989	62.02710846
IAPP	rs116908055	40.86285334
IAPP	rs2242663	768.5956712
IAPP	rs368719209	31.6741761
IAPP	rs117025069	104.591192
IAPP	rs80300795	43.02136586
IAPP	rs825038	87.1616129
IAPP	rs7964859	879.054106
IAPP	rs7955091	35.20402462
IAPP	rs140624338	117.8805179
IAPP	rs11111306	30.28757564
IAPP	rs956886	37.36529206
IAPP	rs148608463	29.81329922
IAPP	rs55707100	99.84793383
IAPP	rs704	89.2511168
IAPP	rs111959800	30.13780026
IAPP	rs8100978	43.50231054
IAPP	rs1260326	88.94033611
IAPP	rs645040	40.11190604
IAPP	rs1355538	47.92498568
IAPP	rs75853426	38.18373119
IAPP	rs41280463	42.86212774
IAPP	rs9639523	35.52367519
IAPP	rs28601761	33.10040938
F11	rs1117743	30.86942373
F11	rs3756008	1102.057736
F11	rs34930947	763.9196802
F11	rs62350309	107.1446823
F11	rs71640056	32.62430214
F11	rs55707100	37.76091012
F11	rs77262773	31.9454398
F11	rs62128132	31.66517867
F11	rs1260326	35.79084437
F11	rs114450524	80.35302168
F11	rs11718570	1148.276605
F11	rs76438938	456.4986535
F11	rs76541243	39.62760808
F11	rs148968975	59.93711615
F11	rs270153	40.96246725
F11	rs35496818	32.45726815
F11	rs9268832	33.17111235
COL18A1	rs150169381	56.59393635
COL18A1	rs11909509	274.5396838
COL18A1	rs17004785	110.631685
COL18A1	rs11701176	47.73049375
COL18A1	rs6652	34.37094467
COL18A1	rs77924615	37.09910915
COL18A1	rs5112	78.72445005
TIMP1	rs12040132	39.46541456
TIMP1	rs2511241	98.37345958

TIMP1	rs2571392	88.36826899
TIMP1	rs6993770	62.14972774
PLAT	rs2020921	653.585099
PLAT	rs4619189	44.22951741
PLAT	rs113414093	41.53610953
PLAT	rs62353834	34.26626545
PLAT	rs12191720	30.01541848
PLAT	rs2227631	38.73441272
PLAT	rs113900202	81.85983282
PLAT	rs35104055	92.41725421
PLAT	rs78848436	51.51634264
PLAT	rs34785256	40.82027023
PLAT	rs117948944	54.68356065
PLAT	rs80043413	47.0670347
PROK1	rs10922098	95.54521719
PROK1	rs6457457	30.24655859
PROK1	rs1846190	37.76787931
TIMP2	rs9894212	68.11248163
TIMP2	rs243841	86.69038789
TIMP2	rs75609019	36.0791439
TIMP2	rs77542162	34.98072537
TIMP2	rs1260326	59.42531384
TIMP2	rs11130306	79.10286531
TIMP2	rs1029239	31.29560799
TGFB1	rs10922094	248.7424792
TGFB1	rs12403425	30.29652324
TGFB1	rs6542680	67.55977443
TGFB1	rs1706435	56.43750667
TGFB1	rs444921	64.50304592
TGFB1	rs28471056	44.78475949
C5	rs1035029	183.9526877
C5	rs9775994	41.21117637
C5	rs10801551	30.72192025
C5	rs17408255	35.97722466
C5	rs8078686	72.77532688
C5	rs780094	56.56777085
APOE	rs73036465	76.83435582
APOE	rs80140611	44.39711418
APOE	rs71352239	778.9012672
APOE	rs76904536	40.43459037
APOE	rs148023052	75.13102128
APOE	rs34631405	34.41081187
APOE	rs964184	139.1522737
APOE	rs1532085	52.89276029
APOE	rs261332	37.37827566
APOE	rs780093	62.20790553
APOE	rs2668207	124.6303271
BDNF	rs10501089	66.52177591
BDNF	rs10822155	74.57485286
BDNF	rs4905998	31.21048803
BDNF	rs61658003	42.52056028
BDNF	rs75967349	31.69324507
BDNF	rs6993770	47.93175381
CTSG	rs10922094	172.060845
CTSG	rs77238211	33.48231263
CTSG	rs6542680	109.4630921
CTSG	rs180977001	31.82047038
CTSG	rs1355538	32.39195889

CTSG	rs9501600	56.48327564
CXCL16	rs35383686	413.2585045
CXCL16	rs5818982	241.8902172
CXCL16	rs41307944	120.2558393
CXCL16	rs748113	103.4415518
CXCL16	rs1250568	54.95176045
CXCL16	rs653178	138.7551239
CXCL16	rs995298	51.70258792
CXCL16	rs9913146	41.84287897
CXCL16	rs77542162	149.1754665
CXCL16	rs75819996	70.1506594
CXCL16	rs273507	35.86835787
CXCL16	rs10183338	296.45897
CXCL16	rs1157083	44.6291528
CXCL16	rs1519504	41.57623595
CXCL16	rs9712275	38.3458593
CXCL16	rs6031305	44.02741514
CXCL16	rs9869609	125.7334234
CXCL16	rs4682809	65.78915105
CXCL16	rs55975201	31.8794701
CXCL16	rs3819306	46.85416995
CXCL16	rs368959394	32.07359841
FGF8	rs10922098	603.9589217
FGF8	rs1047286	47.61055081
FGF8	rs2686395	92.48034854
FGF8	rs444921	153.4367723
FGF8	rs28471056	71.38224613
FGF8	rs9272526	31.1610237
PLA2G2E	rs10922098	168.0768262
PLA2G2E	rs1355538	33.21574165
PLA2G2E	rs28573154	61.42707419
PLA2G2E	rs7774739	54.20285999
APCS	rs148734859	70.99913861
APCS	rs146174215	520.8616753
APCS	rs28383573	377.4591922
APCS	rs469772	34.01018647
APCS	rs113724571	33.63193915
APCS	rs2842700	64.4824843
APCS	rs77428900	30.81062118
APCS	rs11840614	34.251094
APCS	rs4665972	69.3605498
KIT	rs4864902	30.7075036
KIT	rs4512628	31.01596483
KIT	rs10922098	467.49562
KIT	rs56376779	47.18482602
KIT	rs56043070	58.63430599
KIT	rs363281	40.16481645
KIT	rs10444039	32.31482525
KIT	rs61913605	50.25692963
KIT	rs8051733	35.17157772
KIT	rs17758695	49.69167714
KIT	rs78744187	274.5708023
KIT	rs1571732	64.70291999
KIT	rs67525224	40.68141014
KIT	rs12489516	51.54343732
KIT	rs1707652	82.29034722
KIT	rs13095431	75.2899553
KIT	rs444921	122.4627222

KIT	rs9469195	70.69498305
KIT	rs80189144	37.09000251
KIT	rs10962594	34.90610755
KIT	rs62543565	112.8736659
KIT	rs10118718	34.50019862
TIMP3	rs75127320	53.34148885
TIMP3	rs9619260	180.5332729
TIMP3	rs111284156	825.9752997
TIMP3	rs732446	986.0072926
TIMP3	rs73156459	44.00677319
TIMP3	rs3788507	1212.621932
TIMP3	rs4420793	65.80374086
TIMP3	rs117555418	243.9343362
TIMP3	rs11580302	30.38374724
TIMP3	rs7080386	62.41541307
TIMP3	rs113661219	39.90594687
TIMP3	rs78621438	41.78526575
TIMP3	rs5995069	49.25373878
GFRA3	rs10922094	274.2271868
GFRA3	rs6542680	103.092943
GFRA3	rs2686395	51.74103824
GFRA3	rs9501600	63.25874735
GFRA3	rs1846190	30.65522638
GFRA2	rs34466420	170.1547999
GFRA2	rs4612333	1057.790652
GFRA2	rs192923146	168.4797651
GFRA2	rs117190054	57.82798698
GFRA2	rs149112525	31.00411912
GFRA2	rs8192335	83.06519134
GFRA2	rs61105246	35.30364089
GFRA2	rs112771035	40.25174279
GFRA2	rs73021415	44.53139712
GFRA2	rs117633773	31.35607739
GFRA2	rs12945299	100.1491055
GFRA2	rs143501783	51.85175425
GFRA2	rs77542162	34.72743328
GFRA2	rs4760	41.95905433
GFRA2	rs681343	32.21288599
GFRA2	rs2007125	33.74181672
GFRA2	rs1257169	34.57571002
GFRA2	rs844159	36.14010591
GFRA2	rs115216147	53.78433088
GFRA2	rs9469141	67.67598751
GFRA2	rs28653112	85.02867549
GFRA2	rs2856822	31.84605812
GFRA2	rs7857270	43.76176488
CCL21	rs10972211	164.2639841
CCL21	rs10814194	43.14403084
CCL21	rs677106	31.00100416
CCL21	rs35350651	59.15398672
CCL21	rs9551444	34.34400708
CCL21	rs7212344	32.74929109
CCL21	rs9948057	31.70009056
CCL21	rs8107162	44.1454875
CCL21	rs6715061	64.58109921
CCL21	rs6077870	30.52949521
CCL21	rs6017250	38.15391267
CCL21	rs757024	100.0189482

CCL21	rs141300831	36.91196932
CCL21	rs80252447	40.07511519
CCL21	rs74765117	82.35434807
CCL21	rs148360766	215.1863077
CCL21	rs113349704	197.1239463
CCL21	rs11914615	1341.372012
CCL21	rs2600961	96.97685601
CCL21	rs116356943	453.5227643
CCL21	rs114194685	33.42307266
CCL21	rs192289769	30.53915637
CCL21	rs6871748	39.00589663
CCL21	rs17764730	62.5138756
CCL21	rs1264347	32.19678199
CCL21	rs11755562	40.30455879
CCL21	rs41268940	29.7349107
CCL21	rs144706539	36.71391183
CCL21	rs576123	29.89630298
HMGB1	rs10922094	458.2107157
HMGB1	rs1354034	113.5383231
HMGB1	rs1707652	73.31675176
HMGB1	rs9501600	106.031349
HMGB1	rs35209896	36.09344659
POMC	rs10922094	32.98345649
POMC	rs12721028	144.4383947
POMC	rs11216161	100.9895617
POMC	rs75275400	44.49426255
POMC	rs7412	83.87887996
POMC	rs11720167	81.2995565
CFI	rs13117504	562.0023159
CFI	rs4355391	78.11969745
CFI	rs12504835	46.1528585
CFI	rs6855357	32.70618859
CFI	rs4665972	56.84429282
IGFBP3	rs55932586	141.4874073
IGFBP3	rs145188037	1093.253536
IGFBP3	rs144318789	54.69159377
IGFBP3	rs10254413	41.00585592
IGFBP3	rs700752	161.3510944
IGFBP3	rs11042543	29.85485105
IGFBP3	rs201862321	49.42857407
IGFBP3	rs113339946	55.92260691
IGFBP3	rs143008659	57.4589354
IGFBP3	rs74002495	152.2650843
IGFBP3	rs147400256	97.47451604
IGFBP3	rs139482112	198.4033275
IGFBP3	rs117651825	43.56598771
IGFBP3	rs184995649	87.90761056
IGFBP3	rs77589580	102.382975
IGFBP3	rs145410566	30.65772599
IGFBP3	rs77542162	35.15846119
IGFBP3	rs4234797	69.47442106
IGFBP3	rs12540611	41.31564486
CCL2	rs12075	85.85617979
CCL2	rs6542680	42.35263816
CCL2	rs4490403	34.34283722
CCL2	rs1799864	110.512371
MMP9	rs2250889	68.8204096
MMP9	rs6692187	30.0518462

MMP9	rs4660129	39.2777148
MMP9	rs11253518	62.93070333
MMP9	rs117842293	43.1758332
MMP9	rs146509086	40.1466378
MMP9	rs191708354	40.11155405
MMP9	rs74704910	79.33940812
MMP9	rs2511241	1198.588898
MMP9	rs112657228	30.2899788
MMP9	rs57792815	34.57700299
MMP9	rs921651	56.66972708
MMP9	rs8066582	99.73634251
MMP9	rs12613605	33.60785504
MMP9	rs4264815	43.80873296
MMP9	rs2524087	47.77867511
MMP9	rs9264921	61.85738503
MMP9	rs9266642	38.7621037
MMP9	rs72992130	33.57765682
MMP9	rs2158799	56.43767781
MPO	rs146512685	69.974718
MPO	rs117405202	95.57821931
MPO	rs62081796	65.38735627
MPO	rs34097845	806.0648378
MPO	rs190383363	508.789624
MPO	rs3738187	171.8260481
MPO	rs2477639	44.30756299
MPO	rs757081	178.9342823
MPO	rs117937075	44.99706942
MPO	rs139196635	46.80306093
MPO	rs76428106	115.1719412
MPO	rs1263375	31.59655677
MPO	rs2071408	30.86244766
MPO	rs28733475	34.01876606
MPO	rs9898547	29.8517704
MPO	rs145433937	407.9298348
MPO	rs373759006	291.0558755
MPO	rs77542162	34.2807074
MPO	rs56757486	31.16043928
MPO	rs2974751	50.45863633
MPO	rs12712881	74.73486254
MPO	rs2698197	30.08663166
MPO	rs3790316	82.2737072
MPO	rs55771110	117.8313347
MPO	rs13100173	36.61670095
MPO	rs1687481	39.01561775
MPO	rs34198449	69.70679735
MPO	rs144317085	34.89372366
MPO	rs6931332	53.1821882
MPO	rs3104368	38.37099904
MPO	rs11155297	40.63252121
MPO	rs6962012	45.83258291
MPO	rs10107630	115.1225527
ROR1	rs11447165	54.99333115
ROR1	rs61765448	766.7891081
ROR1	rs114735138	81.30564295
ROR1	rs12117461	40.13313629
ROR1	rs10158500	51.84694367
ROR1	rs72704449	89.90598186
ROR1	rs143616286	33.29207717

ROR1	rs11630455	32.9694509
ROR1	rs2725405	30.6099881
ROR1	rs33950747	60.44969301
ROR1	rs1260326	68.40594796
ROR1	rs13108218	34.34861095
VEGFA	rs7692	37.59644986
VEGFA	rs9472159	1151.661253
VEGFA	rs1740073	46.21387796
VEGFA	rs10761739	38.67446527
VEGFA	rs6993770	98.14114558
VEGFA	rs11789392	56.37796002
ANGPT2	rs1968586	217.9332478
ANGPT2	rs2256628	43.82566387
ANGPT2	rs61776478	39.54244815
ANGPT2	rs142029184	41.74568621
ANGPT2	rs35257264	77.42334963
ANGPT2	rs56115403	66.56710851
ANGPT2	rs143501783	43.68525063
ANGPT2	rs1035002	35.27595938
ANGPT2	rs10935480	35.03479306
ANGPT2	rs6601299	36.50583026
ANGPT2	rs187099314	172.2442302
ANGPT2	rs62576042	32.80648949
TNFRSF8	rs10922098	582.1557737
TNFRSF8	rs11569415	68.57466591
TNFRSF8	rs2686395	96.77264099
TNFRSF8	rs9501600	149.8210037
TNFRSF8	rs35209896	40.25853604
CST3	rs6114209	628.05532
CST3	rs73102389	102.5690102
CST3	rs78996714	35.12638458
CST3	rs2129775	31.44313104
CST3	rs77924615	45.00594104
CST3	rs9992101	33.28742019
CST3	rs62435145	30.92414413
CST3	rs10253736	29.85950651
TYRO3	rs2588323	157.0810208
TYRO3	rs10622699	31.33711142
TYRO3	rs10922098	425.92696
TYRO3	rs704	33.28214109
TYRO3	rs1707652	85.54440673
TYRO3	rs555007	55.12926439
TYRO3	rs453821	116.0010689
TYRO3	rs10901252	63.33712874
EIF5	rs203687	41.62095339
EIF5	rs1354034	69.39000477
EIF5	rs6800964	49.6414413
EFNA5	rs2410942	71.01904789
EFNA5	rs12514540	239.5171283
EFNA5	rs78705678	49.49424932
EFNA5	rs2477642	65.01685119
EFNA5	rs11646097	40.63197466
EFNA5	rs77924615	43.98365767
EFNA5	rs1260326	51.26771118
ERBB3	rs2292238	163.6069862
ERBB3	rs147233090	40.04902039
ERBB3	rs8078686	44.6354369
ERBB3	rs1260326	118.4590216

ERBB3	rs56088330	144.0865145
ERBB4	rs10922098	62.43282557
ERBB4	rs117265578	34.79597555
ERBB4	rs9902706	34.14232425
ERBB4	rs13208583	31.41420808
IL6ST	rs113121863	33.97595664
IL6ST	rs7730934	857.9098434
IL6ST	rs116753755	74.79581067
IL6ST	rs111379648	62.45179462
IL6ST	rs12766994	30.71046081
IL6ST	rs78689694	115.8055226
IL6ST	rs117633773	45.83332449
IL6ST	rs55714927	157.1907242
IL6ST	rs143501783	80.20566668
IL6ST	rs77542162	33.40005582
IL6ST	rs615355	35.10635668
IL6ST	rs2008174	39.67072413
IL6ST	rs73133996	258.4558002
IL6ST	rs11920688	31.73048856
IL6ST	rs11921267	32.0532577
IL6ST	rs150328104	38.98017303
IL6ST	rs143693231	55.38127234
IL6ST	rs113608206	45.87376791
IL6ST	rs116703374	123.805344
IL6ST	rs5011432	32.60876438
IL6ST	rs635634	393.8202371
IL6ST	rs41307428	73.5000221
IL10RB	rs4455239	75.43320985
IL10RB	rs10922098	574.1830578
IL10RB	rs1047286	33.76561876
IL10RB	rs2686395	86.98284898
IL10RB	rs9501600	135.9129247
IL10RB	rs35209896	35.20659951
IL12RB1	rs429774	84.71058817
IL12RB1	rs10922098	284.9145839
IL12RB1	rs1707652	38.34887332
IL12RB1	rs9501600	75.93427242
IL12RB1	rs35209896	35.96066613
LAYN	rs4938792	39.49329714
LAYN	rs1451915	32.75199695
LAYN	rs10922098	712.9883193
LAYN	rs7513100	31.67748421
LAYN	rs11569415	64.75985344
LAYN	rs2686395	136.5703763
LAYN	rs444921	173.659413
LAYN	rs28471056	73.40757071
LTBR	rs72748420	46.18458545
LTBR	rs1329424	1331.524996
LTBR	rs3900909	60.38625084
LTBR	rs10801660	39.23325924
LTBR	rs1047286	46.61936938
LTBR	rs1707652	201.9704902
LTBR	rs2161196	29.88265356
LTBR	rs1265941	123.9735708
LTBR	rs550671	319.0134269
LTBR	rs112164276	44.02692537
LTBR	rs41548618	48.55308659
MRC1	rs112163346	50.18795942

MRC1	rs73597929	90.04015879
MRC1	rs34036394	2854.796953
MRC1	rs692719	6336.612202
MRC1	rs200708988	3144.537119
MRC1	rs114558332	51.72932237
MRC1	rs72786098	111.8063261
MRC1	rs148087583	48.54660214
MRC1	rs117583381	99.24200244
MRC1	rs138488415	33.26793024
MRC1	rs117412763	39.66406414
MRC1	rs61801021	54.47208826
MRC1	rs5030924	42.69148197
MRC1	rs4934028	36.97945479
MRC1	rs12945299	60.46204224
MRC1	rs62165726	29.86951051
MRC1	rs17830904	111.2251489
MRC1	rs8140836	34.25468362
MRC1	rs463250	34.02245726
MRC1	rs76636990	57.02822824
MRC1	rs71534526	40.02575125
MRC1	rs35139284	65.09944511
MRC1	rs36229731	41.9669869
MRC1	rs7865362	34.99905952
MST1R	rs114009540	32.21415222
MST1R	rs10922098	903.5700647
MST1R	rs3900909	50.95340802
MST1R	rs1047286	60.9118641
MST1R	rs1707652	131.1038204
MST1R	rs555007	100.9612588
MST1R	rs550671	225.4442288
MST1R	rs9272526	38.67728147
CDH3	rs10922094	182.4192844
CDH3	rs10140610	112.6320107
CDH3	rs10143099	47.42652926
CDH3	rs55870921	35.58887558
CDH3	rs113954333	113.3576509
CDH3	rs11080055	936.7169254
CDH3	rs1260326	75.95360311
CDH3	rs56210800	38.1773454
CDH3	rs140431012	38.18847501
CDH3	rs1706435	58.31141091
CDH3	rs2731672	32.93043253
CDH3	rs550671	70.22981985
CDH3	rs9469195	35.22522586
PRKCA	rs12601617	35.97465701
PRKCA	rs61762372	90.78340291
PRKCA	rs35340377	33.72788625
PRKCA	rs342200	31.82637472
ICAM3	rs10922098	536.9082644
ICAM3	rs11569415	59.4887207
ICAM3	rs2686395	73.49375719
ICAM3	rs550671	130.1937721
ICAM3	rs9270570	34.90363176
ICAM3	rs9469195	64.41862478
PLAUR	rs1001581	46.89735263
PLAUR	rs2302524	511.2627939
PLAUR	rs4251824	140.9576871
PLAUR	rs12045503	41.94987302

PLAUR	rs61761603	41.40284994
PLAUR	rs2227576	250.9073843
PLAUR	rs2511241	33.38047286
PLAUR	rs171270	33.35938949
PLAUR	rs78689694	94.89853058
PLAUR	rs3184504	30.64380302
PLAUR	rs62061425	153.1638216
PLAUR	rs41556717	39.16374435
PLAUR	rs143501783	60.22974495
PLAUR	rs9948409	46.79662492
PLAUR	rs147779538	46.35152994
PLAUR	rs72957552	256.1259461
PLAUR	rs6717518	32.71480143
PLAUR	rs2470854	57.71702009
PLAUR	rs9268581	32.58965366
PLAUR	rs8176741	89.69894004
TNFRSF1A	rs4149584	214.4036594
TNFRSF1A	rs72704449	49.52050753
TNFRSF1A	rs4776168	30.55361315
TNFRSF1A	rs77924615	48.49971337
TNFRSF1A	rs8067774	30.11658107
TNFRSF1A	rs2452170	59.00359142
TNFRSF1A	rs4511696	57.19916295
TNFRSF1A	rs368611	32.08279672
TNFRSF1A	rs9271929	31.47573666
TNFRSF1A	rs3762055	153.8985362
TNFRSF1A	rs9411463	63.09390683
NTRK3	rs56160094	38.75612279
NTRK3	rs1461214	242.1503955
NTRK3	rs28735437	326.1800854
NTRK3	rs4887400	54.65267572
NTRK3	rs12280645	70.65506187
NTRK3	rs181707610	41.36492653
NTRK3	rs79445108	85.25807025
NTRK3	rs4476322	38.23246781
NTRK3	rs914187	144.6830526
TNFRSF17	rs11570136	236.9715234
TNFRSF17	rs12758915	35.88942861
TNFRSF17	rs2532744	68.06755841
TNFRSF17	rs3803286	83.73852024
TNFRSF17	rs34562254	198.5065084
TNFRSF17	rs17334923	31.21867577
TNFRSF17	rs894872	34.03704211
TNFRSF17	rs72939458	42.67969047
TNFRSF17	rs6728684	46.7135027
TNFRSF17	rs4851257	34.5205968
TNFRSF17	rs10048885	36.67538092
TNFRSF17	rs4690163	69.62755207
TNFRSF17	rs1418708	33.92976942
TNFRSF17	rs115736104	47.91218922
TNFRSF17	rs1140809	54.79466846
TNFRSF17	rs2647071	41.96059555
TNFRSF17	rs510432	35.60019219
TNFRSF17	rs78377363	33.06071483
TNFRSF17	rs3778754	45.01784531
EGFR	rs17172402	74.99625735
EGFR	rs13244925	150.1813399
EGFR	rs17172451	43.97278725

EGFR	rs333947	31.53283407
EGFR	rs12280645	63.82728808
EGFR	rs79297632	36.16649508
EGFR	rs79009224	46.02901864
EGFR	rs140118846	52.68805924
EGFR	rs181707610	85.35880484
HGF	rs2214827	158.4973202
HGF	rs2511241	200.8549833
HGF	rs11065979	29.90453064
HGF	rs16844364	42.36016396
C3	rs10408682	46.24493539
C3	rs139329922	31.09473347
C3	rs12975366	33.24468132
MIA	rs111613270	1077.09209
MIA	rs117030537	99.19123755
MIA	rs74746550	2838.182194
MIA	rs150890087	631.5696184
MIA	rs184589612	953.9422694
MIA	rs117613652	61.50855263
MIA	rs34262842	40.6015637
MIA	rs77542162	76.63018141
MIA	rs191048002	38.49904755
MIA	rs60530219	43.06672444
MIA	rs11668176	48.28026734
MIA	rs76157475	168.7551349
MIA	rs10019833	30.30627984
PLA2G2A	rs145437966	133.0788577
PLA2G2A	rs55929612	37.44351043
PLA2G2A	rs114986549	1028.753207
PLA2G2A	rs56066017	439.2141226
PLA2G2A	rs2063054	964.825247
PLA2G2A	rs11379884	110.1412868
PLA2G2A	rs28610043	74.69047591
PLA2G2A	rs115817391	48.71070899
PLA2G2A	rs76972837	36.78694395
PLA2G2A	rs12034502	68.7790708
PLA2G2A	rs61729512	33.62037267
PLA2G2A	rs112001035	172.6178224
OSM	rs72748420	36.93687064
OSM	rs114009540	38.01211887
OSM	rs1329424	1105.083605
OSM	rs3900909	47.784547
OSM	rs1047286	79.08335739
OSM	rs1707652	156.0614766
OSM	rs1265941	100.2651606
OSM	rs550671	266.7307158
OSM	rs112164276	33.22507225
OSM	rs41548618	34.60809063
PECAM1	rs10922098	738.3725342
PECAM1	rs3900909	36.75185994
PECAM1	rs11569415	37.11459523
PECAM1	rs2686395	128.5068171
PECAM1	rs444921	193.6766899
PECAM1	rs9270570	55.87881033
PECAM1	rs9469195	81.32997215
PSPN	rs10922098	114.6862967
PSPN	rs415620	31.55435019
PSPN	rs4713498	39.44044226

PF4	rs1398015	75.59276667
PF4	rs11574452	72.97783784
PF4	rs10822155	161.5351462
PF4	rs113661219	30.15718074
PF4	rs1071960	32.78959066
PF4	rs342200	29.90241992
PF4	rs6993770	50.49868632
PROS1	rs6809958	62.34353184
PROS1	rs1260326	38.78894597
PROS1	rs184624754	36.55694939
PROS1	rs72848256	50.49408384
PROS1	rs2306956	44.70844849
PROS1	rs1623718	40.40152653
PROS1	rs10982156	492.167599
PROS1	rs72750242	38.22131447
TNFRSF13B	rs10922098	406.8776446
TNFRSF13B	rs1707652	71.20385861
TNFRSF13B	rs9267139	33.87220251
TNFRSF13B	rs28573154	101.704785
TNFRSF13B	rs7774739	74.75975807
TNFRSF13B	rs9271766	48.61975134
CCL25	rs80188016	63.56069635
CCL25	rs181528492	177.0911771
CCL25	rs62126622	99.85935499
CCL25	rs17438280	64.39025722
CCL25	rs12980552	1010.355118
CCL25	rs78039161	525.2858039
CCL25	rs4804273	228.5554893
CCL25	rs59141216	119.8668524
CCL25	rs214068	315.7353145
CCL25	rs12279699	61.37794972
CCL25	rs12718462	41.60187968
CCL25	rs11216221	31.11615039
CCL25	rs72654473	39.65159437
CCL25	rs601338	379.1631651
CCL25	rs12983058	37.7301564
CCL25	rs16854775	30.86245172
CCL25	rs1501908	125.0028944
CCL25	rs4841427	50.8141207
CCL25	rs28601761	41.19861453
CCL25	rs635634	99.69701399
SERPINA7	rs35910103	31.08679758
SERPINA7	rs1519125	39.40250671
SERPINA7	rs1169288	129.4483472
SERPINA7	rs10416310	31.12607938
SERPINA7	rs1800961	35.81040353
SERPINA7	rs523118	39.37537315
SERPINA7	rs804267	33.47231441
SERPINA7	rs10110738	62.41372168
TNFSF18	rs10922098	634.2438564
TNFSF18	rs2686395	143.4459313
TNFSF18	rs555007	71.04733731
TNFSF18	rs550671	165.6273053
TNFSF18	rs9269234	30.66172482
AIMP1	rs10922094	93.50163818
AIMP1	rs7920986	29.92755181
AIMP1	rs71352239	62.27569569
AIMP1	rs5167	110.9914793

AIMP1	rs780093	38.51526001
AIMP1	rs1619994	83.81524326
AIMP1	rs11755689	39.08903197
CSF3R	rs10922103	238.7121403
CSF3R	rs6542680	292.4382071
CSF3R	rs1707652	43.85626072
CSF3R	rs9501600	70.48812426
IL37	rs72748420	36.51085284
IL37	rs1329424	1090.428488
IL37	rs3900909	46.36719319
IL37	rs1047286	61.34428705
IL37	rs1707652	165.8785843
IL37	rs555007	103.3228953
IL37	rs550671	273.3441029
IL37	rs41548618	38.81685949
IL37	rs9272526	52.44966598
MICA	rs184375406	1266.297724
MICA	rs114561028	161.28208
MICA	rs55640410	181.3844131
MICA	rs116633868	4960.821437
MICA	rs189334061	624.5287123
MICA	rs181785842	590.8081289
MICA	rs2254620	936.8505136
MICA	rs4713467	976.9744857
MICA	rs116834573	41.98503515
MICA	rs943572	45.13929392
MICA	rs145042412	30.88859167
MICA	rs1362076	260.5438755
MICA	rs146809058	382.8150688
MICA	rs9468618	471.2320402
MICA	rs9258375	944.3936501
MICA	rs117660163	572.0429504
MICA	rs4713602	608.9010523
MICA	rs72896287	42.61593061
MICA	rs9462199	35.51051547
MICA	rs2842636	36.766528
MICA	rs138939507	34.78507206
MICA	rs635634	119.2778777
POR	rs7223	60.07978741
POR	rs59882870	318.6825705
POR	rs7310615	41.12294216
POR	rs3747207	55.81031919
POR	rs3129752	37.01568965
POR	rs74380254	31.59658687
NANOG	rs10922098	66.03620218
NANOG	rs11651658	33.08412668
NANOG	rs415620	30.29147989
NANOG	rs7774739	30.71463896
NCR2	rs10922098	126.4681747
NCR2	rs1555405	30.75534738
NCR2	rs1355538	39.08936815
NCR2	rs9501600	53.7456988
SIGLEC6	rs8103083	47.76481494
SIGLEC6	rs56411232	207.985173
SIGLEC6	rs117496758	74.03751818
SIGLEC6	rs185254	1367.103755
SIGLEC6	rs2864097	1368.977349
SIGLEC6	rs45441795	272.7008588

SIGLEC6	rs78135490	68.6647454
SIGLEC6	rs34449583	50.40379038
SIGLEC6	rs79722030	63.89764014
SIGLEC6	rs2155311	37.46820425
SIGLEC6	rs76428106	39.21593402
SIGLEC6	rs34924625	32.12683371
SIGLEC6	rs112578870	32.62399432
SIGLEC6	rs35930480	43.33984061
SIGLEC6	rs138620872	57.3786617
SIGLEC6	rs3111414	32.8900961
SIGLEC7	rs2691243	64.30508686
SIGLEC7	rs1039405	322.5529859
SIGLEC7	rs140185670	1304.563216
SIGLEC7	rs12462100	33.8710836
SIGLEC7	rs113842511	44.49966644
SIGLEC7	rs78689694	51.81726839
SIGLEC7	rs35350651	49.03065366
SIGLEC7	rs55714927	78.21969981
SIGLEC7	rs143501783	46.89263265
SIGLEC7	rs2160725	52.84060484
SIGLEC7	rs62165726	50.38767227
SIGLEC7	rs34211178	38.62588665
SHH	rs9333592	44.20680617
SHH	rs10225292	112.964867
SHH	rs1864163	41.61226671
SHH	rs1065853	302.6988696
SHH	rs149622866	34.72840736
SHH	rs114006326	49.85714228
APOA1	rs518734	122.7244247
APOA1	rs117192879	339.4333242
APOA1	rs79879928	438.4278693
APOA1	rs117674052	34.99626608
APOA1	rs142498563	77.00965537
APOA1	rs183444525	202.8437533
APOA1	rs587017	35.38024238
APOA1	rs77998935	433.5311965
APOA1	rs371267418	911.1877734
APOA1	rs117620934	265.1462673
APOA1	rs146163219	32.32234897
APOA1	rs35080326	41.34871001
APOA1	rs3794693	280.0517043
APOA1	rs35909200	510.2166985
APOA1	rs4665972	42.67939373
GDF5	rs368465	53.23060733
GDF5	rs6496605	95.5581442
GDF5	rs72780913	32.42546016
GDF5	rs704	403.1964964
GDF5	rs1442	89.92163044
C3	rs10408682	65.4461686
C3	rs10922094	216.585328
C3	rs12589344	30.23627318
C3	rs12975366	33.79029984
C3	rs72548024	33.20482305
C3	rs2242572	92.14391042
C3	rs9272068	46.32045299
FAM107A	rs10922098	453.4775929
FAM107A	rs1706435	76.96465344
FAM107A	rs9267139	44.49749732

FAM107A	rs3891366	137.2092962
FAM107A	rs7774739	104.0913614
FAM107A	rs9271766	53.67550376
FGF20	rs10922098	31.83576454
FGF20	rs1671214	38.98095035
FGF20	rs13208583	31.11875162
CCL1	rs687628	30.63372677
CCL1	rs114558472	30.27722822
CCL1	rs72748420	39.99954049
CCL1	rs1329424	1128.654616
CCL1	rs3900909	58.2240133
CCL1	rs10801660	36.94053983
CCL1	rs13469	31.68541444
CCL1	rs1047286	61.19396356
CCL1	rs111696008	30.36354862
CCL1	rs1707652	192.3437488
CCL1	rs1265941	105.3935871
CCL1	rs550671	277.0468656
CCL1	rs9269234	38.31390367
IGFBP1	rs3828998	89.15641047
IGFBP1	rs4970836	129.1565422
IGFBP1	rs4665972	80.41130139
IL16	rs146949219	37.79914205
IL16	rs62035267	39.66025268
IL16	rs142332135	979.6998602
IL16	rs72746169	468.798132
IL16	rs17875585	82.32503872
IL16	rs4778640	2639.737033
IL16	rs140626393	61.91003688
IL16	rs140102925	53.10059311
IL16	rs76390521	140.2289222
IL16	rs140684257	122.7950754
IL16	rs66962766	36.3711092
IL16	rs28590669	45.66134196
IL16	rs12342201	70.56091721
LTF	rs146097568	371.3265545
LTF	rs6414426	393.2154849
LTF	rs9884011	110.3522779
LTF	rs3917932	38.65734574
LTF	rs2074038	131.7495263
LTF	rs2511241	62.49892606
LTF	rs921651	44.8098451
LTF	rs8066582	69.17058497
LTF	rs243587	36.7511825
LTF	rs145965071	56.90786259
LTF	rs77000231	32.48406689
LTF	rs6803805	41.25917502
LTF	rs2158799	32.90588079
LTF	rs1870027	48.09597278
LTF	rs116412579	55.55601306
LTF	rs35440906	37.70896686
CCL3L1	rs854469	45.62390215
CCL3L1	rs72748420	31.16440027
CCL3L1	rs10922098	914.3680291
CCL3L1	rs3900909	38.66087567
CCL3L1	rs1047286	60.62064447
CCL3L1	rs2686395	152.6700486
CCL3L1	rs3099847	32.87143957

CCL3L1	rs1265941	93.4341913
CCL3L1	rs550671	226.7008918
CCL3L1	rs9276606	38.22221803
MMP3	rs964184	114.8459959
MMP3	rs113403132	33.27425514
MMP3	rs71352239	487.5641399
MMP3	rs204477	41.96327058
MMP3	rs148023052	60.18058357
MMP3	rs1260326	59.23769428
MMP3	rs1707652	64.50623363
MMP7	rs56857975	1137.503252
MMP7	rs67702963	195.8631494
MMP7	rs71480491	95.30316759
MMP7	rs6694034	104.2026387
MMP7	rs595151	35.51806241
MMP7	rs72981067	32.47583641
MMP7	rs61729512	38.70293212
MMP7	rs77924615	100.7172143
MMP7	rs12986064	57.69526889
PPBP	rs10822155	120.7075556
PPBP	rs7172696	33.87746012
PPBP	rs12468189	29.91368128
PPBP	rs13412535	39.28696786
PPBP	rs4541868	56.2278725
PPBP	rs77265585	32.05455285
APOB	rs563290	51.80197517
APOB	rs11591147	38.60423089
APOB	rs12740374	168.5222742
APOB	rs1546954	30.18510705
APOB	rs964184	40.54295019
APOB	rs723967	33.0575438
APOB	rs73013176	40.81174859
APOB	rs77140532	80.43305405
APOB	rs17800819	37.21184406
APOB	rs147711004	122.947587
APOB	rs7412	924.5977471
APOB	rs10412986	55.5802838
APOB	rs111696008	37.01169684
APOB	rs667920	33.59283227
APOB	rs11720167	304.5066065
APOB	rs3863087	126.9210419
APOB	rs148077724	31.96431751
APOB	rs139276089	168.1548223
APOB	rs1801020	294.5101887
ACVR1B	rs10922098	125.5706571
ACVR1B	rs62269916	32.01442425
ACVR1B	rs1355538	35.49042232
ANGPT1	rs72686539	32.69300093
ANGPT1	rs10127790	43.27422905
ANGPT1	rs10822155	49.0984626
ANGPT1	rs11451044	45.82954897
ANGPT1	rs61978204	29.87627392
ANGPT1	rs1522745	33.27950201
ANGPT1	rs113661219	63.05871888
ANGPT1	rs6993770	141.3892777
AGRP	rs34123523	651.6573011
AGRP	rs34693926	383.7036837
AGRP	rs10922098	40.48376101

AGRP	rs7310615	30.39082674
AGRP	rs147784124	64.61868704
AGRP	rs138439096	50.84132776
AGRP	rs7189440	30.83965486
AGRP	rs77542162	334.858232
AGRP	rs72631343	64.60079908
AGRP	rs45541434	30.65516966
AGRP	rs12693095	44.69168015
AGRP	rs9878990	36.11573942
AGRP	rs4976689	31.88906975
AGRP	rs9270551	44.09978858
AGRP	rs9272544	67.86898255
BCAM	rs12461127	65.97210023
BCAM	rs28399656	3194.732638
BCAM	rs117644736	115.1185453
BCAM	rs78675029	58.82961915
BCAM	rs150908522	187.8932928
BCAM	rs112382785	64.97552985
BCAM	rs77825069	37.94190842
BCAM	rs34666101	54.08169543
BCAM	rs2477669	37.43406786
BCAM	rs78705678	240.7835783
BCAM	rs2477642	255.2966123
BCAM	rs11085824	65.93640801
BCAM	rs139645865	44.70217242
BCAM	rs746063	40.21261438
BCAM	rs7610449	31.23498876
BCAM	rs6856326	82.73941784
BCAM	rs9271273	29.83377983
BCAM	rs6592965	32.06331306
BCAM	rs10901252	861.2843272
BCAM	rs62576042	50.96941762
BCAM	rs117446824	30.86629282
CDH5	rs3760043	40.95958327
CDH5	rs11649312	309.5118792
CDH5	rs61801011	50.54376269
CDH5	rs35166255	71.73681084
CDH5	rs7310615	31.99711694
CDH5	rs183857418	32.49891106
CDH5	rs117877056	97.88091909
CDH5	rs117241334	146.1277895
CDH5	rs111903631	98.17905581
CDH5	rs117325225	39.53813678
CDH5	rs71352729	47.28983551
CDH5	rs1260326	51.10112446
CDH5	rs78058190	38.22667749
CDH5	rs62578578	159.4265374
CDH5	rs72759422	126.0449781
CDH5	rs2073926	479.4442504
CDH5	rs12378537	4665.997439
CDH5	rs3124761	243.9079209
CDH5	rs117446824	104.4216089
COMMD7	rs10922094	31.22741377
COMMD7	rs4980320	44.48643317
COMMD7	rs7113761	36.70100964
COMMD7	rs1354034	80.17845475
COMMD7	rs1355538	57.10385434
COMMD7	rs1209879	132.0357012

EDA	rs10922098	366.3373905
EDA	rs2686395	54.38754678
EDA	rs1265941	52.03121662
EDA	rs550671	118.9778313
CX3CL1	rs62037109	75.00497112
CX3CL1	rs147834361	47.97973948
CX3CL1	rs72748420	35.49409411
CX3CL1	rs114009540	32.05262948
CX3CL1	rs1329424	959.3936879
CX3CL1	rs3900909	49.07494455
CX3CL1	rs2230203	42.96773372
CX3CL1	rs1707652	151.4143715
CX3CL1	rs1265941	89.13862488
CX3CL1	rs550671	235.4727596
CX3CL1	rs9276606	44.38283784
SPINT1	rs41277680	49.25022822
SPINT1	rs17658212	422.9251462
SPINT1	rs76769476	43.95852483
SPINT1	rs12067564	96.33679621
SPINT1	rs4443857	948.8775584
SPINT1	rs10789520	177.5193157
SPINT1	rs138965969	36.20185197
SPINT1	rs10922098	166.6198573
SPINT1	rs78705678	49.32935337
SPINT1	rs2477642	40.29075837
SPINT1	rs2010604	29.82944667
SPINT1	rs62061425	51.01752321
SPINT1	rs708686	63.89791172
SPINT1	rs2419900	61.26228428
SPINT1	rs12629034	92.2881778
SPINT1	rs34070949	122.5792237
SPINT1	rs13071075	45.61206662
SPINT1	rs6445035	34.7191776
SPINT1	rs9501393	33.83205363
SPINT1	rs10991614	40.72744865
SPINT1	rs9411365	57.58982661
KLK11	rs62115743	12306.14733
KLK11	rs62117662	414.9419663
KLK11	rs113853183	118.145236
KLK11	rs77924615	33.45154159
KLK11	rs146449966	49.97326639
KLK11	rs73057414	84.04428401
KLK11	rs8102024	47.39958423
KLK11	rs78599127	41.16110771
KLK11	rs62193071	155.8748499
KLK11	rs150571294	43.52006547
KLK11	rs419677	34.83730799
KLK4	rs10922098	102.3298233
KLK4	rs6542680	100.9722733
KLK4	rs9501600	32.99885742
KLK8	rs45533637	51.82151824
KLK8	rs143815637	33.42762384
KLK8	rs2739412	31.6079987
KLK8	rs10407580	410.46117
KLK8	rs74705037	1145.746558
KLK8	rs144461714	217.6785737
KLK8	rs145673783	50.15563804
KLK8	rs10922094	84.1573194

KLK8	rs117778189	44.9858474
KLK8	rs121908120	38.81981527
KLK8	rs6445035	34.41141097
KLK8	rs13208583	30.3599788
LCN2	rs3814526	52.24037797
LCN2	rs3917932	65.69076537
LCN2	rs3014874	37.92133083
LCN2	rs2606725	31.06210083
LCN2	rs1555405	30.99239263
LCN2	rs77924615	30.39324591
LCN2	rs6503533	151.0925942
LCN2	rs74172675	34.66187734
LCN2	rs35795243	35.00365786
LCN2	rs11670056	42.52265725
LCN2	rs1048740	29.92788653
LCN2	rs56185965	38.74740519
LCN2	rs2712431	54.74402127
LCN2	rs1680789	32.65902251
LCN2	rs6870370	56.85927367
LCN2	rs57221392	77.73936869
LCN2	rs607138	34.3589908
LCN2	rs58005484	37.51650463
LCN2	rs11769630	89.72613783
LCN2	rs7846314	63.76129339
LCN2	rs13277237	39.15275264
MET	rs10435378	244.8628082
MET	rs145179853	34.48813864
MET	rs143779007	37.75977145
MET	rs138121767	51.07443373
MET	rs10761784	45.42806918
MET	rs2343551	63.35181944
MET	rs174562	47.64911301
MET	rs11603123	74.11323002
MET	rs11045856	64.09941954
MET	rs1041316	57.35070695
MET	rs139974673	37.26857976
MET	rs72784938	74.97023011
MET	rs55714927	31.3345983
MET	rs77542162	156.4084276
MET	rs76860122	78.66586564
MET	rs3827211	62.27397434
MET	rs1604038	52.41824753
MET	rs13226546	74.06184669
MET	rs146798134	62.63085487
MET	rs635634	1034.432534
MET	rs117210485	42.38233621
MMP17	rs10922098	548.8075659
MMP17	rs11569415	35.49298928
MMP17	rs6542680	45.93232216
MMP17	rs35677470	30.05420602
MMP17	rs1707652	96.01772668
MMP17	rs3130955	30.86245571
MMP17	rs9501600	139.9850818
MMP17	rs35209896	41.16244748
TNFSF4	rs10922094	118.317069
TNFSF4	rs1706435	30.59053624
TNFSF4	rs9501600	44.36417264
SPINT2	rs7254947	1055.800652

SPINT2	rs6508781	2452.623808
SPINT2	rs145684392	7148.539817
SPINT2	rs151168828	103.1299432
SPINT2	rs112885443	245.9277655
SPINT2	rs56404782	32.62740921
SPINT2	rs8191365	33.0452588
SPINT2	rs76499006	64.10738508
SPINT2	rs2918367	158.4425209
SPINT2	rs111320482	43.9465705
SPINT2	rs6993770	42.5789822
TIE1	rs1098783	118.8575704
TIE1	rs7549876	585.1312872
TIE1	rs77165808	76.70652802
TIE1	rs7554123	206.485231
TIE1	rs116668474	203.0930199
TIE1	rs72686784	70.41166094
TIE1	rs2477664	65.73769774
TIE1	rs2461161	61.81493685
TIE1	rs35166255	40.54772617
TIE1	rs7295693	42.34671013
TIE1	rs7310615	32.59523336
TIE1	rs73493038	35.76836029
TIE1	rs878381	97.14798382
TIE1	rs55714927	41.27990635
TIE1	rs7207683	72.54687392
TIE1	rs191218269	62.94911717
TIE1	rs183857418	70.81451884
TIE1	rs142335773	61.28513621
TIE1	rs117877056	191.642555
TIE1	rs117241334	250.449754
TIE1	rs111903631	188.3246011
TIE1	rs117325225	73.48521685
TIE1	rs71352729	62.33081457
TIE1	rs12496730	766.3273927
TIE1	rs13071075	39.49957629
TIE1	rs111472656	37.83292141
TIE1	rs9298506	83.03583242
TIE1	rs62578578	124.2165936
TIE1	rs139075413	63.73086706
TIE1	rs72759422	80.71081978
TIE1	rs2073926	531.7624378
TIE1	rs12378537	3971.242417
TIE1	rs117446824	38.67534167
AIF1	rs142684094	48.35139222
AIF1	rs2261033	244.5417277
AIF1	rs10922094	145.897243
AIF1	rs1354034	30.94515747
AIF1	rs143933760	35.33914829
AIF1	rs11759846	65.60125095
AIF1	rs4631576	44.9560763
C5	rs928406	39.22227816
C5	rs17220750	212.4045688
C5	rs10766303	33.48723568
C5	rs374087027	32.61155318
C5	rs11868058	54.4104773
C5	rs8178824	77.5761484
C5	rs72613567	35.62243505
C5	rs2596481	40.76203189

C5	rs10819449	41.11281935
CHEK1	rs10922098	580.9735305
CHEK1	rs2686395	103.8237653
CHEK1	rs555007	72.18442771
CHEK1	rs453821	158.5515066
CHEK1	rs9269234	30.71390018
MAPK3	rs4427827	102.2917911
MAPK3	rs57149692	854.0277483
MAPK3	rs139529748	41.89152756
MAPK3	rs10876550	30.99069232
MAPK3	rs117198781	37.72453092
MAPK3	rs451689	32.82981873
HDAC8	rs10922098	382.3196647
HDAC8	rs71352239	48.64267621
HDAC8	rs72952553	30.06585308
HDAC8	rs1707652	205.5320164
HDAC8	rs28573154	102.1284181
HDAC8	rs7774739	69.92371747
HDAC8	rs9271766	36.65940922
KPNA2	rs1443264	36.08687128
KPNA2	rs10799536	45.22274031
KPNA2	rs2477639	42.08274414
KPNA2	rs757081	170.6774571
KPNA2	rs2283255	30.78755261
KPNA2	rs7113443	30.44009656
KPNA2	rs2606725	48.5930102
KPNA2	rs117317284	53.11260101
KPNA2	rs78169818	34.43197477
KPNA2	rs76428106	127.9532017
KPNA2	rs6573880	38.05102445
KPNA2	rs202117421	33.85337614
KPNA2	rs2071408	40.7919067
KPNA2	rs7500966	45.85713479
KPNA2	rs1864163	48.88469973
KPNA2	rs117405202	77.39569111
KPNA2	rs35892171	460.8086334
KPNA2	rs147850744	173.8750161
KPNA2	rs7212738	169.4905835
KPNA2	rs143977287	42.84020907
KPNA2	rs145433937	315.1558503
KPNA2	rs4545885	223.4719836
KPNA2	rs75380888	61.7861337
KPNA2	rs138839548	70.58734697
KPNA2	rs61242663	54.97527718
KPNA2	rs8102236	84.04794888
KPNA2	rs6544659	73.38665866
KPNA2	rs3790316	96.20633353
KPNA2	rs2305637	178.1383943
KPNA2	rs13100173	32.22911543
KPNA2	rs1687481	69.77511284
KPNA2	rs34198449	106.0380026
KPNA2	rs183264458	39.74735249
KPNA2	rs3130978	35.62336203
KPNA2	rs601148	40.70380898
KPNA2	rs6796	60.93671046
KPNA2	rs10107630	143.8640795
MAP2K1	rs34591673	47.42554725
MAP2K1	rs200596294	36.85846154

MAP2K1	rs4980320	33.57715171
MAP2K1	rs1354034	80.59632381
RAD51	rs145439832	31.92861541
RAD51	rs114558472	34.79978617
RAD51	rs72748420	41.37348118
RAD51	rs1329424	1358.12526
RAD51	rs3900909	62.84797154
RAD51	rs10801660	40.67501633
RAD51	rs9572914	30.8974019
RAD51	rs1047286	39.61320225
RAD51	rs111696008	34.63395705
RAD51	rs1707652	243.6400995
RAD51	rs73169117	30.64380218
RAD51	rs1265941	124.9773048
RAD51	rs550671	353.4646044
RAD51	rs112164276	51.64763502
RAD51	rs41548618	51.51566277
RAD51	rs3747843	31.18646549
TOP1	rs10922094	129.5978025
TOP1	rs543798	53.04575614
TOP1	rs9501600	32.6995496
YES1	rs10922094	377.0263275
YES1	rs1707652	62.47821905
YES1	rs550671	104.4529507
YES1	rs28471056	40.22231148
SERPINA3	rs10142728	59.43585361
SERPINA3	rs61976127	582.3228598
SERPINA3	rs75498941	83.83824033
C7	rs10941528	932.8471466
C7	rs4957147	348.2568795
C7	rs1838496	893.5667833
C7	rs72746986	97.12970861
C7	rs424535	37.63744812
C7	rs2436670	45.49425267
C7	rs2461161	52.89917124
C7	rs13186153	30.26029264
C7	rs71610306	120.5291991
C7	rs137939567	47.44012948
C7	rs11959586	30.22353717
C7	rs75134610	104.4004537
C7	rs139626890	37.26814383
C7	rs17829979	59.40234852
C7	rs183910448	50.67744035
C7	rs115418828	45.03408846
CTF1	rs10922098	276.2692621
CTF1	rs62269916	40.70679341
CTF1	rs1706435	73.51839803
CTF1	rs28573154	81.79930076
CTF1	rs7774739	60.12351097
CCL28	rs7703662	45.01743562
CCL28	rs139708128	35.38485719
CCL28	rs10886430	55.72658948
CCL28	rs73000929	200.3278253
CCL28	rs12829502	93.89908956
CCL28	rs11451044	265.554258
CCL28	rs10861891	49.74735649
CCL28	rs17712391	35.88968238
CCL28	rs10166836	660.7226543

CCL28	rs72956630	47.26026876
CCL28	rs17537823	31.93157757
CCL28	rs11701223	63.40559287
CCL28	rs3792386	30.47546599
CCL28	rs9842051	63.78004912
CCL28	rs274555	40.46140258
CCL28	rs1064701	36.55695389
CCL28	rs7758845	65.17339941
CCL28	rs10980560	31.26943344
CCL28	rs576123	226.2280511
CCL28	rs11534419	32.01278206
CCL14	rs76492376	68.84442325
CCL14	rs2928997	57.25829333
CCL14	rs11654215	146.3073205
CCL14	rs76190126	1009.784569
CCL14	rs4796153	41.76627571
CCL14	rs13962	53.00344386
CCL14	rs66707022	53.5649968
CCL14	rs182108045	41.95842786
CCL14	rs7218931	38.6783913
CCL14	rs6993770	29.95514327
IL4	rs10922098	89.20021545
IL4	rs6542680	35.21342144
IL4	rs415620	35.17489671
IL4	rs7774739	30.86245561
MDK	rs59980817	34.05973585
MDK	rs77817298	122.9187052
MDK	rs1434282	50.42728577
MDK	rs7073753	32.01978862
MDK	rs10769570	52.35328196
MDK	rs77565778	39.79210481
MDK	rs7137828	40.30052187
MDK	rs6915422	39.14364913
MDK	rs16873402	33.9750169
CCL23	rs4796109	283.4544038
CCL23	rs78655233	297.1781676
CCL23	rs41341749	755.0971229
CCL23	rs7207185	435.7722486
CCL23	rs1984769	44.53709924
CCL23	rs12951344	34.77052303
CCL23	rs62407169	39.18298754
SERPINE1	rs6963607	38.45536914
SERPINE1	rs10953324	67.55577735
SERPINE1	rs4759076	32.57639687
SERPINE1	rs1354034	44.12895997
SERPINE1	rs114694170	48.02080511
SERPINE1	rs6993770	92.03719042
SERPINE1	rs60757417	38.20006026
ARTN	rs10922098	47.31293016
ARTN	rs7080536	44.79362505
ARTN	rs77774564	90.553256
ARTN	rs77065856	32.91114278
CYCS	rs10772424	62.18209789
CYCS	rs8105192	263.8268028
CYCS	rs12975366	64.6694322
CYCS	rs3020644	36.96837659
CYCS	rs6458	41.79542008
CYP3A4	rs11591147	34.82786996

CYP3A4	rs602633	42.51970236
CYP3A4	rs10922094	45.78258088
CYP3A4	rs964184	148.9499431
CYP3A4	rs3764261	51.22474742
CYP3A4	rs6511720	57.63357834
CYP3A4	rs7412	121.5966137
CYP3A4	rs62122484	73.89575605
CYP3A4	rs4665972	51.56474929
CYP3A4	rs1707652	44.87037863
CYP3A4	rs11755689	43.79422487
CYP3A4	rs10096633	52.05112077
CYP3A4	rs112875651	46.74337038
NBL1	rs4911994	41.15227218
NBL1	rs12408663	37.38840943
NBL1	rs72704449	30.32621228
NBL1	rs10922098	34.67609239
NBL1	rs117042741	58.48521842
NBL1	rs35763136	55.7344363
NBL1	rs77924615	45.00325952
NBL1	rs62061425	44.46912541
NBL1	rs4002471	36.86949182
CFD	rs3746136	30.34099402
CFD	rs28591229	33.69157336
CFD	rs10469327	203.7739277
CFD	rs187211507	88.99460367
CFD	rs147416397	44.62949176
CFD	rs74520045	60.01352176
CFD	rs117778730	48.47702908
CFD	rs116940656	36.68568084
CFD	rs10922098	508.8137559
CFD	rs3900909	36.33144298
CFD	rs704	64.172231
CFD	rs6542680	39.3059277
CFD	rs1486236	128.142546
CFD	rs1707652	71.9099398
CFD	rs1357134	69.89984401
CFD	rs453821	108.9297383
CFD	rs28471056	55.66546681
GHR	rs17619781	66.00678473
GHR	rs4610468	461.3920018
GHR	rs140651910	35.18850943
GHR	rs10454866	54.8665811
GHR	rs429358	46.67456165
GHR	rs1260326	57.33890251
GHR	rs115231505	31.42306609
GHR	rs56088330	51.66599013
GHR	rs28601761	71.06304165
GHR	rs10758693	30.24460089
PLA2G10	rs1329424	269.9229394
PLA2G10	rs1355538	83.57430184
PLA2G10	rs2075798	37.28800583
PLA2G10	rs453821	89.89634495
IGFBP4	rs4890114	47.48902454
IGFBP4	rs2867105	29.84151528
IGFBP4	rs11127048	41.71949008
IGFBP4	rs79149713	40.83593012
IGF1	rs10922098	216.376117
IGF1	rs1113480	31.04734907

IGF1	rs201862321	44.64224243
IGF1	rs17205365	31.59988067
IGF1	rs74004837	92.33760591
IGF1	rs373942084	56.75843254
IGF1	rs11647990	40.89563117
IGF1	rs9930725	87.94160226
IGF1	rs9806893	58.88587259
IGF1	rs2686395	45.1042818
IGF1	rs9874429	31.18557977
IGF1	rs7774739	31.96263555
IGF1	rs7742632	61.52105831
IGF1	rs924140	40.71410397
CFP	rs70620	259.3312483
CFP	rs11188446	40.53340428
CFP	rs2305280	38.41075554
CFP	rs2230199	31.89113051
CFP	rs7439493	34.94810634
CFP	rs9332739	170.3753032
CFP	rs114382848	151.7047949
PROC	rs61185143	315.0657311
PROC	rs111339203	37.28647234
PROC	rs660240	74.60572156
PROC	rs4665972	56.08175208
PROC	rs74726059	49.47886143
PROC	rs77425280	173.7605654
PROC	rs75531392	33.85010035
PROC	rs35291022	1383.997308
PROC	rs112021223	773.0134937
PROC	rs73109575	110.0926628
PROC	rs6028703	40.82295107
PTHLH	rs33092	42.58069646
PTHLH	rs11049365	188.3784238
PTHLH	rs7301752	59.27423932
PTHLH	rs10922098	150.1026193
PTHLH	rs2231495	144.8875975
PTHLH	rs1355538	30.35142238
CLEC11A	rs117143947	39.05487594
CLEC11A	rs116919905	37.58575642
CLEC11A	rs41275788	963.2993388
CLEC11A	rs1975032	193.5966399
CLEC11A	rs9970784	33.46116567
CLEC11A	rs17345153	35.04576973
CLEC11A	rs76263184	33.36139418
CLEC11A	rs76239001	41.23946181
CLEC11A	rs112725054	51.81115964
CLEC11A	rs201862321	105.396461
CLEC11A	rs76428106	39.00352821
CLEC11A	rs17075907	36.54465801
CLEC11A	rs6573880	40.28272285
CLEC11A	rs8006994	44.723999
CLEC11A	rs36102158	58.41589749
CLEC11A	rs3830847	143.1848469
CLEC11A	rs11043	31.84003634
CLEC11A	rs116853700	47.27451463
CLEC11A	rs139675927	33.34632723
CLEC11A	rs62180989	37.47647896
CLEC11A	rs2236252	38.2998698
CLEC11A	rs2305637	79.73476923

CLEC11A	rs150918492	47.46556324
CLEC11A	rs28436034	30.7920701
CLEC11A	rs2380606	30.2963171
CLEC11A	rs10107630	76.85810479
CLEC11A	rs11144926	31.12082159
VCAM1	rs55971447	33.89833723
VCAM1	rs10922094	169.8562241
VCAM1	rs7086627	70.46671834
VCAM1	rs10902228	46.44675385
VCAM1	rs3184504	240.2364154
VCAM1	rs62061425	55.03383739
VCAM1	rs62165726	168.3165102
VCAM1	rs1257205	35.55594412
VCAM1	rs1375493	38.85726004
VCAM1	rs9358948	35.62383429
VCAM1	rs3093949	56.36949168
VCAM1	rs200697214	29.96240248
VCAM1	rs76359966	30.45984792
VCAM1	rs41556615	74.57886265
VCAM1	rs187099314	78.23459172
AREG	rs4980320	40.03166036
AREG	rs35979828	31.08750048
AREG	rs1354034	39.04148196
BMP7	rs6123685	42.46303426
BMP7	rs4911994	67.28821982
BMP7	rs12770498	31.90158787
BMP7	rs11236200	45.10032135
BMP7	rs11607100	162.6340951
BMP7	rs35763136	137.8963846
BMP7	rs35887873	30.80841452
BMP7	rs55714927	55.79594485
BMP7	rs1354034	157.7456414
BMP7	rs1355538	51.33912376
BMP7	rs12208776	61.0678775
CD36	rs117334659	40.05541652
CD36	rs6961069	396.1928025
CD36	rs7896518	52.64238112
CD36	rs73000929	36.87746087
CD36	rs11064077	61.81207648
CD36	rs594793	53.42860264
CD36	rs2063439	43.66651294
CD36	rs7618405	142.8005864
CD36	rs11914713	36.87655715
CD36	rs13195816	44.05520205
CD36	rs78724130	73.72954234
CD36	rs6993770	85.81992408
CD36	rs11244024	45.3059952
CD36	rs11244038	951.2836322
CD36	rs11244084	147.6744007
CD36	rs3094377	36.97161252
CNTN1	rs7970063	68.54616042
CNTN1	rs4548696	456.699285
CNTN1	rs1838343	577.7035577
CNTN1	rs4575367	48.55424365
CNTN1	rs7303010	51.91433783
CNTN1	rs112753780	45.82017478
CNTN1	rs140118846	30.32173114
CNTN1	rs181707610	49.40181969

CNTN1	rs8060374	40.25436369
CNTN1	rs61261356	885.8134627
CNTN1	rs112474515	30.40582042
CNTN1	rs112502880	41.51076915
CNTN1	rs9378478	30.84103131
CNTN1	rs1814170	75.86534142
DSG1	rs72748420	43.4037963
DSG1	rs1329424	1163.448396
DSG1	rs3900909	54.77637501
DSG1	rs10801660	34.88046909
DSG1	rs1047286	52.49810877
DSG1	rs2686395	177.6656831
DSG1	rs1265941	106.3312901
DSG1	rs550671	281.3688957
DSG1	rs9269234	43.13709685
EDAR	rs17190813	167.9872137
EDAR	rs2378139	36.2462337
EDAR	rs11900990	301.6449851
EDAR	rs260693	288.4819549
EDAR	rs77304476	1342.927314
EDAR	rs13395699	64.83629209
EDAR	rs11441787	37.09331347
EDAR	rs35340377	32.84957636
EDAR	rs7073753	92.60226712
EDAR	rs1769758	31.43188554
EDAR	rs12767683	173.8388606
EDAR	rs652963	34.94500528
EDAR	rs1555405	34.75453303
EDAR	rs1820994	53.93973656
EDAR	rs74035509	94.83087541
EDAR	rs71395304	42.73905367
EDAR	rs1867852	105.959532
EDAR	rs62158765	30.64037844
EDAR	rs7575040	72.65528702
EDAR	rs78909033	37.73486438
EDAR	rs114694170	112.9029464
EDAR	rs342284	36.10853404
EDAR	rs6993770	292.9071284
EDAR	rs74458759	103.8596051
CXCL5	rs112776817	60.69462455
CXCL5	rs352045	327.9281071
CXCL5	rs10305748	32.344288
CXCL5	rs572515	242.7607977
CXCL5	rs10822155	84.10871064
CXCL5	rs11569393	39.81101175
CXCL5	rs1619994	46.96123535
CXCL5	rs9501393	64.27875364
CXCL5	rs7774739	46.88177186
LGALS4	rs883394	43.60890371
LGALS4	rs203688	32.61559049
LGALS4	rs576123	52.27312134
CXCL1	rs62309626	259.9232273
CXCL1	rs5859374	225.168301
CXCL1	rs115214168	928.1245449
CXCL1	rs11944104	328.2843005
CXCL1	rs140569632	464.8780279
CXCL1	rs10017141	88.13716386
CXCL1	rs10922098	35.11898259

CXCL1	rs11417460	51.82877386
CXCL1	rs712295	30.04041102
CXCL1	rs151107944	31.80209345
CXCL1	rs954645	29.95181123
CXCL1	rs900792	40.57659019
CXCL1	rs16845887	96.39624744
CXCL1	rs144828812	58.12271145
CXCL1	rs72651015	58.50608667
CXCL1	rs114448914	79.63597572
CXCL1	rs114087287	42.61181095
IL1R1	rs11885528	95.16442211
IL1R1	rs6706048	440.5602634
IL1R1	rs2540319	54.43411483
IL1R1	rs61830291	63.81787803
IL1R1	rs78705678	59.98386942
IL1R1	rs2477642	65.82863049
IL1R1	rs12945299	41.47426641
IL1R1	rs4580257	52.71170649
IL1R1	rs11125916	69.75107487
IL1R1	rs6728769	163.7938197
IL1R1	rs55709272	480.5857787
IL1R1	rs67525224	113.9723406
IL1R1	rs9864515	37.92816729
IL1R1	rs12496730	786.3531823
IL1R1	rs13071075	47.52716743
IL1R1	rs2524098	42.54398736
IL1R1	rs9483788	39.00112509
IL1R1	rs10901252	138.8042231
IL1R1	rs3094379	47.53224302
IL17RA	rs73376136	136.4384025
IL17RA	rs7290590	151.8266064
IL17RA	rs117511368	518.9999568
IL17RA	rs118166666	280.5707176
IL17RA	rs112401805	307.070744
IL17RA	rs2241044	6779.296526
IL17RA	rs7287672	870.3266335
IL17RA	rs5747024	230.9502816
IL17RA	rs5748995	37.72886091
IL17RA	rs56344867	51.28030397
IL18RAP	rs4851010	91.62697842
IL18RAP	rs10922098	182.8021032
IL18RAP	rs1355538	40.60017993
IL18RAP	rs28573154	63.29072515
IL18RAP	rs7774739	47.87182493
IL18RAP	rs9271766	30.3064658
IL1RL2	rs4850995	39.79775839
IL1RL2	rs112437560	36.69672892
IL1RL2	rs3917265	374.1434465
IL1RL2	rs10922098	81.30151229
IL1RL2	rs11417460	33.95663366
IL1RL2	rs2244608	43.87236411
JAM2	rs10922098	169.506273
JAM2	rs1355538	46.63868178
JAM2	rs9501600	58.07623762
JAM2	rs10901252	53.56464776
JAM3	rs4980320	40.0573898
JAM3	rs10876550	29.94939076
JAM3	rs1354034	38.86347795

LSAMP	rs112204698	54.80186254
LSAMP	rs115352526	63.07839975
LSAMP	rs13064391	32.5382019
LSAMP	rs1541871	312.1657162
LSAMP	rs141638402	120.4070838
LSAMP	rs13077639	30.77915259
LSAMP	rs72704449	58.09658713
LSAMP	rs61899363	30.17871354
LSAMP	rs11220464	33.9086509
LSAMP	rs12945299	55.80890596
LSAMP	rs4760	65.61534005
LSAMP	rs1260326	50.03170082
LSAMP	rs697742	34.6756283
LSAMP	rs3128981	41.6848661
MBL2	rs10823734	76.63044524
MBL2	rs10437383	278.2092706
MBL2	rs11002760	561.4780478
MBL2	rs12768073	1062.160762
MBL2	rs7475006	1005.000404
MBL2	rs150524062	94.25425828
MBL2	rs3942920	32.84554959
MBL2	rs2261695	179.9923667
MBL2	rs11595864	34.06864973
MBL2	rs10764055	61.61242122
MBL2	rs117944916	34.22293157
MBL2	rs113953295	30.80221058
MBL2	rs1916565	186.4907527
MBL2	rs34529023	123.9637946
MBL2	rs10825251	85.01359492
MBL2	rs117917923	73.40775849
MBL2	rs117573752	146.1004996
MBL2	rs61850494	53.37553617
MBL2	rs2578062	41.09057403
MBL2	rs10996306	47.57444705
MBL2	rs11519274	49.17518708
MBL2	rs1260326	86.59860742
MBL2	rs71503158	31.06969199
MBL2	rs2073925	49.43781476
MBL2	rs576123	851.988297
NCR3	rs697742	110.0592086
NCR3	rs6922431	296.3970344
NCR3	rs34579055	52.38643241
NCR3	rs55740958	86.87590801
NCR3	rs10922098	45.9922089
NCR3	rs3184504	49.38369305
PDCD1LG2	rs2026911	38.08966813
PDCD1LG2	rs143662700	40.02495794
PDCD1LG2	rs10975134	941.8241401
PDCD1LG2	rs7855354	214.8676188
PDCD1LG2	rs78168277	43.20127157
PDCD1LG2	rs1354034	31.87151176
PDCD1LG2	rs12496730	239.3584558
PDCD1LG2	rs9438	295.1384603
PDCD1LG2	rs8176743	77.7249567
PTPN1	rs10922094	119.3261371
PTPN1	rs185009887	30.84112025
PTPN1	rs1706435	80.97590239
PTPN1	rs3891366	36.68126808

SIGLEC9	rs2072689	915.1470193
SIGLEC9	rs12984853	7725.330888
SIGLEC9	rs34815972	6832.502573
SIGLEC9	rs11084067	206.338663
SIGLEC9	rs7258020	57.91778493
SIGLEC9	rs8101887	116.2651952
SIGLEC9	rs78410648	90.57092676
SIGLEC9	rs55714927	30.5941098
SIGLEC9	rs117524660	38.30321927
SIGLEC9	rs12493830	235.2777029
SIGLEC9	rs6905297	32.38157423
SIGLEC9	rs9272359	32.12075549
TGFBR3	rs59178722	114.3500388
TGFBR3	rs11801868	50.05259076
TGFBR3	rs4926870	34.5695337
TGFBR3	rs12733512	123.7937712
TGFBR3	rs61747728	73.72326828
TGFBR3	rs78705678	88.7868658
TGFBR3	rs2477642	81.70639492
TGFBR3	rs789744	29.81573785
TGFBR3	rs77924615	37.78917212
TGFBR3	rs229048	108.5294788
TGFBR3	rs113047164	181.0002831
TGFBR3	rs76788865	44.8936065
SERPINF2	rs11078596	306.0996501
SERPINF2	rs370199414	51.71274097
SERPINF2	rs72805763	31.15687899
SERPINF2	rs144211463	47.36691615
SERPINF2	rs2244608	41.9949678
SERPINF2	rs780093	34.81706043
FGF2	rs13115901	3202.366482
FGF2	rs13126167	360.622478
FGF2	rs1546249	145.4569993
FGF2	rs112777738	55.9879755
FGF2	rs7529925	33.21602769
FGF2	rs13062635	33.26814007
FGF2	rs142982609	63.16388648
FGF2	rs150013145	65.72312118
FGF2	rs6838706	91.57006694
FGF2	rs6993770	80.18012877
FGF2	rs3780420	31.34942504
CAST	rs6679677	60.00014751
CAST	rs2295616	41.48538561
CAST	rs735665	50.58410124
CAST	rs1958988	53.65025762
CAST	rs17580	47.65855134
CAST	rs1059216	150.2330816
CAST	rs2857383	56.25980485
CAST	rs2753516	33.14469201
CAST	rs11862962	33.95070884
CAST	rs62149378	36.74899075
CAST	rs72836320	33.53365075
CAST	rs567189	282.7684286
CAST	rs614004	47.35749655
CAST	rs79385566	36.82604209
CAST	rs6891054	40.44198087
CAST	rs139199587	58.85681608
CAST	rs116446171	75.53788576

CAST	rs1264457	77.1198081
CAST	rs2854003	34.2633845
CAST	rs1826962	37.68142099
CAST	rs2511737	75.63423812
CCL23	rs4796109	413.0913634
CCL23	rs117627558	436.6599521
CCL23	rs16971802	1211.269521
CCL23	rs41505146	148.8729082
CCL23	rs9906092	54.06997398
CCL23	rs1984769	54.64478246
CCL23	rs17664516	44.88626193
CCL23	rs55777621	31.7100232
CD209	rs34840745	35.78424229
CD209	rs73488470	39.93798023
CD209	rs55806425	65.69765749
CD209	rs10411016	87.24171381
CD209	rs4804774	1417.323275
CD209	rs629301	137.3645922
CD209	rs7956510	30.07963519
CD209	rs2986616	71.08440133
CD209	rs10441802	34.61034951
CD209	rs62578600	45.82265089
CD209	rs8193007	62.63054895
CD209	rs2073926	690.6479042
CD209	rs12378537	4238.826029
CD209	rs11244084	968.2425215
CD209	rs143037947	104.9223851
CD209	rs3094377	332.9941401
CD209	rs10821524	51.37633175
CD209	rs36093130	49.38003423
CD209	rs139562936	60.79921058
CLEC4M	rs10922094	358.3319116
CLEC4M	rs12366520	54.04343051
CLEC4M	rs1707652	143.3361683
CLEC4M	rs1144709	44.70396434
CLEC4M	rs550671	139.8262347
CLEC4M	rs9469195	70.38804593
CLEC4M	rs74444206	31.69630988
LGALS2	rs5756736	672.1878176
LGALS2	rs7511061	170.4070001
LGALS2	rs144402192	59.24273141
LGALS2	rs3184504	31.95778543
LGALS2	rs704	29.95692641
LGALS2	rs6542680	89.33277184
GFAP	rs10922098	35.21276461
GFAP	rs1141720	44.64967862
GFAP	rs71549272	31.39019236
IL19	rs61801021	41.73951444
IL19	rs146457388	64.91748224
IL19	rs193165623	64.91210774
IL19	rs80063315	49.37631274
IL19	rs114288983	281.5420388
IL19	rs4850046	5459.871714
IL19	rs4423609	250.8421673
IL19	rs80220851	159.6566235
IL19	rs72769401	62.56703434
IL19	rs2198531	122.4007623
IL19	rs189700862	39.91625372

IL19	rs698092	59.48339484
IL1B	rs146457388	77.43043805
IL1B	rs17441745	75.0475897
IL1B	rs6542680	1246.679802
IL1B	rs138936322	72.96311105
IL1B	rs145976248	31.74629674
IL1B	rs3105782	39.11808784
CXCL11	rs11729174	36.94422289
CXCL11	rs71657382	89.36085423
CXCL11	rs11939003	964.9674346
CXCL11	rs72860111	155.6392698
CXCL11	rs6822200	57.16448536
CXCL11	rs12075	78.26766862
CXCL11	rs10822155	168.2379321
CXCL11	rs12767683	241.4901218
CXCL11	rs595585	44.11455096
CXCL11	rs6580981	42.42086271
CXCL11	rs3184504	150.8228109
CXCL11	rs4783187	59.87169886
CXCL11	rs114694170	47.59587078
CXCL11	rs55792153	34.17024423
CXCL11	rs9501587	34.61827588
CXCL11	rs406012	86.40141565
CXCL11	rs342287	34.94048578
CXCL11	rs6993770	88.59017047
CXCL11	rs35140383	44.96202545
CXCL11	rs111425801	37.20210946
CXCL11	rs117150187	32.13360219
CCL3	rs144476978	32.56022884
CCL3	rs854636	918.8587189
CCL3	rs12950560	118.6457396
CCL3	rs1065853	33.0045987
CCL3	rs62140208	52.08553775
CCL3	rs6029125	35.28756547
CCL3	rs9272780	30.14727765
MRC2	rs12938171	43.25669276
MRC2	rs150930997	116.4789174
MRC2	rs146385050	377.5094022
MRC2	rs111736142	371.7214211
MRC2	rs181242111	38.61910283
MRC2	rs692719	130.166952
MRC2	rs34534206	134.3409695
MRC2	rs35887873	125.1608057
MRC2	rs55714927	124.837816
MRC2	rs143501783	66.78697575
MRC2	rs117336153	56.15960901
MRC2	rs1260326	43.96050591
MB	rs16865583	33.59865278
MB	rs6464165	31.11324164
MB	rs12898	103.2784843
MB	rs621279	38.2361093
SPARC	rs145436966	30.39521005
SPARC	rs715440	33.13511178
SPARC	rs12468189	31.03982231
SPARC	rs1354034	59.77695145
SPARC	rs5008180	38.21323671
SPARC	rs6993770	53.44182541
CCL18	rs2526333	130.9679018

CCL18	rs2735476	1192.717927
CCL18	rs12450552	605.5045947
CCL18	rs76731769	47.46661187
CCL18	rs114282886	31.49143724
CCL18	rs11640599	44.29535113
CCL18	rs36105864	31.9775753
CCL18	rs75223909	69.09483509
CCL18	rs1065853	59.39332621
CCL18	rs71408075	87.13069765
CCL18	rs7570219	183.1352605
CCL18	rs56003568	49.67383673
CCL18	rs16989081	33.06282019
CCL18	rs115216147	58.07025513
CCL18	rs9369324	30.80619789
CCL18	rs72759286	41.9719956
PTN	rs78084707	32.5177348
PTN	rs320682	558.1771939
PTN	rs62490463	95.88661007
PTN	rs9458012	112.7358592
PTN	rs576123	38.00295574
RETN	rs117916280	97.40687114
RETN	rs137964046	192.9607482
RETN	rs117029024	554.6415757
RETN	rs3745368	873.0990933
RETN	rs112736139	41.34856402
RETN	rs419102	38.89422706
RETN	rs3917932	37.01984045
RETN	rs141094656	55.68258541
RETN	rs200008520	38.28848938
RETN	rs2074038	38.38384495
RETN	rs3859194	66.0716252
RETN	rs11078930	139.3356159
RETN	rs13390874	81.88315135
RETN	rs112694524	43.07675075
RETN	rs4811689	101.5253822
RETN	rs243596	54.28336959
RETN	rs56185965	37.38789523
RETN	rs150918492	75.06053358
RETN	rs1144700	34.78365884
RETN	rs139719552	34.90636135
RETN	rs149110519	304.0228336
RETN	rs138604055	42.66234143
RETN	rs2168589	42.31539923
RETN	rs445	33.35167736
RETN	rs10103048	66.65024515
PRSS1	rs62470638	58.63640529
PRSS1	rs61200828	34.62033006
PRSS1	rs35311109	34.22350186
PRSS1	rs1164032	37.18684647
PRSS1	rs4655303	31.79423034
PRSS1	rs2163150	54.3419016
PRSS1	rs8022656	53.41239043
PRSS1	rs77924615	33.34209652
PRSS1	rs8051037	132.7605523
PRSS1	rs533406	190.5268772
PRSS1	rs889573	44.55279968
PRSS1	rs56850260	83.15693182
PRSS1	rs12969748	34.51534405

PRSS1	rs679574	54.16735136
PRSS1	rs12975366	31.68802178
PRSS1	rs66473811	53.25658582
PRSS1	rs71606161	32.95204673
PRSS1	rs6812832	41.59146267
PRSS1	rs1591805	84.65775712
PRSS1	rs4733612	69.45487857
PRSS1	rs1633754	32.98433963
PRSS1	rs635634	94.59788896
VWF	rs2283335	181.6920604
VWF	rs199765497	109.4256781
VWF	rs201862321	45.31682092
VWF	rs868876	42.14790318
VWF	rs2523468	33.9724893
VWF	rs566028	37.71656842
VWF	rs72759424	32.1094785
VWF	rs2073925	87.69586413
VWF	rs576123	763.0525972
FASLG	rs4934436	70.0498765
FASLG	rs11065979	57.67743539
FASLG	rs17580	43.64496436
FASLG	rs4683097	88.93293949
FASLG	rs184852236	402.9439842
FASLG	rs73074378	1404.817827
FASLG	rs193190126	102.1573465
FASLG	rs115261880	306.6158203
FASLG	rs115680081	38.90426689
FASLG	rs148999878	94.01615975
FASLG	rs112238071	61.85397693
FASLG	rs184102325	230.126383
FASLG	rs147660324	45.33953947
FASLG	rs113685194	153.6591326
FASLG	rs9266329	40.39725626
HP	rs2288489	957.6323294
HP	rs34400689	4094.425624
HP	rs61452478	1098.997328
HP	rs146493111	33.71542077
HP	rs113611363	115.8765481
HP	rs1837095	30.03899158
HP	rs142504786	36.36957652
HP	rs190319586	31.62174327
HP	rs76671618	32.27767101
HP	rs78117461	41.11310532
HP	rs111506490	65.08513044
HP	rs2287970	83.17263183
HP	rs2168608	1183.38733
HP	rs71388958	117.2640686
HP	rs6564524	190.1026804
HP	rs12920453	57.86478205
HP	rs72789148	100.1559091
HP	rs146712620	53.48504696
HP	rs12975366	98.39954335
HP	rs1260326	46.5050665
TNFSF13B	rs80291289	33.08695496
TNFSF13B	rs113469631	57.83874959
TNFSF13B	rs6492108	34.81835177
TNFSF13B	rs10922103	64.06593291
TNFSF13B	rs3184504	54.62445405

TNFSF13B	rs11552708	98.23095336
TNFSF13B	rs62078112	30.35377591
TNFSF13B	rs73165139	43.37756944
TNFSF13B	rs686250	63.40590908
C9	rs115096222	37.87703546
C9	rs34803949	117.2313854
C9	rs114472213	118.3984788
C9	rs116351960	97.66903857
C9	rs62358178	163.5810457
C9	rs628745	29.81133027
C9	rs4393147	35.72824208
C9	rs78525930	33.67468525
C9	rs13409371	44.3878155
C9	rs13048321	39.75184632
C9	rs116601579	35.995195
C9	rs116659723	38.43502357
C9	rs9268557	32.86651138
FGF5	rs10922094	47.2640203
FGF5	rs6542680	172.4529619
FGF5	rs2073044	40.1723396
LGALS3	rs56110021	37.88194159
LGALS3	rs76790102	2286.300957
LGALS3	rs141229230	1370.44631
LGALS3	rs74050939	2200.179499
LGALS3	rs117293406	83.57992659
LGALS3	rs112771035	51.72739437
LGALS3	rs1169283	82.67874223
LGALS3	rs2701189	55.96980272
LGALS3	rs79288025	32.51185982
LGALS3	rs74326663	30.31867107
LGALS3	rs17855739	90.54935663
LGALS3	rs778972	31.08854991
LGALS3	rs516246	142.3684487
LGALS3	rs12986064	32.35334082
LGALS3	rs13217434	36.54407384
LGALS3	rs11769828	93.65401698
IL2	rs1260326	71.8447796
IL2	rs139276089	44.0567988
IL2	rs2731673	69.9798585
IL2	rs3129773	33.40036162
IL2	rs799169	35.9222977
IL13	rs10922098	235.8903419
IL13	rs6445035	57.66466993
IL13	rs2242572	72.927558
IL13	rs9272068	31.99152076
IL18BP	rs10158500	31.42793213
IL18BP	rs61747728	52.97351767
IL18BP	rs652534	30.68882453
IL18BP	rs3184504	126.4189076
IL18BP	rs12945299	79.47340656
IL18BP	rs143501783	43.95437909
IL18BP	rs9276601	36.75281996
IL18BP	rs8176743	111.8686326
LBP	rs45589832	2750.72856
LBP	rs2232616	767.3590018
LBP	rs73095812	451.435848
LBP	rs4148008	33.8488495
LBP	rs11696919	31.13998781

LBP	rs78037996	33.81752438
LBP	rs112552581	29.95803763
LBP	rs147273341	36.10296915
LBP	rs149644685	366.6610253
LBP	rs16985185	185.3270548
LBP	rs76462512	83.75044622
LBP	rs75092000	34.21072015
LBP	rs76460856	53.91401225
LBP	rs117767378	70.02233009
LBP	rs1707652	66.22292527
F10	rs8002283	36.84673955
F10	rs3211757	339.1781179
F10	rs5960	162.0949424
F10	rs10761737	42.38800597
F10	rs1260326	55.71392996
F10	rs139189391	39.02187409
F10	rs211833	29.79111422
F10	rs184624754	56.9214371
F10	rs6946689	39.26016455
F10	rs2954021	42.99418515
F10	rs2306956	51.82886735
F10	rs1623718	68.90840764
F10	rs11793434	40.67749527
F10	rs113354603	740.0395315
F10	rs10982820	32.78816477
PGF	rs203688	71.26668482
PGF	rs118062631	31.37514925
PGF	rs188302747	31.86478681
RARRES2	rs883138	723.9166158
RARRES2	rs12673713	44.12252177
RARRES2	rs138246410	32.2136675
RARRES2	rs115713183	30.79633682
RARRES2	rs11642015	41.24426281
RARRES2	rs2087727	50.46265187
RARRES2	rs138480141	31.01464117
RARRES2	rs147906938	54.87972618
RARRES2	rs9501602	40.76515103
RARRES2	rs111675168	30.45138604
EDA2R	rs10922094	50.49729319
EDA2R	rs6542680	150.5992219
EDA2R	rs28455809	35.85524433
AURKA	rs10922098	306.1896315
AURKA	rs11569415	40.35145388
AURKA	rs1707652	107.8814692
AURKA	rs2242572	81.60649234
AURKA	rs9501602	42.41071137
DIABLO	rs704	56.16614553
DIABLO	rs55749024	39.70259616
DIABLO	rs11932097	30.32742947
DIABLO	rs1718860	157.1450718
DIABLO	rs9274470	35.77842118
CD4	rs61916271	38.78823559
CD4	rs10922098	99.67374709
CD4	rs7137828	30.62911718
CD4	rs28672722	38.06665197
IL2RA	rs12722497	86.09829326
IL2RA	rs9268659	46.23039882
IL2RA	rs72508422	35.94745141

TNFRSF1B	rs2301258	170.8051361
TNFRSF1B	rs61747728	60.69404638
TNFRSF1B	rs3184504	45.19065813
TNFRSF1B	rs77924615	37.25406639
TNFRSF1B	rs7207683	46.41073937
TNFRSF1B	rs13108218	30.22051206
TNFRSF1B	rs3093949	79.75660498
TNFRSF1B	rs41547217	33.6266828
CD33	rs117711212	65.68602409
CD33	rs67621164	225.6779524
CD33	rs1846934	1177.469492
CD33	rs2459147	4533.320718
CD33	rs71358836	218.1214713
CD33	rs62114983	41.25024011
CD33	rs6509563	152.3152747
CD33	rs12459518	30.91294357
CD33	rs76920575	41.10619245
CD33	rs16983230	97.09679266
CD33	rs10823375	74.25253045
CD33	rs73021419	35.41795397
CD33	rs55714927	122.1656529
CD33	rs17880604	58.80946116
CD33	rs13059624	77.84680022
CD33	rs11920688	35.45314547
CD33	rs12055445	37.93030622
CD33	rs1042133	33.18421526
ADAMTS5	rs59317497	106.4181455
ADAMTS5	rs2830493	49.09804676
ADAMTS5	rs151064	2714.640473
ADAMTS5	rs2830627	40.82971784
ADAMTS5	rs10922098	93.88288346
ADAMTS5	rs145764536	32.87110516
IDUA	rs148339463	58.56810326
IDUA	rs114480470	121.0761377
IDUA	rs56083715	929.1398341
IDUA	rs748650	2640.363831
IDUA	rs6659116	30.43492479
IDUA	rs3999089	46.75614238
IDUA	rs825038	43.28875943
IDUA	rs7964859	568.4209043
IDUA	rs140624338	69.0319239
IDUA	rs2244608	30.8408056
IDUA	rs139974673	101.2371456
IDUA	rs56252442	46.14833016
IDUA	rs200210321	47.55973497
IDUA	rs10414043	57.96204111
IDUA	rs1883711	44.03903712
IDUA	rs4678322	41.00010031
IDUA	rs141617671	93.40696334
IDUA	rs143583733	77.65372666
IDUA	rs10009405	34.59514538
IDUA	rs185408056	30.39887485
IDUA	rs4698935	40.29130325
METAP2	rs4980320	47.3633147
METAP2	rs10405535	111.2630281
METAP2	rs141714582	49.10562347
METAP2	rs78909033	59.20000904
METAP2	rs1354034	115.4438048

METAP2	rs4541868	40.19442586
APP	rs439826	78.60821901
APP	rs10822155	85.42030136
APP	rs2229498	222.5559886
APP	rs61658003	46.04890484
APP	rs1071960	29.7238537
ARSB	rs11743362	276.0514482
ARSB	rs142106284	43.98805792
ARSB	rs1329424	226.5502285
ARSB	rs2231495	183.9199153
ARSB	rs555007	33.59525801
ARSB	rs389512	68.36581173
NAAA	rs72865578	91.11888558
NAAA	rs143385852	47.40538098
NAAA	rs80283376	315.2190596
NAAA	rs66646795	946.9424462
NAAA	rs74617719	605.9886048
NAAA	rs71659312	922.6655456
NAAA	rs72655528	144.8042183
NAAA	rs11728135	59.65877158
NAAA	rs12727056	41.11415907
NAAA	rs220040	34.75010203
NAAA	rs3956912	54.9400147
NAAA	rs34539738	48.64774241
NAAA	rs187229204	38.88641584
NAAA	rs76391683	42.2476105
NAAA	rs11110781	58.05249554
NAAA	rs78386135	41.11863338
NAAA	rs2251298	1261.78475
NAAA	rs150043343	104.9031962
NAAA	rs7980436	451.416512
NAAA	rs73176937	62.84982393
NAAA	rs17092065	60.73368862
NAAA	rs113170157	36.08394058
NAAA	rs150844304	61.24896582
NAAA	rs373973590	33.96450071
NAAA	rs2696466	44.29156445
NAAA	rs150268548	60.09847573
NAAA	rs429358	66.10378427
NAAA	rs11668882	66.00256199
NAAA	rs1476698	38.73679659
NAAA	rs738409	43.55545586
NAAA	rs1711171	91.87872601
NAAA	rs144366191	44.11343354
NAAA	rs190823261	40.89843684
NAAA	rs77689210	36.48643961
NAAA	rs13124954	96.25340849
NAAA	rs141832481	39.76103224
NAAA	rs9991188	34.16701423
NAAA	rs139366576	78.30540041
NAAA	rs4471106	45.82954845
NAAA	rs55874246	48.41480368
ADAMTS13	rs111568723	41.20743649
ADAMTS13	rs75653292	246.1821563
ADAMTS13	rs145007118	39.60448854
ADAMTS13	rs41302905	994.1204421
ADAMTS13	rs62575963	979.5689292
ADAMTS13	rs111587591	916.4446099

ADAMTS13	rs138661900	50.87620702
ADAMTS13	rs2510238	110.8405896
ADAMTS13	rs117572863	31.64442181
ADAMTS13	rs112814955	56.10562088
ADAMTS13	rs1389065	35.03108973
ADAMTS13	rs12318001	31.12898321
ADAMTS13	rs9957318	45.22406064
ADAMTS13	rs56254331	34.5258598
ADAMTS13	rs3747207	41.22744174
ADAMTS13	rs1433210	61.23050136
ADAMTS13	rs6837794	34.85319968
ADAMTS13	rs2927608	74.61736481
ADAMTS13	rs7739314	31.53599358
ADAMTS13	rs111517155	50.18641253
CTSC	rs10922098	569.2939485
CTSC	rs11064498	43.8166454
CTSC	rs1707652	103.4607424
CTSC	rs550671	151.5070818
CTSC	rs28471056	66.2408108
CTSC	rs9272526	37.84076987
CTSA	rs826404	30.58667951
CTSA	rs7924036	97.79819021
CTSA	rs4883201	97.09726908
CTSA	rs35076711	33.46943007
CTSA	rs825038	64.60358746
CTSA	rs78386135	31.96299204
CTSA	rs7964859	855.3883637
CTSA	rs140624338	89.7854527
CTSA	rs2127868	38.7077334
CTSA	rs147233090	36.83983609
CTSA	rs1354034	34.83886732
CTSA	rs56055826	37.00762752
CTSA	rs78947501	57.1616732
CTSA	rs114694170	43.89950549
CTSA	rs4358518	35.15618659
CTSA	rs2563253	33.89184478
CTSA	rs9639523	33.97985883
CTSA	rs3929643	35.9597421
CTSA	rs6993770	165.4764077
CTSS	rs72692873	1228.409561
CTSS	rs6655975	511.8211678
CTSS	rs12139264	95.5651196
CTSS	rs10494247	30.10364498
CTSS	rs139020547	35.27636241
CTSS	rs1332327	51.81136972
CTSS	rs4919333	35.42312365
CTSS	rs12291142	34.74943459
CTSS	rs10896415	94.30932751
CTSS	rs2511241	91.20271916
CTSS	rs4883201	200.1558228
CTSS	rs876374	32.24095941
CTSS	rs12602829	53.82439711
CTSS	rs9890097	38.17773841
CTSS	rs7209589	30.52702933
CTSS	rs3865331	40.27530845
CTSS	rs67873985	32.07067457
CTSS	rs6727677	36.33874546
CTSS	rs9272207	35.60246392

CTSS	rs9457811	65.26360752
CTSS	rs576123	61.53159941
ENTPD1	rs10922098	756.5261623
ENTPD1	rs1047286	70.78791168
ENTPD1	rs2686395	104.6205735
ENTPD1	rs2075798	88.04951084
ENTPD1	rs550671	209.5382566
ENTPD1	rs9269234	33.23108261
ENTPD1	rs9272526	38.26506579
F7	rs79539941	58.6702658
F7	rs2317136	161.2362376
F7	rs497845	966.9364079
F7	rs80238657	808.7306245
F7	rs559054	266.4853733
F7	rs149440031	714.5135563
F7	rs7232	32.64369315
F7	rs371891062	52.22718355
F7	rs1260326	64.84635172
F7	rs112021223	79.8471194
F7	rs11904888	141.726884
C2	rs9468883	2494.17354
C2	rs4360170	2878.97398
C2	rs75802100	709.7076867
C2	rs71495289	30.01593691
C2	rs78705678	77.19841711
C2	rs2461161	78.46705278
C2	rs4418728	34.31963116
C2	rs139974673	33.89070702
C2	rs17569397	34.41173477
C2	rs199573340	59.39547985
C2	rs149492436	33.21778293
C2	rs34442159	95.54588419
C2	rs17184086	934.4342031
C2	rs9277939	215.5938359
C2	rs4616984	621.7360069
C2	rs1978042	176.1597477
C2	rs12663476	132.3107504
C2	rs150581397	176.5749989
C2	rs114391929	74.61783893
C2	rs111257239	36.00664479
C2	rs4240624	34.58207599
TMPRSS15	rs10922098	172.6224422
TMPRSS15	rs6542680	66.83075905
TMPRSS15	rs1355538	33.29040005
TMPRSS15	rs2072634	43.40757926
TMPRSS15	rs415620	62.02087467
TMPRSS15	rs7774739	55.19057044
WFIKKN1	rs55798945	313.1328367
WFIKKN1	rs116914290	119.6400874
WFIKKN1	rs10922098	29.93553645
WFIKKN1	rs3184504	47.09944229
WFIKKN1	rs4432538	57.38318401
WFIKKN1	rs34579055	40.9030627
WFIKKN1	rs116793716	62.68367682
WFIKKN1	rs6910859	30.39587098
CNDP2	rs10922098	679.934465
CNDP2	rs1047286	39.93938724
CNDP2	rs1707652	109.1639546

CNDP2	rs3130955	33.32322801
CNDP2	rs9501600	165.4391375
CNDP2	rs35209896	42.43991803
GP6	rs78187393	35.63803998
GP6	rs1671152	1056.218986
GP6	rs10409775	31.03874494
GP6	rs12981737	35.95494454
GP6	rs185490651	29.98275751
GP6	rs35340377	34.20305163
GP6	rs1354034	51.01752207
GP6	rs6993770	38.24097719
GP6	rs77265585	36.84199697
GNLY	rs75124059	68.09251713
GNLY	rs7595080	230.0582406
GNLY	rs1130866	282.069131
GNLY	rs3024798	1173.055271
GNLY	rs56054119	159.2391751
GNLY	rs191008487	137.6877072
GNLY	rs183053322	90.08020957
GNLY	rs607268	56.09188465
GNLY	rs10732976	43.08770157
GNLY	rs2497304	48.39944456
GNLY	rs61872201	47.51854237
GNLY	rs61751507	196.3778402
GNLY	rs10427049	40.96054574
GNLY	rs13028852	100.6403575
GNLY	rs113974174	110.6813301
GNLY	rs116052219	32.9118151
GNLY	rs142630510	63.89009855
GNLY	rs201045742	32.65711029
GNLY	rs6543485	71.79766585
GNLY	rs13017718	37.66593469
GNLY	rs4141634	30.26809352
GNLY	rs7620997	49.05171394
GNLY	rs3732477	34.68657984
GNLY	rs27295	102.0703363
GNLY	rs62407606	43.80614229
GNLY	rs713032	385.5190514
GNLY	rs28520918	52.74140228
GNLY	rs34718458	307.4727565
GNLY	rs79507667	61.96354083
GNLY	rs11993233	42.24283992
GNLY	rs10780319	32.4550936
GNLY	rs7040705	84.71239896
HAPLN1	rs191703636	109.2089209
HAPLN1	rs173053	59.56726297
HAPLN1	rs10039191	165.8011644
HAPLN1	rs10922098	45.49789495
HAPLN1	rs224916	32.01012351
HAPLN1	rs2544663	38.64485792
HAPLN1	rs10071051	62.14124494
IDE	rs1329424	180.8013528
IDE	rs1707652	108.8658186
IDE	rs9501600	54.08334913
IDS	rs12132412	40.43023059
IDS	rs10922098	42.06610773
IDS	rs10778149	162.6688663
IDS	rs7964859	398.6392891

IDS	rs7148704	33.01416037
IDS	rs9346804	46.8251808
IDS	rs9365124	42.84822024
KLK12	rs3745540	281.2055764
KLK12	rs72748420	39.87918885
KLK12	rs1329424	1272.268106
KLK12	rs3900909	62.22567915
KLK12	rs10801660	37.07238444
KLK12	rs61821527	33.08134405
KLK12	rs144579409	32.29230295
KLK12	rs1047286	44.28246501
KLK12	rs1707652	228.0092238
KLK12	rs2161196	30.20447618
KLK12	rs1265941	116.6644893
KLK12	rs550671	307.0855593
KLK12	rs112164276	50.0791756
KLK12	rs41548618	47.05287715
KLK12	rs3747843	29.85076164
KREMEN2	rs1329424	161.776834
KREMEN2	rs1706435	39.49413197
KREMEN2	rs28573154	53.09073419
KREMEN2	rs7774739	38.44846682
LTA4H	rs34665243	37.19838754
LTA4H	rs1329424	180.2013738
LTA4H	rs11720167	42.24251926
LTA4H	rs9501600	41.48524162
LYVE1	rs4910063	49.10229367
LYVE1	rs60362241	59.36789173
LYVE1	rs76318574	143.147017
LYVE1	rs72861085	68.78116526
LYVE1	rs12289747	58.5913564
LYVE1	rs114527818	454.4233097
LYVE1	rs333947	30.57607469
LYVE1	rs2394668	51.7340446
LYVE1	rs10769263	48.75355593
LYVE1	rs79376486	68.60212894
LYVE1	rs11216906	30.7090861
LYVE1	rs112771035	798.2590728
LYVE1	rs75071241	109.946792
LYVE1	rs35257264	57.19280717
LYVE1	rs35743748	44.56827627
LYVE1	rs1955512	112.8873958
LYVE1	rs117633773	54.17883899
LYVE1	rs12945299	209.4013661
LYVE1	rs143501783	88.57862998
LYVE1	rs12449331	39.94968111
LYVE1	rs12978875	32.8669901
LYVE1	rs7601767	37.50153008
LYVE1	rs61516276	75.90901218
LYVE1	rs10678265	39.22895694
LYVE1	rs7266553	96.60976721
LYVE1	rs9832668	31.51918039
LYVE1	rs2470870	56.83307205
LYVE1	rs9826842	39.34644566
LYVE1	rs1453869	33.79373956
LYVE1	rs2797335	59.00806895
LYVE1	rs2671424	34.42342788
LYVE1	rs199980439	35.88230143

LYVE1	rs7865362	273.6464213
LYVE1	rs56157435	42.25947103
LYVE1	rs700801	60.07749926
LYVE1	rs7856589	32.73445947
ASAH2	rs79439167	49.49148832
ASAH2	rs146622748	1208.386829
ASAH2	rs200610304	267.3612769
ASAH2	rs4935128	3795.96038
ASAH2	rs182892473	77.50006114
ASAH2	rs11005387	194.5474112
ASAH2	rs12763713	110.14666
ASAH2	rs116910690	60.24509646
ASAH2	rs182350853	172.8054169
ASAH2	rs144264152	164.3085974
ASAH2	rs12032634	56.57914824
ASAH2	rs203688	50.7454607
ASAH2	rs72797107	37.89397461
ASAH2	rs1628107	38.595534
ASAH2	rs11204288	65.12576394
ASAH2	rs35764600	118.9578381
ASAH2	rs35196633	45.24316004
ASAH2	rs11045856	78.13019612
ASAH2	rs12319043	38.70887275
ASAH2	rs8078686	74.67951352
ASAH2	rs77262773	57.18898559
ASAH2	rs34164492	57.37227105
ASAH2	rs34060476	33.38800399
ASAH2	rs7828742	69.54068198
NID1	rs144669411	35.17116703
NID1	rs143743775	40.58829679
NID1	rs17554536	67.91935571
NID1	rs58074293	379.9361736
NID1	rs642905	30.65644545
NID1	rs75519859	42.34842492
NID1	rs927826	329.4898339
NID1	rs4311960	37.89248327
NID1	rs7073753	41.84773663
NID1	rs1671152	32.94507056
NID1	rs548630	31.95187763
PIGR	rs748844	287.0318445
PIGR	rs34019521	31.78396501
PIGR	rs61418148	36.31432715
PIGR	rs708686	39.00423002
PIGR	rs2524080	58.42998065
PIGR	rs9398073	34.20307517
PIGR	rs6583438	32.40985579
PIGR	rs113745074	118.6804911
PIGR	rs75444660	38.80343803
RET	rs2795496	1101.436935
RET	rs752978	334.0095833
RET	rs2946992	29.92514899
RET	rs113834925	33.56571303
RET	rs116883658	57.17482484
RET	rs10909012	148.2133037
RET	rs71496658	47.49975683
RET	rs4278528	47.66571931
SFRP1	rs12681760	181.6421977
SFRP1	rs62642270	127.3815333

SFRP1	rs72643819	344.7597598
SFRP1	rs2990223	41.35042023
SFRP1	rs12051272	54.86850615
SFRP1	rs1981431	48.20173068
SFRP1	rs1053924	45.5094788
SFRP1	rs6994498	32.00879615
SFRP1	rs7817574	76.23566317
SFRP1	rs635634	145.3429023
ACP5	rs10403399	634.3817234
ACP5	rs5851	99.54512114
ACP5	rs61801013	49.19554539
ACP5	rs7910087	38.3988397
ACP5	rs4919420	40.66484307
ACP5	rs139196635	87.23338601
ACP5	rs80253746	35.29669514
ACP5	rs2073333	41.64906626
ACP5	rs77095127	37.41098924
ACP5	rs12459949	32.47194977
ACP5	rs76513382	35.50008571
ACP5	rs199602723	53.66734932
ACP5	rs34007487	49.77062217
ACP5	rs3780495	53.64924109
CCDC80	rs9845240	122.0563803
CCDC80	rs6797920	45.29157139
CCDC80	rs55714927	60.54022362
CCDC80	rs143501783	42.31355821
CCDC80	rs767298	43.90519739
CCDC80	rs2142306	130.3588216
CCDC80	rs2978006	67.22689851
CCDC80	rs73669307	62.55549406
CCDC80	rs10819325	33.61736043
WFIKKN2	rs1460212	86.01841374
WFIKKN2	rs36088946	219.1055078
WFIKKN2	rs4794183	1347.420638
WFIKKN2	rs9908461	32.95825099
WFIKKN2	rs2215316	183.4062658
WFIKKN2	rs12939752	40.74011308
WFIKKN2	rs72704449	30.50709032
WFIKKN2	rs9972653	37.03753182
WFIKKN2	rs71354106	54.92199445
WFIKKN2	rs4132385	38.9465501
ACAN	rs12441312	76.92382701
ACAN	rs34949187	173.184811
ACAN	rs10922098	65.11851483
ACAN	rs12153751	40.23718154
ACAN	rs9272799	39.18832154
ACAN	rs11144945	36.43033506
TGFBI	rs76221198	37.03239474
TGFBI	rs138605513	68.04702786
TGFBI	rs6892697	971.9609438
TGFBI	rs17750709	77.03603341
TGFBI	rs188992974	101.2536519
TGFBI	rs34199389	141.6435164
TGFBI	rs699	136.1937189
TGFBI	rs78705678	37.90383495
TGFBI	rs2393986	54.56049111
TGFBI	rs2255531	36.79404267
TGFBI	rs12952730	38.90115772

TGFBI	rs10052528	84.55959777
TGFBI	rs76926676	38.13060891
TGFBI	rs7810066	85.18967747
BGN	rs2902364	80.57080475
BGN	rs7942947	31.47267678
BGN	rs4981022	55.7461668
BGN	rs12318001	61.11972147
BGN	rs35878488	397.2435474
BGN	rs12444004	1035.39286
BGN	rs9922607	271.6233328
BGN	rs7203880	133.4508965
BGN	rs150018437	35.4914032
BGN	rs140493165	75.12461081
BGN	rs11641833	50.11973788
BGN	rs704	189.382461
BGN	rs8075656	30.52434504
BGN	rs2686395	40.25183144
BGN	rs11711157	71.77969264
BGN	rs61216603	48.15753102
BGN	rs9327541	42.83399298
BGN	rs6987335	35.5033798
C1R	rs113448098	492.8847582
C1R	rs139449440	30.88202991
C1R	rs7970720	992.0994088
C1R	rs75767107	79.29739371
C1R	rs2242511	31.88771364
C1R	rs183893251	32.35187643
C1R	rs28362950	223.1109769
C1R	rs7948571	64.10008453
C1R	rs150196765	34.4671308
C1R	rs61984231	45.57788671
C1R	rs12433460	417.1433413
CD109	rs200559724	130.1460621
CD109	rs7763514	289.3648187
CD109	rs76081669	130.778913
CD109	rs9350500	2985.940804
CD109	rs139277266	115.364198
CD109	rs28823351	95.58943381
CD109	rs138578493	367.6041061
CD109	rs61801021	42.43044559
CD109	rs506317	38.07589285
CD109	rs36040496	39.82634476
CD109	rs78705678	91.84820864
CD109	rs2477642	101.591399
CD109	rs55714927	47.32088983
CD109	rs2732617	31.93886753
CD109	rs868876	36.98434287
CD109	rs72961725	32.24700222
CD109	rs79495888	31.63681141
CD109	rs141698681	78.62785487
CD109	rs140072581	50.33042362
CD109	rs61313163	47.53323551
CD109	rs113501304	58.95626746
CD109	rs76232494	36.9427444
CD109	rs141909149	177.7175963
CD109	rs140383761	59.40753478
CD109	rs188774024	41.68816573
CD109	rs117247182	34.22163299

CD109	rs143249580	50.36796071
CD109	rs10241439	32.37645614
CD109	rs62578578	49.25230485
CD109	rs8176693	868.4119097
CD109	rs739468	279.4398024
CD109	rs10123394	29.88055924
FCER2	rs78903647	35.75750128
FCER2	rs12971319	934.3803261
FCER2	rs12980031	851.8627164
FCER2	rs79083213	123.9765794
FCER2	rs4948492	49.9479903
FCER2	rs12413053	41.13915033
FCER2	rs3809088	36.44960269
FCER2	rs11614785	31.68541484
FCER2	rs73217470	30.32397514
FCER2	rs2969921	38.85579831
FCER2	rs8030299	35.22797709
FCER2	rs13333054	39.35365789
FCER2	rs12944882	97.38776639
FCER2	rs55737395	39.78997065
FCER2	rs1883832	46.83071512
FCER2	rs73165110	63.60231096
FCER2	rs113286101	41.27516282
FCER2	rs717255	40.6439721
FCER2	rs700588	31.80979197
FCER2	rs77018303	30.24727577
FCER2	rs58889466	45.97992117
FCER2	rs1986997	76.83019064
FCER2	rs77114005	88.79472606
FCER2	rs1318232	50.4262952
FCER2	rs9387400	109.1107191
FCER2	rs7840212	51.68555907
CD48	rs41266895	117.0631876
CD48	rs114547897	483.4804969
CD48	rs1980606	662.0493662
CD48	rs6427541	96.65276703
CD48	rs10908821	61.92737698
CD48	rs188662236	37.58135507
CD48	rs78689694	45.05496158
CD48	rs3184504	154.4401292
CD48	rs12945299	147.3595342
CD48	rs143501783	49.06913698
CD48	rs112058117	45.22467795
CD48	rs2659005	66.57226586
CD48	rs1260326	33.21224755
CD48	rs9810602	30.96441624
CD48	rs13108218	29.84169079
CD48	rs148844907	32.24805042
CD48	rs451681	38.56388488
CD5L	rs4971154	964.2896851
CD5L	rs113224872	110.9329942
CD5L	rs115231584	44.38113975
CD5L	rs11265044	62.14830363
CD5L	rs16830188	130.4862199
CD5L	rs7918238	38.82705425
CD5L	rs3184504	111.3137753
CD5L	rs1134590	31.58826058
CD5L	rs72637119	36.72532861

CD5L	rs34824568	83.96450397
CD5L	rs116891509	42.68337027
CD5L	rs9930957	57.66906676
CD5L	rs9901675	41.17963959
CD5L	rs2532362	31.52725065
CD5L	rs2950706	35.49484803
CD5L	rs139015452	39.92224636
CD5L	rs13043452	61.72991816
CD5L	rs872071	34.76450706
CD5L	rs1264457	117.58758
CD5L	rs2769	42.37623576
CD5L	rs9270152	74.47479661
CD5L	rs34281918	31.91234371
CD5L	rs12527949	29.95507274
CD5L	rs3807307	69.39051342
CD5L	rs73169649	72.89265589
CD5L	rs2436888	29.91834107
CNTN2	rs16854023	153.3354764
CNTN2	rs79763013	68.05608311
CNTN2	rs2802840	924.2398799
CNTN2	rs3903399	1089.944934
CNTN2	rs12067235	822.3124374
CNTN2	rs12119834	116.8499355
CNTN2	rs1409153	35.12497578
CNTN2	rs6663303	59.48339408
CNTN2	rs111528448	55.99081035
CNTN2	rs2477639	88.12496
CNTN2	rs692440	54.77059372
CNTN2	rs35527562	47.85060916
CNTN2	rs2012725	31.58878827
CNTN2	rs4894803	35.60019111
CNTN2	rs1294414	88.41662055
CNTN2	rs635634	42.25537414
CNTN4	rs115510911	39.90417648
CNTN4	rs9878112	78.02286122
CNTN4	rs13071423	523.4111154
CNTN4	rs115370013	180.5232632
CNTN4	rs1949342	30.09540577
CNTN4	rs975334	65.53594027
CNTN4	rs1499126	193.583766
CNTN4	rs163352	707.5505444
CNTN4	rs10922098	61.48660545
CNTN4	rs35887873	152.6515792
CNTN4	rs181707610	34.55343192
CNTN4	rs55714927	49.72293568
CNTN4	rs1850507	39.45470964
CNTN5	rs4528296	785.4605327
CNTN5	rs2123691	35.68066695
CNTN5	rs10922098	192.4525034
CNTN5	rs4754290	71.73700147
CNTN5	rs11223396	90.09417193
CNTN5	rs4325882	31.86490543
CNTN5	rs415620	61.84333041
CNTN5	rs7774739	51.43258481
CNTN5	rs74671299	30.1940736
CST7	rs74784851	56.54827526
CST7	rs112412739	115.3151284
CST7	rs6083533	36.53641991

CST7	rs148902838	170.9488941
CST7	rs974647	1076.283207
CST7	rs150749053	2972.988768
CST7	rs6115172	495.6139783
CST7	rs34345105	102.6321157
CST7	rs113422568	69.1247256
CST7	rs2511241	79.99270175
CST7	rs35979828	43.37626025
CST7	rs79634769	33.18610985
CST7	rs139196635	29.83425319
CST7	rs3184504	44.27190025
CST7	rs4783187	32.96753754
CST7	rs9898547	125.3998704
CST7	rs62146272	33.81312804
CST7	rs926646	32.01482316
CST7	rs80186620	32.21829705
CST7	rs4815175	55.61305724
CST7	rs113822376	221.7335626
CST7	rs111478778	163.2130724
CST7	rs71240113	54.42496369
CST7	rs34938560	176.4907074
CST7	rs36154728	185.0100131
CST7	rs35231432	227.8359624
CST7	rs35060861	137.2987524
CST7	rs35065709	90.24594927
CST7	rs144481686	144.2041925
CST7	rs6142023	81.86552701
CST7	rs117753689	73.17718723
CST7	rs142309628	56.06423761
CST7	rs111262642	94.63603242
CST7	rs150077408	75.51420007
CST7	rs189733686	36.70787847
CST7	rs75092000	37.04841669
CST7	rs6993770	136.0593779
DLL4	rs35748882	47.6770302
DLL4	rs72704449	42.34459426
DLL4	rs117241334	35.27388226
DLL4	rs8176743	768.158616
DLL4	rs62576042	43.65209653
FCGR2A	rs114014204	214.958627
FCGR2A	rs150752639	483.5653243
FCGR2A	rs34307129	362.1701377
FCGR2A	rs56241622	229.8301748
FCGR2A	rs4233370	148.0162771
FCGR2A	rs144449952	154.2298274
FCGR2A	rs114844178	4778.909783
FCGR2A	rs138293152	1028.70882
FCGR2A	rs113727631	690.1511867
FCGR2A	rs34436026	398.7224023
FCGR2A	rs61801160	383.9157905
FCGR2A	rs17452514	182.4152779
FCGR2A	rs72702031	36.97377003
FCGR2A	rs145300976	30.40056515
FCGR2A	rs34676060	52.11685996
FCGR2A	rs114499445	57.60350671
FCGR2A	rs55714927	77.12557877
FCGR2B	rs202146582	361.9638684
FCGR2B	rs114820463	177.5076684

FCGR2B	rs150752639	158.4339934
FCGR2B	rs116769960	992.1701331
FCGR2B	rs35951159	54.10739919
FCGR2B	rs148483621	1055.323874
FCGR2B	rs114797774	5116.932622
FCGR2B	rs41297690	676.7250692
FCGR2B	rs115264949	1306.739486
FCGR2B	rs115002999	200.5380817
FCGR2B	rs424288	5141.926313
FCGR2B	rs141762637	1355.278459
FCGR2B	rs72554670	927.9532339
FCGR2B	rs114265063	89.47918295
FCGR2B	rs113350478	70.06877499
FCGR2B	rs145264017	67.31772066
FCGR3B	rs148765873	44.20047602
FCGR3B	rs150752639	30.80715754
FCGR3B	rs139826170	35.26515652
FCGR3B	rs71639066	82.86592177
FCGR3B	rs139121685	54.57990104
FCGR3B	rs12029217	938.6088869
FCGR3B	rs56238128	369.345627
FCGR3B	rs116332947	66.68588731
FCGR3B	rs74779882	69.99603851
FCGR3B	rs9420589	56.12532894
FCGR3B	rs11229527	30.96748005
FCGR3B	rs78689694	60.61746947
FCGR3B	rs3184504	137.3471136
FCGR3B	rs374229803	64.76145411
FCGR3B	rs58102398	167.0928575
FCGR3B	rs61984254	33.75995356
FCGR3B	rs3088145	56.59615273
FCGR3B	rs12945299	109.226572
FCGR3B	rs143501783	51.95470498
FCGR3B	rs9303277	67.97144366
FCGR3B	rs11650022	32.0882542
FCGR3B	rs7207683	38.97214943
FCGR3B	rs681343	38.45478601
FCGR3B	rs3747207	66.11334318
FCGR3B	rs5028132	56.43863156
FCGR3B	rs2028392	187.4834164
FCGR3B	rs13071075	48.99722623
FCGR3B	rs11920688	81.55374233
FCGR3B	rs6866614	50.68132803
FCGR3B	rs509227	72.39185866
FCGR3B	rs375004483	76.3488606
FCGR3B	rs9270601	42.35219413
FCGR3B	rs9282052	57.51428118
FCGR3B	rs9277935	34.31964914
FCGR3B	rs7789913	95.23724366
FCGR3B	rs12350739	36.62351506
FCGR1A	rs1265564	34.79356858
FCGR1A	rs6542680	140.3037938
FCGR1A	rs5844513	241.9870617
FCGR1A	rs35677470	42.4029162
FCN2	rs7846761	94.8821196
FCN2	rs183486037	1268.783836
FCN2	rs2989725	332.0479286
FCN2	rs55646420	124.3670133

FCN2	rs7858399	62.8892173
FCN2	rs72550870	38.57698398
FCN2	rs572515	108.1133322
FCN2	rs12286277	536.002723
FCN2	rs10891719	61.04678461
FCN2	rs33998096	35.25697399
GFRA1	rs4550150	463.8358085
GFRA1	rs6018653	58.83795999
GFRA1	rs56819515	32.09978343
GFRA1	rs6457457	84.35776479
GFRA1	rs6458	116.4520445
GFRA1	rs3117229	37.46355056
GFRA1	rs10251094	40.04976135
GFRA1	rs2954038	44.90995189
SERPIND1	rs140854257	43.57798884
SERPIND1	rs2549034	76.29737056
SERPIND1	rs55776462	63.94943775
SERPIND1	rs7202724	160.9128303
SERPIND1	rs217184	576.4360598
SERPIND1	rs12975366	53.57514742
SERPIND1	rs1260326	90.92776488
SERPIND1	rs13244614	33.31717065
IGFBP7	rs139826977	150.6080028
IGFBP7	rs76023148	213.7369559
IGFBP7	rs11133472	159.5601111
IGFBP7	rs10013945	968.6769911
IGFBP7	rs77542162	59.62764881
IGFBP7	rs5839310	31.53872641
IGFBP7	rs36221780	34.98223654
IGFBP7	rs145552690	36.33992431
IGFBP7	rs143745420	52.57891289
IGFBP7	rs146357856	31.80447228
IGFBP7	rs77202557	40.19045837
IGFBP7	rs57760582	60.38986939
IGFBP7	rs3129781	115.1779111
LRIG3	rs189955853	63.61849457
LRIG3	rs74711935	609.1292626
LRIG3	rs61096689	67.39006428
LRIG3	rs2293659	770.4068544
LRIG3	rs61921897	52.0174112
LRIG3	rs146511124	136.5279765
LRIG3	rs140344867	1350.949896
LRIG3	rs141381480	634.0274962
LRIG3	rs78705678	46.91943607
LRIG3	rs2477642	39.79525337
LRIG3	rs79363674	164.938588
LRIG3	rs11170974	40.88935688
LRIG3	rs117378724	31.35913212
LRIG3	rs149127747	384.6255643
LRIG3	rs150937490	629.0245377
LRIG3	rs12810354	963.2282761
LRIG3	rs9668323	61.08746535
LRIG3	rs35646256	388.1453579
LRIG3	rs635634	43.66275171
LRP8	rs7546246	456.6209345
LRP8	rs1342970	85.79640447
LRP8	rs78689694	32.29581371
LRP8	rs117633773	36.88236272

LRP8	rs55714927	208.86074
LRP8	rs143501783	68.05053169
LRP8	rs1245369	144.4717359
LRP8	rs12529130	32.16835049
LRP8	rs79202843	100.5614311
LRP8	rs115625939	251.3789145
LRP8	rs635634	193.8798751
LY9	rs7515941	86.34948097
LY9	rs7555137	1229.820465
LY9	rs72712829	447.9454852
LY9	rs11265550	128.142059
LY9	rs332398	48.06926936
LY9	rs660240	34.51381292
LY9	rs6427670	38.10354738
LY9	rs3184504	141.2048748
LY9	rs3078012	32.88425252
LY9	rs12945299	114.590823
LY9	rs143501783	55.43266659
LY9	rs2725405	72.53202337
LY9	rs34243886	31.50235027
LY9	rs4823075	33.08361068
LY9	rs9861501	41.47998644
LY9	rs4705862	36.09727182
LY9	rs9267084	46.16100232
LY9	rs3117037	35.25587302
LY9	rs3181356	37.17967621
MATN2	rs11783428	95.51853995
MATN2	rs4489302	41.6360375
MATN2	rs34302865	100.2803134
MATN2	rs78548919	175.6508481
MATN2	rs72704449	37.5333949
MATN2	rs77542162	54.05516363
MATN2	rs2685411	31.33320497
CADM1	rs11215406	306.6975068
CADM1	rs10158500	61.40345617
CADM1	rs12133018	30.03569797
CADM1	rs72704449	72.76842504
CADM1	rs4149083	42.29143817
CADM1	rs77542162	29.78078252
CADM1	rs2659005	35.96264123
NTN4	rs10777719	75.73429561
NTN4	rs17288108	456.8295191
NTN4	rs1025607	32.5312978
NTN4	rs2660888	52.2563473
NTN4	rs10797801	49.81060993
NTN4	rs11612228	44.41097332
NTN4	rs1800127	33.64170273
NTN4	rs77542162	63.5797416
NTN4	rs71352239	33.79716645
NTN4	rs75892962	53.90737395
NTN4	rs6121571	87.85459035
NTN4	rs10068965	31.19021849
NTN4	rs9274470	31.99577787
PGLYRP1	rs2072563	482.0747409
PGLYRP1	rs11667174	45.75987182
PGLYRP1	rs13346756	32.24876987
PGLYRP1	rs2511241	179.0309876
PGLYRP1	rs2012811	33.29850733

PGLYRP1	rs3859194	33.30366652
PGLYRP1	rs2270401	63.32466713
PGLYRP1	rs11881751	31.90414949
PGLYRP1	rs28498283	45.57540841
PGLYRP1	rs1043160	81.77877897
PGLYRP1	rs34119183	79.01506776
PGLYRP1	rs9269958	200.1120215
PGLYRP1	rs1794269	303.9534201
PGLYRP1	rs114349958	35.24938107
RGMB	rs61048056	297.8394549
RGMB	rs10066211	54.93705073
RGMB	rs40263	56.24320707
RGMB	rs9328003	68.10994906
RGMB	rs72704449	72.55622565
RGMB	rs78705678	70.65853357
RGMB	rs2477642	85.663178
RGMB	rs77924615	60.06839472
RGMB	rs1260326	47.4127246
RGMB	rs368611	30.76470462
RGMB	rs11135015	34.97014935
RGMB	rs11244049	40.41067476
TFPI	rs181756447	38.32207921
TFPI	rs6700487	40.25380753
TFPI	rs1209731	75.29428447
TFPI	rs974793	436.9400462
TFPI	rs7533223	32.07320007
TFPI	rs6127	152.0264354
TFPI	rs7894992	59.42795003
TFPI	rs10159712	148.4861505
TFPI	rs145500693	29.89546682
TFPI	rs17580	37.78058751
TFPI	rs77262773	34.86087431
TFPI	rs6018653	31.44094195
TFPI	rs115005201	55.92953461
TFPI	rs74999184	85.04792586
TFPI	rs116334170	42.25532603
TFPI	rs112875651	42.91306279
TFPI	rs112164771	203.4619847
THBS2	rs9346538	37.48525382
THBS2	rs148674511	69.05680553
THBS2	rs71574910	61.09959547
THBS2	rs186322434	113.6111939
THBS2	rs73034020	1187.29729
THBS2	rs12664015	807.1339875
THBS2	rs148506905	94.10713971
THBS2	rs139841116	163.5248548
THBS2	rs6456172	57.63448283
THBS2	rs10417163	34.56636367
THBS2	rs116793716	30.0012777
THBS4	rs10069277	100.0237574
THBS4	rs2404710	121.4350454
THBS4	rs13167730	602.1301591
THBS4	rs12986064	39.98691306
THBS4	rs11899888	35.62676237
THBS4	rs3890787	34.02787141
ABL2	rs13332649	40.01644481
ABL2	rs6542680	35.4671452
ABL2	rs3131297	38.17959568

ACY1	rs148804382	509.0309168
ACY1	rs780093	52.76318897
ACY1	rs3747207	47.30109086
ACY1	rs79442098	34.17877181
ACY1	rs1808831	198.4560662
ACY1	rs140864960	159.4077463
ACY1	rs75425541	70.39102065
ACY1	rs28601761	60.5232059
AURKB	rs10305748	35.91720581
AURKB	rs4512628	32.85307486
AURKB	rs114009540	32.91463452
AURKB	rs1329424	916.6195778
AURKB	rs35351085	46.18458589
AURKB	rs11569415	44.50880094
AURKB	rs1707652	144.0807582
AURKB	rs1144709	38.50451684
AURKB	rs1265941	97.31110596
AURKB	rs550671	224.9742452
AURKB	rs112164276	30.7544507
BMP1	rs1126931	71.76087739
BMP1	rs1260326	65.13446672
BMP1	rs13108218	32.71643773
BMP1	rs9266224	35.3614523
CAMK2B	rs12292693	39.21686232
CAMK2B	rs1354034	36.21300381
CAMK2B	rs450269	48.30519308
CA6	rs191693468	84.35806329
CA6	rs11121209	310.7301603
CA6	rs116775713	263.6466338
CA6	rs72641551	928.3402576
CA6	rs35994272	147.616573
CA6	rs12034435	55.84222027
CA6	rs12059915	49.39766116
CA6	rs1891216	92.2285591
CA6	rs836144	60.50226982
CA6	rs16920648	107.8081694
CA6	rs28376342	30.64486317
CA6	rs969587	68.75709925
CA6	rs9939224	43.16538961
CA6	rs2607276	30.4255127
CA6	rs601338	67.92920159
CA6	rs11902236	55.69567692
CA6	rs2419900	93.71532374
CA6	rs11902072	33.78398044
CA7	rs572515	44.83594497
CA7	rs7148049	45.01659821
CA7	rs9902706	37.27255973
CA7	rs6542680	47.18616567
CA7	rs2858879	32.77520643
CD207	rs10922098	186.0476023
CD207	rs1355538	30.87776518
CD207	rs7774739	44.78155663
CD207	rs7742632	51.59958386
CHRDL1	rs333947	34.29279095
CHRDL1	rs10922098	36.34382233
CHRDL1	rs2477664	32.57128436
CHRDL1	rs2461161	30.80929595
CHRDL1	rs6487543	35.01505644

CHRDL1	rs2569491	48.30147378
CHRDL1	rs760715	61.78246381
CHRDL1	rs9458012	33.58065191
CTSV	rs4645657	124.3067732
CTSV	rs28825490	222.7303255
CTSV	rs10981890	407.1170225
CTSV	rs116989414	36.08574166
CTSV	rs112597538	59.28456546
CTSV	rs4917908	29.90392394
CTSV	rs2695294	206.8488857
CTSV	rs2259820	98.47862533
CTSV	rs7493181	31.85487277
CTSV	rs2729805	33.49651847
CTSV	rs11649114	49.24102783
CTSV	rs11085799	31.26941732
CTSV	rs7603621	53.17615007
CTSV	rs1736147	40.76984315
CTSV	rs5030873	71.08363813
CTSV	rs9349232	39.17753804
CTSV	rs9370162	172.802897
CTSV	rs7793437	35.77508069
CTSV	rs45467892	36.72671813
CTSV	rs9299274	31.61845851
CTSV	rs576123	63.70031723
DKK4	rs10888208	168.3741027
DKK4	rs141099634	330.3766764
DKK4	rs9415731	126.3452675
DKK4	rs72795135	142.2712107
DKK4	rs7101062	160.003625
DKK4	rs75580312	480.1244822
DKK4	rs150513777	373.7265879
DKK4	rs117565303	49.68558563
DKK4	rs61850494	39.48514256
DKK4	rs145591269	32.53117289
DKK4	rs10822155	181.6321226
DKK4	rs34416903	31.96621523
DKK4	rs9905408	297.0359314
DKK4	rs113661219	33.89877364
DKK4	rs12468189	32.49628636
DKK4	rs6993770	186.2290776
ECM1	rs35987241	2703.254502
ECM1	rs112088857	1152.499888
ECM1	rs12119409	600.5487683
ECM1	rs185236008	217.9115722
ECM1	rs3811409	69.92833045
ECM1	rs1289670	44.96776241
ECM1	rs4411121	40.67793606
ECM1	rs74818062	34.2479868
ECM1	rs116567297	70.36744138
ECM1	rs4606261	67.06203606
ECM1	rs11577274	75.23538818
ECM1	rs13796	36.25273404
ECM1	rs12124306	30.91707932
ECM1	rs77262773	43.2870894
ECM1	rs41285751	34.13324537
FETUB	rs12497361	66.76666991
FETUB	rs77283793	32.58245234
FETUB	rs11716033	835.6186763

FETUB	rs62292821	58.75944769
FETUB	rs78719817	31.71322535
FETUB	rs7540115	34.33563287
FETUB	rs3999089	123.1531226
FETUB	rs55707100	170.7987564
FETUB	rs56139860	33.45260003
FETUB	rs61690552	44.84979653
FETUB	rs112779928	32.09371733
FETUB	rs12650033	31.22454756
FETUB	rs2954021	155.9419951
GZMH	rs368465	161.2154948
GZMH	rs6445180	31.48544705
GZMH	rs9501600	47.87686127
HCK	rs10922098	82.55722805
HCK	rs415620	43.00255297
HCK	rs4713498	46.36398161
IL17RD	rs62251090	126.1857716
IL17RD	rs9862503	952.49051
IL17RD	rs142146699	292.1675484
IL17RD	rs10922098	292.556646
IL17RD	rs116097686	37.82749251
IL17RD	rs79257513	50.9246358
IL17RD	rs77470761	29.86603261
IL17RD	rs115623261	186.21858
IL17RD	rs112835310	106.0272777
IL17RD	rs1707652	39.15086382
IL17RD	rs9501600	86.82064234
KLK7	rs4802763	988.679232
KLK7	rs1829971	119.0817108
KLK7	rs71333926	44.14125992
KLK7	rs80293268	57.86200738
KLK7	rs61729512	169.6312543
KLK7	rs7305932	87.84137728
KLK7	rs12986064	270.7092615
KLK7	rs9272505	34.31456648
PRKCI	rs4980320	44.52919941
PRKCI	rs78909033	34.5751545
PRKCI	rs1354034	61.65476312
LYN	rs12280510	56.92856722
LYN	rs185009887	32.86377882
LYN	rs1354034	46.1134441
PAK3	rs4512628	41.59004122
PAK3	rs1329424	1153.464523
PAK3	rs7513100	52.37313732
PAK3	rs1047286	38.48877593
PAK3	rs111696008	30.33283176
PAK3	rs1707652	175.9239843
PAK3	rs3099847	30.09462205
PAK3	rs1265941	108.7763573
PAK3	rs550671	286.4150643
PAK3	rs78897911	36.23194003
PAK3	rs9276606	43.60179617
SERPINA5	rs6119	676.5697936
SERPINA5	rs4905225	237.7216431
SERPINA5	rs780093	32.54843954
SERPINA5	rs2304595	57.61454485
SERPINA5	rs1801020	57.69004262
PIK3CG	rs342294	35.09312483

PIK3CG	rs72748418	31.16109247
PIK3CG	rs10922098	560.8950653
PIK3CG	rs146453555	81.85502917
PIK3CG	rs11569415	39.0438015
PIK3CG	rs1707652	117.7594069
PIK3CG	rs550671	138.7198452
PIK3CG	rs9270570	30.09002483
PIK3CG	rs9469195	56.47930561
PIK3CG	rs4541868	52.33713327
PLK1	rs572515	106.1324419
PLK1	rs7742632	34.32398851
PLK1	rs34028938	36.31734817
REN	rs116468241	60.91428369
REN	rs12739352	32.09319355
REN	rs112938492	65.79900792
REN	rs3014620	150.5037888
REN	rs12123481	63.51119562
REN	rs4920187	38.89357198
REN	rs7483477	33.97388522
REN	rs72932727	45.75990384
REN	rs34788166	41.48168687
STAB2	rs144704969	33.22381871
STAB2	rs1329424	999.4404123
STAB2	rs3900909	48.74858649
STAB2	rs1047286	38.77515006
STAB2	rs2686395	174.034353
STAB2	rs1265941	101.0434708
STAB2	rs444921	272.5405813
STAB2	rs9269234	43.6644448
PTPN2	rs10922098	321.0313867
PTPN2	rs9864801	46.92685328
PTPN2	rs9501600	80.1075828
PTPN2	rs587495	31.20746016
TPSB2	rs72773434	40.20757253
TPSB2	rs146039313	105.1940374
TPSB2	rs12928519	42.04221079
TPSB2	rs12933924	1123.520759
TPSB2	rs2235634	9713.518795
TPSB2	rs148413829	163.9984439
TPSB2	rs72775484	207.0751689
TPSB2	rs140532679	577.6378737
TPSB2	rs731046	536.515565
TPSB2	rs141716219	322.1066125
TPSB2	rs58293579	208.5451086
TPSB2	rs115427247	154.7340877
TPSB2	rs16856110	79.36782851
TPSB2	rs56043070	76.96469321
TPSB2	rs78705678	33.00559733
TPSB2	rs35693349	51.99567629
TPSB2	rs146112168	49.76784896
TPSB2	rs138144869	64.5170894
TPSB2	rs17758695	32.65298991
TPSB2	rs78744187	250.3861709
TPSB2	rs56344893	130.322829
TPSB2	rs36059907	62.5709762
TPSB2	rs35930348	32.14586187
TPSB2	rs9871203	51.3986322
TPSB2	rs28455789	34.95681867

UFC1	rs4980320	31.4509411
UFC1	rs11451044	33.40004
UFC1	rs1354034	69.62170463
BCL2A1	rs10509186	51.50346097
BCL2A1	rs17580	40.17507045
BCL2A1	rs111696008	32.08711783
BCL2A1	rs1355538	140.617982
BCL2A1	rs28919269	29.78955194
BCL2A1	rs28374650	31.14782133
BCL2A1	rs6993770	37.38877028
IBSP	rs2616262	134.815037
IBSP	rs4658046	40.11899068
IBSP	rs117801489	63.75267019
IBSP	rs58973023	50.69058494
IBSP	rs56225285	32.82218709
IBSP	rs13426965	39.22323088
IBSP	rs703869	35.99206288
IBSP	rs6894139	50.19956897
IBSP	rs6477	32.27619418
IBSP	rs635634	76.36695367
CAMK1D	rs1451915	33.40020533
CAMK1D	rs10922098	762.5428644
CAMK1D	rs10922361	35.14890174
CAMK1D	rs704	53.29749981
CAMK1D	rs1047286	66.42586431
CAMK1D	rs2686395	89.20821543
CAMK1D	rs550671	178.0454711
CAMK1D	rs9270570	43.18082989
CAMK1D	rs9469195	80.47616248
CAMK2D	rs6836139	30.33806193
CAMK2D	rs9971407	44.6543173
CAMK2D	rs1354034	31.77481793
CAMK2D	rs342298	46.60951454
CA13	rs317935	121.8121684
CA13	rs76996041	1099.145877
CA13	rs4266652	302.308241
CA13	rs1534040	789.5021229
CA13	rs35340377	32.09681834
CA13	rs35979828	33.74167422
CA13	rs1354034	32.39551711
CA13	rs9864801	66.48335025
CA13	rs76230412	59.83762235
CA13	rs148157999	119.2832285
CA13	rs79480792	32.37826422
CA13	rs62532765	47.68133962
CA13	rs190922269	43.73414397
CA13	rs77265585	30.40880248
TNFSF8	rs10982299	41.04287626
TNFSF8	rs1006026	280.8239078
TNFSF8	rs37454	58.84827937
TNFSF8	rs10922098	307.1179457
TNFSF8	rs78689694	42.20510242
TNFSF8	rs3184504	60.34064114
TNFSF8	rs1555405	33.27716185
TNFSF8	rs117633773	50.10921783
TNFSF8	rs55714927	169.2208268
TNFSF8	rs143501783	81.9467373
TNFSF8	rs681343	45.08022788

TNFSF8	rs2062059	32.8896871
TNFSF8	rs697357	39.4167882
TNFSF8	rs9810602	75.11527891
TNFSF8	rs9501600	56.96619708
CMA1	rs10922098	313.5673217
CMA1	rs11569415	30.33354271
CMA1	rs1706435	54.48342922
CMA1	rs9501600	75.35610685
CSNK2A1	rs528298	42.15281217
CSNK2A1	rs3811444	34.14047426
CSNK2A1	rs11082513	31.25138827
CSNK2A1	rs67036916	34.94184632
EPHA1	rs10272702	83.00403982
EPHA1	rs11772895	921.8306785
EPHA1	rs73154244	414.0633654
EPHA1	rs10243226	209.7008211
EPHA1	rs10922094	228.8102609
EPHA1	rs41285751	51.84654217
EPHA1	rs451652	44.73423819
EPHA1	rs1181753	52.55129852
EPHA1	rs62484383	108.1225339
EPHA1	rs17286117	176.6769682
EPHA1	rs146670385	122.2363019
EPHA1	rs2158751	55.96180024
EPHA3	rs10922098	40.0172148
EPHA3	rs150476910	30.59486006
EPHA3	rs1846190	31.26670476
FN1	rs7580569	59.34802465
FN1	rs138259153	95.87373937
FN1	rs1250258	372.5162233
FN1	rs4674006	63.88789584
FN1	rs188982853	30.07646802
FN1	rs7761068	33.23457844
FN1	rs7580569	70.97975838
FN1	rs138259153	105.1004456
FN1	rs1250259	362.9233476
FN1	rs4674006	65.96594527
FN1	rs10922094	32.61417622
FN1	rs10784951	30.10365657
FN1	rs3130955	30.0855197
FN1	rs7761068	38.3332427
FLT3	rs10733084	32.08879458
FLT3	rs10922098	756.4429376
FLT3	rs3900909	32.54602338
FLT3	rs11569393	37.01382451
FLT3	rs1707652	106.4836179
FLT3	rs1265941	72.12502294
FLT3	rs444921	194.9301753
FSTL3	rs56014328	57.57015937
FSTL3	rs10409425	30.04469943
FSTL3	rs7250835	75.71238671
FSTL3	rs72704449	38.48685636
FSTL3	rs77924615	39.9009181
GZMA	rs199507411	144.3921136
GZMA	rs142909603	419.5826814
GZMA	rs139268409	583.0148104
GZMA	rs80166608	74.95606217
GZMA	rs1860545	31.00458096

GZMA	rs7314318	37.60022444
GZMA	rs79634769	47.59571215
GZMA	rs139196635	50.02571973
GZMA	rs7310615	163.6711672
GZMA	rs12986064	94.62494888
GZMA	rs12151752	33.05327699
GZMA	rs13063578	38.99400936
GZMA	rs680242	30.72634346
GZMA	rs116818570	54.92065631
GZMA	rs113437464	87.1401362
GZMA	rs116522067	133.145594
GZMA	rs142021463	134.9679026
GZMA	rs142390300	75.92596197
GZMA	rs181076930	32.95306938
GZMA	rs17710827	36.85239379
GZMA	rs375751680	32.07924363
GZMA	rs9264638	784.9100851
GZMA	rs2844557	141.6318241
GZMA	rs148046661	113.7268569
GZMA	rs3130163	43.62690274
GZMA	rs10813948	44.85238418
GZMA	rs10982456	49.69552587
IL15RA	rs8177639	44.69168008
IL15RA	rs10922098	258.3329714
IL15RA	rs1355538	46.25954461
IL15RA	rs9501600	65.17086597
IL15RA	rs557011	31.11490828
CXCL8	rs79237872	30.0034133
CXCL8	rs12075	69.61127867
CXCL8	rs10922094	207.8302779
CXCL8	rs1706435	36.90617245
CXCL8	rs41285751	34.99450491
CXCL8	rs3130235	43.34106764
INSR	rs4804368	65.1502635
INSR	rs150960915	43.00424733
INSR	rs7080169	43.76204125
INSR	rs9420589	33.72007735
INSR	rs10836486	32.63995539
INSR	rs11605349	59.65326864
INSR	rs2859660	41.1139867
INSR	rs7310615	192.3017985
INSR	rs11574938	354.405814
INSR	rs9936992	129.6815323
INSR	rs55714927	64.3565211
INSR	rs143501783	38.83726265
INSR	rs58745116	99.38398051
INSR	rs387057	76.34789351
INSR	rs4458292	73.83410796
INSR	rs3133023	86.86437479
INSR	rs760462	374.8013828
INSR	rs6414436	42.98433173
INSR	rs157572	36.57540324
INSR	rs17710008	43.0197568
INSR	rs10962668	61.19118583
INSR	rs11244016	121.1090588
INSR	rs186670880	34.18386466
INSR	rs576123	1255.275861
INSR	rs10901256	298.4756402

INSR	rs55978893	62.35205649
INSR	rs2502742	48.58568704
INSR	rs72762820	45.42832523
SERPINA4	rs12590755	33.60990698
SERPINA4	rs138642808	61.349999
SERPINA4	rs141091603	2462.030308
SERPINA4	rs1884080	323.4414076
SERPINA4	rs1884082	107.1358881
SERPINA4	rs113713641	162.6168884
SERPINA4	rs7964859	30.42595867
SERPINA4	rs2264778	58.24086359
SERPINA4	rs1743492	57.55587072
SERPINA4	rs1230164	38.65235214
SERPINA4	rs73023864	30.57012349
KLK6	rs114558472	33.2217958
KLK6	rs72748420	44.72572646
KLK6	rs1329424	1307.531167
KLK6	rs3900909	56.9997112
KLK6	rs10801660	42.45185671
KLK6	rs1047286	37.66289922
KLK6	rs111696008	30.91090417
KLK6	rs1707652	244.3182071
KLK6	rs73169117	31.21167026
KLK6	rs1265941	119.9842384
KLK6	rs550671	331.1903952
KLK6	rs112164276	48.67012027
KLK6	rs41548618	45.76509439
LYN	rs12289048	51.5525509
LYN	rs185009887	31.93934201
LYN	rs1354034	44.13738545
POSTN	rs8002559	130.075619
POSTN	rs35842206	69.4794015
POSTN	rs4941866	79.45394837
POSTN	rs9532075	454.0037582
POSTN	rs9594222	163.0776209
POSTN	rs185853091	38.17237675
POSTN	rs1609860	108.3533714
POSTN	rs4981022	39.49574698
PDGFRB	rs72830245	164.821406
PDGFRB	rs216151	1020.046593
PDGFRB	rs246392	7139.967982
PDGFRB	rs72832182	7246.317213
PDGFRB	rs13357922	215.4277142
PDGFRB	rs36007018	296.1518693
PDGFRB	rs141907998	116.4515732
PDGFRB	rs73283796	73.05202642
PDGFRB	rs75515047	35.68144587
BCAN	rs2365715	560.3436644
BCAN	rs4661063	37.34723861
BCAN	rs2227160	43.70909563
RPS6KA3	rs4980320	30.90863541
RPS6KA3	rs11451044	79.76221949
RPS6KA3	rs1354034	33.12873925
SELE	rs3917390	179.8813969
SELE	rs646776	170.0092223
SELE	rs117603193	122.5071416
SELE	rs35166255	236.9466706
SELE	rs2244608	50.247986

SELE	rs62061425	47.07415392
SELE	rs8071235	32.88698212
SELE	rs3760775	132.5768816
SELE	rs1585488	83.95854185
SELE	rs62576476	64.15096836
SELE	rs141668780	175.164731
SELE	rs7868232	1058.359527
SELE	rs75212266	172.543761
SELE	rs7857390	1079.715053
SELE	rs28699275	94.68275877
SELE	rs55710199	396.0441588
SELE	rs2519992	50.00645661
SELE	rs4545169	35.0181539
STK16	rs4512628	30.1454907
STK16	rs10922098	902.8924761
STK16	rs7513100	33.60299645
STK16	rs704	43.51008746
STK16	rs1047286	41.5988456
STK16	rs1707652	155.8092536
STK16	rs1265941	82.68269715
STK16	rs550671	213.2623703
STK16	rs9269234	36.55616287
BIRC5	rs1451915	31.18343075
BIRC5	rs10922098	643.3015452
BIRC5	rs11569415	43.86756682
BIRC5	rs10208529	30.67637021
BIRC5	rs2686395	101.1134105
BIRC5	rs1265941	80.69978698
BIRC5	rs453821	178.8068445
THBS1	rs12906549	34.55931362
THBS1	rs35340377	32.57587973
THBS1	rs7080386	117.4136309
THBS1	rs715440	40.48001565
THBS1	rs1671152	47.50532691
THBS1	rs1071960	34.58476412
THBS1	rs114694170	37.91228787
THBS1	rs6993770	131.6699293
NTRK1	rs10922103	41.45394856
NTRK1	rs146457388	33.81655244
NTRK1	rs17441745	32.75618752
NTRK1	rs6542680	477.9608428
NTRK1	rs80220851	29.9506923
AGT	rs2071404	413.2868673
AGT	rs2354846	41.29135897
AGT	rs7956099	70.88111708
AGT	rs139974673	45.04009558
AGT	rs780093	81.28013668
AGT	rs13234378	67.13390922
B2M	rs3184504	96.14831533
B2M	rs77924615	40.94467962
B2M	rs74439742	31.31601832
B2M	rs9259225	104.0188879
B2M	rs17198860	41.36282155
B2M	rs9266141	337.6183492
B2M	rs34115112	174.8607758
B2M	rs9391787	70.17481083
FGF1	rs572515	145.1543136
FGF1	rs41285751	53.38107584

FGF1	rs415620	47.6902004
CXCL13	rs10849448	35.78509125
CXCL13	rs7310615	38.96620407
CXCL13	rs12166583	67.2473599
CXCL13	rs2596569	47.23711533
CXCL13	rs4367411	37.47964654
CAT	rs2746626	31.0502437
CAT	rs2755171	98.42206286
CAT	rs769218	483.9087207
CAT	rs12239179	32.07026123
CAT	rs111366006	33.66769825
CAT	rs76016104	32.5488374
CAT	rs73123625	75.30314937
CNTF	rs10922098	192.736497
CNTF	rs117265578	31.30740656
CNTF	rs1355538	36.46864651
CNTF	rs28573154	74.55894628
CNTF	rs7774739	60.37082322
FGF17	rs572515	281.0761159
FGF17	rs1355538	50.49999605
FGF17	rs9501600	79.82342879
FGF17	rs35209896	34.38072405
CXCL6	rs1534450	232.5787836
CXCL6	rs10031141	6722.582563
CXCL6	rs10021230	293.5893965
CXCL6	rs77858271	84.65149143
CXCL6	rs653178	58.75664788
CXCL6	rs116608301	63.68466788
CXCL6	rs116280909	73.96873106
CXCL6	rs116119339	82.83417096
CXCL6	rs79190329	172.5433319
CXCL6	rs72653982	51.52566109
CXCL6	rs72647456	70.38820241
CXCL6	rs9494145	39.99376393
IFNA2	rs10922098	519.6986895
IFNA2	rs1707652	98.24211025
IFNA2	rs3130955	33.17935923
IFNA2	rs9501600	155.8910923
IFNA2	rs35209896	58.85768693
IL17B	rs6887452	59.43576416
IL17B	rs10922098	127.5498742
IL17B	rs11755689	38.97342498
IL17B	rs11794541	32.9349016
HAMP	rs4774514	43.39496653
HAMP	rs34523089	31.2076858
HAMP	rs516316	42.18126748
HAMP	rs855791	50.9321742
HAMP	rs885122	108.2818806
HAMP	rs55925606	85.10615895
CCL22	rs1123507	57.2023182
CCL22	rs140133389	37.31522222
CCL22	rs289750	70.53087964
CCL22	rs71375907	257.8779797
CCL22	rs9921051	233.6094877
CCL22	rs62037104	644.1641426
CCL22	rs192297333	36.33664321
CCL22	rs10886437	69.16639878
CCL22	rs35350651	42.54746132

CCL22	rs77542162	453.9801472
CCL22	rs72631343	109.490936
CCL22	rs11697497	37.26753243
CCL22	rs11129978	76.79828825
CCL22	rs6808893	51.51440412
CCL22	rs7774739	30.58887503
CCL22	rs9276255	35.189051
CCL22	rs60066732	39.26754146
CCL22	rs913914	30.92698311
CXCL12	rs10793518	45.49227146
CXCL12	rs266085	48.06755153
CXCL12	rs1023264	238.7725229
CXCL12	rs11208704	39.81097563
CXCL12	rs2393989	30.18788259
CXCL12	rs10762477	48.07745799
CXCL12	rs3184504	34.42910427
CXCL12	rs77542162	232.6746014
CXCL12	rs72631343	53.47625086
CXCL12	rs13015258	36.76353748
CXCL12	rs299654	40.84518565
CXCL12	rs17138667	73.87254586
CXCL12	rs115841185	30.00476817
CXCL12	rs10123570	31.14831802
CPB2	rs2573284	211.4872863
CPB2	rs7325308	2709.419631
CPB2	rs17647647	364.7712577
CPB2	rs12867023	60.50821845
CPB2	rs78064641	75.4485531
CPB2	rs11220463	34.73163415
CPB2	rs7337310	41.37950262
CPB2	rs73178358	42.42155554
CPB2	rs146690802	38.09212897
CPB2	rs9524538	35.6765557
CPB2	rs1260326	52.65036837
CCL17	rs223895	271.7538193
CCL17	rs28695417	624.5727735
CCL17	rs17098260	50.68890951
CCL17	rs10886430	1036.668143
CCL17	rs10788007	31.04092479
CCL17	rs3785155	34.29265475
CCL17	rs148393806	34.57723366
CCL17	rs150210110	33.93526263
CCL17	rs115952894	874.1494838
CCL17	rs139617453	37.69588819
CCL17	rs35216408	212.7930964
CCL17	rs13078687	99.05904341
CCL17	rs35574803	42.23211024
CCL17	rs4343578	32.20258411
CCL17	rs111265267	63.55937245
CCL17	rs11718659	32.14104644
CCL17	rs151193446	154.3135255
CCL17	rs2228467	647.4472745
CCL17	rs74597484	39.72621937
CCL17	rs4683159	33.86105
CCL17	rs6993770	142.321016
CCL17	rs60757417	33.98194899
TGFB3	rs10922094	97.80605087
TGFB3	rs9902706	38.05411679

TGFB3	rs7625980	82.46200479
TGFB3	rs28672722	47.14284151
DKK1	rs72795135	143.2205141
DKK1	rs7101062	155.0561316
DKK1	rs75580312	467.924526
DKK1	rs150513777	371.1175835
DKK1	rs4648825	31.51522893
DKK1	rs10888208	167.4817617
DKK1	rs141099634	322.5109687
DKK1	rs9415731	130.8406688
DKK1	rs61851099	52.09061041
DKK1	rs61850494	44.68846192
DKK1	rs145591269	30.4827431
DKK1	rs10761739	166.4441896
DKK1	rs34416903	33.69218694
DKK1	rs1801690	85.68666544
DKK1	rs113661219	33.48170392
DKK1	rs12468189	29.81329941
DKK1	rs6993770	206.0000154
ADIPOQ	rs149434699	62.13520599
ADIPOQ	rs113659044	44.65057825
ADIPOQ	rs199695767	109.0694293
ADIPOQ	rs143257534	700.8732867
ADIPOQ	rs13066690	50.36642137
ADIPOQ	rs1538742	52.10573908
ADIPOQ	rs2477664	36.41894775
ADIPOQ	rs7118490	39.9671446
ADIPOQ	rs59436957	43.09898146
ADIPOQ	rs117195351	30.65351801
ADIPOQ	rs1187415	69.10931115
ADIPOQ	rs56823429	83.17364281
ADIPOQ	rs12445207	98.95083413
ADIPOQ	rs4805881	87.08193803
ADIPOQ	rs2581790	35.01769242
ADIPOQ	rs67435657	31.55842767
ADIPOQ	rs5013866	32.74929083
ADIPOQ	rs115177000	30.79113416
ADIPOQ	rs4896469	30.84167922
ADIPOQ	rs2954038	35.99273821
SERPINA1	rs148597299	55.51567876
SERPINA1	rs72702354	1072.13104
SERPINA1	rs117562583	55.2326261
SERPINA1	rs4905172	530.1359345
SERPINA1	rs112963922	536.323186
SERPINA1	rs17825092	46.95810113
SERPINA1	rs7160243	70.45813501
SERPINA1	rs17091144	177.0637327
SERPINA1	rs660240	239.6518311
SERPINA1	rs10761787	44.23606772
SERPINA1	rs145322110	30.46103346
SERPINA1	rs150172846	38.32144741
SERPINA1	rs2402227	51.25766152
SERPINA1	rs704	78.14923284
SERPINA1	rs1260326	57.14462766
AHSG	rs80069596	34.41823269
AHSG	rs78963288	65.33125727
AHSG	rs34295758	32.86865416
AHSG	rs11414733	129.0193984

AHSG	rs7647776	2209.657302
AHSG	rs822395	89.65979732
AHSG	rs10922094	101.2606083
AHSG	rs3775327	30.55130127
AHSG	rs550671	54.53753985
BSG	rs10922098	94.16561105
BSG	rs429358	31.32084283
BSG	rs6542680	101.8411356
BSG	rs114115200	103.9931193
BSG	rs3863087	35.17434161
BSG	rs415620	33.7941478
BSG	rs28374650	32.68170155
BMP10	rs10922098	45.98666108
BMP10	rs146457388	31.28969046
BMP10	rs6542680	319.0212683
BMP10	rs13217434	31.73794295
CAMK1	rs12730935	67.06406612
CAMK1	rs56168465	86.40921158
CAMK1	rs56084204	1283.67383
CAMK1	rs9332591	6744.60452
CAMK1	rs3917727	351.2301662
CAMK1	rs71635640	130.4088469
CAMK1	rs12402584	142.8272166
CAMK1	rs113258252	41.51053609
CAMK1	rs148302954	108.5341481
CAMK1	rs138517625	47.10886353
CAMK1	rs61826722	40.33826401
CAMK1	rs3908047	35.32738822
CAMK1	rs10159712	44.26032806
CAMK1	rs61751507	109.8453002
CAMK1	rs149925556	34.1936198
CASP3	rs111667591	327.2735109
CASP3	rs10428373	237.3768432
CASP3	rs35340377	39.10161795
CASP3	rs4980320	32.10883113
CASP3	rs4541868	48.96595384
CASP3	rs77265585	32.56388791
CHIT1	rs149820561	95.18675465
CHIT1	rs115402627	105.0523337
CHIT1	rs3766562	1192.410164
CHIT1	rs3766561	1223.746786
CHIT1	rs3862944	808.8794437
CHIT1	rs148541330	564.1090889
CHIT1	rs114066783	209.0264695
CHIT1	rs10793756	68.83146326
CHIT1	rs143841225	31.08254716
CHIT1	rs190770651	35.82596304
CHIT1	rs116201986	136.5996374
CHIT1	rs79991585	42.24577095
CHIT1	rs5785544	58.50435622
CHIT1	rs10405357	30.4063814
CLEC7A	rs203687	42.2095226
CLEC7A	rs6542680	182.9322716
CLEC7A	rs13217434	38.69102262
MASP1	rs698082	99.22312148
MASP1	rs10922098	196.4364864
MASP1	rs4938107	73.90093221
MASP1	rs1486236	182.4076366

MASP1	rs6445035	42.58851932
MASP1	rs550671	70.78512406
MASP1	rs9469195	42.68747594
MASP1	rs57136797	171.791261
DKK3	rs78528738	54.62340288
DKK3	rs10734190	315.3379172
DKK3	rs11022114	2278.543613
DKK3	rs17384316	30.7645625
DKK3	rs61801013	65.4948489
DKK3	rs196205	38.52031562
DKK3	rs139804748	31.66828923
DKK3	rs79435523	43.4488973
DKK3	rs17548461	37.62025042
DKK3	rs60478155	60.71506856
DKK3	rs1609860	217.7822643
DKK3	rs4981022	166.9697124
DKK3	rs188547348	77.78874123
DKK3	rs57326739	29.72150106
DKK3	rs1404421	55.83630079
DKK3	rs635634	71.46820243
GNS	rs1690278	31.31803739
GNS	rs660240	81.10498091
GNS	rs2559851	51.6112221
GNS	rs35530272	40.77054486
GNS	rs6542680	48.94411537
GNS	rs55776147	40.60557271
GNS	rs576123	121.4464905
HGFAC	rs3775067	967.985894
HGFAC	rs3752439	4613.165682
HGFAC	rs116737061	4763.644774
HGFAC	rs2983535	87.707396
HGFAC	rs114185131	317.0530071
HGFAC	rs73198499	160.2253874
HGFAC	rs141866277	47.29891367
HGFAC	rs1047891	82.93153714
HGFAC	rs75176598	76.66247015
HGFAC	rs142962885	324.6165491
HGFAC	rs35574323	123.258173
HGFAC	rs147126736	189.3289614
HGFAC	rs147006571	99.60630206
HGFAC	rs4841133	42.40487795
IL22RA1	rs10922098	212.4979916
IL22RA1	rs6542680	154.3465205
IL22RA1	rs2686395	77.96442606
IL22RA1	rs444921	68.73247385
IL22RA1	rs34927613	49.37022712
LGMN	rs148659834	183.3430643
LGMN	rs2236264	192.0840962
LGMN	rs75747979	39.80450803
LGMN	rs10922098	36.50597032
LGMN	rs2250644	34.07976235
LGMN	rs4561280	30.96834532
LGMN	rs4883201	352.847002
LGMN	rs117317284	70.77072769
LGMN	rs11111000	65.77730908
LGMN	rs35036246	32.60575535
LGMN	rs35677470	33.18030884
LGMN	rs6993770	54.28427025

MMEL1	rs10922098	114.8371062
MMEL1	rs1355538	36.97643026
MMEL1	rs9501600	30.84618453
MAP2K2	rs117359758	31.7430781
MAP2K2	rs72661955	112.1156717
MAP2K2	rs528218	248.4065681
MAP2K2	rs559054	2615.247647
MAP2K2	rs9604093	74.12607747
MAP2K2	rs8006631	67.82558038
MAP2K2	rs4439696	1066.937795
MAP2K2	rs77879682	33.27072735
MAP2K2	rs75806984	63.34122608
MAP2K2	rs79575982	38.58910113
MAP2K2	rs78200457	59.58830922
MAP2K2	rs150718850	30.29512567
MAP2K2	rs9304953	80.04258025
MAP2K2	rs187211507	63.23805918
MAP2K2	rs141399595	41.27452167
MAP2K2	rs698098	58.46189181
CDC42BPB	rs10922098	146.0183859
CDC42BPB	rs12465887	30.43756196
CDC42BPB	rs1355538	42.10131888
CDC42BPB	rs454212	63.08951234
CDC42BPB	rs28471056	31.02698201
LRPAP1	rs34084581	61.75428647
LRPAP1	rs2699435	79.59223933
LRPAP1	rs1800493	416.3796575
LRPAP1	rs117654599	38.87259075
LRPAP1	rs115989817	40.71585174
LRPAP1	rs116491682	43.65397894
LRPAP1	rs115000510	145.579904
LRPAP1	rs116834013	30.98504804
LRPAP1	rs115208272	94.27229371
LRPAP1	rs12190821	110.160021
LRPAP1	rs113969967	264.0209563
LRPAP1	rs115103442	1019.665446
LRPAP1	rs116633440	5020.071843
LRPAP1	rs9264480	1404.173744
LRPAP1	rs190253656	955.8269873
LRPAP1	rs73741546	602.4058761
LRPAP1	rs4713748	52.12041349
LRPAP1	rs115661362	171.7099357
LRPAP1	rs74906171	129.0598687
LRPAP1	rs114441543	142.7088962
LRPAP1	rs77960115	195.9101762
LRPAP1	rs139503432	34.22270755
KDR	rs34231037	1076.834696
KDR	rs2305948	411.3693485
KDR	rs6823788	61.22172143
KDR	rs144426992	37.18888894
KDR	rs61830291	39.46157692
KDR	rs16921960	41.19206922
KDR	rs112564808	30.22735631
KDR	rs117603193	40.4242569
KDR	rs35166255	190.8885017
KDR	rs143501783	36.1374883
KDR	rs149151704	34.40874489
KDR	rs4073039	30.72732045

KDR	rs34211178	367.9242742
KDR	rs62297731	35.87639471
KDR	rs35297343	36.54891941
KDR	rs187570181	44.9674254
KDR	rs8192585	61.08638294
KDR	rs35783704	30.5465136
KDR	rs2491103	31.68739423
KDR	rs112471944	30.54719131
KDR	rs576123	927.4886334
KDR	rs4962125	141.6540135
KDR	rs55978893	41.45997036
MFRP	rs6542680	124.238044
MFRP	rs111329206	68.64351035
MFRP	rs6458	40.63281912
A2M	rs226379	128.7551614
A2M	rs142799933	47.49498183
A2M	rs35161452	30.11535785
A2M	rs80177247	40.44365289
A2M	rs145912452	38.90747783
A2M	rs190543502	102.3106928
A2M	rs6440008	50.66823327
A2M	rs10014102	61.68296153
A2M	rs9312345	39.89409016
A2M	rs1973612	886.1179748
A2M	rs2731672	346.2135925
A2M	rs205757	37.28214256
PLG	rs3127580	318.3721106
PLG	rs142126734	130.0463302
PLG	rs11751347	598.3752245
PLG	rs10945703	295.0740078
PLG	rs62435485	137.9109649
PLG	rs71565799	29.79462507
PLG	rs10858851	107.3250994
PLG	rs12986064	41.89078253
PLG	rs1260326	63.58092809
PLG	rs9898	44.83828982
PLG	rs72848256	58.99937853
PLG	rs3116990	39.86605269
PLG	rs17088106	66.65413122
PLG	rs34778552	42.53067591
CDKN1B	rs111909987	81.9226407
CDKN1B	rs57865714	52.43285464
CDKN1B	rs10820606	441.062153
CDKN1B	rs147602960	33.98401921
PYY	rs62080325	88.36074014
PYY	rs3802841	106.9925377
PYY	rs681343	30.74300448
PYY	rs56092644	90.44599866
PYY	rs1942142	48.42023002
PYY	rs576123	72.56859655
CSF1	rs10922094	74.14941915
CSF1	rs6542680	211.891336
CSF1	rs13217434	32.63520903
TEK	rs116998311	50.63452537
TEK	rs76985127	43.29028666
TEK	rs682632	496.1715842
TEK	rs77688991	56.79611649
TEK	rs116960624	32.76222989

TEK	rs11605349	59.85733504
TEK	rs4796771	34.87996426
TEK	rs117877056	64.49943612
TEK	rs117241334	94.70868214
TEK	rs111903631	62.9526038
TEK	rs1050518	285.8507381
TEK	rs9266689	78.87518802
TEK	rs9267211	85.65857203
TEK	rs1910731	53.95698511
TEK	rs62578578	72.22974859
TEK	rs11244038	1082.576952
TEK	rs11522319	36.1343894
TEK	rs9286383	64.87573549
TEK	rs117446824	40.49139775
ANGPTL4	rs116843064	151.9091569
ANGPTL4	rs146457388	66.13385107
ANGPTL4	rs188023612	34.64816008
ANGPTL4	rs17441745	36.16032309
ANGPTL4	rs6542680	748.8880013
ANGPTL4	rs80220851	37.82487349
ANGPTL4	rs28374650	35.95420847
CDH2	rs10922098	141.0847892
CDH2	rs114115200	49.50984575
CDH2	rs444921	60.20920013
CDH2	rs28471056	36.67767005
CA3	rs192681919	91.25407223
CA3	rs1387065	33.09199861
CA3	rs4266652	78.27530736
CA3	rs2072696	436.9657872
CA3	rs112126364	102.0053001
CA3	rs193257820	69.48298003
CA3	rs72749982	82.218231
CA3	rs3811444	151.022346
CA3	rs113711572	45.35414979
CA3	rs543798	69.0213146
CA3	rs56387760	43.82352501
CA3	rs11085825	51.46136708
CA3	rs1047891	79.91911733
CA3	rs11097789	34.91219925
CA3	rs1405212	34.81003209
CA3	rs1331309	81.1274567
CA3	rs60757417	38.59283487
CST5	rs2424590	995.69931
CST5	rs6076134	1225.288149
CST5	rs73092950	271.1969139
CST5	rs4091660	56.81005077
CST5	rs12032424	33.61496048
CST5	rs13190	47.03243463
CST5	rs17711539	53.04620346
CST5	rs7302130	100.5509024
CST5	rs140998362	39.51222615
CST5	rs1998764	36.64999374
CST5	rs34230357	41.74020897
CST5	rs67020211	82.11489039
CST5	rs7215833	38.56601467
CST5	rs681343	114.6568389
CST5	rs6047101	47.24888899
CST5	rs7270493	79.48638523

CST5	rs149982525	65.4150033
CST5	rs137912604	174.9940048
CST5	rs35065709	34.10937133
CST5	rs17343651	46.10634321
CST5	rs78744669	46.09419307
CST5	rs140615158	37.03650347
CST5	rs117753689	40.03789651
CST5	rs17753046	37.41416219
CST5	rs35023929	42.67491888
CST5	rs78887924	35.2158796
CST5	rs17637318	41.41516527
CST5	rs3749751	63.98058144
ESM1	rs58538591	49.10780174
ESM1	rs4242051	245.8692417
ESM1	rs1363864	99.00918426
ESM1	rs11208704	39.80800646
ESM1	rs7080536	30.17349235
ESM1	rs7310615	50.37123664
ESM1	rs4802271	30.6011839
ESM1	rs445810	32.93769592
ESM1	rs111916003	37.90600212
ESM1	rs3135363	37.96123343
ESM1	rs635634	39.02776136
ESM1	rs7028191	43.77392923
EPHA5	rs151144506	42.92474906
EPHA5	rs2122718	50.24823086
EPHA5	rs62300408	128.6959874
EPHA5	rs72704449	31.875734
EPHA5	rs3917538	32.11206936
FGFR3	rs10922098	297.6296872
FGFR3	rs11569415	31.79120193
FGFR3	rs1355538	73.55578772
FGFR3	rs453821	73.43108874
FGFR3	rs28471056	44.59177251
FGR	rs3737801	274.8901015
FGR	rs11581624	60.87246514
FGR	rs61787564	31.82168231
FGR	rs9971407	31.99248645
FGR	rs450269	40.99804036
MAPKAPK3	rs4980320	35.43577831
MAPKAPK3	rs2182483	30.38023347
MAPKAPK3	rs1354034	46.31093169
MAPKAPK3	rs342200	39.48080432
MAPK8	rs1997365	40.18541673
MAPK8	rs11451044	33.873229
MAPK8	rs79377825	70.26364418
MAPK8	rs1354034	58.5415329
MAPK8	rs6851072	150.8283765
MAPK8	rs10984506	33.59054457
TLR2	rs112595928	40.31809965
TLR2	rs10922098	57.7845847
TLR2	rs17580	132.7853442
UFM1	rs79923112	113.497219
UFM1	rs17397332	36.00114388
UFM1	rs1354034	44.53783089
ZAP70	rs10305748	32.39372629
ZAP70	rs10922098	782.2983635
ZAP70	rs3900909	39.51250291

ZAP70	rs1047286	50.52715647
ZAP70	rs6445035	147.6475528
ZAP70	rs3863087	67.09682655
ZAP70	rs3828879	42.09093355
ZAP70	rs1265941	77.83676095
ZAP70	rs444921	234.1255302
ZAP70	rs78897911	31.47852891
DYNLRB1	rs140797967	80.14742592
DYNLRB1	rs114584644	40.70014747
DYNLRB1	rs1338394	51.36146513
DYNLRB1	rs11204523	1180.821943
DYNLRB1	rs79322592	323.4054684
DYNLRB1	rs11417460	33.68812893
DYNLRB1	rs7926172	58.51716011
DYNLRB1	rs1354034	297.6998854
DYNLRB1	rs342292	34.18460937
DNAJB1	rs11085896	40.60784622
DNAJB1	rs35340377	44.54589028
DNAJB1	rs4980320	36.89650174
DNAJB1	rs762960	31.69677207
DNAJB1	rs9612021	83.36534099
DNAJB1	rs1354034	80.41014379
NACA	rs72882960	30.85567539
NACA	rs35979828	36.48946654
NACA	rs1354034	72.8339862
NACA	rs7009435	37.65524117
PRDX1	rs114488034	75.86449406
PRDX1	rs115822551	144.9525037
PRDX1	rs183380628	39.95393704
PRDX1	rs115874207	67.26486976
PRDX1	rs61774410	116.946047
PRDX1	rs1355538	45.38143256
ACP1	rs113106463	5327.177883
ACP1	rs4854353	4945.559504
ACP1	rs71392506	7905.938371
ACP1	rs1046207	402.4215333
ACP1	rs2685226	132.9368564
ACP1	rs17042314	264.061158
ACP1	rs11895897	260.7527685
ACP1	rs7576574	59.46108469
ACP1	rs9677202	100.4582037
ACP1	rs79857466	122.0531013
ACP1	rs11885419	39.49916326
RPS7	rs10922098	85.11170973
RPS7	rs11602951	77.04481418
RPS7	rs11615667	33.33689865
RPS7	rs1037590	88.9817829
RPS7	rs35550784	31.89657859
RPS7	rs1354034	359.8942129
SBDS	rs79344818	191.8433384
SBDS	rs4980320	32.21407085
SBDS	rs1354034	57.12120559
TPO	rs1451915	31.57403054
TPO	rs10922098	658.494009
TPO	rs1047286	68.565522
TPO	rs2686395	110.3606679
TPO	rs3130955	33.93548334
TPO	rs9501600	190.0580174

TPO	rs35209896	46.38754246
UBE2L3	rs4980320	44.32169122
UBE2L3	rs35979828	32.17265244
UBE2L3	rs1354034	37.70435994
DYNLL1	rs10801548	31.73592888
DYNLL1	rs7546015	212.9901362
DYNLL1	rs79341730	63.4069671
DYNLL1	rs147690220	47.73283638
DYNLL1	rs1706435	30.94663324
DYNLL1	rs9271573	30.39378437
KPNB1	rs4980320	35.2519118
KPNB1	rs10876550	30.98501363
KPNB1	rs1354034	61.32044162
KPNB1	rs342200	31.4094632
LMNB1	rs10922098	95.32185156
LMNB1	rs1355538	32.68326738
LMNB1	rs415620	34.59404846
LMNB1	rs4713498	37.64944487
NAGK	rs2902577	40.15609925
NAGK	rs146010065	93.55601755
NAGK	rs11680831	433.6443123
NAGK	rs1265564	34.08661197
NAGK	rs7412	33.29002029
NAGK	rs1354034	30.36145218
NAGK	rs6884904	30.79782341
NAGK	rs9274470	31.1977845
PGAM1	rs76789850	30.84038726
PGAM1	rs61751507	159.6017624
PGAM1	rs704	31.45748725
PDXP	rs4980320	41.99985993
PDXP	rs35979828	32.4798384
PDXP	rs1354034	36.94010481
PSMD7	rs1355538	30.91707798
PSMD7	rs7009435	34.60897132
PSMD7	rs17430287	34.82978155
SKP1	rs146457388	53.66533794
SKP1	rs6542680	345.8124326
SKP1	rs80220851	32.09152132
SNX4	rs10908505	30.35479472
SNX4	rs1354034	75.97919304
SNX4	rs114694170	41.5397025
SNX4	rs342292	63.35731594
SNX4	rs2645984	165.3678842
SNX4	rs8193003	40.79279813
HSPA1A	rs562047	110.1301889
HSPA1A	rs9269271	36.1150769
HSPA1A	rs28672722	126.4403855
HSPA1A	rs10922098	44.32343884
HSPA1A	rs11646715	35.71346671
HSPA1A	rs72801474	47.43979967
HSPA1A	rs3130169	36.74806996
AGER	rs7746336	148.9252444
AGER	rs2844456	982.0409294
AGER	rs76359966	110.3200687
AGER	rs79451346	179.0848841
AGER	rs28893547	587.296359
AGER	rs1569419	187.6952489
AGER	rs7530075	80.4042254

AGER	rs28551116	30.04571659
AGER	rs1057239	31.98913706
AGER	rs2477664	43.55943979
AGER	rs2461161	43.39526906
AGER	rs7920682	30.30487585
AGER	rs10824665	49.37921003
AGER	rs35705950	56.89022878
AGER	rs17762402	41.17374647
AGER	rs7306778	242.6239225
AGER	rs141603060	31.04864781
AGER	rs149363012	59.31387501
AGER	rs199728188	52.29908256
AGER	rs7962469	43.59867057
AGER	rs10735335	38.12818346
AGER	rs2393329	37.6530866
AGER	rs9600022	49.54110987
AGER	rs56049626	147.1810568
AGER	rs148898768	35.84715286
AGER	rs10138671	523.790363
AGER	rs4924456	38.35366834
AGER	rs34562714	33.50394743
AGER	rs12906898	35.79887997
AGER	rs4886410	48.32154965
AGER	rs35005610	38.54394395
AGER	rs56332871	47.28232777
AGER	rs78442819	30.11170028
AGER	rs62055069	30.1423749
AGER	rs374138354	79.7177963
AGER	rs79730878	90.61269187
AGER	rs7212311	50.24052921
AGER	rs2852746	47.40445556
AGER	rs12461753	39.19976767
AGER	rs6745864	41.74168326
AGER	rs2463097	48.94453043
AGER	rs6045291	61.17670151
AGER	rs34979081	31.69677115
AGER	rs4685105	48.91912679
AGER	rs7613427	33.39373291
AGER	rs7616330	57.14425217
AGER	rs200773131	36.61338936
AGER	rs116592029	39.24055229
AGER	rs117048263	58.79438776
AGER	rs73093253	47.36985526
AGER	rs35210512	79.49597084
AGER	rs12501006	142.5337978
AGER	rs4975932	33.32485167
AGER	rs33443	42.43038965
AGER	rs4700418	30.53633766
AGER	rs6897262	31.62599889
AGER	rs1294430	71.88654119
AGER	rs7744366	87.47166166
AGER	rs143857717	63.93089232
AGER	rs140250893	47.77269417
AGER	rs12201133	107.0704863
AGER	rs73135307	42.98888615
AGER	rs149438404	59.25646099
AGER	rs6961094	44.70035808
AGER	rs111510768	37.67871348

AGER	rs4734869	272.4472509
AGER	rs7868554	32.74849574
AGER	rs11243421	507.1671417
BPI	rs34939349	90.16463257
BPI	rs6069524	1116.802727
BPI	rs11698530	922.0688011
BPI	rs117353696	67.14740632
BPI	rs187806568	103.4169041
BPI	rs199913299	202.1066545
BPI	rs117744914	78.35772181
BPI	rs2511241	266.2847834
BPI	rs9898547	41.45485346
BPI	rs77990716	33.63195293
BPI	rs6037243	46.37998278
BPI	rs140465583	50.92123307
BPI	rs117799040	52.12804425
BPI	rs2668202	107.8016976
C6	rs10067548	129.6775368
C6	rs77444140	385.1869549
C6	rs436077	349.2090329
C6	rs316724	46.84917252
C6	rs10922098	220.1817725
C6	rs56929569	34.68702274
C6	rs199967197	69.19258929
C6	rs61319746	33.11855833
C6	rs114815454	37.49768679
C6	rs181071768	32.81884645
C6	rs9501600	65.31380985
CCL24	rs10922098	394.1603035
CCL24	rs76715859	33.14515597
CCL24	rs1355538	49.95331541
CCL24	rs9501600	92.60799737
CCL24	rs557011	36.76970923
CFB	rs114400264	914.7332766
CFB	rs2072634	933.2651132
CFB	rs114382848	1135.349078
CFB	rs3129888	388.7221681
CFB	rs6937167	67.04608623
CFB	rs12133641	40.97225014
CFB	rs47219	296.7004363
CFB	rs200227895	34.47709523
CFB	rs2230199	33.50851086
CFB	rs4665382	42.19542142
CFB	rs7439493	31.02881602
CFB	rs12207143	29.95365244
CFB	rs78222565	32.15347318
CFB	rs111696429	89.7675686
CFB	rs3117013	36.55177499
CFB	rs181369017	119.0958464
CFB	rs111445649	33.16912671
FN1	rs7580569	59.6654287
FN1	rs138259153	79.08806431
FN1	rs1250258	461.5522581
FN1	rs4674006	74.54866794
FST	rs10922094	101.1724669
FST	rs1355538	30.14212659
FST	rs28573154	34.56297889
GZMB	rs78564162	99.17242773

GZMB	rs10922094	75.25744183
GZMB	rs75475328	74.68247392
GZMB	rs367562546	40.77471415
GZMB	rs1071649	211.7126939
GZMB	rs62395441	406.6025835
GZMB	rs9271636	47.15567017
IL25	rs141363286	35.55864615
IL25	rs2508490	707.2825638
IL25	rs11160923	467.4677816
IL25	rs146796125	34.2024403
IL7	rs10922098	61.69081792
IL7	rs2393326	32.84925063
IL7	rs6993770	32.56516584
CXCL10	rs6532086	72.51193131
CXCL10	rs3184504	126.3458066
CXCL10	rs144219691	29.90858298
CXCL10	rs113341849	69.05153967
CXCL10	rs113587264	32.65705314
CXCL10	rs115214168	31.26956849
CXCL10	rs9273366	36.0069136
XCL1	rs10922098	190.3795839
XCL1	rs1706435	39.5358759
XCL1	rs2284179	35.47971018
XCL1	rs415620	55.95611034
XCL1	rs7774739	57.49641484
NTF3	rs4766352	55.90317563
NTF3	rs148590960	117.2182393
NTF3	rs3782581	63.14790234
NTF3	rs1461214	43.76951958
NTF3	rs12324332	87.07152481
NTF3	rs6566810	40.45318249
NTF3	rs6542680	41.06512702
NTF3	rs10868235	76.28043512
PAPPA	rs1234212	29.90650834
PAPPA	rs35659770	33.77172858
PAPPA	rs10792093	123.7702483
PAPPA	rs35350651	56.3427182
PAPPA	rs7323613	59.58846179
PAPPA	rs12431998	67.08254649
PAPPA	rs35829912	30.10774202
PAPPA	rs3785496	34.15304598
PAPPA	rs4807601	178.6567156
PAPPA	rs78198540	155.5118976
PAPPA	rs1260326	40.17434912
PAPPA	rs2694616	225.5449383
PAPPA	rs13001714	40.20607268
PAPPA	rs193607	64.24832291
PAPPA	rs17354348	49.45982755
PAPPA	rs2242892	42.26239583
PAPPA	rs11701383	41.2598889
PAPPA	rs2305637	127.0851284
PAPPA	rs13089722	368.4318684
PAPPA	rs56003704	37.62411999
PAPPA	rs10068466	31.80277258
PAPPA	rs9348865	36.62647087
PAPPA	rs210795	34.7015231
PAPPA	rs28371059	30.328422
PAPPA	rs13256023	149.273039

PAPPA	rs7840212	65.09105537
PDGFB	rs10822155	76.99477567
PDGFB	rs715440	42.96463671
PDGFB	rs4965869	41.32418963
PDGFB	rs61658003	52.24348114
PDGFB	rs68066031	102.7510622
PDGFB	rs6993770	85.2543907
PDGFB	rs10820606	39.56399511
PLG	rs10922098	654.9556045
PLG	rs1047286	38.97696273
PLG	rs1707652	80.43606479
PLG	rs444921	127.9634734
PLG	rs9270570	43.48157696
PLG	rs9469195	61.52997614
PLG	rs3127580	319.5582005
PLG	rs117152889	63.88180982
PLG	rs11751347	480.6532854
PLG	rs10945703	214.9826295
PLG	rs62435485	124.0559537
PLG	rs117444691	32.32051587
PLG	rs2532434	52.6959005
PLG	rs12986064	32.66645097
PLG	rs1260326	61.28812004
PLG	rs9898	35.06038315
PLG	rs72848256	35.36426597
KLKB1	rs13129009	30.87852087
KLKB1	rs2292428	1105.547121
KLKB1	rs3822055	280.1230824
KLKB1	rs75749843	67.6707618
KLKB1	rs1839746	70.2591544
KLKB1	rs182853123	56.85960841
KLKB1	rs1260326	116.6393485
KLKB1	rs7627243	36.83243684
KLKB1	rs11718570	1082.707951
KLKB1	rs115613078	420.5697281
KLKB1	rs6801612	59.71274182
KLKB1	rs67563526	31.27459733
SERPINA3	rs56317088	59.86577468
SERPINA3	rs10149025	65.33708467
SERPINA3	rs17753556	940.9683774
SERPINA3	rs75498941	123.3182351
SERPINA3	rs77262773	40.70604416
SERPINA3	rs118137024	59.47082004
SERPINA3	rs34434863	29.76114377
SELP	rs17349460	152.2156466
SELP	rs9332575	996.7925112
SELP	rs6425520	52.5249764
SELP	rs10850001	30.40108739
SELP	rs655029	32.49330632
SELP	rs6993770	47.20489534
SELP	rs77265585	34.27273671
SELP	rs635634	363.5429107
TNC	rs74742617	105.8334018
TNC	rs36118932	189.0893229
TNC	rs144359615	1336.703164
TNC	rs10817700	1265.460975
TNC	rs10759806	511.2701222
TNC	rs78705678	51.04356113

TNC	rs2477642	41.13011319
TNC	rs1316361	45.1039994
TNC	rs9668337	35.86282369
TNC	rs760715	74.37479658
TNC	rs138529507	78.14418171
TNC	rs2685417	846.3619002
TNC	rs116933711	41.68781229
TNC	rs79098169	57.59368121
TNC	rs76104930	40.90635817
TNC	rs10983509	123.0422008
TNC	rs2722830	76.92350408
TGFB2	rs6542680	106.8559616
TGFB2	rs2858879	31.89369792
TGFB2	rs211161	38.66151429
TGFB2	rs3778754	30.15740138
F2	rs3136516	180.0437093
F2	rs34105209	51.62654818
F2	rs7232	30.65194905
F2	rs1260326	62.90457832
F2	rs5030062	153.9208272
F2	rs2292423	558.3403018
F2	rs2963846	56.31974849
F2	rs62386961	30.07890221
F2	rs139281525	46.56948751
F2	rs6875461	145.9998415
F2	rs4976691	1370.821454
F2	rs185903163	86.12247876
F2	rs6880928	33.23248457
F2	rs112260799	48.94127995
F2	rs112164771	39.28041019
PLAU	rs150026080	431.6838333
PLAU	rs2633298	950.2946507
PLAU	rs192913987	121.4254378
PLAU	rs11000927	51.02813354
PLAU	rs10922098	117.5751939
PLAU	rs12570183	44.76416827
PLAU	rs75350427	54.02608212
PLAU	rs147611419	329.4243151
PLAU	rs7924152	64.10461231
PLAU	rs78290332	64.92556721
PLAU	rs78689694	49.41692655
PLAU	rs55714927	86.02272194
PLAU	rs143501783	32.13253422
PLAU	rs9948409	74.2501307
PLAU	rs2302524	271.6809832
PLAU	rs36229204	210.1393839
PLAU	rs2470854	35.06717407
PLAU	rs147906938	36.46554721
PLAU	rs8176743	41.14477671
CFH	rs77834171	46.69136371
CFH	rs70620	711.2086905
CFH	rs78265616	65.76168531
MMP2	rs171498	124.1428602
MMP2	rs75609019	50.64988807
MMP2	rs77542162	41.56679373
MMP2	rs1260326	58.97962516
MMP2	rs11130306	87.97703703
TF	rs8177245	280.8504321

TF	rs2690093	37.69691427
TF	rs80215559	118.3842947
EIF4EBP2	rs10822155	38.82697871
EIF4EBP2	rs9306429	32.77270764
EIF4EBP2	rs2367289	97.55959614
PGD	rs201225440	70.52087595
PGD	rs72867469	74.99158535
PGD	rs35979828	41.85061168
PGD	rs1354034	31.66230922
PGD	rs4734879	30.02573228
AKR7A2	rs61766662	305.7680012
AKR7A2	rs11594179	45.2483463
AKR7A2	rs4980320	37.80496503
AKR7A2	rs1354034	39.25670214
AKR7A2	rs342200	45.74262785
AKR7A2	rs6993770	125.9163278
AKR1A1	rs41269067	977.2975237
AKR1A1	rs150080554	217.9755925
AKR1A1	rs113151279	254.124982
AKR1A1	rs141228413	1044.389679
AKR1A1	rs3001722	142.6687969
AKR1A1	rs72673097	51.89243604
AKR1A1	rs1738050	138.2893469
AKR1A1	rs75339918	206.0238221
AKR1A1	rs188254809	542.2883201
AKR1A1	rs181255685	93.58077107
AKR1A1	rs1354034	38.23443534
AKR1A1	rs4541868	40.33703035
CFL1	rs10922098	36.75127152
CFL1	rs5167	96.60006297
CFL1	rs1706417	64.50523214
EPS15L1	rs4980320	42.00871299
EPS15L1	rs10876550	38.47713186
EPS15L1	rs185009887	30.2392341
EPS15L1	rs1354034	58.75471209
FER	rs13178668	81.97358309
FER	rs4980320	36.4738769
FER	rs6224	57.5090776
FER	rs1354034	31.91370642
SYNCRIP	rs72748420	33.42579891
SYNCRIP	rs1329424	762.1191574
SYNCRIP	rs3900909	35.55774249
SYNCRIP	rs1354034	48.36708866
SYNCRIP	rs1707652	121.9725665
SYNCRIP	rs550671	196.2900607
SYNCRIP	rs9270570	37.23932235
SYNCRIP	rs9469195	73.66047429
EIF4G2	rs4980320	45.69180704
EIF4G2	rs1354034	102.5634788
EIF4G2	rs342200	42.56203439
IGF1R	rs80262698	39.97040326
IGF1R	rs35359680	34.19467833
IGF1R	rs72752895	174.7890216
IGF1R	rs78705678	55.9766208
IGF1R	rs2477642	61.98471163
IGF1R	rs78689694	56.61825543
IGF1R	rs11605349	55.07188238
IGF1R	rs11574938	42.68203085

IGF1R	rs12945299	78.64425977
IGF1R	rs116852327	69.9840668
IGF1R	rs2838737	44.32514657
IGF1R	rs709821	29.95204934
IGF1R	rs146798134	34.29514385
IGF1R	rs635634	724.6244238
IGF1R	rs41307428	88.95234583
IL1RL1	rs79400648	1053.545563
IL1RL1	rs11123911	1016.498583
IL1RL1	rs72627435	4867.729436
IL1RL1	rs74909818	299.6280943
IL1RL1	rs148378006	389.0972539
IL1RL1	rs192790767	62.58074158
IL1RL1	rs151312446	241.5167741
IL1RL1	rs3915279	104.5715289
IL1RL1	rs12757307	36.44331896
IL1RL1	rs672806	205.8103249
IL1RL1	rs35166255	450.0189967
IL1RL1	rs117926781	38.8860072
IL1RL1	rs527629	35.45423323
IL1RL1	rs7176022	30.40179649
IL1RL1	rs35598617	73.0334438
IL1RL1	rs55714927	130.7964323
IL1RL1	rs307626	30.8310166
IL1RL1	rs143501783	47.62805221
IL1RL1	rs9906366	34.3898061
IL1RL1	rs1186706	50.32680742
IL1RL1	rs70958912	49.50837952
IL1RL1	rs79182285	72.27212204
IL1RL1	rs10199430	31.83859926
IL1RL1	rs149935614	40.65416135
IL1RL1	rs113281683	35.5163633
IL1RL1	rs146792612	62.69991867
IL1RL1	rs114411783	149.60804
IL1RL1	rs142469916	41.49252509
IL1RL1	rs143783875	79.56468938
IL1RL1	rs117777073	87.2715513
IL1RL1	rs78717642	49.89954383
IL1RL1	rs113280910	49.38989236
IL1RL1	rs9611177	44.63887593
IL1RL1	rs7627757	33.85678636
IL1RL1	rs9842051	77.1035649
IL1RL1	rs6837794	33.99094074
IL1RL1	rs855658	31.46951668
IL1RL1	rs4841437	31.00924206
IL1RL1	rs635634	136.7877713
LCMT1	rs10922098	141.9749807
LCMT1	rs4980320	32.30021392
LCMT1	rs5030044	255.2210952
LCMT1	rs28573154	47.26219926
LCMT1	rs7774739	32.88461039
L1CAM	rs3902968	47.46116144
L1CAM	rs2477639	46.30269323
L1CAM	rs11007130	29.90987771
L1CAM	rs11220463	54.14472715
L1CAM	rs35257264	31.58598517
L1CAM	rs55714927	86.46518959
L1CAM	rs17880604	39.2165591

L1CAM	rs1260326	47.13570913
L1CAM	rs10928231	36.6390114
L1CAM	rs12470153	34.79665457
L1CAM	rs35915962	30.32213761
L1CAM	rs4894808	37.90993153
L1CAM	rs35003579	34.01635118
L1CAM	rs849133	83.9865705
L1CAM	rs1204371	39.57372658
L1CAM	rs4644350	55.85909318
L1CAM	rs78409522	40.67308704
L1CAM	rs635634	529.9900982
NUDCD3	rs306995	74.17458142
NUDCD3	rs1354034	56.6069741
NUDCD3	rs1355538	77.81163442
PA2G4	rs10913644	77.0907297
PA2G4	rs4980320	35.70462972
PA2G4	rs35979828	39.30800213
PA2G4	rs10490468	66.19615811
PA2G4	rs78909033	87.51244279
PA2G4	rs1354034	69.13914717
PA2G4	rs9494145	34.78454511
PON1	rs7546015	90.47328715
PON1	rs1355538	39.21575289
PON1	rs9501600	52.96362618
PFDN5	rs1329424	233.119528
PFDN5	rs117848237	30.43573204
PFDN5	rs10405535	75.36192745
PFDN5	rs141714582	39.39075635
PFDN5	rs1706435	53.71513354
PFDN5	rs391165	66.70553522
PFDN5	rs34927613	32.3380915
GPI	rs11611373	62.08547226
GPI	rs75036712	62.66648553
GPI	rs1354034	43.9468794
GPI	rs342200	30.88537642
PEBP1	rs11068663	122.834133
PEBP1	rs192243378	108.1423668
PEBP1	rs4766891	128.5110643
PEBP1	rs73217917	2215.813031
PEBP1	rs77598459	37.5793273
PEBP1	rs76027544	170.025475
PEBP1	rs61945816	110.9662786
PEBP1	rs185465848	43.0970194
PEBP1	rs1354034	29.85243657
P4HB	rs181355252	45.47485853
P4HB	rs62079523	232.8582516
P4HB	rs10922094	67.26212446
P4HB	rs74090714	50.30165002
P4HB	rs9469079	32.17141285
P4HB	rs28374650	42.77477787
PSMA2	rs1451915	30.03703959
PSMA2	rs10922098	539.7044835
PSMA2	rs11569415	37.84098097
PSMA2	rs2686395	112.3021839
PSMA2	rs9501600	126.888282
PSMA2	rs72508422	32.01495985
PSMA2	rs587495	43.88292372
RBM39	rs10922094	33.08420593

RBM39	rs6542680	67.90611128
RBM39	rs3129752	31.885473
NAPA	rs1984983	53.0136593
NAPA	rs1354034	36.30872129
NAPA	rs342200	31.15213099
SPHK1	rs12939873	47.26784396
SPHK1	rs114602896	57.93689735
SPHK1	rs35340377	40.22354509
SPHK1	rs4759076	41.07107718
SPHK1	rs11082304	38.02412931
SPHK1	rs342292	53.0507447
SPON1	rs75495212	53.08344586
SPON1	rs1819084	100.4484468
SPON1	rs7116230	1001.207994
SPON1	rs11023122	206.9557996
SPON1	rs78705678	34.53585093
SPON1	rs77542162	142.0467026
SPON1	rs492602	63.06477283
SPON1	rs2534660	50.66439119
SPON1	rs9274510	30.4616243
SPON1	rs11244049	62.26766832
TK1	rs10922098	374.7261909
TK1	rs11569415	59.00332311
TK1	rs1707652	93.35873402
TK1	rs3130955	35.33366024
TK1	rs9501600	176.1333186
TK1	rs35209896	51.62156812
LCORL	rs10922094	168.1422151
LCORL	rs1355538	35.03078475
LCORL	rs391165	53.47981091
LCORL	rs28471056	38.45417651
DCTPP1	rs11640950	38.02795916
DCTPP1	rs1984769	37.4921012
DCTPP1	rs78705678	103.3284337
DCTPP1	rs2477642	127.9061746
DCTPP1	rs137927952	31.93481673
DCTPP1	rs11111634	37.41517794
DCTPP1	rs17090693	73.04722452
DCTPP1	rs1042153	38.09275271
CST2	rs77709598	60.3549778
CST2	rs6137875	240.6041988
CST2	rs62208869	931.1611251
CST2	rs6049023	1002.034372
CST2	rs2424610	221.6916344
CST2	rs6132718	34.65052431
CST2	rs6427943	131.2773127
CST2	rs55733944	32.8112699
CST2	rs836124	54.34143522
CST2	rs11611410	201.167806
CST2	rs200029065	70.09051587
CST2	rs848097	52.1873992
CST2	rs34230357	67.47775109
CST2	rs67020211	129.124928
CST2	rs681343	77.34599306
CST2	rs73125764	78.64173144
CST2	rs141264356	31.28205154
CST2	rs113166859	36.04134989
CST2	rs62213165	59.15354837

CST2	rs4246778	72.88676798
CST2	rs10435096	33.43982183
BOC	rs76491164	41.66279211
BOC	rs66819830	57.05121317
BOC	rs4682481	580.2267191
BOC	rs9866486	35.74460677
BOC	rs112168484	54.24195461
BOC	rs1260326	102.3089533
BOC	rs12621136	34.22495677
BOC	rs9612041	40.7285893
BOC	rs78753351	47.68647832
BOC	rs7793710	63.66503517
BOC	rs112875651	30.04763792
BOC	rs635634	84.21842014
CLEC1B	rs11053499	57.15336778
CLEC1B	rs659928	312.7457439
CLEC1B	rs10761739	64.78831384
CLEC1B	rs3792386	30.27276027
CLEC1B	rs114694170	37.38081309
CLEC1B	rs6993770	56.99734551
CRP	rs145663198	48.9028921
CRP	rs2808628	350.0267436
CRP	rs16826069	29.73601977
CRP	rs11208691	168.974935
CRP	rs61812598	156.7647719
CRP	rs9738226	327.3421432
CRP	rs2239222	34.26076824
CRP	rs79391862	53.05839896
CRP	rs17616063	78.96023221
CRP	rs75746043	37.6181419
CRP	rs8104548	62.36992886
CRP	rs429358	556.2410046
CRP	rs1260326	34.51214487
CRP	rs6734238	46.82321794
CRP	rs1800961	39.8998007
CRP	rs9268557	44.51676778
ICAM1	rs78374606	108.9590447
ICAM1	rs12611271	1035.538731
ICAM1	rs149481337	368.2877401
ICAM1	rs35660845	16555.93074
ICAM1	rs3093033	231.6425878
ICAM1	rs35186095	8288.490991
ICAM1	rs10427017	81.1131538
ICAM1	rs6603148	43.39031837
ICAM1	rs62118265	136.0345989
ICAM1	rs75410819	152.4488313
ICAM1	rs140614696	100.7138327
ICAM1	rs635634	471.4365687
DAPK2	rs12903543	33.81090312
DAPK2	rs55986634	1023.03287
DAPK2	rs79227106	58.34038577
DAPK2	rs71394560	33.37468742
DAPK2	rs11594179	31.83851248
DAPK2	rs4759076	59.03696959
DAPK2	rs1354034	38.09081257
DYRK3	rs1451915	29.99551178
DYRK3	rs10922098	545.2938623
DYRK3	rs6445035	88.8335721

DYRK3	rs555007	63.55443605
DYRK3	rs453821	164.745005
DYRK3	rs9272526	34.07747034
GDF15	rs11670016	128.3801786
GDF15	rs12462034	1078.352921
GDF15	rs8105118	577.6899087
GDF15	rs7251232	81.43853123
GDF15	rs114681259	72.49747187
GDF15	rs77985679	56.6037508
GDF15	rs115605748	29.98928804
DHH	rs4980320	42.73727454
DHH	rs11451044	30.04474172
DHH	rs1354034	33.39443143
IFNL2	rs368465	53.12956068
IFNL2	rs7183	35.0361818
IFNL2	rs6542680	38.16299295
IFNL2	rs9272371	31.13697031
MST1	rs183938645	6200.076647
MST1	rs77440492	366.1534669
MST1	rs116285936	1318.573948
MST1	rs147118406	778.1091335
MST1	rs7633243	459.7779468
MST1	rs73074821	619.8377917
MST1	rs191534350	736.9340531
MST1	rs1762486	62.64264298
MST1	rs1260326	35.25251333
MST1	rs6771444	44.03237886
MST1	rs199753792	39.7109662
MST1	rs13082427	37.87885066
MST1	rs9846752	48.16540256
MST1	rs77597703	38.85580176
MST1	rs1907581	64.39000077
MST1	rs9863890	186.2394351
MST1	rs73075540	1016.229032
MST1	rs115194087	311.3918959
MST1	rs144275493	948.106153
MST1	rs185820072	132.4661498
MST1	rs137985341	419.8870629
MST1	rs187630010	5383.883336
MST1	rs114976310	335.1301081
MST1	rs114964512	986.4570307
MST1	rs79312973	802.3818237
MST1	rs140642246	302.5680242
MST1	rs74320783	114.9569389
MST1	rs11715674	45.77406556
MST1	rs9847267	48.47657998
SLPI	rs6104034	173.984966
SLPI	rs7205804	37.48529156
SLPI	rs5112	117.7215177
SLPI	rs12986064	48.65328418
SLPI	rs1355538	30.12713163
SLPI	rs72813185	32.71589556
SLPI	rs3130060	31.15471174
BCL2L1	rs72748420	30.36474211
BCL2L1	rs10922098	665.304782
BCL2L1	rs3900909	31.12514984
BCL2L1	rs1047286	36.01836138
BCL2L1	rs1707652	145.7236669

BCL2L1	rs555007	69.83009297
BCL2L1	rs550671	187.9082922
BCL2L1	rs9272526	30.44610352
COLEC11	rs72773101	58.08623472
COLEC11	rs115785583	105.9195062
COLEC11	rs78062064	972.5774812
COLEC11	rs10183939	6683.817012
COLEC11	rs11123637	447.2882561
COLEC11	rs111625037	78.69515006
COLEC11	rs61801021	68.12733487
COLEC11	rs1031101	269.3617712
COLEC11	rs114986725	36.31449886
COLEC11	rs10175354	77.06478514
COLEC11	rs1486236	107.7572479
COLEC11	rs73886131	65.53226141
COLEC11	rs72682450	166.8090564
COLEC11	rs9693053	88.72439623
COLEC11	rs10858300	95.98023097
ENPP7	rs117151992	50.06637779
ENPP7	rs80071684	290.8952133
ENPP7	rs6565654	1114.708056
ENPP7	rs113286331	753.1842375
ENPP7	rs147002317	275.7580697
ENPP7	rs183833760	139.7437331
ENPP7	rs72846544	295.2616517
ENPP7	rs144360067	166.4596032
ENPP7	rs74000618	44.33597362
ENPP7	rs7217011	99.77372605
ENPP7	rs2477664	33.862507
ENPP7	rs692719	30.54748325
ENPP7	rs10509186	71.28201028
ENPP7	rs111522557	46.75651772
ENPP7	rs6130613	33.7645963
ENTPD3	rs4512628	36.00769363
ENTPD3	rs10922098	844.8460992
ENTPD3	rs7513100	36.9767261
ENTPD3	rs1047286	90.5819856
ENTPD3	rs2686395	116.2144014
ENTPD3	rs555007	84.33760245
ENTPD3	rs550671	204.7431755
ENTPD3	rs9272526	35.0708234
ENTPD5	rs150243994	54.94140049
ENTPD5	rs142343302	313.4898071
ENTPD5	rs112109204	243.6927363
ENTPD5	rs150185151	73.96510837
ENTPD5	rs28458356	1044.525696
ENTPD5	rs10922098	100.1878465
ENTPD5	rs2477664	41.91749712
ENTPD5	rs2461161	42.16740548
ENTPD5	rs10843403	74.48605925
ENTPD5	rs2244608	65.33133126
ENTPD5	rs1205042	43.86807479
ENTPD5	rs145561224	125.2784096
ENTPD5	rs12433287	35.18074927
ENTPD5	rs201241015	37.51077704
ENTPD5	rs17580	35.69980327
ENTPD5	rs112963922	37.38344223
ENTPD5	rs9302635	43.02886862

ENTPD5	rs8178824	54.49490795
ENTPD5	rs7236989	41.47217627
ENTPD5	rs4806498	30.17330713
ENTPD5	rs1707652	43.8341834
ENTPD5	rs16860640	29.99618055
ENTPD5	rs55663055	73.90840598
ENTPD5	rs149062332	765.6812359
ENTPD5	rs13068263	103.8007683
ENTPD5	rs41285751	42.0316792
ENTPD5	rs147390813	48.83488612
ENTPD5	rs33951980	32.9965163
FCRL3	rs12404207	52.02029984
FCRL3	rs74818440	63.46062776
FCRL3	rs72702697	95.57073229
FCRL3	rs1133427	918.438302
FCRL3	rs115423724	759.0922291
FCRL3	rs11264868	208.9838596
FCRL3	rs191679185	34.49125212
FCRL3	rs2213948	57.71781871
FCRL3	rs572515	98.10289319
FCRL3	rs9864801	29.72014434
FCRL3	rs415620	30.10825334
LRRTM3	rs10922094	63.99637794
LRRTM3	rs2106003	32.15876531
LRRTM3	rs6542680	93.64101685
LRRTM3	rs557011	30.41076943
LRRTM3	rs211161	31.52382735
LRRTM3	rs3778754	31.42872308
MFGE8	rs4932442	174.807374
MFGE8	rs34239095	813.2131588
MFGE8	rs1550473	63.97307953
MFGE8	rs76829020	54.02775555
MFGE8	rs12274522	31.19636335
MFGE8	rs7232	300.7886579
MFGE8	rs964184	174.7992754
MFGE8	rs261290	97.17155298
MFGE8	rs473224	48.90562764
MFGE8	rs1260326	54.66456522
MFGE8	rs11720167	84.15515121
MFGE8	rs3863087	29.96229039
MFGE8	rs35103650	30.08115581
MFGE8	rs11134475	36.44097624
MFGE8	rs35062987	38.63005471
MFGE8	rs41270871	29.88775124
MFGE8	rs998584	37.57920074
MFGE8	rs7841189	38.51779363
MFGE8	rs2980888	46.81257427
PCSK7	rs61904945	98.10169037
PCSK7	rs76202632	34.79976939
PCSK7	rs575280	1140.447363
PCSK7	rs7126214	1089.238894
PCSK7	rs117594337	419.5954422
PCSK7	rs536648	844.1256816
PCSK7	rs73018313	291.6984317
PCSK7	rs7949751	72.91811143
PCSK7	rs11825535	107.7498847
PCSK7	rs10892330	62.66333005
PCSK7	rs12211087	237.6542862

PCSK7	rs1634771	113.4863164
PCSK7	rs143867680	41.86576715
PDPK1	rs4980320	32.69163634
PDPK1	rs185009887	31.97104539
PDPK1	rs1354034	52.56783573
PDPK1	rs342293	38.22274118
SIGLEC1	rs34058426	46.47170972
SIGLEC1	rs143959838	36.5705111
SIGLEC1	rs143501783	30.33773714
SIGLEC1	rs62165726	29.95293093
SPARCL1	rs13150578	1011.024508
SPARCL1	rs13144424	738.5683509
SPARCL1	rs76370957	196.8973823
SPARCL1	rs9799552	39.29453493
SPARCL1	rs2616261	91.10762586
SPARCL1	rs2035675	94.38207857
SPARCL1	rs77681972	30.32554903
SPARCL1	rs146139118	33.92876136
SPARCL1	rs72655742	47.08689826
SPARCL1	rs217184	638.8468356
SPARCL1	rs72791162	46.72853216
SPARCL1	rs9937452	46.74731791
SPARCL1	rs77262773	144.5630474
SPARCL1	rs11085810	30.34319738
SPARCL1	rs11903631	32.87692501
SPARCL1	rs145316268	88.17730268
SPARCL1	rs896656	102.8428933
SPARCL1	rs13102162	54.6228823
SPARCL1	rs28367727	37.91573381
SPARCL1	rs7844805	33.55663305
SPHK2	rs443527	59.72228265
SPHK2	rs10922094	261.6343082
SPHK2	rs1707652	54.5058344
SPHK2	rs9266642	30.05309794
SPHK2	rs555007	43.02555169
SPHK2	rs550671	87.16018937
CHST15	rs6588740	116.7582928
CHST15	rs4379785	79.34019351
CHST15	rs73183043	89.70286055
CHST15	rs77542162	127.3619061
CHST15	rs6506033	38.97206855
CHST15	rs28873836	253.6910015
CHST15	rs907868	32.39306436
CHST15	rs72492332	49.40223587
CHST15	rs3129724	34.47918925
CHST15	rs146798134	37.97039471
CHST15	rs635634	869.8831101
CHST15	rs41307428	51.74324304
TGM3	rs6047520	72.89991074
TGM3	rs214830	2330.070372
TGM3	rs73892471	70.85376969
TGM3	rs144337455	43.23363602
TGM3	rs80278967	89.80469188
TGM3	rs34739718	59.08457133
TGM3	rs190547981	30.72527826
TGM3	rs1071742	50.70332012
TGM3	rs204879	80.20216643
TGM3	rs57760582	241.9616528

TGM3	rs3129781	672.4930302
TGM3	rs12662536	51.56255359
TPM2	rs72748420	45.78989832
TPM2	rs1329424	1296.41964
TPM2	rs3900909	61.44839391
TPM2	rs10801660	41.0031385
TPM2	rs1047286	70.63793733
TPM2	rs1707652	207.1336245
TPM2	rs1265941	118.4300522
TPM2	rs550671	317.1864485
TPM2	rs112164276	40.7051885
TPM2	rs41548618	41.84162047
ZAP70	rs72748420	41.74549792
ZAP70	rs114009540	40.64472532
ZAP70	rs1329424	1193.536041
ZAP70	rs3900909	57.13438265
ZAP70	rs1047286	90.48609973
ZAP70	rs1707652	191.0828943
ZAP70	rs3099847	33.76607732
ZAP70	rs1265941	102.6047436
ZAP70	rs550671	273.0717948
ZAP70	rs9276606	38.99944372
SERPING1	rs143045302	170.693106
SERPING1	rs11229039	1294.701435
SERPING1	rs117960683	414.279408
SERPING1	rs147960245	187.8953987
SERPING1	rs117358897	57.37205863
SERPING1	rs117279020	53.96937168
SERPING1	rs835803	48.25180595
SERPING1	rs11604846	89.70772135
SERPING1	rs143597074	182.5980855
SERPING1	rs9795516	77.11854747
SERPING1	rs11229778	111.7188443
SERPING1	rs556693	62.74463066
SERPING1	rs61898772	57.03785347
SERPING1	rs17156580	35.4109668
SERPING1	rs373096669	32.58001407
SERPING1	rs12422724	80.78852268
SERPING1	rs3184504	39.2298522
SERPING1	rs17580	63.5304155
SERPING1	rs77542162	124.2612213
C3	rs11085197	119.9577715
C3	rs10922098	303.1722068
C3	rs12975366	37.12221406
C3	rs9501393	57.77184683
C3	rs72848261	36.80133535
FGF7	rs4980320	37.35943374
FGF7	rs10876550	30.59977857
FGF7	rs1354034	36.31203392
MMP12	rs2186990	50.6008046
MMP12	rs118009855	100.972926
MMP12	rs470530	2919.690839
MMP12	rs368148236	709.9705869
MMP12	rs114888379	147.6635837
MMP12	rs2332969	31.16684487
MMP12	rs147023841	66.85431702
MMP12	rs4755031	39.83691919
MMP12	rs73543030	79.43664165

MMP12	rs61729512	68.1202913
MMP12	rs12975366	73.17225173
NCAM1	rs61902388	284.848554
NCAM1	rs11214489	619.5464933
NCAM1	rs2288158	677.1374492
NCAM1	rs333947	68.97384656
NCAM1	rs9425316	37.80028973
NCAM1	rs142597063	31.50364685
NCAM1	rs1318331	36.0337141
NCAM1	rs705705	36.1027229
NCAM1	rs78091364	36.67620923
NCAM1	rs41455646	45.60020066
NCAM1	rs181707610	74.19616333
NCAM1	rs1028883	47.75725992
NCAM1	rs11074065	53.2157785
NCAM1	rs41463547	29.83544315
NCAM1	rs35996069	52.70762447
NCAM1	rs148404036	81.87663407
NCAM1	rs143966156	85.90570618
NCAM1	rs117896937	47.58455209
NCAM1	rs1260326	59.95435544
NCAM1	rs11130306	38.10539655
NCAM1	rs145887694	33.79222645
NCAM1	rs79953286	34.7287438
NCAM1	rs4959098	52.22529125
PDGFA	rs145726150	50.03947325
PDGFA	rs150821630	38.46288755
PDGFA	rs145793648	31.93767976
PDGFA	rs3811444	69.02843198
PDGFA	rs10822155	85.99769349
PDGFA	rs113661219	32.15370343
PDGFA	rs12468189	31.78306443
PDGFA	rs199772122	32.18777576
PDGFA	rs6993770	128.5670134
CLEC11A	rs77578935	88.73684417
CLEC11A	rs117120439	64.18183288
CLEC11A	rs148097549	35.48298551
CLEC11A	rs117083993	945.2687041
CLEC11A	rs34734775	48.36180972
CLEC11A	rs139404143	30.24045699
CLEC11A	rs148851768	36.9016434
CLEC11A	rs76239001	45.8331658
CLEC11A	rs112906159	61.00078834
CLEC11A	rs78004438	62.25548446
CLEC11A	rs4981022	124.0896229
CLEC11A	rs188547348	77.67695755
CLEC11A	rs76428106	41.81773589
CLEC11A	rs17075907	33.77551217
CLEC11A	rs6573880	36.74346073
CLEC11A	rs11621300	42.7671729
CLEC11A	rs36102158	53.35496702
CLEC11A	rs3830847	142.639867
CLEC11A	rs11043	31.31058807
CLEC11A	rs116853700	47.46667921
CLEC11A	rs139675927	38.62819198
CLEC11A	rs62180989	37.39040818
CLEC11A	rs2595507	40.22175535
CLEC11A	rs2236252	37.82351836

CLEC11A	rs62242981	30.88126668
CLEC11A	rs2305637	90.83431448
CLEC11A	rs6775011	48.0015135
CLEC11A	rs28436034	31.21274638
CLEC11A	rs10107630	82.85530354
CLEC11A	rs11144926	33.80761842
PRSS22	rs3094789	42.65004945
PRSS22	rs7204669	702.8621342
PRSS22	rs76110063	51.60544199
PRSS22	rs111497883	32.0979922
PRSS22	rs78705678	65.50212504
PRSS22	rs2477642	69.13958197
PRSS22	rs73183043	52.21398423
PRSS22	rs56344427	29.97259162
PRSS22	rs28817896	37.8934002
PRSS22	rs1049163	29.74796157
BST1	rs144572272	4071.503022
BST1	rs80094095	183.7000747
BST1	rs76892788	938.4942907
BST1	rs10755215	5596.802432
BST1	rs73801515	439.993231
BST1	rs55768074	271.1587471
BST1	rs13133332	298.7143523
BST1	rs34656952	35.43735803
BST1	rs12280645	139.7825224
BST1	rs55714927	46.72277851
BST1	rs116852327	32.03655309
BST1	rs4760	298.3614847
BST1	rs116210165	33.73057719
BST1	rs144827449	29.82285466
BST1	rs16890557	127.0923249
BST1	rs142983596	210.2124758
BST1	rs143786529	118.8855852
BST1	rs74695642	99.14889883
CBX5	rs1451915	38.2700627
CBX5	rs10922098	829.0179317
CBX5	rs7513100	33.412903
CBX5	rs1047286	59.5615851
CBX5	rs1707652	171.4683169
CBX5	rs1265941	81.15132411
CBX5	rs550671	222.1558656
CBX5	rs145704749	31.65532351
CDON	rs61029109	59.92767799
CDON	rs7120521	2841.776766
CDON	rs562672	123.7684074
CDON	rs657225	88.27336479
CDON	rs78705678	29.9377865
CDON	rs143487718	41.94031435
CDON	rs116852327	31.519646
CDON	rs187099314	31.81265403
CLU	rs602633	29.88047266
CLU	rs10922098	112.9728743
CLU	rs75849644	47.51684173
CLU	rs1126605	140.1409789
CLU	rs704	163.6288982
CLU	rs6073971	71.1033672
CLU	rs56210800	52.59477655
CLU	rs140431012	52.48281016

CLU	rs2668202	52.29160655
CLU	rs147390813	57.64048721
CLU	rs4713498	34.53068019
COL23A1	rs203688	84.89531009
COL23A1	rs75327431	32.86500688
COL23A1	rs444921	31.95911759
PPBP	rs7080386	128.6924149
PPBP	rs4541868	58.6315264
PPBP	rs77265585	31.10694872
ADGRE2	rs117798403	53.59012927
ADGRE2	rs375122042	122.703273
ADGRE2	rs116910289	70.06809736
ADGRE2	rs888875	173.6212499
ADGRE2	rs12973667	1390.67561
ADGRE2	rs145145957	63.83594474
ADGRE2	rs144196512	67.81514071
ADGRE2	rs117033566	34.70189724
ADGRE2	rs2477664	84.02842406
ADGRE2	rs2461161	51.77017936
ADGRE2	rs117633773	32.2358543
ADGRE2	rs12945299	79.6530256
ADGRE2	rs143501783	34.96403835
ADGRE2	rs11665758	37.63160458
ADGRE2	rs28396306	31.66219749
FLRT1	rs572515	53.36633353
FLRT1	rs4980320	39.74597487
FLRT1	rs1354034	30.51048963
FUT3	rs80124263	68.2109426
FUT3	rs75136459	130.7908272
FUT3	rs28721898	2237.154806
FUT3	rs3894326	922.5368996
FUT3	rs77315797	85.00551073
FUT3	rs138022188	96.56218681
FUT3	rs146505276	87.23731313
FUT3	rs61003547	73.68761982
FUT3	rs75437292	49.74482745
FUT3	rs528298	110.0252813
FUT3	rs601338	384.428723
FUT3	rs1355538	34.41798095
FUT5	rs189922342	68.20282548
FUT5	rs144449404	103.4369594
FUT5	rs4807031	233.0883209
FUT5	rs73920020	142.1267184
FUT5	rs145405806	265.9278459
FUT5	rs117032075	1091.595284
FUT5	rs11878862	3056.183705
FUT5	rs10402728	107.9151097
FUT5	rs140538827	51.59502603
FUT5	rs4914939	47.88786672
FUT5	rs4845623	38.37582585
FUT5	rs2477664	83.29628212
FUT5	rs2461161	54.11082485
FUT5	rs9738365	92.95625466
FUT5	rs10771887	52.79170565
FUT5	rs2393775	166.3716031
FUT5	rs55780081	34.1413798
FUT5	rs374087027	30.2526826
FUT5	rs4264433	42.44824408

FUT5	rs147632697	55.57443814
FUT5	rs10401969	37.56996391
FUT5	rs504963	60.93475408
FUT5	rs1800961	36.13274974
FUT5	rs1800759	33.67894705
FUT5	rs28601761	32.4699454
ADGRG5	rs10922098	353.2647395
ADGRG5	rs1707652	81.63321843
ADGRG5	rs438999	79.42007241
ADGRG5	rs9272068	42.54057121
IL34	rs145439832	31.00401836
IL34	rs72748420	40.68185645
IL34	rs1329424	1375.535311
IL34	rs3900909	65.19977555
IL34	rs10801660	40.91705973
IL34	rs1047286	42.35325555
IL34	rs1707652	183.8606943
IL34	rs1265941	130.5795791
IL34	rs550671	348.9079488
IL34	rs9269234	48.04318076
IL34	rs3747843	30.44294918
KIRREL3	rs10922098	63.23097692
KIRREL3	rs62269916	29.84730858
KIRREL3	rs13208583	36.96416365
KYNU	rs7575509	47.21819407
KYNU	rs10206294	134.4701319
KYNU	rs7607734	2030.866776
KYNU	rs3795872	132.2579291
KYNU	rs1697422	32.12859339
KYNU	rs368465	154.5220303
KYNU	rs1706435	61.97712322
KYNU	rs9501600	46.29276098
NXPH1	rs587255	139.607407
NXPH1	rs12085435	82.87812952
NXPH1	rs10922098	647.7624783
NXPH1	rs1552948	90.13498313
NXPH1	rs13412535	387.1540695
NXPH1	rs116898372	31.23908474
NXPH1	rs2686395	87.41691036
NXPH1	rs2242572	157.6431902
NXPH1	rs9501602	56.67218734
NXPH1	rs6993770	51.94356455
PLCG1	rs753381	70.03246149
PLCG1	rs10922098	393.8829319
PLCG1	rs704	47.31572537
PLCG1	rs11569415	49.23515145
PLCG1	rs12478841	37.58394071
PLCG1	rs1706435	65.86021469
PLCG1	rs1265941	48.39283451
PLCG1	rs550671	116.0858215
PLXNC1	rs113937085	31.30530767
PLXNC1	rs73218896	182.2994325
PLXNC1	rs270862	217.8032336
PLXNC1	rs11107427	301.5912618
PLXNC1	rs115651556	6432.330953
PLXNC1	rs117699264	73.39551943
PLXNC1	rs75674989	1006.892953
PLXNC1	rs12829636	327.8533183

PLXNC1	rs12231228	105.3086777
PLXNC1	rs141186127	49.42513736
PLXNC1	rs12302104	342.9478806
PLXNC1	rs61801011	32.76161492
PLXNC1	rs7937122	43.48435223
PLXNC1	rs10893501	102.9007489
PLXNC1	rs11108494	69.13551173
PLXNC1	rs117077998	31.87775979
PLXNC1	rs7310615	46.71910349
PLXNC1	rs117633773	41.32194608
PLXNC1	rs55714927	123.5708563
PLXNC1	rs116852327	63.76024622
PLXNC1	rs9271573	34.5422809
SH2D1A	rs704	37.77567567
SH2D1A	rs146457388	37.34866709
SH2D1A	rs17441745	30.655299
SH2D1A	rs6542680	508.6269711
SH2D1A	rs80220851	33.64599691
SH2D1A	rs3129752	31.15738005
SLITRK5	rs4113280	46.56677607
SLITRK5	rs12877225	209.3333615
SLITRK5	rs4502088	202.6349781
SLITRK5	rs72704449	30.49156359
SLITRK5	rs2477642	53.47584895
SLITRK5	rs2461139	36.43036743
SLITRK5	rs2461164	33.69504177
SLITRK5	rs2497824	35.11057911
SLITRK5	rs116953572	31.2873691
SLITRK5	rs59023732	31.73308935
SLITRK5	rs9523205	55.44821788
SLITRK5	rs12945299	62.46511738
SLITRK5	rs199500	30.52111842
SLITRK5	rs681343	40.73037635
SORCS2	rs2857383	45.52046812
SORCS2	rs704	120.8735343
SORCS2	rs56210800	38.39301352
SORCS2	rs149468457	38.48798692
SORCS2	rs144094035	33.09848989
SORCS2	rs67817769	125.6212813
SORCS2	rs8227	168.610049
PPY	rs1493694	76.01975731
PPY	rs4427508	63.87758985
PPY	rs9421775	114.2606494
PPY	rs2670543	268.0507438
PPY	rs2163150	731.2939652
PPY	rs363226	36.01379334
PPY	rs7994281	31.93318885
PPY	rs73174306	362.6549088
PPY	rs72730671	46.70931472
PPY	rs9368222	47.83100822
PPY	rs2326451	78.96031698
PPY	rs141999981	32.86887951
PPY	rs111609211	36.84328665
HIBADH	rs10922098	101.8768408
HIBADH	rs12147435	75.95066619
HIBADH	rs9501393	35.05614419
HIBADH	rs67399078	34.32404817
LTA	rs147906938	30.61615921

LTA	rs10922094	82.39451616
LTA	rs3782677	35.65186925
LTA	rs6542680	52.23045019
EPB41	rs204074	61.0414368
EPB41	rs12403568	41.90921558
EPB41	rs10443899	47.92751002
HSD17B1	rs572515	113.597258
HSD17B1	rs1355538	34.80802988
HSD17B1	rs415620	29.83583812
APOD	rs10922098	729.4203267
APOD	rs34967494	37.04671327
APOD	rs35351085	38.50089111
APOD	rs1707652	144.7332623
APOD	rs444921	195.8538445
APOD	rs9270570	54.35253502
APOD	rs9469195	89.92354623
IL3	rs10922098	87.09381032
IL3	rs1355538	43.2434514
IL3	rs686250	34.53166758
PPIB	rs10922103	170.5533076
PPIB	rs146457388	31.75430925
PPIB	rs6542680	399.5124479
PPIB	rs1706435	32.97501841
PPIB	rs9501600	57.10634803
TFF3	rs2839493	117.2234851
TFF3	rs142554955	56.68821691
TFF3	rs7283656	32.89382109
TFF3	rs10923385	30.04313151
TFF3	rs4764723	34.1147486
TFF3	rs77924615	30.39113765
TFF3	rs885928	68.72596453
TFF3	rs2523483	250.8343893
TFF3	rs2596463	385.7287034
TFF3	rs6926082	146.3356869
TFF3	rs115272033	43.64846846
AFM	rs72858937	434.7402983
AFM	rs150577204	97.25341517
AFM	rs1391790	50.68606738
AFM	rs4575545	48.24728284
AFM	rs273486	35.02395598
AFM	rs11554159	45.77178986
AFM	rs429358	31.25017825
AFM	rs143052621	31.56057184
AFM	rs77708623	32.30137187
AFM	rs142287165	73.433759
OLFM4	rs10922098	67.27305136
OLFM4	rs686250	32.00627199
OLFM4	rs13208583	32.96399073
SMPDL3A	rs28385609	791.330762
SMPDL3A	rs9401605	31.76878946
SMPDL3A	rs10778152	168.95035
SMPDL3A	rs140624338	40.41618884
SMPDL3A	rs146457388	40.18743033
SMPDL3A	rs6542680	431.9695122
SMPDL3A	rs80220851	36.30113936
SMPDL3A	rs523118	42.46303243
GSN	rs16910132	32.7982516
GSN	rs10985149	231.3311678

GSN	rs10818526	212.9477103
GSN	rs6478516	68.88684169
GSN	rs139301169	146.9573799
GSN	rs11054040	32.37509676
GSN	rs2361797	38.90085198
GSN	rs111520336	58.84356959
GSN	rs148425777	56.29164108
LPO	rs56044323	58.27238006
LPO	rs8178290	919.5389168
LPO	rs149925361	34.9068295
LPO	rs5821248	57.98684408
LPO	rs6427943	72.45647056
LPO	rs1150258	51.47383892
LPO	rs2461155	40.94884179
LPO	rs2477639	189.4807929
LPO	rs692440	144.183096
LPO	rs2421743	39.1710748
LPO	rs3906864	74.69709731
LPO	rs4423274	60.03937922
LPO	rs12019136	555.7190341
LPO	rs187995217	39.66312138
LPO	rs34262244	64.97197687
LPO	rs3760923	33.14658484
LPO	rs516246	333.4735819
LPO	rs61739245	112.233626
LPO	rs79773931	35.34973535
LPO	rs62418808	354.6310107
LPO	rs73769418	35.96616314
LPO	rs141280132	39.75122358
LPO	rs954050	58.3120529
COL8A1	rs56091027	34.91004585
COL8A1	rs9268560	61.32982878
COL8A1	rs4367411	38.43187617
ITIH4	rs77347777	97.22036843
ITIH4	rs12478841	35.25609914
ITIH4	rs6807194	43.15160017
ITIH4	rs1042445	30.10020652
ITIH4	rs9391786	32.87876419
SELL	rs139621801	40.72143817
SELL	rs6136	186.7736948
SELL	rs75776028	3575.189787
SELL	rs57110660	230.5730772
SELL	rs17350144	72.70119421
SELL	rs10922098	35.20561857
SELL	rs4649425	30.07213833
SELL	rs240537	31.41416453
SELL	rs7310615	119.2042695
SELL	rs76428106	112.6439973
SELL	rs12945299	47.66941701
SELL	rs143501783	36.96504461
SELL	rs9898547	91.67270136
SELL	rs36084354	38.16310631
SELL	rs11125915	73.60083853
SELL	rs62246503	32.69023637
SELL	rs34890930	44.13480366
SELL	rs218265	29.73351337
SELL	rs9271593	36.20497427
SELL	rs6796	42.6108006

SELL	rs1982094	42.80752684
EPHA2	rs36086195	269.7330675
EPHA2	rs2920115	151.7944054
EPHA2	rs145075169	59.81733787
EPHA2	rs150337494	142.7629476
EPHA2	rs72648451	38.46121168
EPHA2	rs116652944	53.45256209
EPHA2	rs72704449	39.57423612
EPHA2	rs2638281	37.31949175
EPHA2	rs9276171	43.07360479
EPHA2	rs6954724	32.21209311
EPHA2	rs11244049	31.54773639
CSF3	rs117443470	34.61345059
CSF3	rs145790346	32.64939109
CSF3	rs111780744	52.13152841
CSF3	rs77241309	135.5139546
CSF3	rs73045691	5253.963177
CSF3	rs138759462	65.02193069
CSF3	rs62118471	90.07475422
CSF3	rs184227198	38.72274486
CSF3	rs6445035	89.12173719
GPC3	rs2633317	76.84939192
GPC3	rs35448	34.94865172
GPC3	rs72694391	31.08025574
GPC3	rs77924615	72.53856131
GPC3	rs3786872	50.73958695
GPC3	rs760715	29.8121163
GPC3	rs3127580	186.077729
GPC3	rs3120137	224.8407846
GPC3	rs112842440	35.66604787
GPC3	rs3798938	31.58581377
GPC3	rs67760235	31.31400068
GPC3	rs139033671	108.0888487
BMPR2	rs10922098	224.7260974
BMPR2	rs1706435	91.80275235
BMPR2	rs6457457	51.33141329
BMPR2	rs3891366	77.95312304
NTRK2	rs1624327	55.9551774
NTRK2	rs333947	31.99979978
NTRK2	rs78705678	66.48086475
NTRK2	rs2477642	62.14013808
NTRK2	rs871843	35.28586746
NTRK2	rs56344867	98.63632163
NTRK2	rs181707610	47.35868338
NTRK2	rs1260326	75.9463946
NTRK2	rs2008174	32.41093659
NTRK2	rs7716761	33.6124693
VEGFA	rs2841642	64.49332633
VEGFA	rs2759270	57.87820576
VEGFA	rs78076125	52.94990582
VEGFA	rs4426996	193.8148553
VEGFA	rs7739450	4272.453177
VEGFA	rs9296424	119.5493896
VEGFA	rs7080386	137.7359647
VEGFA	rs12444373	38.78814204
VEGFA	rs114694170	52.80530132
VEGFA	rs1460580	31.72195432
VEGFA	rs6993770	241.0504355

VEGFA	rs7852409	95.35581207
VEGFA	rs11789392	380.7935312
ANG	rs4456427	74.66964968
ANG	rs71410230	62.82205261
ANG	rs148025035	58.313566
ANG	rs72659937	522.7905621
ANG	rs11624221	1261.702118
ANG	rs11623233	338.6769649
ANG	rs4982411	188.0100098
ANG	rs150844304	48.78012425
ANG	rs1059541	38.77319783
ANG	rs645040	51.61794033
F9	rs61962166	31.15021215
F9	rs1260326	109.5264613
F9	rs13244614	31.94691951
F10	rs3211757	315.6260222
F10	rs5960	167.8770844
F10	rs10761737	41.33973234
F10	rs4575545	30.61097422
F10	rs780093	56.19061495
F10	rs139189391	33.8442284
F10	rs184624754	58.30132865
F10	rs13244614	37.04775273
F10	rs2954021	39.61062971
F10	rs16927141	41.31696845
F10	rs2306956	53.33473387
F10	rs3762055	739.7171306
F10	rs76455359	32.25701052
F10	rs72750242	40.89454038
GDF2	rs10922098	103.7527847
GDF2	rs7080386	41.62608894
GDF2	rs2229498	39.25402228
GDF2	rs35887873	37.46850799
GDF2	rs1956713	96.91447624
GDF2	rs68066031	50.55569193
GDF2	rs2395329	36.0404297
GDF2	rs7848055	259.6435672
CCL7	rs73276641	33.22293276
CCL7	rs190850301	105.6789586
CCL7	rs74832623	1323.048505
CCL7	rs80219280	859.1269197
CCL7	rs41274294	353.7979855
CCL7	rs144618566	56.33286642
CCL7	rs117627996	672.2759993
CCL7	rs182223589	321.8725268
CCL7	rs151166933	70.54310549
CCL7	rs74359781	103.148289
CCL7	rs12075	176.7716443
CCL7	rs13208583	35.66617475
GCG	rs7300312	31.38255418
GCG	rs62066051	47.43015743
GCG	rs77542162	34.99120964
GCG	rs601338	125.1899979
GCG	rs576123	33.51875814
COTL1	rs704	38.00902167
COTL1	rs429358	195.004812
COTL1	rs35136575	42.87852165
COTL1	rs1619994	103.2892732

COTL1	rs6993770	32.73248703
F5	rs115977231	58.41995319
F5	rs199817936	149.044038
F5	rs61806985	1151.370681
F5	rs2239853	222.8144724
F5	rs4654748	34.8810916
F5	rs28360431	30.33259918
F5	rs7894992	141.2228452
F5	rs10159712	247.4652826
F5	rs10437425	30.89001388
F5	rs55731794	32.1817106
F5	rs1051317	46.98806003
F5	rs149062332	32.02874423
F5	rs2270190	33.5756745
F5	rs3909111	34.13956113
F5	rs60378094	48.7005725
F5	rs28366220	61.52050333
F5	rs560530	125.0332455
ENG	rs7037926	245.7493034
ENG	rs10819316	30.21216359
ENG	rs71497415	145.3503076
ENG	rs112564808	37.42692333
ENG	rs117603193	40.94881284
ENG	rs35166255	195.3022446
ENG	rs55714927	47.70722666
ENG	rs4760	577.036165
ENG	rs6141486	41.07742433
ENG	rs11944995	31.97806568
ENG	rs9402686	58.43947367
ENG	rs113599685	77.57178397
ENG	rs149024872	139.0940165
ENG	rs111888671	44.36968545
ENG	rs12378537	1011.461266
ENG	rs3094379	850.0509625
ENG	rs34250484	63.39509421
ENG	rs139562936	54.61738748
LGALS8	rs2393989	30.5359271
LGALS8	rs199772122	41.42390792
LGALS8	rs635634	389.8283891
GOT1	rs12146727	31.16640809
GOT1	rs12858483	57.38881032
GOT1	rs55704412	45.2638406
GOT1	rs2232710	560.6046707
GOT1	rs55821276	44.93030025
GOT1	rs17580	227.5037188
GOT1	rs116925319	35.69230235
GOT1	rs4803791	30.80912612
CCL16	rs142649371	50.82556757
CCL16	rs78280900	215.2174684
CCL16	rs78270565	37.21865813
CCL16	rs4251784	413.6091207
CCL16	rs113132408	1254.46634
CCL16	rs4795104	4471.639642
CCL16	rs139778553	233.4987133
CCL16	rs72829262	127.7413622
CCL16	rs6505427	93.93768862
CCL16	rs148663776	40.46706774
CCL16	rs1047891	42.52769273

CCL16	rs2268078	38.84258429
CCL16	rs4682863	37.26341244
CCL16	rs56826489	34.18605489
LYZ	rs142603793	44.02421053
LYZ	rs7486911	111.7443822
LYZ	rs6581886	2759.3978
LYZ	rs17225272	338.7092624
LYZ	rs1152971	121.7562321
LYZ	rs79146545	35.21622221
LYZ	rs147295417	53.55684527
LYZ	rs61754227	70.92668892
LYZ	rs34663796	35.42445749
LYZ	rs147332539	85.30340756
LYZ	rs3184504	51.80570795
LYZ	rs76428106	70.08551213
LYZ	rs150918492	59.4106609
LYZ	rs28500514	53.17842967
CCL19	rs1810819	31.75460388
CCL19	rs6679677	54.28405614
CCL19	rs35350651	62.981558
CCL19	rs77542162	37.86768119
CCL19	rs62293990	144.6523672
CCL19	rs115991275	41.73631943
CCL19	rs189005954	104.6822752
CCL19	rs12529318	887.8694903
CCL19	rs140070075	54.52174758
CCL19	rs9269562	228.9331346
CCL19	rs2071571	30.13635586
CCL19	rs41270506	61.04719401
AMH	rs10769124	34.81741302
AMH	rs192777257	311.8801344
AMH	rs112189508	207.2605514
AMH	rs16914574	442.6933743
AMH	rs67183304	53.06711883
AMH	rs143666677	1246.132106
AMH	rs117627469	151.1227872
AMH	rs56385952	59.38386433
AMH	rs1619994	42.32595398
MMP1	rs7938172	35.81467728
MMP1	rs2846703	217.0305678
MMP1	rs514921	1139.957247
MMP1	rs11225454	891.2448661
MMP1	rs6591003	141.2241511
MMP1	rs117342740	63.37252357
MMP1	rs117695939	152.0577819
MMP1	rs7940393	121.9080888
MMP1	rs10908505	110.1802613
MMP1	rs1434282	36.99332295
MMP1	rs35340377	46.79346815
MMP1	rs11190141	31.87897803
MMP1	rs12767683	43.63834824
MMP1	rs138804703	55.98852175
MMP1	rs144817684	42.21541646
MMP1	rs12793021	41.1062156
MMP1	rs79977579	30.25249134
MMP1	rs114694170	47.57265023
MMP1	rs7774377	76.20122687
MMP1	rs6993770	139.3683096

MMP1	rs10820606	91.30482628
MMP1	rs11243920	33.30233244
SHBG	rs372452045	170.6387238
SHBG	rs858519	426.4881756
SHBG	rs55662831	103.2871947
SHBG	rs9435441	35.77325915
SHBG	rs7920036	119.6388844
SHBG	rs79391862	63.04425655
SHBG	rs8023580	39.61214337
SHBG	rs12185242	71.50761217
SHBG	rs1260326	54.50583737
SHBG	rs2273618	31.48536525
SHBG	rs523118	53.40107724
SHBG	rs7039458	37.92126066
STC1	rs13262489	84.47789753
STC1	rs646776	196.713194
STC1	rs80282103	43.59511418
STC1	rs35393359	87.5614249
STC1	rs77797953	36.65699305
STC1	rs2058727	108.9311461
STC1	rs11072526	43.4854063
STC1	rs10901252	158.9675709
F3	rs4148325	104.1659795
F3	rs6445180	44.49705103
F3	rs2284179	52.42984795
F3	rs415620	60.69727999
F3	rs7774739	58.84977877
F3	rs11373170	41.32028411
EREG	rs10822155	30.37942437
EREG	rs1354034	60.4481559
EREG	rs10820606	34.31596014
AGR2	rs10922098	217.8321672
AGR2	rs1706435	100.3589504
AGR2	rs6457457	52.29973519
AGR2	rs9501393	77.55332064
ANXA2	rs78548957	185.9029003
ANXA2	rs6494153	104.8930494
ANXA2	rs7173250	89.22320634
ANXA2	rs3743268	995.3290669
ANXA2	rs340029	165.3559312
ANXA2	rs11434024	35.17104477
ANXA2	rs8039327	49.85607894
ANXA2	rs429358	159.8562756
ANXA2	rs450269	35.0481794
CDNF	rs79688957	102.4384426
CDNF	rs17340890	67.34309004
CDNF	rs79088080	78.0830794
CDNF	rs61738953	1306.065627
CDNF	rs146460991	184.6950569
CDNF	rs116919909	36.34938549
CDNF	rs1340143	30.81281408
CDNF	rs77924615	30.02560308
ERAP1	rs11135458	1169.086684
ERAP1	rs10075736	5873.063679
ERAP1	rs76200405	926.2042778
ERAP1	rs72777613	5252.489469
ERAP1	rs174528	38.65548982
ERAP1	rs72774481	30.30943859

ERAP1	rs78174795	61.53463387
ERAP1	rs66941665	35.42772194
ERAP1	rs17530412	32.68182156
ERAP1	rs1422251	68.12543183
ERAP1	rs2115459	101.2326497
ERAP1	rs10474346	64.58157197
ERAP1	rs12054781	160.6988132
ERAP1	rs10066356	359.4201344
ERAP1	rs111755236	307.3298925
ERAP1	rs10066211	45.83823316
ERAP1	rs370889487	44.17575987
ERAP1	rs114068443	53.31969056
ERAP1	rs6871839	144.472048
ERAP1	rs12187061	30.07872913
ERAP1	rs75705738	130.3624158
ERAP1	rs62355918	33.05084106
ERAP1	rs62377124	70.56063488
ERAP1	rs17132981	29.95700097
ERAP1	rs35652172	40.03931883
ERAP1	rs17485031	32.58439795
C1QBP	rs10922094	108.0590383
C1QBP	rs73400343	30.75859802
C1QBP	rs964184	42.92118425
C1QBP	rs12146727	53.30651703
C1QBP	rs4665383	34.81385411
C1QBP	rs111696008	35.66721207
C1QBP	rs111602331	31.95935869
C1QBP	rs1707652	162.5862548
C1QBP	rs11755689	54.76694801
CAPG	rs950362	2288.787198
CAPG	rs62162757	503.9703603
CAPG	rs199714103	85.5687963
CAPG	rs78642823	35.21042308
CAPG	rs7602538	35.01420911
CAPG	rs140827550	61.87777483
CAPG	rs141579448	50.95092714
CAPG	rs141265062	37.2608405
CA1	rs112708469	166.4014354
CA1	rs75881014	312.5895597
CA1	rs3811444	102.8025355
CA1	rs12591119	108.0540027
CA1	rs4895441	51.6506676
CA1	rs144251614	77.02217807
CA1	rs180748753	146.4997182
CA1	rs117472412	30.29078691
CTSZ	rs144349863	214.889789
CTSZ	rs127432	54.95148721
CTSZ	rs6070664	951.517175
CTSZ	rs77816791	46.70204379
CTSZ	rs6070808	44.23661003
CTSZ	rs41271951	50.91927025
CTSZ	rs72704449	40.81360023
CTSZ	rs116965114	29.84726727
CTSZ	rs632198	37.28204879
CTSZ	rs825038	82.61364604
CTSZ	rs4764822	1051.708277
CTSZ	rs140624338	105.7200684
CTSZ	rs56272941	35.15279854

CTSZ	rs339969	35.12878602
CTSZ	rs150268548	63.34025093
CTSZ	rs429358	46.60100825
CTSZ	rs4678361	56.17047403
CTSZ	rs8192589	44.22571792
CTSZ	rs9346805	30.51827781
DBNL	rs56665454	72.22069396
DBNL	rs1354034	30.65432393
DBNL	rs2731672	169.1973305
DPT	rs72705884	314.5811924
DPT	rs580360	1067.98697
DPT	rs151250811	118.1956838
DPT	rs115717655	166.8829084
DPT	rs151241919	220.6424314
DPT	rs111731543	51.854374
DPT	rs115574519	39.69446367
DPT	rs78705678	34.41510232
DPT	rs1931782	45.35206343
PI3	rs6073532	59.07262347
PI3	rs62206429	996.7452321
PI3	rs73619208	38.15521786
PI3	rs2297201	38.09480208
PI3	rs3818838	30.96001725
PI3	rs12211087	30.82448284
PI3	rs7012744	50.58277794
ESD	rs199670525	50.83032739
ESD	rs79668640	73.01847944
ESD	rs2298087	669.8714933
ESD	rs203687	36.93457298
FCAR	rs10922098	129.3308844
FCAR	rs415620	40.21133679
FCAR	rs7774739	33.68567819
FCAR	rs147168394	34.16661004
FGFR4	rs6542680	83.28100211
FGFR4	rs9259928	43.35857471
FGFR4	rs66962766	31.22973149
FGFR4	rs2002777	79.51483307
FGG	rs4220	240.6161896
FGG	rs12730935	58.00825625
FGG	rs12749237	31.8019838
FGG	rs2477664	37.71356649
FGG	rs2461161	30.10364469
FGG	rs7718024	53.26502948
FGG	rs2523558	38.35068225
GP1BA	rs117107082	46.02593204
GP1BA	rs72835078	291.897632
GP1BA	rs238241	90.91865583
GP1BA	rs117240565	42.61276413
GP1BA	rs10633030	39.22744006
GP1BA	rs1434282	63.16162978
GP1BA	rs10876913	39.72902919
GP1BA	rs3184504	273.8333786
GP1BA	rs1555405	66.97828607
GP1BA	rs17834140	33.84435528
GP1BA	rs62175229	49.64105818
GP1BA	rs56407378	43.17899228
GP1BA	rs201009932	35.14683832
GP1BA	rs78565404	54.78267959

GP1BA	rs114694170	42.81334569
GP1BA	rs28590669	40.887089
GP1BA	rs11759553	85.64247086
GP1BA	rs819441	34.71890231
GP1BA	rs11998274	36.67317626
GP1BA	rs6993770	147.842997
GP1BA	rs12005199	140.1587472
GP1BA	rs10758659	48.16175279
GPC5	rs111426655	41.19815548
GPC5	rs1330064	564.0510576
GPC5	rs139282387	1238.770298
GPC5	rs1819421	235.4991234
GPC5	rs1926624	54.87826132
GPC5	rs2671676	31.93392825
GRN	rs5848	539.7754379
GRN	rs11102684	54.2335376
GRN	rs187542824	92.18172563
GRN	rs35271870	1422.969773
GRN	rs141521383	38.36768752
GRN	rs35358959	1029.779399
GRN	rs147668562	54.07932589
GRN	rs72687286	31.18406521
GRN	rs35645512	36.32580424
GRN	rs145803034	37.31100087
GRN	rs7869	467.7789245
GRN	rs10762487	136.4240606
GRN	rs4397962	30.53285664
GRN	rs72802342	108.8796367
GRN	rs12945299	60.86745306
GRN	rs7210348	57.02813134
GRN	rs117010051	67.65554441
GRN	rs140183833	54.28542633
GRN	rs35600988	40.31897369
GRN	rs73725598	94.7052987
GRN	rs13240039	32.41514787
GRN	rs8176743	53.54507483
HPGD	rs12146727	60.58871787
HPGD	rs17580	457.1776956
HPGD	rs533617	58.29188294
HPGD	rs1355538	70.99568461
HPGD	rs11755689	29.93397418
HRG	rs6444134	449.086612
HRG	rs7627243	693.5675207
HRG	rs66965282	15253.47693
HRG	rs5030063	78.18682192
HRG	rs147208860	88.25873223
HRG	rs77278673	102.608466
HRG	rs1727	81.86883626
HRG	rs78875622	40.05671478
HRG	rs138892217	37.49349217
HRG	rs10937331	49.32964256
LGALS3BP	rs9916673	47.61509024
LGALS3BP	rs3826311	363.5386527
LGALS3BP	rs3740688	53.15814656
LGALS3BP	rs664034	50.28695764
LGALS3BP	rs4149056	30.59577887
LGALS3BP	rs117519814	38.7709247
LGALS3BP	rs143501783	33.83237347

LGALS3BP	rs2777897	33.68038749
LGALS3BP	rs12975366	52.77735192
LGALS3BP	rs1257168	31.08250579
LGALS3BP	rs755240	108.2981353
LGALS3BP	rs13043452	324.8050155
LGALS3BP	rs34061452	31.00164172
LGALS3BP	rs1126670	34.36588759
LGALS3BP	rs115216147	91.58842157
LGALS3BP	rs11745587	66.81107491
LGALS3BP	rs9264185	31.64652688
LGALS3BP	rs377133153	37.26626144
LGALS3BP	rs62503162	31.55476661
LGALS3BP	rs989221	118.9443272
LGALS3BP	rs73665251	421.6917139
LGALS3BP	rs1433289	56.44761529
LGALS3BP	rs112593770	71.37821849
LGALS3BP	rs8176743	82.72749469
SCGB2A1	rs10922094	36.87652063
SCGB2A1	rs10822155	62.91934254
SCGB2A1	rs1706435	47.94933135
SCGB2A1	rs6993770	61.7788049
MMP14	rs4658046	67.37086894
MMP14	rs709932	74.39262415
MMP14	rs11846452	40.89372385
MMP14	rs9268581	44.56216824
MMP14	rs77411399	31.09946488
MAPK13	rs10447396	156.3425782
MAPK13	rs203688	167.715806
MAPK13	rs2848407	43.5039756
SOD2	rs9326002	34.32135978
SOD2	rs10922098	397.2955285
SOD2	rs372434515	44.25867645
SOD2	rs1047286	40.9277184
SOD2	rs12975366	33.76436874
SOD2	rs1154988	32.50510927
SOD2	rs1707652	85.79902711
SOD2	rs2913230	64.24290457
SOD2	rs478253	78.26882558
SOD2	rs4976691	1037.971698
SOD2	rs4976704	98.58489524
SOD2	rs112305384	30.57397982
SOD2	rs3130955	37.18490695
SOD2	rs9501600	264.7309761
SOD2	rs9268744	47.3857532
SOD2	rs12009548	30.02937541
MSN	rs10922094	53.95808432
MSN	rs12292693	190.5261
MSN	rs1355538	45.59679363
CLIC1	rs1354034	42.6851917
CLIC1	rs1619994	57.66904763
CLIC1	rs710449	30.5530561
PLA2G7	rs71566584	76.91601545
PLA2G7	rs115054514	125.3272897
PLA2G7	rs11591147	72.53082646
PLA2G7	rs553741	31.07304473
PLA2G7	rs599839	91.35787571
PLA2G7	rs10922098	52.49001777
PLA2G7	rs12026141	60.43592084

PLA2G7	rs174576	53.34284677
PLA2G7	rs964184	68.03607908
PLA2G7	rs11057840	32.58984669
PLA2G7	rs17248720	98.97591744
PLA2G7	rs10629382	73.93512961
PLA2G7	rs429358	505.2008559
PLA2G7	rs144311893	152.352348
PLA2G7	rs4803776	39.57219826
PLA2G7	rs934197	60.06084241
PLA2G7	rs62182252	68.97865921
PLA2G7	rs34648639	31.67203949
PLA2G7	rs111696008	111.5268717
PLA2G7	rs144202860	73.45873409
PLA2G7	rs114115200	1052.359156
PLA2G7	rs3863087	388.8918293
PLA2G7	rs148077724	134.4242729
PLA2G7	rs111266377	88.08182495
PLA2G7	rs147033786	78.05764607
PLA2G7	rs9881140	40.95080819
PLA2G7	rs6556405	40.90623543
PLA2G7	rs3094204	39.21590488
PLA2G7	rs6474	83.76168165
PLA2G7	rs11571943	32.21136151
PLA2G7	rs139434889	135.8205476
PLA2G7	rs116824493	145.9387993
PLA2G7	rs72891103	53.50595663
PLA2G7	rs35705010	93.96278009
PLA2G7	rs143372081	36.79537383
PLA2G7	rs142933011	35.48459487
PLA2G7	rs17145750	31.64002453
PRDX5	rs10922098	105.0833331
PRDX5	rs114115200	35.03666434
PRDX5	rs28573154	38.26721377
UCHL1	rs72702354	1252.939644
UCHL1	rs1243160	36.33797497
UCHL1	rs12437339	72.54570015
UCHL1	rs56252683	121.4731462
UCHL1	rs2232613	39.78268288
UCHL1	rs1354034	30.8923571
UCHL1	rs1619994	42.54336418
RB1	rs10922098	475.3489845
RB1	rs1047286	45.92902107
RB1	rs10208529	34.94793025
RB1	rs1707652	102.0579592
RB1	rs9267139	42.86459197
RB1	rs9501393	128.4656002
RB1	rs7774739	86.85265468
RB1	rs9271766	39.8118713
RPS3	rs1976848	45.42678928
RPS3	rs112963922	171.4021902
RPS3	rs71366466	46.14037796
RPS3	rs1354034	214.4598536
RPS3	rs3129755	32.94610903
CD163	rs144788908	101.4275605
CD163	rs11054859	105.1803117
CD163	rs2099684	34.43878594
CD163	rs10838705	43.31590221
CD163	rs112771035	63.22390159

CD163	rs73021423	138.7141347
CD163	rs11820880	45.0710188
CD163	rs73600961	38.68295528
CD163	rs3184504	60.0414699
CD163	rs4788460	86.87859252
CD163	rs117902156	71.53312884
CD163	rs11571364	113.6292873
CD163	rs55714927	648.8645645
CD163	rs112723993	88.42299173
CD163	rs79744308	30.15868158
CD163	rs681343	90.7403862
CD163	rs114475253	30.82605358
CD163	rs55924807	39.85189108
CD163	rs62165726	739.9492439
CD163	rs9677256	109.7006679
CD163	rs192992318	43.35517462
CD163	rs10208580	35.39549834
CD163	rs3747207	37.23696245
CD163	rs9860747	78.76117043
CD163	rs1139490	63.68721786
CD163	rs1444087	35.71570704
CD163	rs115216147	137.2969537
CD163	rs1226013	29.86458875
CD163	rs9272779	51.55092563
CD163	rs28817896	48.0700466
CD163	rs1042133	56.41540567
CD163	rs13203302	56.38990351
CD163	rs11153164	42.07755294
CD163	rs869	129.8756321
CD163	rs1571262	31.04966724
CD163	rs7037849	54.03245089
FAP	rs188235382	48.19972086
FAP	rs76713183	74.90402925
FAP	rs10490425	544.77187
FAP	rs78722278	134.7849903
FAP	rs79900435	44.75723914
FAP	rs506317	32.30170757
FAP	rs35864030	32.05136763
FAP	rs12916255	42.95651224
FAP	rs62018183	101.8920572
FAP	rs740516	94.35806816
FAP	rs2668202	45.31690948
FAP	rs3020582	69.92191414
SIRT2	rs72773851	71.05866704
SIRT2	rs75357403	71.92642254
SIRT2	rs76157261	52.80218235
SIRT2	rs113889195	915.8715582
SIRT2	rs181764777	63.94870768
SIRT2	rs704	52.1525566
SIRT2	rs112106670	33.95069673
SIRT2	rs9905408	594.6783068
SIRT2	rs1873388	923.3946062
SIRT2	rs6779670	65.38588788
SIRT2	rs819085	46.85036733
SPTAN1	rs10922098	126.8328956
SPTAN1	rs1355538	29.72564182
SPTAN1	rs415620	31.05601078
SPTAN1	rs7774739	30.13386616

PRSS2	rs62479377	49.46042507
PRSS2	rs7383917	84.61960969
PRSS2	rs1799886	563.9060886
PRSS2	rs139172780	119.6601733
PRSS2	rs35311109	30.78048031
PRSS2	rs3768234	31.55432026
PRSS2	rs1164032	59.43196261
PRSS2	rs507695	49.95753356
PRSS2	rs2638493	36.44305824
PRSS2	rs10135188	30.09486115
PRSS2	rs72802342	377.0283549
PRSS2	rs533406	74.8730583
PRSS2	rs56850260	88.33120251
PRSS2	rs62052254	30.45601989
PRSS2	rs3110649	35.74729558
PRSS2	rs2277710	44.88875543
PRSS2	rs838133	54.21815129
PRSS2	rs17032925	66.11013712
PRSS2	rs6842549	51.95666656
PRSS2	rs71624710	53.01372345
PRSS2	rs2842895	46.56014557
PRSS2	rs2534791	36.25248315
PRSS2	rs9482772	59.21074356
PRSS2	rs13438309	50.55645224
PRSS2	rs193098612	59.27017792
PRSS2	rs748208	36.94908096
PRSS2	rs8176743	52.45754863
TYMS	rs10922098	30.88579159
TYMS	rs1985791	114.2369311
TYMS	rs12160212	34.9900367
TYMS	rs67399078	34.40874655
TNFAIP6	rs115633223	95.48349233
TNFAIP6	rs113697916	61.76226206
TNFAIP6	rs16829593	1163.255618
TNFAIP6	rs4664356	2975.361033
TNFAIP6	rs17270394	223.3526099
TNFAIP6	rs2298318	46.28260109
TNFAIP6	rs1013495	37.82169792
TNFAIP6	rs9568476	48.35930336
TNFAIP6	rs1260326	39.79242713
TNFAIP6	rs139808240	34.04145655
TNFAIP6	rs189716947	39.83265001
TNFAIP6	rs11275578	133.7052438
TNFAIP6	rs146828432	36.50517479
TNFAIP6	rs28430325	35.81317789
TNFAIP6	rs66960403	44.05992672
TNFAIP6	rs3774355	124.6934684
TNFAIP6	rs6768289	586.2505873
TNFAIP6	rs55753501	44.79604926
TNFAIP6	rs2524136	35.58846035
TNFAIP6	rs42122	49.8353683
TNFAIP6	rs9649546	35.45864988
TNFAIP6	rs4263799	38.30010774
TNFAIP6	rs4604455	35.38704379
TNFAIP6	rs1475718	31.45417899
CD274	rs1411262	746.781296
CD274	rs6476985	33.93071331
CD274	rs12892823	98.91107319

CD274	rs3806157	40.84346647
ICOSLG	rs56124762	75.18345986
ICOSLG	rs7261193	31.76913842
ICOSLG	rs3130169	72.39802147
ICOSLG	rs12675298	60.74242749
ICOSLG	rs146118510	38.1155657
CD226	rs10922094	43.64133824
CD226	rs6542680	154.867345
CD226	rs2232613	128.2342459
CD226	rs1706435	44.97654766
CD244	rs114594620	99.91877666
CD244	rs113896482	33.97740046
CD244	rs10774624	44.42031298
CD300C	rs62064540	61.48010155
CD300C	rs117027254	150.42603
CD300C	rs72844326	912.9554251
CD300C	rs145767181	77.32353648
CD300C	rs58440339	131.9367158
CD300C	rs76081401	68.86784662
CD300C	rs11230289	43.93943936
CD300C	rs78689694	81.12110949
CD300C	rs11105271	987.1931446
CD300C	rs4842674	527.95877
CD300C	rs5799932	172.3683433
CD300C	rs78124338	30.31599262
CD300C	rs139524107	46.36446845
CD300C	rs35415187	48.49955089
CD300C	rs3184504	60.48237017
CD300C	rs117902156	33.22605755
CD300C	rs12945299	153.3728592
CD300C	rs143501783	48.56154353
CD300C	rs11888817	262.0030946
CD300C	rs9852537	31.84295488
CD300C	rs35234681	90.50381685
CD300C	rs6846475	37.32826241
CD300C	rs368611	36.59965043
CD300C	rs13205436	67.95608716
CD300C	rs1010366	38.78903963
CD55	rs148838761	46.34652796
CD55	rs6666604	967.3065812
CD55	rs10863437	97.45401426
CD55	rs148327582	42.49856384
CD55	rs72704449	52.53657146
CD55	rs12945299	119.61537
CD55	rs143501783	55.01589683
CD55	rs3814995	42.03856965
CD55	rs13108218	31.15203058
CD55	rs9269271	39.47767772
CD55	rs116334170	30.85511401
CD55	rs767298	34.33473087
CD55	rs2142306	59.06834798
CD55	rs2978006	54.01453503
TNFRSF6B	rs10922098	38.66610753
TNFRSF6B	rs6542680	34.1101273
TNFRSF6B	rs3129754	38.80354796
EPHB6	rs10243942	74.26915959
EPHB6	rs116887927	57.93115674
EPHB6	rs8177107	612.008815

EPHB6	rs61747728	86.15235987
EPHB6	rs77924615	59.53497115
EPHB6	rs13108218	35.33028834
EPHB6	rs145312664	41.67497525
EPHB6	rs11984489	29.95335819
IL1RAPL2	rs10922098	111.3360257
IL1RAPL2	rs9864801	48.30989953
IL1RAPL2	rs9501393	38.57218534
IL1RAPL2	rs7774739	30.98084826
IL22RA2	rs71560620	38.1943666
IL22RA2	rs375503263	101.7896072
IL22RA2	rs76488192	51.34735115
IL22RA2	rs77551490	160.7629122
IL22RA2	rs17175050	62.49447161
IL22RA2	rs74648690	42.25075105
IL22RA2	rs6600249	171.8693127
IL22RA2	rs79882224	61.66448622
IL22RA2	rs139795227	35.19379588
IL22RA2	rs184409863	214.5590705
IL22RA2	rs191615076	205.5517313
IL22RA2	rs752806	81.84961922
IL22RA2	rs10849448	150.3306045
IL22RA2	rs763948	30.96595534
IL22RA2	rs7310615	191.5911796
IL22RA2	rs74940255	31.30569991
IL22RA2	rs7334951	222.6943855
IL22RA2	rs9554263	63.07500935
IL22RA2	rs11641991	40.25977044
IL22RA2	rs12232384	51.63641263
IL22RA2	rs55714927	30.63989089
IL22RA2	rs4969405	59.70317694
IL22RA2	rs75489957	39.65758554
IL22RA2	rs3111414	54.22832152
IL22RA2	rs5831754	34.80578967
IL22RA2	rs4991877	30.1128077
IL22RA2	rs7287486	85.79865026
IL22RA2	rs10013613	103.5931311
IL22RA2	rs10519515	30.166492
IL22RA2	rs9269272	107.1767707
IL22RA2	rs1846190	159.780105
IL22RA2	rs28383461	38.70877436
IL22RA2	rs111470468	40.41786358
IL23R	rs4980320	33.19384452
IL23R	rs2176862	53.34614322
IL23R	rs34762726	640.5744696
IL23R	rs1354034	38.79617901
IL7R	rs10058572	91.20033093
IL7R	rs10922098	116.8670158
IL7R	rs6542680	33.22085057
IL7R	rs1706435	45.8790135
IL7R	rs415620	45.73300466
IL7R	rs7774739	36.45386417
IL7R	rs28688825	31.41212169
LILRB1	rs187157427	59.32270775
LILRB1	rs79870162	98.25157894
LILRB1	rs79026716	179.162367
LILRB1	rs117886241	1376.187558
LILRB1	rs7246707	970.9743629

LILRB1	rs371668305	681.6307785
LILRB1	rs10423751	331.491195
LILRB1	rs202218989	53.86669485
LILRB1	rs117868499	110.7657436
LILRB1	rs191514554	72.91883087
LILRB1	rs10823375	133.8705502
LILRB1	rs73021415	43.5137667
LILRB1	rs2240866	31.73605058
LILRB1	rs289747	29.8867205
LILRB1	rs117587884	51.09991505
LILRB1	rs154042	60.51259476
LILRB1	rs56115403	76.26203985
LILRB1	rs143501783	39.99341434
LILRB1	rs55755628	60.2156073
LILRB1	rs62165726	92.02343268
LILRB1	rs27291	34.5454218
LILRB1	rs73404772	49.61574082
LILRB1	rs9468618	53.57854974
LILRB1	rs28565959	97.6622613
LILRB1	rs9263773	285.0122897
LILRB1	rs17408553	519.7105253
LILRB1	rs709053	769.8724102
LILRB1	rs35064159	29.76618196
LILRB1	rs7865362	36.97848731
LILRB1	rs576123	34.09293277
LILRB2	rs73062693	179.1425974
LILRB2	rs71365450	1225.148525
LILRB2	rs117421142	59.75211721
LILRB2	rs3855678	6221.270304
LILRB2	rs1761463	1353.653759
LILRB2	rs11673154	49.05209122
LILRB2	rs11670723	30.78895645
LILRB2	rs4020189	50.67851296
LILRB2	rs34764559	101.6940371
LILRB2	rs6688272	73.91788294
LILRB2	rs10823375	52.13455444
LILRB2	rs55714927	47.33708905
LILRB2	rs9265651	80.76943531
LILRB2	rs9267084	150.1857097
LILRB2	rs3116976	31.96230579
JAG1	rs2423507	65.30339327
JAG1	rs1051412	30.68869717
JAG1	rs11220463	32.9867805
JAG1	rs55714927	63.91274465
JAG1	rs77542162	52.20639774
JAG1	rs10935478	59.46061689
JAG1	rs842999	39.7253803
JAG1	rs635634	298.7073837
JAG1	rs41307428	101.4231913
KIR2DL4	rs149314836	153.6882481
KIR2DL4	rs146426067	40.64493164
KIR2DL4	rs377155038	33.10982961
KIR2DL4	rs12976350	938.0399028
KIR2DL4	rs2916049	297.8336164
KIR2DL4	rs374763999	112.2038305
KIR2DL4	rs203687	54.60723382
KIR2DL4	rs1976848	60.92220986
KIR2DL4	rs7310615	40.49906263

KIR2DL4	rs1046321	98.00010256
KIR2DL4	rs2298429	33.26493694
KIR2DL4	rs1354034	244.8372215
KLRF1	rs10922098	138.7799102
KLRF1	rs1355538	42.08347699
KLRF1	rs415620	43.14270732
KLRF1	rs7774739	39.52433869
KLRF1	rs188302747	34.65825582
SCARB2	rs10922098	53.67869538
SCARB2	rs6542680	43.39234415
SCARB2	rs16881504	29.93528762
MICB	rs9265336	558.6925362
MICB	rs151341076	104.0970267
MICB	rs76874043	33.5977508
MICB	rs7310615	67.26254779
MICB	rs12895651	233.9496667
MICB	rs12945299	32.42278554
MICB	rs143501783	35.90681574
MICB	rs10935480	143.3660335
MICB	rs16888397	71.77379768
MICB	rs189521594	78.00761265
MICB	rs75782365	1066.833951
MICB	rs148589791	1018.458263
MICB	rs9469195	1175.351858
MICB	rs1057373	264.2200527
MICB	rs6457710	255.930063
MICB	rs3800443	38.37883164
MICB	rs45519938	30.32435997
MICB	rs6939608	40.67287125
MICB	rs635634	109.2254188
CD200R1	rs62263730	187.0008924
CD200R1	rs10922098	50.59367498
CD200R1	rs7137828	31.6610084
CD200R1	rs12496730	37.66839091
CD200R1	rs211161	29.91013438
CD200R1	rs576123	32.91220193
RTN4R	rs62222320	70.11219144
RTN4R	rs175206	928.455715
RTN4R	rs701424	789.7546535
RTN4R	rs117680284	53.39911531
RTN4R	rs111410527	67.45165704
RTN4R	rs72704449	33.46340246
RTN4R	rs11821656	31.23073952
RTN4R	rs17580	31.90949028
RTN4R	rs149624078	41.92905508
RTN4R	rs118147862	34.85276637
RTN4R	rs5112	57.19016398
RTN4R	rs1291921	30.0510802
RTN4R	rs28709106	32.91352091
RTN4R	rs9271375	44.88529599
NOTCH2	rs415620	42.61658341
NOTCH2	rs7774739	42.88733065
NOTCH2	rs188302747	41.41754618
NOTCH1	rs9411207	119.9277868
NOTCH1	rs17250281	36.07614177
NOTCH1	rs35406997	32.46842035
NOTCH1	rs78705678	53.24891914
NOTCH1	rs2477642	50.6739703

NOTCH1	rs35166255	43.03246112
NOTCH1	rs3184504	46.43740574
NOTCH1	rs1260326	49.47869249
NOTCH1	rs10901252	939.4785112
NOTCH1	rs62576042	66.31853017
NOTCH3	rs10417239	78.87752558
NOTCH3	rs4657078	71.94267703
NOTCH3	rs2477664	38.03723988
NOTCH3	rs186511240	45.74327528
NOTCH3	rs2279373	65.95076091
NOTCH3	rs17580	47.68204557
NOTCH3	rs150844304	32.58381966
NOTCH3	rs55714927	32.268801
NOTCH3	rs4968845	30.27027679
NOTCH3	rs1260326	36.7247548
NOTCH3	rs116298992	36.49011609
NOTCH3	rs2906140	44.31713871
NOTCH3	rs2237582	109.5124623
NOTCH3	rs635634	48.78092848
NRCAM	rs1034826	48.4760921
NRCAM	rs4657078	41.00929472
NRCAM	rs2461155	90.00577413
NRCAM	rs2477639	271.2872012
NRCAM	rs692440	202.2162442
NRCAM	rs11465428	43.64202361
NRCAM	rs635634	134.074415
NRXN3	rs118164450	34.23073345
NRXN3	rs34121288	341.1438325
NRXN3	rs72704449	44.25154299
NRXN3	rs78689694	39.21185732
NRXN3	rs17598373	117.8543277
NRXN3	rs4540997	140.832019
NRXN3	rs2940174	30.32520003
NRXN3	rs12945299	148.3935848
NRXN3	rs17880604	64.15011005
NRXN3	rs1260326	59.82286874
NRXN3	rs4234797	48.03675377
NRXN3	rs368611	29.91458889
NRXN3	rs71549279	69.03722093
NRXN3	rs116334170	33.39858105
CD200	rs7619941	65.71827029
CD200	rs10922098	83.46151844
CD200	rs6542680	41.5559329
CD200	rs635634	350.5243089
PRLR	rs10922098	207.2600136
PRLR	rs1926446	34.21427472
PRLR	rs704	42.54853022
PRLR	rs1706435	52.54539444
PRLR	rs453821	66.74593362
PRLR	rs28471056	34.49703942
ROBO2	rs6766424	34.14629194
ROBO2	rs78705678	77.17115555
ROBO2	rs2477642	65.20401281
ROBO2	rs77262773	46.40598104
ROBO2	rs1260326	30.72882609
ROBO2	rs78908809	64.51461114
ROBO2	rs150130692	46.24785226
ROBO2	rs34172220	40.47757536

ROBO2	rs5030044	102.22345
SEMA6B	rs10404223	121.6449428
SEMA6B	rs73920854	29.92244481
SEMA6B	rs55993371	52.76591576
SEMA6B	rs34666101	72.91962413
SEMA6B	rs78705678	190.1836868
SEMA6B	rs2477642	204.26572
SEMA6B	rs3184504	62.97278437
SEMA6B	rs56093546	45.34428262
SEMA6B	rs183857418	36.88385266
SEMA6B	rs117877056	93.43823336
SEMA6B	rs117241334	94.71653043
SEMA6B	rs111903631	71.63824769
SEMA6B	rs55954186	67.94277881
SEMA6B	rs10935473	108.2717326
SEMA6B	rs3128932	31.34464972
SEMA6B	rs11784862	34.64037455
SEMA6B	rs76324624	31.75320118
SEMA6B	rs2013075	972.6258894
SEMA6B	rs62576042	94.53920371
SEMA6B	rs117854892	37.39269334
ICAM5	rs9749467	90.7804272
ICAM5	rs140787039	69.63997756
ICAM5	rs3093035	1160.34053
ICAM5	rs8101473	4578.660945
ICAM5	rs145530718	84.70414443
ICAM5	rs10417443	159.8703415
ICAM5	rs72658879	146.8404898
ICAM5	rs2116877	30.19512654
ICAM5	rs78689694	39.29184737
ICAM5	rs75806286	30.28856829
ICAM5	rs77542162	401.870764
ICAM5	rs9916644	146.4904699
ICAM5	rs1275922	31.10262608
ICAM5	rs12501006	31.00048721
ICAM5	rs146798134	51.96774425
ICAM5	rs635634	1116.635645
SLAMF6	rs12137007	410.8919802
SLAMF6	rs145748959	49.3729792
SLAMF6	rs10922094	32.21147127
SLAMF6	rs3184504	57.65411023
SLAMF6	rs12945886	38.72687155
SCARF1	rs141910927	49.47533246
SCARF1	rs116929249	69.66638823
SCARF1	rs144086249	33.55227926
SCARF1	rs13342837	1284.855585
SCARF1	rs57017972	733.9171932
SCARF1	rs75695549	35.8296431
SCARF1	rs35340377	29.82575668
SCARF1	rs10850001	39.76856522
SCARF1	rs6993770	77.75226727
SCARF1	rs75179845	35.87710231
TNFRSF19	rs61947047	141.6507891
TNFRSF19	rs11701489	46.66716465
TNFRSF19	rs3827211	82.41431656
IL27RA	rs141425154	40.37028842
IL27RA	rs11667546	4389.736176
IL27RA	rs56361194	54.71735533

IL27RA	rs34317732	596.1527879
IL27RA	rs6511928	171.1099848
IL27RA	rs144126569	55.37492436
IL27RA	rs113020163	60.02455443
IL27RA	rs3184504	116.2253419
IL27RA	rs12945299	52.33253097
IL27RA	rs72759422	32.05398196
IL27RA	rs10901252	882.9065072
IL27RA	rs62576042	90.359216
TGFBR2	rs10922098	317.0723156
TGFBR2	rs1706435	44.11694353
TGFBR2	rs6457457	35.57162881
TGFBR2	rs9501393	51.91760739
HAVCR2	rs72801156	49.18036563
HAVCR2	rs466781	974.4671285
HAVCR2	rs30118	81.5842507
HAVCR2	rs62388507	286.6731404
HAVCR2	rs61747728	41.55174502
HAVCR2	rs950802	56.00287124
HAVCR2	rs56093546	48.03897491
HAVCR2	rs7713768	93.2003581
HAVCR2	rs958444	74.73205004
UNC5C	rs33936765	42.63737045
UNC5C	rs17383694	404.4102776
UNC5C	rs199995337	66.26283066
UNC5C	rs61747728	56.78329419
UNC5C	rs5801771	30.54700902
UNC5C	rs12922822	37.15639327
UNC5C	rs11702475	79.67983957
UNC5C	rs368611	35.6311132
PDE7A	rs10922098	150.6417106
PDE7A	rs9864801	30.63968726
PDE7A	rs451652	40.1293966
PDE7A	rs4713498	47.24502821
NMT1	rs1053733	31.81370822
NMT1	rs35340377	35.08269545
NMT1	rs11417460	29.78756195
NMT1	rs1354034	87.19953274
PPID	rs17843929	840.7815377
PPID	rs112970794	79.29222733
PPID	rs1354034	50.17573367
PPID	rs74736025	29.97106072
PPID	rs7693847	32.19919341
PPID	rs116461890	47.67787266
GCKR	rs1260326	106.4829999
GCKR	rs12621136	35.69001705
GCKR	rs572515	232.3083072
GCKR	rs1355538	52.35125575
GCKR	rs453821	58.03390423
GCKR	rs3129773	35.69335199
PDK1	rs35783685	35.38798079
PDK1	rs35022330	437.800523
PDK1	rs12622141	39.55316023
PDK1	rs10922094	102.405703
PDK1	rs704	47.94371566
PDK1	rs1706435	43.52701229
IMPDH1	rs7073753	33.39229159
IMPDH1	rs1976848	62.10229985

IMPDH1	rs76294435	31.24072732
IMPDH1	rs2144987	110.0435966
IMPDH1	rs2896439	939.4686922
IMPDH1	rs1190233	69.98157832
IMPDH1	rs11082304	32.55408586
IMPDH1	rs139406187	60.90883366
IMPDH1	rs7587319	111.6553756
IMPDH1	rs78909033	2040.800094
IMPDH1	rs35788092	74.65559181
IMPDH1	rs1354034	354.3429414
IMPDH1	rs342293	77.12680382
PCSK9	rs11591147	529.2701408
PCSK9	rs472495	330.1763588
PCSK9	rs7525503	96.92612868
PCSK9	rs147720706	34.11149635
PCSK9	rs80098465	138.932075
PCSK9	rs3999089	39.53680495
PCSK9	rs174564	74.96223862
PCSK9	rs77303550	36.06973182
PCSK9	rs112107114	32.56965528
PCSK9	rs58542926	50.13956378
PCSK9	rs1367117	55.56741974
PCSK9	rs4665972	37.24997467
PCSK9	rs28601761	31.87151243
NR1D1	rs10922098	41.52320026
NR1D1	rs6542680	75.19715917
NR1D1	rs13409225	41.11325858
NR1D1	rs147168394	29.760415
PPIE	rs12074147	1222.246288
PPIE	rs200232278	40.80687356
PPIE	rs822856	31.00427164
PPIE	rs34124744	42.67040337
PPIE	rs7073753	36.15326251
PPIE	rs4980320	39.55025019
PPIE	rs1354034	141.3629652
PPIE	rs342200	31.58913646
MAP2K4	rs16841344	34.70961115
MAP2K4	rs144052318	37.02761592
MAP2K4	rs4631704	1158.386072
MAP2K4	rs1043897	98.02166098
MAP2K4	rs1727	80.86026325
MAP2K4	rs4334315	30.93848951
MAP2K4	rs907866	98.96960267
MAP2K4	rs75129524	32.62461446
MAP2K4	rs111611525	52.41745832
MAP2K4	rs878095	255.7490891
MAP2K4	rs8179931	435.4426772
MAP2K4	rs66965282	11327.6681
MAP2K4	rs5030073	714.3907222
MAP2K4	rs2889825	114.3727676
MAP2K4	rs1574648	74.13875271
MAP2K4	rs1348638	35.36097702
PDE2A	rs10922098	394.0358152
PDE2A	rs1803274	33.38082676
PDE2A	rs9501600	103.3974154
STK17B	rs10922098	243.692139
STK17B	rs6445035	37.54918936
STK17B	rs9501600	60.77679371

IMPDH2	rs4980320	64.6722177
IMPDH2	rs118028900	39.79155077
IMPDH2	rs3742446	489.1341829
IMPDH2	rs7158166	47.41595188
IMPDH2	rs7587319	33.96754573
IMPDH2	rs78909033	446.2765737
IMPDH2	rs1354034	106.2444245
IMPDH2	rs67036916	37.71514201
PDE11A	rs1043314	63.62301321
PDE11A	rs75327431	31.29060469
PDE11A	rs2588512	30.09540476
PDE11A	rs3864033	33.76599359
PDE4D	rs10922098	88.34702075
PDE4D	rs1619994	38.68535551
PDE4D	rs145269503	36.49439698
PDE4D	rs1265941	75.66023733
PDE4D	rs550671	179.0972002
PDE4D	rs148766553	32.04372721
PDE5A	rs75135627	297.0768154
PDE5A	rs9307476	212.6473802
PDE5A	rs2389874	528.264015
PDE5A	rs28646193	44.21166571
PDE5A	rs7073753	46.57598639
PDE5A	rs76215025	35.92255936
PDE5A	rs139534073	66.02433153
ABL2	rs10922098	176.3325534
ABL2	rs1355538	45.1301665
ABL2	rs453821	65.74157394
ABL2	rs28471056	40.8572127
BCAR3	rs10922098	109.7493327
BCAR3	rs6542680	60.26783227
BCAR3	rs12478841	33.23296241
BCAR3	rs686250	46.16659701
SHC1	rs4980320	39.74187271
SHC1	rs11451044	30.03933983
SHC1	rs185009887	34.12106631
SHC1	rs1354034	101.7702558
VAV1	rs36097961	68.29044762
VAV1	rs4980320	32.57478273
VAV1	rs1354034	41.10685863
CCL11	rs4795886	46.44304939
CCL11	rs1989357	194.8689648
CCL11	rs8026803	31.99771904
CCL11	rs9932617	39.31406637
CCL11	rs17639910	36.78679523
CCL11	rs1671176	31.26541063
CCL11	rs2228467	697.3527618
CCL11	rs2280565	94.56193406
CCL11	rs9882305	33.83854316
CCL11	rs7616215	166.7431738
CCL11	rs11755562	33.15127356
CCL11	rs6970107	284.8661977
CCL11	rs826727	36.85536367
TNNT2	rs12038333	212.3145141
TNNT2	rs41285751	42.18790757
TNNT2	rs147390813	61.91275861
TNNT2	rs116334170	55.16685219
F2	rs3136516	150.7580755

F2	rs2930196	45.97888536
F2	rs7232	33.39046454
F2	rs150611042	35.3980456
ANXA6	rs10922094	30.09105777
ANXA6	rs10822155	130.9935188
ANXA6	rs113661219	34.49614759
ANXA6	rs1398015	57.2302533
ANXA6	rs11574452	69.06331254
ANXA6	rs6993770	57.12760674
CD86	rs2681406	51.25206164
CD86	rs150649461	43.44110446
CD86	rs1613078	83.01687651
CD86	rs73021441	52.37703657
CD86	rs3184504	61.84657061
CD86	rs76428106	55.63289073
CD86	rs143501783	33.59841857
CD86	rs13063578	34.36090802
S100A9	rs3014874	32.77316087
S100A9	rs8066582	39.95361848
S100A9	rs28383233	32.73798858
CASP10	rs10822155	29.91943999
CASP10	rs1619994	55.22768589
CASP10	rs66489920	43.80457104
CPE	rs10922098	442.9167921
CPE	rs11569415	45.82570405
CPE	rs429358	110.8074261
CPE	rs1707652	153.4703088
CPE	rs550671	121.7185653
CPE	rs28471056	63.48122755
CKAP2	rs10922094	284.0299268
CKAP2	rs12026141	30.16897414
CKAP2	rs964184	95.34852934
CKAP2	rs41290120	32.43217609
CKAP2	rs8106813	92.64701043
CKAP2	rs4666000	46.6976297
CKAP2	rs111696008	41.10771121
CKAP2	rs144202860	32.98859979
CKAP2	rs1618069	30.60574721
CKAP2	rs6445035	254.000993
CKAP2	rs3863087	99.46017602
CKAP2	rs73169103	36.42465534
CKAP2	rs550671	82.54202882
CKAP2	rs34927613	63.99638816
CKAP2	rs2057681	183.9907652
CKAP2	rs854572	32.01246036
CKAP2	rs78810414	39.89612996
CPNE1	rs78732698	1178.11411
CPNE1	rs6120992	4994.484011
CPNE1	rs79102969	359.6870372
CPNE1	rs74963480	31.84584349
CPNE1	rs139335456	58.34316067
CPNE1	rs143250645	100.0184445
CPNE1	rs79967377	72.60732122
CPNE1	rs188768522	34.46042282
CPNE1	rs76221656	286.3661913
CPNE1	rs147799064	223.3477687
CPNE1	rs73111607	65.02416325
CPNE1	rs677870	163.6393418

CPNE1	rs79743642	146.629508
CPNE1	rs113252750	128.3308088
CPNE1	rs79324137	44.68018729
CCNB1	rs10922098	203.2647752
CCNB1	rs1619994	41.2320075
CCNB1	rs28573154	62.21839949
CCNB1	rs7774739	49.26754965
DLL1	rs13193455	41.05572672
DLL1	rs9356631	137.7895447
DLL1	rs72704449	47.61347102
DLL1	rs2837988	46.10927903
DLL1	rs114434032	96.40018497
DLL1	rs9468931	92.30851705
DLL1	rs9266225	39.94831012
DLL1	rs7762054	33.15203052
DLL1	rs11244049	80.15677477
GPC6	rs2393989	31.0492583
GPC6	rs1535692	47.70872521
GPC6	rs12370	35.4324405
GPC6	rs55972004	34.88642661
GPC6	rs17580	458.155161
GPC6	rs112963922	148.6286714
GPC6	rs111397271	53.21532
GPC6	rs5030049	38.69650392
IL1RN	rs4848312	208.6486318
IL1RN	rs55709272	503.663105
IL1RN	rs139133059	44.39534522
IL1RN	rs6489859	31.67473988
IL1RN	rs8047587	30.47230048
IL1RN	rs28455809	33.16261983
TNFSF14	rs113790324	36.3995694
TNFSF14	rs413141	225.0906974
TNFSF14	rs2511241	48.2576735
NLGN4X	rs10922094	45.42585346
NLGN4X	rs831429	34.40066082
NLGN4X	rs201862321	34.55947286
NLGN4X	rs2367289	32.62596635
OMD	rs141960185	45.69735906
OMD	rs333947	43.62857159
OMD	rs11601603	31.39359014
OMD	rs147712570	46.45341994
OMD	rs148977521	41.89728786
OMD	rs112725054	31.29334968
OMD	rs73177940	34.29560903
OMD	rs117801489	663.3920864
OMD	rs12581309	80.21605597
OMD	rs9594738	48.16071096
OMD	rs9033	35.89269689
OMD	rs704	106.863671
OMD	rs74814272	31.17229845
OMD	rs141555794	35.72675121
OMD	rs11130306	54.00594124
OMD	rs115832719	50.86342881
OMD	rs11726170	30.93790103
OMD	rs2844572	39.62913796
PIM1	rs10922098	170.7437103
PIM1	rs112963922	67.00596227
PIM1	rs1355538	44.21024559

PIM1	rs33972313	43.44165523
PIM1	rs9501600	33.42981537
SEMA3E	rs17284837	923.177289
SEMA3E	rs112429006	497.6603604
SEMA3E	rs4577905	560.5863053
SEMA3E	rs118161729	4242.842908
SEMA3E	rs2722997	10186.44478
SEMA3E	rs302121	1364.198994
SEMA3E	rs117816758	44.56458287
SEMA3E	rs148081846	94.66606055
SEMA3E	rs149394237	71.119191
SEMA3E	rs41313381	32.33883561
SEMA3E	rs61801013	37.05671946
SEMA3E	rs12563740	31.81201033
SEMA3E	rs78705678	50.44877679
SEMA3E	rs2477642	76.37694211
SEMA3E	rs7940060	36.9749574
SEMA3E	rs114522670	49.53130555
SEMA3E	rs11709377	81.09341432
SEMA3E	rs368364462	79.1251617
SEMA3E	rs3761986	41.60378484
SEMA3E	rs9389269	67.33956531
SEMA3E	rs1733609	307.5485082
SEMA3E	rs34779581	384.2009129
SEMA3E	rs199749831	36.18651612
SEMA3E	rs79983584	76.72736966
SEMA3E	rs149656509	421.6917031
SEMA3E	rs4727237	212.2925537
SEMA3E	rs141830128	71.31291599
SEMA3E	rs10102164	35.9685954
SEMA3E	rs5893860	43.23623306
SET	rs2667684	30.45805096
SET	rs10499638	29.90738503
SET	rs700753	126.0007722
TNFRSF13C	rs704	64.58178415
TNFRSF13C	rs141622900	35.07218413
TNFRSF13C	rs5167	388.1827692
TNFRSF13C	rs4665383	42.64423897
TNFRSF13C	rs1706435	96.27202559
FAS	rs1998629	75.91547628
FAS	rs9658742	90.31096403
FAS	rs7911226	692.8527063
FAS	rs61853122	34.86379053
FAS	rs72704449	30.89740803
FAS	rs351973	39.58634569
FAS	rs2242660	49.13823982
FAS	rs35508382	233.4290858
FAS	rs9273258	93.23978709
LEPR	rs184647339	37.51361121
LEPR	rs72679406	967.7054471
LEPR	rs4468199	4078.963326
LEPR	rs6678033	21282.29422
LEPR	rs72667434	294.9378901
LEPR	rs7530150	60.81879058
LEPR	rs147254080	311.1897563
LEPR	rs72667283	30.47989697
LEPR	rs72676194	71.32763305
LEPR	rs6679356	159.8077431

LEPR	rs78618931	125.8395521
LEPR	rs12036274	81.80264694
LEPR	rs151042095	95.80882103
LEPR	rs116548914	52.61387366
LEPR	rs72673653	50.92565513
LEPR	rs12081633	33.80014301
LEPR	rs77902262	34.37334785
LEPR	rs4950017	30.03784077
LEPR	rs602633	31.52392251
LEPR	rs7949566	34.97711239
LEPR	rs2393775	36.42393948
LEPR	rs112963922	30.92083288
LEPR	rs1126464	29.8523996
LEPR	rs72808882	32.46057676
LEPR	rs1260326	172.0576387
LEPR	rs909341	29.89046233
LEPR	rs760715	44.39238598
LEPR	rs28478252	33.99290634
LEPR	rs149062332	34.4762328
LEPR	rs7012814	30.51372757
TNFRSF21	rs6458544	43.72016799
TNFRSF21	rs6458555	176.2199032
TNFRSF21	rs660240	55.66455014
TNFRSF21	rs12133018	33.99627433
TNFRSF21	rs61747728	92.69560102
TNFRSF21	rs112771035	46.77085402
TNFRSF21	rs142029184	34.4224901
TNFRSF21	rs2298475	85.97660085
TNFRSF21	rs77924615	36.22908949
TNFRSF21	rs12945299	73.66019829
TNFRSF21	rs143501783	59.66320913
TNFRSF21	rs1260326	48.76916019
TNFRSF21	rs13108218	37.98757676
TNFRSF21	rs885945	48.84456433
CDH15	rs10922098	163.6992358
CDH15	rs6445035	49.89999676
CDH15	rs41285751	55.90176591
CDH15	rs451652	43.38449288
CD27	rs954439	32.24528241
CD27	rs4234593	47.10730453
CD27	rs55719415	57.82413202
CD27	rs7627243	202.5021704
CD27	rs66965282	4666.266913
CD27	rs822370	142.9380332
CD27	rs79574829	31.67861403
SIRPA	rs140057736	119.4324817
SIRPA	rs112299692	78.77847561
SIRPA	rs73071702	37.88717311
SIRPA	rs117498650	99.27738875
SIRPA	rs6078982	188.4438217
SIRPA	rs1884564	4764.927895
SIRPA	rs6081084	7377.641667
SIRPA	rs3828016	430.6284456
SIRPA	rs17312424	253.0744245
SIRPA	rs6115555	78.41372987
SIRPA	rs145057011	33.28085869
SIRPA	rs6051869	44.13668665
SIRPA	rs142902318	32.38081289

SIRPA	rs635634	57.60918195
FABP3	rs1421085	61.56299327
FABP3	rs34674929	33.31916957
FABP3	rs2197423	68.23754404
FABP3	rs114740093	30.81176178
FABP3	rs72961007	32.34171506
FABP3	rs76370550	35.68894476
FABP3	rs57697972	112.4203622
TNNI2	rs583104	106.7933089
TNNI2	rs11599353	32.32723421
TNNI2	rs10833719	36.31613724
TNNI2	rs1815739	35.1059434
TNNI2	rs11651658	32.1822519
TNNI2	rs1799938	42.68428851
TNNI2	rs1976353	200.2915652
TNNI2	rs3887575	131.9081385
TNNI2	rs4089292	171.4013172
TNNI2	rs6467	30.38776998
NPPA	rs145488887	48.03033479
NPPA	rs10922098	46.32899212
NPPA	rs146457388	42.5467259
NPPA	rs6542680	506.5506464
NPPA	rs11755689	44.36550496
ALCAM	rs9834384	54.67588477
ALCAM	rs35067598	40.35038485
ALCAM	rs77228368	175.5718407
ALCAM	rs11719305	57.87737005
ALCAM	rs78689694	45.01902897
ALCAM	rs11075299	61.15802107
ALCAM	rs12945299	71.17222132
ALCAM	rs143501783	37.51227795
ALCAM	rs77262773	52.28610999
ALCAM	rs281377	69.72212894
ALCAM	rs3747207	37.78858228
ALCAM	rs3129960	47.93190958
ALCAM	rs10901252	63.61488342
ASGR1	rs434325	32.31495534
ASGR1	rs55714927	121.4177483
ASGR1	rs7528419	103.2327351
ASGR1	rs2622935	151.4784816
ASGR1	rs5021452	36.80677666
CNDP1	rs17806420	160.4431531
CNDP1	rs17817077	1098.184584
CNDP1	rs12326826	396.5200475
CNDP1	rs148000502	137.4821617
CNDP1	rs11873408	67.26261204
CNDP1	rs150844304	60.64704713
CNDP1	rs149624078	47.80867984
CNDP1	rs149274613	39.73985756
CNDP1	rs75621351	34.18283494
CNDP1	rs8102710	1088.305696
CNDP1	rs71167713	50.92287918
CNDP1	rs17771170	40.77859662
CNDP1	rs1154988	106.0236423
CNDP1	rs28601761	40.97225046
COLEC12	rs1539790	170.1339612
COLEC12	rs72865631	61.29991481
COLEC12	rs493130	121.0586447

COLEC12	rs374164158	36.63227549
COLEC12	rs56344867	65.4867227
COLEC12	rs181707610	35.01221711
COLEC12	rs12480076	53.46688849
COLEC12	rs755240	387.3656908
COLEC12	rs117435358	214.5589199
COLEC12	rs34058426	666.7776677
COLEC12	rs150141224	62.3644787
COLEC12	rs78873101	60.79519978
COLEC12	rs62217531	110.6158585
COLEC12	rs1386931	40.3165203
COLEC12	rs78273381	90.03362408
COLEC12	rs374407247	153.3713268
COLEC12	rs4834240	51.71399912
COLEC12	rs9271368	34.82143244
COLEC12	rs709822	340.5124299
COLEC12	rs7818169	33.69476142
CST1	rs6106592	144.6339604
CST1	rs2424519	90.00097854
CST1	rs2424592	1137.688264
CST1	rs2070856	2429.355991
CST1	rs61685000	91.50438474
CST1	rs836124	32.30021526
CST1	rs17742991	140.3185194
CST1	rs2774035	43.07105413
CST1	rs34230357	45.88284352
CST1	rs67020211	83.34133828
CST1	rs681343	61.96658904
CST1	rs112865649	59.84775092
CST1	rs1571251	44.01458562
CST1	rs62213165	45.80724736
CST1	rs55739757	37.56024581
CST1	rs4246778	41.4789211
FCN3	rs147810594	950.8585088
FCN3	rs140858042	205.5892269
FCN3	rs79832171	389.1587336
FCN3	rs72550870	244.5969797
FCN3	rs311428	41.71306789
FCN3	rs41284319	129.9060575
FCN3	rs1938402	104.9925958
FCN3	rs150665026	107.1379433
FCN3	rs74538145	51.10260049
FCN3	rs144943189	66.60495985
FCN3	rs116498837	174.9326522
FCN3	rs74409324	38.77698562
FCN3	rs3901907	66.10171198
FCN3	rs757081	145.1507417
FCN3	rs11606706	33.93271231
FCN3	rs368207242	56.11673884
FCN3	rs139801914	351.9663116
FCN3	rs9965948	47.03835106
FCN3	rs780093	36.4406011
FCN3	rs1486236	80.41463907
FCN3	rs35677470	38.60175836
FCN3	rs3774275	57.04621113
FCN3	rs9298506	392.364109
FCN3	rs7873100	50.76562244
GAS1	rs2150719	78.35179707

GAS1	rs4878043	101.4815844
GAS1	rs2477664	33.89759631
GAS1	rs140545159	46.27489529
GAS1	rs4795734	33.61659898
GAS1	rs1260326	34.61590264
GAS1	rs9992101	42.80332331
GAS1	rs2853950	32.8753208
GAS1	rs635634	46.26388123
HS6ST1	rs78029512	40.42993917
HS6ST1	rs35182715	58.04028615
HS6ST1	rs840870	351.5426158
HS6ST1	rs66476363	31.5598865
HS6ST1	rs660240	68.09392168
HS6ST1	rs78705678	46.77932108
HS6ST1	rs2477642	43.19132224
HS6ST1	rs3184504	31.15828055
HS6ST1	rs55993634	74.50690271
HS6ST1	rs704	252.798846
HS6ST1	rs77262773	78.67151374
HS6ST1	rs907866	66.43457547
HS6ST1	rs56210800	42.19402171
HS6ST1	rs17282078	43.09425882
HS6ST1	rs1861877	30.36028492
HS6ST1	rs141236281	36.34645095
HS6ST1	rs35914490	57.07827179
HS6ST1	rs146224174	48.34171349
HS6ST1	rs113688927	85.25018266
HS6ST1	rs4947328	35.36531847
HS6ST1	rs143867680	36.94123367
HS6ST1	rs6952090	38.55041788
IL17RC	rs10922098	32.97957027
IL17RC	rs697357	31.66257679
IL17RC	rs6458	66.99624404
PRKCG	rs56826489	46.62245082
PRKCG	rs148766553	38.49741659
PRKCG	rs6458	77.51875611
FOLH1	rs10700622	33.22021688
FOLH1	rs67833256	263.4786137
FOLH1	rs61885329	279.8356286
FOLH1	rs28629977	41.67091266
FOLH1	rs10897485	45.26258838
FOLH1	rs11624282	87.84368388
FOLH1	rs34664313	35.89623986
FOLH1	rs9953884	39.72175752
FOLH1	rs7658706	44.78686015
CCL5	rs4239252	600.8798827
CCL5	rs4796184	30.64354314
CCL5	rs111887239	40.89913387
CCL5	rs1926231	30.88482723
CCL5	rs176833	47.83182518
CCL5	rs34283456	31.31787013
CCL5	rs7073753	184.7383459
CCL5	rs1654446	39.52730888
CCL5	rs1354034	36.85090526
CCL5	rs9494145	30.01483506
CCL5	rs6993770	33.42206273
RASA1	rs704	288.8982379
RASA1	rs1045300	43.0721902

RASA1	rs1354034	31.19454702
RGMA	rs11074135	386.613608
RGMA	rs113520033	40.61379462
RGMA	rs7171384	31.64395333
RGMA	rs8035095	63.35096199
RGMA	rs660240	69.954737
RGMA	rs72704449	36.06860403
RGMA	rs9318187	35.12838934
RGMA	rs2167031	30.11169062
RGMA	rs77542162	49.89680639
RGMA	rs429358	165.6305726
RGMA	rs1260326	30.63044441
RGMA	rs6779998	31.0260396
RGMA	rs7047617	33.42562887
RGMA	rs2236291	482.0994935
RGMA	rs138398219	33.78924277
RPS3A	rs1976848	54.78397206
RPS3A	rs11553699	33.20593733
RPS3A	rs71366466	67.91695848
RPS3A	rs1354034	236.7310846
ICAM2	rs12146727	62.1578449
ICAM2	rs3184504	89.84016141
ICAM2	rs438811	50.12249599
ICAM2	rs6099309	71.54763584
ICAM2	rs1706435	47.37548329
ICAM2	rs635634	234.6388356
SLAMF7	rs17370655	36.91488042
SLAMF7	rs352686	1111.048251
SLAMF7	rs34158391	1107.172519
SLAMF7	rs75213764	742.7859138
SLAMF7	rs6702875	90.81208124
SLAMF7	rs116205652	325.5713719
SLAMF7	rs16830188	60.7322944
SLAMF7	rs115877710	389.9620092
SLAMF7	rs12079579	72.13118243
SLAMF7	rs190841461	50.88460493
SLAMF7	rs10849448	31.05676732
SLAMF7	rs6580642	33.00407321
SLAMF7	rs11160704	39.5107236
SLAMF7	rs3812999	35.62171354
SLAMF7	rs9901675	31.86345211
SLAMF7	rs61764072	31.57250893
SLAMF7	rs2725405	44.54770743
SLAMF7	rs123698	61.12879288
SLAMF7	rs1512226	31.27877519
SLAMF7	rs1448187	48.96333681
SLAMF7	rs5997579	69.60614753
SLAMF7	rs11710456	118.46603
SLAMF7	rs7754000	35.51048095
SLAMF7	rs1611589	50.59149737
SLAMF7	rs9257052	60.46848208
SLAMF7	rs3134969	29.82423623
SLAMF7	rs62447171	40.47738571
SLAMF7	rs3807307	38.17724796
SLAMF7	rs13297246	38.61224439
SLAMF7	rs2067583	31.28510167
SPOCK2	rs1245548	527.3878582
SPOCK2	rs35801422	31.12877592

SPOCK2	rs10157710	121.1931333
SPOCK2	rs72820511	115.816175
SPOCK2	rs141343804	47.06914752
SPOCK2	rs138646309	43.51669382
SPOCK2	rs80263240	37.81669569
SPOCK2	rs143760180	30.75704058
SPOCK2	rs192127052	36.85872876
SPOCK2	rs11055134	53.9509784
SPOCK2	rs80279219	33.18718053
SPOCK2	rs10166630	33.39013289
SPOCK2	rs11722445	47.37658643
SPOCK2	rs13210483	41.86206854
SPOCK2	rs2040748	72.55142243
SPOCK2	rs2071278	37.37550952
SNRPF	rs10922098	84.80540719
SNRPF	rs17580	47.18904864
SNRPF	rs11845244	32.02264381
SNRPF	rs34562807	30.48141322
SNRPF	rs429358	613.4222536
SNRPF	rs35136575	122.264455
SNRPF	rs72952553	31.62670735
SNRPF	rs11720167	213.9784078
SNRPF	rs3863087	88.18502948
SNRPF	rs73169103	31.86017009
SNRPF	rs11755689	34.31476257
CTSD	rs12574439	33.90882502
CTSD	rs199527622	36.03536479
CTSD	rs59724260	94.90285811
CTSD	rs4752744	124.3033333
CTSD	rs55861089	646.3294266
CTSD	rs11824983	48.13897001
CTSD	rs7964859	271.2352501
CTSD	rs140624338	37.60960874
CTSD	rs11127048	34.60011971
CTSD	rs28615248	30.21030143
EGF	rs4355391	44.75747637
EGF	rs115294968	195.1974584
EGF	rs369610634	94.82988185
EGF	rs7073753	39.3021638
EGF	rs75890625	86.61918469
EGF	rs77107029	36.13235113
EGF	rs6993770	31.38885361
TNFRSF18	rs10922098	296.8931657
TNFRSF18	rs9267139	29.72517086
TNFRSF18	rs28573154	137.0296087
TNFRSF18	rs7774739	90.93709879
TNFRSF18	rs9271689	60.82096128
FGFR1	rs62505473	126.2819865
FGFR1	rs57128498	34.69075444
FGFR1	rs688627	44.5394092
FGFR1	rs602633	32.6799368
FGFR1	rs7952602	51.24263775
FGFR1	rs12945299	80.09859289
FGFR1	rs17880604	61.65346483
FGFR1	rs1260326	43.63885719
FGFR1	rs62217923	112.9706808
FGFR1	rs2837988	177.230743
FGFR1	rs62276455	143.2593772

FGFR1	rs2462226	67.75895044
FGFR1	rs11920688	66.13198604
FGFR1	rs7643766	76.84613908
FGFR1	rs3823381	29.83816925
FGFR1	rs635634	141.464876
FGFR1	rs117210485	46.59333108
TNFRSF10B	rs10922094	43.41880854
TNFRSF10B	rs6542680	321.5375413
TNFRSF10B	rs11755689	46.46591544
NRP1	rs3006692	38.76549674
NRP1	rs142268798	58.89862769
NRP1	rs2383984	1035.683265
NRP1	rs189444333	73.9000299
NRP1	rs7555577	105.4239809
NRP1	rs10864726	424.3713599
NRP1	rs1043897	50.78441191
NRP1	rs146107972	34.29301865
NRP1	rs187621637	37.37579516
NRP1	rs148255421	46.27647189
NRP1	rs41556717	31.62337584
NRP1	rs143501783	43.21594094
NRP1	rs77542162	48.48378909
NRP1	rs1260326	68.35577587
NRP1	rs1883711	32.839805
NRP1	rs185081604	70.65572129
NRP1	rs28601761	31.16085471
FGL1	rs76714504	183.6872109
FGL1	rs71526143	78.44158479
FGL1	rs6986061	1002.487748
FGL1	rs417661	2502.436309
FGL1	rs924058	180.1634592
FGL1	rs17127314	68.29123597
FGL1	rs72713144	29.77375115
FGL1	rs56383622	36.04357437
FGL1	rs1837095	38.20070945
FGL1	rs17408255	59.05881849
FGL1	rs731170	44.28192479
FGL1	rs4665972	91.42938477
FGL1	rs62499724	34.61668911
FGL1	rs17487121	55.30273304
TCN2	rs5763749	72.01588055
TCN2	rs73881478	2848.247937
TCN2	rs7364237	302.9550423
TCN2	rs7286054	977.3498759
TCN2	rs3788427	45.39011914
TCN2	rs61729512	33.56705401
TCN2	rs112648662	109.504631
TCN2	rs116211475	1018.998696
TCN2	rs2241590	232.3919376
TCN2	rs62117477	536.9061184
TCN2	rs80275520	203.4305586
TCN2	rs12986064	56.82306631
TCN2	rs2205771	96.97944345
TCN2	rs113274966	358.7815549
TCN2	rs41307235	84.39737027
TCN2	rs180827560	38.33928764
TCN2	rs635634	51.5666764
MINPP1	rs59980852	133.6160054

MINPP1	rs2285170	166.4941156
MINPP1	rs2477664	64.41861573
MINPP1	rs2461161	64.46004845
MINPP1	rs17476364	30.38464638
MINPP1	rs543798	56.18970067
MINPP1	rs74717647	32.65626212
MINPP1	rs16860640	35.05093001
MINPP1	rs9854418	34.44860463
MINPP1	rs3864105	722.7626474
MINPP1	rs6444195	58.00873368
MINPP1	rs8176743	520.3392455
ZPLD1	rs10922094	163.8281949
ZPLD1	rs1706435	52.1546101
ZPLD1	rs9501600	62.94296776
PDIA5	rs16833133	39.08589665
PDIA5	rs17212030	44.17198241
PDIA5	rs9873067	185.8725709
PDIA5	rs3804749	9220.274788
PDIA5	rs62263781	145.7283177
PDIA5	rs144827616	83.86041516
PDIA5	rs2588511	33.32613524
PDIA5	rs138872539	33.83673331
PDIA5	rs143633454	62.62411893
PDIA5	rs6793326	50.0853648
PDIA5	rs62267604	66.58616514
CD5	rs78689694	161.6090504
CD5	rs1866767	38.64342567
CD5	rs117902156	46.82460342
CD5	rs11571364	119.5277996
CD5	rs55714927	353.6043797
CD5	rs112723993	63.85440391
CD5	rs2232528	37.79862942
CD5	rs2569444	408.9534587
CD5	rs33978622	52.01515369
CD5	rs150824684	58.45781662
CD5	rs73066161	58.79195218
CD5	rs117524660	59.27987316
CD5	rs2593704	41.26686027
CD5	rs3747207	32.29495023
CD5	rs1374898	43.83062372
CD5	rs17018841	68.19836783
CD5	rs9824576	56.14777162
CD5	rs36195304	50.73565614
CD5	rs4858926	60.29416437
CD5	rs2172259	45.00473337
CD5	rs62269703	199.8314954
CD5	rs112655189	37.78524314
CD5	rs3804623	962.6930919
CD5	rs74890720	887.3981481
CD5	rs13071075	63.53088536
CD5	rs11920688	87.00679875
CD5	rs11718075	115.6848283
CD5	rs114747602	116.8876205
CD5	rs114978758	144.1511197
CD5	rs111472656	154.5595849
CD5	rs9273067	150.1572735
CD5	rs2858879	87.13843482
CD5	rs13209376	31.86391892

CD5	rs4841132	33.06273349
CD5	rs10121987	79.95935938
CD5	rs146798134	30.12471911
CD5	rs635634	369.952529
GREM2	rs116135446	44.57635324
GREM2	rs682479	51.32350859
GREM2	rs2393791	41.96711383
GREM2	rs72706230	30.37576652
GREM2	rs73376930	128.2866835
GREM2	rs35166538	54.36053131
GREM2	rs4686731	66.04927863
GREM2	rs1541614	35.4000956
GREM2	rs138871952	2188.38292
GREM2	rs13068263	271.0496999
GREM2	rs2052768	82.7962395
GREM2	rs2271872	55.54472321
PCYOX1L	rs7310615	33.51906277
PCYOX1L	rs6884904	32.32994029
PCYOX1L	rs6472812	82.5878653
PGLYRP2	rs61747527	44.99298518
PGLYRP2	rs1122713	36.03268677
PGLYRP2	rs10419330	40.34211119
PGLYRP2	rs78677793	1034.526629
PGLYRP2	rs1273532	104.8955675
PGLYRP2	rs11078597	36.95584708
PGLYRP2	rs12609794	37.33464564
PGLYRP2	rs59950280	54.90410883
RNASE10	rs10922098	169.9685584
RNASE10	rs6542680	39.73293898
RNASE10	rs1706435	35.14133106
RNASE10	rs6457457	42.40003751
RNASE10	rs28573154	53.35255582
HPSE	rs61751211	136.2904005
HPSE	rs11732892	192.0470885
HPSE	rs4883201	181.5496021
HPSE	rs1795861	30.59509694
HPSE	rs15951	92.17571998
HPSE	rs9494145	31.57340007
HPSE	rs6993770	102.4432232
MFNG	rs56249362	292.1187896
MFNG	rs7533384	30.38165434
MFNG	rs80132192	30.15993214
MFNG	rs7754518	39.20515092
FAM171A1	rs12411755	32.60101945
FAM171A1	rs10922098	105.0374089
FAM171A1	rs9501600	32.6082705
FAM171A1	rs1846190	38.27155491
DEFB108B	rs572515	37.38857784
DEFB108B	rs73926056	29.90495181
DEFB108B	rs67399078	33.15173872
HINT2	rs10922098	112.862734
HINT2	rs1355538	40.62186026
HINT2	rs28573154	47.66824863
HINT2	rs7774739	39.9269624
CRH	rs10922103	69.44051426
CRH	rs6542680	31.28629163
CRH	rs9501600	55.34931512
FAM172A	rs13165400	50.2654942

FAM172A	rs10922098	38.23708356
FAM172A	rs9272780	30.26389777
NPFF	rs371834260	42.95874451
NPFF	rs8075695	76.16916016
NPFF	rs241790	943.882256
NPFF	rs17719944	238.6755566
NPFF	rs16964261	156.9663936
NPFF	rs77263366	68.07667871
NPFF	rs6505168	36.88838555
NPFF	rs7412	208.0364233
NPFF	rs12478841	32.38053253
NPFF	rs56210800	37.39731
NPFF	rs149468457	37.45822443
NPFF	rs1619994	45.13362191
NPFF	rs872914	47.52180709
NPFF	rs406936	74.22434685
FAM3B	rs60265870	827.1123062
FAM3B	rs114002959	53.30797729
FAM3B	rs60975870	41.20539935
FAM3B	rs73370115	74.4091851
FAM3B	rs2559851	47.86981355
FAM3B	rs731541	39.36555391
FAM3B	rs708686	149.0837122
FAM3B	rs62265615	37.4896182
FAM3B	rs56210800	36.18323673
FAM3B	rs635634	126.2228374
PAM	rs58786975	1256.302124
PAM	rs62370439	1302.853487
PAM	rs148194384	299.6156893
PAM	rs6596528	6012.857105
PAM	rs58117256	124.137177
PAM	rs333947	35.03405645
PAM	rs78705678	108.3282401
PAM	rs2477642	88.15009668
PAM	rs142196190	29.91877871
PAM	rs7247412	29.78985264
PAM	rs12986064	66.53386129
PAM	rs689122	50.45245365
PAM	rs186487066	29.78737581
PAM	rs6878991	37.90182852
PAM	rs28550273	42.79293913
PAM	rs148623446	91.33134698
PAM	rs150915429	116.9593174
PAM	rs57512238	95.45448256
PAM	rs35719897	953.3550452
PAM	rs149533656	165.8837643
PAM	rs62368375	213.5521176
PAM	rs72809624	50.78894281
PAM	rs885122	103.83255
PAM	rs62406303	35.70985558
THSD1	rs73199769	95.73894247
THSD1	rs74543965	2461.824399
THSD1	rs10529386	470.4586988
THSD1	rs61957359	67.80136348
THSD1	rs144876910	43.99686623
THSD1	rs11603123	120.3755935
THSD1	rs140147078	195.4000252
THSD1	rs146184955	82.5488477

THSD1	rs73485835	169.6901882
THSD1	rs60363555	126.4315701
THSD1	rs76518888	48.07281354
THSD1	rs3205136	163.997176
THSD1	rs600038	2857.933231
THSD1	rs117608058	79.38346827
THSD1	rs117541350	51.30762717
THSD1	rs139562936	37.40355282
CD300LF	rs10922094	140.6962462
CD300LF	rs58068569	30.18293323
CD300LF	rs9864801	48.89570906
CD300LF	rs9501393	43.41441181
CD300LF	rs7774739	34.98412895
CTRC	rs7080386	104.6712334
CTRC	rs1398015	45.6374494
CTRC	rs11574452	60.84171236
CTRC	rs342200	31.11322587
CTRC	rs6993770	49.88755528
GNRH1	rs4465978	52.24368512
GNRH1	rs113567935	41.54130852
GNRH1	rs75097760	68.78141147
GNRH1	rs75400206	49.02585557
GNRH1	rs140678946	45.5929766
SEMA3G	rs115483359	41.11722695
SEMA3G	rs13091025	472.884481
SEMA3G	rs115990971	71.03773743
SEMA3G	rs333947	40.85671238
SEMA3G	rs10904974	45.32993992
SEMA3G	rs2947595	83.06044207
SEMA3G	rs2477664	300.839567
SEMA3G	rs2461161	313.5673287
SEMA3G	rs2066985	39.909801
SEMA3G	rs55714927	124.8403697
SEMA3G	rs143501783	47.58003676
SEMA3G	rs117241334	31.06445176
SEMA3G	rs2008174	129.087654
SEMA3G	rs141686191	35.10062729
SEMA3G	rs12487573	46.83868652
SEMA3G	rs113245157	117.3896337
SEMA3G	rs115087321	43.54021836
SEMA3G	rs9859898	61.53578844
SEMA3G	rs10513546	45.21070399
SEMA3G	rs12496329	39.36133192
SEMA3G	rs9270016	30.91061618
SEMA3G	rs1010881	36.50690746
SEMA3G	rs8176743	221.8488708
CD300A	rs6501688	46.36768767
CD300A	rs9797243	57.71572735
CD300A	rs1320040	126.4625804
CD300A	rs7225955	127.0524072
CD300A	rs2706525	5006.68599
CD300A	rs59728170	441.5331545
CD300A	rs1996710	34.47977734
CD300A	rs117162506	126.2360409
CD300A	rs1838095	30.50611728
CD300A	rs78689694	47.61320158
CD300A	rs10858824	67.25189021
CD300A	rs142807125	64.88452508

CD300A	rs10858852	927.6892071
CD300A	rs2722224	159.5064583
CD300A	rs11495145	32.43148167
CD300A	rs7310615	47.49331979
CD300A	rs12601859	35.31483456
CD300A	rs11571364	42.40484402
CD300A	rs12945299	118.7125581
CD300A	rs62624498	34.65902715
CD300A	rs11888817	166.426394
CD300A	rs115931	38.32918558
CD300A	rs13205436	36.7454025
MLN	rs112742857	130.5767116
MLN	rs73408232	219.4818833
MLN	rs2296329	1273.858466
MLN	rs13209147	786.8892246
MLN	rs9380392	117.9455933
MLN	rs7516442	42.37984587
MLN	rs826404	61.40165194
MLN	rs1041309	101.7594372
MLN	rs10906953	68.32583461
MLN	rs2579674	35.52497702
MLN	rs10994838	31.9446653
MLN	rs4536103	38.417334
MLN	rs2366040	29.78437197
MLN	rs17358295	35.1633537
MLN	rs148524560	31.72163583
MLN	rs4943547	125.3927743
MLN	rs74395814	31.7001158
MLN	rs11855098	54.38991012
MLN	rs141716219	44.7272405
MLN	rs150325021	30.02569309
MLN	rs323031	32.14855837
MLN	rs62066052	63.67759536
MLN	rs10503013	38.2864946
MLN	rs492602	108.8169085
MLN	rs9808155	59.03317196
MLN	rs36007556	66.60132908
MLN	rs16862260	634.1664732
MLN	rs56796398	68.15674975
MLN	rs1028373	165.791961
MLN	rs73256185	41.44044882
MLN	rs74183487	36.30398401
MLN	rs4285017	31.09353047
MLN	rs815233	32.77681764
MLN	rs12521989	58.83408737
MLN	rs56092644	77.71721732
MLN	rs2206734	238.12502
MLN	rs116491682	45.17483057
MLN	rs169503	71.00904914
MLN	rs375703547	47.08204177
MLN	rs679332	83.0604253
MLN	rs4236771	55.86906577
MLN	rs1107080	234.8153728
MLN	rs2810491	40.4531964
CRTAC1	rs7086776	79.13631999
CRTAC1	rs145222503	47.23035968
CRTAC1	rs7078809	155.255314
CRTAC1	rs684225	3017.333217

CRTAC1	rs36182127	220.4899259
CRTAC1	rs71486144	134.2294049
CRTAC1	rs117781346	61.43187712
CRTAC1	rs116009432	31.94960847
CRTAC1	rs17468083	66.50164417
CRTAC1	rs12258909	57.51315979
CRTAC1	rs7644310	197.7867119
CRTAC1	rs13068263	62.56714965
CRTAC1	rs6775048	3435.586366
CRTAC1	rs143893205	108.5638557
CRTAC1	rs74477803	41.35194166
POFUT1	rs76143353	1212.844774
POFUT1	rs73114148	33.5022548
POFUT1	rs75106956	90.04981352
POFUT1	rs2588511	37.23818637
POFUT1	rs118102836	33.71210745
POFUT1	rs17861054	68.56695567
POFUT1	rs111376709	33.0995794
POFUT1	rs117296985	45.78425752
POFUT1	rs75570998	188.5233357
POFUT1	rs1768917	73.2228886
POFUT1	rs76055770	30.05372121
POFUT1	rs79498267	72.32390797
POFUT1	rs9985266	32.13344785
POFUT1	rs2598290	40.35396817
POFUT1	rs4240624	50.47052957
MFAP4	rs11658402	38.26807138
MFAP4	rs144628398	31.11787335
MFAP4	rs139356332	590.7165845
MFAP4	rs12757307	38.77082418
MFAP4	rs333947	36.89732699
MFAP4	rs61801021	60.71325656
MFAP4	rs117731563	47.98244728
MFAP4	rs373724912	31.34616862
MFAP4	rs74814272	44.55399009
MFAP4	rs144881474	161.1828197
MFAP4	rs186189054	100.3280006
MFAP4	rs376663529	38.82375313
MFAP4	rs141359357	72.84431499
MFAP4	rs74580987	37.25746818
MFAP4	rs930948	72.71333912
MFAP4	rs141459037	30.04891843
MFAP4	rs34958	41.29104721
MFAP4	rs114208897	30.57865959
MFAP4	rs1050529	39.38216155
MFAP4	rs9270317	45.48218723
MFAP4	rs635634	74.19923658
NTNG1	rs114774918	2560.157064
NTNG1	rs111996264	192.1475007
NTNG1	rs34038473	3954.911173
NTNG1	rs17447113	1123.752721
NTNG1	rs34434455	63.35575271
NTNG1	rs77833233	56.65901858
NTNG1	rs75579988	30.45751482
NTNG1	rs116829178	49.27191969
NTNG1	rs149313233	32.99699074
NTNG1	rs138537624	248.9756681
NTNG1	rs113858120	155.1342458

NTNG1	rs138024970	113.6729839
NTNG1	rs80247948	122.7010398
NTNG1	rs150892436	81.43425358
NTNG1	rs115069191	743.219152
NTNG1	rs114610219	34.70366477
NTNG1	rs9567612	208.1565601
COLGALT1	rs4808666	238.6558604
COLGALT1	rs10426931	32.61961321
COLGALT1	rs35745856	32.10789174
COLGALT1	rs4847398	40.32219318
COLGALT1	rs144577567	32.47784632
COLGALT1	rs115744844	215.1352398
COLGALT1	rs4340676	103.8827838
COLGALT1	rs6796	559.645171
COLGALT1	rs13225119	32.09001058
COLGALT1	rs35385182	43.99736653
COLGALT1	rs3752617	48.06115831
COLGALT1	rs2169387	51.69217361
NETO1	rs10922098	199.5372702
NETO1	rs6445035	42.2713031
NETO1	rs2242572	48.09210024
NETO1	rs9272068	34.3935322
NETO1	rs6993770	29.87440794
LCN8	rs67295569	34.13282277
LCN8	rs1965605	45.80783595
LCN8	rs1355538	30.21224427
LCN8	rs3129773	45.26166481
RNASE4	rs1756548	84.56491618
RNASE4	rs12588573	1305.038038
RNASE4	rs11624594	67.55579874
RNASE4	rs10138732	56.69128012
RNASE4	rs10135366	57.53952908
RNASE4	rs77924615	30.80361553
RNASE6	rs7158129	109.7957868
RNASE6	rs8006024	3960.29354
RNASE6	rs4572299	1173.039909
RNASE6	rs17097726	131.8729758
RNASE6	rs117219627	331.1811996
RNASE6	rs2282036	46.07237298
RNASE6	rs75106782	72.11204158
RNASE6	rs3184504	30.60146028
RNASE6	rs77924615	31.18908322
RNASE6	rs66962766	38.08778562
RNASE6	rs9272209	53.23104527
CTRB2	rs117274901	248.6927344
CTRB2	rs72798674	1096.474573
CTRB2	rs113400434	916.4143472
CTRB2	rs149263900	315.9698095
CTRB2	rs73605166	839.255918
CTRB2	rs148793797	415.2468756
CTRB2	rs112716238	130.987736
CTRB2	rs35311109	34.89271962
CTRB2	rs11166273	31.88798187
CTRB2	rs2641346	40.61484784
CTRB2	rs17580	145.4104884
CTRB2	rs112963922	81.96822682
CTRB2	rs71387088	40.33657914
CTRB2	rs199954217	54.77902137

CTRB2	rs372466280	33.44845148
CTRB2	rs76708035	31.45305966
CTRB2	rs117187218	44.65617081
CTRB2	rs75213011	236.2541633
CTRB2	rs79500103	99.98213164
CTRB2	rs117069519	45.76499819
CTRB2	rs118003726	48.98023333
CTRB2	rs117323918	62.69403216
CTRB2	rs117445981	32.12298187
CTRB2	rs1126464	37.1221183
CTRB2	rs8099628	29.78198425
CTRB2	rs17032925	31.95718197
CTRB2	rs13405197	29.98356563
CTRB2	rs10013413	31.29085708
CTRB2	rs11724862	35.06937693
CTRB2	rs9491696	37.62329777
CTRB2	rs11244049	43.43819953
PSG4	rs113797028	99.35574503
PSG4	rs7259457	2663.282076
PSG4	rs142376868	58.09487606
PSG4	rs138744737	65.79017916
PSG4	rs151145534	50.3487143
PSG4	rs192292781	1111.347184
PSG4	rs147075006	82.78697864
PSG4	rs112059502	177.3686438
PSG4	rs139960875	64.91438726
PSG4	rs1329424	32.72995819
PSG4	rs149958555	32.3634019
PSG4	rs73033341	37.72563939
PSG4	rs148053437	50.41827659
PSG4	rs111403605	121.3546432
PSG4	rs62119458	179.5505763
PSG4	rs145949601	221.4873228
PSG7	rs117356953	167.0073366
PSG7	rs140272367	29.81276178
PSG7	rs10922094	29.83551212
TMX3	rs7112154	73.09933395
TMX3	rs34105209	30.62140792
TMX3	rs6597	134.833774
TMX3	rs117676160	54.38104558
TMX3	rs117316091	41.09669959
TMX3	rs113907437	51.56824846
TMX3	rs6501599	185.6506199
TMX3	rs72854796	112.9473061
TMX3	rs56112103	942.4206489
TMX3	rs72816916	58.15375101
TMX3	rs113432335	77.52985969
TMX3	rs147067275	114.8083098
TMX3	rs112483799	87.43844516
TMX3	rs12202796	45.29086828
TMX3	rs14365	33.94953433
TMX3	rs7781312	112.0883056
ENDOU	rs148824401	30.49586351
ENDOU	rs11168215	58.80038787
ENDOU	rs10922098	49.41791054
ST3GAL1	rs9643300	948.091776
ST3GAL1	rs62520289	54.96609918
ST3GAL1	rs583104	36.55823404

ST3GAL1	rs2477664	60.81574829
ST3GAL1	rs2461161	38.39609223
ST3GAL1	rs28873836	161.041668
ST3GAL1	rs4672497	72.95481596
ST3GAL1	rs635634	64.89382415
F13B	rs111430107	58.95512591
F13B	rs75109228	107.9783034
F13B	rs1412636	1116.924521
F13B	rs79844463	44.62203786
F13B	rs199560872	35.52765427
F13B	rs7532568	66.37115642
F13B	rs78024727	33.11575148
F13B	rs73079476	37.47064253
F13B	rs1958770	30.00093826
F13B	rs12590165	31.08323065
F13B	rs1047891	43.03208504
F13B	rs4220	34.35398812
F13B	rs5982	31.91626891
TRH	rs10922094	79.39505742
TRH	rs7292000	53.42940416
TRH	rs9864801	32.95746541
TRH	rs557011	44.41962601
SOD3	rs201718821	799.2044071
SOD3	rs2695234	1145.208477
SOD3	rs800454	520.5976085
SOD3	rs4510458	231.9130918
SOD3	rs10015837	82.41355378
SOD3	rs62410642	40.17173669
SOD3	rs4697596	52.34878831
SOD3	rs333947	37.30228452
SOD3	rs78705678	55.09342878
SOD3	rs2477642	64.85564349
SOD3	rs56344867	35.63229123
SOD3	rs181707610	34.3780467
SOD3	rs10805206	31.16401543
SOD3	rs4696870	71.9936727
SOD3	rs78842674	48.48011145
SOD3	rs112992178	90.38084107
SOD3	rs184693375	130.2289062
PF4V1	rs75038538	67.86905141
PF4V1	rs7658970	1353.1002
PF4V1	rs10922098	70.75028249
PF4V1	rs6445180	30.08378609
PF4V1	rs74706296	36.71483657
PF4V1	rs72658540	45.16261461
PF4V1	rs200815906	33.30746736
DEFB106A	rs528298	394.6647221
DEFB106A	rs6445035	95.57201045
DEFB106A	rs3863087	38.92973356
DEFB106A	rs3099847	34.97887958
DEFB106A	rs9501600	79.71212227
SEMA3B	rs1329424	172.8472002
SEMA3B	rs1355538	42.33834255
SEMA3B	rs6457457	37.92075649
SEMA3B	rs7742632	48.55686316
CTRB1	rs117414814	54.99742237
CTRB1	rs149031578	45.08938648
CTRB1	rs149935681	975.9814723

CTRB1	rs9928744	333.3118888
CTRB1	rs6564267	93.2407507
CTRB1	rs327197	35.88964358
CTRB1	rs10922094	40.84867945
CTRB1	rs6686515	45.04046804
CTRB1	rs77177732	29.84620595
CTRB1	rs507695	41.37108557
CTRB1	rs10500579	30.8068817
CTRB1	rs533406	49.89976036
CTRB1	rs56850260	61.40915034
CTRB1	rs6835370	45.30766384
CTRB1	rs9482772	69.32047318
CTRB1	rs1018941	35.06432139
CTRB1	rs6977665	29.83489201
CTRB1	rs635634	41.16158375
ASIP	rs293724	1093.912641
ASIP	rs572515	62.76059851
ASIP	rs142181090	30.31821435
ASIP	rs7361512	36.74258495
ASIP	rs367964394	37.41405587
ASIP	rs138963020	143.5054832
ASIP	rs371977919	584.9561652
ASIP	rs137981944	111.2612044
ASIP	rs74402782	57.95055508
ASIP	rs78202849	56.37696222
ASIP	rs208804	33.98347311
ASIP	rs34832185	30.50727649
OBP2B	rs10901246	977.3711003
OBP2B	rs147980161	71.88232792
OBP2B	rs76523956	41.95041505
OBP2B	rs7857413	1323.353133
OBP2B	rs615364	1296.49088
OBP2B	rs7856882	168.4867192
OBP2B	rs11103374	84.73193486
VASN	rs757593	73.82347481
VASN	rs1337526	32.0544039
VASN	rs72704449	65.04071893
VASN	rs7096909	40.29138448
VASN	rs77262773	107.7992431
VASN	rs1260326	60.72902752
VASN	rs113804124	30.92430498
VASN	rs35332062	35.33442075
PRKCSH	rs11557488	136.3241104
PRKCSH	rs76572368	43.02626011
PRKCSH	rs116986544	53.5643728
PRKCSH	rs115701654	60.41946575
PRKCSH	rs485073	35.25146173
PRKCSH	rs1355538	38.05083578
CBLN4	rs78604342	73.10872355
CBLN4	rs601279	37.13736804
CBLN4	rs7351418	312.8592171
CBLN4	rs6024417	290.3252685
CBLN4	rs1036491	187.3744299
CBLN4	rs35887873	84.0543384
CBLN4	rs7684253	142.3126638
CBLN4	rs1277294	31.99248619
DEFB112	rs3184504	32.57639682
DEFB112	rs11846842	31.83136653

DEFB112	rs12434146	170.1562582
DEFB112	rs147103055	55.18690378
DEFB112	rs61995551	1091.46626
DEFB112	rs11847228	93.30315063
DEFB112	rs151256582	217.5442064
DEFB112	rs3016930	32.68237963
TUFT1	rs10794998	36.0170376
TUFT1	rs1706435	35.95410276
TUFT1	rs6993770	31.946294
CRISPLD2	rs144746688	66.27941822
CRISPLD2	rs2326394	470.763608
CRISPLD2	rs60910901	720.3131144
CRISPLD2	rs72799568	348.6668193
CRISPLD2	rs75567869	51.21627166
CRISPLD2	rs9970896	39.91078637
CRISPLD2	rs11253518	46.99099367
CRISPLD2	rs117842293	38.30916088
CRISPLD2	rs146509086	45.15572337
CRISPLD2	rs191708354	44.92694794
CRISPLD2	rs74704910	95.92465242
CRISPLD2	rs2511241	1176.850403
CRISPLD2	rs921651	39.29955793
CRISPLD2	rs8066582	75.53887111
CRISPLD2	rs139082193	37.3010611
CRISPLD2	rs9265807	40.03422403
CRISPLD2	rs9265977	79.26388608
CRISPLD2	rs2158799	35.92010709
TNXB	rs114382848	8511.004101
TNXB	rs6446	2839.418935
TNXB	rs9269547	504.6382519
TNXB	rs28622052	408.5699783
TNXB	rs2493136	29.92037478
TNXB	rs78705678	35.34445874
TNXB	rs12280645	85.64261747
TNXB	rs55714927	62.44722966
TNXB	rs143501783	29.82828292
TNXB	rs77542162	49.79549347
TNXB	rs34842930	32.5589181
TNXB	rs1442	107.877703
TNXB	rs12529322	76.47175882
TNXB	rs199573340	82.60686814
TNXB	rs115054151	205.3017314
TNXB	rs17184514	1233.228872
TNXB	rs183006581	56.07853277
TNXB	rs141244319	38.66646914
TNXB	rs1555686	1095.768216
TNXB	rs77988055	185.0746615
TNXB	rs79798018	282.5042185
TNXB	rs76384554	55.687592
TNXB	rs2685417	37.78427912
CLEC3B	rs76951547	47.66113036
CLEC3B	rs3216800	463.941472
CLEC3B	rs4910023	46.60454151
CLEC3B	rs77262773	37.53175248
CLEC3B	rs342507	30.39768898
OLFM1	rs11103515	46.74434591
OLFM1	rs57136797	182.6608785
OLFM1	rs11103667	125.368742

OLFM1	rs34793466	37.7512355
OLFM1	rs681343	35.84916781
OLFM1	rs118118711	30.05695622
OLFM1	rs9268581	35.75148703
OLFM1	rs140753685	57.0824666
OLFM1	rs635634	169.0933073
GZMM	rs114537924	47.9541868
GZMM	rs10922094	37.24940401
GZMM	rs3184504	43.99041979
GZMM	rs68037221	52.72300061
ELK3	rs3766744	113.1725981
ELK3	rs10633030	38.91275439
ELK3	rs146587041	61.29301449
ELK3	rs72692819	86.8099739
ELK3	rs6494730	38.82364571
ELK3	rs10820606	53.076183
LEAP2	rs757537	919.3972445
LEAP2	rs7717377	145.8175701
LEAP2	rs13360043	47.81655892
LEAP2	rs152784	61.27529955
LEAP2	rs10761737	33.67006162
LEAP2	rs150844304	35.91863817
LEAP2	rs1260326	147.5712435
LEAP2	rs113414093	37.8157182
LEAP2	rs10068638	38.22964518
LEAP2	rs185081604	71.50231009
LEAP2	rs2954038	44.12744261
LEAP2	rs150611042	74.04770963
IFNL3	rs62122869	35.86872065
IFNL3	rs7254729	30.93207949
IFNL3	rs3132487	42.21760439
IFNL3	rs34115112	83.93196686
IFNL3	rs115938440	96.72790576
IFNL3	rs9271710	35.18408137
IFNL3	rs239933	39.62432972
IFNA6	rs10922098	155.0907779
IFNA6	rs415620	41.15707774
IFNA6	rs4713498	45.66230303
IFNA6	rs67399078	34.80412806
CSAG1	rs10922098	30.93176559
CSAG1	rs76705436	30.43647396
CSAG1	rs139492403	35.37435878
CSAG1	rs77939743	111.9863708
CSAG1	rs10171839	1204.414448
CSAG1	rs146775047	49.54524982
PRCP	rs11233045	35.92369101
PRCP	rs2229437	371.9769342
PRCP	rs188172480	94.26272151
PRCP	rs34769119	38.19096139
PRCP	rs2461155	40.57047265
PRCP	rs2477639	160.853198
PRCP	rs692440	93.46838841
PRCP	rs74538877	34.36378401
PRCP	rs36014724	36.92811686
PRCP	rs61942123	30.01657933
PRCP	rs80300795	49.95054792
PRCP	rs825038	51.19627952
PRCP	rs7964859	580.7278933

PRCP	rs140624338	189.0273993
PRCP	rs28698773	31.74064016
PRCP	rs3116995	36.84451672
PRCP	rs6924387	56.73485832
PRCP	rs9298506	322.9542958
PRCP	rs10813949	62.39231188
PRCP	rs11142435	37.58289848
INSL3	rs10922098	483.6683998
INSL3	rs704	43.90809846
INSL3	rs11569415	51.03961605
INSL3	rs1707652	146.3168435
INSL3	rs9266729	37.87542333
INSL3	rs9267139	37.29762949
INSL3	rs28573154	129.2181582
INSL3	rs7774739	82.56555694
INSL3	rs9271683	30.32265862
SBSN	rs10922094	194.1885099
SBSN	rs697356	46.12979476
SBSN	rs9501393	69.6116179
SBSN	rs7774739	41.3266998
SBSN	rs601020	30.13176222
SPAG11A	rs1451915	39.19589138
SPAG11A	rs10922094	627.2903645
SPAG11A	rs11569415	43.56011198
SPAG11A	rs1707652	119.1636803
SPAG11A	rs3130955	30.86245571
SPAG11A	rs9501600	152.3806914
SPAG11A	rs35209896	46.31867532
FCRL1	rs4971125	43.86692373
FCRL1	rs7546031	939.0728
FCRL1	rs188948098	91.24491454
FCRL1	rs12138588	89.55625723
FCRL1	rs41264463	57.84866083
FCRL1	rs4948492	166.0668487
FCRL1	rs12784975	43.97357214
FCRL1	rs76428106	55.02365055
FCRL1	rs73217470	34.18870366
FCRL1	rs73609086	36.01852929
FCRL1	rs2867429	29.94805997
FCRL1	rs9939427	53.48908212
FCRL1	rs12945299	75.30661924
FCRL1	rs907091	80.42260756
FCRL1	rs77262773	33.09663703
FCRL1	rs55803162	83.1666015
FCRL1	rs76529516	36.39129968
FCRL1	rs4494728	37.64302142
FCRL1	rs7266148	33.74698432
FCRL1	rs73165110	130.8632524
FCRL1	rs113286101	91.18252537
FCRL1	rs10212190	43.15958666
FCRL1	rs13104150	41.69662675
FCRL1	rs717255	65.37627509
FCRL1	rs160045	49.2077814
FCRL1	rs77018303	42.06920538
FCRL1	rs58889466	86.42287782
FCRL1	rs6911448	57.65625674
FCRL1	rs1611284	53.89542997
FCRL1	rs113871699	36.62689444

FCRL1	rs1293245	37.74422958
FCRL1	rs10956485	91.04822912
SPINK6	rs7723023	153.748966
SPINK6	rs75666474	93.34099512
SPINK6	rs17599360	1028.475811
SPINK6	rs6884063	3347.505726
SPINK6	rs2161431	165.5592212
SPINK6	rs114240396	36.94382113
SPINK6	rs369422405	43.56911505
C1GALT1C1	rs2477664	67.05060016
C1GALT1C1	rs2461161	72.46611579
C1GALT1C1	rs35141421	51.91329342
C1GALT1C1	rs681125	55.9639522
C1GALT1C1	rs601338	76.61729437
C1GALT1C1	rs12986064	44.57527486
C1GALT1C1	rs1049709	40.81011226
C1GALT1C1	rs9639031	389.0595783
C1GALT1C1	rs1638216	33.24491423
C1GALT1C1	rs146798134	61.88749898
C1GALT1C1	rs635634	1000.404823
C1GALT1C1	rs11516156	40.74322549
TREML2	rs57368372	232.6476067
TREML2	rs991762	1004.445567
TREML2	rs6918969	425.9542157
TREML2	rs9471533	163.7028274
TREML2	rs9471595	57.51396592
TREML2	rs140136183	33.68026632
TREML2	rs61747728	39.37560555
TREML2	rs7310615	91.6652556
TREML2	rs1555405	36.14001532
TREML2	rs1361387	35.21686235
TREML2	rs1126504	38.78027989
TREML2	rs6920211	46.10485864
SEMA4D	rs117475749	989.4764761
SEMA4D	rs62547211	33.60242827
SEMA4D	rs371163291	901.3627797
SEMA4D	rs79903267	2473.255545
SEMA4D	rs3211698	46.29190054
SEMA4D	rs143192156	268.4148545
SEMA4D	rs117738937	311.9694727
SEMA4D	rs986425	33.5500846
SEMA4D	rs3002598	54.69510065
SEMA4D	rs2477639	142.5415768
SEMA4D	rs692440	99.96968749
SEMA4D	rs7310615	180.8008798
SEMA4D	rs1354034	42.461632
SEMA4D	rs2071805	32.12359281
SEMA4D	rs117268309	98.44326787
SEMA4D	rs117995385	46.25517954
KRTDAP	rs10922098	228.0298561
KRTDAP	rs4448834	43.36732575
KRTDAP	rs111696008	31.3124793
KRTDAP	rs2232613	85.55131484
KRTDAP	rs6445035	199.8862039
KRTDAP	rs3863087	76.81474571
KRTDAP	rs73169103	30.73021072
KRTDAP	rs9501393	79.29015039
KRTDAP	rs7774739	50.13887937

KRTDAP	rs73216598	38.98265009
KRTDAP	rs2237582	435.8973954
KRTDAP	rs854572	61.79384224
ROBO1	rs73103884	45.09108226
ROBO1	rs3773233	485.4430649
ROBO1	rs73111654	81.4832913
ROBO1	rs331172	88.58428814
ROBO1	rs7639769	100.9512189
ROBO1	rs34172220	132.1925425
ROBO1	rs11121245	75.80917429
ROBO1	rs10919615	35.5178722
ROBO1	rs78705678	118.1571941
ROBO1	rs2477642	101.6718696
ROBO1	rs78539764	29.71983269
ROBO1	rs12945299	37.09172844
ROBO1	rs2686395	78.60358497
ROBO1	rs1003879	41.86456046
ROBO1	rs10901252	47.95283116
ACP6	rs2985722	99.84973114
ACP6	rs201940212	46.83424357
ACP6	rs150956916	289.2941294
ACP6	rs202051594	566.5445421
ACP6	rs145317206	961.250552
ACP6	rs137961621	40.98019425
ACP6	rs143333817	1408.377796
ACP6	rs116523799	1026.731584
ACP6	rs116161634	330.5796964
ACP6	rs148127209	141.9200565
ACP6	rs76390540	562.9711199
ACP6	rs59326551	188.8242388
ACP6	rs116509906	195.5824726
ACP6	rs112053014	53.71626111
ACP6	rs17229389	48.91561625
ACP6	rs77953932	115.0017221
ACP6	rs12124243	93.29497433
ACP6	rs139616980	77.03459443
ACP6	rs838522	305.2742725
ACP6	rs1891031	198.2980004
ACP6	rs190942314	254.3102027
ACP6	rs61789667	37.10420146
ACP6	rs148969065	148.8257945
ACP6	rs11486019	147.7236183
ACP6	rs143771029	69.038782
ACP6	rs150990548	36.79263974
ACP6	rs112044625	67.78992206
ACP6	rs12073794	66.87534841
PI15	rs10922098	644.1631746
PI15	rs35351085	32.74024193
PI15	rs1707652	119.0200638
PI15	rs1265941	81.152346
PI15	rs550671	164.8875815
ASAH1	rs3766744	111.3466495
ASAH1	rs10633030	37.28911503
ASAH1	rs146587041	61.82948285
ASAH1	rs72692819	86.6231402
ASAH1	rs6494730	37.909724
ASAH1	rs11082304	29.93397012
ASAH1	rs10820606	50.81085153

CLPS	rs7763946	457.9494587
CLPS	rs370033786	118.0251526
CLPS	rs79476859	93.59002168
CLPS	rs2766575	2508.114499
CLPS	rs372084948	123.3956349
CLPS	rs10922094	30.18492036
CLPS	rs77472583	38.39086479
CLPS	rs8051363	51.40030744
CLPS	rs9275884	42.77060927
CLPS	rs10947428	53.29831922
CLPS	rs2451340	69.8922623
CLPS	rs190927521	63.07428807
CLPS	rs115985672	32.48350133
CLPS	rs2467071	78.78492547
INSL6	rs10922098	93.63161543
INSL6	rs415620	34.41527735
INSL6	rs4713498	45.75718977
PAPPA2	rs10922098	117.9477185
PAPPA2	rs6457457	32.83305938
PAPPA2	rs9501393	40.43020623
KLK9	rs10922098	152.1993641
KLK9	rs3820897	37.47980417
KLK9	rs11720167	52.26292508
KLK9	rs9501393	51.01752891
KLK9	rs7774739	39.8279771
SPAG11B	rs17580	42.35959631
SPAG11B	rs1265184	52.31788873
SPAG11B	rs4713488	68.61227516
SPAG11B	rs2734325	383.8052651
SPAG11B	rs79451346	46.49076849
SPAG11B	rs4841655	33.36747741
DEFB104A	rs35989515	167.8812172
DEFB104A	rs62488803	86.79838168
DEFB104A	rs11786478	714.7255895
DEFB104A	rs146143496	69.30836811
DEFB104A	rs6695321	51.43618928
DEFB104A	rs77262773	36.26345442
DEFB104A	rs149454038	156.1152934
GALNT2	rs10922098	55.37729567
GALNT2	rs17020993	40.3424765
GALNT2	rs78912960	35.92920066
DEFB121	rs4883201	36.72584971
DEFB121	rs72701845	31.9550985
DEFB121	rs61751211	267.7961435
DEFB121	rs6993770	51.29286337
BID	rs151268390	90.29200043
BID	rs11120218	45.93625786
BID	rs1354034	32.67872726
C3	rs201063729	89.2112051
C3	rs2230199	121.0984383
C3	rs10801559	113.5510116
CD70	rs10922098	201.7017355
CD70	rs1355538	42.42890719
CD70	rs28573154	61.65084041
CD70	rs7774739	48.91620134
TDGF1	rs57126329	1051.002842
TDGF1	rs36023124	1272.079292
TDGF1	rs34000233	4972.69706

TDGF1	rs141531713	908.3522125
TDGF1	rs3184504	33.27516684
TDGF1	rs35429	38.70066215
TDGF1	rs113414093	46.85279244
TDGF1	rs3763575	45.09024841
TDGF1	rs4678494	39.87446344
TDGF1	rs191270734	115.914579
TDGF1	rs113326507	150.1270159
TDGF1	rs73083221	57.35407131
TDGF1	rs9863890	45.67341839
TDGF1	rs149184033	69.10998908
TDGF1	rs73068222	194.5827852
TDGF1	rs76886896	117.4264113
TDGF1	rs6802320	366.9241155
TDGF1	rs7612148	241.4201739
TDGF1	rs7614338	39.35752856
TDGF1	rs188788627	72.49470714
TDGF1	rs11715209	38.39911139
TDGF1	rs242212	43.40507912
TDGF1	rs3762056	38.31091472
TDGF1	rs635634	61.18339778
EPO	rs221790	69.39029823
EPO	rs653178	37.34194342
EPO	rs189143685	41.06161102
EPO	rs117507076	41.23505287
EPO	rs78744187	33.45731226
EPO	rs77552793	47.35905929
EPO	rs855791	63.53347964
EPO	rs1619994	42.07367812
EPO	rs218264	63.52482793
EPO	rs351742	44.0070289
EPO	rs9402686	508.743209
EPO	rs4895447	31.92249261
GDNF	rs10922094	103.0261832
GDNF	rs6457457	32.52745719
GDNF	rs1846190	31.45468949
GDNF	rs211161	32.32031302
IFNGR1	rs72975557	31.70901568
IFNGR1	rs9376267	59.70359222
IFNGR1	rs7080536	208.9004855
IFNGR1	rs8176743	81.0288994
IL9	rs151133658	30.12820724
IL9	rs7702138	96.44705662
IL9	rs10922098	29.98922647
IL9	rs11451044	74.19676195
IL9	rs9494145	30.5217103
LIFR	rs146222431	30.47840271
LIFR	rs16903989	229.4375777
LIFR	rs34411312	97.94176729
LIFR	rs368433	37.6955366
LIFR	rs78705678	33.00005735
LIFR	rs2477642	32.7994244
LIFR	rs35166255	44.29187434
LIFR	rs62061425	64.38909318
LIFR	rs116852327	30.6488127
LIFR	rs72740108	49.14795071
LIFR	rs146780831	34.60255796
LIFR	rs371029813	50.54382786

LIFR	rs115445305	40.68171095
LIFR	rs79630295	34.64698259
LIFR	rs3205136	209.3206836
LIFR	rs600038	998.7992931
LIFR	rs28444941	31.94409339
LIFR	rs117608058	40.31619243
LIFR	rs7855899	32.27517002
EHMT2	rs12045422	43.1195043
EHMT2	rs55711441	31.51161834
EHMT2	rs74502455	35.57627406
EHMT2	rs102275	52.90491123
EHMT2	rs1601935	88.9343128
EHMT2	rs1800588	86.1204678
EHMT2	rs3764261	192.9417332
EHMT2	rs35495893	45.27503604
EHMT2	rs112001035	83.01445995
EHMT2	rs2156552	32.5774474
EHMT2	rs8106813	38.80361772
EHMT2	rs780093	45.33410619
EHMT2	rs55881728	43.55968006
EHMT2	rs111696008	66.32791377
EHMT2	rs6073971	46.05378736
EHMT2	rs11720167	459.2720823
EHMT2	rs3863087	155.6422864
EHMT2	rs73169103	35.80883263
EHMT2	rs6829364	37.02667645
EHMT2	rs7674623	121.5939813
EHMT2	rs185081604	57.63885245
EHMT2	rs4128744	32.8548127
S100A12	rs3014874	521.9774501
S100A12	rs61803119	108.9876215
S100A12	rs146588776	64.08855113
S100A12	rs704	46.47746877
S100A12	rs9898547	31.97897942
S100A12	rs62143194	31.27102844
S100A12	rs9271573	30.17051089
HAAO	rs1253593	33.49475298
HAAO	rs2862296	38.23500007
HAAO	rs143771088	96.09738898
HAAO	rs79642555	44.22792651
HAAO	rs11190375	1059.532927
HAAO	rs704	93.81915283
HAAO	rs4665972	29.85143247
HAAO	rs4687374	55.66957597
HAAO	rs35838665	168.6838591
HAAO	rs4075427	50.38602321
HAAO	rs6797360	175.3382182
HAAO	rs6806282	1024.093197
HAAO	rs9868646	136.6850508
HAAO	rs147278314	30.72942774
HAAO	rs111886030	32.31888041
HAAO	rs28455809	38.85444451
ARG1	rs2781668	125.5932539
ARG1	rs7756633	40.08939732
ARG1	rs10922098	166.8254831
ARG1	rs8014529	283.3834852
ARG1	rs2668202	39.51134408
ARG1	rs9501600	62.23979574

DCTN2	rs61678916	30.78714826
DCTN2	rs73262897	52.50846731
DCTN2	rs1126605	196.9341839
DCTN2	rs2017737	39.18281518
DCTN2	rs11755689	31.10412541
EIF4H	rs4980320	34.15691431
EIF4H	rs10876550	35.36803986
EIF4H	rs1354034	90.94950449
PSME1	rs3742500	41.17997791
PSME1	rs28399664	37.84695709
PSME1	rs7412	500.3284487
PSME1	rs1354034	30.4446906
TNF	rs10922094	47.04788399
TNF	rs11536949	34.73388132
TNF	rs13208583	29.86531968
TNFSF12	rs7209977	971.5657013
TNFSF12	rs12600749	187.201011
TNFSF12	rs8073033	51.9848938
TNFSF12	rs10822155	44.69213801
TNFSF12	rs2127870	68.60198744
THPO	rs11248395	31.87420438
THPO	rs16978104	29.8006911
THPO	rs17758695	34.81401012
THPO	rs1706417	40.42237603
THPO	rs9389278	34.27153517
THPO	rs6993770	52.86796774
PTH	rs6127099	205.0394468
PTH	rs219771	38.59706527
PTH	rs10051765	104.5717107
SST	rs13379369	66.17085269
SST	rs2753517	33.93270326
SST	rs6757263	59.92052115
SST	rs55636108	41.38860698
SST	rs62469224	35.46713363
DMKN	rs11882238	41.38329167
DMKN	rs704	193.1265788
DMKN	rs385982	129.4813816
DMKN	rs75627662	978.1153564
DMKN	rs190921611	65.48660616
DMKN	rs111696008	40.98258022
DMKN	rs11720167	285.2604189
DMKN	rs3863087	101.3161659
DMKN	rs148077724	33.3992152
DMKN	rs73169103	35.24013281
TNFSF14	rs1047286	55.43494655
TNFSF14	rs10922098	512.0340251
TNFSF14	rs1707652	120.3613782
TNFSF14	rs3891366	134.8199766
TNFSF14	rs7774739	91.11133489
TNFSF14	rs28645249	42.21298349
UTS2	rs72748420	43.83757758
UTS2	rs1329424	1285.198027
UTS2	rs3900909	59.57478415
UTS2	rs10801660	37.86617623
UTS2	rs2686395	242.4874356
UTS2	rs1265941	130.2598097
UTS2	rs550671	333.5119731
UTS2	rs9269234	49.83182084

NELL2	rs72704449	36.13667449
NELL2	rs77542162	64.53607423
NELL2	rs2685417	33.14780389
NELL2	rs8796	67.09452107
NELL2	rs34302865	87.99829091
RLN3	rs572515	139.4718559
RLN3	rs1355538	43.47411523
RLN3	rs6457457	31.26509285
RLN3	rs9501393	38.13032188
CRHBP	rs2069643	138.3850426
CRHBP	rs7736104	3435.789136
CRHBP	rs34574997	82.46641056
CRHBP	rs56326322	116.4008845
CRHBP	rs34478888	67.90202019
CRHBP	rs74514452	66.95520005
CRHBP	rs12912009	56.91613572
CRHBP	rs11739254	33.54887347
CRHBP	rs6887176	33.30700053
CRHBP	rs6924387	31.75095393
CRHBP	rs9298506	266.2094854
PTPRS	rs55763631	143.7947495
PTPRS	rs151162242	38.65298252
PTPRS	rs73069021	39.30486848
PTPRS	rs1260326	86.4547055
PTPRS	rs13108218	64.83697664
PTPRS	rs61442444	77.68455667
PTPRS	rs147315241	33.23026019
NXPH3	rs10922094	267.9127704
NXPH3	rs964184	118.6179596
NXPH3	rs113747960	33.85884924
NXPH3	rs1260326	50.22117618
NXPH3	rs1355538	81.75846421
NXPH3	rs550671	85.19983972
NXPH3	rs28471056	63.32248383
NXPH3	rs78810414	75.48238013
PIP	rs8177171	341.1209788
PIP	rs73170678	4369.409749
PIP	rs6962934	156.4166014
PIP	rs137860941	42.59569353
PIP	rs74920406	39.21632647
PIP	rs28549287	66.15416028
PIP	rs6678914	30.82623471
PIP	rs10876635	50.42356167
PIP	rs681343	316.3635683
PIP	rs2227411	55.92678217
PIP	rs10479093	42.45812296
PIP	rs186410432	33.51157526
PIP	rs150747152	72.99718609
PIP	rs6969058	83.37522897
PIP	rs148389263	37.50852608
PIP	rs17169742	106.9210791
ERP44	rs10127790	42.16891556
ERP44	rs10822155	65.00427294
ERP44	rs715440	35.63685374
ERP44	rs113661219	36.06124217
ERP44	rs114587946	32.72502759
ERP44	rs11896749	45.45105422
ERP44	rs143997020	31.59773682

ERP44	rs6993770	166.6452635
SUMF2	rs77444140	77.41007448
SUMF2	rs11749466	56.00437527
SUMF2	rs72801474	38.0428811
SUMF2	rs562047	88.63970271
SUMF2	rs72848256	72.78005958
SUMF2	rs3116995	48.46453287
PCOLCE2	rs13086711	207.4510225
PCOLCE2	rs35351308	347.7859644
PCOLCE2	rs9829564	1395.684861
PCOLCE2	rs113856318	526.4130794
PCOLCE2	rs1353887	90.7891325
PCOLCE2	rs6440169	168.8589995
PCOLCE2	rs61729512	47.87997261
PCOLCE2	rs3751151	41.78402422
PCOLCE2	rs12952154	42.93678524
PCOLCE2	rs117755721	114.4152716
PCOLCE2	rs374087027	32.47604944
PCOLCE2	rs4794048	46.55142086
PCOLCE2	rs12975366	138.9261531
PCOLCE2	rs138500392	41.3288884
PCOLCE2	rs1364076	41.05439189
PCOLCE2	rs2844811	30.30848308
PCOLCE2	rs9271383	33.01194908
PCOLCE2	rs9388764	46.01855753
PCOLCE2	rs73669307	31.5433086
CHRDL2	rs80086566	59.25631846
CHRDL2	rs117464057	33.87023229
CHRDL2	rs6592590	331.2061544
CHRDL2	rs34695217	1145.67311
CHRDL2	rs140198958	45.70821149
CHRDL2	rs2365384	42.84156438
CHRDL2	rs78689694	69.12849655
CHRDL2	rs10858853	30.34157576
CHRDL2	rs138903858	64.9742112
CHRDL2	rs142705448	32.27969682
CHRDL2	rs72837687	36.83651551
CELA1	rs373579	38.52734495
CELA1	rs200250001	38.81486021
CELA1	rs78705678	78.46050747
CELA1	rs2477642	73.87979271
CELA1	rs12288924	45.89046774
CELA1	rs9300169	37.25065754
CELA1	rs55714927	35.95700524
CELA1	rs760715	77.95855678
CELA1	rs114115200	32.67484357
CELA1	rs78912080	33.44097291
CELA1	rs28552890	29.85464952
CELA1	rs1871808	62.69069525
CELA1	rs4732746	93.41202045
CELA1	rs2726951	990.9156042
CELA1	rs56289258	30.36918011
CELA1	rs7045062	69.48396477
CELA1	rs10739407	127.2680741
CELA1	rs58090448	180.4348956
CELA1	rs117157109	1090.294119
CELA1	rs7470092	761.1327691
CELA1	rs12348583	90.01438944

CELA1	rs112463699	195.6393534
CELA1	rs7044769	53.64235802
CELA1	rs80312042	75.76793859
PCSK2	rs76831210	32.83279187
PCSK2	rs34124994	35.04323183
PCSK2	rs10820967	178.996714
MAMDC2	rs10922094	146.8336477
MAMDC2	rs6542680	47.62010161
MAMDC2	rs6457457	35.74529158
MAMDC2	rs9501393	45.36054304
TP53	rs646776	71.41767612
TP53	rs2276060	65.1704175
TP53	rs77620124	84.72140254
TP53	rs17580	48.93498021
TP53	rs165694	108.0882448
TP53	rs9611729	30.88661428
TP53	rs17713196	210.5054852
TP53	rs3863087	97.41920596
MAP2K3	rs8074866	31.7101921
MAP2K3	rs572515	79.22839876
MAP2K3	rs6445035	60.375733
MAP2K3	rs28573154	31.01089221
MAP2K3	rs4541868	30.39077239
TP53	rs10922098	497.2955512
TP53	rs10127894	31.60597058
TP53	rs5167	187.1635393
TP53	rs56317765	45.12650046
TP53	rs2232613	670.4475745
TP53	rs11906988	70.13818908
TP53	rs117247004	41.93086456
TP53	rs1707652	191.9061508
TP53	rs9266642	29.97309465
TP53	rs9267139	40.80980532
TP53	rs9501393	141.8204492
TP53	rs7774739	89.60373686
TP53	rs9271766	44.76123805
PSAP	rs6480555	998.5962552
PSAP	rs113654122	202.0561351
PSAP	rs1530804	43.68275351
PSAP	rs10822159	33.95463895
PSAP	rs111290178	48.69947689
PSAP	rs174584	31.47217628
PSAP	rs4883201	60.26536419
PSAP	rs825038	37.86359276
PSAP	rs11111026	264.354866
PSAP	rs2127868	38.77804561
PSAP	rs56255430	33.89202077
PSAP	rs2868346	74.54089157
PSAP	rs139596721	34.97763792
PSAP	rs2347784	32.05359376
PSAP	rs1194179	41.66198
PSAP	rs1293316	148.6102916
PSAP	rs6993770	109.5547582
IFNA5	rs10922098	612.8912538
IFNA5	rs3900909	30.22157228
IFNA5	rs1047286	51.52010222
IFNA5	rs2686395	125.5449324
IFNA5	rs1265941	62.4718588

IFNA5	rs550671	147.478386
CGB2	rs10633030	33.74586783
CGB2	rs10822155	81.25838935
CGB2	rs1654447	38.84185205
CGB2	rs1398015	36.12734074
CGB2	rs6993770	84.88093617
PDZD11	rs1329424	70.81826856
PDZD11	rs1355538	33.99456877
PDZD11	rs4713498	34.27423806
ADPGK	rs10922094	36.60648128
ADPGK	rs3184504	37.5647766
ADPGK	rs12986064	33.21514175
ADPGK	rs6881988	71.43062817
GUCA2B	rs11210603	150.4578647
GUCA2B	rs115897087	50.67767698
GUCA2B	rs141640396	66.29145102
GUCA2B	rs12145751	34.58747107
GUCA2B	rs150627305	53.39294877
GUCA2B	rs139712698	32.70324876
GUCA2B	rs56086072	39.07844835
KLK10	rs73060995	60.61685879
KLK10	rs61729813	180.332299
KLK10	rs3760731	116.972142
KLK10	rs149957772	35.6274369
KLK10	rs79031312	41.42609328
KLK10	rs74717060	1250.542594
KLK10	rs374962818	4161.265027
KLK10	rs149201050	136.6523567
KLK10	rs148108087	39.90137847
KLK10	rs4659007	39.93178307
VIT	rs113274571	2362.807168
VIT	rs72783126	114.997689
VIT	rs2072528	915.3041111
VIT	rs114702953	95.333725
VIT	rs116816240	145.6945662
VIT	rs4670750	36.55022965
VIT	rs7512012	103.2072742
VIT	rs34752898	84.12238551
VIT	rs2142306	42.95593002
VIT	rs11783413	61.81303906
FGFRL1	rs10922098	90.19183036
FGFRL1	rs704	52.02967292
FGFRL1	rs9501393	42.13452602
FGFRL1	rs7774739	31.3411326
RSPO1	rs10922098	89.034233
RSPO1	rs1355538	30.21629378
RSPO1	rs4713498	34.27423806
SIRPB1	rs76137931	69.60974304
SIRPB1	rs183218916	96.70708563
SIRPB1	rs62187494	688.0560533
SIRPB1	rs17791824	1190.577557
SIRPB1	rs73081790	1070.403644
SIRPB1	rs55650624	1375.790492
SIRPB1	rs117284274	4462.355358
SIRPB1	rs79039084	135.7572573
SIRPB1	rs34442420	81.68613298
SIRPB1	rs117149764	104.150924
SIRPB1	rs113484100	41.88809873

SIRPB1	rs10853015	30.61162907
SIRPB1	rs117245515	71.16265151
SIRPB1	rs62405651	29.8190163
SCGB3A1	rs7728174	32.05642365
SCGB3A1	rs307803	209.553532
SCGB3A1	rs382266	139.8580171
SCGB3A1	rs28601634	32.94610619
SCGB3A1	rs4970836	50.95615893
SCGB3A1	rs17257455	36.54204646
SCGB3A1	rs2990223	167.5185676
SCGB3A1	rs72927368	87.00294831
SCGB3A1	rs11032734	40.44506384
SCGB3A1	rs704735	262.1201014
SCGB3A1	rs10836312	689.2000878
SCGB3A1	rs3741240	473.2925746
SCGB3A1	rs7122541	41.08349671
SCGB3A1	rs186618572	30.07520425
SCGB3A1	rs2238133	84.45224817
SCGB3A1	rs771997	42.10814586
SCGB3A1	rs712333	37.84511674
SCGB3A1	rs10520801	37.21402616
SCGB3A1	rs1420533	38.47252789
SCGB3A1	rs56228609	65.35249883
SCGB3A1	rs16948226	31.04249966
SCGB3A1	rs77519145	60.85112562
SCGB3A1	rs61703905	86.99604719
SCGB3A1	rs4671448	102.2127144
SCGB3A1	rs61739245	34.92381532
SCGB3A1	rs1877593	43.0768565
SCGB3A1	rs2686410	131.3271734
SCGB3A1	rs1515496	93.34121502
SCGB3A1	rs11741572	30.87766451
SCGB3A1	rs17678708	49.56447038
SCGB3A1	rs11747035	31.0307846
SCGB3A1	rs2431320	249.9254687
SCGB3A1	rs2250292	44.48529957
SCGB3A1	rs34249863	108.575484
SCGB3A1	rs117102789	43.14600385
CPXM1	rs6136492	51.02787761
CPXM1	rs144337455	66.04607163
CPXM1	rs67367303	1071.885877
CPXM1	rs2326055	208.8573806
CPXM1	rs73088759	139.962898
CPXM1	rs618047	90.8825795
CPXM1	rs6427756	46.33186073
CPXM1	rs9807368	36.25032188
CPXM1	rs113661219	34.67119077
CPXM1	rs154513	48.5974874
CPXM1	rs2596540	53.02958905
CPXM1	rs6993770	109.5124651
CPXM1	rs296849	30.15105803
CGREF1	rs144669411	37.51898746
CGREF1	rs143743775	35.63031703
CGREF1	rs147519678	35.012963
CGREF1	rs35592937	65.84789766
CGREF1	rs58074293	364.515855
CGREF1	rs75426726	41.0057562
CGREF1	rs74671302	43.02795258

CGREF1	rs75519859	44.17644174
CGREF1	rs927826	328.1687935
CGREF1	rs4311960	32.0958981
CGREF1	rs7080386	47.1189585
CGREF1	rs72840032	52.51110453
CGREF1	rs117761748	72.67597758
CGREF1	rs17124884	54.82194761
CGREF1	rs1671152	32.49367059
CGREF1	rs548630	32.07978913
IL17RB	rs3766744	104.2721035
IL17RB	rs10633030	38.71438194
IL17RB	rs146587041	56.80755661
IL17RB	rs72692819	79.95046949
IL17RB	rs6494730	35.39577879
IL17RB	rs10820606	48.98079939
FURIN	rs10922098	33.9495715
FURIN	rs4639017	33.14608918
FURIN	rs562047	51.76183397
FURIN	rs28672722	75.94907005
FURIN	rs3130169	33.11030623
MCEMP1	rs1047286	45.61801053
MCEMP1	rs10801548	34.65968145
MCEMP1	rs10922098	858.0271427
MCEMP1	rs35351085	37.46471261
MCEMP1	rs1354034	37.74364077
MCEMP1	rs1707652	172.6709201
MCEMP1	rs1265941	90.99275264
MCEMP1	rs550671	234.0191812
MCEMP1	rs9269234	37.5798126
MLEC	rs3811444	46.68941798
MLEC	rs4980320	32.12964695
MLEC	rs1354034	30.43641313
ARHGAP36	rs10922098	55.07900847
ARHGAP36	rs964184	79.05954326
ARHGAP36	rs67769959	30.90595989
ARHGAP36	rs6445035	96.19749691
ARHGAP36	rs3863087	57.44631945
ARHGAP36	rs686250	45.83142053
ARHGAP36	rs10096633	38.53356996
CLSTN3	rs7137828	48.73570388
CLSTN3	rs78909033	31.2861962
CLSTN3	rs3129754	43.60104819
OIT3	rs75634068	30.96988251
OIT3	rs7310615	45.64442666
OIT3	rs3129754	42.1502896
C1QTNF1	rs7538475	60.91441688
C1QTNF1	rs6657811	977.9669136
C1QTNF1	rs12023150	58.28481909
C1QTNF1	rs114003685	151.7882865
C1QTNF1	rs7869	230.0338157
C1QTNF1	rs10762487	64.24160752
C1QTNF1	rs2289702	51.96709249
C1QTNF1	rs72802342	50.86227968
C1QTNF1	rs12945299	36.23435652
C1QTNF1	rs7210348	37.23596285
C1QTNF1	rs5848	316.3859064
C1QTNF1	rs117010051	41.22283729
C1QTNF1	rs140183833	38.28871668

C1QTNF1	rs73725598	53.87929763
C1QTNF1	rs943616	30.77406649
C1QTNF1	rs8176693	34.00260011
PLBD1	rs1474868	40.92429683
PLBD1	rs10922094	63.54058152
PLBD1	rs11569415	40.35596192
PCDHGA10	rs2831443	37.96188677
PCDHGA10	rs2831516	31.71790346
PCDHGA10	rs11909120	43.49899383
PCDHGA10	rs2740592	117.4691487
PCDHGA10	rs1571262	32.68592249
DNASE1L2	rs72801474	49.53237492
DNASE1L2	rs562047	105.9205641
DNASE1L2	rs184358099	41.96609476
DNASE1L2	rs28672722	88.08795661
DNASE1L2	rs62522552	32.90002222
BMP4	rs10822155	71.4688731
BMP4	rs188400754	35.17763755
BMP4	rs1398015	34.3764027
BMP4	rs16850360	40.53568075
BMP4	rs6993770	43.60350878
NPNT	rs9760233	42.0702326
NPNT	rs34712979	452.4522205
NPNT	rs10025052	61.12120328
NPNT	rs5112	51.01752296
NPNT	rs7580569	57.71774777
NPNT	rs138259153	48.83026913
NPNT	rs1250258	482.6402809
NPNT	rs4674006	107.7504423
APOC2	rs10922094	84.11619998
APOC2	rs34275670	41.66192598
APOC2	rs11845244	166.7766529
APOC2	rs2007528	93.13096483
APOC2	rs9501600	32.68336056
CD79B	rs114115200	64.10517448
CD79B	rs555007	45.17272677
CD79B	rs550671	95.99919506
INSRR	rs61808983	79.69784694
INSRR	rs572515	88.76253064
INSRR	rs3129882	32.72597794
HEPHL1	rs10922094	564.4558925
HEPHL1	rs11720167	118.8370733
HEPHL1	rs3863087	39.05461905
HEPHL1	rs9266774	36.12922797
HEPHL1	rs555007	61.88717434
HEPHL1	rs444921	155.3978503
CELA3B	rs10922094	178.077667
CELA3B	rs1707652	56.76894917
CELA3B	rs6457457	32.54270371
CELA3B	rs3891366	47.67645806
POMGNT2	rs62248848	94.15532128
POMGNT2	rs476336	1010.147489
POMGNT2	rs140119162	40.29086618
POMGNT2	rs34542110	46.86440488
POMGNT2	rs12037485	49.69277699
POMGNT2	rs2285176	59.56009171
POMGNT2	rs2477639	63.69807283
POMGNT2	rs692440	57.36594271

POMGNT2	rs34124994	53.76982468
POMGNT2	rs2193005	93.96356579
POMGNT2	rs7310615	56.65669798
POMGNT2	rs149181595	51.30254902
POMGNT2	rs117747069	31.99878562
POMGNT2	rs62108438	30.90843043
POMGNT2	rs1653769	30.47033241
POMGNT2	rs132665	45.5689254
POMGNT2	rs35011079	58.04001831
POMGNT2	rs142957768	137.5052618
POMGNT2	rs17750042	965.5683275
POMGNT2	rs1994812	37.55120987
POMGNT2	rs635634	125.6055435
DEFB128	rs10922094	449.2102108
DEFB128	rs1707652	83.53324427
DEFB128	rs6457457	81.66225921
DEFB128	rs3891366	128.8435997
TAPBPL	rs117443665	30.17796851
TAPBPL	rs182944391	161.1458202
TAPBPL	rs56983450	926.7071722
TAPBPL	rs140187376	1141.248869
TAPBPL	rs10849454	1125.774758
TAPBPL	rs1045553	5535.932435
TAPBPL	rs117244485	313.7083376
TAPBPL	rs12370543	65.82363157
TAPBPL	rs11611373	276.0063836
TAPBPL	rs11063208	78.41123165
TAPBPL	rs11831822	85.36697758
TAPBPL	rs6488340	243.2181528
TAPBPL	rs146840578	57.7013554
TAPBPL	rs504963	31.80802295
TAPBPL	rs116455037	35.15491868
TAPBPL	rs1233421	80.99837542
TAPBPL	rs2735049	1016.991511
TAPBPL	rs9468618	145.1128437
TAPBPL	rs139803620	407.0114804
TAPBPL	rs2517686	157.9835516
TAPBPL	rs60120766	89.99236828
TAPBPL	rs805296	50.58577645
TXNDC15	rs329122	68.66868153
TXNDC15	rs3733897	2512.061431
TXNDC15	rs651249	48.81679085
TXNDC15	rs16830188	74.85831578
TXNDC15	rs8178824	45.78600062
TXNDC15	rs1468512	31.21592455
TXNDC15	rs1633915	31.30557164
TXNDC15	rs35668054	40.24852382
TXNDC15	rs2289235	43.12142023
TXNDC15	rs10065637	60.77076551
TXNDC15	rs59307279	41.29645911
TXNDC15	rs27302	50.64769637
TXNDC15	rs28377047	39.21272836
TXNDC15	rs76520335	65.22661417
TXNDC15	rs149670015	77.74500051
TXNDC15	rs76035204	65.02631223
TXNDC15	rs9468618	69.07553056
TXNDC15	rs9260332	59.15400487
TXNDC15	rs2517531	339.9645627

TXNDC15	rs16898823	1117.585893
TXNDC15	rs9265463	908.5984924
TXNDC15	rs112253087	109.8803142
TXNDC15	rs2284179	59.10877251
TXNDC15	rs28383417	192.3716041
TXNDC15	rs116078841	98.03976661
TXNDC15	rs73169668	54.61703631
FMOD	rs56035601	54.46761877
FMOD	rs16851364	242.1457487
FMOD	rs139343579	38.77181456
FMOD	rs139774444	31.36590918
FMOD	rs78242118	38.89775258
FMOD	rs78705678	36.0343384
FMOD	rs1052454	38.74894202
FMOD	rs147712570	33.38397681
FMOD	rs36136188	38.14201632
FMOD	rs117801489	430.6326111
FMOD	rs12581309	46.35319518
FMOD	rs9033	41.33738911
FMOD	rs11128309	34.35750215
CSTL1	rs4980320	46.79658421
CSTL1	rs1354034	141.3593909
CSTL1	rs4868149	29.92168941
DEAF1	rs10922094	161.6712386
DEAF1	rs12146727	37.720417
DEAF1	rs10208529	30.81804834
DEAF1	rs1706435	92.43267885
DEAF1	rs28573154	58.01694883
DEAF1	rs7774739	40.37456503
DEAF1	rs66489920	34.68910351
ANGPTL7	rs10922094	61.72980246
ANGPTL7	rs4980320	31.01106341
ANGPTL7	rs1354034	57.47108241
ANGPTL7	rs3917538	40.8402582
YBX2	rs10922094	70.03045469
YBX2	rs72865316	97.78575266
YBX2	rs10772354	32.26105036
YBX2	rs9894813	53.79355797
YBX2	rs1037590	219.7984752
YBX2	rs11082513	30.17601191
YBX2	rs4820091	37.49697656
YBX2	rs1354034	470.7864219
SEMG2	rs2305201	39.19910601
SEMG2	rs6416180	29.77302932
SEMG2	rs138057831	63.71205813
SEMG2	rs183901897	79.68534918
SEMG2	rs8008285	927.7510887
SEMG2	rs8004581	891.6435413
SEMG2	rs34709808	100.5053939
SEMG2	rs74930943	57.58310658
SEMG2	rs11648186	32.28245141
SEMG2	rs112001035	190.8074312
SEMG2	rs145989992	30.55538416
SEMG2	rs7644664	34.15735274
SEMG2	rs732194	43.24722414
XXYLT1	rs12635047	189.4635511
XXYLT1	rs12695049	574.20188
XXYLT1	rs2641721	59.35706785

XXYLT1	rs72704449	43.71546501
XXYLT1	rs78539764	31.86938599
XXYLT1	rs12986064	80.57512294
LYZL2	rs10922098	33.37002802
LYZL2	rs4980320	63.73875164
LYZL2	rs1037590	47.95335336
LYZL2	rs1354034	222.025675
LYZL2	rs114647468	41.29906464
LYZL2	rs114115200	68.09392103
ADAMTSL2	rs11534419	164.4844891
ADAMTSL2	rs56355857	59.50504858
ADAMTSL2	rs1079783	36.05429726
ADAMTSL2	rs2027962	63.63277659
ADAMTSL2	rs61830291	92.47985095
ADAMTSL2	rs217184	38.21599971
ADAMTSL2	rs77262773	60.48360566
ADAMTSL2	rs56254331	69.18657618
MANBA	rs6841750	77.79218358
MANBA	rs228619	1203.126887
MANBA	rs17216959	404.8067169
MANBA	rs73183043	132.4358198
MANBA	rs1242103	105.3918545
MANBA	rs61991664	46.74713796
MANBA	rs7188856	103.6869185
MANBA	rs146978714	44.20650181
MANBA	rs17031475	92.70892057
MANBA	rs56402844	36.21106659
MANBA	rs34115112	44.91879108
MANBA	rs2187668	50.47161458
MANBA	rs8191835	34.71260065
MANBA	rs1293303	53.3914905
MANBA	rs635634	34.04602616
TLL1	rs1903176	45.95588714
TLL1	rs139413362	29.93848531
TLL1	rs61801825	44.60045288
TLL1	rs78705678	46.20594614
TLL1	rs2461161	42.91668668
TLL1	rs1805482	31.19302955
TLL1	rs653178	32.42634364
TLL1	rs601338	119.7666677
TLL1	rs66782572	45.90639283
TLL1	rs13088856	35.23676347
TLL1	rs3815916	36.96477132
TLL1	rs78526834	31.68541479
TLL1	rs8176743	669.0097927
TLL1	rs739468	70.32868402
WFDC3	rs3766744	116.0895001
WFDC3	rs10633030	35.48675677
WFDC3	rs146587041	64.03794759
WFDC3	rs72692819	85.51915954
WFDC3	rs6494730	36.3166227
WFDC3	rs10820606	50.85327527
VWA1	rs13302914	160.3278488
VWA1	rs78717314	982.5000661
VWA1	rs3843248	140.8481537
VWA1	rs12731916	97.9249498
VWA1	rs6700884	39.54453082
VWA1	rs2678943	47.43234168

VWA1	rs61762080	34.0015194
VWA1	rs28546127	93.12939391
VWA1	rs77262773	46.03954508
VWA1	rs7636224	73.05031932
VWA1	rs149062332	99.59442516
VWA1	rs147906938	32.89773381
VWA1	rs112164771	39.69210485
CCDC126	rs74702905	31.39780529
CCDC126	rs9986726	48.42932362
CCDC126	rs113556001	52.84224141
CCDC126	rs73287817	1017.338383
CCDC126	rs7779633	77.3076668
CCDC126	rs12026141	50.12700178
CCDC126	rs12446515	91.01935936
CCDC126	rs28873836	36.5280671
CCDC126	rs11690221	30.61415376
CCDC126	rs111696008	74.25532065
CCDC126	rs144202860	31.89650419
CCDC126	rs6065906	121.783688
CCDC126	rs1707652	594.0413771
CCDC126	rs371809862	37.65480316
CCDC126	rs3926704	38.16932763
CCDC126	rs17138649	35.87630573
CCDC126	rs17138656	86.38291144
CCDC126	rs112853430	50.20479689
CCDC126	rs2575876	39.33067427
CCDC126	rs9411365	35.14612869
NPS	rs145306329	80.79615298
NPS	rs6864210	63.79158602
NPS	rs4376276	228.5171412
NPS	rs13165115	42.32125134
NPS	rs41546317	33.43262642
NPS	rs147440497	38.95018902
HSP90B1	rs1165593	5648.460401
HSP90B1	rs11111878	897.56718
HSP90B1	rs2374394	414.1834346
HSP90B1	rs4964294	113.7898182
HSP90B1	rs2374409	30.95717355
HSP90B1	rs787418	39.79091343
HSP90B1	rs1196837	158.7089009
HSP90B1	rs12578779	52.95373688
HSP90B1	rs34642982	52.71262766
GPHA2	rs10922098	603.8561061
GPHA2	rs892145	93.40382285
GPHA2	rs1707652	92.12798142
GPHA2	rs2075798	70.29424644
GPHA2	rs550671	170.628842
GPHA2	rs78897911	32.99000905
GPHA2	rs73726194	32.56640773
LONP1	rs3766744	115.7281666
LONP1	rs10633030	36.04321597
LONP1	rs146587041	62.32631721
LONP1	rs72692819	85.31283309
LONP1	rs6494730	34.5276883
LONP1	rs10820606	50.98495152
DEFB107A	rs10922098	93.5084085
DEFB107A	rs6542680	37.31446426
DEFB107A	rs6457457	33.85328756

DEFB107A	rs9501393	41.86041434
TP53I13	rs1434282	89.57333509
TP53I13	rs10822155	74.78704185
TP53I13	rs79880068	34.38230401
TP53I13	rs113661219	32.51188107
TP53I13	rs7776054	31.69022103
TP53I13	rs342299	94.44780495
TP53I13	rs6993770	85.5051418
TP53I13	rs5978964	47.50360751
PILRA	rs45454594	1198.760194
PILRA	rs117009535	74.00765231
PILRA	rs199974872	280.6123993
PILRA	rs76265464	173.1996808
PILRA	rs75126895	443.4829711
PILRA	rs12705068	1405.531733
PILRA	rs371165331	1074.465787
PILRA	rs62482204	865.3661026
PILRA	rs149744179	210.3480635
PILRA	rs78690606	530.462605
PILRA	rs73405382	1080.601896
PILRA	rs117078492	130.2296516
PILRA	rs55867908	606.8601826
PILRA	rs117123505	259.0294692
PILRA	rs143364347	495.4498975
PILRA	rs28853072	40.3475041
PILRA	rs41547217	35.98576874
PILRA	rs9276171	29.99701248
PILRA	rs146216542	37.1564805
PILRA	rs117117117	44.21082281
PILRA	rs7811014	39.83101726
PILRA	rs79858296	42.82281902
PILRA	rs12670513	162.1371362
PILRA	rs117850863	1324.259878
PILRA	rs712846	247.1241065
PILRA	rs186710281	67.11770179
PILRA	rs139360617	93.94182002
PILRA	rs73203019	43.72801598
PILRA	rs6466518	38.05788292
PILRA	rs1206383	31.84931018
C1QL1	rs71373512	38.4202138
C1QL1	rs7225162	618.365531
C1QL1	rs34497316	148.1830825
C1QL1	rs10922098	110.0411913
C1QL1	rs2808466	60.59251168
C1QL1	rs4266889	296.2260016
C1QL1	rs2477642	30.45207581
C1QL1	rs12766994	31.62044857
C1QL1	rs3784535	31.4770269
C1QL1	rs1706435	35.12709919
C1QL1	rs4336251	41.28857281
PSG2	rs10922098	331.8922562
PSG2	rs1047286	35.88569419
PSG2	rs114115200	79.15099892
PSG2	rs550671	78.75157584
PSG2	rs206018	36.28334658
PSG2	rs73403125	31.13223619
INHBC	rs3809113	124.2213265
INHBC	rs141214108	37.01494074

INHBC	rs62037312	29.7383673
INHBC	rs429358	57.46581057
INHBC	rs1260326	39.17595303
ADGRF5	rs6922527	403.3370287
ADGRF5	rs57362802	1346.5412
ADGRF5	rs4711861	494.2095464
ADGRF5	rs72660908	117.8708856
ADGRF5	rs61801011	48.76569325
ADGRF5	rs34666101	37.58210798
ADGRF5	rs78705678	69.92309923
ADGRF5	rs2477642	72.81598352
ADGRF5	rs11603123	45.46602963
ADGRF5	rs2377058	34.17821964
ADGRF5	rs117633773	31.68814555
ADGRF5	rs55714927	95.76533522
ADGRF5	rs143501783	33.00961386
ADGRF5	rs7738764	124.0596605
ADGRF5	rs75822890	149.8889657
ADGRF5	rs13193969	30.80622071
ADGRF5	rs79666796	118.9049114
ADGRF5	rs142359253	62.69855256
ADGRF5	rs137894154	31.85217388
ADGRF5	rs113328087	31.83046384
ADGRF5	rs6597573	46.65447313
ADGRF5	rs72759422	55.52866108
ADGRF5	rs141668780	137.9985679
ADGRF5	rs35434910	947.5883805
ADGRF5	rs17150482	1012.371822
ADGRF5	rs34250484	118.502366
ADGRF5	rs116960840	47.44213016
ADGRF5	rs79028752	34.07283263
DEFB135	rs10922094	348.6181224
DEFB135	rs1355538	70.31843072
DEFB135	rs1144709	43.29450373
DEFB135	rs1265941	82.51250728
DEFB135	rs550671	179.348202
DEFB135	rs112164276	33.98529752
OAF	rs11217526	104.7014089
OAF	rs1349689	967.9787776
OAF	rs61898281	771.0404717
OAF	rs61898760	118.7551138
OAF	rs146589617	35.23467315
OAF	rs7483806	46.91248233
OAF	rs77825069	34.79555294
OAF	rs4418728	29.73847315
OAF	rs74550804	49.12605267
OAF	rs41297901	38.37004545
OAF	rs112725054	31.67473994
OAF	rs201862321	65.47116332
OAF	rs1260326	80.63436517
CPN2	rs116253218	43.62728428
CPN2	rs112109292	38.18254162
CPN2	rs7635813	69.12114957
CPN2	rs140950702	114.7180148
CPN2	rs6774800	1175.464346
CPN2	rs138073243	117.4677184
CPN2	rs116803428	49.87485684
CPN2	rs188600680	65.74768919

CPN2	rs77439828	30.09110945
CPN2	rs9832860	29.86322328
CPN2	rs61751507	86.05128401
CPN2	rs200022366	37.66302905
CPN2	rs113329541	59.47698049
CPN2	rs72842004	44.60143467
CPN2	rs369031766	86.95343567
CPN2	rs12952423	74.95122277
CPN2	rs8065748	285.4293956
CPN2	rs1242299	1085.410539
CPN2	rs55885265	135.4218985
CPN2	rs17878945	105.3757964
CPN2	rs78817988	29.85307044
CPN2	rs2706759	31.69441532
CPN2	rs780093	58.60004531
CPN2	rs56210800	51.82172785
CPN2	rs149468457	52.13954422
CPN2	rs34442410	30.89337227
CPN2	rs6975495	38.73911134
CPN2	rs28601761	52.57728301
GKN2	rs143227779	138.8628127
GKN2	rs17602973	91.07454747
GKN2	rs13008230	3116.709395
GKN2	rs13387607	107.6722656
GKN2	rs140464556	334.777959
GKN2	rs74738594	43.81567857
GKN2	rs76823104	94.38658384
GKN2	rs2990223	193.3929115
GKN2	rs71469854	334.2741865
GKN2	rs80334578	65.53530478
GKN2	rs117107041	30.49556213
GKN2	rs34953075	30.84180518
GKN2	rs492602	435.3294528
GKN2	rs12713743	55.30066527
GKN2	rs28421599	29.79712206
GKN2	rs3129773	38.81387447
GKN2	rs2013075	33.72135769
IFNA16	rs572515	52.31075401
IFNA16	rs1555405	39.71147656
IFNA16	rs6445035	34.08146053
IFNA16	rs550671	34.53258404
IFNA16	rs67399078	35.87230541
MMP19	rs7296597	105.6396322
MMP19	rs6581105	42.24949142
MMP19	rs112771035	322.8728536
MMP19	rs11220505	92.72875883
MMP19	rs34286028	40.89647324
MMP19	rs71455286	88.65897478
MMP19	rs17124370	31.34150874
MMP19	rs12945299	46.48797616
MMP19	rs72853625	29.8887877
MMP19	rs7650880	65.1934472
MMP19	rs4958935	32.52814547
PCYOX1	rs11899723	965.6586404
PCYOX1	rs141913319	37.82832544
PCYOX1	rs2305144	761.7807073
PCYOX1	rs11689828	439.9225452
PCYOX1	rs2904815	34.21488583

PCYOX1	rs602633	35.2157665
PCYOX1	rs7917260	43.68769335
PCYOX1	rs74563318	42.0432447
PCYOX1	rs99780	65.07646943
PCYOX1	rs117498846	29.96121502
PCYOX1	rs1801689	36.28068215
PCYOX1	rs7251858	30.45558651
PCYOX1	rs6511720	52.59492308
PCYOX1	rs58542926	359.6960077
PCYOX1	rs5117	87.72379437
PCYOX1	rs533617	68.73353728
PCYOX1	rs4665985	43.55966518
PCYOX1	rs137906029	30.28576311
PCYOX1	rs62133344	86.93452209
PCYOX1	rs11898122	81.31336909
PCYOX1	rs150759843	182.4050168
PCYOX1	rs72622637	103.0196575
PCYOX1	rs4849902	32.25160918
PCYOX1	rs2618566	31.8671019
PCYOX1	rs6065904	52.40379114
PCYOX1	rs2894211	38.62856497
PCYOX1	rs28601761	37.66664644
FAM20A	rs12941038	255.3629405
FAM20A	rs929477	192.7947296
FAM20A	rs117753190	81.20957138
FAM20A	rs72631343	31.04214603
FAM20A	rs10922094	46.7376278
FAM20A	rs2622935	76.33018413
FAM20A	rs7964859	85.77750103
FAM20A	rs35495893	55.00517051
FAM20A	rs72654473	41.14943125
FAM20A	rs28873836	82.85762121
FAM20A	rs1260326	63.39226309
FAM20A	rs34632463	32.17748067
FAM20A	rs147906938	39.1946131
FAM20A	rs3116995	30.91430349
FAM20A	rs150231592	74.81209538
C14orf93	rs587255	751.5030039
C14orf93	rs41286852	70.00522661
C14orf93	rs61808983	54.79288894
C14orf93	rs742127	37.24608296
C14orf93	rs704	52.28436074
C14orf93	rs5117	90.62291575
C14orf93	rs5112	49.45186139
C14orf93	rs1260326	45.00270747
C14orf93	rs2071006	75.62860183
MFAP5	rs35705950	31.95645685
MFAP5	rs12342201	71.20875582
MFAP5	rs62578121	46.09228385
MFAP5	rs117294446	70.29974633
MFAP5	rs149685171	42.74075714
ADAMTS6	rs10922094	62.58596762
ADAMTS6	rs2956634	98.19249575
ADAMTS6	rs4639017	31.81650683
PSG3	rs369150044	190.6120579
PSG3	rs8105365	3795.771642
PSG3	rs143700556	1248.987705
PSG3	rs4028444	310.4020598

PSG3	rs35078440	573.1262836
PSG3	rs117473000	97.64193021
PSG3	rs368465	48.63818008
PSG3	rs115810579	45.48861394
PSG3	rs45537140	86.2278039
PSG3	rs73036465	182.9602697
PSG3	rs73048316	58.20510509
PSG3	rs41289514	29.94690178
PSG3	rs35945249	35.53676644
PTX3	rs10922098	464.2130788
PTX3	rs76004643	30.62380241
PTX3	rs1707652	144.716617
PTX3	rs3099847	33.10392791
PTX3	rs1265941	63.00919504
PTX3	rs550671	148.8422318
SEMA3C	rs917191	169.1225632
SEMA3C	rs7796714	58.10513142
SEMA3C	rs34601069	48.98970044
SEMA3C	rs2436670	69.36120508
SEMA3C	rs2461161	81.35218143
SEMA3C	rs3184504	72.97281148
SEMA3C	rs2760994	37.14756851
SEMA3C	rs635634	41.04446897
ASPN	rs1201042	63.48478766
ASPN	rs10046898	1050.158701
ASPN	rs369954270	254.9843307
ASPN	rs333947	44.31678543
ASPN	rs2229498	47.02783465
ASPN	rs35887873	120.1206148
ASPN	rs181707610	48.05437382
ASPN	rs2073333	110.3622441
ASPN	rs55714927	89.30069765
ASPN	rs143501783	32.38553801
ASPN	rs868876	33.79097023
ASPN	rs3199918	31.3807979
ASPN	rs73505430	41.8561333
ASPN	rs10821516	32.70670417
ASPN	rs67012539	93.95803777
ASPN	rs12378270	60.91753279
LILRB4	rs826404	34.45640133
LILRB4	rs1227041	42.10756672
LILRB4	rs41281334	132.3839749
LILRB4	rs12413995	1017.549323
LILRB4	rs12245196	272.8393775
LILRB4	rs2675660	49.56610948
LILRB4	rs78704481	31.05271718
LILRB4	rs4883201	50.32262885
LILRB4	rs825038	30.14989415
LILRB4	rs11111026	236.1655437
LILRB4	rs56255430	37.90407836
LILRB4	rs6073958	43.23868168
LILRB4	rs697357	30.07881531
LILRB4	rs139596721	36.92827248
LILRB4	rs1873425	37.51574038
LILRB4	rs1194178	39.04092183
LILRB4	rs1293316	176.139695
LILRB4	rs6993770	105.3392043
ARHGDIA	rs10922098	194.7209905

ARHGDIA	rs9864801	32.21682936
ARHGDIA	rs9501600	58.09977907
HAPLN4	rs112253053	105.7931747
HAPLN4	rs10922098	50.62481161
HAPLN4	rs6542680	32.73779318
PSG6	rs10922094	127.7147001
PSG6	rs1355538	37.11504844
PSG6	rs9501600	56.37893791
PSG6	rs35209896	31.1994073
PCDHGA1	rs12975234	34.2360662
PCDHGA1	rs76081751	41.16078849
PCDHGA1	rs117216419	37.62097194
PCDHGA1	rs117090502	196.510118
PCDHGA1	rs117453984	1275.250723
PCDHGA1	rs75394930	927.0153824
PCDHGA1	rs112052262	1297.797223
PCDHGA1	rs140776740	655.2916164
PCDHGA1	rs34408465	675.0137105
PCDHGA1	rs144494943	31.47936358
PCDHGA1	rs111984974	665.3947904
PCDHGA1	rs2354283	4242.016361
PCDHGA1	rs140064353	384.0500372
PCDHGA1	rs10411716	277.3947314
PCDHGA1	rs17656836	211.9940446
PCDHGA1	rs11668974	199.8817153
PCDHGA1	rs12460062	149.4033819
PCDHGA1	rs117530850	49.40855557
RPN1	rs7310615	29.93952926
RPN1	rs62115758	65.94805568
RPN1	rs1551556	932.5808118
RPN1	rs2864138	285.5734174
RPN1	rs3829655	956.8856607
RPN1	rs10413815	195.9993252
RPN1	rs77298770	136.3226664
RPN1	rs35764467	39.90426127
RPN1	rs12978451	42.63182509
APOC3	rs964184	291.2066844
APOC3	rs141469619	52.20794657
APOC3	rs10889353	87.70873228
APOC3	rs144009388	33.42387193
APOC3	rs34895872	40.2380511
APOC3	rs139076649	36.33969793
APOC3	rs261332	38.16111258
APOC3	rs56228609	107.1400002
APOC3	rs2156552	47.74997928
APOC3	rs58542926	34.8700795
APOC3	rs5112	69.38971033
APOC3	rs1042034	49.75044708
APOC3	rs1260326	195.4255318
APOC3	rs62182214	33.41743182
APOC3	rs111696008	36.43054885
APOC3	rs6029143	30.52037739
APOC3	rs6445035	342.876351
APOC3	rs3863087	144.9943123
APOC3	rs148077724	39.12493701
APOC3	rs73169103	45.49300543
APOC3	rs4704826	79.99317337
APOC3	rs28752924	30.05738286

APOC3	rs9267542	36.19996154
APOC3	rs9271636	81.60416002
APOC3	rs116189786	85.88419567
APOC3	rs61010704	73.79221744
APOC3	rs4921915	34.9357699
APOC3	rs2954021	40.25861065
APOC3	rs2740488	33.70409762
TIMP4	rs35932350	30.36785409
TIMP4	rs151094541	44.63566454
TIMP4	rs2600261	1036.826154
TIMP4	rs17029006	449.7434908
TIMP4	rs434460	72.16390955
TIMP4	rs11708734	86.60405778
TIMP4	rs56120972	53.44543682
TIMP4	rs7133378	48.42565572
TIMP4	rs12986064	36.51276097
TIMP4	rs10184004	30.72251084
TIMP4	rs13423088	48.16886604
TIMP4	rs5994626	49.64337739
TIMP4	rs9368696	34.31322473
TIMP4	rs114133813	78.39566198
TIMP4	rs9469266	47.17942755
QRFP	rs10922098	412.5038605
QRFP	rs1707652	91.32176502
QRFP	rs2075798	45.10299734
QRFP	rs453821	117.1168625
POGLUT1	rs6807888	45.1670785
POGLUT1	rs9860775	613.756137
POGLUT1	rs2873968	78.5665404
POGLUT1	rs2588511	34.78040511
TAC4	rs1451915	31.06515907
TAC4	rs10922098	547.2503117
TAC4	rs1047286	49.11130561
TAC4	rs2686395	148.4710681
TAC4	rs550671	149.0499851
TAC4	rs28471056	61.4202901
LY6G6D	rs67399078	34.65138279
LY6G6D	rs10922098	113.7045727
LY6G6D	rs6445180	31.55988538
FBLN1	rs1040402	304.6259762
FBLN1	rs71313011	48.52333771
FBLN1	rs136743	66.1834831
FBLN1	rs333947	32.3698461
FBLN1	rs35406997	40.09222155
FBLN1	rs56344867	39.56220837
FBLN1	rs1260326	52.84473392
FBLN1	rs74814272	30.75999809
FBLN1	rs183784507	39.46098639
FBLN1	rs115578531	33.46439894
FBLN1	rs11130306	64.67477444
FBLN1	rs141359357	43.64664007
FBLN1	rs145887694	48.17090786
FBLN1	rs73215978	30.39181621
FBLN1	rs2442722	37.79154185
CFHR4	rs11588705	1056.833678
CFHR4	rs74950062	1038.855527
CFHR4	rs12071084	87.2382576
CFHR4	rs74897622	937.6691301

CFHR4	rs12029494	1283.606064
CFHR4	rs72732292	483.3882349
CFHR4	rs112488423	580.5787601
CFHR4	rs75024683	789.5947818
CFHR4	rs139088313	615.8130899
CFHR4	rs17618402	218.0432688
CFHR4	rs74420582	352.6234421
CFHR4	rs34453819	30.3511616
CFHR4	rs78576220	40.90116704
CFHR4	rs635796	64.08405444
CFHR4	rs521366	56.3703699
CFHR4	rs232300	100.3910469
CFHR4	rs140137211	34.37285518
CFHR4	rs115231001	39.3792199
CFHR4	rs116443069	43.0551535
CFHR4	rs138366774	206.4554531
CFHR4	rs80104950	53.72981462
CFHR4	rs74475713	58.13391991
CFHR4	rs144949872	99.65175717
CFHR4	rs79141044	49.29354579
CFHR4	rs1169286	35.76821322
CFHR4	rs80028711	40.905456
CFHR4	rs56411757	46.01598425
PMEL	rs2550671	32.01917869
PMEL	rs2069398	868.0689538
PMEL	rs34060703	248.9745675
PMEL	rs2461155	30.52957459
PMEL	rs2477639	155.5945716
PMEL	rs692440	111.9967981
PMEL	rs3829241	31.16281484
PMEL	rs1126809	47.62869418
PMEL	rs10655	49.00588751
PMEL	rs12447797	49.43016511
PMEL	rs117204628	92.77032341
PMEL	rs11076664	436.481325
PMEL	rs11668064	30.47203661
PMEL	rs1205312	84.07998099
PMEL	rs34255600	38.25135765
PMEL	rs183671	55.64829993
PMEL	rs6873160	43.19516676
PMEL	rs113959851	91.95424318
PMEL	rs849134	49.10591606
PMEL	rs140367586	62.74850319
PMEL	rs1204371	102.7802488
PMEL	rs2120470	134.8048756
PMEL	rs9297881	31.1646197
PMEL	rs10962596	79.129611
PMEL	rs12350739	258.0550819
BPIFA1	rs10922098	413.143241
BPIFA1	rs1047286	35.08270764
BPIFA1	rs2686395	98.77033219
BPIFA1	rs9266766	30.6022676
BPIFA1	rs2075798	60.72593452
BPIFA1	rs453821	118.6783478
IGLON5	rs148108087	125.9657305
IGLON5	rs140230420	36.67390664
IGLON5	rs12035995	31.02086915
IGLON5	rs78705678	64.38509612

IGLON5	rs2477642	72.10490195
IGLON5	rs1260326	30.48104579
IGLON5	rs11691872	29.88583506
IGLON5	rs2143083	37.0514871
CANT1	rs1706435	33.62256559
CANT1	rs449962	42.41919076
CANT1	rs56826489	87.61111673
CANT1	rs148766553	78.49134789
CANT1	rs6458	179.571301
CANT1	rs145981697	43.88979911
CANT1	rs12378445	81.76632986
IGLL1	rs139571703	1331.682535
IGLL1	rs140174	806.4165211
IGLL1	rs118124522	93.074383
IGLL1	rs144261409	73.4652333
IGLL1	rs3761420	36.50851896
IGLL1	rs11164801	35.1568292
IGLL1	rs1538971	75.12205698
IGLL1	rs2493136	65.91174313
IGLL1	rs4245595	209.3032723
IGLL1	rs78754886	30.05548205
IGLL1	rs79755767	45.88710376
IGLL1	rs76428106	71.90937122
IGLL1	rs73217470	69.14567637
IGLL1	rs9939427	150.3095404
IGLL1	rs72809277	30.88555772
IGLL1	rs11658278	114.0619306
IGLL1	rs9953268	32.83963448
IGLL1	rs55737395	195.1232224
IGLL1	rs73028280	37.15466674
IGLL1	rs76529516	79.4175261
IGLL1	rs4549071	58.1266117
IGLL1	rs62237291	49.34444029
IGLL1	rs6001558	191.4462754
IGLL1	rs9811380	44.52157585
IGLL1	rs717255	96.67908498
IGLL1	rs700589	113.8637051
IGLL1	rs75141179	59.47833609
IGLL1	rs111672628	192.4009793
IGLL1	rs139439683	33.29750777
IGLL1	rs138385964	35.84890252
IGLL1	rs57743756	83.33369594
IGLL1	rs116968179	36.98063129
IGLL1	rs147878351	30.63262788
IGLL1	rs2266194	42.5046418
IGLL1	rs1433577	163.7067707
IGLL1	rs1687391	63.38094085
CPZ	rs871363	70.11271784
CPZ	rs13127468	70.20454708
CPZ	rs572515	61.8551826
CPZ	rs557011	31.11660924
DLK1	rs74621727	96.47524619
DLK1	rs188825923	52.27773772
DLK1	rs34616122	121.4600554
DLK1	rs12588718	3172.553981
DLK1	rs55996894	65.39912456
DLK1	rs12881023	73.37885789
DLK1	rs7099549	38.58023516

DLK1	rs6416180	32.16763776
DLK1	rs11648186	29.75225903
DLK1	rs112001035	202.4015734
DLK1	rs732194	40.37000517
RS1	rs10922098	120.5632542
RS1	rs1355538	36.9978168
RS1	rs9501393	55.92196548
RS1	rs7774739	38.94010449
TMED10	rs77924615	41.76809863
TMED10	rs79879530	56.82608263
TMED10	rs6114138	59.52949678
TMED10	rs2405392	3246.381017
TMED10	rs78516764	136.3907186
TMED10	rs2224232	73.01255088
TMED10	rs145313608	68.22969806
TMED10	rs147470985	34.02585373
TMED10	rs189662746	37.49918296
TMED10	rs187657447	32.82636411
NCAM2	rs2826608	52.5317629
NCAM2	rs11911765	495.437621
NCAM2	rs34399466	3978.608385
NCAM2	rs17806256	52.51042261
NCAM2	rs2827034	130.0311416
NCAM2	rs117729196	104.2818859
NCAM2	rs61261356	321.6966825
NCAM2	rs4760	59.0461924
NCAM2	rs169475	31.00095478
NCAM2	rs13128006	30.58727711
RNF128	rs10922098	273.9157442
RNF128	rs2686395	96.32343313
RNF128	rs62395845	104.3694017
RNF128	rs2284179	47.01436114
RNF128	rs415620	82.87477785
RNF128	rs7774739	68.33831206
RNF128	rs74671299	32.54223395
RNF128	rs73403125	32.69385599
GDF10	rs10922098	154.9999212
GDF10	rs9864801	36.79334
GDF10	rs453821	53.70328699
MGP	rs75226989	36.97418517
MGP	rs10846071	733.4380541
MGP	rs7135211	880.6164011
MGP	rs11830258	85.38590728
MGP	rs704	80.17503793
MGP	rs77542162	340.5573105
MGP	rs72631343	104.0664169
MGP	rs141622900	36.76668761
MGP	rs9857914	40.92810006
NPTX2	rs13437914	44.03072916
NPTX2	rs13308423	172.020308
NPTX2	rs970741	355.6326041
NPTX2	rs2040443	37.16942935
NPTX2	rs964184	124.0674821
NPTX2	rs71352239	155.7875584
NPTX2	rs1707652	119.9820907
NPTX2	rs73407207	36.4945583
NPTX2	rs35597441	44.40333302
NPTX2	rs143669824	67.54028856

NPTX2	rs4407894	32.65876989
DUSP13	rs4746262	331.7225229
DUSP13	rs10922098	59.53113197
DUSP13	rs28479572	88.19086998
DUSP13	rs1707652	51.70734061
DUSP13	rs2299258	295.9142574
DUSP13	rs369836640	74.73979027
TRIL	rs221187	68.46116817
TRIL	rs10922098	692.4144659
TRIL	rs3900909	35.74737274
TRIL	rs2477664	53.89083737
TRIL	rs2461161	38.9452095
TRIL	rs704	259.8489202
TRIL	rs1707652	164.0109291
TRIL	rs7630163	31.61829758
TRIL	rs1144709	38.32776771
TRIL	rs1265941	88.5323511
TRIL	rs550671	215.5828157
EXTL2	rs10305748	31.98789665
EXTL2	rs10922098	510.4546177
EXTL2	rs28658508	32.48827878
EXTL2	rs704	99.18814436
EXTL2	rs11569393	31.43676965
EXTL2	rs10208529	30.42943666
EXTL2	rs114115200	106.3005909
EXTL2	rs3863087	31.02509841
EXTL2	rs453821	121.2057201
EXTL2	rs28471056	42.84245716
DNASE2B	rs572515	32.07010858
DNASE2B	rs4977155	60.05221563
DNASE2B	rs601020	35.0124035
PLBD2	rs10922098	279.3229911
PLBD2	rs1706435	70.88452937
PLBD2	rs9501600	77.28099053
KIAA2013	rs9567566	55.12888027
KIAA2013	rs7987805	139.4179529
KIAA2013	rs7995412	1053.354543
KIAA2013	rs34289921	183.0831293
KIAA2013	rs17281886	57.9660402
KIAA2013	rs7328313	58.93701272
KIAA2013	rs1375663	59.89349359
KIAA2013	rs1050628	32.51673442
PRLH	rs10922098	111.1002478
PRLH	rs1260326	35.55110945
PRLH	rs56210800	31.56556883
PRLH	rs17282078	31.66891415
PRLH	rs1355538	39.19654027
PRLH	rs9501393	48.24269529
PRLH	rs9272068	33.05996353
NELL1	rs77979656	69.67182547
NELL1	rs1826940	142.4854487
NELL1	rs139961786	954.6604811
NELL1	rs2163149	169.3227132
NELL1	rs7115020	299.4833536
NELL1	rs11820583	57.24052217
NELL1	rs2990223	34.5070874
NELL1	rs2477642	38.22789632
NELL1	rs616941	61.01864196

NELL1	rs139099371	86.43140133
NELL1	rs11027982	30.07474658
NELL1	rs7123489	56.36946635
NELL1	rs2298475	93.54640617
NELL1	rs2291925	70.58921851
NELL1	rs34716573	96.5550974
NELL1	rs7298766	507.6557519
NELL1	rs192828744	31.78260509
NELL1	rs139223084	41.43742108
NELL1	rs2898820	39.43388407
NELL1	rs55854536	31.06594477
NELL1	rs9806761	32.94144063
NELL1	rs55714927	47.26459576
NELL1	rs61543910	91.65209946
NELL1	rs2587699	30.38466587
NELL1	rs863678	53.66734786
NELL1	rs35436189	33.89878451
NELL1	rs1290785	31.99711622
NELL1	rs1468906	49.22603428
NELL1	rs72711359	32.78383404
NELL1	rs7770526	67.64105029
NELL1	rs13230625	39.65114336
NELL1	rs71303418	37.11145701
NELL1	rs7033145	31.58666661
PRNP	rs10922098	360.2978543
PRNP	rs1707652	75.33797465
PRNP	rs1265941	39.18251813
PRNP	rs444921	101.6539859
TMPRSS11D	rs10922098	102.1638658
TMPRSS11D	rs62251293	30.05945017
TMPRSS11D	rs73089342	31.4978946
TMPRSS11D	rs11709525	795.0406402
TMPRSS11D	rs6793992	55.32992424
TMPRSS11D	rs184430520	31.65202188
TMPRSS11D	rs415620	36.90221832
TMPRSS11D	rs7774739	32.1875313
VSTM2L	rs10922094	125.9277201
VSTM2L	rs6542680	66.39925193
VSTM2L	rs9864801	30.70751904
VSTM2L	rs147906938	43.07376487
ICAM4	rs5030377	161.5437949
ICAM4	rs188966040	126.4835995
ICAM4	rs78103613	40.84684507
ICAM4	rs11806510	69.29324822
ICAM4	rs2375113	408.5640103
ICAM4	rs2375278	1115.674503
ICAM4	rs3754148	56.20548454
ICAM4	rs6703730	87.5373256
ICAM4	rs148642368	31.02316064
ICAM4	rs116703053	34.40588509
ICAM4	rs11576522	251.1404821
ICAM4	rs1946842	66.03059637
ICAM4	rs3184504	36.58263316
ICAM4	rs588136	30.7050578
ICAM4	rs55714927	31.19048316
ICAM4	rs7245091	51.84221894
ICAM4	rs148201697	68.5155926
ICAM4	rs184174081	51.17538966

ICAM4	rs7606173	38.2457075
ICAM4	rs4342150	51.16507842
ICAM4	rs9266141	32.01302176
ICAM4	rs697742	32.29765817
ICAM4	rs9402685	62.58229116
ICAM4	rs146798134	41.4696248
ICAM4	rs635634	782.1089501
ICAM4	rs117210485	47.70517192
SERPINA12	rs1885065	62.83917376
SERPINA12	rs55911632	226.4234176
SERPINA12	rs113958256	1275.756837
SERPINA12	rs11160190	1338.125563
SERPINA12	rs10137923	259.0499366
SERPINA12	rs59233955	44.1055409
SERPINA12	rs10922098	220.5164235
SERPINA12	rs339969	45.18816356
SERPINA12	rs704	58.22958748
SERPINA12	rs6445035	52.96380819
SERPINA12	rs9501600	56.74642488
CFAP45	rs10922098	72.59411417
CFAP45	rs2232613	66.19861174
CFAP45	rs9864801	44.77797276
CFAP45	rs9268581	30.81772057
STOML2	rs506317	52.94194093
STOML2	rs35524242	156.4101481
STOML2	rs67086575	30.20274304
STOML2	rs1355538	60.34671078
STOML2	rs73216598	156.9811628
STOML2	rs2237582	1393.485157
STOML2	rs854572	198.5753346
STOML2	rs2706869	53.56252542
STOML2	rs2299269	68.34265879
ENPP5	rs75953810	58.3143004
ENPP5	rs62401884	284.4154262
ENPP5	rs79601106	193.1677641
ENPP5	rs6906620	3504.81601
ENPP5	rs34405250	429.0260629
ENPP5	rs7760667	32.98740522
ENPP5	rs181707610	31.58657315
ENPP5	rs1260326	37.43419871
ENPP5	rs13041788	34.451222
ENPP5	rs2144677	31.38280523
ENPP5	rs116232742	34.47236587
ENPP5	rs80266997	121.7672789
ENPP5	rs2281445	148.8263084
ENPP5	rs77315792	75.59821272
ENPP5	rs181582240	136.254068
ENPP5	rs142373835	46.10756566
ENPP5	rs7770561	38.97311235
ENPP5	rs77653596	30.37686927
ENPP5	rs117865293	32.26890824
ENPP5	rs709821	47.06463047
LRRC15	rs2630231	36.83858839
LRRC15	rs145643822	73.44274182
LRRC15	rs1972456	965.9886044
LRRC15	rs1770636	68.00037091
LRRC15	rs78705678	96.19149452
LRRC15	rs2477642	90.25508569

LRRC15	rs7966590	66.21652113
LRRC15	rs9594738	76.73009044
LRRC15	rs884205	31.10324639
LRRC15	rs11726170	35.02450032
LRRC15	rs2062375	49.94221334
COLEC10	rs7844871	49.3102847
COLEC10	rs10922098	407.5494491
COLEC10	rs3820897	226.6186567
COLEC10	rs111696008	53.85538797
COLEC10	rs144202860	31.45287467
COLEC10	rs6445035	298.3117214
COLEC10	rs3863087	122.7743409
COLEC10	rs73169103	31.19384268
COLEC10	rs9267139	35.57392259
COLEC10	rs9501393	147.4907443
COLEC10	rs7774739	98.92490551
COLEC10	rs9271766	39.5801529
COL13A1	rs1995651	30.81398277
COL13A1	rs60095937	41.4046226
COL13A1	rs117272793	53.83689827
COL13A1	rs139482112	42.11753898
COL13A1	rs2854746	34.13547736
COL13A1	rs700752	203.8796767
LRRTM4	rs1922804	38.12646839
LRRTM4	rs10922094	57.67028722
LRRTM4	rs62119261	30.56976322
LRRTM4	rs73045691	2098.775206
LRRTM4	rs77660932	52.29908717
LRRTM4	rs1049739	42.72042752
LRRTM4	rs1355538	61.9383223
ART4	rs117782398	126.3523376
ART4	rs11055919	217.6647079
ART4	rs1344560	49.83918242
ART4	rs12318199	999.3399724
ART4	rs12828195	240.8836434
ART4	rs191368549	527.4680092
ART4	rs79740319	959.5428564
ART4	rs74699037	427.0130661
ART4	rs150620413	100.9159467
ART4	rs10846071	31653.26634
ART4	rs146778457	109.1683227
ART4	rs34851273	115.899742
ART4	rs138585600	317.0087281
ART4	rs7301830	744.1358792
ART4	rs73053131	84.76899776
ART4	rs71457202	61.88104982
ART4	rs1355538	56.6686954
LAMA4	rs10922098	90.98867368
LAMA4	rs75327431	30.38630907
LAMA4	rs12995461	31.44529988
LAMA4	rs77347777	40.63910898
TNMD	rs10922094	356.1460712
TNMD	rs1707652	85.54467942
TNMD	rs9267139	35.49157312
TNMD	rs9501393	99.56142005
TNMD	rs7774739	72.84658937
PZP	rs34022782	150.6698382
PZP	rs226399	983.9729148

PZP	rs3782676	3696.326014
PZP	rs4271437	686.037199
PZP	rs117006594	168.4510113
PZP	rs12810163	48.55308257
PZP	rs7075901	88.83270584
PZP	rs55680021	30.32729695
PZP	rs75718176	34.32406877
PZP	rs11045856	30.52423879
PZP	rs79391862	76.83151864
PZP	rs374087027	38.14800811
PZP	rs7222851	53.96829487
PZP	rs2297441	30.20976256
PZP	rs61791721	45.5238792
PZP	rs10013413	30.79904679
PZP	rs62385693	37.88129993
ADAM11	rs7901010	42.00587517
ADAM11	rs12572683	77.40663638
ADAM11	rs12763796	102.5722209
ADAM11	rs429358	133.0376214
ADAM11	rs17441745	30.57832474
ADAM11	rs6542680	607.1256601
ADAM11	rs12475572	33.63351595
ADAM11	rs1706435	43.24478815
LRFN5	rs1451915	38.63712921
LRFN5	rs10922098	756.5798526
LRFN5	rs7513100	32.11804675
LRFN5	rs1047286	67.55106867
LRFN5	rs4665987	31.09233958
LRFN5	rs1707652	149.0372108
LRFN5	rs3099847	33.21663596
LRFN5	rs1265941	75.9251004
LRFN5	rs550671	193.2604018
SAYSD1	rs10922098	241.6793156
SAYSD1	rs1355538	34.6343499
SAYSD1	rs453821	56.82946023
FAM174A	rs10922098	132.5068401
FAM174A	rs74611276	53.1792438
FAM174A	rs112291863	55.03722525
FAM174A	rs141857316	79.7263235
FAM174A	rs78932638	131.6581001
FAM174A	rs139873289	155.2674953
FAM174A	rs117342095	55.49354379
FAM174A	rs12254574	2487.359816
FAM174A	rs77835145	71.79506365
FAM174A	rs4933164	95.34864673
FAM174A	rs7951764	54.97836934
FAM174A	rs1706435	61.41611601
FAM174A	rs9501600	37.78069064
APCDD1	rs368465	176.1457768
APCDD1	rs9864801	49.90683108
APCDD1	rs28573154	63.70170741
APCDD1	rs3129773	47.16372936
RELL2	rs10922098	293.0941586
RELL2	rs2073453	148.1672883
RELL2	rs1707652	66.83879828
RELL2	rs9501393	65.6589979
RELL2	rs7774739	35.84790209
NDNF	rs10922098	243.3722674

NDNF	rs1355538	50.8049348
NDNF	rs444921	91.96277679
NDNF	rs28471056	42.98827595
IGFALS	rs76440160	46.89888909
IGFALS	rs139698013	31.52895918
IGFALS	rs11647990	103.8775858
IGFALS	rs35391516	203.1586501
IGFALS	rs139482112	223.883094
IGFALS	rs77589580	119.2510755
IGFALS	rs61754523	67.10818834
IGFALS	rs12484700	37.54656789
IGFALS	rs1863622	68.36764536
IGFALS	rs4699726	39.56567227
IGFALS	rs2854746	339.2490613
IGFALS	rs856557	31.33543199
KISS1	rs72673550	159.7821537
KISS1	rs113272037	57.71377581
KISS1	rs682479	1337.871209
KISS1	rs12085435	1339.864892
KISS1	rs116094092	131.2093657
KISS1	rs12133084	44.90165538
KISS1	rs17457811	45.37999367
KISS1	rs62073286	109.1946076
KISS1	rs75452426	72.81964801
KISS1	rs78087381	173.9349205
KISS1	rs143258091	1205.477577
KISS1	rs148840367	5946.023884
KISS1	rs973753	173.6615664
KISS1	rs149395572	177.6377671
KISS1	rs61760359	50.42033683
KISS1	rs9906360	83.59295462
KISS1	rs138694429	30.90368431
KISS1	rs138772443	37.27399942
KISS1	rs908830	276.5896192
CNP	rs12602950	82.3978434
CNP	rs4980320	35.33490734
CNP	rs342200	43.70638237
BRICD5	rs12710562	157.7308321
BRICD5	rs59502684	59.94632438
BRICD5	rs1354034	83.64947163
FCRL6	rs11585836	210.1596484
FCRL6	rs115041442	31.75221354
FCRL6	rs10922098	432.1750727
FCRL6	rs11569415	38.45808601
FCRL6	rs2686395	85.0312753
FCRL6	rs9266729	40.2692144
FCRL6	rs9267139	57.22897517
FCRL6	rs28573154	156.0449434
FCRL6	rs7774739	109.3730602
FCRL6	rs9271766	65.98388784
LINGO1	rs62009067	95.04722246
LINGO1	rs10158500	58.52095731
LINGO1	rs17423152	30.40922945
LINGO1	rs10922098	233.5906302
LINGO1	rs2477664	77.13319126
LINGO1	rs2461161	57.01920245
LINGO1	rs10762694	62.81767877
LINGO1	rs215226	41.13249959

LINGO1	rs1033415	48.231869
LINGO1	rs1706435	65.48904347
LINGO1	rs1467206	42.81064847
LINGO1	rs7756673	43.27203055
LINGO1	rs28573154	55.45600163
LINGO1	rs7774739	34.6668515
LINGO1	rs4413619	44.45612584
LINGO1	rs11153801	32.14026567
RLN2	rs10922098	389.8858658
RLN2	rs1047286	40.37073589
RLN2	rs6445035	177.9611477
RLN2	rs3863087	66.44645348
RLN2	rs1144709	54.5251465
RLN2	rs1265941	91.60011253
RLN2	rs453821	212.560893
RLN2	rs112164276	41.91728561
APLN	rs10922098	86.41741134
APLN	rs2806894	31.34311931
APLN	rs9562634	437.0621469
APLN	rs9469077	30.16061621
WNT11	rs10922098	303.8213959
WNT11	rs1706435	77.59822072
WNT11	rs7774739	70.07957382
WNT11	rs7742632	80.81123389
WNT11	rs112692477	30.00392947
CHST12	rs4720987	41.64667396
CHST12	rs6952433	430.8053679
CHST12	rs10922098	66.26197536
CHST12	rs3184504	46.61082483
CHST12	rs704	35.72637115
CHST12	rs111696008	38.38503392
CHST12	rs17713196	307.4408026
CHST12	rs3863087	124.7030476
CHST12	rs4713460	29.86423845
CHST12	rs635634	253.9614501
PNLIPRP1	rs12249434	77.71209246
PNLIPRP1	rs2915766	313.2334012
PNLIPRP1	rs35311109	50.74274343
PNLIPRP1	rs140379829	46.47510002
PNLIPRP1	rs9919157	41.26195798
PNLIPRP1	rs6685892	52.04369417
PNLIPRP1	rs72704449	37.13489586
PNLIPRP1	rs10922098	54.3717602
PNLIPRP1	rs4494220	32.88613421
PNLIPRP1	rs111871876	45.56630559
PNLIPRP1	rs507695	62.42075152
PNLIPRP1	rs8022656	53.11096613
PNLIPRP1	rs16950178	52.42424863
PNLIPRP1	rs72802342	1283.849191
PNLIPRP1	rs1153021	33.34688613
PNLIPRP1	rs144395013	64.02314201
PNLIPRP1	rs1126464	76.22706885
PNLIPRP1	rs9931889	36.21223478
PNLIPRP1	rs3110649	33.68949299
PNLIPRP1	rs2277710	29.86362354
PNLIPRP1	rs9976812	35.17577062
PNLIPRP1	rs66473811	38.74150456
PNLIPRP1	rs11724862	59.73002105

PNLIPRP1	rs2534792	49.51943274
PNLIPRP1	rs1591805	56.75289669
PNLIPRP1	rs7803628	36.87156138
PNLIPRP1	rs13438309	40.59471315
PNLIPRP1	rs748208	46.65883173
PNLIPRP1	rs576123	74.08140097
DEFB1	rs11137060	56.05680148
DEFB1	rs6988626	114.0960506
DEFB1	rs2741108	1003.812197
DEFB1	rs2702884	270.3012914
DEFB1	rs2741712	85.69909096
DEFB1	rs112142883	68.03525687
DEFB1	rs4439150	39.58392783
DEFB1	rs4418728	39.75756975
DEFB1	rs9738226	62.94326758
DEFB1	rs147233090	79.55490715
DEFB1	rs79391862	42.38456807
DEFB1	rs374087027	34.56718241
DEFB1	rs62074055	39.29700492
DEFB1	rs1260326	186.8873625
DEFB1	rs73207243	39.23041067
DEFB1	rs11713634	342.0423575
DEFB1	rs75303792	34.48762653
DEFB1	rs9326982	259.2663568
DEFB1	rs6968170	138.3379914
DEFB1	rs6990769	42.54391166
DEFB1	rs28601761	63.73016548
NTN1	rs2518026	35.63857678
NTN1	rs12950996	88.84648102
NTN1	rs9914641	2496.12772
NTN1	rs8071180	775.9860231
NTN1	rs17743659	91.4710014
NTN1	rs75040456	31.85517378
NTN1	rs148639577	49.42507035
NTN1	rs8081902	37.89001219
NTN1	rs6018652	35.40258075
NTN1	rs2467071	62.03975183
NTN1	rs28601761	33.42334524
UBB	rs10922094	151.2538541
UBB	rs34253288	30.06368027
UBB	rs1355538	35.00652703
UBB	rs9501600	52.10934707
CD47	rs572515	42.19396206
CD47	rs4980320	45.11515763
CD47	rs1354034	32.76542387
CD47	rs1355538	30.40581777
LRP11	rs71568262	54.91749069
LRP11	rs141534993	163.8955754
LRP11	rs524161	3076.828824
LRP11	rs34238099	324.2884768
LRP11	rs9397451	1080.967737
LRP11	rs12191283	414.4032139
LRP11	rs789818	45.98459465
LRP11	rs74972795	115.3146638
LRP11	rs72704449	41.17121778
LRP11	rs147662563	33.13599569
LRP11	rs77332707	32.896487
LRP11	rs13218716	129.1753219

LRP11	rs11984489	30.88009017
TFRC	rs1574539	32.00714414
TFRC	rs10922094	185.4155773
TFRC	rs71515351	40.4981628
TFRC	rs10900219	109.6684523
TFRC	rs3741983	31.79234239
TFRC	rs8047587	40.47368643
TFRC	rs12461546	76.36458875
TFRC	rs2413450	35.03889261
TFRC	rs9864801	34.59169162
TFRC	rs580921	37.39947489
TFRC	rs13217434	61.1858597
TFRC	rs12210538	44.57920384
TFRC	rs576123	55.56125786
C1orf162	rs10922098	405.1637109
C1orf162	rs72840032	249.8552516
C1orf162	rs8192297	41.62095439
C1orf162	rs704	72.62098215
C1orf162	rs1047286	261.7528582
C1orf162	rs1260326	67.69365674
C1orf162	rs116347702	79.03542334
C1orf162	rs116749582	40.6820277
C1orf162	rs148104039	34.44155117
C1orf162	rs9501600	110.6293168
C1orf162	rs35209896	36.71056959
B3GAT3	rs71490375	34.98243802
B3GAT3	rs11389683	52.00198678
B3GAT3	rs12794886	991.3026201
B3GAT3	rs7104492	40.38251307
B3GAT3	rs68023366	35.15335282
B3GAT3	rs4698746	39.07805156
PCBD2	rs111655578	42.76169976
PCBD2	rs1641679	31.26897859
PCBD2	rs827241	320.927976
PCBD2	rs7606173	31.6203307
LRRTM2	rs34811565	87.85962627
LRRTM2	rs6664906	140.2046093
LRRTM2	rs72704449	30.35506288
LRRTM2	rs12278345	32.79149934
LRRTM2	rs62061425	50.35120414
LRRTM2	rs33950747	94.45937082
LRRTM2	rs635634	67.69451772
LRRTM2	rs41307428	41.2753852
MGAT2	rs7519	265.2002346
MGAT2	rs76209120	30.45547888
MGAT2	rs28549287	46.92129454
MGAT2	rs7964859	270.8994717
MGAT2	rs139974673	58.8108127
MGAT2	rs429358	33.00658283
MGAT2	rs12467476	33.04812476
MGAT2	rs2593704	53.59951842
MGAT2	rs1490750	33.69919583
CD164L2	rs1451915	33.18433154
CD164L2	rs10922098	536.726943
CD164L2	rs1047286	42.50939471
CD164L2	rs1707652	91.13072645
CD164L2	rs1265941	71.53927352
CD164L2	rs550671	140.5137259

CCK	rs7080536	234.2887655
CCK	rs709932	39.44845785
CCK	rs112001035	31.0984457
CCK	rs77542162	55.96111806
CCK	rs516246	199.5550338
CCK	rs56092644	304.3770427
CCK	rs2281686	31.66478479
CCK	rs576123	84.58467294
HBZ	rs2858050	914.7782674
HBZ	rs78369087	106.3363381
HBZ	rs10903015	674.6272867
HBZ	rs117082297	57.01031735
HBZ	rs8642	172.3815261
HBZ	rs59548273	33.46159697
HBZ	rs704	611.4879114
HBZ	rs62108438	36.25006967
HBZ	rs7606173	179.7681632
HBZ	rs114115200	64.51753374
HBZ	rs3863087	35.46236277
HBZ	rs635243	59.82180263
GFRAL	rs12529201	255.382199
GFRAL	rs72988065	269.9582571
GFRAL	rs6925652	123.8973303
GFRAL	rs10922098	49.89943038
GFRAL	rs35166255	83.76972995
GFRAL	rs117241334	36.39941474
GFRAL	rs111903631	31.46310583
GFRAL	rs73133996	47.31748134
GFRAL	rs622871	31.70251031
GFRAL	rs6906645	41.74710982
GFRAL	rs143350267	29.83965105
GFRAL	rs78514778	34.13662517
GFRAL	rs75179845	341.0435267
GFRAL	rs62576042	35.72103034
B4GALT3	rs10922098	161.4893293
B4GALT3	rs6542680	48.73216568
B4GALT3	rs6457457	45.41987418
B4GALT3	rs3891366	41.23668807
PLOD2	rs12495057	100.0924487
PLOD2	rs10876550	37.9788933
PLOD2	rs7964859	51.47822595
PLOD2	rs187553507	47.13968433
PLOD2	rs576123	41.1050585
SNX8	rs2398666	144.688959
SNX8	rs1465226	45.18973634
SNX8	rs342200	42.0610771
NDST1	rs1560661	49.31655527
NDST1	rs6863373	258.2418669
NDST1	rs7964859	237.5125633
NDST1	rs140624338	43.5405259
NDST1	rs9554252	30.16220164
NDST1	rs950419	29.79837901
NDST1	rs77542162	88.56102101
NDST1	rs13107325	58.94438562
NDST1	rs10055076	30.78023395
NDST1	rs2272662	32.75195368
NDST1	rs635634	128.4126334
ST8SIA3	rs10922098	31.67233176

ST8SIA3	rs4977155	35.36543351
ST8SIA3	rs41547217	40.21345179
ST8SIA6	rs45462798	36.77221385
ST8SIA6	rs10922098	87.28726366
ST8SIA6	rs6542680	33.59794665
ST8SIA6	rs697357	30.93069277
ST8SIA6	rs1042663	35.16288128
ITGA5	rs10922098	598.1200291
ITGA5	rs1047286	37.09613585
ITGA5	rs1707652	125.5318633
ITGA5	rs9267139	47.09147516
ITGA5	rs3891366	146.1167269
ITGA5	rs7774739	103.7438771
ITGA5	rs9271766	39.77154564
MRPL34	rs10922098	136.1711461
MRPL34	rs6542680	40.06169532
MRPL34	rs114115200	36.79485691
MRPL34	rs9501600	43.79078119
PMEPA1	rs10922098	159.3557135
PMEPA1	rs150820726	40.34364974
PMEPA1	rs11720167	56.54578035
PMEPA1	rs9501393	42.57297904
PMEPA1	rs17205128	30.02614228
C1orf43	rs10922094	69.66731561
C1orf43	rs1965605	53.58780368
C1orf43	rs41547217	36.64073571
JPH1	rs145300538	71.3091692
JPH1	rs1894693	41.08328916
JPH1	rs6672595	163.874623
JPH1	rs6127	55.54911037
JPH1	rs10922098	78.62988109
JPH1	rs10159712	52.49041642
JPH1	rs6544941	32.8172649
JPH1	rs9501393	59.61317275
JPH1	rs9271739	42.65114933
SUMF1	rs682479	227.2524589
SUMF1	rs10922098	36.32862571
SUMF1	rs2472297	30.09671395
SUMF1	rs1065853	78.57384625
SUMF1	rs1707652	60.61577721
SUMF1	rs149062332	200.4250394
SUMF1	rs6457457	66.5480015
SUMF1	rs6458	105.2423546
SUMF1	rs9269187	36.47754151
SUMF1	rs2106727	53.71823541
SUMF1	rs10982050	81.38885265
SUMF1	rs7862602	304.990974
ST3GAL6	rs149517005	953.0260726
ST3GAL6	rs74890720	1187.954948
ST3GAL6	rs13071075	256.1657694
ST3GAL6	rs201898290	8096.200672
ST3GAL6	rs150360848	969.450472
ST3GAL6	rs139117241	33.36323388
ST3GAL6	rs12436465	60.82154875
ST3GAL6	rs374229803	32.71047662
ST3GAL6	rs28873836	215.1728887
ST3GAL6	rs7433284	53.4628593
ST3GAL6	rs141240008	31.02703958

ST3GAL6	rs9842169	44.65318018
ST3GAL6	rs147080039	155.9369078
ST3GAL6	rs77011240	216.0873951
ST3GAL6	rs116589363	66.1846605
ST3GAL6	rs146499133	90.2231857
ST3GAL6	rs9836217	139.5903079
ST3GAL6	rs76239915	154.9786047
ST3GAL6	rs76902616	35.04273235
ST3GAL6	rs80266383	95.80499059
ST3GAL6	rs9310128	67.46909888
ST3GAL6	rs74413799	227.5022995
ST3GAL6	rs116778313	37.55973322
ST3GAL6	rs7613334	48.29070479
ST3GAL6	rs75344916	119.4743774
ST3GAL6	rs115658481	122.8511588
ST3GAL6	rs73155483	157.1375723
ST3GAL6	rs73150411	226.6085549
ST3GAL6	rs75126986	195.0179866
ST3GAL6	rs113764913	51.79663318
ST3GAL6	rs1264708	46.92002665
ST3GAL6	rs3130975	29.9732991
ST3GAL6	rs113354603	42.11941512
ASIC4	rs10922098	164.2335697
ASIC4	rs1355538	50.14506406
ASIC4	rs28573154	66.70805141
ASIC4	rs7774739	51.24983804
SNX1	rs141730302	143.196305
SNX1	rs77404765	218.074429
SNX1	rs10922098	111.0304038
SNX1	rs1354034	57.65145674
SNX1	rs1619994	31.24860065
SNX1	rs28573154	32.39133285
SNX1	rs3748136	135.2005783
IGFL3	rs72748420	35.72102902
IGFL3	rs114009540	37.95023393
IGFL3	rs1329424	1134.36157
IGFL3	rs3900909	52.35570358
IGFL3	rs704	60.2027704
IGFL3	rs1047286	59.01703937
IGFL3	rs111696008	34.69618886
IGFL3	rs2686395	228.5905563
IGFL3	rs3099847	34.28370002
IGFL3	rs1265941	103.5110356
IGFL3	rs550671	278.5832754
IGFL3	rs78897911	31.44336403
IGFL3	rs9276606	43.55163612
CNTNAP2	rs62498270	172.6511297
CNTNAP2	rs62504964	1113.765538
CNTNAP2	rs114425483	280.7615446
CNTNAP2	rs146967864	45.35515583
CNTNAP2	rs74954603	63.55233205
CNTNAP2	rs117849203	58.02094218
CNTNAP2	rs117462905	97.2523571
CNTNAP2	rs10922098	91.45063211
CNTNAP2	rs2477639	97.0830354
CNTNAP2	rs692440	79.12772059
CNTNAP2	rs1654439	32.42119479
CNTNAP2	rs760715	35.84389296

CNTNAP2	rs1355538	40.50813011
CNTNAP2	rs9501600	34.76549085
CNTNAP2	rs6979099	38.91583621
STX1B	rs10922098	180.3466618
STX1B	rs114115200	61.14561795
STX1B	rs9267139	31.67274948
STX1B	rs9501393	66.65710441
STX1B	rs7774739	54.77242386
WFDC5	rs35456522	42.81659589
WFDC5	rs10922098	464.9284216
WFDC5	rs1047286	37.01020137
WFDC5	rs1707652	94.42806681
WFDC5	rs6457457	73.77912053
WFDC5	rs28573154	129.3864061
SNAPIN	rs572515	201.4701716
SNAPIN	rs62269916	30.84550988
SNAPIN	rs11720167	74.67951053
SNAPIN	rs3863087	37.01743838
SNAPIN	rs550671	73.94109735
SNAPIN	rs28471056	44.46832293
IGSF8	rs12408242	247.9457187
IGSF8	rs12408203	52.21998719
IGSF8	rs6542680	53.19915103
IGSF8	rs1486236	86.72865966
IGSF8	rs698082	59.95250556
HS3ST3B1	rs17669311	35.71928057
HS3ST3B1	rs10922098	320.0044906
HS3ST3B1	rs1355538	44.25665238
HS3ST3B1	rs453821	126.2351704
HS3ST3B1	rs28471056	53.45546096
HS3ST3B1	rs9272526	33.29899482
SDF2L1	rs73166641	40.24351084
SDF2L1	rs10922098	62.67409392
SDF2L1	rs200210321	109.9287636
SDF2L1	rs676210	93.21428238
SDF2L1	rs41556417	51.90202687
SDF2L1	rs114029601	83.73468862
FUT9	rs10922098	176.8181217
FUT9	rs185009887	30.86727609
FUT9	rs704	423.5126537
FUT9	rs1706435	61.73809024
FUT9	rs9501393	56.59555404
FUT9	rs7774739	40.47150388
HBD	rs114009540	30.28296666
HBD	rs10922098	793.9557719
HBD	rs3900909	40.08059948
HBD	rs704	60.89179636
HBD	rs1047286	54.27068685
HBD	rs2686395	144.7040467
HBD	rs3099847	32.01170208
HBD	rs1265941	98.19729408
HBD	rs550671	217.3513016
HBD	rs9276606	35.98720495
C3orf18	rs10922098	893.487309
C3orf18	rs3900909	40.50704076
C3orf18	rs111696008	30.2421168
C3orf18	rs2686395	203.2229292
C3orf18	rs1265941	101.2667516

C3orf18	rs550671	241.5840307
C3orf18	rs9276606	39.34029667
RAB26	rs6685931	29.8421522
RAB26	rs10799536	100.6507392
RAB26	rs2477639	33.18979719
RAB26	rs757081	115.6410927
RAB26	rs2606724	29.83265708
RAB26	rs117317284	40.33018305
RAB26	rs76428106	100.2313044
RAB26	rs11629297	37.40246434
RAB26	rs28678897	49.15040929
RAB26	rs72786786	57.79732247
RAB26	rs768053	33.50467083
RAB26	rs117405202	62.22959068
RAB26	rs181252	93.71647394
RAB26	rs35892171	373.2972076
RAB26	rs147850744	121.0195833
RAB26	rs76312221	121.6206358
RAB26	rs145433937	282.6042656
RAB26	rs4545885	145.9378053
RAB26	rs147414880	65.3588018
RAB26	rs138839548	51.2736571
RAB26	rs62132278	45.00423214
RAB26	rs444053	45.77874481
RAB26	rs6544659	72.12097506
RAB26	rs6080761	76.97630144
RAB26	rs4383561	33.90764857
RAB26	rs2305637	123.7161465
RAB26	rs1687481	50.23247241
RAB26	rs6775011	90.6268078
RAB26	rs1707652	80.70408947
RAB26	rs34591574	87.24389497
RAB26	rs1264457	119.1690811
RAB26	rs3763305	33.12389271
RAB26	rs10107630	106.5048984
ASPH	rs16927830	49.87931786
ASPH	rs112800350	132.1579972
ASPH	rs17372735	30.17299836
ASPH	rs79634769	85.12970739
GALNT10	rs10922094	100.5739801
GALNT10	rs6542680	36.56157858
GALNT10	rs6770895	30.4159575
GALNT10	rs415620	38.08120421
COL25A1	rs10922098	151.0854208
COL25A1	rs28573154	64.13624556
COL25A1	rs7774739	41.98868971
COL25A1	rs2073044	43.95631681
CD72	rs10972306	51.42806918
CD72	rs34791102	207.0379612
CD72	rs10922094	241.7497295
CD72	rs3184504	42.8773667
CD72	rs1555405	30.07760876
CD72	rs114115200	30.60821573
CD72	rs4713498	39.15983944
CD72	rs3116995	69.29476003
KCNIP4	rs10922098	151.8490552
KCNIP4	rs1355537	39.02297343
KCNIP4	rs6457457	31.75008264

KCNIP4	rs28573154	46.31297267
LILRB5	rs185606799	88.2516266
LILRB5	rs393799	147.3056372
LILRB5	rs117575899	135.8527954
LILRB5	rs71365433	972.8745263
LILRB5	rs2361808	970.1645219
LILRB5	rs387805	6878.333697
LILRB5	rs373052198	233.5375627
LILRB5	rs140244810	73.1684403
LILRB5	rs73060927	37.48699205
LILRB5	rs12371	102.4381066
LILRB5	rs59862691	51.65508388
LILRB5	rs11084405	58.58259821
TSR3	rs1451915	30.35365022
TSR3	rs10922098	549.2223144
TSR3	rs1047286	39.38671652
TSR3	rs1707652	145.5311434
TSR3	rs1265941	63.28297253
TSR3	rs550671	160.0180948
TSR3	rs9276606	35.3890831
SEMA7A	rs78994380	92.39442059
SEMA7A	rs1984769	48.26776415
SEMA7A	rs61843998	39.16087264
SEMA7A	rs36040496	76.97601769
SEMA7A	rs2477669	32.24116512
SEMA7A	rs78705678	195.8275709
SEMA7A	rs2477642	220.3409869
SEMA7A	rs7310615	124.1985118
SEMA7A	rs55714927	76.15726518
SEMA7A	rs281377	37.47390372
SEMA7A	rs1427407	36.08281379
SEMA7A	rs3864105	226.1487299
SEMA7A	rs4686841	31.42377223
SEMA7A	rs3117037	33.52200282
SEMA7A	rs4873804	32.01259711
SEMA7A	rs10901252	165.0729379
SEMA7A	rs3094379	32.95050165
BNIP3	rs10922098	39.67536398
BNIP3	rs1354034	46.14051409
BNIP3	rs6444156	93.58489444
C20orf173	rs1451915	36.25023786
C20orf173	rs1329424	921.2506524
C20orf173	rs7513100	37.66250336
C20orf173	rs12026141	29.96243328
C20orf173	rs704	578.690297
C20orf173	rs111696008	41.51462729
C20orf173	rs1707652	284.3728932
C20orf173	rs62277557	37.14169355
C20orf173	rs1265941	94.33389513
C20orf173	rs444921	276.3158038
C20orf173	rs112164276	37.4352618
C20orf173	rs41548618	48.09718232
CEACAM19	rs10922098	398.3047441
CEACAM19	rs2686395	111.3643827
CEACAM19	rs9266722	34.62290726
CEACAM19	rs2242572	113.6769913
CEACAM19	rs9501602	56.53215939
CEACAM19	rs9271529	33.63654004

ADAM23	rs77497798	48.86683001
ADAM23	rs79387475	948.3364394
ADAM23	rs141950547	788.7072865
ADAM23	rs143223375	100.5291014
ADAM23	rs13016266	907.277353
ADAM23	rs144317120	1128.510113
ADAM23	rs75962834	80.29266494
ADAM23	rs78652356	394.5451196
ADAM23	rs12476420	64.61662522
ADAM23	rs115009864	38.85262882
ADAM23	rs72956375	90.95416299
ADAM23	rs62188621	203.2930312
ADAM23	rs35196633	105.4124079
ADAM23	rs11045856	250.2388893
ADAM23	rs12475054	44.78396361
ADAM23	rs28515959	36.12181412
ADAM23	rs1294437	39.8202816
ADAM23	rs62578578	33.9274985
ADAM23	rs8176743	557.4773801
ADAM23	rs3124761	131.9922328
NEGR1	rs140320901	41.03087205
NEGR1	rs1026566	218.4537905
NEGR1	rs12133018	34.86069584
NEGR1	rs72704449	62.77296341
NEGR1	rs77924615	37.93674036
NEGR1	rs2725405	31.1670328
NEGR1	rs4760	47.5258624
NEGR1	rs1260326	41.13613852
CLEC2D	rs10922098	339.0773477
CLEC2D	rs1706435	72.57175604
CLEC2D	rs453821	106.2530174
CLEC2D	rs35426875	55.46278716
LILRA6	rs7253537	156.4818112
LILRA6	rs429533	117.7156641
LILRA6	rs182476546	304.79137
LILRA6	rs112312277	5797.79178
LILRA6	rs145888232	6457.723703
LILRA6	rs73053436	1044.755642
LILRA6	rs1802057	8726.838704
LILRA6	rs10416260	288.0091402
LILRA6	rs62122550	174.6822697
LILRA6	rs35429338	34.2527375
LILRA6	rs7080536	73.88712716
LILRA6	rs6509727	47.02878279
VAMP8	rs10922098	332.4537706
VAMP8	rs1706435	88.61154484
VAMP8	rs9267139	40.93005851
VAMP8	rs28573154	86.19429556
VAMP8	rs7774739	53.69220992
VAMP8	rs9271766	31.50269503
TSNARE1	rs10922094	561.0179529
TSNARE1	rs3900909	31.46621962
TSNARE1	rs704	53.56784795
TSNARE1	rs1707652	109.0477889
TSNARE1	rs550671	162.8225368
TSNARE1	rs28471056	70.9487794
TSNARE1	rs9272526	32.07263396
MAN1B1	rs7514261	35.58175384

MAN1B1	rs17580	36.54895807
MAN1B1	rs1355538	68.75872628
MAN1B1	rs28573154	73.41229848
MAN1B1	rs34927613	55.36778042
MAN1B1	rs2237582	127.3860064
MAN1B1	rs854572	30.48157807
ECEL1	rs10922098	146.7707949
ECEL1	rs1706435	34.8527648
ECEL1	rs4713498	46.33199902
BTN3A1	rs9467711	30.54525128
BTN3A1	rs10922098	294.2869583
BTN3A1	rs117841447	31.15637832
BTN3A1	rs1047286	37.3200365
BTN3A1	rs2686395	48.74457546
BTN3A1	rs9501600	89.4897227
B3GNT6	rs10922098	459.7207581
B3GNT6	rs114115200	87.79934885
B3GNT6	rs6457457	46.68970243
B3GNT6	rs28573154	60.21075708
MATN4	rs144840755	82.22284543
MATN4	rs9784191	729.4853866
MATN4	rs139633476	51.44691194
MATN4	rs74174963	38.46706171
MATN4	rs78705678	59.1400345
MATN4	rs2477642	43.07606892
MATN4	rs12332693	33.34447401
MATN4	rs576123	34.5400043
CDSN	rs12075	232.9125617
CDSN	rs3917900	277.5155013
CDSN	rs3136676	713.7197592
CDSN	rs147683529	42.23029468
CDSN	rs76811287	65.71975637
CDSN	rs188400754	34.49230232
CDSN	rs2228468	166.2033011
CDSN	rs6774629	48.92257114
CDSN	rs1799864	48.13260971
CDSN	rs9275924	37.208146
CDSN	rs9276401	39.16221398
CDSN	rs11778934	39.71699036
CDSN	rs2737246	140.5333261
SYT11	rs71628688	66.13587871
SYT11	rs13412535	58.2900706
SYT11	rs3792386	63.47622508
SYT11	rs6993770	63.69937576
GALNT1	rs2622935	85.64944049
GALNT1	rs11519061	46.1639968
GALNT1	rs1706435	39.67543973
GALNT1	rs147906938	47.17335614
GALNT1	rs2647074	29.78640583
GALNT1	rs2057681	86.98596759
PDILT	rs10922098	136.0554629
PDILT	rs6445035	39.95404402
PDILT	rs74290525	51.96152442
PDILT	rs415620	62.88230312
PDILT	rs7774739	63.8809752
PDILT	rs67322126	34.42024649
RMDN1	rs79117756	51.25067916
RMDN1	rs4961042	137.0814953

RMDN1	rs4464946	914.3830015
RMDN1	rs369540210	172.8438561
RMDN1	rs2943179	44.03451941
RMDN1	rs4980320	40.20644574
RMDN1	rs1354034	83.43003457
RMDN1	rs6473547	30.11262912
RMDN1	rs76090154	56.33086217
FSTL5	rs10922098	98.92535516
FSTL5	rs1355538	40.71303718
FSTL5	rs9469077	31.06305623
CABP7	rs10733084	30.38500741
CABP7	rs10922098	288.7669237
CABP7	rs12147435	34.75574971
CABP7	rs704	210.5949895
CABP7	rs483082	45.82685686
CABP7	rs1706435	66.25742293
CABP7	rs872914	66.67302641
CABP7	rs550671	72.43317977
DNAJB11	rs55663055	34.23940194
DNAJB11	rs9841006	227.2716166
DNAJB11	rs112656750	33.19914965
DNAJB11	rs1275009	34.29493658
DNAJB11	rs78947501	30.33936957
DNAJB11	rs113342772	52.61685377
DNAJB11	rs2181802	62.4810015
DNAJB11	rs1619716	435.3177139
DNAJB11	rs41556417	272.1150593
DNAJB11	rs498422	38.77719652
DNAJB11	rs148513395	39.71983747
DNAJB11	rs6993770	51.67111391
SEMG1	rs28588150	46.69134492
SEMG1	rs61769371	52.26352587
SEMG1	rs72673550	272.399475
SEMG1	rs186126632	97.01924307
SEMG1	rs11206920	104.3064957
SEMG1	rs113272037	126.7319747
SEMG1	rs6696924	5083.917552
SEMG1	rs856843	234.4472974
SEMG1	rs114114065	462.5856758
SEMG1	rs155303	101.9855118
SEMG1	rs61768457	83.95226496
SEMG1	rs12239208	44.64114481
SEMG1	rs351973	30.38354041
SEMG1	rs4665972	32.53468087
SEMG1	rs138772443	71.49328508
SEMG1	rs17244005	47.92144867
SEMG1	rs7850844	697.3948205
SEMG1	rs369806509	42.75578722
SERPINI2	rs10922098	241.1752059
SERPINI2	rs1706435	69.08499566
SERPINI2	rs2242572	85.31369605
SERPINI2	rs9501602	42.41366857
SYT7	rs10922098	75.9493568
SYT7	rs6542680	34.95442906
SYT7	rs415620	41.07293183
SYT7	rs7774739	38.34506964
SYT7	rs188302747	33.14048299
MRPL52	rs10922098	168.8920064

MRPL52	rs1355538	48.67616952
MRPL52	rs6457457	36.69722288
MRPL52	rs28573154	52.21066638
IL21	rs10922098	56.98070561
IL21	rs11064498	66.94203165
IL21	rs7651933	35.18582754
IL21	rs9263707	32.79026205
IL21	rs2523611	67.27690764
IL21	rs7887	679.5271428
IL21	rs57718550	111.3633522
IL21	rs17207986	368.9142694
IL21	rs9270787	149.46379
APOA2	rs71631868	81.44239736
APOA2	rs757081	35.63859307
APOA2	rs9630213	41.10870243
APOA2	rs174619	96.06492781
APOA2	rs2509956	113.9526402
APOA2	rs2509962	1309.517609
APOA2	rs1058678	241.7021589
APOA2	rs185241069	30.16489797
APOA2	rs12986064	38.9318788
APOA2	rs61732279	57.17120519
VWA2	rs80113039	174.5621776
VWA2	rs34668207	457.1183575
VWA2	rs35893988	358.8875285
VWA2	rs148990087	90.80547628
VWA2	rs117719887	96.79880199
VWA2	rs117358628	70.23558601
VWA2	rs61751507	81.92342667
VWA2	rs116925860	57.37784984
VWA2	rs77064159	40.18636366
VWA2	rs28999107	32.74881268
VWA2	rs3184504	33.60842883
VWA2	rs73307089	31.14139421
VWA2	rs117340335	50.39141841
VWA2	rs241771	924.6250919
VWA2	rs9890678	54.15168214
VWA2	rs111987450	44.18303478
VWA2	rs13088856	62.07495995
VWA2	rs7635813	40.1542675
VWA2	rs11711157	666.4277631
VWA2	rs4974528	142.7253449
VWA2	rs147154500	34.29069093
C1QL4	rs10922094	177.5249775
C1QL4	rs1355538	39.27386564
C1QL4	rs9501600	55.78209953
C1QL4	rs35209896	33.26079347
CELA2A	rs78880003	133.9406052
CELA2A	rs35311109	31.52614193
CELA2A	rs10922098	41.82752081
CELA2A	rs1429830	53.78492961
CELA2A	rs75593565	138.6343183
CELA2A	rs79934953	35.42602284
CELA2A	rs28365941	49.4717913
CELA2A	rs72802358	54.68483336
CELA2A	rs533406	240.2363962
CELA2A	rs8058009	68.07226016
CELA2A	rs112188338	41.83884455

CELA2A	rs17032925	96.87741927
CELA2A	rs6812832	49.2728378
CELA2A	rs9482770	43.62087455
CELA2A	rs77643237	145.2255949
CELA2A	rs7023365	53.58978541
MGAT4B	rs374413309	86.4868889
MGAT4B	rs2288382	46.07287689
MGAT4B	rs77037744	43.38120145
MGAT4B	rs72824579	918.7521778
MGAT4B	rs74399127	197.3275936
MGAT4B	rs4700871	139.5328851
MGAT4B	rs78705678	41.80710089
MGAT4B	rs2461161	43.29704085
MGAT4B	rs12306780	128.9604141
MGAT4B	rs1260326	45.61106579
MGAT4B	rs1619994	56.58134173
CPN1	rs10922098	392.3781868
CPN1	rs1707652	94.89243925
CPN1	rs453821	105.9681831
CPN1	rs28471056	47.52662759
KAZALD1	rs77908245	115.3220433
KAZALD1	rs150026080	162.5171773
KAZALD1	rs2633320	204.2438613
KAZALD1	rs964184	242.3226798
KAZALD1	rs141469619	37.76584079
KAZALD1	rs2414577	96.27493182
KAZALD1	rs2070895	76.49780918
KAZALD1	rs3816117	184.4607864
KAZALD1	rs344773	34.18673203
KAZALD1	rs75425987	59.25163318
KAZALD1	rs5112	97.86560855
KAZALD1	rs673548	112.2298615
KAZALD1	rs11127048	32.58085202
KAZALD1	rs1707652	51.41407978
KAZALD1	rs10096633	89.52046547
KAZALD1	rs2954038	56.93753547
ITIH3	rs6802320	1253.002229
ITIH3	rs74320783	2959.47657
ITIH3	rs3774518	456.3299887
ITIH3	rs77825069	48.29935538
ITIH3	rs10922098	30.35948058
ITIH3	rs1025647	30.1962226
ITIH3	rs1070073	38.38145176
ITIH3	rs4981022	57.6891929
ITIH3	rs7633684	31.49825057
ITIH3	rs146133599	36.51468723
ITIH3	rs142131136	48.20206597
ITIH3	rs35142429	94.13937753
ITIH3	rs62246138	123.5781511
ITIH3	rs140300210	32.10262418
ITIH3	rs142044921	73.40316893
ITIH3	rs148704782	367.3192891
ITIH3	rs11130393	72.88462373
ITIH3	rs111989250	155.3723987
ITIH3	rs9841474	32.77136547
ITIH3	rs1495741	40.5181311
ITIH3	rs112875651	59.42443973
PDIA4	rs10922094	170.8521136

PDIA4	rs6542680	47.4487422
PDIA4	rs1355538	56.78078386
PDIA4	rs453821	77.81398185
PDIA4	rs35426875	52.25225796
WFDC8	rs41452950	211.9649536
WFDC8	rs617283	211.835121
WFDC8	rs7310615	35.78705874
NGRN	rs10922098	94.6100913
NGRN	rs1355538	34.47994876
NGRN	rs550671	44.23830799
C4BPB	rs528298	90.4697301
C4BPB	rs9864801	41.40465195
C4BPB	rs56218451	33.10183805
C4BPB	rs9267920	32.65385013
FUT10	rs10105155	356.2776954
FUT10	rs146637002	154.743107
FUT10	rs4733407	4055.788869
FUT10	rs7838624	959.4960233
FUT10	rs2250984	60.7150672
FUT10	rs61801011	40.9670457
FUT10	rs78705678	66.69479523
FUT10	rs2477642	89.78528516
FUT10	rs2288369	41.01918962
FUT10	rs73183043	46.5086105
FUT10	rs12945886	34.2962039
FUT10	rs10225433	30.31749657
FUT10	rs11774682	107.4011308
FUT10	rs10088815	192.4046093
FUT10	rs78930932	448.5998806
FUT10	rs79558899	47.41308032
FUT10	rs2186296	91.52446729
FUT10	rs7830964	104.1173918
FUT10	rs189826607	39.91636303
FUT10	rs635634	371.146228
PCDHA4	rs6679677	57.78879266
PCDHA4	rs2295616	36.3180206
PCDHA4	rs10737300	32.3251114
PCDHA4	rs10863663	31.74813927
PCDHA4	rs17578693	31.3138493
PCDHA4	rs11039197	34.83559434
PCDHA4	rs3802843	31.24568407
PCDHA4	rs735665	57.64980769
PCDHA4	rs1953209	48.02885418
PCDHA4	rs112763553	54.10613415
PCDHA4	rs1059216	175.3966418
PCDHA4	rs7500719	51.52577575
PCDHA4	rs4608411	33.4940701
PCDHA4	rs12989427	37.32479307
PCDHA4	rs3810560	56.85465469
PCDHA4	rs567189	495.0232364
PCDHA4	rs79395339	33.20683373
PCDHA4	rs614004	55.92603946
PCDHA4	rs6845263	29.83324284
PCDHA4	rs247559	38.87816013
PCDHA4	rs6891054	47.18886276
PCDHA4	rs139199587	56.74354967
PCDHA4	rs116446171	105.9976386
PCDHA4	rs1264457	110.4029914

PCDHA4	rs3180380	47.88891895
PCDHA4	rs9270152	50.52122509
PCDHA4	rs28383344	67.42983248
PCDHA4	rs3789210	30.05059898
PCDHA4	rs1110701	38.40004877
PCDHA4	rs3807306	30.43111564
PCDHA4	rs2511737	95.44389901
H6PD	rs139911739	148.0075866
H6PD	rs10779706	32.68773484
H6PD	rs74937107	1124.670798
H6PD	rs62642596	590.1897116
H6PD	rs72643830	123.4498122
H6PD	rs57853394	140.7375568
H6PD	rs11585670	53.19503273
H6PD	rs995628	61.75198429
H6PD	rs10843403	558.49353
H6PD	rs8178824	82.58130927
H6PD	rs1790861	30.79315341
H6PD	rs2230203	34.55746057
H6PD	rs56255430	181.0802007
H6PD	rs2239944	32.00319014
H6PD	rs111959800	39.04977855
H6PD	rs10184054	57.275974
H6PD	rs62140549	51.10990342
H6PD	rs35166538	89.60851081
H6PD	rs202064714	50.01966927
H6PD	rs76624319	196.931841
H6PD	rs73178183	139.4979554
H6PD	rs146361217	79.77752764
H6PD	rs144749939	972.9466532
H6PD	rs374317914	701.0606625
H6PD	rs77188638	250.6592309
H6PD	rs74315900	43.68008376
H6PD	rs60056504	91.03029544
H6PD	rs3130285	975.775268
H6PD	rs73403125	994.9652529
H6PD	rs57760582	265.948409
H6PD	rs35209896	77.32302909
H6PD	rs1570759	59.47979925
H6PD	rs8176693	36.57242368
LACRT	rs10747715	41.06937832
LACRT	rs74920406	31.04339222
LACRT	rs28549287	60.40088798
LACRT	rs681343	408.0794196
LACRT	rs2227411	48.67220796
LACRT	rs1834904	30.0279364
LACRT	rs148254553	33.44786931
LACRT	rs149642283	52.56831909
LACRT	rs78537273	140.6821919
LACRT	rs150556893	60.32700715
LACRT	rs151086022	308.2334845
LACRT	rs4647288	702.326414
LACRT	rs75553531	51.71019817
LACRT	rs73154254	57.96255429
LACRT	rs71523930	141.49781
LACRT	rs10245373	86.76712016
TMEM132C	rs1356014	93.66865588
TMEM132C	rs7973997	798.9175211

TMEM132C	rs1683696	152.4894027
TMEM132C	rs10773540	43.78440164
TMEM132C	rs117965239	878.851859
TMEM132C	rs1996253	149.6468294
TMEM132C	rs12768219	141.1518644
TMEM132C	rs73021419	117.8135844
TMEM132C	rs56115403	73.69345275
TMEM132C	rs41556717	47.97788471
TMEM132C	rs143501783	50.30142247
TMEM132C	rs77542162	49.13578726
TMEM132C	rs2008174	158.8353835
TMEM132C	rs3827380	29.90716073
TMEM132C	rs1328899	37.69580346
NFASC	rs12144762	56.94070119
NFASC	rs16854533	428.8466821
NFASC	rs6656887	1310.874016
NFASC	rs72755552	83.96311758
NFASC	rs36040496	54.4857748
NFASC	rs2477669	43.31867982
NFASC	rs78705678	187.6726392
NFASC	rs2477642	198.5393925
NFASC	rs635634	57.7492501
IFNA14	rs368465	99.84654746
IFNA14	rs28573154	38.05919507
IFNA14	rs7774739	30.96815364
CEACAM7	rs190119408	31.89228579
CEACAM7	rs12035228	40.28367642
CEACAM7	rs2027368	1049.651134
CEACAM7	rs2477981	228.2597613
CEACAM7	rs141924829	51.53956519
CEACAM7	rs150215601	100.4492121
CEACAM7	rs116582102	51.27613525
CEACAM7	rs141323461	74.40144016
CEACAM7	rs12884037	56.08661974
GP5	rs1466733	149.600898
GP5	rs115202389	37.2490839
GP5	rs10893507	66.41248627
GP5	rs1555405	31.38973616
GP5	rs838136	36.73654626
GP5	rs3792386	113.7713437
GP5	rs77265585	37.41271981
CHST3	rs10922094	167.30051
CHST3	rs6445035	59.63423049
CHST3	rs1056429	30.46005608
CHST3	rs9501600	97.7468823
IFNLR1	rs12751091	32.05501941
IFNLR1	rs139958347	540.5615119
IFNLR1	rs417688	53.65580204
IFNLR1	rs368465	52.17407592
IFNLR1	rs34976781	30.1050006
PCDHAC1	rs10922098	31.67291017
PCDHAC1	rs2851584	74.72581309
PCDHAC1	rs1355538	33.14128083
NPTN	rs2477642	30.57124497
NPTN	rs58973023	37.68131478
NPTN	rs59661818	37.19755028
NPTN	rs8010344	36.08853299
NPTN	rs635634	59.94158889

DNAJC15	rs10922098	51.43765188
DNAJC15	rs74798440	30.23542723
DNAJC15	rs13208583	31.69611512
FAM20B	rs143123965	30.66344287
FAM20B	rs67498652	93.81005119
FAM20B	rs78705678	102.0559054
FAM20B	rs2477642	118.7558771
FAM20B	rs174528	41.88824831
FAM20B	rs681343	37.02364056
FAM20B	rs4672497	125.8413478
FAM20B	rs78654883	47.76055531
FAM20B	rs635634	167.6005355
SYN3	rs10922098	191.0102919
SYN3	rs9864801	50.65191783
SYN3	rs9501393	59.47841627
SYN3	rs7774739	49.73480677
SEMA6C	rs10922094	37.25855451
SEMA6C	rs964184	51.5861016
SEMA6C	rs71352239	105.8967352
SEMA6C	rs11720167	116.4577403
SEMA6C	rs3863087	57.98756577
SEMA6C	rs28374650	34.27263514
RFNG	rs10922098	111.9586717
RFNG	rs438811	99.25148681
RFNG	rs5112	40.1424211
RFNG	rs11720167	85.60901532
RFNG	rs3863087	29.95647147
RFNG	rs453821	64.92500901
RFNG	rs28471056	44.79889806
FBP1	rs7857240	29.76979729
FBP1	rs4294246	31.31599389
FBP1	rs2460137	138.1897114
FBP1	rs1983127	38.04162525
FBP1	rs4408267	38.86987977
FBP1	rs7305678	43.07888752
FBP1	rs12975366	63.89426656
FBP1	rs1260326	41.3619934
FBP1	rs1476029	33.25898491
FBP1	rs3747207	77.85026923
FBP1	rs148046661	48.21615544
FBP1	rs28601761	37.65419865
MGAT4C	rs56299996	41.13174638
MGAT4C	rs1329424	37.22445329
MGAT4C	rs17580	55.37659006
APLP1	rs2229498	199.365886
APLP1	rs112906159	31.98854643
APLP1	rs831428	38.80457767
APLP1	rs372462064	40.75081643
APLP1	rs150382930	30.99390864
APLP1	rs34826779	32.87785011
RNASE1	rs75124405	66.29265887
RNASE1	rs2002078	158.958505
RNASE1	rs199717548	59.83885297
RNASE1	rs12897030	592.5152126
RNASE1	rs188152481	63.18988483
RNASE1	rs142822958	39.64604934
RNASE1	rs2129775	37.62610116
RNASE1	rs72733906	33.90897593

RNASE1	rs77924615	34.73999104
RNASE1	rs434215	41.44839089
RNASE1	rs76509141	30.18650181
ATP1B2	rs858519	1017.306298
ATP1B2	rs1050541	185.9686032
ATP1B2	rs34873228	99.30389477
ATP1B2	rs61776478	37.18740325
ATP1B2	rs105290	58.45500866
ATP1B2	rs11220463	61.80469077
ATP1B2	rs3759752	31.12276611
ATP1B2	rs726679	33.92359733
ATP1B2	rs2413593	98.68357173
ATP1B2	rs480330	33.05937298
ATP1B2	rs635634	246.1739966
CKAP4	rs10922098	350.002249
CKAP4	rs6445035	56.84895249
CKAP4	rs9501600	100.1864268
CKAP4	rs35209896	36.21270364
S100A13	rs102275	83.64047574
S100A13	rs525028	37.67966162
S100A13	rs2722757	125.5137979
S100A13	rs55923289	993.4185816
S100A13	rs11668861	4065.361443
S100A13	rs2075649	6548.279326
S100A13	rs344793	147.2286341
S100A13	rs6509222	80.44364272
S100A13	rs143864025	39.88989789
S100A13	rs8099992	30.92570131
S100A13	rs149857247	112.3323763
S100A13	rs7819706	60.27183233
TRAT1	rs10922098	44.40908817
TRAT1	rs7080386	39.52947849
TRAT1	rs11574452	32.40144255
TRAT1	rs6993770	36.41836368
MMGT1	rs10922094	172.8213414
MMGT1	rs1707652	67.87729659
MMGT1	rs453821	69.95125007
MMGT1	rs28471056	33.07091438
COCH	rs117309559	355.9022218
COCH	rs7159420	1105.367148
COCH	rs142183746	54.49444846
COCH	rs76539348	34.00538937
COCH	rs4414033	41.5098979
COCH	rs1434282	103.8065016
COCH	rs10822155	58.25049549
COCH	rs1769758	62.24473439
COCH	rs73000929	110.2833971
COCH	rs12825574	39.7342824
COCH	rs3759217	31.4753088
COCH	rs4759076	233.4828908
COCH	rs11171914	43.72591795
COCH	rs61269268	62.11883821
COCH	rs1057881	50.37810333
COCH	rs11451825	31.62526659
COCH	rs11083114	32.82768914
COCH	rs60377712	42.43382277
COCH	rs4671605	49.14626855
COCH	rs7618405	30.4337494

COCH	rs11708890	33.0463488
COCH	rs114694170	167.4126846
COCH	rs10045921	34.29583702
COCH	rs154513	31.31787129
COCH	rs11154799	34.2006631
COCH	rs432002	32.69325032
COCH	rs342299	90.848791
COCH	rs690686	86.82670992
COCH	rs1460580	47.6824251
COCH	rs6993770	518.4308215
COCH	rs35140383	30.11379654
ST6GALNAC6	rs72769820	85.53093067
ST6GALNAC6	rs72616671	33.0144577
ST6GALNAC6	rs10770	53.16799327
ST6GALNAC6	rs77089732	42.18023872
TMPRSS11A	rs10922098	139.6215573
TMPRSS11A	rs6457457	51.18372017
TMPRSS11A	rs28573154	68.13367543
P2RX6	rs10922098	135.9265119
P2RX6	rs1355538	44.29988106
P2RX6	rs28573154	48.69266643
P2RX6	rs7774739	40.26306011
COLGALT2	rs10922098	129.4073175
COLGALT2	rs6542680	43.09371083
COLGALT2	rs9864801	34.4444175
COLGALT2	rs415620	31.50264683
VSTM4	rs10922098	745.5212635
VSTM4	rs3900909	31.92349882
VSTM4	rs1047286	55.3523707
VSTM4	rs111696008	31.76702808
VSTM4	rs2686395	153.4071532
VSTM4	rs3099847	30.74454763
VSTM4	rs1265941	82.97153996
VSTM4	rs453821	193.6308293
VSTM4	rs9276606	30.7109161
IGFLR1	rs12459073	1013.012534
IGFLR1	rs35090101	427.8823059
IGFLR1	rs145086956	150.317309
IGFLR1	rs144347438	47.49471799
IGFLR1	rs118146634	136.9713875
IGFLR1	rs7310615	79.67336234
IGFLR1	rs75572774	39.21900397
IGFLR1	rs1050628	188.9016139
IGFLR1	rs9267211	152.7530348
CELF2	rs4658046	37.06986296
CELF2	rs35677470	65.8856435
CELF2	rs9271573	37.85114687
FREM2	rs10922098	176.4874661
FREM2	rs1706435	69.20215052
FREM2	rs28573154	69.13729208
FREM2	rs7774739	51.35212989
FREM2	rs9271766	33.322854
GORAB	rs10922098	259.4543238
GORAB	rs2232613	48.31337607
GORAB	rs1707652	82.48645202
GORAB	rs28573154	89.19626901
GORAB	rs7774739	59.18950725
GORAB	rs9271683	38.62333401

BCL2L10	rs10922098	260.2332437
BCL2L10	rs1707652	69.56964582
BCL2L10	rs1265941	39.69399936
BCL2L10	rs453821	91.50413368
C1QTNF3	rs840390	239.766744
C1QTNF3	rs374113375	91.1507423
C1QTNF3	rs602633	34.77868134
C1QTNF3	rs506317	34.43077944
C1QTNF3	rs10915983	36.34806322
C1QTNF3	rs11190970	43.18766596
C1QTNF3	rs77262773	32.2322491
C1QTNF3	rs11900031	32.74266799
PTH2	rs10922098	121.9719286
PTH2	rs6445035	38.52854379
PTH2	rs7774739	30.77775013
PTH2	rs28455809	47.52628995
PTH2	rs4077151	29.99108214
PRG2	rs2493133	59.18293588
PRG2	rs7310615	42.49268162
PRG2	rs2816618	31.31368568
PRG2	rs10165349	90.5488734
PRG2	rs4328821	84.93272922
PRG2	rs2266161	30.88779746
PRG2	rs41547217	35.221515
PRG2	rs13256023	40.02551615
CHST14	rs10922094	64.06787561
CHST14	rs6542680	33.83192375
CHST14	rs4713498	30.97644447
SERPINA9	rs11850199	360.0141201
SERPINA9	rs72692880	31.71526069
SERPINA9	rs78420694	64.70082464
SERPINA9	rs138939460	53.5097699
SERPINA9	rs113448098	56.5169809
SERPINA9	rs12146727	212.7703857
SERPINA9	rs11621145	78.6426606
SERPINA9	rs704	64.81998062
SPTLC2	rs10922098	379.6693694
SPTLC2	rs1707652	96.70885581
SPTLC2	rs9501600	106.8729981
SPTLC2	rs35209896	48.39313541
LRRC32	rs1320646	55.71493831
LRRC32	rs12045955	39.18134073
LRRC32	rs36040496	47.02236101
LRRC32	rs78705678	123.4498059
LRRC32	rs2477642	159.2573291
LRRC32	rs12019136	32.78333355
LRRC32	rs15052	31.94854308
LRRC32	rs117241334	37.29507116
LRRC32	rs38029	33.56994082
LRRC32	rs34794906	159.6668288
LRRC32	rs28752924	106.7775593
LRRC32	rs9272273	41.72473799
LRRC32	rs77164813	30.98867903
LRRC32	rs681470	37.41818098
LRRC32	rs2013075	943.7121882
LRRC32	rs149181677	121.6703762
LRRC32	rs36222279	65.66982012
ANK2	rs79043624	30.20165892

ANK2	rs12757091	105.8759038
ANK2	rs72737690	95.80536724
ANK2	rs142479568	38.91260299
ANK2	rs1340239	529.4714192
ANK2	rs55741360	920.2689263
ANK2	rs12040899	184.0675926
ANK2	rs61827272	51.9030181
ANK2	rs1554678	51.80243487
ANK2	rs146608551	104.5937883
ANK2	rs77176058	51.58246201
ANK2	rs114827829	51.26467849
ANK2	rs6542680	41.69233371
ANK2	rs9864801	30.3910182
CRELD1	rs57153389	166.6266905
CRELD1	rs142444285	102.0423168
CRELD1	rs279546	4964.014742
CRELD1	rs114286445	337.2850754
CRELD1	rs144363827	491.1857748
CRELD1	rs34684179	887.9532571
CRELD1	rs143087448	9604.471712
CRELD1	rs17537486	681.7801813
CRELD1	rs140614750	375.7206447
CRELD1	rs142299532	263.1603875
CRELD1	rs1180047	148.9915608
CRELD1	rs77602078	48.93671779
CRELD1	rs4684712	106.4385763
CRELD1	rs9268560	33.04590117
CRELD1	rs10124390	31.54927754
LMAN2	rs12132412	65.14776246
LMAN2	rs1914886	31.17385437
LMAN2	rs704	378.5280944
LMAN2	rs372434515	45.56033287
LMAN2	rs1260326	69.47258847
LMAN2	rs2427291	33.91865577
LMAN2	rs56210800	233.8030271
LMAN2	rs140431012	233.8707519
LMAN2	rs13086079	35.14494381
LMAN2	rs7625980	80.92405551
MYBPC1	rs3817552	85.76186846
MYBPC1	rs17617337	60.79083727
MYBPC1	rs4275631	121.2357671
MYBPC1	rs56678255	35.67005964
MYBPC1	rs9318187	83.70348095
MYBPC1	rs55890226	30.87711217
MYBPC1	rs1801690	50.72022214
MYBPC1	rs2244492	36.6263452
MYBPC1	rs875097	32.90561745
NPPB	rs10864508	31.96797722
NPPB	rs2233019	40.83940597
NPPB	rs198379	965.877583
NPPB	rs1970152	105.2643817
NPPB	rs760189	121.688137
NPPB	rs3740293	61.87569254
NPPB	rs2234962	66.89139832
NBR1	rs9971407	48.62632685
NBR1	rs1354034	50.91765907
NBR1	rs4639017	40.93117879
NBR1	rs111311909	30.49576265

NBR1	rs2524280	30.91377067
FMR1	rs11601665	38.96590532
FMR1	rs7114094	32.56022021
FMR1	rs10876550	40.30022383
FMR1	rs185009887	31.41389639
FMR1	rs1354034	90.15081978
FMR1	rs342200	34.48921425
ITGB6	rs10922094	100.6761612
ITGB6	rs8099840	41.94883246
ITGB6	rs9501600	39.16423919
ITGB6	rs78897911	30.23590525
ITGB6	rs74671299	32.87764294
ITGB6	rs73403125	32.64688794
STX2	rs10922094	149.2608987
STX2	rs201028957	39.96545948
STX2	rs145496844	1305.522376
STX2	rs2104066	84.79348152
STX2	rs55698602	165.0295734
STX2	rs72704666	87.12414293
STX2	rs1706435	57.70076527
STX2	rs28573154	101.9274428
STX2	rs7774739	58.50086264
STX2	rs9271766	32.79136888
FATE1	rs10922098	101.5756447
FATE1	rs6542680	68.00456928
FATE1	rs415620	37.89775011
FATE1	rs28374650	32.93899308
RNF148	rs10922098	107.0986947
RNF148	rs4977155	40.37952912
RNF148	rs415620	35.19175387
RNF148	rs67399078	33.03585258
CISD1	rs10922098	185.2575415
CISD1	rs1706435	67.01000949
CISD1	rs9501600	56.8636607
CISD1	rs73403125	32.21834325
NDUFB11	rs1697422	35.44462463
NDUFB11	rs10922098	327.2170735
NDUFB11	rs1555405	32.92368869
NDUFB11	rs704	66.28357717
NDUFB11	rs11569415	32.03119213
NDUFB11	rs12478841	42.1684663
NDUFB11	rs1707652	66.63128226
NDUFB11	rs6444156	35.07375346
NDUFB11	rs9501393	99.40003788
NDUFB11	rs7774739	62.41240144
NDUFB11	rs9271683	29.97417751
NDUFV2	rs10922094	105.7111253
NDUFV2	rs1355538	30.21341627
NDUFV2	rs4713498	32.41475425
SFRP2	rs1451915	38.55136561
SFRP2	rs10922098	805.4661365
SFRP2	rs704	57.44037657
SFRP2	rs11080290	30.07256423
SFRP2	rs1047286	30.03713291
SFRP2	rs1707652	138.1281635
SFRP2	rs1265941	88.95069332
SFRP2	rs550671	214.724561
SFRP2	rs9269234	31.63492403

CLEC4D	rs10922098	569.3410637
CLEC4D	rs35351085	30.41398345
CLEC4D	rs1047286	51.31205917
CLEC4D	rs1707652	138.8815571
CLEC4D	rs3828879	30.43415887
CLEC4D	rs550671	162.0065643
CLEC4D	rs28471056	61.46287235
HTATIP2	rs11025525	166.9409414
HTATIP2	rs10922094	114.0045389
HTATIP2	rs79836542	55.27888326
HTATIP2	rs113370836	59.41207847
HTATIP2	rs149127312	135.233392
HTATIP2	rs34941420	1168.935939
HTATIP2	rs75475095	66.28966015
HTATIP2	rs11848829	903.9308284
HTATIP2	rs79123775	30.07098209
HTATIP2	rs72719202	209.9245559
HTATIP2	rs75141912	35.19725863
HTATIP2	rs1706435	45.52123235
HTATIP2	rs1042663	40.63307516
HTATIP2	rs9272068	33.21861399
HTATIP2	rs6993770	77.51613586
ITGB5	rs10922098	350.0262035
ITGB5	rs6445035	66.62514314
ITGB5	rs9501600	82.06176085
ITGB5	rs78897911	30.1484328
ITGB5	rs74671299	33.99576553
ITGB5	rs73403125	32.45905671
CHKB	rs117438528	59.17313486
CHKB	rs117265627	69.21552202
CHKB	rs141909966	280.2091331
CHKB	rs34939255	85.97824185
CHKB	rs793074	134.9699216
CHKB	rs73442381	90.03995552
SNN	rs10922098	323.8711643
SNN	rs4148325	91.89994178
SNN	rs438999	88.36065064
SNN	rs9501602	39.79504182
CDH11	rs1451915	30.91551987
CDH11	rs10922098	735.8080496
CDH11	rs704	30.83939863
CDH11	rs1047286	36.33415839
CDH11	rs12478841	34.49287138
CDH11	rs1707652	116.529572
CDH11	rs3099847	30.15647513
CDH11	rs1265941	75.67873584
CDH11	rs550671	203.4674333
CDH11	rs9276606	34.07489186
ITM2A	rs10922094	432.2962218
ITM2A	rs1354034	80.54295802
ITM2A	rs1707652	75.60131003
ITM2A	rs7746553	53.74142871
ITM2A	rs2242572	106.6293337
ZP4	rs10922094	92.08194457
ZP4	rs111696008	34.47342367
ZP4	rs1707652	173.9757737
ZP4	rs2284179	35.70562108
ZP4	rs11755689	46.19903459

CLPSL2	rs10922098	336.2426168
CLPSL2	rs12478841	42.13342256
CLPSL2	rs1707652	73.00980907
CLPSL2	rs6457457	68.10268832
CLPSL2	rs9501393	96.96059465
CLPSL2	rs6451	35.53451833
SH3BP2	rs181345578	45.22978179
SH3BP2	rs374899290	110.878646
SH3BP2	rs1877721	1000.070954
SH3BP2	rs4980320	35.32406941
SH3BP2	rs1354034	121.9463519
SH3BP2	rs935973	31.07194739
UNC5B	rs72704449	76.0042639
UNC5B	rs77924615	36.73945344
UNC5B	rs11702475	68.20336897
UNC5B	rs265021	36.00067928
UNC5B	rs112323541	390.1507475
UNC5B	rs11931784	60.15972425
UNC5B	rs987383	45.79776016
UNC5B	rs1633065	30.57923653
UNC5B	rs11203609	30.57250823
CHST11	rs7295941	30.71585045
CHST11	rs11112069	213.360687
CHST11	rs79213114	59.60967346
CHST11	rs1650127	988.1704177
CHST11	rs146776981	49.78544358
CHST11	rs856595	139.2738738
CHST11	rs10922098	62.06492512
CHST11	rs1983127	33.12270602
CHST11	rs11216161	63.65726221
CHST11	rs113661219	33.11959225
CHST11	rs7606173	55.33980285
CHST11	rs6993770	86.8857446
CHST11	rs576123	282.1865831
EDDM3B	rs10922094	73.09512845
EDDM3B	rs6542680	40.94547317
EDDM3B	rs9501393	31.04655813
EDDM3B	rs7774739	33.16774555
KNG1	rs55663055	57.8627667
KNG1	rs3733160	32.14871649
KNG1	rs2304456	764.9511667
KNG1	rs76438938	702.6391177
KNG1	rs36219760	47.79928219
KNG1	rs1260326	37.53790311
EFNB3	rs1780322	30.89400921
EFNB3	rs704	423.026776
EFNB3	rs28624101	32.52886963
CLEC2B	rs10922094	89.37139724
CLEC2B	rs9501393	39.62519451
CLEC2B	rs7774739	37.60285897
LILRA5	rs148825612	45.15130645
LILRA5	rs611435	30.96474252
LILRA5	rs62133140	173.1610882
LILRA5	rs431001	2645.800252
LILRA5	rs141001499	52.6152579
LILRA5	rs1834699	36.81955438
LILRA5	rs2070902	150.9907686
LILRA5	rs80123037	45.20019481

LILRA5	rs6695321	281.9919578
LILRA5	rs12408496	51.89049639
LILRA5	rs76428106	39.69954021
LILRA5	rs1892548	37.41510396
LILRA5	rs8033923	46.14653315
LILRA5	rs56013073	40.22496513
LILRA5	rs1449263	36.39338726
LILRA5	rs913678	42.6240923
LILRA5	rs2237085	33.52968296
LILRA5	rs9264683	30.21755531
LILRA5	rs10980797	56.57756396
PRDX4	rs10922094	139.2199123
PRDX4	rs6542680	148.9440627
PRDX4	rs1707652	47.6872064
PRDX4	rs415620	45.79307413
PRDX4	rs7774739	38.97588634
LIF	rs10922098	58.21777024
LIF	rs6542680	104.6526897
LIF	rs3129752	38.87165866
CCDC90B	rs10922098	163.6928516
CCDC90B	rs6542680	41.22492483
CCDC90B	rs9864801	37.15945612
CCDC90B	rs9501600	42.06104122
KLRC3	rs10922094	76.79468052
KLRC3	rs140748737	37.40392949
KLRC3	rs77508077	91.74670424
KLRC3	rs59779517	44.15807099
KLRC3	rs41324748	180.6253753
KLRC3	rs117517474	32.30417163
KLRC3	rs4251784	143.4744096
KLRC3	rs112930619	914.8489242
KLRC3	rs146038760	923.5910987
KLRC3	rs150951362	1126.602189
KLRC3	rs1355538	41.06342502
KLRC3	rs609061	436.8538734
KLRC3	rs9501602	138.5095921
KLRC3	rs2395195	94.37262469
KLRC3	rs241438	66.57795748
CD79A	rs41290289	63.3222494
CD79A	rs1555405	35.939773
CD79A	rs3917545	132.9827343
CCDC167	rs528298	362.2320941
CCDC167	rs1707652	83.6261106
CCDC167	rs550671	96.55641376
CCDC167	rs28471056	46.21471877
CHST1	rs10922094	131.1407338
CHST1	rs1355538	41.47131667
CHST1	rs9501393	40.55272194
CHST1	rs7774739	30.96901801
GOSR1	rs144704969	32.72665966
GOSR1	rs10922098	515.1028037
GOSR1	rs1555405	30.82007388
GOSR1	rs72952553	29.92035885
GOSR1	rs1707652	133.1491672
GOSR1	rs9267139	48.99613611
GOSR1	rs9501393	136.9926352
GOSR1	rs7774739	93.53516846
GOSR1	rs9271766	38.62285615

B4GALT7	rs75331352	474.0742737
B4GALT7	rs188745628	54.56527094
B4GALT7	rs187579051	577.8932783
B4GALT7	rs59195598	521.5277137
B4GALT7	rs749462	319.472674
B4GALT7	rs80019554	157.1355579
B4GALT7	rs6589598	57.36096921
B4GALT7	rs59595881	86.55318198
B4GALT7	rs112560688	89.75604685
B4GALT7	rs138932207	185.7179211
B4GALT7	rs2544220	126.8003606
B4GALT7	rs6993770	80.34633242
GLCE	rs74497467	31.98397314
GLCE	rs1553254	185.5165702
GLCE	rs12708499	960.4362532
GLCE	rs1109980	504.8384537
GLCE	rs17315418	91.46105162
GLCE	rs305028	45.90281002
GLCE	rs7173843	75.85923711
GLCE	rs2927396	38.78055096
GLCE	rs2471884	51.01752386
GLCE	rs217184	30.60911189
GLCE	rs679574	267.8495181
GLCE	rs76518888	53.34395117
GLCE	rs11244016	84.49978265
GLCE	rs576123	3098.499738
GLCE	rs9411492	254.1547713
GLCE	rs117541350	58.39982833
GLCE	rs139562936	60.31894374
SPATA9	rs2857334	44.50358431
SPATA9	rs9905408	262.7828471
SPATA9	rs7505052	32.89989894
SPATA9	rs78937964	32.22228583
SPATA9	rs9271590	45.74187559
SPATA9	rs9484759	173.9012207
C1QTNF5	rs9640	531.0230651
C1QTNF5	rs587985	100.8547038
C1QTNF5	rs2461139	114.3590984
C1QTNF5	rs2461164	109.606533
C1QTNF5	rs2461161	55.44042216
C1QTNF5	rs2497824	58.1232108
C1QTNF5	rs4876	40.20451081
C1QTNF5	rs11039297	55.17788164
C1QTNF5	rs61897977	50.25140427
C1QTNF5	rs112972638	214.9816935
C1QTNF5	rs7304836	107.4904619
C1QTNF5	rs117981338	121.9775162
C1QTNF5	rs27289	165.6759459
C1QTNF5	rs2089855	370.7832124
C1QTNF5	rs792975	89.84951914
C1QTNF5	rs546388	49.32831864
C1QTNF5	rs3132487	30.55594036
C1QTNF5	rs2853975	108.1221667
C1QTNF5	rs1130117	35.25065413
C1QTNF5	rs10901262	44.66358657
IGFBPL1	rs10922098	160.5046617
IGFBPL1	rs1355538	29.92572188
IGFBPL1	rs415620	38.89366787

IGFBPL1	rs7774739	34.45564869
GBP6	rs4512628	30.46849687
GBP6	rs114009540	31.94930433
GBP6	rs1329424	950.3958696
GBP6	rs35351085	41.59195146
GBP6	rs704	97.75219614
GBP6	rs1047286	39.90247013
GBP6	rs1707652	156.7449863
GBP6	rs3099847	39.04964869
GBP6	rs1265941	103.4549699
GBP6	rs550671	248.216131
GBP6	rs78897911	30.83881936
GBP6	rs9276606	45.50876389
C1D	rs10922098	219.6415147
C1D	rs1355538	49.7854542
C1D	rs550671	53.0926079
C1D	rs28471056	32.62540971
ST6GALNAC2	rs72748420	36.85111064
ST6GALNAC2	rs114009540	40.90566203
ST6GALNAC2	rs1329424	1147.311519
ST6GALNAC2	rs3900909	52.18658395
ST6GALNAC2	rs1047286	66.11136809
ST6GALNAC2	rs1707652	202.0952226
ST6GALNAC2	rs3099847	35.77708473
ST6GALNAC2	rs1265941	107.0324848
ST6GALNAC2	rs550671	281.9722083
ST6GALNAC2	rs78897911	30.85455539
ST6GALNAC2	rs9276606	45.14022749
ABHD12	rs10922098	84.38130874
ABHD12	rs415620	36.47274809
ABHD12	rs7774739	33.65757079
DCLK3	rs4241819	33.02096568
DCLK3	rs375109452	38.73781539
DCLK3	rs10985149	32.29652271
ESAM	rs78934954	32.54570117
ESAM	rs79288090	139.9144191
ESAM	rs58648710	55.15073201
ESAM	rs11219769	455.2041402
ESAM	rs117102510	141.1121116
ESAM	rs35730907	63.56599633
ESAM	rs72704449	33.76080598
ESAM	rs113735401	35.81614157
ESAM	rs6993770	35.53793093
ESAM	rs8176743	166.0367723
SERF1A	rs10922098	220.1960459
SERF1A	rs1355538	49.73442574
SERF1A	rs6457457	51.1883306
SERF1A	rs28573154	75.41290172
PSG11	rs10922098	447.2077896
PSG11	rs1047286	35.55425009
PSG11	rs2686395	109.2224631
PSG11	rs555007	69.27133044
PSG11	rs550671	153.7521945
QPCT	rs79685402	37.31964549
QPCT	rs9309007	194.4805133
QPCT	rs4384764	575.6751117
QPCT	rs139035930	60.31636486
QPCT	rs16830188	50.30713206

QPCT	rs61747728	57.45190138
QPCT	rs2948711	35.05218419
QPCT	rs2477664	117.4562274
QPCT	rs2461161	114.3102521
QPCT	rs10896045	299.6361717
QPCT	rs76428106	37.08190317
QPCT	rs9901675	34.0815079
QPCT	rs34562254	60.90433409
QPCT	rs3859194	34.14724377
QPCT	rs2227319	48.85823663
QPCT	rs2659008	43.63428339
QPCT	rs182664662	30.41957582
QPCT	rs74262625	50.32182994
QPCT	rs9880192	52.16623783
QPCT	rs2518136	85.43738625
QPCT	rs2596570	47.06829564
QPCT	rs3129724	35.44392823
QPCT	rs73169671	74.02163387
QPCT	rs10107630	44.32638197
COX4I2	rs72748420	33.71885137
COX4I2	rs114009540	35.46332903
COX4I2	rs1329424	1099.877191
COX4I2	rs3900909	52.9701909
COX4I2	rs1047286	32.53643275
COX4I2	rs111696008	35.95565054
COX4I2	rs1707652	204.3536446
COX4I2	rs1265941	109.1151803
COX4I2	rs550671	291.8101813
COX4I2	rs145704749	41.96205741
ANKRD46	rs10922094	173.4054848
ANKRD46	rs1619994	51.11063957
ANKRD46	rs9501393	48.53064759
ANKRD46	rs7774739	43.04379652
SCO1	rs10922098	30.78501393
SCO1	rs114115200	88.16984033
SCO1	rs150241543	44.11309756
NCR3LG1	rs10305748	31.04316688
NCR3LG1	rs10922094	362.0437606
NCR3LG1	rs1706435	76.71948352
NCR3LG1	rs9501393	90.11329346
NCR3LG1	rs7774739	58.97436989
FAM151A	rs1410896	69.03243272
FAM151A	rs300275	222.1634558
FAM151A	rs7545297	734.3617967
FAM151A	rs79726097	32.45650822
FAM151A	rs4927191	36.02813823
FAM151A	rs952574	30.02978892
FAM151A	rs36086195	45.07953422
FAM151A	rs5030938	45.55366752
FAM151A	rs11830764	35.86798667
FAM151A	rs7970695	95.72592023
FAM151A	rs7150997	96.23939035
FAM151A	rs339969	37.34201039
FAM151A	rs4794058	101.4279369
FAM151A	rs4940691	43.99840164
FAM151A	rs55865752	30.22795924
FAM151A	rs4835263	44.11761022
FAM151A	rs10642257	39.65724441

NTS	rs35338528	188.7718589
NTS	rs9579135	34.98467086
NTS	rs2301151	44.47898922
NTS	rs35106244	31.88941943
NTS	rs3746778	56.85498151
NTS	rs6815054	71.70473286
NTS	rs9297994	32.38104199
PCDHGC3	rs4654748	56.78944213
PCDHGC3	rs1867585	29.85513455
PCDHGC3	rs57096150	39.67529835
PCDHGC3	rs35253049	53.06039813
PCDHGC3	rs117062157	32.16717931
PCDHGC3	rs241790	984.3532296
PCDHGC3	rs17719944	235.194711
PCDHGC3	rs16964261	207.6688341
PCDHGC3	rs543798	76.68903209
PCDHGC3	rs62066840	53.43521115
PCDHGC3	rs117978071	69.78028642
PCDHGC3	rs75048991	70.82725344
PCDHGC3	rs1260326	68.53660647
PCDHGC3	rs2427291	34.23356128
PCDHGC3	rs62265615	90.48979259
PCDHGC3	rs56210800	89.90327328
PCDHGC3	rs7658970	38.1874618
HERC4	rs10922094	424.0293765
HERC4	rs1555405	29.9816128
HERC4	rs12478841	30.68409397
HERC4	rs1706435	89.81527506
HERC4	rs550671	137.0228167
HERC4	rs28471056	63.00688108
HERC4	rs9272526	30.61813975
ROR2	rs4604505	168.5467696
ROR2	rs10820918	297.8583277
ROR2	rs76590235	65.44111561
ROR2	rs72704449	37.52706573
ROR2	rs34654815	42.94131392
PGRMC1	rs2251124	40.34475046
PGRMC1	rs6742078	33.06234898
PGRMC1	rs7625980	42.05983759
SIGLEC8	rs572515	481.0753053
SIGLEC8	rs17580	214.7087891
SIGLEC8	rs704	36.43904086
SIGLEC8	rs1047286	35.65025343
SIGLEC8	rs697357	75.9357018
SIGLEC8	rs9266766	33.47672684
SIGLEC8	rs2075798	54.59270897
SIGLEC8	rs550671	150.1920518
DNAJC30	rs2960972	31.10722129
DNAJC30	rs11544049	398.7526401
DNAJC30	rs1355538	42.77947803
DNAJC30	rs149784651	34.30311373
ST6GALNAC1	rs10305748	31.50741786
ST6GALNAC1	rs10922098	484.0921289
ST6GALNAC1	rs1047286	52.74193827
ST6GALNAC1	rs1707652	100.437128
ST6GALNAC1	rs1265941	58.3590372
ST6GALNAC1	rs453821	110.1605018
TMEM132A	rs118094284	121.2229539

TMEM132A	rs6591624	1139.772256
TMEM132A	rs75210230	77.79072915
TMEM132A	rs175111	304.8260532
TMEM132A	rs174472	134.9163633
TMEM132A	rs11037987	37.02337674
TMEM132A	rs79473175	31.59746694
TMEM132A	rs1488682	67.42604742
TMEM132A	rs72903900	30.80970149
TMEM132A	rs183627396	43.0847589
TMEM132A	rs117456719	38.18307977
TMEM132A	rs36130299	67.1641463
TMEM132A	rs74956044	33.09182935
TMEM132A	rs77542162	52.51700009
TMEM132A	rs1260326	47.66064708
TMEM132A	rs8176743	54.45338965
PLEK	rs1867312	204.5255751
PLEK	rs12992698	80.40834291
PLEK	rs1354034	32.75379778
SEC61B	rs10922098	106.3544508
SEC61B	rs9864801	39.78715949
SEC61B	rs415620	38.54016758
SEC61B	rs7774739	32.68387179
SEC61B	rs147168394	32.61808759
CYP3A4	rs115041442	31.30329501
CYP3A4	rs76418562	31.84308537
CYP3A4	rs1451915	33.16502864
CYP3A4	rs10922098	588.0247268
CYP3A4	rs1047286	35.72294133
CYP3A4	rs111696008	29.88682694
CYP3A4	rs2686395	103.4207272
CYP3A4	rs550671	160.6421271
CYP3A4	rs28471056	56.35815016
PSMA1	rs10922098	718.2840074
PSMA1	rs3900909	37.57254872
PSMA1	rs1555405	32.75991837
PSMA1	rs704	107.8061259
PSMA1	rs1047286	35.61007068
PSMA1	rs111696008	31.23367338
PSMA1	rs1707652	138.1592085
PSMA1	rs3828879	34.74284891
PSMA1	rs1265941	78.7222769
PSMA1	rs453821	192.6485558
PSMA1	rs9276578	30.25668131
PARP16	rs10922094	146.7550071
PARP16	rs964184	44.58752364
PARP16	rs704	43.98122508
PARP16	rs12478841	36.17584181
PARP16	rs1619994	85.83203869
PARP16	rs5030049	63.37405351
PARP16	rs6457457	43.78601583
PARP16	rs9501393	61.7135555
SPTLC1	rs5112	30.70197915
SPTLC1	rs17713196	119.1926133
SPTLC1	rs3863087	50.27609809
SPTLC1	rs17777164	32.02093572
SPTLC1	rs116370523	42.95762822
COA3	rs10922098	675.5909779
COA3	rs3900909	37.12329028

COA3	rs1047286	42.42477523
COA3	rs2686395	112.8473145
COA3	rs1265941	80.0300821
COA3	rs453821	170.6646337
DPP10	rs10922094	70.96056008
DPP10	rs6542680	33.01857106
DPP10	rs3129773	29.80136222
UGT1A6	rs143066237	56.86537825
UGT1A6	rs58817302	50.60856708
UGT1A6	rs887829	756.028076
UGT1A6	rs10822156	46.31655312
UGT1A6	rs7139079	50.75420699
UGT1A6	rs112963922	54.01365096
UGT1A6	rs7238784	30.52691077
MRAP	rs10922098	227.5518379
MRAP	rs6542680	40.85911241
MRAP	rs9864801	33.32908915
MRAP	rs453821	63.38819107
MRAP	rs28675927	44.34949332
DLG3	rs10908505	128.2475069
DLG3	rs10922098	39.33036014
DLG3	rs114694170	367.9651475
DLG3	rs74360467	35.78407825
DLG3	rs111432261	30.4842659
DLG3	rs9494145	32.24760447
DLG3	rs342295	43.90324532
DLG3	rs34826779	88.78863359
DLG3	rs4962034	91.15530988
MDM1	rs2294081	41.91436861
MDM1	rs3824949	199.8413125
MDM1	rs10839067	34.01596508
MDM1	rs45488900	31.43383252
MDM1	rs1354034	77.57482167
MDM1	rs342292	45.70214857
VAMP3	rs10922098	109.9598938
VAMP3	rs9864801	31.49050039
VAMP3	rs415620	41.22638943
VAMP3	rs4713498	38.27710381
VAMP3	rs67399078	31.43812799
HP	rs71391409	960.6441762
HP	rs12925078	1403.095136
HP	rs112291945	377.5476073
HP	rs77080601	603.7432022
HP	rs188257868	280.357136
HP	rs1837095	31.77390007
HP	rs142504786	32.8972104
HP	rs200914232	51.79834894
HP	rs78175040	31.62047251
HP	rs78581868	44.19838848
HP	rs75708868	45.4705097
HP	rs2168608	1036.171177
HP	rs1818437	142.523633
HP	rs67632703	91.77070249
HP	rs12975366	97.06068625
HP	rs1260326	46.53626429
LRFN1	rs1451915	38.65325739
LRFN1	rs10922098	677.9973859
LRFN1	rs1047286	45.08642111

LRFN1	rs111696008	31.26286037
LRFN1	rs2686395	124.2193585
LRFN1	rs4293988	29.83452826
LRFN1	rs555007	67.4444594
LRFN1	rs550671	163.5772606
LRFN1	rs9276606	30.60088144
SCN4B	rs10922098	639.745131
SCN4B	rs35351085	32.62280101
SCN4B	rs1047286	63.70756763
SCN4B	rs11720167	99.87564116
SCN4B	rs3863087	40.06124452
SCN4B	rs550671	172.0059043
SCN4B	rs9270570	42.7077851
SCN4B	rs9469195	70.47966995
S100A7	rs34961571	1221.326293
S100A7	rs3014824	2798.263082
S100A7	rs115200978	4031.378974
S100A7	rs35402075	228.6663834
S100A7	rs78987818	32.92595736
S100A7	rs78024109	69.02604638
S100A7	rs12117121	43.91543636
S100A7	rs150207136	55.74584987
S100A7	rs143962097	68.56387857
S100A7	rs189959042	124.1909889
S100A7	rs10639543	67.32654231
S100A7	rs74566616	67.97073714
S100A7	rs12048392	59.47803209
S100A7	rs79364659	52.9815987
CPD	rs10922098	276.803667
CPD	rs1707652	113.1089159
CPD	rs9501600	68.07369022
AMY1A	rs140026476	245.262792
AMY1A	rs116274910	52.28498918
AMY1A	rs6676813	4597.231817
AMY1A	rs114922930	890.3614822
AMY1A	rs4434842	962.8478836
AMY1A	rs141392442	213.0335137
AMY1A	rs2317401	228.265825
AMY1A	rs181103540	37.25930015
AMY1A	rs145121189	553.2002267
AMY1A	rs151015881	31.63239487
AMY1A	rs78282816	75.70752897
AMY1A	rs115456582	47.7372074
AMY1A	rs74374280	43.67685568
AMY1A	rs61816384	132.3581506
AMY1A	rs112063273	37.30387094
AMY1A	rs148587593	42.33316094
AMY1A	rs145950874	145.1188163
AMY1A	rs77538122	31.65396179
AMY1A	rs7556372	98.23195695
AMY1A	rs78705678	36.12676548
AMY1A	rs2227296	304.98888
AMY1A	rs34858945	88.99155389
AMY1A	rs4423274	84.21706314
AMY1A	rs77645215	46.59403433
AMY1A	rs10130078	37.41078971
AMY1A	rs848104	87.72828361
AMY1A	rs601338	139.049855

AMY1A	rs13186320	43.32479974
AMY1A	rs1482685	111.4015635
AMY1A	rs888686	76.81455559
AMY1A	rs12111617	40.03850232
AMY1A	rs635634	42.68933573
ST8SIA2	rs10922094	132.7317537
ST8SIA2	rs4713498	39.91066804
ST8SIA2	rs67399078	45.361725
FJX1	rs474415	159.0154435
FJX1	rs10768174	465.4832337
FJX1	rs12574547	159.9525666
FJX1	rs12576371	58.96581157
FJX1	rs643785	36.6153424
FJX1	rs629301	713.6096529
FJX1	rs11110316	31.7016814
FJX1	rs7964859	208.1063738
FJX1	rs140624338	35.88486572
FJX1	rs11065979	65.82929904
FJX1	rs143875230	38.22616261
FJX1	rs3116995	44.43368432
ADM	rs10922094	149.6504223
ADM	rs1706435	37.95104231
ADM	rs28573154	44.41776007
ADM	rs28471056	30.59237978
SEMA4C	rs56065109	198.8235245
SEMA4C	rs704	148.7792989
SEMA4C	rs17261391	42.59604724
SEMA4C	rs76385116	85.45512095
SEMA4C	rs111735822	47.80123011
SEMA4C	rs72836497	33.57103631
SEMA4C	rs144069380	96.83091721
SEMA4C	rs114993002	126.7568264
SEMA4C	rs112907540	74.71975674
SEMA4C	rs115334553	572.9535137
SEMA4C	rs181277928	502.9907786
SEMA4C	rs115978648	572.7323492
SEMA4C	rs184888994	105.0822552
SEMA4C	rs79270856	83.81430449
SEMA4C	rs193261272	33.46147918
SEMA4C	rs9264669	33.95388313
SEMA4C	rs187099314	182.7089525
SPINT3	rs6017583	1269.421321
SPINT3	rs2200587	1049.667023
SPINT3	rs197674	80.75373292
SPINT3	rs112379552	55.85640604
TPST1	rs7811214	936.0666786
TPST1	rs117456949	133.849421
TPST1	rs28549287	38.24244721
TPST1	rs4846923	43.06224758
TPST1	rs2074311	31.56597558
TPST1	rs174528	34.79832925
TPST1	rs2622935	30.91915908
TPST1	rs12438878	41.88325161
TPST1	rs11075921	49.98137227
TPST1	rs704	69.03854628
TPST1	rs1800961	33.52100662
TPST1	rs56210800	67.48829415
TPST1	rs17282078	68.23399735

TPST1	rs1880431	39.19716643
TPST1	rs75757921	43.61488409
TPST1	rs11773474	33.40940527
TPST1	rs73130138	31.50425885
TPST1	rs73124197	69.95706057
TPST1	rs138004235	56.24681655
TPST1	rs887011	34.1764731
TPST1	rs73132551	51.78229908
TPST1	rs113919461	139.2468103
TPST1	rs74476624	94.07062518
TPST1	rs200379036	84.95959829
TPST1	rs28601761	71.8280794
TPST1	rs72773785	31.89414878
EXOSC1	rs368465	77.02659477
EXOSC1	rs2808466	37.15923868
EXOSC1	rs6542680	40.84416755
EXOSC1	rs1706435	32.83174486
EXOSC1	rs6457457	33.90882973
EXOSC1	rs557011	31.40287619
SYT8	rs10922098	149.1367152
SYT8	rs1706435	54.45502646
SYT8	rs78725664	31.50137412
SYT8	rs28573154	49.00235906
SYT8	rs7774739	39.35375207
ADAM22	rs139090951	95.99224856
ADAM22	rs80069206	39.86980814
ADAM22	rs12535512	1012.624408
ADAM22	rs35844181	571.8382215
ADAM22	rs12280645	37.02169982
ADAM22	rs7305932	72.70786703
ADAM22	rs12975366	245.9402864
ADAM22	rs62471676	84.72886438
ADAM22	rs11983946	40.37718426
ADAM22	rs887461	43.36990513
NDRG4	rs10922094	477.5225764
NDRG4	rs35351085	31.40767453
NDRG4	rs10208529	30.0918848
NDRG4	rs1707652	125.4203322
NDRG4	rs9267139	33.41739379
NDRG4	rs9501393	112.4519083
NDRG4	rs7774739	76.34289359
NDRG4	rs9271683	36.97003088
CUZD1	rs10922098	125.8246615
CUZD1	rs74607435	33.44638638
CUZD1	rs199956232	40.50719016
CUZD1	rs429358	193.9014117
CUZD1	rs1707652	89.87773766
CUZD1	rs6457457	36.28679072
CUZD1	rs28573154	38.11138492
KIR3DL3	rs572515	74.71677564
KIR3DL3	rs9864801	35.32718444
KIR3DL3	rs9501393	33.19767292
SEMA6A	rs3984965	122.8009753
SEMA6A	rs11956300	30.1667587
SEMA6A	rs607350	66.41445297
SEMA6A	rs61801013	52.46910177
SEMA6A	rs61843998	67.76603889
SEMA6A	rs36040496	72.13020946

SEMA6A	rs78705678	247.5033308
SEMA6A	rs2477642	290.4615902
SEMA6A	rs35166255	31.64478392
SEMA6A	rs55714927	39.1753379
SEMA6A	rs7369864	64.08790092
SEMA6A	rs4857414	61.84462345
SEMA6A	rs9271325	41.91766972
SEMA6A	rs10901252	443.3994026
SEMA6A	rs3094379	171.5527061
GLTPD2	rs4523990	57.52485316
GLTPD2	rs8078118	493.8558993
GLTPD2	rs2304969	335.5455313
GLTPD2	rs150866751	38.97544938
GLTPD2	rs10740131	43.93293555
GLTPD2	rs77232416	31.64086594
GLTPD2	rs7232	290.184839
GLTPD2	rs10858851	104.6419395
GLTPD2	rs1169288	31.88308269
GLTPD2	rs147233090	72.75453498
GLTPD2	rs339969	35.99253134
GLTPD2	rs1260326	52.76752948
GLTPD2	rs1800961	31.40619246
GLTPD2	rs149062332	255.9410292
GLTPD2	rs13068263	36.73474184
GLTPD2	rs35491981	42.40141098
GLTPD2	rs62440901	34.0946208
GLTPD2	rs28601761	60.31562869
GLTPD2	rs1107080	62.2716965
BTNL9	rs10922094	85.4064694
BTNL9	rs1355538	42.41467898
BTNL9	rs3135461	74.48913473
BTNL9	rs10901252	47.78153205
SLAMF1	rs1801133	88.73361456
SLAMF1	rs11076612	36.31690416
SLAMF1	rs6458	67.58069716
SLAMF1	rs7749543	30.30113555
ITIH1	rs352157	1059.703104
ITIH1	rs36012032	258.4825196
ITIH1	rs62251870	170.0725639
ITIH1	rs57031794	35.07766542
ITIH1	rs150119278	34.0984212
ITIH1	rs112727502	101.5551844
ITIH1	rs77938199	453.7415747
ITIH1	rs114419941	50.00699534
ITIH1	rs1727	61.54629662
ITIH1	rs7970695	38.43321398
ITIH1	rs372434515	40.63853218
ITIH1	rs1260326	40.50774043
ITIH1	rs77819425	51.55605364
ITIH1	rs149100084	51.74935966
ITIH1	rs187630010	39.39506338
ITIH1	rs35703936	103.461539
ITIH1	rs114938769	74.56248287
ITIH1	rs62257485	53.42369262
ITIH1	rs1056522	238.3367845
ITIH1	rs2286276	39.5948105
ITIH1	rs28601761	36.57405815
SCG3	rs2470193	2676.998213

SCG3	rs34989450	118.278013
SCG3	rs76034856	58.08441175
SCG3	rs72704449	37.55118813
SCG3	rs8031813	77.70791274
SCG3	rs117739068	101.7638383
SCG3	rs11070971	36.64386898
SCG3	rs77924615	43.72216457
SCG3	rs1260326	33.28473845
SCG3	rs55879786	55.79067856
CDH7	rs12132412	46.8953733
CDH7	rs1569631	36.83566412
CDH7	rs1591734	255.9544492
CDH7	rs61808983	600.7482427
CDH7	rs41272457	90.33405441
CDH7	rs2239853	541.010658
CDH7	rs7894992	207.5824184
CDH7	rs10159712	568.6028685
CDH7	rs10437425	70.30185671
CDH7	rs11012153	50.56155773
CDH7	rs17580	32.02325394
CDH7	rs594793	45.21650401
CDH7	rs62140549	86.31847366
CDH7	rs149062332	35.1121233
CDH7	rs2270190	55.10597066
CDH7	rs3909111	31.79983239
CDH7	rs60378094	78.55765796
CDH7	rs28366220	118.8596069
CDH7	rs560530	226.2595618
POTEG	rs572515	115.2749393
POTEG	rs1801690	36.63733278
POTEG	rs1355538	42.35058299
POTEG	rs9501393	51.75619947
POTEG	rs7774739	39.21712094
POTEG	rs67399078	31.96909046
POTEM	rs10922098	69.15737269
POTEM	rs415620	52.3689278
POTEM	rs7774739	39.82242953
CRTAM	rs7925381	93.76690331
CRTAM	rs2370794	687.6454726
CRTAM	rs61904391	32.01296836
CRTAM	rs186822928	40.04603038
CRTAM	rs118117028	151.7995082
CRTAM	rs111925642	126.8832769
CRTAM	rs3184504	102.3046422
CRTAM	rs12945299	47.18908444
CRTAM	rs112058117	36.59076791
CRTAM	rs6752770	39.19941953
CRTAM	rs13063578	116.1269488
CRTAM	rs62401070	58.08215243
CRTAM	rs9468618	43.73930847
CRTAM	rs181816207	99.43978484
CRTAM	rs148844907	128.2751558
CRTAM	rs41268928	75.31261631
CRTAM	rs138826033	69.00650874
CRTAM	rs28986428	59.16825819
CRTAM	rs2075533	71.67321585
ART3	rs9993150	117.6318532
ART3	rs4859610	755.1706805

ART3	rs6856609	150.1510568
ART3	rs11728135	45.68807132
ART3	rs7681822	31.40955428
ART3	rs72704449	87.29072731
ART3	rs9318186	55.05999
ART3	rs540	46.68668454
ART3	rs77924615	60.02446782
ART3	rs1260326	43.00417017
ART3	rs9818393	30.94235497
ART3	rs75457531	30.4871074
ART3	rs12654812	36.24781458
ART3	rs737954	35.33586636
ART3	rs4962237	32.59613241
B3GNT2	rs10638897	44.61672585
B3GNT2	rs34012279	150.136973
B3GNT2	rs4073090	388.3986606
B3GNT2	rs78187929	30.79204936
B3GNT2	rs61804164	55.37949901
B3GNT2	rs78705678	135.7099651
B3GNT2	rs2477642	123.9047519
B3GNT2	rs10437655	39.65399768
B3GNT2	rs12986210	98.20857412
B3GNT2	rs11083616	915.5024337
B3GNT2	rs55997514	76.15041938
B3GNT2	rs4803517	63.90534215
B3GNT2	rs62110998	45.48042458
B3GNT2	rs281377	200.3686398
B3GNT2	rs9862378	33.01943142
B3GNT2	rs605783	37.53196287
B3GNT2	rs635634	449.778028
B3GNT2	rs11244083	55.06623545
B3GALT6	rs6697886	38.74917498
B3GALT6	rs10922094	148.6091098
B3GALT6	rs1706435	39.97496278
B3GALT6	rs28573154	37.95906675
B3GALT6	rs7774739	34.63160223
B3GALT6	rs6993770	32.05833179
MRPL32	rs11208685	33.35297605
MRPL32	rs10922098	56.01122796
MRPL32	rs9271642	33.81388186
PCDHGC5	rs10922094	81.9019148
PCDHGC5	rs1355538	30.35783351
PCDHGC5	rs147906938	34.7374731
NAALAD2	rs855791	61.42152639
NAALAD2	rs112667192	41.60322438
NAALAD2	rs75858866	60.79464373
NAALAD2	rs74357662	1150.578265
NAALAD2	rs185357408	266.8604649
NAALAD2	rs1105878	133.0982713
NAALAD2	rs73219477	29.78685728
NAALAD2	rs12488908	60.4117852
NAALAD2	rs147023857	39.79982476
NAALAD2	rs202240117	44.04109363
NAALAD2	rs144861591	587.6894252
NAALAD2	rs73391153	29.96921781
NAALAD2	rs7196	42.85267034
NAALAD2	rs73403125	32.13105247
C1orf226	rs34924625	49.1144996

C1orf226	rs73579049	31.72854886
C1orf226	rs12924886	56.1996964
C1orf226	rs117051919	123.0786426
C1orf226	rs116929468	29.76983367
C1orf226	rs211836	37.85087036
C1orf226	rs2523492	33.89717635
C1orf226	rs71534519	49.08644118
C1orf226	rs1687391	156.8185286
C1orf226	rs116965083	40.98014585
IQCF1	rs1697422	38.55034584
IQCF1	rs10922098	322.5613681
IQCF1	rs10208529	45.14455989
IQCF1	rs56210800	35.79543444
IQCF1	rs140431012	35.79543444
IQCF1	rs1355538	69.54396369
IQCF1	rs9501393	93.03476181
IQCF1	rs7774739	56.76821814
IQCF1	rs67965605	34.24395077
ASTL	rs368465	79.82601564
ASTL	rs6542680	48.48436373
ASTL	rs6457457	31.73296021
VWC2L	rs10922094	43.98790629
VWC2L	rs1355538	31.67841991
VWC2L	rs6457457	46.45958017
VWC2L	rs9501393	36.48432891
ENTPD1	rs7072953	315.2080392
ENTPD1	rs189315256	48.11235526
ENTPD1	rs28378465	71.06189978
ENTPD1	rs35617250	53.43182883
ENTPD1	rs78705678	45.10771756
ENTPD1	rs2477642	42.95741017
ENTPD1	rs78689694	30.21535646
ENTPD1	rs12945299	96.48868216
ENTPD1	rs143501783	46.96557186
ENTPD1	rs3787938	45.86504211
ENTPD1	rs35098355	136.0804832
ENTPD1	rs11920688	42.43530573
ENTPD1	rs2623449	58.39718382
ENTPD1	rs4639017	34.05052181
ENTPD1	rs9272310	86.47036014
ENTPD1	rs4713570	29.9486014
ENTPD1	rs1042133	58.11233514
ENTPD1	rs187099314	134.9644041
ENTPD1	rs3094379	33.35927281
TMPRSS5	rs10922098	180.0351246
TMPRSS5	rs1355538	38.69474732
TMPRSS5	rs6457457	45.62845089
TMPRSS5	rs9501393	60.31989776
MXRA7	rs11870168	59.95881311
MXRA7	rs9896226	149.1063658
MXRA7	rs9900613	1127.345396
MXRA7	rs77929614	59.98124617
MXRA7	rs77924615	33.56990018
MXRA7	rs8075793	34.72356814
MXRA7	rs1355538	39.71887861
MXRA7	rs72813185	30.71777664
MXRA7	rs184358099	30.79038929
MXRA7	rs66962766	34.12993033

DNAJB12	rs9416018	92.39066171
DNAJB12	rs77924615	38.10179206
DNAJB12	rs2974355	225.0939382
CTSB	rs4557666	77.55611229
CTSB	rs35657205	135.4997839
CTSB	rs73201446	914.1261727
CTSB	rs56213203	958.8830766
CTSB	rs144176129	71.65950146
CTSB	rs1293308	726.8230571
CTSB	rs55751927	35.70771959
CTSB	rs7869	44.94250564
CTSB	rs7130812	31.89964093
CTSB	rs7964859	122.631187
CTSB	rs73001065	45.17478556
CTSB	rs9272154	41.00814439
CTSB	rs113853613	49.97295569
CTSB	rs4307365	101.3580784
CTSB	rs75528075	52.81161599
CTSB	rs137869729	46.476393
HRK	rs572515	234.5795832
HRK	rs6542680	36.80377435
HRK	rs1707652	49.78936614
HRK	rs9501393	70.53962354
HRK	rs7774739	55.20352618
HRK	rs9271766	30.76807507
SURF1	rs1591734	99.6259088
SURF1	rs61808983	386.3566305
SURF1	rs6670678	282.8499136
SURF1	rs7894992	95.44702768
SURF1	rs10159712	251.4372075
SURF1	rs10437425	35.96701795
SURF1	rs11012153	37.32039766
SURF1	rs117606766	48.53491779
SURF1	rs548589	54.43169876
SURF1	rs2956634	876.7585143
SURF1	rs11024589	30.113483
SURF1	rs146593335	39.39288683
SURF1	rs964184	56.91142539
SURF1	rs71352239	190.9956953
SURF1	rs12053441	34.80569756
SURF1	rs2668202	80.00676087
SURF1	rs3130685	60.62626136
SURF1	rs41268896	160.8395222
SURF1	rs370635202	137.2977759
SURF1	rs9269271	36.21745555
MANEA	rs2501992	1136.767531
MANEA	rs17711202	5721.667371
MANEA	rs34463787	9326.617296
MANEA	rs9403054	3760.022168
MANEA	rs72704449	34.29630086
MANEA	rs76935248	30.6768269
MANEA	rs9922160	29.97240104
MANEA	rs12975366	55.43566282
MANEA	rs2460698	35.94299648
MANEA	rs12201004	32.14803176
MANEA	rs72895471	42.77241845
MANEA	rs4552704	91.51483163
MANEA	rs6937033	301.5799207

MANEA	rs74726105	329.3461121
MANEA	rs55785722	38.95623359
MANEA	rs1451341	126.7067614
MANEA	rs76285575	144.0239906
MANEA	rs9373797	33.35540988
MANEA	rs463854	35.23770105
MANEA	rs6993770	33.61274079
MANEA	rs635634	57.00182693
DNAJC4	rs10922098	204.5811518
DNAJC4	rs1706435	65.7755148
DNAJC4	rs550671	69.5886652
DNAJC4	rs28645249	37.64797184
FOXRED1	rs10922098	383.6835312
FOXRED1	rs34698754	30.13884129
FOXRED1	rs1047286	36.54670009
FOXRED1	rs2686395	114.9596824
FOXRED1	rs9267139	34.76440321
FOXRED1	rs3891366	113.5793108
FOXRED1	rs7774739	73.74887348
VSIG2	rs11867511	36.11052105
VSIG2	rs7214750	1229.066592
VSIG2	rs4791070	251.1990534
VSIG2	rs113485183	60.50549179
VSIG2	rs77311729	34.2905033
VSIG2	rs9271636	54.99360548
STMN3	rs4759076	75.03630988
STMN3	rs6436128	33.35308939
STMN3	rs1354034	105.3861762
TPST2	rs2283824	302.211504
TPST2	rs77332738	30.91728799
TPST2	rs2622935	133.1102995
TPST2	rs6416742	29.85143252
TPST2	rs35495893	46.69699184
TPST2	rs2844622	32.61045423
TPST2	rs72673751	38.24048251
TPST2	rs635634	357.5109312
SPINK5	rs11168015	55.08798732
SPINK5	rs1422994	207.4731675
SPINK5	rs78924983	33.43062374
SPINK5	rs77924615	32.594558
SPINK5	rs55714927	35.56114026
SPINK5	rs11751495	37.16773097
SPINK5	rs7823957	30.21421704
DNAJC18	rs4977155	76.8815568
DNAJC18	rs7576170	32.41483859
DNAJC18	rs67399078	41.98449466
DRGX	rs7546274	29.79191292
DRGX	rs61801570	61.51002668
DRGX	rs111605355	38.34165672
DRGX	rs10800475	38.10308843
DRGX	rs10800507	411.7135029
DRGX	rs61826205	1244.410069
DRGX	rs6427276	123.8738768
DRGX	rs2050151	284.9357682
DRGX	rs1602244	77.62763203
DRGX	rs12734872	34.10028441
DRGX	rs528298	56.51410266
C1orf198	rs56148170	143.7598276

C1orf198	rs4414033	32.67504578
C1orf198	rs10876550	31.10558266
FSHB	rs572515	149.8607233
FSHB	rs114115200	33.56421427
FSHB	rs9501393	62.16969391
FSHB	rs7774739	37.56651749
NSDHL	rs188023612	51.0361224
NSDHL	rs17441745	48.41606308
NSDHL	rs6542680	950.8723389
NSDHL	rs80220851	56.43279796
NSDHL	rs3105782	29.95629035
NSDHL	rs28366281	56.42132775
FAM177A1	rs113731836	808.4454984
FAM177A1	rs76204064	1029.697524
FAM177A1	rs79036977	69.99860011
FAM177A1	rs74654111	66.82716859
FAM177A1	rs2956634	50.68611837
FAM177A1	rs139884204	52.66026406
FAM177A1	rs9646145	63.00594827
FAM177A1	rs4553500	71.73503452
FAM177A1	rs117981331	93.45112151
FAM177A1	rs9939224	151.7673201
FAM177A1	rs116843064	31.69985185
FAM177A1	rs72654473	73.13067745
FAM177A1	rs34953075	59.68689011
FAM177A1	rs679574	956.0474791
FAM177A1	rs35320397	43.72756938
FAM177A1	rs1822200	30.28054698
FAM177A1	rs635634	246.3662022
FAM177A1	rs41307428	39.36190331
LEMD1	rs188066772	31.38042692
LEMD1	rs80182784	59.66910562
LEMD1	rs10922094	75.62887597
LEMD1	rs4639017	32.31971046
SPINK9	rs9731301	88.42854711
SPINK9	rs2664904	96.54799853
SPINK9	rs61730306	35.61105629
SPINK9	rs117268623	34.76943367
SPINK9	rs13250710	121.0762473
SPINK9	rs9297543	39.3804894
COMP	rs12974746	609.245732
COMP	rs115860110	33.40710096
COMP	rs4233367	106.4588548
COMP	rs78705678	163.1215485
COMP	rs2461161	131.9319075
COMP	rs117205342	31.38932255
COMP	rs181707610	31.78127795
COMP	rs55714927	54.68159246
COMP	rs150174856	41.39766196
COMP	rs12609373	30.73516654
COMP	rs11899888	34.69312048
COMP	rs7649094	34.86724033
COMP	rs9851987	73.85311593
KIR3DL3	rs2317230	35.53966484
KIR3DL3	rs10922098	80.61838095
KIR3DL3	rs55650306	30.28267548
KIR3DL3	rs1706435	33.94274977
KIR3DL3	rs79473311	68.46442999

LGALS1	rs113448098	66.85089205
LGALS1	rs7183	212.8820618
LGALS1	rs34295723	45.53919206
LGALS1	rs2237582	37.93021492
FTMT	rs4666000	33.47810844
FTMT	rs7752746	49.22852191
FTMT	rs138215943	75.65395128
FTMT	rs60194987	46.44639569
FTMT	rs2023473	988.4321592
FTMT	rs28752924	628.9254941
FTMT	rs9469051	996.1284791
FTMT	rs62404825	420.4220671
FTMT	rs8192569	130.6006066
FTMT	rs114446488	171.3053509
FTMT	rs1465651	124.7582985
DGCR2	rs10922098	50.53676301
DGCR2	rs34240868	30.05471392
DGCR2	rs6542680	35.68787938
THPO	rs10922098	137.2651777
THPO	rs1707652	89.63675405
THPO	rs9267655	37.86768536
THPO	rs4713498	52.39209774
THPO	rs6993770	62.505254
EVA1B	rs10922098	247.6010109
EVA1B	rs11720167	47.63566242
EVA1B	rs1042663	55.95711247
PVR	rs10922094	63.70003392
PVR	rs6792369	155.1089115
PVR	rs9501393	59.29268617
PVR	rs7774739	42.06119786
PATE4	rs71467539	54.99034254
PATE4	rs665677	196.2236982
PATE4	rs10922098	50.30772026
SYT9	rs10922098	96.82326553
SYT9	rs4711283	34.3479041
SYT9	rs71549272	50.82874997
KCNE3	rs10922094	215.685034
KCNE3	rs2686395	68.04443051
KCNE3	rs3828879	38.00134759
KCNE3	rs550671	54.17285424
CD3E	rs4654748	44.9678353
CD3E	rs646441	36.85186895
CD3E	rs1591734	116.9241241
CD3E	rs9332701	580.6437059
CD3E	rs6677374	322.0822241
CD3E	rs3917765	44.60994403
CD3E	rs60642321	36.59866727
CD3E	rs10764161	152.4898264
CD3E	rs10159712	415.6525543
CD3E	rs10437425	52.56904159
CD3E	rs11012153	32.04695153
CD3E	rs594793	35.61262925
CD3E	rs1051317	77.08779025
CD3E	rs3934504	30.23661693
CD3E	rs9257052	31.08293672
CD3E	rs34568880	50.55012982
CD3E	rs28366220	113.6080622
CD3E	rs34958241	232.5270164

CD3E	rs9271669	93.63186639
CD3E	rs80189144	34.43936256
PCDHB7	rs10922098	321.2325961
PCDHB7	rs1047286	32.44567247
PCDHB7	rs697356	61.51132049
PCDHB7	rs555007	45.98943609
PCDHB7	rs550671	91.23658318
TMEM70	rs2317231	54.30011611
TMEM70	rs73073557	76.07633526
TMEM70	rs10406069	57.16550572
TMEM70	rs16864367	62.4562795
TMEM70	rs1265888	63.12198635
TMEM70	rs116240177	77.80280063
TMEM70	rs4707609	58.08261641
SPACA3	rs704	54.79584476
SPACA3	rs1126477	216.9144058
SPACA3	rs28895267	77.85637494
SPACA3	rs60378094	49.94611791
MSMP	rs10758322	55.07559549
MSMP	rs35451914	36.28361166
MSMP	rs34315214	58.72421299
MSMP	rs7902581	98.97759427
MSMP	rs72979233	233.3749873
MSMP	rs9316871	56.72265615
MSMP	rs71149134	35.52127793
MSMP	rs2247261	36.77462423
MSMP	rs34125642	35.39896489
MSMP	rs59345518	33.26900314
MSMP	rs6547787	59.26003987
MSMP	rs78862061	52.34716795
MSMP	rs2130499	40.28150671
MSMP	rs77249799	34.68514744
MSMP	rs10270210	43.99040109
MSMP	rs7836359	31.13386114
MSMP	rs4740734	45.90776488
MSMP	rs1598307	187.6851316
MSMP	rs515380	191.2625117
PLD5	rs2304200	31.38759302
PLD5	rs371314787	40.35545573
PLD5	rs557011	33.96443337
ITM2B	rs660240	43.5903983
ITM2B	rs1321257	32.7156467
ITM2B	rs964184	240.6855539
ITM2B	rs141469619	29.9715068
ITM2B	rs3816117	129.5496616
ITM2B	rs78606665	72.5120598
ITM2B	rs116843064	59.59131865
ITM2B	rs2231935	97.75260062
ITM2B	rs7412	138.7884032
ITM2B	rs34810028	30.80328625
ITM2B	rs520354	270.1825671
ITM2B	rs10189499	29.99821061
ITM2B	rs111696008	85.15384885
ITM2B	rs144202860	45.16260529
ITM2B	rs113414093	30.43052417
ITM2B	rs1618069	44.76322426
ITM2B	rs12485327	32.09919232
ITM2B	rs6445035	669.6085394

ITM2B	rs3863087	261.8627102
ITM2B	rs148077724	69.79059106
ITM2B	rs111266377	57.96452904
ITM2B	rs9271543	32.95092023
ITM2B	rs9272195	45.61105151
ITM2B	rs10096633	133.4710105
ITM2B	rs2954038	61.19669236
RNF13	rs929130	114.97243
RNF13	rs704	112.5716971
RNF13	rs59123177	59.02802589
C1orf210	rs10922094	33.28213997
C1orf210	rs773316	35.7750172
C1orf210	rs10138174	39.60931771
CISD2	rs368465	94.30817718
CISD2	rs9864801	31.9071084
CISD2	rs6457457	33.89256434
CISD2	rs9501393	31.05548273
LIPN	rs17109158	102.4372415
LIPN	rs117983161	193.972201
LIPN	rs2576155	1248.928843
LIPN	rs1238449	371.619765
LIPN	rs913042	660.897665
LIPN	rs9970784	46.88599111
LIPN	rs265492	35.72413068
LIPN	rs7918518	76.75821833
LIPN	rs186488150	167.6738982
LIPN	rs7095217	260.0507693
LIPN	rs78866702	115.8100948
LIPN	rs200227455	37.45885656
LIPN	rs142253602	42.60395064
LIPN	rs28475163	32.45780083
LIPN	rs10403676	39.59378644
LIPN	rs3746638	30.11517612
LIPN	rs6065904	31.55279588
LIPN	rs11720167	190.5246869
LIPN	rs3863087	67.6952398
TBXAS1	rs10922098	46.66925346
TBXAS1	rs11632987	96.31162152
TBXAS1	rs7172696	51.36223012
TBXAS1	rs704	30.57480033
TBXAS1	rs68066031	91.16282951
TBXAS1	rs28455809	32.87901696
SPON2	rs73217579	52.99626029
SPON2	rs11510471	950.9364572
SPON2	rs139033323	180.9877544
SPON2	rs7186908	37.00966119
ADM2	rs8142906	181.9440574
ADM2	rs16830188	31.36265911
ADM2	rs74093681	36.34475501
ADM2	rs10135066	87.00374105
ADM2	rs75397818	56.99517965
ADM2	rs9271378	34.16187416
IL10RA	rs10922098	210.589238
IL10RA	rs1707652	34.64094152
IL10RA	rs2266161	29.90695693
IL10RA	rs9501393	61.61074274
IL10RA	rs7774739	44.20714738
SSR1	rs10922094	86.33403917

SSR1	rs1260326	38.1801644
SSR1	rs1250259	109.1240889
SSR1	rs147906938	47.26087755
CDHR3	rs10922098	41.31959794
CDHR3	rs7084817	171.5072158
CDHR3	rs71515486	36.68174058
CDHR3	rs112130428	49.51810214
CDHR3	rs2284351	75.7139589
EPHB2	rs28455953	942.864177
EPHB2	rs6686906	133.3380198
EPHB2	rs72655556	36.70847627
EPHB2	rs417688	30.35567888
EPHB2	rs72704449	38.88154778
EPHB2	rs7952602	34.84798789
EPHB2	rs12945299	51.3736654
EPHB2	rs143501783	38.06381419
GXYLT1	rs117903281	293.6583414
GXYLT1	rs75586953	732.2292222
GXYLT1	rs113355147	44.85911493
GXYLT1	rs7960537	62.59547822
GXYLT1	rs190407775	2432.65516
GXYLT1	rs61940179	55.76642463
GXYLT1	rs7302324	36.45999009
GXYLT1	rs28549287	37.54294756
GXYLT1	rs10883451	35.14457996
GXYLT1	rs97384	42.40415941
GXYLT1	rs118141779	120.6500432
GXYLT1	rs183286794	65.37149795
GXYLT1	rs139485922	102.7810316
GXYLT1	rs144355501	134.6800802
GXYLT1	rs2706333	37.725094
GXYLT1	rs77827196	102.7941936
GXYLT1	rs825038	99.56217051
GXYLT1	rs4764822	1199.922978
GXYLT1	rs140624338	115.9309599
GXYLT1	rs56272941	38.14545349
GXYLT1	rs956886	40.25973486
GXYLT1	rs139974673	84.66169381
GXYLT1	rs2532240	44.92243953
GXYLT1	rs891488	32.88227647
GXYLT1	rs351973	31.75163464
GXYLT1	rs8736	102.4816722
GXYLT1	rs1711171	30.61802393
FLT1	rs56728557	32.80861539
FLT1	rs7098181	44.8530611
FLT1	rs10513546	45.07593485
FLT1	rs114694170	40.7044274
FLT1	rs10807291	42.52414226
FLT1	rs13212731	37.54682899
FLT1	rs4426996	96.51348286
FLT1	rs7739450	1383.37714
FLT1	rs9472175	53.05639984
FLT1	rs6993770	88.66641626
FLT1	rs2375981	128.1319243
ITIH5	rs12573122	48.27433445
ITIH5	rs191234395	129.6724337
ITIH5	rs12765303	74.55258897
ITIH5	rs10752113	947.6615569

ITIH5	rs7902103	1084.281352
ITIH5	rs79051267	52.20603974
ITIH5	rs118063690	60.36853084
ITIH5	rs74124181	49.27496708
ITIH5	rs35801422	33.65145861
ITIH5	rs10789510	63.18340249
ITIH5	rs61830291	39.43598901
ITIH5	rs78705678	181.8277435
ITIH5	rs2477642	173.4382324
ITIH5	rs137927952	63.25515691
ITIH5	rs7952602	31.76935275
ITIH5	rs28616296	66.00960245
ITIH5	rs34975687	32.86612833
ITIH5	rs760715	116.1482221
ITIH5	rs2413595	36.61333896
ITIH5	rs576123	37.26975691
CHGB	rs36054946	68.2162199
CHGB	rs2163150	34.35860996
CHGB	rs77924615	38.77030863
CHGB	rs2848657	41.17438118
CHGB	rs73970948	31.50953381
CHGB	rs71148170	29.95011297
CHGB	rs2668202	55.71097616
CHGB	rs611349	60.88987665
CHGB	rs79535801	39.14074275
CHGB	rs55748670	157.1843614
CHGB	rs71510684	118.1865389
FUT8	rs77221370	39.84703935
FUT8	rs141178967	88.61772564
FUT8	rs151070677	1081.425392
FUT8	rs116965662	1163.405495
FUT8	rs190679746	3217.69054
FUT8	rs77515250	685.6505071
FUT8	rs141929124	203.5265474
FUT8	rs147253319	103.2765868
FUT8	rs11845739	333.7150743
FUT8	rs12590951	314.8784739
FUT8	rs11620883	33.57335328
FUT8	rs117180235	42.2339854
FUT8	rs1205087	41.47511471
FUT8	rs2735832	29.8347647
FUT8	rs2002739	32.16529074
FUT8	rs12986064	40.8766934
FUT8	rs113661219	29.74437499
FUT8	rs6993770	99.66622565
ICAM5	rs16982090	44.76536168
ICAM5	rs62104333	81.1705102
ICAM5	rs9749467	97.85373015
ICAM5	rs78862777	74.50520529
ICAM5	rs3093035	1090.438619
ICAM5	rs8101473	4373.513413
ICAM5	rs10417443	159.0663819
ICAM5	rs72658879	140.5921307
ICAM5	rs78689694	46.10696461
ICAM5	rs77542162	336.9415257
ICAM5	rs6502002	130.1652646
ICAM5	rs77293962	55.16695099
ICAM5	rs146798134	29.9063607

ICAM5	rs635634	802.7716216
ICAM5	rs118112075	31.39541414
SIGLEC14	rs2062655	45.34088538
SIGLEC14	rs112062715	163.342895
SIGLEC14	rs3810110	914.4306585
SIGLEC14	rs2864138	418.4344829
SIGLEC14	rs3829655	1288.472186
SIGLEC14	rs8112223	362.3094683
SIGLEC14	rs12462691	169.6778576
SIGLEC14	rs62110409	112.4928941
SIGLEC14	rs55952958	53.77368527
SIGLEC14	rs3184504	39.07998636
ALK	rs10922098	121.3893122
ALK	rs1707652	63.63860714
ALK	rs550671	43.74390626
PTPRJ	rs79905736	70.08513191
PTPRJ	rs78239375	40.90650748
PTPRJ	rs191507130	119.9263467
PTPRJ	rs72896749	29.86432071
PTPRJ	rs61889535	33.71087876
PTPRJ	rs61889920	32.77481126
PTPRJ	rs55976823	99.32701124
PTPRJ	rs12282853	60.6387049
PTPRJ	rs72914272	56.21903555
PTPRJ	rs117807766	29.83435749
PTPRJ	rs78689694	41.95589416
PTPRJ	rs143959838	31.07664059
NOTUM	rs149820034	42.44667166
NOTUM	rs113928649	249.1359837
NOTUM	rs72861729	34.83179332
NOTUM	rs181242111	29.8452891
NOTUM	rs56404419	30.8316743
NOTUM	rs1260326	121.6805567
NOTUM	rs853966	40.6070839
NOTUM	rs6975495	61.95735784
NOTUM	rs11781006	31.13424575
POMGNT1	rs572515	134.3802764
POMGNT1	rs451652	33.39471789
POMGNT1	rs4713498	44.41997753
UXS1	rs7599703	57.02759901
UXS1	rs78870678	164.916565
UXS1	rs4980320	33.38271346
UXS1	rs1149607	119.70045
UXS1	rs11553699	39.13417018
UXS1	rs13412535	79.34630641
UXS1	rs4699154	31.62583467
UXS1	rs150614636	32.1434214
UXS1	rs2206964	51.99924698
UXS1	rs9494145	36.42388219
UXS1	rs199772122	75.48061207
UXS1	rs6993770	74.69918038
UXS1	rs651007	33.20350699
STOM	rs10922094	78.69138025
STOM	rs964184	35.15556449
STOM	rs438811	49.57589497
STOM	rs34762726	80.85919717
STOM	rs11720167	95.70034024
STOM	rs3863087	37.30942406

TMPO	rs7080536	36.82568152
TMPO	rs653178	36.31084757
TMPO	rs1354034	52.50700654
HS3ST3A1	rs7086057	131.2948949
HS3ST3A1	rs4980320	30.21224494
HS3ST3A1	rs4963	75.96134644
ARSK	rs62365862	88.7421128
ARSK	rs10922098	77.20637341
ARSK	rs10822155	46.58738182
ARSK	rs12813762	182.0280501
ARSK	rs4883201	43.57795446
ARSK	rs7964859	216.658913
ARSK	rs140624338	37.62309974
ARSK	rs34762726	35.08261945
ARSK	rs1354034	56.25580427
ARSK	rs1355538	37.95464449
ARSK	rs114300458	33.07175301
ARSK	rs139102439	35.14497858
ARSK	rs13208583	30.94138462
IL31RA	rs10922098	116.2262508
IL31RA	rs17580	30.60887156
IL31RA	rs9501393	43.11738806
IL31RA	rs28675927	38.16233556
STX7	rs3813356	201.8967834
STX7	rs5029990	34.90876903
STX7	rs342292	32.15555169
PEAR1	rs114346493	42.93295679
PEAR1	rs140477301	41.10383961
PEAR1	rs4661012	498.8940888
PEAR1	rs17490626	46.1410404
PEAR1	rs60843925	45.20221285
PEAR1	rs12795076	58.38387155
PEAR1	rs2230283	44.88338721
PEAR1	rs7310615	130.6355773
PEAR1	rs1555405	40.51675713
PEAR1	rs56115403	40.77788595
PEAR1	rs41556717	30.66584966
PEAR1	rs2413593	37.32173829
PEAR1	rs1354034	61.26503093
PEAR1	rs7776054	37.39052938
PEAR1	rs12005199	49.75931836
PEAR1	rs62578578	43.98435993
PEAR1	rs75179845	758.736448
PEAR1	rs3124761	103.9202203
APOH	rs75003668	2371.741323
APOH	rs9912634	235.8810537
APOH	rs111481700	133.8466304
APOH	rs145128839	33.71031013
GPNMB	rs140966178	89.98000944
GPNMB	rs10250602	714.673664
GPNMB	rs79914289	167.5522958
GPNMB	rs75988976	48.32067996
GPNMB	rs12576761	31.92254145
GPNMB	rs56344867	80.29702999
GPNMB	rs12945299	30.49635291
GPNMB	rs77262773	30.86922156
GPNMB	rs116357733	35.12862816
GPNMB	rs12203592	36.34924178

GPNMB	rs702814	59.63747286
GPNMB	rs2737212	44.8489638
GPNMB	rs12350739	33.16535263
DNAJC10	rs34121158	73.91996412
DNAJC10	rs72898732	92.93655187
DNAJC10	rs79382622	34.02209987
DNAJC10	rs140791170	46.96289728
DNAJC10	rs150883685	84.99628047
DNAJC10	rs139019195	34.49493013
DNAJC10	rs2523480	58.52841409
SSMEM1	rs11417460	31.42732769
SSMEM1	rs4980320	41.89441069
SSMEM1	rs1354034	48.49253908
SSMEM1	rs61749657	116.9148527
SSMEM1	rs342292	40.30764636
SSMEM1	rs77265585	30.27415133
LILRA4	rs117408808	35.96537507
LILRA4	rs8105624	1078.601556
LILRA4	rs7253911	241.0365039
LILRA4	rs11881487	121.8453244
LILRA4	rs143411334	63.61914636
LILRA4	rs111269305	30.24187756
LILRA4	rs34974314	49.26101775
LILRA4	rs2251746	73.67094485
LILRA4	rs4233366	56.90659989
LILRA4	rs2343306	41.46157994
LILRA4	rs3184504	51.73096596
LILRA4	rs76428106	131.7921966
LILRA4	rs62045817	33.2038322
LILRA4	rs1396210	31.32575031
LILRA4	rs4849402	120.2516131
LILRA4	rs144317085	43.84941963
LILRA4	rs1846190	30.36288876
LILRA4	rs6796	45.94533326
LILRA4	rs876036	396.5182046
PEX14	rs684016	40.53990564
PEX14	rs28629977	31.59561128
PEX14	rs12444636	32.75076139
PEX14	rs4503880	54.65151864
PEX14	rs4547811	45.78106571
PEX14	rs34115226	30.31999677
TNFRSF11B	rs28670194	37.11639797
TNFRSF11B	rs4242582	65.52427227
TNFRSF11B	rs80089906	96.12864484
TNFRSF11B	rs2465403	332.4498149
TNFRSF11B	rs79598313	62.65652677
TNFRSF11B	rs660240	34.9900912
TNFRSF11B	rs190543502	50.80969142
TNFRSF11B	rs704	255.9855204
TNFRSF11B	rs77542162	50.62966408
TNFRSF11B	rs1800961	67.68304623
TNFRSF11B	rs738409	29.98400864
TNFRSF11B	rs56210800	43.57035351
TNFRSF11B	rs17282078	43.85715424
TNFRSF11B	rs149062332	105.4993955
TNFRSF11B	rs1800759	51.7333776
TNFRSF11B	rs148663274	33.04358459
SULF2	rs3741414	472.9370565

SULF2	rs55852335	36.03374225
SULF2	rs12721051	40.10593203
SULF2	rs11127048	32.12892688
SULF2	rs697356	55.11032045
SULF2	rs7614709	40.07924873
PRRG1	rs10922098	125.4887292
PRRG1	rs6445035	70.96333323
PRRG1	rs9267139	38.42795135
PRRG1	rs453821	55.65319582
HYAL1	rs116482870	159.5183346
HYAL1	rs367723330	45.26157461
HYAL1	rs6479877	30.54680376
HYAL1	rs7232	46.74447532
HYAL1	rs7311982	97.33718724
HYAL1	rs11050145	44.51841982
HYAL1	rs11075921	38.31468922
HYAL1	rs6811535	32.80258904
SPRED1	rs10922098	108.0587246
SPRED1	rs7080536	57.18706824
SPRED1	rs4977155	32.77512693
SPRED1	rs9501393	44.24004247
SPRED1	rs7774739	40.42940818
ZNF843	rs10922098	97.31537791
ZNF843	rs1355538	32.43948371
ZNF843	rs211161	30.81266883
TFF3	rs2839493	78.0227095
TFF3	rs4764723	30.10001779
TFF3	rs2129775	32.36577625
TFF3	rs9258405	30.9118881
TFF3	rs2523541	188.8973269
TFF3	rs2524277	284.674625
TFF3	rs2734588	159.3748441
TFF3	rs13204736	34.63472966
ADH4	rs2851274	173.2139314
ADH4	rs5030941	33.51674667
ADH4	rs4408267	43.81452251
ADH4	rs3747207	64.21079685
ADH4	rs2954038	50.59218188
ADH4	rs4360309	30.18788341
SIGIRR	rs10922098	30.73393765
SIGIRR	rs12257143	129.953817
SIGIRR	rs3802589	46.14603999
SIGIRR	rs148645177	31.43218964
SIGIRR	rs73064834	44.16847105
SIGIRR	rs186797403	43.82736874
SIGIRR	rs114076668	40.00906695
SIGIRR	rs62242377	66.47229628
SIGIRR	rs77563378	108.2874181
SIGIRR	rs187979386	66.05429601
SIGIRR	rs148834251	101.1230373
SIGIRR	rs140750015	47.74600321
SIGIRR	rs4974107	467.1359502
SIGIRR	rs146456061	90.98080258
SIGIRR	rs62261068	1200.76471
SIGIRR	rs150933254	587.4669693
SIGIRR	rs143836348	188.6803117
SIGIRR	rs17477867	809.3717918
SIGIRR	rs2633726	277.4114182

SIGIRR	rs114541510	31.26398228
SIGIRR	rs6766712	343.0611489
DPEP2	rs116008080	56.83655363
DPEP2	rs368324587	250.7956418
DPEP2	rs141039314	253.9295453
DPEP2	rs6145876	101.5881391
DPEP2	rs1984769	33.38487158
DPEP2	rs2936613	39.38924837
DPEP2	rs4766578	46.5105747
DPEP2	rs9927130	30.29562604
DPEP2	rs12945299	50.99731225
DPEP2	rs143501783	42.00050231
DPEP2	rs4760	40.185971
DPEP2	rs13062108	32.37287454
DPEP2	rs76044721	63.35230463
DPEP2	rs41338844	84.03904922
DPEP2	rs13083728	225.3051293
DPEP2	rs143479101	233.8226735
DPEP2	rs71301894	149.9036004
DPEP2	rs71308053	55.09234158
DPEP2	rs191798511	82.67354015
DPEP2	rs115283850	72.44004006
DPEP2	rs73167083	44.08134715
DPEP2	rs66530140	36.77171503
DPEP2	rs1801020	127.7893061
DPEP2	rs146798134	63.27006957
DPEP2	rs635634	1124.40728
ETNK1	rs10922098	95.60706701
ETNK1	rs1355538	37.03451188
ETNK1	rs4713488	41.4392819
ETNK1	rs7774739	34.31830071
TCP11	rs10922098	706.2715047
TCP11	rs35351085	30.94250918
TCP11	rs28944192	48.73913911
TCP11	rs704	965.83112
TCP11	rs11080290	39.87894489
TCP11	rs111696008	30.9832895
TCP11	rs6445035	218.5384558
TCP11	rs3863087	80.85756115
TCP11	rs73169117	31.60668826
TCP11	rs3099847	31.47153336
TCP11	rs1265941	84.89989511
TCP11	rs550671	196.4281845
TCP11	rs116334170	30.01350179
TCP11	rs74374003	37.22709513
TCP11	rs854536	119.5375125
GRAMD1C	rs4682146	299.4285265
GRAMD1C	rs58358607	56.80666675
GRAMD1C	rs1697422	30.54751199
GRAMD1C	rs10922098	65.18581026
GRAMD1C	rs4795730	29.77772108
GRAMD1C	rs704	621.9828397
GRAMD1C	rs148931761	44.03457512
GRAMD1C	rs78441035	96.2632644
GRAMD1C	rs56209585	98.4125119
GRAMD1C	rs1355538	44.12656553
GRAMD1C	rs28374650	31.2262326
PTPRU	rs183909650	107.1244922

PTPRU	rs12402615	54.68817961
PTPRU	rs2179795	228.7293054
PTPRU	rs145329681	31.72498636
PTPRU	rs149176316	37.05071108
PTPRU	rs1984769	34.29430009
PTPRU	rs200250001	53.94444286
PTPRU	rs78705678	136.818491
PTPRU	rs2477642	126.8638313
PTPRU	rs1869026	33.33049861
PAPOLG	rs11227136	136.2024672
PAPOLG	rs704	93.36843076
PAPOLG	rs6445826	31.55023923
GPX7	rs111943091	323.1571382
GPX7	rs79235526	927.332267
GPX7	rs835344	1085.050532
GPX7	rs150323612	67.83001556
GPX7	rs114842128	41.96349206
GPX7	rs4926743	60.22908353
GPX7	rs77126389	38.31328184
GPX7	rs2588511	32.73178412
GPX7	rs6993770	150.2696008
DPP7	rs371274645	47.39940253
DPP7	rs2811789	113.3517771
DPP7	rs2176675	1028.193031
DPP7	rs147058434	102.7840902
DPP7	rs10747050	336.4154418
DPP7	rs77298262	41.36815745
DPP7	rs72763283	35.1771154
DPP7	rs35275521	40.53270679
DPP7	rs1242103	48.02861414
DPP7	rs1293303	32.88169829
DPP7	rs146403924	124.447933
PRSS57	rs117024528	68.61273925
PRSS57	rs34668346	1045.228778
PRSS57	rs28641712	73.91575436
PRSS57	rs139651162	145.9325329
PRSS57	rs75840974	38.43155495
PRSS57	rs74253480	75.29400144
PRSS57	rs7310615	42.31245538
PRSS57	rs1555405	30.55186428
PRSS57	rs4334224	31.31735673
PRSS57	rs140956807	39.85622774
PRSS57	rs14142	31.12632158
PRSS57	rs2305637	43.9541047
PRSS57	rs11718130	36.70544817
PRSS57	rs144317085	38.90314362
PRSS57	rs7705526	35.00221308
PRSS57	rs41286279	43.78488777
PRSS57	rs59846247	51.9708888
PRSS57	rs138231254	266.4441195
PRSS57	rs3177747	798.130879
PRSS57	rs169504	59.37456479
PRSS57	rs6993770	36.01667162
PRSS57	rs409801	31.70280017
SIGLEC12	rs1106357	39.09751832
SIGLEC12	rs2461838	35.10562699
SIGLEC12	rs2304885	36.61116625
SIGLEC12	rs704	1084.53772

SIGLEC12	rs11868058	38.6234597
SIGLEC12	rs1260326	66.28741356
SIGLEC12	rs76706776	39.42871356
SIGLEC12	rs56210800	167.2695954
SIGLEC12	rs17282078	167.4931034
SIGLEC12	rs799169	30.83322567
SCN2B	rs506317	50.19900266
SCN2B	rs72773841	64.04045317
SCN2B	rs35524242	828.1057164
SCN2B	rs927914	126.2182991
SCN2B	rs113010247	90.72918598
SCN2B	rs942929	31.73246597
SCN2B	rs11907335	30.23449463
SCN2B	rs4485709	38.67908292
SCN2B	rs2300149	291.7866844
SCN2B	rs777488	87.06994981
SCN2B	rs244452	74.43116262
OXT	rs6047800	34.11248221
OXT	rs62205566	40.27526453
OXT	rs6115677	202.592909
OXT	rs6115776	2208.444914
OXT	rs11087565	53.76997022
OXT	rs10922094	42.26968717
OXT	rs10822186	32.9623781
OXT	rs28601761	50.7688395
NCR1	rs4343402	49.92554911
NCR1	rs16985962	35.16821059
NCR1	rs77046104	1078.214644
NCR1	rs11880295	222.0062139
NCR1	rs10922098	30.21739118
NCR1	rs3184504	147.9575683
NCR1	rs196904	34.33657199
NCR1	rs875063	90.43293207
UST	rs7738724	80.01502761
UST	rs6920109	211.1372985
UST	rs7747823	67.72746785
UST	rs61803025	30.13660213
UST	rs2477639	148.4334443
UST	rs692440	94.8642764
UST	rs34124994	34.03752432
UST	rs3184504	89.00296619
UST	rs17849626	42.25090282
UST	rs3130304	49.46530193
UST	rs587495	38.46963861
UST	rs2342859	33.03656456
UST	rs66962214	51.34782509
UST	rs6796	432.7637233
C1orf115	rs10922098	294.330937
C1orf115	rs697356	63.53347608
C1orf115	rs6457457	64.81544474
C1orf115	rs28573154	86.06229533
TNFRSF1B	rs2301258	386.2007669
TNFRSF1B	rs5746001	55.47905931
TNFRSF1B	rs61747728	73.57299178
TNFRSF1B	rs3184504	56.73394509
TNFRSF1B	rs77924615	40.37352834
TNFRSF1B	rs7207683	49.14590047
TNFRSF1B	rs1260326	29.99023241

TNFRSF1B	rs13108218	37.8808696
TNFRSF1B	rs368611	32.21340293
TNFRSF1B	rs34321847	30.17645041
TNFRSF1B	rs3093949	98.40233879
DAG1	rs62061425	38.18031288
DAG1	rs73135583	69.29625085
DAG1	rs2075823	32.72068571
CDHR1	rs10922098	113.6619284
CDHR1	rs17580	564.5547145
CDHR1	rs28573154	43.73422223
CDHR1	rs7774739	36.7488749
NGFR	rs187320701	32.66214019
NGFR	rs72926572	107.0375118
NGFR	rs190160503	83.52020697
NGFR	rs60241046	31.40027411
NGFR	rs7256279	32.48599708
NGFR	rs76288224	29.75391125
LHB	rs150873161	42.45243056
LHB	rs7409311	1214.544001
LHB	rs11669977	102.7856798
LHB	rs10410923	34.55461955
LDOC1	rs10922098	40.73003419
LDOC1	rs113964757	41.29554553
LDOC1	rs2956479	30.48553289
LDOC1	rs919791	170.5725948
LDOC1	rs415620	30.34986251
LDOC1	rs4713498	34.25287517
DLK1	rs10922098	325.2574939
DLK1	rs1707652	95.08205046
DLK1	rs9267139	31.71675628
DLK1	rs28573154	105.4814856
DLK1	rs7774739	70.70641542
MAPKAPK5	rs12132412	32.75156099
MAPKAPK5	rs72840032	82.55221357
MAPKAPK5	rs9577463	30.62395482
MAPKAPK5	rs4907591	42.87786697
MAPKAPK5	rs111797939	53.42547764
MAPKAPK5	rs142469240	54.0062217
MAPKAPK5	rs138781102	33.51783883
MAPKAPK5	rs190324353	57.49978829
MAPKAPK5	rs72821015	39.45040292
MAPKAPK5	rs201234732	69.74929073
MAPKAPK5	rs62075078	60.32498252
MAPKAPK5	rs11655538	210.2398699
MAPKAPK5	rs140390732	107.2237221
MAPKAPK5	rs2004584	361.5149602
MAPKAPK5	rs4239214	4732.018671
MAPKAPK5	rs144924964	1231.017316
MAPKAPK5	rs117451747	442.8436299
MAPKAPK5	rs117348742	203.6616166
MAPKAPK5	rs16964261	831.0434894
MAPKAPK5	rs142216432	80.81962835
MAPKAPK5	rs78119120	136.5857149
MAPKAPK5	rs137940962	68.31423455
MAPKAPK5	rs74602839	58.90366527
MAPKAPK5	rs111278137	34.31176926
MAPKAPK5	rs73045691	1090.485493
MAPKAPK5	rs204483	317.4986719

MAPKAPK5	rs113354603	155.0466119
SPAST	rs10922094	90.22233534
SPAST	rs149468457	29.82064049
SPAST	rs9501393	47.73188699
SPAST	rs7774739	30.17795374
LRRC74A	rs10922098	726.9533446
LRRC74A	rs3900909	32.6659093
LRRC74A	rs1047286	54.95465747
LRRC74A	rs2686395	153.0333382
LRRC74A	rs555007	79.34502503
LRRC74A	rs444921	184.2002159
LRRC74A	rs9272526	34.29310527
COX7A1	rs34124994	32.68368647
COX7A1	rs62269916	32.71998272
COX7A1	rs151242509	123.407649
COX7A1	rs138711481	45.93998631
COX7A1	rs2817203	157.3950011
COX7A1	rs2760180	158.6057892
COX7A1	rs17243234	73.0602343
COX7A1	rs3131337	31.62991739
COX7A1	rs3129015	41.69490245
RNASE2	rs2233859	333.3918619
RNASE2	rs10922094	81.24468935
RNASE2	rs147168394	35.82955184
RNASE2	rs6796	31.81874426
QSOX2	rs111589804	61.85315313
QSOX2	rs11103367	139.9589981
QSOX2	rs1886296	970.4365203
QSOX2	rs17476364	30.7873264
QSOX2	rs61858556	42.58246716
QSOX2	rs10876168	151.9857316
QSOX2	rs7965213	55.17761082
QSOX2	rs10850001	35.96512565
QSOX2	rs10849820	55.68854144
QSOX2	rs8887	31.21216495
QSOX2	rs56397034	68.79330306
QSOX2	rs6511291	37.55629377
QSOX2	rs56102089	30.59628237
QSOX2	rs34130413	51.98205883
QSOX2	rs4672497	280.9136846
QSOX2	rs9392385	33.0996222
QSOX2	rs2923444	58.65689044
QSOX2	rs12349897	36.32659317
QSOX2	rs76518888	157.0901107
QSOX2	rs7044834	150.5442318
QSOX2	rs7849542	1142.05606
QSOX2	rs1179011	941.5941993
QSOX2	rs139562936	142.6024475
RARRES1	rs62278958	154.5546299
RARRES1	rs2693531	115.4797004
RARRES1	rs146361965	1099.753639
RARRES1	rs183235455	29662.36834
RARRES1	rs2595260	584.380199
RARRES1	rs1434282	33.51102091
RARRES1	rs10822155	30.71559243
RARRES1	rs4759076	62.0322083
RARRES1	rs10176736	32.27365059
RARRES1	rs2976828	29.74877876

RARRES1	rs1687481	56.11920332
RARRES1	rs9857086	32.19371302
RARRES1	rs1532207	52.66743489
RARRES1	rs13079479	31.87019674
RARRES1	rs13077355	70.02095944
RARRES1	rs145138743	40.55850332
RARRES1	rs138609313	126.0995354
RARRES1	rs114334418	939.8798772
RARRES1	rs62277159	1389.027559
RARRES1	rs62278751	939.5667059
RARRES1	rs62270393	608.6728963
RARRES1	rs71314064	655.1621533
RARRES1	rs150004903	65.30261312
RARRES1	rs75275706	87.93385374
RARRES1	rs12492447	163.2630111
RARRES1	rs139652760	129.1822285
RARRES1	rs115106820	70.72750545
RARRES1	rs138017839	42.87294105
RARRES1	rs80253876	29.8710217
RARRES1	rs111756965	34.20503524
RARRES1	rs114694170	35.59363656
RARRES1	rs6993770	93.99620626
GRP	rs10922098	234.7437227
GRP	rs1706435	34.27173748
GRP	rs1042445	83.53306346
GRP	rs438999	45.40120379
GRP	rs28455809	44.94680782
CYTL1	rs200042473	40.67701994
CYTL1	rs151316626	77.72415674
CYTL1	rs56724509	109.5501082
CYTL1	rs10031270	2635.441506
CYTL1	rs77924615	58.91746525
FASN	rs62078747	335.3487163
FASN	rs4980320	52.39919688
FASN	rs11451044	32.62350345
FASN	rs342292	46.65584255
FASN	rs72673751	38.5085893
BOLA2	rs113889195	50.41284089
BOLA2	rs12435340	39.45381752
BOLA2	rs557011	31.53446315
IGF1	rs61802335	33.98948029
IGF1	rs79874727	64.7297875
IGF1	rs111736413	39.22072743
IGF1	rs76359966	31.58980751
IGF1	rs41556615	60.08157781
RSPO2	rs1329424	109.2913348
RSPO2	rs1707652	51.21308359
RSPO2	rs9501393	37.46540691
RSPO2	rs9272068	31.479249
CTNNB1	rs114009540	42.44615554
CTNNB1	rs1329424	992.8159456
CTNNB1	rs3900909	54.80025494
CTNNB1	rs1047286	47.22084703
CTNNB1	rs2686395	206.4358186
CTNNB1	rs73169117	29.80962187
CTNNB1	rs555007	104.5912048
CTNNB1	rs550671	262.1220805
CTNNB1	rs41548618	38.86176822

CTNNB1	rs9272526	56.18320381
RSPO3	rs853974	102.7060821
RSPO3	rs9285458	849.8275832
RSPO3	rs6770670	52.94175319
RSPO3	rs142707166	46.35417364
RSPO3	rs6932834	37.47918317
NTM	rs7111143	59.72421381
NTM	rs1793768	45.86844861
NTM	rs10894353	1014.449798
NTM	rs147824634	995.2572907
NTM	rs11825002	129.124928
NTM	rs487303	31.2706553
NTM	rs7118465	81.73574788
NTM	rs1544300	34.84670876
NTM	rs78705678	174.6690347
NTM	rs2477642	165.6216139
NTM	rs540	51.43145259
NTM	rs1260326	81.56394989
AXIN2	rs61802335	117.730733
AXIN2	rs10922098	179.3726435
AXIN2	rs148480125	50.2749694
AXIN2	rs112197784	35.02409991
AXIN2	rs139712292	121.6037186
AXIN2	rs149191976	102.8278929
AXIN2	rs12883601	1308.807751
AXIN2	rs79867344	106.3883942
AXIN2	rs140391681	181.9420491
AXIN2	rs145723740	56.60557106
AXIN2	rs150855374	33.00235647
AXIN2	rs150238887	32.71528329
AXIN2	rs78122038	31.27217594
AXIN2	rs2242572	35.13171895
GHRL	rs10922098	72.92481673
GHRL	rs140271398	31.11022788
GHRL	rs9469077	37.37510505
GHRL	rs4921701	34.7710342
GHRL	rs1940249	44.69712428
SNCA	rs2119785	32.64716667
SNCA	rs3811444	58.9882521
SNCA	rs10822155	41.77511526
SNCA	rs1354034	35.92549663
SNCA	rs342200	34.3444538
BMP6	rs12751502	70.46419805
BMP6	rs34586497	64.71140102
BMP6	rs1894254	68.24074149
BMP6	rs663499	44.43621227
BMP6	rs10793092	74.90107045
BMP6	rs61389091	431.0104088
BMP6	rs35763136	372.7364946
BMP6	rs7927540	41.77394576
BMP6	rs113747960	33.77435224
BMP6	rs10861032	41.72768741
BMP6	rs201862321	66.43040063
BMP6	rs55714927	93.01928345
BMP6	rs143501783	47.69852192
BMP6	rs145079924	48.07289079
BMP6	rs1033415	30.81777259
BMP6	rs635634	53.98237921

RSPO4	rs75904281	127.4746923
RSPO4	rs6140899	66.66650799
RSPO4	rs62187552	44.6754635
RSPO4	rs368465	43.34804921
RSPO4	rs6542680	34.06632747
RSPO4	rs62154858	30.028836
RSPO4	rs9458012	36.68414674
CTSH	rs117753864	236.4553479
CTSH	rs75299846	740.2934158
CTSH	rs36073954	471.2057914
CTSH	rs11638020	983.735373
CTSH	rs56225615	162.4432205
CTSH	rs62013198	4246.67098
CTSH	rs78788913	180.8981872
CTSH	rs117857171	55.91572229
CTSH	rs78705678	72.09173288
CTSH	rs2477642	64.4996253
CTSH	rs61991538	41.96665775
CTSH	rs143494606	33.98027464
CTSH	rs78641362	44.09084381
CTSH	rs148156457	59.05582236
CTSH	rs148311323	47.64047543
CTSH	rs148978553	107.2737819
CTSH	rs138963977	204.6516862
CTSH	rs4310815	36.25357803
CTSH	rs113776771	55.67419965
CTSH	rs146769105	43.43278069
CTSH	rs8042072	32.06187968
CTSH	rs75555084	39.06028958
CTSH	rs62120372	42.64411538
CTSH	rs55954186	40.42494813
CTSH	rs5029990	57.65858866
CTSH	rs9274664	45.26428306
CTSH	rs576123	36.17339082
KLK3	rs2659051	40.71637567
KLK3	rs2739466	29.92363856
KLK3	rs10993994	49.48568067
IGFBP2	rs4674100	36.49485245
IGFBP2	rs7248104	34.01062704
IGFBP2	rs1260326	51.82913309
IGFBP2	rs1154988	44.8487197
IGFBP2	rs4976033	29.72212079
IGFBP2	rs11153382	30.31837896
CHGA	rs150344128	33.15168879
CHGA	rs35252916	31.58223581
CHGA	rs729940	839.1043997
CHGA	rs137906439	47.9063237
CHGA	rs74863902	30.2683314
CHGA	rs12801747	62.41502547
CHGA	rs16854319	35.51109272
CHGA	rs56092644	57.29490287
CHGA	rs17092104	120.5635903
CHGA	rs2976384	32.66314885
MMP10	rs187376428	131.6751656
MMP10	rs371427075	325.9736667
MMP10	rs1320633	928.1345234
MMP10	rs35622132	315.058211
MMP10	rs150266249	81.82735686

MMP10	rs60661469	50.32983554
MMP10	rs61910583	31.66068657
MMP10	rs145452310	45.74620068
MMP10	rs675999	35.51642754
MMP10	rs681343	247.153411
MMP10	rs9271669	37.93118743
MMP10	rs9271726	38.12835357
EFEMP1	rs13424210	140.543334
EFEMP1	rs9309272	282.4472194
EFEMP1	rs72704449	37.22176581
EFEMP1	rs77262773	36.29305034
EFEMP1	rs2070634	38.96528852
LEP	rs55872725	71.00974507
LEP	rs7240682	30.4592727
LEP	rs10184004	45.39645887
LEP	rs12154627	44.59938583
DMKN	rs10922094	158.6523104
DMKN	rs111696008	36.92491201
DMKN	rs1707652	170.6823103
DMKN	rs9501393	52.73734641
DMKN	rs7774739	40.11496858
PLVAP	rs13379369	194.8223631
PLVAP	rs2753516	94.57669238
PLVAP	rs78818281	115.04859
PLVAP	rs71534519	37.01668238
ARL8B	rs10922098	65.11002915
ARL8B	rs9864801	46.9385667
ARL8B	rs9501393	33.78228991
SPINK14	rs10922098	181.9943273
SPINK14	rs704	32.34179255
SPINK14	rs1707652	74.16898311
SPINK14	rs2534681	41.95287456
SPINK14	rs9501600	116.3887255
SPINK14	rs80163833	49.28494092
CDCP1	rs10922094	67.7781413
CDCP1	rs704	130.285945
CDCP1	rs35004449	42.18762994
CDCP1	rs1706435	35.00400684
CDCP1	rs28573154	46.55474673
CDCP1	rs7774739	44.81813702
TMEM9	rs2842700	93.07836319
TMEM9	rs780093	36.29102307
TMEM9	rs138498518	30.34359263
TMEM9	rs140666611	30.83668366
TMEM9	rs62259021	41.45191319
TMEM9	rs181227722	126.0939281
TMEM9	rs77539534	41.82485086
TMEM9	rs77608513	149.9422772
TMEM9	rs146473837	183.5719186
TMEM9	rs114658296	73.59470621
TMEM9	rs149389924	55.62829945
TMEM9	rs115667089	85.1174467
TMEM9	rs3131643	68.26123183
TMEM9	rs34115112	42.88130803
TMEM9	rs112164771	275.3920941
LRP1	rs10922098	36.03376359
LRP1	rs704	136.3700931
LRP1	rs111304729	30.46412571

LRP1	rs111842802	37.68299734
GPNMB	rs3114714	39.31951986
GPNMB	rs35540007	58.02294807
GPNMB	rs58856240	2066.610798
GPNMB	rs858257	284.0888133
GPNMB	rs117012150	68.37139581
GPNMB	rs143026417	113.2481633
GPNMB	rs174564	64.68502898
GPNMB	rs77262773	29.95918241
GPNMB	rs111696008	31.99687498
GPNMB	rs11720167	214.2154073
GPNMB	rs3863087	66.04736997
GPNMB	rs146916275	34.15303278
ERMAP	rs10922098	34.35989948
ERMAP	rs73216598	55.9205923
ERMAP	rs2057681	455.1700555
ERMAP	rs854572	66.86026977
RNF128	rs10922098	549.9259644
RNF128	rs1707652	118.5640028
RNF128	rs555007	61.87164466
RNF128	rs444921	158.1493709
RNF128	rs9272526	32.42231567
ZPBP	rs10922098	527.3094881
ZPBP	rs1707652	147.7996797
ZPBP	rs1265941	67.581713
ZPBP	rs550671	163.0170954
ZPBP	rs9276606	32.99210613
LRRTM4	rs10922098	350.1275101
LRRTM4	rs1355538	65.53296491
LRRTM4	rs3828879	33.0157381
LRRTM4	rs1265941	51.89426953
LRRTM4	rs453821	103.6893951
DNAJC4	rs4654748	36.87955148
DNAJC4	rs10922098	558.5717403
DNAJC4	rs11569415	42.51228275
DNAJC4	rs12478841	38.75943781
DNAJC4	rs1707652	93.19691913
DNAJC4	rs1265941	67.18947553
DNAJC4	rs550671	151.3017069
ADAM10	rs10922098	367.7780205
ADAM10	rs1707652	78.32847646
ADAM10	rs9267139	33.10574232
ADAM10	rs9501393	92.82234973
ADAM10	rs7774739	68.49322337
OLFML3	rs4381184	104.777826
OLFML3	rs17531384	34.47288428
OLFML3	rs61830291	40.20050772
OLFML3	rs2436670	33.63287218
OLFML3	rs80215761	42.19547139
OLFML3	rs7951028	53.52586418
OLFML3	rs77262773	45.933649
OLFML3	rs56254331	41.84562991
OLFML3	rs1448110	43.48621803
OLFML3	rs760715	93.9522966
OLFML3	rs2413595	40.60035053
OLFML3	rs10068965	38.76804029
OLFML3	rs34691781	31.20950053
PKDCC	rs11902166	73.45999833

PKDCC	rs78947001	35.79135228
PKDCC	rs114009540	33.41112946
PKDCC	rs10922098	680.2418782
PKDCC	rs3900909	33.12787754
PKDCC	rs1047286	46.5014096
PKDCC	rs12478841	29.91783608
PKDCC	rs1707652	136.8147778
PKDCC	rs555007	83.92135623
PKDCC	rs550671	175.8835789
PKDCC	rs9272526	38.12039085
TRAPPC5	rs10922098	215.2958708
TRAPPC5	rs9864801	54.79867919
TRAPPC5	rs555007	35.53284484
TRAPPC5	rs453821	64.80003271
PGRMC2	rs114009540	30.73383048
PGRMC2	rs10922098	457.0090531
PGRMC2	rs1047286	62.16299413
PGRMC2	rs4665987	34.22141301
PGRMC2	rs1707652	101.1369239
PGRMC2	rs1265941	50.97761698
PGRMC2	rs550671	132.880482
TMEM106B	rs11980425	48.67220703
TMEM106B	rs3173615	193.1883424
TMEM106B	rs10922098	85.35046524
TMEM106B	rs10751648	40.37597493
TMEM106B	rs9864801	32.74531774
TMEM106B	rs3129773	31.07036388
GAL3ST1	rs145439832	31.5775496
GAL3ST1	rs72748420	53.29361579
GAL3ST1	rs1329424	2181.373433
GAL3ST1	rs3900909	67.75940113
GAL3ST1	rs10801660	41.2036214
GAL3ST1	rs61821527	35.72775863
GAL3ST1	rs704	427.9844356
GAL3ST1	rs201063729	31.4462693
GAL3ST1	rs111696008	42.53596535
GAL3ST1	rs144202860	29.83006508
GAL3ST1	rs76315187	31.40251752
GAL3ST1	rs2686395	380.3205779
GAL3ST1	rs73169117	39.63600448
GAL3ST1	rs1265941	146.177866
GAL3ST1	rs444921	386.2315334
GAL3ST1	rs41291806	55.37237404
GAL3ST1	rs41548618	60.67776694
GPC1	rs142340362	134.7950042
GPC1	rs74578793	1126.861813
GPC1	rs142981046	35.59814305
GPC1	rs140037101	98.23647922
GPC1	rs150687841	222.0127583
GPC1	rs10922098	52.94021684
GPC1	rs117225800	68.98320792
GPC1	rs79100463	44.86185828
GPC1	rs61872852	102.9806858
GPC1	rs11196413	110.0385035
GPC1	rs117850703	40.43006874
GPC1	rs138375658	72.4643684
GPC1	rs62623669	49.34922231
GPC1	rs73006377	57.24197408

GPC1	rs115510766	70.94769624
GPC1	rs737939	34.49389022
GPC1	rs140029094	32.3987534
GPC1	rs7708299	212.7714681
GPC1	rs4434408	120.6392303
CRIM1	rs10922098	154.693458
CRIM1	rs1355538	44.89527416
CRIM1	rs6457457	31.25007391
CRIM1	rs9501393	39.20822309
GALNT11	rs10922098	410.4571056
GALNT11	rs1707652	86.14277565
GALNT11	rs9267139	39.45903951
GALNT11	rs28573154	113.8300674
GALNT11	rs7774739	81.60734318
GALNT11	rs9271766	40.09961464
CD74	rs10922094	117.282478
CD74	rs1355538	55.89691487
CD74	rs550671	49.01212339
CD74	rs28645249	30.08714635
CD1D	rs10922094	178.1445425
CD1D	rs1355538	50.195028
CD1D	rs9501393	63.59601372
CD1D	rs7774739	41.84118629
CD1D	rs75185583	38.87592762
NMU	rs572515	50.32147576
NMU	rs6489859	32.05429407
NMU	rs6542680	58.2052828
NMU	rs686250	32.98827677
PCDHA7	rs2935539	43.60358126
PCDHA7	rs61751507	169.0158583
PCDHA7	rs2753506	30.69351435
PCDHA7	rs149013447	41.46978792
PCDHA7	rs9271636	101.3742525
A4GALT	rs137139	183.4787767
A4GALT	rs5758884	931.1222804
A4GALT	rs2064465	179.4321595
A4GALT	rs61880646	64.41498817
A4GALT	rs3740702	31.31777079
A4GALT	rs41474450	62.85948353
A4GALT	rs72902478	39.84834875
A4GALT	rs117100086	100.8506616
A4GALT	rs71454336	43.79895722
A4GALT	rs6591491	39.93480444
A4GALT	rs116898071	41.05896041
A4GALT	rs562744	31.82805026
A4GALT	rs116920273	281.1714356
A4GALT	rs573790	1090.311308
A4GALT	rs76673655	81.45752315
A4GALT	rs76006758	57.6543833
A4GALT	rs6073952	73.81928211
A4GALT	rs117018441	41.34552399
A4GALT	rs576123	84.5298457
DCUN1D5	rs10922094	79.71487606
DCUN1D5	rs964184	105.9484625
DCUN1D5	rs2414577	32.16209353
DCUN1D5	rs62120575	65.65545888
DCUN1D5	rs142042446	1262.174225
DCUN1D5	rs117310449	109.0872405

DCUN1D5	rs8106813	1135.976144
DCUN1D5	rs76856627	81.34991729
DCUN1D5	rs143539524	124.2896014
DCUN1D5	rs190529845	36.32036133
DCUN1D5	rs10420758	33.14723001
DCUN1D5	rs138999114	31.58435647
DCUN1D5	rs780093	64.94419303
DCUN1D5	rs1707652	47.93575105
DCUN1D5	rs11755689	35.8434857
LILRA5	rs146170601	65.53237727
LILRA5	rs80047999	67.27353535
LILRA5	rs12232829	160.763659
LILRA5	rs1645788	949.9941153
LILRA5	rs2885686	46.76530548
LILRA5	rs188411739	44.5718852
LILRA5	rs150649461	31.10287068
LILRA5	rs77535019	32.67409952
LILRA5	rs2070902	148.7534826
LILRA5	rs80123037	40.58597305
LILRA5	rs6695321	153.4728591
LILRA5	rs7310615	37.04292361
LILRA5	rs76428106	47.92858943
LILRA5	rs1892548	42.55899324
LILRA5	rs2562750	48.37698522
LILRA5	rs11870683	31.32572372
LILRA5	rs56013073	36.25354367
LILRA5	rs62110762	31.10294068
LILRA5	rs35723031	33.1842862
LILRA5	rs913678	43.6760061
LILRA5	rs509227	31.4813972
LILRA5	rs9264683	32.10961511
LILRA5	rs877544	57.05407691
ECE1	rs72748420	30.39030482
ECE1	rs10922098	804.173744
ECE1	rs3900909	39.38051901
ECE1	rs704	43.96682606
ECE1	rs1047286	51.90481565
ECE1	rs1707652	187.8549644
ECE1	rs1265941	91.01640862
ECE1	rs550671	222.901684
ECE1	rs9276606	39.43860118
EMILIN3	rs367558006	173.2143768
EMILIN3	rs11472459	391.9541091
EMILIN3	rs6072300	234.0983272
EMILIN3	rs61730990	35.42831518
EMILIN3	rs61739314	973.2782312
EMILIN3	rs73267471	92.90143795
EMILIN3	rs6129887	53.86262178
EMILIN3	rs138006680	35.80754154
EMILIN3	rs143378550	36.01315139
ERLIN1	rs10922098	447.4196265
ERLIN1	rs704	92.464356
ERLIN1	rs1047286	34.87660567
ERLIN1	rs12478841	31.55593266
ERLIN1	rs114115200	80.66920449
ERLIN1	rs3863087	31.30016252
ERLIN1	rs9267139	42.4635184
ERLIN1	rs3891366	120.5073632

ERLIN1	rs7774739	86.76283981
ERLIN1	rs9271766	35.75689186
NOG	rs71387447	38.44621434
NOG	rs76164057	1264.259838
NOG	rs8065342	164.0146705
NOG	rs4141420	35.89479191
NOG	rs12136856	85.56299462
NOG	rs10822155	32.20309556
NOG	rs11187019	36.52025679
NOG	rs7475335	269.987946
NOG	rs73000929	73.39225642
NOG	rs35979828	116.4635249
NOG	rs2249219	32.66551622
NOG	rs78909033	110.9916778
NOG	rs7622284	37.95175697
NOG	rs4699156	40.66809406
NOG	rs114694170	352.4268435
NOG	rs62428833	44.69460125
NOG	rs2759385	29.89111873
NOG	rs342293	172.647502
NOG	rs60757417	106.4267187
NOG	rs114968084	48.95378538
PCDH10	rs10922098	30.42520784
PCDH10	rs4846913	133.454631
PCDH10	rs704	254.5890737
PCDH10	rs75215268	34.39083519
PCDH10	rs139253162	106.5759827
PCDH10	rs147815265	50.03917479
PCDH10	rs145964362	146.3863472
PCDH10	rs9858594	1127.271976
PCDH10	rs6797321	17572.97194
PCDH10	rs114397899	422.1719025
PCDH10	rs257108	94.42908584
PCDH10	rs73189768	213.9959197
PCDH10	rs9816257	33.02385916
PCDH10	rs79362041	89.14383909
ARMC5	rs10922098	290.8961474
ARMC5	rs2686395	75.4091304
ARMC5	rs9267139	34.15816043
ARMC5	rs3891366	110.3246551
ARMC5	rs7774739	84.78134614
ARMC5	rs9271766	45.1115329
FAM171B	rs6742943	55.48167655
FAM171B	rs1123176	298.0744589
FAM171B	rs10922094	35.56984395
FAM171B	rs4917	75.19451193
FAM171B	rs12216891	35.73071461
DPEP1	rs141328593	338.2937914
DPEP1	rs145843803	933.2083094
DPEP1	rs28414505	486.1193497
DPEP1	rs12918760	56.89036873
DPEP1	rs143028323	1207.487336
DPEP1	rs62067101	183.8798123
DPEP1	rs140499892	59.75344943
DPEP1	rs112126489	176.2387489
DPEP1	rs144557636	110.9327895
DPEP1	rs145163232	34.70451627
DPEP1	rs181073811	109.687105

DPEP1	rs78689694	38.49144523
DPEP1	rs55714927	66.20345007
DPEP1	rs143501783	43.08560752
DPEP1	rs10989527	41.72482396
TFRC	rs3827554	74.13727962
TFRC	rs113026442	37.16762612
TFRC	rs73592285	43.72137205
TFRC	rs2471884	501.0602787
TFRC	rs543798	32.86543237
TFRC	rs78899080	30.3877334
TFRC	rs8887	36.68491554
TFRC	rs855791	232.473139
TFRC	rs35762167	35.98051705
TFRC	rs2879280	145.907287
TFRC	rs2906082	32.95110567
TFRC	rs144861591	225.5221619
TFRC	rs9405046	47.86724166
TFRC	rs6592965	51.23797759
TFRC	rs9801017	43.14158688
TFRC	rs635634	86.54264164
SNX1	rs10922098	32.94998491
SNX1	rs28395407	31.07714026
SNX1	rs11709525	35.06793145
SNX1	rs67399078	34.48780641
PSAPL1	rs28685850	36.59575836
PSAPL1	rs139414812	34.31700203
PSAPL1	rs35791045	2499.296489
PSAPL1	rs62277606	154.894188
PSAPL1	rs62277617	194.4656476
PSAPL1	rs149415322	57.44959759
PSAPL1	rs73216627	49.77286945
PSAPL1	rs2285780	41.32961094
PSAPL1	rs34560261	52.63502353
PSAPL1	rs28409139	84.88641091
PSAPL1	rs12053317	37.78644088
PSAPL1	rs12185764	74.47505297
PSAPL1	rs310762	103.4742371
OLFML1	rs10922098	151.377173
OLFML1	rs1355538	49.14593632
OLFML1	rs438999	62.42881301
PILRA	rs969427	50.77789567
PILRA	rs12127364	1026.944469
PILRA	rs115955722	34.40044168
PILRA	rs10922098	74.46304818
PILRA	rs28573154	35.18813636
ESRP1	rs368465	39.54629044
ESRP1	rs6542680	134.0429451
ESRP1	rs28573154	40.77466458
ESRP1	rs7774739	40.36138238
TNFSF10	rs10922098	108.3932437
TNFSF10	rs6542680	72.14364281
TNFSF10	rs1355538	36.4012979
TNFSF10	rs9501600	49.40687707
TNFSF10	rs557011	35.48349522
MFAP3L	rs10922098	35.13611703
MFAP3L	rs113747960	29.85015062
MFAP3L	rs1706435	79.57233375
MTX2	rs10922098	78.72339965

MTX2	rs112969279	30.44504989
MTX2	rs3129882	34.35682842
C1S	rs73262897	1205.840446
C1S	rs373096669	666.1402696
C1S	rs78749261	58.87895815
C1S	rs145124748	49.96383483
C1S	rs181314422	81.38871362
C1S	rs61984231	44.78190864
C1S	rs12433460	249.9920094
CILP2	rs2315282	103.740584
CILP2	rs34416533	56.74291323
CILP2	rs78705678	43.46885421
CILP2	rs2461161	37.30880608
CILP2	rs80215761	37.87606527
CILP2	rs2679120	34.22341055
GRAMD1C	rs7622601	41.00368269
GRAMD1C	rs2712355	1285.099001
GRAMD1C	rs55990517	259.6904975
GRAMD1C	rs2462782	98.67318366
GRAMD1C	rs73224542	203.1541581
GRAMD1C	rs10922098	40.29543471
GRAMD1C	rs704	551.324916
GRAMD1C	rs114712881	135.4497587
GRAMD1C	rs4306850	62.64207172
GRAMD1C	rs78441035	206.864623
GRAMD1C	rs4271907	46.37202335
GRAMD1C	rs10644920	31.13182526
GRAMD1C	rs116587920	30.72156527
GRAMD1C	rs3129773	30.8702685
MYRF	rs10922098	567.7565902
MYRF	rs12403425	30.01101985
MYRF	rs704	30.4544214
MYRF	rs11569415	44.336306
MYRF	rs10208529	30.50964998
MYRF	rs2686395	94.53510129
MYRF	rs9267139	49.25281835
MYRF	rs3891366	161.0336241
MYRF	rs7774739	106.7593624
MYRF	rs9271766	50.54152633
PLA2G2C	rs10922098	149.3910787
PLA2G2C	rs147906938	40.91322754
PLA2G2C	rs62404084	29.96048364
SUN3	rs10922098	48.82158937
SUN3	rs373459330	132.8522231
SUN3	rs4977155	90.39602084
SUN3	rs9271573	32.98444446
SUN3	rs4842181	37.5167689
SUN3	rs7041446	376.6677469
CLEC4A	rs10922098	320.5094855
CLEC4A	rs2232710	42.23584907
CLEC4A	rs704	42.09579902
CLEC4A	rs10208529	29.89307885
CLEC4A	rs1707652	93.16133587
CLEC4A	rs28573154	101.9291906
CLEC4A	rs7774739	57.98270001
CLEC4A	rs9271766	37.99425728
CA11	rs2303147	289.0661102
CA11	rs226379	102.2732104

CA11	rs190543502	32.3569126
CA11	rs6440008	32.01695281
CENPW	rs1780322	32.96786595
CENPW	rs10922098	98.37064328
CENPW	rs56210800	32.24852155
CENPW	rs149468457	32.30913544
CENPW	rs9501393	34.23008863
QPCTL	rs140684051	171.8569535
QPCTL	rs3916837	106.0531648
QPCTL	rs142299722	270.6941587
QPCTL	rs17850756	1018.532311
QPCTL	rs759299	44.69280237
QPCTL	rs881376	30.2381254
QPCTL	rs147233090	107.6796204
QPCTL	rs61690552	41.77238754
QPCTL	rs78834848	50.76593339
QPCTL	rs74544477	66.6154029
QPCTL	rs28493806	34.77849039
NPM1	rs10922098	268.5568126
NPM1	rs704	33.69976667
NPM1	rs1707652	83.20143429
NPM1	rs550671	110.6510646
NPM1	rs34927613	59.23686313
SEC61G	rs10922094	32.43138149
SEC61G	rs6542680	31.67696242
SEC61G	rs67399078	32.19273364
CLN5	rs700363	157.6817432
CLN5	rs10922098	91.36245071
CLN5	rs6542680	38.71710447
CLN5	rs9501393	37.69974603
NRXN2	rs10922098	321.0776501
NRXN2	rs704	44.83509586
NRXN2	rs1707652	84.97952954
NRXN2	rs9266766	31.88398429
NRXN2	rs2242572	117.5987018
NRXN2	rs9501602	60.40962946
EVA1C	rs34028712	74.28255892
EVA1C	rs704	33.57908898
EVA1C	rs6542680	271.8750859
EVA1C	rs686250	33.61274476
YTHDC1	rs10922094	29.85590832
YTHDC1	rs1265564	30.05183468
YTHDC1	rs6542680	40.91269633
CACNA2D3	rs147660324	45.84691746
CACNA2D3	rs9878608	697.7896723
CACNA2D3	rs148477941	74.95598214
CACNA2D3	rs13062287	84.38553896
CACNA2D3	rs78258914	424.8910631
CACNA2D3	rs6693520	32.78233129
CACNA2D3	rs3213563	681.1861759
CACNA2D3	rs79941266	38.13665962
CACNA2D3	rs74502455	34.73728423
CACNA2D3	rs705705	55.09409578
CACNA2D3	rs181707610	42.97712642
CACNA2D3	rs11158602	66.68027923
CACNA2D3	rs67328311	31.73416773
CACNA2D3	rs1260326	40.72757693
CACNA2D3	rs13389409	40.67621738

CACNA2D3	rs9977504	44.65546618
CACNA2D3	rs11404982	38.01543104
CACNA2D3	rs9840584	69.25639647
CACNA2D3	rs115399595	32.66988022
CACNA2D3	rs145666159	31.08321768
NUDC	rs4980320	45.10405874
NUDC	rs6580980	39.91259422
NUDC	rs1354034	116.1269514
NUDC	rs342200	43.87330271
SMIM9	rs10922094	188.0652871
SMIM9	rs10784960	593.5822749
SMIM9	rs145554313	59.22379511
SMIM9	rs10208529	33.87930364
SMIM9	rs111696008	31.90634646
SMIM9	rs6445035	106.4407751
SMIM9	rs3863087	39.2841941
SMIM9	rs9501393	56.59697528
SMIM9	rs7774739	40.17624769
SMIM9	rs28601761	114.901444
CCPG1	rs10922098	225.807638
CCPG1	rs1354034	34.48095486
CCPG1	rs1355538	55.16684338
CCPG1	rs9501393	62.90603133
CCPG1	rs7774739	44.3048257
TMEM132B	rs3825381	316.9856499
TMEM132B	rs4657078	43.12842834
TMEM132B	rs506317	40.9823292
TMEM132B	rs2477664	41.20593481
TMEM132B	rs201862321	34.45307049
TMEM132B	rs148111729	675.6046298
TMEM132B	rs117375431	62.61940287
TMEM132B	rs55851595	748.0296251
TMEM132B	rs2844572	47.36173786
TMEM132B	rs635634	40.73568021
CPVL	rs4661012	54.81943193
CPVL	rs41556615	47.02366002
CPVL	rs9270317	31.42063676
HNRNPAB	rs1059367	70.62667432
HNRNPAB	rs192599142	33.35430311
HNRNPAB	rs2856354	37.20625935
HNRNPAB	rs1354034	125.2585708
HNRNPAB	rs59358791	30.43028374
HNRNPAB	rs1580881	33.05061417
LRRC37A2	rs10922094	131.808503
LRRC37A2	rs9864801	45.47087642
LRRC37A2	rs9501393	48.31383999
UGT1A8	rs10922098	33.53740842
UGT1A8	rs80167373	35.46795052
UGT1A8	rs13208583	31.3972358
NEO1	rs139976086	47.82658152
NEO1	rs8040099	95.25288364
NEO1	rs62016793	425.8390316
NEO1	rs7528419	159.6870372
NEO1	rs35166255	34.30355084
NEO1	rs11636866	35.70257667
NEO1	rs74603062	51.80072238
NEO1	rs75806286	36.15693952
NEO1	rs4791810	40.74974667

NEO1	rs10929580	36.67675271
NEO1	rs6000553	62.48619552
NEO1	rs144861591	84.90409779
NEO1	rs12216891	97.59401046
NEO1	rs3124761	39.11577057
COX6C	rs1106357	50.36026062
COX6C	rs12039757	34.81418953
COX6C	rs1591734	141.1181044
COX6C	rs9332701	671.7339494
COX6C	rs2239853	340.2230793
COX6C	rs10764161	130.7500496
COX6C	rs10159712	407.8777533
COX6C	rs10437425	55.44488117
COX6C	rs11012183	30.25526027
COX6C	rs594793	41.4847541
COX6C	rs62140549	73.77200497
COX6C	rs34568880	34.98470991
COX6C	rs28366220	85.73238952
COX6C	rs34958241	154.326147
COX6C	rs9271669	70.43239814
KCNMB3	rs602633	43.26137821
KCNMB3	rs964184	158.2728899
KCNMB3	rs3764261	79.41565971
KCNMB3	rs116843064	43.10566136
KCNMB3	rs7412	123.0781874
KCNMB3	rs11695134	41.35422793
KCNMB3	rs679899	655.157501
KCNMB3	rs74717300	80.32059473
KCNMB3	rs77804075	31.74182345
KCNMB3	rs111696008	53.15090523
KCNMB3	rs571513	47.10568398
KCNMB3	rs12485327	30.35366847
KCNMB3	rs6445035	403.3022768
KCNMB3	rs3863087	146.7699505
KCNMB3	rs148077724	51.02152992
KCNMB3	rs111266377	42.30654109
KCNMB3	rs9272195	39.29245129
KCNMB3	rs10096633	85.97165599
LRTM2	rs10801548	69.7826694
LRTM2	rs1329424	950.2543172
LRTM2	rs35351085	51.938086
LRTM2	rs6445035	128.5354285
LRTM2	rs3863087	43.57129293
LRTM2	rs1265941	115.7823224
LRTM2	rs550671	289.2210734
LRTM2	rs9269234	57.09693111
LRTM2	rs3117229	35.22312569
UGT2A1	rs368465	37.25352674
UGT2A1	rs10822155	42.55844329
UGT2A1	rs704	31.61116091
UGT2A1	rs113661219	33.65698315
UGT2A1	rs13412535	109.6762701
UGT2A1	rs6993770	86.96835849
KCNE5	rs192123799	48.1823193
KCNE5	rs10922098	168.5142943
KCNE5	rs9427493	63.52453332
KCNE5	rs2794457	36.46454211
KCNE5	rs2233726	33.24294212

KCNE5	rs146724217	38.92799577
KCNE5	rs7358384	140.3370944
KCNE5	rs117801489	40.27846486
KCNE5	rs150849265	29.77796579
KCNE5	rs9501600	54.127286
KCNE5	rs3853	44.85588252
SIGLEC11	rs12127364	971.239726
SIGLEC11	rs115886838	36.72548046
SIGLEC11	rs10922094	144.9706126
SIGLEC11	rs6542680	36.89122789
SIGLEC11	rs1355538	36.98052002
SIGLEC11	rs9501600	38.94397857
STIM1	rs1869085	124.9048876
STIM1	rs7934995	38.79848425
STIM1	rs4460801	35.02099073
STIM1	rs704	62.30772758
STIM1	rs1354034	34.38113123
PTK2B	rs10922094	38.37466361
PTK2B	rs964184	31.22045059
PTK2B	rs3816117	30.23543635
PTK2B	rs12992267	156.6853057
PTK2B	rs4665985	40.83675535
PTK2B	rs114115200	202.2123046
PTK2B	rs3863087	74.54114114
PTK2B	rs74615535	40.77665563
TMCC3	rs964184	143.5978532
TMCC3	rs2043085	30.72190775
TMCC3	rs60170459	42.77601944
TMCC3	rs78986976	45.56764645
TMCC3	rs769449	970.1057623
TMCC3	rs35136575	487.7144743
TMCC3	rs57355367	76.42279566
TMCC3	rs111411487	47.8175874
TMCC3	rs1260326	80.52928425
TMCC3	rs114115200	146.2264678
TMCC3	rs3863087	57.89246568
TMCC3	rs73169103	31.13592731
TMCC3	rs72848256	34.03394582
TMCC3	rs2954038	32.52177493
GALNT16	rs11629094	184.184881
GALNT16	rs1303451	67.31446679
GALNT16	rs12100668	408.5637505
GALNT16	rs9796392	31.6568196
GALNT16	rs3184504	87.99017536
GALNT16	rs148795948	33.68777658
RRM2B	rs28999668	363.0488383
RRM2B	rs4980320	43.43612648
RRM2B	rs1354034	100.4097387
RRM2B	rs2343592	31.48310887
BDP1	rs10922098	302.140866
BDP1	rs12995461	34.06559563
BDP1	rs1355538	51.52389487
BDP1	rs9501393	93.14180203
BDP1	rs7774739	59.28609645
BDP1	rs9271766	33.92501511
ENTPD6	rs6050446	208.9492991
ENTPD6	rs12085435	49.35994667
ENTPD6	rs617283	49.3439935

ENTPD6	rs10922094	105.0983645
ENTPD6	rs1706435	61.55197518
ENTPD6	rs9267139	29.77977283
ENTPD6	rs28573154	70.03657874
ENTPD6	rs7774739	65.718187
ABCC6	rs10922094	44.27625572
ABCC6	rs6542680	31.8999768
ABCC6	rs1355538	31.47983881
MRPL21	rs964184	69.15521323
MRPL21	rs4665383	55.50913906
MRPL21	rs6445035	136.9930639
MRPL21	rs3863087	45.69193831
MRPL21	rs2135058	30.78124325
MRPL21	rs74615535	40.25255993
NR1H4	rs10922098	401.0645917
NR1H4	rs1707652	185.3330222
NR1H4	rs9267139	37.44483553
NR1H4	rs9501393	117.4935929
NR1H4	rs7774739	87.98071895
NR1H4	rs9271766	39.43511615
CLCA2	rs10922098	127.8145437
CLCA2	rs704	37.11727699
CLCA2	rs438811	366.8309495
CLCA2	rs5112	89.30270298
CLCA2	rs72784378	33.57845614
CLCA2	rs6445035	102.0984954
CLCA2	rs3863087	36.83989016
CLCA2	rs11755689	45.42968534
CSPG4	rs10922098	279.5618808
CSPG4	rs1355538	59.21239042
CSPG4	rs9267139	30.43702469
CSPG4	rs9501393	75.53852958
CSPG4	rs7774739	50.82286736
MAP4K1	rs1329424	182.1858001
MAP4K1	rs11902299	62.53468746
MAP4K1	rs10183955	36.07138022
MAP4K1	rs4671944	285.1837008
MAP4K1	rs78496188	988.2999103
MAP4K1	rs1978974	51.82430909
MAP4K1	rs2576707	133.2399425
MAP4K1	rs1354034	47.86040029
MAP4K1	rs28573154	51.05487702
MAP4K1	rs7774739	35.63741721
GLT8D1	rs190119408	32.62516606
GLT8D1	rs144786001	42.39826921
GLT8D1	rs12035228	30.75344432
GLT8D1	rs72748418	39.74545325
GLT8D1	rs114009540	32.60815186
GLT8D1	rs10801548	245.7662927
GLT8D1	rs1329424	4361.333237
GLT8D1	rs35351085	124.4074118
GLT8D1	rs4497244	45.75759058
GLT8D1	rs1706435	42.41775777
GLT8D1	rs28573154	51.39578547
GLT8D1	rs7774739	37.3184748
SCARF2	rs738087	248.6201592
SCARF2	rs72704449	41.41746356
SCARF2	rs78689694	35.68089876

SCARF2	rs112153377	31.12388622
SCARF2	rs635634	83.2277731
SCARF2	rs41307428	33.55011572
CHL1	rs13077895	707.6484562
CHL1	rs466892	253.8262288
CHL1	rs6442794	118.1237522
CHL1	rs11129766	146.9848696
CHL1	rs10490842	43.55854113
CHL1	rs7937122	33.11972197
CHL1	rs2066985	97.74292351
CHL1	rs2057417	31.17850169
CHL1	rs12945886	56.05564642
CHL1	rs7253490	34.85345371
CHL1	rs17016909	47.87600878
CHL1	rs2454673	181.8728194
CHL1	rs958650	29.97154026
CHL1	rs11920688	99.10206124
ADAM17	rs10922098	252.8086261
ADAM17	rs1355538	66.74602831
ADAM17	rs6457457	39.94805671
ADAM17	rs9501393	64.47756242
ERAP2	rs3811952	79.62122849
ERAP2	rs7709763	1173.043651
ERAP2	rs888713	1288.460871
ERAP2	rs113007670	156.1890644
ERAP2	rs113728742	320.8159176
ERAP2	rs144063264	286.7056219
ERAP2	rs62376423	330.6621043
ERAP2	rs114499253	6863.474151
ERAP2	rs7705528	765.64987
ERAP2	rs150497532	32.06726199
ERAP2	rs79728738	99.42220318
ERAP2	rs7310615	31.90086424
ERAP2	rs75059312	33.75836596
ERAP2	rs35950258	32.75290083
ERAP2	rs16940186	45.16455316
ERAP2	rs36120431	31.7692706
ERAP2	rs112672253	32.68662722
ERAP2	rs116790552	39.04731579
ERAP2	rs113448956	51.69510831
ERAP2	rs78036869	67.96116975
ERAP2	rs72771211	42.82146313
ERAP2	rs76872289	86.10902422
ERAP2	rs115680316	59.13917738
ERAP2	rs146840726	69.27613094
ERAP2	rs13153882	145.0483628
ERAP2	rs6815	479.8688624
ERAP2	rs116575206	262.6788978
ERAP2	rs10066211	55.31291089
ERAP2	rs10041142	45.26574795
ERAP2	rs185880938	72.65523672
ERAP2	rs114530311	105.2851352
ERAP2	rs62357017	35.09731909
ERAP2	rs152558	141.6772173
ERAP2	rs80332069	52.41416011
ERAP2	rs78768201	33.28371906
ERAP2	rs191446308	50.09993228
ERAP2	rs72782129	58.03131987

ERAP2	rs77989040	37.91872379
ERAP2	rs62373640	31.52640271
ERAP2	rs13165038	37.30721051
ERAP2	rs9495450	30.18140021
ERAP2	rs187099314	82.27110937
RPS6KB1	rs10922094	442.2769925
RPS6KB1	rs6512033	926.4945947
RPS6KB1	rs78464136	42.5125403
RPS6KB1	rs2686395	78.00452388
RPS6KB1	rs9267139	52.79772382
RPS6KB1	rs550671	119.7922542
RPS6KB1	rs9271766	38.25190952
VTI1B	rs12292693	48.08976324
VTI1B	rs185009887	31.84341762
VTI1B	rs1354034	68.33931162
DUSP26	rs10922098	213.8146028
DUSP26	rs71352239	53.44687336
DUSP26	rs10208529	36.56930238
DUSP26	rs1707652	129.2325747
DUSP26	rs444921	60.89224231
DUSP26	rs34927613	33.00033652
CD14	rs114928388	80.70308638
CD14	rs5744454	472.1296692
CD14	rs7411767	51.63564255
CD14	rs10922098	76.50796347
CD14	rs2477664	78.93527467
CD14	rs2461161	81.70502703
CD14	rs1377416	30.04763835
CD14	rs9878990	34.28610818
CD14	rs6457457	34.90032171
CD14	rs635634	59.15843344
RIPK2	rs160438	51.06072531
RIPK2	rs4980320	37.93354096
RIPK2	rs1354034	41.24855441
NRXN1	rs964184	79.48454667
NRXN1	rs62087820	35.25652268
NRXN1	rs73051460	38.49113019
NRXN1	rs387190	101.3515202
NRXN1	rs62119281	155.8469213
NRXN1	rs394353	95.17605712
NRXN1	rs283813	300.0335352
NRXN1	rs73045691	57932.93494
NRXN1	rs77660932	663.9472801
NRXN1	rs1049739	292.4396287
NRXN1	rs8101216	65.84455676
NRXN1	rs117629091	70.44867286
NRXN1	rs2678379	47.09288784
NRXN1	rs111696008	40.87915754
NRXN1	rs11720167	477.950013
NRXN1	rs3863087	163.4078321
NRXN1	rs148077724	69.23130041
NRXN1	rs111266377	38.4248693
NRXN1	rs147033786	34.17647393
NRXN1	rs4704826	58.37567316
FCRL4	rs113219259	107.8131047
FCRL4	rs34296440	189.8065431
FCRL4	rs59788339	1076.255005
FCRL4	rs12039758	937.0692192

FCRL4	rs1412330	1025.374026
FCRL4	rs9787108	96.92911757
FCRL4	rs139610741	45.84551551
FCRL4	rs12124727	56.24681835
FCRL4	rs4606261	34.93364466
FCRL4	rs191615076	46.6602858
FCRL4	rs10910830	32.27380515
FCRL4	rs61751627	168.1281782
FCRL4	rs76616066	47.04048228
FCRL4	rs857680	60.86135166
FCRL4	rs12126715	71.77122451
FCRL4	rs10788619	35.75536677
FCRL4	rs9419741	32.86946072
FCRL4	rs1407	60.4419287
FCRL4	rs58036957	31.5786129
FCRL4	rs8052064	30.41273273
FCRL4	rs79129533	31.95277298
FCRL4	rs9675122	89.81651177
FCRL4	rs78269692	38.43728046
FCRL4	rs1432296	33.62464592
FCRL4	rs1736961	39.56176133
FCRL4	rs2516456	40.02726889
FCRL4	rs4959105	41.16220708
FCRL4	rs148317837	39.72072993
FCRL4	rs2511739	33.37954064
FCRL4	rs77328788	98.3071742
COL15A1	rs7851057	328.8706453
COL15A1	rs56997979	35.01580588
COL15A1	rs16918163	298.1925359
COL15A1	rs1031755	34.29493763
COL15A1	rs77924615	62.06363912
COL15A1	rs9992101	35.87381329
COL15A1	rs62435145	36.3100317
COL15A1	rs10253736	31.79219243
COL15A1	rs1360098	47.673039
CD200R1L	rs1065853	252.6625832
CD200R1L	rs144094035	29.83466545
CD200R1L	rs116751091	34.24372907
CD200R1L	rs67399078	95.11037482
THBS3	rs80204403	58.10775538
THBS3	rs72704117	138.7675457
THBS3	rs61732802	58.11956775
THBS3	rs72697252	32.40809852
THBS3	rs139293838	51.05078072
THBS3	rs113851011	29.75653089
THBS3	rs200250001	31.34389572
THBS3	rs78705678	89.57886861
THBS3	rs2477642	82.21121152
THBS3	rs56344867	60.14543809
THBS3	rs3184504	42.32740801
THBS3	rs55714927	80.11116279
THBS3	rs143501783	36.90651282
THBS3	rs2277998	45.36778033
THBS3	rs12974746	30.74454326
THBS3	rs11748596	139.0778668
THBS3	rs2290471	42.39503284
GOLM1	rs113968521	108.6918271
GOLM1	rs7854118	315.4489375

GOLM1	rs112567181	79.11781667
GOLM1	rs201823050	55.69122142
GOLM1	rs11216257	33.5123973
GOLM1	rs12945299	33.3639997
GOLM1	rs708686	29.83137493
GOLM1	rs4011541	48.18999695
GOLM1	rs368565	1047.883792
GOLM1	rs111277780	110.0888215
GOLM1	rs45518331	31.54843979
GOLM1	rs3115574	49.88201888
GOLM1	rs787891	48.54882059
GOLM1	rs375994438	962.7955535
GOLM1	rs176694	371.062024
GOLM1	rs28446901	880.5437716
GOLM1	rs150730585	46.73688261
GOLM1	rs618439	43.31322011
GOLM1	rs139562936	56.94962851
SERAC1	rs10922098	409.0049038
SERAC1	rs704	47.60143236
SERAC1	rs1707652	83.81699213
SERAC1	rs6457457	77.68296861
SERAC1	rs3891366	120.7695727
SCUBE1	rs11704700	84.35315276
SCUBE1	rs6003130	39.63520973
SCUBE1	rs5759270	46.92061627
SCUBE1	rs695786	198.7503292
SCUBE1	rs2068888	30.45095775
SCUBE1	rs7974902	90.41392189
SCUBE1	rs77542162	30.989589
SCUBE1	rs117579730	71.35776898
SCUBE1	rs1260326	75.97907761
SCUBE1	rs6018653	36.27883975
SCUBE1	rs42122	88.08516034
SCUBE1	rs73137074	33.00796663
SCUBE1	rs28601761	62.10687676
ATP1B3	rs4512628	34.30418079
ATP1B3	rs10922098	800.05628
ATP1B3	rs35351085	39.14066264
ATP1B3	rs1047286	42.76467406
ATP1B3	rs2686395	162.0602445
ATP1B3	rs1265941	83.17882546
ATP1B3	rs550671	203.39424
ATP1B3	rs9269234	37.9927701
RIPK2	rs7625980	136.3021416
RIPK2	rs368870400	94.09439352
RIPK2	rs376911700	40.76852915
SLAMF8	rs17111540	33.27493079
SLAMF8	rs13379369	36.72884487
SLAMF8	rs1518722	30.6429567
SLAMF8	rs144272926	107.7687058
SLAMF8	rs72490085	921.2166646
SLAMF8	rs4089292	372.8473951
SLAMF8	rs2580539	124.3034004
SLAMF8	rs28784889	323.7246132
SLAMF8	rs12615758	70.29251368
SLAMF8	rs73169674	70.58418025
HS3ST4	rs10922094	59.72560656
HS3ST4	rs6542680	30.52058655

HS3ST4	rs3828879	31.80528071
CEACAM3	rs10922098	199.5411392
CEACAM3	rs1707652	114.0885816
CEACAM3	rs9501393	66.08214059
CEACAM3	rs7774739	55.17218196
SLC3A1	rs10922098	341.3869523
SLC3A1	rs704	45.73861416
SLC3A1	rs2230203	31.7868223
SLC3A1	rs1355538	68.82281064
SLC3A1	rs9501393	81.10409744
SLC3A1	rs7774739	62.16172969
SERPINA11	rs7156347	55.44036862
SERPINA11	rs72631635	716.5705325
SERPINA11	rs9671989	123.9501121
SERPINA11	rs77403344	51.75846832
SERPINA11	rs59233955	43.6840333
SERPINA11	rs144507412	33.20165311
SERPINA11	rs10740134	64.24609045
SERPINA11	rs375452507	34.74654032
SERPINA11	rs34717890	64.99689748
SERPINA11	rs4665972	60.70573896
SERPINA11	rs12468558	40.8385892
SERPINA11	rs149062332	78.27633677
SERPINA11	rs4484649	48.80248596
MARCO	rs10922098	359.8082414
MARCO	rs704	107.9106845
MARCO	rs1047286	33.59904412
MARCO	rs41338844	41.77762581
MARCO	rs142275326	109.5108745
MARCO	rs140678946	96.32357304
MARCO	rs11714342	34.21633912
MARCO	rs1355538	63.74352167
MARCO	rs9501393	99.76812516
MARCO	rs7774739	55.10995884
MARCO	rs35058673	49.21426179
LDLRAD4	rs72865316	62.14126411
LDLRAD4	rs9907259	61.55343739
LDLRAD4	rs1354034	299.717838
LDLRAD4	rs686250	46.85563272
PLXNA1	rs12695485	42.48679276
PLXNA1	rs4679138	828.8037375
PLXNA1	rs79118580	34.32675975
PLXNA1	rs1984769	36.29773678
PLXNA1	rs2477639	77.85624545
PLXNA1	rs692440	39.3796466
PLXNA1	rs56344867	68.39298255
PLXNA1	rs181707610	30.67902692
PLXNA1	rs1042704	152.3723884
PLXNA1	rs8011224	59.27692857
PLXNA1	rs55714927	49.22608267
PLXNA1	rs1260326	48.03652285
PLXNA1	rs141511547	31.28598631
PLXNA1	rs9270747	48.05472246
PRRG1	rs10922098	224.413378
PRRG1	rs2856876	42.18771659
PRRG1	rs1707652	83.91951816
PRRG1	rs6457457	43.19502606
PRRG1	rs3891366	64.29012115

PRPF6	rs10922094	306.5799689
PRPF6	rs2686395	55.94436183
PRPF6	rs28573154	87.29141447
PRPF6	rs7774739	70.50124884
PRG3	rs61886960	96.2380753
PRG3	rs1234212	30.57449008
PRG3	rs117024075	31.96007386
PRG3	rs4766578	103.8538472
PRG3	rs7323613	103.9573705
PRG3	rs78198540	78.19603408
PRG3	rs2694616	294.1629565
PRG3	rs13020553	42.12872238
PRG3	rs17354348	68.64329953
PRG3	rs2242886	37.75935928
PRG3	rs11701383	34.86712946
PRG3	rs13089722	250.3764473
PRG3	rs56003704	41.68433319
PRG3	rs10068466	33.53964288
PRG3	rs12174396	55.62439258
PRG3	rs78819516	31.97131188
PRG3	rs1870028	58.42136976
PRG3	rs513973	36.17960736
PRG3	rs13256023	138.6921663
PRG3	rs7840212	80.54250802
PRG3	rs57993781	55.95838553
NR1H2	rs10922098	290.677132
NR1H2	rs3824988	90.61140515
NR1H2	rs7948571	31.51743548
NR1H2	rs9567607	53.29199313
NR1H2	rs704	372.0648931
NR1H2	rs12478841	48.83598824
NR1H2	rs1707652	105.4239777
NR1H2	rs550671	98.17146912
NR1H2	rs9469195	63.40413488
LCT	rs12614226	250.0645743
LCT	rs77854115	628.3998581
LCT	rs78918822	542.0273347
LCT	rs13426918	399.9053994
LCT	rs12477662	134.137588
LCT	rs78705678	72.02814062
LCT	rs2477642	35.57705839
LCT	rs11195909	30.17107466
LCT	rs10846742	33.70105964
LCT	rs2216812	30.02269603
LCT	rs616114	440.8648264
LCT	rs141079659	31.95138405
LCT	rs11564538	29.77501182
LCT	rs34953075	74.43273904
LCT	rs368565	1061.711972
LCT	rs142430894	37.36637553
LCT	rs479980	1219.920391
LCT	rs139040889	70.7147341
LCT	rs374187016	73.76482218
LCT	rs145169212	48.69485324
LCT	rs150543594	41.61378197
LCT	rs4705877	56.92298152
LCT	rs9270334	42.45710468
LCT	rs3129747	48.87245244

LCT	rs635634	377.0068724
LCT	rs41307428	53.32178215
PCDH10	rs72715776	279.6203445
PCDH10	rs138664559	37.15269622
PCDH10	rs143830859	169.8108705
PCDH10	rs61747728	44.40590317
PCDH10	rs77924615	32.92866991
PCDH10	rs12475061	35.38113602
HAVCR1	rs5872468	107.4366469
HAVCR1	rs4704821	1030.860128
HAVCR1	rs452223	314.0550918
HAVCR1	rs149116516	32.53788869
HAVCR1	rs10138174	29.74850242
HAVCR1	rs261290	45.43345069
HAVCR1	rs77924615	121.5943624
HAVCR1	rs34931250	103.9519456
HAVCR1	rs77262773	44.30756434
HAVCR1	rs321098	30.50804948
HAVCR1	rs593637	37.47310148
HAVCR1	rs116253556	34.87645464
HAVCR1	rs150767969	42.98517035
DEFB132	rs72748420	43.2654717
DEFB132	rs1329424	1258.445436
DEFB132	rs3900909	56.70697955
DEFB132	rs10801660	40.74011197
DEFB132	rs704	39.84745337
DEFB132	rs11080290	31.27685402
DEFB132	rs1047286	50.31566623
DEFB132	rs4665987	30.2584313
DEFB132	rs111696008	32.17619666
DEFB132	rs1707652	207.6559855
DEFB132	rs3099847	41.81118804
DEFB132	rs1265941	117.1612345
DEFB132	rs550671	333.0832053
DEFB132	rs9276606	49.01284255
AHSP	rs4512628	30.90861582
AHSP	rs10922098	618.2199027
AHSP	rs965344	44.20105424
AHSP	rs111696008	36.45870258
AHSP	rs1707652	187.1935346
AHSP	rs550671	170.3007387
AHSP	rs9469195	65.67914451
BTNL8	rs2387717	359.9741692
BTNL8	rs76546858	65.73769426
BTNL8	rs10922098	30.66613967
BTNL8	rs601338	126.1718858
BTNL8	rs6542680	31.31148359
NLGN2	rs10922094	73.71604246
NLGN2	rs6542680	33.6408326
NLGN2	rs3129773	35.6221752
MTPAP	rs10922094	331.903086
MTPAP	rs704	98.54751952
MTPAP	rs1707652	75.39052686
MTPAP	rs9267139	31.77034195
MTPAP	rs28573154	87.71179838
MTPAP	rs7774739	58.36361726
ROS1	rs1451915	33.53770814
ROS1	rs10922098	618.505088

ROS1	rs7513100	30.65300911
ROS1	rs11569415	37.77978211
ROS1	rs1707652	113.2827242
ROS1	rs1265941	65.94368721
ROS1	rs453821	151.7017302
JTB	rs10922098	382.9836627
JTB	rs10777170	116.2107902
JTB	rs2256952	89.7631547
JTB	rs1707652	102.1703315
JTB	rs9501393	81.87478345
JTB	rs7774739	53.43489351
JTB	rs9271766	32.12893016
JTB	rs11244049	32.83276856
TOR1AIP1	rs4654748	54.05229596
TOR1AIP1	rs1591734	213.4275168
TOR1AIP1	rs61808983	609.2488607
TOR1AIP1	rs41272457	69.85781122
TOR1AIP1	rs2187955	428.3310708
TOR1AIP1	rs7894992	190.7535746
TOR1AIP1	rs10159712	452.6181519
TOR1AIP1	rs10437425	58.56347982
TOR1AIP1	rs11012153	42.57974192
TOR1AIP1	rs704	273.497459
TOR1AIP1	rs594793	42.83595444
TOR1AIP1	rs6544941	69.29667277
TOR1AIP1	rs9257052	50.98453628
TOR1AIP1	rs1071649	35.70205933
TOR1AIP1	rs1265764	42.53330584
TOR1AIP1	rs28366220	119.7150834
TOR1AIP1	rs34958241	224.5355411
TOR1AIP1	rs9271669	92.45491615
TOR1AIP1	rs35814008	30.83307964
EPDR1	rs10922094	147.3041512
EPDR1	rs1355538	50.47960898
EPDR1	rs9469077	35.02921466
TFIP11	rs10922098	624.9646273
TFIP11	rs1047286	39.49793824
TFIP11	rs1707652	129.58827
TFIP11	rs2242572	159.7401131
TFIP11	rs9501602	74.31610339
TFIP11	rs9271529	32.26671285
CNTNAP5	rs10922098	354.8520478
CNTNAP5	rs4795434	33.47301412
CNTNAP5	rs1707652	71.13342164
CNTNAP5	rs453821	86.8146523
CNTNAP5	rs9469195	36.03051422
SLC27A2	rs10922094	92.26713733
SLC27A2	rs1354034	37.24025789
SLC27A2	rs697356	41.54491714
CRIP2	rs10922098	50.44041816
CRIP2	rs188530181	136.0011043
CRIP2	rs26515	113.3035538
RAPGEF5	rs114009540	30.43472541
RAPGEF5	rs10922098	817.8883672
RAPGEF5	rs3900909	39.6381692
RAPGEF5	rs1047286	39.99211443
RAPGEF5	rs1707652	137.7922942
RAPGEF5	rs1265941	89.25387179

RAPGEF5	rs550671	211.2038333
RAPGEF5	rs145704749	34.68113731
IGSF9B	rs10922098	115.2161739
IGSF9B	rs6542680	53.47730442
IGSF9B	rs9864801	39.63703353
IGSF9B	rs1042663	35.83773772
ARHGEF10	rs188303944	66.02017786
ARHGEF10	rs10468017	59.85242974
ARHGEF10	rs6083	75.29533834
ARHGEF10	rs4665383	42.59238218
RNF215	rs10922094	30.82421446
RNF215	rs4980320	33.50074198
RNF215	rs185009887	42.10005599
RNF215	rs1354034	38.12429097
RNF215	rs9864801	34.45303992
JAKMIP3	rs10922094	98.05140172
JAKMIP3	rs216311	45.30233386
JAKMIP3	rs1884547	140.6527452
JAKMIP3	rs11714743	36.82425811
JAKMIP3	rs201699955	136.3316554
JAKMIP3	rs6802320	1011.831145
JAKMIP3	rs3774558	72.43612231
JAKMIP3	rs13319916	40.04141929
JAKMIP3	rs71301892	62.45823148
JAKMIP3	rs697356	49.0335412
JAKMIP3	rs9501600	37.88290974
FAXDC2	rs61808983	43.87936206
FAXDC2	rs10922098	194.3207187
FAXDC2	rs1706435	51.20363661
FAXDC2	rs9501393	51.4404305
FAXDC2	rs7774739	41.8352535
PENK	rs181978437	37.29363357
PENK	rs138079906	84.63155473
PENK	rs11781555	183.4300478
PENK	rs13263968	973.0522877
PENK	rs2610035	447.7889345
PENK	rs117021828	191.8734117
PENK	rs112747675	97.04010368
PENK	rs72683129	52.9808368
PENK	rs4633781	56.4846992
PENK	rs7658601	213.6663254
PENK	rs9275052	39.94953384
PENK	rs112363235	54.6379116
PENK	rs200336971	29.941829
PENK	rs28408880	31.01781217
PENK	rs139381640	34.46892191
PENK	rs576123	43.00873209
MAN1A2	rs147472694	79.3745623
MAN1A2	rs1998921	32.8119913
MAN1A2	rs12117281	136.9320465
MAN1A2	rs73013841	495.8072243
MAN1A2	rs4452991	40.24494568
MAN1A2	rs28549707	45.34951683
MAN1A2	rs139783923	34.3948235
MAN1A2	rs11122449	41.27551827
MAN1A2	rs2947595	51.2075
MAN1A2	rs2477664	228.057263
MAN1A2	rs2461161	248.7753422

MAN1A2	rs2354447	34.69130836
MAN1A2	rs77542162	45.11841332
MAN1A2	rs28873836	124.8894478
MAN1A2	rs463250	30.46533482
MAN1A2	rs17138656	37.94098998
MAN1A2	rs3097671	36.65825497
MAN1A2	rs635634	201.5712322
SNTB1	rs4980320	46.62233342
SNTB1	rs12819689	38.64116785
SNTB1	rs1354034	84.83480987
SNTB1	rs114694170	46.96022751
SNTB1	rs342200	42.14258585
SLC30A3	rs4512628	34.25145731
SLC30A3	rs10922098	740.0157049
SLC30A3	rs35351085	32.40860033
SLC30A3	rs1047286	60.09932764
SLC30A3	rs111696008	32.13758318
SLC30A3	rs1707652	150.2334735
SLC30A3	rs1265941	83.94337997
SLC30A3	rs550671	215.8325925
SLC30A3	rs9276606	54.11806317
SEL1L2	rs1329424	967.99907
SEL1L2	rs3900909	45.83779976
SEL1L2	rs10801660	30.75418776
SEL1L2	rs704	74.36388117
SEL1L2	rs11080290	31.65364529
SEL1L2	rs1047286	40.52930596
SEL1L2	rs111696008	29.7801262
SEL1L2	rs1707652	222.2395933
SEL1L2	rs73169117	34.98165458
SEL1L2	rs3099847	38.99620089
SEL1L2	rs1265941	100.0073107
SEL1L2	rs550671	264.697283
SEL1L2	rs9276606	41.86359286
NHLRC3	rs10922098	69.83479388
NHLRC3	rs2731171	37.38816696
NHLRC3	rs11845244	90.3444212
NHLRC3	rs73216598	80.15121984
NHLRC3	rs2057681	830.2854704
NHLRC3	rs854572	99.87054847
NHLRC3	rs2299269	36.42408541
ANGPTL1	rs11581618	42.05088109
ANGPTL1	rs71630204	81.6181708
ANGPTL1	rs10922098	427.215389
ANGPTL1	rs117516437	60.39143726
ANGPTL1	rs10929302	63.16232501
ANGPTL1	rs2686395	75.24323944
ANGPTL1	rs9267139	50.07933199
ANGPTL1	rs454212	108.722883
CLEC4C	rs151250036	227.6130644
CLEC4C	rs75808010	1360.800678
CLEC4C	rs11055473	4215.873472
CLEC4C	rs7312313	806.6642288
CLEC4C	rs117812534	244.7518943
CLEC4C	rs141613637	66.74256944
CLEC4C	rs7129402	181.5965555
CLEC4C	rs73267361	40.89284201
CLEC4C	rs17804845	145.9415991

CLEC4C	rs11051491	53.81770133
CLEC4C	rs3184504	30.10982139
CLEC4C	rs76428106	162.7045147
CLEC4C	rs9508005	32.65632677
CLEC4C	rs62045817	42.11248656
CLEC4C	rs2331413	39.82986104
CLEC4C	rs199970514	31.6256574
CLEC4C	rs11125837	31.52114898
CLEC4C	rs2661797	63.23927073
CLEC4C	rs13012495	42.54832571
CLEC4C	rs1837369	83.93691346
CLEC4C	rs77852018	33.54245331
CLEC4C	rs7684212	48.29388652
CLEC4C	rs17216465	73.91231579
CLEC4C	rs6818511	175.7788164
CLEC4C	rs7762909	59.18330721
CLEC4C	rs3104368	38.06992616
CLEC4C	rs6796	59.23258899
CLEC4C	rs876037	356.3171808
CLEC4C	rs2395906	35.79057481
CLEC4C	rs10971418	44.8918842
CLEC4C	rs7873862	50.84539789
SYT5	rs10922098	120.5139037
SYT5	rs76525968	32.25330111
SYT5	rs113448098	71.38692593
SYT5	rs12146727	225.3641527
SYT5	rs12897751	59.19693922
SYT5	rs12897067	30.21639249
SYT5	rs916547	57.97437775
SYT5	rs4713498	39.68029064
C5orf46	rs35340377	42.08536406
C5orf46	rs7073753	30.1864213
C5orf46	rs4980320	37.90916245
C5orf46	rs814131	69.01758988
C5orf46	rs6445035	133.6407893
C5orf46	rs3863087	45.90439328
C5orf46	rs4541868	76.09123214
ZFYVE27	rs17736427	30.57327612
ZFYVE27	rs72701845	62.7480342
ZFYVE27	rs1354034	31.55075665
ZFYVE27	rs61751211	337.8359615
ZFYVE27	rs11732892	55.19636541
ZFYVE27	rs72654977	30.71797831
ARMC10	rs424772	45.72838917
ARMC10	rs1619994	56.94034307
ARMC10	rs9501393	36.2052095
SYT17	rs572515	45.80607798
SYT17	rs10822155	32.93227594
SYT17	rs557011	30.10605172
JPH4	rs10922098	72.41803479
JPH4	rs28362944	36.58147733
JPH4	rs12146727	29.88390883
JPH4	rs9379919	35.29958372
JPH4	rs9262696	349.0591439
JPH4	rs1634781	43.20109116
JPH4	rs41548618	44.49323997
JPH4	rs9269967	184.6219794
JPH4	rs28986789	62.99716671

UMODL1	rs10922098	160.2058203
UMODL1	rs10208529	29.73585408
UMODL1	rs147906938	41.97828169
ADAMDEC1	rs368465	51.13002482
ADAMDEC1	rs6542680	74.22013789
ADAMDEC1	rs16850360	32.86965296
HEPACAM2	rs10922098	274.1385918
HEPACAM2	rs2686395	62.27484744
HEPACAM2	rs6457457	61.35261273
HEPACAM2	rs28573154	91.8495361
HEPACAM2	rs6451	31.25686014
PSD2	rs10922094	114.4830161
PSD2	rs12026141	54.29055764
PSD2	rs2068888	31.65992793
PSD2	rs964184	120.7284282
PSD2	rs200662911	63.47878713
PSD2	rs112450640	77.26523641
PSD2	rs78986976	141.4534798
PSD2	rs73045691	3661.638612
PSD2	rs113708952	61.21500306
PSD2	rs10411862	135.9830495
PSD2	rs2871768	37.87120144
PSD2	rs673548	48.92215374
PSD2	rs780093	33.23412746
PSD2	rs62182810	45.25916353
PSD2	rs111696008	96.62594795
PSD2	rs144202860	45.9879169
PSD2	rs687339	81.14631836
PSD2	rs62290472	34.14874815
PSD2	rs76315187	58.70870551
PSD2	rs114115200	815.8926689
PSD2	rs3863087	375.2313149
PSD2	rs73169103	75.01045679
PSD2	rs6874202	108.6115233
PSD2	rs453821	32.18972303
PSD2	rs28471056	45.6737212
FUT2	rs10922098	135.2207258
FUT2	rs6445035	87.86810443
FUT2	rs550671	66.45575426
FUT2	rs28471056	38.74043418
MASP1	rs10922098	165.1041693
MASP1	rs7774739	32.21763565
MASP1	rs7742632	44.83522585
NT5DC3	rs954921	443.3554858
NT5DC3	rs74517500	317.7690612
NT5DC3	rs1165681	37.53954722
SLC26A5	rs375046	58.95677198
SLC26A5	rs3129773	31.75042142
SLC26A5	rs13208583	30.2741994
FLRT3	rs10922098	306.319131
FLRT3	rs1047286	34.74943492
FLRT3	rs6542680	41.17699927
FLRT3	rs1706435	76.52357313
FLRT3	rs444921	81.64634973
FLRT3	rs28471056	46.00915052
CCL26	rs572515	245.5719345
CCL26	rs12403425	29.91321131
CCL26	rs11720167	53.66467137

CCL26	rs9501600	58.54896992
CCL26	rs557011	30.90084901
CSRP3	rs4980320	31.5707282
CSRP3	rs342200	44.04089559
CSRP3	rs7801889	67.20505562
MMP8	rs117557731	195.5734956
MMP8	rs35231465	632.9401484
MMP8	rs10922094	44.55897252
MMP8	rs75422278	39.73624217
MMP8	rs115512583	35.3657799
MMP8	rs78425657	36.09942227
MMP8	rs181016664	29.91981026
MMP8	rs1465528	49.20590678
MMP8	rs8075668	79.6528583
MMP8	rs429358	475.8087558
MMP8	rs6960617	230.2013754
MMP8	rs10640497	60.49410749
MMP8	rs66595754	69.88286426
MMP8	rs331	31.40639331
MMP8	rs307736	62.87365333
MMP8	rs4961341	31.49163693
PGM1	rs7535245	30.25713849
PGM1	rs61765292	125.2943713
PGM1	rs10922098	123.5716835
PGM1	rs1355538	53.31187799
PGM1	rs9501393	42.83518608
PGM1	rs28471056	31.57872731
DSCAM	rs6517602	49.64521647
DSCAM	rs78111814	362.6746079
DSCAM	rs146320719	64.07978443
DSCAM	rs10922098	137.5439463
DSCAM	rs2477642	60.98375053
DSCAM	rs2461139	43.77782763
DSCAM	rs2461164	39.64786845
DSCAM	rs1355538	41.70705427
DSCAM	rs147906938	41.92995441
DSCAM	rs9272780	34.41545539
MUC1	rs1451915	30.6977874
MUC1	rs10922098	685.7793906
MUC1	rs704	338.9760761
MUC1	rs111696008	31.83418008
MUC1	rs1707652	169.123292
MUC1	rs1265941	79.33981187
MUC1	rs550671	176.8542712
MUC1	rs9276606	36.37904666
FAM3B	rs150417632	51.32255946
FAM3B	rs73224189	931.0735932
FAM3B	rs434411	607.8974912
FAM3B	rs140935232	58.91887681
FAM3B	rs111752961	265.1354483
FAM3B	rs982870	30.86934385
FAM3B	rs117041662	195.161069
FAM3B	rs2559851	93.77721435
FAM3B	rs77924615	30.16986795
FAM3B	rs731541	44.44193147
FAM3B	rs708686	201.0314159
FAM3B	rs492602	111.4215368
FAM3B	rs67940439	56.76949007

FAM3B	rs635634	380.4999042
LRP1	rs10922098	43.46521317
LRP1	rs35887873	33.21305593
LRP1	rs62061425	32.13024648
LRP1	rs78810414	37.38083222
IFNAR1	rs2834027	252.1802939
IFNAR1	rs67172970	122.6127372
IFNAR1	rs117031240	62.19355795
IFNAR1	rs58886971	3729.929333
IFNAR1	rs139340327	1310.960987
IFNAR1	rs183019916	461.9909847
IFNAR1	rs12627454	340.5325209
IFNAR1	rs12720356	108.9155044
IFNAR1	rs2409426	51.45487142
IFNAR1	rs117718349	230.8136656
IFNAR1	rs73210910	57.09793057
IFNAR1	rs635634	89.95609806
TFF1	rs220250	37.84180215
TFF1	rs225358	1039.018225
TFF1	rs117396825	154.5946001
TFF1	rs2990223	158.5234807
TFF1	rs71469854	143.2761747
TFF1	rs80334578	43.53173468
TFF1	rs143227779	36.12998088
TFF1	rs12990985	371.8578187
TFF1	rs62133344	853.7688033
TFF1	rs13387607	107.1696699
TFF1	rs79188300	54.39430784
TFF1	rs1047891	71.45318021
TFF1	rs13141329	38.79566853
TFF1	rs10094872	31.81936993
TFF1	rs10817564	35.96319555
HMGN1	rs73182299	149.8736458
HMGN1	rs146093390	40.99729563
HMGN1	rs4686788	141.4338612
HMGN1	rs7627277	3050.019742
HMGN1	rs1501299	37.43175997
CXCL9	rs2276886	60.12642506
CXCL9	rs7137828	77.19116852
CXCL9	rs113341849	33.23150171
CXCL9	rs3129882	35.74327537
CXCL9	rs9270555	41.71478897
TFF2	rs77505241	112.6878087
TFF2	rs225313	37.70654224
TFF2	rs2990223	91.76572173
TFF2	rs71469854	110.2873788
TFF2	rs80334578	47.01863269
TFF2	rs10675603	30.3691248
TFF2	rs9535108	78.8112955
TFF2	rs12945299	57.22041136
TFF2	rs1797886	94.1868568
TFF2	rs9878990	30.91851335
TFF2	rs113722899	30.89165742
TFF2	rs2585183	47.82837964
LGALS7	rs10822155	34.67786669
LGALS7	rs533617	47.38361277
LGALS7	rs34762726	34.02359296
LGALS7	rs1619994	38.21879964

LGALS7	rs6993770	30.45732347
LGALS9	rs9896600	205.9152176
LGALS9	rs10922098	316.1949945
LGALS9	rs516246	46.95348575
LGALS9	rs2686395	59.57672708
LGALS9	rs9267139	32.01482663
LGALS9	rs9501393	62.14258534
LGALS9	rs7774739	42.20372606
UBE2G2	rs10922094	64.31377107
UBE2G2	rs429358	48.3674845
UBE2G2	rs409632	31.26557851
UBE2G2	rs9501342	31.39915837
UBE2G2	rs28474889	127.5227524
UBE2G2	rs148766553	71.79286761
UBE2G2	rs6458	261.8864835
UBE2G2	rs9273325	59.39426928
UBE2G2	rs12378445	58.7485968
POMC	rs72797924	73.43655695
POMC	rs139403245	54.12149683
POMC	rs2180234	147.4223668
POMC	rs34501420	35.5969333
POMC	rs28664725	30.8891145
POMC	rs8045875	247.8644796
POMC	rs877127	61.52207577
POMC	rs145530718	120.9092265
POMC	rs718928	84.5987285
POMC	rs4075810	32.38058208
POMC	rs12616772	34.14697751
POMC	rs8126001	89.84558264
POMC	rs27289	31.52577197
POMC	rs1529866	32.61745308
POMC	rs71024759	79.83863531
POMC	rs11774829	39.68754952
CRYZL1	rs9436221	39.84041305
CRYZL1	rs964184	224.8088248
CRYZL1	rs141469619	41.24853072
CRYZL1	rs2043085	35.2900512
CRYZL1	rs17715069	58.01791848
CRYZL1	rs483082	975.7610166
CRYZL1	rs5112	639.7810014
CRYZL1	rs57355367	53.47154707
CRYZL1	rs55797612	31.72490252
CRYZL1	rs676210	35.52752624
CRYZL1	rs780093	94.5131972
CRYZL1	rs1707652	67.8744104
CRYZL1	rs78810414	52.54990543
CRYZL1	rs2954038	36.37690875
SERPINF1	rs59096313	961.5913129
SERPINF1	rs6503063	78.03656754
SERPINF1	rs72704449	40.55946723
SERPINF1	rs72817385	74.46885567
SERPINF1	rs74673621	51.16529938
SERPINF1	rs245446	31.14460506
CTSF	rs1791679	444.3295037
CTSF	rs116675228	37.87648704
CTSF	rs117945386	31.83495877
CTSF	rs146936815	29.98913828
CTSF	rs80300795	31.50336381

CTSF	rs825038	71.81905694
CTSF	rs7964859	868.251815
CTSF	rs7955091	31.15005136
CTSF	rs140624338	127.0700637
CTSF	rs956886	41.72552404
CTSF	rs2244608	31.10885789
CTSF	rs147233090	109.2967036
CTSF	rs429358	46.28644089
CTSF	rs8736	36.36360926
CTSF	rs1260326	153.1855332
CTSF	rs61791757	46.40134191
CTSF	rs189319377	69.12622358
CTSF	rs147762032	55.32095954
CTSF	rs138343081	31.17903617
CTSF	rs139366576	38.90964051
CTSF	rs4722551	38.60068298
CTSF	rs28601761	39.36604568
FTCD	rs117167824	30.4443333
FTCD	rs7717	104.2492451
FTCD	rs61735836	2877.824413
FTCD	rs113030590	145.1875436
FTCD	rs2154576	149.5359042
FTCD	rs12165290	34.19661703
FTCD	rs3747207	38.78416164
FTCD	rs2980880	33.12947426
USP25	rs572515	88.04403564
USP25	rs9971407	42.74306334
USP25	rs1707652	32.95225863
USP25	rs114898621	40.85803058
USP25	rs7741329	49.34899311
USP25	rs146224174	416.4218019
USP25	rs3021366	997.3854824
USP25	rs2284179	50.15006367
USP25	rs2228296	72.10909932
USP25	rs143330132	399.1674715
USP25	rs143867680	896.0876344
USP25	rs55928678	64.59424036
PLXNB2	rs73183310	182.7146946
PLXNB2	rs5771217	302.0119193
PLXNB2	rs11703790	919.5299707
PLXNB2	rs28553432	221.7613715
PLXNB2	rs148201653	45.72399165
PLXNB2	rs61801013	130.9865673
PLXNB2	rs1277443	32.66962235
PLXNB2	rs34666101	135.8060302
PLXNB2	rs2477669	55.11957318
PLXNB2	rs78705678	417.4323267
PLXNB2	rs2477642	469.9047973
PLXNB2	rs868876	38.56975672
PLXNB2	rs1260326	69.56822471
PLXNB2	rs142982411	30.66399034
PLXNB2	rs62259782	30.1328929
PLXNB2	rs1265889	40.56932385
PLXNB2	rs635634	88.16382747
EPHB3	rs10922094	75.84646476
EPHB3	rs4774178	52.58863438
EPHB3	rs3814923	348.9257852
EPHB3	rs16559	30.47480717

EPHB3	rs3129882	32.18667242
ITPRIPL1	rs11258928	40.76641416
ITPRIPL1	rs72782898	137.9887381
ITPRIPL1	rs3740120	597.9935152
ITPRIPL1	rs11259286	45.74017787
ITPRIPL1	rs2488087	43.54821625
ITPRIPL1	rs1354034	59.56539928
ITPRIPL1	rs6993770	39.6301258
CTRL	rs10922094	522.6790958
CTRL	rs11569415	38.29931244
CTRL	rs1354034	116.6376433
CTRL	rs1707652	92.50238321
CTRL	rs9267139	44.49118131
CTRL	rs28573154	155.9010964
CTRL	rs7774739	108.8391048
CTRL	rs9271766	58.83883623
TFPI2	rs62466701	45.39282801
TFPI2	rs4657078	45.44196291
TFPI2	rs10922098	220.1115679
TFPI2	rs11039122	40.82440102
TFPI2	rs962656	35.79008057
TFPI2	rs704	73.30638194
TFPI2	rs1873400	87.69631954
TFPI2	rs6445035	154.0665946
TFPI2	rs3863087	41.50966942
TFPI2	rs9501393	74.10154907
TFPI2	rs7774739	64.81859677
TFPI2	rs140753685	33.93396451
TWSG1	rs12969243	38.91548945
TWSG1	rs62087497	137.0311761
TWSG1	rs145735407	48.99971091
TWSG1	rs12945299	34.03952279
TWSG1	rs12944480	30.03541601
TWSG1	rs148599443	30.73050429
PLXDC1	rs111986733	36.20067349
PLXDC1	rs4432615	49.56109457
PLXDC1	rs62247063	56.76902829
PLXDC1	rs115881877	44.57363666
PLXDC1	rs55757623	30.50040017
PLXDC1	rs113567935	61.64449242
PLXDC1	rs77347777	444.091366
PLXDC1	rs740757	45.68081886
PLXDC1	rs35793532	299.4038387
PLXDC1	rs76880066	61.1462668
PLXDC1	rs116749582	123.3654969
PLXDC1	rs4253305	75.44644556
PLXDC1	rs1801020	304.4289334
ACP2	rs111296493	42.68146168
ACP2	rs112848587	39.57948278
ACP2	rs117836448	48.08125984
ACP2	rs74077264	61.04703289
ACP2	rs78111684	61.74356497
ACP2	rs11627377	1007.575094
ACP2	rs112963922	335.7365786
ACP2	rs2097580	225.4432623
ACP2	rs4774178	65.27578571
SIRPG	rs6043409	311.8613451
SIRPG	rs10922098	393.6344236

SIRPG	rs1707652	115.3919431
SIRPG	rs9267139	34.02889836
SIRPG	rs9501393	127.226849
SIRPG	rs7774739	83.7216175
SIRPG	rs9271766	45.5871359
LYG1	rs117261070	47.83797607
LYG1	rs17112491	455.2774712
LYG1	rs12100747	36.28766155
PPT1	rs6681804	254.7783911
PPT1	rs4660386	320.6322158
PPT1	rs4883201	61.26856626
PPT1	rs79634769	51.56186579
PPT1	rs140279936	45.07599731
PPT1	rs72952553	30.38527689
PPT1	rs6445035	146.0603159
PPT1	rs3863087	41.62095333
PPT1	rs9269273	38.29971473
PPT1	rs6993770	58.75609919
TMEM9	rs4915486	62.52383641
TMEM9	rs115880416	30.2071587
TMEM9	rs1293303	30.32040527
MAN2B2	rs62297711	30.29258071
MAN2B2	rs4277850	48.51504269
MAN2B2	rs57410633	53.86552903
MAN2B2	rs4346716	2817.1572
MAN2B2	rs79493386	317.3190574
MAN2B2	rs150335747	110.1777026
MAN2B2	rs73213615	240.7890718
MAN2B2	rs73199928	113.8296942
MAN2B2	rs67321746	55.79198845
MAN2B2	rs10922098	97.506988
MAN2B2	rs113448098	42.00650245
MAN2B2	rs10849546	161.8295782
MAN2B2	rs7964859	153.8422268
MAN2B2	rs483465	34.50274214
MAN2B2	rs9501393	46.95142676
MAN2B2	rs11155297	176.6976806
ABO	rs787891	91.15636209
ABO	rs7033492	1032.805042
ABO	rs56302081	5399.899813
ABO	rs176694	1029.381126
ABO	rs4962160	591.0746699
ABO	rs11522324	329.104397
ABO	rs56296048	60.69479481
ABO	rs139562936	153.5359226
ABO	rs10922098	36.71727602
ABO	rs2313155	60.12973941
FCER1A	rs687628	42.43740542
FCER1A	rs114009540	34.19286607
FCER1A	rs10922098	775.7140065
FCER1A	rs3900909	36.65347021
FCER1A	rs11078072	31.91448498
FCER1A	rs1047286	145.2586574
FCER1A	rs1707652	89.76166583
FCER1A	rs542418	38.64216874
FCER1A	rs550671	185.6613658
FCER1A	rs9270570	31.72082293
FCER1A	rs9469195	71.73481047

IL17C	rs10922098	291.2785606
IL17C	rs1355538	57.69664242
IL17C	rs9501393	77.45038155
IL17C	rs7774739	52.99604801
IL17C	rs35058673	34.28911253
NPTX1	rs183833760	34.00605154
NPTX1	rs181350939	59.77142333
NPTX1	rs116946917	114.8272885
NPTX1	rs142109116	122.2104967
NPTX1	rs79744555	2520.736271
NPTX1	rs75855189	44.24180332
NPTX1	rs7503460	70.56216414
NPTX1	rs56886956	34.72699693
NPTX1	rs78265569	32.42272212
NPTX1	rs55794721	37.31252675
NPTX1	rs1891058	83.96217163
NPTX1	rs2436670	55.21732477
NPTX1	rs2461161	49.2551368
NPTX1	rs2497824	40.02802391
NPTX1	rs214098	31.02742475
NPTX1	rs7937122	71.51061843
NPTX1	rs62061425	38.70677145
NPTX1	rs72827584	32.44594479
NPTX1	rs3800326	33.49079444
NPTX1	rs9258540	72.26542386
NPTX1	rs16896742	493.4416649
NPTX1	rs1050517	40.78969541
NPTX1	rs635634	62.57959858
SIAE	rs78778622	196.4614672
SIAE	rs2808466	56.65448213
SIAE	rs10761785	47.47870821
SIAE	rs34533614	55.05156908
SIAE	rs302644	29.75484454
SIAE	rs61269268	31.74107611
SIAE	rs1271816	54.39117756
SIAE	rs8100156	33.97467542
SIAE	rs12191772	52.36290051
SIAE	rs10812628	75.24438238
CTSO	rs2334114	336.7373943
CTSO	rs70954081	221.0113906
CTSO	rs660240	57.20241356
CTSO	rs2461155	31.55361072
CTSO	rs34202988	34.85992354
CTSO	rs692719	29.77187498
CTSO	rs2417942	41.64856061
CTSO	rs35757085	81.16699266
CTSO	rs11111026	231.356225
CTSO	rs200210321	147.5865145
CTSO	rs1618069	78.97865751
CTSO	rs1707652	65.96317289
CTSO	rs9346804	115.2275256
GLIPR1	rs12422952	112.6808261
GLIPR1	rs10922098	36.80605945
GLIPR1	rs12608549	37.84800196
GLIPR1	rs9271642	32.95344839
TREM1	rs75839893	44.00556513
TREM1	rs149063339	35.60426366
TREM1	rs991762	147.9516087

TREM1	rs4394214	373.0532271
TREM1	rs6914090	1137.704882
TREM1	rs1853833	62.89600103
TREM1	rs2395780	38.05267465
TREM1	rs2180976	31.76221628
TREM1	rs2511241	71.26043417
TREM1	rs921651	44.7233806
TREM1	rs8066582	62.6018296
CPA4	rs192805484	285.0527243
CPA4	rs10260496	237.7503288
CPA4	rs9641869	933.520868
CPA4	rs78283599	9841.08525
CPA4	rs139185474	249.8814874
CPA4	rs117327347	1162.89607
CPA4	rs111576843	117.9128434
CPA4	rs78924983	40.48862695
CPA4	rs113344850	35.21563844
CPA4	rs17583841	39.52040556
CPA4	rs146499629	54.18975174
CPA4	rs74632709	42.65548092
CPA4	rs145492054	46.68585458
CPA4	rs1033063	33.06807607
CPA4	rs117925228	39.43597753
CPA4	rs138970916	65.88033242
CPA4	rs76284662	59.53819287
CPA4	rs10276126	105.561622
CPA4	rs145375923	72.14379791
STIM1	rs10922098	885.6684737
STIM1	rs3900909	37.51707991
STIM1	rs11569393	30.21026024
STIM1	rs2686395	194.6286646
STIM1	rs1265941	88.40384884
STIM1	rs550671	216.8840531
STIM1	rs41548618	31.33014373
CTSL	rs7310615	43.02642786
CTSL	rs201227162	108.9800666
CTSL	rs7258020	174.8135049
CTSL	rs75818312	934.3133339
CTSL	rs10402333	962.6070195
CTSL	rs3829657	193.5122179
CTSL	rs2218886	847.3142833
CTSL	rs11665816	286.1574349
CTSL	rs139531640	473.7448063
CTSL	rs8103573	58.17583626
CPA2	rs1697422	35.89232133
CPA2	rs10922098	183.0030762
CPA2	rs75327431	34.71980114
CPA2	rs704	133.3975788
CPA2	rs8105779	55.12301005
CPA2	rs12478841	34.12994114
CPA2	rs1706420	109.4501529
CPA2	rs9501600	68.23561122
CPA2	rs35209896	31.41184334
EPYC	rs2802492	56.61355912
EPYC	rs10900074	59.25421633
EPYC	rs12773941	30.67744238
EPYC	rs35350651	38.64591025
EPYC	rs77542162	504.8506958

EPYC	rs72631343	111.4926321
EPYC	rs7412	30.84827231
EPYC	rs148242830	34.39498917
EPYC	rs10497223	37.59473379
EPYC	rs13015258	93.44143255
EPYC	rs75232675	35.47776922
EPYC	rs2648312	31.61932514
EPYC	rs66478236	30.88216943
CRISP2	rs114986602	38.80733199
CRISP2	rs74987156	245.5203701
CRISP2	rs115534907	923.3208417
CRISP2	rs369990071	1233.81849
CRISP2	rs113979133	1351.955971
CRISP2	rs200698058	57.51705963
CRISP2	rs10993994	46.40396611
CRISP2	rs185683664	143.9079155
CRISP2	rs1755785	32.18560572
CRISP2	rs2412640	55.36052371
CRISP2	rs2905421	46.32470814
CRISP2	rs3851028	48.85660787
CRISP2	rs9287979	98.09486446
CRISP2	rs114502964	43.31552493
CRISP2	rs2709396	132.9763045
CRISP2	rs2424904	49.16469288
CRISP2	rs13046908	42.70933245
CRISP2	rs78192758	45.0414347
CRISP2	rs17216646	65.99431234
CRISP2	rs112050018	55.76061852
CRISP2	rs76237797	193.3999417
CRISP2	rs17659760	339.9735569
CRISP2	rs12200464	68.05562537
CRISP2	rs36179854	46.52592171
CRISP2	rs16881244	144.1990828
CRISP2	rs6926242	82.67605645
CRISP2	rs114446310	42.19562849
CRISP2	rs1779748	50.97990968
CRISP2	rs9351779	31.47806075
CRISP2	rs370708443	45.03983815
CRISP2	rs59322	37.4362223
CRISP2	rs377580726	79.52028071
CRISP2	rs112951321	90.68071194
CRISP2	rs62461227	224.0647174
CRISP2	rs147050987	30.18296286
CRISP2	rs6997943	97.55233802
CRISP2	rs10119214	32.52607468
CD44	rs1555405	30.08391393
CD44	rs114115200	74.74151228
CD44	rs9501393	33.92616429
SPR	rs10922098	173.553034
SPR	rs1355538	69.37116033
SPR	rs28573154	36.29697913
FKBP7	rs4894018	1007.656819
FKBP7	rs79146658	198.5424876
FKBP7	rs2588511	39.9840712
FKBP7	rs142459065	71.9288846
FKBP7	rs182191955	122.299713
FKBP7	rs148560548	156.3332287
FKBP7	rs147039679	47.66832889

MFAP2	rs151160967	37.23110068
MFAP2	rs10749619	162.07954
MFAP2	rs11587728	36.58614301
MFAP2	rs4920605	909.5788698
MFAP2	rs34976145	83.30685355
MFAP2	rs7966067	30.95249668
MFAP2	rs1499813	33.9445067
MFAP2	rs7614709	278.6326604
PTPRD	rs6695915	37.9135569
PTPRD	rs2477664	44.93536785
PTPRD	rs2461161	37.47923756
PTPRD	rs2173651	31.64223098
PTPRD	rs112557952	37.10423422
PTPRD	rs4807017	29.73750863
PTPRD	rs1260326	34.2607695
B3GNT8	rs12985721	310.6474886
B3GNT8	rs12461895	1196.771403
B3GNT8	rs73047241	644.4136727
B3GNT8	rs35377912	248.5160182
B3GNT8	rs73045078	528.7993911
B3GNT8	rs138358776	51.09974314
B3GNT8	rs7310615	32.15415709
B3GNT8	rs4932141	63.65431299
B3GNT8	rs28569158	527.212201
B3GNT8	rs112938613	51.13279399
B3GNT8	rs76574898	37.59856269
B3GNT8	rs113212587	59.13327033
B3GNT8	rs593990	37.26465875
B3GNT8	rs138788715	35.27060672
B3GNT8	rs113971049	92.28573913
B3GNT8	rs373009504	83.77897728
B3GNT8	rs140150342	121.9558024
B3GNT8	rs58572634	63.20746441
B3GNT8	rs75179845	36.11195265
GADD45GIP1	rs147403462	31.24823605
GADD45GIP1	rs528298	522.7604722
GADD45GIP1	rs1355538	71.05082603
GADD45GIP1	rs9501600	73.49945328
DEFB118	rs10922098	121.9899511
DEFB118	rs6542680	68.98149316
DEFB118	rs9501600	38.35009244
CD8B	rs114009540	30.28568809
CD8B	rs10922098	808.5314339
CD8B	rs3900909	45.84509133
CD8B	rs1047286	37.89611578
CD8B	rs4665987	32.04911419
CD8B	rs1707652	166.9298257
CD8B	rs1265941	86.18155144
CD8B	rs550671	237.8546607
CD8B	rs145704749	34.93501146
AZGP1	rs1128097	35.94836321
AZGP1	rs2525546	719.8427628
AZGP1	rs72704449	65.78695445
AZGP1	rs10922098	84.52043317
AZGP1	rs1727	48.76246821
AZGP1	rs150844304	77.04596238
AZGP1	rs77924615	42.28826197
AZGP1	rs201747090	30.56166091

AZGP1	rs6535576	30.74428997
AZGP1	rs10051765	34.0384846
AZGP1	rs4713498	34.56333331
AZGP1	rs9272122	30.92257248
CBLN1	rs62029101	123.274782
CBLN1	rs27817	319.3064816
CBLN1	rs117959836	115.0754073
CBLN1	rs114398277	274.590562
CBLN1	rs79190220	72.4371428
CBLN1	rs72792061	233.771444
CBLN1	rs111546471	74.77467191
CBLN1	rs66619583	44.01244918
CBLN1	rs215226	44.97939075
CBLN1	rs648997	33.29062066
CBLN1	rs7988061	53.63659086
CBLN1	rs10133638	47.37771298
CBLN1	rs10143150	51.73669031
CBLN1	rs2439377	31.28875079
CBLN1	rs34353956	44.05892939
CBLN1	rs193630	153.2206928
CBLN1	rs141605360	59.81860158
CBLN1	rs201909614	176.2639966
CBLN1	rs138804090	116.3525404
CBLN1	rs139529748	56.06889898
CBLN1	rs149119631	118.8399571
CBLN1	rs147643545	80.7489802
CBLN1	rs11867137	81.73422428
CBLN1	rs117462763	410.1852065
CBLN1	rs112669843	35.48872808
CBLN1	rs201794935	68.63064197
CBLN1	rs12443595	163.0593439
CBLN1	rs149966451	47.85329077
CBLN1	rs1566044	76.60759193
CBLN1	rs11644731	75.07326707
CBLN1	rs2647998	41.8161539
CBLN1	rs17201113	30.55034555
CBLN1	rs9674555	33.03856085
CBLN1	rs6504180	46.43507219
CBLN1	rs868876	30.91968468
CBLN1	rs5112	219.5921853
CBLN1	rs2950790	74.13082469
CBLN1	rs2413593	324.5529655
CBLN1	rs6852182	87.96674221
CBLN1	rs7679276	159.8504063
CBLN1	rs72836800	47.33190173
CBLN1	rs7740107	37.72113146
PSG5	rs113631324	172.1356336
PSG5	rs150252870	106.8860761
PSG5	rs10418846	1235.061399
PSG5	rs28657936	866.4097341
PSG5	rs3810408	637.9169434
PSG5	rs10922098	83.5117062
PSG5	rs12026141	33.79236179
PSG5	rs964184	67.77295604
PSG5	rs8108272	45.11007986
PSG5	rs143650967	29.97297504
PSG5	rs11673647	57.90117218
PSG5	rs2965160	46.03933885

PSG5	rs157582	821.1146618
PSG5	rs1041968	31.13211
PSG5	rs62183912	46.54999752
PSG5	rs111696008	59.94506846
PSG5	rs144202860	66.10858734
PSG5	rs62290472	46.39424845
PSG5	rs11720167	635.4872966
PSG5	rs3863087	256.4667392
PSG5	rs73169103	59.7547629
MUL1	rs2511241	41.17067611
MUL1	rs2516979	35.04419001
MUL1	rs2105284	95.01130107
MUL1	rs141395633	303.0517176
MUL1	rs6014745	177.9533365
MUL1	rs5030062	52.24180203
MUL1	rs41556615	74.05212616
MUL1	rs9275602	51.69331006
WFDC1	rs168646	42.73471827
WFDC1	rs62050665	1076.478097
WFDC1	rs58268746	288.7614929
WFDC1	rs13337314	206.4114689
WFDC1	rs1149168	65.84860374
WFDC1	rs11637184	89.14240338
WFDC1	rs28426984	44.74141926
WFDC1	rs13412535	519.0908468
LRRC25	rs1451915	31.24464447
LRRC25	rs10922098	631.7126627
LRRC25	rs2686395	140.4037523
LRRC25	rs2242572	167.1463316
LRRC25	rs9501602	76.73925416
LRRC25	rs241438	30.86245561
NMB	rs77510589	89.01166053
NMB	rs35660015	183.4192619
NMB	rs11633788	271.9765335
NMB	rs10922094	46.67454425
NMB	rs17580	34.2363207
NMB	rs34931250	71.35366565
NMB	rs77262773	49.71878597
NMB	rs11720167	120.7515282
NMB	rs3863087	52.55402883
NMB	rs1558917	59.26361168
RCN1	rs531576	226.1066462
RCN1	rs10922098	120.7787055
RCN1	rs3842763	39.0758941
RCN1	rs8078686	32.53644762
RCN1	rs11127048	30.81757794
RCN1	rs697356	31.01474485
RCN1	rs41285751	32.84620212
RCN1	rs147390813	50.50363095
ITIH2	rs150119278	156.6548236
ITIH2	rs10795548	80.81698703
ITIH2	rs78189039	984.813643
ITIH2	rs7342066	350.9393693
ITIH2	rs10905252	162.0779796
ITIH2	rs2787154	90.39640046
ITIH2	rs661955	37.72677305
ITIH2	rs12413578	40.89817331
ITIH2	rs112725054	35.90965743

ITIH2	rs831428	32.50645701
ITIH2	rs201862321	46.73986688
ITIH2	rs1056522	64.10576114
ITIH2	rs190284	66.53693303
LYPD1	rs10922098	190.4511042
LYPD1	rs6445035	74.45695214
LYPD1	rs453821	83.96538697
LYPD1	rs28675927	37.13500897
TREML1	rs74467546	114.9549126
TREML1	rs144384520	206.2836368
TREML1	rs140136183	45.34046262
TREML1	rs826404	60.98449367
TREML1	rs572515	32.9631036
TREML1	rs7080386	48.02181357
TREML1	rs7774739	32.58854534
TREML1	rs188302747	36.75519124
TREML1	rs114926417	33.51004595
TREML1	rs111638498	70.40228191
TREML1	rs342294	44.44193019
TREML1	rs6993770	46.45668872
TREML1	rs3183928	47.23176592
ERP27	rs10922098	78.83807728
ERP27	rs9267655	32.79862788
ERP27	rs8176743	30.65555159
PSG9	rs145216547	118.1081749
PSG9	rs7247496	250.6673763
PSG9	rs77043841	513.912786
PSG9	rs10407046	42.7473921
PSG9	rs10922094	32.11308284
PSG9	rs80142449	45.2476902
PSG9	rs117875851	42.63996608
PSG9	rs117199551	52.11729326
TAC1	rs10272589	97.70003268
TAC1	rs17168679	107.0566348
TAC1	rs3779470	360.2428405
TAC1	rs117081694	30.04487152
TAC1	rs3184504	70.20077963
TAC1	rs1108109	33.76840628
TAC1	rs11684299	43.57625668
TAC1	rs4432538	84.92407106
TAC1	rs557011	35.03439534
TAC1	rs17431071	34.20824242
FKBP14	rs4980320	39.40225835
FKBP14	rs123698	31.20670141
FKBP14	rs2588511	44.29829243
FKBP14	rs1354034	69.52494743
FKBP14	rs1619994	35.20147423
FKBP14	rs11792848	30.62309534
PDGFD	rs10791612	71.68410772
PDGFD	rs7104359	156.2411633
PDGFD	rs2515082	244.5609201
PDGFD	rs11226172	935.6168541
PDGFD	rs34377578	30.26609048
PDGFD	rs7927668	30.81430726
PDGFD	rs146414450	49.31357631
PDGFD	rs9807368	32.92781536
PDGFD	rs342200	146.4181166
PDGFD	rs1460580	30.62391927

PDGFD	rs6993770	294.7771921
IL2RB	rs228953	138.906482
IL2RB	rs72704449	39.84149696
IL2RB	rs8177683	61.43123681
IL2RB	rs7897395	39.18383117
IL2RB	rs3184504	456.3424585
IL2RB	rs11574938	93.37415933
IL2RB	rs9936992	44.03185761
IL2RB	rs58745116	97.85039496
IL2RB	rs2659005	36.63609689
IL2RB	rs17035364	32.3928119
IL2RB	rs4848370	90.9883757
IL2RB	rs4458292	78.18717088
IL2RB	rs3133023	76.94825262
IL2RB	rs3788150	187.9015109
IL2RB	rs9996712	36.71406565
IL2RB	rs2517819	58.2612583
IL2RB	rs3132612	30.17534633
WFDC13	rs10822155	106.1220121
WFDC13	rs1398015	42.4459329
WFDC13	rs16850360	42.45638695
WFDC13	rs6993770	44.51483888
C1RL	rs116896271	78.69350995
C1RL	rs7956150	689.2482292
C1RL	rs140272193	46.34566757
C1RL	rs12230154	154.3477963
C1RL	rs79195731	258.1213118
C1RL	rs181314422	390.4761274
C1RL	rs11229075	72.78971723
C1RL	rs7973257	323.2345647
C1RL	rs12825069	205.5880108
C1RL	rs74533811	203.0276495
C1RL	rs148439198	62.47967068
C1RL	rs8178824	40.36828111
C1RL	rs9264443	30.59092173
FSTL4	rs17683306	45.36529115
FSTL4	rs17642974	39.02441369
FSTL4	rs10922098	161.9743034
FSTL4	rs1355538	32.17193582
FSTL4	rs9501600	38.89827585
CREG1	rs1229429	122.77155
CREG1	rs7513428	369.6181961
CREG1	rs34601069	61.54734089
CREG1	rs2436670	36.14128261
CREG1	rs2461161	53.30902327
CREG1	rs117483006	68.21653496
CREG1	rs35076711	47.90669266
CREG1	rs10860761	146.5803511
CREG1	rs10128996	993.4566384
CREG1	rs11111203	91.49846593
CREG1	rs12313323	48.6759457
CREG1	rs56255430	96.29177767
CREG1	rs429358	43.01064479
CREG1	rs34894639	64.1746568
CREG1	rs1293303	48.4403466
DLK2	rs2125739	382.8031022
DLK2	rs72704449	58.85769663
DLK2	rs77924615	63.14848836

DLK2	rs1260326	54.72670564
DLK2	rs2013075	36.7395831
EDIL3	rs10434639	55.70935687
EDIL3	rs10073440	35.80809657
EDIL3	rs3184504	52.93956139
EDIL3	rs34931250	100.3142422
EDIL3	rs56210800	103.616589
EDIL3	rs17282078	103.7089909
EDIL3	rs1803274	91.84120888
EDIL3	rs3863087	32.85179211
EDIL3	rs35062987	35.06705161
CT55	rs10922098	251.2139822
CT55	rs9864801	62.3700062
CT55	rs550671	83.55862451
CT55	rs28471056	42.93889142
EYS	rs10922098	137.5022178
EYS	rs1355538	32.95822807
EYS	rs9267139	30.05389966
EYS	rs28573154	44.52333176
LRRC4C	rs6485156	54.55451073
LRRC4C	rs7941452	418.2315824
LRRC4C	rs113039972	30.06429566
LRRC4C	rs13208583	30.62127103
GGH	rs118104630	34.57606815
GGH	rs77291996	1204.413303
GGH	rs201996075	453.2839935
GGH	rs7459882	147.7563404
GGH	rs191353675	46.91654889
GGH	rs4883263	71.49411494
GGH	rs7314249	40.01525799
GGH	rs825038	45.64718072
GGH	rs10860794	640.3314904
GGH	rs75127467	47.10616962
GGH	rs17532371	32.81737529
GGH	rs147233090	42.91221563
GGH	rs6499609	47.09144468
GGH	rs28654257	30.61360805
GGH	rs73001065	34.26813998
GGH	rs12975366	100.9299024
GGH	rs1394094	44.02610205
GGH	rs9266196	37.35509993
GGH	rs3116995	37.12154218
GGH	rs12547350	93.29875853
GGH	rs76279395	41.13678457
MPZL2	rs72748418	35.69552121
MPZL2	rs10801548	74.70032084
MPZL2	rs1329424	1395.60551
MPZL2	rs35351085	49.09551911
MPZL2	rs704	139.1922686
MPZL2	rs71335276	31.94576667
MPZL2	rs111696008	36.15969157
MPZL2	rs2686395	155.5933769
MPZL2	rs555007	109.728408
MPZL2	rs444921	334.4261053
MPZL2	rs41548618	44.95630613
MPZL2	rs9272526	54.03423701
NNAT	rs1451915	39.33903525
NNAT	rs10922098	789.6169967

NNAT	rs7513100	38.48644732
NNAT	rs1707652	118.3619988
NNAT	rs1265941	77.15439392
NNAT	rs550671	208.8333957
NNAT	rs78897911	32.28793657
KITLG	rs4634925	53.73441354
KITLG	rs61747728	37.46370152
KITLG	rs150844304	45.15032866
KITLG	rs1532085	47.08652173
KITLG	rs77924615	31.81435854
KITLG	rs12446515	68.88667303
KITLG	rs55661503	37.97332095
KITLG	rs61703366	53.23632947
KITLG	rs6547409	32.2063899
KITLG	rs6073949	60.83945257
KITLG	rs2868346	527.6941185
KITLG	rs34211178	39.13609711
KITLG	rs442177	37.14425517
KITLG	rs705379	33.6025001
KITLG	rs10991229	32.03805978
KITLG	rs77391147	37.08668642
KITLG	rs3824477	69.9128109
KITLG	rs2575876	74.54558917
KITLG	rs8176743	37.91414906
FIBCD1	rs10922098	346.0278417
FIBCD1	rs11569415	39.72412142
FIBCD1	rs2686395	69.16500517
FIBCD1	rs555007	64.16102037
FIBCD1	rs550671	120.8638811
SMIM10	rs10922094	115.7394925
SMIM10	rs6445035	59.84204553
SMIM10	rs547921	43.25687137
PLA2G12B	rs3829126	118.5855376
PLA2G12B	rs7087762	31.84483584
PLA2G12B	rs583169	32.6512137
PLA2G12B	rs74586295	61.30959771
PLA2G12B	rs112172017	75.3486499
PLA2G12B	rs4970834	2172.384965
PLA2G12B	rs2603592	29.87890938
PLA2G12B	rs2642438	46.47812496
PLA2G12B	rs2792703	58.83770371
PLA2G12B	rs56228609	66.68099526
PLA2G12B	rs35136575	40.72504268
PLA2G12B	rs4665972	84.06741665
PLA2G12B	rs9260557	73.07762743
PLA2G12B	rs662	199.5728117
PLA2G12B	rs854572	75.34412249
CHI3L2	rs112260552	47.80890343
CHI3L2	rs11556868	51.79528202
CHI3L2	rs1451915	30.62385503
CHI3L2	rs10922098	608.9552197
CHI3L2	rs704	53.061236
CHI3L2	rs111696008	33.88861393
CHI3L2	rs11536949	69.46168108
CHI3L2	rs1707652	239.1746178
CHI3L2	rs73169117	36.448964
CHI3L2	rs555007	68.34604166
CHI3L2	rs550671	165.3127717

CHI3L2	rs9269234	32.40947032
CAMP	rs7518497	47.27983553
CAMP	rs6677052	29.94412615
CAMP	rs7523510	31.7574216
CAMP	rs12762973	33.23637963
CAMP	rs2478232	39.21183457
CAMP	rs2074038	291.7465763
CAMP	rs964184	153.5741546
CAMP	rs12180	58.20116863
CAMP	rs9551444	40.24116725
CAMP	rs2414577	36.92724173
CAMP	rs3935281	57.68413598
CAMP	rs35362691	54.29784504
CAMP	rs60642791	118.2209098
CAMP	rs34216592	3506.887808
CAMP	rs4147974	376.7767759
CAMP	rs17703558	356.5711995
CAMP	rs2521350	32.16374985
CAMP	rs149524607	67.22917891
CAMP	rs2041037	43.38830728
CAMP	rs6507939	37.62193915
CAMP	rs8102236	30.82284664
CAMP	rs5117	33.14709532
CAMP	rs5112	60.01375741
CAMP	rs848613	40.42918436
CAMP	rs111696008	41.1459907
CAMP	rs417463	94.69284098
CAMP	rs2305637	74.01739415
CAMP	rs148874483	91.70079148
CAMP	rs150918492	51.28603545
CAMP	rs1707652	300.2563967
CAMP	rs1471251	34.13086078
CAMP	rs4619875	31.48139755
CAMP	rs13165628	35.96980195
CAMP	rs2301594	32.36226179
CAMP	rs199842489	31.35823005
CAMP	rs73747624	72.29546571
CAMP	rs2734934	47.07985438
CAMP	rs41556417	73.93602057
CAMP	rs141874749	92.99016729
CAMP	rs71549250	30.28924159
CAMP	rs11769630	149.6308784
CAMP	rs1982094	45.73766037
GAA	rs34336446	127.9538003
GAA	rs7211532	42.85868638
GAA	rs145961846	72.4554591
GAA	rs2304850	1153.187995
GAA	rs77319086	62.82066408
GAA	rs11656949	162.7639581
GAA	rs2672896	48.38129855
GAA	rs8071514	80.20064544
GAA	rs2477639	86.45360795
GAA	rs692440	56.76537458
GAA	rs10745925	110.4851055
GAA	rs56255430	36.9215183
GAA	rs9271273	34.34477276
TTC9B	rs10922098	35.12108723
TTC9B	rs12589444	197.3721896

TTC9B	rs1434579	283.8352899
TTC9B	rs111696008	35.82135356
TTC9B	rs11720167	177.4338557
TTC9B	rs3863087	60.55195113
PCSK1N	rs10922094	64.36876005
PCSK1N	rs72904397	35.25541063
PCSK1N	rs10692241	63.29359973
PCSK1N	rs7108689	378.2703794
PCSK1N	rs141454471	499.6605993
PCSK1N	rs72922318	1075.8639
PCSK1N	rs202076380	1243.058038
PCSK1N	rs118111537	469.0841279
PCSK1N	rs140172352	2390.295597
PCSK1N	rs114823028	208.7875915
PCSK1N	rs78724679	46.89242753
PCSK1N	rs1355538	46.85009396
PCSK1N	rs11752007	35.52338327
PCSK1N	rs137893789	43.08329363
MGAT4A	rs10922098	649.4051198
MGAT4A	rs12478841	32.86559411
MGAT4A	rs1707652	123.0768215
MGAT4A	rs542418	31.23997464
MGAT4A	rs550671	179.8707958
MGAT4A	rs9469195	66.37975134
CPQ	rs55671178	162.5129681
CPQ	rs72680193	739.35775
CPQ	rs142439242	4064.558804
CPQ	rs117990540	342.6770004
CPQ	rs79884132	44.8120432
CPQ	rs111581606	43.88104353
CPQ	rs10860761	270.5693616
CPQ	rs10128996	1225.142966
CPQ	rs116908064	63.06418716
CPQ	rs11111203	104.1616366
CPQ	rs11111347	32.88569289
CPQ	rs73176937	41.95378165
CPQ	rs149181595	43.34255546
CPQ	rs73001065	76.93456372
CPQ	rs75627662	30.17240443
CPQ	rs34330586	71.78157573
CPQ	rs13135092	36.45388331
CPQ	rs72729610	32.10784447
CPQ	rs143346386	38.04499446
CPQ	rs111695502	62.02494058
CPQ	rs145704203	83.99179199
CPQ	rs147016589	39.29717744
CPQ	rs149794999	38.30290079
CPQ	rs3134374	73.54562296
CPQ	rs116876243	100.1001607
CPQ	rs2272662	43.27807442
GALP	rs139011716	62.59722263
GALP	rs10922094	104.377001
GALP	rs9501393	34.12844306
TPSAB1	rs117712465	136.9685771
TPSAB1	rs117690000	73.32813679
TPSAB1	rs73491534	72.14454412
TPSAB1	rs117717200	278.5432448
TPSAB1	rs188566928	200.2335583

TPSAB1	rs34777355	7928.173843
TPSAB1	rs78306371	6323.619457
TPSAB1	rs181292568	142.6042561
TPSAB1	rs150298197	155.6525132
TPSAB1	rs141196266	343.912157
TPSAB1	rs117492134	34.79463743
TPSAB1	rs79658155	855.7852561
TPSAB1	rs118073880	100.8610974
TPSAB1	rs118102263	69.5888472
TPSAB1	rs184695141	50.64731524
TPSAB1	rs115340020	177.0496426
TPSAB1	rs114730474	34.35433856
TPSAB1	rs16856110	67.51895846
TPSAB1	rs2884425	40.76355044
TPSAB1	rs56043070	115.7372805
TPSAB1	rs78705678	42.24143169
TPSAB1	rs2477642	47.64724389
TPSAB1	rs7127546	96.91982886
TPSAB1	rs140216362	106.8426963
TPSAB1	rs72801130	34.95188751
TPSAB1	rs62082093	30.01968421
TPSAB1	rs17758695	60.7649988
TPSAB1	rs78744187	331.5642029
TPSAB1	rs56344893	176.8555255
TPSAB1	rs36059907	81.20102824
TPSAB1	rs35930348	50.1624971
TPSAB1	rs10196335	41.8171213
TPSAB1	rs12695310	81.32526033
TPSAB1	rs68082716	48.1784511
TPSAB1	rs2961914	35.13290152
TPSAB1	rs716719	44.79604654
ACVR2B	rs10922098	183.028729
ACVR2B	rs6542680	32.48627598
ACVR2B	rs9501600	34.12720026
CPM	rs1908671	125.1461284
CPM	rs56369151	81.28607594
CPM	rs78705678	58.71572218
CPM	rs2477642	48.4049201
CPM	rs10822143	29.95262418
CPM	rs1129555	31.89435482
CPM	rs7480474	38.74399463
CPM	rs2244608	37.27817804
CPM	rs79391862	93.61346761
CPM	rs487766	38.79488992
CPM	rs1077834	37.86585748
CPM	rs12945299	42.28166077
CPM	rs5112	42.92700732
CPM	rs8736	58.24485515
CPM	rs11087520	56.30042558
CPM	rs760904	74.14885556
CPM	rs9379685	35.43329974
CPM	rs2954021	126.8295856
DGCR6	rs10922098	329.3732786
DGCR6	rs2686395	73.49387398
DGCR6	rs9267139	30.48761647
DGCR6	rs3891366	89.44274577
DGCR6	rs7774739	65.03039809
C4BPA	rs8942	1199.974193

C4BPA	rs75279995	59.41107484
C4BPA	rs41274770	63.28021684
C4BPA	rs61821320	59.00234008
C4BPA	rs4418728	32.20814633
C4BPA	rs1260326	63.79149947
C4BPA	rs9271537	46.70706143
C4BPA	rs112164771	70.42010792
UMOD	rs78689694	134.9319984
UMOD	rs10894808	30.98051853
UMOD	rs117633773	52.55486132
UMOD	rs55714927	280.8054096
UMOD	rs143501783	104.5518677
UMOD	rs1060461	37.37207294
UMOD	rs10426	48.43323687
UMOD	rs2691261	41.67885176
UMOD	rs2691273	1050.567404
UMOD	rs2280806	131.8740453
UMOD	rs34616839	77.30363443
UMOD	rs2593704	34.75360971
UMOD	rs1374898	43.94636411
UMOD	rs17018841	53.2662971
UMOD	rs6777831	37.19486608
UMOD	rs71617905	31.55881947
UMOD	rs151078915	35.30454691
UMOD	rs73132003	48.48441096
UMOD	rs146900894	57.04568768
UMOD	rs78661192	44.84627309
UMOD	rs202137963	66.45417738
UMOD	rs3749260	436.8416374
UMOD	rs3804622	3693.637909
UMOD	rs13071075	60.98358054
UMOD	rs11920688	61.09607251
UMOD	rs114598716	38.71352534
UMOD	rs189392979	101.3885174
UMOD	rs77050848	91.83151306
UMOD	rs56162907	34.06554341
UMOD	rs9269272	57.16866696
UMOD	rs9273394	119.585111
UMOD	rs9987289	32.3271361
UMOD	rs10121987	65.91817156
UMOD	rs635634	320.0415752
CAV2	rs10922098	477.2809797
CAV2	rs1555405	31.40190165
CAV2	rs1047286	34.54770375
CAV2	rs1707652	106.2901811
CAV2	rs2075798	58.77480821
CAV2	rs453821	131.8364188
FAS	rs717576	79.47614657
FAS	rs7911226	760.0670558
FAS	rs61854887	60.743656
FAS	rs72704449	34.22854342
FAS	rs77663942	59.57476913
FAS	rs351973	43.62819244
FAS	rs41548618	31.03815958
FAS	rs202092176	232.9148954
FAS	rs9272263	153.8200691
LMAN2	rs4131289	125.6304732
LMAN2	rs72704449	90.54549457

LMAN2	rs77924615	59.34981756
LMAN2	rs368611	37.37941919
LMAN2	rs6879657	172.2828576
LMAN2	rs2347784	53.12330048
METTL24	rs4484552	183.3019791
METTL24	rs12189608	44.64696895
METTL24	rs9384738	128.0085424
METTL24	rs9486977	51.41918672
METTL24	rs1405530	32.83174644
NUDT9	rs10033280	48.594565
NUDT9	rs28529046	159.7842935
NUDT9	rs8178824	61.05103754
NUDT9	rs1354034	50.41958223
NUDT9	rs149062332	66.0271885
DSG2	rs148666188	58.42090999
DSG2	rs1020839	64.81247768
DSG2	rs9304098	540.3404386
DSG2	rs147145691	89.29356632
DSG2	rs75426668	42.4226913
DSG2	rs150816167	52.2183602
DSG2	rs11078597	30.66317865
DSG2	rs117225497	35.03475102
DSG2	rs708686	80.43722465
DSG2	rs1231281	32.86925278
DSG2	rs6905288	54.48092355
DSG2	rs537879	217.7006493
DSG2	rs62578578	58.28908494
DSG2	rs10901252	697.1704338
DSG2	rs149181677	54.33275377
DSG2	rs9286383	53.71005152
GHRH	rs10922098	278.0347734
GHRH	rs111696008	34.02695071
GHRH	rs1706435	84.72947571
GHRH	rs2075798	45.39542511
GHRH	rs453821	99.04141625
GHRH	rs9272526	32.99254504
CXCL17	rs10922098	448.2824451
CXCL17	rs2230203	33.08339052
CXCL17	rs1707652	129.3773492
CXCL17	rs9267139	36.38020813
CXCL17	rs9501393	134.5628407
CXCL17	rs7774739	92.20452023
RAB27A	rs10922094	44.78153467
RAB27A	rs11856676	158.4668743
RAB27A	rs8039725	42.77481625
RAB27A	rs114115200	33.03674254
APOL1	rs140668164	52.19314753
APOL1	rs139361990	119.5248113
APOL1	rs11190328	1268.069474
APOL1	rs11190354	854.1831328
APOL1	rs73336163	88.30738498
APOL1	rs147343606	241.0163034
APOL1	rs117104675	52.18786155
APOL1	rs4968317	31.94019267
APOL1	rs5167	87.02880927
APOL1	rs4665972	40.74316082
APOL1	rs57796598	33.50785934
APOL1	rs67024748	61.40934713

APOL1	rs35838665	190.9752604
APOL1	rs4075427	57.49275795
APOL1	rs4626036	209.5819525
APOL1	rs6806282	1237.821417
APOL1	rs9868646	260.4756008
APOL1	rs147278314	39.28733773
TMEM38B	rs628357	90.37230994
TMEM38B	rs12614	848.9267485
TMEM38B	rs57718550	109.6579849
TMEM38B	rs10456062	89.3387375
TMEM38B	rs74671299	60.31497651
NXPH2	rs4950806	58.38697591
NXPH2	rs10750096	357.2355296
NXPH2	rs11216221	45.06567068
NXPH2	rs3816117	124.9520093
NXPH2	rs11668738	36.32773939
NXPH2	rs429358	87.1686231
NXPH2	rs55881728	91.45796797
NXPH2	rs111696008	172.9242863
NXPH2	rs144202860	88.36211278
NXPH2	rs887829	50.58465292
NXPH2	rs1291921	35.40088882
NXPH2	rs114115200	1148.838539
NXPH2	rs3863087	472.3467012
NXPH2	rs148077724	49.82942727
NXPH2	rs73169103	73.65978358
NXPH2	rs150456509	35.83733395
NXPH2	rs35173225	40.34544353
NXPH2	rs2740488	41.52134545
AIFM1	rs10922094	42.50639913
AIFM1	rs1180658	50.58862476
AIFM1	rs704	36.64181951
AIFM1	rs1265941	70.81914396
AIFM1	rs453821	208.9565684
ITM2C	rs10922098	101.9277665
ITM2C	rs4977155	30.55355237
ITM2C	rs1706435	33.82013533
ITM2C	rs9501600	41.19332218
SPATA31D4	rs10922094	85.63148168
SPATA31D4	rs9864801	62.39583853
SPATA31D4	rs9501600	82.45282501
SPATA31D4	rs587495	36.86401249
PTK7	rs148886793	47.91201294
PTK7	rs3828755	105.3105597
PTK7	rs34775765	41.48778979
PTK7	rs78705678	159.4255176
PTK7	rs2477642	153.7464822
PTK7	rs12475061	107.3859456
PTK7	rs1260326	53.28867866
PTK7	rs7716761	36.0648267
PTK7	rs62621812	110.5555556
CLPSL1	rs4133213	33.99751613
CLPSL1	rs73104854	30.98934591
CLPSL1	rs56037182	63.77994487
CLPSL1	rs2284878	484.530499
CLPSL1	rs45589832	1110.093161
CLPSL1	rs8116471	87.18116411
CLPSL1	rs6123893	188.3354882

CLPSL1	rs2867239	45.84440561
CLPSL1	rs117477036	47.71371727
CLPSL1	rs1707652	136.5502394
WBP1L	rs10922098	115.8995085
WBP1L	rs7651933	38.69262483
WBP1L	rs9501393	31.33354158
EDDM3A	rs1988608	65.87115353
EDDM3A	rs528298	40.49357592
EDDM3A	rs9864801	41.20341915
EDDM3A	rs9501393	50.49209557
EDDM3A	rs7774739	37.73477735
B3GALT1	rs1329424	56.67268171
B3GALT1	rs6542680	47.67559985
B3GALT1	rs2379887	30.25812691
GZMK	rs3797235	31.38151572
GZMK	rs10471928	310.6111508
GZMK	rs6693121	62.05891711
GZMK	rs1265564	74.20616726
GZMK	rs34177255	44.13399559
GZMK	rs6746608	39.54340628
GZMK	rs2110690	29.94337302
GZMK	rs12054477	32.89873669
GZMK	rs11567694	31.82936425
GZMK	rs6926028	63.61159047
GZMK	rs2853944	242.9349713
GZMK	rs9273143	74.09852837
GZMK	rs3181348	47.50142666
EMID1	rs10922094	57.91386002
EMID1	rs9501393	34.64642668
EMID1	rs67399078	34.86960386
MANSC1	rs118164843	31.76402078
MANSC1	rs3825259	755.4011116
MANSC1	rs11054925	31.13122026
MANSC1	rs12035995	37.00583186
MANSC1	rs61747728	64.58389077
MANSC1	rs1059091	40.03201088
MANSC1	rs34656952	62.26862952
MANSC1	rs73017394	176.7312989
MANSC1	rs11610473	32.16570164
MANSC1	rs12945299	41.49729832
MANSC1	rs56378716	43.2712303
MANSC1	rs77542162	38.41713753
MANSC1	rs62132278	96.57962797
MANSC1	rs601338	52.50948712
MANSC1	rs1260326	60.85868851
MANSC1	rs55954186	38.32831645
MANSC1	rs183113686	29.94157859
MANSC1	rs2011962	38.29533707
MANSC1	rs812561	33.75034128
MANSC1	rs370635202	33.35113572
MANSC1	rs2978916	37.80412869
MANSC1	rs11783134	30.77280069
MANSC1	rs600038	77.13078837
PAWR	rs10506806	34.41881584
PAWR	rs2360962	55.11673298
PAWR	rs4759076	71.80734694
PAWR	rs114694170	69.80743896
PAWR	rs342200	44.42027254

FCAMR	rs13379369	38.33565287
FCAMR	rs35495893	37.37589775
FCAMR	rs17784127	86.72032971
FCAMR	rs34665982	32.2619375
FCAMR	rs9273429	61.78627566
BIN1	rs2404176	32.3978997
BIN1	rs35103166	242.349097
BIN1	rs10922094	50.76669746
BIN1	rs35586905	34.9413357
BIN1	rs1706435	32.81787164
BIN1	rs28636516	36.58436965
MNX1	rs572515	178.7482559
MNX1	rs1555405	31.18121781
MNX1	rs1355538	54.84595448
MNX1	rs9501600	58.02614109
SYT2	rs10922098	164.8028941
SYT2	rs1619994	42.3961595
SYT2	rs9501600	40.46442995
MANSC4	rs73294067	95.82466019
MANSC4	rs11049091	1043.076312
MANSC4	rs61915452	415.6521716
MANSC4	rs11049245	96.37338275
MANSC4	rs10843188	69.36076822
MANSC4	rs203688	40.14458525
MANSC4	rs76785061	75.58490506
MANSC4	rs7307323	112.7480922
MANSC4	rs145142574	30.09568901
MANSC4	rs4439576	31.87179757
MANSC4	rs34793466	32.42068394
MANSC4	rs12055445	59.8403351
PRRG4	rs12879997	38.76805373
PRRG4	rs17580	55.88742667
PRRG4	rs146525713	75.4889517
PRRG4	rs78912960	32.60826983
LAMC2	rs111875294	136.391113
LAMC2	rs147108848	407.6937992
LAMC2	rs12146099	941.1453885
LAMC2	rs2301876	36.62890322
LAMC2	rs12737538	242.0844291
LAMC2	rs148981091	71.10327991
LAMC2	rs77338610	71.56592721
LAMC2	rs10911697	44.03606987
LAMC2	rs2180480	44.53923079
LAMC2	rs692719	34.97123784
LAMC2	rs34534206	48.0947076
LAMC2	rs12457684	29.80662977
LAMC2	rs12458632	51.7751733
LAMC2	rs28817896	36.70735417
LAMC2	rs1049087	48.14320255
LAMC2	rs9276757	33.34712916
LAMC2	rs9411365	32.81652899
PRPF6	rs1329424	916.2091842
PRPF6	rs3900909	49.85780987
PRPF6	rs10801660	32.84017605
PRPF6	rs11078072	32.73689784
PRPF6	rs111696008	43.12387808
PRPF6	rs2686395	230.6968869
PRPF6	rs1265941	89.0710481

PRPF6	rs550671	233.2788835
PRPF6	rs41291806	31.75548014
RNF24	rs11208704	30.03110813
RNF24	rs10922098	126.6545874
RNF24	rs111696008	36.81818018
RNF24	rs56317765	44.04583759
RNF24	rs2232613	933.0479452
RNF24	rs11906988	60.16483733
RNF24	rs2092494	37.65283014
RNF24	rs6029138	43.5057505
RNF24	rs114115200	182.2330781
RNF24	rs3863087	70.86551539
RNF24	rs147906938	37.7080515
CLMP	rs10922098	101.3020995
CLMP	rs1355538	34.76526286
CLMP	rs9268560	33.49605112
COX7A2L	rs4657078	48.44661054
COX7A2L	rs3794468	38.11280145
COX7A2L	rs2006166	198.5973409
COX7A2L	rs704	66.41331294
COX7A2L	rs80094848	30.76470906
COX7A2L	rs9269129	32.94522494
COX7A2L	rs2395199	49.86772993
SLC30A5	rs74181972	150.2635381
SLC30A5	rs1619994	37.10748118
SLC30A5	rs10646379	29.76467554
SLC30A5	rs342293	66.28320348
B4GALT2	rs148707035	36.41967442
B4GALT2	rs3762423	576.883181
B4GALT2	rs115268037	59.8159103
B4GALT2	rs333947	34.83042157
B4GALT2	rs78705678	93.99654191
B4GALT2	rs2477642	93.91505453
B4GALT2	rs7232	52.60947339
B4GALT2	rs1126605	73.5326489
B4GALT2	rs34163180	55.3850248
B4GALT2	rs28873836	166.0860425
B4GALT2	rs2770808	38.02052604
B4GALT2	rs576123	119.8370997
PIANP	rs71450153	40.61366956
PIANP	rs11064321	536.3473437
PIANP	rs77924615	57.89385897
NUP210L	rs369905	41.20792203
NUP210L	rs111696008	48.03178946
NUP210L	rs11720167	282.2647121
NUP210L	rs3863087	92.92228351
NUP210L	rs17201417	43.07055792
NUP210L	rs77734842	36.81640445
PRR27	rs10922094	350.8768717
PRR27	rs12146727	62.46620901
PRR27	rs13469	47.39319328
PRR27	rs2230203	37.49812092
PRR27	rs1707652	81.75528472
PRR27	rs6457457	65.84667523
PRR27	rs28573154	90.51780014
ZNF275	rs78364821	62.09958701
ZNF275	rs12478841	35.27379936
ZNF275	rs62269916	31.09981508

TIGIT	rs73238029	83.40741933
TIGIT	rs3184504	32.65932852
TIGIT	rs41547217	37.78630349
PDGFRL	rs7013924	59.030363
PDGFRL	rs2188020	80.90970474
PDGFRL	rs117482439	1959.585899
PDGFRL	rs79088892	197.184601
PDGFRL	rs11709846	168.5254447
PDGFRL	rs6441990	965.8413346
PDGFRL	rs77014442	34.98781857
PDGFRL	rs34964126	37.51071304
PDGFRL	rs150356904	54.32768248
PDGFRL	rs7814472	48.14248927
IGSF3	rs113856068	169.3720811
IGSF3	rs41276576	32.90713409
IGSF3	rs1411623	40.1376191
IGSF3	rs113414299	39.6556865
IGSF3	rs200250001	115.6137624
IGSF3	rs78705678	455.9539079
IGSF3	rs2477642	503.6970222
IGSF3	rs55714927	52.84269364
IGSF3	rs1260326	36.86363832
IGSF3	rs4704040	32.18384056
IGSF3	rs2442731	29.91858817
IGSF3	rs7889	36.04409001
IGSF3	rs635634	251.033835
MMP16	rs35889496	59.27256844
MMP16	rs10922098	87.61672627
MMP16	rs58205855	29.97911317
MMP16	rs1355538	32.85860586
MMRN2	rs10922098	274.3718521
MMRN2	rs4821102	38.45204296
MMRN2	rs1707652	135.0064207
MMRN2	rs2242572	68.78388126
GHDC	rs10922098	184.8541111
GHDC	rs9902706	43.77040075
GHDC	rs9501600	47.33347644
CD96	rs800255	32.27686352
CD96	rs6457457	81.08662582
CD96	rs148766553	44.47116967
CD96	rs6458	134.7354835
CD96	rs191795965	45.84404961
CD96	rs73216598	55.92633841
CD96	rs2057681	462.3810722
CD96	rs854572	91.98055024
FAF2	rs117014988	30.06736239
FAF2	rs7113761	95.34208536
FAF2	rs1354034	40.40152608
MSH2	rs10922098	79.59888195
MSH2	rs13379369	145.531283
MSH2	rs11846842	68.07481426
MSH2	rs5167	196.8958444
MSH2	rs11720167	50.13624635
MSH2	rs444921	29.94661004
MSH2	rs67399078	33.84084201
EIF4E2	rs4980320	36.40763579
EIF4E2	rs4759076	31.68352044
EIF4E2	rs1354034	66.12884771

EIF4E2	rs9864801	30.62226191
GSTM3	rs187542824	299.0170178
GSTM3	rs143379394	76.05441316
GSTM3	rs148631275	38.16832797
GSTM3	rs116276872	143.8972523
GSTM3	rs116211032	61.42712099
GSTM3	rs116532364	772.4083127
GSTM3	rs115450622	974.8372862
GSTM3	rs36210087	392.43418
GSTM3	rs147668562	131.2178319
GSTM3	rs112852158	1199.628939
GSTM3	rs72705270	273.3385482
GSTM3	rs10776701	62.32798266
GSTM3	rs11102126	35.9455235
GSTM3	rs112591404	37.28101455
GSTM3	rs1707654	72.4384674
YBX1	rs2739240	70.32773315
YBX1	rs11053902	71.01476155
YBX1	rs78151684	49.33867739
YBX1	rs1046321	214.3674307
YBX1	rs11082513	36.42189227
YBX1	rs6084653	47.62935292
YBX1	rs4820091	63.75088873
YBX1	rs1354034	364.0189872
EZR	rs12365171	52.02353225
EZR	rs185009887	31.11227765
EZR	rs1354034	33.67387486
NQO2	rs17135727	241.8830948
NQO2	rs115311554	1156.523587
NQO2	rs114141799	129.756999
NQO2	rs62391549	4897.823176
NQO2	rs56336390	928.3378364
NQO2	rs62391551	706.9009544
NQO2	rs146932567	77.15914553
NQO2	rs1077782	419.6487851
NQO2	rs17548252	35.7412251
NQO2	rs79087434	88.32071771
NQO2	rs116168350	49.94218425
NQO2	rs78705678	35.12087996
NQO2	rs2461161	42.89301831
NQO2	rs143586648	37.18732988
RPS4X	rs1976848	74.26136933
RPS4X	rs10876550	39.39954421
RPS4X	rs1037590	112.8579831
RPS4X	rs1354034	442.8368583
IDO1	rs6474193	47.90360399
IDO1	rs7137828	40.75013645
IDO1	rs808726	33.00686764
IDO1	rs7634877	46.08520514
IDO1	rs116761496	36.58309358
IDO1	rs35351750	182.58999
IDO1	rs13086611	1128.223607
IDO1	rs6784807	87.77132765
IDO1	rs41547217	39.76725484
HNRNPF	rs4980320	35.21668631
HNRNPF	rs1354034	80.0646323
HNRNPF	rs342200	38.06774233
RIPPLY1	rs10922094	134.5940621

RIPPLY1	rs1706435	37.21839856
RIPPLY1	rs28573154	53.34445583
RIPPLY1	rs7774739	46.6916013
DNER	rs7577541	59.74676844
DNER	rs35032874	221.8627717
DNER	rs67240011	31.09295704
DNER	rs12052464	117.6669069
DNER	rs62193248	454.8228098
DNER	rs181707610	40.02617131
DNER	rs780094	45.71618761
DNER	rs55879786	48.86557863
NLGN2	rs7226204	50.06468771
NLGN2	rs114576150	230.7563061
NLGN2	rs61801011	55.83518477
NLGN2	rs831428	45.71345277
NLGN2	rs201862321	70.35120239
NLGN2	rs7559416	105.4128561
NLGN2	rs635634	39.45514774
RNF149	rs10922098	635.9023598
RNF149	rs35351085	33.93153249
RNF149	rs1047286	44.11036479
RNF149	rs111696008	35.00633322
RNF149	rs1707652	153.074675
RNF149	rs9266642	36.76461013
RNF149	rs555007	78.45607361
RNF149	rs550671	190.7436281
RNF149	rs9276606	39.79017318
RNF150	rs10922094	87.17832455
RNF150	rs4977155	98.59414416
RNF150	rs9501393	35.11966752
RNF150	rs2395175	38.69726483
TMEM52B	rs10922098	75.75056983
TMEM52B	rs17580	56.4103313
TMEM52B	rs704	313.0862383
TMEM52B	rs61146270	215.7978829
TMEM52B	rs4822348	34.05483204
TMEM52B	rs28573154	49.25653947
TMEM52B	rs62404084	43.26932913
HERPUD2	rs10754199	109.9833605
HERPUD2	rs41556615	38.35827754
HERPUD2	rs73161871	32.79787467
HERPUD2	rs2929937	32.16613349
DNAJC11	rs10922094	116.2608528
DNAJC11	rs6542680	102.8821851
DNAJC11	rs1355538	34.9087397
DNAJC11	rs415620	52.89660518
DNAJC11	rs7774739	46.21083983
DNAJC11	rs28688825	43.16348193
IER3	rs10922098	594.2315454
IER3	rs35351085	31.13550156
IER3	rs11569415	35.27846334
IER3	rs1707652	109.4300771
IER3	rs1265941	83.24732994
IER3	rs550671	156.5429253
IER3	rs9269234	32.77350755
SYNE2	rs61074241	30.42661827
SYNE2	rs185009887	33.76587935
SYNE2	rs1354034	37.79322007

SYNE2	rs1355538	52.43398628
BRSK2	rs34434067	35.56955781
BRSK2	rs4255564	314.3501583
BRSK2	rs1329424	125.6765187
BRSK2	rs4980320	31.75163703
BRSK2	rs1354034	34.2180294
BRSK2	rs697356	31.8595099
BRSK2	rs6457457	31.6504746
BRSK2	rs28573154	51.08112196
IGDCC4	rs111302521	72.43172136
IGDCC4	rs144180812	284.8512658
IGDCC4	rs184453313	971.2680661
IGDCC4	rs191350199	303.3175744
IGDCC4	rs8034057	595.9316104
IGDCC4	rs72741403	70.40229028
IGDCC4	rs7164579	42.49657915
IGDCC4	rs77659620	130.9377394
IGDCC4	rs112502880	33.08632231
IGDCC4	rs780094	38.72541933
IGDCC4	rs182820178	29.79948906
CEL	rs148690761	195.5103743
CEL	rs72767846	52.1655781
CEL	rs10491534	74.15066134
CEL	rs113529953	1041.061142
CEL	rs2073926	284.7145437
CEL	rs117119759	164.4745944
CEL	rs886136	41.65952585
CEL	rs342214	57.17954462
CEL	rs3780420	43.79623253
CEL	rs79100721	115.8229156
NDUFB8	rs10922094	204.0700827
NDUFB8	rs1707652	103.2271788
NDUFB8	rs28573154	66.62608478
NDUFB8	rs7774739	48.71916488
NDUFB8	rs9271766	32.31968841
PNPLA2	rs11227136	52.6406039
PNPLA2	rs704	40.27447635
PNPLA2	rs6542680	31.86693286
PNPLA2	rs1354034	50.64133417
PNPLA2	rs576123	46.55010974
SEMA4B	rs61804164	62.69299969
SEMA4B	rs28452703	57.37151407
SEMA4B	rs7358384	96.76989855
SEMA4B	rs117801489	30.15619812
SEMA4B	rs730086	31.38909432
SEMA4B	rs907891	42.15921646
TNRC6B	rs10922094	35.45854242
TNRC6B	rs4980320	33.03754249
TNRC6B	rs1354034	51.98216379
ARHGAP1	rs2968478	54.20215056
ARHGAP1	rs1354034	38.34134661
ARHGAP1	rs201682171	33.50860463
ARHGAP1	rs7836609	30.58695155
ARHGAP1	rs4734879	107.6511399
ARHGAP1	rs6559693	31.18792958
ISOC1	rs2526256	386.264332
ISOC1	rs2526206	339.05164
ISOC1	rs2577431	339.5275509

ISOC1	rs369763546	325.3526463
ISOC1	rs181912748	33.68903676
ISOC1	rs1354034	37.00645465
ISOC1	rs113794264	131.2003379
ISOC1	rs76212014	63.83446515
ISOC1	rs342200	33.95958743
NUP210	rs10922098	332.0808919
NUP210	rs1355538	69.37157346
NUP210	rs9267139	32.6693404
NUP210	rs28573154	86.544101
NUP210	rs7774739	56.18226854
NUP210	rs9271766	34.60012325
DHFR	rs10922094	58.96869333
DHFR	rs10774378	91.15523545
DHFR	rs216858	136.9763392
DHFR	rs542993	2253.510352
DHFR	rs11539945	38.18905309
DHFR	rs483082	29.74629391
DHFR	rs1355538	52.81935331
DHFR	rs147906938	33.9315378
DHFR	rs28479464	44.55870544
DHFR	rs576123	145.494867
FHIT	rs2687899	83.45499739
FHIT	rs11130790	45.63453905
FHIT	rs7611232	59.1243005
SULT2A1	rs62129966	395.1863829
SULT2A1	rs377618587	53.83718182
SULT2A1	rs3747207	35.45164516
SULT2A1	rs112875651	35.58077278
VAV3	rs1706435	31.22001949
VAV3	rs1064588	36.75025701
VAV3	rs9266221	30.47195891
VAV3	rs28474889	90.39352488
VAV3	rs6458	177.2098408
VAV3	rs9275553	59.55708443
VAV3	rs10982054	57.30137863
G3BP2	rs4980320	40.44042381
G3BP2	rs185009887	32.16778226
G3BP2	rs1354034	68.63541552
ALDH1A3	rs368465	47.53161617
ALDH1A3	rs7080386	155.9411291
ALDH1A3	rs892090	58.08043986
ALDH1A3	rs610077	34.92251476
ALDH1A3	rs1071960	29.88115473
ALDH1A3	rs6993770	150.7203273
ALDH1A3	rs10820606	31.00394796
DCK	rs10922094	216.2077677
DCK	rs1354034	34.00671845
DCK	rs9864801	48.72092562
DCK	rs9501600	59.97784886
NQO1	rs141233052	100.7208028
NQO1	rs35463268	124.9220667
NQO1	rs4783718	1213.043624
NQO1	rs12149517	1275.115951
NQO1	rs138475217	43.40692892
NQO1	rs142621498	33.48790409
NQO1	rs74990318	305.2617035
NQO1	rs1429430	116.7646494

NQO1	rs111346926	46.14118766
NQO1	rs143380183	43.88269708
NQO1	rs5996419	135.6440611
NQO1	rs1707652	37.67462608
SMAD1	rs34180190	36.21783358
SMAD1	rs1354034	41.91761043
SMAD1	rs114694170	39.79320604
SMAD1	rs7702439	30.51767916
SMAD1	rs342293	57.87687616
SMAD1	rs4541868	50.39182994
TIRAP	rs8177399	321.9717841
TIRAP	rs12574844	49.7545034
TIRAP	rs4937125	39.00604252
TIRAP	rs342200	66.481141
TIRAP	rs16873418	37.08039488
CTNNB1	rs35340377	32.95840651
CTNNB1	rs10876550	31.46964475
CTNNB1	rs185992068	33.89072114
CTNNB1	rs150195415	39.68919915
CTNNB1	rs79780354	53.35013231
CTNNB1	rs149683334	69.62259015
CTNNB1	rs111632808	117.6889578
CTNNB1	rs116148318	36.51072744
CTNNB1	rs2018523	2567.435459
CTNNB1	rs1347640	245.1962332
CTNNB1	rs62356994	595.5212382
CTNNB1	rs1276194	122.9962112
CTNNB1	rs1697093	114.8418394
CTNNB1	rs6993770	31.07830706
CTNNB1	rs4962034	42.71191823
ACTN2	rs61752479	30.20231165
ACTN2	rs17617337	55.78879075
ACTN2	rs56678255	30.09406715
ACTN2	rs10833719	154.9695459
ACTN2	rs9318187	86.29704953
ACTN2	rs2289292	34.83012932
ACTN2	rs7211380	35.53515888
ACTN2	rs1799938	53.50197657
ACTN2	rs138536547	30.07840958
ACTN2	rs12463674	52.66368918
ACTN2	rs523206	30.01928344
ACTN2	rs2800280	31.30302287
CCNH	rs2230641	95.61629655
CCNH	rs10922094	77.0649117
CCNH	rs3129754	30.01694919
IFIT2	rs10922098	45.56365598
IFIT2	rs964184	43.08612532
IFIT2	rs71352239	212.2415956
IFIT2	rs144202860	31.79534902
IFIT2	rs11720167	128.2622125
IFIT2	rs3863087	56.1187056
DHPS	rs10922098	158.3345476
DHPS	rs1047286	32.14104757
DHPS	rs11720167	31.16197619
DHPS	rs28573154	47.13131782
SAT1	rs10922094	57.16094112
SAT1	rs1355538	37.28119048
SAT1	rs13208583	30.19450357

UBE2B	rs10922094	89.34798627
UBE2B	rs4977155	40.30072146
UBE2B	rs9501600	37.00871099
UBE2B	rs2395175	35.13819195
FBP2	rs10761339	47.32842344
FBP2	rs573212	249.2277504
FBP2	rs10922098	389.9105238
FBP2	rs2686395	81.39876998
FBP2	rs1265941	49.33133394
FBP2	rs444921	114.3507422
NFKB1	rs230539	62.54862132
NFKB1	rs11601507	36.8648168
NFKB1	rs10876550	32.57024494
NFKB1	rs3915182	316.6691685
NFKB1	rs4004781	158.2269556
NFKB1	rs4089292	45.10292591
NFKB1	rs144883301	29.84477933
CTNNA2	rs10922094	107.5841373
CTNNA2	rs6542680	118.4870277
CTNNA2	rs6457457	31.46554103
CTNNA2	rs28573154	38.65832364
ESRRA	rs10922098	160.8627952
ESRRA	rs1706435	64.13666101
ESRRA	rs9501393	61.06385614
ESRRA	rs7774739	40.31741804
CDKN2B	rs10922094	166.0142559
CDKN2B	rs2232710	226.7527784
CDKN2B	rs1555405	31.11038567
CDKN2B	rs190712692	39.25461092
CDKN2B	rs1707652	84.58741599
CDKN2B	rs28573154	97.63700872
CDKN2B	rs7774739	68.16271525
CDKN2B	rs9271689	39.27957479
THOC1	rs10922094	254.3110145
THOC1	rs72637119	51.82894986
THOC1	rs704	32.85676129
THOC1	rs1355538	42.99410543
THOC1	rs9501600	77.57524831
ALDOC	rs28944190	54.91858953
ALDOC	rs141921160	247.9043457
ALDOC	rs3747207	33.00984952
TDO2	rs11105305	31.30139628
TDO2	rs73183457	31.11577994
TDO2	rs1581846	520.4384444
TDO2	rs1260326	57.62214568
TDO2	rs3129773	37.49460693
TDO2	rs62432338	42.73033666
TDO2	rs3127580	254.4159272
TDO2	rs71296989	98.07480148
TDO2	rs3124784	77.28903468
TDO2	rs187158158	220.8729066
TDO2	rs150683617	128.7532623
TDO2	rs112839059	300.8323259
TDO2	rs115174830	42.89780348
GLO1	rs79058690	67.20847351
GLO1	rs9462450	124.2520652
GLO1	rs9357282	42.30621142
PPIL1	rs12190328	123.1704916

PPIL1	rs114403297	92.8413617
PPIL1	rs148054374	1284.121436
PPIL1	rs79276891	169.6878202
PPIL1	rs373381440	474.4323824
PPIL1	rs115916910	84.56441833
PPIL1	rs114058208	46.70171897
PPIL1	rs144717024	29.90074677
PPIL1	rs76644067	31.20801884
PPIL1	rs45519938	132.8741383
XRCC4	rs374001978	42.62031361
XRCC4	rs7736592	158.0859044
XRCC4	rs16831329	49.91991026
XRCC4	rs2015255	32.20751559
RBBP6	rs10922098	159.573697
RBBP6	rs1355538	58.95176652
RBBP6	rs28573154	50.01121026
RBBP6	rs7774739	32.88752992
OAZ1	rs112385133	39.82130515
OAZ1	rs35340377	38.16564204
OAZ1	rs11451044	87.08218233
OAZ1	rs114694170	47.15158022
OAZ1	rs342292	110.5137997
OAZ1	rs6993770	32.80032562
MYO6	rs12292693	77.16360978
MYO6	rs342293	74.28974414
MYO6	rs6993770	39.13363206
ERCC4	rs10922098	50.72418879
ERCC4	rs147653240	30.32605754
ERCC4	rs34240868	48.41189676
FOXG1	rs10922098	355.7284743
FOXG1	rs59228823	33.14659693
FOXG1	rs1707652	63.02334997
FOXG1	rs36076454	30.56547348
FOXG1	rs9267139	32.49975941
FOXG1	rs28573154	93.46988218
FOXG1	rs7774739	62.16004554
FOXG1	rs72714690	30.4514143
MCM6	rs572515	136.0169425
MCM6	rs1707652	62.38665813
MCM6	rs6457457	48.13116771
MCM6	rs28573154	52.68607524
ARID1A	rs10922094	273.3695461
ARID1A	rs7321465	29.9704214
ARID1A	rs1706435	34.11244305
ARID1A	rs555007	33.98830194
ARID1A	rs550671	71.61993371
KIF1C	rs528298	45.99880241
KIF1C	rs28573154	29.81639515
KIF1C	rs3129773	32.67690953
NEFH	rs10922098	419.4308885
NEFH	rs2686395	82.17956205
NEFH	rs555007	55.5820826
NEFH	rs550671	128.1298692
EGLN1	rs73102039	76.63113408
EGLN1	rs146276525	202.8023066
EGLN1	rs113722620	227.8074079
EGLN1	rs114459700	448.3817624
EGLN1	rs61835449	38.72228605

EGLN1	rs821726	50.42748595
EGLN1	rs1329424	121.7622385
EGLN1	rs140898720	49.77368552
EGLN1	rs115894679	64.12022415
EGLN1	rs58762588	158.2237576
EGLN1	rs1354034	38.01809793
EGLN1	rs1619994	30.16337449
EGLN1	rs9501600	33.68896779
EGLN1	rs342290	47.31572515
SPOCK3	rs6834264	127.0580441
SPOCK3	rs75897620	395.0066297
SPOCK3	rs1023875	55.46037121
SPOCK3	rs17599599	2858.302603
SPOCK3	rs77904897	41.20513509
SPOCK3	rs13143783	811.3382699
SPOCK3	rs62333599	201.0913432
SPOCK3	rs116614155	151.0345674
SPOCK3	rs1434282	44.54933009
SPOCK3	rs670179	39.49469245
SPOCK3	rs34924625	38.73246839
SPOCK3	rs112578870	44.41403766
SPOCK3	rs371763418	48.76120642
SPOCK3	rs138620872	92.35265225
SPOCK3	rs704	94.04808002
SPOCK3	rs11668974	41.85371352
SPOCK3	rs2556099	35.9768857
SPOCK3	rs181329862	45.32022315
SPOCK3	rs17504449	120.4951153
SPOCK3	rs192358371	360.1980543
SPOCK3	rs6902010	37.23941719
SPOCK3	rs342239	46.68296765
SPOCK3	rs67647638	30.55231015
SPOCK3	rs6993770	125.8955876
SPOCK3	rs7030887	37.04894316
SPOCK3	rs60757417	73.57053554
TNKS	rs10922094	93.67047672
TNKS	rs6445035	31.78872365
TNKS	rs6457457	32.40779402
TNKS	rs557011	39.39243108
MTAP	rs10509186	48.89215657
MTAP	rs1126605	37.00171479
MTAP	rs75327431	32.74672869
MTAP	rs79391862	35.35188124
MTAP	rs62269916	31.67453097
MTAP	rs697356	45.55791612
MTAP	rs151242509	203.7623104
MTAP	rs144693508	53.05342845
MTAP	rs138711481	102.3178653
MTAP	rs2817203	268.1348021
MTAP	rs2760180	266.5066746
MTAP	rs17243234	135.1722239
MTAP	rs11965121	31.23575644
MTAP	rs13217620	34.63871939
RRBP1	rs1976848	32.60916917
RRBP1	rs11227136	89.49581797
RRBP1	rs10876550	33.45384887
RRBP1	rs1354034	84.82009126
LRRC4B	rs181596081	79.05538964

LRRC4B	rs12974169	40.99687487
LRRC4B	rs35518299	52.29958824
LRRC4B	rs10922098	64.68774755
LRRC4B	rs2686395	37.56525428
LRRC4B	rs9501600	43.31218532
CALB1	rs2573278	112.8373702
CALB1	rs9534312	804.9649465
CALB1	rs12867023	38.02246448
CALB1	rs116911825	34.51225458
SOS1	rs10922094	83.25920647
SOS1	rs6445035	34.95408093
SOS1	rs4713498	34.93394671
S100P	rs572515	39.73067308
S100P	rs1797077	47.86152909
S100P	rs17580	71.58831231
S100P	rs1801701	167.0611053
S100P	rs2237582	53.84606028
TNS4	rs368465	31.53172358
TNS4	rs146457388	37.16187208
TNS4	rs6542680	426.6913064
TNS4	rs80220851	29.83394903
TNS4	rs697357	29.84115008
TNS4	rs11755689	42.76277132
GABBR2	rs10922094	201.8244868
GABBR2	rs2686395	62.79672003
GABBR2	rs9501393	58.06601289
GABBR2	rs7774739	33.50606797
GABBR2	rs9271683	32.81602874
KRT1	rs641621	72.20338278
KRT1	rs147540582	75.73833459
KRT1	rs113543611	96.85940741
KRT1	rs186618212	41.69579118
KRT1	rs117484558	76.77310227
KRT1	rs111494201	43.30518712
KRT1	rs7240884	58.14396548
PITPNA	rs10922098	224.7097608
PITPNA	rs174533	40.15188822
PITPNA	rs11720167	116.7164776
PITPNA	rs3863087	48.6603914
PITPNA	rs4955716	33.49519643
PITPNA	rs9501600	86.38562587
LNX1	rs10922098	358.7902632
LNX1	rs2686395	64.9932905
LNX1	rs1265941	46.13279925
LNX1	rs444921	100.7998418
GJA1	rs368465	84.82598945
GJA1	rs6542680	49.40453764
GJA1	rs9501600	29.77640975
DUSP28	rs112548426	485.9315034
DUSP28	rs75383755	34.36477118
DUSP28	rs1925950	73.79102002
DUSP28	rs4980320	34.73620541
DUSP28	rs35344581	44.01931017
DUSP28	rs1354034	60.0489085
DUSP28	rs342200	35.81937571
PCDHB1	rs10922098	399.9773615
PCDHB1	rs11569415	30.94189241
PCDHB1	rs1707652	102.0811227

PCDHB1	rs9267139	48.06926335
PCDHB1	rs28573154	145.3506045
PCDHB1	rs7774739	104.9253728
PCDHB1	rs9271766	41.47916437
NXT1	rs10774624	29.71705136
NXT1	rs7632706	50.99207871
NXT1	rs7626301	862.0953055
NXT1	rs266723	85.26936165
NXT1	rs79480970	31.3209842
TOPBP1	rs10922094	47.95578827
TOPBP1	rs697356	57.45142087
TOPBP1	rs557011	39.41565649
LAG3	rs11064323	52.17215037
LAG3	rs3782735	229.4261737
LAG3	rs660240	87.62331513
LAG3	rs6667260	32.91714708
LAG3	rs77526450	37.92880725
LAG3	rs3184504	136.7545012
LAG3	rs77542162	94.25209015
LAG3	rs4951984	39.15345749
LAG3	rs115216147	114.8866698
LAG3	rs13165038	42.64648321
LAG3	rs2523948	31.98490453
LAG3	rs9264949	72.67662004
LAG3	rs189117034	59.12192156
LAG3	rs138826033	43.50435077
LAG3	rs62447171	30.20550392
NPIPB3	rs1451915	37.1069278
NPIPB3	rs10922098	804.3392934
NPIPB3	rs7513100	32.37728903
NPIPB3	rs704	96.99086942
NPIPB3	rs1047286	40.8382027
NPIPB3	rs111696008	33.22331709
NPIPB3	rs2686395	174.9523659
NPIPB3	rs1265941	76.21673926
NPIPB3	rs453821	211.6039449
NPIPB3	rs9276606	43.01595346
RNF148	rs368465	85.58411303
RNF148	rs6542680	33.44983223
RNF148	rs28672722	32.27673542
ADAM15	rs10922098	130.5886652
ADAM15	rs1706435	39.51060347
ADAM15	rs74290525	39.07693645
ADAM15	rs415620	47.50062151
ADAM15	rs7774739	42.88374327
RNF114	rs10922098	128.1260412
RNF114	rs9864801	52.89936578
RNF114	rs188302747	37.15015072
TMEM59L	rs1329424	315.5436797
TMEM59L	rs1555405	30.04868937
TMEM59L	rs1355538	80.87077999
TMEM59L	rs28573154	94.97378348
TMEM59L	rs7774739	62.49615427
TMEM59L	rs9271766	41.79681167
TMEM59L	rs2057681	91.56366136
TBC1D5	rs4980320	36.0997497
TBC1D5	rs10876550	31.5458325
TBC1D5	rs12465887	30.92203097

TBC1D5	rs1354034	34.13931962
TBC1D5	rs1619994	50.09321634
CDHR5	rs12360820	44.48416555
CDHR5	rs10922094	399.2903267
CDHR5	rs1047286	34.26094099
CDHR5	rs71352239	34.25293685
CDHR5	rs1707652	120.4075687
CDHR5	rs9267139	49.14098346
CDHR5	rs28573154	113.7892205
CDHR5	rs7774739	82.50676306
CDHR5	rs9271766	42.3960262
PCDHB10	rs10922098	123.8422099
PCDHB10	rs964184	29.75365276
PCDHB10	rs5117	106.5857612
PCDHB10	rs5112	64.75243285
PCDHB10	rs4665985	35.12948062
PCDHB10	rs1706435	94.62044871
PCDHB10	rs451652	30.26285357
PCDHB10	rs4713498	38.34017548
MYZAP	rs4774967	76.79931846
MYZAP	rs10922098	197.8868879
MYZAP	rs1564943	29.98443392
MYZAP	rs1619994	52.49001785
MYZAP	rs7774739	40.54263888
MYZAP	rs3129882	63.61311608
MYZAP	rs76359966	41.86412998
MYZAP	rs74671299	45.32946777
SLC22A16	rs3820296	48.02845935
SLC22A16	rs77141449	32.66550134
SLC22A16	rs1591734	105.5120641
SLC22A16	rs61808983	669.3474197
SLC22A16	rs6670678	434.5095025
SLC22A16	rs7894992	167.3636824
SLC22A16	rs10159712	422.2312471
SLC22A16	rs10437425	46.88391241
SLC22A16	rs11012153	41.69284579
SLC22A16	rs17580	33.92221474
SLC22A16	rs594793	44.87209897
SLC22A16	rs6544941	66.61215278
SLC22A16	rs9257052	46.21700322
SLC22A16	rs34283380	41.56468382
SLC22A16	rs28366220	112.0681756
SLC22A16	rs34958241	235.7234067
SLC22A16	rs9271669	115.8930432
UBXN4	rs10922098	36.94046751
UBXN4	rs12478841	36.09721132
UBXN4	rs1355538	30.64636022
UBXN4	rs6986061	34.33375107
UBXN4	rs117815264	32.40994288
DLL3	rs12757377	36.33709538
DLL3	rs9943101	31.65357095
DLL3	rs72748418	40.87176466
DLL3	rs10801548	214.4448923
DLL3	rs1329424	3611.404396
DLL3	rs35351085	60.38218879
DLL3	rs12739875	43.03516362
DLL3	rs34855206	33.93153179
DLL3	rs7080386	106.519689

DLL3	rs4239252	362.1509622
DLL3	rs111887239	34.69232945
DLL3	rs775824	33.67181583
DLL3	rs6993770	36.49466444
AMIGO1	rs76755323	59.18287433
AMIGO1	rs1141720	32.41739751
AMIGO1	rs41547217	35.21312299
AMIGO1	rs4713555	46.27109495
PRSS35	rs10922098	407.8506447
PRSS35	rs10208529	32.99436783
PRSS35	rs1707652	64.93541716
PRSS35	rs9501393	103.4744775
PRSS35	rs7774739	61.73120968
PRSS35	rs9271766	31.2070525
PRSS35	rs57136797	55.22583202
YIPF6	rs10922098	357.7416608
YIPF6	rs1355538	78.56653738
YIPF6	rs1265941	43.27333401
YIPF6	rs453821	101.6716748
NPW	rs72775463	52.75280653
NPW	rs117550430	289.2374899
NPW	rs12933410	3795.803636
NPW	rs2432380	80.48544295
NPW	rs140216362	56.85171281
NPW	rs7397189	40.81834403
NPW	rs140118846	34.59132942
NPW	rs181707610	35.82625473
NPW	rs17580	45.51594892
NPW	rs339969	62.10115488
NPW	rs11078596	55.40628856
NPW	rs7405889	48.23625271
NPW	rs374087027	38.65206903
NPW	rs77262773	36.56981804
NPW	rs28616221	33.73269373
NPW	rs281377	42.18763169
NPW	rs1260326	55.84479341
NPW	rs17050272	85.90592603
NPW	rs762861	58.99357693
NPW	rs1800760	34.90578244
NPW	rs995104	30.32101541
NPW	rs71557726	34.08972695
LRRC24	rs80167373	85.83564941
LRRC24	rs6542680	31.78766401
LRRC24	rs7614981	30.63818077
ZNF264	rs10922098	156.0862709
ZNF264	rs1355538	42.90364035
ZNF264	rs6457457	36.88048844
ZNF264	rs9501393	46.84848591
ATP4B	rs572515	106.5496405
ATP4B	rs1707652	50.56281067
ATP4B	rs6457457	30.64612182
ATP4B	rs9501393	41.32942321
IRF6	rs11605332	30.02436459
IRF6	rs9864801	36.06336747
IRF6	rs342292	30.14702547

Table S5: Interacting residues between all candidate drugs and the target protein

Drugs	Target	Interacting residues
Agarose	EGFR	Chain A :LEU718 GLY719 SER720 GLY721 PHE723 VAL726 TYR727 ALA743 ILE744 LYS745 CYS775 MET790 GLN791 LEU792 MET793 PRO794 PHE795 GLY796 CYS797 LEU799 ASP800 TYR801 GLU804 ARG841 ASN842 LEU844 VAL845 THR854 ASP855 PHE856
Agarose	PLG	Chain A: PHE587 CYS588 HIS603 CYS604 GLU606 LYS607 ASP735 SER736 CYS737 GLN738 GLY739 ASP740 SER741 THR759 SER760 TRP761 GLY762 LEU763 GLY764 CYS765 ALA766 LYS770 PRO771 GLY772 VAL773 TYR774
Agarose	MMP3	Chain A:VAL102 ASN103 MET143 SER145 PHE146 ALA147 VAL148 ARG149 GLU150 HIS151 GLY152 ASP153 ILE174 ASP177 HIS179<br>Chain B: VAL102 ASN103 GLU139 MET143 ILE144 SER145 PHE146 ALA147 VAL148 ARG149 GLU150 HIS151 GLY152 ASP153 ILE174 ASP177 ALA178 HIS179
Agarose	LTF	Chain A: VAL11 SER12 PRO14 GLU15 ASP40 PRO42 VAL57 THR58 LEU59 ASP60 PHE63 GLU80 ARG121 PHE183 SER184 SER185 GLN186 GLU187 PRO188 TYR189 PHE190 SER191 TYR192 SER193 GLY194 ALA195 HIS253 GLY294 GLN295 LYS296 ASP297 LEU298 LEU299 PHE300 LYS301
clofibrate	EGFR	Chain A :LEU718 GLY719 SER720 PHE723 VAL726 ALA743 LYS745 CYS775 MET790 GLN791 LEU792 MET793 PHE795 GLY796 CYS797 ASP800 ARG841 ASN842 LEU844 THR854 PHE856
clofibrate	PLG	Chain A: PHE587 CYS588 ALA602 HIS603 GLU606 ARG644 LYS645 ASP646 ARG719 GLU724 ASP735 SER736 CYS737 GLN738 GLY739 ASP740 SER741 THR759 SER760 TRP761 GLY762 GLY764 CYS765 GLY772 VAL773 TYR774
clofibrate	APOA1	Chain A: VAL21 LEU22 LYS23 ASP24 SER25 GLY26 ARG27 ASP28 TYR29 VAL30 GLN32 TRP50 LYS94 VAL97 GLN98 TYR100 LEU101 PHE104 GLN105
ascorbic acid	EGFR	Chain A :LYS745 GLU762 VAL765 MET766 ALA767 VAL769 VAL774 CYS775 ARG776 LEU777 LEU788 MET790 LEU828 LEU833 HIS835 ILE853 THR854 ASP855 PHE856 GLY857 ARG858 ALA859 LYS860 LEU861
ascorbic acid	PLG	Chain A: PHE587 CYS588 HIS603 CYS604 LYS607 ASP735 SER736 CYS737 GLN738 GLY739 ASP740 SER741 GLY742 THR759 SER760 TRP761 GLY762 LEU763 GLY764 CYS765 ALA766 GLY772 VAL773 TYR774
ascorbic acid	APOA1	Chain A: VAL19 ASP20 VAL21 LYS23 ASP24 TRP50 ASP51 SER52 VAL53 THR54 SER55 THR56 PHE57 LYS59
ascorbic acid	TAGLN	Chain A: GLN37 CYS38 GLY39 ASP41 VAL42 SER63 LYS64

		LEU65 VAL66 ASN67 SER68 LEU69 TYR70 PRO71 ASP72 LYS75 VAL79 GLY143 HIS144 ARG146
6-aminohexanoic acid	EGFR	Chain A:LEU718 GLY719 SER720 PHE723 VAL726 ALA743 LYS745 CYS775 ARG776 MET790 GLN791 LEU792 MET793 PRO794 PHE795 GLY796 CYS797 ARG841 LEU844 VAL845 THR854 ASP855 PHE856
6-aminohexanoic acid	PLG	Chain A: PHE587 CYS588 HIS603 ASP646 ASP735 SER736 CYS737 GLN738 GLY739 ASP740 SER741 THR759 SER760 TRP761 GLY762 LEU763 GLY764 CYS765 ALA766 GLY772 VAL773 TYR774
tetracycline	EGFR	Chain A:ALA722 PHE723 GLY724 LYS745 LEU747 ARG748 PRO753 LYS754 GLU758 ILE759 ASP761 GLU762 ALA763 VAL765 MET766 ILE780 VAL786 LEU788 LEU833 ARG836 ASP837 ASP855 PHE856 GLY857 ARG858 ALA859 LYS860 LEU861 GLY874 LYS875
tetracycline	MMP3	Chain A: TYR104 LYS110 ALA147 VAL148 ARG149 GLU150 HIS151 ASP183<br>Chain B: VAL102 ASN103 PRO106 ASP107 LEU108 GLU139 MET143 ILE144 SER145 ALA147 HIS151 GLY152 ASP153 TYR168 GLY173 ILE174 ASP177 A LA178 HIS179 GLN185
tetracycline	TAGLN	Chain A: MET1 ALA2 ASN3 LYS4 GLY5 PRO6 SER7 SER11 ARG12 GLN15 SER16 GLU19 ARG49 LEU50 GLN53 LYS57 PHE118 GLU119 GLY120 LYS121 ASP122

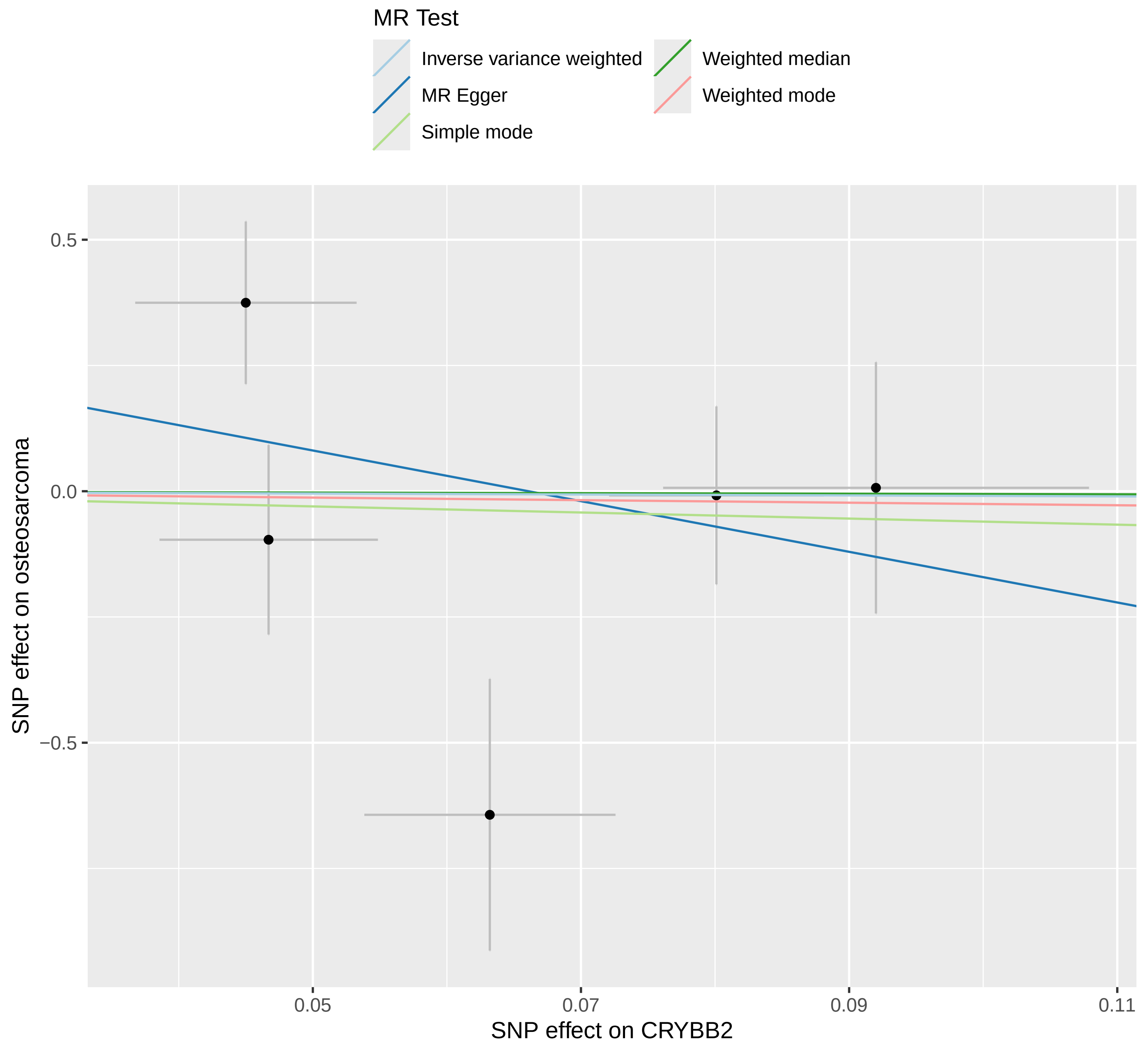

MR Test
Inverse variance weighted
MR Egger
Simple mode
Weighted median
Weighted mode
0.5
0.0
−0.5
SNP effect on osteosarcoma
0.05
0.07
0.09
0.11
SNP effect on CRYBB2

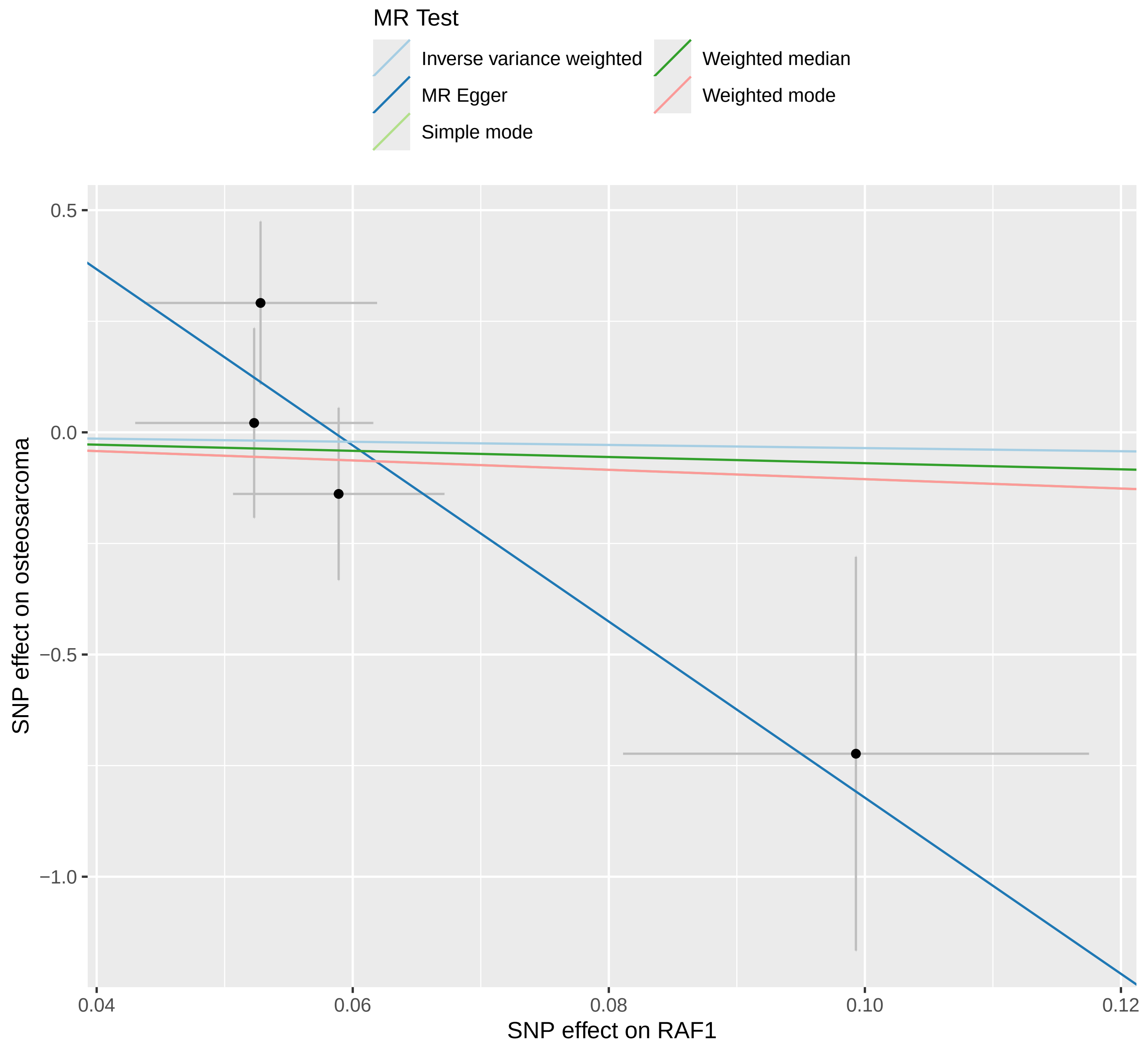
MR Test
Inverse variance weighted
MR Egger
Simple mode
Weighted median
Weighted mode
0.5
0.0
−0.5
−1.0
0.04
0.06
0.08
0.10
0.12
SNP effect on RAF1
SNP effect on osteosarcoma

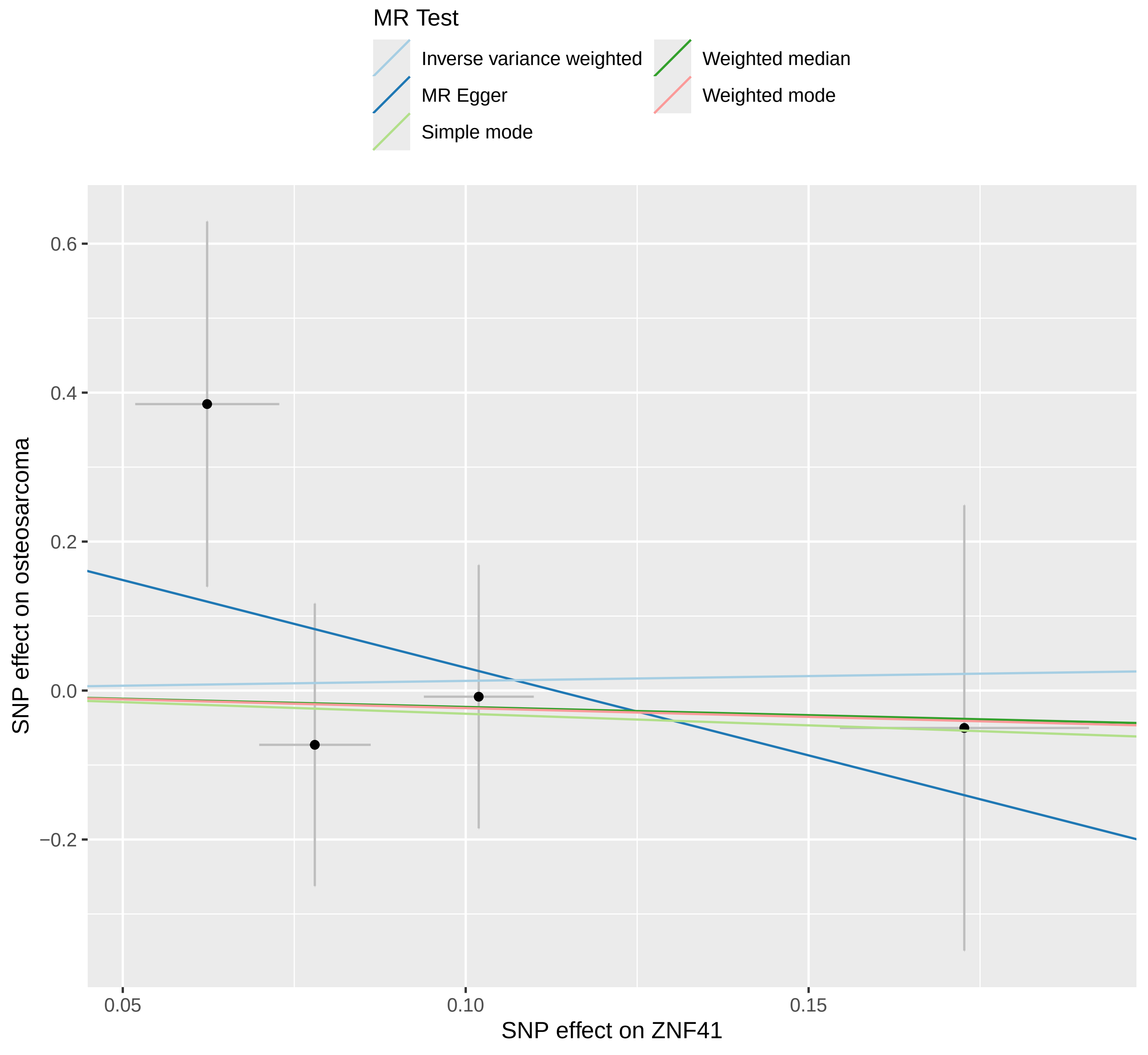

MR Test
Inverse variance weighted
MR Egger
Simple mode
Weighted median
Weighted mode
0.6
0.4
0.2
0.0
−0.2
0.05
0.10
0.15
SNP effect on osteosarcoma
SNP effect on ZNF41

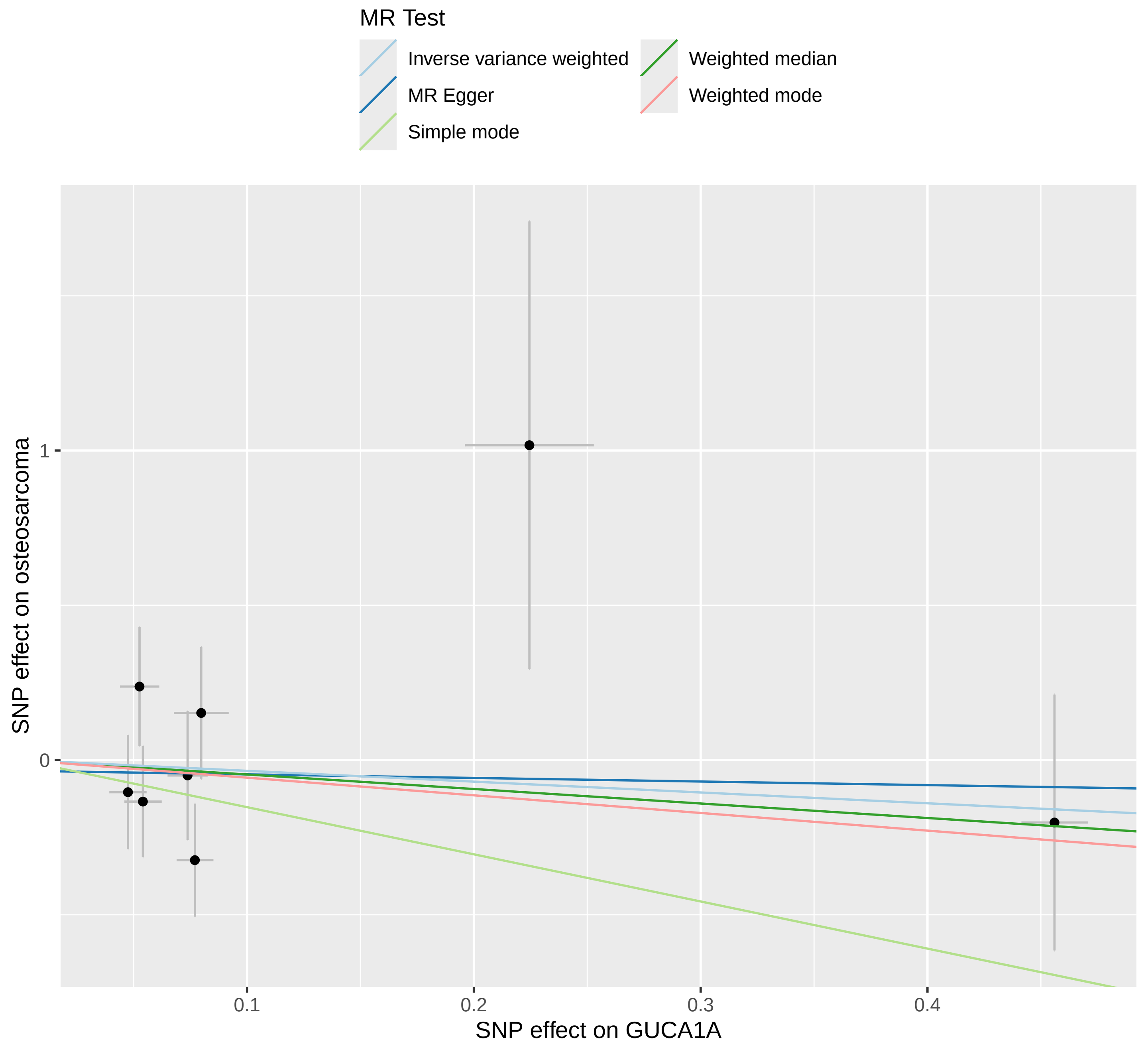
MR Test
Inverse variance weighted
MR Egger
Simple mode
Weighted median
Weighted mode
1
0
0.1
0.2
0.3
0.4
SNP effect on GUCA1A
SNP effect on osteosarcoma

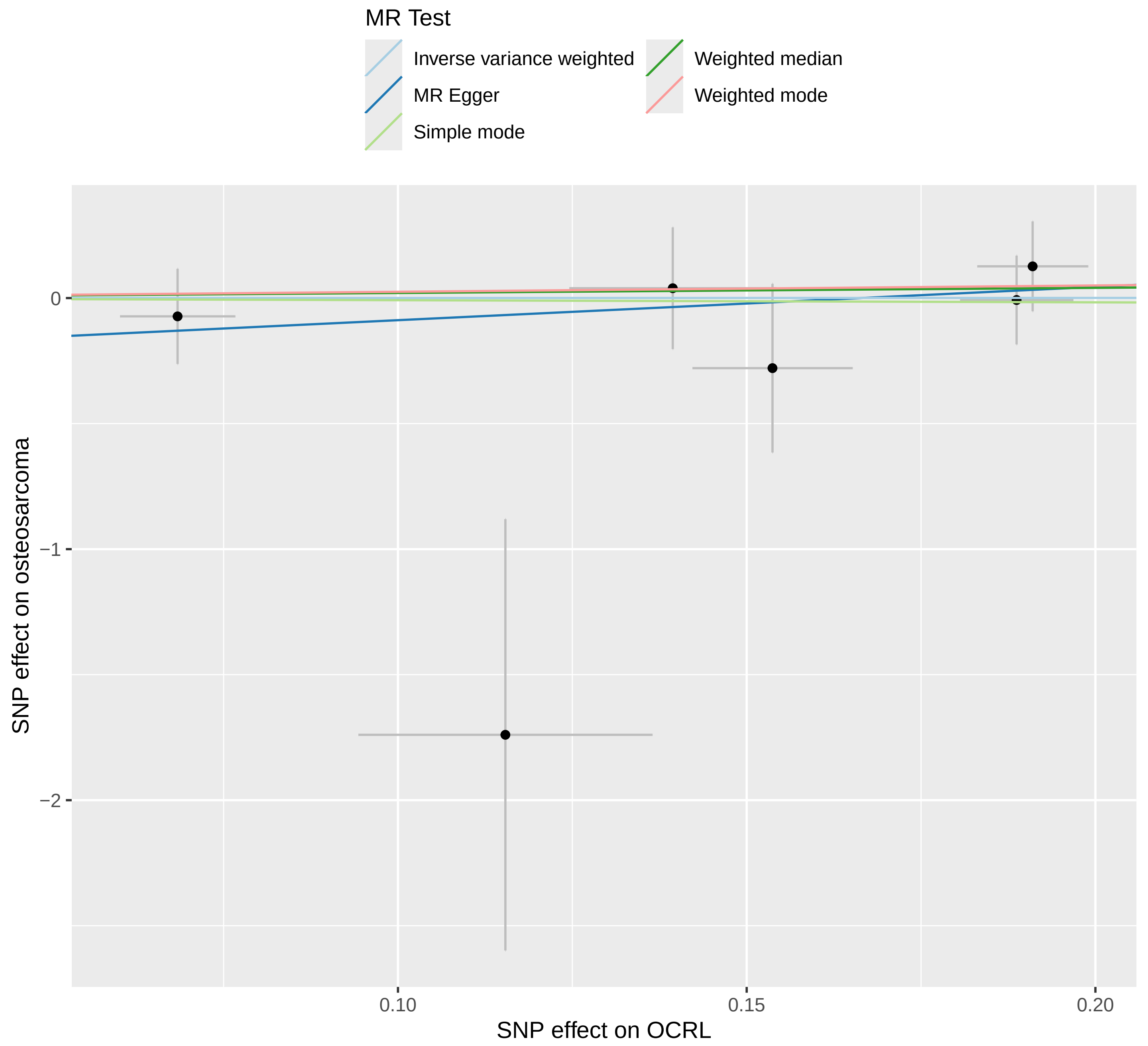

MR Test
Inverse variance weighted
MR Egger
Simple mode
Weighted median
Weighted mode
0
−1
−2
0.10
0.15
0.20
SNP effect on osteosarcoma
SNP effect on OCRL

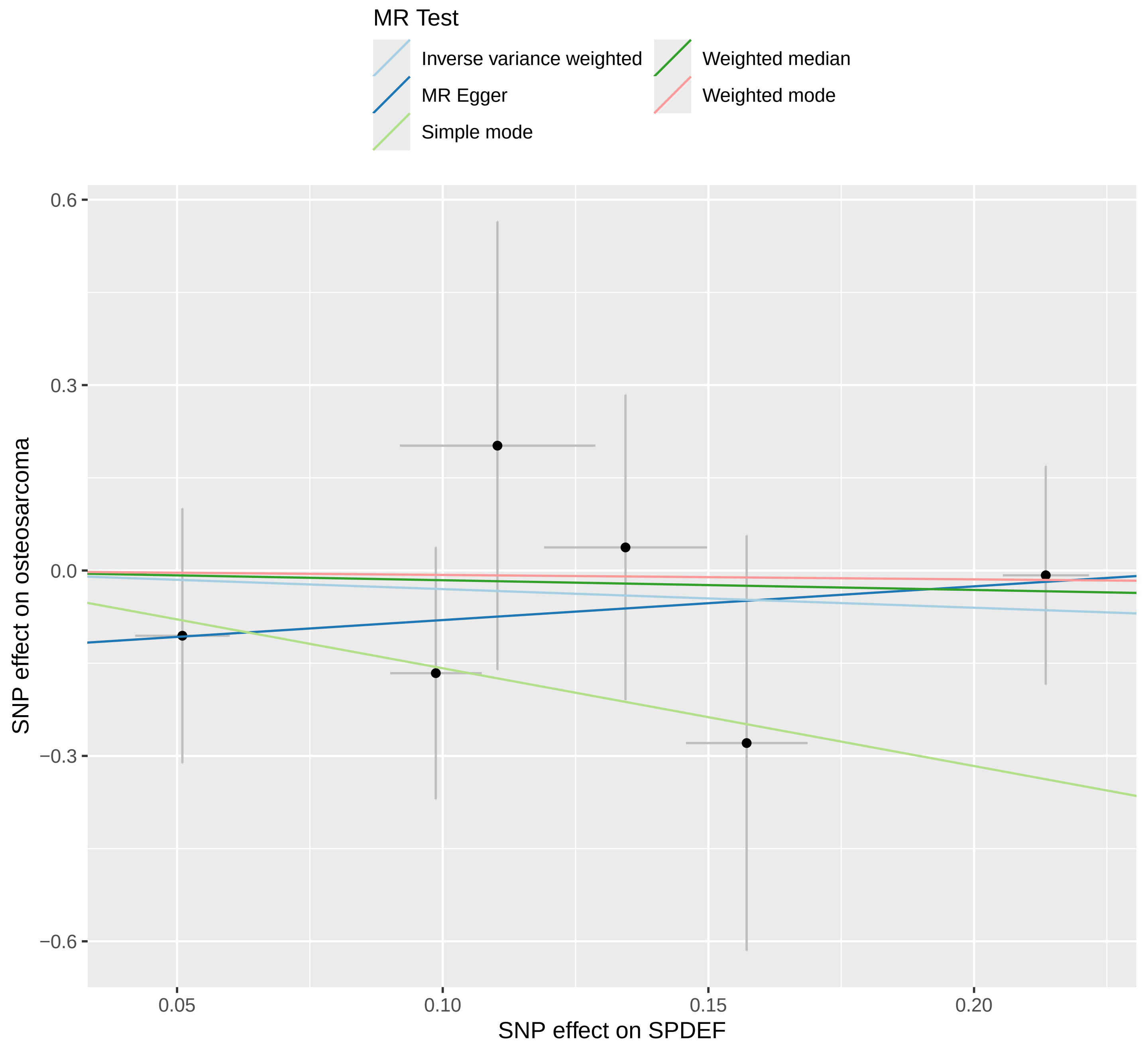

MR Test
Inverse variance weighted
MR Egger
Simple mode
Weighted median
Weighted mode
0.6
0.3
0.0
−0.3
−0.6
SNP effect on osteosarcoma
0.05
0.10
0.15
0.20
SNP effect on SPDEF

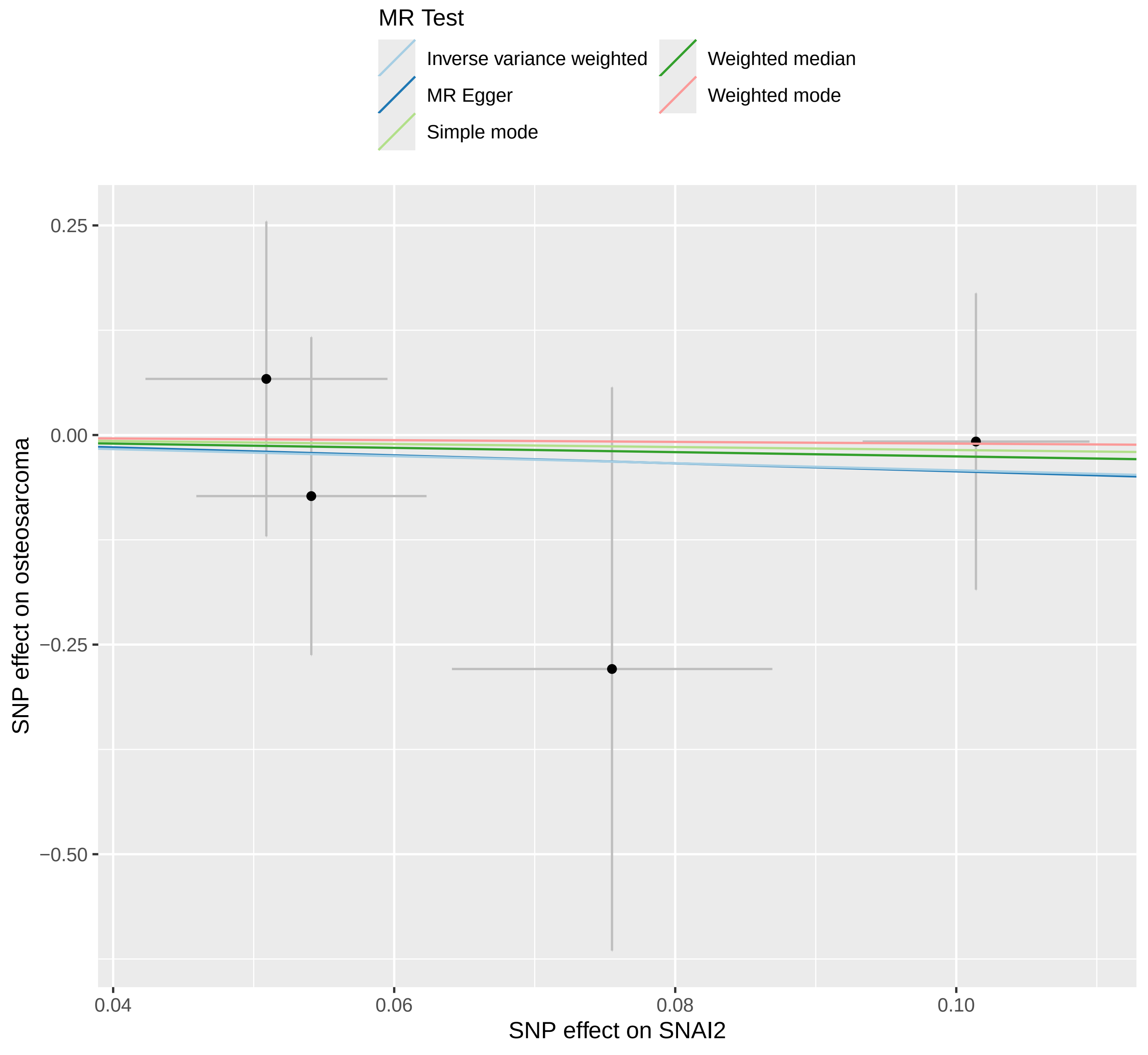

MR Test
Inverse variance weighted
MR Egger
Simple mode
Weighted median
Weighted mode
0.25
0.00
−0.25
−0.50
0.04
0.06
0.08
0.10
SNP effect on osteosarcoma
SNP effect on SNAI2

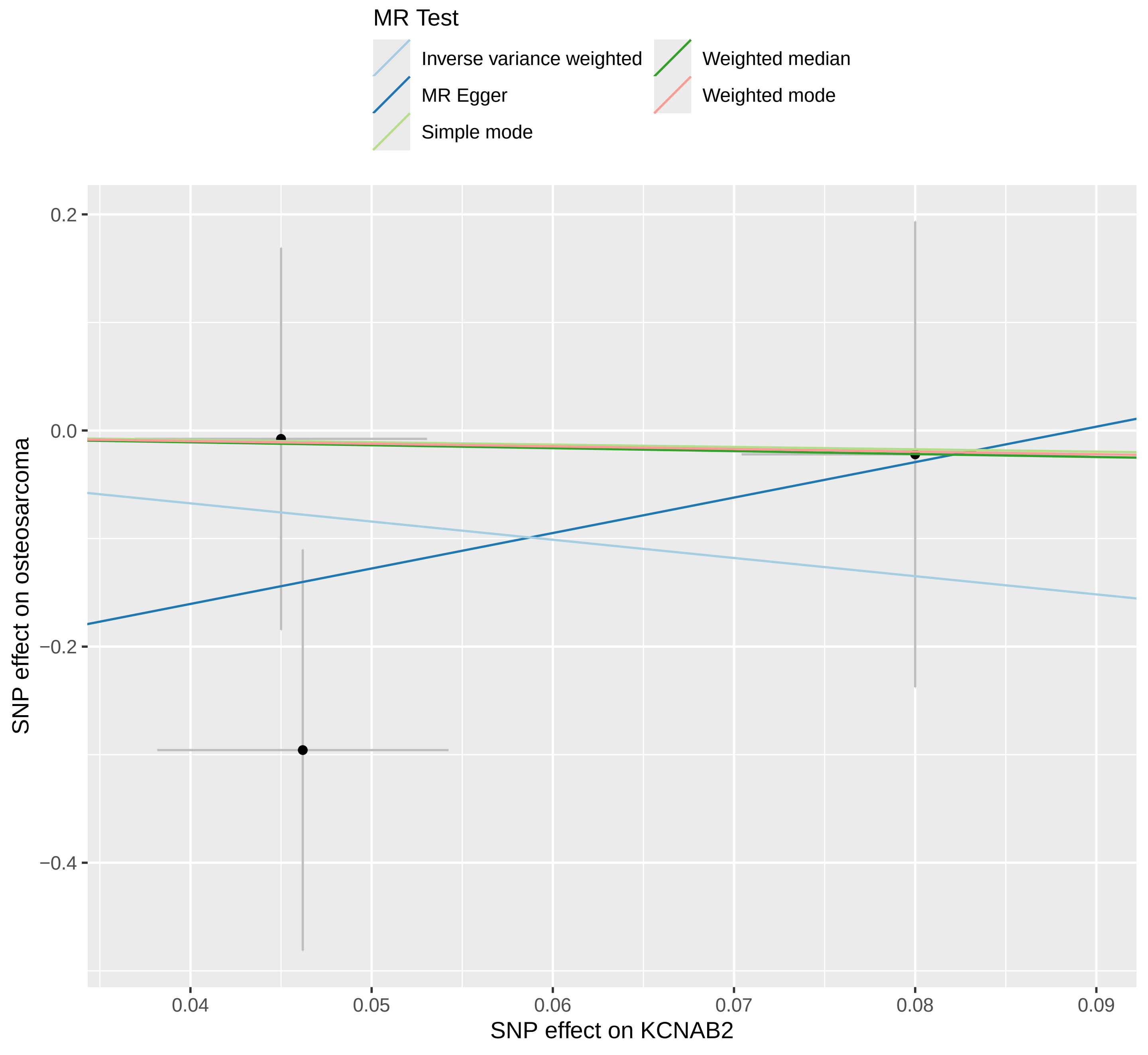

MR Test
Inverse variance weighted
MR Egger
Simple mode
Weighted median
Weighted mode
0.2
0.0
−0.2
−0.4
0.04
0.05
0.06
0.07
0.08
0.09
SNP effect on KCNAB2
SNP effect on osteosarcoma

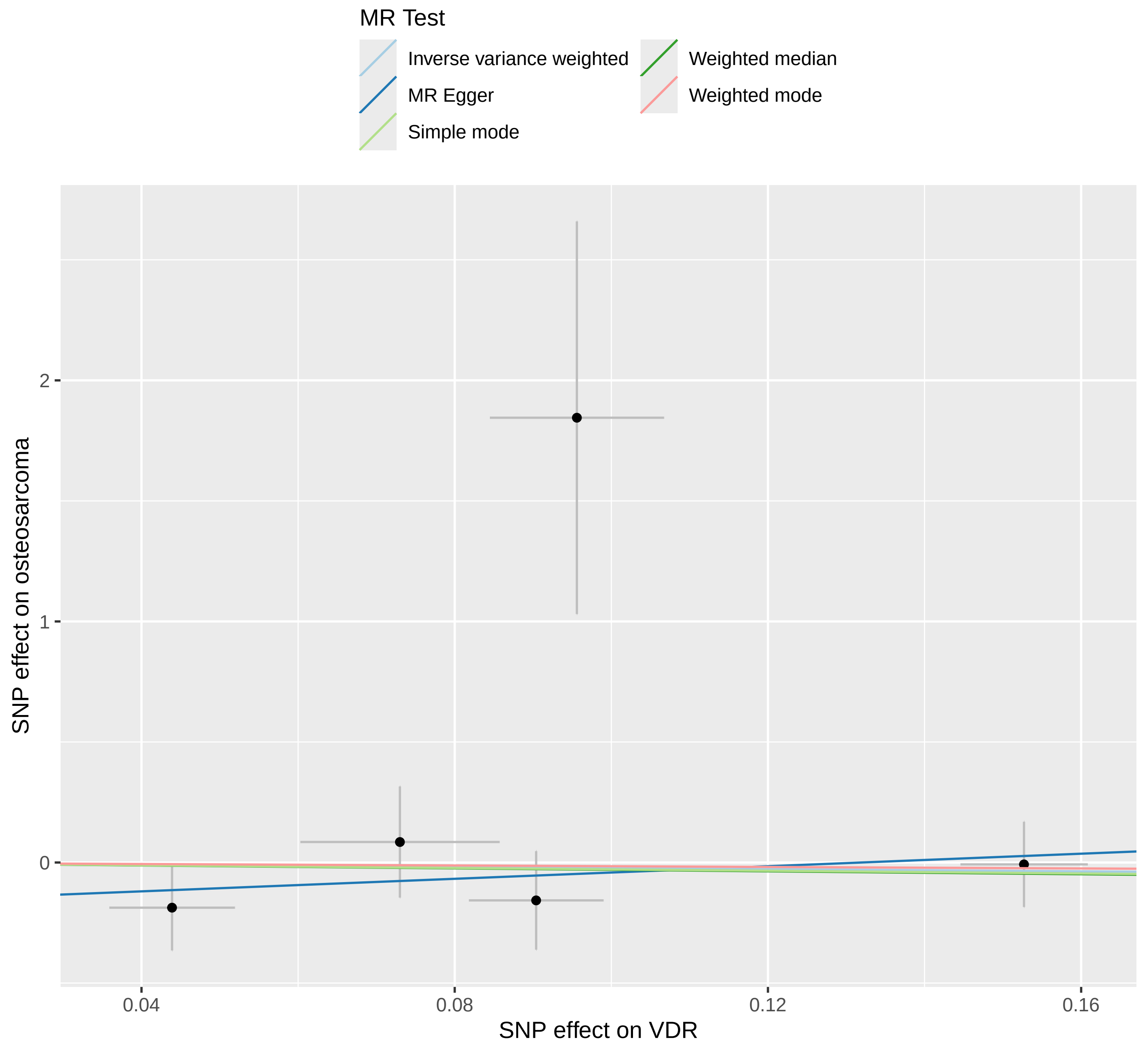

MR Test
Inverse variance weighted
MR Egger
Simple mode
Weighted median
Weighted mode
SNP effect on osteosarcoma
0
1
2
0.04
0.08
0.12
0.16
SNP effect on VDR

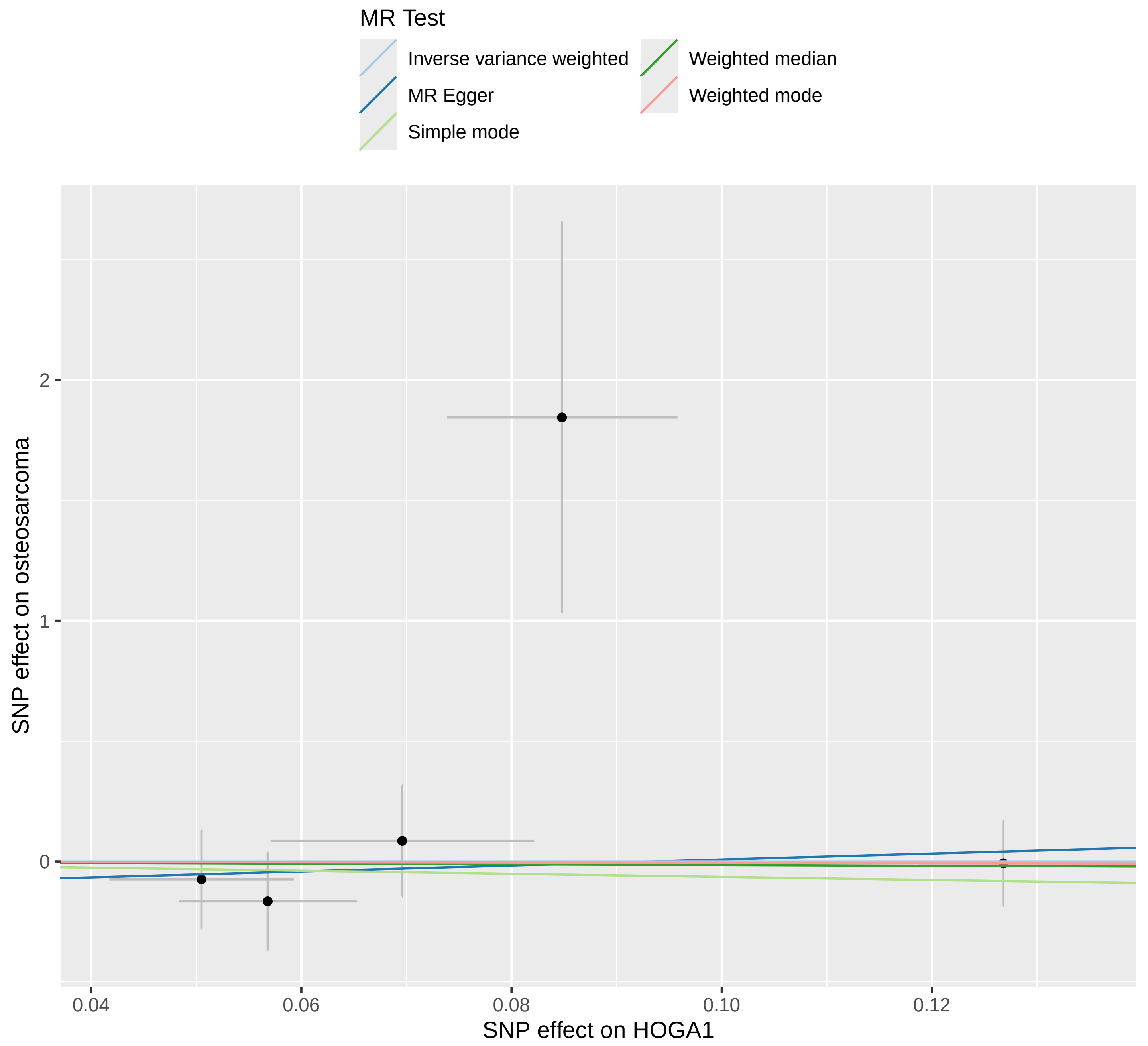
MR Test
Inverse variance weighted
MR Egger
Simple mode
Weighted median
Weighted mode
SNP effect on osteosarcoma
2
1
0
0.04
0.06
0.08
0.10
0.12
SNP effect on HOGA1

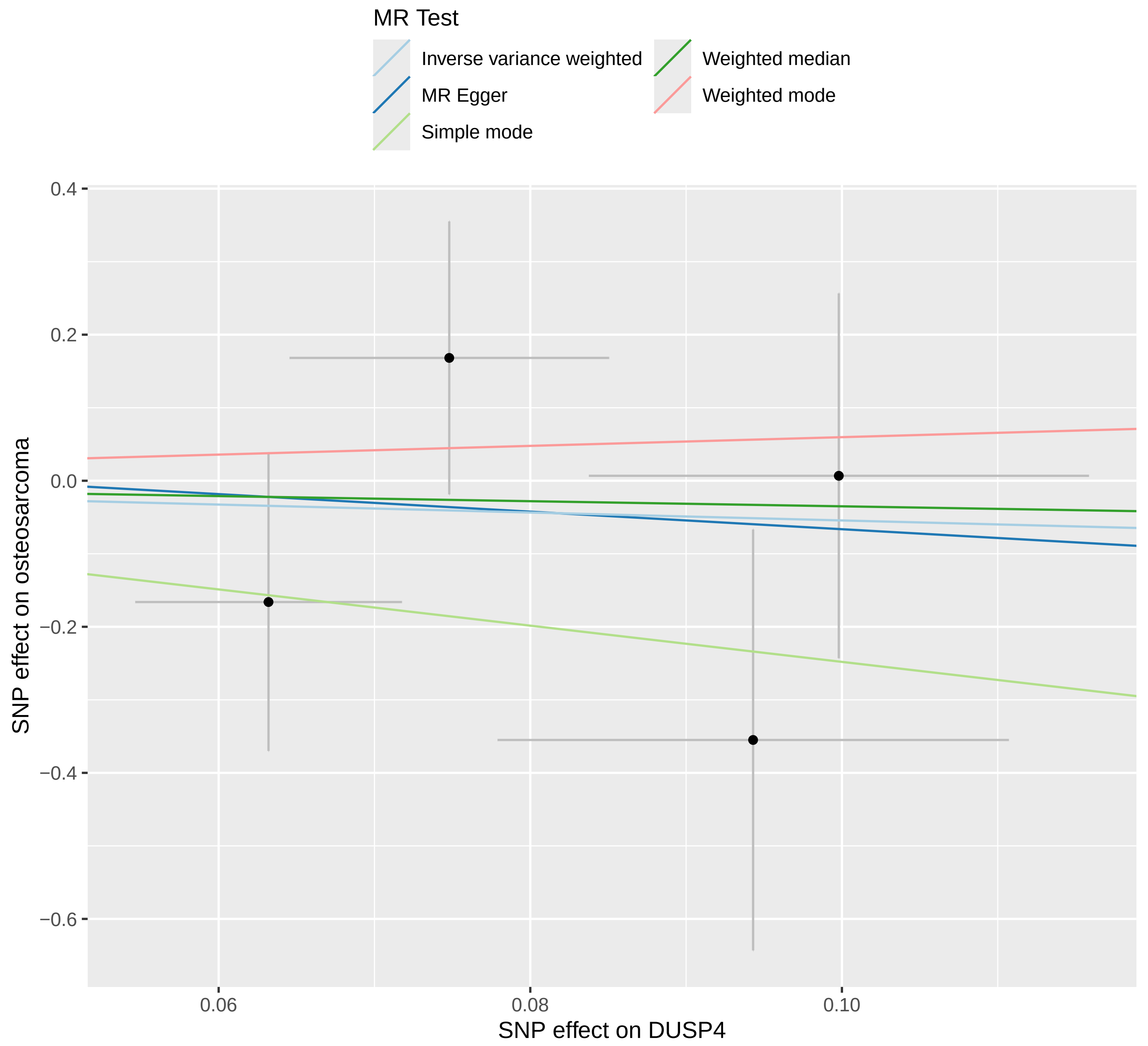
MR Test
Inverse variance weighted
MR Egger
Simple mode
Weighted median
Weighted mode
0.4
0.2
0.0
−0.2
−0.4
−0.6
0.06
0.08
0.10
SNP effect on osteosarcoma
SNP effect on DUSP4

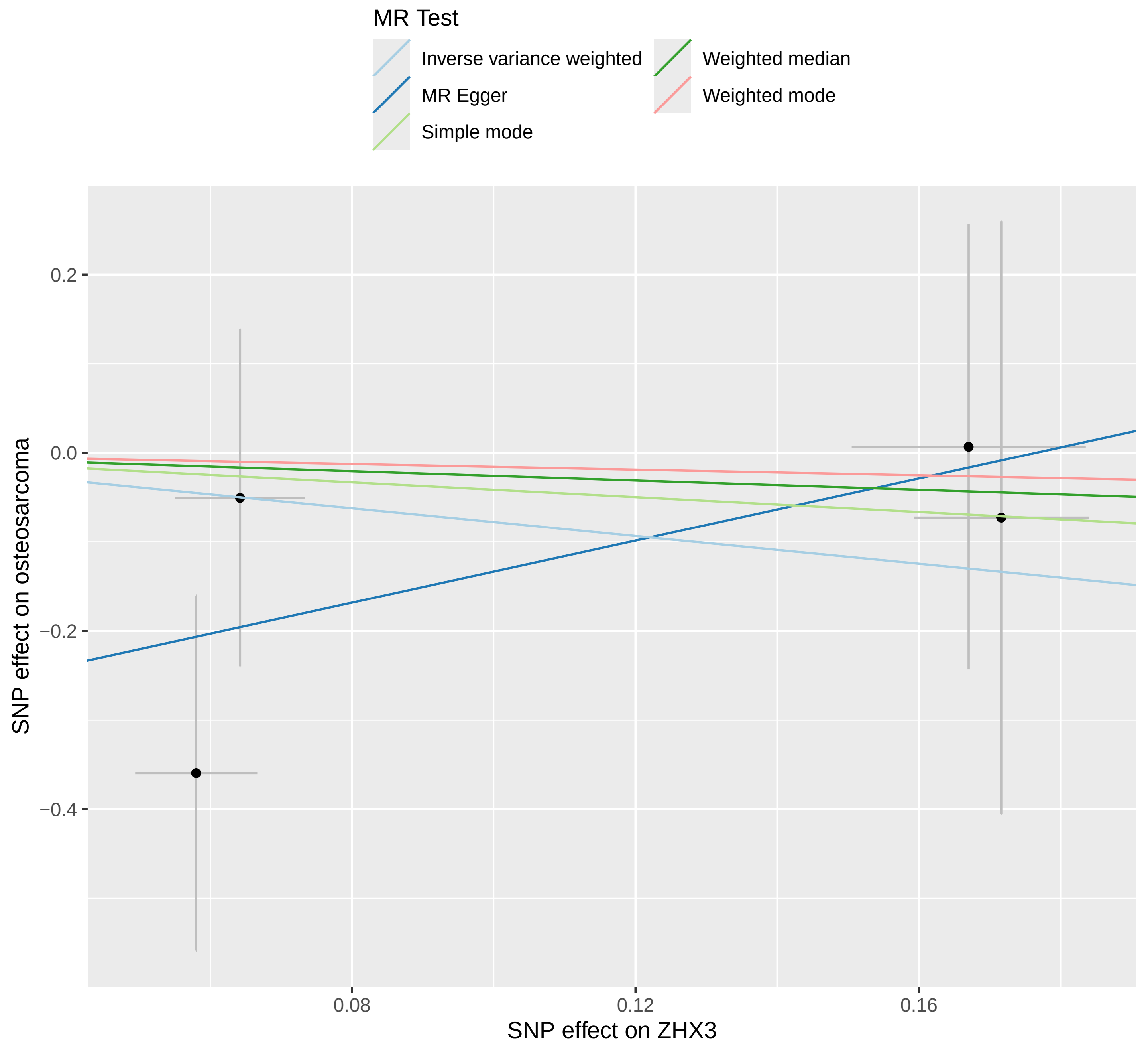
MR Test
Inverse variance weighted
MR Egger
Simple mode
Weighted median
Weighted mode
0.2
0.0
−0.2
−0.4
SNP effect on osteosarcoma
0.08
0.12
0.16
SNP effect on ZHX3

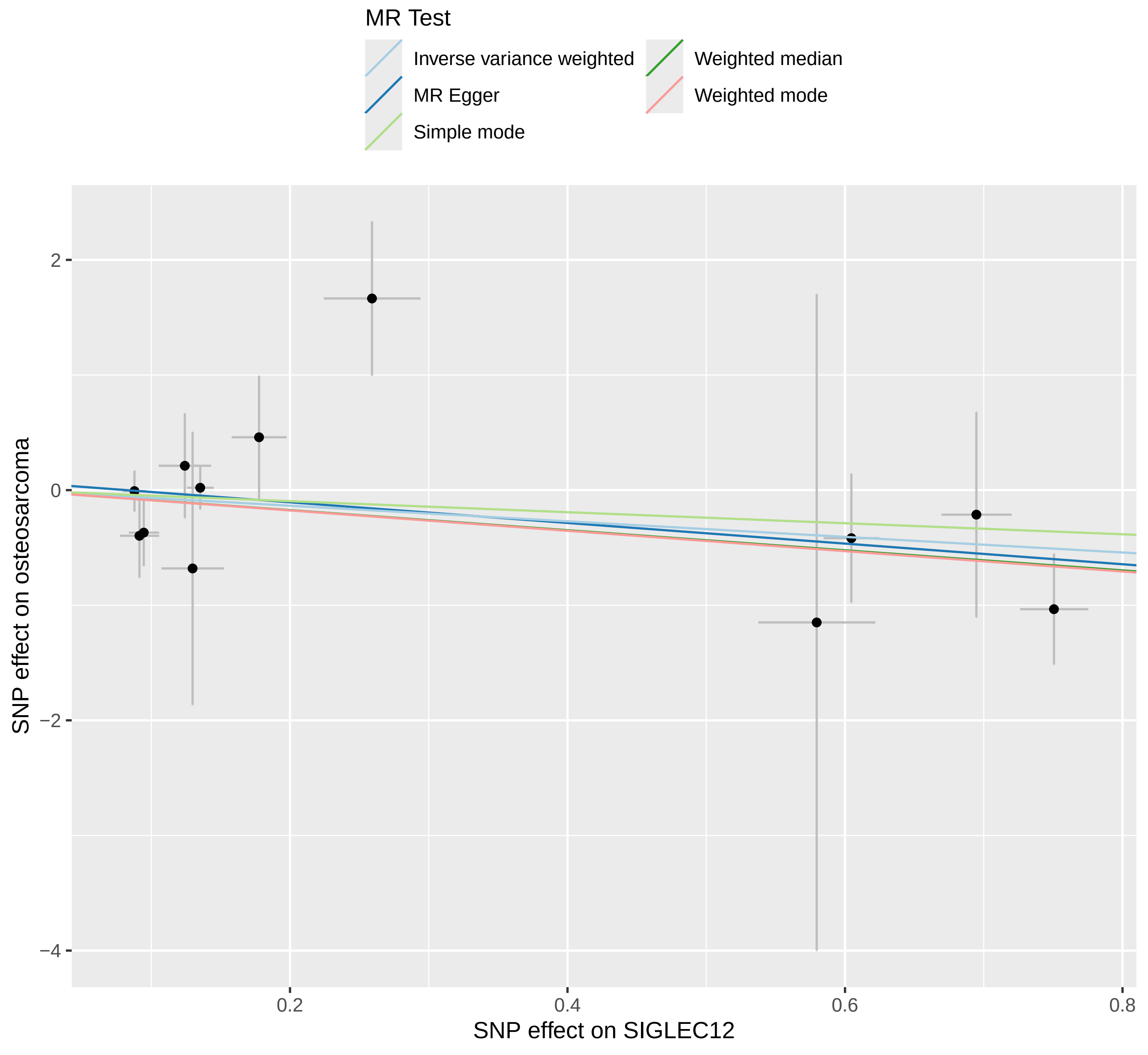
MR Test
Inverse variance weighted
MR Egger
Simple mode
Weighted median
Weighted mode
SNP effect on osteosarcoma
2
0
−2
−4
0.2
0.4
0.6
0.8
SNP effect on SIGLEC12

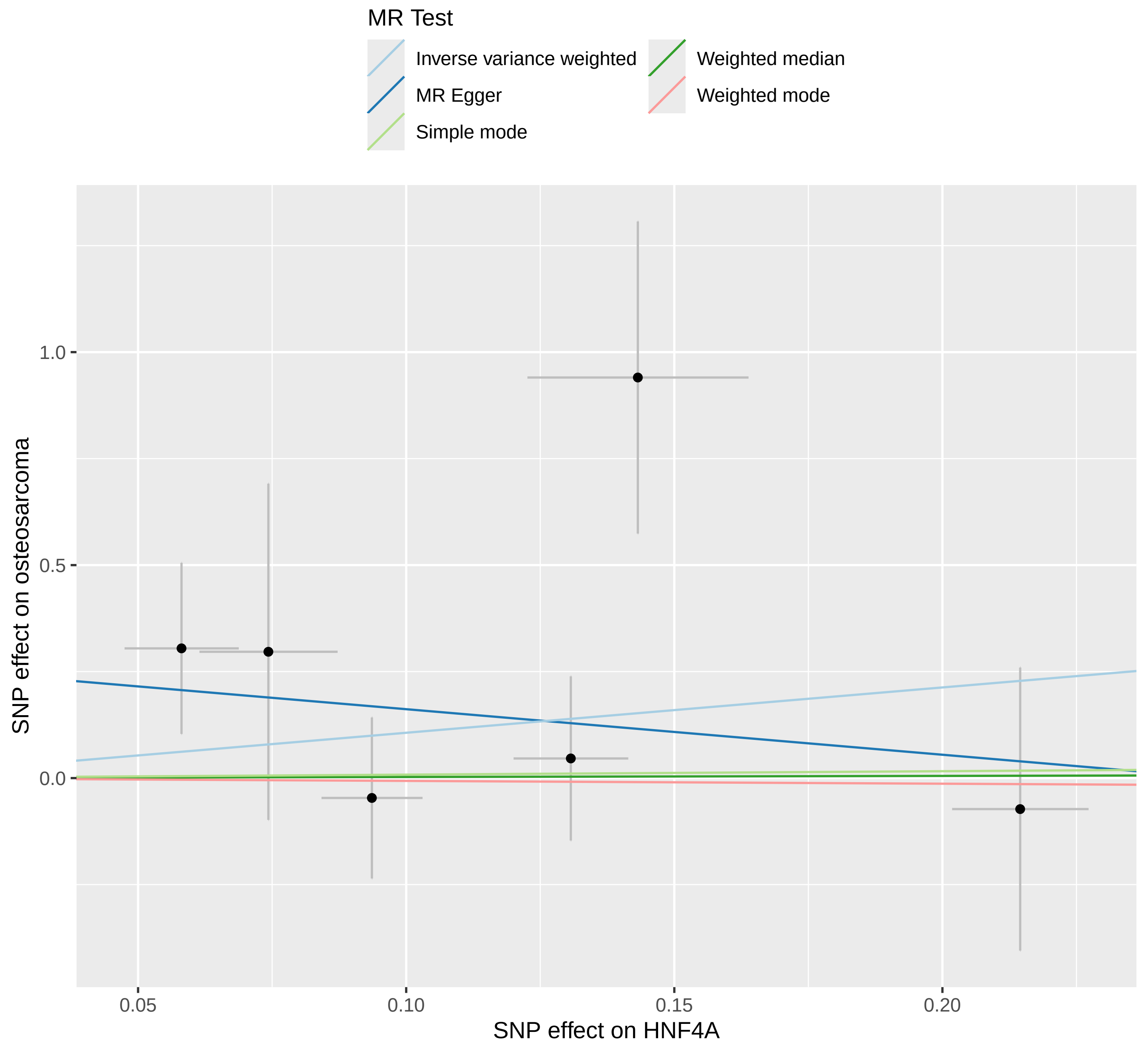
MR Test
Inverse variance weighted
MR Egger
Simple mode
Weighted median
Weighted mode
1.0
0.5
0.0
0.05
0.10
0.15
0.20
SNP effect on osteosarcoma
SNP effect on HNF4A

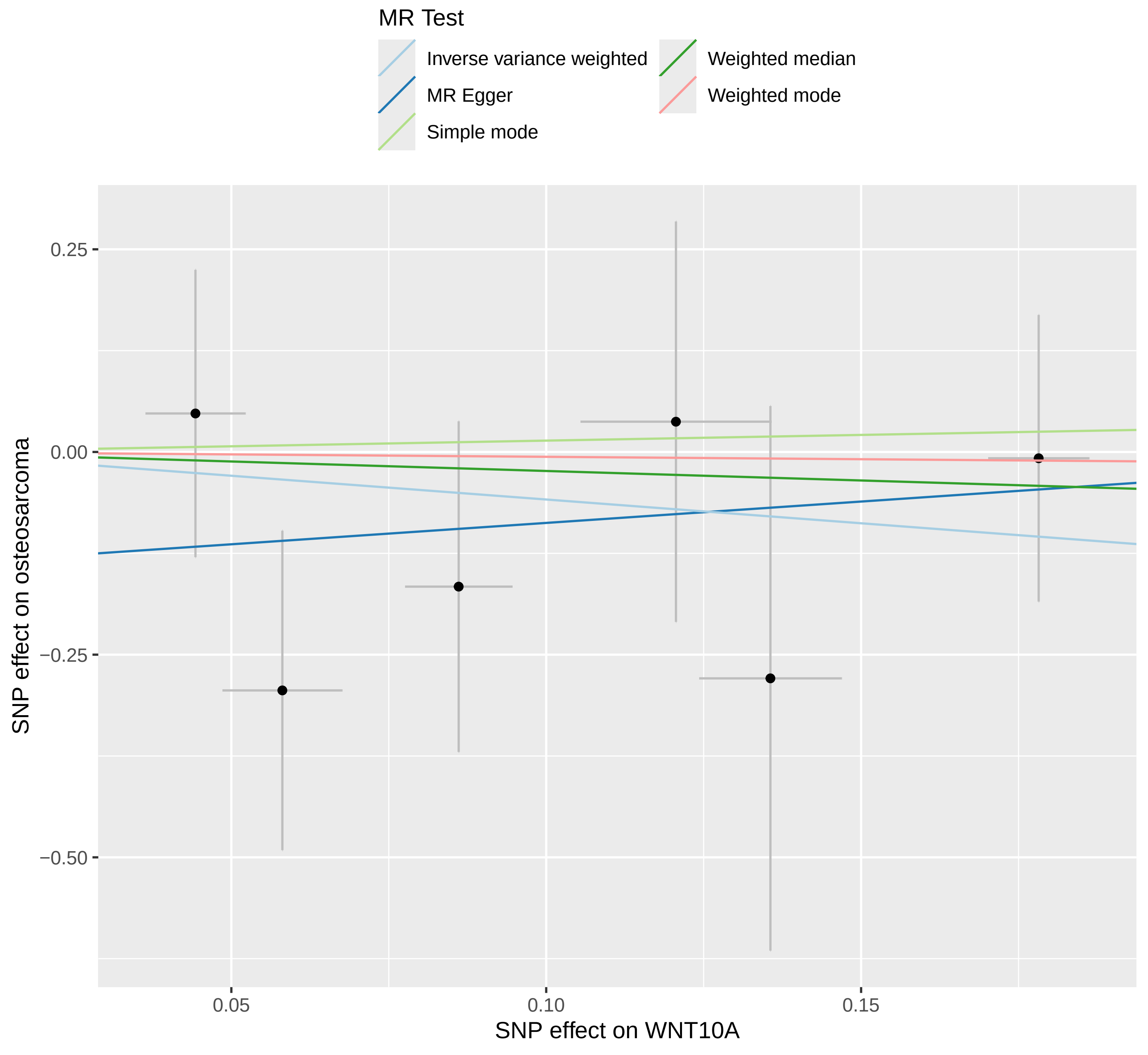

MR Test
Inverse variance weighted
MR Egger
Simple mode
Weighted median
Weighted mode
0.25
0.00
−0.25
−0.50
0.05
0.10
0.15
SNP effect on osteosarcoma
SNP effect on WNT10A

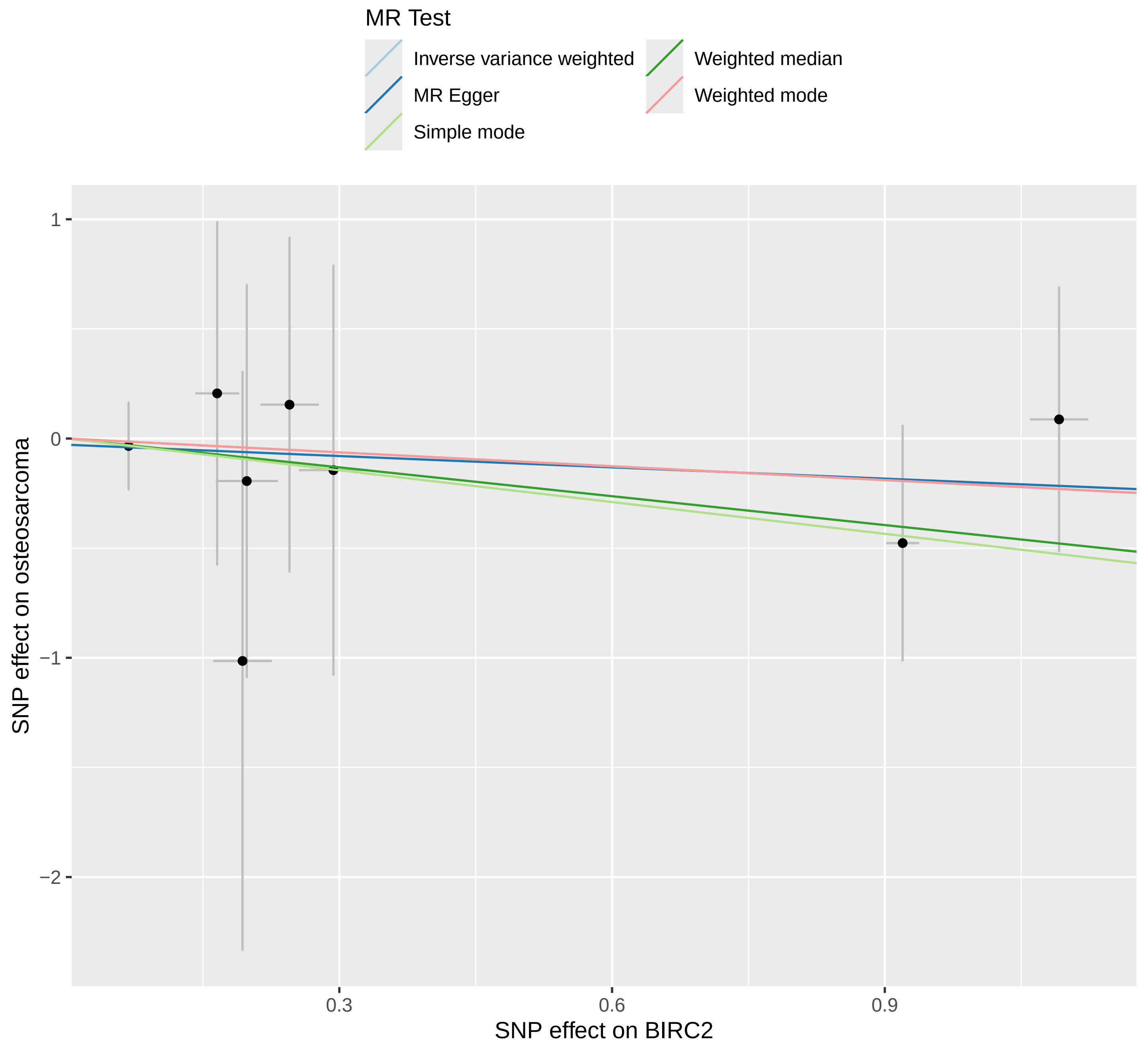
MR Test
Inverse variance weighted
MR Egger
Simple mode
Weighted median
Weighted mode
1
0
−1
−2
0.3
0.6
0.9
SNP effect on osteosarcoma
SNP effect on BIRC2

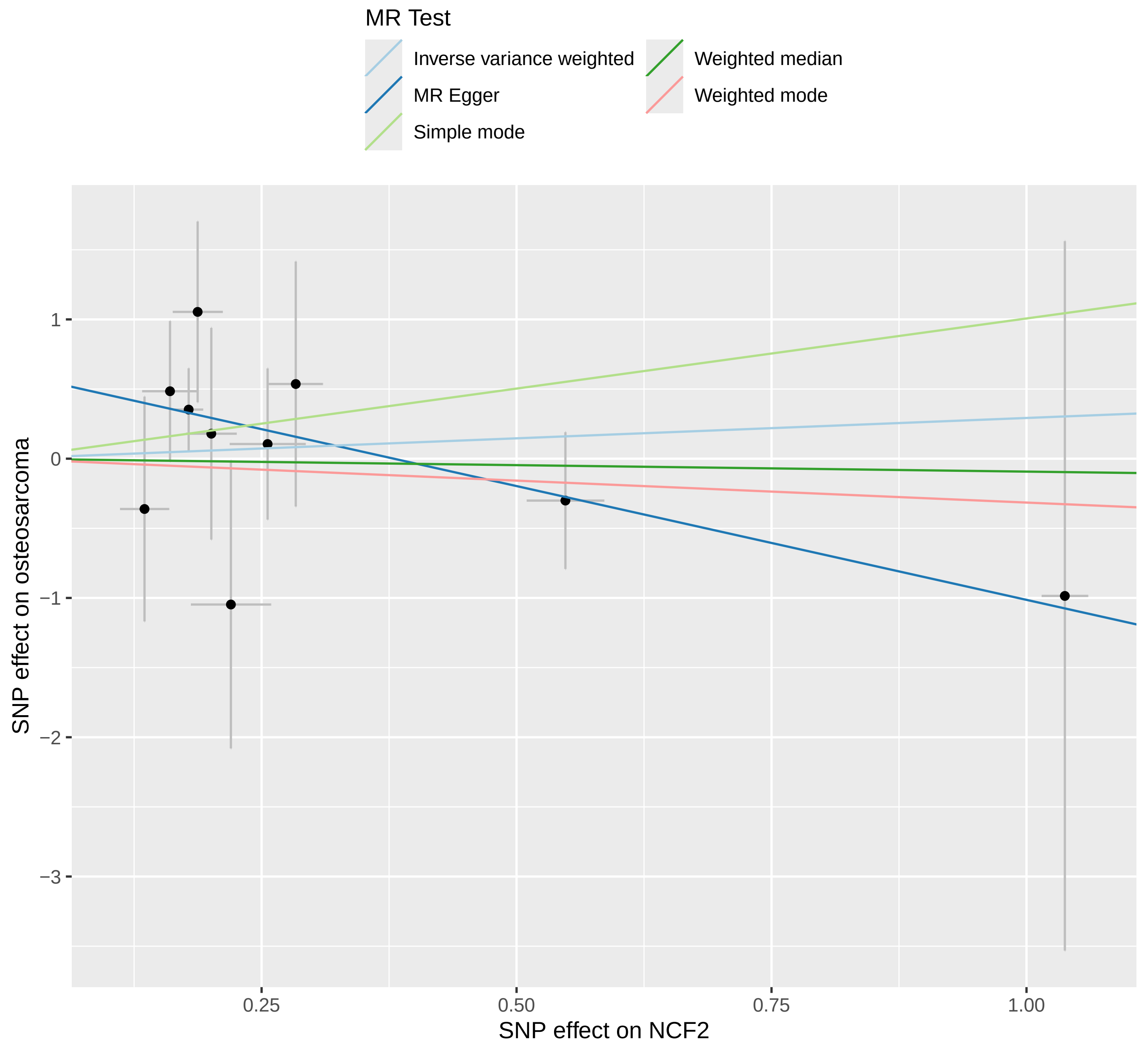

MR Test
Inverse variance weighted
MR Egger
Simple mode
Weighted median
Weighted mode
1
0
−1
−2
−3
SNP effect on osteosarcoma
0.25
0.50
0.75
1.00
SNP effect on NCF2

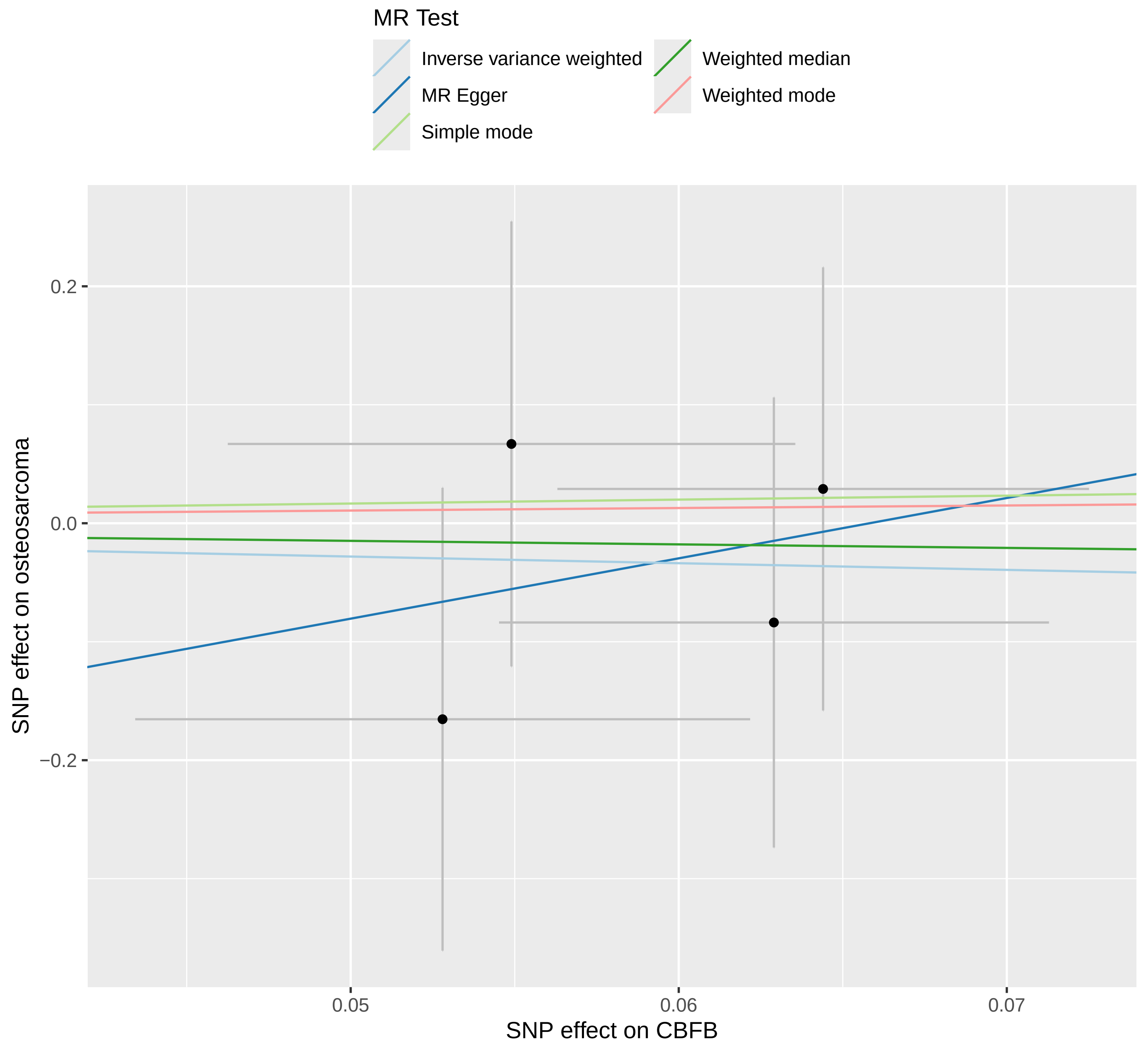
MR Test
Inverse variance weighted
MR Egger
Simple mode
Weighted median
Weighted mode
0.2
0.0
−0.2
SNP effect on osteosarcoma
0.05
0.06
0.07
SNP effect on CBFB

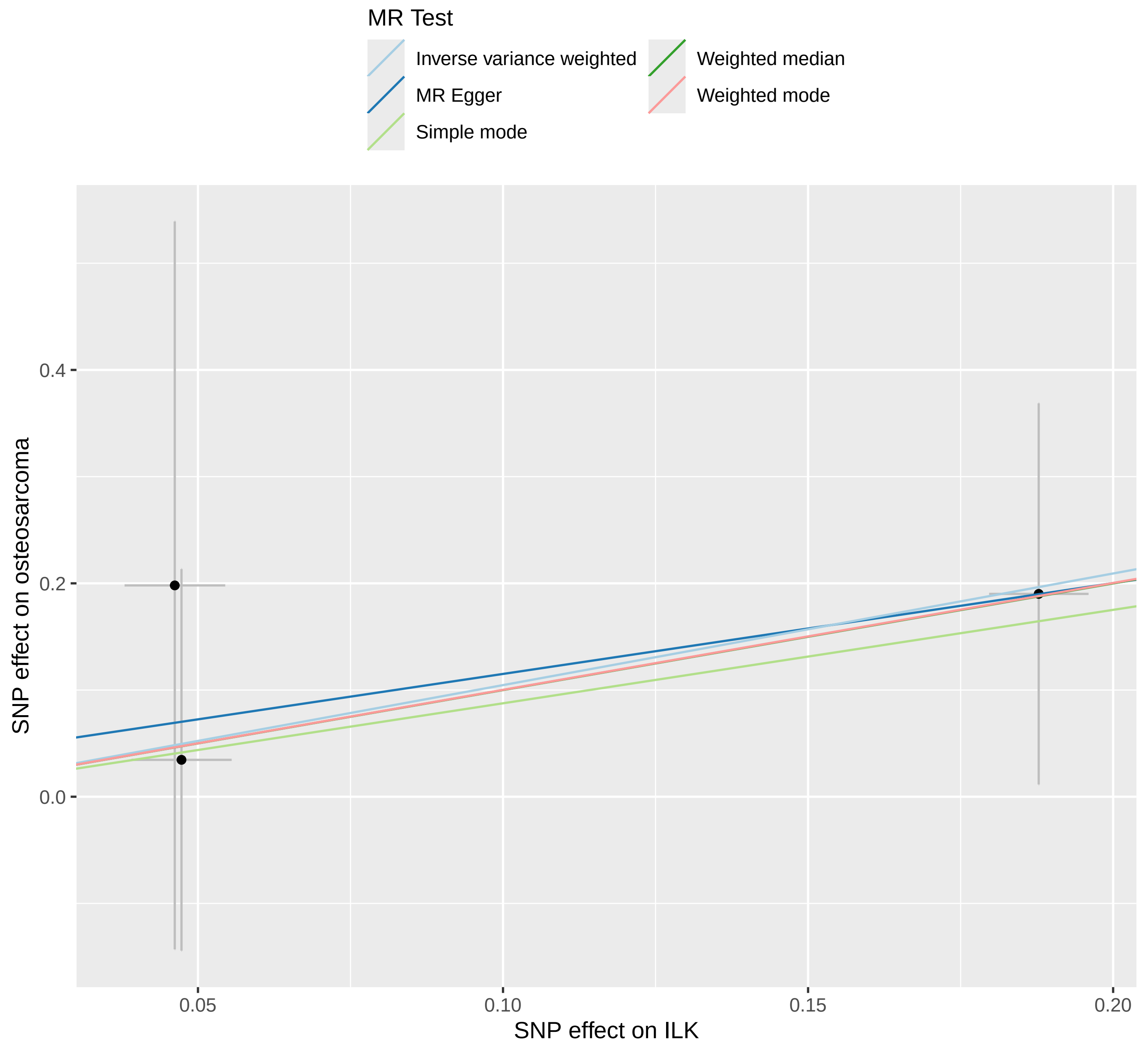

MR Test
Inverse variance weighted
MR Egger
Simple mode
Weighted median
Weighted mode
0.4
0.2
0.0
SNP effect on osteosarcoma
0.05
0.10
0.15
0.20
SNP effect on ILK

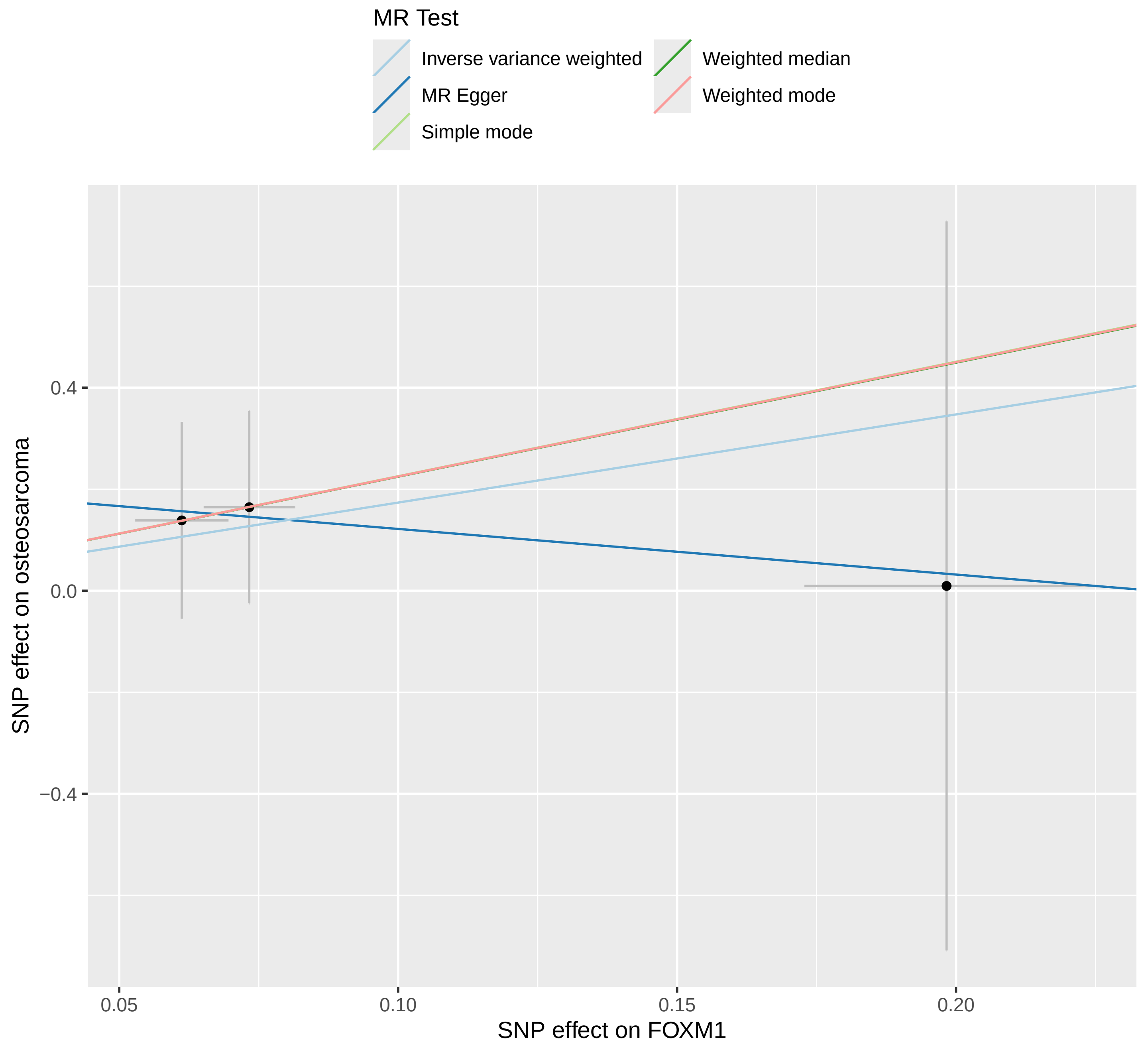

MR Test
Inverse variance weighted
MR Egger
Simple mode
Weighted median
Weighted mode
0.4
0.0
−0.4
0.05
0.10
0.15
0.20
SNP effect on osteosarcoma
SNP effect on FOXM1

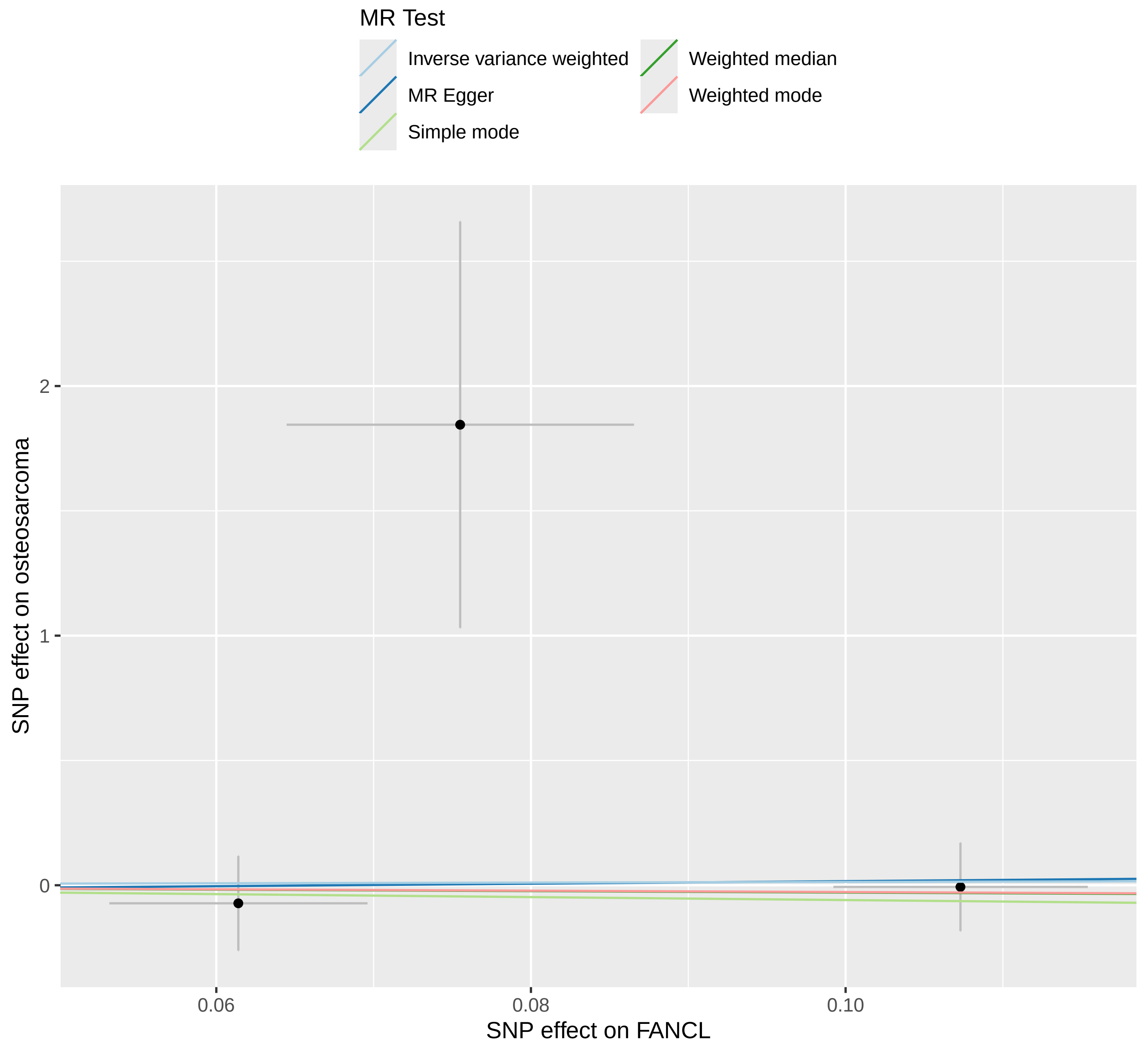
MR Test
Inverse variance weighted
MR Egger
Simple mode
Weighted median
Weighted mode
SNP effect on osteosarcoma
2
1
0
0.06
0.08
0.10
SNP effect on FANCL

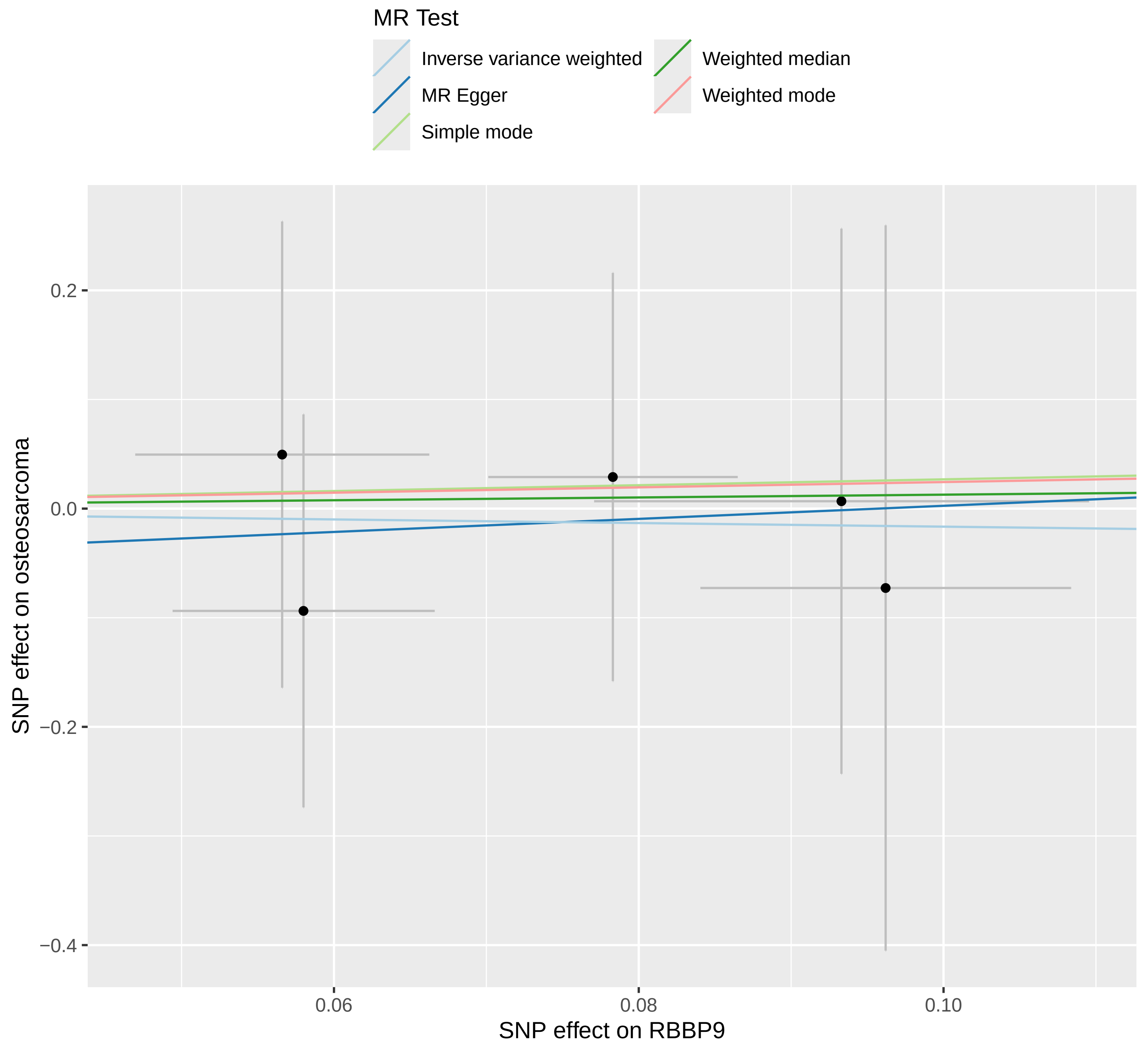

MR Test
Inverse variance weighted
MR Egger
Simple mode
Weighted median
Weighted mode
0.2
0.0
−0.2
−0.4
0.06
0.08
0.10
SNP effect on osteosarcoma
SNP effect on RBBP9

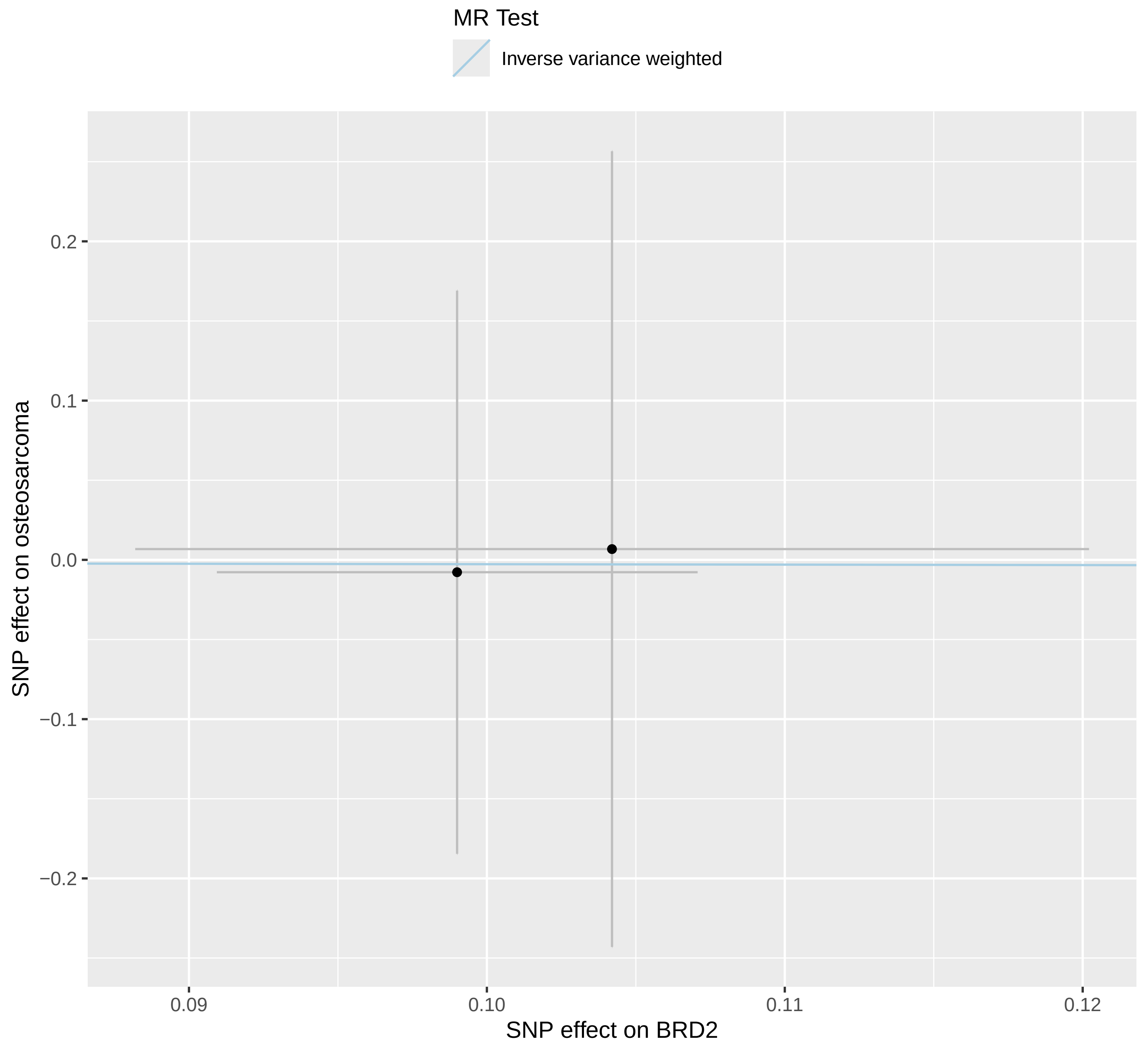
MR Test
Inverse variance weighted
0.2
0.1
0.0
−0.1
−0.2
SNP effect on osteosarcoma
0.09
0.10
0.11
0.12
SNP effect on BRD2

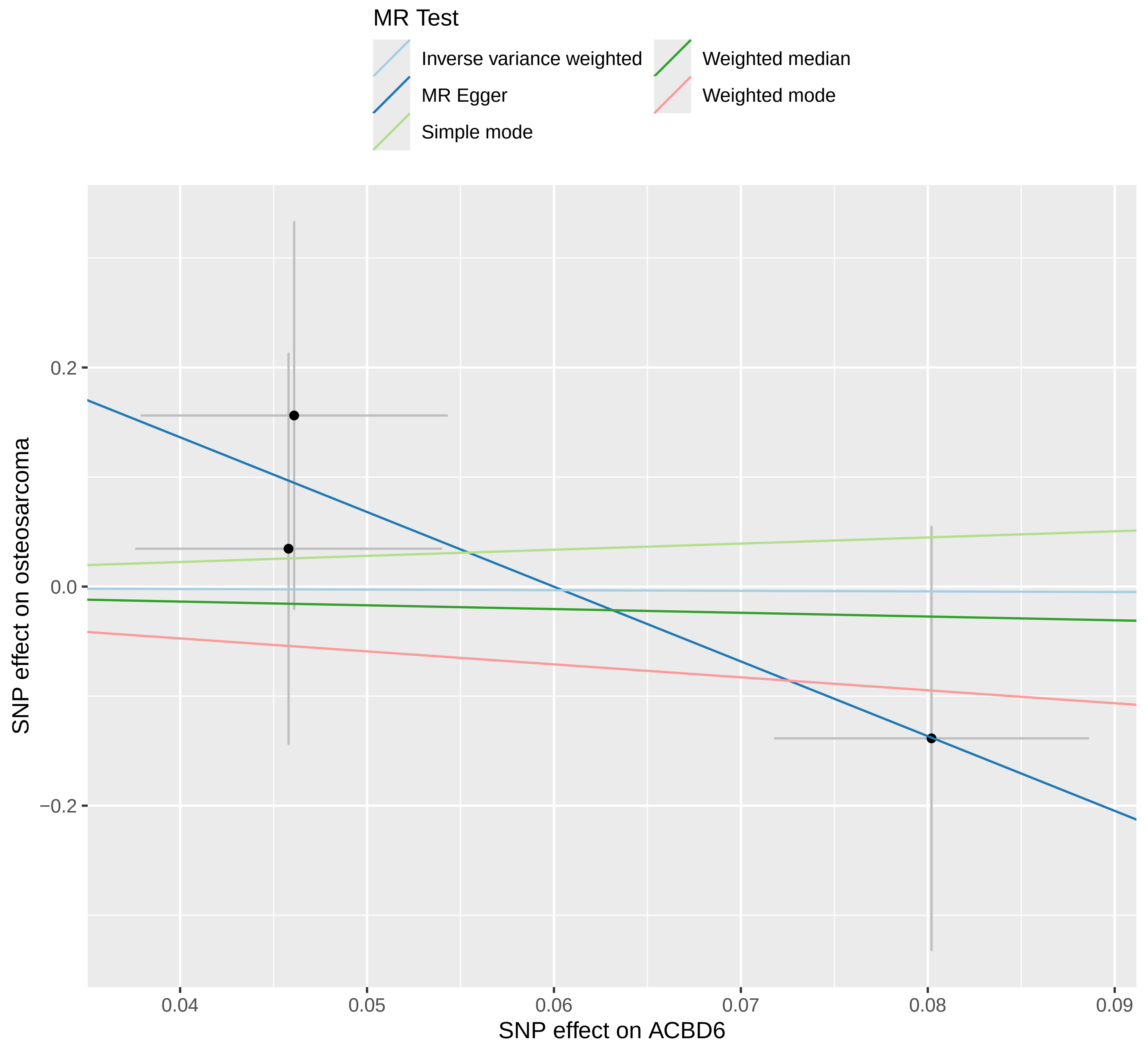

MR Test
Inverse variance weighted
MR Egger
Simple mode
Weighted median
Weighted mode
0.2
0.0
−0.2
SNP effect on osteosarcoma
0.04
0.05
0.06
0.07
0.08
0.09
SNP effect on ACBD6

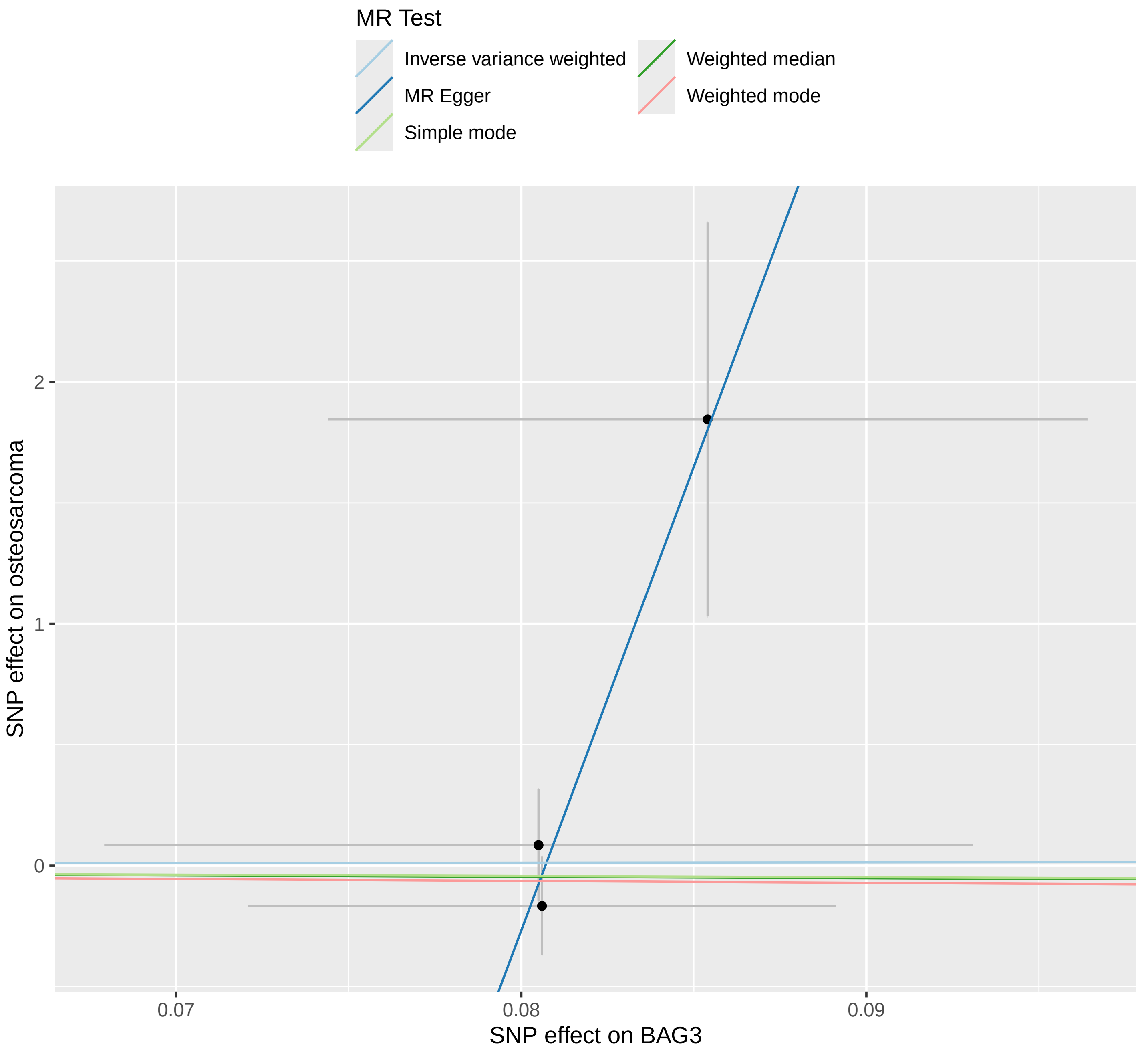
MR Test
Inverse variance weighted
MR Egger
Simple mode
Weighted median
Weighted mode
2
1
0
0.07
0.08
0.09
SNP effect on osteosarcoma
SNP effect on BAG3

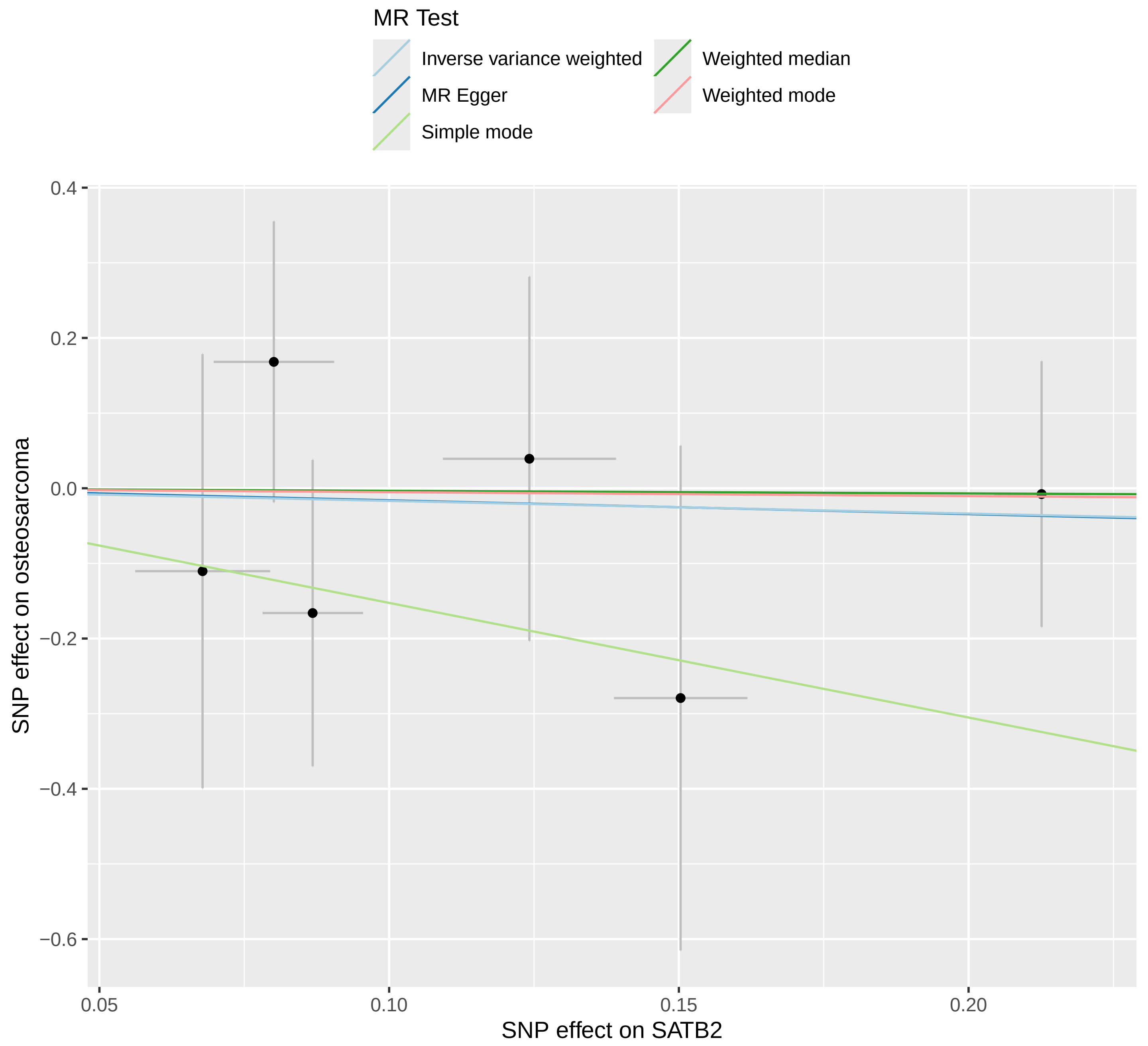

MR Test
Inverse variance weighted
MR Egger
Simple mode
Weighted median
Weighted mode
0.4
0.2
0.0
−0.2
−0.4
−0.6
0.05
0.10
0.15
0.20
SNP effect on osteosarcoma
SNP effect on SATB2

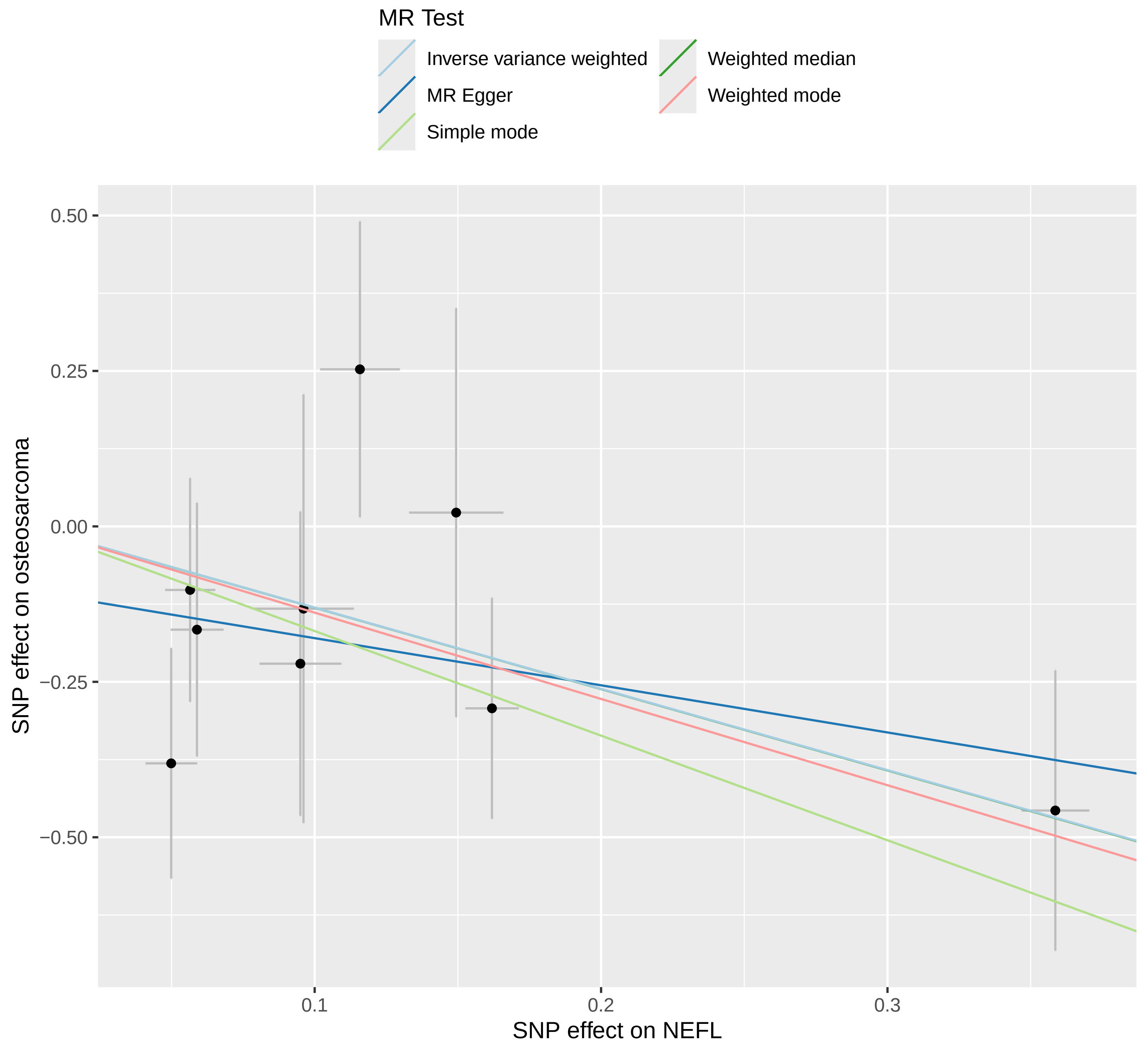
MR Test
Inverse variance weighted
MR Egger
Simple mode
Weighted median
Weighted mode
0.50
0.25
0.00
−0.25
−0.50
0.1
0.2
0.3
SNP effect on osteosarcoma
SNP effect on NEFL

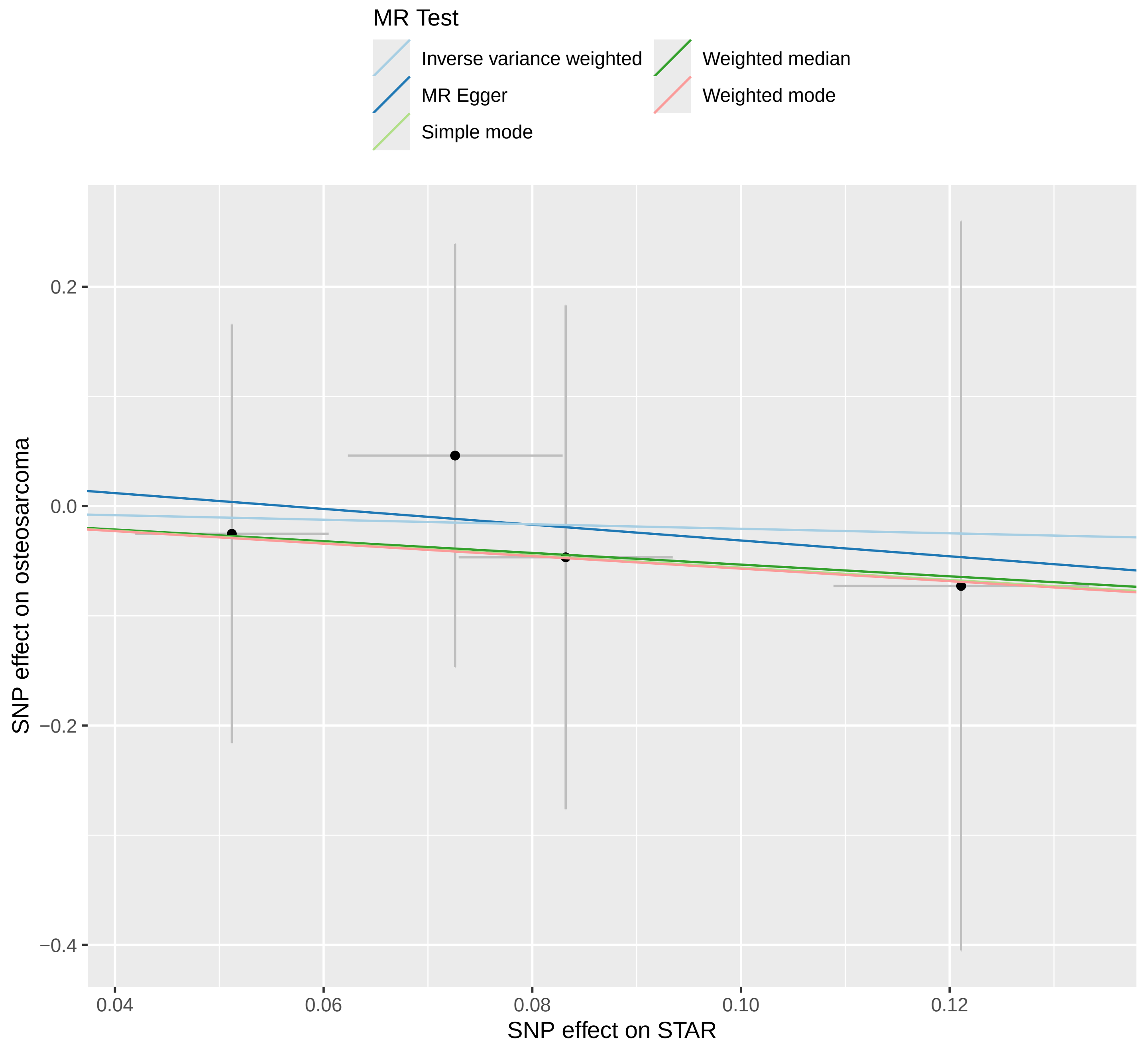

MR Test
Inverse variance weighted
MR Egger
Simple mode
Weighted median
Weighted mode
0.2
0.0
−0.2
−0.4
SNP effect on osteosarcoma
0.04
0.06
0.08
0.10
0.12
SNP effect on STAR

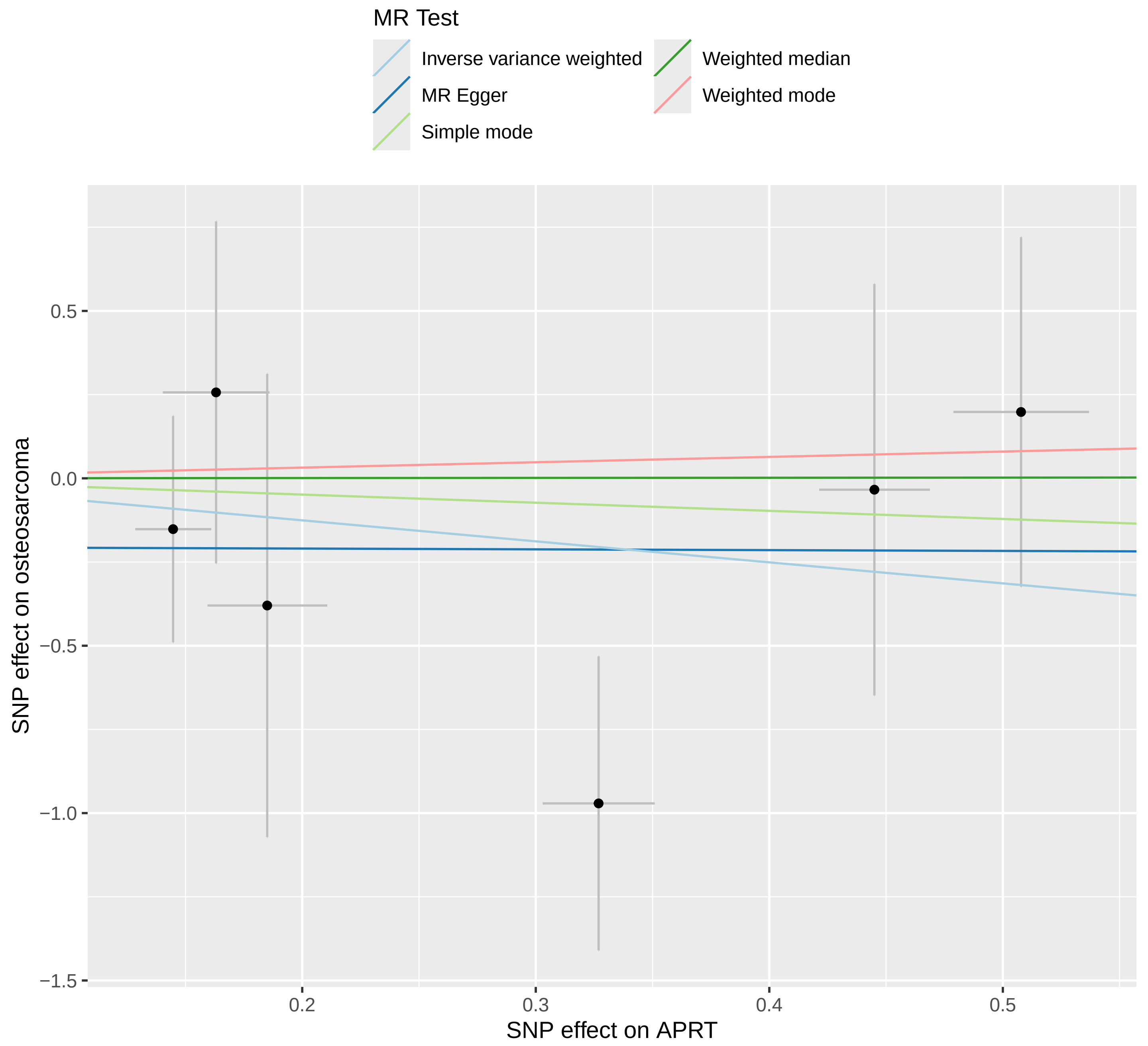
MR Test
Inverse variance weighted
MR Egger
Simple mode
Weighted median
Weighted mode
0.5
0.0
−0.5
−1.0
−1.5
SNP effect on osteosarcoma
0.2
0.3
0.4
0.5
SNP effect on APRT

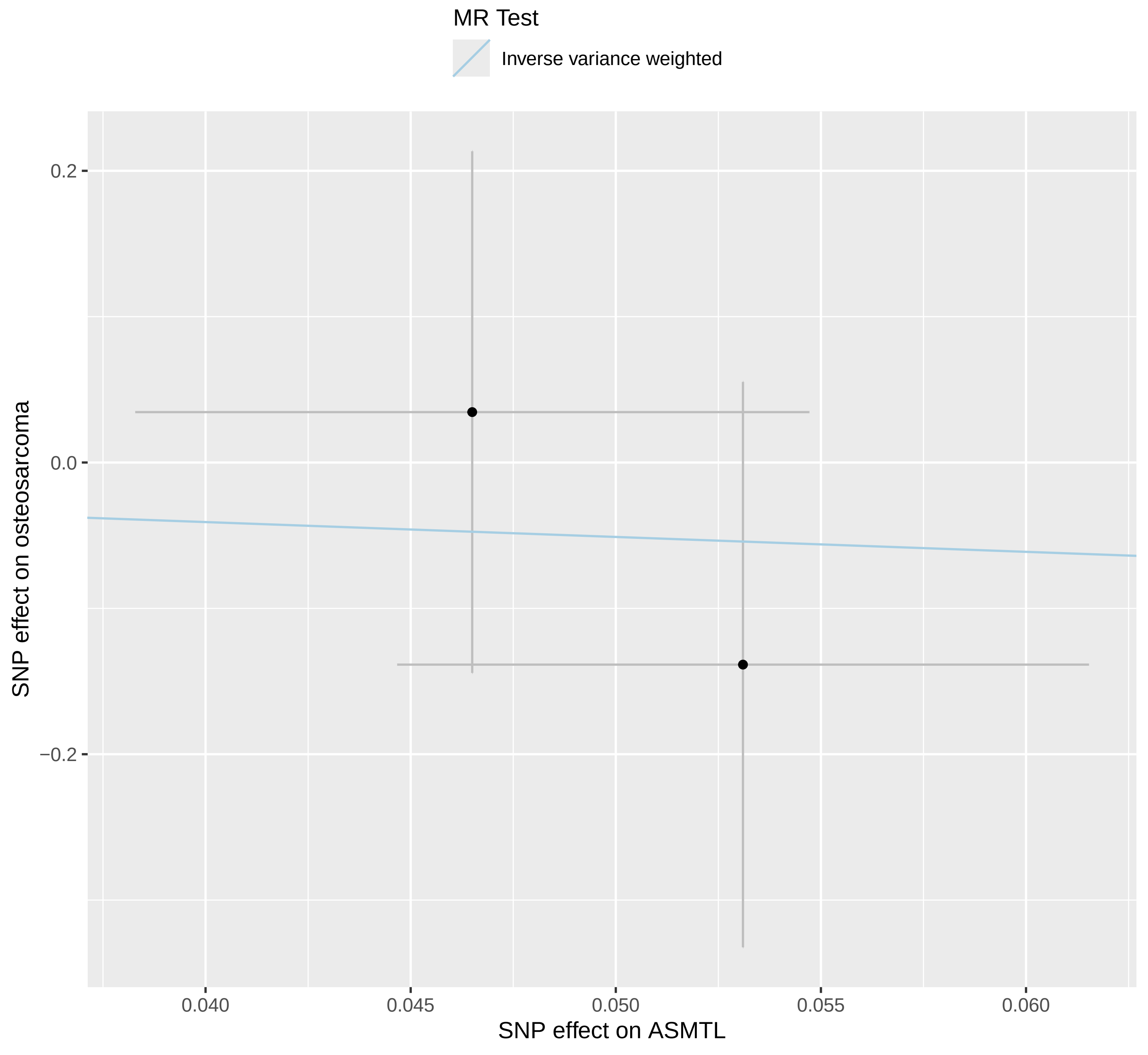
MR Test
Inverse variance weighted
0.2
0.0
−0.2
SNP effect on osteosarcoma
0.040
0.045
0.050
0.055
0.060
SNP effect on ASMTL

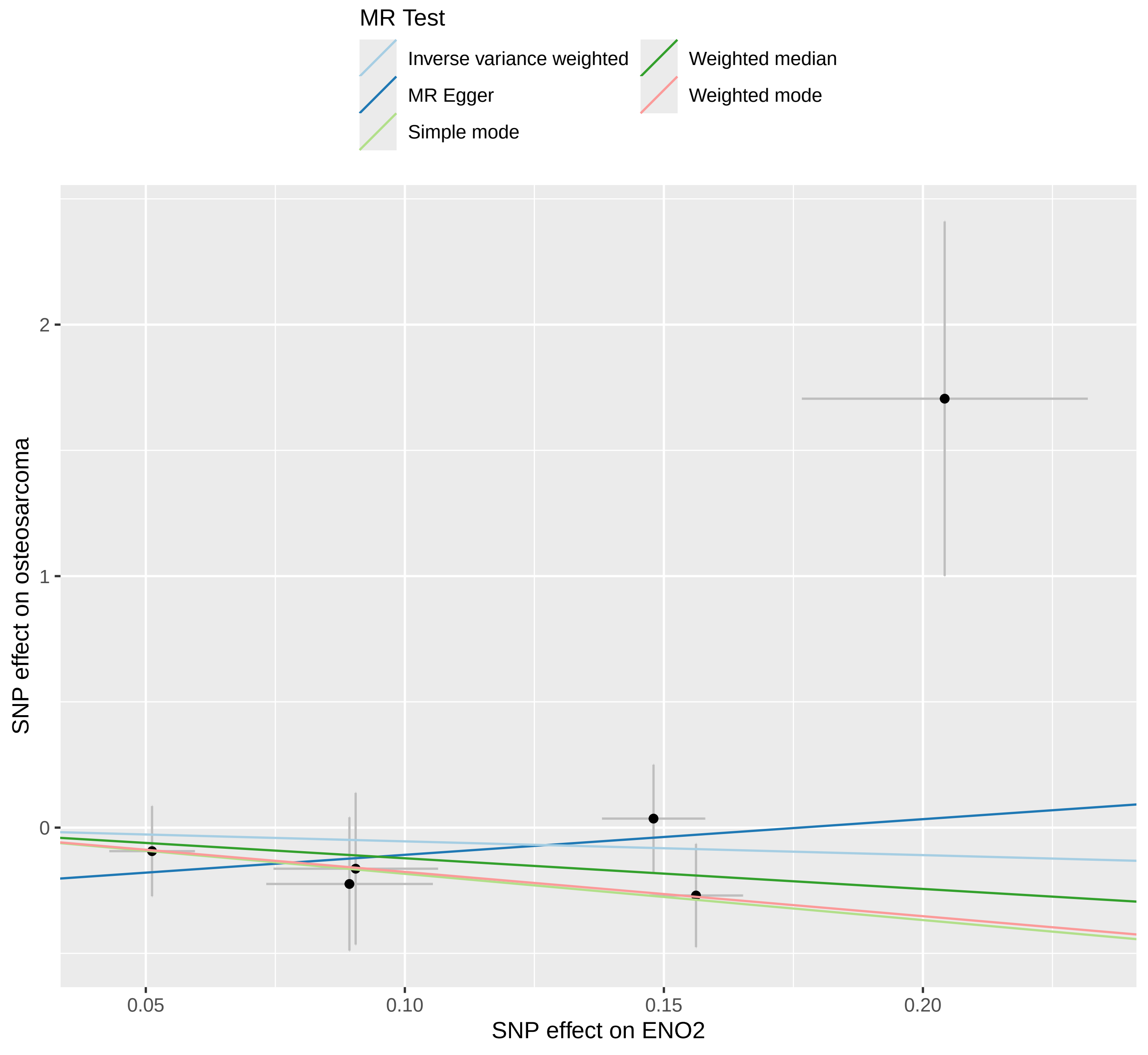

MR Test
Inverse variance weighted
MR Egger
Simple mode
Weighted median
Weighted mode
2
1
0
0.05
0.10
0.15
0.20
SNP effect on osteosarcoma
SNP effect on ENO2

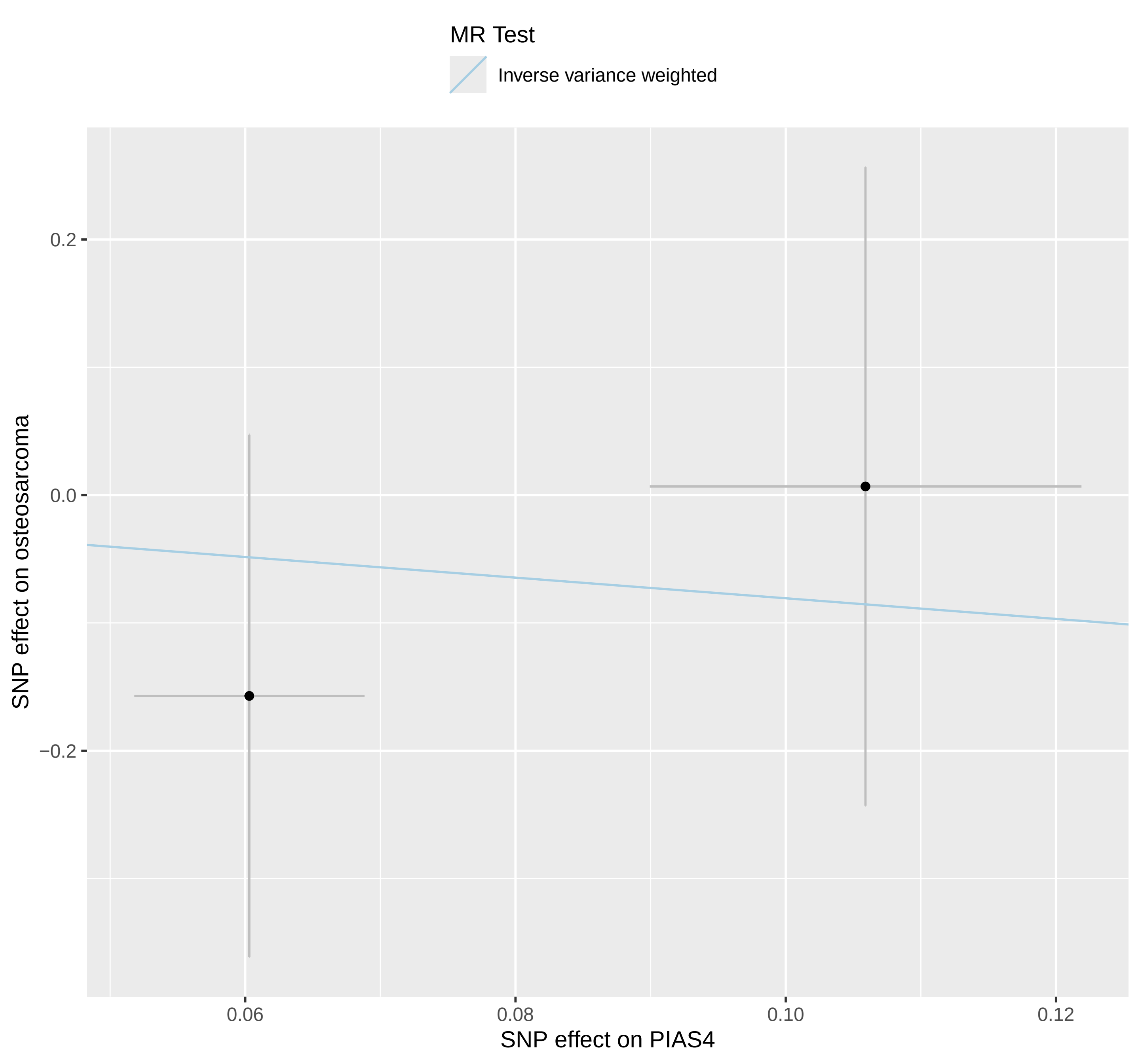
MR Test
Inverse variance weighted
0.2
0.0
−0.2
SNP effect on osteosarcoma
0.06
0.08
0.10
0.12
SNP effect on PIAS4

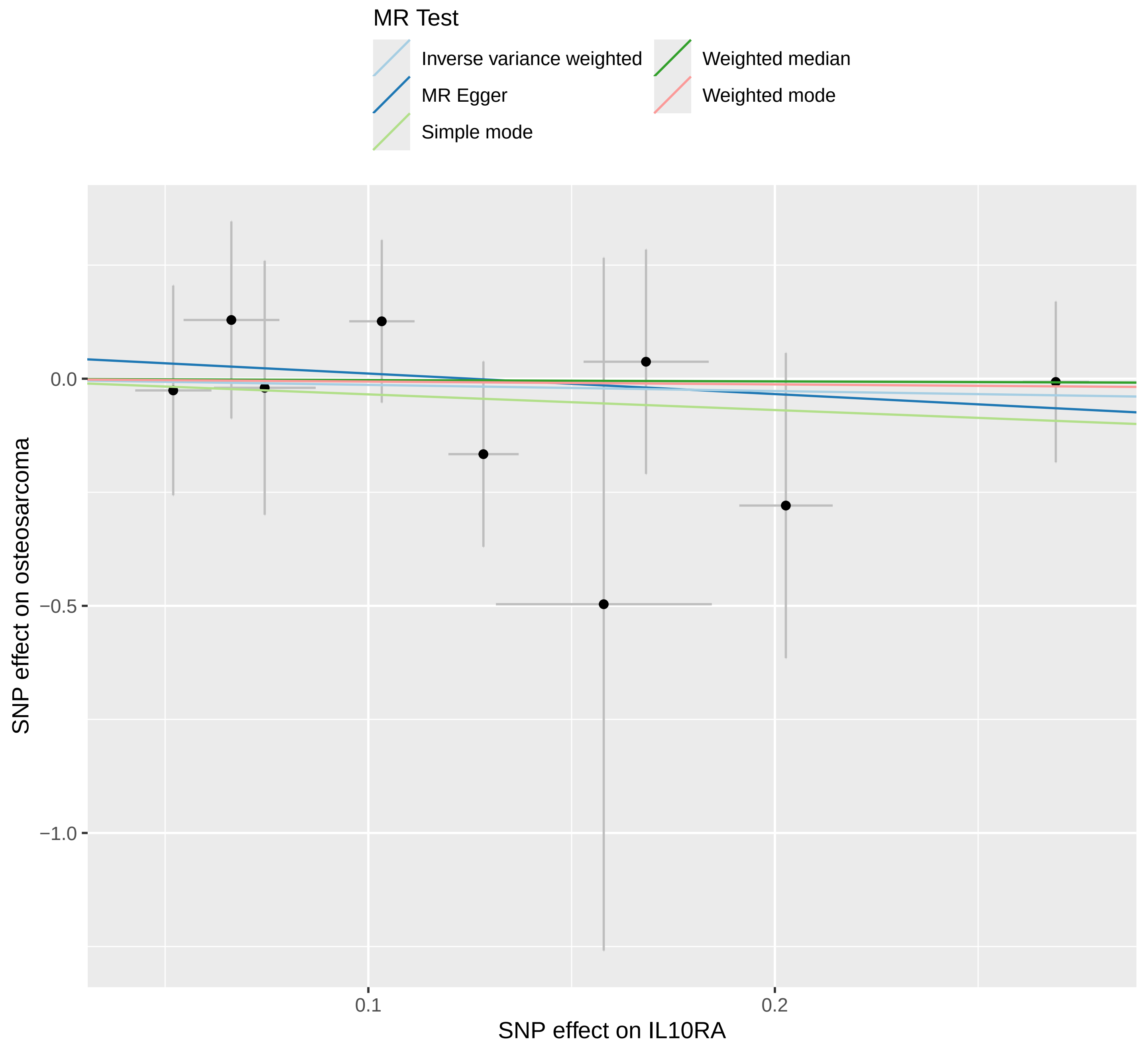
MR Test
Inverse variance weighted
MR Egger
Simple mode
Weighted median
Weighted mode
0.0
−0.5
−1.0
0.1
0.2
SNP effect on osteosarcoma
SNP effect on IL10RA

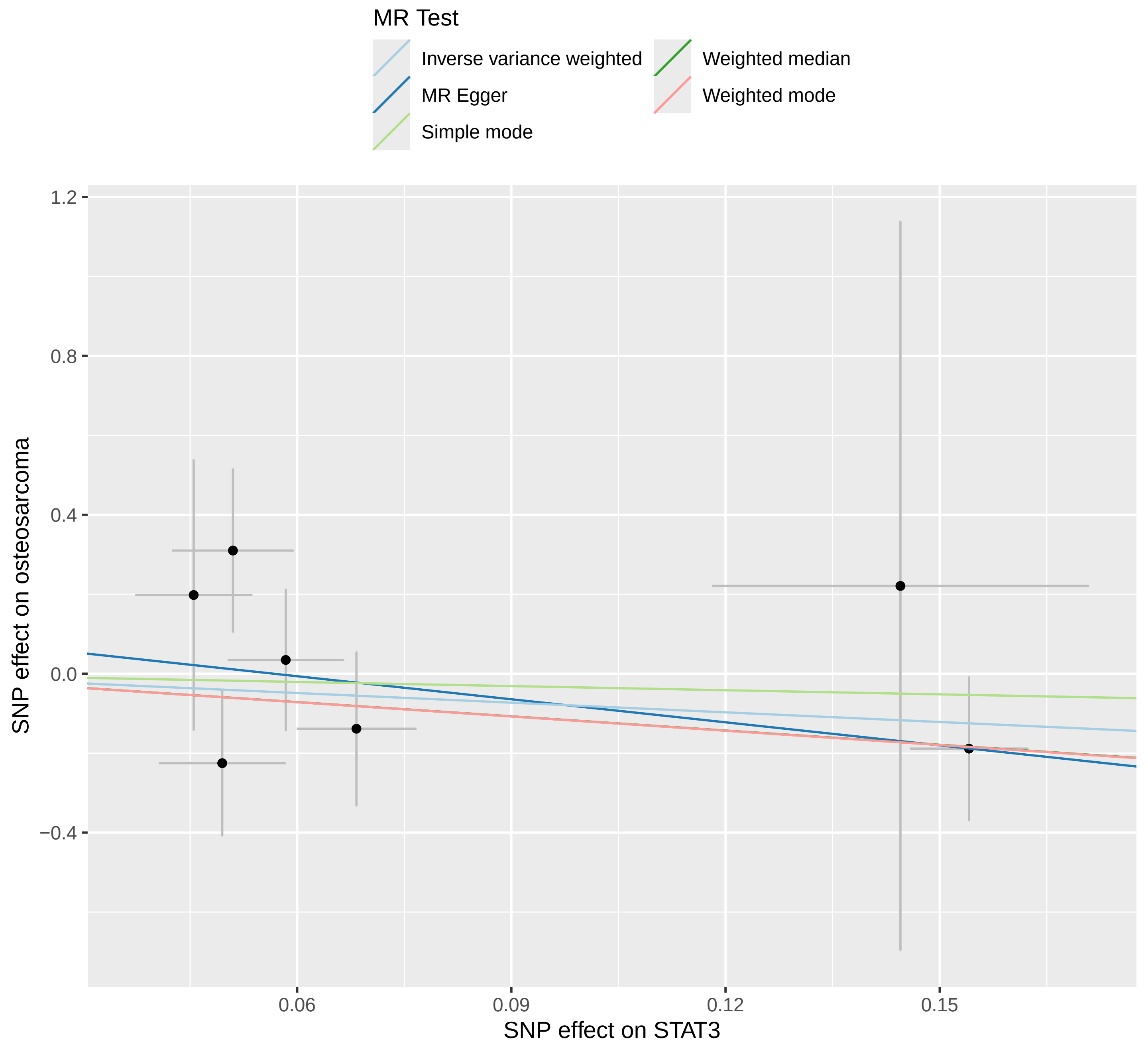
MR Test
Inverse variance weighted
MR Egger
Simple mode
Weighted median
Weighted mode
1.2
0.8
0.4
0.0
−0.4
SNP effect on osteosarcoma
0.06
0.09
0.12
0.15
SNP effect on STAT3

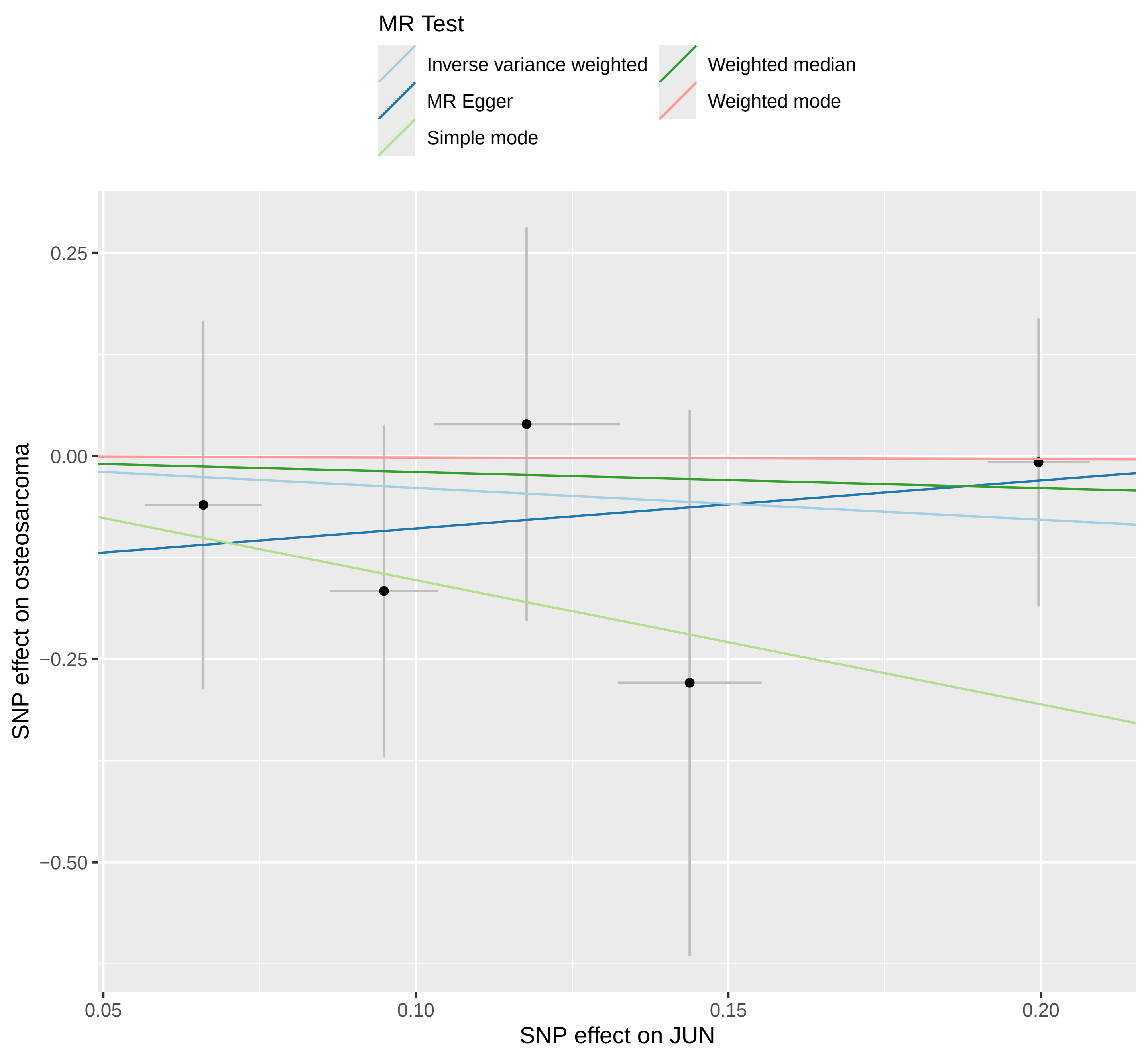

MR Test
Inverse variance weighted
MR Egger
Simple mode
Weighted median
Weighted mode
0.25
0.00
−0.25
−0.50
SNP effect on osteosarcoma
0.05
0.10
0.15
0.20
SNP effect on JUN

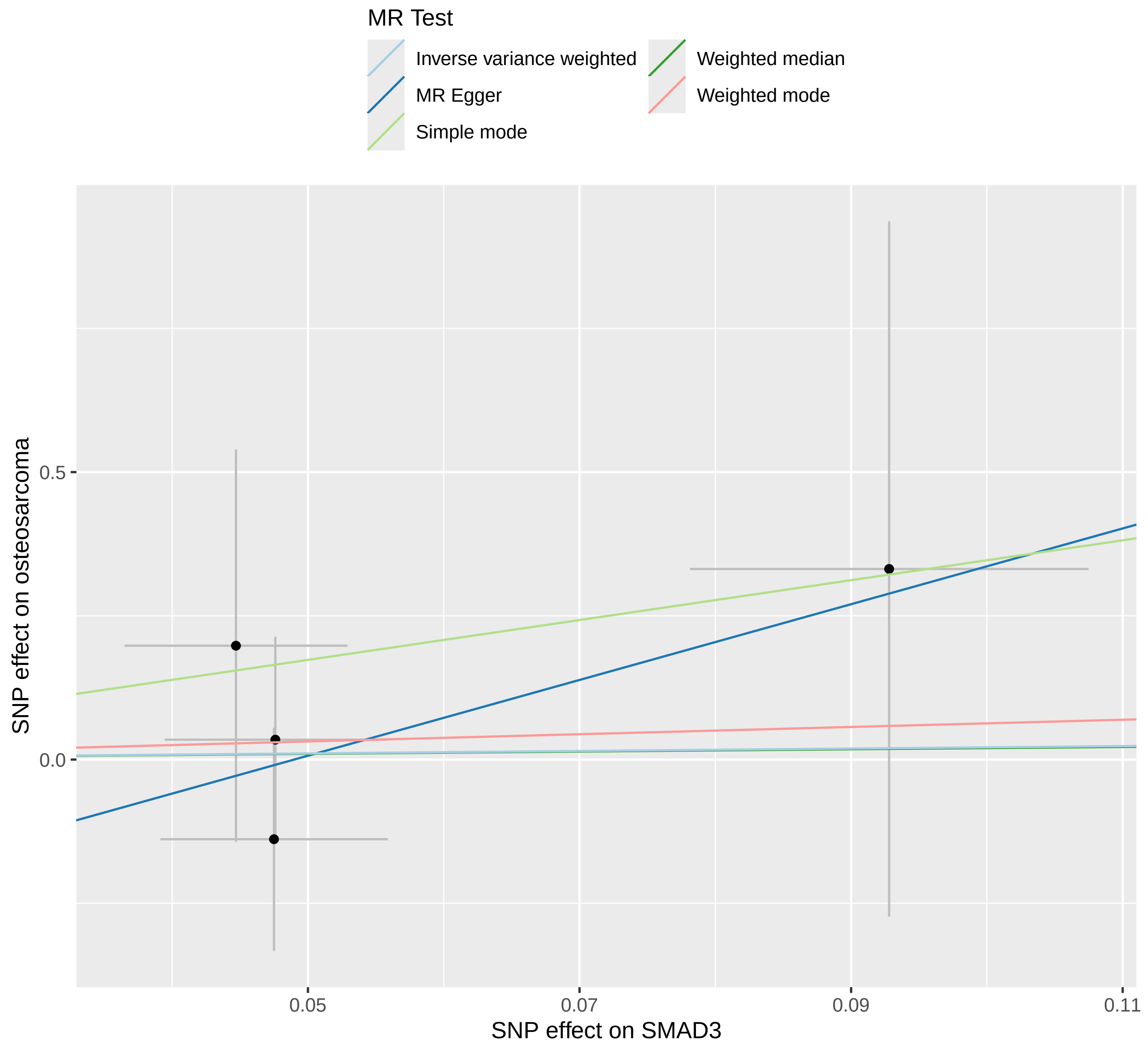
MR Test
Inverse variance weighted
MR Egger
Simple mode
Weighted median
Weighted mode
0.5
0.0
0.05
0.07
0.09
0.11
SNP effect on SMAD3
SNP effect on osteosarcoma

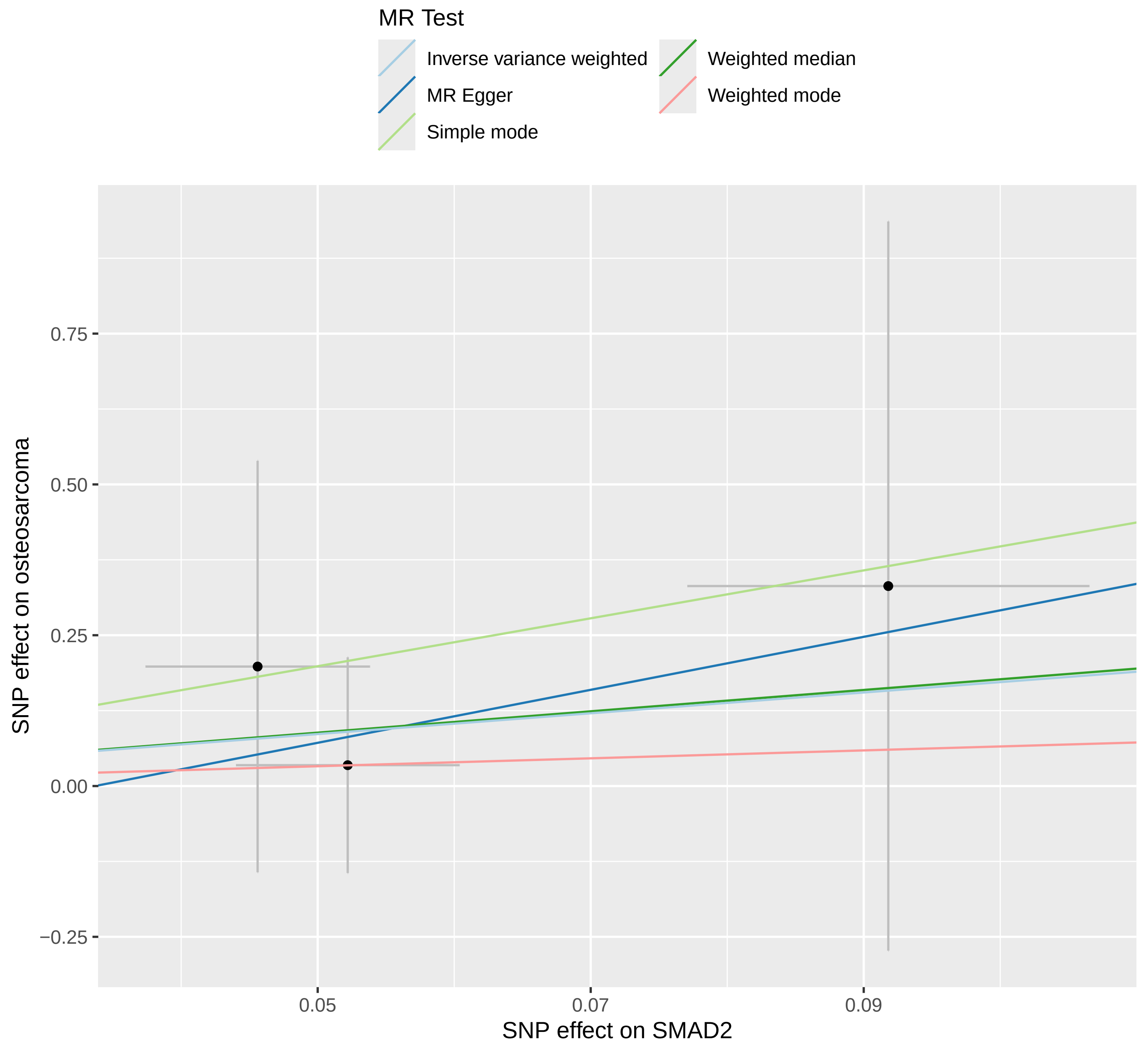
MR Test
Inverse variance weighted
MR Egger
Simple mode
Weighted median
Weighted mode
0.75
0.50
0.25
0.00
−0.25
0.05
0.07
0.09
SNP effect on SMAD2
SNP effect on osteosarcoma

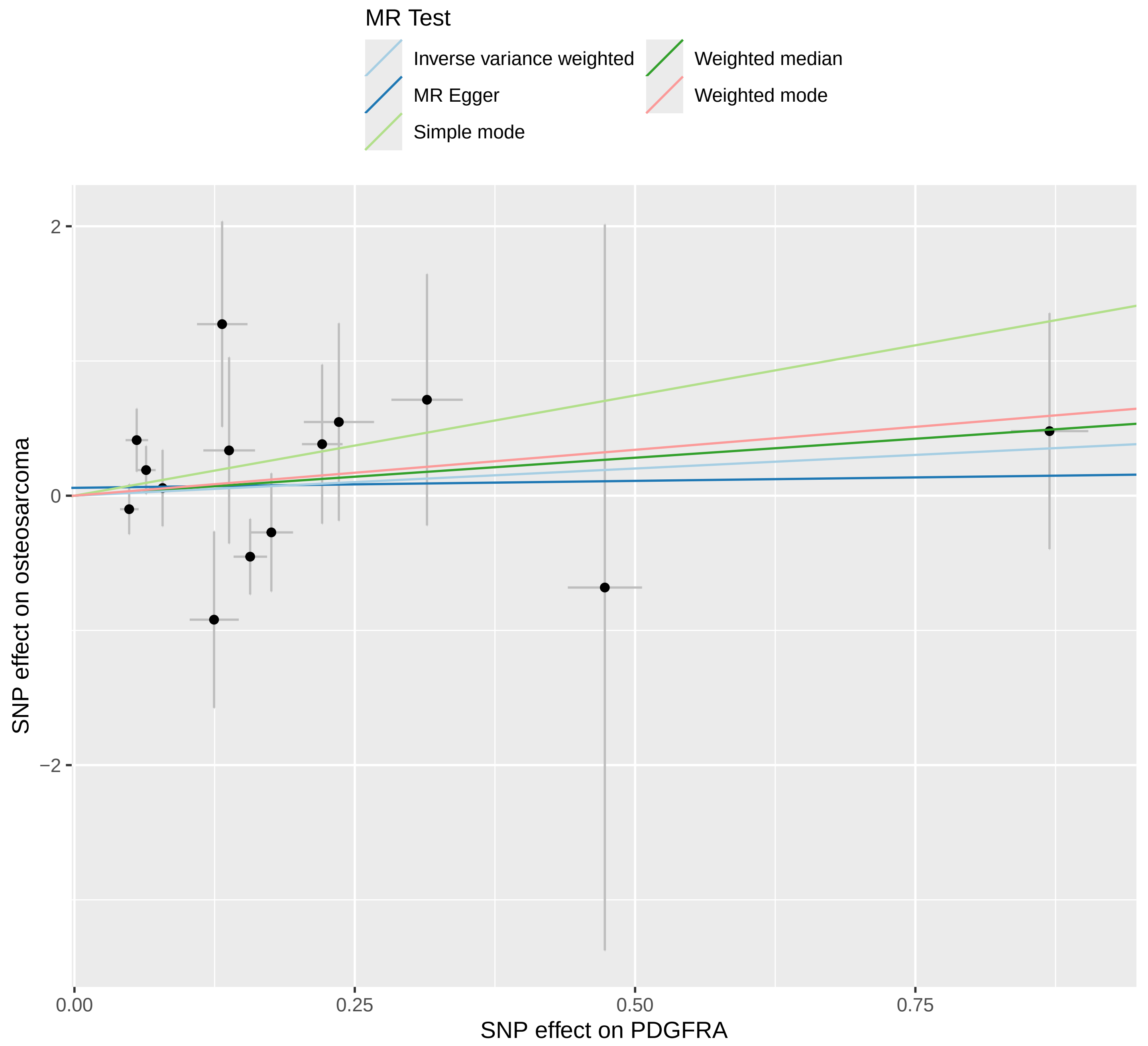
MR Test
Inverse variance weighted
MR Egger
Simple mode
Weighted median
Weighted mode
2
0
−2
0.00
0.25
0.50
0.75
SNP effect on PDGFRA
SNP effect on osteosarcoma

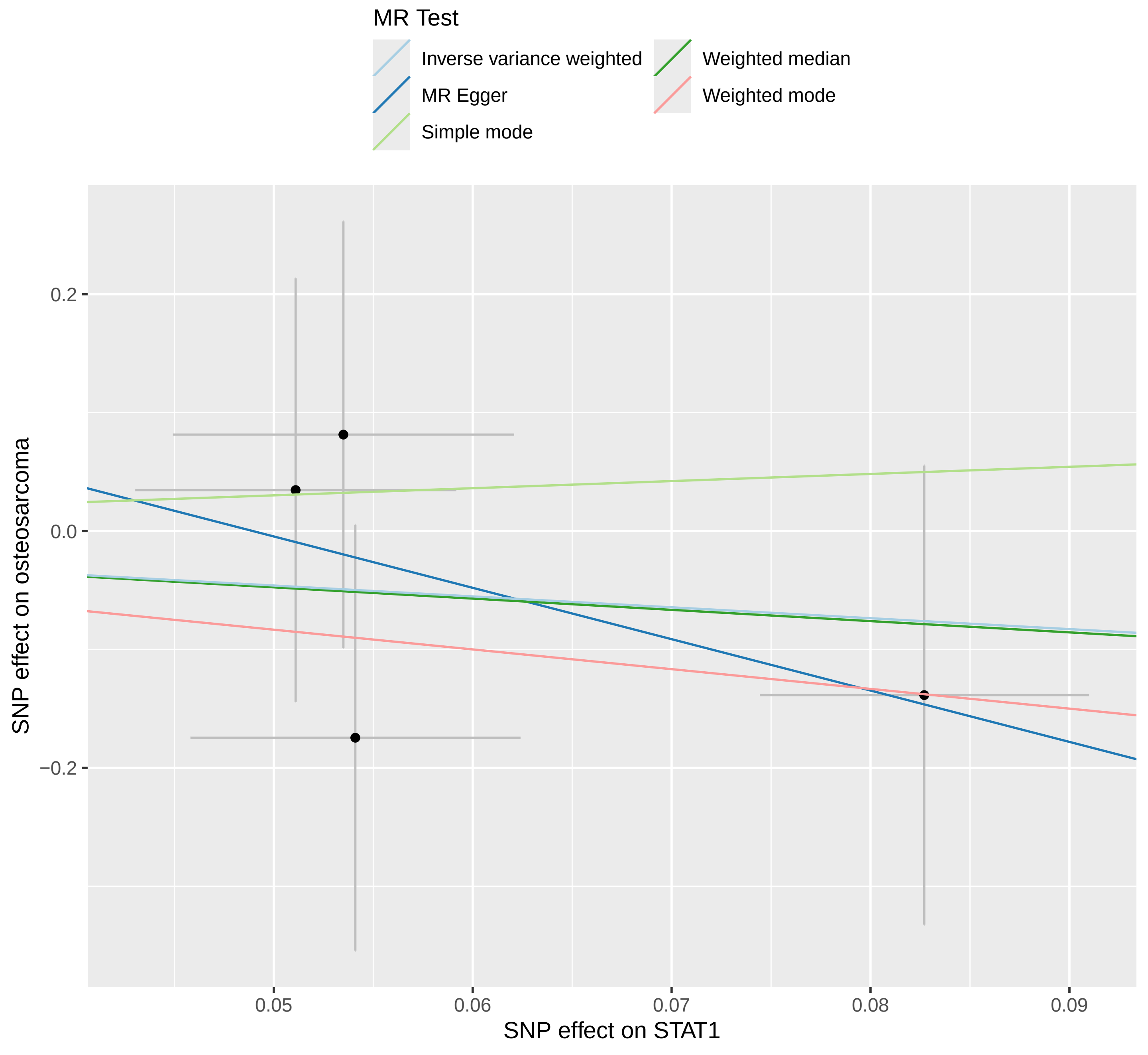

MR Test
Inverse variance weighted
MR Egger
Simple mode
Weighted median
Weighted mode
0.2
0.0
−0.2
0.05
0.06
0.07
0.08
0.09
SNP effect on STAT1
SNP effect on osteosarcoma

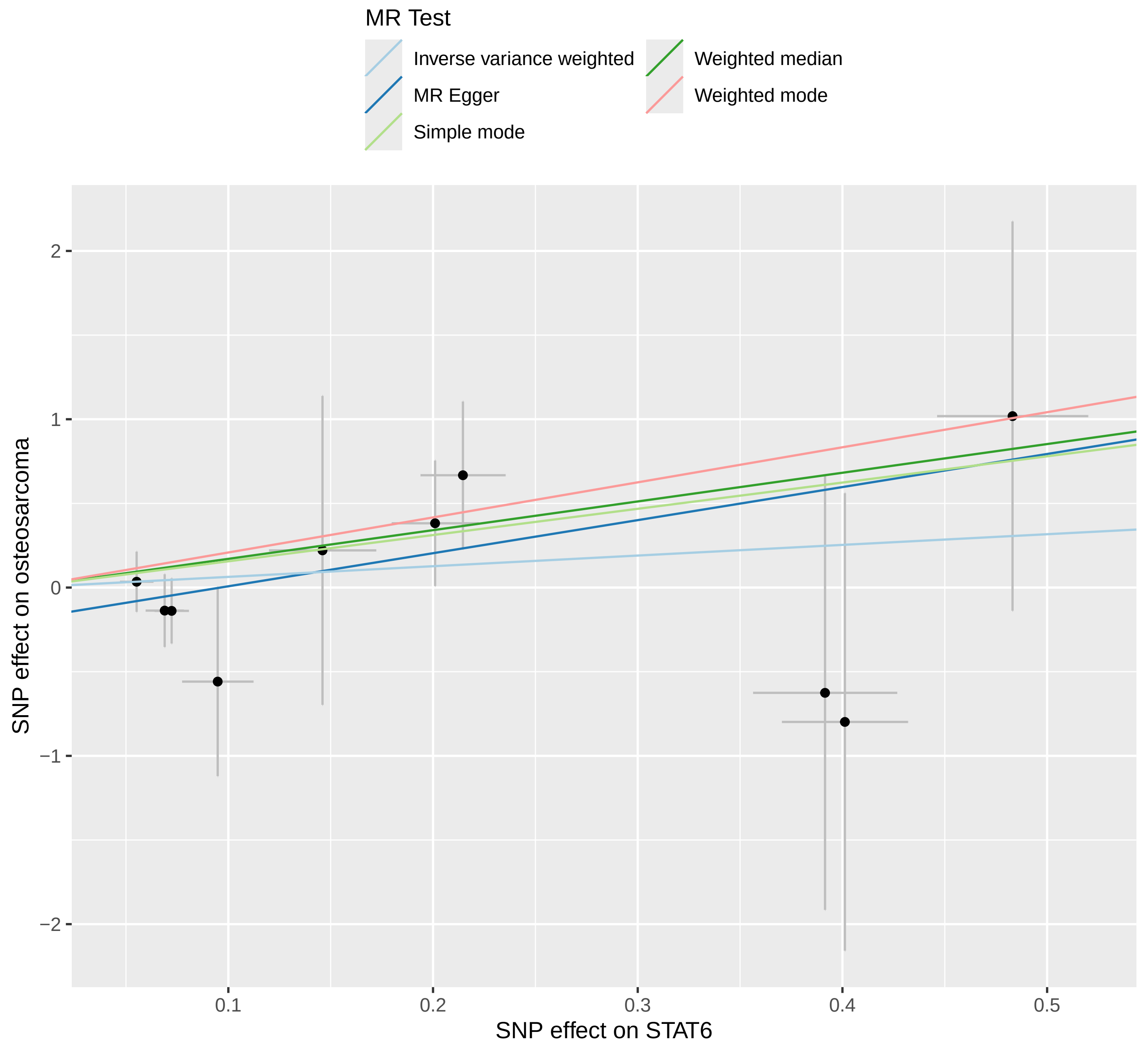
MR Test
Inverse variance weighted
MR Egger
Simple mode
Weighted median
Weighted mode
SNP effect on osteosarcoma
SNP effect on STAT6
2
1
0
−1
−2
0.1
0.2
0.3
0.4
0.5

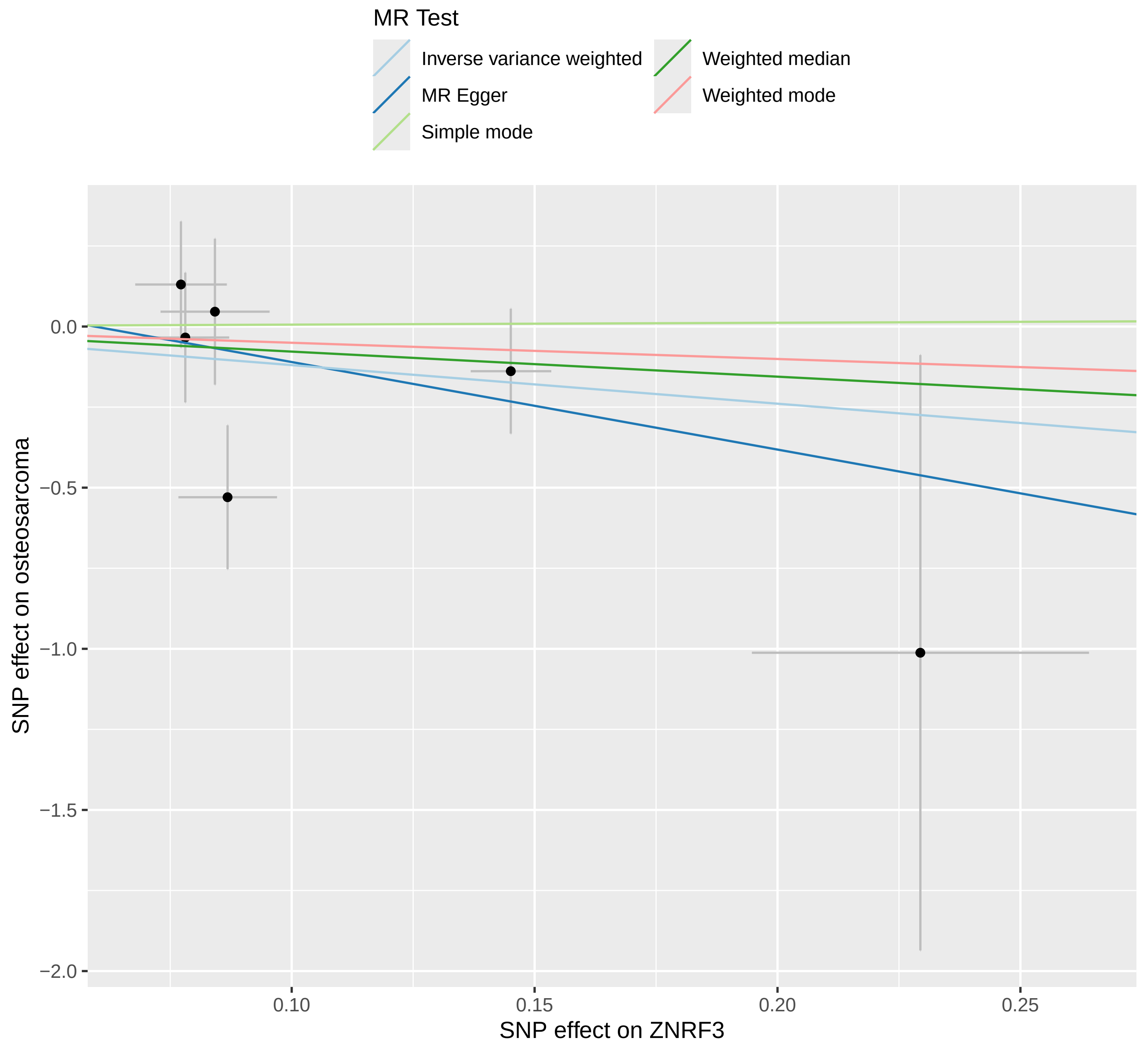
MR Test
Inverse variance weighted
MR Egger
Simple mode
Weighted median
Weighted mode
0.0
−0.5
−1.0
−1.5
−2.0
0.10
0.15
0.20
0.25
SNP effect on osteosarcoma
SNP effect on ZNRF3

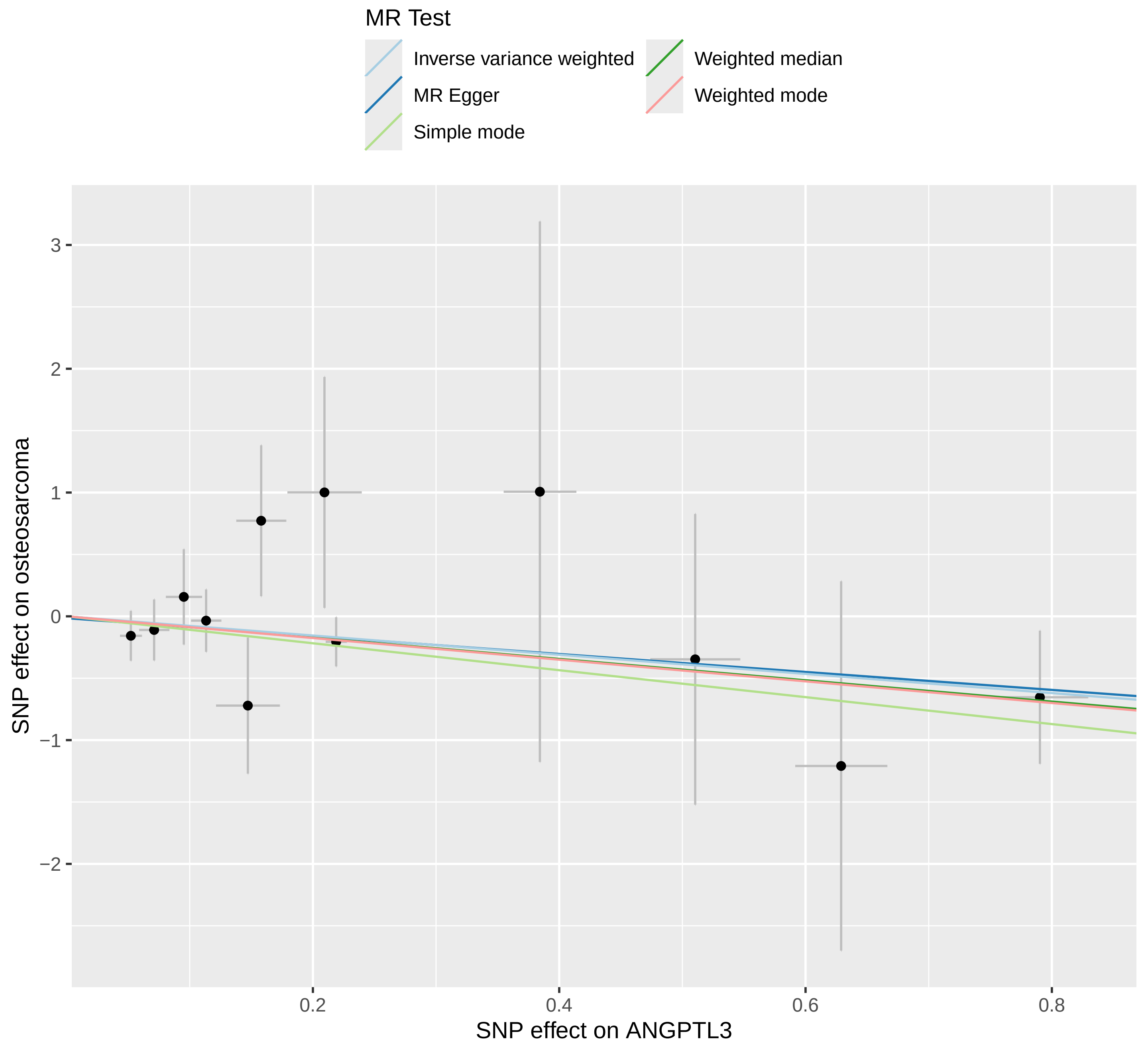
MR Test
Inverse variance weighted
MR Egger
Simple mode
Weighted median
Weighted mode
3
2
1
0
−1
−2
SNP effect on osteosarcoma
0.2
0.4
0.6
0.8
SNP effect on ANGPTL3

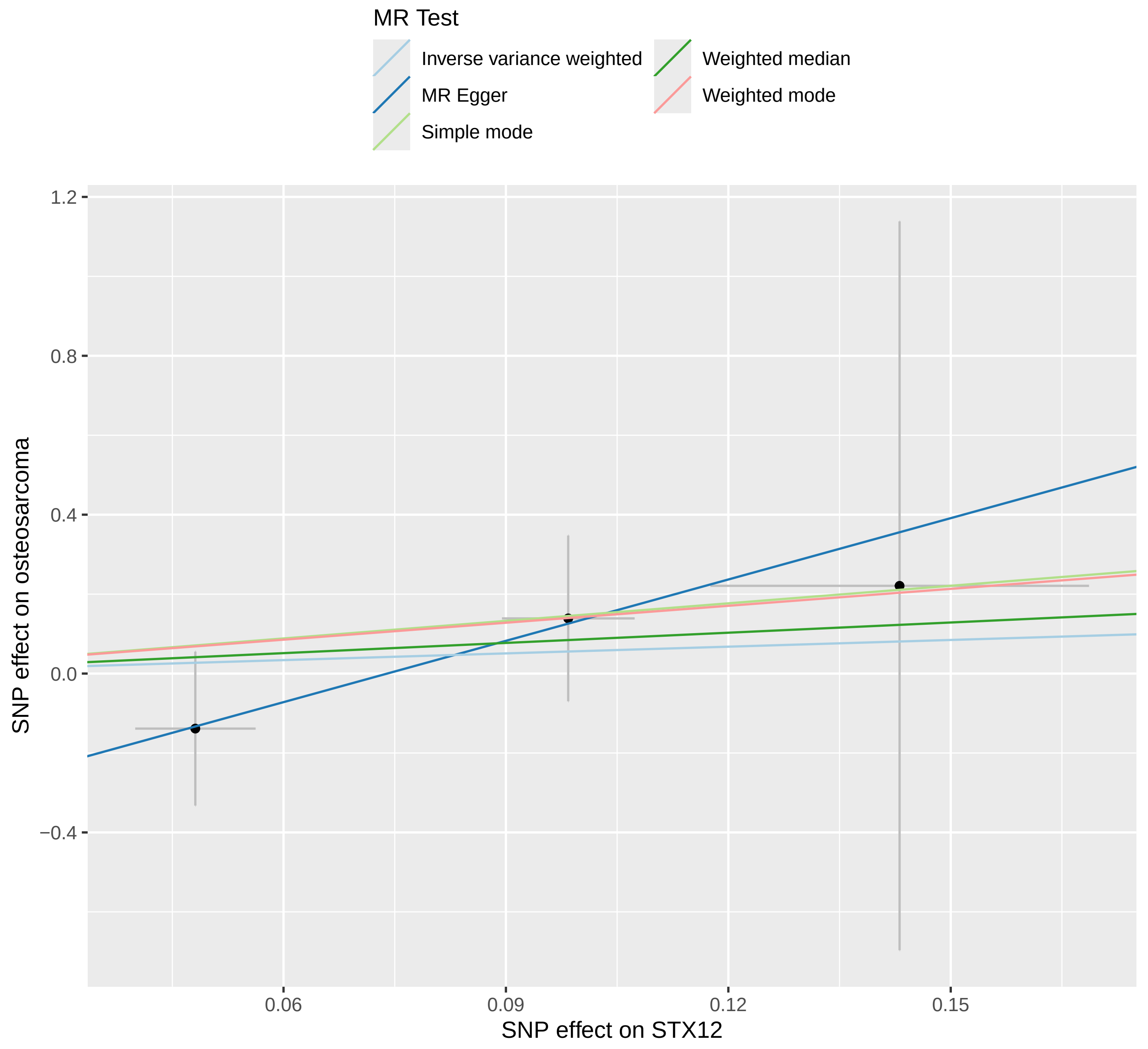
MR Test
Inverse variance weighted
MR Egger
Simple mode
Weighted median
Weighted mode
1.2
0.8
0.4
0.0
−0.4
SNP effect on osteosarcoma
0.06
0.09
0.12
0.15
SNP effect on STX12

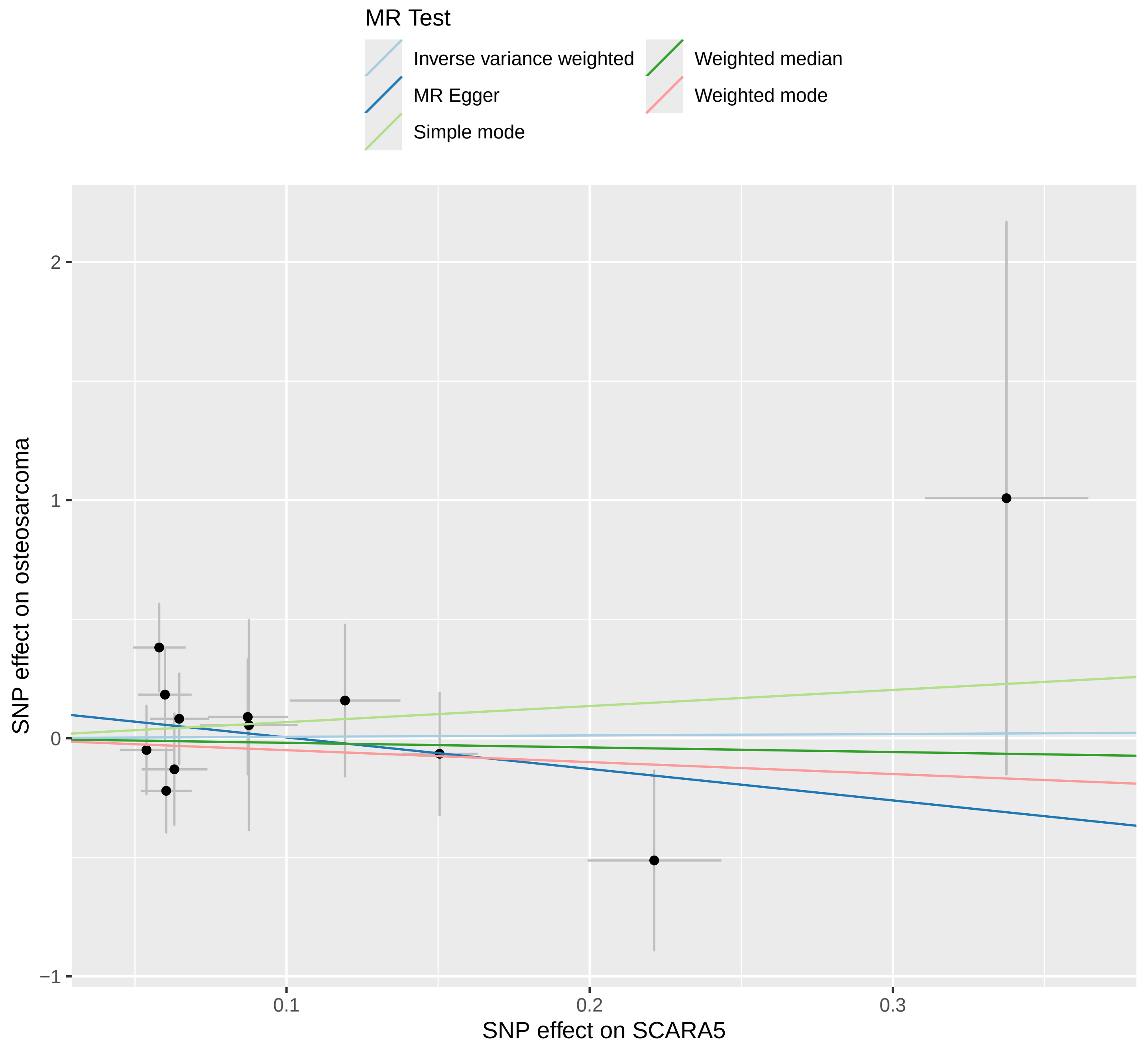

MR Test
Inverse variance weighted
MR Egger
Simple mode
Weighted median
Weighted mode
2
1
0
−1
SNP effect on osteosarcoma
0.1
0.2
0.3
SNP effect on SCARA5

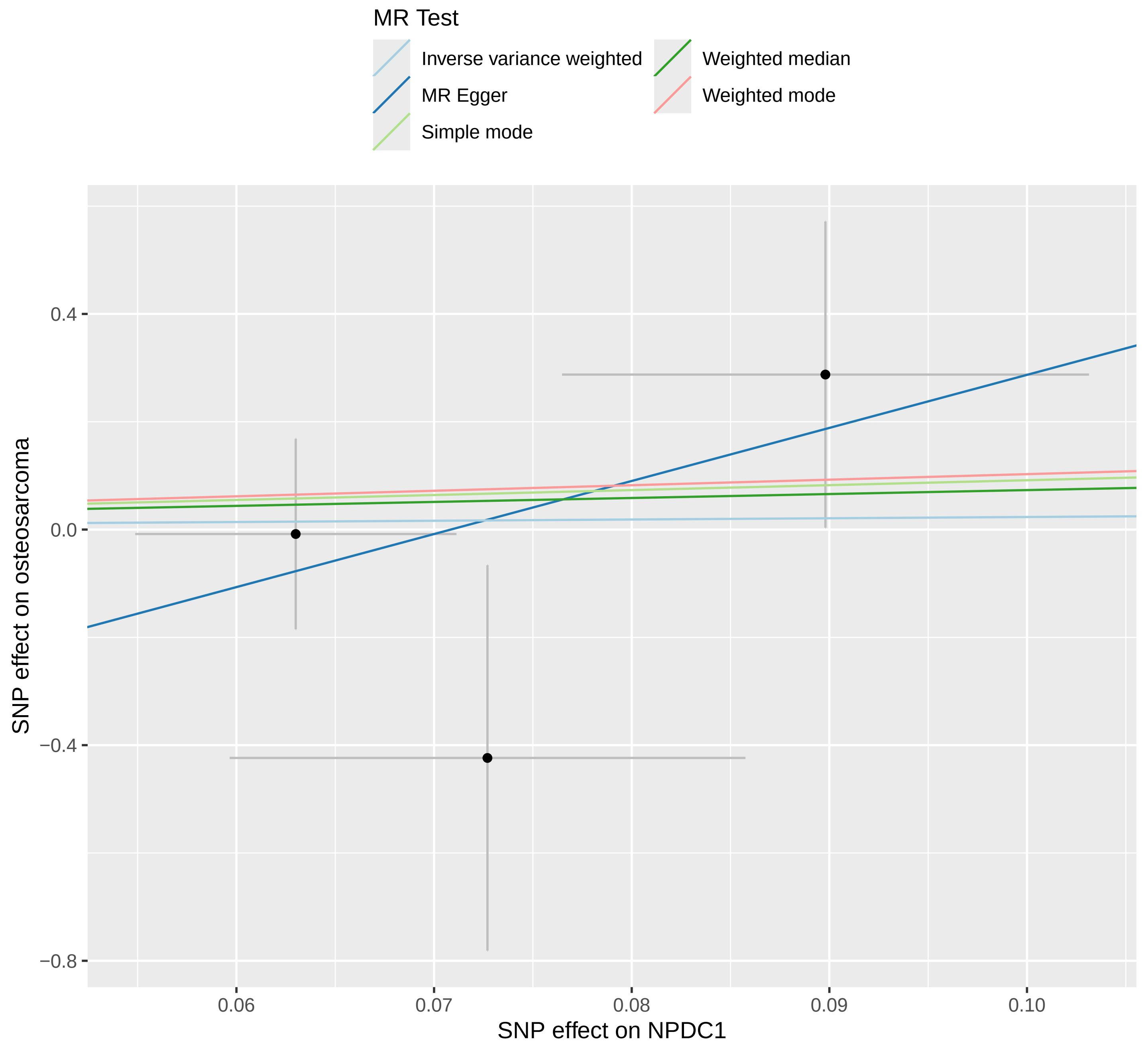

MR Test
Inverse variance weighted
MR Egger
Simple mode
Weighted median
Weighted mode
0.4
0.0
−0.4
−0.8
SNP effect on osteosarcoma
0.06
0.07
0.08
0.09
0.10
SNP effect on NPDC1

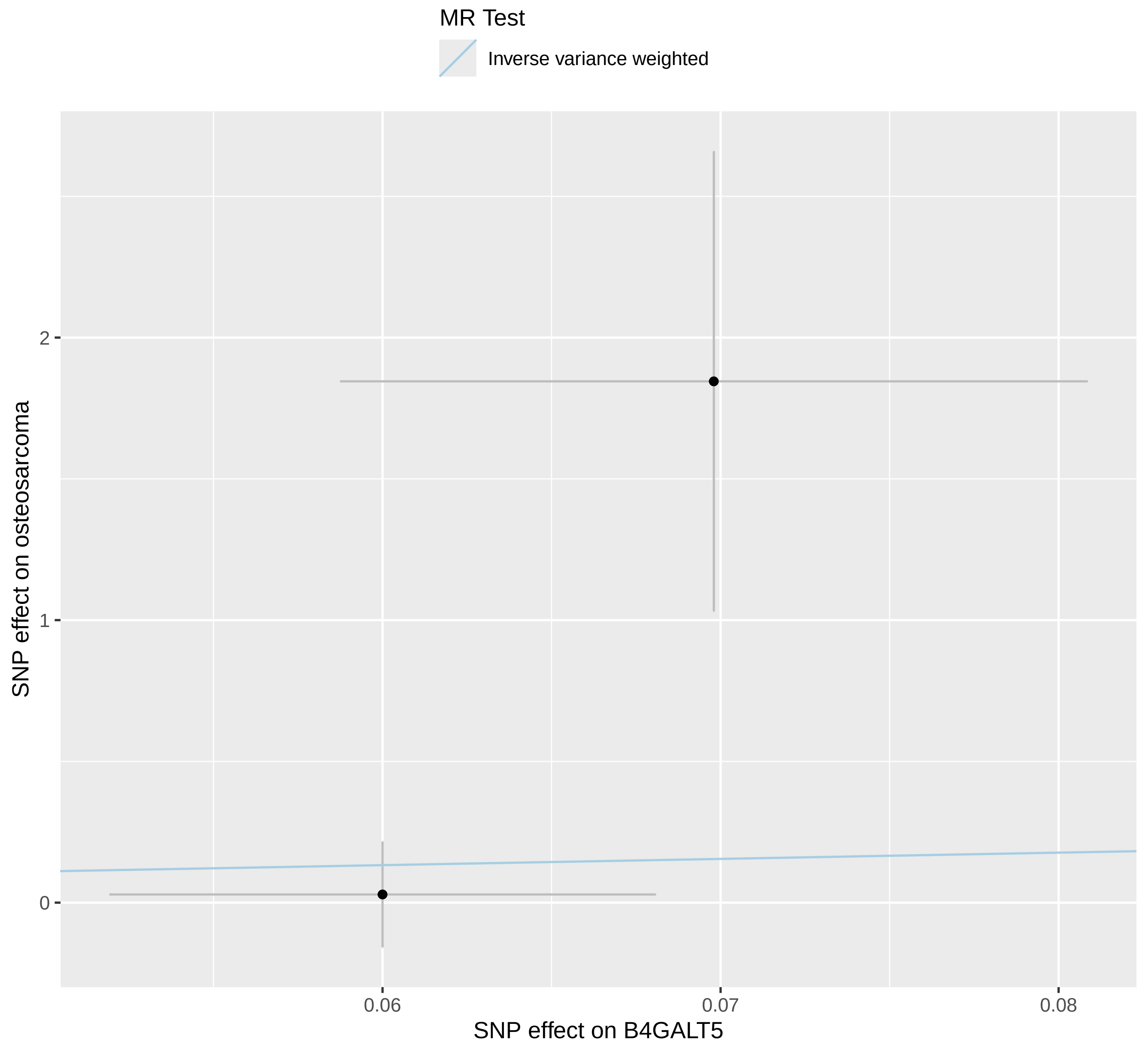
MR Test
Inverse variance weighted
SNP effect on osteosarcoma
2
1
0
0.06
0.07
0.08
SNP effect on B4GALT5

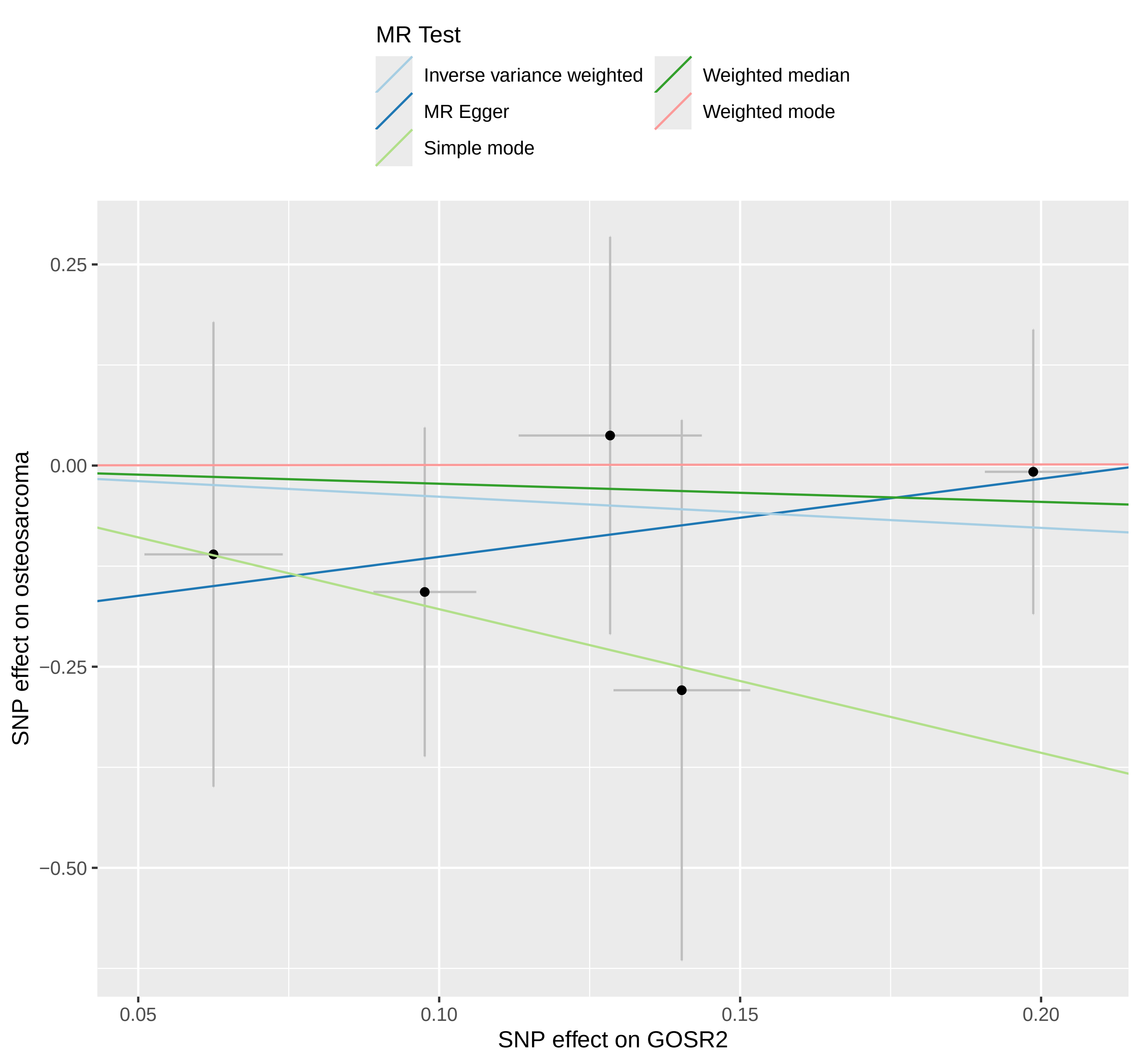
MR Test
Inverse variance weighted
MR Egger
Simple mode
Weighted median
Weighted mode
0.25
0.00
−0.25
−0.50
0.05
0.10
0.15
0.20
SNP effect on osteosarcoma
SNP effect on GOSR2

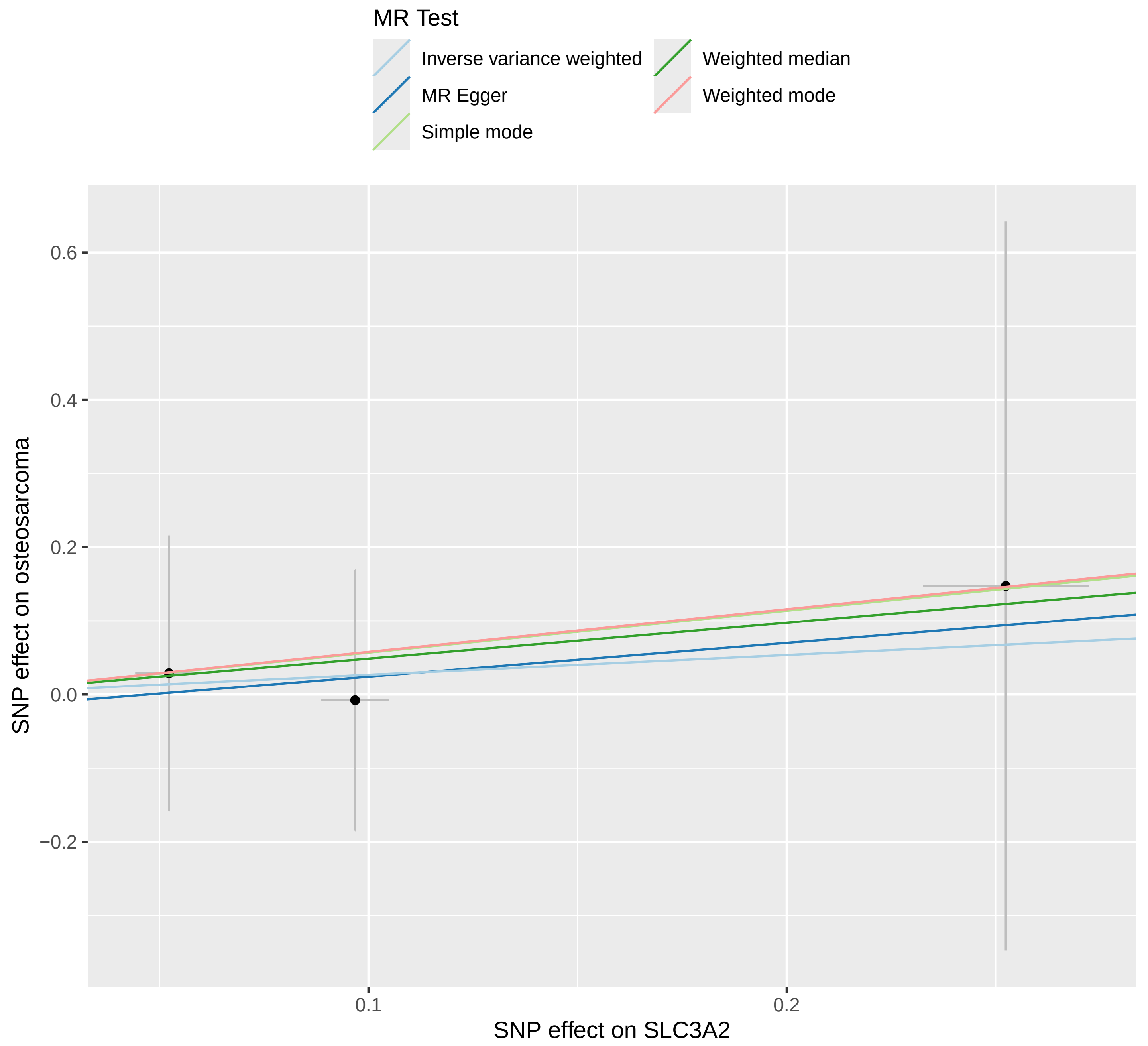

MR Test
Inverse variance weighted
MR Egger
Simple mode
Weighted median
Weighted mode
0.6
0.4
0.2
0.0
−0.2
0.1
0.2
SNP effect on SLC3A2
SNP effect on osteosarcoma

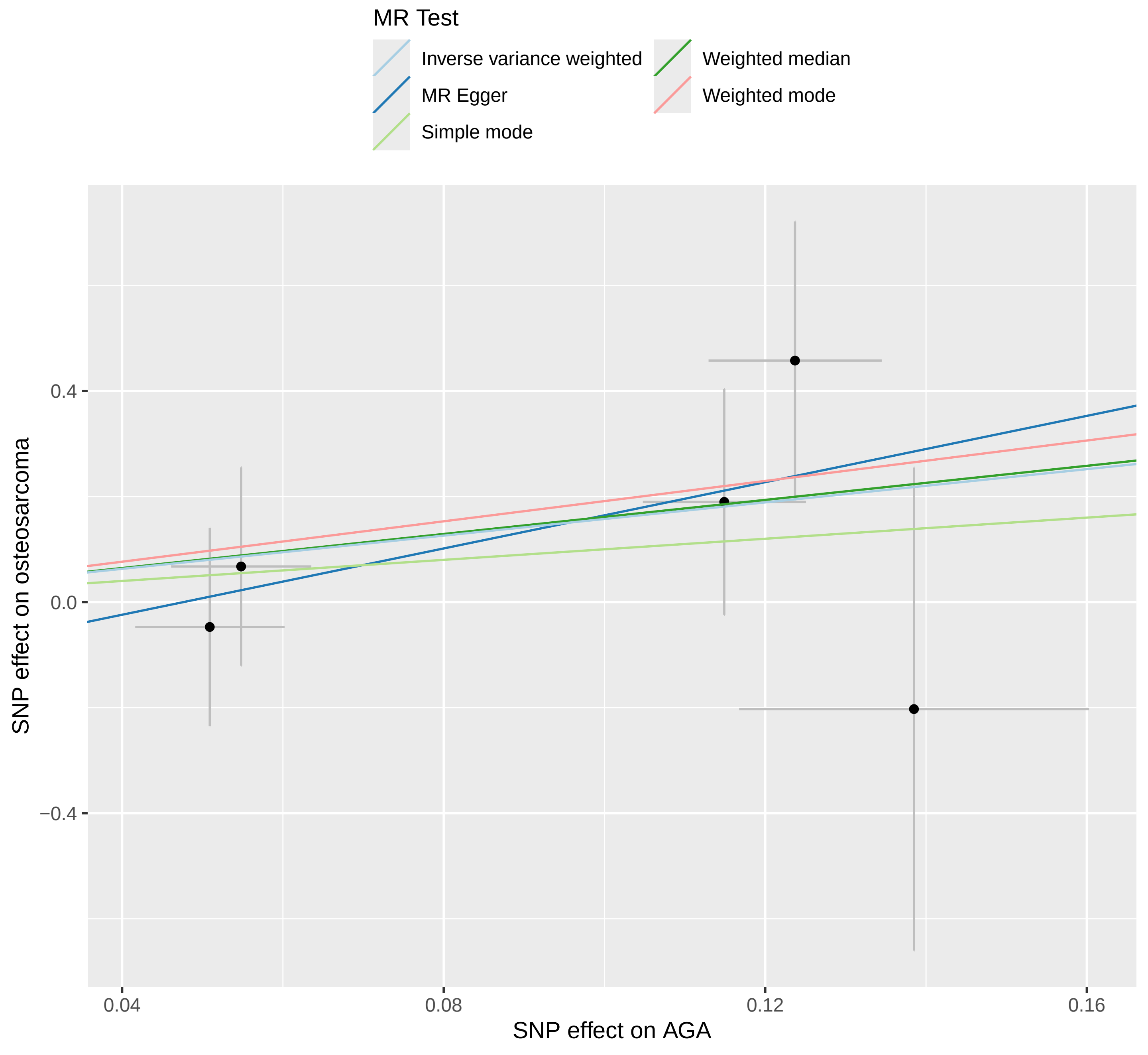

MR Test
Inverse variance weighted
MR Egger
Simple mode
Weighted median
Weighted mode
SNP effect on osteosarcoma
0.4
0.0
−0.4
0.04
0.08
0.12
0.16
SNP effect on AGA

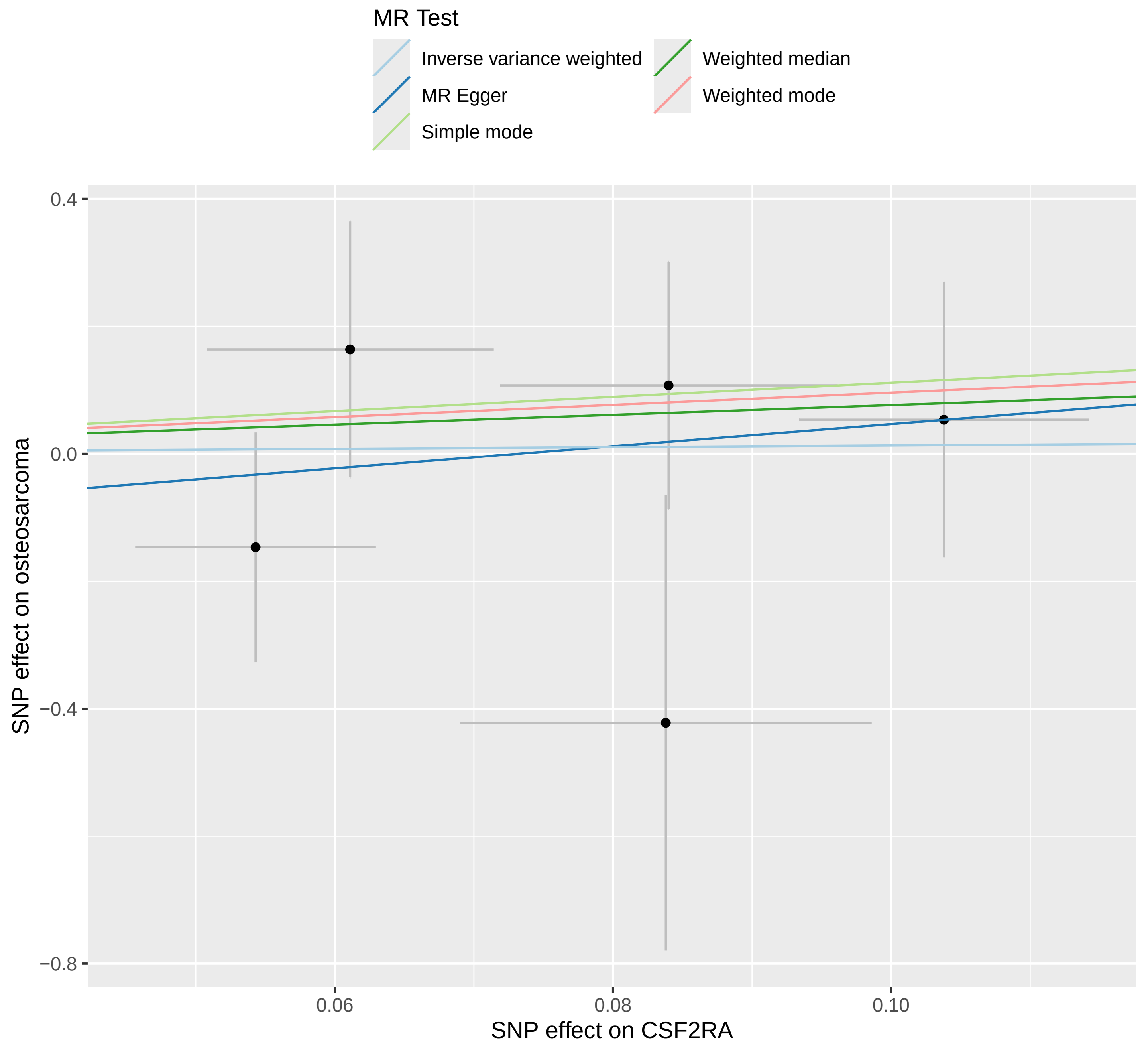

MR Test
Inverse variance weighted
MR Egger
Simple mode
Weighted median
Weighted mode
0.4
0.0
−0.4
−0.8
0.06
0.08
0.10
SNP effect on osteosarcoma
SNP effect on CSF2RA

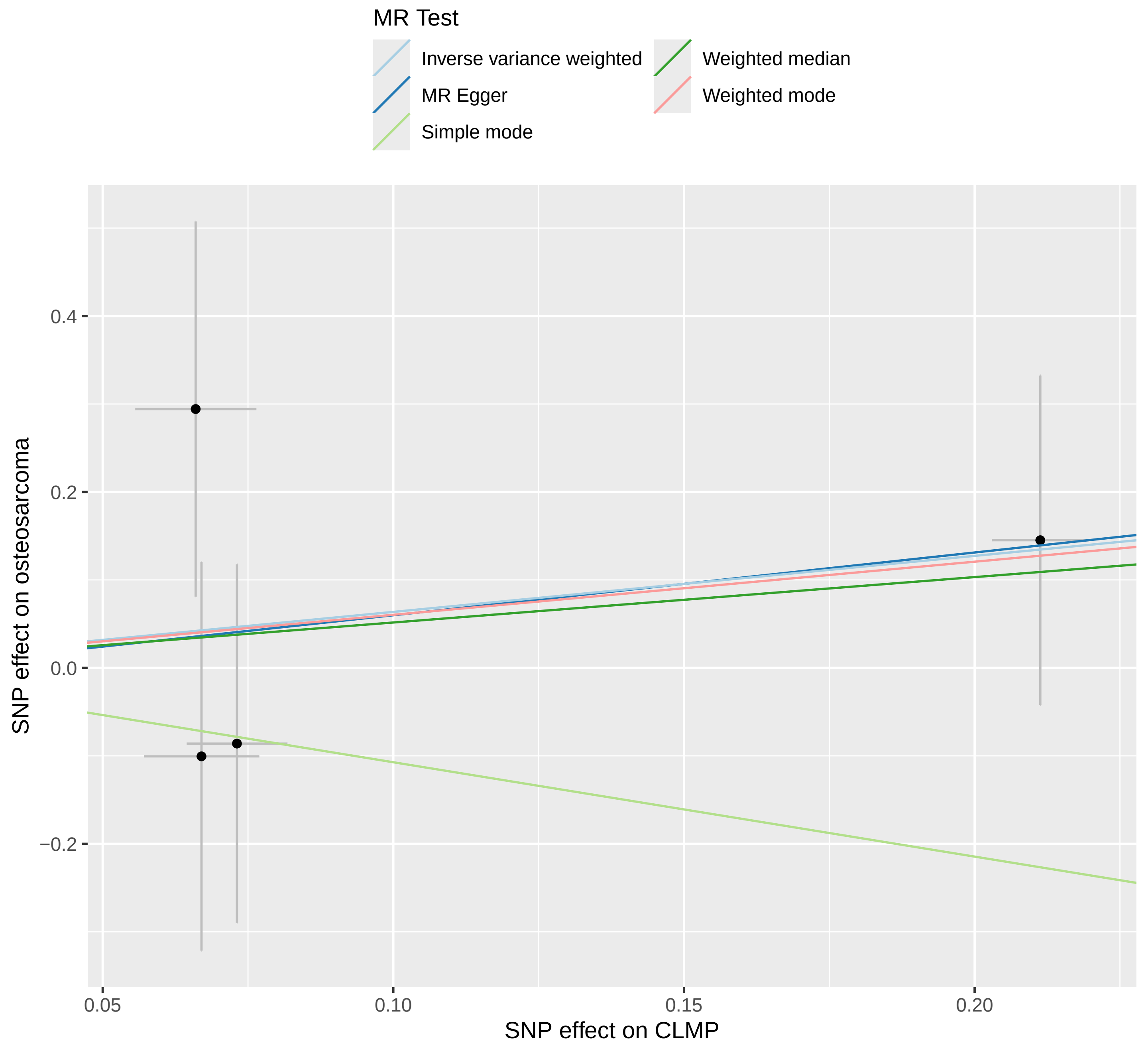
MR Test
Inverse variance weighted
MR Egger
Simple mode
Weighted median
Weighted mode
0.4
0.2
0.0
−0.2
SNP effect on osteosarcoma
0.05
0.10
0.15
0.20
SNP effect on CLMP

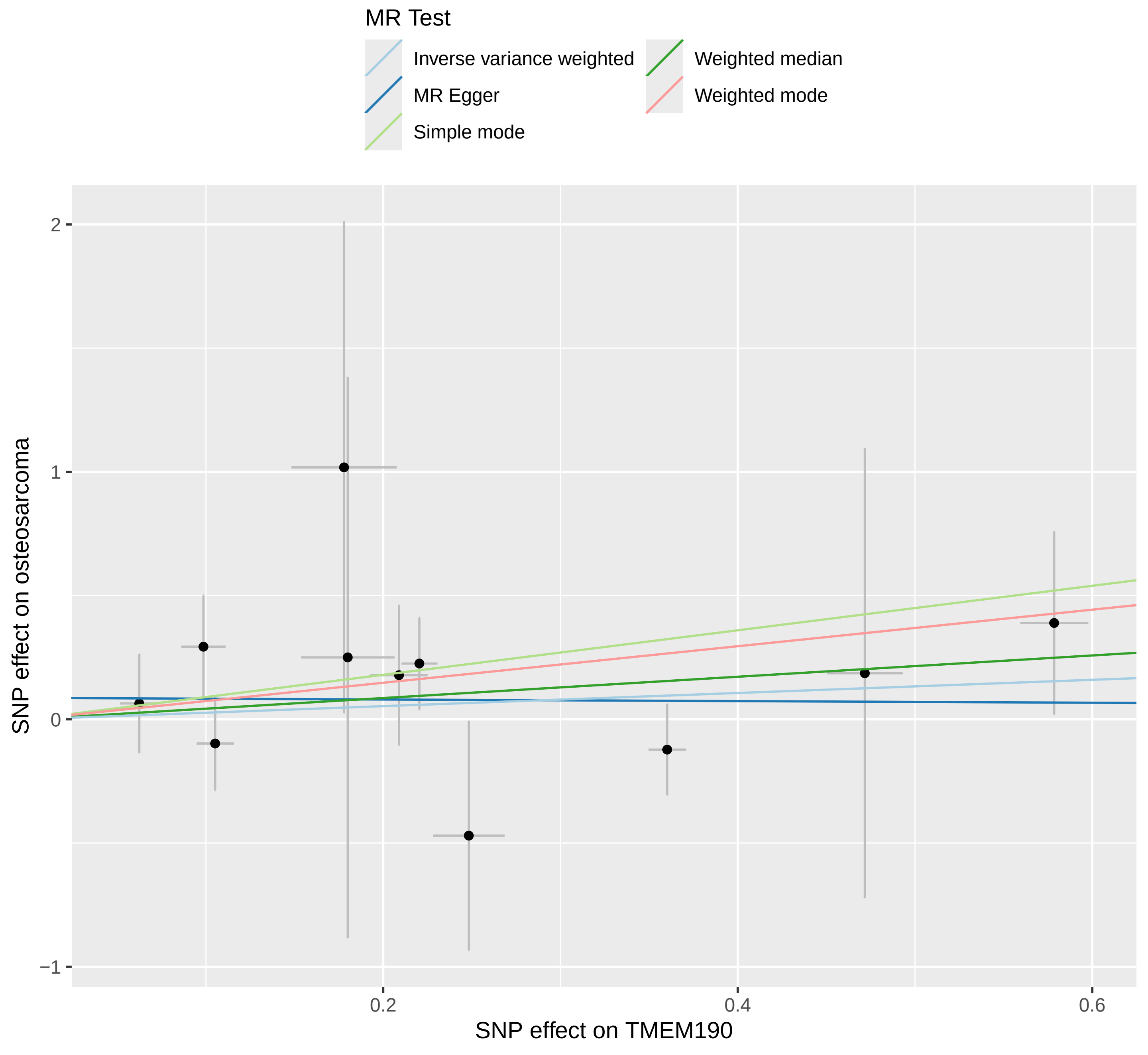

MR Test
Inverse variance weighted
MR Egger
Simple mode
Weighted median
Weighted mode
2
1
0
−1
0.2
0.4
0.6
SNP effect on osteosarcoma
SNP effect on TMEM190

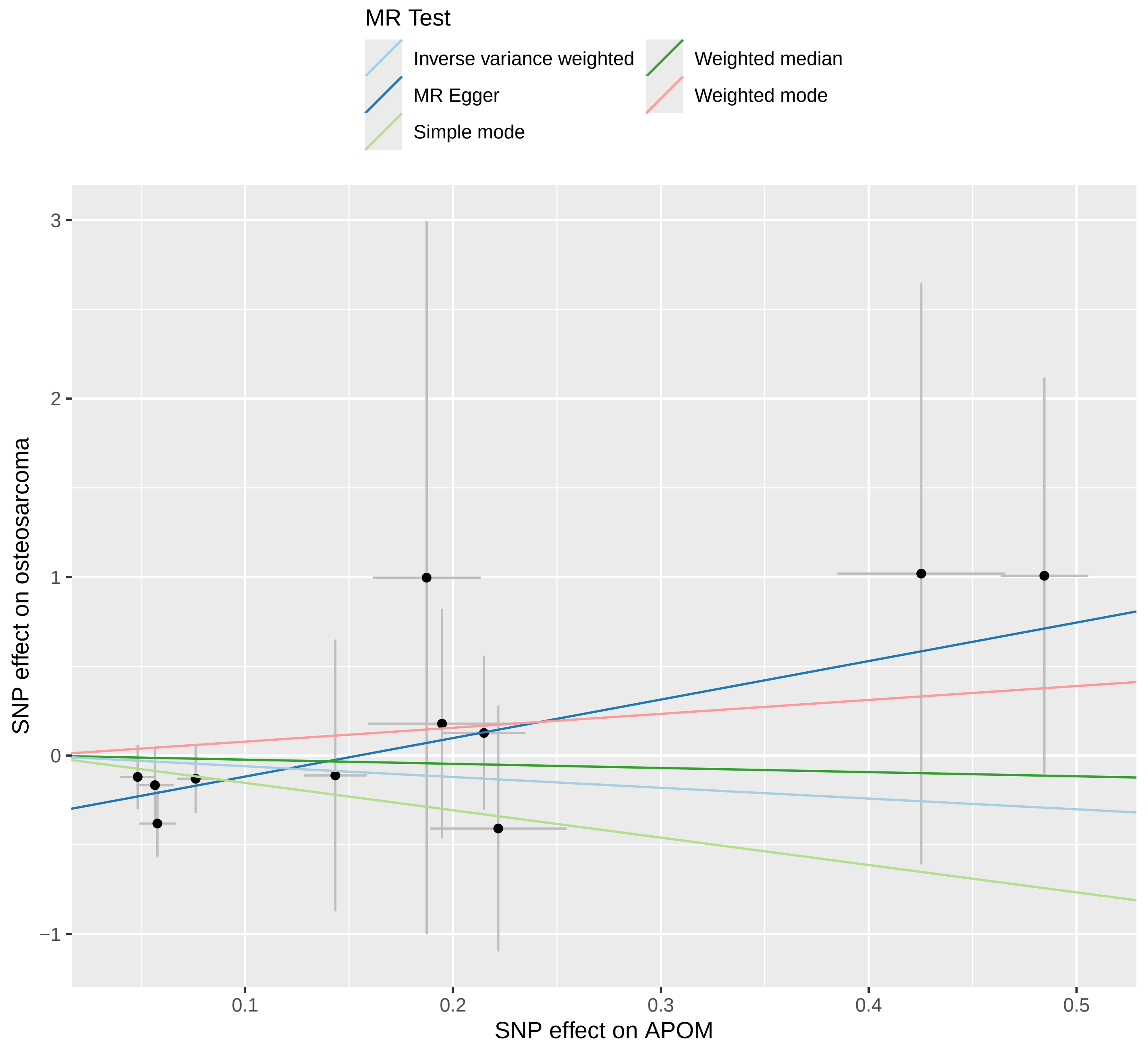
MR Test
Inverse variance weighted
MR Egger
Simple mode
Weighted median
Weighted mode
SNP effect on osteosarcoma
SNP effect on APOM
3
2
1
0
−1
0.1
0.2
0.3
0.4
0.5

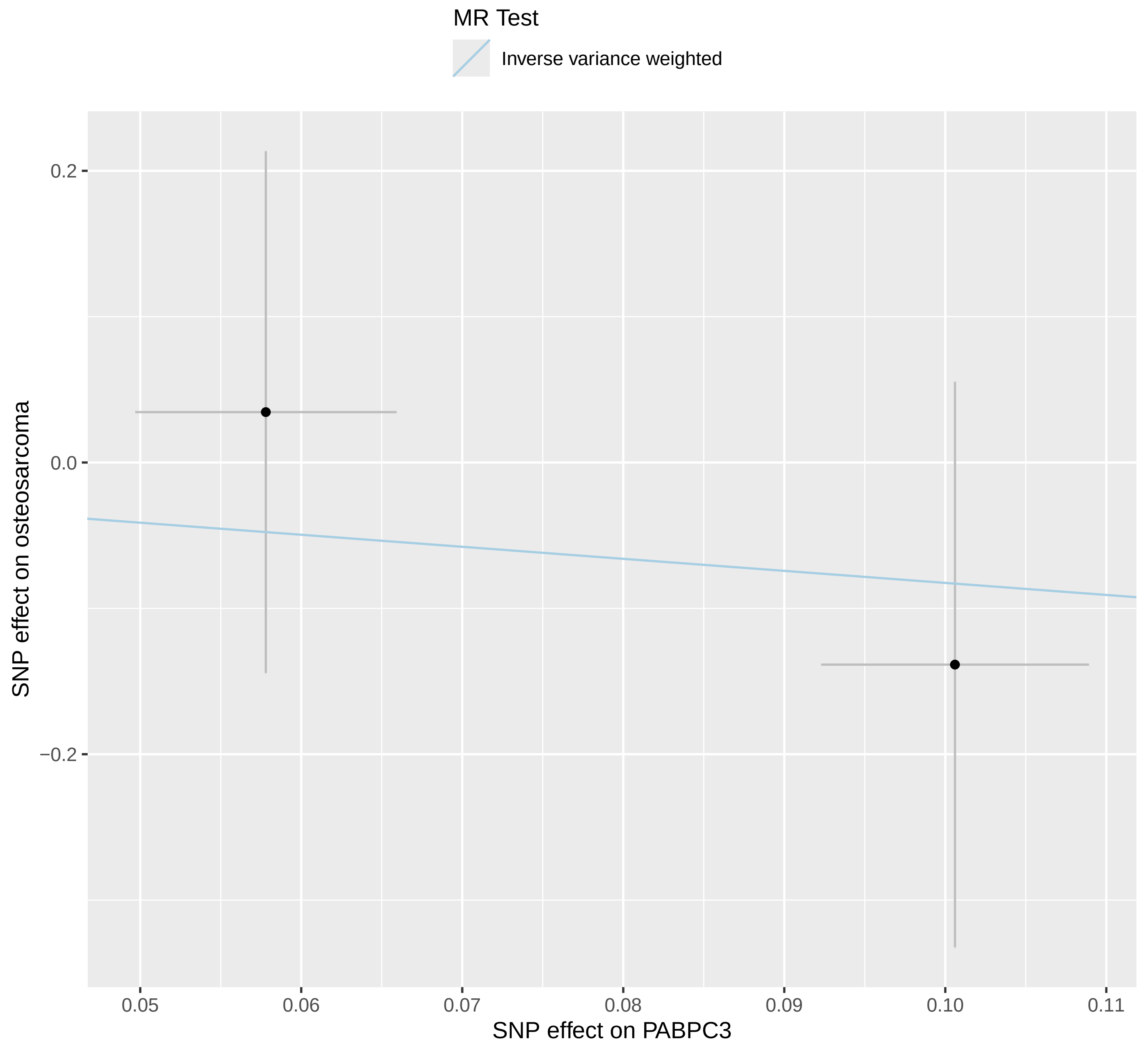

MR Test
Inverse variance weighted
0.2
0.0
−0.2
0.05
0.06
0.07
0.08
0.09
0.10
0.11
SNP effect on osteosarcoma
SNP effect on PABPC3

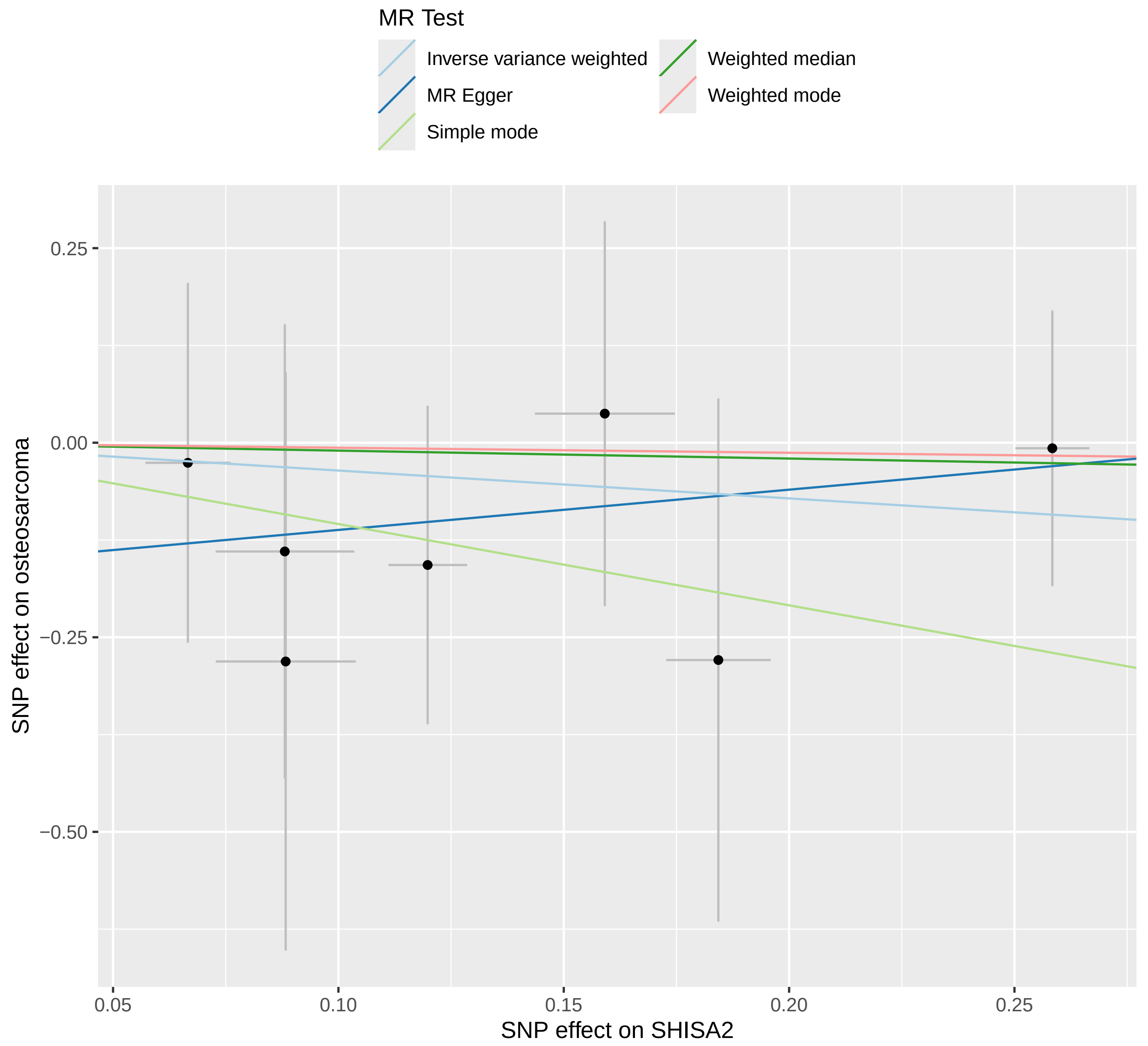
MR Test
Inverse variance weighted
MR Egger
Simple mode
Weighted median
Weighted mode
0.25
0.00
−0.25
−0.50
0.05
0.10
0.15
0.20
0.25
SNP effect on SHISA2
SNP effect on osteosarcoma

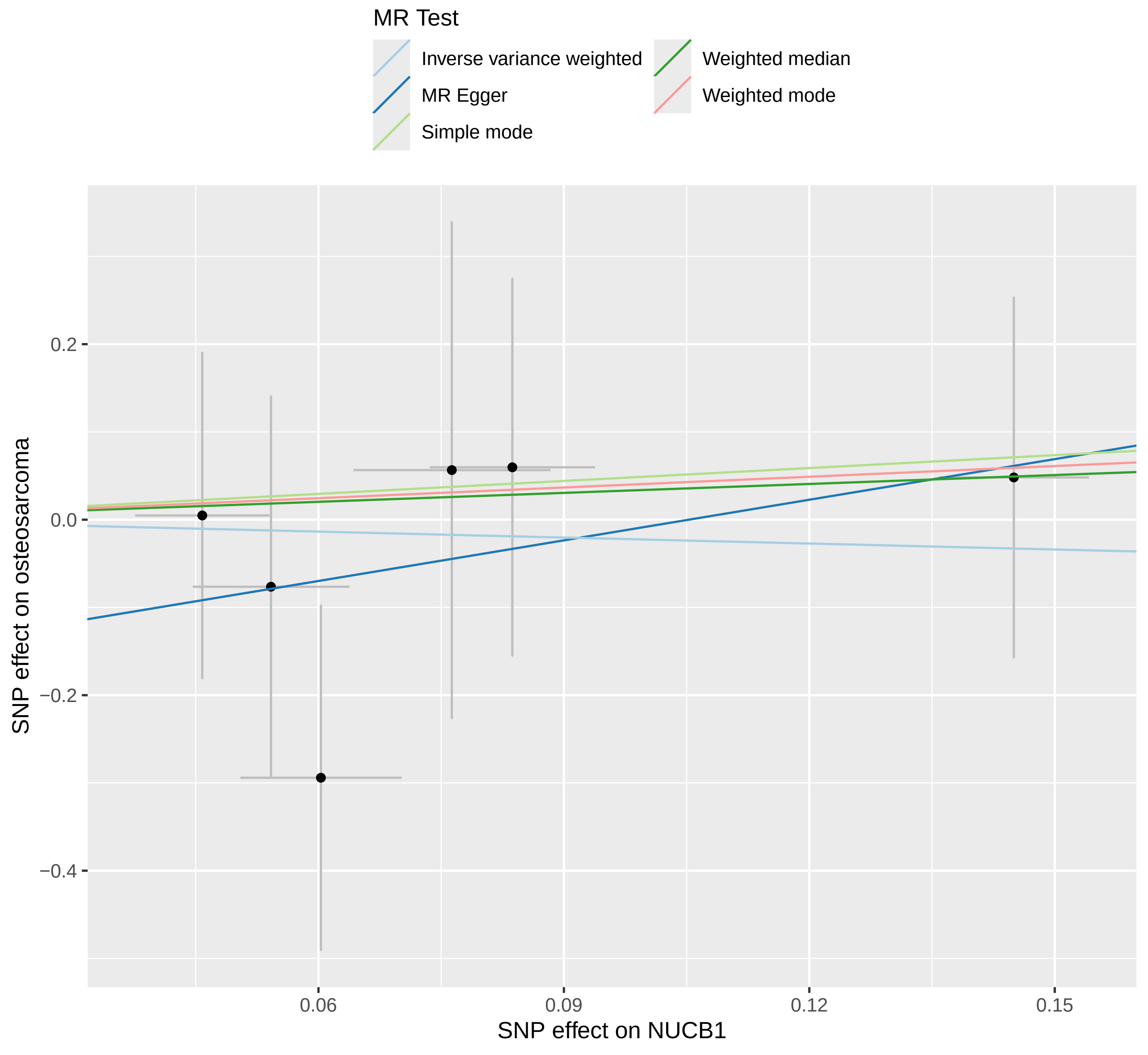
MR Test
Inverse variance weighted
MR Egger
Simple mode
Weighted median
Weighted mode
SNP effect on osteosarcoma
0.2
0.0
−0.2
−0.4
0.06
0.09
0.12
0.15
SNP effect on NUCB1

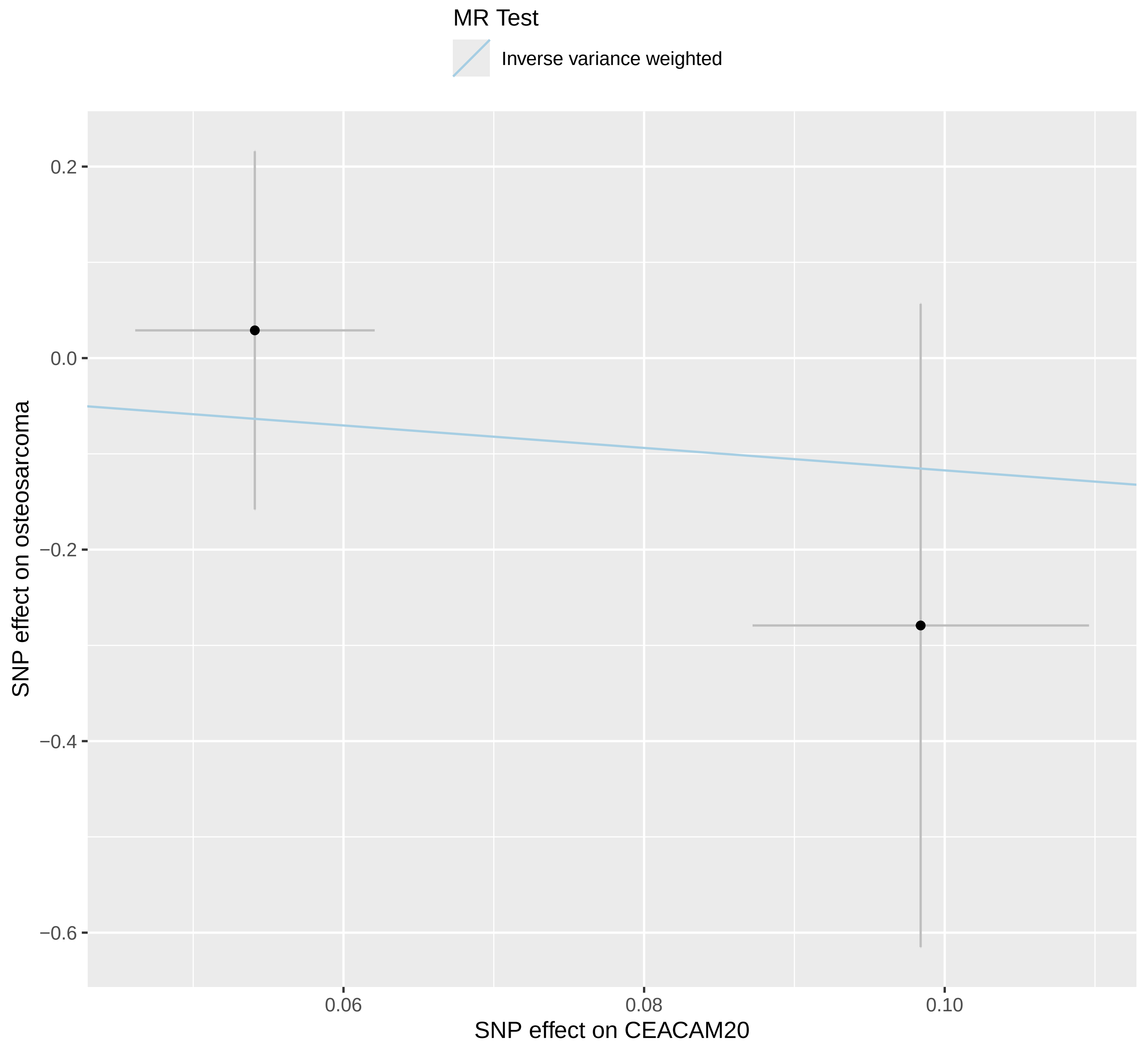
MR Test
Inverse variance weighted
0.2
0.0
−0.2
−0.4
−0.6
SNP effect on osteosarcoma
0.06
0.08
0.10
SNP effect on CEACAM20

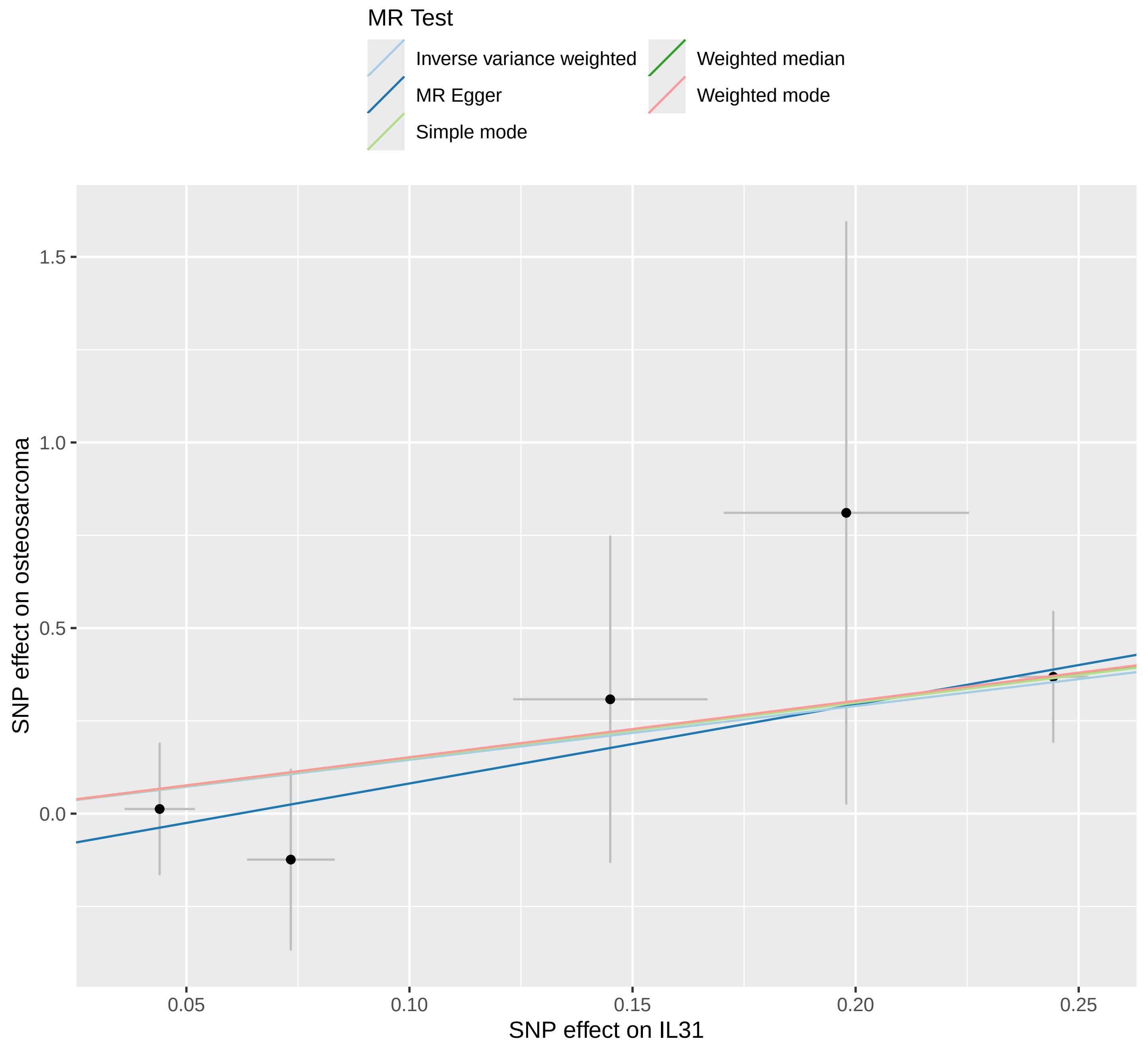

MR Test
Inverse variance weighted
MR Egger
Simple mode
Weighted median
Weighted mode
SNP effect on osteosarcoma
1.5
1.0
0.5
0.0
0.05
0.10
0.15
0.20
0.25
SNP effect on IL31

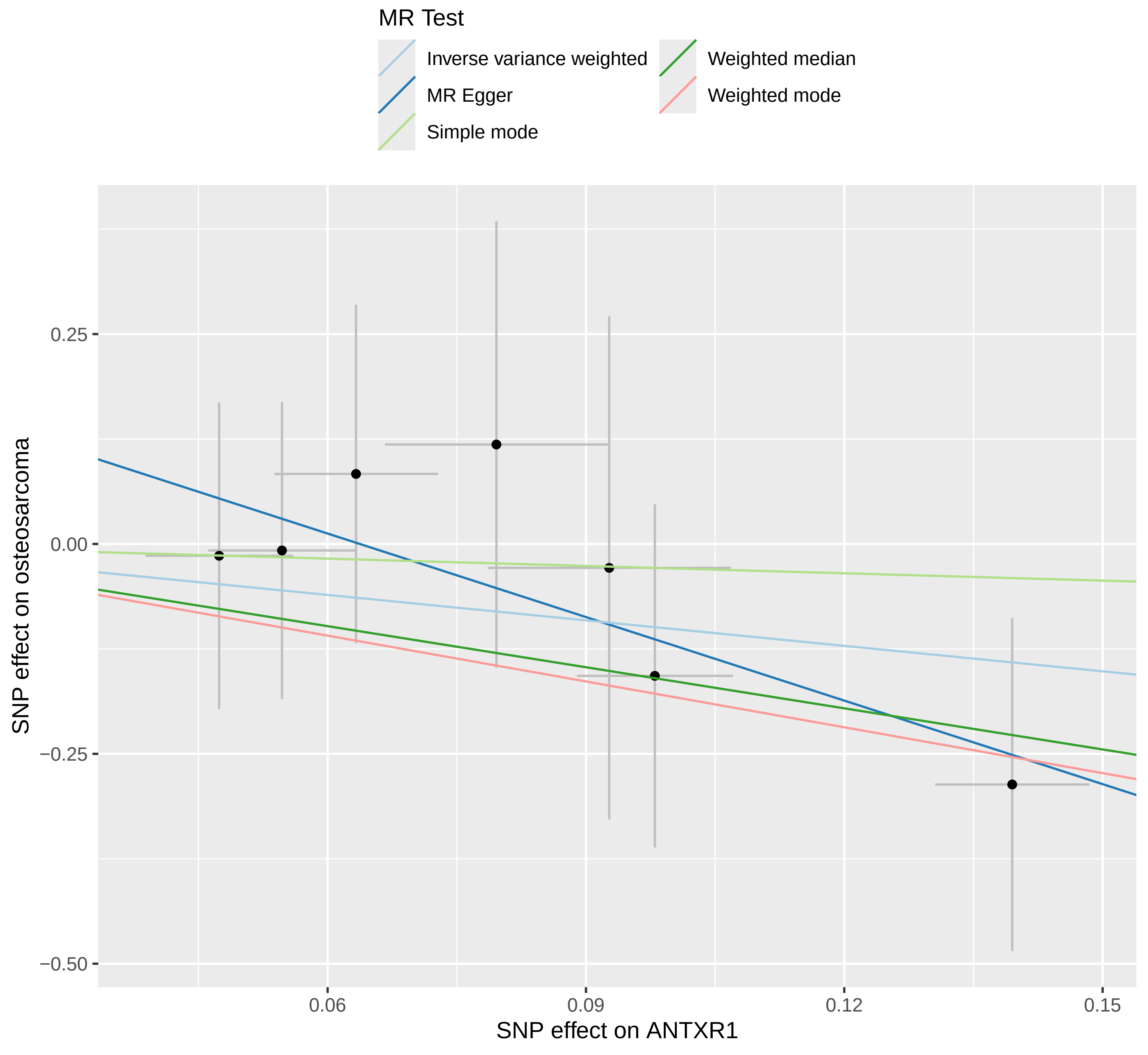
MR Test
Inverse variance weighted
MR Egger
Simple mode
Weighted median
Weighted mode
0.25
0.00
−0.25
−0.50
0.06
0.09
0.12
0.15
SNP effect on osteosarcoma
SNP effect on ANTXR1

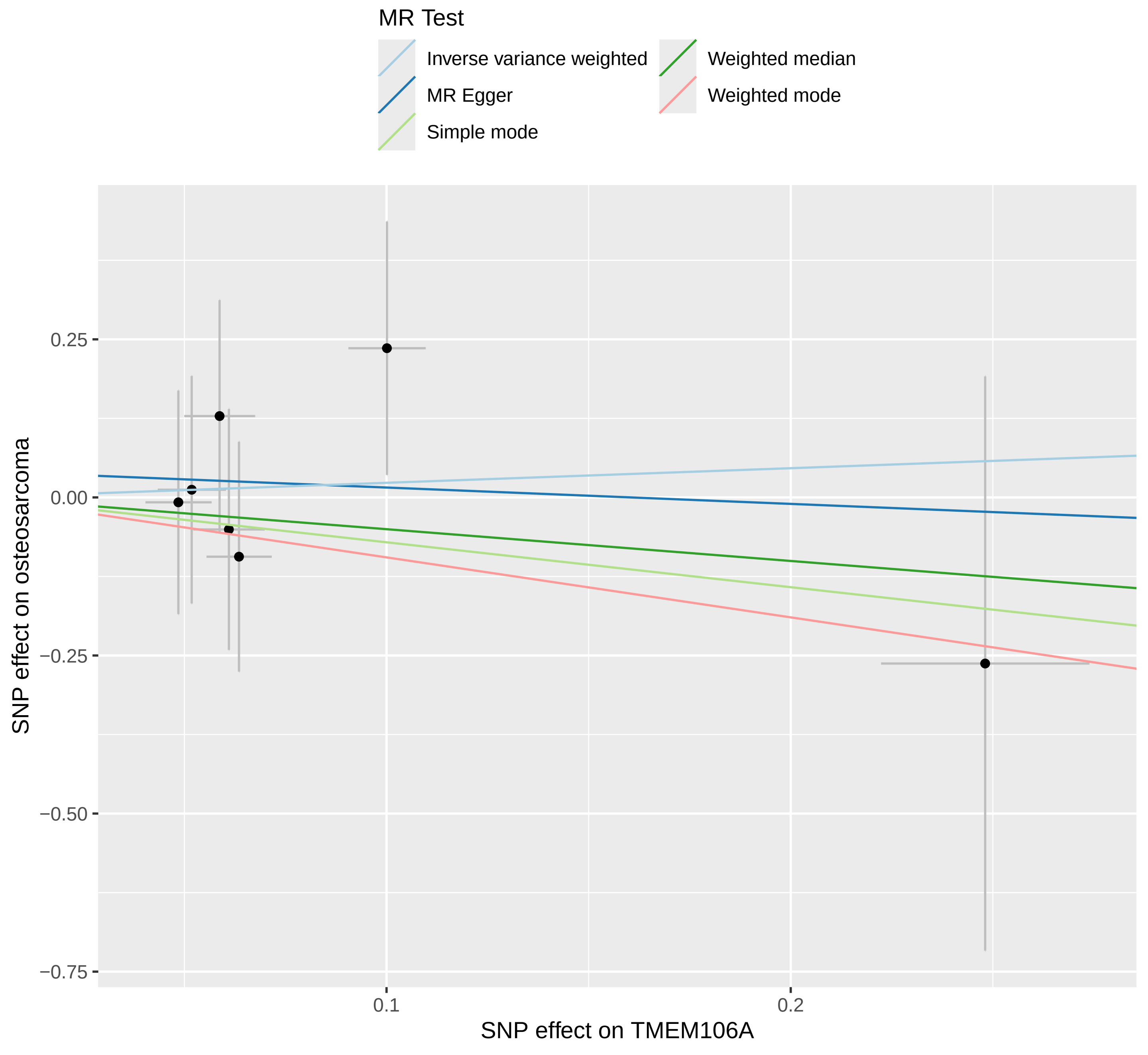
MR Test
Inverse variance weighted
MR Egger
Simple mode
Weighted median
Weighted mode
SNP effect on osteosarcoma
0.25
0.00
−0.25
−0.50
−0.75
0.1
0.2
SNP effect on TMEM106A

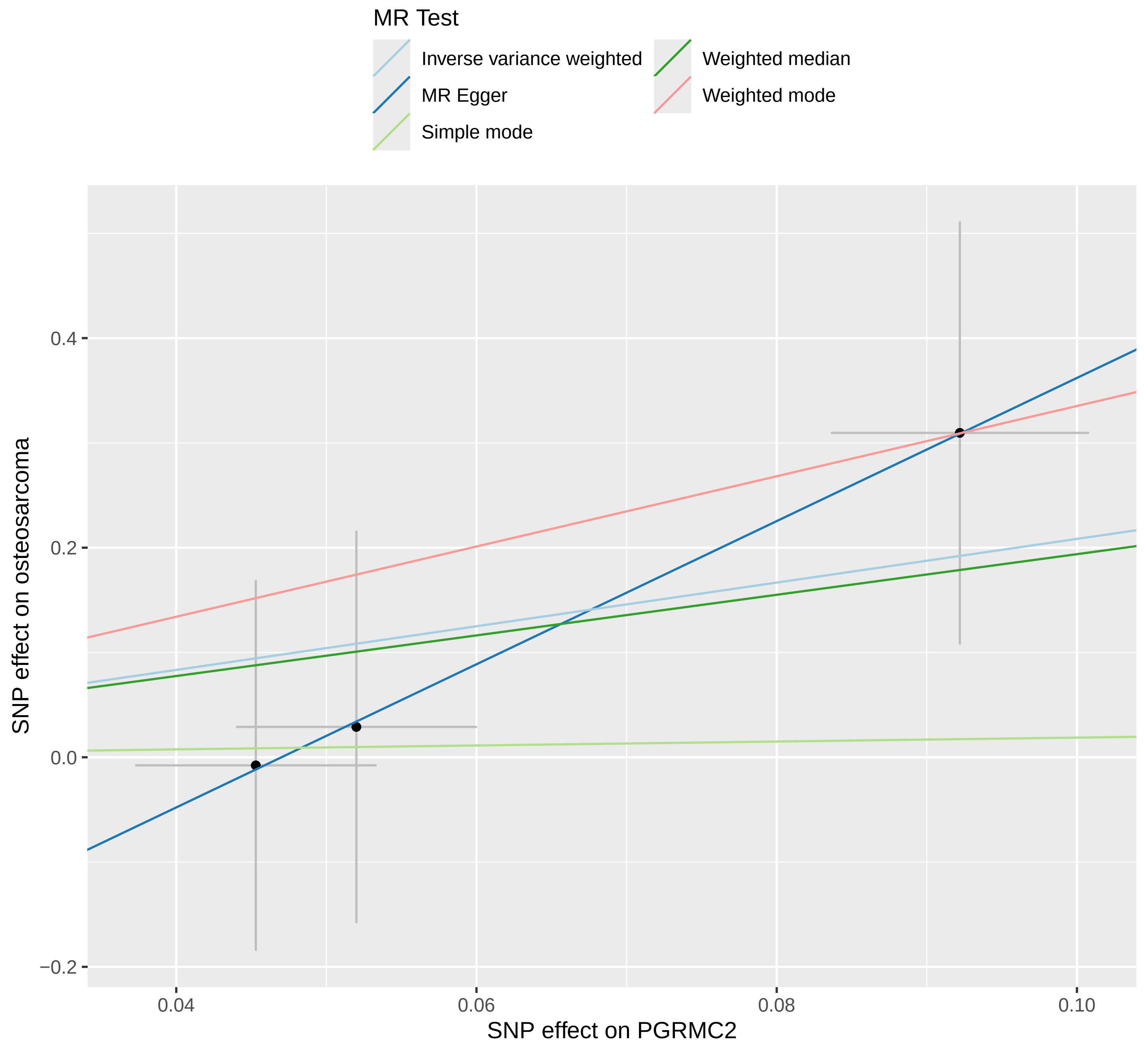
MR Test
Inverse variance weighted
MR Egger
Simple mode
Weighted median
Weighted mode
SNP effect on osteosarcoma
0.4
0.2
0.0
−0.2
0.04
0.06
0.08
0.10
SNP effect on PGRMC2

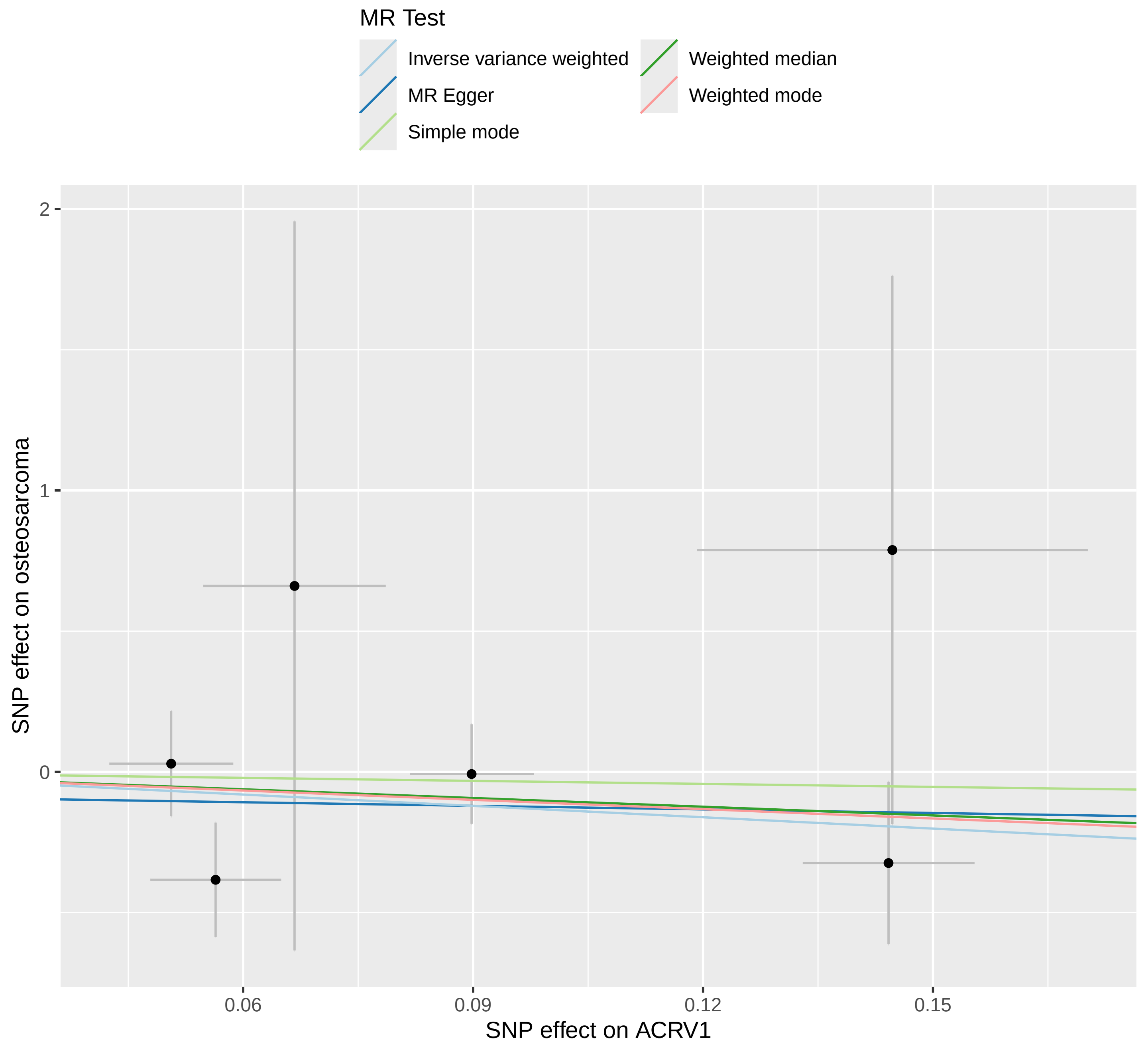
MR Test
Inverse variance weighted
MR Egger
Simple mode
Weighted median
Weighted mode
SNP effect on osteosarcoma
2
1
0
0.06
0.09
0.12
0.15
SNP effect on ACRV1

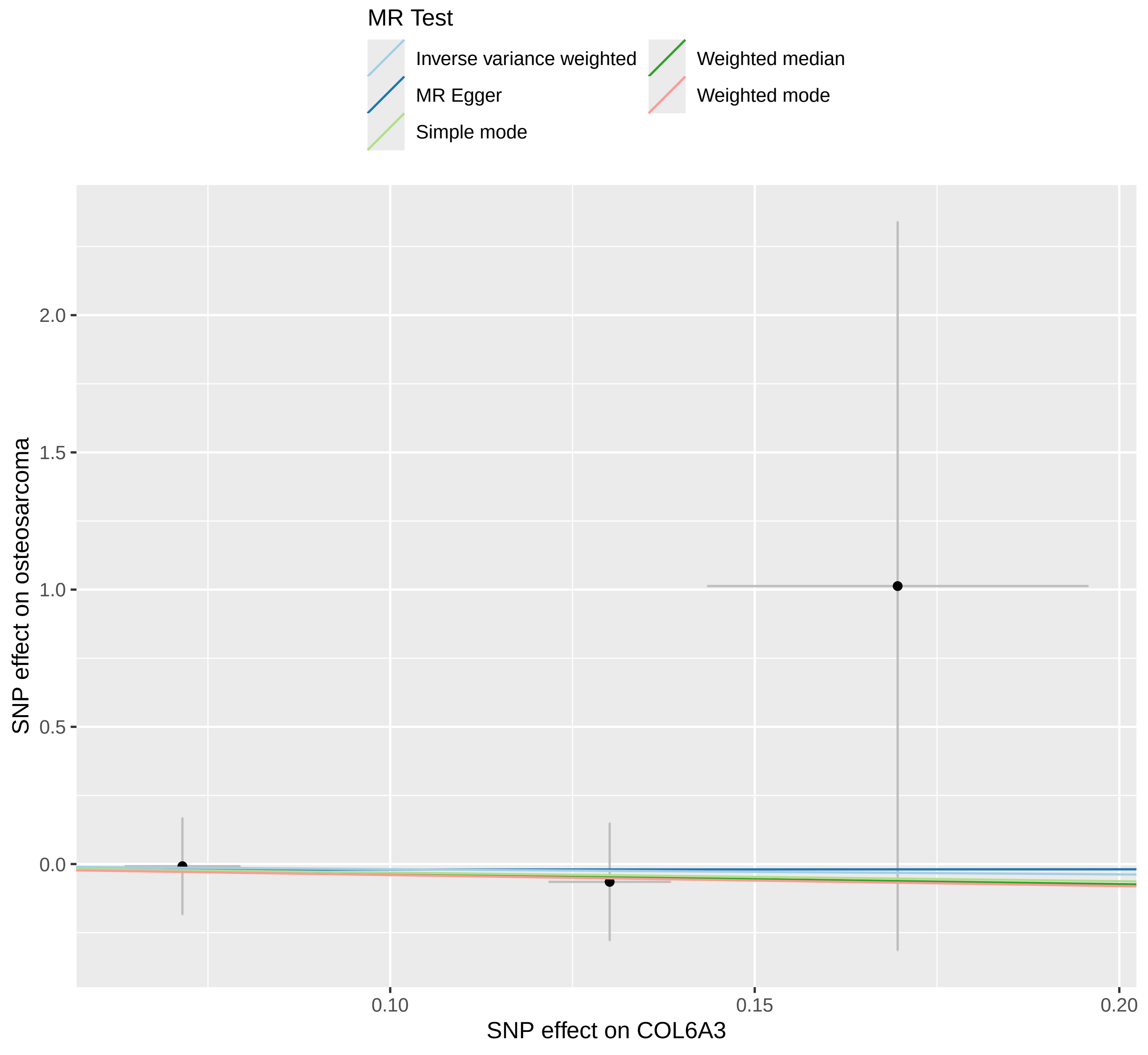

MR Test
Inverse variance weighted
MR Egger
Simple mode
Weighted median
Weighted mode
SNP effect on osteosarcoma
2.0
1.5
1.0
0.5
0.0
0.10
0.15
0.20
SNP effect on COL6A3

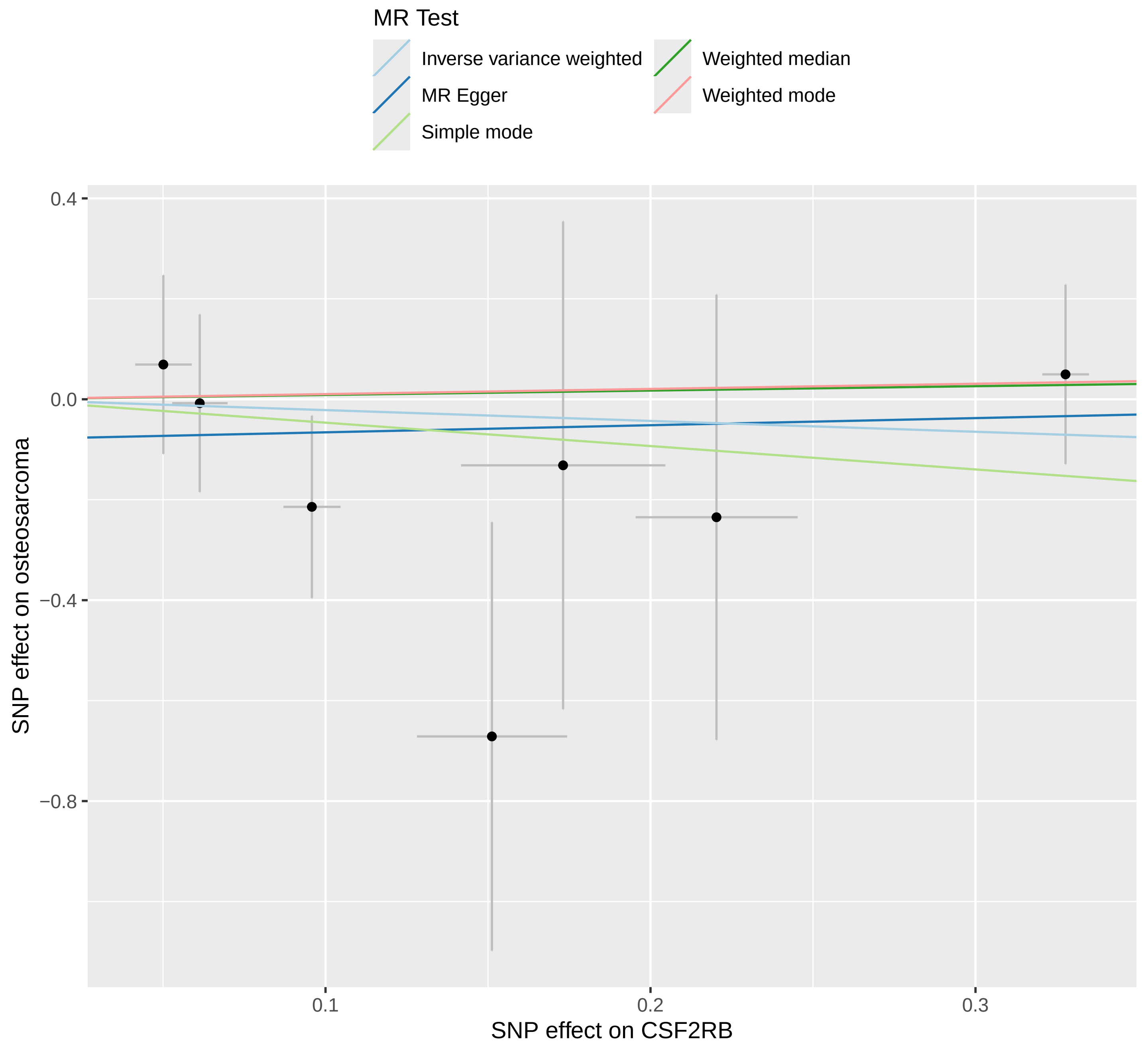

MR Test
Inverse variance weighted
MR Egger
Simple mode
Weighted median
Weighted mode
0.4
0.0
−0.4
−0.8
0.1
0.2
0.3
SNP effect on osteosarcoma
SNP effect on CSF2RB

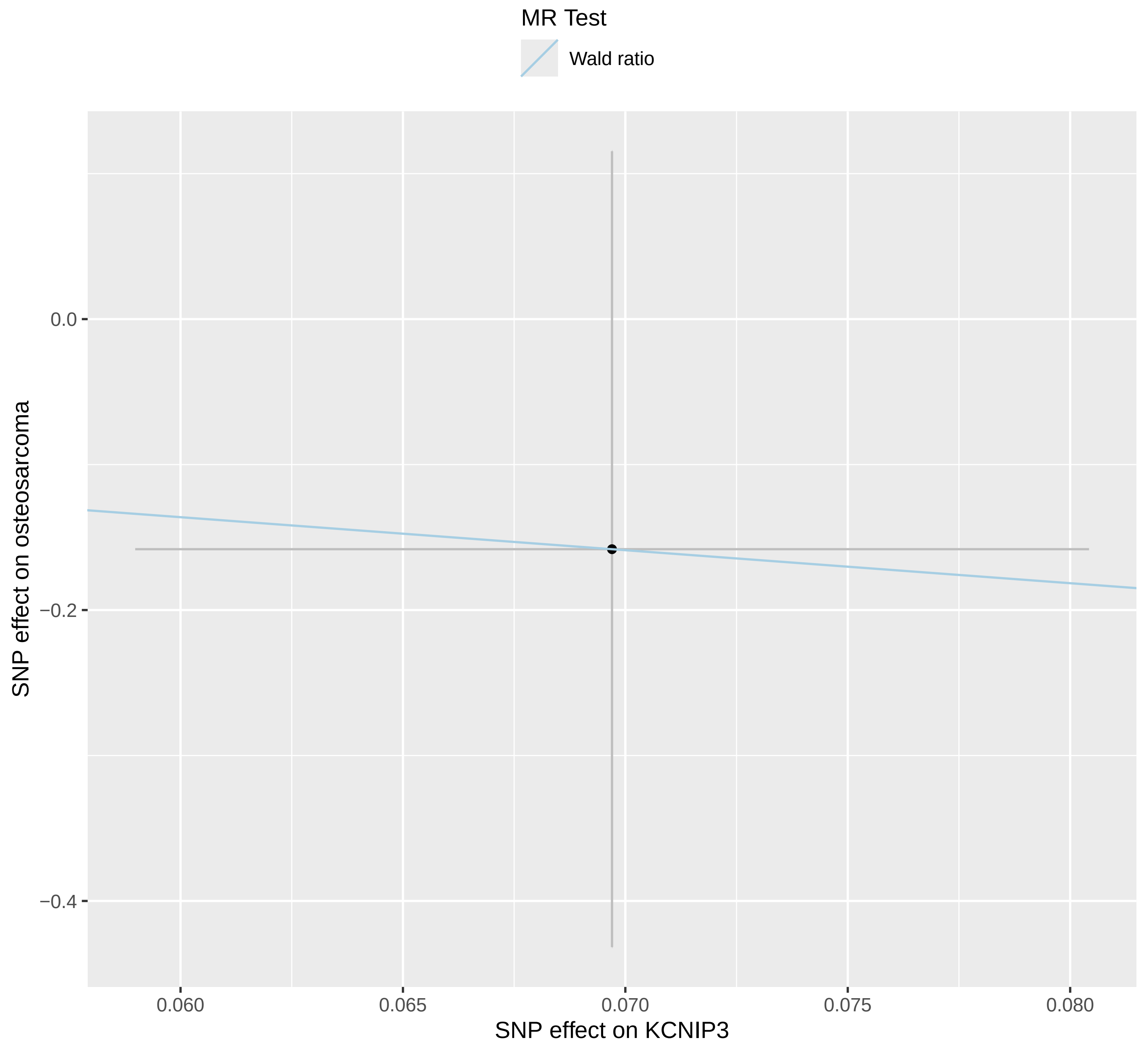
MR Test
Wald ratio
0.0
−0.2
−0.4
SNP effect on osteosarcoma
0.060
0.065
0.070
0.075
0.080
SNP effect on KCNIP3

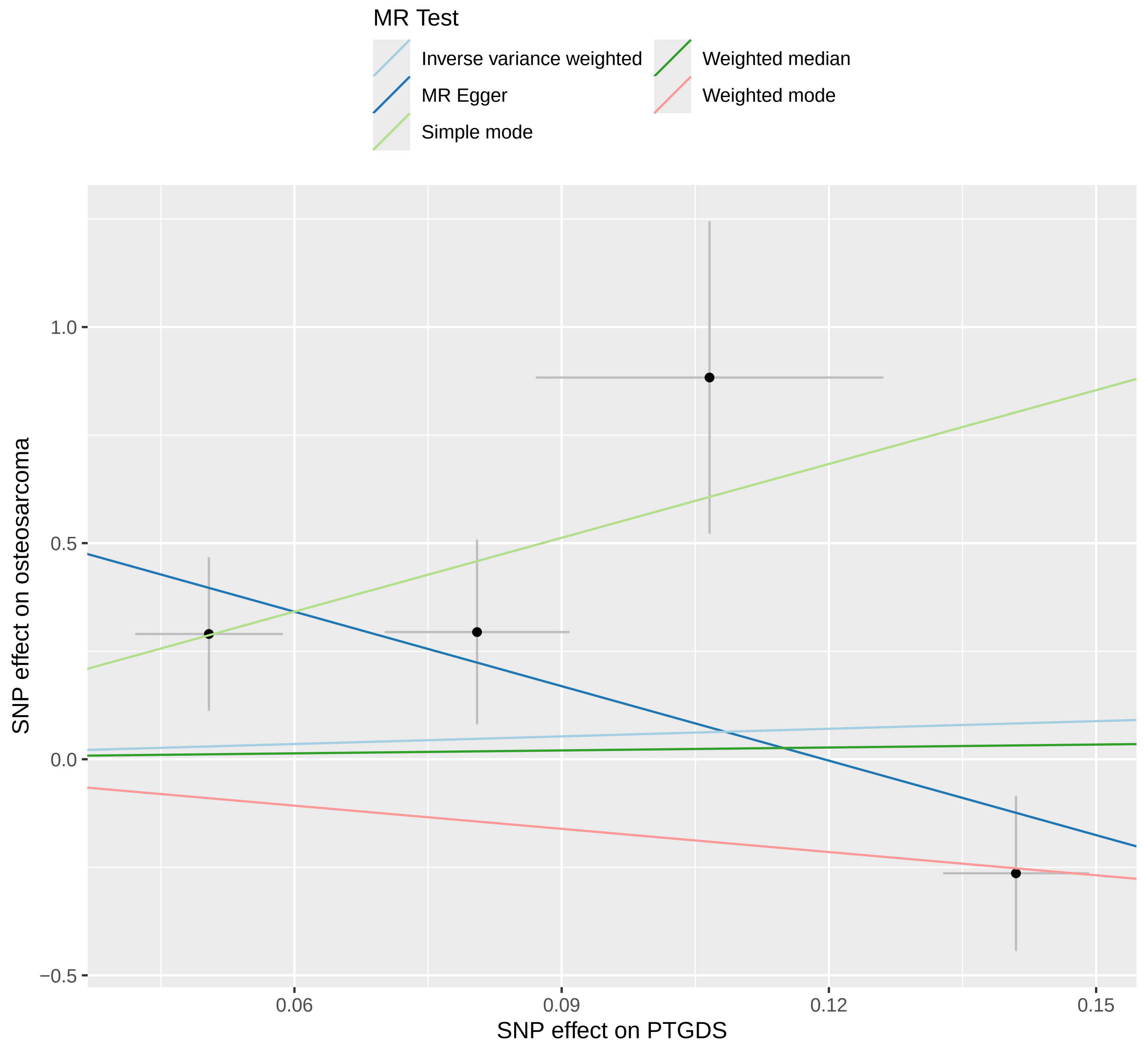
MR Test
Inverse variance weighted
MR Egger
Simple mode
Weighted median
Weighted mode
1.0
0.5
0.0
−0.5
0.06
0.09
0.12
0.15
SNP effect on PTGDS
SNP effect on osteosarcoma

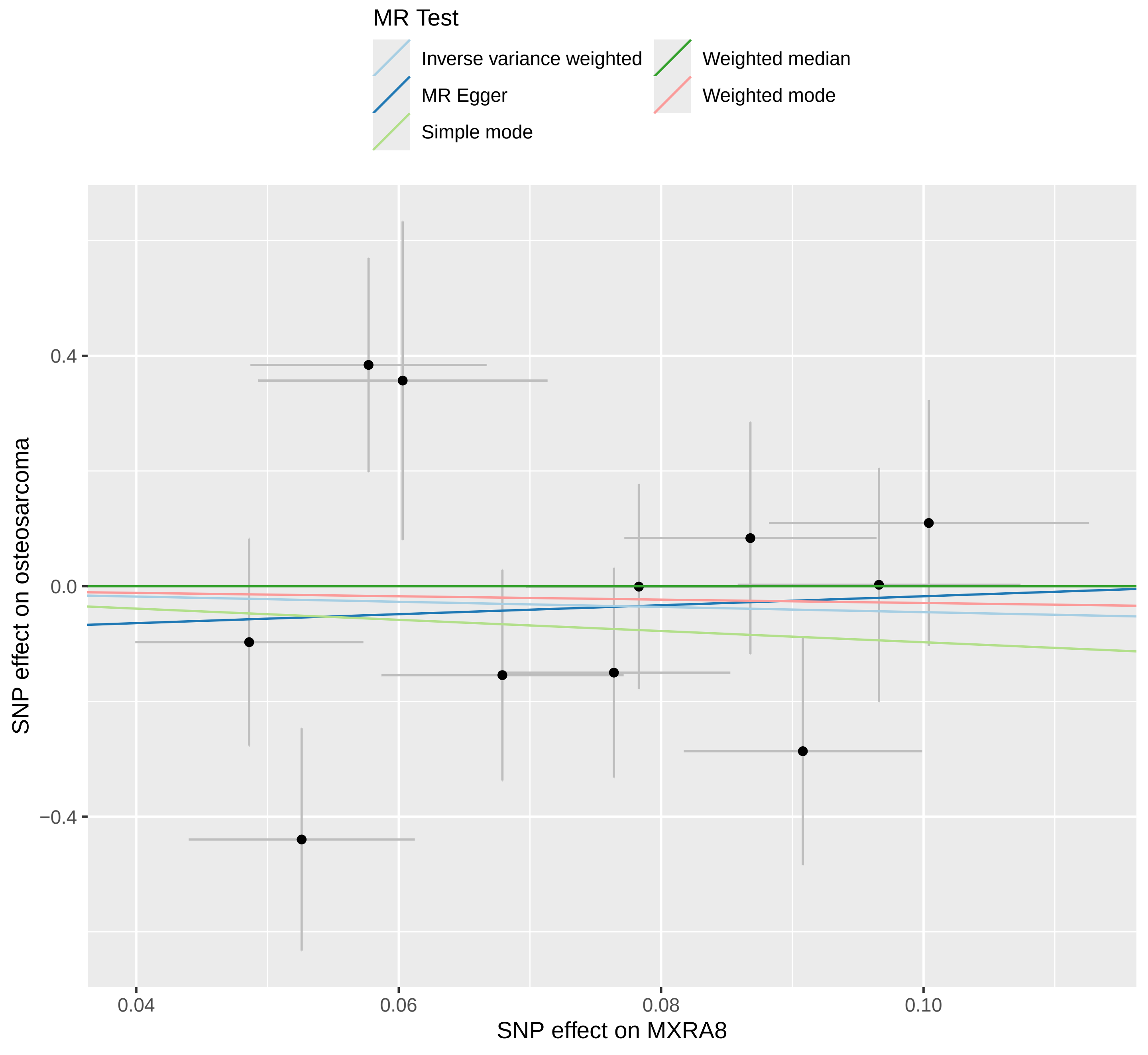

MR Test
Inverse variance weighted
MR Egger
Simple mode
Weighted median
Weighted mode
0.4
0.0
−0.4
SNP effect on osteosarcoma
0.04
0.06
0.08
0.10
SNP effect on MXRA8

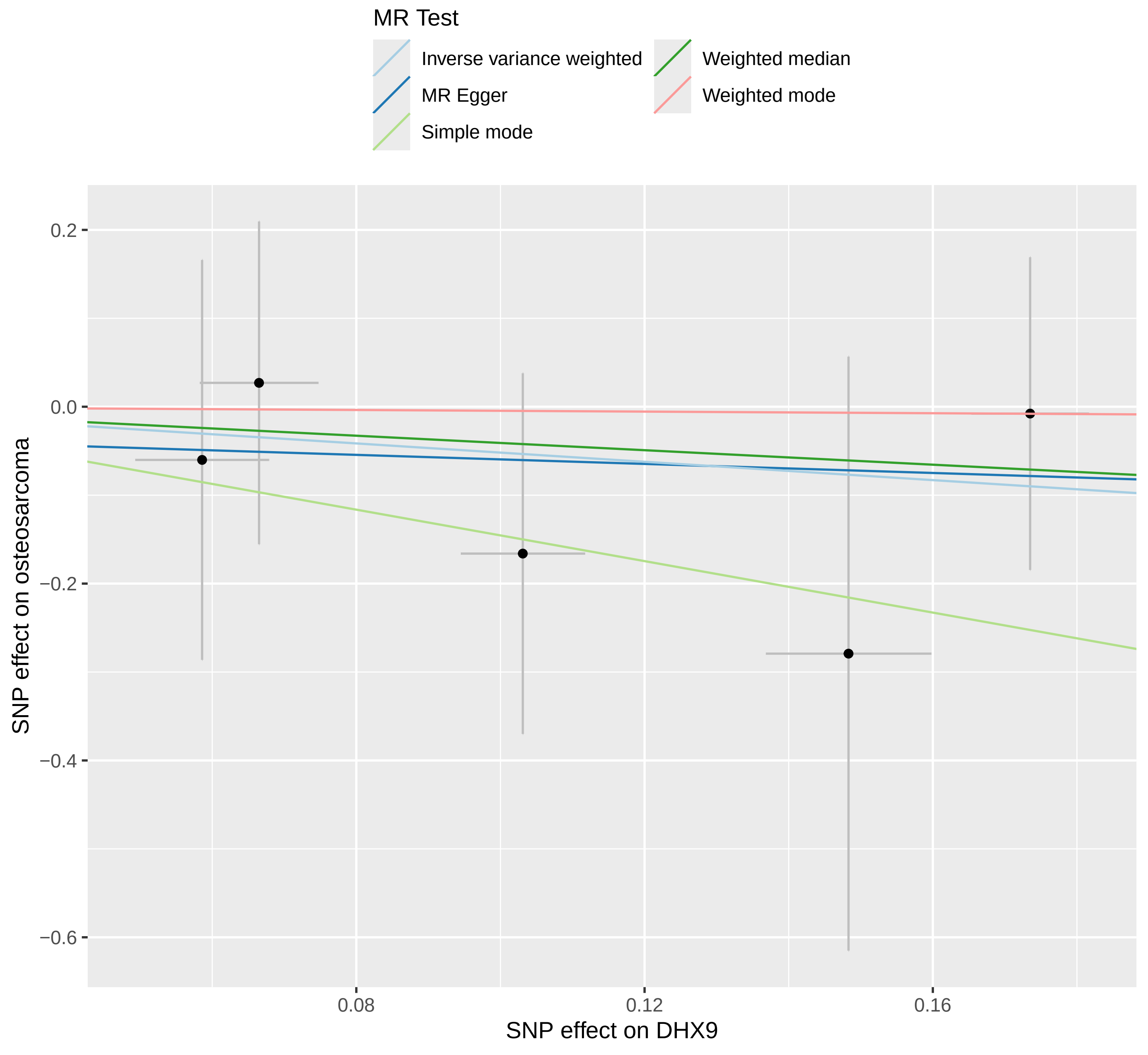

MR Test
Inverse variance weighted
MR Egger
Simple mode
Weighted median
Weighted mode
0.2
0.0
−0.2
−0.4
−0.6
SNP effect on osteosarcoma
0.08
0.12
0.16
SNP effect on DHX9

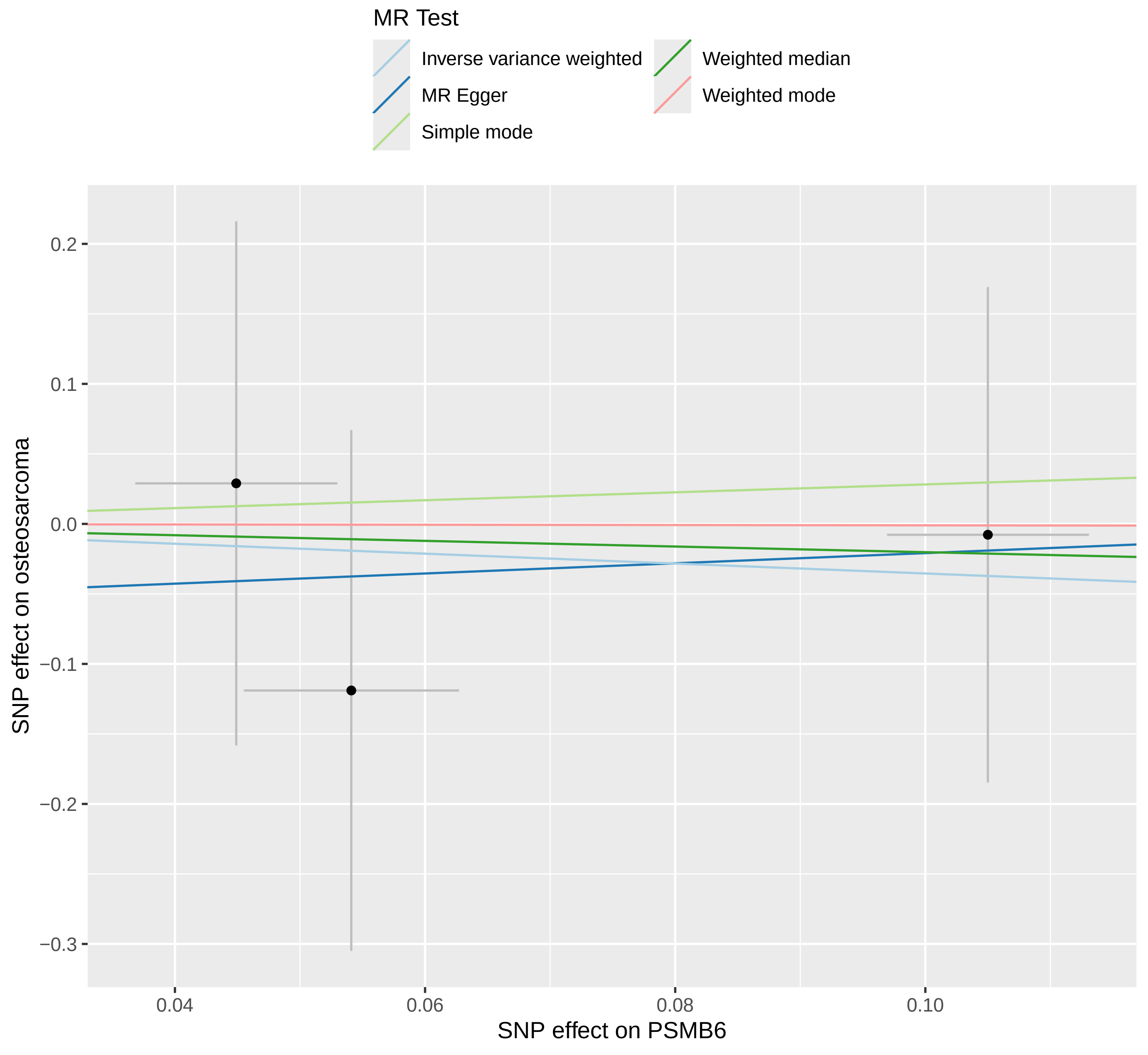

MR Test
Inverse variance weighted
MR Egger
Simple mode
Weighted median
Weighted mode
0.2
0.1
0.0
−0.1
−0.2
−0.3
SNP effect on osteosarcoma
0.04
0.06
0.08
0.10
SNP effect on PSMB6

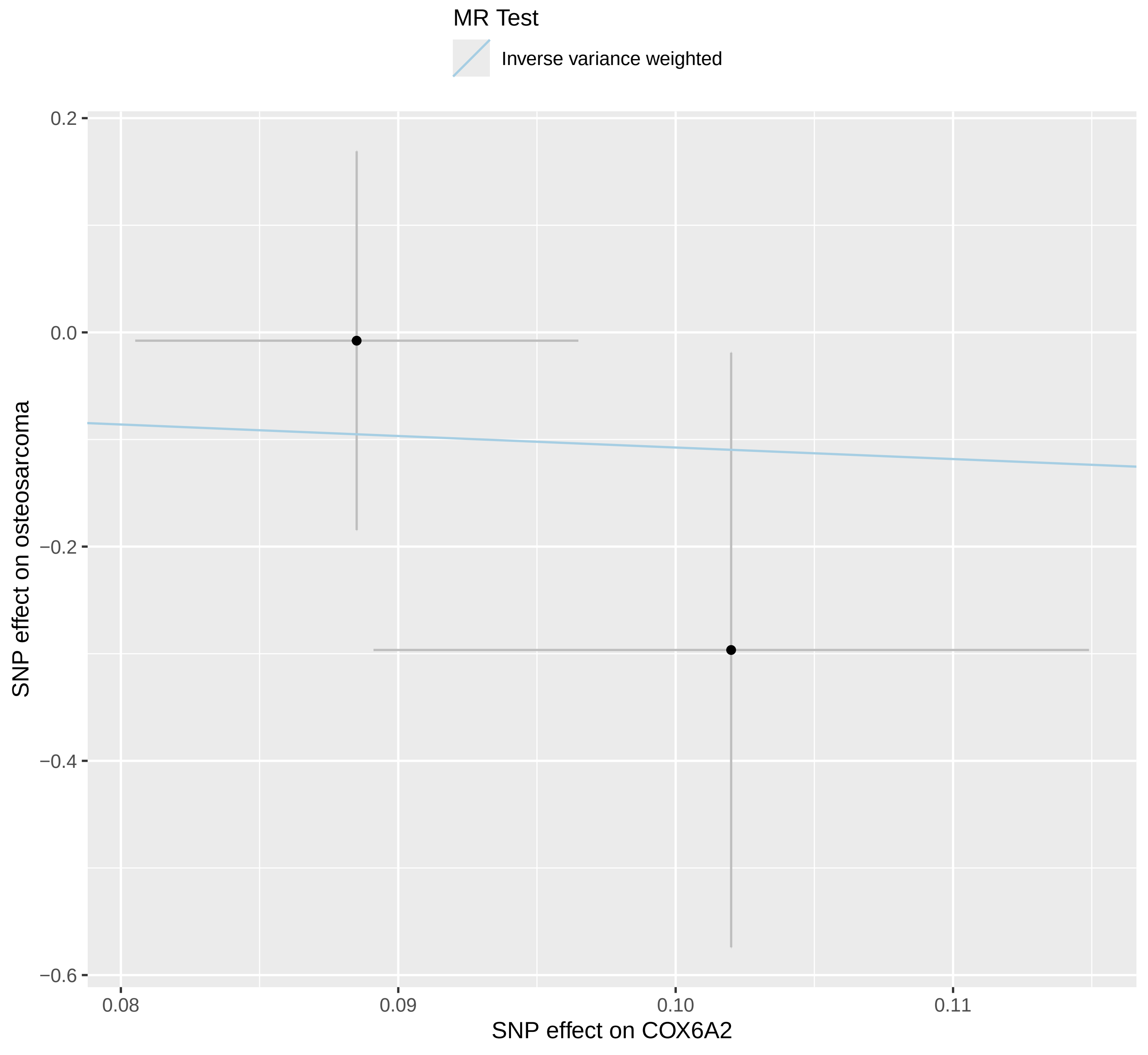
MR Test
Inverse variance weighted
0.2
0.0
−0.2
−0.4
−0.6
0.08
0.09
0.10
0.11
SNP effect on COX6A2
SNP effect on osteosarcoma

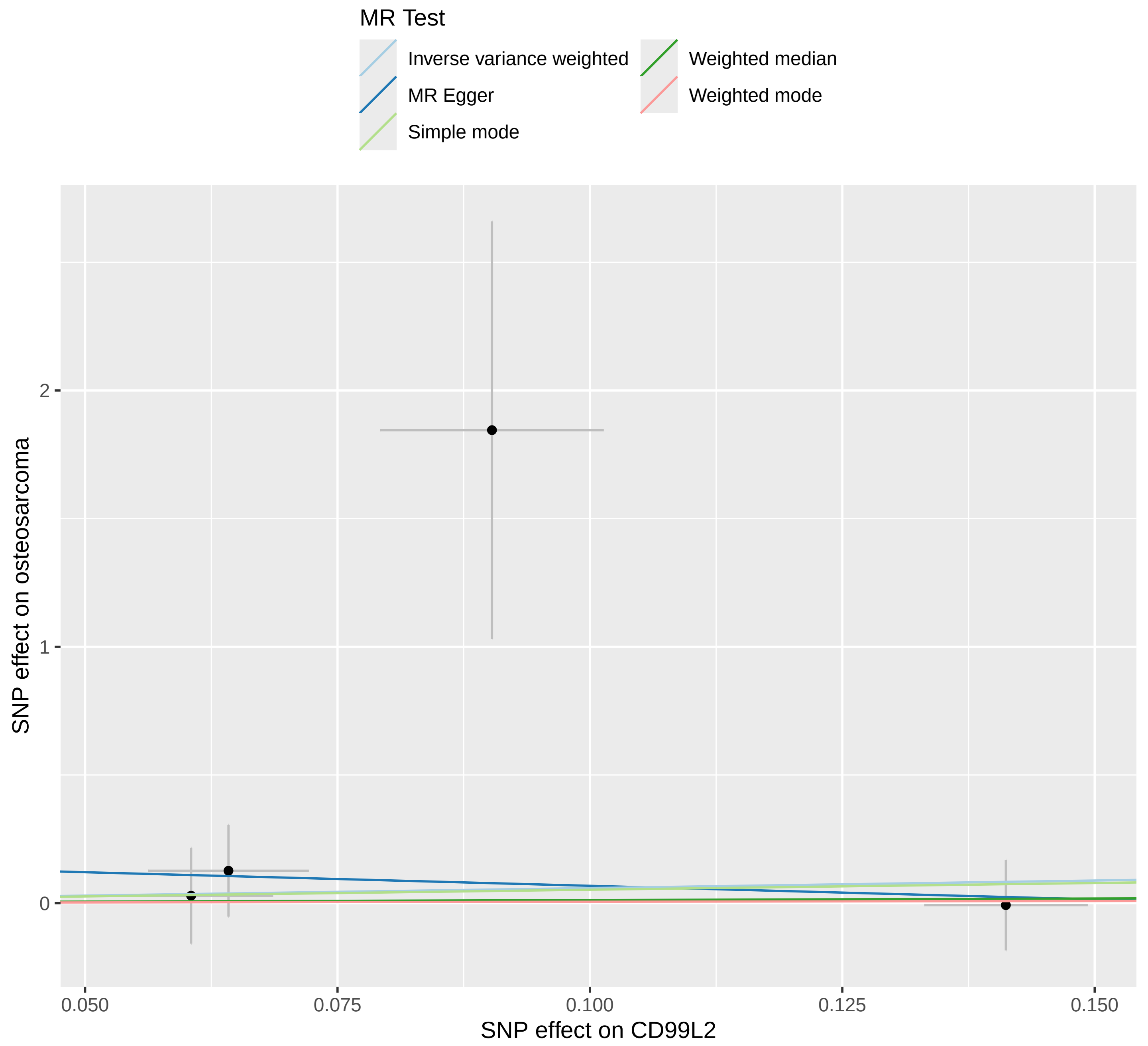

MR Test
Inverse variance weighted
MR Egger
Simple mode
Weighted median
Weighted mode
SNP effect on osteosarcoma
2
1
0
0.050
0.075
0.100
0.125
0.150
SNP effect on CD99L2

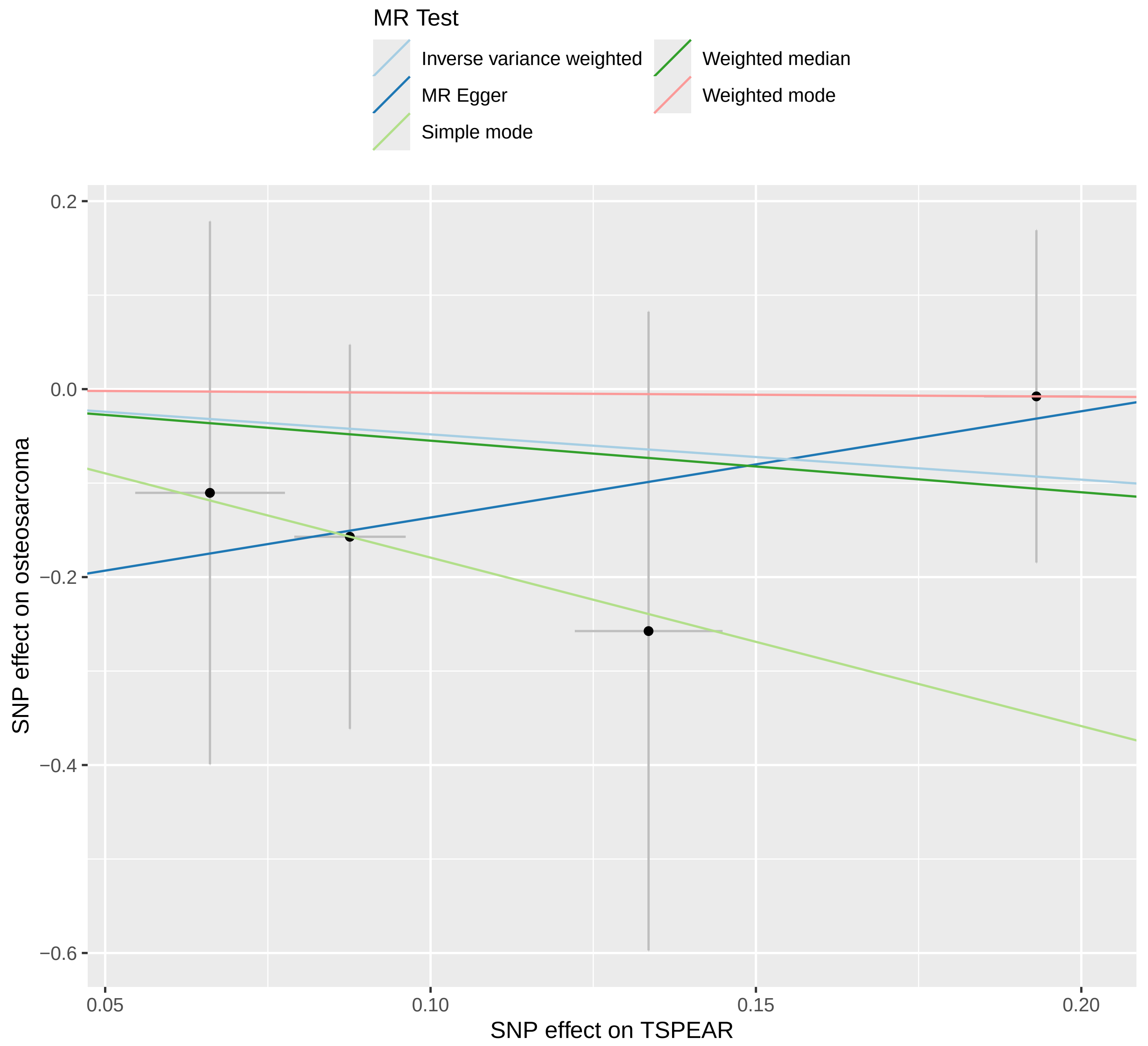
MR Test
Inverse variance weighted
MR Egger
Simple mode
Weighted median
Weighted mode
0.2
0.0
−0.2
−0.4
−0.6
SNP effect on osteosarcoma
0.05
0.10
0.15
0.20
SNP effect on TSPEAR

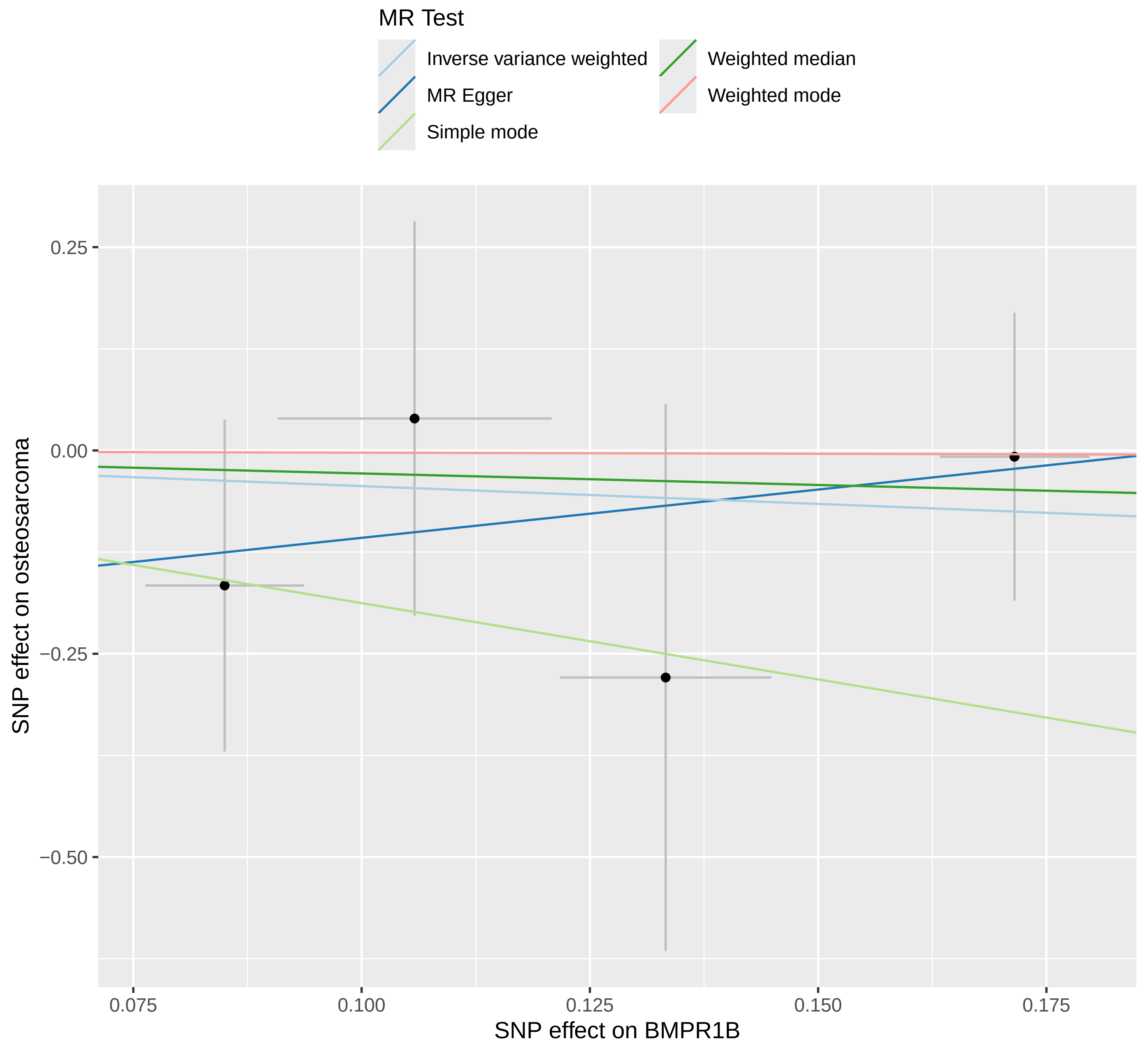
MR Test
Inverse variance weighted
MR Egger
Simple mode
Weighted median
Weighted mode
0.25
0.00
−0.25
−0.50
0.075
0.100
0.125
0.150
0.175
SNP effect on BMPR1B
SNP effect on osteosarcoma

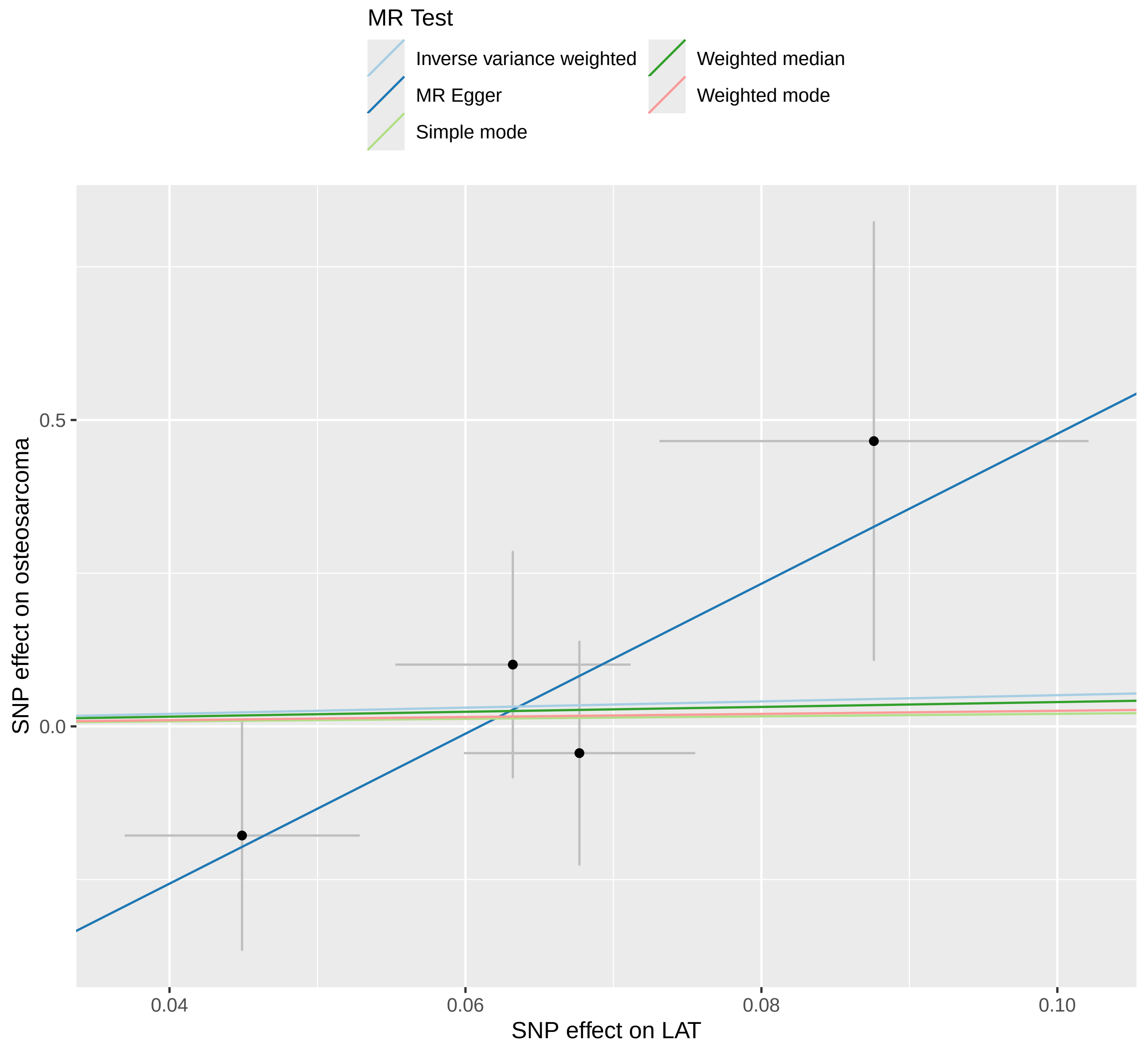
MR Test
Inverse variance weighted
MR Egger
Simple mode
Weighted median
Weighted mode
0.5
0.0
SNP effect on osteosarcoma
0.04
0.06
0.08
0.10
SNP effect on LAT

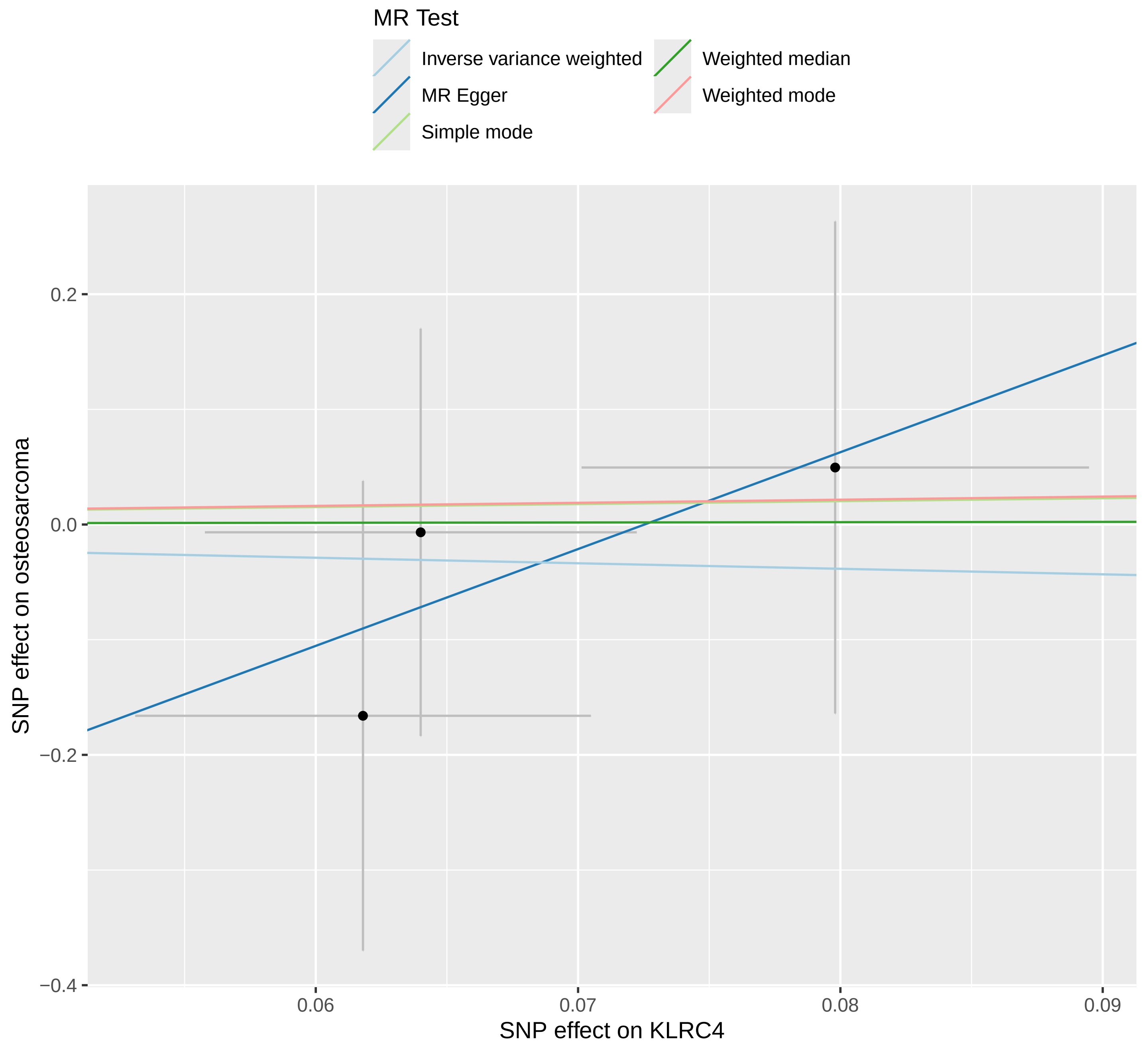
MR Test
Inverse variance weighted
MR Egger
Simple mode
Weighted median
Weighted mode
0.2
0.0
−0.2
−0.4
SNP effect on osteosarcoma
0.06
0.07
0.08
0.09
SNP effect on KLRC4

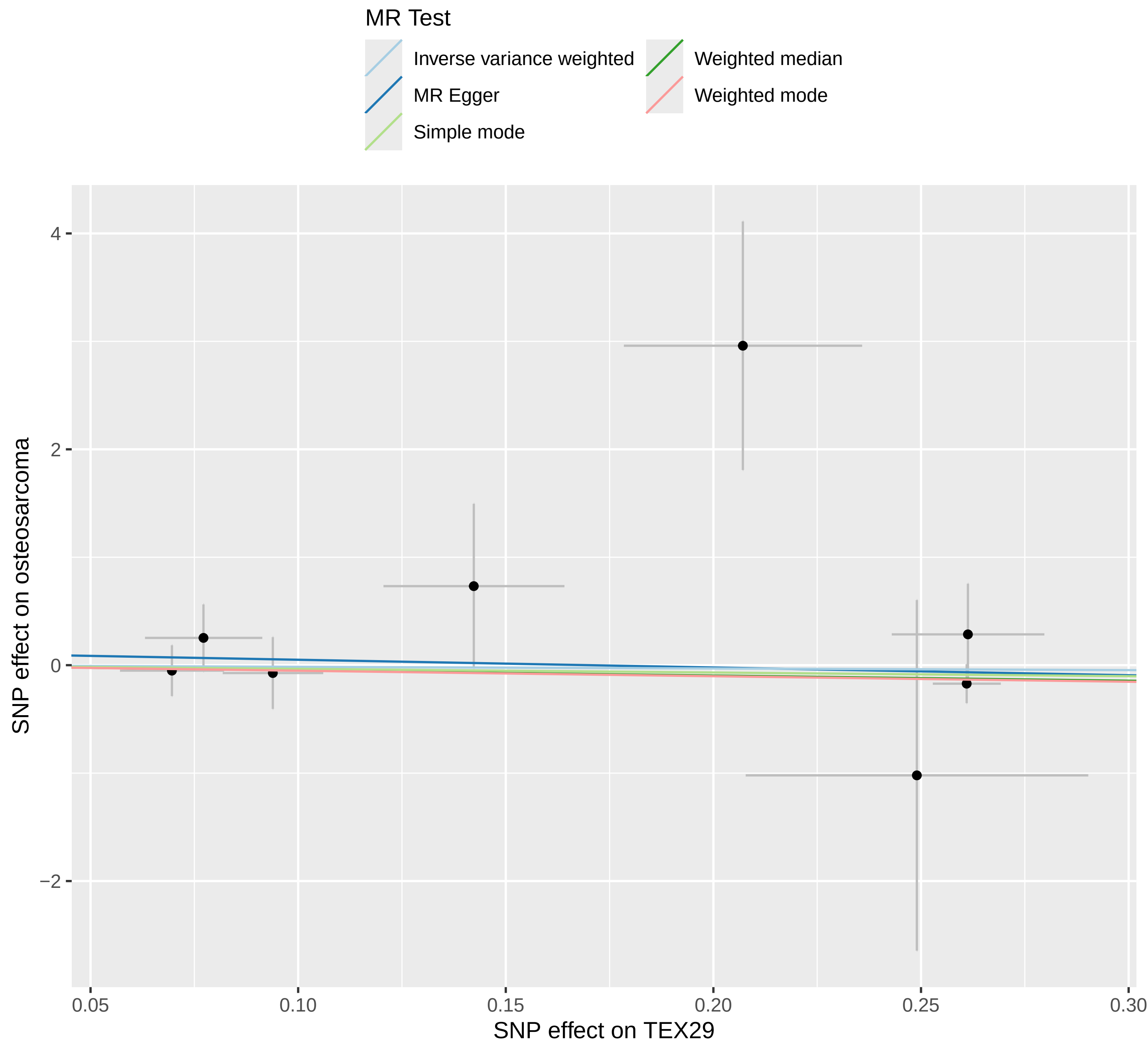
MR Test
Inverse variance weighted
MR Egger
Simple mode
Weighted median
Weighted mode
4
2
0
−2
SNP effect on osteosarcoma
0.05
0.10
0.15
0.20
0.25
0.30
SNP effect on TEX29

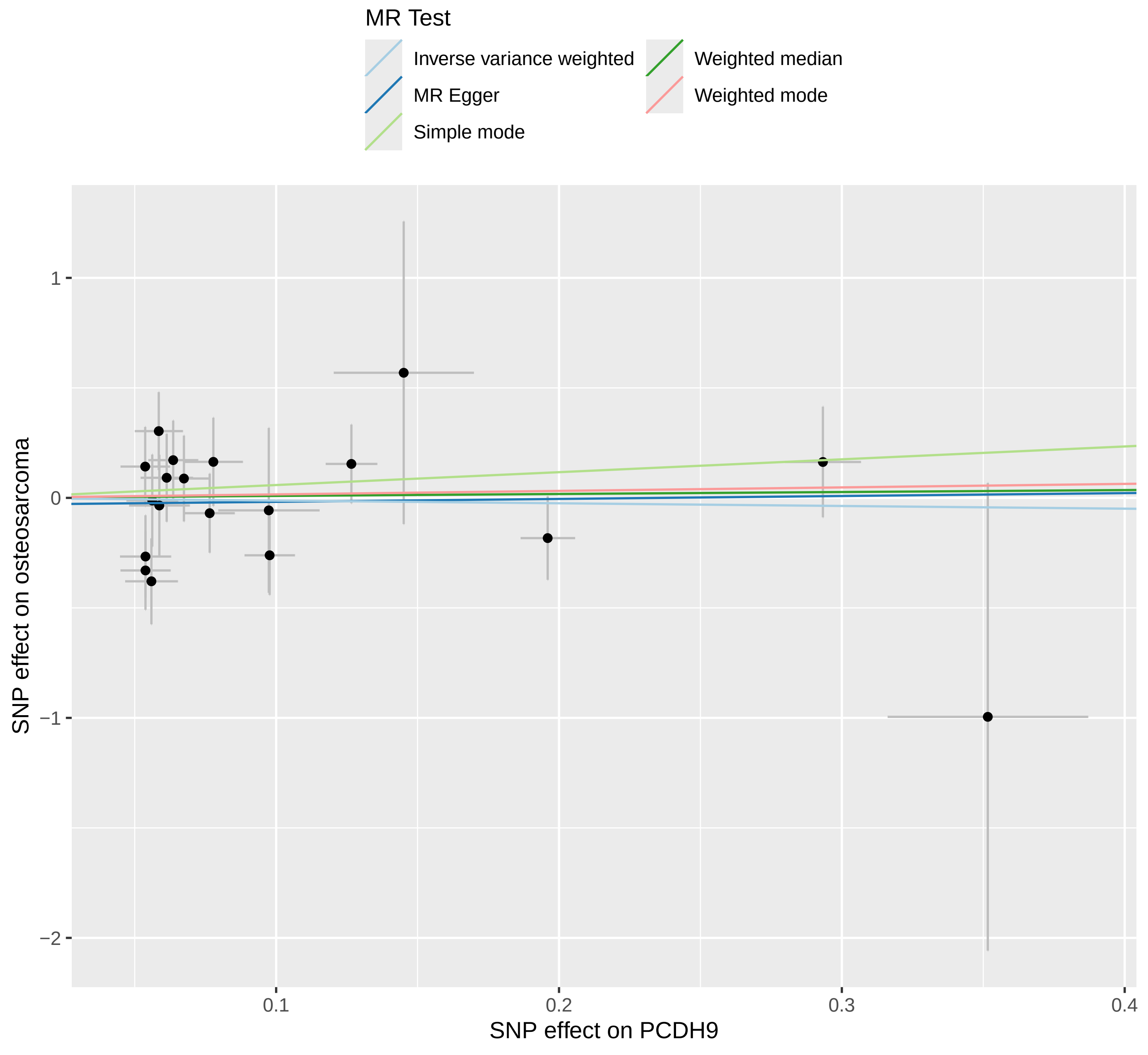

MR Test
Inverse variance weighted
MR Egger
Simple mode
Weighted median
Weighted mode
1
0
−1
−2
0.1
0.2
0.3
0.4
SNP effect on osteosarcoma
SNP effect on PCDH9

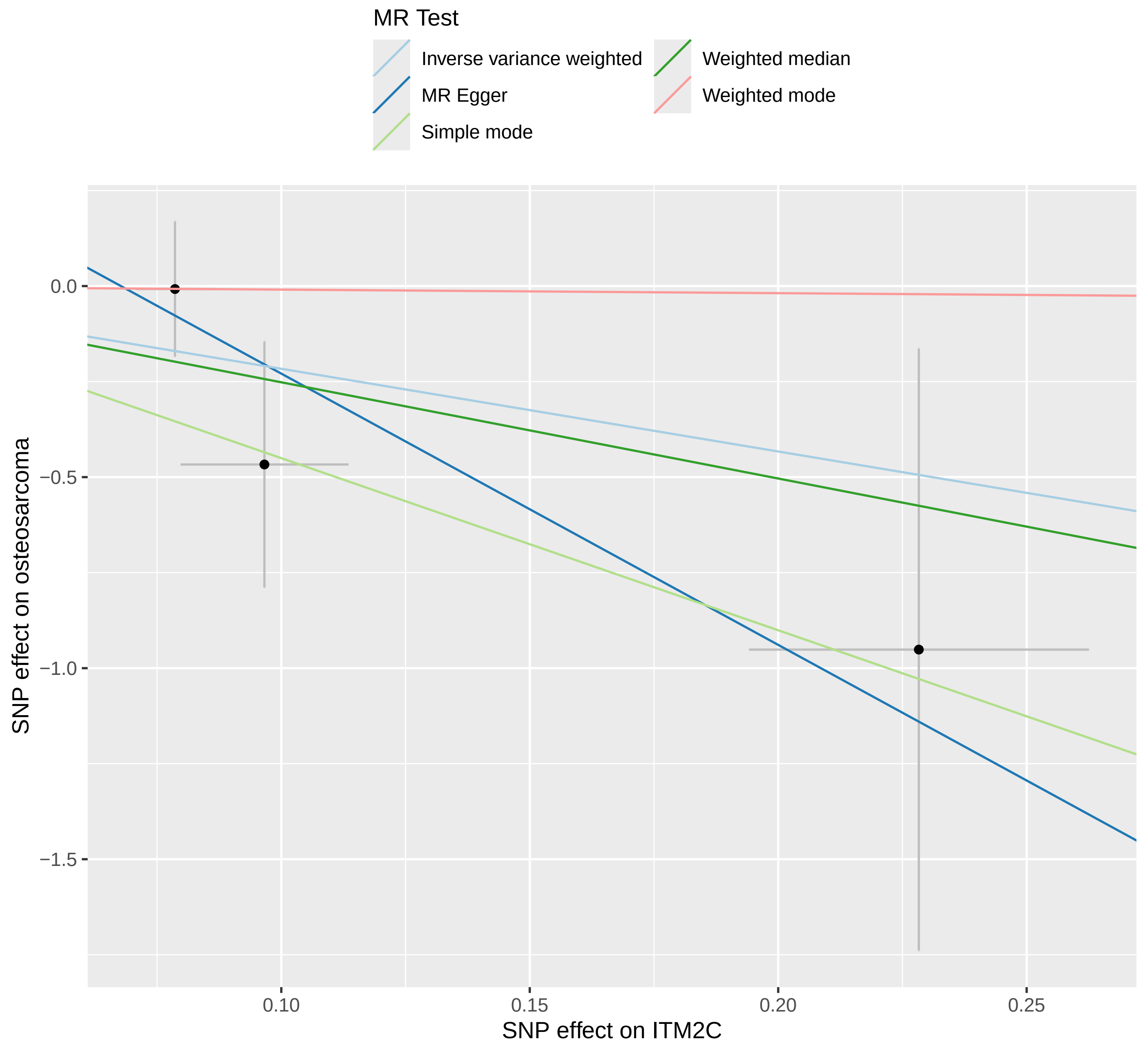

MR Test
Inverse variance weighted
MR Egger
Simple mode
Weighted median
Weighted mode
0.0
−0.5
−1.0
−1.5
SNP effect on osteosarcoma
0.10
0.15
0.20
0.25
SNP effect on ITM2C

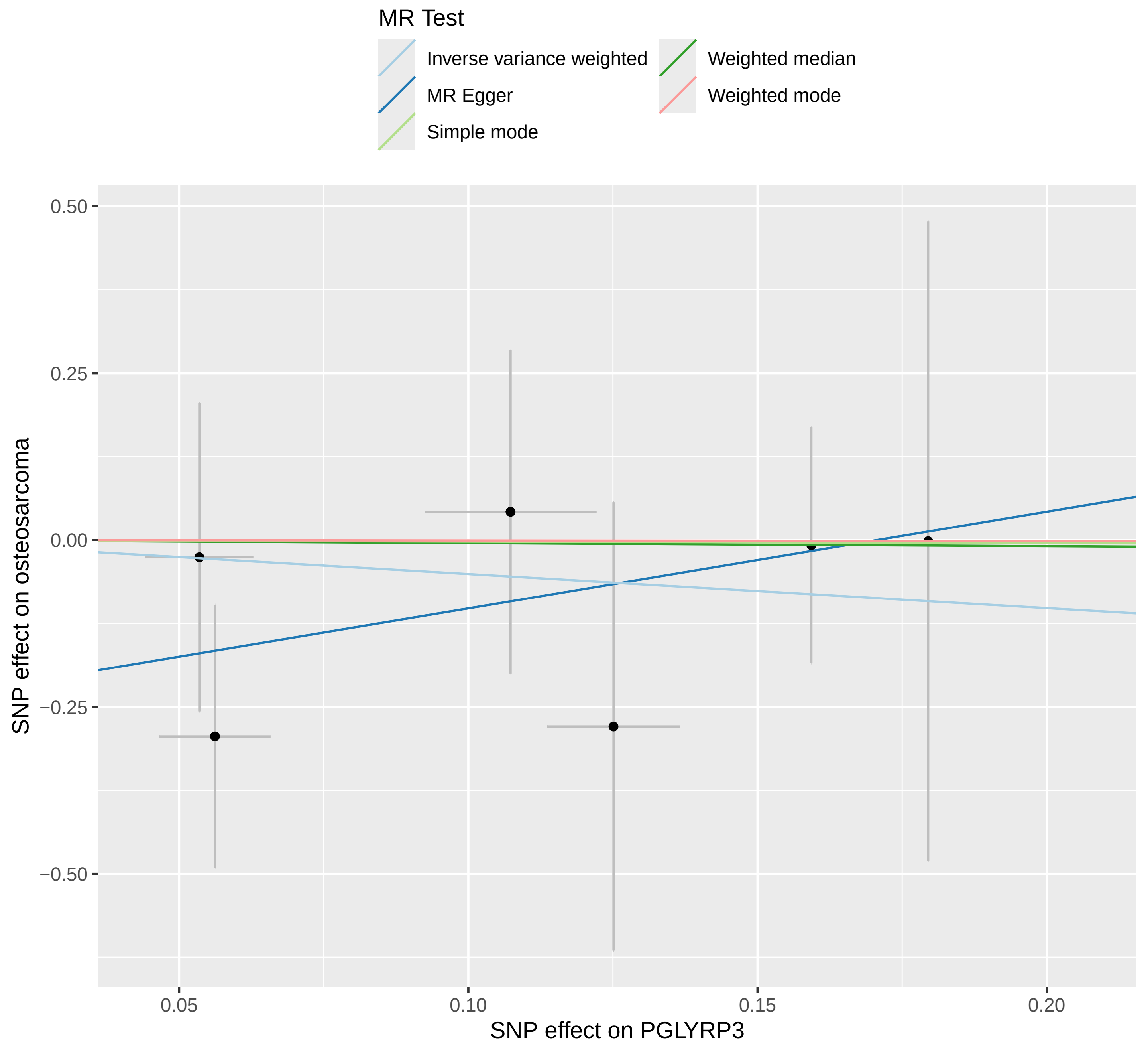
MR Test
Inverse variance weighted
MR Egger
Simple mode
Weighted median
Weighted mode
0.50
0.25
0.00
−0.25
−0.50
SNP effect on osteosarcoma
0.05
0.10
0.15
0.20
SNP effect on PGLYRP3

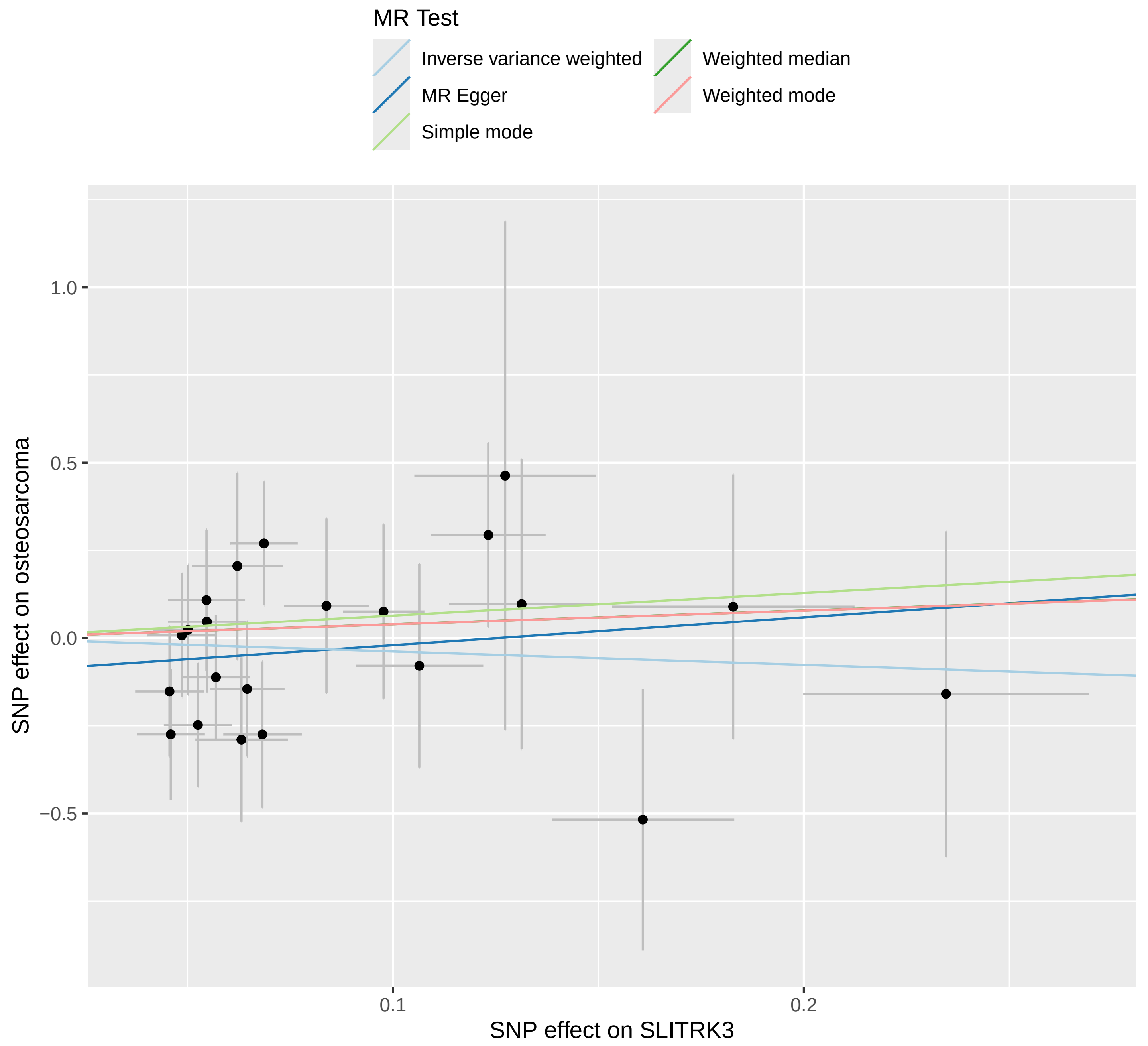
MR Test
Inverse variance weighted
MR Egger
Simple mode
Weighted median
Weighted mode
1.0
0.5
0.0
−0.5
0.1
0.2
SNP effect on osteosarcoma
SNP effect on SLITRK3

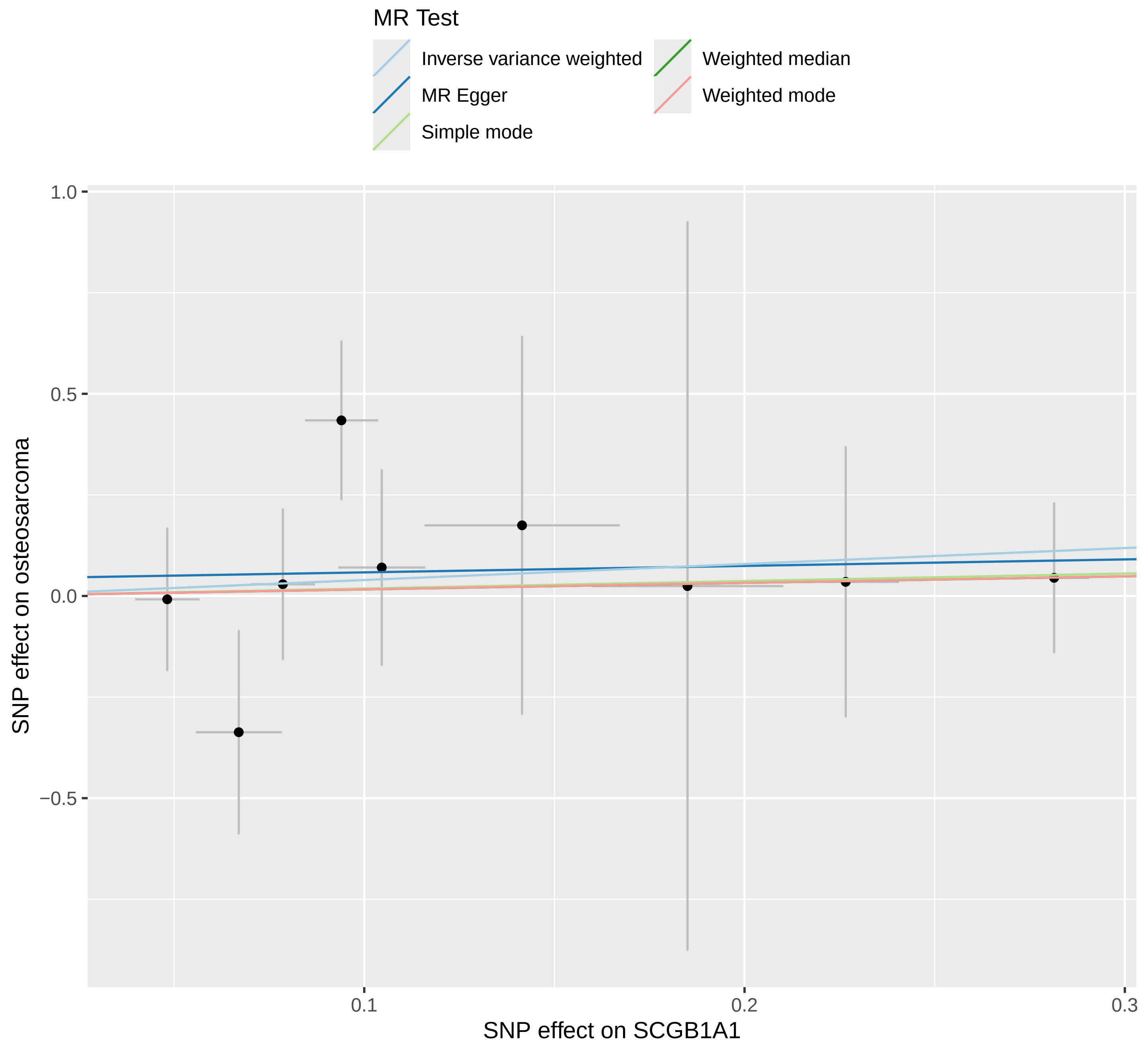

MR Test
Inverse variance weighted
MR Egger
Simple mode
Weighted median
Weighted mode
1.0
0.5
0.0
−0.5
SNP effect on osteosarcoma
0.1
0.2
0.3
SNP effect on SCGB1A1

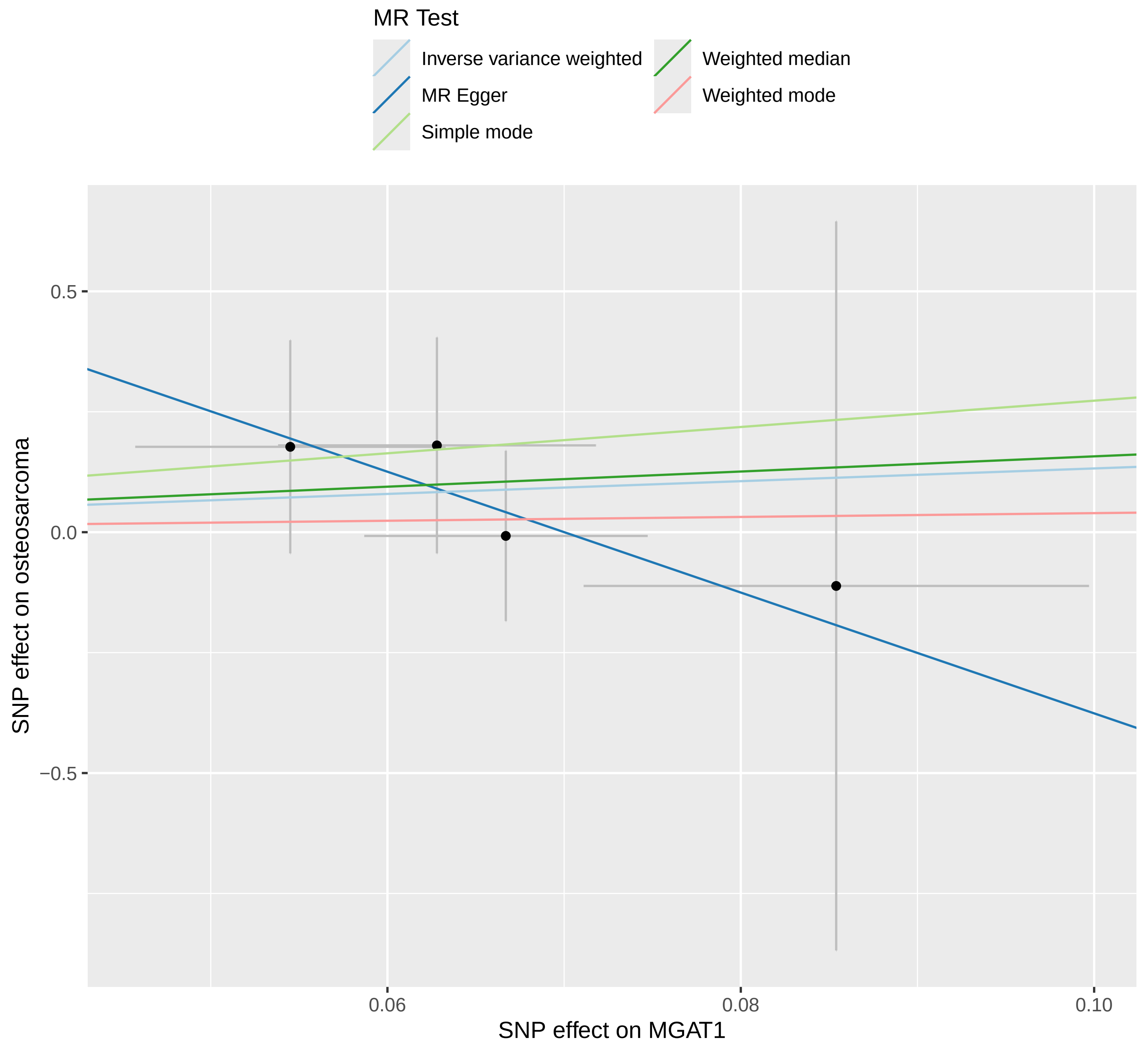

MR Test
Inverse variance weighted
MR Egger
Simple mode
Weighted median
Weighted mode
0.5
0.0
−0.5
SNP effect on osteosarcoma
0.06
0.08
0.10
SNP effect on MGAT1

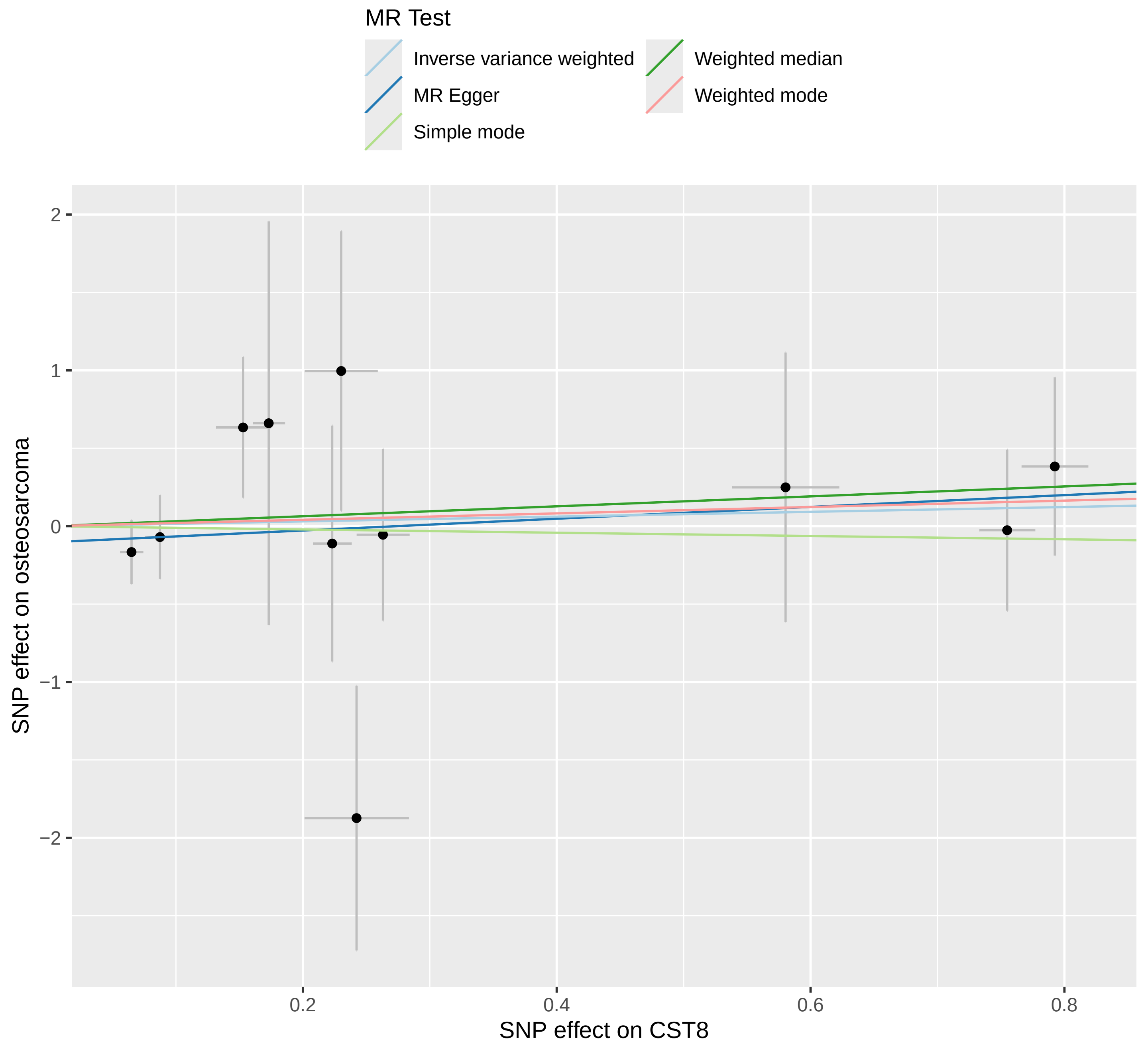
MR Test
Inverse variance weighted
MR Egger
Simple mode
Weighted median
Weighted mode
2
1
0
−1
−2
SNP effect on osteosarcoma
0.2
0.4
0.6
0.8
SNP effect on CST8

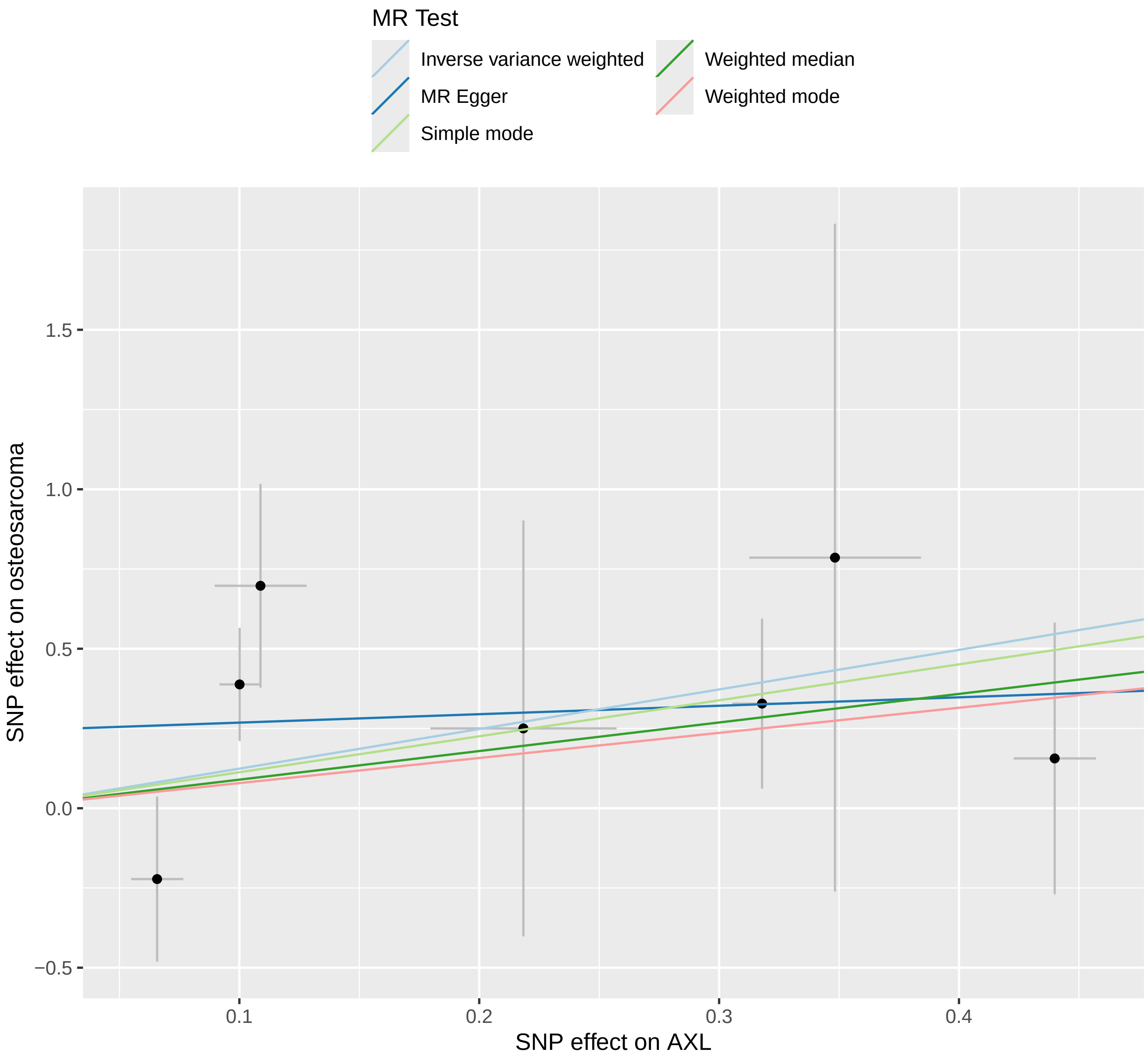

MR Test
Inverse variance weighted
MR Egger
Simple mode
Weighted median
Weighted mode
1.5
1.0
0.5
0.0
−0.5
SNP effect on osteosarcoma
0.1
0.2
0.3
0.4
SNP effect on AXL

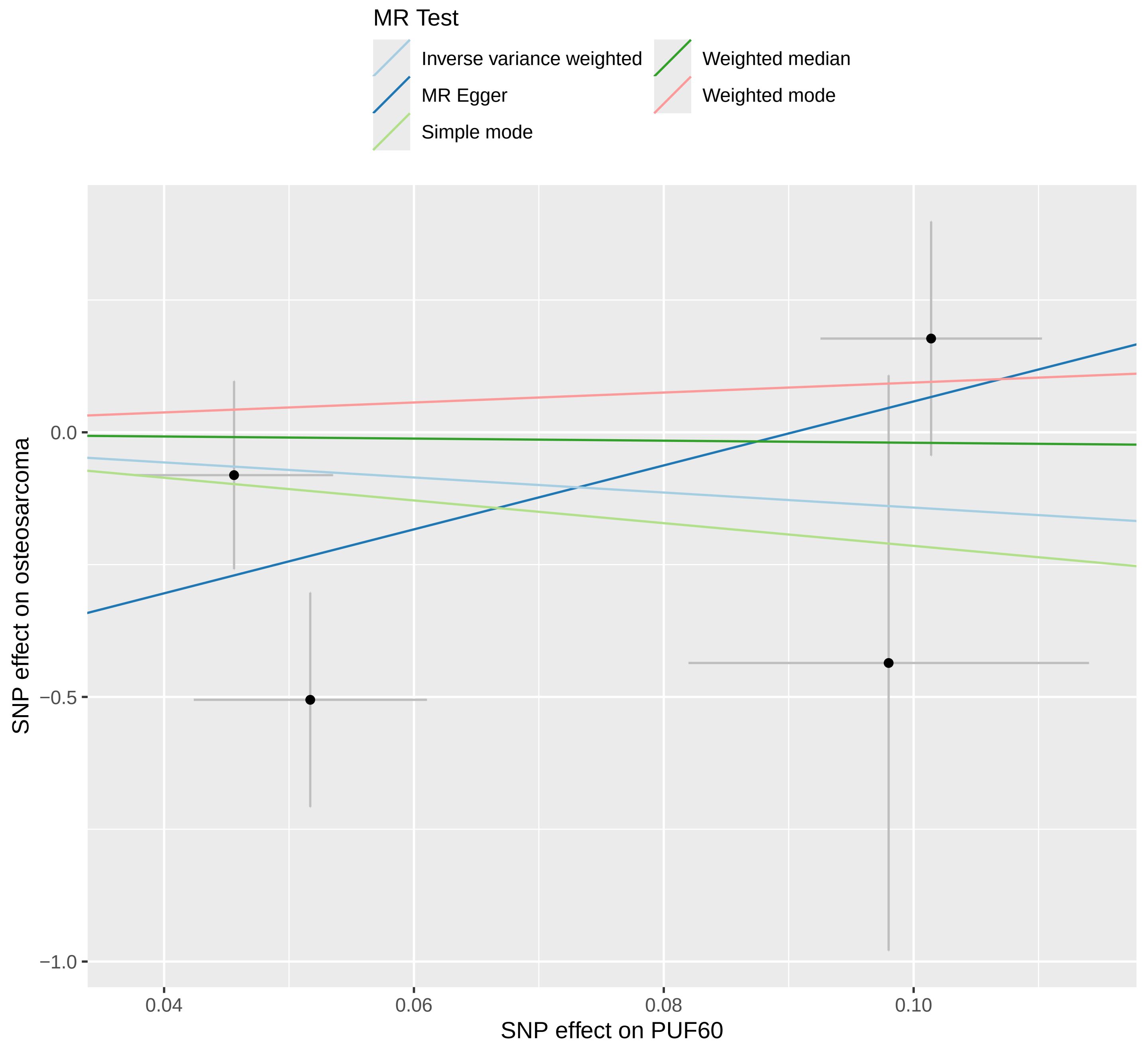

MR Test
Inverse variance weighted
MR Egger
Simple mode
Weighted median
Weighted mode
0.0
−0.5
−1.0
0.04
0.06
0.08
0.10
SNP effect on osteosarcoma
SNP effect on PUF60

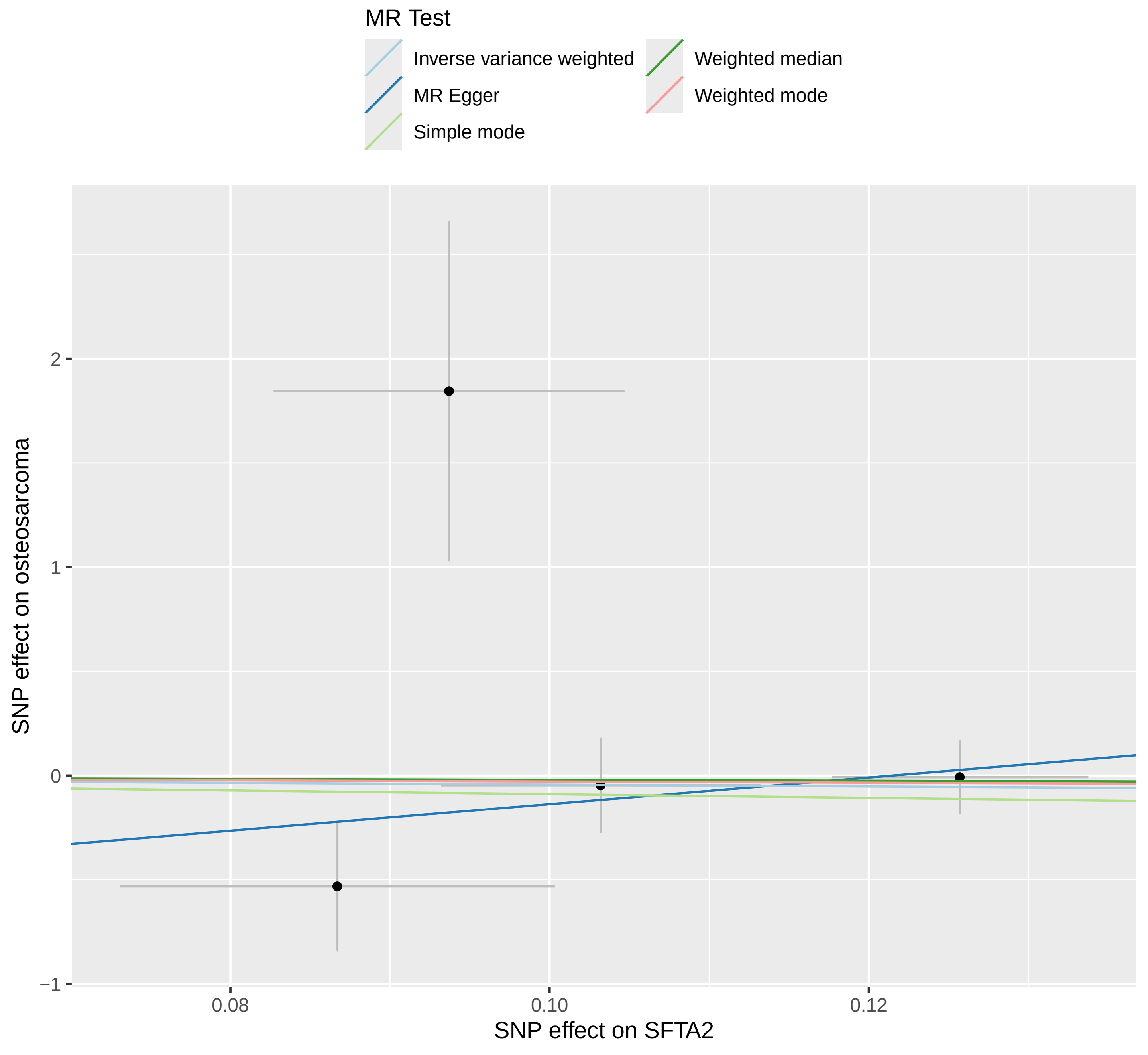
MR Test
Inverse variance weighted
MR Egger
Simple mode
Weighted median
Weighted mode
2
1
0
−1
0.08
0.10
0.12
SNP effect on osteosarcoma
SNP effect on SFTA2

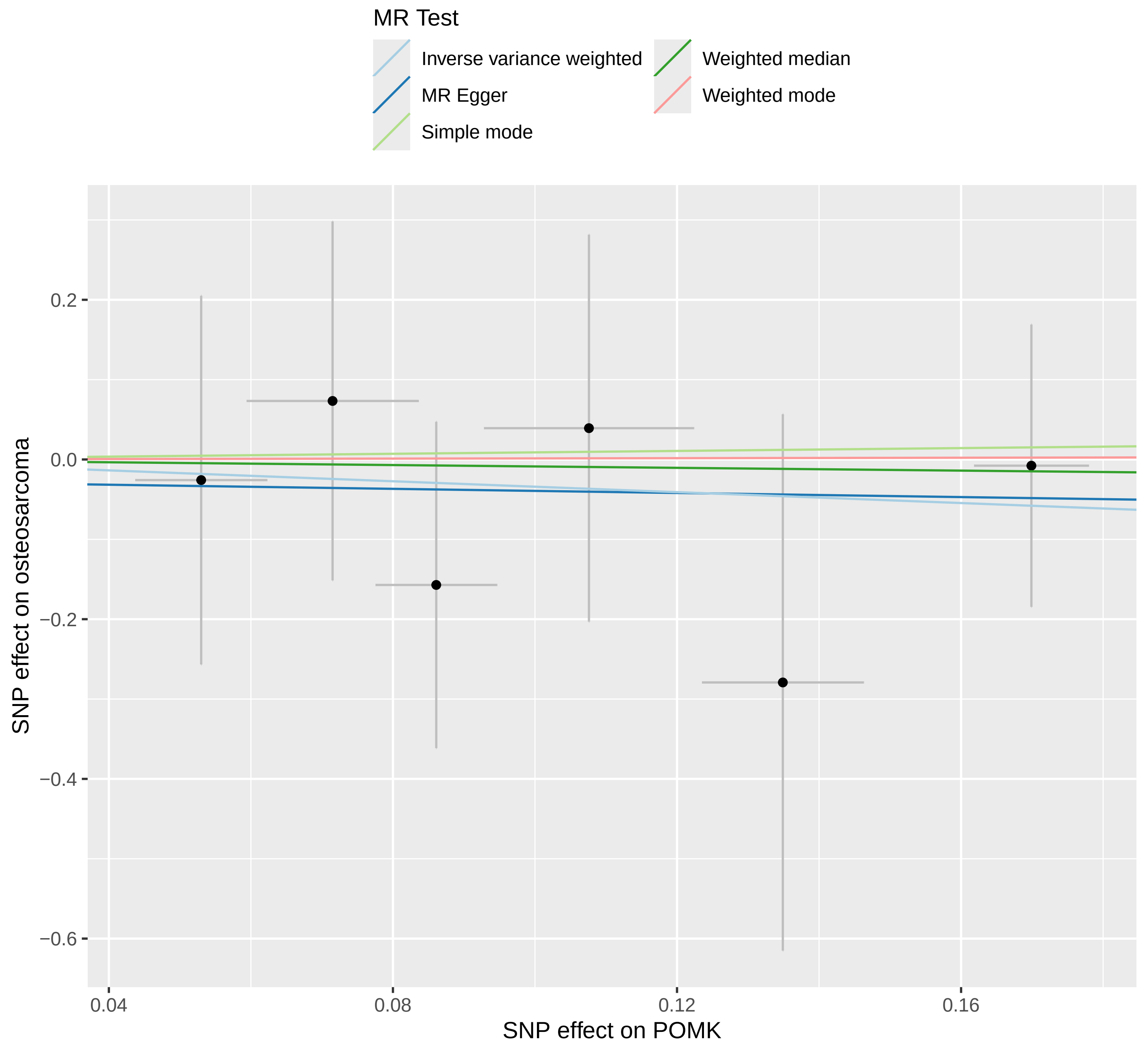

MR Test
Inverse variance weighted
MR Egger
Simple mode
Weighted median
Weighted mode
0.2
0.0
−0.2
−0.4
−0.6
SNP effect on osteosarcoma
0.04
0.08
0.12
0.16
SNP effect on POMK

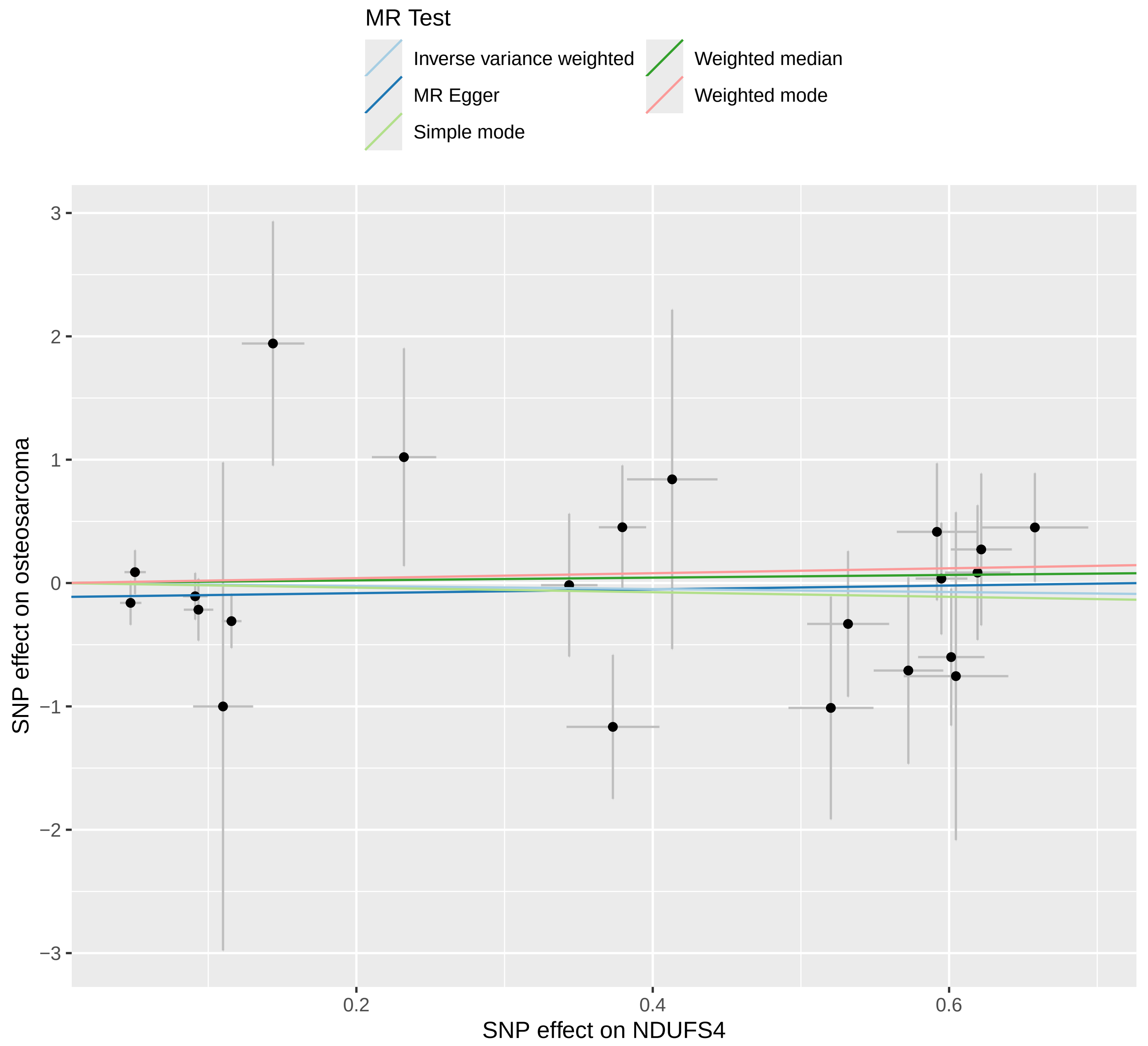
MR Test
Inverse variance weighted
MR Egger
Simple mode
Weighted median
Weighted mode
3
2
1
0
−1
−2
−3
0.2
0.4
0.6
SNP effect on osteosarcoma
SNP effect on NDUFS4

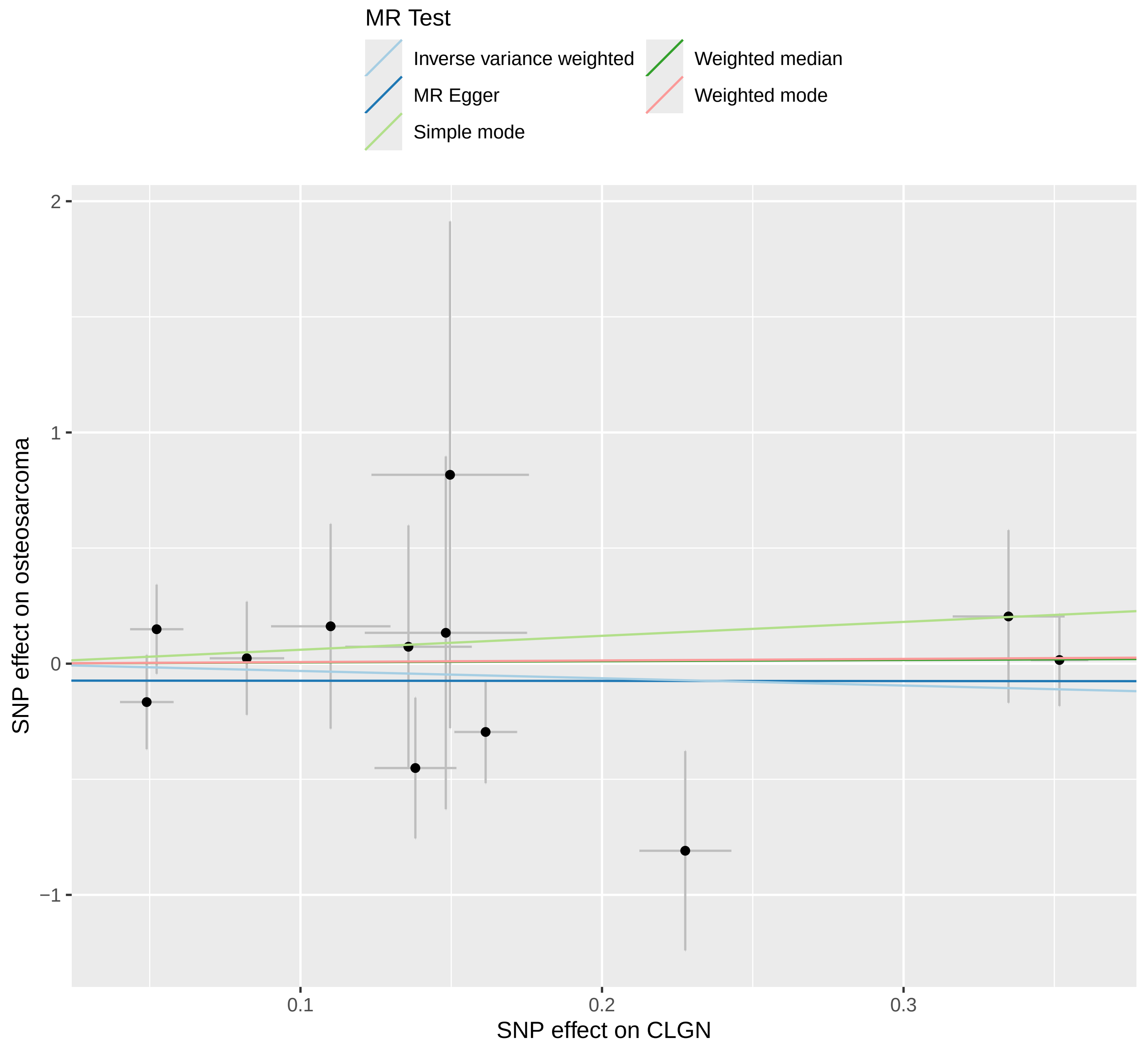

MR Test
Inverse variance weighted
MR Egger
Simple mode
Weighted median
Weighted mode
2
1
0
−1
0.1
0.2
0.3
SNP effect on osteosarcoma
SNP effect on CLGN

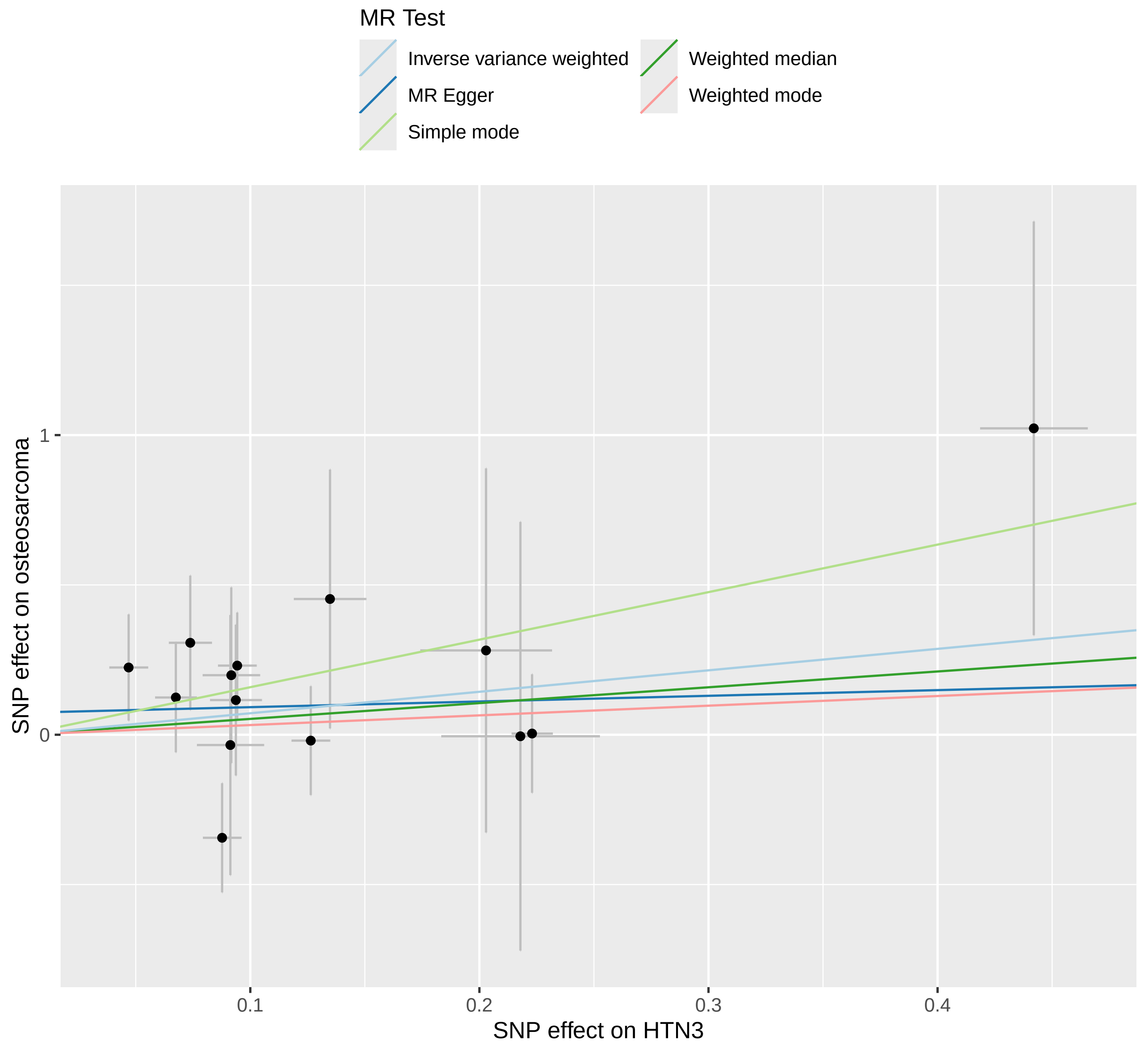
MR Test
Inverse variance weighted
MR Egger
Simple mode
Weighted median
Weighted mode
SNP effect on osteosarcoma
1
0
0.1
0.2
0.3
0.4
SNP effect on HTN3

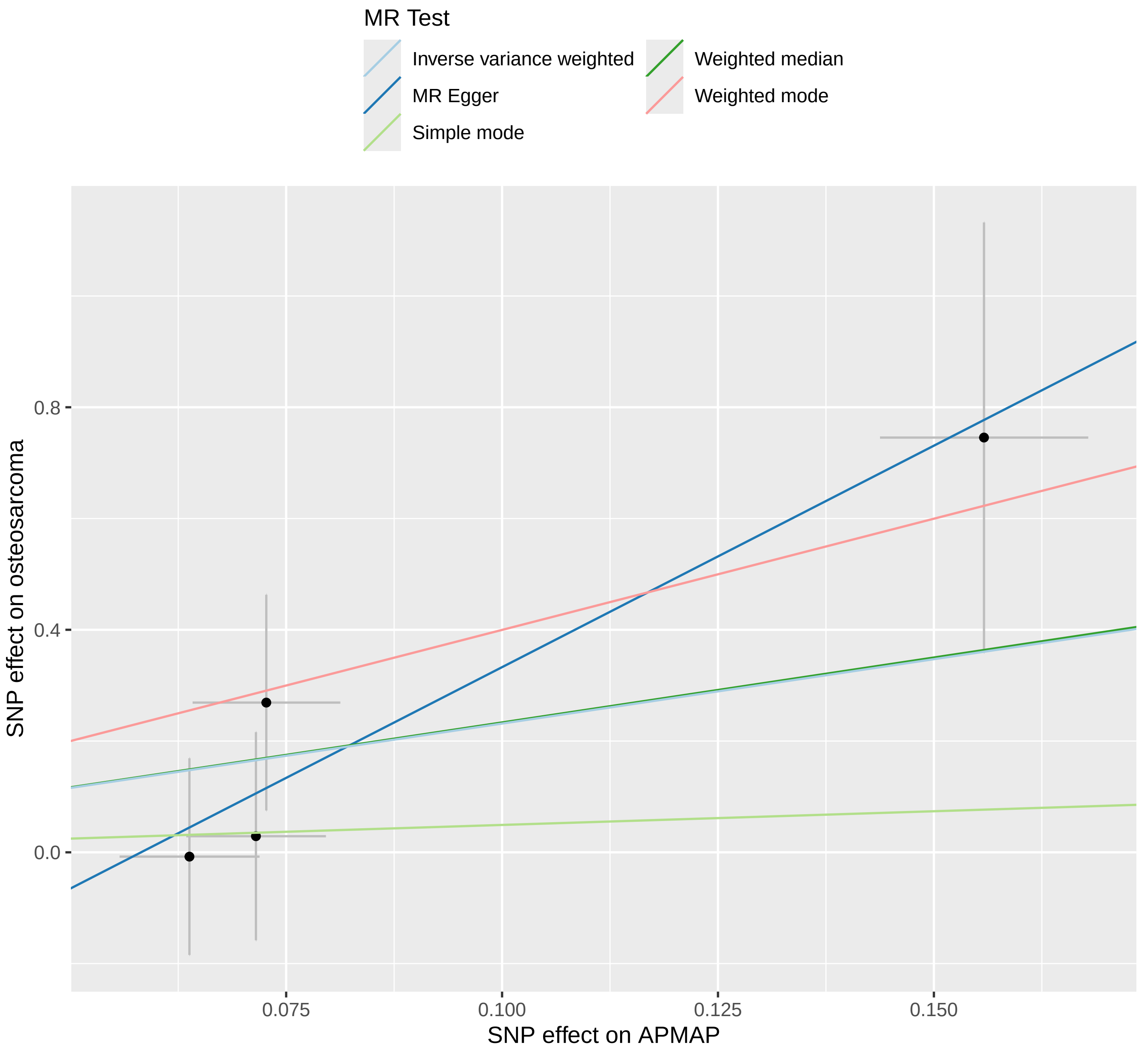
MR Test
Inverse variance weighted
MR Egger
Simple mode
Weighted median
Weighted mode
SNP effect on osteosarcoma
0.8
0.4
0.0
0.075
0.100
0.125
0.150
SNP effect on APMAP

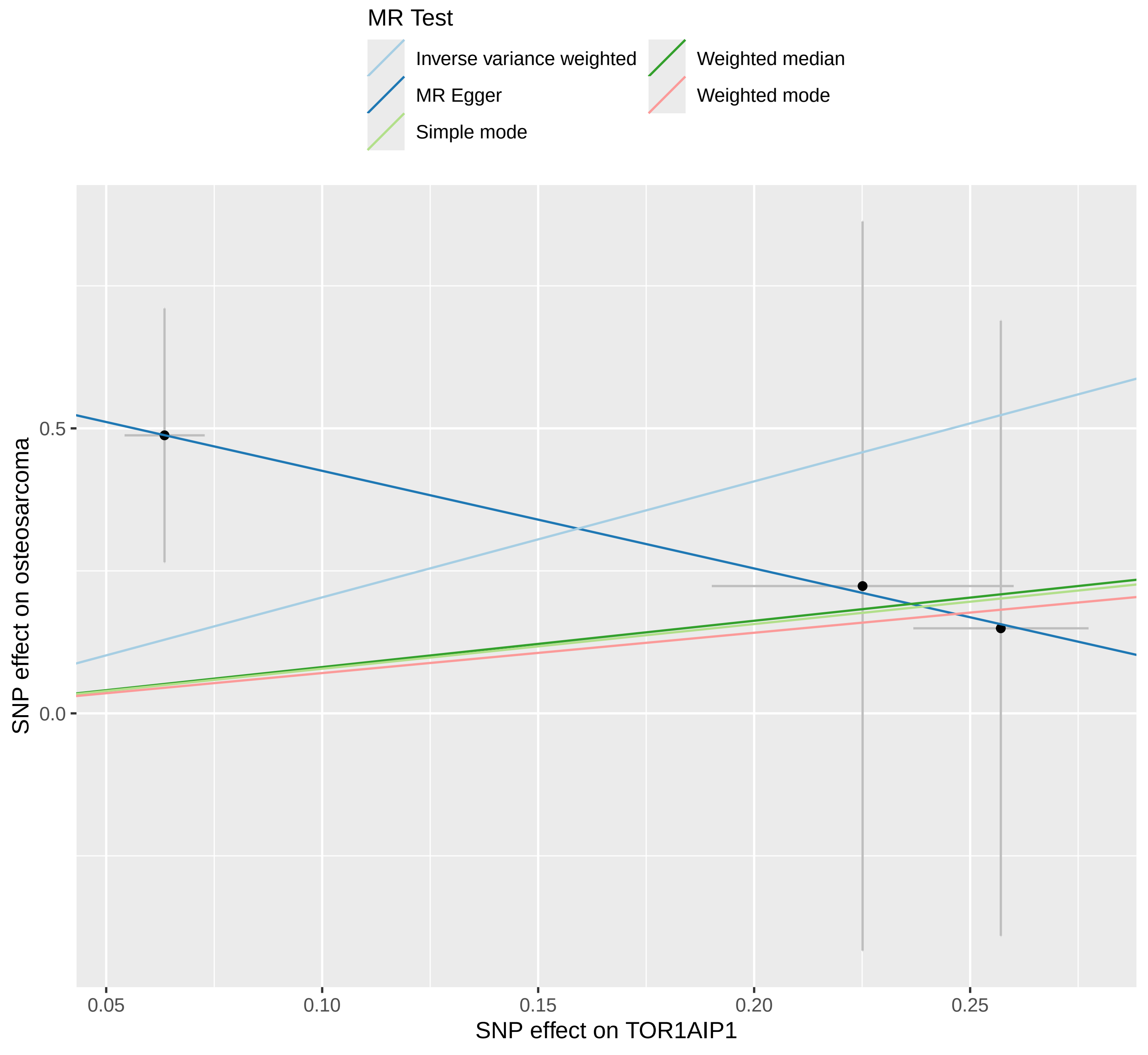
MR Test
Inverse variance weighted
MR Egger
Simple mode
Weighted median
Weighted mode
0.5
0.0
SNP effect on osteosarcoma
0.05
0.10
0.15
0.20
0.25
SNP effect on TOR1AIP1

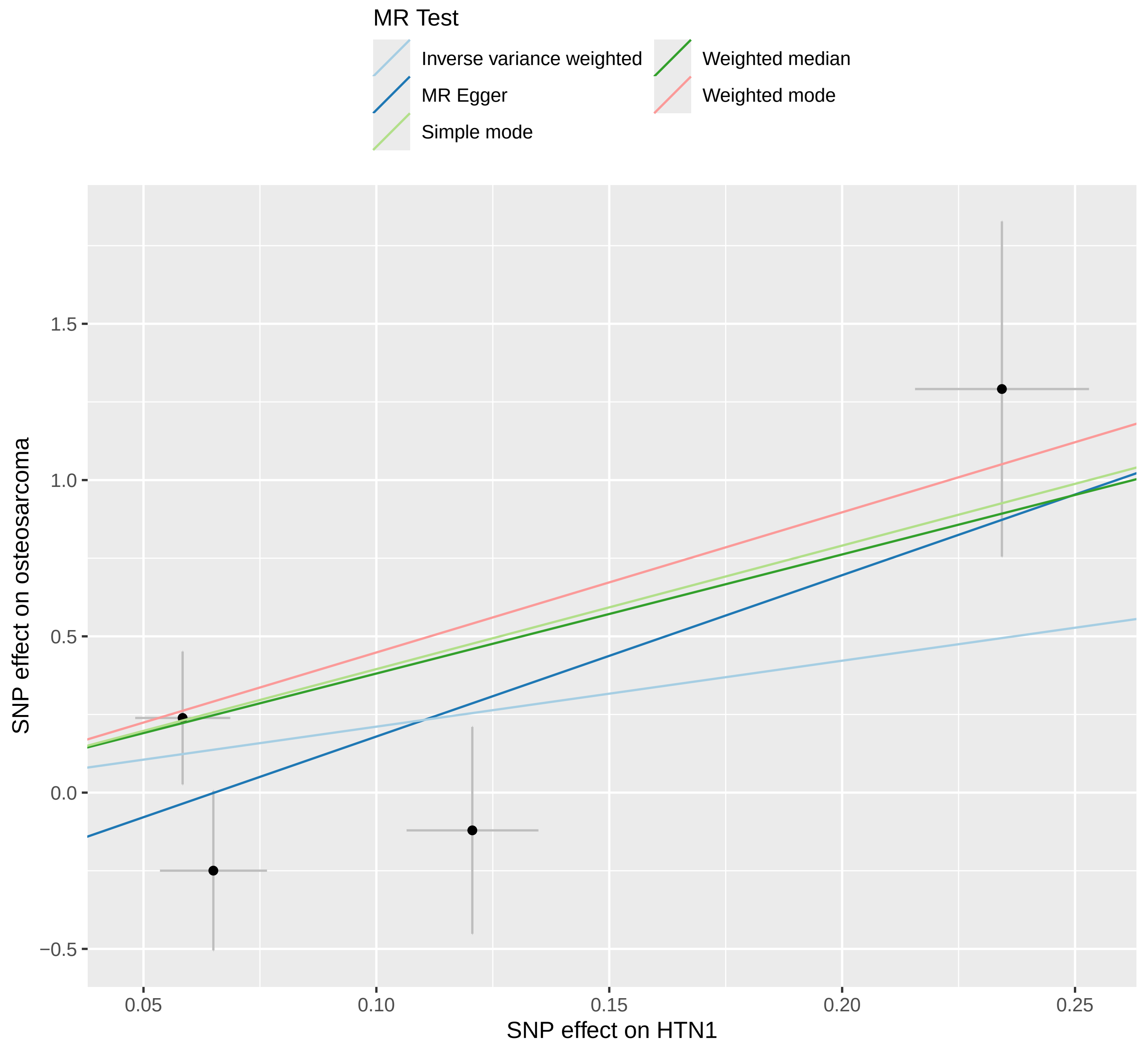
MR Test
Inverse variance weighted
MR Egger
Simple mode
Weighted median
Weighted mode
SNP effect on osteosarcoma
1.5
1.0
0.5
0.0
−0.5
0.05
0.10
0.15
0.20
0.25
SNP effect on HTN1

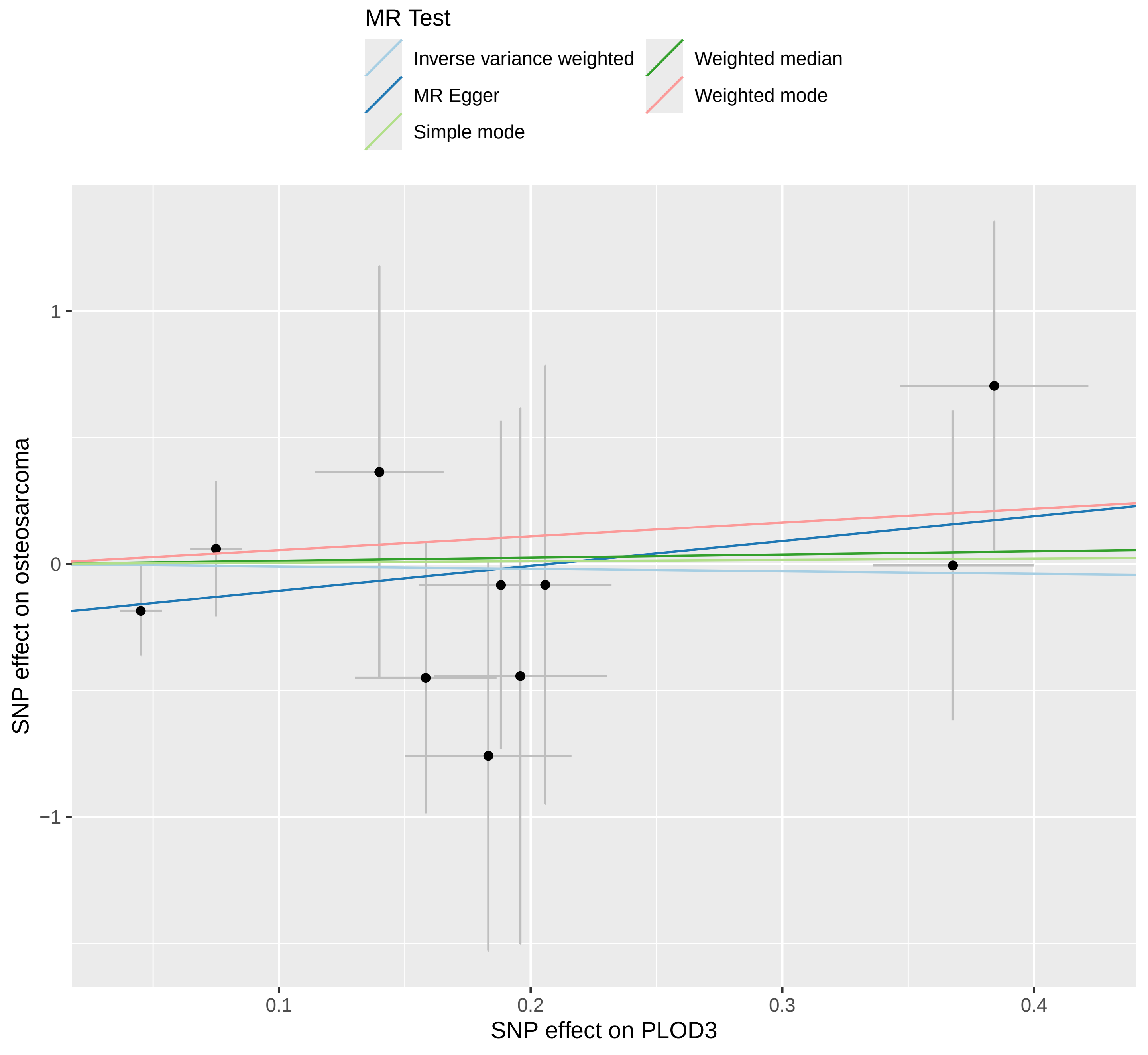
MR Test
Inverse variance weighted
MR Egger
Simple mode
Weighted median
Weighted mode
1
0
−1
0.1
0.2
0.3
0.4
SNP effect on PLOD3
SNP effect on osteosarcoma

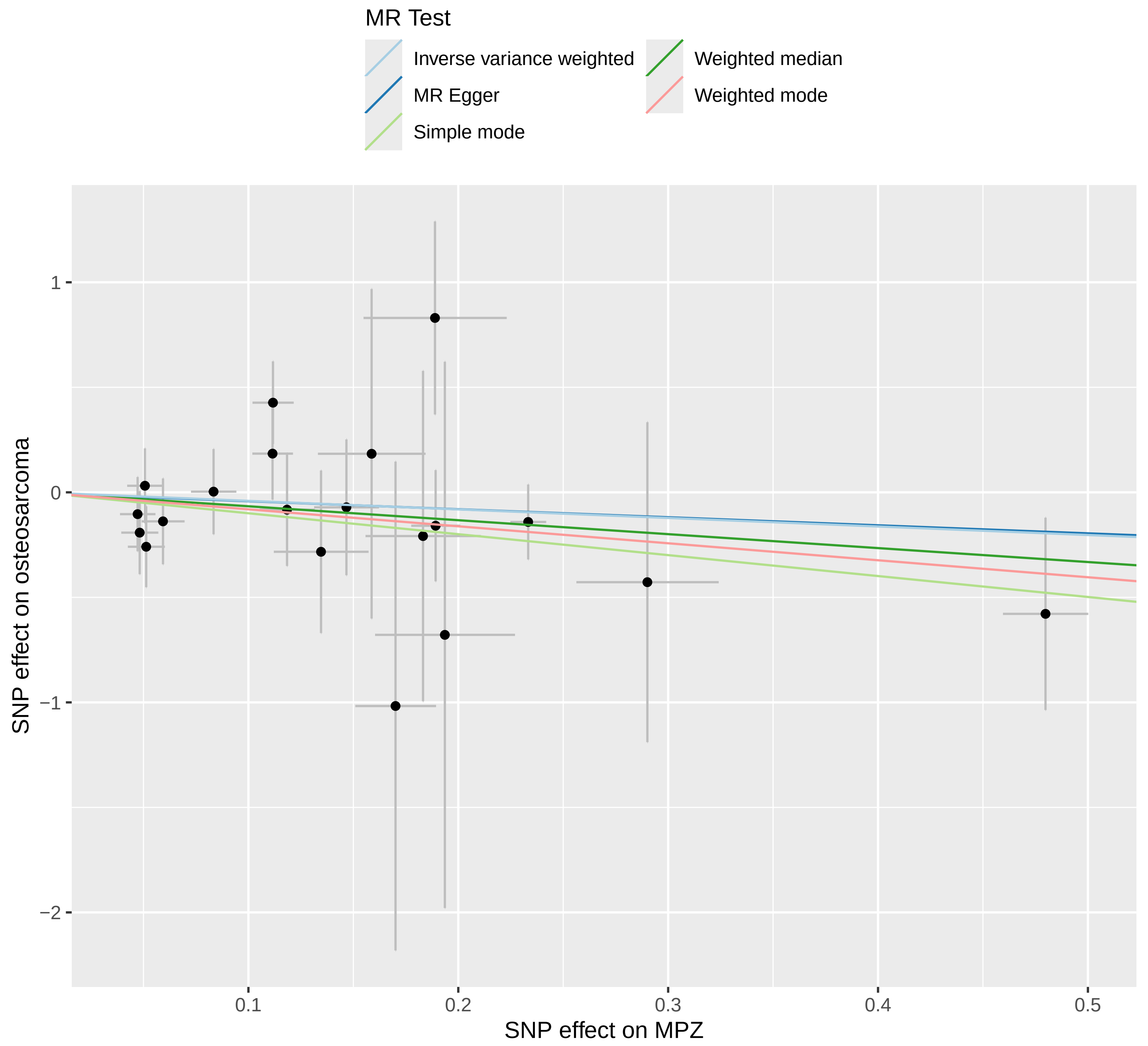
MR Test
Inverse variance weighted
MR Egger
Simple mode
Weighted median
Weighted mode
SNP effect on osteosarcoma
SNP effect on MPZ
1
0
−1
−2
0.1
0.2
0.3
0.4
0.5

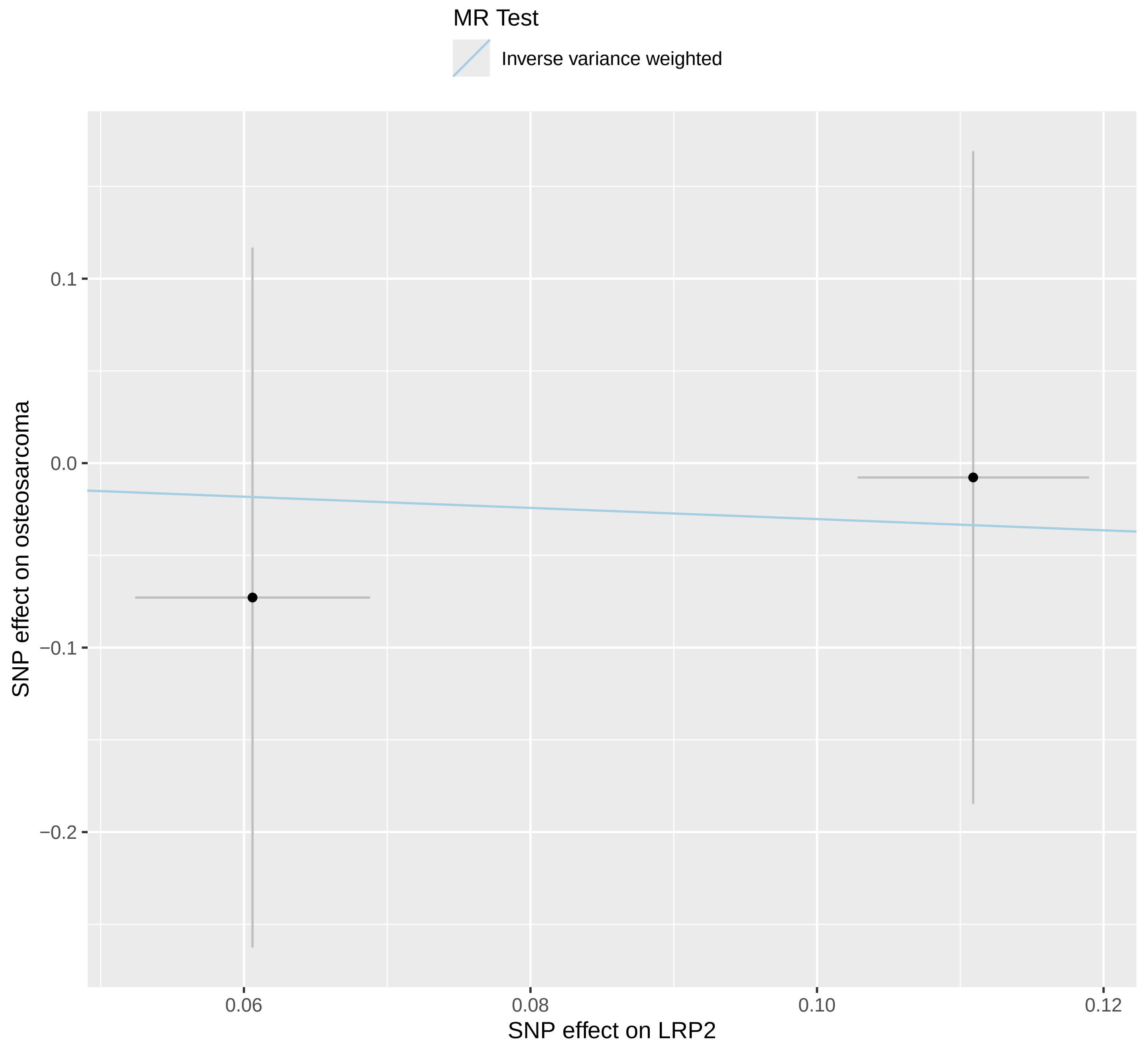
MR Test
Inverse variance weighted
0.1
0.0
−0.1
−0.2
0.06
0.08
0.10
0.12
SNP effect on LRP2
SNP effect on osteosarcoma

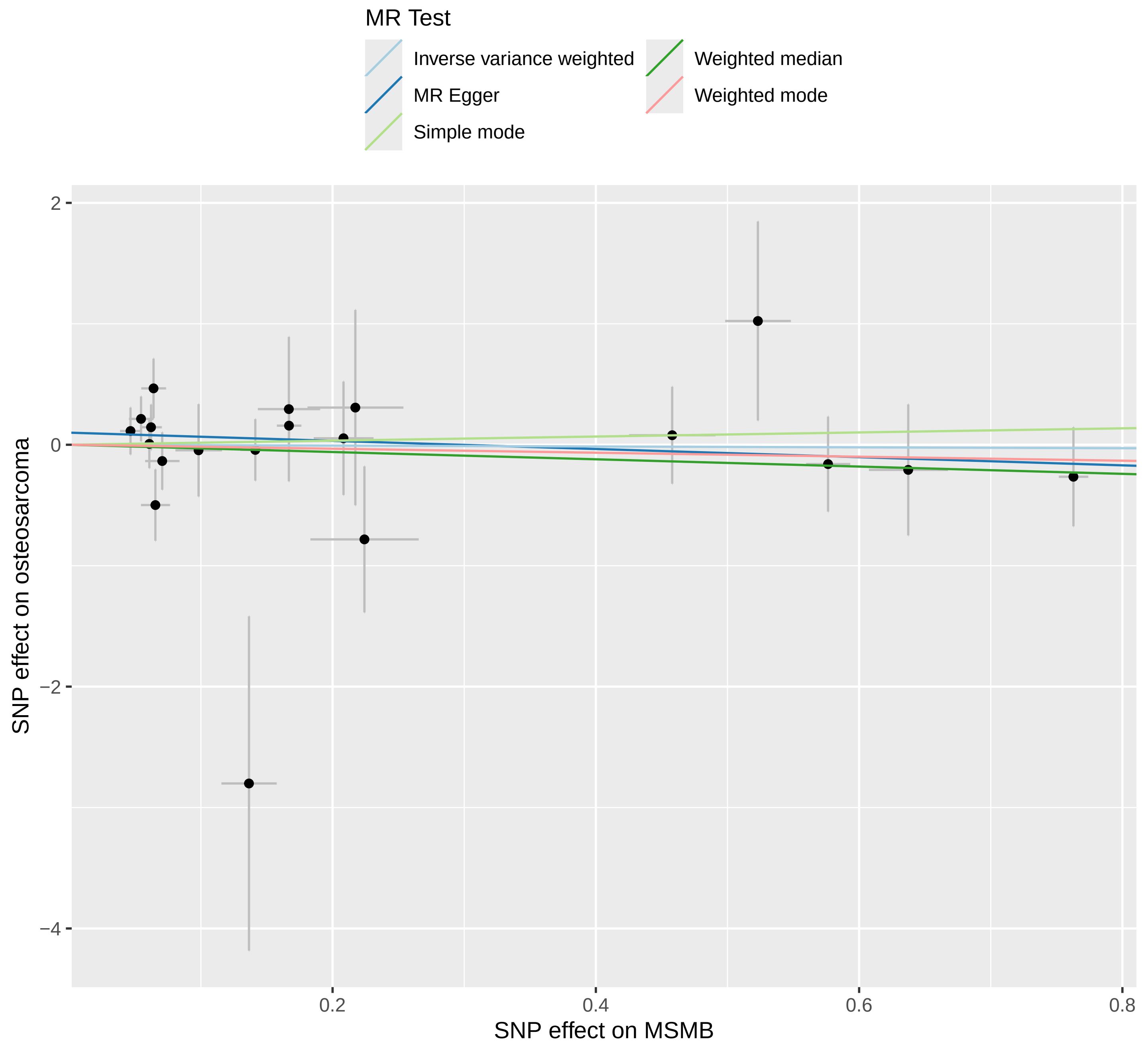

MR Test
Inverse variance weighted
MR Egger
Simple mode
Weighted median
Weighted mode
2
0
−2
−4
0.2
0.4
0.6
0.8
SNP effect on osteosarcoma
SNP effect on MSMB

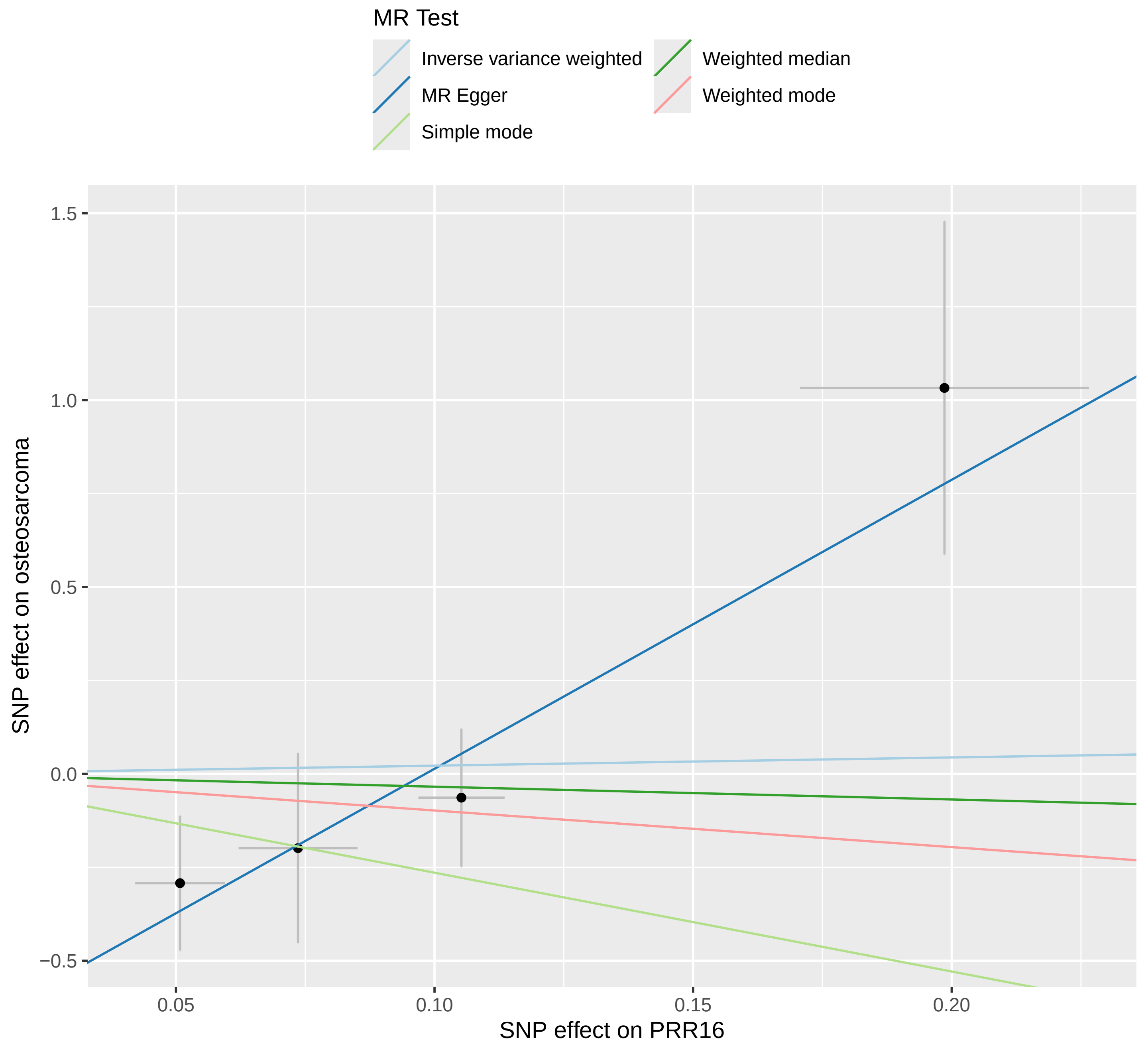
MR Test
Inverse variance weighted
MR Egger
Simple mode
Weighted median
Weighted mode
1.5
1.0
0.5
0.0
−0.5
SNP effect on osteosarcoma
0.05
0.10
0.15
0.20
SNP effect on PRR16

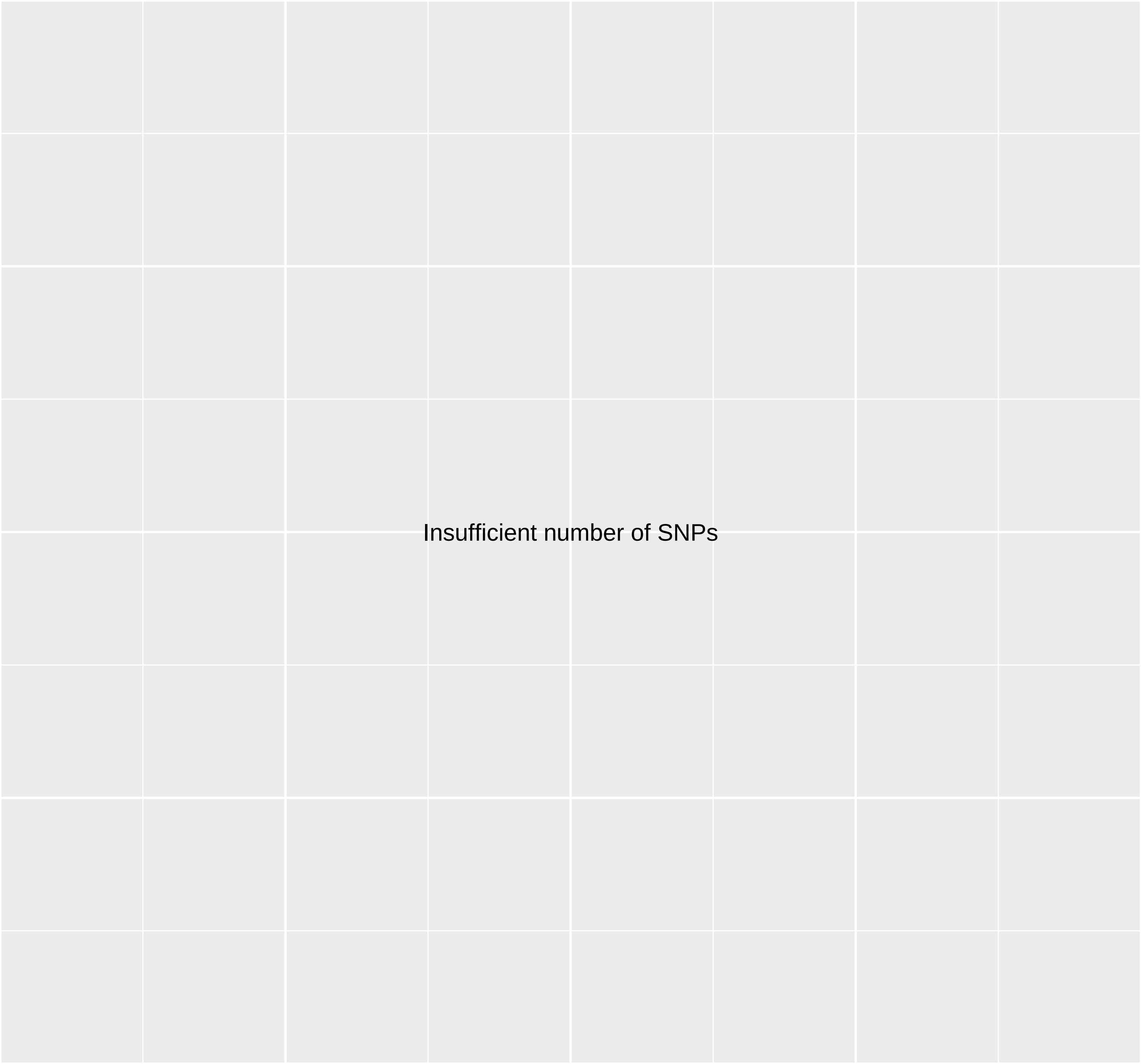
Insufficient number of SNPs

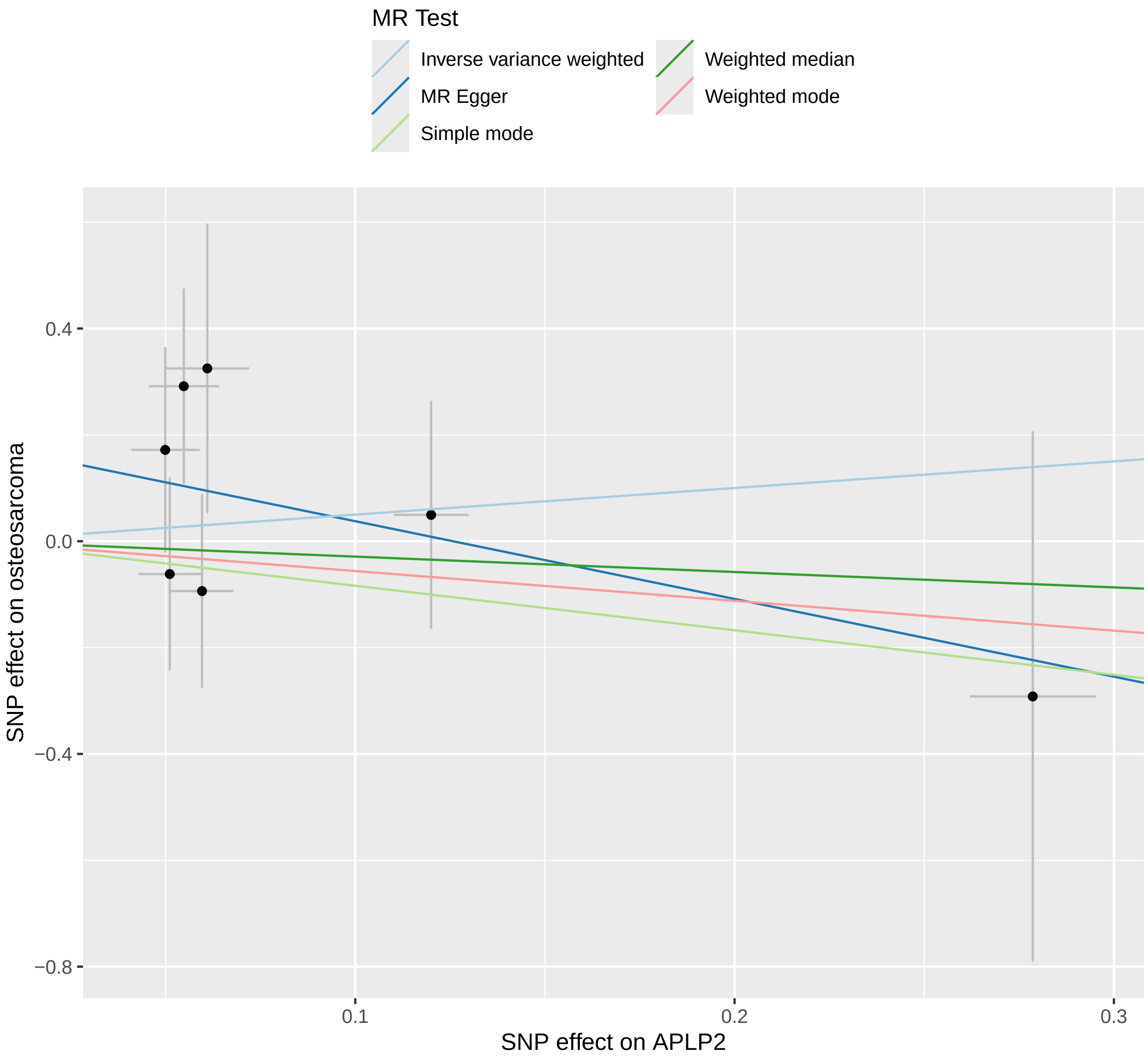
MR Test
Inverse variance weighted
MR Egger
Simple mode
Weighted median
Weighted mode
0.4
0.0
−0.4
−0.8
SNP effect on osteosarcoma
0.1
0.2
0.3
SNP effect on APLP2

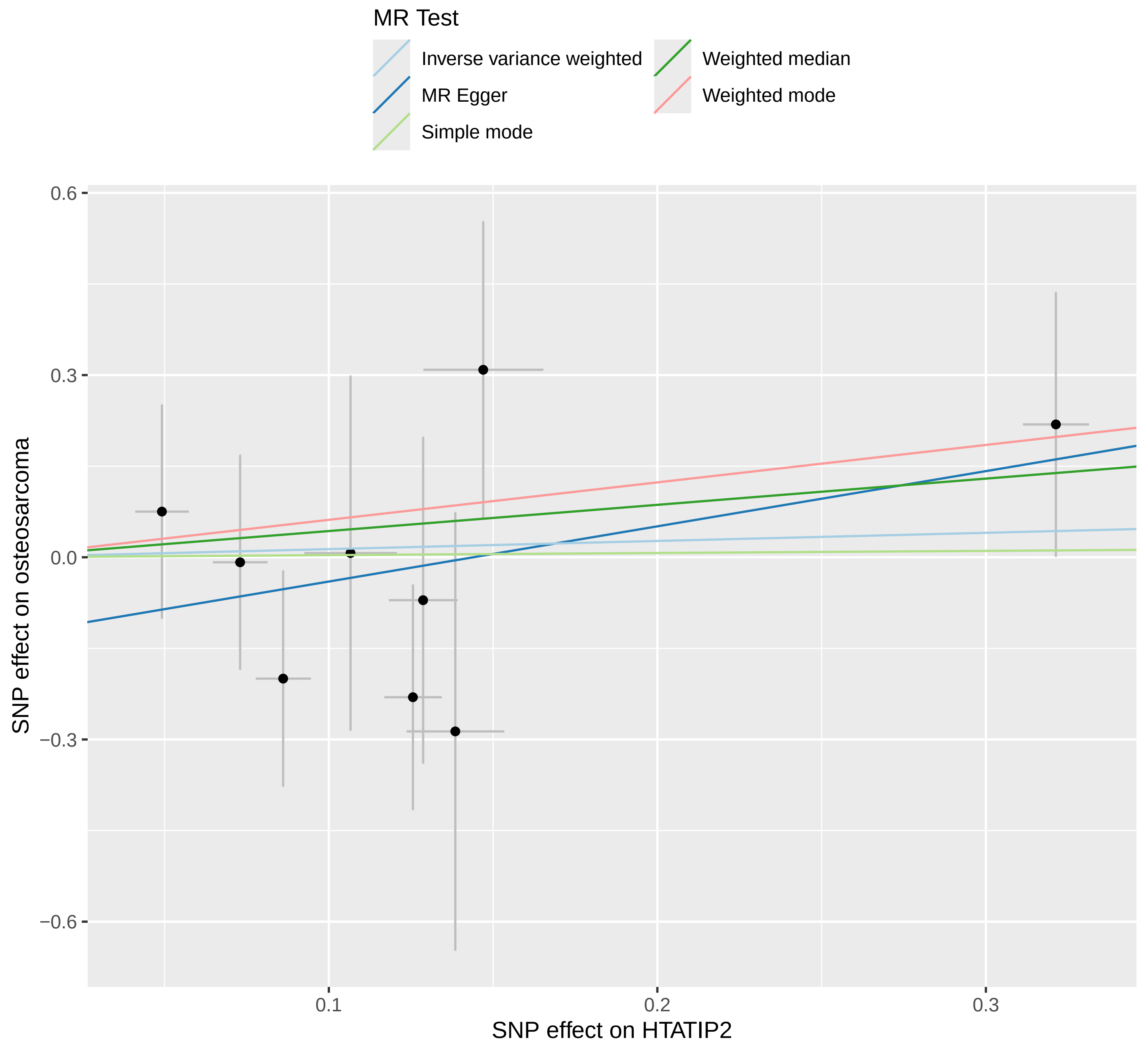
MR Test
Inverse variance weighted
MR Egger
Simple mode
Weighted median
Weighted mode
0.6
0.3
0.0
−0.3
−0.6
0.1
0.2
0.3
SNP effect on osteosarcoma
SNP effect on HTATIP2

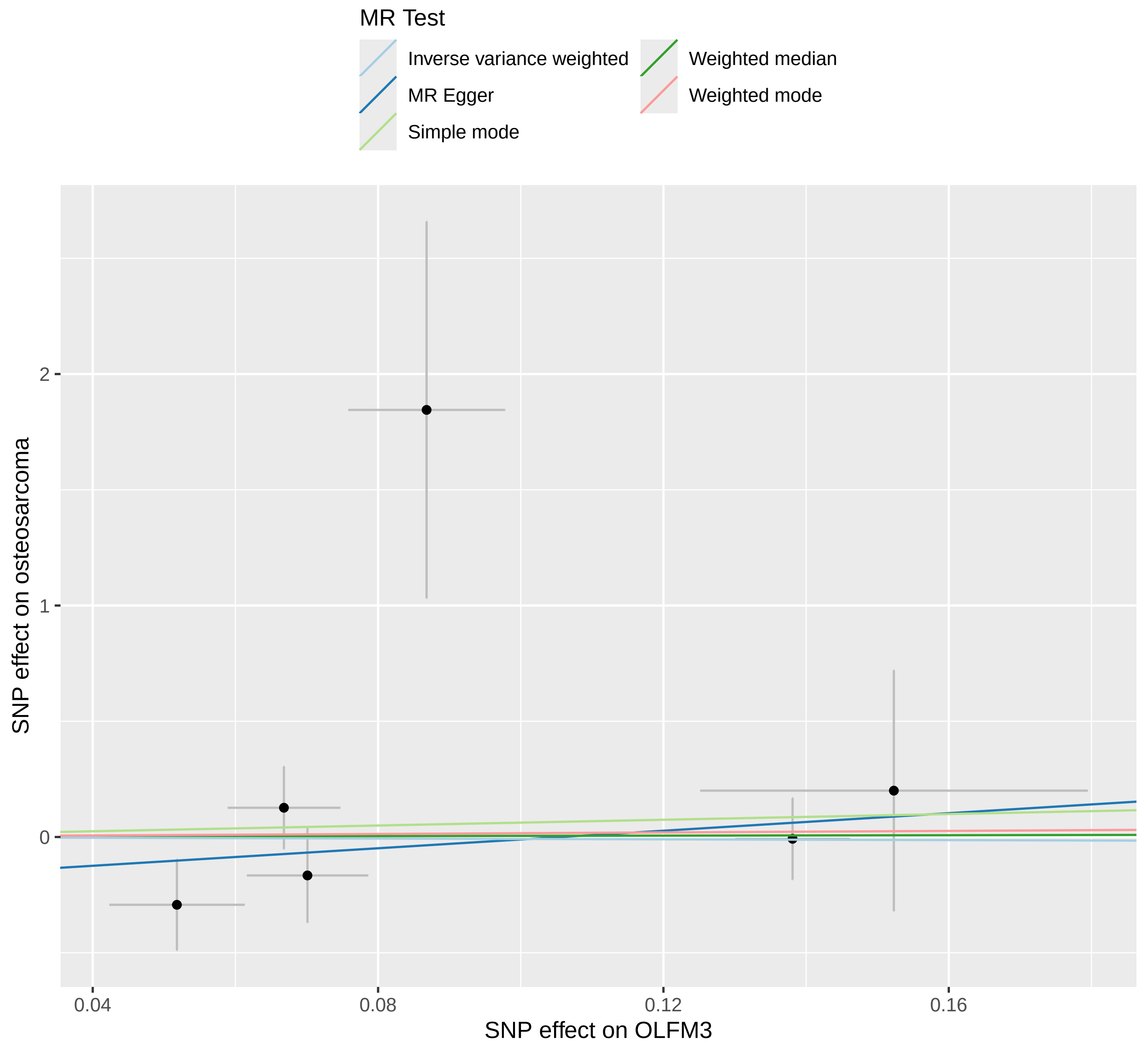

MR Test
Inverse variance weighted
MR Egger
Simple mode
Weighted median
Weighted mode
2
1
0
0.04
0.08
0.12
0.16
SNP effect on osteosarcoma
SNP effect on OLFM3

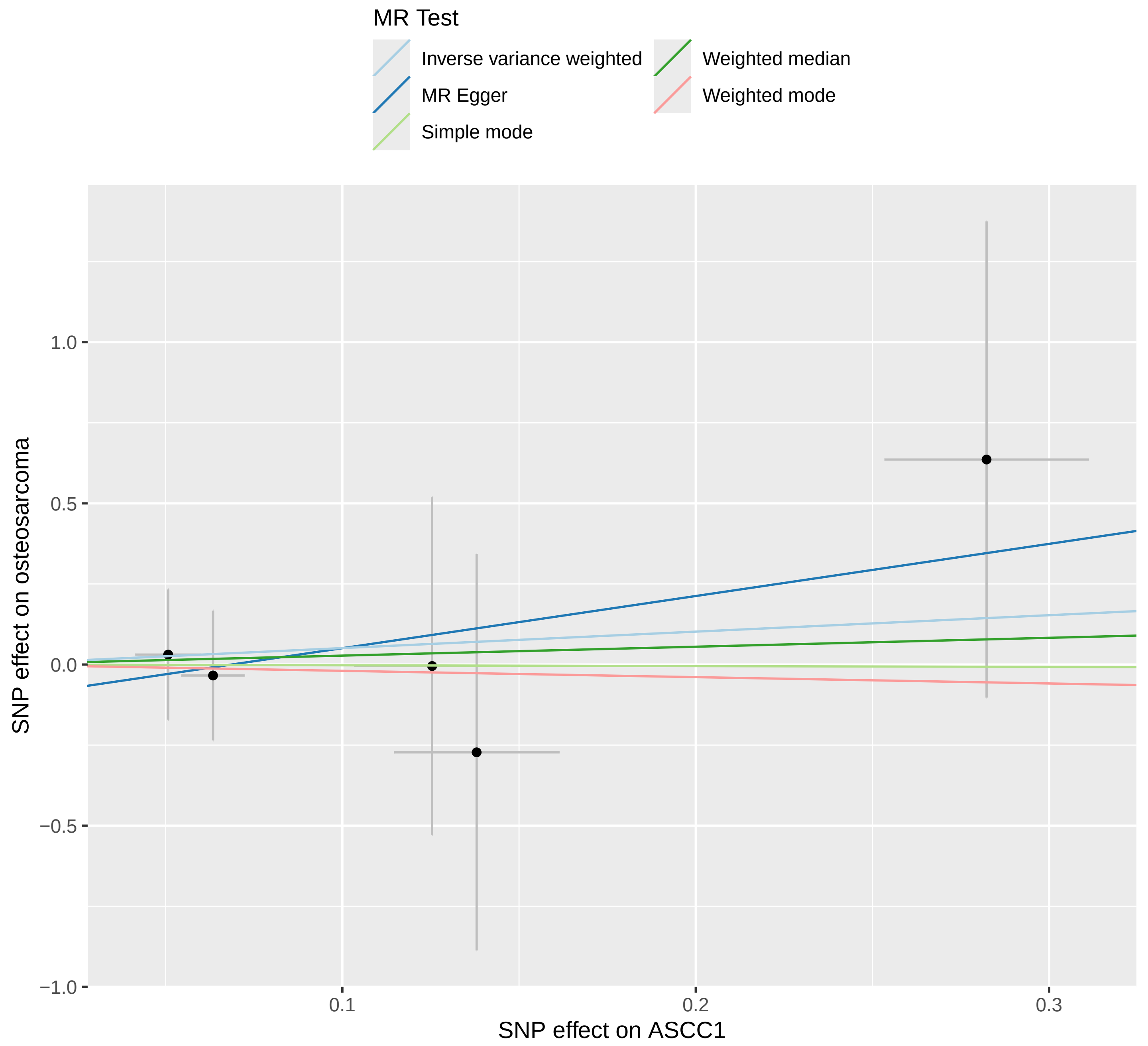
MR Test
Inverse variance weighted
MR Egger
Simple mode
Weighted median
Weighted mode
1.0
0.5
0.0
−0.5
−1.0
0.1
0.2
0.3
SNP effect on osteosarcoma
SNP effect on ASCC1

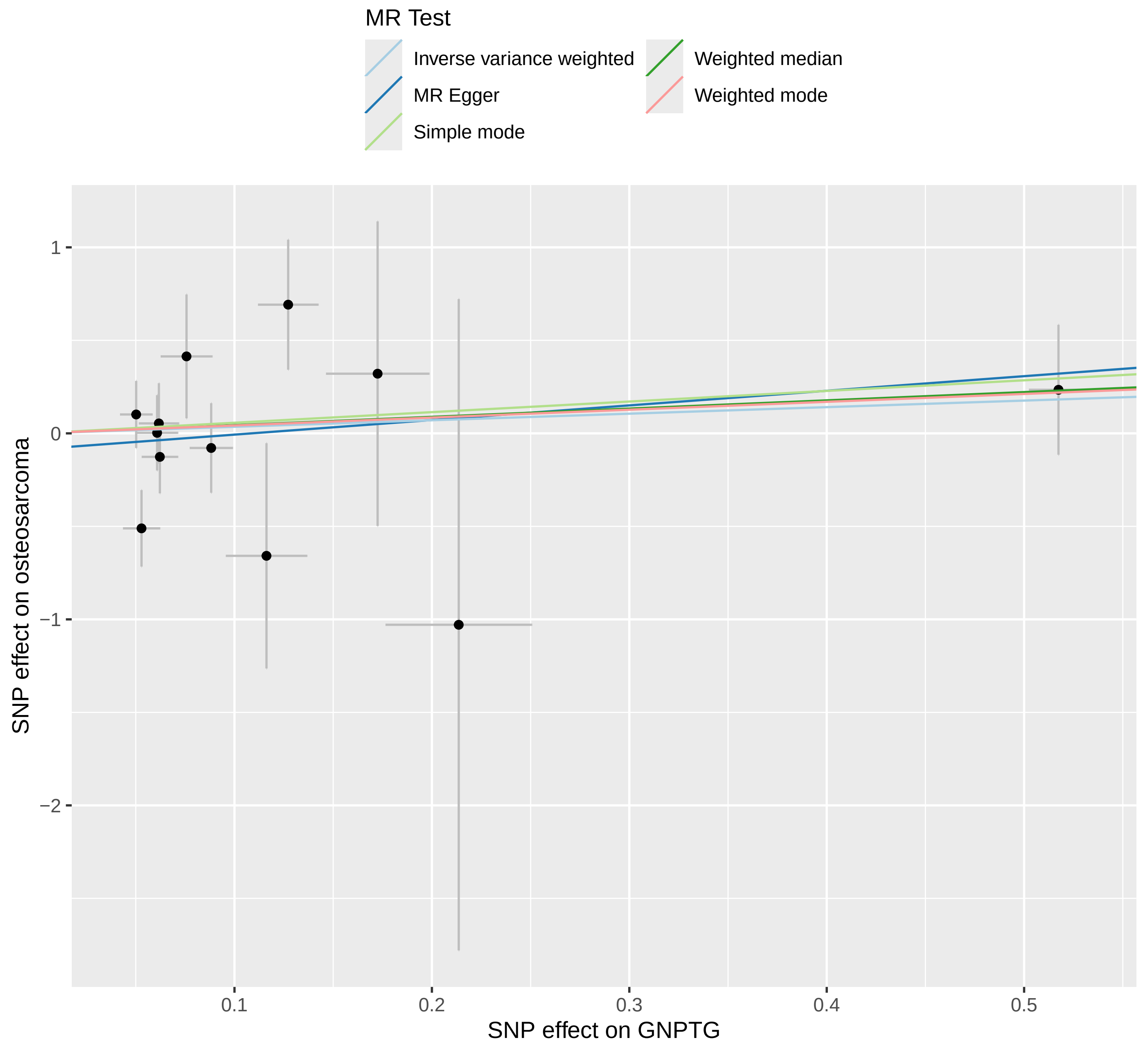
MR Test
Inverse variance weighted
MR Egger
Simple mode
Weighted median
Weighted mode
1
0
−1
−2
0.1
0.2
0.3
0.4
0.5
SNP effect on osteosarcoma
SNP effect on GNPTG

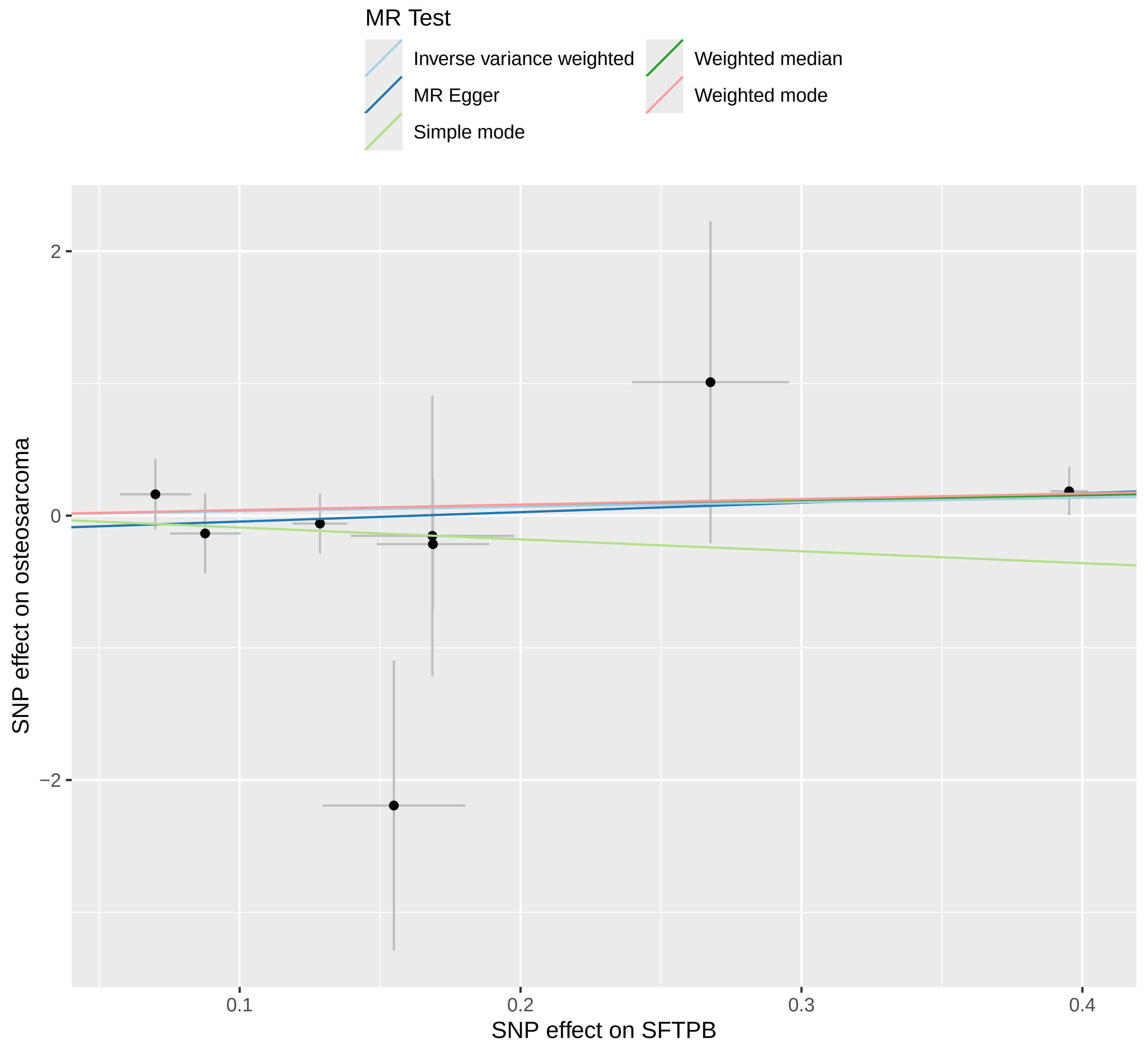

MR Test
Inverse variance weighted
MR Egger
Simple mode
Weighted median
Weighted mode
2
0
−2
0.1
0.2
0.3
0.4
SNP effect on osteosarcoma
SNP effect on SFTPB

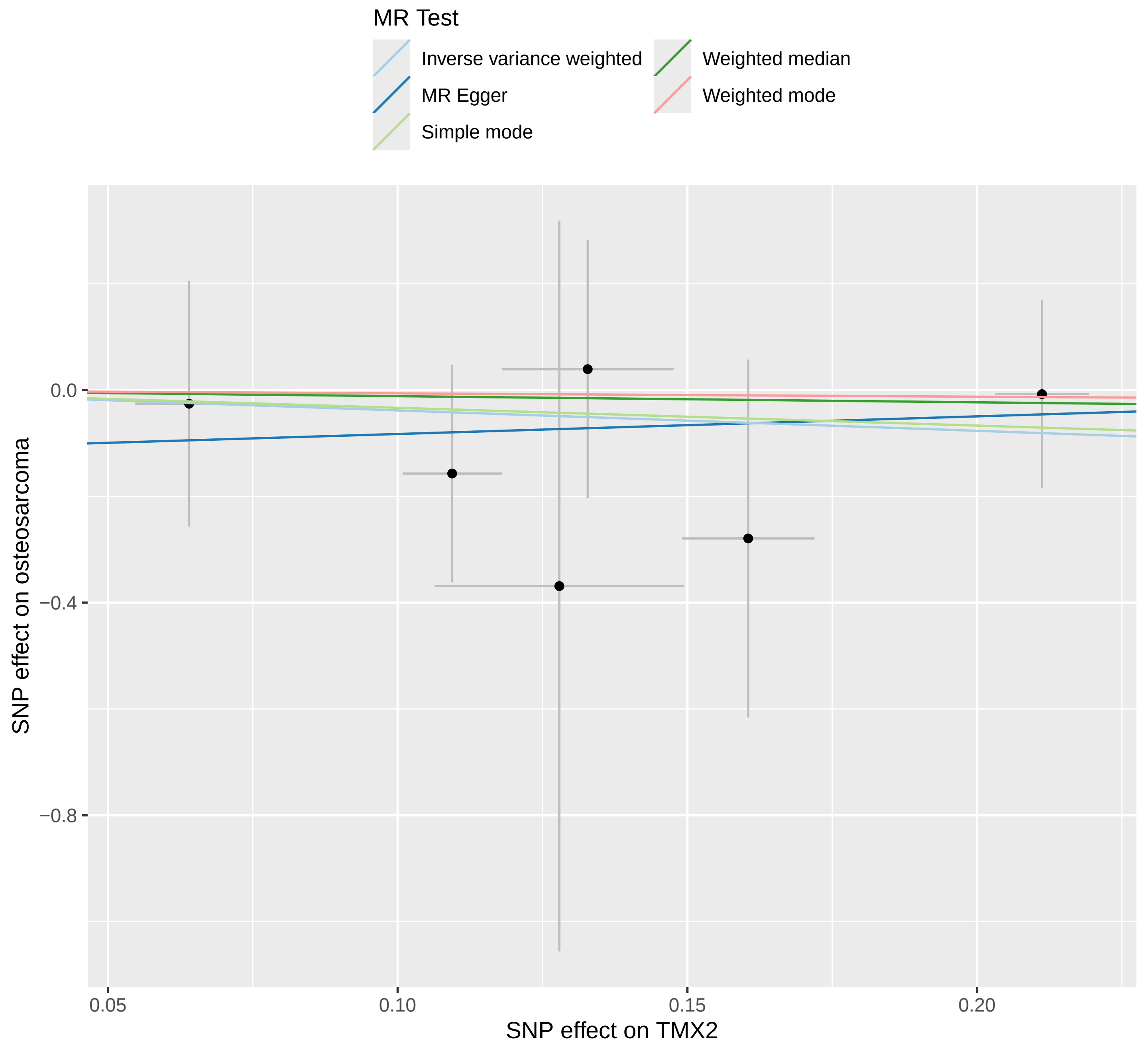
MR Test
Inverse variance weighted
MR Egger
Simple mode
Weighted median
Weighted mode
0.0
−0.4
−0.8
SNP effect on osteosarcoma
0.05
0.10
0.15
0.20
SNP effect on TMX2

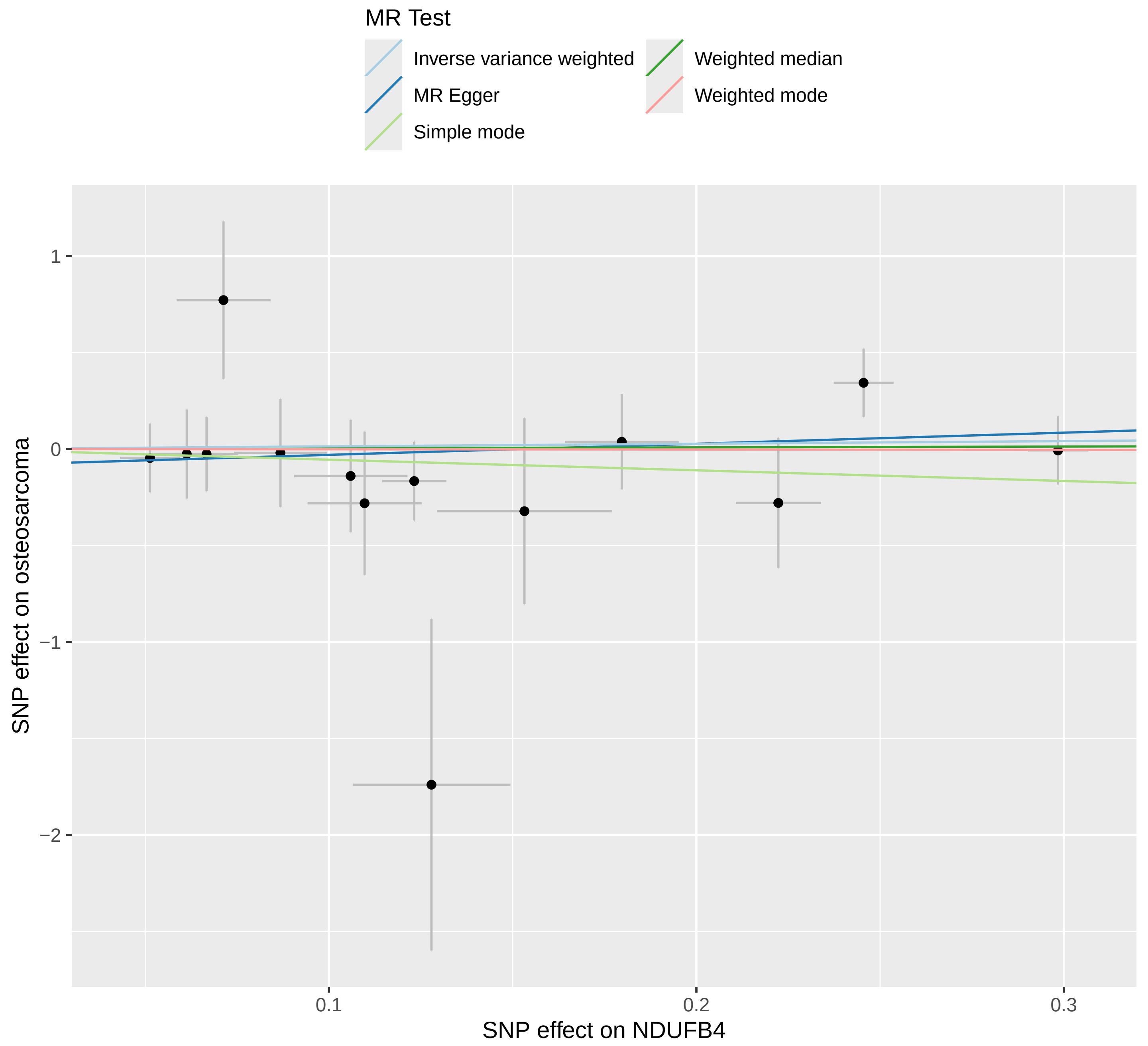
MR Test
Inverse variance weighted
MR Egger
Simple mode
Weighted median
Weighted mode
SNP effect on osteosarcoma
1
0
−1
−2
0.1
0.2
0.3
SNP effect on NDUFB4

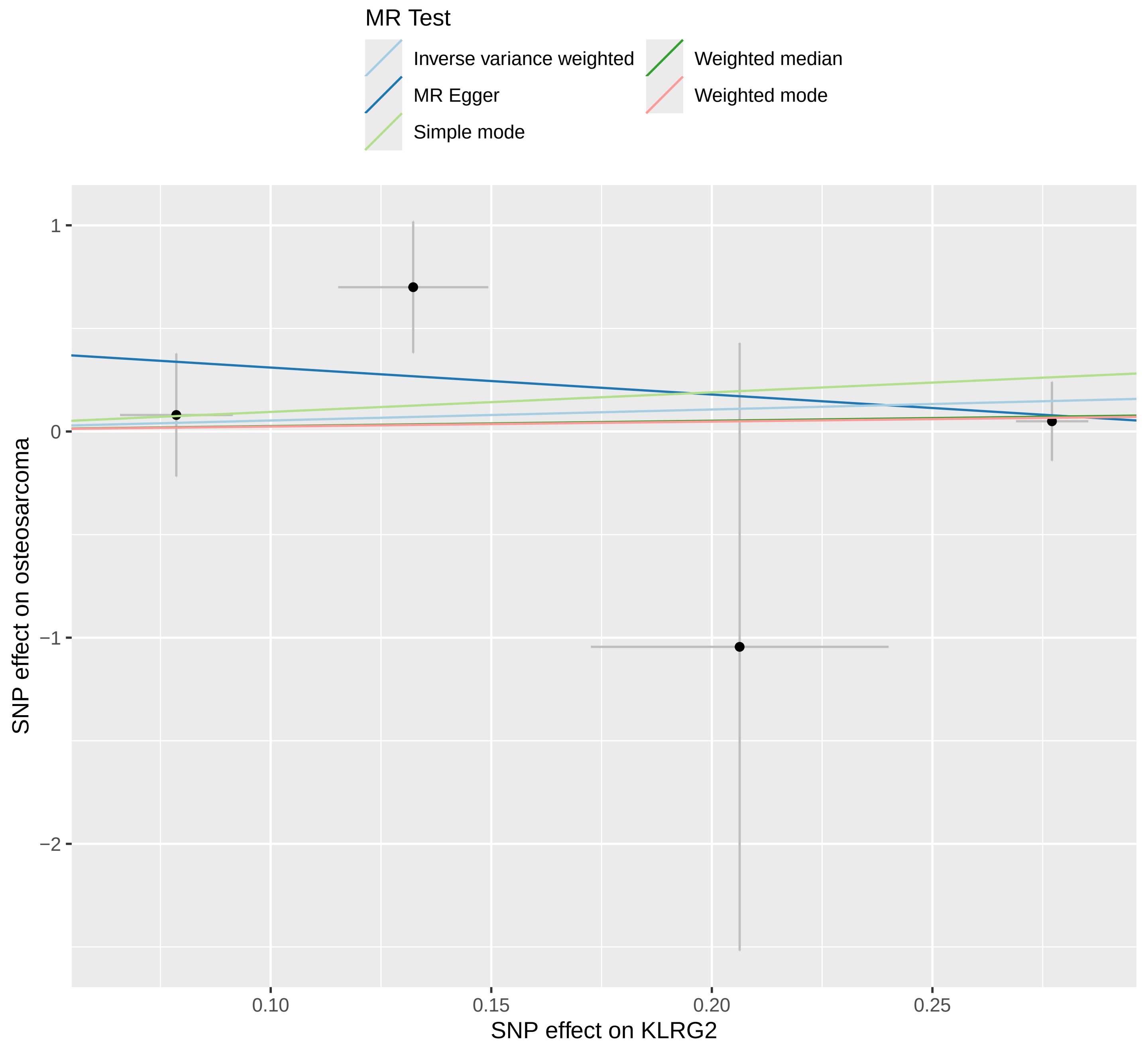
MR Test
Inverse variance weighted
MR Egger
Simple mode
Weighted median
Weighted mode
1
0
−1
−2
0.10
0.15
0.20
0.25
SNP effect on KLRG2
SNP effect on osteosarcoma

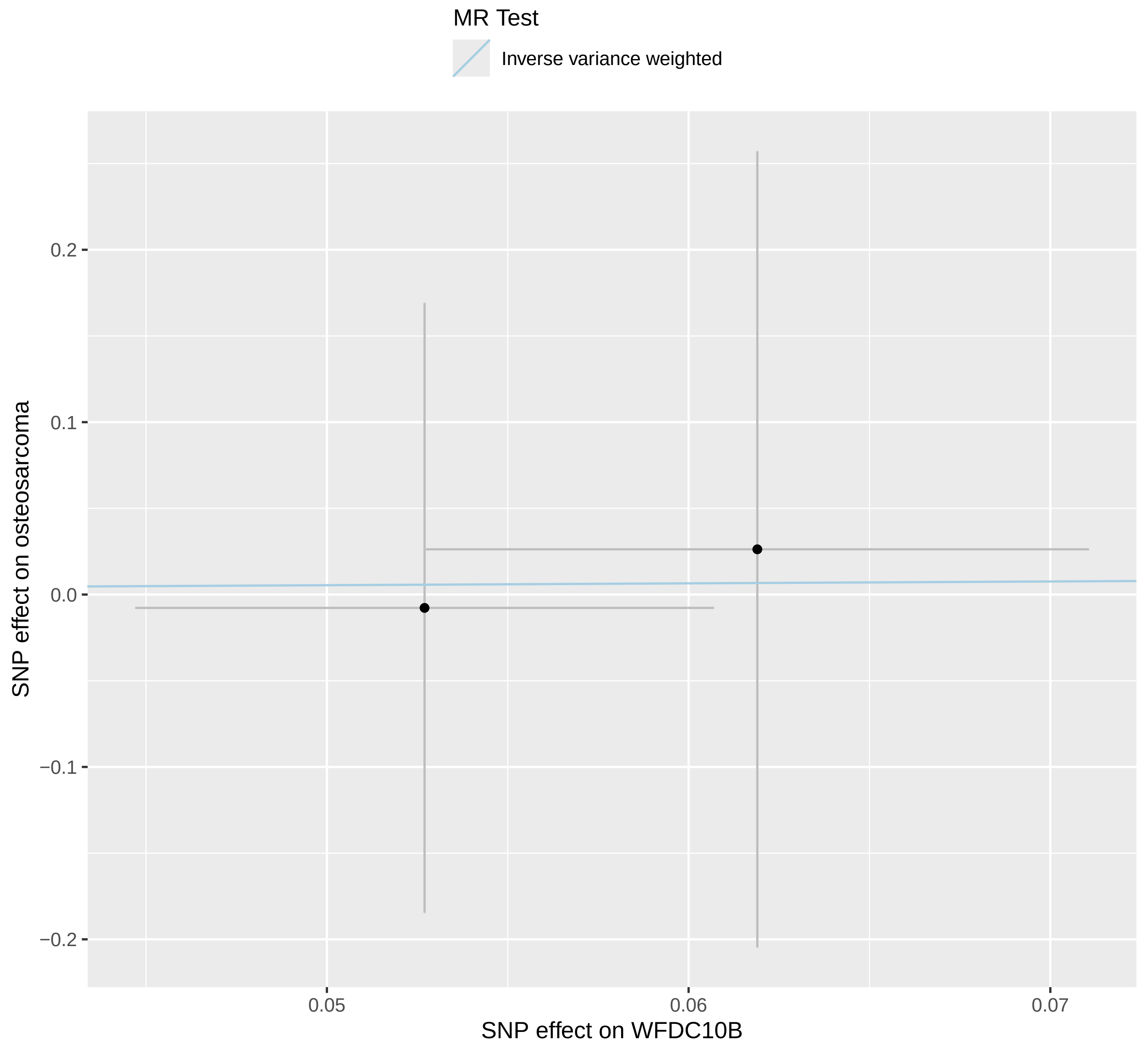

MR Test
Inverse variance weighted
0.2
0.1
0.0
−0.1
−0.2
SNP effect on osteosarcoma
0.05
0.06
0.07
SNP effect on WFDC10B

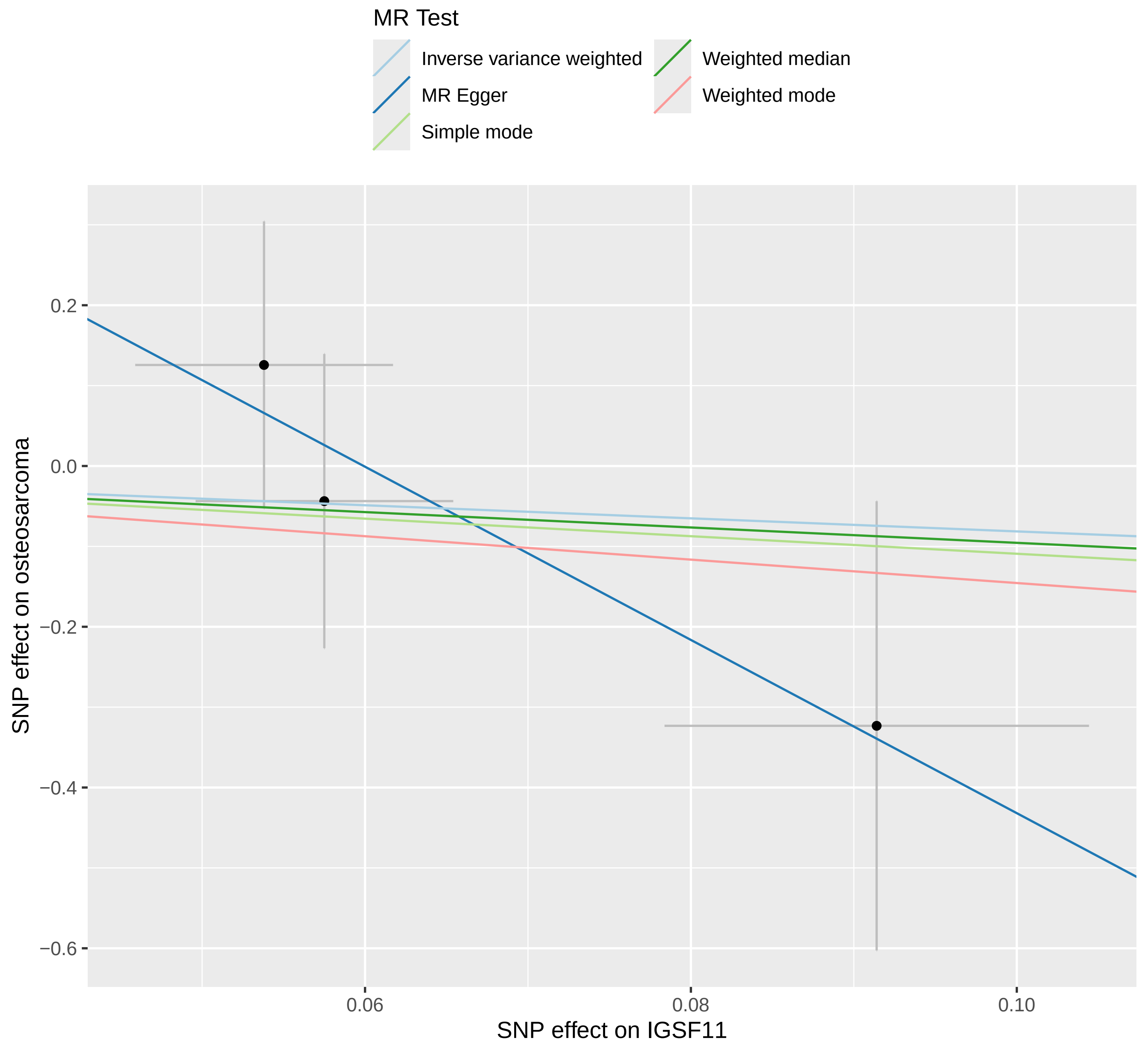
MR Test
Inverse variance weighted
MR Egger
Simple mode
Weighted median
Weighted mode
0.2
0.0
−0.2
−0.4
−0.6
SNP effect on osteosarcoma
0.06
0.08
0.10
SNP effect on IGSF11

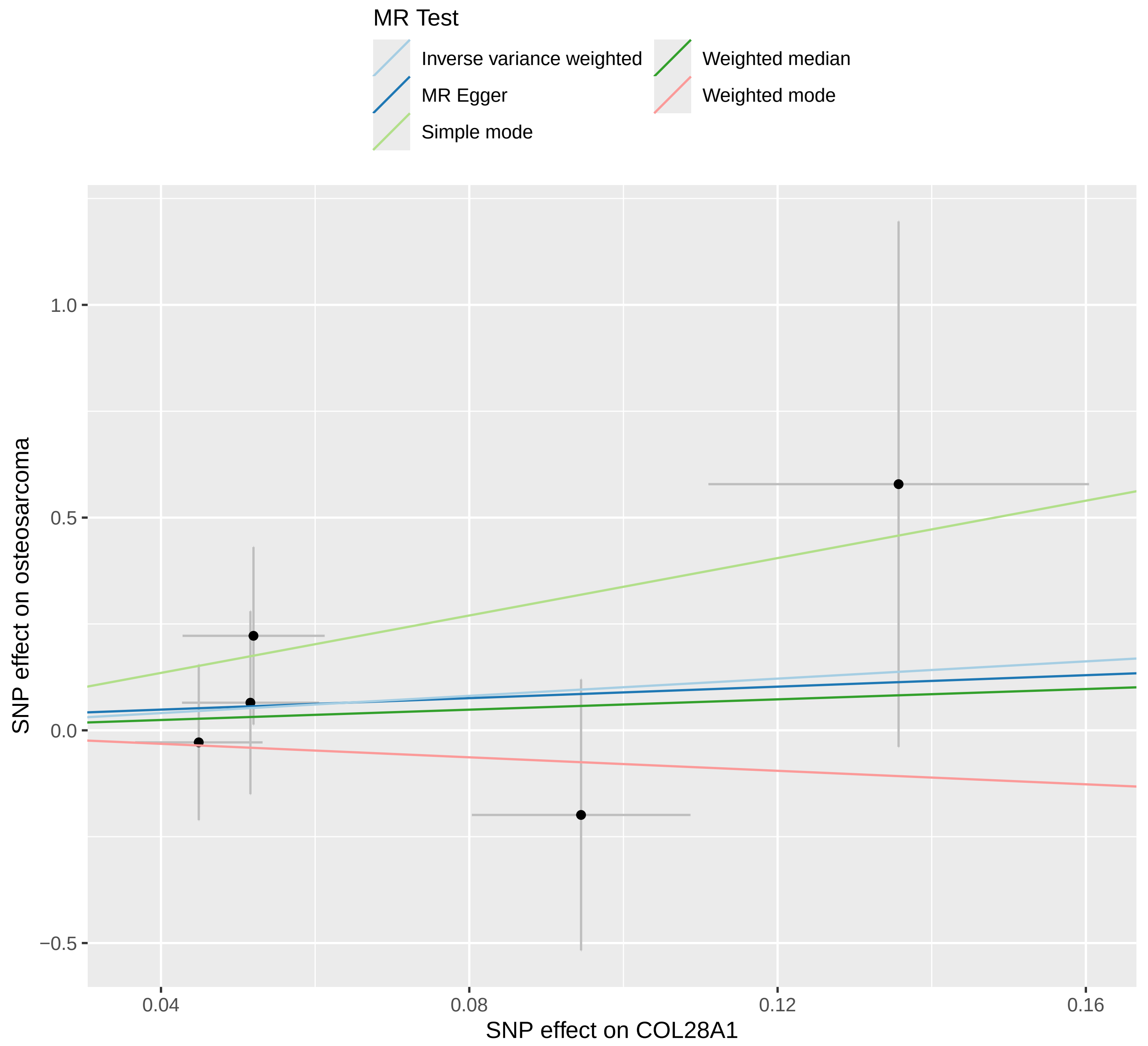

MR Test
Inverse variance weighted
MR Egger
Simple mode
Weighted median
Weighted mode
1.0
0.5
0.0
−0.5
0.04
0.08
0.12
0.16
SNP effect on osteosarcoma
SNP effect on COL28A1

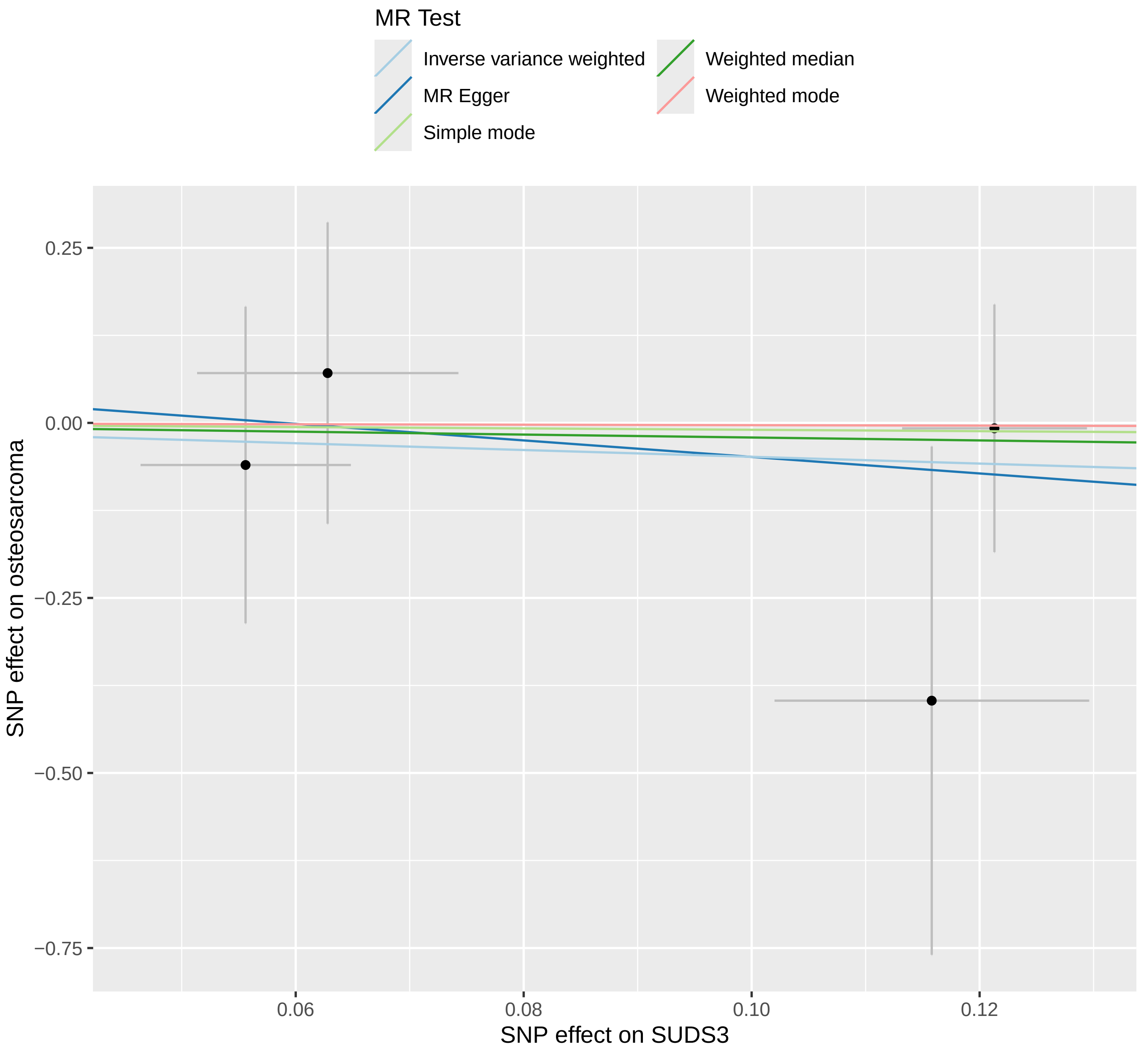
MR Test
Inverse variance weighted
MR Egger
Simple mode
Weighted median
Weighted mode
0.25
0.00
−0.25
−0.50
−0.75
SNP effect on osteosarcoma
0.06
0.08
0.10
0.12
SNP effect on SUDS3

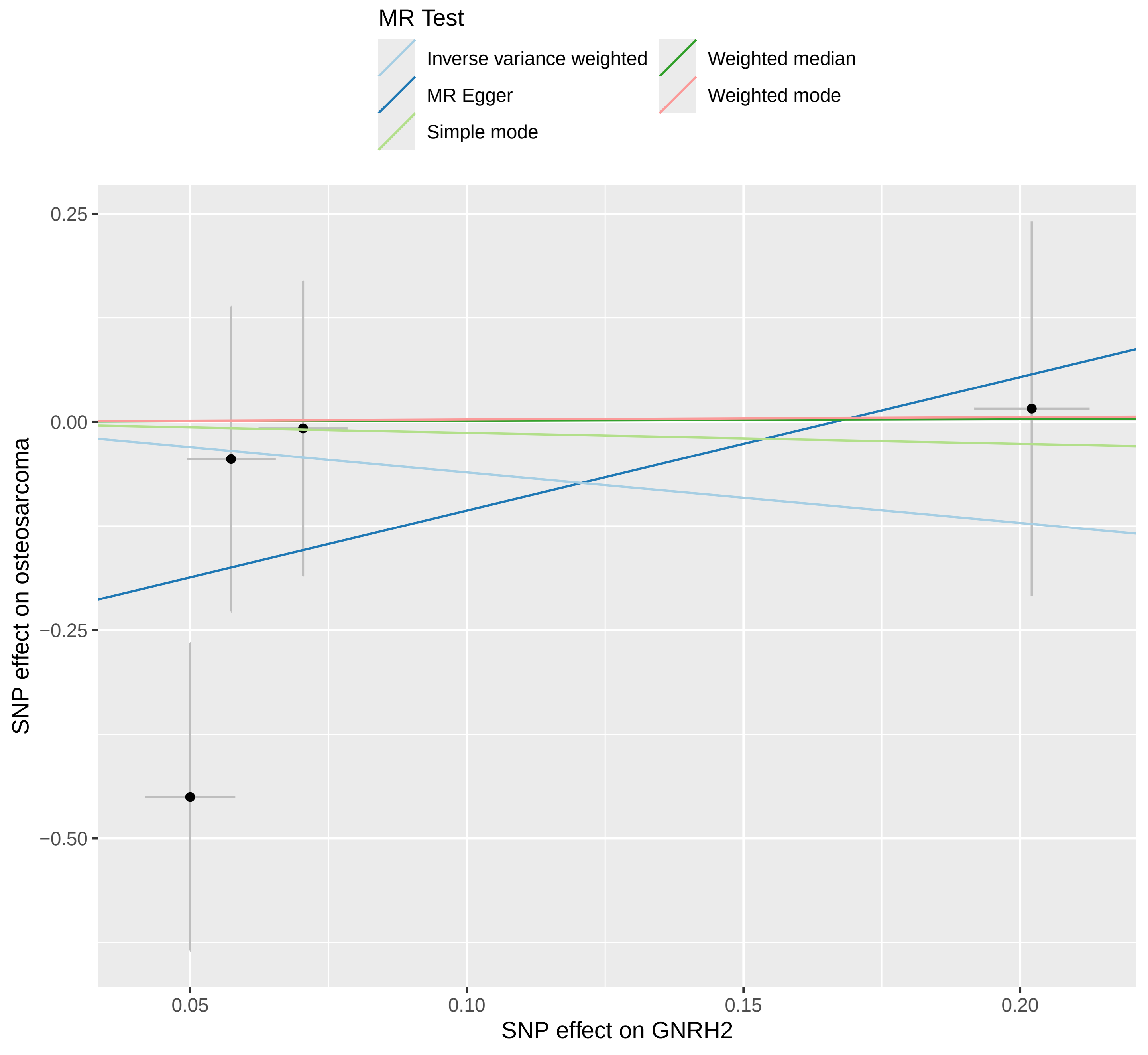
MR Test
Inverse variance weighted
MR Egger
Simple mode
Weighted median
Weighted mode
0.25
0.00
−0.25
−0.50
SNP effect on osteosarcoma
0.05
0.10
0.15
0.20
SNP effect on GNRH2

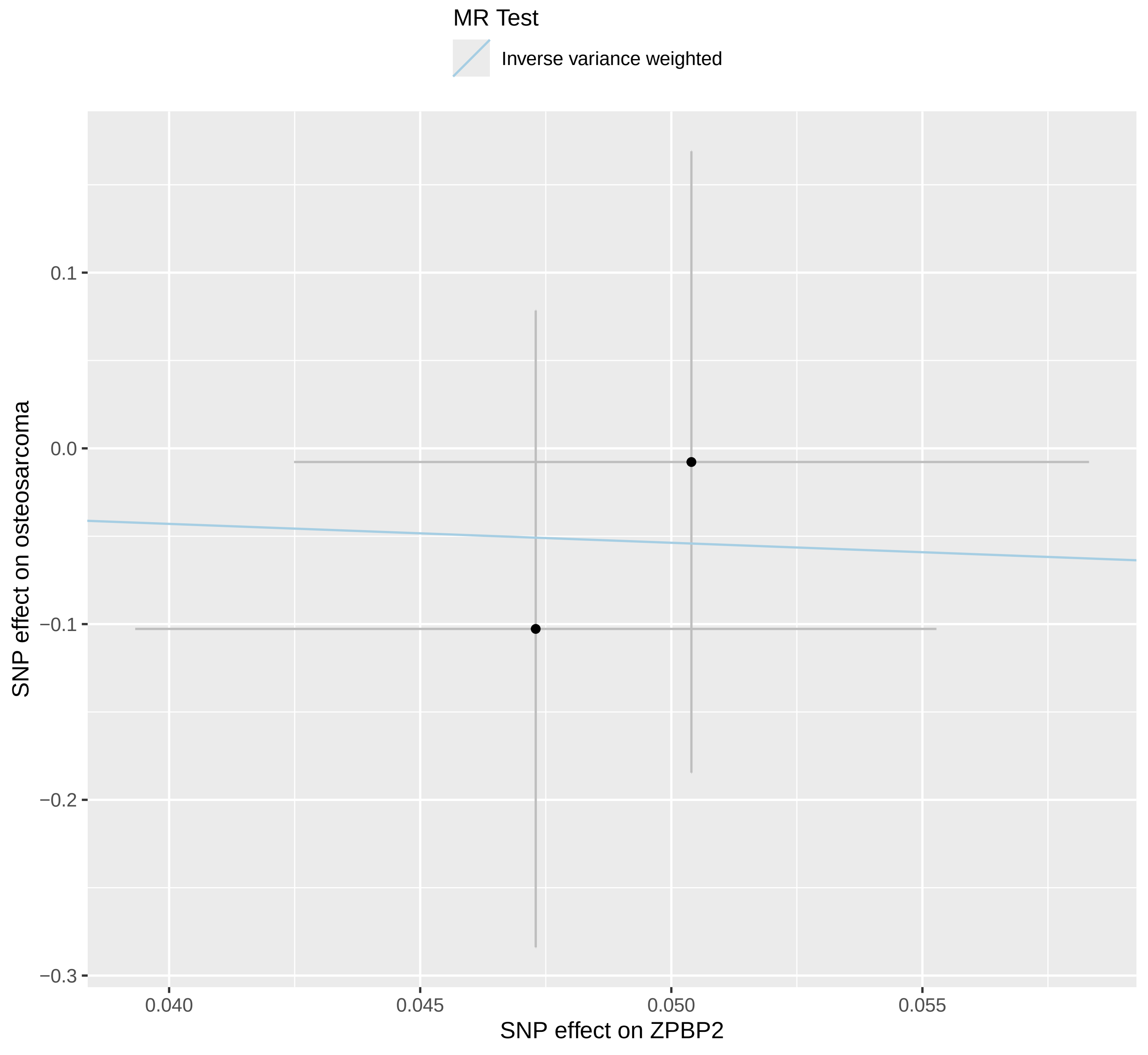

MR Test
Inverse variance weighted
0.1
0.0
−0.1
−0.2
−0.3
0.040
0.045
0.050
0.055
SNP effect on ZPBP2
SNP effect on osteosarcoma

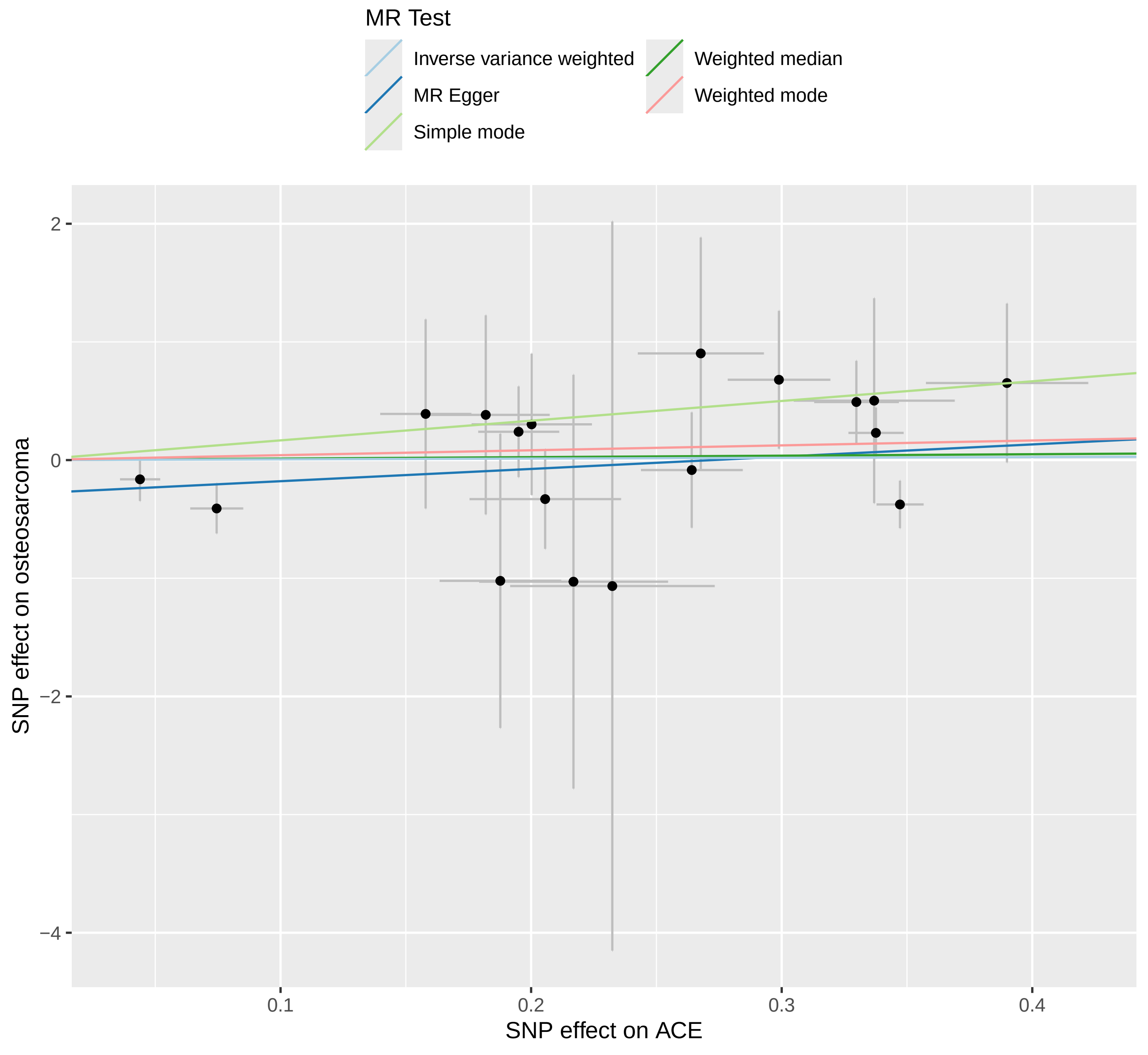
MR Test
Inverse variance weighted
MR Egger
Simple mode
Weighted median
Weighted mode
2
0
−2
−4
SNP effect on osteosarcoma
0.1
0.2
0.3
0.4
SNP effect on ACE

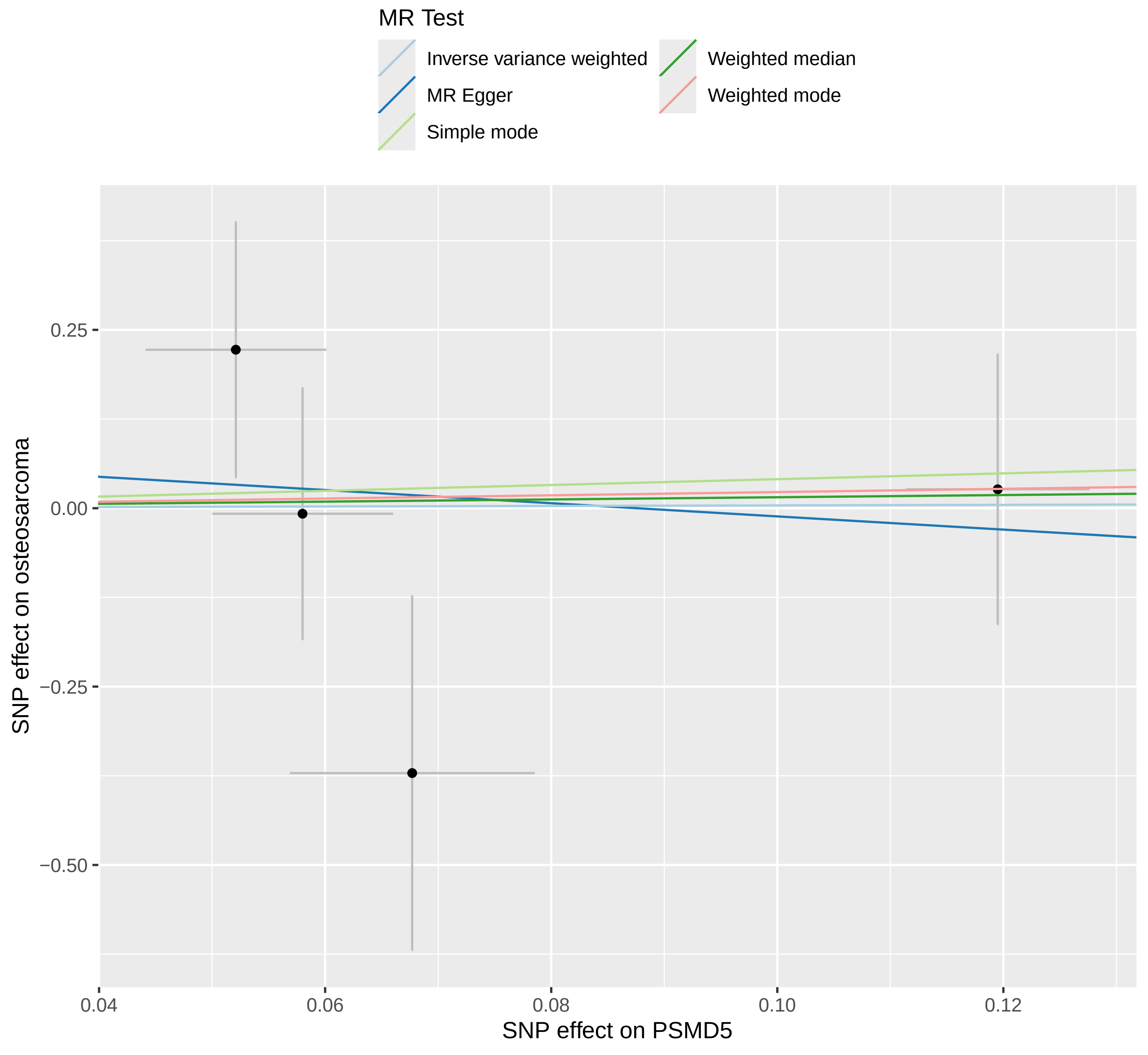
MR Test
Inverse variance weighted
MR Egger
Simple mode
Weighted median
Weighted mode
0.25
0.00
−0.25
−0.50
SNP effect on osteosarcoma
0.04
0.06
0.08
0.10
0.12
SNP effect on PSMD5

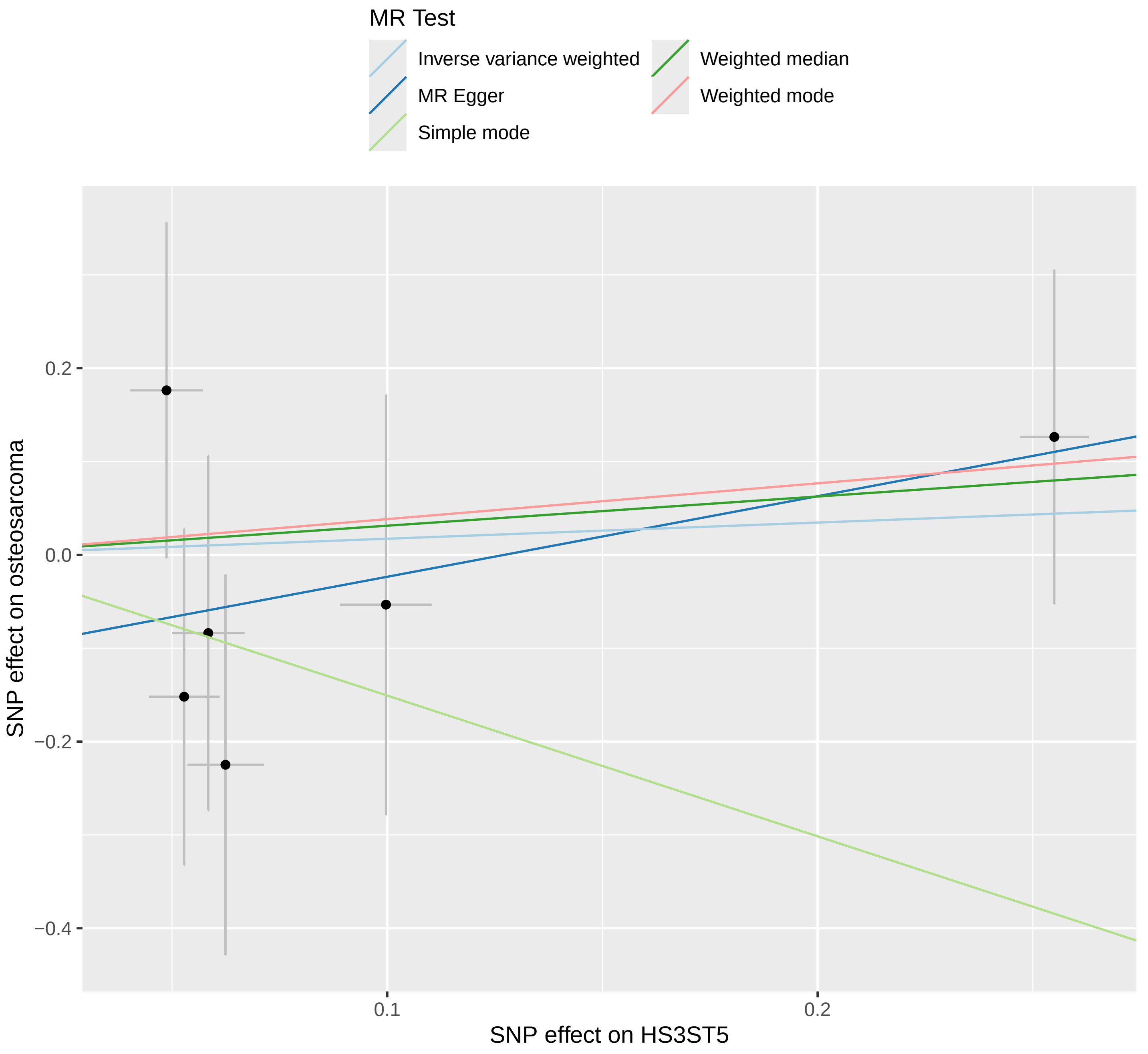

MR Test
Inverse variance weighted
MR Egger
Simple mode
Weighted median
Weighted mode
0.2
0.0
−0.2
−0.4
SNP effect on osteosarcoma
0.1
0.2
SNP effect on HS3ST5

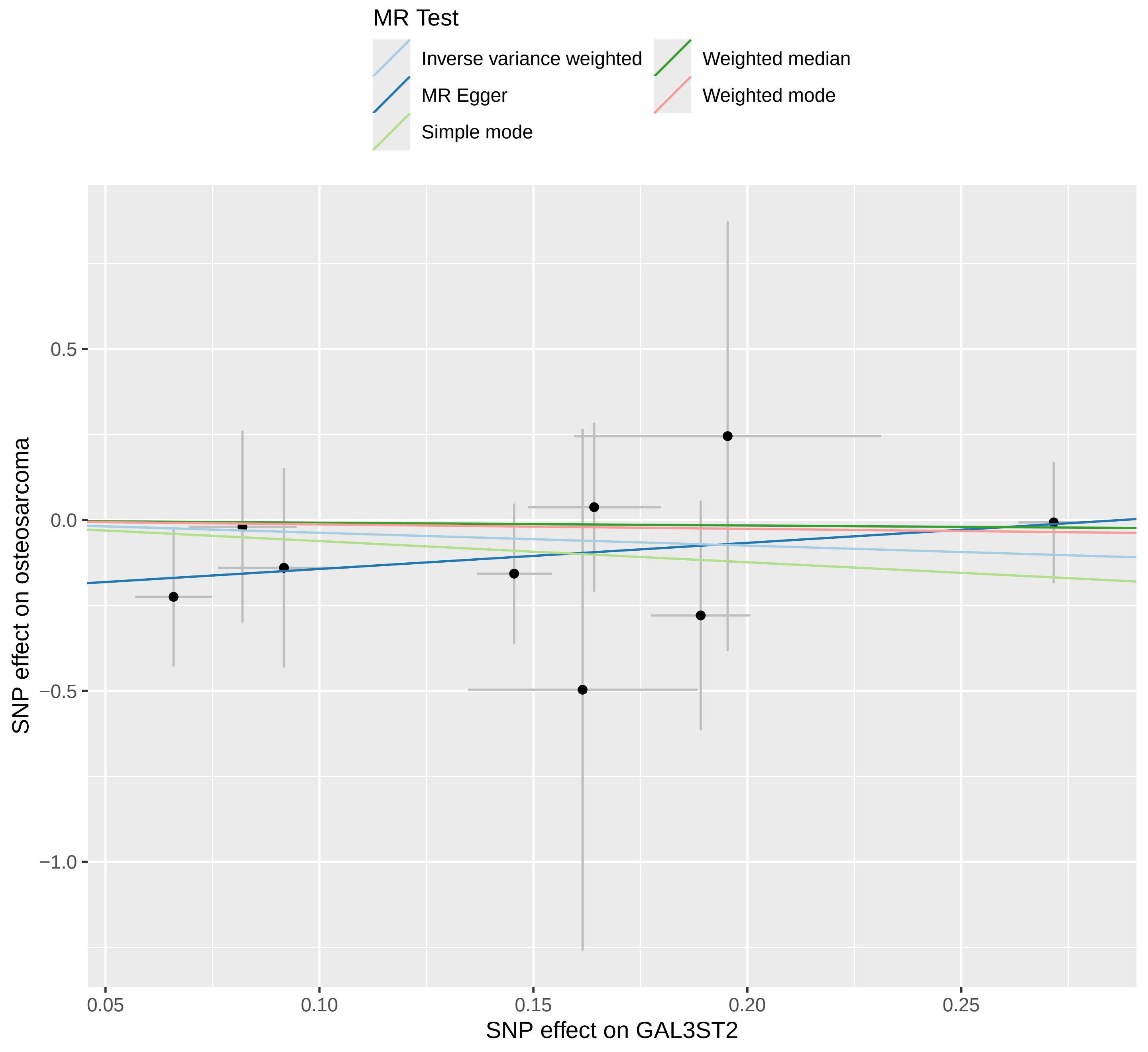

MR Test
Inverse variance weighted
MR Egger
Simple mode
Weighted median
Weighted mode
0.5
0.0
−0.5
−1.0
SNP effect on osteosarcoma
0.05
0.10
0.15
0.20
0.25
SNP effect on GAL3ST2

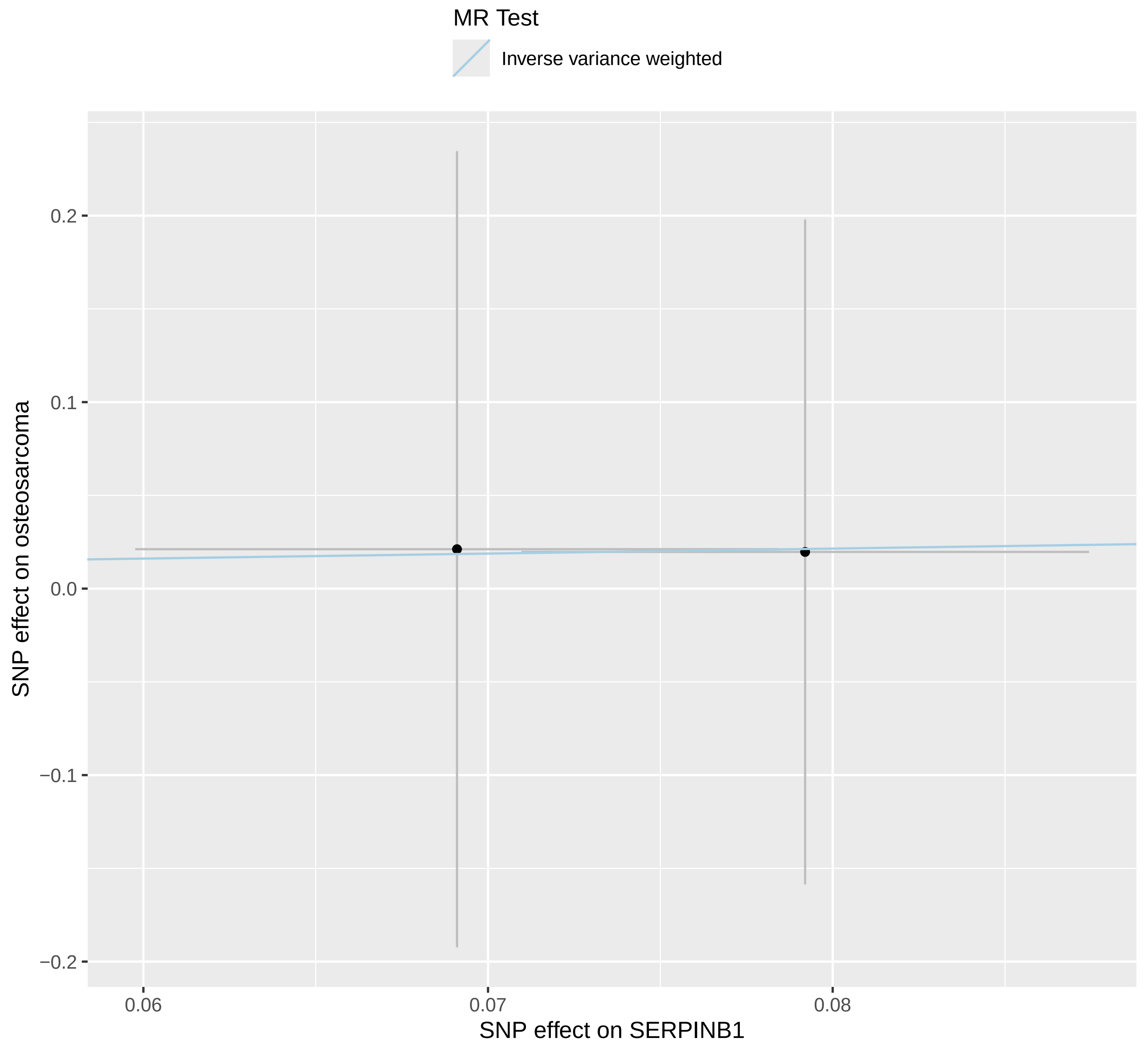
MR Test
Inverse variance weighted
SNP effect on osteosarcoma
0.2
0.1
0.0
−0.1
−0.2
0.06
0.07
0.08
SNP effect on SERPINB1

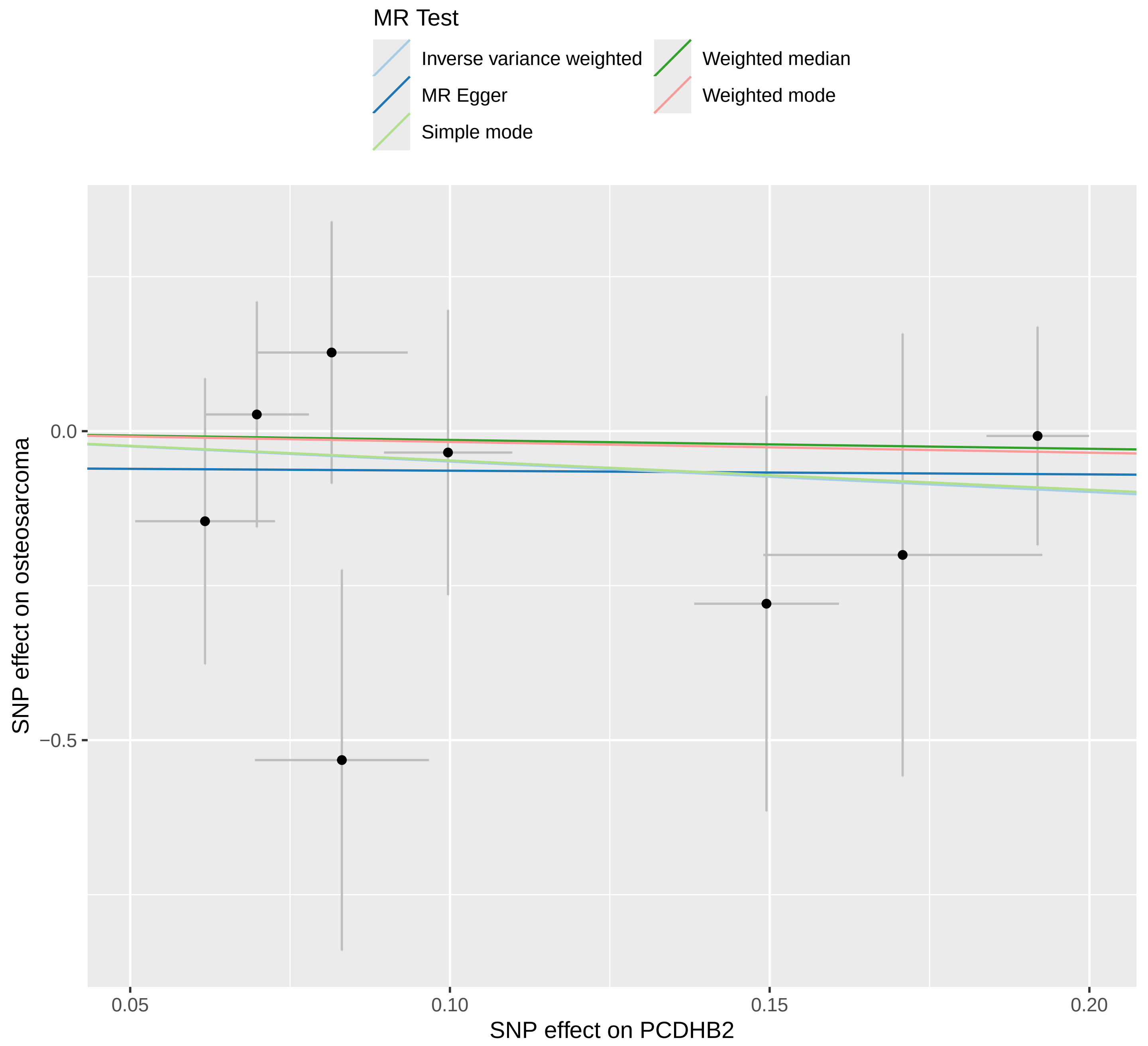
MR Test
Inverse variance weighted
MR Egger
Simple mode
Weighted median
Weighted mode
0.0
−0.5
SNP effect on osteosarcoma
0.05
0.10
0.15
0.20
SNP effect on PCDHB2

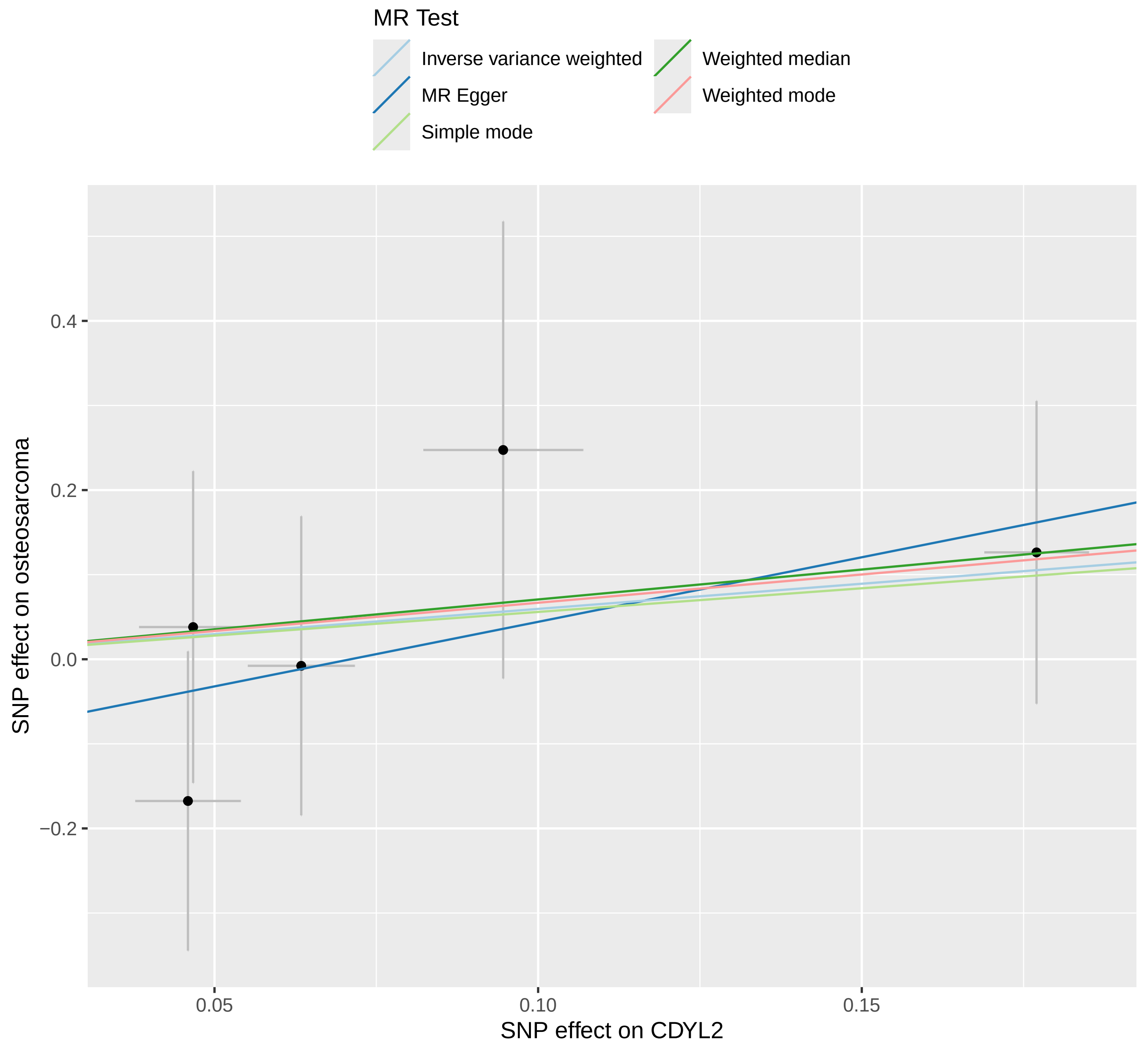
MR Test
Inverse variance weighted
MR Egger
Simple mode
Weighted median
Weighted mode
SNP effect on osteosarcoma
0.4
0.2
0.0
−0.2
0.05
0.10
0.15
SNP effect on CDYL2

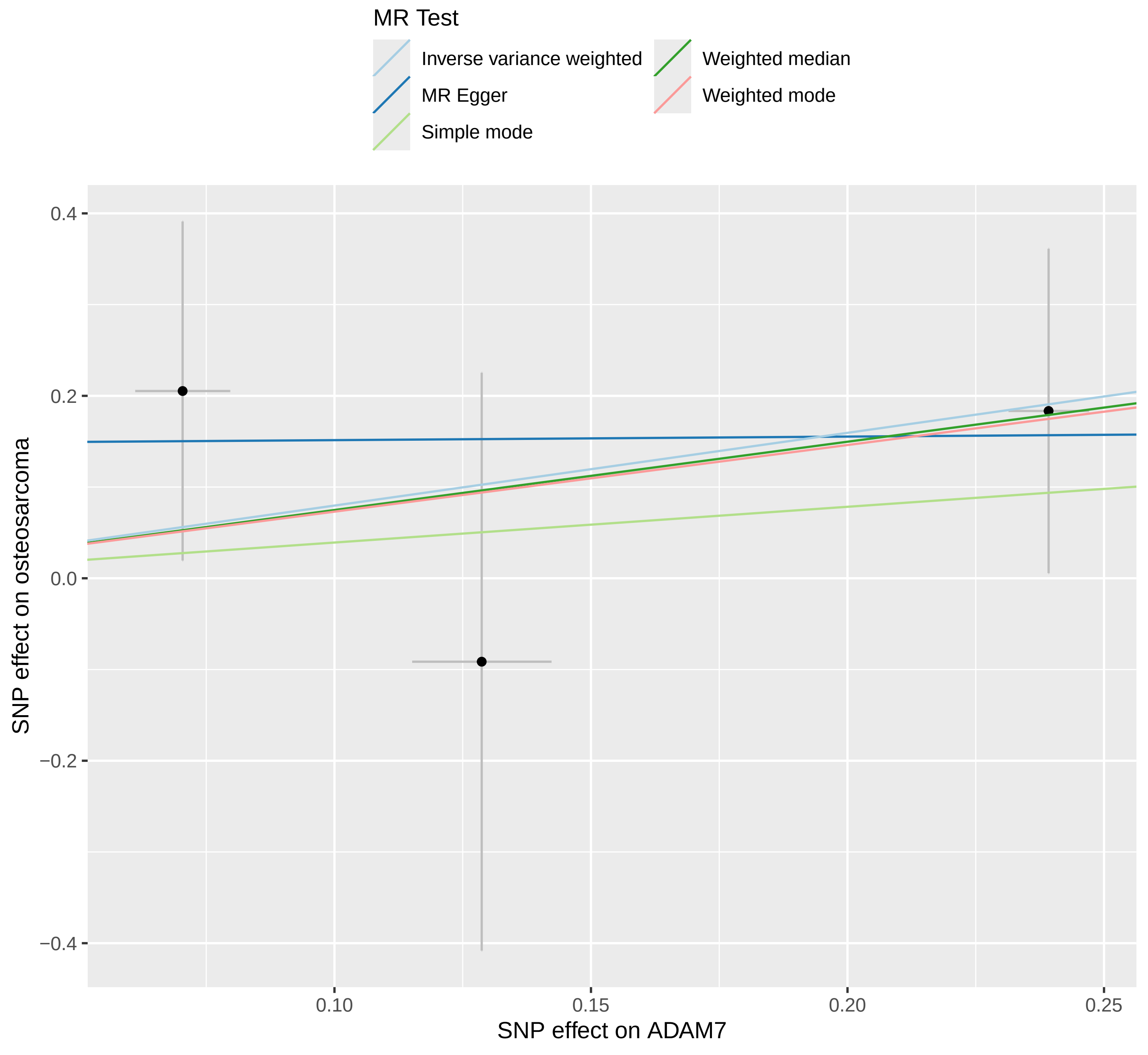
MR Test
Inverse variance weighted
MR Egger
Simple mode
Weighted median
Weighted mode
0.4
0.2
0.0
−0.2
−0.4
SNP effect on osteosarcoma
0.10
0.15
0.20
0.25
SNP effect on ADAM7

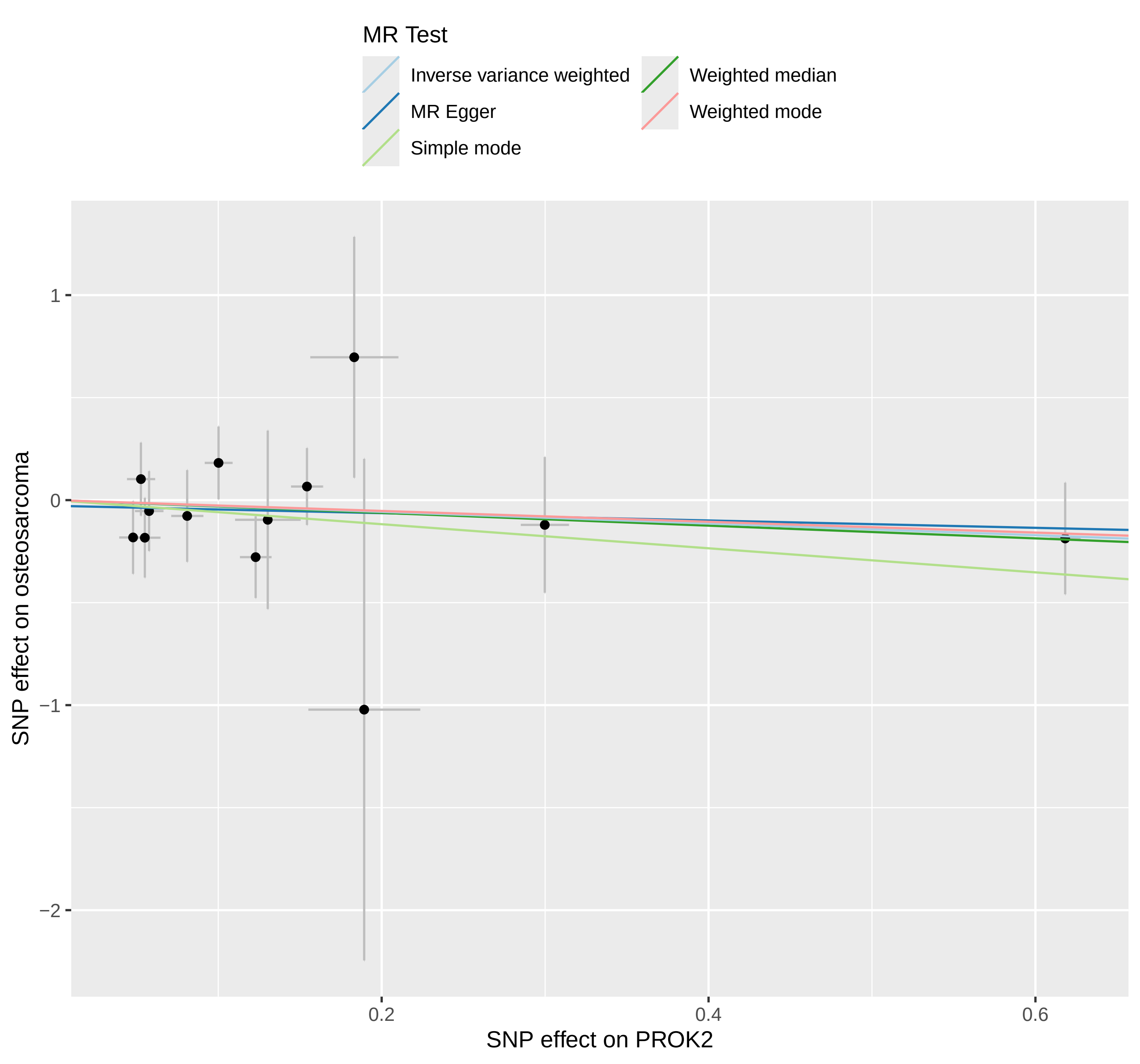

MR Test
Inverse variance weighted
MR Egger
Simple mode
Weighted median
Weighted mode
1
0
−1
−2
SNP effect on osteosarcoma
0.2
0.4
0.6
SNP effect on PROK2

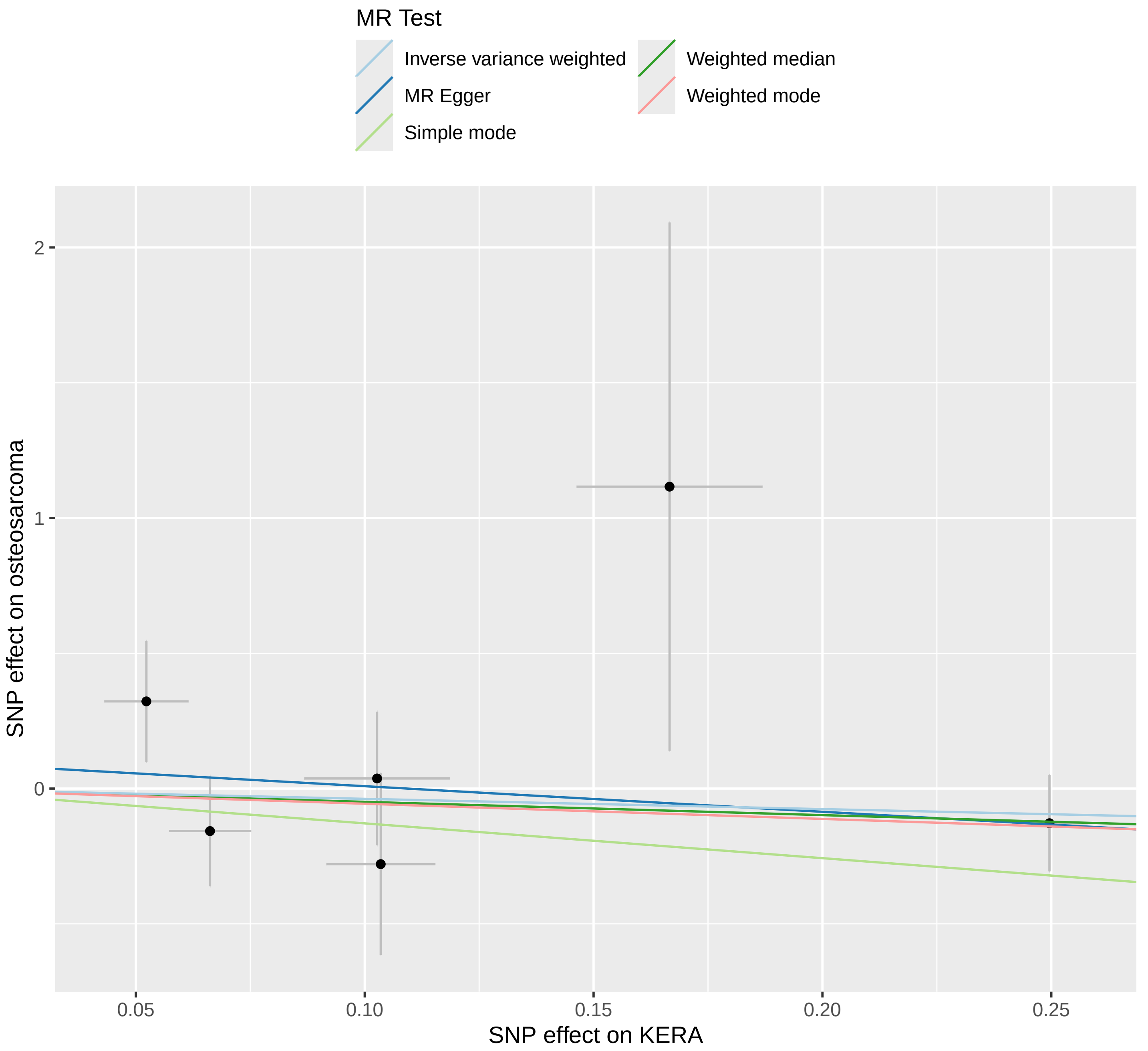
MR Test
Inverse variance weighted
MR Egger
Simple mode
Weighted median
Weighted mode
SNP effect on osteosarcoma
2
1
0
0.05
0.10
0.15
0.20
0.25
SNP effect on KERA

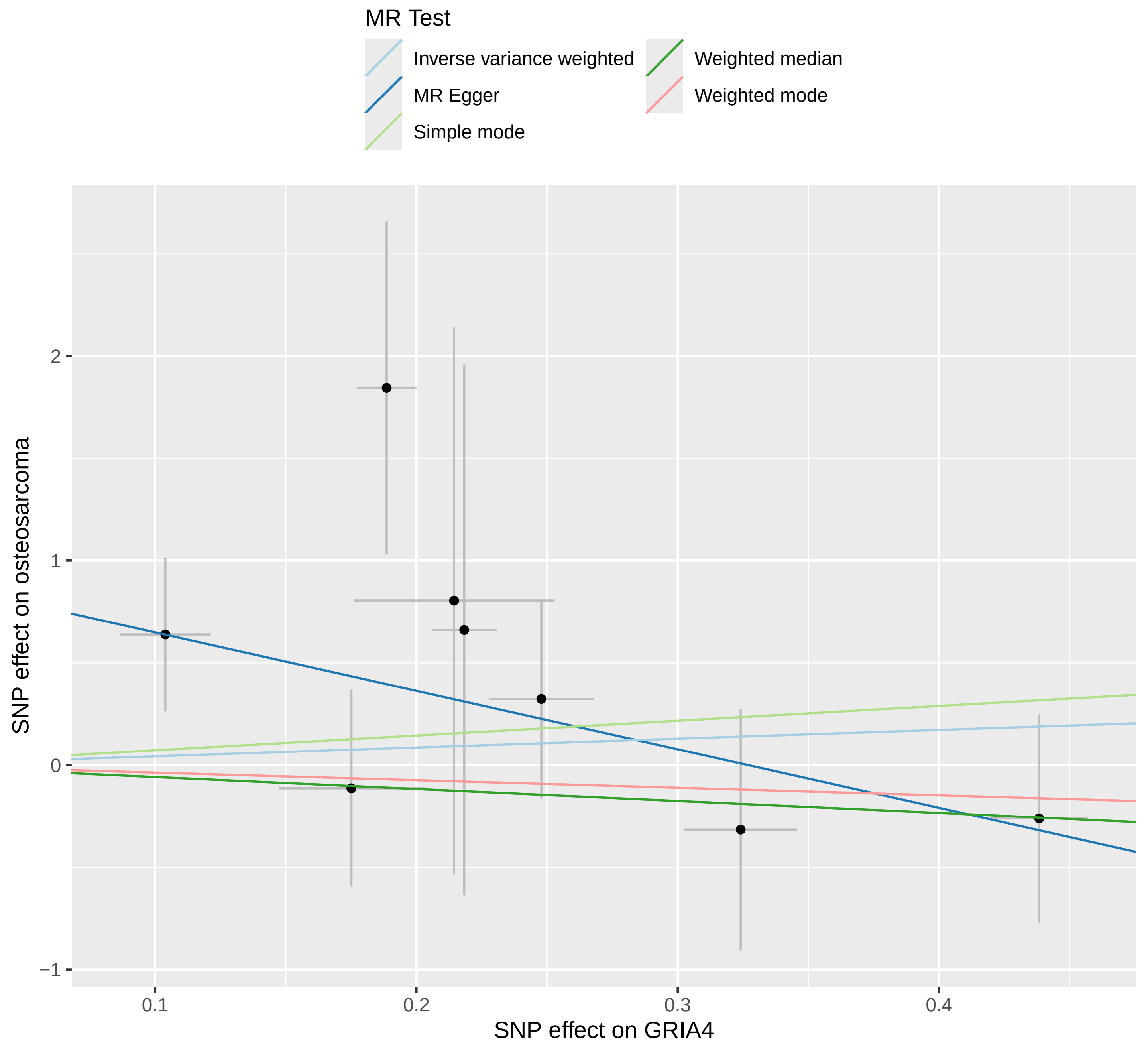
MR Test
Inverse variance weighted
MR Egger
Simple mode
Weighted median
Weighted mode
SNP effect on osteosarcoma
SNP effect on GRIA4
2
1
0
−1
0.1
0.2
0.3
0.4

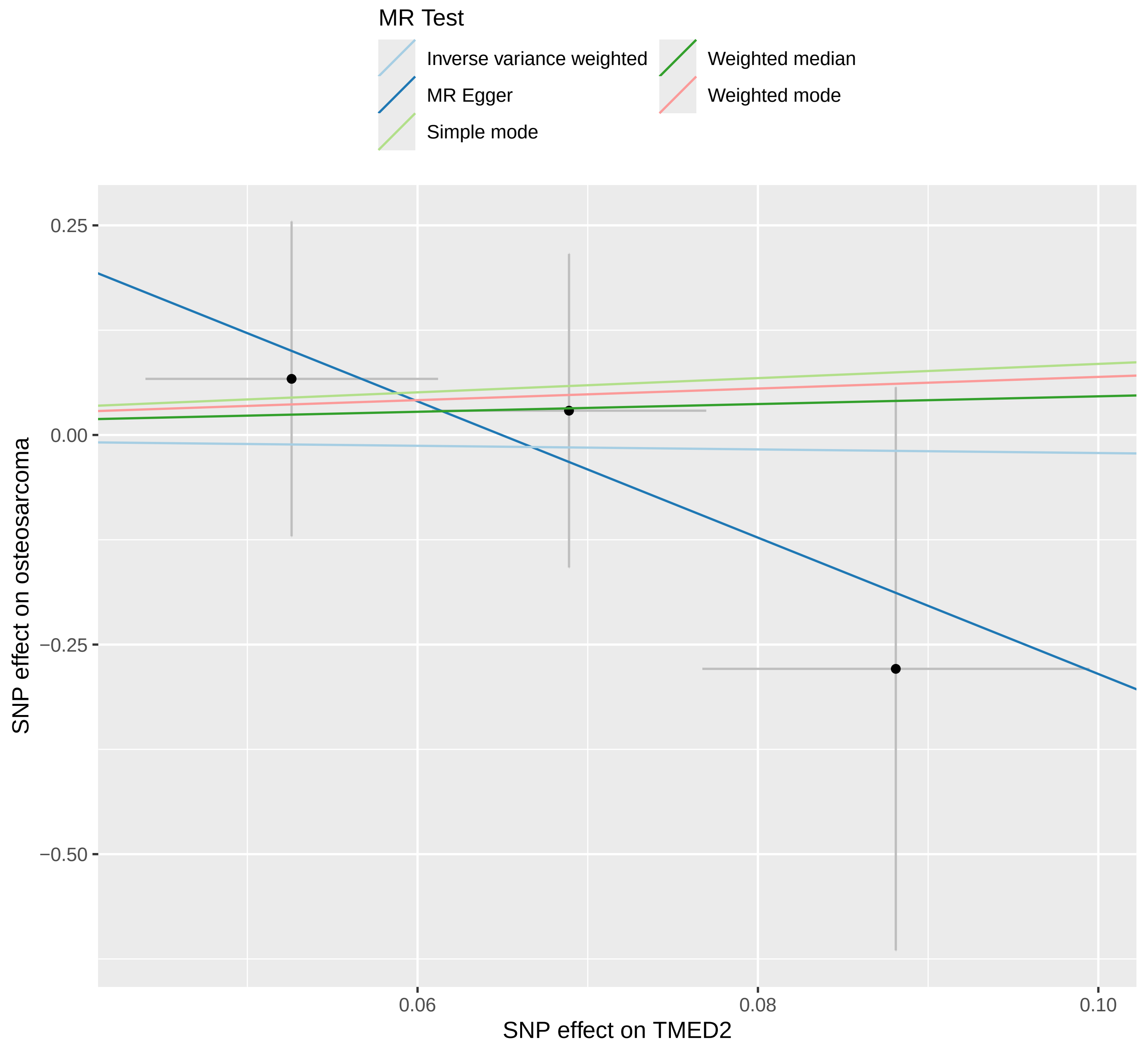
MR Test
Inverse variance weighted
MR Egger
Simple mode
Weighted median
Weighted mode
0.25
0.00
−0.25
−0.50
0.06
0.08
0.10
SNP effect on osteosarcoma
SNP effect on TMED2

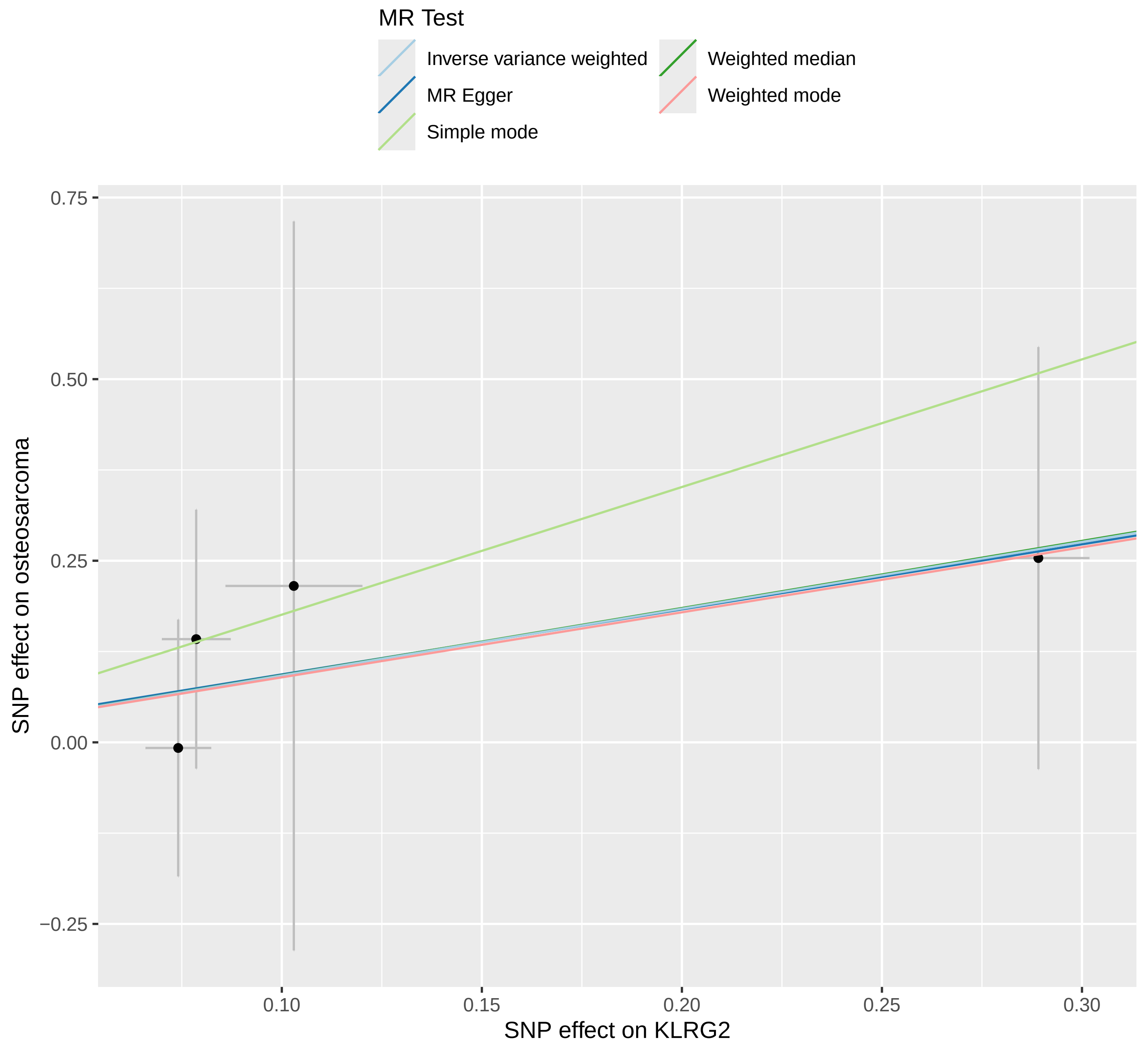

MR Test
Inverse variance weighted
MR Egger
Simple mode
Weighted median
Weighted mode
0.75
0.50
0.25
0.00
−0.25
SNP effect on osteosarcoma
0.10
0.15
0.20
0.25
0.30
SNP effect on KLRG2

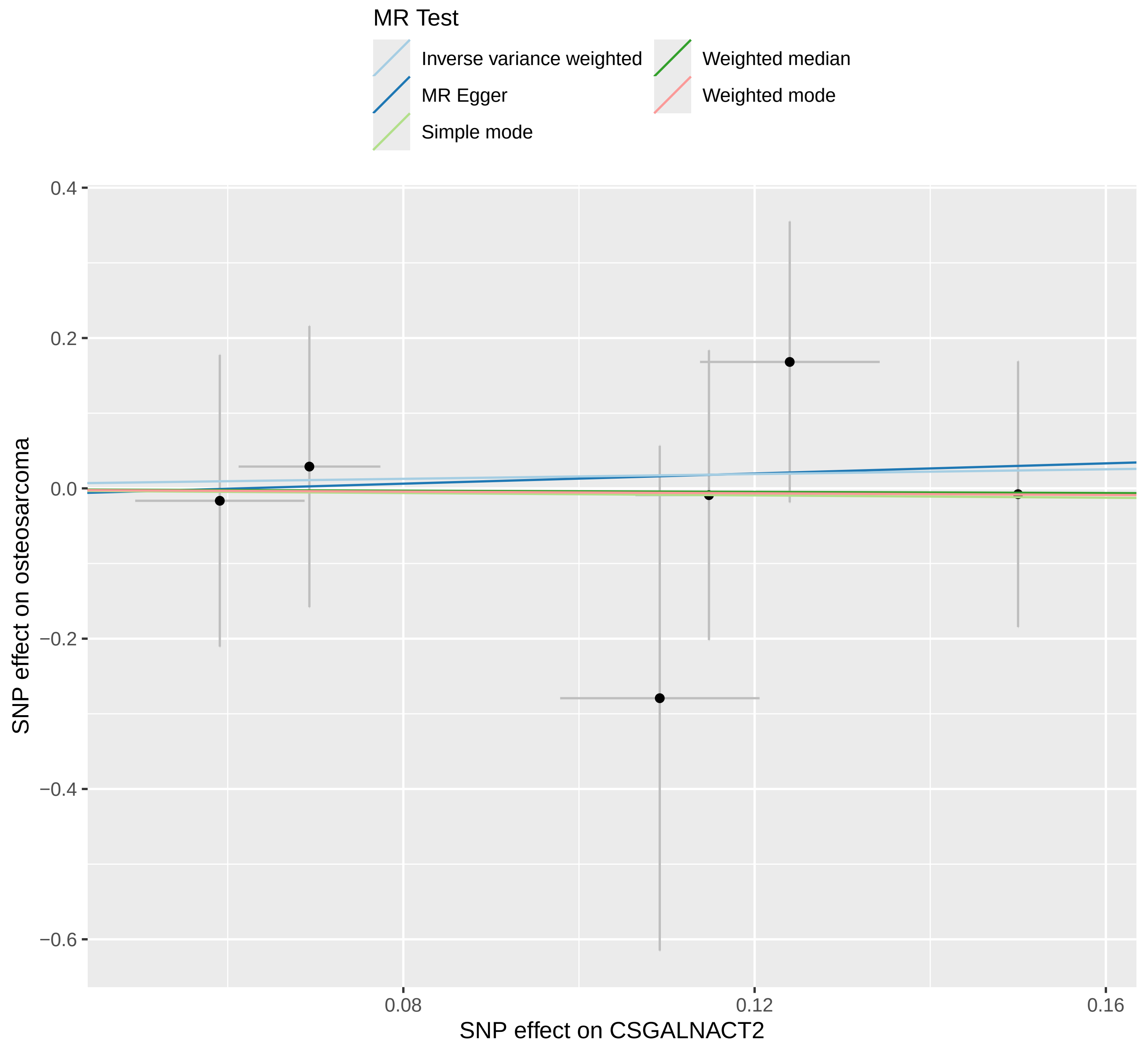

MR Test
Inverse variance weighted
MR Egger
Simple mode
Weighted median
Weighted mode
0.4
0.2
0.0
−0.2
−0.4
−0.6
SNP effect on osteosarcoma
0.08
0.12
0.16
SNP effect on CSGALNACT2

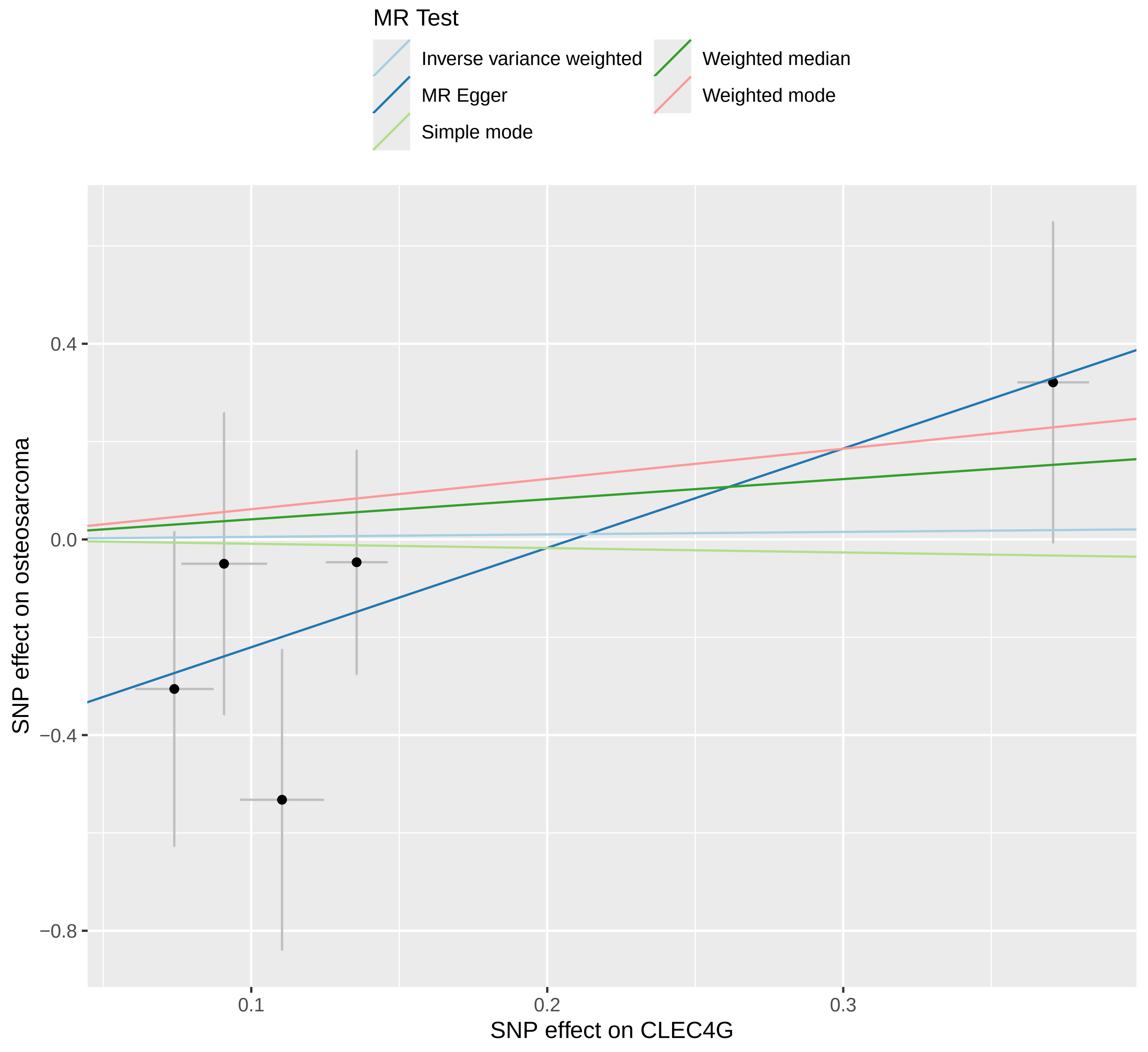

MR Test
Inverse variance weighted
MR Egger
Simple mode
Weighted median
Weighted mode
0.4
0.0
−0.4
−0.8
0.1
0.2
0.3
SNP effect on osteosarcoma
SNP effect on CLEC4G

Insufficient number of SNPs

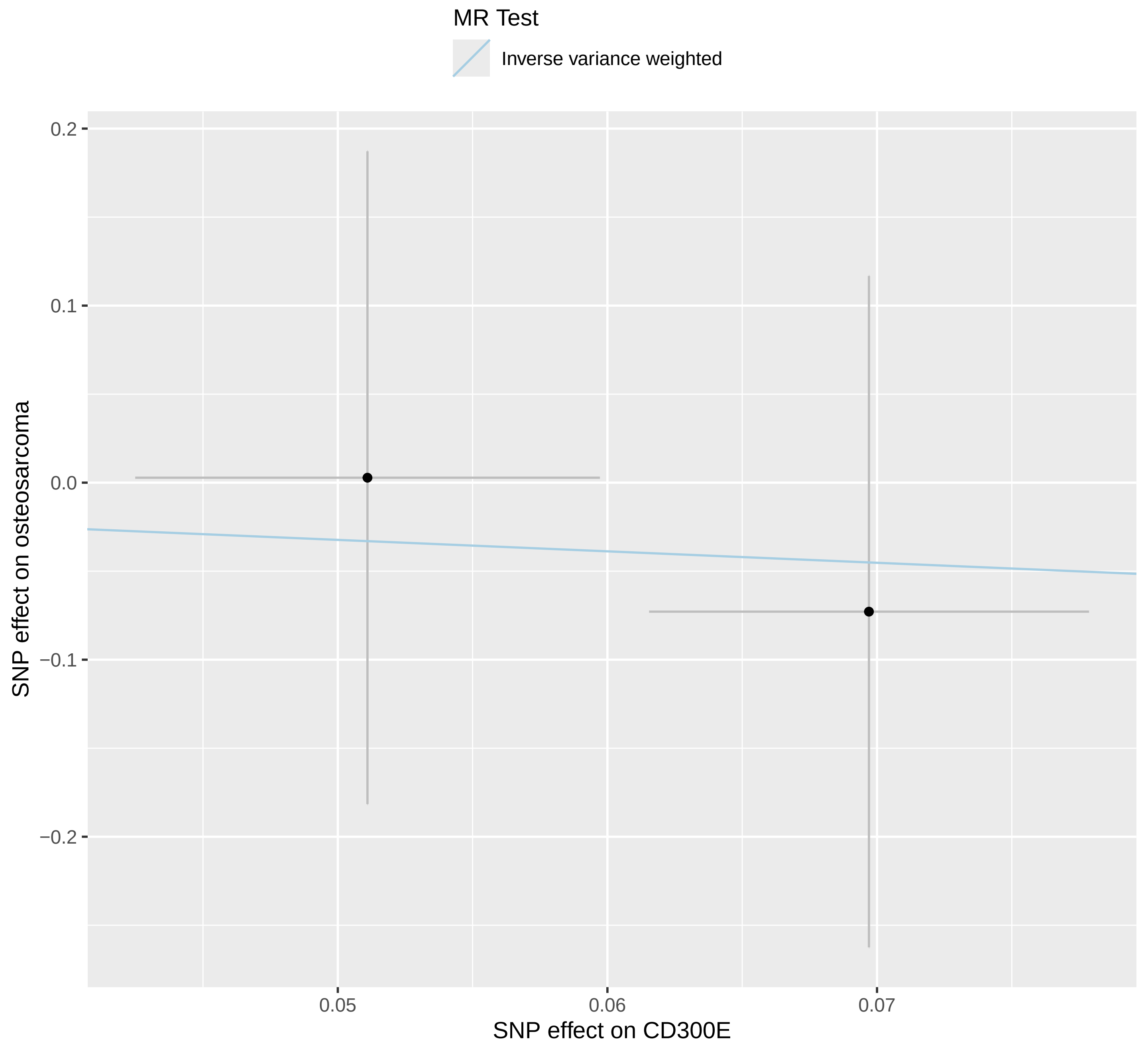
MR Test
Inverse variance weighted
0.2
0.1
0.0
−0.1
−0.2
0.05
0.06
0.07
SNP effect on osteosarcoma
SNP effect on CD300E

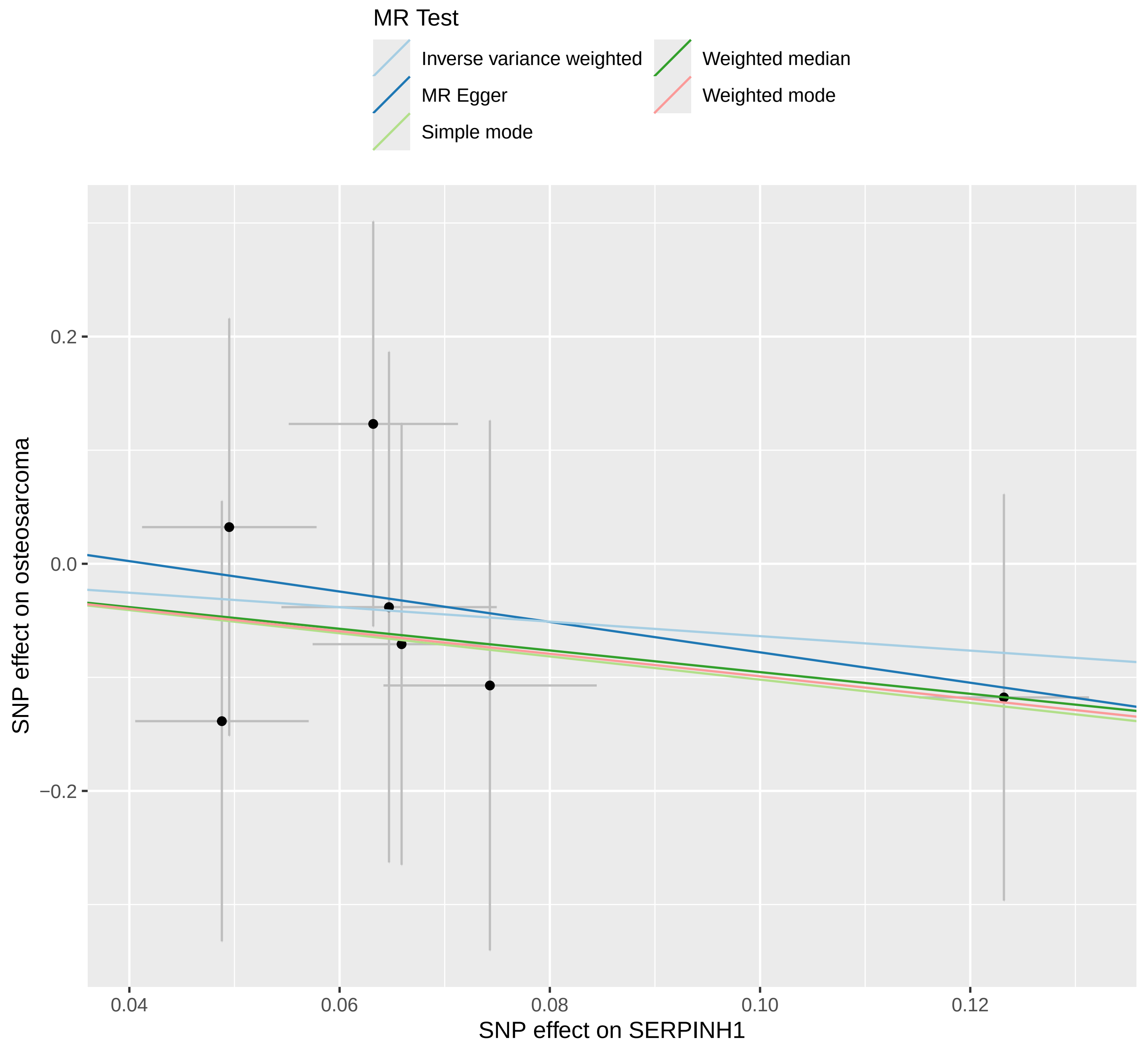
MR Test
Inverse variance weighted
MR Egger
Simple mode
Weighted median
Weighted mode
0.2
0.0
−0.2
0.04
0.06
0.08
0.10
0.12
SNP effect on osteosarcoma
SNP effect on SERPINH1

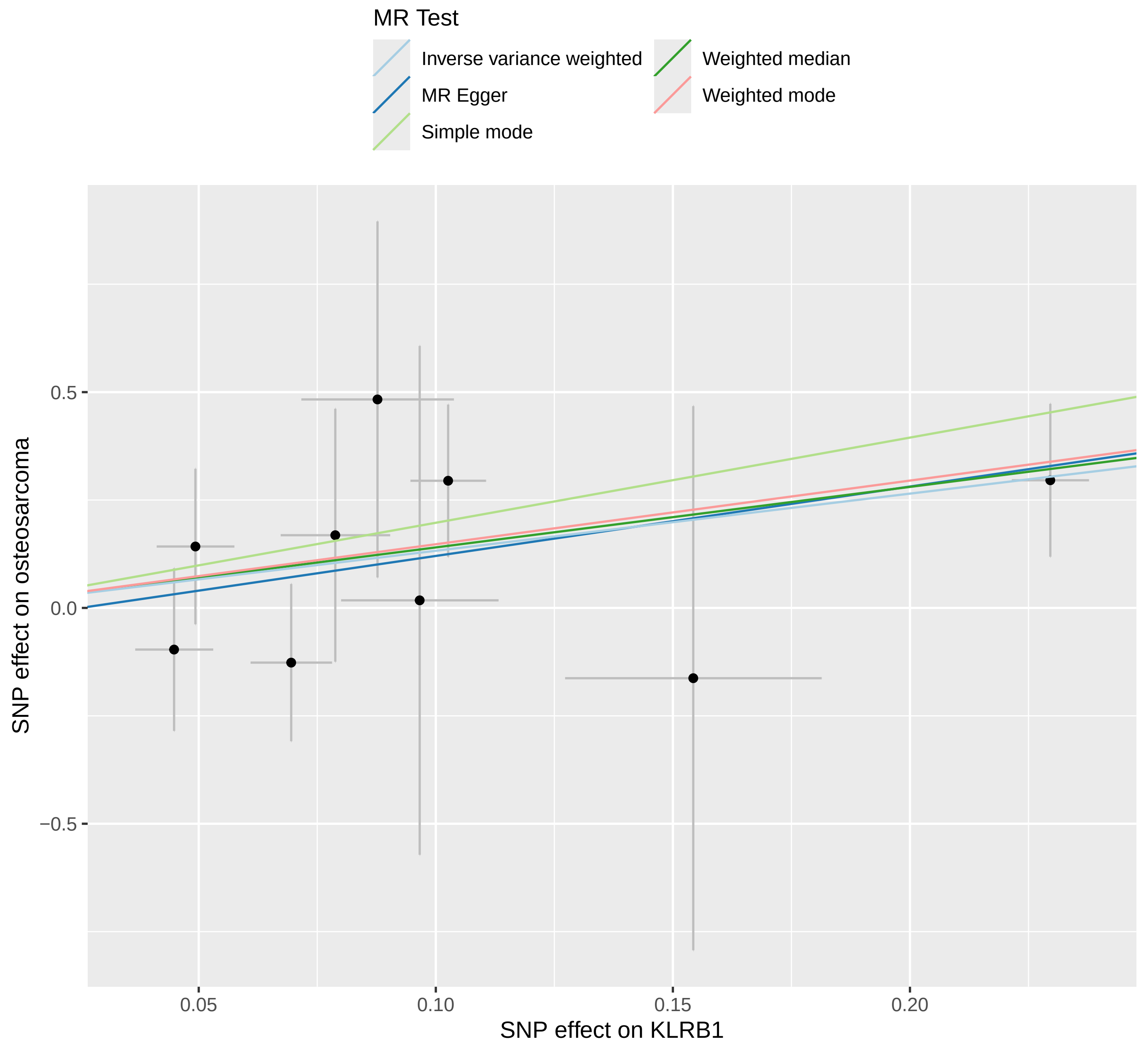
MR Test
Inverse variance weighted
MR Egger
Simple mode
Weighted median
Weighted mode
SNP effect on osteosarcoma
0.5
0.0
−0.5
0.05
0.10
0.15
0.20
SNP effect on KLRB1

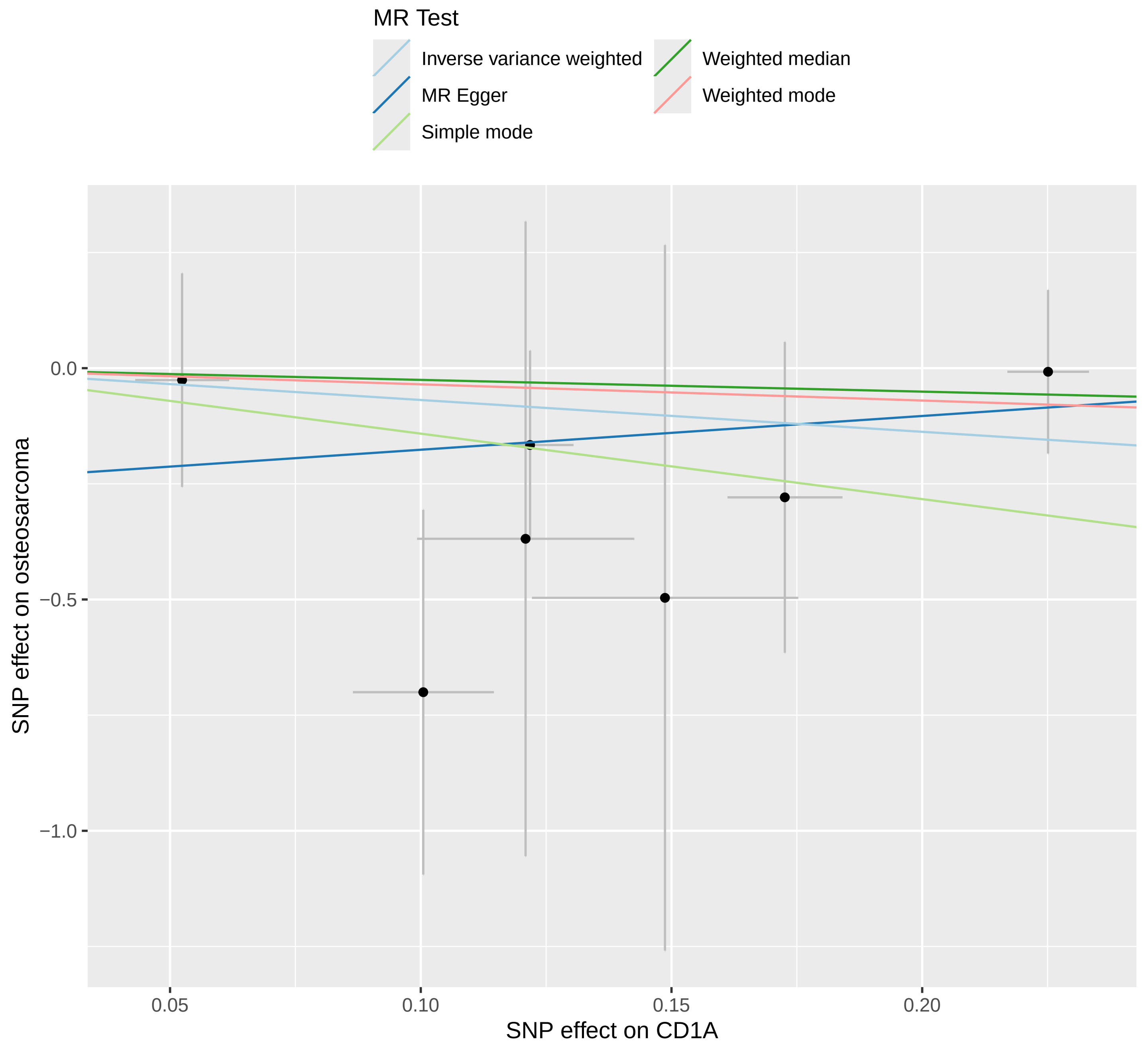

MR Test
Inverse variance weighted
MR Egger
Simple mode
Weighted median
Weighted mode
0.0
−0.5
−1.0
0.05
0.10
0.15
0.20
SNP effect on CD1A
SNP effect on osteosarcoma

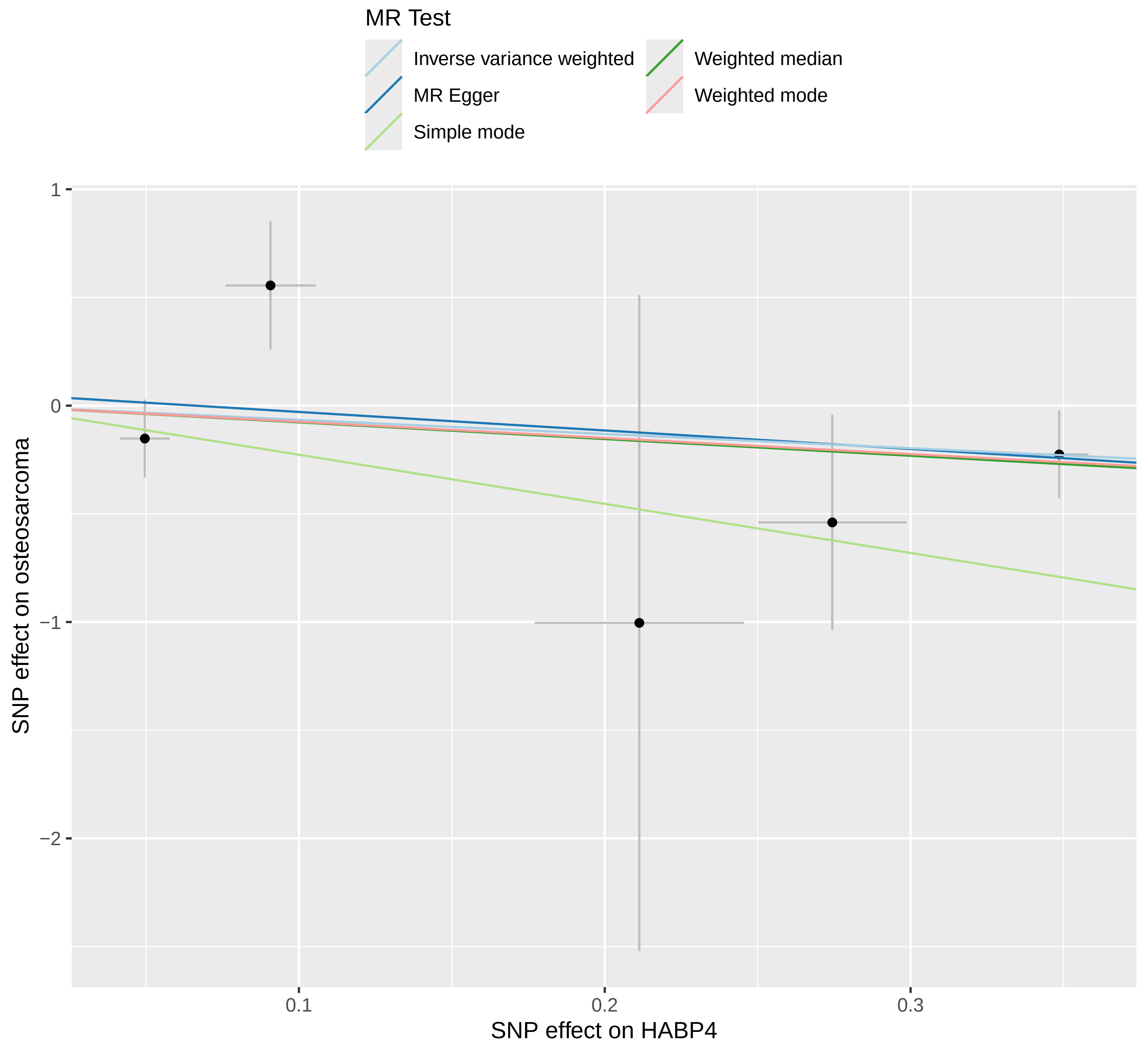
MR Test
Inverse variance weighted
MR Egger
Simple mode
Weighted median
Weighted mode
1
0
−1
−2
0.1
0.2
0.3
SNP effect on osteosarcoma
SNP effect on HABP4

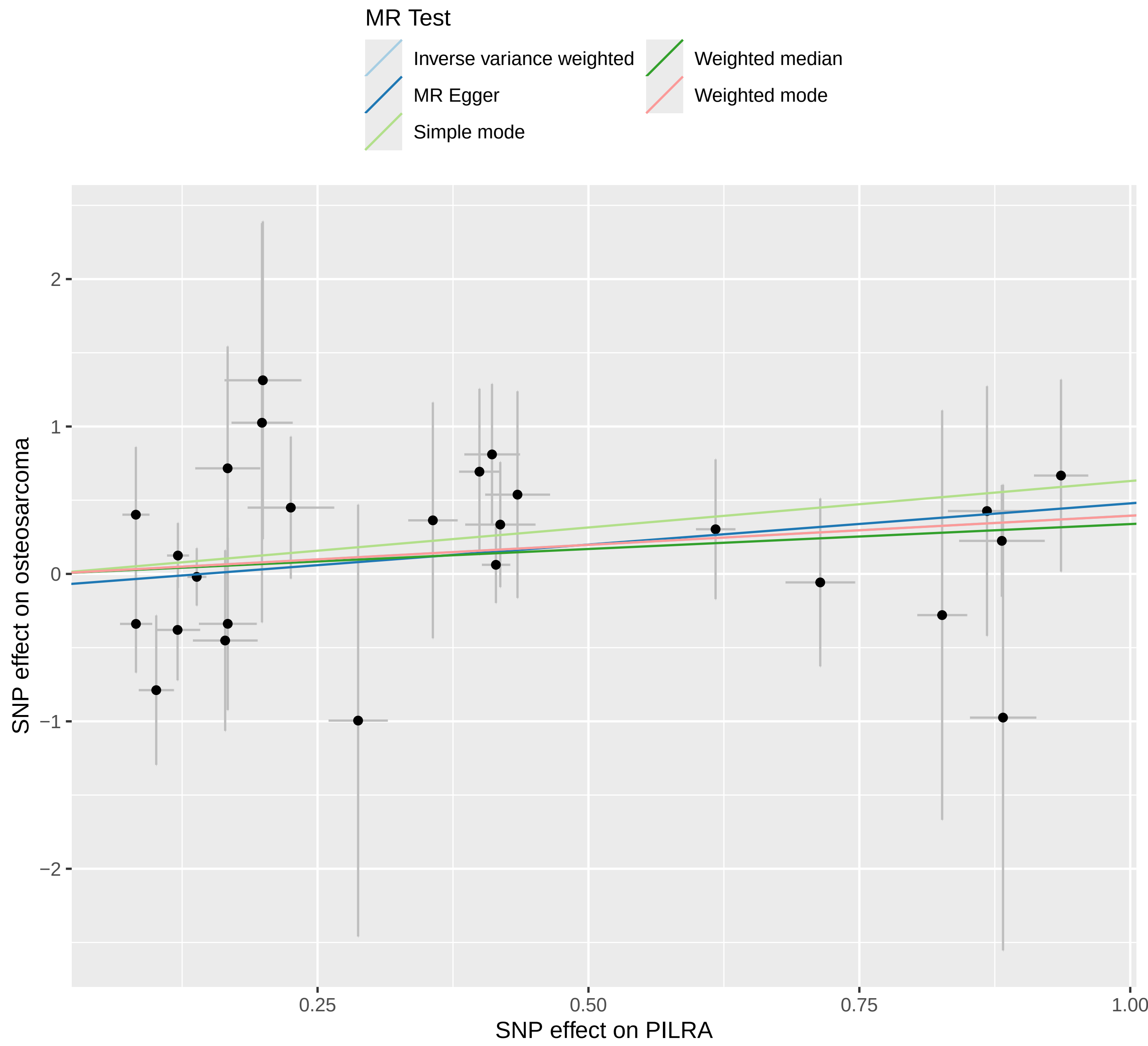

MR Test
Inverse variance weighted
MR Egger
Simple mode
Weighted median
Weighted mode
SNP effect on osteosarcoma
2
1
0
−1
−2
0.25
0.50
0.75
1.00
SNP effect on PILRA

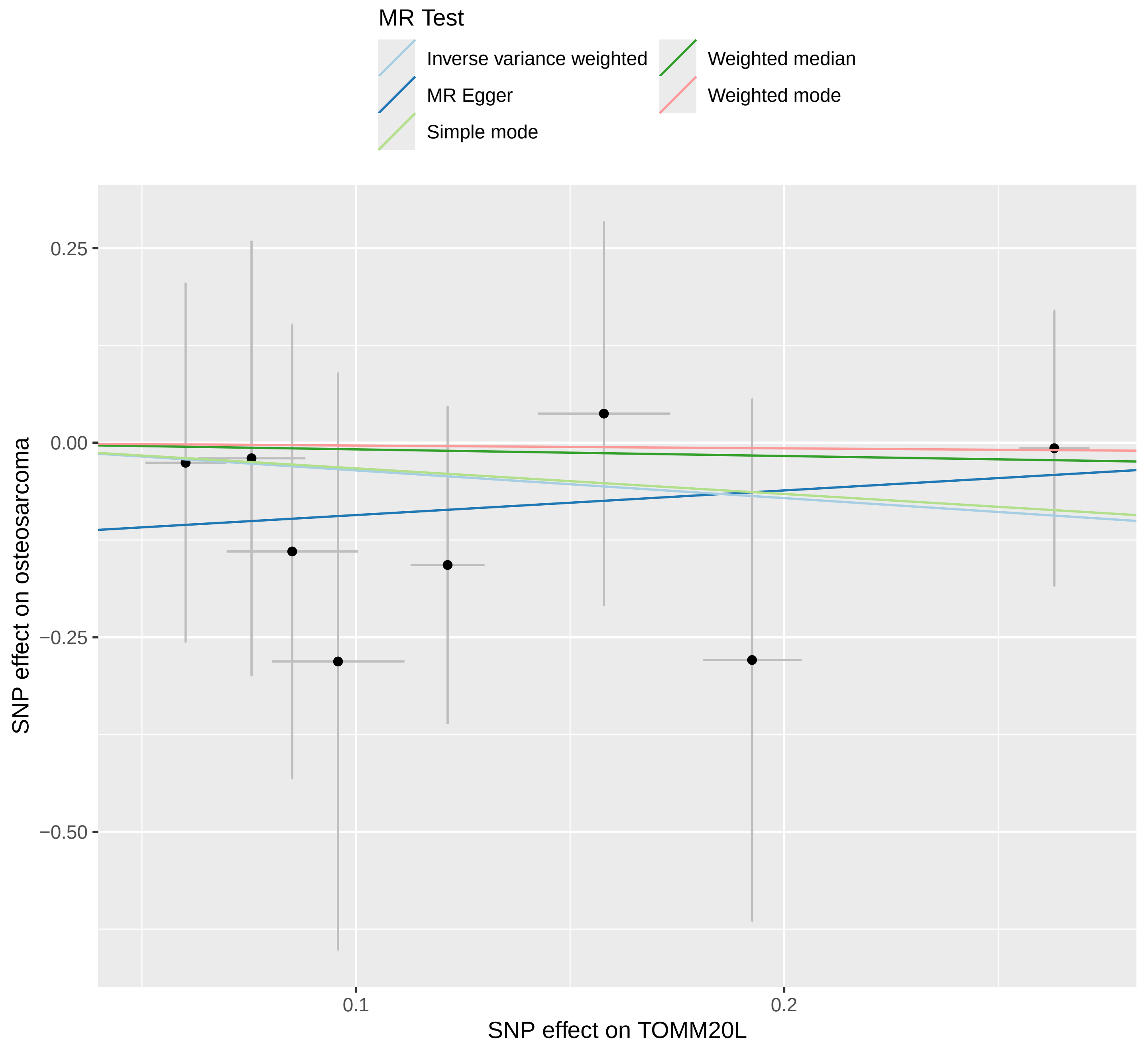
MR Test
Inverse variance weighted
MR Egger
Simple mode
Weighted median
Weighted mode
0.25
0.00
−0.25
−0.50
0.1
0.2
SNP effect on osteosarcoma
SNP effect on TOMM20L

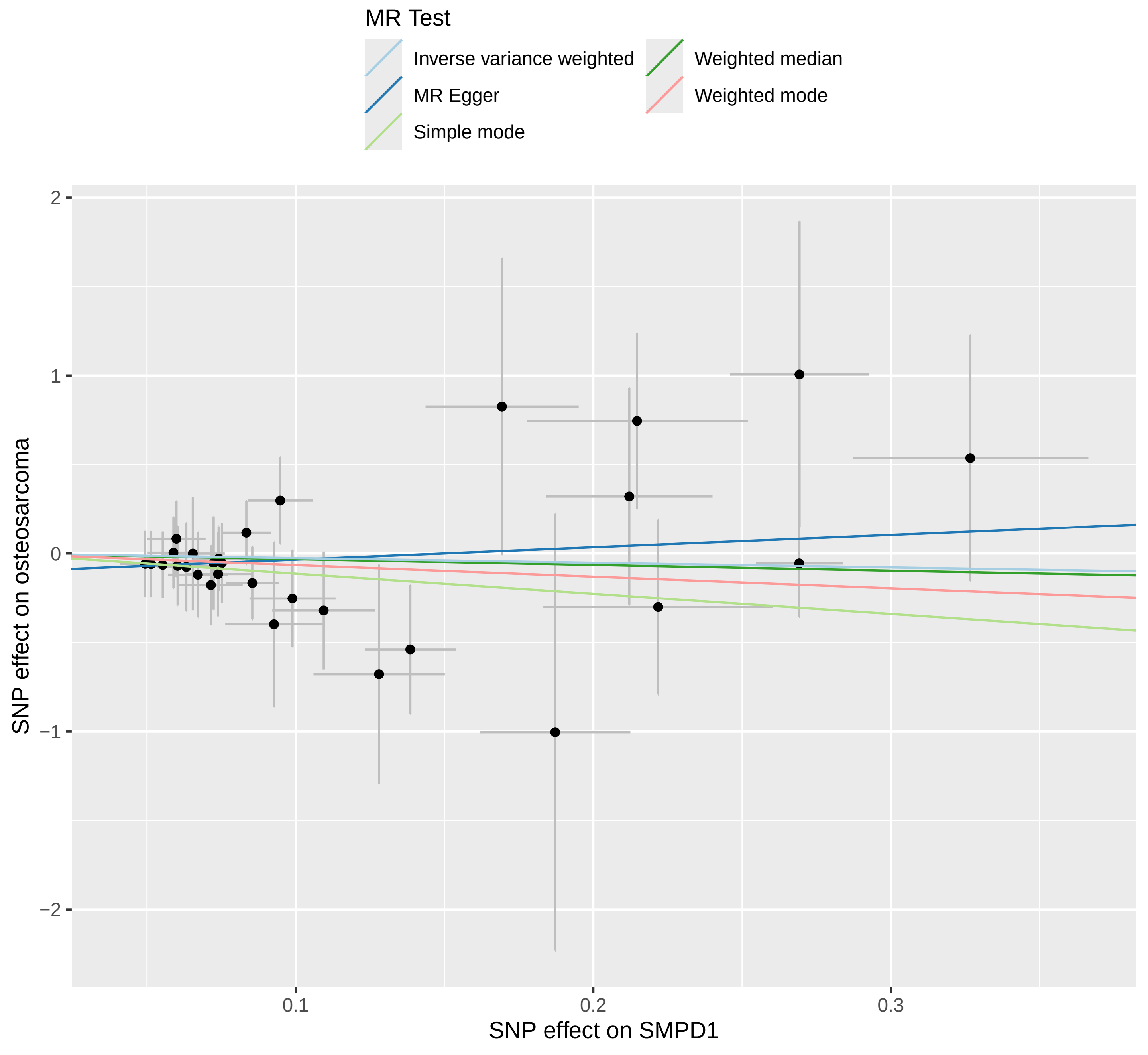

MR Test
Inverse variance weighted
MR Egger
Simple mode
Weighted median
Weighted mode
2
1
0
−1
−2
0.1
0.2
0.3
SNP effect on osteosarcoma
SNP effect on SMPD1

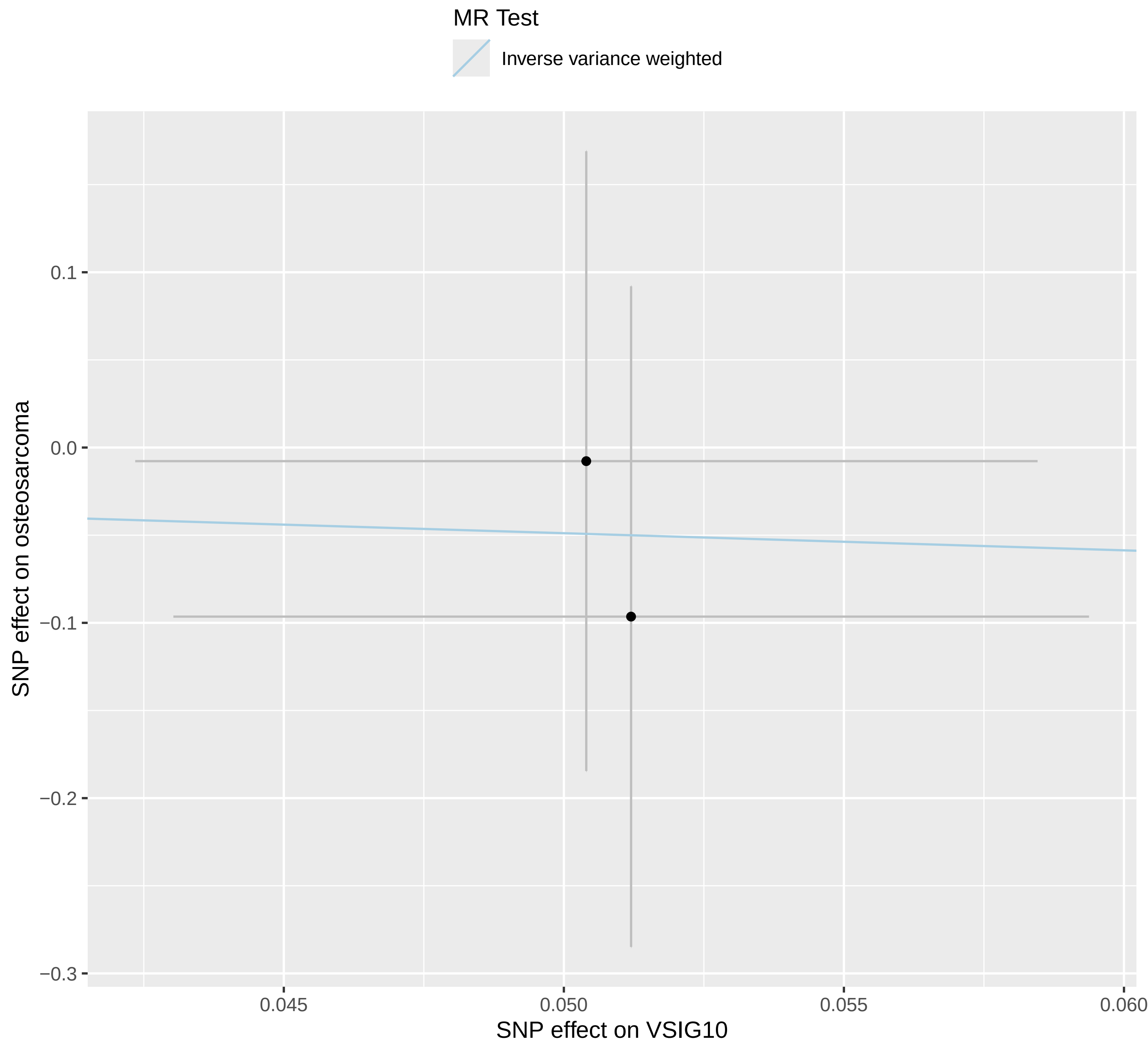
MR Test
Inverse variance weighted
0.1
0.0
−0.1
−0.2
−0.3
0.045
0.050
0.055
0.060
SNP effect on VSIG10
SNP effect on osteosarcoma

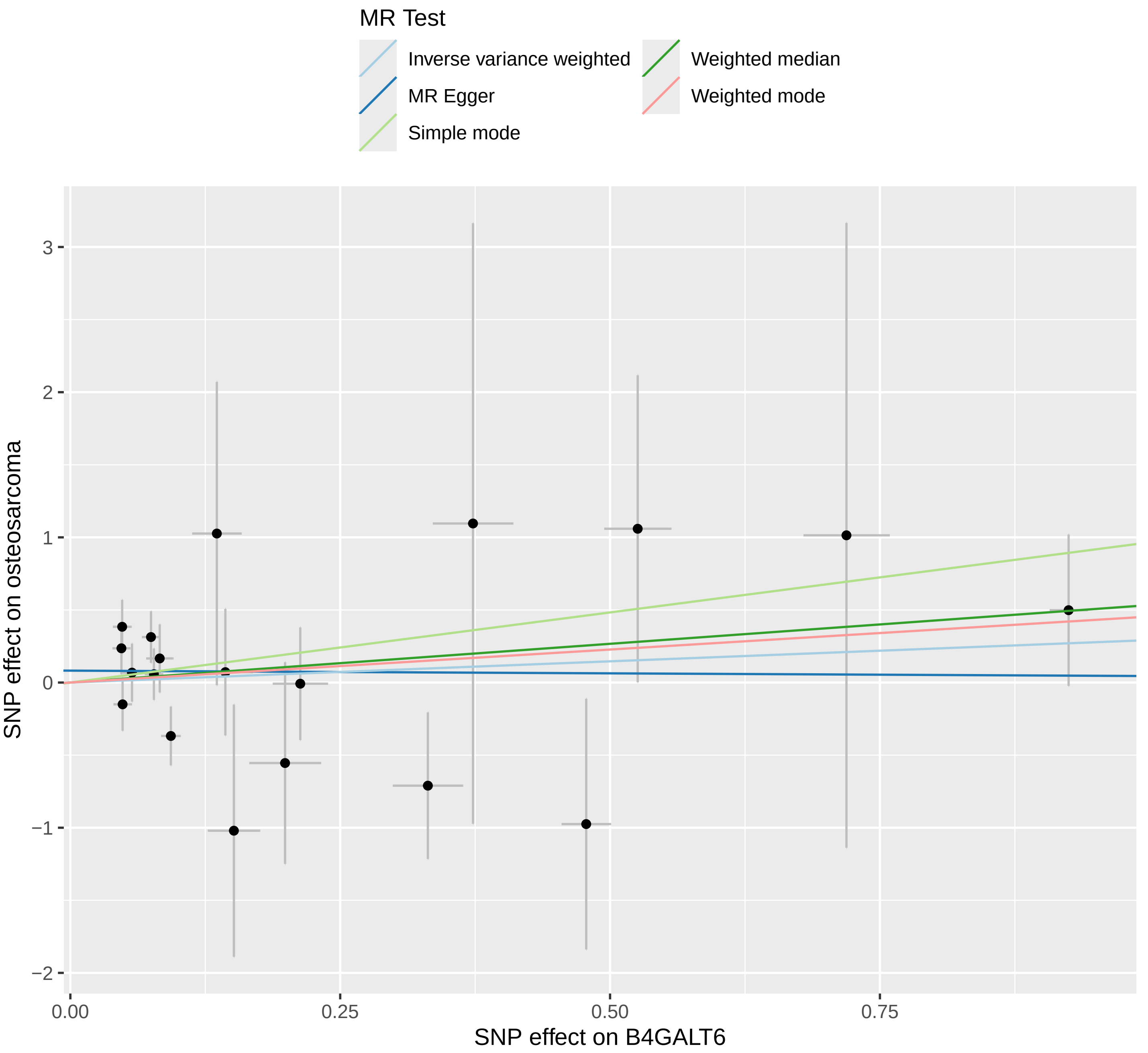
MR Test
Inverse variance weighted
MR Egger
Simple mode
Weighted median
Weighted mode
3
2
1
0
−1
−2
0.00
0.25
0.50
0.75
SNP effect on osteosarcoma
SNP effect on B4GALT6

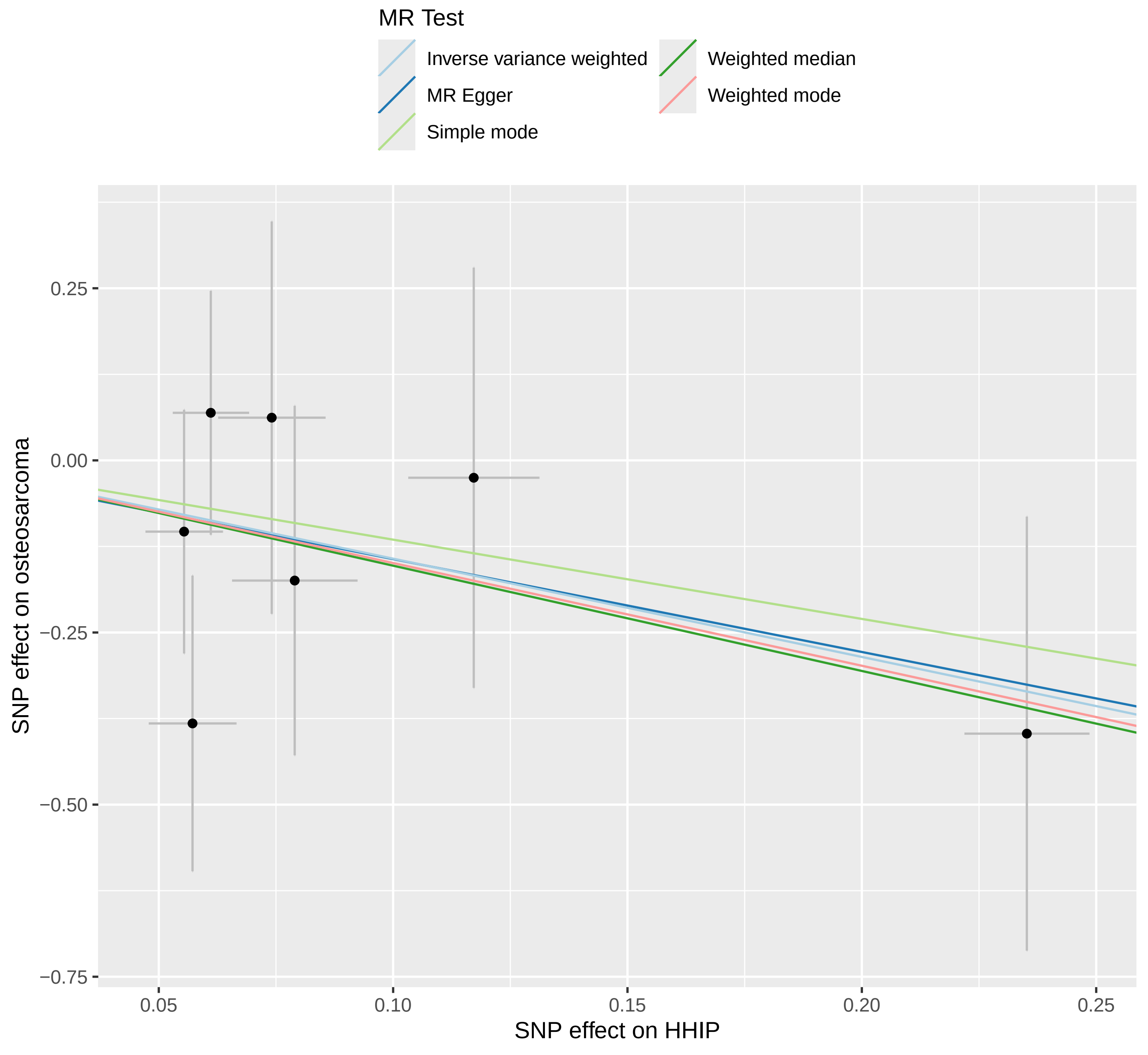
MR Test
Inverse variance weighted
MR Egger
Simple mode
Weighted median
Weighted mode
0.25
0.00
−0.25
−0.50
−0.75
0.05
0.10
0.15
0.20
0.25
SNP effect on osteosarcoma
SNP effect on HHIP

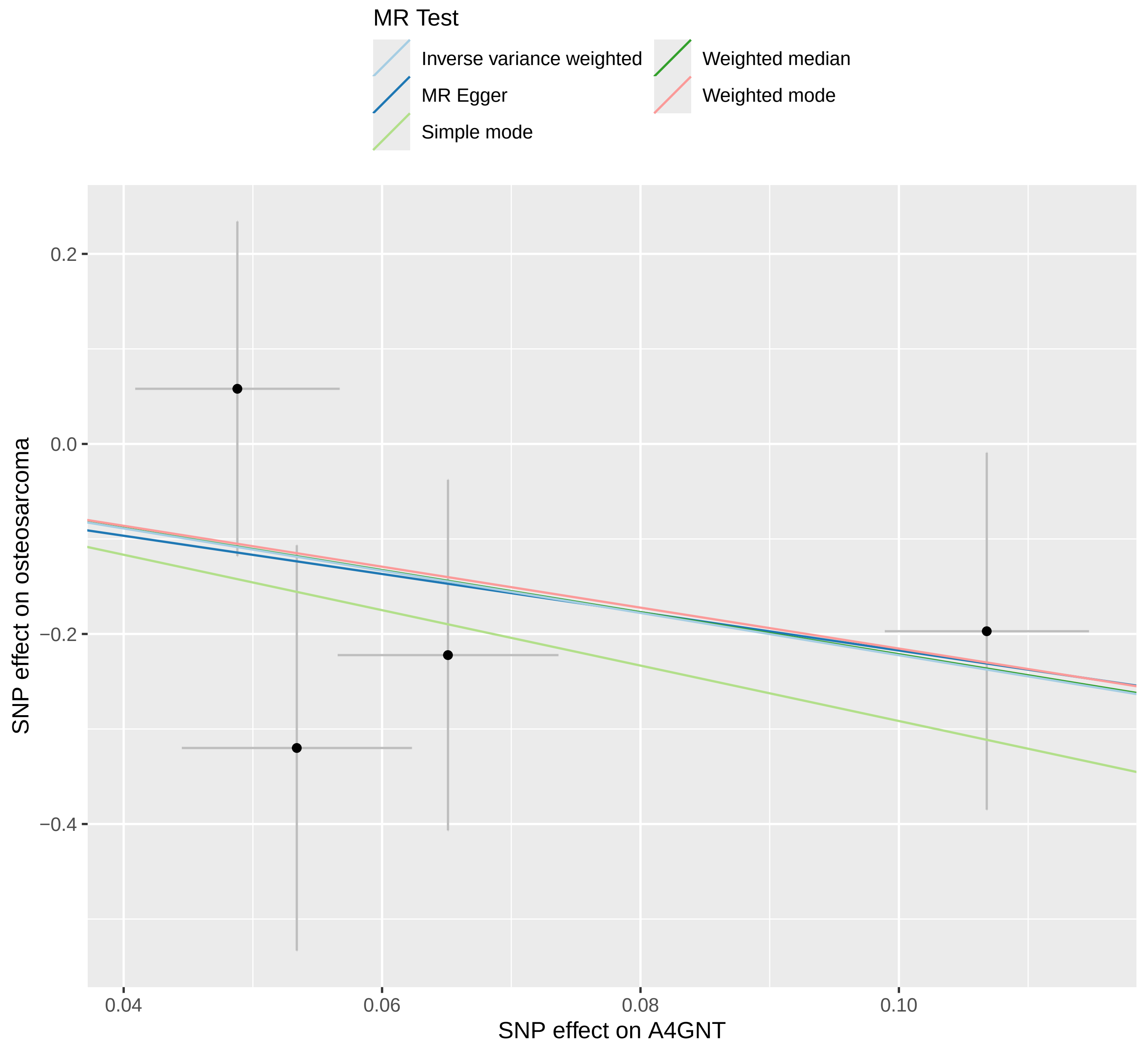
MR Test
Inverse variance weighted
MR Egger
Simple mode
Weighted median
Weighted mode
0.2
0.0
−0.2
−0.4
SNP effect on osteosarcoma
0.04
0.06
0.08
0.10
SNP effect on A4GNT

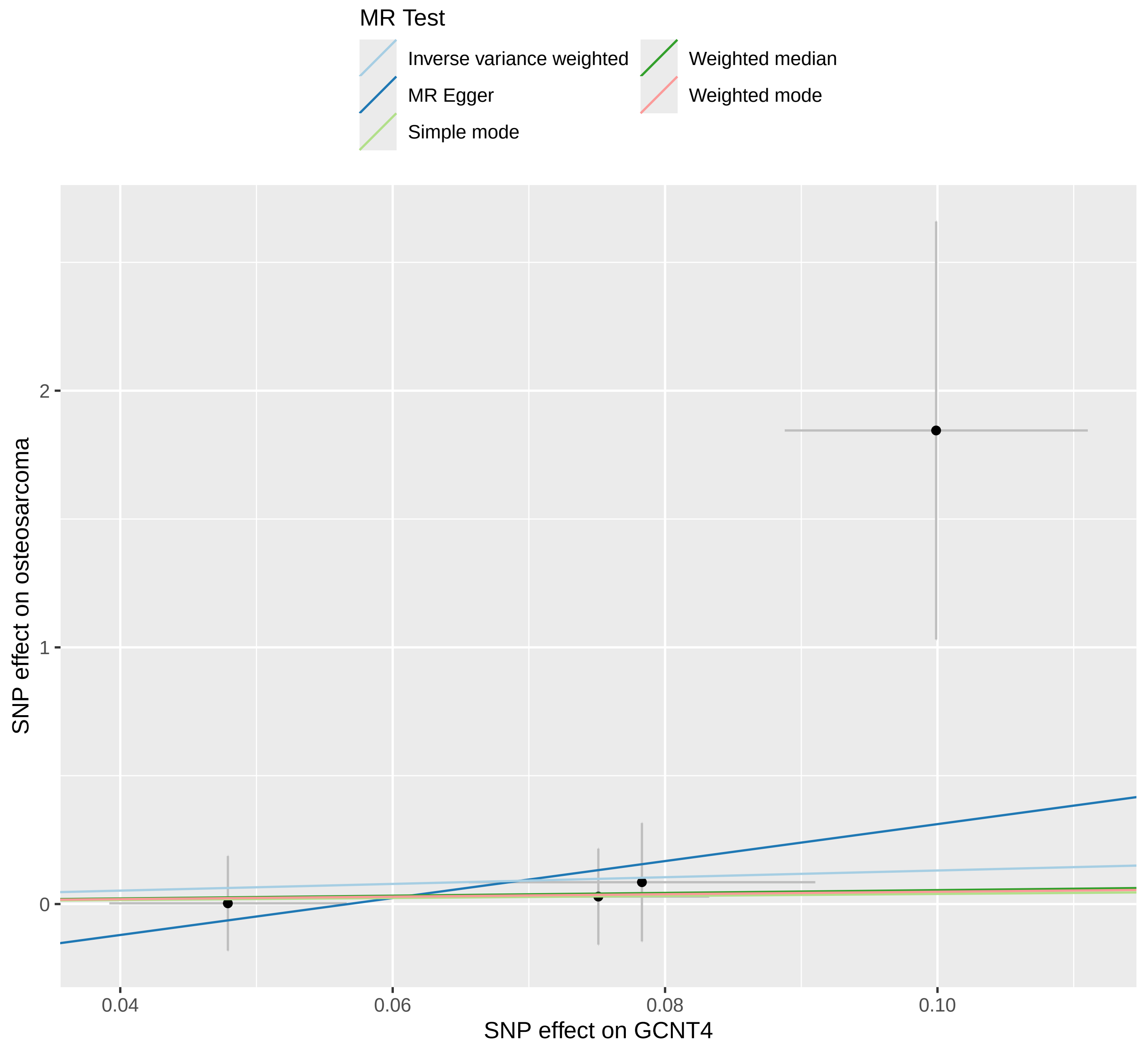

MR Test
Inverse variance weighted
MR Egger
Simple mode
Weighted median
Weighted mode
SNP effect on osteosarcoma
2
1
0
0.04
0.06
0.08
0.10
SNP effect on GCNT4

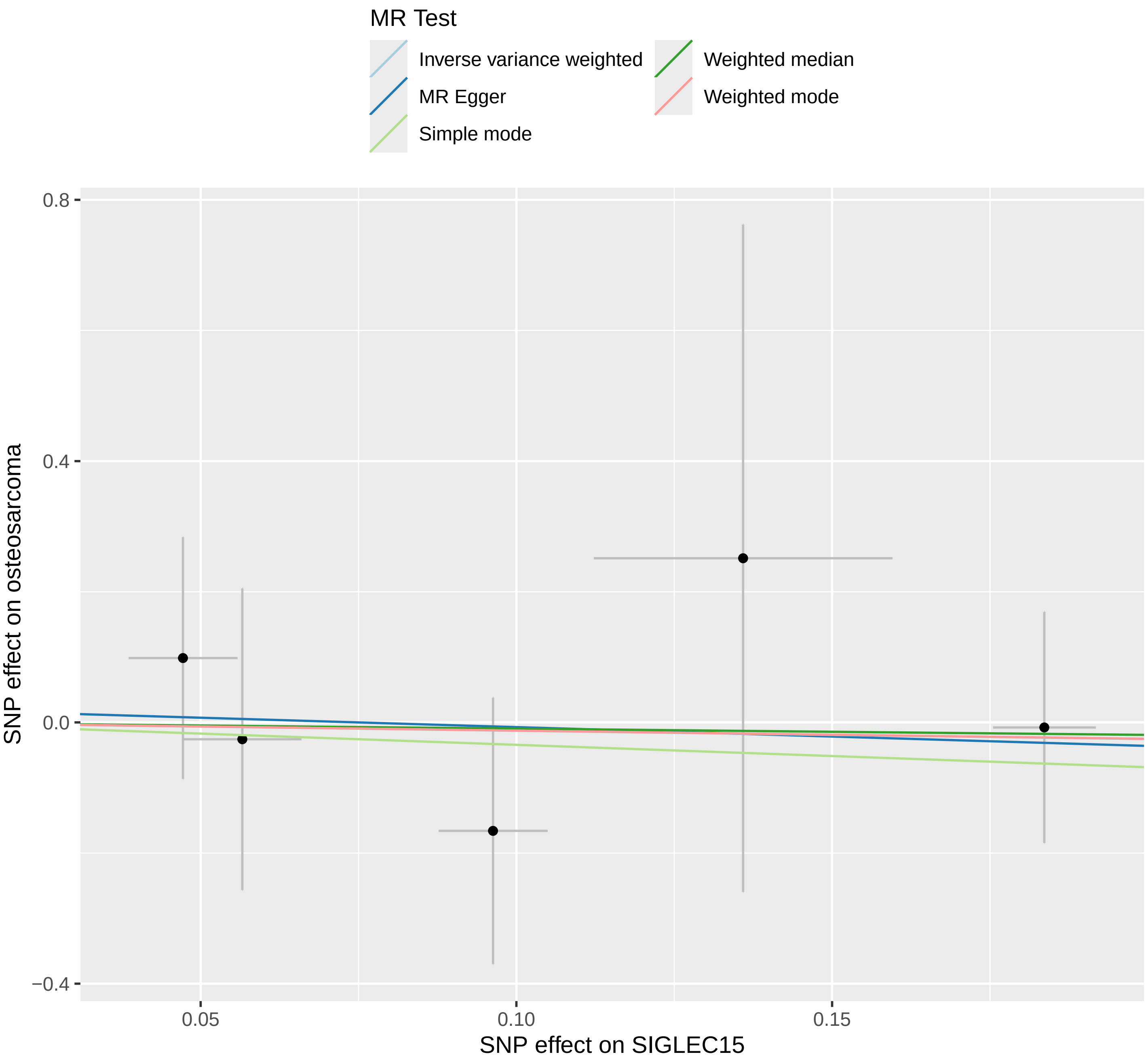
MR Test
Inverse variance weighted
MR Egger
Simple mode
Weighted median
Weighted mode
0.8
0.4
0.0
−0.4
0.05
0.10
0.15
SNP effect on osteosarcoma
SNP effect on SIGLEC15

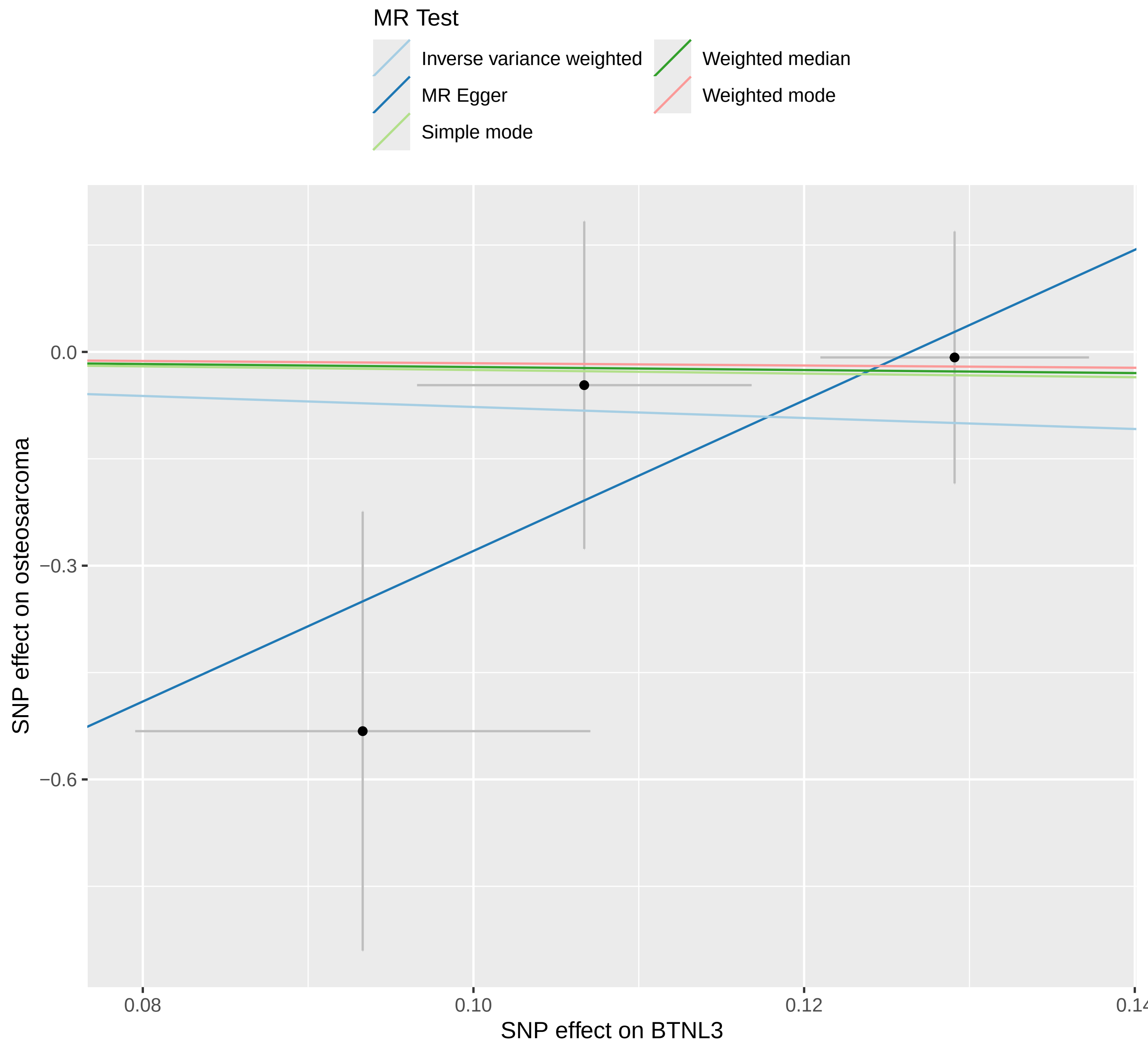
MR Test
Inverse variance weighted
MR Egger
Simple mode
Weighted median
Weighted mode
0.0
−0.3
−0.6
0.08
0.10
0.12
SNP effect on osteosarcoma
SNP effect on BTNL3

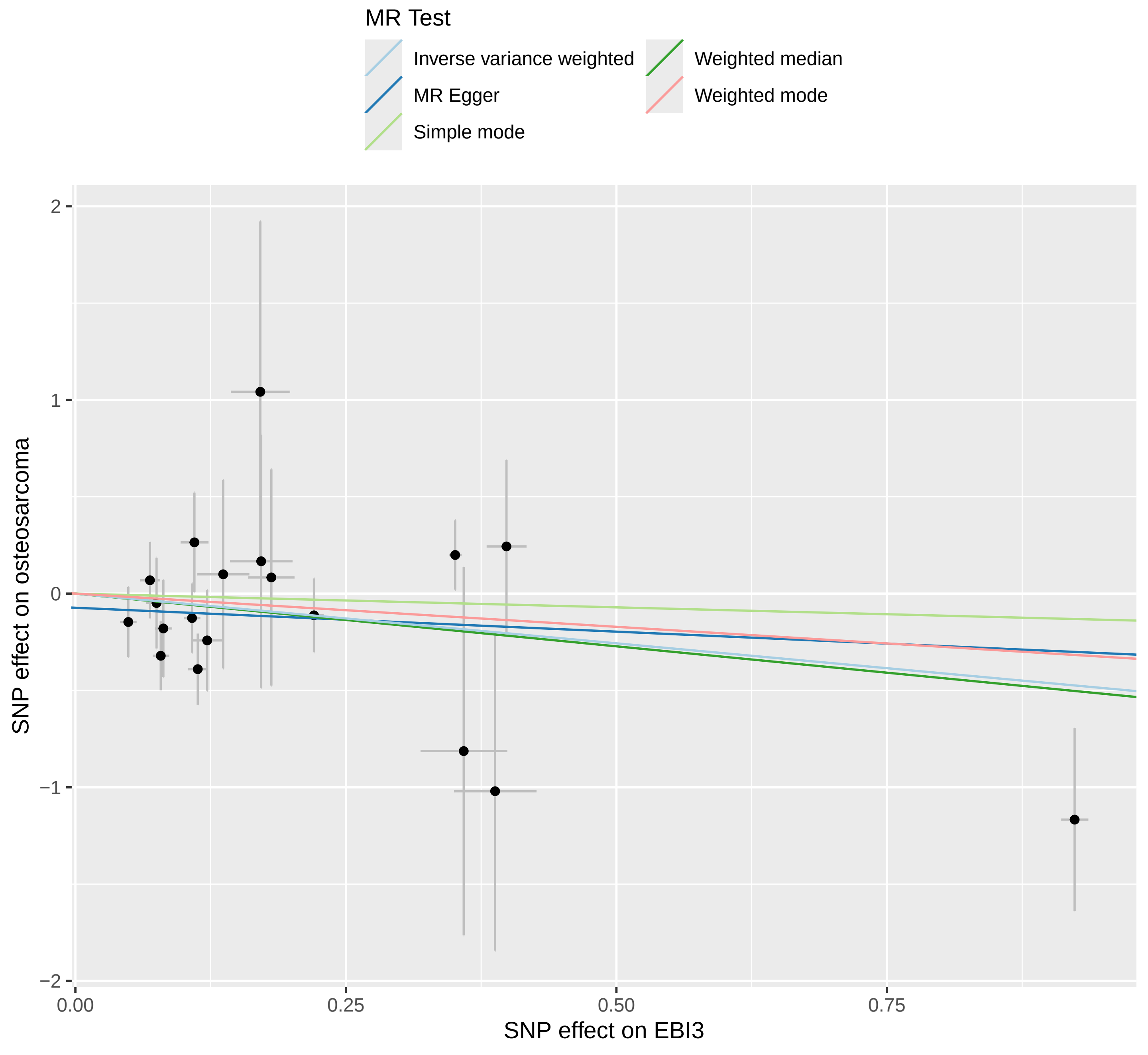
MR Test
Inverse variance weighted
MR Egger
Simple mode
Weighted median
Weighted mode
SNP effect on osteosarcoma
SNP effect on EBI3
2
1
0
−1
−2
0.00
0.25
0.50
0.75

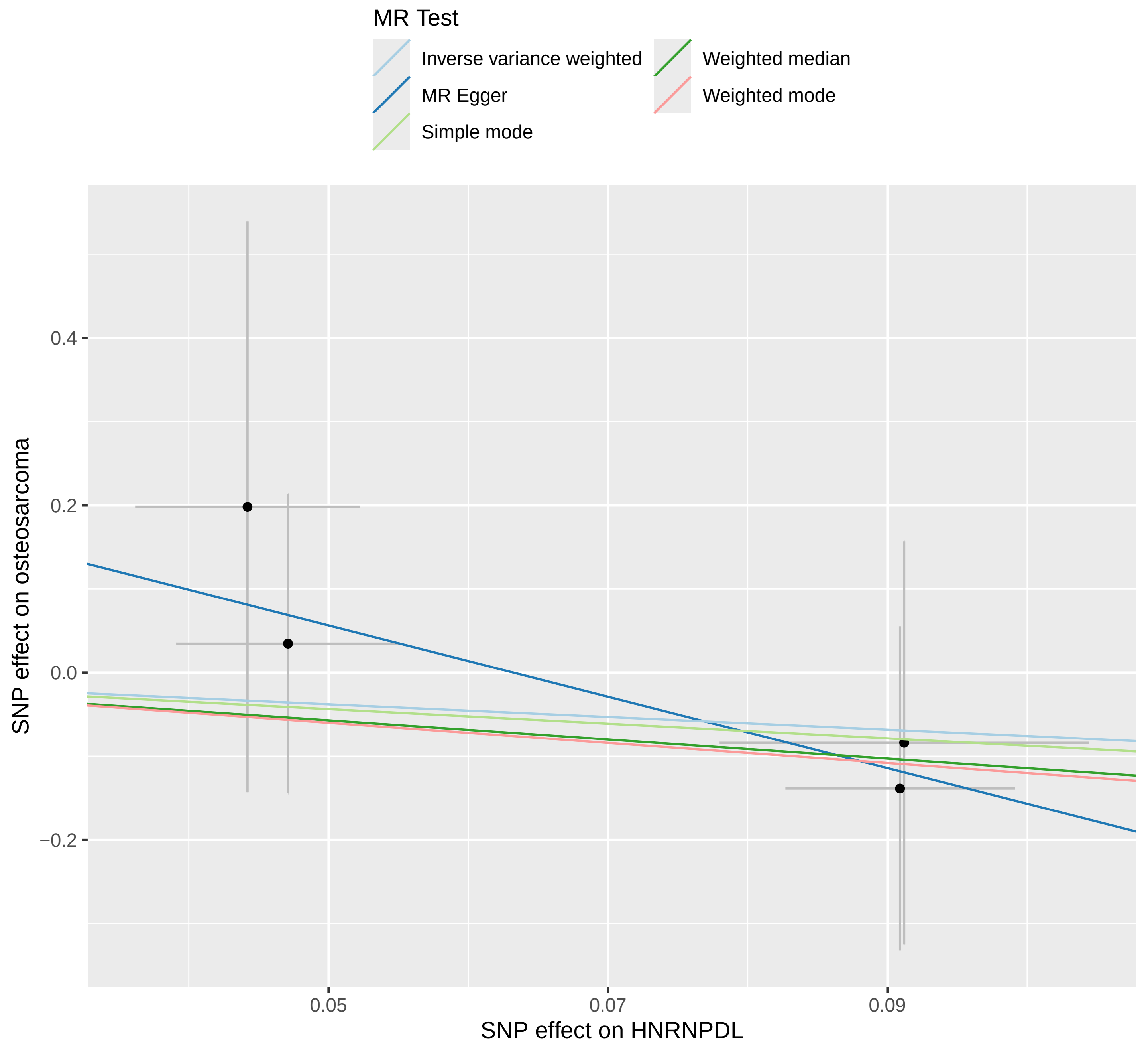
MR Test
Inverse variance weighted
MR Egger
Simple mode
Weighted median
Weighted mode
0.4
0.2
0.0
−0.2
SNP effect on osteosarcoma
0.05
0.07
0.09
SNP effect on HNRNPDL

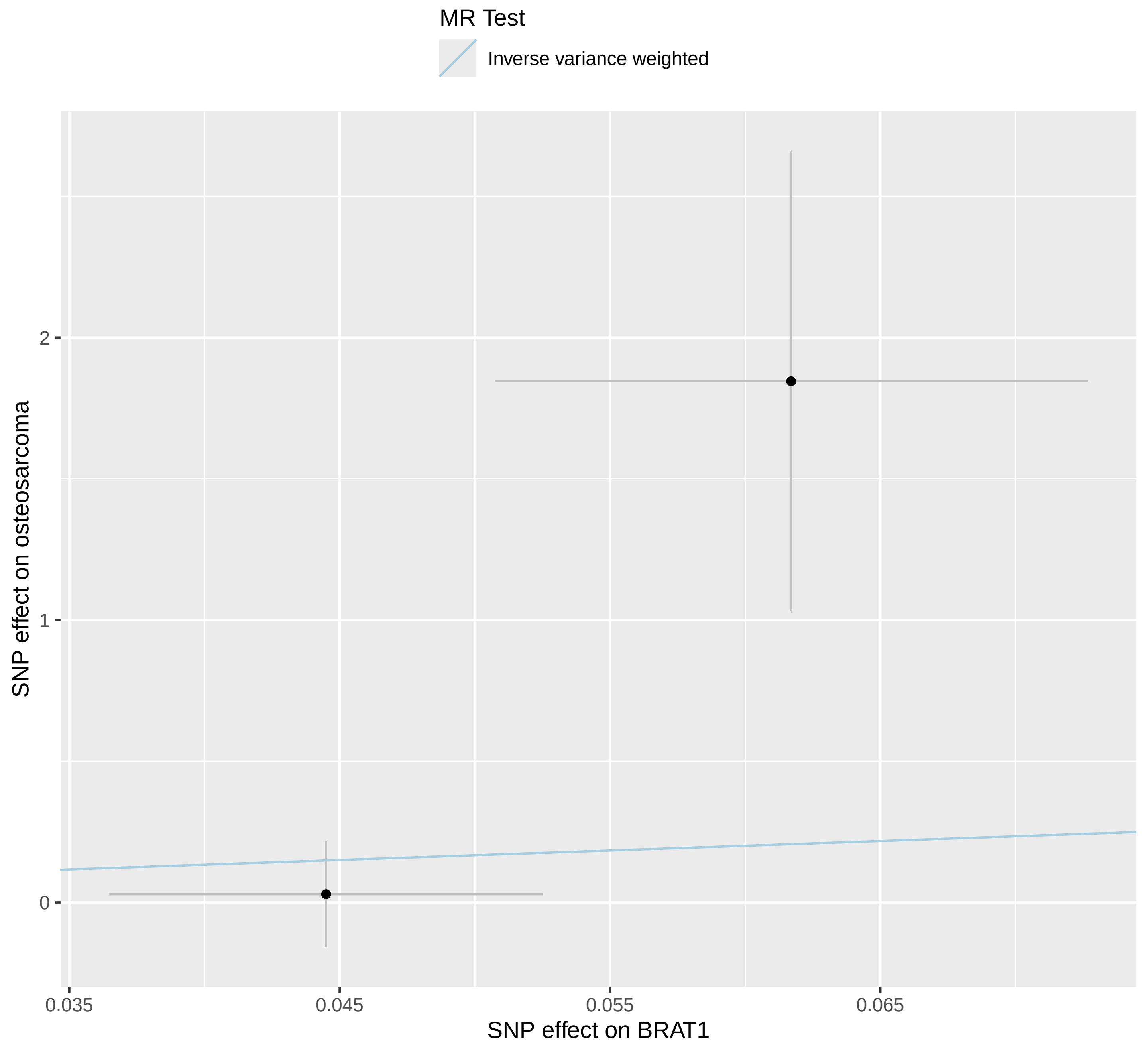
MR Test
Inverse variance weighted
SNP effect on osteosarcoma
2
1
0
0.035
0.045
0.055
0.065
SNP effect on BRAT1

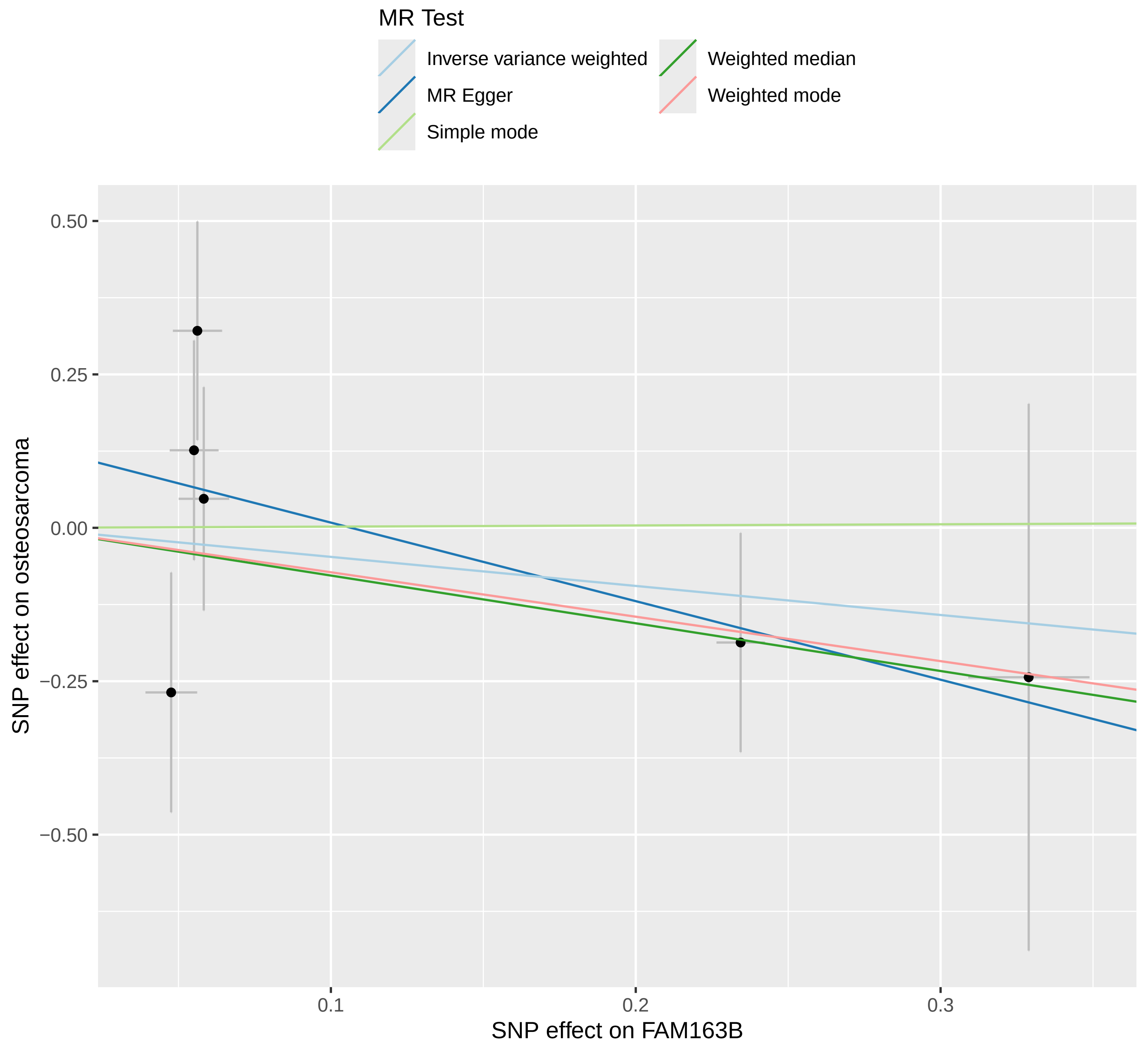
MR Test
Inverse variance weighted
MR Egger
Simple mode
Weighted median
Weighted mode
0.50
0.25
0.00
−0.25
−0.50
SNP effect on osteosarcoma
0.1
0.2
0.3
SNP effect on FAM163B

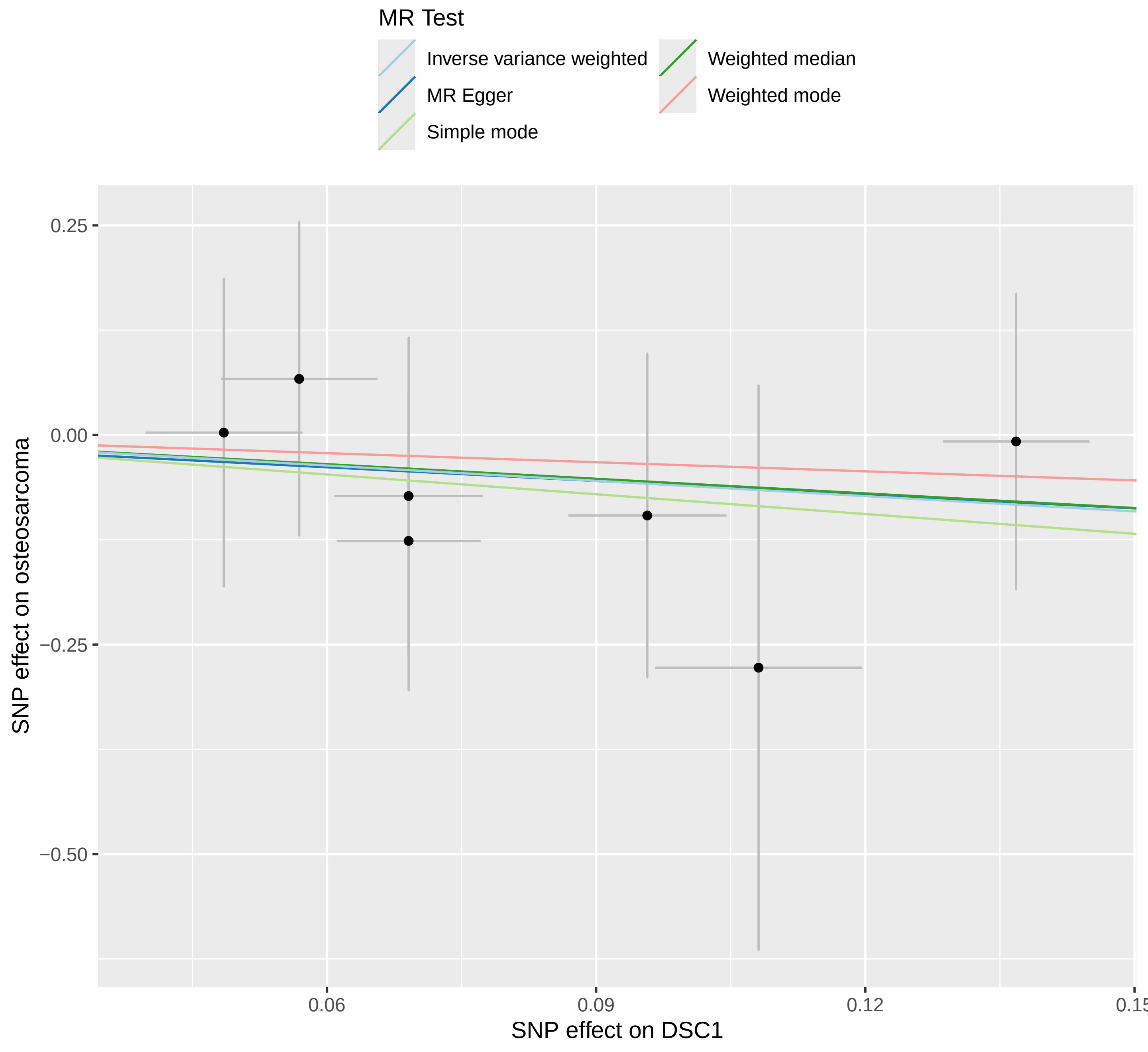
MR Test
Inverse variance weighted
MR Egger
Simple mode
Weighted median
Weighted mode
0.25
0.00
−0.25
−0.50
0.06
0.09
0.12
0.15
SNP effect on osteosarcoma
SNP effect on DSC1

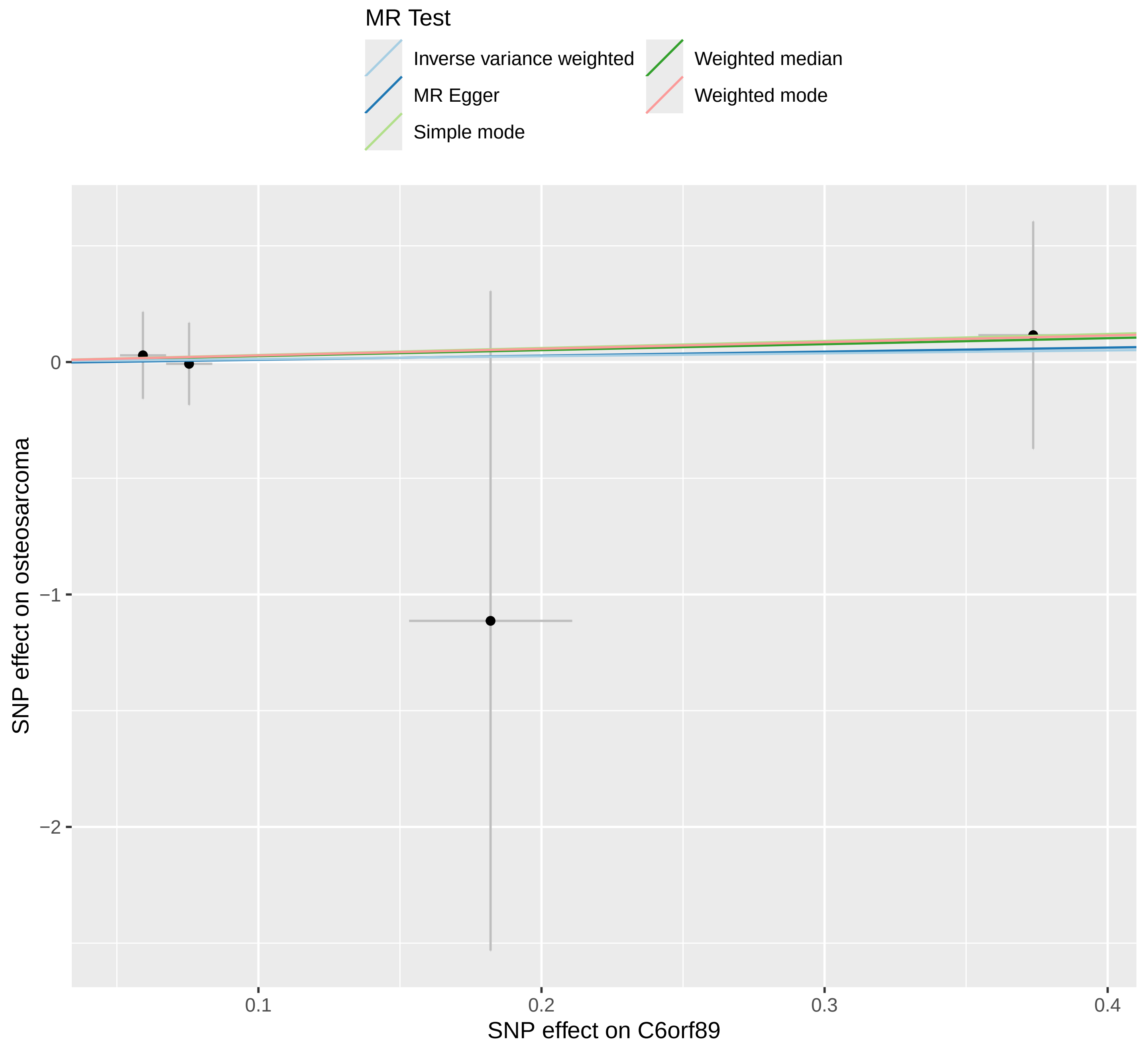
MR Test
Inverse variance weighted
MR Egger
Simple mode
Weighted median
Weighted mode
0
−1
−2
0.1
0.2
0.3
0.4
SNP effect on C6orf89
SNP effect on osteosarcoma

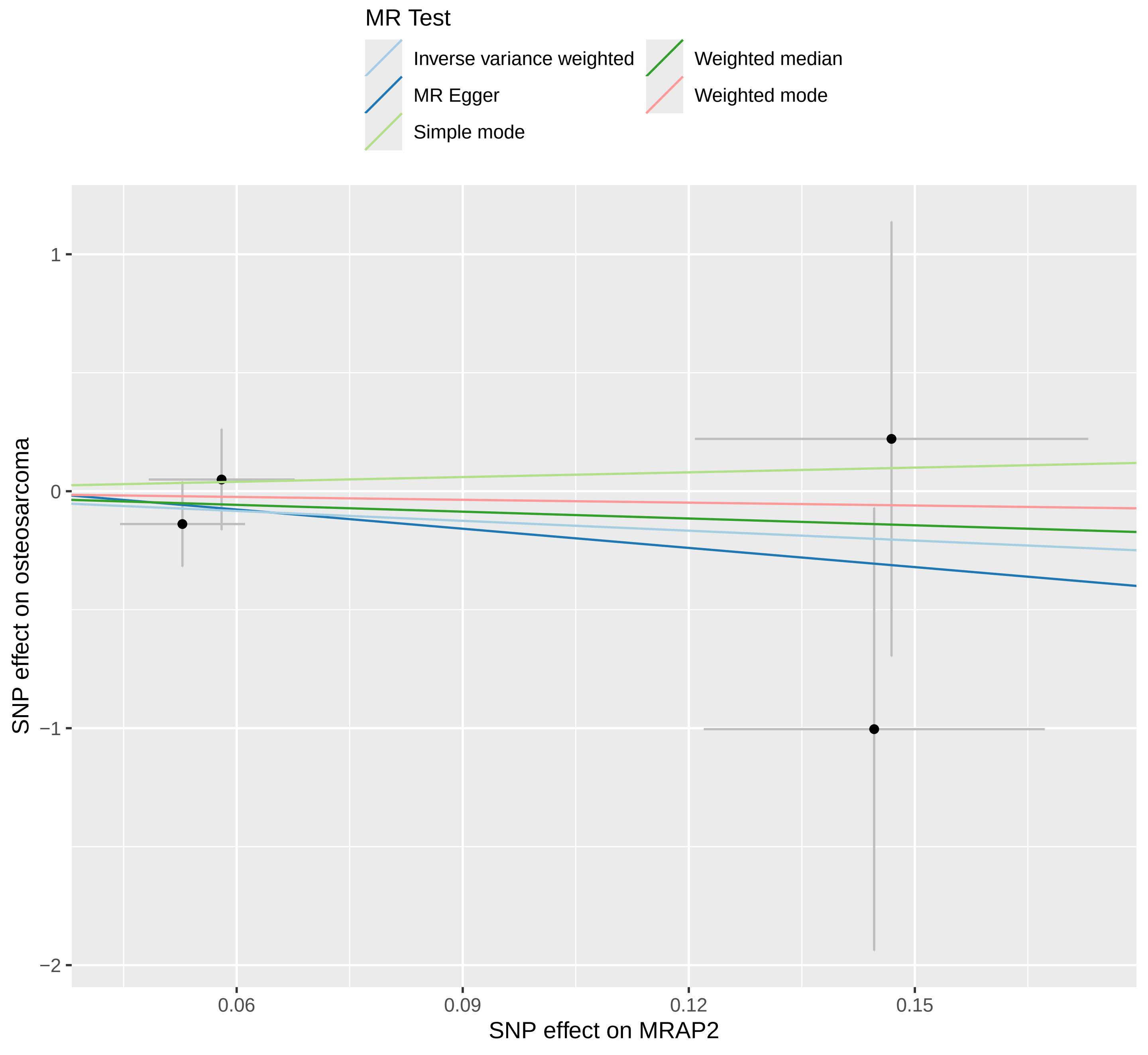

MR Test
Inverse variance weighted
MR Egger
Simple mode
Weighted median
Weighted mode
SNP effect on osteosarcoma
1
0
−1
−2
0.06
0.09
0.12
0.15
SNP effect on MRAP2

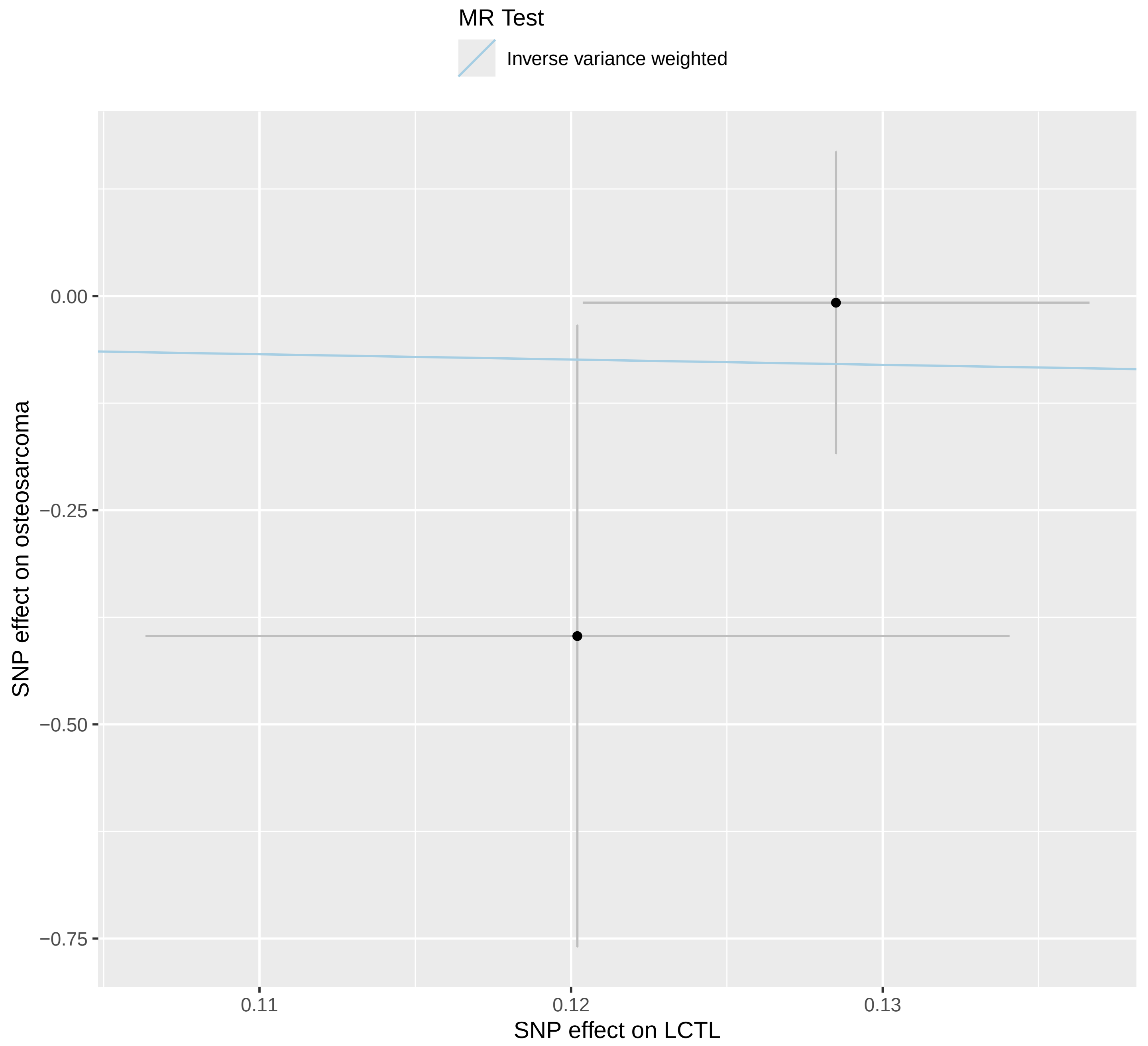

MR Test
Inverse variance weighted
0.00
−0.25
−0.50
−0.75
SNP effect on osteosarcoma
0.11
0.12
0.13
SNP effect on LCTL

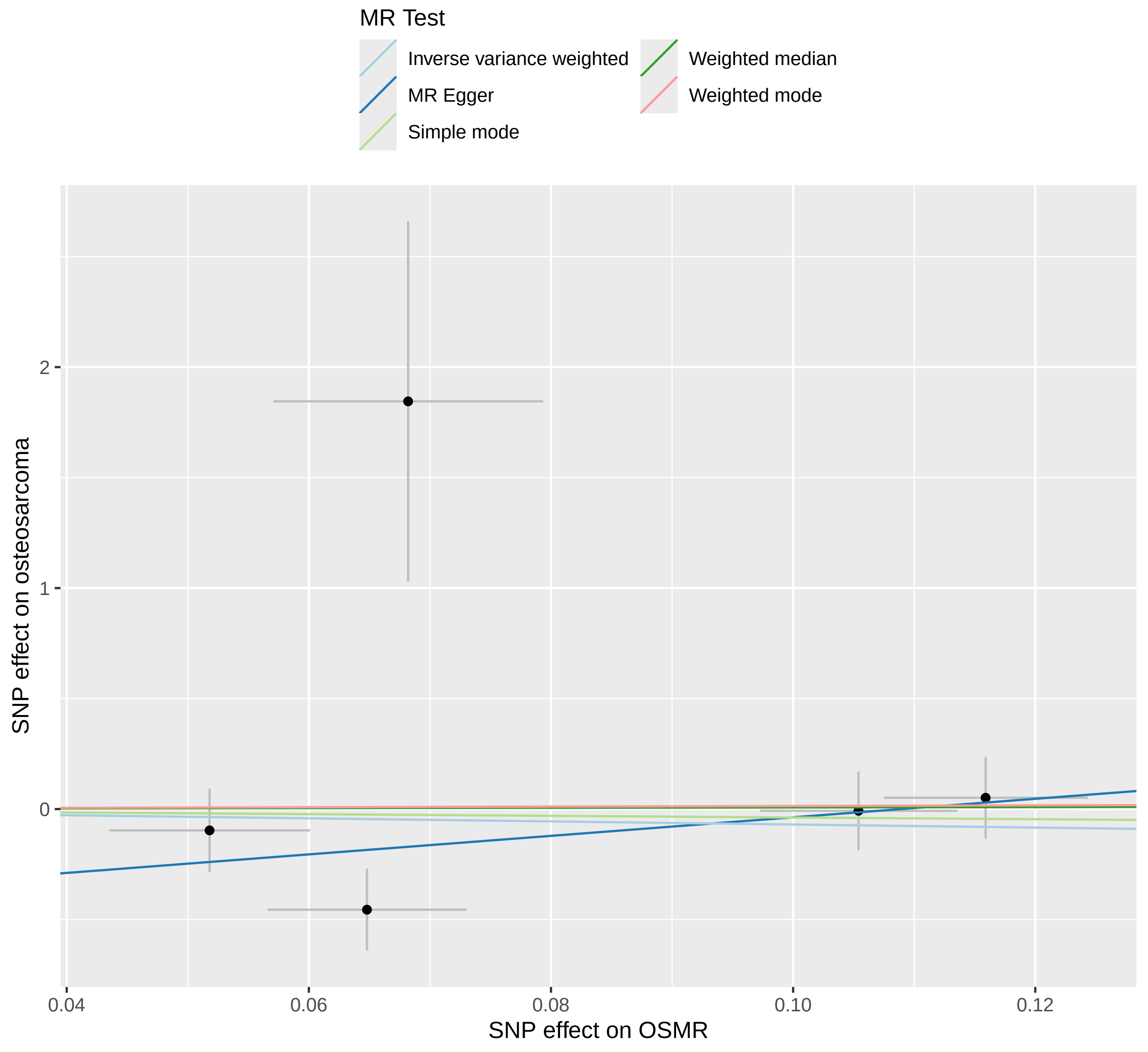

MR Test
Inverse variance weighted
MR Egger
Simple mode
Weighted median
Weighted mode
2
1
0
SNP effect on osteosarcoma
0.04
0.06
0.08
0.10
0.12
SNP effect on OSMR

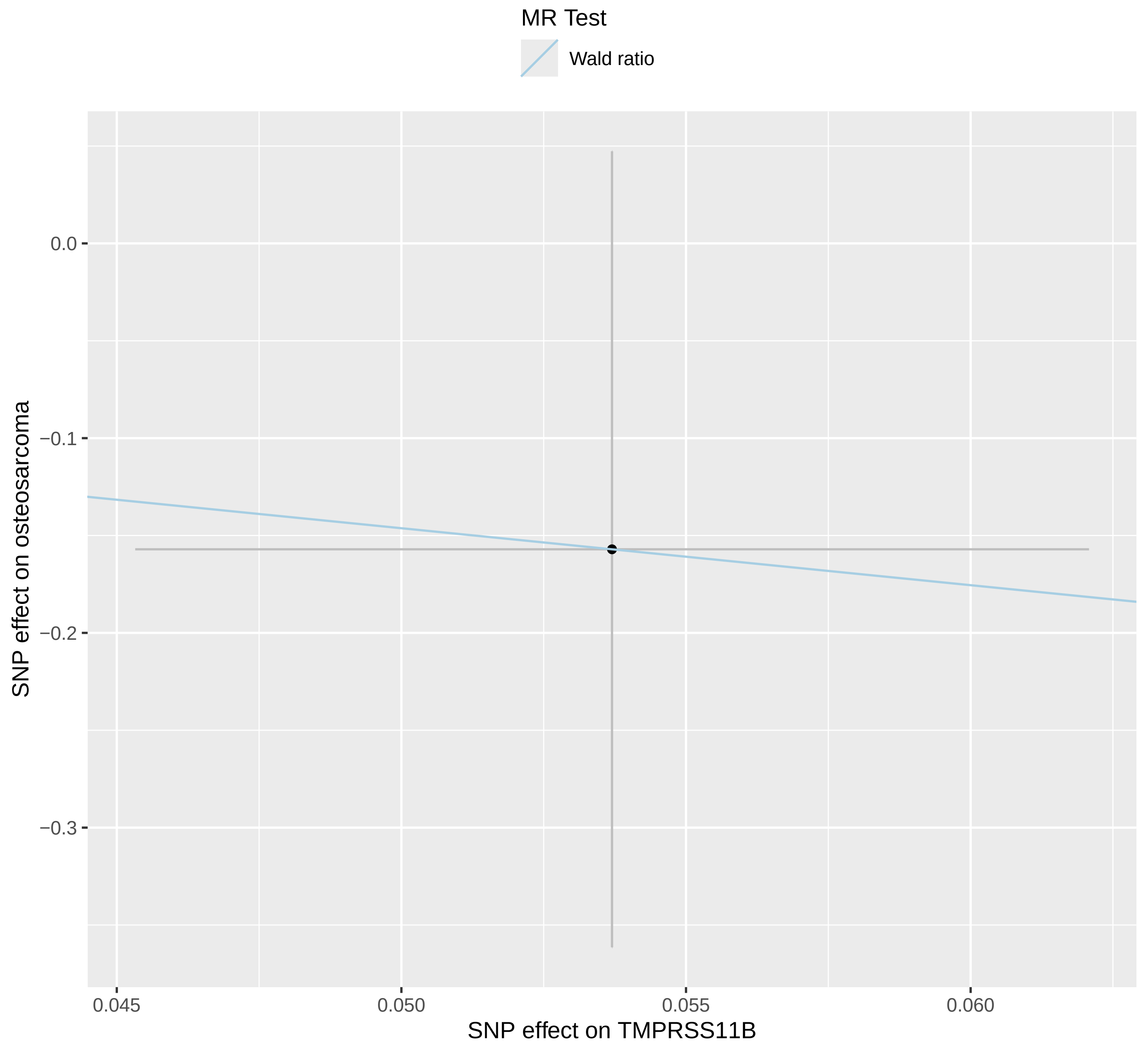
MR Test
Wald ratio
SNP effect on osteosarcoma
0.0
−0.1
−0.2
−0.3
0.045
0.050
0.055
0.060
SNP effect on TMPRSS11B

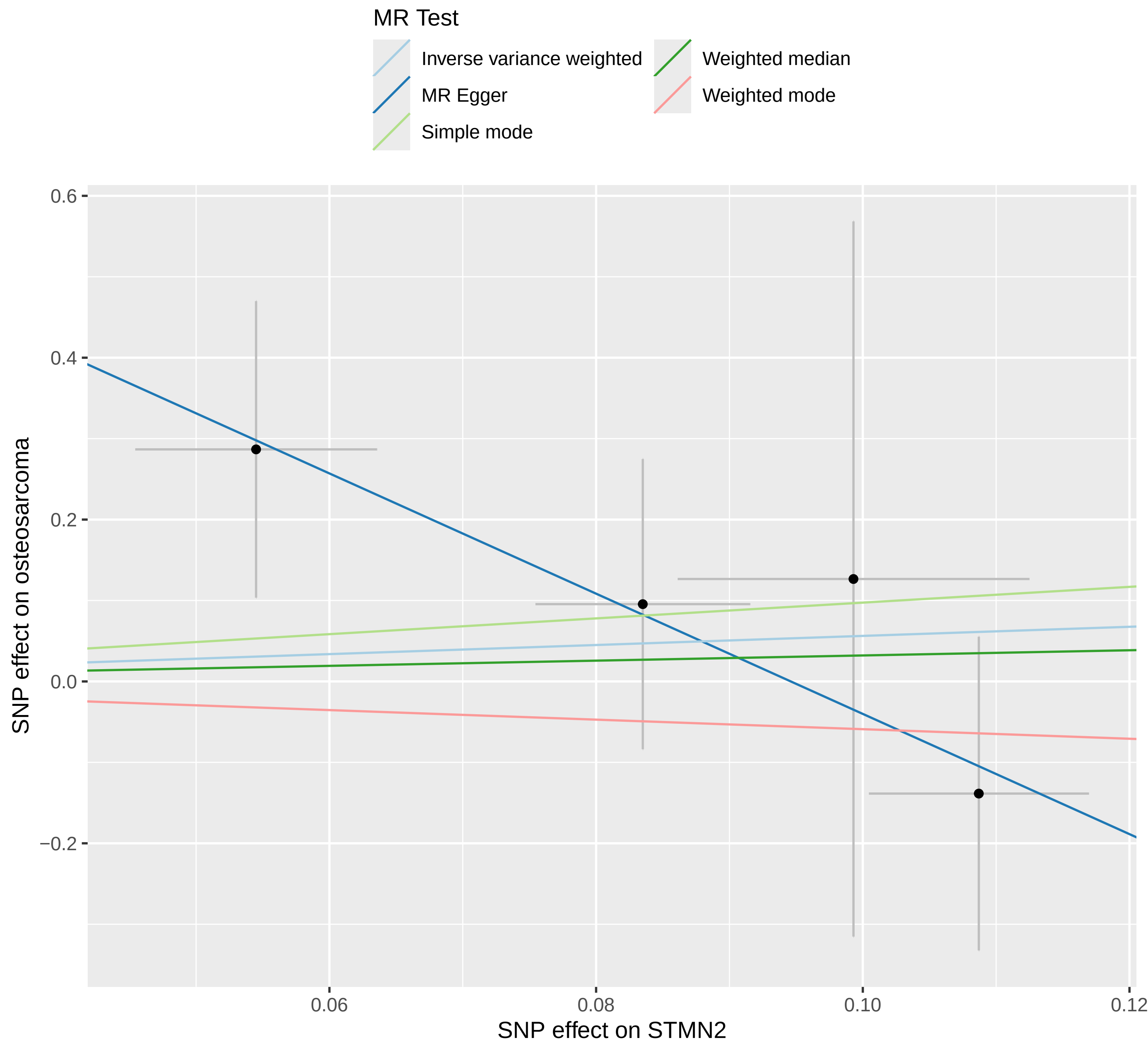
MR Test
Inverse variance weighted
MR Egger
Simple mode
Weighted median
Weighted mode
0.6
0.4
0.2
0.0
−0.2
SNP effect on osteosarcoma
0.06
0.08
0.10
0.12
SNP effect on STMN2

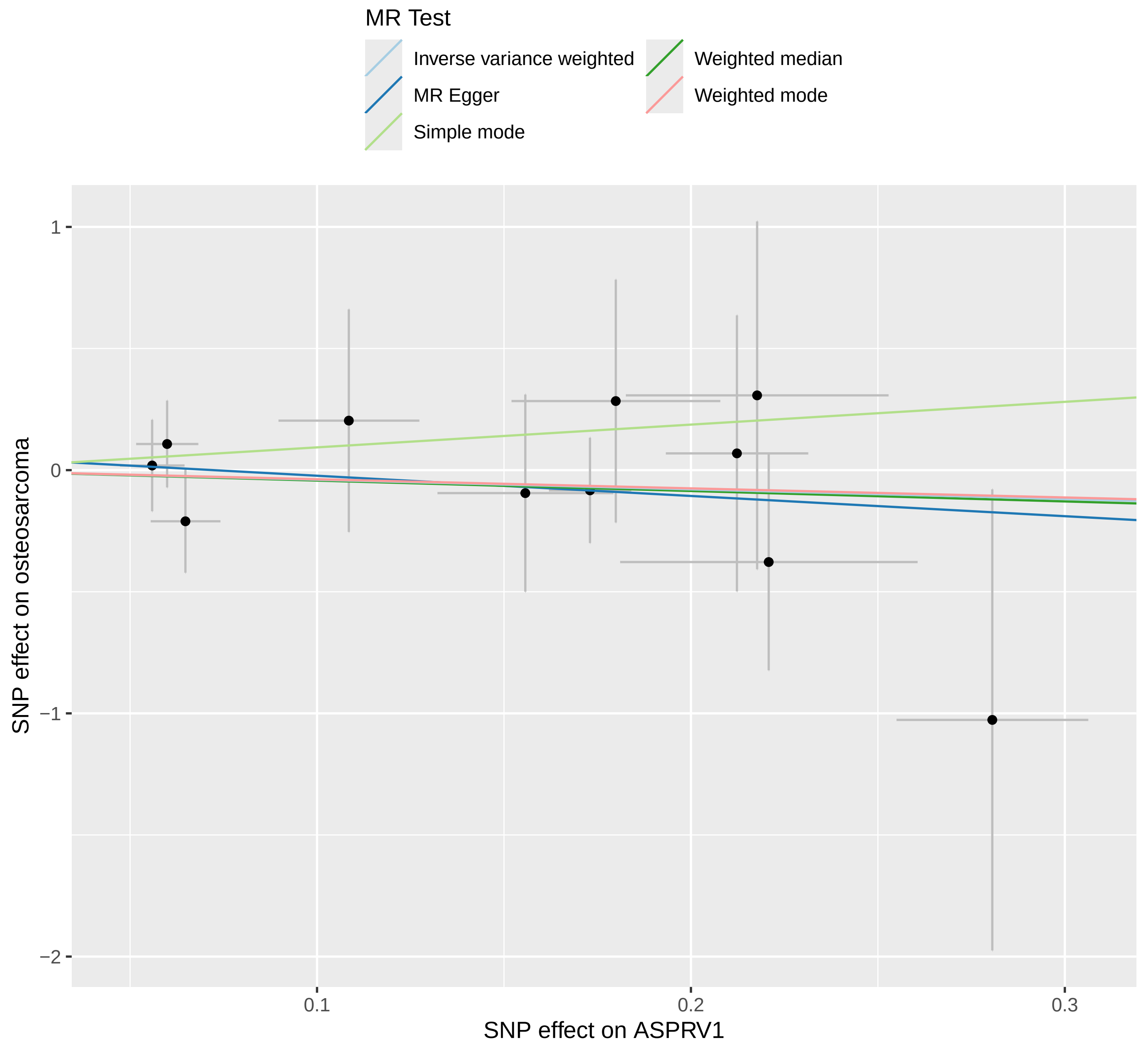
MR Test
Inverse variance weighted
MR Egger
Simple mode
Weighted median
Weighted mode
1
0
−1
−2
0.1
0.2
0.3
SNP effect on osteosarcoma
SNP effect on ASPRV1

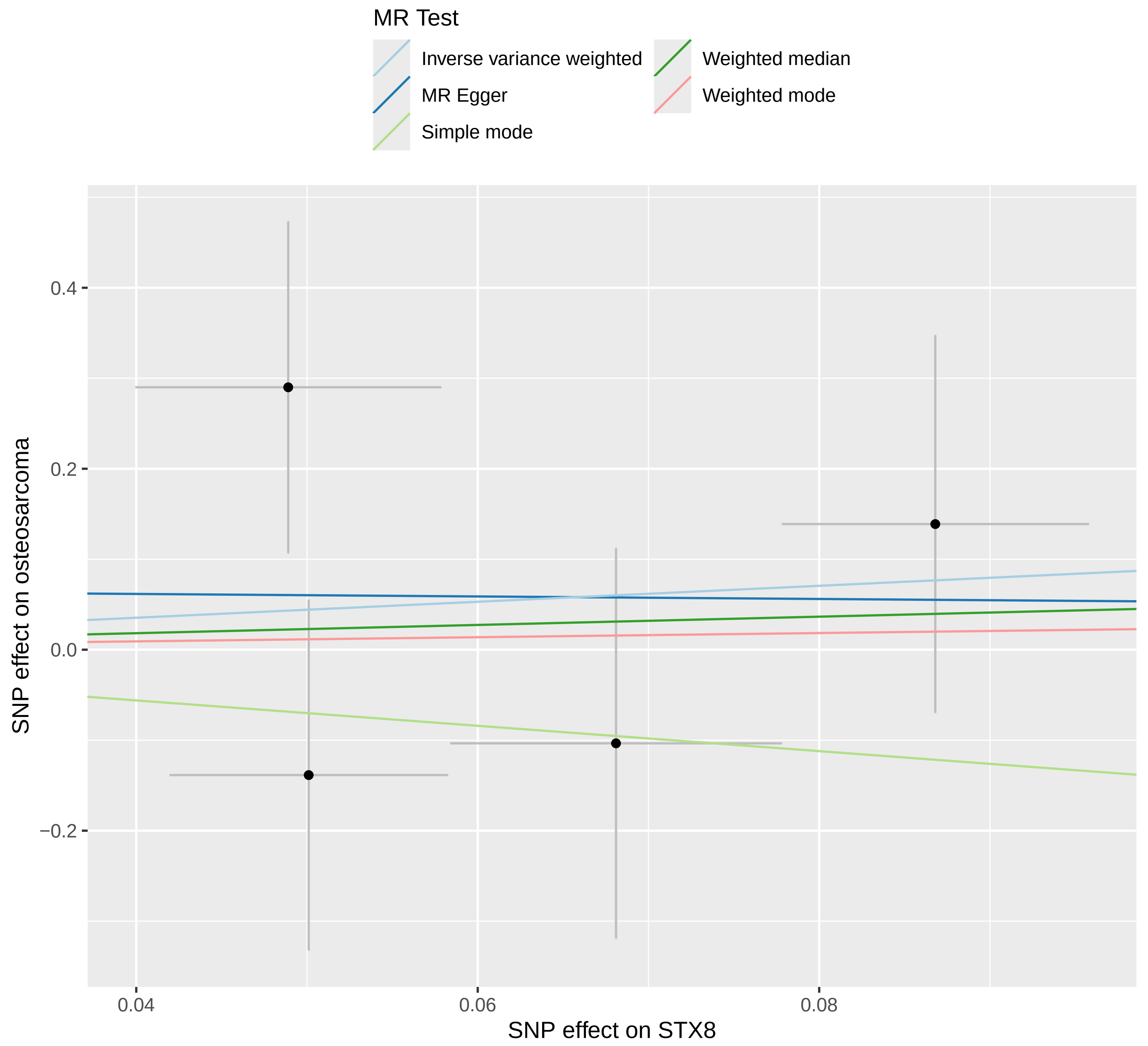

MR Test
Inverse variance weighted
MR Egger
Simple mode
Weighted median
Weighted mode
SNP effect on osteosarcoma
0.4
0.2
0.0
−0.2
0.04
0.06
0.08
SNP effect on STX8

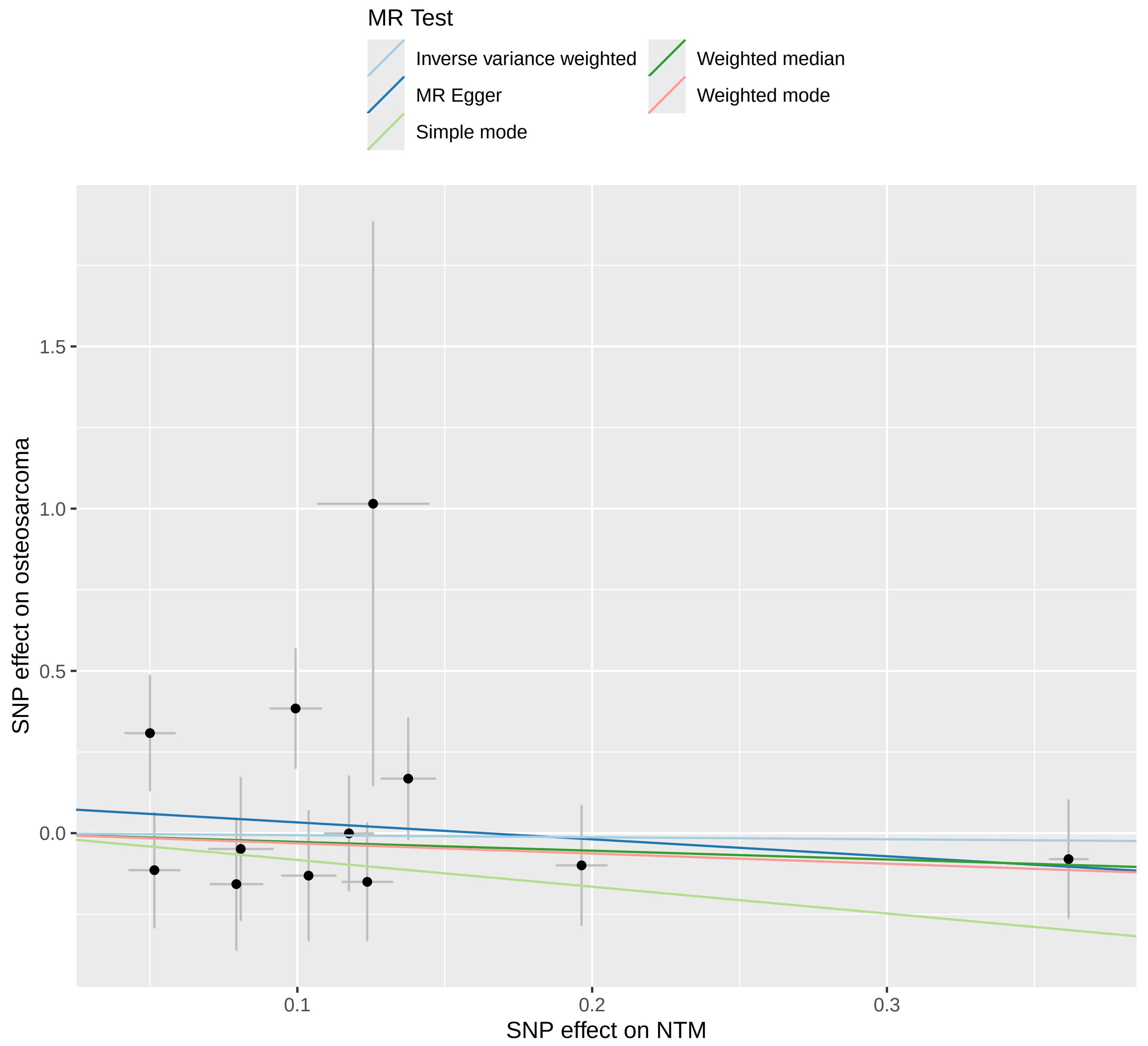

MR Test
Inverse variance weighted
MR Egger
Simple mode
Weighted median
Weighted mode
1.5
1.0
0.5
0.0
0.1
0.2
0.3
SNP effect on osteosarcoma
SNP effect on NTM

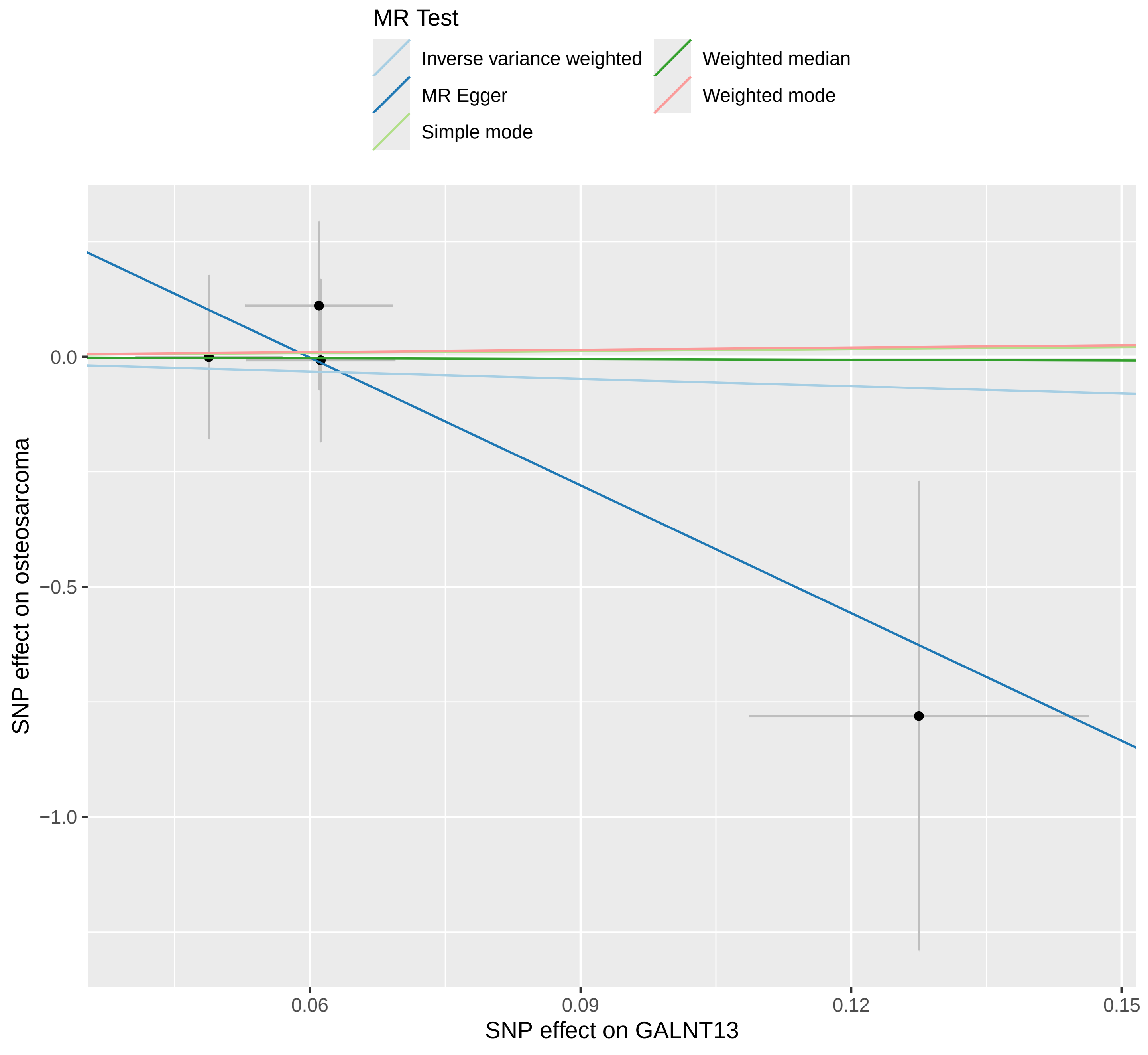
MR Test
Inverse variance weighted
MR Egger
Simple mode
Weighted median
Weighted mode
0.0
−0.5
−1.0
0.06
0.09
0.12
0.15
SNP effect on GALNT13
SNP effect on osteosarcoma

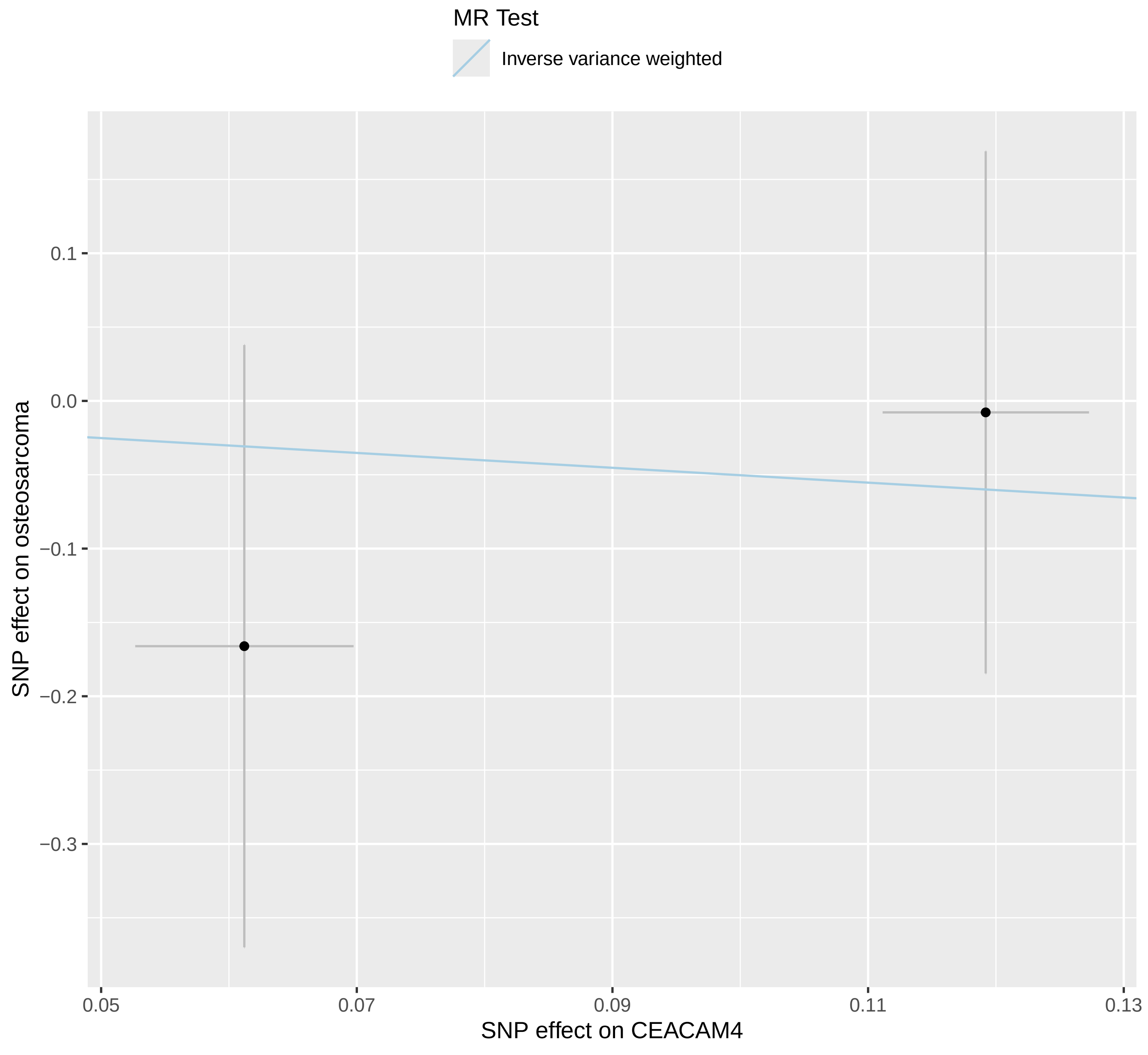
MR Test
Inverse variance weighted
0.1
0.0
−0.1
−0.2
−0.3
SNP effect on osteosarcoma
0.05
0.07
0.09
0.11
0.13
SNP effect on CEACAM4

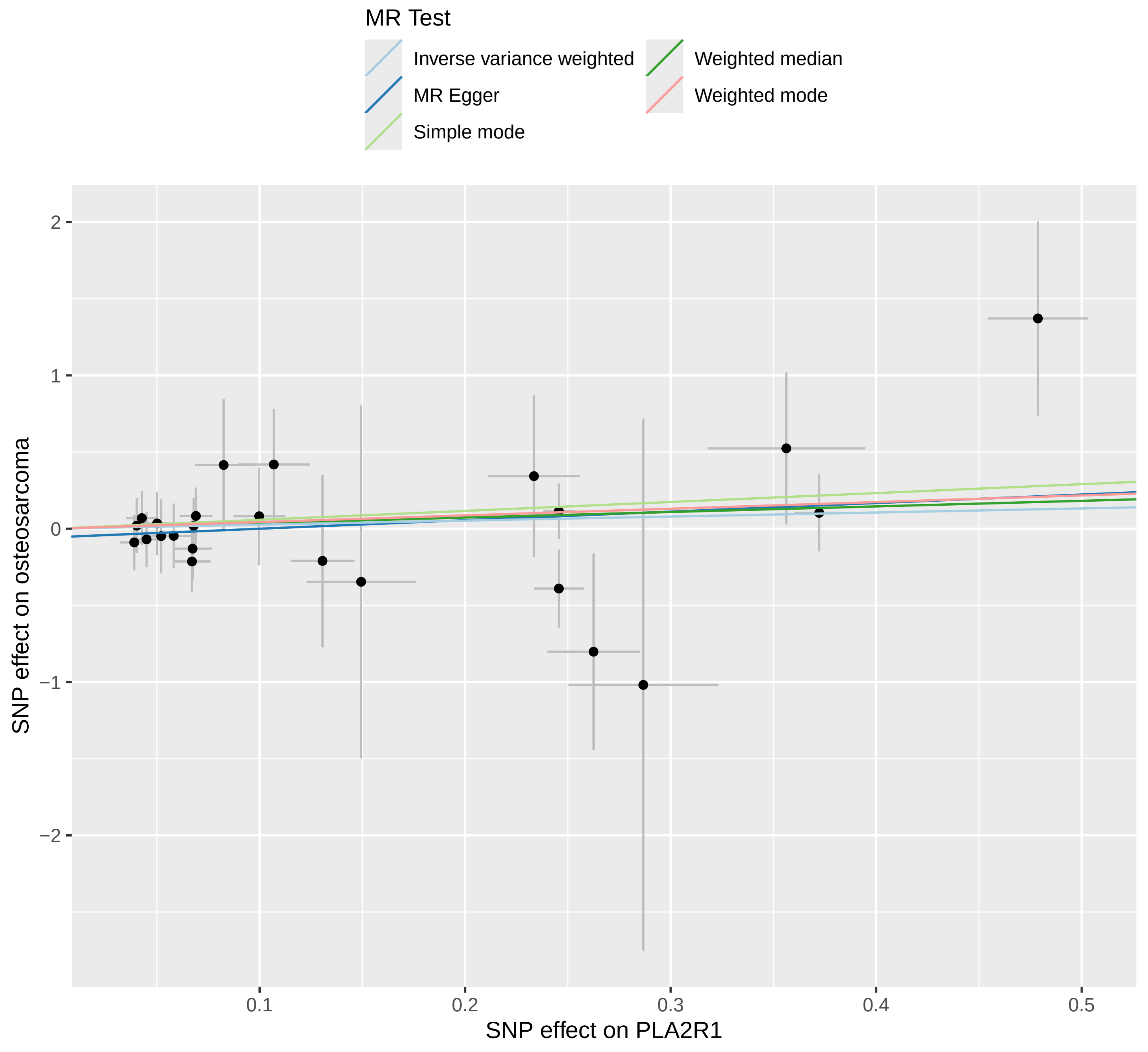

MR Test
Inverse variance weighted
MR Egger
Simple mode
Weighted median
Weighted mode
2
1
0
−1
−2
0.1
0.2
0.3
0.4
0.5
SNP effect on PLA2R1
SNP effect on osteosarcoma

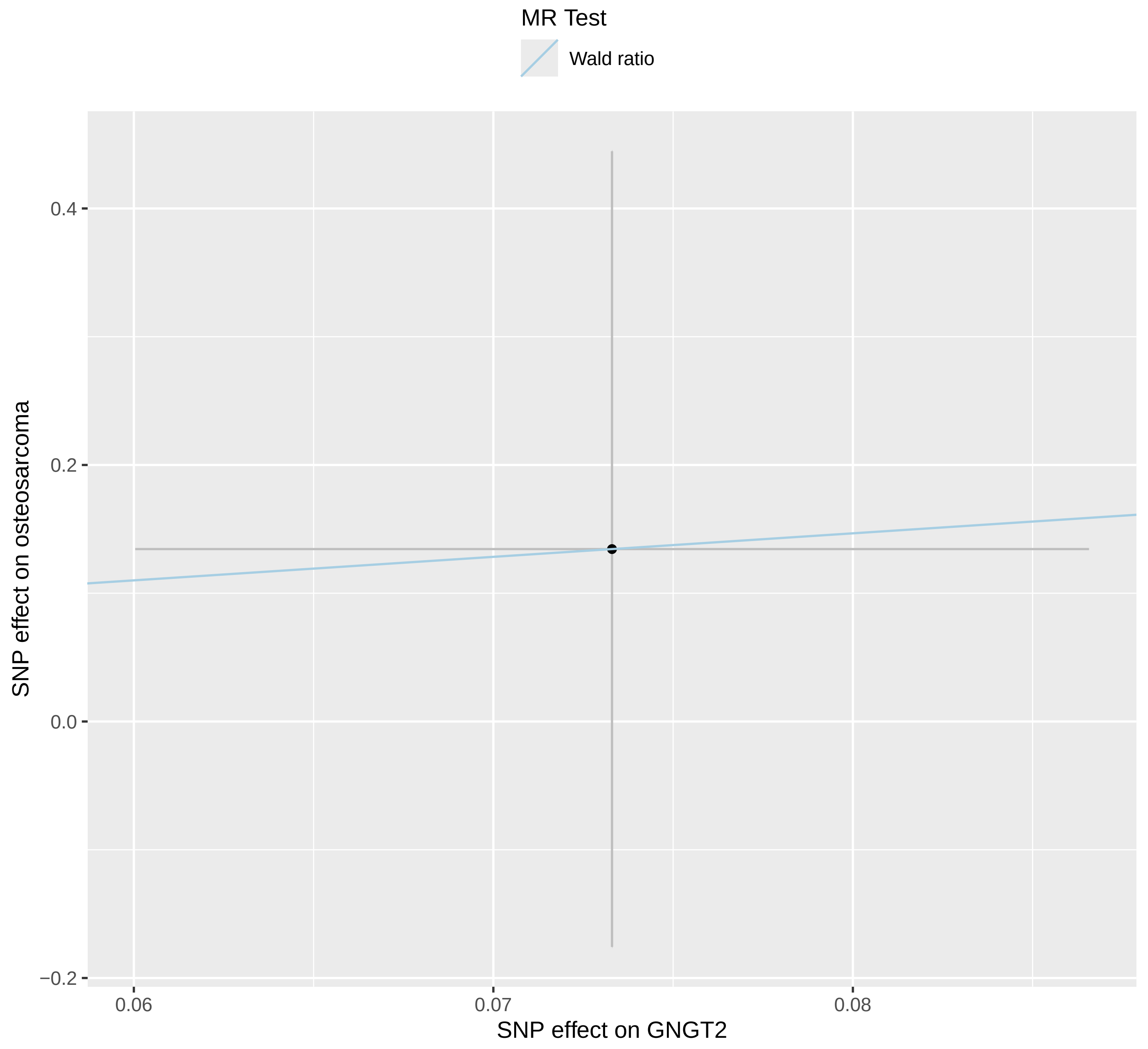

MR Test
Wald ratio
0.4
0.2
0.0
−0.2
0.06
0.07
0.08
SNP effect on osteosarcoma
SNP effect on GNGT2

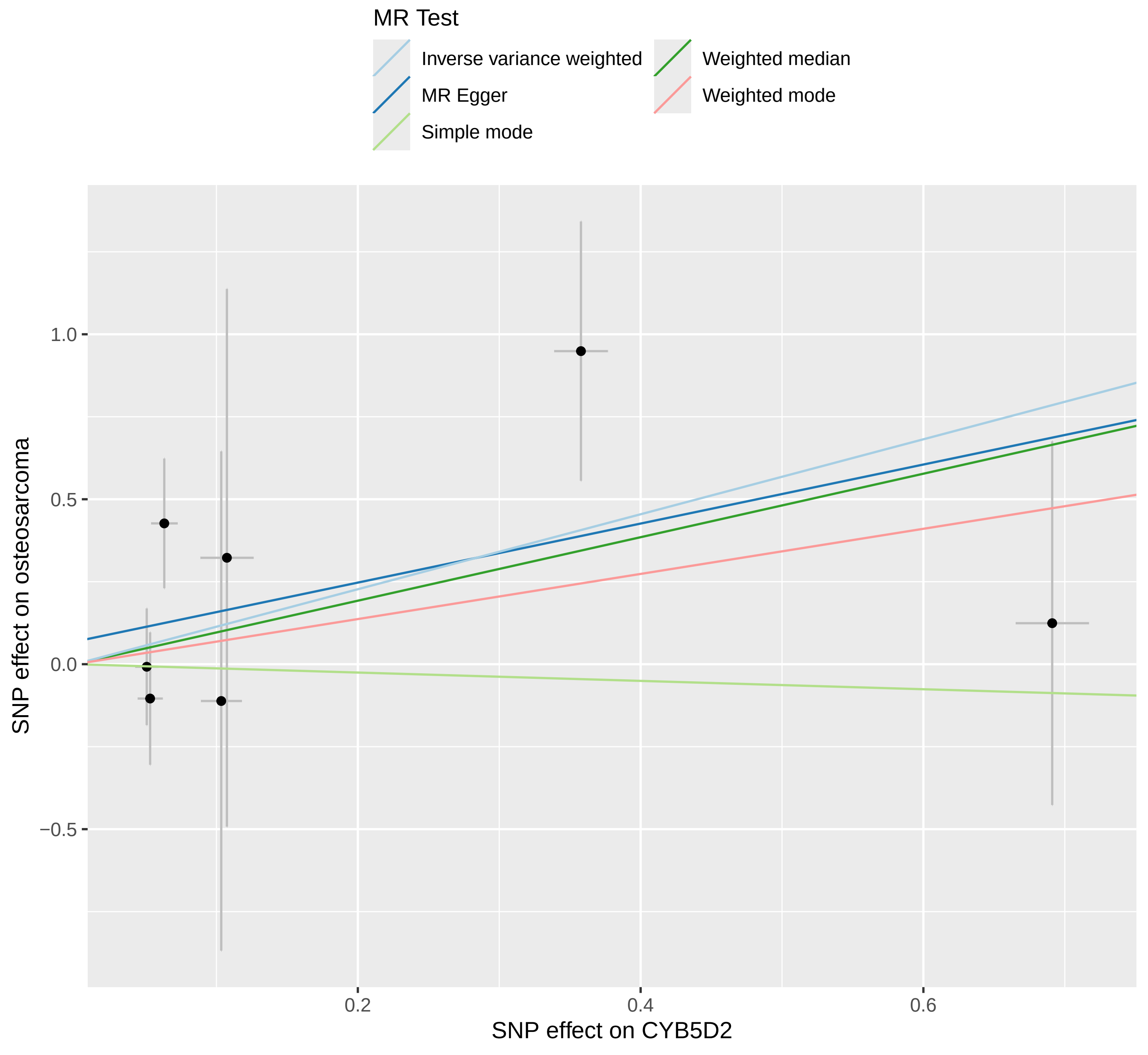
MR Test
Inverse variance weighted
MR Egger
Simple mode
Weighted median
Weighted mode
SNP effect on osteosarcoma
1.0
0.5
0.0
−0.5
0.2
0.4
0.6
SNP effect on CYB5D2

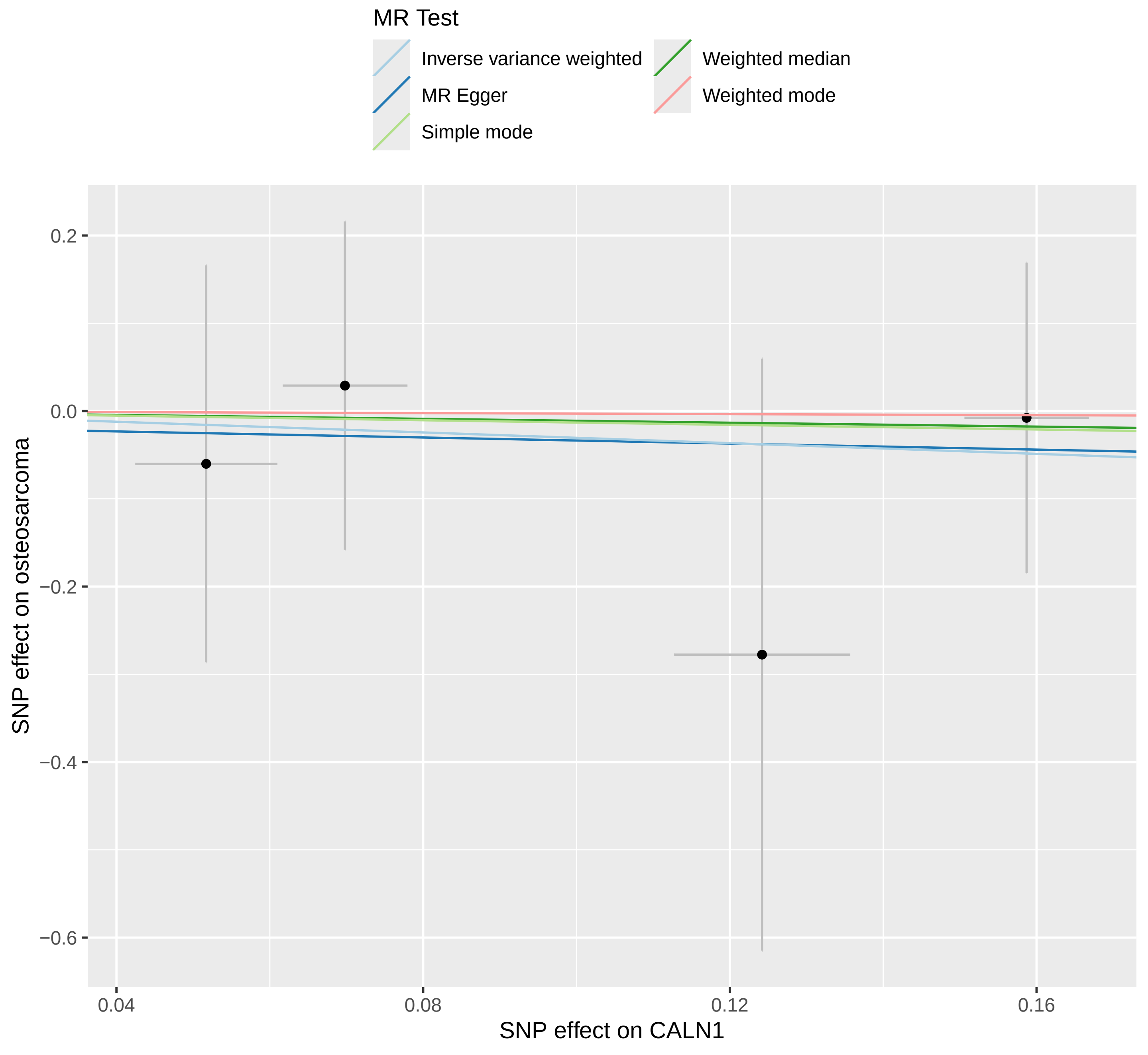
MR Test
Inverse variance weighted
MR Egger
Simple mode
Weighted median
Weighted mode
0.2
0.0
−0.2
−0.4
−0.6
SNP effect on osteosarcoma
0.04
0.08
0.12
0.16
SNP effect on CALN1

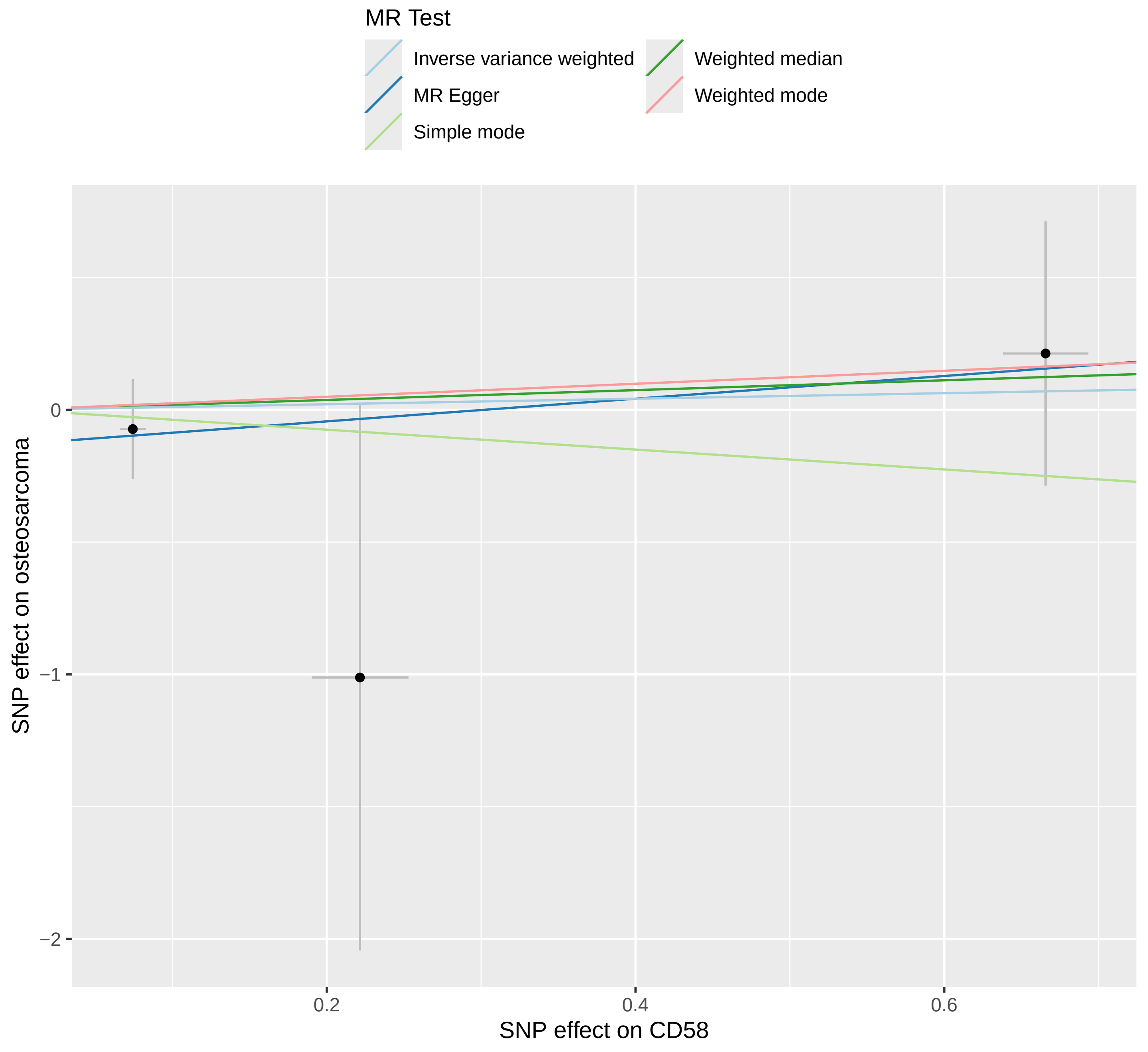
MR Test
Inverse variance weighted
MR Egger
Simple mode
Weighted median
Weighted mode
0
−1
−2
0.2
0.4
0.6
SNP effect on osteosarcoma
SNP effect on CD58

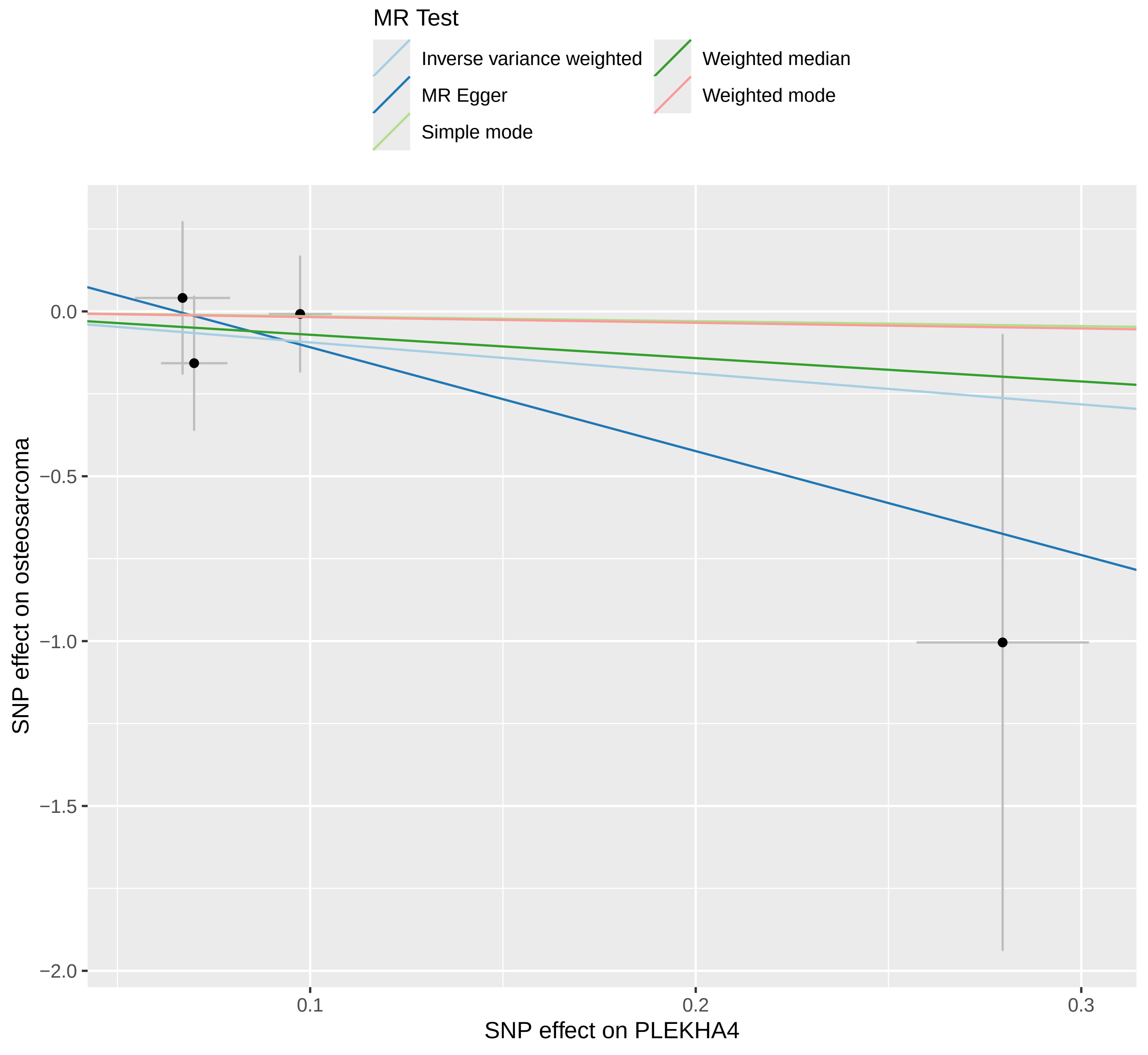

MR Test
Inverse variance weighted
MR Egger
Simple mode
Weighted median
Weighted mode
SNP effect on osteosarcoma
0.0
−0.5
−1.0
−1.5
−2.0
0.1
0.2
0.3
SNP effect on PLEKHA4

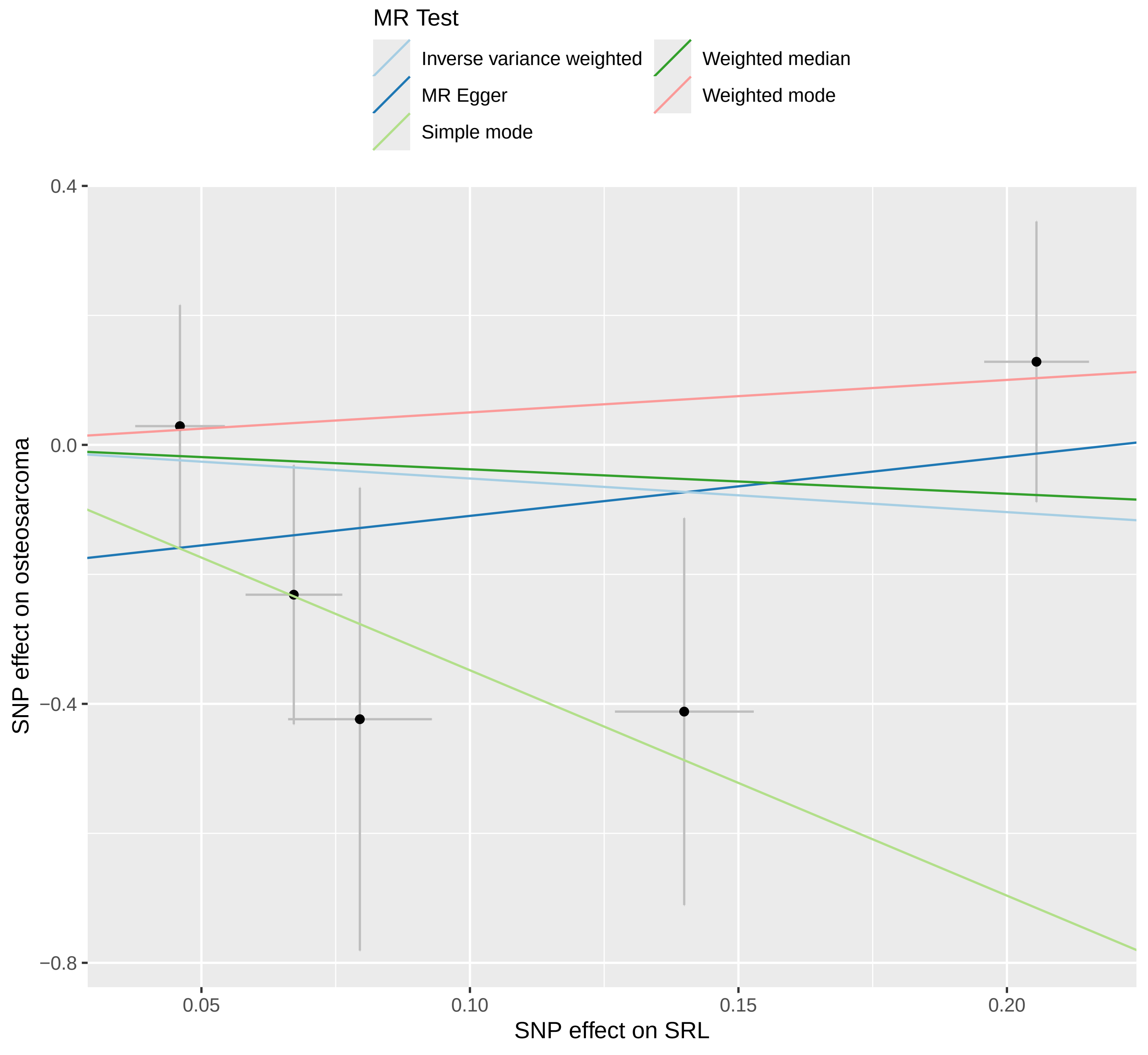
MR Test
Inverse variance weighted
MR Egger
Simple mode
Weighted median
Weighted mode
0.4
0.0
−0.4
−0.8
0.05
0.10
0.15
0.20
SNP effect on osteosarcoma
SNP effect on SRL

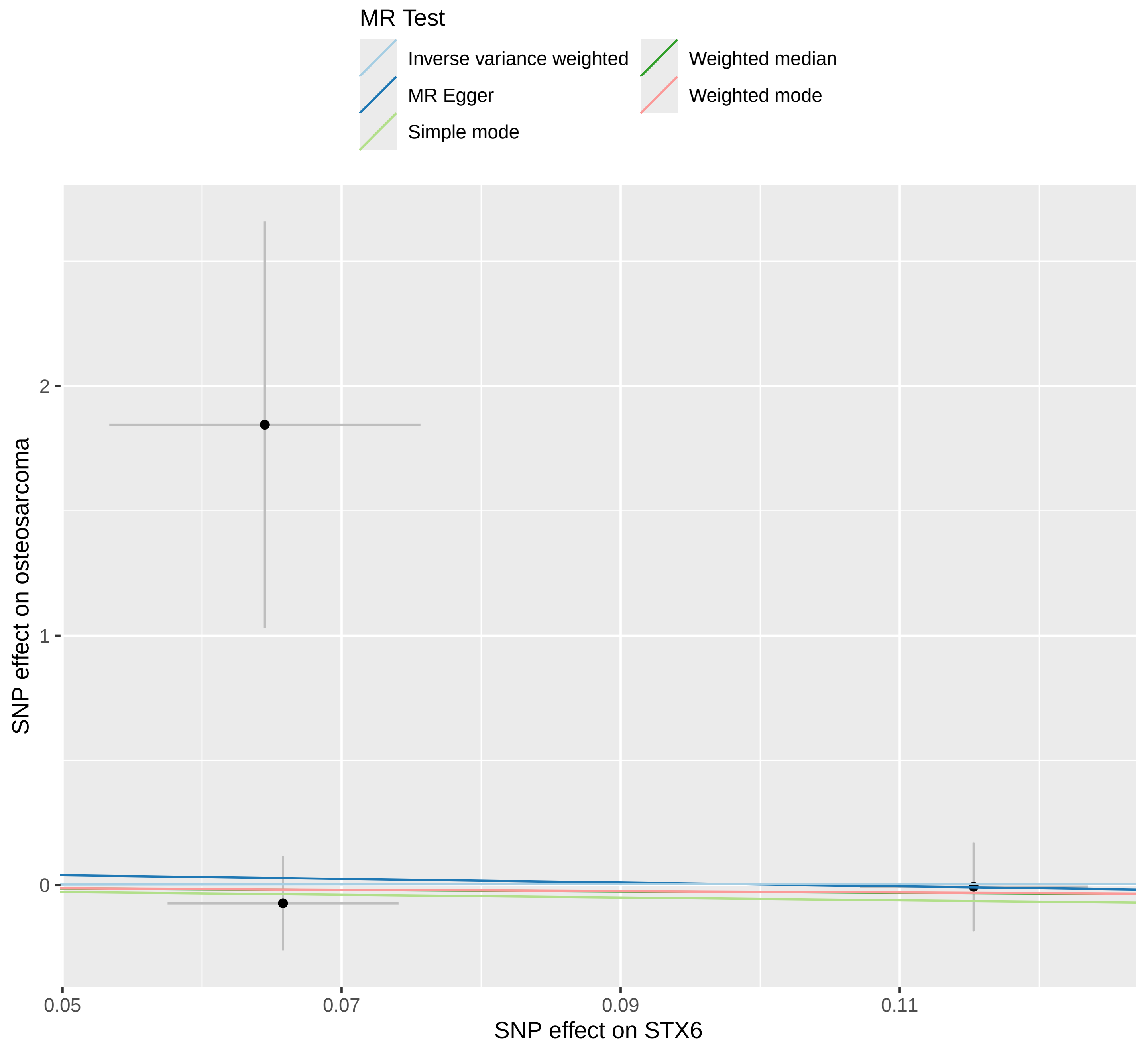
MR Test
Inverse variance weighted
MR Egger
Simple mode
Weighted median
Weighted mode
SNP effect on osteosarcoma
2
1
0
0.05
0.07
0.09
0.11
SNP effect on STX6

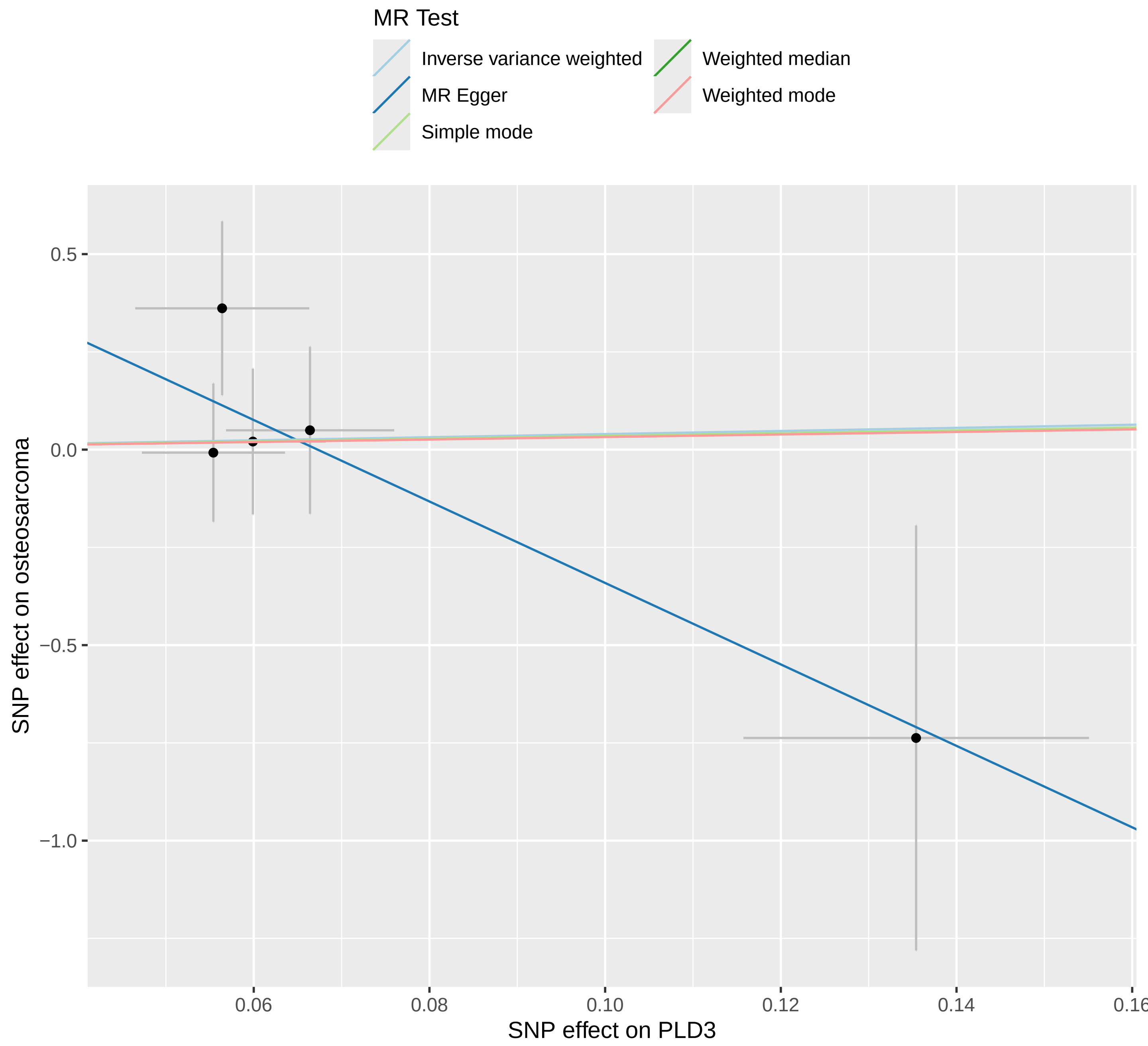

MR Test
Inverse variance weighted
MR Egger
Simple mode
Weighted median
Weighted mode
0.5
0.0
−0.5
−1.0
0.06
0.08
0.10
0.12
0.14
0.16
SNP effect on osteosarcoma
SNP effect on PLD3

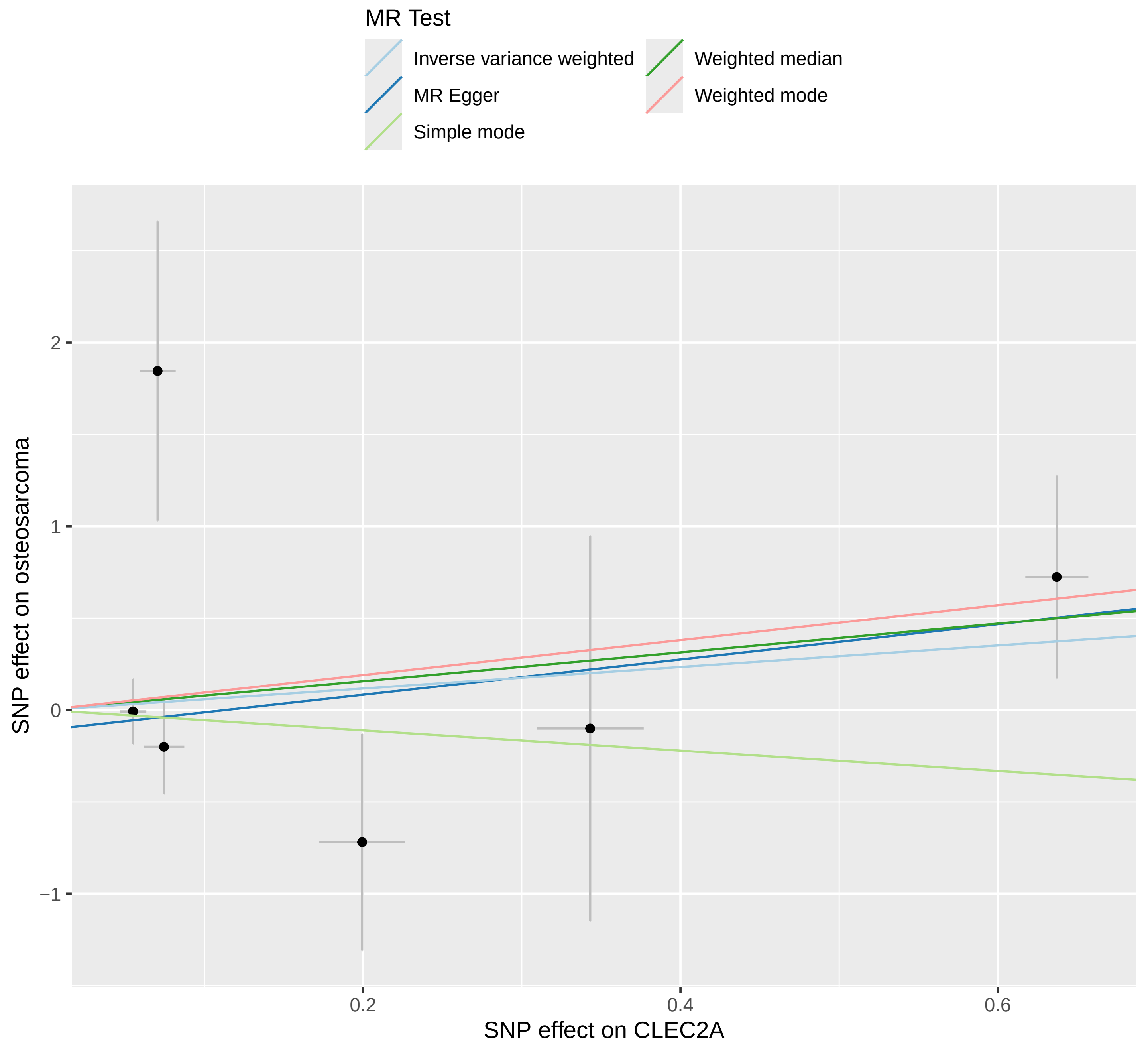

MR Test
Inverse variance weighted
MR Egger
Simple mode
Weighted median
Weighted mode
SNP effect on osteosarcoma
2
1
0
−1
0.2
0.4
0.6
SNP effect on CLEC2A

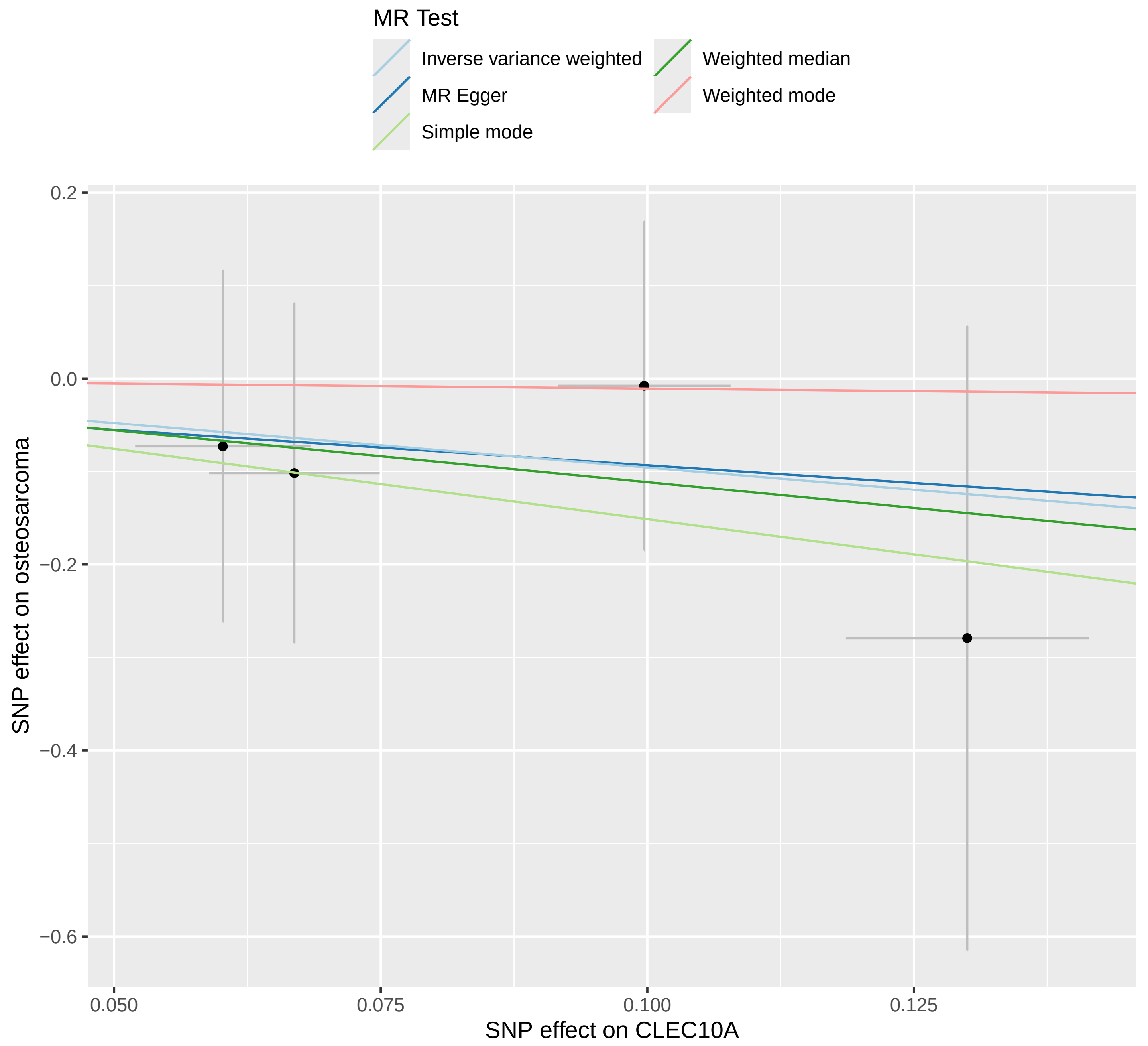

MR Test
Inverse variance weighted
MR Egger
Simple mode
Weighted median
Weighted mode
0.2
0.0
−0.2
−0.4
−0.6
SNP effect on osteosarcoma
0.050
0.075
0.100
0.125
SNP effect on CLEC10A

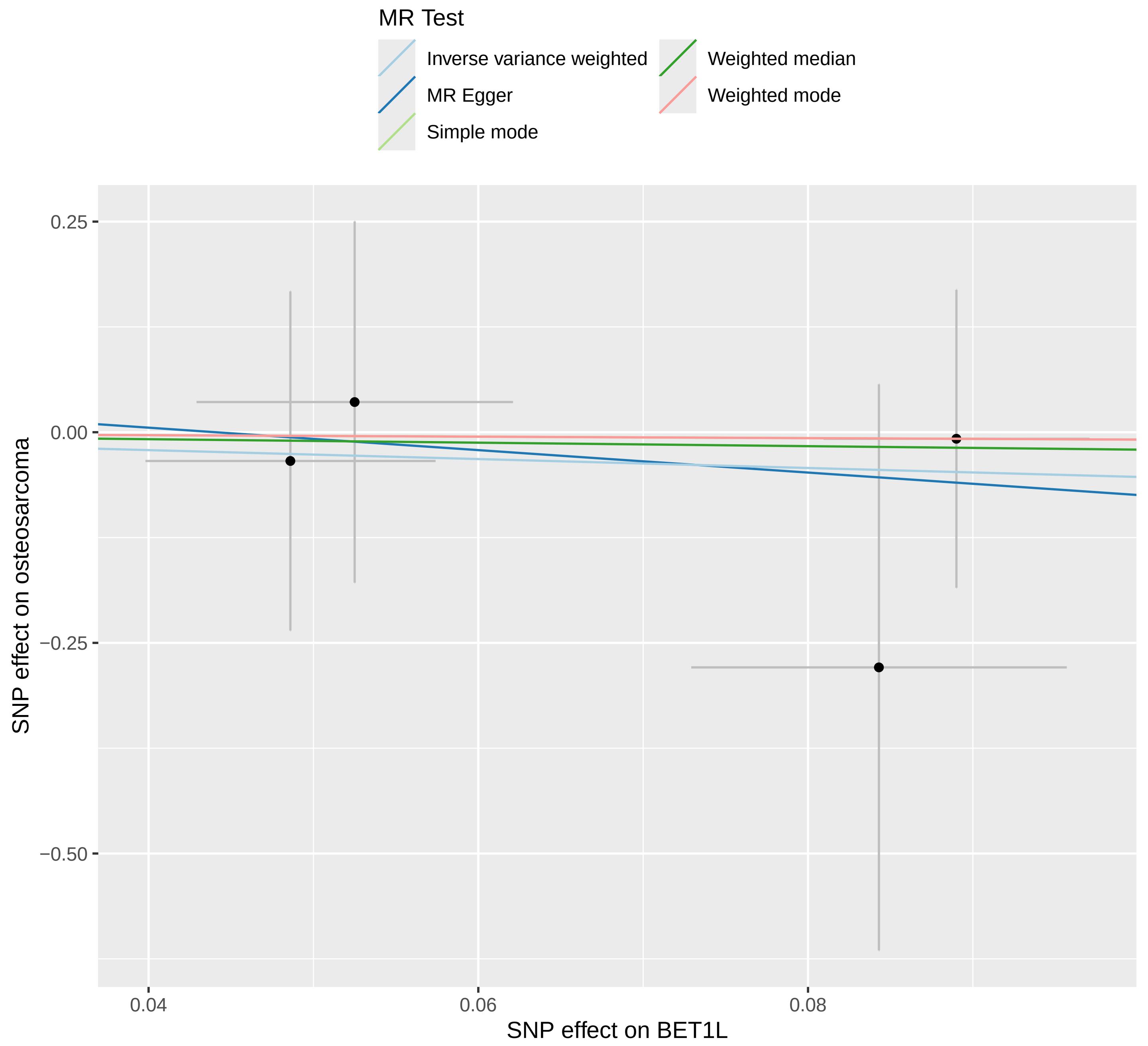
MR Test
Inverse variance weighted
MR Egger
Simple mode
Weighted median
Weighted mode
0.25
0.00
−0.25
−0.50
SNP effect on osteosarcoma
0.04
0.06
0.08
SNP effect on BET1L

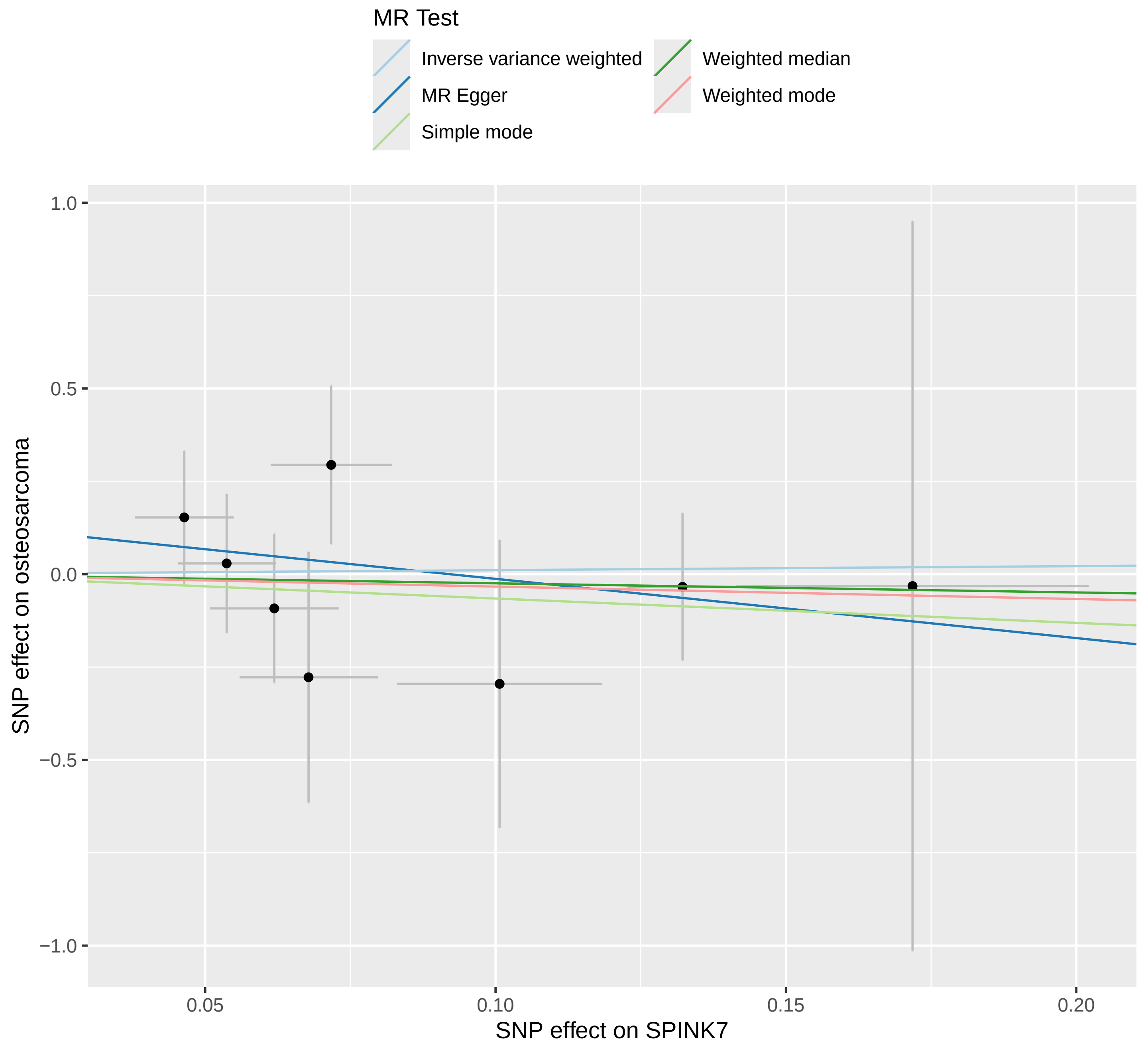
MR Test
Inverse variance weighted
MR Egger
Simple mode
Weighted median
Weighted mode
1.0
0.5
0.0
−0.5
−1.0
SNP effect on osteosarcoma
0.05
0.10
0.15
0.20
SNP effect on SPINK7

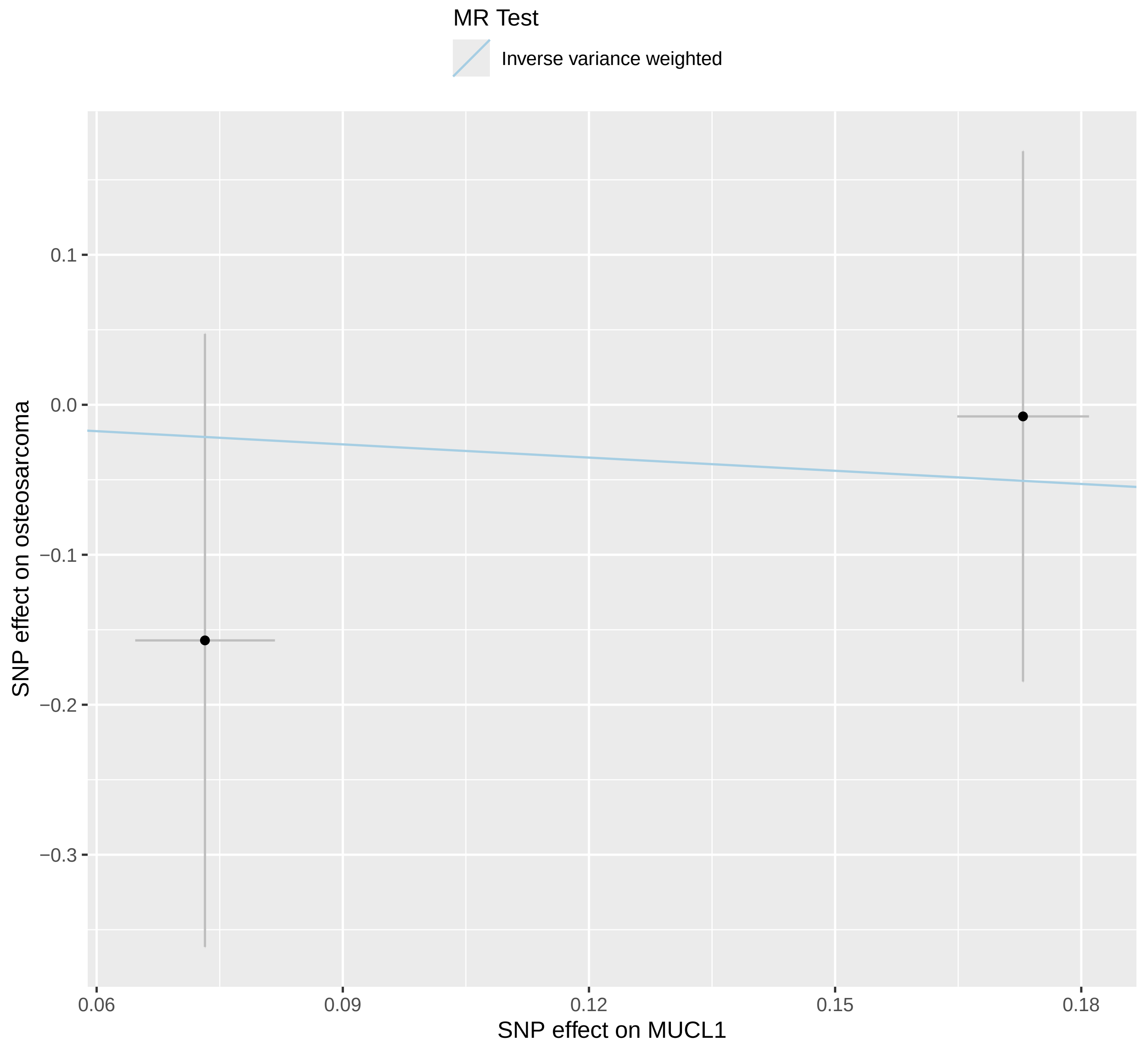
MR Test
Inverse variance weighted
SNP effect on osteosarcoma
0.1
0.0
−0.1
−0.2
−0.3
0.06
0.09
0.12
0.15
0.18
SNP effect on MUCL1

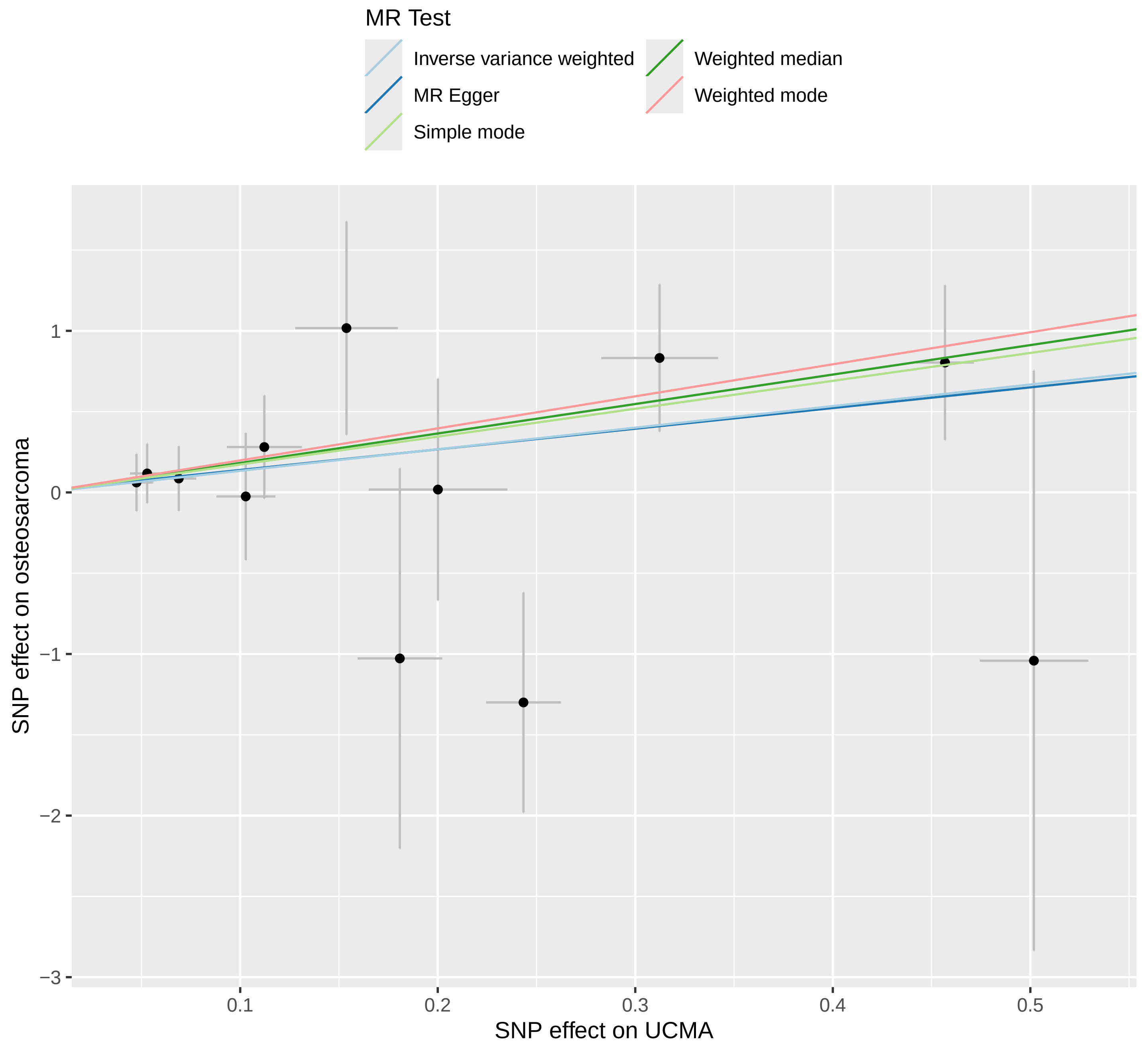
MR Test
Inverse variance weighted
MR Egger
Simple mode
Weighted median
Weighted mode
SNP effect on osteosarcoma
SNP effect on UCMA
1
0
−1
−2
−3
0.1
0.2
0.3
0.4
0.5

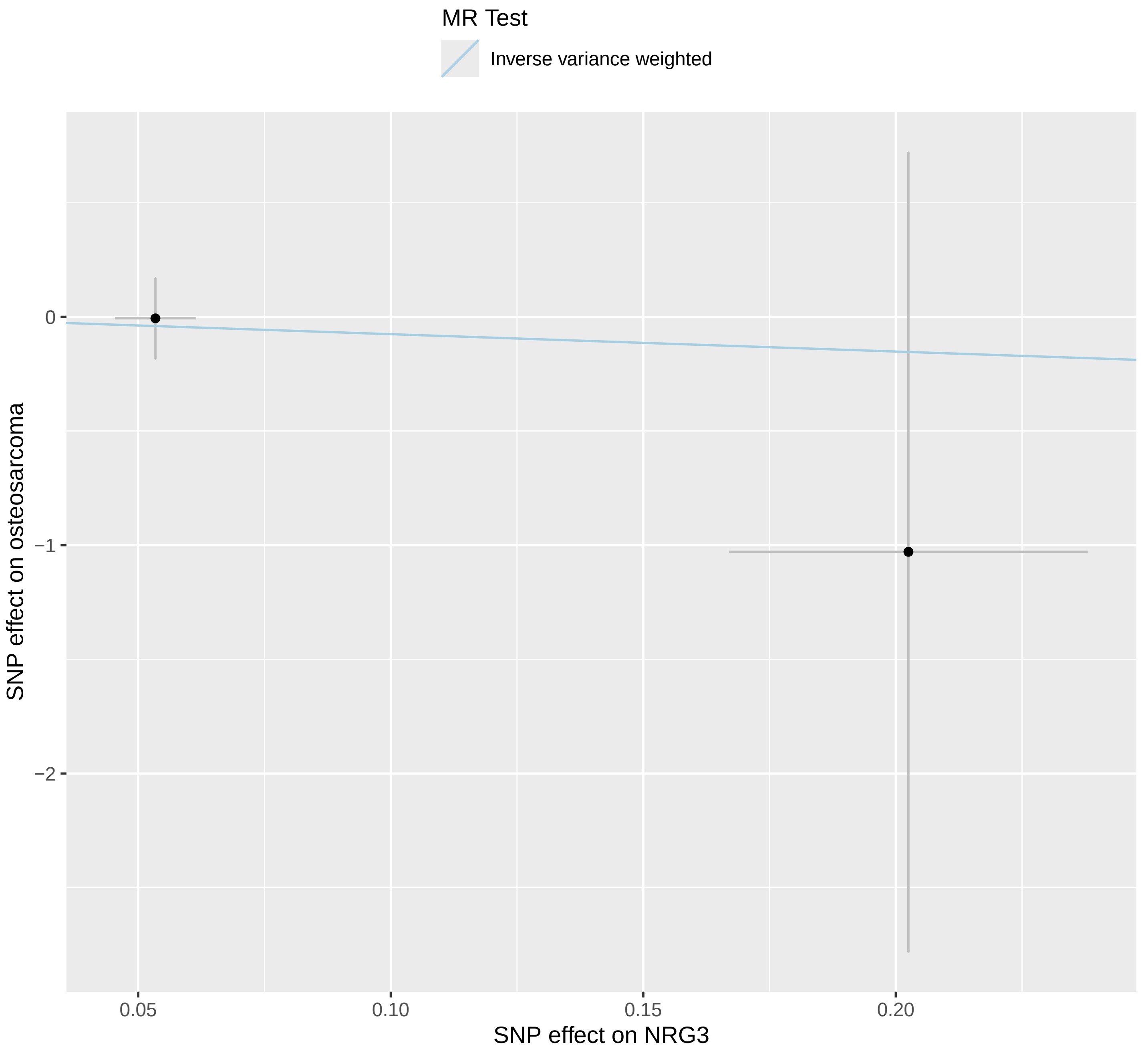
MR Test
Inverse variance weighted
0
−1
−2
0.05
0.10
0.15
0.20
SNP effect on NRG3
SNP effect on osteosarcoma

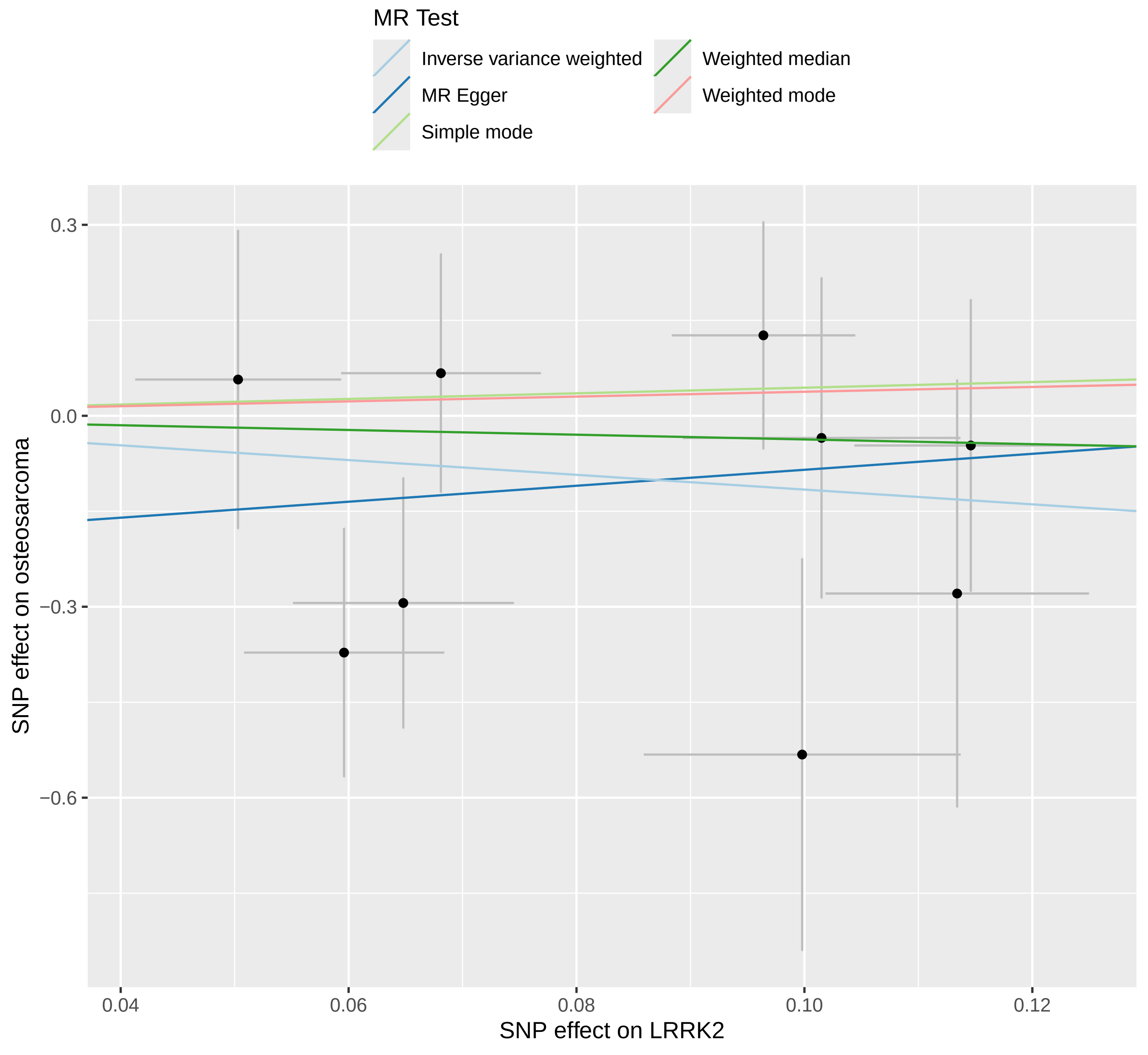
MR Test
Inverse variance weighted
MR Egger
Simple mode
Weighted median
Weighted mode
0.3
0.0
−0.3
−0.6
SNP effect on osteosarcoma
0.04
0.06
0.08
0.10
0.12
SNP effect on LRRK2

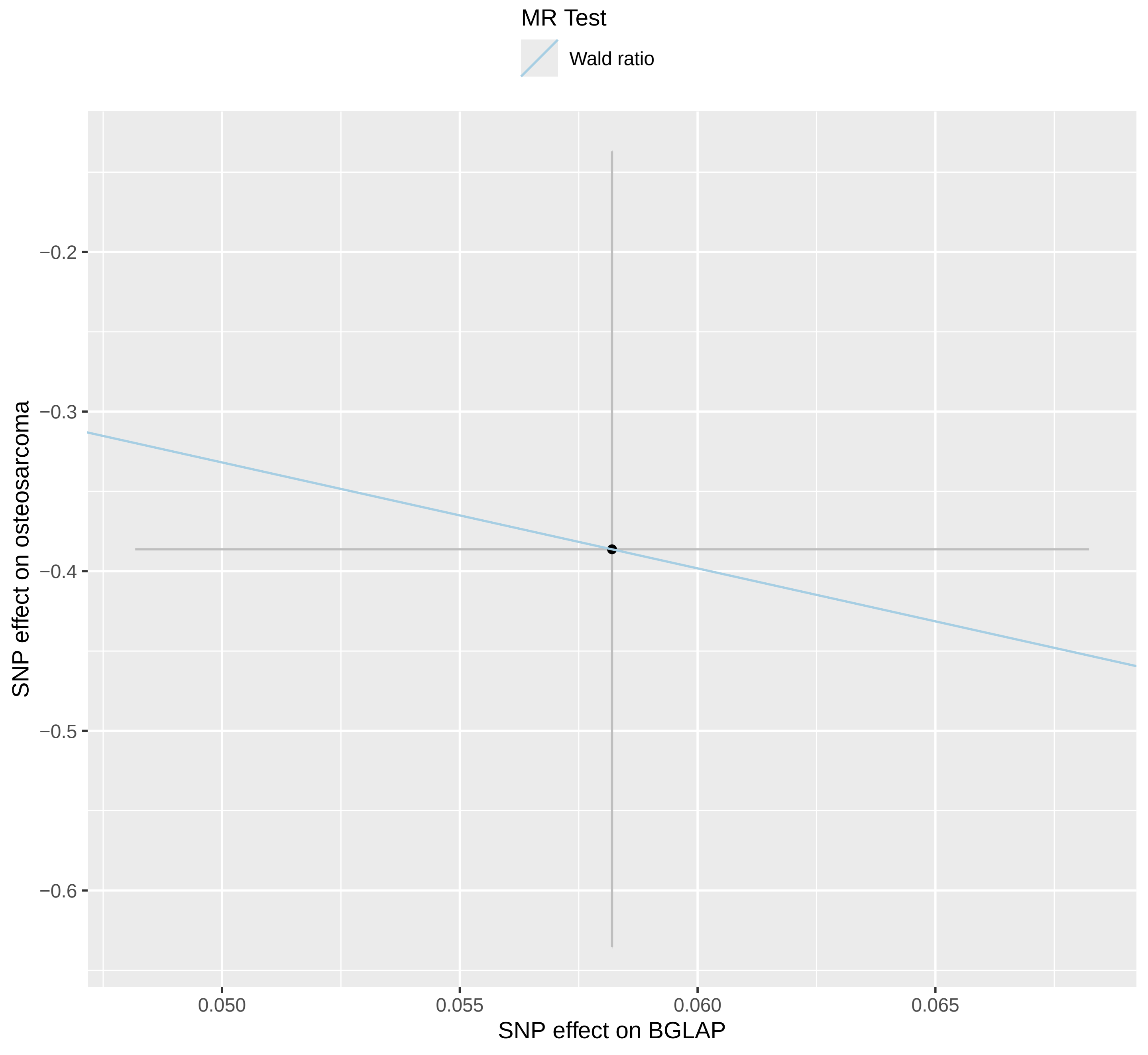
MR Test
Wald ratio
−0.2
−0.3
−0.4
−0.5
−0.6
SNP effect on osteosarcoma
0.050
0.055
0.060
0.065
SNP effect on BGLAP

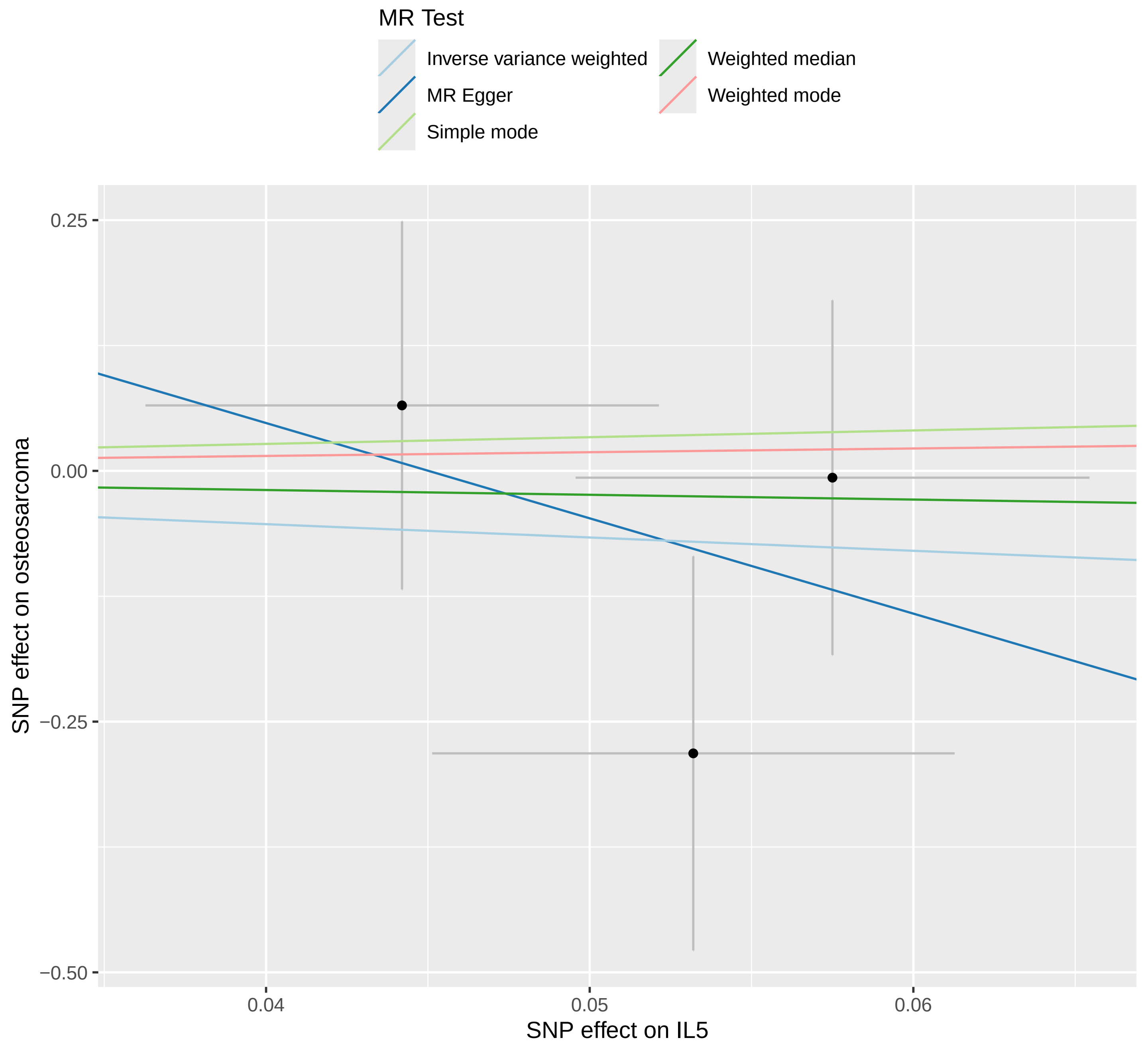
MR Test
Inverse variance weighted
MR Egger
Simple mode
Weighted median
Weighted mode
0.25
0.00
−0.25
−0.50
0.04
0.05
0.06
SNP effect on osteosarcoma
SNP effect on IL5

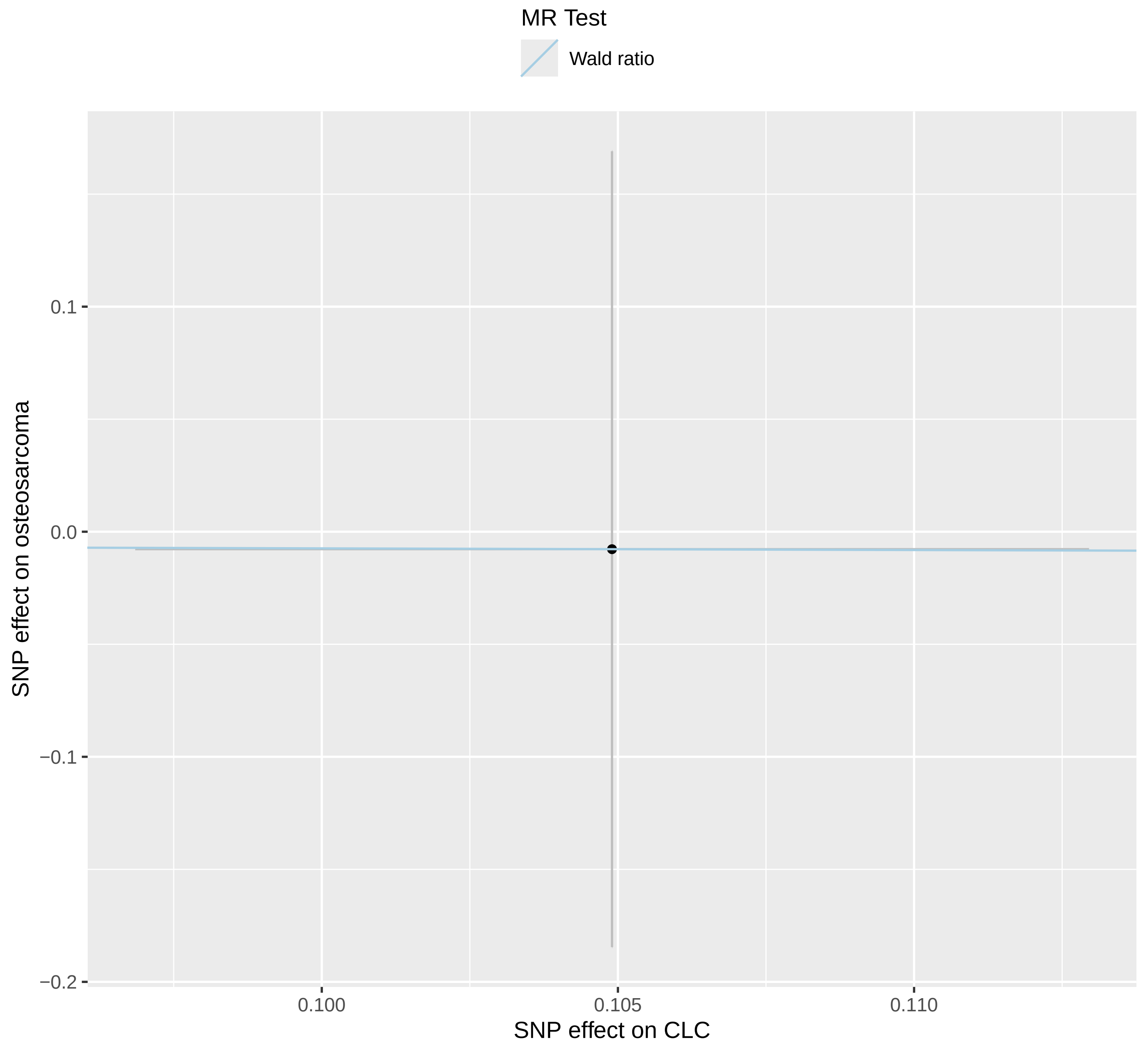
MR Test
Wald ratio
0.1
0.0
−0.1
−0.2
0.100
0.105
0.110
SNP effect on CLC
SNP effect on osteosarcoma

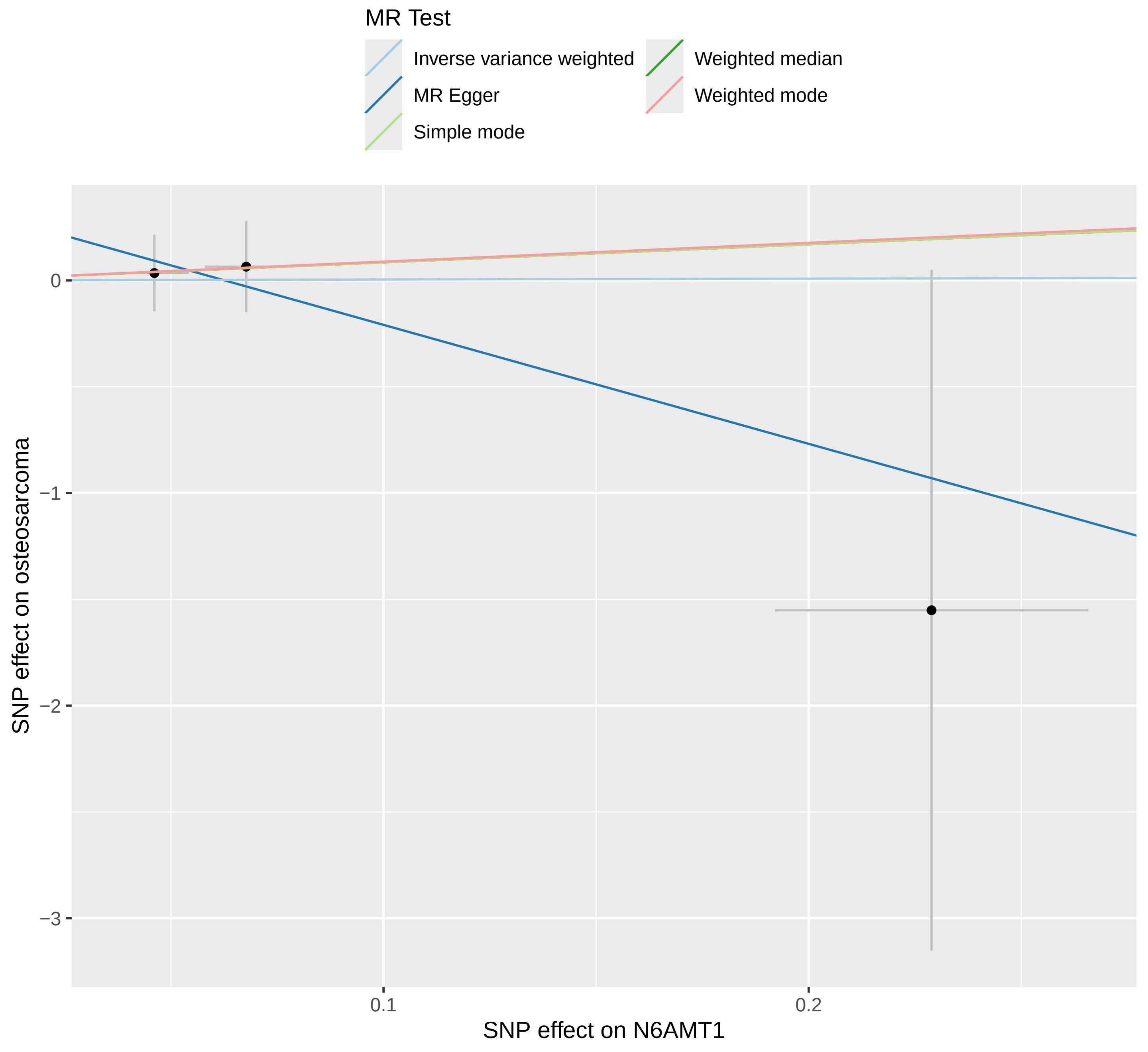
MR Test
Inverse variance weighted
MR Egger
Simple mode
Weighted median
Weighted mode
0
−1
−2
−3
0.1
0.2
SNP effect on osteosarcoma
SNP effect on N6AMT1

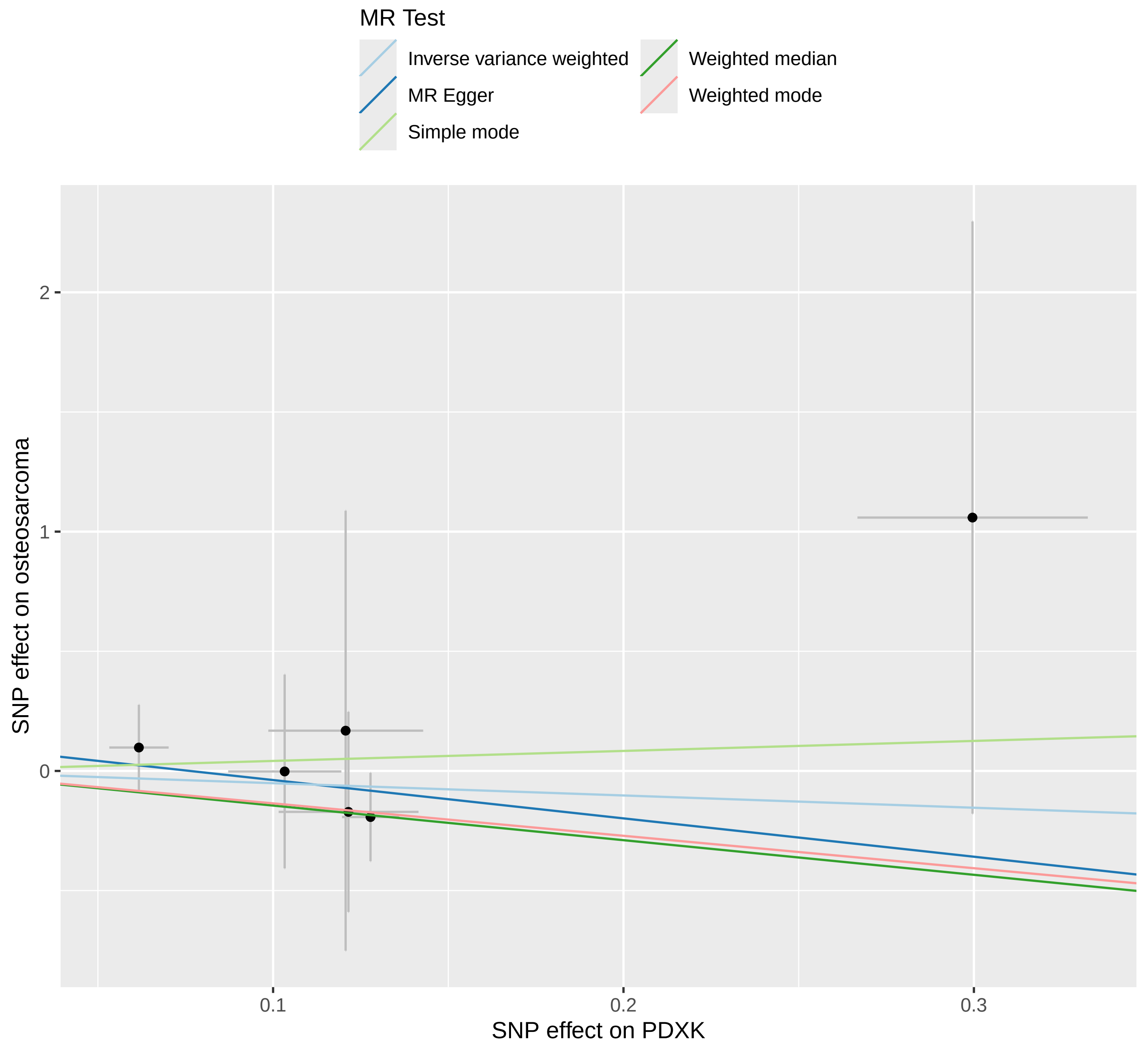
MR Test
Inverse variance weighted
MR Egger
Simple mode
Weighted median
Weighted mode
SNP effect on osteosarcoma
2
1
0
0.1
0.2
0.3
SNP effect on PDXK

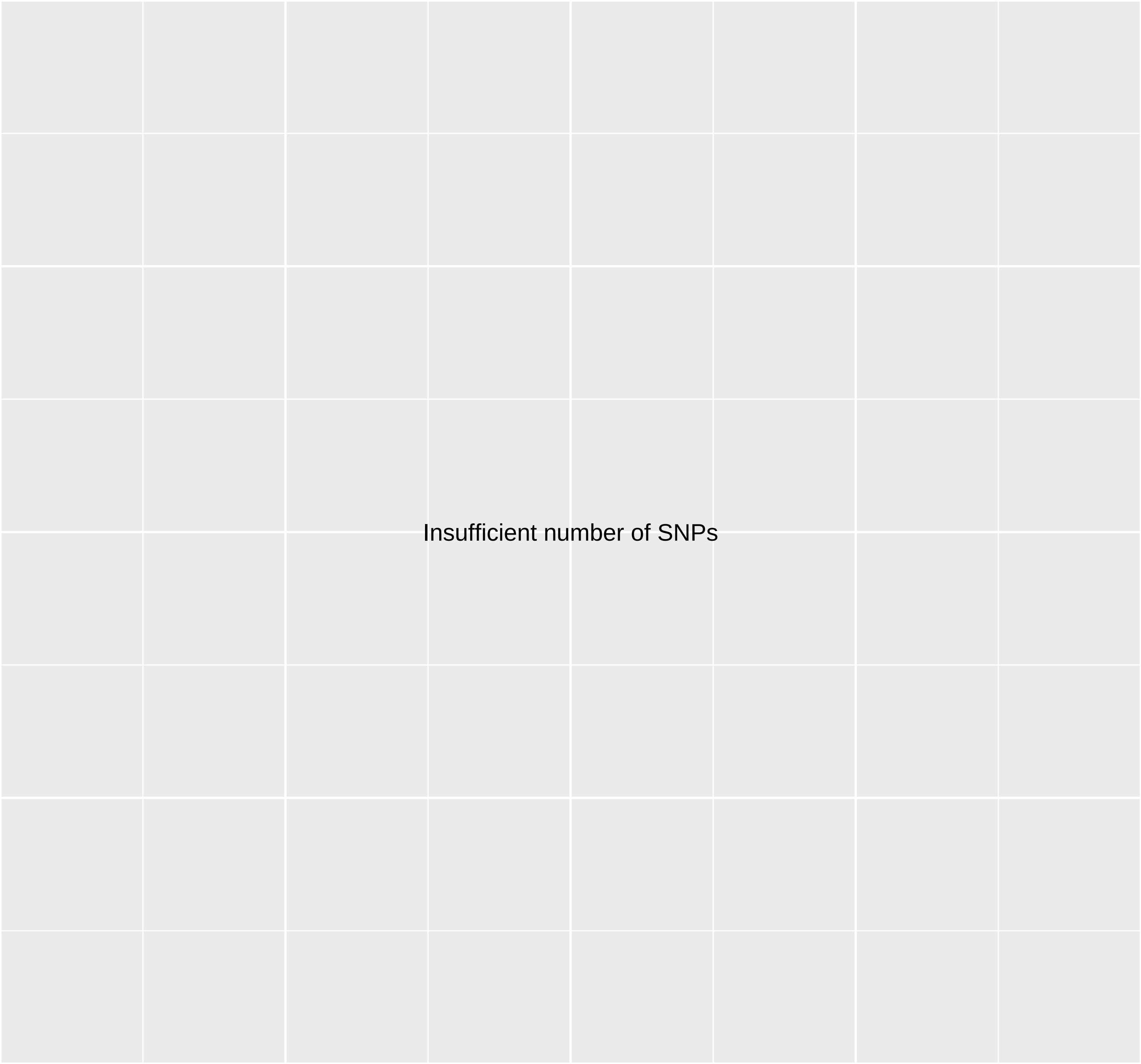
Insufficient number of SNPs

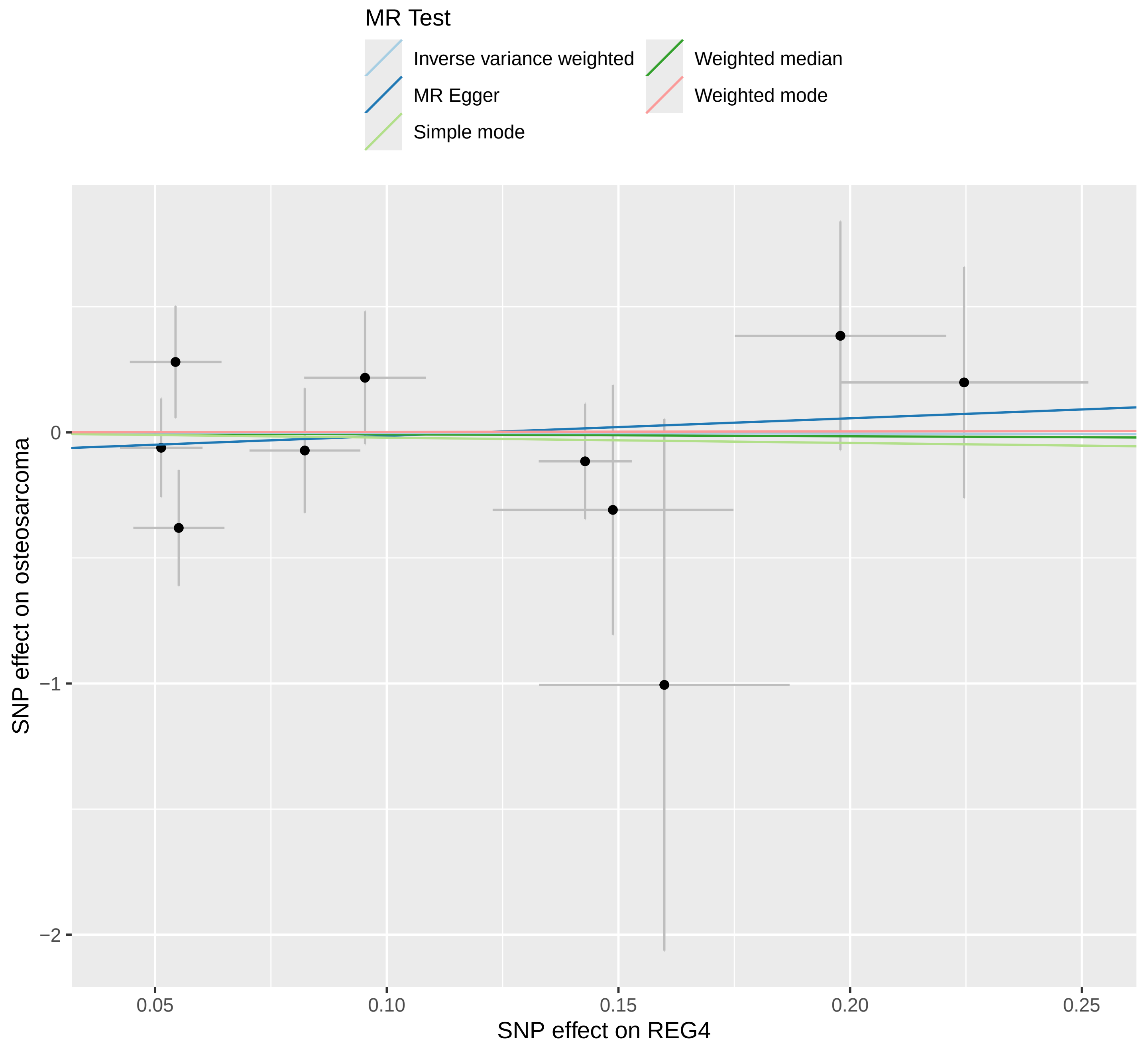
MR Test
Inverse variance weighted
MR Egger
Simple mode
Weighted median
Weighted mode
SNP effect on osteosarcoma
0
−1
−2
0.05
0.10
0.15
0.20
0.25
SNP effect on REG4

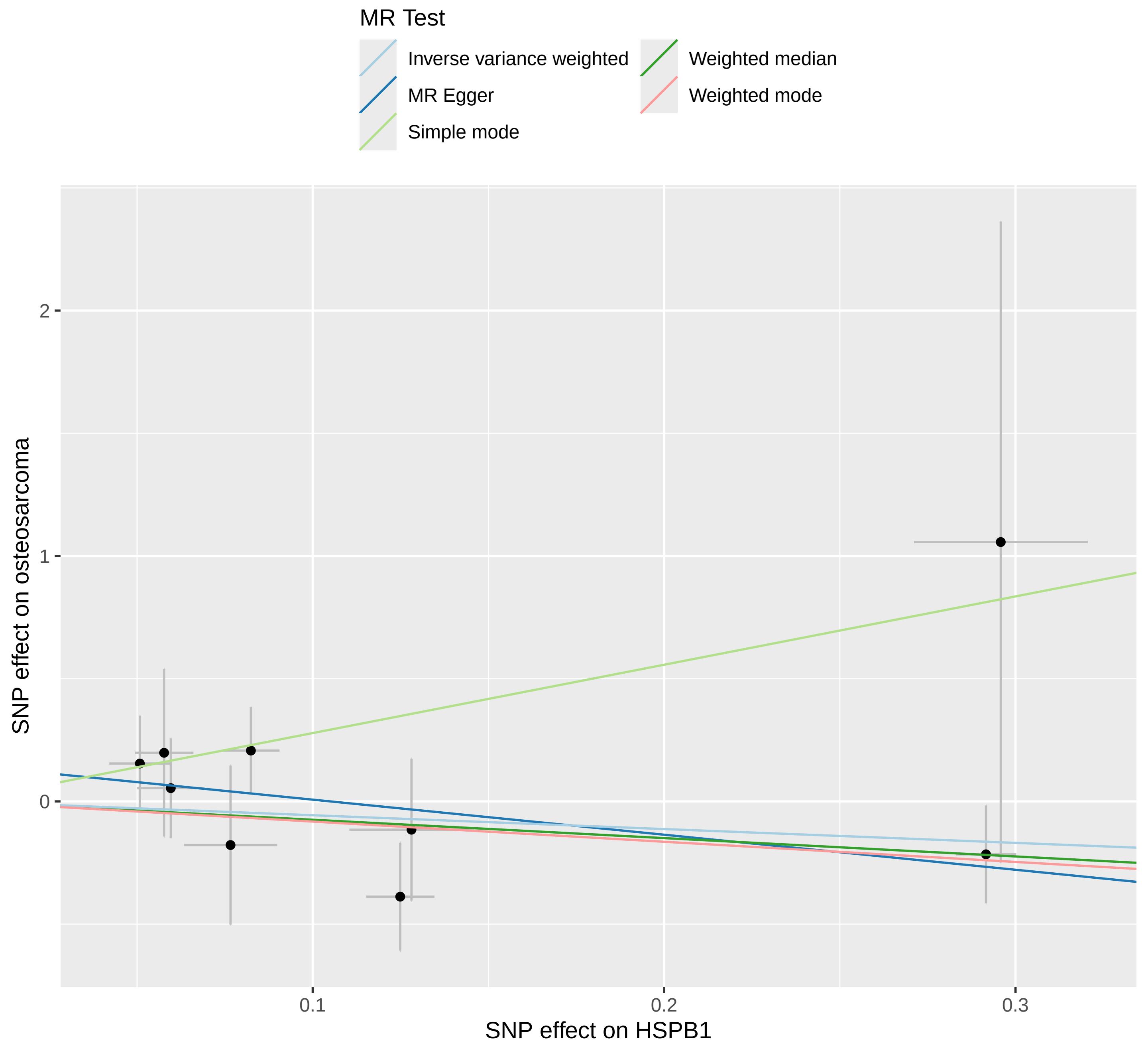
MR Test
Inverse variance weighted
MR Egger
Simple mode
Weighted median
Weighted mode
2
1
0
SNP effect on osteosarcoma
0.1
0.2
0.3
SNP effect on HSPB1

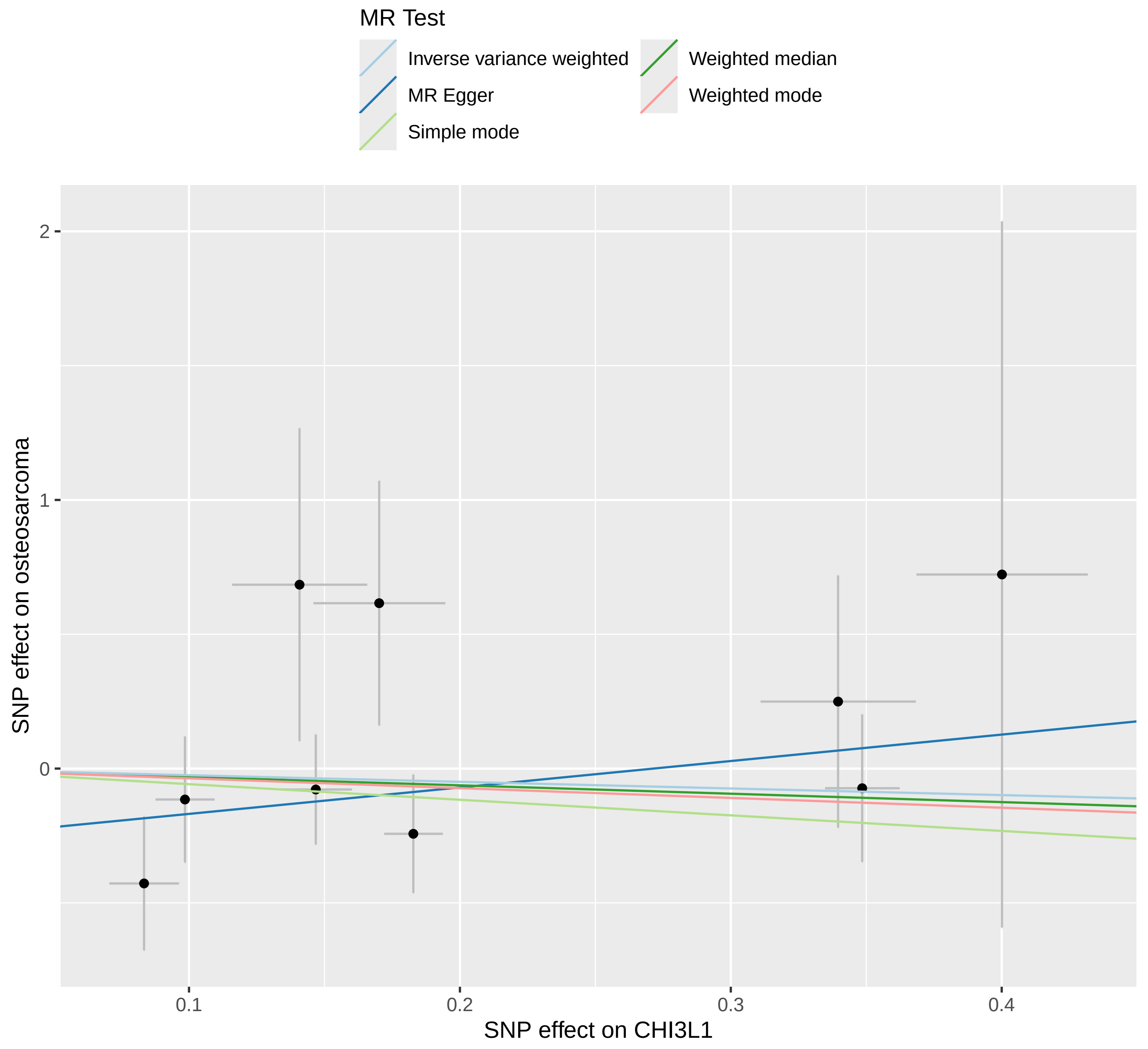

MR Test
Inverse variance weighted
MR Egger
Simple mode
Weighted median
Weighted mode
2
1
0
0.1
0.2
0.3
0.4
SNP effect on CHI3L1
SNP effect on osteosarcoma

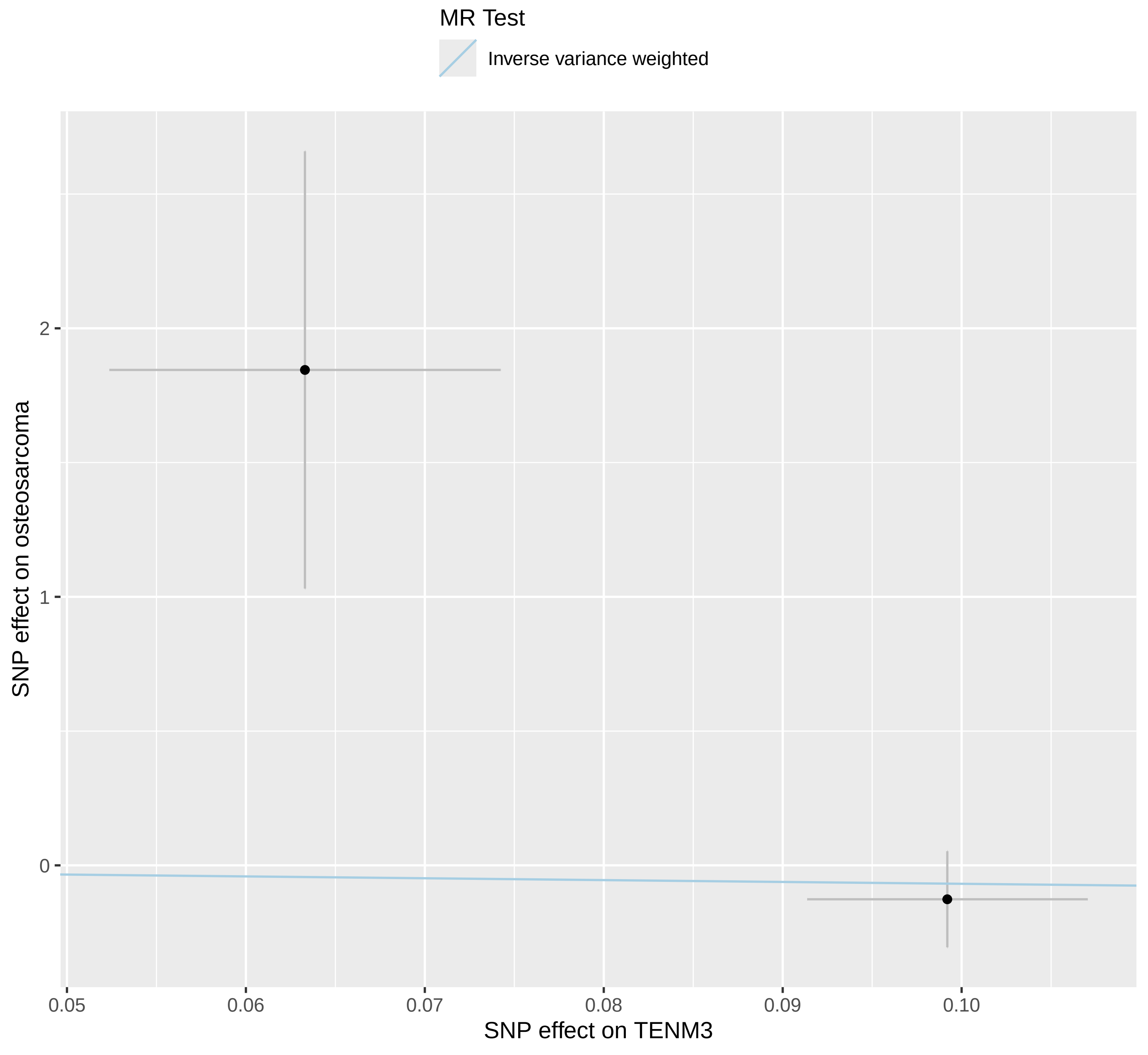

MR Test
Inverse variance weighted
SNP effect on osteosarcoma
2
1
0
0.05
0.06
0.07
0.08
0.09
0.10
SNP effect on TENM3

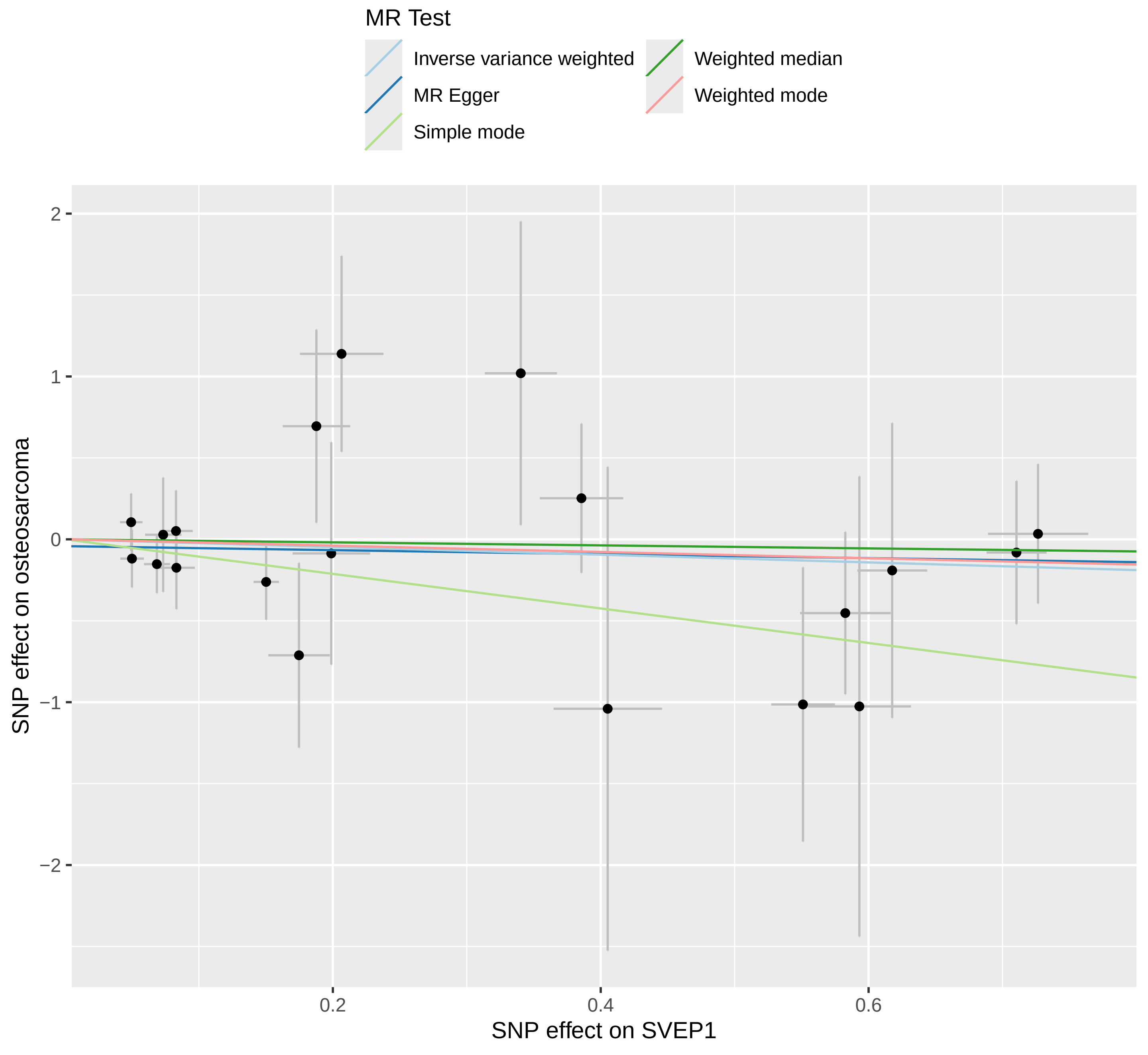
MR Test
Inverse variance weighted
MR Egger
Simple mode
Weighted median
Weighted mode
SNP effect on osteosarcoma
SNP effect on SVEP1
2
1
0
−1
−2
0.2
0.4
0.6

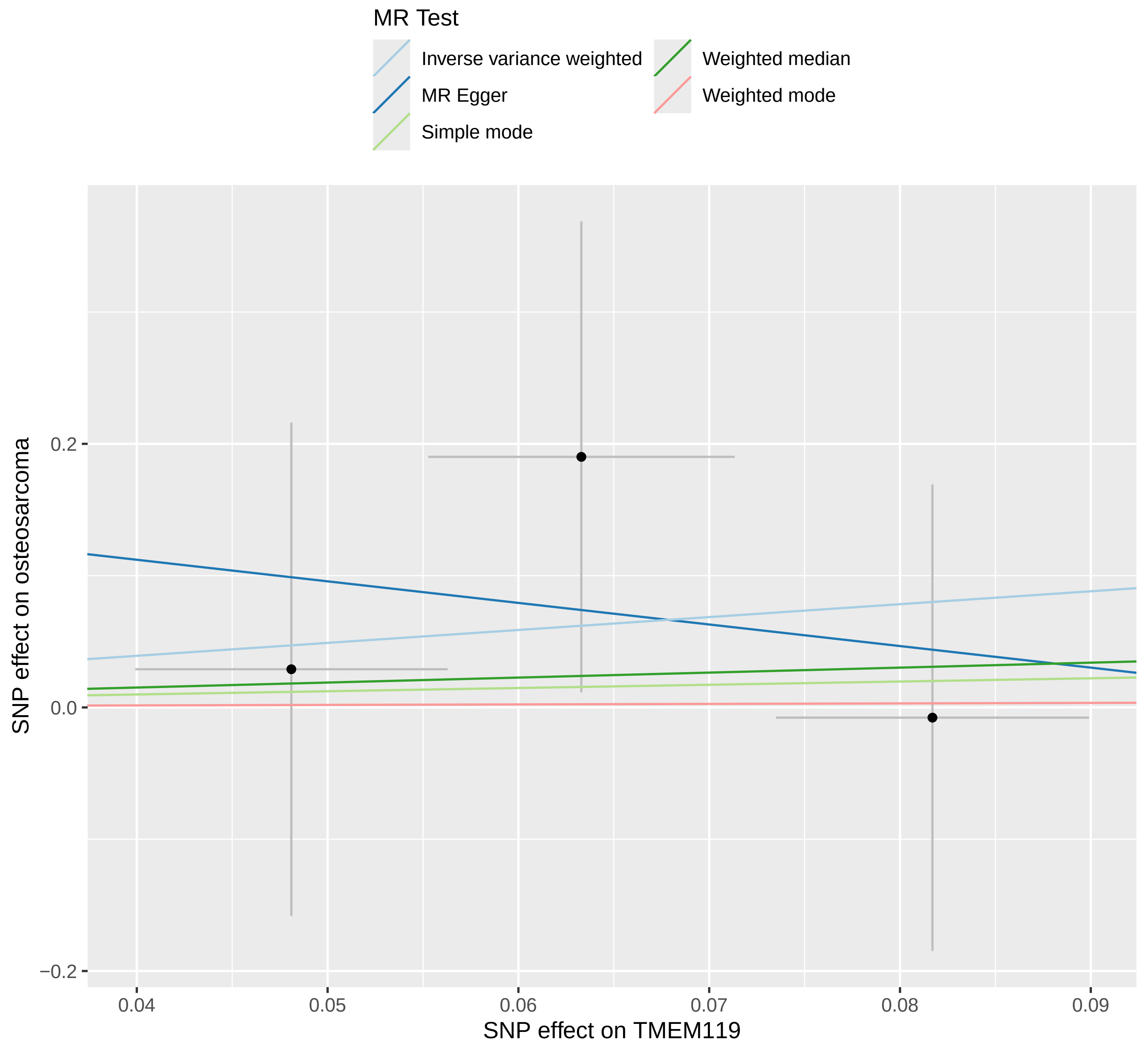

MR Test
Inverse variance weighted
MR Egger
Simple mode
Weighted median
Weighted mode
SNP effect on osteosarcoma
0.2
0.0
−0.2
0.04
0.05
0.06
0.07
0.08
0.09
SNP effect on TMEM119

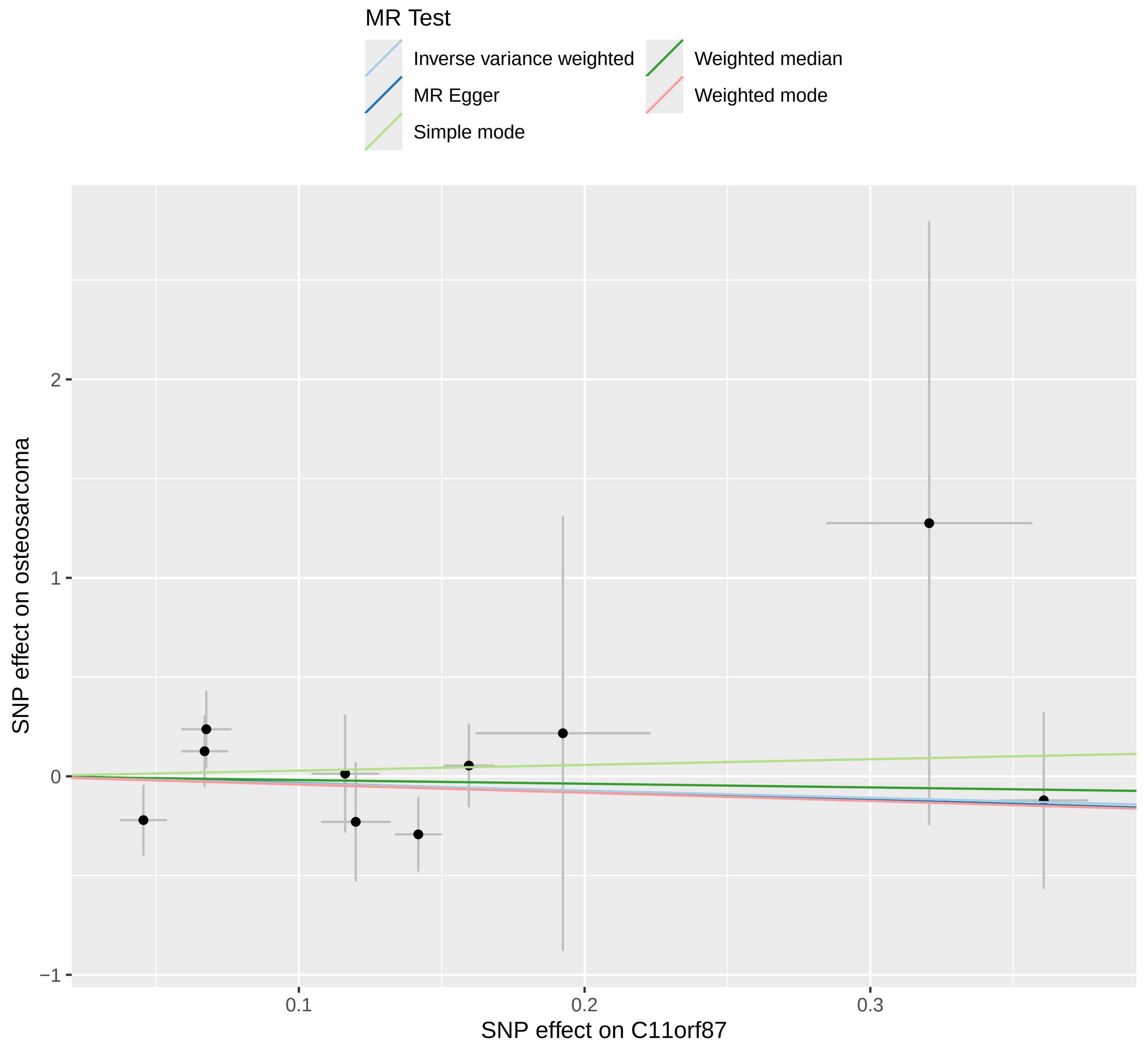

MR Test
Inverse variance weighted
MR Egger
Simple mode
Weighted median
Weighted mode
SNP effect on osteosarcoma
2
1
0
−1
0.1
0.2
0.3
SNP effect on C11orf87

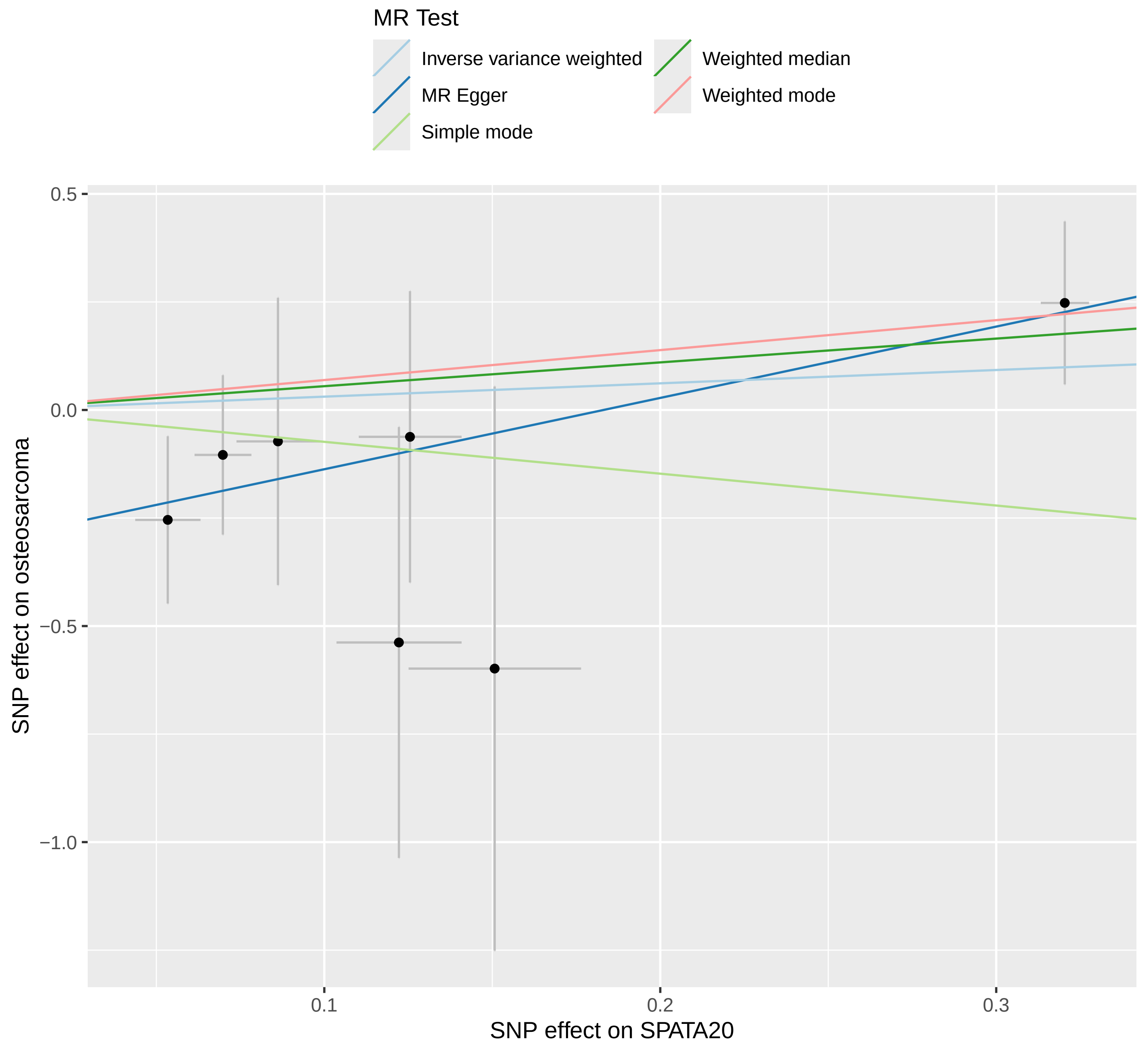
MR Test
Inverse variance weighted
MR Egger
Simple mode
Weighted median
Weighted mode
0.5
0.0
−0.5
−1.0
SNP effect on osteosarcoma
0.1
0.2
0.3
SNP effect on SPATA20

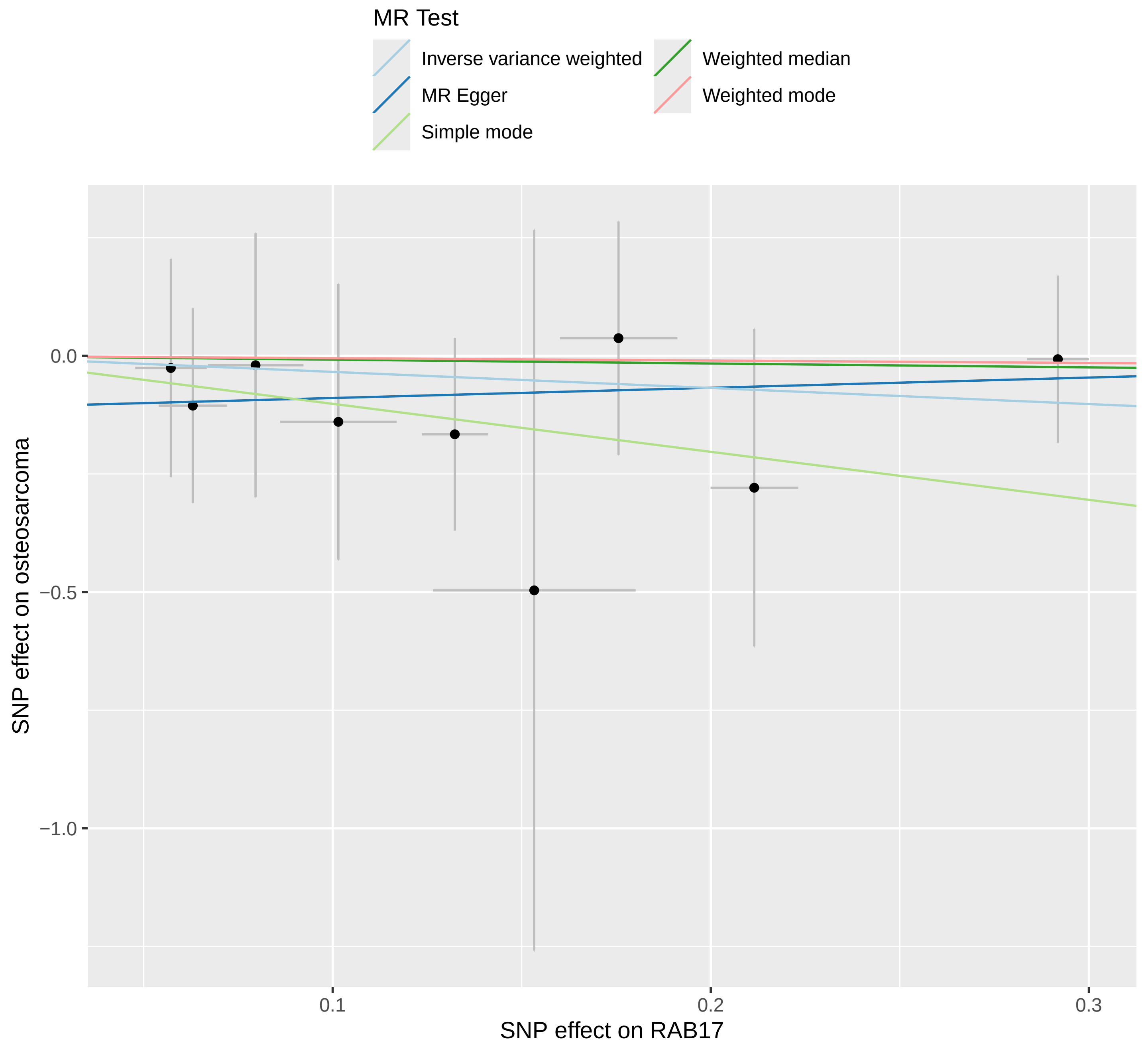
MR Test
Inverse variance weighted
MR Egger
Simple mode
Weighted median
Weighted mode
0.0
−0.5
−1.0
SNP effect on osteosarcoma
0.1
0.2
0.3
SNP effect on RAB17

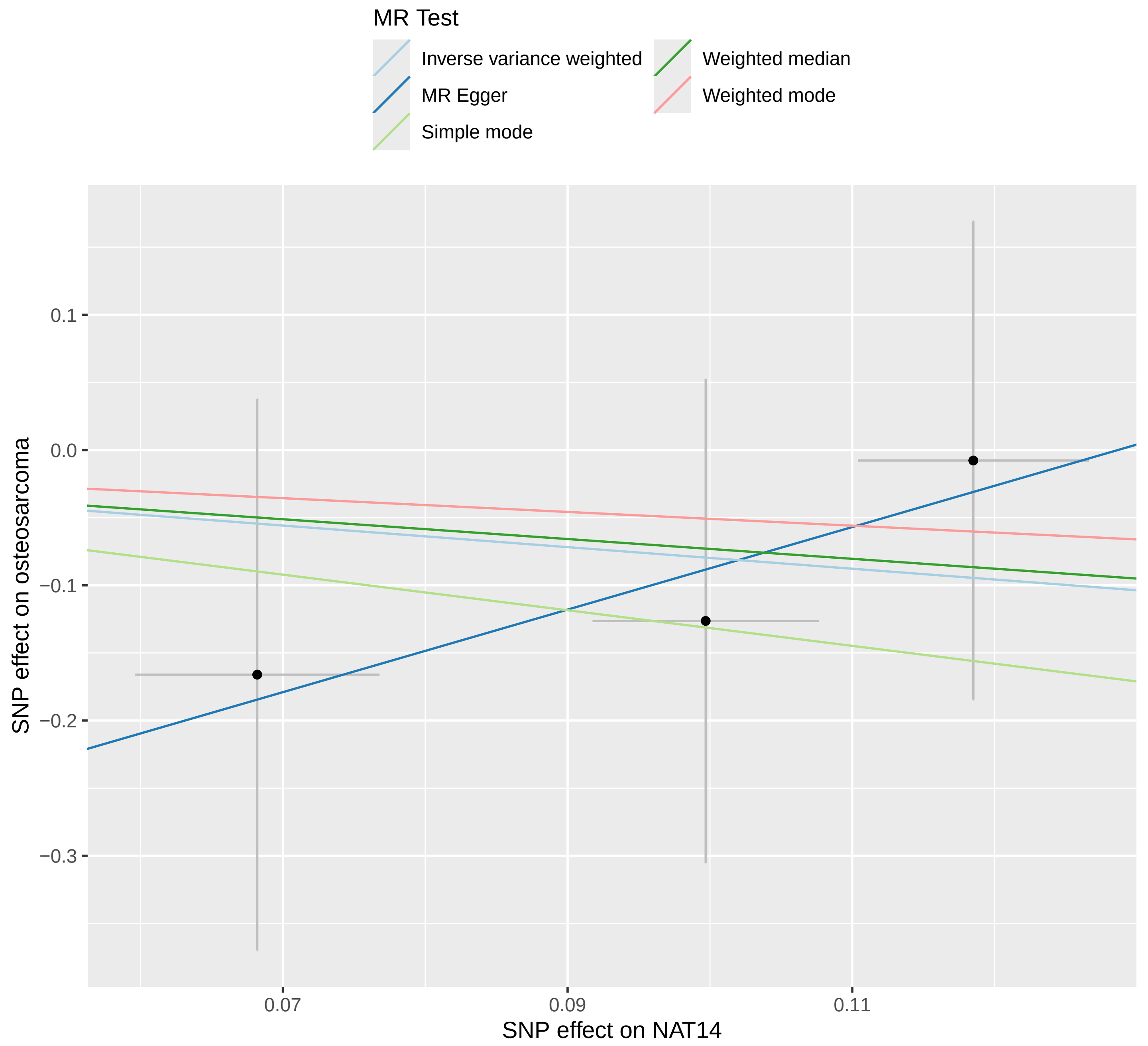

MR Test
Inverse variance weighted
MR Egger
Simple mode
Weighted median
Weighted mode
0.1
0.0
−0.1
−0.2
−0.3
0.07
0.09
0.11
SNP effect on NAT14
SNP effect on osteosarcoma

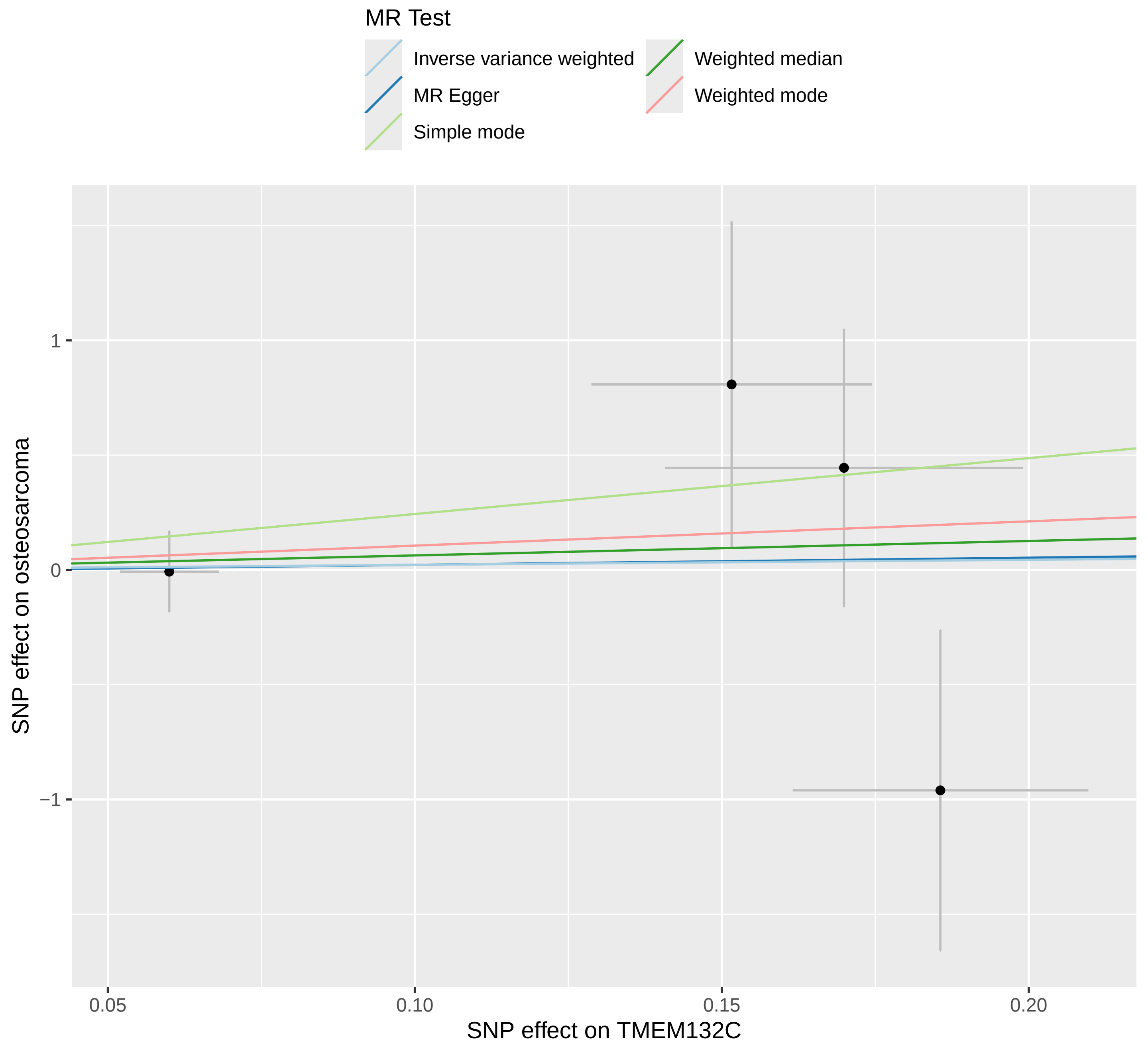

MR Test
Inverse variance weighted
MR Egger
Simple mode
Weighted median
Weighted mode
SNP effect on osteosarcoma
1
0
−1
0.05
0.10
0.15
0.20
SNP effect on TMEM132C

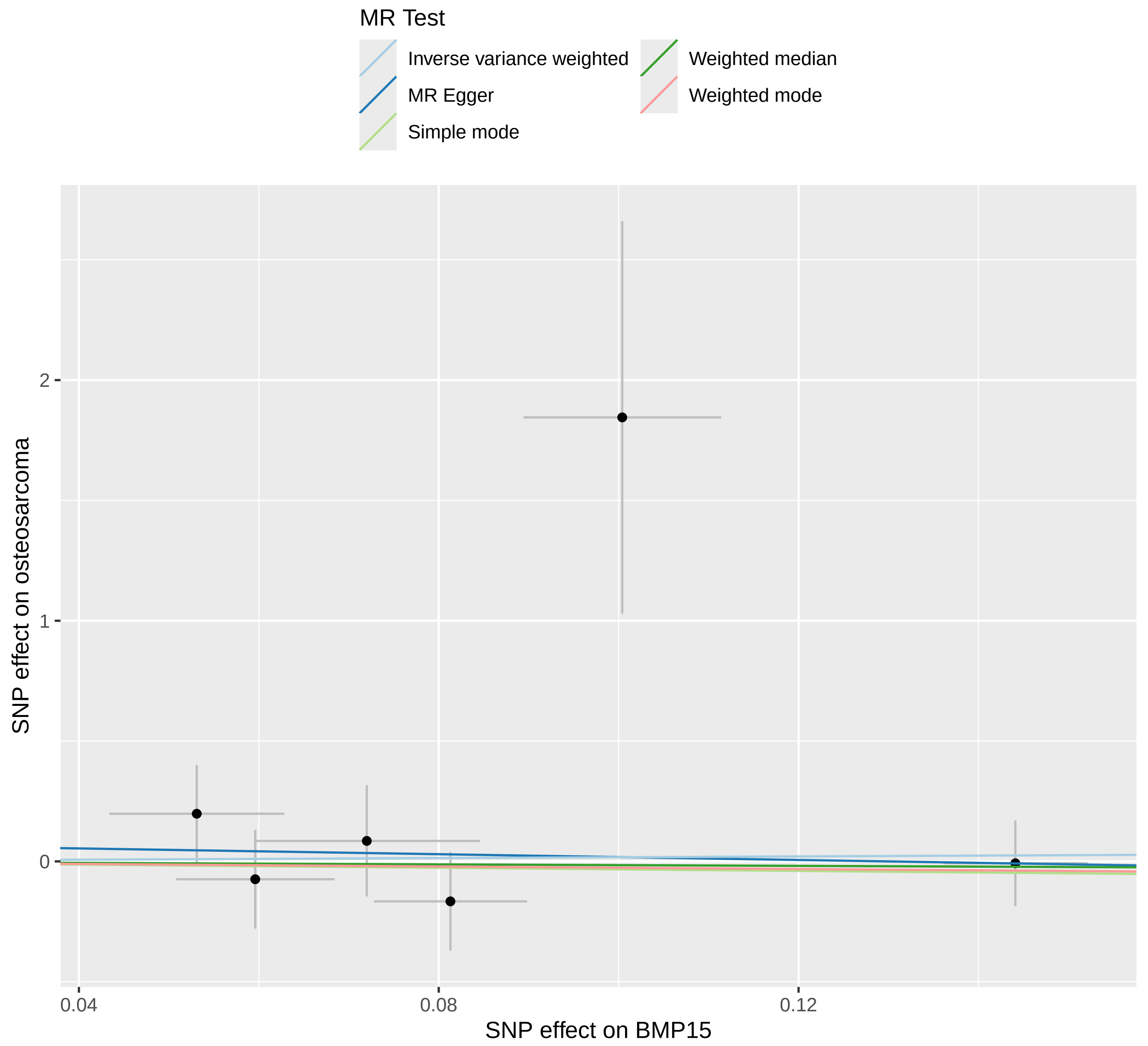
MR Test
Inverse variance weighted
MR Egger
Simple mode
Weighted median
Weighted mode
SNP effect on osteosarcoma
2
1
0
0.04
0.08
0.12
SNP effect on BMP15

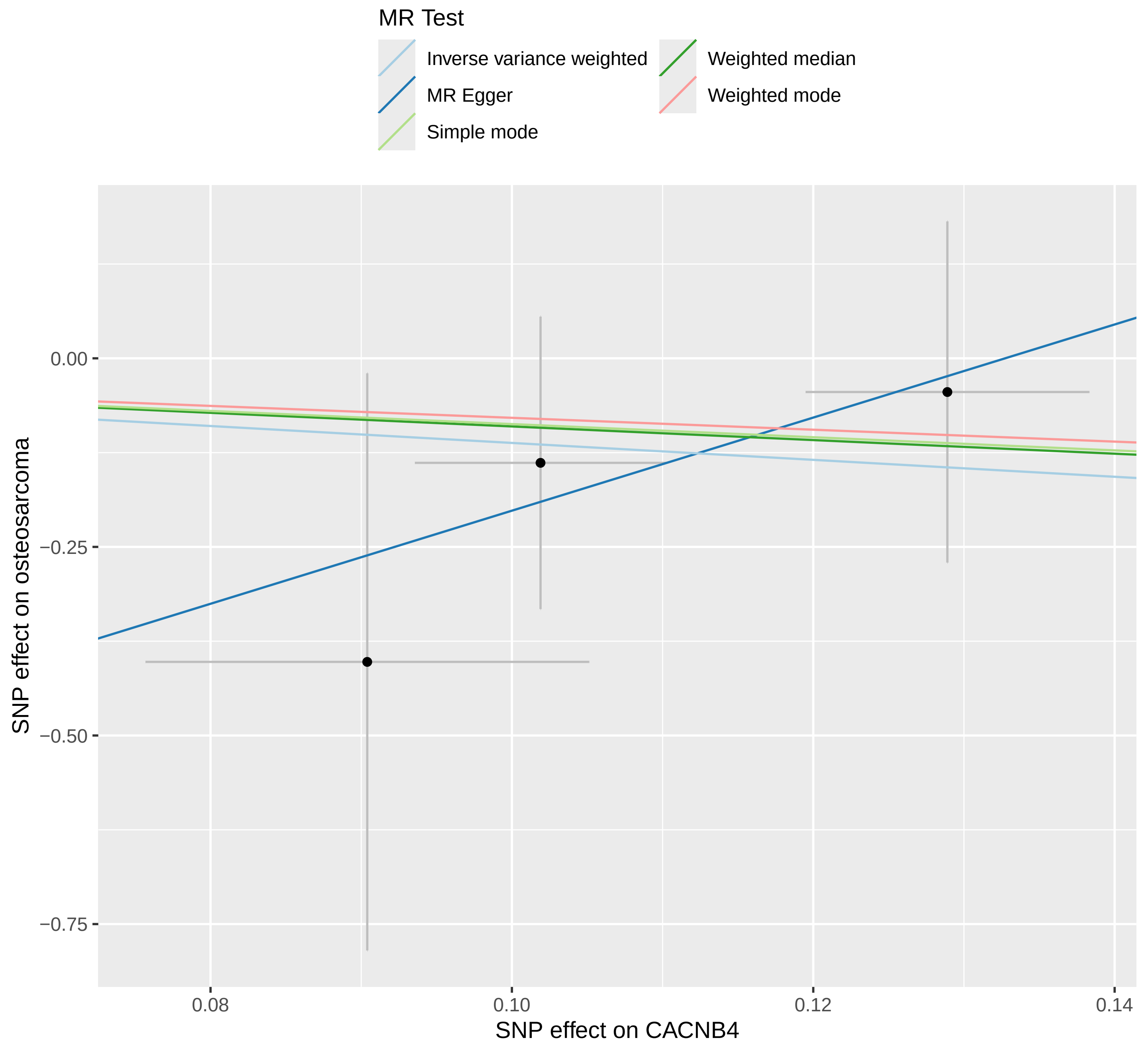

MR Test
Inverse variance weighted
MR Egger
Simple mode
Weighted median
Weighted mode
0.00
−0.25
−0.50
−0.75
SNP effect on osteosarcoma
0.08
0.10
0.12
0.14
SNP effect on CACNB4

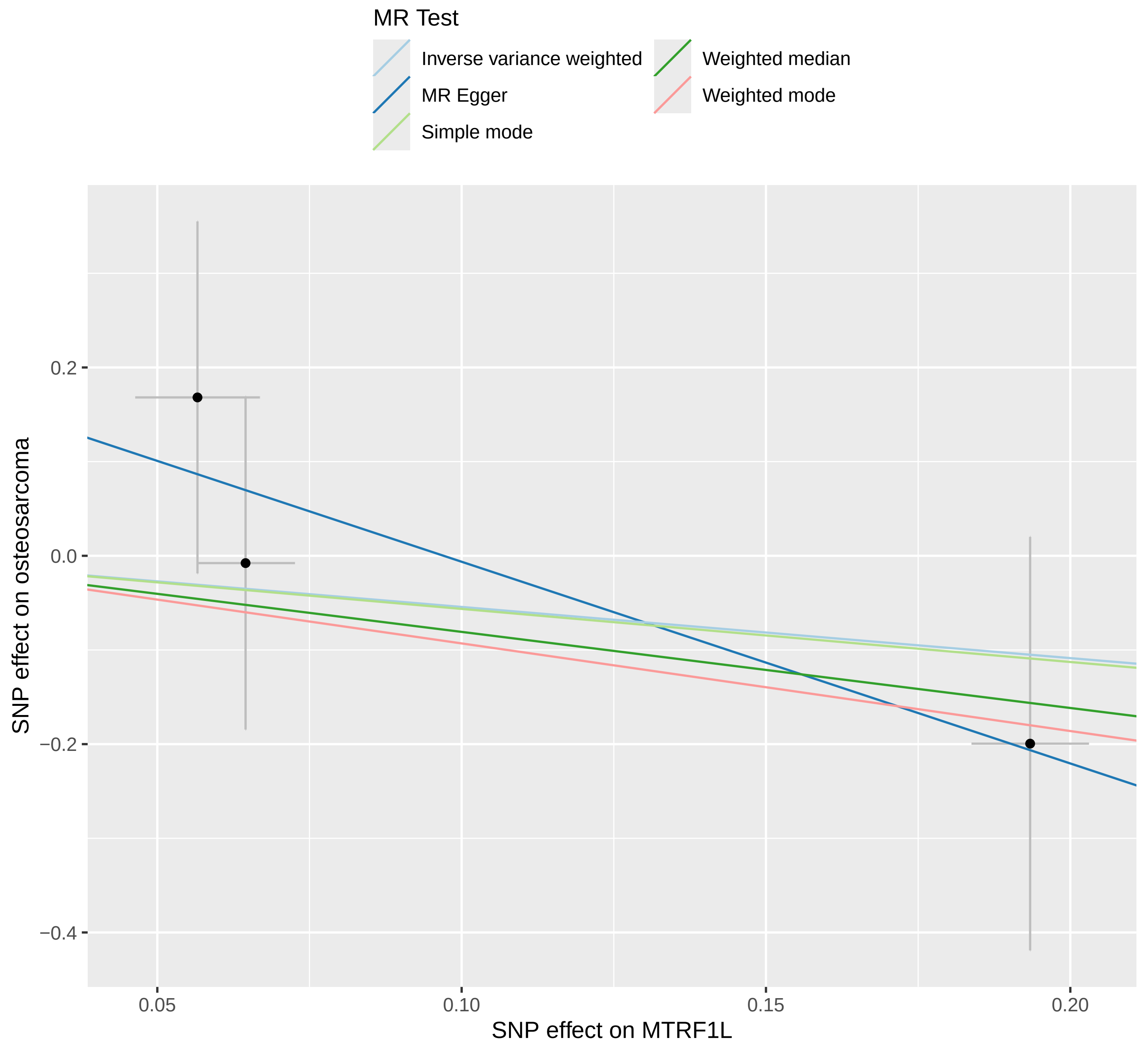

MR Test
Inverse variance weighted
MR Egger
Simple mode
Weighted median
Weighted mode
0.2
0.0
−0.2
−0.4
SNP effect on osteosarcoma
0.05
0.10
0.15
0.20
SNP effect on MTRF1L

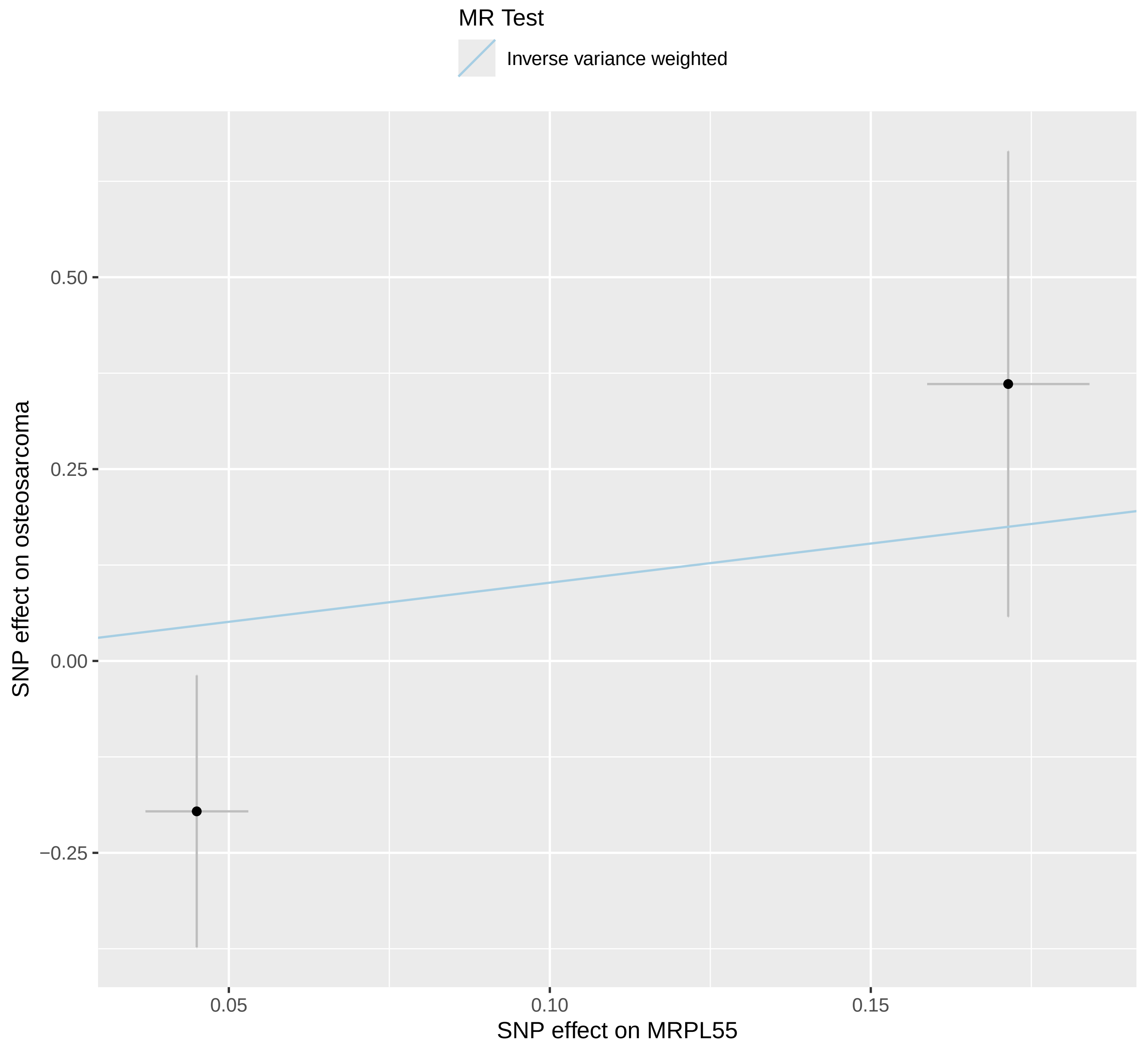
MR Test
Inverse variance weighted
0.50
0.25
0.00
−0.25
0.05
0.10
0.15
SNP effect on MRPL55
SNP effect on osteosarcoma

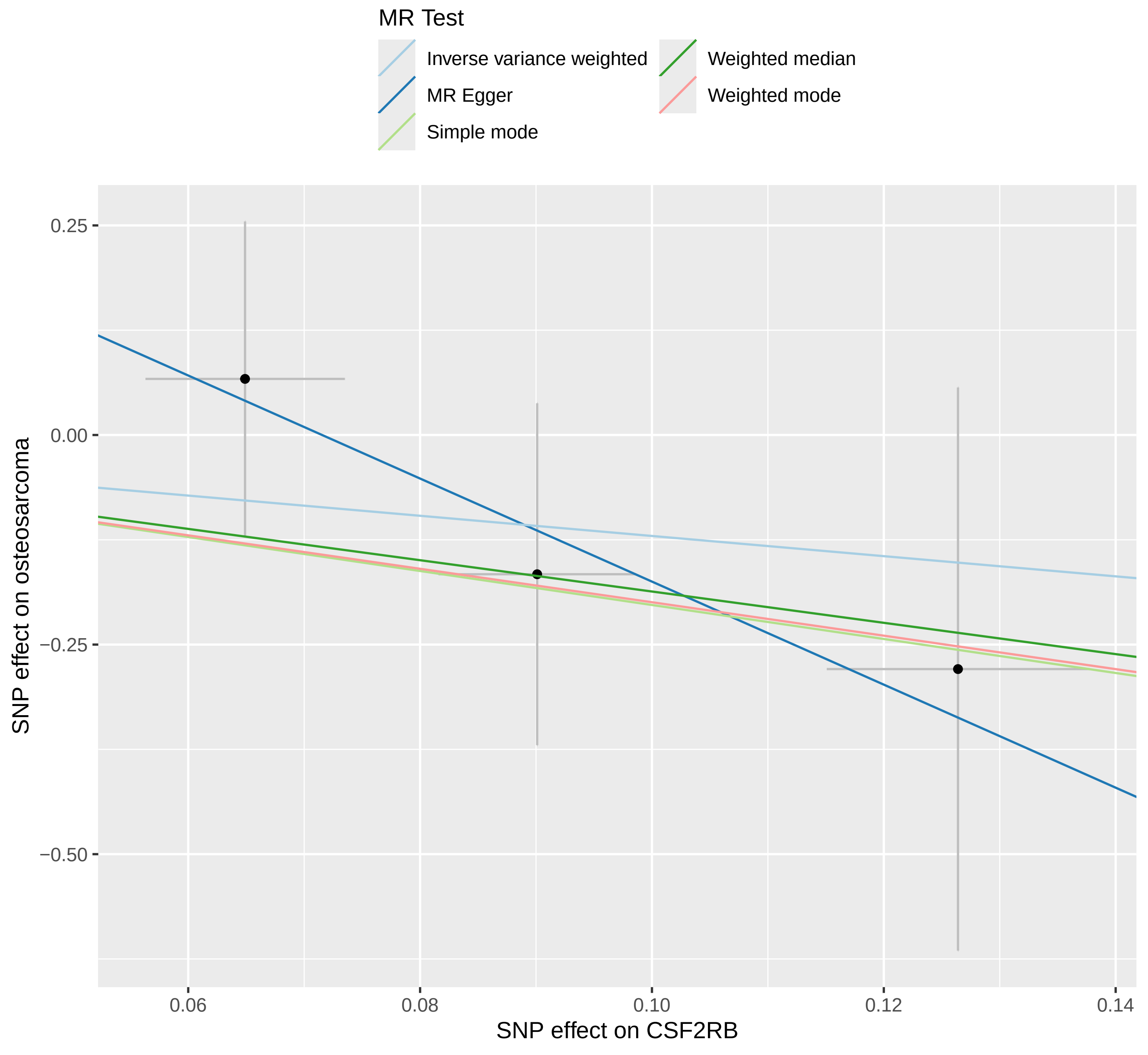
MR Test
Inverse variance weighted
MR Egger
Simple mode
Weighted median
Weighted mode
0.25
0.00
−0.25
−0.50
0.06
0.08
0.10
0.12
0.14
SNP effect on CSF2RB
SNP effect on osteosarcoma

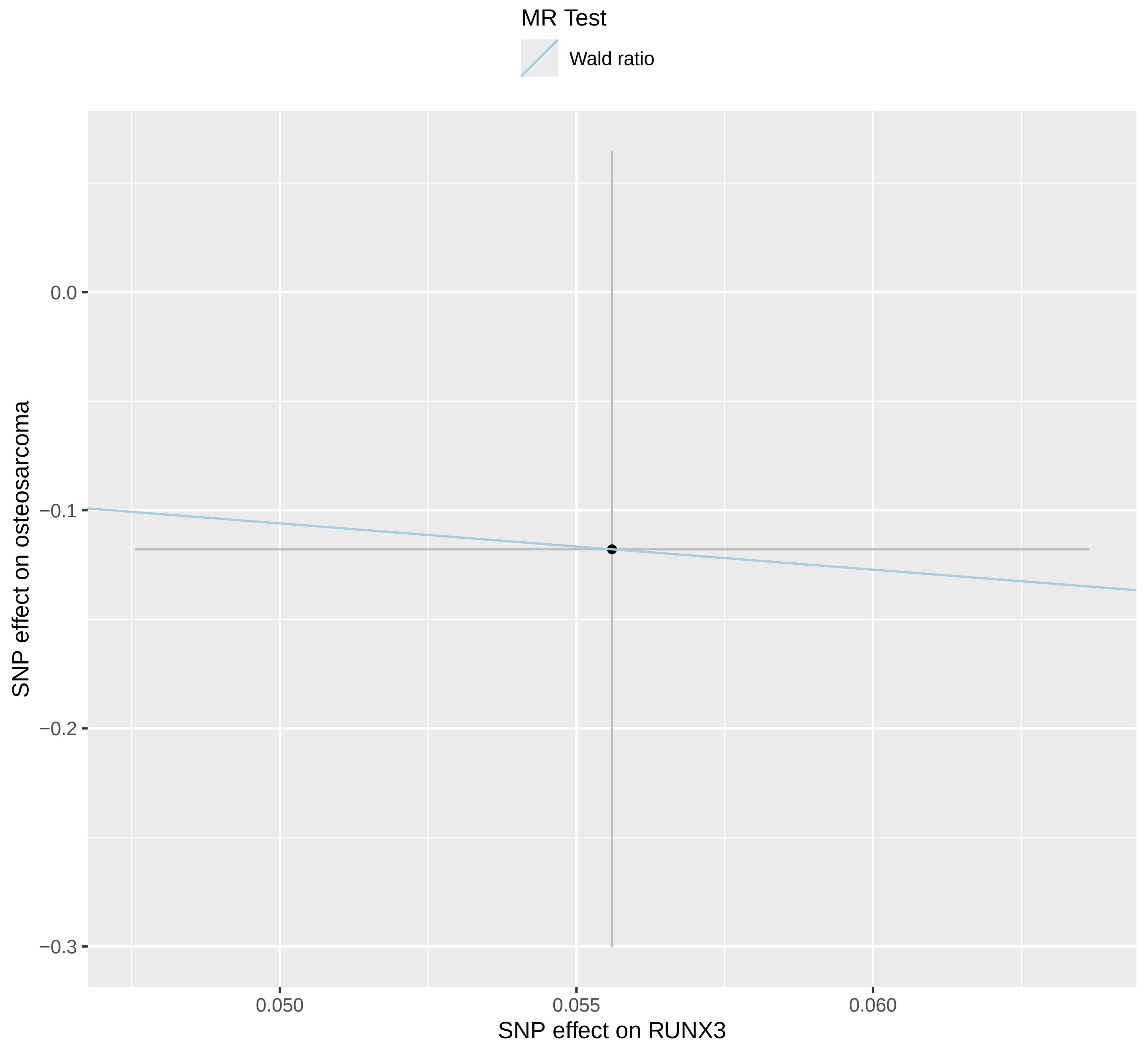
MR Test
Wald ratio
SNP effect on osteosarcoma
0.0
−0.1
−0.2
−0.3
0.050
0.055
0.060
SNP effect on RUNX3

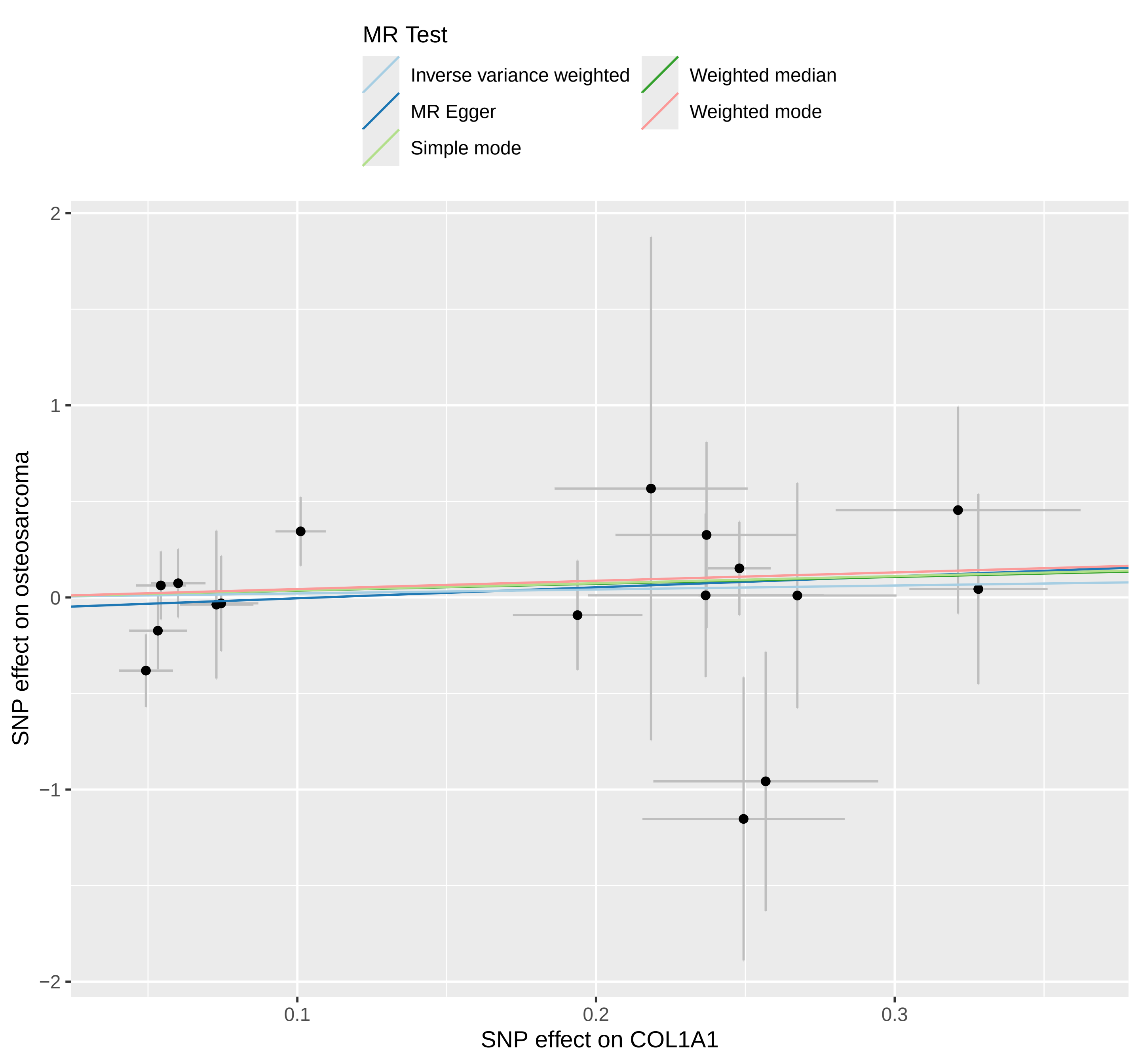
MR Test
Inverse variance weighted
MR Egger
Simple mode
Weighted median
Weighted mode
SNP effect on osteosarcoma
2
1
0
−1
−2
0.1
0.2
0.3
SNP effect on COL1A1

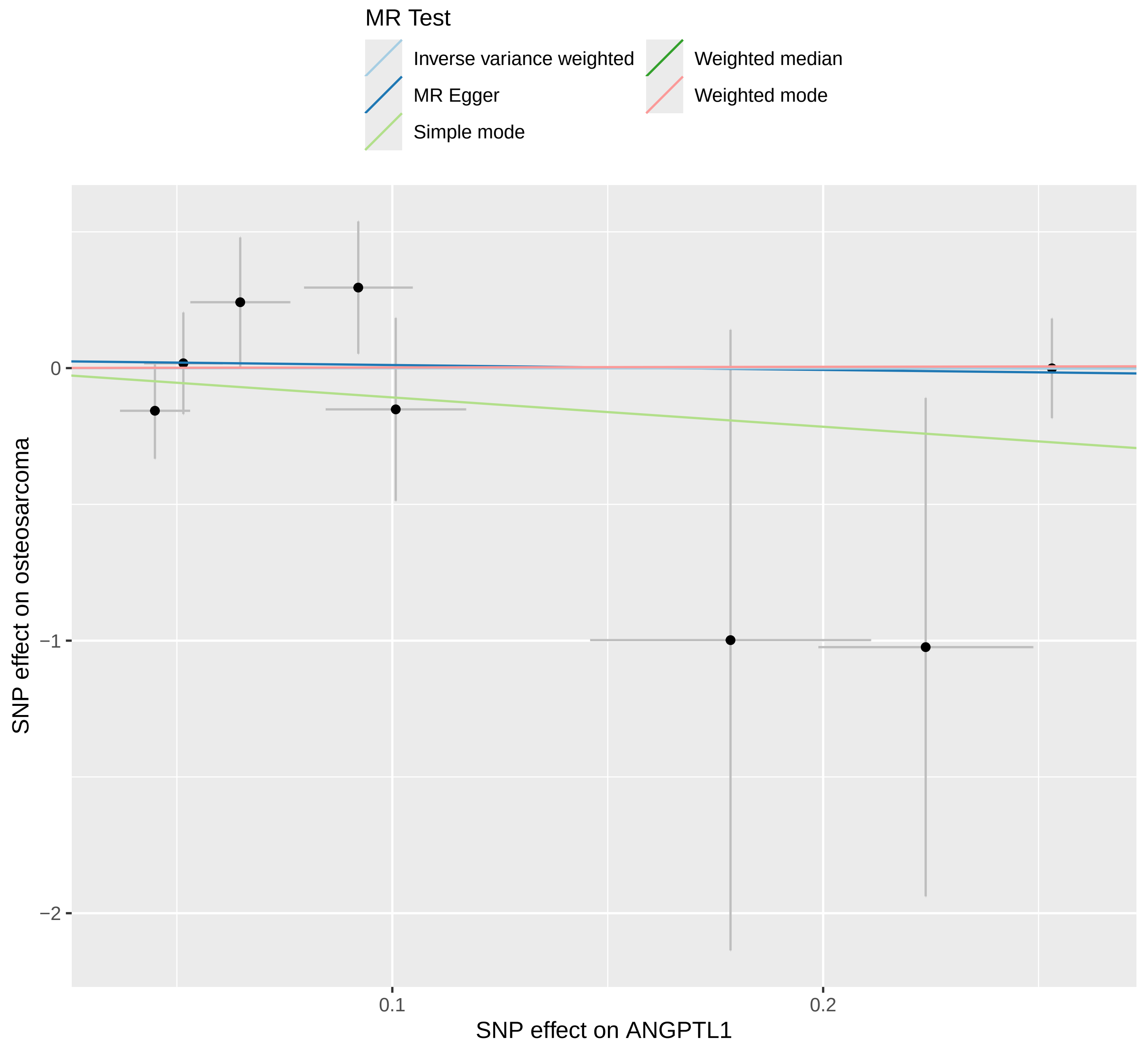
MR Test
Inverse variance weighted
MR Egger
Simple mode
Weighted median
Weighted mode
0
−1
−2
0.1
0.2
SNP effect on osteosarcoma
SNP effect on ANGPTL1

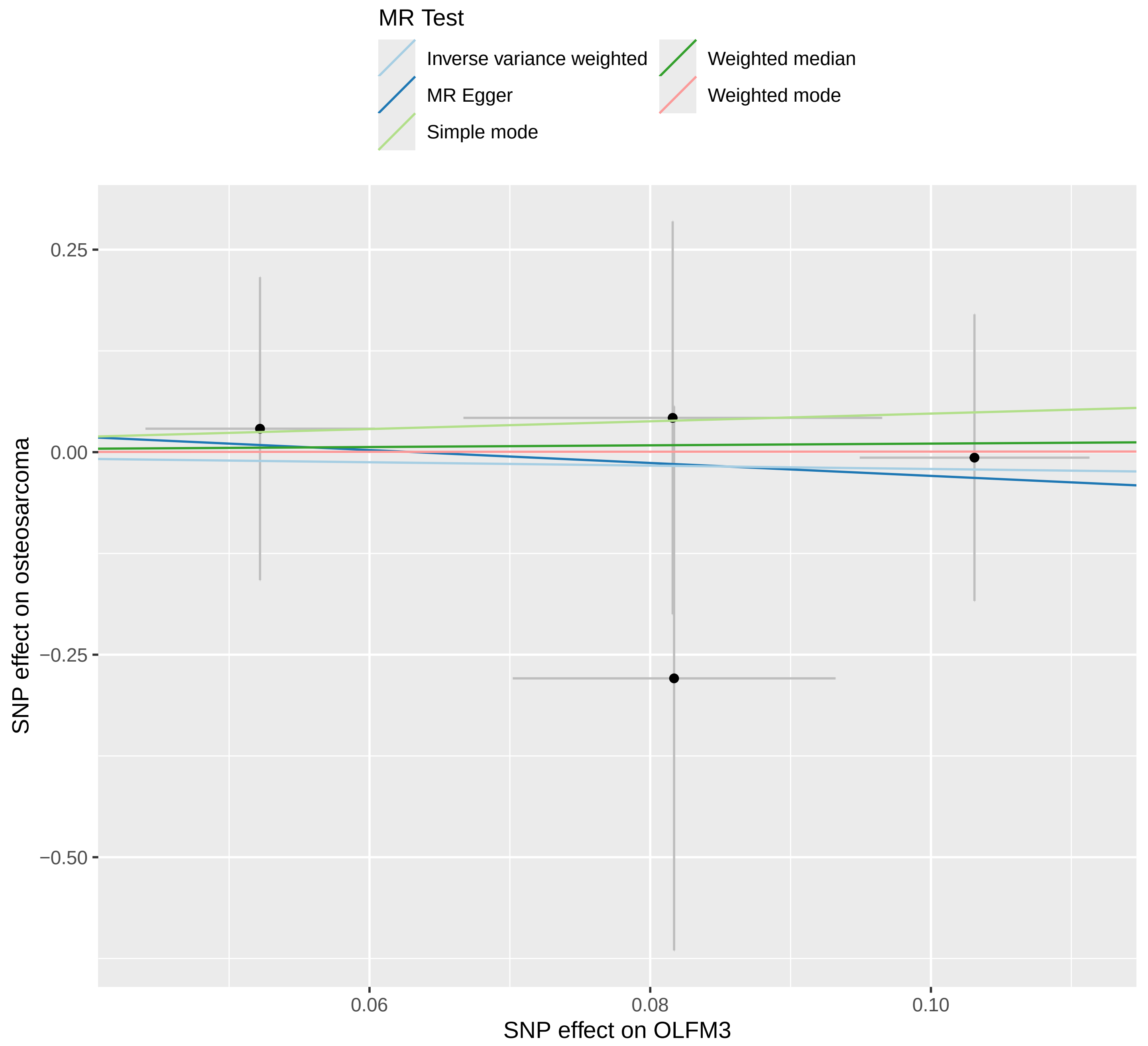

MR Test
Inverse variance weighted
MR Egger
Simple mode
Weighted median
Weighted mode
SNP effect on osteosarcoma
0.25
0.00
−0.25
−0.50
0.06
0.08
0.10
SNP effect on OLFM3

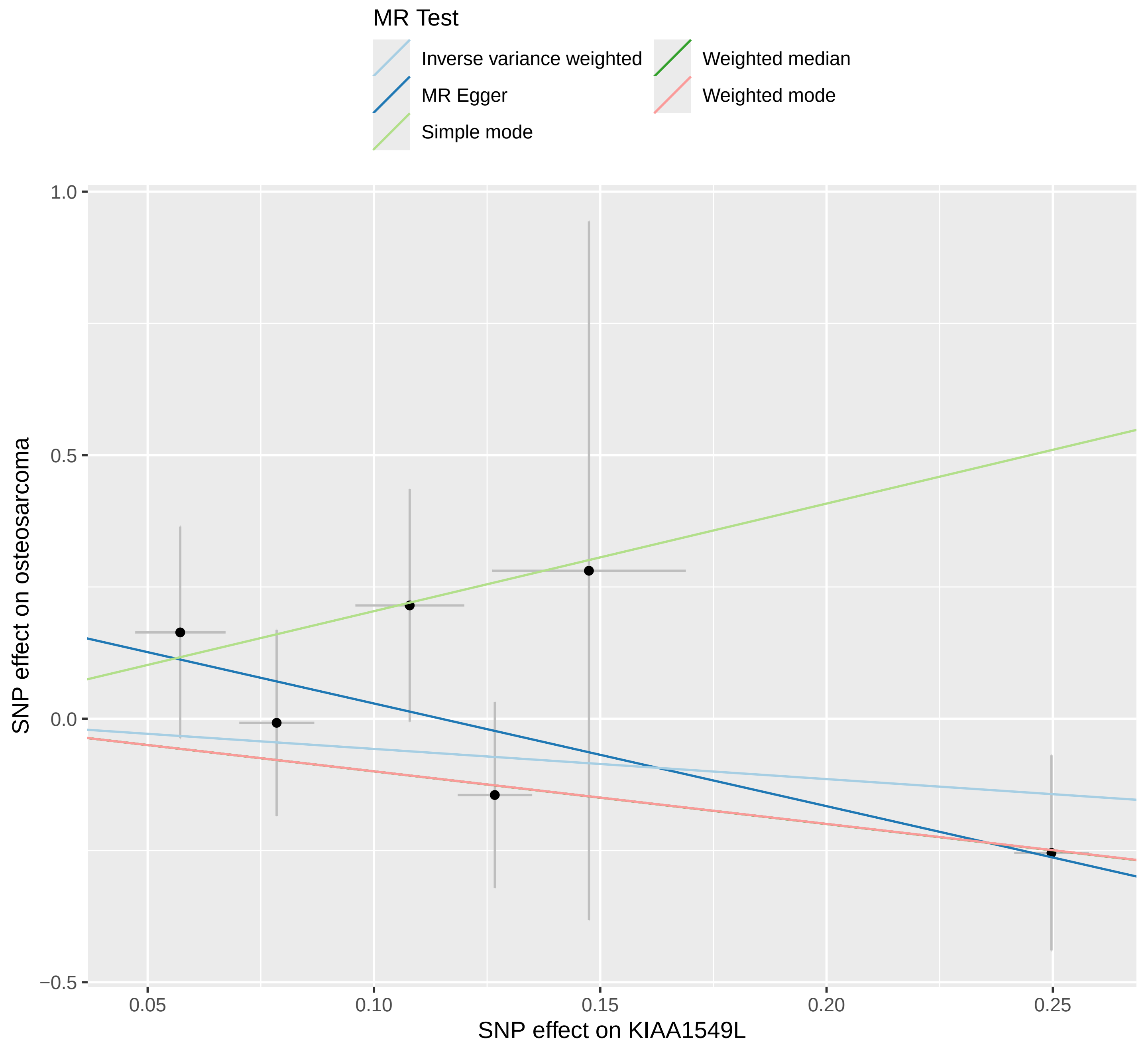

MR Test
Inverse variance weighted
MR Egger
Simple mode
Weighted median
Weighted mode
1.0
0.5
0.0
−0.5
0.05
0.10
0.15
0.20
0.25
SNP effect on osteosarcoma
SNP effect on KIAA1549L

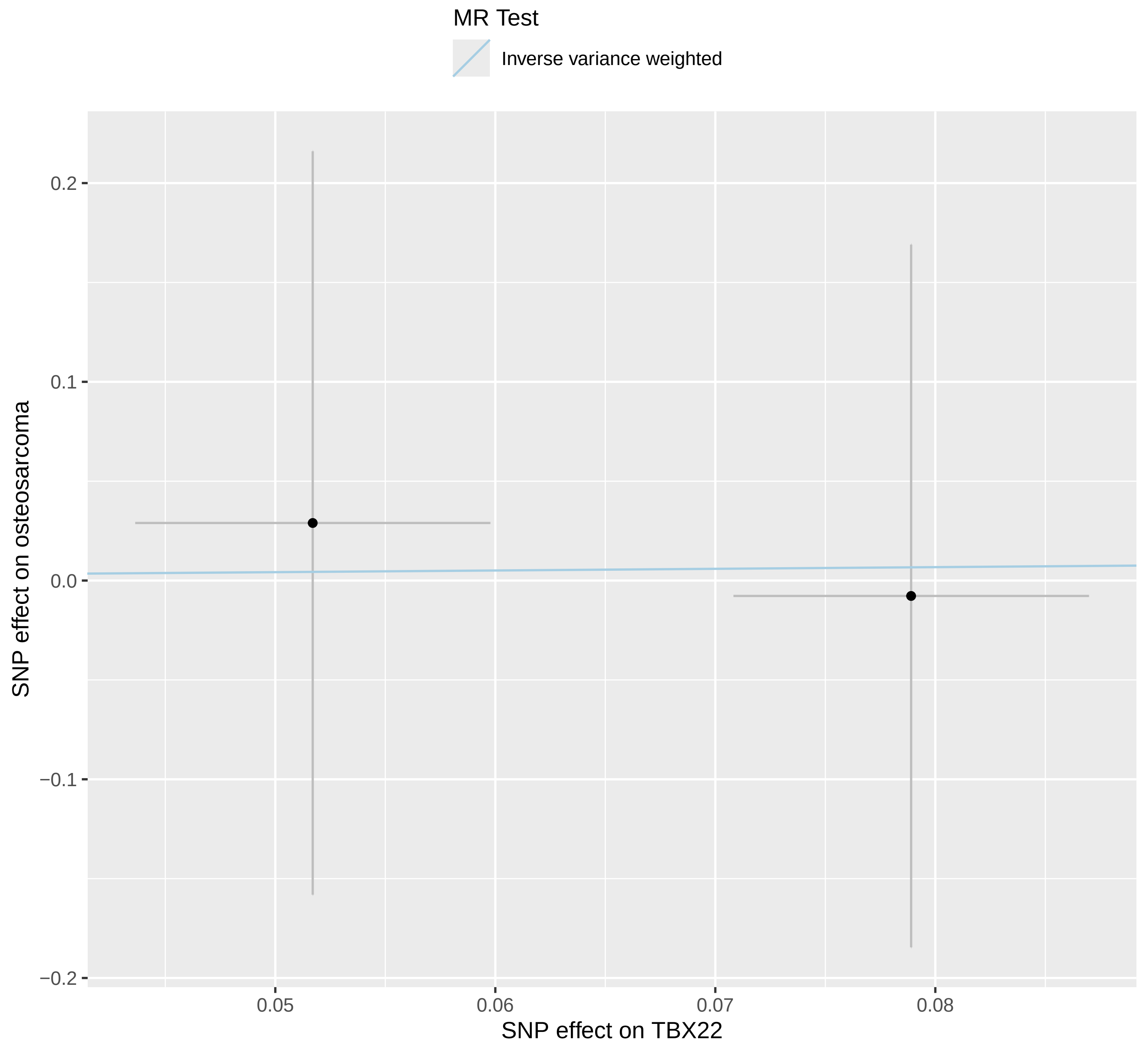
MR Test
Inverse variance weighted
0.2
0.1
0.0
−0.1
−0.2
0.05
0.06
0.07
0.08
SNP effect on TBX22
SNP effect on osteosarcoma

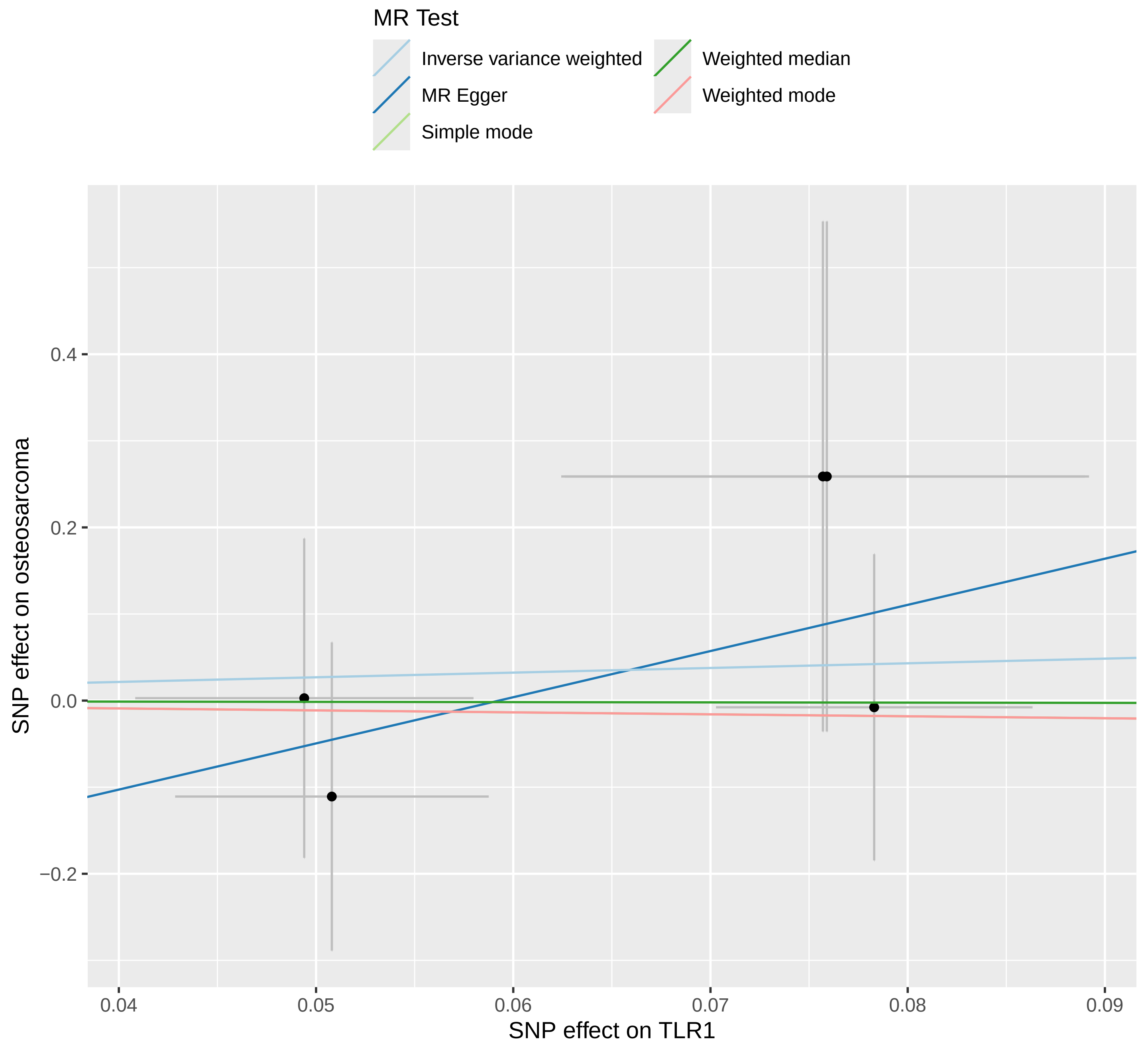

MR Test
Inverse variance weighted
MR Egger
Simple mode
Weighted median
Weighted mode
0.4
0.2
0.0
−0.2
SNP effect on osteosarcoma
0.04
0.05
0.06
0.07
0.08
0.09
SNP effect on TLR1

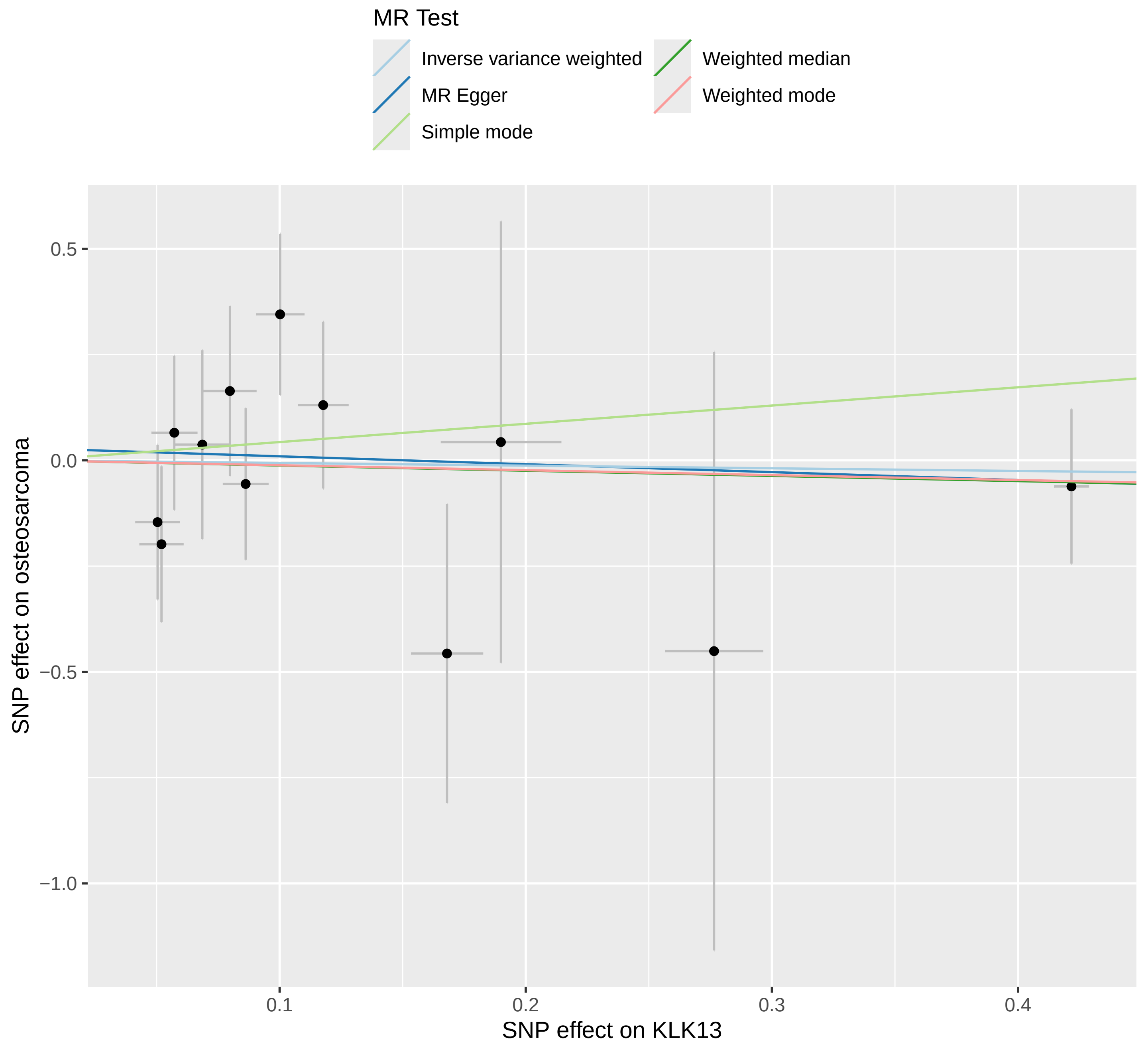
MR Test
Inverse variance weighted
Weighted median
MR Egger
Weighted mode
Simple mode
0.5
0.0
−0.5
−1.0
0.1
0.2
0.3
0.4
SNP effect on osteosarcoma
SNP effect on KLK13

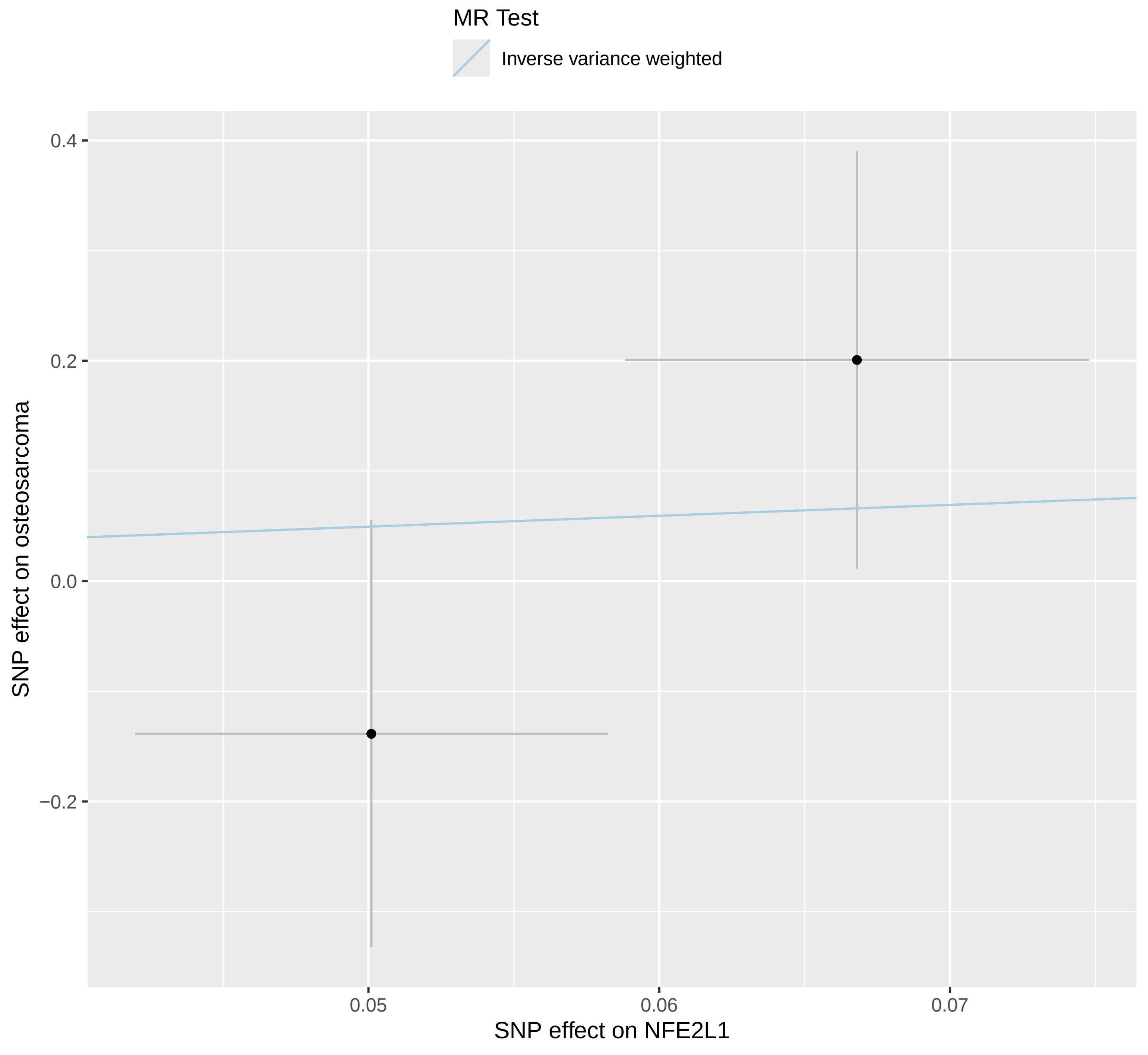

MR Test
Inverse variance weighted
0.4
0.2
0.0
−0.2
0.05
0.06
0.07
SNP effect on NFE2L1
SNP effect on osteosarcoma

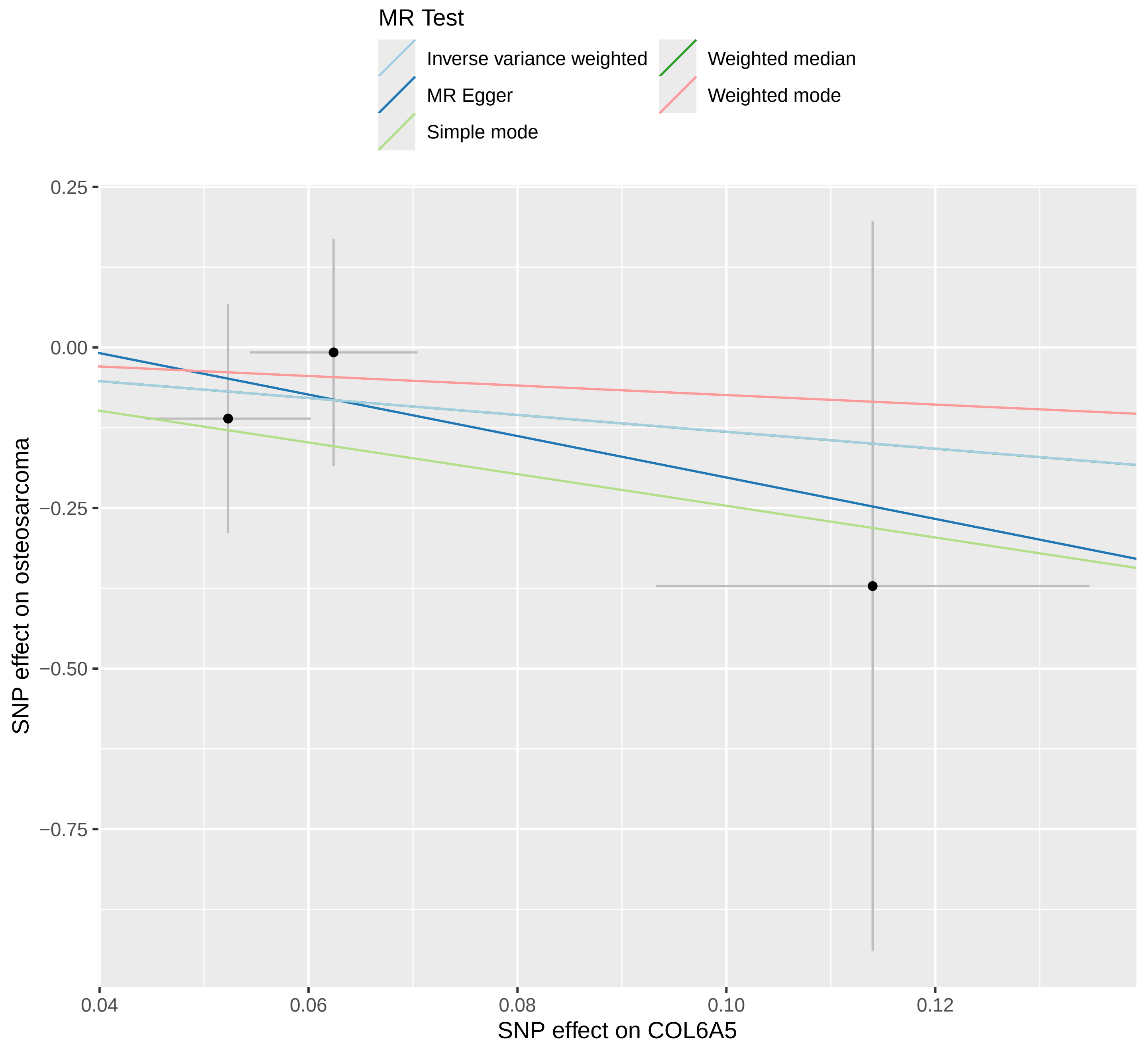

MR Test
Inverse variance weighted
MR Egger
Simple mode
Weighted median
Weighted mode
0.25
0.00
−0.25
−0.50
−0.75
SNP effect on osteosarcoma
0.04
0.06
0.08
0.10
0.12
SNP effect on COL6A5

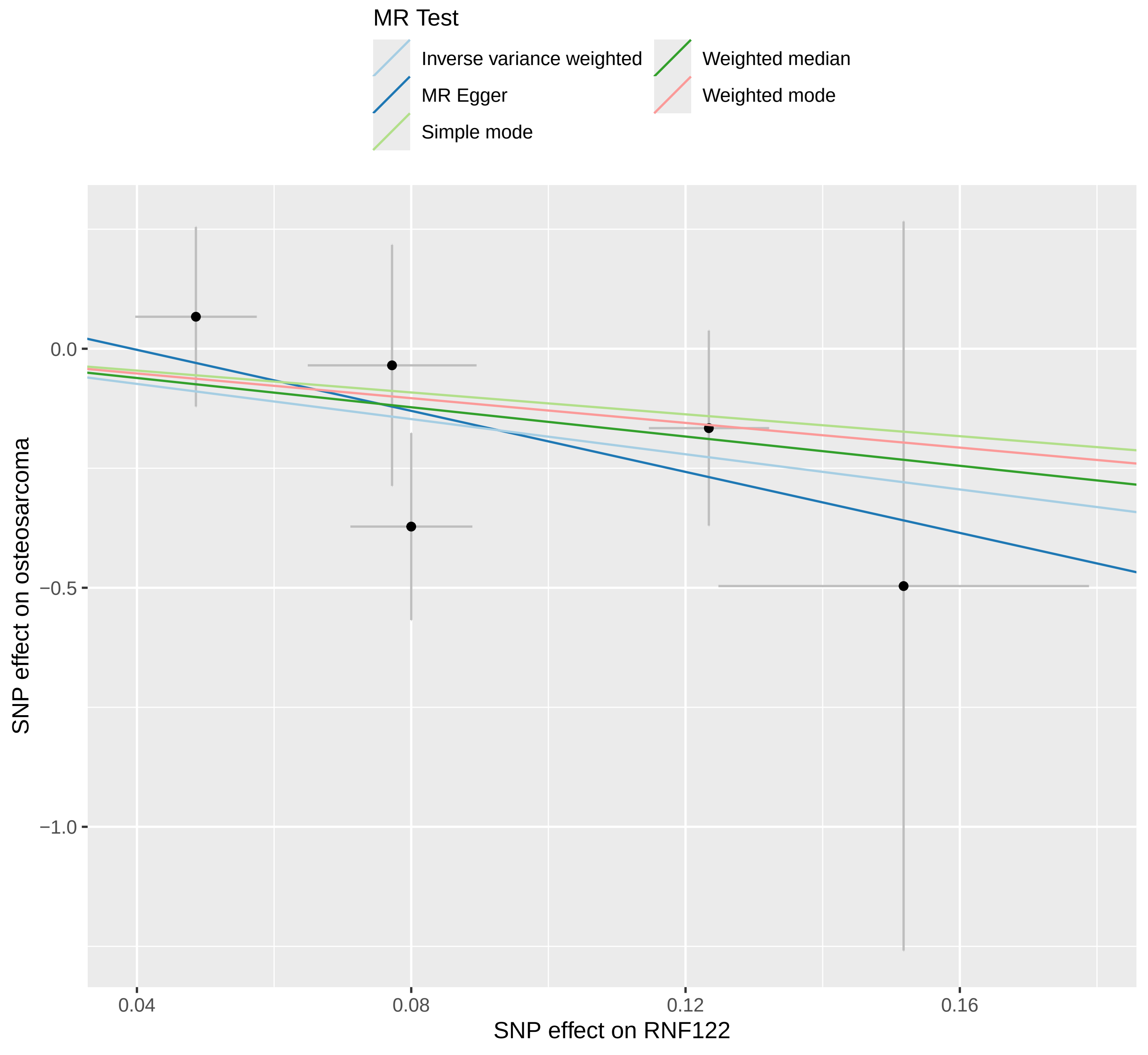
MR Test
Inverse variance weighted
MR Egger
Simple mode
Weighted median
Weighted mode
0.0
−0.5
−1.0
SNP effect on osteosarcoma
0.04
0.08
0.12
0.16
SNP effect on RNF122

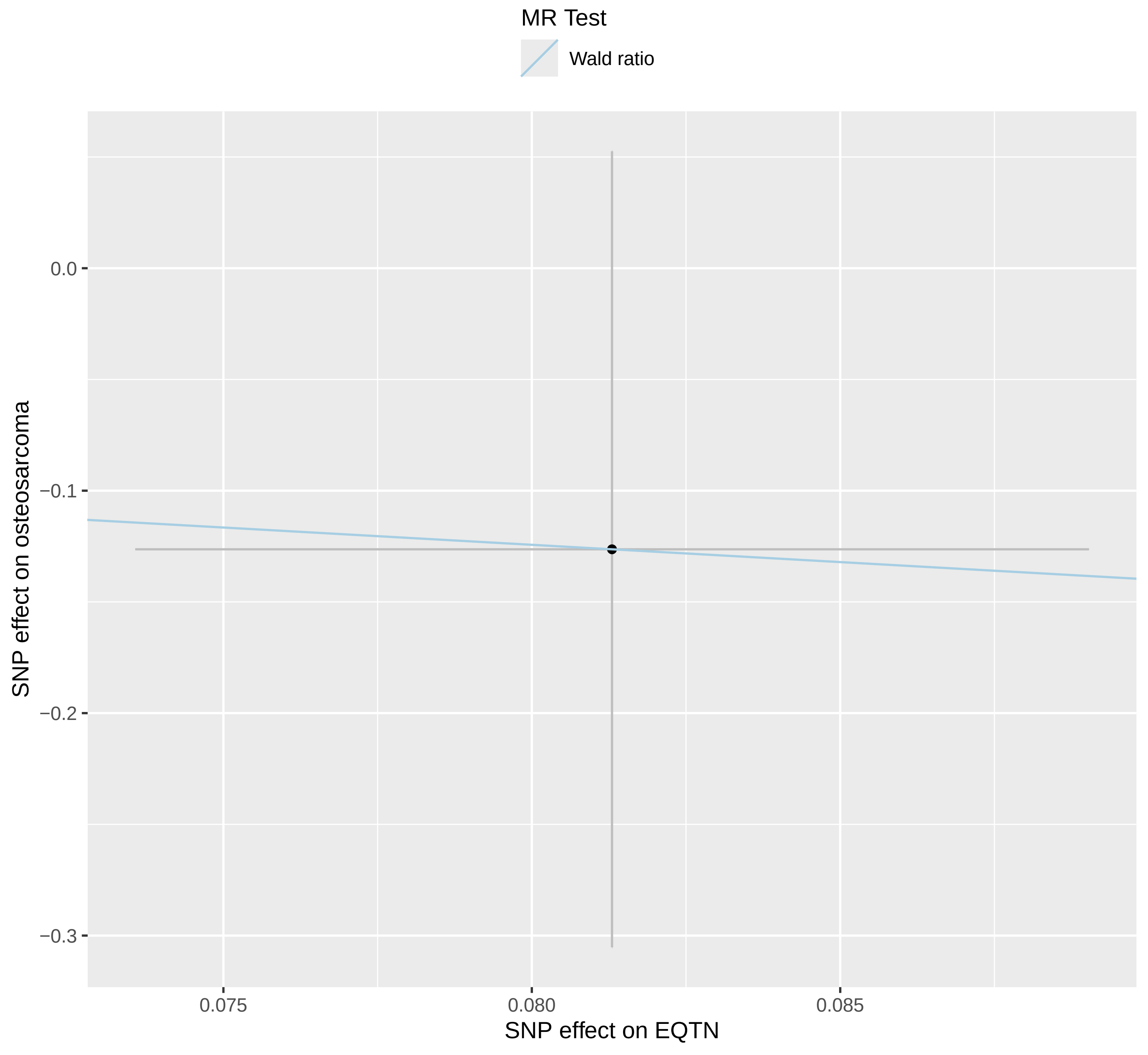

MR Test
Wald ratio
SNP effect on osteosarcoma
0.0
−0.1
−0.2
−0.3
0.075
0.080
0.085
SNP effect on EQTN

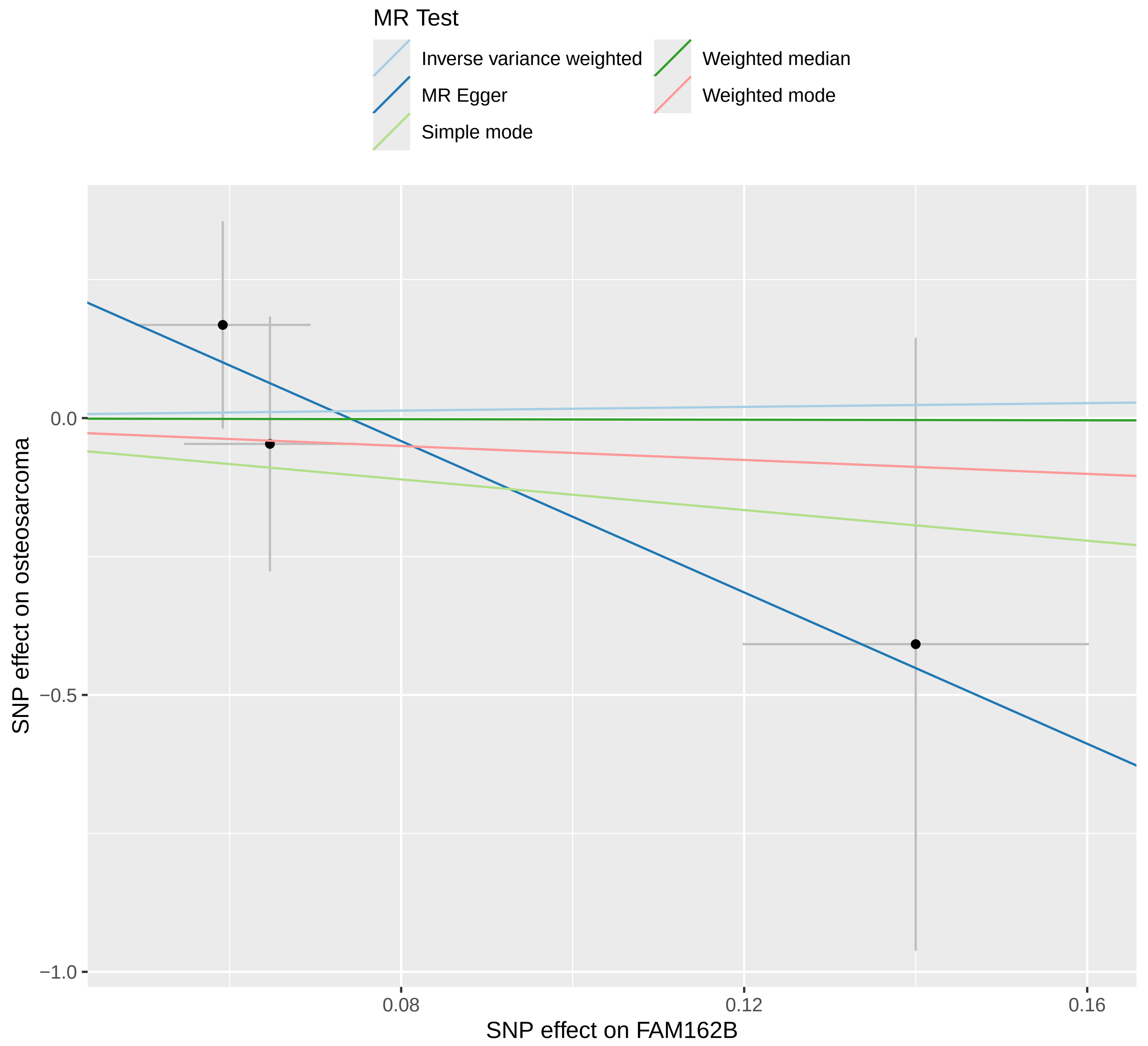
MR Test
Inverse variance weighted
MR Egger
Simple mode
Weighted median
Weighted mode
0.0
−0.5
−1.0
0.08
0.12
0.16
SNP effect on osteosarcoma
SNP effect on FAM162B

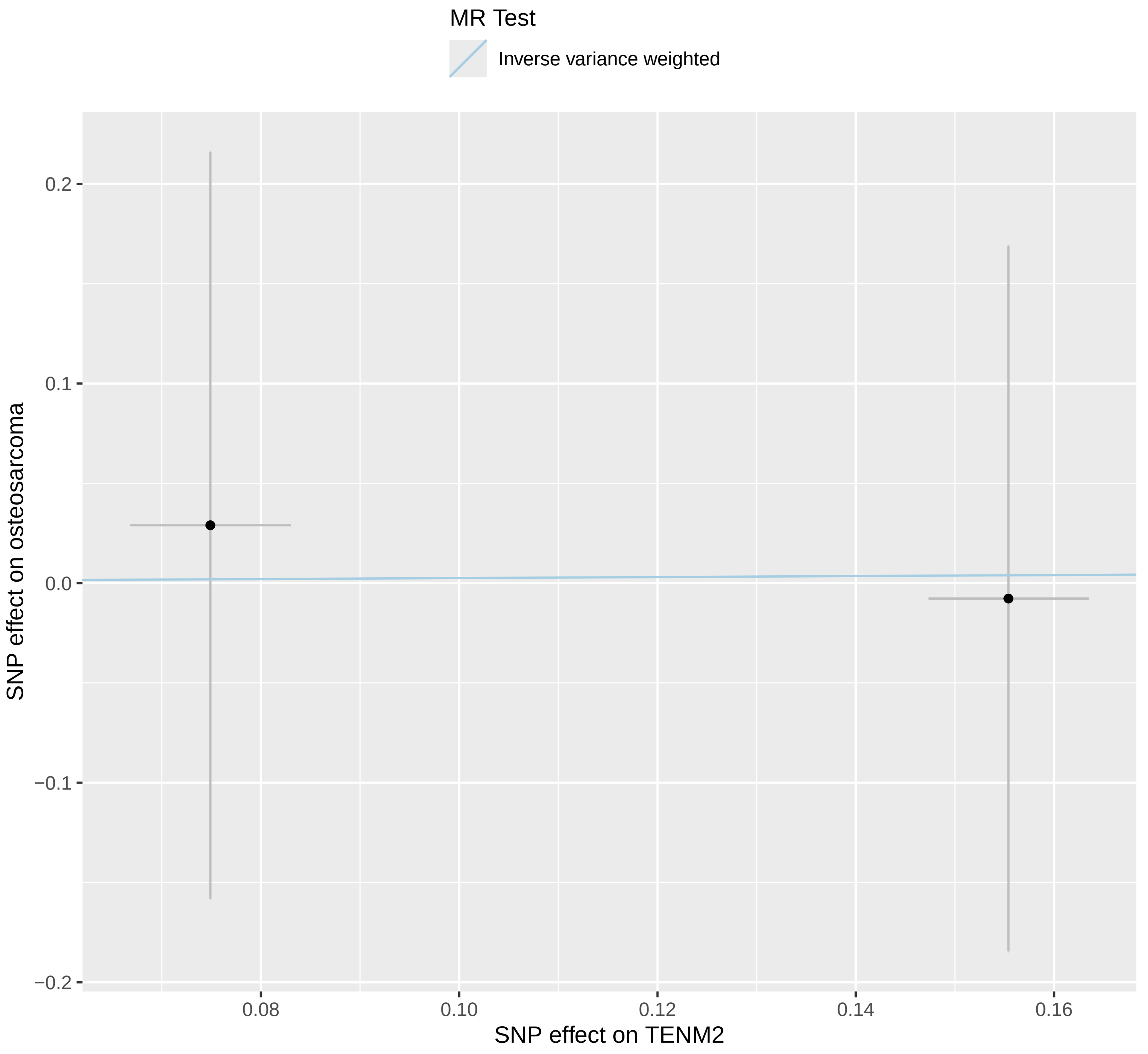
MR Test
Inverse variance weighted
SNP effect on osteosarcoma
0.2
0.1
0.0
−0.1
−0.2
0.08
0.10
0.12
0.14
0.16
SNP effect on TENM2

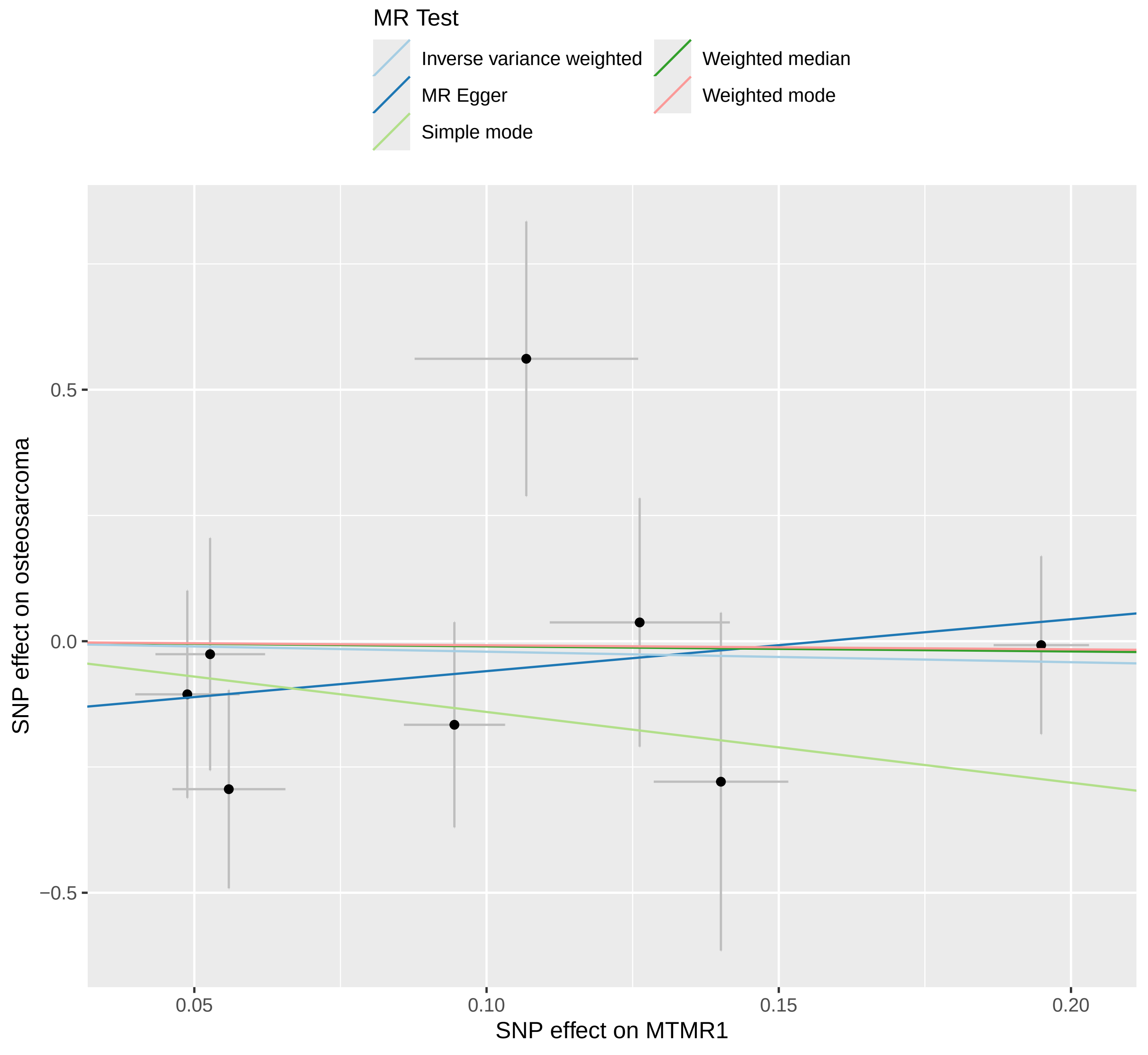
MR Test
Inverse variance weighted
MR Egger
Simple mode
Weighted median
Weighted mode
0.5
0.0
−0.5
SNP effect on osteosarcoma
0.05
0.10
0.15
0.20
SNP effect on MTMR1

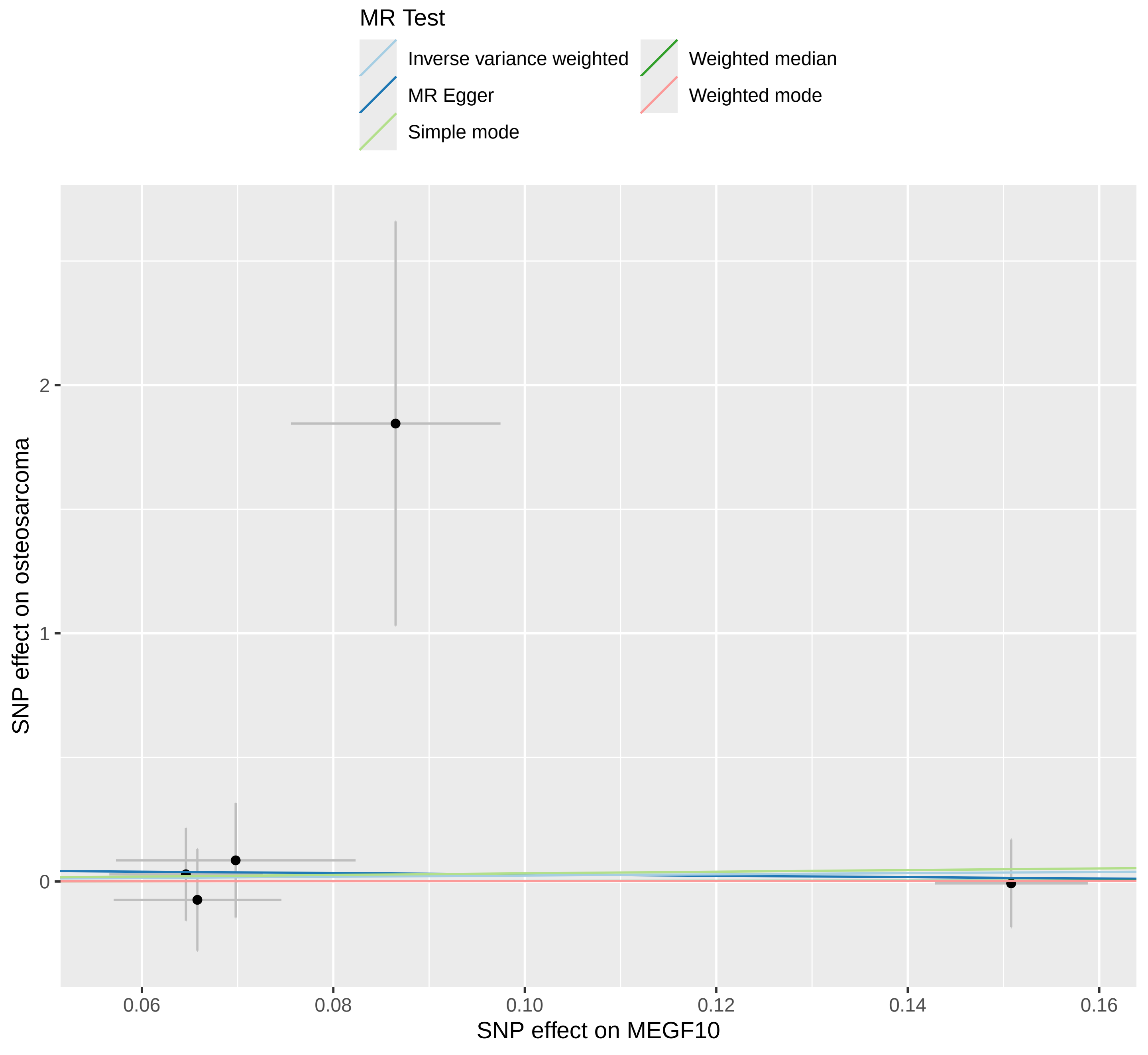
MR Test
Inverse variance weighted
MR Egger
Simple mode
Weighted median
Weighted mode
SNP effect on osteosarcoma
0
1
2
0.06
0.08
0.10
0.12
0.14
0.16
SNP effect on MEGF10

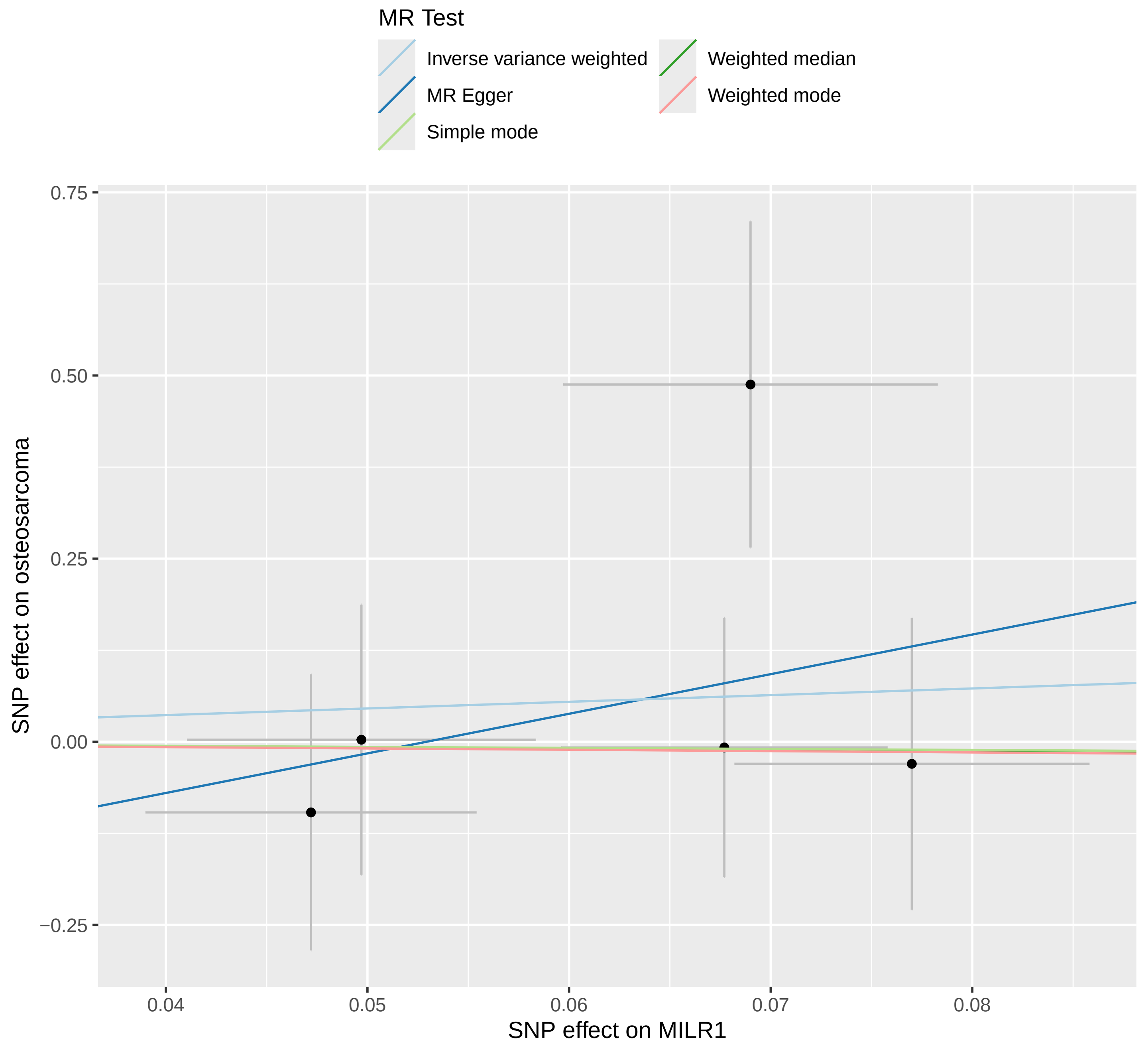

MR Test
Inverse variance weighted
MR Egger
Simple mode
Weighted median
Weighted mode
SNP effect on osteosarcoma
0.75
0.50
0.25
0.00
−0.25
0.04
0.05
0.06
0.07
0.08
SNP effect on MILR1

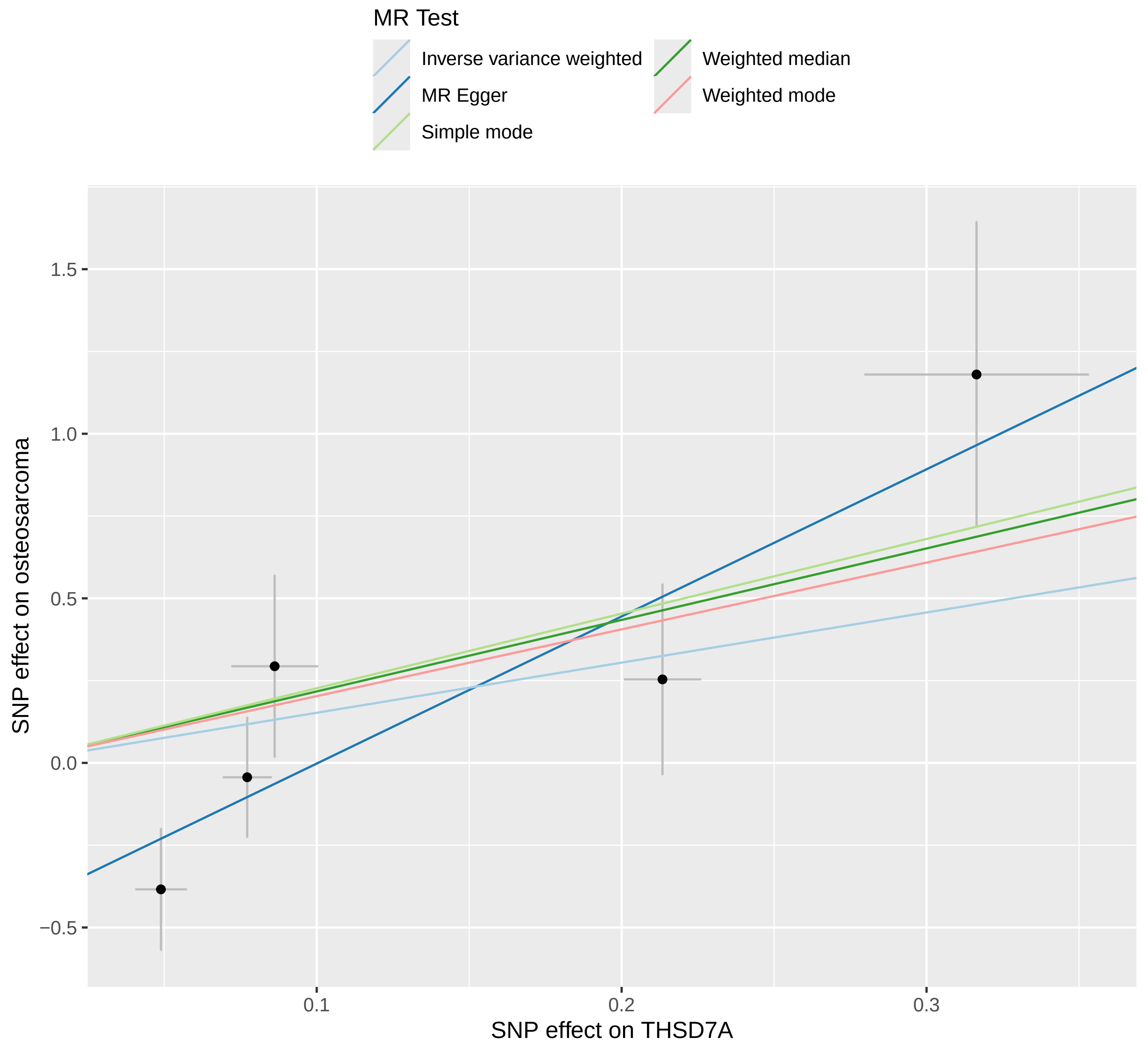
MR Test
Inverse variance weighted
MR Egger
Simple mode
Weighted median
Weighted mode
1.5
1.0
0.5
0.0
−0.5
0.1
0.2
0.3
SNP effect on osteosarcoma
SNP effect on THSD7A

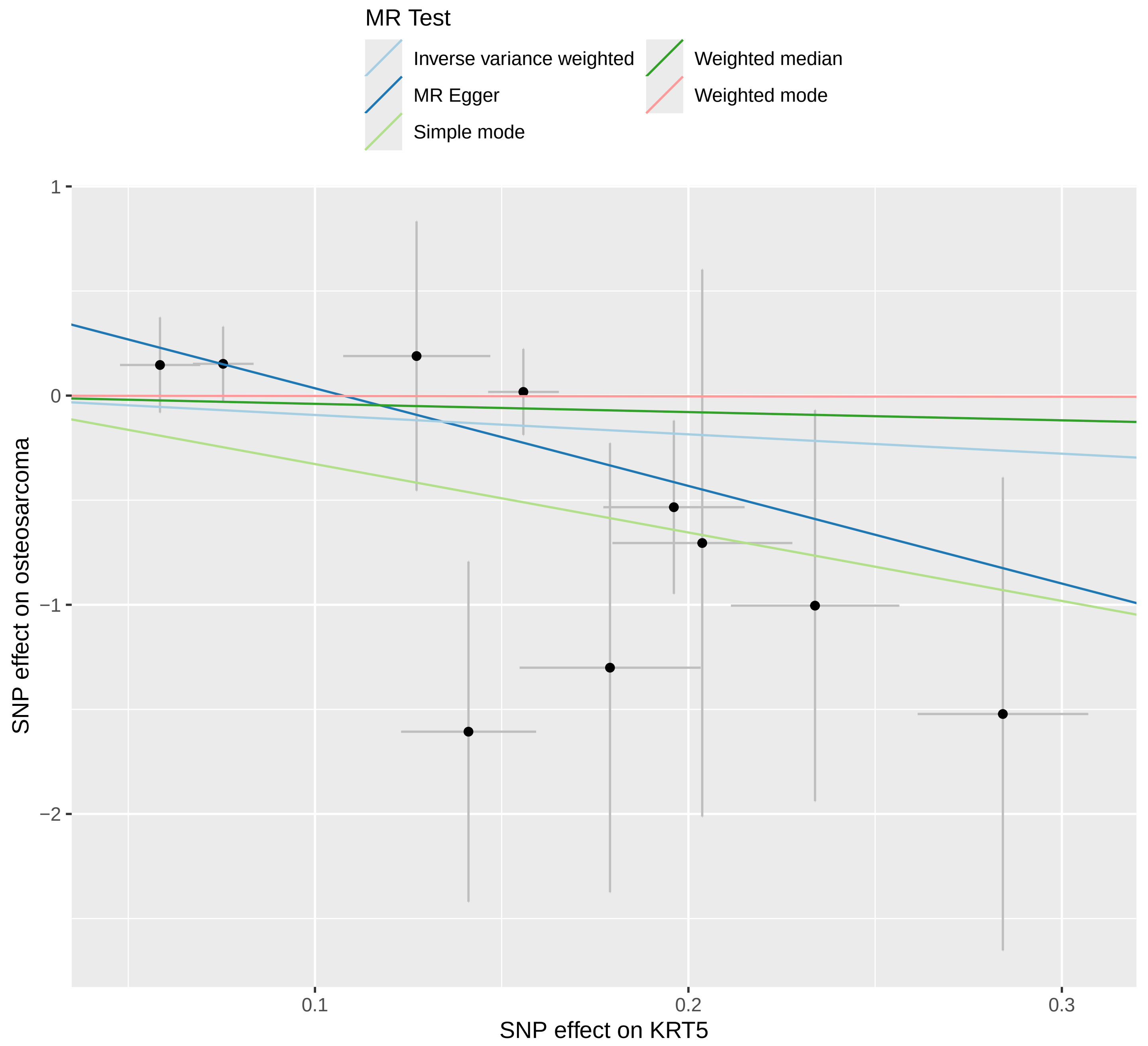

MR Test
Inverse variance weighted
MR Egger
Simple mode
Weighted median
Weighted mode
SNP effect on osteosarcoma
SNP effect on KRT5
1
0
−1
−2
0.1
0.2
0.3

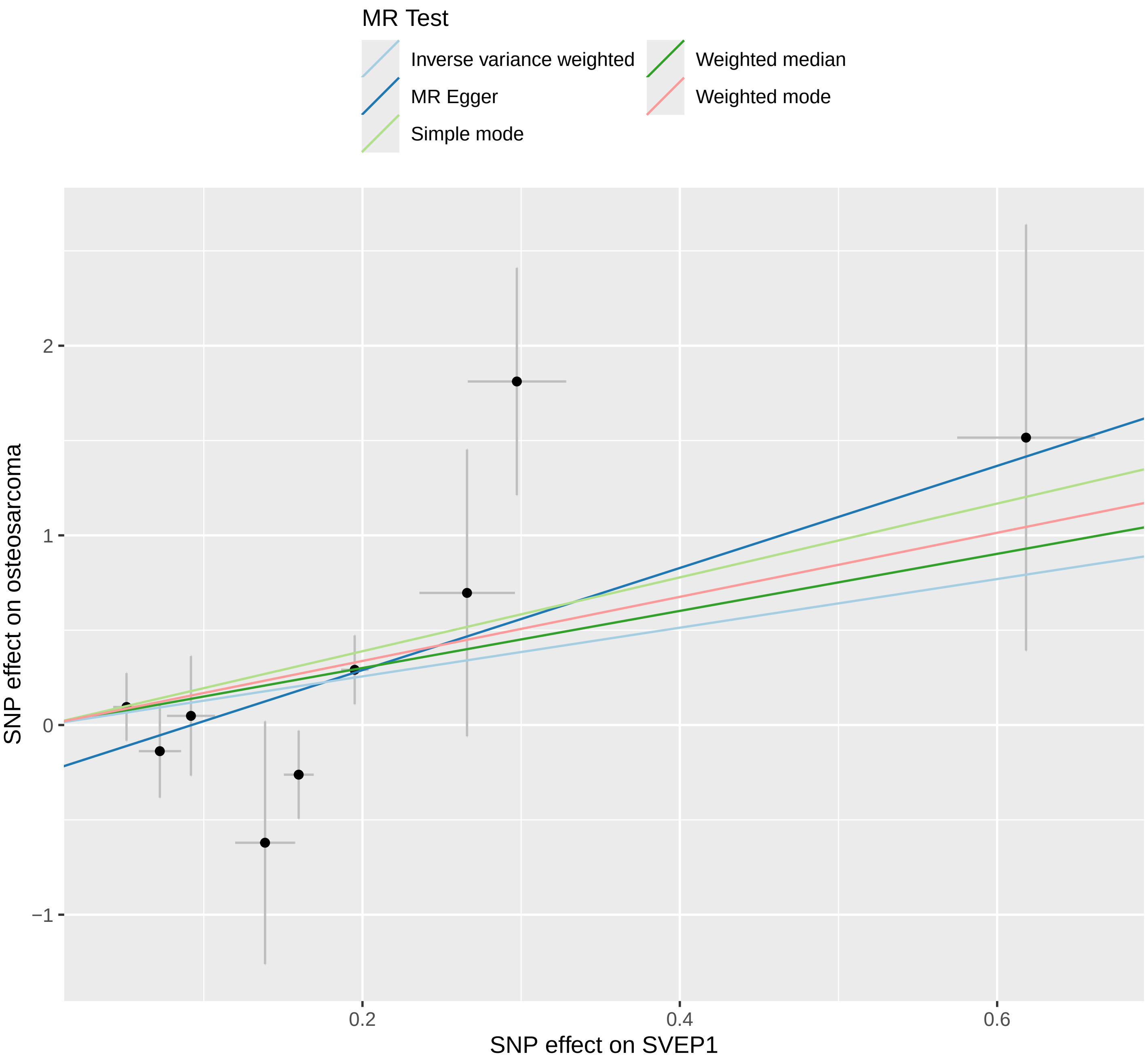
MR Test
Inverse variance weighted
MR Egger
Simple mode
Weighted median
Weighted mode
SNP effect on osteosarcoma
2
1
0
−1
0.2
0.4
0.6
SNP effect on SVEP1

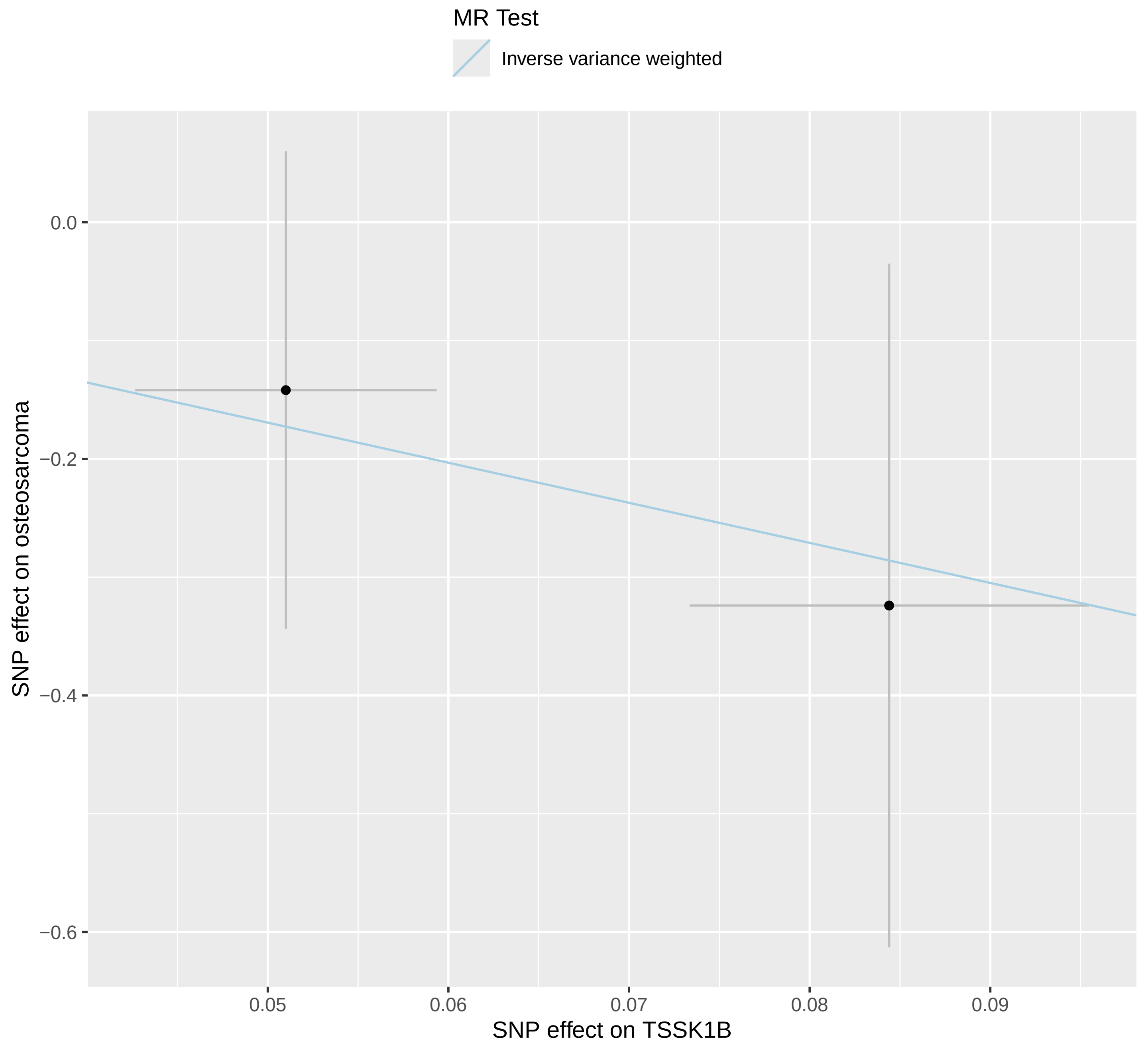
MR Test
Inverse variance weighted
0.0
−0.2
−0.4
−0.6
0.05
0.06
0.07
0.08
0.09
SNP effect on osteosarcoma
SNP effect on TSSK1B

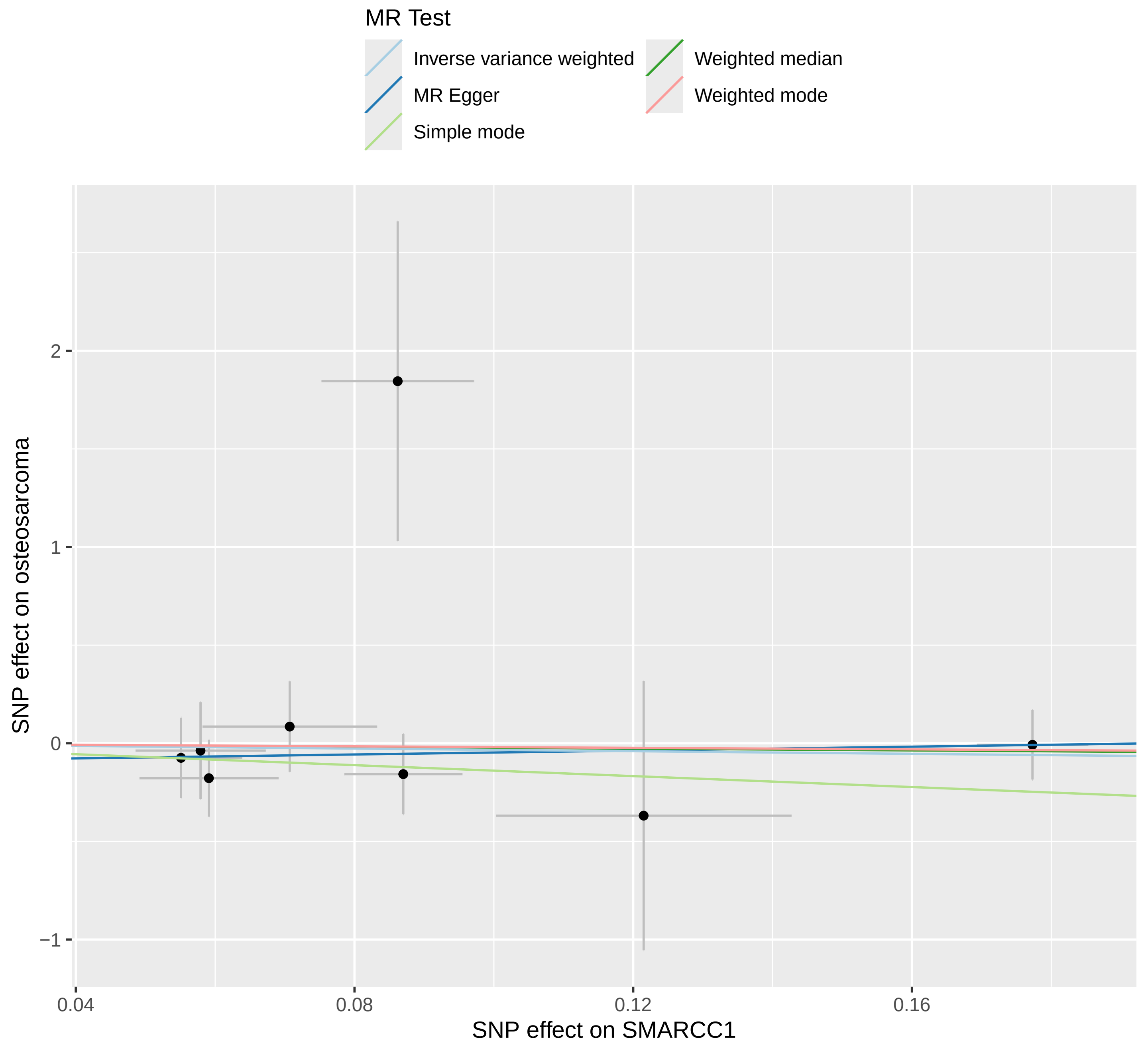

MR Test
Inverse variance weighted
MR Egger
Simple mode
Weighted median
Weighted mode
SNP effect on osteosarcoma
2
1
0
−1
0.04
0.08
0.12
0.16
SNP effect on SMARCC1

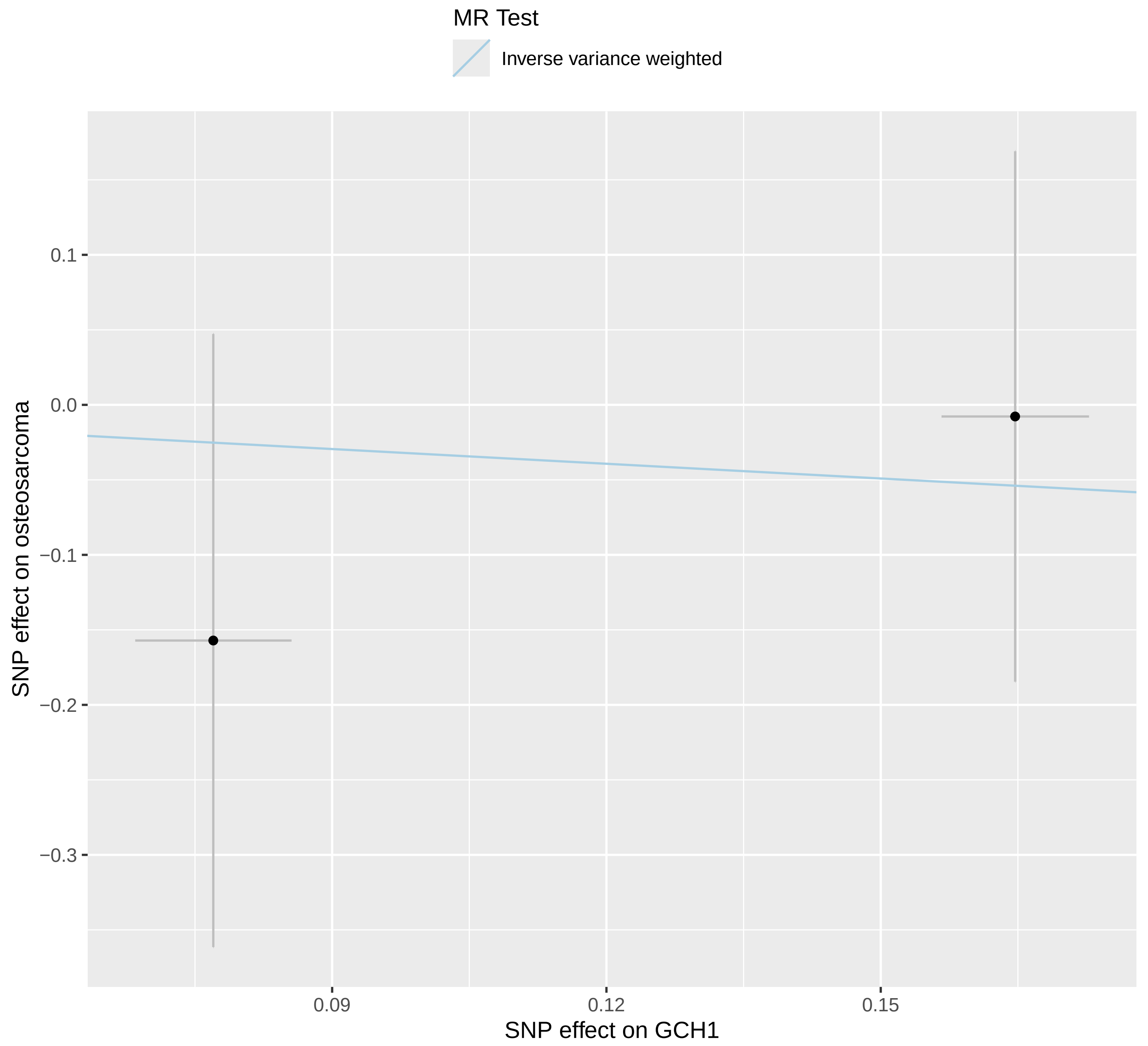
MR Test
Inverse variance weighted
0.1
0.0
−0.1
−0.2
−0.3
0.09
0.12
0.15
SNP effect on osteosarcoma
SNP effect on GCH1

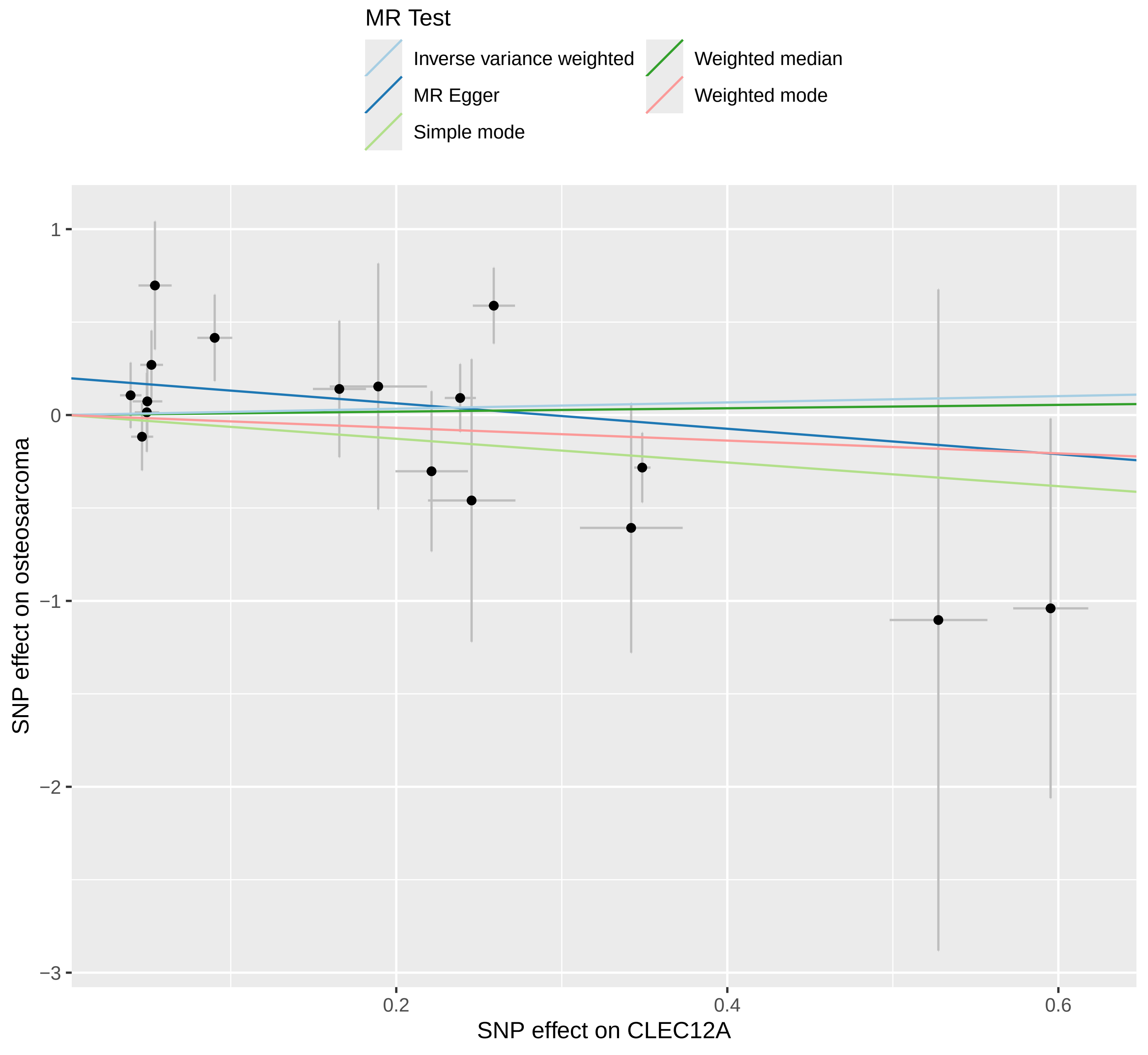
MR Test
Inverse variance weighted
MR Egger
Simple mode
Weighted median
Weighted mode
1
0
−1
−2
−3
0.2
0.4
0.6
SNP effect on osteosarcoma
SNP effect on CLEC12A

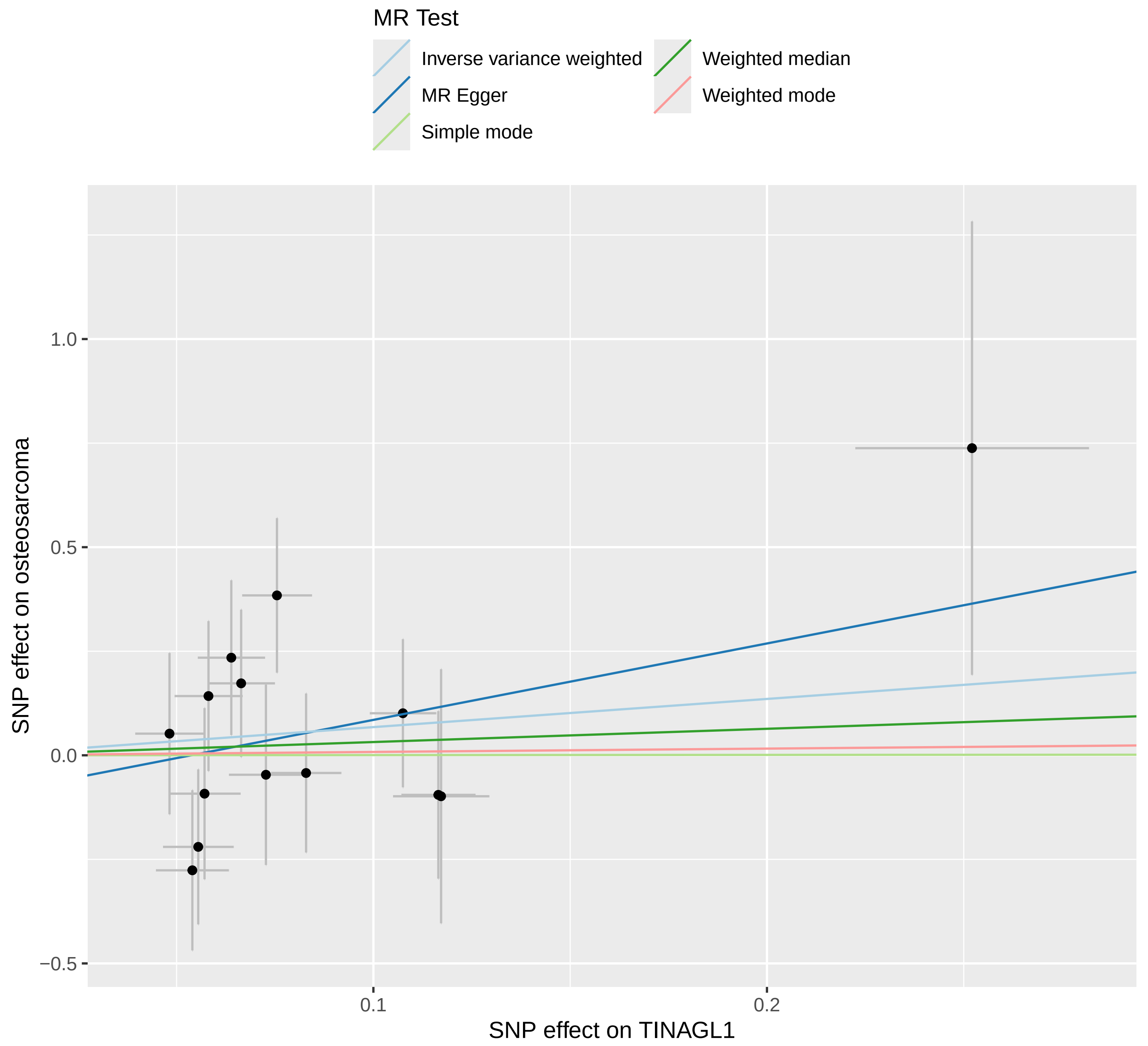
MR Test
Inverse variance weighted
MR Egger
Simple mode
Weighted median
Weighted mode
SNP effect on osteosarcoma
1.0
0.5
0.0
−0.5
0.1
0.2
SNP effect on TINAGL1

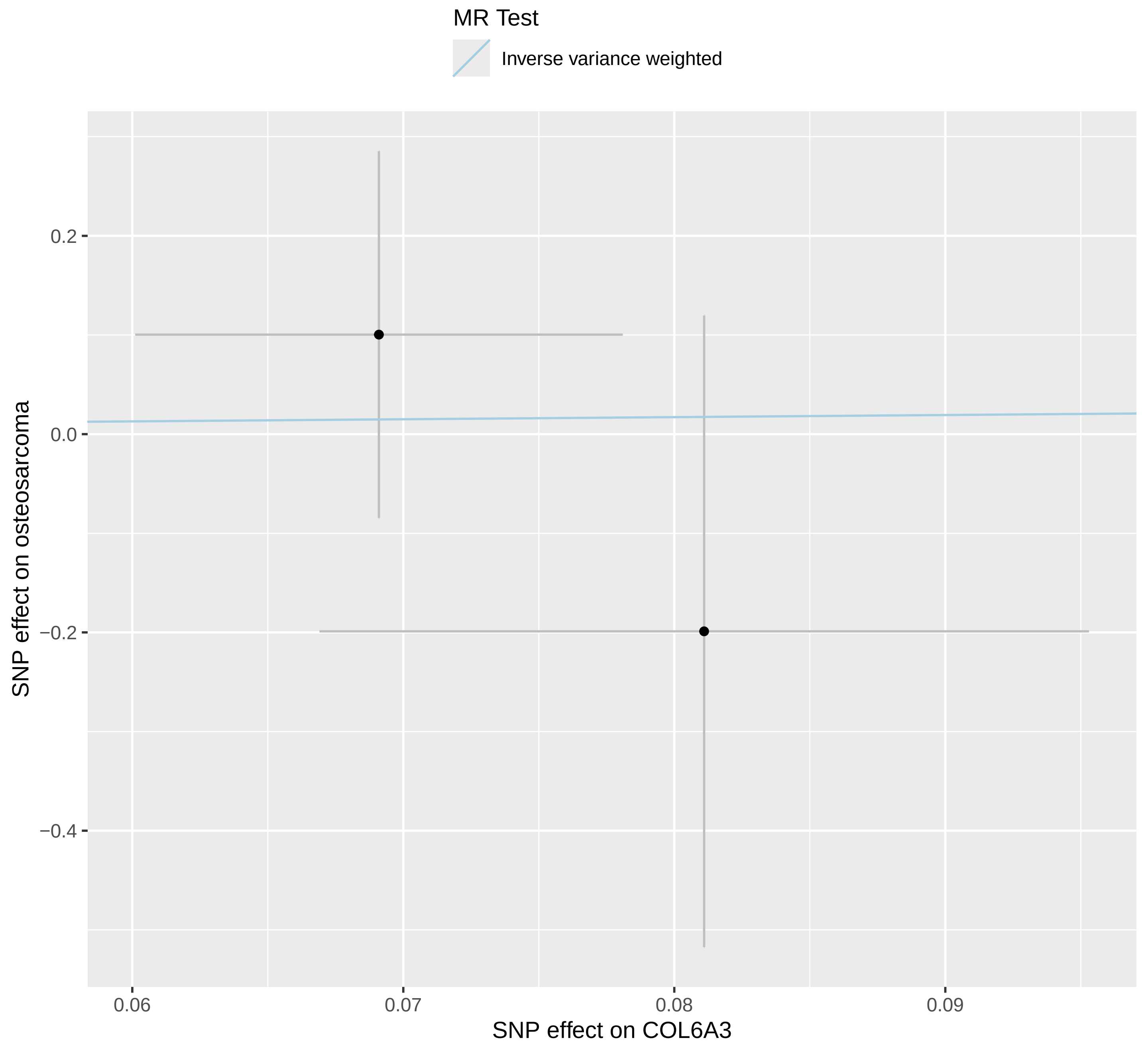
MR Test
Inverse variance weighted
0.2
0.0
−0.2
−0.4
SNP effect on osteosarcoma
0.06
0.07
0.08
0.09
SNP effect on COL6A3

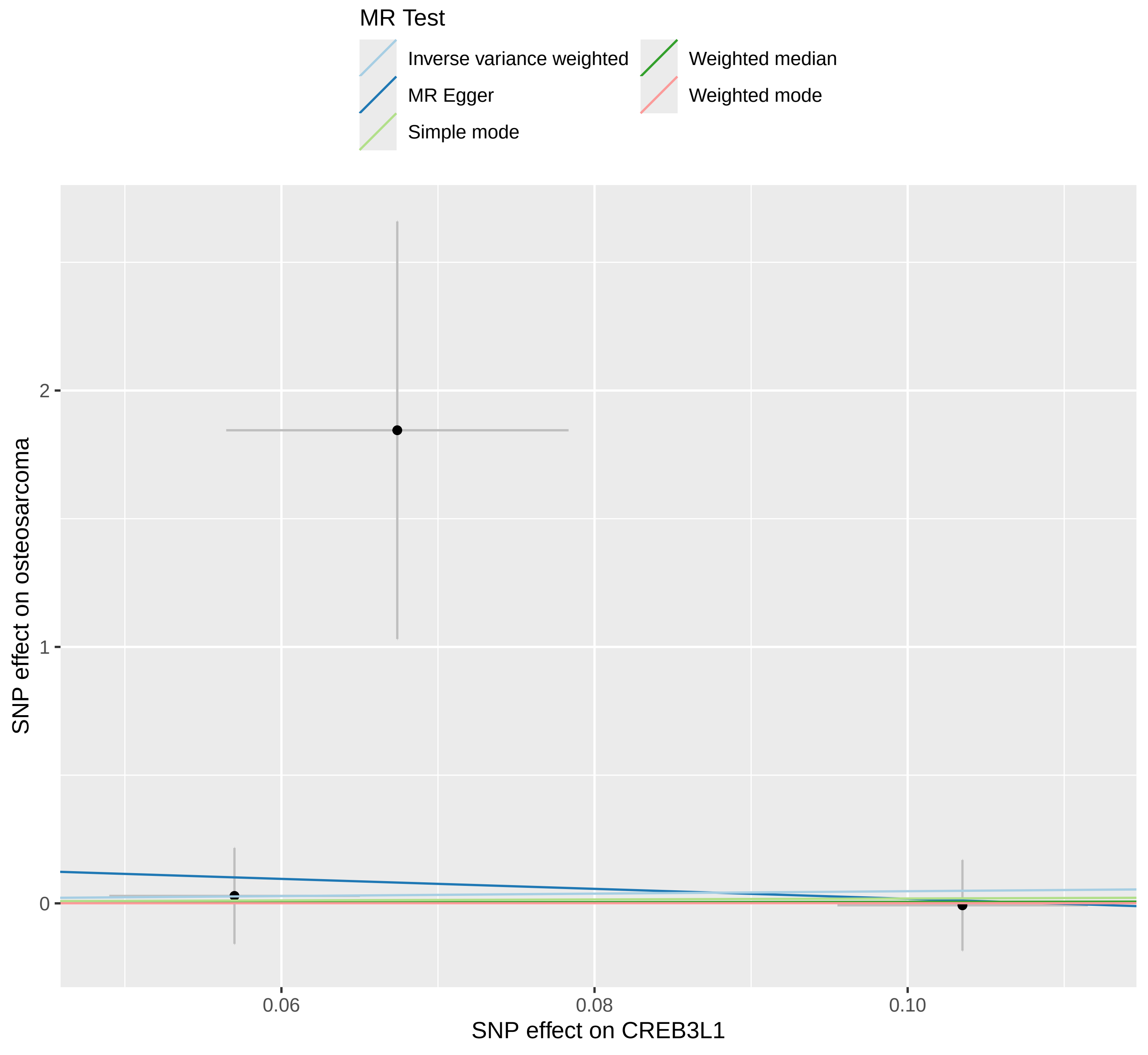

MR Test
Inverse variance weighted
MR Egger
Simple mode
Weighted median
Weighted mode
SNP effect on osteosarcoma
2
1
0
0.06
0.08
0.10
SNP effect on CREB3L1

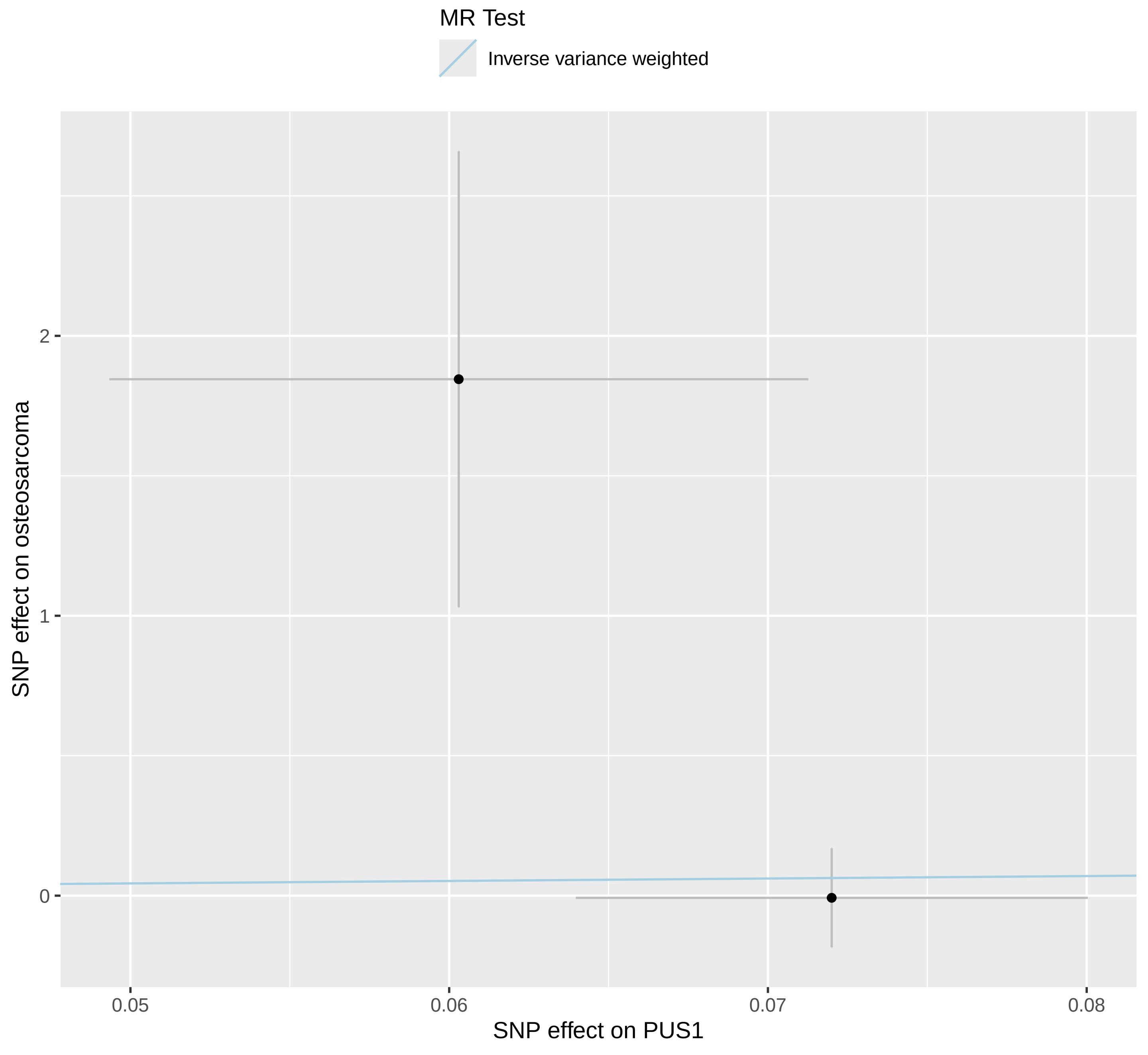

MR Test
Inverse variance weighted
SNP effect on osteosarcoma
2
1
0
0.05
0.06
0.07
0.08
SNP effect on PUS1

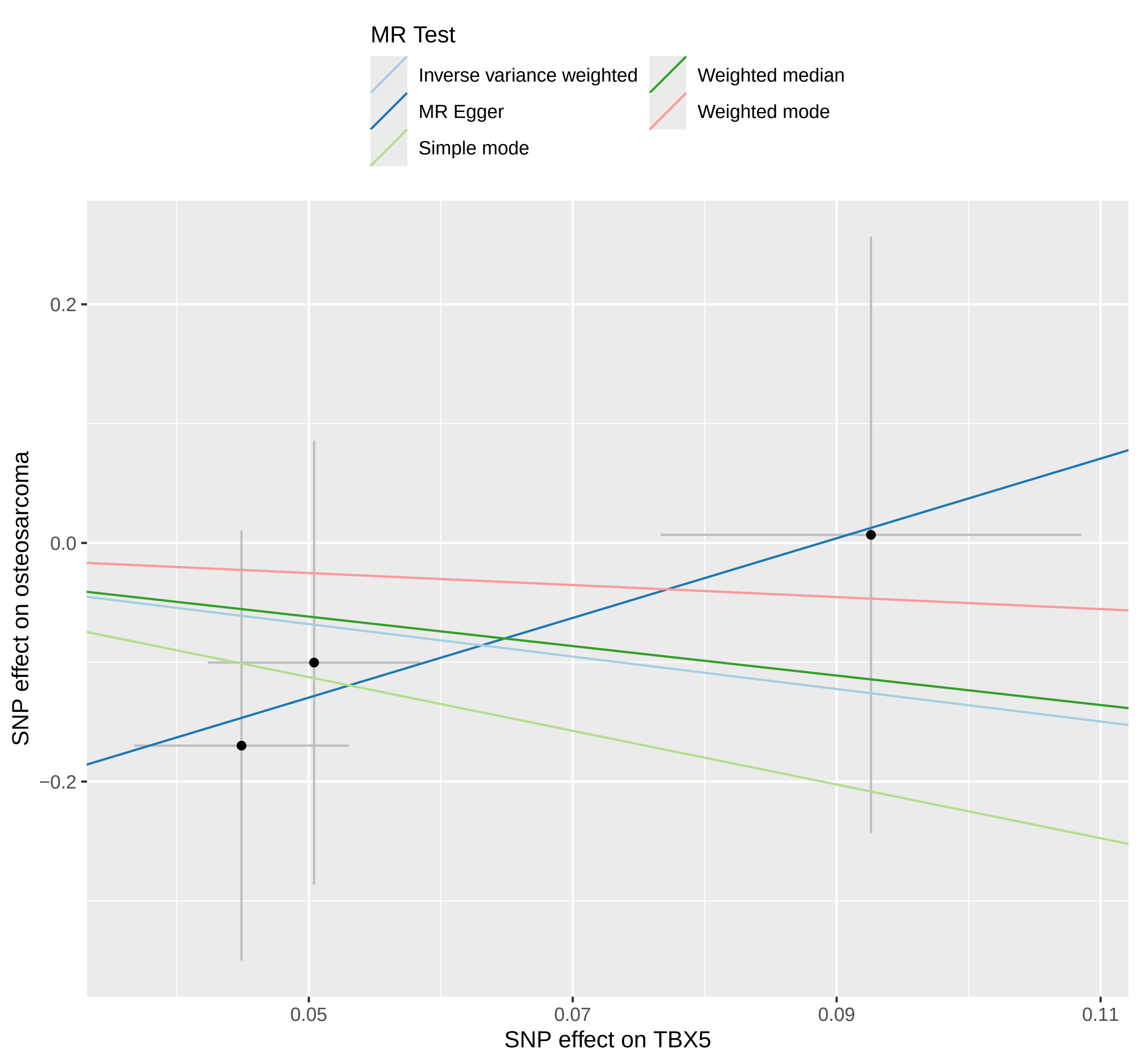
MR Test
Inverse variance weighted
MR Egger
Simple mode
Weighted median
Weighted mode
0.2
0.0
−0.2
SNP effect on osteosarcoma
0.05
0.07
0.09
0.11
SNP effect on TBX5

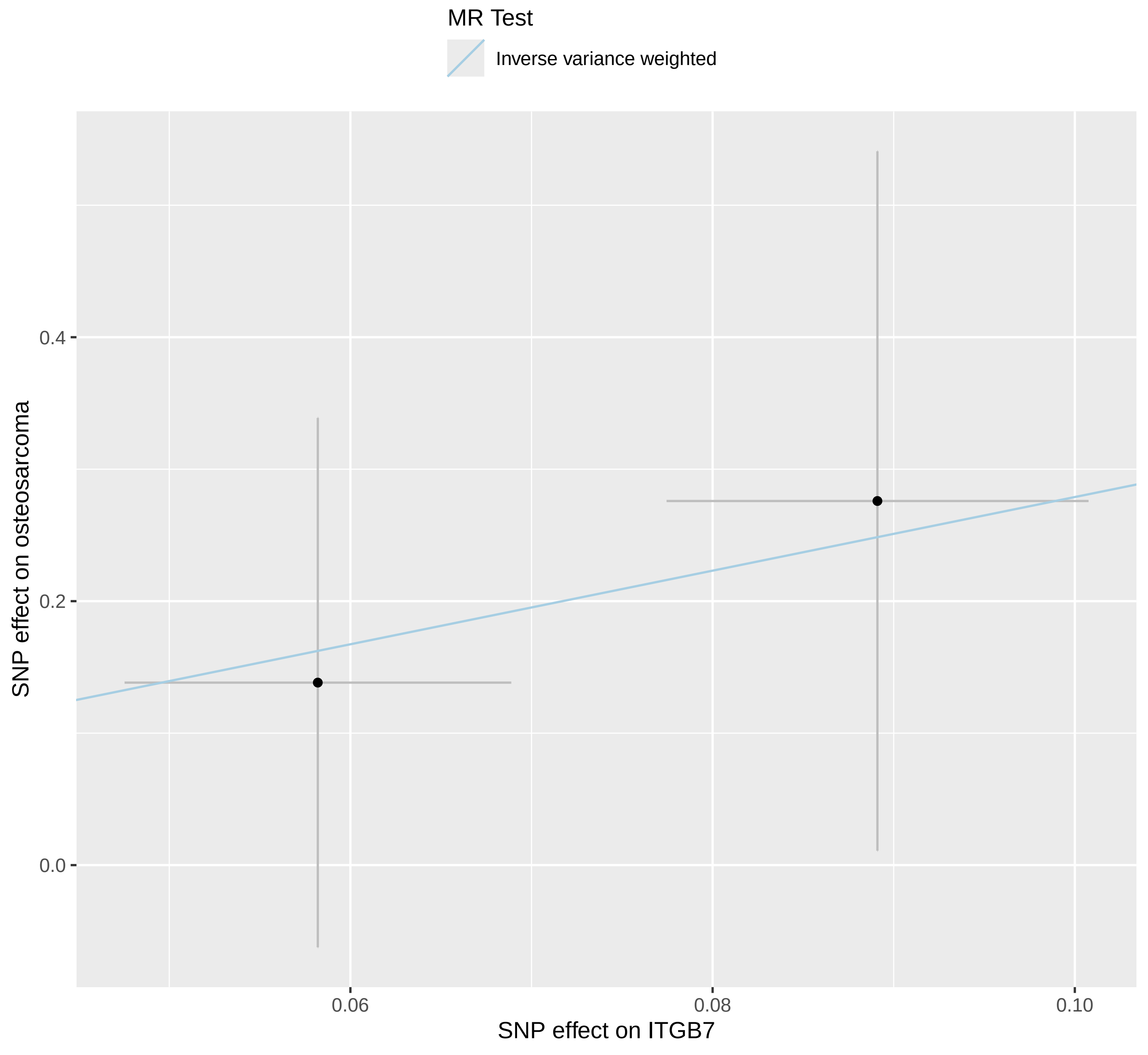
MR Test
Inverse variance weighted
0.4
0.2
0.0
SNP effect on osteosarcoma
0.06
0.08
0.10
SNP effect on ITGB7

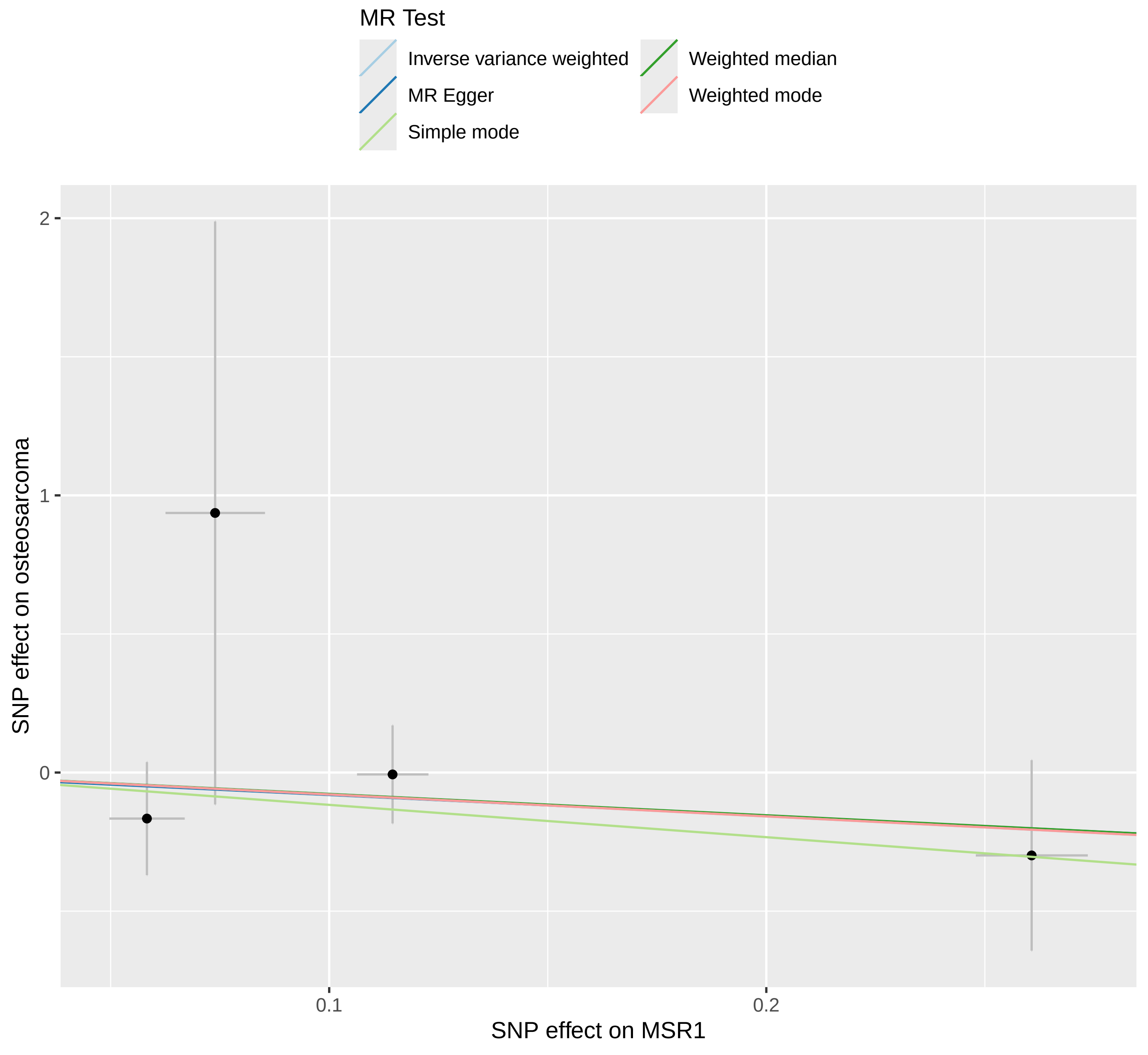
MR Test
Inverse variance weighted
MR Egger
Simple mode
Weighted median
Weighted mode
2
1
0
0.1
0.2
SNP effect on osteosarcoma
SNP effect on MSR1

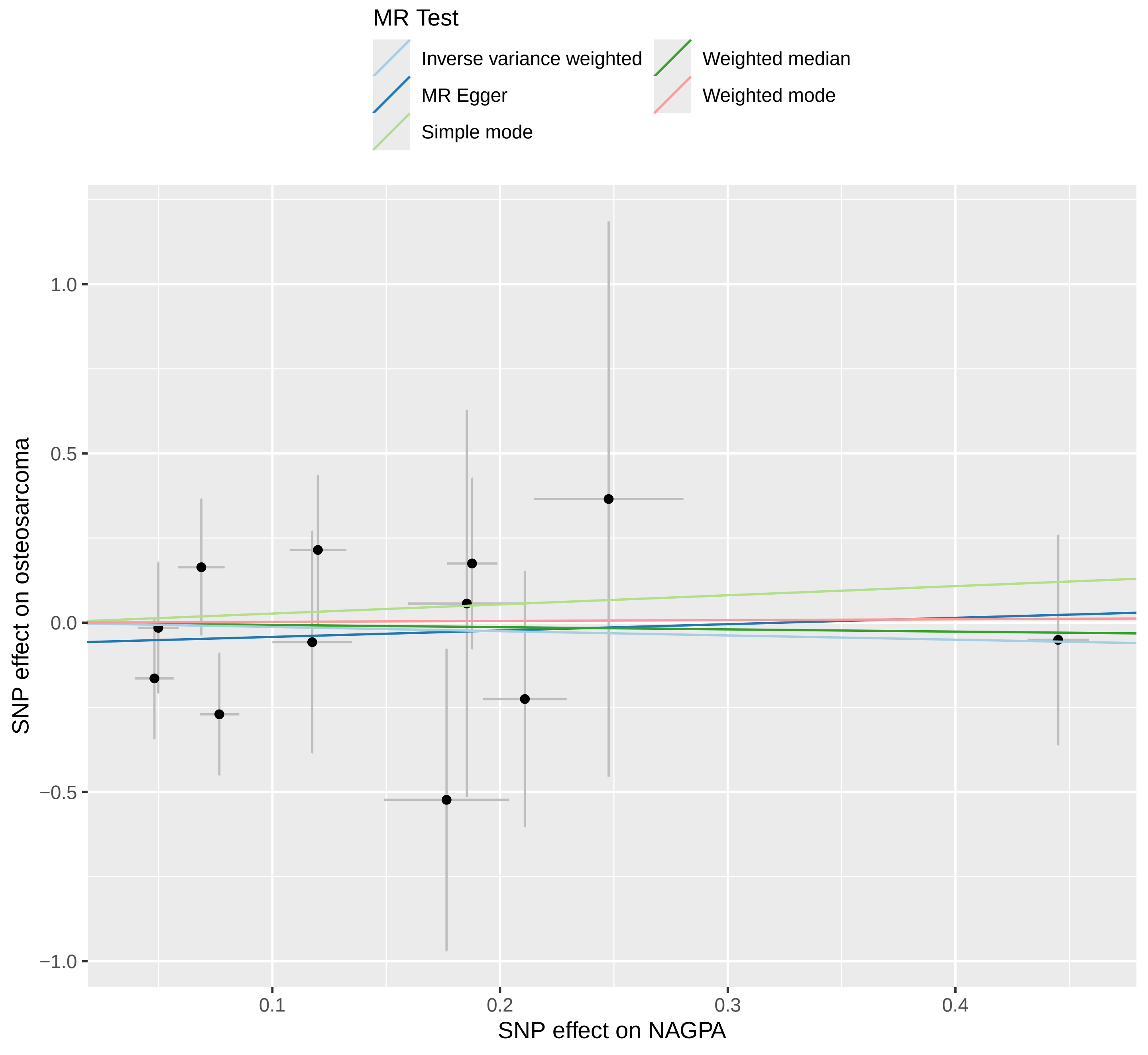
MR Test
Inverse variance weighted
MR Egger
Simple mode
Weighted median
Weighted mode
1.0
0.5
0.0
−0.5
−1.0
0.1
0.2
0.3
0.4
SNP effect on NAGPA
SNP effect on osteosarcoma

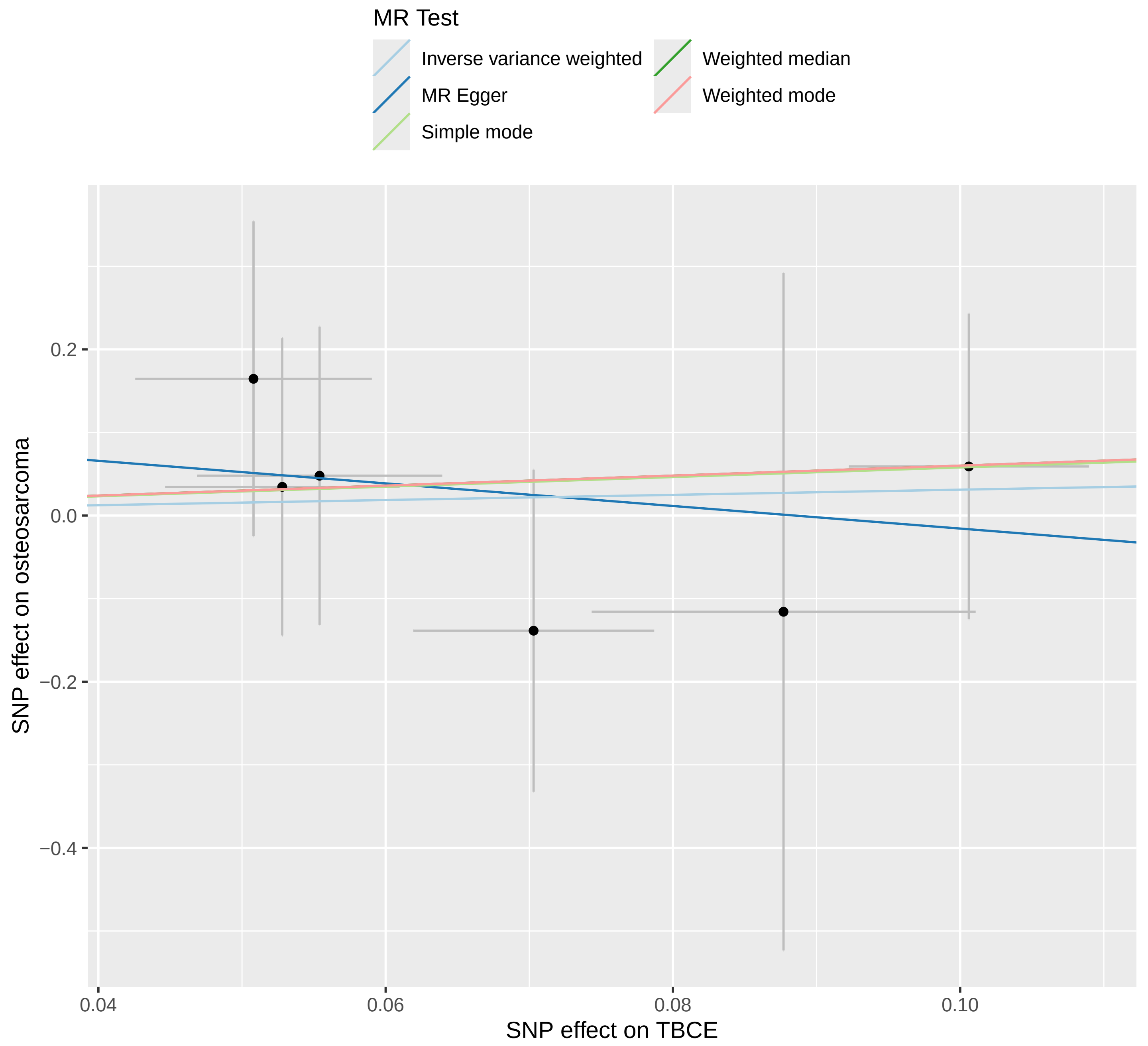

MR Test
Inverse variance weighted
MR Egger
Simple mode
Weighted median
Weighted mode
0.2
0.0
−0.2
−0.4
0.04
0.06
0.08
0.10
SNP effect on osteosarcoma
SNP effect on TBCE

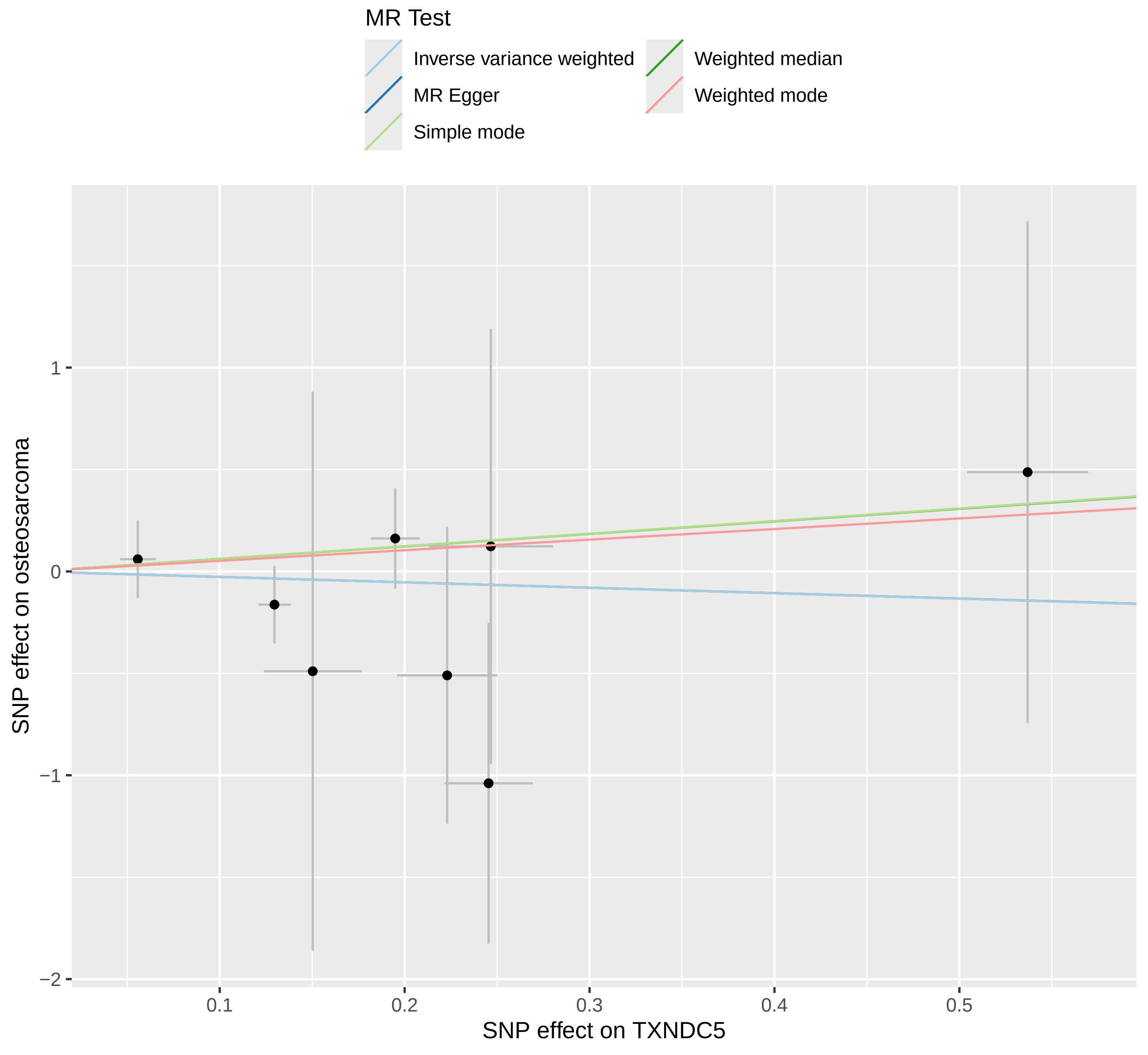

MR Test
Inverse variance weighted
MR Egger
Simple mode
Weighted median
Weighted mode
1
0
−1
−2
SNP effect on osteosarcoma
0.1
0.2
0.3
0.4
0.5
SNP effect on TXNDC5

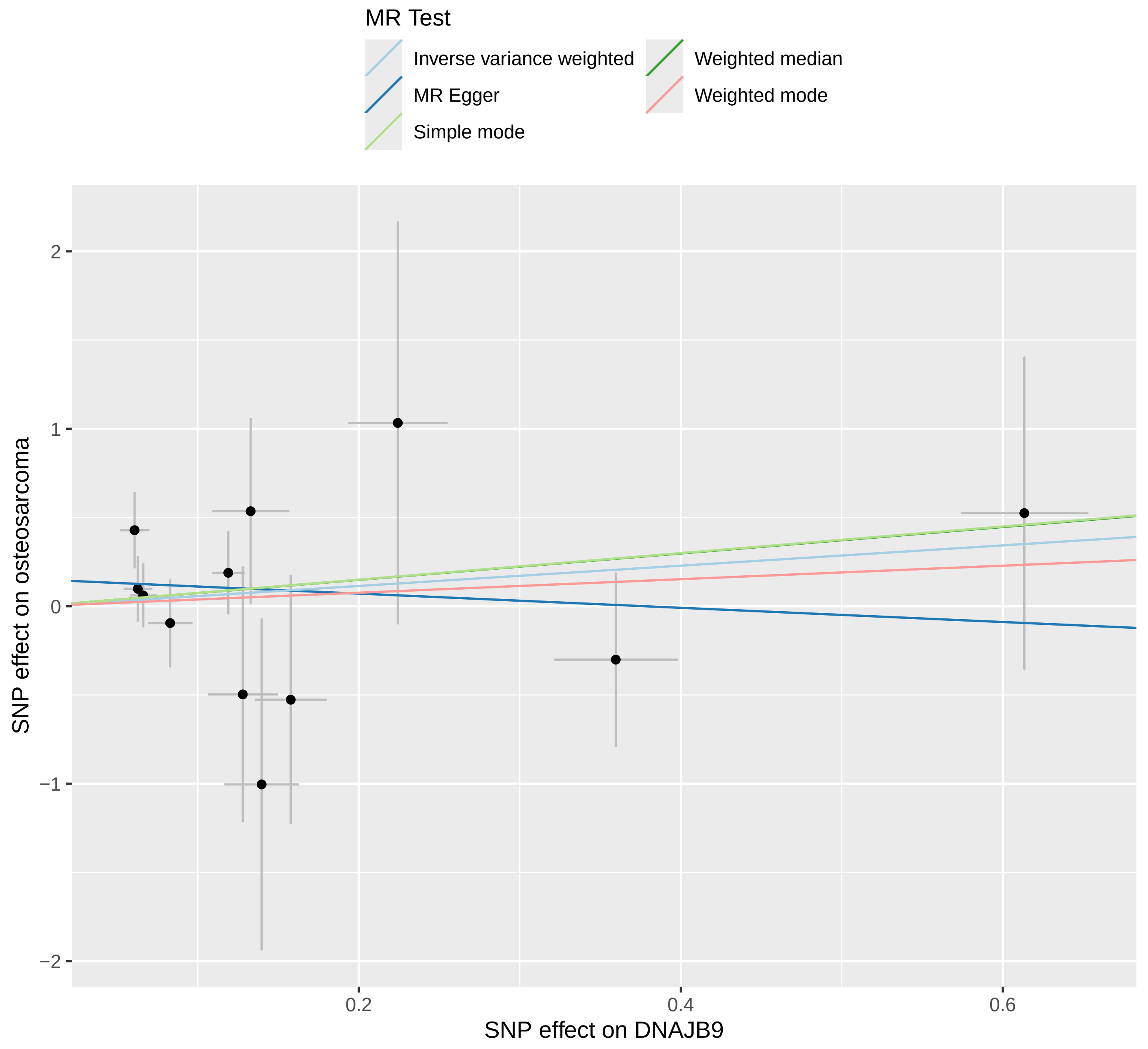

MR Test
Inverse variance weighted
MR Egger
Simple mode
Weighted median
Weighted mode
2
1
0
−1
−2
0.2
0.4
0.6
SNP effect on osteosarcoma
SNP effect on DNAJB9

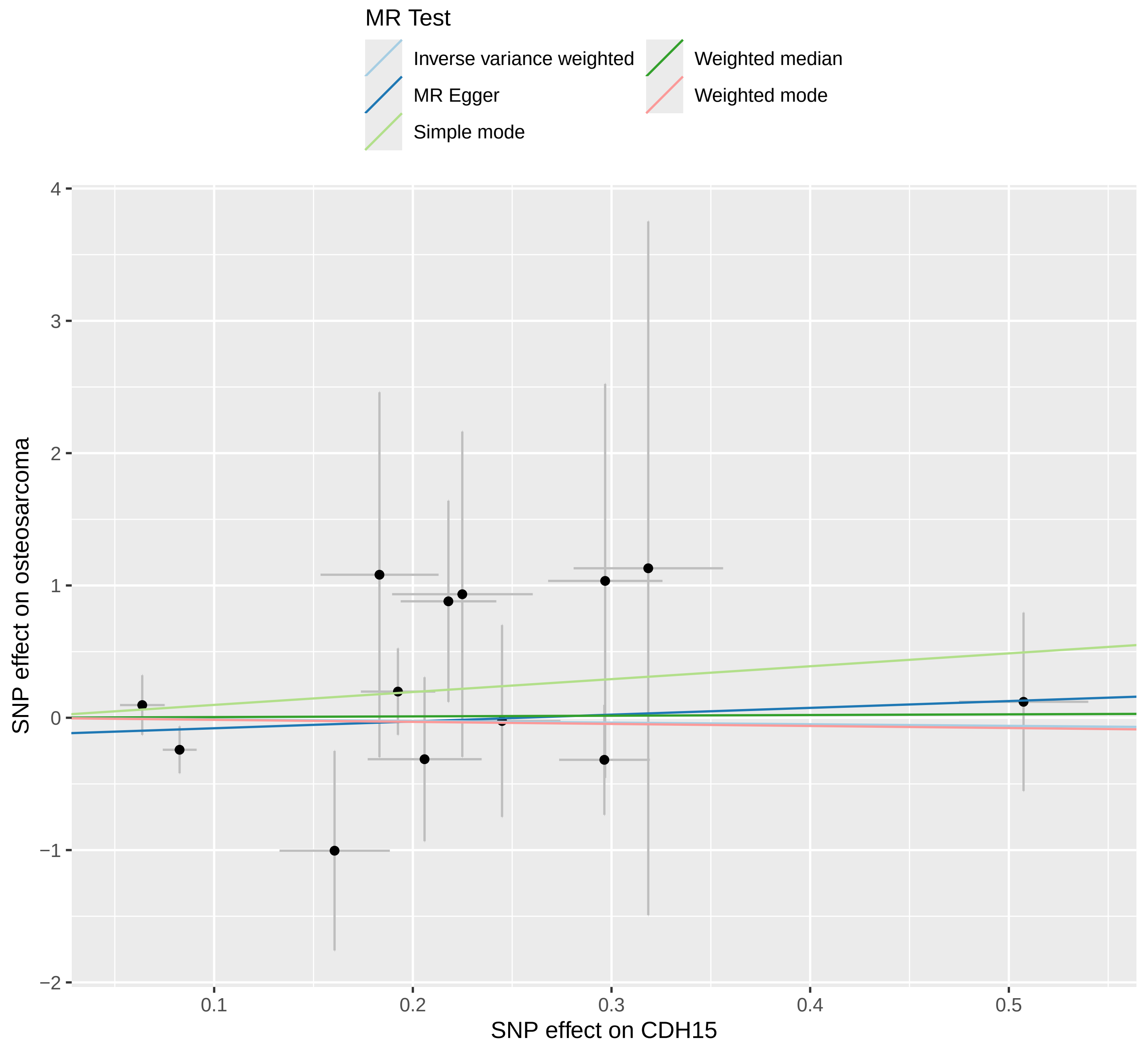
MR Test
Inverse variance weighted
MR Egger
Simple mode
Weighted median
Weighted mode
SNP effect on osteosarcoma
4
3
2
1
0
−1
−2
0.1
0.2
0.3
0.4
0.5
SNP effect on CDH15

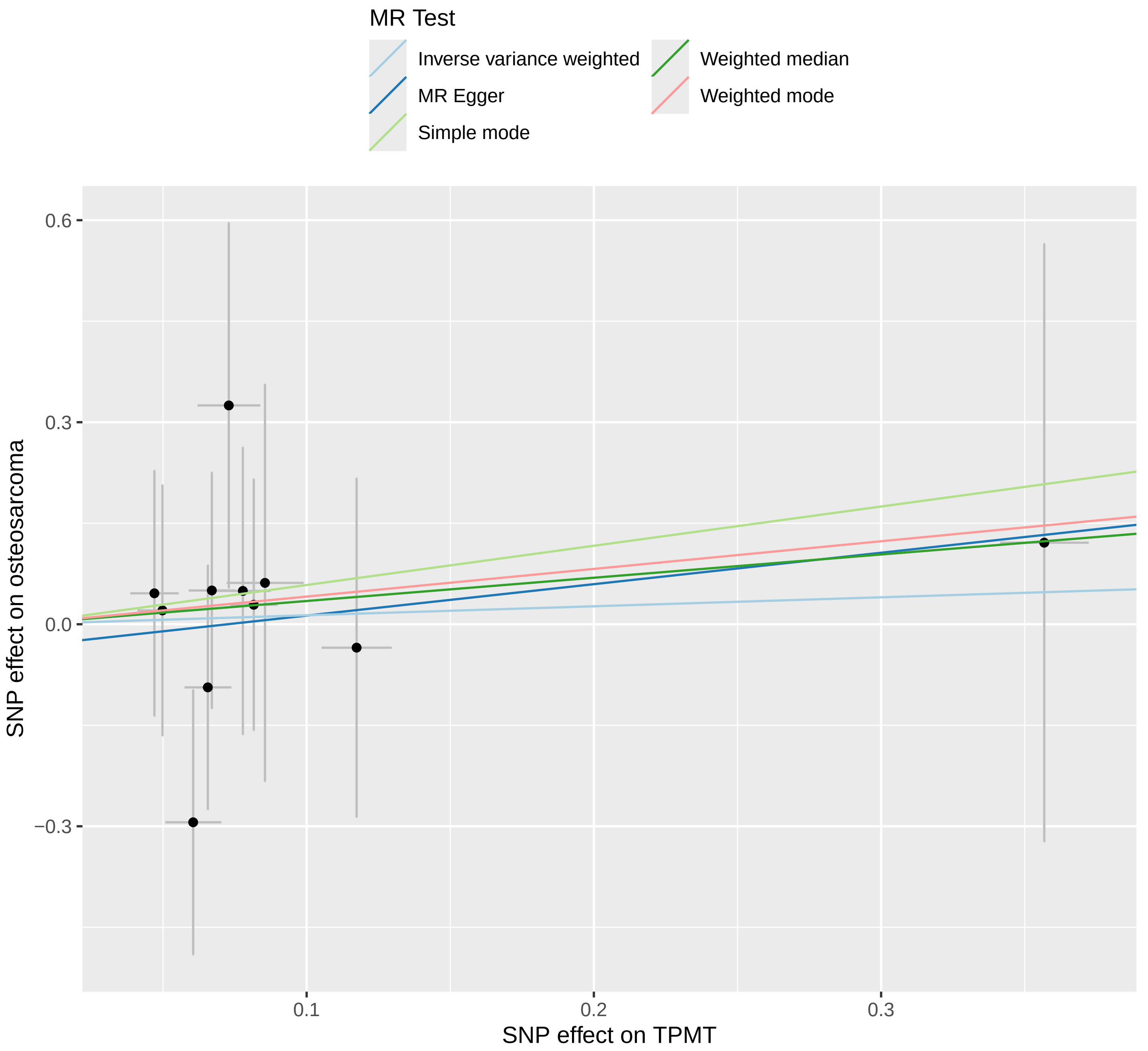

MR Test
Inverse variance weighted
MR Egger
Simple mode
Weighted median
Weighted mode
0.6
0.3
0.0
−0.3
SNP effect on osteosarcoma
0.1
0.2
0.3
SNP effect on TPMT

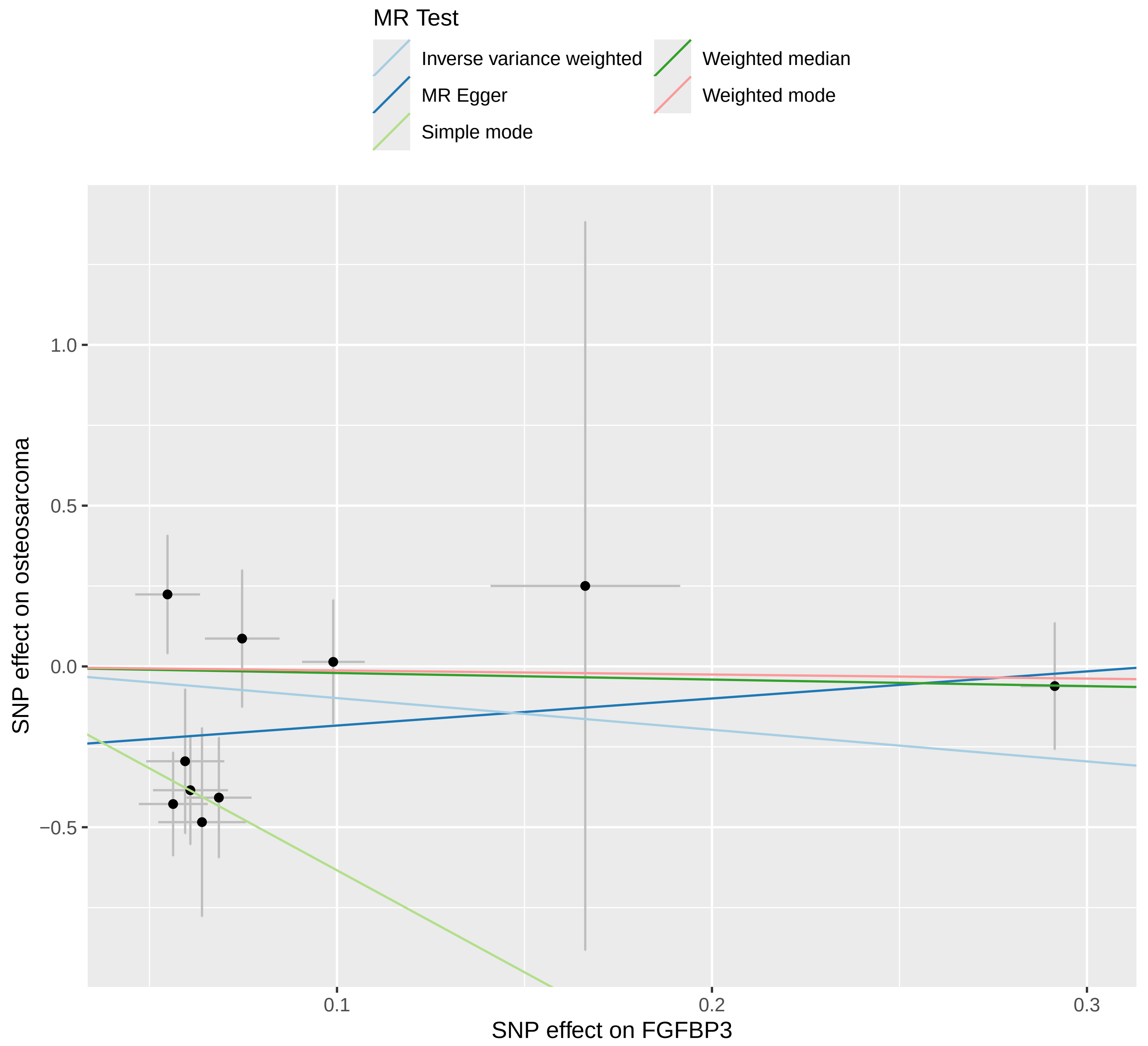
MR Test
Inverse variance weighted
MR Egger
Simple mode
Weighted median
Weighted mode
1.0
0.5
0.0
−0.5
0.1
0.2
0.3
SNP effect on osteosarcoma
SNP effect on FGFBP3

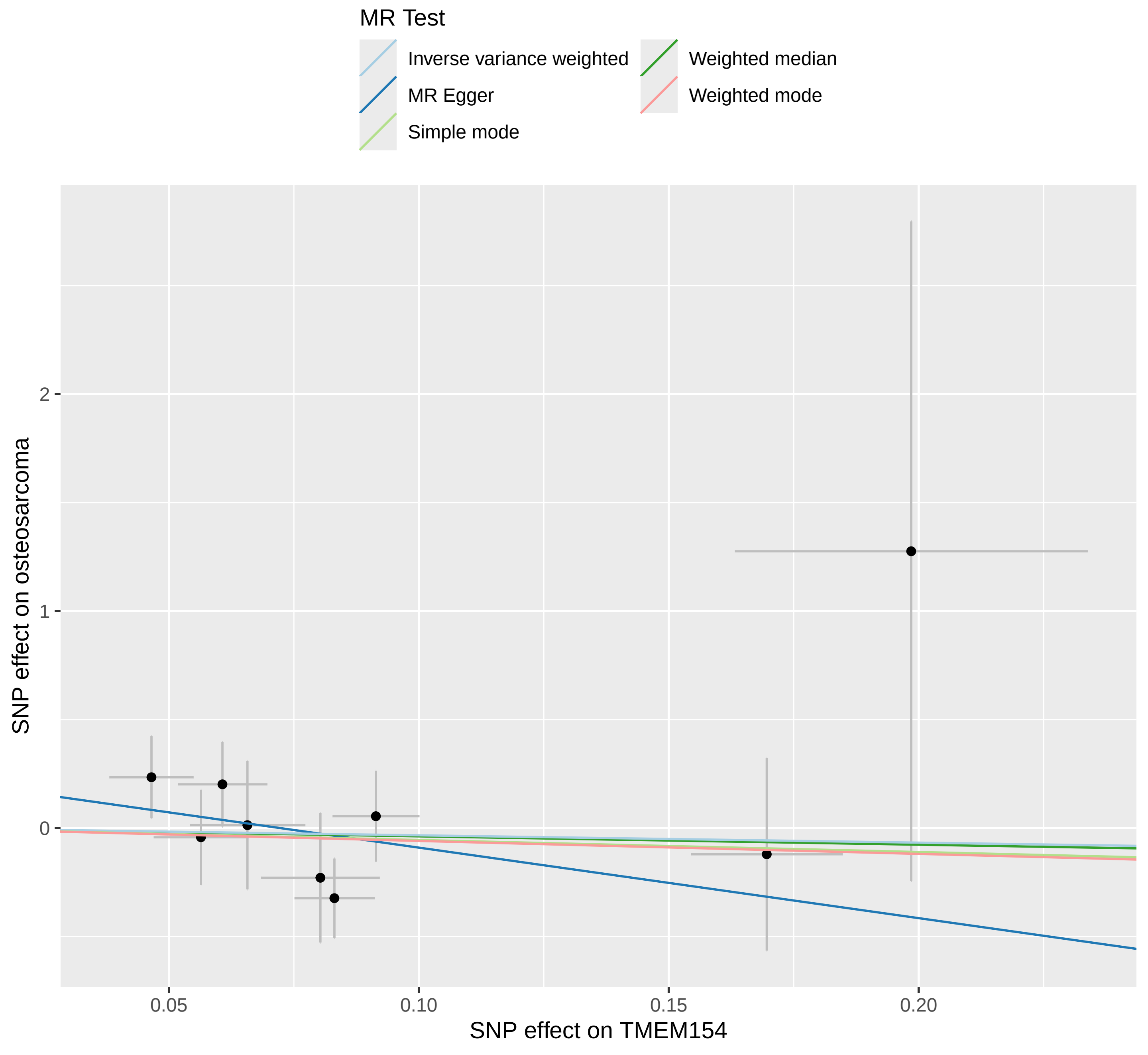

MR Test
Inverse variance weighted
MR Egger
Simple mode
Weighted median
Weighted mode
SNP effect on osteosarcoma
2
1
0
0.05
0.10
0.15
0.20
SNP effect on TMEM154

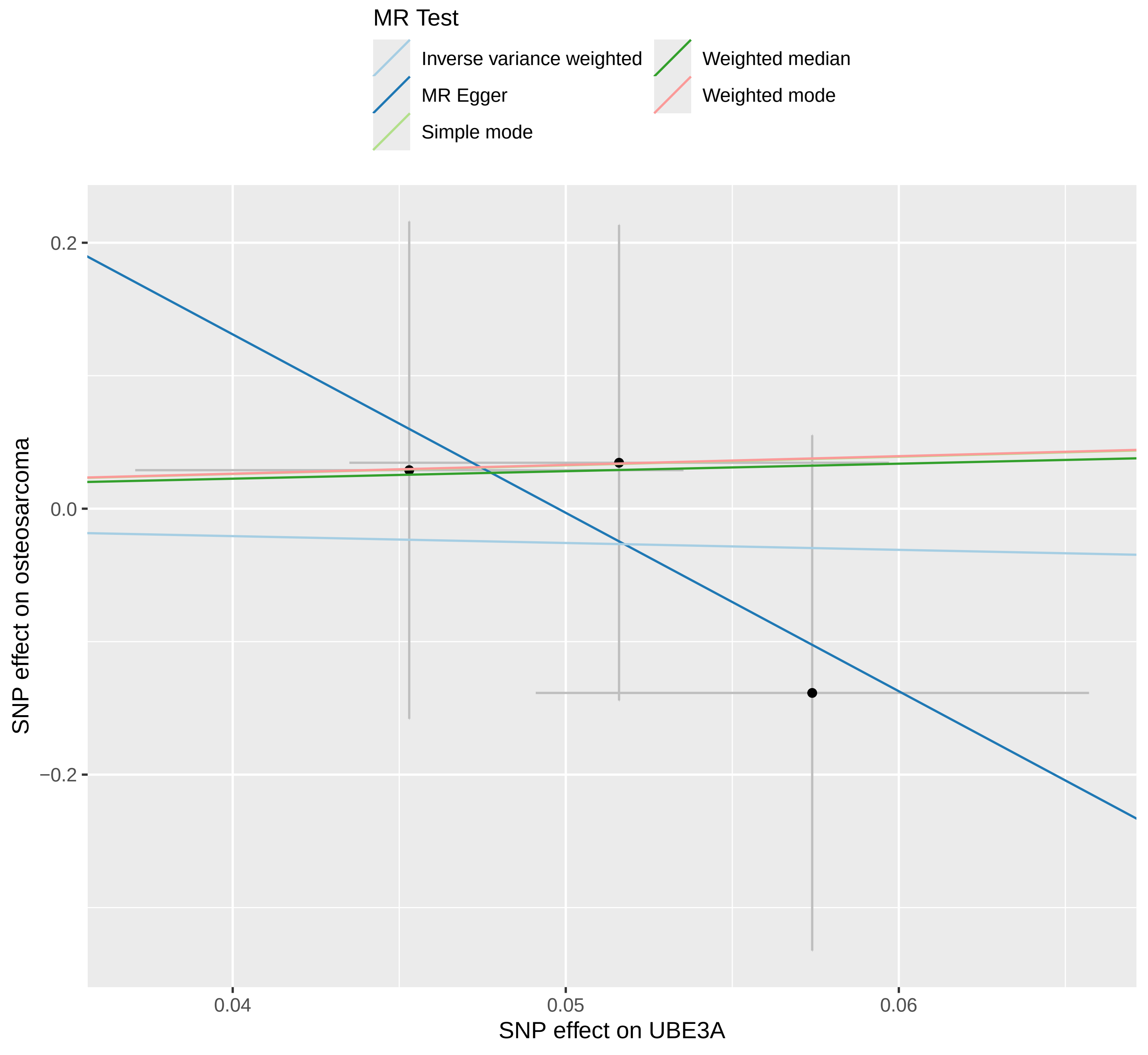

MR Test
Inverse variance weighted
MR Egger
Simple mode
Weighted median
Weighted mode
SNP effect on osteosarcoma
0.2
0.0
−0.2
0.04
0.05
0.06
SNP effect on UBE3A

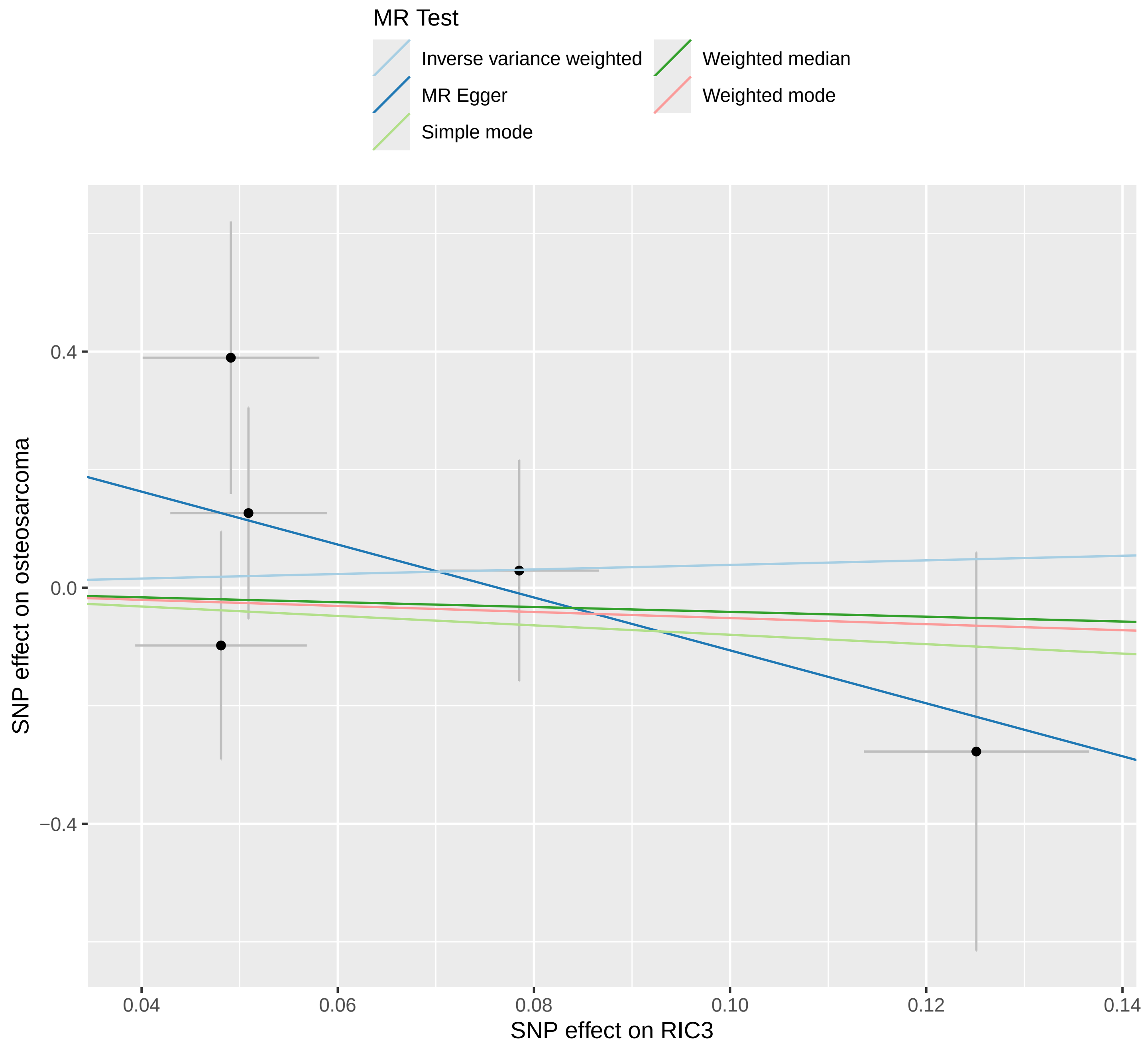
MR Test
Inverse variance weighted
MR Egger
Simple mode
Weighted median
Weighted mode
SNP effect on osteosarcoma
0.4
0.0
−0.4
0.04
0.06
0.08
0.10
0.12
0.14
SNP effect on RIC3

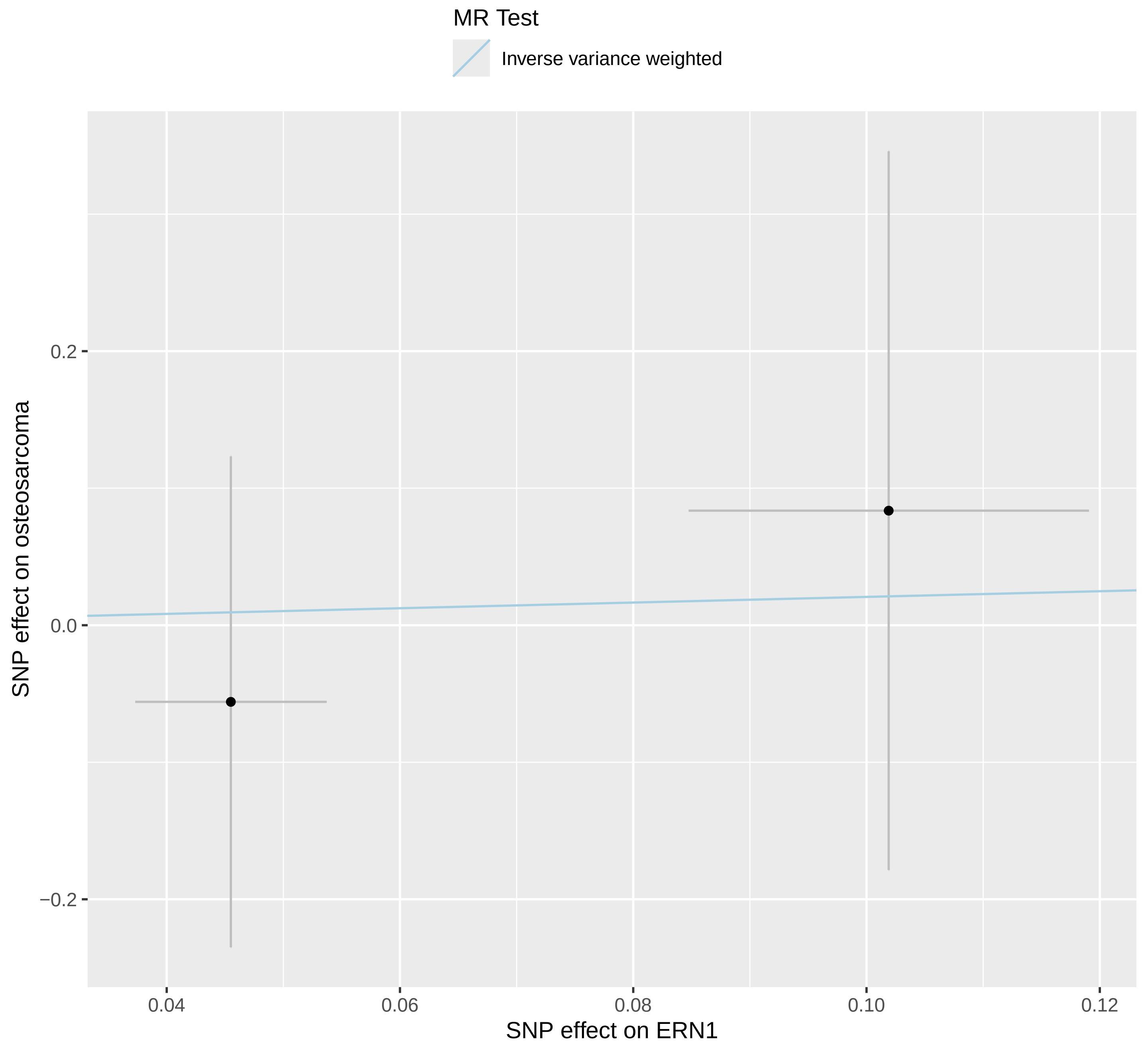
MR Test
Inverse variance weighted
SNP effect on osteosarcoma
0.2
0.0
−0.2
0.04
0.06
0.08
0.10
0.12
SNP effect on ERN1

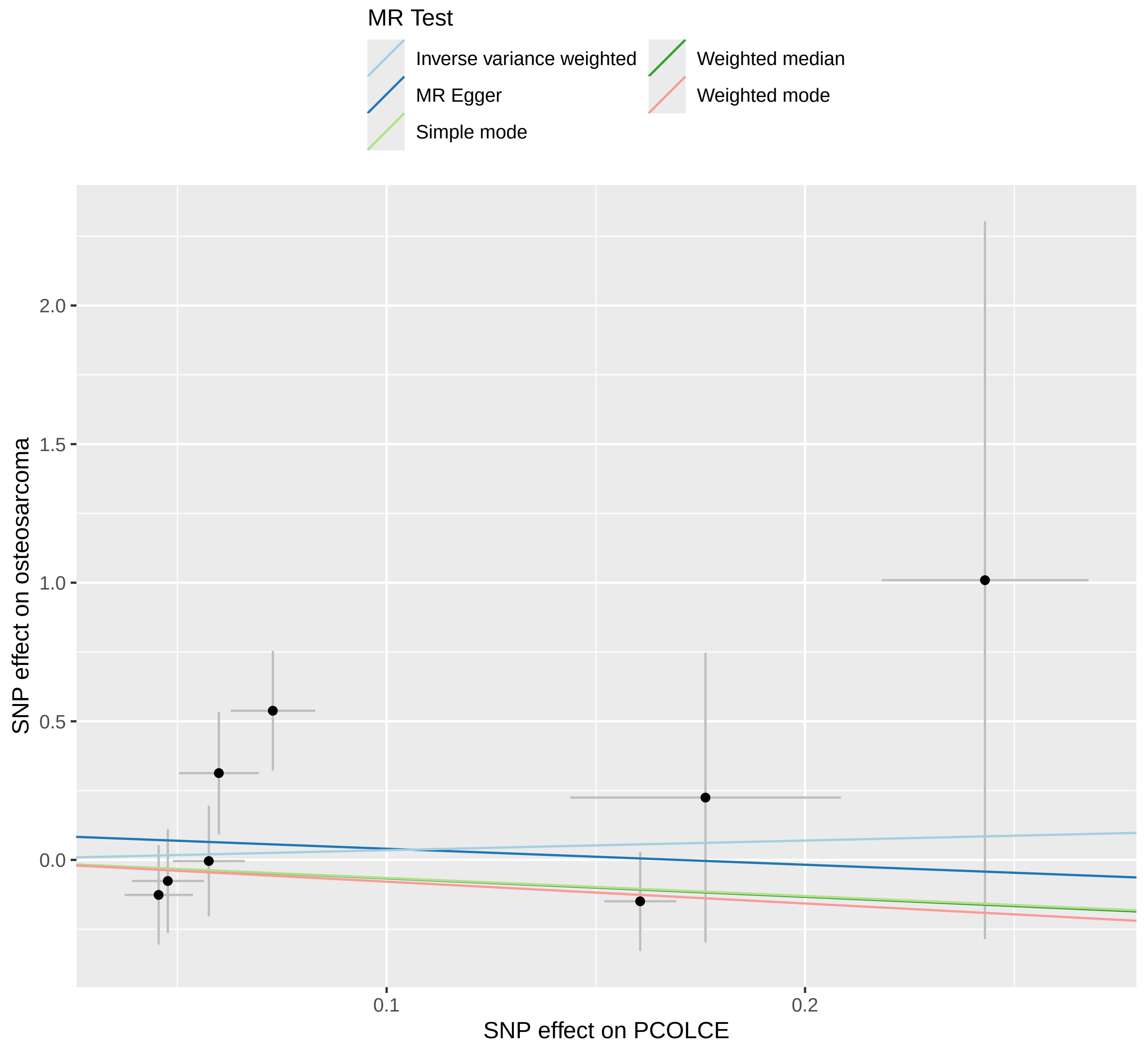

MR Test
Inverse variance weighted
MR Egger
Simple mode
Weighted median
Weighted mode
2.0
1.5
1.0
0.5
0.0
0.1
0.2
SNP effect on osteosarcoma
SNP effect on PCOLCE

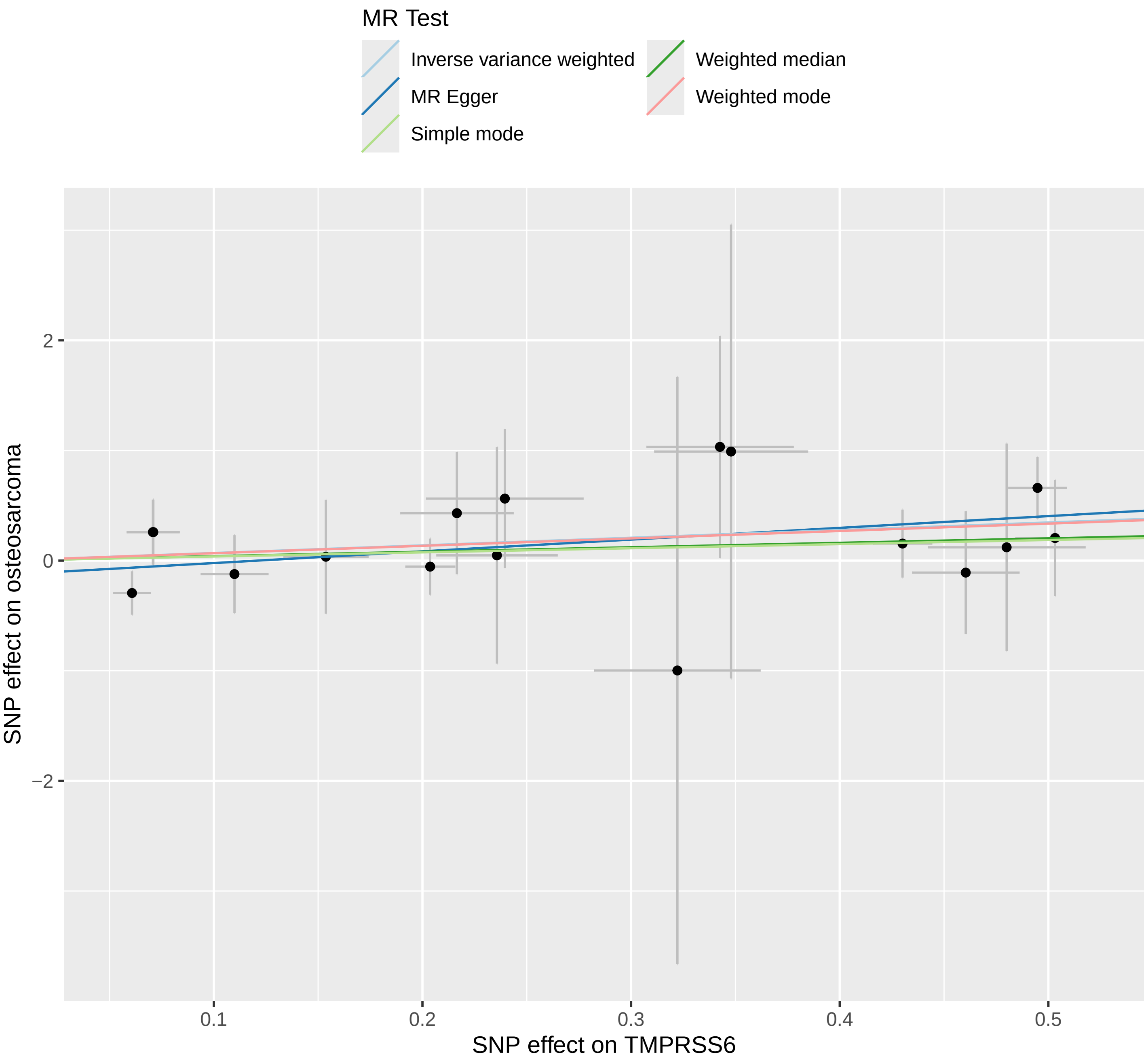

MR Test
Inverse variance weighted
MR Egger
Simple mode
Weighted median
Weighted mode
2
0
−2
0.1
0.2
0.3
0.4
0.5
SNP effect on osteosarcoma
SNP effect on TMPRSS6

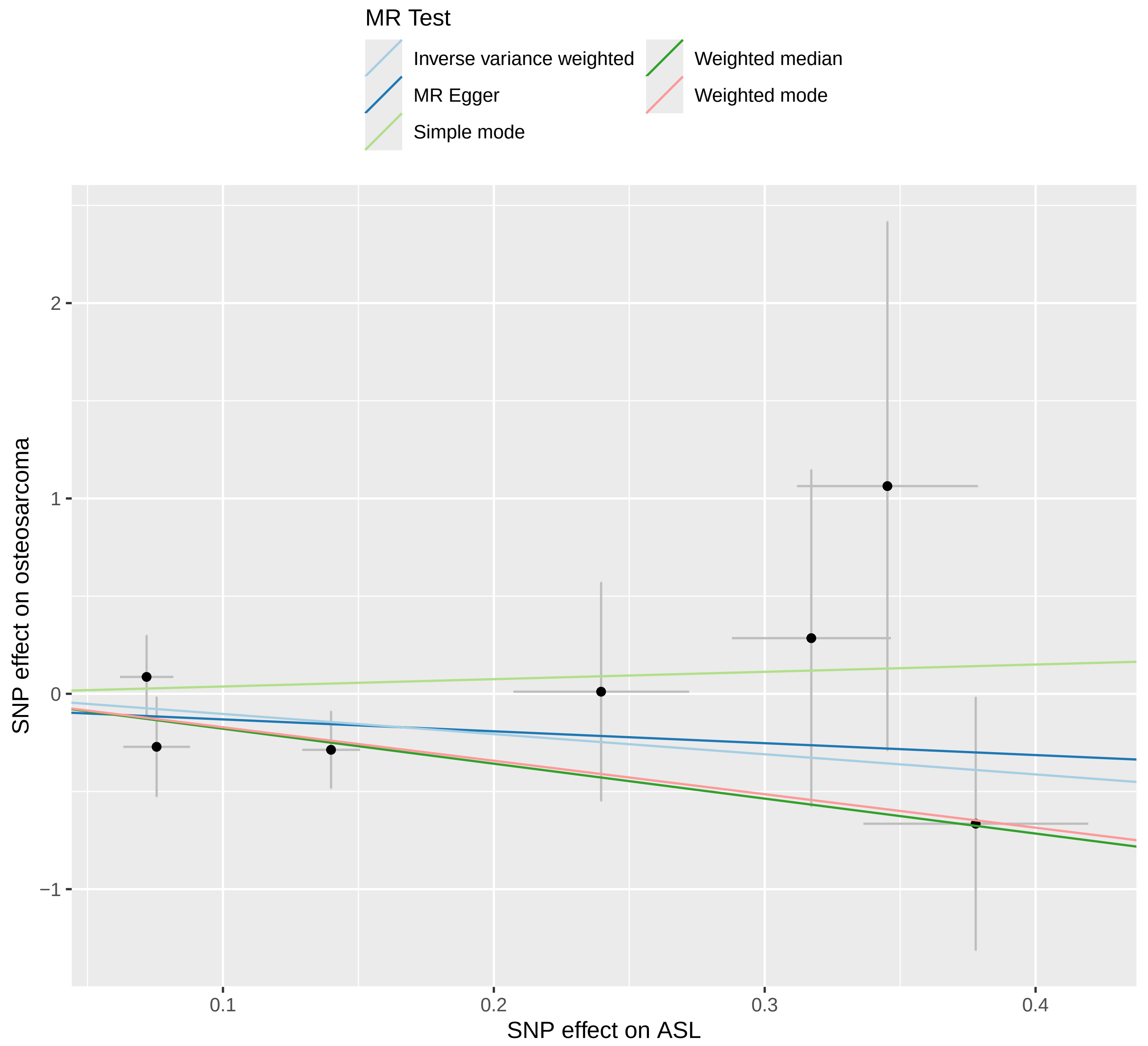
MR Test
Inverse variance weighted
MR Egger
Simple mode
Weighted median
Weighted mode
2
1
0
−1
0.1
0.2
0.3
0.4
SNP effect on ASL
SNP effect on osteosarcoma

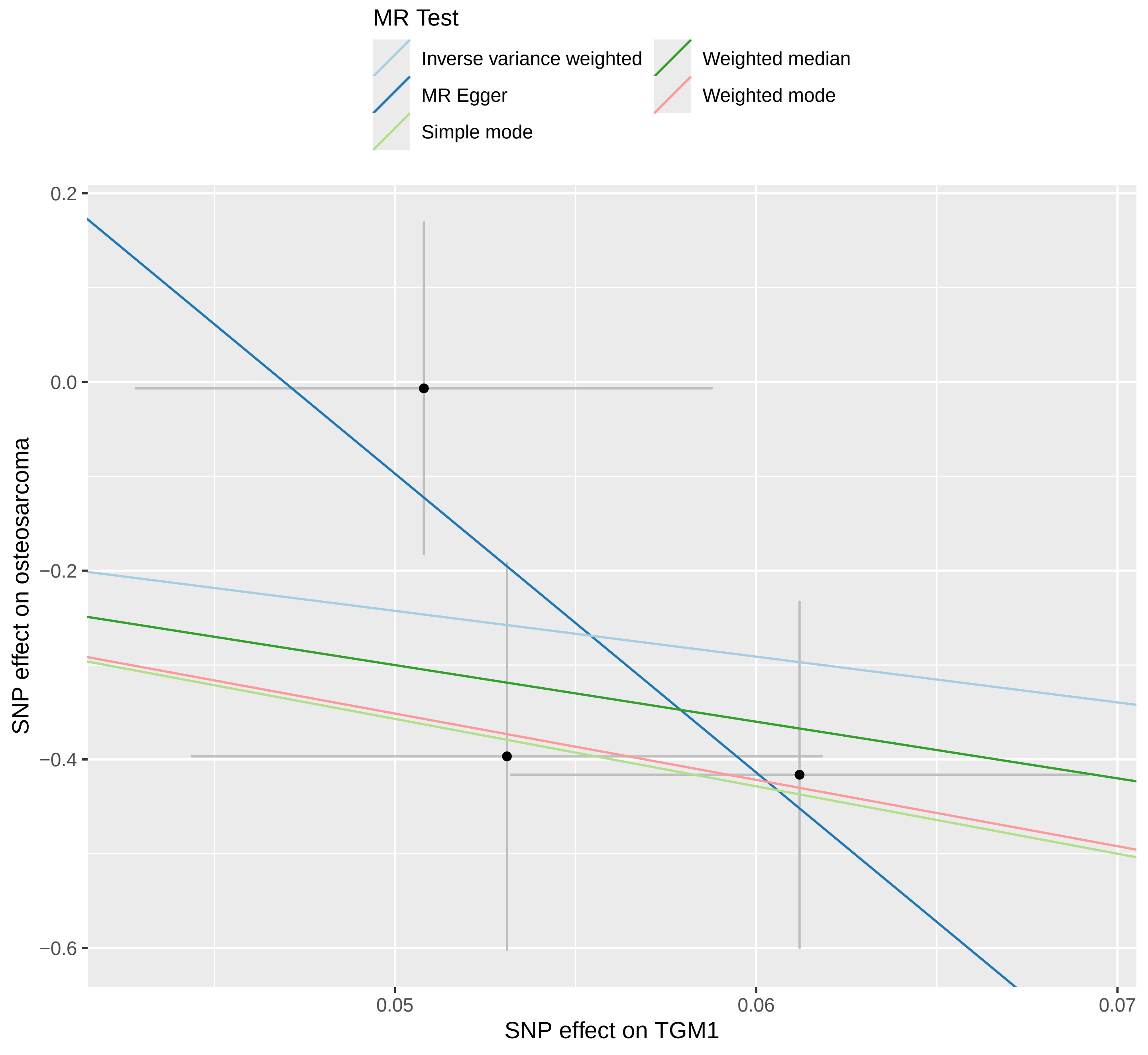

MR Test
Inverse variance weighted
MR Egger
Simple mode
Weighted median
Weighted mode
0.2
0.0
−0.2
−0.4
−0.6
SNP effect on osteosarcoma
0.05
0.06
0.07
SNP effect on TGM1

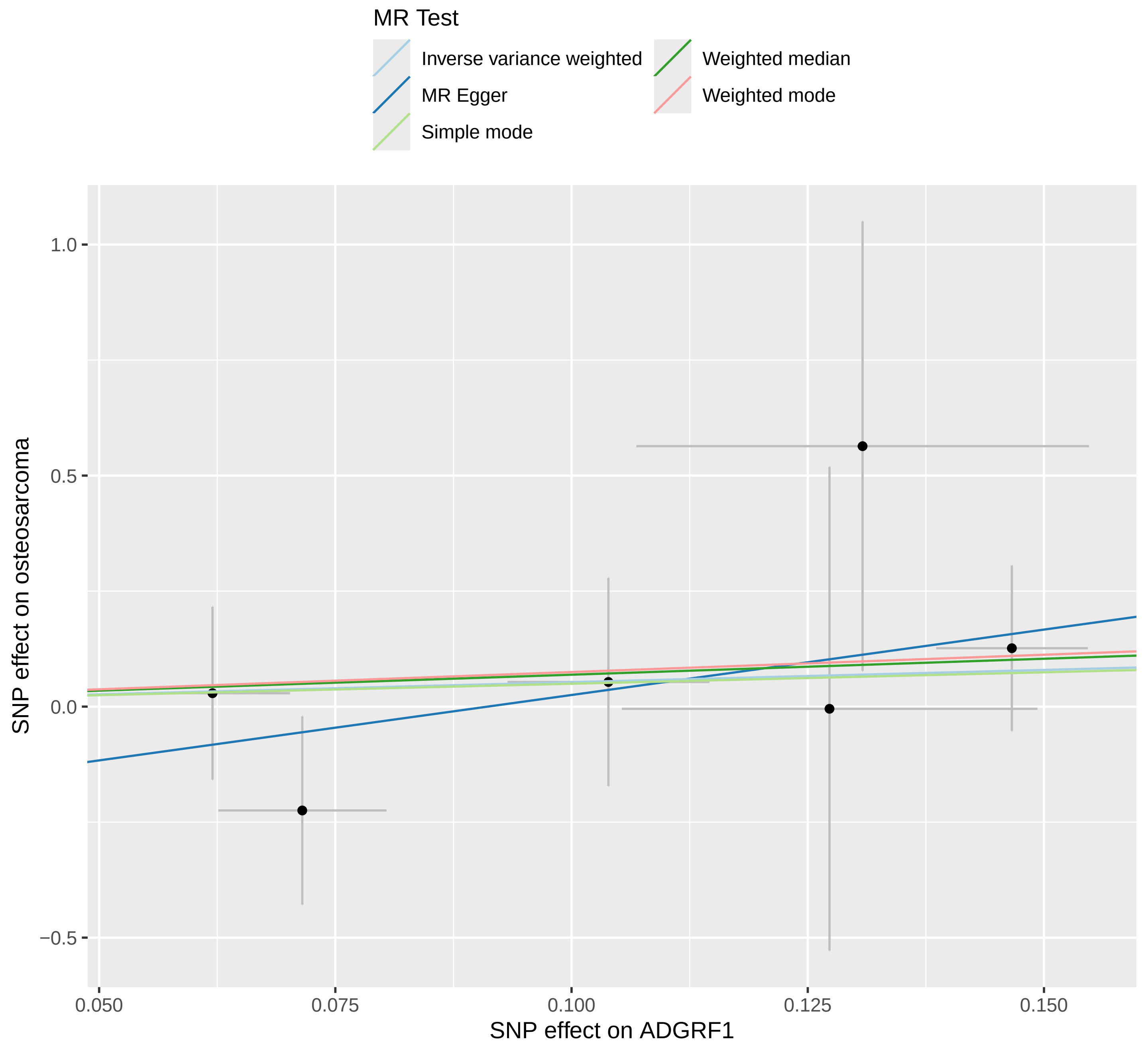
MR Test
Inverse variance weighted
MR Egger
Simple mode
Weighted median
Weighted mode
SNP effect on osteosarcoma
1.0
0.5
0.0
−0.5
0.050
0.075
0.100
0.125
0.150
SNP effect on ADGRF1

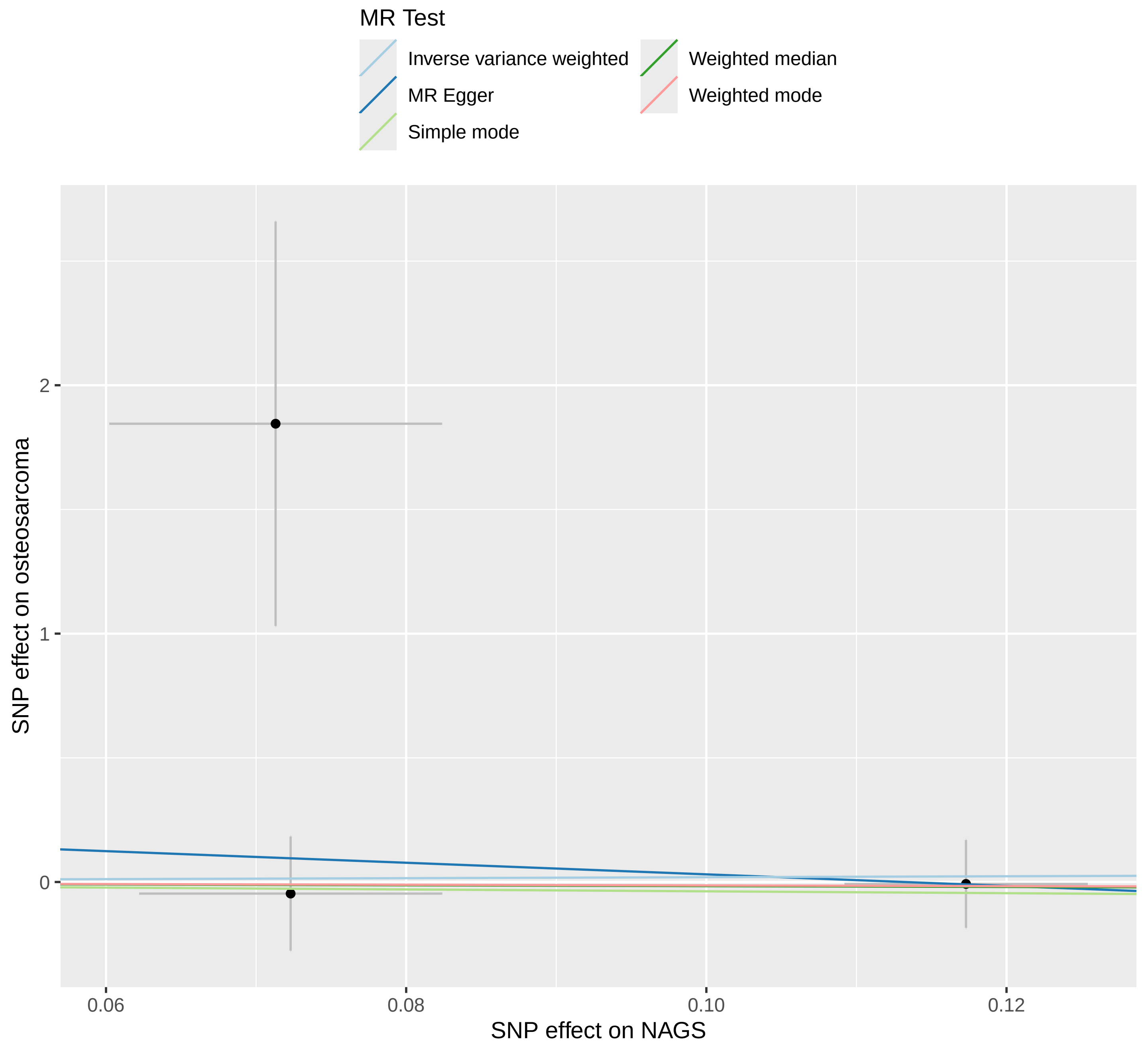

MR Test
Inverse variance weighted
MR Egger
Simple mode
Weighted median
Weighted mode
SNP effect on osteosarcoma
2
1
0
0.06
0.08
0.10
0.12
SNP effect on NAGS

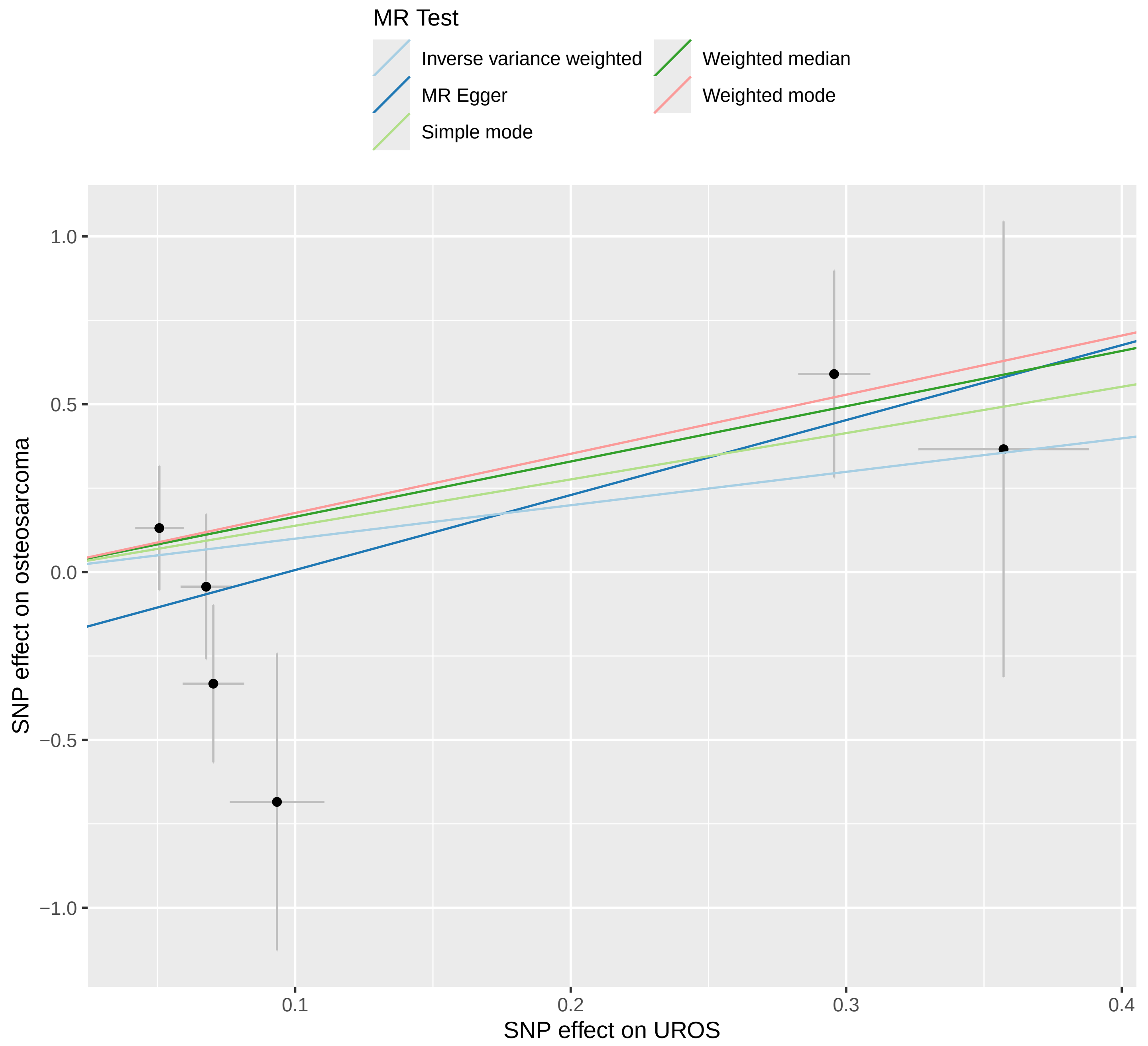

MR Test
Inverse variance weighted
MR Egger
Simple mode
Weighted median
Weighted mode
1.0
0.5
0.0
−0.5
−1.0
0.1
0.2
0.3
0.4
SNP effect on osteosarcoma
SNP effect on UROS

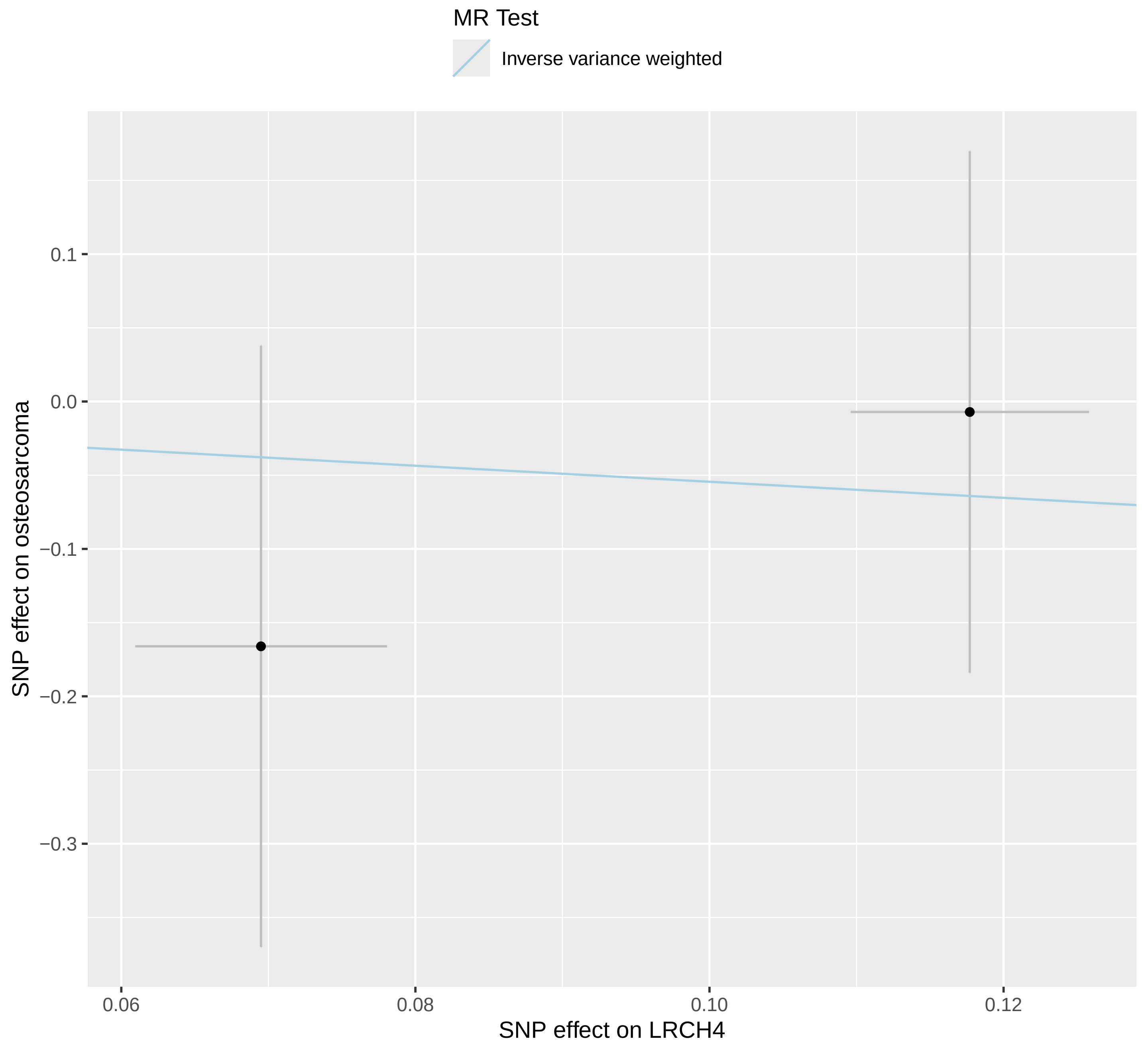

MR Test
Inverse variance weighted
0.1
0.0
−0.1
−0.2
−0.3
0.06
0.08
0.10
0.12
SNP effect on LRCH4
SNP effect on osteosarcoma

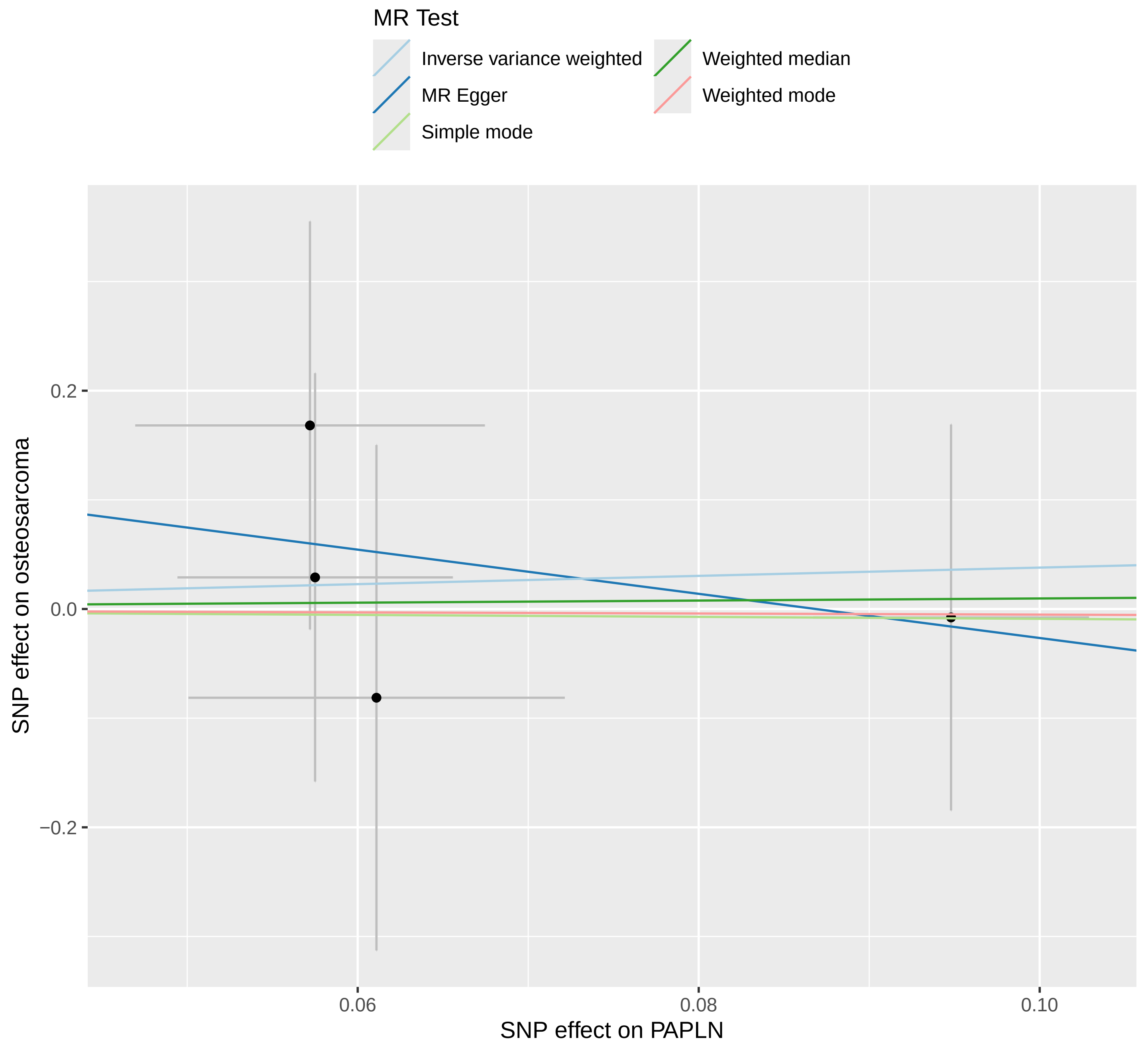
MR Test
Inverse variance weighted
MR Egger
Simple mode
Weighted median
Weighted mode
0.2
0.0
−0.2
0.06
0.08
0.10
SNP effect on osteosarcoma
SNP effect on PAPLN

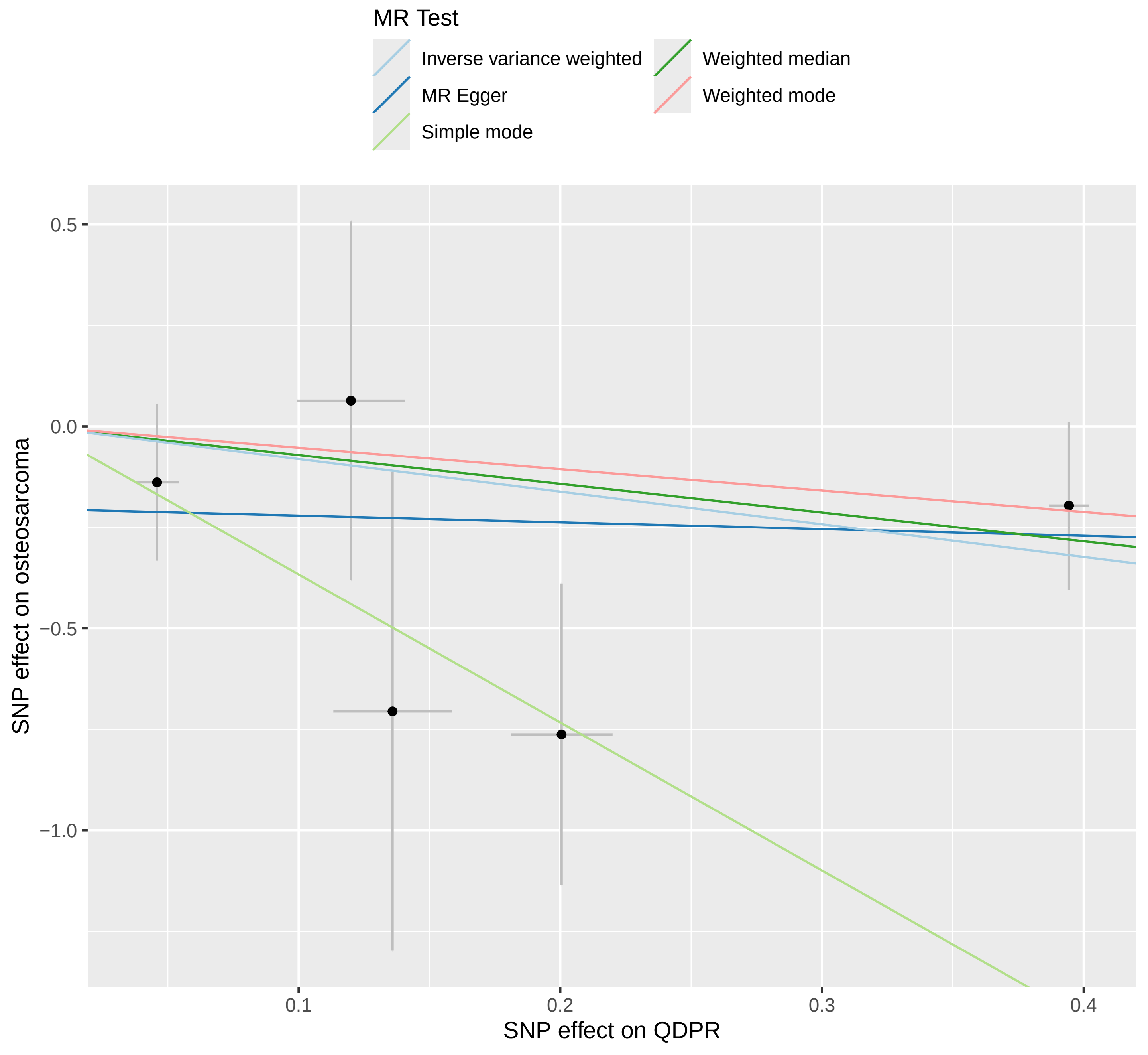

MR Test
Inverse variance weighted
MR Egger
Simple mode
Weighted median
Weighted mode
0.5
0.0
−0.5
−1.0
0.1
0.2
0.3
0.4
SNP effect on osteosarcoma
SNP effect on QDPR

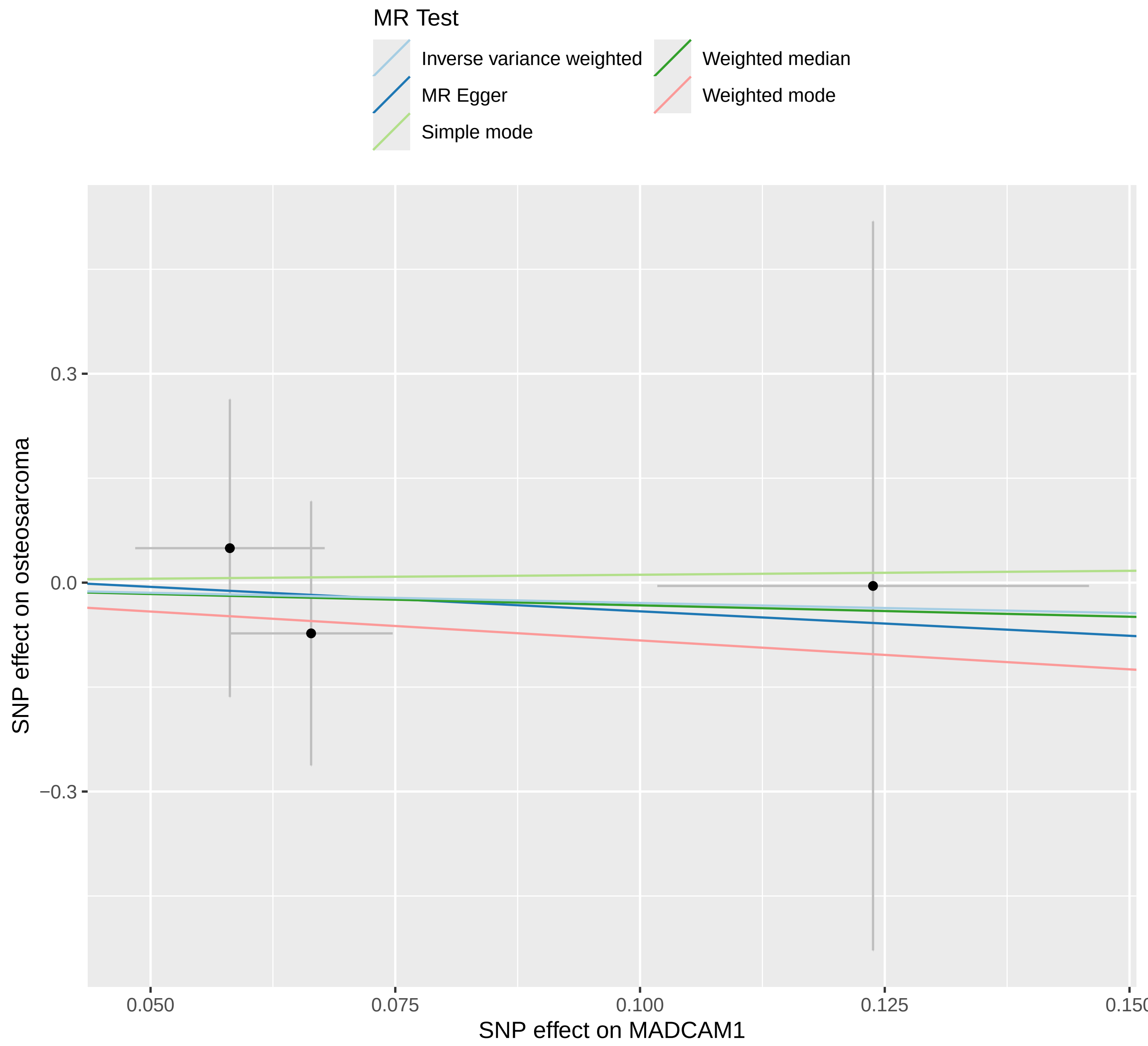

MR Test
Inverse variance weighted
MR Egger
Simple mode
Weighted median
Weighted mode
0.3
0.0
−0.3
SNP effect on osteosarcoma
0.050
0.075
0.100
0.125
0.150
SNP effect on MADCAM1

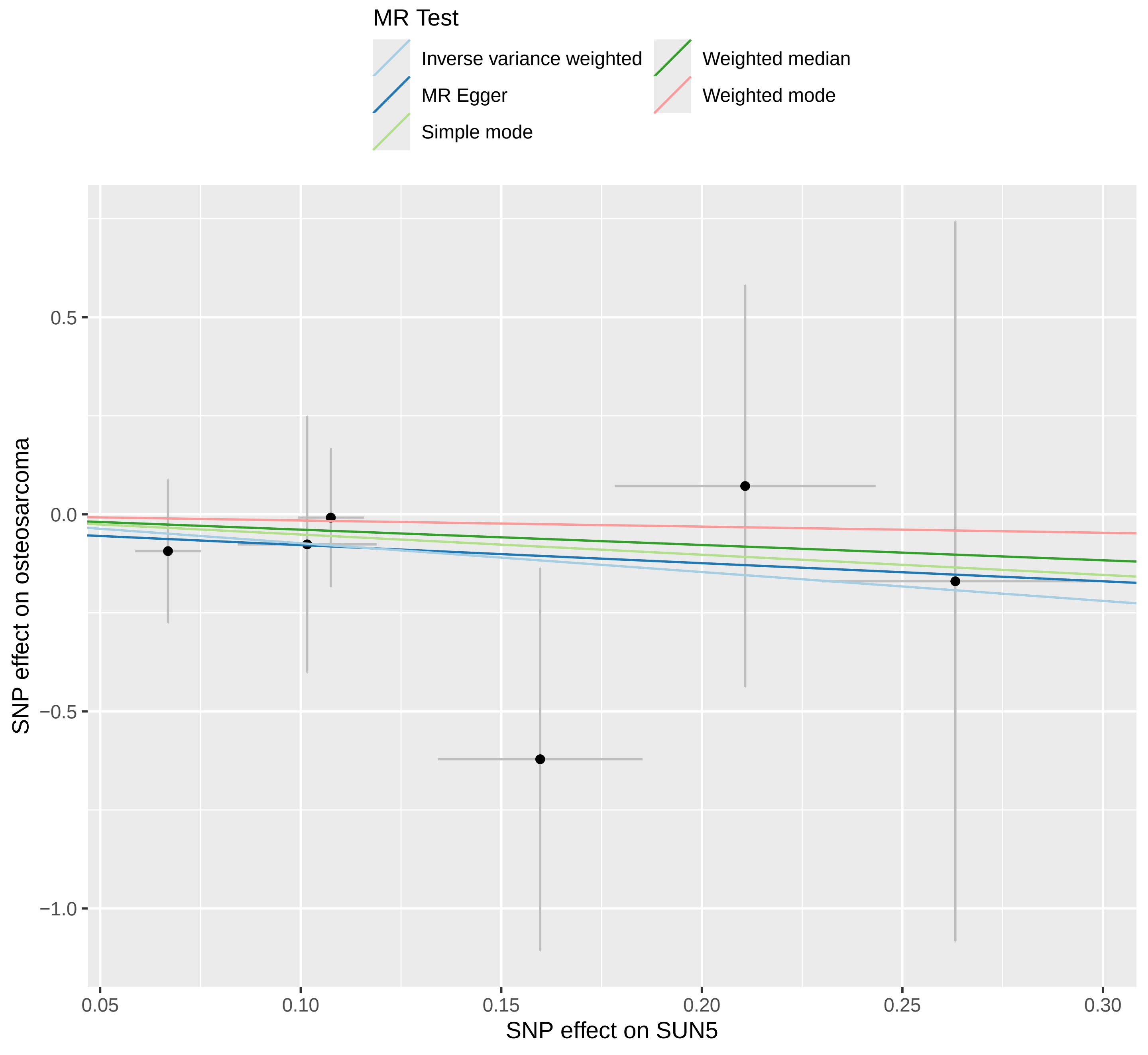
MR Test
Inverse variance weighted
MR Egger
Simple mode
Weighted median
Weighted mode
0.5
0.0
−0.5
−1.0
0.05
0.10
0.15
0.20
0.25
0.30
SNP effect on osteosarcoma
SNP effect on SUN5

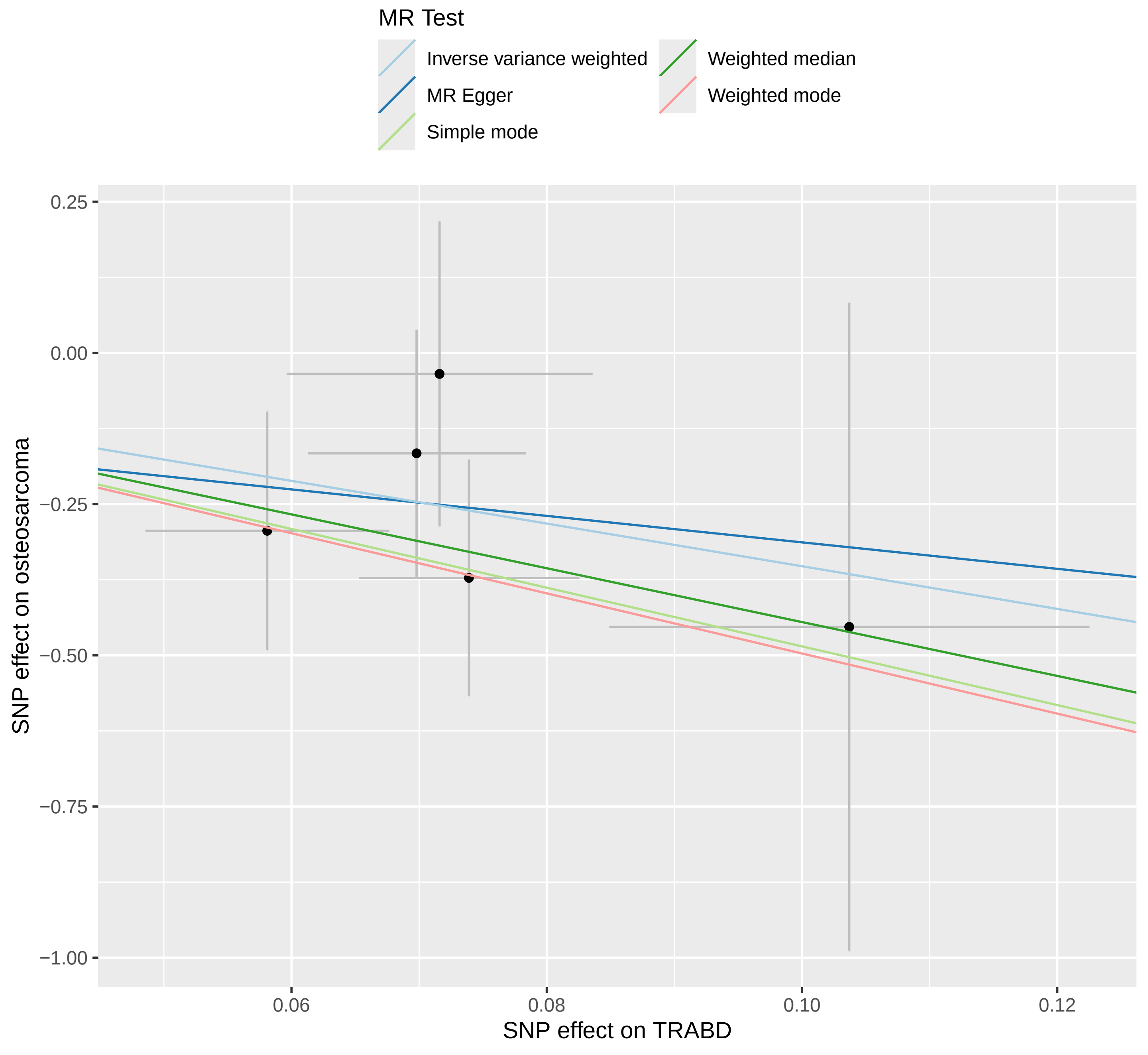
MR Test
Inverse variance weighted
MR Egger
Simple mode
Weighted median
Weighted mode
0.25
0.00
−0.25
−0.50
−0.75
−1.00
0.06
0.08
0.10
0.12
SNP effect on osteosarcoma
SNP effect on TRABD

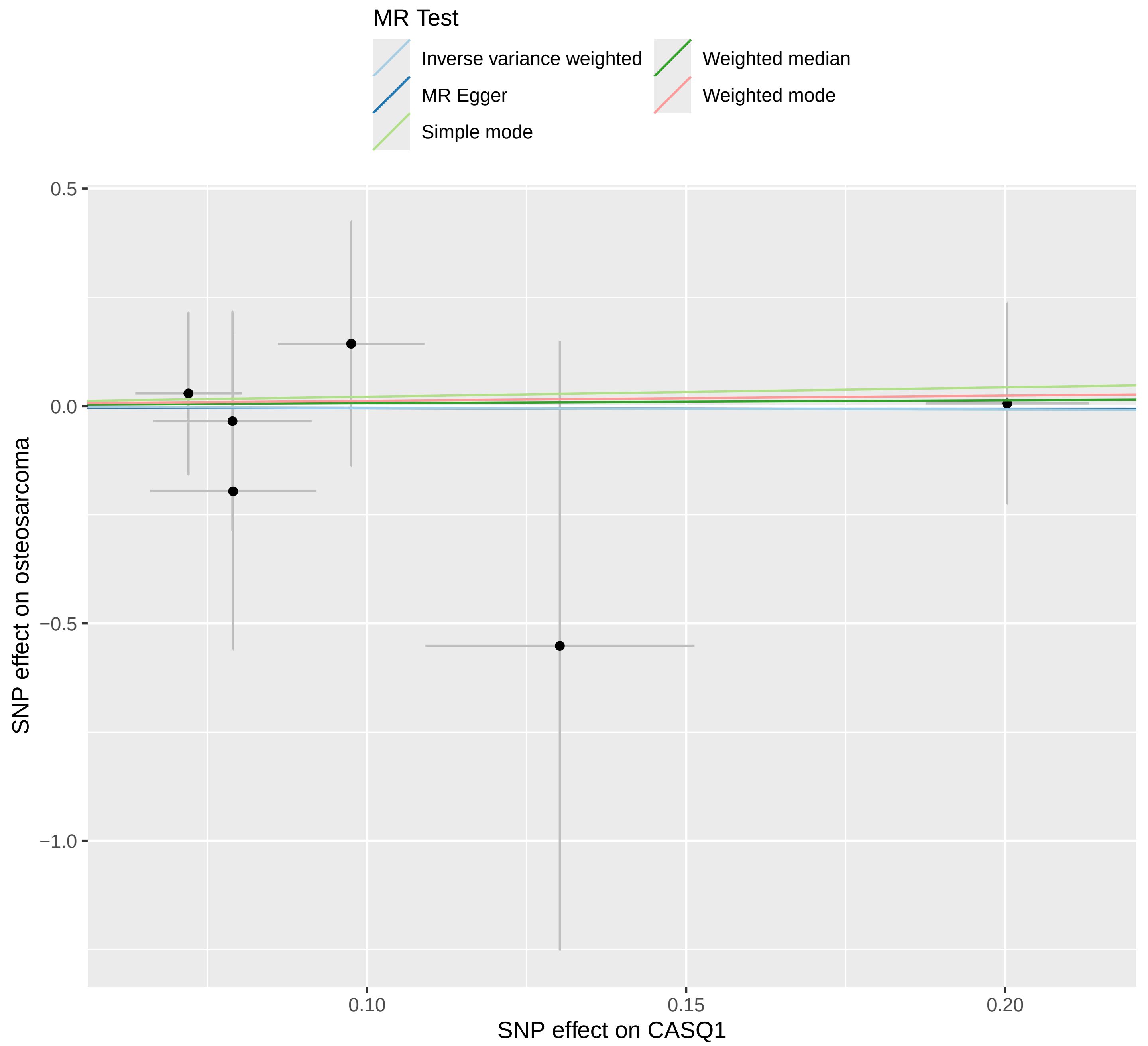

MR Test
Inverse variance weighted
MR Egger
Simple mode
Weighted median
Weighted mode
0.5
0.0
−0.5
−1.0
SNP effect on osteosarcoma
0.10
0.15
0.20
SNP effect on CASQ1

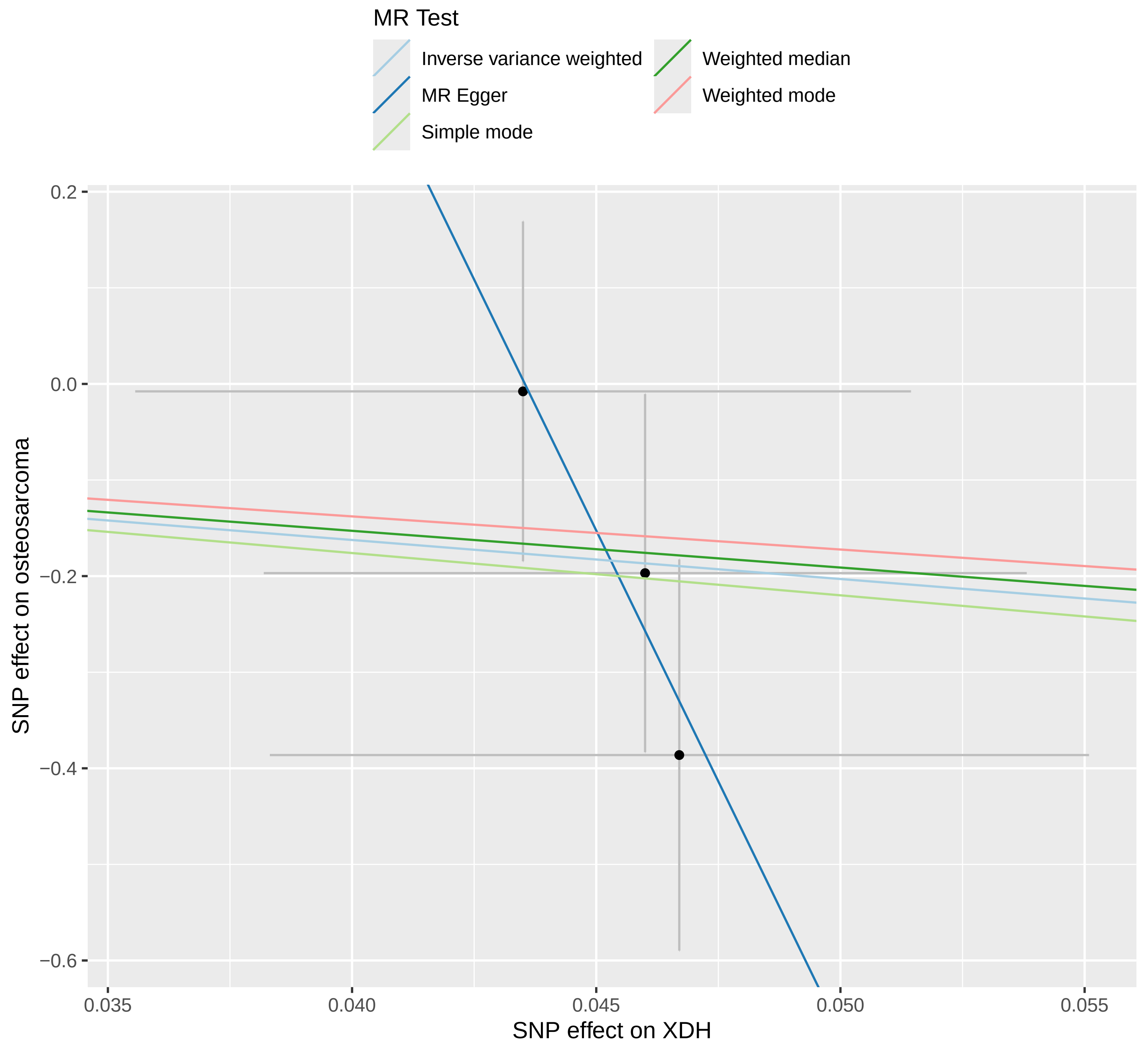
MR Test
Inverse variance weighted
MR Egger
Simple mode
Weighted median
Weighted mode
SNP effect on osteosarcoma
0.2
0.0
−0.2
−0.4
−0.6
0.035
0.040
0.045
0.050
0.055
SNP effect on XDH

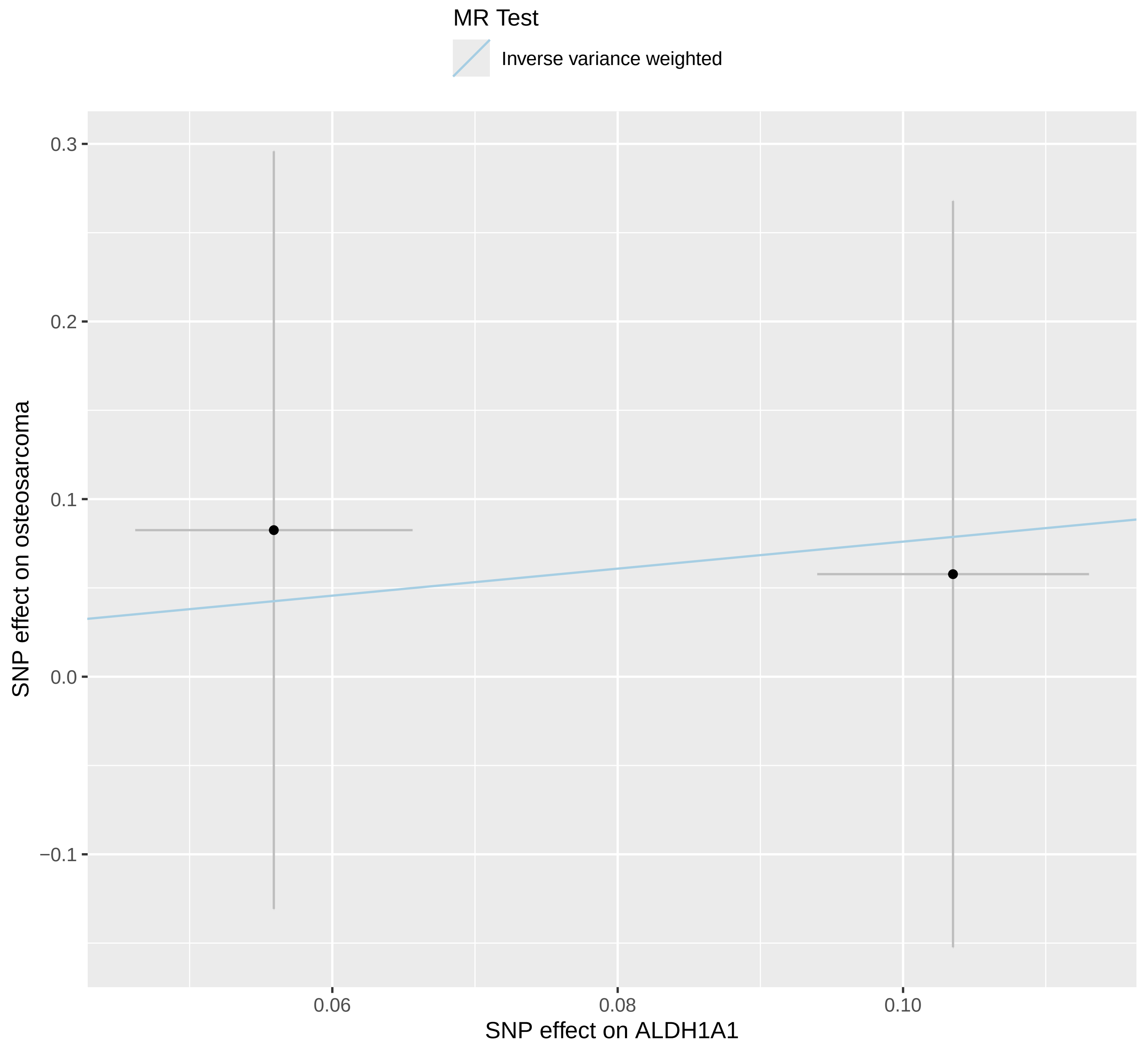

MR Test
Inverse variance weighted
0.3
0.2
0.1
0.0
−0.1
SNP effect on osteosarcoma
0.06
0.08
0.10
SNP effect on ALDH1A1

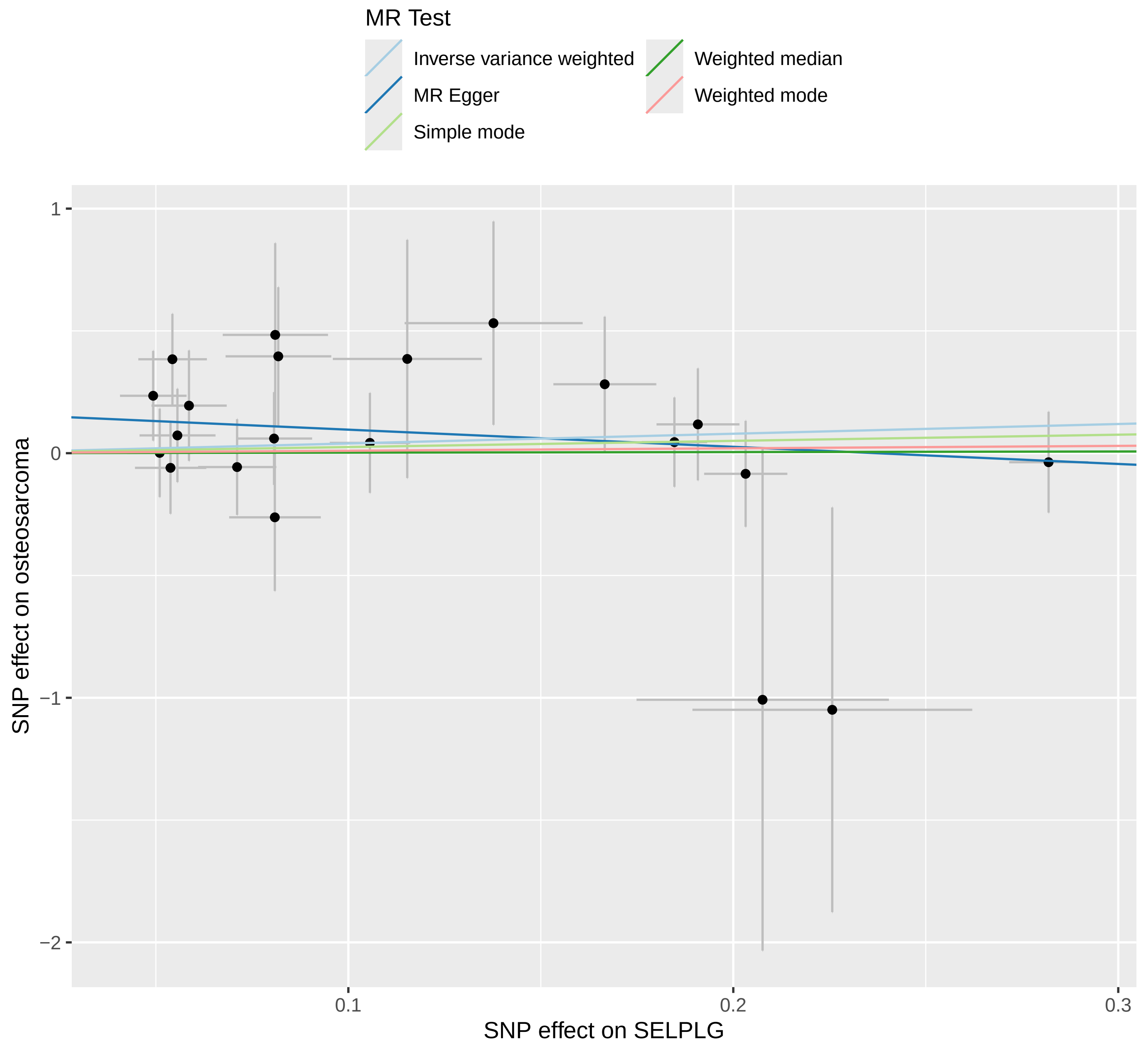

MR Test
Inverse variance weighted
MR Egger
Simple mode
Weighted median
Weighted mode
1
0
−1
−2
0.1
0.2
0.3
SNP effect on osteosarcoma
SNP effect on SELPLG

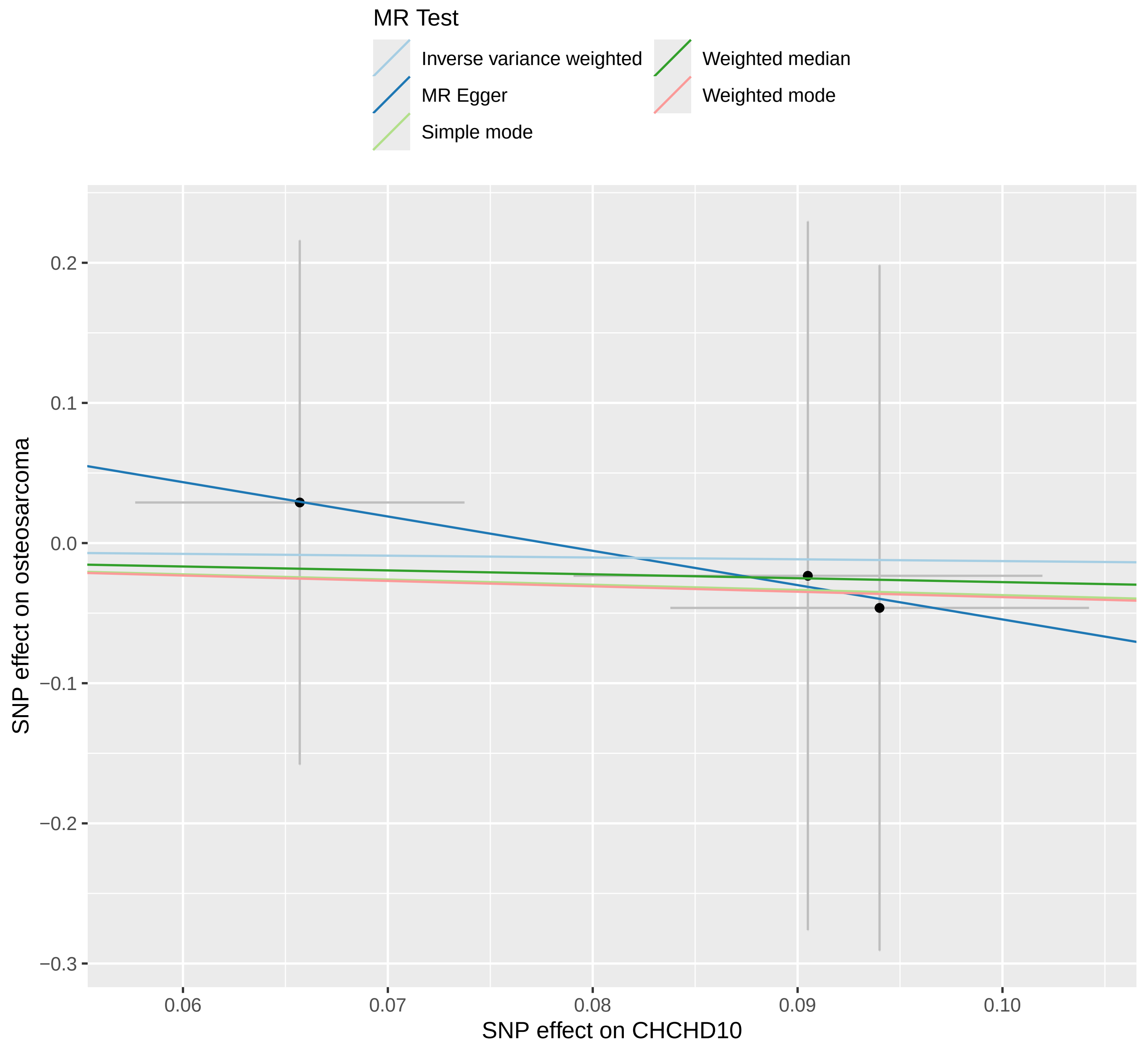

MR Test
Inverse variance weighted
MR Egger
Simple mode
Weighted median
Weighted mode
0.2
0.1
0.0
−0.1
−0.2
−0.3
SNP effect on osteosarcoma
0.06
0.07
0.08
0.09
0.10
SNP effect on CHCHD10

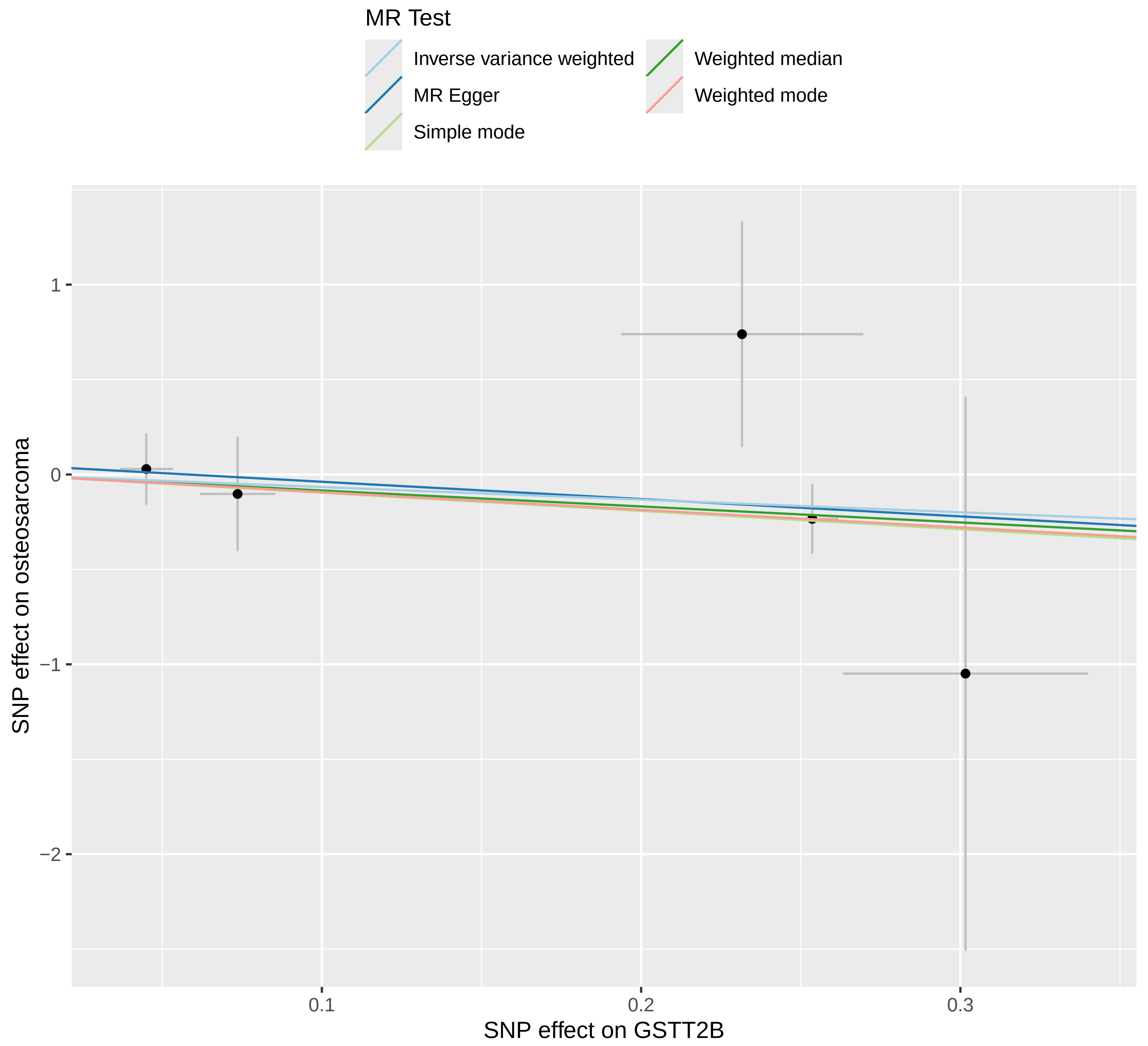

MR Test
Inverse variance weighted
MR Egger
Simple mode
Weighted median
Weighted mode
1
0
−1
−2
0.1
0.2
0.3
SNP effect on osteosarcoma
SNP effect on GSTT2B

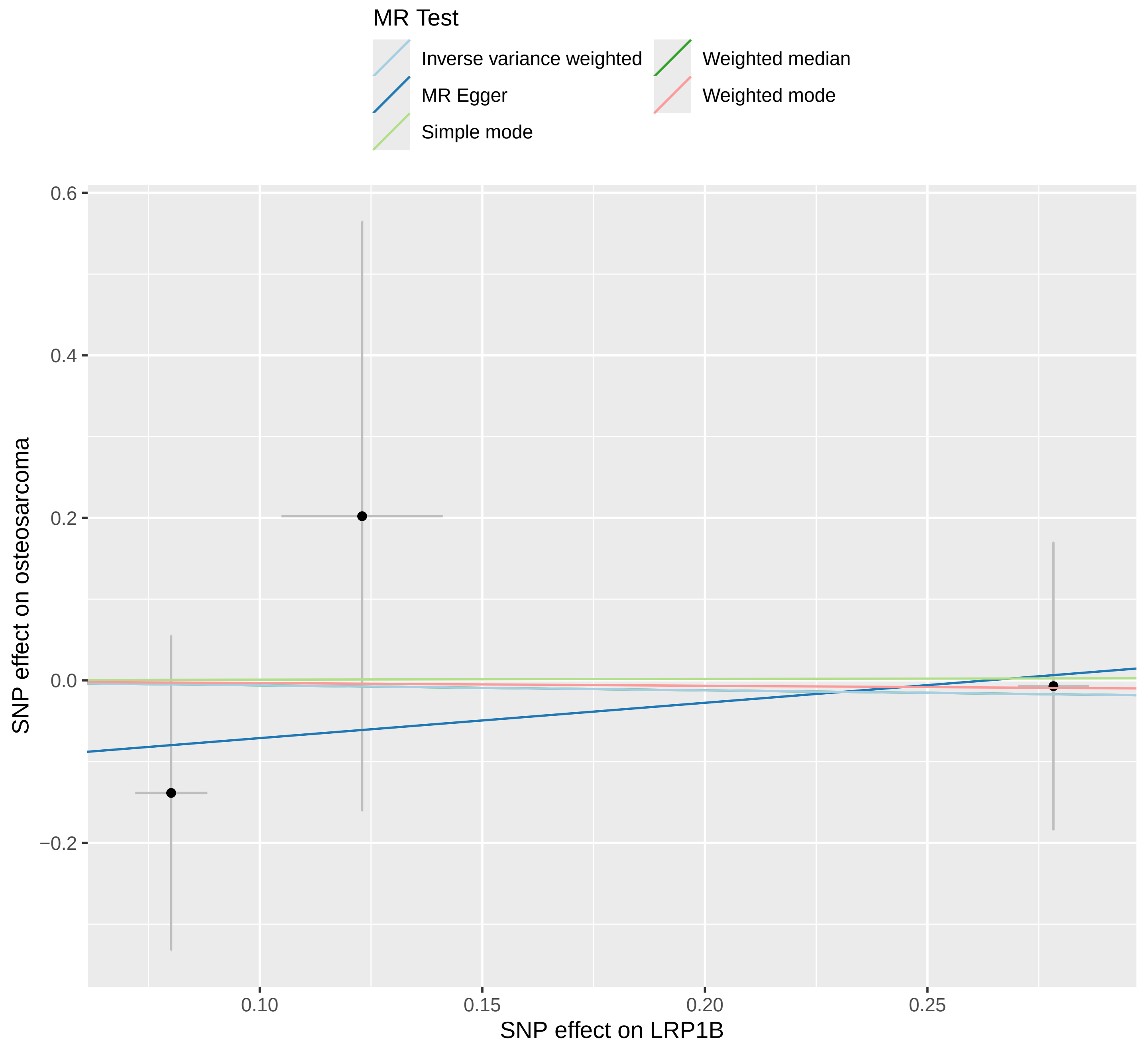
MR Test
Inverse variance weighted
MR Egger
Simple mode
Weighted median
Weighted mode
0.6
0.4
0.2
0.0
−0.2
0.10
0.15
0.20
0.25
SNP effect on osteosarcoma
SNP effect on LRP1B

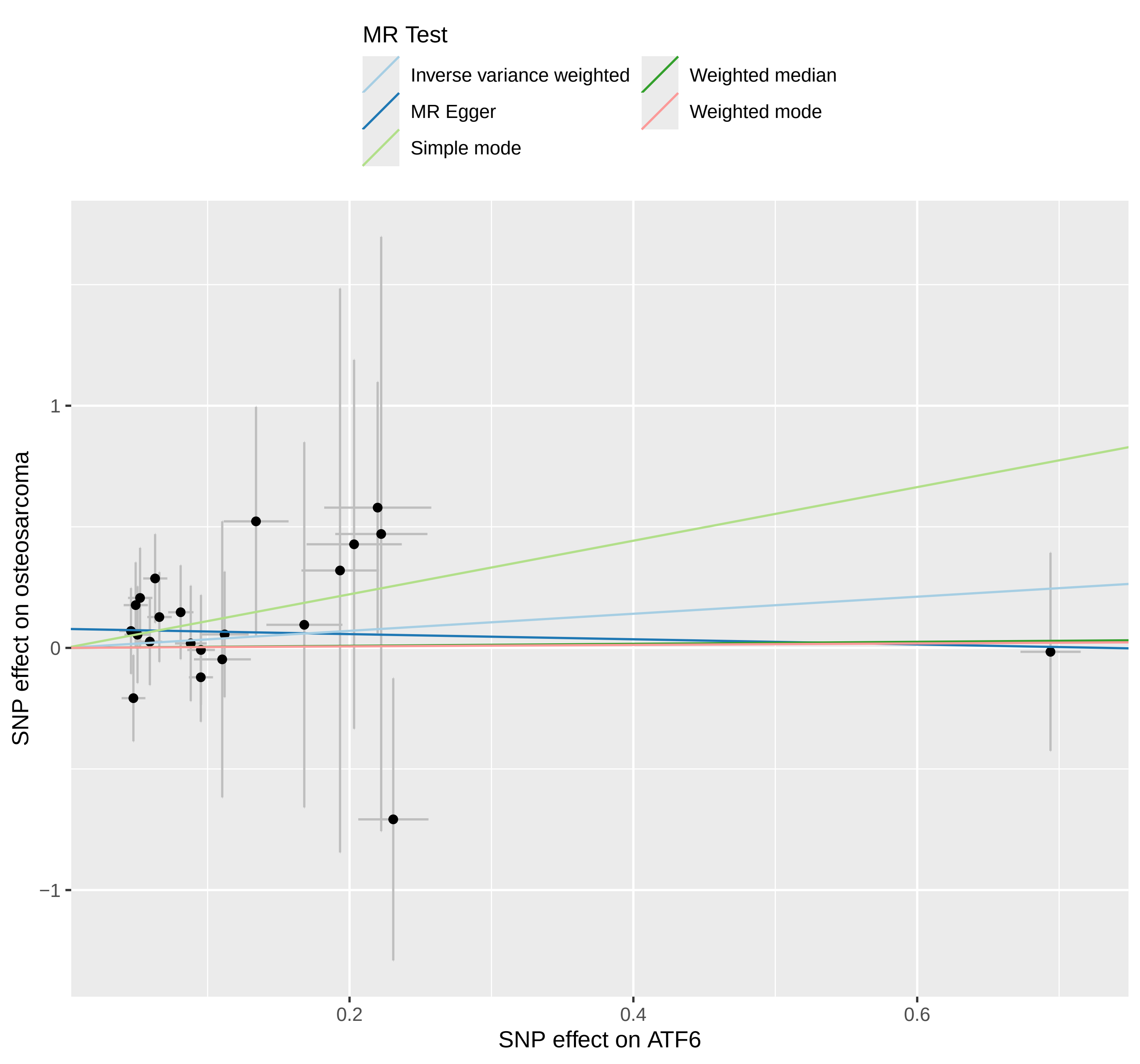

MR Test
Inverse variance weighted
MR Egger
Simple mode
Weighted median
Weighted mode
1
0
−1
0.2
0.4
0.6
SNP effect on osteosarcoma
SNP effect on ATF6

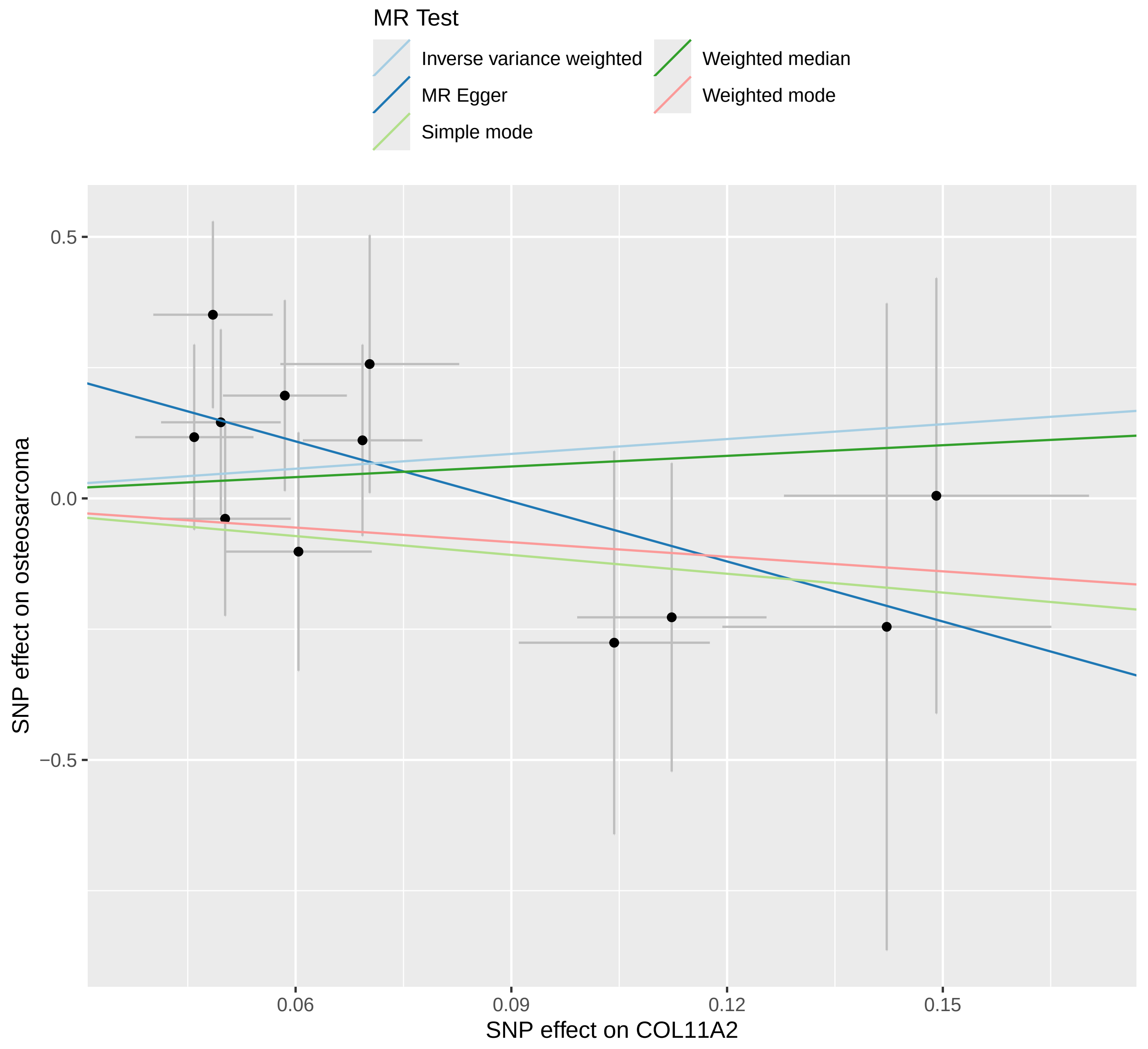

MR Test
Inverse variance weighted
MR Egger
Simple mode
Weighted median
Weighted mode
0.5
0.0
−0.5
0.06
0.09
0.12
0.15
SNP effect on osteosarcoma
SNP effect on COL11A2

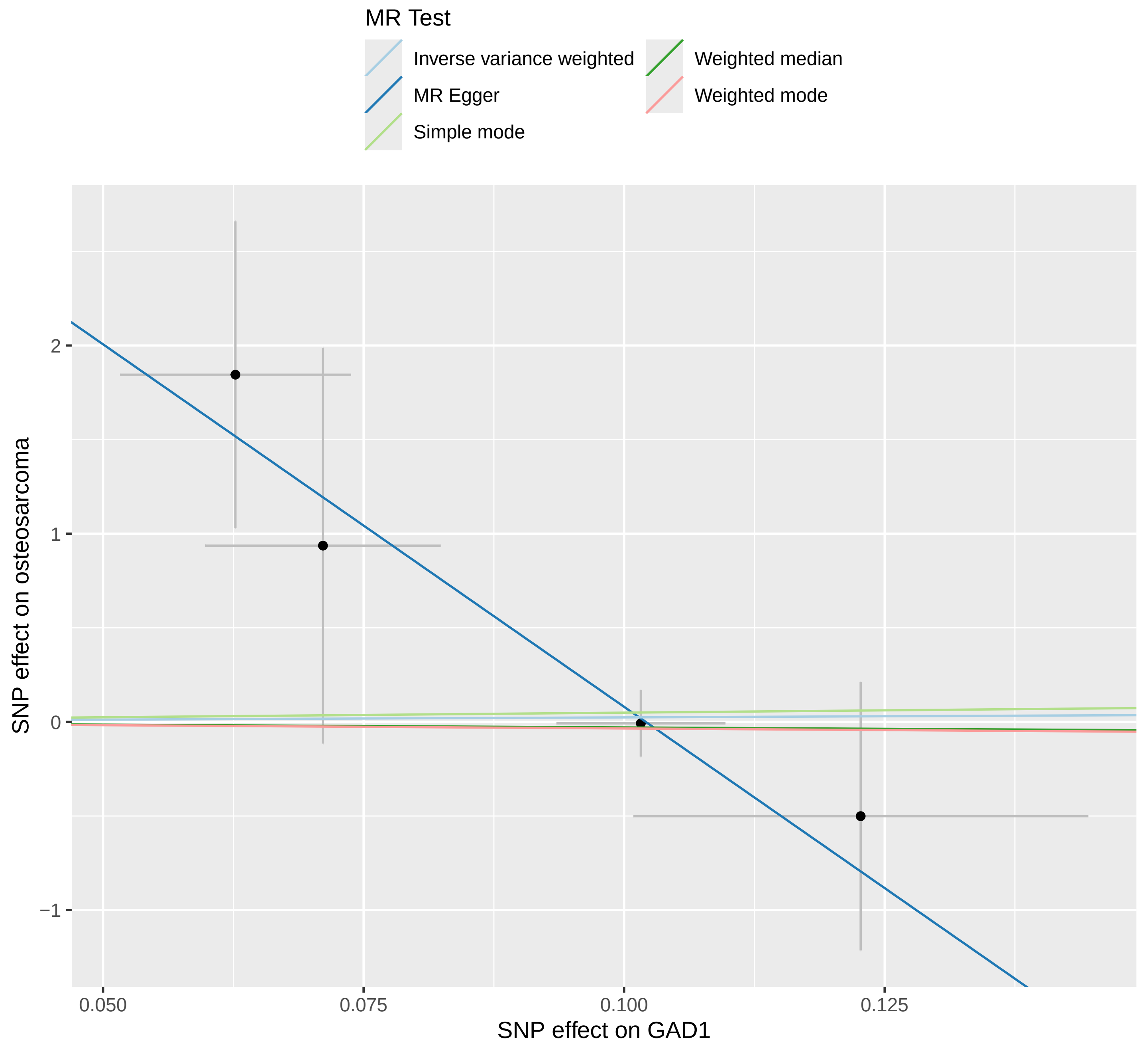
MR Test
Inverse variance weighted
MR Egger
Simple mode
Weighted median
Weighted mode
SNP effect on osteosarcoma
2
1
0
−1
0.050
0.075
0.100
0.125
SNP effect on GAD1

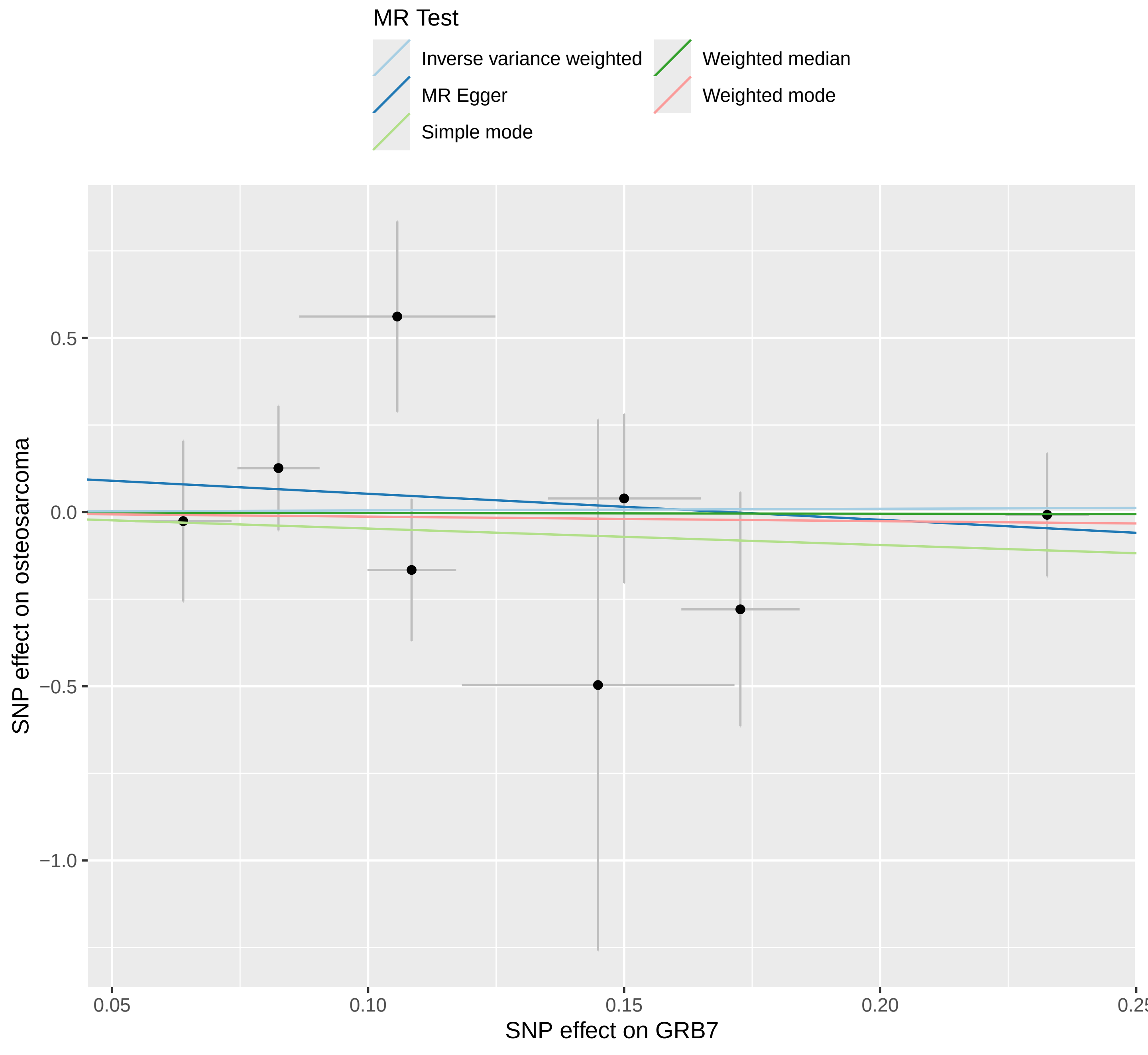

MR Test
Inverse variance weighted
MR Egger
Simple mode
Weighted median
Weighted mode
0.5
0.0
−0.5
−1.0
SNP effect on osteosarcoma
0.05
0.10
0.15
0.20
0.25
SNP effect on GRB7

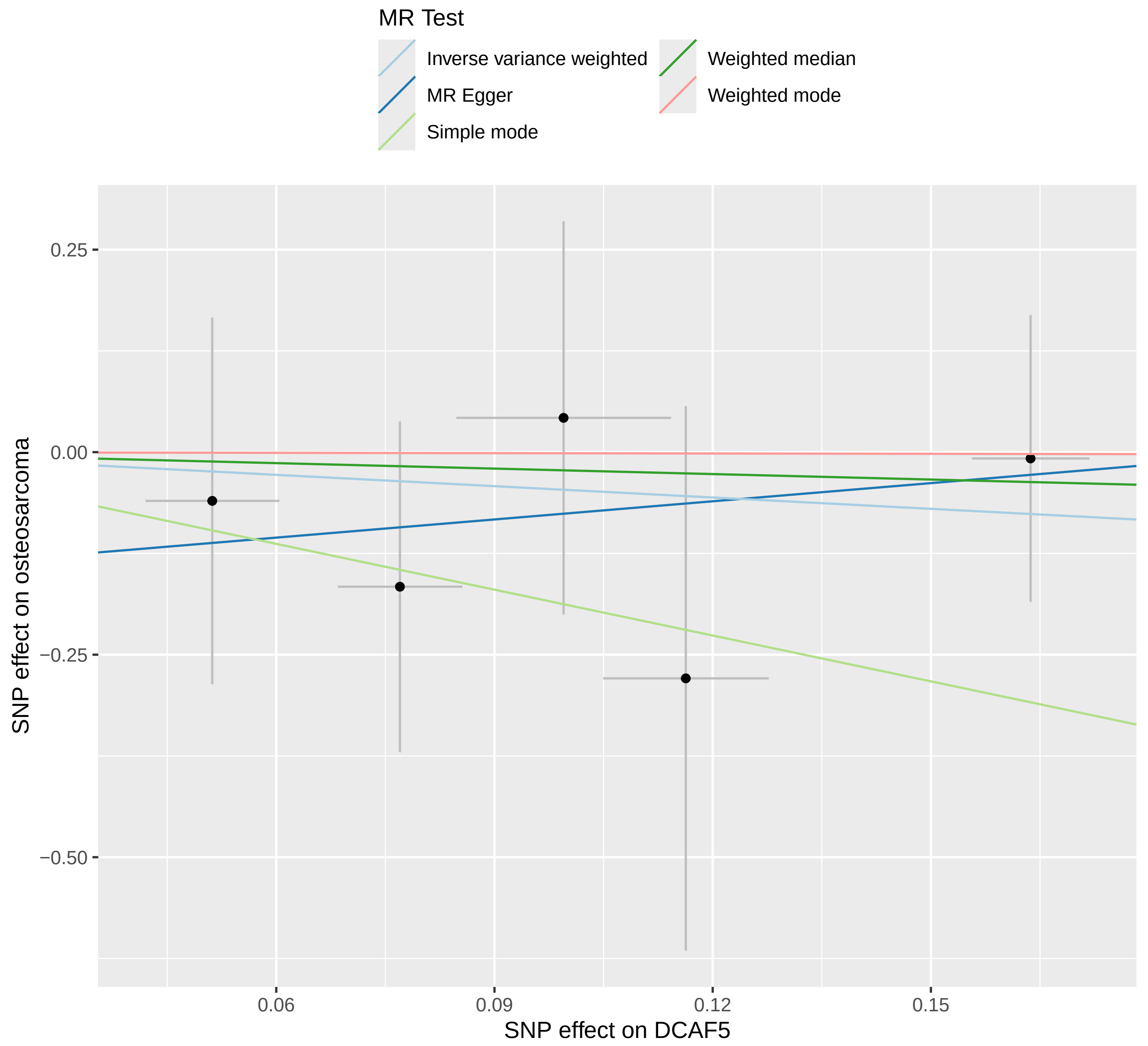
MR Test
Inverse variance weighted
MR Egger
Simple mode
Weighted median
Weighted mode
0.25
0.00
−0.25
−0.50
0.06
0.09
0.12
0.15
SNP effect on DCAF5
SNP effect on osteosarcoma

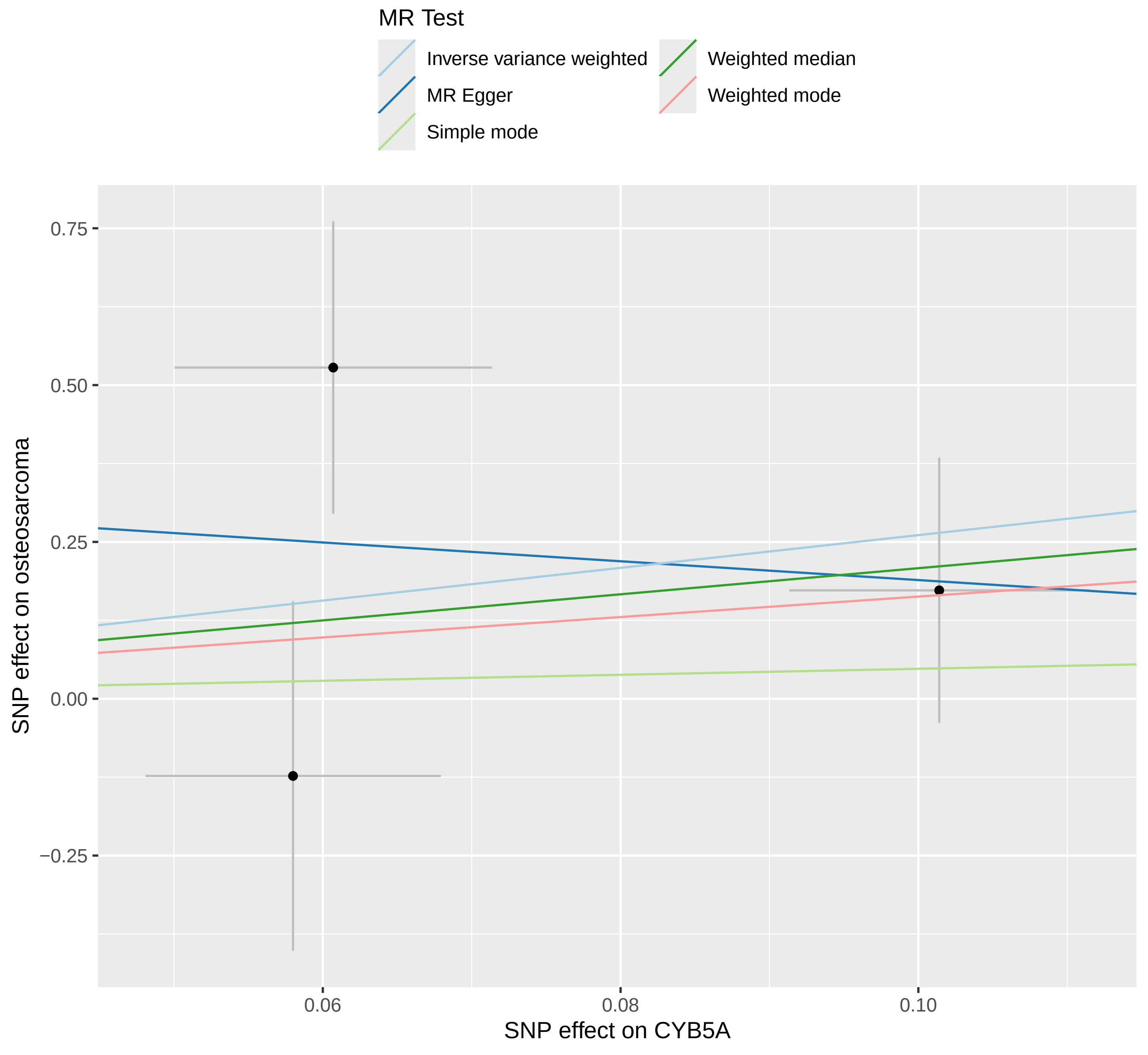
MR Test
Inverse variance weighted
MR Egger
Simple mode
Weighted median
Weighted mode
0.75
0.50
0.25
0.00
−0.25
SNP effect on osteosarcoma
0.06
0.08
0.10
SNP effect on CYB5A

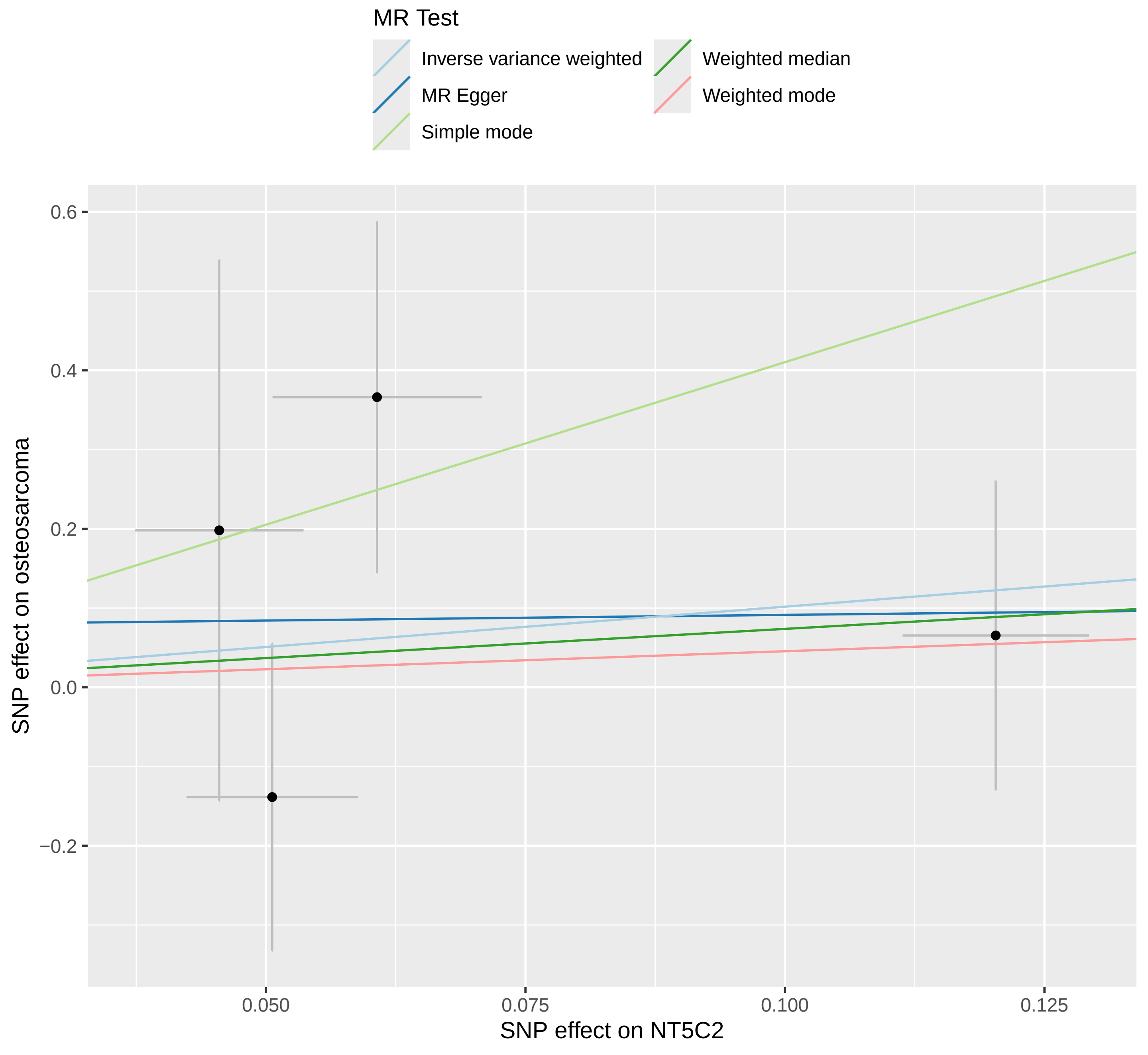

MR Test
Inverse variance weighted
MR Egger
Simple mode
Weighted median
Weighted mode
0.6
0.4
0.2
0.0
−0.2
SNP effect on osteosarcoma
0.050
0.075
0.100
0.125
SNP effect on NT5C2

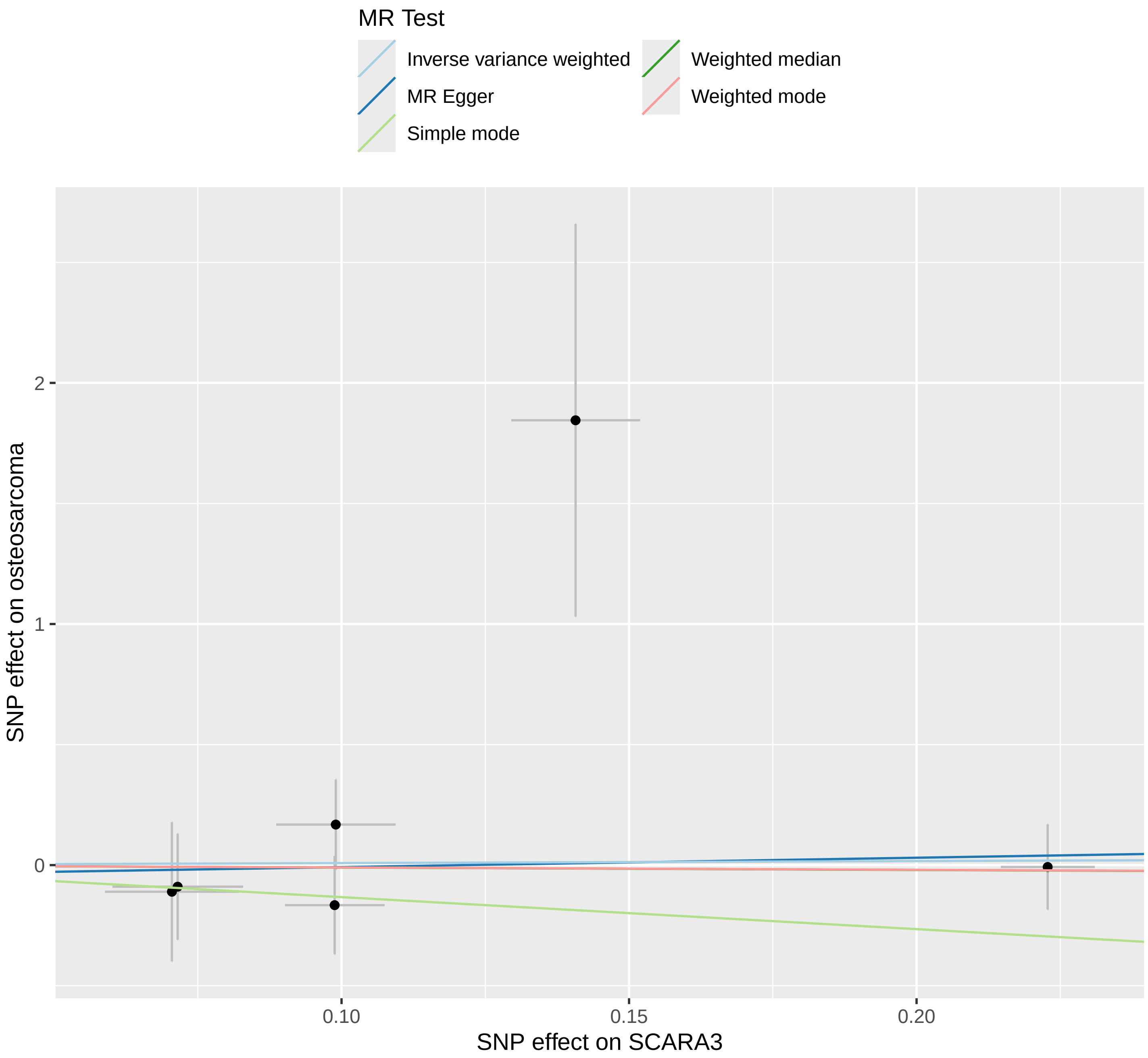
MR Test
Inverse variance weighted
MR Egger
Simple mode
Weighted median
Weighted mode
2
1
0
SNP effect on osteosarcoma
0.10
0.15
0.20
SNP effect on SCARA3

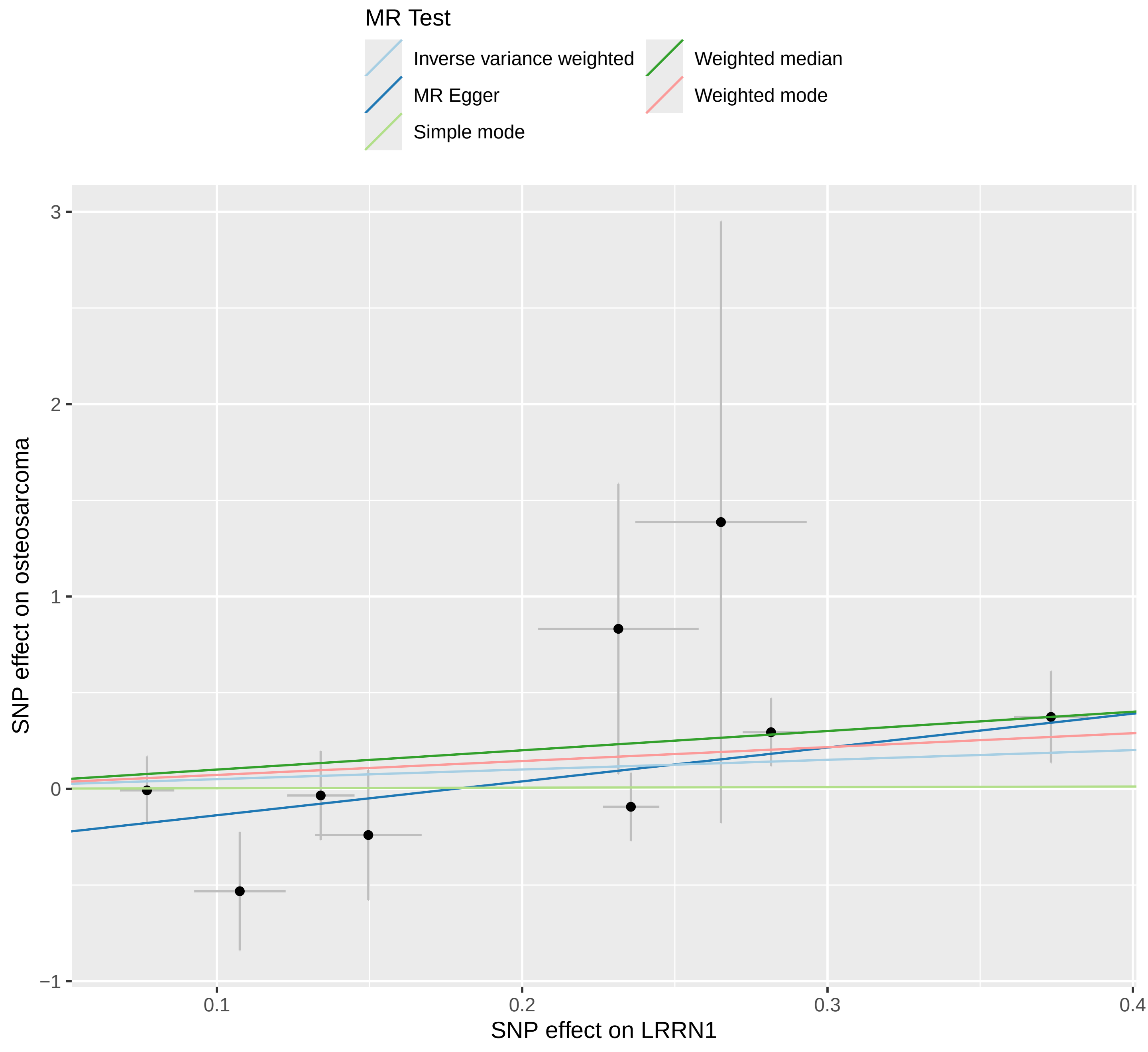

MR Test
Inverse variance weighted
MR Egger
Simple mode
Weighted median
Weighted mode
3
2
1
0
−1
0.1
0.2
0.3
0.4
SNP effect on osteosarcoma
SNP effect on LRRN1

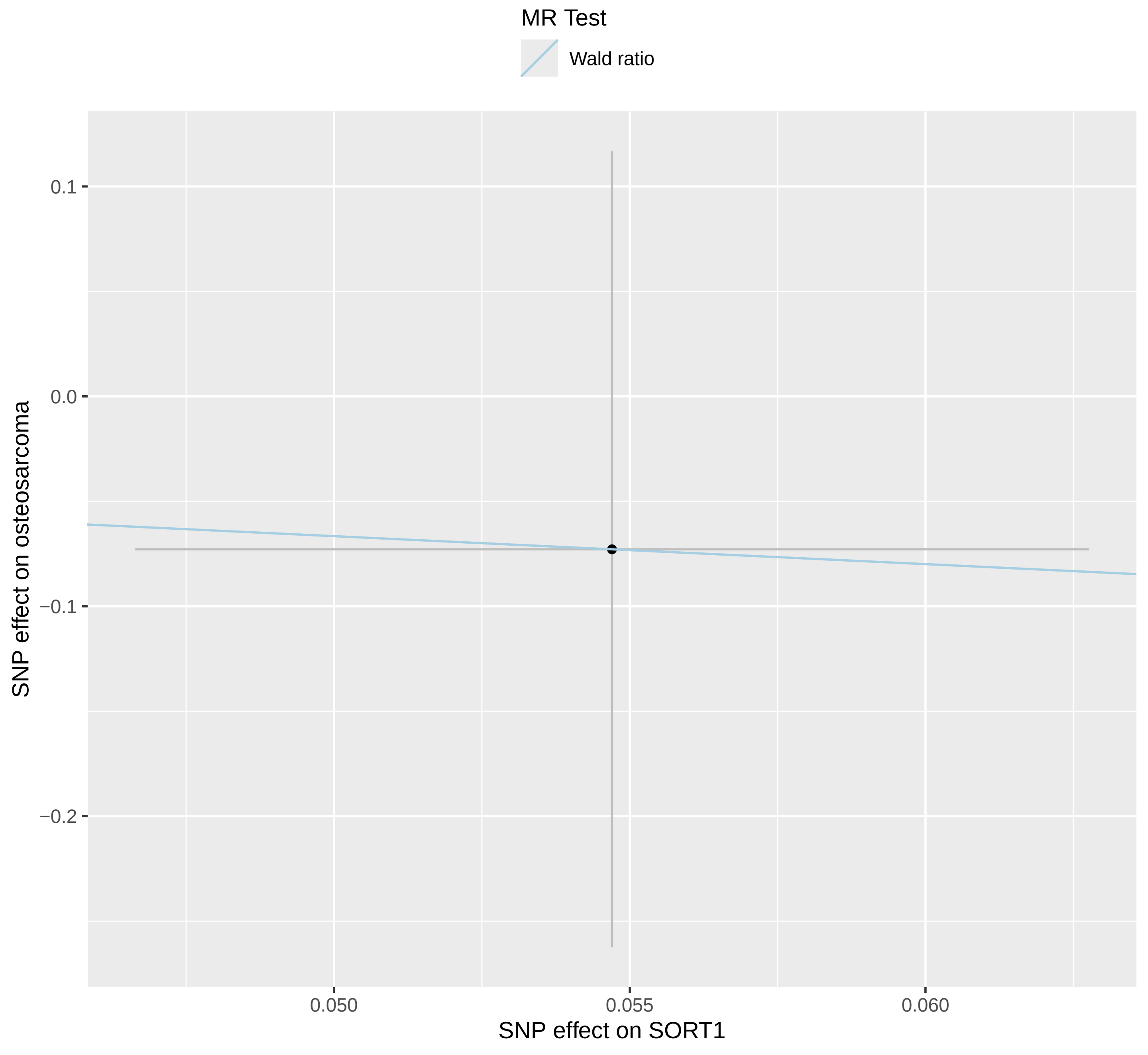

MR Test
Wald ratio
0.1
0.0
−0.1
−0.2
SNP effect on osteosarcoma
0.050
0.055
0.060
SNP effect on SORT1

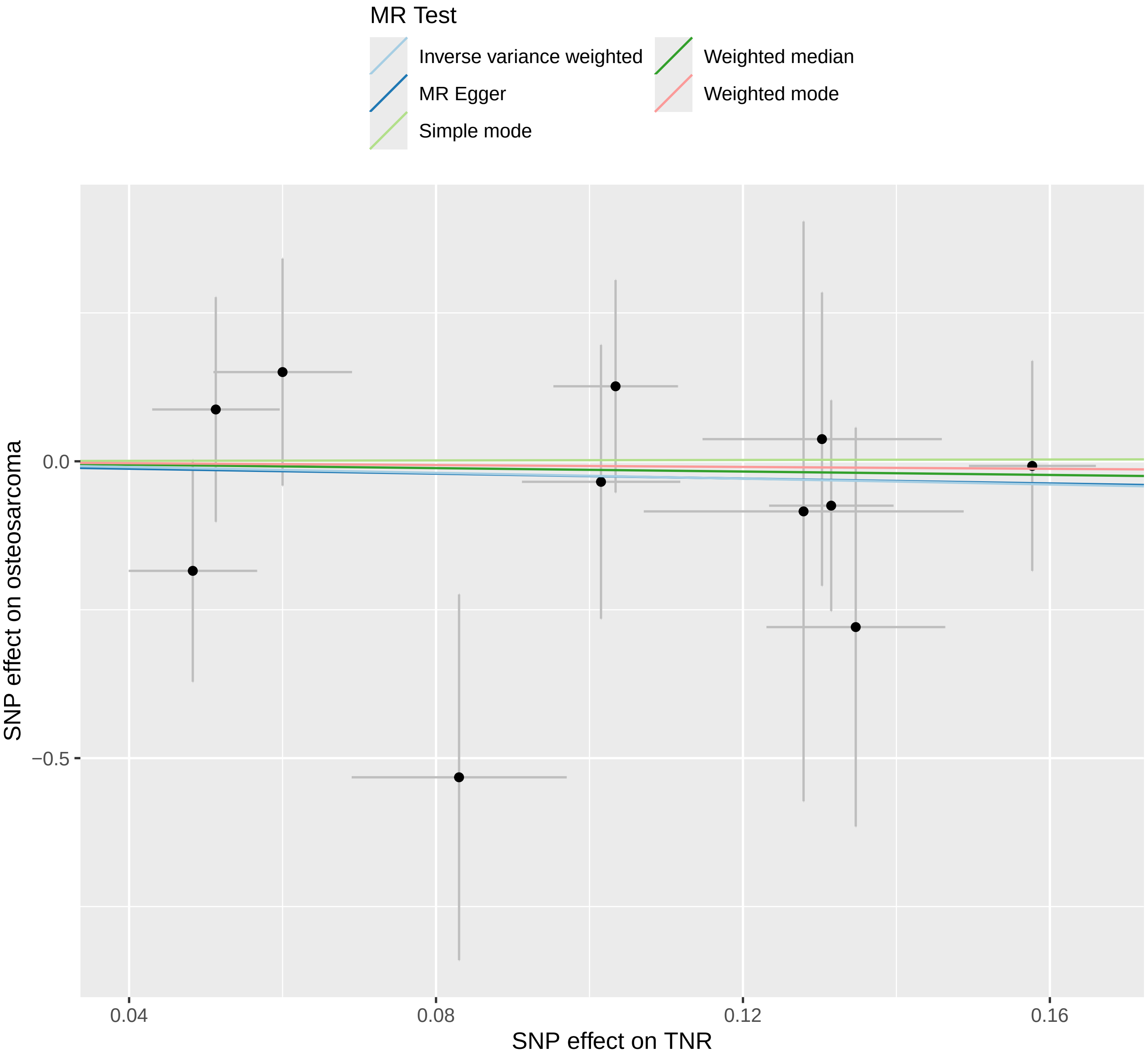

MR Test
Inverse variance weighted
MR Egger
Simple mode
Weighted median
Weighted mode
0.0
−0.5
SNP effect on osteosarcoma
0.04
0.08
0.12
0.16
SNP effect on TNR

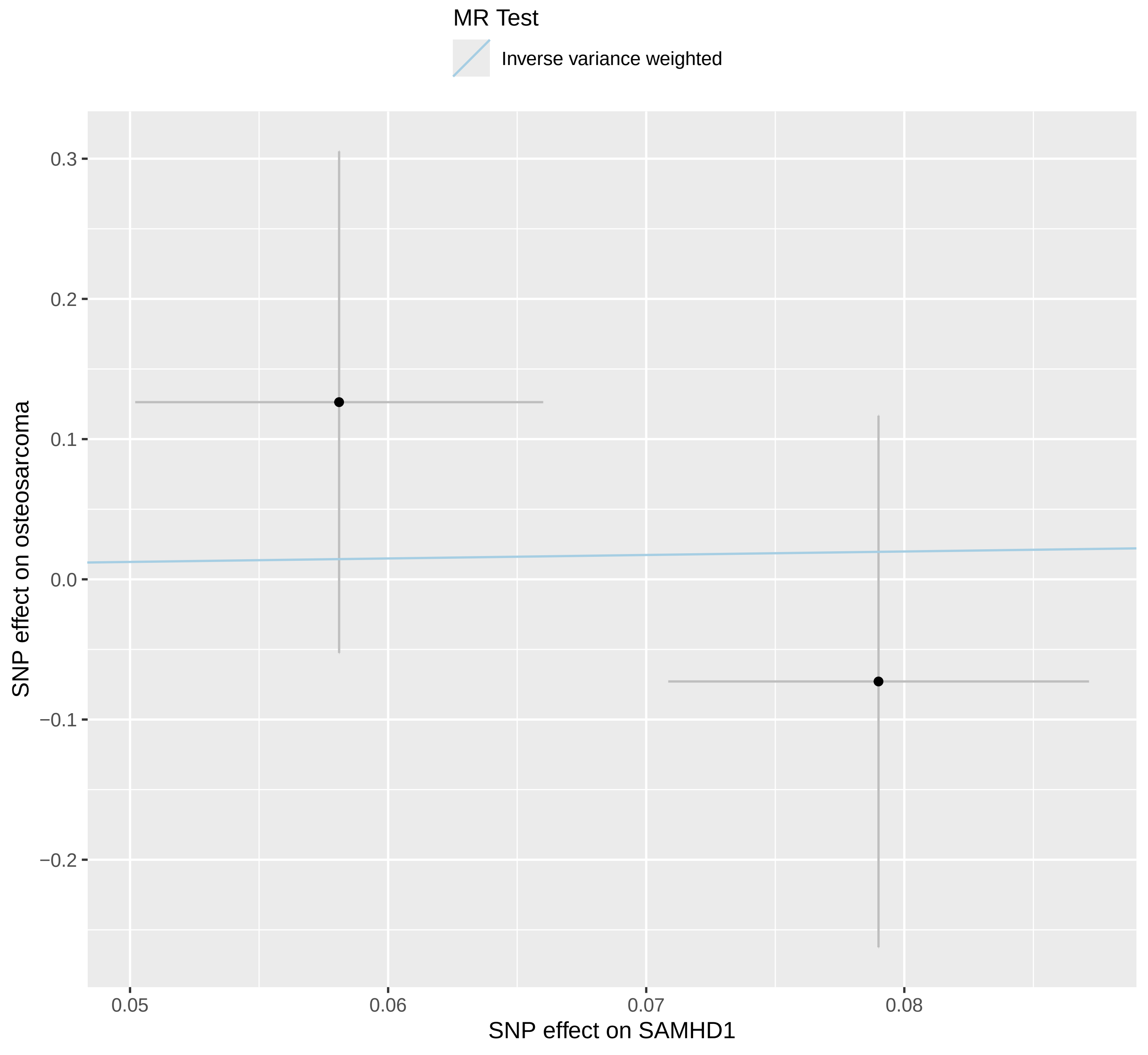

MR Test
Inverse variance weighted
0.3
0.2
0.1
0.0
−0.1
−0.2
SNP effect on osteosarcoma
0.05
0.06
0.07
0.08
SNP effect on SAMHD1

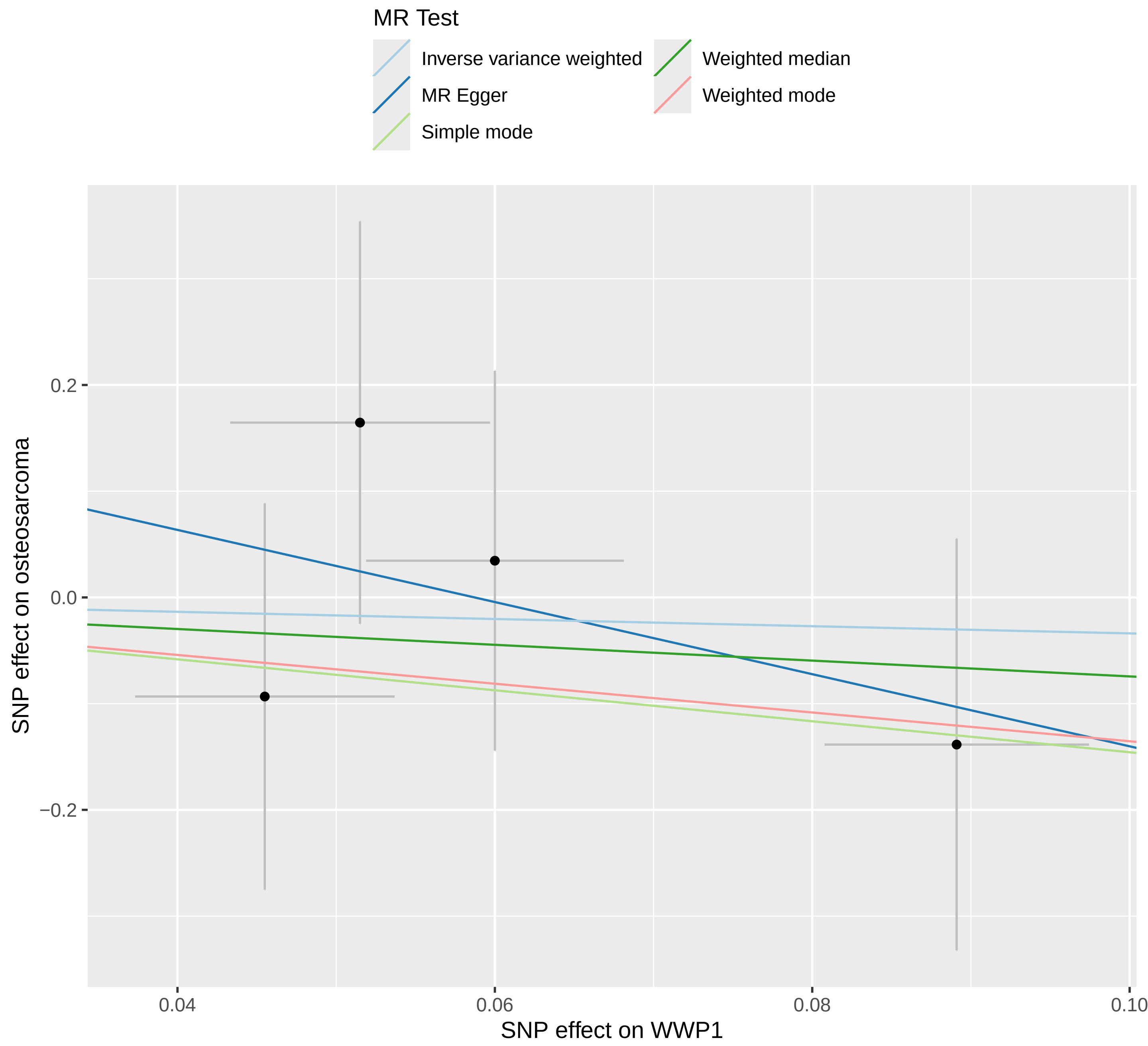
MR Test
Inverse variance weighted
MR Egger
Simple mode
Weighted median
Weighted mode
0.2
0.0
−0.2
0.04
0.06
0.08
0.10
SNP effect on osteosarcoma
SNP effect on WWP1

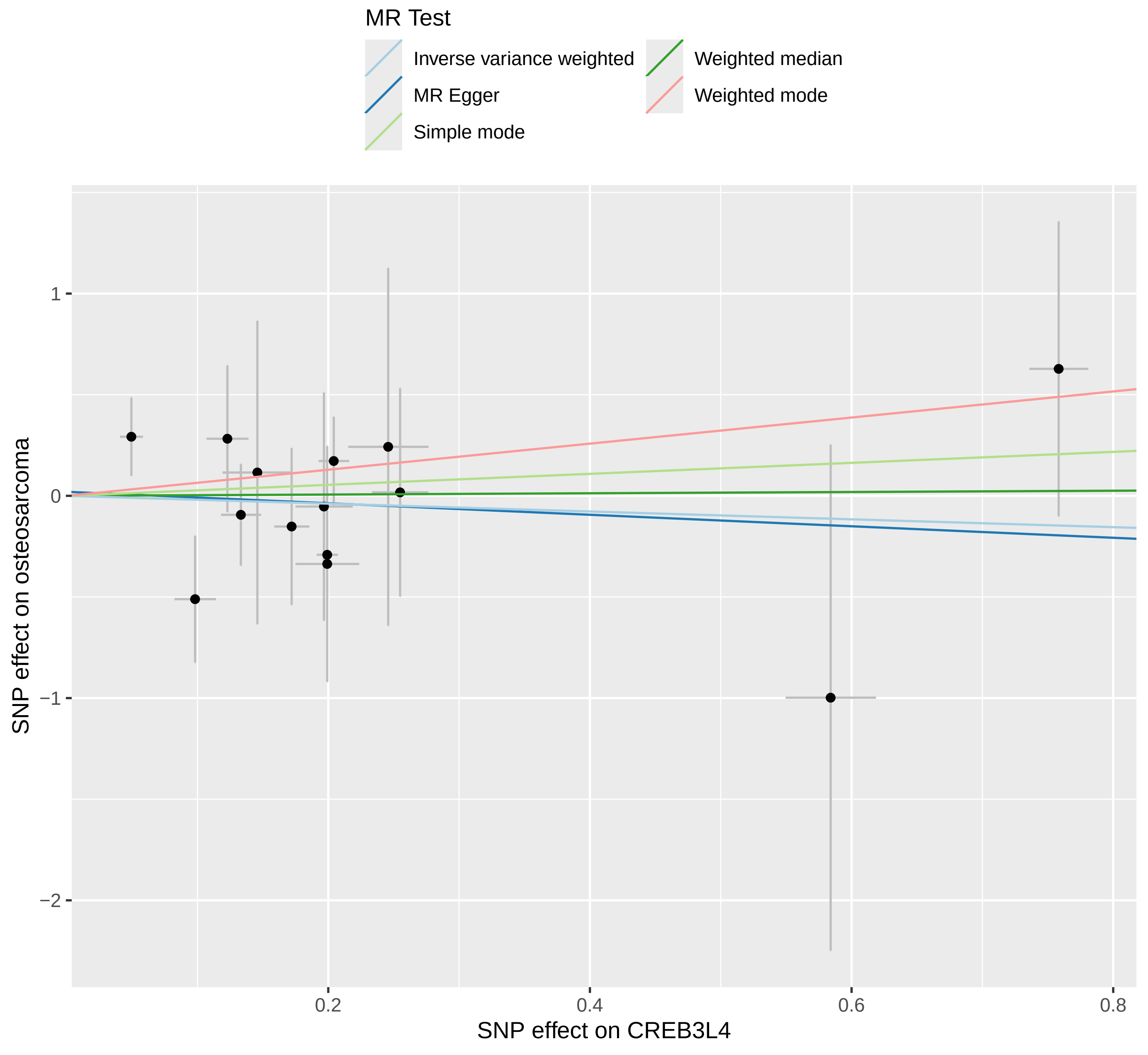
MR Test
Inverse variance weighted
MR Egger
Simple mode
Weighted median
Weighted mode
SNP effect on osteosarcoma
1
0
−1
−2
0.2
0.4
0.6
0.8
SNP effect on CREB3L4

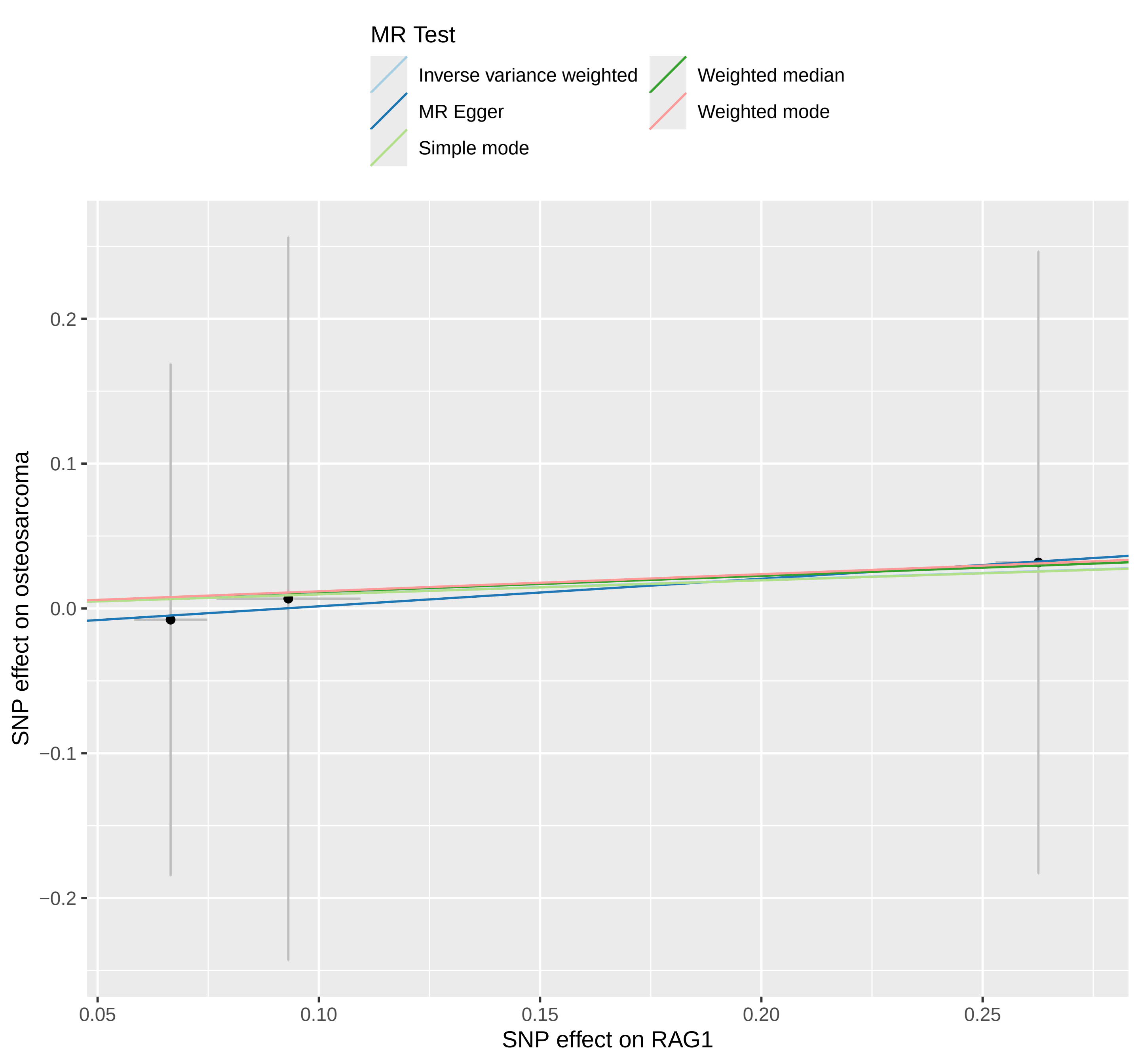
MR Test
Inverse variance weighted
MR Egger
Simple mode
Weighted median
Weighted mode
0.2
0.1
0.0
−0.1
−0.2
SNP effect on osteosarcoma
0.05
0.10
0.15
0.20
0.25
SNP effect on RAG1

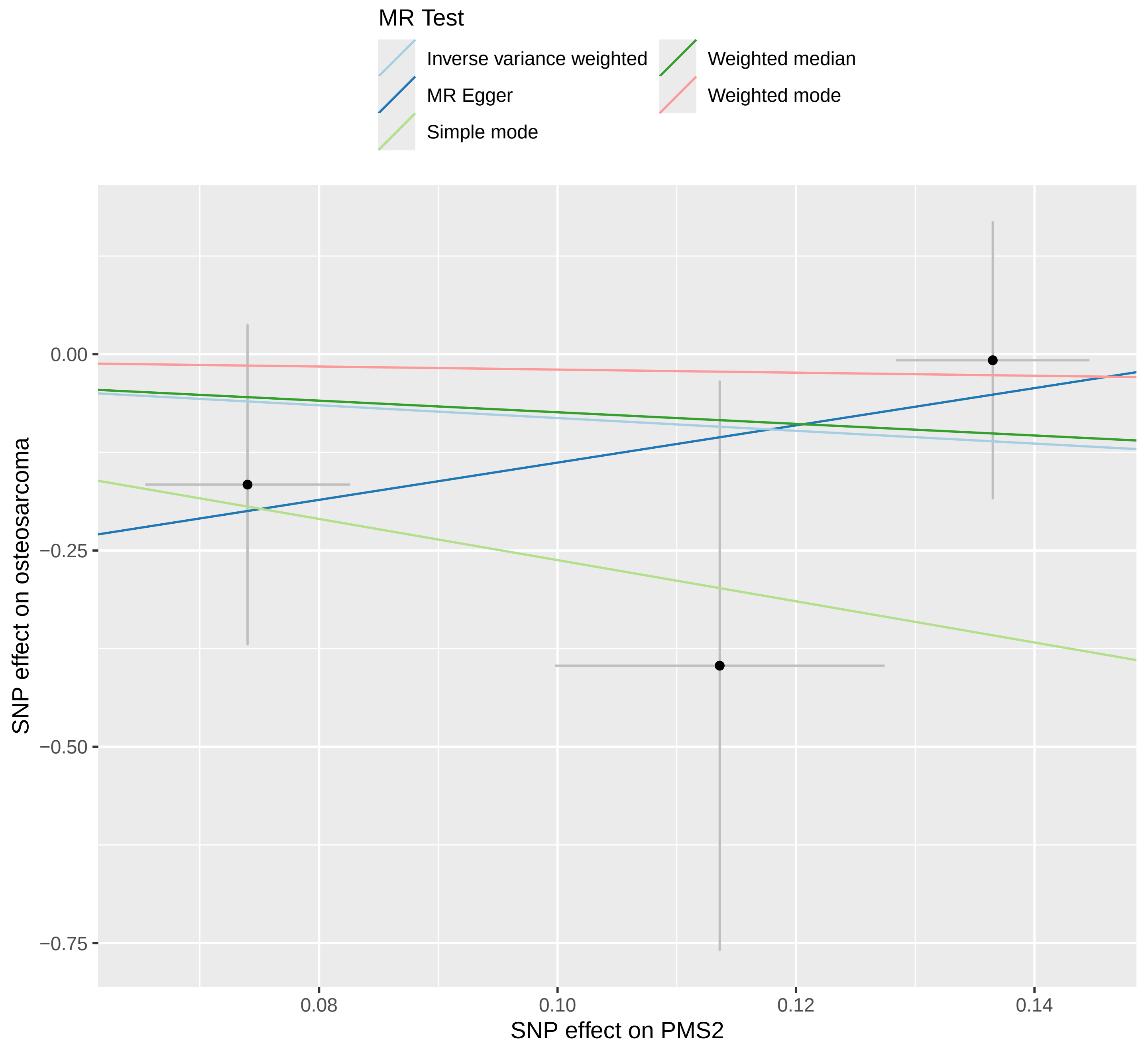
MR Test
Inverse variance weighted
MR Egger
Simple mode
Weighted median
Weighted mode
0.00
−0.25
−0.50
−0.75
0.08
0.10
0.12
0.14
SNP effect on osteosarcoma
SNP effect on PMS2

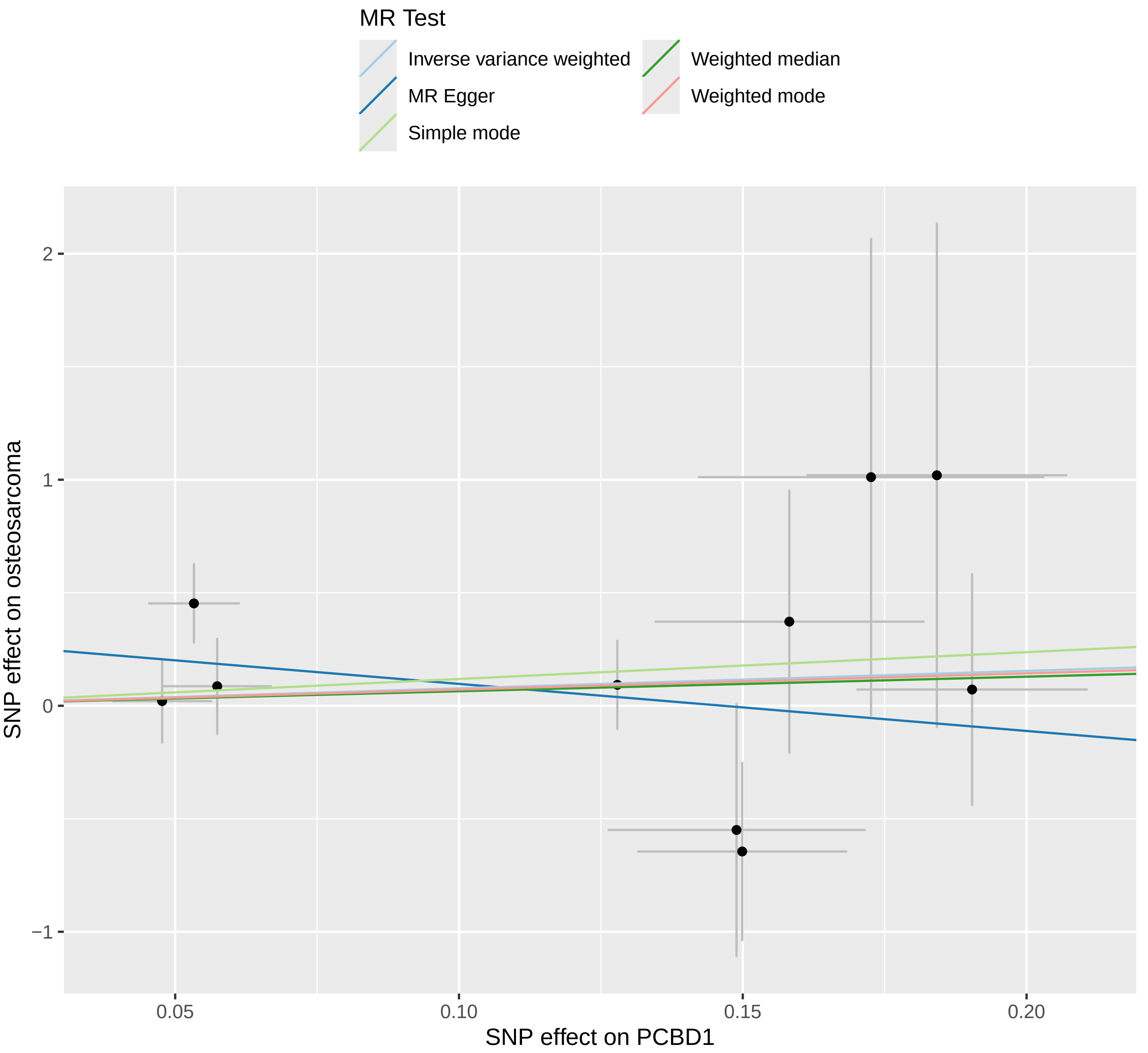
MR Test
Inverse variance weighted
MR Egger
Simple mode
Weighted median
Weighted mode
2
1
0
−1
0.05
0.10
0.15
0.20
SNP effect on osteosarcoma
SNP effect on PCBD1

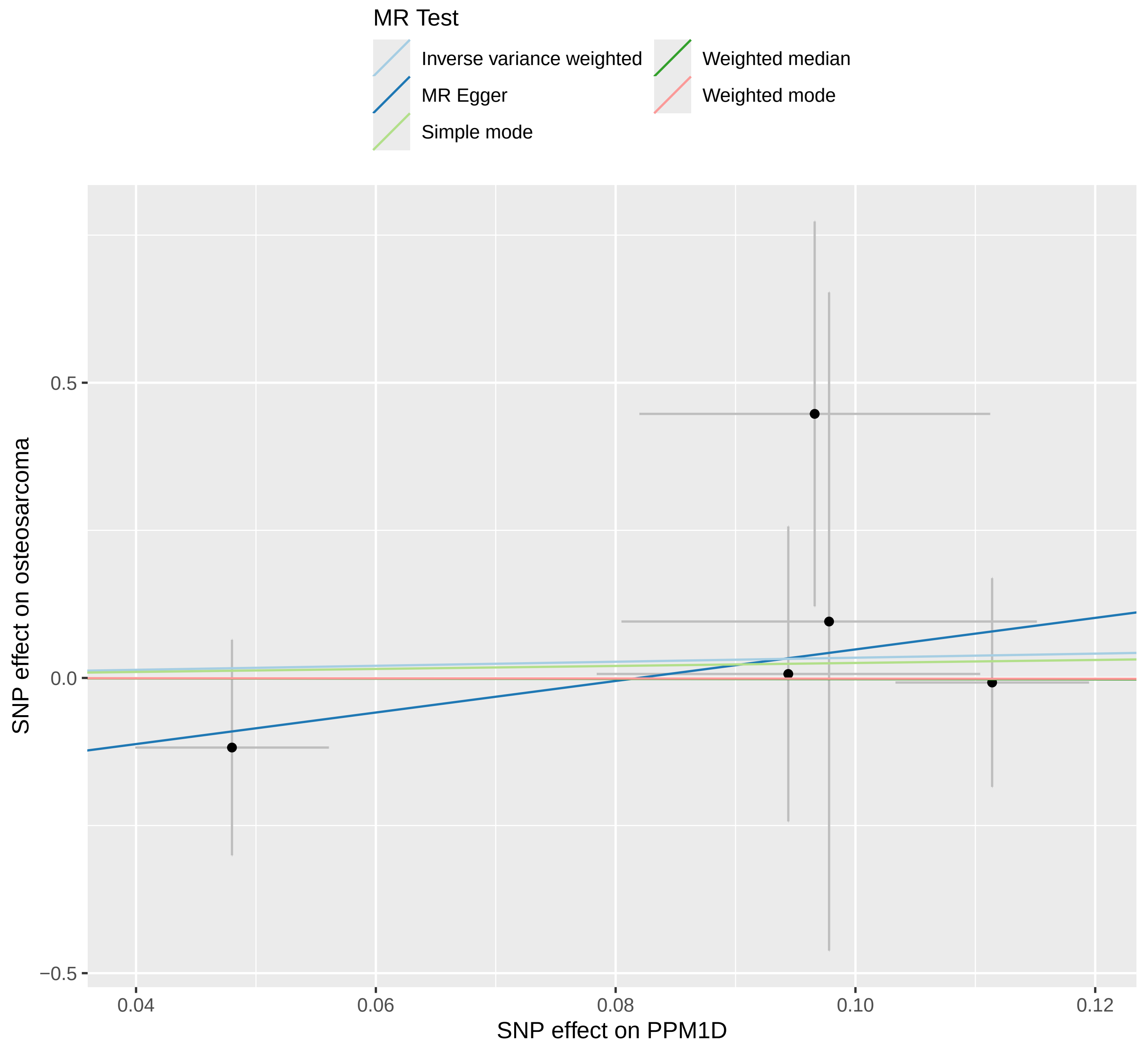

MR Test
Inverse variance weighted
MR Egger
Simple mode
Weighted median
Weighted mode
0.5
0.0
−0.5
0.04
0.06
0.08
0.10
0.12
SNP effect on PPM1D
SNP effect on osteosarcoma

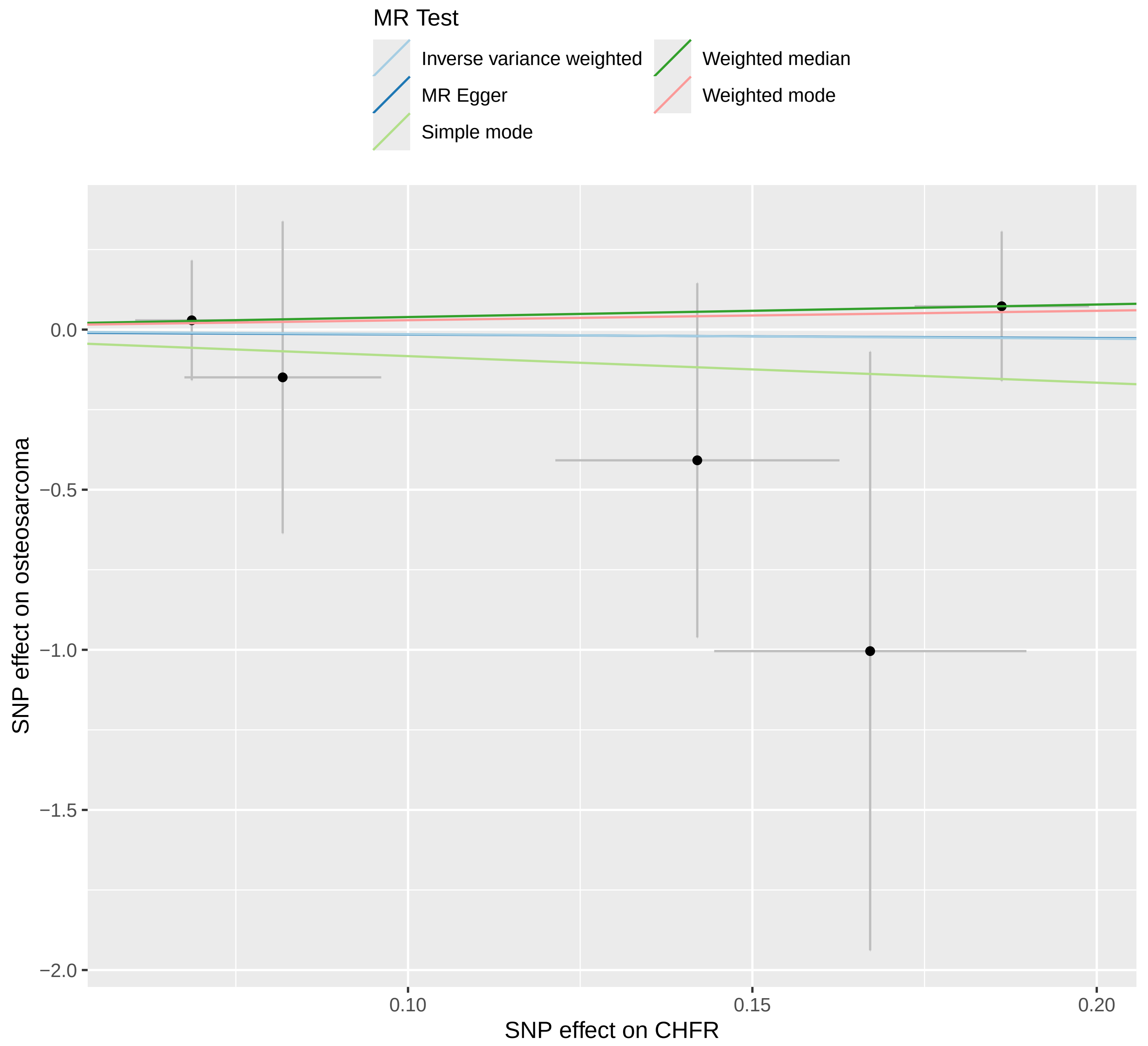

MR Test
Inverse variance weighted
MR Egger
Simple mode
Weighted median
Weighted mode
0.0
−0.5
−1.0
−1.5
−2.0
0.10
0.15
0.20
SNP effect on osteosarcoma
SNP effect on CHFR

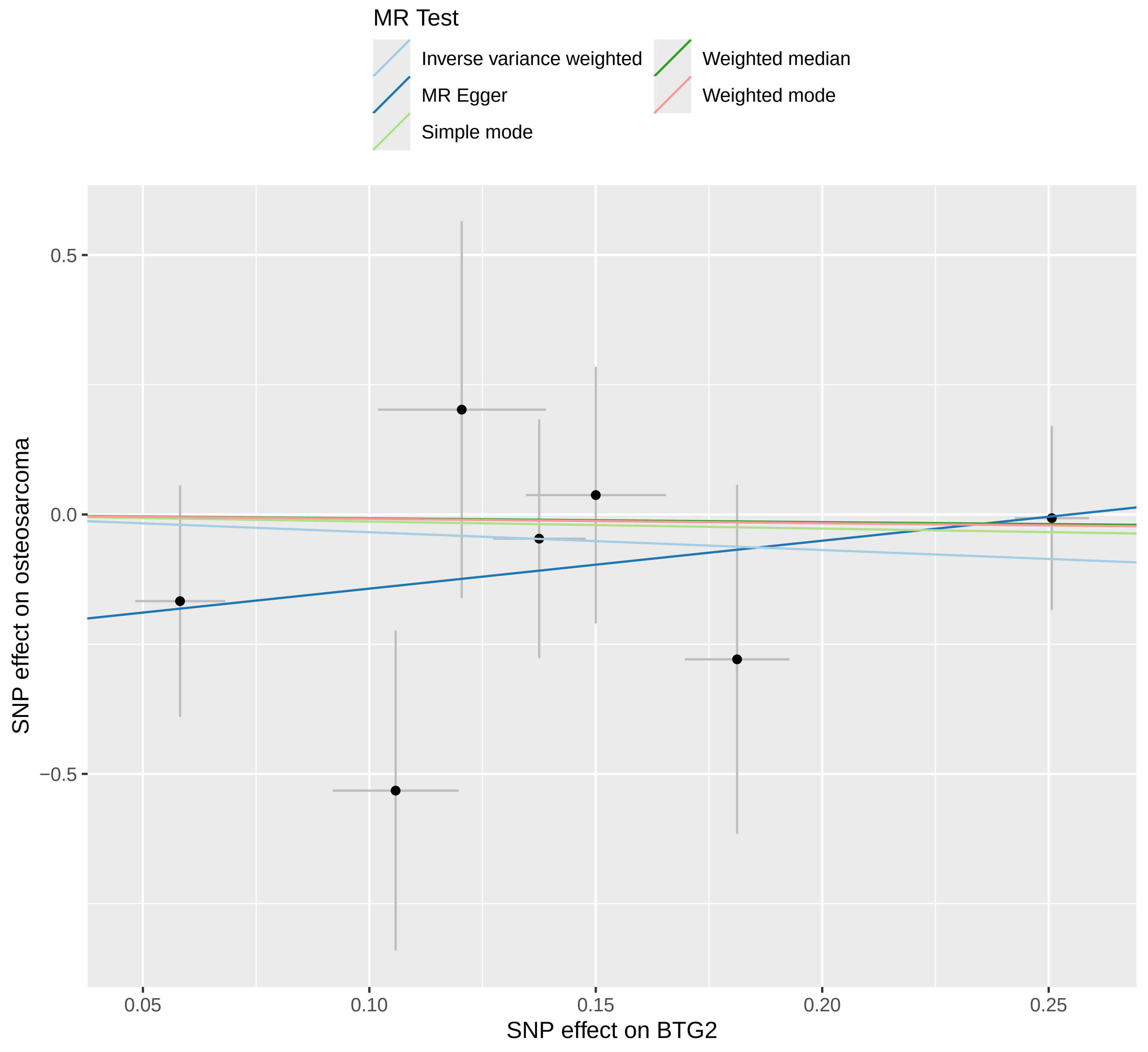
MR Test
Inverse variance weighted
MR Egger
Simple mode
Weighted median
Weighted mode
0.5
0.0
−0.5
0.05
0.10
0.15
0.20
0.25
SNP effect on BTG2
SNP effect on osteosarcoma

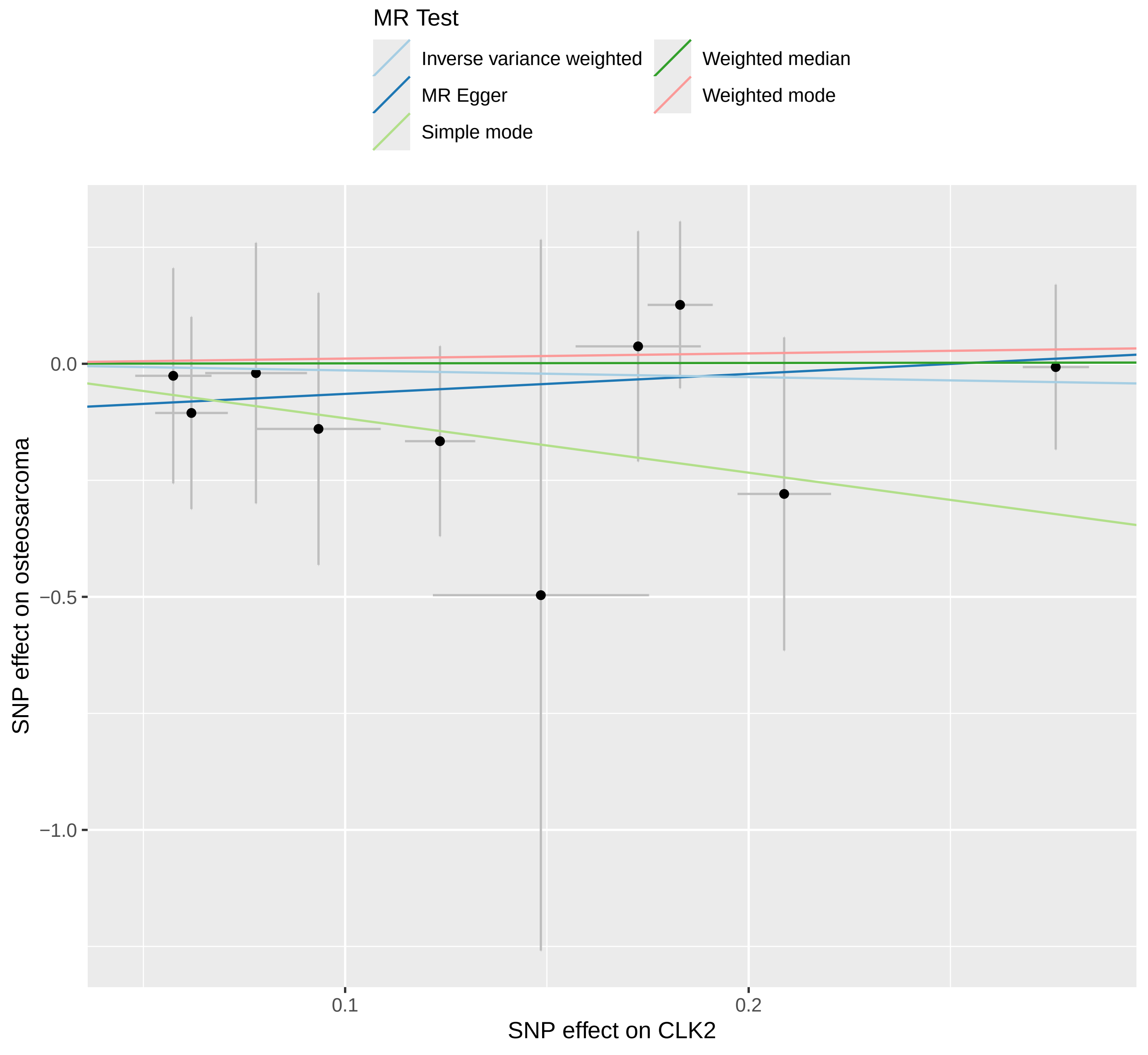
MR Test
Inverse variance weighted
MR Egger
Simple mode
Weighted median
Weighted mode
0.0
−0.5
−1.0
0.1
0.2
SNP effect on osteosarcoma
SNP effect on CLK2

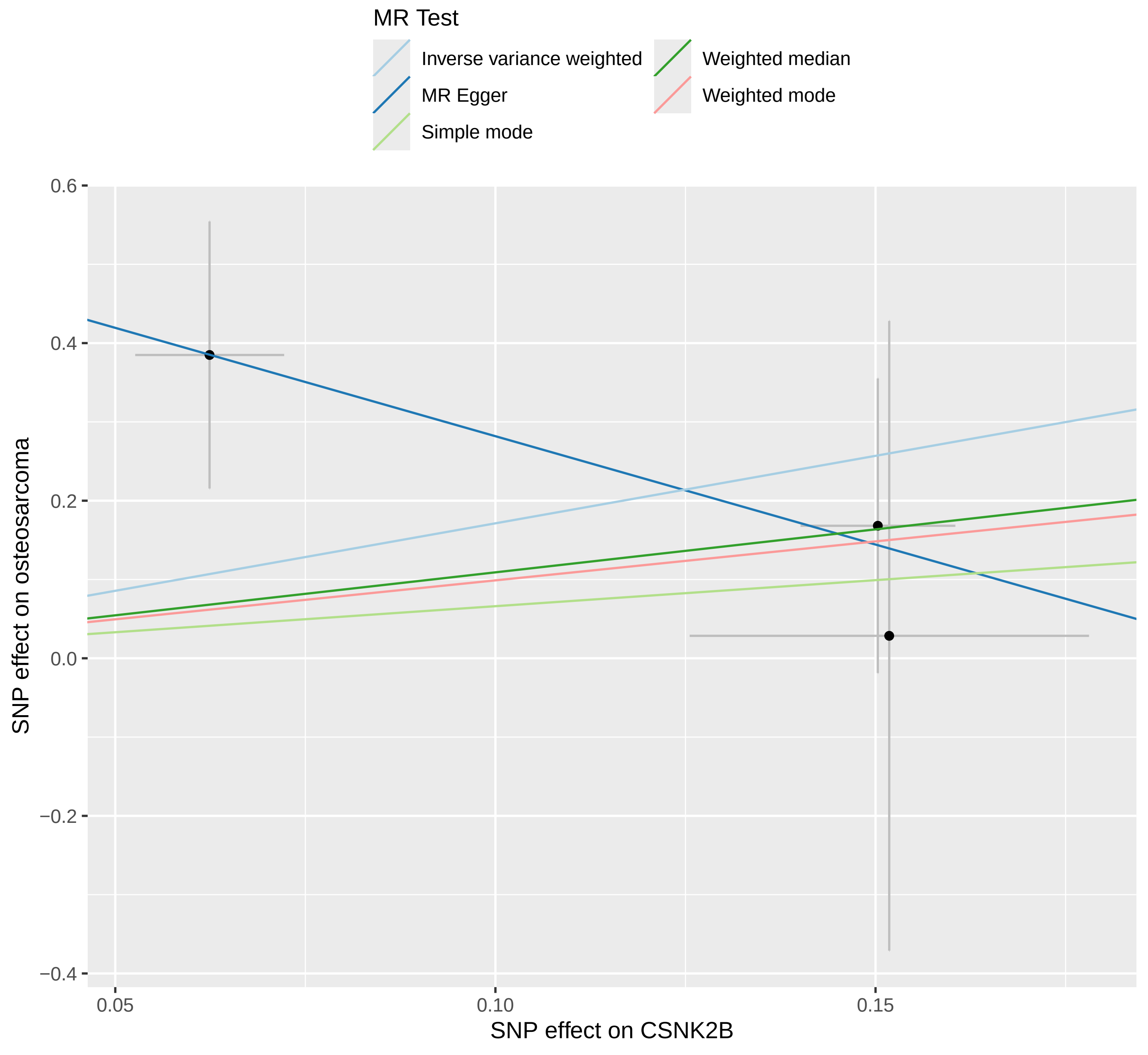
MR Test
Inverse variance weighted
MR Egger
Simple mode
Weighted median
Weighted mode
0.6
0.4
0.2
0.0
−0.2
−0.4
0.05
0.10
0.15
SNP effect on osteosarcoma
SNP effect on CSNK2B

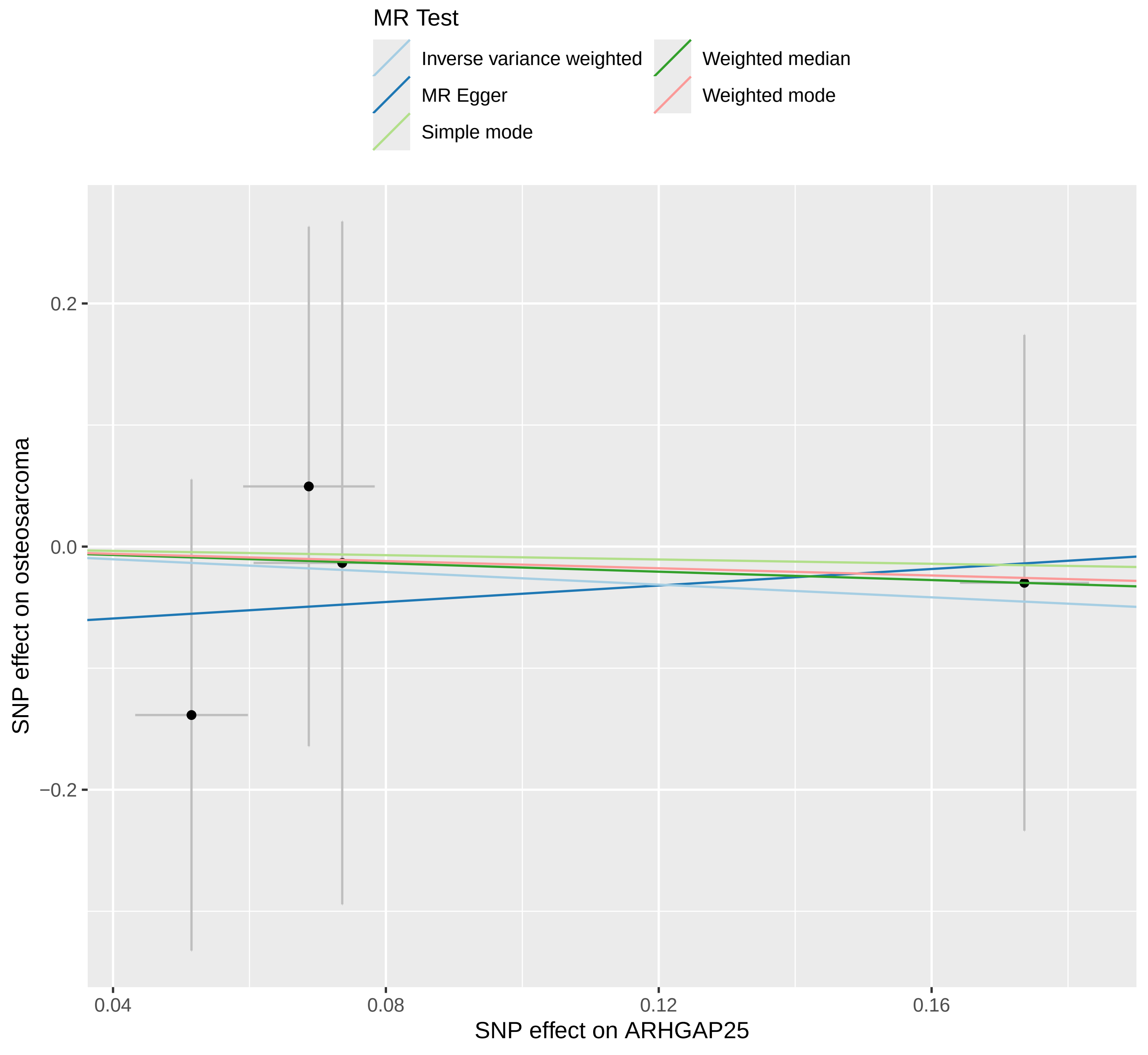

MR Test
Inverse variance weighted
MR Egger
Simple mode
Weighted median
Weighted mode
0.2
0.0
−0.2
SNP effect on osteosarcoma
0.04
0.08
0.12
0.16
SNP effect on ARHGAP25

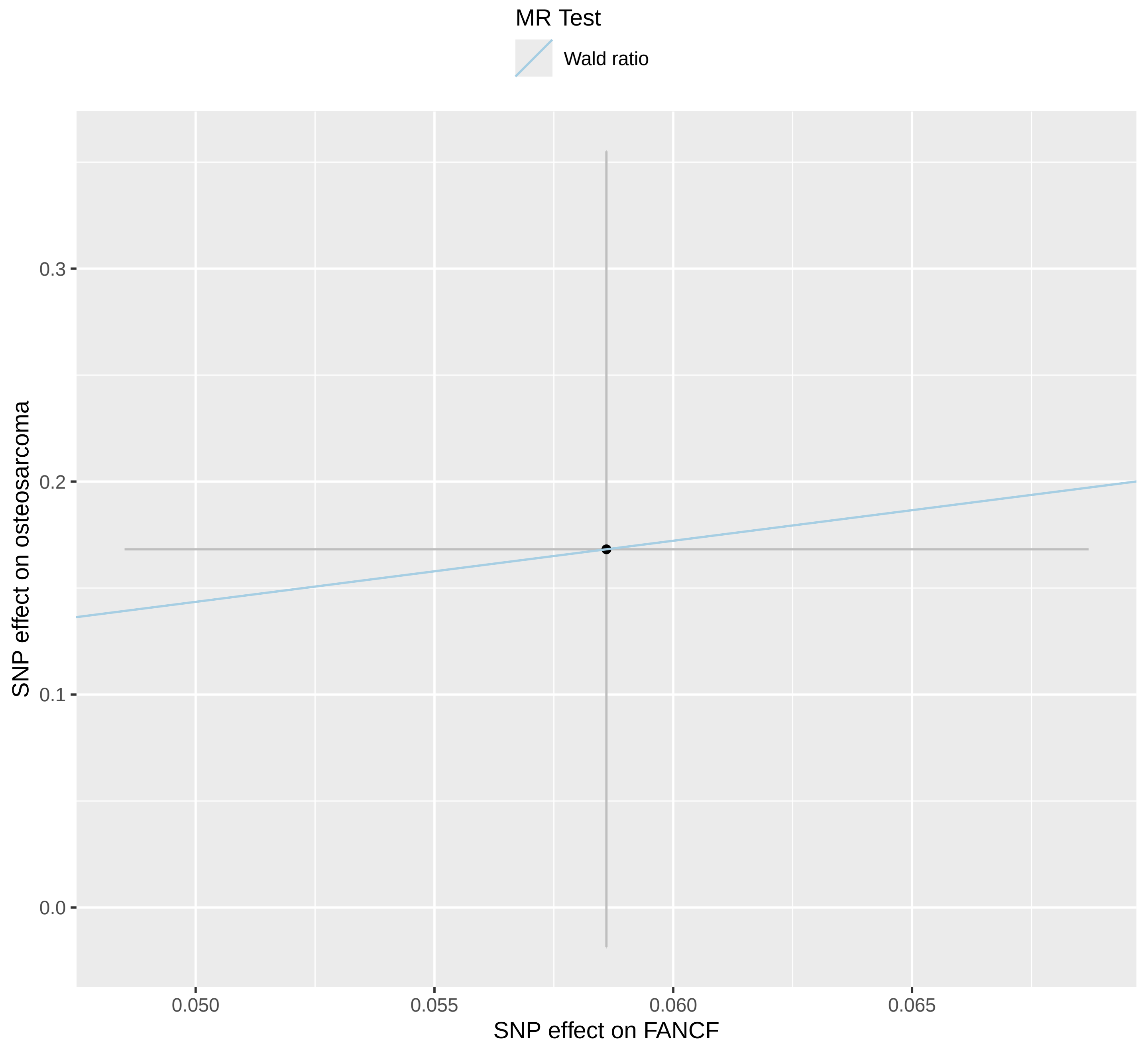
MR Test
Wald ratio
SNP effect on osteosarcoma
0.3
0.2
0.1
0.0
0.050
0.055
0.060
0.065
SNP effect on FANCF

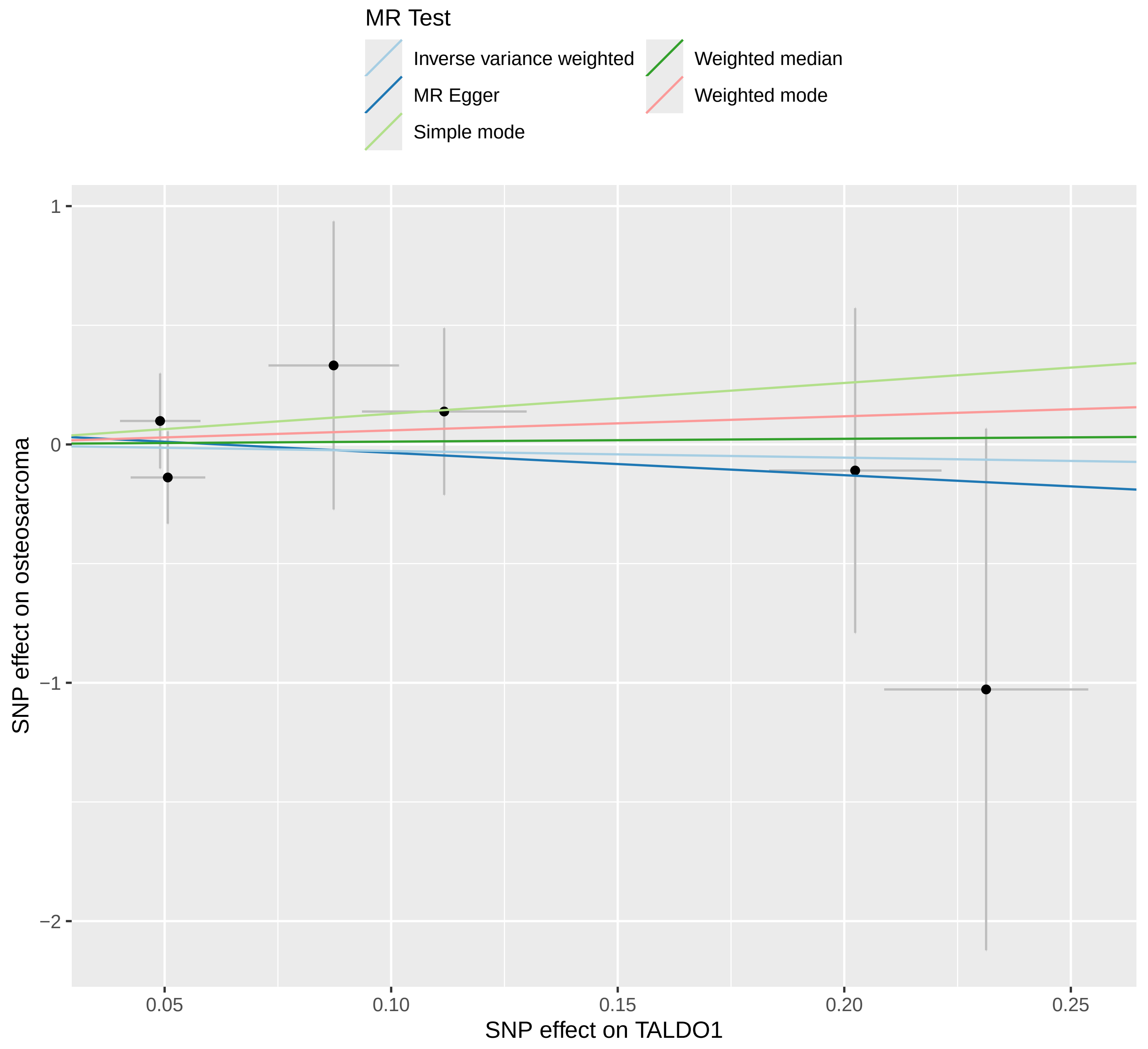

MR Test
Inverse variance weighted
MR Egger
Simple mode
Weighted median
Weighted mode
1
0
−1
−2
0.05
0.10
0.15
0.20
0.25
SNP effect on TALDO1
SNP effect on osteosarcoma

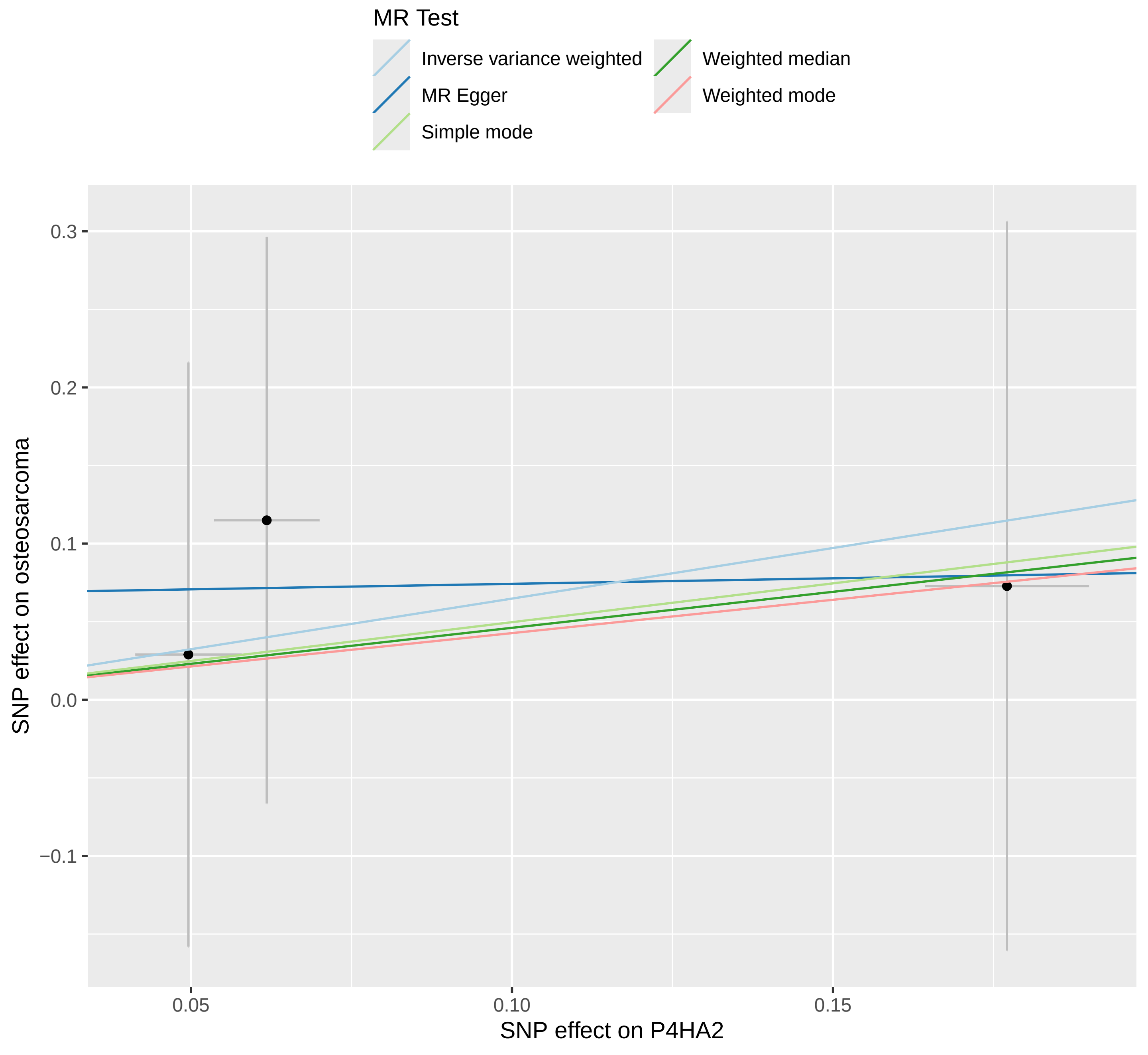

MR Test
Inverse variance weighted
MR Egger
Simple mode
Weighted median
Weighted mode
0.3
0.2
0.1
0.0
−0.1
SNP effect on osteosarcoma
0.05
0.10
0.15
SNP effect on P4HA2

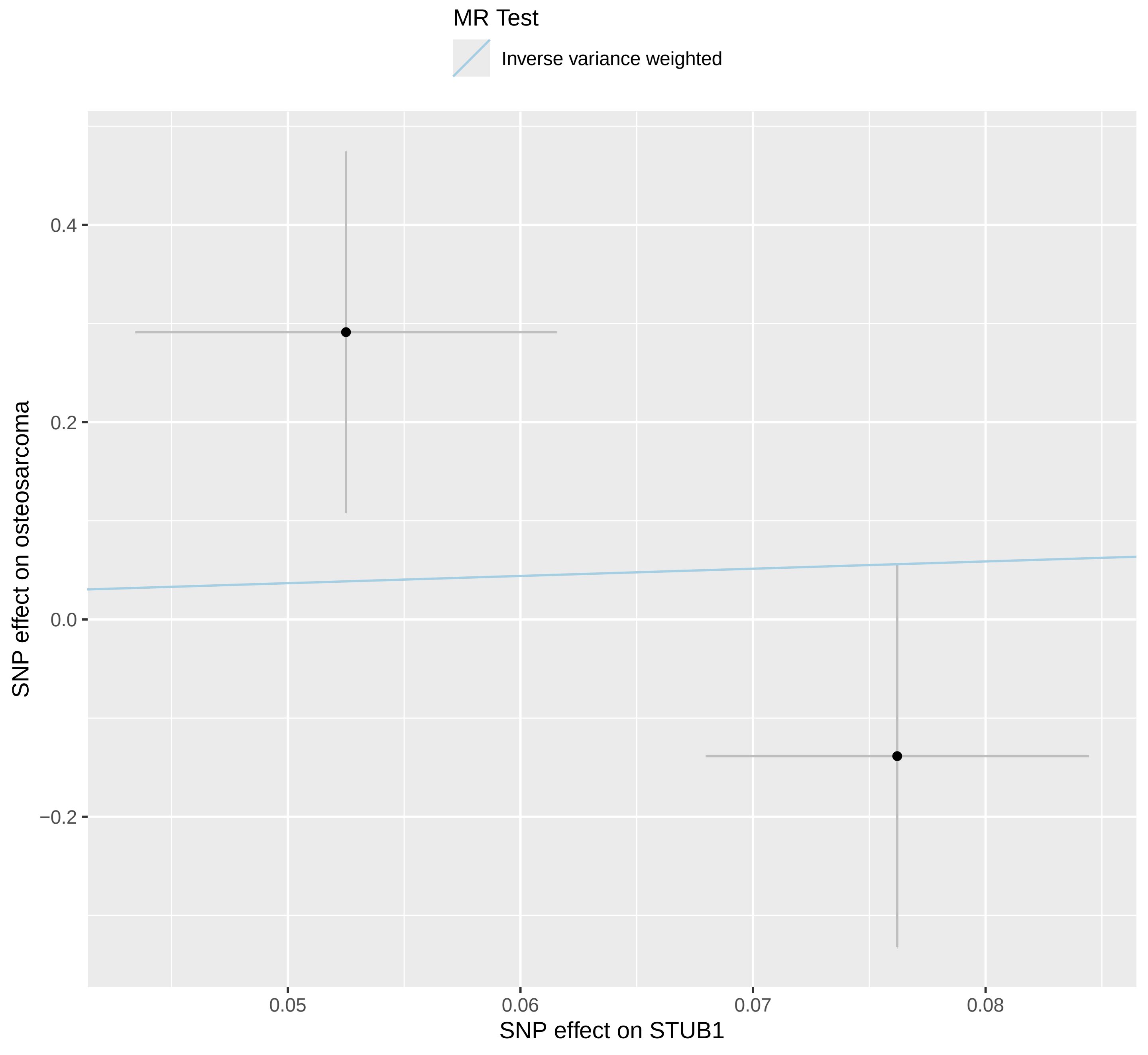

MR Test
Inverse variance weighted
0.4
0.2
0.0
−0.2
SNP effect on osteosarcoma
0.05
0.06
0.07
0.08
SNP effect on STUB1

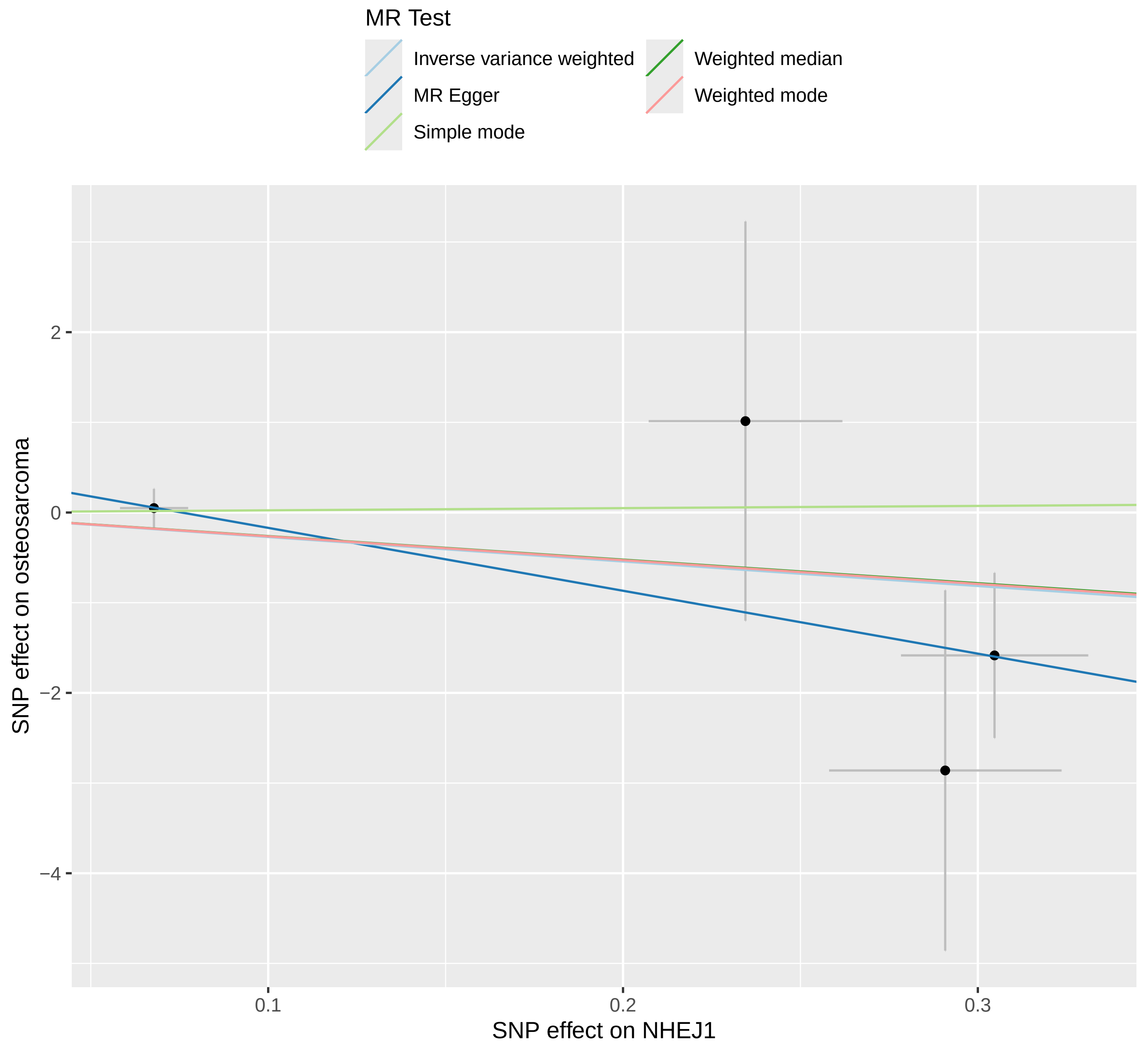
MR Test
Inverse variance weighted
MR Egger
Simple mode
Weighted median
Weighted mode
2
0
−2
−4
0.1
0.2
0.3
SNP effect on NHEJ1
SNP effect on osteosarcoma

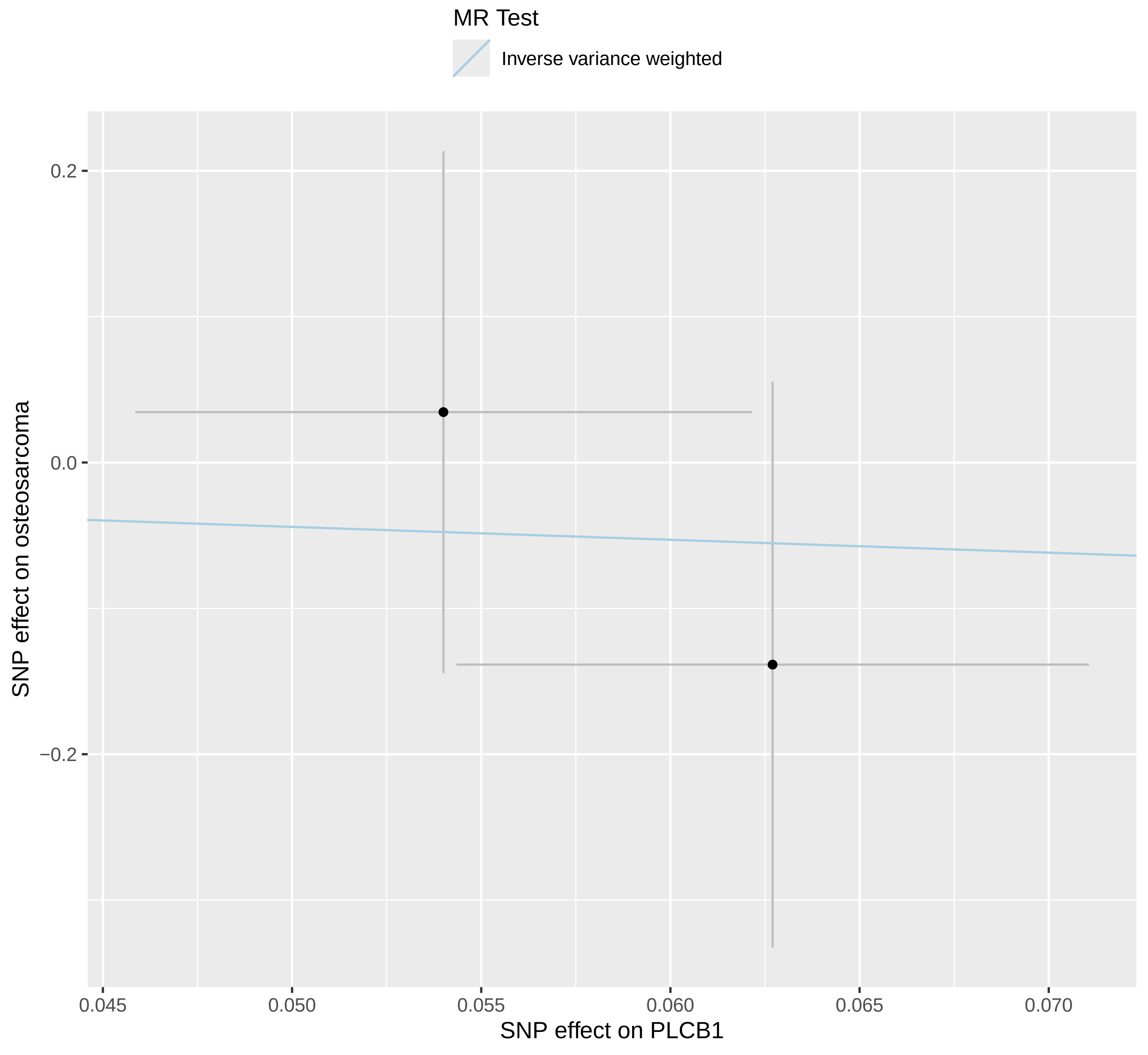

MR Test
Inverse variance weighted
0.2
0.0
−0.2
SNP effect on osteosarcoma
0.045
0.050
0.055
0.060
0.065
0.070
SNP effect on PLCB1

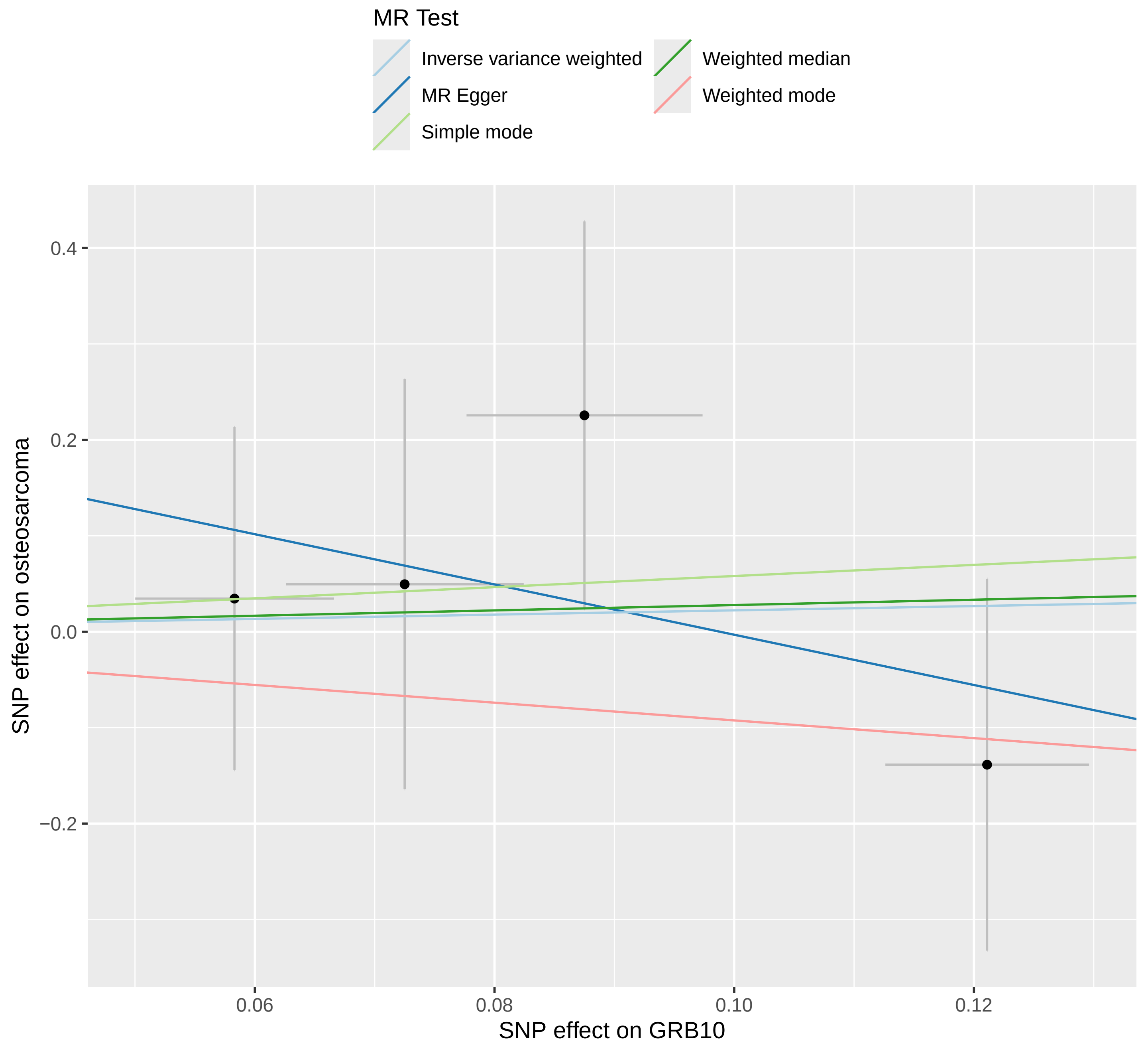
MR Test
Inverse variance weighted
MR Egger
Simple mode
Weighted median
Weighted mode
0.4
0.2
0.0
−0.2
SNP effect on osteosarcoma
0.06
0.08
0.10
0.12
SNP effect on GRB10

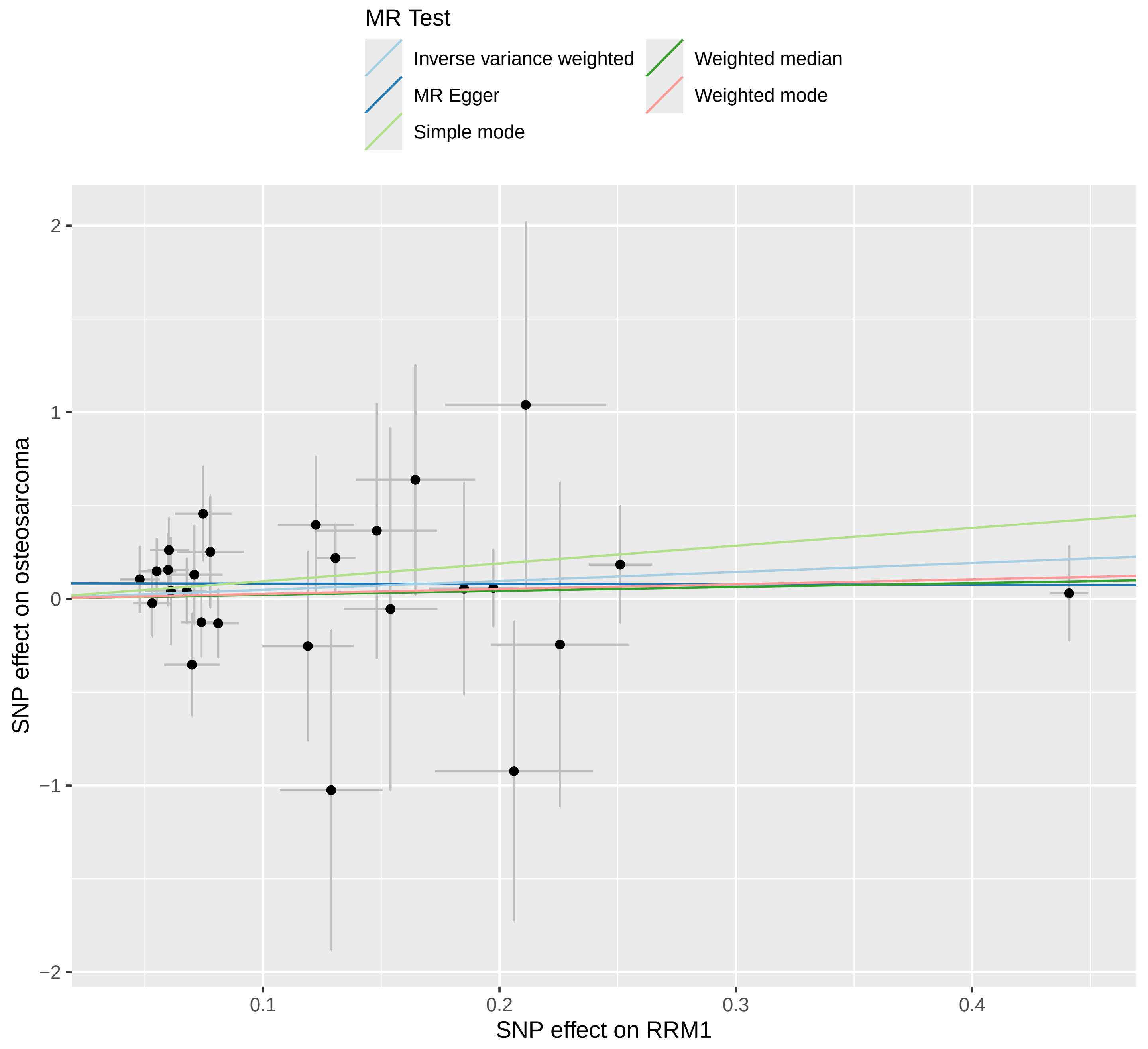
MR Test
Inverse variance weighted
MR Egger
Simple mode
Weighted median
Weighted mode
SNP effect on osteosarcoma
2
1
0
−1
−2
0.1
0.2
0.3
0.4
SNP effect on RRM1

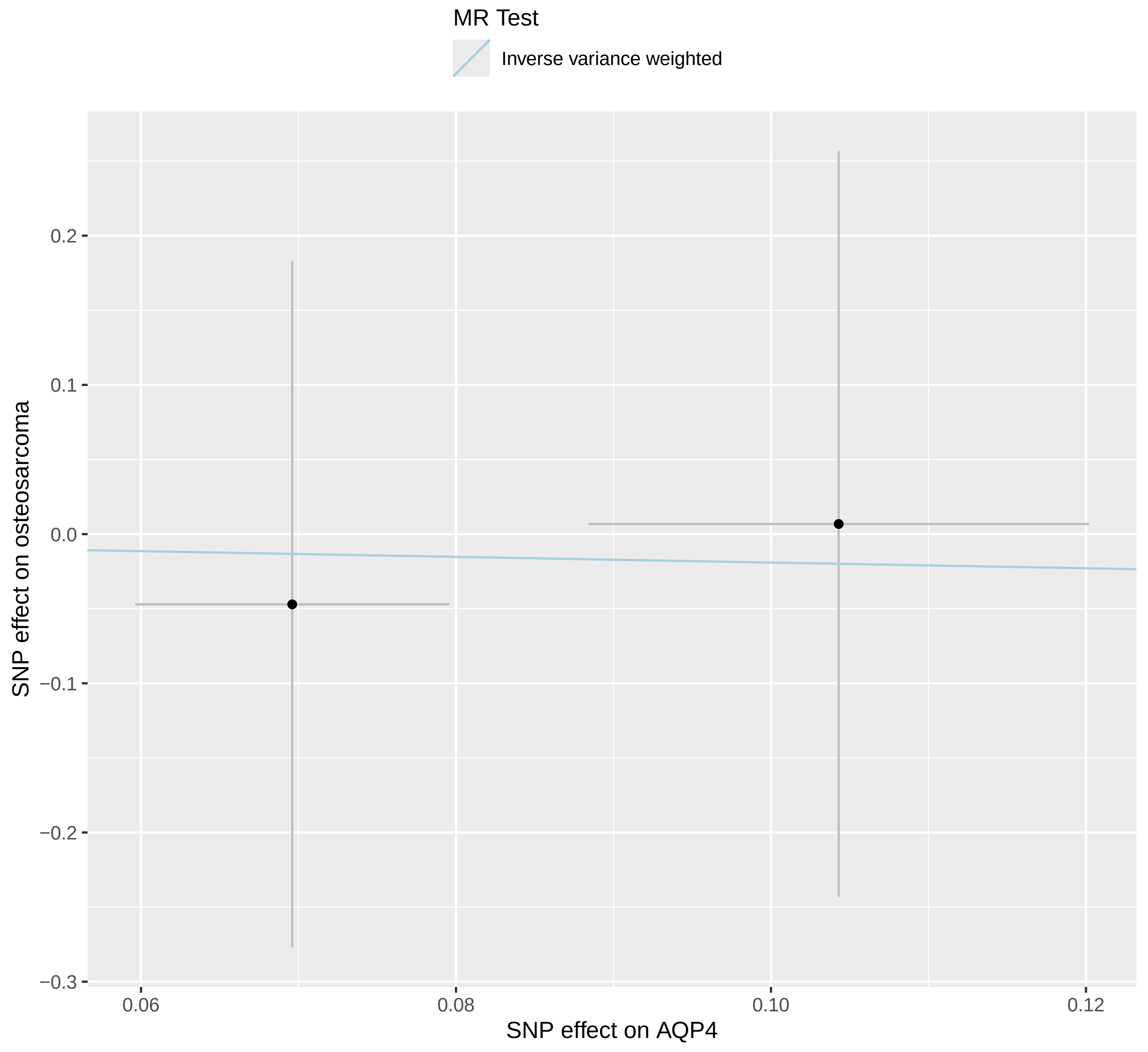

MR Test
Inverse variance weighted
0.2
0.1
0.0
−0.1
−0.2
−0.3
0.06
0.08
0.10
0.12
SNP effect on osteosarcoma
SNP effect on AQP4

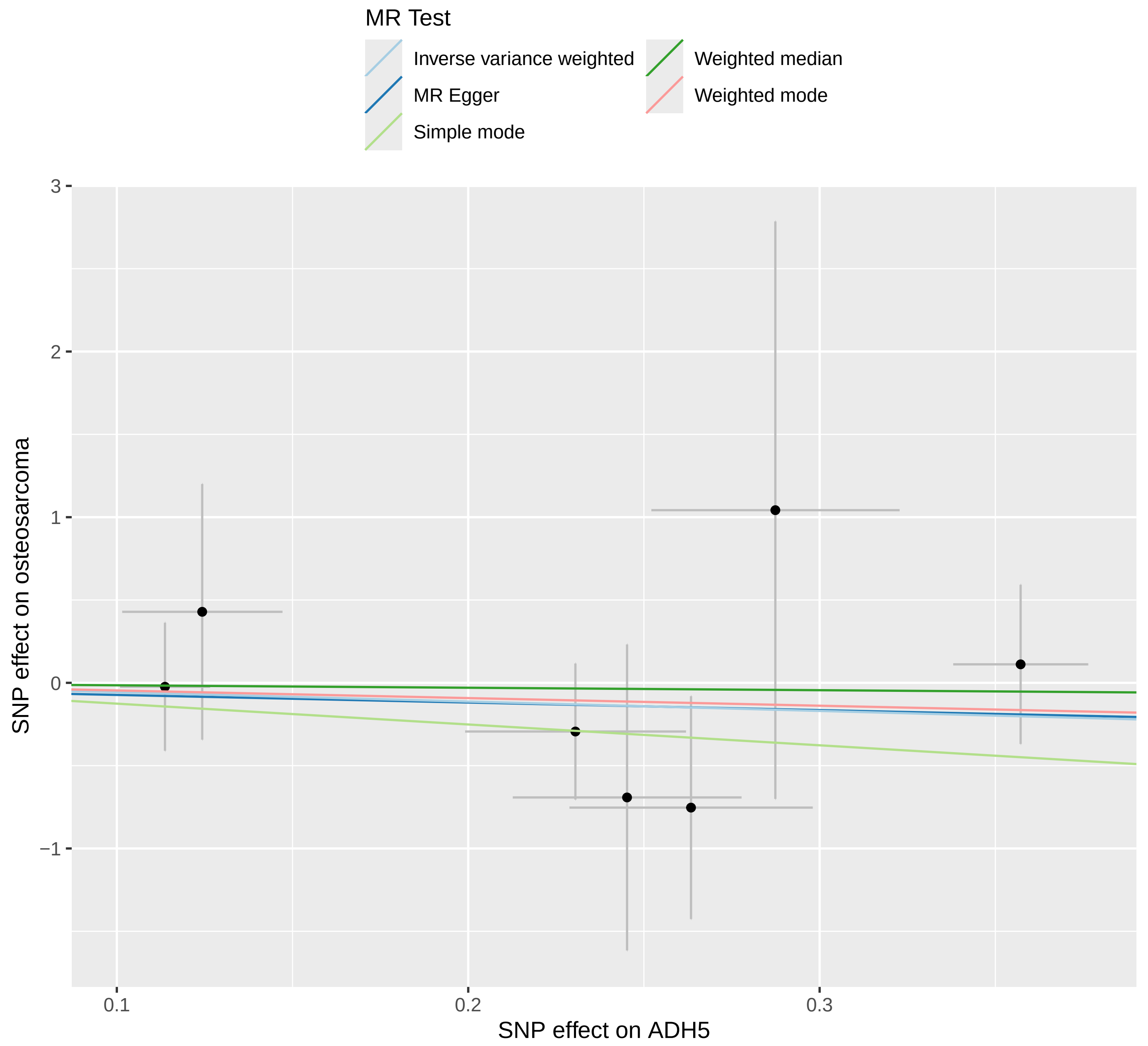

MR Test
Inverse variance weighted
MR Egger
Simple mode
Weighted median
Weighted mode
3
2
1
0
−1
0.1
0.2
0.3
SNP effect on osteosarcoma
SNP effect on ADH5

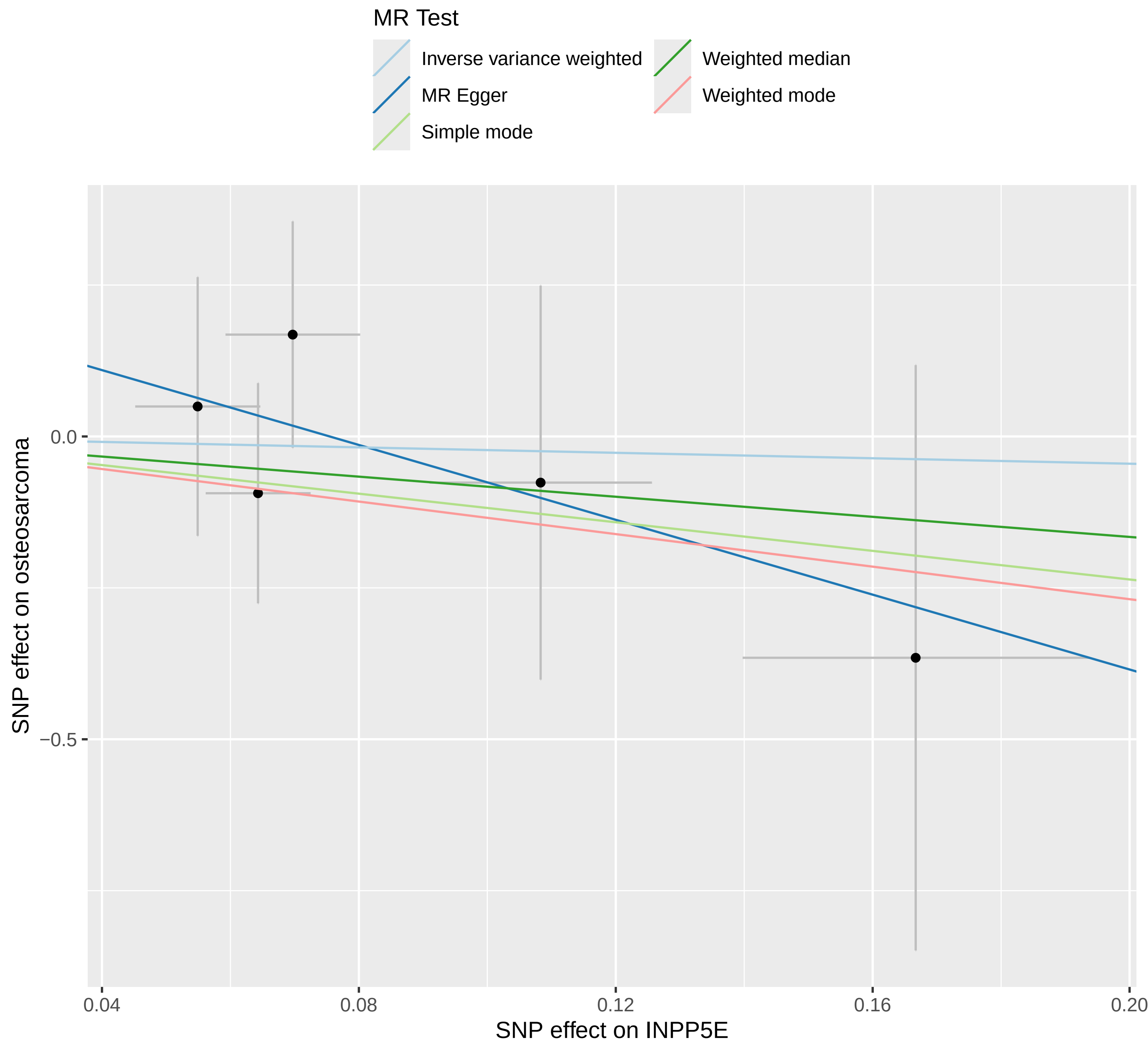
MR Test
Inverse variance weighted
MR Egger
Simple mode
Weighted median
Weighted mode
0.0
−0.5
0.04
0.08
0.12
0.16
0.20
SNP effect on INPP5E
SNP effect on osteosarcoma

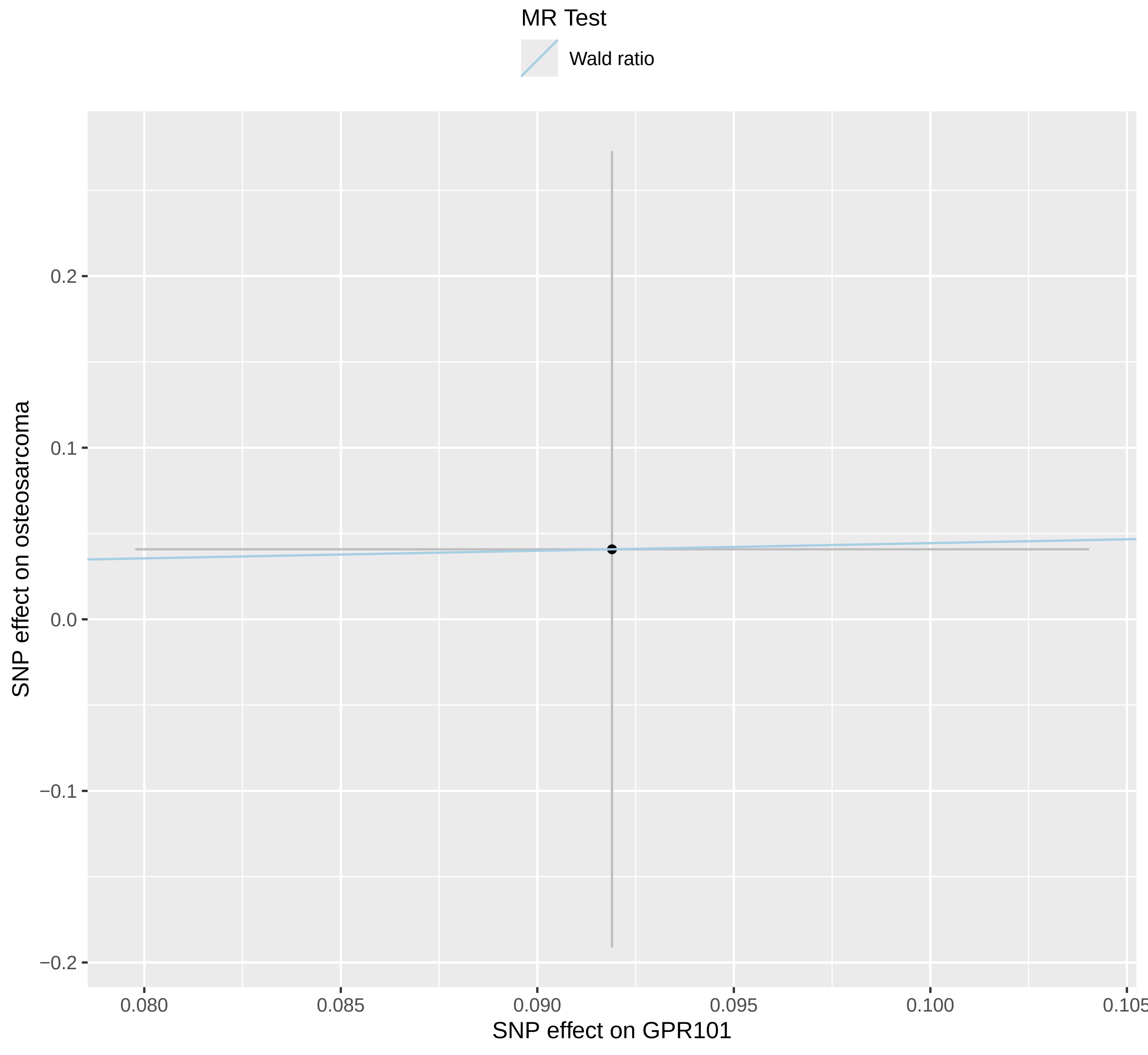

MR Test
Wald ratio
0.2
0.1
0.0
−0.1
−0.2
SNP effect on osteosarcoma
0.080
0.085
0.090
0.095
0.100
0.105
SNP effect on GPR101

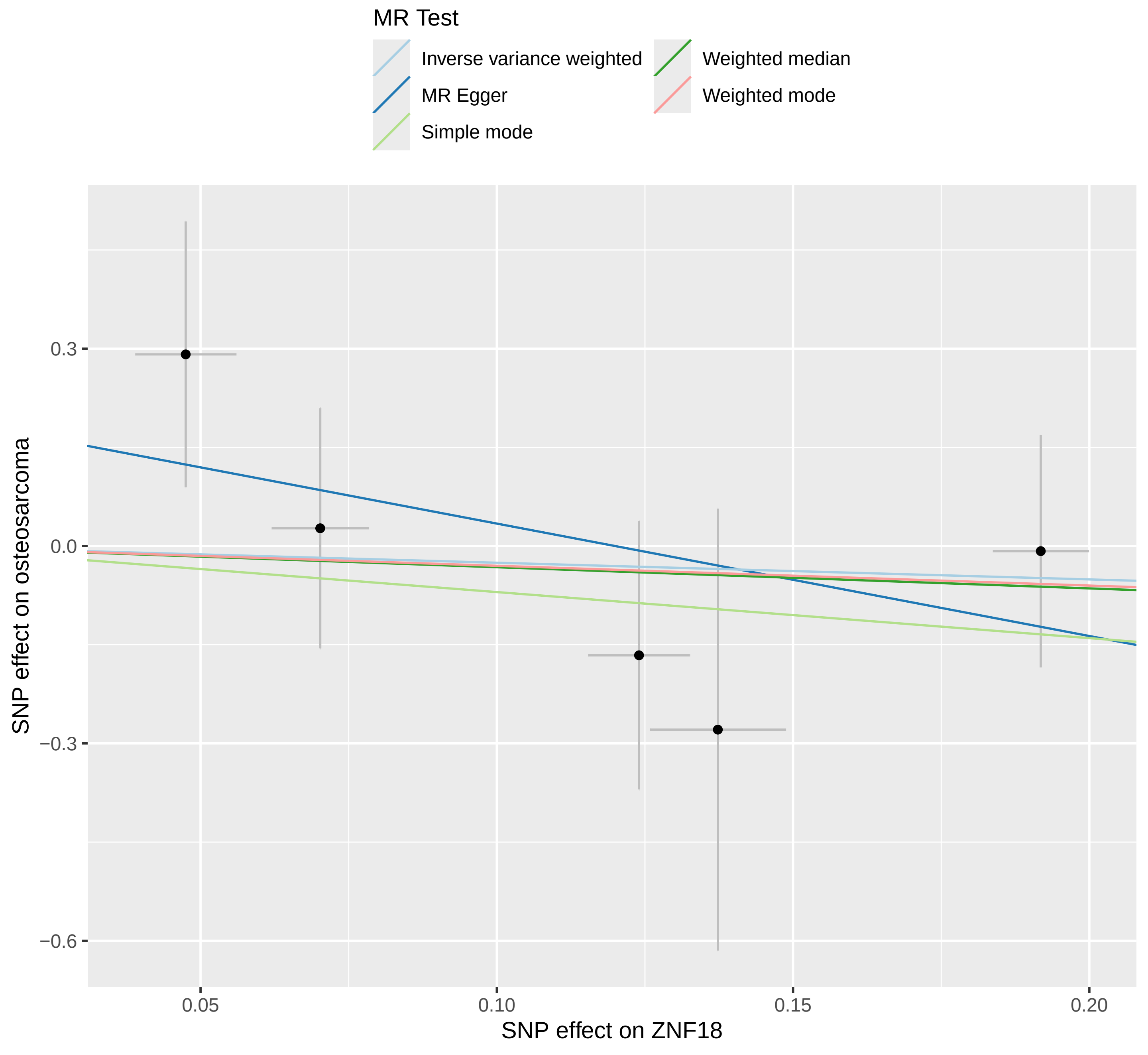

MR Test
Inverse variance weighted
MR Egger
Simple mode
Weighted median
Weighted mode
0.3
0.0
−0.3
−0.6
0.05
0.10
0.15
0.20
SNP effect on osteosarcoma
SNP effect on ZNF18

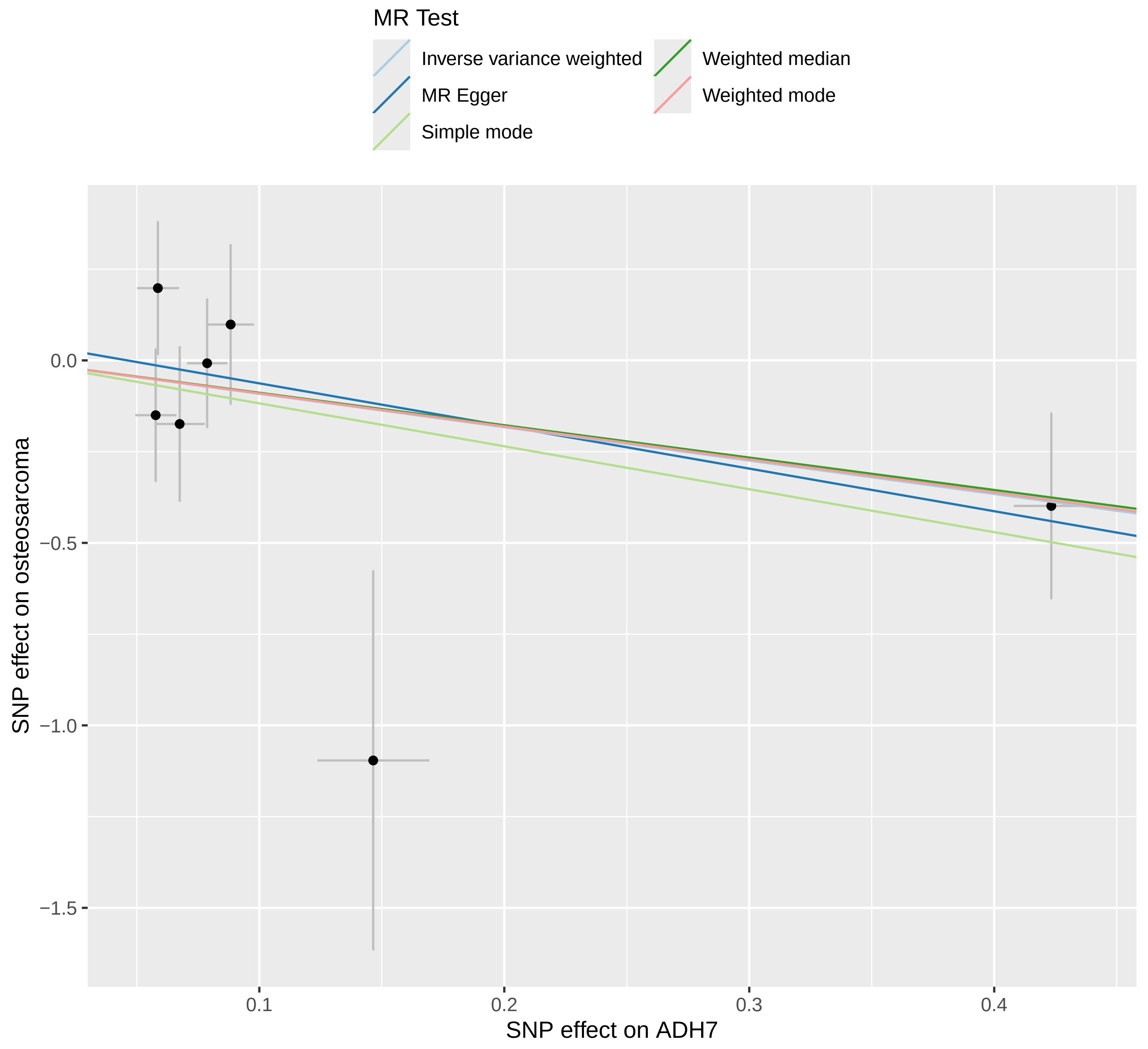

MR Test
Inverse variance weighted
MR Egger
Simple mode
Weighted median
Weighted mode
0.0
−0.5
−1.0
−1.5
SNP effect on osteosarcoma
0.1
0.2
0.3
0.4
SNP effect on ADH7

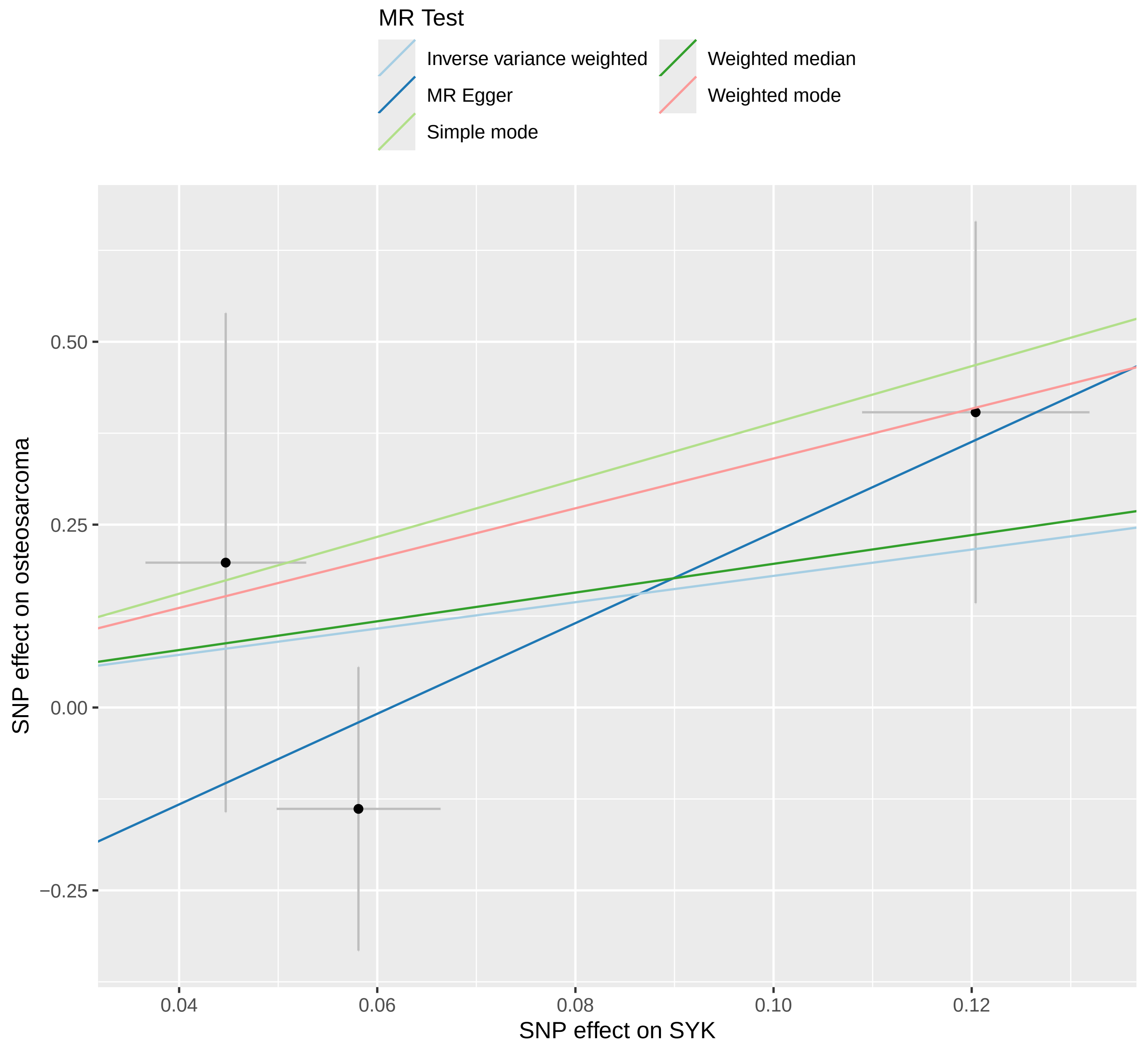
MR Test
Inverse variance weighted
MR Egger
Simple mode
Weighted median
Weighted mode
SNP effect on osteosarcoma
0.50
0.25
0.00
−0.25
0.04
0.06
0.08
0.10
0.12
SNP effect on SYK

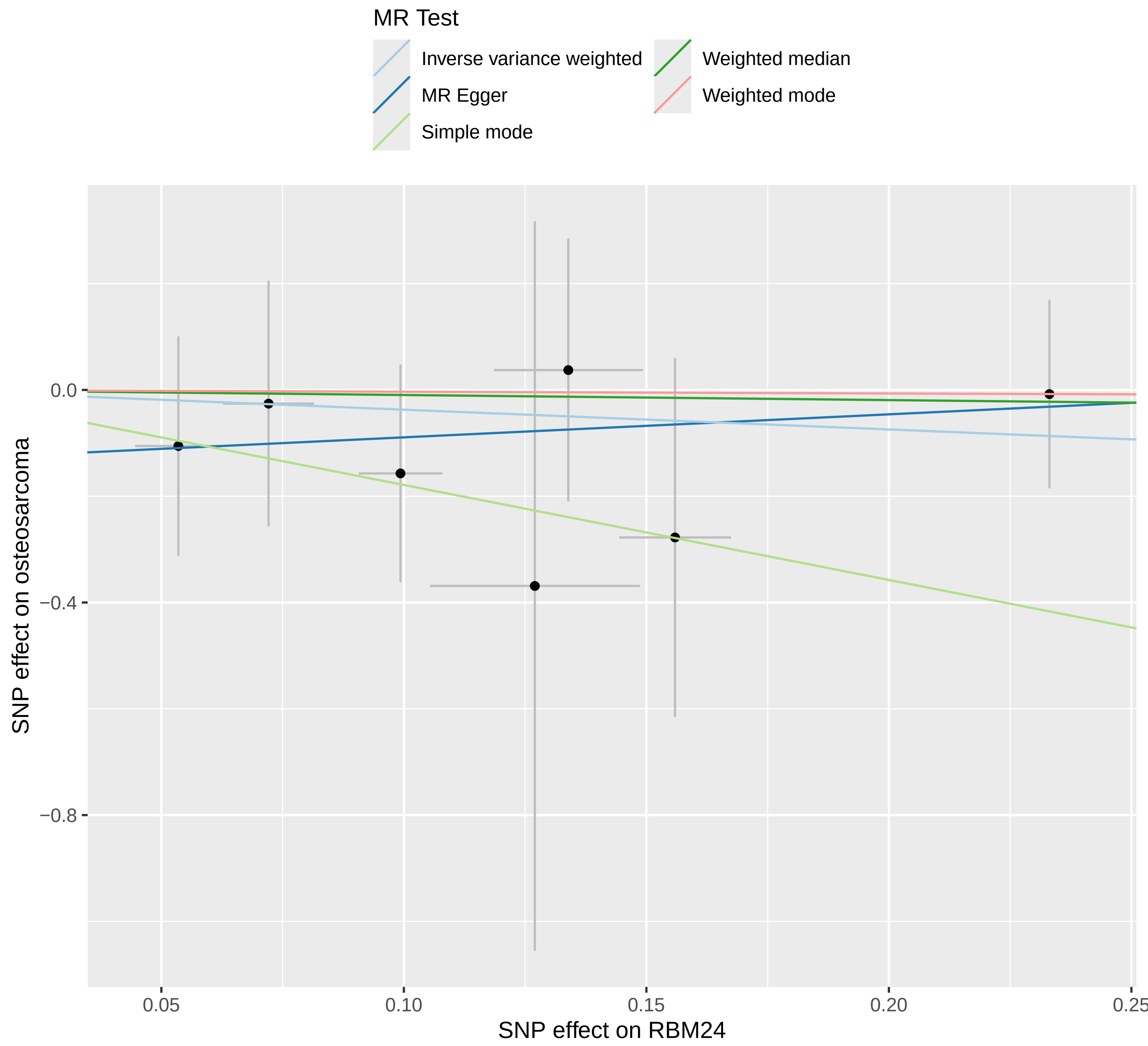

MR Test
Inverse variance weighted
MR Egger
Simple mode
Weighted median
Weighted mode
0.0
−0.4
−0.8
0.05
0.10
0.15
0.20
0.25
SNP effect on osteosarcoma
SNP effect on RBM24

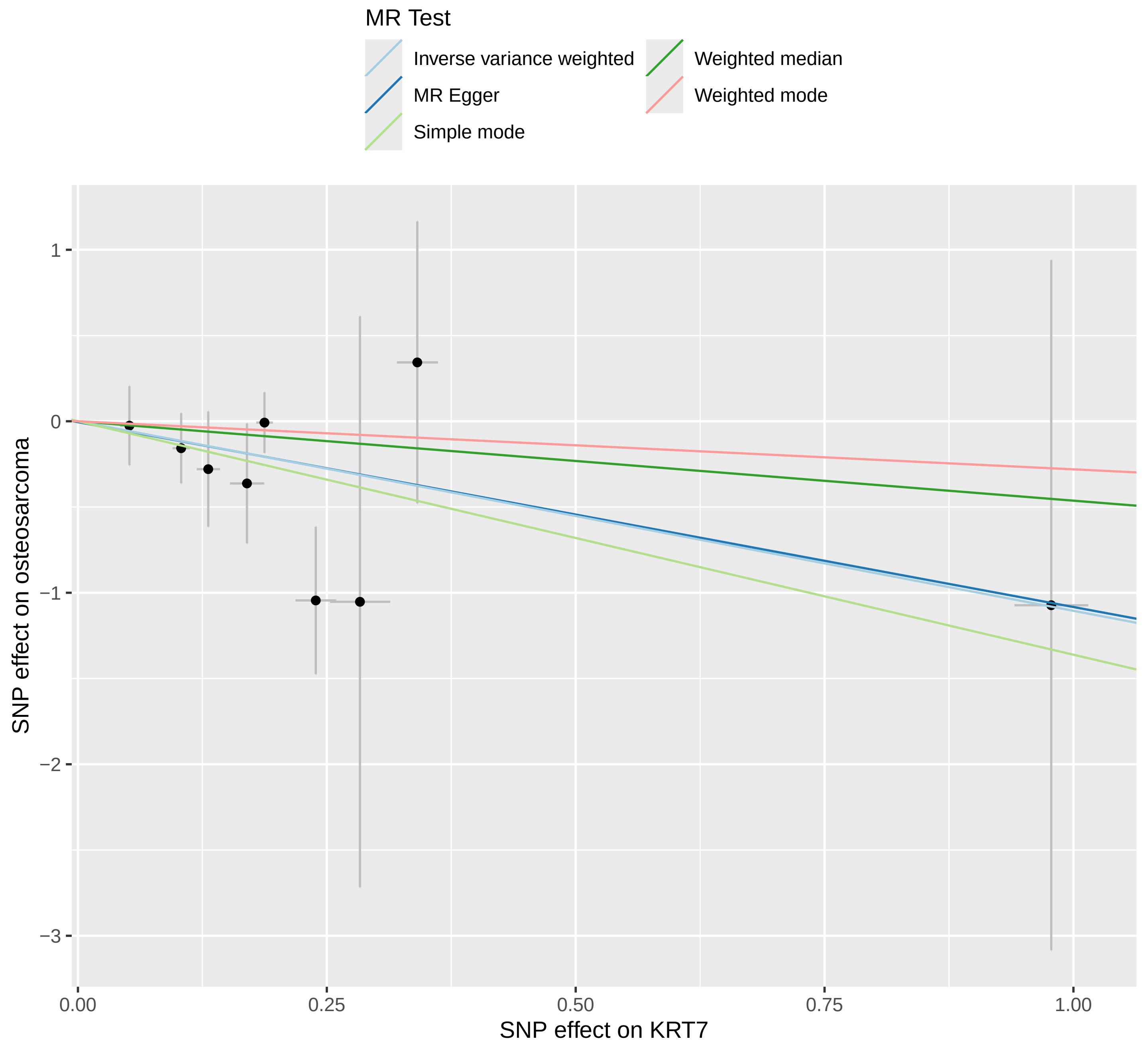
MR Test
Inverse variance weighted
MR Egger
Simple mode
Weighted median
Weighted mode
SNP effect on osteosarcoma
1
0
−1
−2
−3
0.00
0.25
0.50
0.75
1.00
SNP effect on KRT7

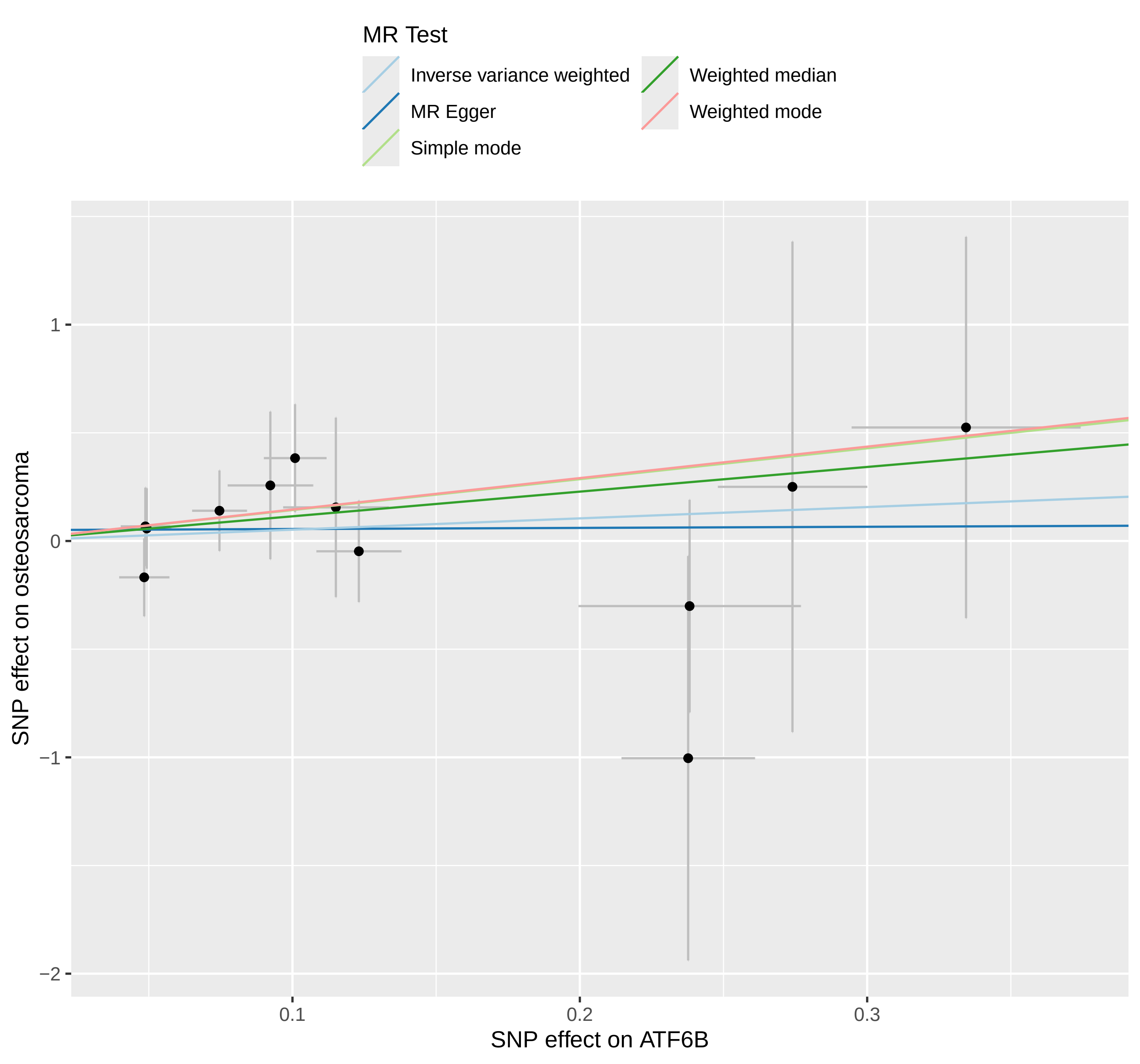

MR Test
Inverse variance weighted
MR Egger
Simple mode
Weighted median
Weighted mode
SNP effect on osteosarcoma
1
0
−1
−2
0.1
0.2
0.3
SNP effect on ATF6B

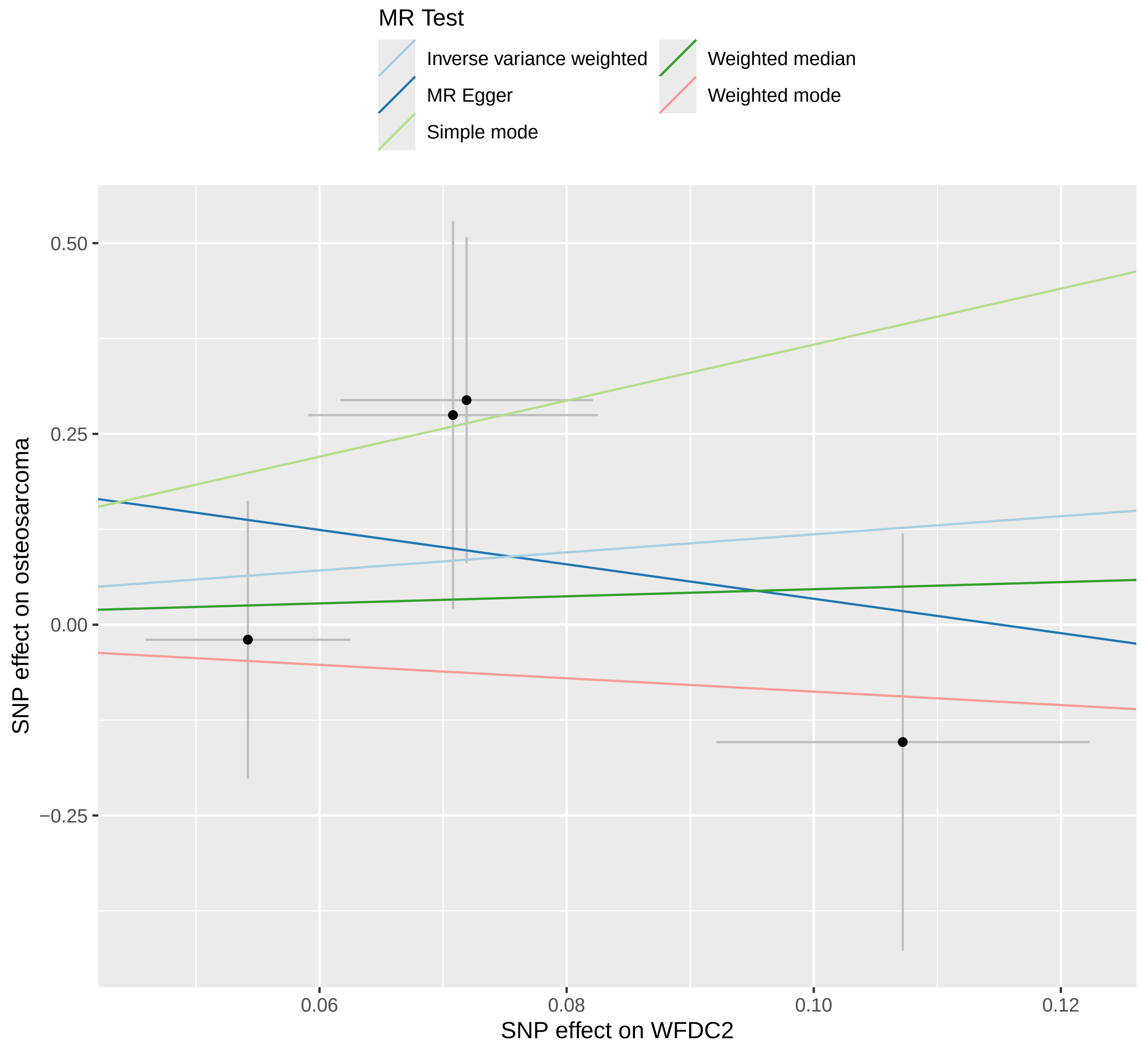
MR Test
Inverse variance weighted
MR Egger
Simple mode
Weighted median
Weighted mode
0.50
0.25
0.00
−0.25
0.06
0.08
0.10
0.12
SNP effect on osteosarcoma
SNP effect on WFDC2

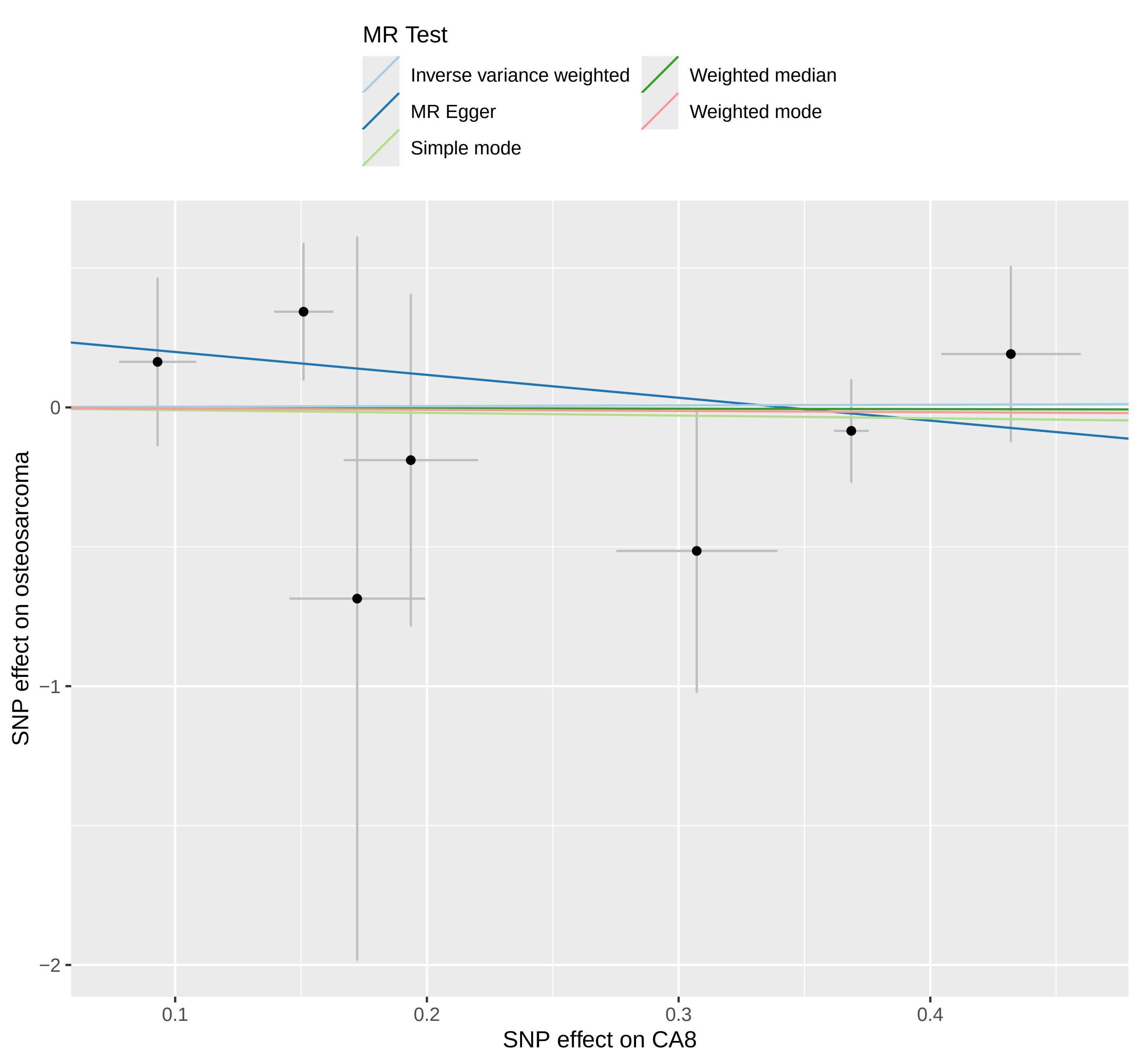
MR Test
Inverse variance weighted
MR Egger
Simple mode
Weighted median
Weighted mode
0
−1
−2
0.1
0.2
0.3
0.4
SNP effect on osteosarcoma
SNP effect on CA8

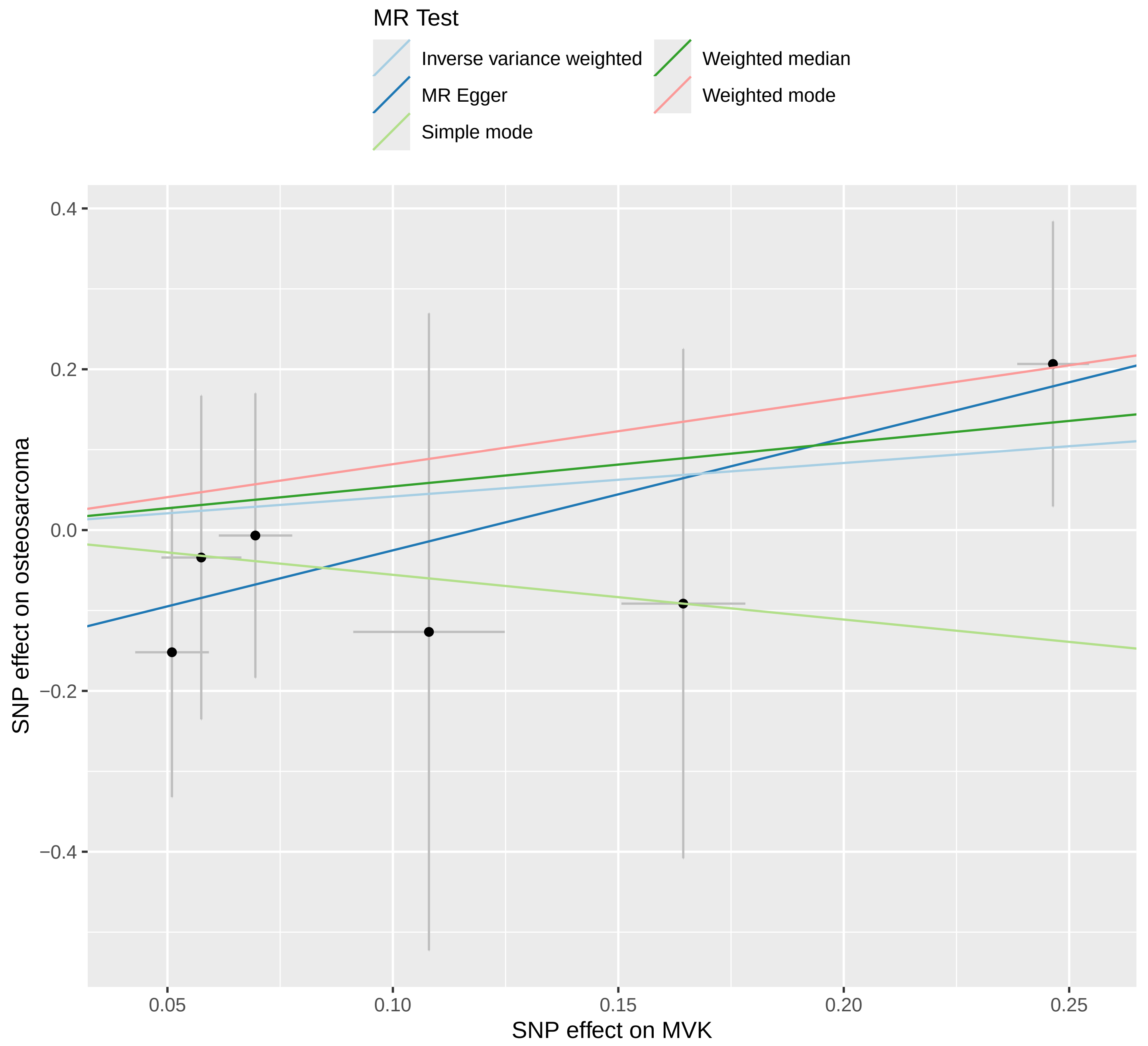

MR Test
Inverse variance weighted
MR Egger
Simple mode
Weighted median
Weighted mode
0.4
0.2
0.0
−0.2
−0.4
SNP effect on osteosarcoma
0.05
0.10
0.15
0.20
0.25
SNP effect on MVK

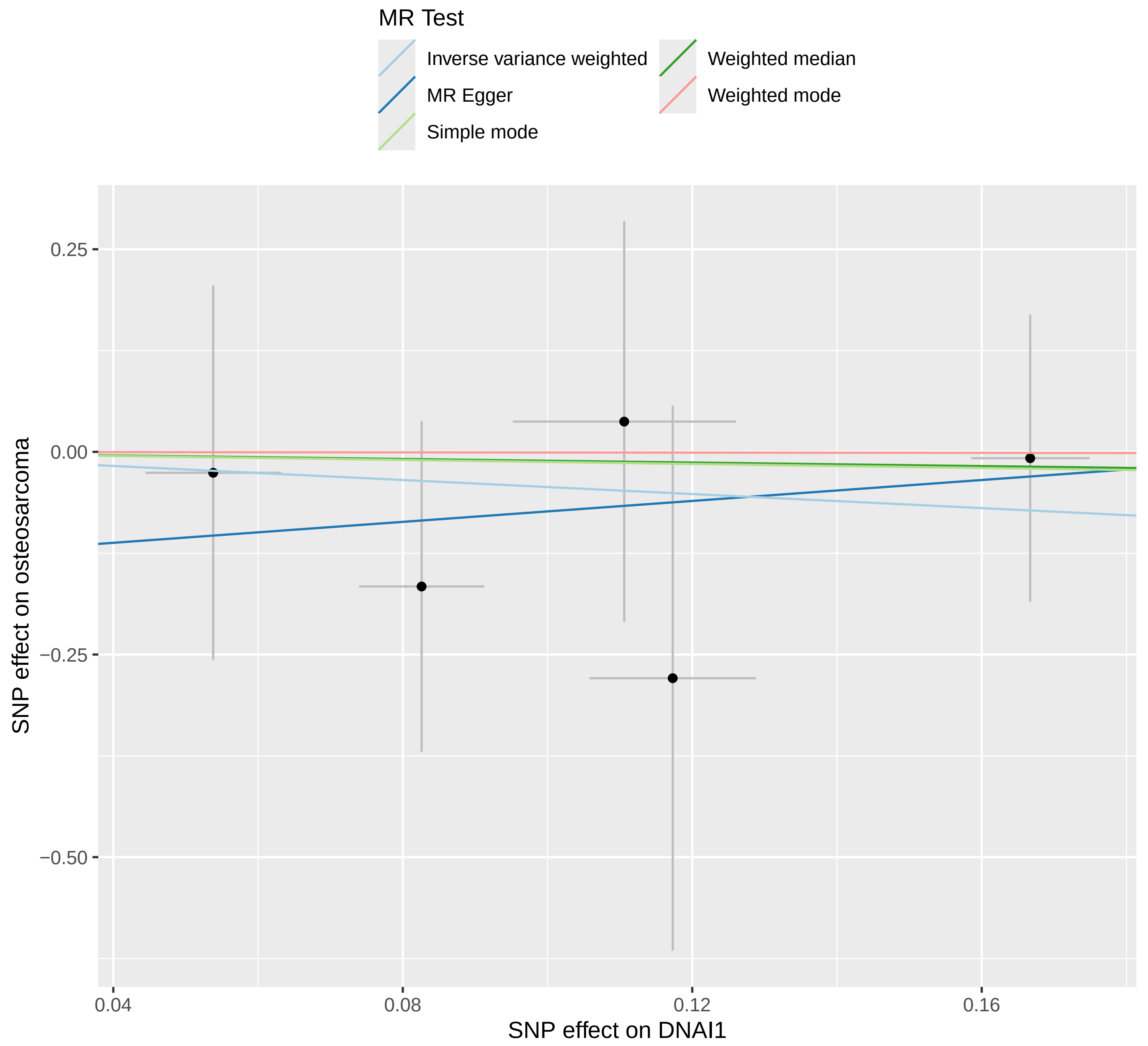
MR Test
Inverse variance weighted
MR Egger
Simple mode
Weighted median
Weighted mode
0.25
0.00
−0.25
−0.50
SNP effect on osteosarcoma
0.04
0.08
0.12
0.16
SNP effect on DNAI1

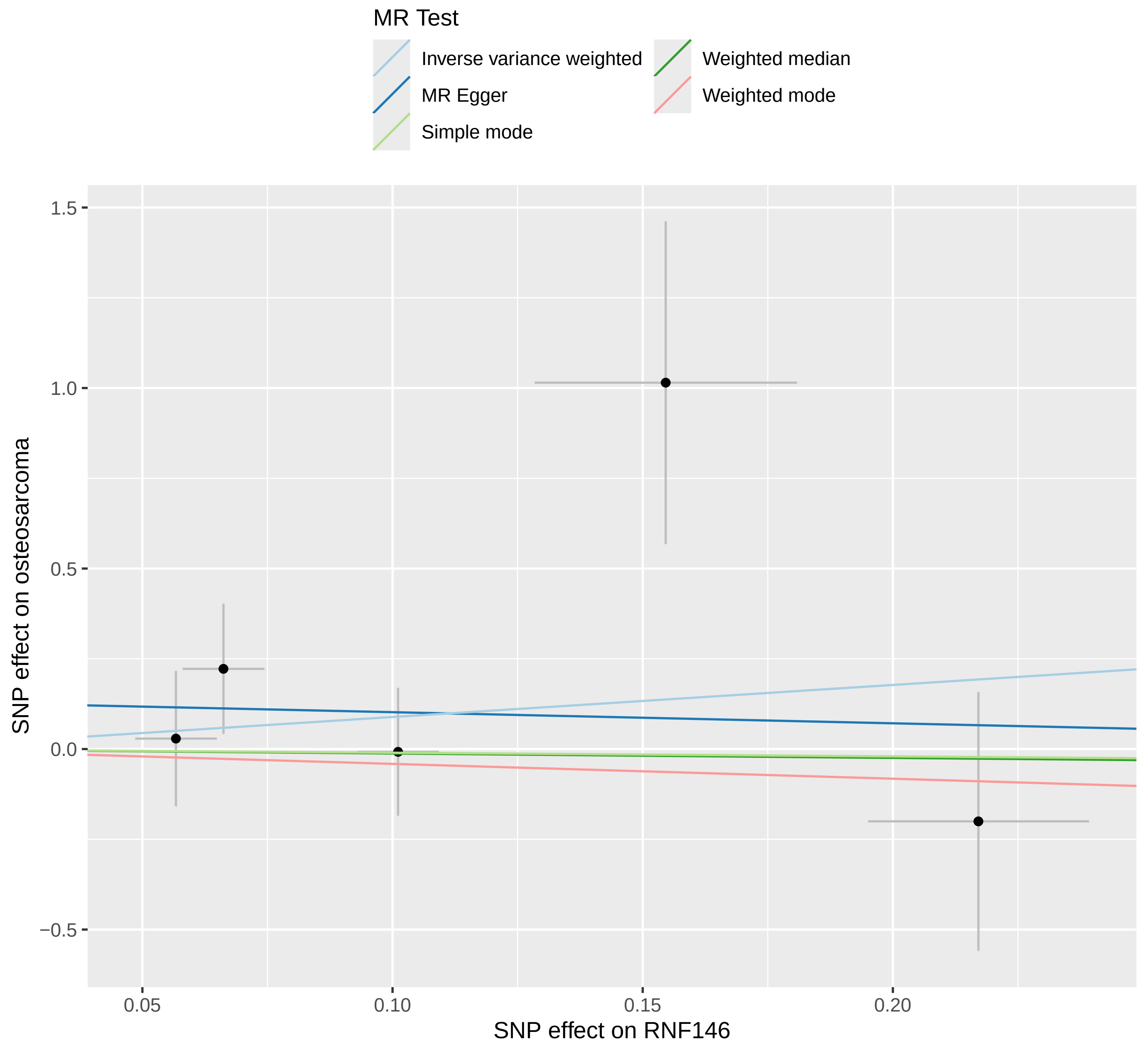
MR Test
Inverse variance weighted
MR Egger
Simple mode
Weighted median
Weighted mode
1.5
1.0
0.5
0.0
−0.5
0.05
0.10
0.15
0.20
SNP effect on osteosarcoma
SNP effect on RNF146

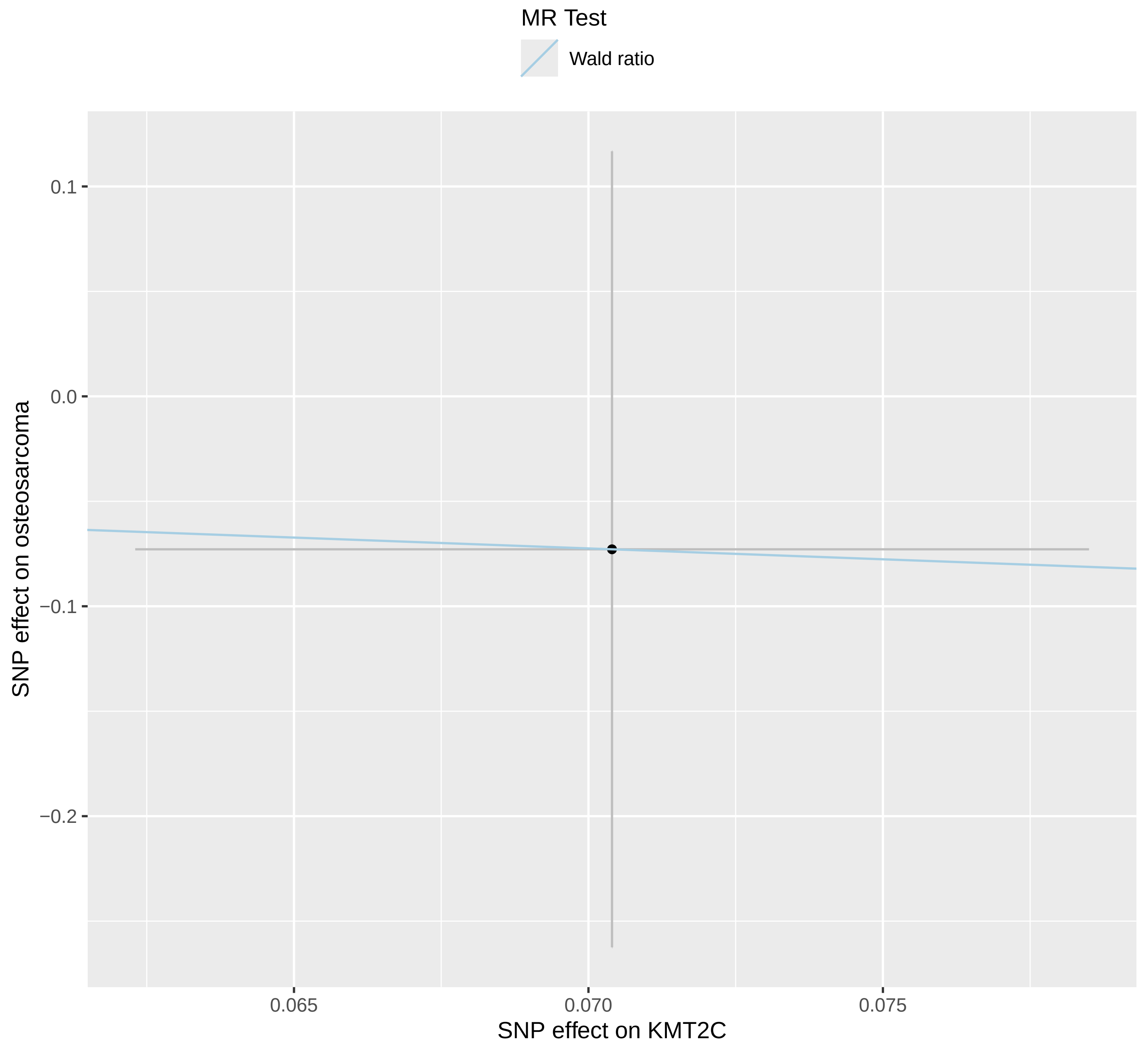

MR Test
Wald ratio
0.1
0.0
−0.1
−0.2
SNP effect on osteosarcoma
0.065
0.070
0.075
SNP effect on KMT2C

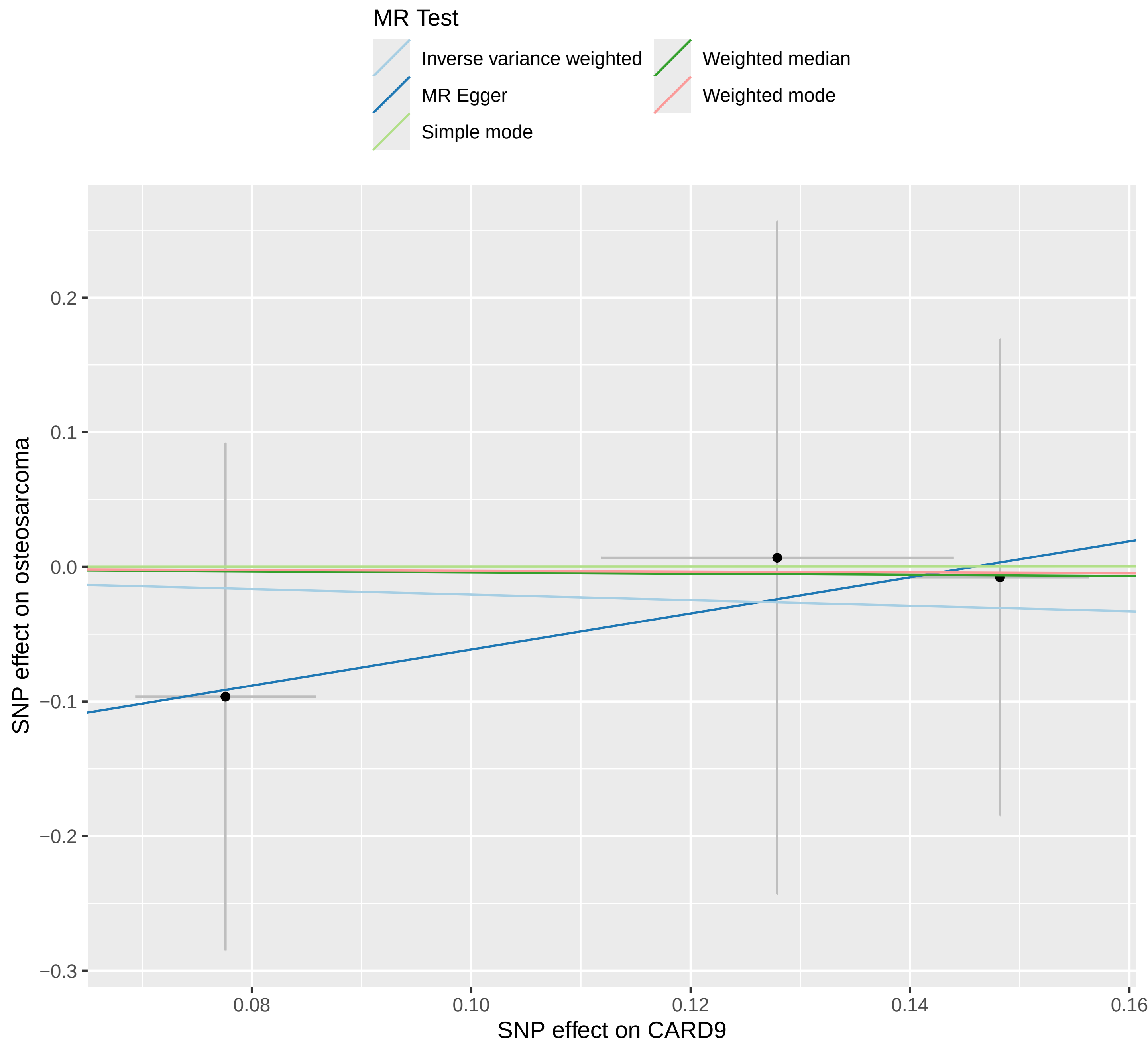

MR Test
Inverse variance weighted
MR Egger
Simple mode
Weighted median
Weighted mode
0.2
0.1
0.0
−0.1
−0.2
−0.3
SNP effect on osteosarcoma
0.08
0.10
0.12
0.14
0.16
SNP effect on CARD9

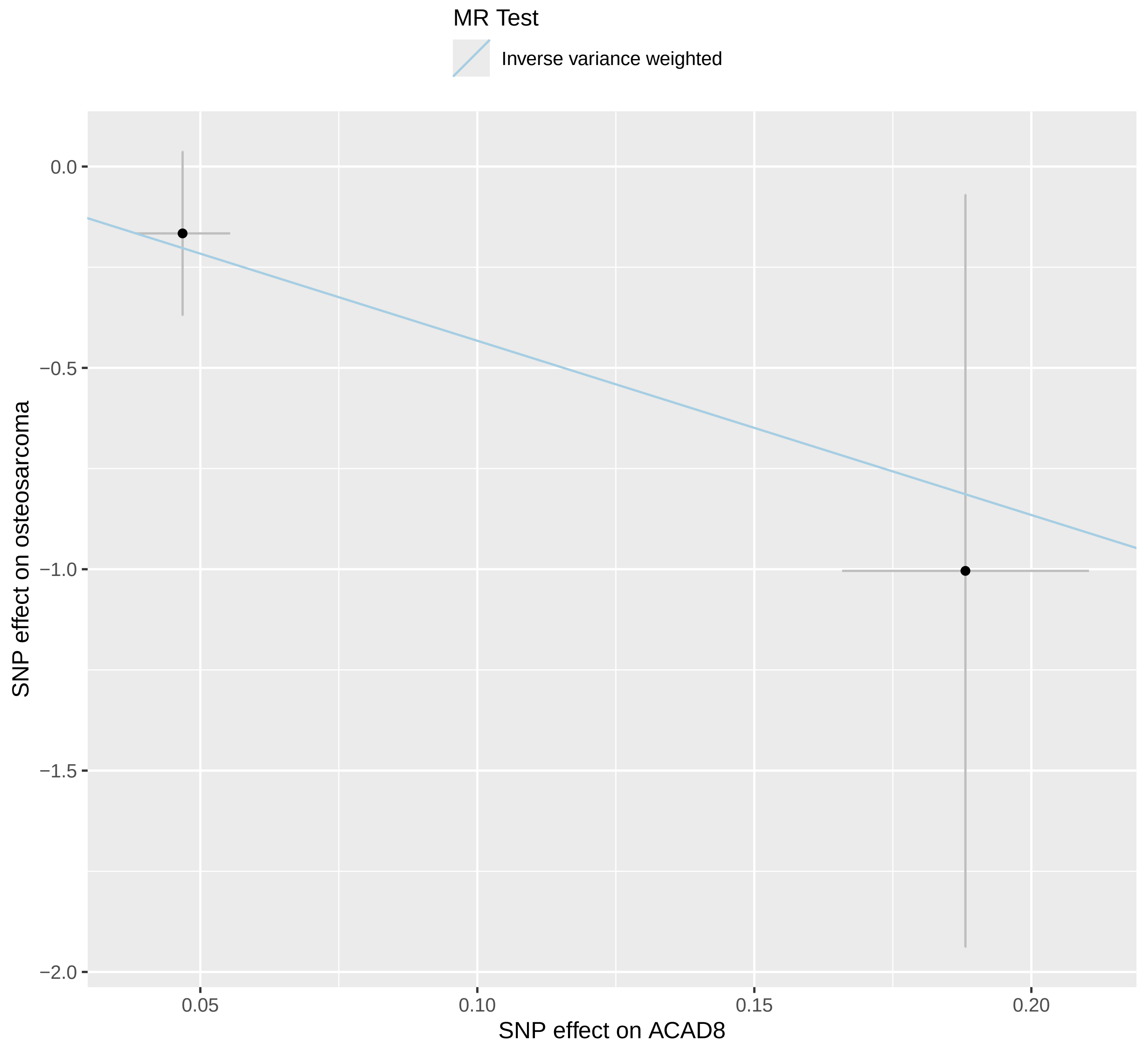
MR Test
Inverse variance weighted
0.0
−0.5
−1.0
−1.5
−2.0
SNP effect on osteosarcoma
0.05
0.10
0.15
0.20
SNP effect on ACAD8

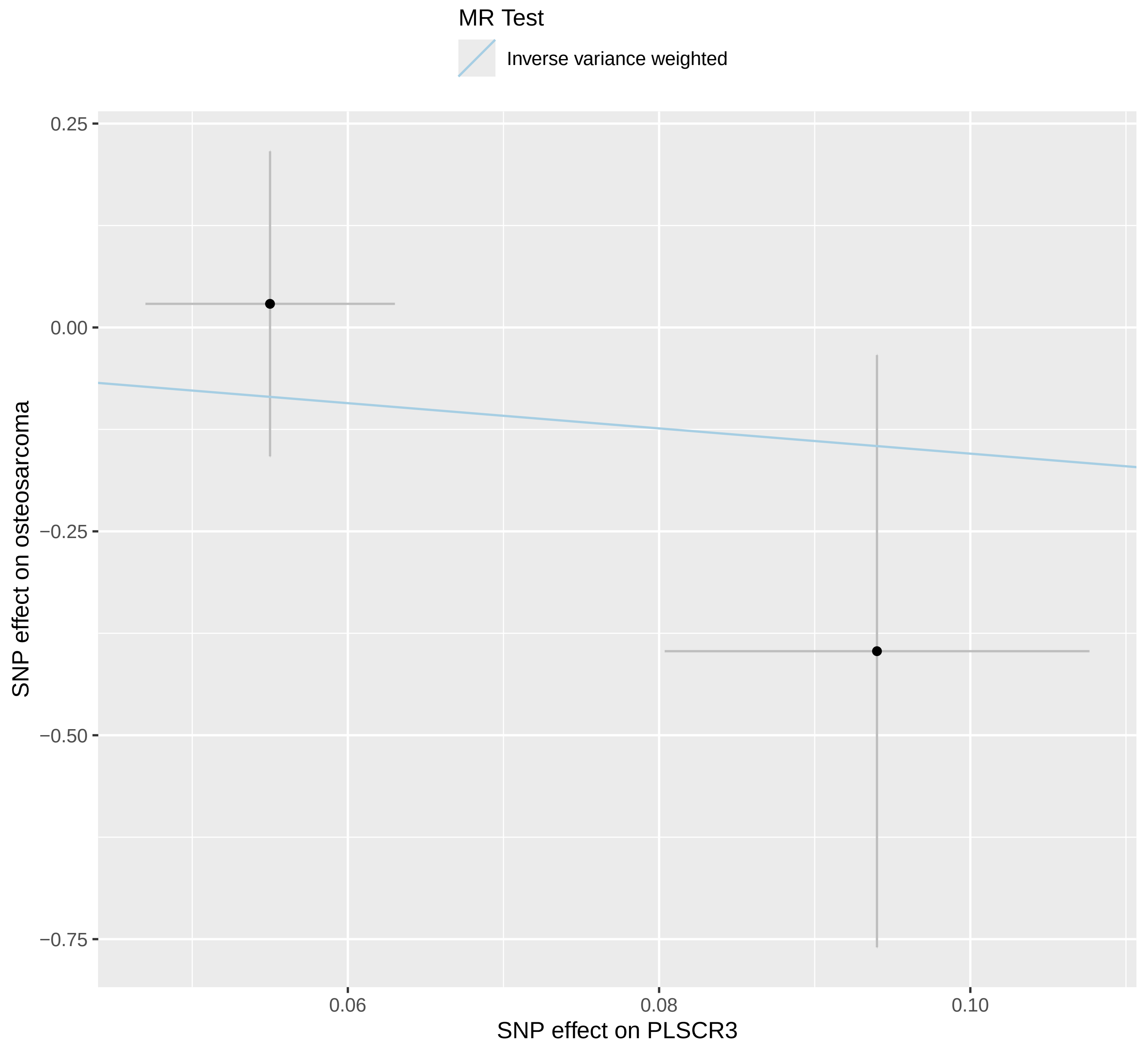
MR Test
Inverse variance weighted
0.25
0.00
−0.25
−0.50
−0.75
SNP effect on osteosarcoma
0.06
0.08
0.10
SNP effect on PLSCR3

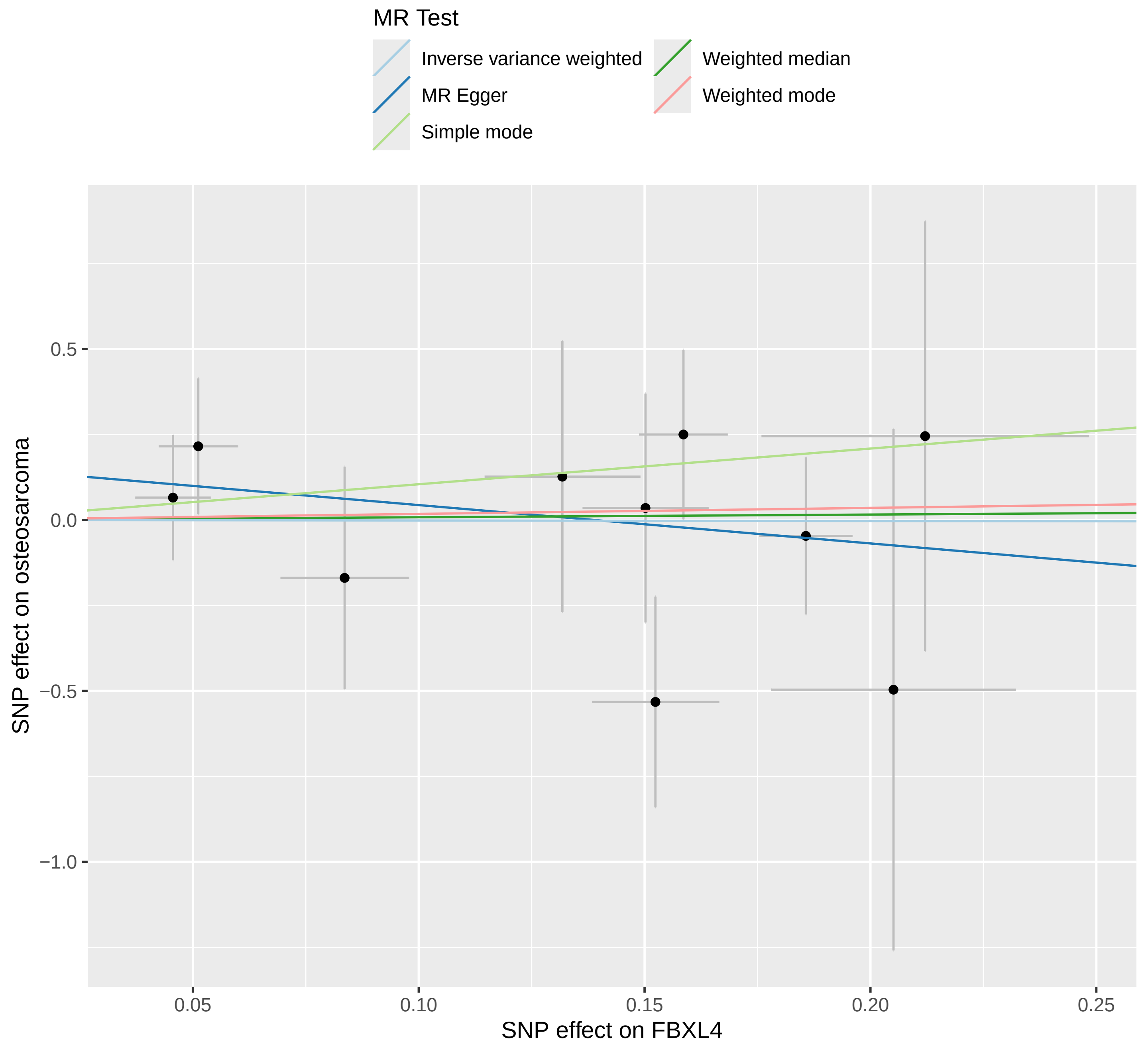
MR Test
Inverse variance weighted
MR Egger
Simple mode
Weighted median
Weighted mode
SNP effect on osteosarcoma
0.5
0.0
−0.5
−1.0
0.05
0.10
0.15
0.20
0.25
SNP effect on FBXL4

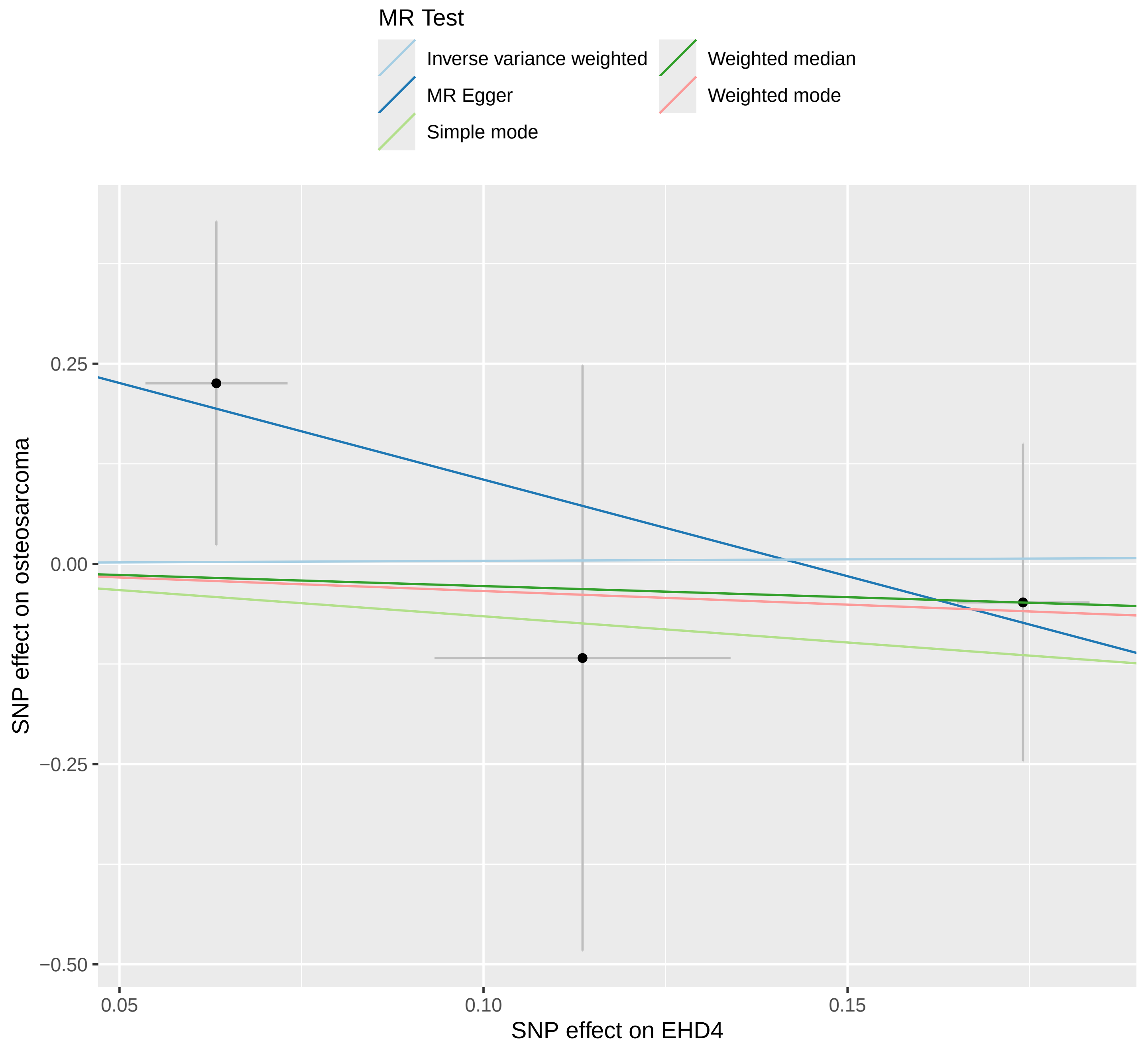
MR Test
Inverse variance weighted
MR Egger
Simple mode
Weighted median
Weighted mode
0.25
0.00
−0.25
−0.50
SNP effect on osteosarcoma
0.05
0.10
0.15
SNP effect on EHD4

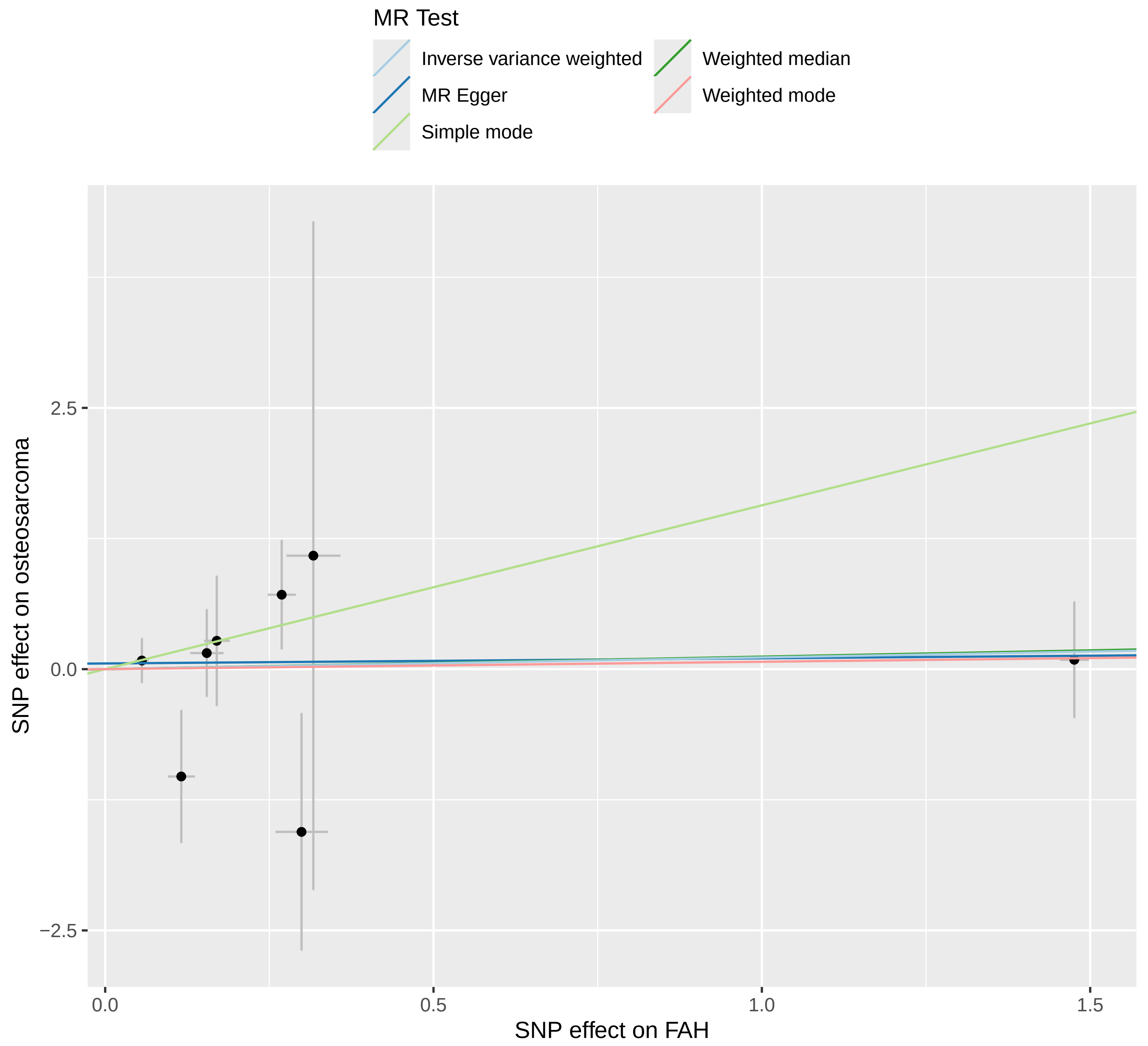

MR Test
Inverse variance weighted
MR Egger
Simple mode
Weighted median
Weighted mode
2.5
0.0
−2.5
0.0
0.5
1.0
1.5
SNP effect on osteosarcoma
SNP effect on FAH

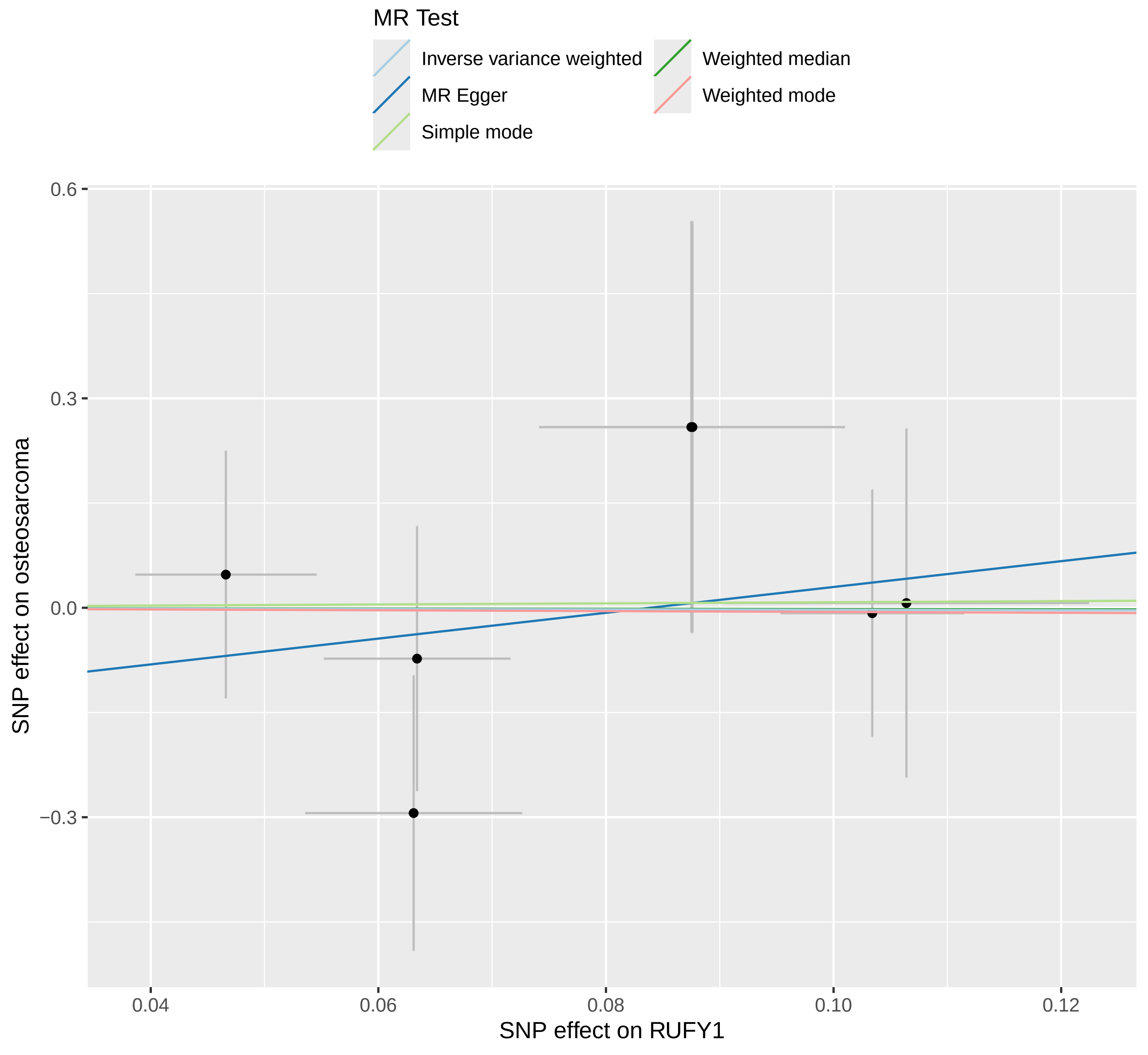
MR Test
Inverse variance weighted
MR Egger
Simple mode
Weighted median
Weighted mode
0.6
0.3
0.0
−0.3
0.04
0.06
0.08
0.10
0.12
SNP effect on osteosarcoma
SNP effect on RUFY1

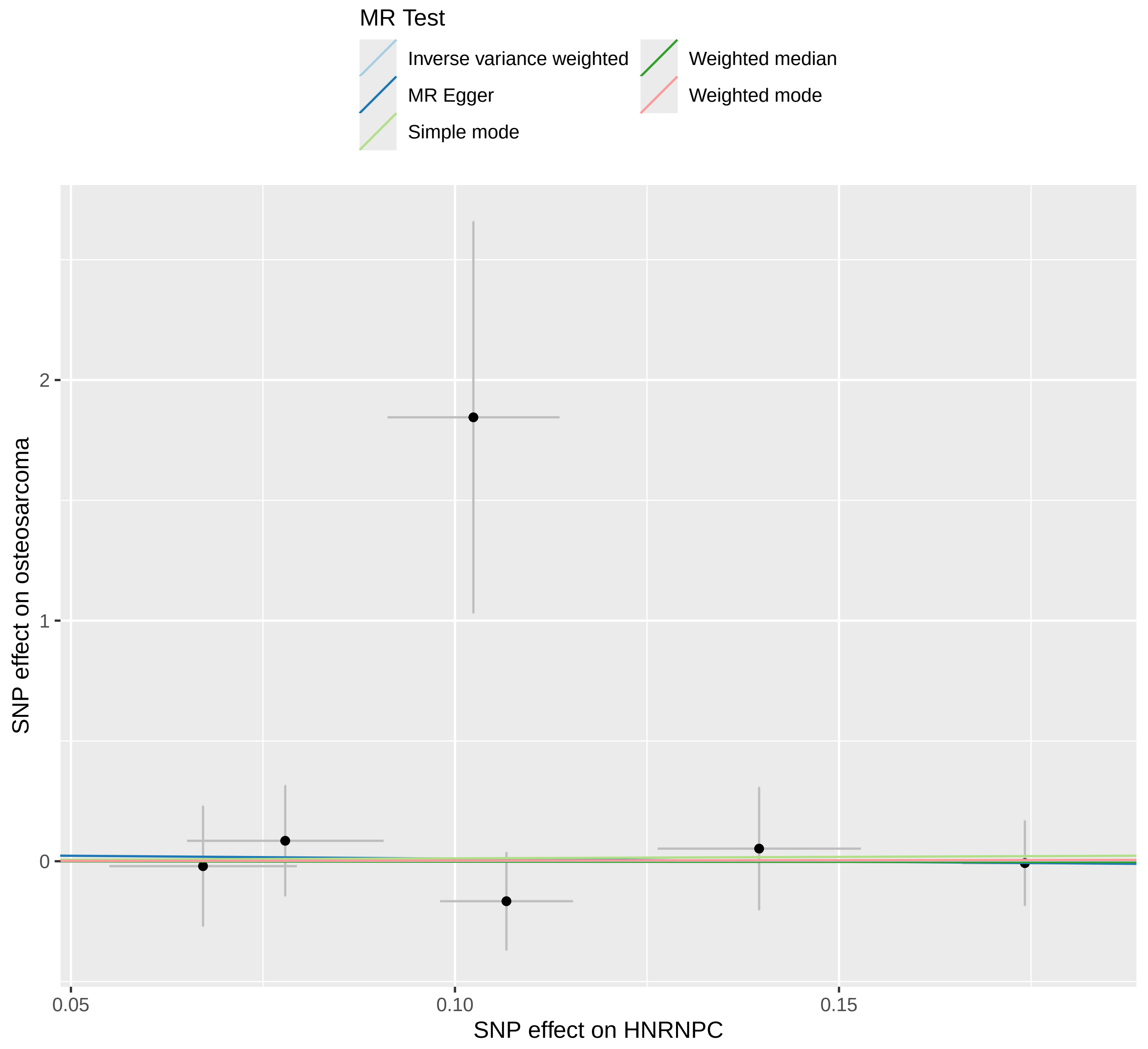
MR Test
Inverse variance weighted
MR Egger
Simple mode
Weighted median
Weighted mode
SNP effect on osteosarcoma
2
1
0
0.05
0.10
0.15
SNP effect on HNRNPC

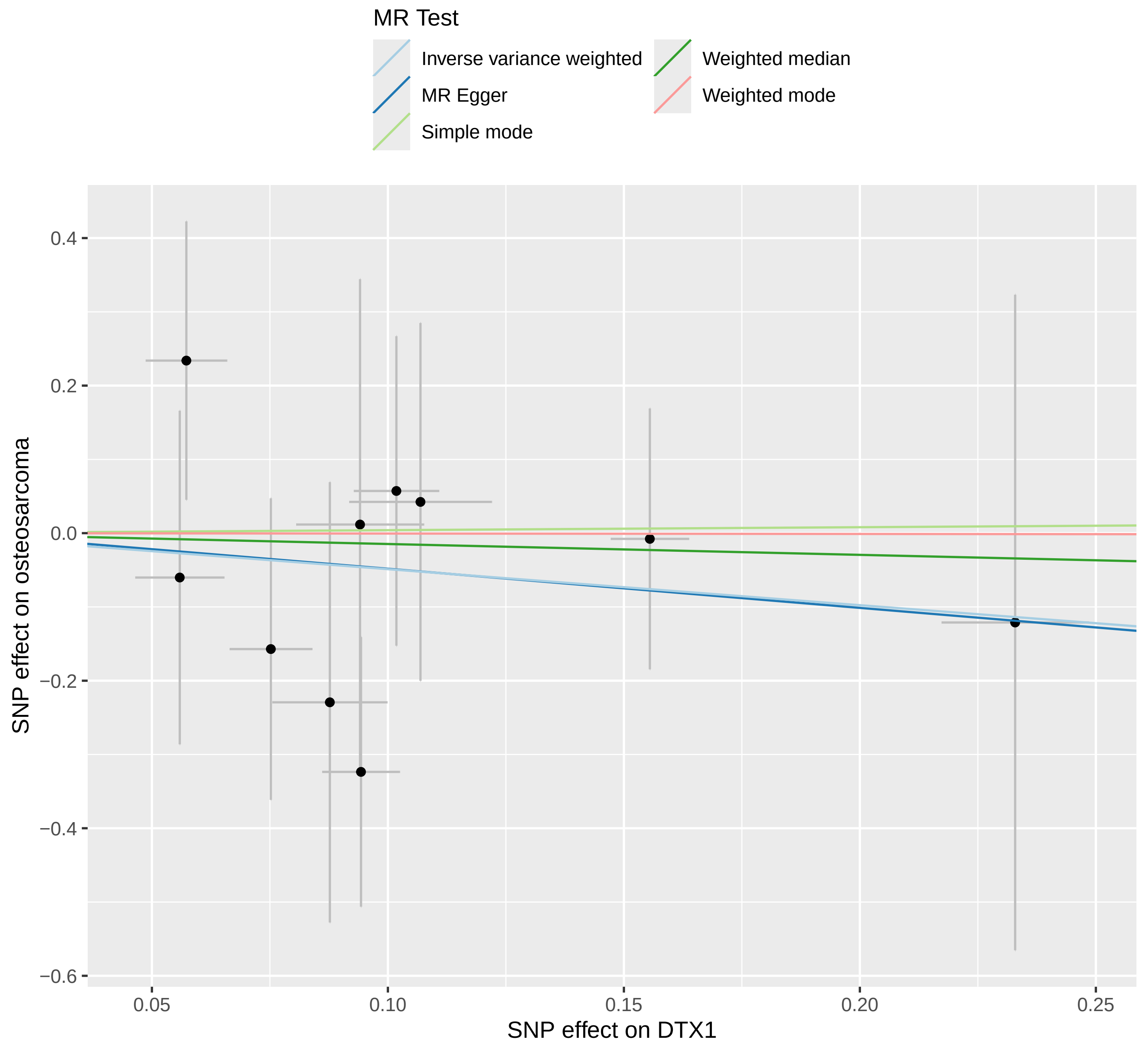

MR Test
Inverse variance weighted
MR Egger
Simple mode
Weighted median
Weighted mode
SNP effect on osteosarcoma
0.4
0.2
0.0
−0.2
−0.4
−0.6
0.05
0.10
0.15
0.20
0.25
SNP effect on DTX1

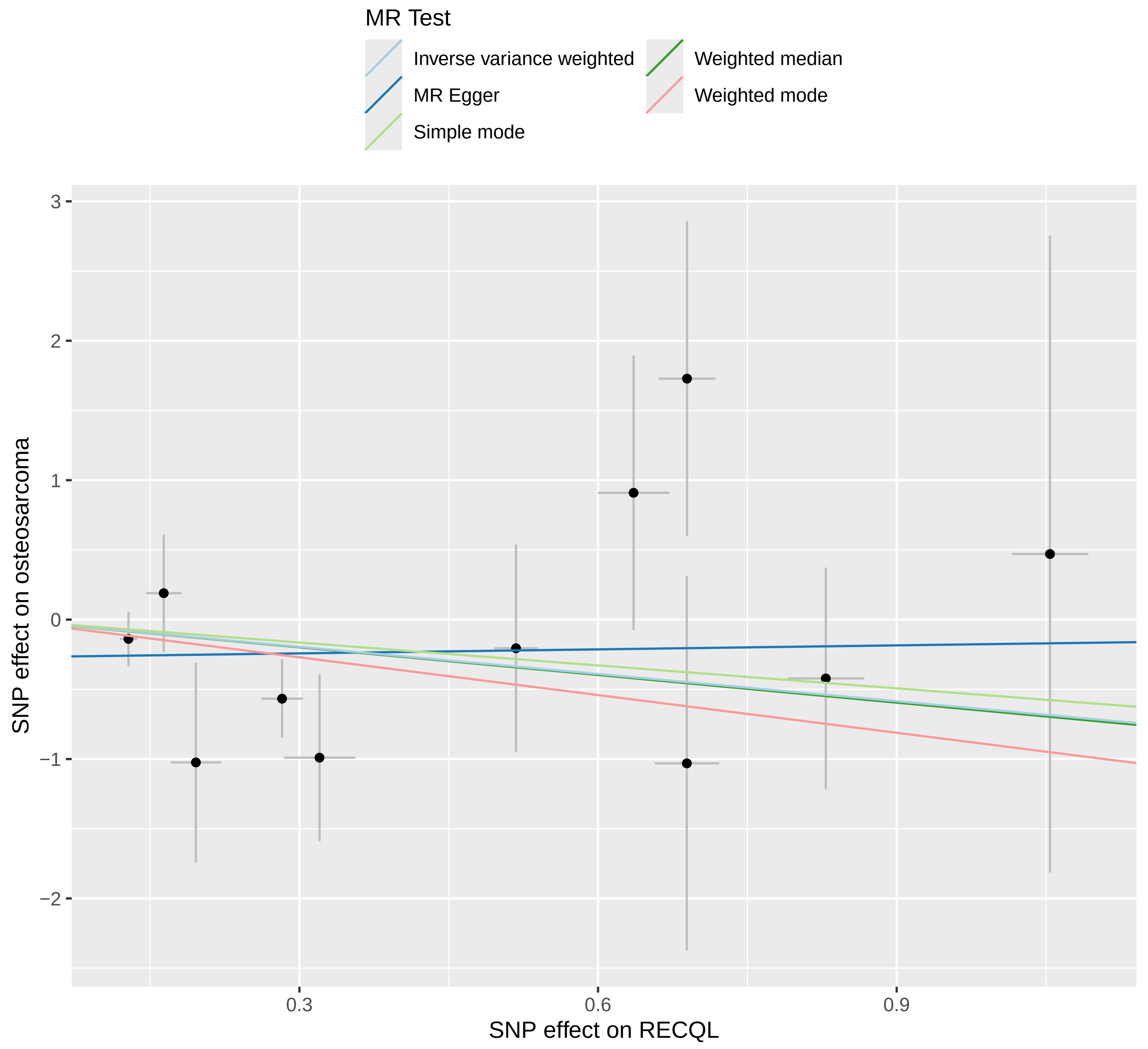

MR Test
Inverse variance weighted
MR Egger
Simple mode
Weighted median
Weighted mode
3
2
1
0
−1
−2
SNP effect on osteosarcoma
0.3
0.6
0.9
SNP effect on RECQL

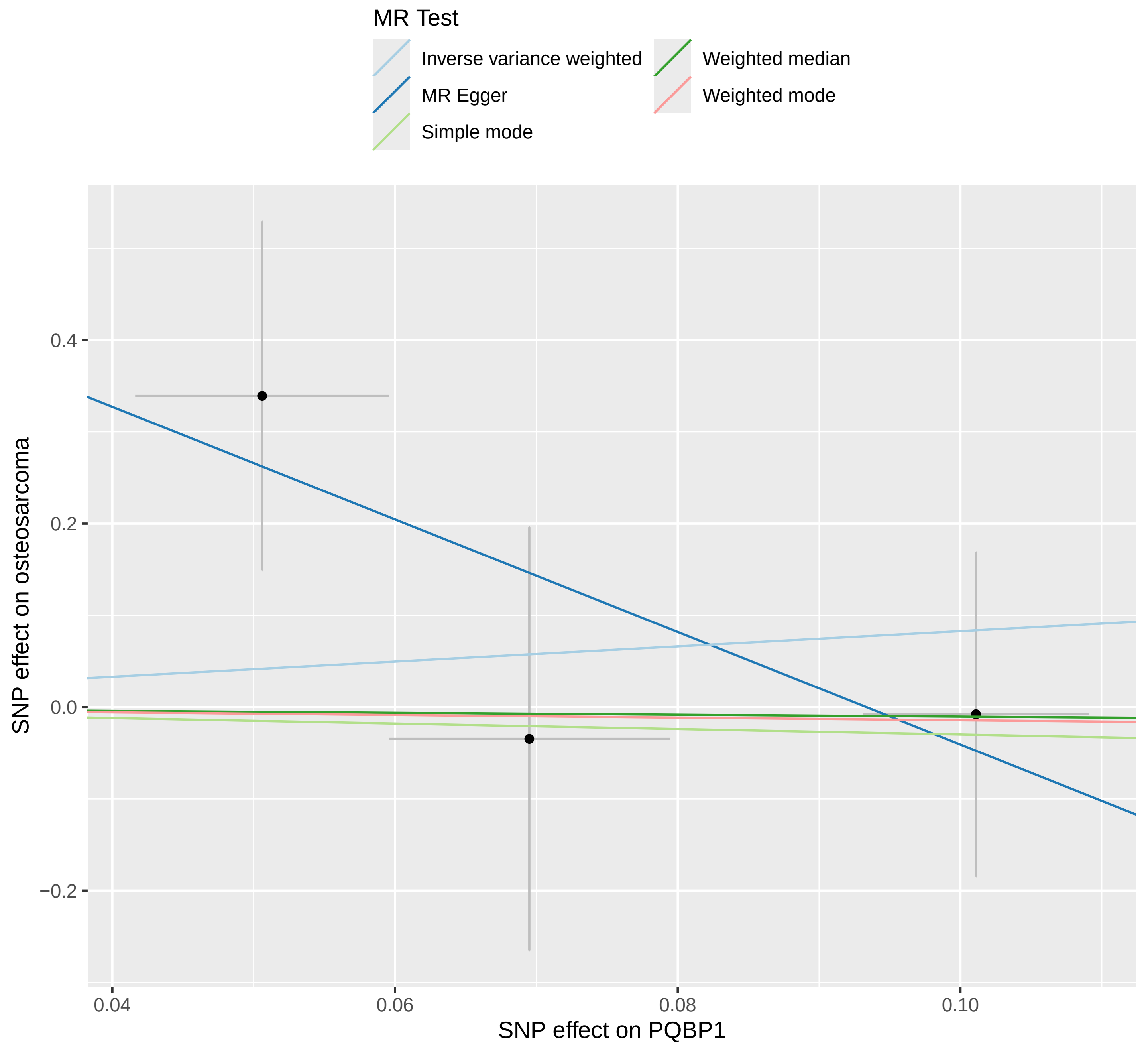
MR Test
Inverse variance weighted
MR Egger
Simple mode
Weighted median
Weighted mode
0.4
0.2
0.0
−0.2
SNP effect on osteosarcoma
0.04
0.06
0.08
0.10
SNP effect on PQBP1

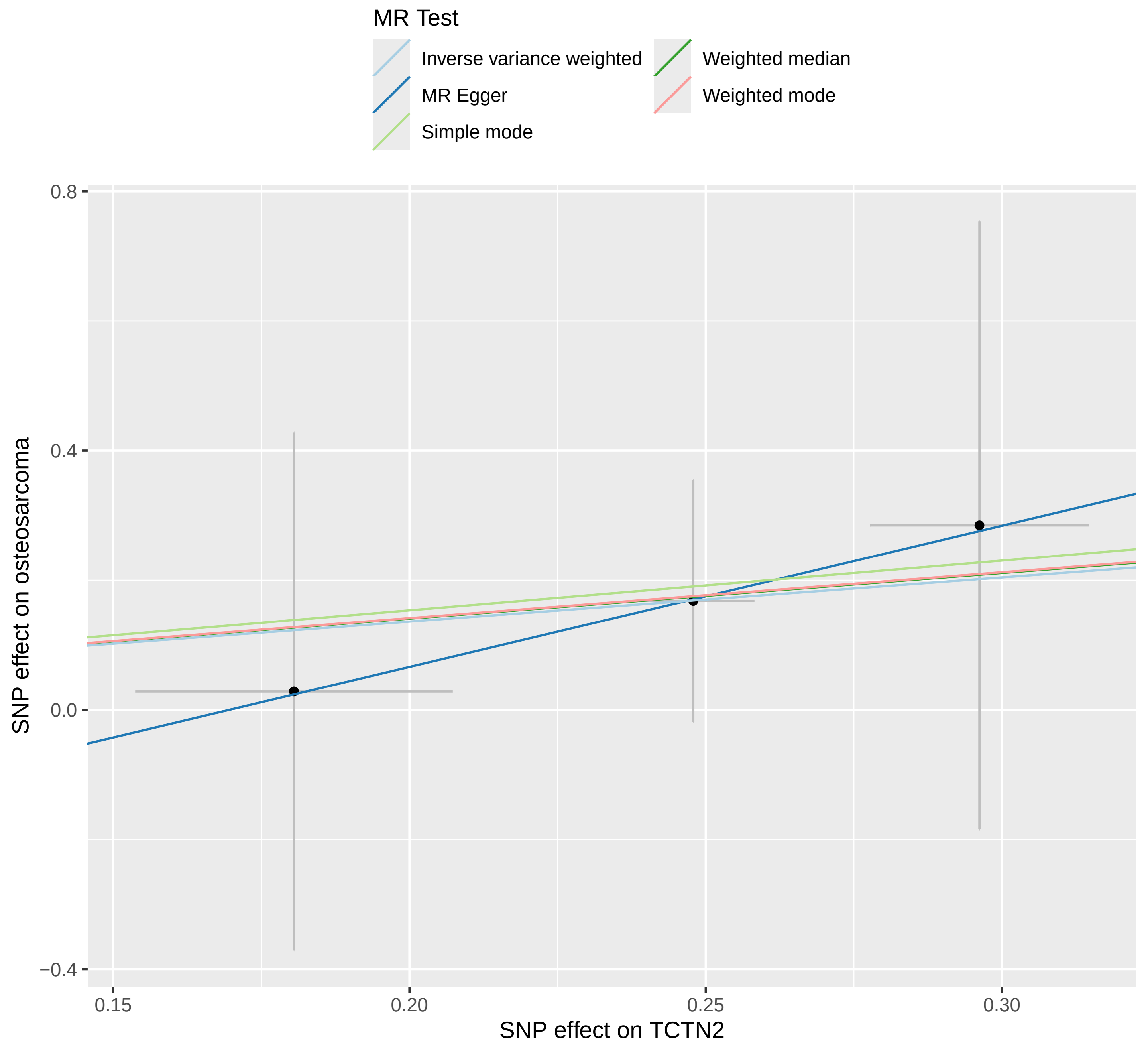
MR Test
Inverse variance weighted
MR Egger
Simple mode
Weighted median
Weighted mode
0.8
0.4
0.0
−0.4
0.15
0.20
0.25
0.30
SNP effect on osteosarcoma
SNP effect on TCTN2

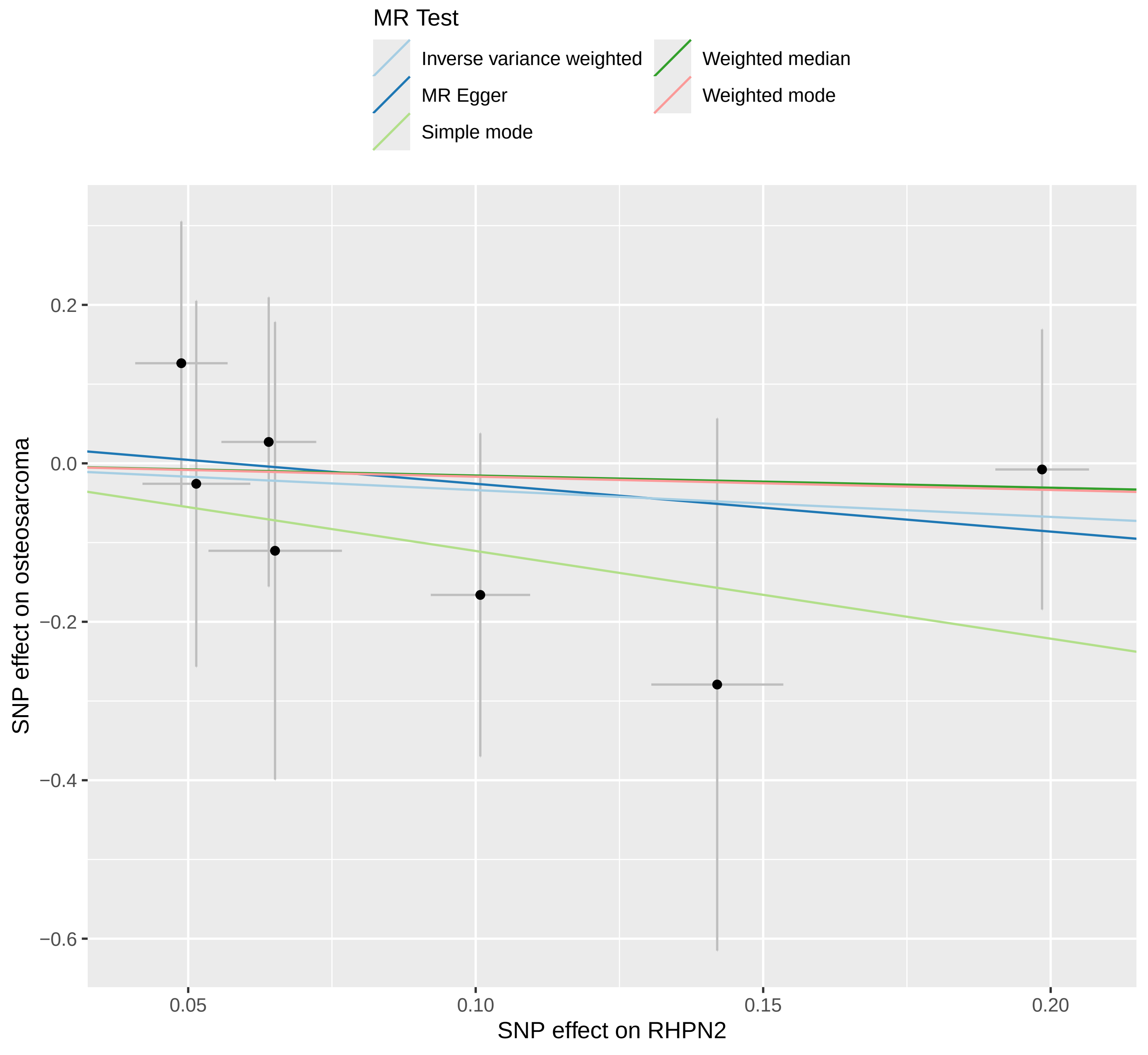
MR Test
Inverse variance weighted
MR Egger
Simple mode
Weighted median
Weighted mode
0.2
0.0
−0.2
−0.4
−0.6
SNP effect on osteosarcoma
0.05
0.10
0.15
0.20
SNP effect on RHPN2

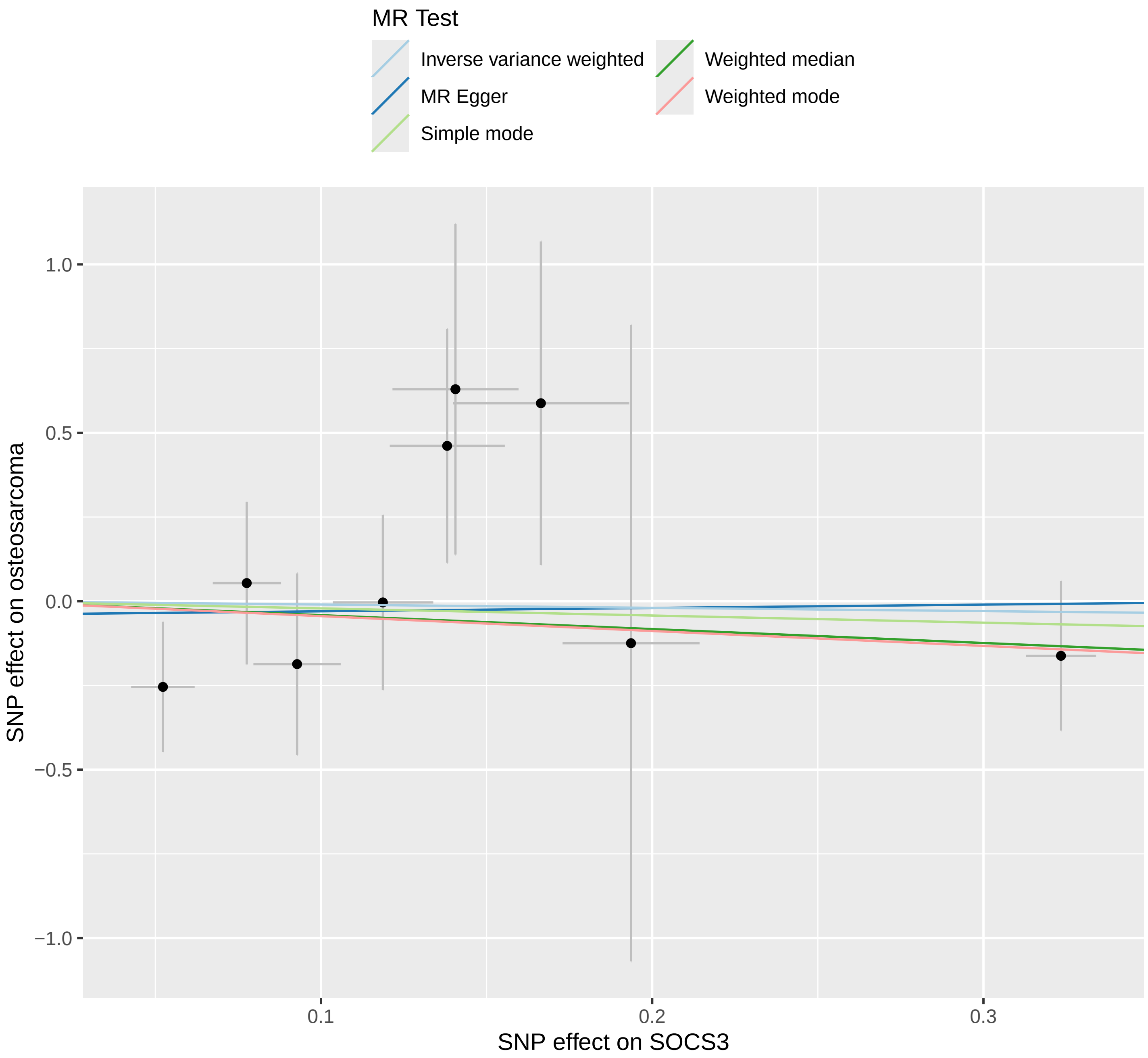
MR Test
Inverse variance weighted
MR Egger
Simple mode
Weighted median
Weighted mode
1.0
0.5
0.0
−0.5
−1.0
SNP effect on osteosarcoma
0.1
0.2
0.3
SNP effect on SOCS3

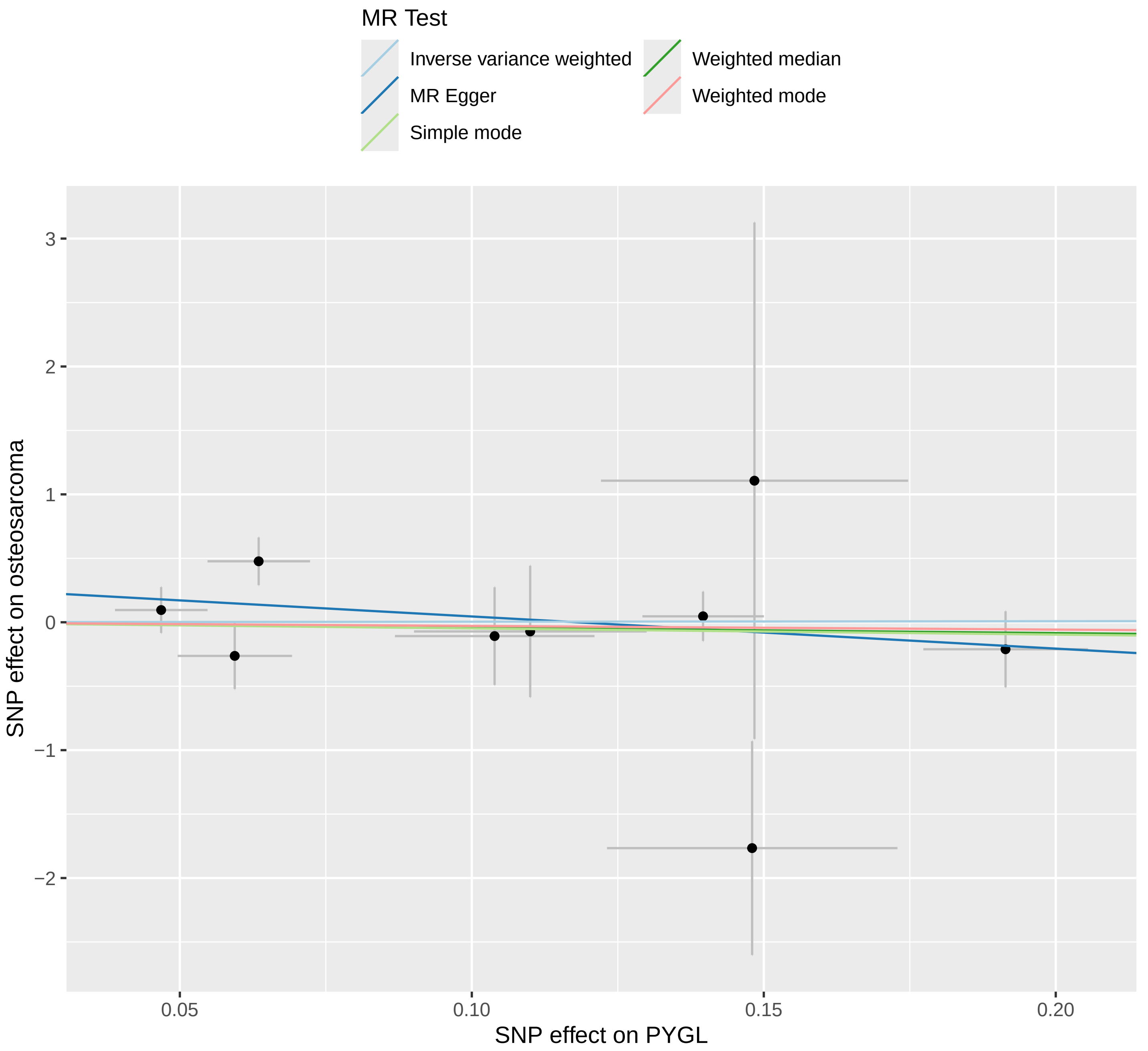
MR Test
Inverse variance weighted
MR Egger
Simple mode
Weighted median
Weighted mode
SNP effect on osteosarcoma
3
2
1
0
−1
−2
0.05
0.10
0.15
0.20
SNP effect on PYGL

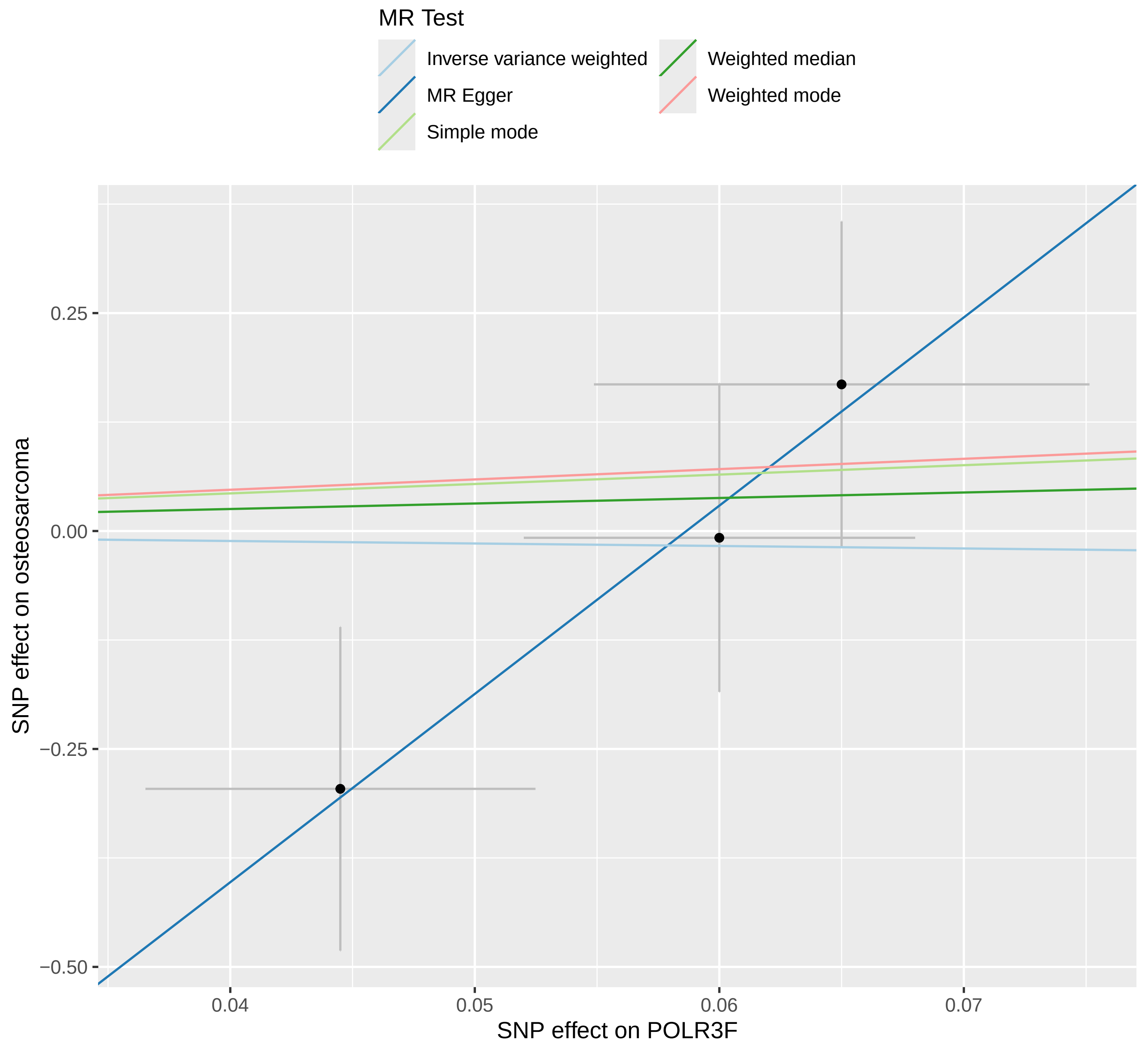
MR Test
Inverse variance weighted
MR Egger
Simple mode
Weighted median
Weighted mode
0.25
0.00
−0.25
−0.50
0.04
0.05
0.06
0.07
SNP effect on osteosarcoma
SNP effect on POLR3F

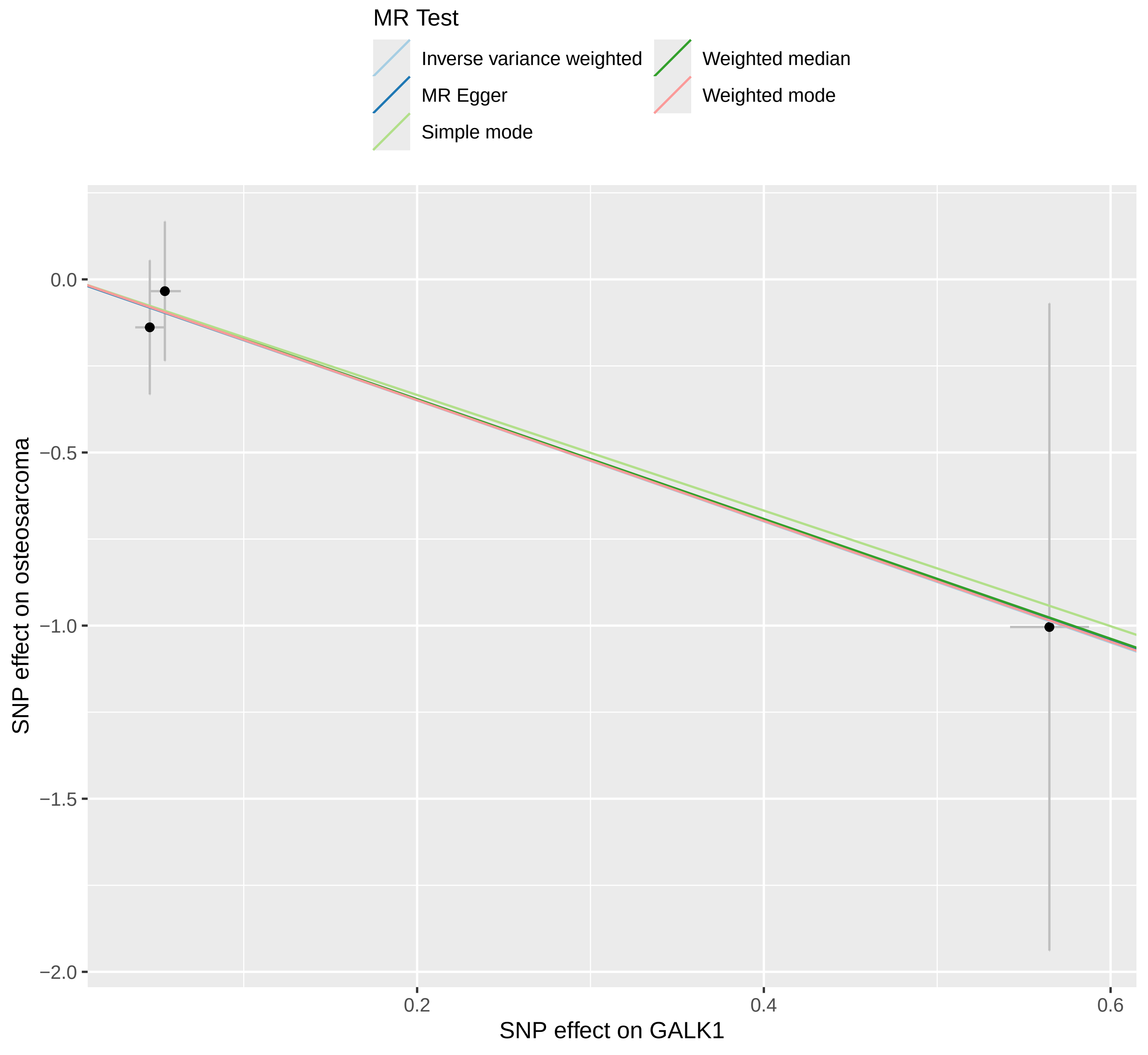

MR Test
Inverse variance weighted
MR Egger
Simple mode
Weighted median
Weighted mode
0.0
−0.5
−1.0
−1.5
−2.0
0.2
0.4
0.6
SNP effect on osteosarcoma
SNP effect on GALK1

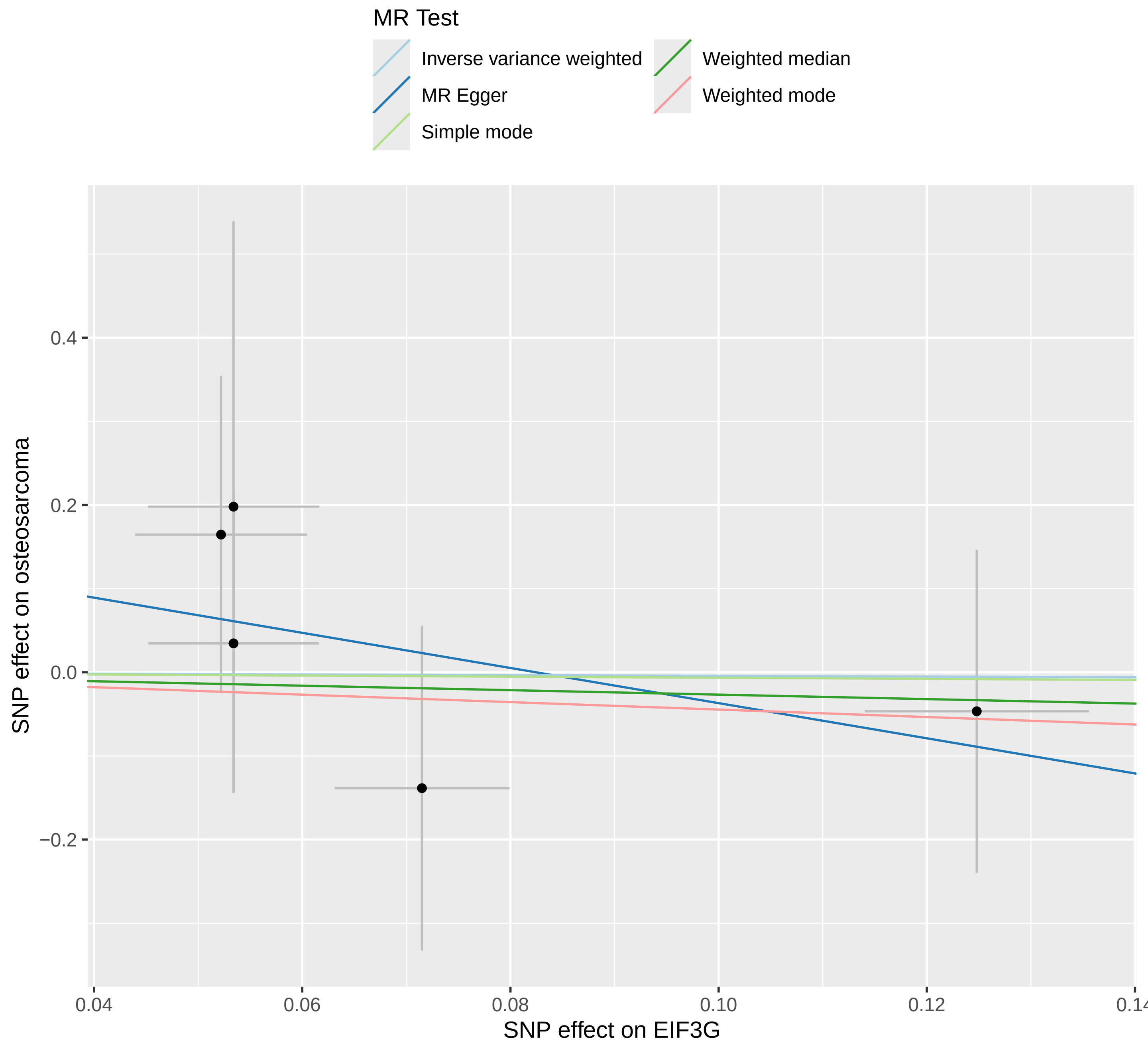
MR Test
Inverse variance weighted
MR Egger
Simple mode
Weighted median
Weighted mode
SNP effect on osteosarcoma
0.4
0.2
0.0
−0.2
0.04
0.06
0.08
0.10
0.12
SNP effect on EIF3G

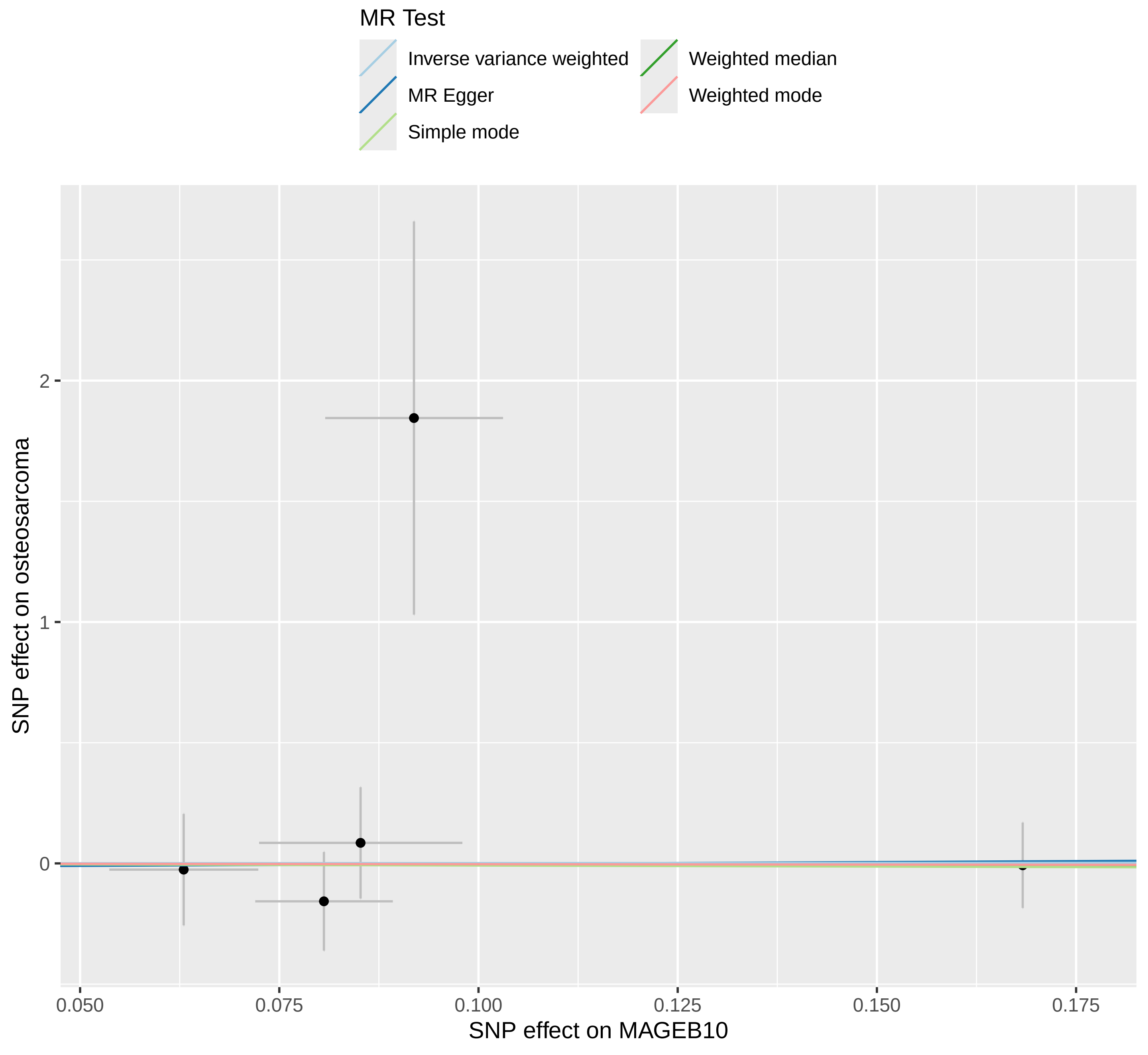
MR Test
Inverse variance weighted
MR Egger
Simple mode
Weighted median
Weighted mode
SNP effect on osteosarcoma
2
1
0
0.050
0.075
0.100
0.125
0.150
0.175
SNP effect on MAGEB10

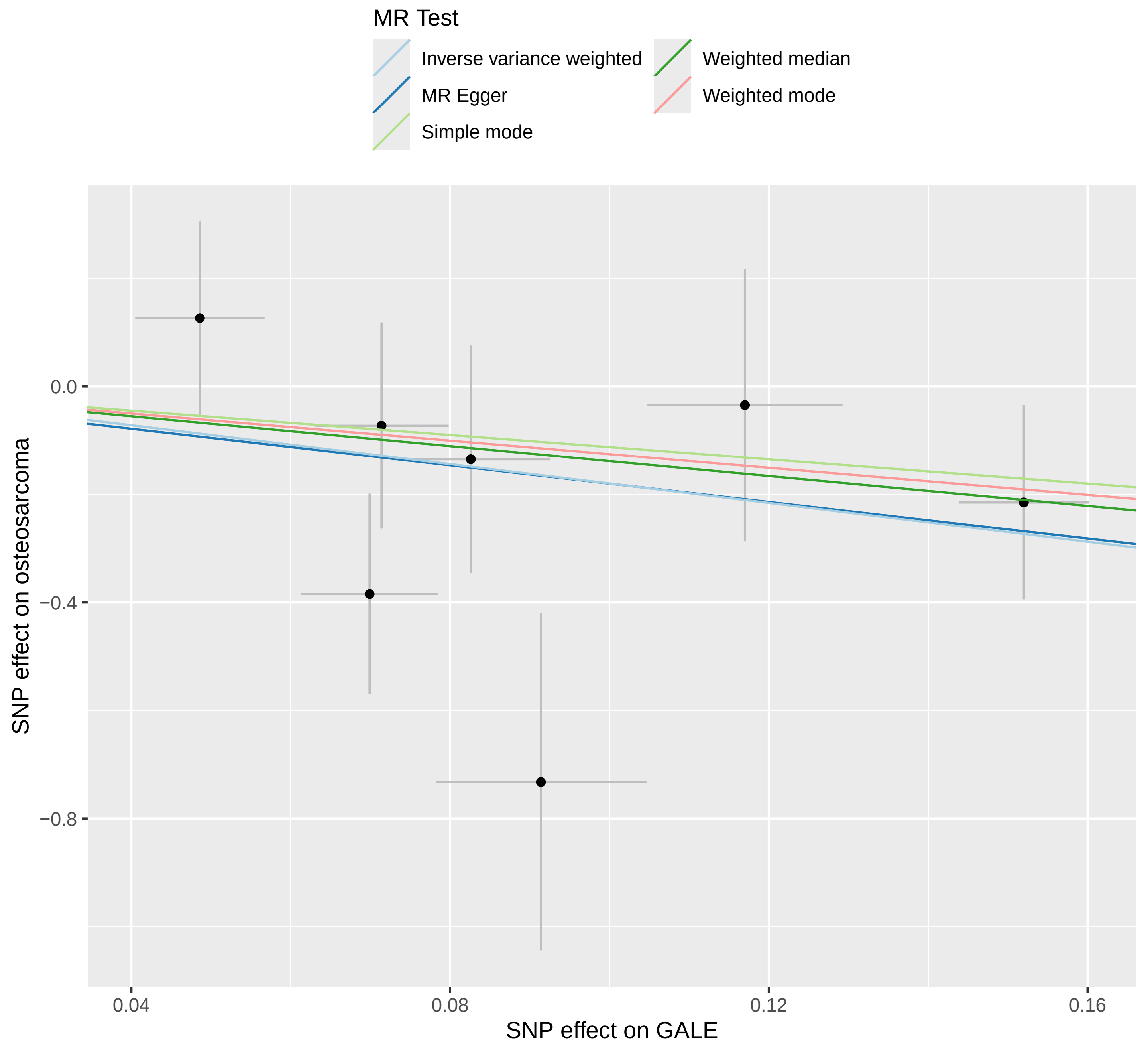

MR Test
Inverse variance weighted
MR Egger
Simple mode
Weighted median
Weighted mode
0.0
−0.4
−0.8
0.04
0.08
0.12
0.16
SNP effect on osteosarcoma
SNP effect on GALE

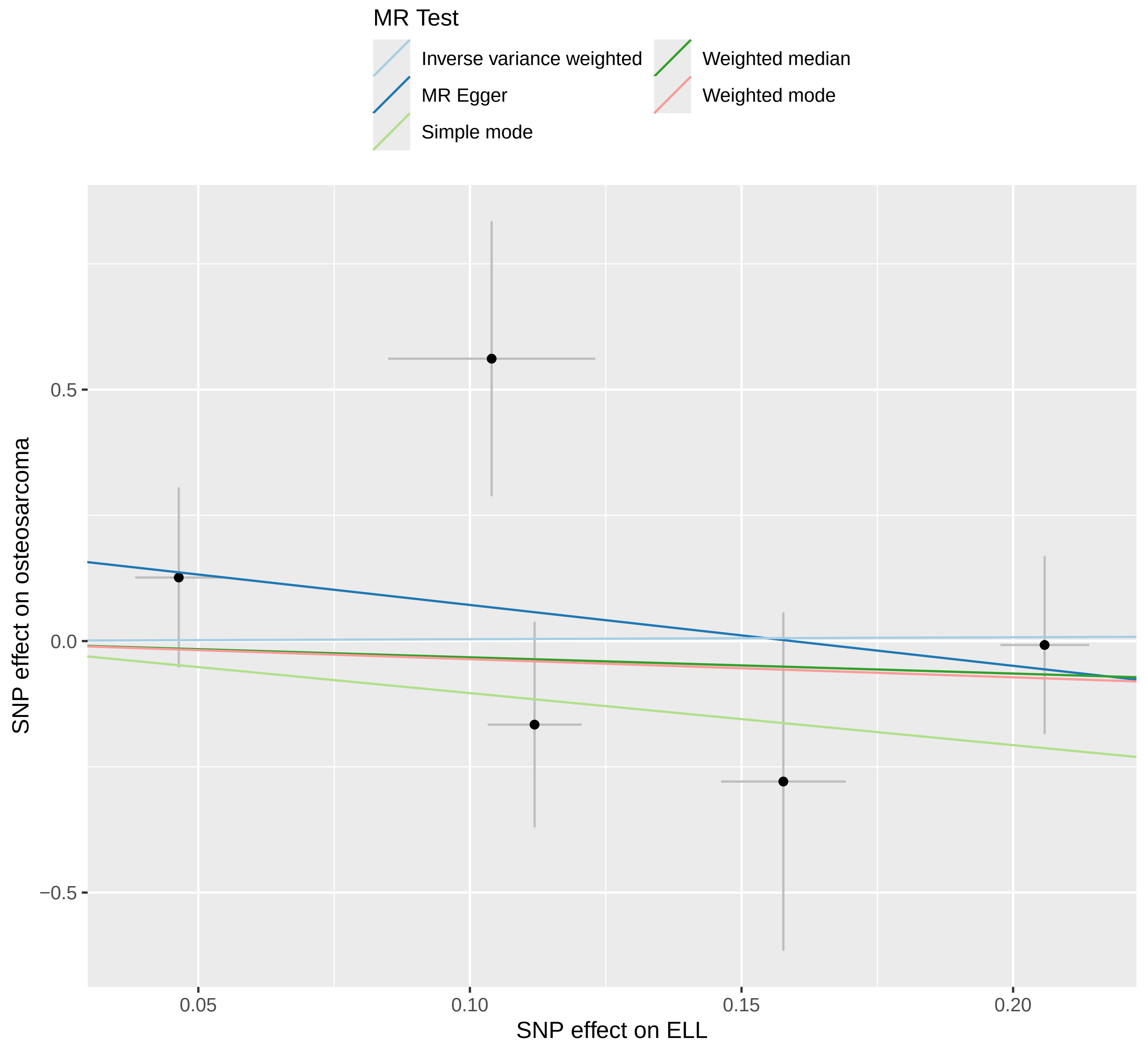
MR Test
Inverse variance weighted
MR Egger
Simple mode
Weighted median
Weighted mode
0.5
0.0
−0.5
SNP effect on osteosarcoma
0.05
0.10
0.15
0.20
SNP effect on ELL

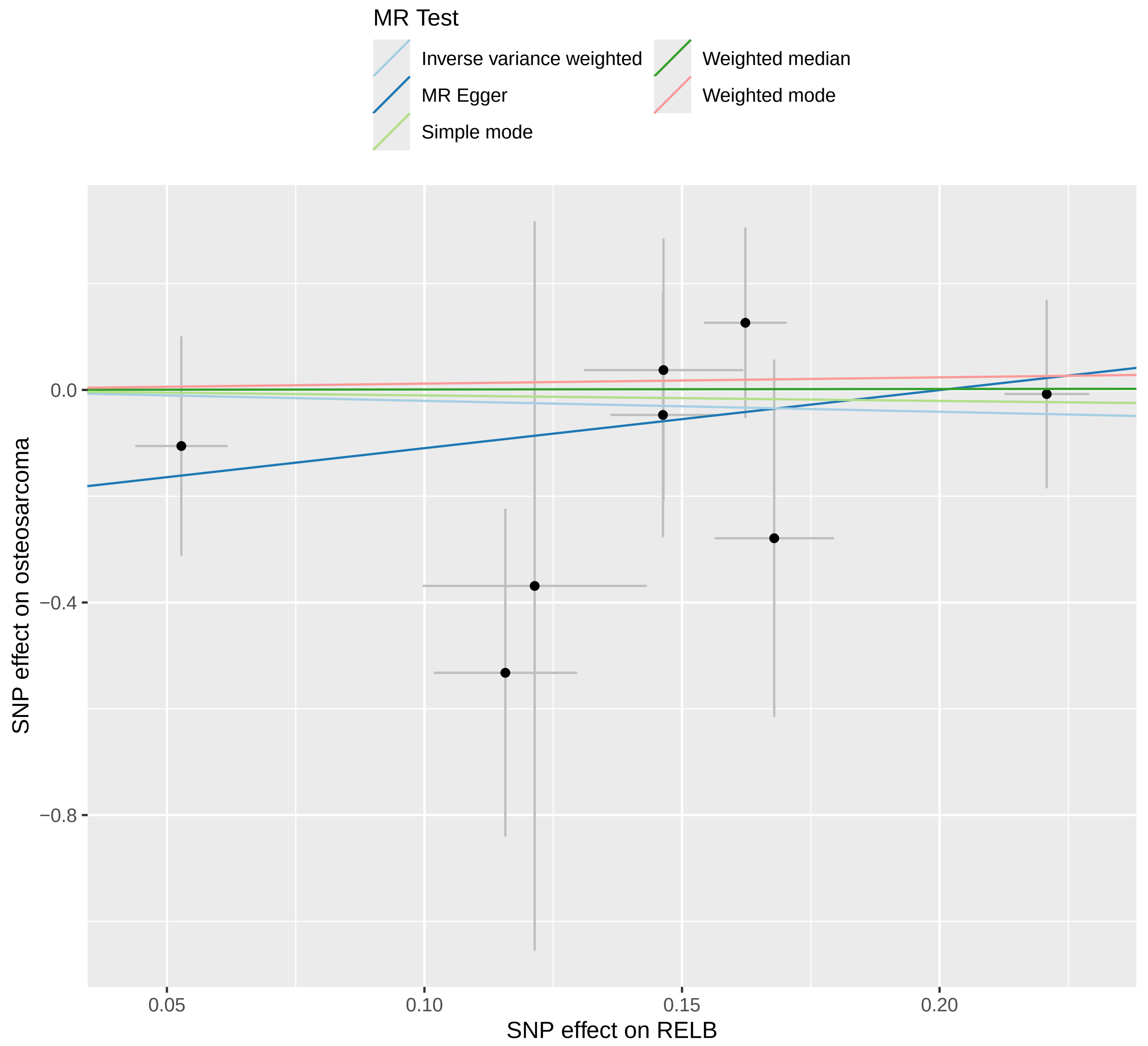
MR Test
Inverse variance weighted
MR Egger
Simple mode
Weighted median
Weighted mode
SNP effect on osteosarcoma
0.0
−0.4
−0.8
0.05
0.10
0.15
0.20
SNP effect on RELB

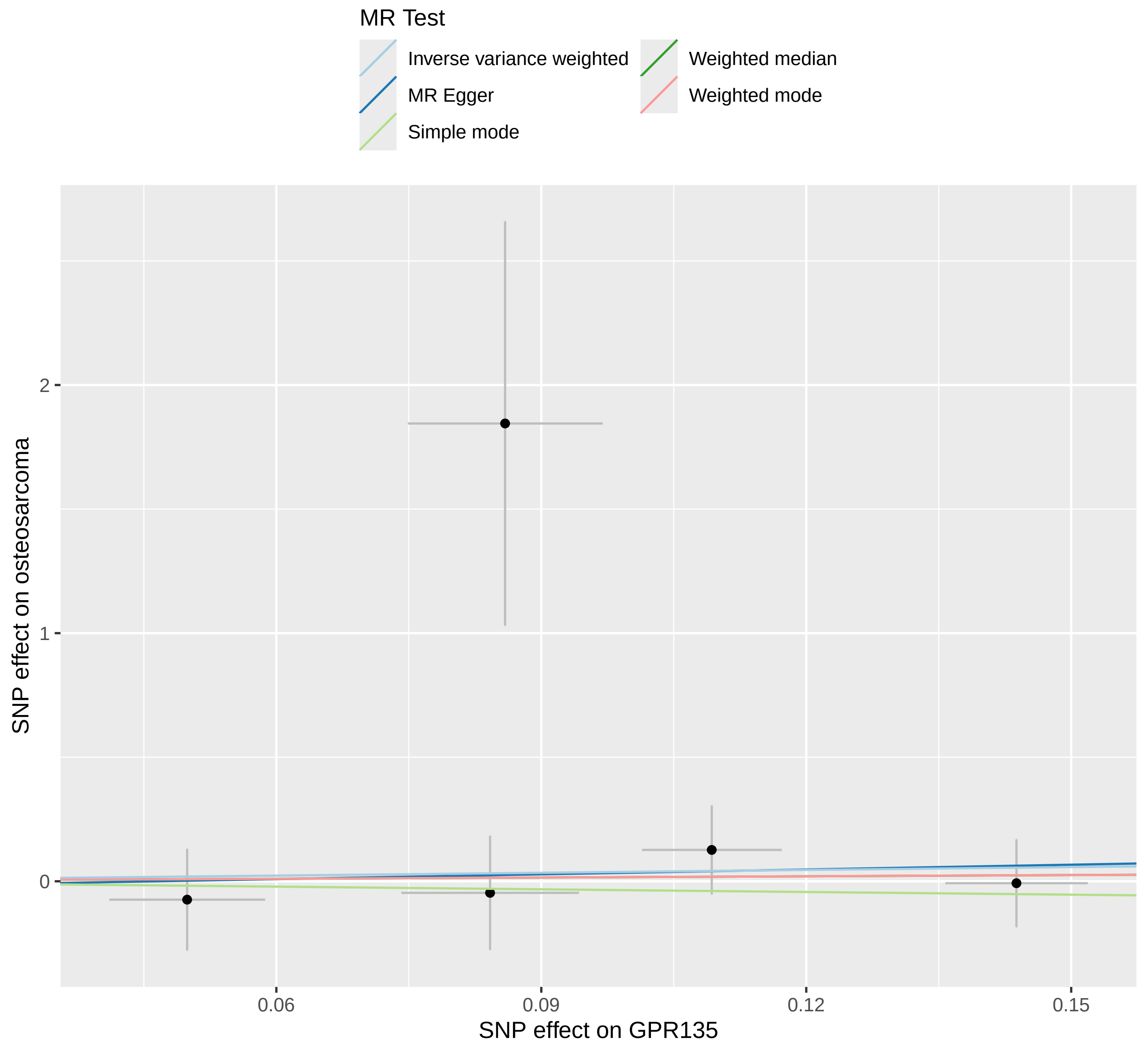

MR Test
Inverse variance weighted
MR Egger
Simple mode
Weighted median
Weighted mode
SNP effect on osteosarcoma
2
1
0
0.06
0.09
0.12
0.15
SNP effect on GPR135

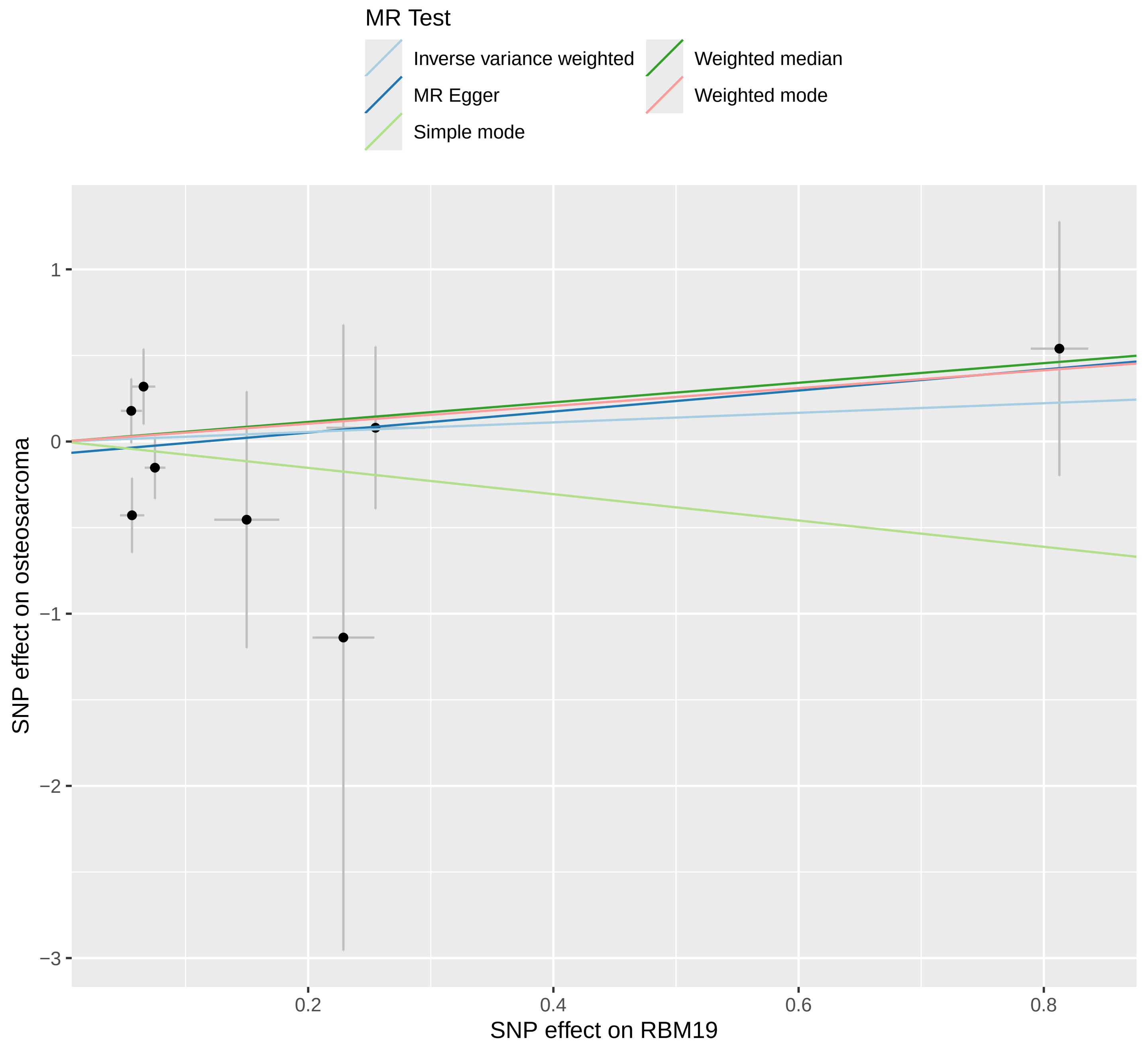
MR Test
Inverse variance weighted
MR Egger
Simple mode
Weighted median
Weighted mode
1
0
−1
−2
−3
0.2
0.4
0.6
0.8
SNP effect on osteosarcoma
SNP effect on RBM19

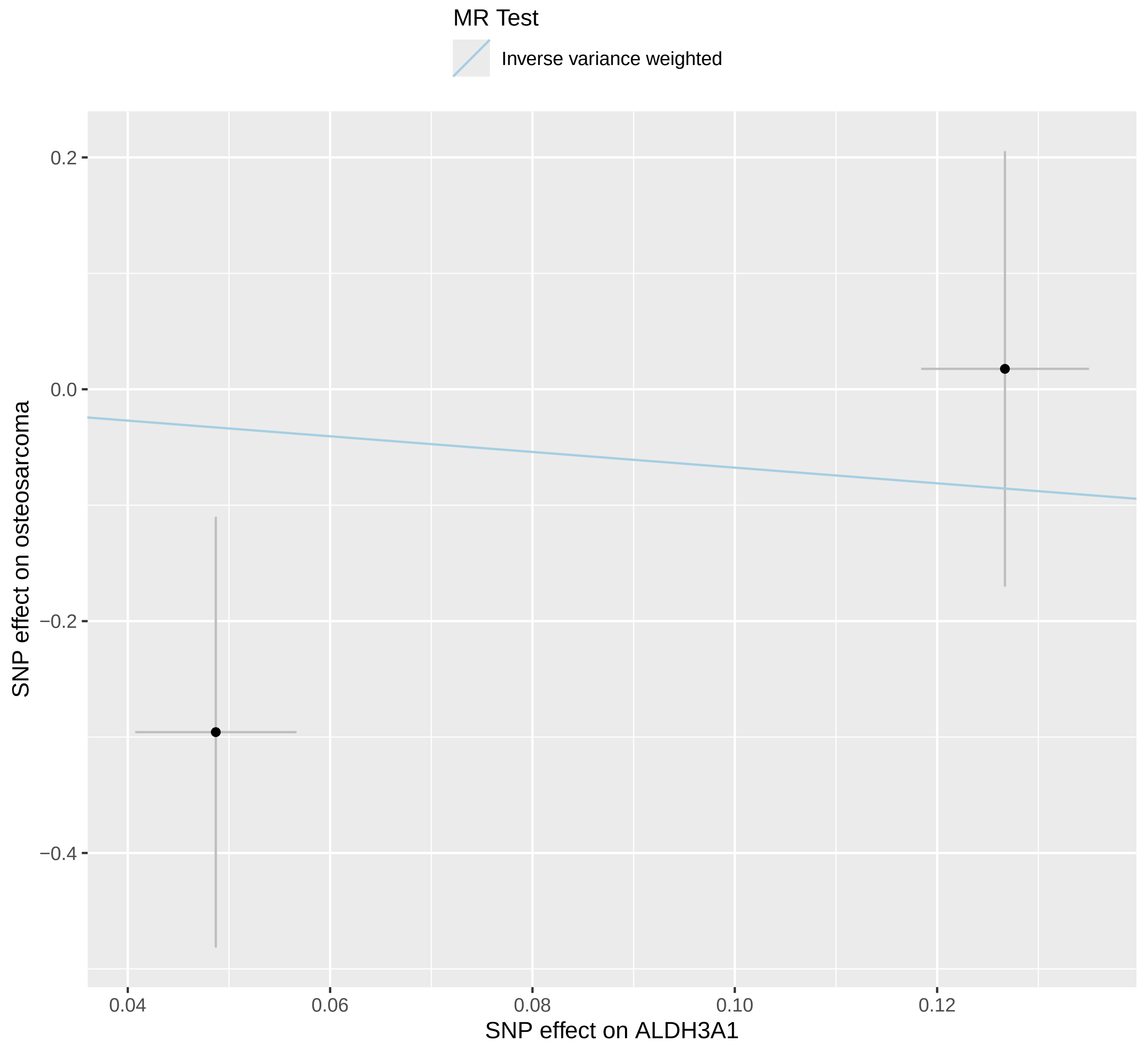

MR Test
Inverse variance weighted
0.2
0.0
−0.2
−0.4
SNP effect on osteosarcoma
0.04
0.06
0.08
0.10
0.12
SNP effect on ALDH3A1

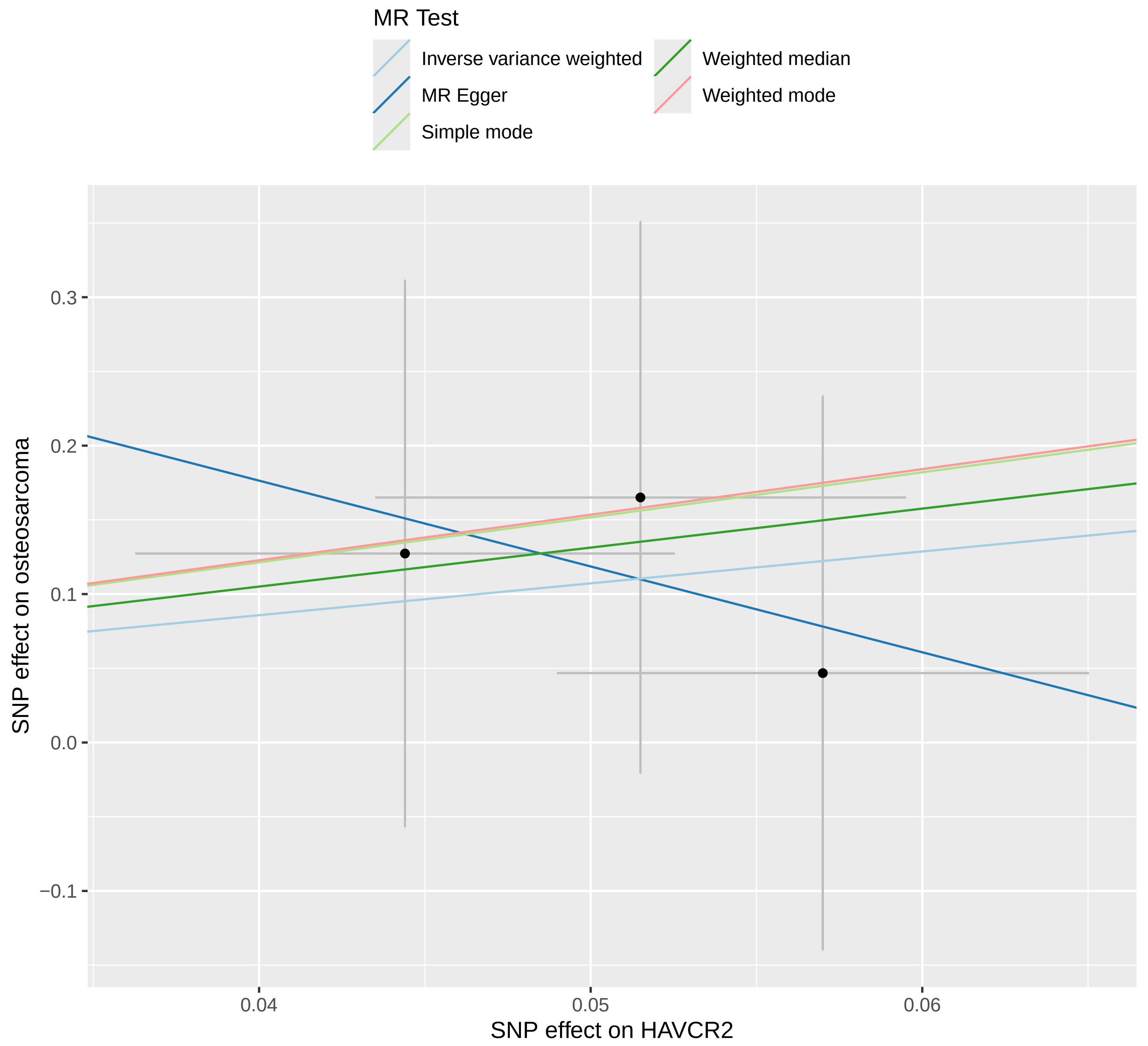
MR Test
Inverse variance weighted
MR Egger
Simple mode
Weighted median
Weighted mode
SNP effect on osteosarcoma
0.3
0.2
0.1
0.0
−0.1
0.04
0.05
0.06
SNP effect on HAVCR2

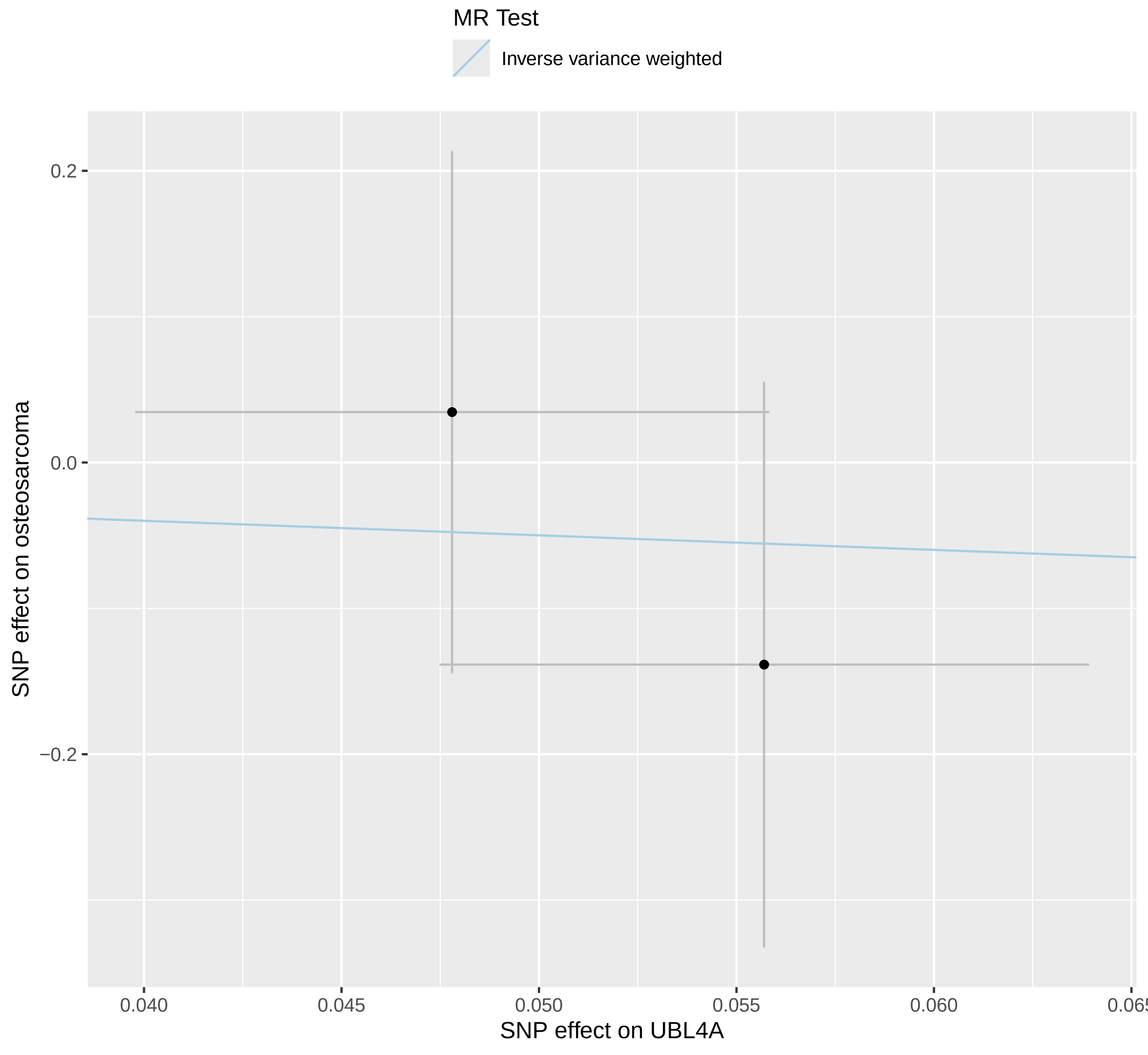
MR Test
Inverse variance weighted
0.2
0.0
−0.2
SNP effect on osteosarcoma
0.040
0.045
0.050
0.055
0.060
SNP effect on UBL4A

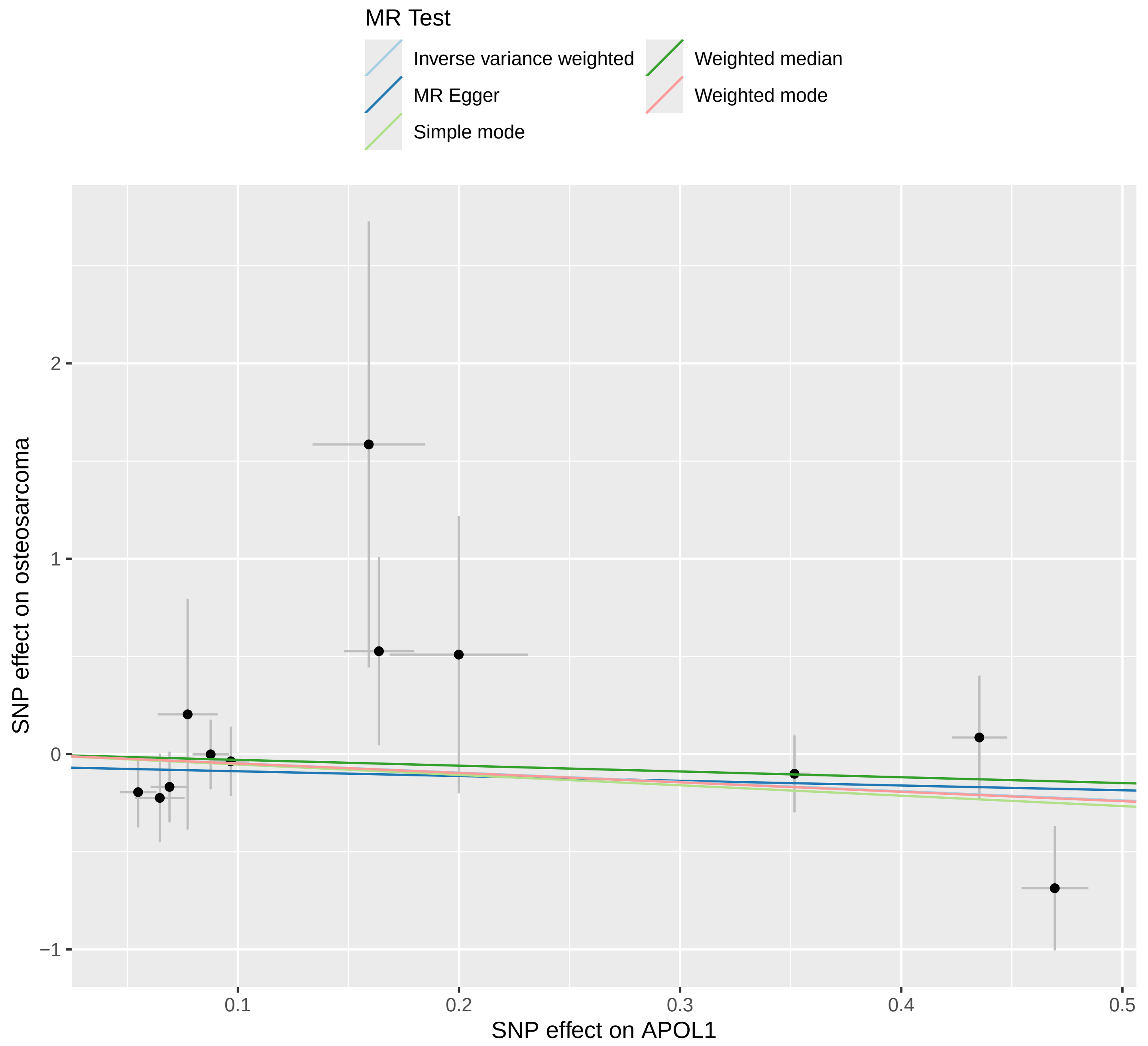

MR Test
Inverse variance weighted
MR Egger
Simple mode
Weighted median
Weighted mode
SNP effect on osteosarcoma
2
1
0
−1
0.1
0.2
0.3
0.4
0.5
SNP effect on APOL1

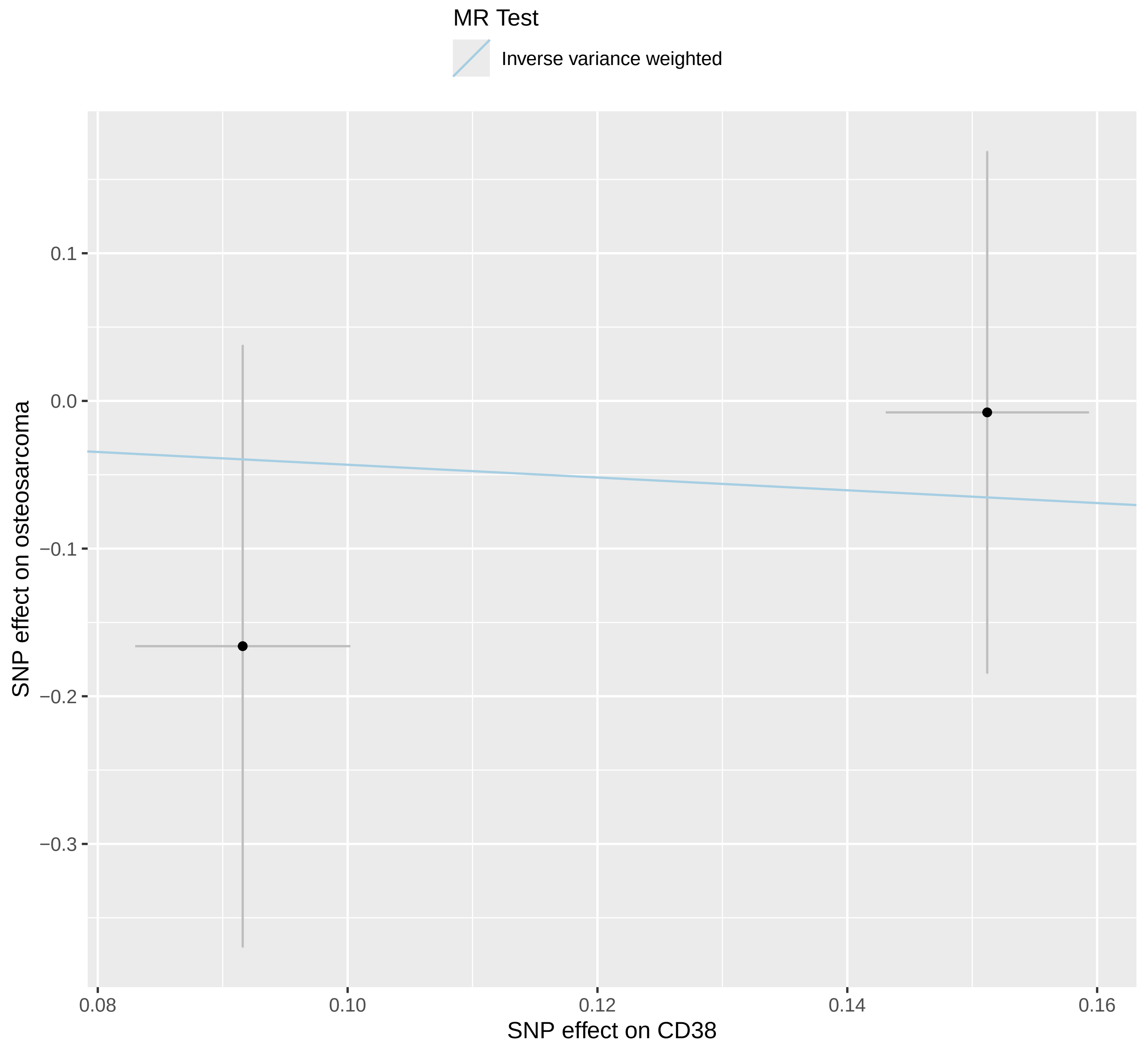

MR Test
Inverse variance weighted
0.1
0.0
−0.1
−0.2
−0.3
SNP effect on osteosarcoma
0.08
0.10
0.12
0.14
0.16
SNP effect on CD38

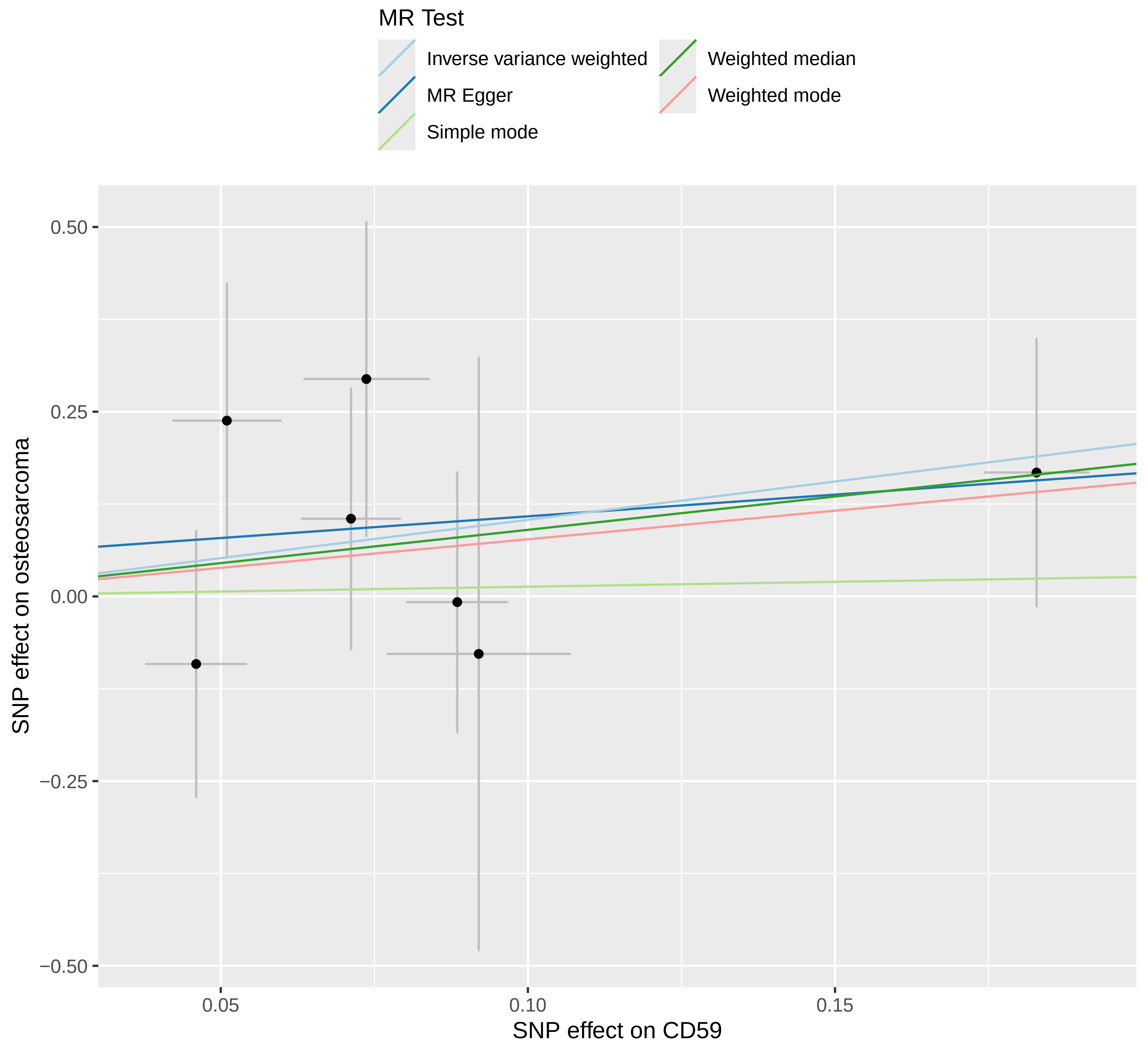
MR Test
Inverse variance weighted
MR Egger
Simple mode
Weighted median
Weighted mode
0.50
0.25
0.00
−0.25
−0.50
SNP effect on osteosarcoma
0.05
0.10
0.15
SNP effect on CD59

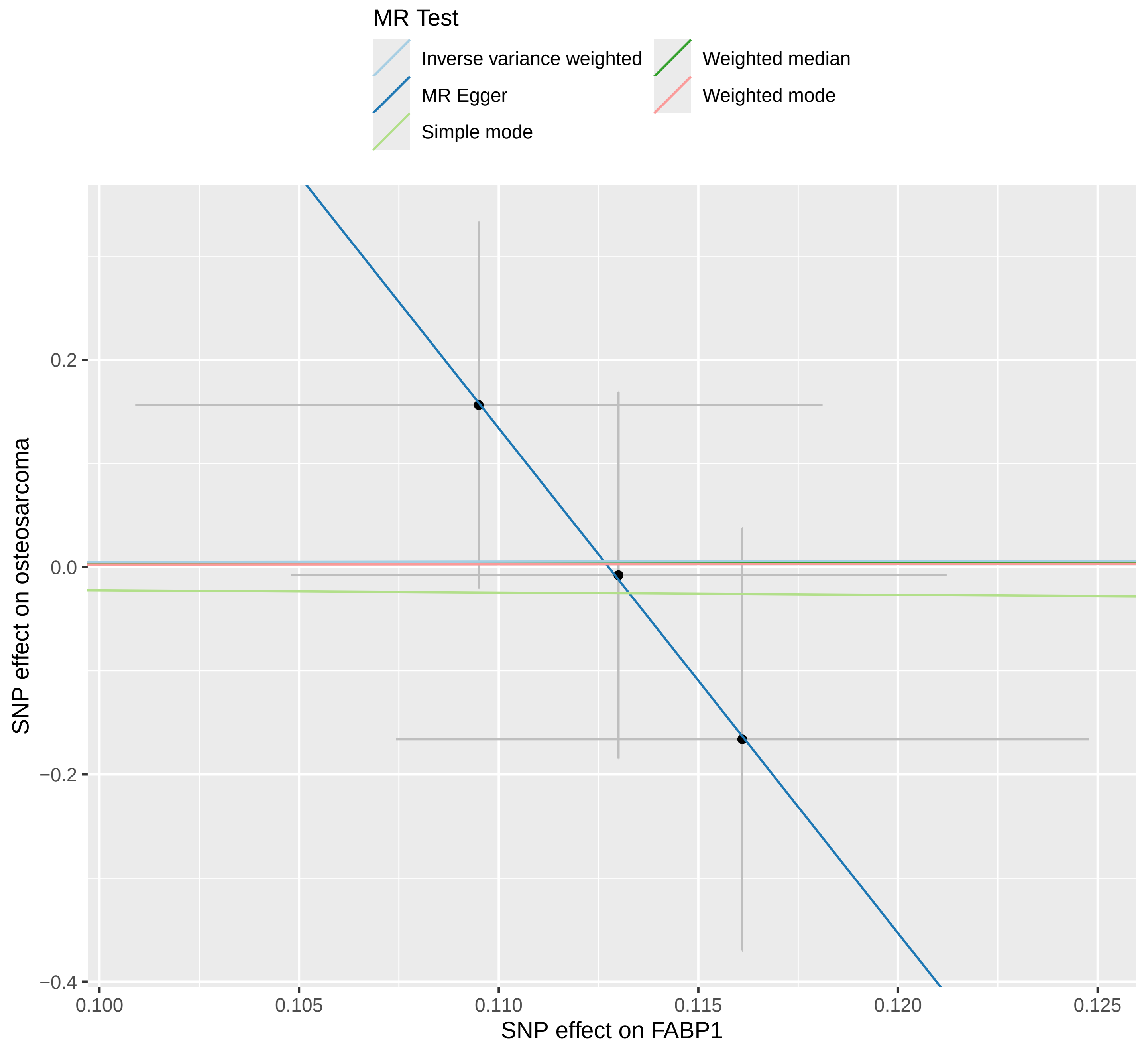

MR Test
Inverse variance weighted
MR Egger
Simple mode
Weighted median
Weighted mode
SNP effect on osteosarcoma
0.2
0.0
−0.2
−0.4
0.100
0.105
0.110
0.115
0.120
0.125
SNP effect on FABP1

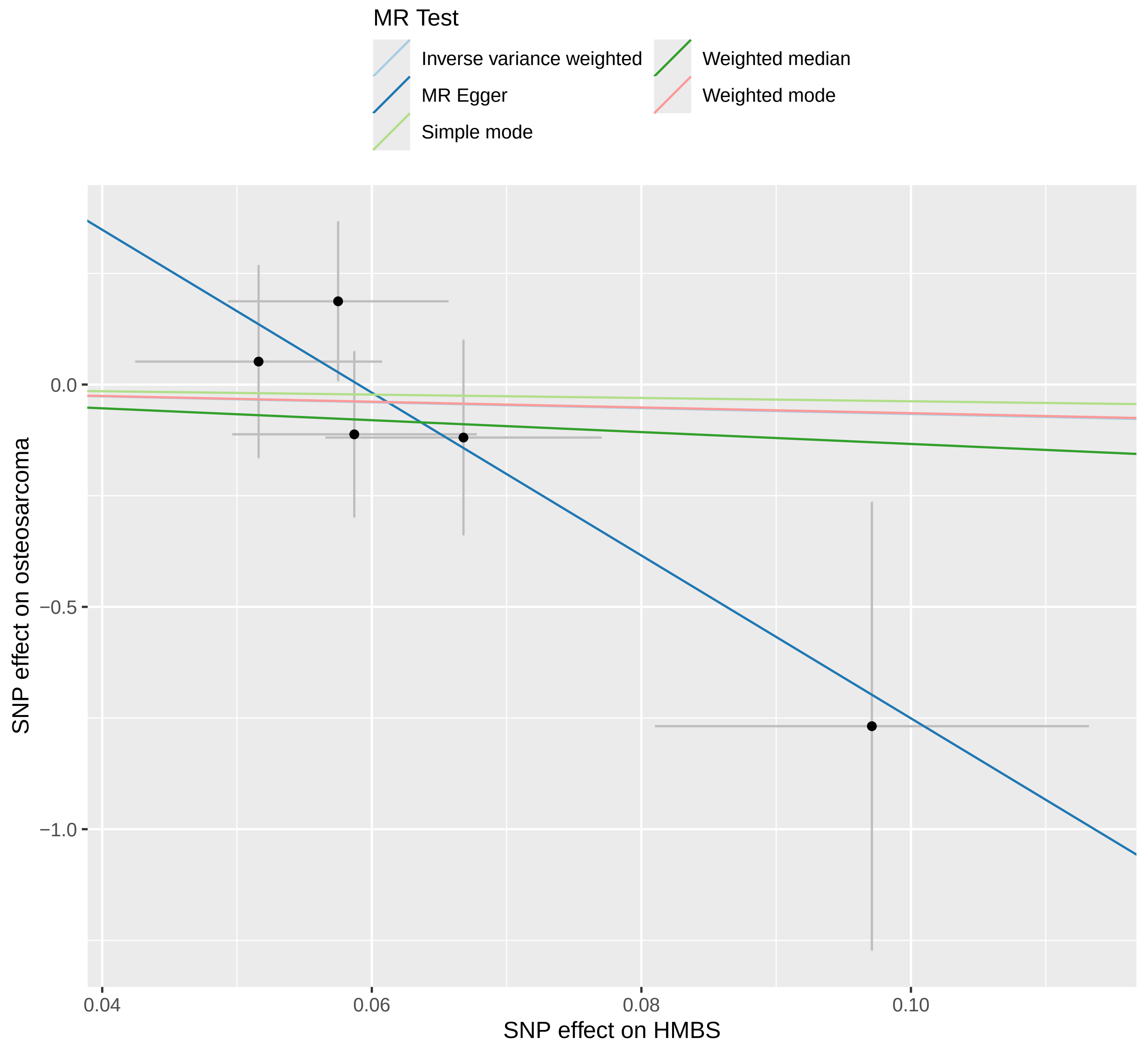
MR Test
Inverse variance weighted
MR Egger
Simple mode
Weighted median
Weighted mode
0.0
−0.5
−1.0
0.04
0.06
0.08
0.10
SNP effect on osteosarcoma
SNP effect on HMBS

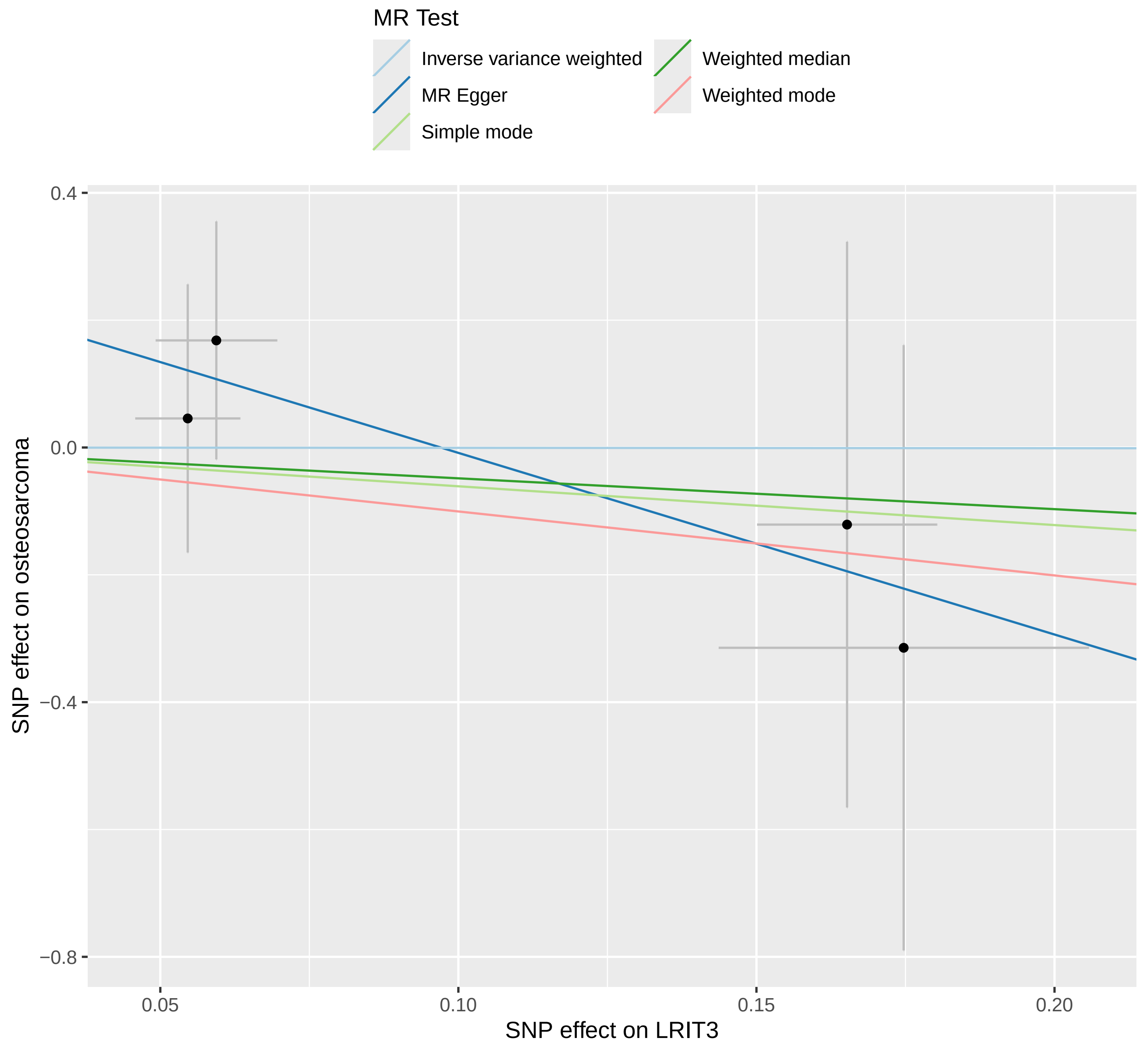

MR Test
Inverse variance weighted
MR Egger
Simple mode
Weighted median
Weighted mode
0.4
0.0
−0.4
−0.8
SNP effect on osteosarcoma
0.05
0.10
0.15
0.20
SNP effect on LRIT3

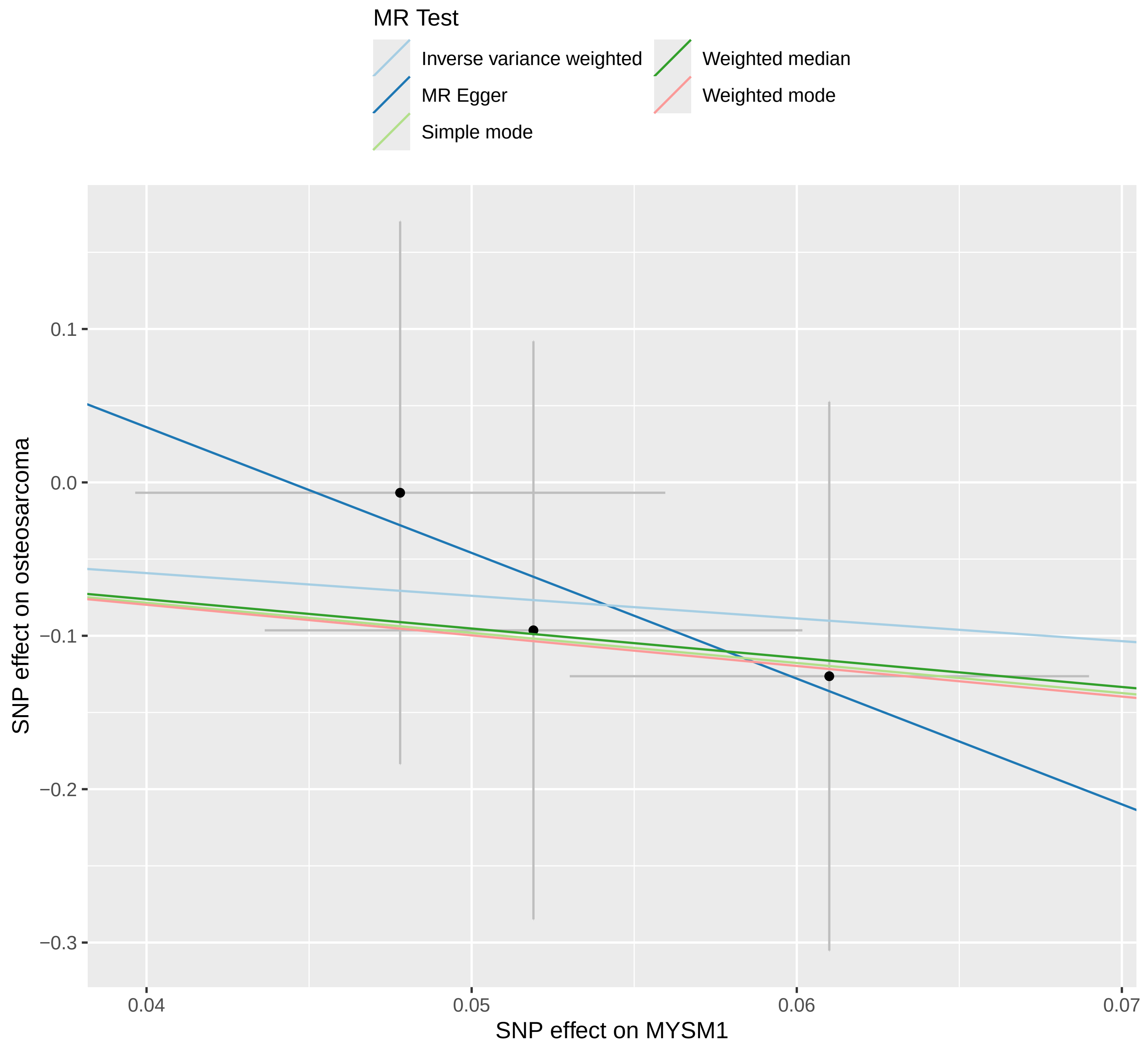
MR Test
Inverse variance weighted
MR Egger
Simple mode
Weighted median
Weighted mode
0.1
0.0
−0.1
−0.2
−0.3
SNP effect on osteosarcoma
0.04
0.05
0.06
0.07
SNP effect on MYSM1

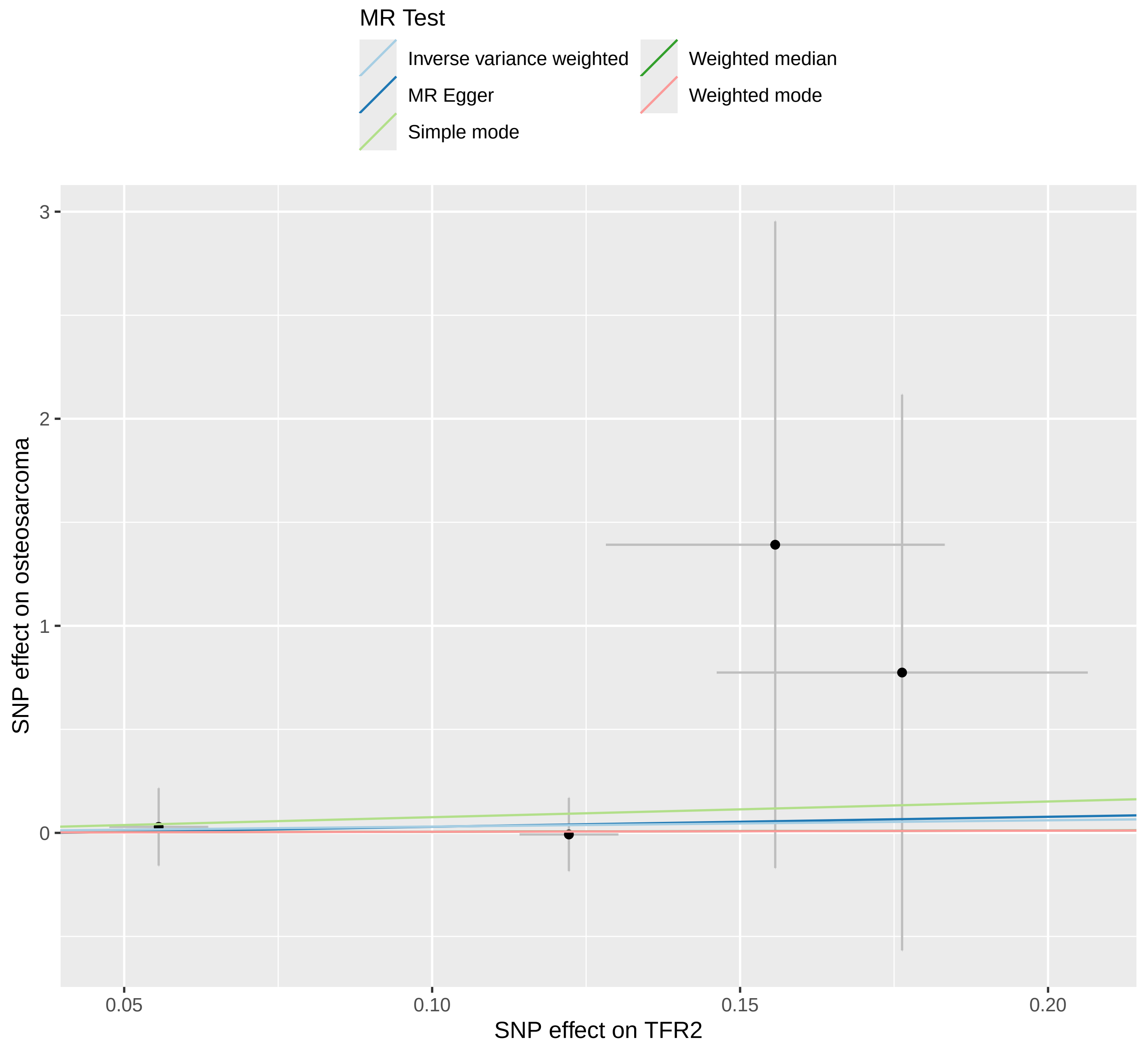

MR Test
Inverse variance weighted
MR Egger
Simple mode
Weighted median
Weighted mode
SNP effect on osteosarcoma
3
2
1
0
0.05
0.10
0.15
0.20
SNP effect on TFR2

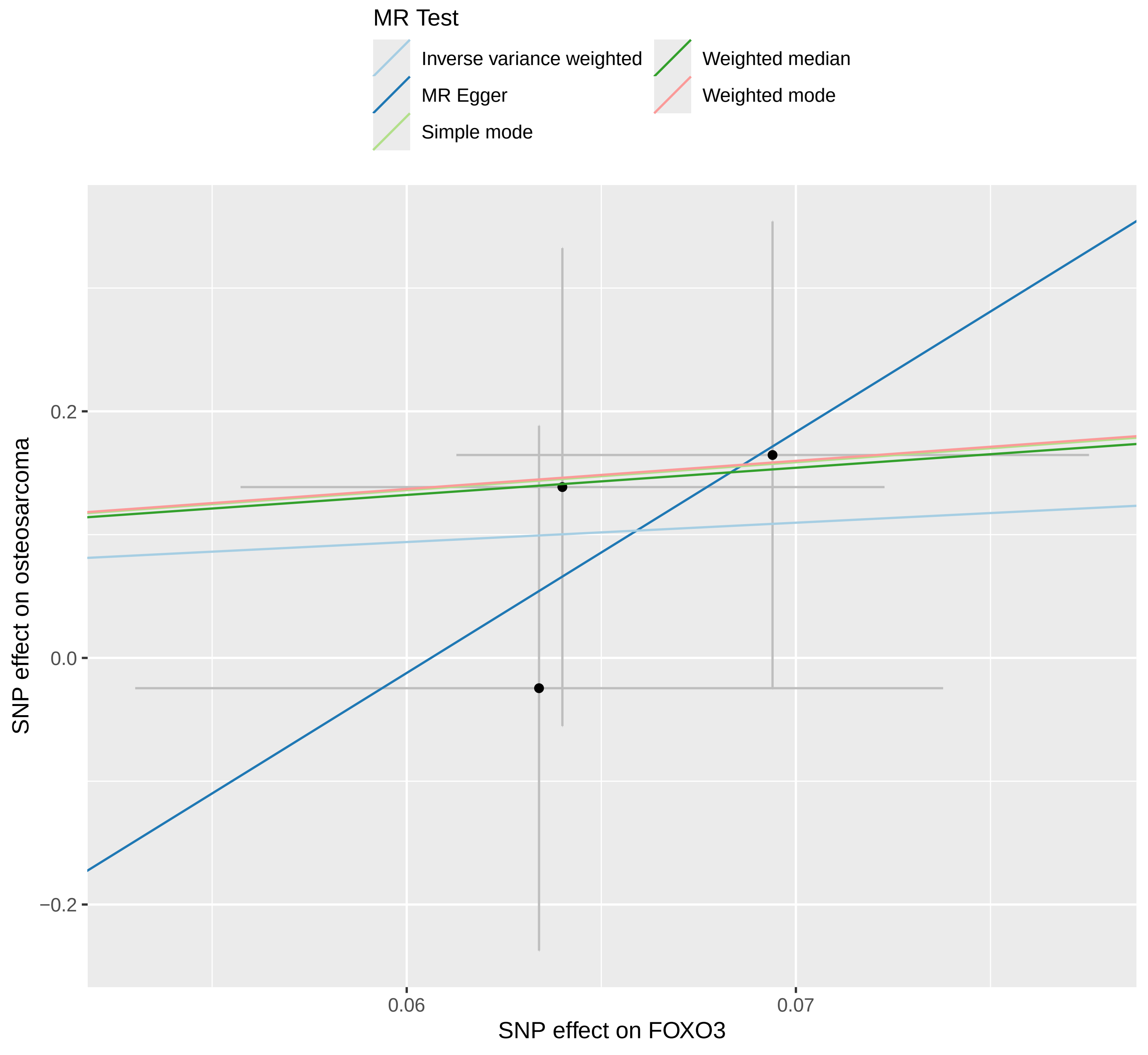

MR Test
Inverse variance weighted
MR Egger
Simple mode
Weighted median
Weighted mode
0.2
0.0
−0.2
0.06
0.07
SNP effect on osteosarcoma
SNP effect on FOXO3

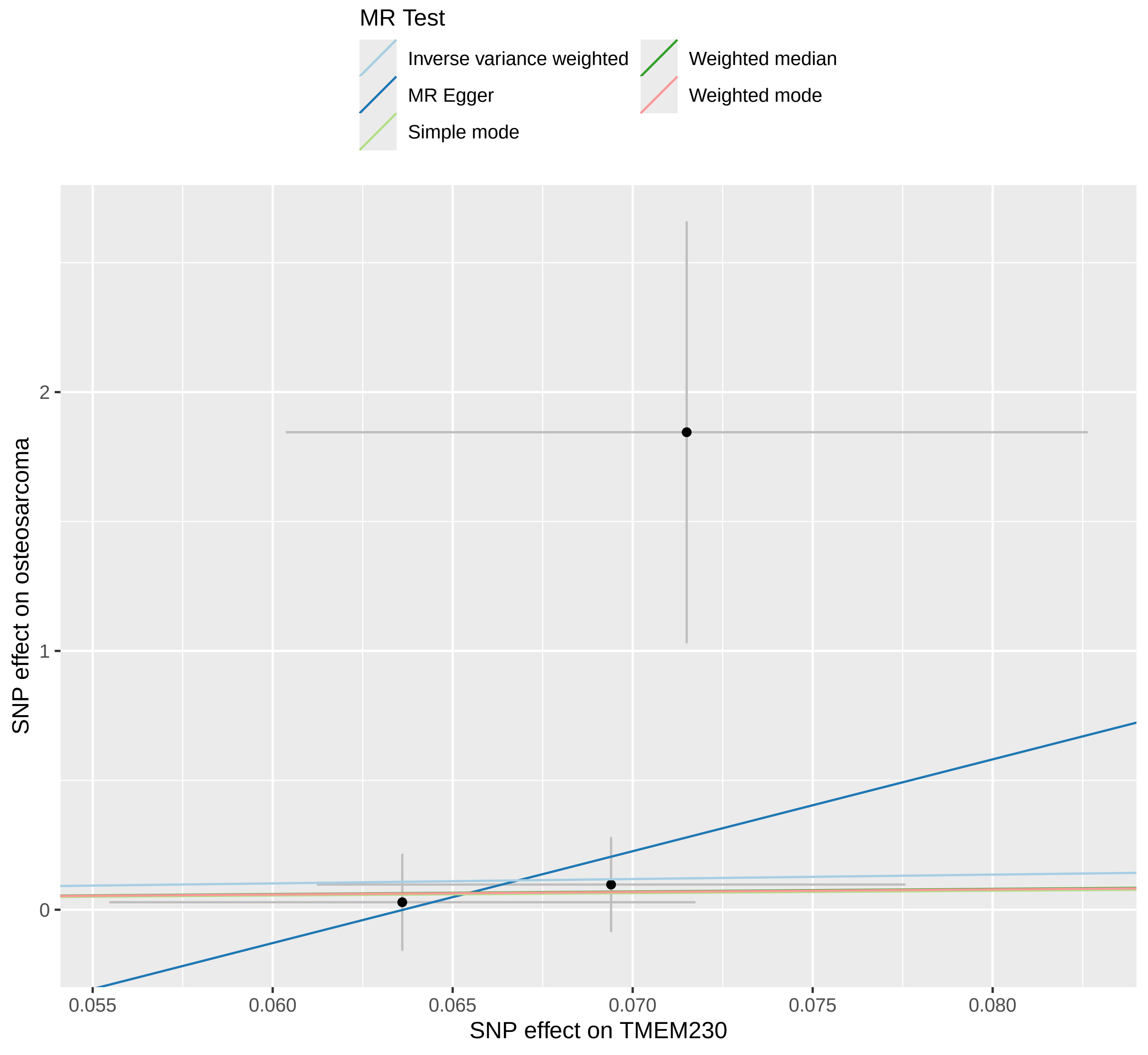

MR Test
Inverse variance weighted
MR Egger
Simple mode
Weighted median
Weighted mode
SNP effect on osteosarcoma
0
1
2
0.055
0.060
0.065
0.070
0.075
0.080
SNP effect on TMEM230

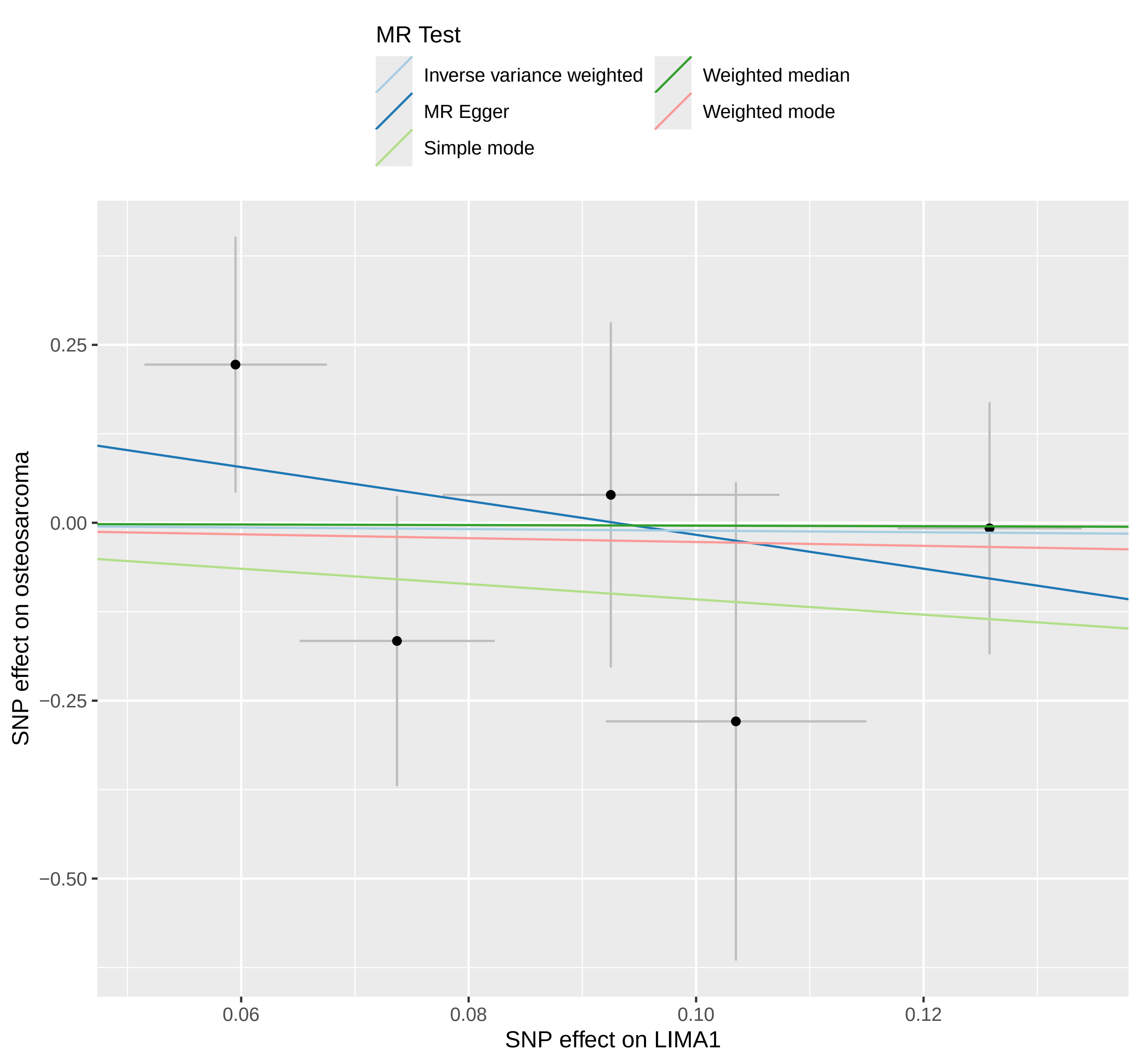
MR Test
Inverse variance weighted
MR Egger
Simple mode
Weighted median
Weighted mode
0.25
0.00
−0.25
−0.50
SNP effect on osteosarcoma
0.06
0.08
0.10
0.12
SNP effect on LIMA1

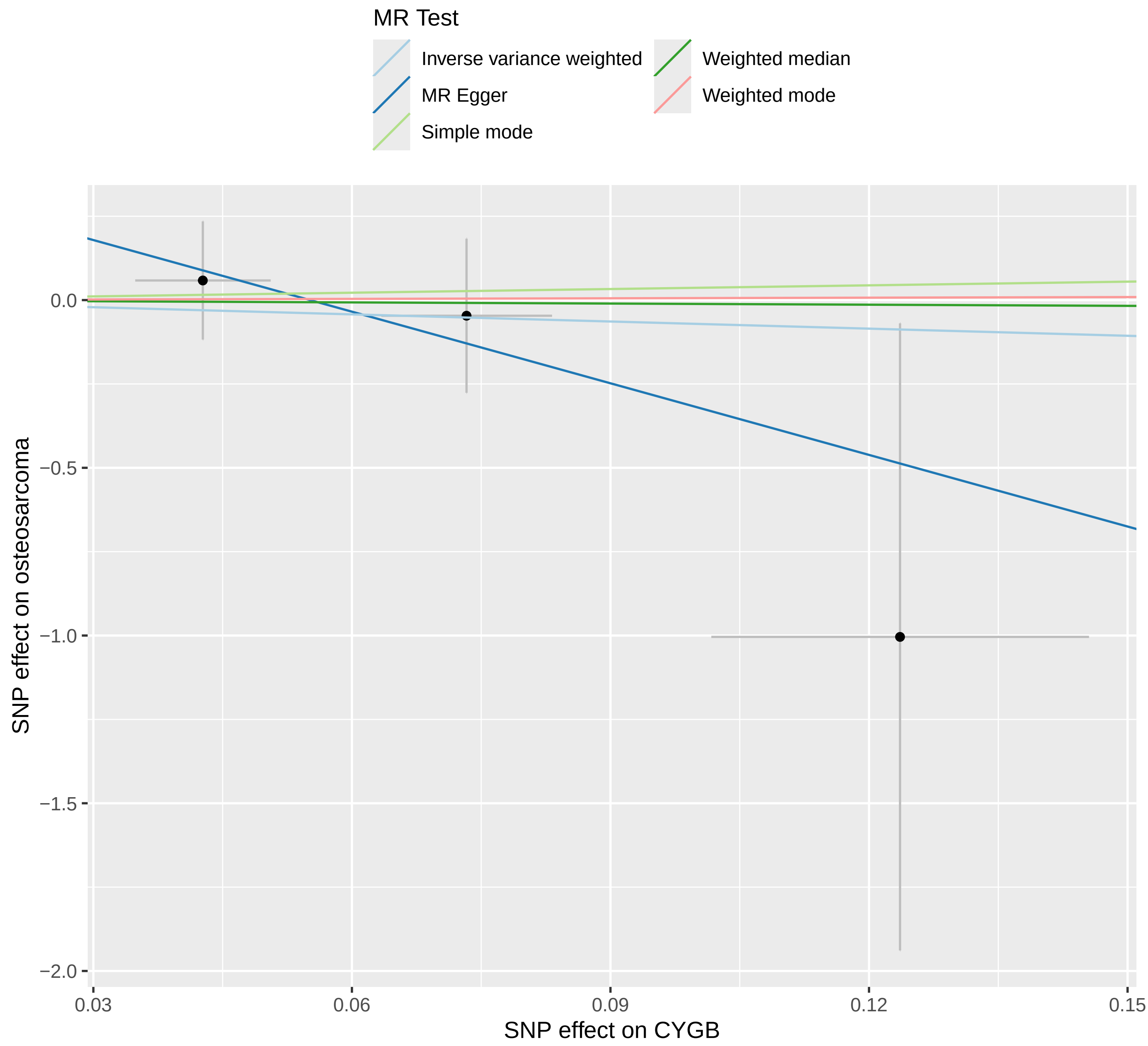

MR Test
Inverse variance weighted
MR Egger
Simple mode
Weighted median
Weighted mode
SNP effect on osteosarcoma
0.0
−0.5
−1.0
−1.5
−2.0
0.03
0.06
0.09
0.12
0.15
SNP effect on CYGB

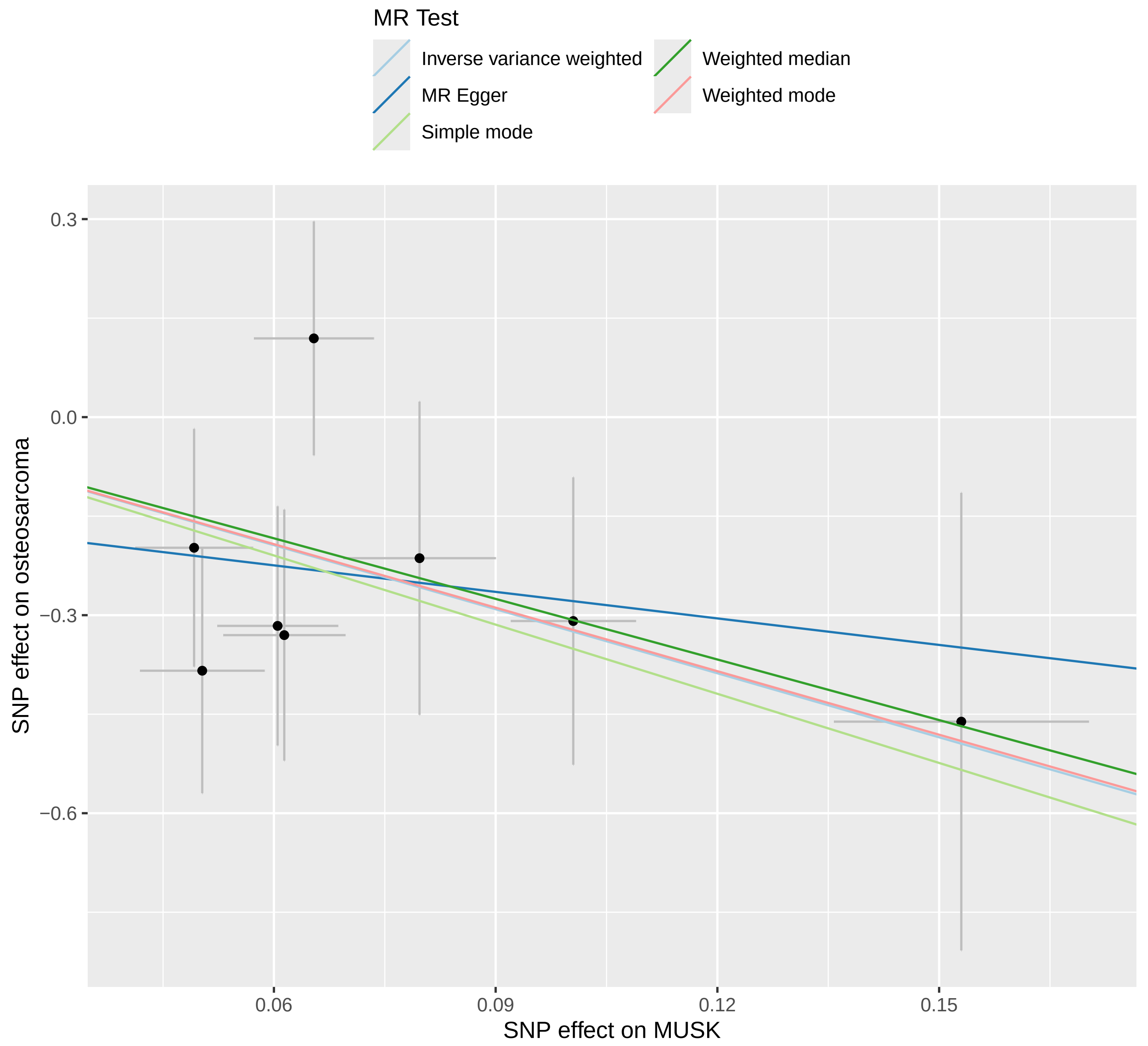

MR Test
Inverse variance weighted
MR Egger
Simple mode
Weighted median
Weighted mode
0.3
0.0
−0.3
−0.6
SNP effect on osteosarcoma
0.06
0.09
0.12
0.15
SNP effect on MUSK

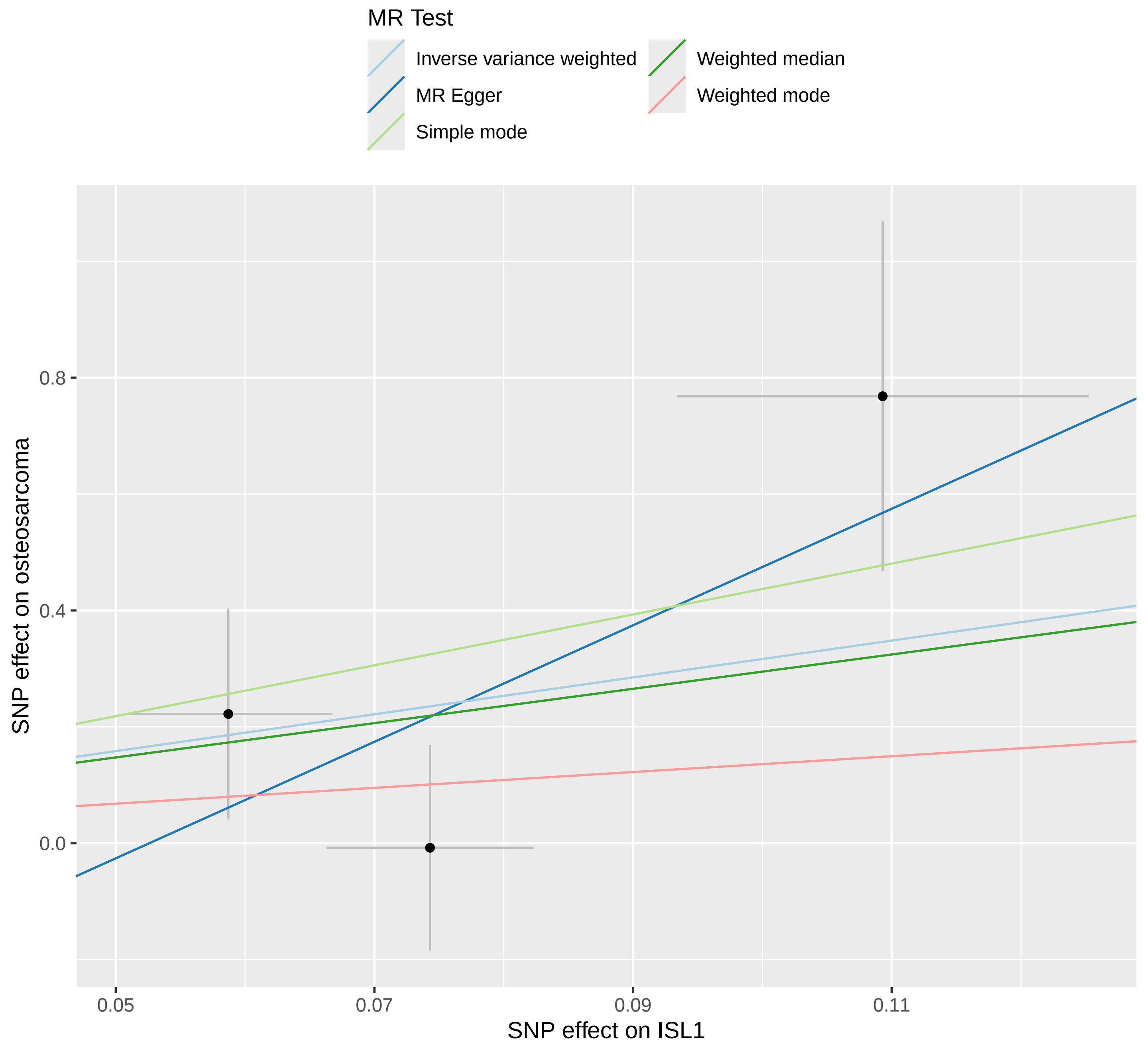
MR Test
Inverse variance weighted
MR Egger
Simple mode
Weighted median
Weighted mode
0.8
0.4
0.0
0.05
0.07
0.09
0.11
SNP effect on osteosarcoma
SNP effect on ISL1

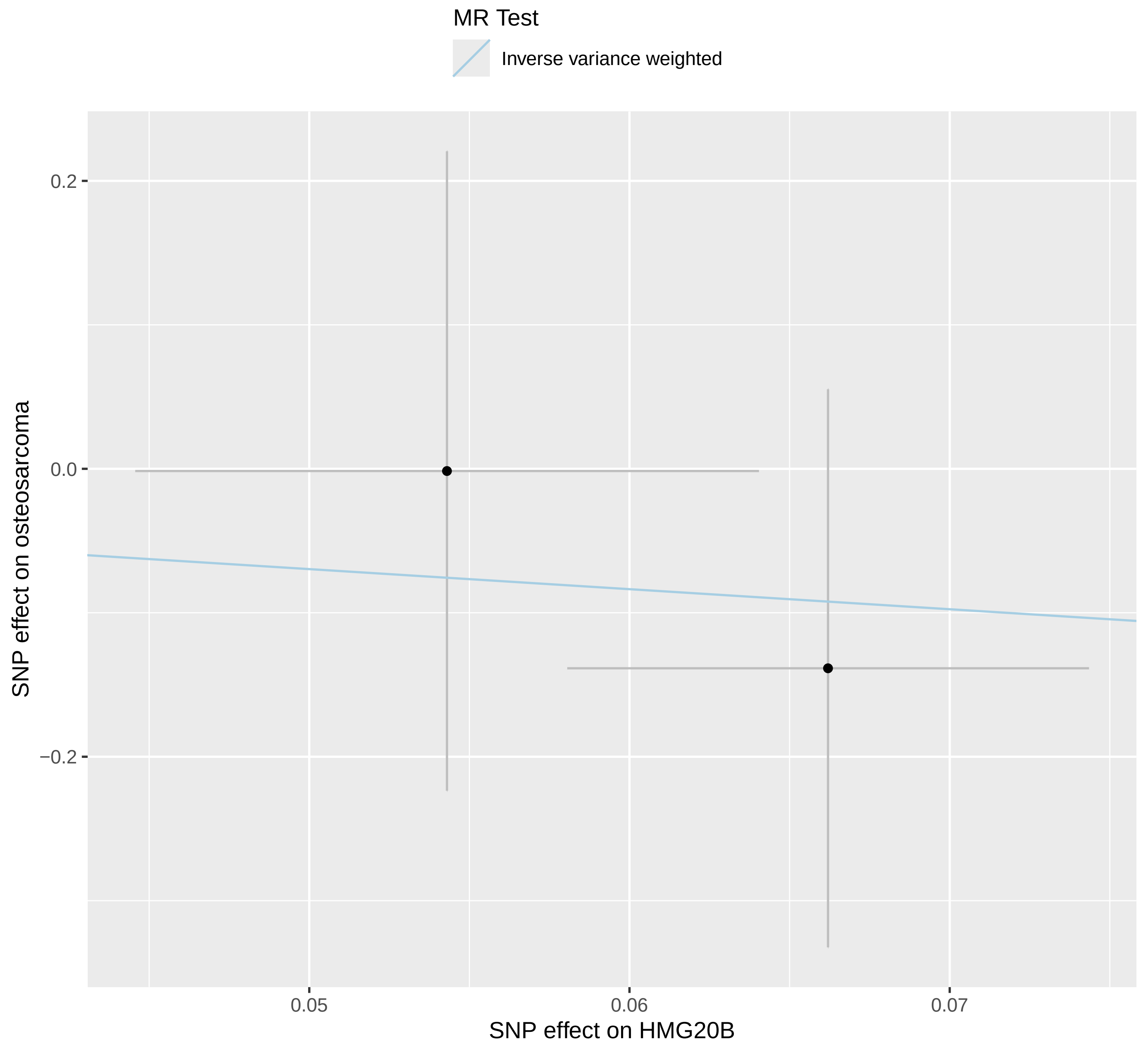

MR Test
Inverse variance weighted
0.2
0.0
−0.2
0.05
0.06
0.07
SNP effect on osteosarcoma
SNP effect on HMG20B

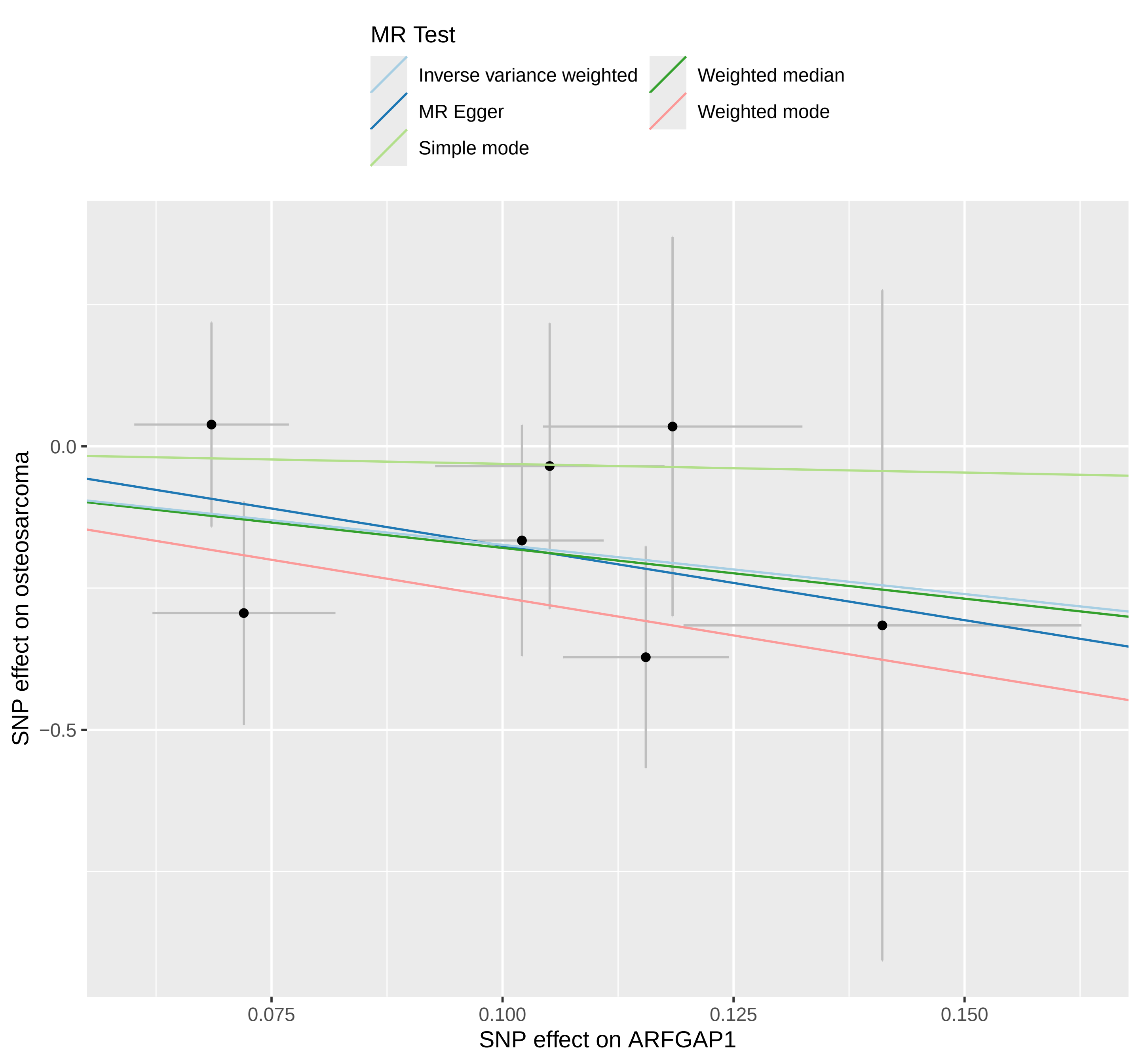

MR Test
Inverse variance weighted
MR Egger
Simple mode
Weighted median
Weighted mode
0.0
−0.5
0.075
0.100
0.125
0.150
SNP effect on ARFGAP1
SNP effect on osteosarcoma

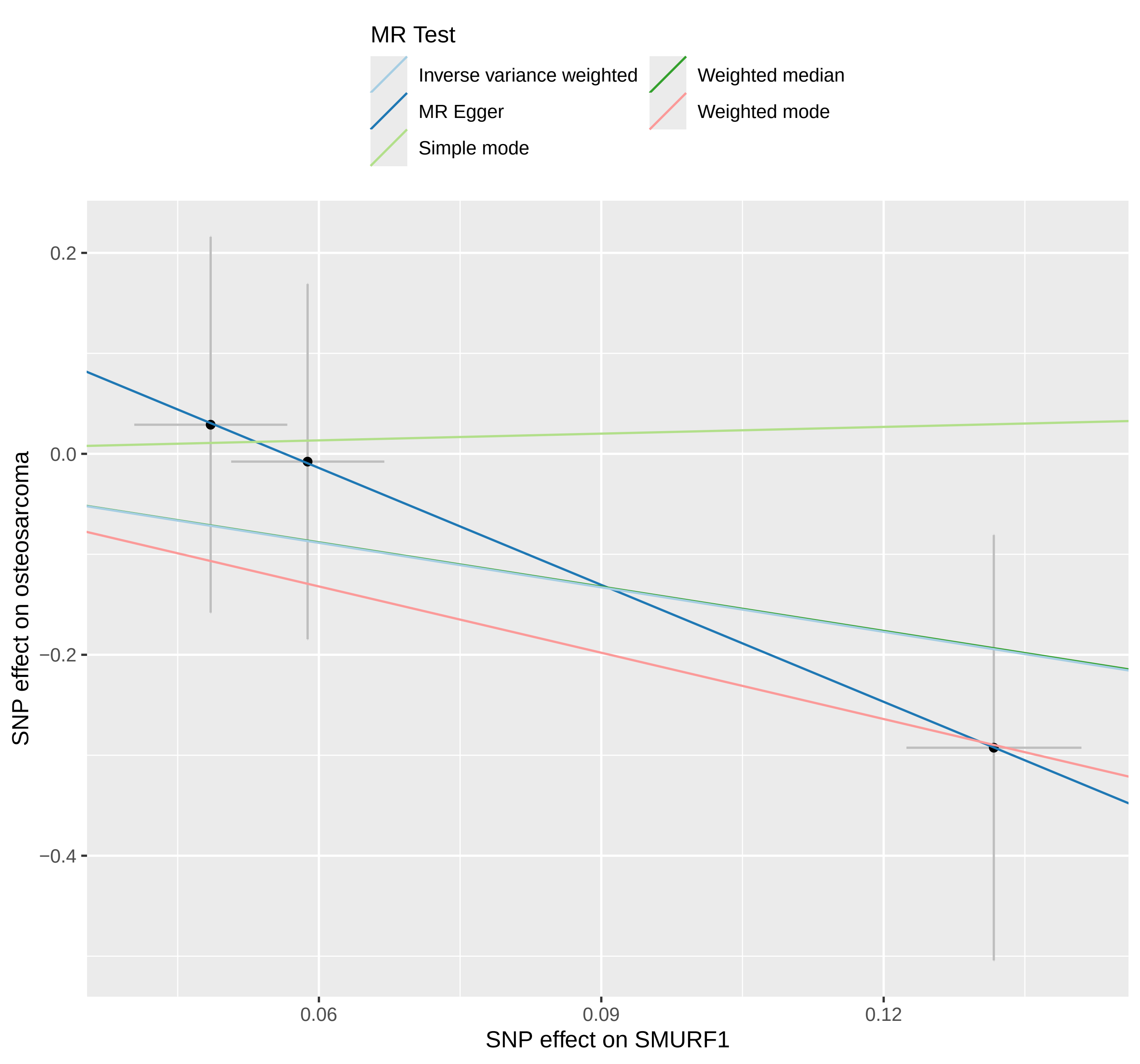
MR Test
Inverse variance weighted
MR Egger
Simple mode
Weighted median
Weighted mode
0.2
0.0
−0.2
−0.4
0.06
0.09
0.12
SNP effect on osteosarcoma
SNP effect on SMURF1

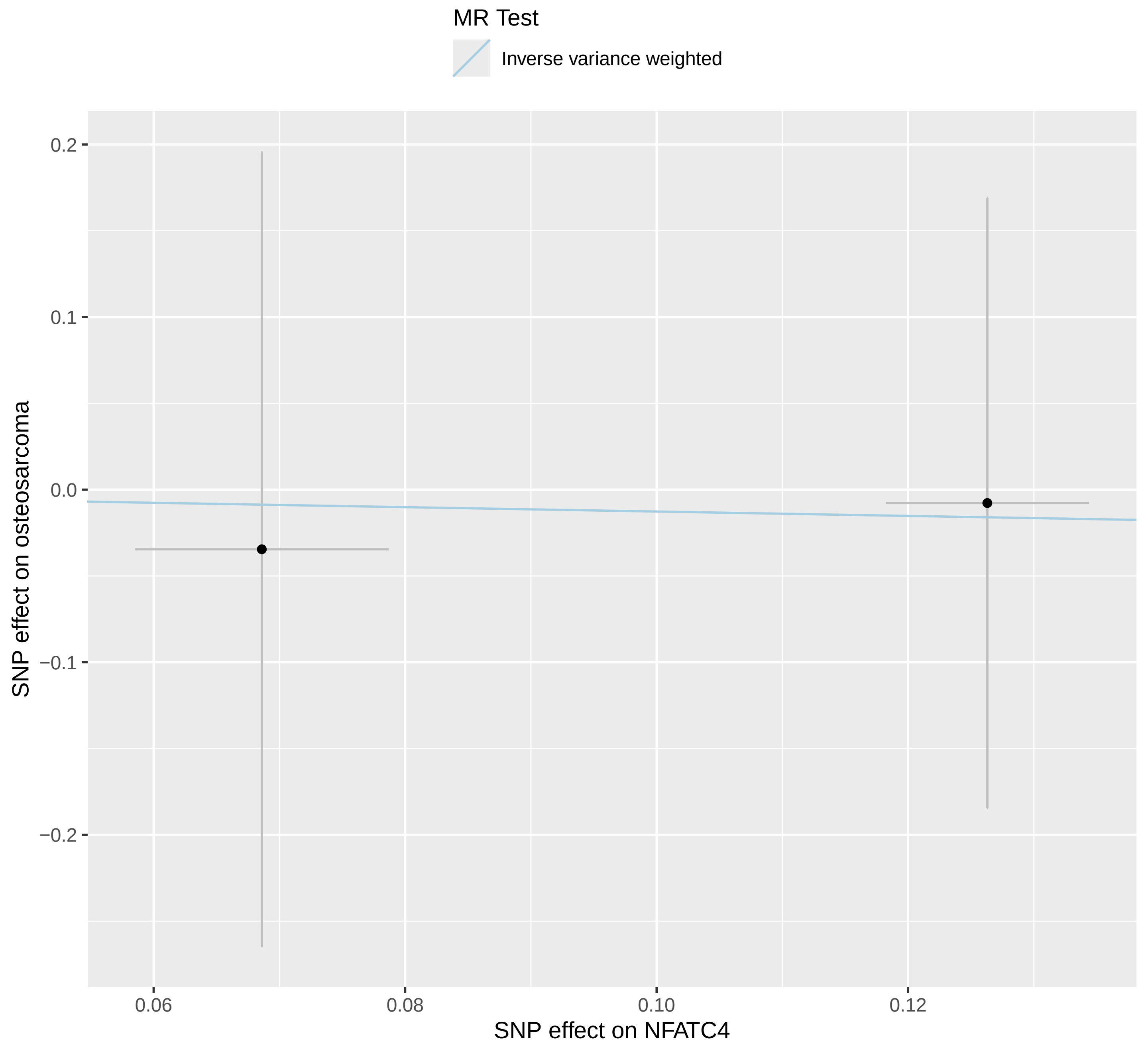

MR Test
Inverse variance weighted
0.2
0.1
0.0
−0.1
−0.2
SNP effect on osteosarcoma
0.06
0.08
0.10
0.12
SNP effect on NFATC4

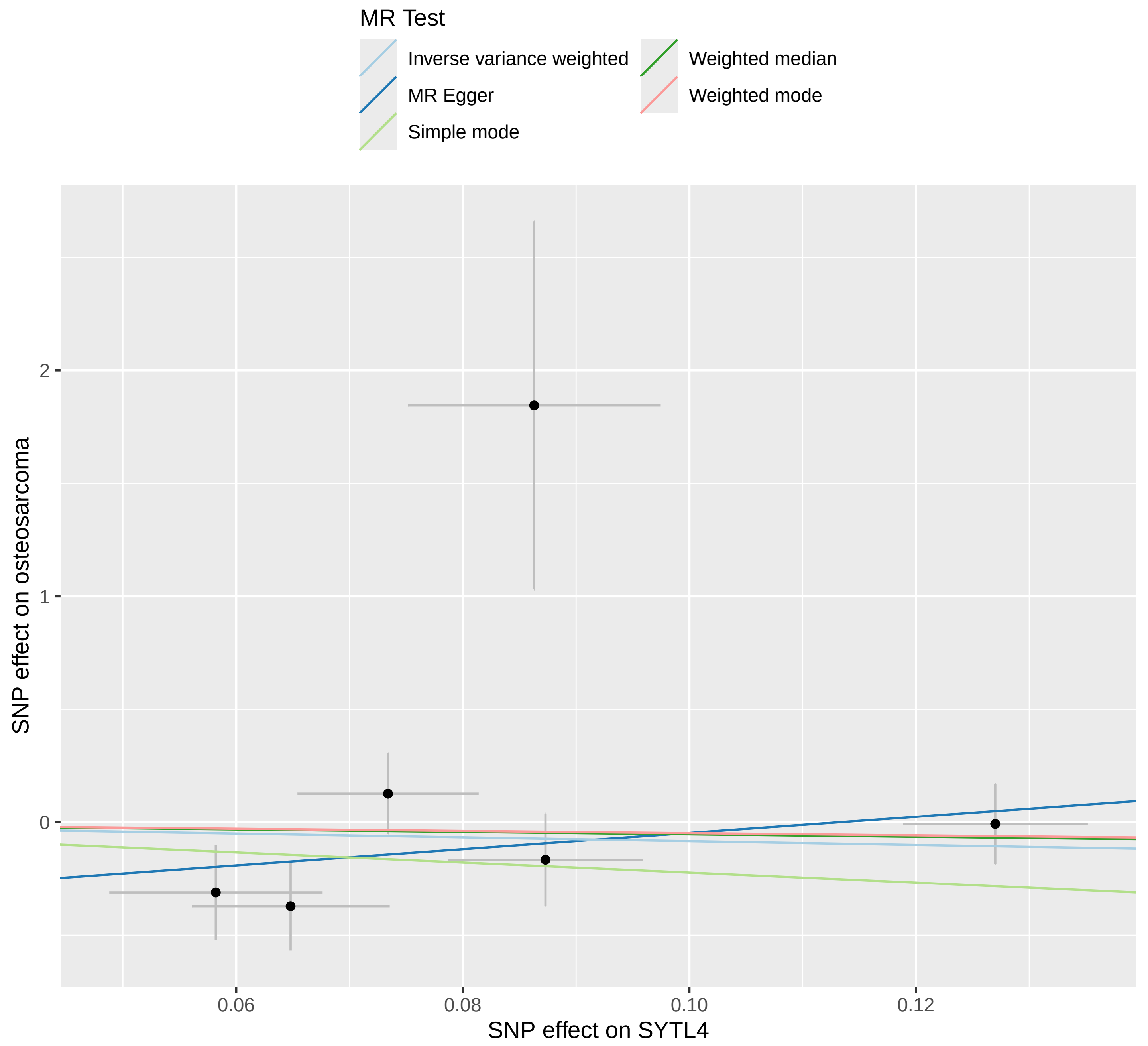

MR Test
Inverse variance weighted
MR Egger
Simple mode
Weighted median
Weighted mode
SNP effect on osteosarcoma
SNP effect on SYTL4
0
1
2
0.06
0.08
0.10
0.12

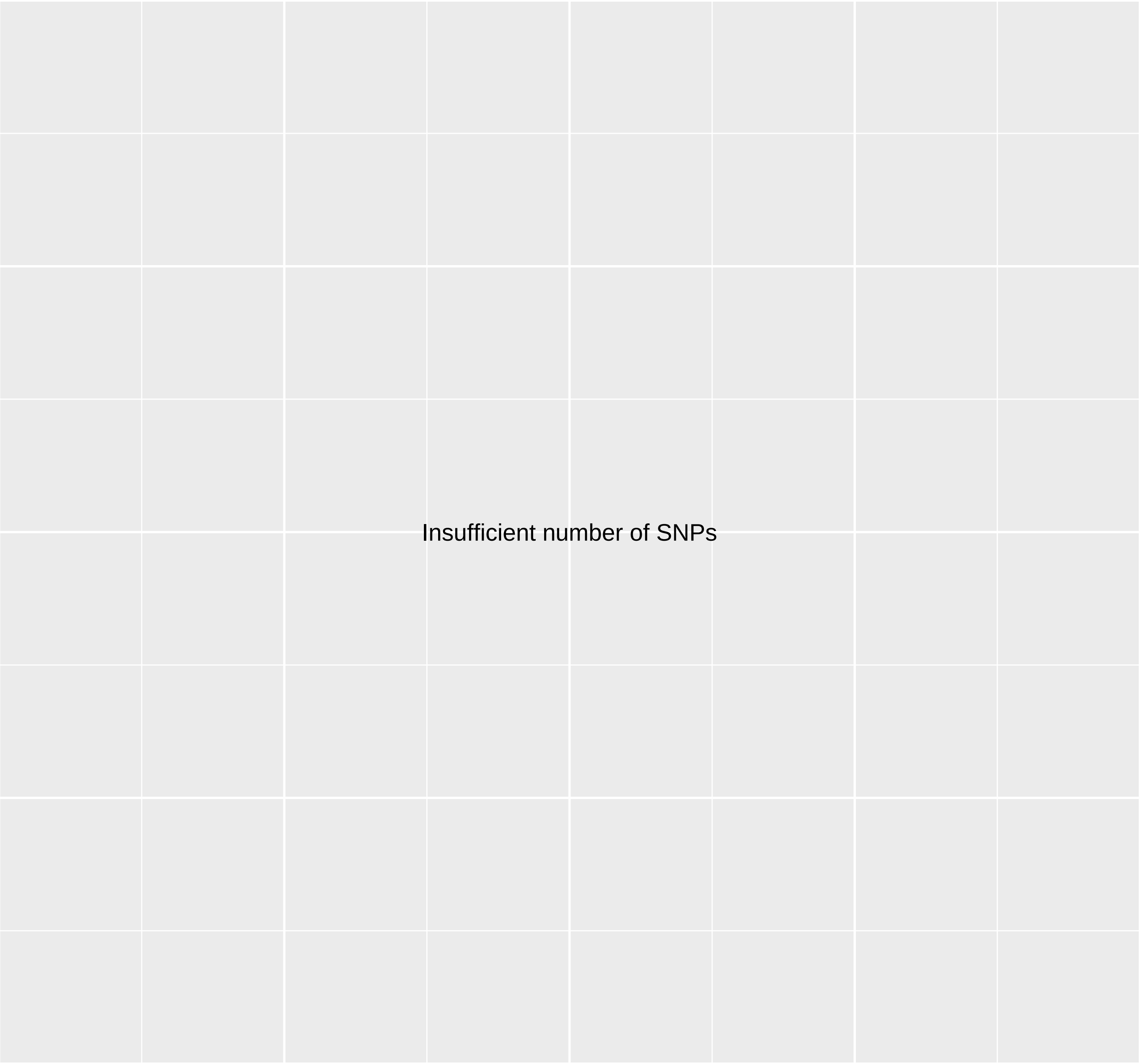
Insufficient number of SNPs

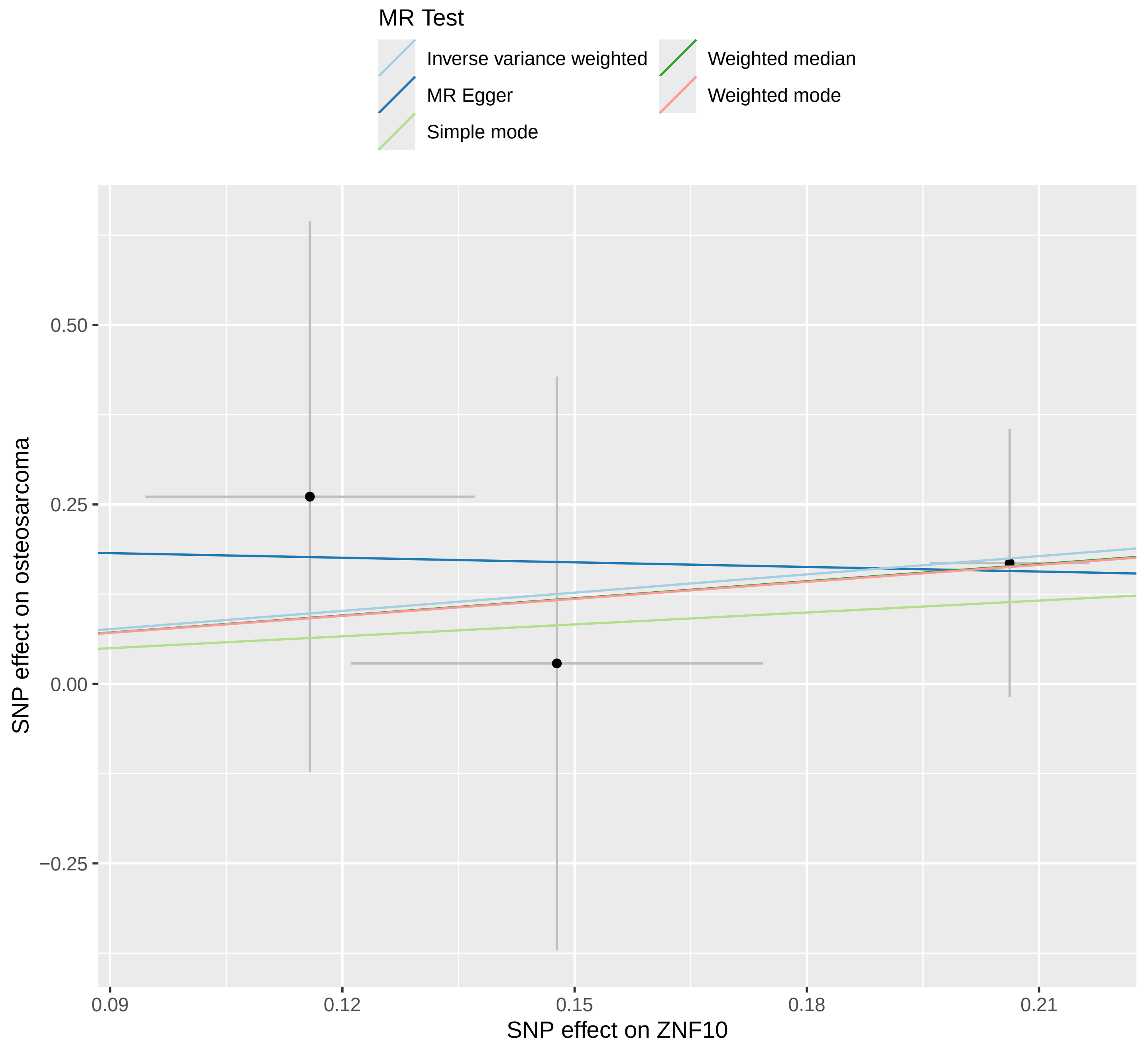

MR Test
Inverse variance weighted
MR Egger
Simple mode
Weighted median
Weighted mode
0.50
0.25
0.00
−0.25
SNP effect on osteosarcoma
0.09
0.12
0.15
0.18
0.21
SNP effect on ZNF10

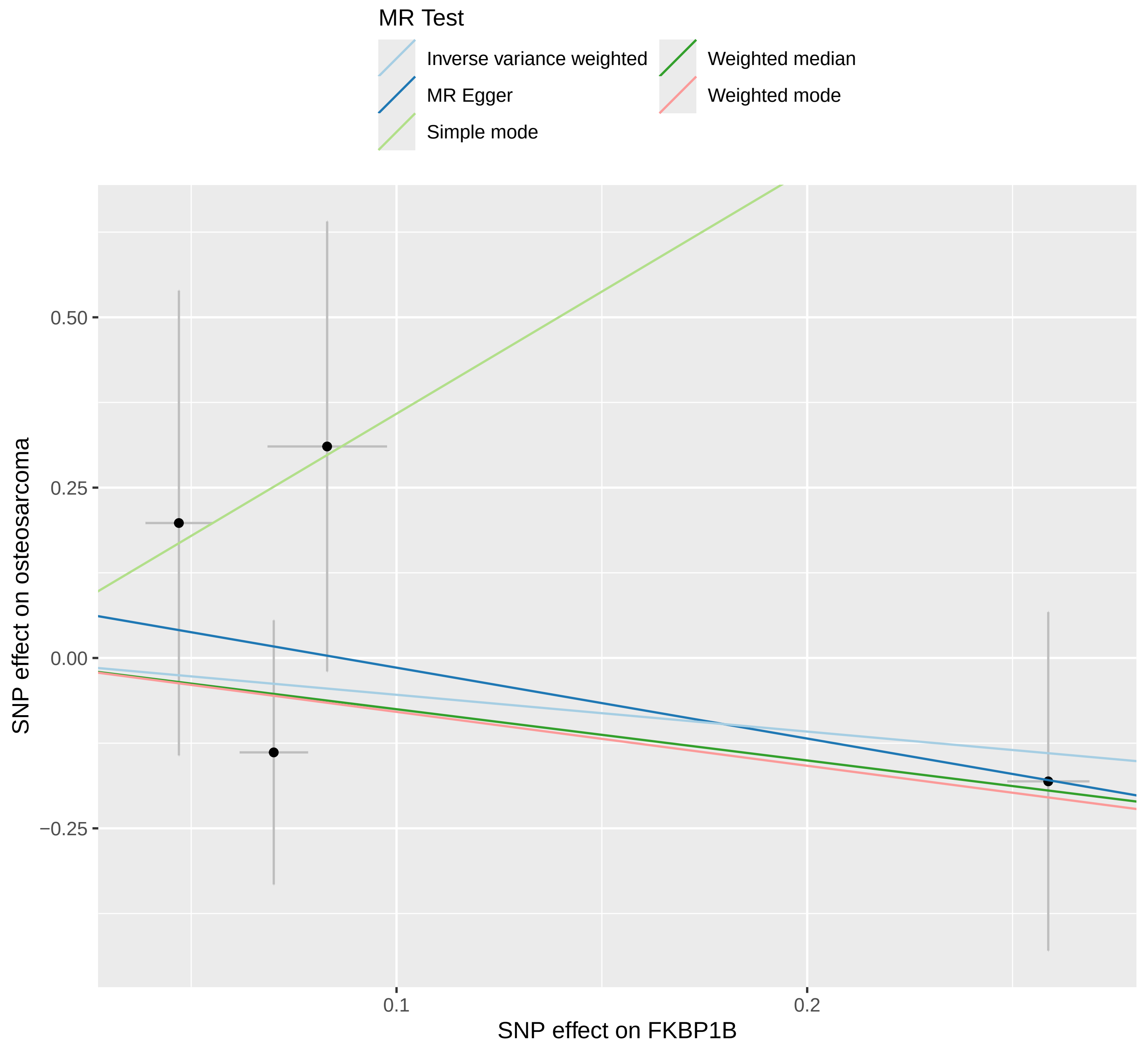
MR Test
Inverse variance weighted
MR Egger
Simple mode
Weighted median
Weighted mode
0.50
0.25
0.00
−0.25
SNP effect on osteosarcoma
0.1
0.2
SNP effect on FKBP1B

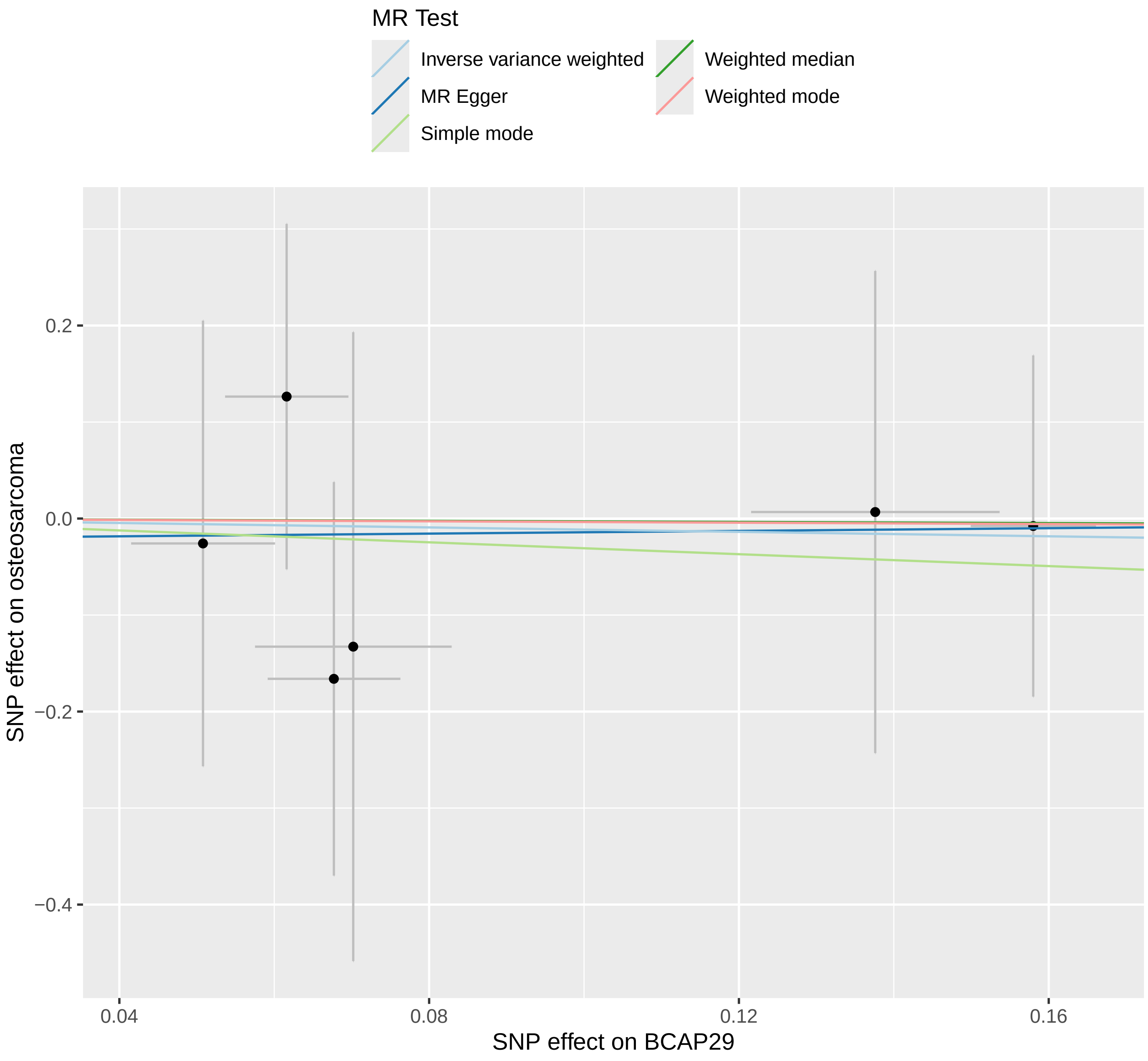

MR Test
Inverse variance weighted
MR Egger
Simple mode
Weighted median
Weighted mode
0.2
0.0
−0.2
−0.4
SNP effect on osteosarcoma
0.04
0.08
0.12
0.16
SNP effect on BCAP29

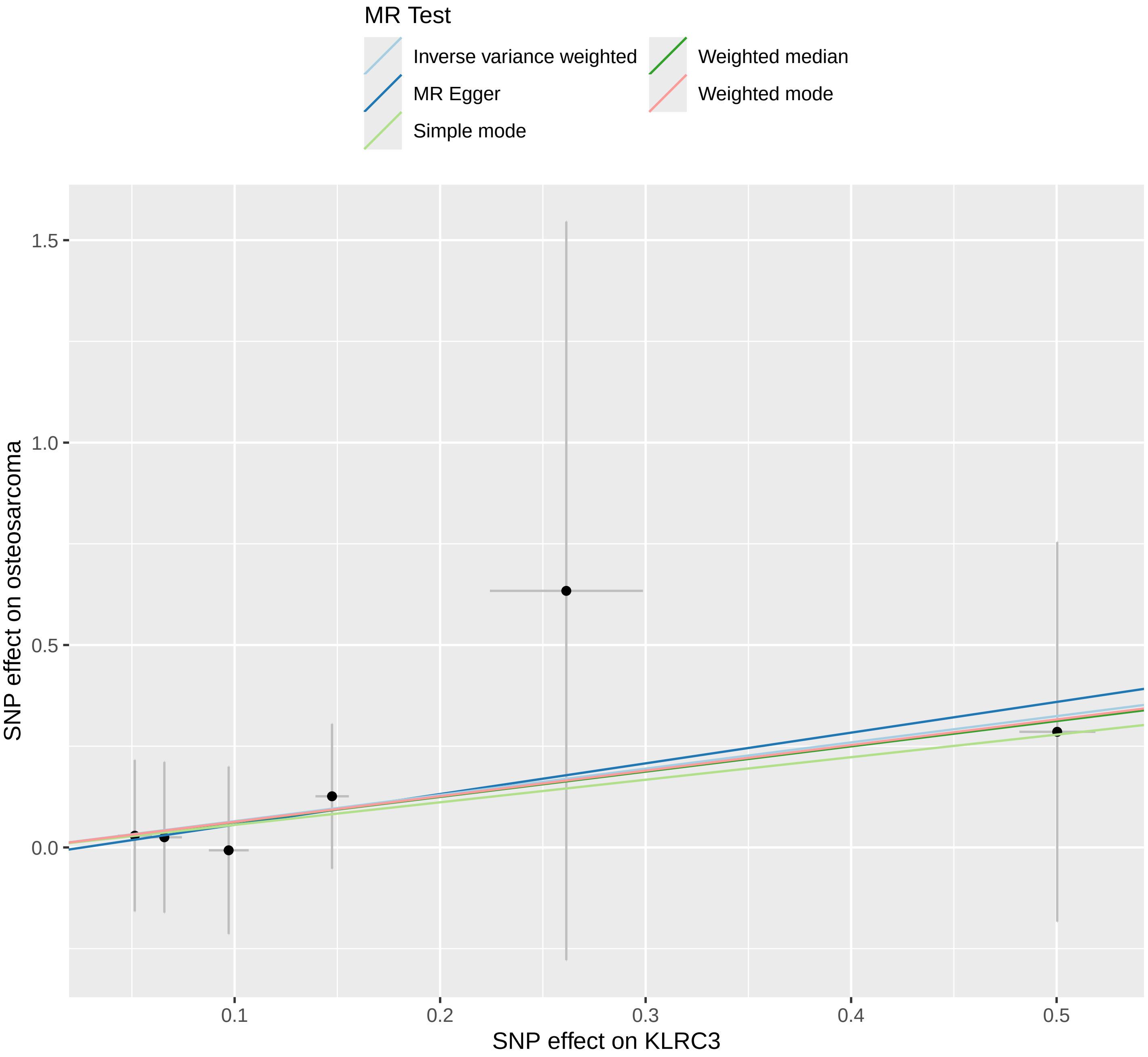
MR Test
Inverse variance weighted
MR Egger
Simple mode
Weighted median
Weighted mode
1.5
1.0
0.5
0.0
SNP effect on osteosarcoma
0.1
0.2
0.3
0.4
0.5
SNP effect on KLRC3

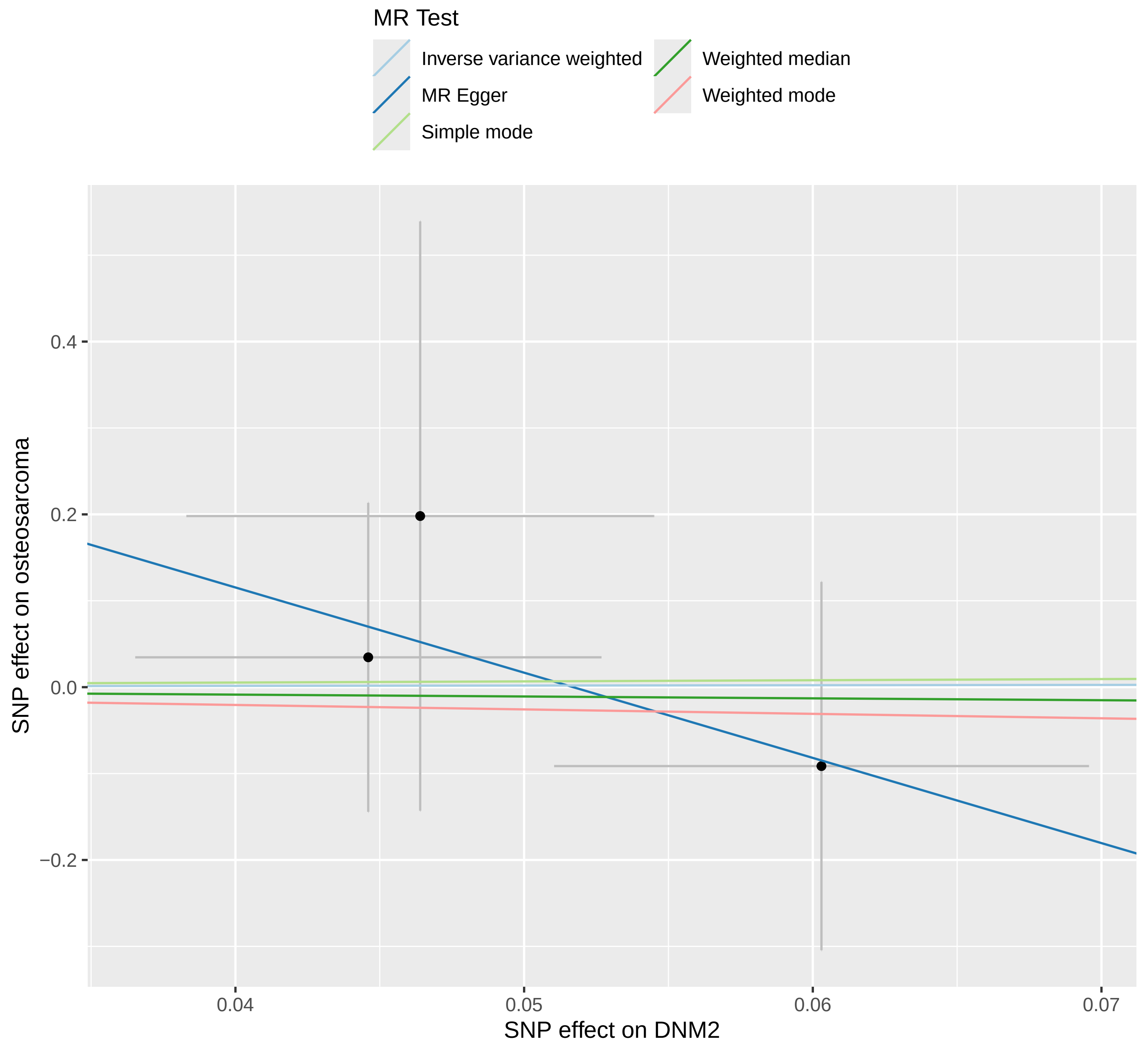

MR Test
Inverse variance weighted
MR Egger
Simple mode
Weighted median
Weighted mode
0.4
0.2
0.0
−0.2
0.04
0.05
0.06
0.07
SNP effect on osteosarcoma
SNP effect on DNM2

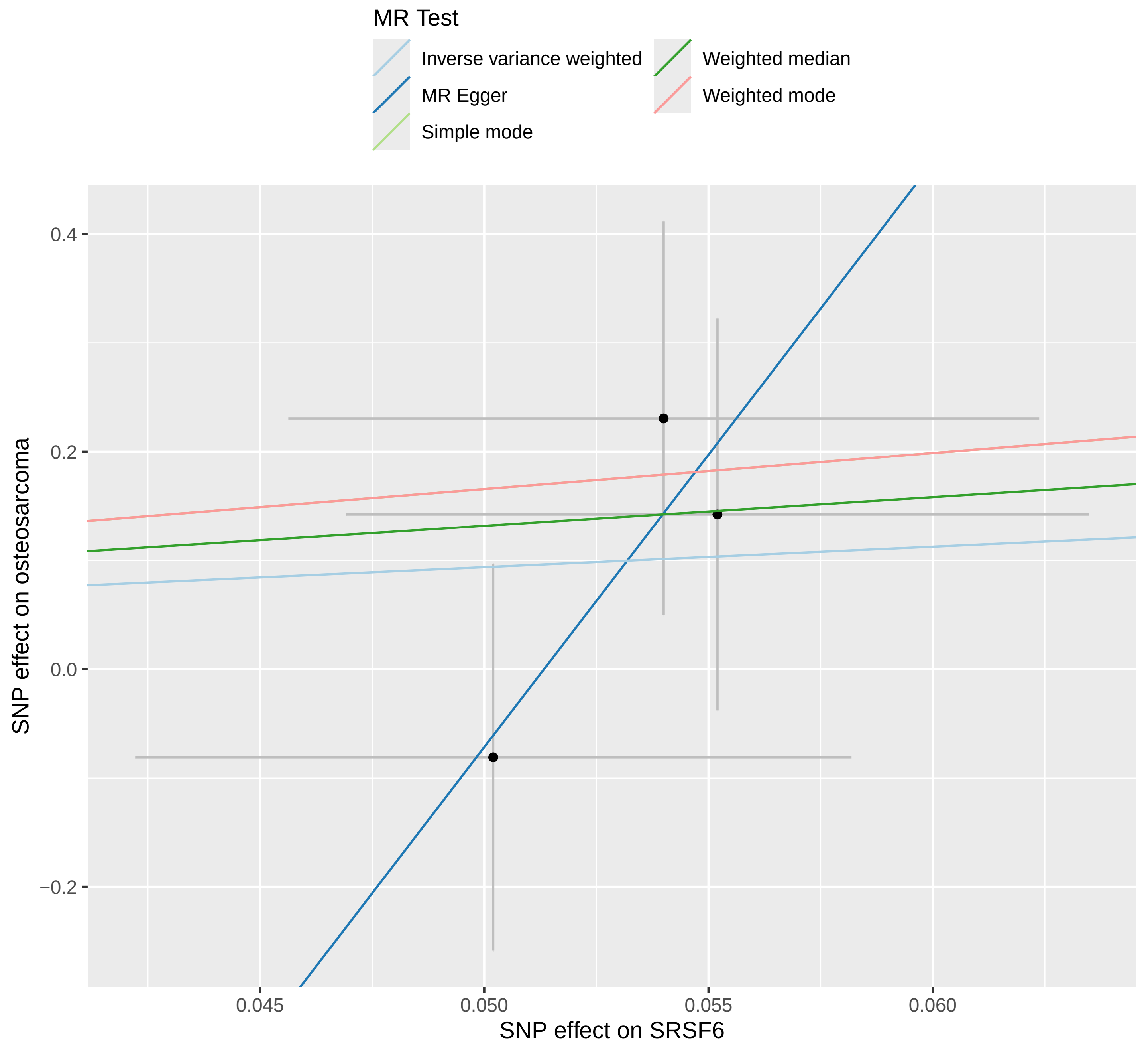
MR Test
Inverse variance weighted
MR Egger
Simple mode
Weighted median
Weighted mode
0.4
0.2
0.0
−0.2
SNP effect on osteosarcoma
0.045
0.050
0.055
0.060
SNP effect on SRSF6

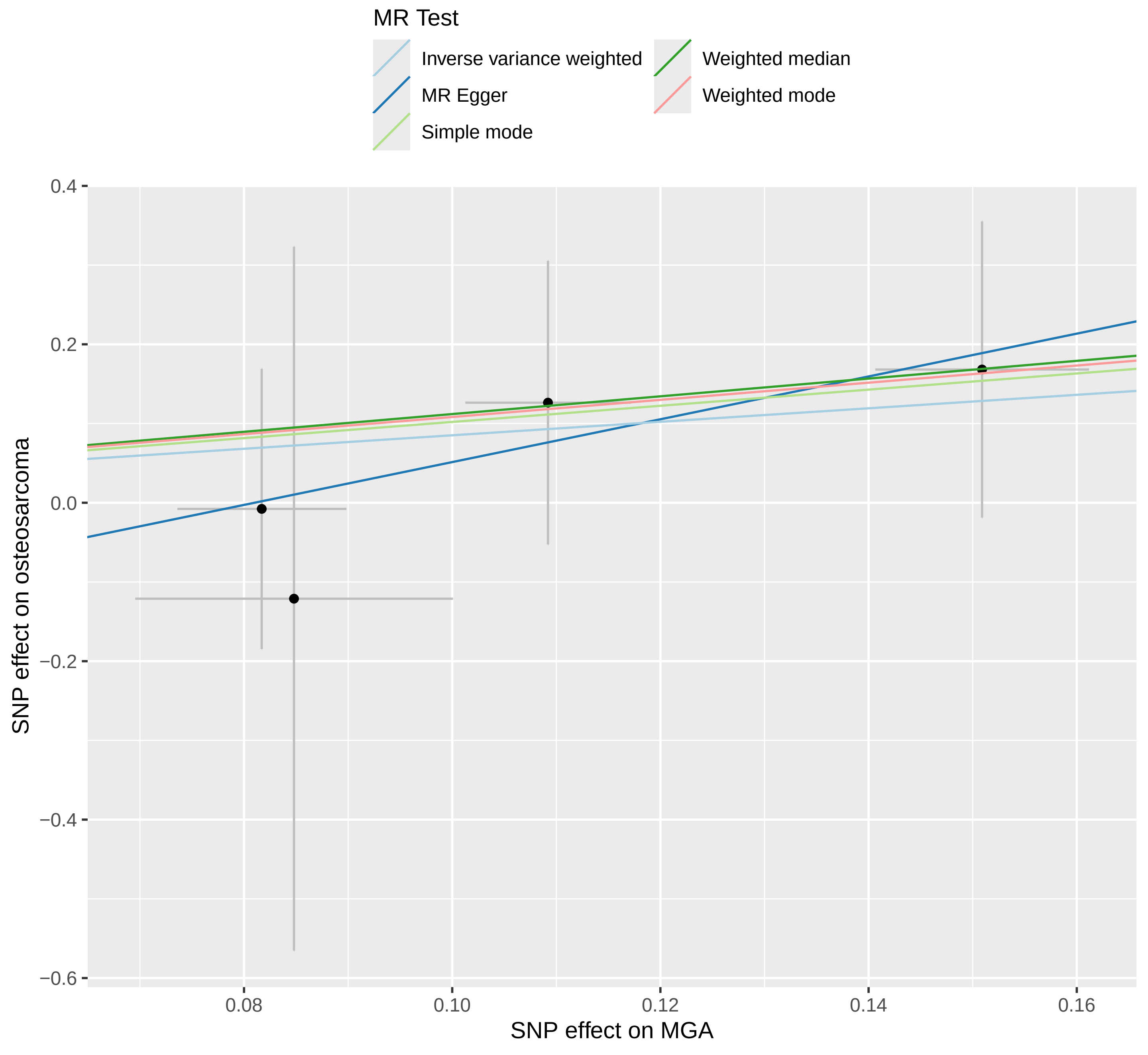
MR Test
Inverse variance weighted
MR Egger
Simple mode
Weighted median
Weighted mode
0.4
0.2
0.0
−0.2
−0.4
−0.6
SNP effect on osteosarcoma
0.08
0.10
0.12
0.14
0.16
SNP effect on MGA

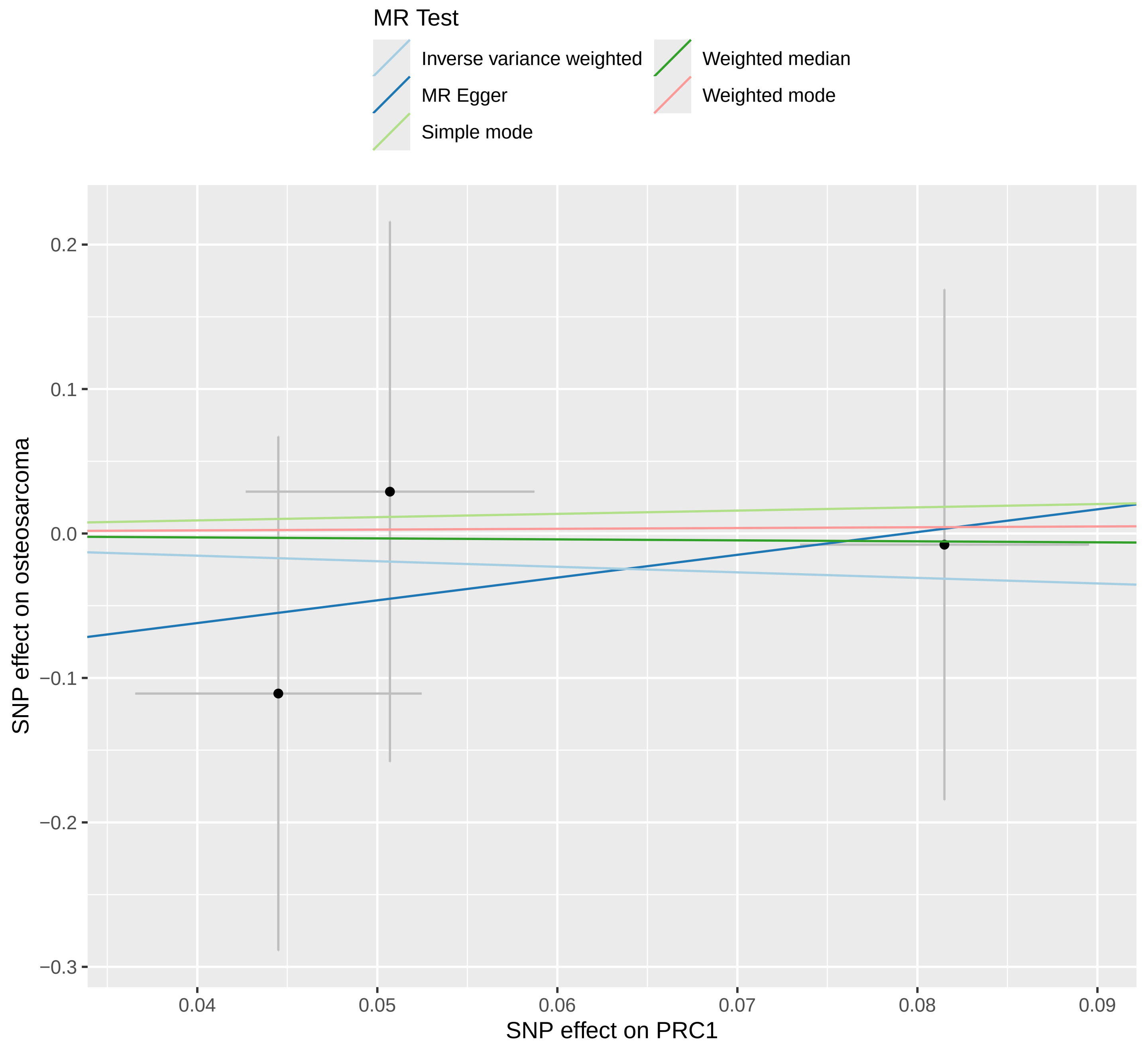

MR Test
Inverse variance weighted
MR Egger
Simple mode
Weighted median
Weighted mode
0.2
0.1
0.0
−0.1
−0.2
−0.3
SNP effect on osteosarcoma
0.04
0.05
0.06
0.07
0.08
0.09
SNP effect on PRC1

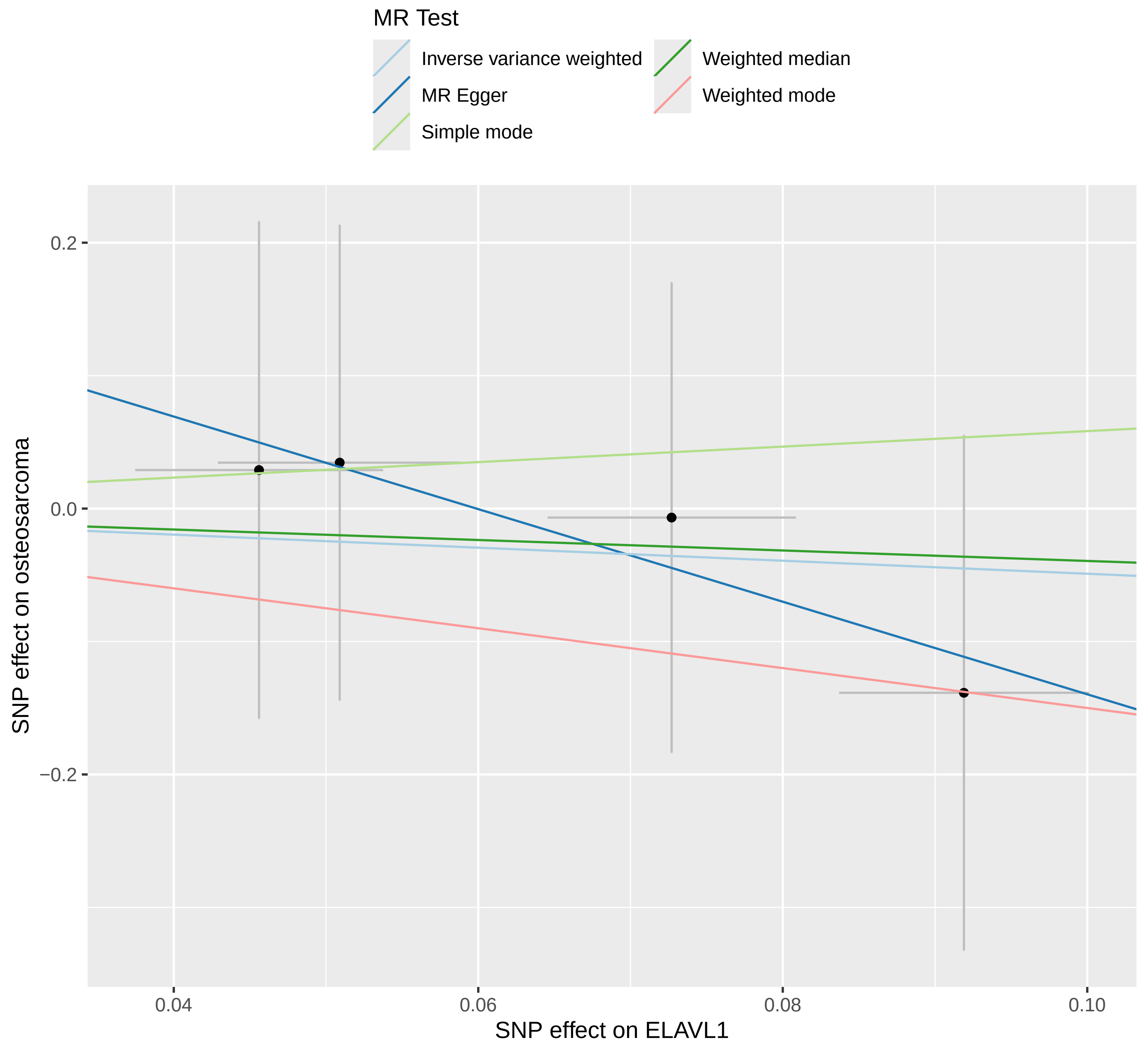
MR Test
Inverse variance weighted
MR Egger
Simple mode
Weighted median
Weighted mode
0.2
0.0
−0.2
0.04
0.06
0.08
0.10
SNP effect on osteosarcoma
SNP effect on ELAVL1

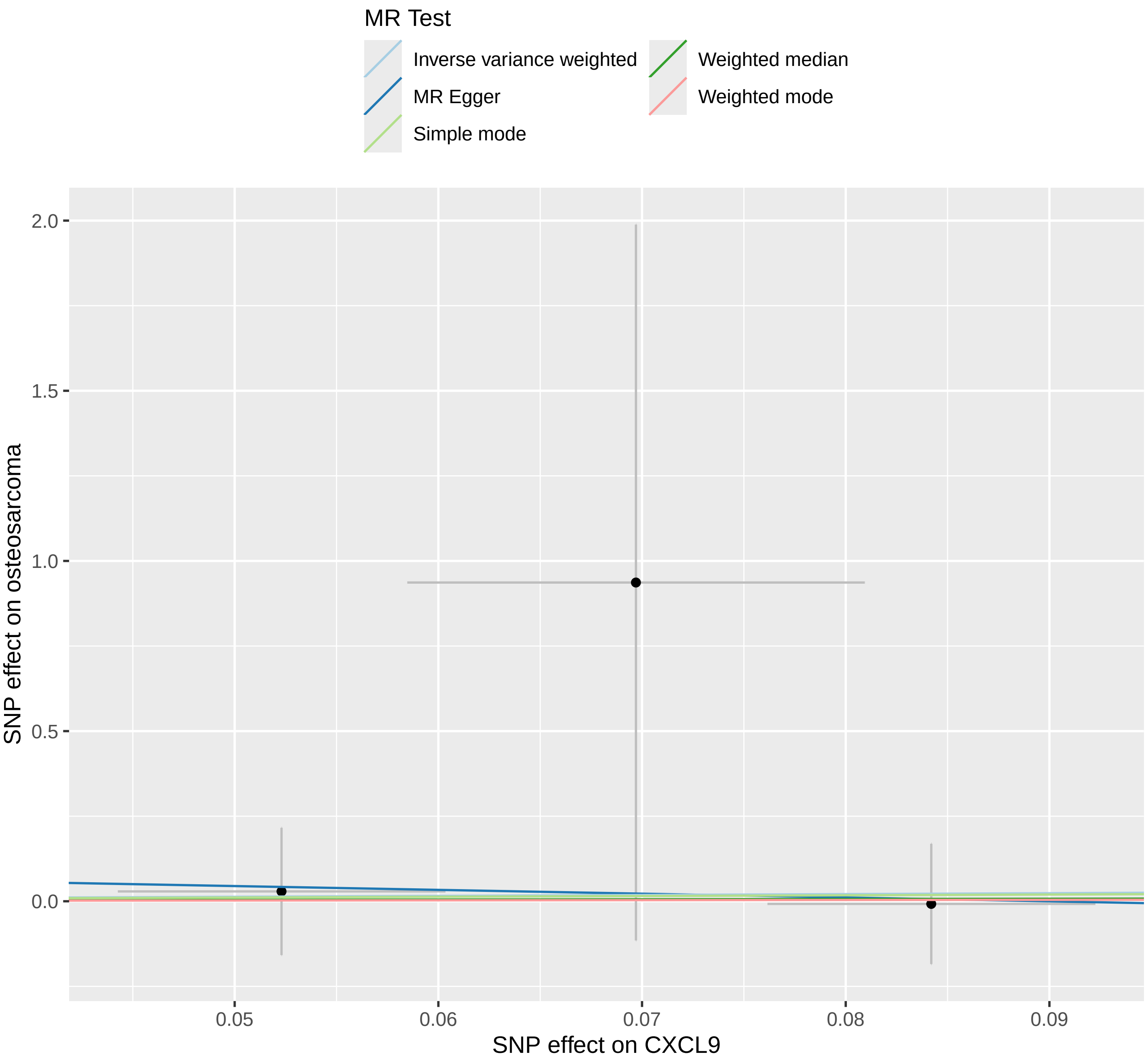
MR Test
Inverse variance weighted
MR Egger
Simple mode
Weighted median
Weighted mode
2.0
1.5
1.0
0.5
0.0
SNP effect on osteosarcoma
0.05
0.06
0.07
0.08
0.09
SNP effect on CXCL9

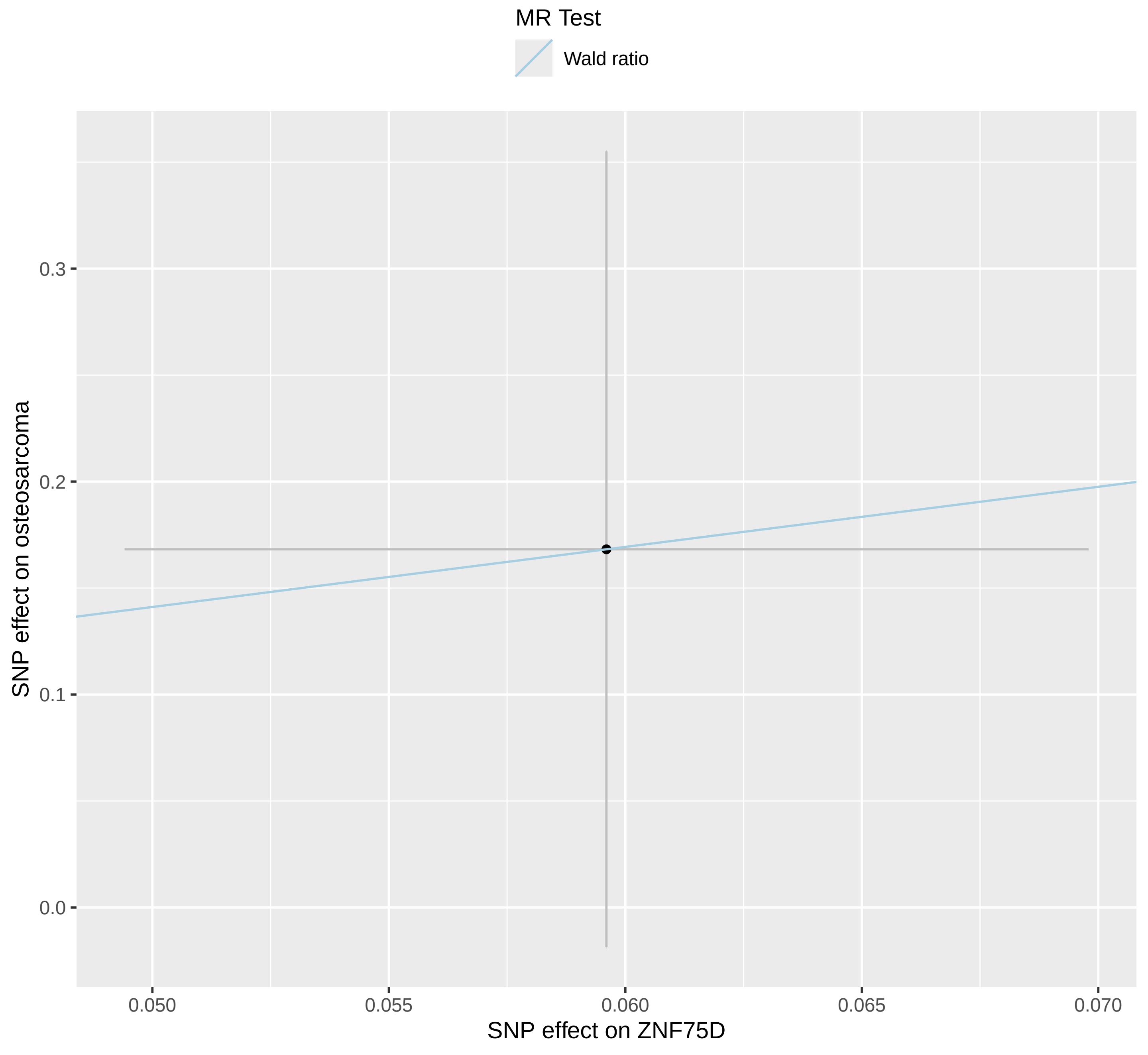
MR Test
Wald ratio
0.3
0.2
0.1
0.0
0.050
0.055
0.060
0.065
0.070
SNP effect on osteosarcoma
SNP effect on ZNF75D

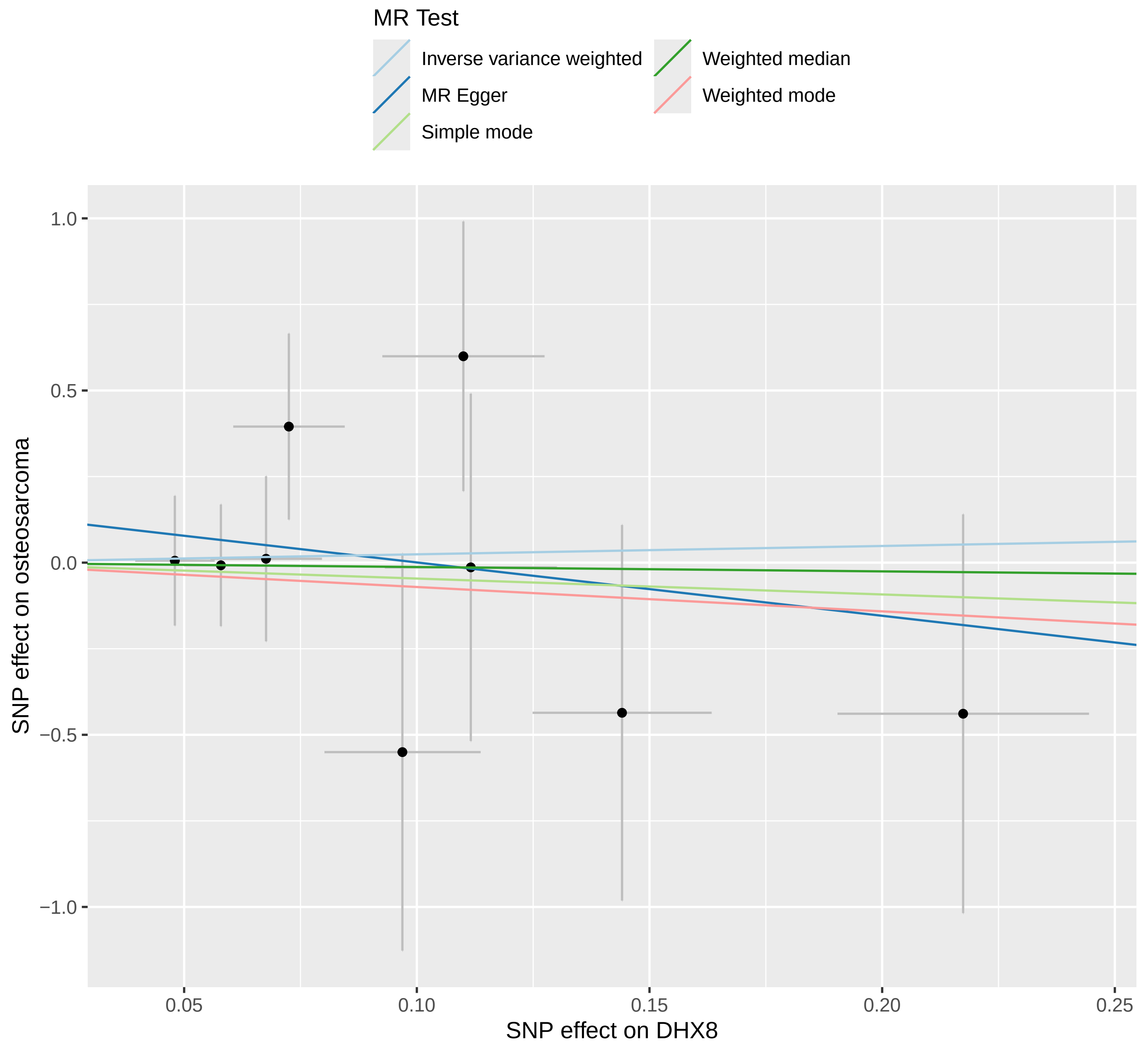
MR Test
Inverse variance weighted
MR Egger
Simple mode
Weighted median
Weighted mode
1.0
0.5
0.0
−0.5
−1.0
SNP effect on osteosarcoma
0.05
0.10
0.15
0.20
0.25
SNP effect on DHX8

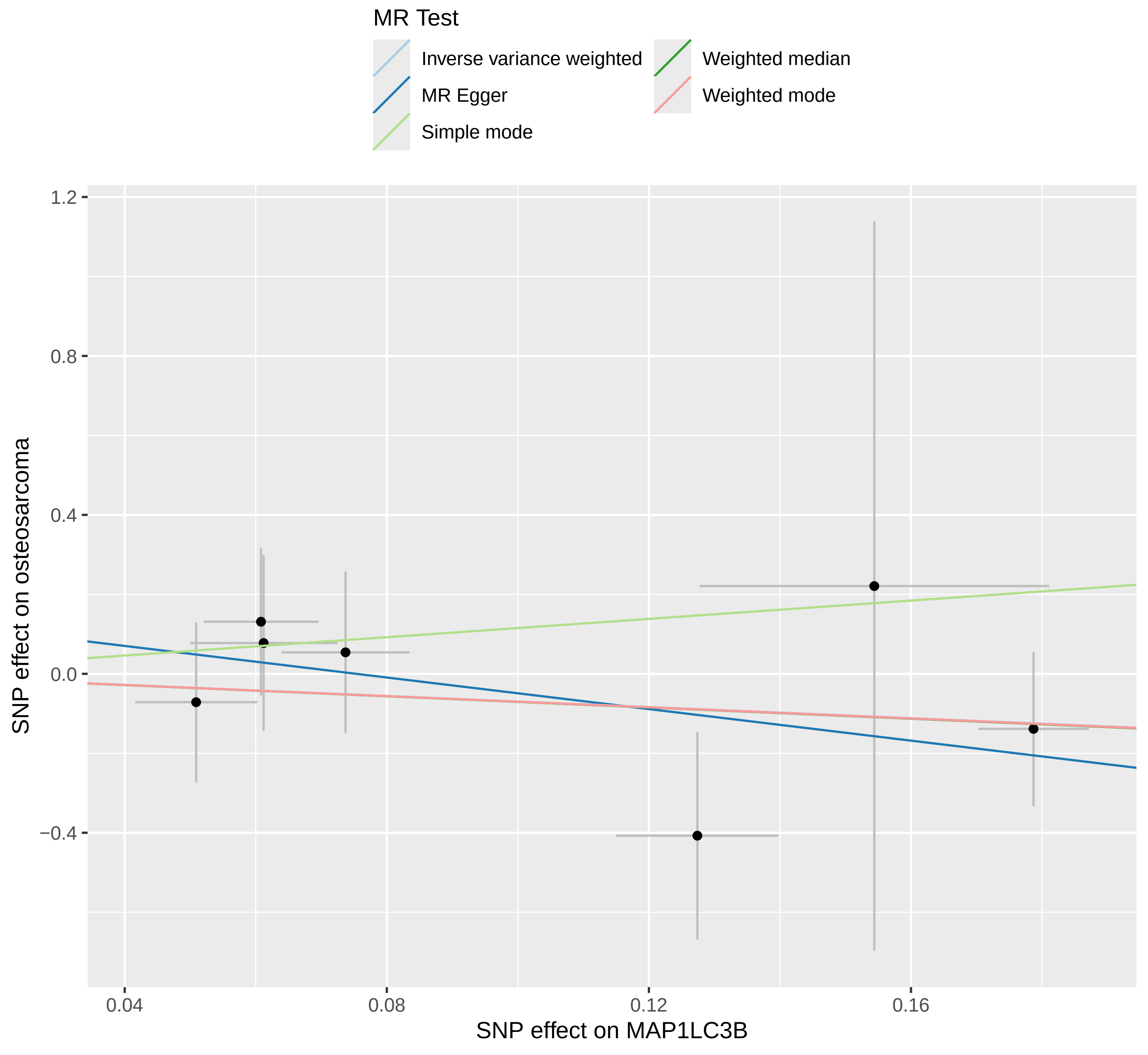

MR Test
Inverse variance weighted
MR Egger
Simple mode
Weighted median
Weighted mode
1.2
0.8
0.4
0.0
−0.4
0.04
0.08
0.12
0.16
SNP effect on osteosarcoma
SNP effect on MAP1LC3B

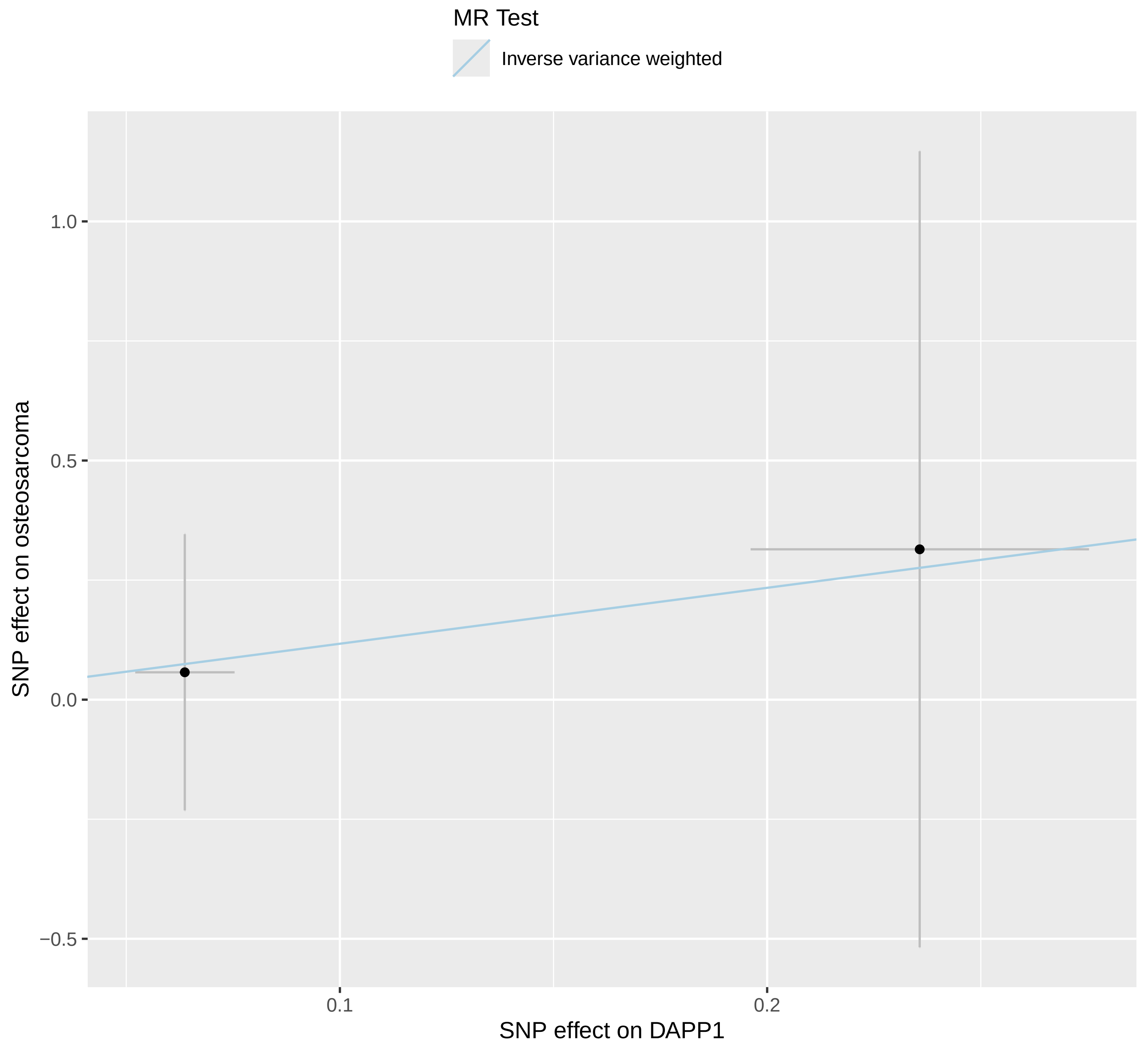
MR Test
Inverse variance weighted
1.0
0.5
0.0
−0.5
0.1
0.2
SNP effect on DAPP1
SNP effect on osteosarcoma

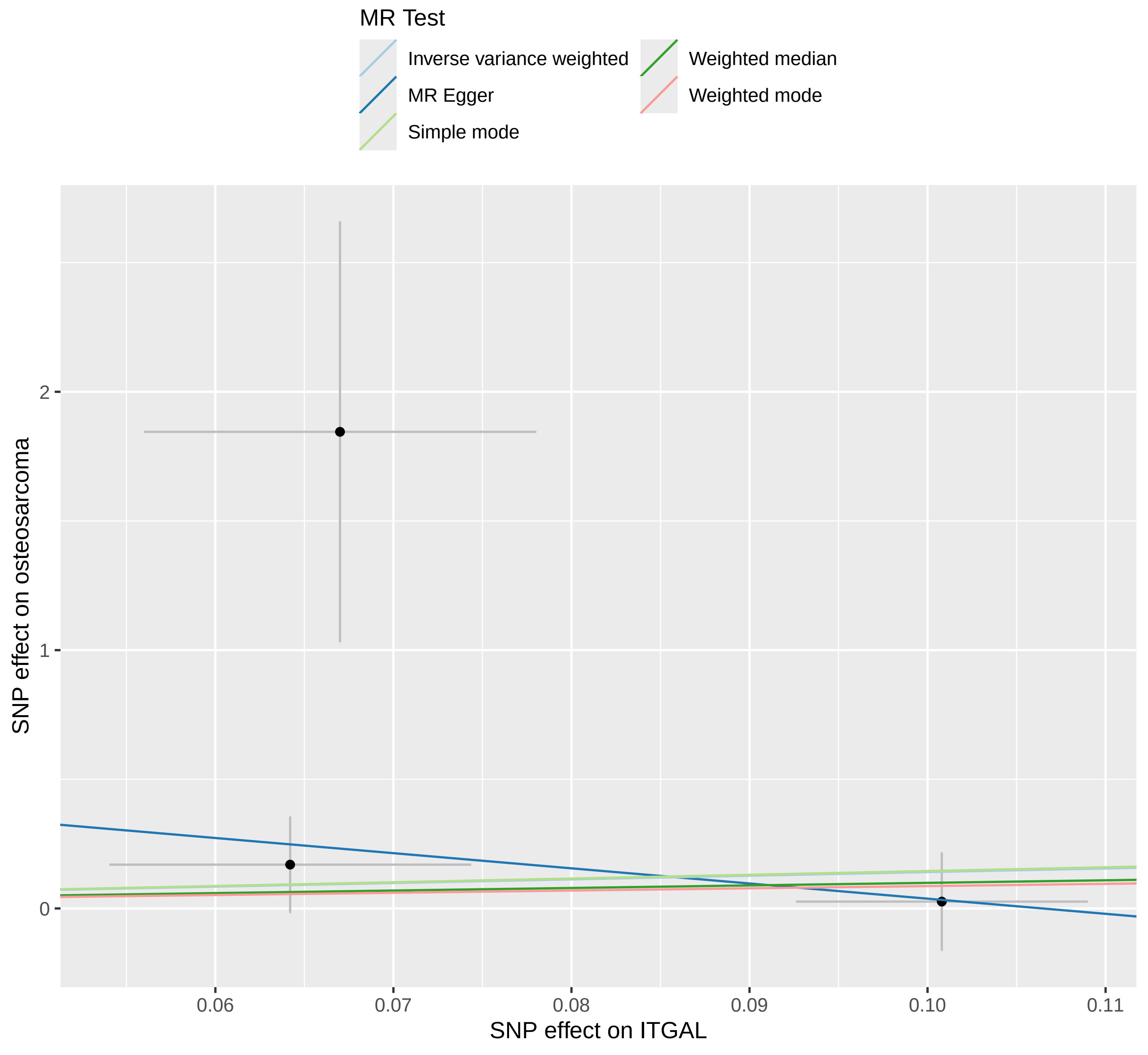
MR Test
Inverse variance weighted
MR Egger
Simple mode
Weighted median
Weighted mode
SNP effect on osteosarcoma
2
1
0
0.06
0.07
0.08
0.09
0.10
0.11
SNP effect on ITGAL

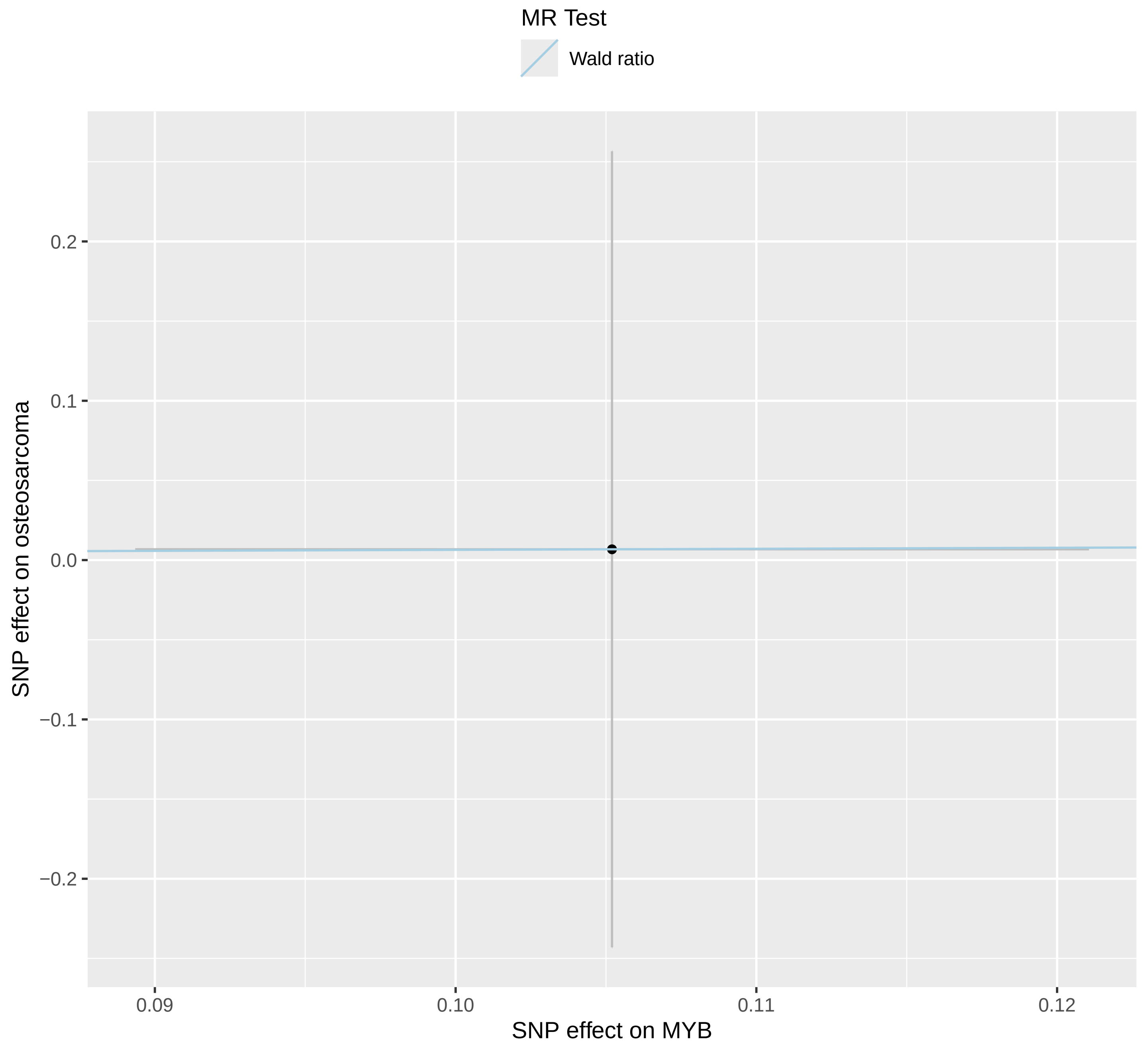
MR Test
Wald ratio
SNP effect on osteosarcoma
0.2
0.1
0.0
−0.1
−0.2
0.09
0.10
0.11
0.12
SNP effect on MYB

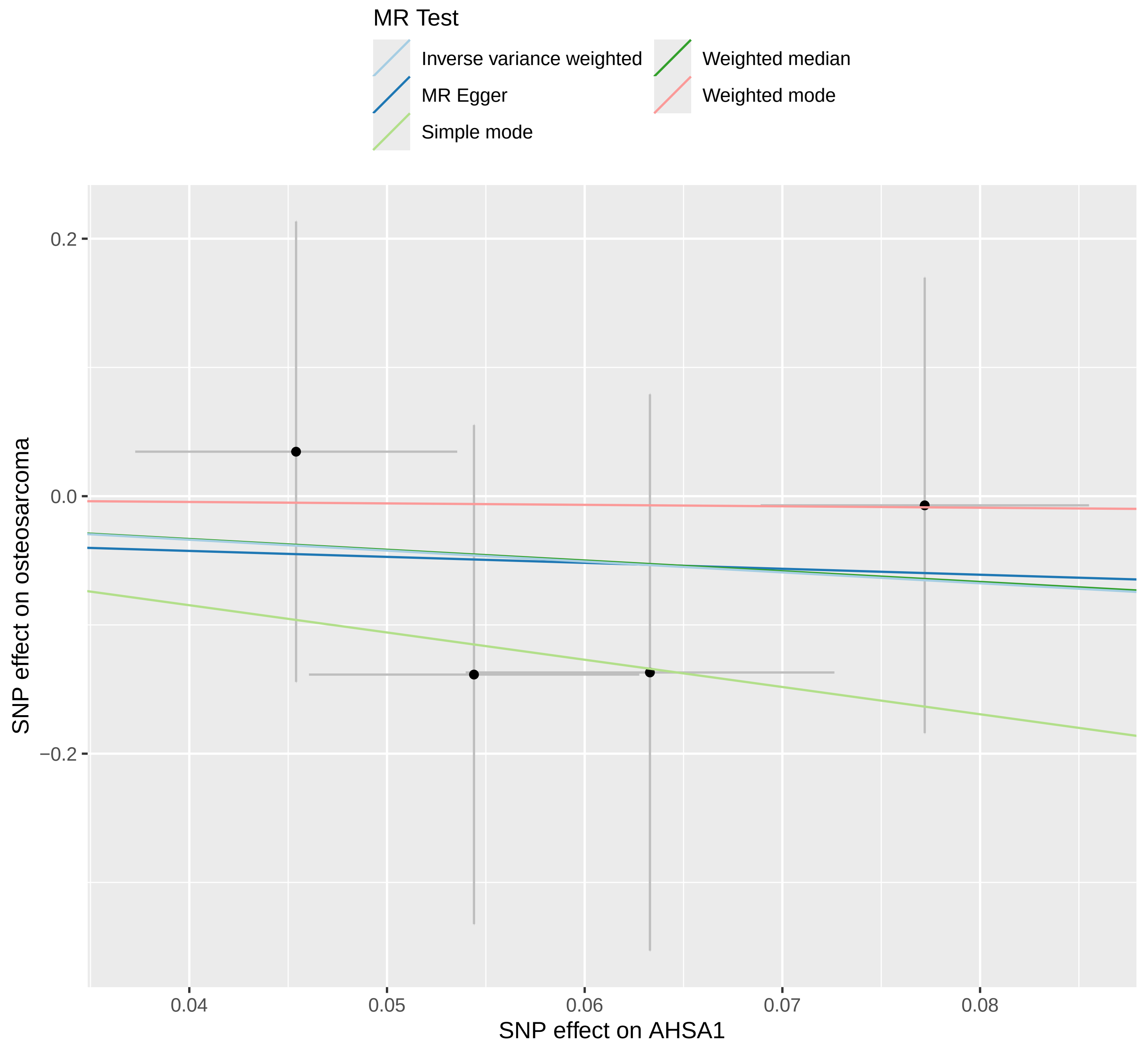
MR Test
Inverse variance weighted
MR Egger
Simple mode
Weighted median
Weighted mode
0.2
0.0
−0.2
SNP effect on osteosarcoma
0.04
0.05
0.06
0.07
0.08
SNP effect on AHSA1

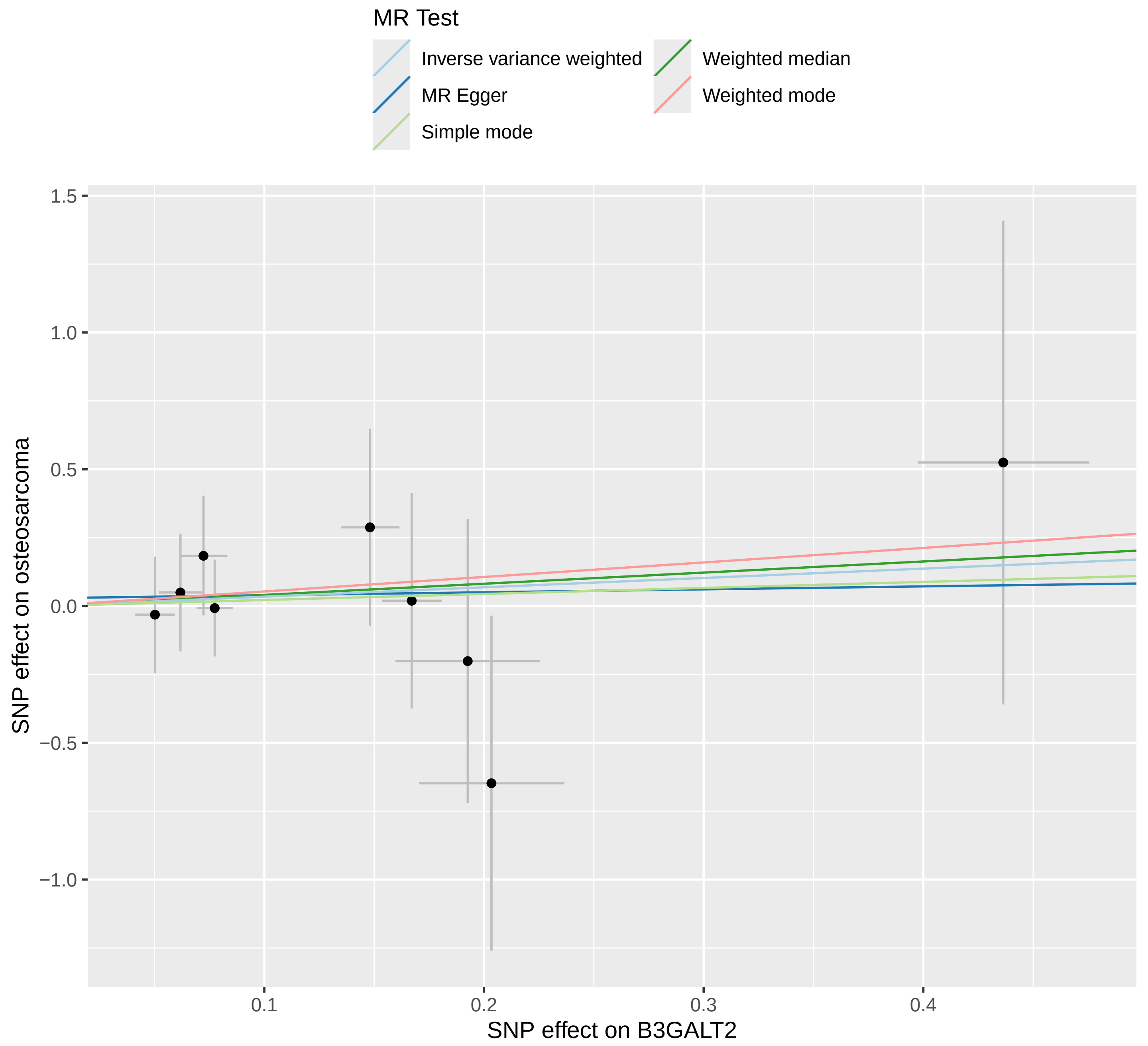
MR Test
Inverse variance weighted
MR Egger
Simple mode
Weighted median
Weighted mode
1.5
1.0
0.5
0.0
−0.5
−1.0
0.1
0.2
0.3
0.4
SNP effect on B3GALT2
SNP effect on osteosarcoma

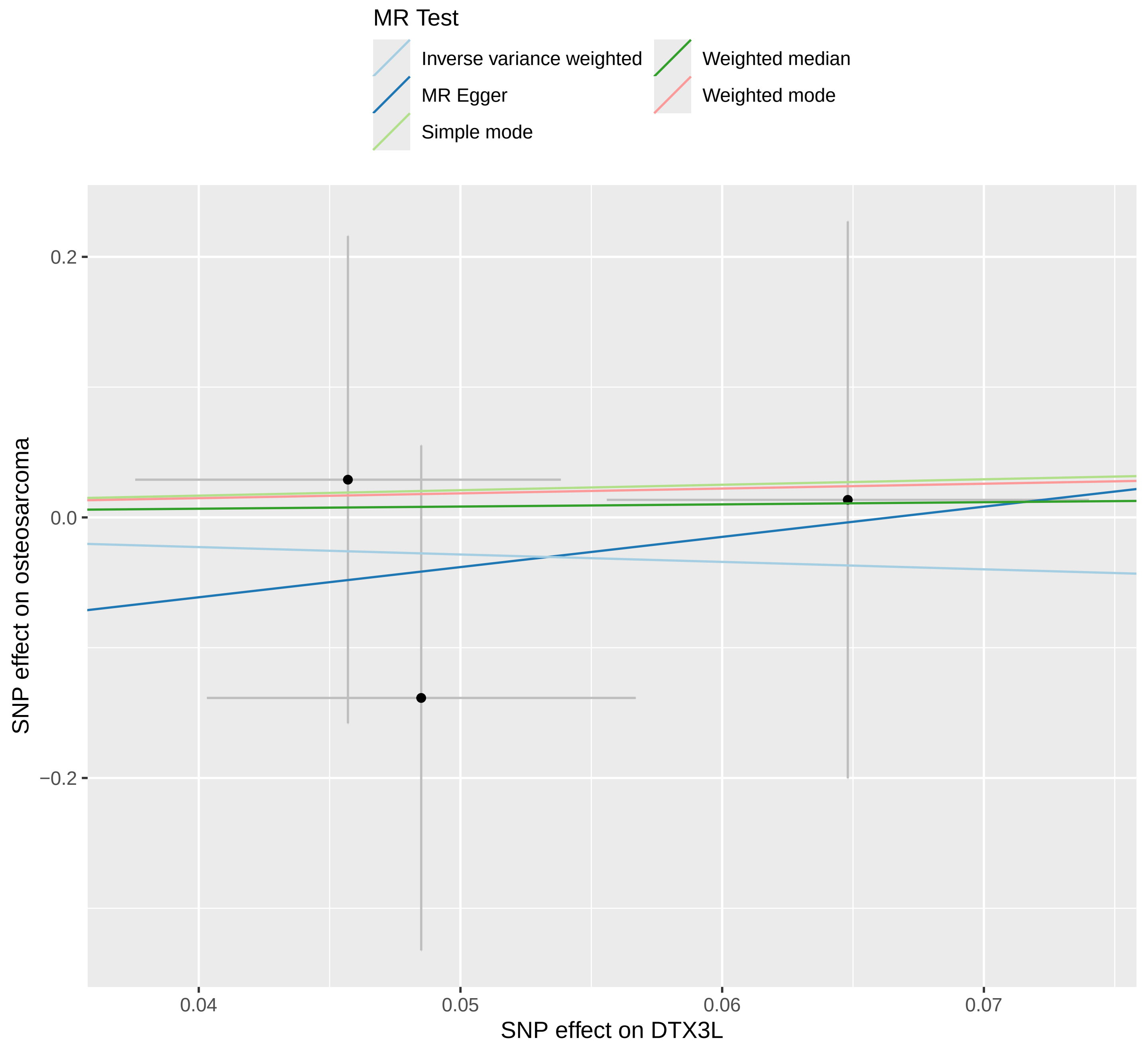

MR Test
Inverse variance weighted
MR Egger
Simple mode
Weighted median
Weighted mode
0.2
0.0
−0.2
SNP effect on osteosarcoma
0.04
0.05
0.06
0.07
SNP effect on DTX3L

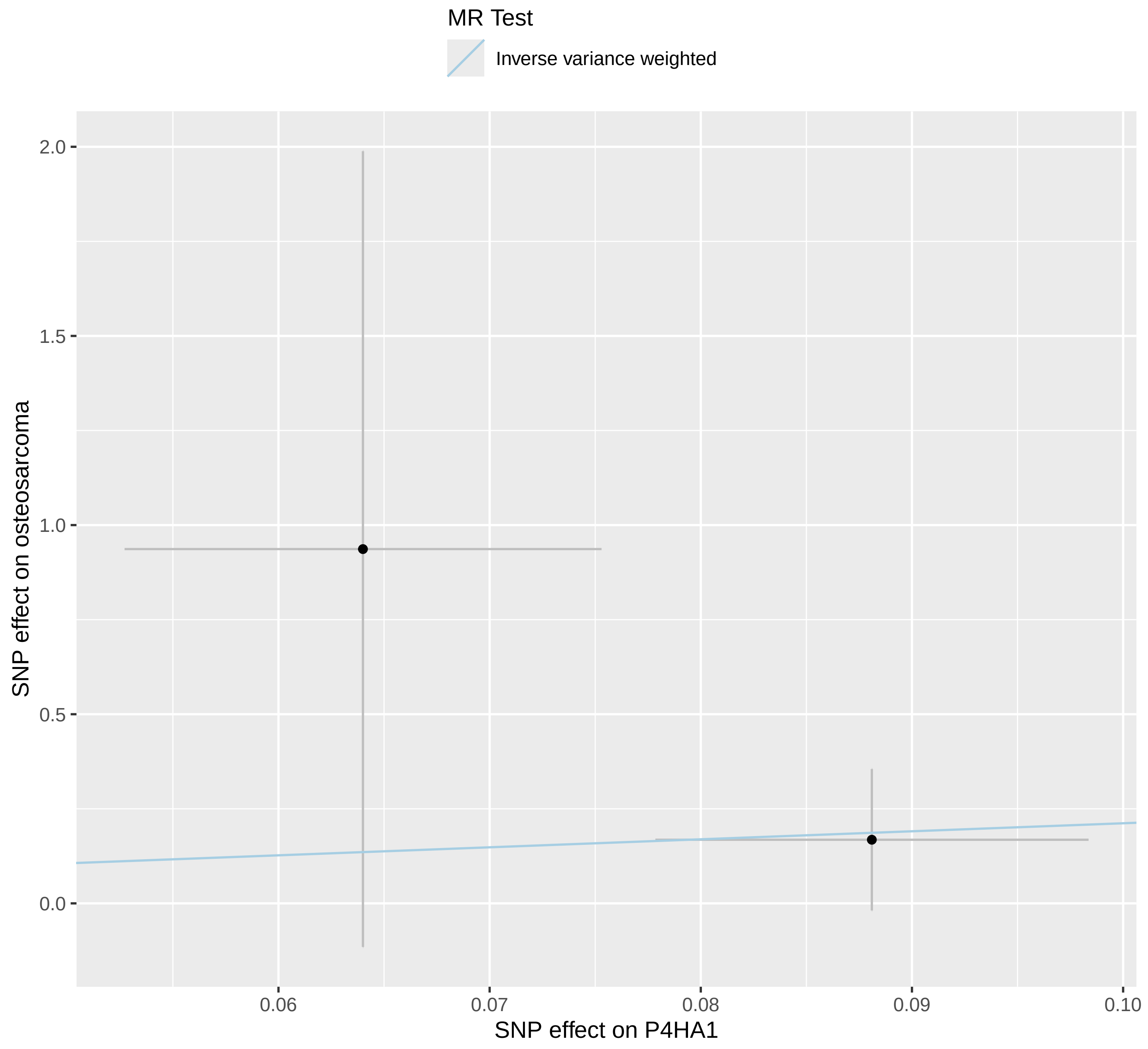
MR Test
Inverse variance weighted
2.0
1.5
1.0
0.5
0.0
0.06
0.07
0.08
0.09
0.10
SNP effect on P4HA1
SNP effect on osteosarcoma

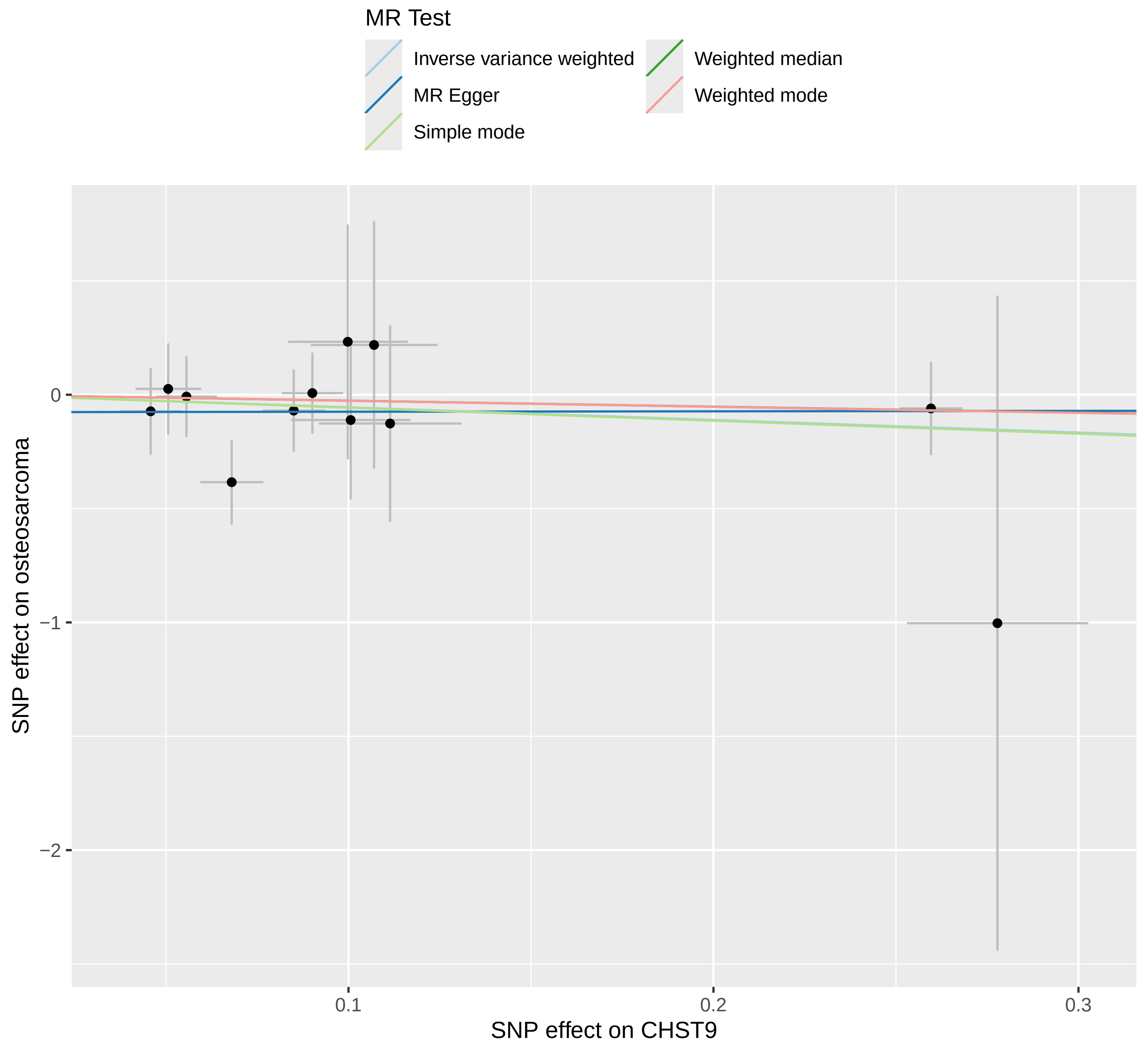

MR Test
Inverse variance weighted
MR Egger
Simple mode
Weighted median
Weighted mode
0
−1
−2
0.1
0.2
0.3
SNP effect on osteosarcoma
SNP effect on CHST9

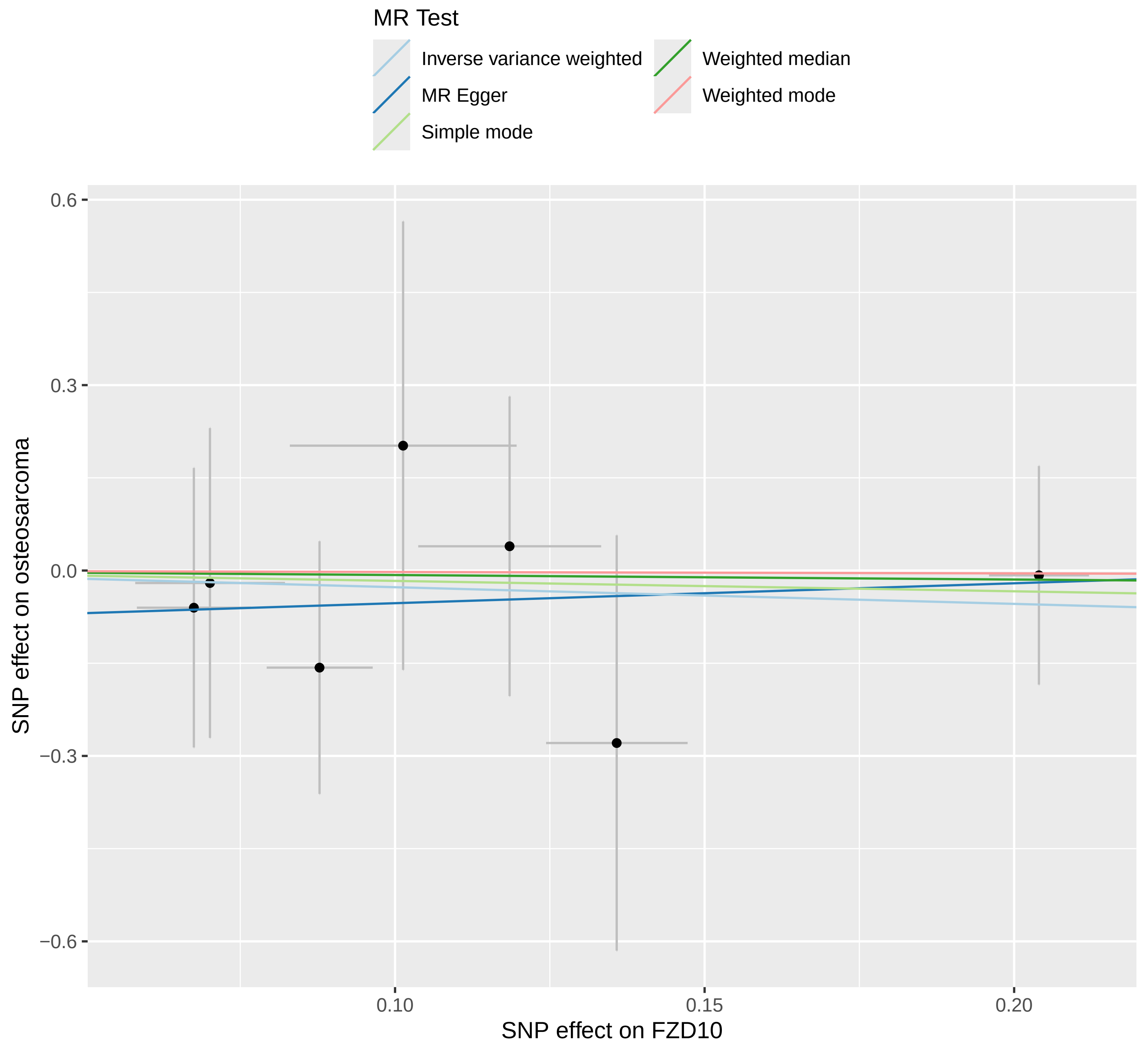

MR Test
Inverse variance weighted
MR Egger
Simple mode
Weighted median
Weighted mode
0.6
0.3
0.0
−0.3
−0.6
0.10
0.15
0.20
SNP effect on osteosarcoma
SNP effect on FZD10

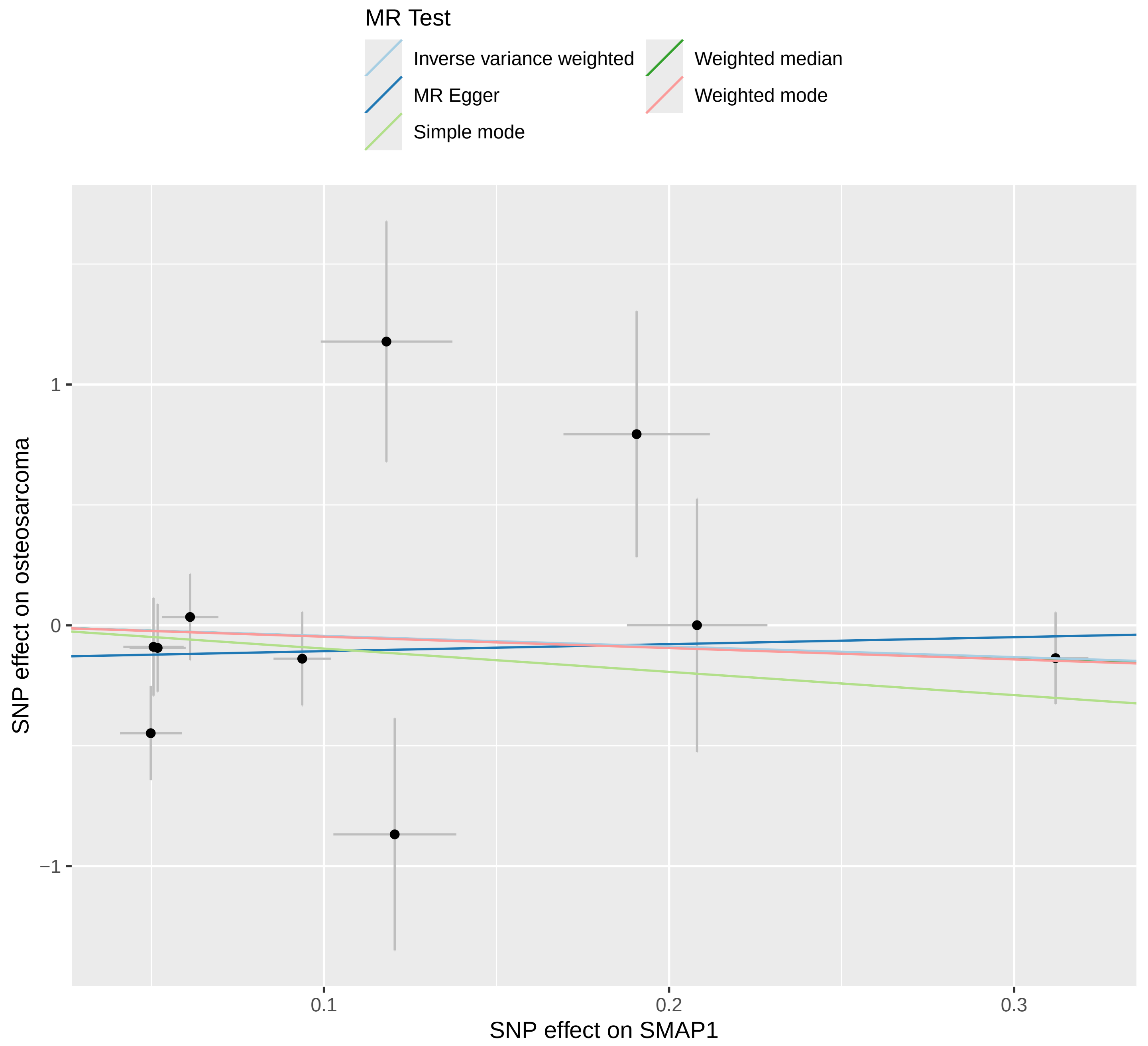

MR Test
Inverse variance weighted
MR Egger
Simple mode
Weighted median
Weighted mode
1
0
−1
0.1
0.2
0.3
SNP effect on osteosarcoma
SNP effect on SMAP1

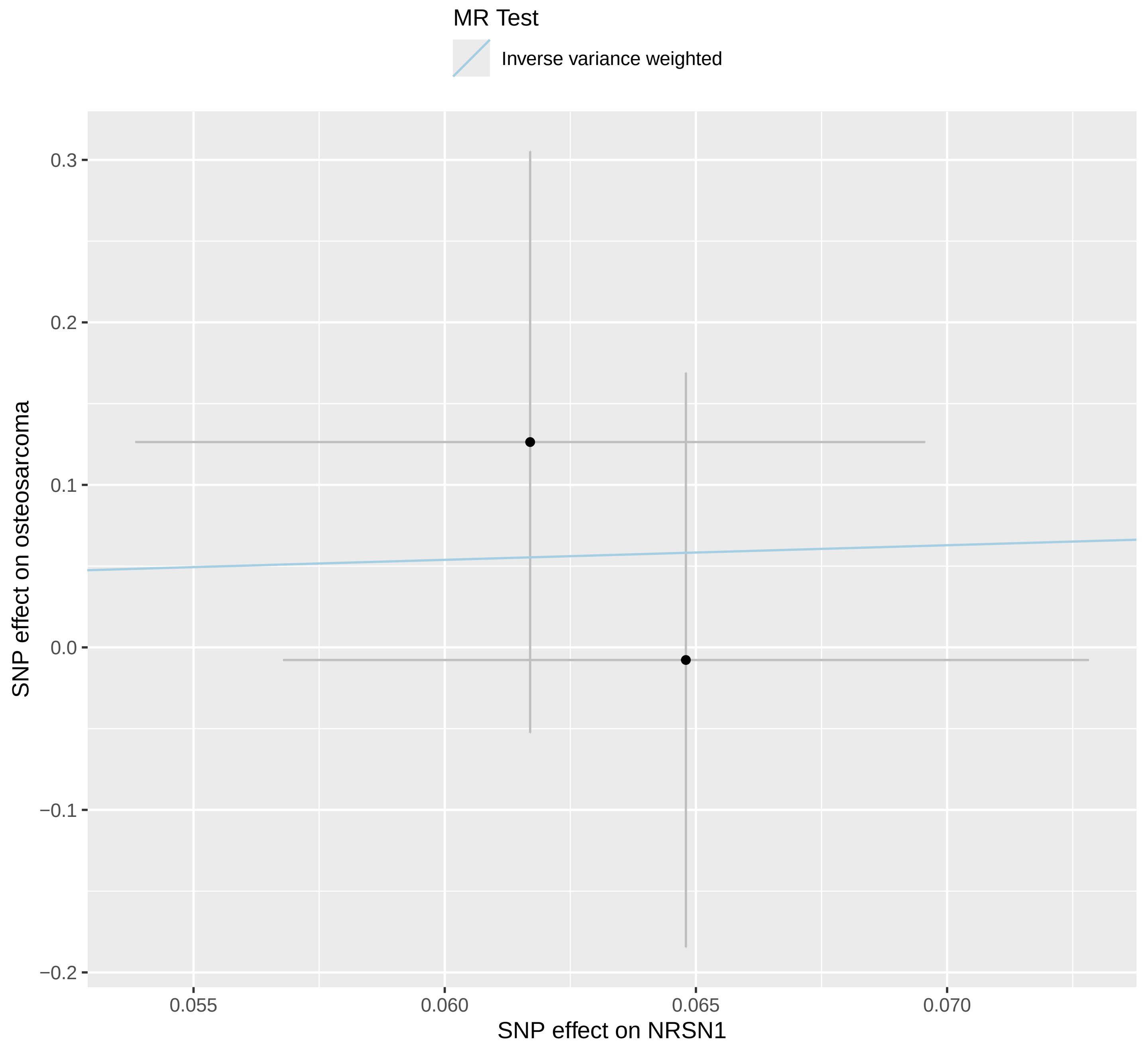

MR Test
Inverse variance weighted
0.3
0.2
0.1
0.0
−0.1
−0.2
SNP effect on osteosarcoma
0.055
0.060
0.065
0.070
SNP effect on NRSN1

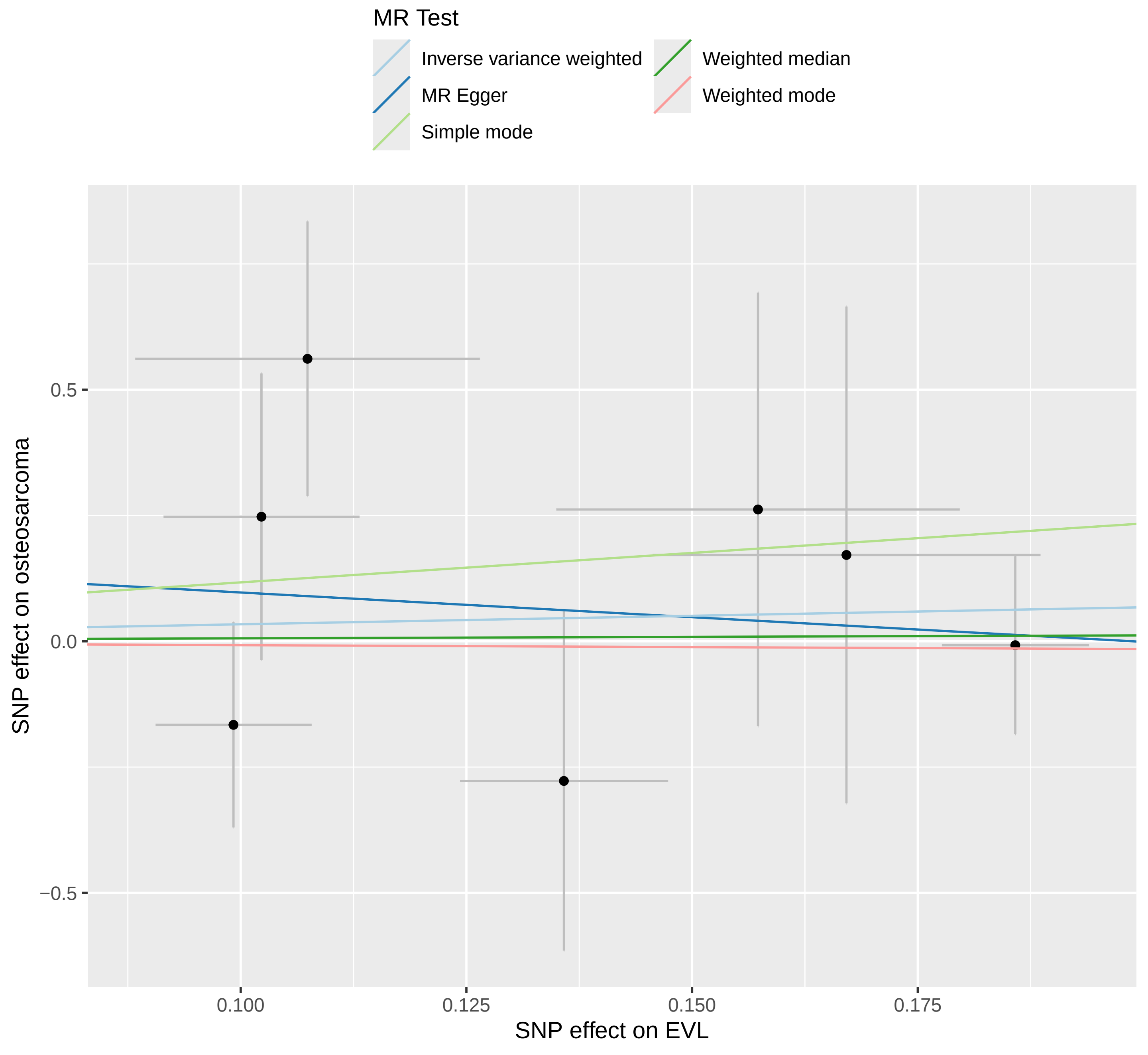
MR Test
Inverse variance weighted
MR Egger
Simple mode
Weighted median
Weighted mode
0.5
0.0
−0.5
0.100
0.125
0.150
0.175
SNP effect on osteosarcoma
SNP effect on EVL

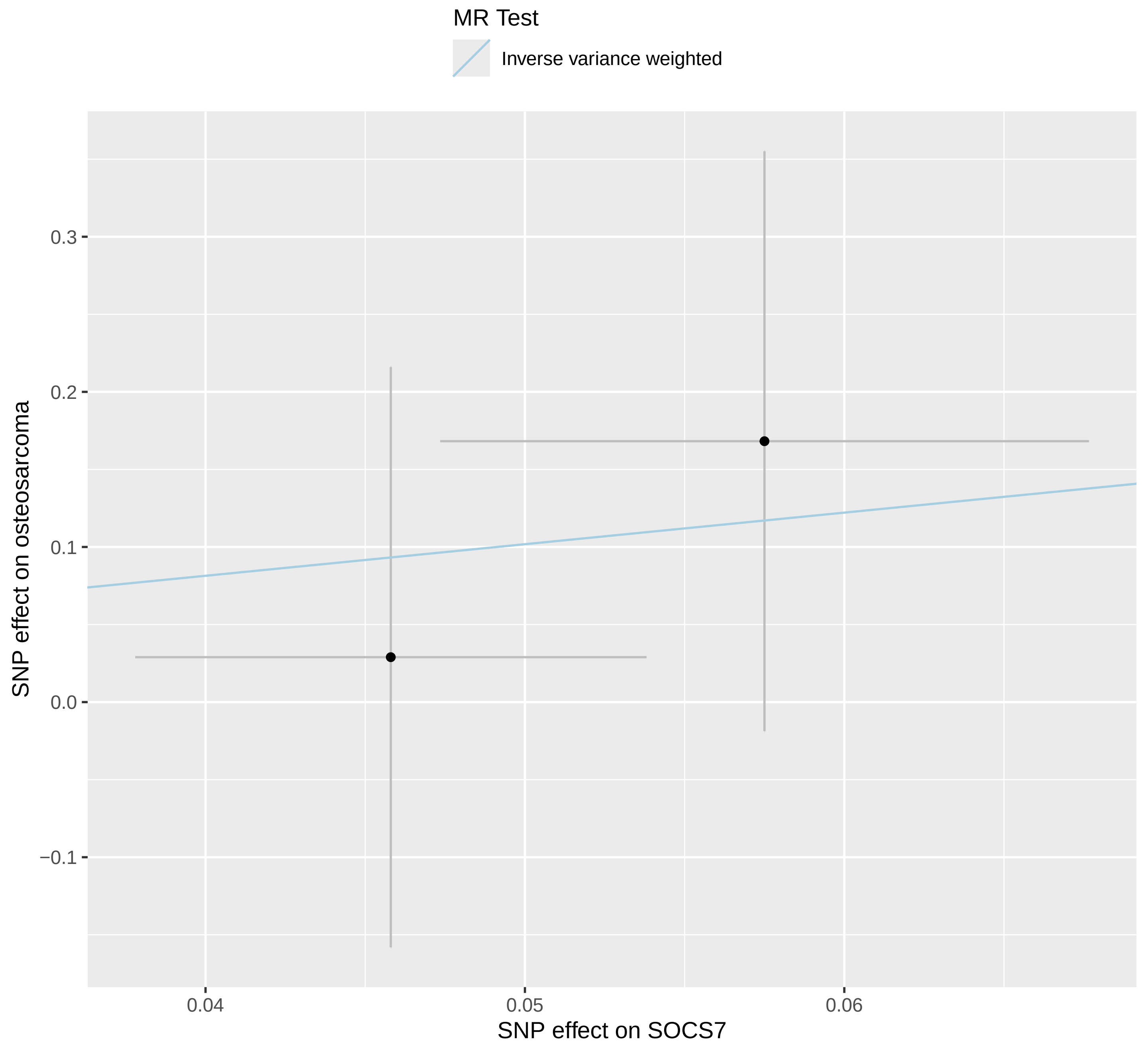
MR Test
Inverse variance weighted
SNP effect on osteosarcoma
0.3
0.2
0.1
0.0
−0.1
0.04
0.05
0.06
SNP effect on SOCS7

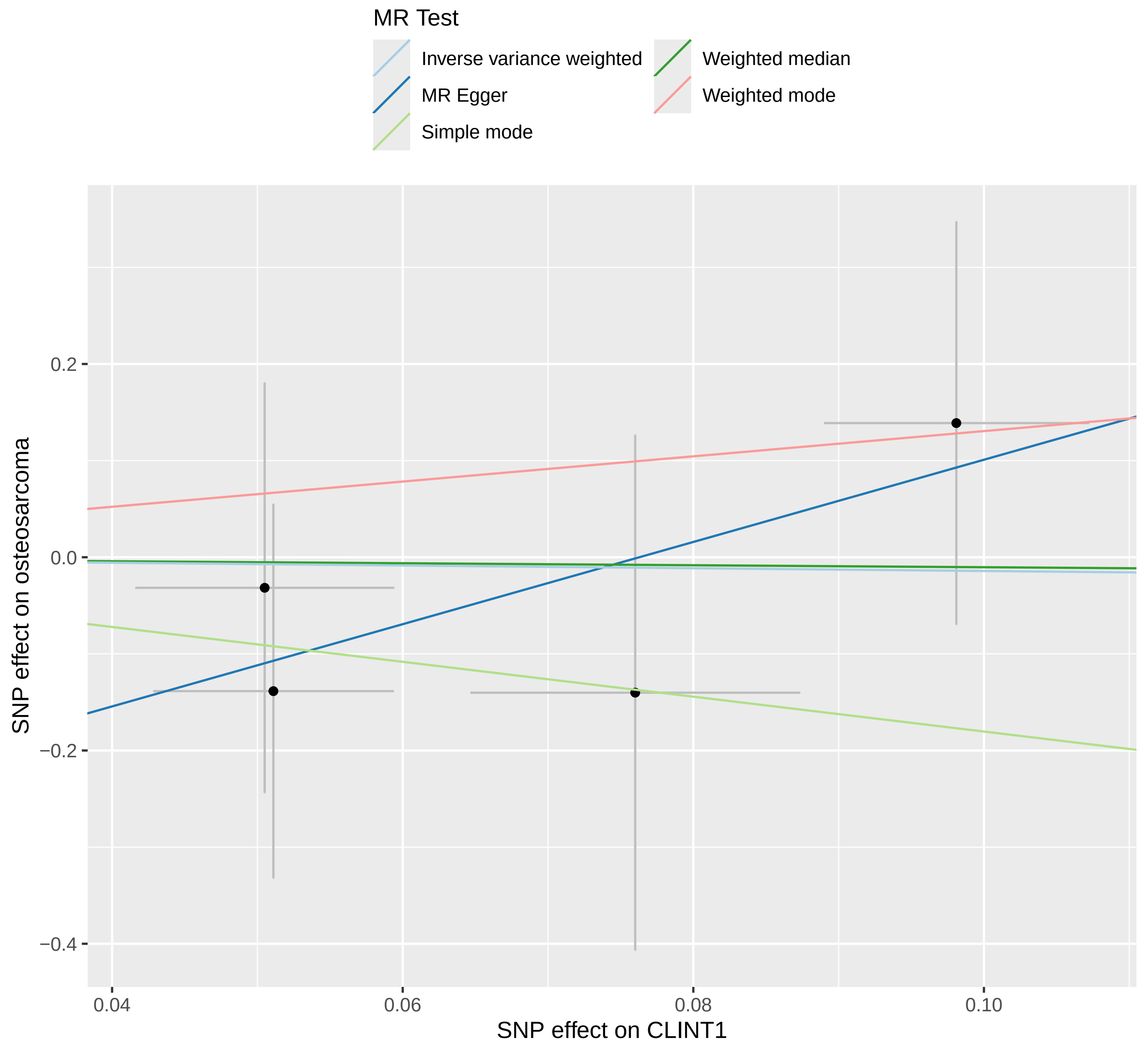

MR Test
Inverse variance weighted
MR Egger
Simple mode
Weighted median
Weighted mode
0.2
0.0
−0.2
−0.4
SNP effect on osteosarcoma
0.04
0.06
0.08
0.10
SNP effect on CLINT1

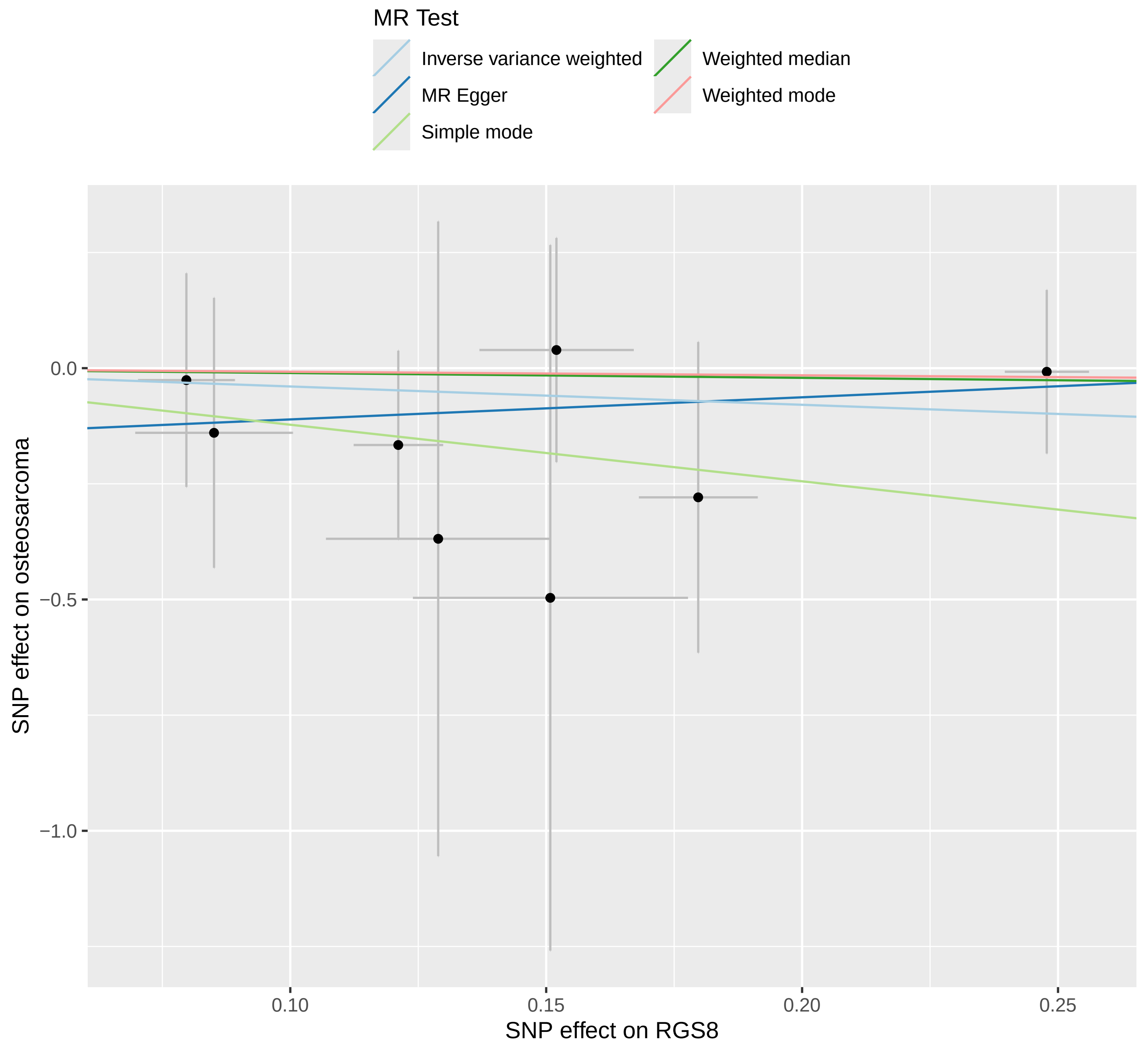
MR Test
Inverse variance weighted
MR Egger
Simple mode
Weighted median
Weighted mode
0.0
−0.5
−1.0
0.10
0.15
0.20
0.25
SNP effect on osteosarcoma
SNP effect on RGS8

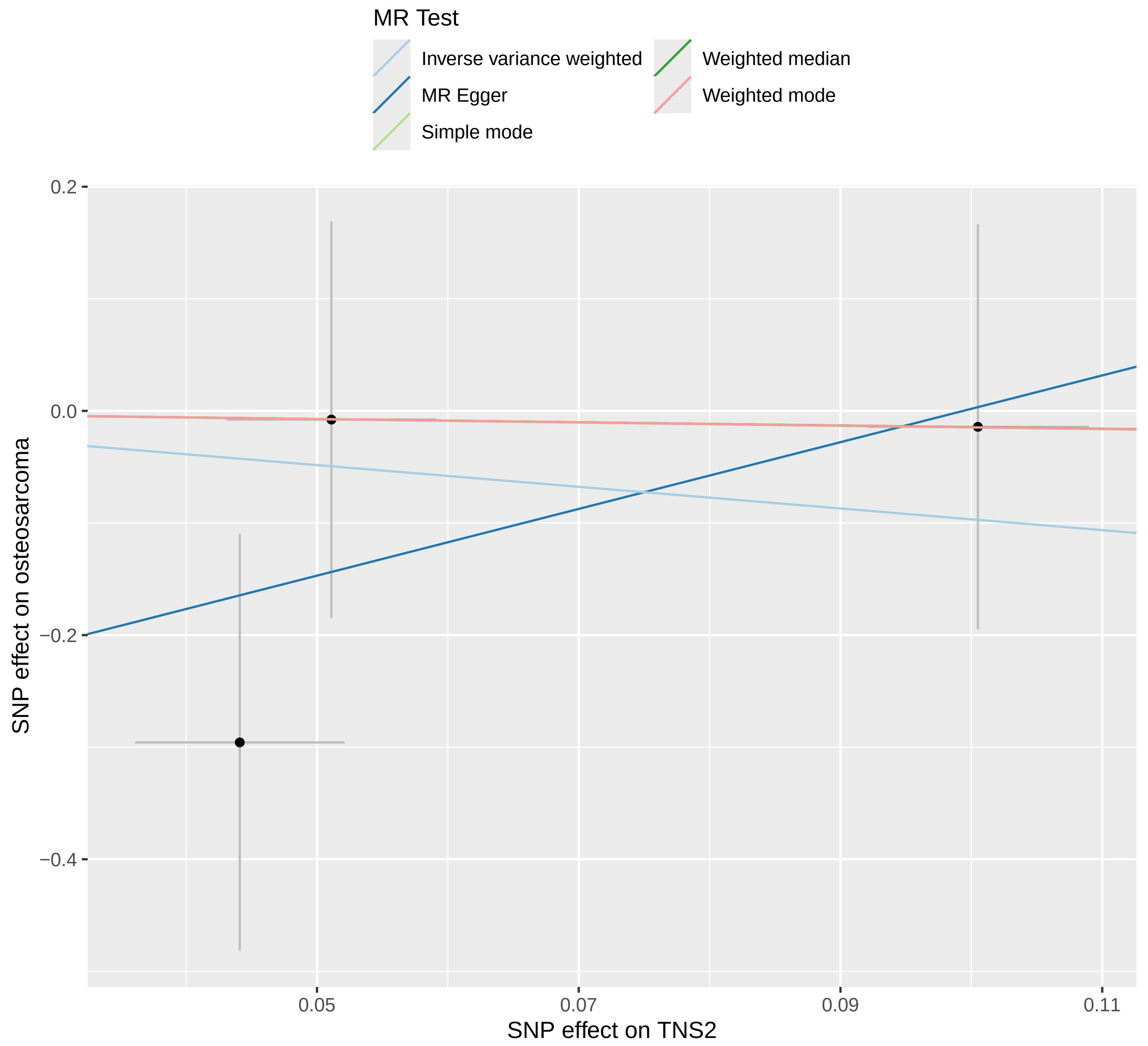

MR Test
Inverse variance weighted
MR Egger
Simple mode
Weighted median
Weighted mode
0.2
0.0
−0.2
−0.4
0.05
0.07
0.09
0.11
SNP effect on TNS2
SNP effect on osteosarcoma

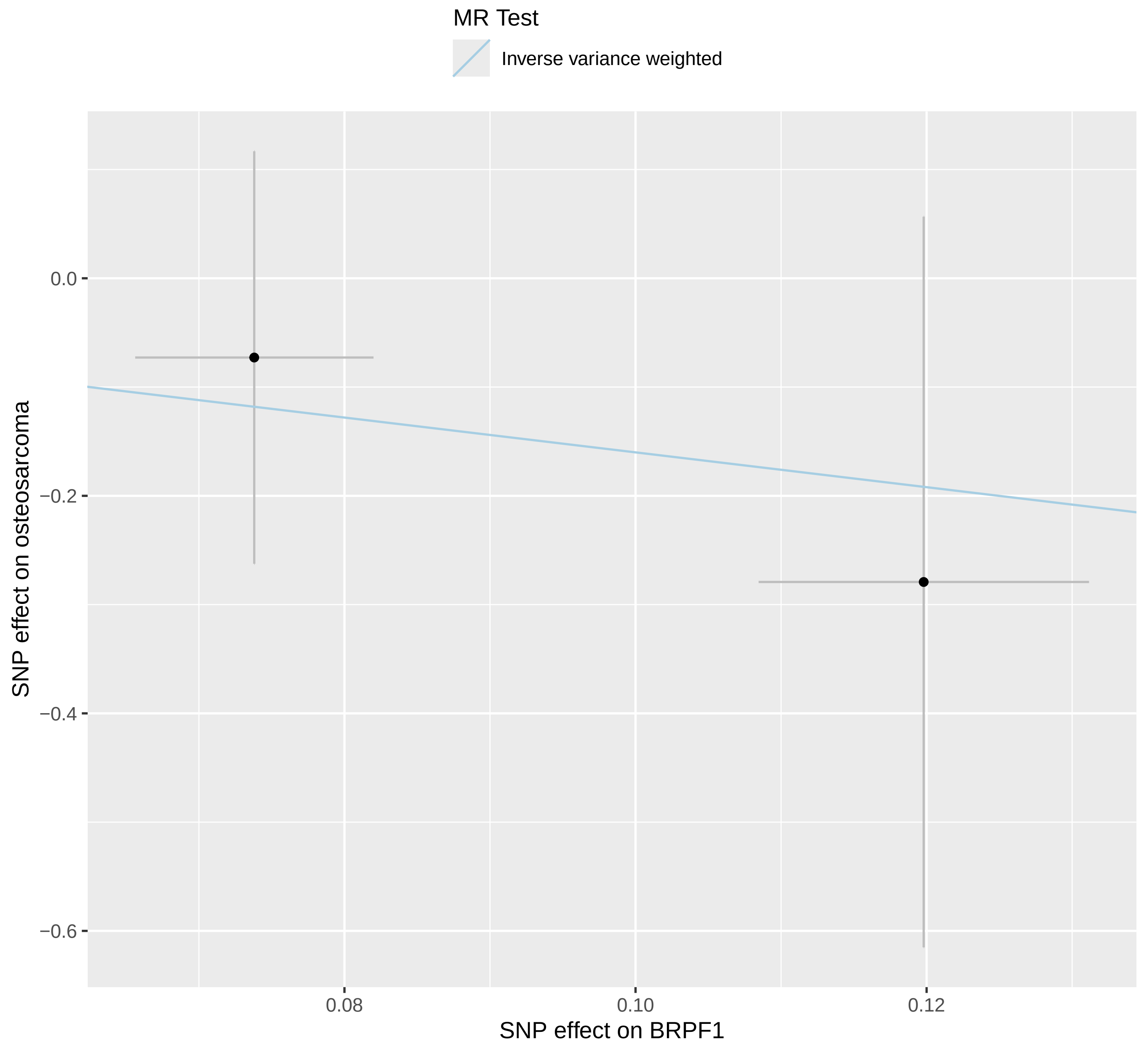
MR Test
Inverse variance weighted
0.0
−0.2
−0.4
−0.6
SNP effect on osteosarcoma
0.08
0.10
0.12
SNP effect on BRPF1

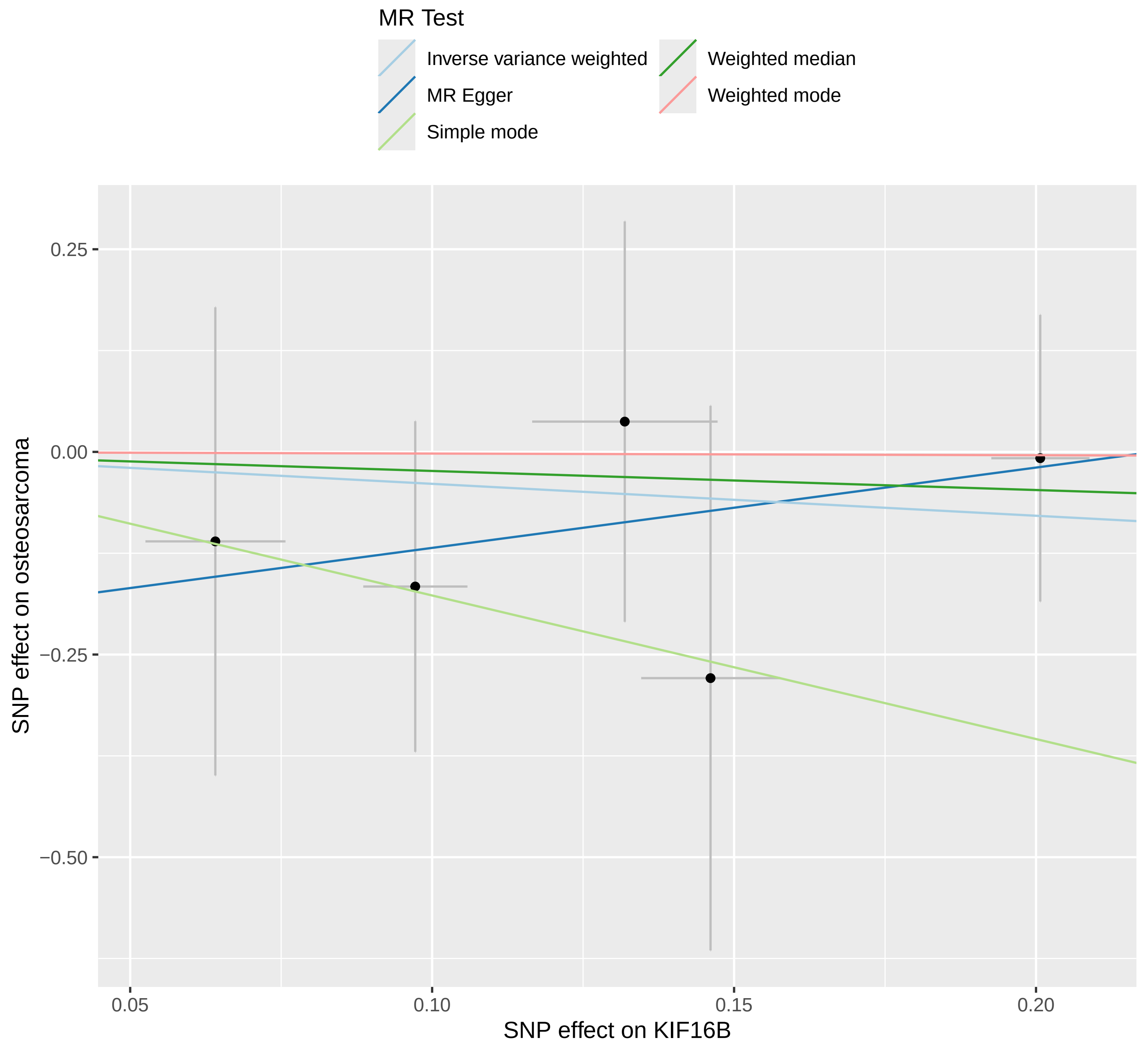
MR Test
Inverse variance weighted
MR Egger
Simple mode
Weighted median
Weighted mode
0.25
0.00
−0.25
−0.50
0.05
0.10
0.15
0.20
SNP effect on osteosarcoma
SNP effect on KIF16B

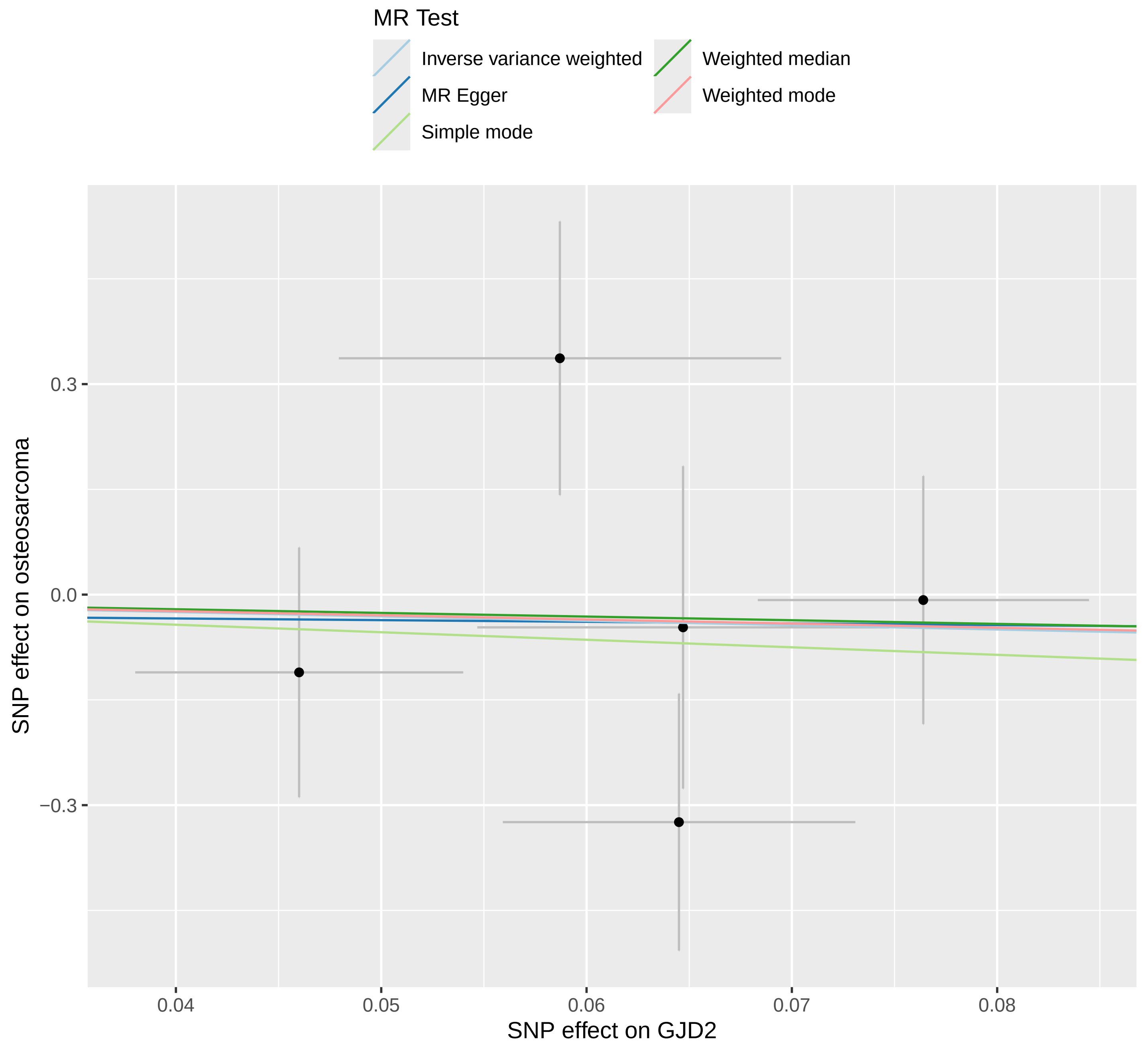
MR Test
Inverse variance weighted
MR Egger
Simple mode
Weighted median
Weighted mode
0.3
0.0
−0.3
SNP effect on osteosarcoma
0.04
0.05
0.06
0.07
0.08
SNP effect on GJD2

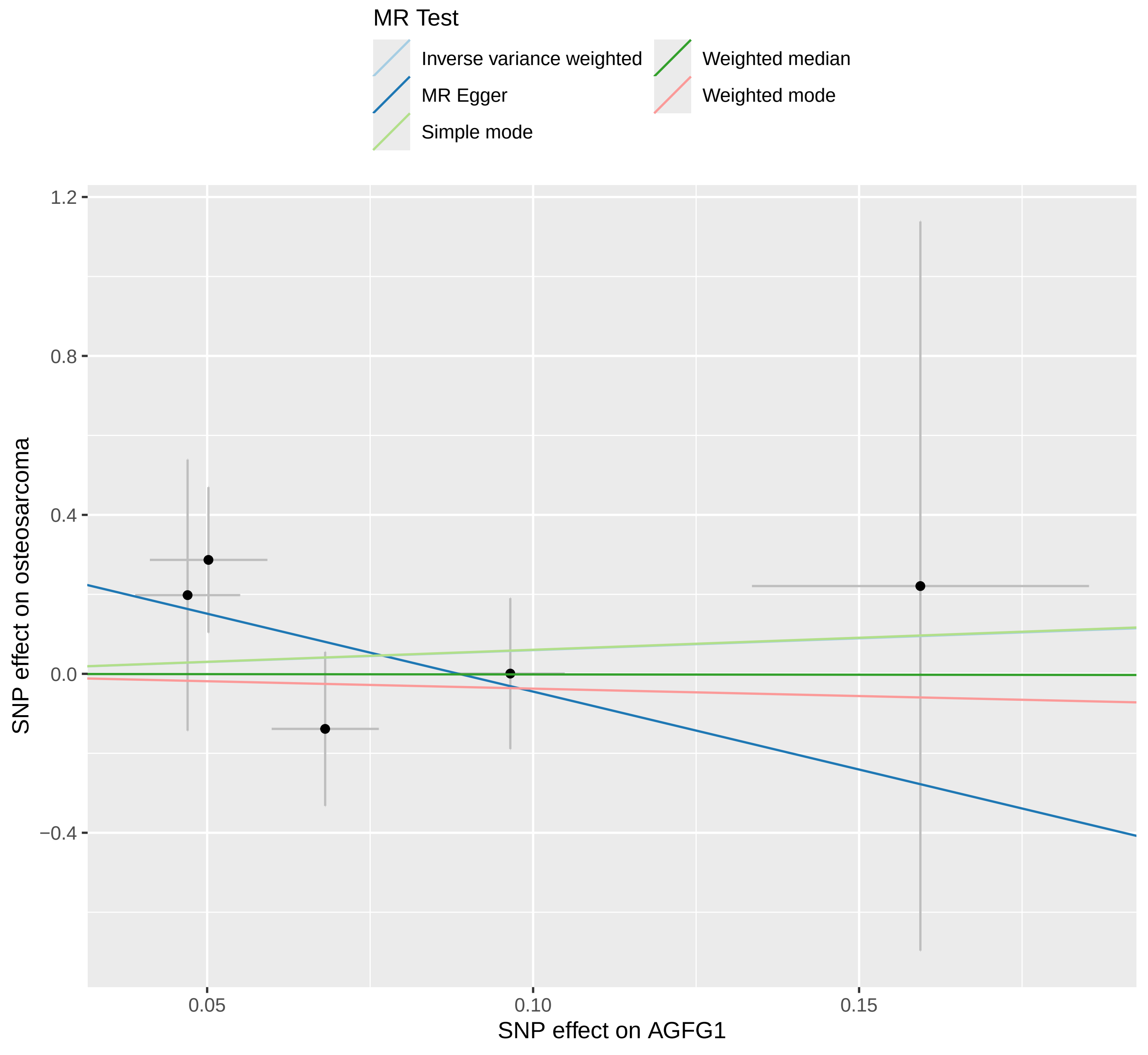

MR Test
Inverse variance weighted
MR Egger
Simple mode
Weighted median
Weighted mode
1.2
0.8
0.4
0.0
−0.4
SNP effect on osteosarcoma
0.05
0.10
0.15
SNP effect on AGFG1

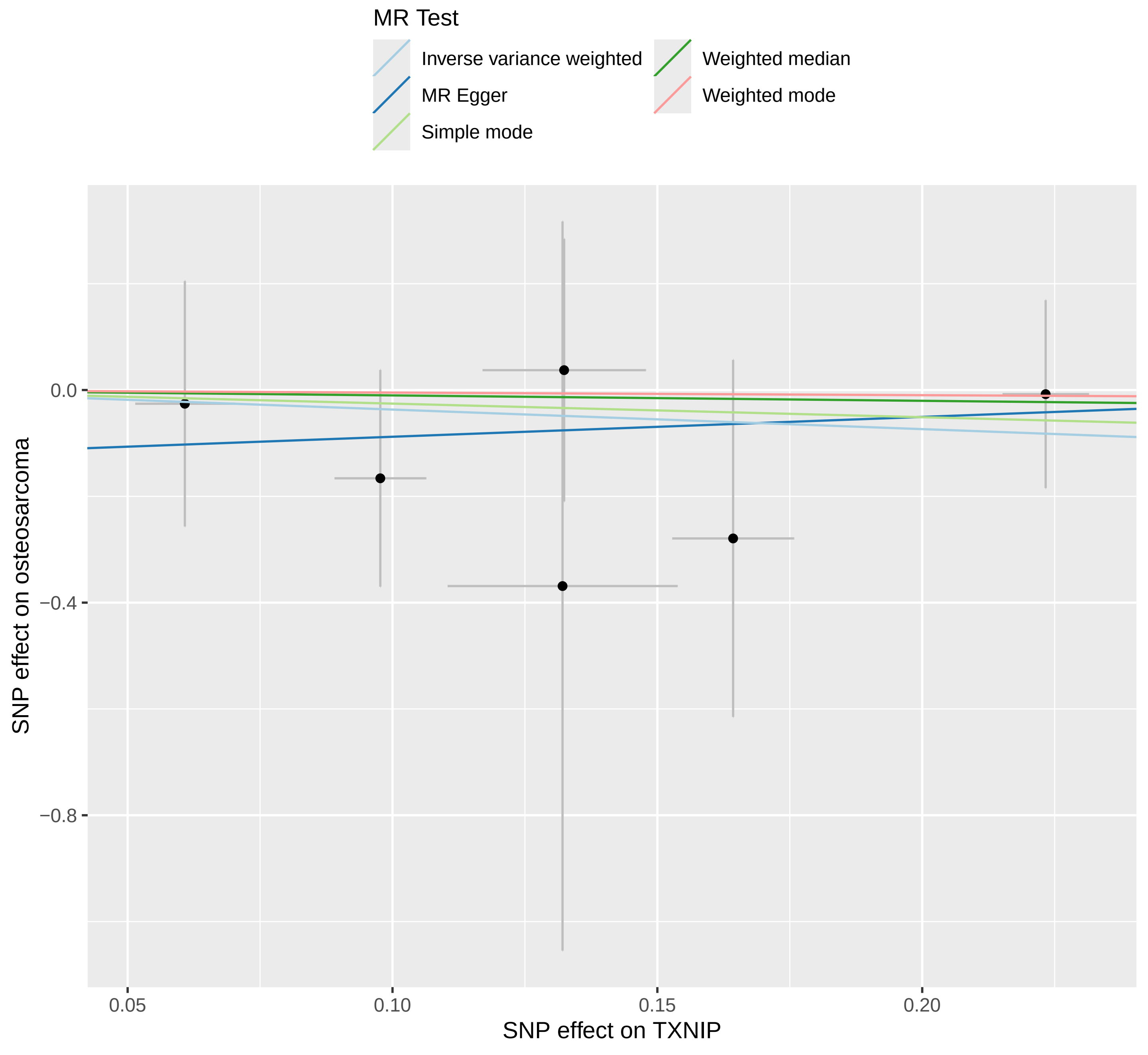
MR Test
Inverse variance weighted
MR Egger
Simple mode
Weighted median
Weighted mode
0.0
−0.4
−0.8
0.05
0.10
0.15
0.20
SNP effect on osteosarcoma
SNP effect on TXNIP

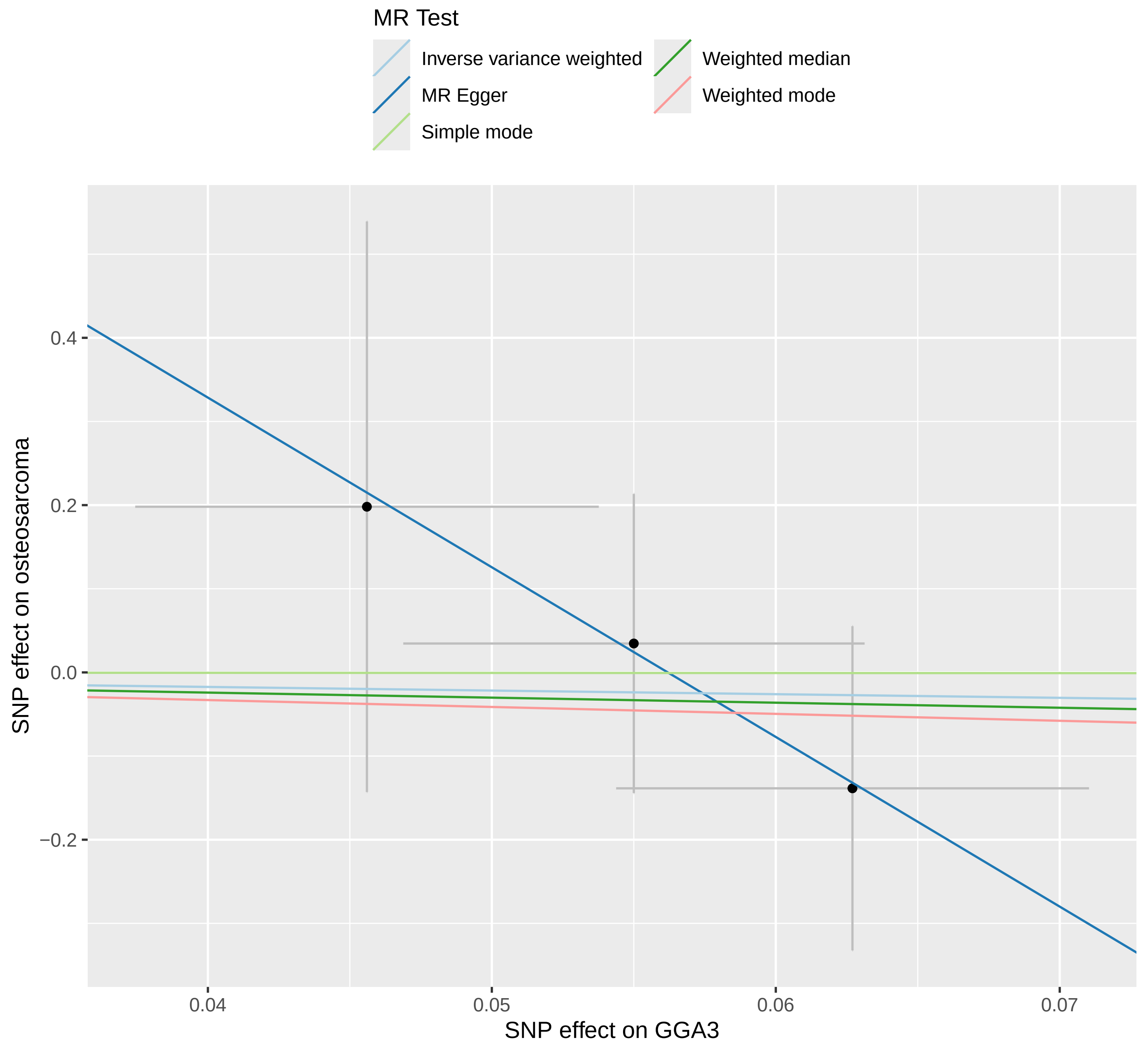

MR Test
Inverse variance weighted
MR Egger
Simple mode
Weighted median
Weighted mode
0.4
0.2
0.0
−0.2
0.04
0.05
0.06
0.07
SNP effect on osteosarcoma
SNP effect on GGA3

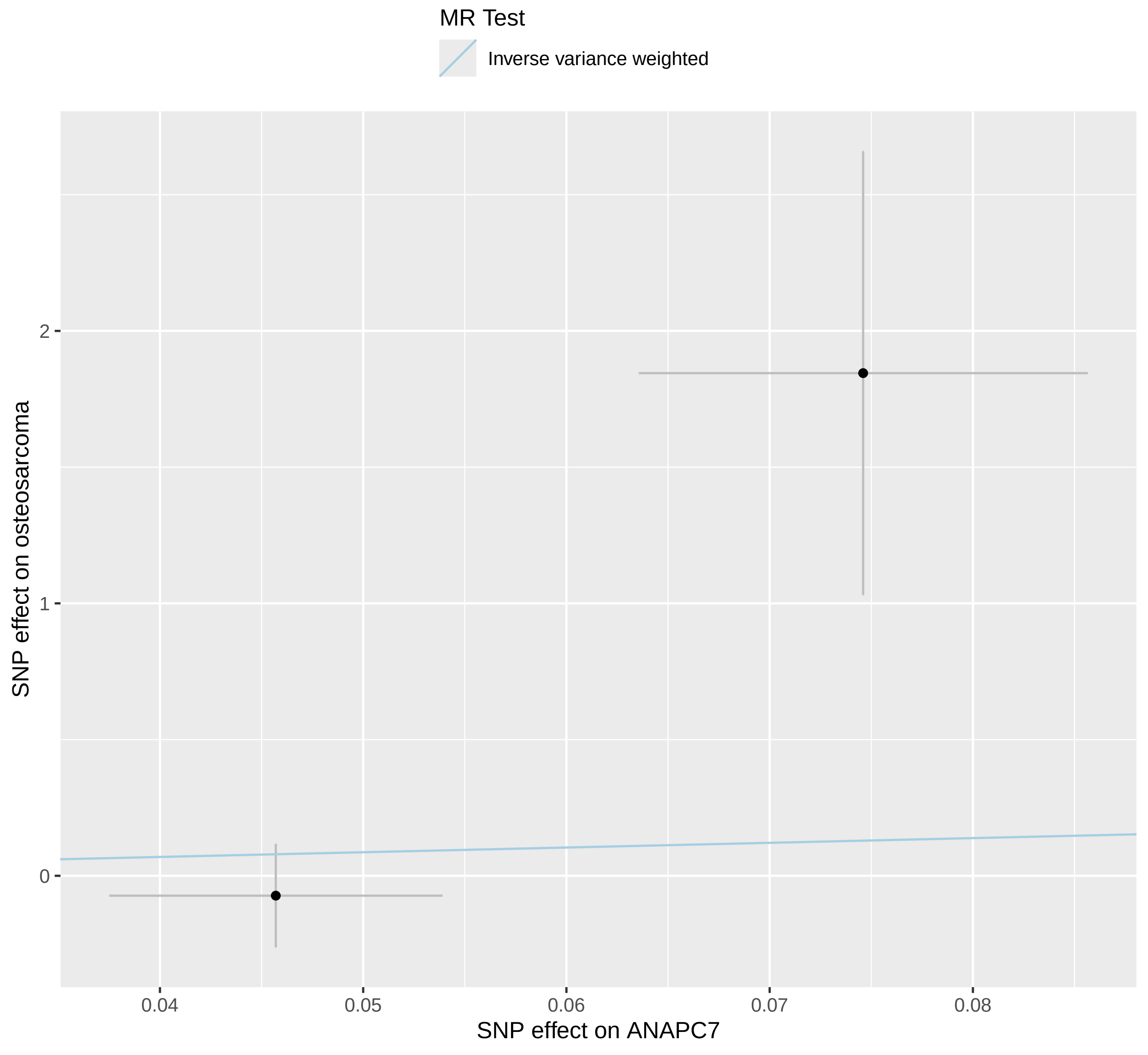

MR Test
Inverse variance weighted
SNP effect on osteosarcoma
2
1
0
0.04
0.05
0.06
0.07
0.08
SNP effect on ANAPC7

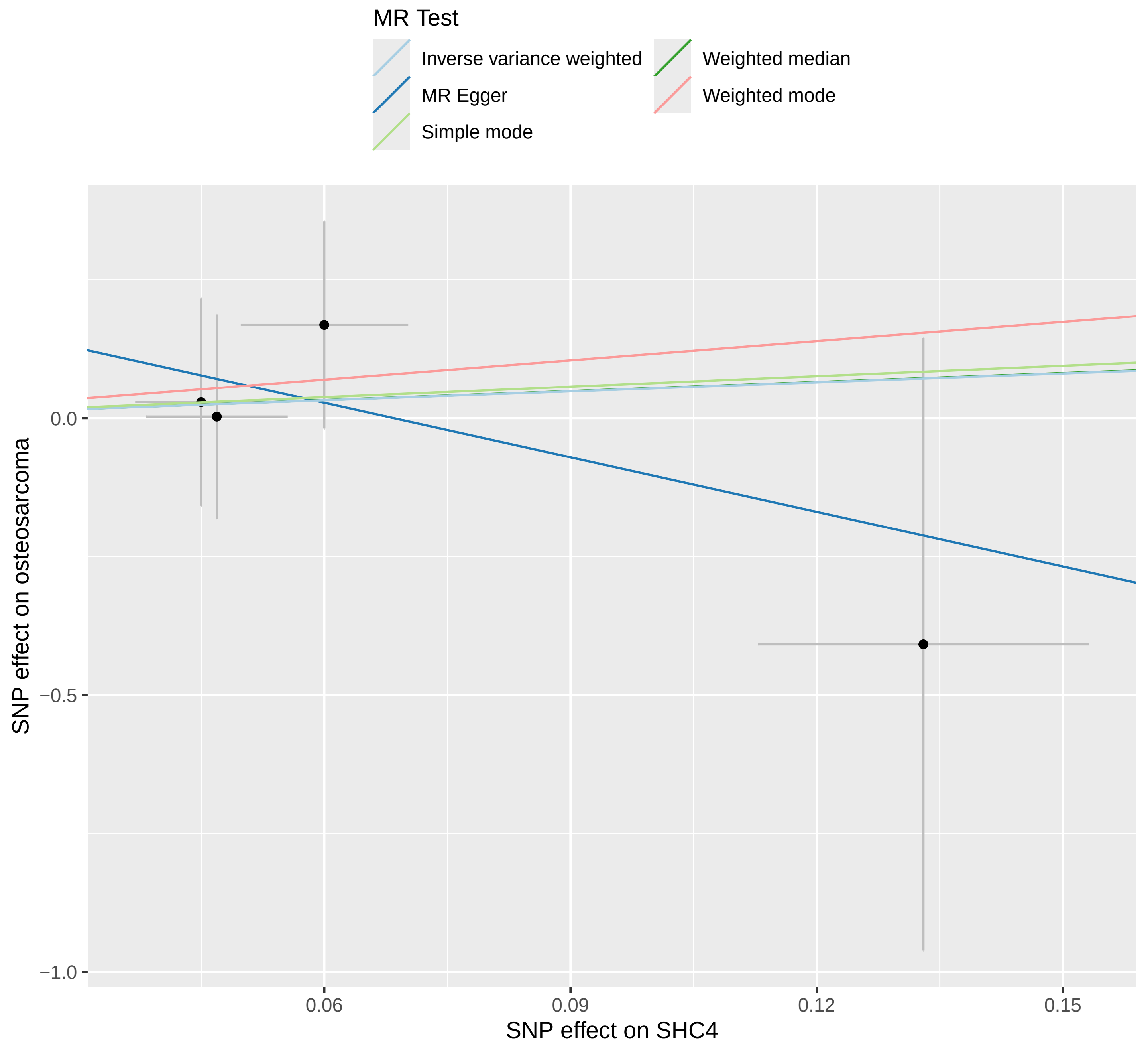

MR Test
Inverse variance weighted
MR Egger
Simple mode
Weighted median
Weighted mode
0.0
−0.5
−1.0
0.06
0.09
0.12
0.15
SNP effect on osteosarcoma
SNP effect on SHC4

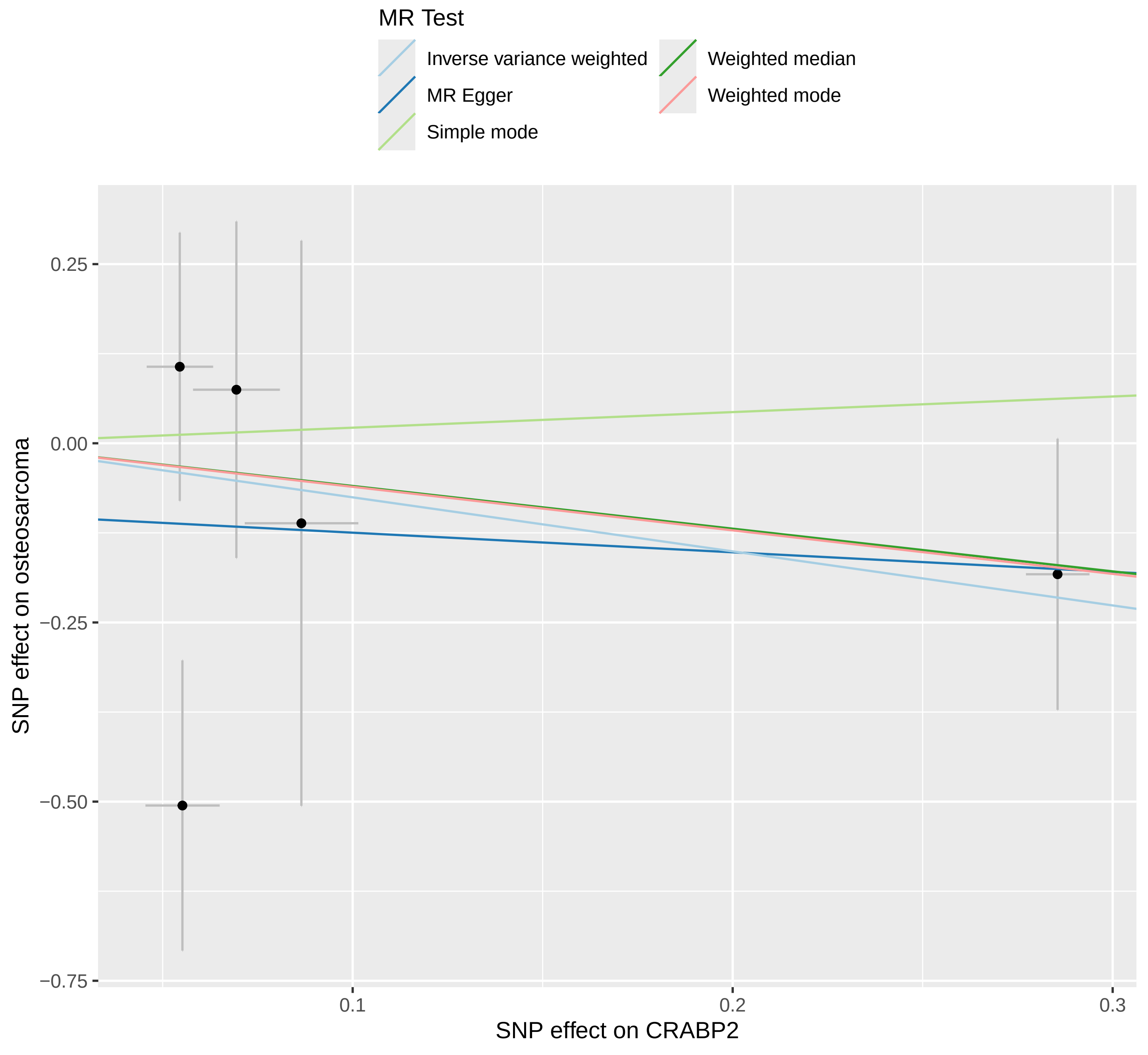
MR Test
Inverse variance weighted
MR Egger
Simple mode
Weighted median
Weighted mode
0.25
0.00
−0.25
−0.50
−0.75
SNP effect on osteosarcoma
0.1
0.2
0.3
SNP effect on CRABP2

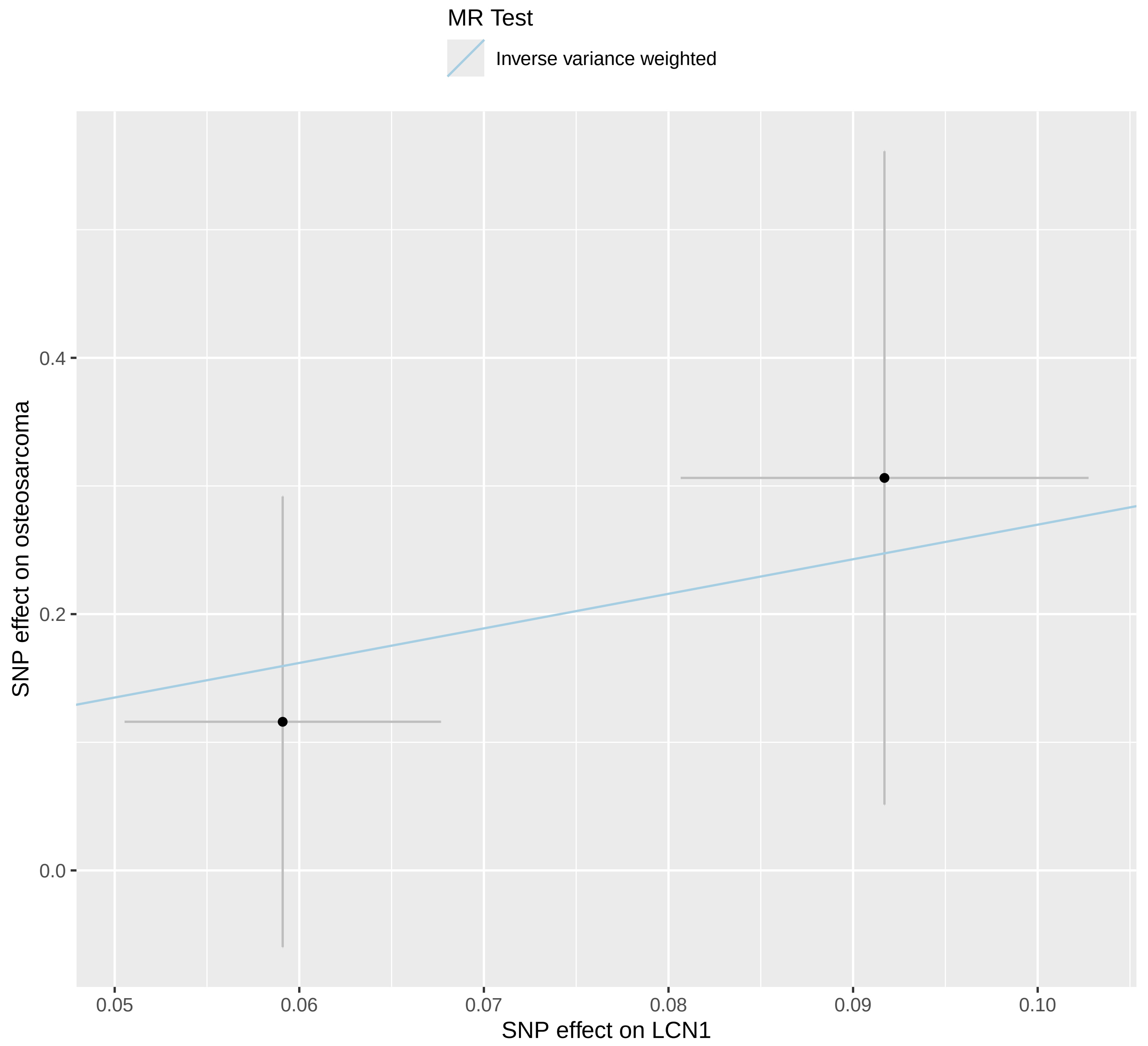

MR Test
Inverse variance weighted
SNP effect on osteosarcoma
0.4
0.2
0.0
0.05
0.06
0.07
0.08
0.09
0.10
SNP effect on LCN1

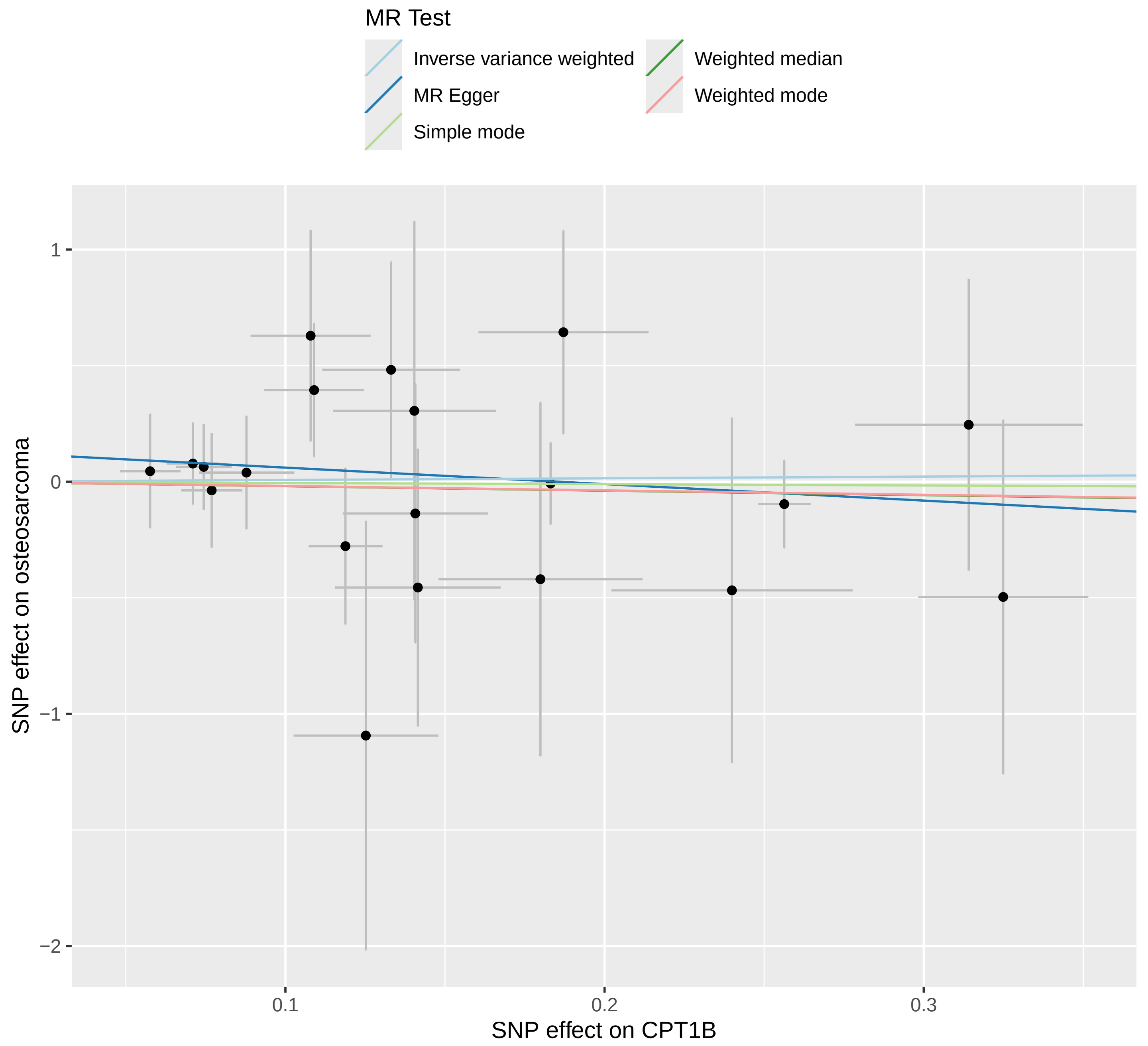

MR Test
Inverse variance weighted
MR Egger
Simple mode
Weighted median
Weighted mode
1
0
−1
−2
0.1
0.2
0.3
SNP effect on osteosarcoma
SNP effect on CPT1B

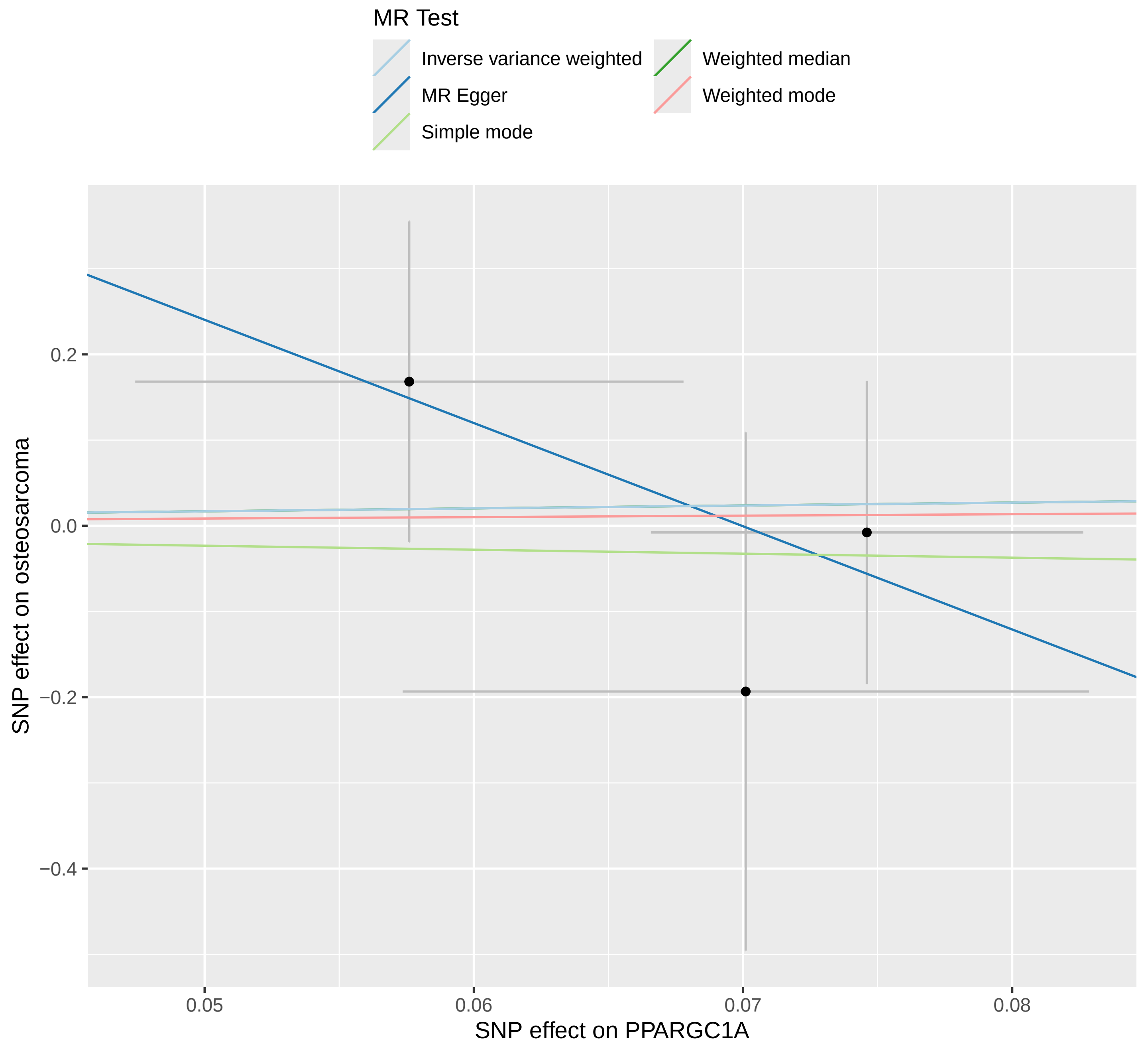

MR Test
Inverse variance weighted
MR Egger
Simple mode
Weighted median
Weighted mode
0.2
0.0
−0.2
−0.4
SNP effect on osteosarcoma
0.05
0.06
0.07
0.08
SNP effect on PPARGC1A

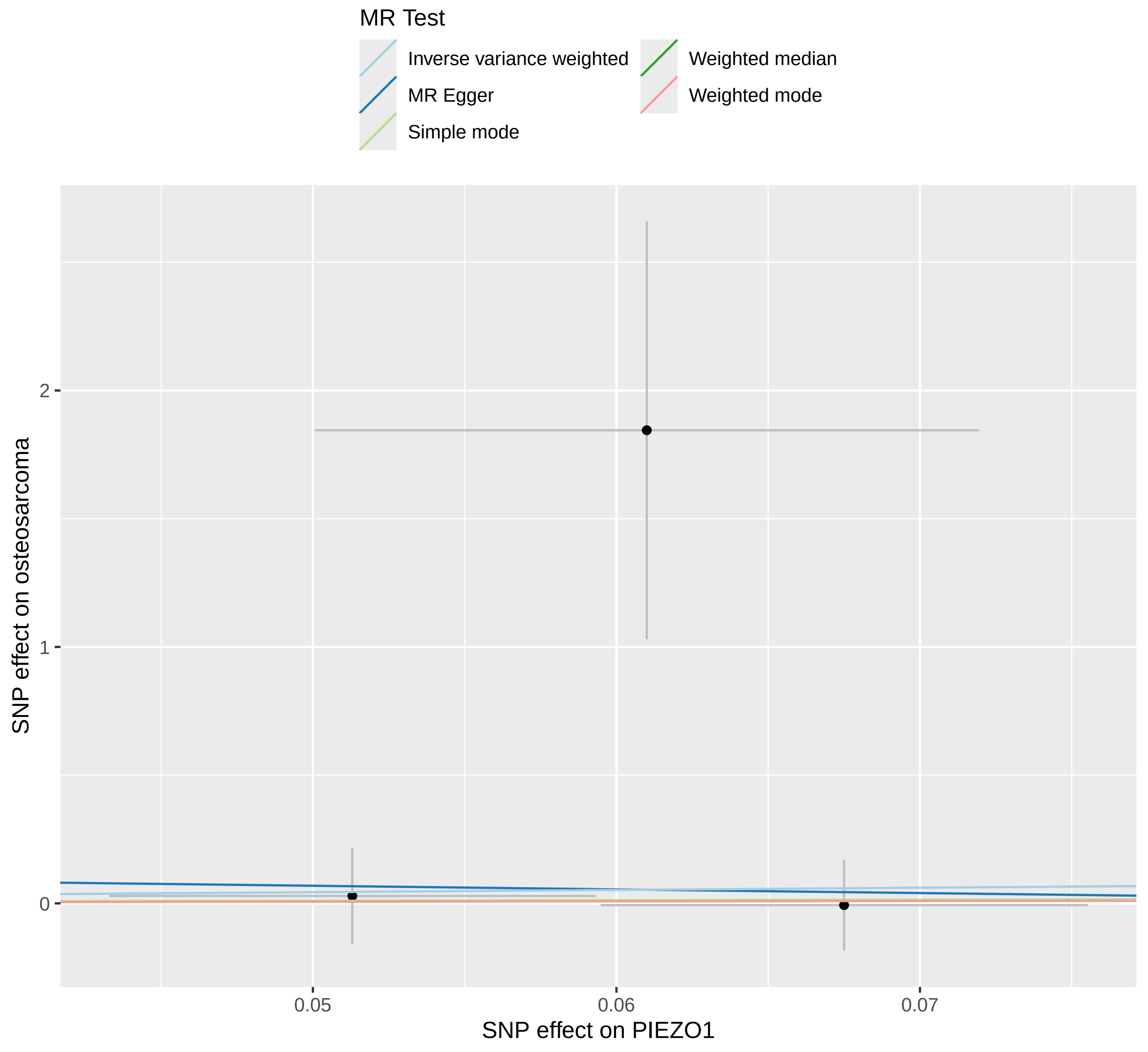
MR Test
Inverse variance weighted
MR Egger
Simple mode
Weighted median
Weighted mode
SNP effect on osteosarcoma
2
1
0
0.05
0.06
0.07
SNP effect on PIEZO1

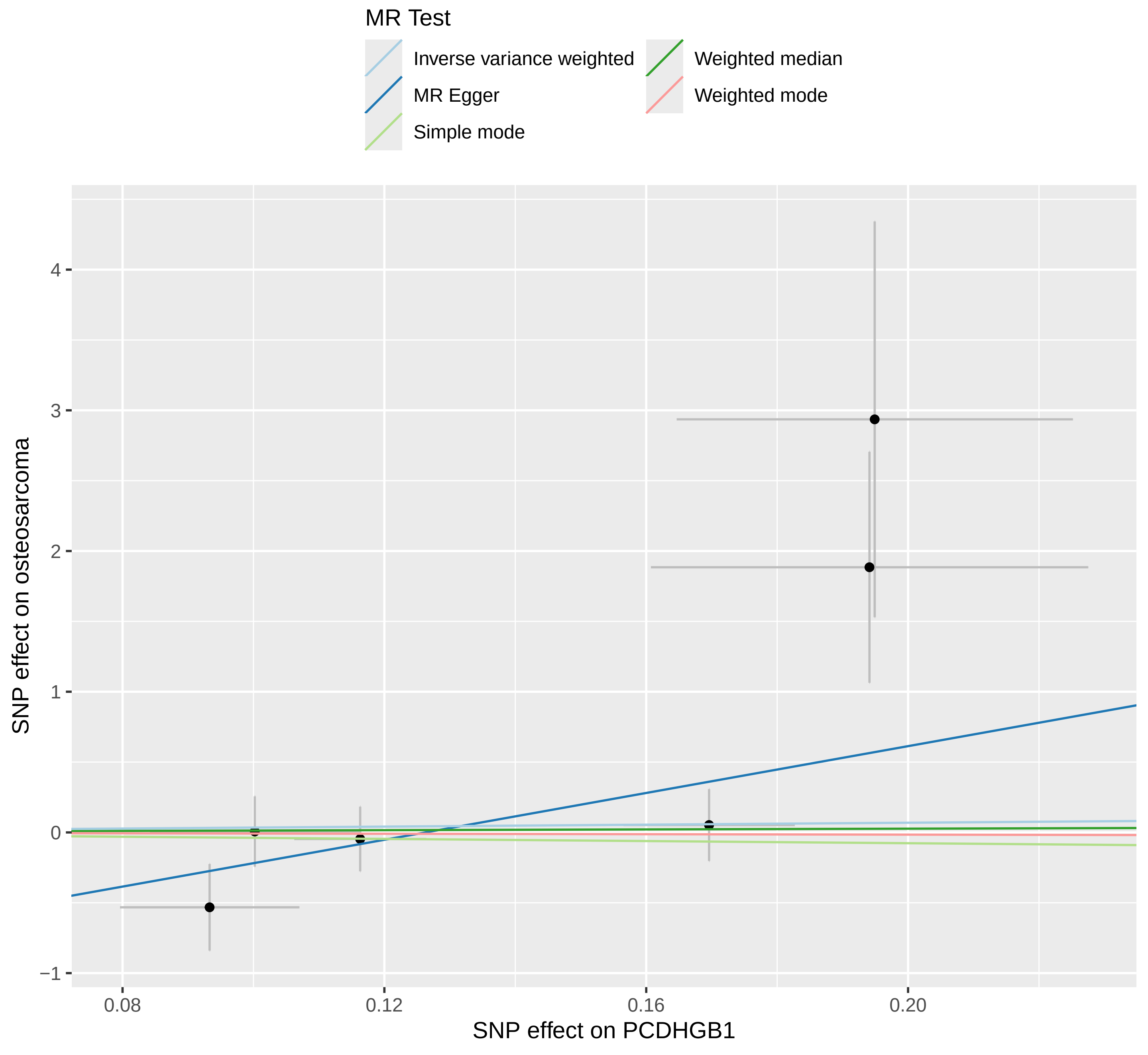
MR Test
Inverse variance weighted
MR Egger
Simple mode
Weighted median
Weighted mode
SNP effect on osteosarcoma
SNP effect on PCDHGB1
4
3
2
1
0
−1
0.08
0.12
0.16
0.20

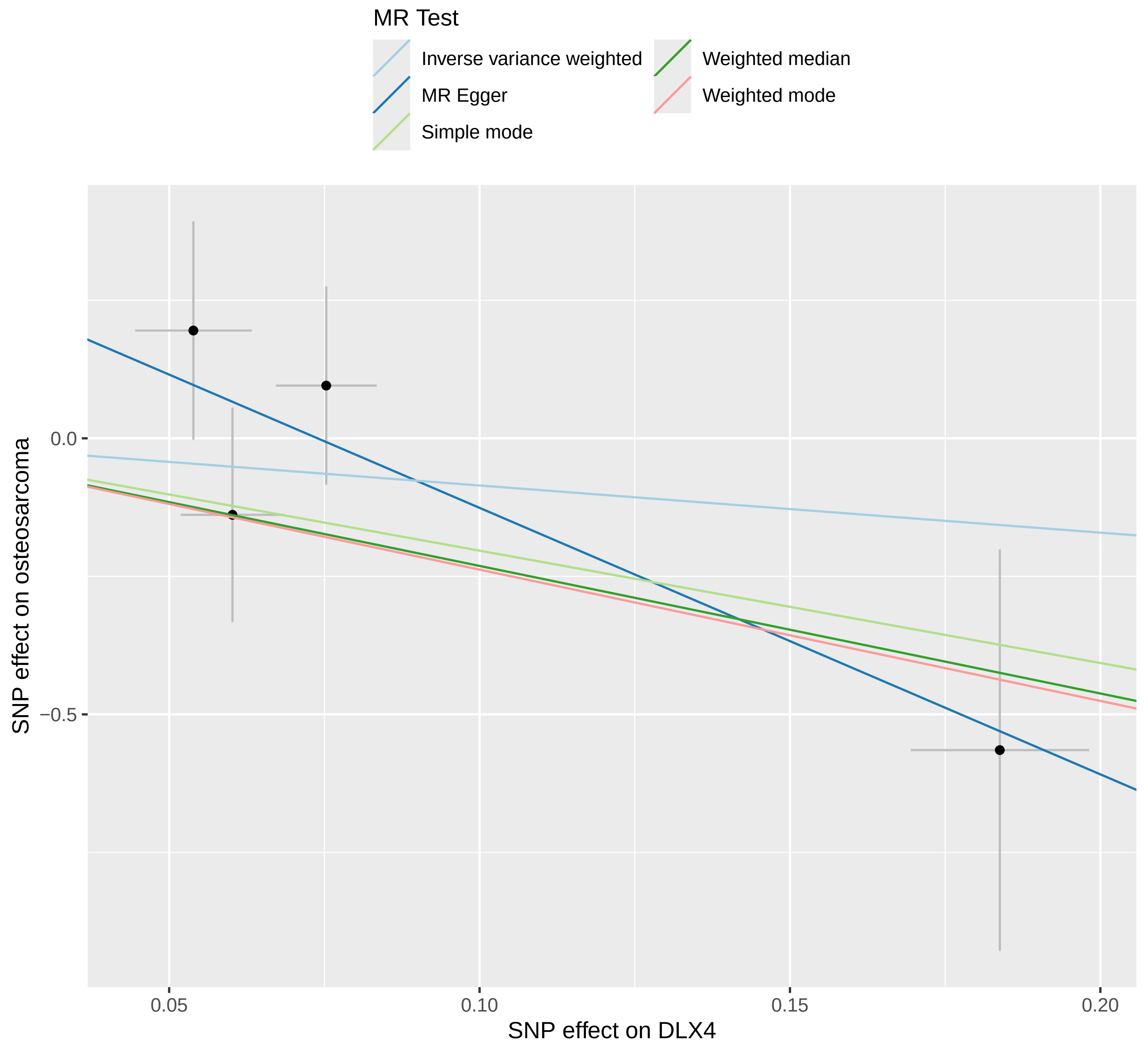
MR Test
Inverse variance weighted
MR Egger
Simple mode
Weighted median
Weighted mode
0.0
−0.5
0.05
0.10
0.15
0.20
SNP effect on osteosarcoma
SNP effect on DLX4

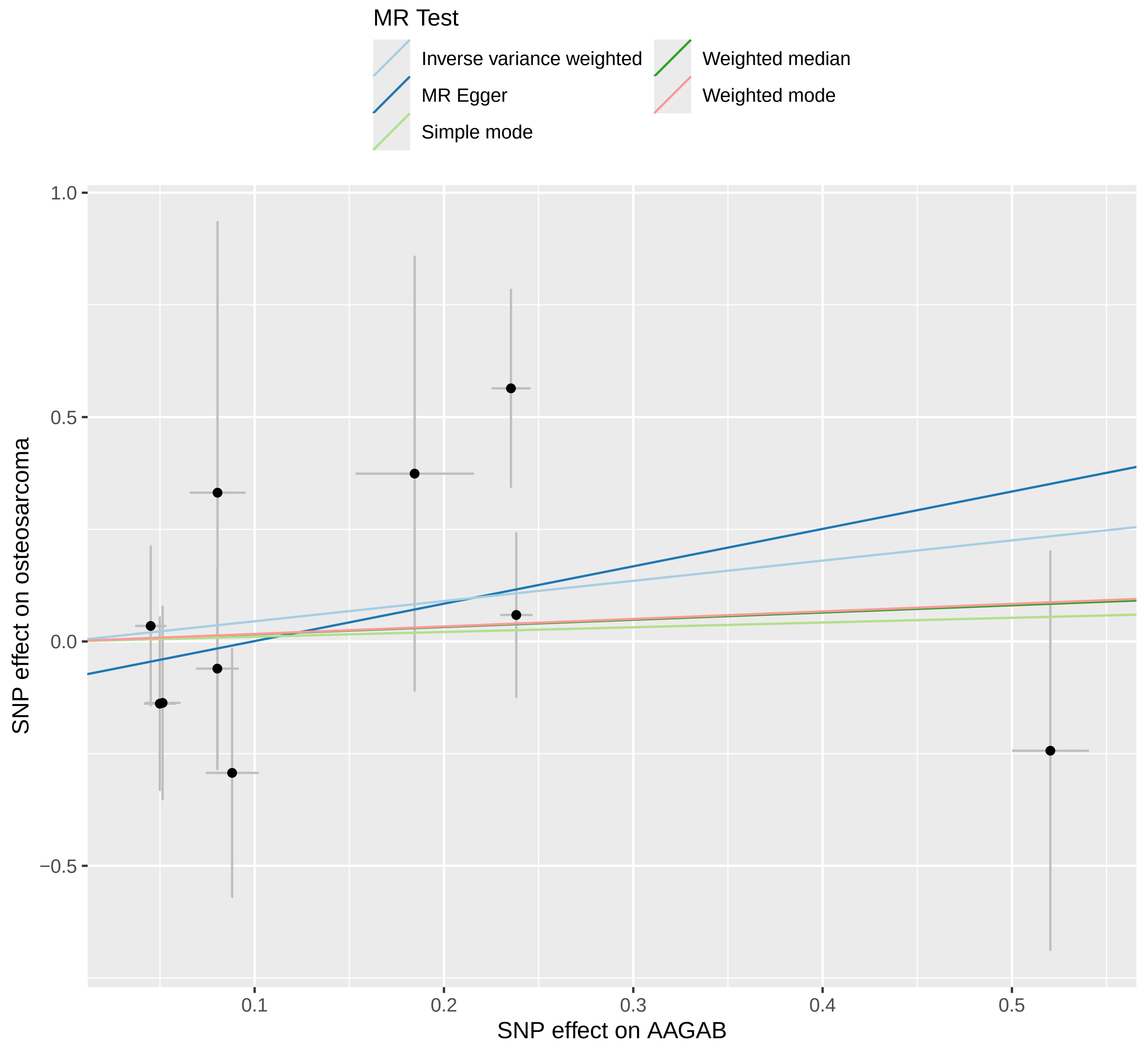

MR Test
Inverse variance weighted
MR Egger
Simple mode
Weighted median
Weighted mode
1.0
0.5
0.0
−0.5
SNP effect on osteosarcoma
0.1
0.2
0.3
0.4
0.5
SNP effect on AAGAB

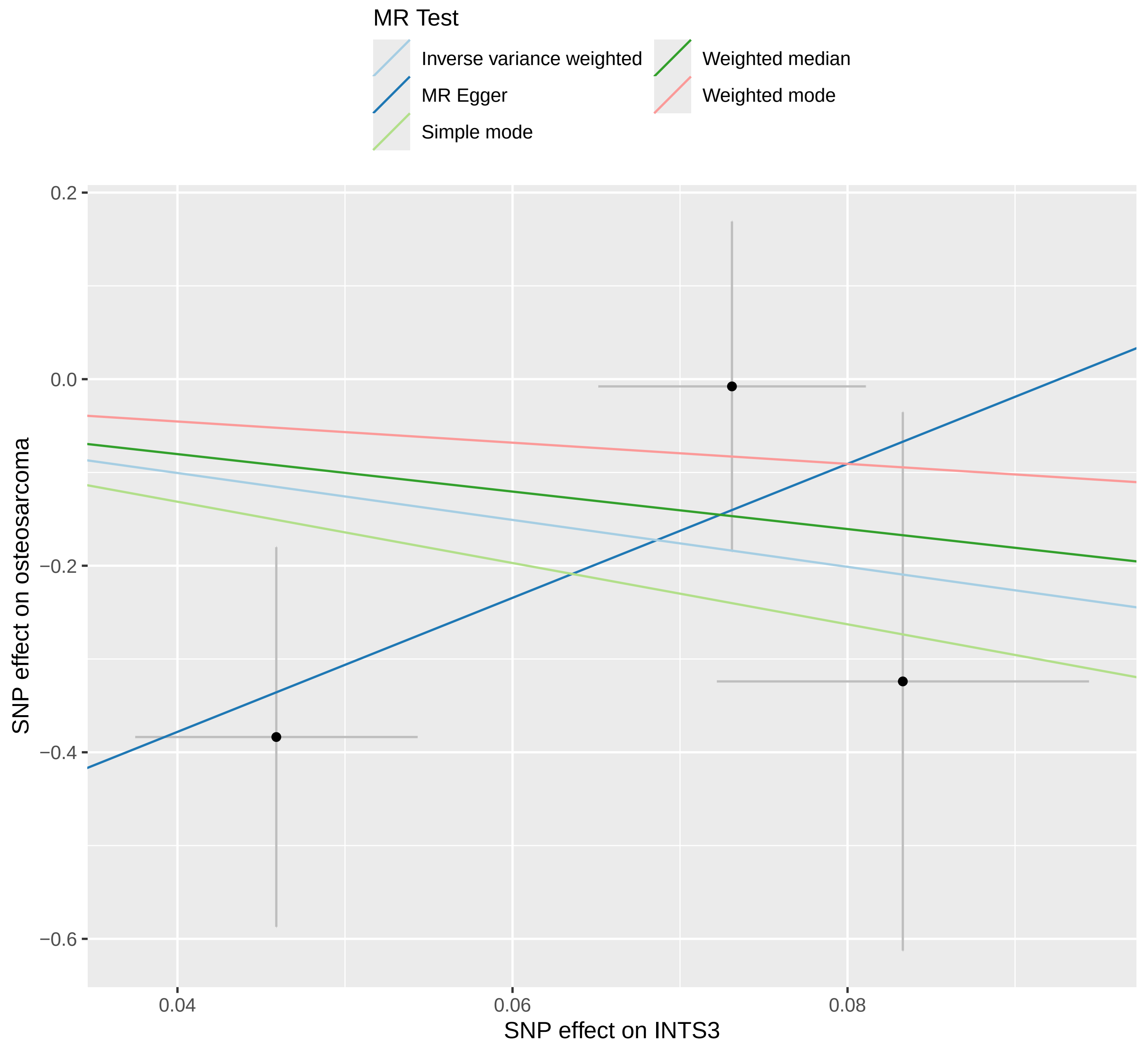
MR Test
Inverse variance weighted
MR Egger
Simple mode
Weighted median
Weighted mode
0.2
0.0
−0.2
−0.4
−0.6
SNP effect on osteosarcoma
0.04
0.06
0.08
SNP effect on INTS3

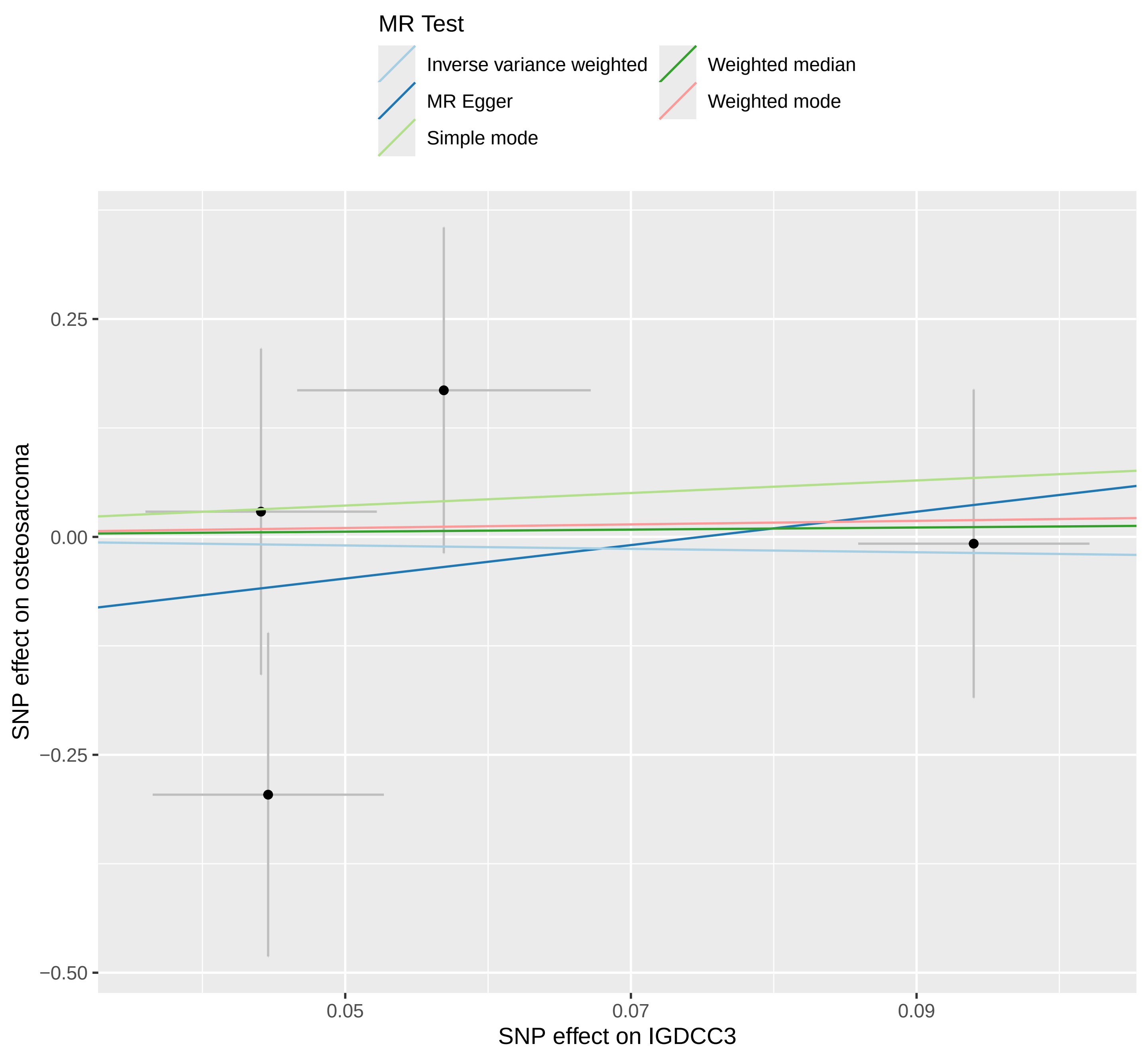
MR Test
Inverse variance weighted
MR Egger
Simple mode
Weighted median
Weighted mode
0.25
0.00
−0.25
−0.50
SNP effect on osteosarcoma
0.05
0.07
0.09
SNP effect on IGDCC3

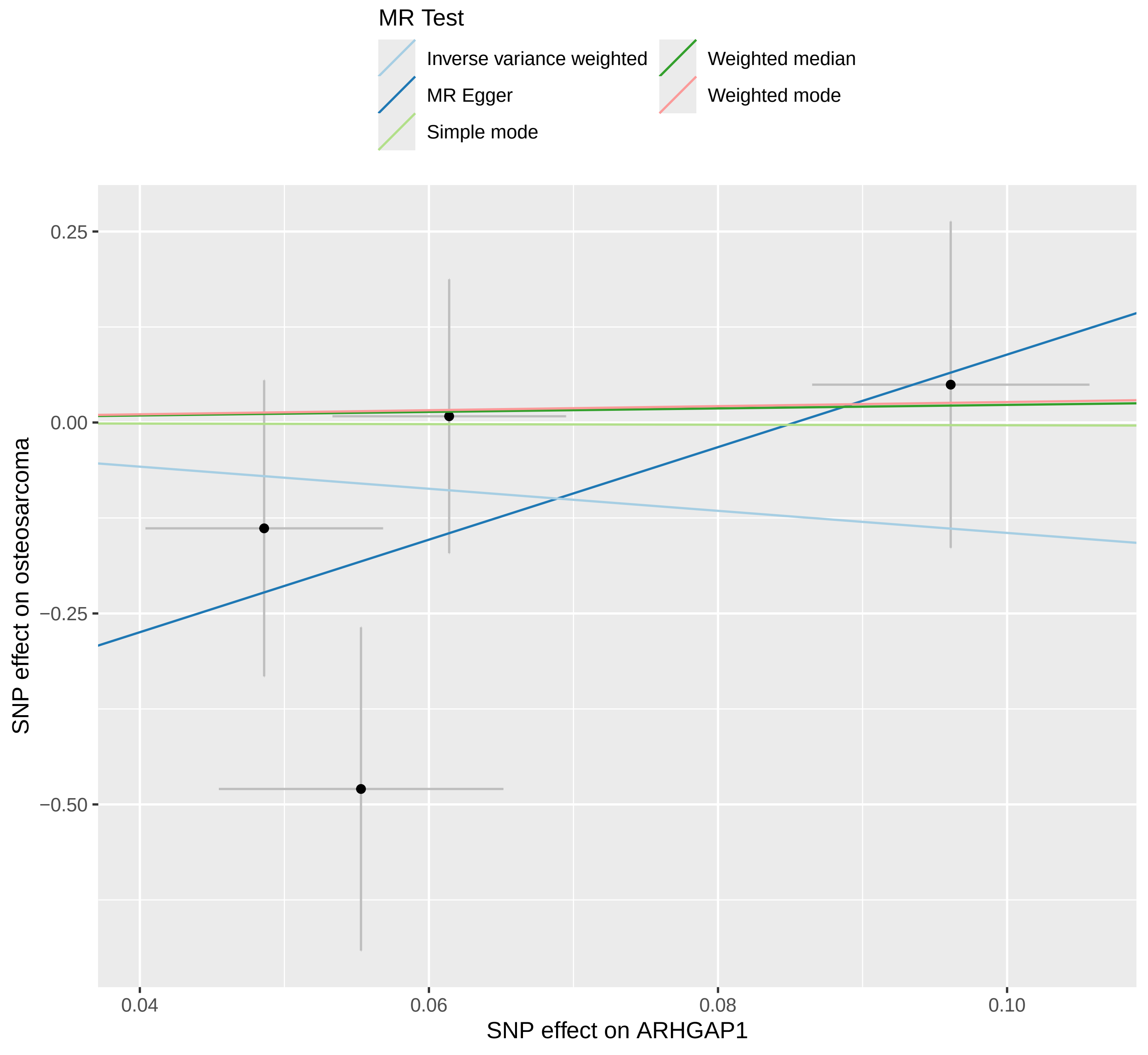

MR Test
Inverse variance weighted
MR Egger
Simple mode
Weighted median
Weighted mode
0.25
0.00
−0.25
−0.50
0.04
0.06
0.08
0.10
SNP effect on osteosarcoma
SNP effect on ARHGAP1

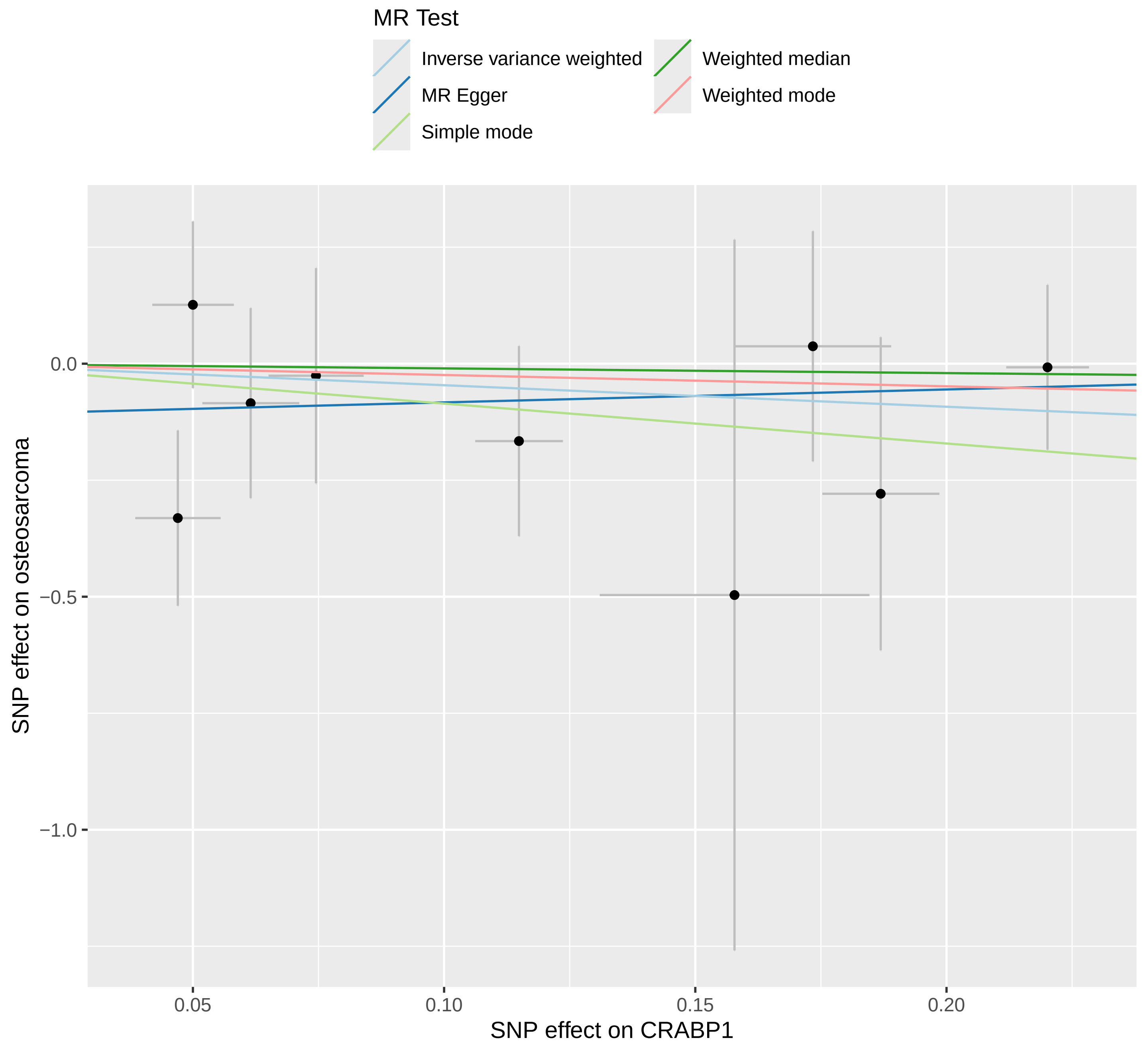
MR Test
Inverse variance weighted
MR Egger
Simple mode
Weighted median
Weighted mode
0.0
−0.5
−1.0
SNP effect on osteosarcoma
0.05
0.10
0.15
0.20
SNP effect on CRABP1

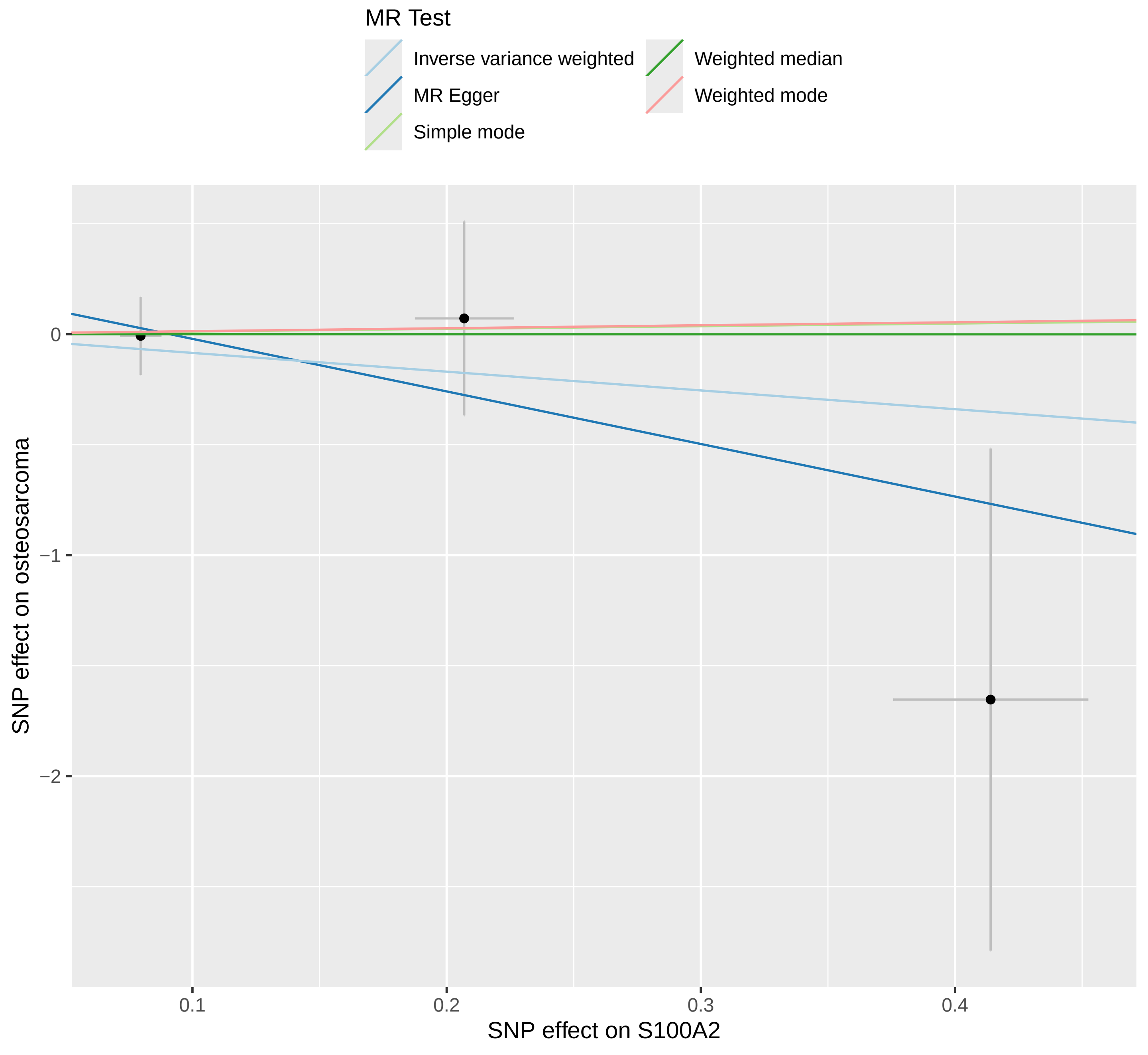

MR Test
Inverse variance weighted
MR Egger
Simple mode
Weighted median
Weighted mode
0
−1
−2
0.1
0.2
0.3
0.4
SNP effect on osteosarcoma
SNP effect on S100A2

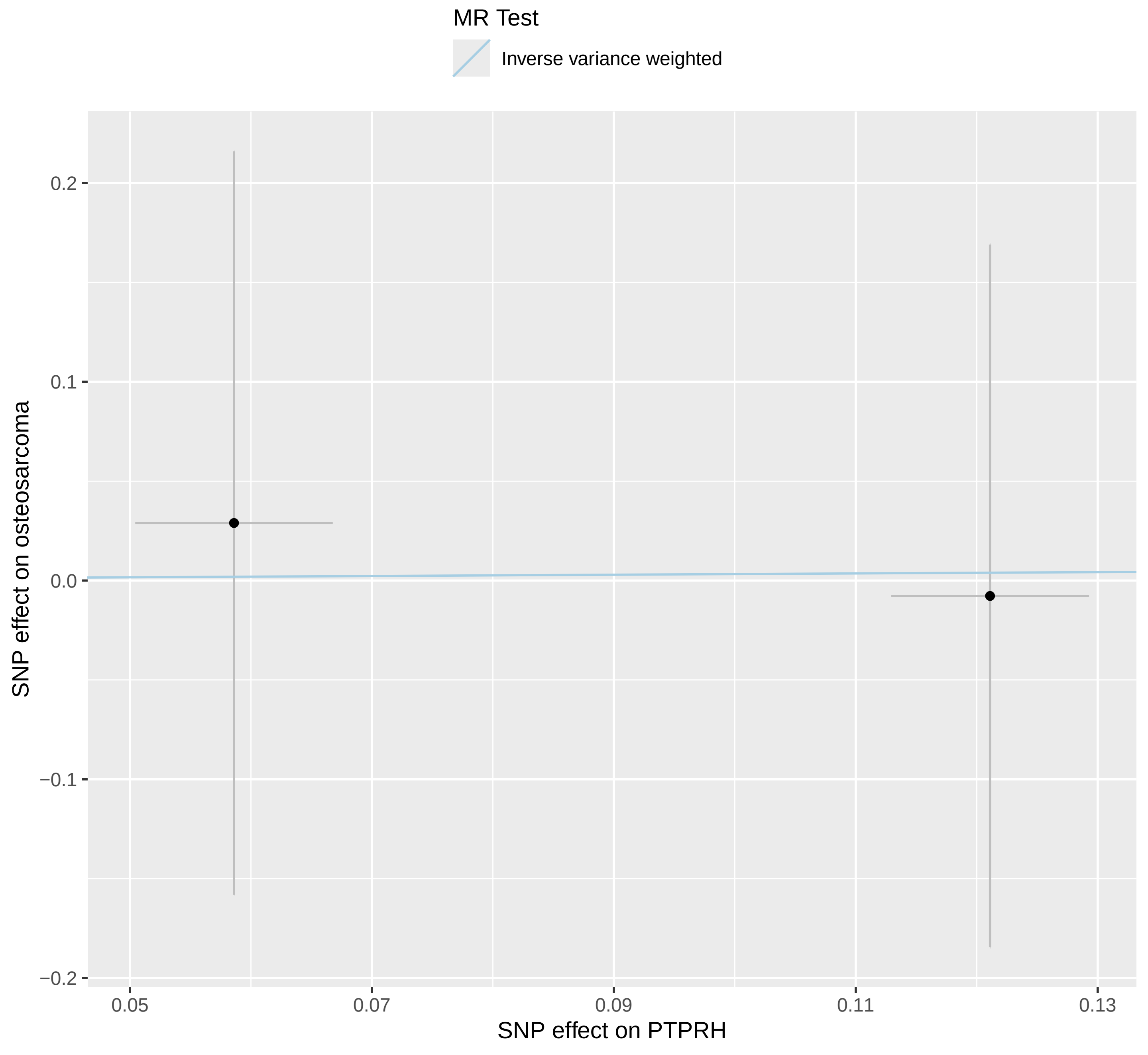

MR Test
Inverse variance weighted
0.2
0.1
0.0
−0.1
−0.2
SNP effect on osteosarcoma
0.05
0.07
0.09
0.11
0.13
SNP effect on PTPRH

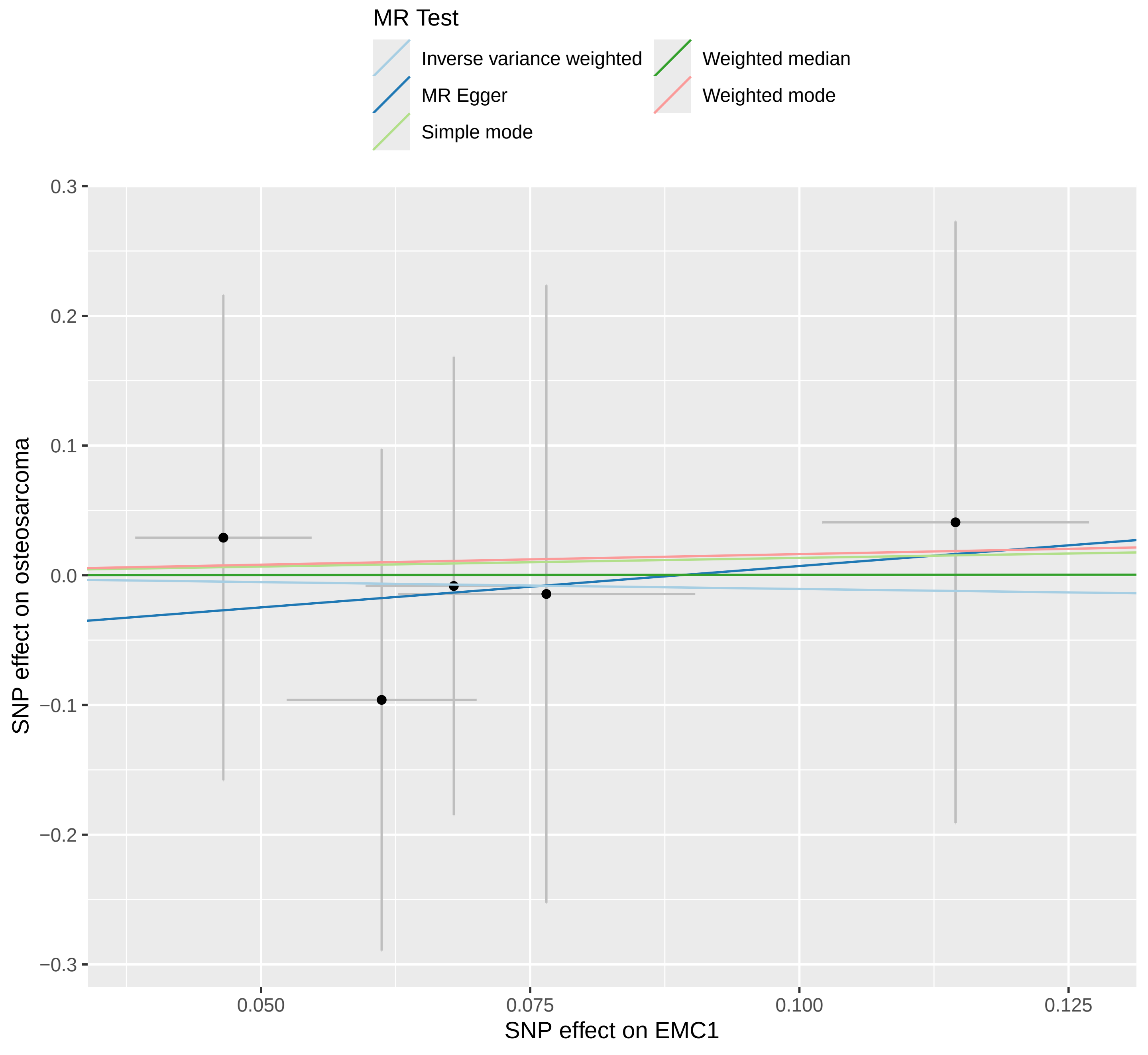
MR Test
Inverse variance weighted
MR Egger
Simple mode
Weighted median
Weighted mode
0.3
0.2
0.1
0.0
−0.1
−0.2
−0.3
SNP effect on osteosarcoma
0.050
0.075
0.100
0.125
SNP effect on EMC1

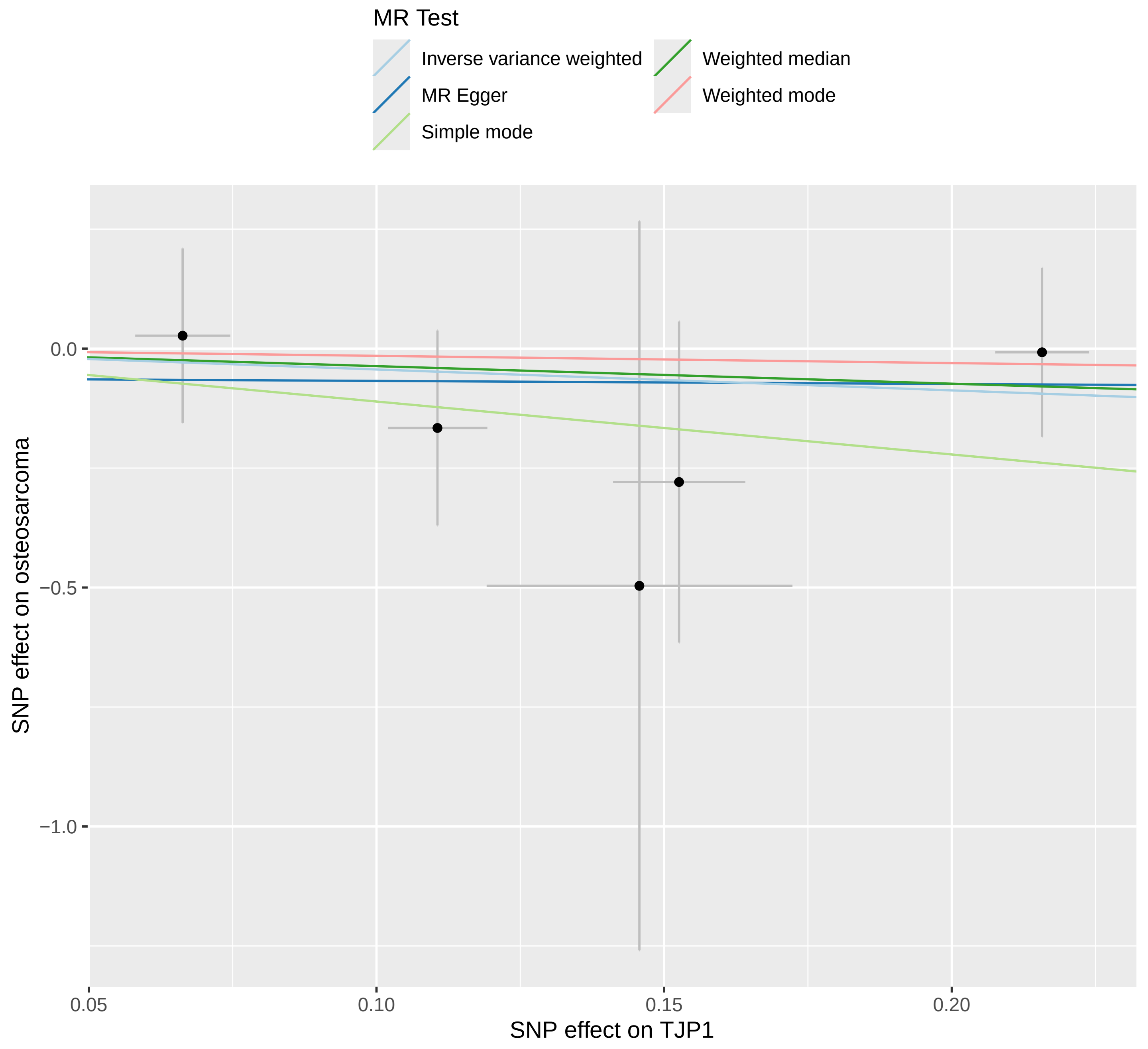
MR Test
Inverse variance weighted
MR Egger
Simple mode
Weighted median
Weighted mode
0.0
−0.5
−1.0
0.05
0.10
0.15
0.20
SNP effect on osteosarcoma
SNP effect on TJP1

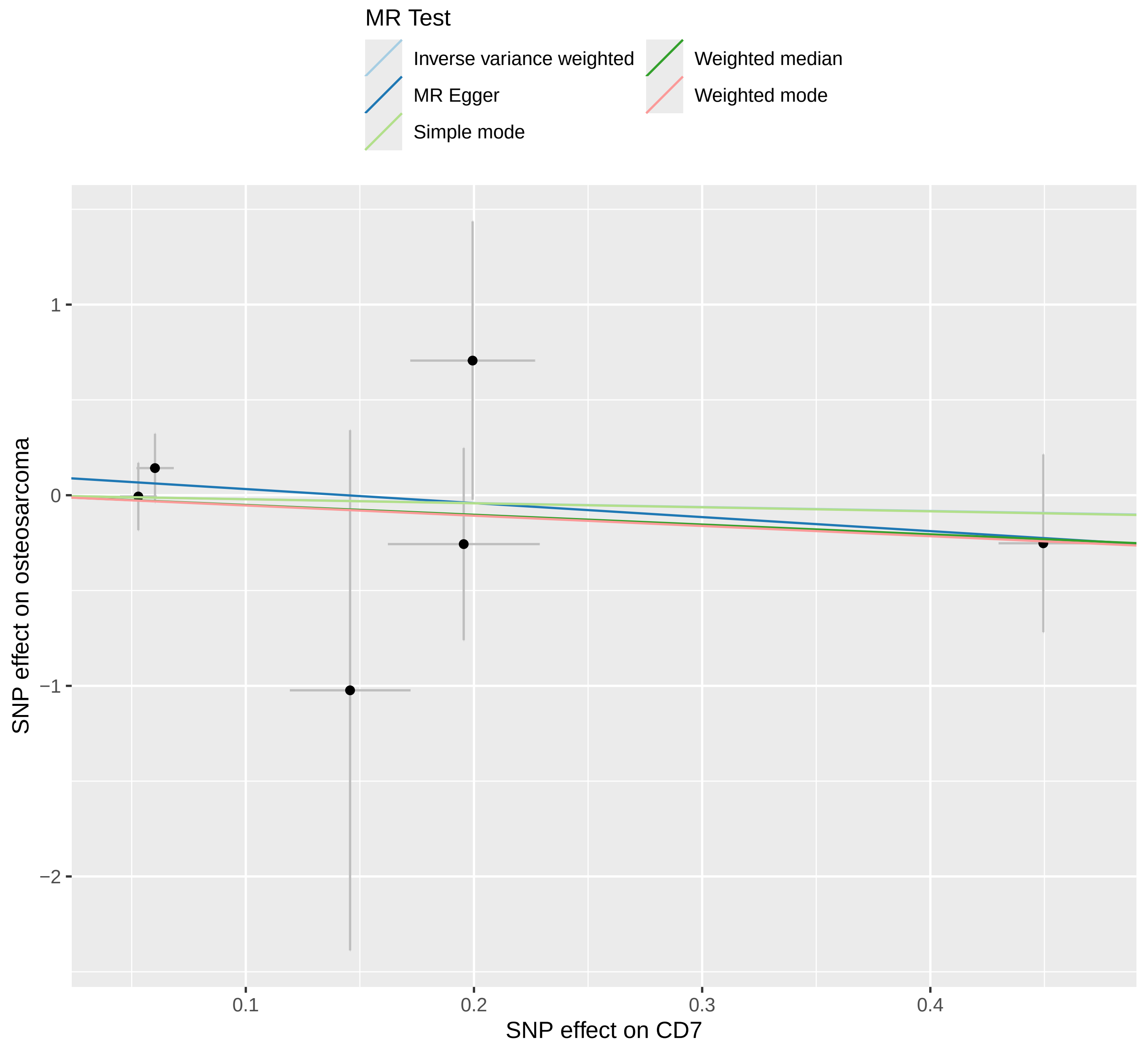

MR Test
Inverse variance weighted
MR Egger
Simple mode
Weighted median
Weighted mode
1
0
−1
−2
0.1
0.2
0.3
0.4
SNP effect on osteosarcoma
SNP effect on CD7

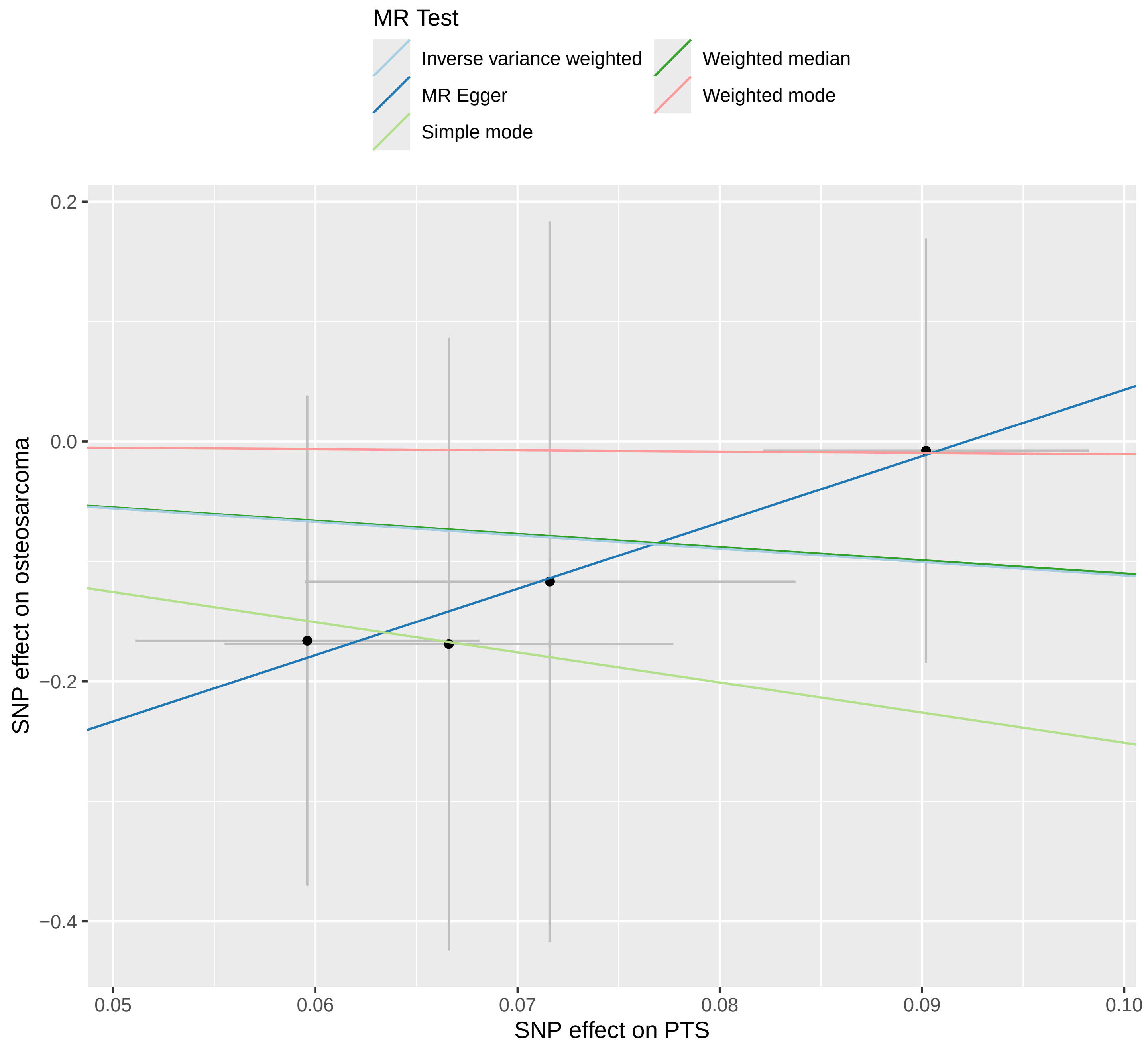
MR Test
Inverse variance weighted
MR Egger
Simple mode
Weighted median
Weighted mode
0.2
0.0
−0.2
−0.4
SNP effect on osteosarcoma
0.05
0.06
0.07
0.08
0.09
0.10
SNP effect on PTS

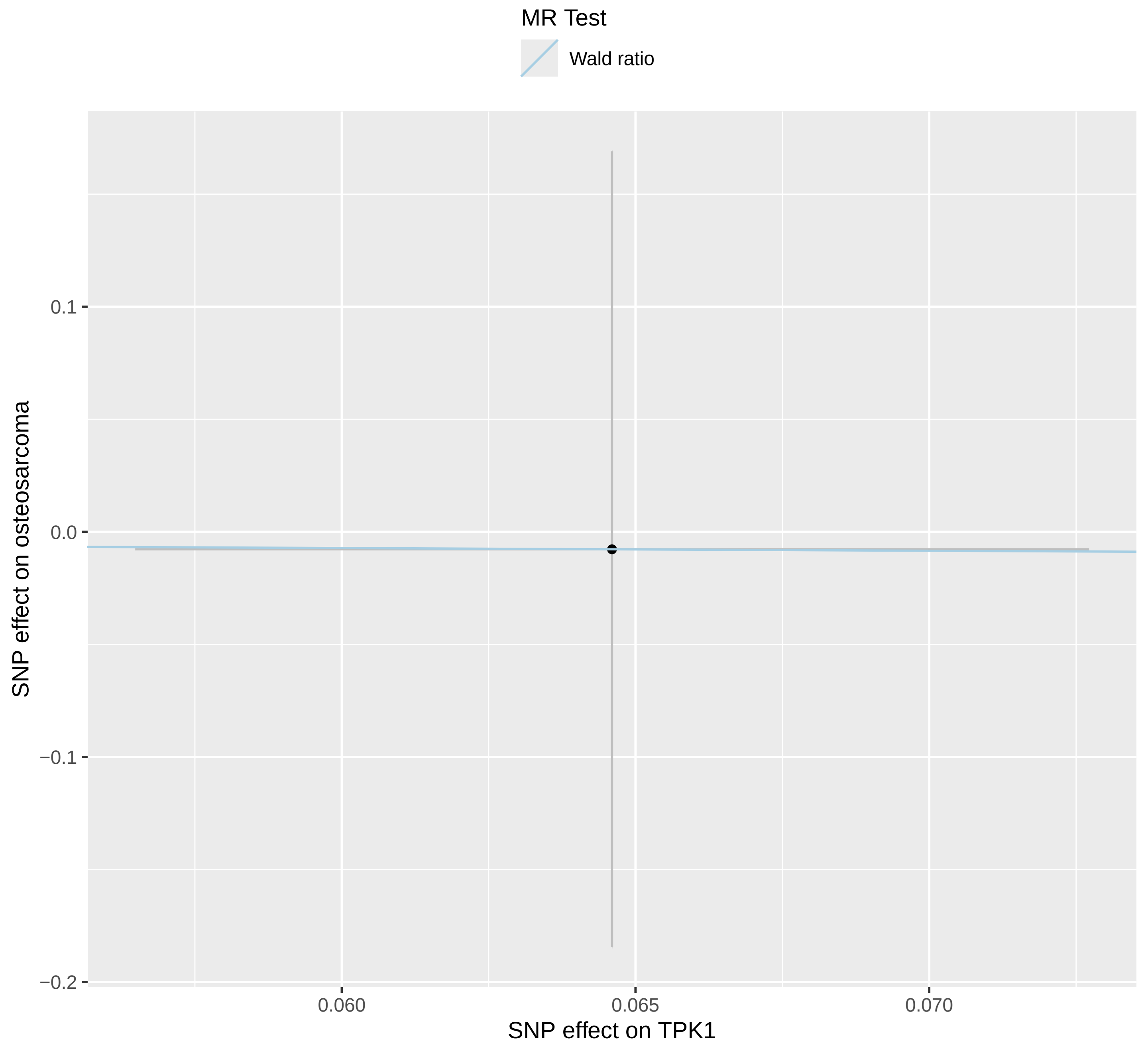
MR Test
Wald ratio
0.1
0.0
−0.1
−0.2
SNP effect on osteosarcoma
0.060
0.065
0.070
SNP effect on TPK1

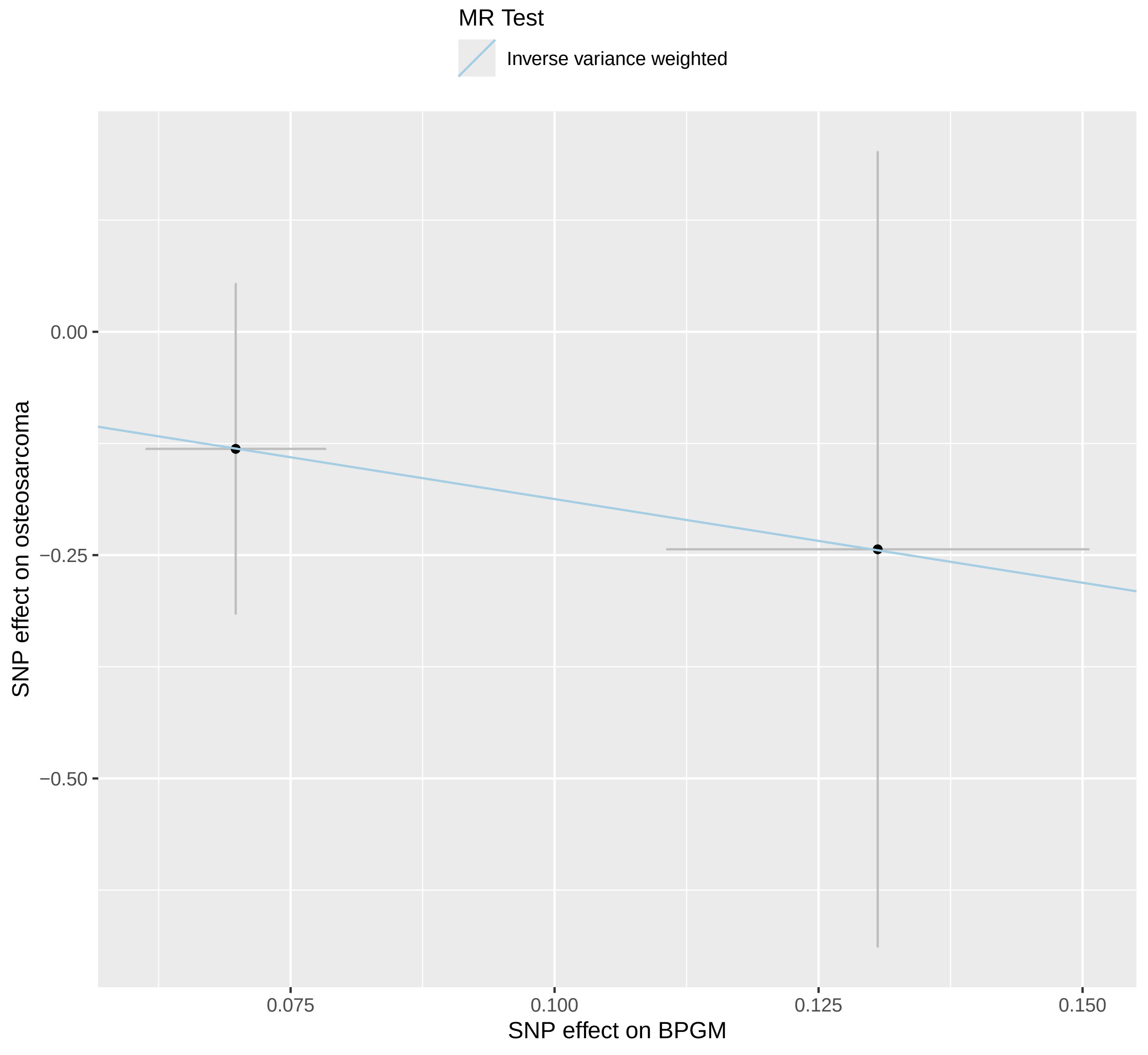

MR Test
Inverse variance weighted
0.00
−0.25
−0.50
SNP effect on osteosarcoma
0.075
0.100
0.125
0.150
SNP effect on BPGM

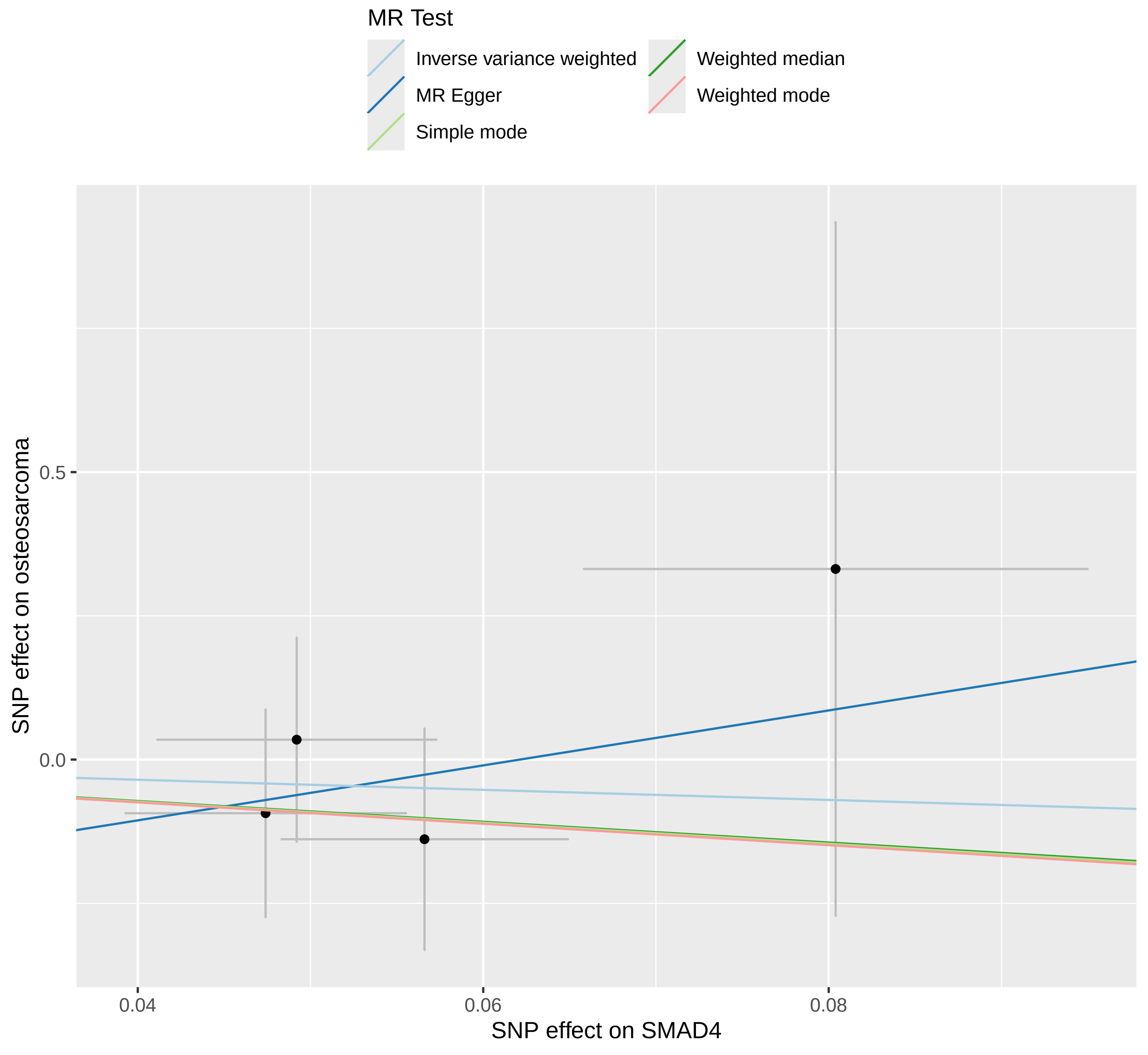
MR Test
Inverse variance weighted
MR Egger
Simple mode
Weighted median
Weighted mode
SNP effect on osteosarcoma
0.5
0.0
0.04
0.06
0.08
SNP effect on SMAD4

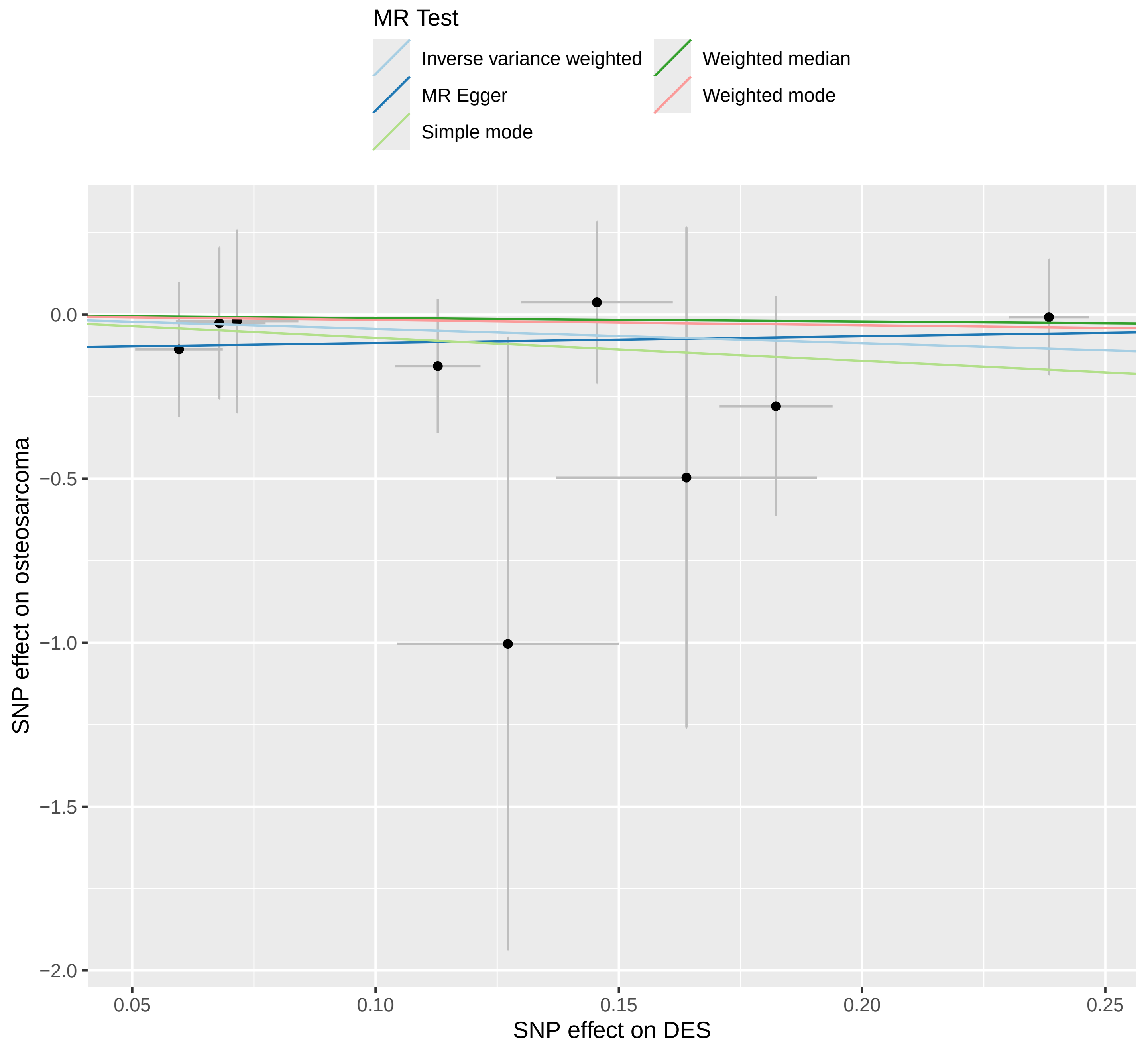
MR Test
Inverse variance weighted
MR Egger
Simple mode
Weighted median
Weighted mode
0.0
−0.5
−1.0
−1.5
−2.0
0.05
0.10
0.15
0.20
0.25
SNP effect on DES
SNP effect on osteosarcoma

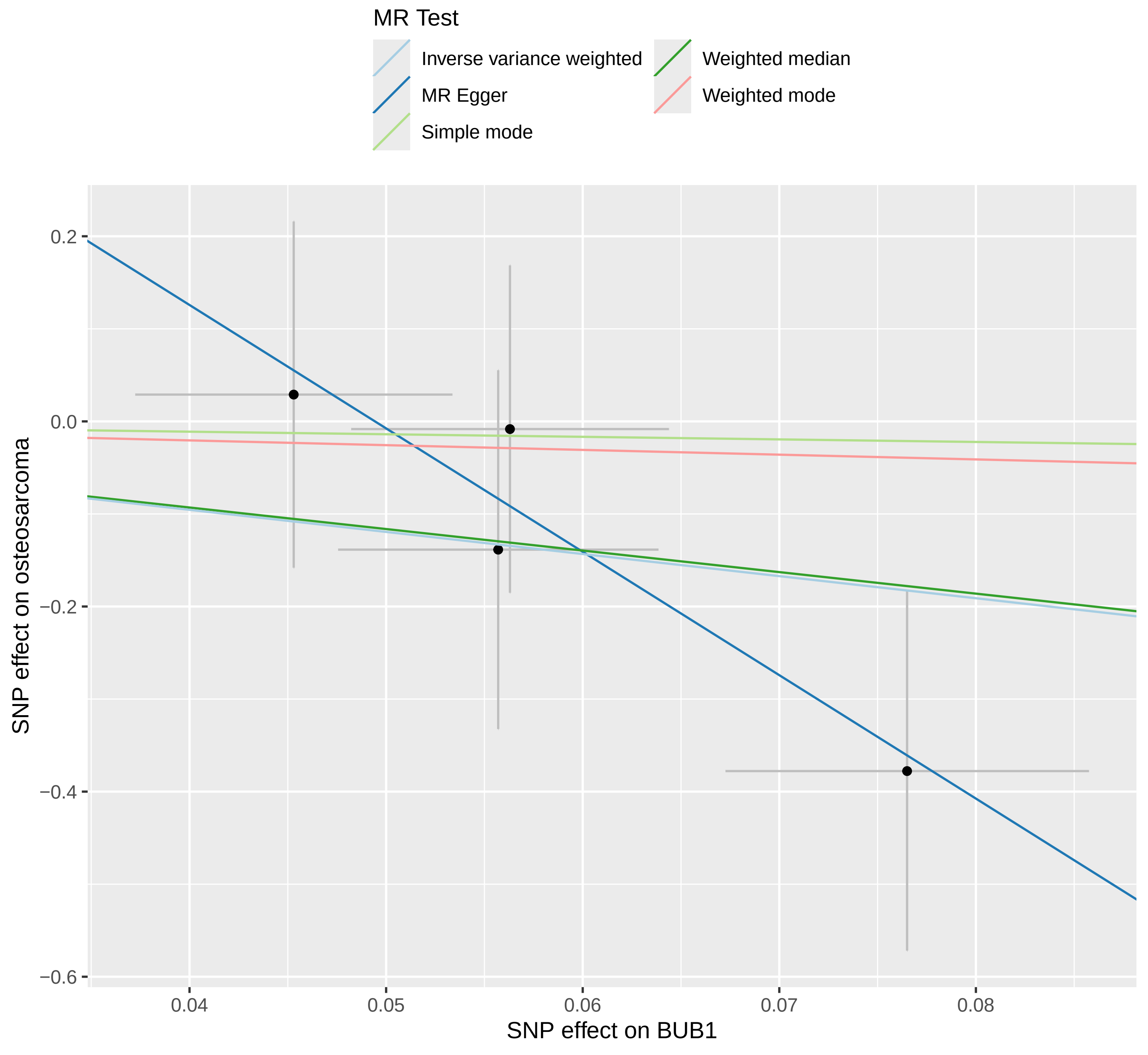

MR Test
Inverse variance weighted
MR Egger
Simple mode
Weighted median
Weighted mode
0.2
0.0
−0.2
−0.4
−0.6
0.04
0.05
0.06
0.07
0.08
SNP effect on osteosarcoma
SNP effect on BUB1

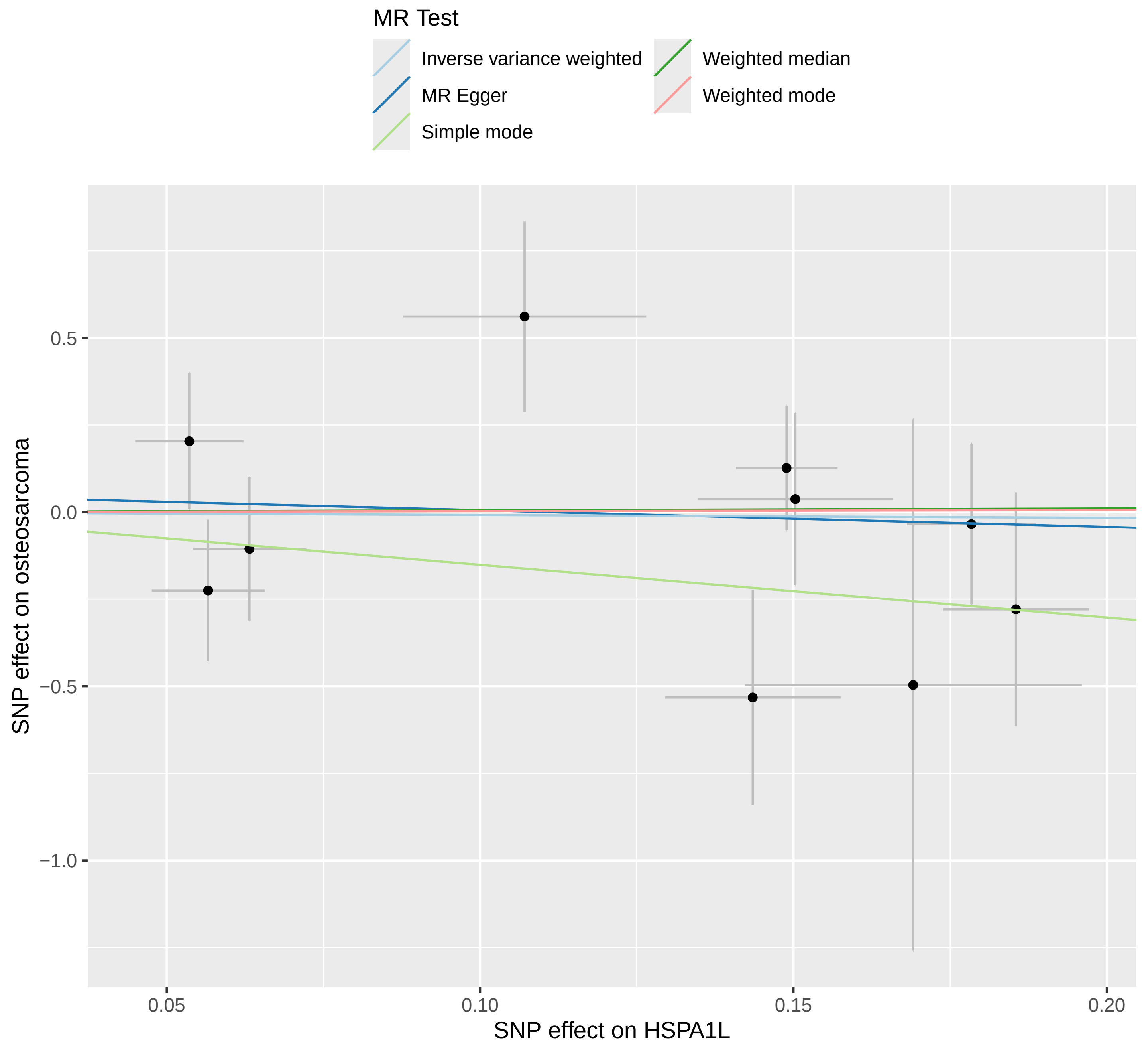

MR Test
Inverse variance weighted
MR Egger
Simple mode
Weighted median
Weighted mode
0.5
0.0
−0.5
−1.0
0.05
0.10
0.15
0.20
SNP effect on HSPA1L
SNP effect on osteosarcoma

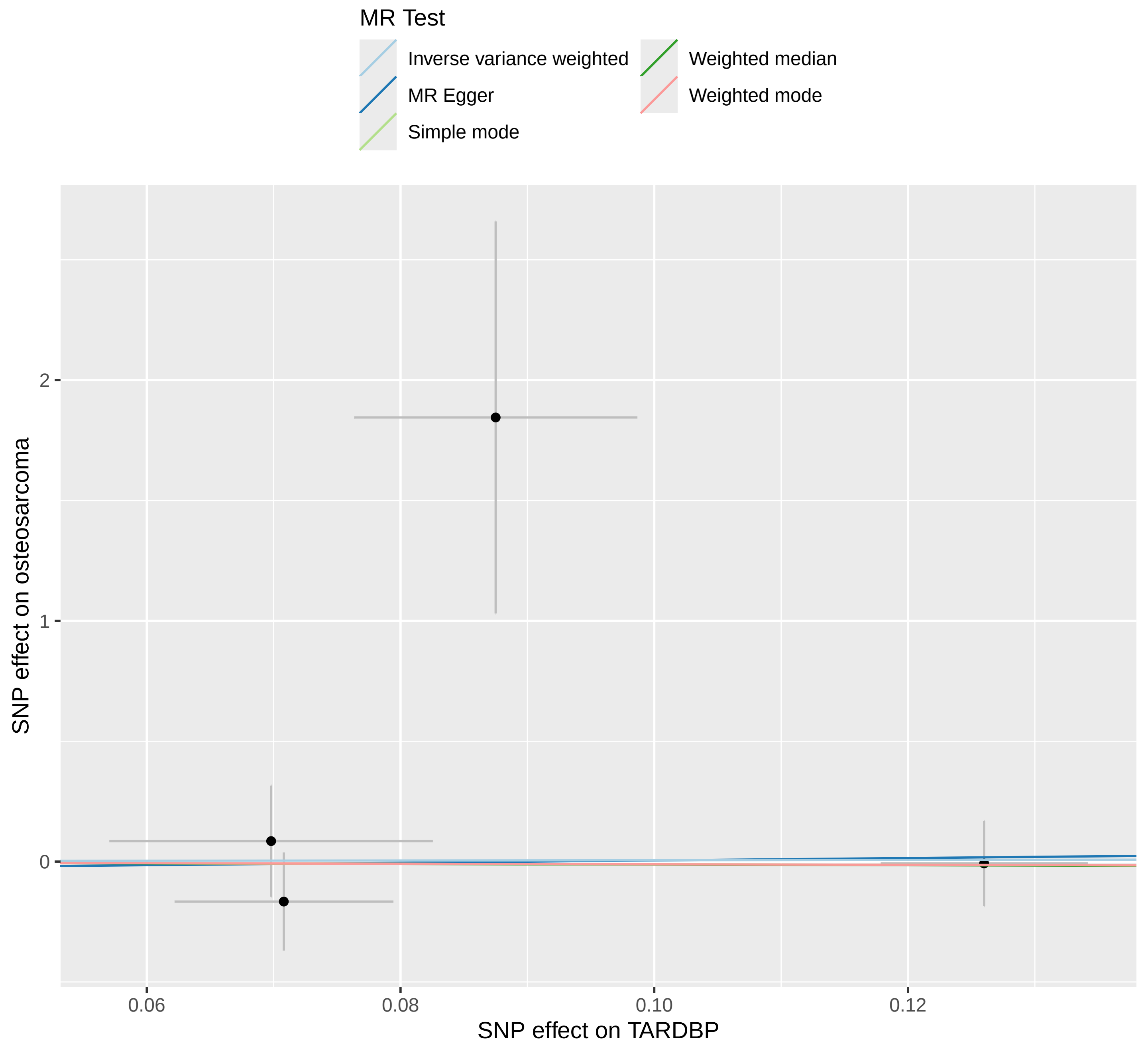
MR Test
Inverse variance weighted
MR Egger
Simple mode
Weighted median
Weighted mode
SNP effect on osteosarcoma
2
1
0
0.06
0.08
0.10
0.12
SNP effect on TARDBP

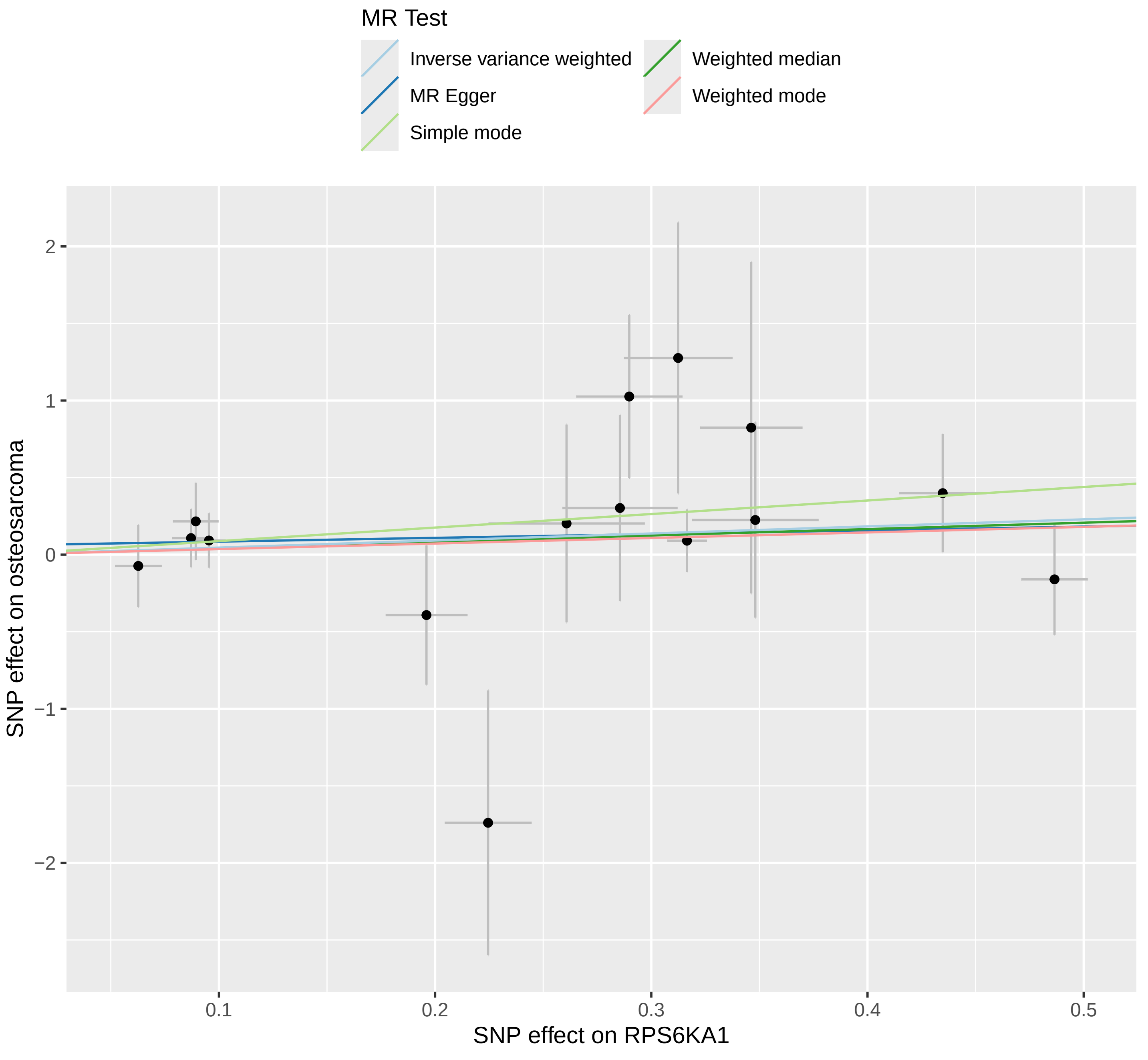
MR Test
Inverse variance weighted
MR Egger
Simple mode
Weighted median
Weighted mode
2
1
0
−1
−2
SNP effect on osteosarcoma
0.1
0.2
0.3
0.4
0.5
SNP effect on RPS6KA1

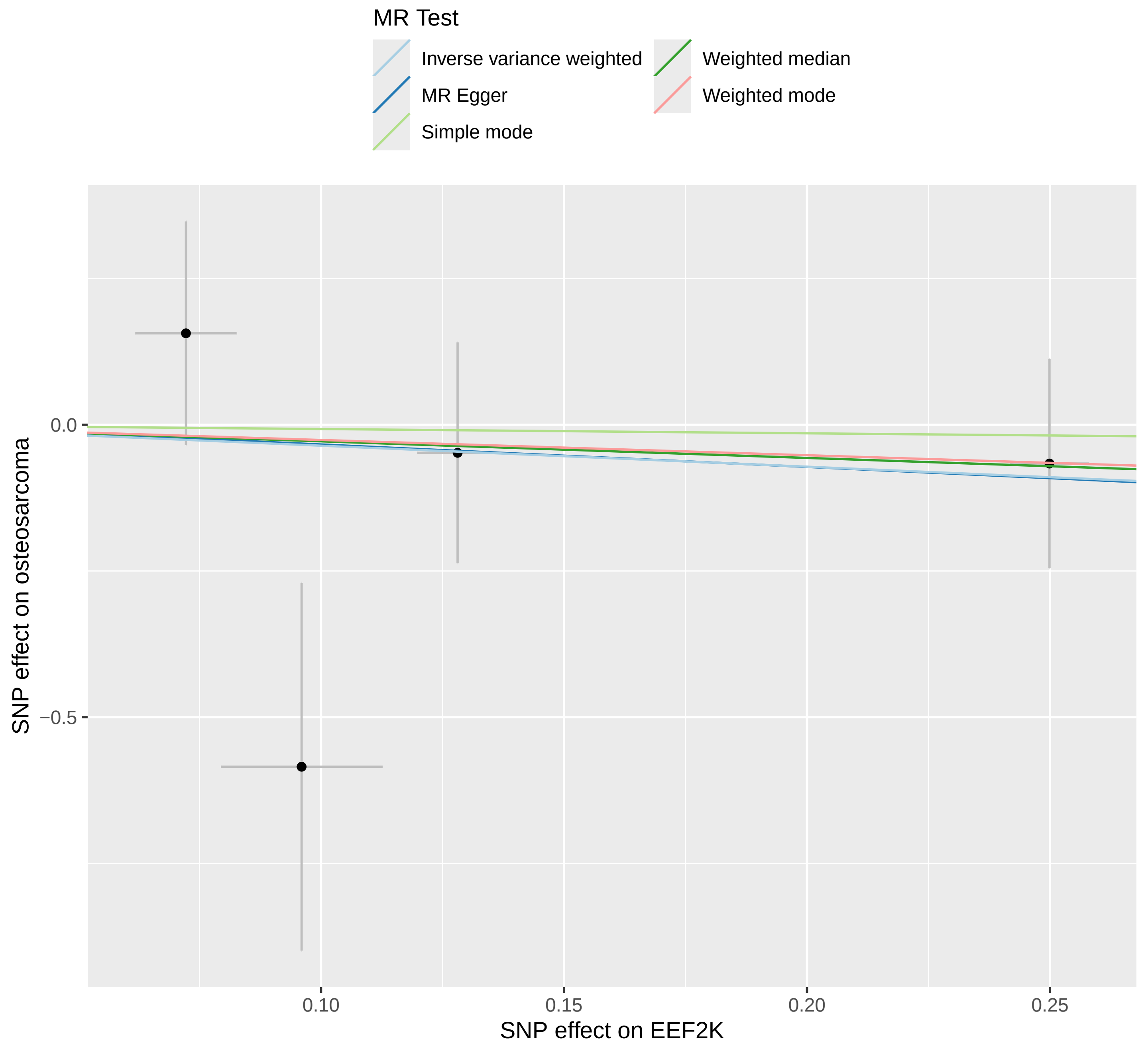
MR Test
Inverse variance weighted
MR Egger
Simple mode
Weighted median
Weighted mode
0.0
−0.5
SNP effect on osteosarcoma
0.10
0.15
0.20
0.25
SNP effect on EEF2K

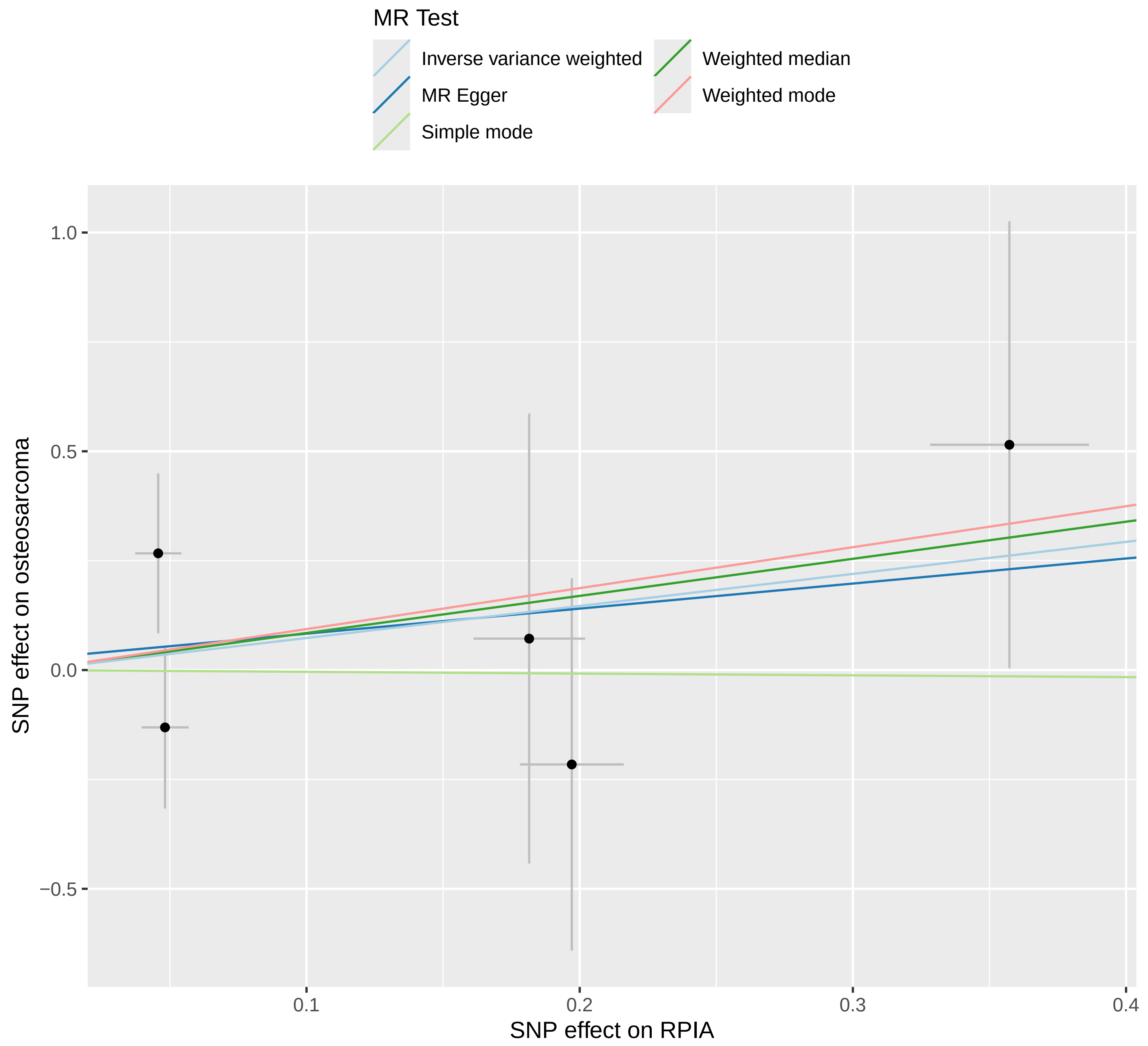
MR Test
Inverse variance weighted
MR Egger
Simple mode
Weighted median
Weighted mode
1.0
0.5
0.0
−0.5
SNP effect on osteosarcoma
0.1
0.2
0.3
0.4
SNP effect on RPIA

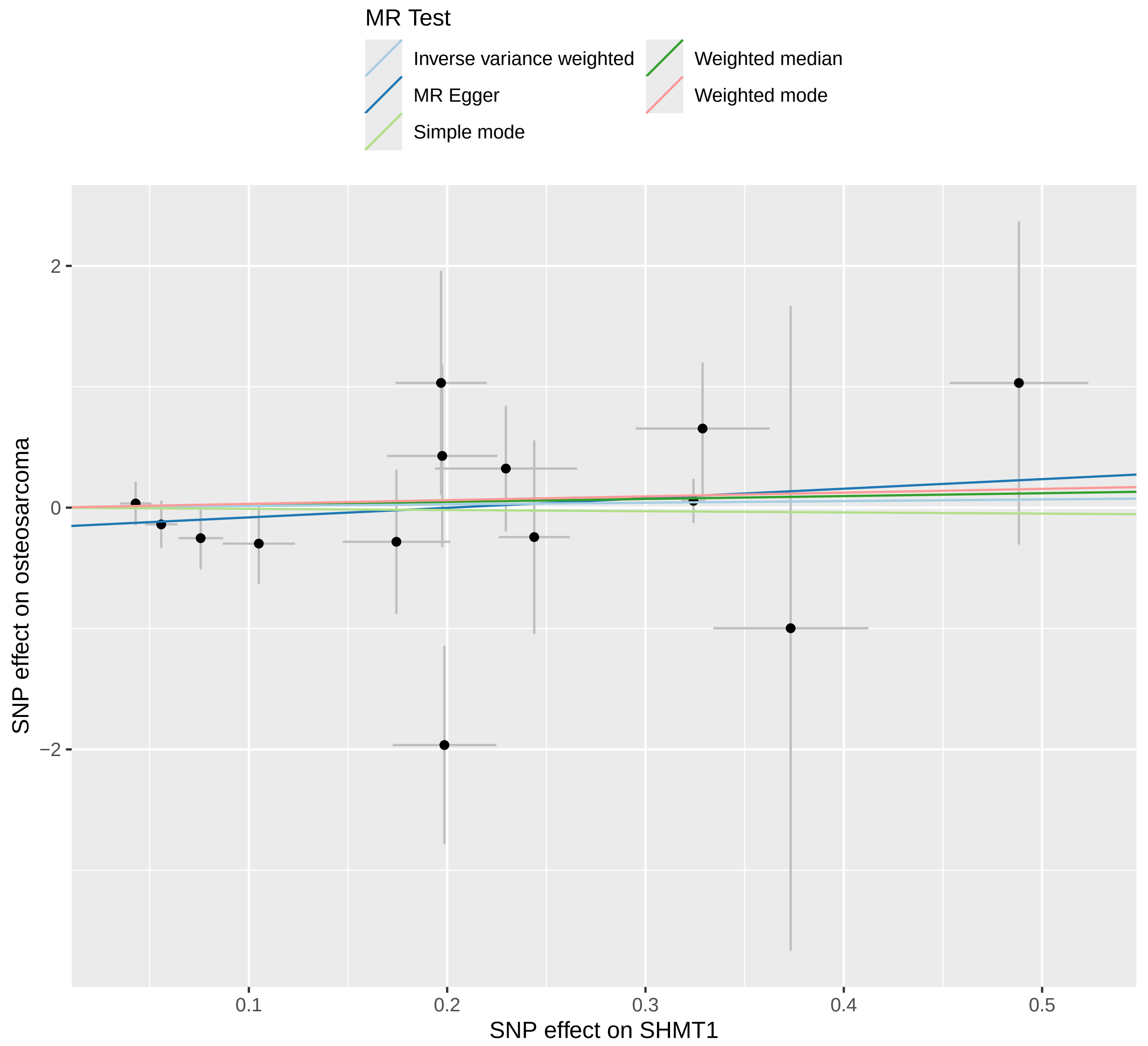

MR Test
Inverse variance weighted
MR Egger
Simple mode
Weighted median
Weighted mode
2
0
−2
0.1
0.2
0.3
0.4
0.5
SNP effect on SHMT1
SNP effect on osteosarcoma

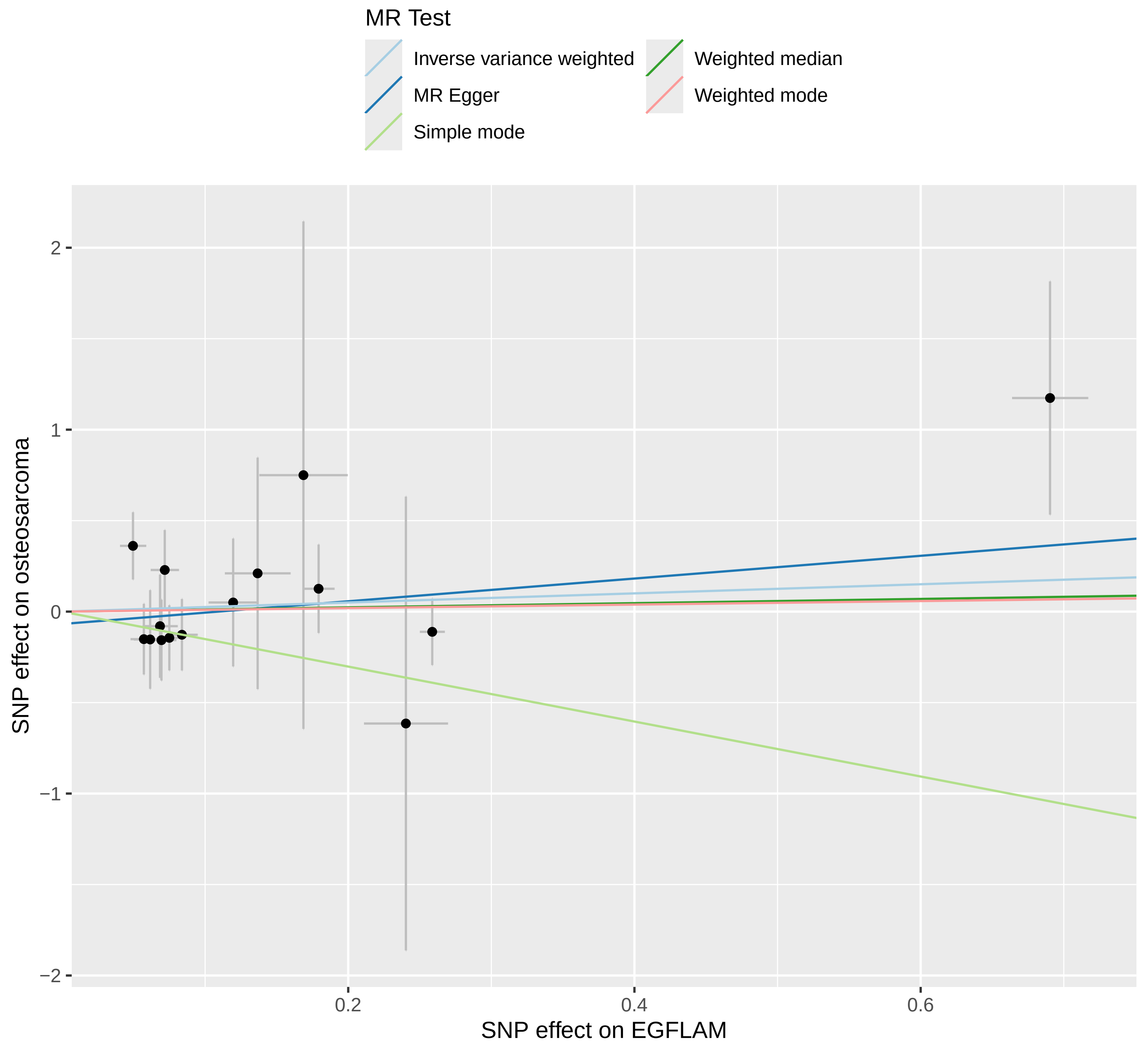
MR Test
Inverse variance weighted
MR Egger
Simple mode
Weighted median
Weighted mode
2
1
0
−1
−2
0.2
0.4
0.6
SNP effect on osteosarcoma
SNP effect on EGFLAM

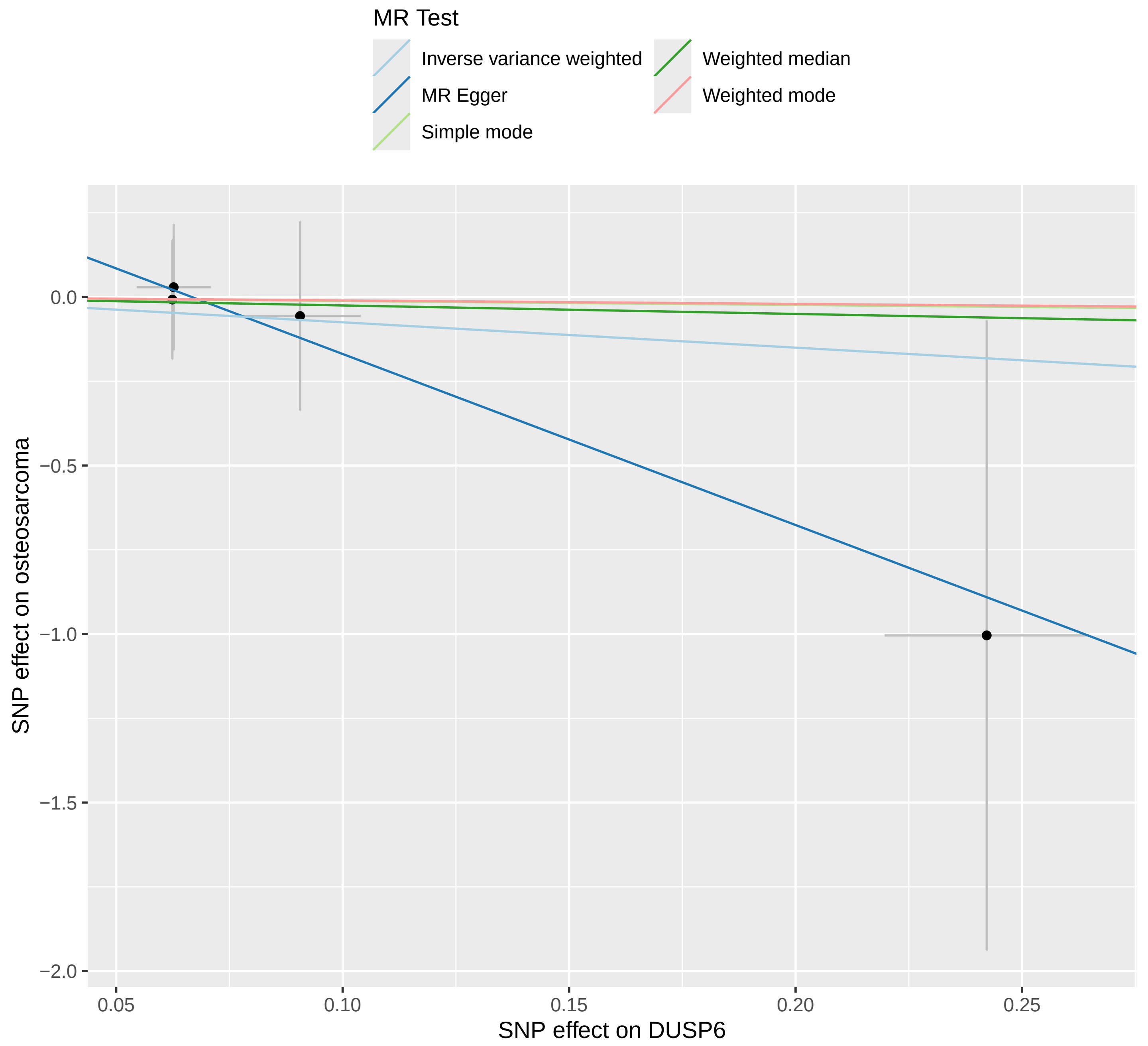

MR Test
Inverse variance weighted
MR Egger
Simple mode
Weighted median
Weighted mode
0.0
−0.5
−1.0
−1.5
−2.0
0.05
0.10
0.15
0.20
0.25
SNP effect on osteosarcoma
SNP effect on DUSP6

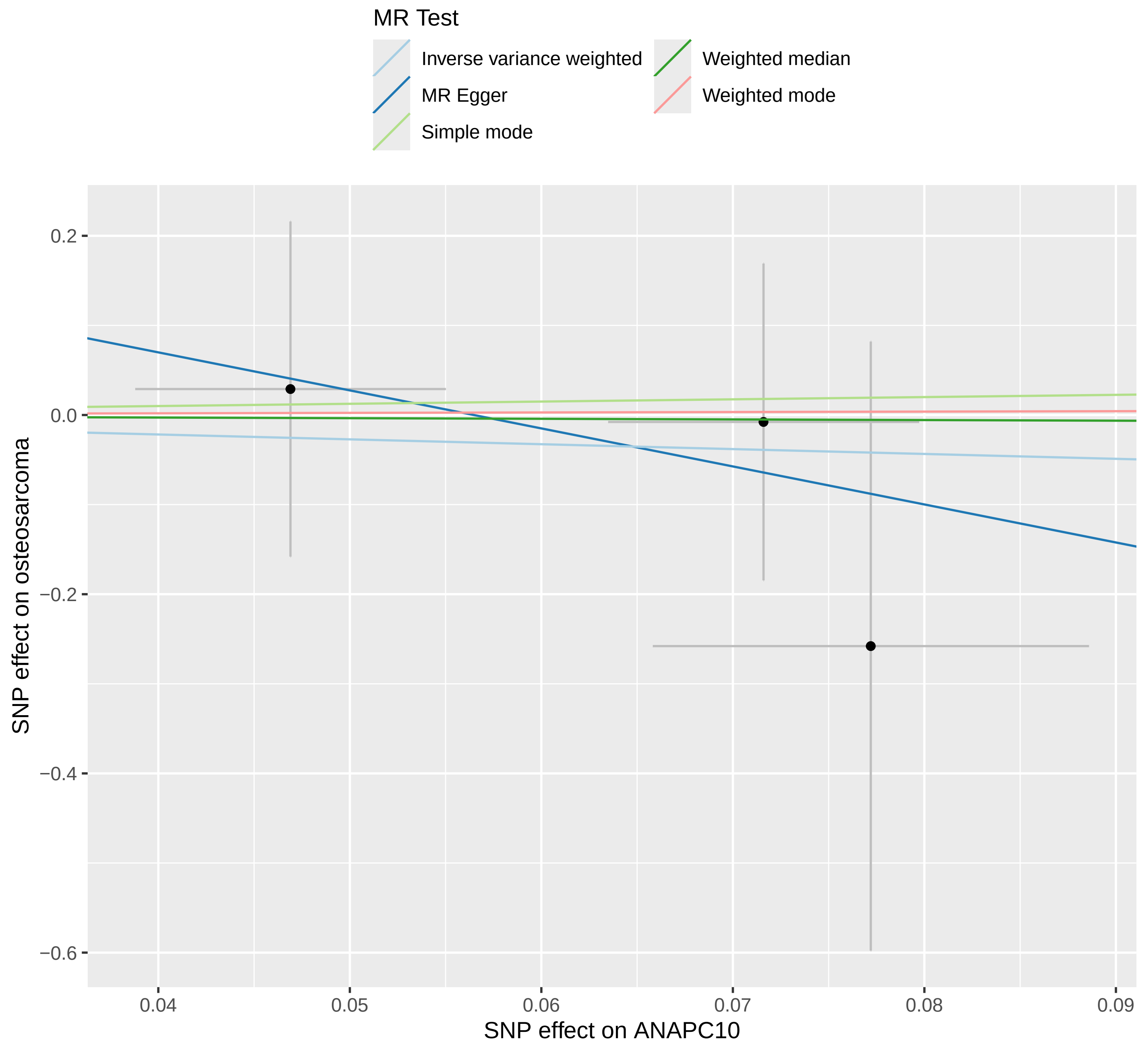
MR Test
Inverse variance weighted
MR Egger
Simple mode
Weighted median
Weighted mode
SNP effect on osteosarcoma
0.2
0.0
−0.2
−0.4
−0.6
0.04
0.05
0.06
0.07
0.08
0.09
SNP effect on ANAPC10

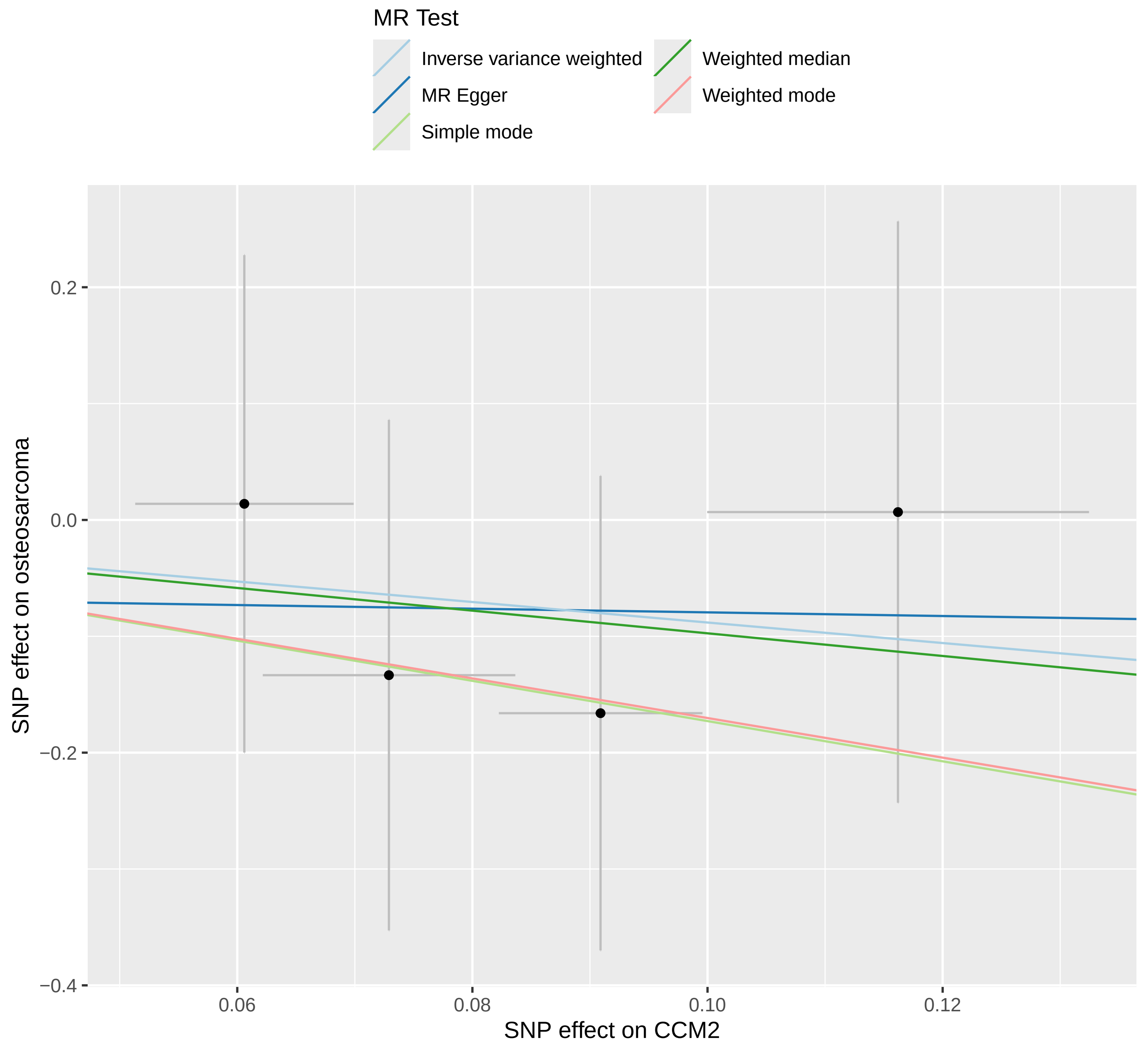
MR Test
Inverse variance weighted
MR Egger
Simple mode
Weighted median
Weighted mode
0.2
0.0
−0.2
−0.4
0.06
0.08
0.10
0.12
SNP effect on osteosarcoma
SNP effect on CCM2

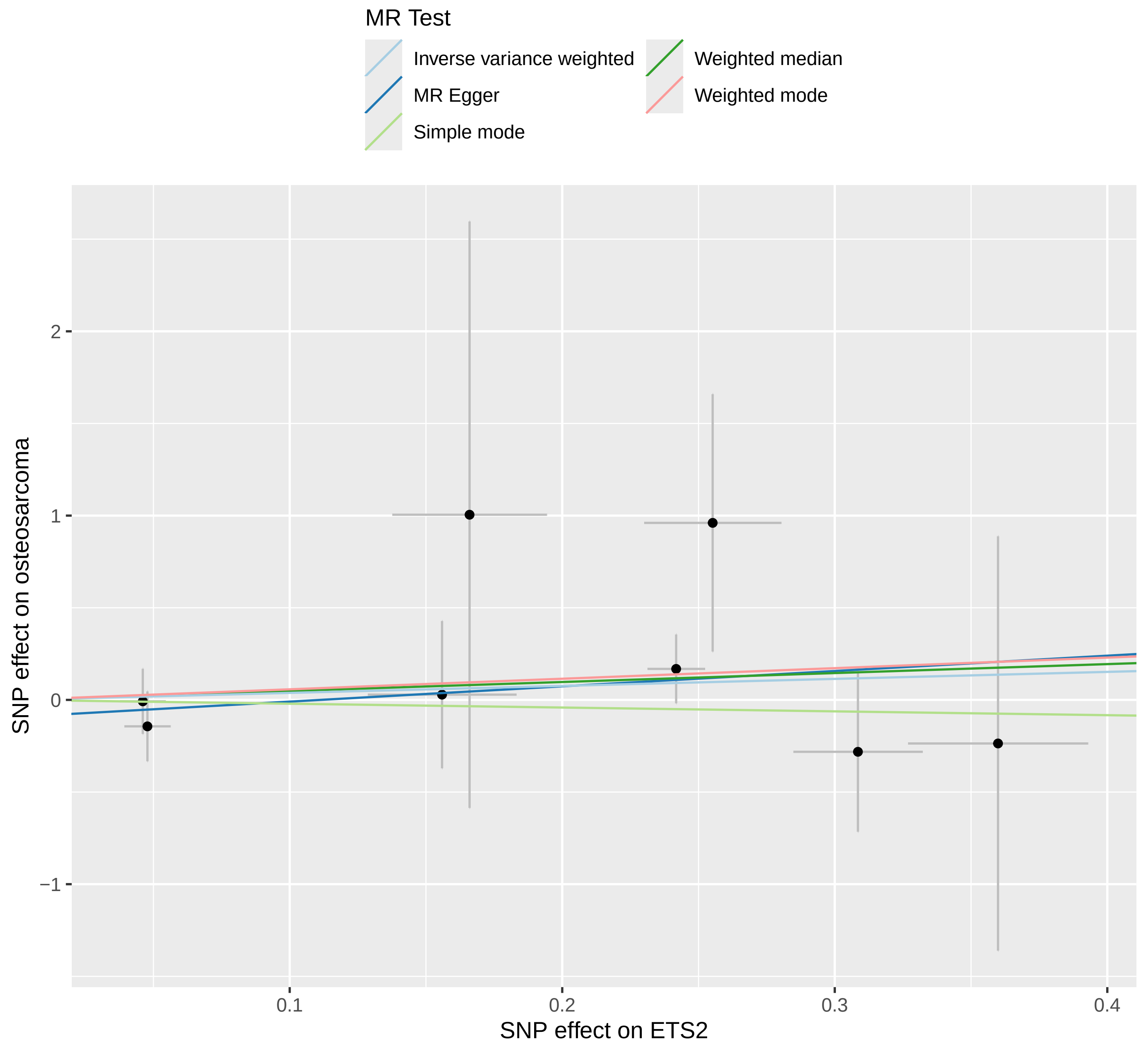
MR Test
Inverse variance weighted
MR Egger
Simple mode
Weighted median
Weighted mode
2
1
0
−1
0.1
0.2
0.3
0.4
SNP effect on osteosarcoma
SNP effect on ETS2

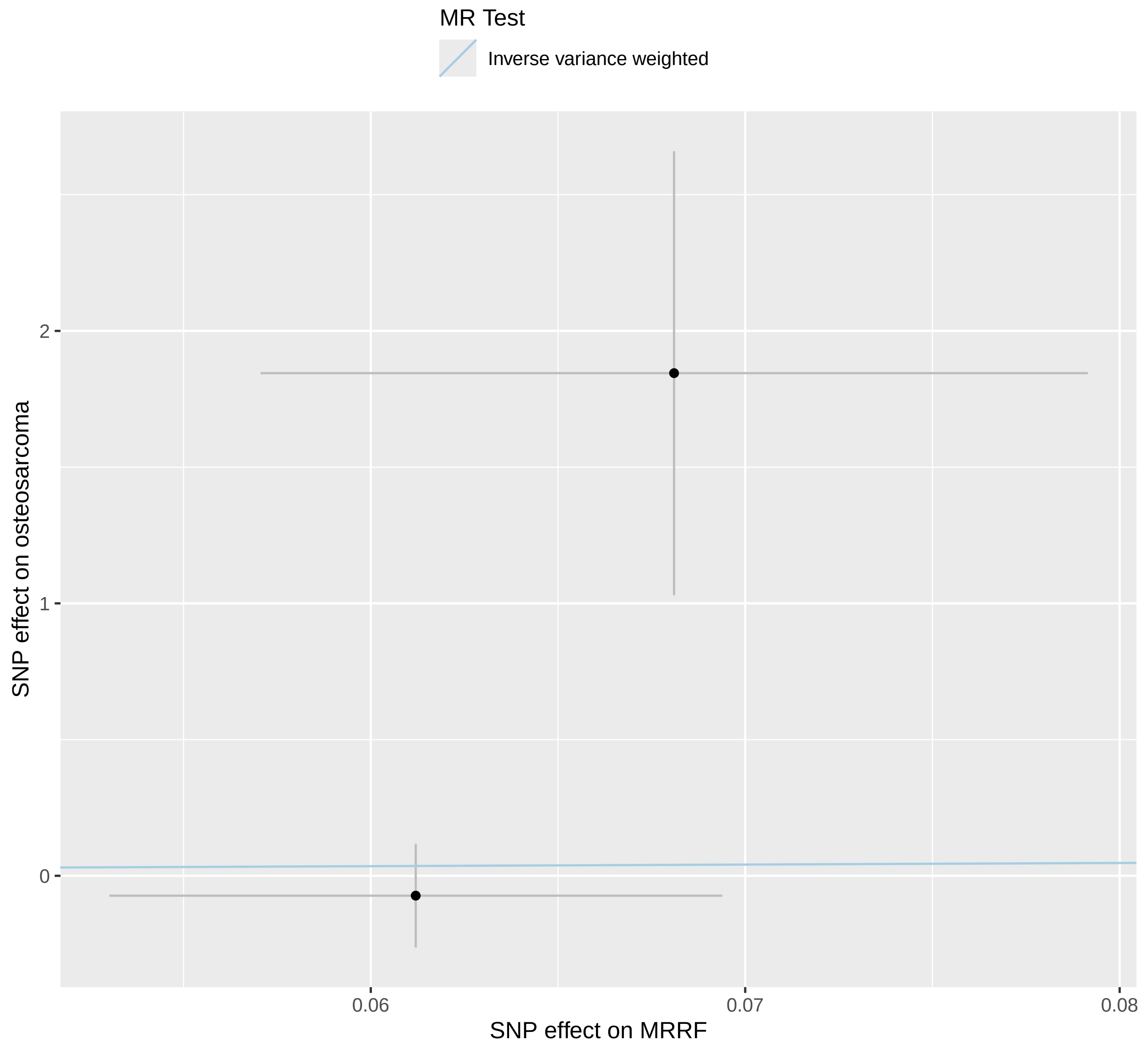
MR Test
Inverse variance weighted
SNP effect on osteosarcoma
2
1
0
0.06
0.07
0.08
SNP effect on MRRF

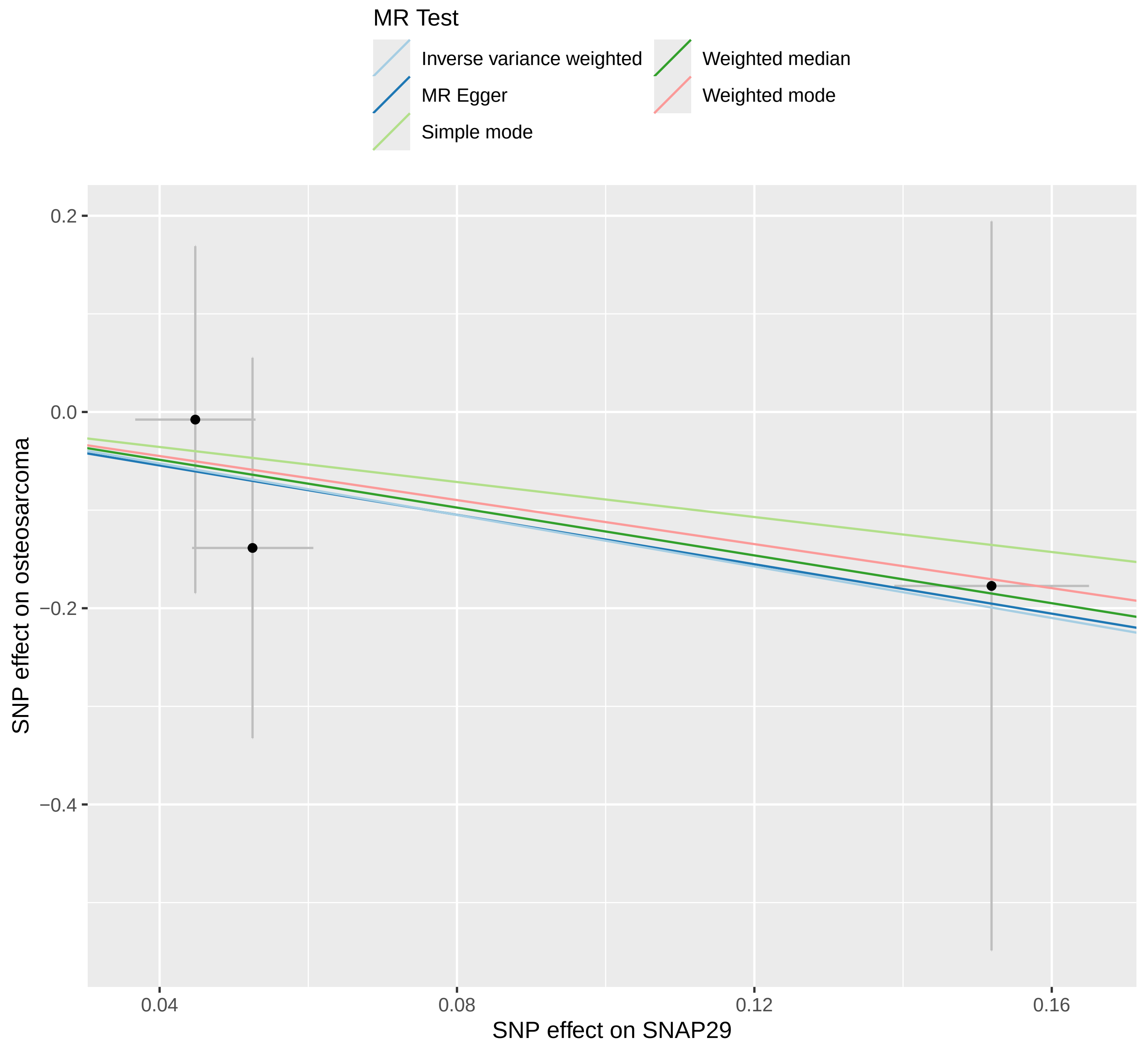

MR Test
Inverse variance weighted
MR Egger
Simple mode
Weighted median
Weighted mode
0.2
0.0
−0.2
−0.4
SNP effect on osteosarcoma
0.04
0.08
0.12
0.16
SNP effect on SNAP29

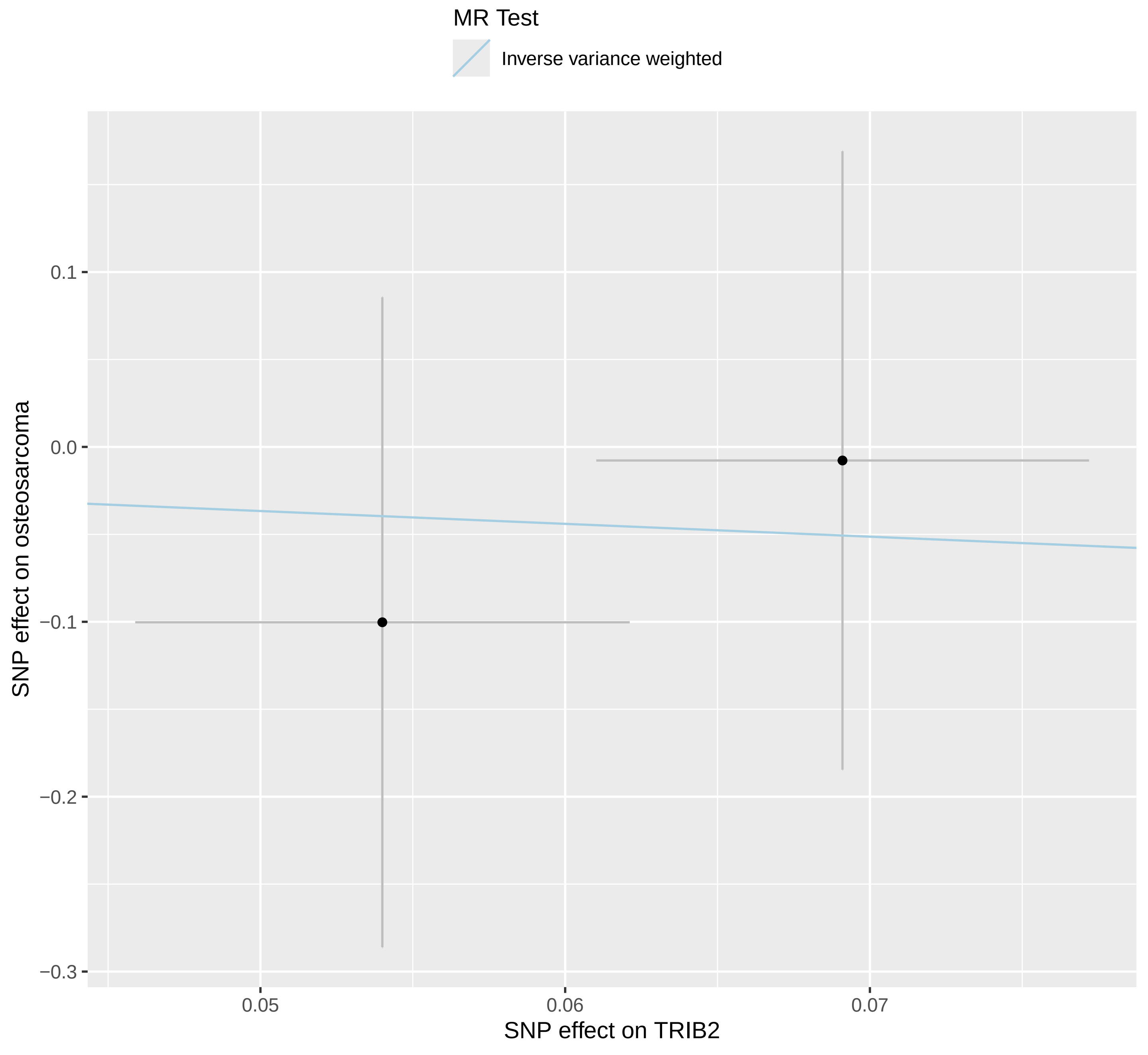
MR Test
Inverse variance weighted
0.1
0.0
−0.1
−0.2
−0.3
0.05
0.06
0.07
SNP effect on osteosarcoma
SNP effect on TRIB2

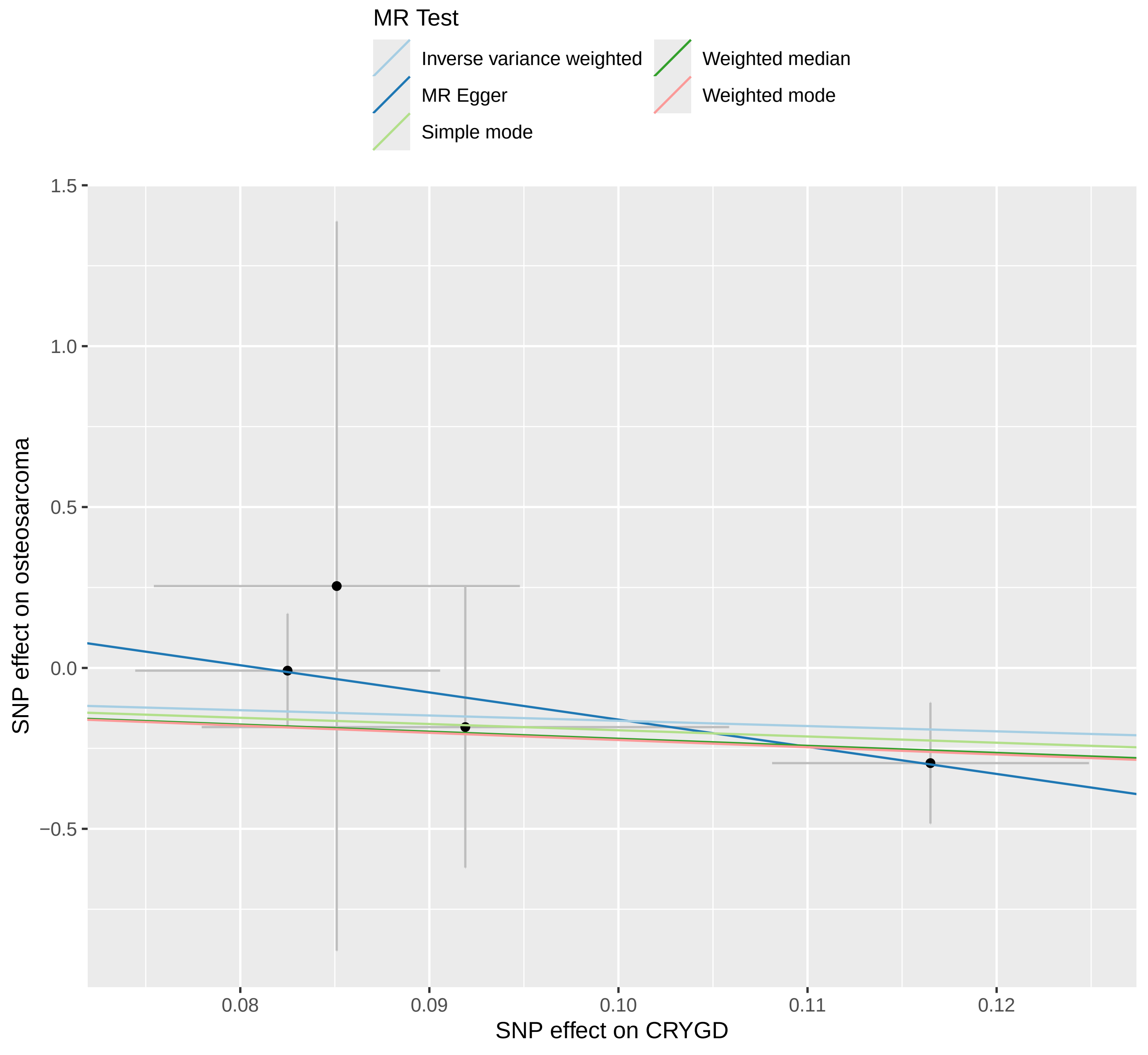

MR Test
Inverse variance weighted
MR Egger
Simple mode
Weighted median
Weighted mode
1.5
1.0
0.5
0.0
−0.5
SNP effect on osteosarcoma
0.08
0.09
0.10
0.11
0.12
SNP effect on CRYGD

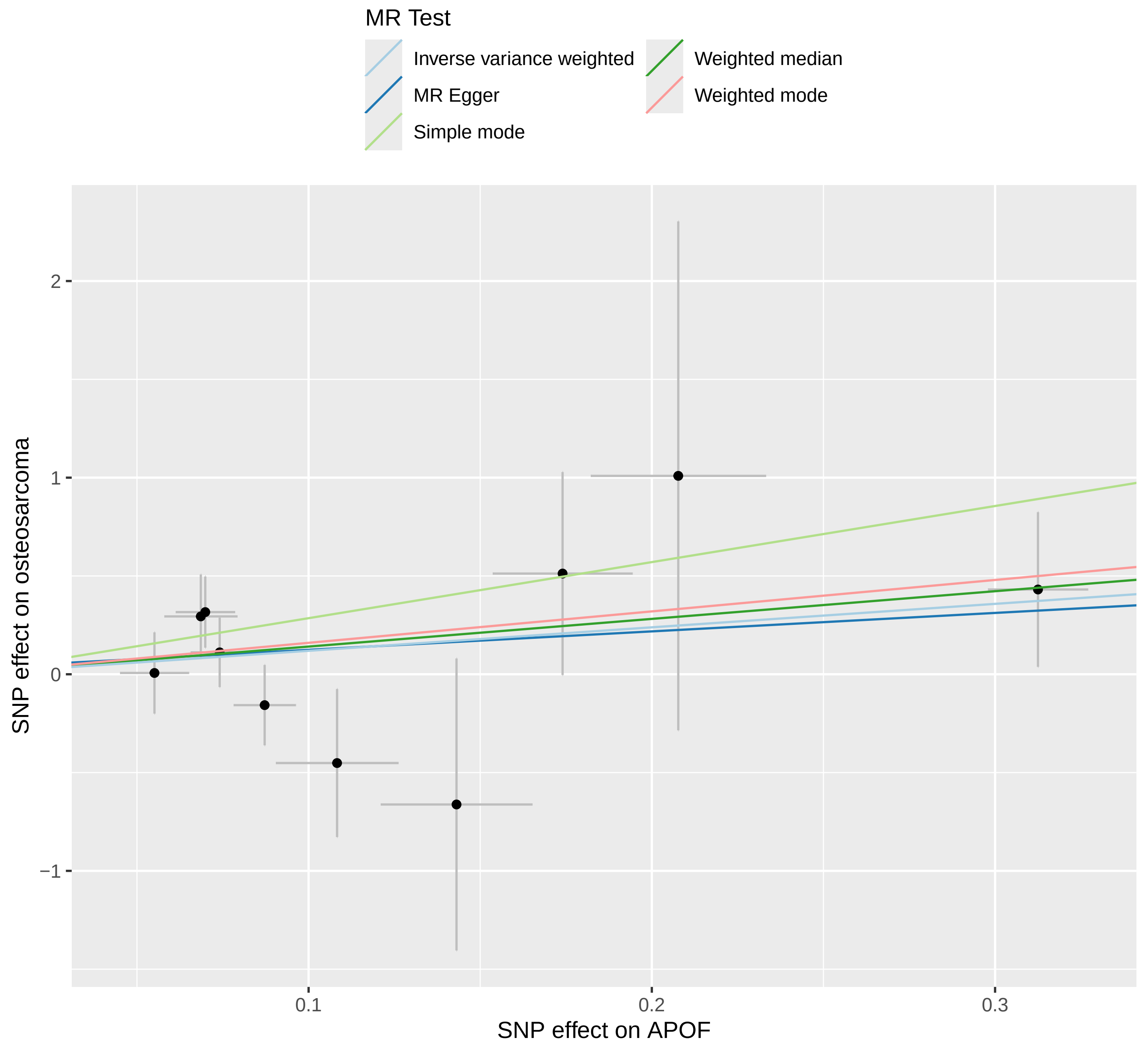
MR Test
Inverse variance weighted
MR Egger
Simple mode
Weighted median
Weighted mode
SNP effect on osteosarcoma
2
1
0
−1
0.1
0.2
0.3
SNP effect on APOF

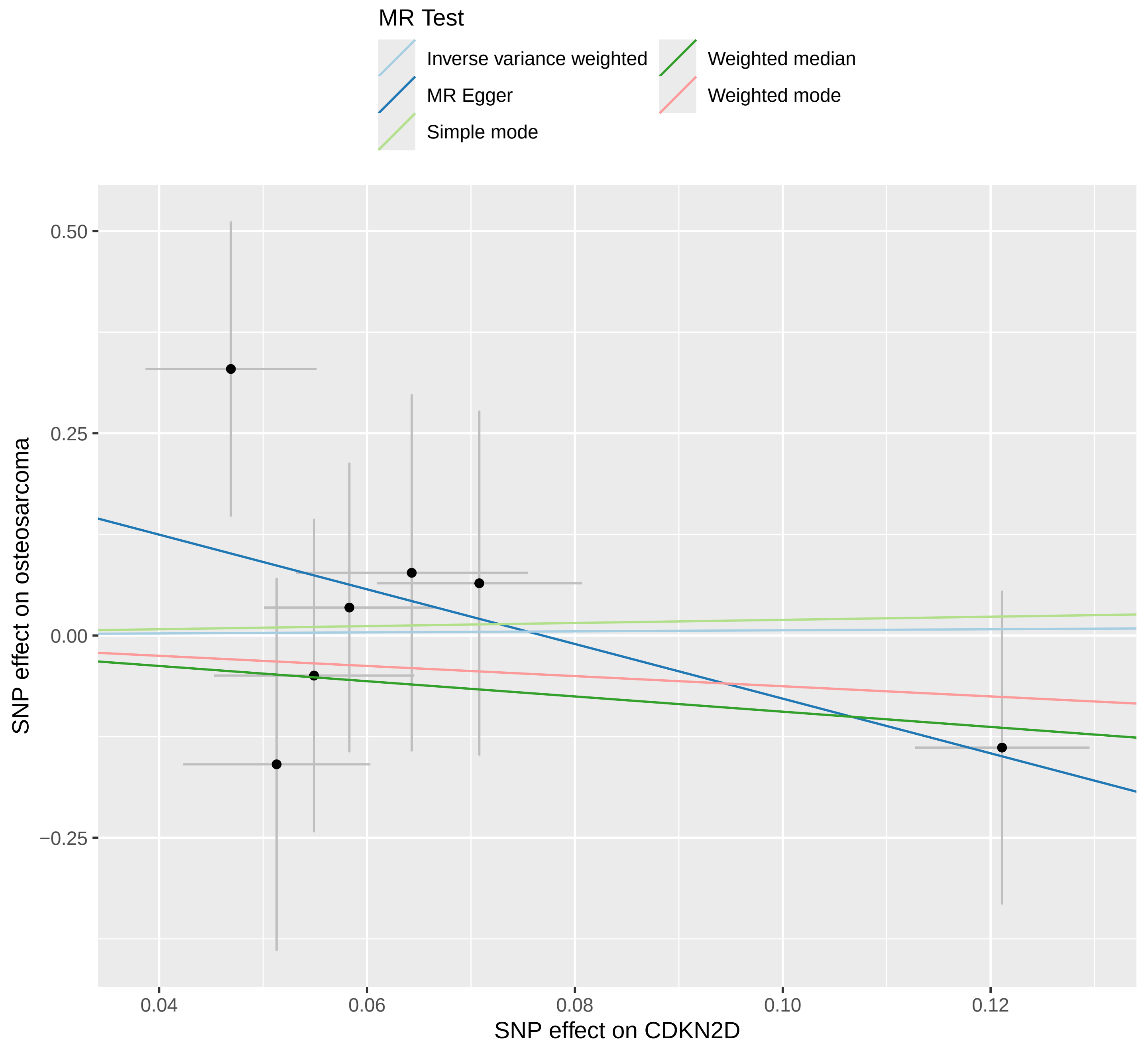

MR Test
Inverse variance weighted
MR Egger
Simple mode
Weighted median
Weighted mode
0.50
0.25
0.00
−0.25
SNP effect on osteosarcoma
0.04
0.06
0.08
0.10
0.12
SNP effect on CDKN2D

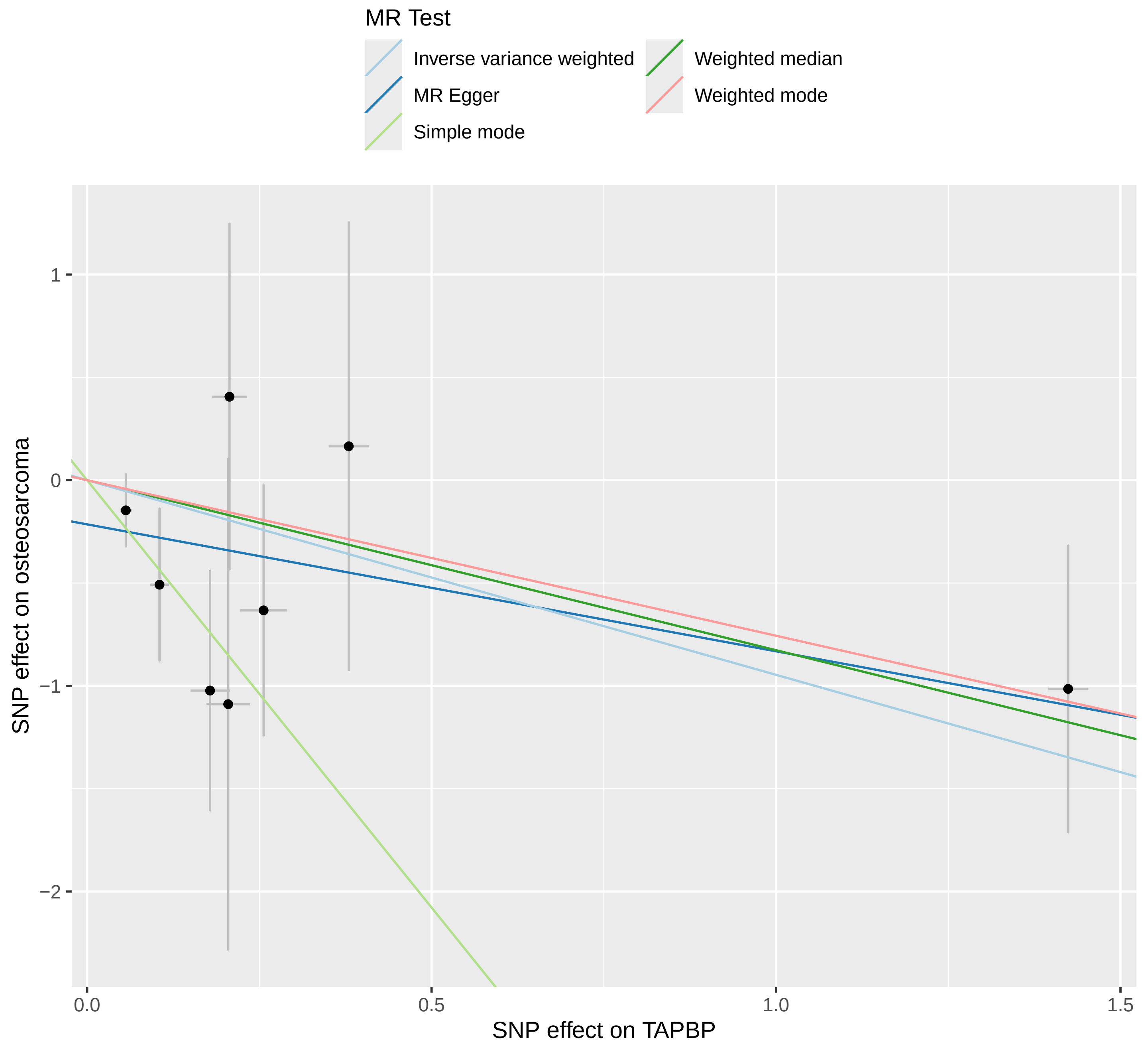

MR Test
Inverse variance weighted
MR Egger
Simple mode
Weighted median
Weighted mode
SNP effect on osteosarcoma
1
0
−1
−2
0.0
0.5
1.0
1.5
SNP effect on TAPBP

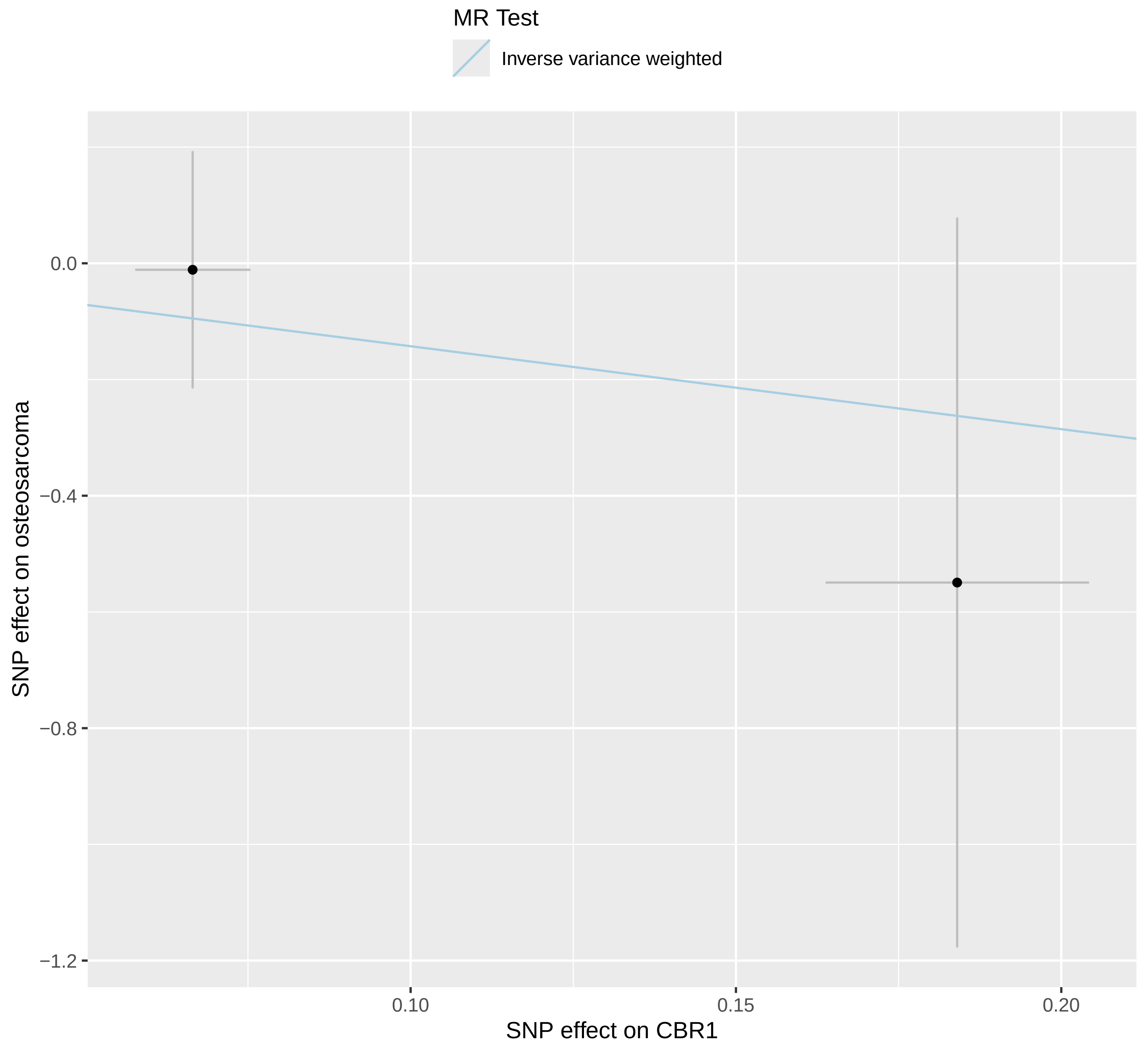
MR Test
Inverse variance weighted
0.0
−0.4
−0.8
−1.2
SNP effect on osteosarcoma
0.10
0.15
0.20
SNP effect on CBR1

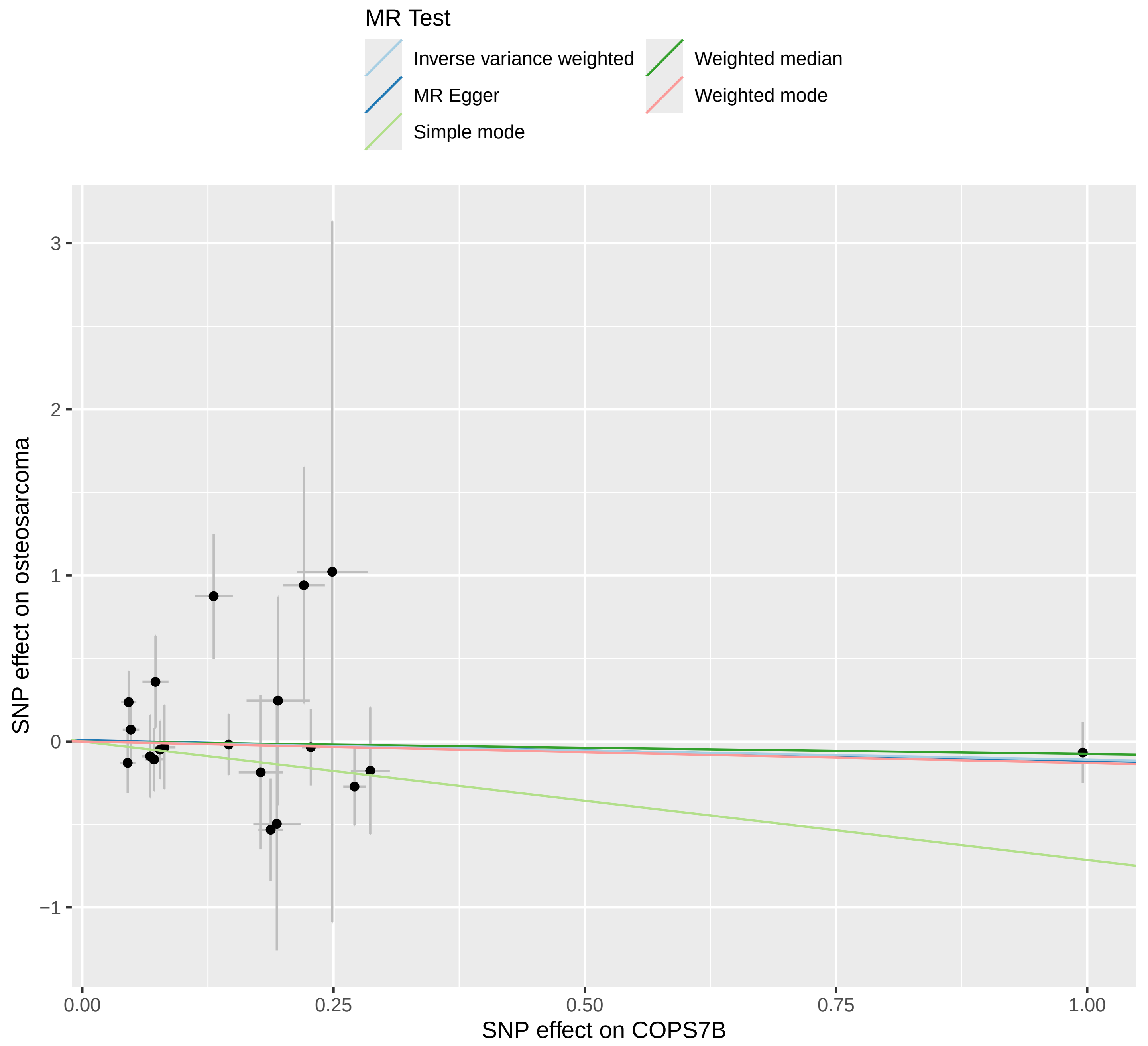

MR Test
Inverse variance weighted
MR Egger
Simple mode
Weighted median
Weighted mode
3
2
1
0
−1
SNP effect on osteosarcoma
0.00
0.25
0.50
0.75
1.00
SNP effect on COPS7B

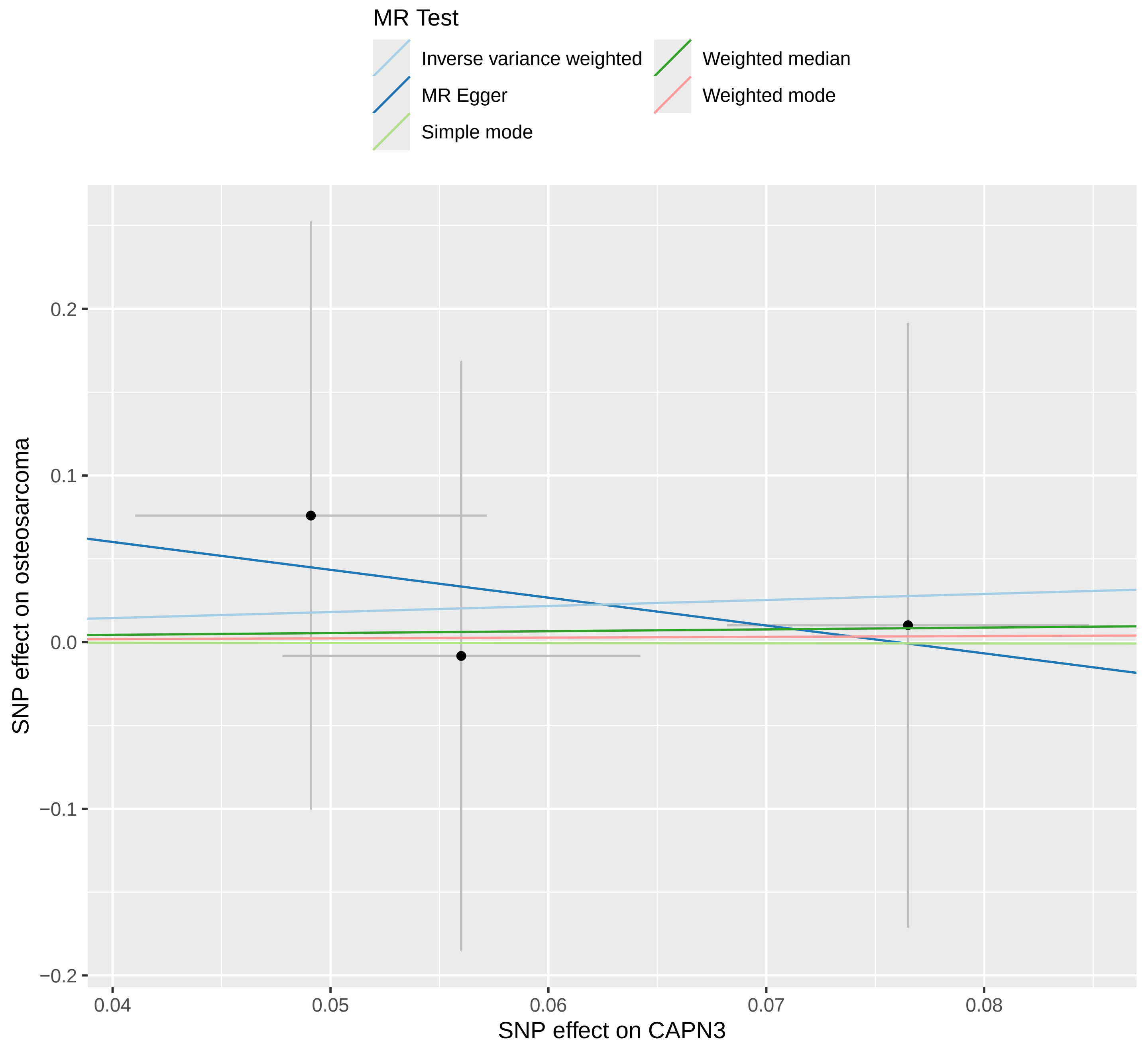

MR Test
Inverse variance weighted
MR Egger
Simple mode
Weighted median
Weighted mode
0.2
0.1
0.0
−0.1
−0.2
SNP effect on osteosarcoma
0.04
0.05
0.06
0.07
0.08
SNP effect on CAPN3

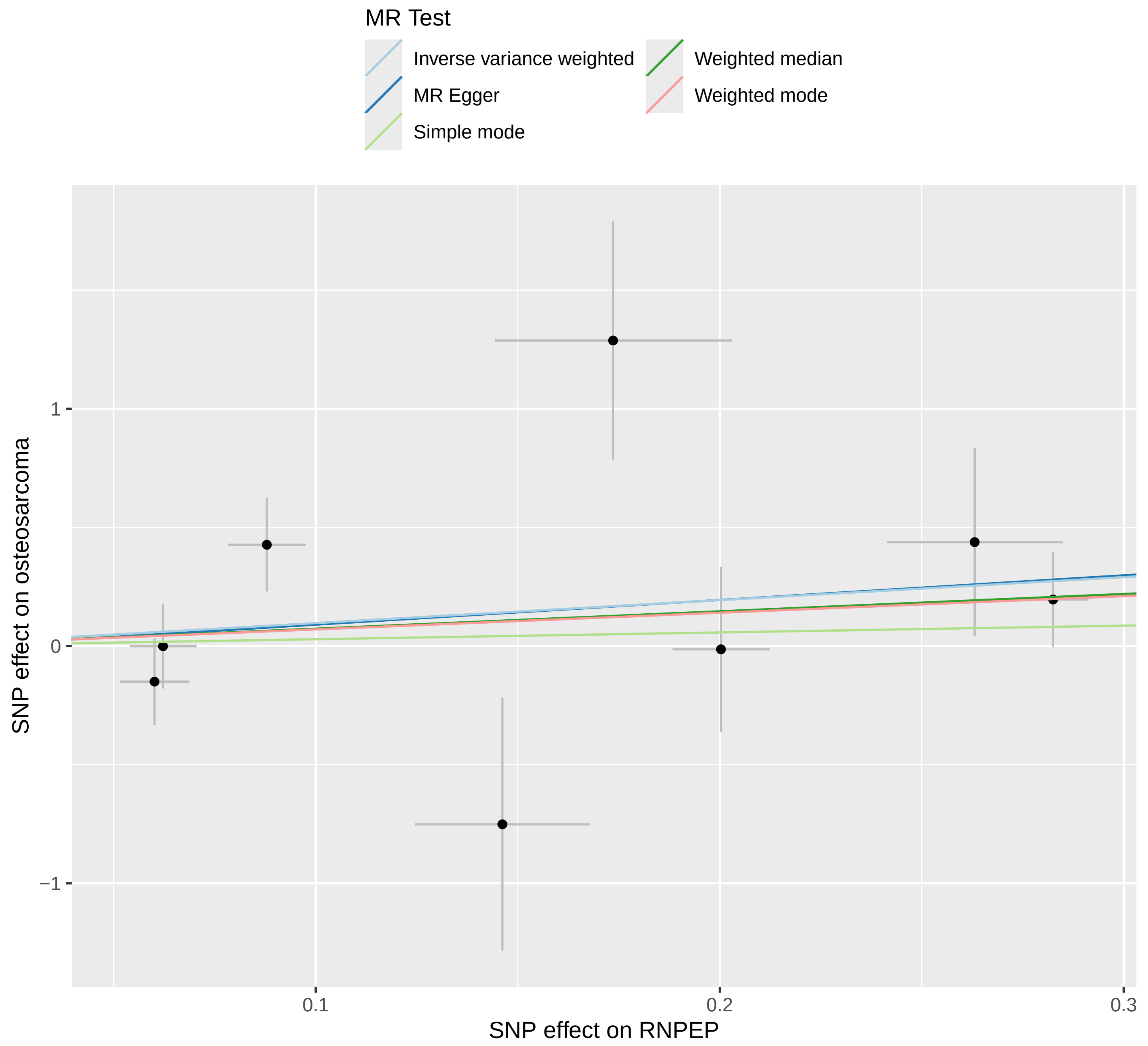

MR Test
Inverse variance weighted
MR Egger
Simple mode
Weighted median
Weighted mode
1
0
−1
0.1
0.2
0.3
SNP effect on osteosarcoma
SNP effect on RNPEP

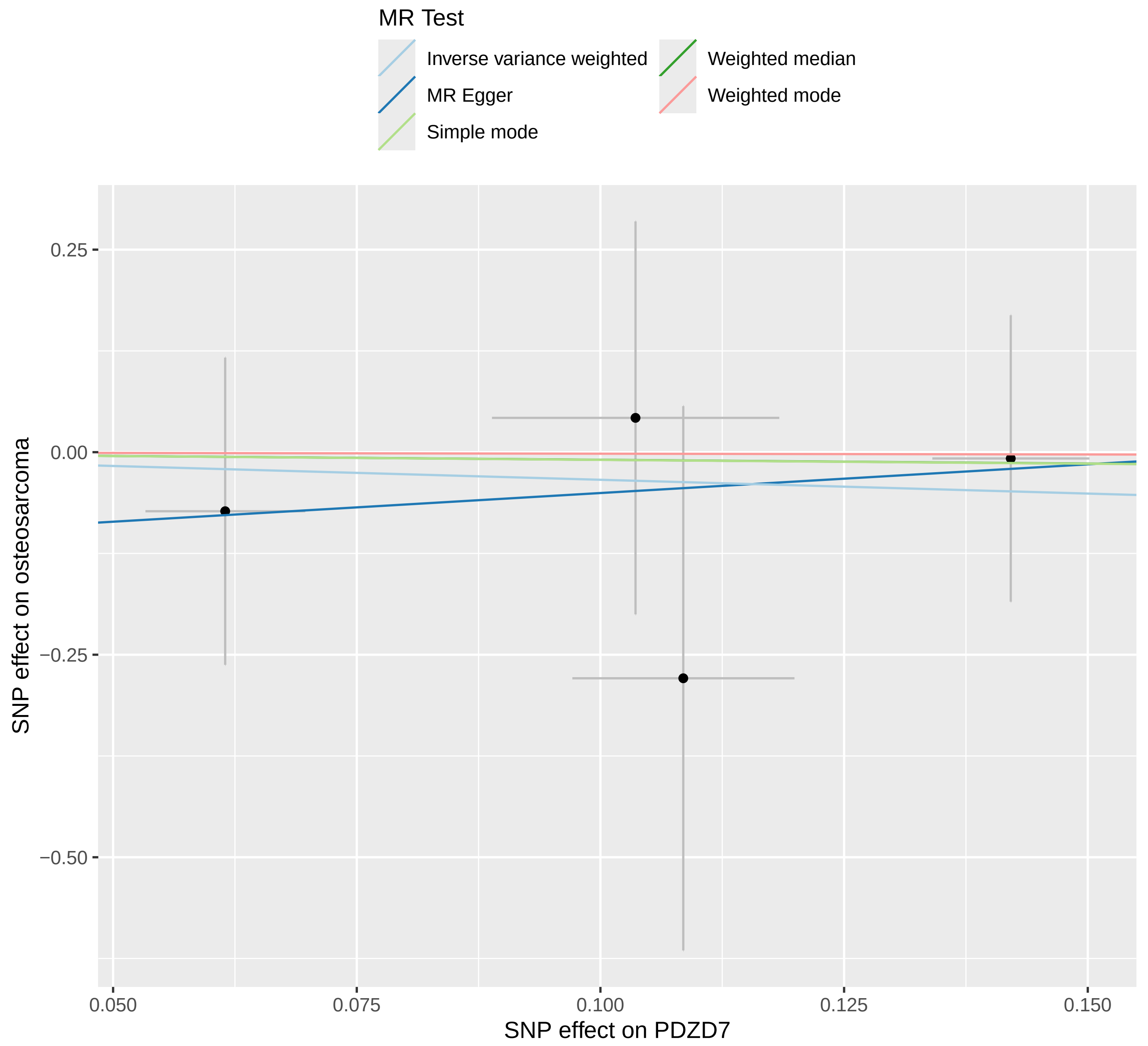
MR Test
Inverse variance weighted
MR Egger
Simple mode
Weighted median
Weighted mode
0.25
0.00
−0.25
−0.50
0.050
0.075
0.100
0.125
0.150
SNP effect on osteosarcoma
SNP effect on PDZD7

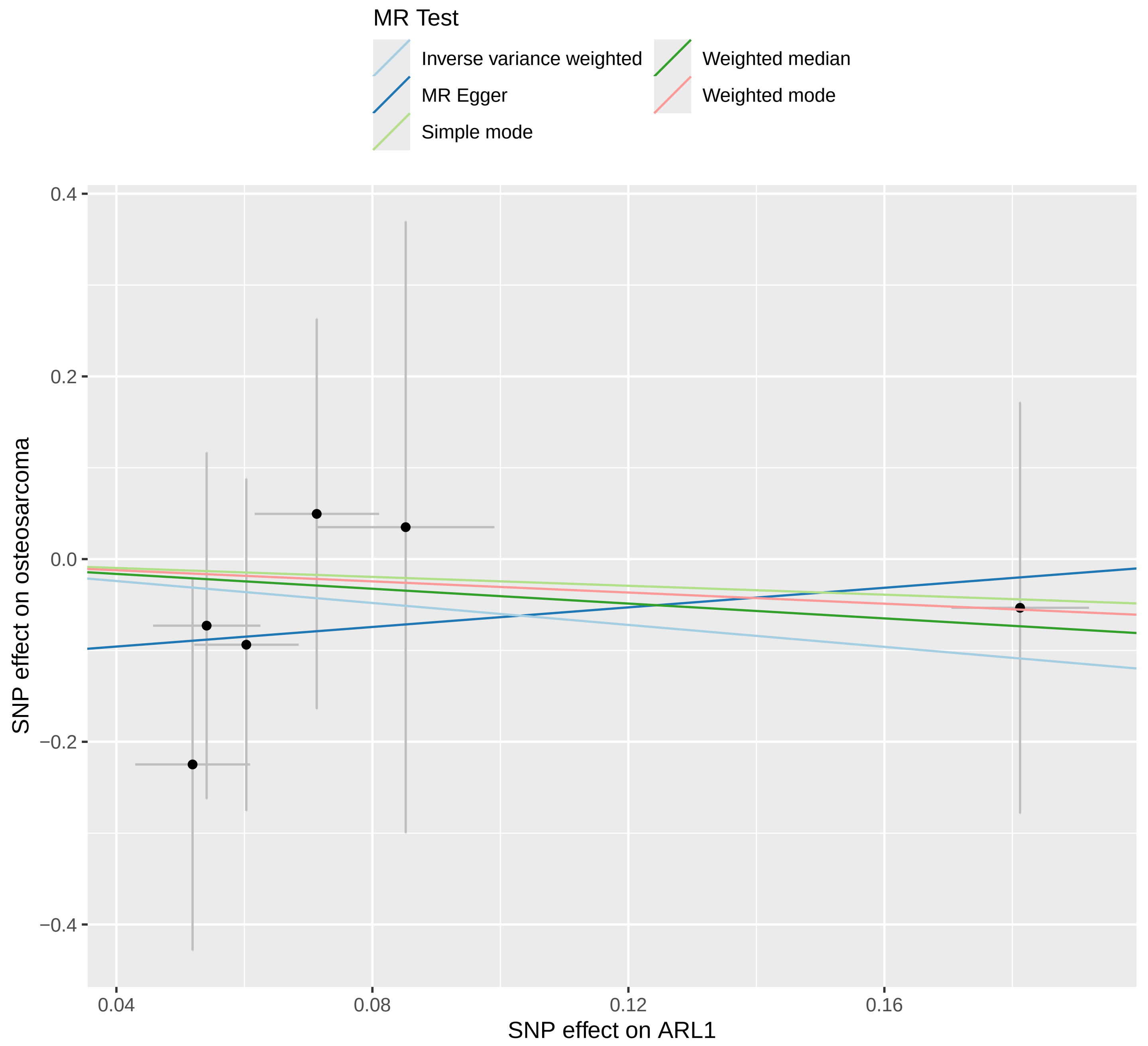
MR Test
Inverse variance weighted
MR Egger
Simple mode
Weighted median
Weighted mode
0.4
0.2
0.0
−0.2
−0.4
SNP effect on osteosarcoma
0.04
0.08
0.12
0.16
SNP effect on ARL1

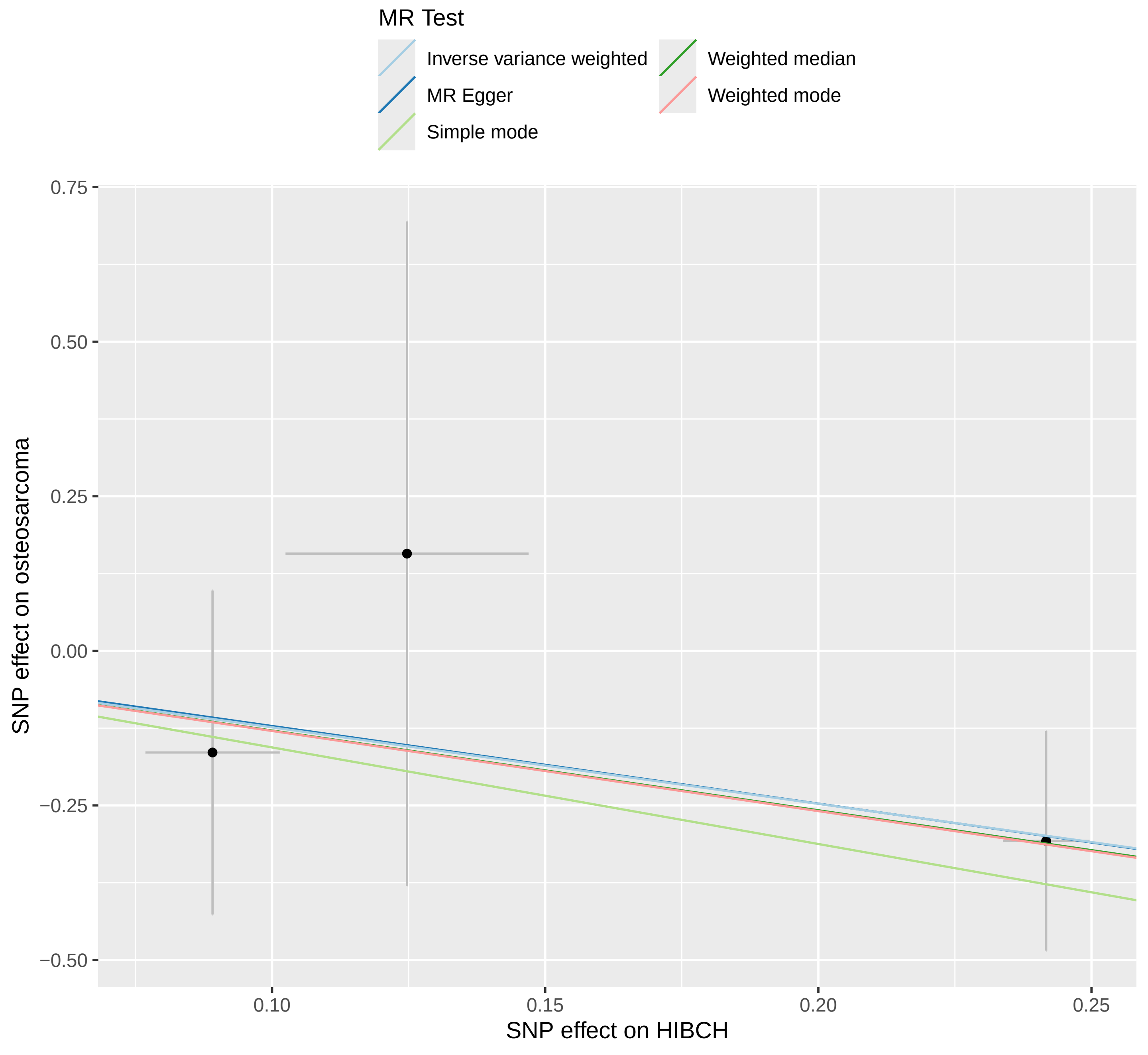
MR Test
Inverse variance weighted
MR Egger
Simple mode
Weighted median
Weighted mode
0.75
0.50
0.25
0.00
−0.25
−0.50
SNP effect on osteosarcoma
0.10
0.15
0.20
0.25
SNP effect on HIBCH

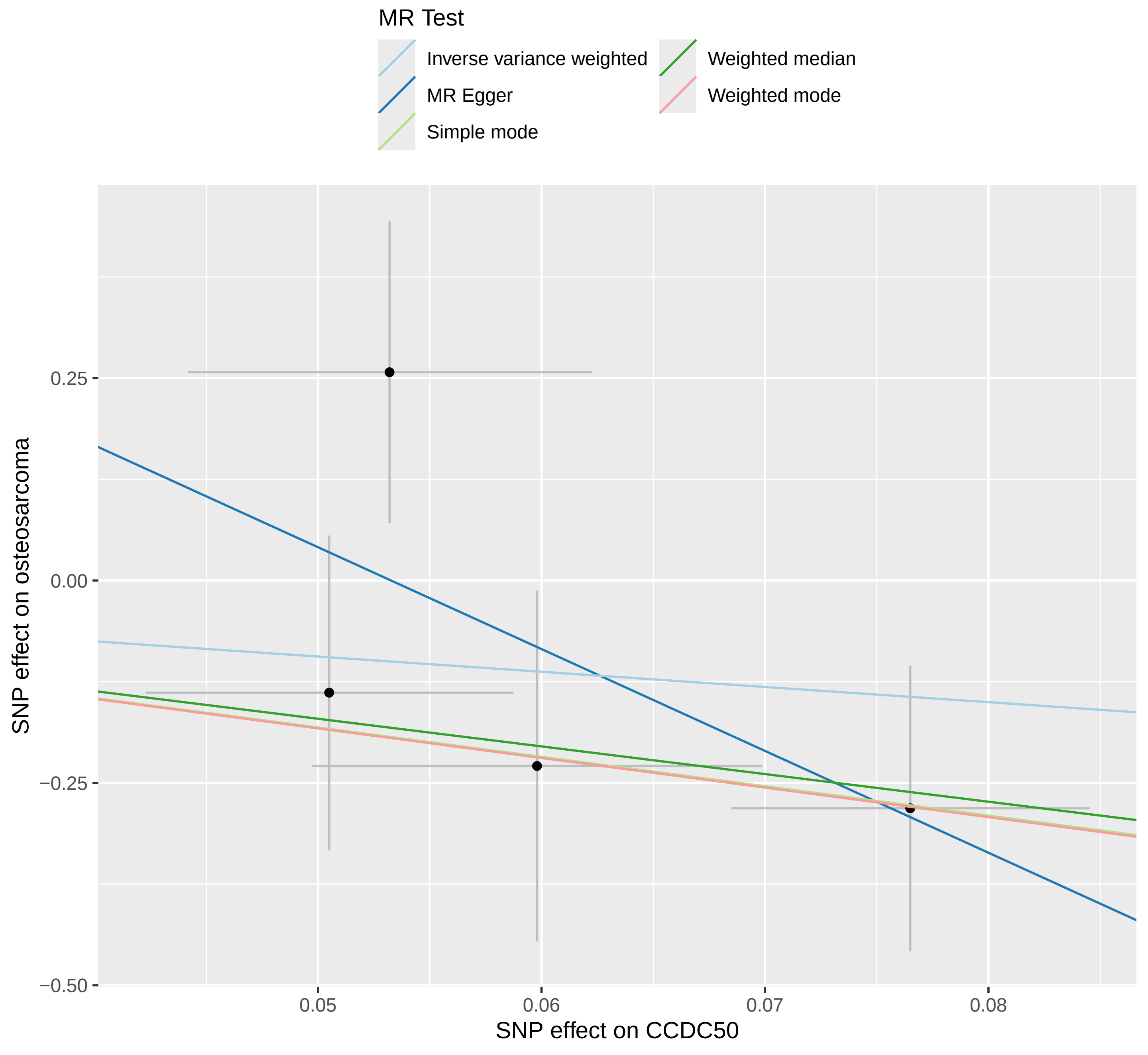

MR Test
Inverse variance weighted
MR Egger
Simple mode
Weighted median
Weighted mode
SNP effect on osteosarcoma
0.25
0.00
−0.25
−0.50
0.05
0.06
0.07
0.08
SNP effect on CCDC50

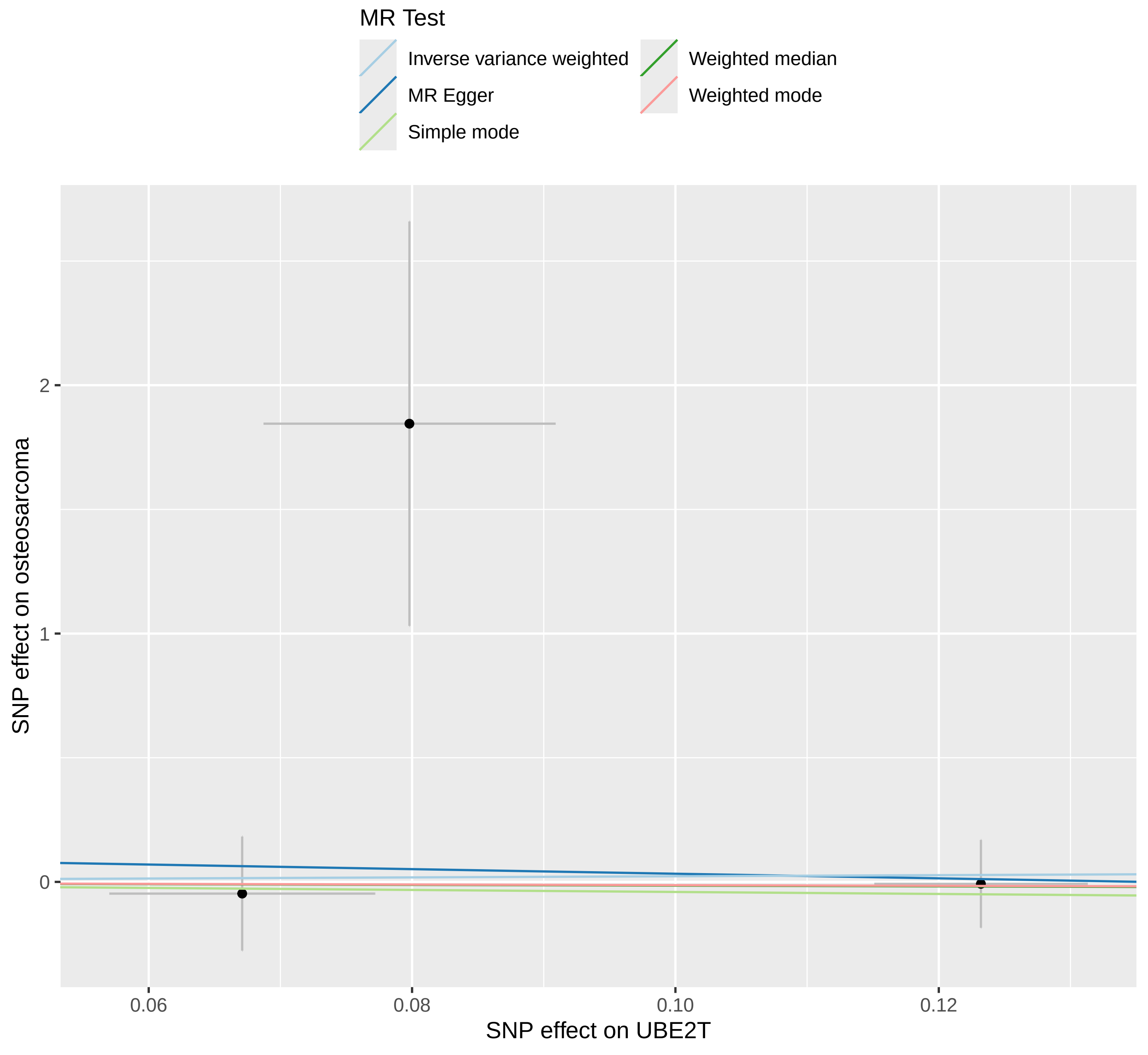
MR Test
Inverse variance weighted
MR Egger
Simple mode
Weighted median
Weighted mode
SNP effect on osteosarcoma
2
1
0
0.06
0.08
0.10
0.12
SNP effect on UBE2T

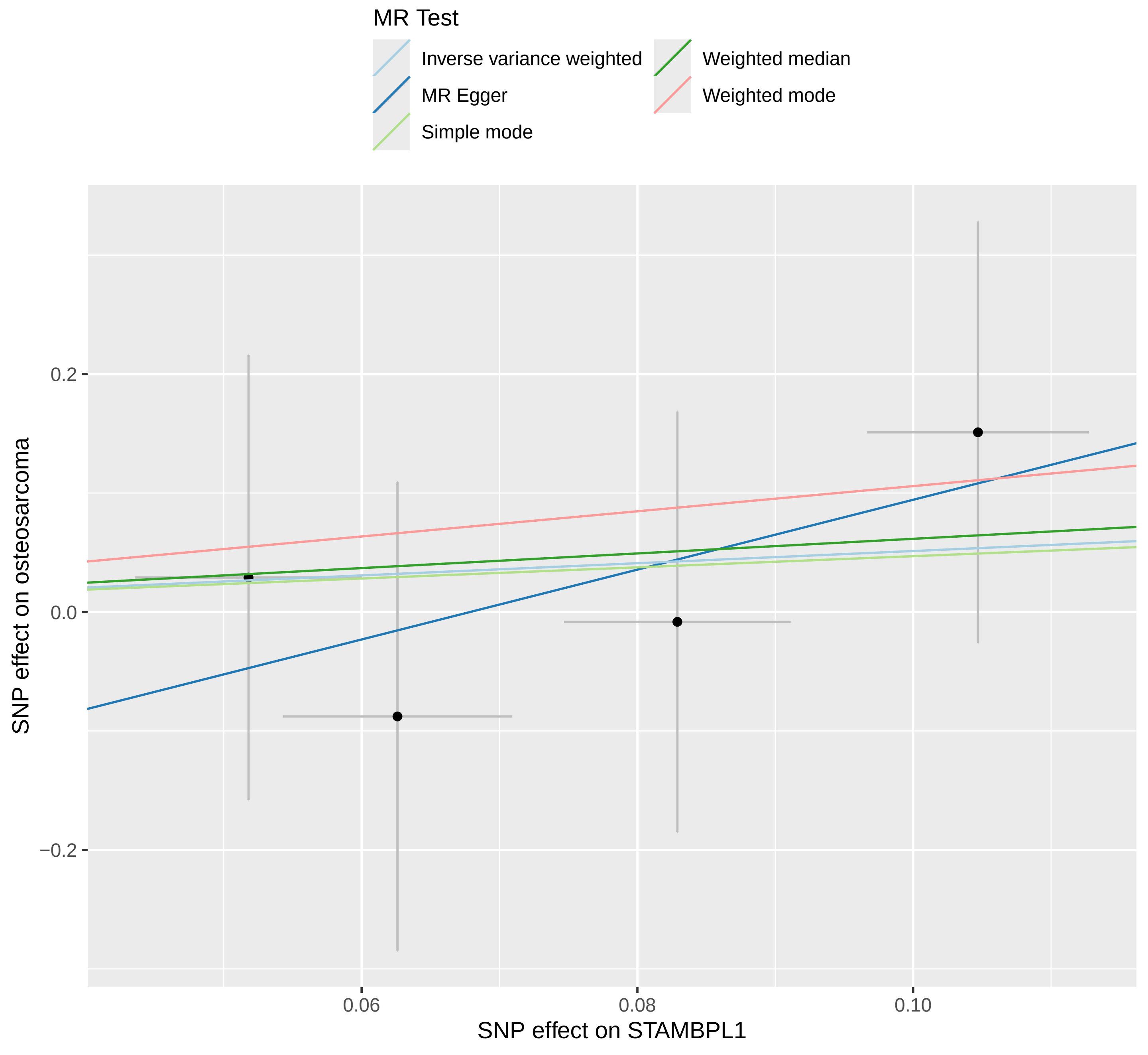

MR Test
Inverse variance weighted
MR Egger
Simple mode
Weighted median
Weighted mode
0.2
0.0
−0.2
SNP effect on osteosarcoma
0.06
0.08
0.10
SNP effect on STAMBPL1

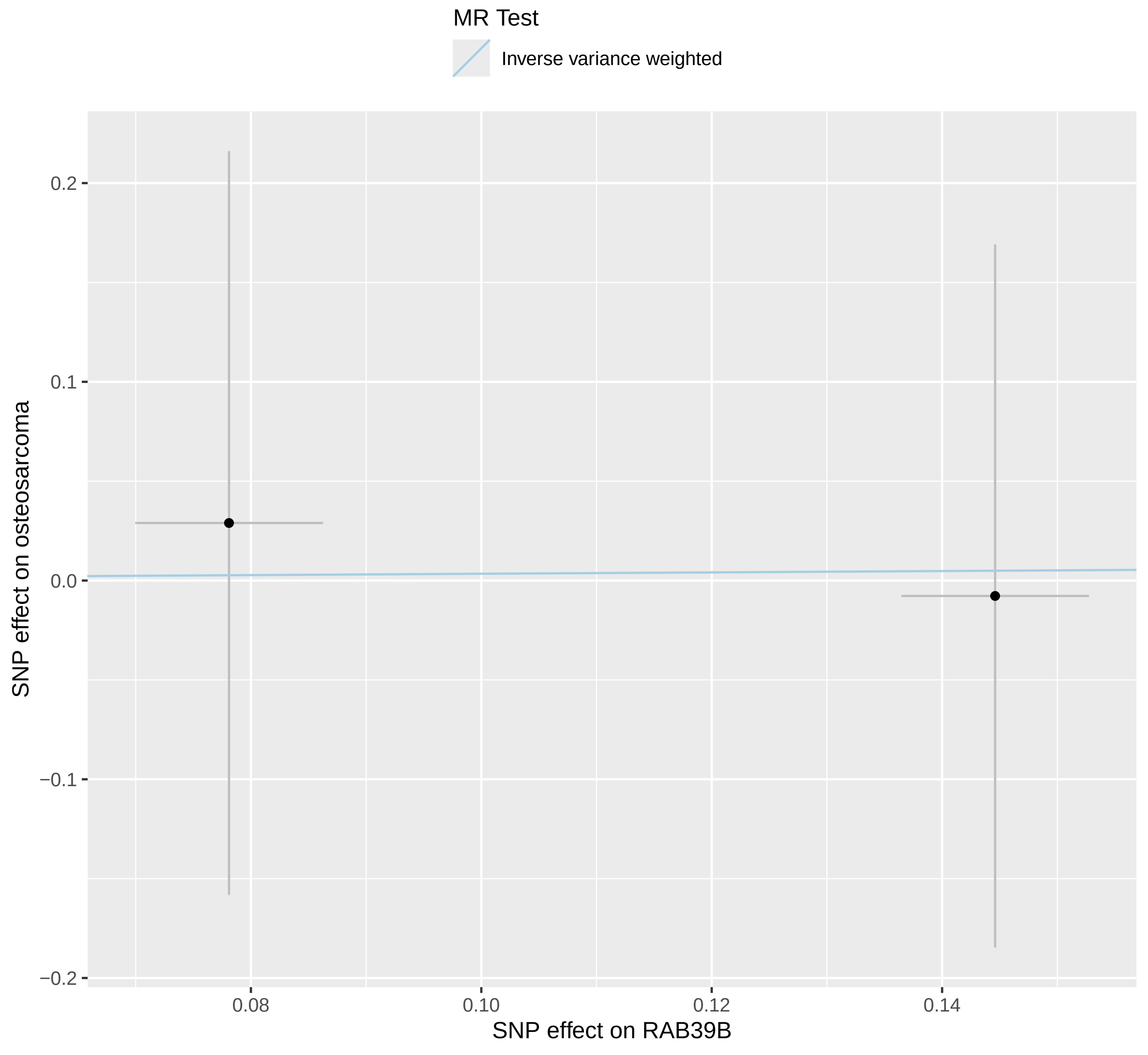
MR Test
Inverse variance weighted
0.2
0.1
0.0
−0.1
−0.2
SNP effect on osteosarcoma
0.08
0.10
0.12
0.14
SNP effect on RAB39B

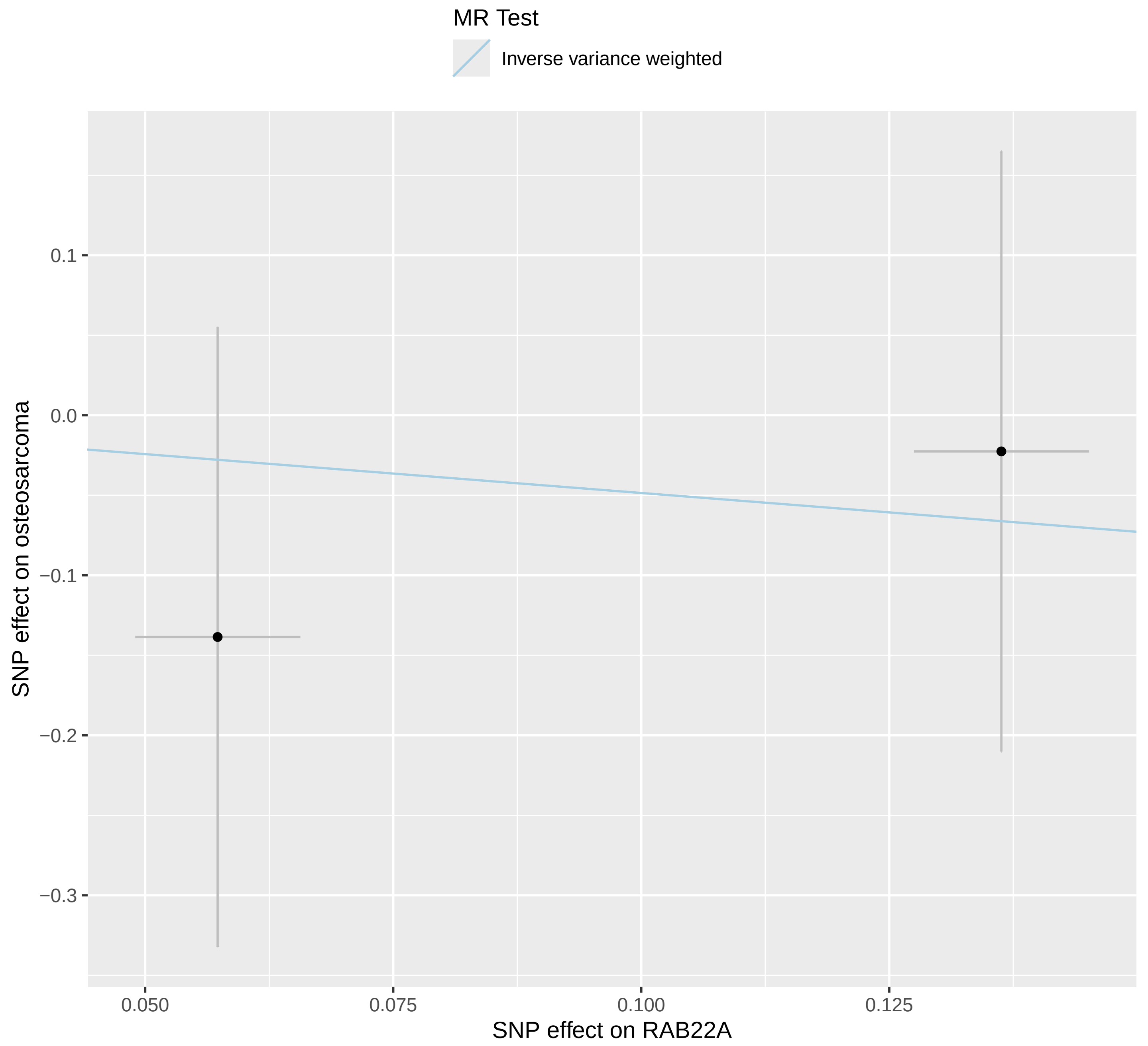
MR Test
Inverse variance weighted
SNP effect on osteosarcoma
0.1
0.0
−0.1
−0.2
−0.3
0.050
0.075
0.100
0.125
SNP effect on RAB22A

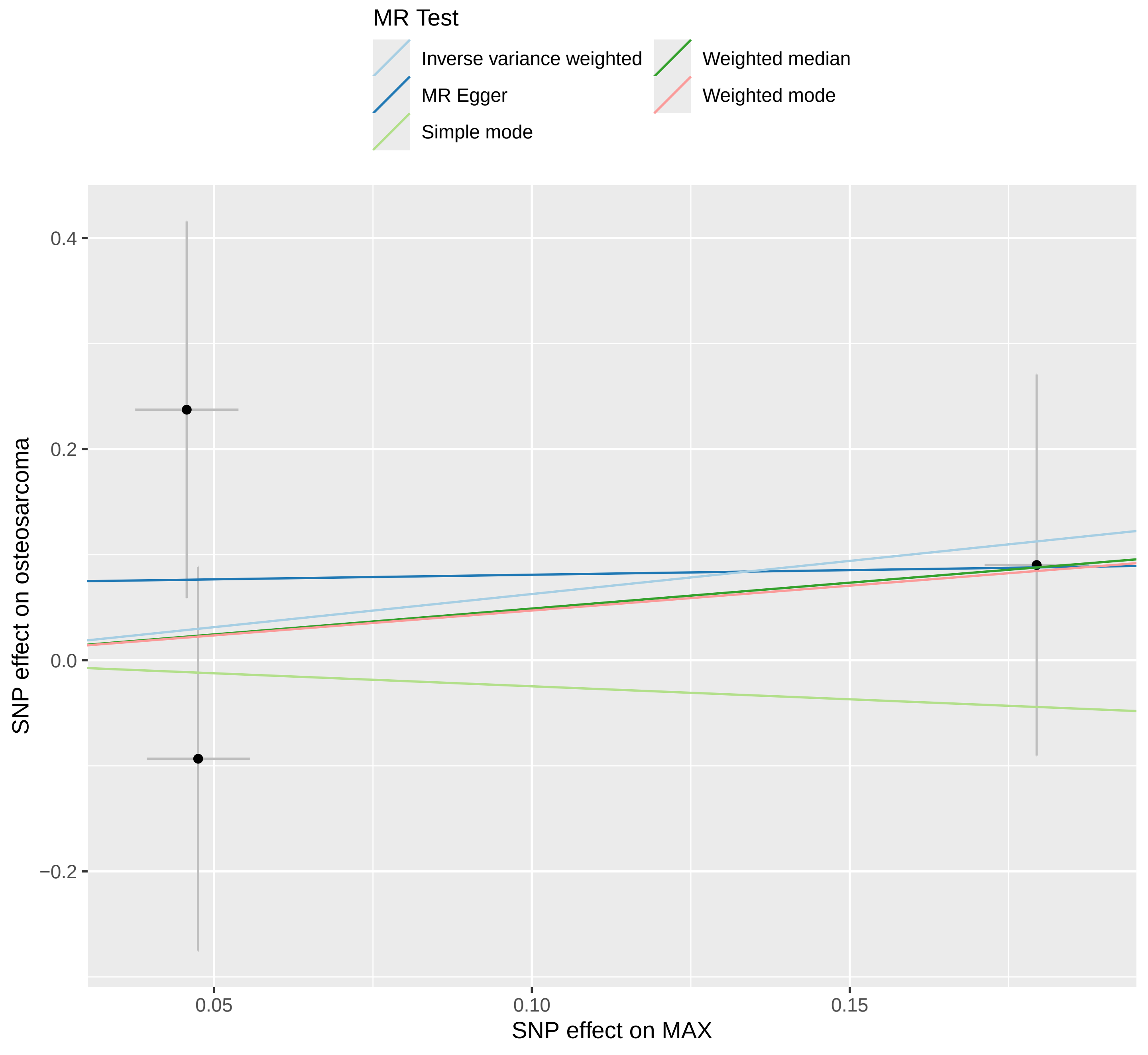
MR Test
Inverse variance weighted
MR Egger
Simple mode
Weighted median
Weighted mode
0.4
0.2
0.0
−0.2
0.05
0.10
0.15
SNP effect on osteosarcoma
SNP effect on MAX

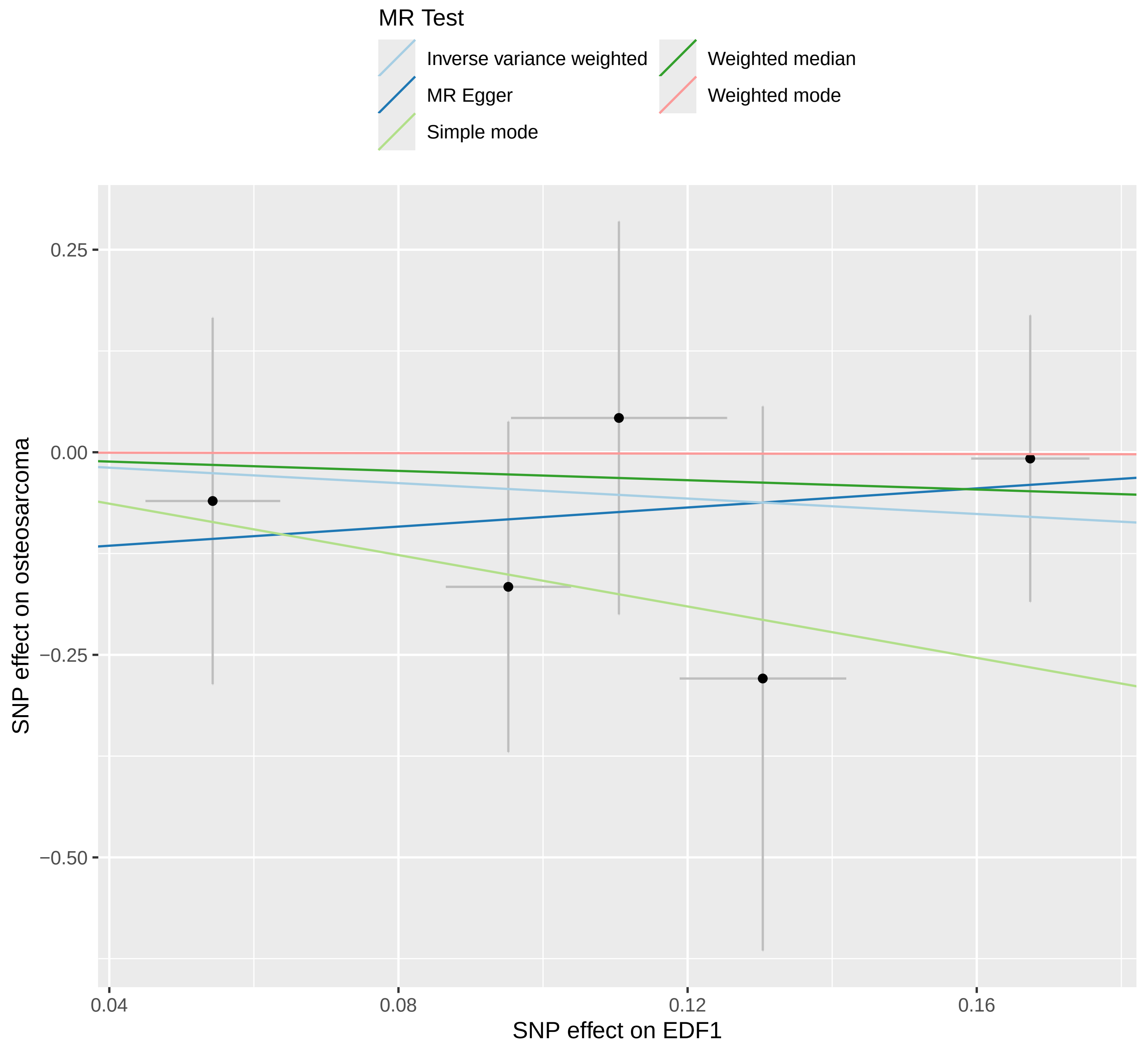
MR Test
Inverse variance weighted
MR Egger
Simple mode
Weighted median
Weighted mode
0.25
0.00
−0.25
−0.50
0.04
0.08
0.12
0.16
SNP effect on EDF1
SNP effect on osteosarcoma

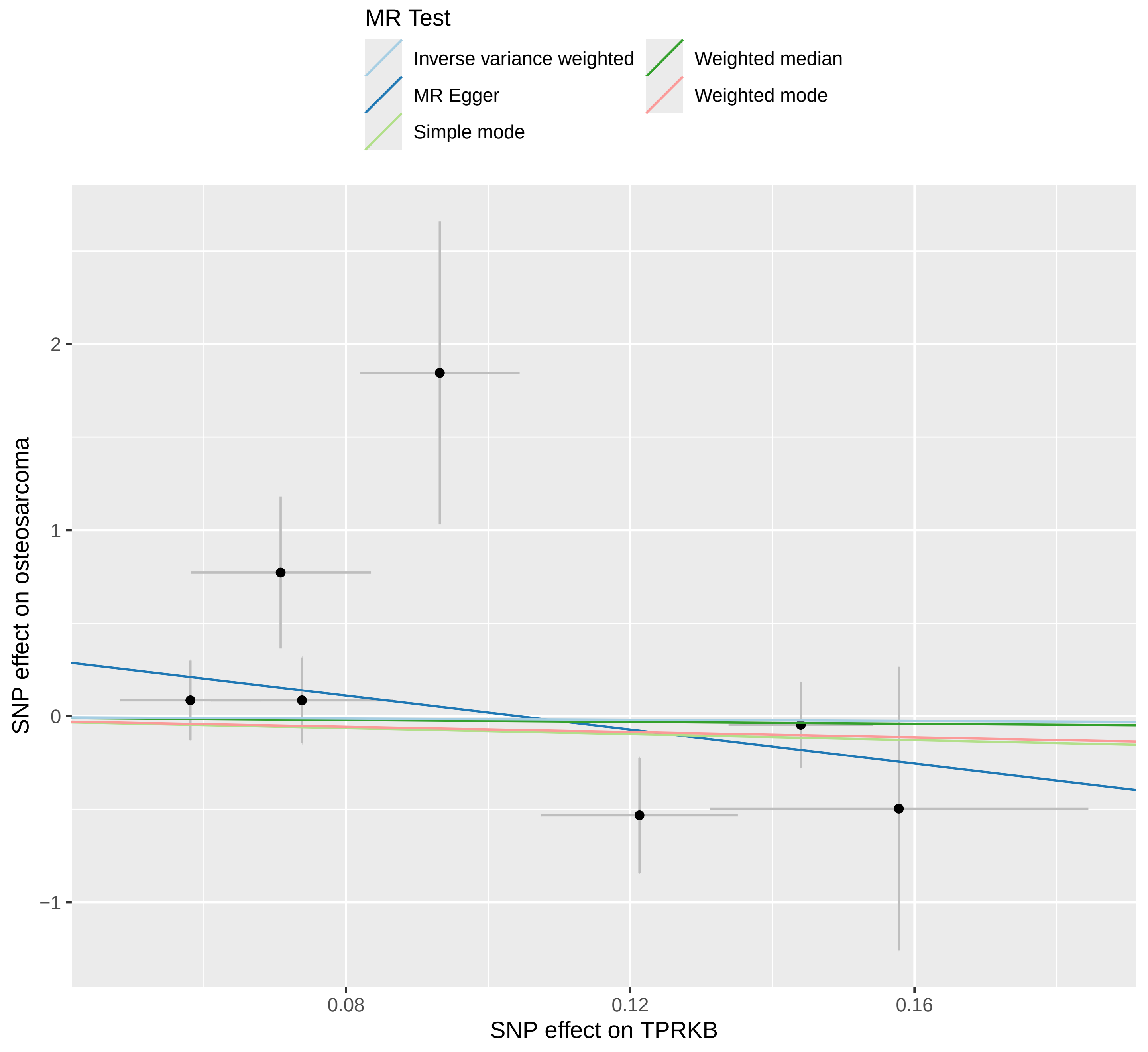
MR Test
Inverse variance weighted
MR Egger
Simple mode
Weighted median
Weighted mode
2
1
0
−1
SNP effect on osteosarcoma
0.08
0.12
0.16
SNP effect on TPRKB

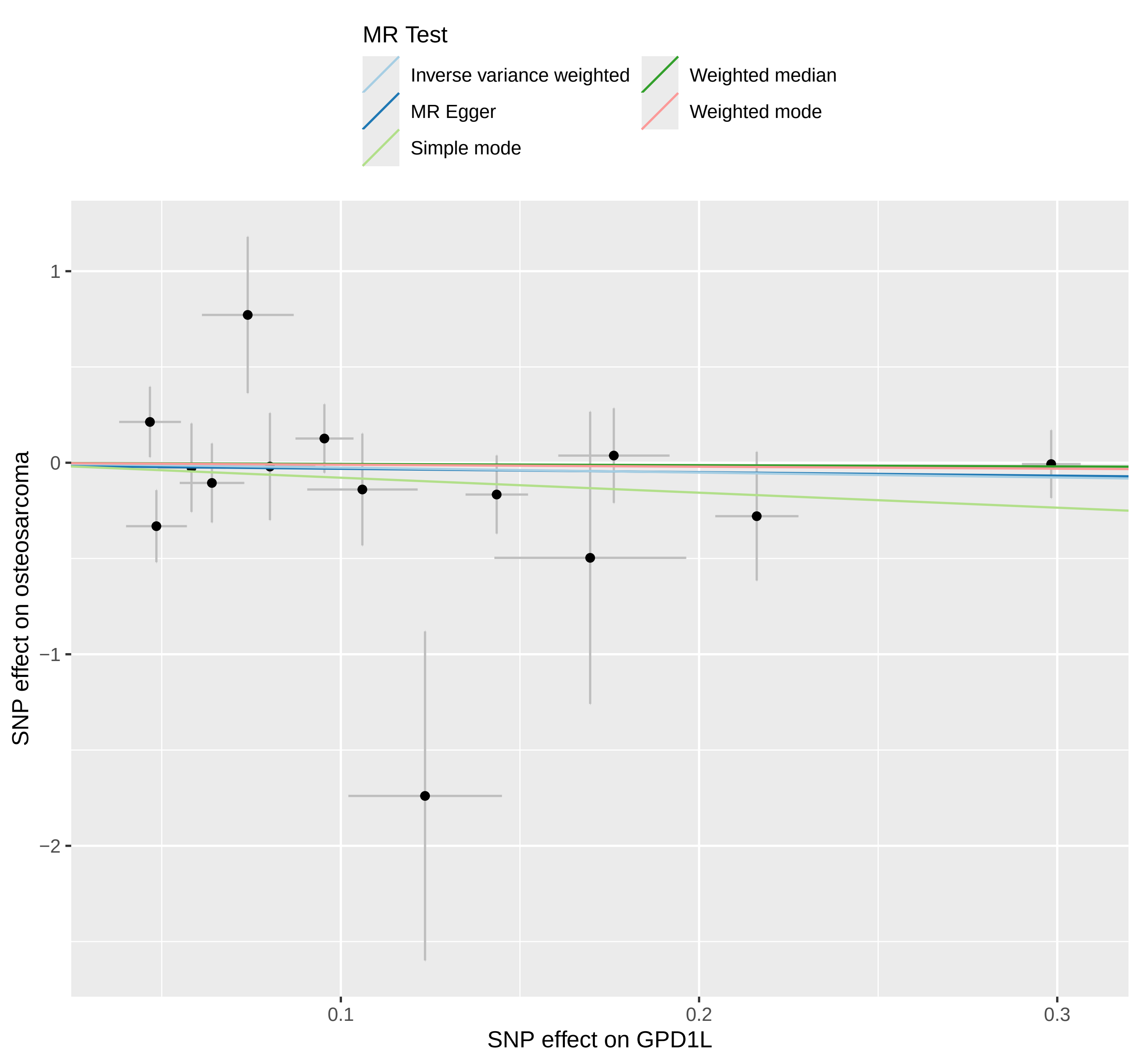

MR Test
Inverse variance weighted
MR Egger
Simple mode
Weighted median
Weighted mode
1
0
−1
−2
0.1
0.2
0.3
SNP effect on osteosarcoma
SNP effect on GPD1L

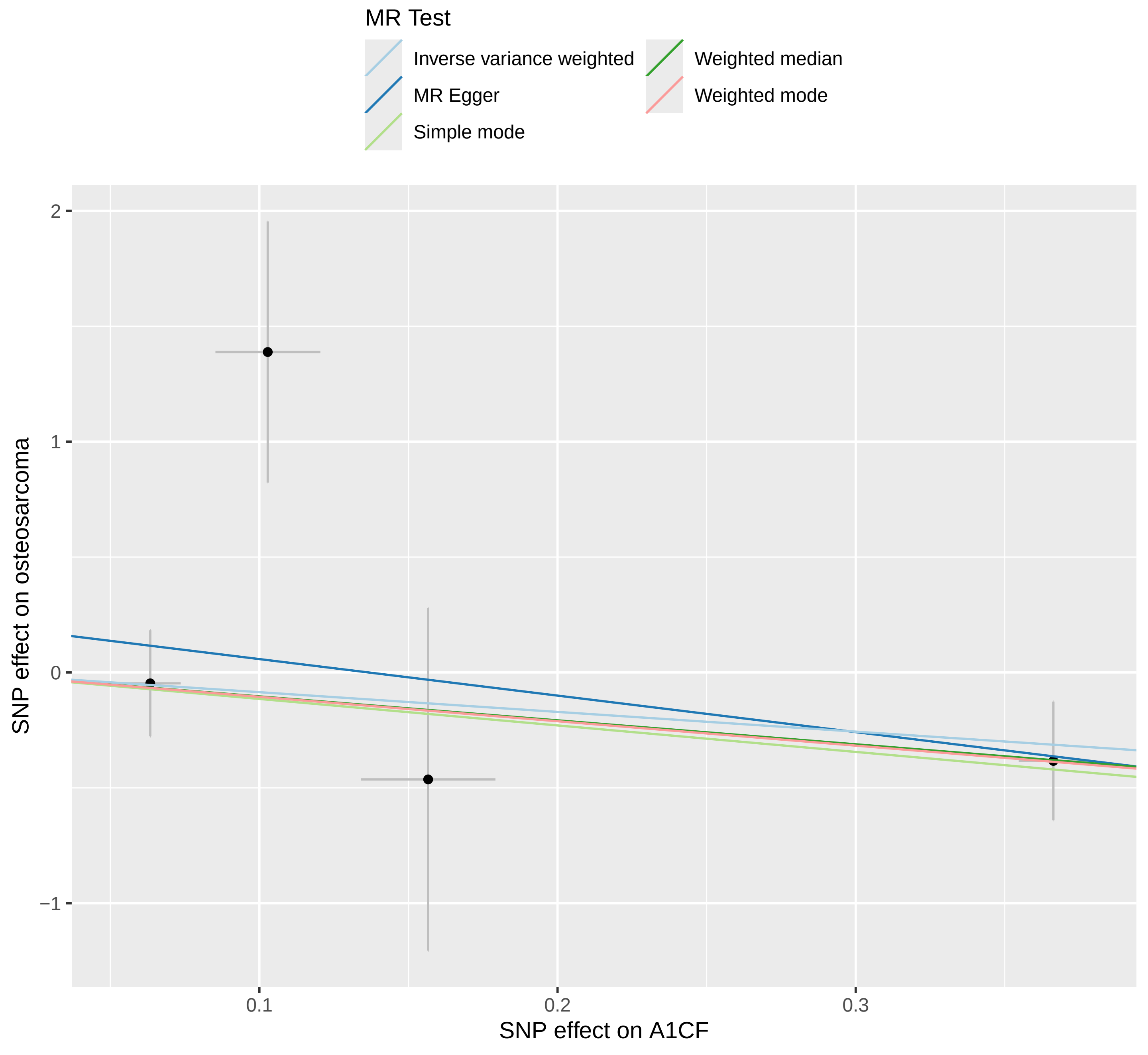
MR Test
Inverse variance weighted
MR Egger
Simple mode
Weighted median
Weighted mode
SNP effect on osteosarcoma
2
1
0
−1
0.1
0.2
0.3
SNP effect on A1CF

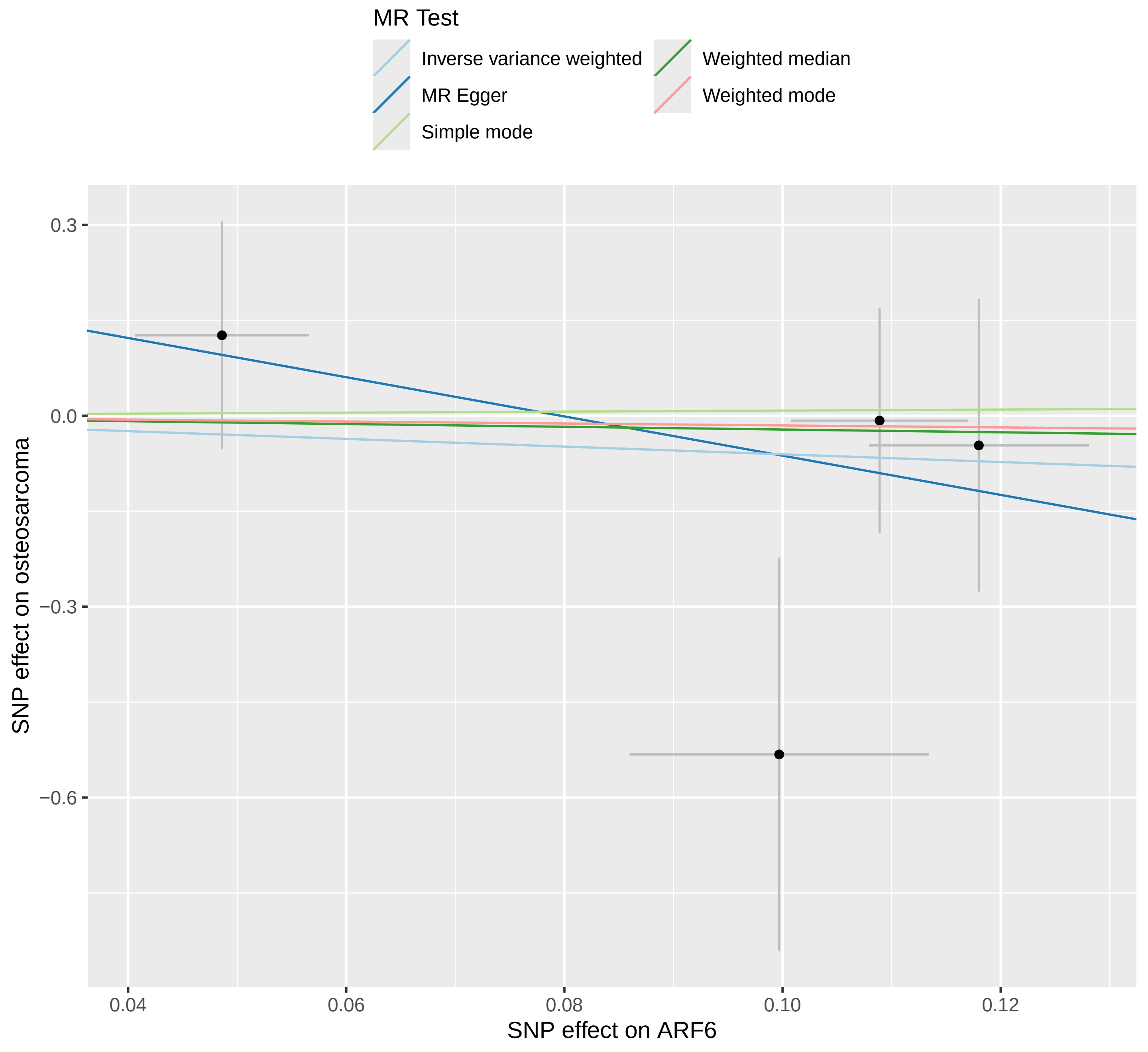
MR Test
Inverse variance weighted
MR Egger
Simple mode
Weighted median
Weighted mode
0.3
0.0
−0.3
−0.6
SNP effect on osteosarcoma
0.04
0.06
0.08
0.10
0.12
SNP effect on ARF6

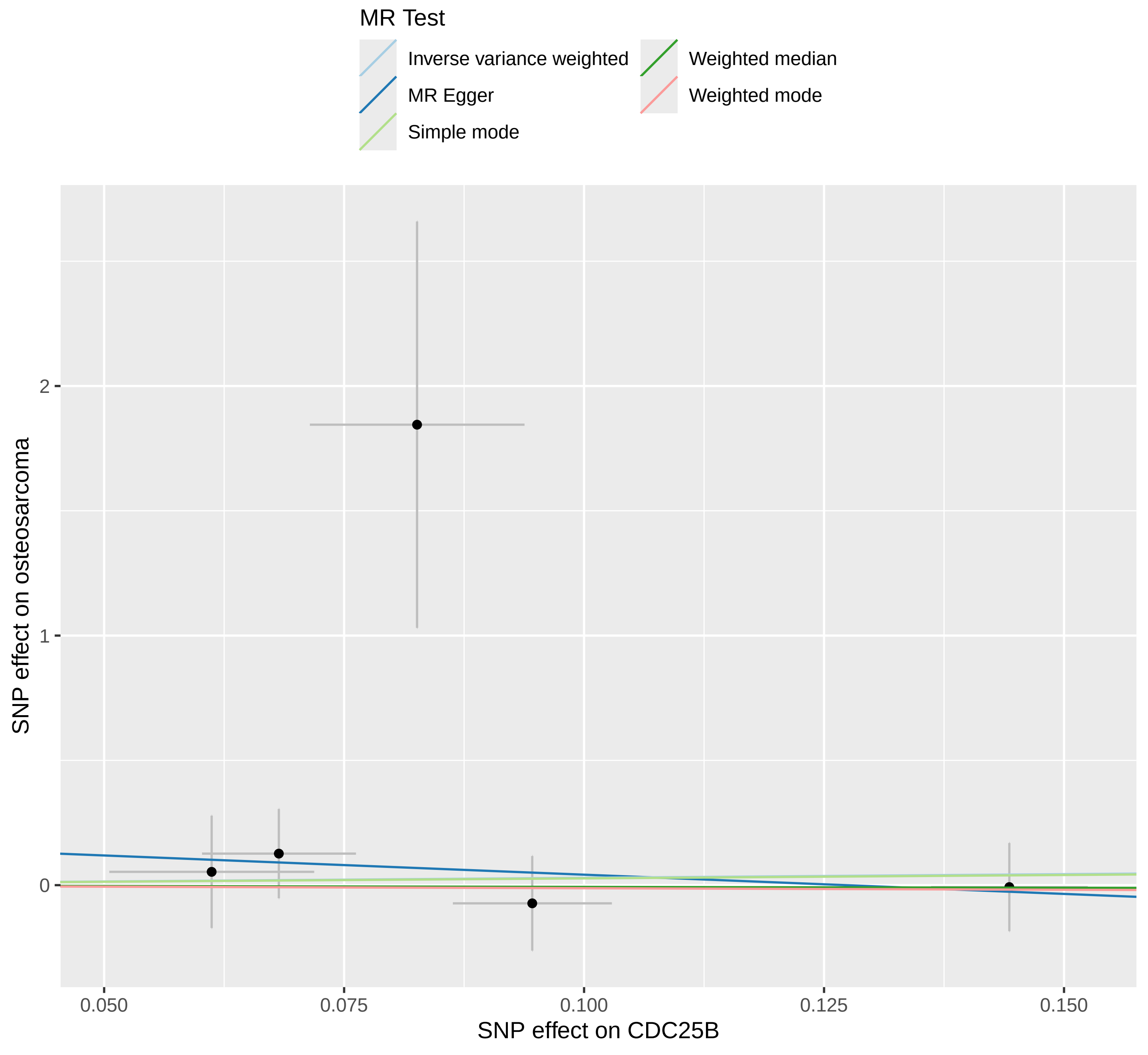

MR Test
Inverse variance weighted
MR Egger
Simple mode
Weighted median
Weighted mode
SNP effect on osteosarcoma
2
1
0
0.050
0.075
0.100
0.125
0.150
SNP effect on CDC25B

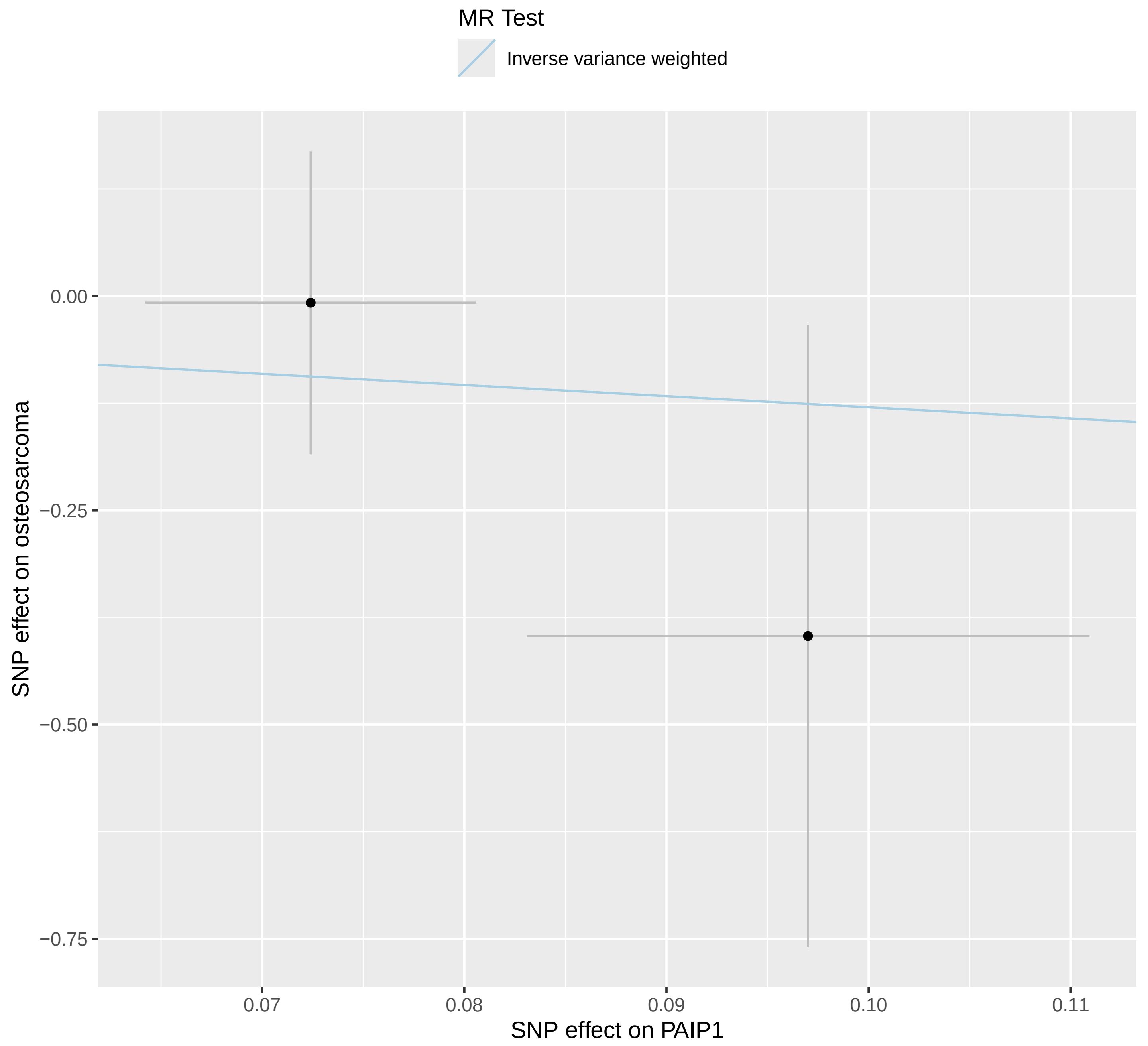

MR Test
Inverse variance weighted
0.00
−0.25
−0.50
−0.75
SNP effect on osteosarcoma
0.07
0.08
0.09
0.10
0.11
SNP effect on PAIP1

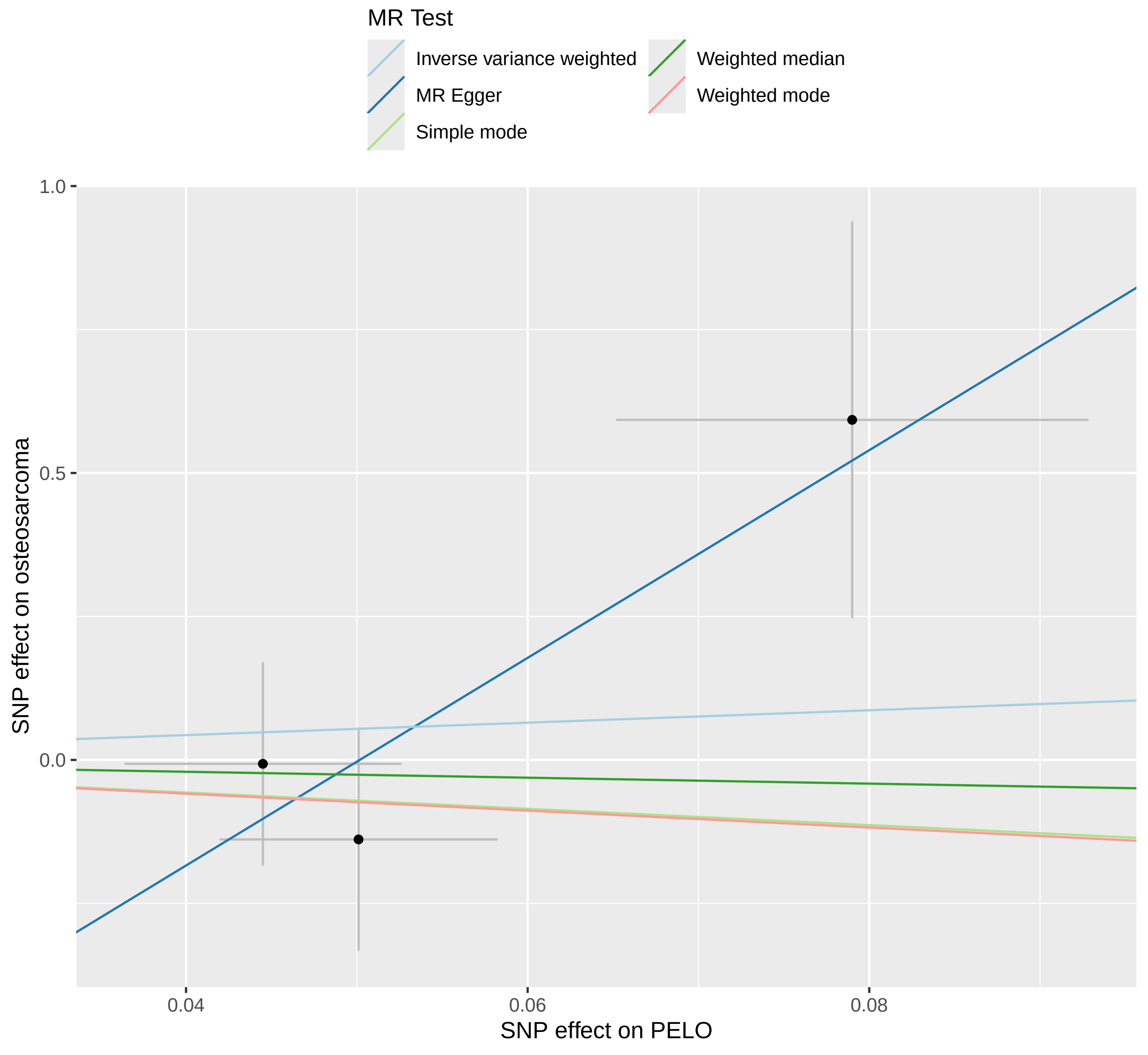
MR Test
Inverse variance weighted
MR Egger
Simple mode
Weighted median
Weighted mode
1.0
0.5
0.0
SNP effect on osteosarcoma
0.04
0.06
0.08
SNP effect on PELO

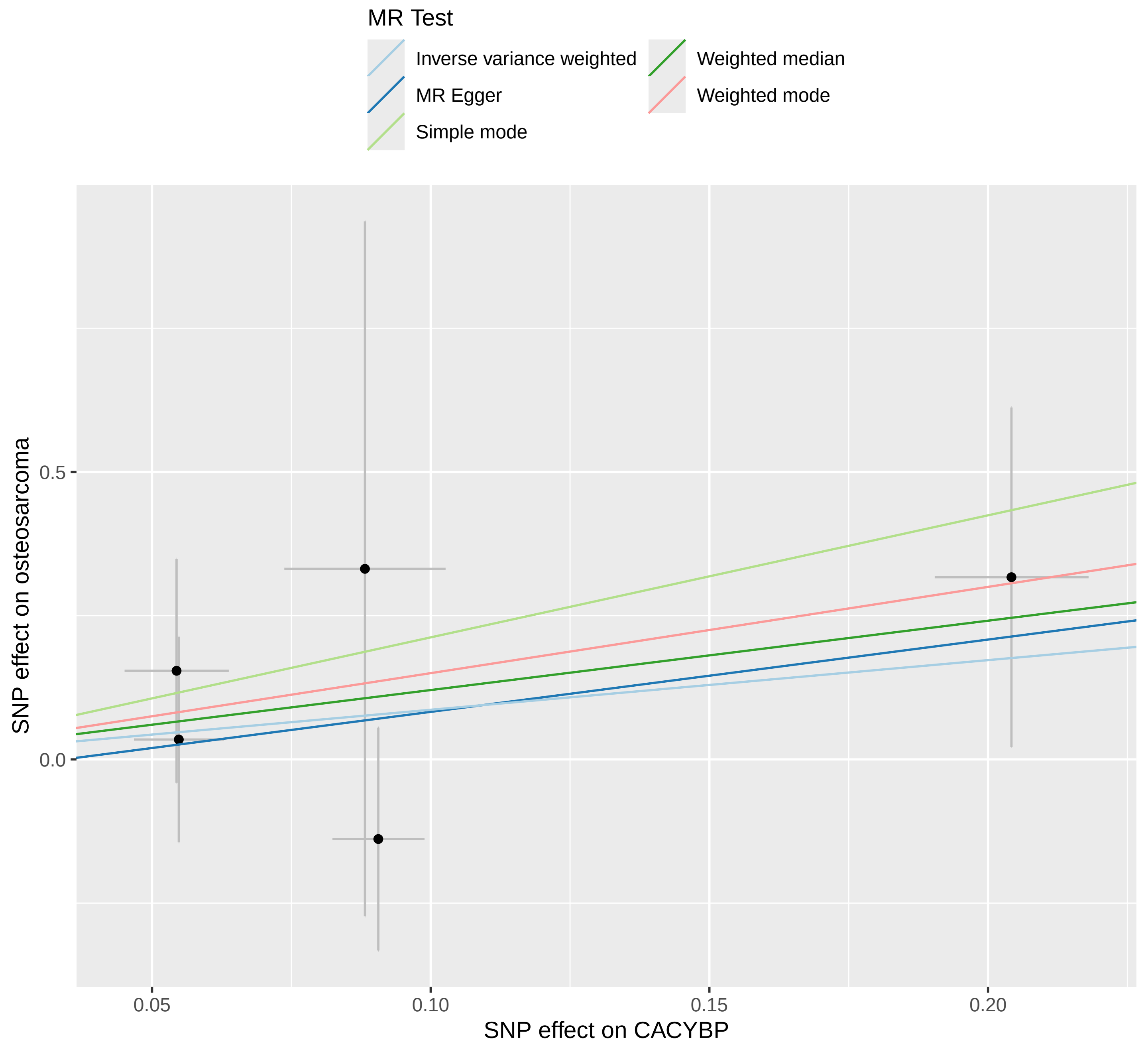

MR Test
Inverse variance weighted
MR Egger
Simple mode
Weighted median
Weighted mode
SNP effect on osteosarcoma
0.5
0.0
0.05
0.10
0.15
0.20
SNP effect on CACYBP

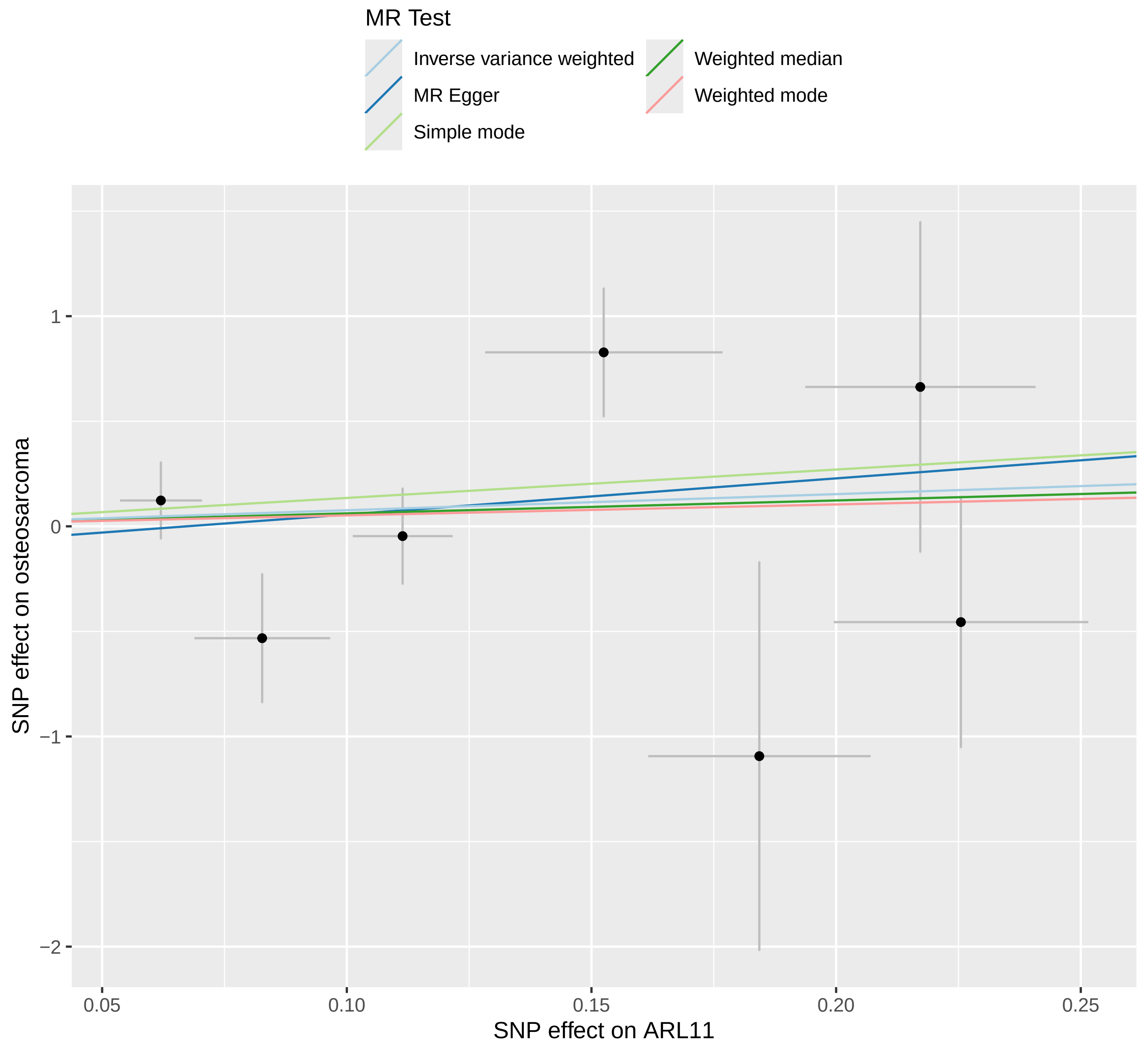
MR Test
Inverse variance weighted
MR Egger
Simple mode
Weighted median
Weighted mode
SNP effect on osteosarcoma
1
0
−1
−2
0.05
0.10
0.15
0.20
0.25
SNP effect on ARL11

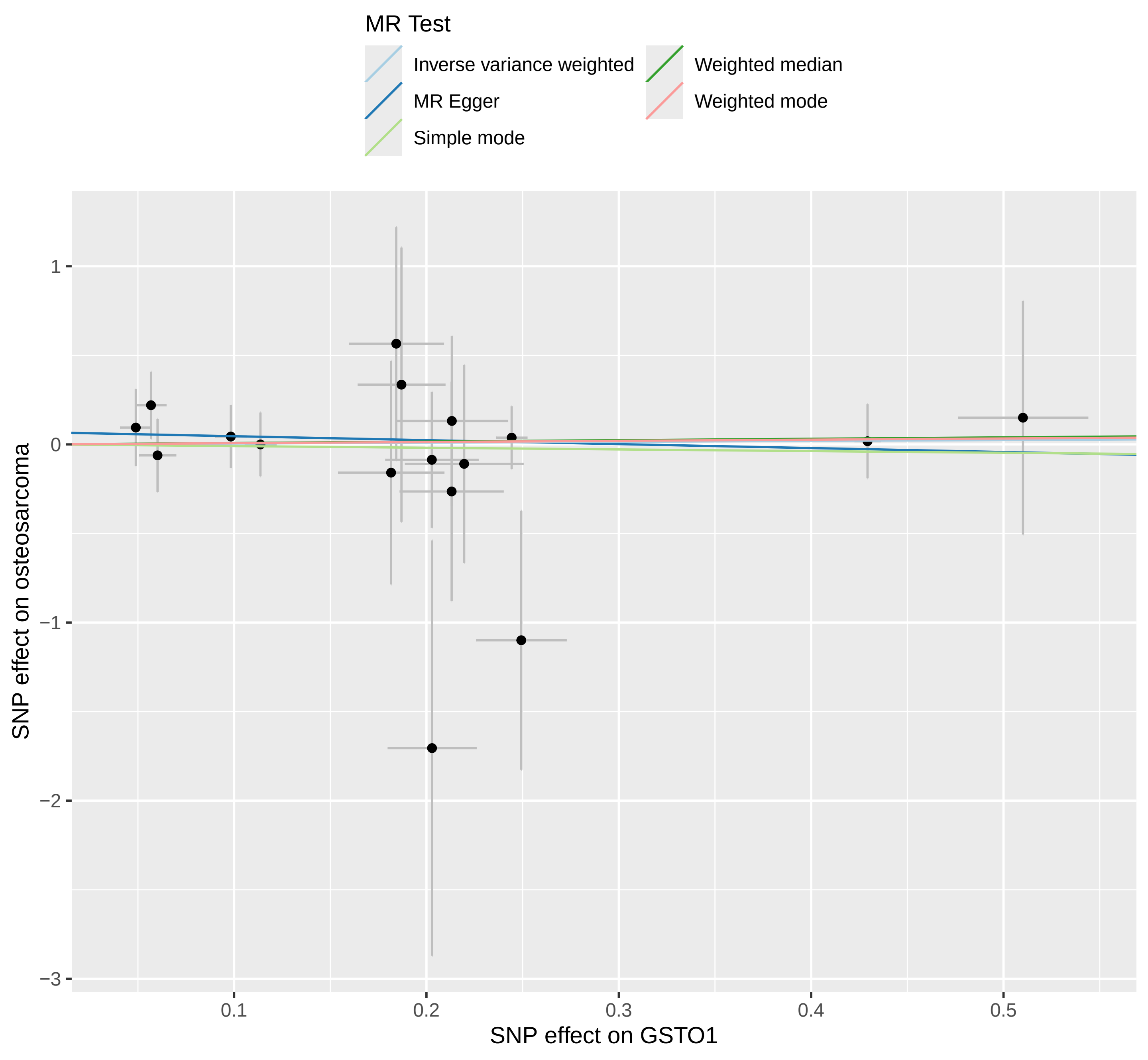
MR Test
Inverse variance weighted
MR Egger
Simple mode
Weighted median
Weighted mode
1
0
−1
−2
−3
0.1
0.2
0.3
0.4
0.5
SNP effect on osteosarcoma
SNP effect on GSTO1

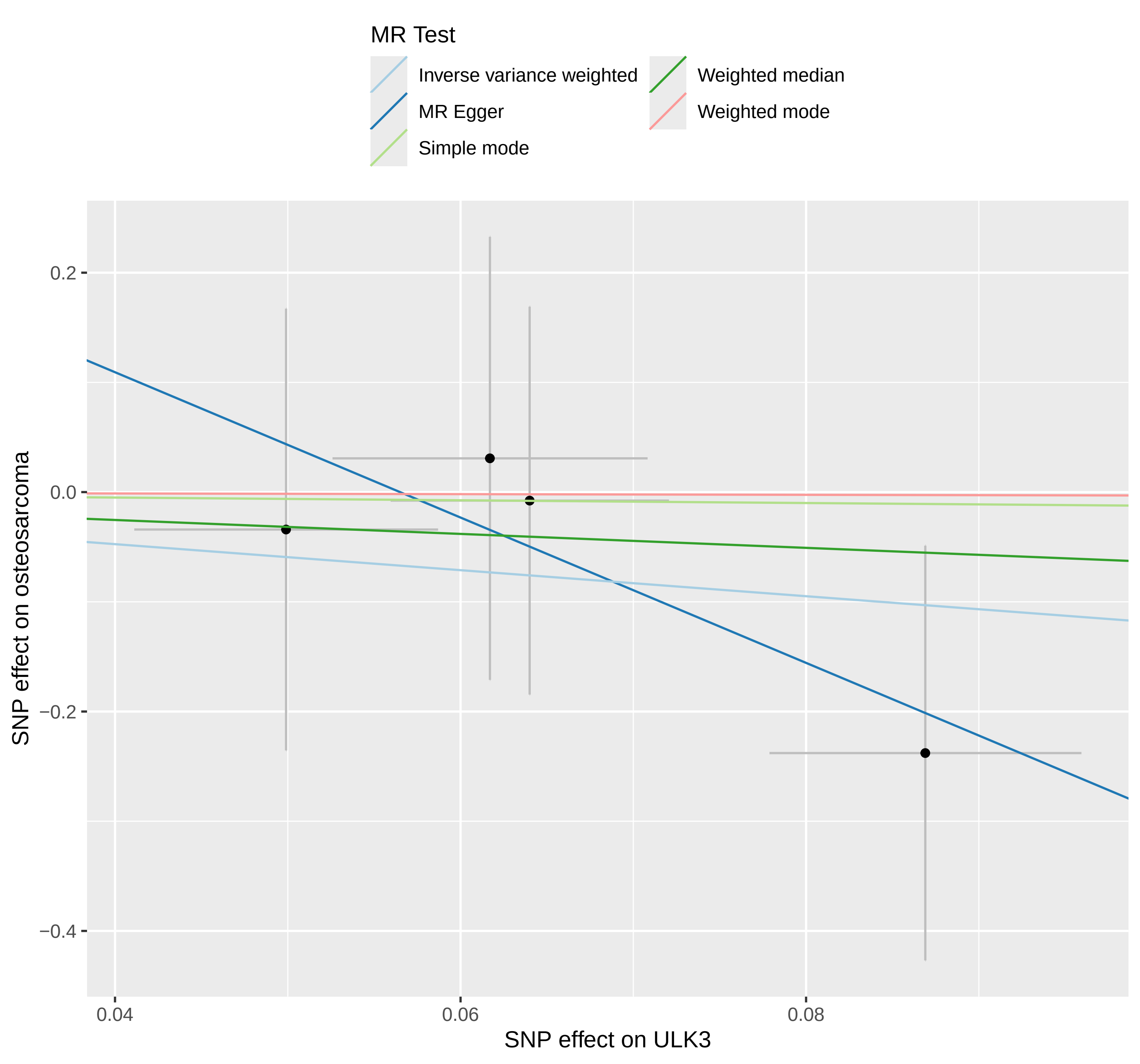
MR Test
Inverse variance weighted
MR Egger
Simple mode
Weighted median
Weighted mode
0.2
0.0
−0.2
−0.4
SNP effect on osteosarcoma
0.04
0.06
0.08
SNP effect on ULK3

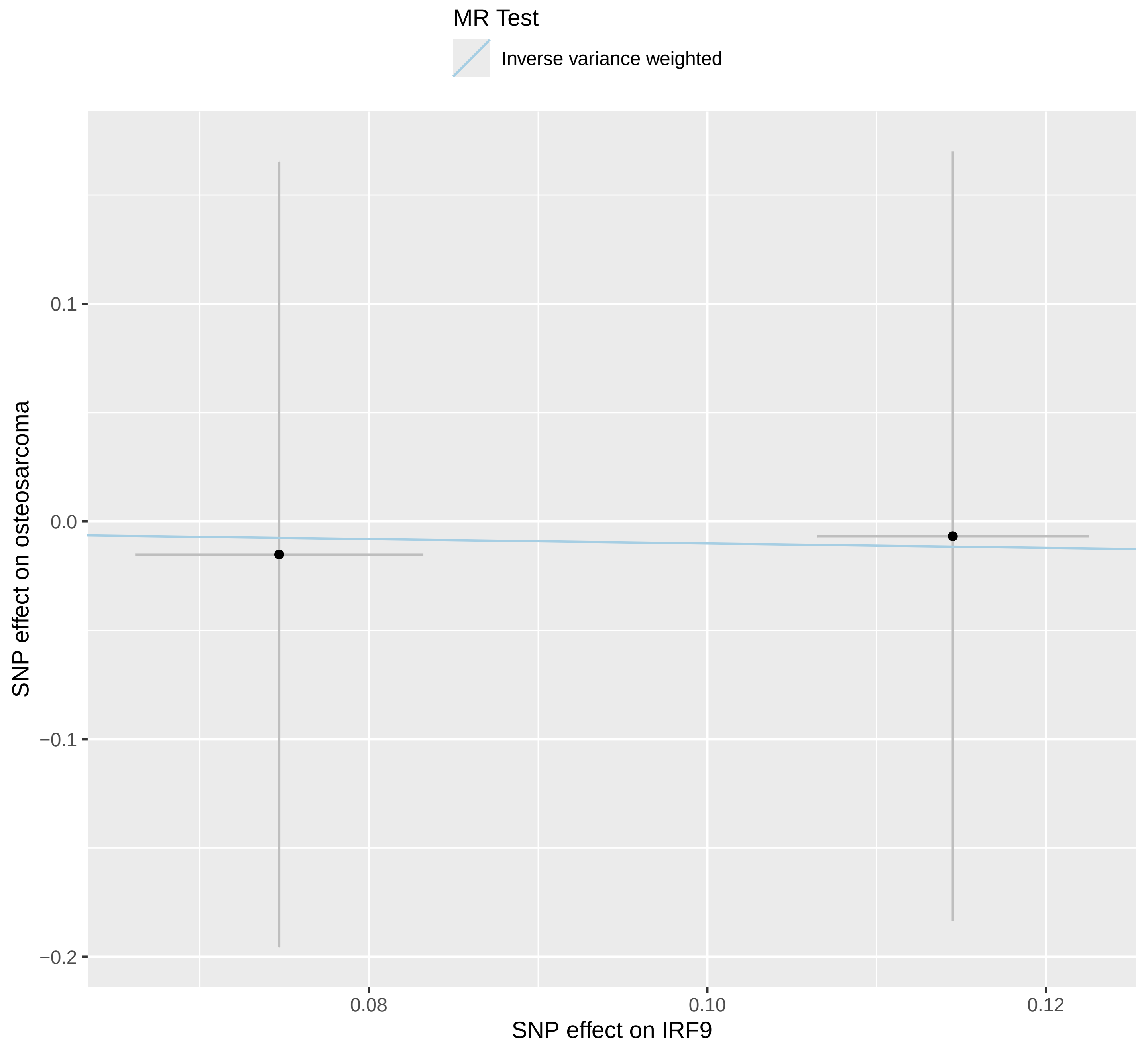

MR Test
Inverse variance weighted
0.1
0.0
−0.1
−0.2
SNP effect on osteosarcoma
0.08
0.10
0.12
SNP effect on IRF9

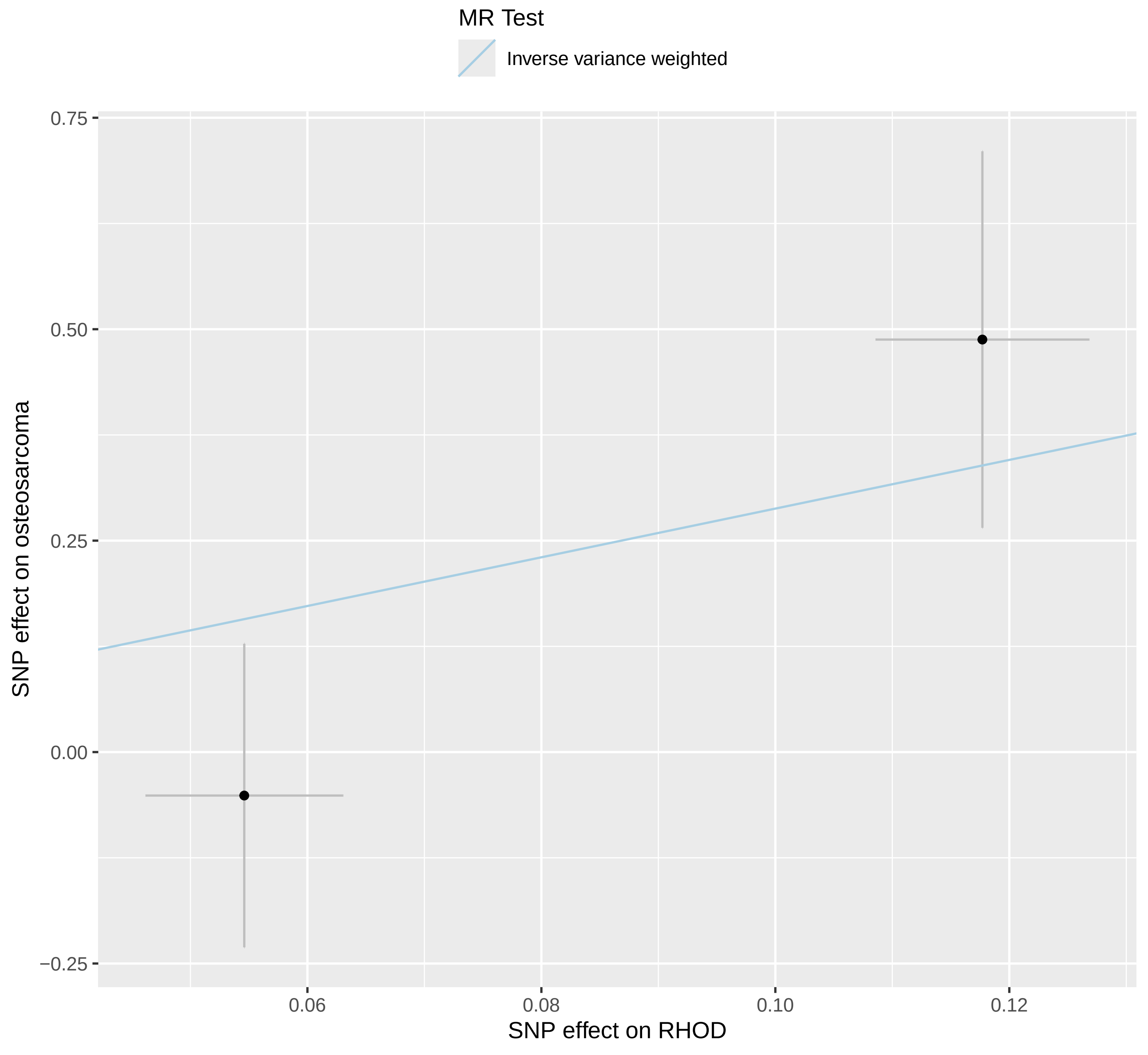
MR Test
Inverse variance weighted
0.75
0.50
0.25
0.00
−0.25
SNP effect on osteosarcoma
0.06
0.08
0.10
0.12
SNP effect on RHOD

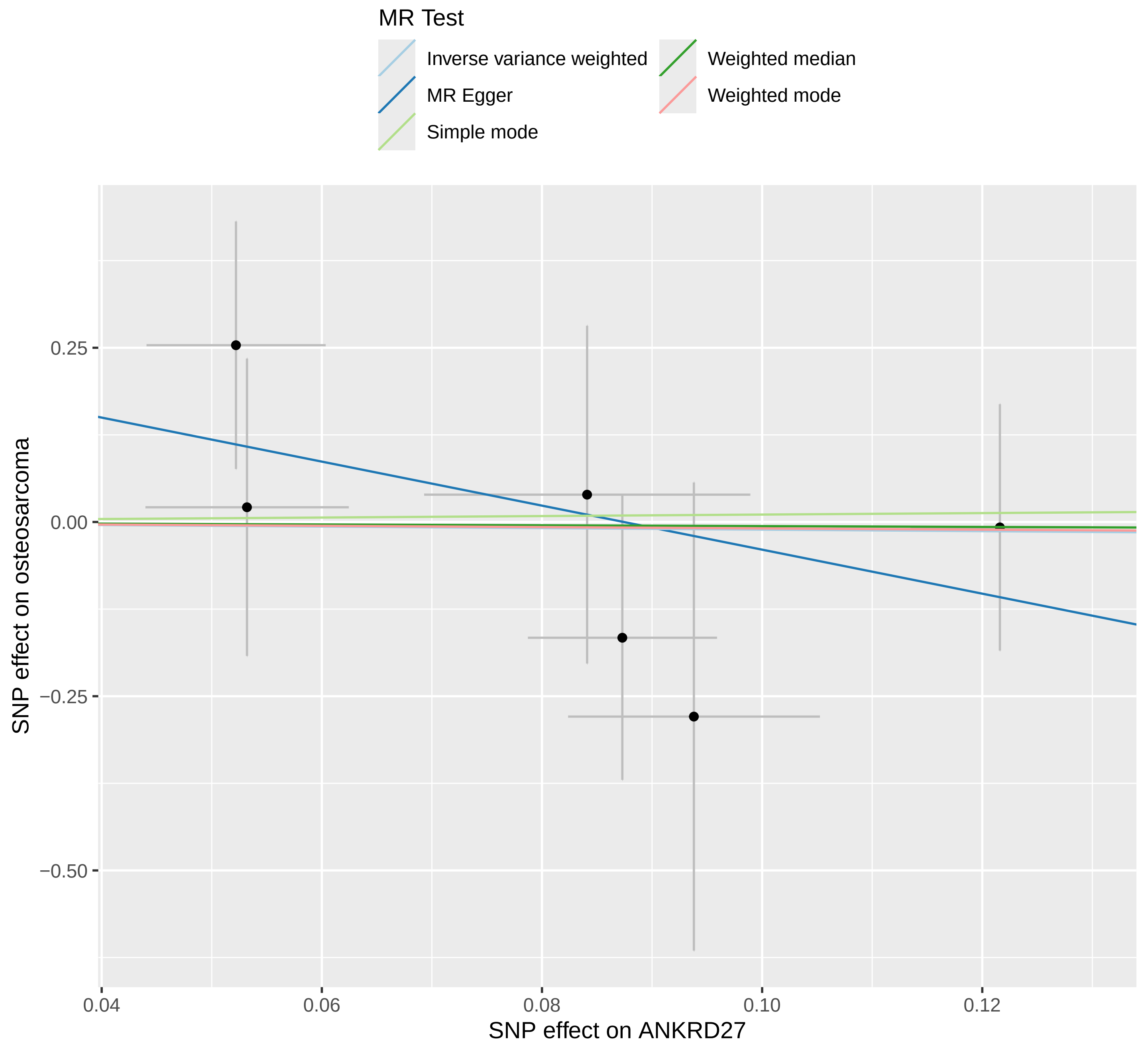
MR Test
Inverse variance weighted
MR Egger
Simple mode
Weighted median
Weighted mode
0.25
0.00
−0.25
−0.50
SNP effect on osteosarcoma
0.04
0.06
0.08
0.10
0.12
SNP effect on ANKRD27

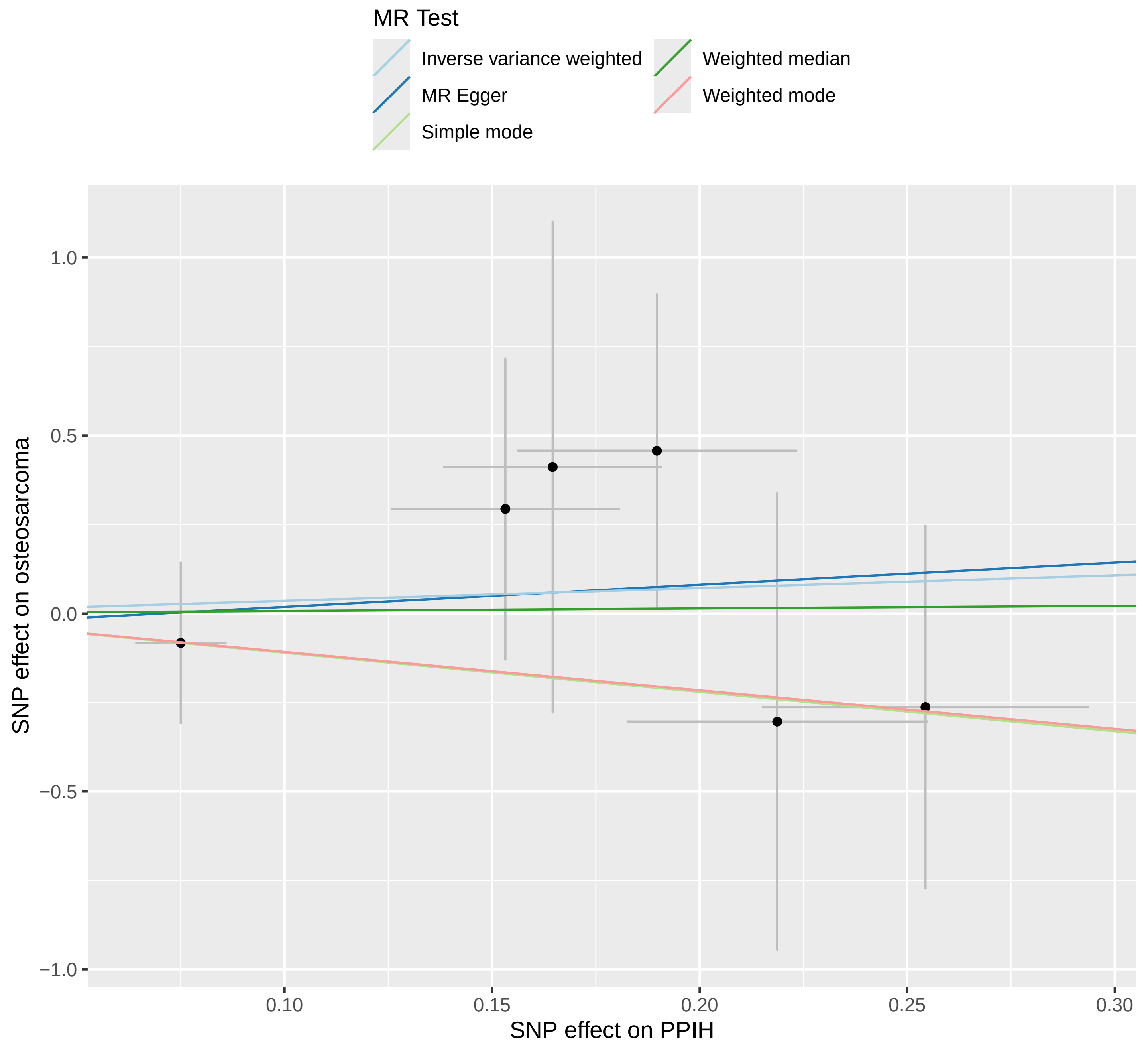
MR Test
Inverse variance weighted
MR Egger
Simple mode
Weighted median
Weighted mode
1.0
0.5
0.0
−0.5
−1.0
SNP effect on osteosarcoma
0.10
0.15
0.20
0.25
0.30
SNP effect on PPIH

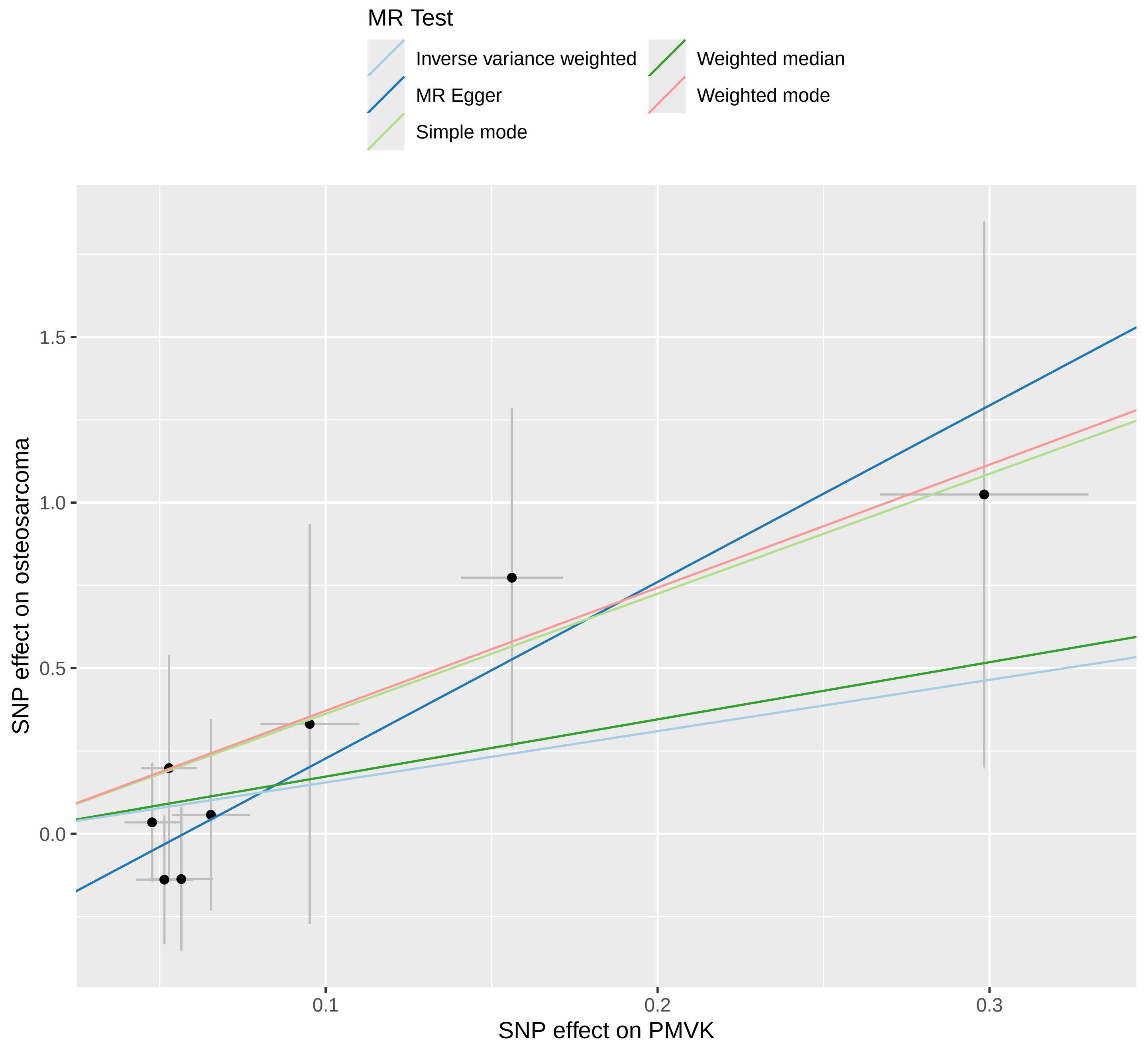
MR Test
Inverse variance weighted
MR Egger
Simple mode
Weighted median
Weighted mode
SNP effect on osteosarcoma
1.5
1.0
0.5
0.0
0.1
0.2
0.3
SNP effect on PMVK

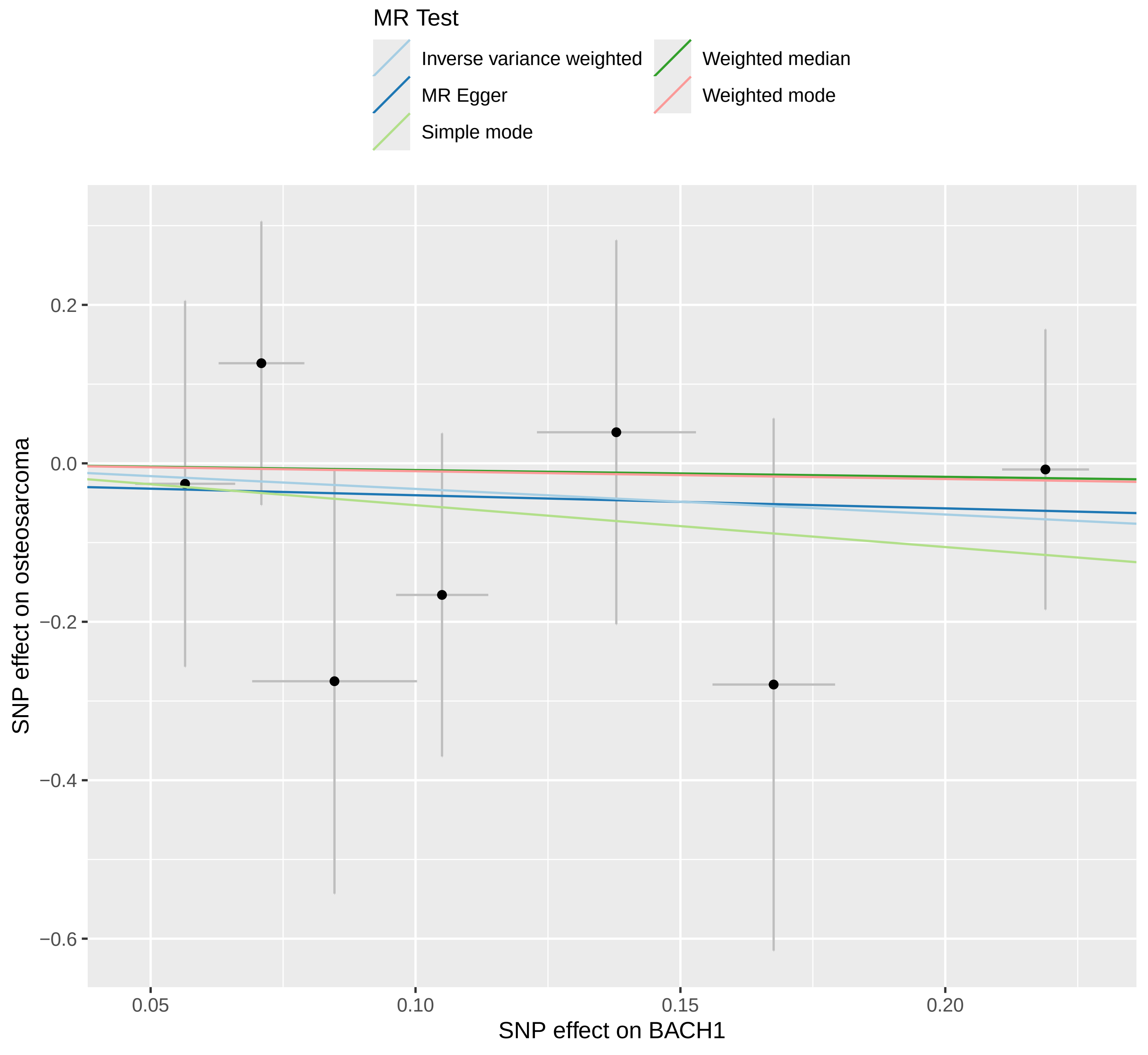
MR Test
Inverse variance weighted
MR Egger
Simple mode
Weighted median
Weighted mode
0.2
0.0
−0.2
−0.4
−0.6
SNP effect on osteosarcoma
0.05
0.10
0.15
0.20
SNP effect on BACH1

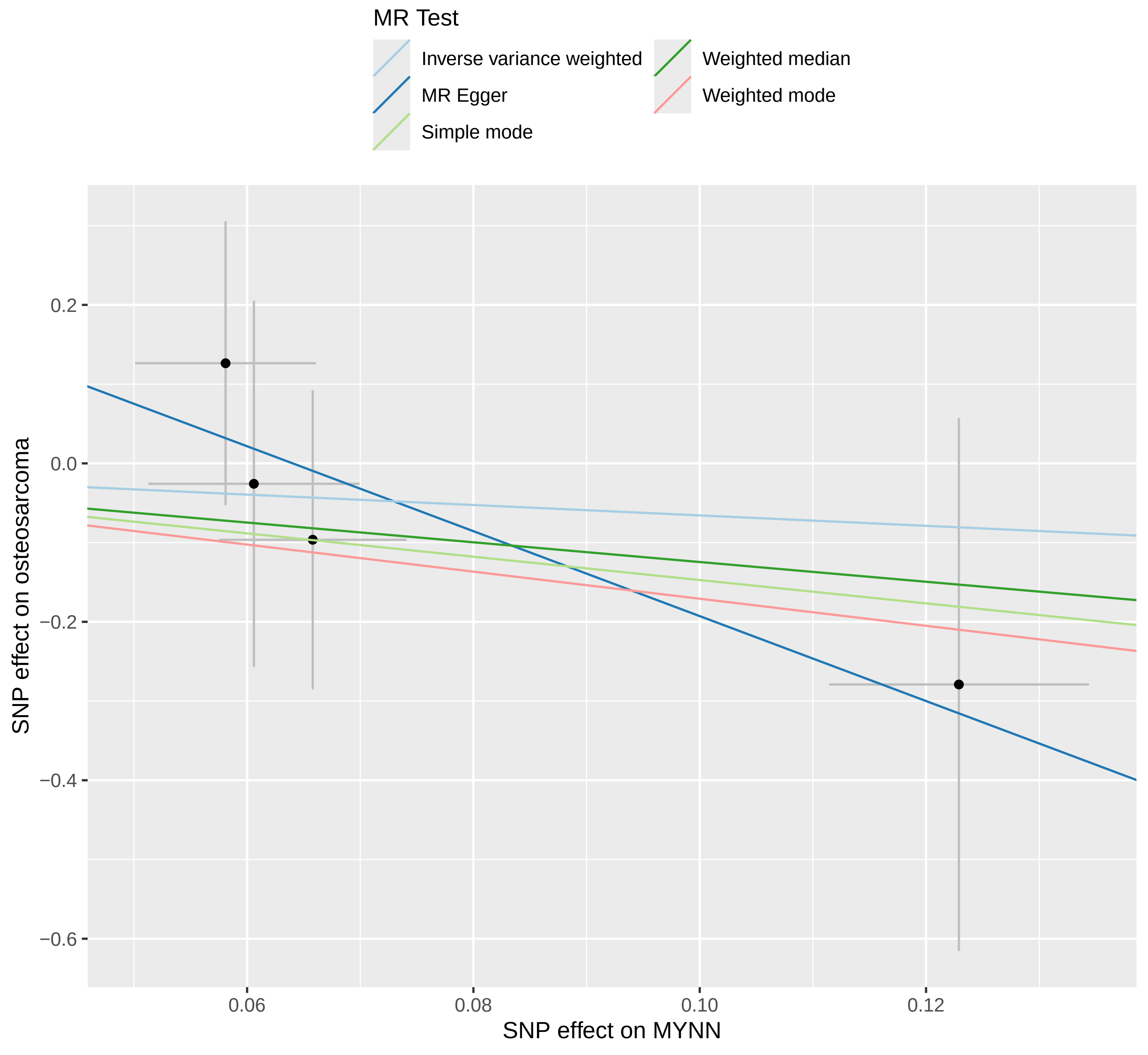

MR Test
Inverse variance weighted
MR Egger
Simple mode
Weighted median
Weighted mode
0.2
0.0
−0.2
−0.4
−0.6
SNP effect on osteosarcoma
0.06
0.08
0.10
0.12
SNP effect on MYNN

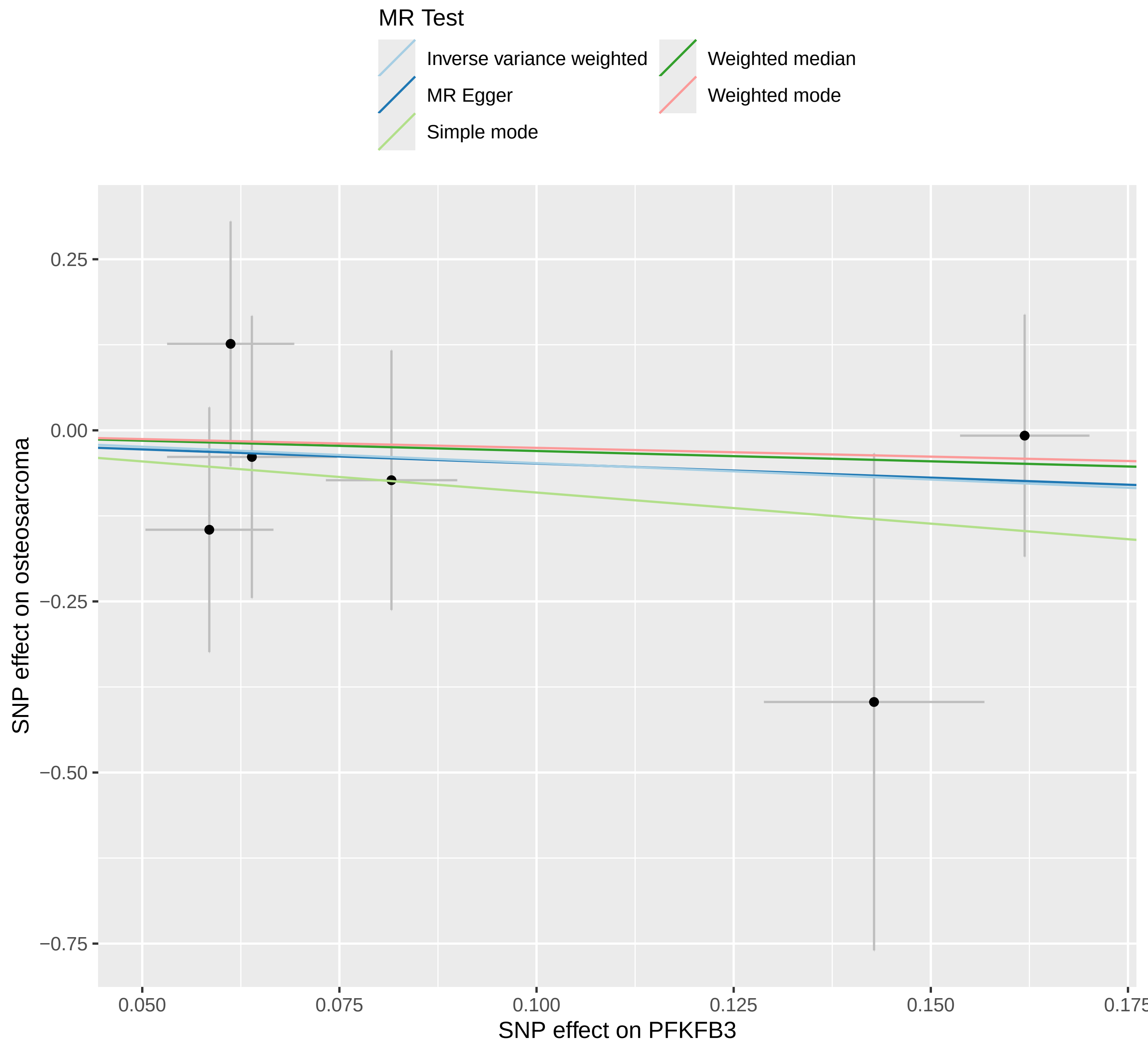
MR Test
Inverse variance weighted
MR Egger
Simple mode
Weighted median
Weighted mode
0.25
0.00
−0.25
−0.50
−0.75
0.050
0.075
0.100
0.125
0.150
0.175
SNP effect on PFKFB3
SNP effect on osteosarcoma

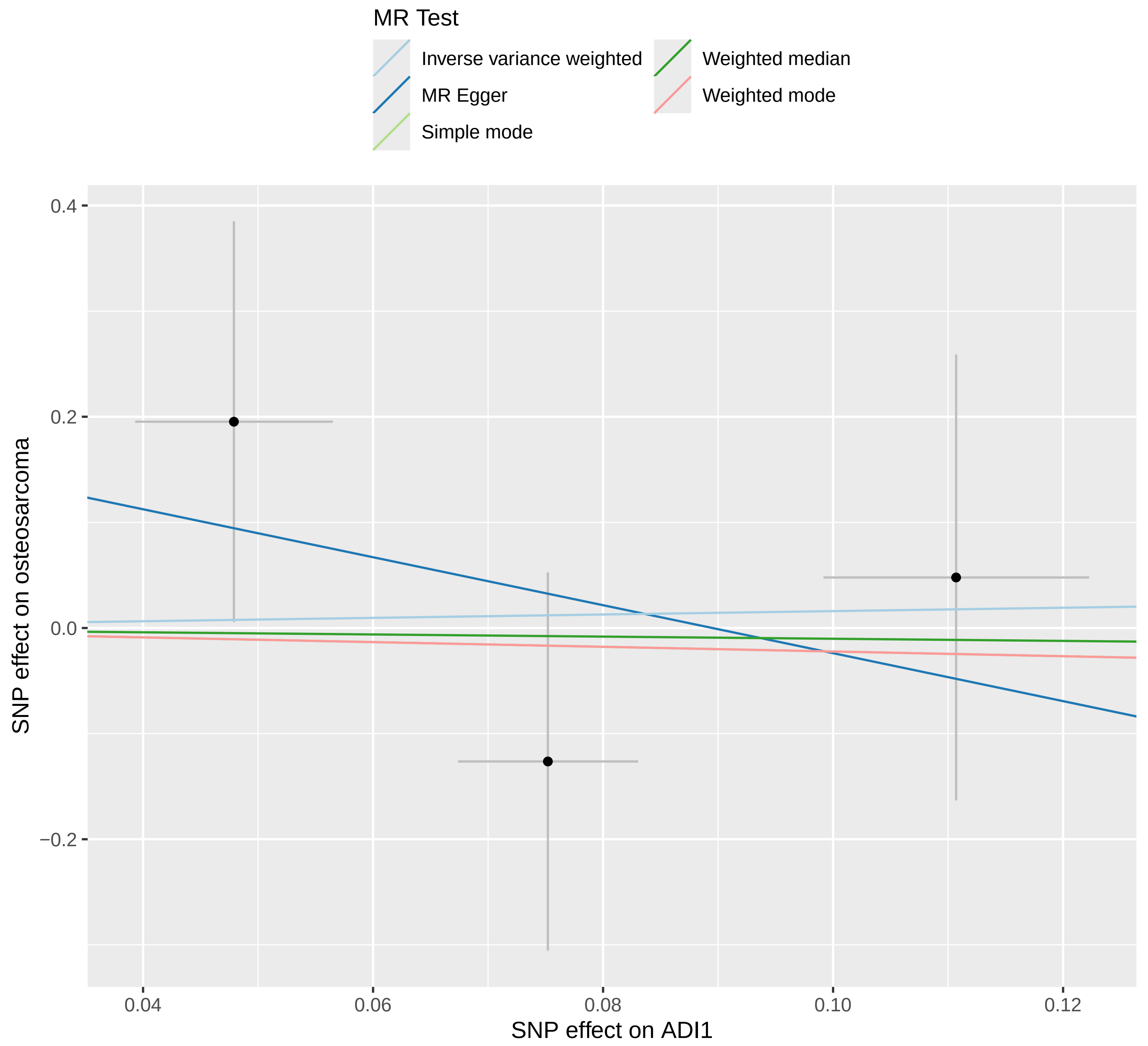
MR Test
Inverse variance weighted
MR Egger
Simple mode
Weighted median
Weighted mode
0.4
0.2
0.0
−0.2
SNP effect on osteosarcoma
0.04
0.06
0.08
0.10
0.12
SNP effect on ADI1

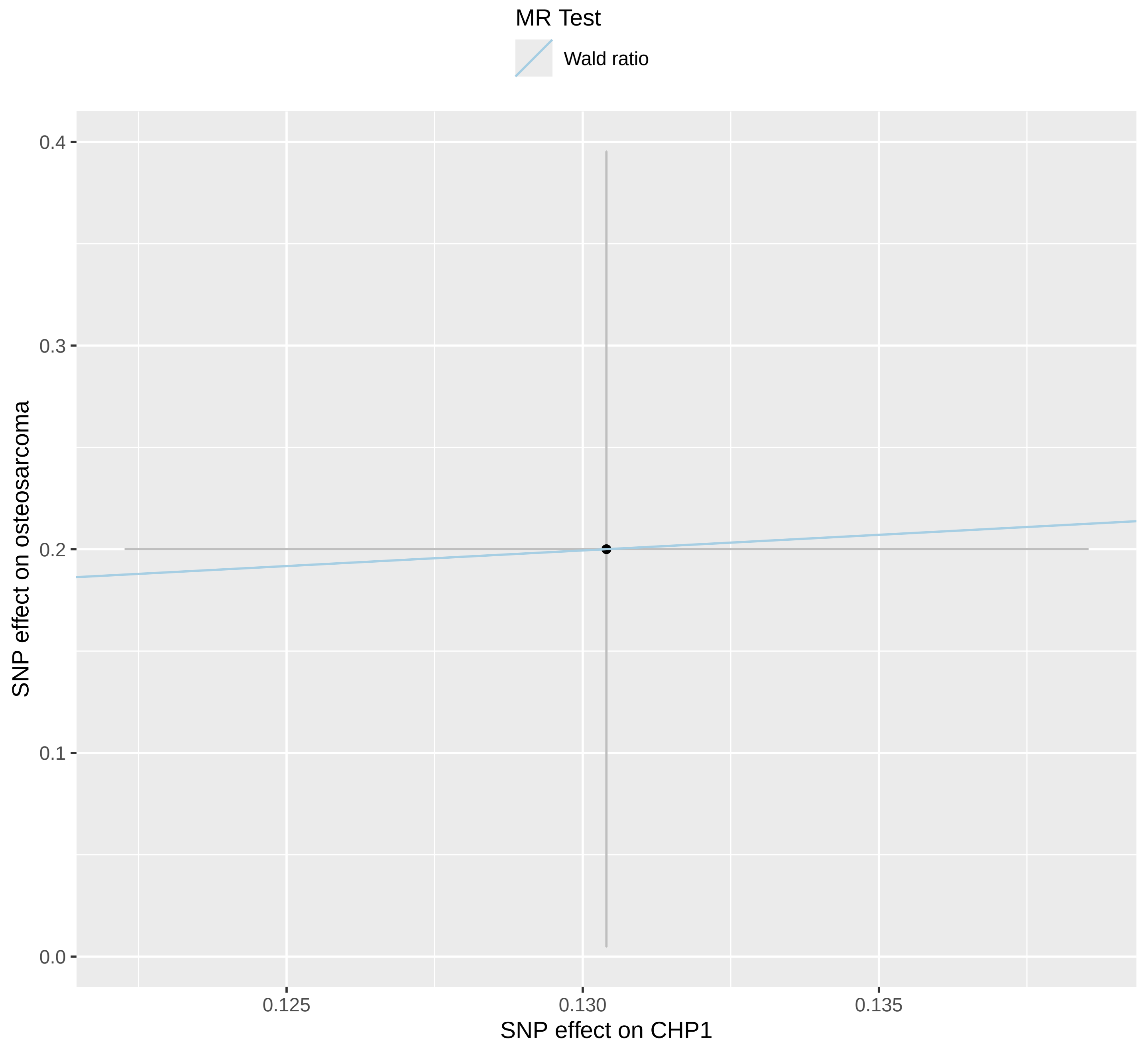
MR Test
Wald ratio
SNP effect on osteosarcoma
0.4
0.3
0.2
0.1
0.0
0.125
0.130
0.135
SNP effect on CHP1

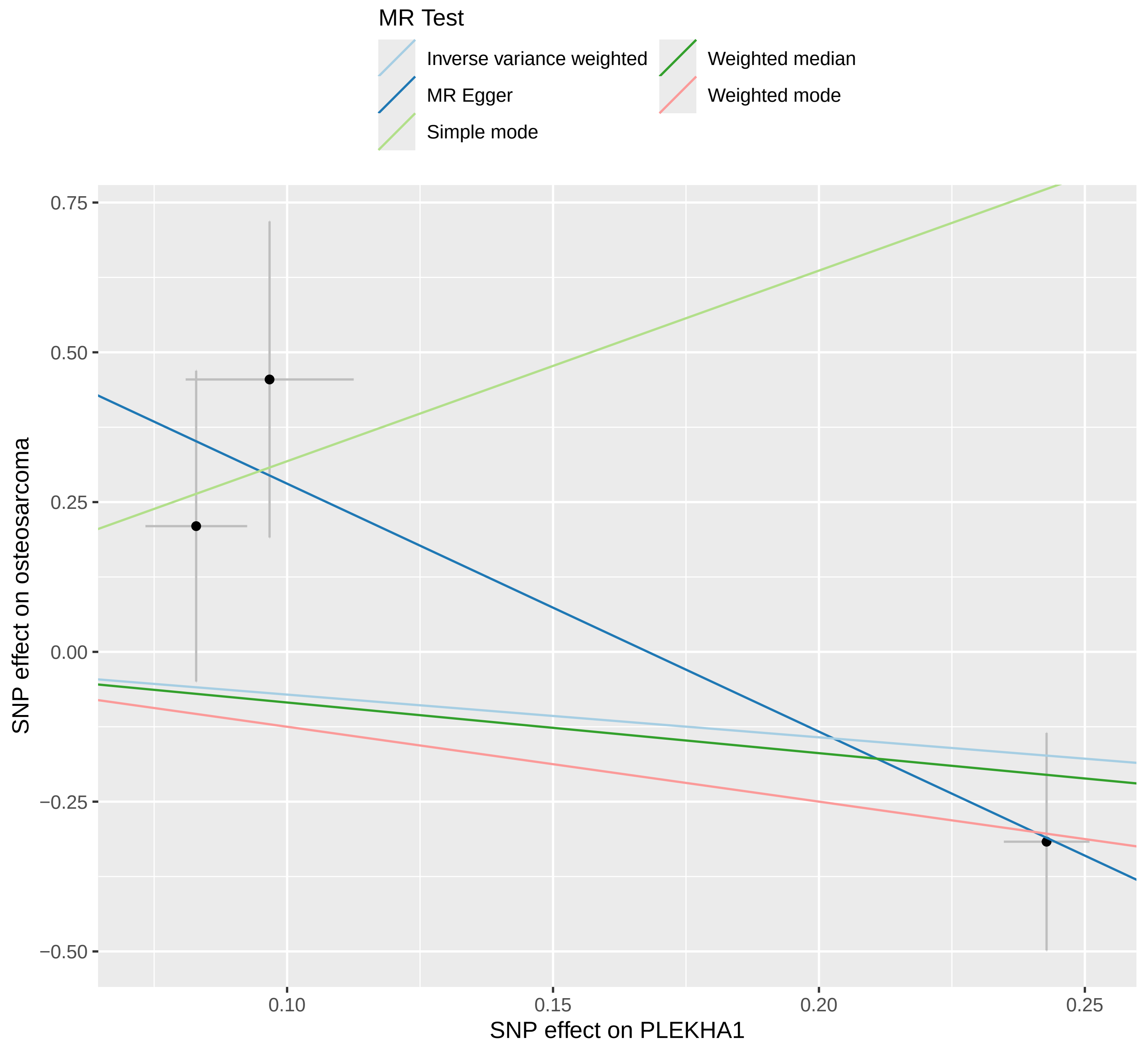
MR Test
Inverse variance weighted
MR Egger
Simple mode
Weighted median
Weighted mode
0.75
0.50
0.25
0.00
−0.25
−0.50
SNP effect on osteosarcoma
0.10
0.15
0.20
0.25
SNP effect on PLEKHA1

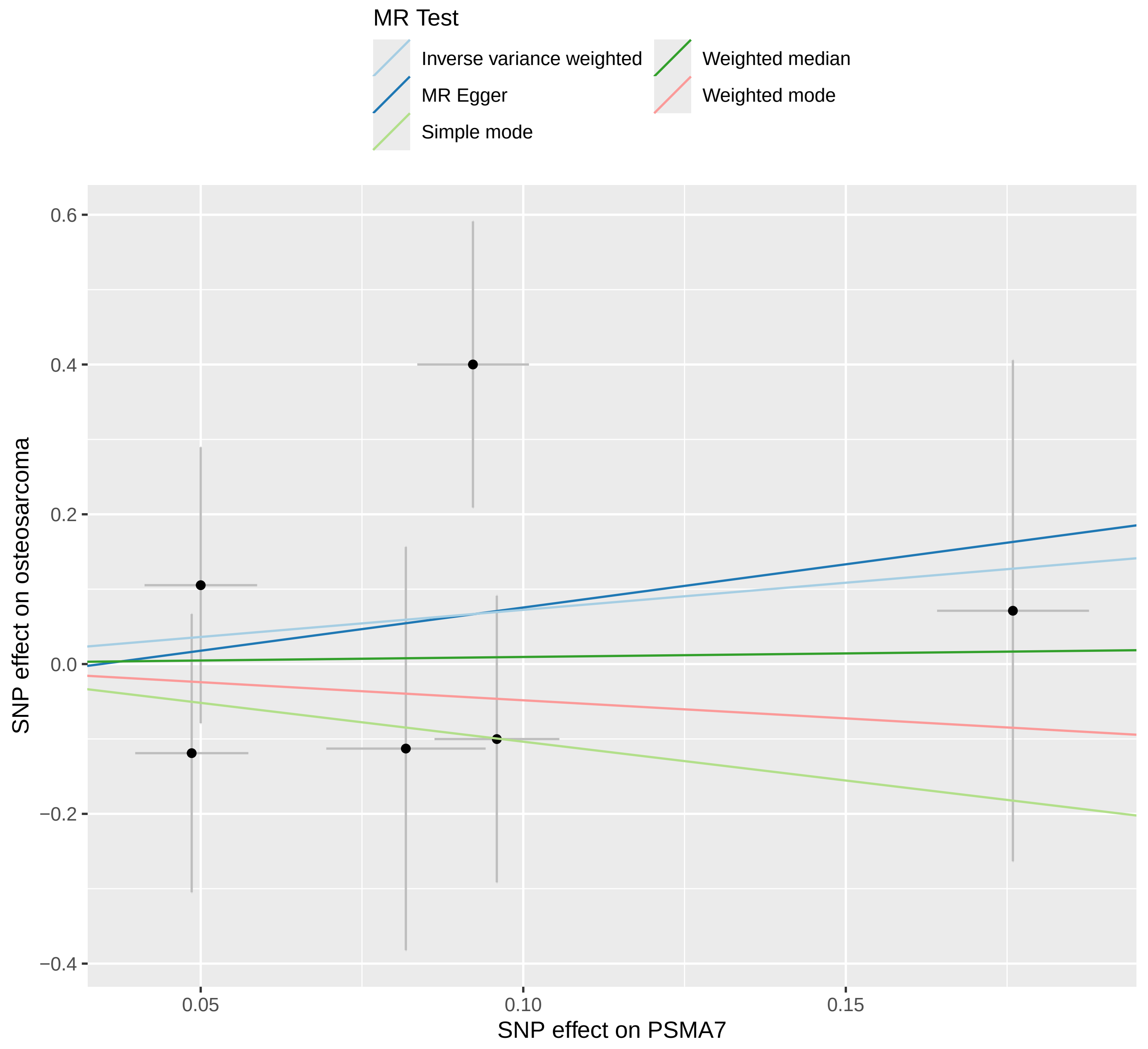

MR Test
Inverse variance weighted
MR Egger
Simple mode
Weighted median
Weighted mode
0.6
0.4
0.2
0.0
−0.2
−0.4
SNP effect on osteosarcoma
0.05
0.10
0.15
SNP effect on PSMA7

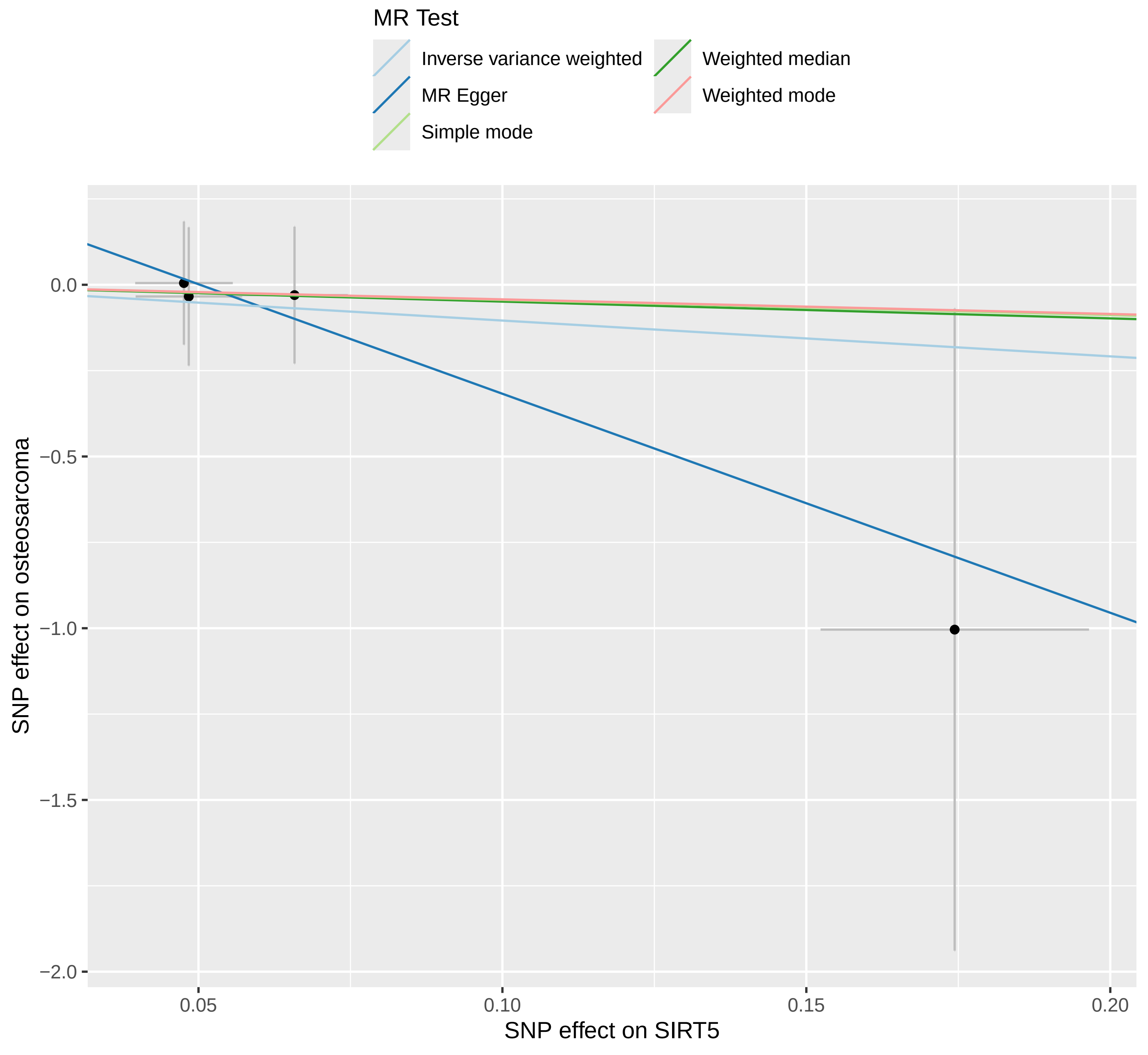
MR Test
Inverse variance weighted
MR Egger
Simple mode
Weighted median
Weighted mode
SNP effect on osteosarcoma
0.0
−0.5
−1.0
−1.5
−2.0
0.05
0.10
0.15
0.20
SNP effect on SIRT5

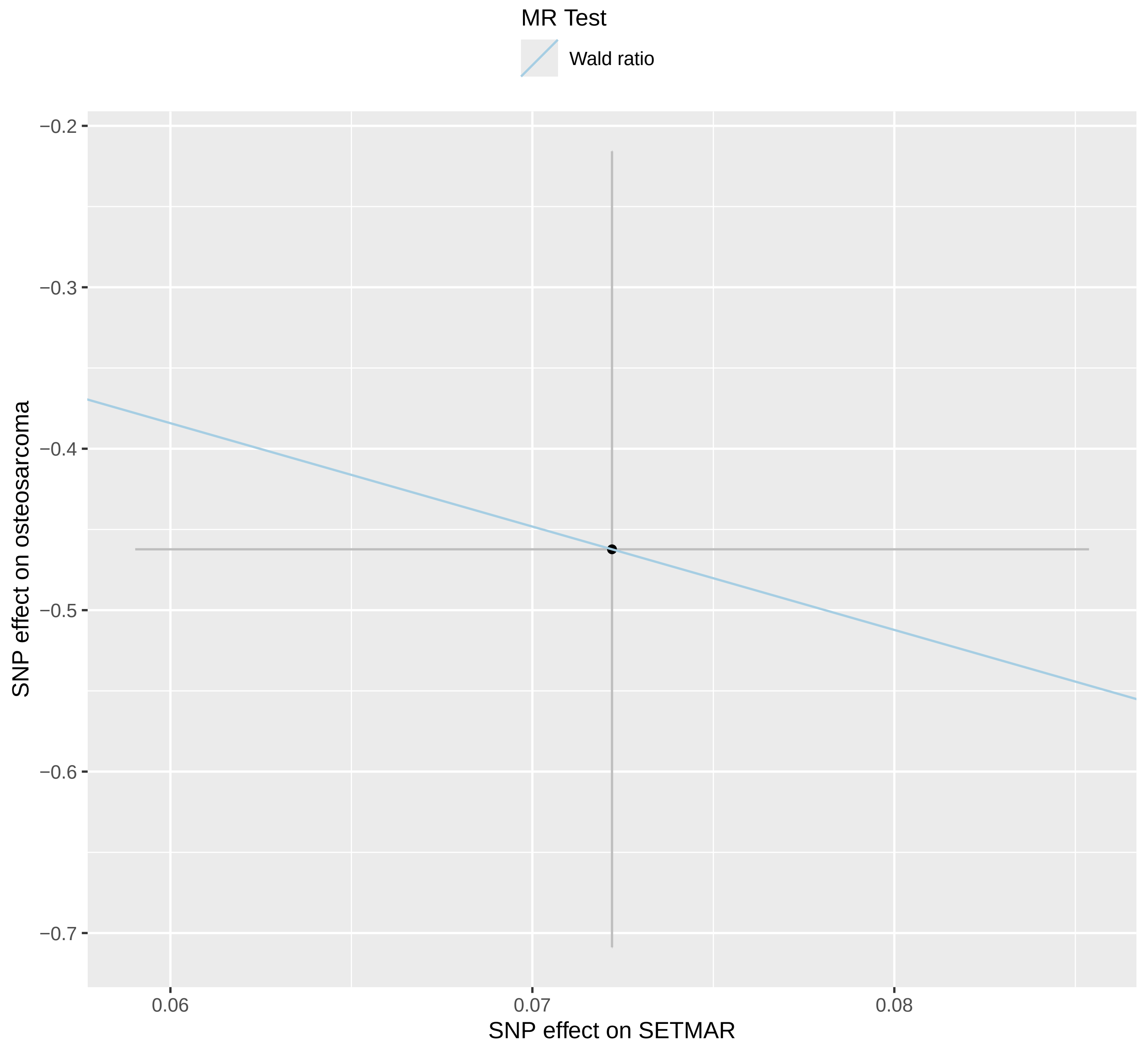

MR Test
Wald ratio
−0.2
−0.3
−0.4
−0.5
−0.6
−0.7
SNP effect on osteosarcoma
0.06
0.07
0.08
SNP effect on SETMAR

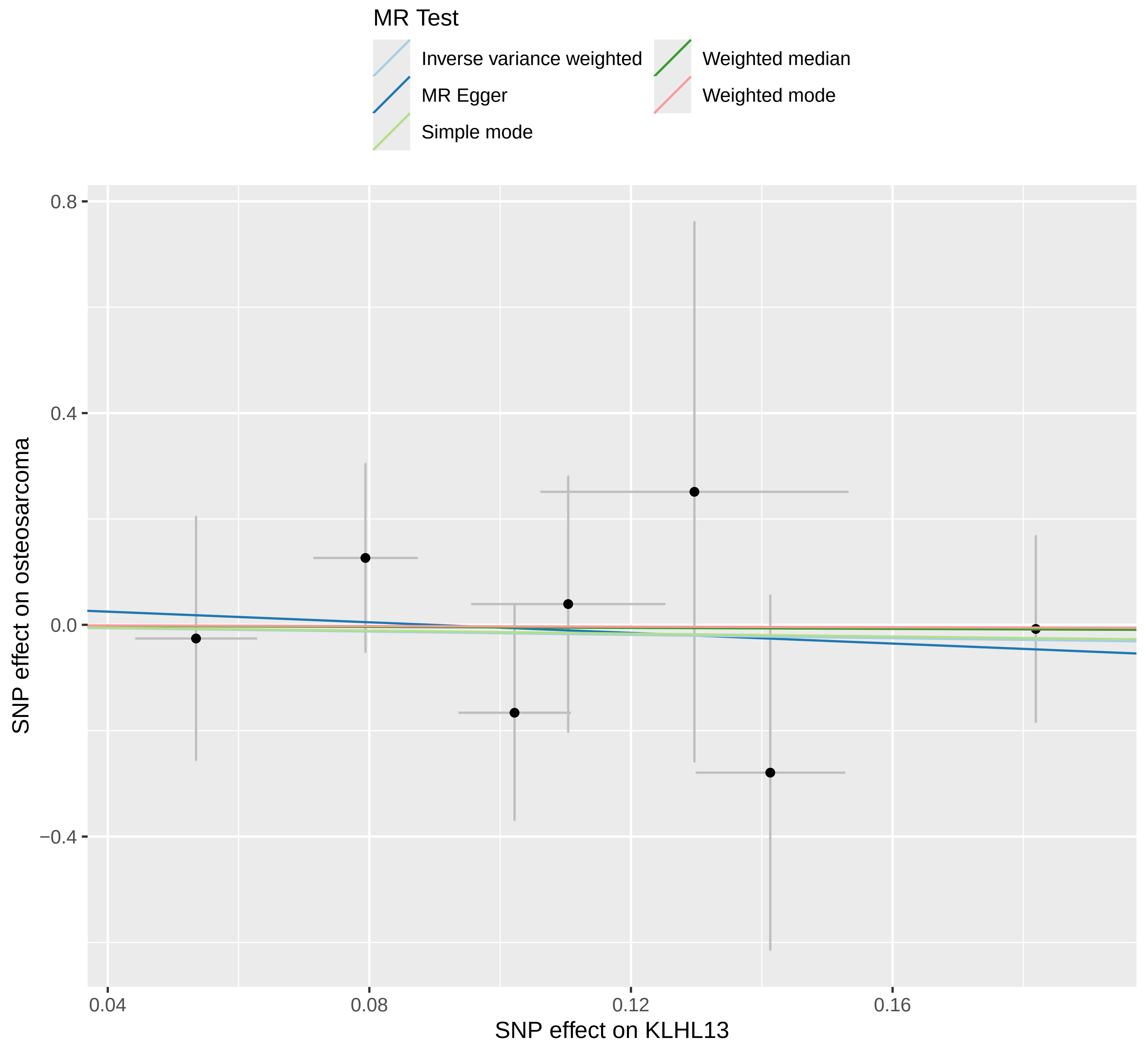
MR Test
Inverse variance weighted
MR Egger
Simple mode
Weighted median
Weighted mode
0.8
0.4
0.0
−0.4
0.04
0.08
0.12
0.16
SNP effect on osteosarcoma
SNP effect on KLHL13

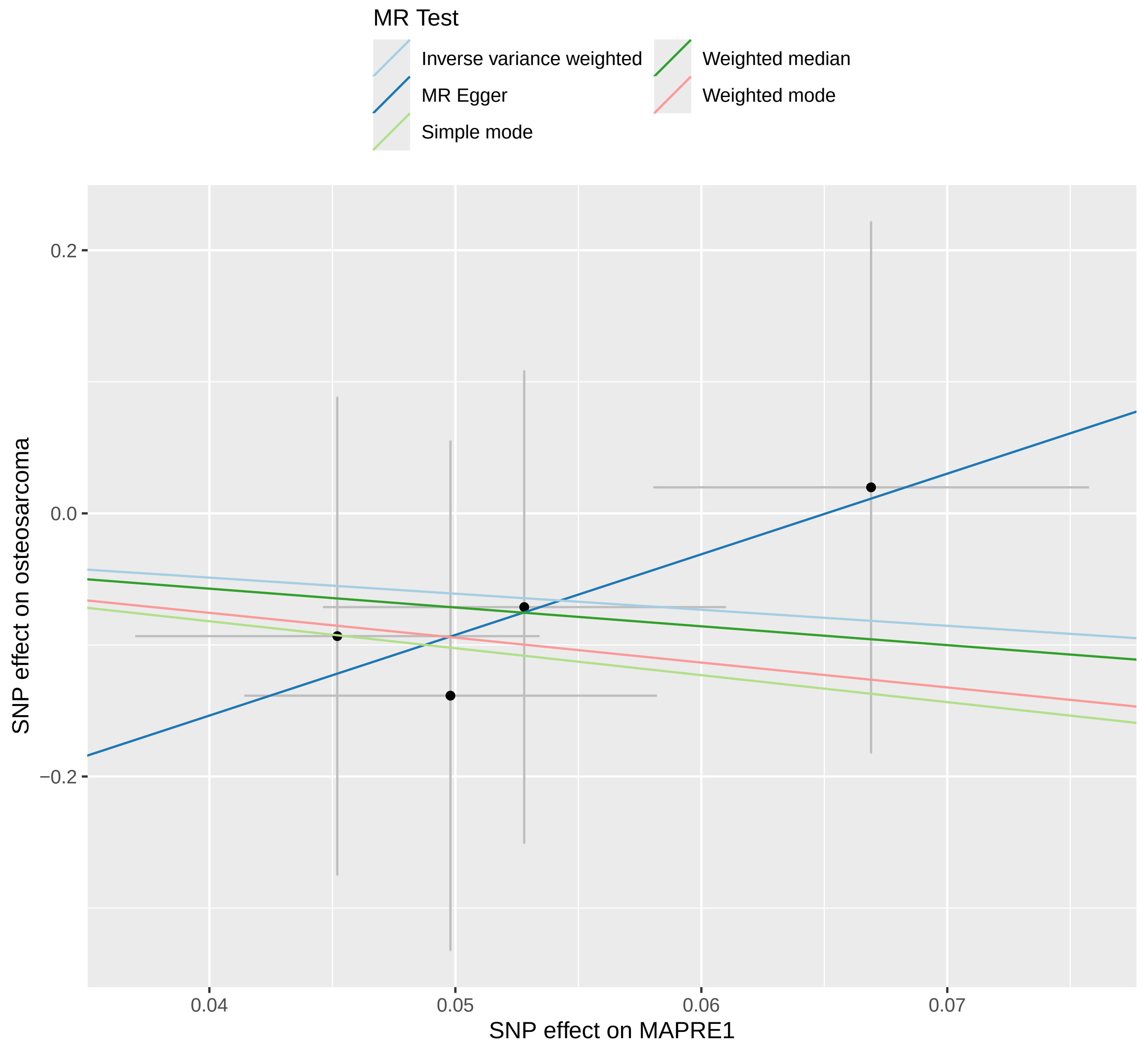
MR Test
Inverse variance weighted
MR Egger
Simple mode
Weighted median
Weighted mode
SNP effect on osteosarcoma
0.2
0.0
−0.2
0.04
0.05
0.06
0.07
SNP effect on MAPRE1

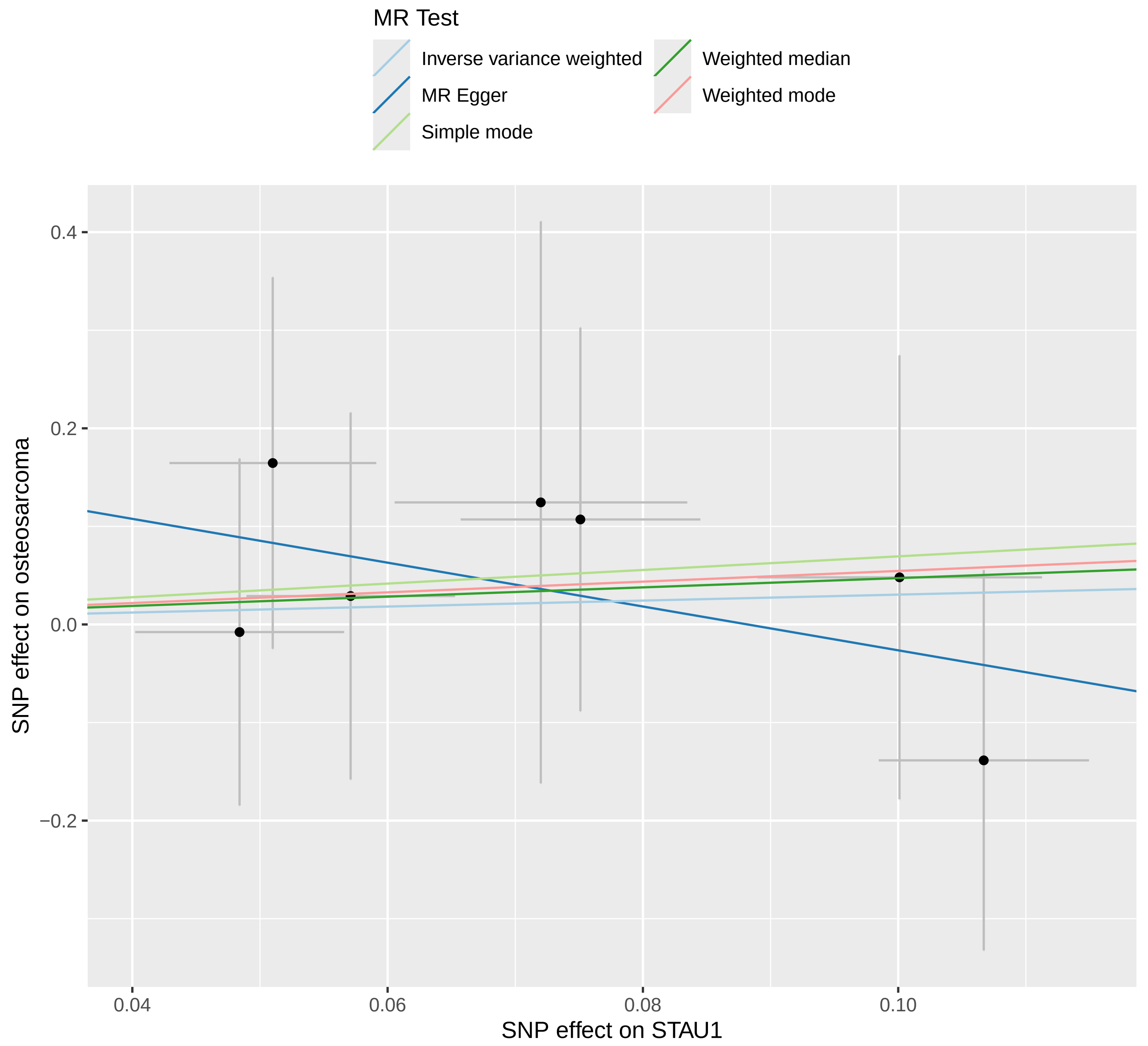
MR Test
Inverse variance weighted
MR Egger
Simple mode
Weighted median
Weighted mode
0.4
0.2
0.0
−0.2
0.04
0.06
0.08
0.10
SNP effect on osteosarcoma
SNP effect on STAU1

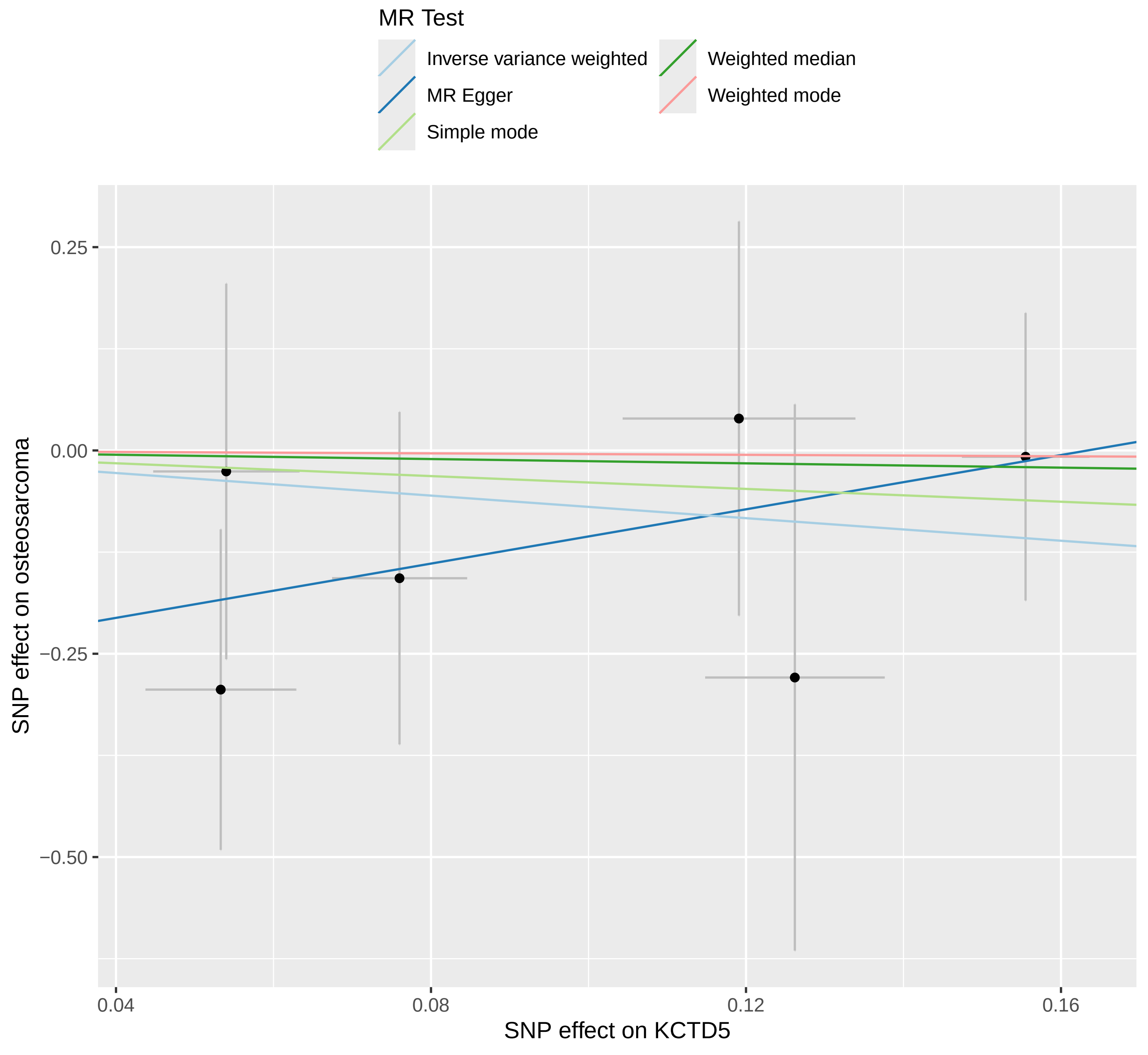
MR Test
Inverse variance weighted
MR Egger
Simple mode
Weighted median
Weighted mode
0.25
0.00
−0.25
−0.50
SNP effect on osteosarcoma
0.04
0.08
0.12
0.16
SNP effect on KCTD5

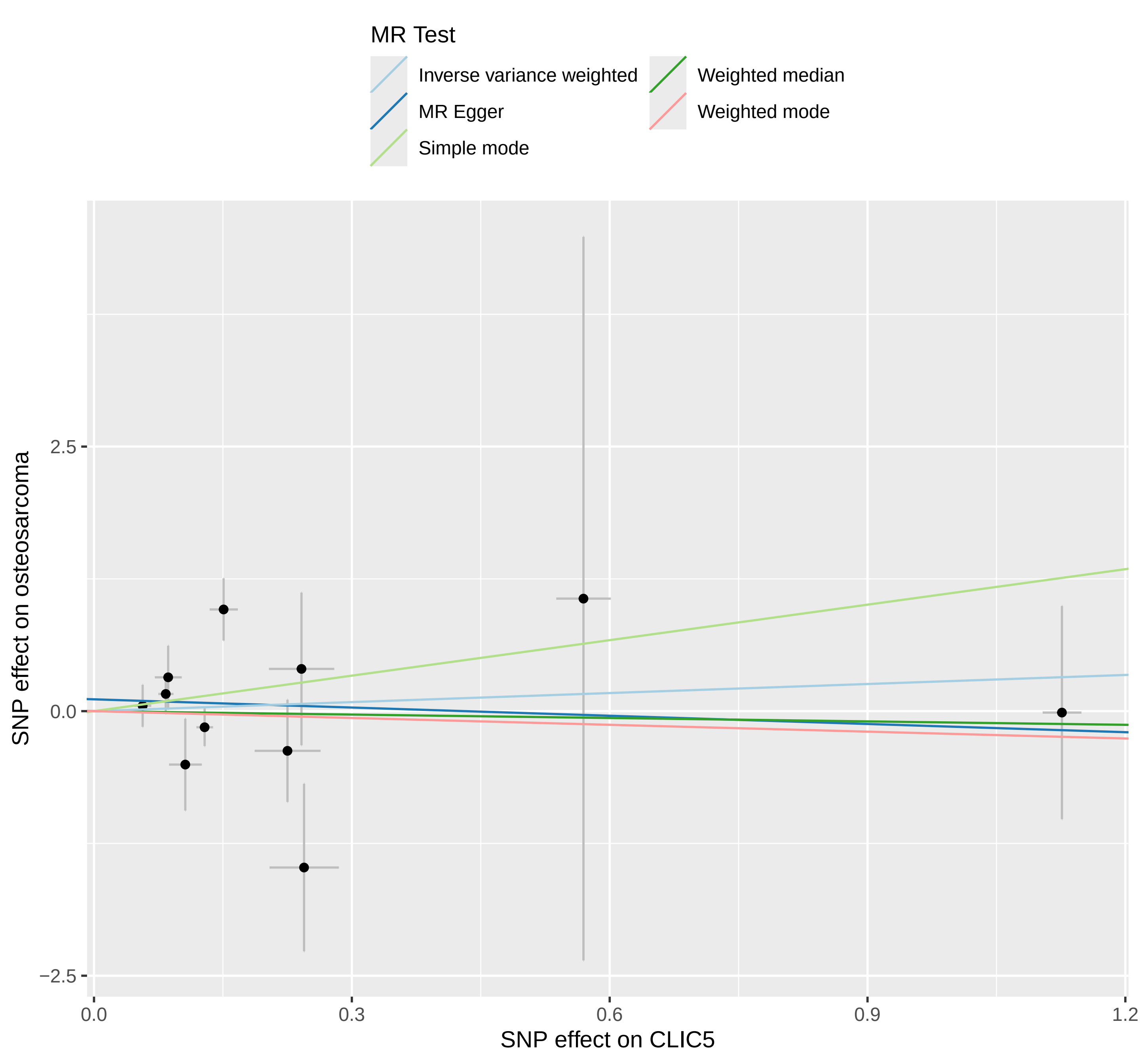
MR Test
Inverse variance weighted
MR Egger
Simple mode
Weighted median
Weighted mode
SNP effect on osteosarcoma
2.5
0.0
−2.5
0.0
0.3
0.6
0.9
1.2
SNP effect on CLIC5

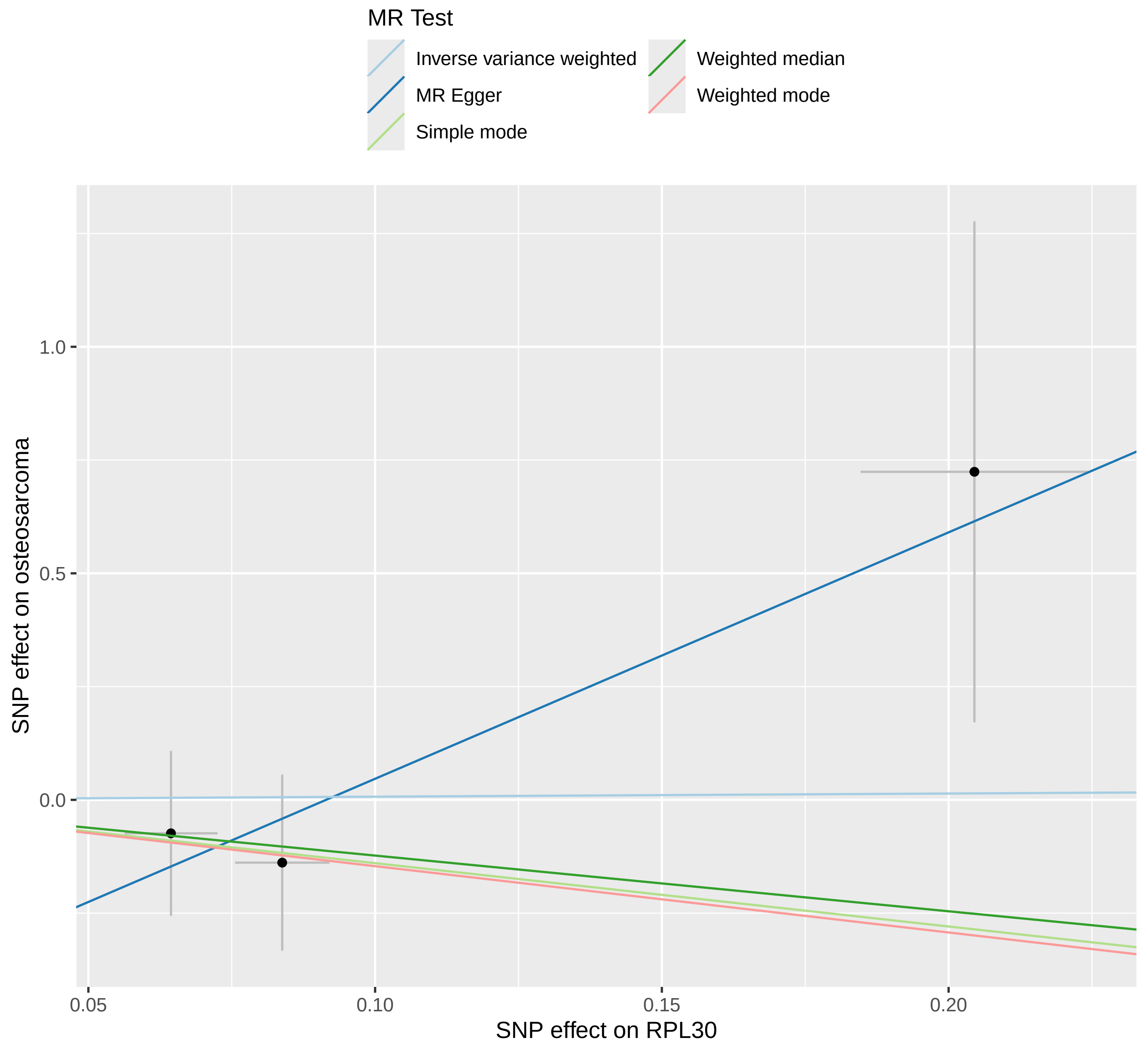
MR Test
Inverse variance weighted
MR Egger
Simple mode
Weighted median
Weighted mode
1.0
0.5
0.0
SNP effect on osteosarcoma
0.05
0.10
0.15
0.20
SNP effect on RPL30

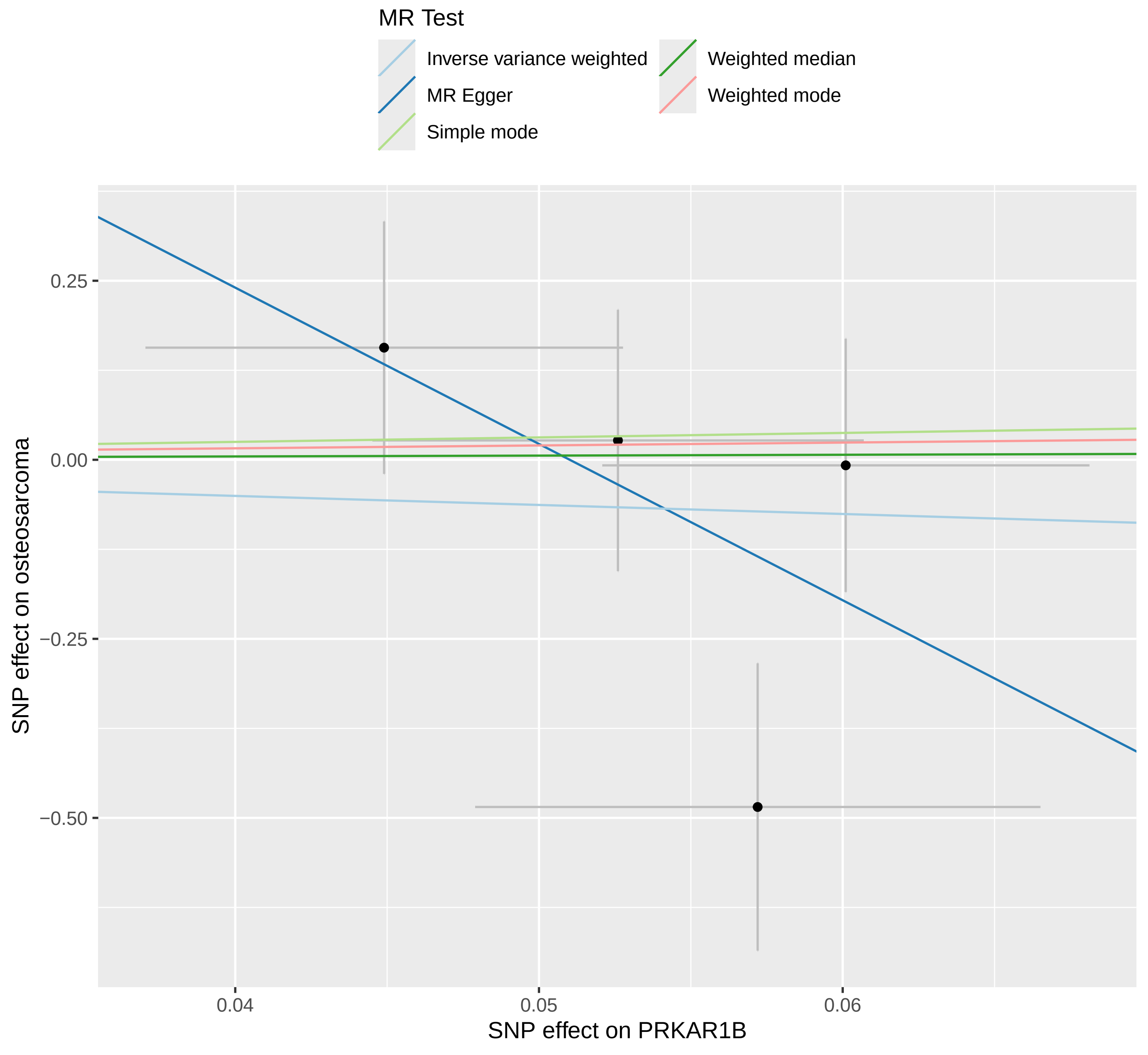
MR Test
Inverse variance weighted
MR Egger
Simple mode
Weighted median
Weighted mode
0.25
0.00
−0.25
−0.50
SNP effect on osteosarcoma
0.04
0.05
0.06
SNP effect on PRKAR1B

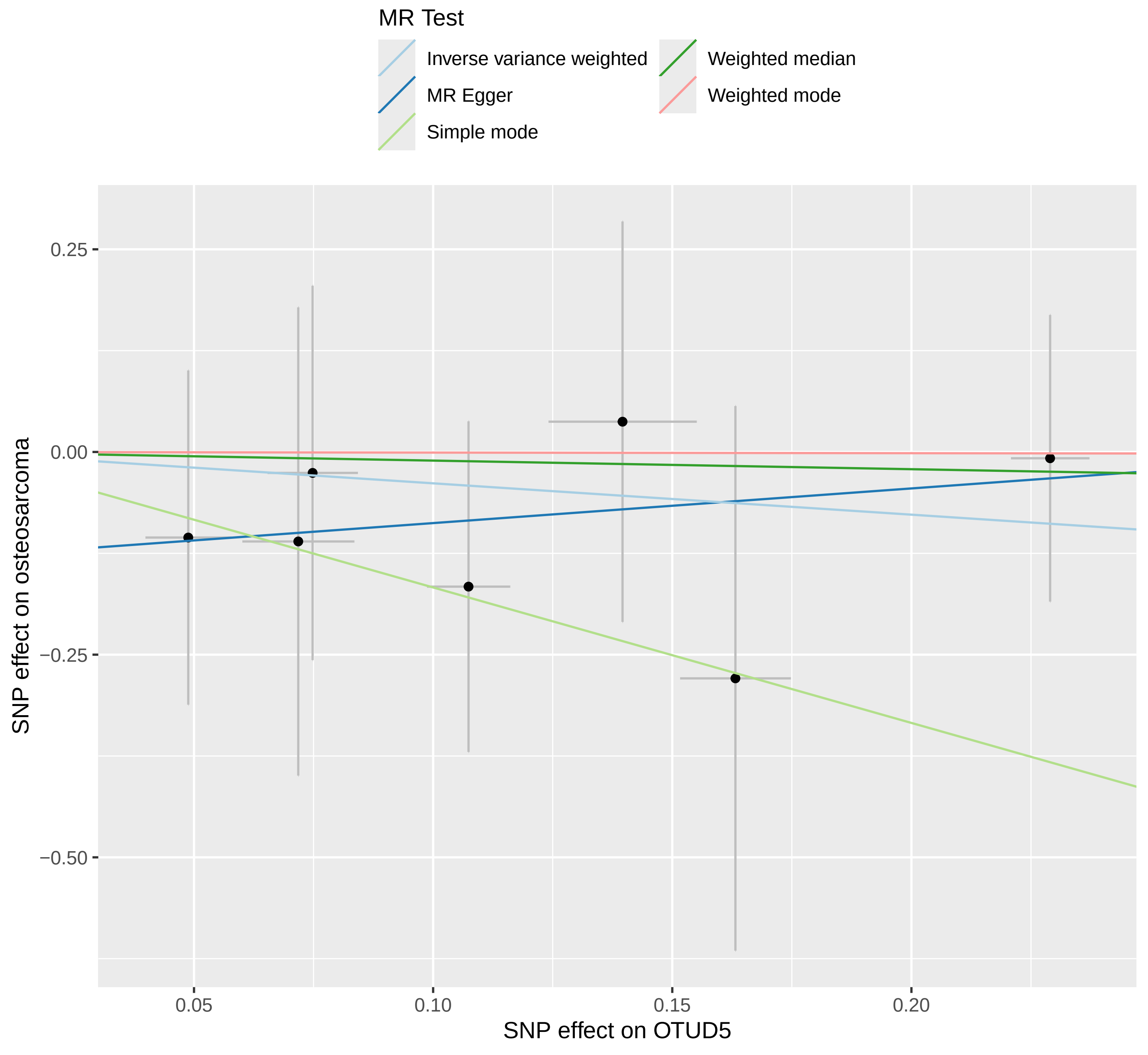
MR Test
Inverse variance weighted
MR Egger
Simple mode
Weighted median
Weighted mode
0.25
0.00
−0.25
−0.50
SNP effect on osteosarcoma
0.05
0.10
0.15
0.20
SNP effect on OTUD5

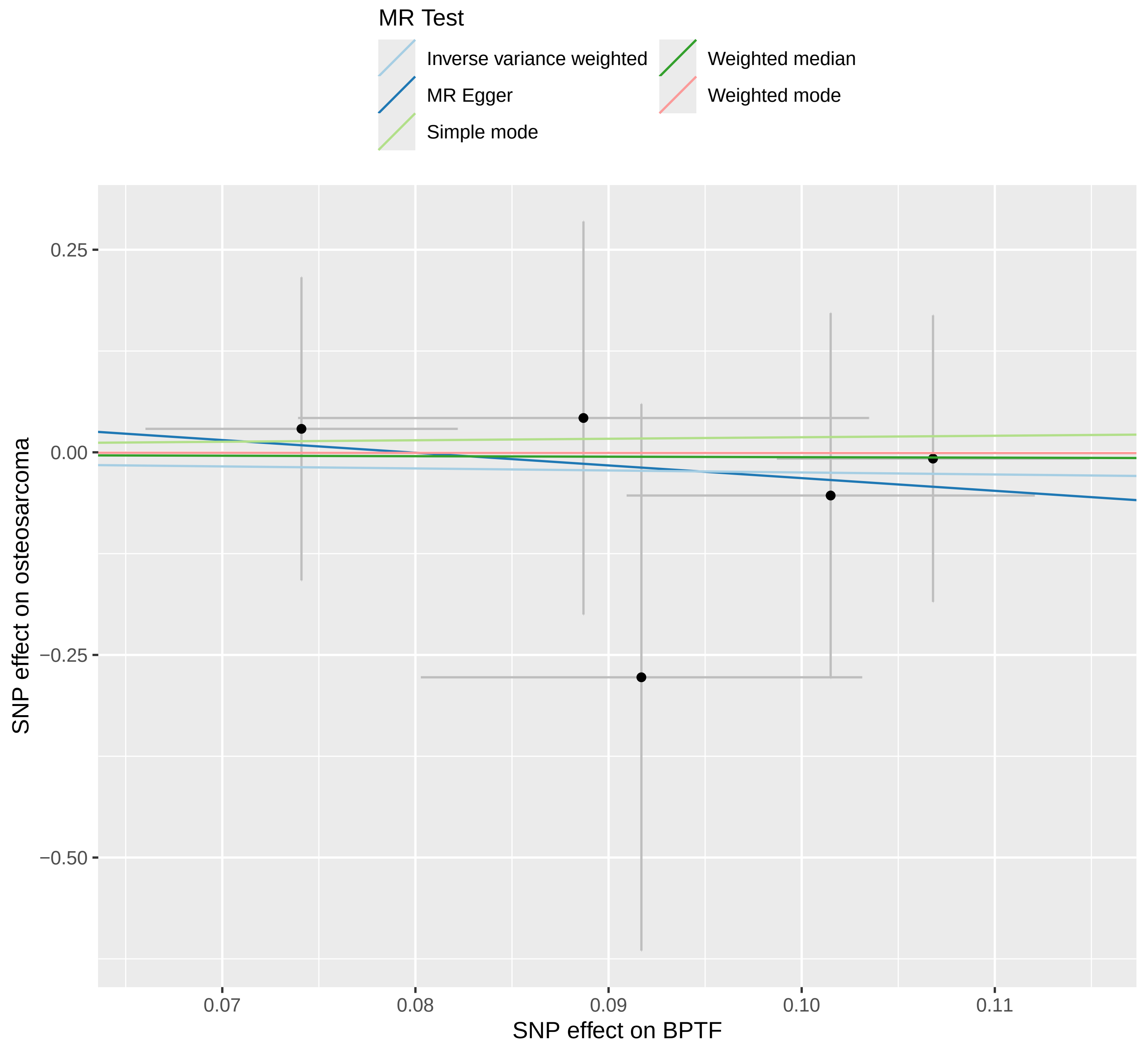
MR Test
Inverse variance weighted
MR Egger
Simple mode
Weighted median
Weighted mode
0.25
0.00
−0.25
−0.50
0.07
0.08
0.09
0.10
0.11
SNP effect on osteosarcoma
SNP effect on BPTF

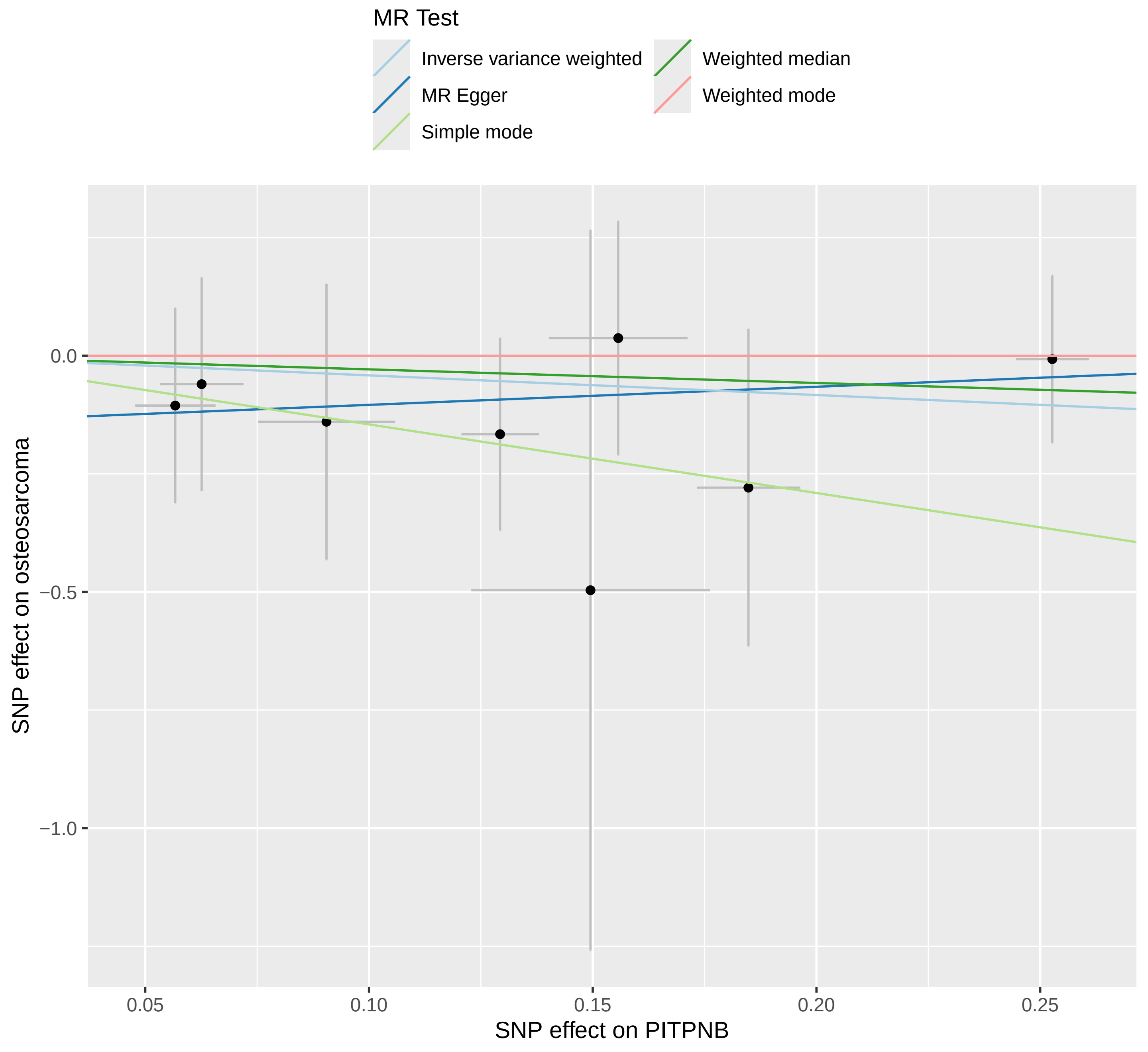

MR Test
Inverse variance weighted
MR Egger
Simple mode
Weighted median
Weighted mode
0.0
−0.5
−1.0
0.05
0.10
0.15
0.20
0.25
SNP effect on osteosarcoma
SNP effect on PITPNB

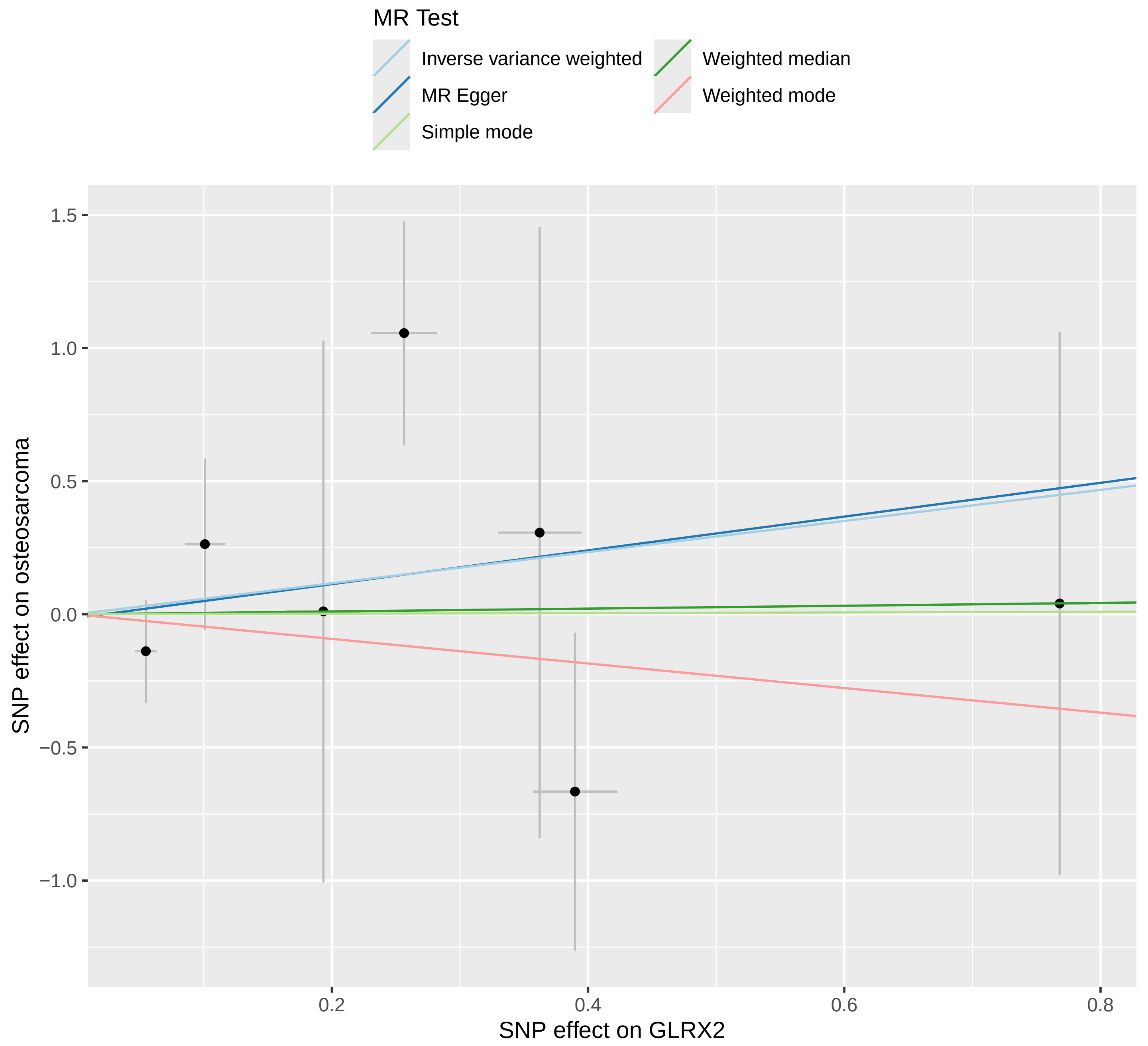
MR Test
Inverse variance weighted
MR Egger
Simple mode
Weighted median
Weighted mode
SNP effect on osteosarcoma
1.5
1.0
0.5
0.0
−0.5
−1.0
0.2
0.4
0.6
0.8
SNP effect on GLRX2

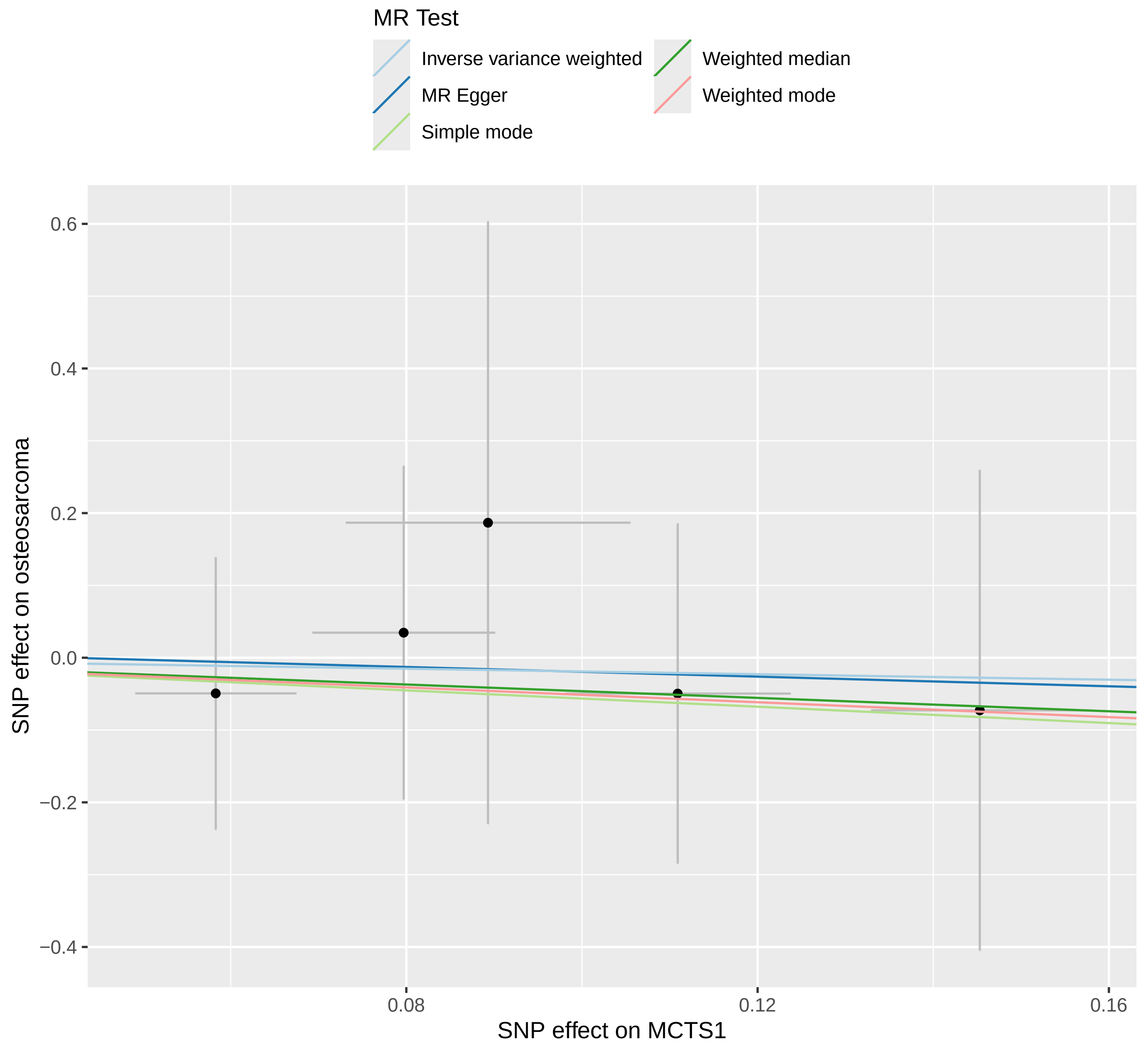

MR Test
Inverse variance weighted
MR Egger
Simple mode
Weighted median
Weighted mode
SNP effect on osteosarcoma
0.6
0.4
0.2
0.0
−0.2
−0.4
0.08
0.12
0.16
SNP effect on MCTS1

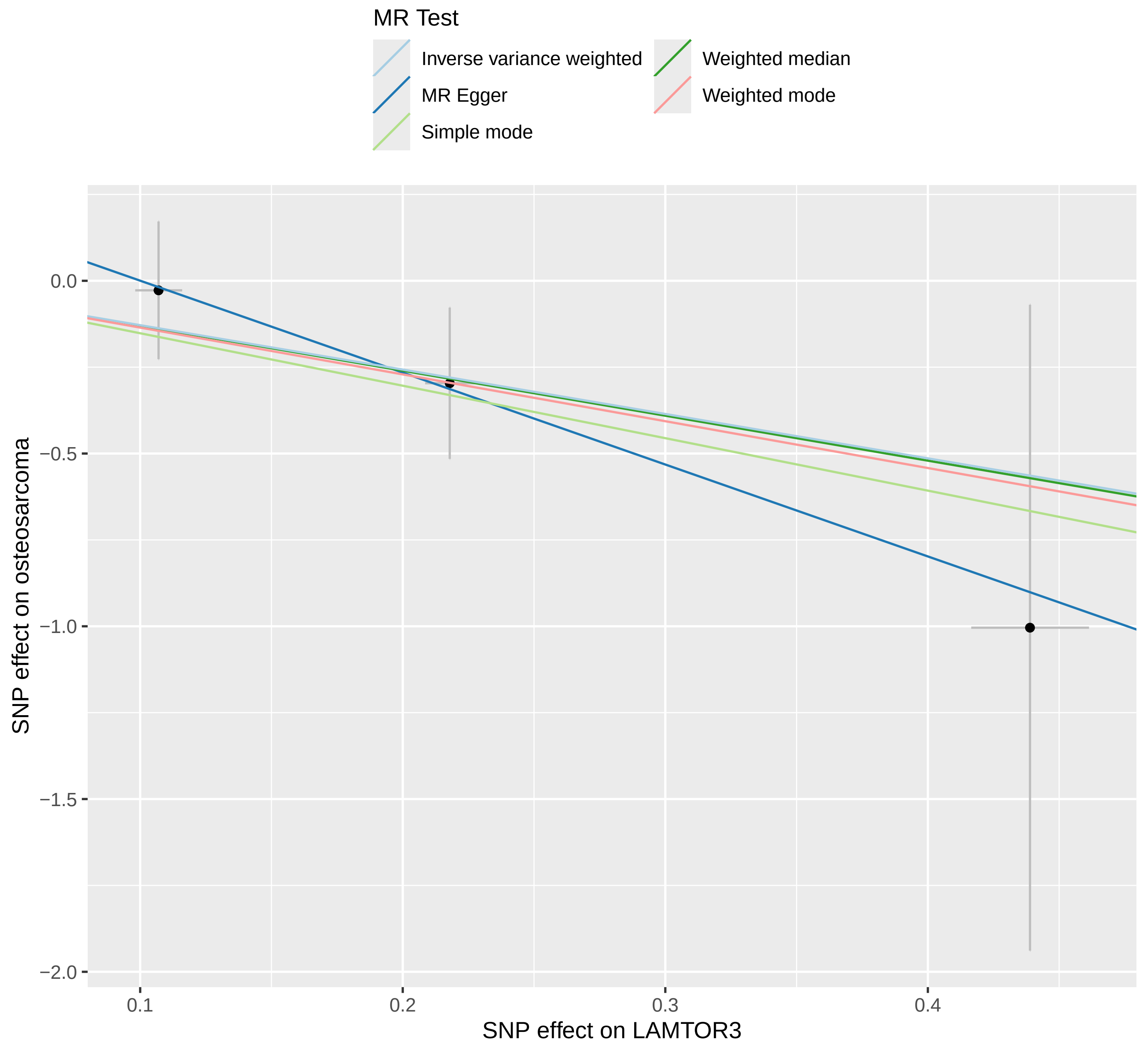
MR Test
Inverse variance weighted
MR Egger
Simple mode
Weighted median
Weighted mode
SNP effect on osteosarcoma
0.0
−0.5
−1.0
−1.5
−2.0
0.1
0.2
0.3
0.4
SNP effect on LAMTOR3

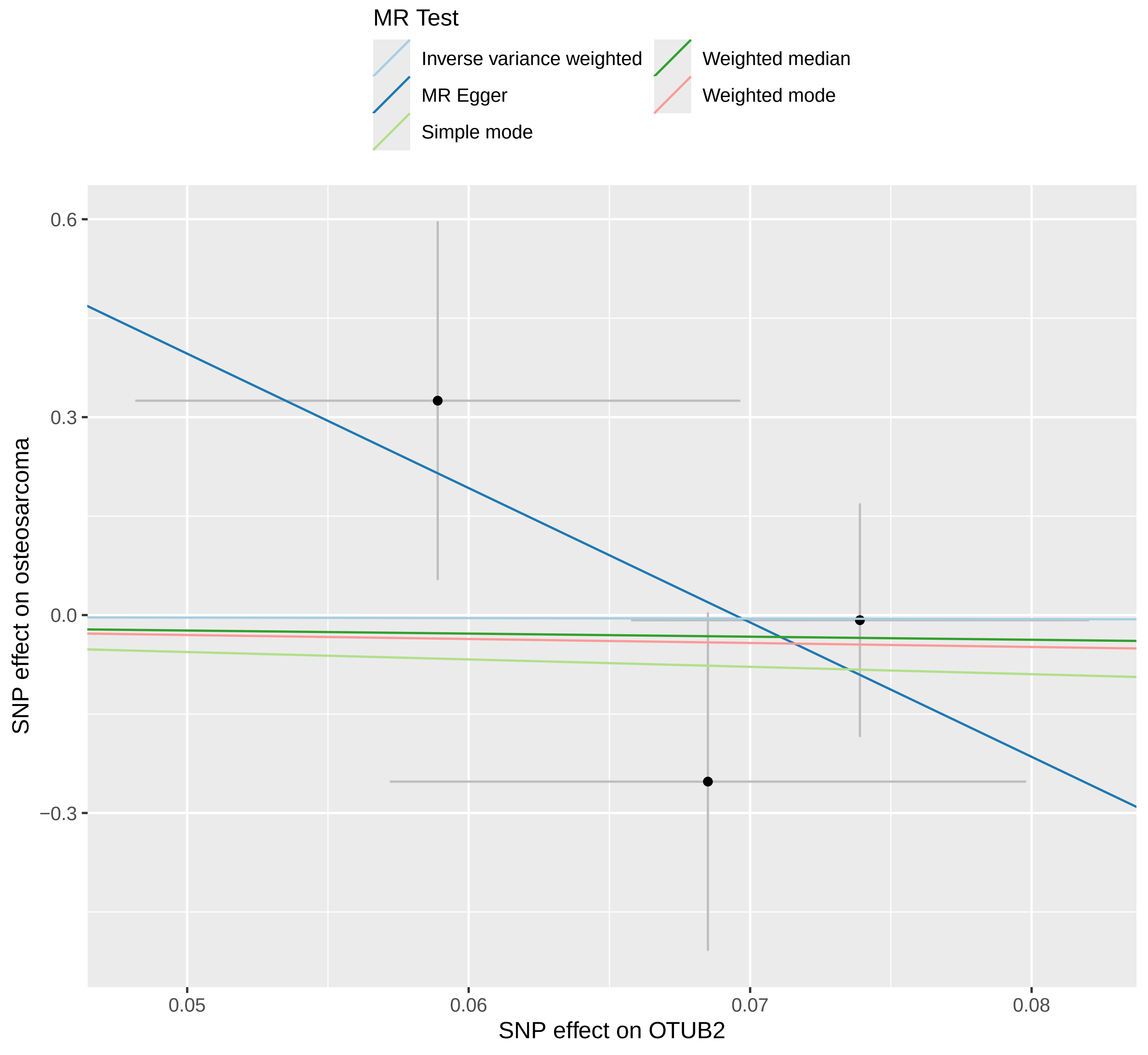
MR Test
Inverse variance weighted
MR Egger
Simple mode
Weighted median
Weighted mode
SNP effect on osteosarcoma
0.6
0.3
0.0
−0.3
0.05
0.06
0.07
0.08
SNP effect on OTUB2

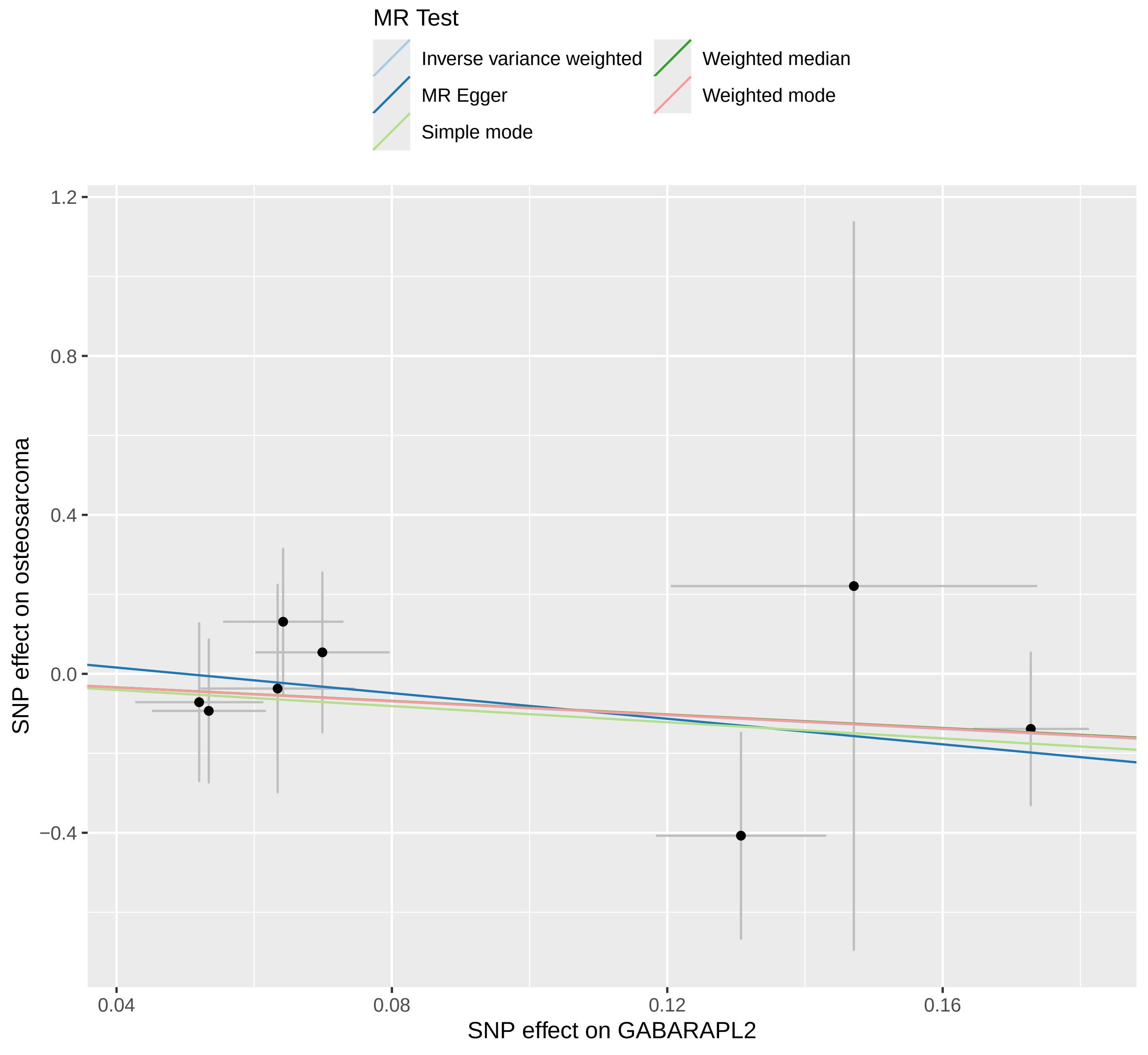
MR Test
Inverse variance weighted
MR Egger
Simple mode
Weighted median
Weighted mode
1.2
0.8
0.4
0.0
−0.4
SNP effect on osteosarcoma
0.04
0.08
0.12
0.16
SNP effect on GABARAPL2

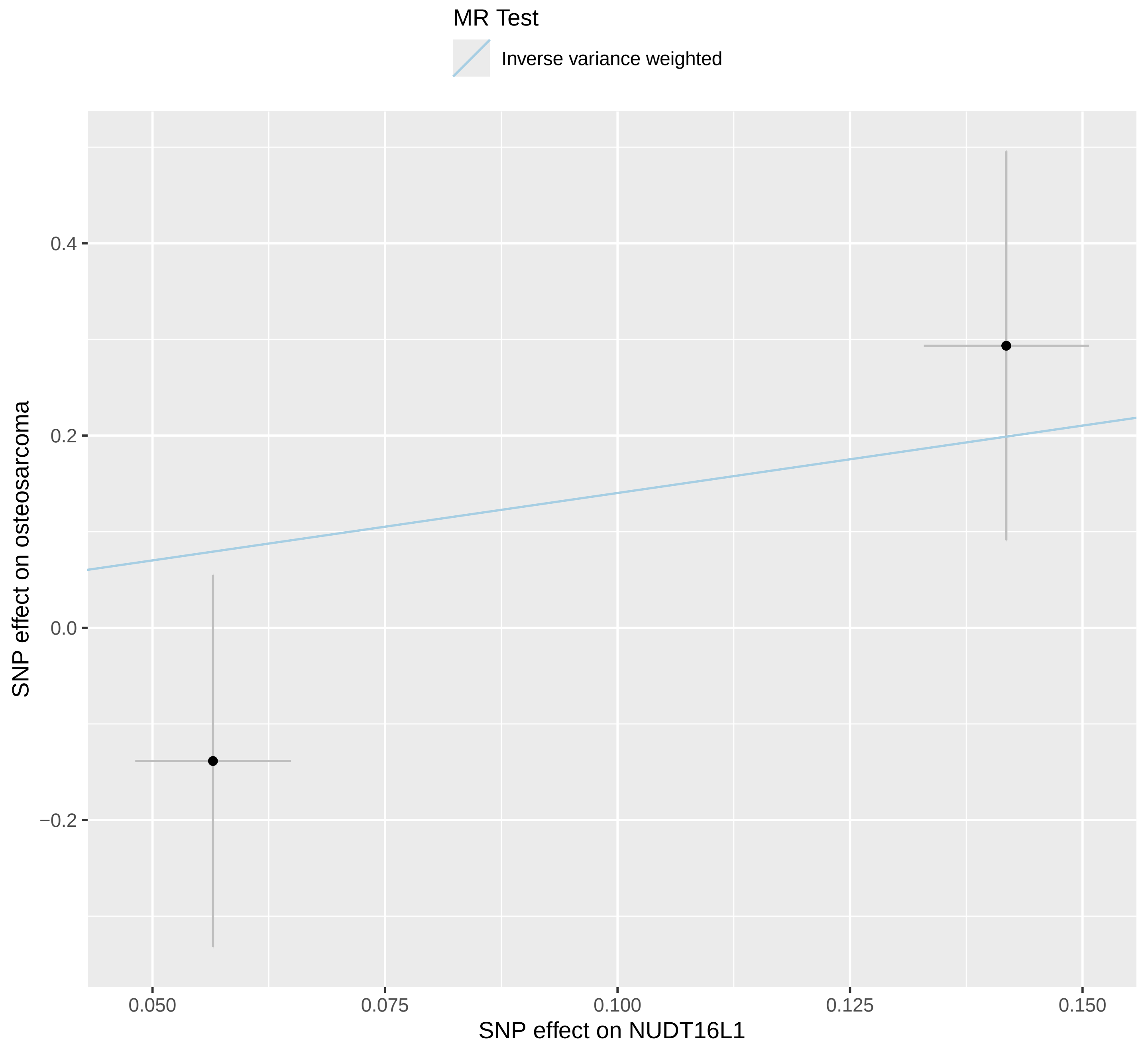
MR Test
Inverse variance weighted
SNP effect on osteosarcoma
0.4
0.2
0.0
−0.2
0.050
0.075
0.100
0.125
0.150
SNP effect on NUDT16L1

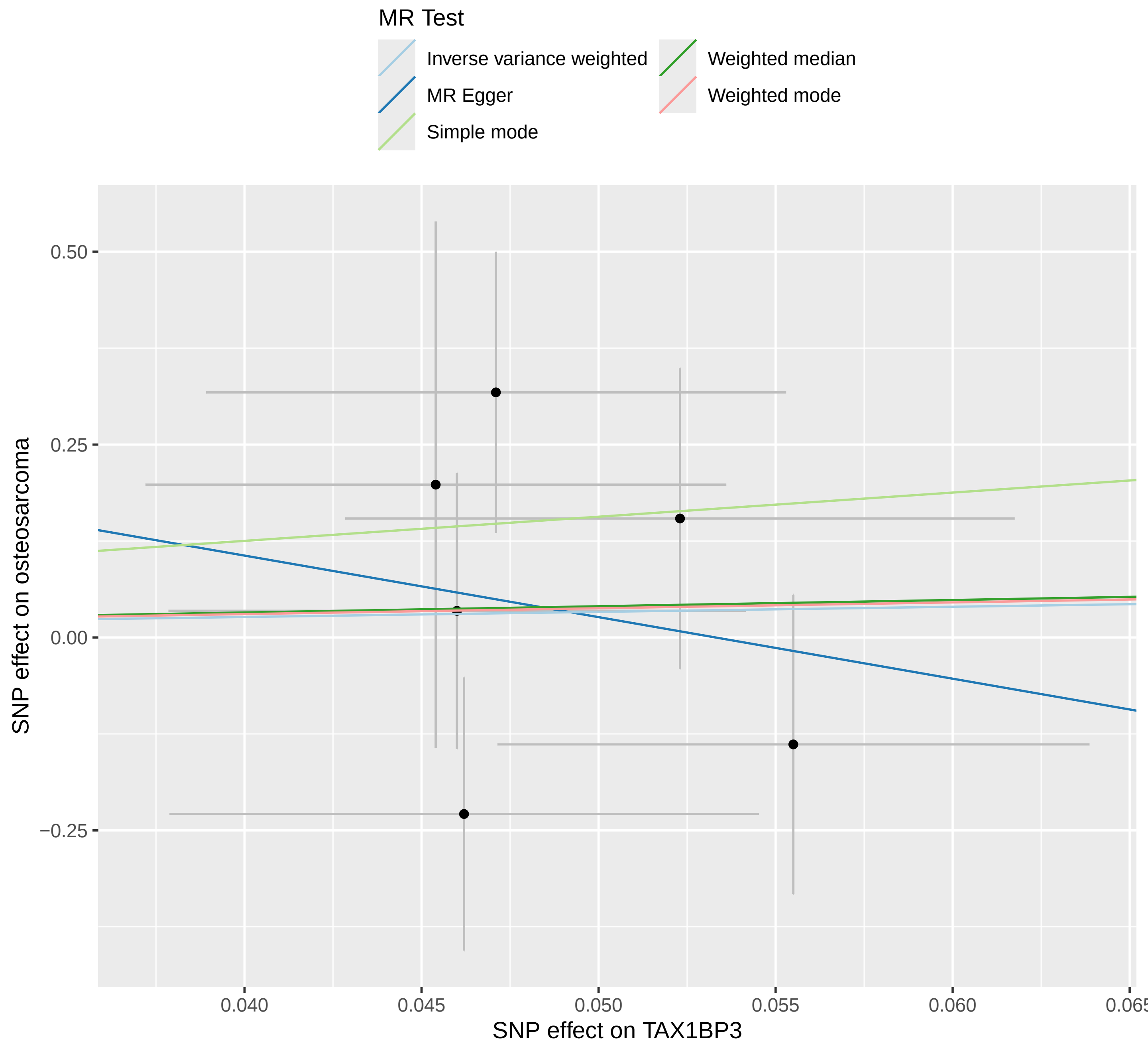
MR Test
Inverse variance weighted
MR Egger
Simple mode
Weighted median
Weighted mode
0.50
0.25
0.00
−0.25
SNP effect on osteosarcoma
0.040
0.045
0.050
0.055
0.060
0.065
SNP effect on TAX1BP3

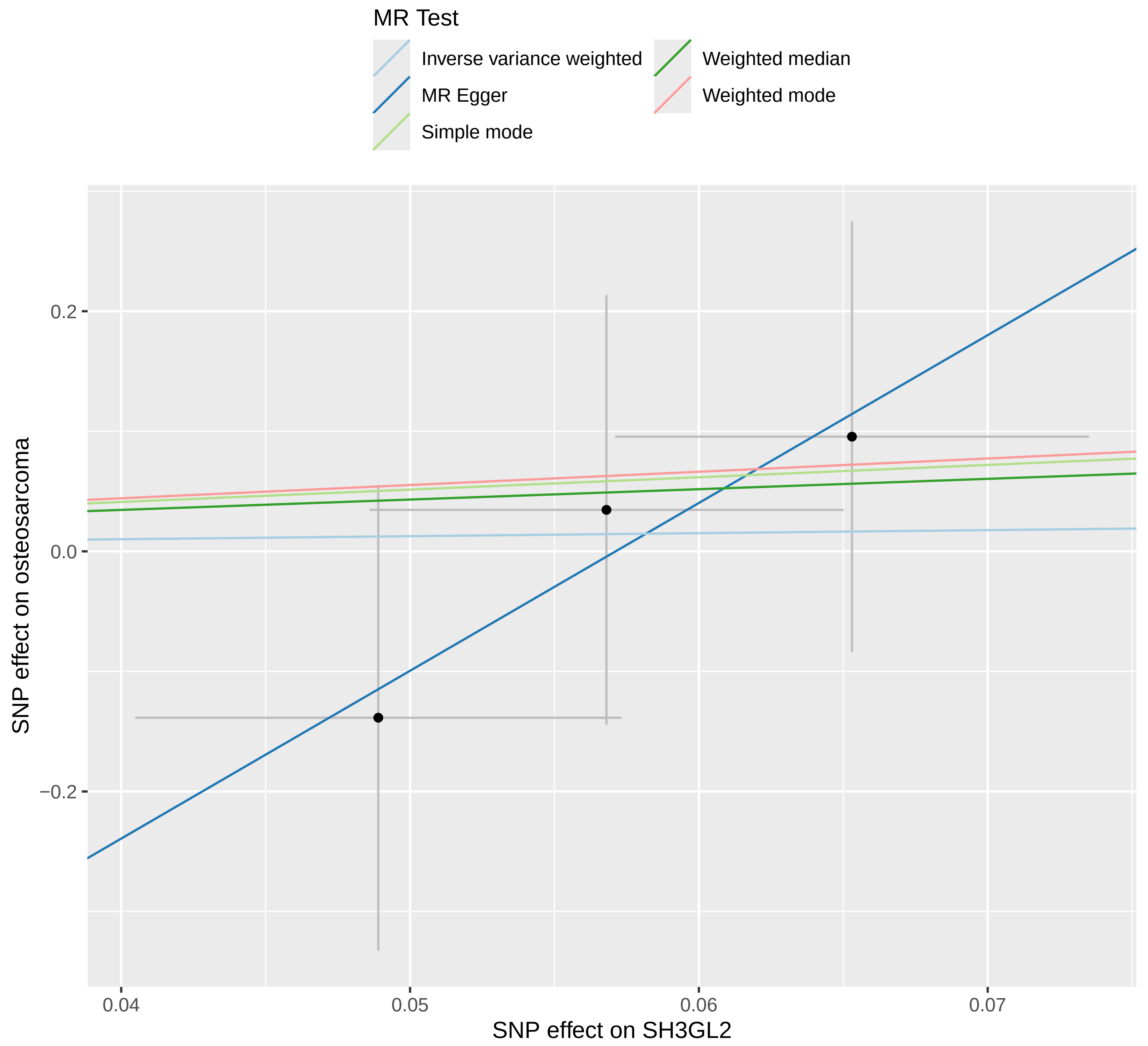
MR Test
Inverse variance weighted
MR Egger
Simple mode
Weighted median
Weighted mode
0.2
0.0
−0.2
SNP effect on osteosarcoma
0.04
0.05
0.06
0.07
SNP effect on SH3GL2

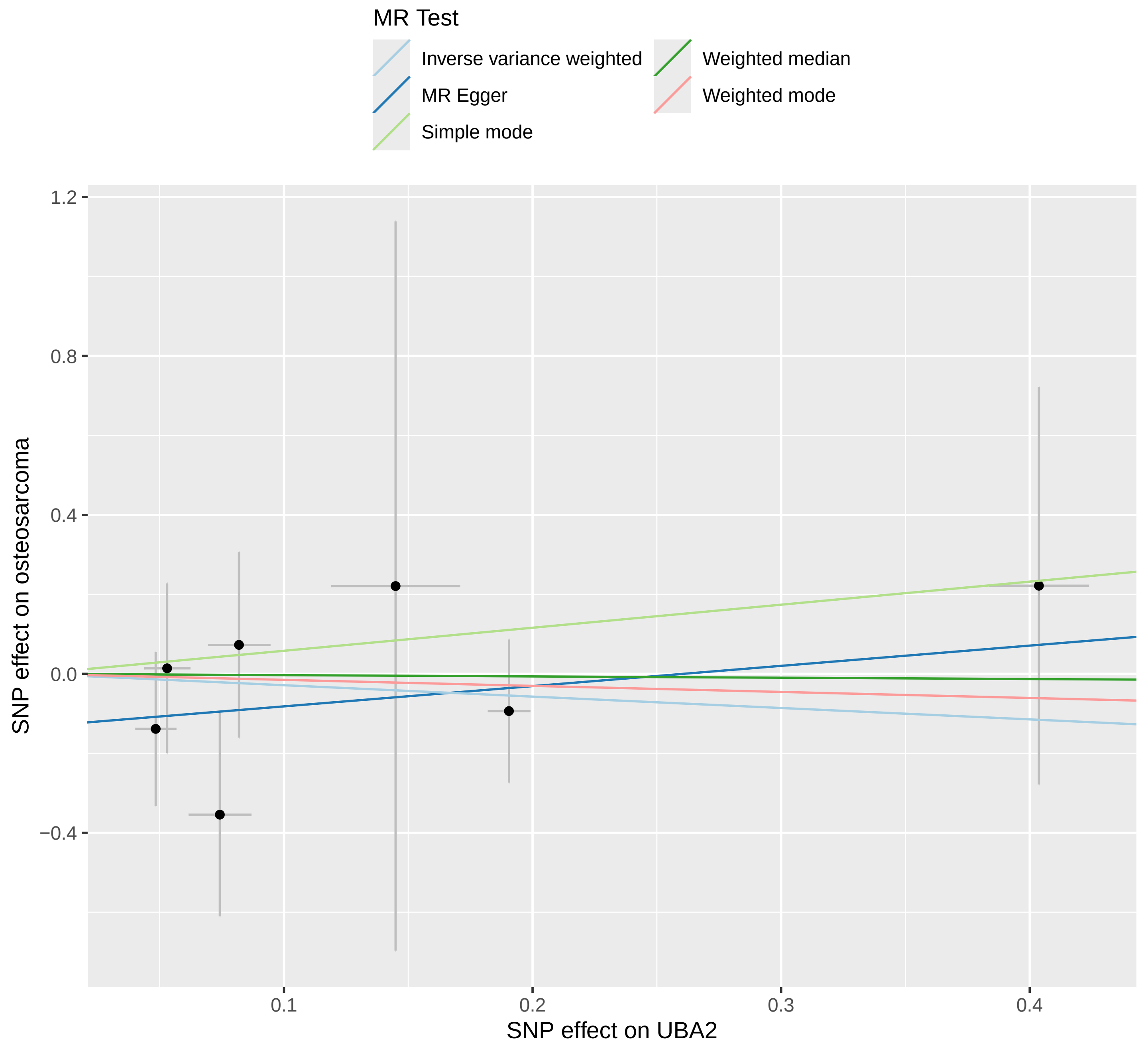
MR Test
Inverse variance weighted
MR Egger
Simple mode
Weighted median
Weighted mode
1.2
0.8
0.4
0.0
−0.4
0.1
0.2
0.3
0.4
SNP effect on osteosarcoma
SNP effect on UBA2

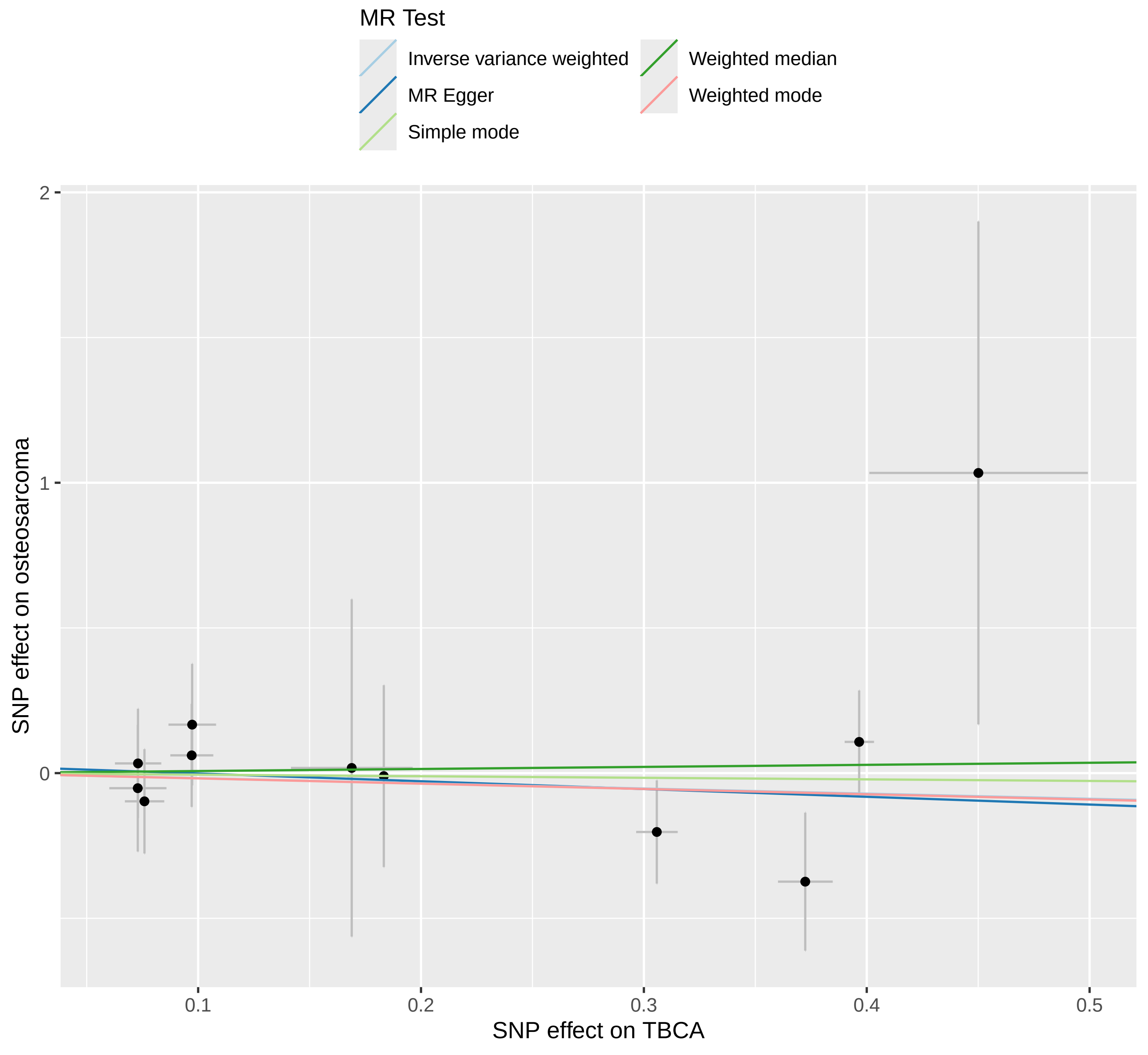
MR Test
Inverse variance weighted
MR Egger
Simple mode
Weighted median
Weighted mode
2
1
0
0.1
0.2
0.3
0.4
0.5
SNP effect on TBCA
SNP effect on osteosarcoma

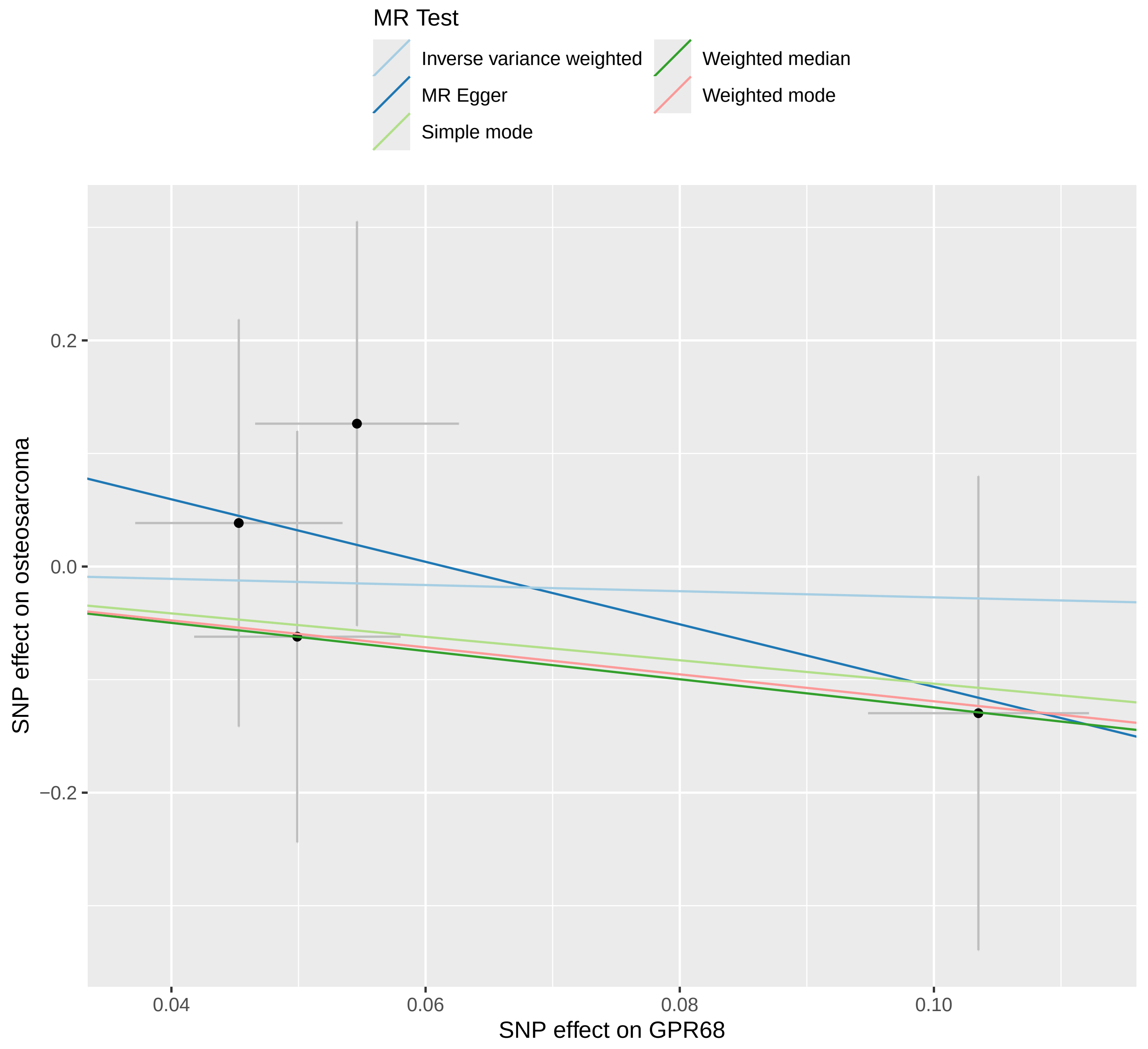
MR Test
Inverse variance weighted
MR Egger
Simple mode
Weighted median
Weighted mode
0.2
0.0
−0.2
SNP effect on osteosarcoma
0.04
0.06
0.08
0.10
SNP effect on GPR68

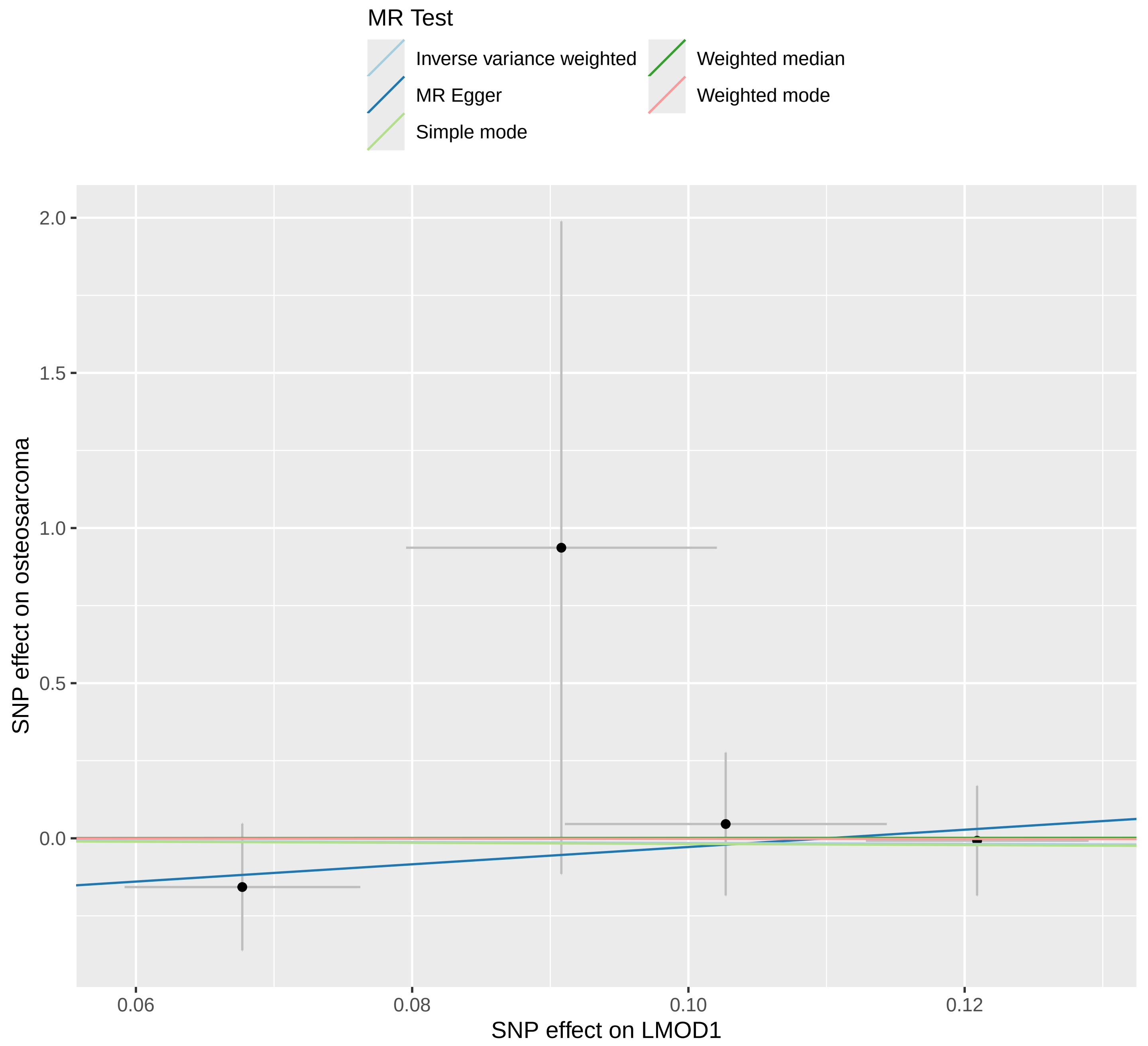
MR Test
Inverse variance weighted
MR Egger
Simple mode
Weighted median
Weighted mode
2.0
1.5
1.0
0.5
0.0
0.06
0.08
0.10
0.12
SNP effect on osteosarcoma
SNP effect on LMOD1

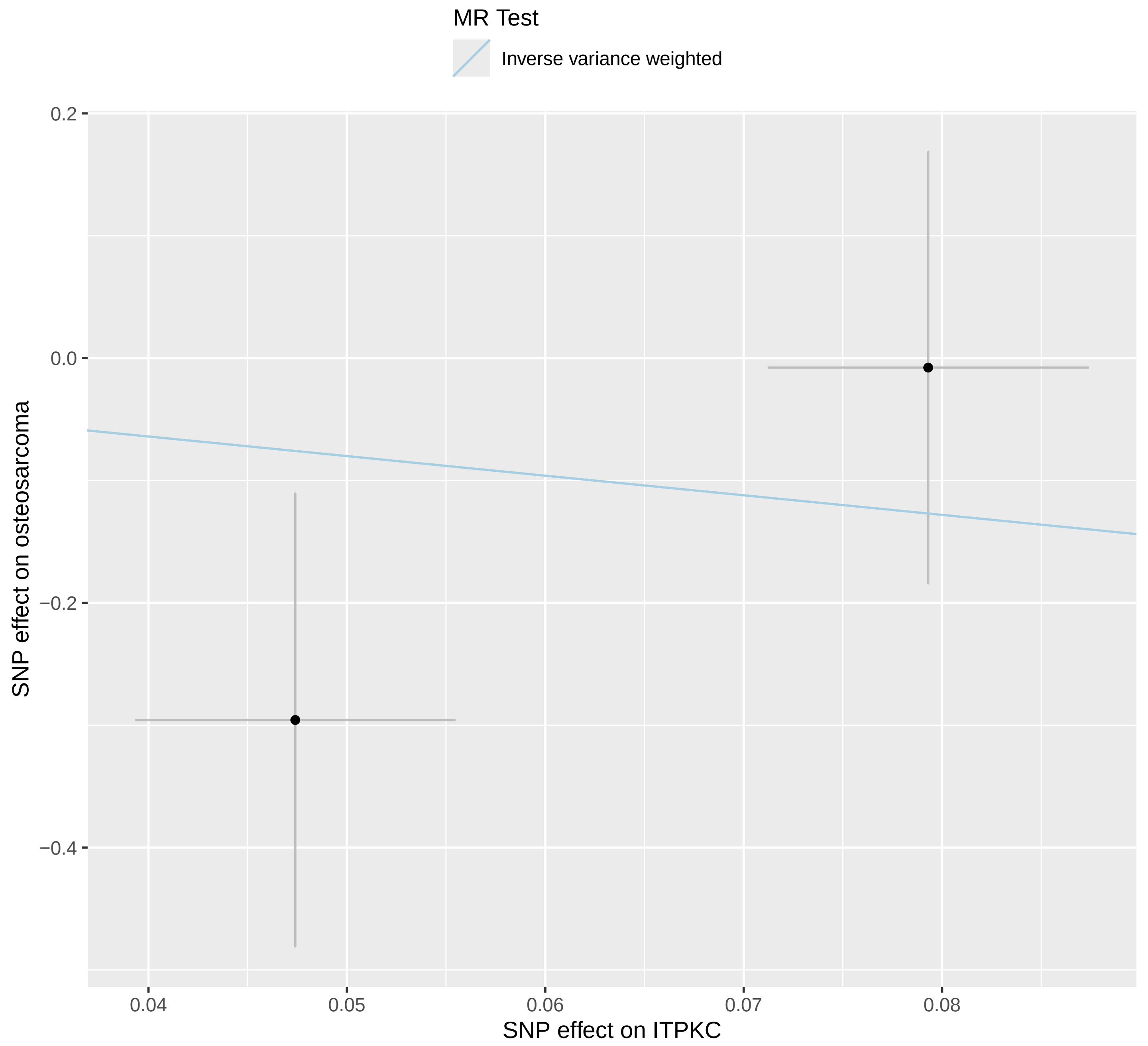
MR Test
Inverse variance weighted
0.2
0.0
−0.2
−0.4
SNP effect on osteosarcoma
0.04
0.05
0.06
0.07
0.08
SNP effect on ITPKC

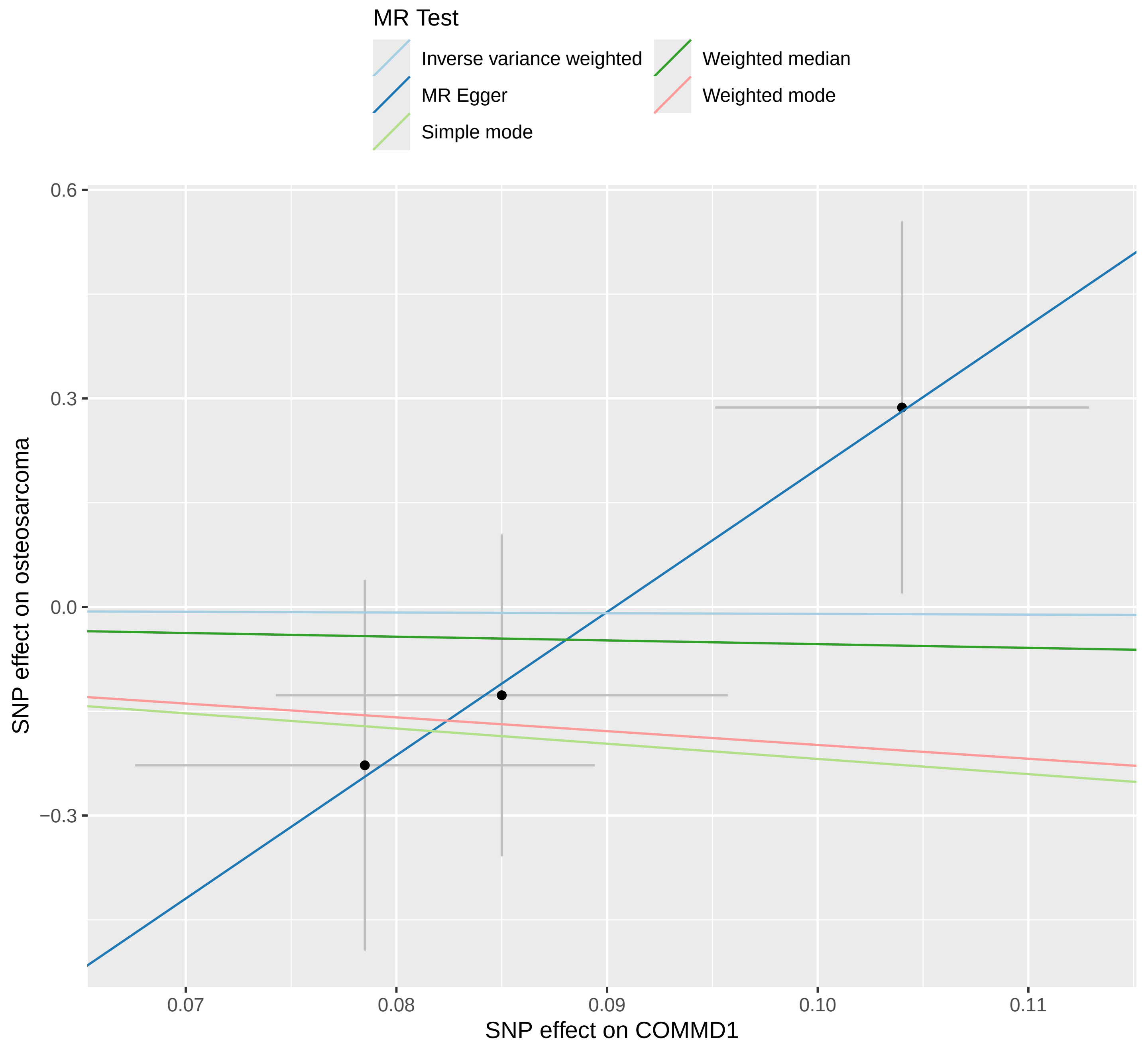
MR Test
Inverse variance weighted
MR Egger
Simple mode
Weighted median
Weighted mode
0.6
0.3
0.0
−0.3
SNP effect on osteosarcoma
0.07
0.08
0.09
0.10
0.11
SNP effect on COMMD1

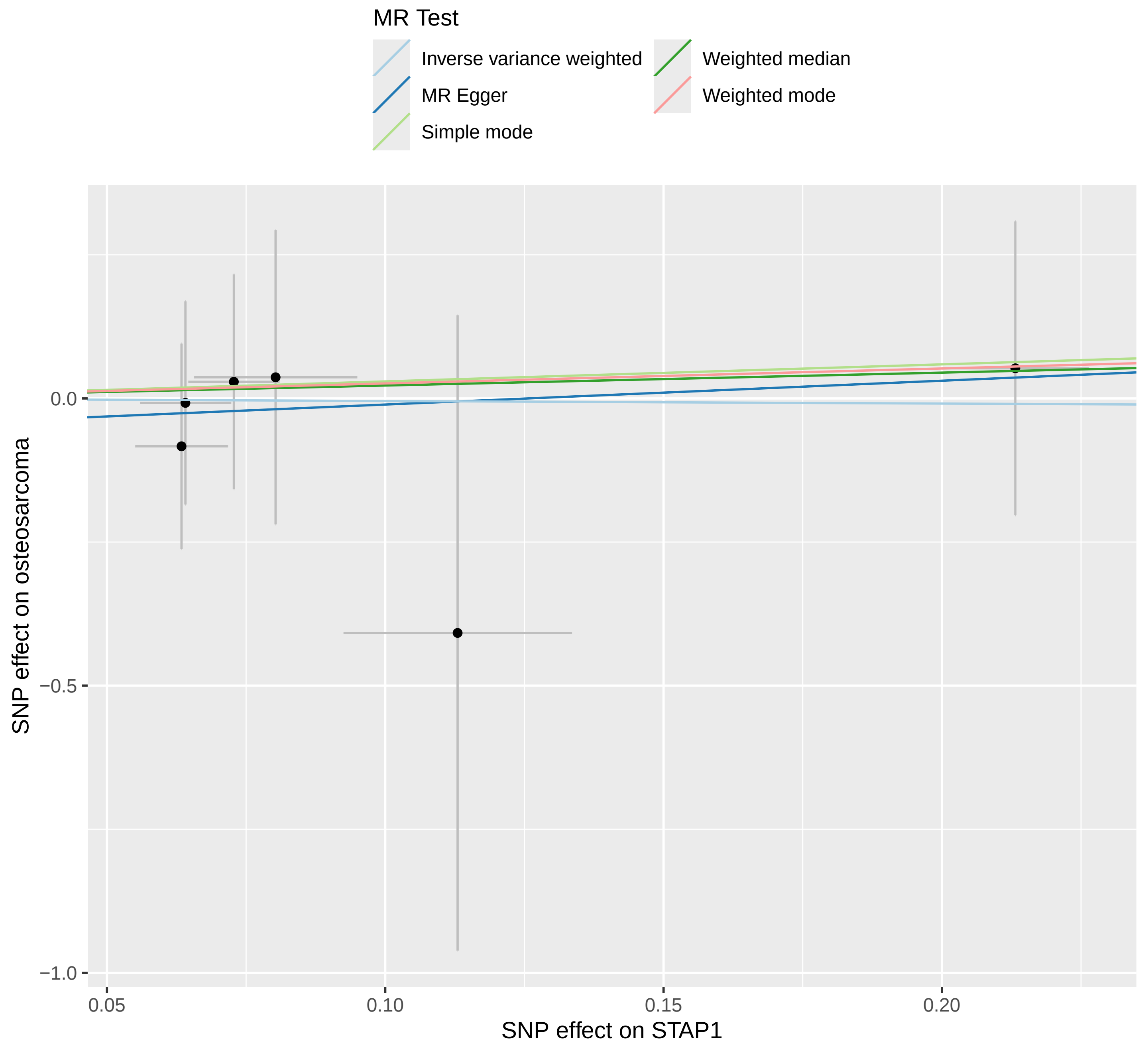
MR Test
Inverse variance weighted
MR Egger
Simple mode
Weighted median
Weighted mode
SNP effect on osteosarcoma
0.0
−0.5
−1.0
0.05
0.10
0.15
0.20
SNP effect on STAP1

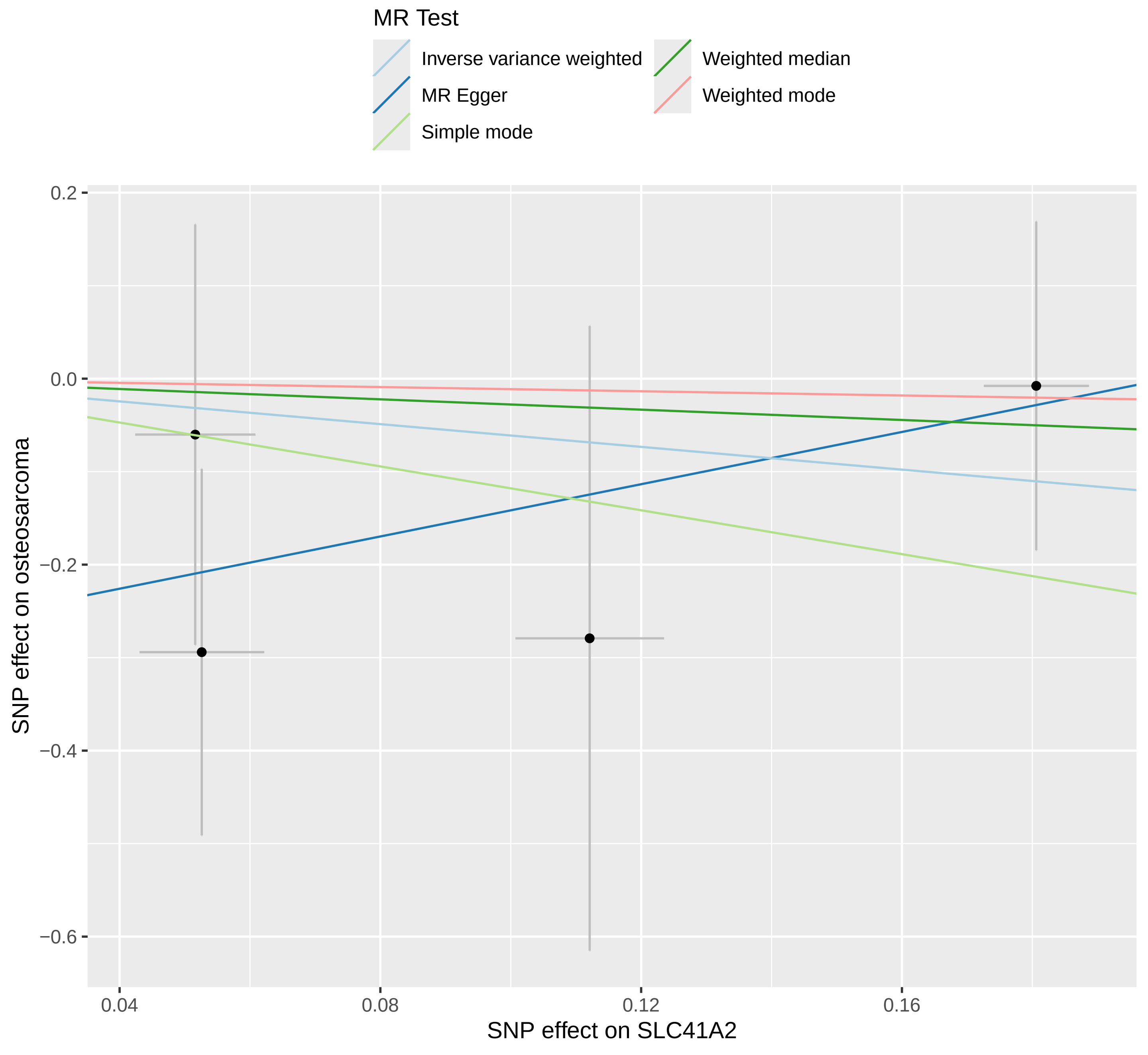
MR Test
Inverse variance weighted
MR Egger
Simple mode
Weighted median
Weighted mode
0.2
0.0
−0.2
−0.4
−0.6
SNP effect on osteosarcoma
0.04
0.08
0.12
0.16
SNP effect on SLC41A2

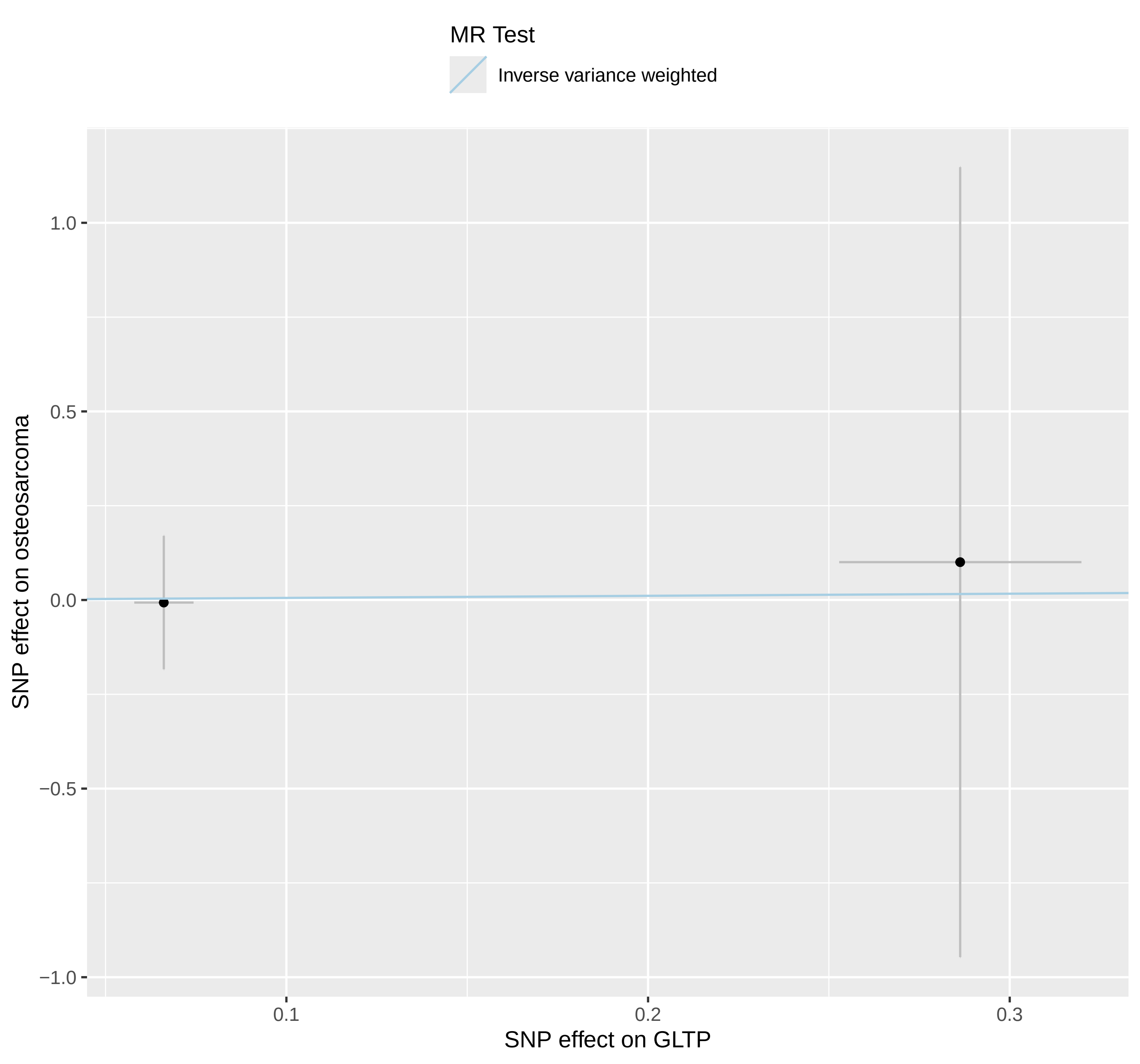
MR Test
Inverse variance weighted
1.0
0.5
0.0
−0.5
−1.0
0.1
0.2
0.3
SNP effect on osteosarcoma
SNP effect on GLTP

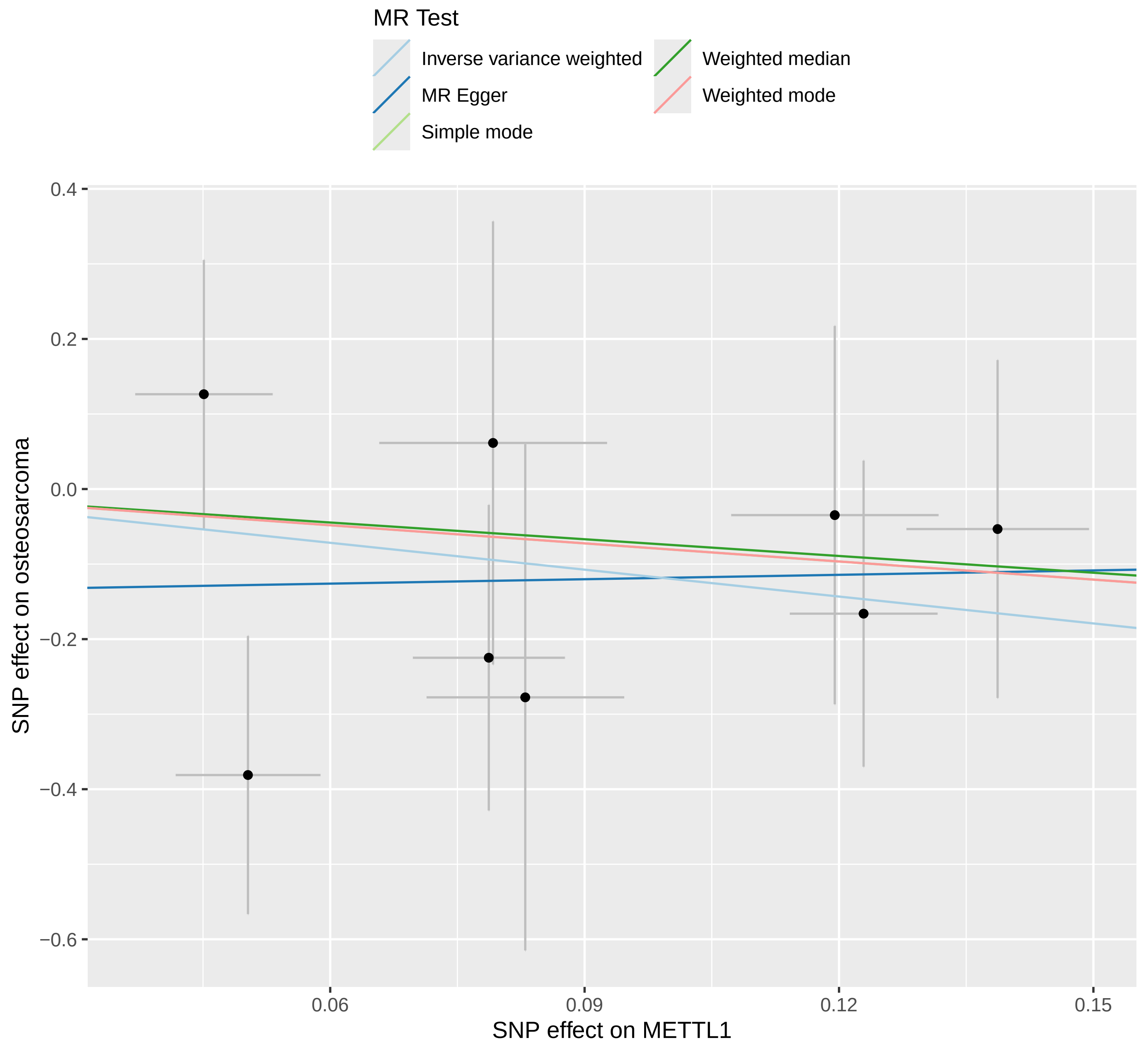

MR Test
Inverse variance weighted
MR Egger
Simple mode
Weighted median
Weighted mode
0.4
0.2
0.0
−0.2
−0.4
−0.6
SNP effect on osteosarcoma
0.06
0.09
0.12
0.15
SNP effect on METTL1

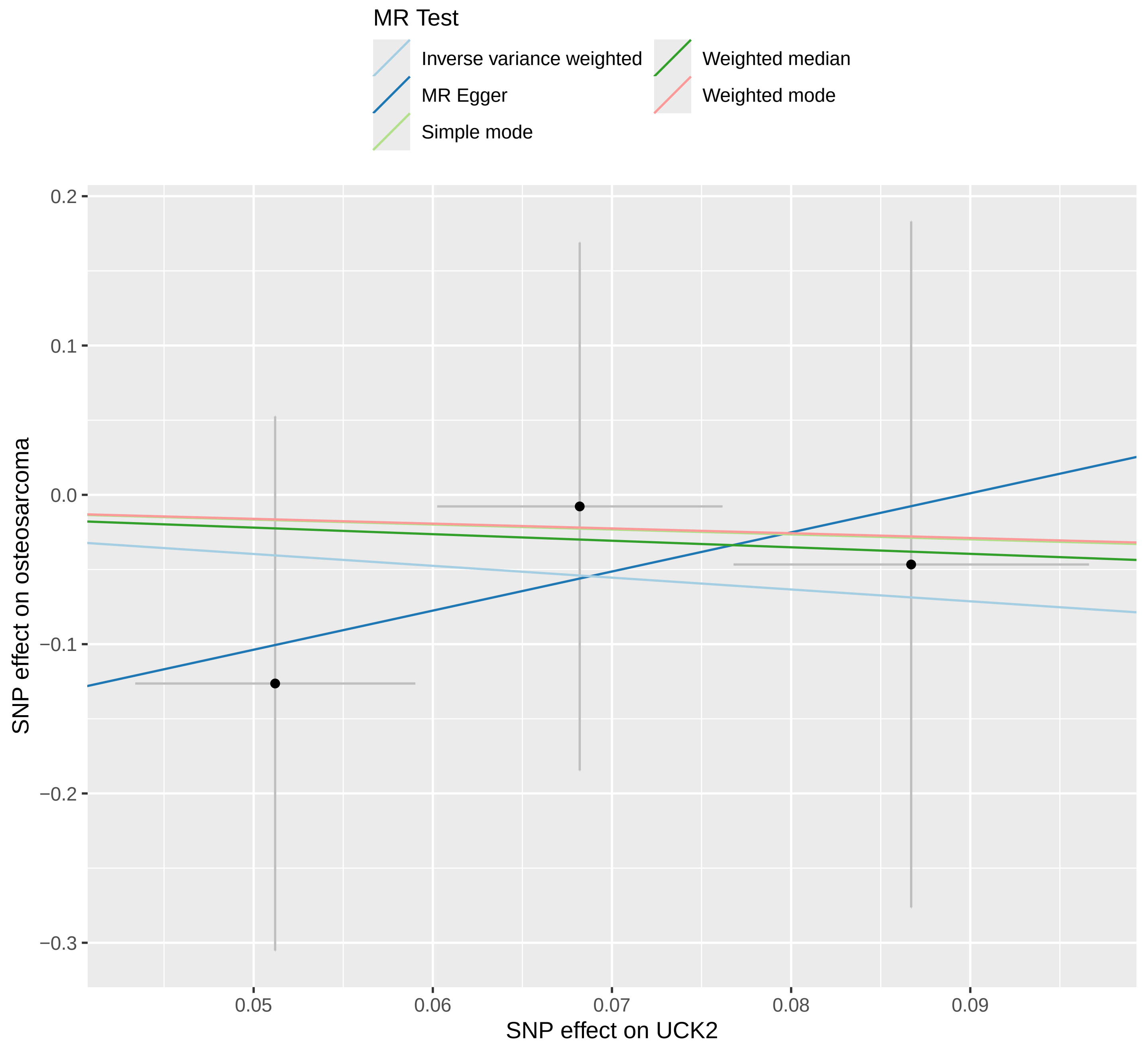
MR Test
Inverse variance weighted
MR Egger
Simple mode
Weighted median
Weighted mode
0.2
0.1
0.0
−0.1
−0.2
−0.3
SNP effect on osteosarcoma
0.05
0.06
0.07
0.08
0.09
SNP effect on UCK2

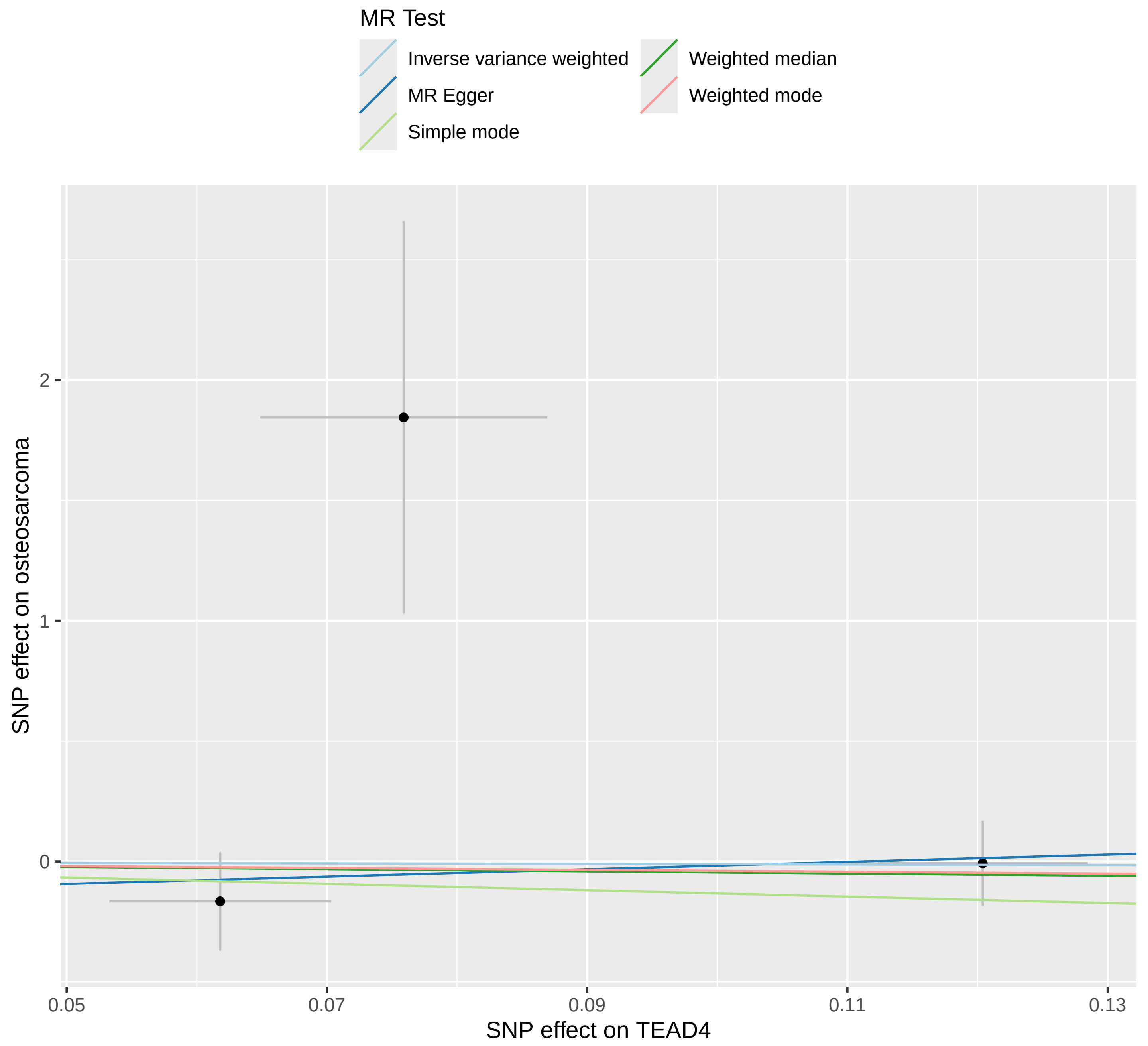
MR Test
Inverse variance weighted
MR Egger
Simple mode
Weighted median
Weighted mode
2
1
0
SNP effect on osteosarcoma
0.05
0.07
0.09
0.11
0.13
SNP effect on TEAD4

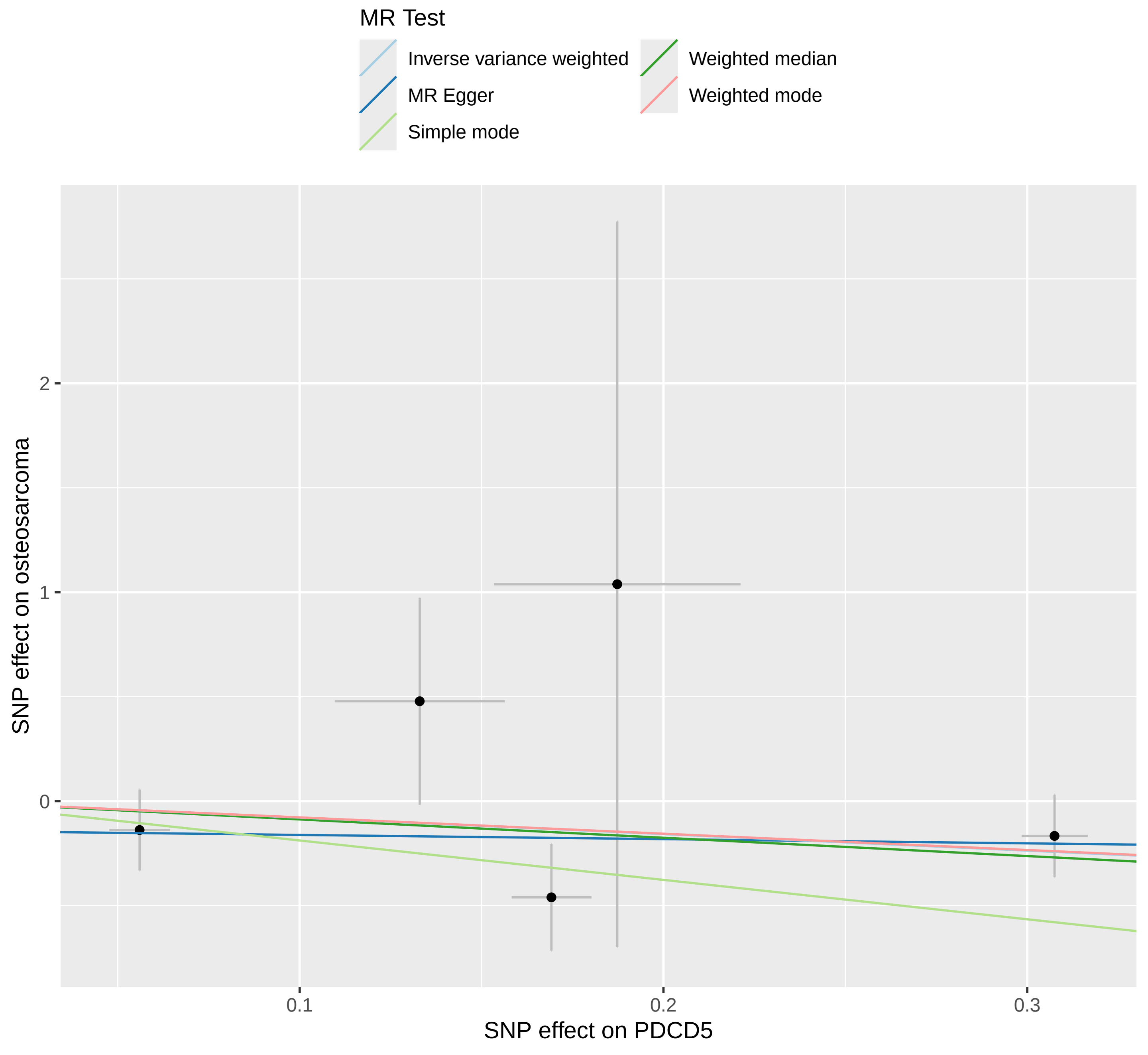
MR Test
Inverse variance weighted
MR Egger
Simple mode
Weighted median
Weighted mode
SNP effect on osteosarcoma
2
1
0
0.1
0.2
0.3
SNP effect on PDCD5

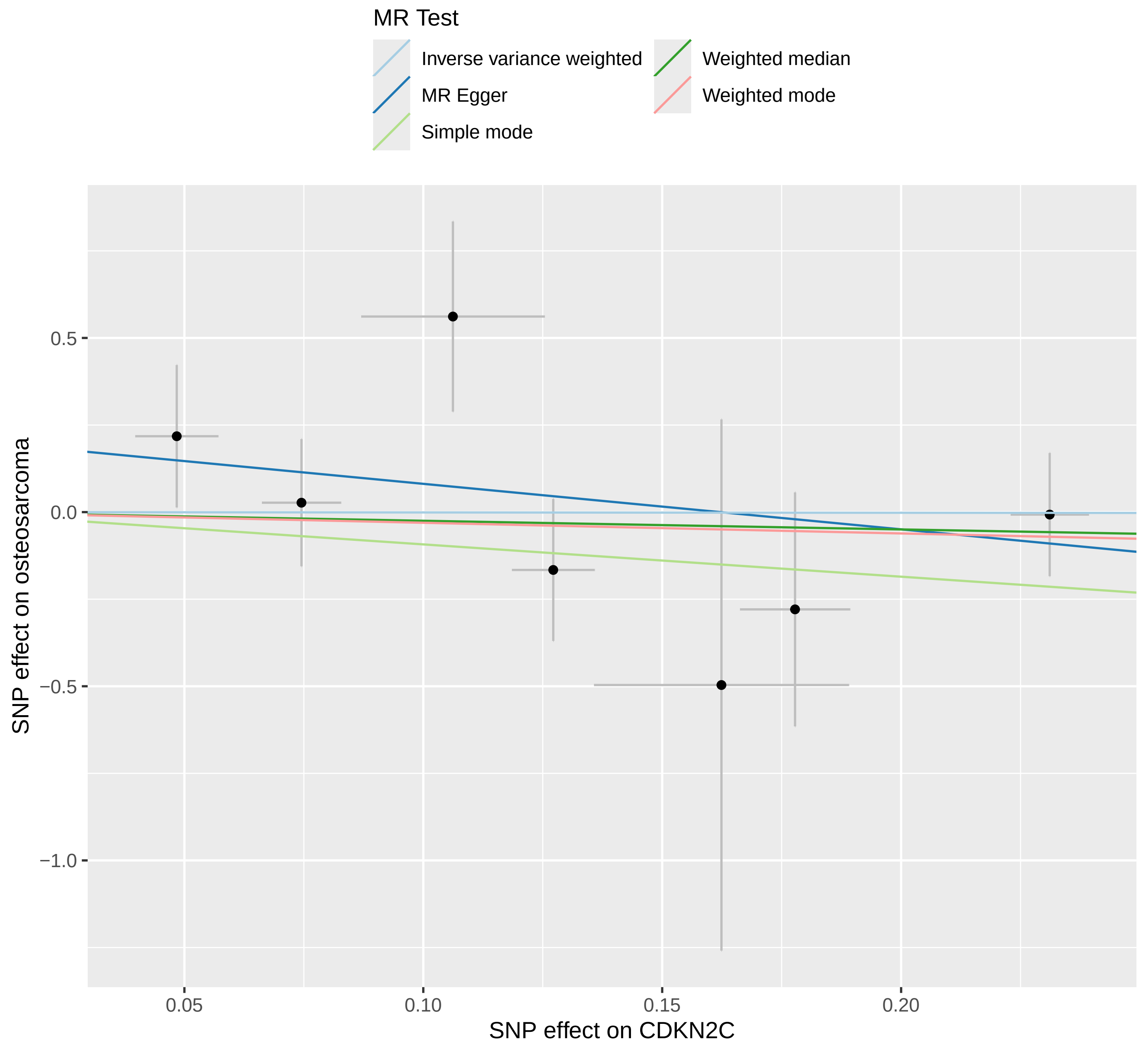

MR Test
Inverse variance weighted
MR Egger
Simple mode
Weighted median
Weighted mode
SNP effect on osteosarcoma
0.5
0.0
−0.5
−1.0
0.05
0.10
0.15
0.20
SNP effect on CDKN2C

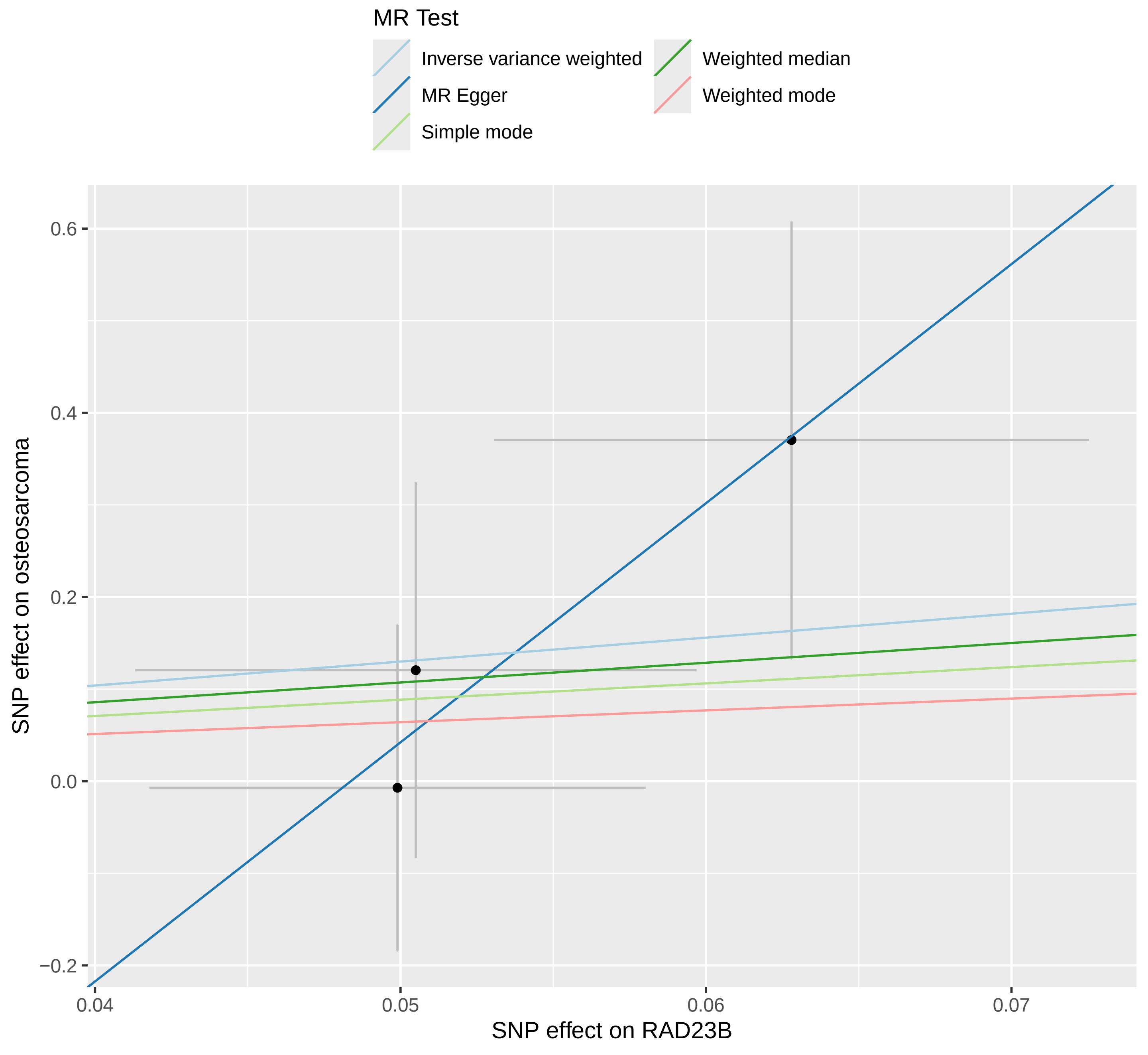
MR Test
Inverse variance weighted
MR Egger
Simple mode
Weighted median
Weighted mode
0.6
0.4
0.2
0.0
−0.2
SNP effect on osteosarcoma
0.04
0.05
0.06
0.07
SNP effect on RAD23B

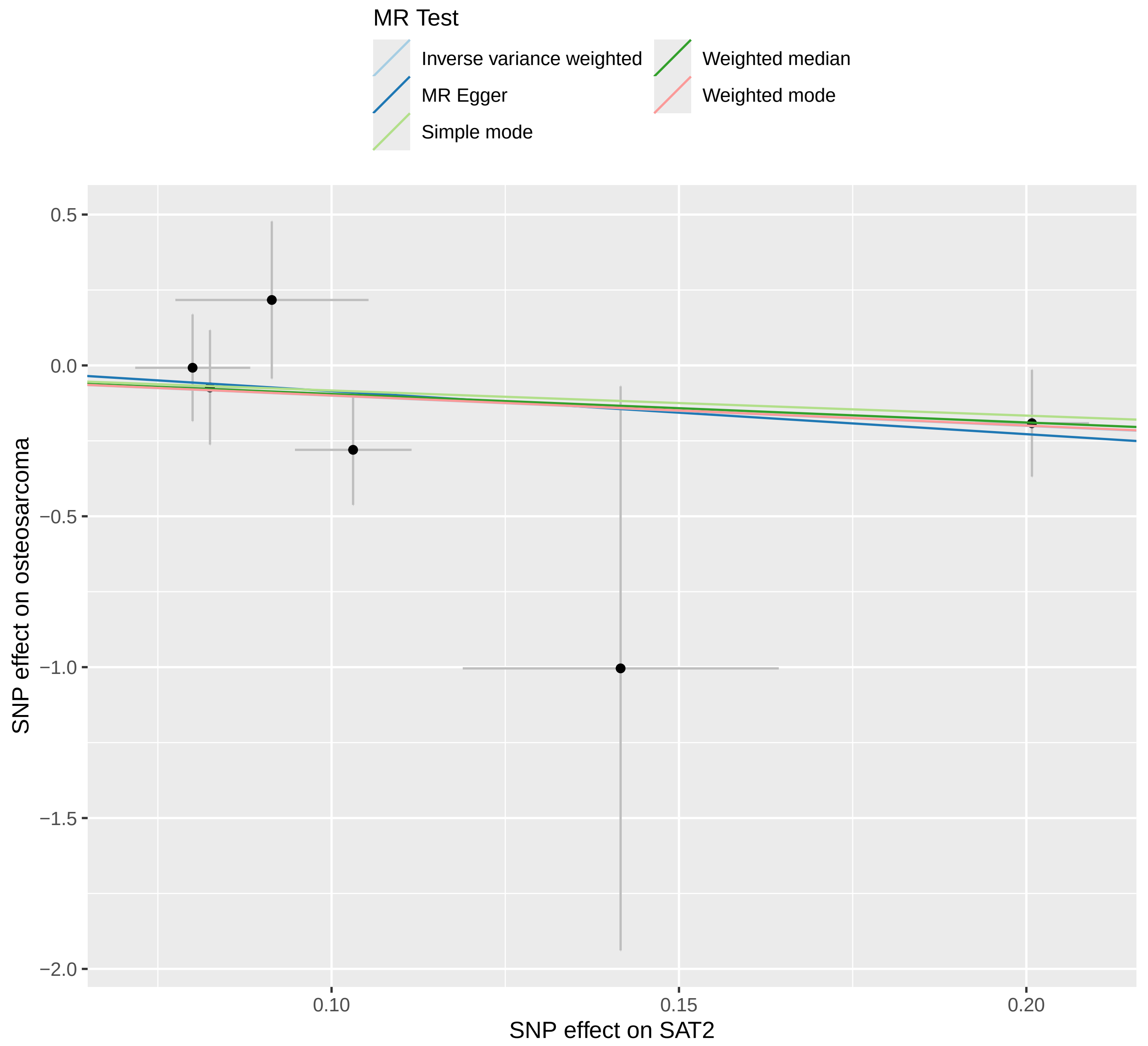
MR Test
Inverse variance weighted
MR Egger
Simple mode
Weighted median
Weighted mode
0.5
0.0
−0.5
−1.0
−1.5
−2.0
0.10
0.15
0.20
SNP effect on osteosarcoma
SNP effect on SAT2

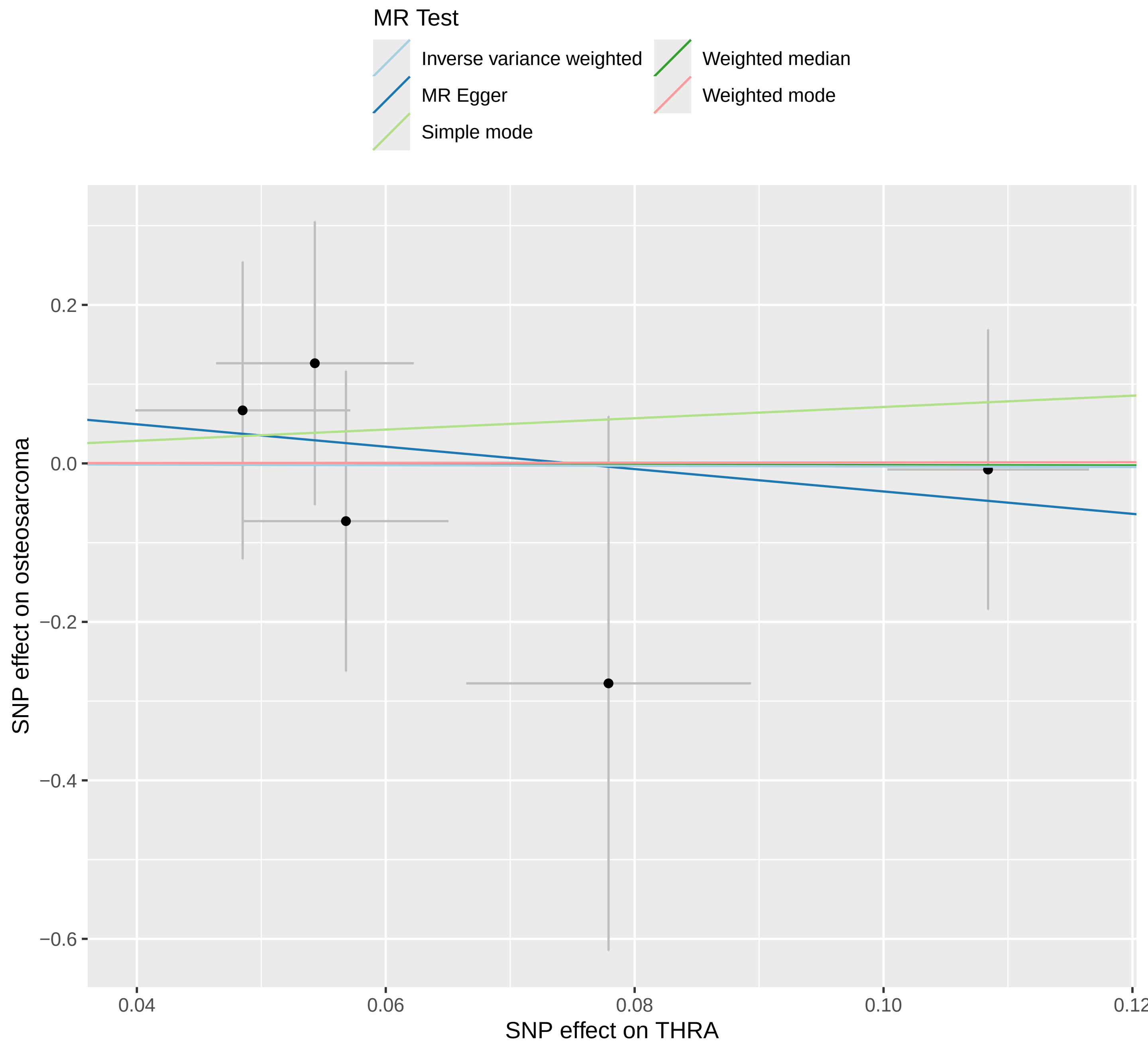
MR Test
Inverse variance weighted
MR Egger
Simple mode
Weighted median
Weighted mode
0.2
0.0
−0.2
−0.4
−0.6
SNP effect on osteosarcoma
0.04
0.06
0.08
0.10
0.12
SNP effect on THRA

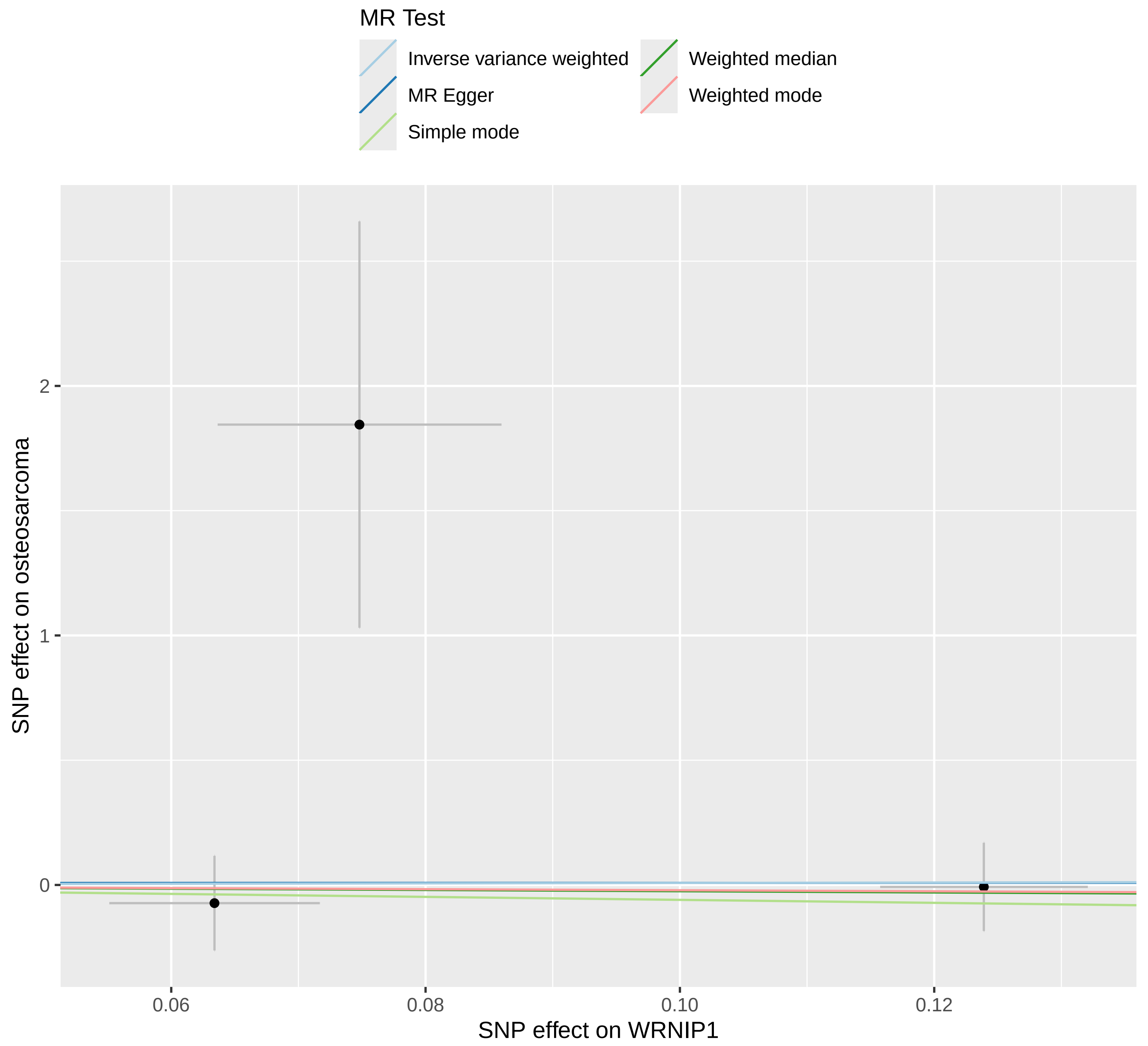
MR Test
Inverse variance weighted
MR Egger
Simple mode
Weighted median
Weighted mode
SNP effect on osteosarcoma
SNP effect on WRNIP1
0
1
2
0.06
0.08
0.10
0.12

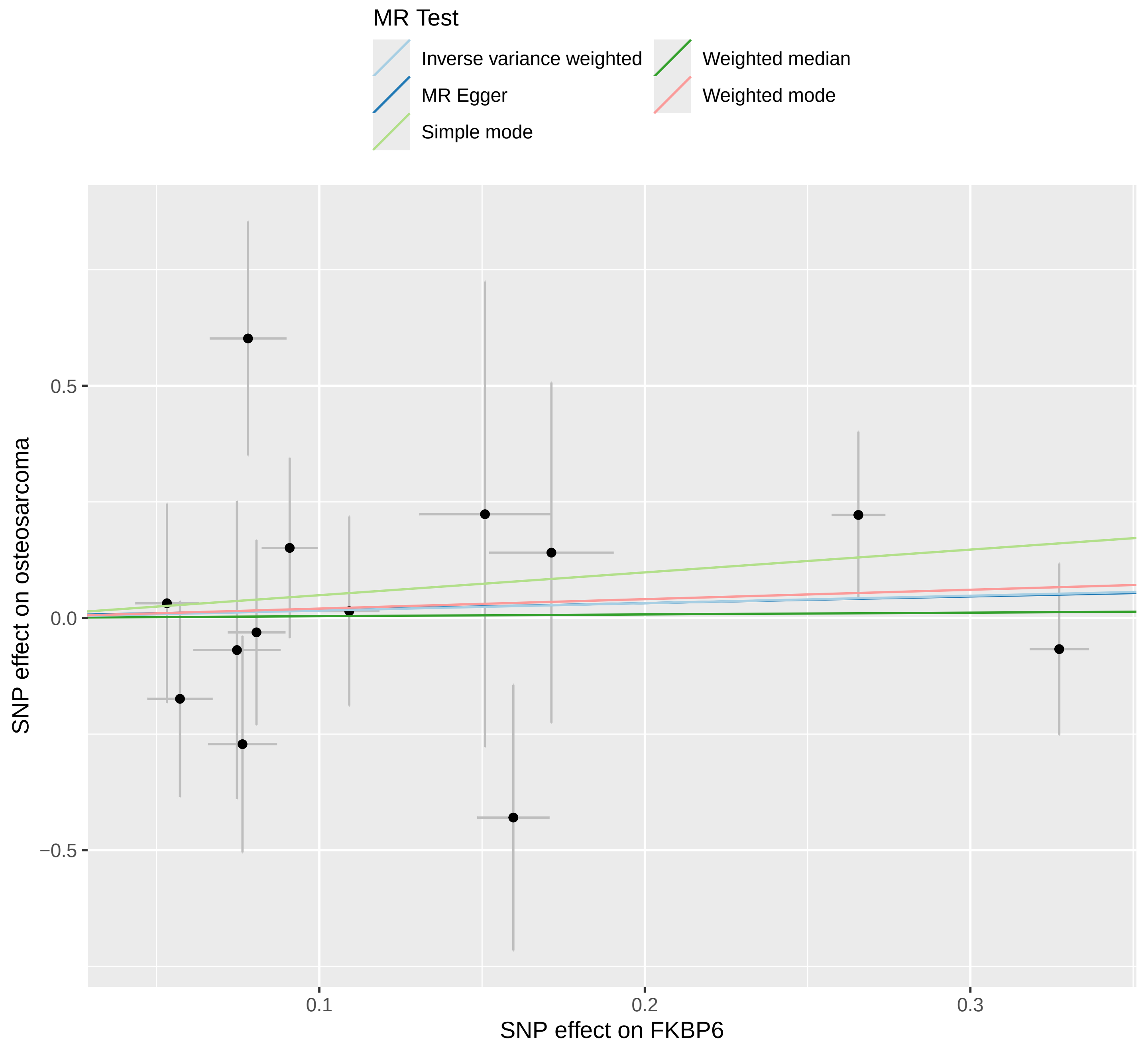

MR Test
Inverse variance weighted
MR Egger
Simple mode
Weighted median
Weighted mode
0.5
0.0
−0.5
0.1
0.2
0.3
SNP effect on osteosarcoma
SNP effect on FKBP6

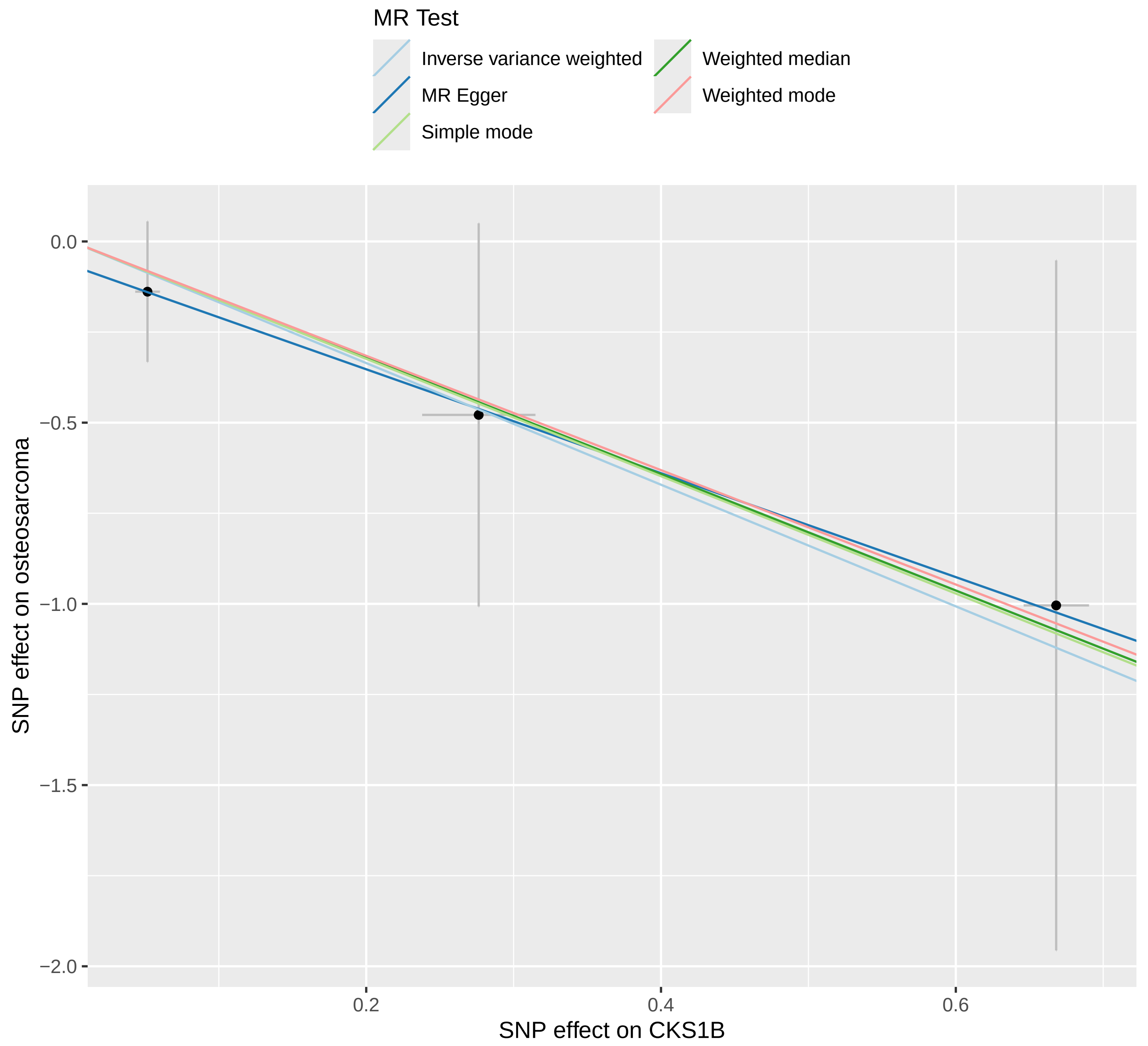
MR Test
Inverse variance weighted
MR Egger
Simple mode
Weighted median
Weighted mode
0.0
−0.5
−1.0
−1.5
−2.0
SNP effect on osteosarcoma
0.2
0.4
0.6
SNP effect on CKS1B

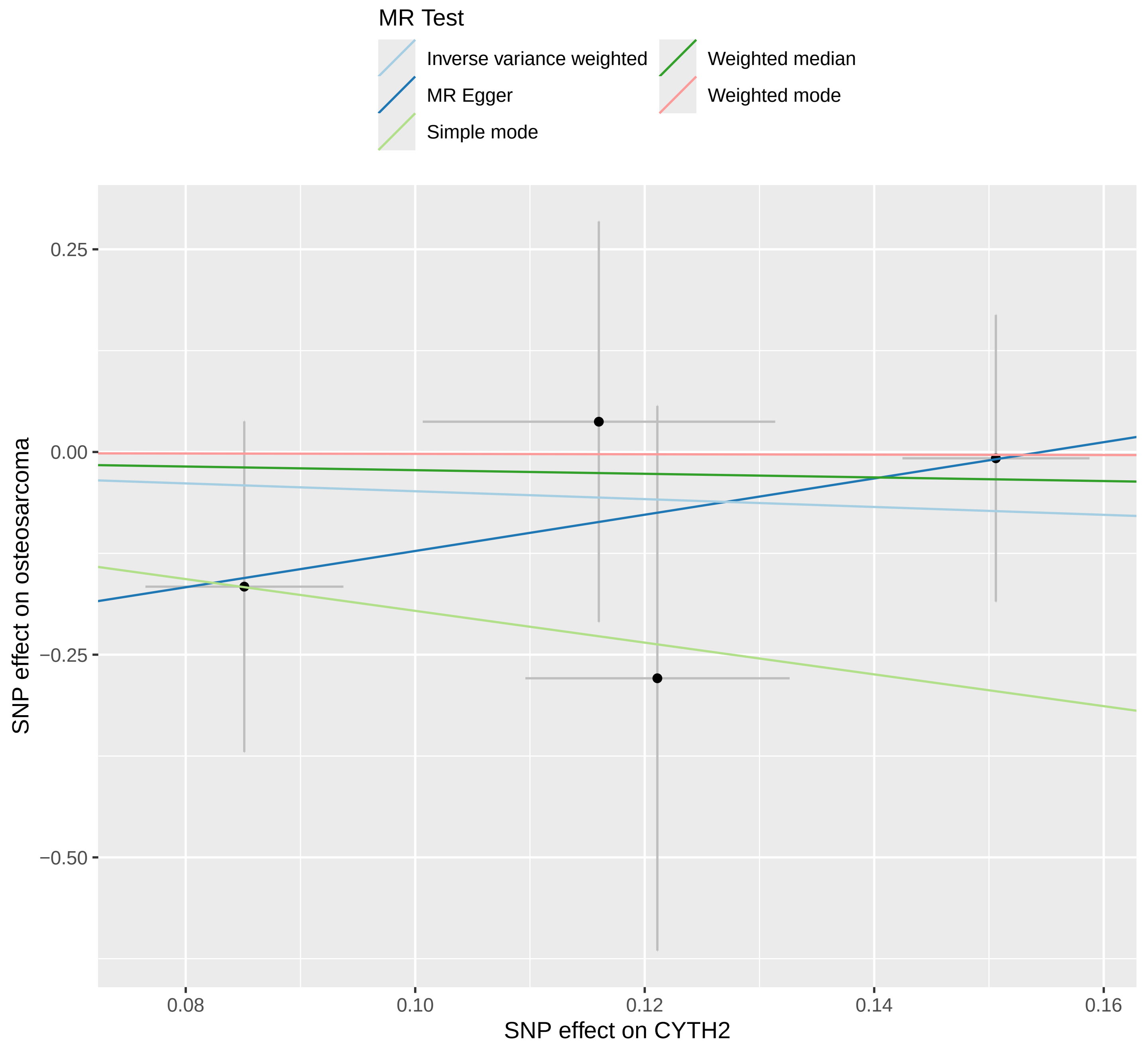
MR Test
Inverse variance weighted
MR Egger
Simple mode
Weighted median
Weighted mode
0.25
0.00
−0.25
−0.50
SNP effect on osteosarcoma
0.08
0.10
0.12
0.14
0.16
SNP effect on CYTH2

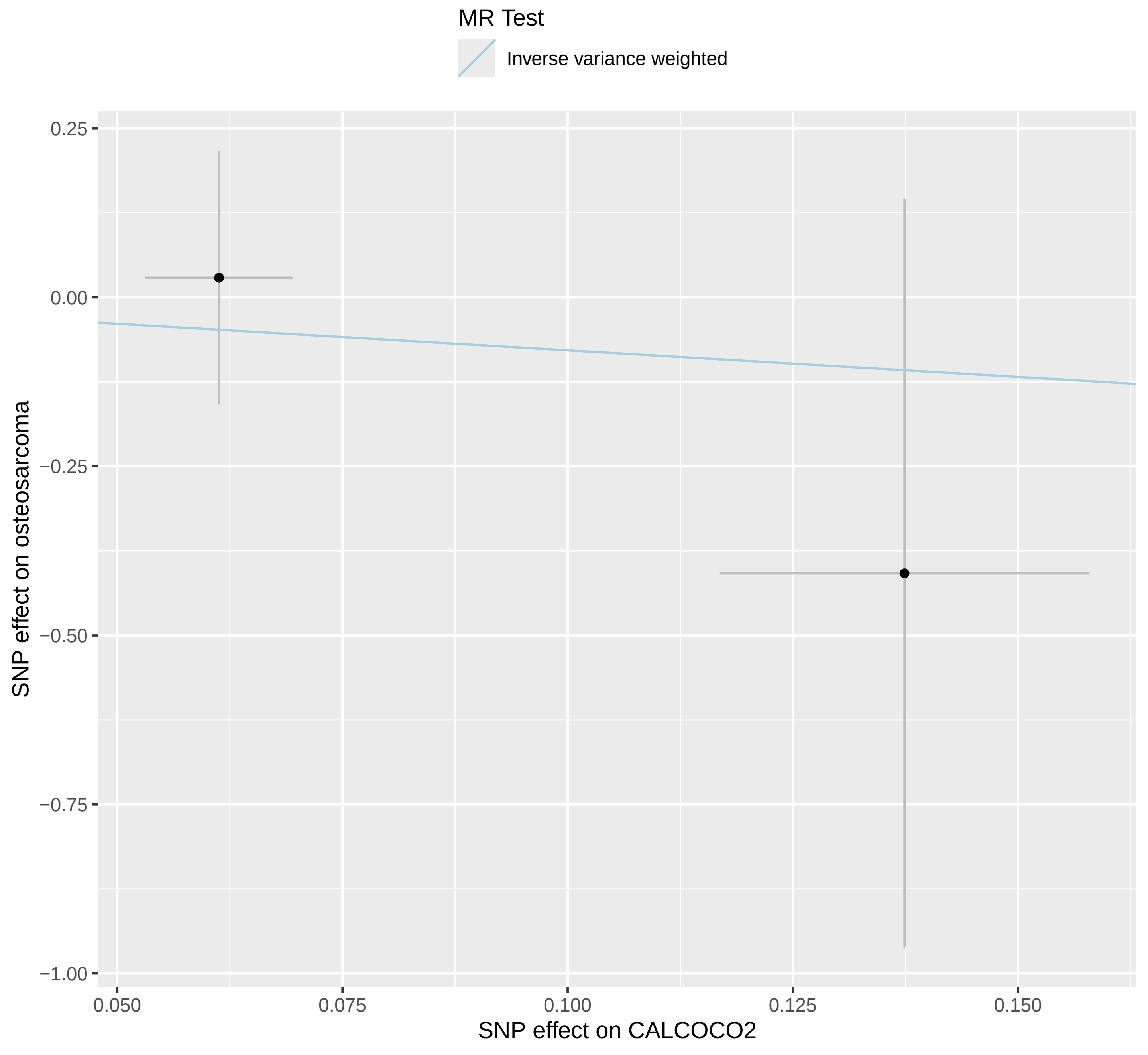
MR Test
Inverse variance weighted
0.25
0.00
−0.25
−0.50
−0.75
−1.00
0.050
0.075
0.100
0.125
0.150
SNP effect on osteosarcoma
SNP effect on CALCOCO2

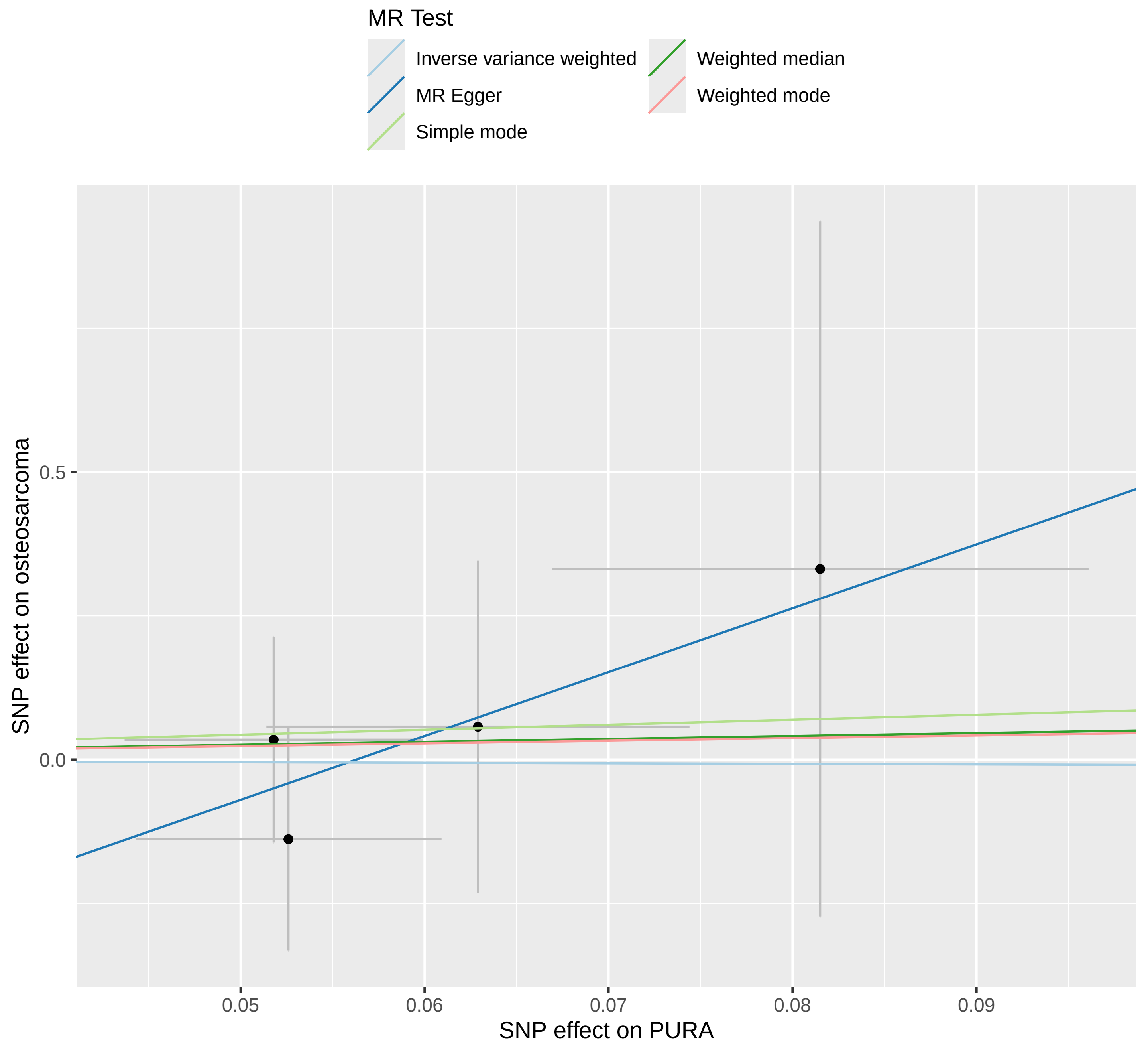

MR Test
Inverse variance weighted
MR Egger
Simple mode
Weighted median
Weighted mode
SNP effect on osteosarcoma
0.5
0.0
0.05
0.06
0.07
0.08
0.09
SNP effect on PURA

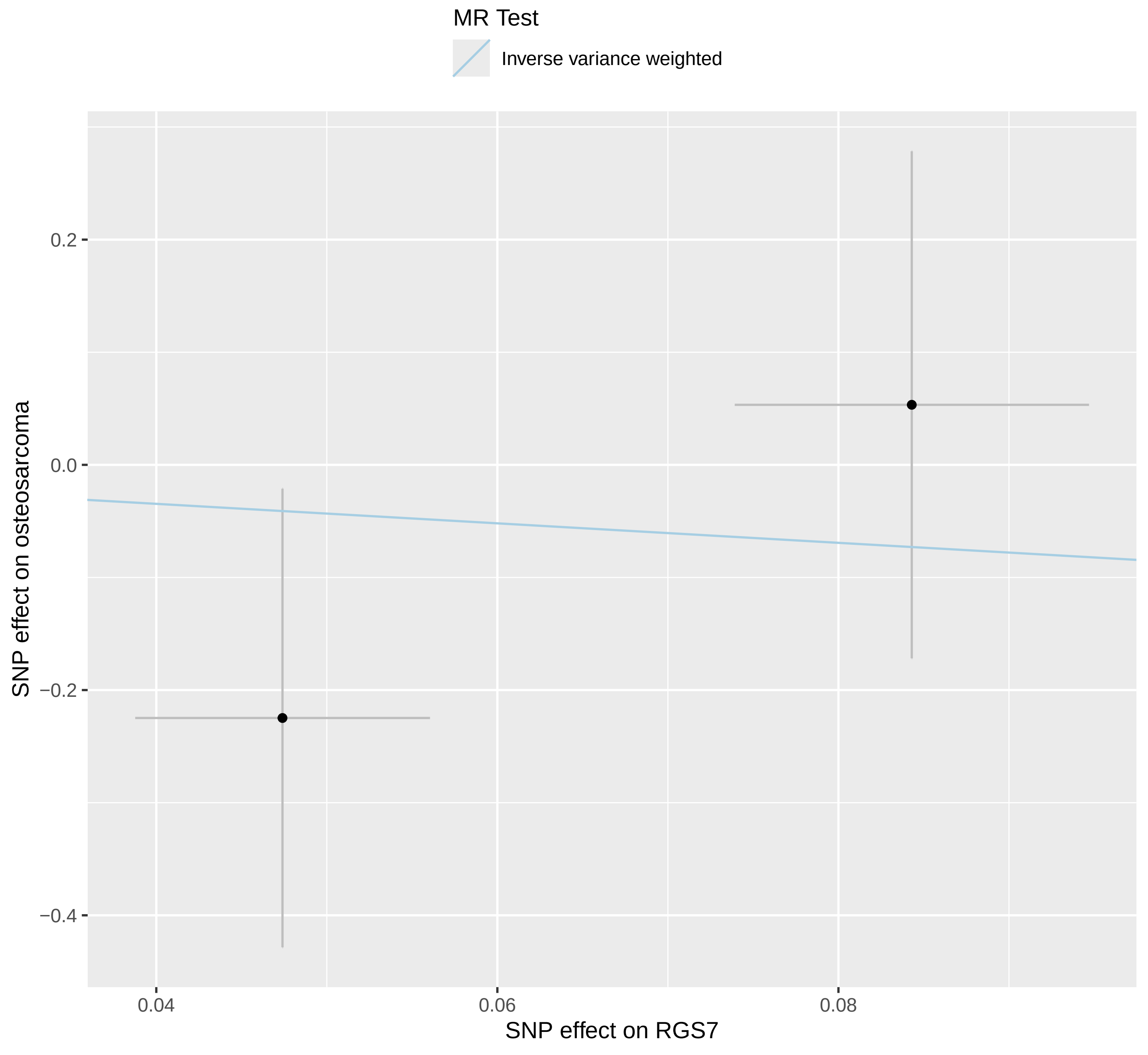

MR Test
Inverse variance weighted
SNP effect on osteosarcoma
0.2
0.0
−0.2
−0.4
0.04
0.06
0.08
SNP effect on RGS7

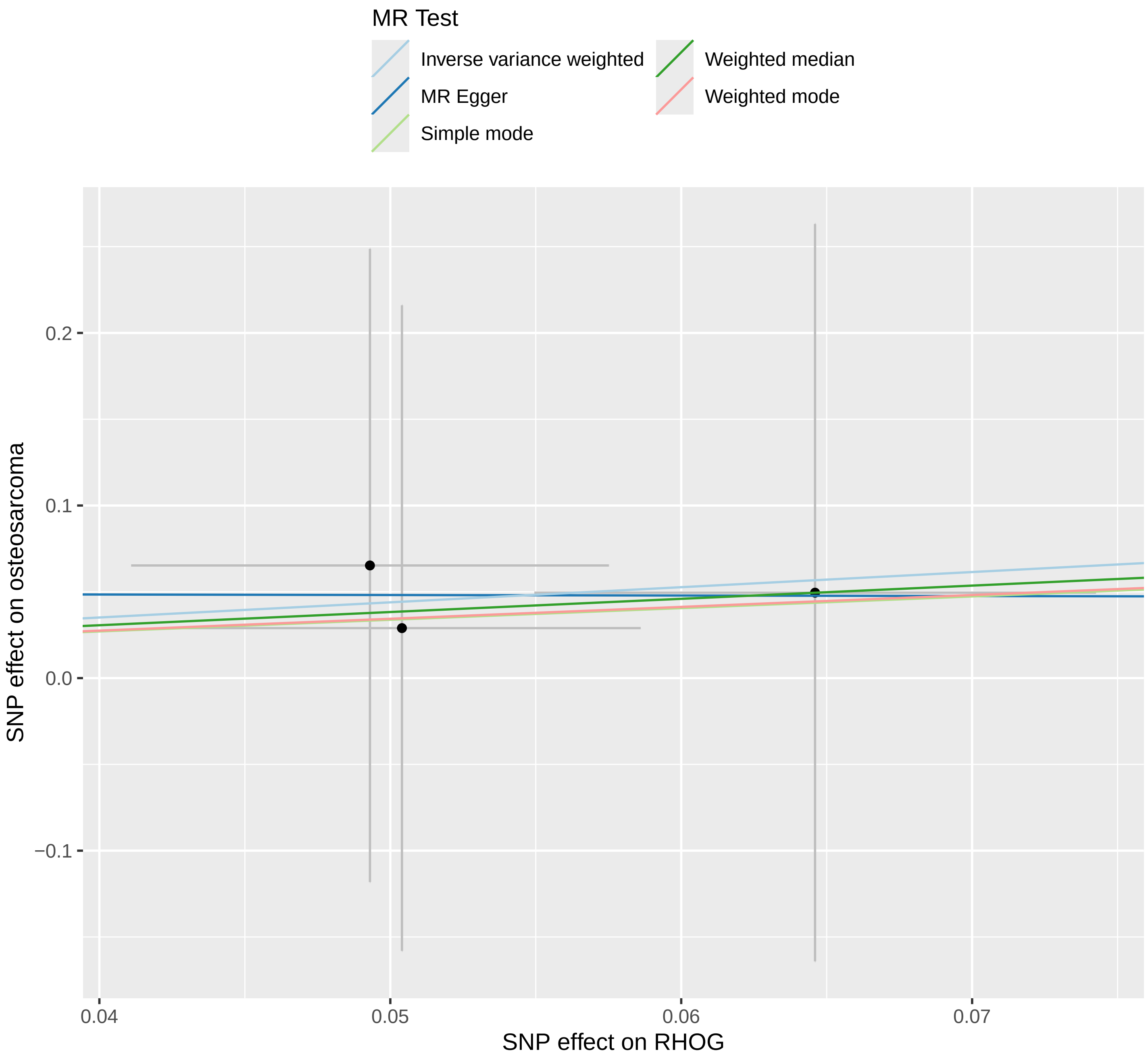
MR Test
Inverse variance weighted
MR Egger
Simple mode
Weighted median
Weighted mode
SNP effect on osteosarcoma
0.2
0.1
0.0
−0.1
0.04
0.05
0.06
0.07
SNP effect on RHOG

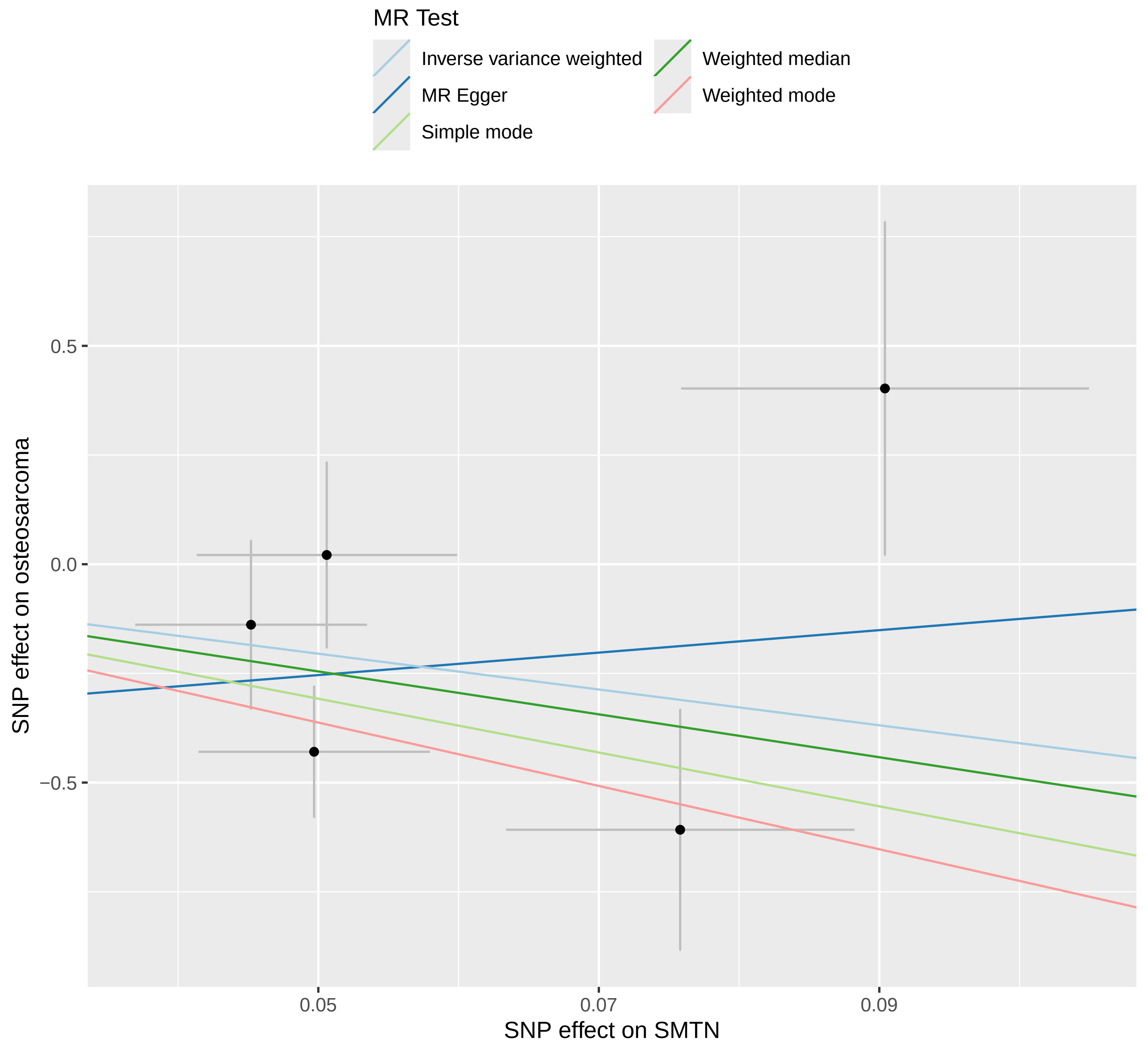
MR Test
Inverse variance weighted
MR Egger
Simple mode
Weighted median
Weighted mode
SNP effect on osteosarcoma
0.5
0.0
−0.5
0.05
0.07
0.09
SNP effect on SMTN

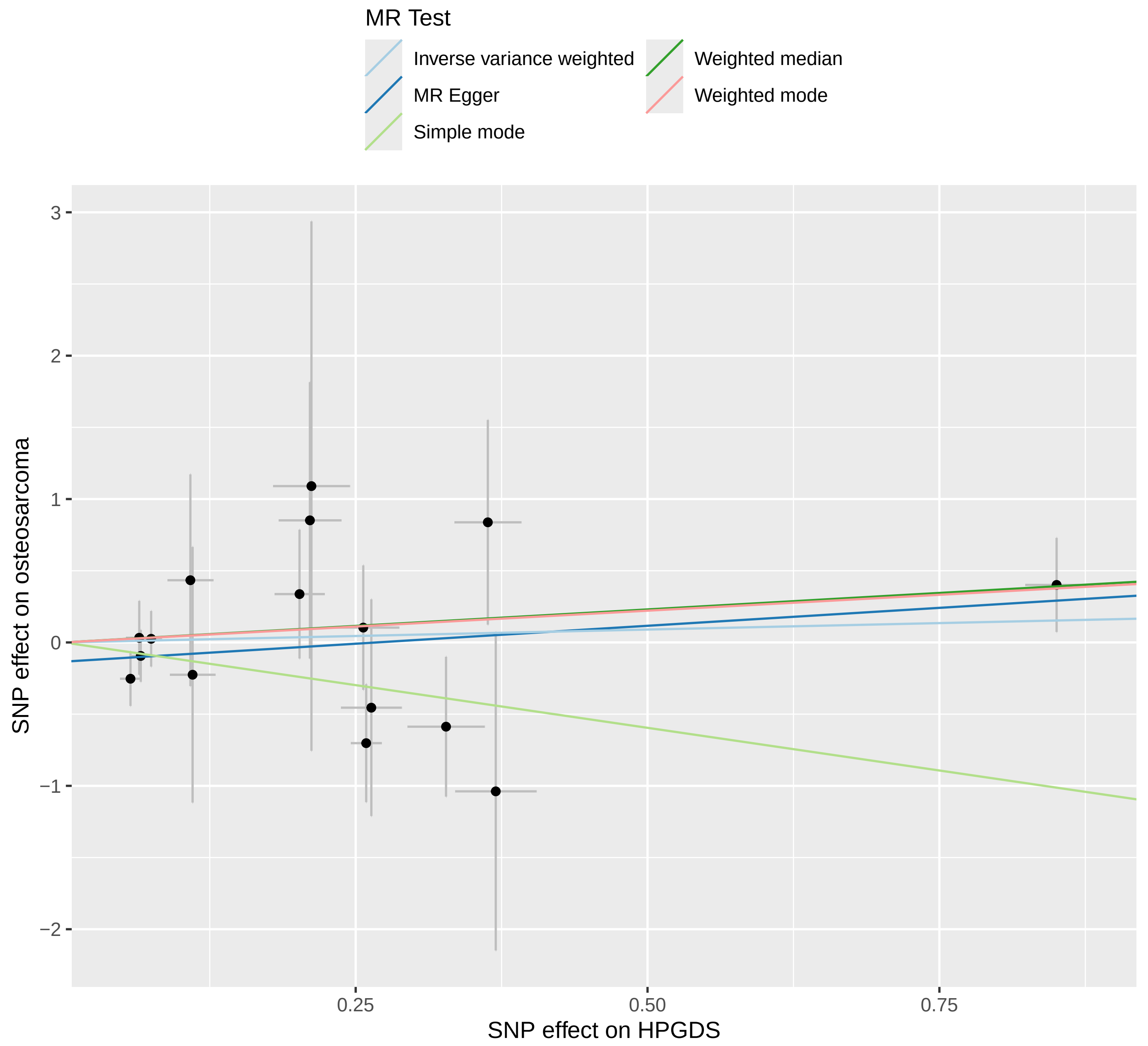

MR Test
Inverse variance weighted
MR Egger
Simple mode
Weighted median
Weighted mode
3
2
1
0
−1
−2
0.25
0.50
0.75
SNP effect on osteosarcoma
SNP effect on HPGDS

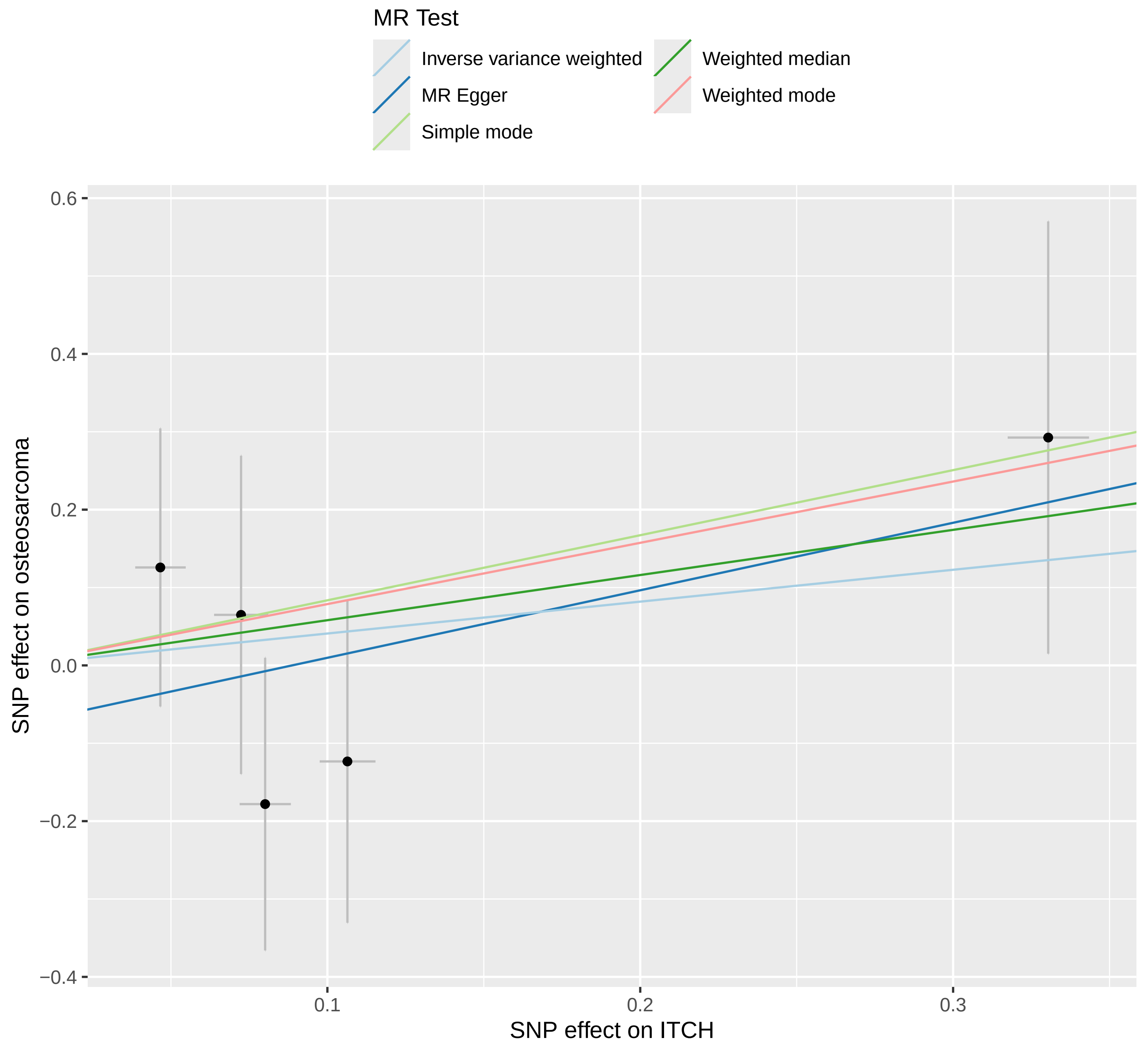

MR Test
Inverse variance weighted
MR Egger
Simple mode
Weighted median
Weighted mode
SNP effect on osteosarcoma
0.6
0.4
0.2
0.0
−0.2
−0.4
0.1
0.2
0.3
SNP effect on ITCH

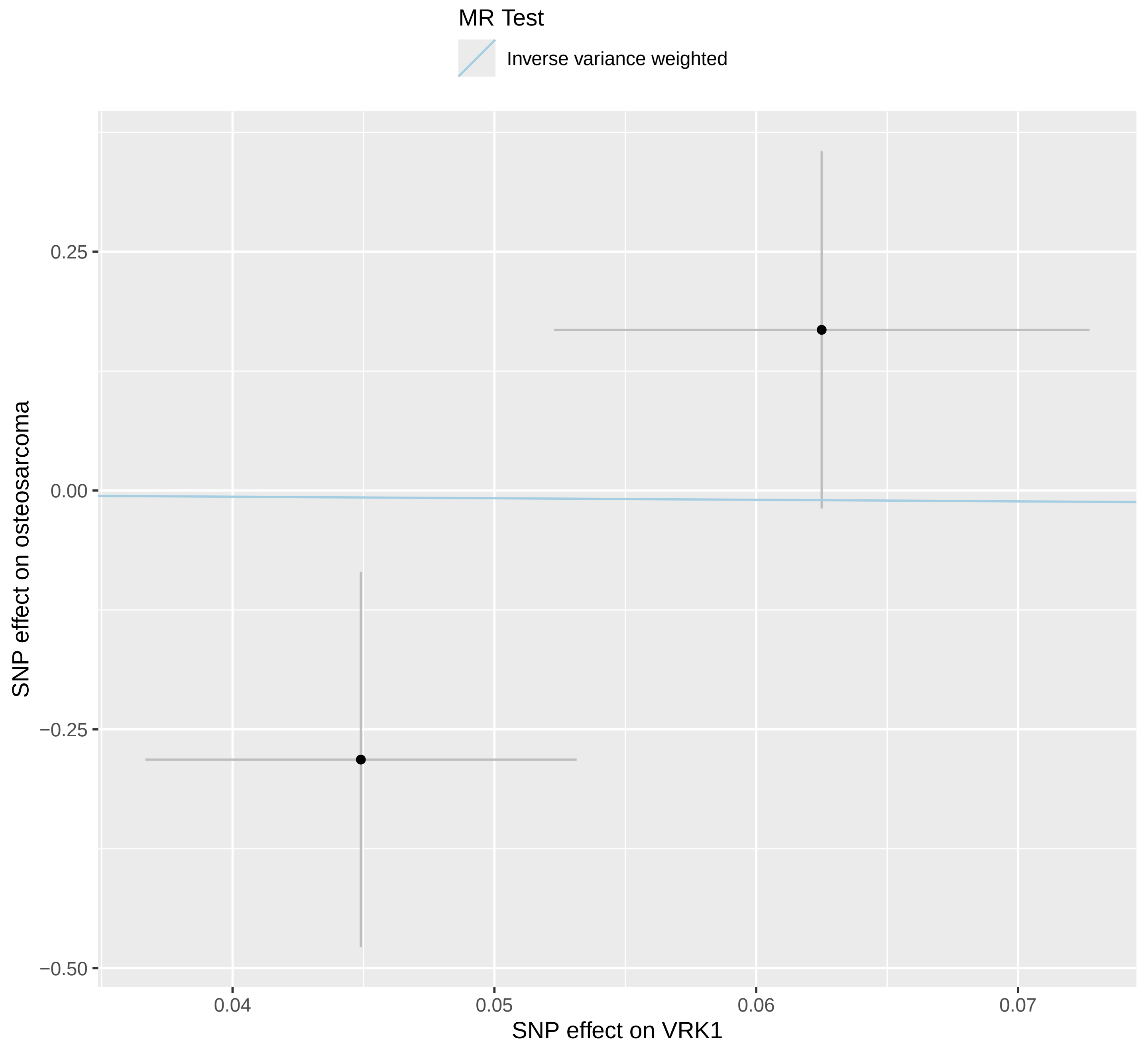
MR Test
Inverse variance weighted
0.25
0.00
−0.25
−0.50
SNP effect on osteosarcoma
0.04
0.05
0.06
0.07
SNP effect on VRK1

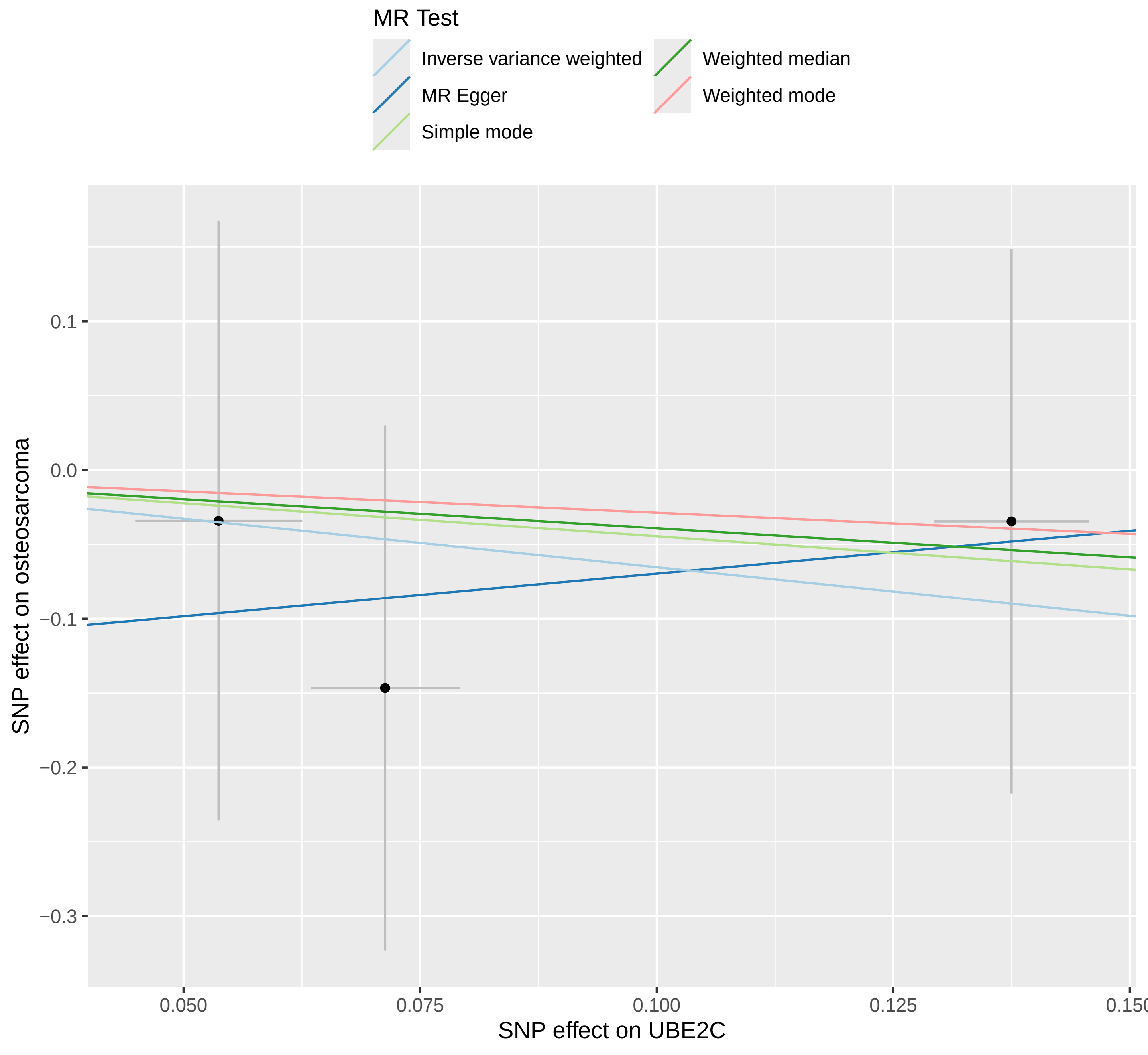
MR Test
Inverse variance weighted
MR Egger
Simple mode
Weighted median
Weighted mode
0.1
0.0
−0.1
−0.2
−0.3
0.050
0.075
0.100
0.125
0.150
SNP effect on UBE2C
SNP effect on osteosarcoma

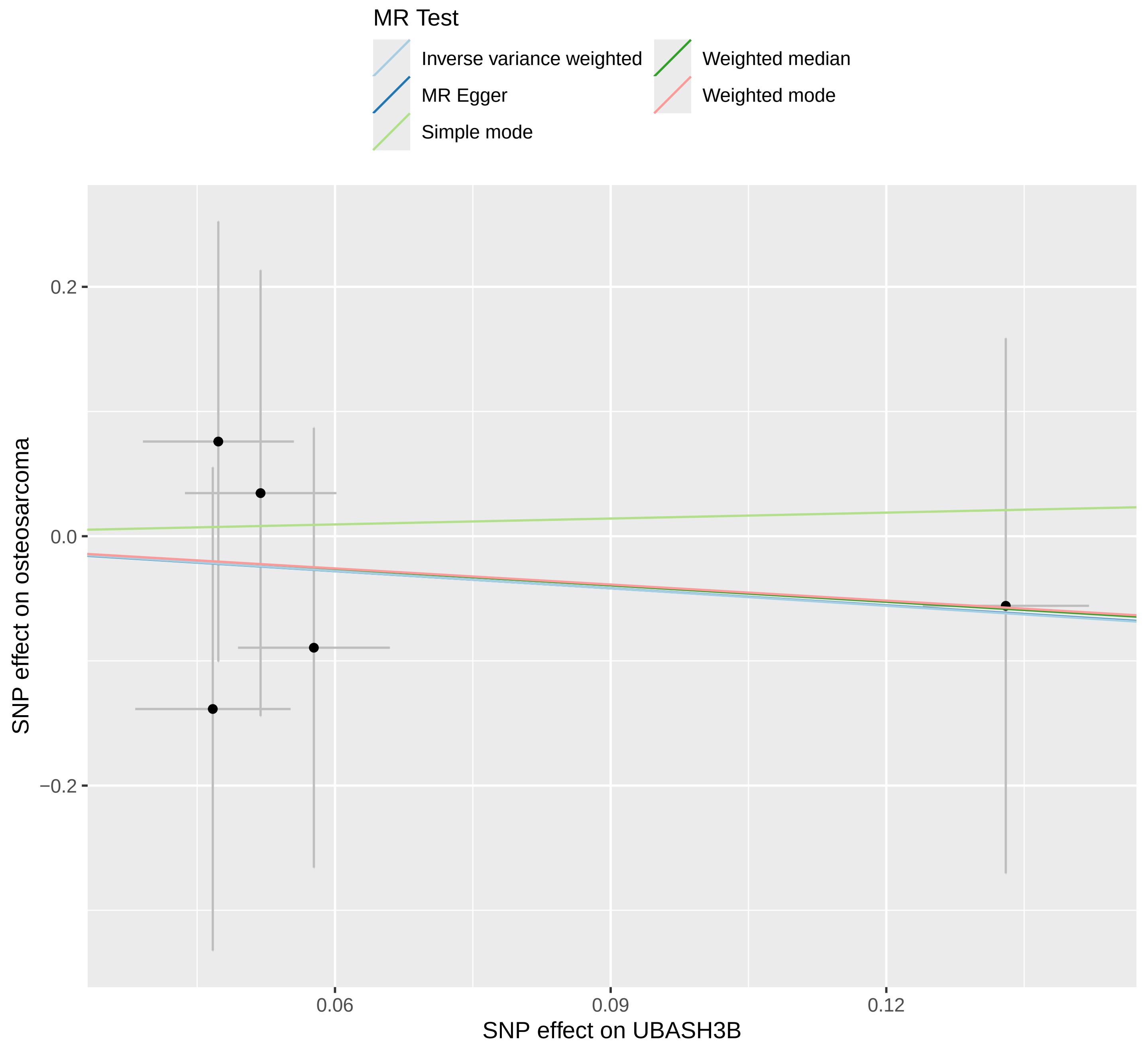

MR Test
Inverse variance weighted
MR Egger
Simple mode
Weighted median
Weighted mode
0.2
0.0
−0.2
0.06
0.09
0.12
SNP effect on osteosarcoma
SNP effect on UBASH3B

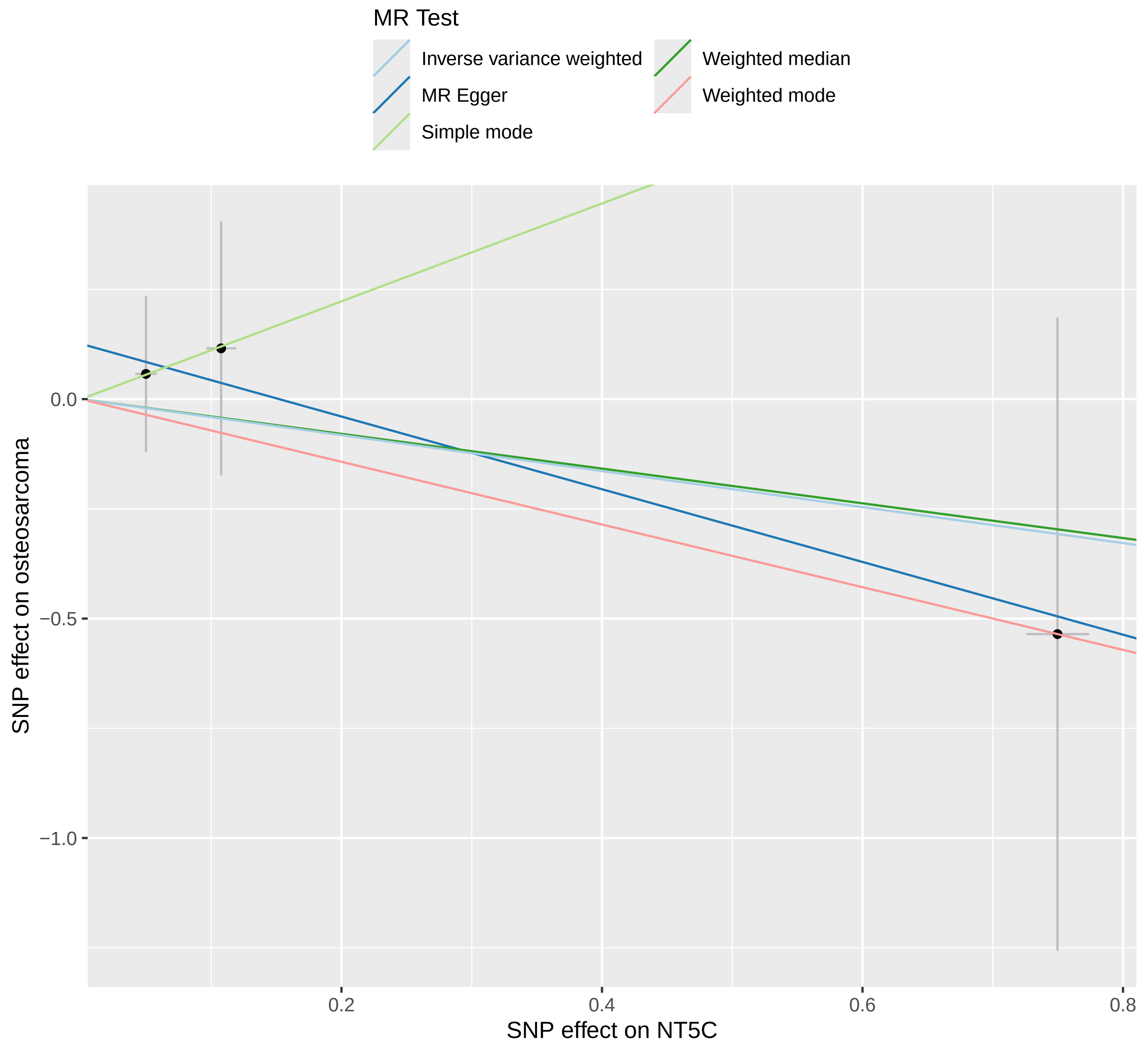
MR Test
Inverse variance weighted
MR Egger
Simple mode
Weighted median
Weighted mode
SNP effect on osteosarcoma
0.0
−0.5
−1.0
0.2
0.4
0.6
0.8
SNP effect on NT5C

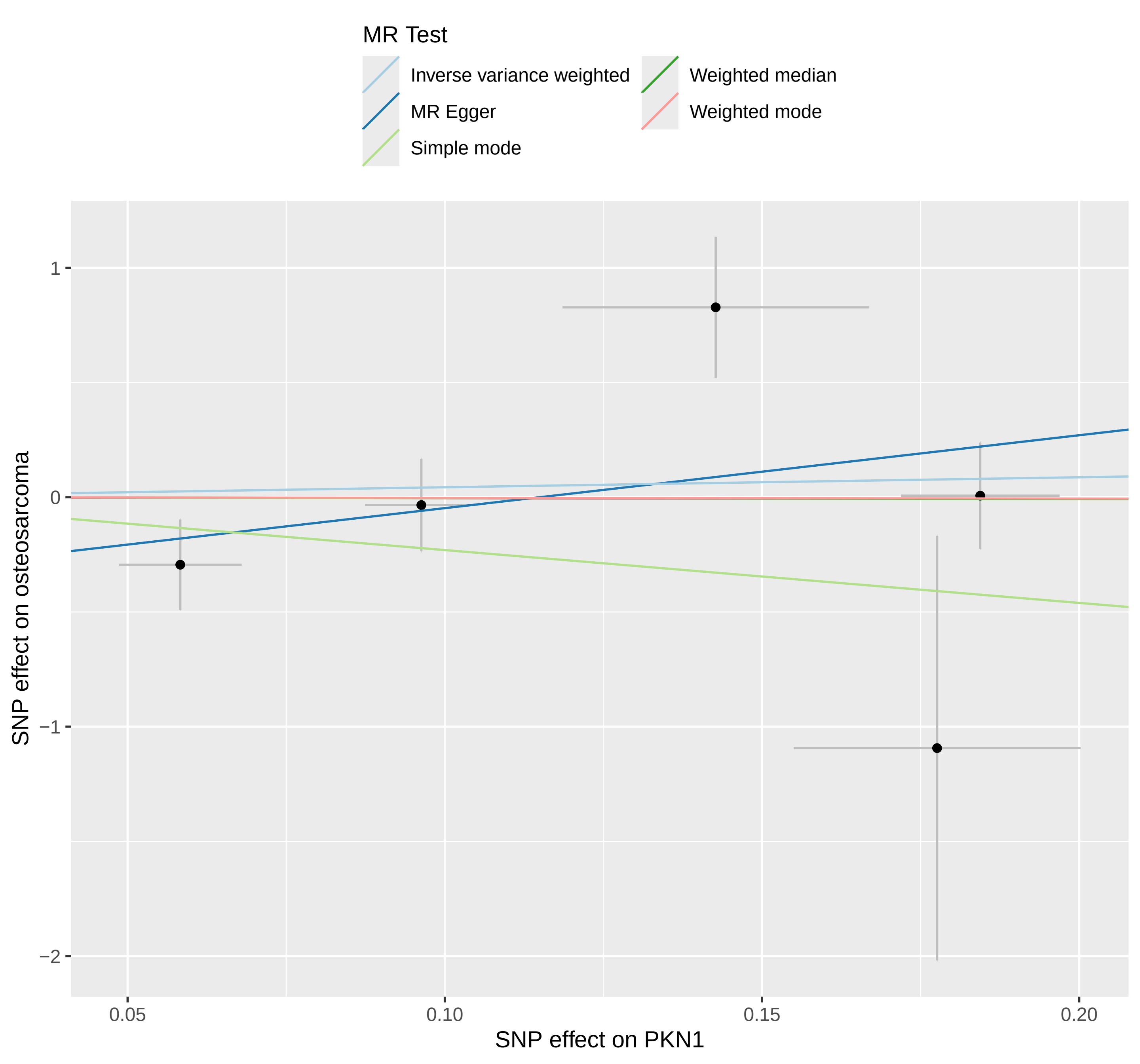

MR Test
Inverse variance weighted
MR Egger
Simple mode
Weighted median
Weighted mode
1
0
−1
−2
0.05
0.10
0.15
0.20
SNP effect on osteosarcoma
SNP effect on PKN1

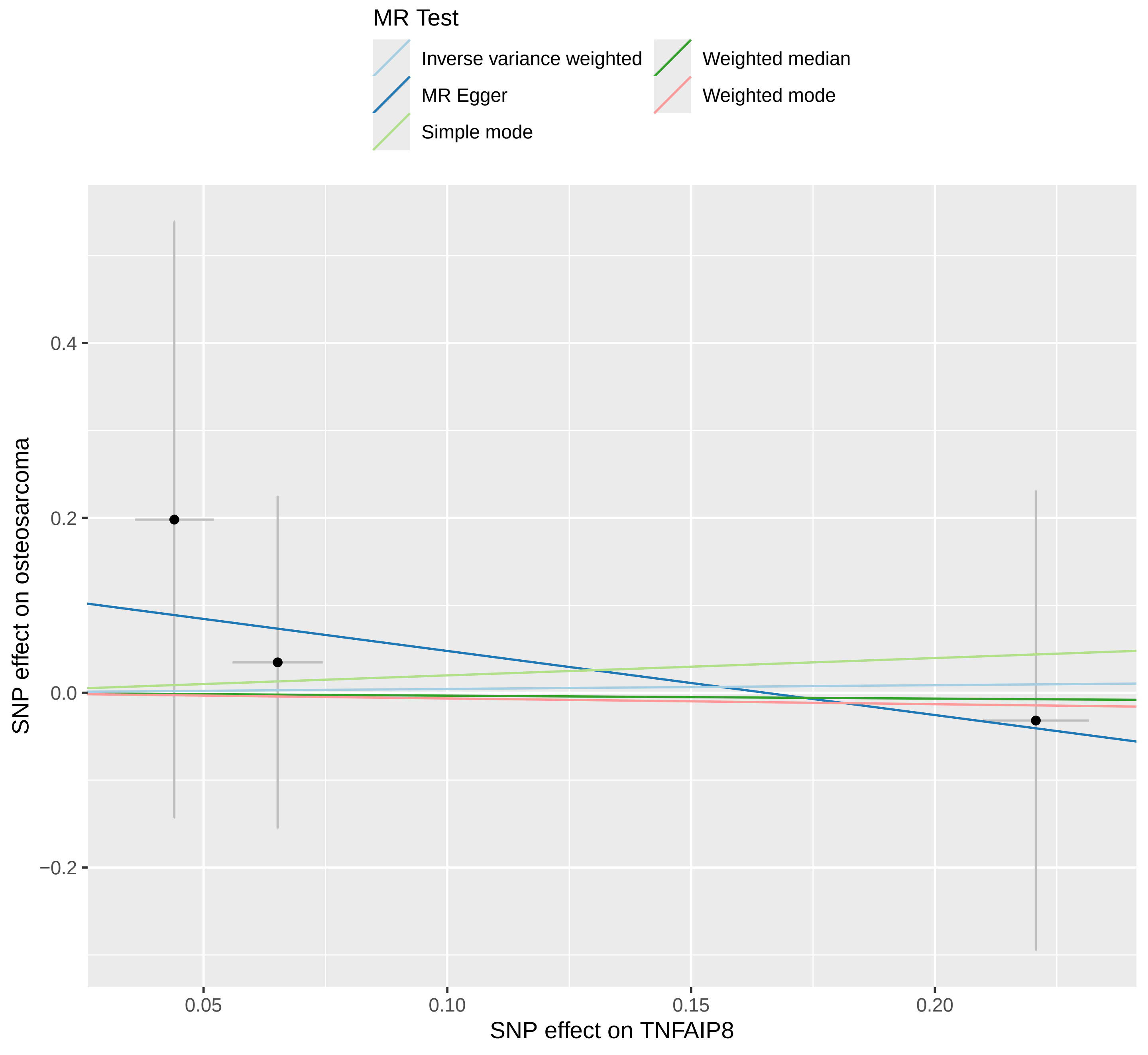
MR Test
Inverse variance weighted
MR Egger
Simple mode
Weighted median
Weighted mode
SNP effect on osteosarcoma
0.4
0.2
0.0
−0.2
0.05
0.10
0.15
0.20
SNP effect on TNFAIP8

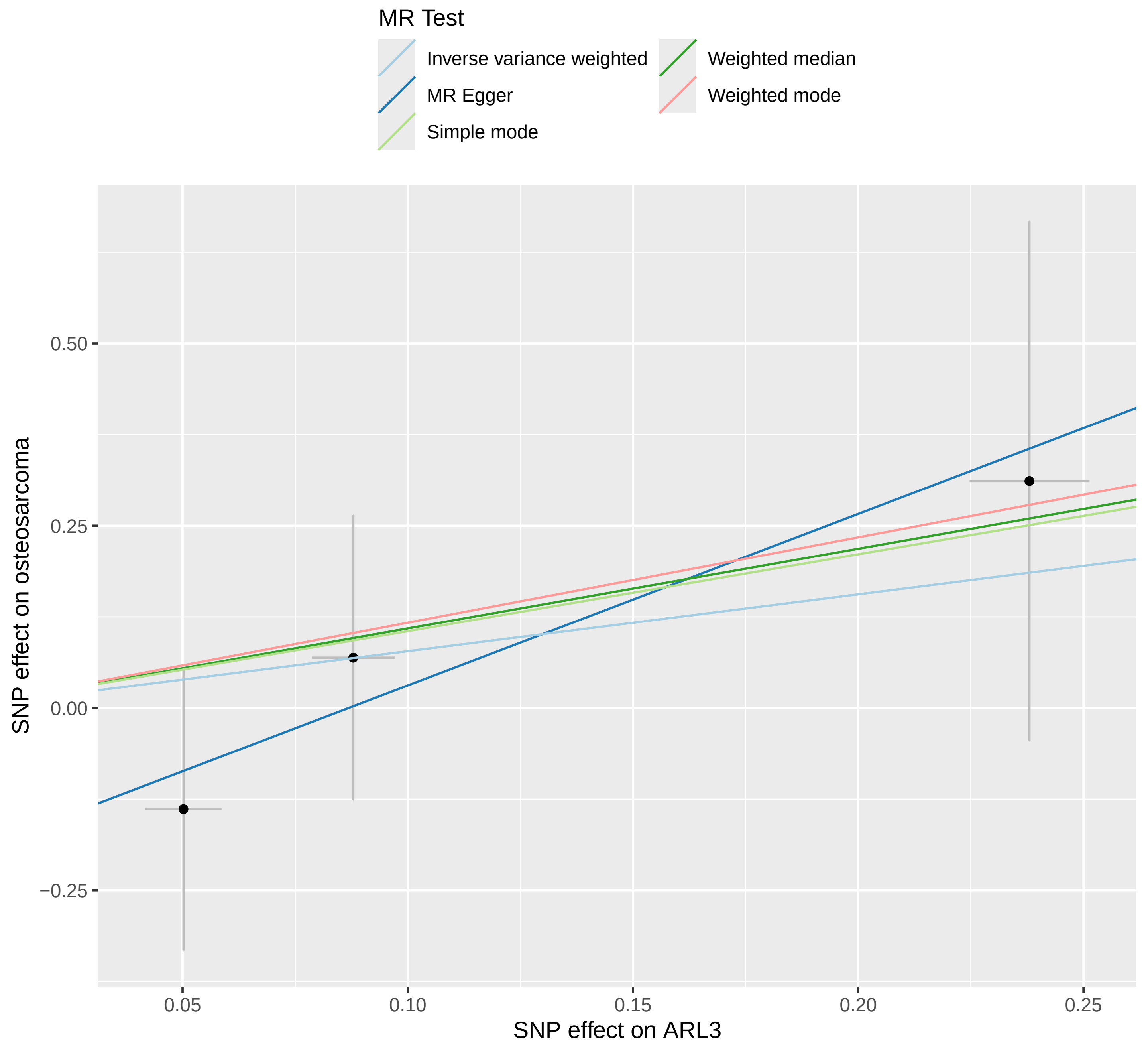

MR Test
Inverse variance weighted
MR Egger
Simple mode
Weighted median
Weighted mode
SNP effect on osteosarcoma
0.50
0.25
0.00
−0.25
0.05
0.10
0.15
0.20
0.25
SNP effect on ARL3

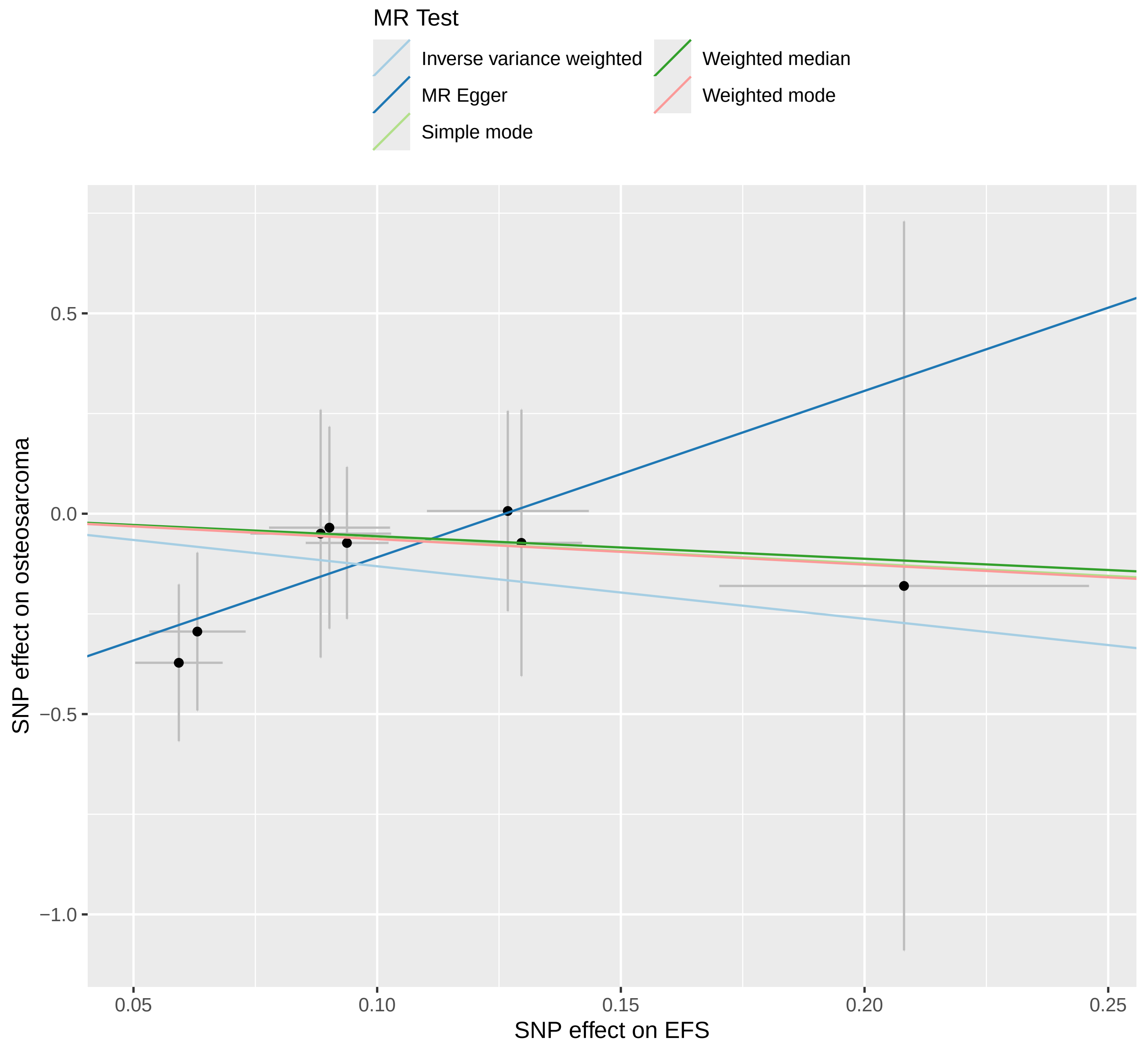
MR Test
Inverse variance weighted
MR Egger
Simple mode
Weighted median
Weighted mode
0.5
0.0
−0.5
−1.0
SNP effect on osteosarcoma
0.05
0.10
0.15
0.20
0.25
SNP effect on EFS

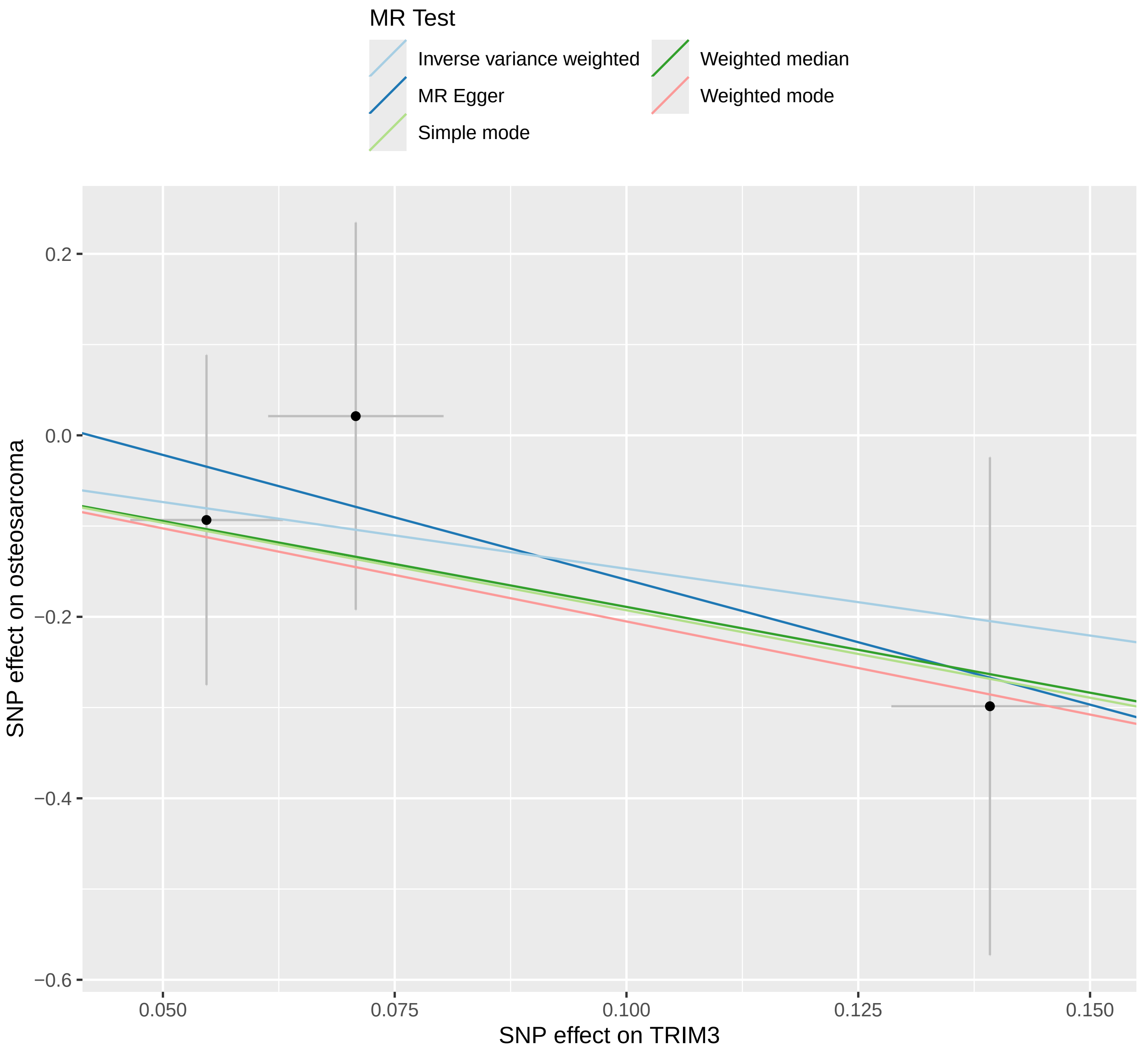

MR Test
Inverse variance weighted
MR Egger
Simple mode
Weighted median
Weighted mode
0.2
0.0
−0.2
−0.4
−0.6
0.050
0.075
0.100
0.125
0.150
SNP effect on TRIM3
SNP effect on osteosarcoma

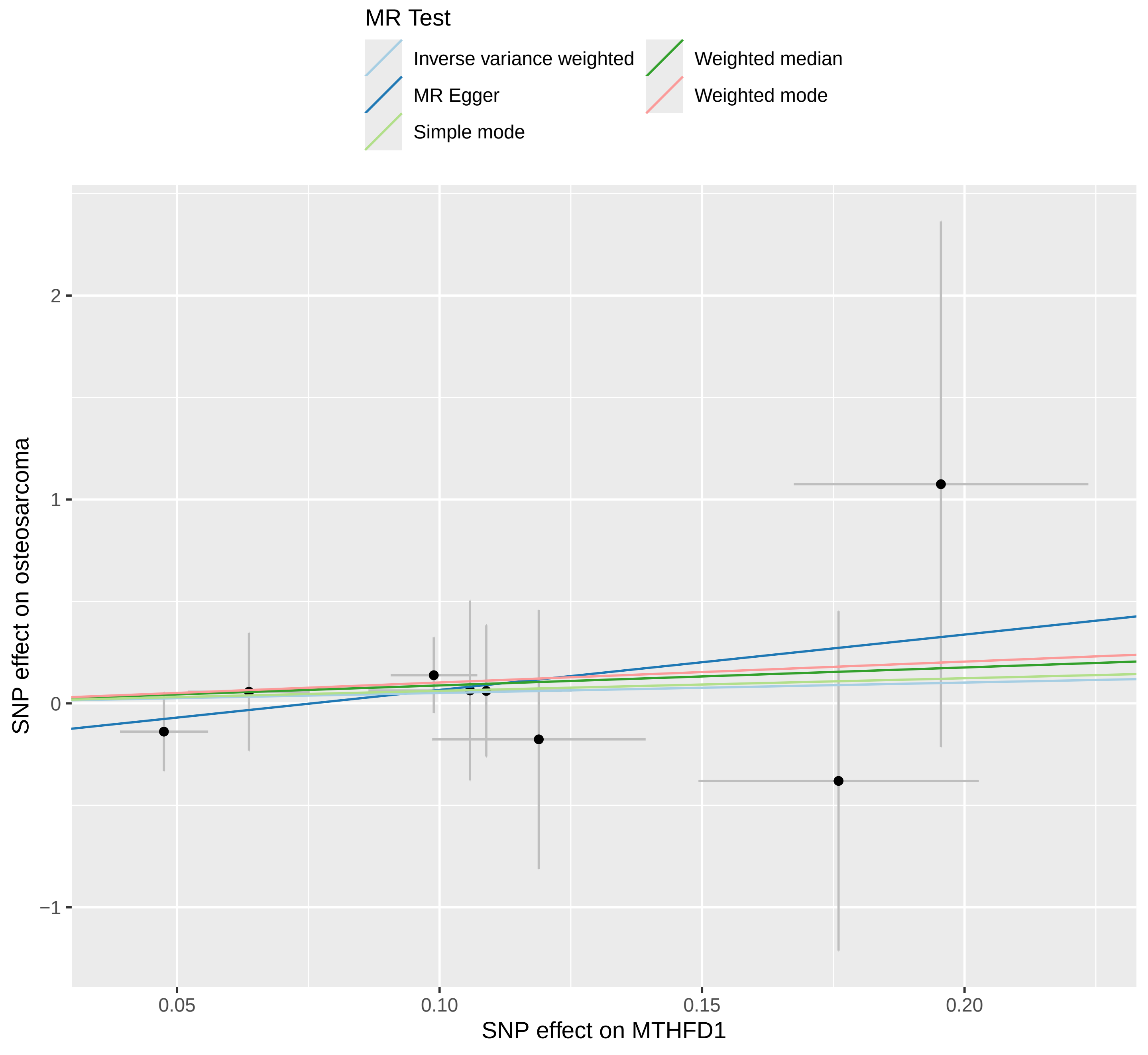
MR Test
Inverse variance weighted
MR Egger
Simple mode
Weighted median
Weighted mode
SNP effect on osteosarcoma
2
1
0
−1
0.05
0.10
0.15
0.20
SNP effect on MTHFD1

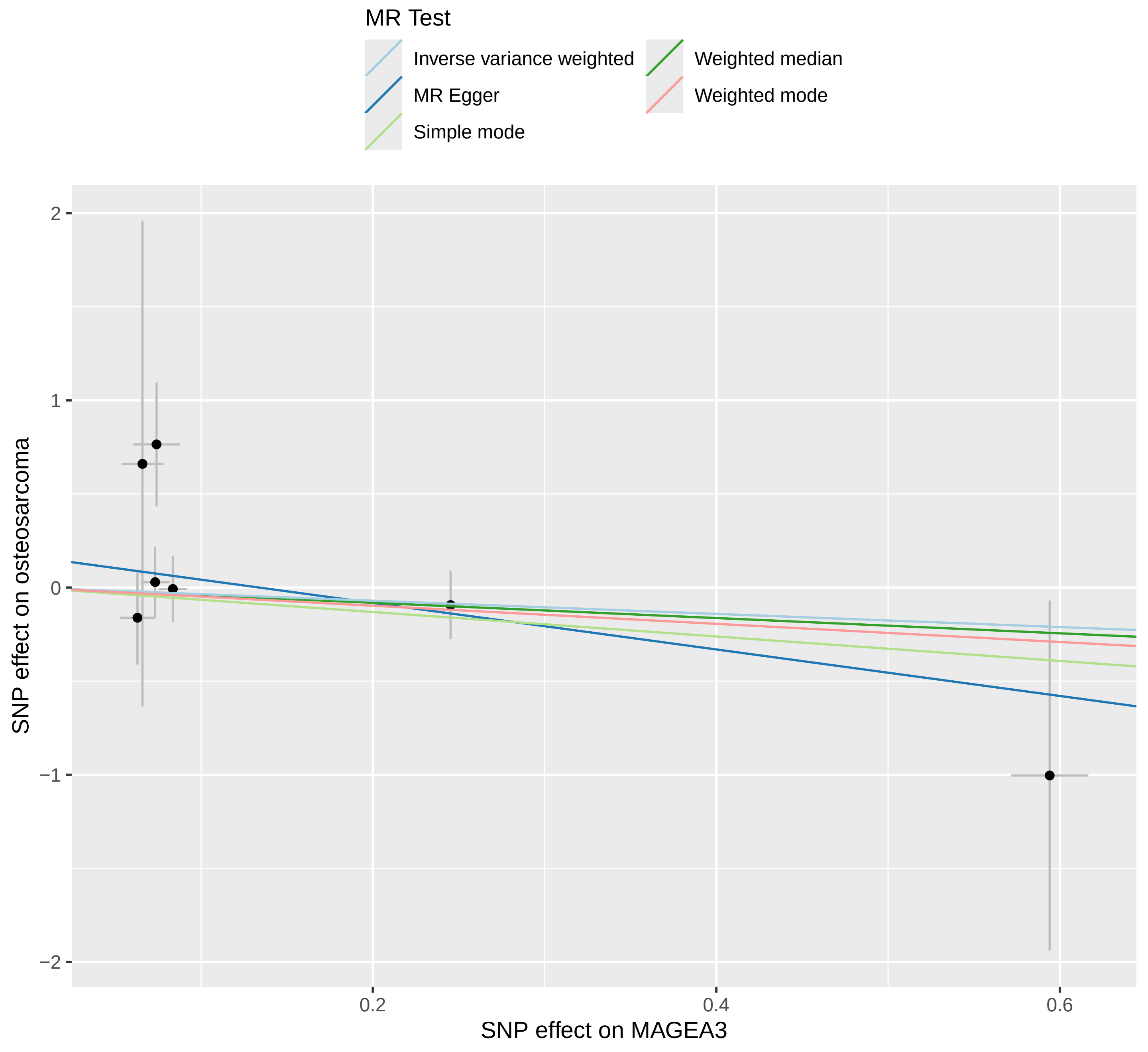

MR Test
Inverse variance weighted
MR Egger
Simple mode
Weighted median
Weighted mode
2
1
0
−1
−2
SNP effect on osteosarcoma
0.2
0.4
0.6
SNP effect on MAGEA3

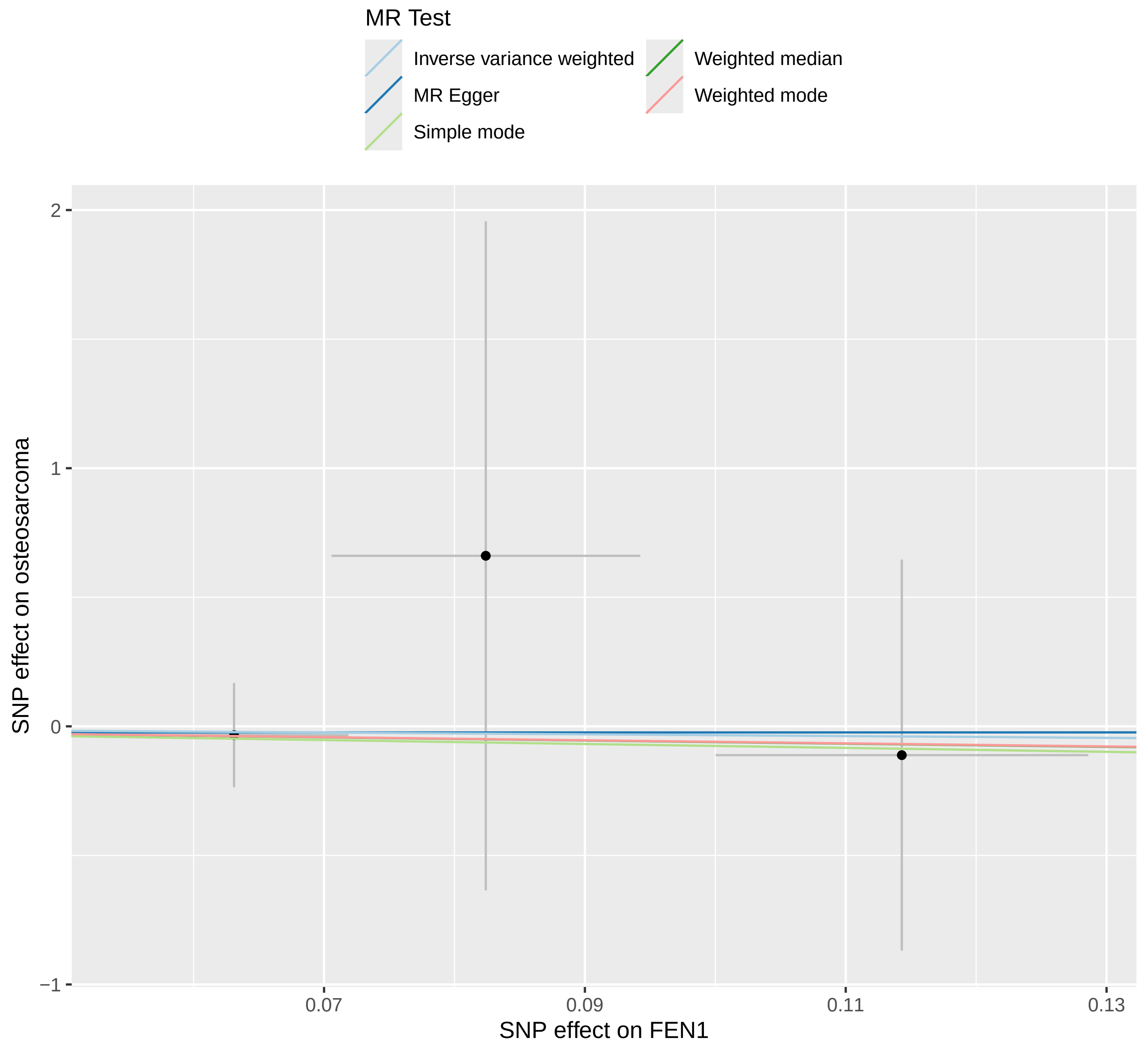

MR Test
Inverse variance weighted
MR Egger
Simple mode
Weighted median
Weighted mode
2
1
0
−1
0.07
0.09
0.11
0.13
SNP effect on osteosarcoma
SNP effect on FEN1

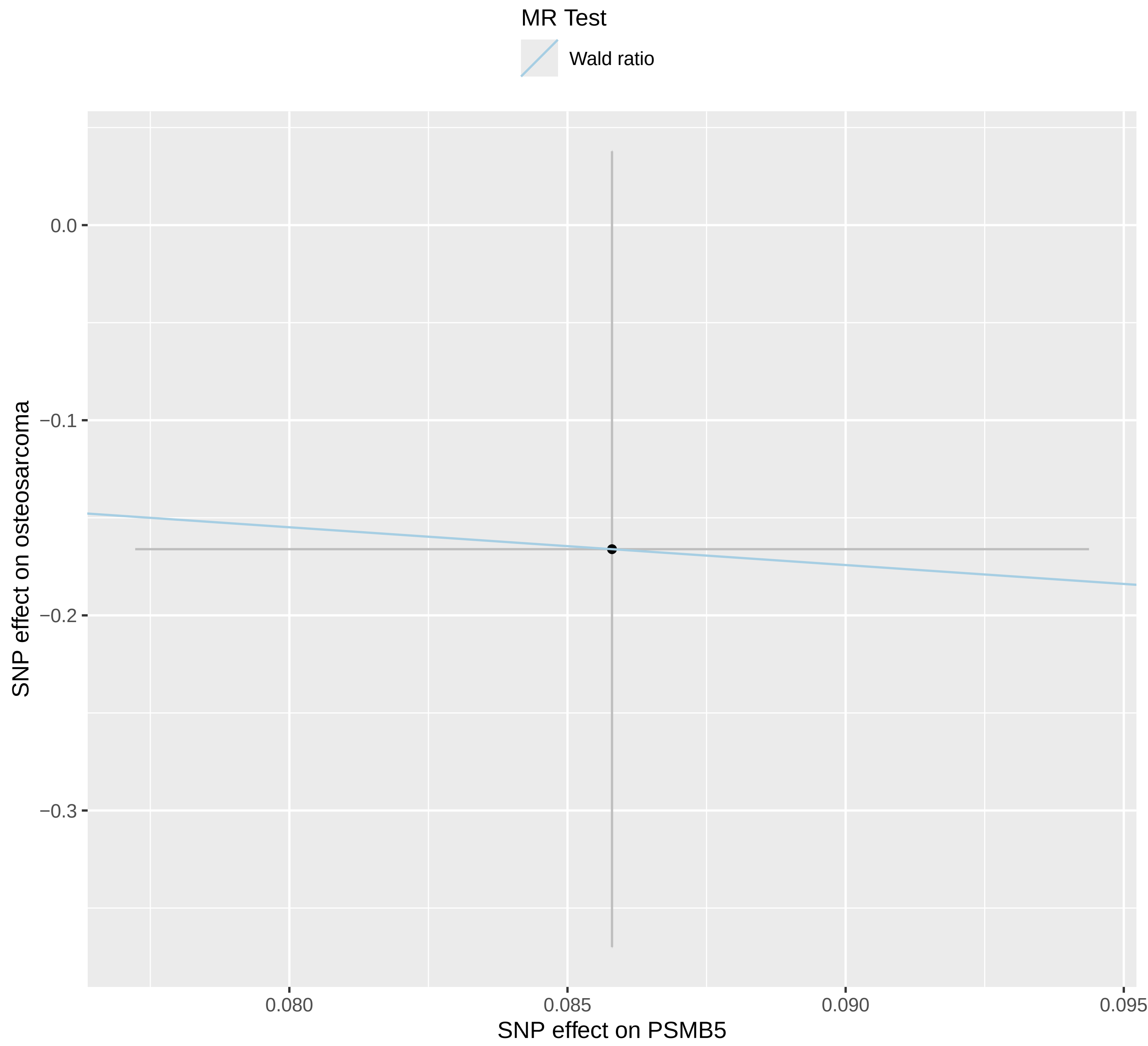
MR Test
Wald ratio
0.0
−0.1
−0.2
−0.3
SNP effect on osteosarcoma
0.080
0.085
0.090
0.095
SNP effect on PSMB5

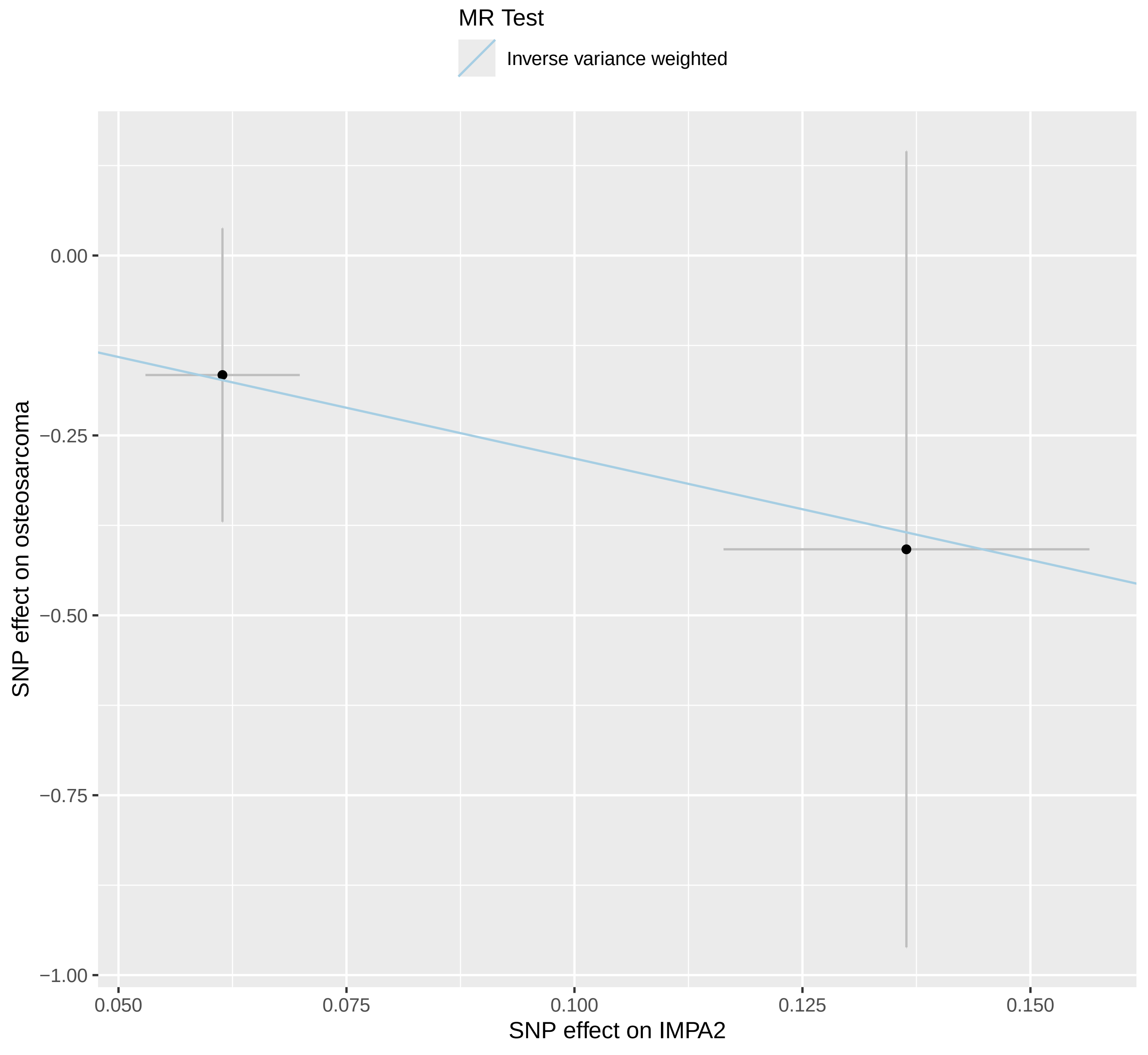

MR Test
Inverse variance weighted
0.00
−0.25
−0.50
−0.75
−1.00
0.050
0.075
0.100
0.125
0.150
SNP effect on osteosarcoma
SNP effect on IMPA2

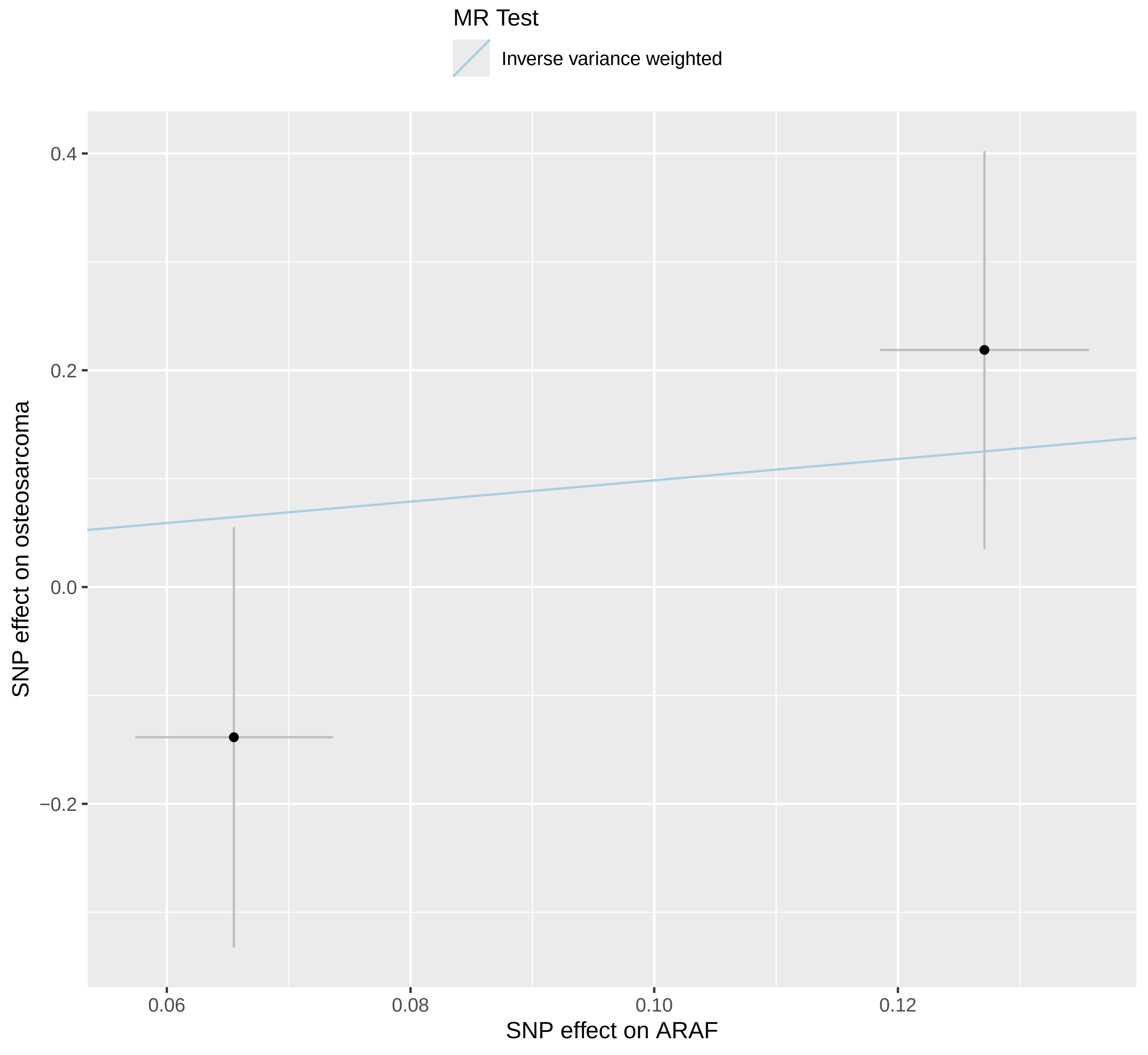

MR Test
Inverse variance weighted
0.4
0.2
0.0
−0.2
SNP effect on osteosarcoma
0.06
0.08
0.10
0.12
SNP effect on ARAF

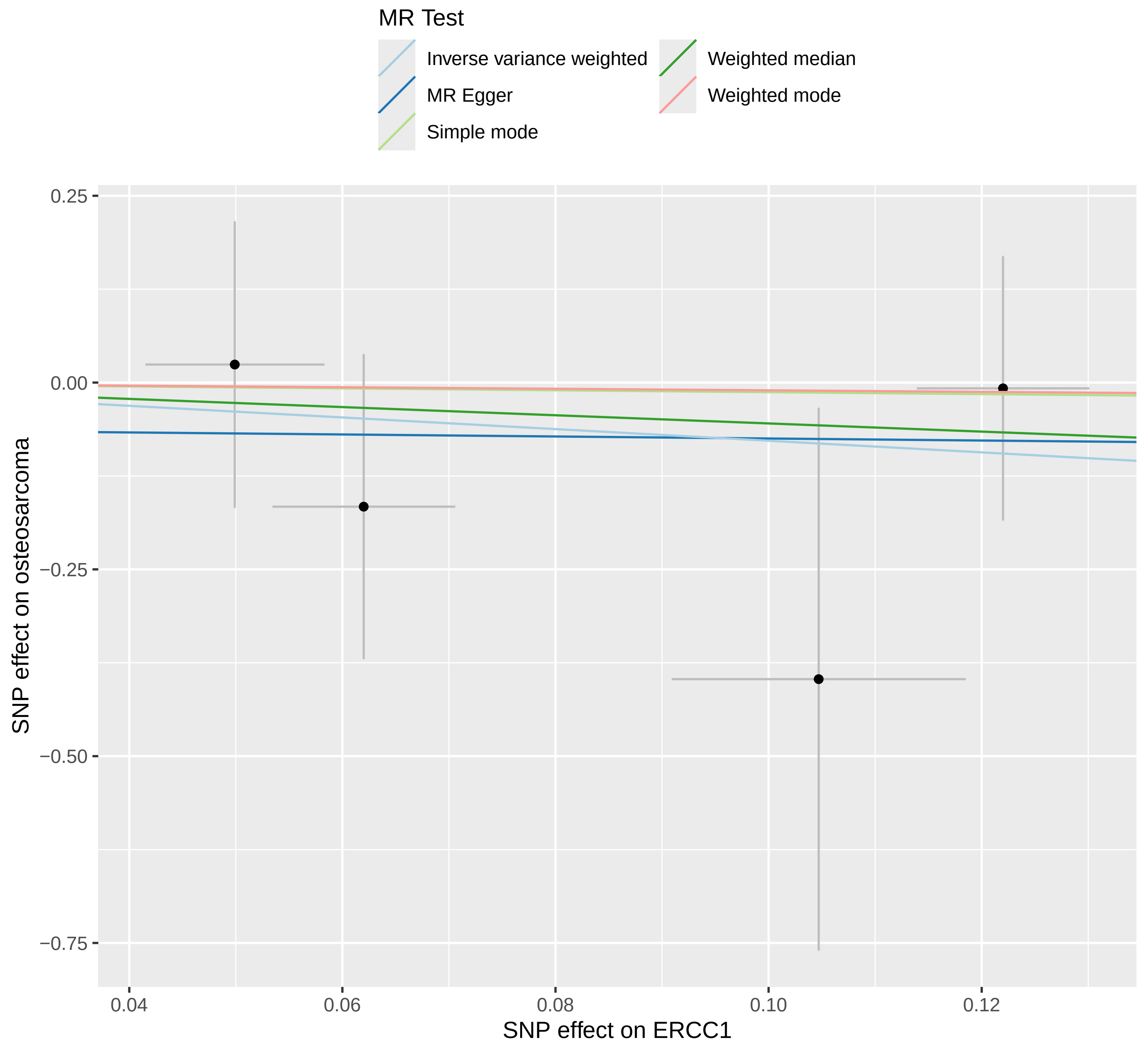

MR Test
Inverse variance weighted
MR Egger
Simple mode
Weighted median
Weighted mode
0.25
0.00
−0.25
−0.50
−0.75
SNP effect on osteosarcoma
0.04
0.06
0.08
0.10
0.12
SNP effect on ERCC1

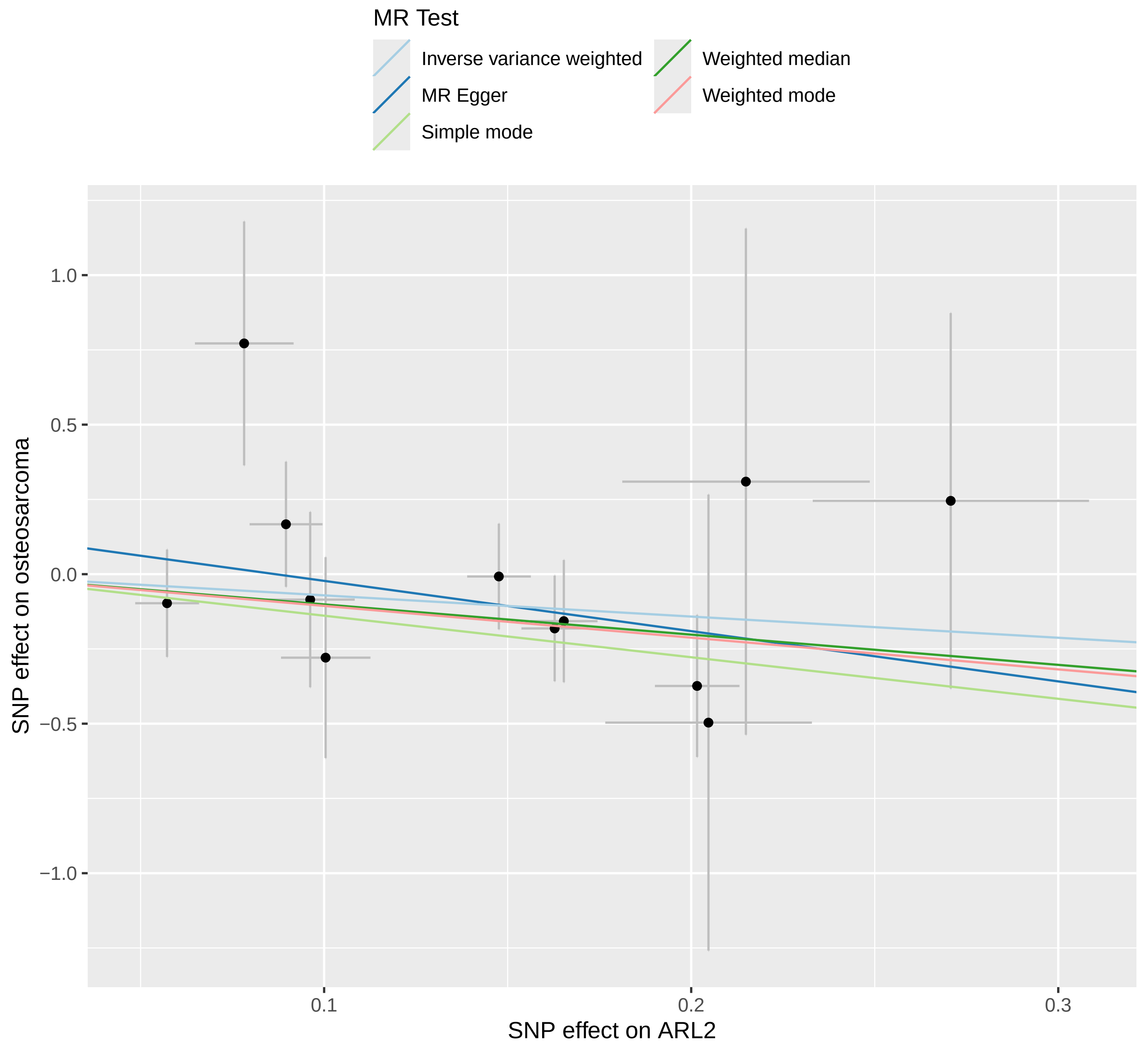
MR Test
Inverse variance weighted
MR Egger
Simple mode
Weighted median
Weighted mode
1.0
0.5
0.0
−0.5
−1.0
SNP effect on osteosarcoma
0.1
0.2
0.3
SNP effect on ARL2

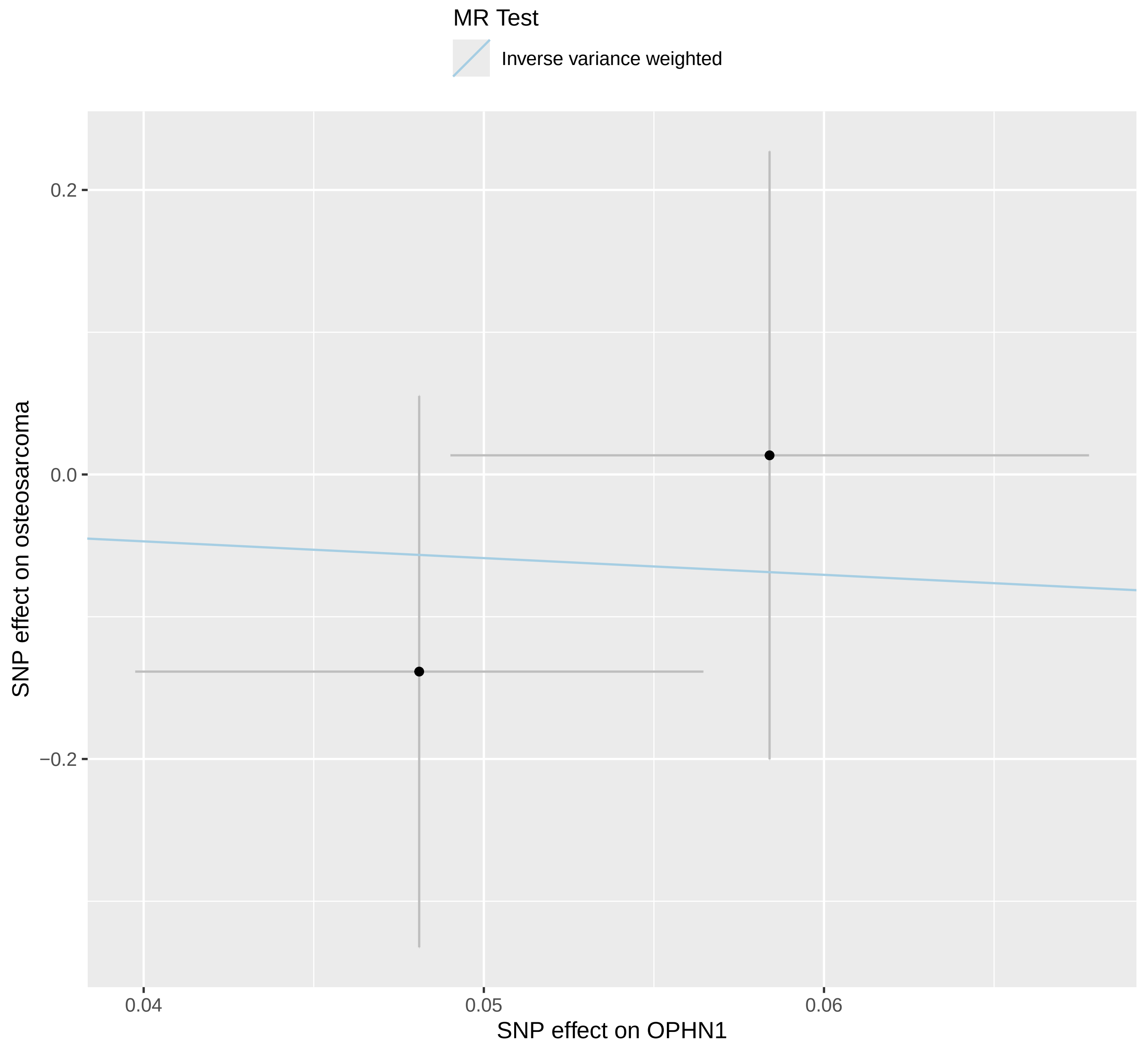
MR Test
Inverse variance weighted
0.2
0.0
−0.2
0.04
0.05
0.06
SNP effect on OPHN1
SNP effect on osteosarcoma

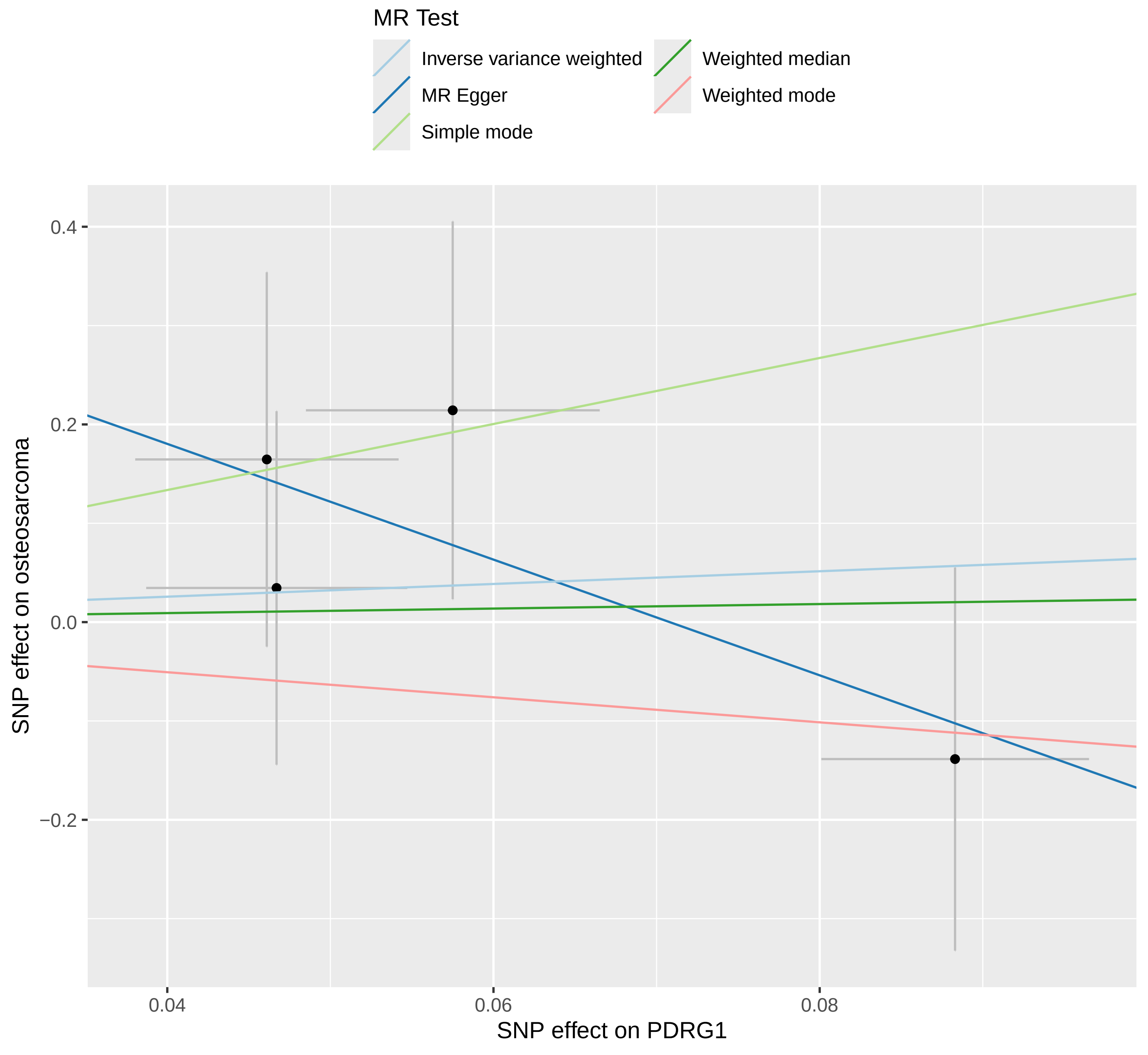
MR Test
Inverse variance weighted
MR Egger
Simple mode
Weighted median
Weighted mode
0.4
0.2
0.0
−0.2
SNP effect on osteosarcoma
0.04
0.06
0.08
SNP effect on PDRG1

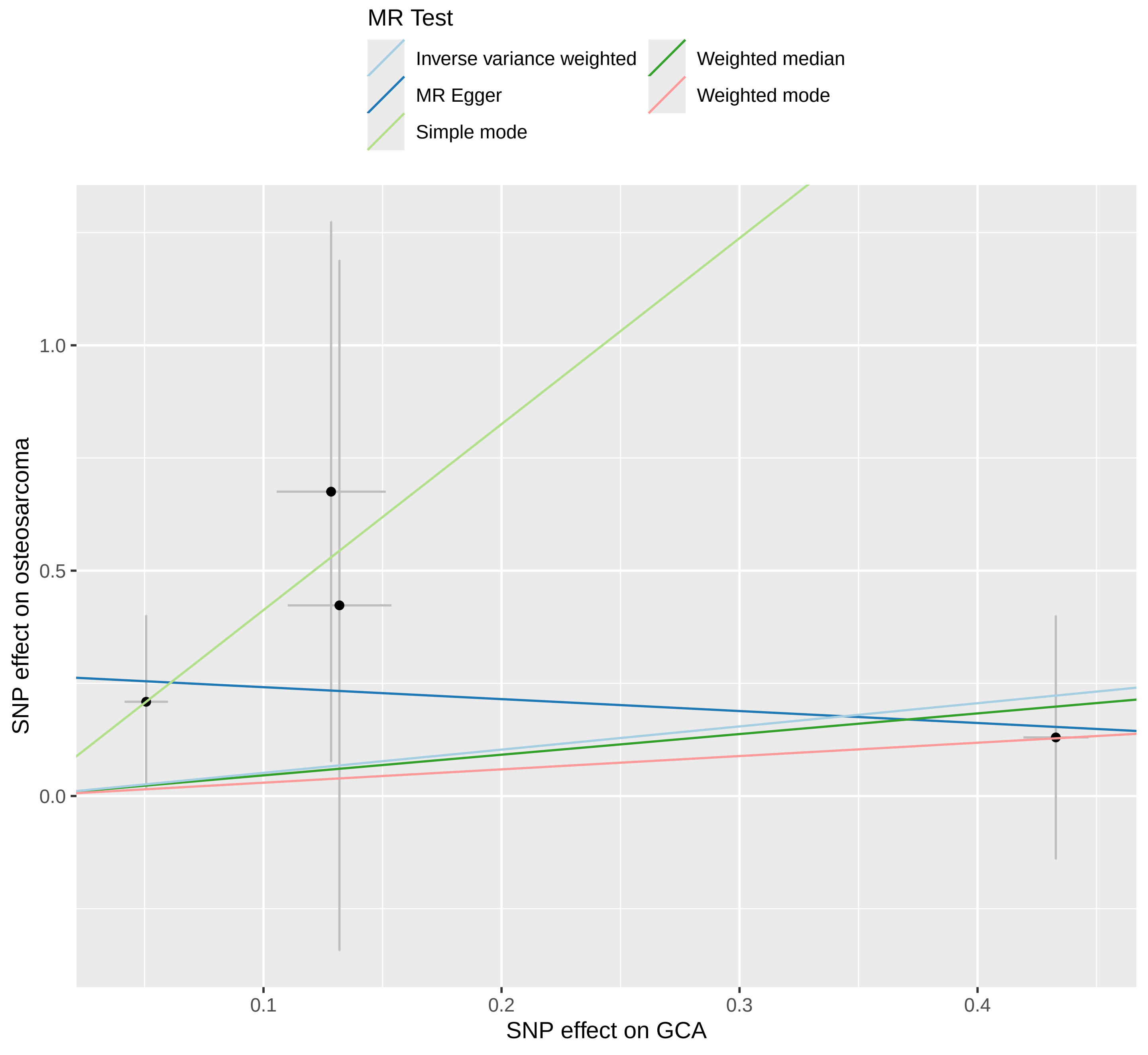
MR Test
Inverse variance weighted
MR Egger
Simple mode
Weighted median
Weighted mode
1.0
0.5
0.0
0.1
0.2
0.3
0.4
SNP effect on osteosarcoma
SNP effect on GCA

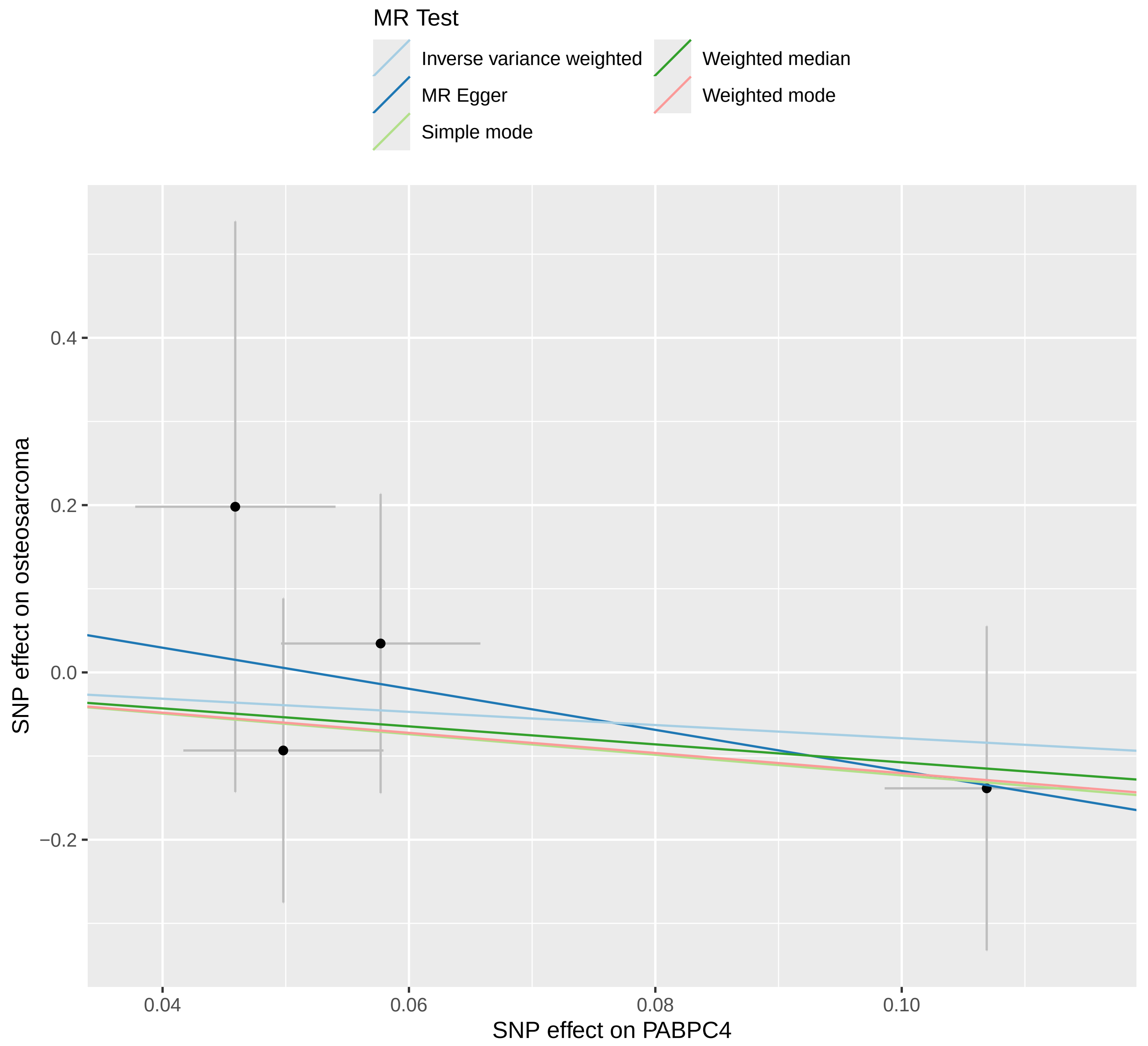
MR Test
Inverse variance weighted
MR Egger
Simple mode
Weighted median
Weighted mode
0.4
0.2
0.0
−0.2
SNP effect on osteosarcoma
0.04
0.06
0.08
0.10
SNP effect on PABPC4

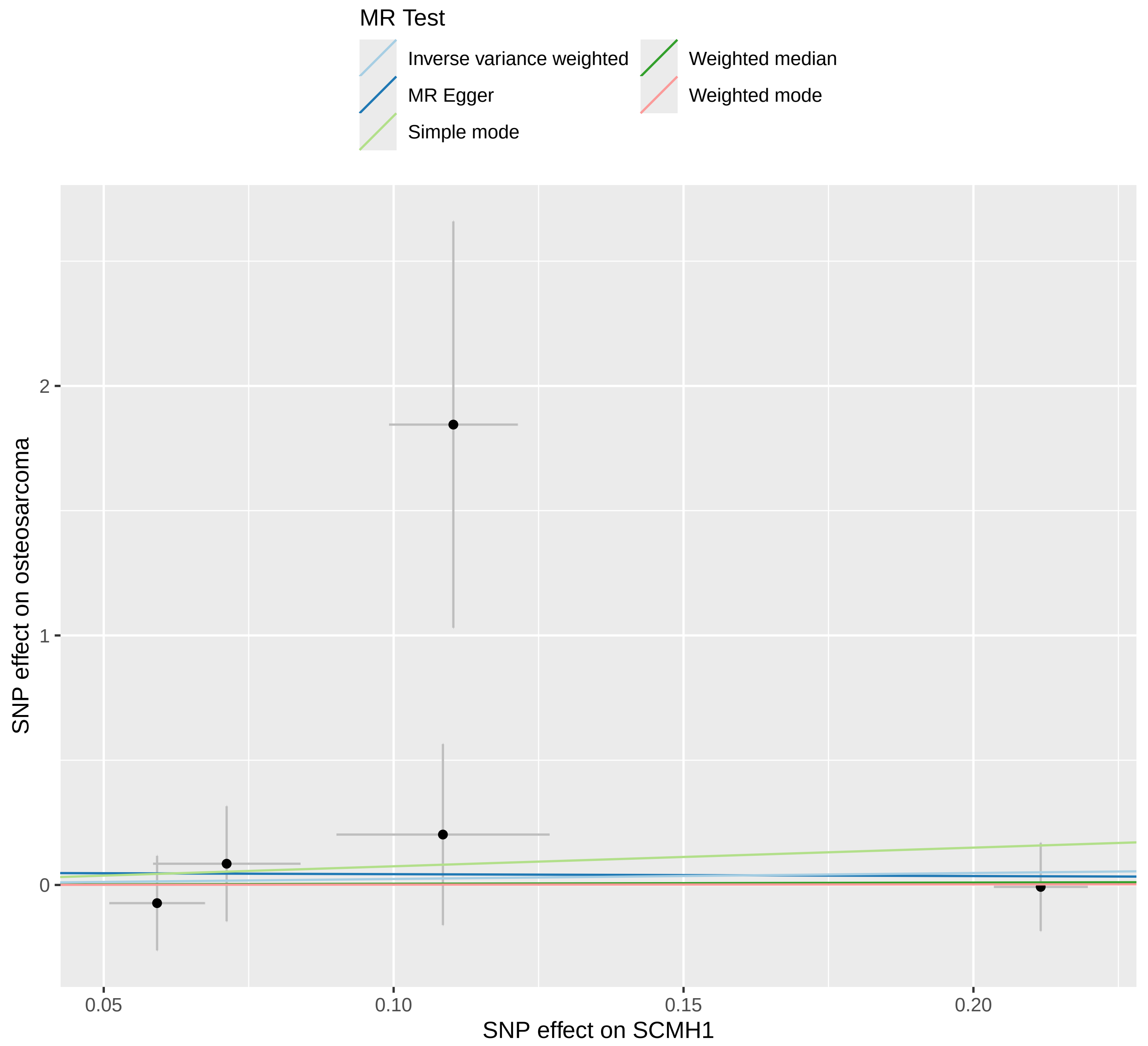
MR Test
Inverse variance weighted
MR Egger
Simple mode
Weighted median
Weighted mode
SNP effect on osteosarcoma
2
1
0
0.05
0.10
0.15
0.20
SNP effect on SCMH1

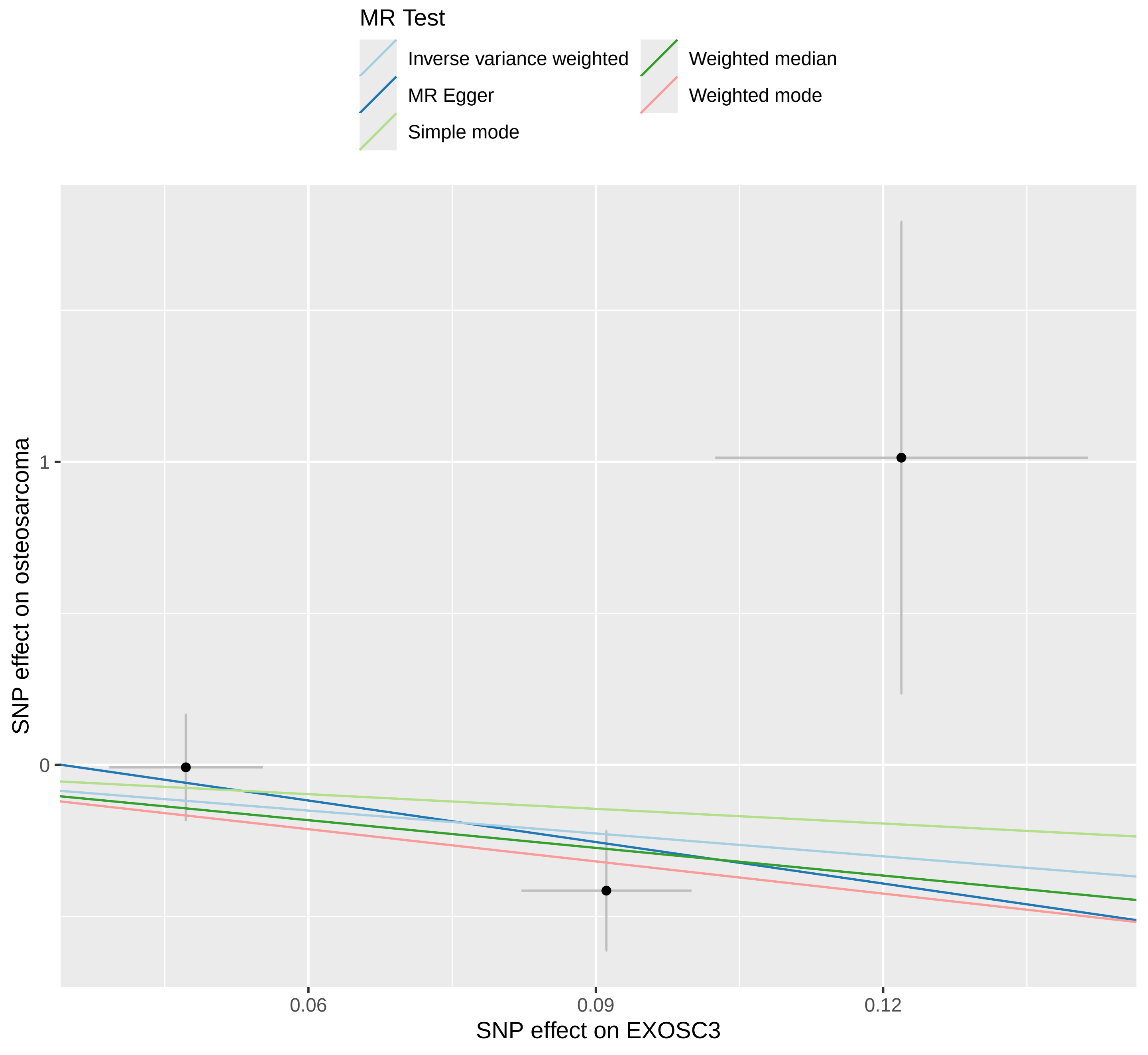

MR Test
Inverse variance weighted
MR Egger
Simple mode
Weighted median
Weighted mode
SNP effect on osteosarcoma
1
0
0.06
0.09
0.12
SNP effect on EXOSC3

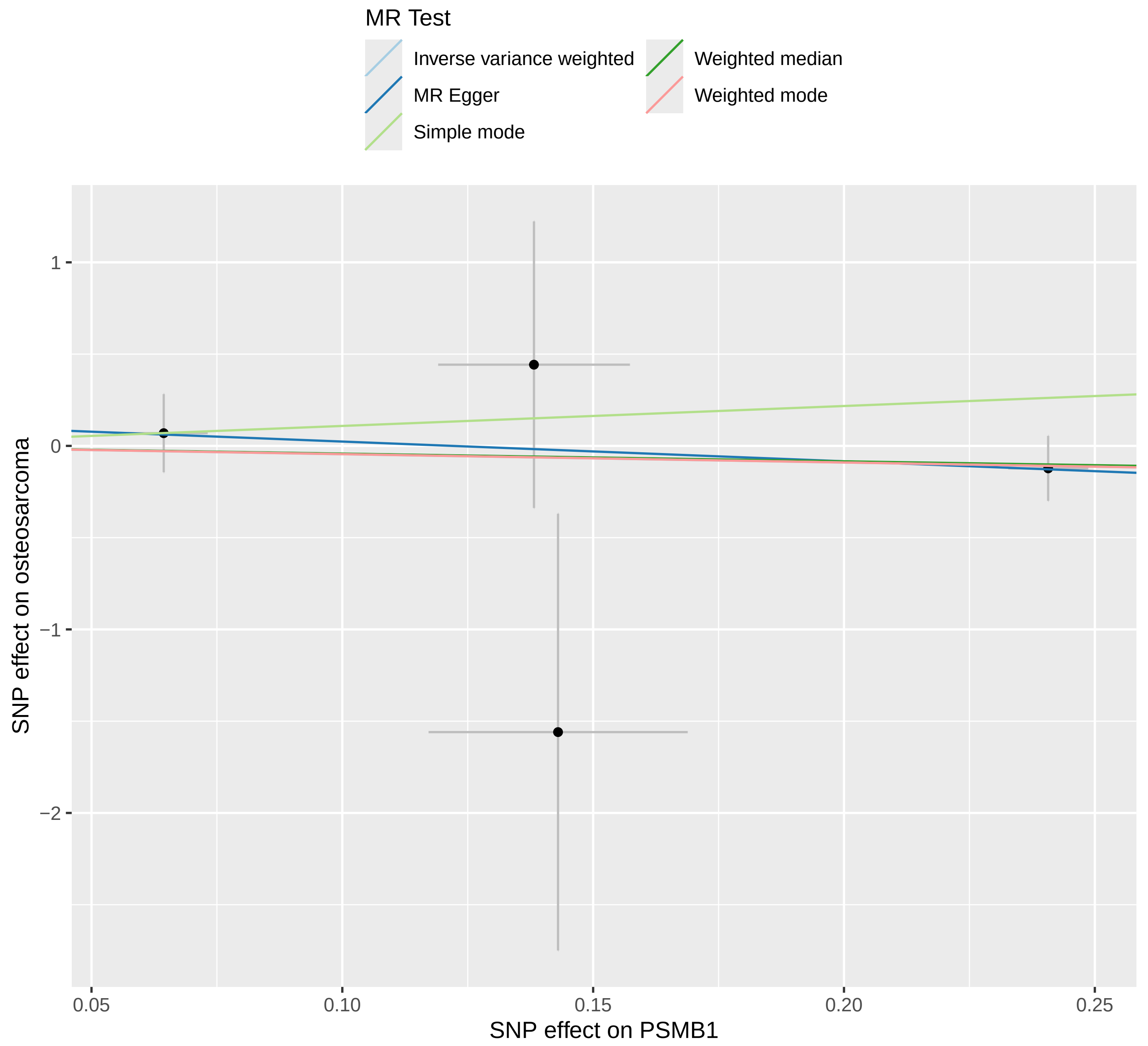
MR Test
Inverse variance weighted
MR Egger
Simple mode
Weighted median
Weighted mode
1
0
−1
−2
SNP effect on osteosarcoma
0.05
0.10
0.15
0.20
0.25
SNP effect on PSMB1

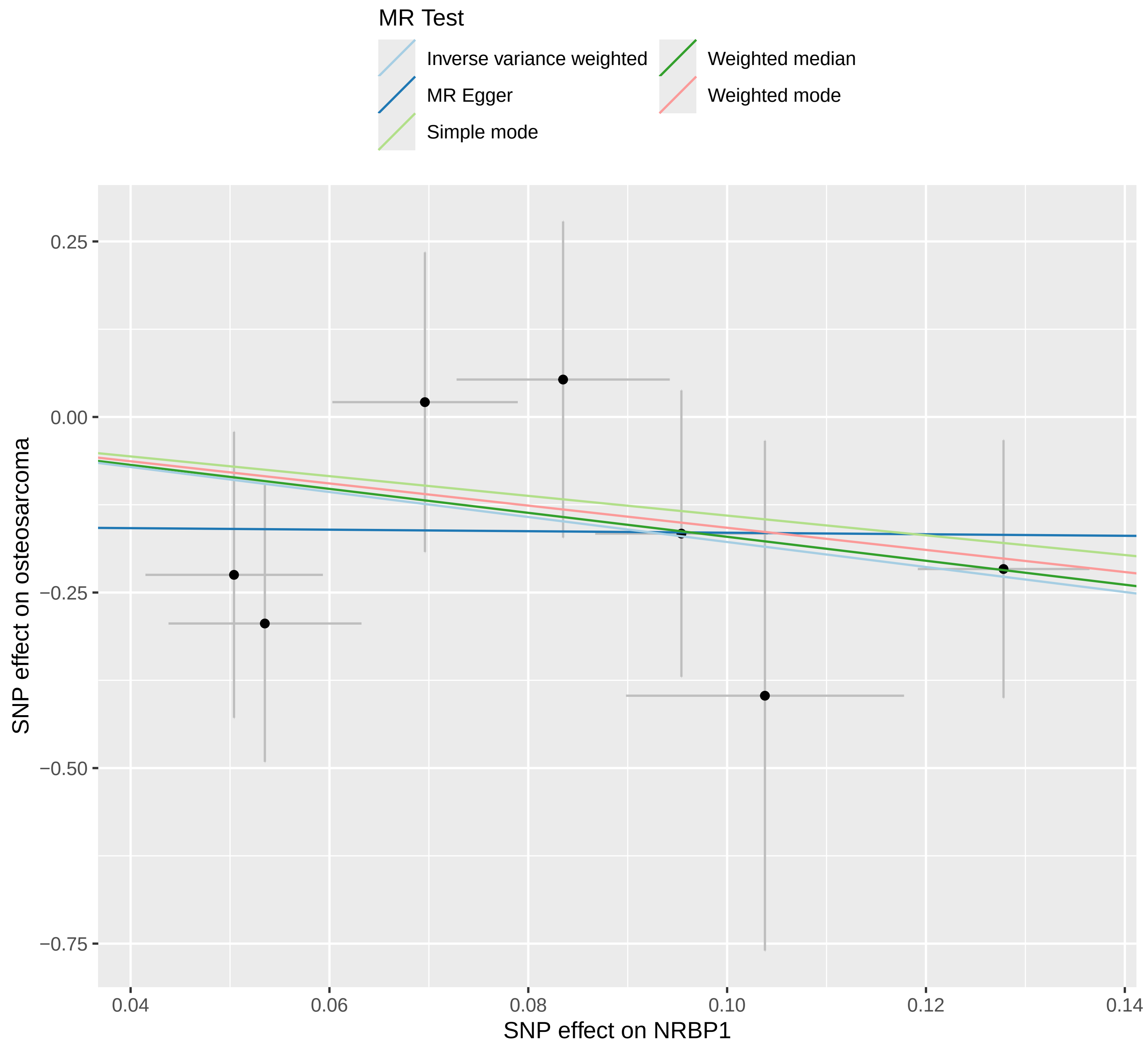

MR Test
Inverse variance weighted
MR Egger
Simple mode
Weighted median
Weighted mode
0.25
0.00
−0.25
−0.50
−0.75
SNP effect on osteosarcoma
0.04
0.06
0.08
0.10
0.12
0.14
SNP effect on NRBP1

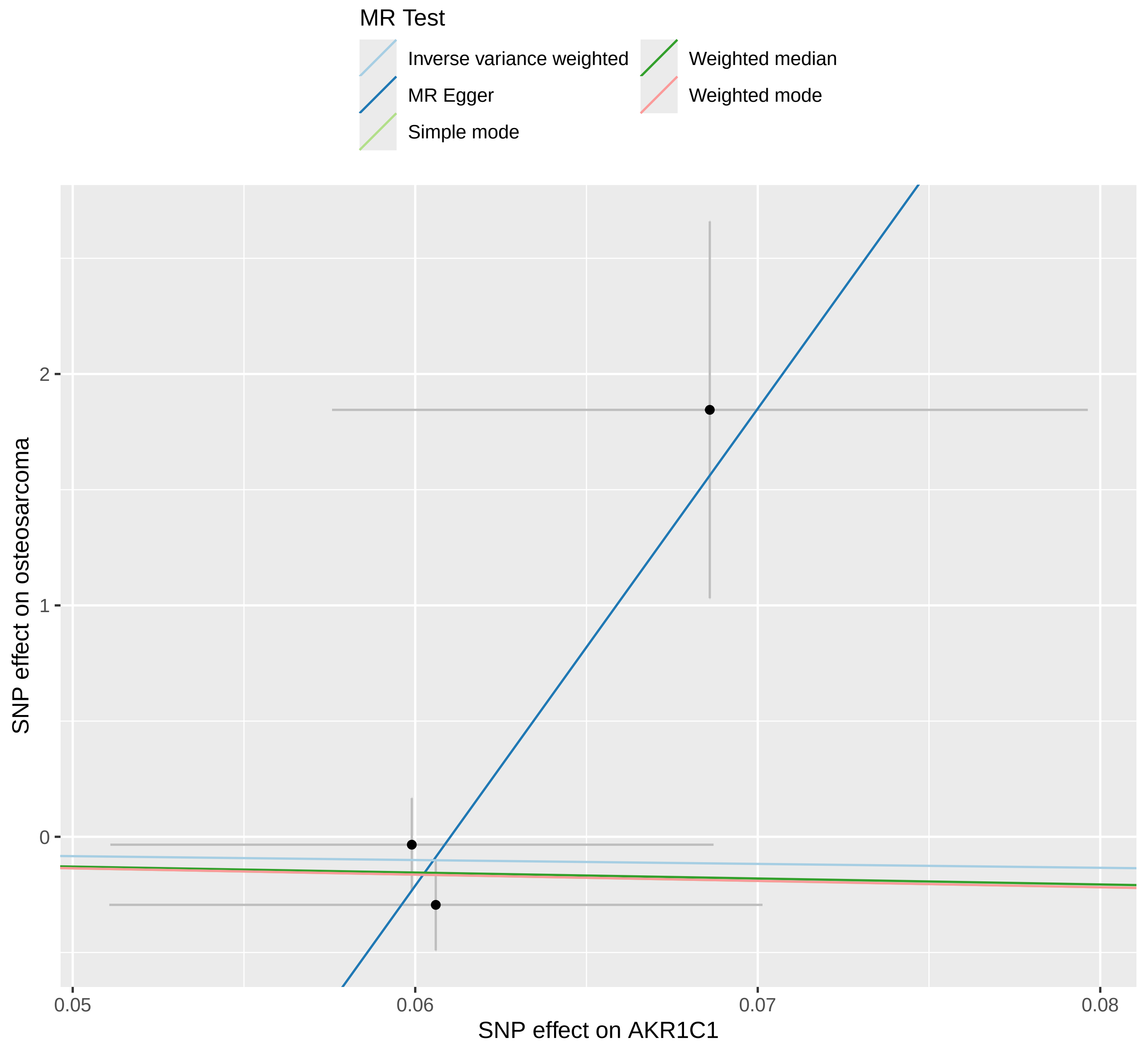

MR Test
Inverse variance weighted
MR Egger
Simple mode
Weighted median
Weighted mode
SNP effect on osteosarcoma
2
1
0
0.05
0.06
0.07
0.08
SNP effect on AKR1C1

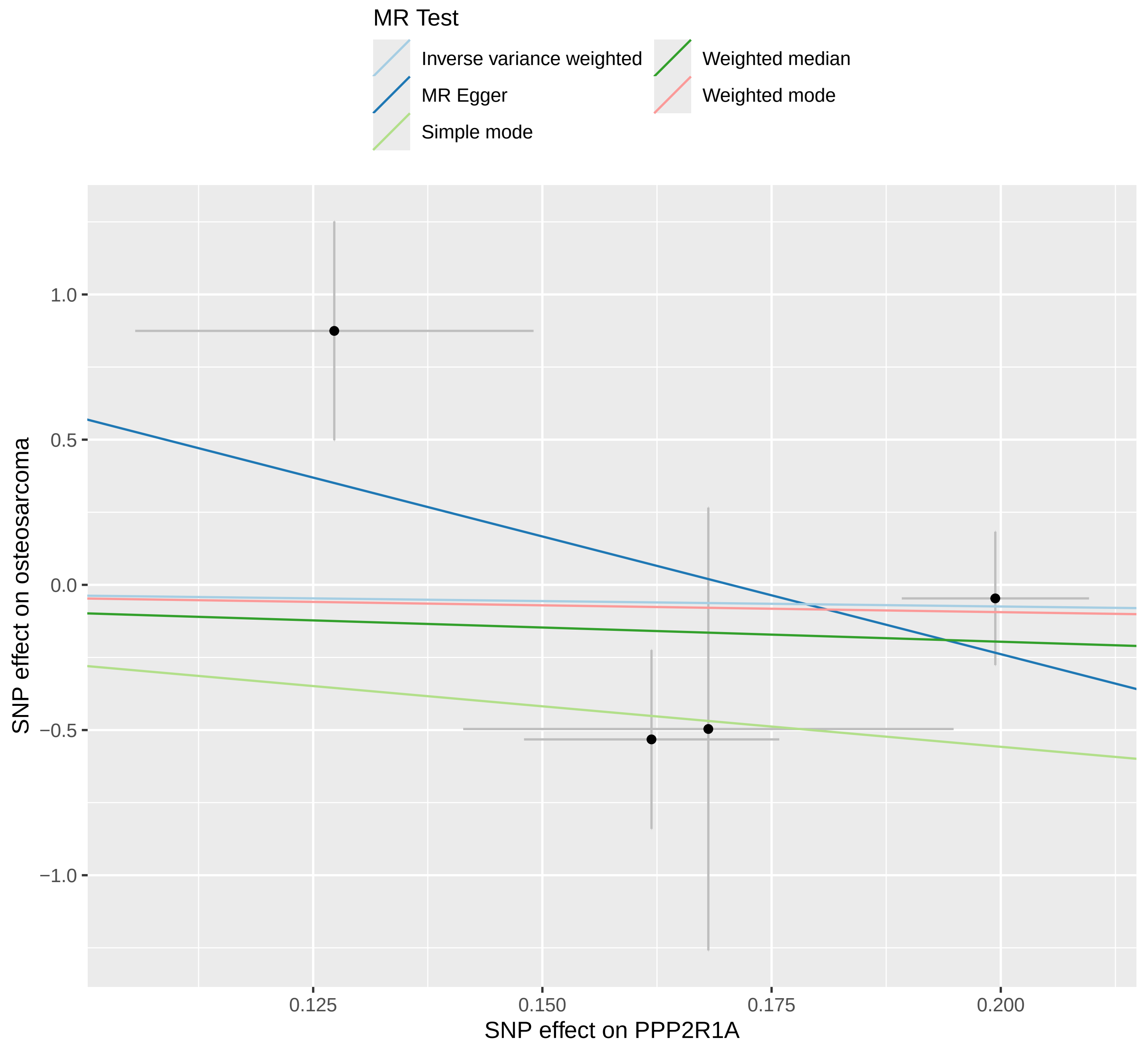
MR Test
Inverse variance weighted
MR Egger
Simple mode
Weighted median
Weighted mode
1.0
0.5
0.0
−0.5
−1.0
0.125
0.150
0.175
0.200
SNP effect on osteosarcoma
SNP effect on PPP2R1A

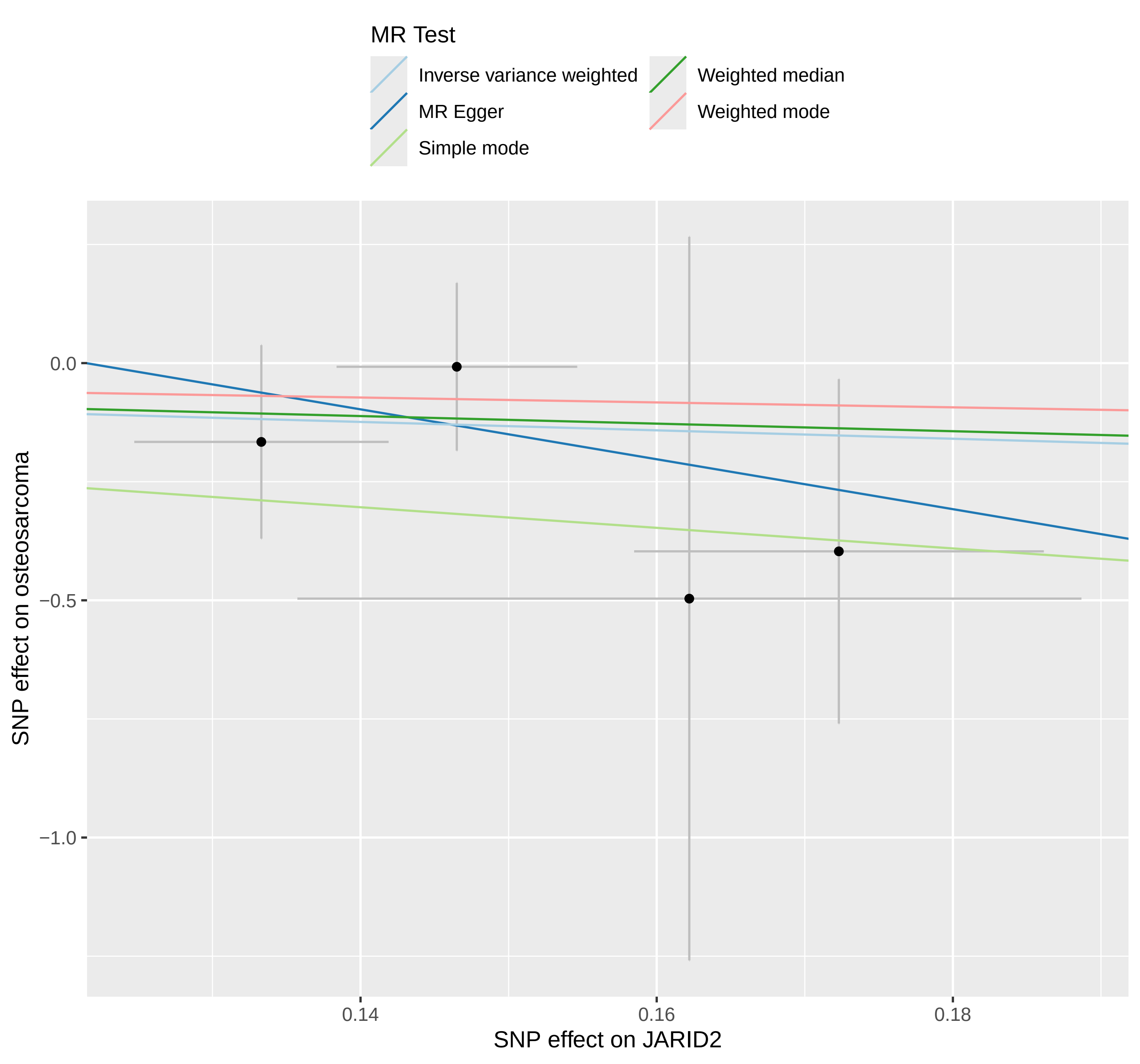
MR Test
Inverse variance weighted
MR Egger
Simple mode
Weighted median
Weighted mode
0.0
−0.5
−1.0
0.14
0.16
0.18
SNP effect on osteosarcoma
SNP effect on JARID2

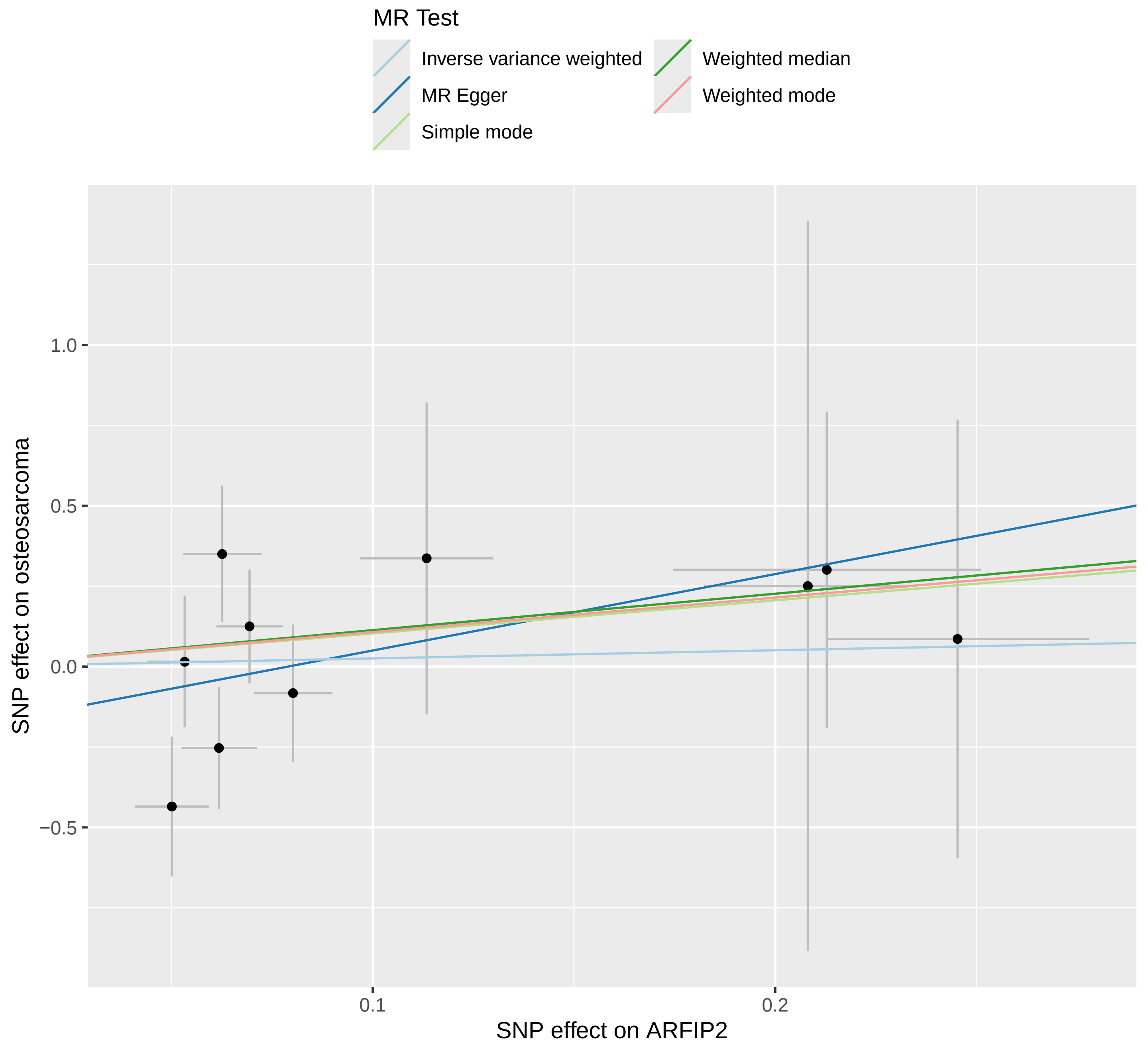
MR Test
Inverse variance weighted
MR Egger
Simple mode
Weighted median
Weighted mode
1.0
0.5
0.0
−0.5
0.1
0.2
SNP effect on osteosarcoma
SNP effect on ARFIP2

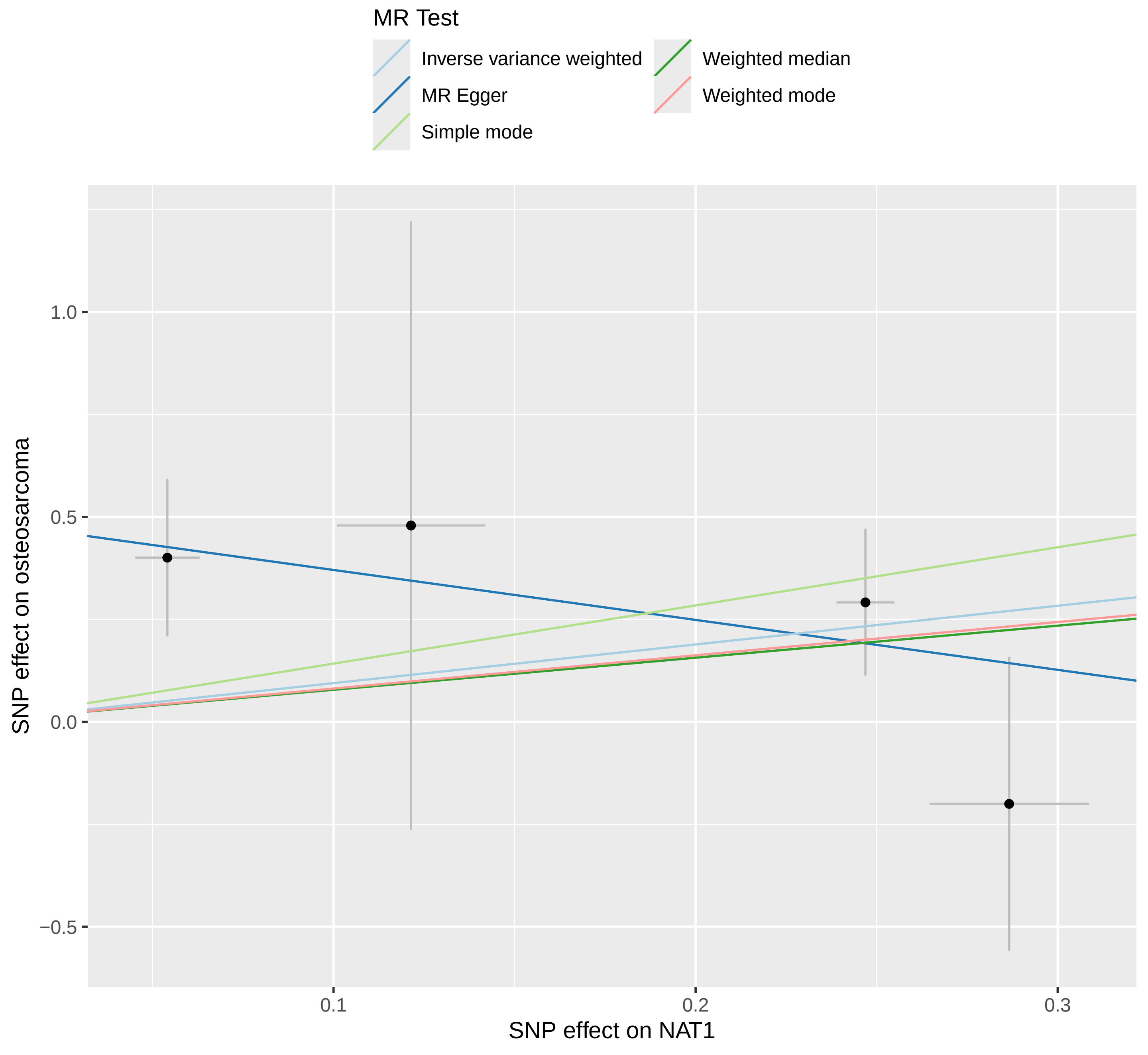
MR Test
Inverse variance weighted
MR Egger
Simple mode
Weighted median
Weighted mode
1.0
0.5
0.0
−0.5
SNP effect on osteosarcoma
0.1
0.2
0.3
SNP effect on NAT1

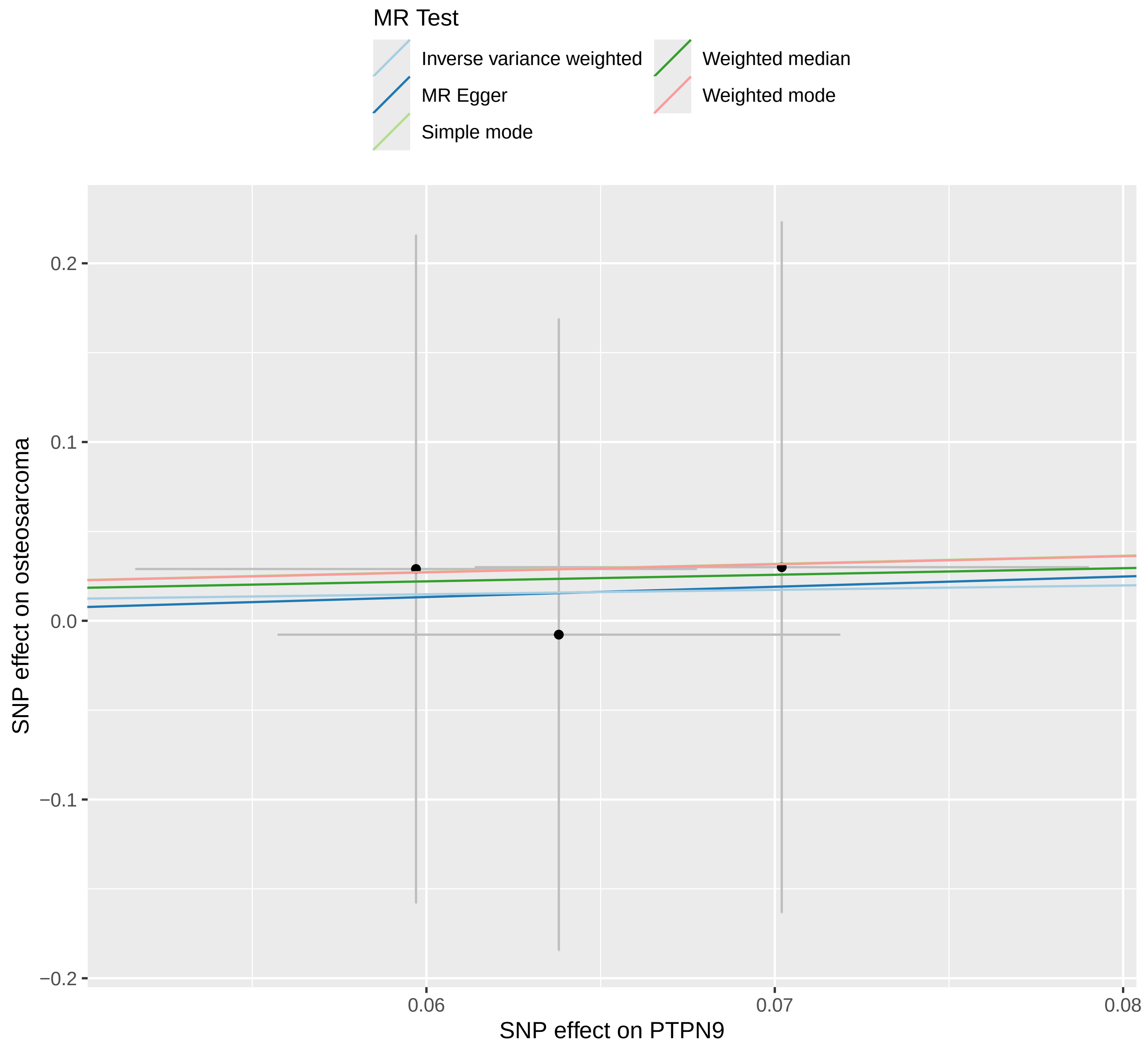
MR Test
Inverse variance weighted
MR Egger
Simple mode
Weighted median
Weighted mode
0.2
0.1
0.0
−0.1
−0.2
0.06
0.07
0.08
SNP effect on osteosarcoma
SNP effect on PTPN9

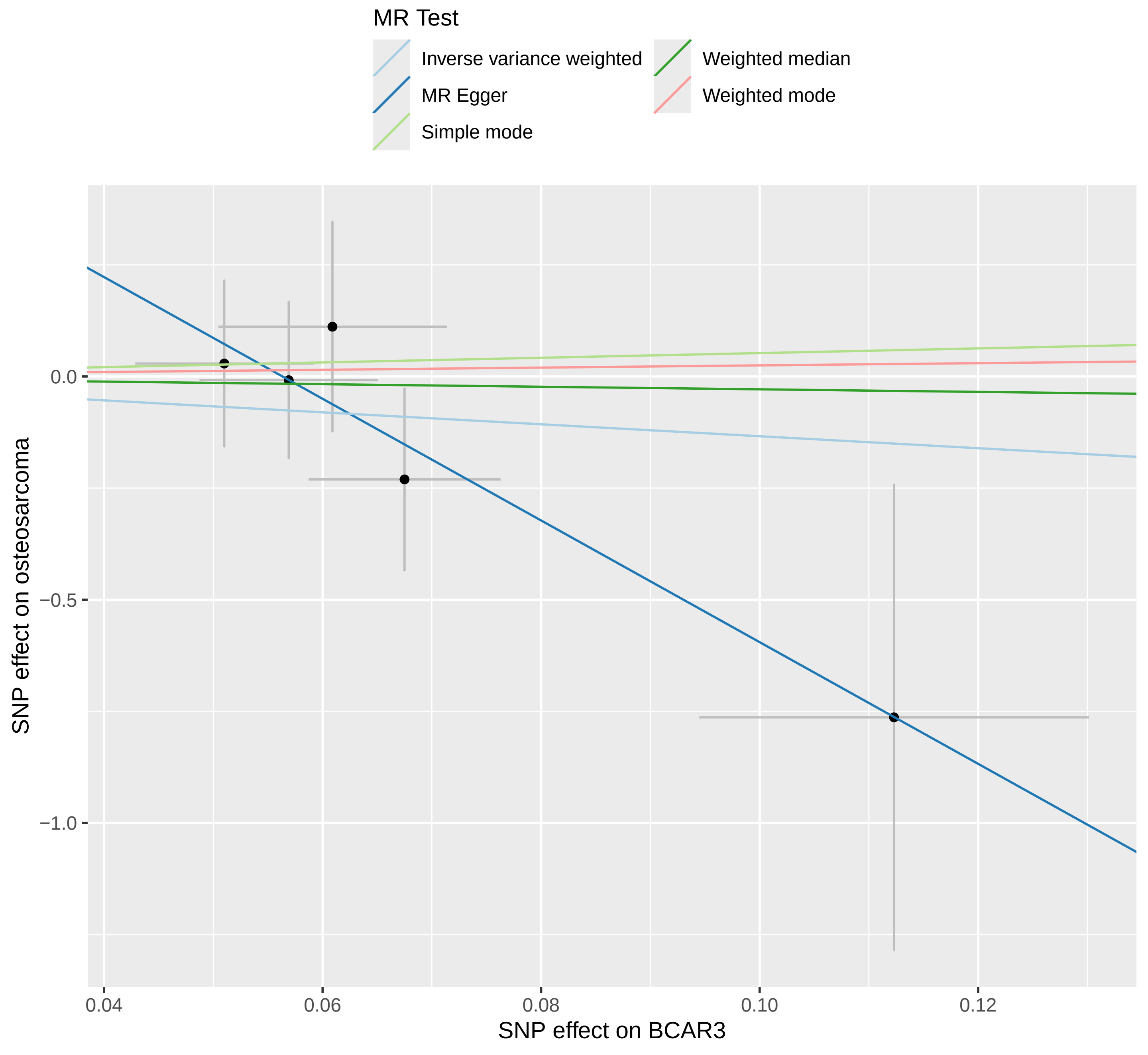
MR Test
Inverse variance weighted
MR Egger
Simple mode
Weighted median
Weighted mode
0.0
−0.5
−1.0
SNP effect on osteosarcoma
0.04
0.06
0.08
0.10
0.12
SNP effect on BCAR3

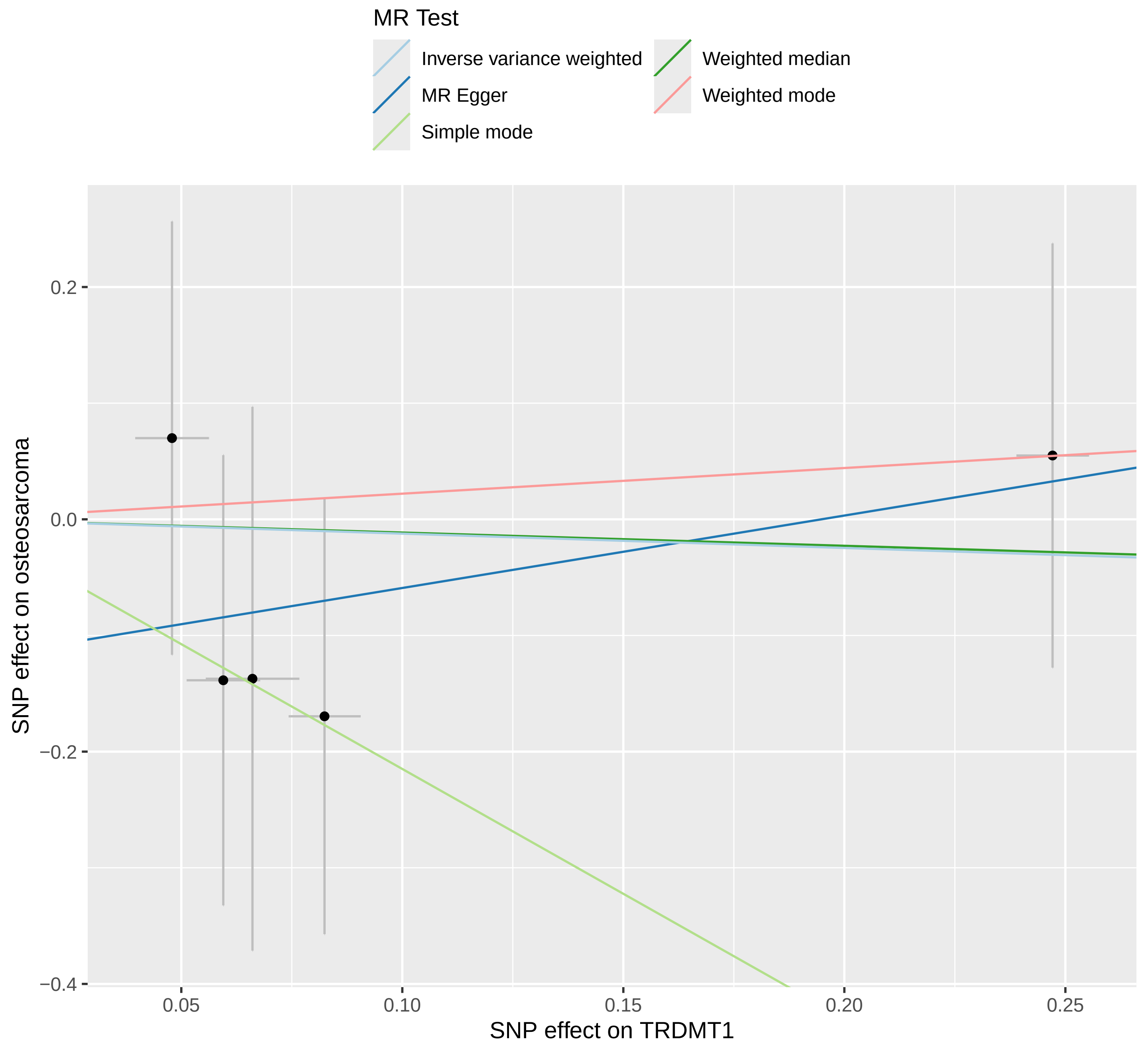
MR Test
Inverse variance weighted
MR Egger
Simple mode
Weighted median
Weighted mode
0.2
0.0
−0.2
−0.4
0.05
0.10
0.15
0.20
0.25
SNP effect on TRDMT1
SNP effect on osteosarcoma

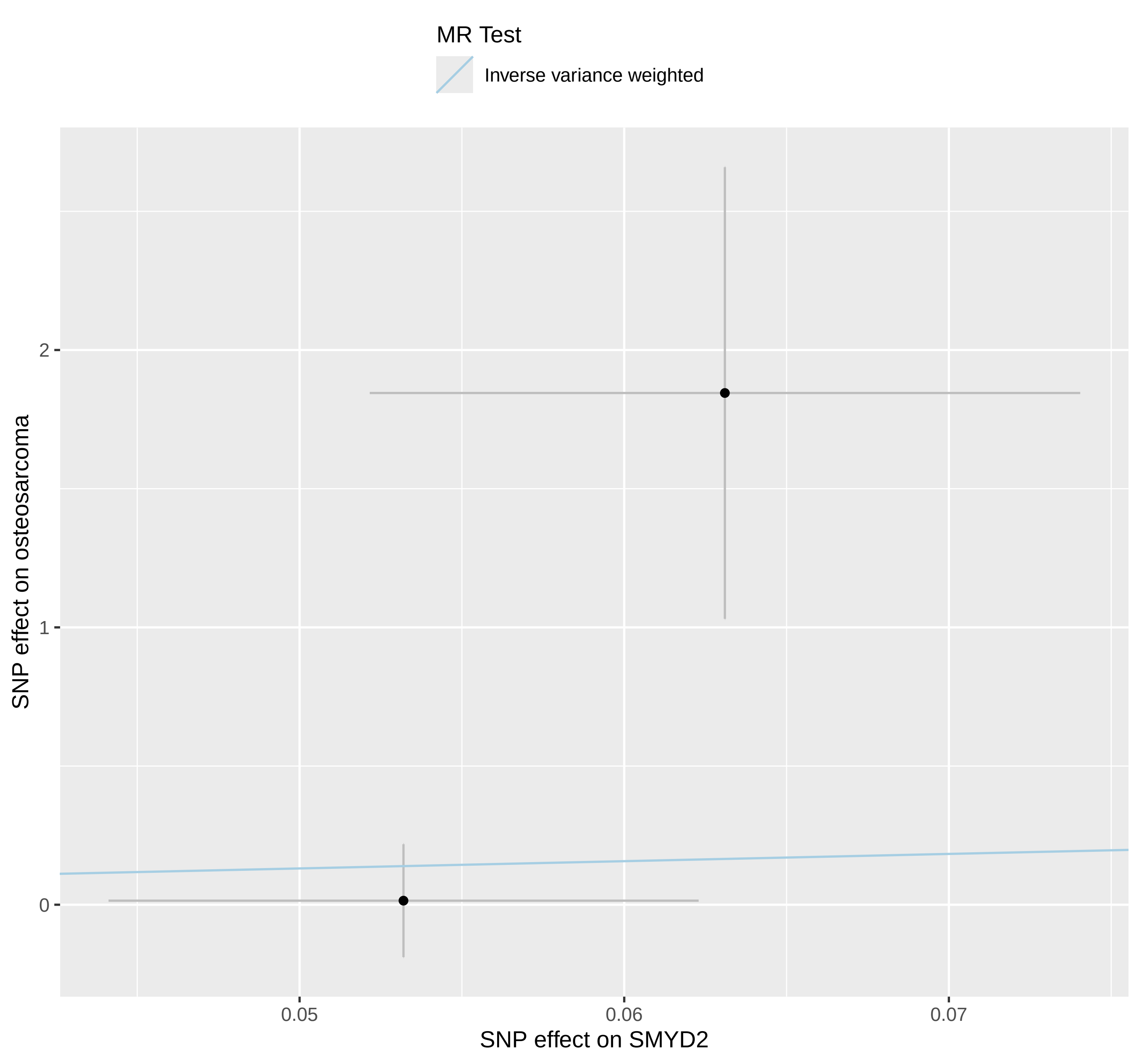
MR Test
Inverse variance weighted
SNP effect on osteosarcoma
SNP effect on SMYD2
0
1
2
0.05
0.06
0.07

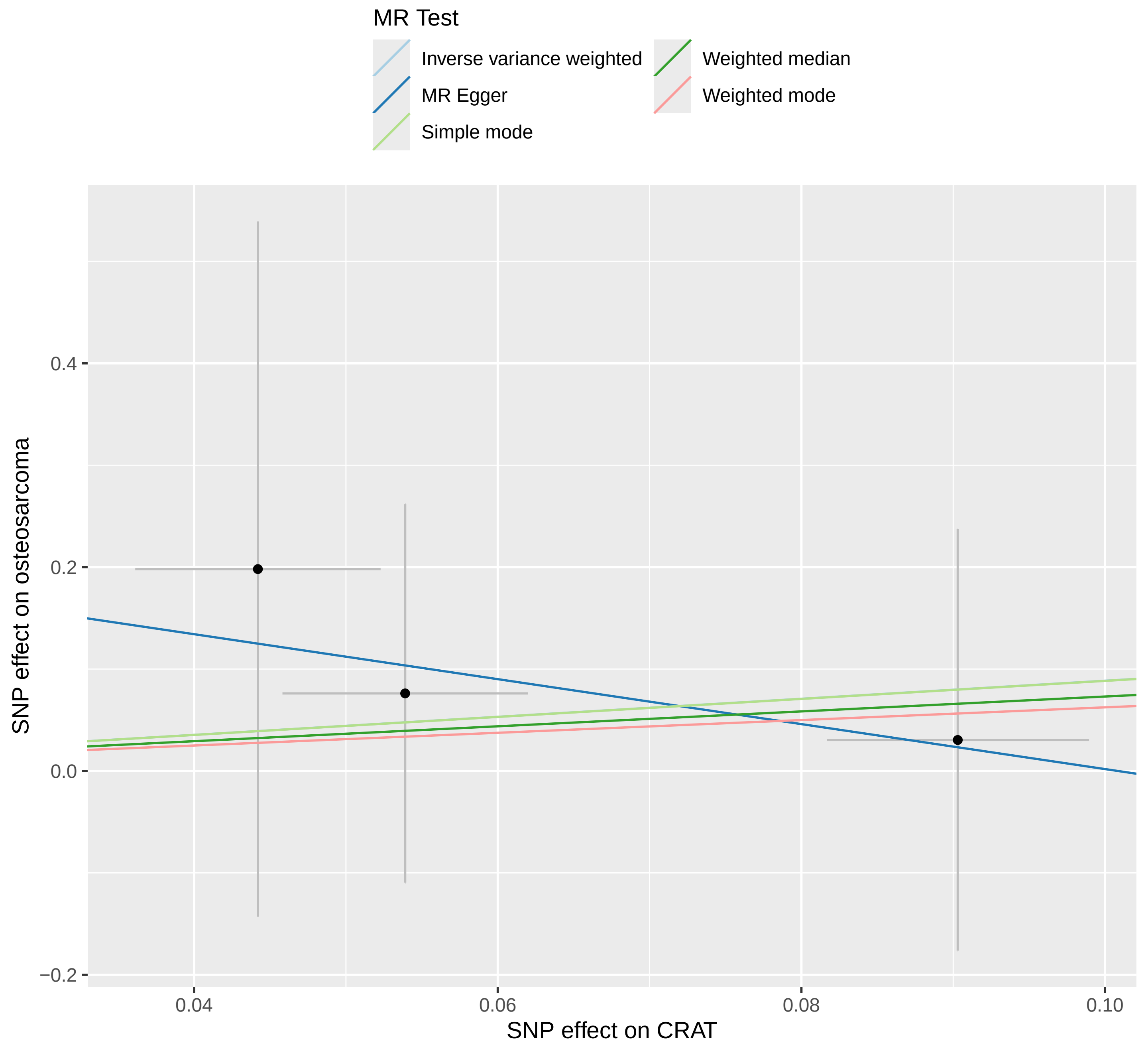
MR Test
Inverse variance weighted
MR Egger
Simple mode
Weighted median
Weighted mode
SNP effect on osteosarcoma
0.4
0.2
0.0
−0.2
0.04
0.06
0.08
0.10
SNP effect on CRAT

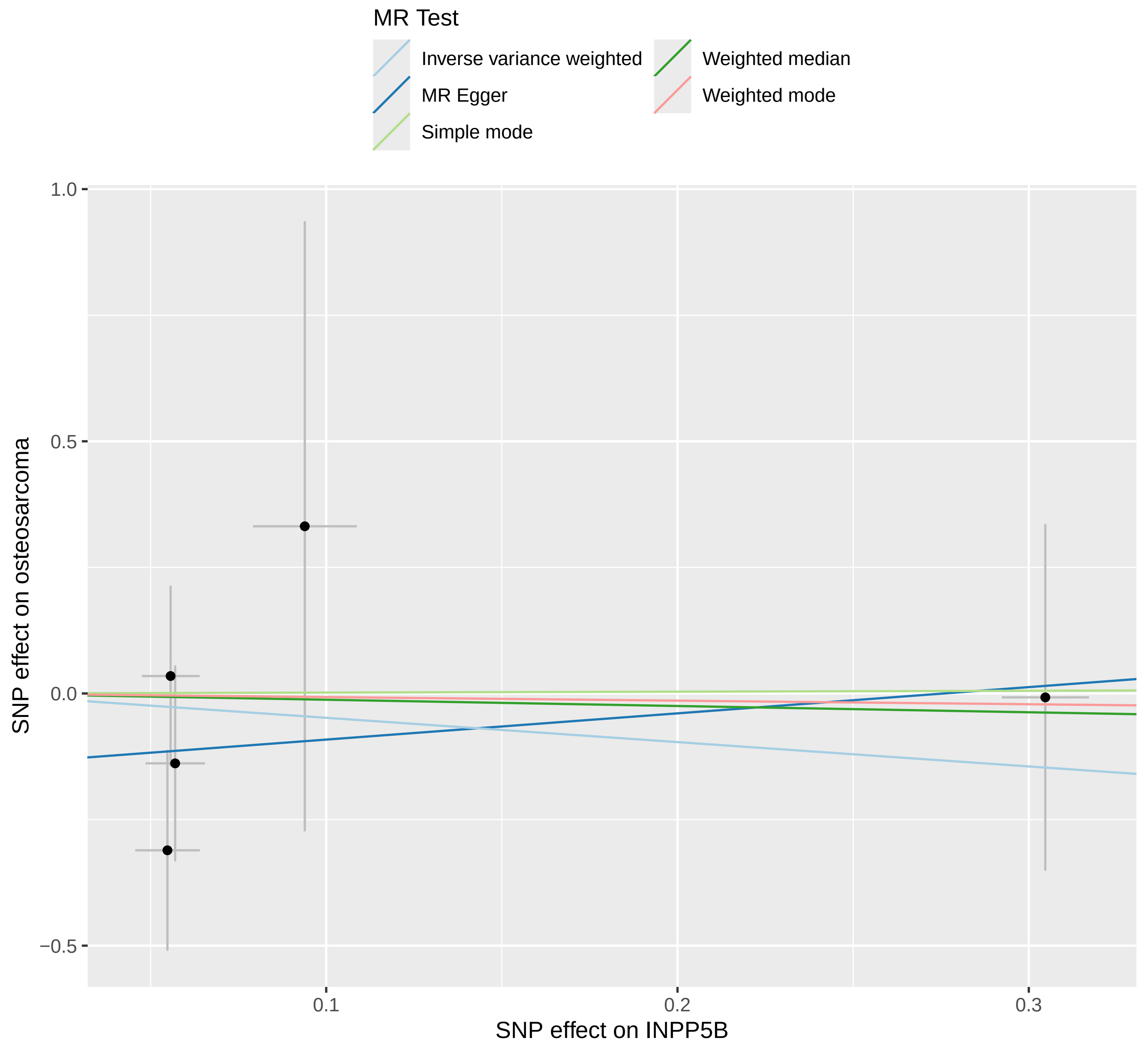
MR Test
Inverse variance weighted
MR Egger
Simple mode
Weighted median
Weighted mode
1.0
0.5
0.0
−0.5
0.1
0.2
0.3
SNP effect on osteosarcoma
SNP effect on INPP5B

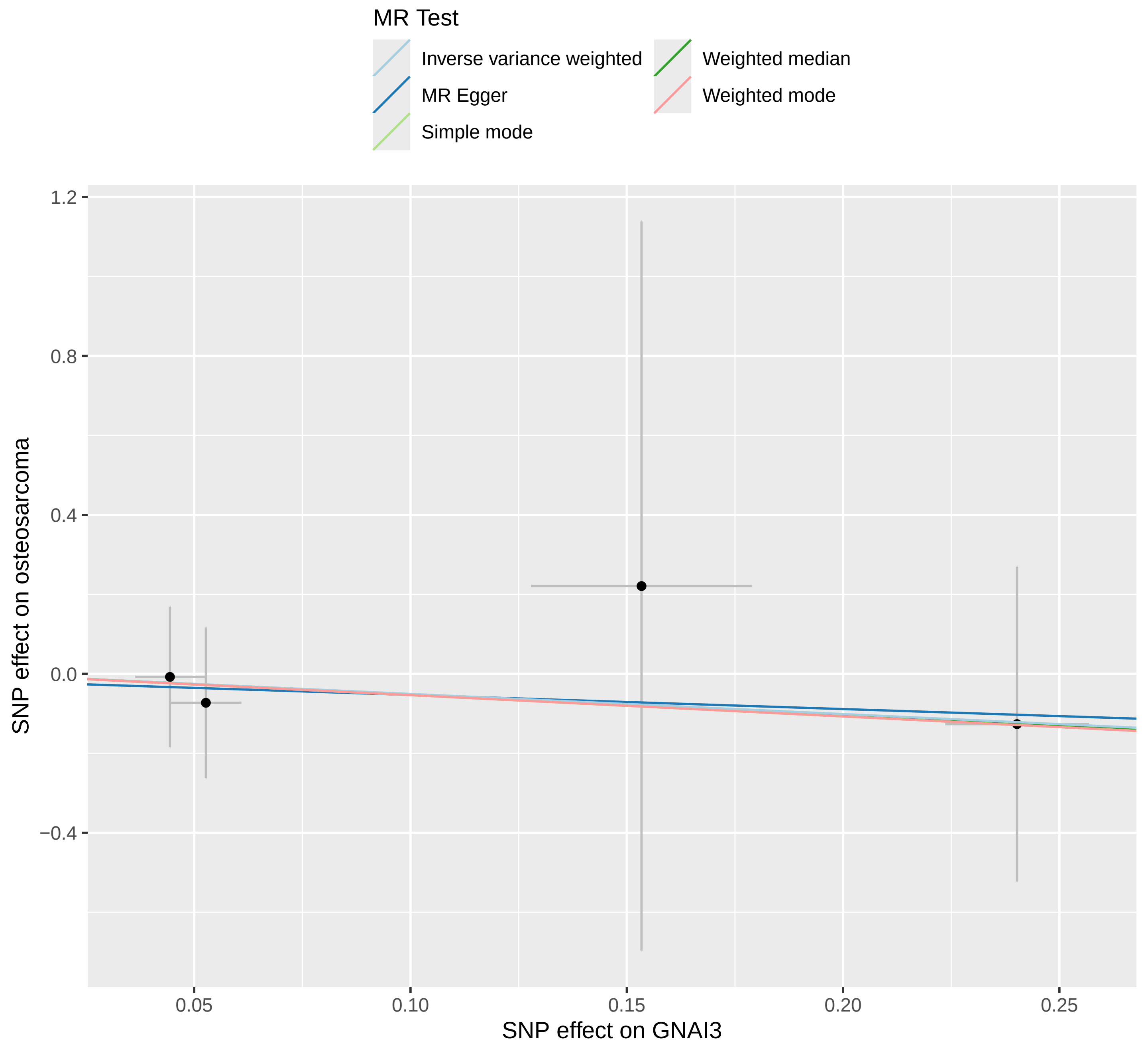
MR Test
Inverse variance weighted
MR Egger
Simple mode
Weighted median
Weighted mode
SNP effect on osteosarcoma
SNP effect on GNAI3
1.2
0.8
0.4
0.0
−0.4
0.05
0.10
0.15
0.20
0.25

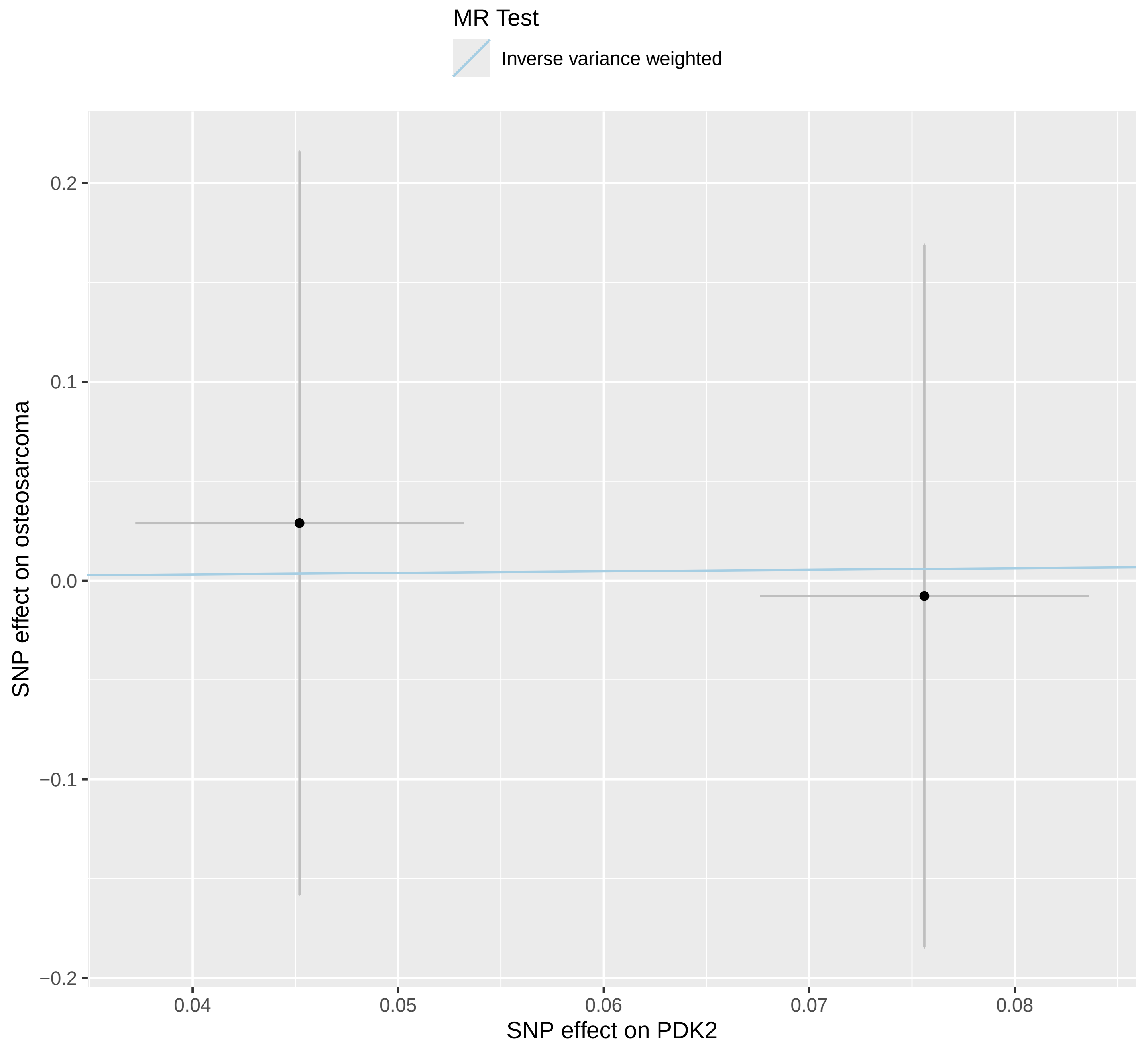
MR Test
Inverse variance weighted
0.2
0.1
0.0
−0.1
−0.2
SNP effect on osteosarcoma
0.04
0.05
0.06
0.07
0.08
SNP effect on PDK2

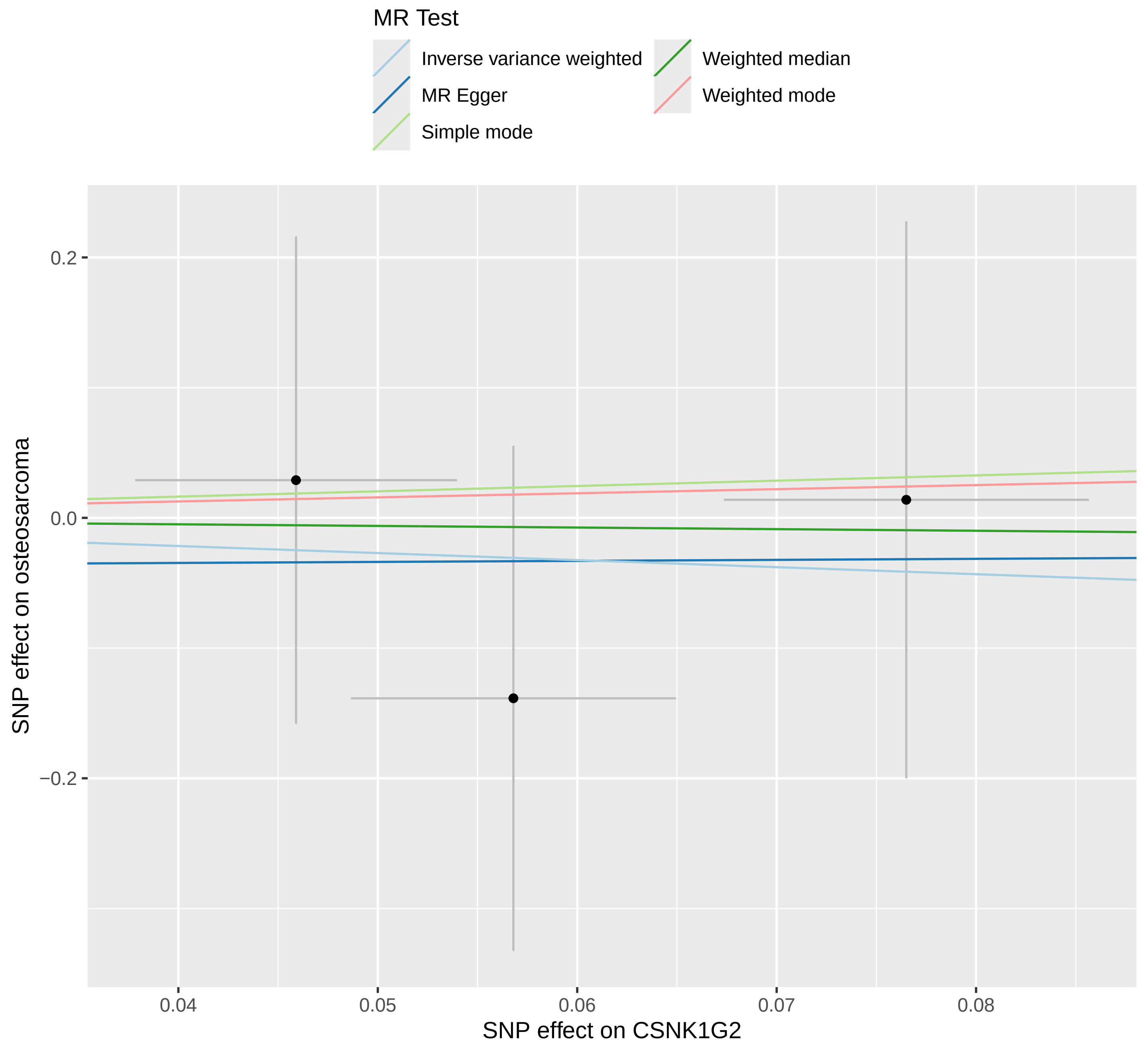

MR Test
Inverse variance weighted
MR Egger
Simple mode
Weighted median
Weighted mode
0.2
0.0
−0.2
0.04
0.05
0.06
0.07
0.08
SNP effect on osteosarcoma
SNP effect on CSNK1G2

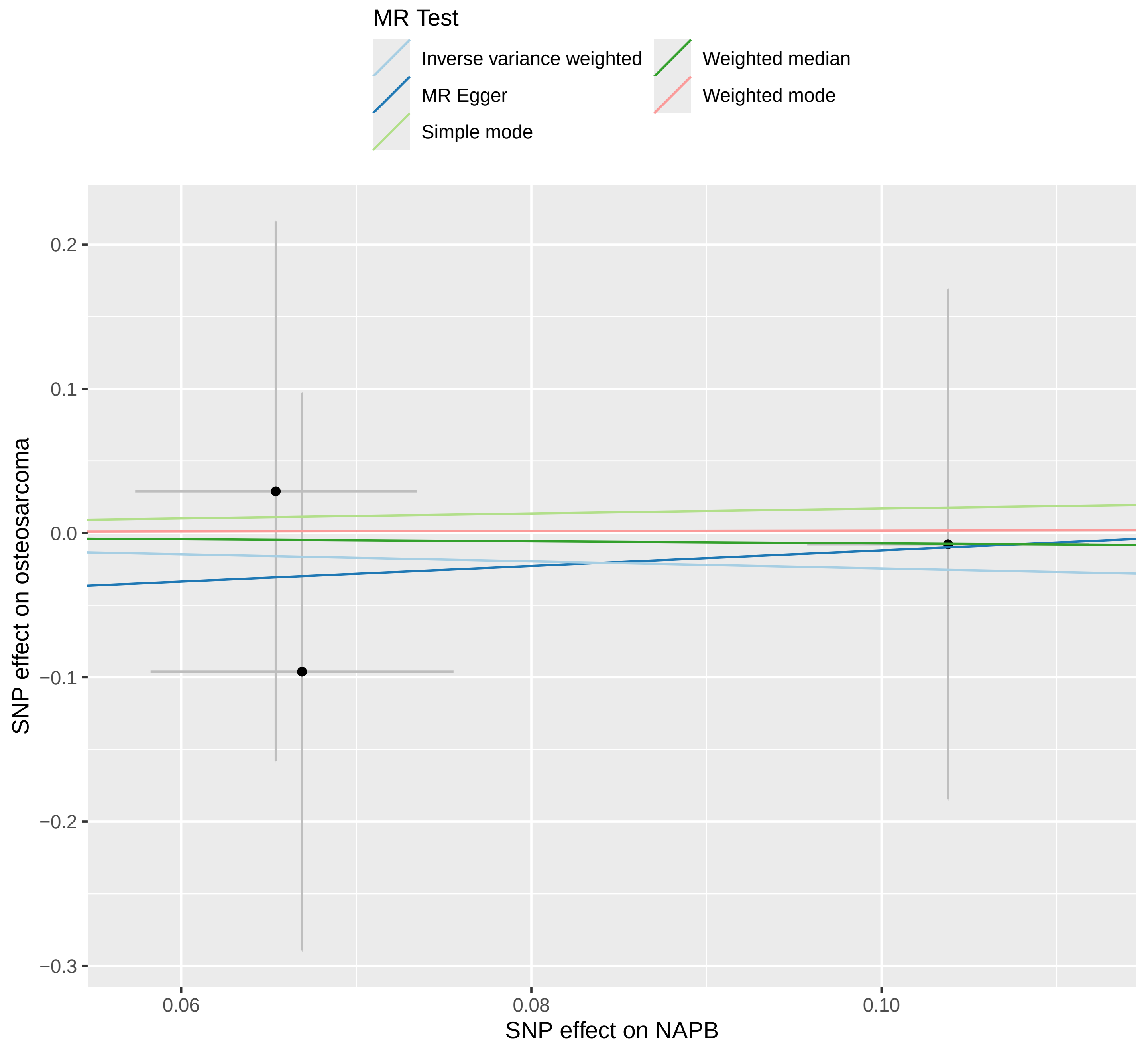

MR Test
Inverse variance weighted
MR Egger
Simple mode
Weighted median
Weighted mode
SNP effect on osteosarcoma
0.2
0.1
0.0
−0.1
−0.2
−0.3
0.06
0.08
0.10
SNP effect on NAPB

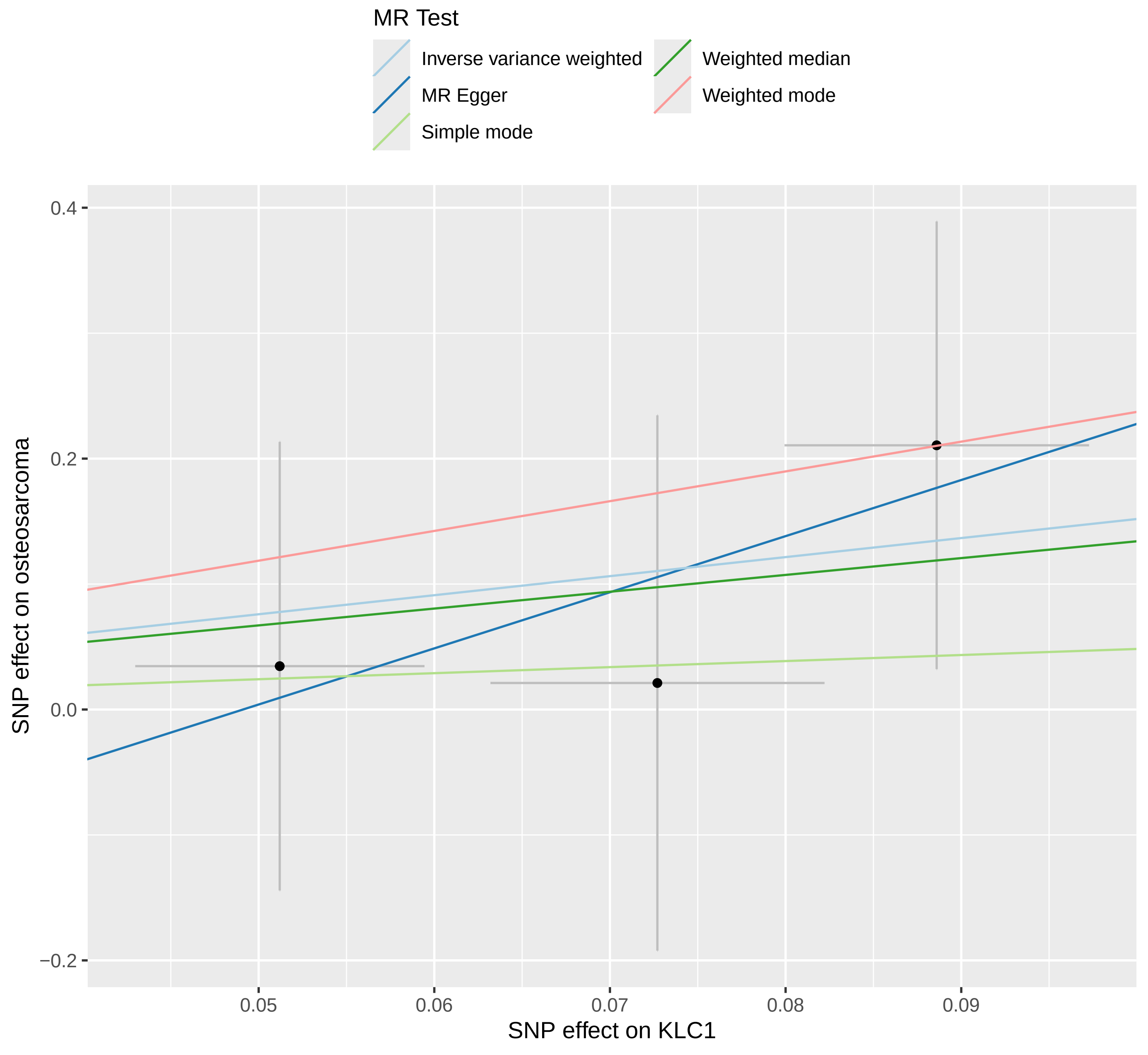
MR Test
Inverse variance weighted
MR Egger
Simple mode
Weighted median
Weighted mode
0.4
0.2
0.0
−0.2
0.05
0.06
0.07
0.08
0.09
SNP effect on osteosarcoma
SNP effect on KLC1

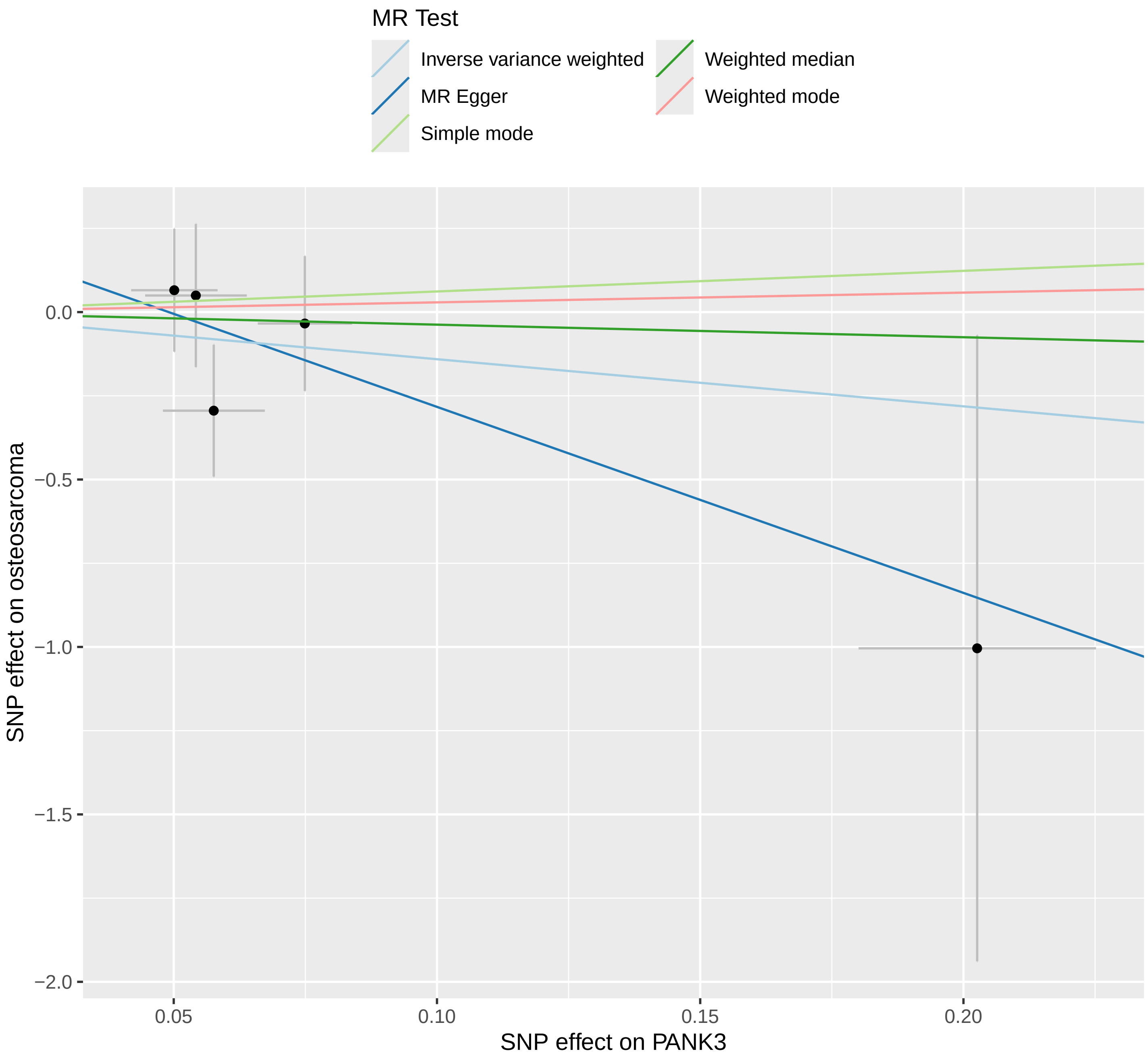
MR Test
Inverse variance weighted
MR Egger
Simple mode
Weighted median
Weighted mode
0.0
−0.5
−1.0
−1.5
−2.0
SNP effect on osteosarcoma
0.05
0.10
0.15
0.20
SNP effect on PANK3

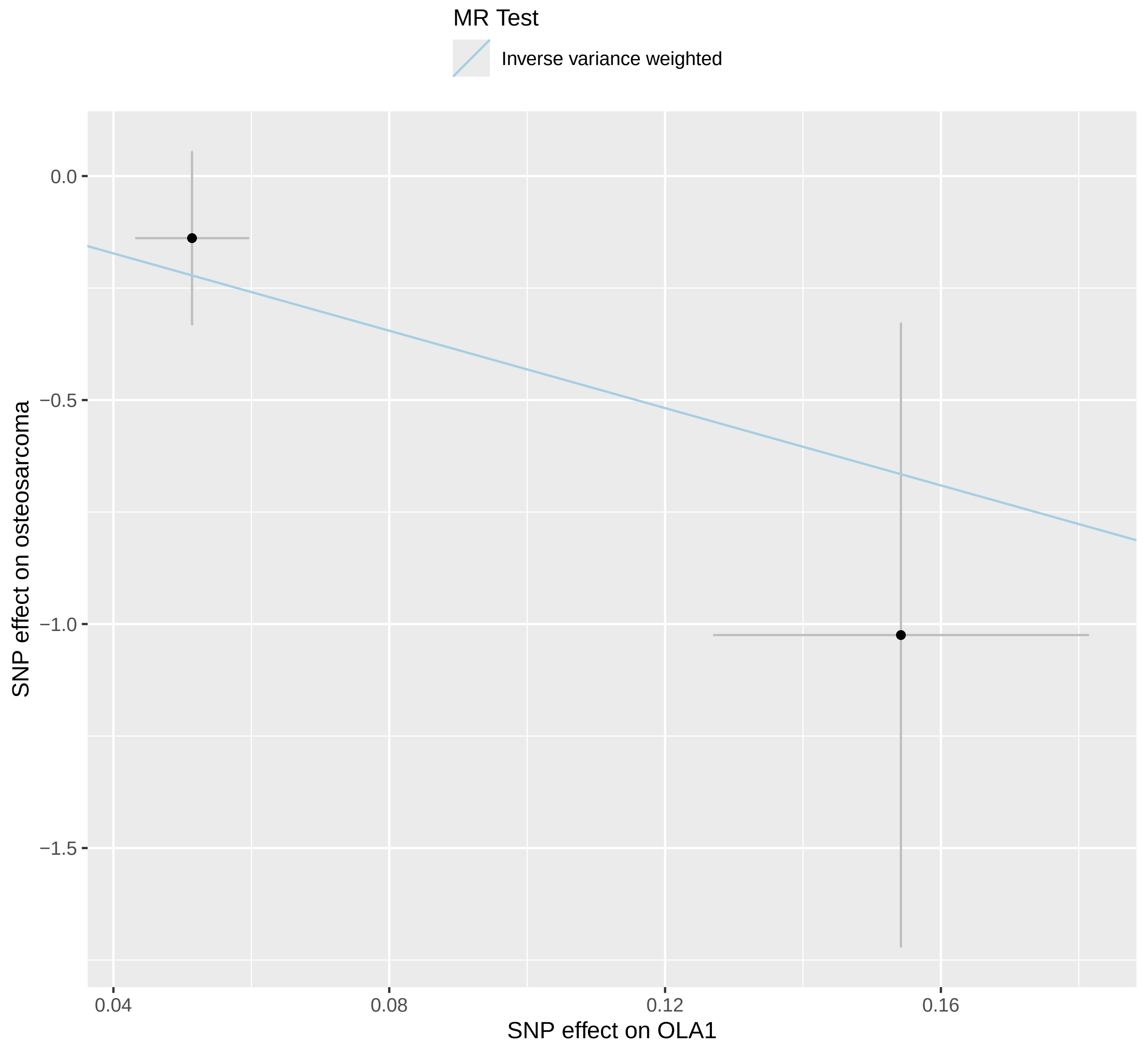
MR Test
Inverse variance weighted
SNP effect on osteosarcoma
SNP effect on OLA1
0.0
−0.5
−1.0
−1.5
0.04
0.08
0.12
0.16

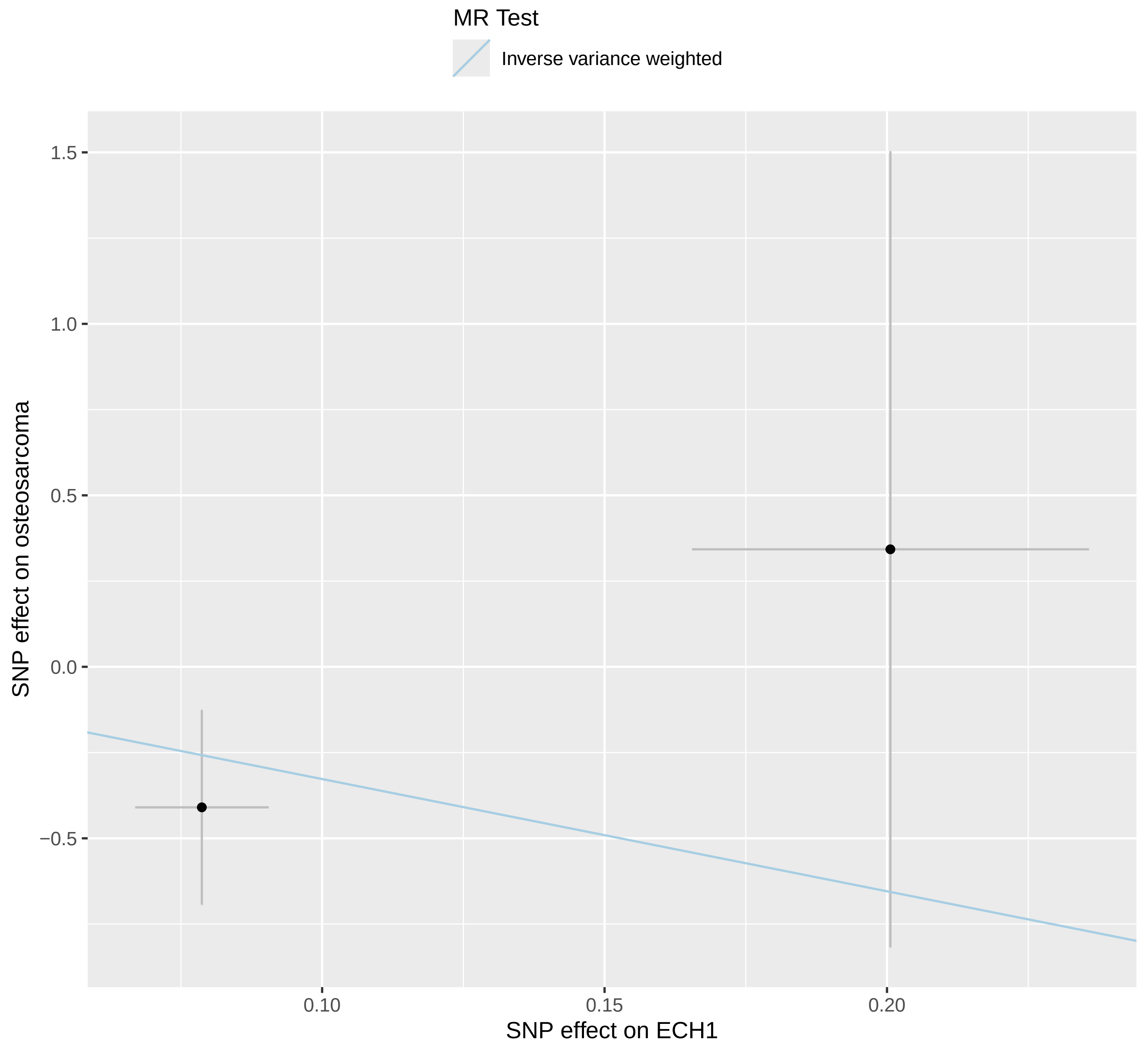
MR Test
Inverse variance weighted
1.5
1.0
0.5
0.0
−0.5
SNP effect on osteosarcoma
0.10
0.15
0.20
SNP effect on ECH1

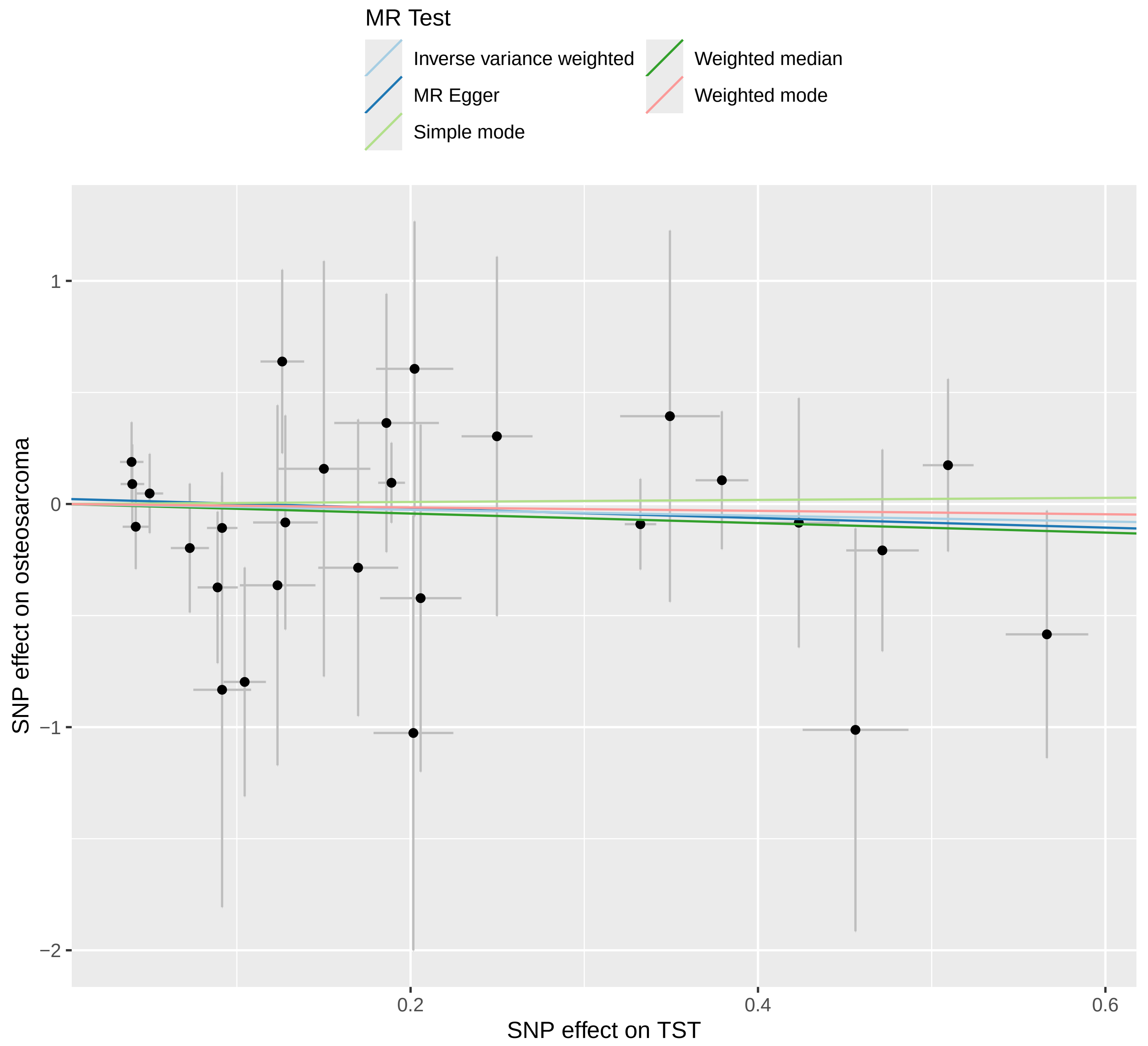
MR Test
Inverse variance weighted
MR Egger
Simple mode
Weighted median
Weighted mode
1
0
−1
−2
0.2
0.4
0.6
SNP effect on osteosarcoma
SNP effect on TST

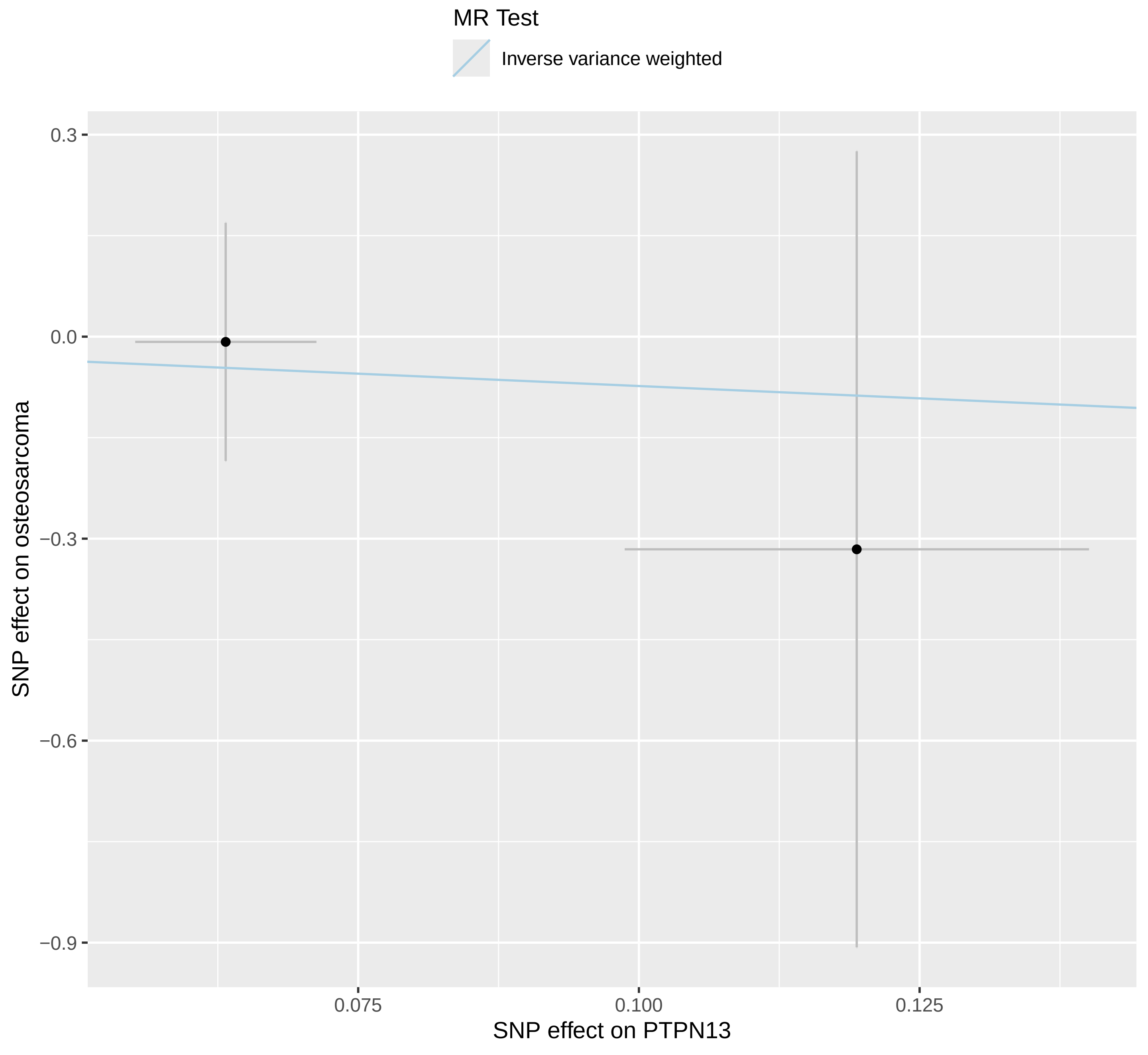

MR Test
Inverse variance weighted
0.3
0.0
−0.3
−0.6
−0.9
SNP effect on osteosarcoma
0.075
0.100
0.125
SNP effect on PTPN13

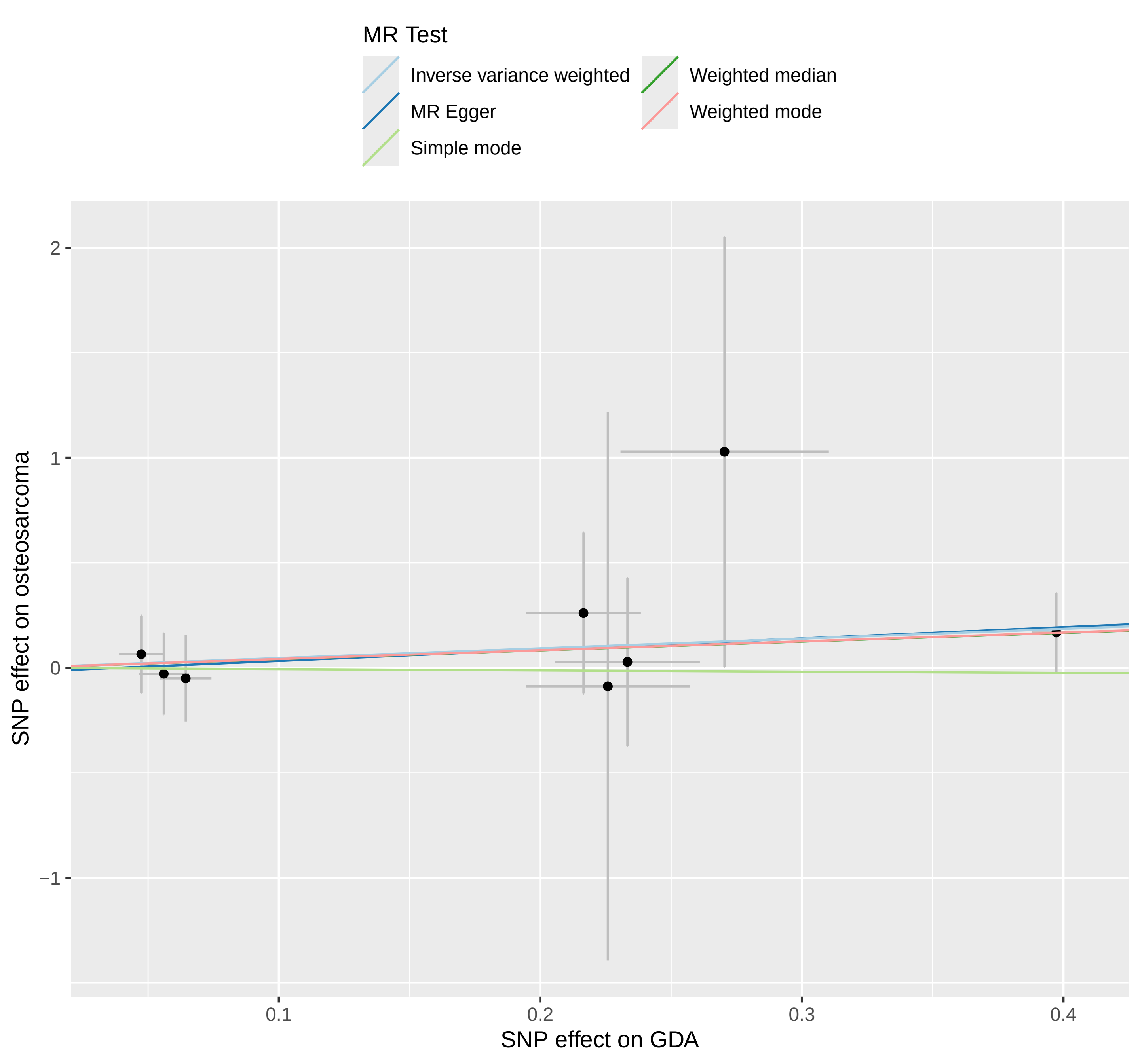
MR Test
Inverse variance weighted
MR Egger
Simple mode
Weighted median
Weighted mode
2
1
0
−1
SNP effect on osteosarcoma
0.1
0.2
0.3
0.4
SNP effect on GDA

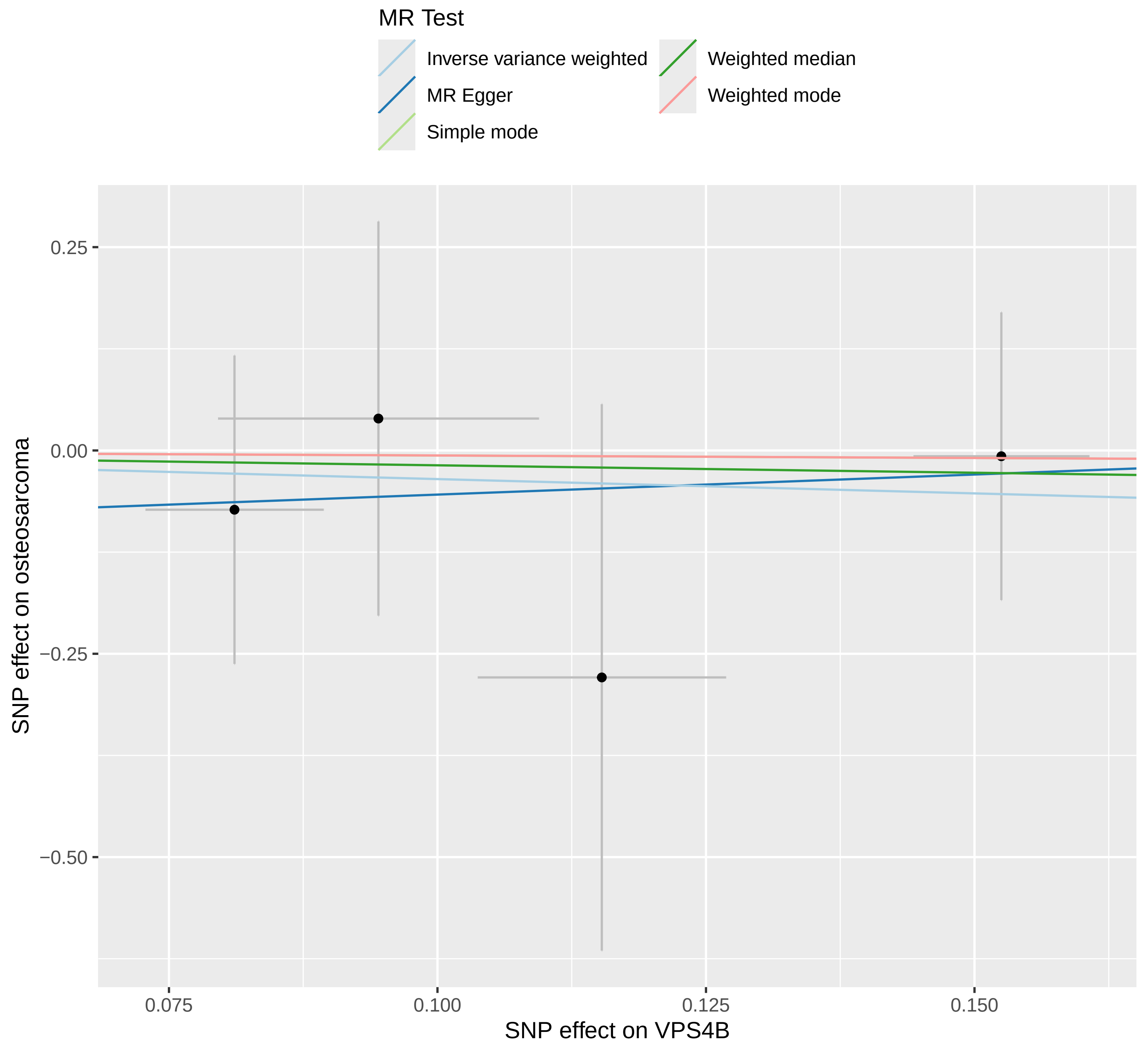
MR Test
Inverse variance weighted
MR Egger
Simple mode
Weighted median
Weighted mode
0.25
0.00
−0.25
−0.50
SNP effect on osteosarcoma
0.075
0.100
0.125
0.150
SNP effect on VPS4B

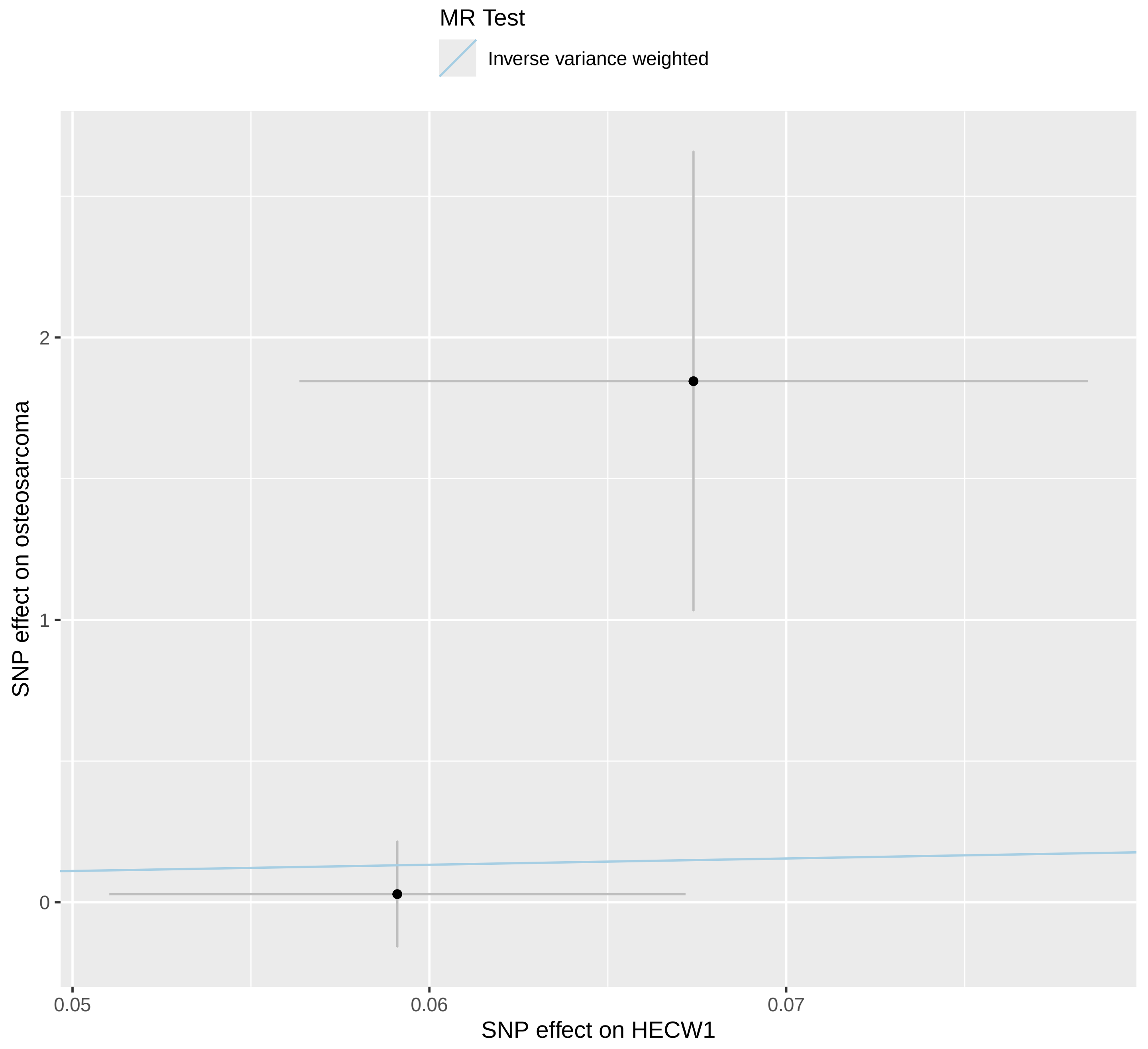

MR Test
Inverse variance weighted
SNP effect on osteosarcoma
2
1
0
0.05
0.06
0.07
SNP effect on HECW1

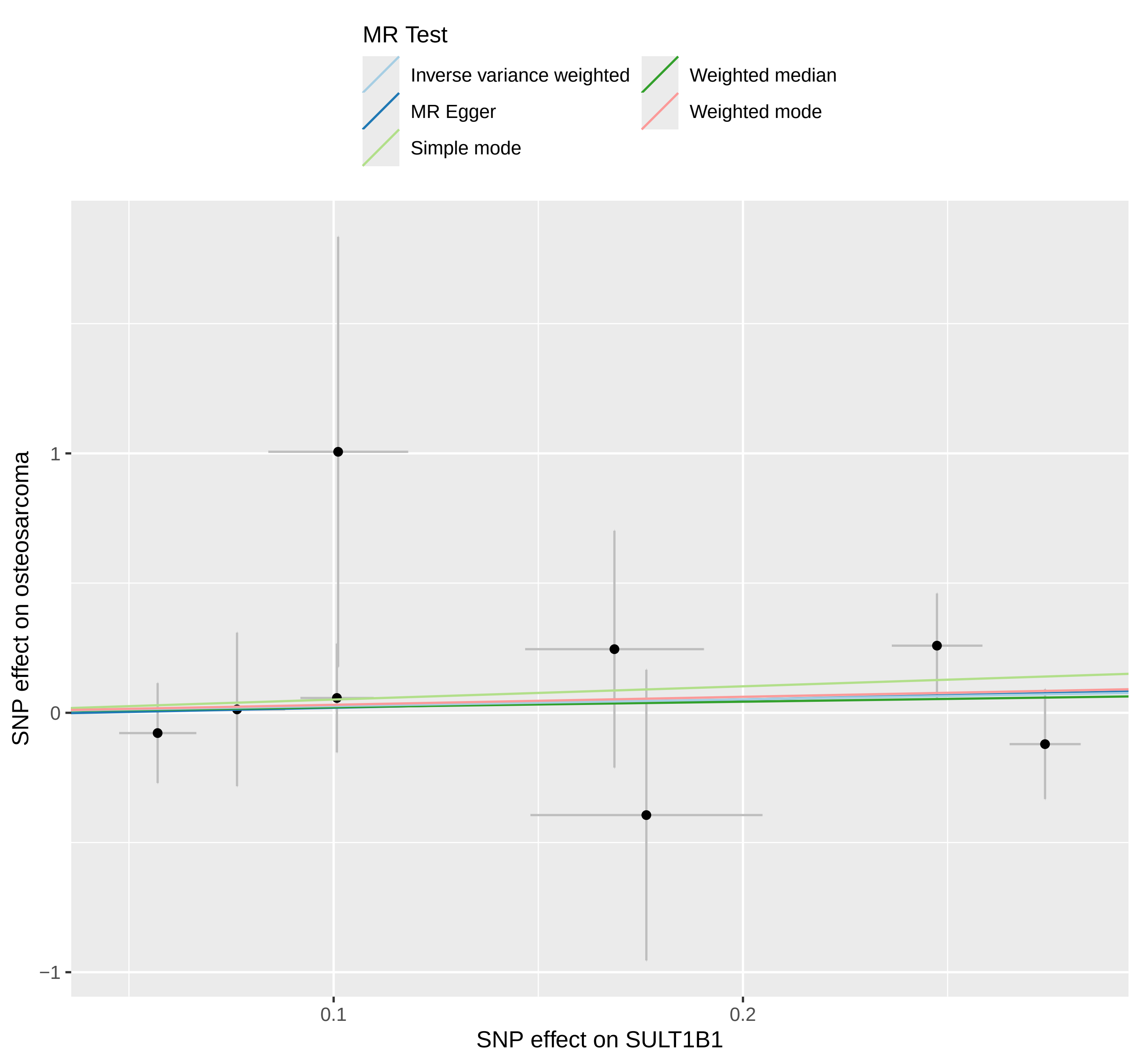

MR Test
Inverse variance weighted
MR Egger
Simple mode
Weighted median
Weighted mode
1
0
−1
0.1
0.2
SNP effect on osteosarcoma
SNP effect on SULT1B1

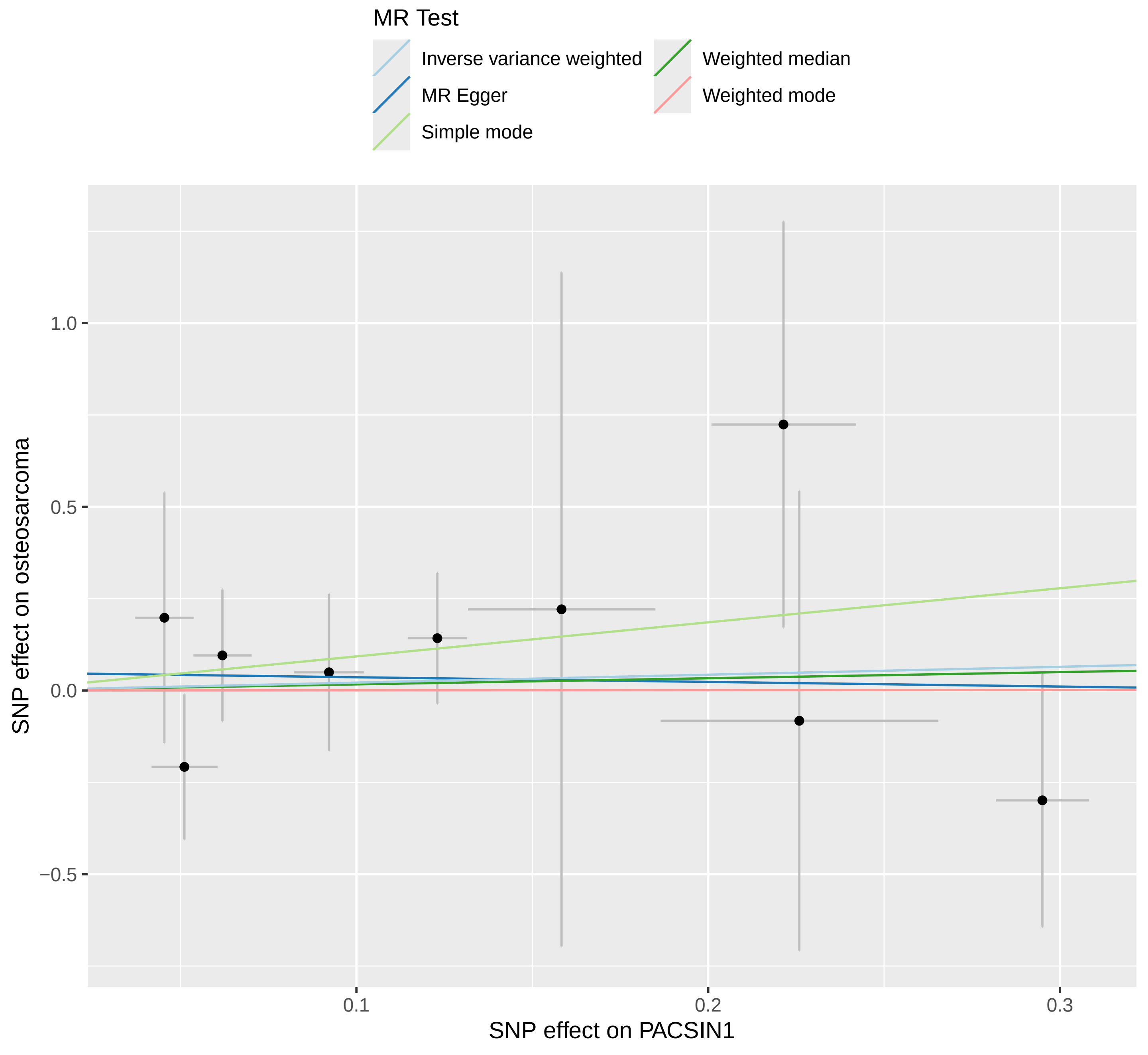

MR Test
Inverse variance weighted
MR Egger
Simple mode
Weighted median
Weighted mode
1.0
0.5
0.0
−0.5
SNP effect on osteosarcoma
0.1
0.2
0.3
SNP effect on PACSIN1

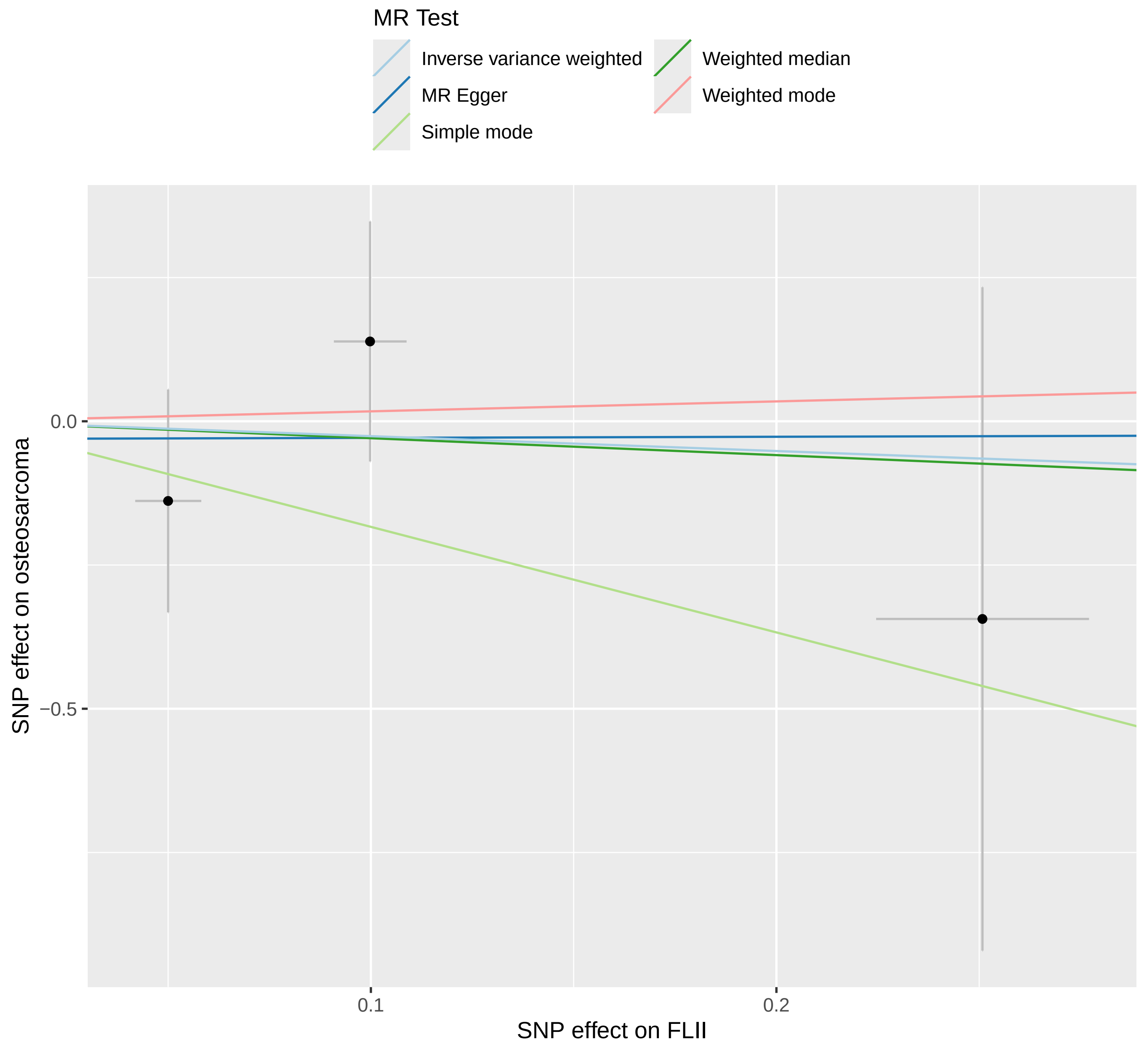
MR Test
Inverse variance weighted
MR Egger
Simple mode
Weighted median
Weighted mode
0.0
−0.5
0.1
0.2
SNP effect on osteosarcoma
SNP effect on FLII

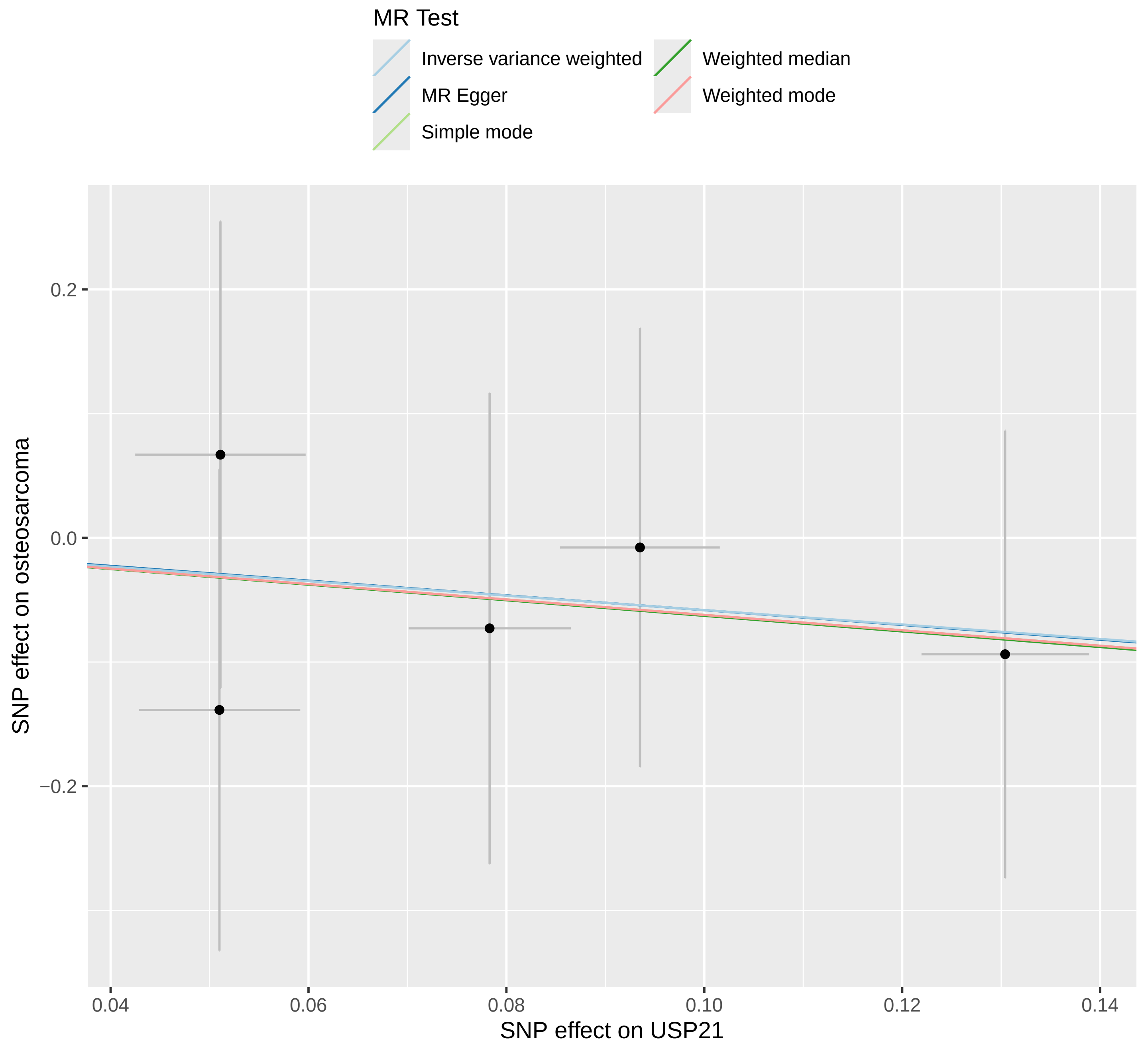

MR Test
Inverse variance weighted
MR Egger
Simple mode
Weighted median
Weighted mode
0.2
0.0
−0.2
SNP effect on osteosarcoma
0.04
0.06
0.08
0.10
0.12
0.14
SNP effect on USP21

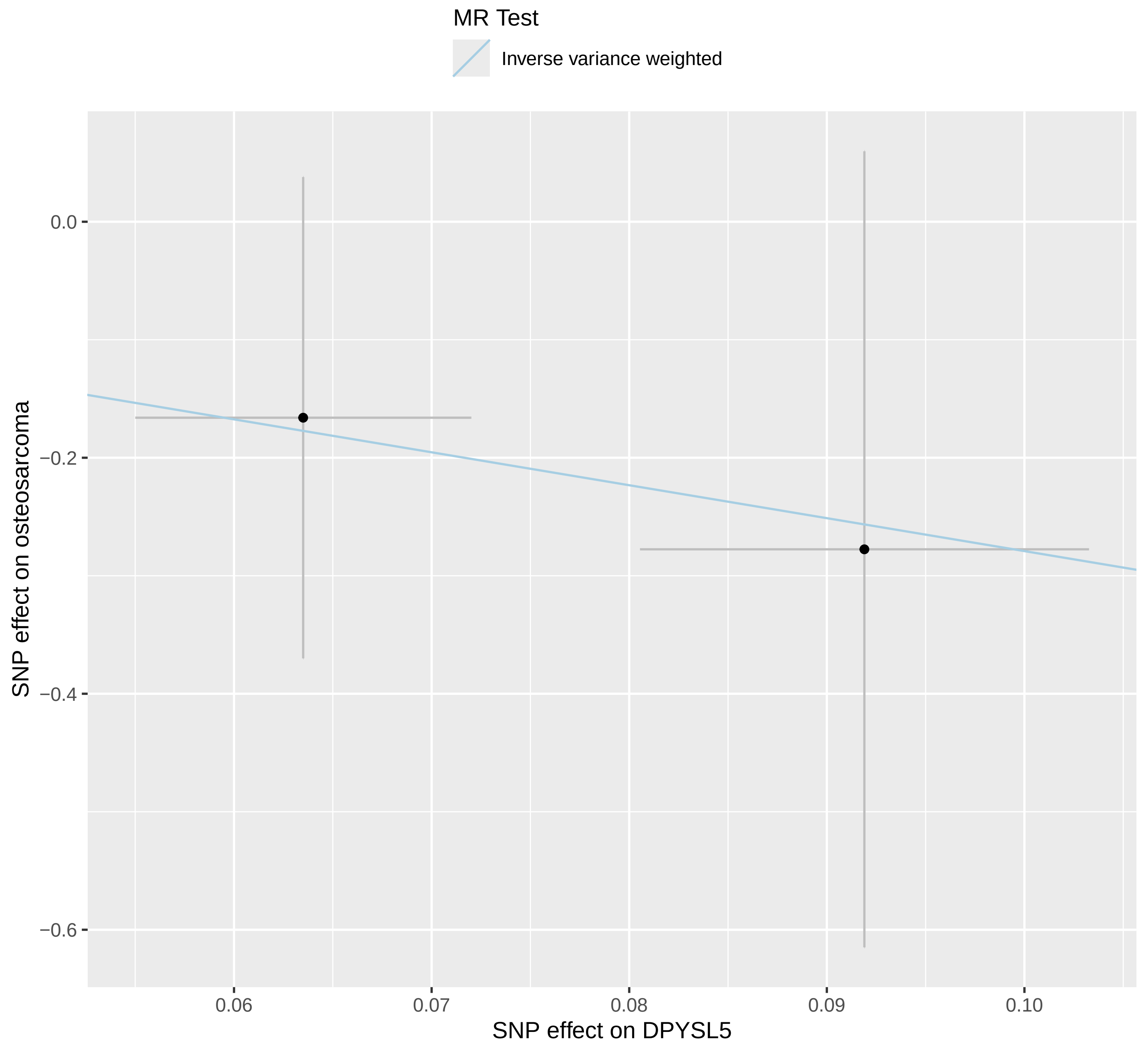
MR Test
Inverse variance weighted
0.0
−0.2
−0.4
−0.6
SNP effect on osteosarcoma
0.06
0.07
0.08
0.09
0.10
SNP effect on DPYSL5

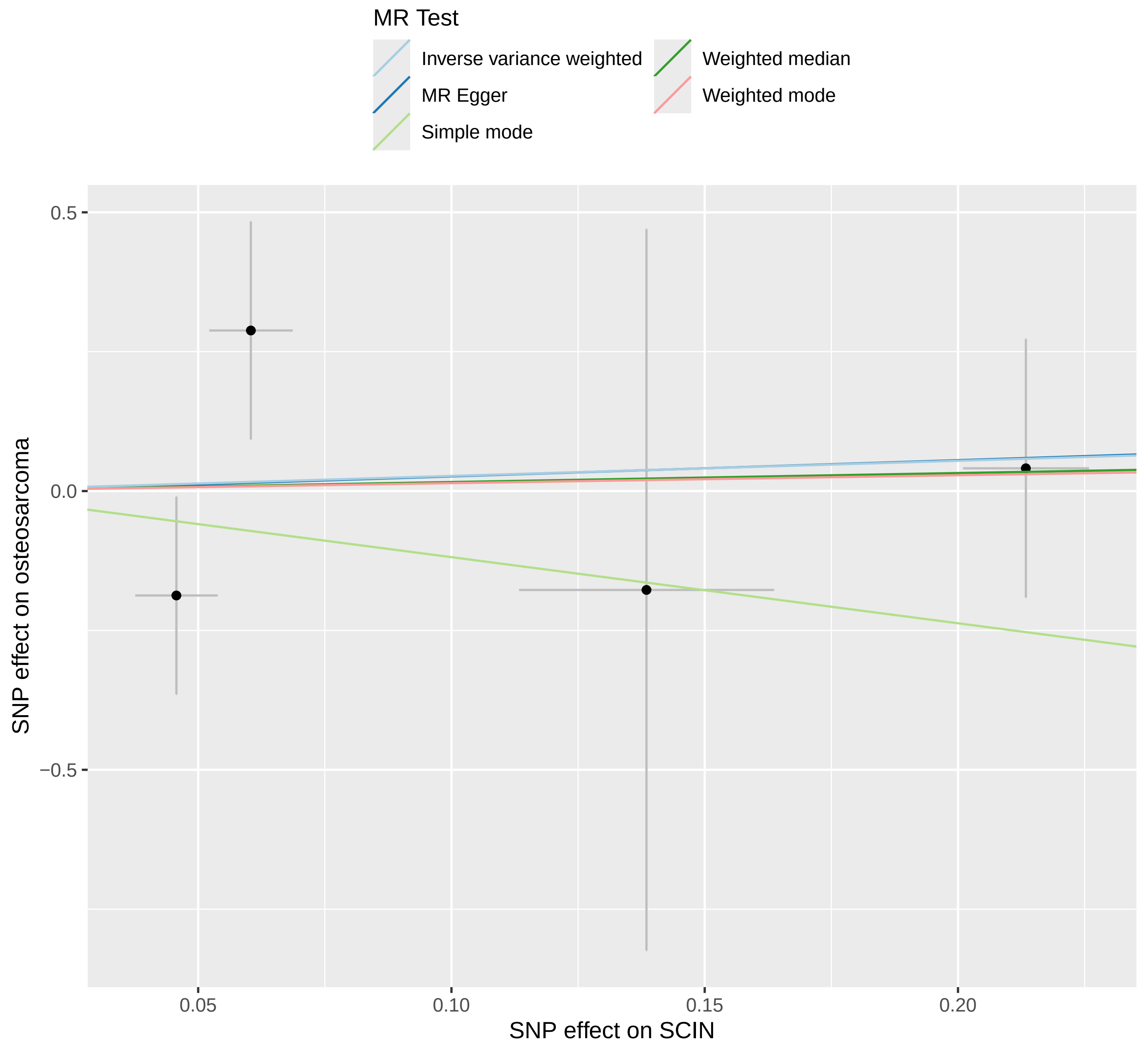

MR Test
Inverse variance weighted
MR Egger
Simple mode
Weighted median
Weighted mode
0.5
0.0
−0.5
SNP effect on osteosarcoma
0.05
0.10
0.15
0.20
SNP effect on SCIN

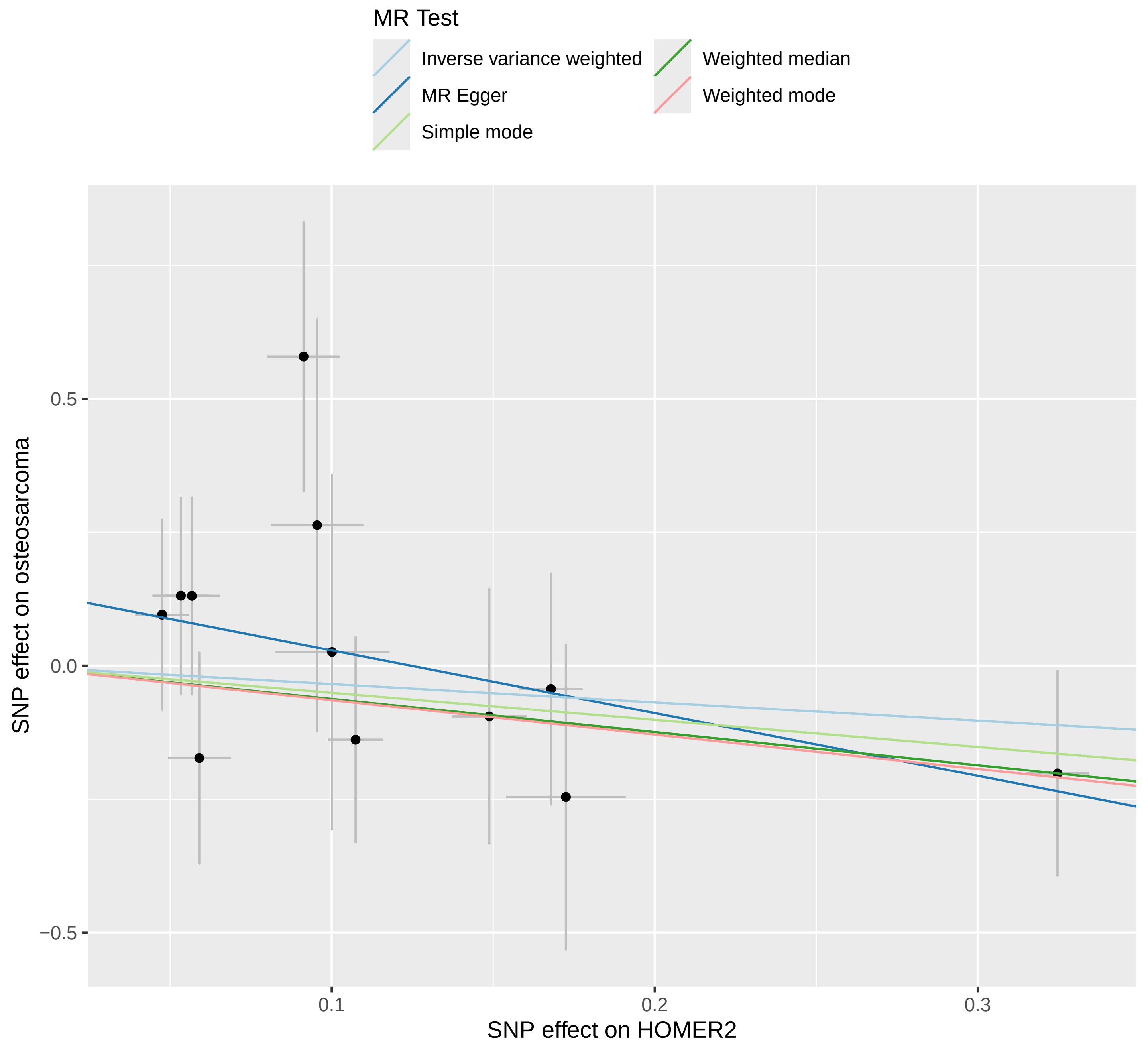

MR Test
Inverse variance weighted
MR Egger
Simple mode
Weighted median
Weighted mode
SNP effect on osteosarcoma
0.5
0.0
−0.5
0.1
0.2
0.3
SNP effect on HOMER2

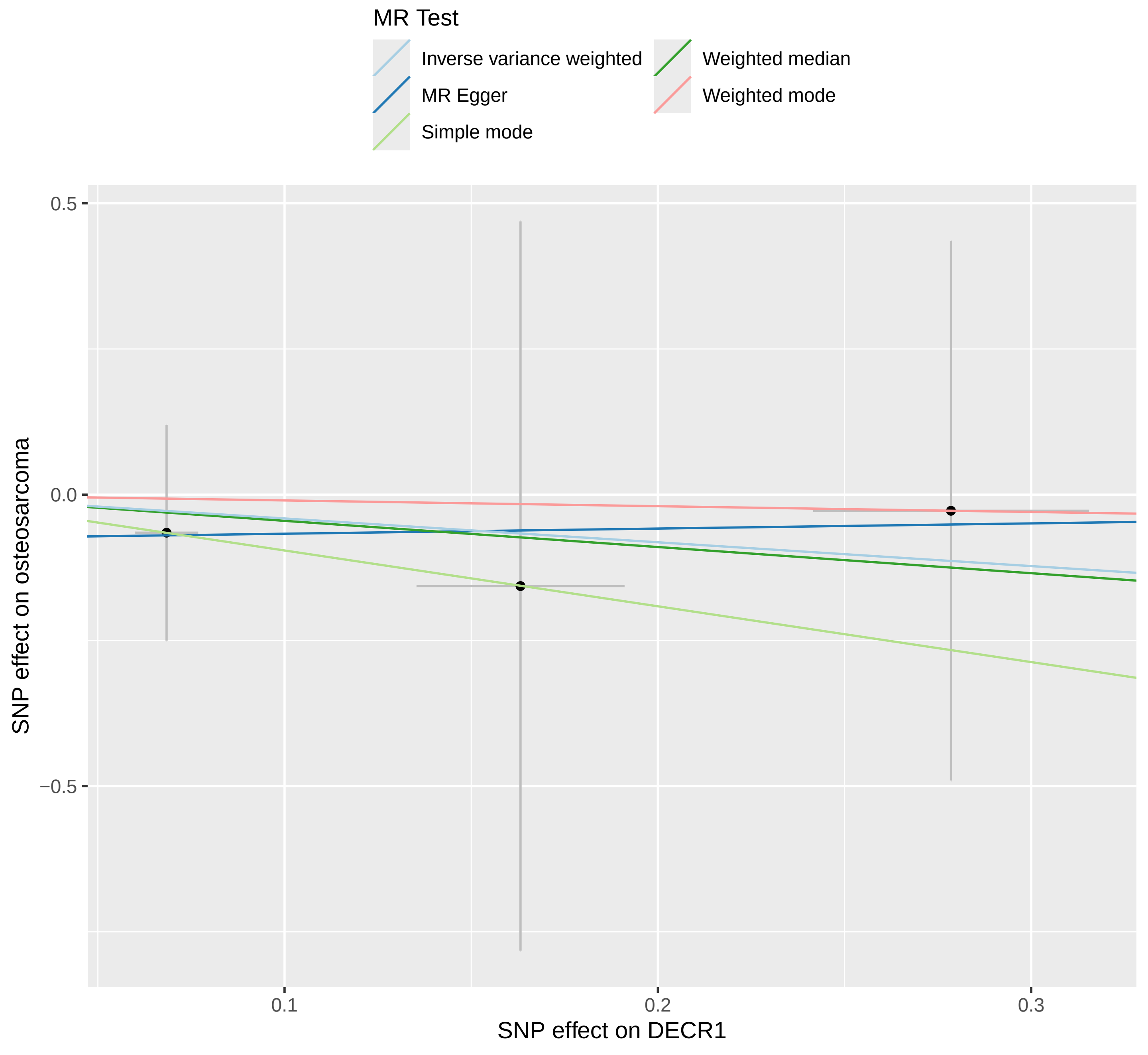
MR Test
Inverse variance weighted
MR Egger
Simple mode
Weighted median
Weighted mode
0.5
0.0
−0.5
0.1
0.2
0.3
SNP effect on osteosarcoma
SNP effect on DECR1

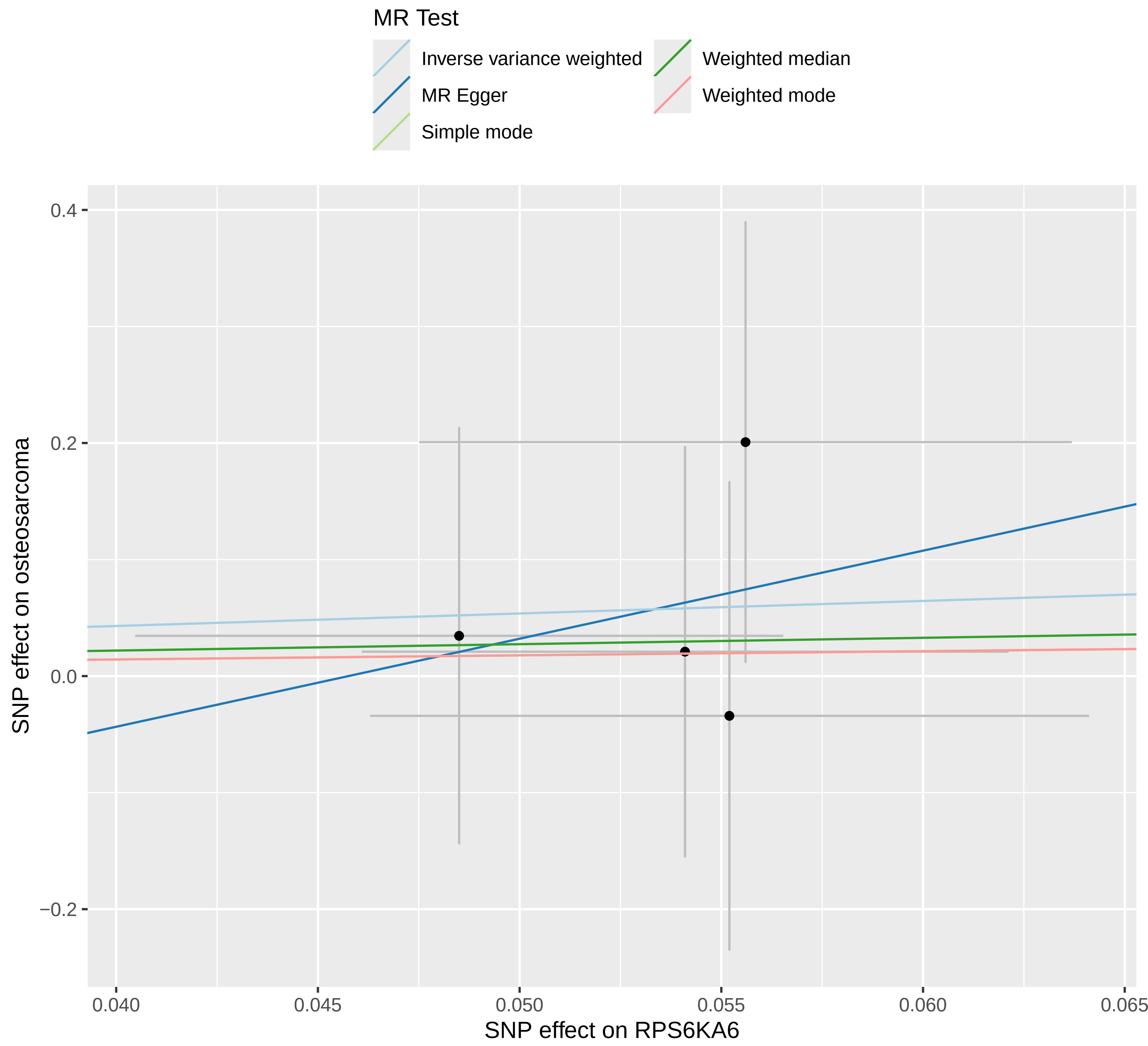

MR Test
Inverse variance weighted
MR Egger
Simple mode
Weighted median
Weighted mode
0.4
0.2
0.0
−0.2
SNP effect on osteosarcoma
0.040
0.045
0.050
0.055
0.060
0.065
SNP effect on RPS6KA6

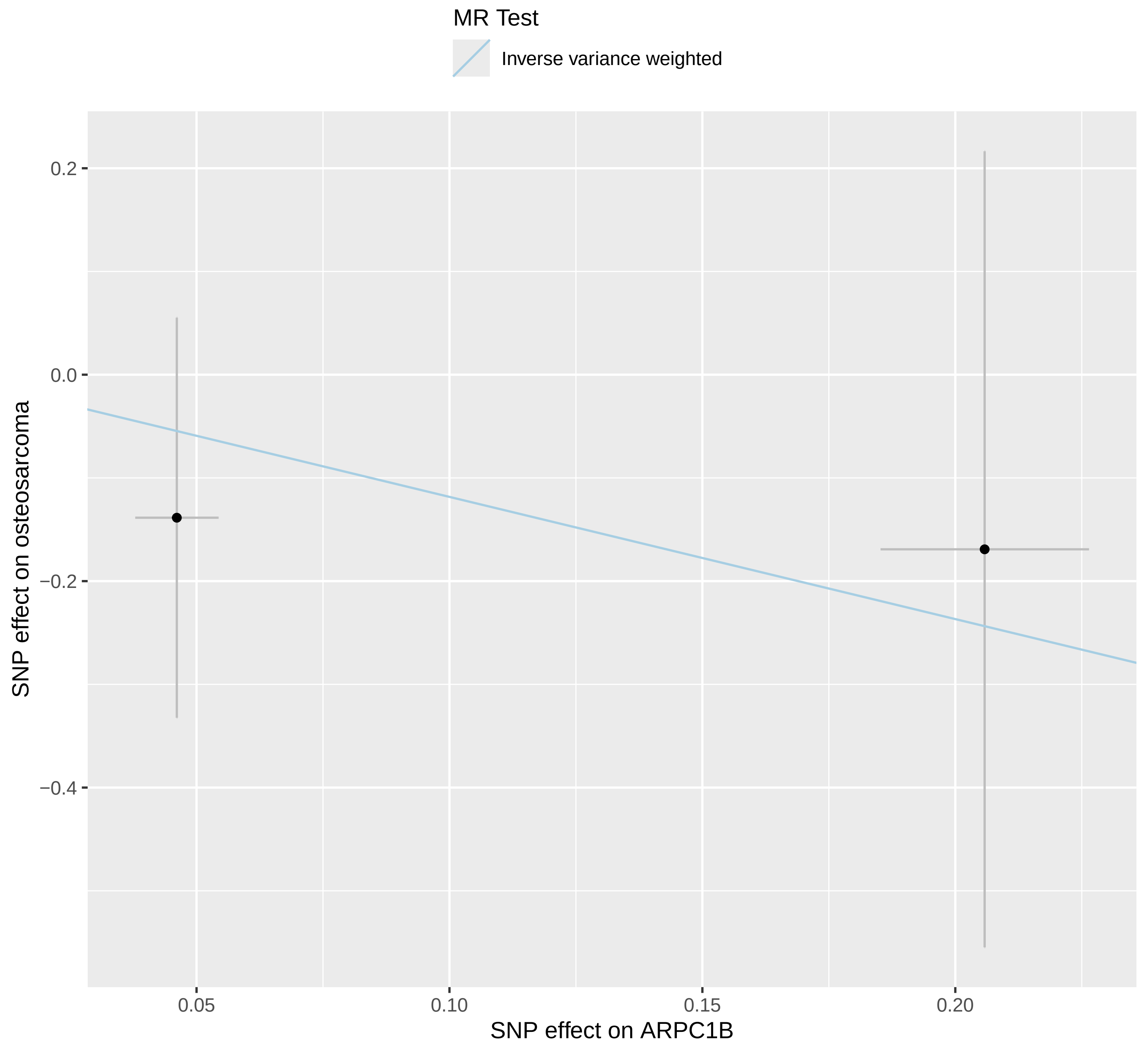
MR Test
Inverse variance weighted
0.2
0.0
−0.2
−0.4
SNP effect on osteosarcoma
0.05
0.10
0.15
0.20
SNP effect on ARPC1B

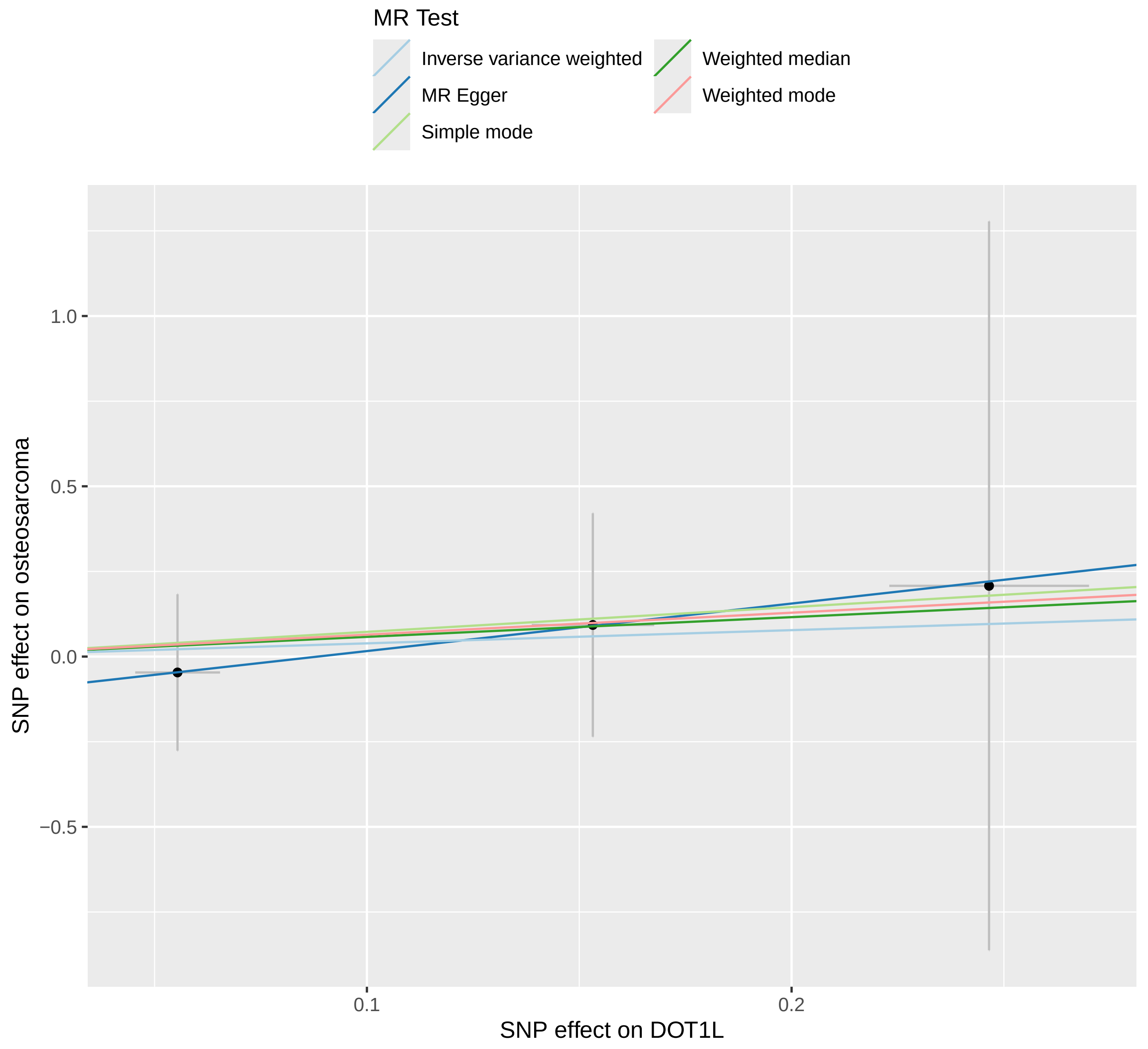
MR Test
Inverse variance weighted
MR Egger
Simple mode
Weighted median
Weighted mode
1.0
0.5
0.0
−0.5
0.1
0.2
SNP effect on osteosarcoma
SNP effect on DOT1L

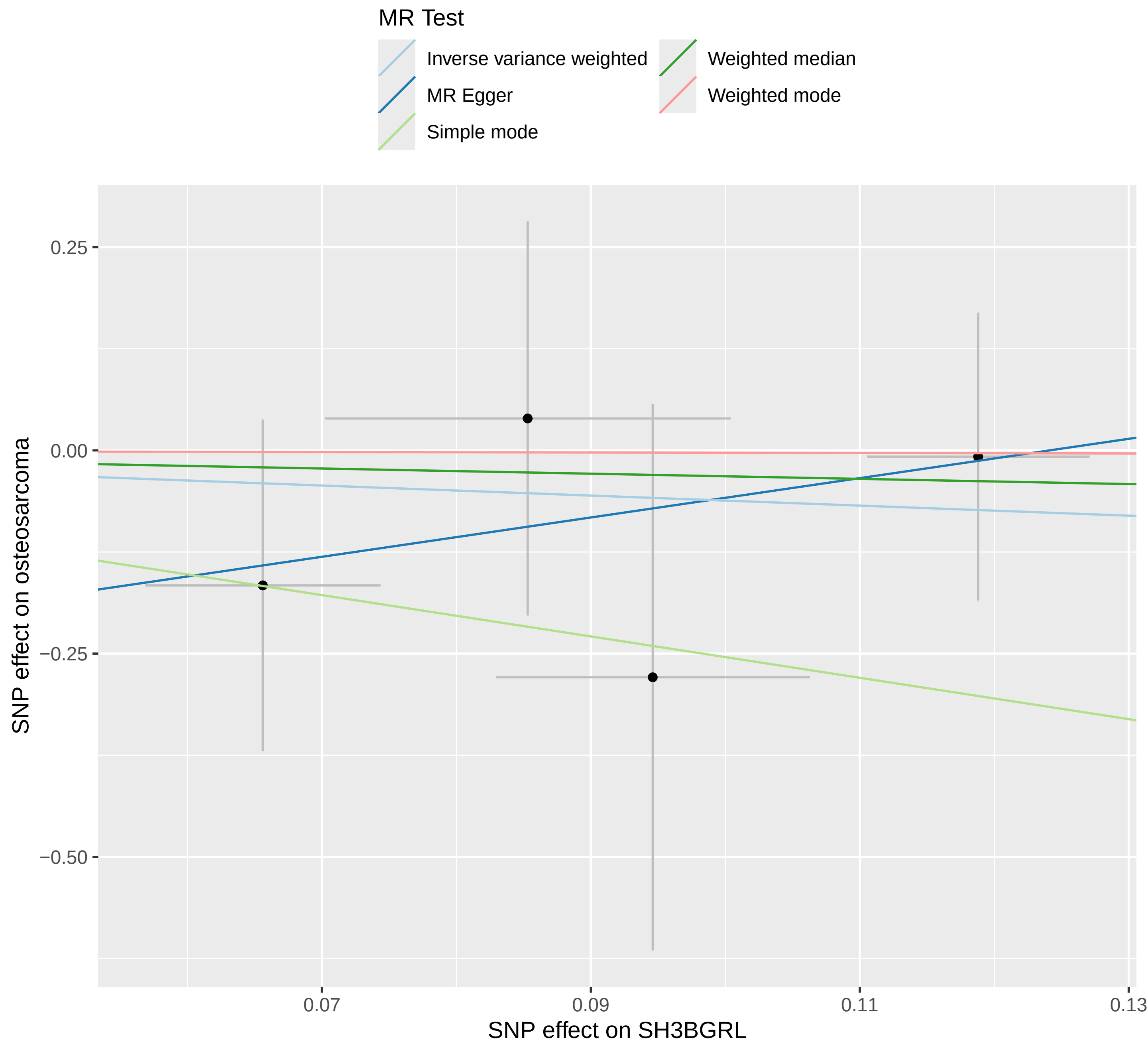

MR Test
Inverse variance weighted
MR Egger
Simple mode
Weighted median
Weighted mode
0.25
0.00
−0.25
−0.50
0.07
0.09
0.11
0.13
SNP effect on osteosarcoma
SNP effect on SH3BGRL

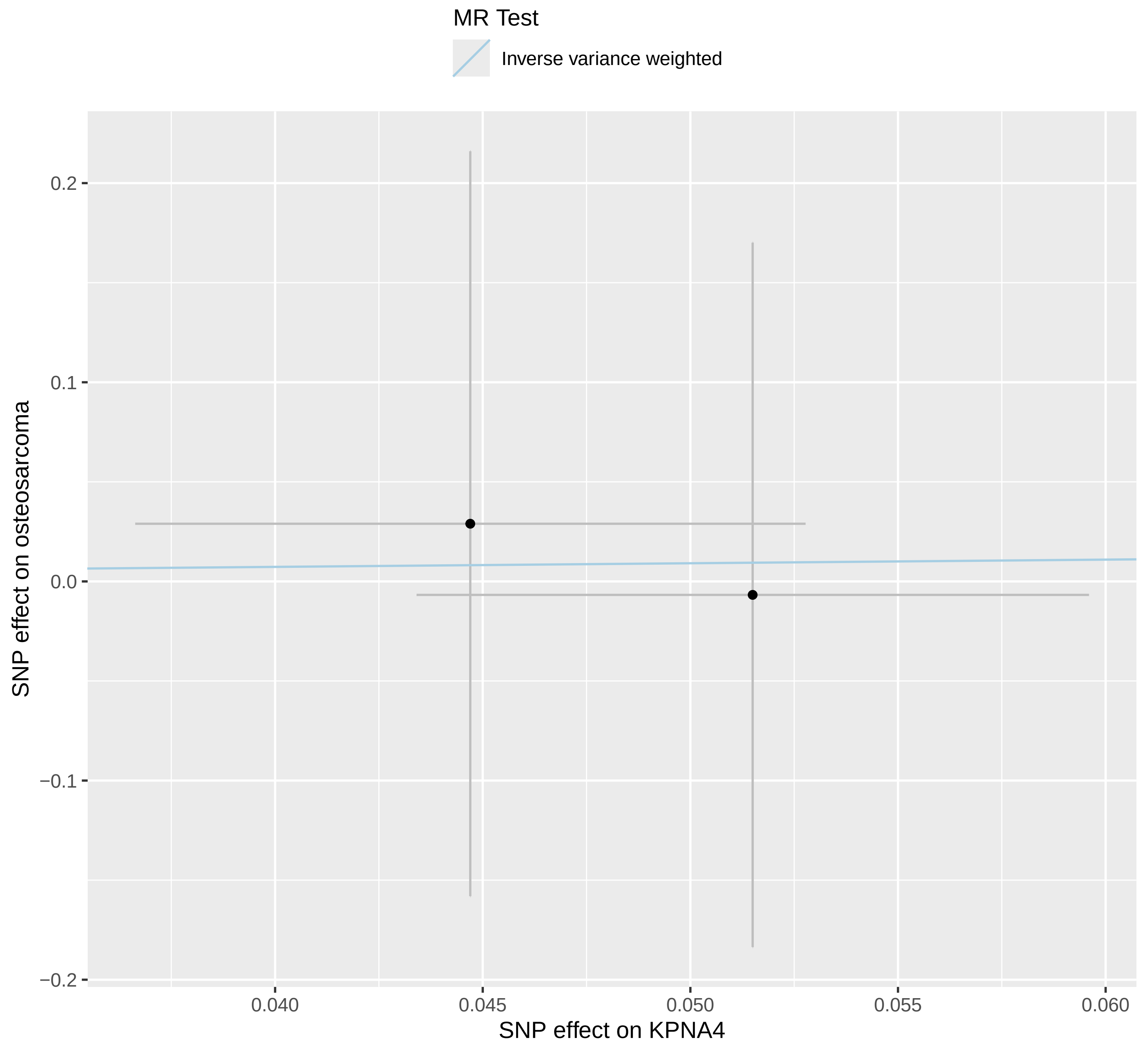
MR Test
Inverse variance weighted
0.2
0.1
0.0
−0.1
−0.2
0.040
0.045
0.050
0.055
0.060
SNP effect on KPNA4
SNP effect on osteosarcoma

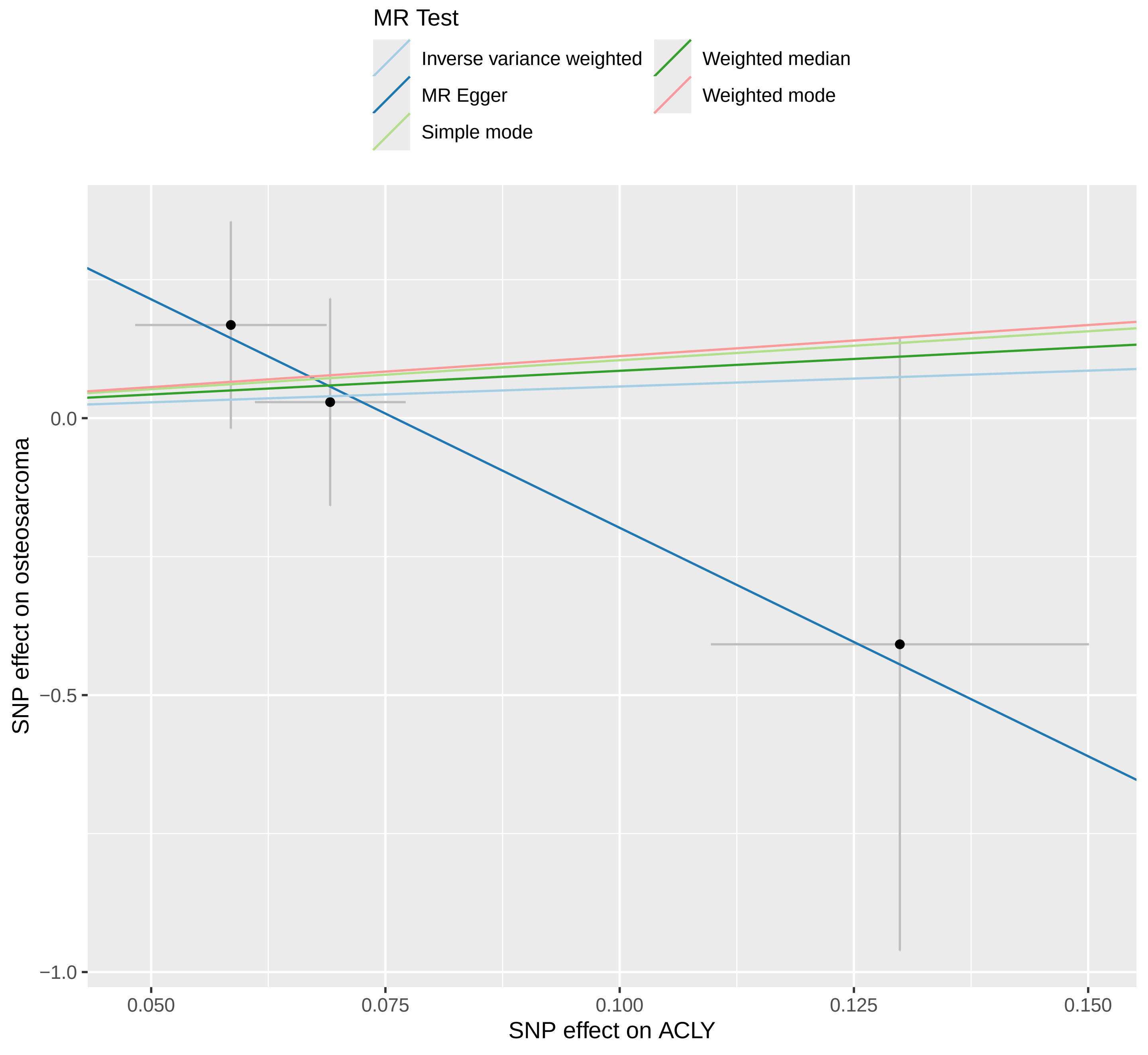
MR Test
Inverse variance weighted
MR Egger
Simple mode
Weighted median
Weighted mode
0.0
−0.5
−1.0
SNP effect on osteosarcoma
0.050
0.075
0.100
0.125
0.150
SNP effect on ACLY

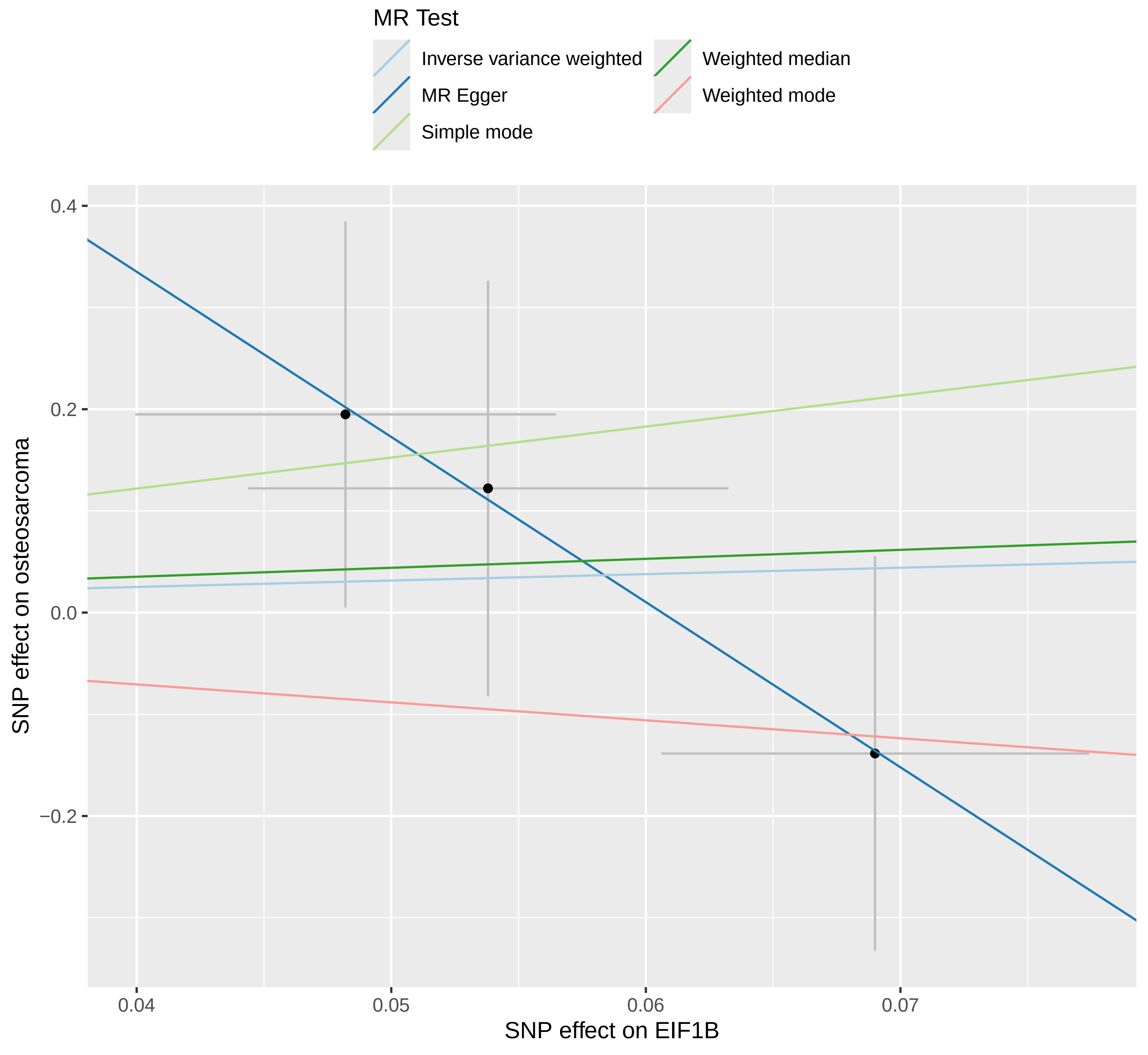
MR Test
Inverse variance weighted
MR Egger
Simple mode
Weighted median
Weighted mode
0.4
0.2
0.0
−0.2
0.04
0.05
0.06
0.07
SNP effect on EIF1B
SNP effect on osteosarcoma

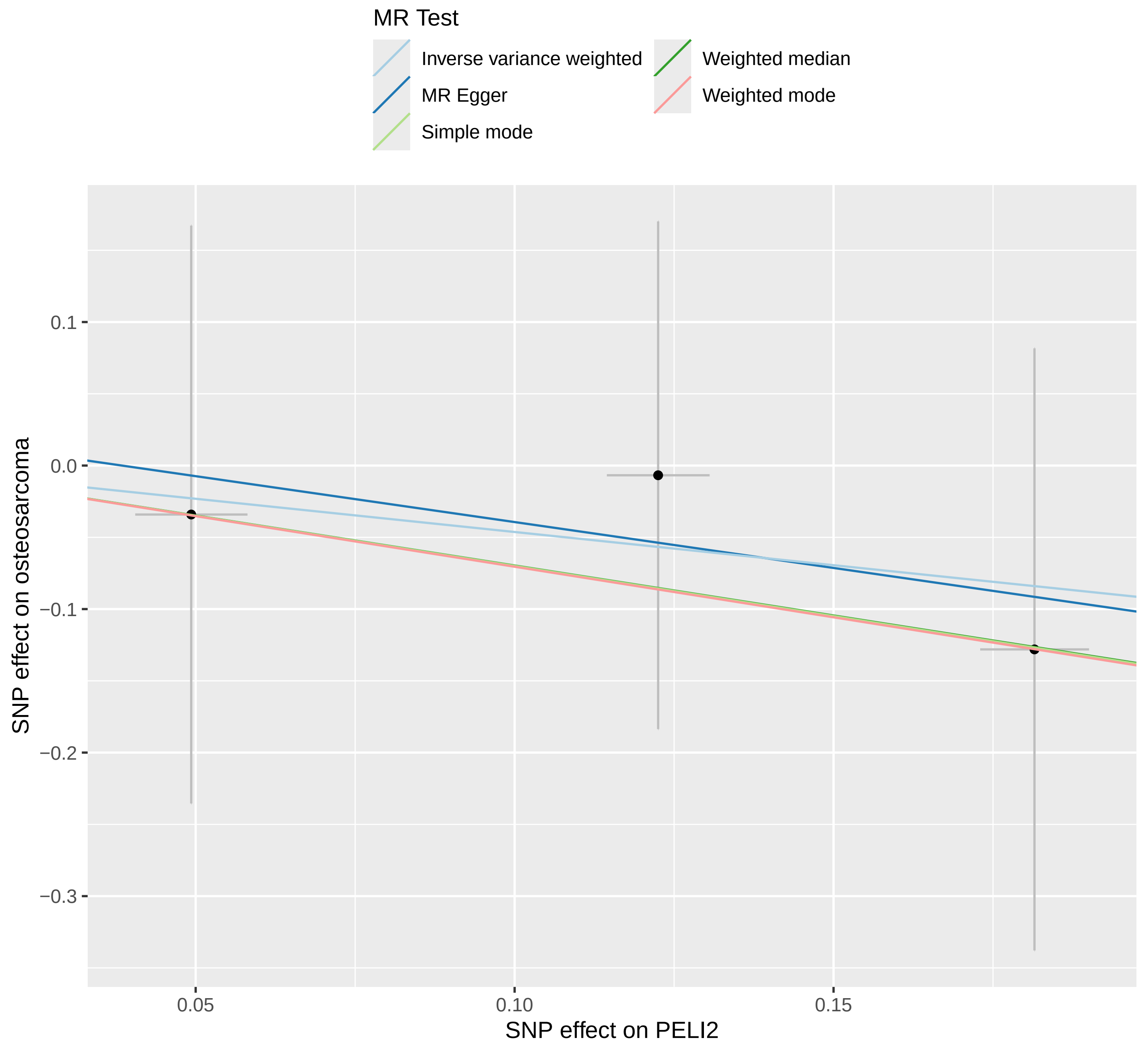

MR Test
Inverse variance weighted
MR Egger
Simple mode
Weighted median
Weighted mode
0.1
0.0
−0.1
−0.2
−0.3
SNP effect on osteosarcoma
0.05
0.10
0.15
SNP effect on PELI2

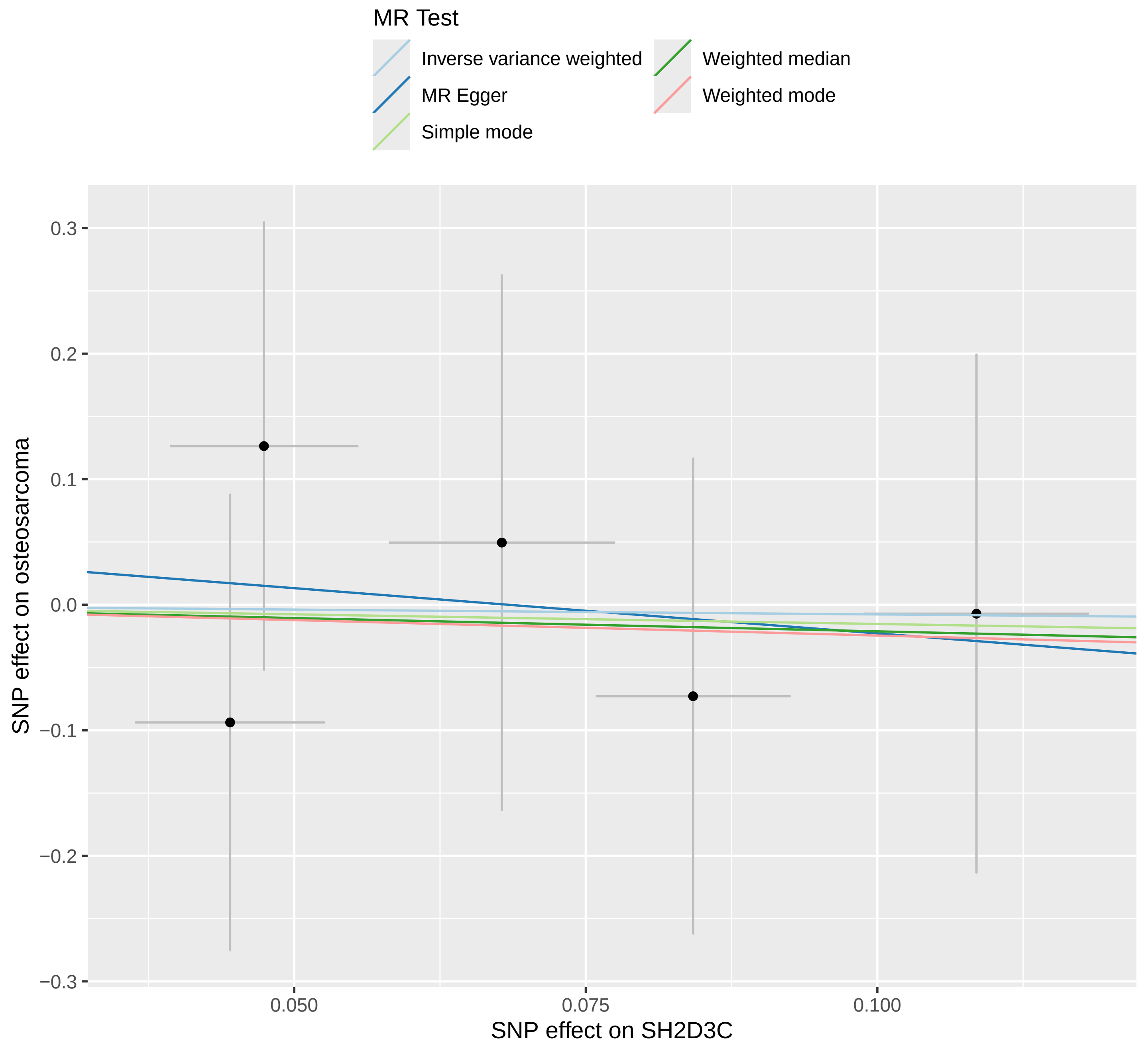
MR Test
Inverse variance weighted
MR Egger
Simple mode
Weighted median
Weighted mode
SNP effect on osteosarcoma
0.3
0.2
0.1
0.0
−0.1
−0.2
−0.3
0.050
0.075
0.100
SNP effect on SH2D3C

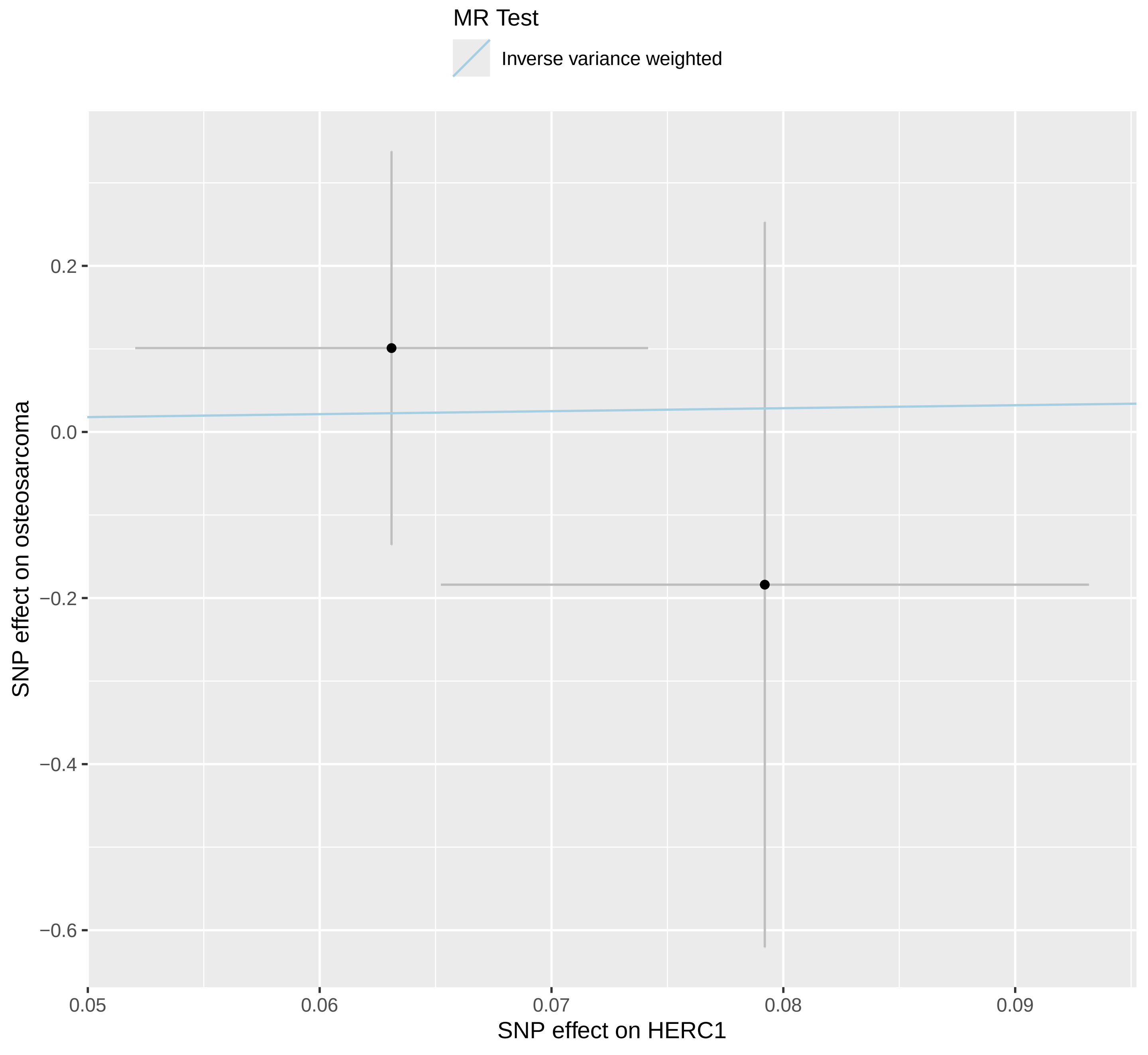
MR Test
Inverse variance weighted
0.2
0.0
−0.2
−0.4
−0.6
SNP effect on osteosarcoma
0.05
0.06
0.07
0.08
0.09
SNP effect on HERC1

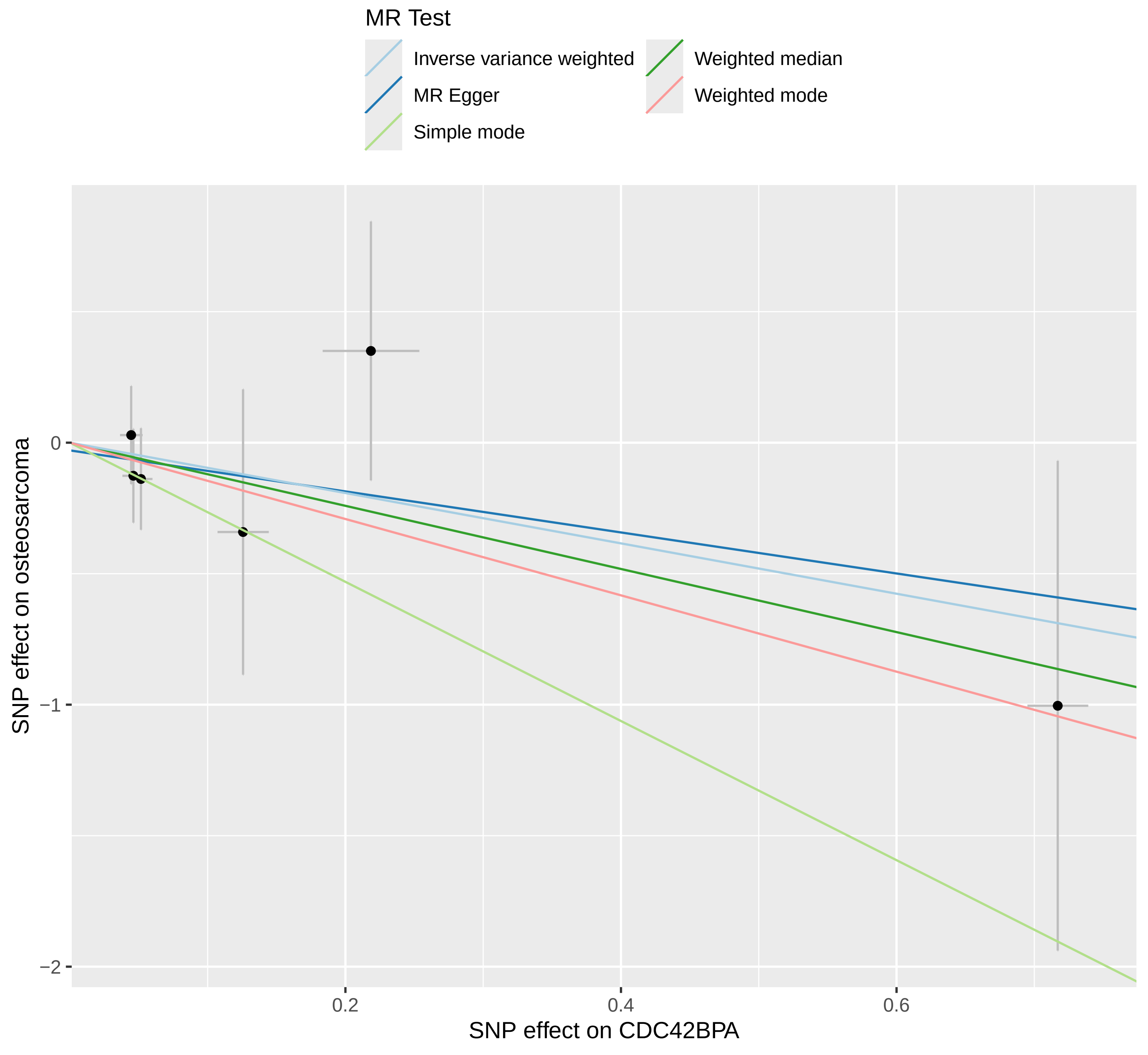
MR Test
Inverse variance weighted
MR Egger
Simple mode
Weighted median
Weighted mode
0
−1
−2
0.2
0.4
0.6
SNP effect on CDC42BPA
SNP effect on osteosarcoma

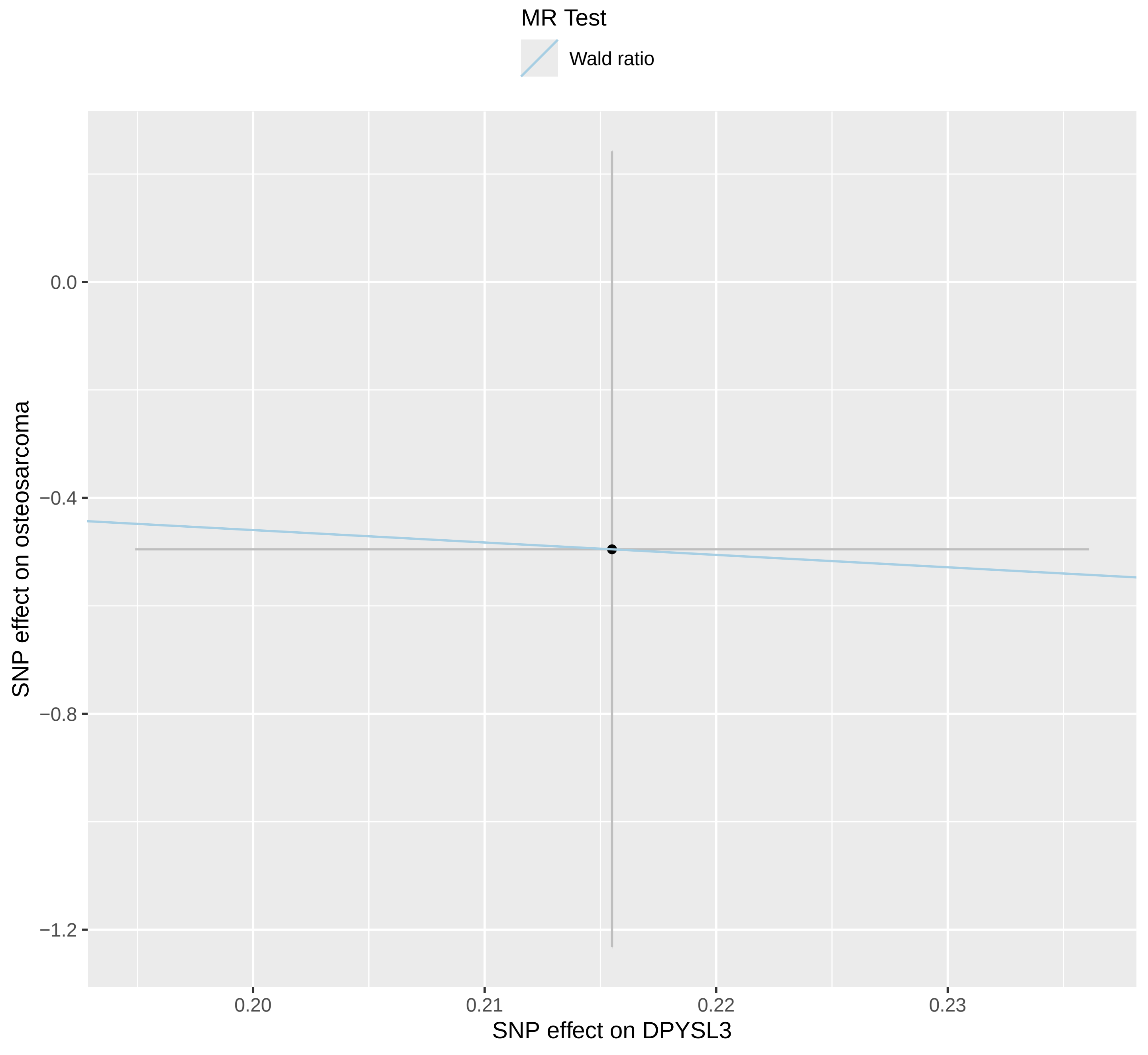
MR Test
Wald ratio
0.0
−0.4
−0.8
−1.2
SNP effect on osteosarcoma
0.20
0.21
0.22
0.23
SNP effect on DPYSL3

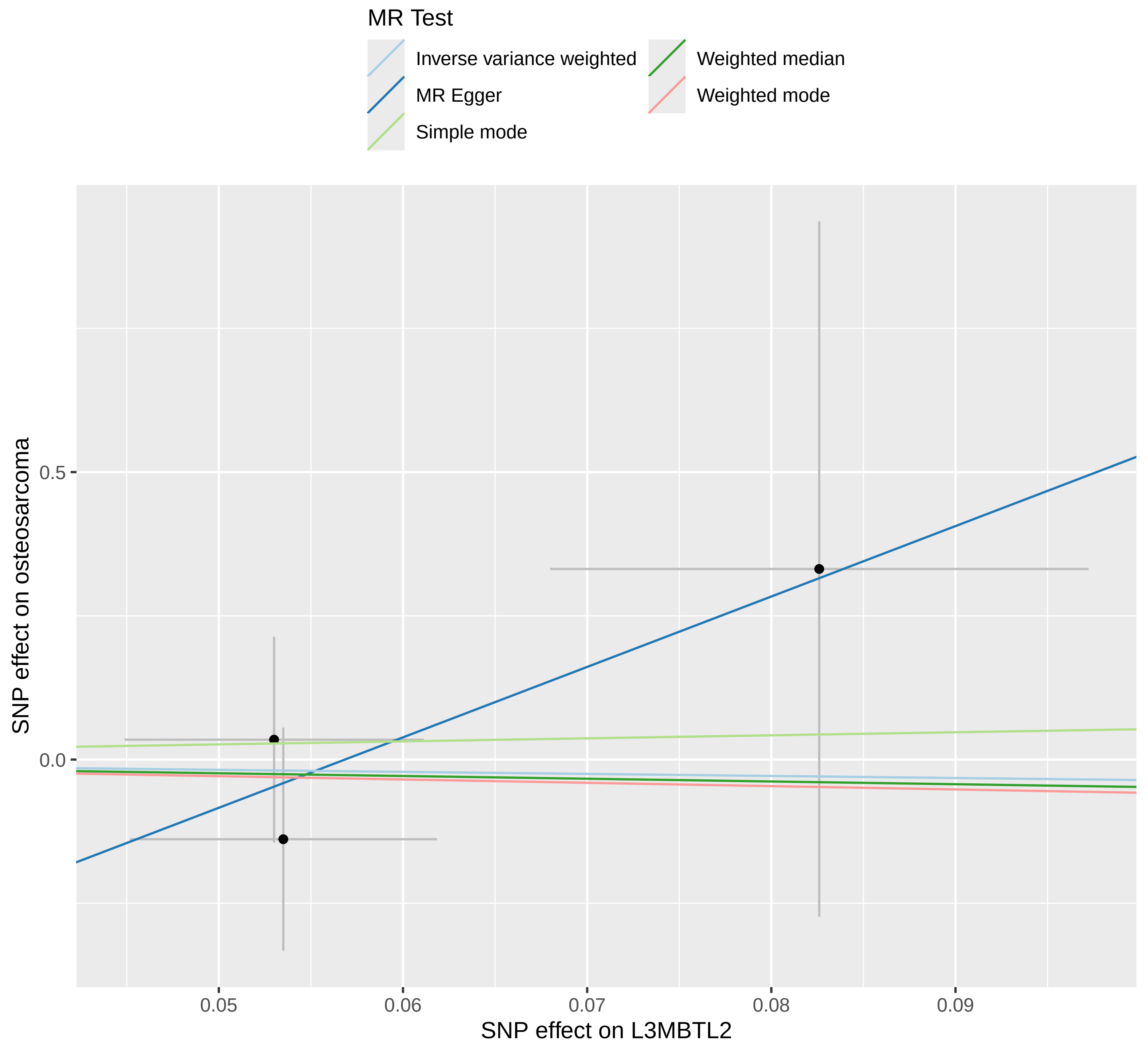
MR Test
Inverse variance weighted
MR Egger
Simple mode
Weighted median
Weighted mode
0.5
0.0
SNP effect on osteosarcoma
0.05
0.06
0.07
0.08
0.09
SNP effect on L3MBTL2

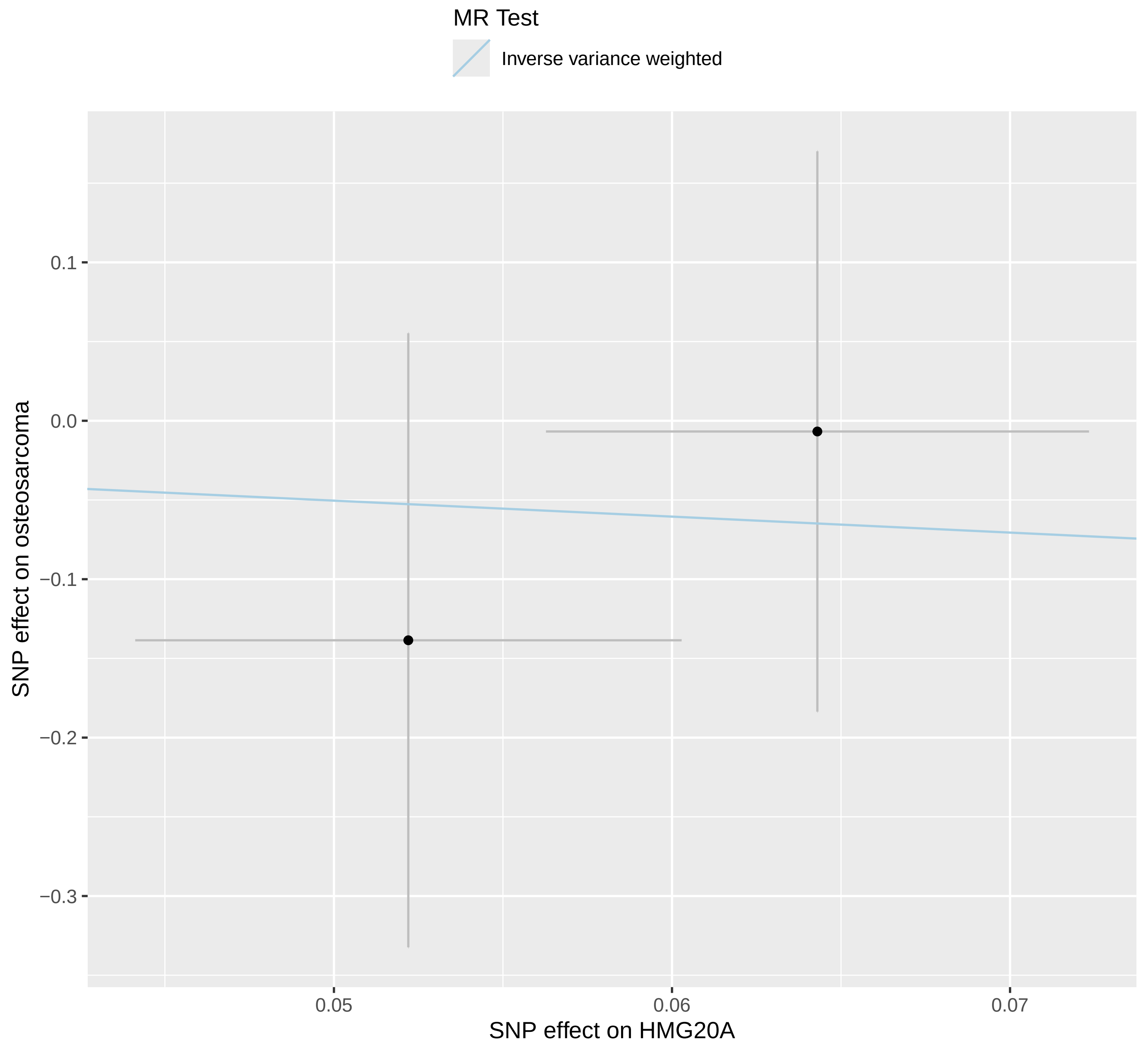
MR Test
Inverse variance weighted
0.1
0.0
−0.1
−0.2
−0.3
SNP effect on osteosarcoma
0.05
0.06
0.07
SNP effect on HMG20A

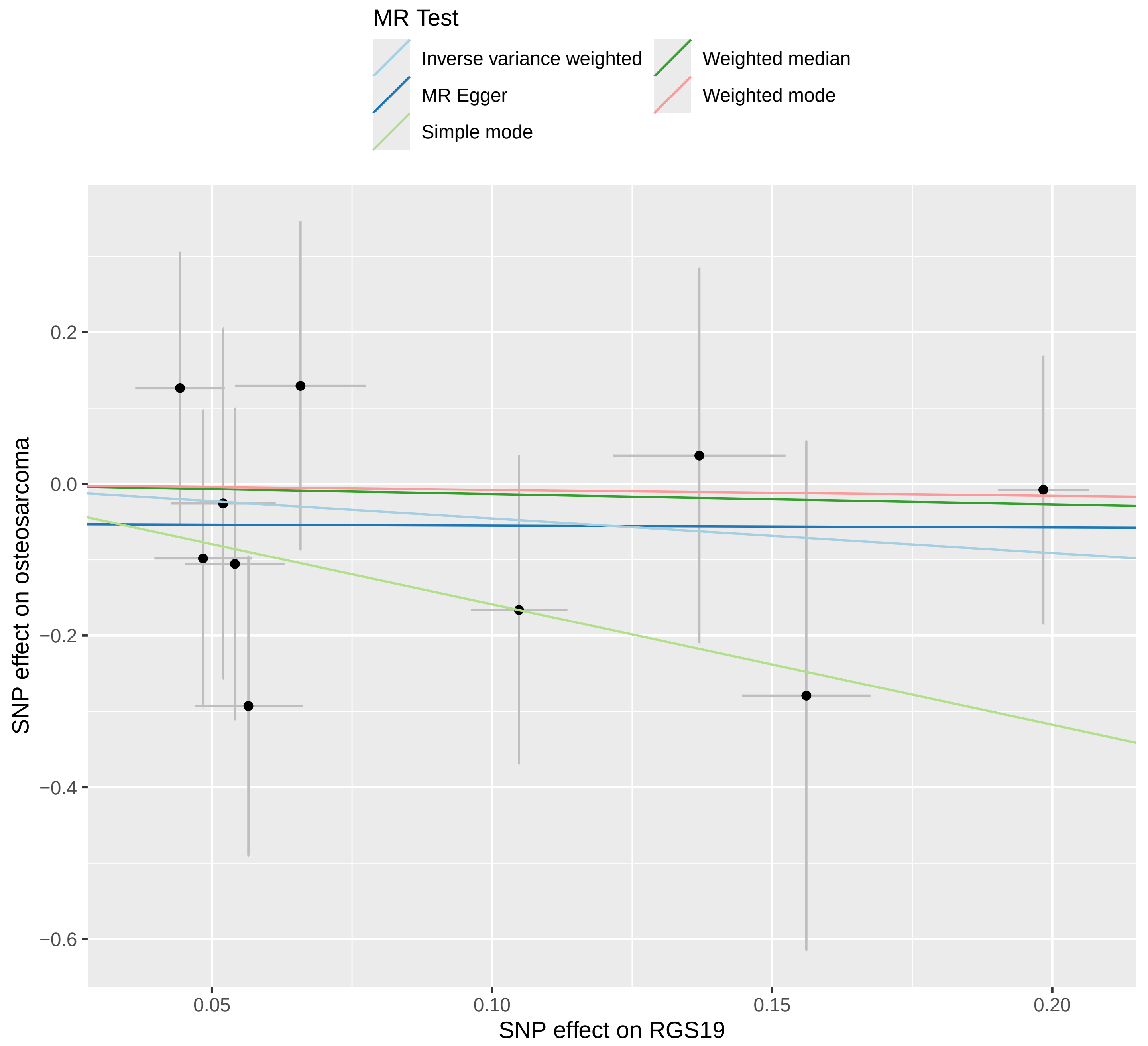
MR Test
Inverse variance weighted
MR Egger
Simple mode
Weighted median
Weighted mode
SNP effect on osteosarcoma
0.2
0.0
−0.2
−0.4
−0.6
0.05
0.10
0.15
0.20
SNP effect on RGS19

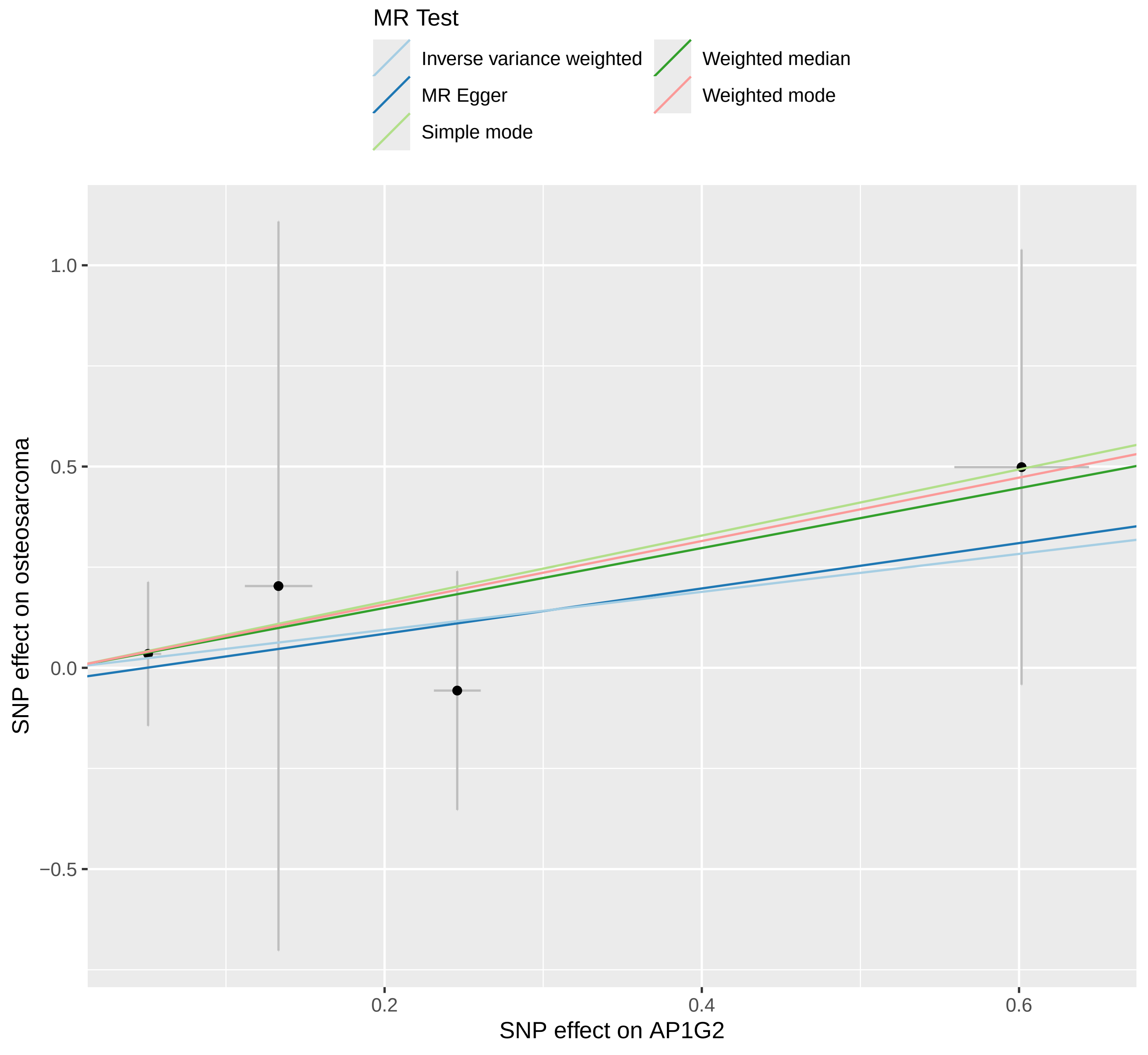

MR Test
Inverse variance weighted
MR Egger
Simple mode
Weighted median
Weighted mode
1.0
0.5
0.0
−0.5
0.2
0.4
0.6
SNP effect on osteosarcoma
SNP effect on AP1G2

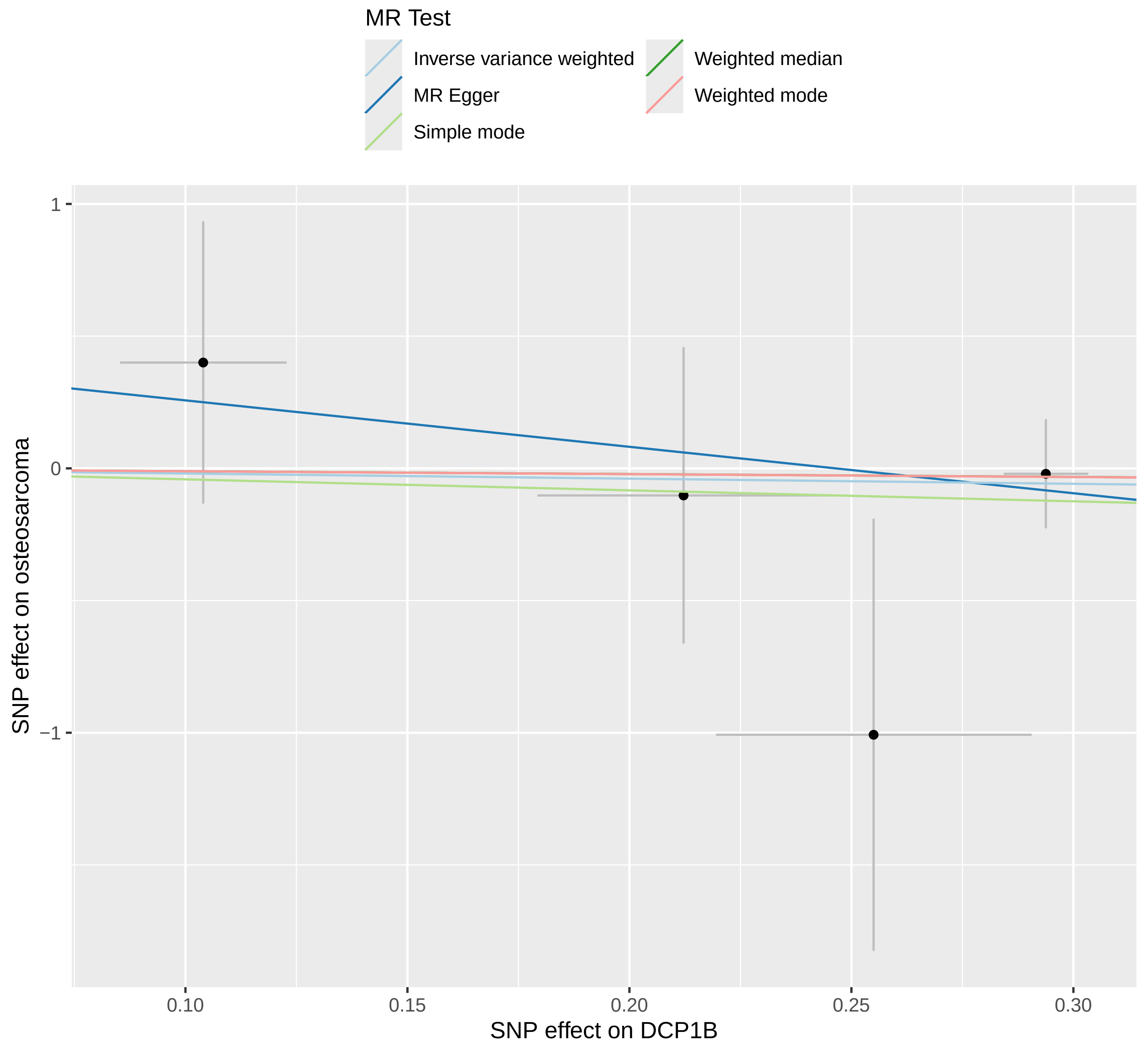

MR Test
Inverse variance weighted
MR Egger
Simple mode
Weighted median
Weighted mode
1
0
−1
0.10
0.15
0.20
0.25
0.30
SNP effect on osteosarcoma
SNP effect on DCP1B

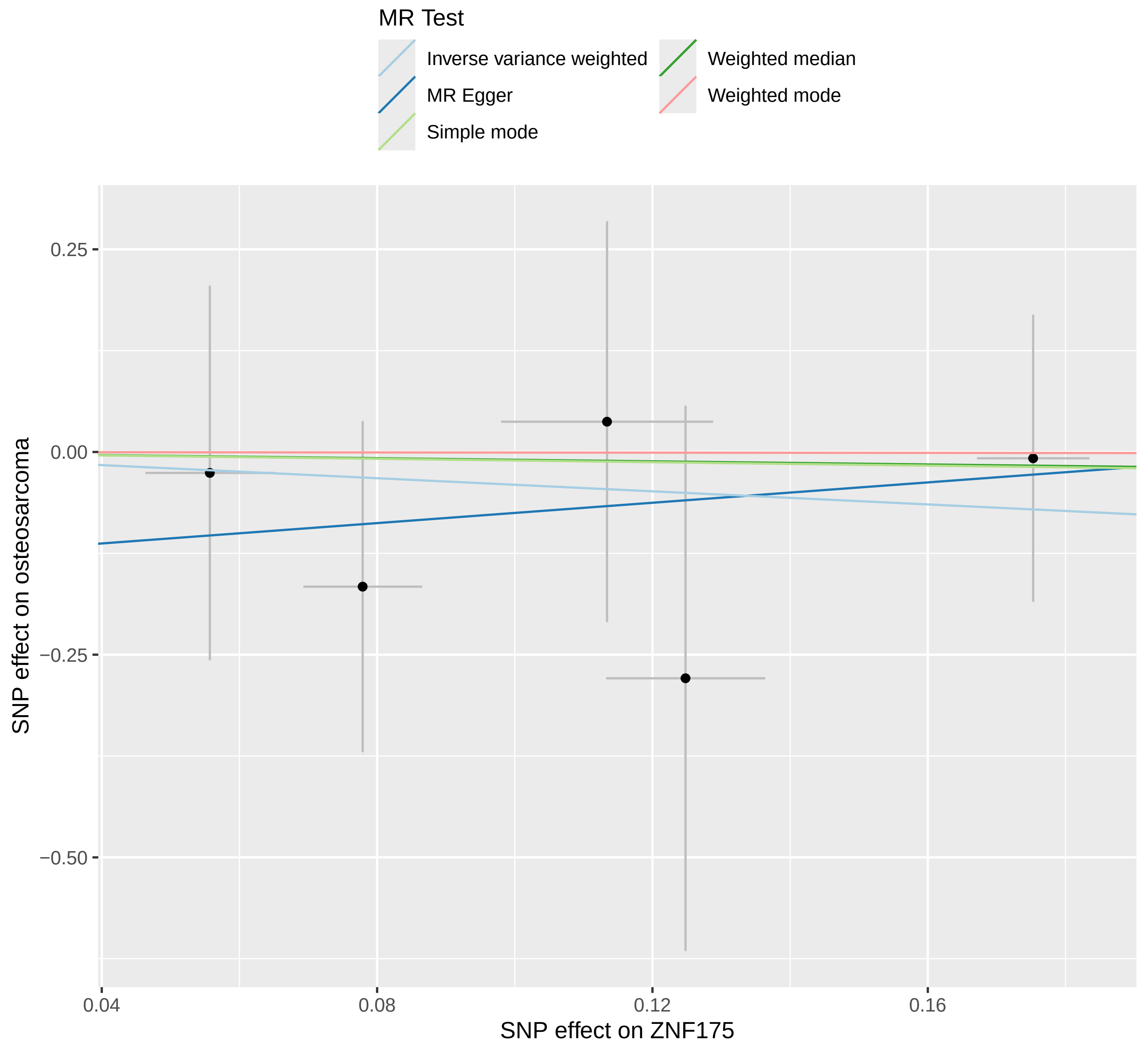

MR Test
Inverse variance weighted
MR Egger
Simple mode
Weighted median
Weighted mode
0.25
0.00
−0.25
−0.50
0.04
0.08
0.12
0.16
SNP effect on ZNF175
SNP effect on osteosarcoma

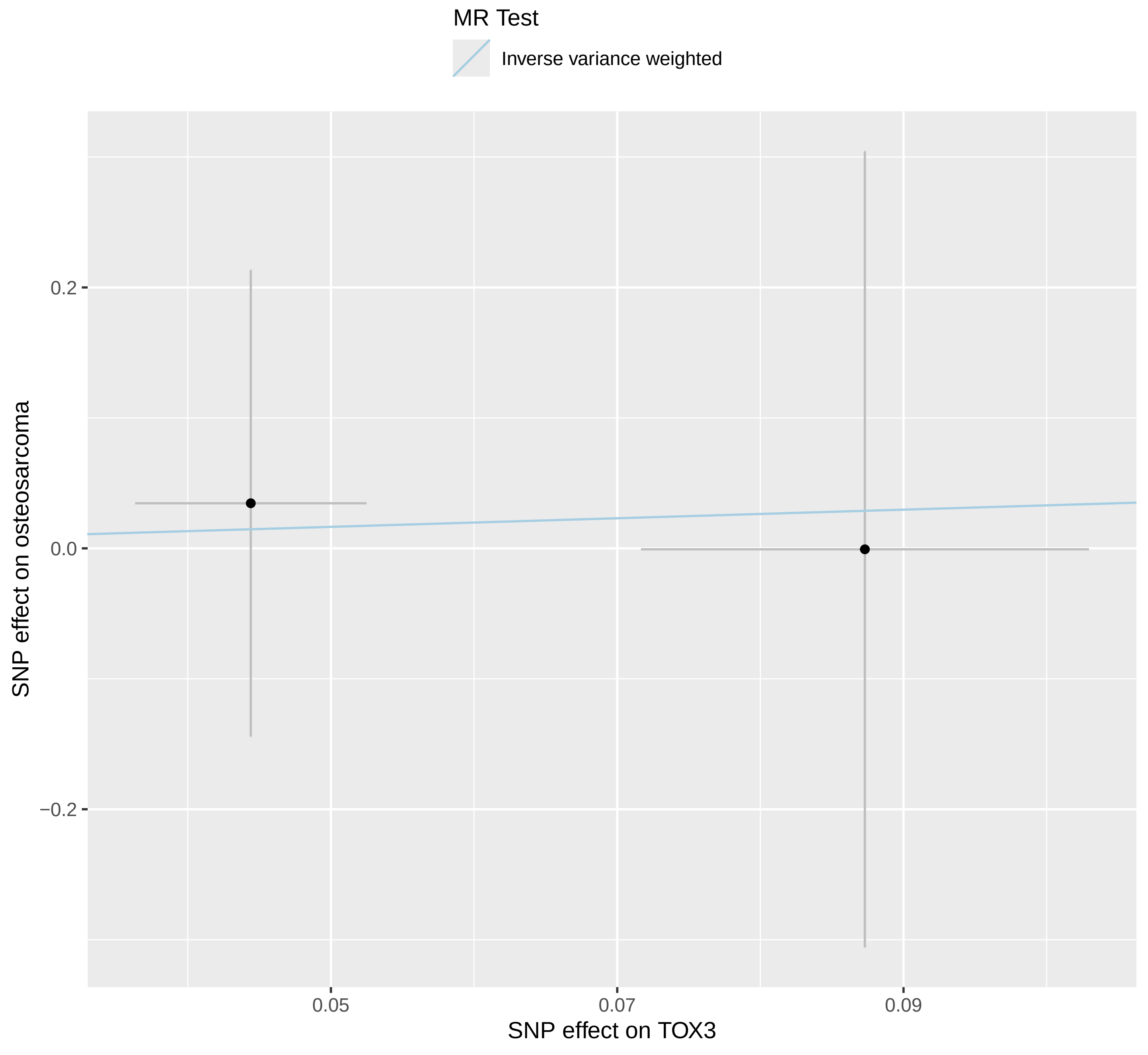
MR Test
Inverse variance weighted
0.2
0.0
−0.2
SNP effect on osteosarcoma
0.05
0.07
0.09
SNP effect on TOX3

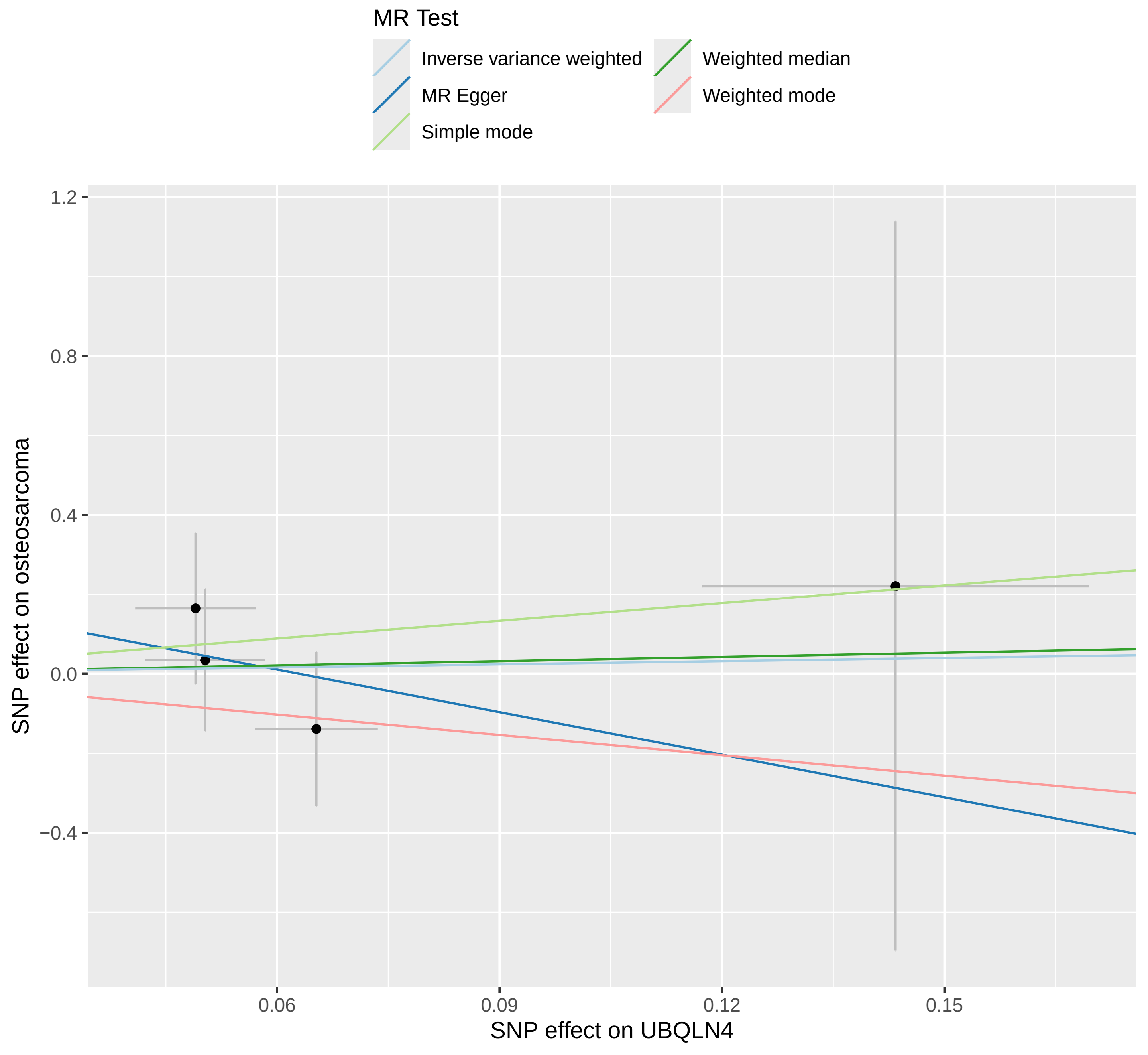
MR Test
Inverse variance weighted
MR Egger
Simple mode
Weighted median
Weighted mode
1.2
0.8
0.4
0.0
−0.4
0.06
0.09
0.12
0.15
SNP effect on UBQLN4
SNP effect on osteosarcoma

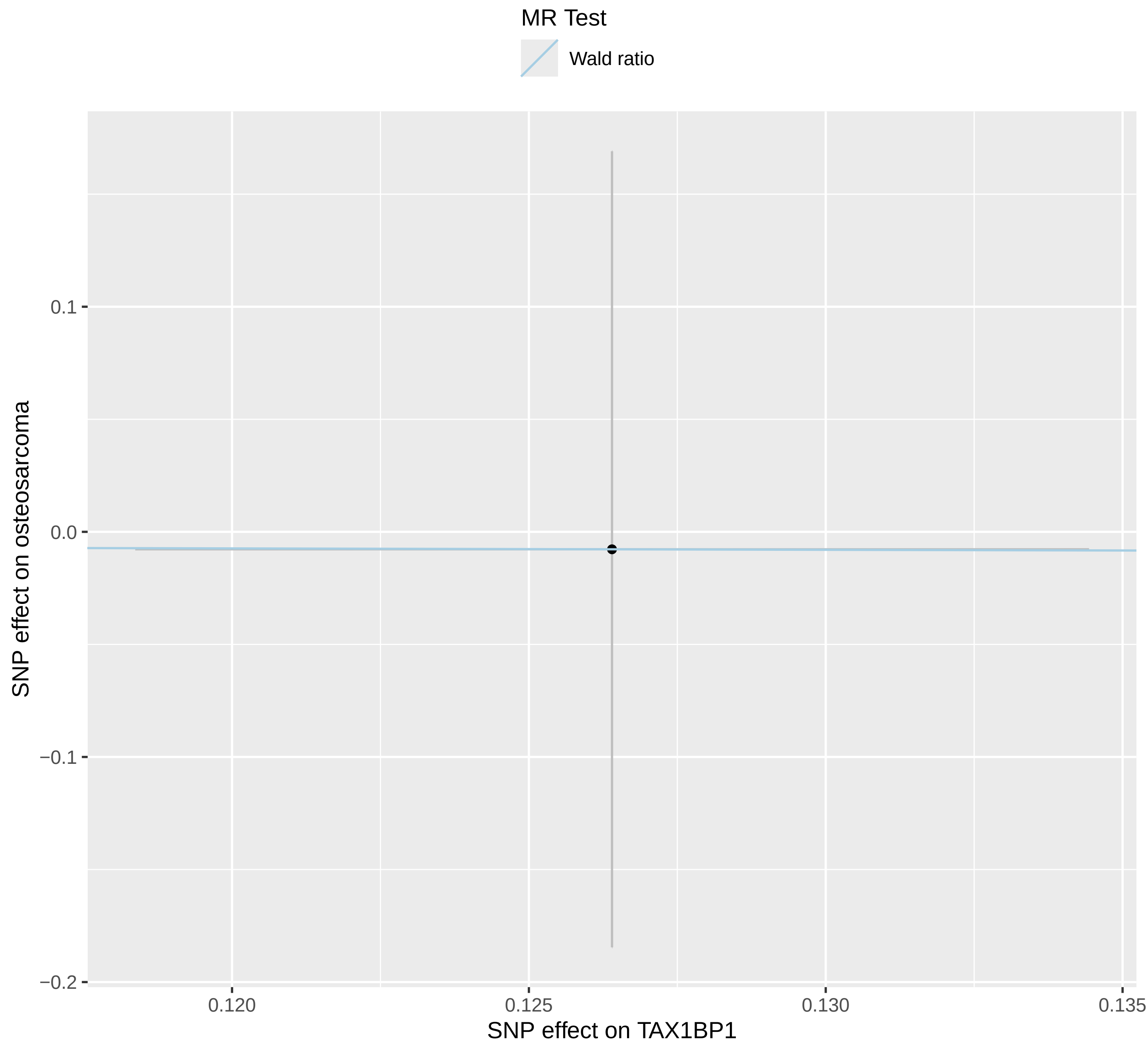

MR Test
Wald ratio
SNP effect on osteosarcoma
0.1
0.0
−0.1
−0.2
0.120
0.125
0.130
0.135
SNP effect on TAX1BP1

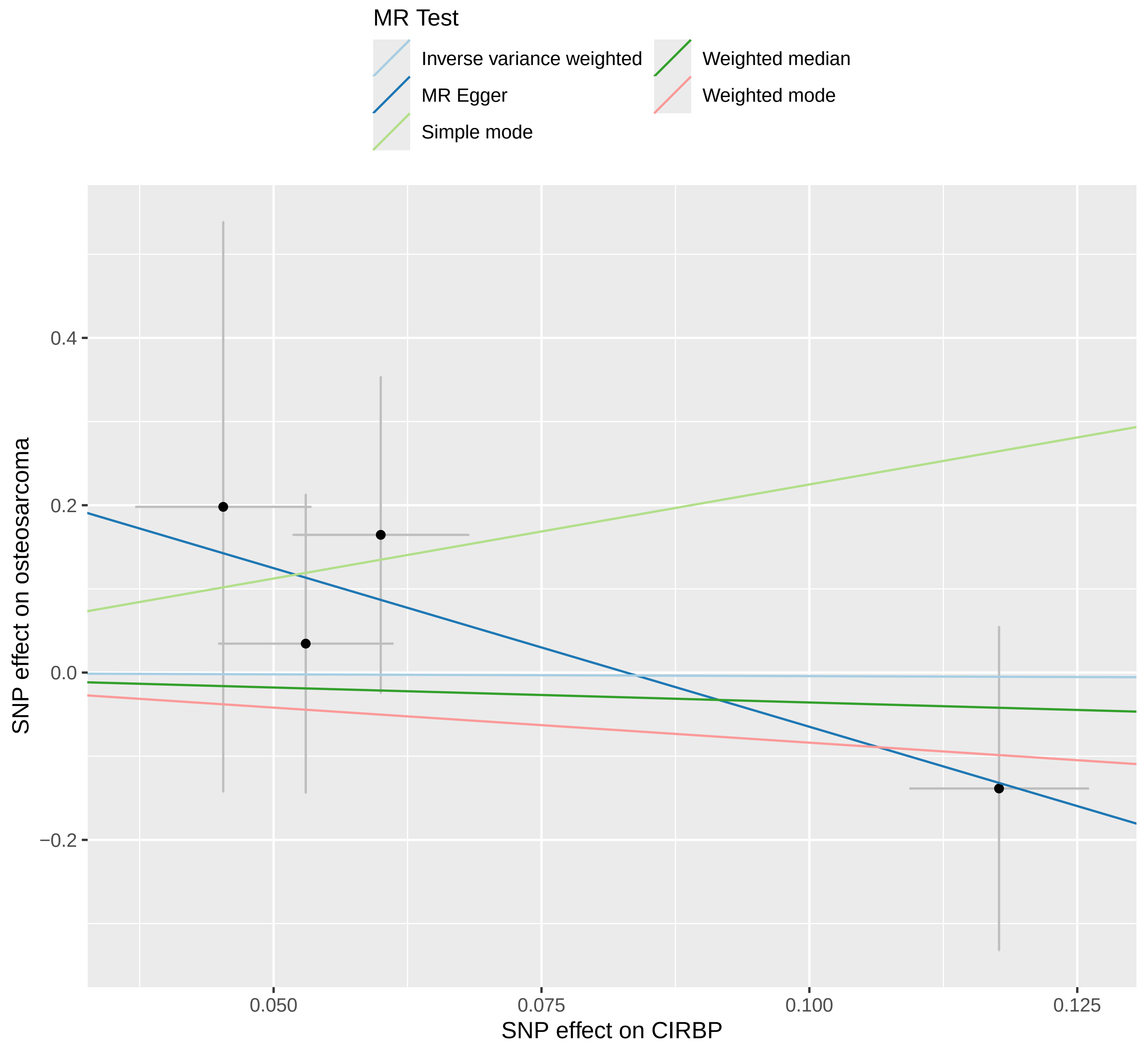
MR Test
Inverse variance weighted
MR Egger
Simple mode
Weighted median
Weighted mode
0.4
0.2
0.0
−0.2
SNP effect on osteosarcoma
0.050
0.075
0.100
0.125
SNP effect on CIRBP

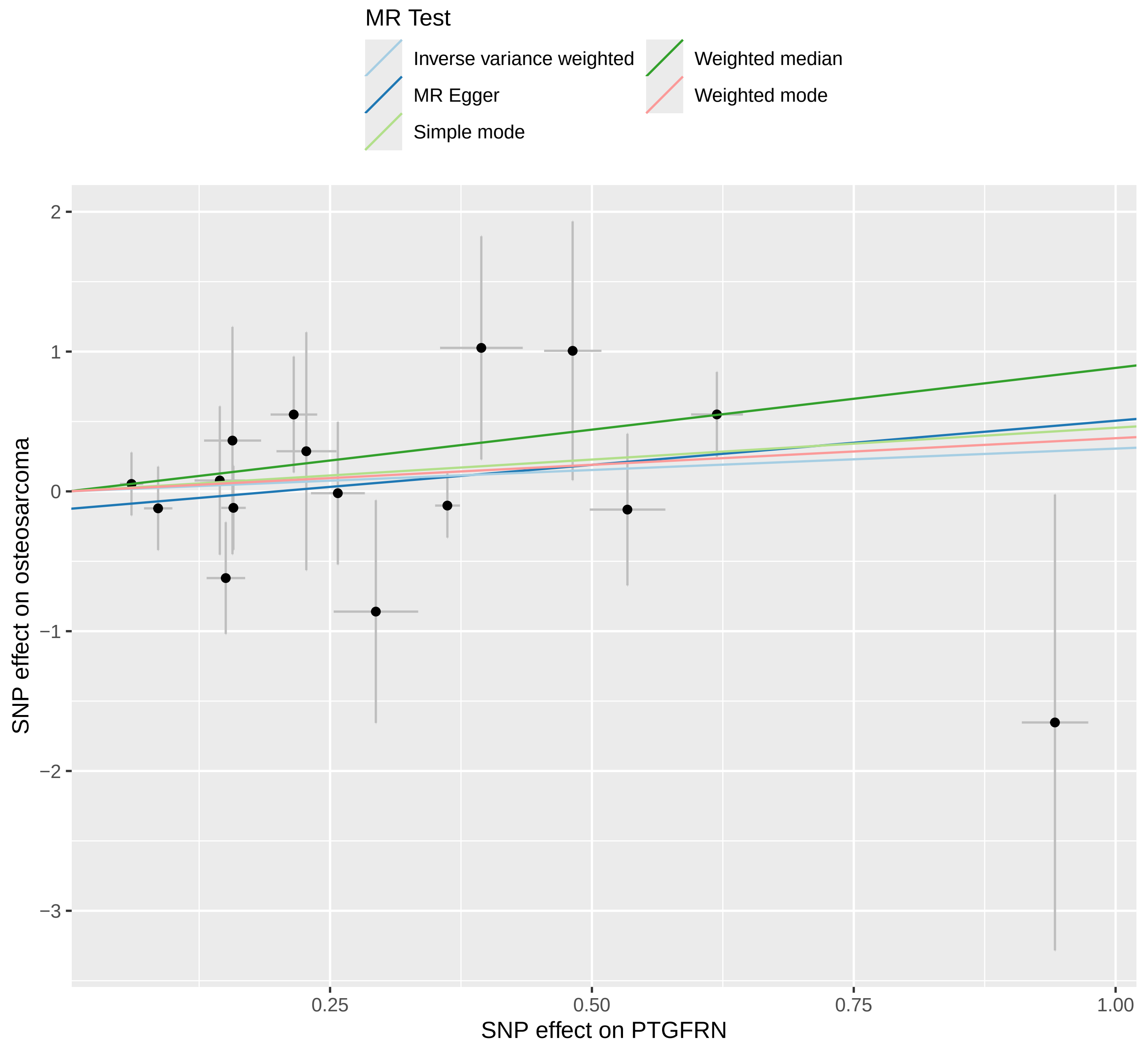
MR Test
Inverse variance weighted
MR Egger
Simple mode
Weighted median
Weighted mode
2
1
0
−1
−2
−3
SNP effect on osteosarcoma
0.25
0.50
0.75
1.00
SNP effect on PTGFRN

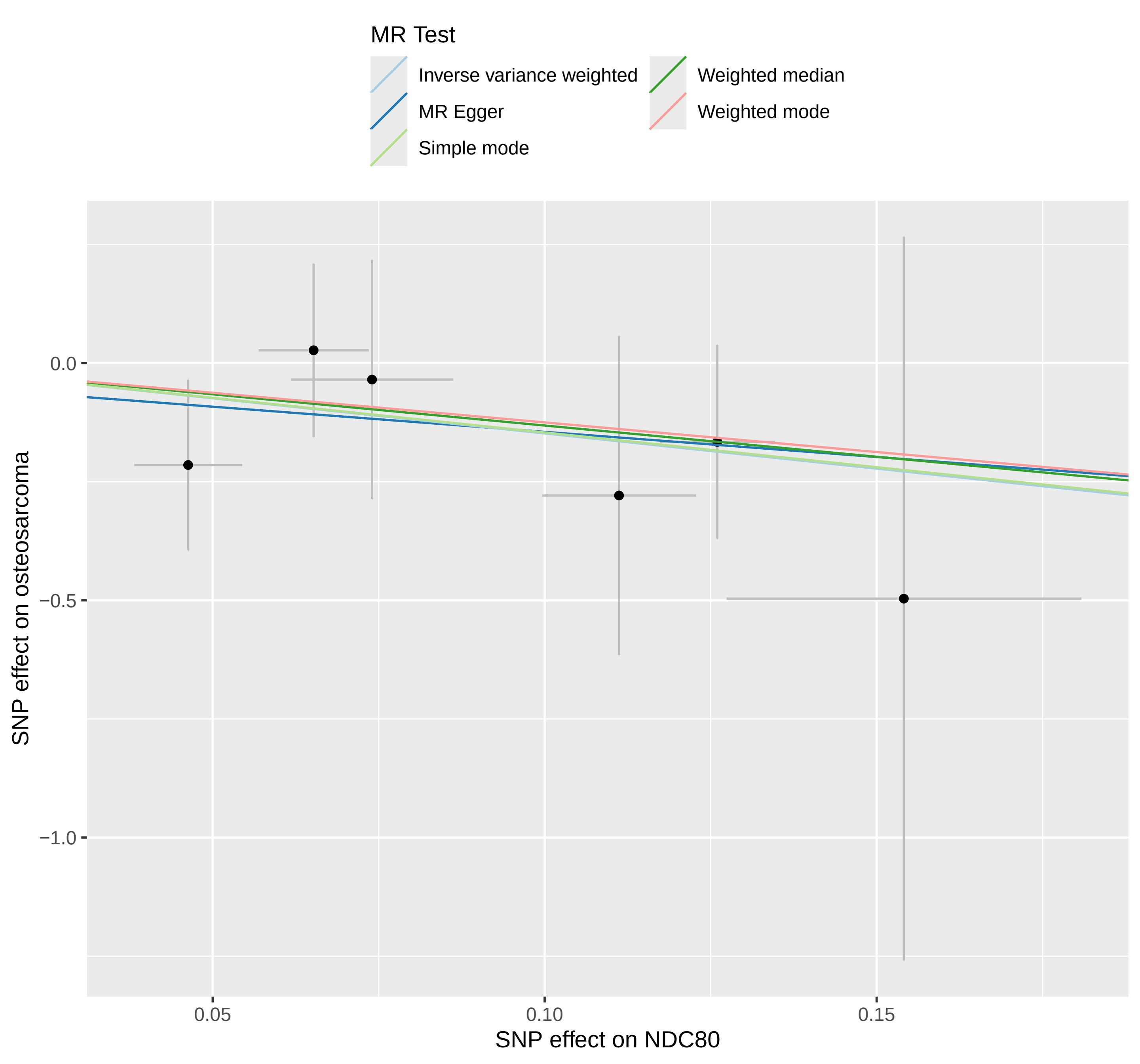

MR Test
Inverse variance weighted
MR Egger
Simple mode
Weighted median
Weighted mode
0.0
−0.5
−1.0
0.05
0.10
0.15
SNP effect on osteosarcoma
SNP effect on NDC80

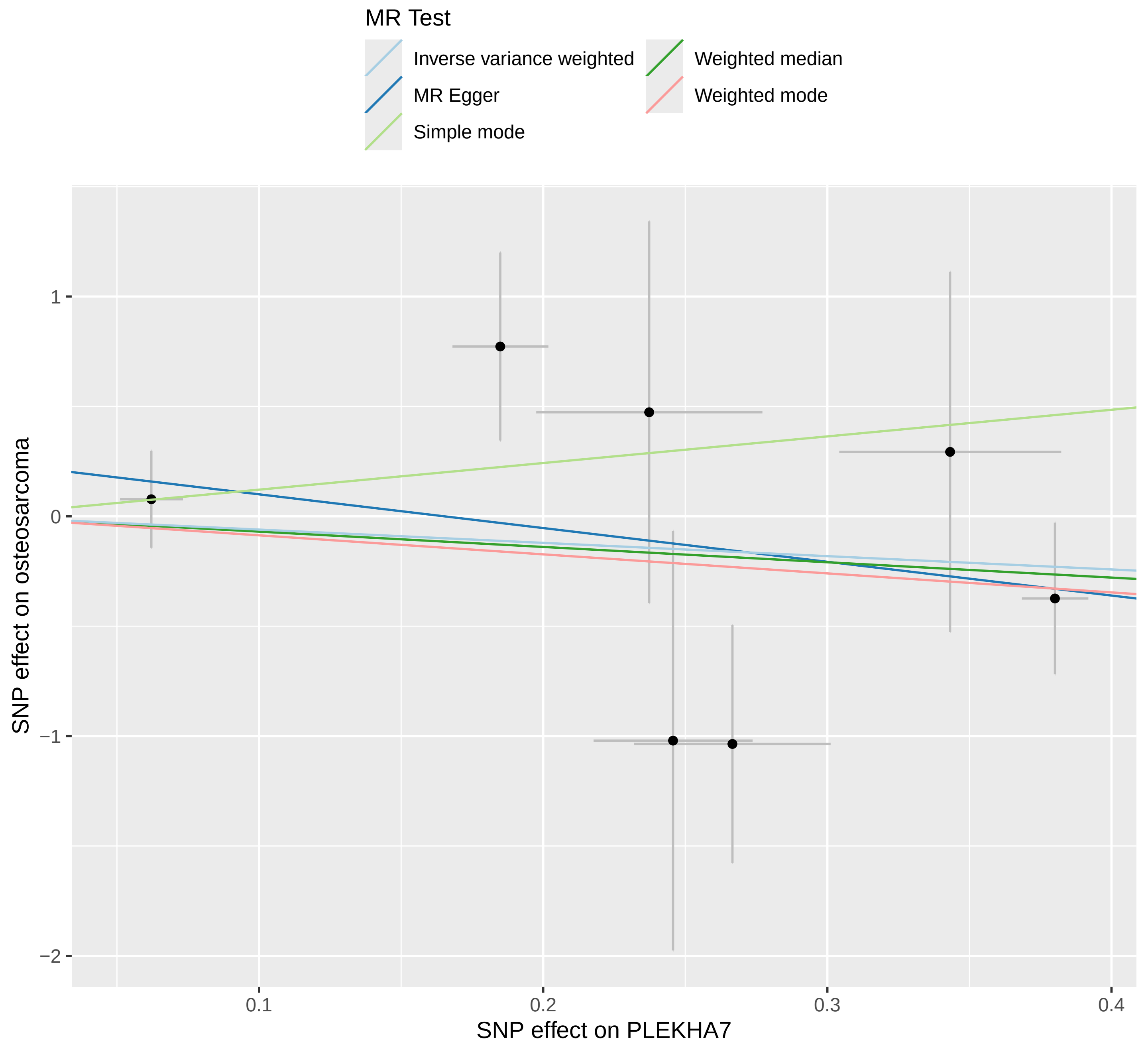
MR Test
Inverse variance weighted
MR Egger
Simple mode
Weighted median
Weighted mode
1
0
−1
−2
0.1
0.2
0.3
0.4
SNP effect on osteosarcoma
SNP effect on PLEKHA7

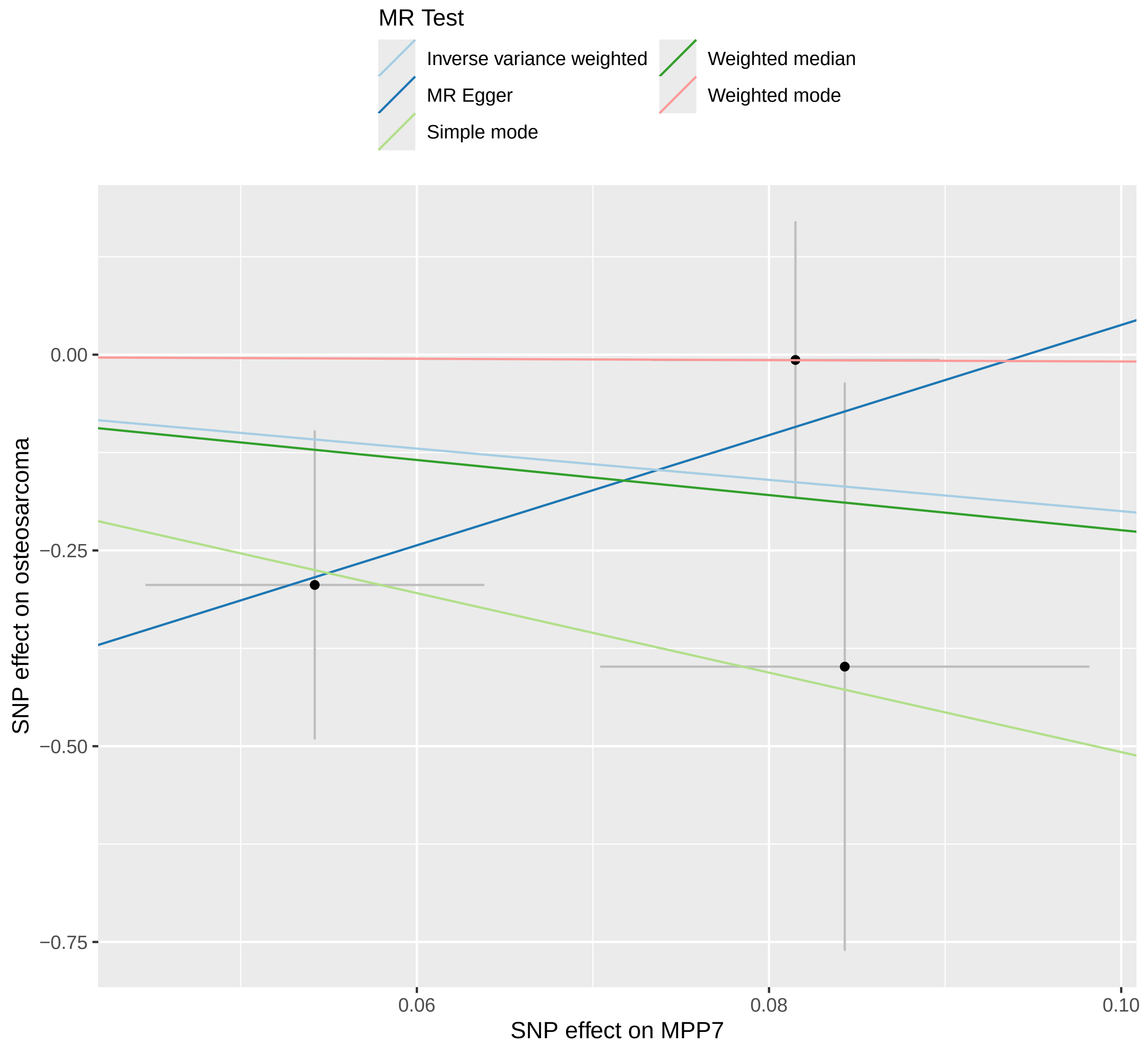
MR Test
Inverse variance weighted
MR Egger
Simple mode
Weighted median
Weighted mode
0.00
−0.25
−0.50
−0.75
0.06
0.08
0.10
SNP effect on osteosarcoma
SNP effect on MPP7

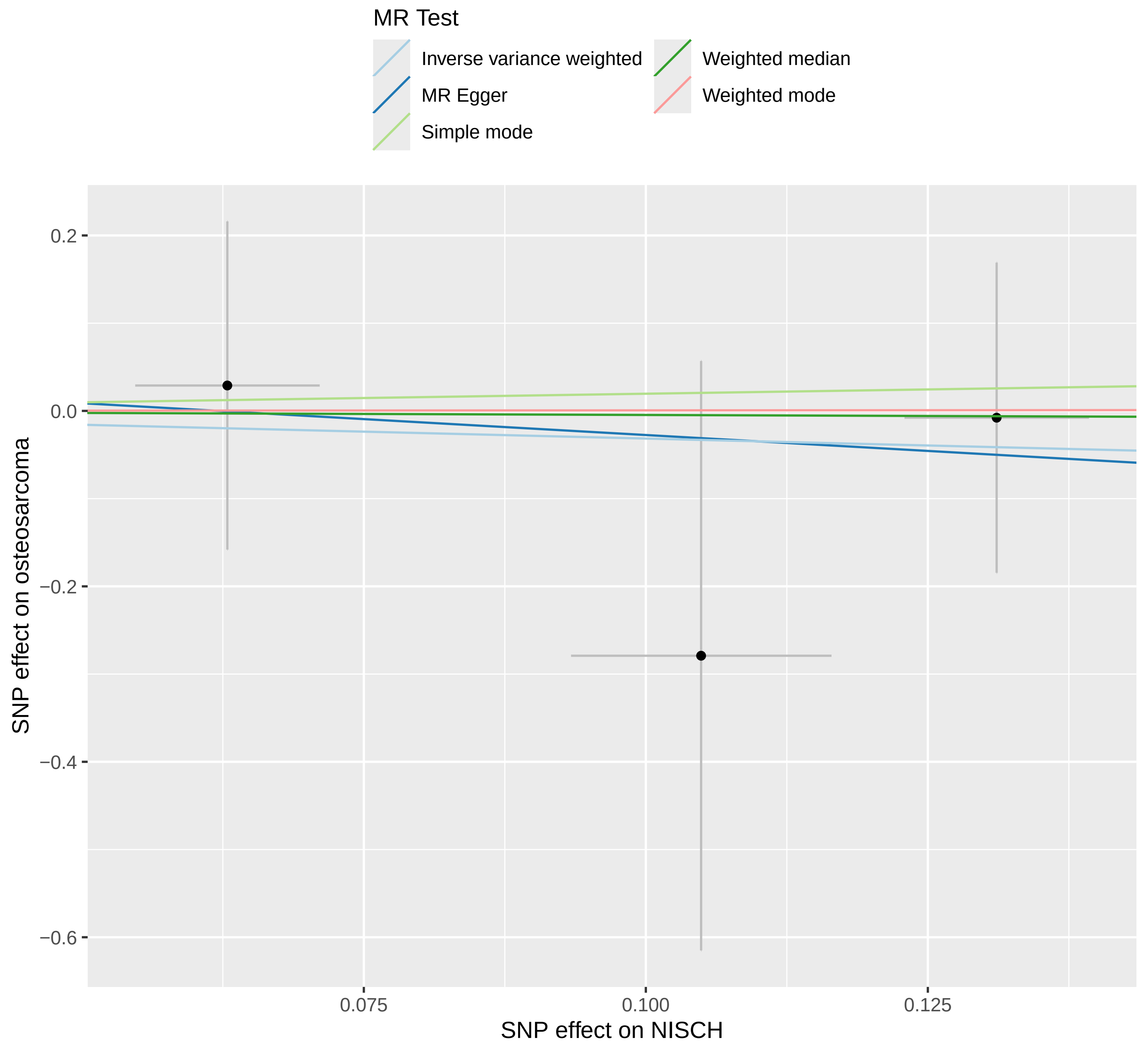
MR Test
Inverse variance weighted
MR Egger
Simple mode
Weighted median
Weighted mode
0.2
0.0
−0.2
−0.4
−0.6
SNP effect on osteosarcoma
0.075
0.100
0.125
SNP effect on NISCH

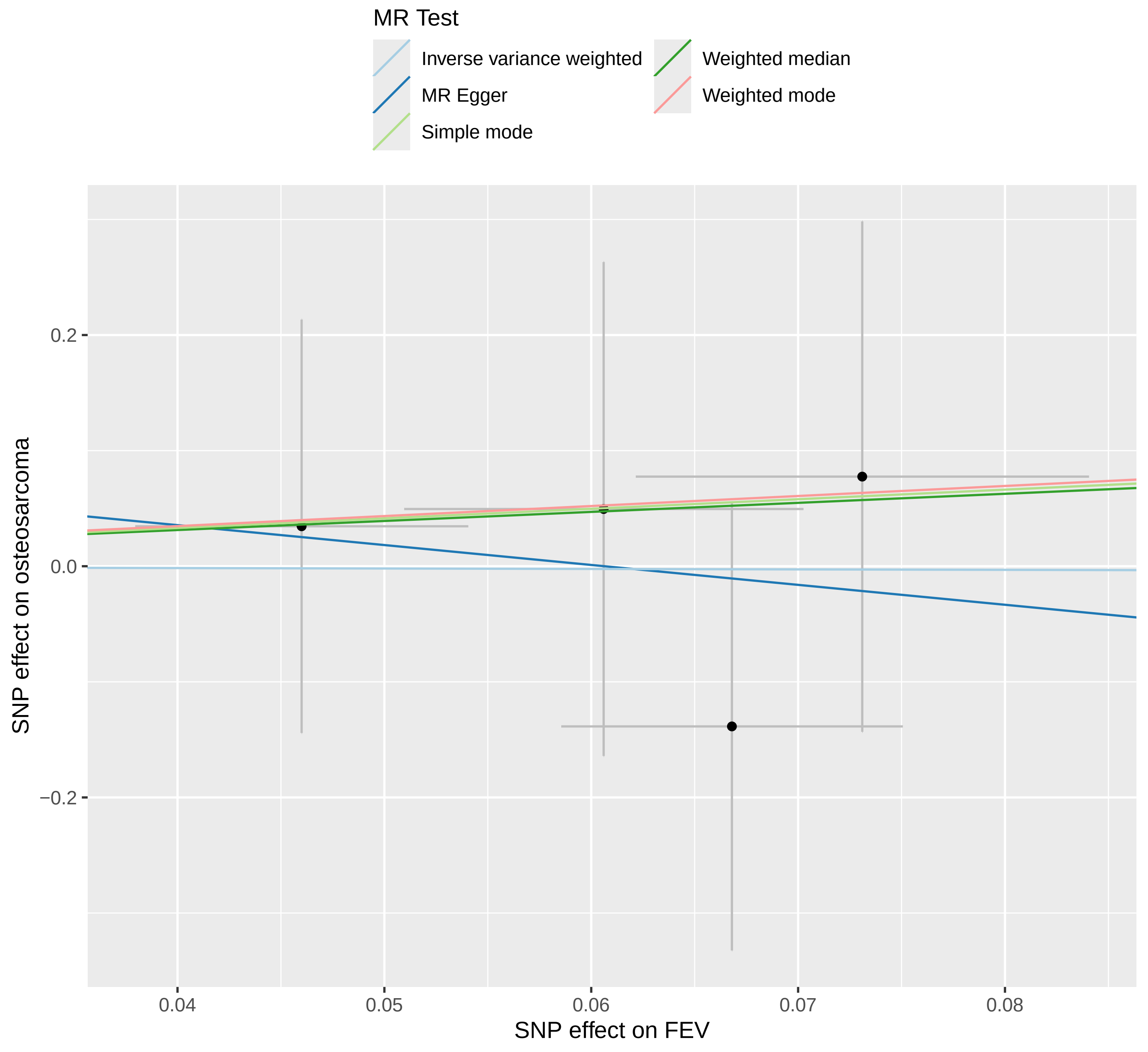

MR Test
Inverse variance weighted
MR Egger
Simple mode
Weighted median
Weighted mode
0.2
0.0
−0.2
SNP effect on osteosarcoma
0.04
0.05
0.06
0.07
0.08
SNP effect on FEV

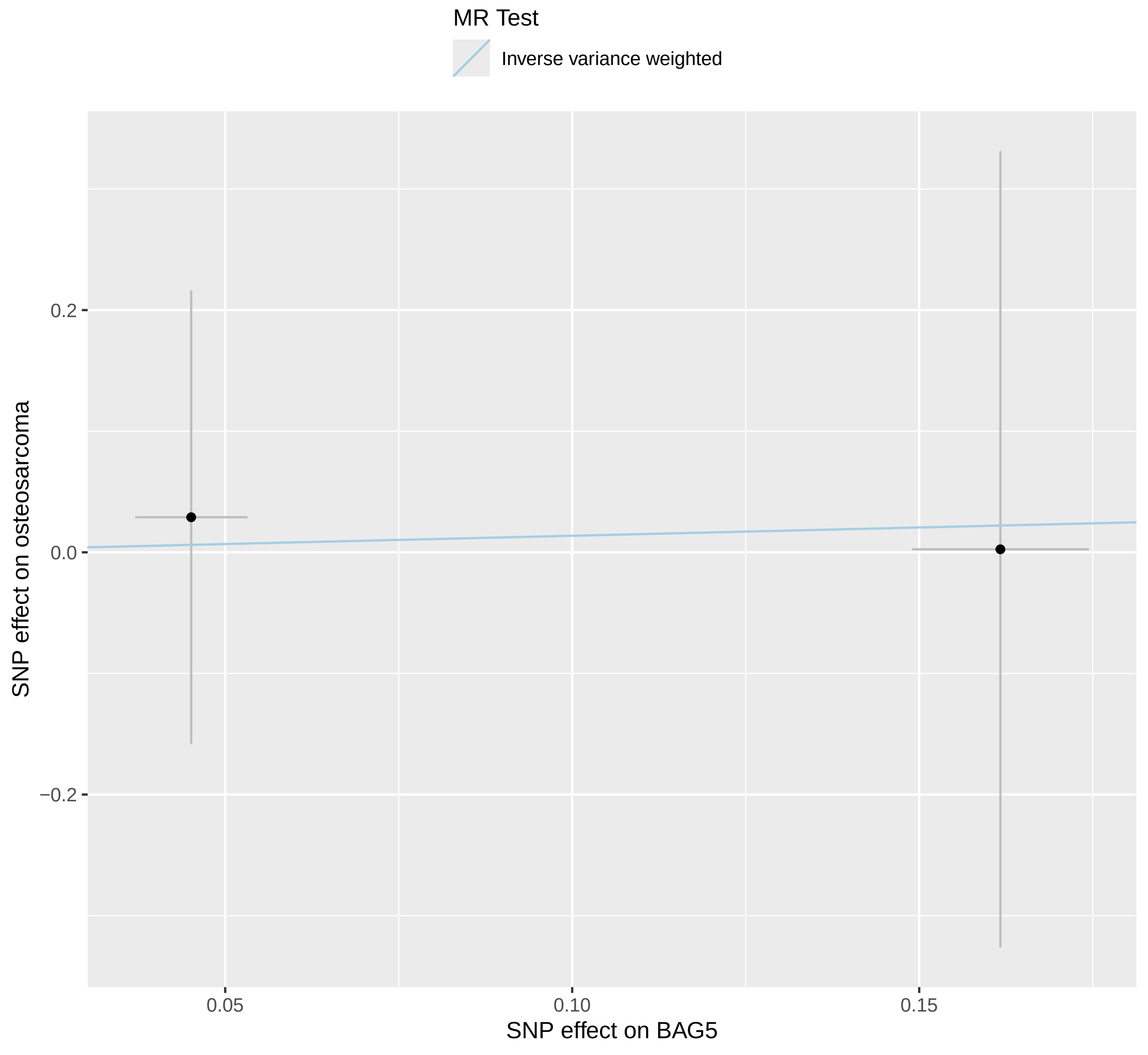
MR Test
Inverse variance weighted
SNP effect on osteosarcoma
0.2
0.0
−0.2
0.05
0.10
0.15
SNP effect on BAG5

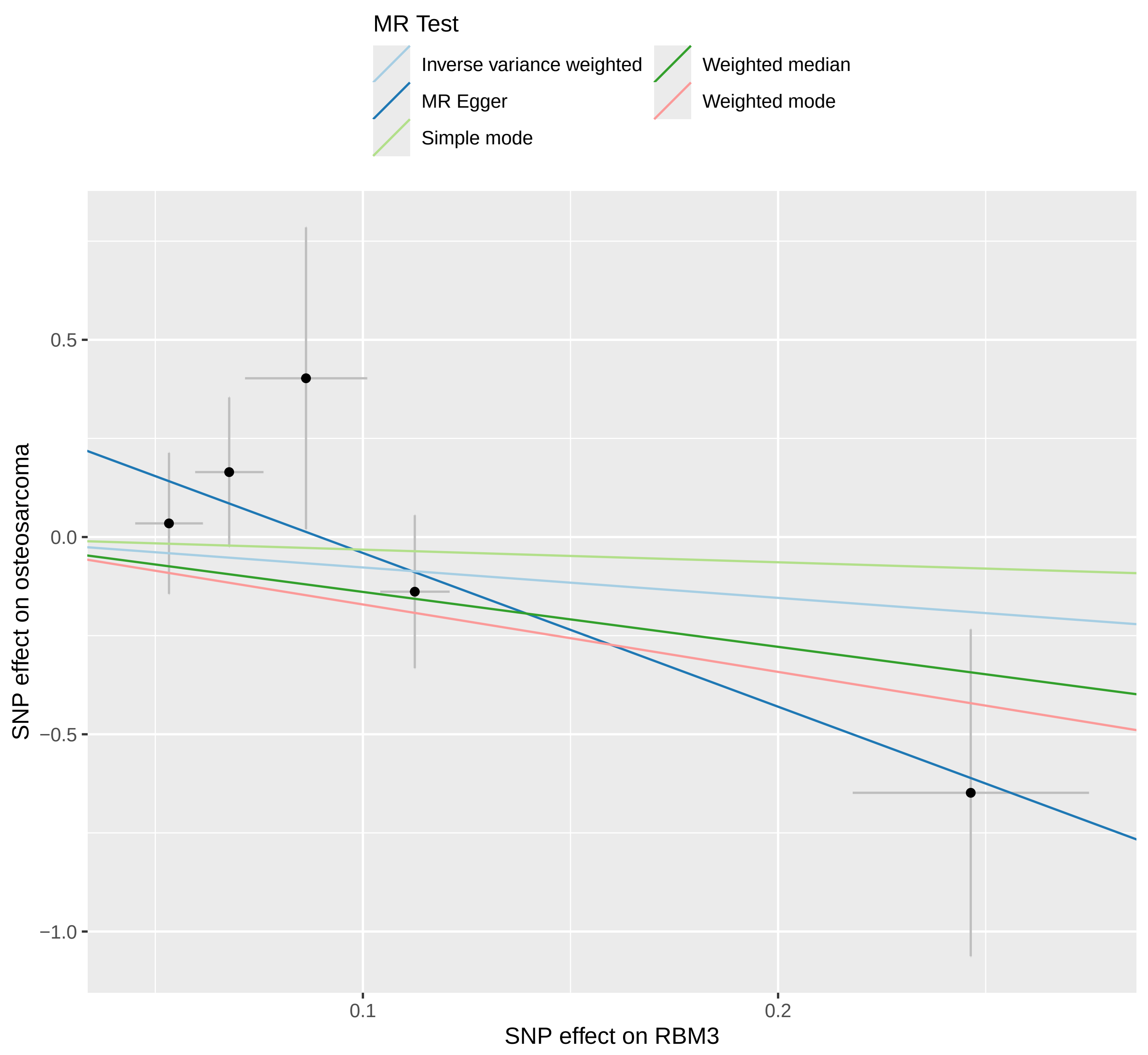
MR Test
Inverse variance weighted
MR Egger
Simple mode
Weighted median
Weighted mode
0.5
0.0
−0.5
−1.0
SNP effect on osteosarcoma
0.1
0.2
SNP effect on RBM3

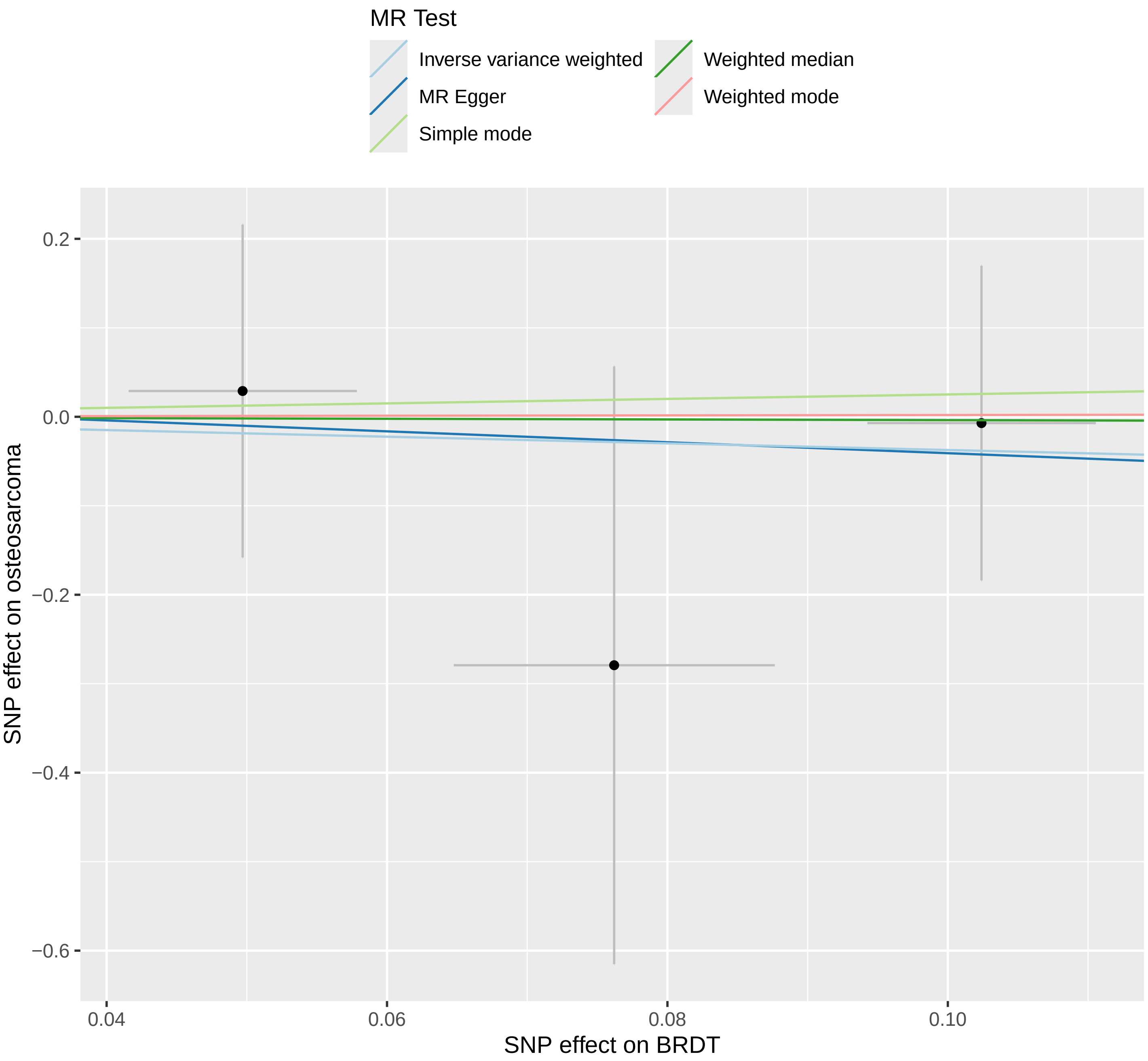
MR Test
Inverse variance weighted
MR Egger
Simple mode
Weighted median
Weighted mode
0.2
0.0
−0.2
−0.4
−0.6
SNP effect on osteosarcoma
0.04
0.06
0.08
0.10
SNP effect on BRDT

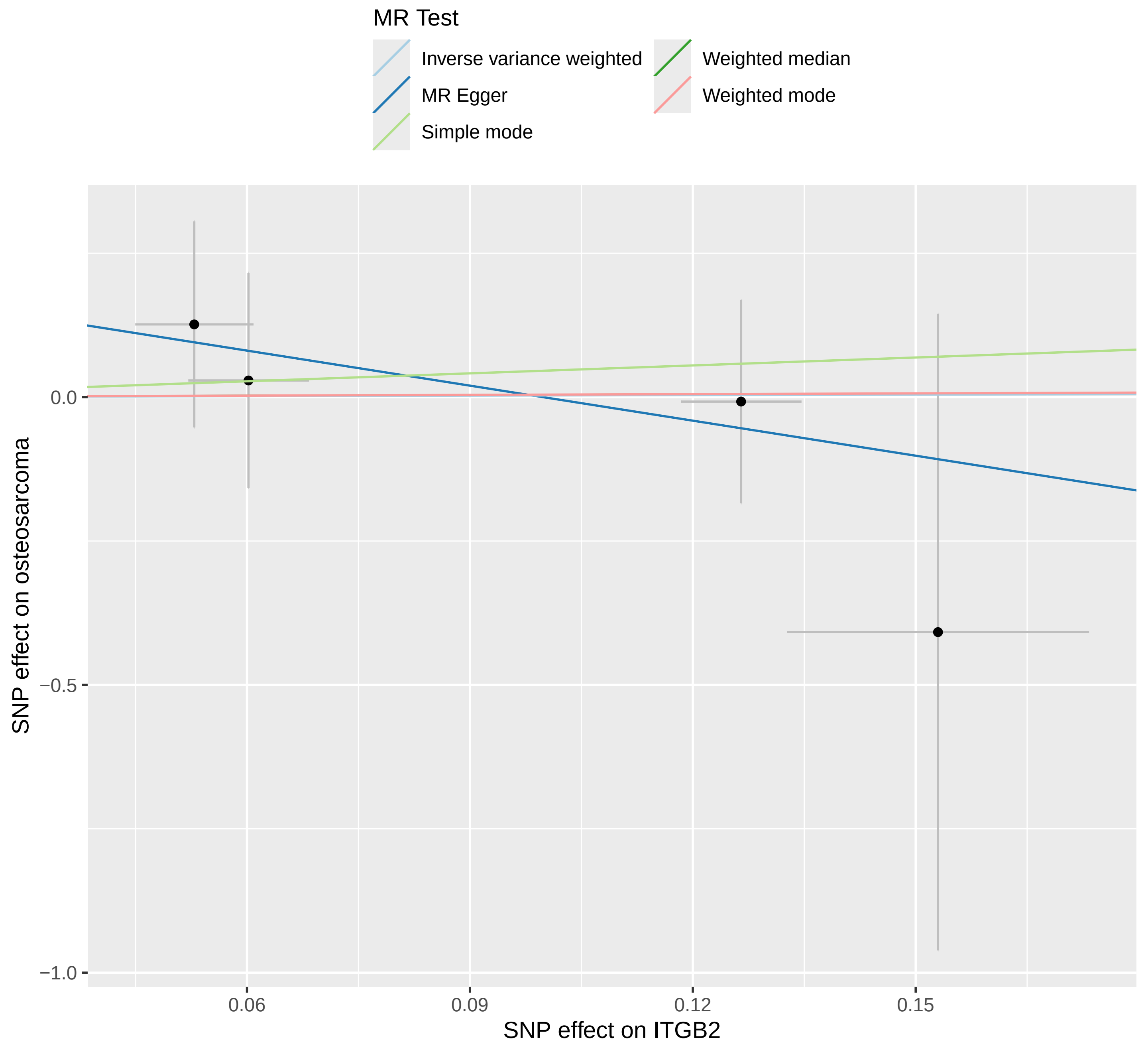

MR Test
Inverse variance weighted
MR Egger
Simple mode
Weighted median
Weighted mode
0.0
−0.5
−1.0
0.06
0.09
0.12
0.15
SNP effect on ITGB2
SNP effect on osteosarcoma

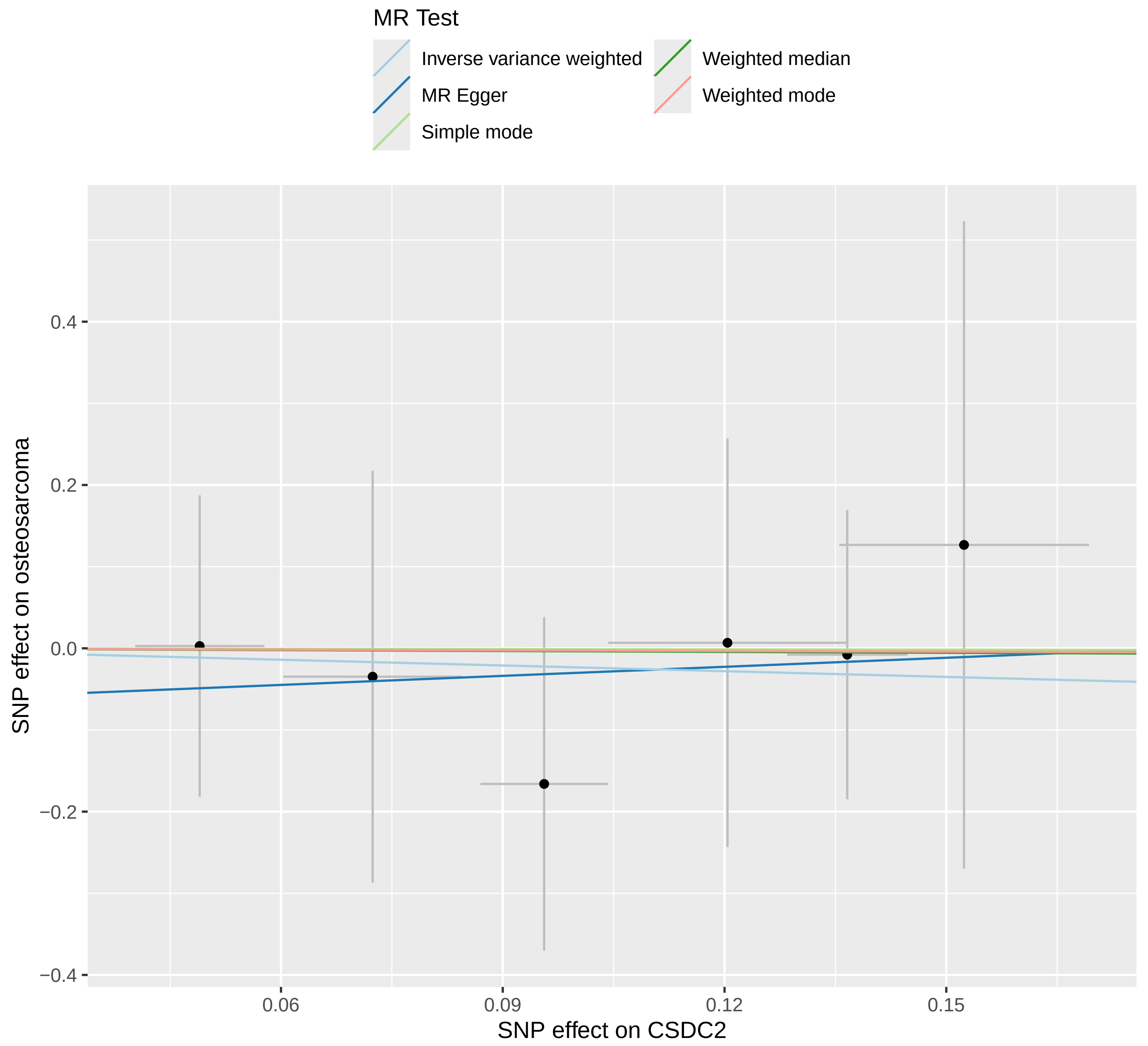
MR Test
Inverse variance weighted
MR Egger
Simple mode
Weighted median
Weighted mode
0.4
0.2
0.0
−0.2
−0.4
SNP effect on osteosarcoma
0.06
0.09
0.12
0.15
SNP effect on CSDC2

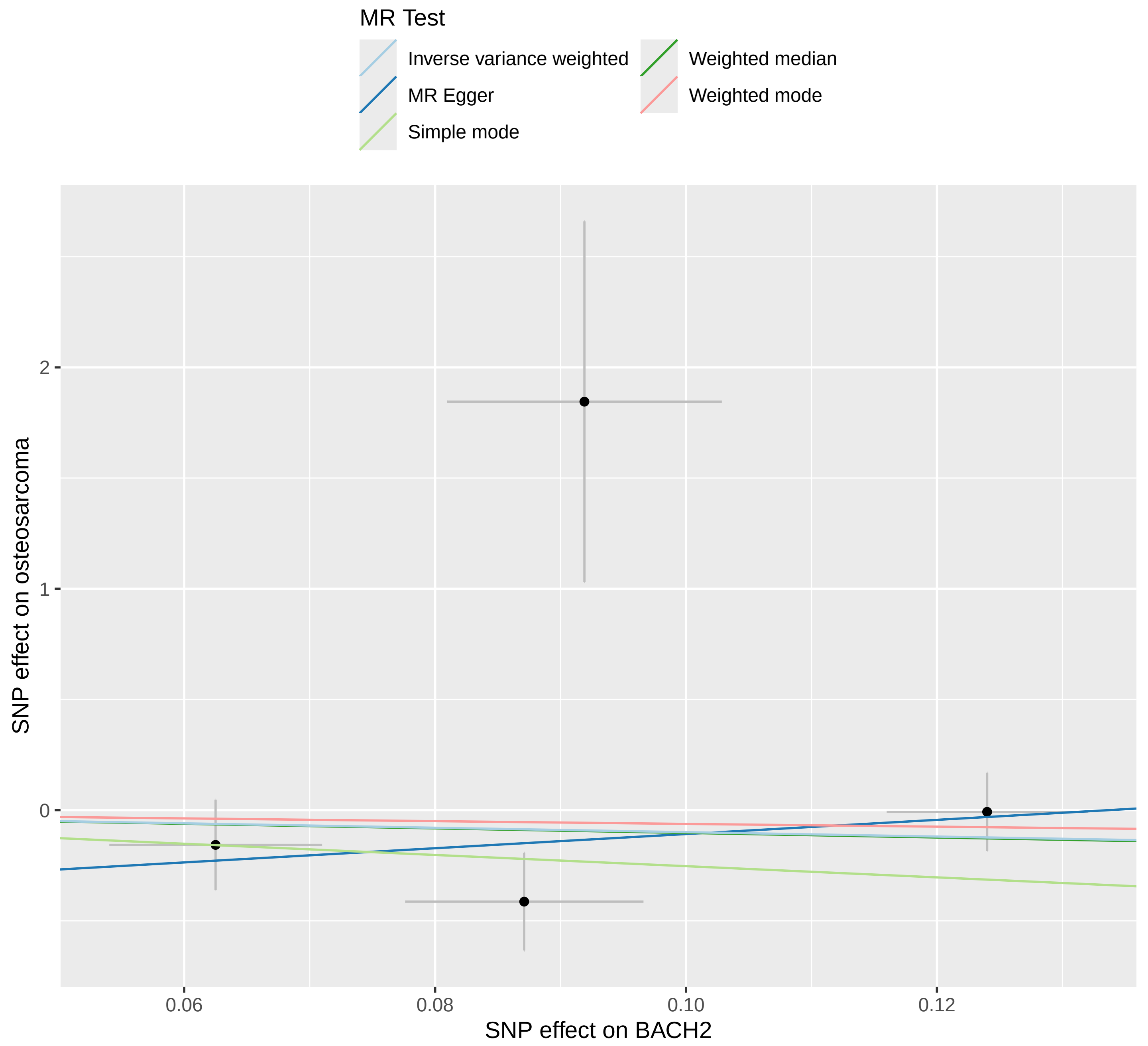
MR Test
Inverse variance weighted
MR Egger
Simple mode
Weighted median
Weighted mode
SNP effect on osteosarcoma
2
1
0
0.06
0.08
0.10
0.12
SNP effect on BACH2

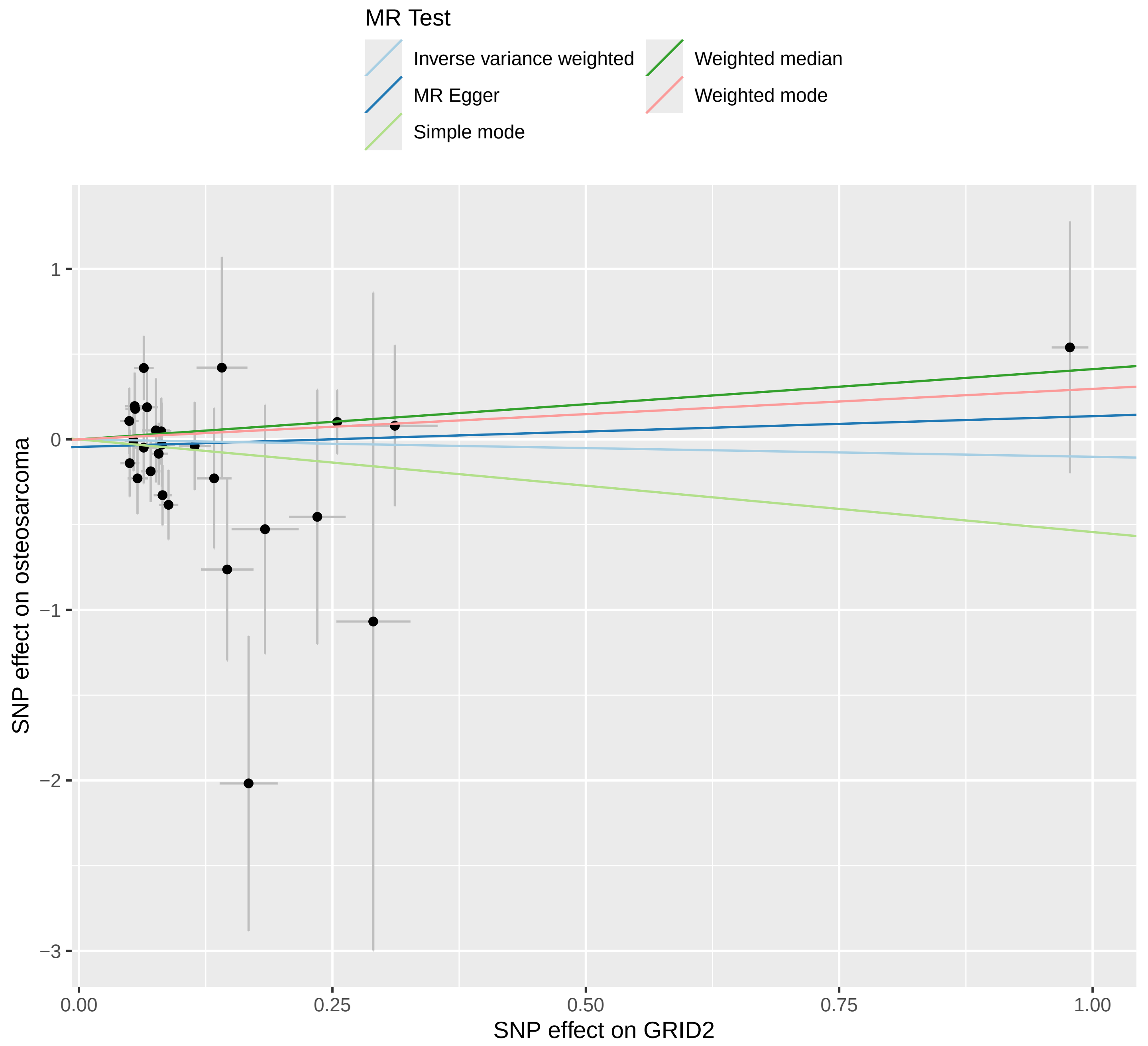

MR Test
Inverse variance weighted
MR Egger
Simple mode
Weighted median
Weighted mode
SNP effect on osteosarcoma
SNP effect on GRID2
1
0
−1
−2
−3
0.00
0.25
0.50
0.75
1.00

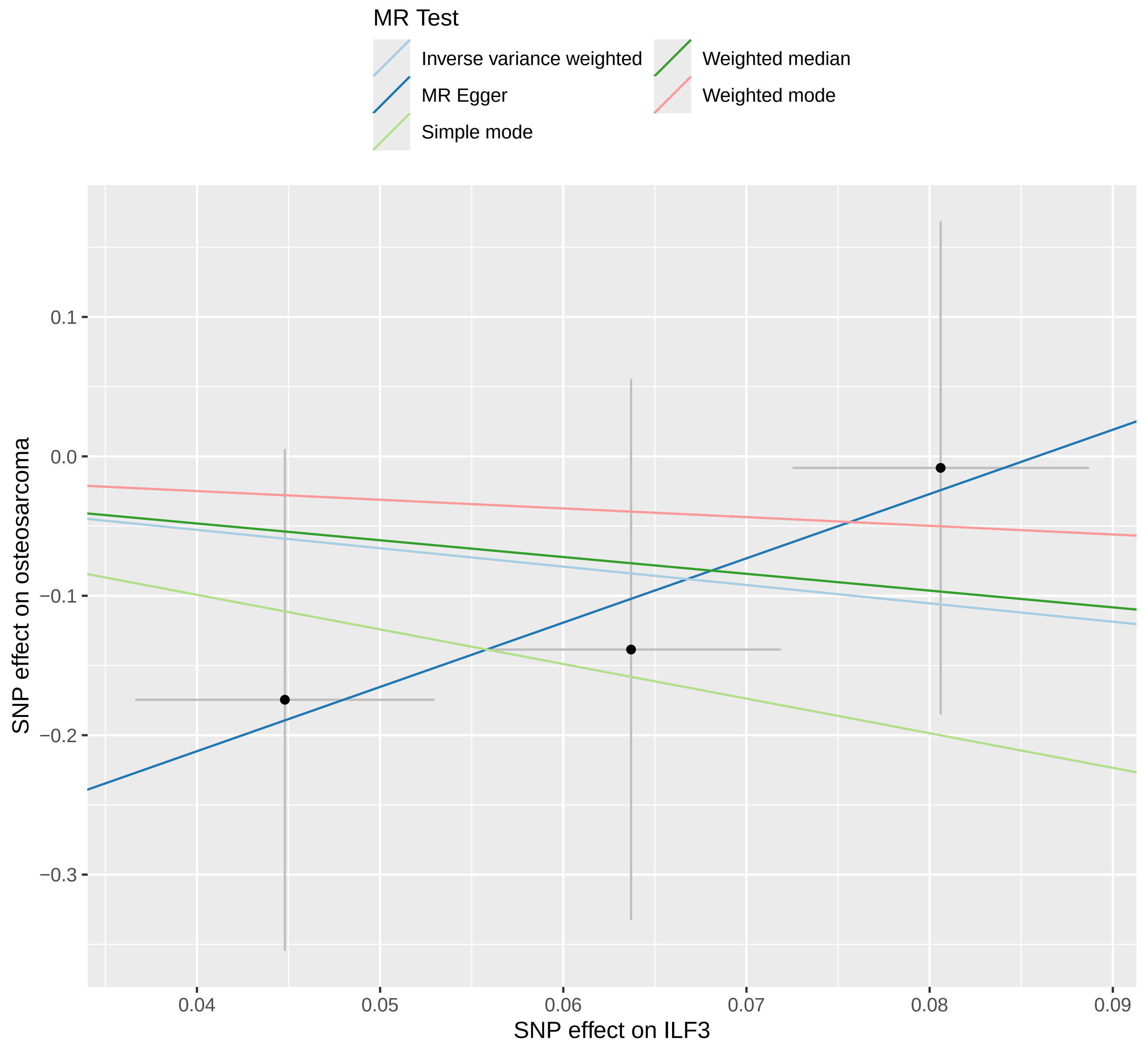

MR Test
Inverse variance weighted
MR Egger
Simple mode
Weighted median
Weighted mode
0.1
0.0
−0.1
−0.2
−0.3
0.04
0.05
0.06
0.07
0.08
0.09
SNP effect on ILF3
SNP effect on osteosarcoma

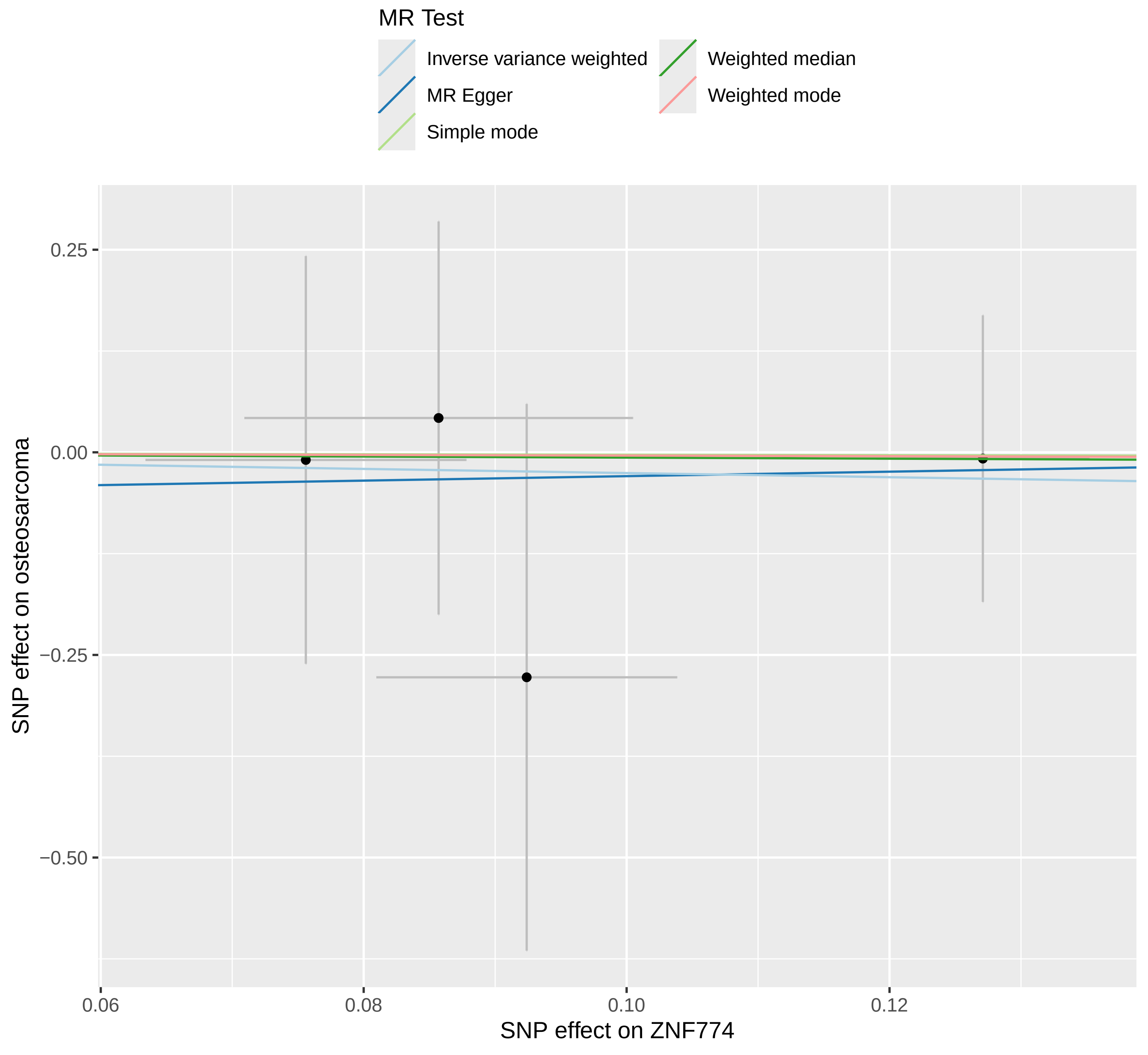

MR Test
Inverse variance weighted
MR Egger
Simple mode
Weighted median
Weighted mode
0.25
0.00
−0.25
−0.50
0.06
0.08
0.10
0.12
SNP effect on osteosarcoma
SNP effect on ZNF774

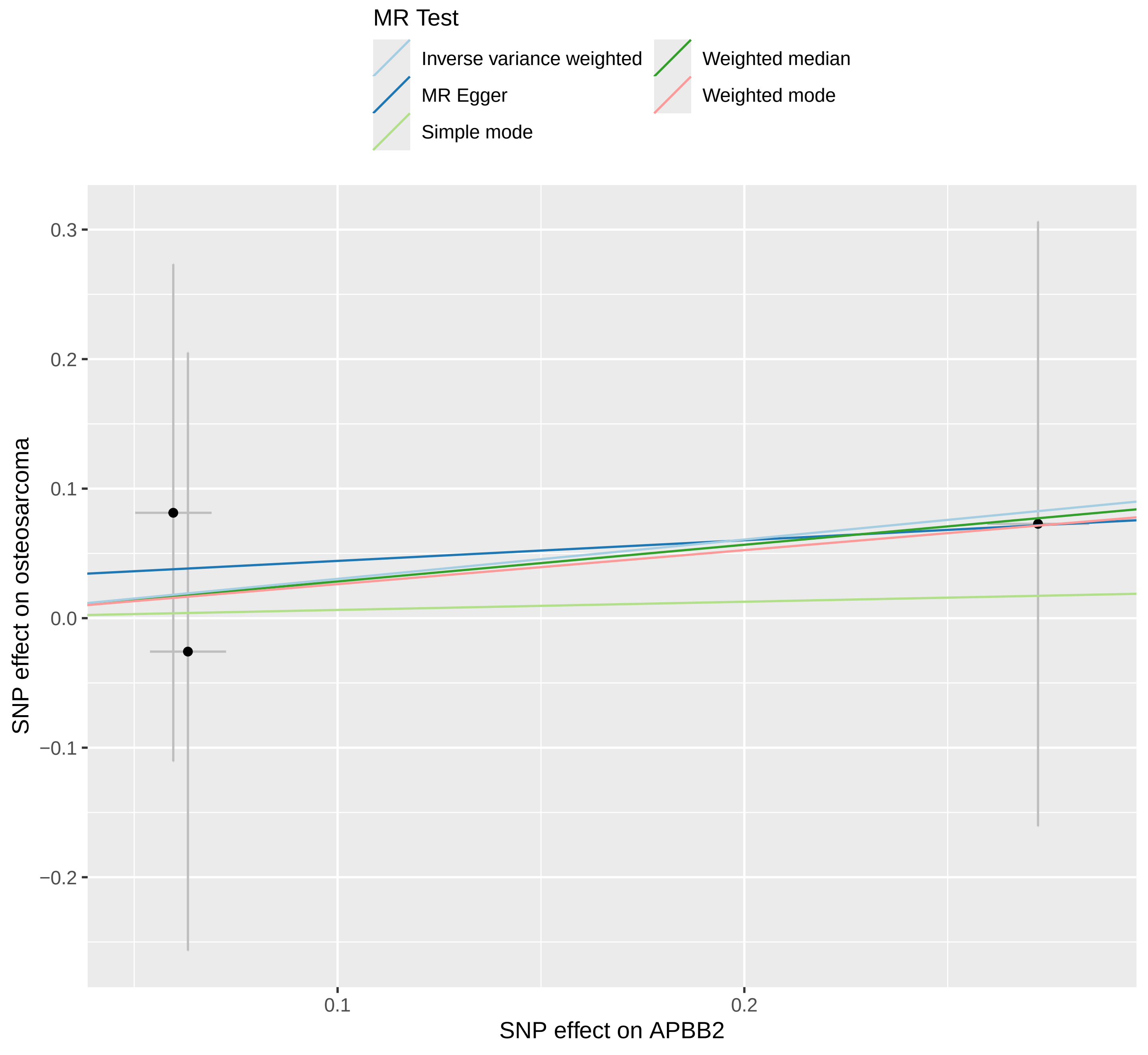
MR Test
Inverse variance weighted
MR Egger
Simple mode
Weighted median
Weighted mode
0.3
0.2
0.1
0.0
−0.1
−0.2
SNP effect on osteosarcoma
0.1
0.2
SNP effect on APBB2

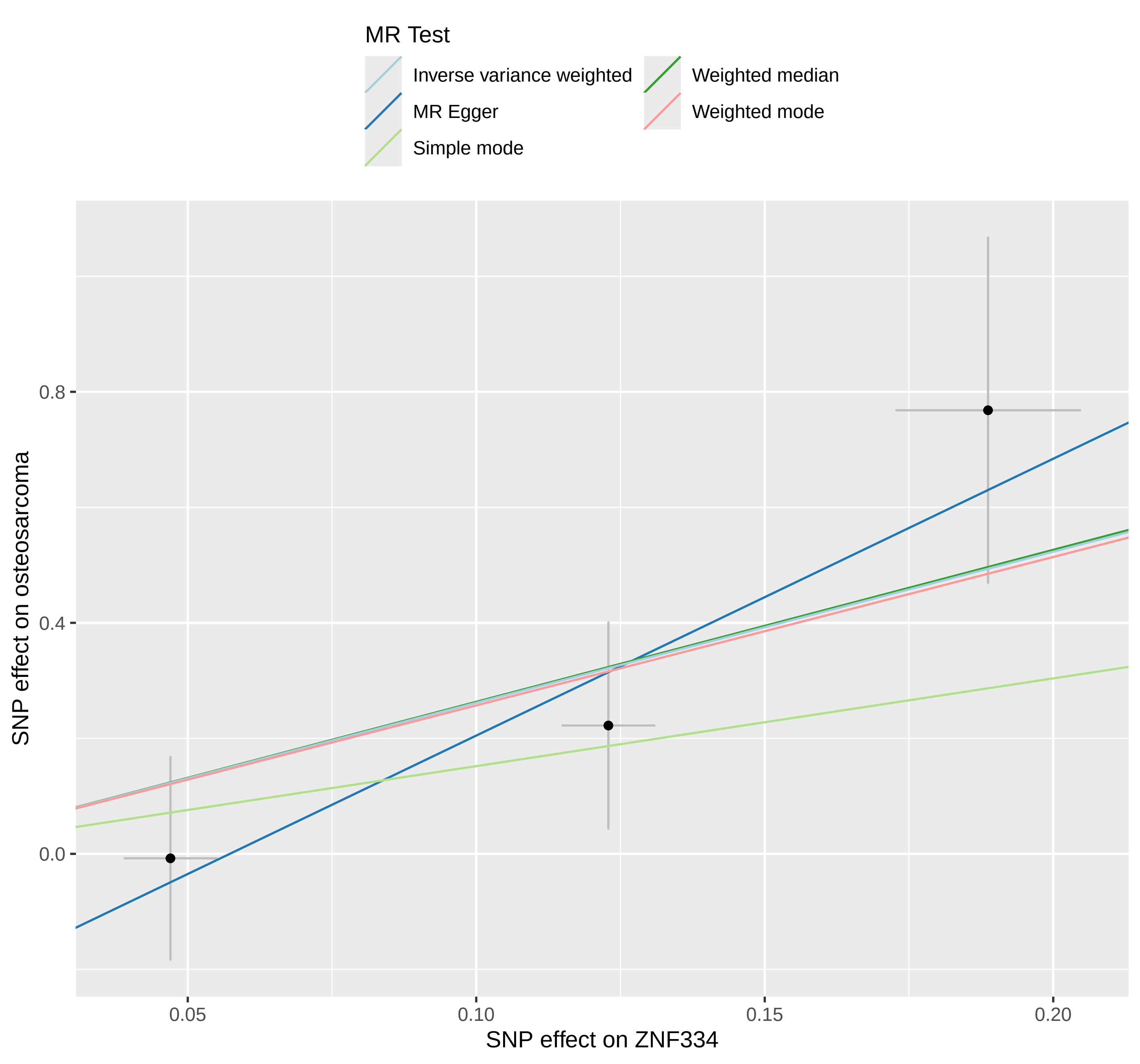
MR Test
Inverse variance weighted
MR Egger
Simple mode
Weighted median
Weighted mode
0.8
0.4
0.0
SNP effect on osteosarcoma
0.05
0.10
0.15
0.20
SNP effect on ZNF334

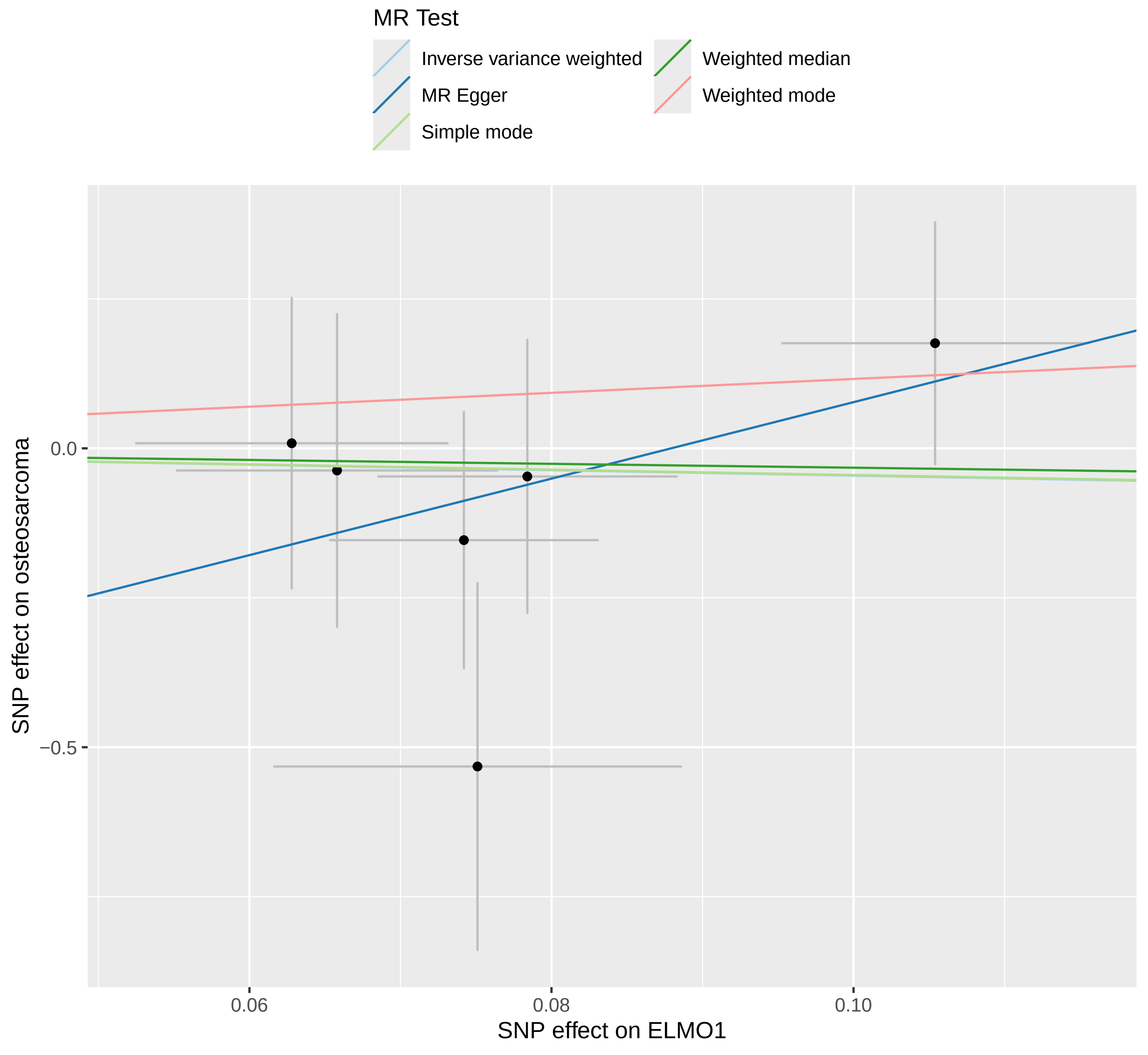

MR Test
Inverse variance weighted
MR Egger
Simple mode
Weighted median
Weighted mode
0.0
−0.5
0.06
0.08
0.10
SNP effect on ELMO1
SNP effect on osteosarcoma

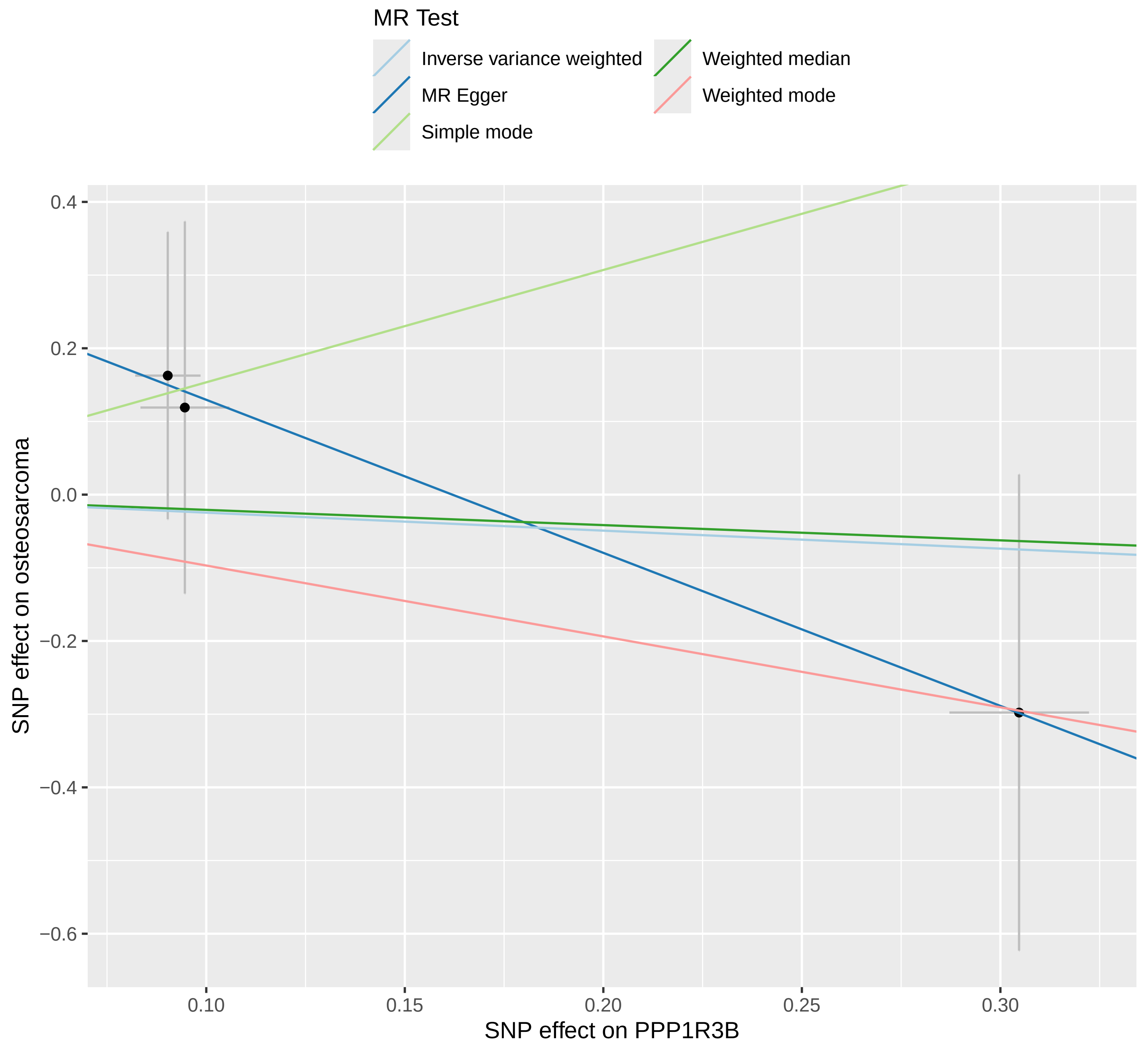
MR Test
Inverse variance weighted
MR Egger
Simple mode
Weighted median
Weighted mode
0.4
0.2
0.0
−0.2
−0.4
−0.6
SNP effect on osteosarcoma
0.10
0.15
0.20
0.25
0.30
SNP effect on PPP1R3B

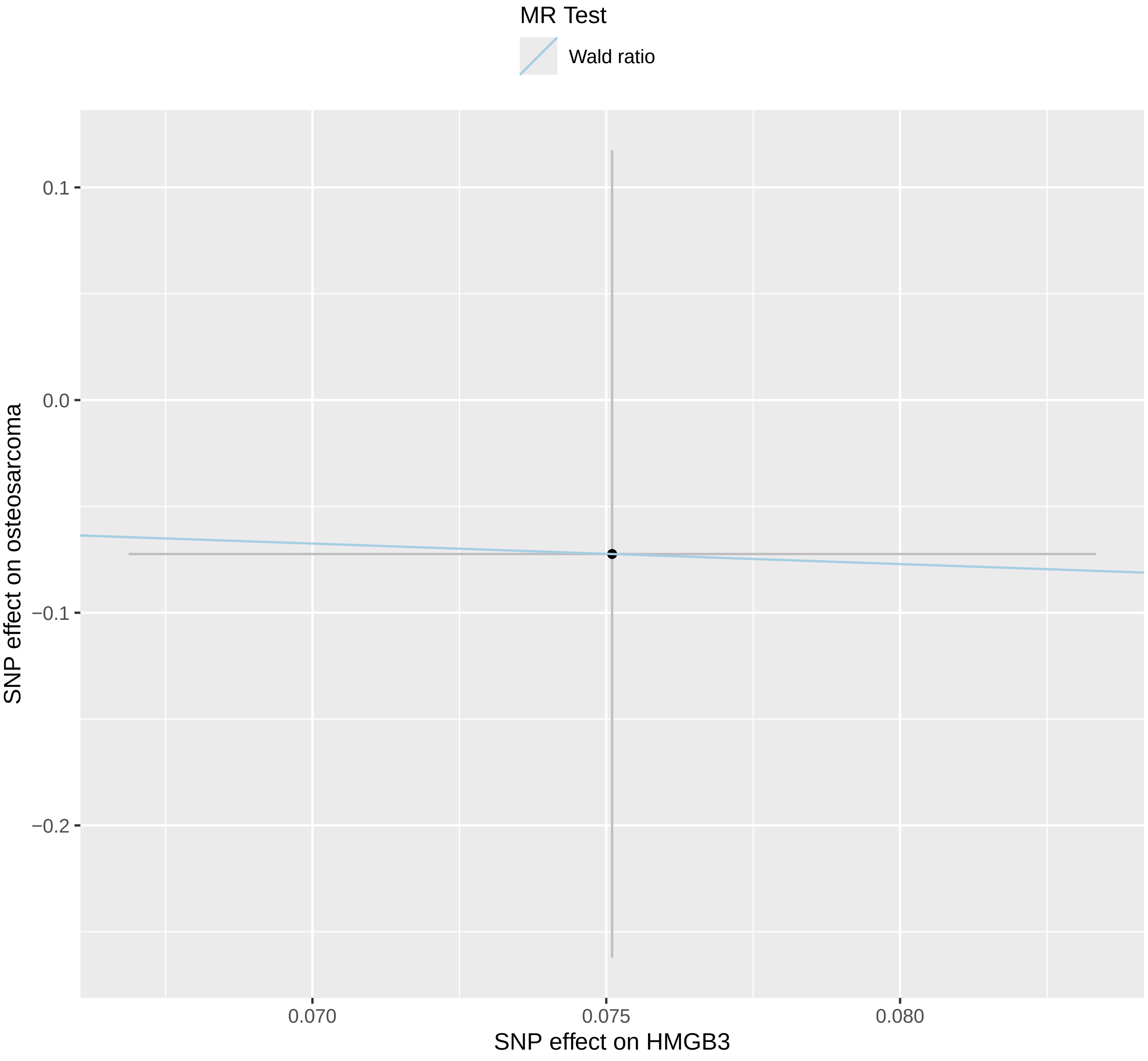
MR Test
Wald ratio
0.1
0.0
−0.1
−0.2
SNP effect on osteosarcoma
0.070
0.075
0.080
SNP effect on HMGB3

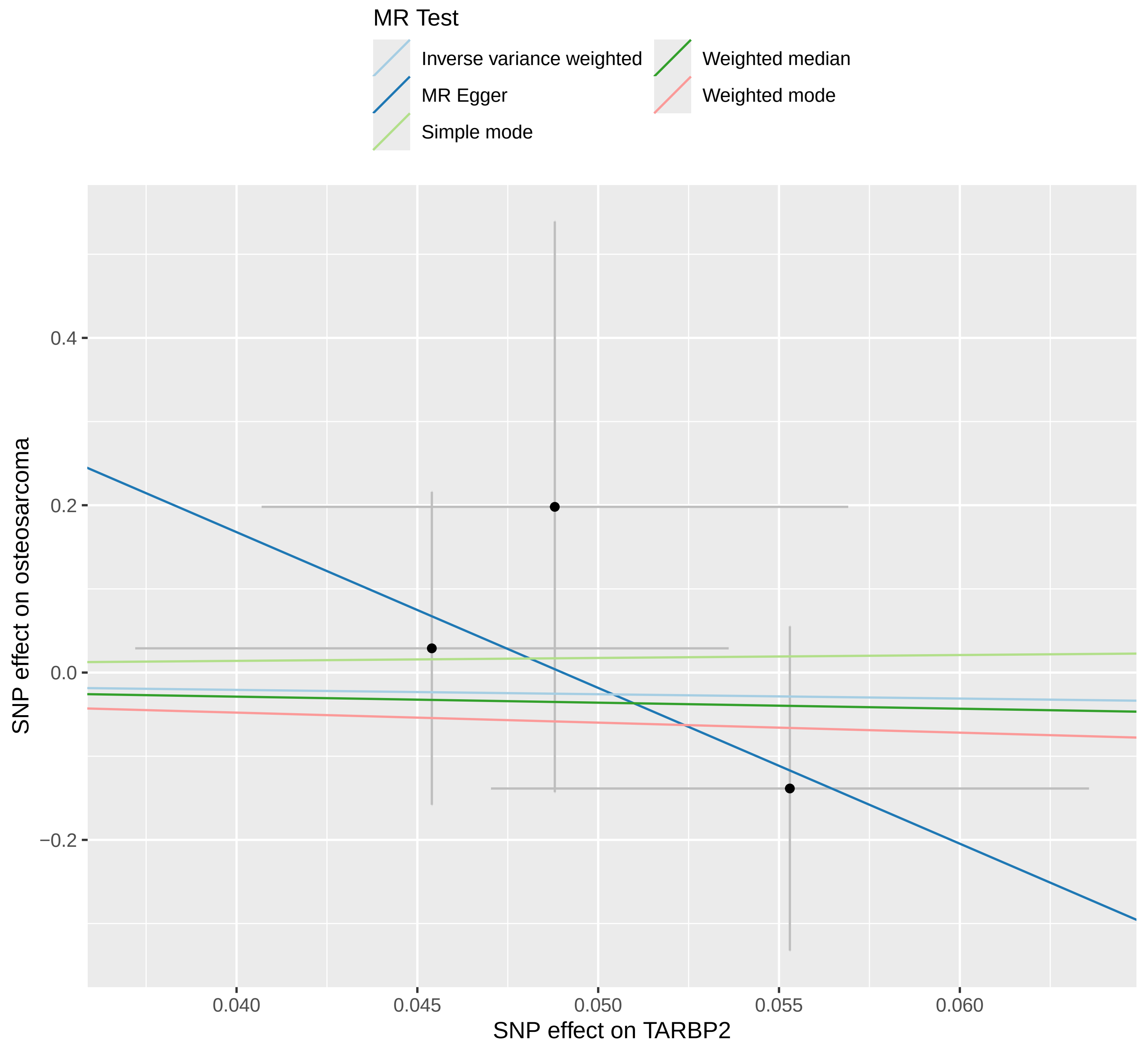
MR Test
Inverse variance weighted
MR Egger
Simple mode
Weighted median
Weighted mode
0.4
0.2
0.0
−0.2
SNP effect on osteosarcoma
0.040
0.045
0.050
0.055
0.060
SNP effect on TARBP2

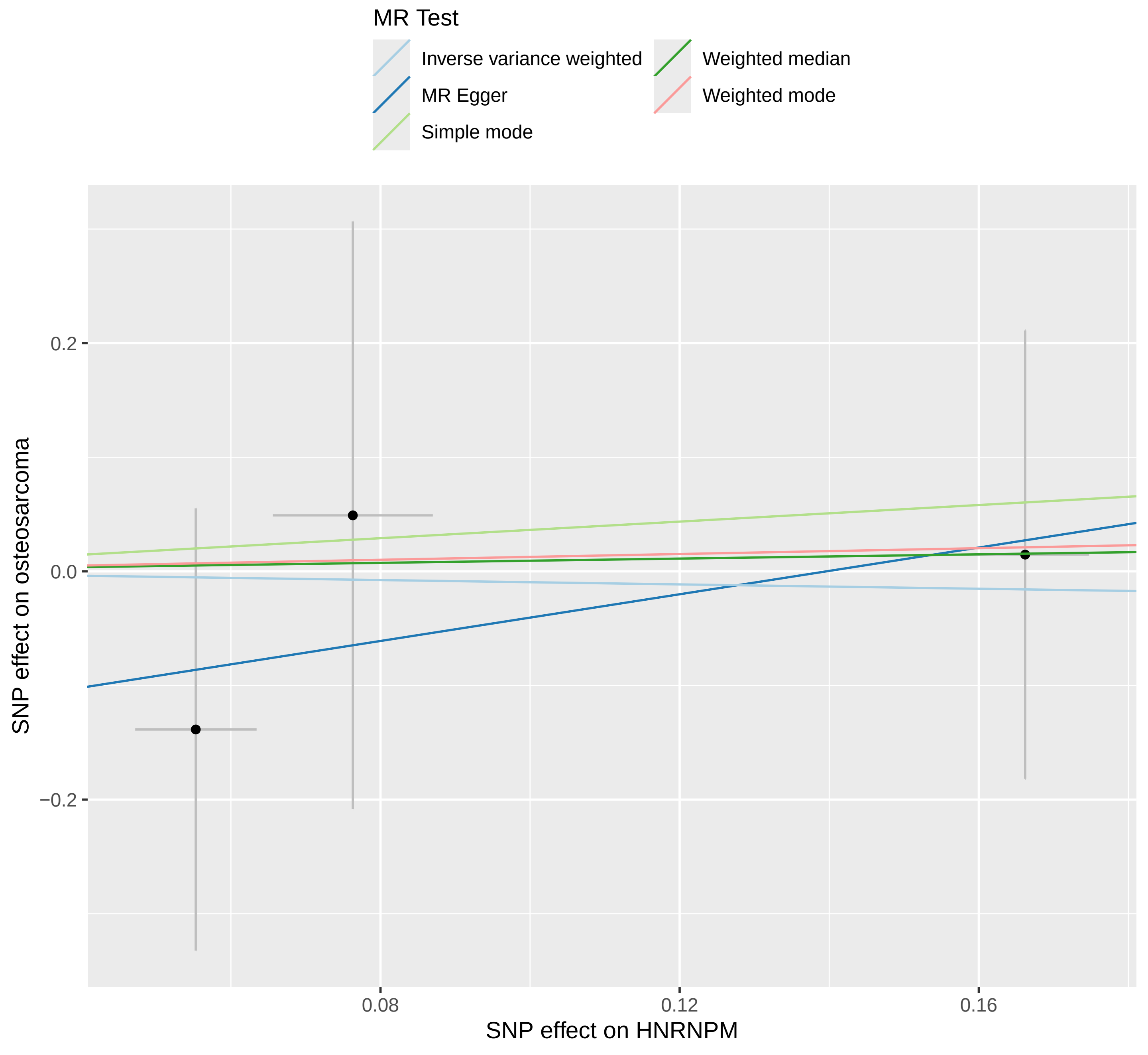
MR Test
Inverse variance weighted
MR Egger
Simple mode
Weighted median
Weighted mode
0.2
0.0
−0.2
SNP effect on osteosarcoma
0.08
0.12
0.16
SNP effect on HNRNPM

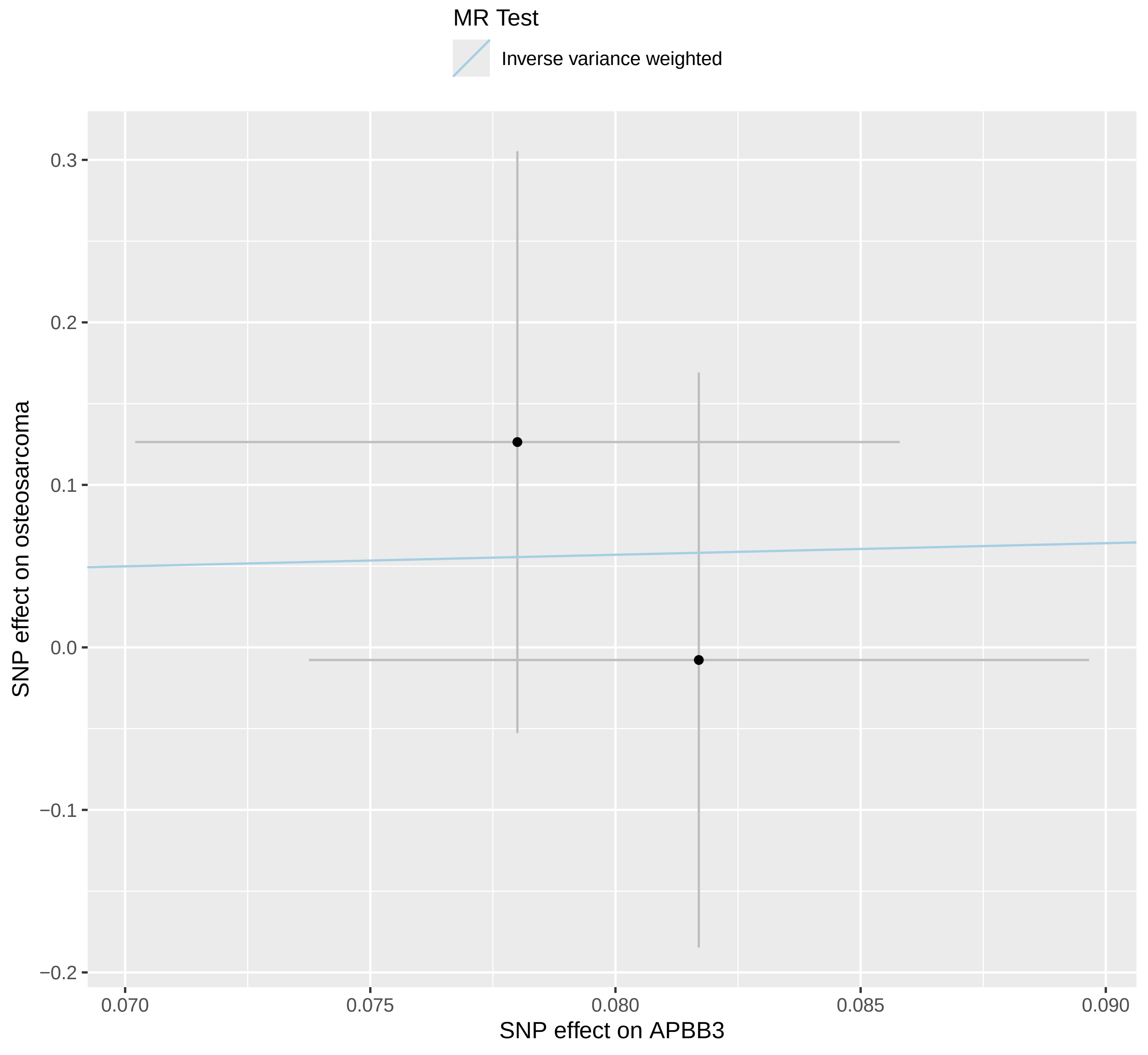
MR Test
Inverse variance weighted
SNP effect on osteosarcoma
0.3
0.2
0.1
0.0
−0.1
−0.2
0.070
0.075
0.080
0.085
0.090
SNP effect on APBB3

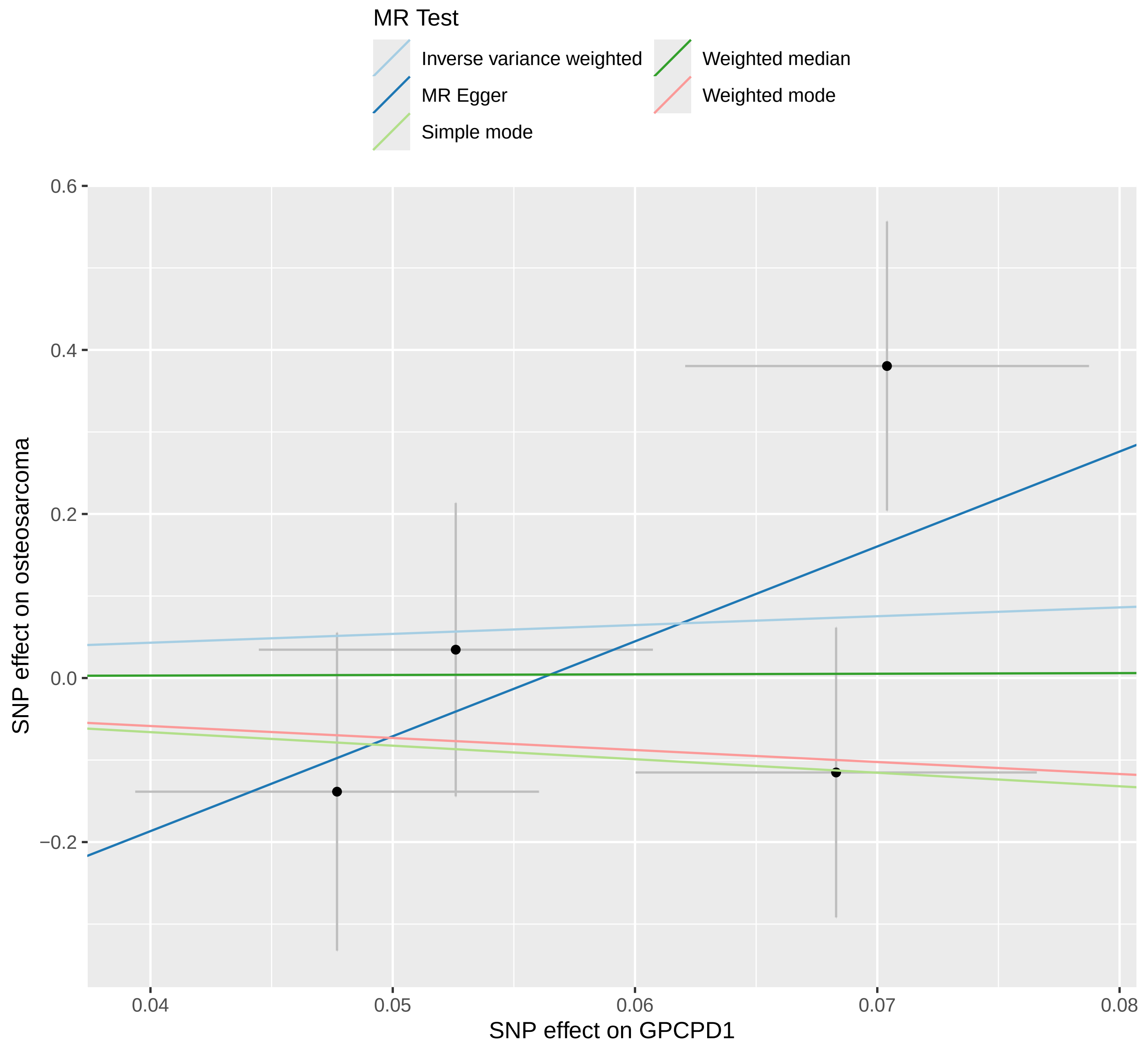
MR Test
Inverse variance weighted
MR Egger
Simple mode
Weighted median
Weighted mode
0.6
0.4
0.2
0.0
−0.2
SNP effect on osteosarcoma
0.04
0.05
0.06
0.07
0.08
SNP effect on GPCPD1

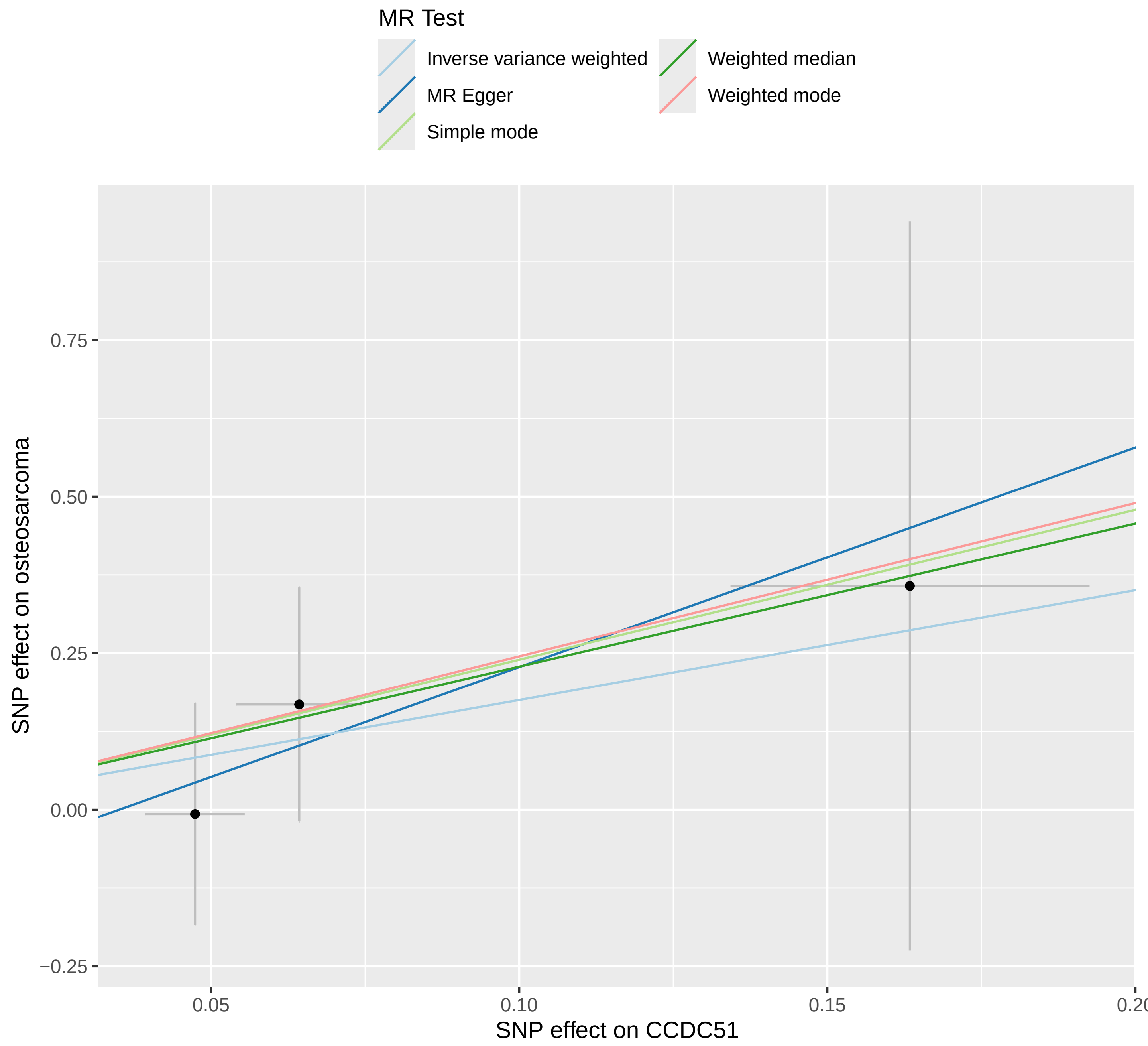
MR Test
Inverse variance weighted
MR Egger
Simple mode
Weighted median
Weighted mode
SNP effect on osteosarcoma
0.75
0.50
0.25
0.00
−0.25
0.05
0.10
0.15
0.20
SNP effect on CCDC51

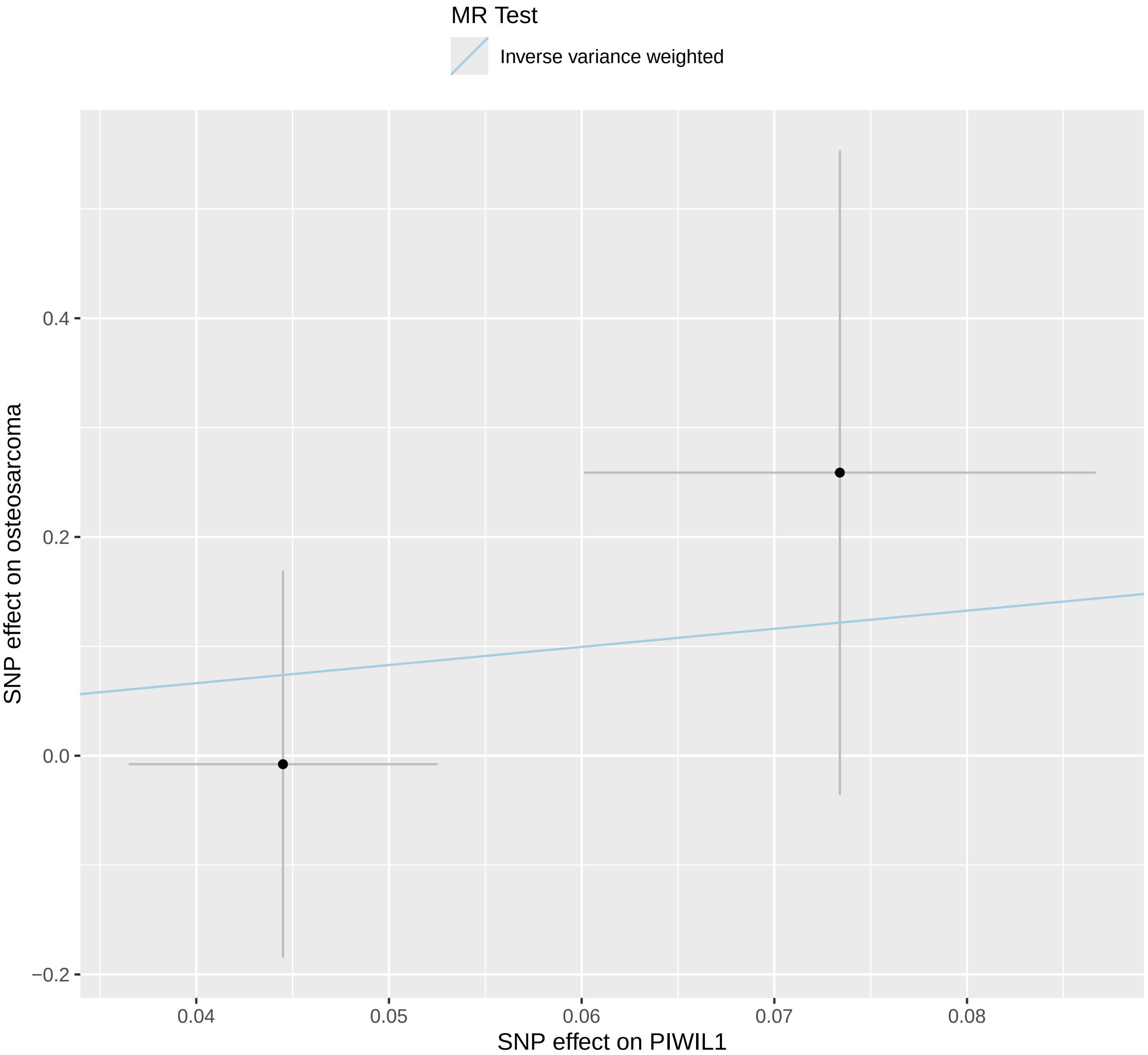
MR Test
Inverse variance weighted
SNP effect on osteosarcoma
0.4
0.2
0.0
−0.2
0.04
0.05
0.06
0.07
0.08
SNP effect on PIWIL1

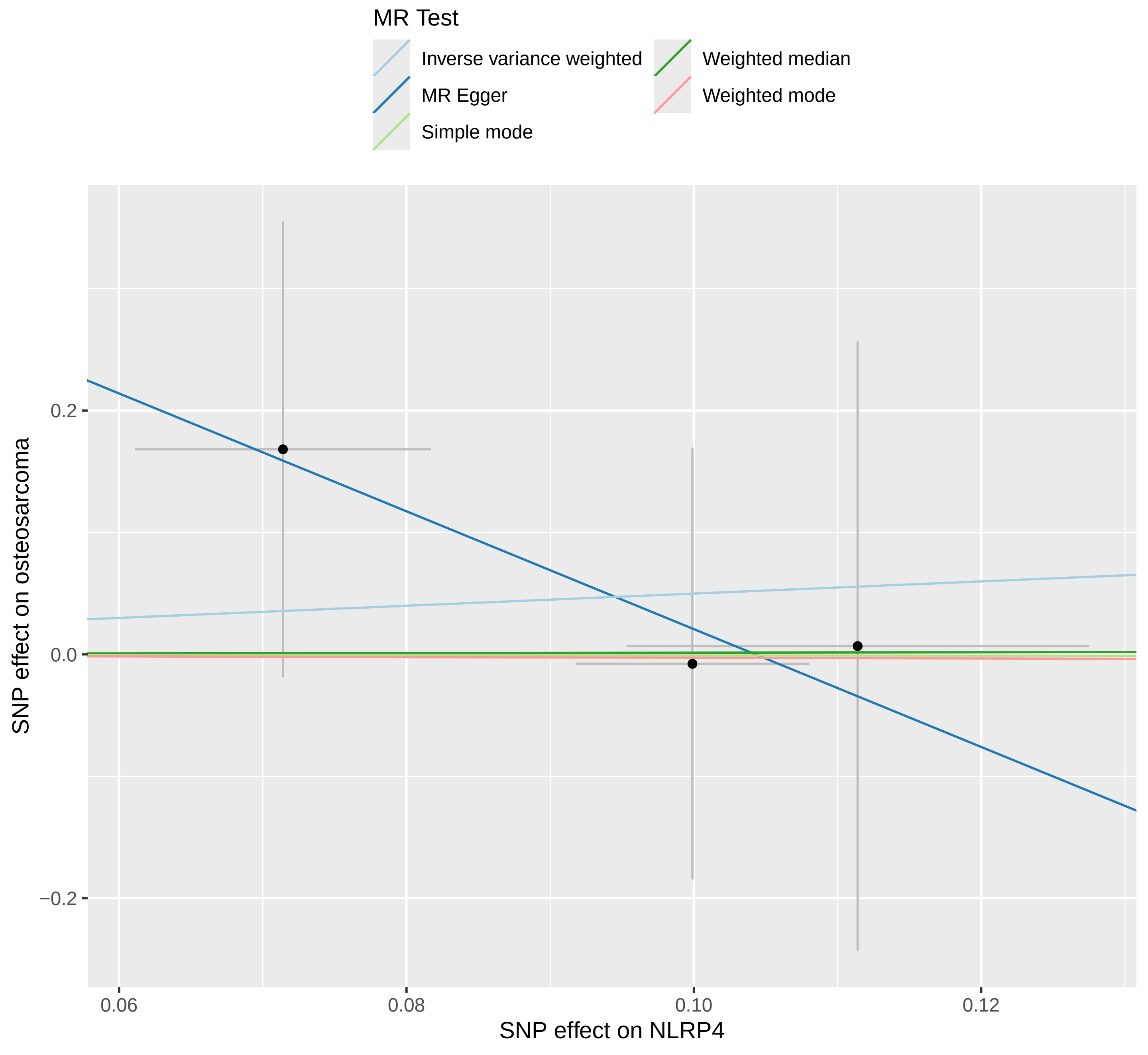

MR Test
Inverse variance weighted
MR Egger
Simple mode
Weighted median
Weighted mode
0.2
0.0
−0.2
SNP effect on osteosarcoma
0.06
0.08
0.10
0.12
SNP effect on NLRP4

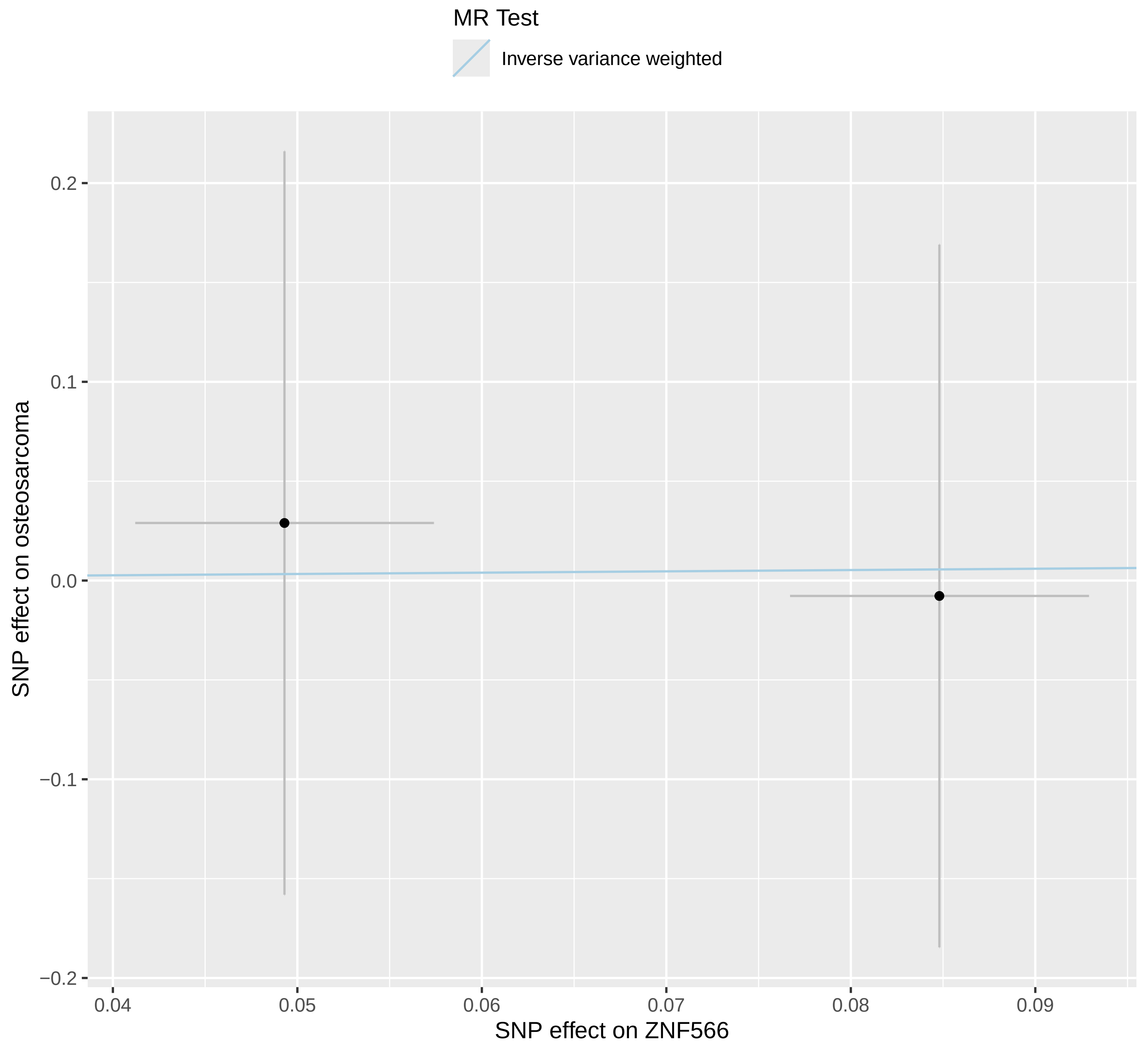

MR Test
Inverse variance weighted
SNP effect on osteosarcoma
0.2
0.1
0.0
−0.1
−0.2
0.04
0.05
0.06
0.07
0.08
0.09
SNP effect on ZNF566

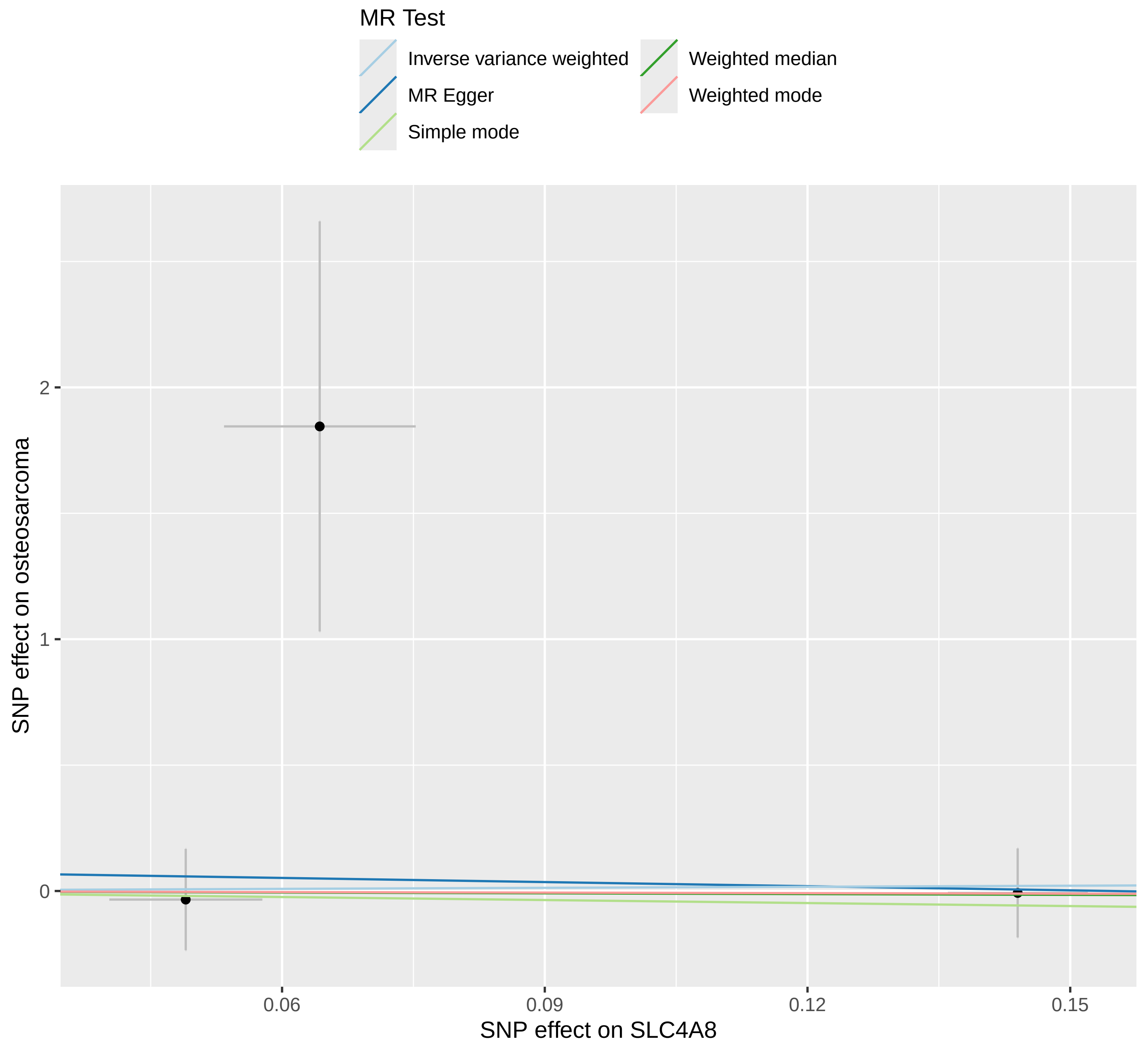

MR Test
Inverse variance weighted
MR Egger
Simple mode
Weighted median
Weighted mode
SNP effect on osteosarcoma
2
1
0
0.06
0.09
0.12
0.15
SNP effect on SLC4A8

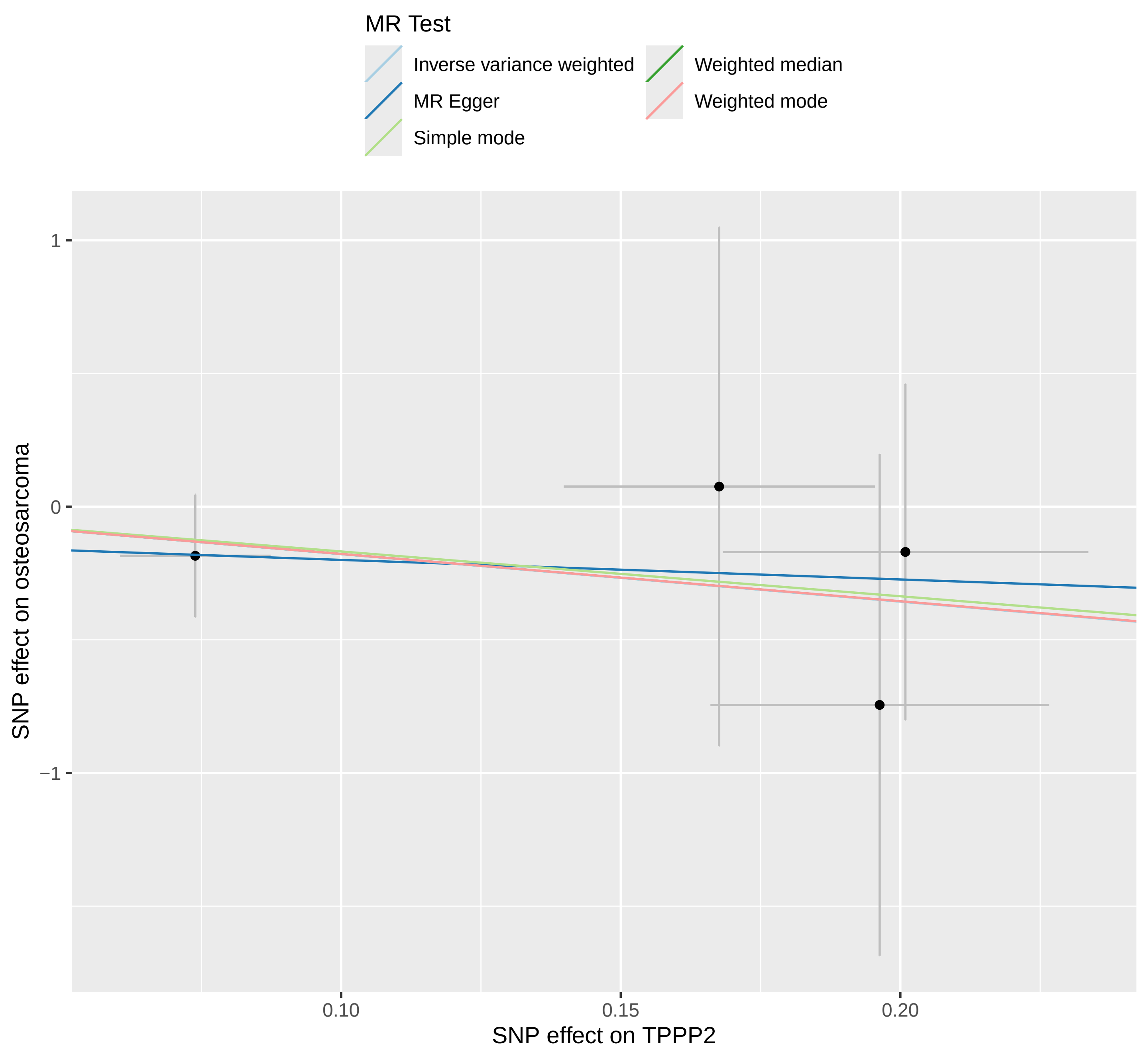
MR Test
Inverse variance weighted
MR Egger
Simple mode
Weighted median
Weighted mode
1
0
−1
0.10
0.15
0.20
SNP effect on TPPP2
SNP effect on osteosarcoma

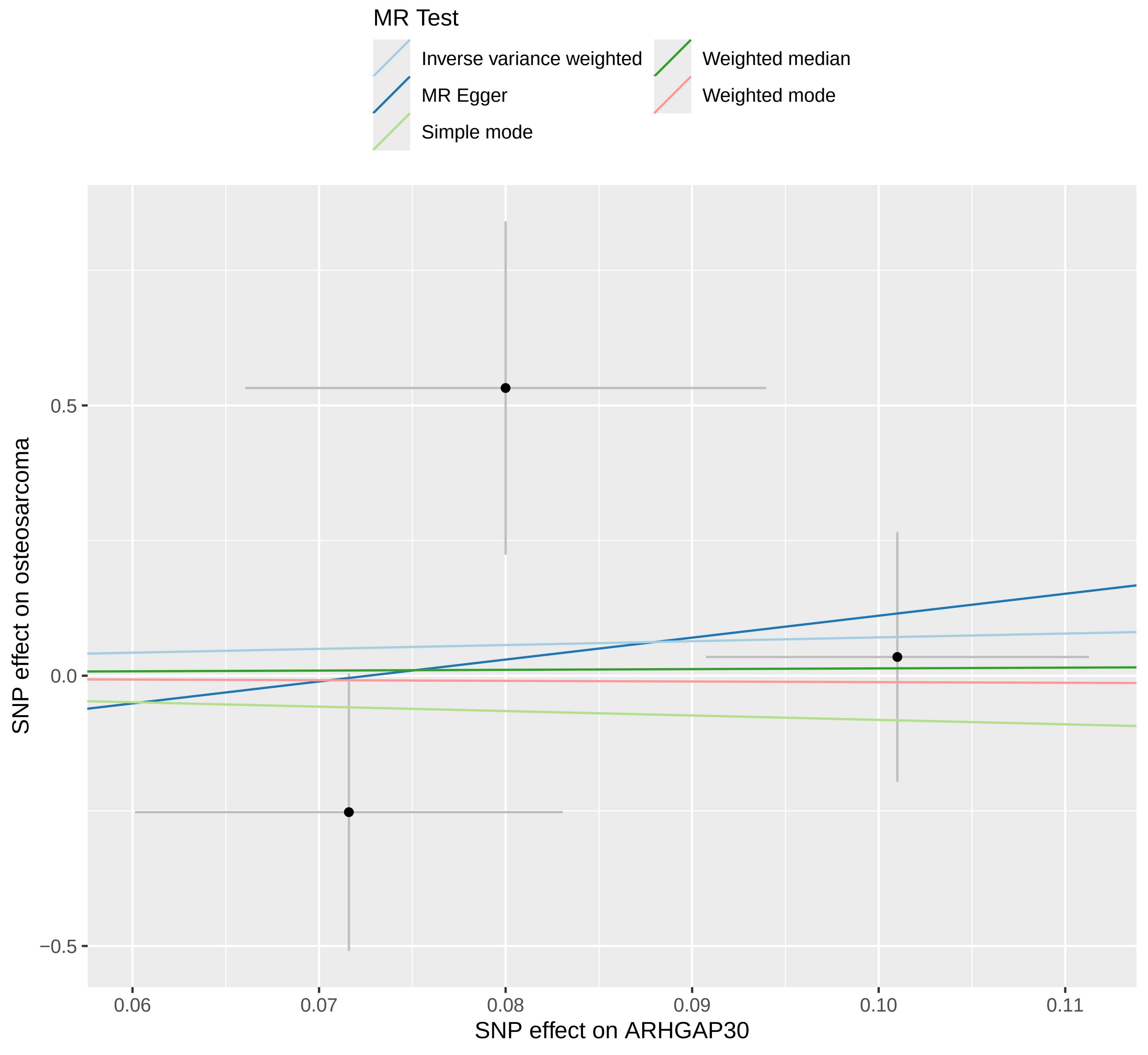

MR Test
Inverse variance weighted
MR Egger
Simple mode
Weighted median
Weighted mode
SNP effect on osteosarcoma
0.5
0.0
−0.5
0.06
0.07
0.08
0.09
0.10
0.11
SNP effect on ARHGAP30

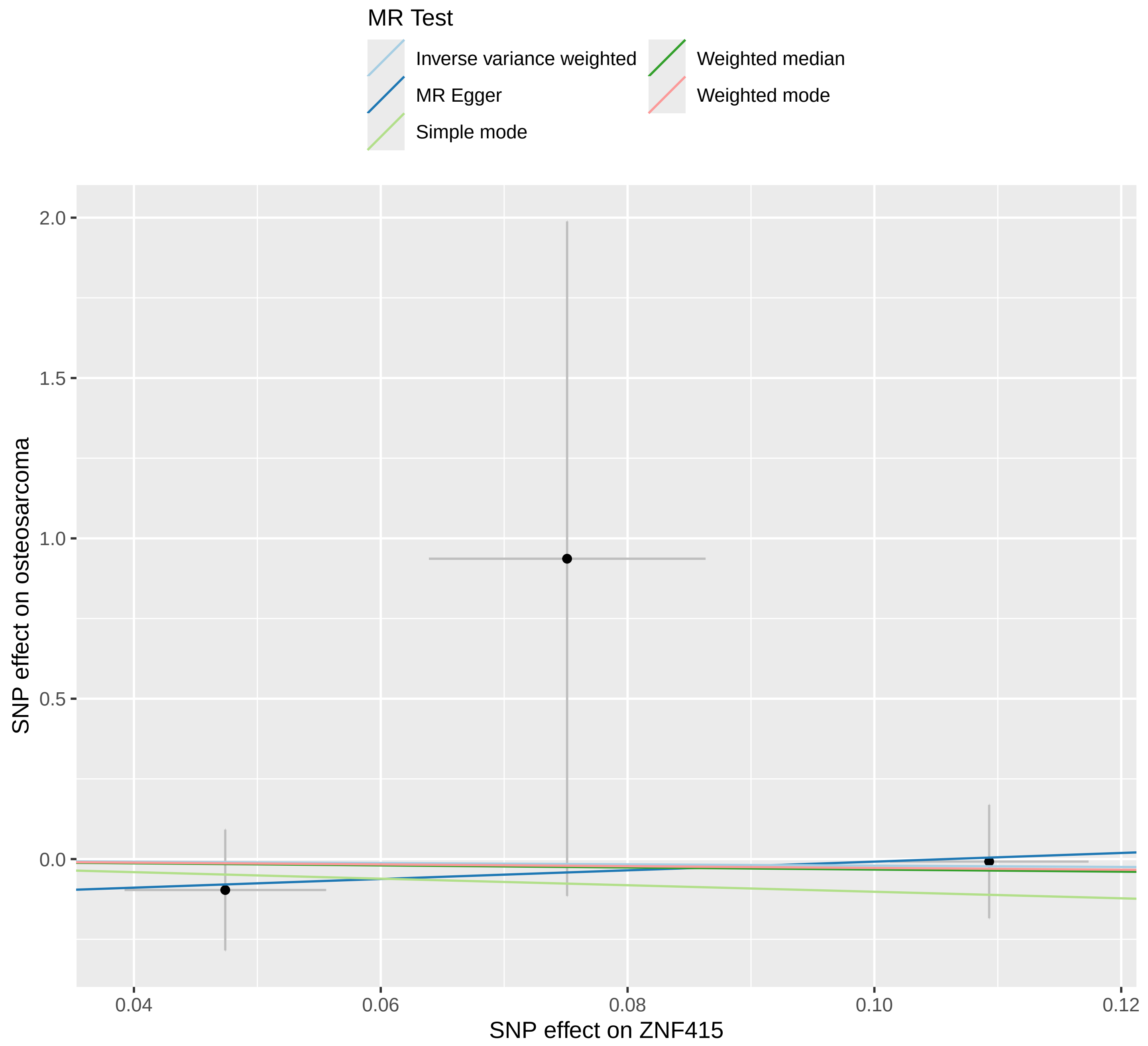

MR Test
Inverse variance weighted
MR Egger
Simple mode
Weighted median
Weighted mode
2.0
1.5
1.0
0.5
0.0
SNP effect on osteosarcoma
0.04
0.06
0.08
0.10
0.12
SNP effect on ZNF415

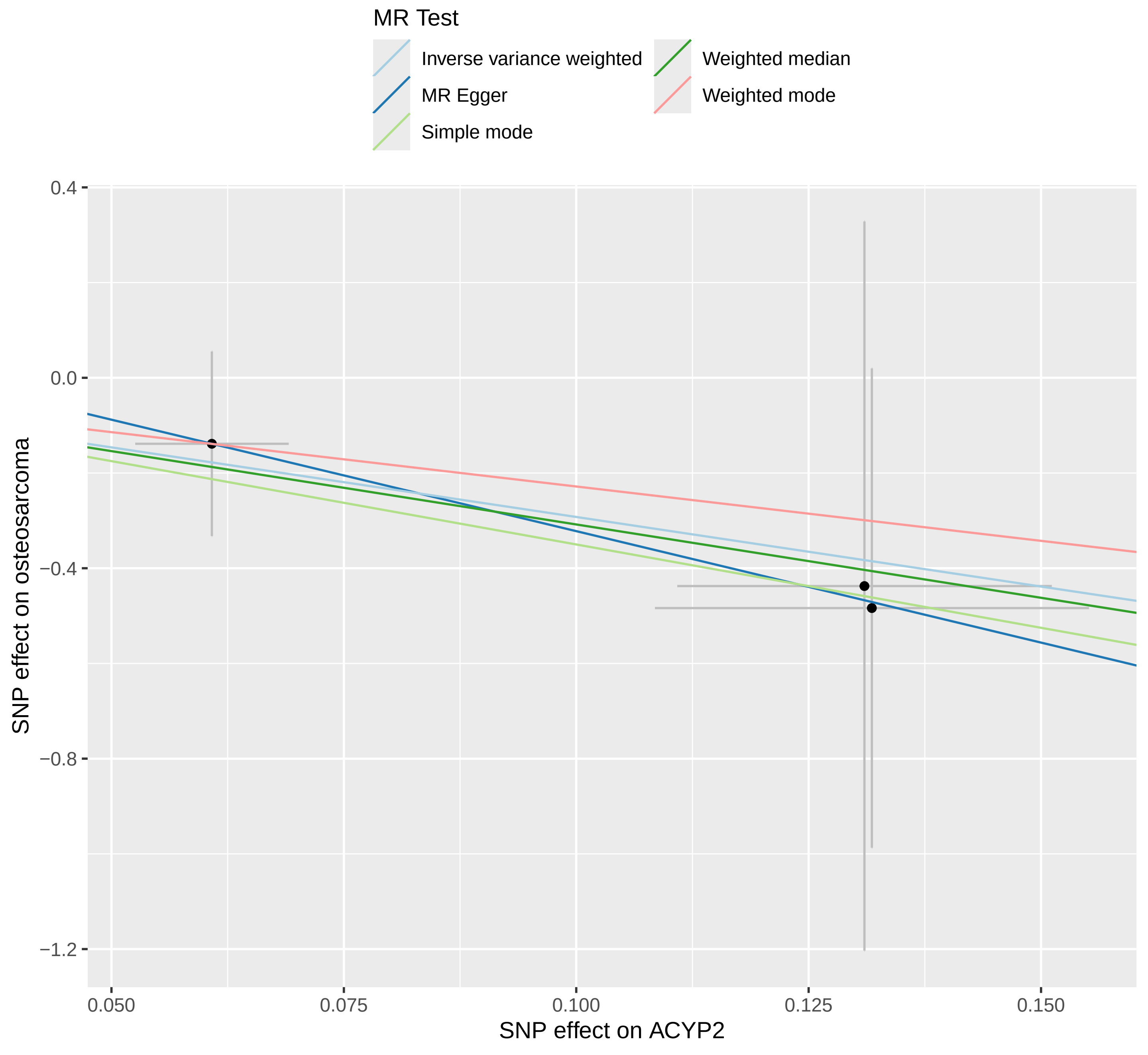
MR Test
Inverse variance weighted
MR Egger
Simple mode
Weighted median
Weighted mode
0.4
0.0
−0.4
−0.8
−1.2
SNP effect on osteosarcoma
0.050
0.075
0.100
0.125
0.150
SNP effect on ACYP2

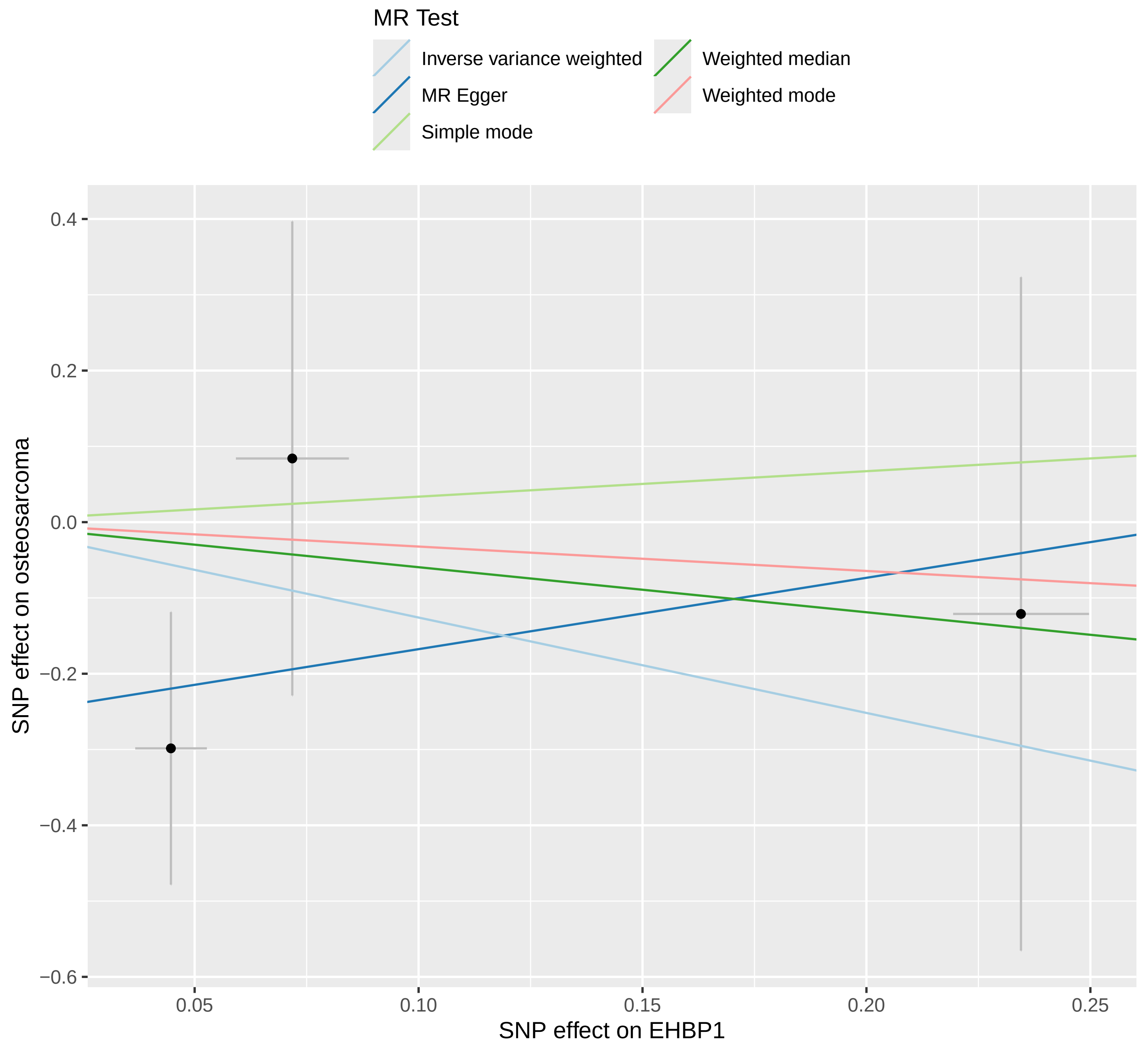
MR Test
Inverse variance weighted
MR Egger
Simple mode
Weighted median
Weighted mode
0.4
0.2
0.0
−0.2
−0.4
−0.6
SNP effect on osteosarcoma
0.05
0.10
0.15
0.20
0.25
SNP effect on EHBP1

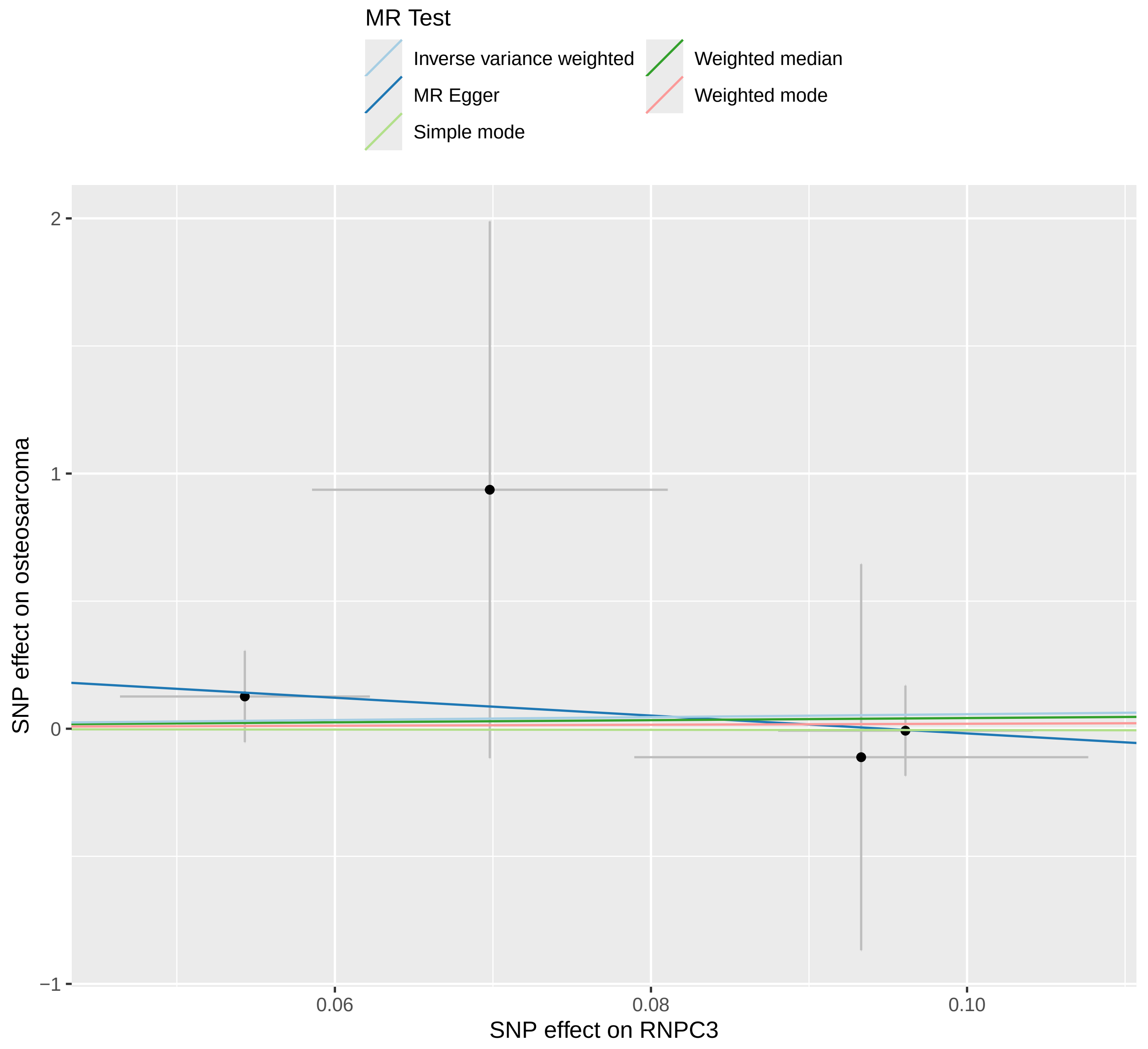
MR Test
Inverse variance weighted
MR Egger
Simple mode
Weighted median
Weighted mode
SNP effect on osteosarcoma
2
1
0
−1
0.06
0.08
0.10
SNP effect on RNPC3

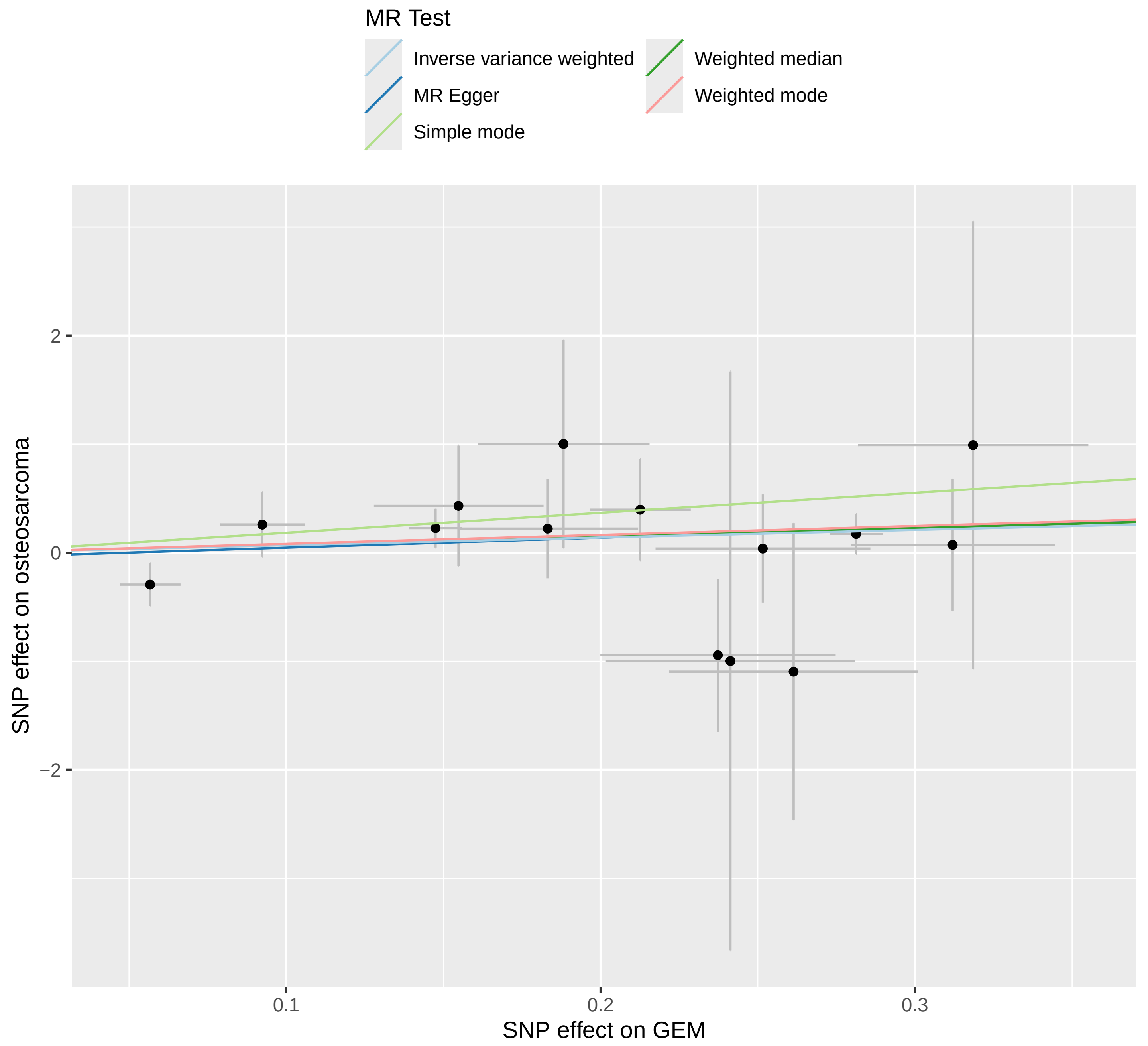

MR Test
Inverse variance weighted
MR Egger
Simple mode
Weighted median
Weighted mode
2
0
−2
0.1
0.2
0.3
SNP effect on osteosarcoma
SNP effect on GEM

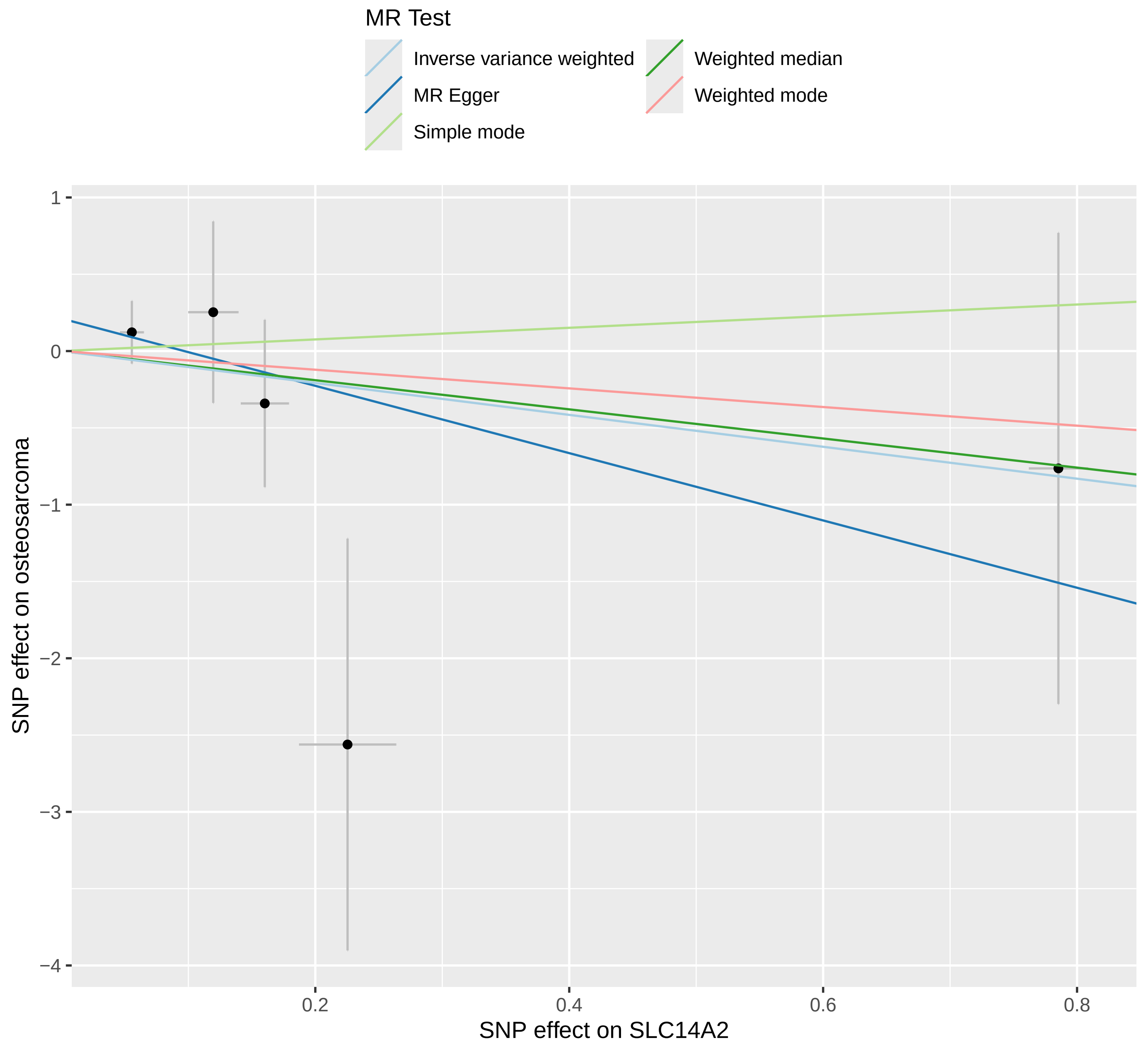
MR Test
Inverse variance weighted
MR Egger
Simple mode
Weighted median
Weighted mode
1
0
−1
−2
−3
−4
SNP effect on osteosarcoma
0.2
0.4
0.6
0.8
SNP effect on SLC14A2

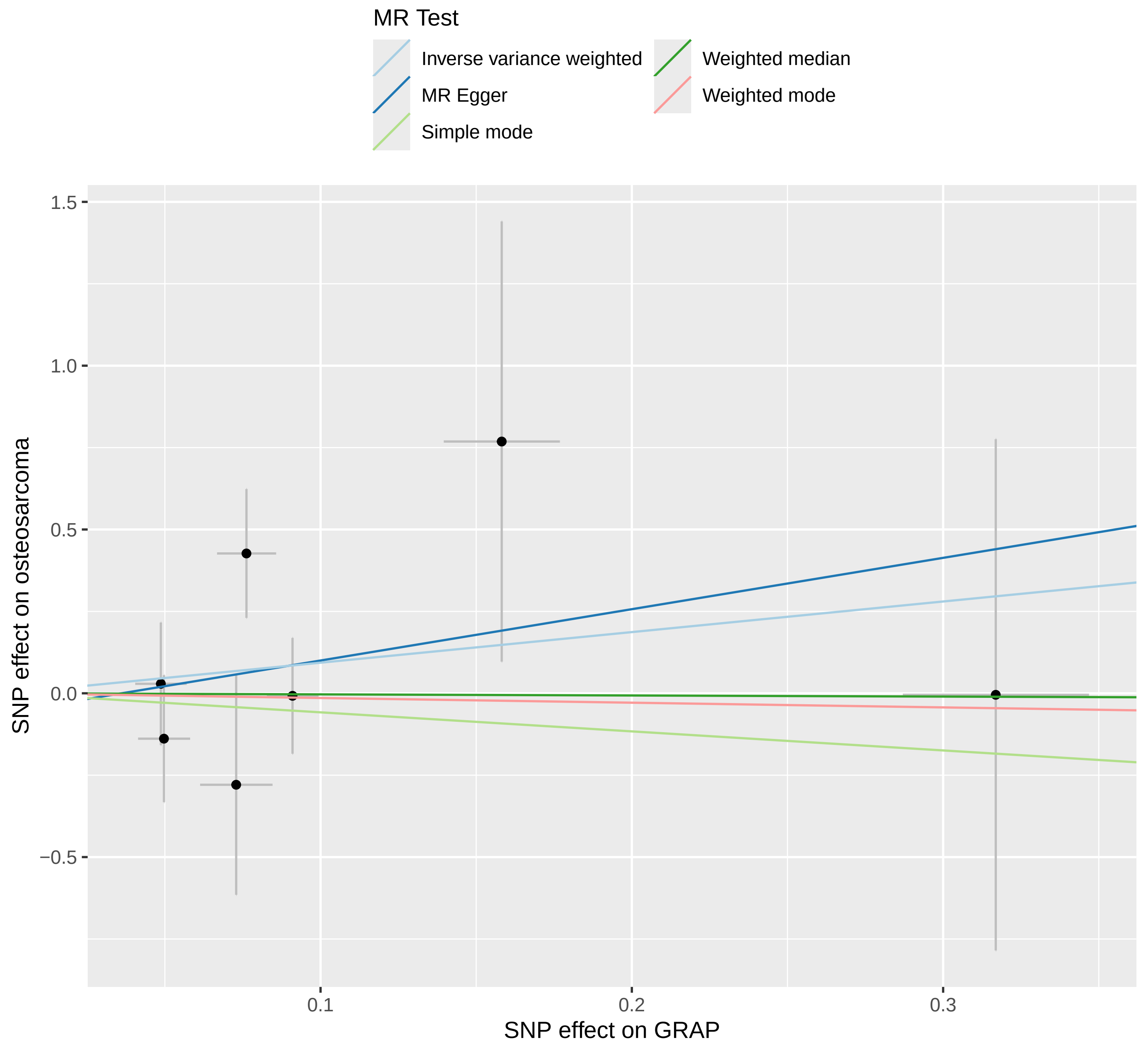

MR Test
Inverse variance weighted
MR Egger
Simple mode
Weighted median
Weighted mode
1.5
1.0
0.5
0.0
−0.5
SNP effect on osteosarcoma
0.1
0.2
0.3
SNP effect on GRAP

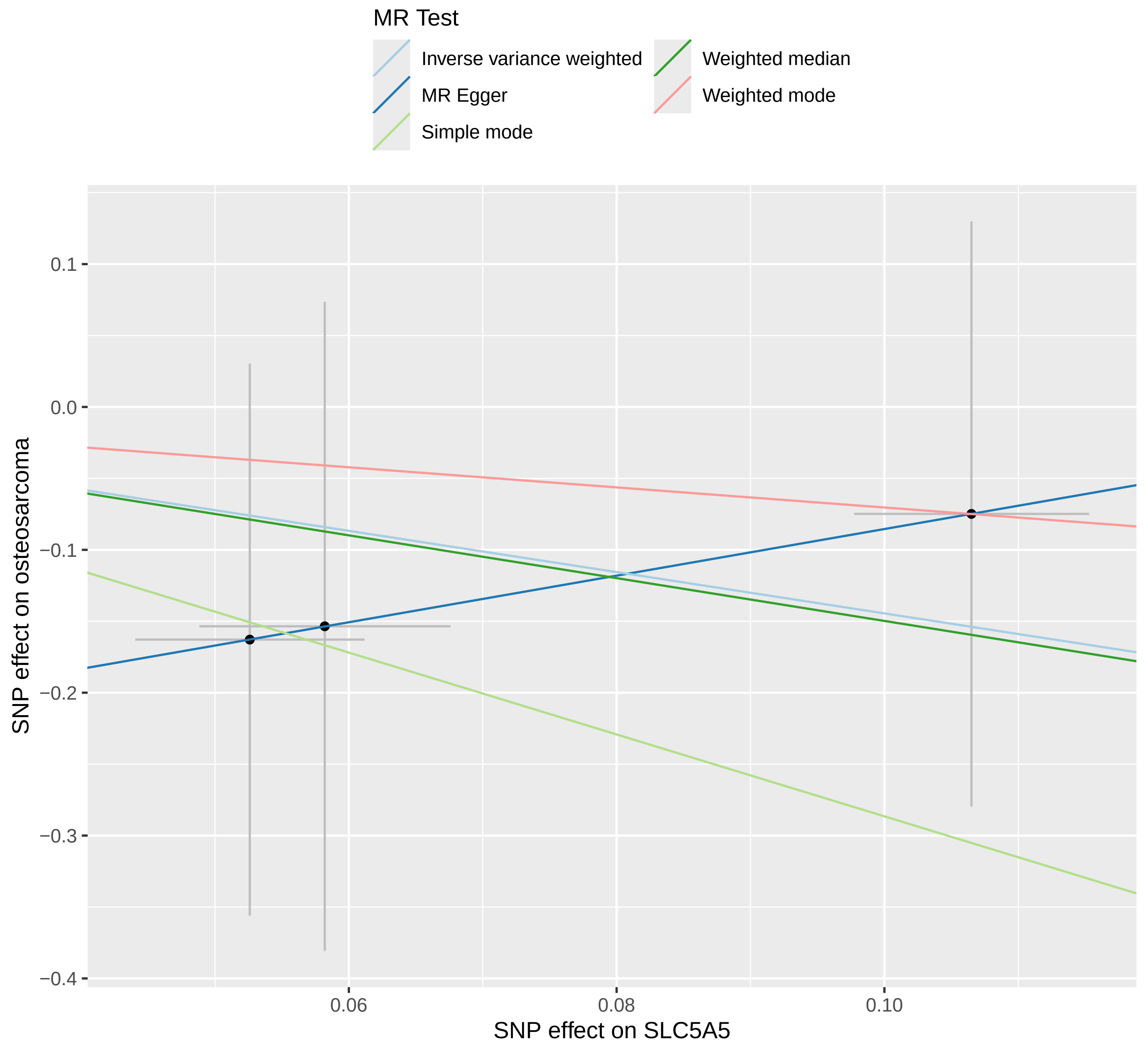
MR Test
Inverse variance weighted
MR Egger
Simple mode
Weighted median
Weighted mode
0.1
0.0
−0.1
−0.2
−0.3
−0.4
SNP effect on osteosarcoma
0.06
0.08
0.10
SNP effect on SLC5A5

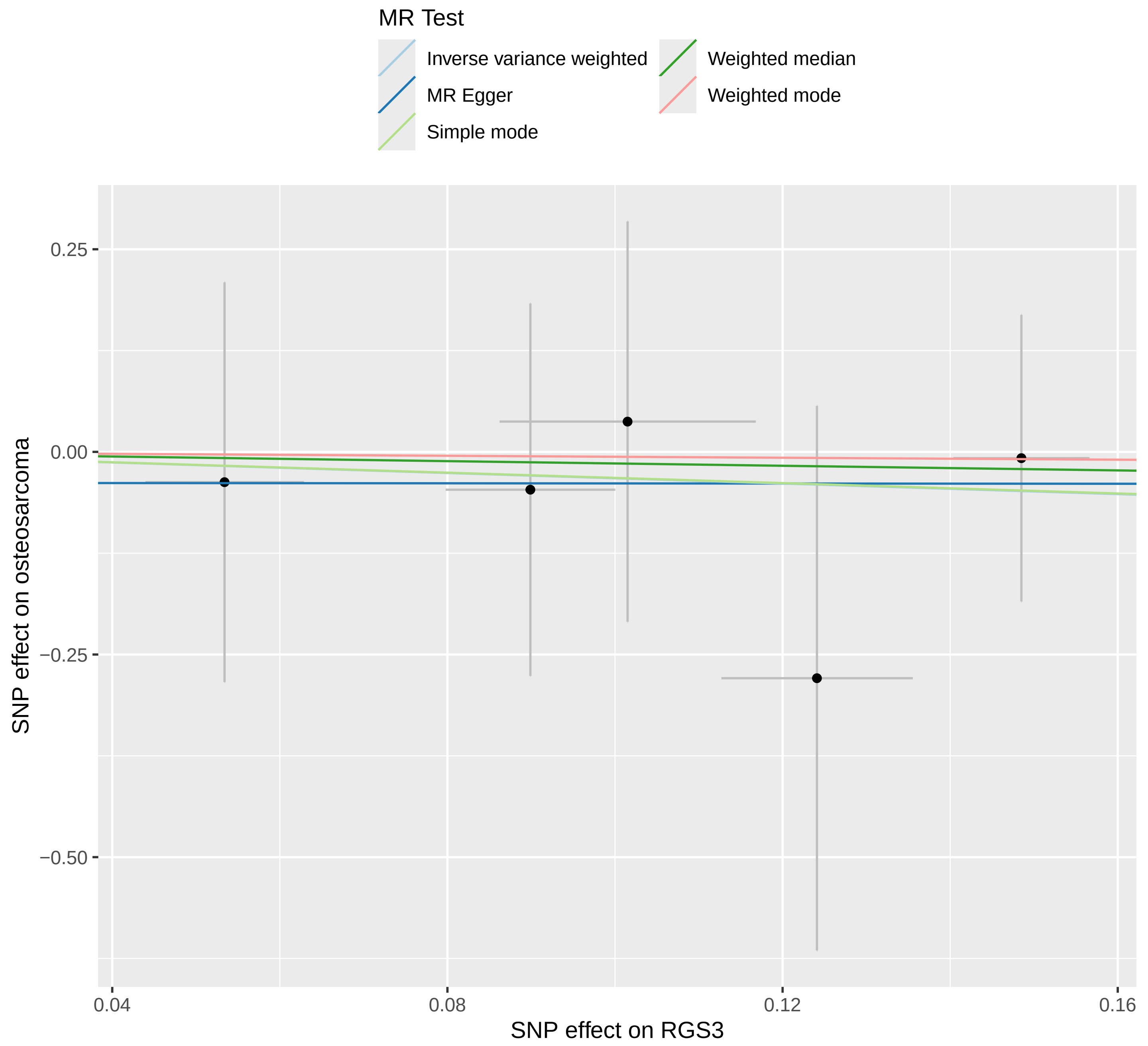

MR Test
Inverse variance weighted
MR Egger
Simple mode
Weighted median
Weighted mode
0.25
0.00
−0.25
−0.50
SNP effect on osteosarcoma
0.04
0.08
0.12
0.16
SNP effect on RGS3

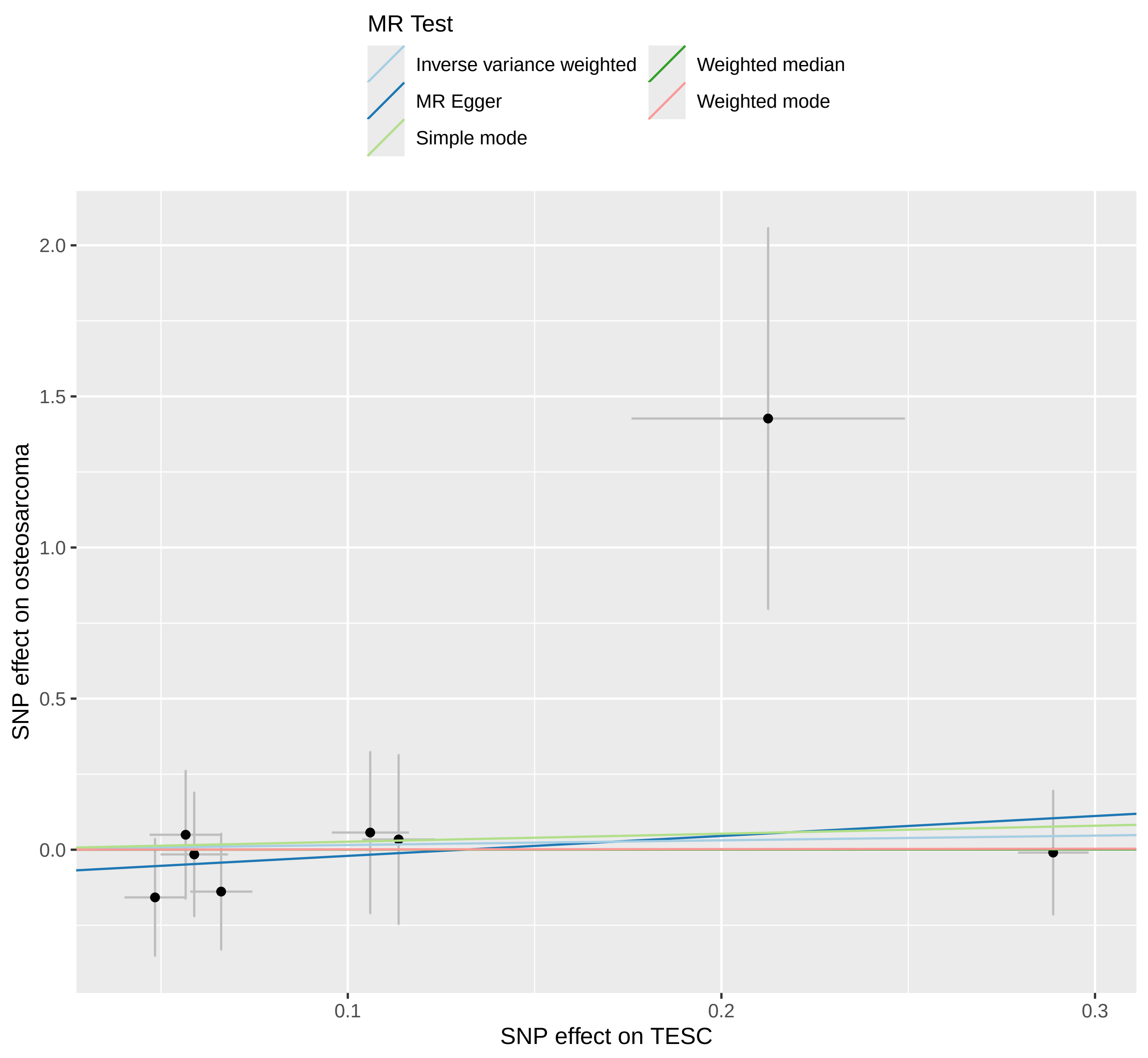
MR Test
Inverse variance weighted
MR Egger
Simple mode
Weighted median
Weighted mode
2.0
1.5
1.0
0.5
0.0
SNP effect on osteosarcoma
0.1
0.2
0.3
SNP effect on TESC

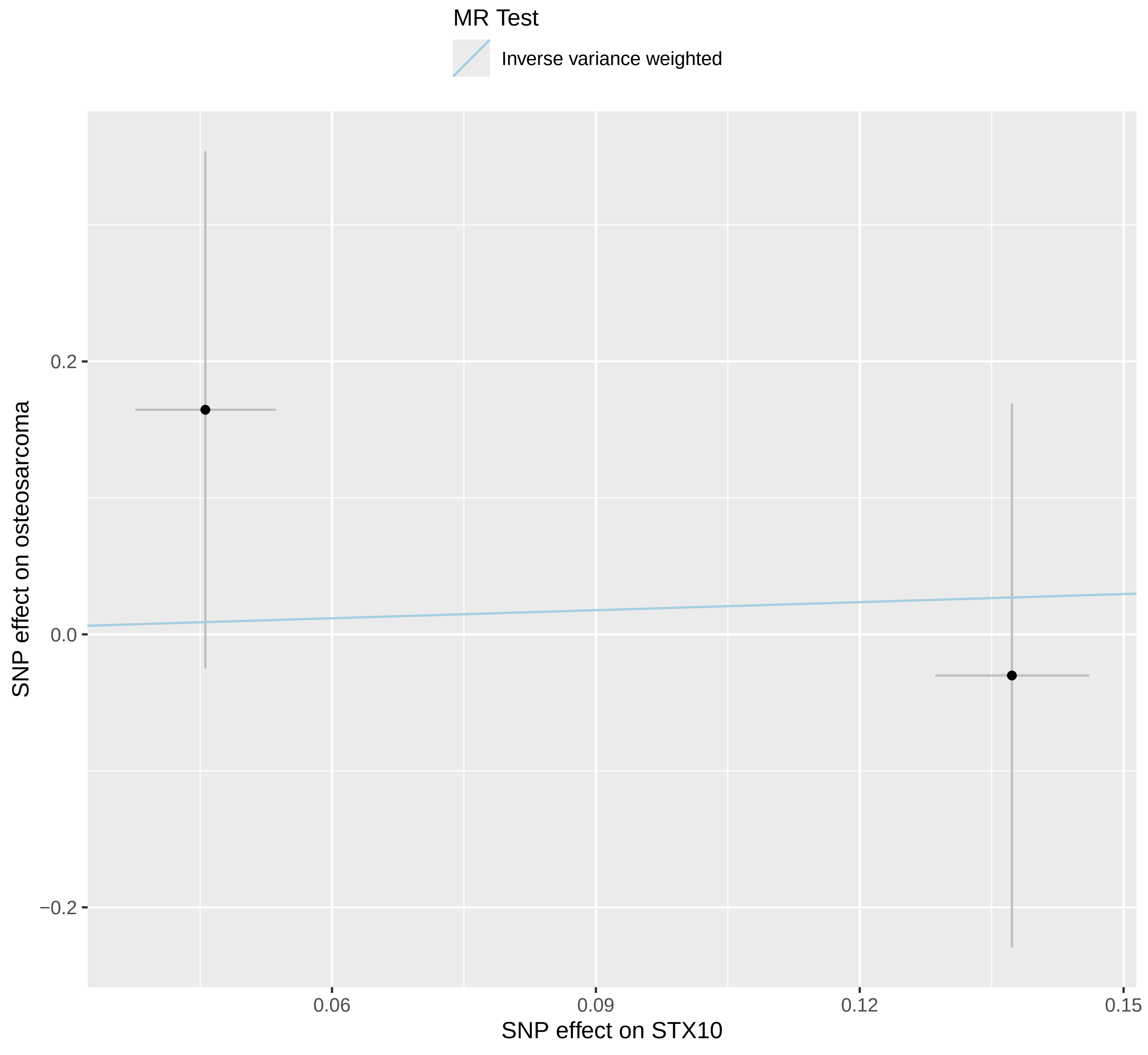

MR Test
Inverse variance weighted
0.2
0.0
−0.2
SNP effect on osteosarcoma
0.06
0.09
0.12
0.15
SNP effect on STX10

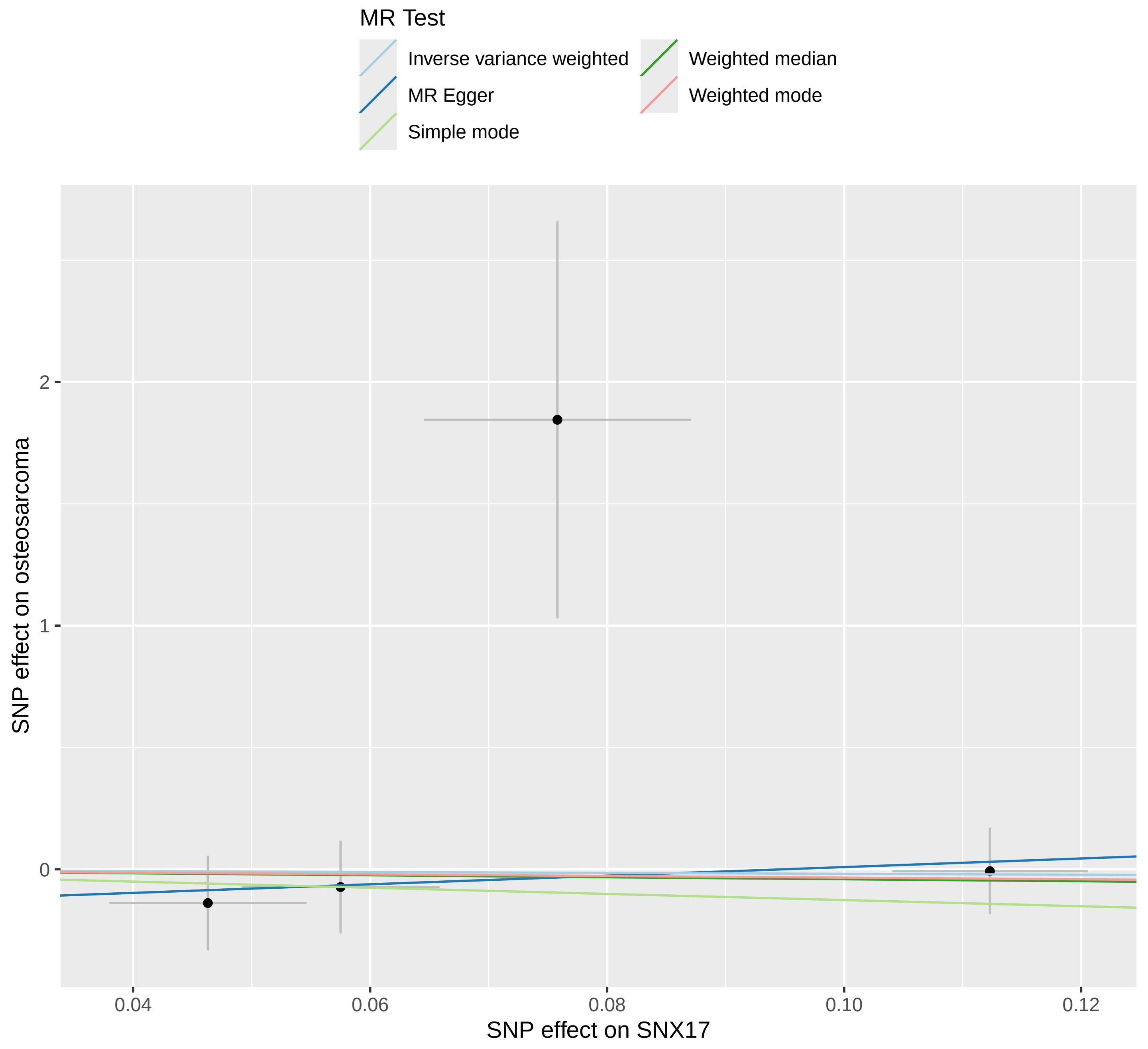
MR Test
Inverse variance weighted
MR Egger
Simple mode
Weighted median
Weighted mode
SNP effect on osteosarcoma
2
1
0
0.04
0.06
0.08
0.10
0.12
SNP effect on SNX17

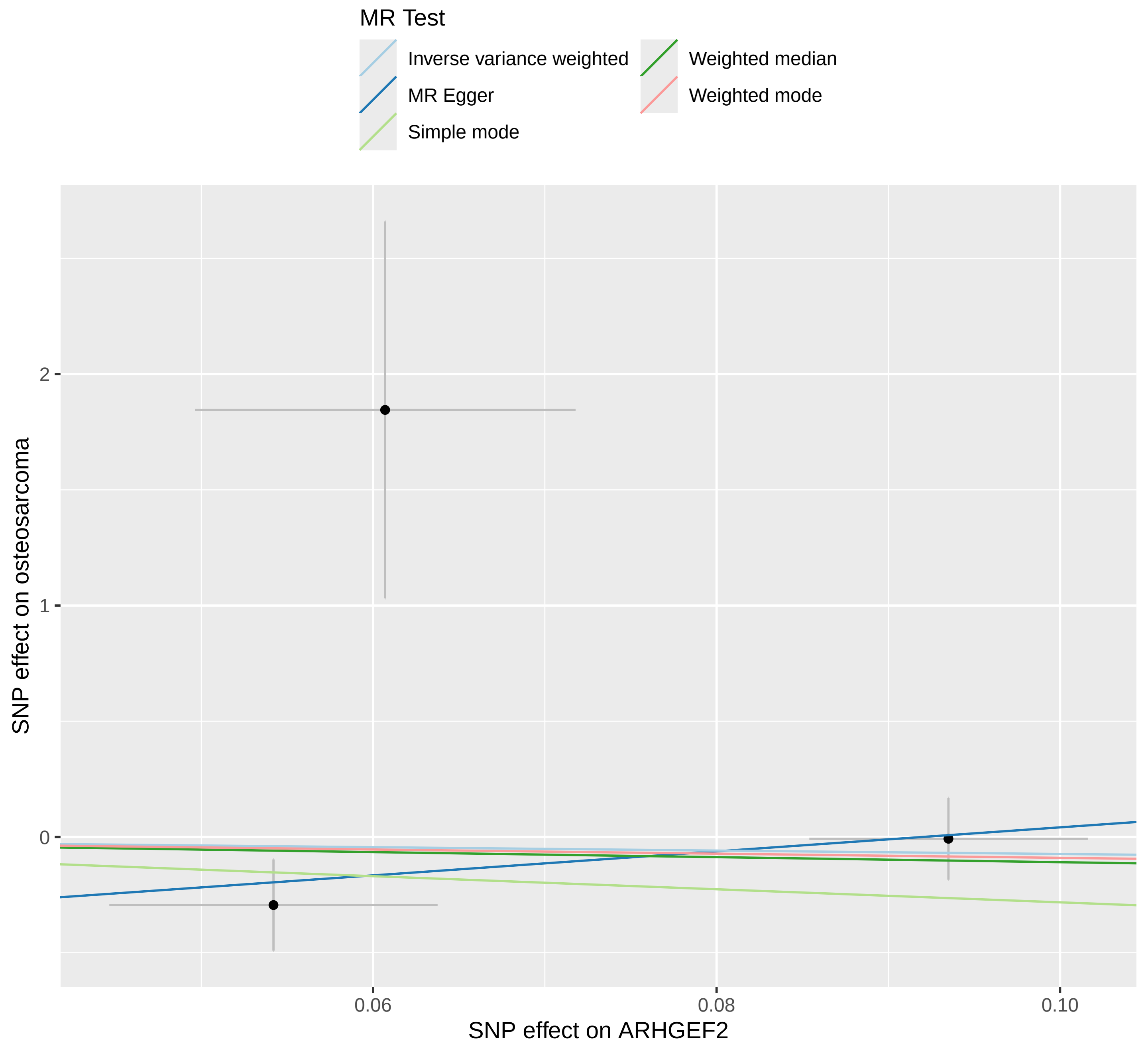
MR Test
Inverse variance weighted
MR Egger
Simple mode
Weighted median
Weighted mode
2
1
0
SNP effect on osteosarcoma
0.06
0.08
0.10
SNP effect on ARHGEF2

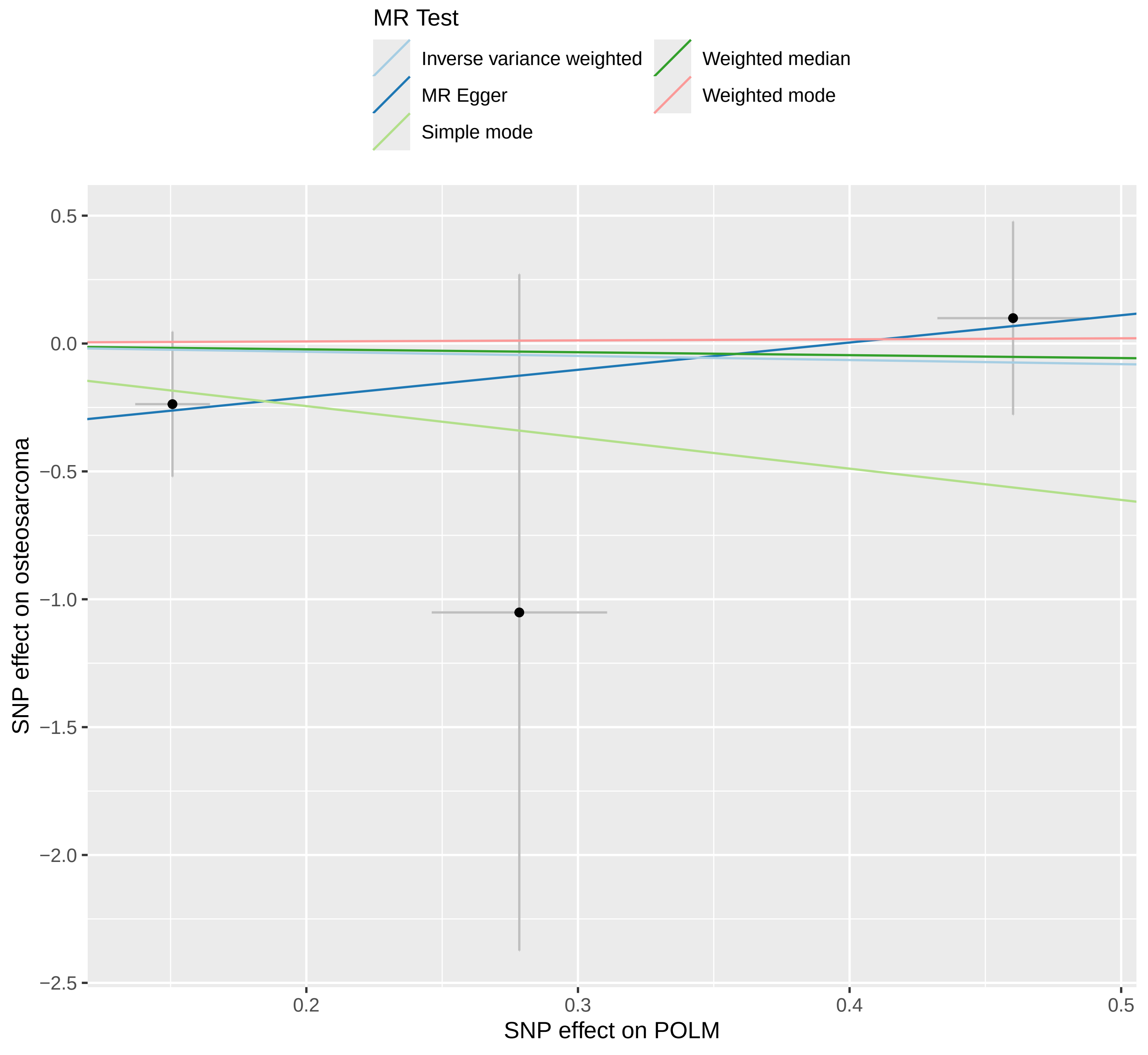

MR Test
Inverse variance weighted
MR Egger
Simple mode
Weighted median
Weighted mode
0.5
0.0
−0.5
−1.0
−1.5
−2.0
−2.5
SNP effect on osteosarcoma
0.2
0.3
0.4
0.5
SNP effect on POLM

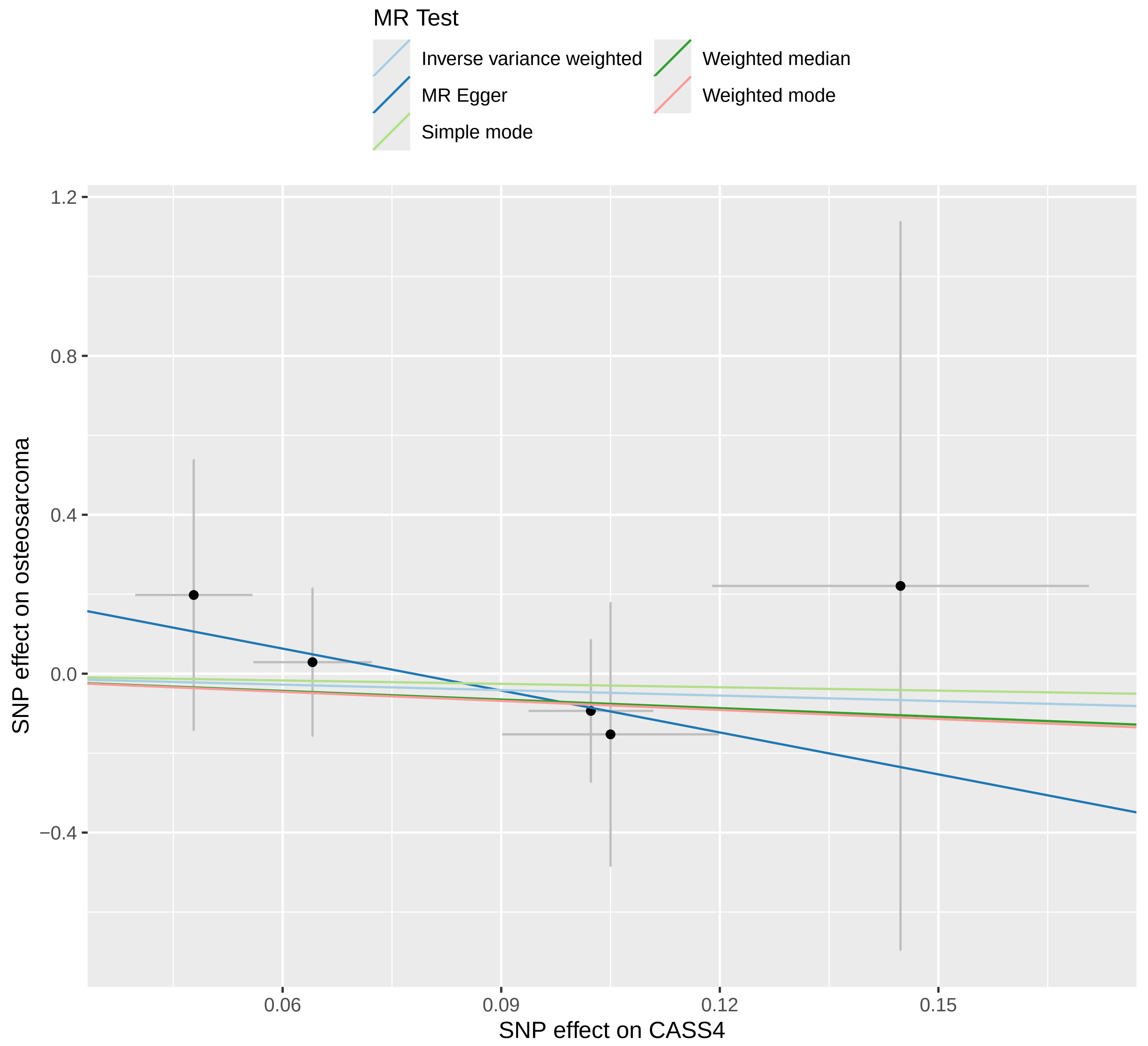
MR Test
Inverse variance weighted
MR Egger
Simple mode
Weighted median
Weighted mode
1.2
0.8
0.4
0.0
−0.4
SNP effect on osteosarcoma
0.06
0.09
0.12
0.15
SNP effect on CASS4

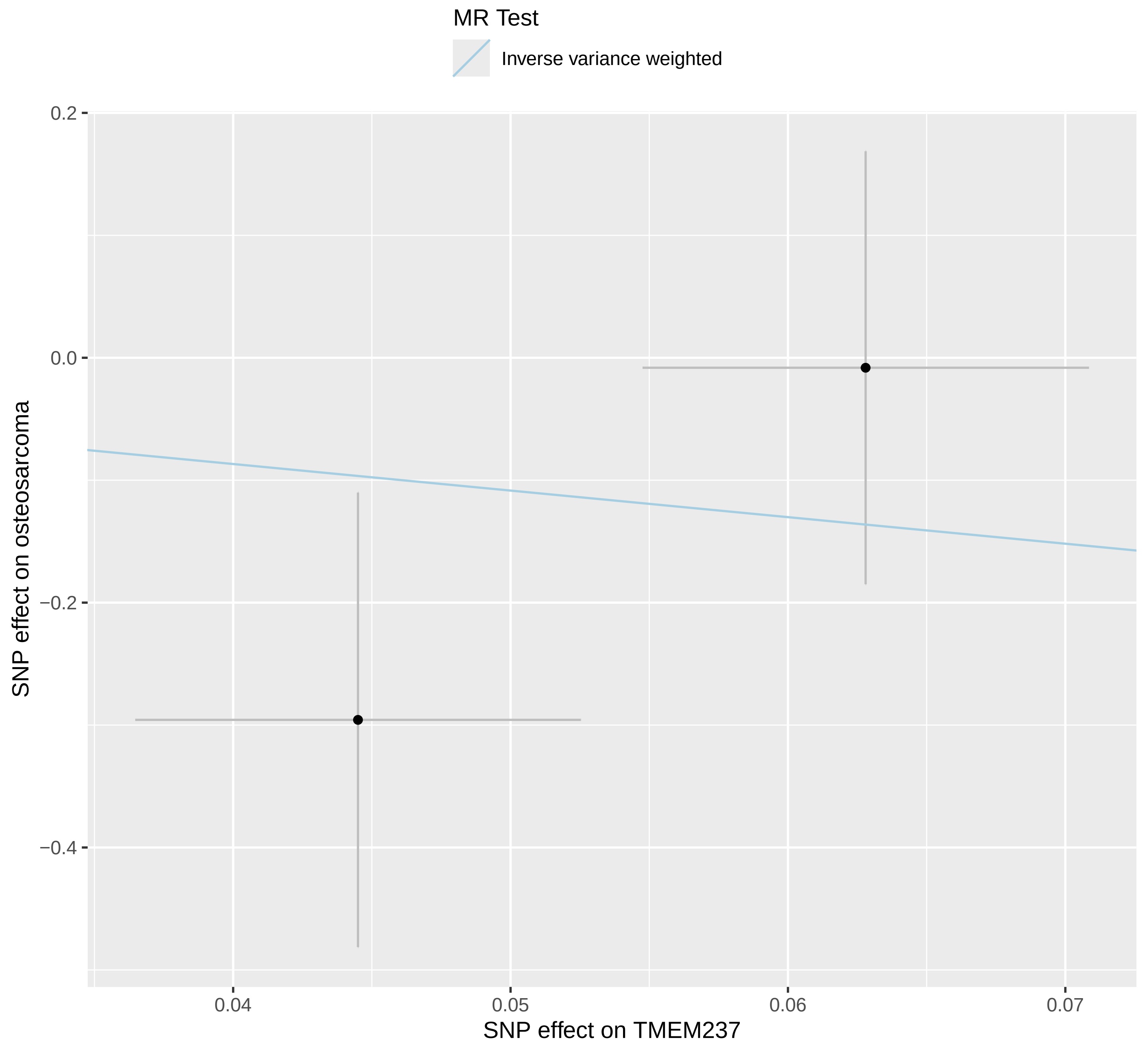

MR Test
Inverse variance weighted
0.2
0.0
−0.2
−0.4
SNP effect on osteosarcoma
0.04
0.05
0.06
0.07
SNP effect on TMEM237

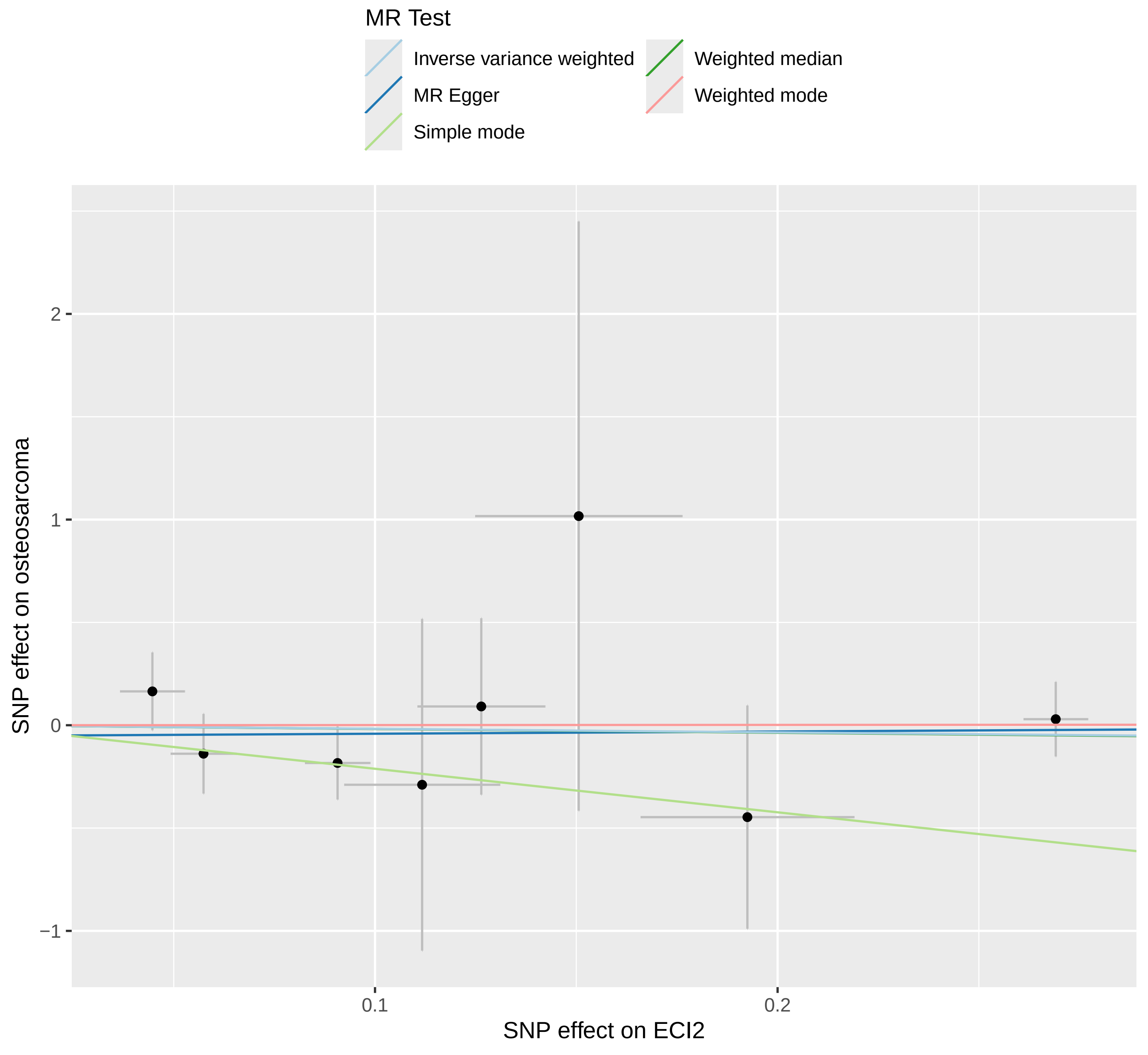

MR Test
Inverse variance weighted
MR Egger
Simple mode
Weighted median
Weighted mode
2
1
0
−1
0.1
0.2
SNP effect on ECI2
SNP effect on osteosarcoma

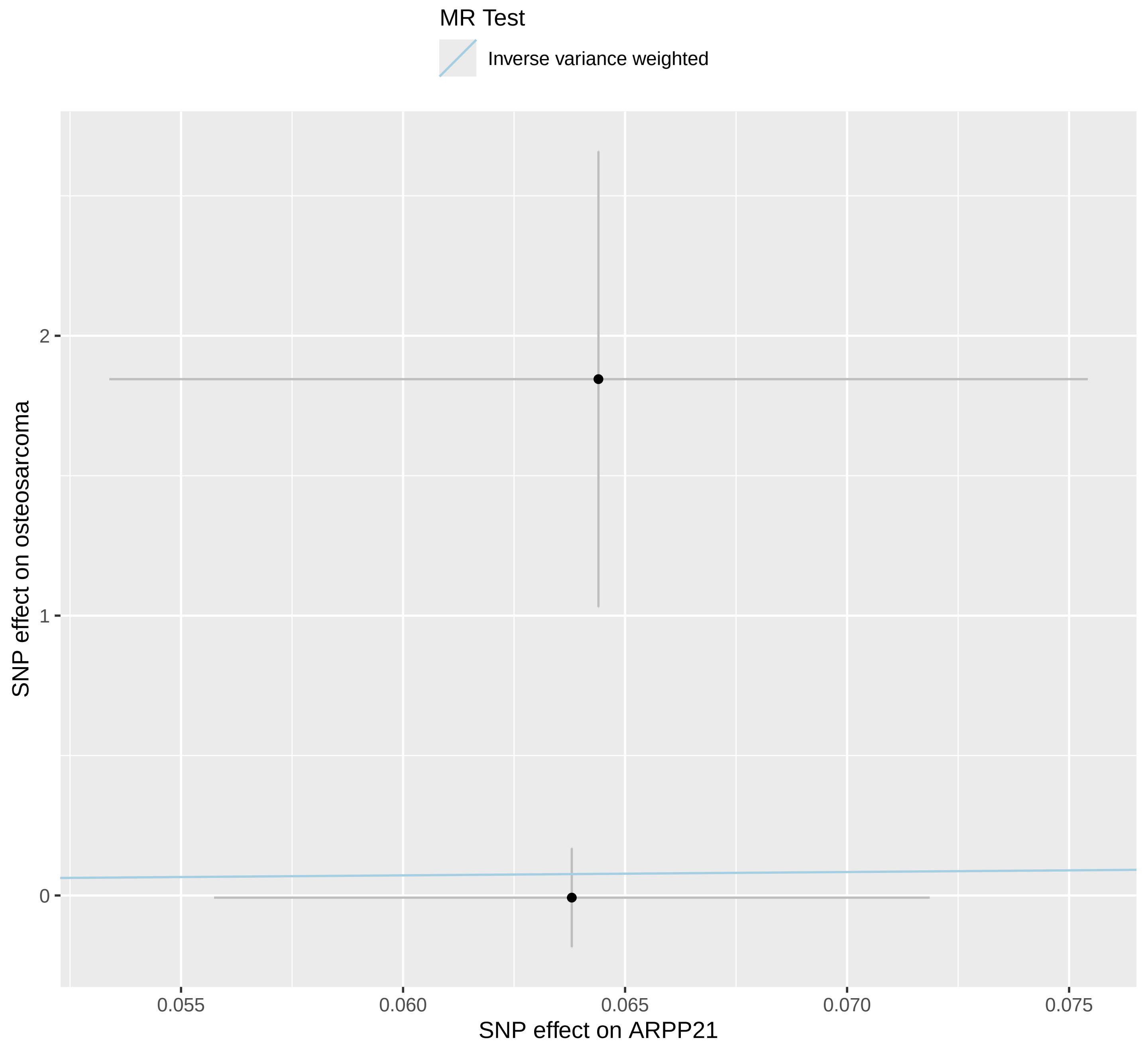

MR Test
Inverse variance weighted
SNP effect on osteosarcoma
2
1
0
0.055
0.060
0.065
0.070
0.075
SNP effect on ARPP21

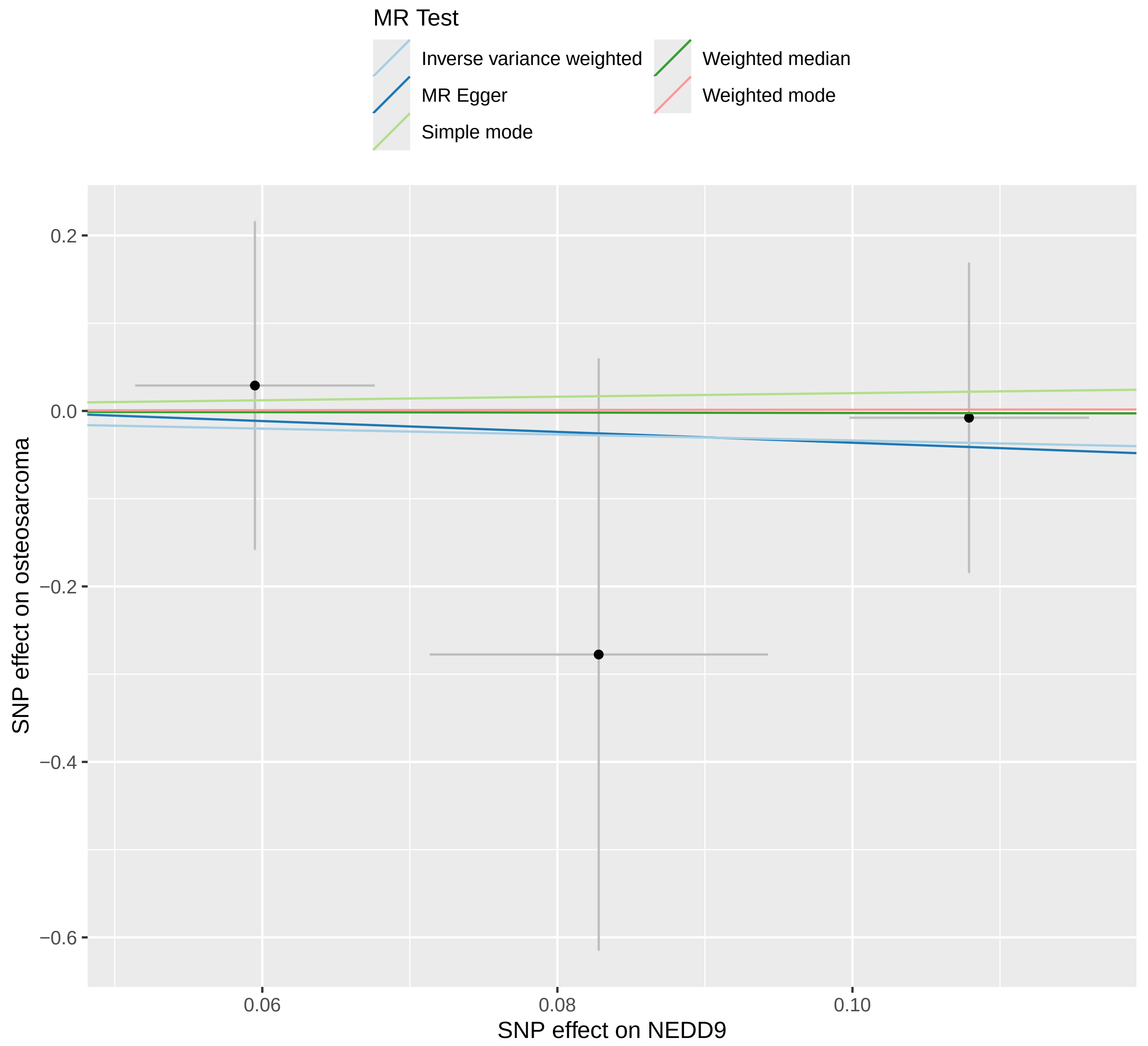

MR Test
Inverse variance weighted
MR Egger
Simple mode
Weighted median
Weighted mode
0.2
0.0
−0.2
−0.4
−0.6
0.06
0.08
0.10
SNP effect on osteosarcoma
SNP effect on NEDD9

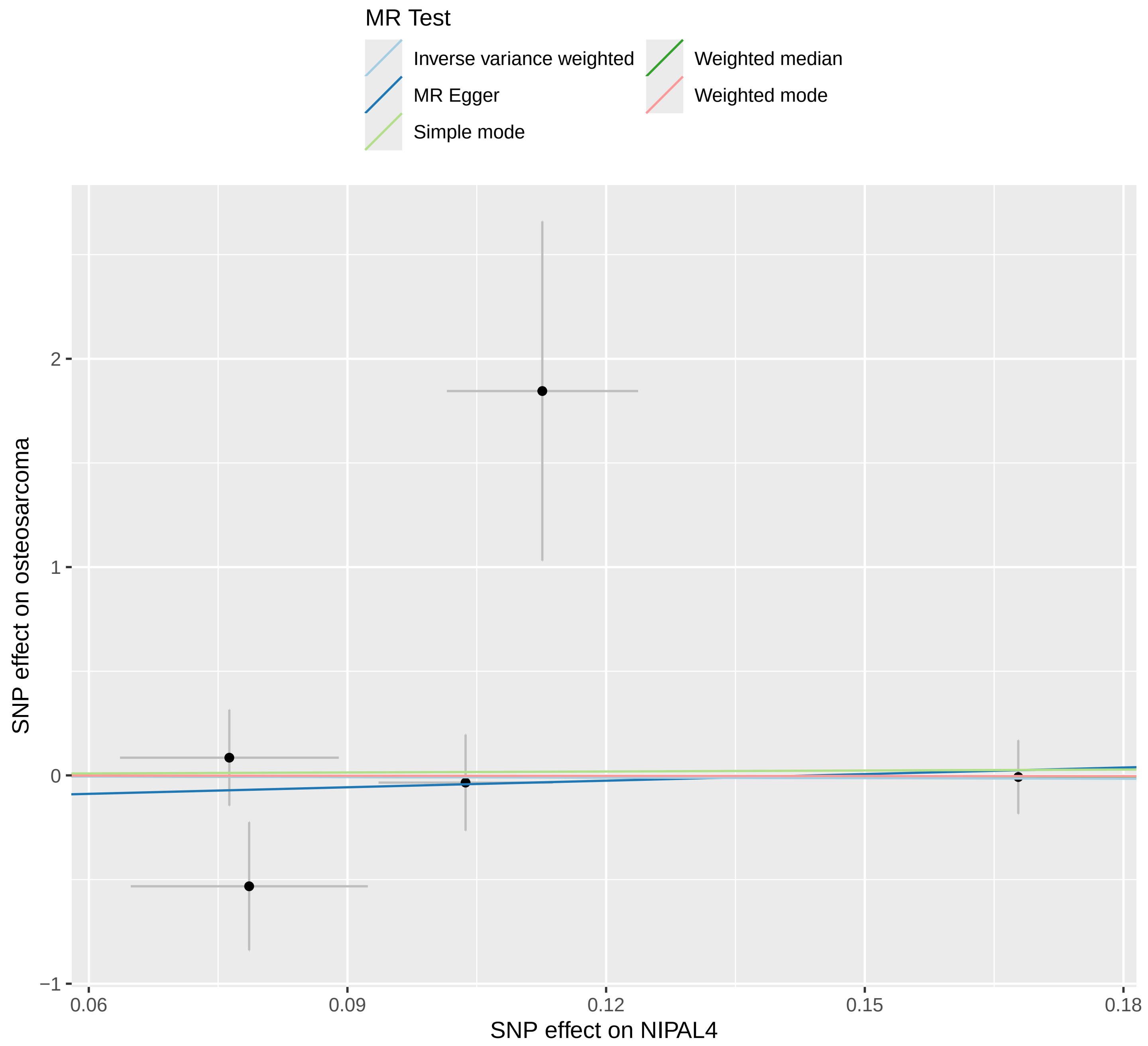
MR Test
Inverse variance weighted
MR Egger
Simple mode
Weighted median
Weighted mode
SNP effect on osteosarcoma
2
1
0
−1
0.06
0.09
0.12
0.15
0.18
SNP effect on NIPAL4

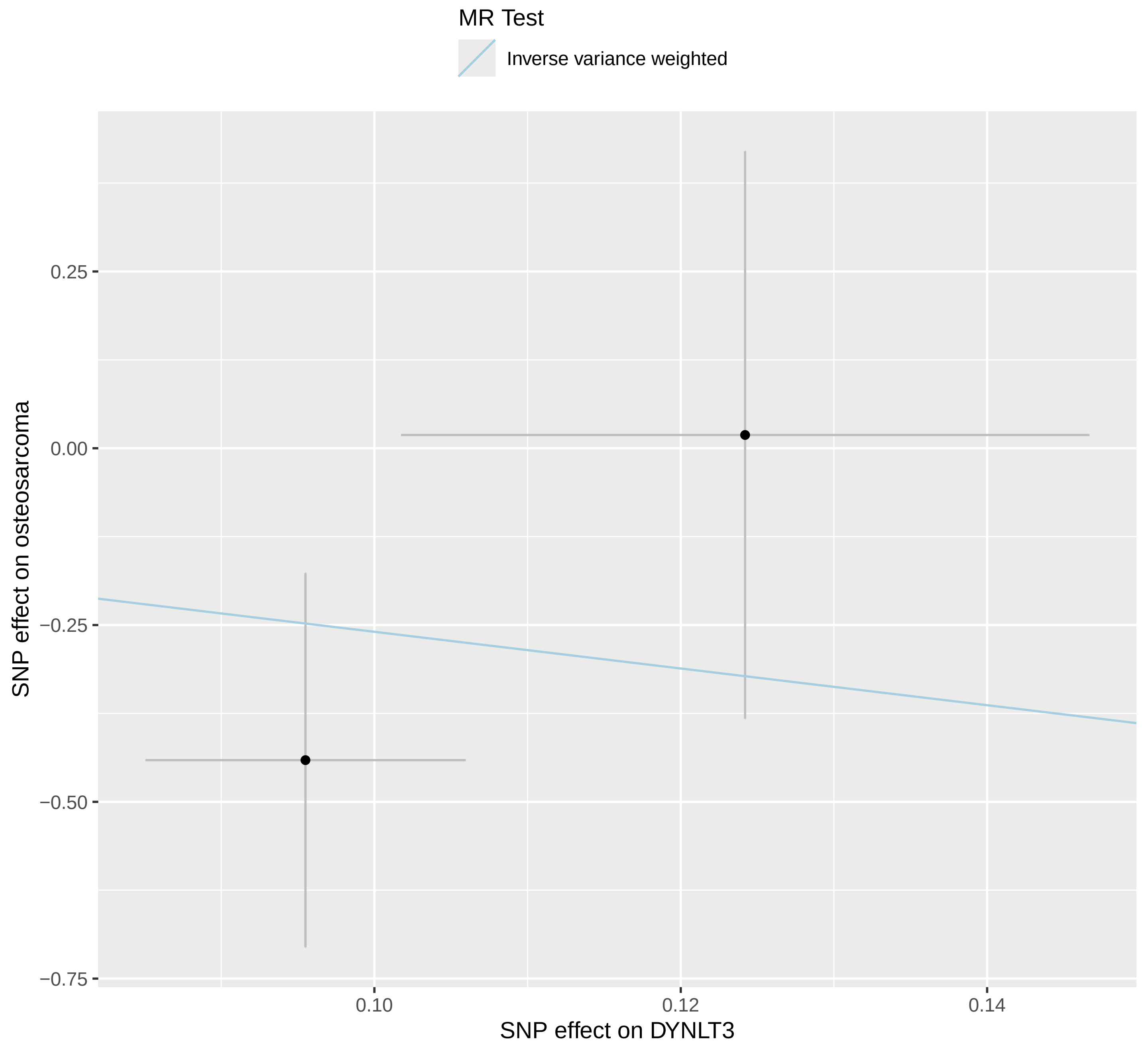
MR Test
Inverse variance weighted
SNP effect on osteosarcoma
0.25
0.00
−0.25
−0.50
−0.75
0.10
0.12
0.14
SNP effect on DYNLT3

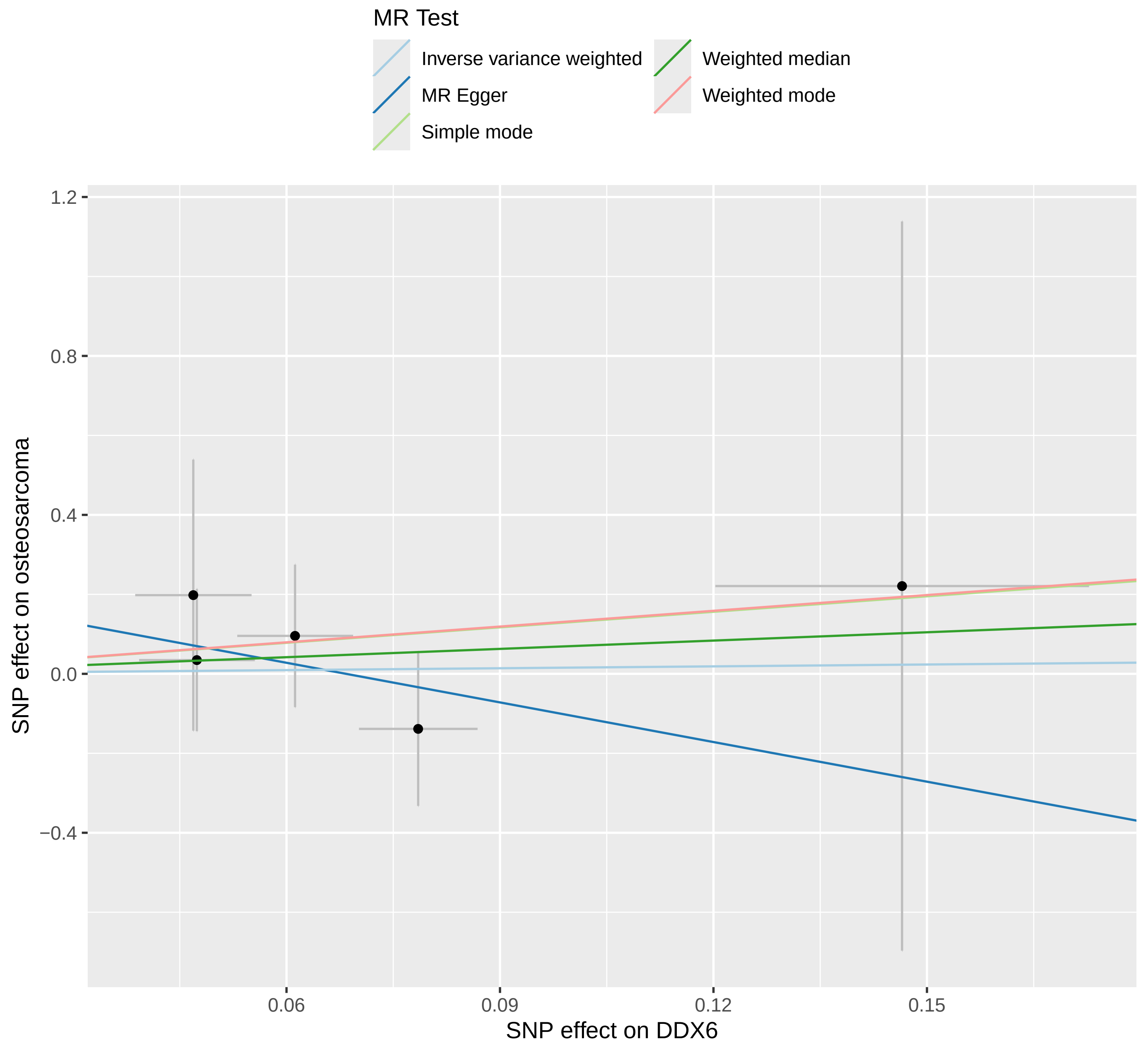

MR Test
Inverse variance weighted
MR Egger
Simple mode
Weighted median
Weighted mode
1.2
0.8
0.4
0.0
−0.4
SNP effect on osteosarcoma
0.06
0.09
0.12
0.15
SNP effect on DDX6

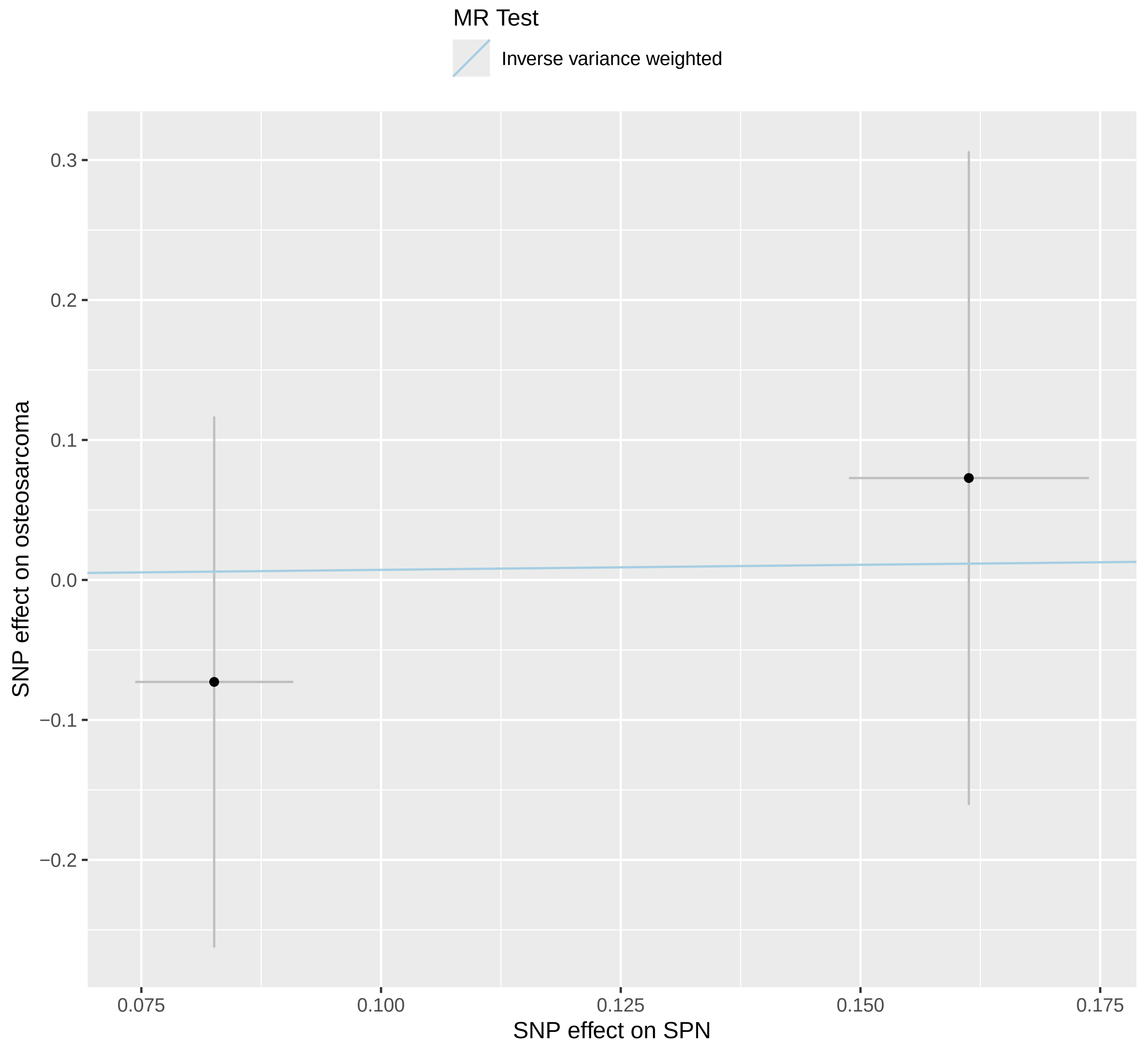
MR Test
Inverse variance weighted
SNP effect on osteosarcoma
0.3
0.2
0.1
0.0
−0.1
−0.2
0.075
0.100
0.125
0.150
0.175
SNP effect on SPN

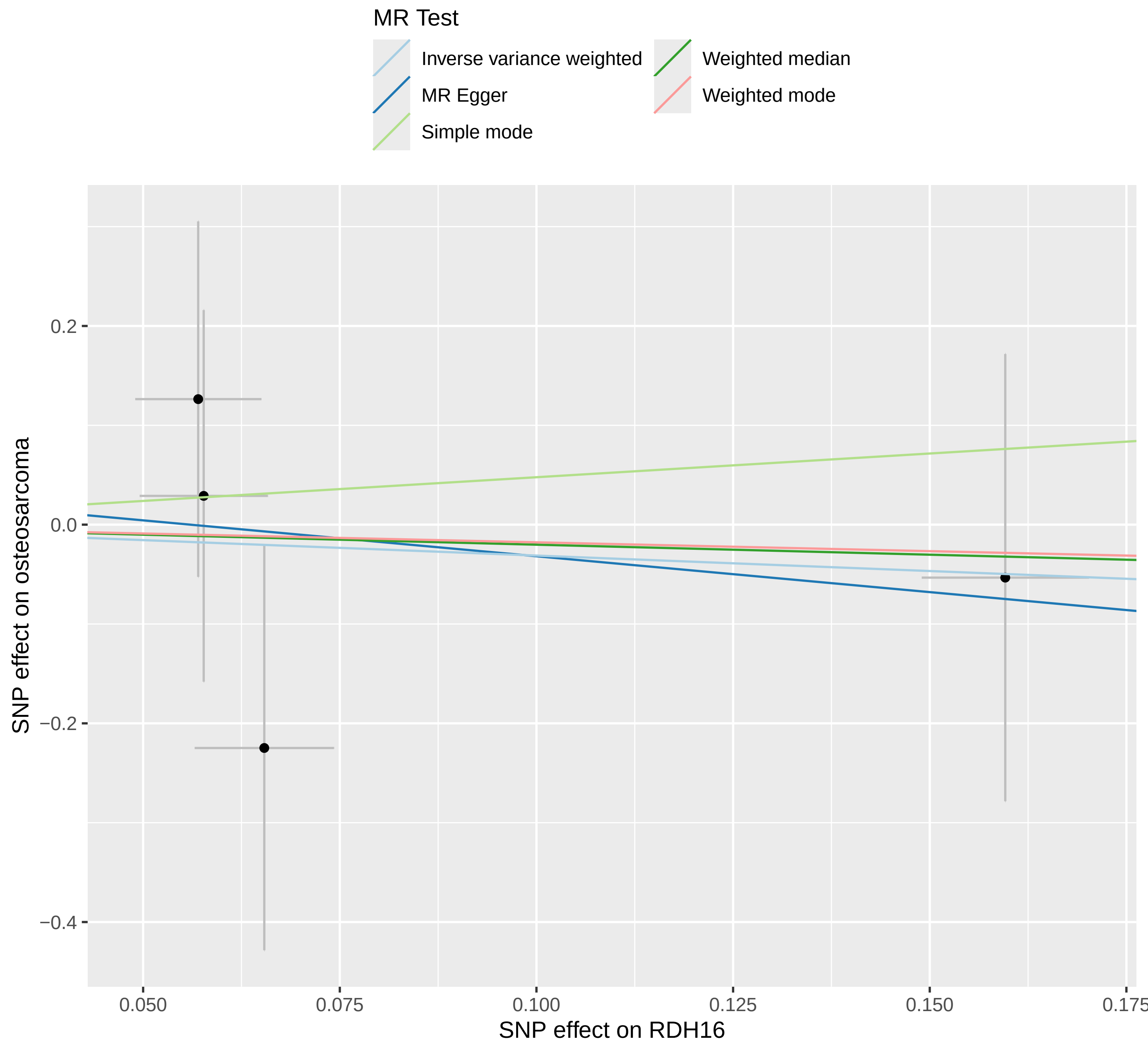
MR Test
Inverse variance weighted
MR Egger
Simple mode
Weighted median
Weighted mode
0.2
0.0
−0.2
−0.4
SNP effect on osteosarcoma
0.050
0.075
0.100
0.125
0.150
0.175
SNP effect on RDH16

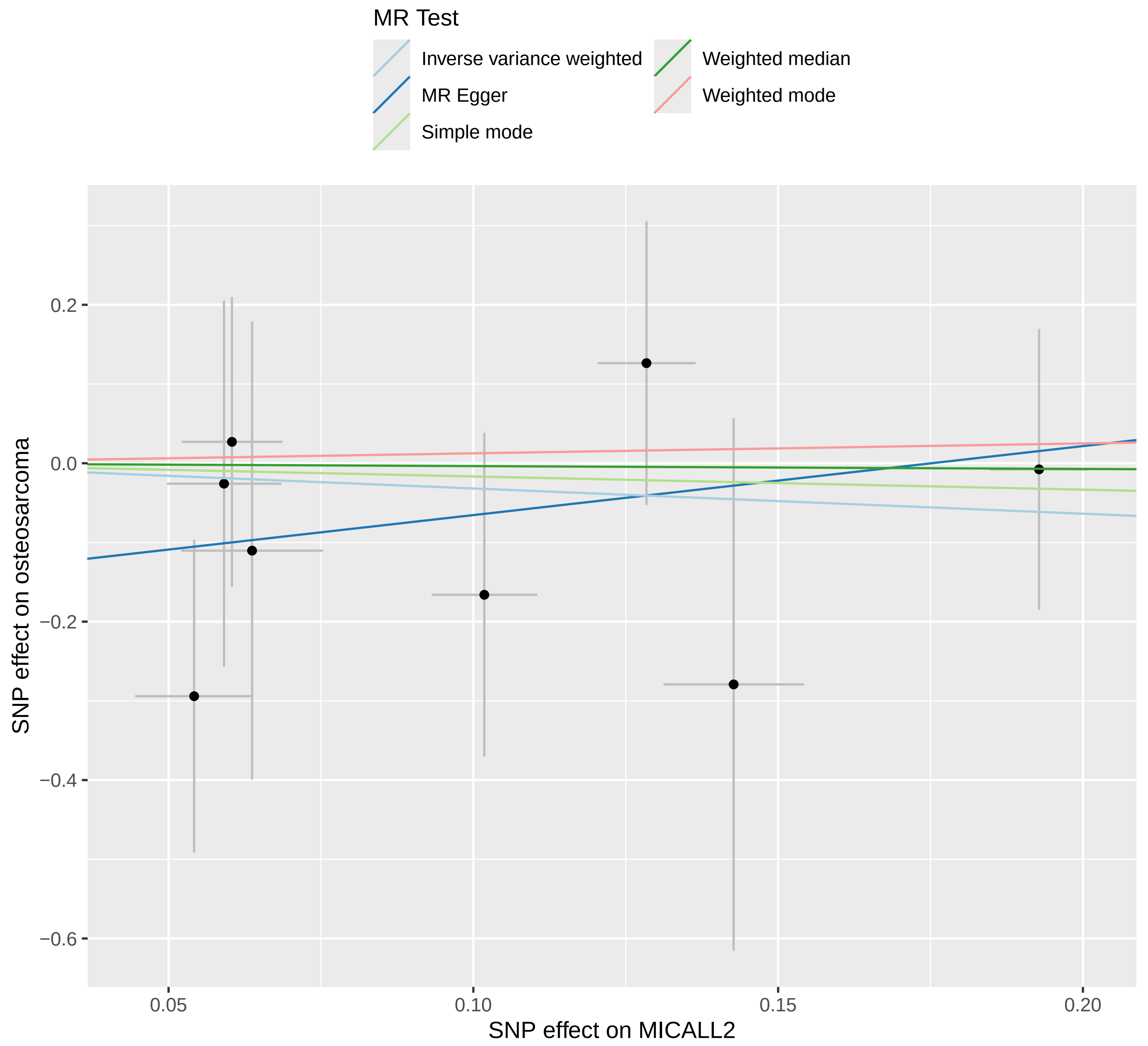

MR Test
Inverse variance weighted
MR Egger
Simple mode
Weighted median
Weighted mode
0.2
0.0
−0.2
−0.4
−0.6
SNP effect on osteosarcoma
0.05
0.10
0.15
0.20
SNP effect on MICALL2

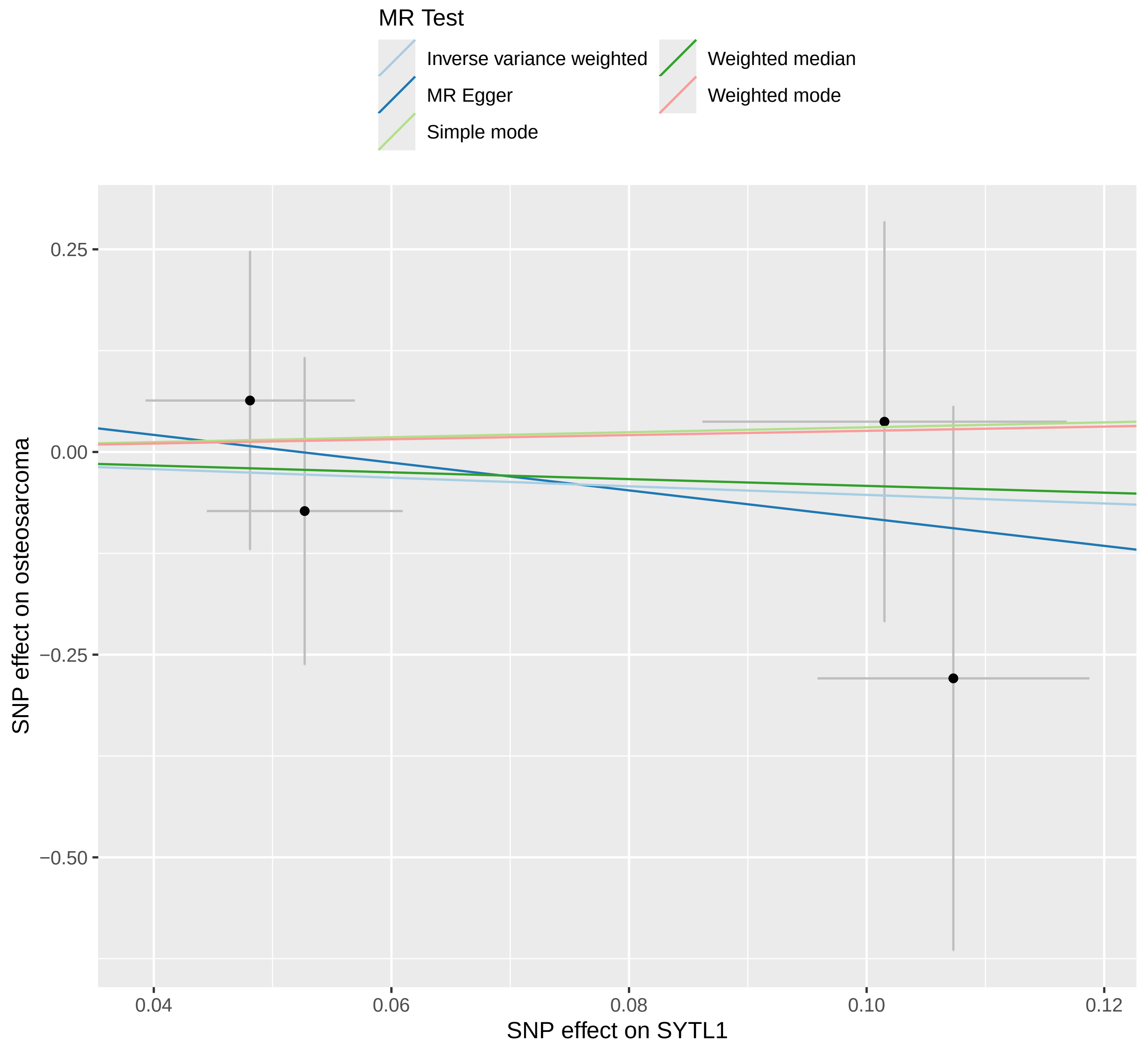
MR Test
Inverse variance weighted
MR Egger
Simple mode
Weighted median
Weighted mode
0.25
0.00
−0.25
−0.50
SNP effect on osteosarcoma
0.04
0.06
0.08
0.10
0.12
SNP effect on SYTL1

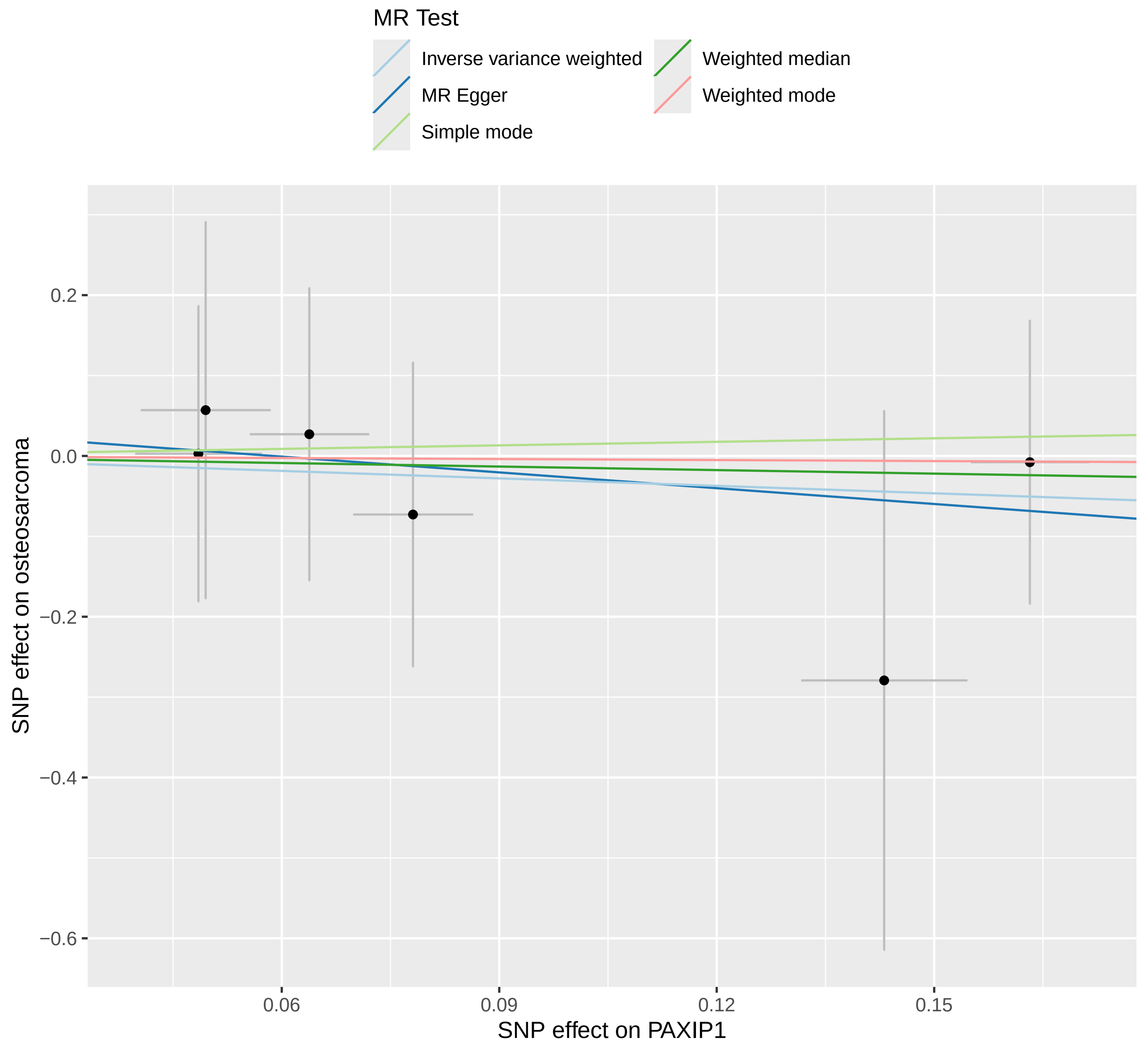

MR Test
Inverse variance weighted
MR Egger
Simple mode
Weighted median
Weighted mode
0.2
0.0
−0.2
−0.4
−0.6
SNP effect on osteosarcoma
0.06
0.09
0.12
0.15
SNP effect on PAXIP1

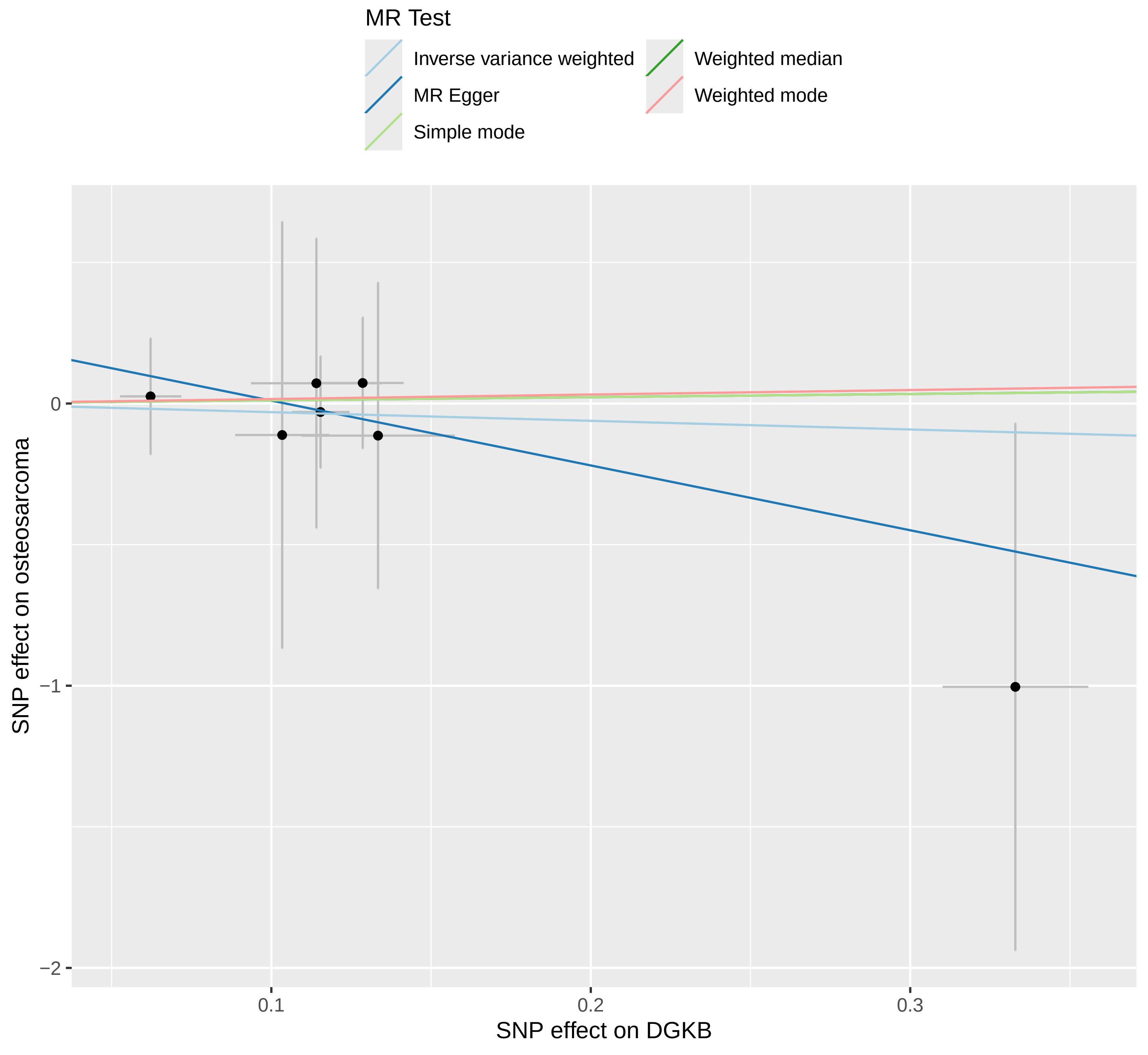
MR Test
Inverse variance weighted
MR Egger
Simple mode
Weighted median
Weighted mode
SNP effect on osteosarcoma
0
−1
−2
0.1
0.2
0.3
SNP effect on DGKB

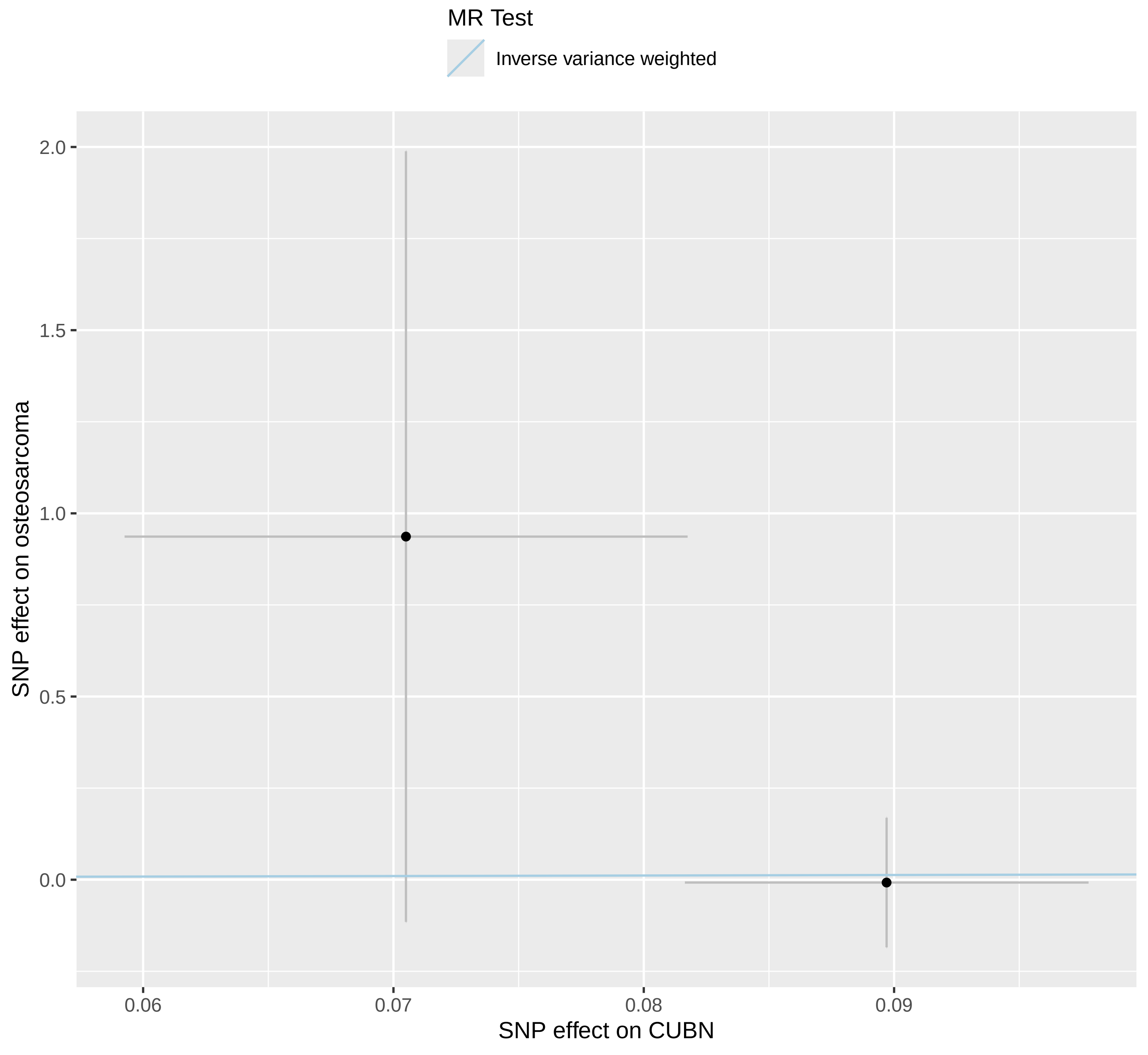

MR Test
Inverse variance weighted
2.0
1.5
1.0
0.5
0.0
0.06
0.07
0.08
0.09
SNP effect on osteosarcoma
SNP effect on CUBN

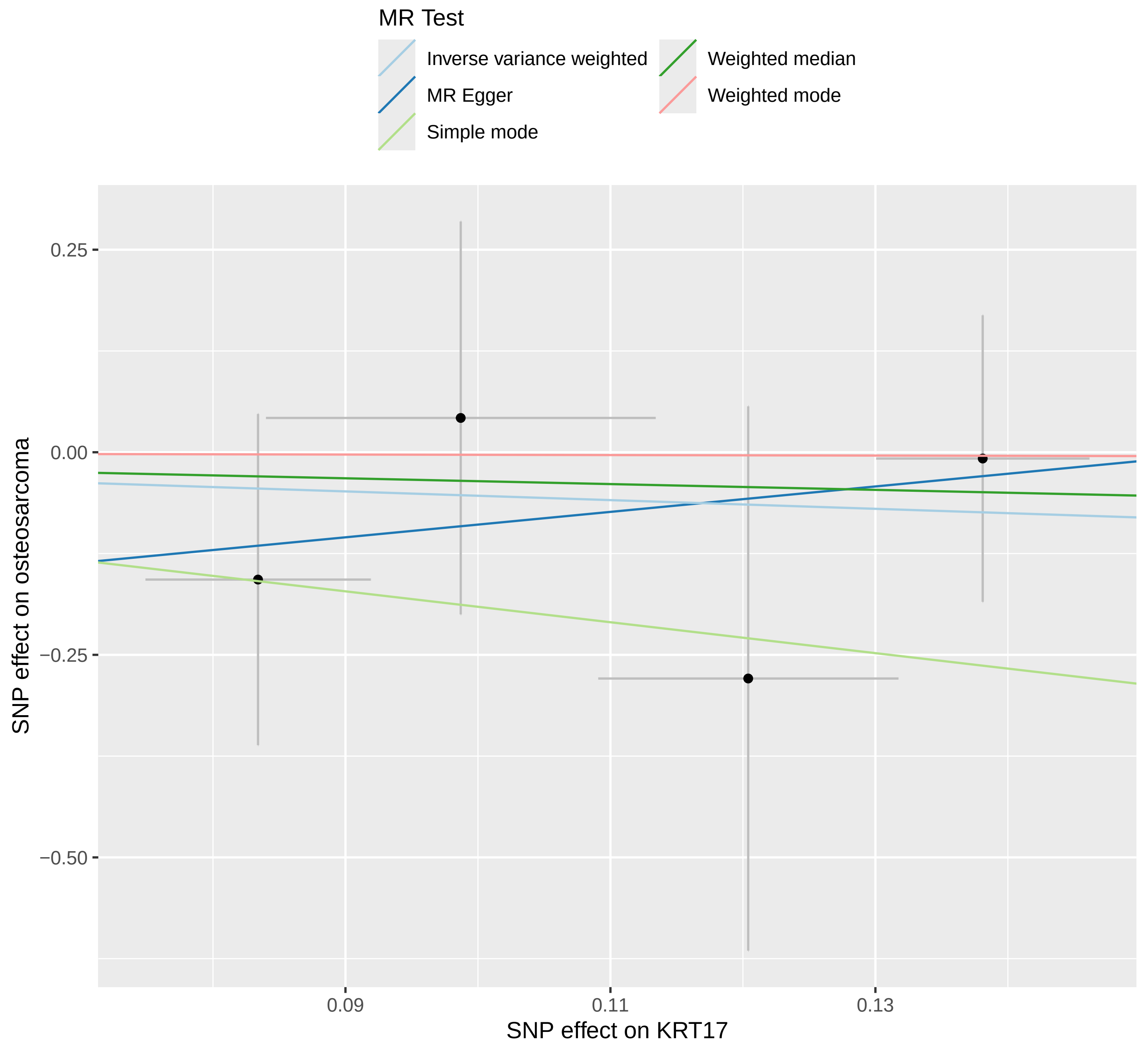

MR Test
Inverse variance weighted
MR Egger
Simple mode
Weighted median
Weighted mode
0.25
0.00
−0.25
−0.50
0.09
0.11
0.13
SNP effect on osteosarcoma
SNP effect on KRT17

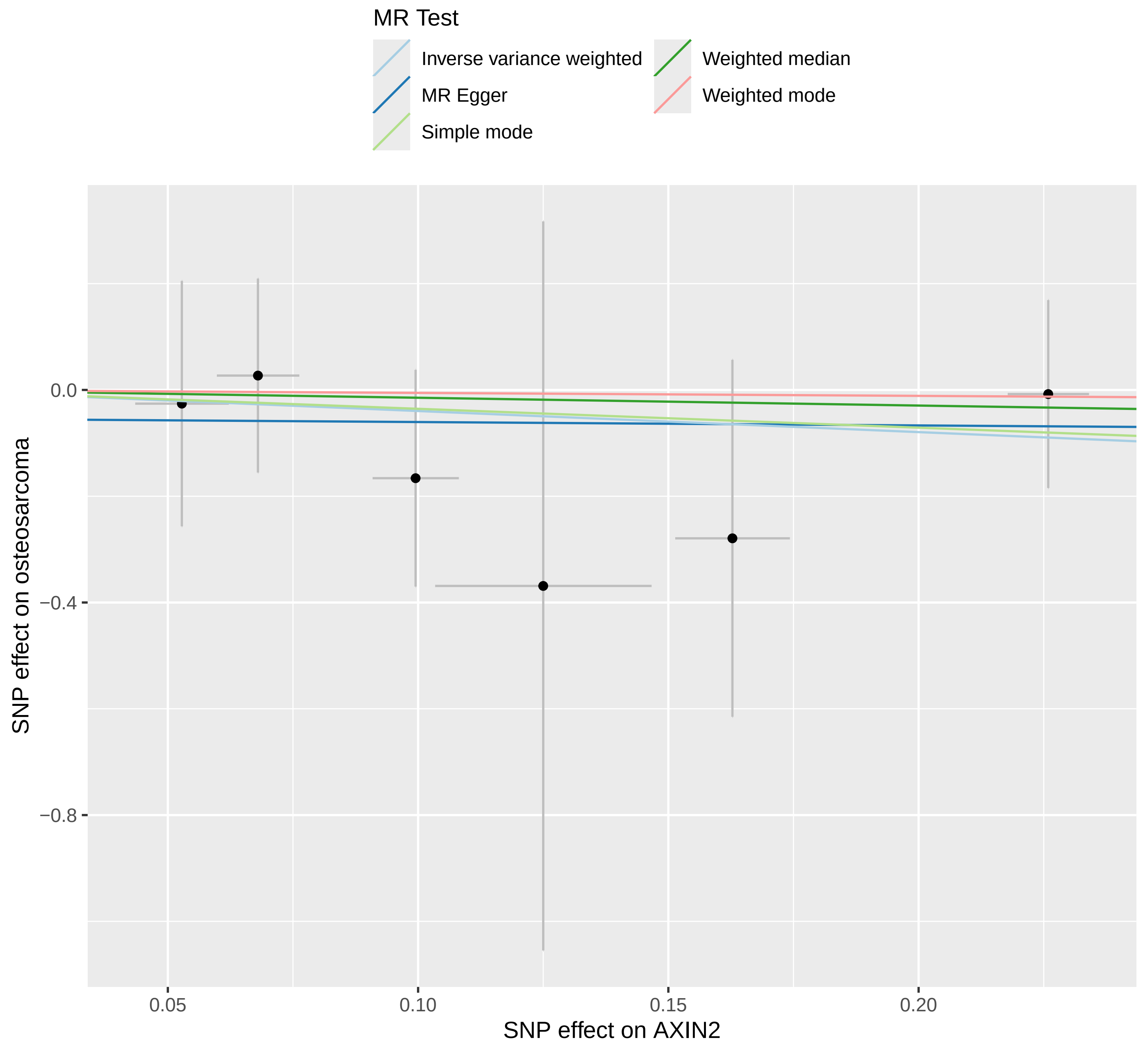
MR Test
Inverse variance weighted
MR Egger
Simple mode
Weighted median
Weighted mode
0.0
−0.4
−0.8
SNP effect on osteosarcoma
0.05
0.10
0.15
0.20
SNP effect on AXIN2

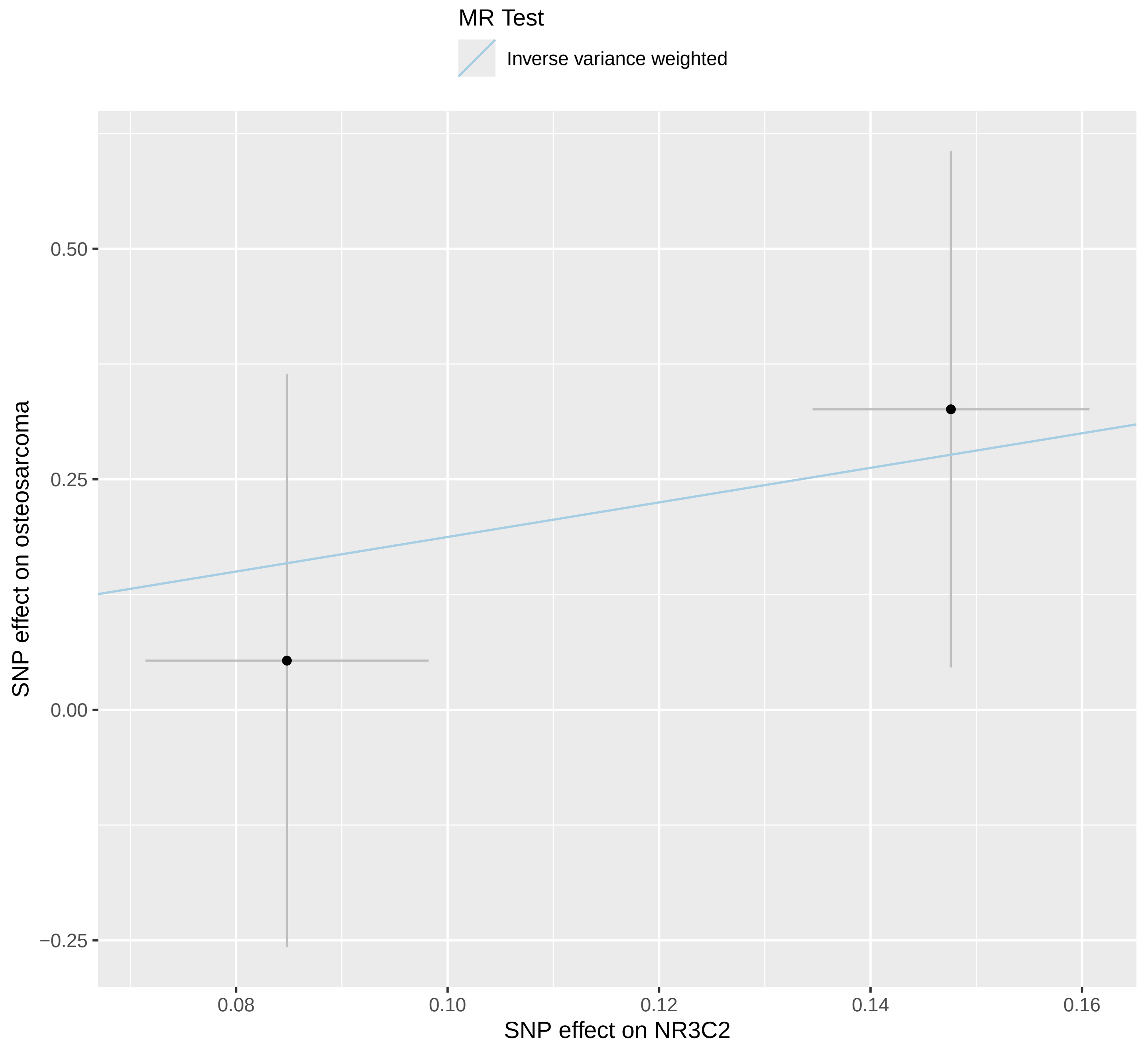

MR Test
Inverse variance weighted
0.50
0.25
0.00
−0.25
SNP effect on osteosarcoma
0.08
0.10
0.12
0.14
0.16
SNP effect on NR3C2

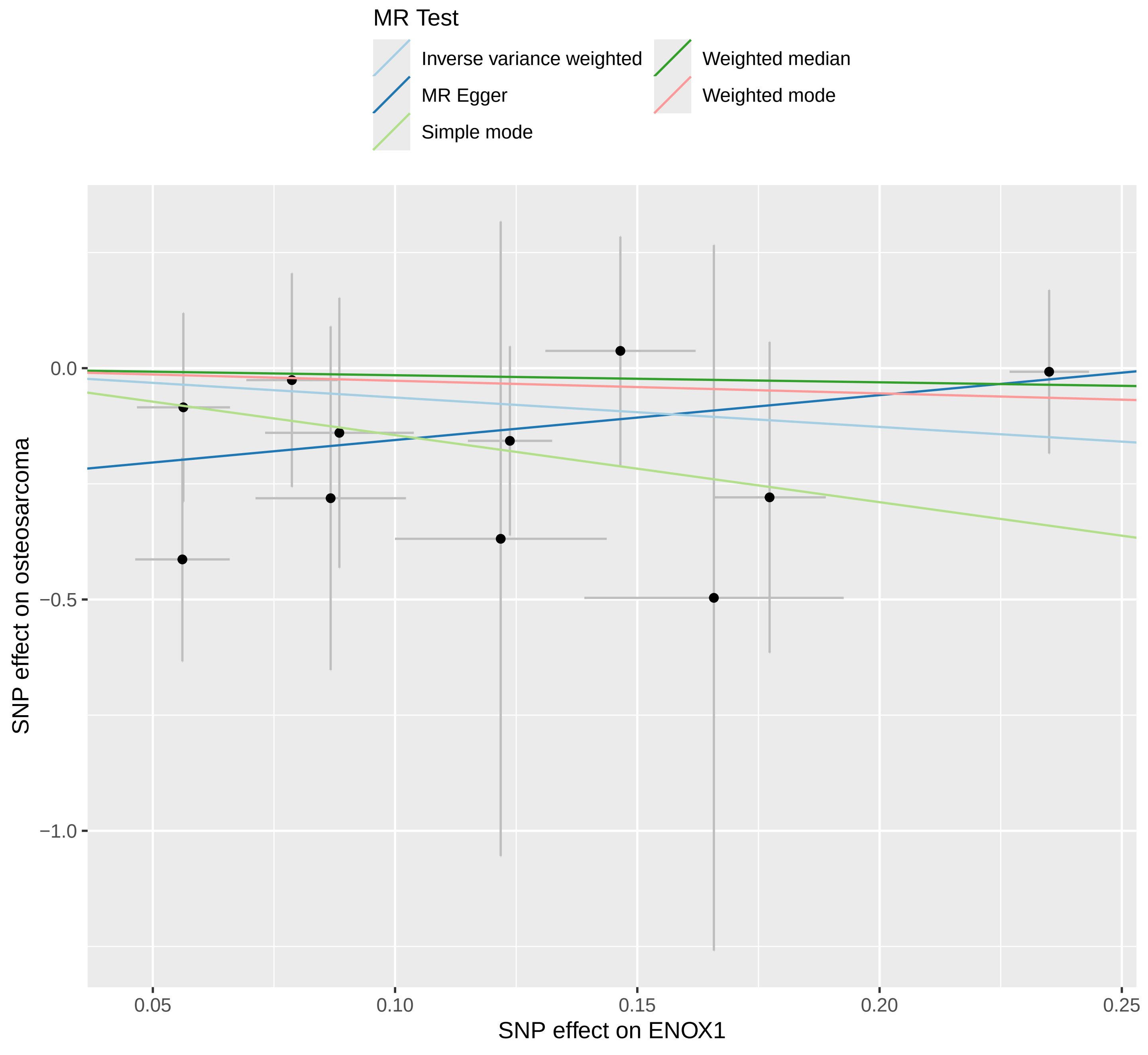

MR Test
Inverse variance weighted
MR Egger
Simple mode
Weighted median
Weighted mode
0.0
−0.5
−1.0
0.05
0.10
0.15
0.20
0.25
SNP effect on ENOX1
SNP effect on osteosarcoma

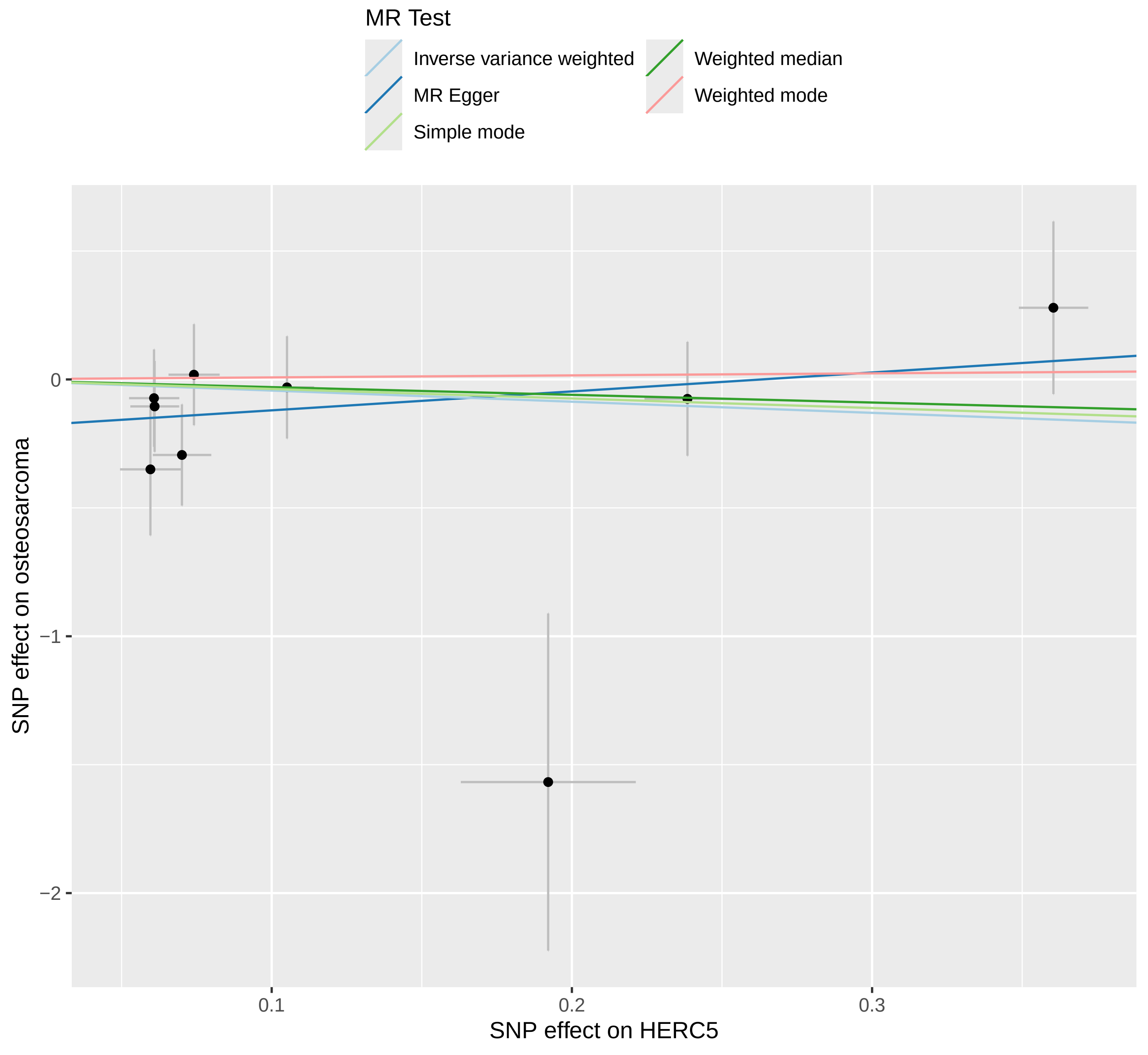
MR Test
Inverse variance weighted
MR Egger
Simple mode
Weighted median
Weighted mode
0
−1
−2
SNP effect on osteosarcoma
0.1
0.2
0.3
SNP effect on HERC5

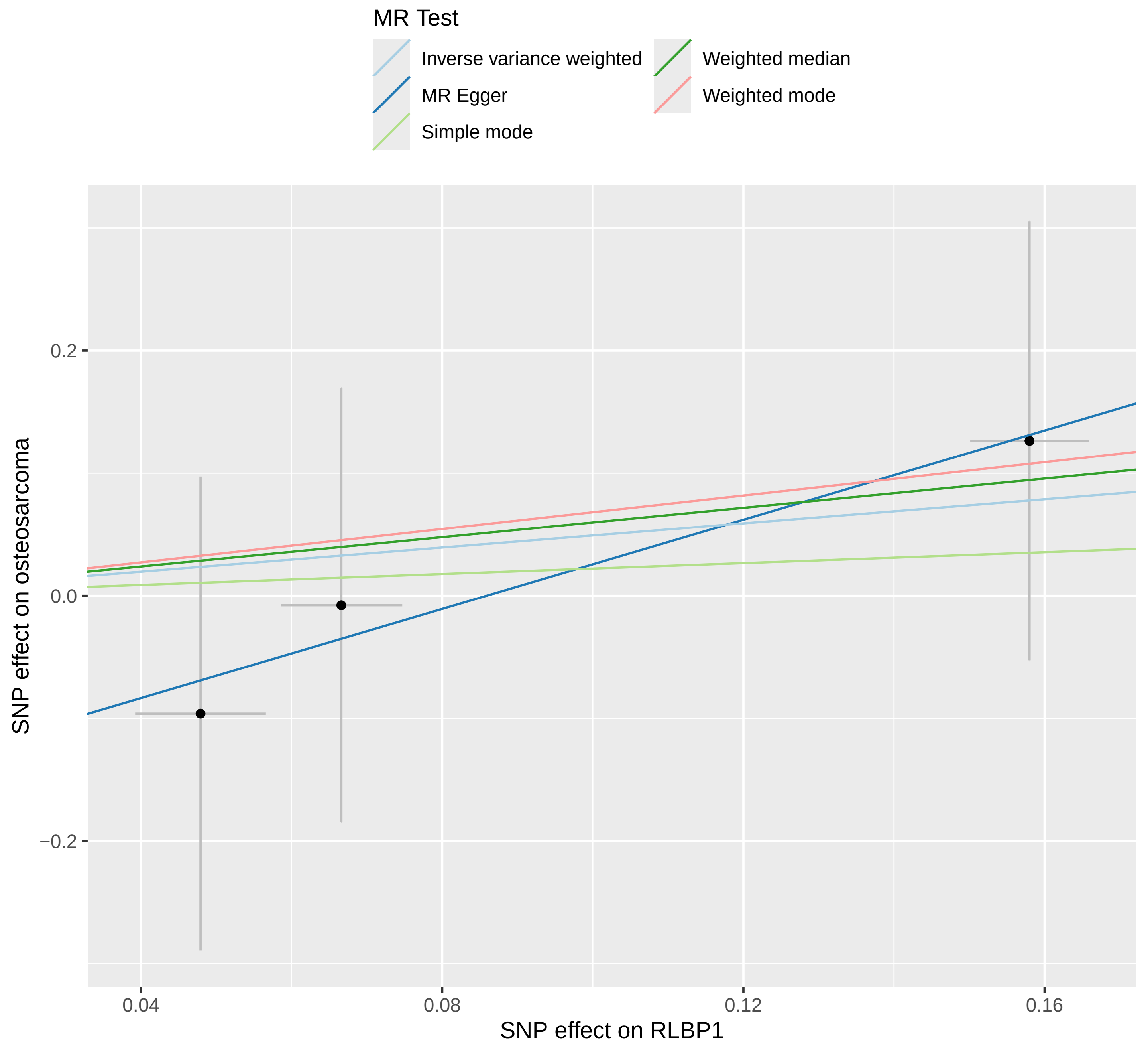
MR Test
Inverse variance weighted
MR Egger
Simple mode
Weighted median
Weighted mode
0.2
0.0
−0.2
SNP effect on osteosarcoma
0.04
0.08
0.12
0.16
SNP effect on RLBP1

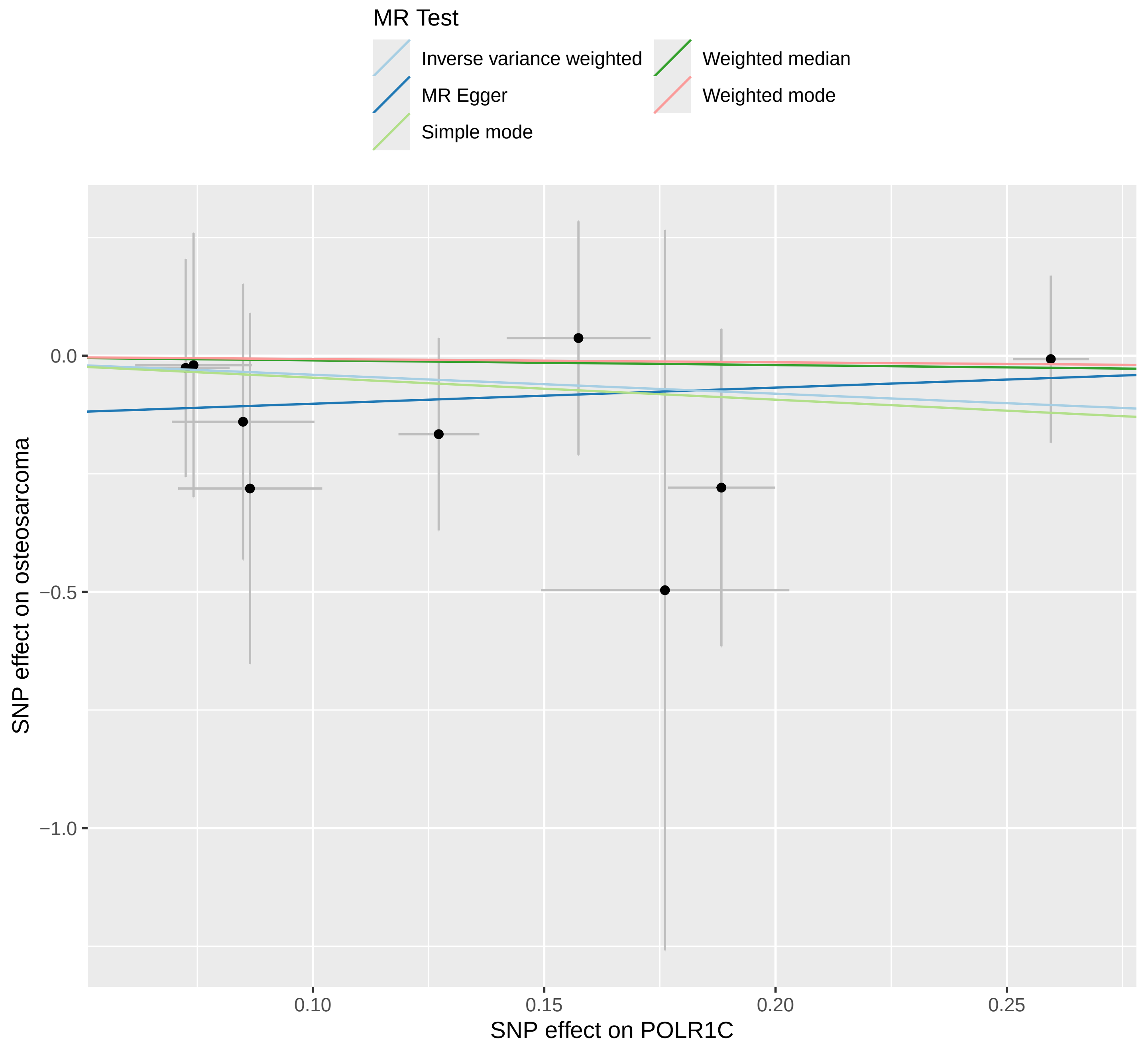

MR Test
Inverse variance weighted
Weighted median
MR Egger
Weighted mode
Simple mode
0.0
−0.5
−1.0
0.10
0.15
0.20
0.25
SNP effect on osteosarcoma
SNP effect on POLR1C

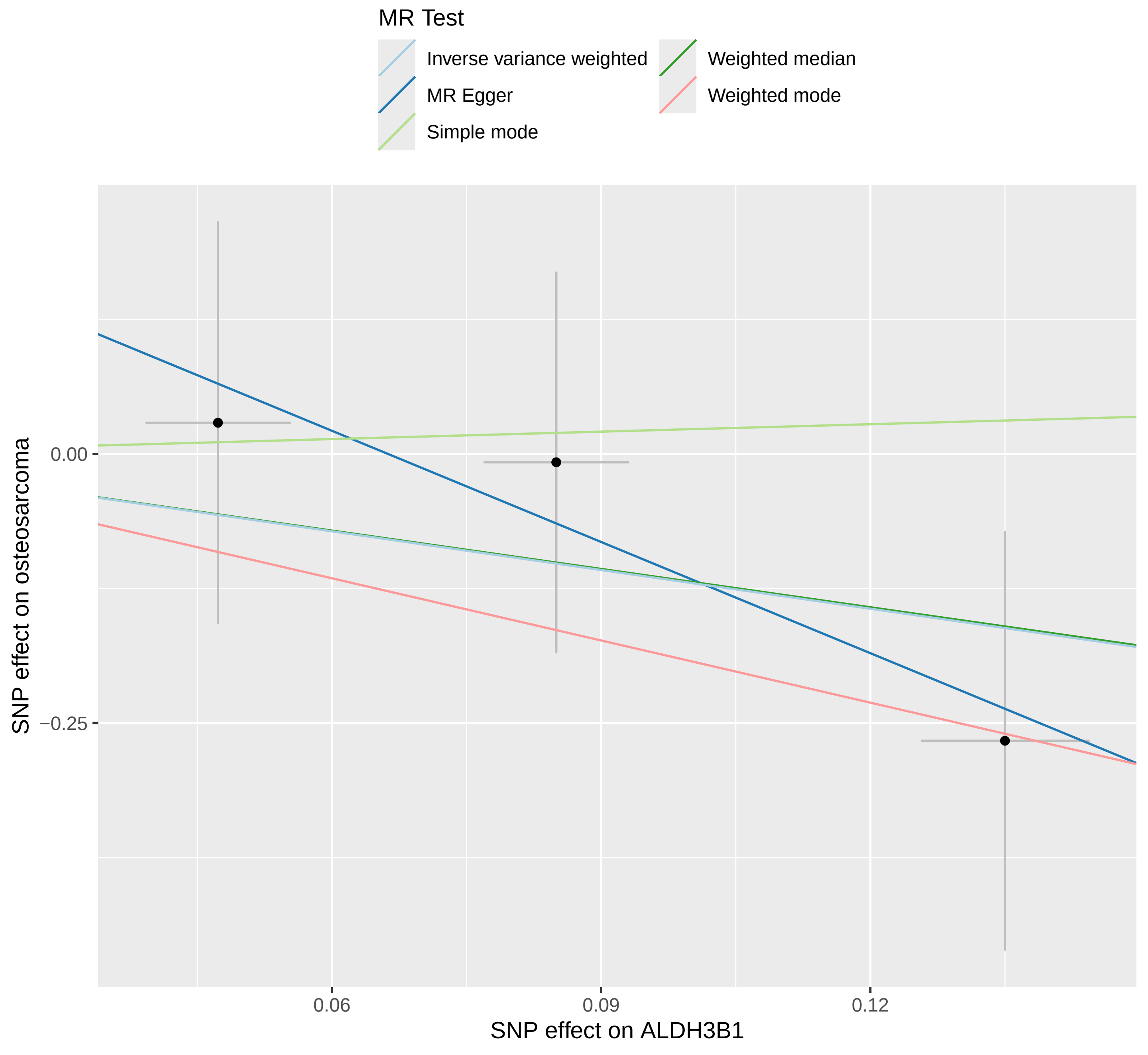
MR Test
Inverse variance weighted
MR Egger
Simple mode
Weighted median
Weighted mode
0.00
−0.25
0.06
0.09
0.12
SNP effect on ALDH3B1
SNP effect on osteosarcoma

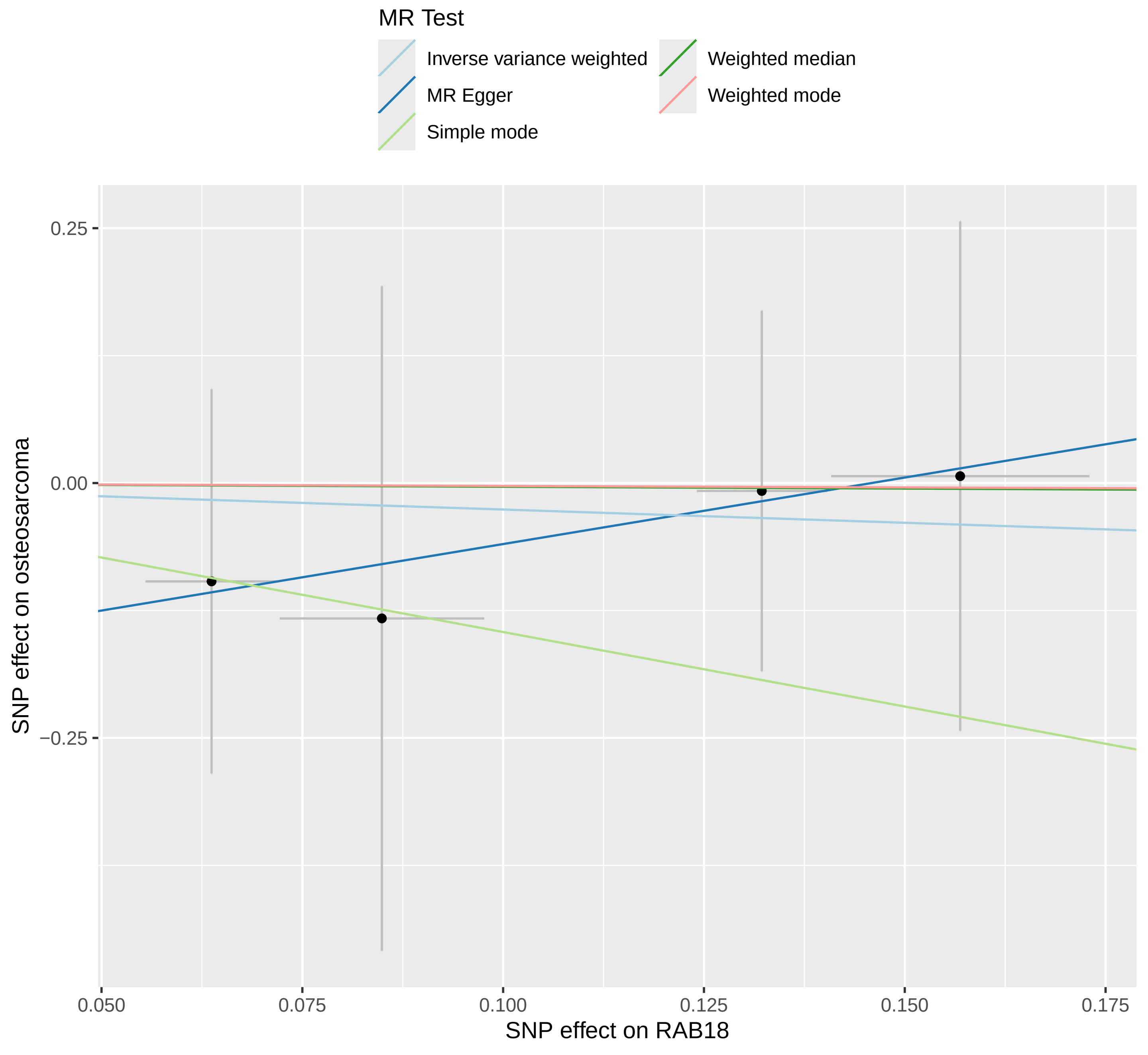

MR Test
Inverse variance weighted
MR Egger
Simple mode
Weighted median
Weighted mode
0.25
0.00
−0.25
0.050
0.075
0.100
0.125
0.150
0.175
SNP effect on osteosarcoma
SNP effect on RAB18

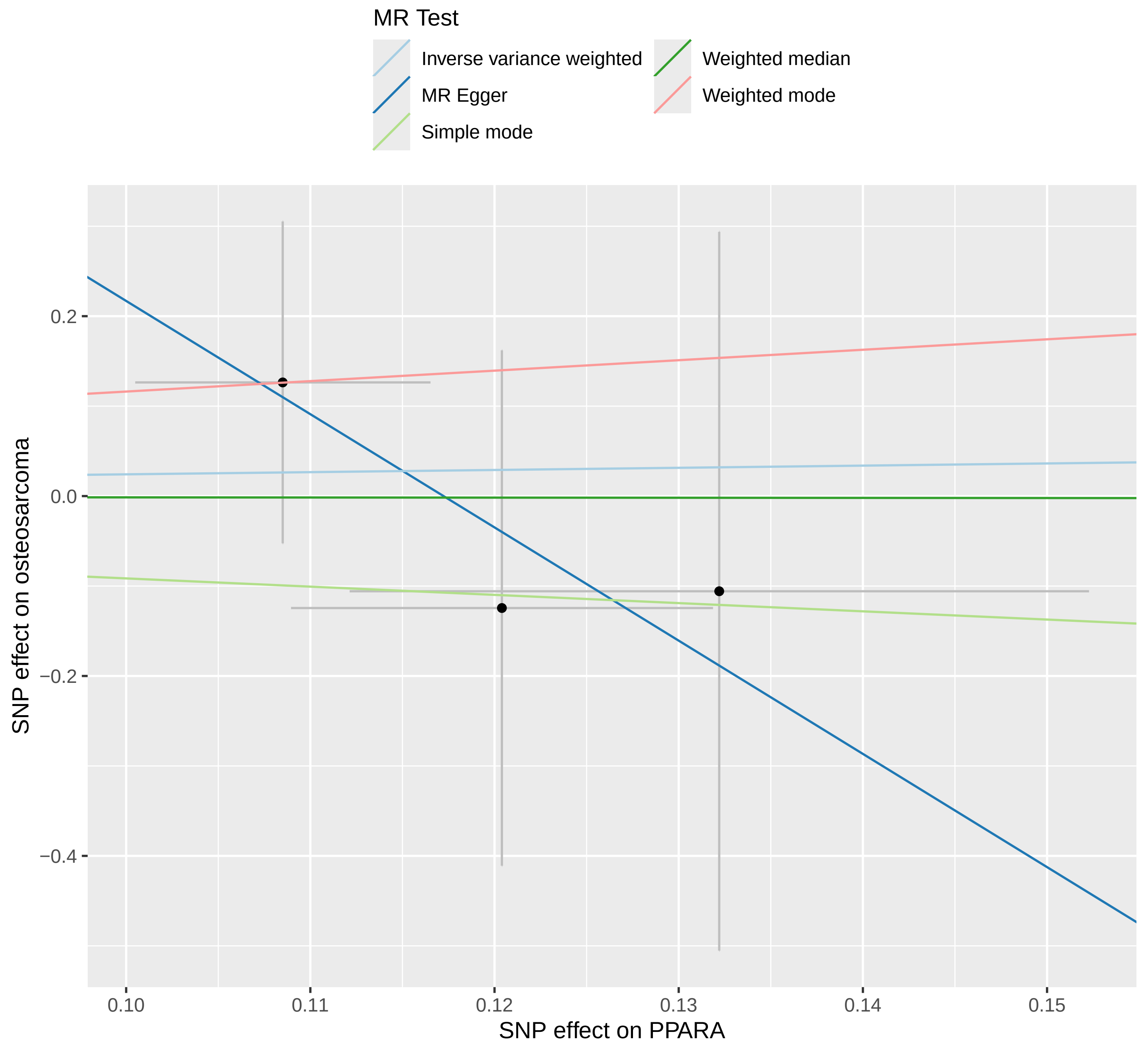
MR Test
Inverse variance weighted
MR Egger
Simple mode
Weighted median
Weighted mode
0.2
0.0
−0.2
−0.4
SNP effect on osteosarcoma
0.10
0.11
0.12
0.13
0.14
0.15
SNP effect on PPARA

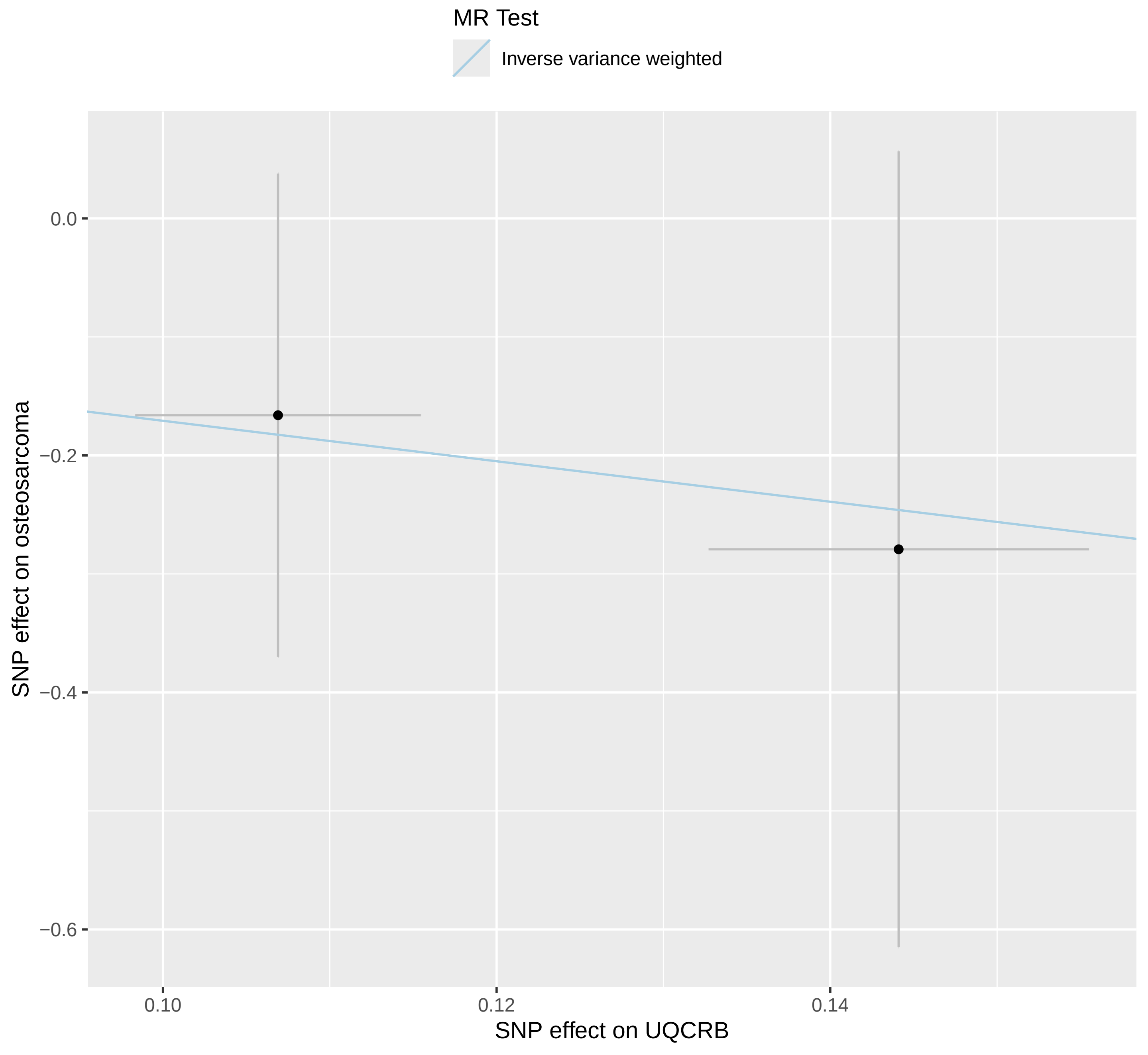

MR Test
Inverse variance weighted
0.0
−0.2
−0.4
−0.6
SNP effect on osteosarcoma
0.10
0.12
0.14
SNP effect on UQCRB

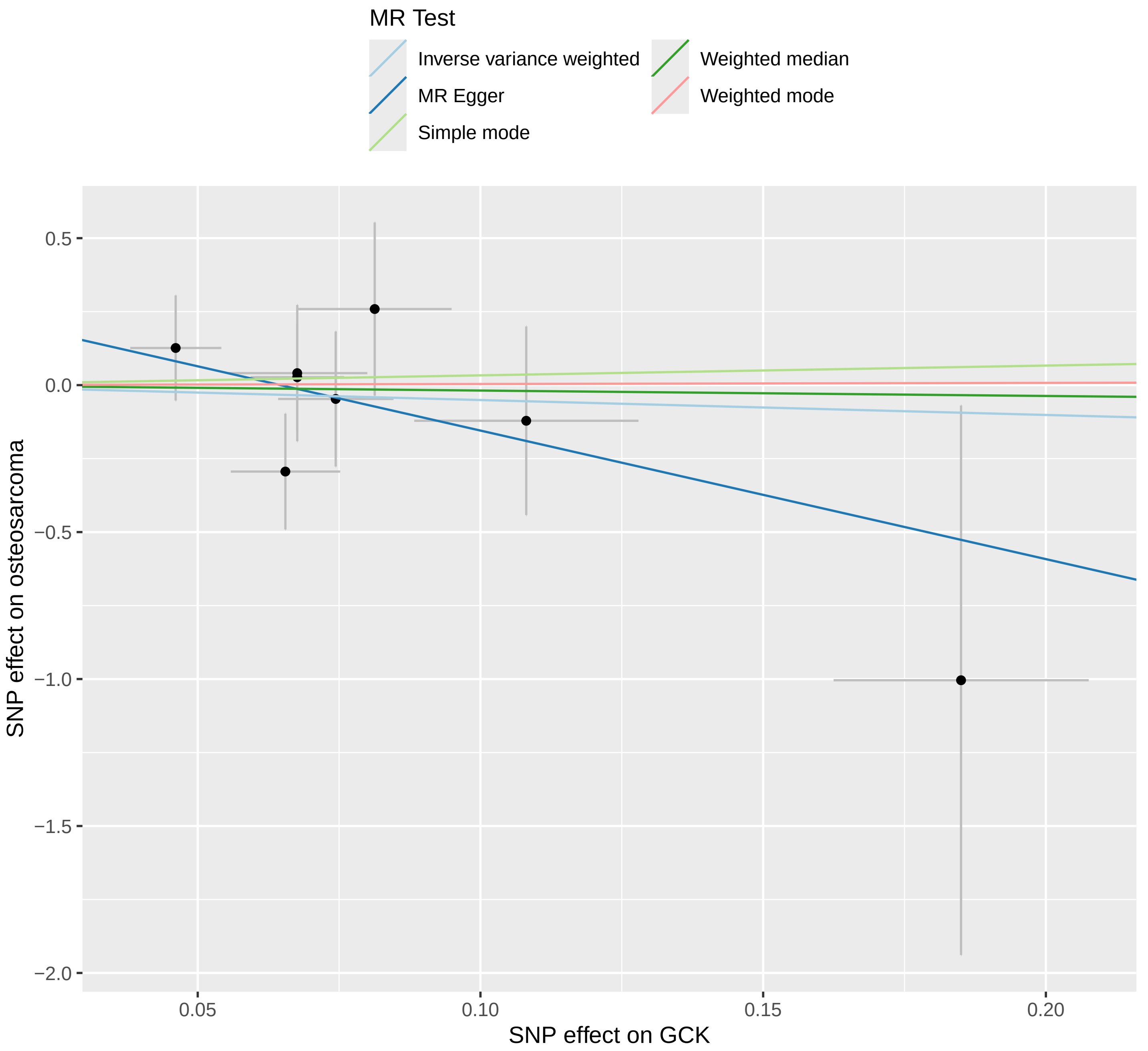
MR Test
Inverse variance weighted
MR Egger
Simple mode
Weighted median
Weighted mode
SNP effect on osteosarcoma
0.5
0.0
−0.5
−1.0
−1.5
−2.0
0.05
0.10
0.15
0.20
SNP effect on GCK

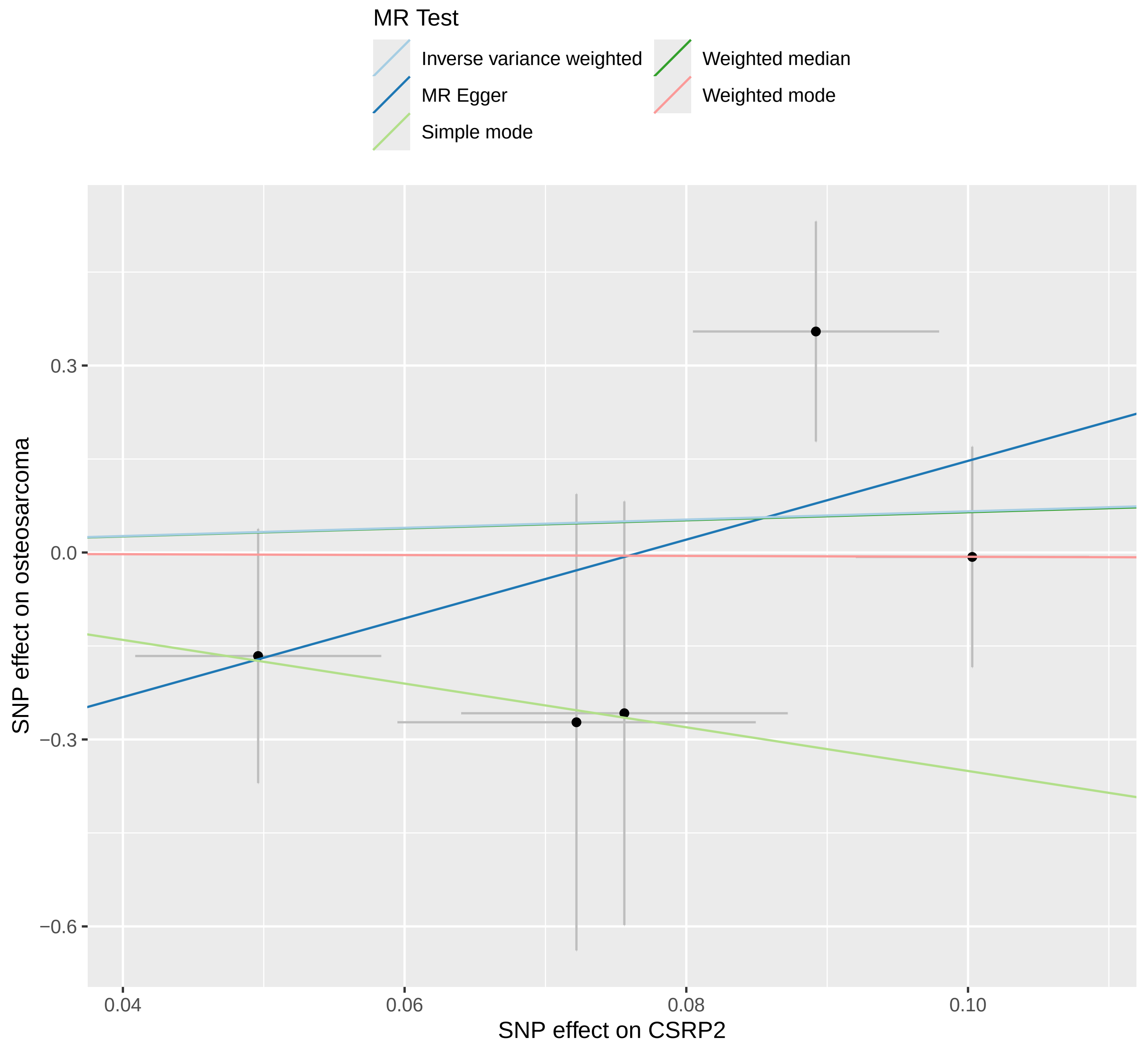
MR Test
Inverse variance weighted
MR Egger
Simple mode
Weighted median
Weighted mode
0.3
0.0
−0.3
−0.6
SNP effect on osteosarcoma
0.04
0.06
0.08
0.10
SNP effect on CSRP2

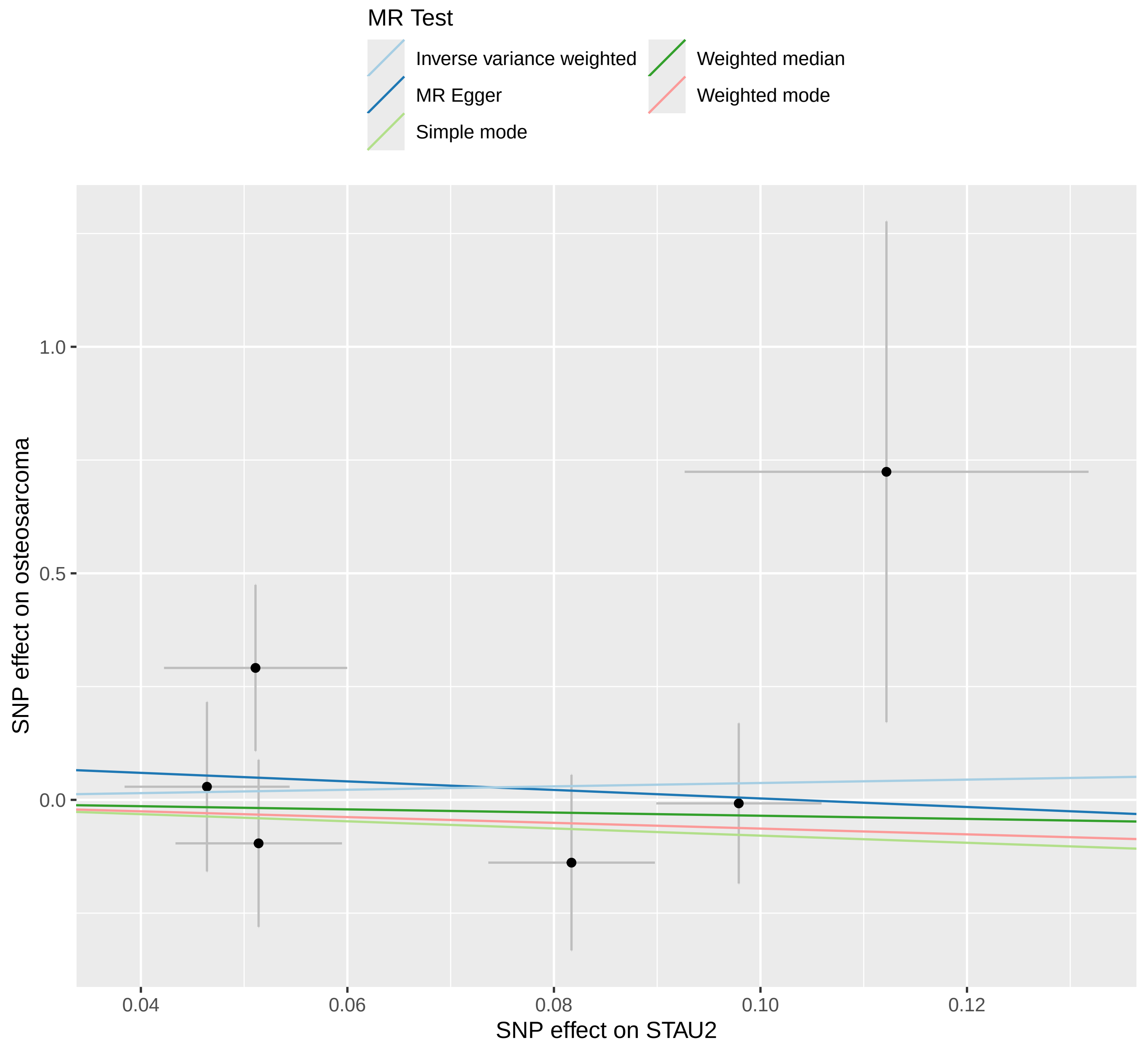

MR Test
Inverse variance weighted
MR Egger
Simple mode
Weighted median
Weighted mode
SNP effect on osteosarcoma
1.0
0.5
0.0
0.04
0.06
0.08
0.10
0.12
SNP effect on STAU2

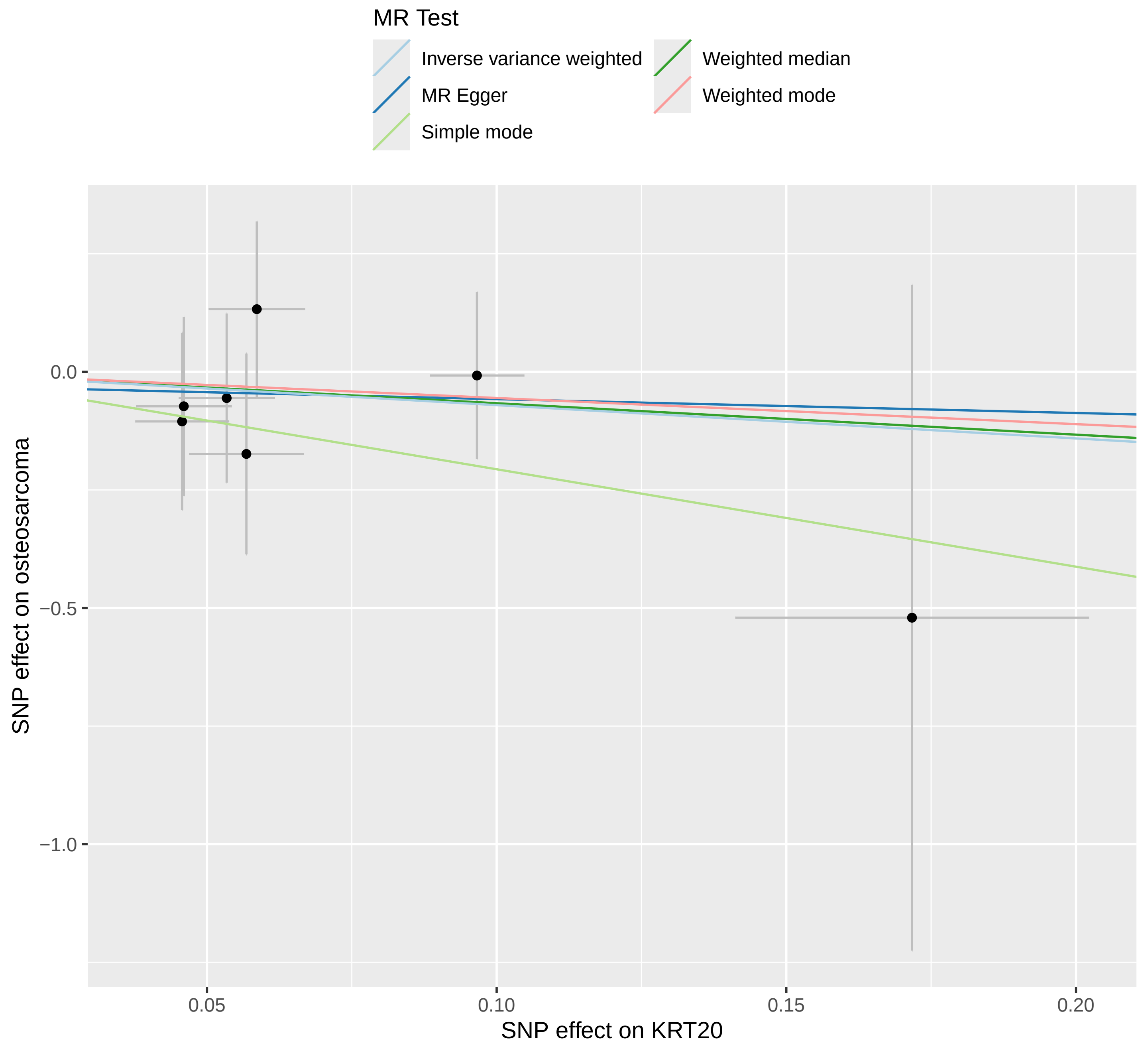
MR Test
Inverse variance weighted
MR Egger
Simple mode
Weighted median
Weighted mode
0.0
−0.5
−1.0
0.05
0.10
0.15
0.20
SNP effect on KRT20
SNP effect on osteosarcoma

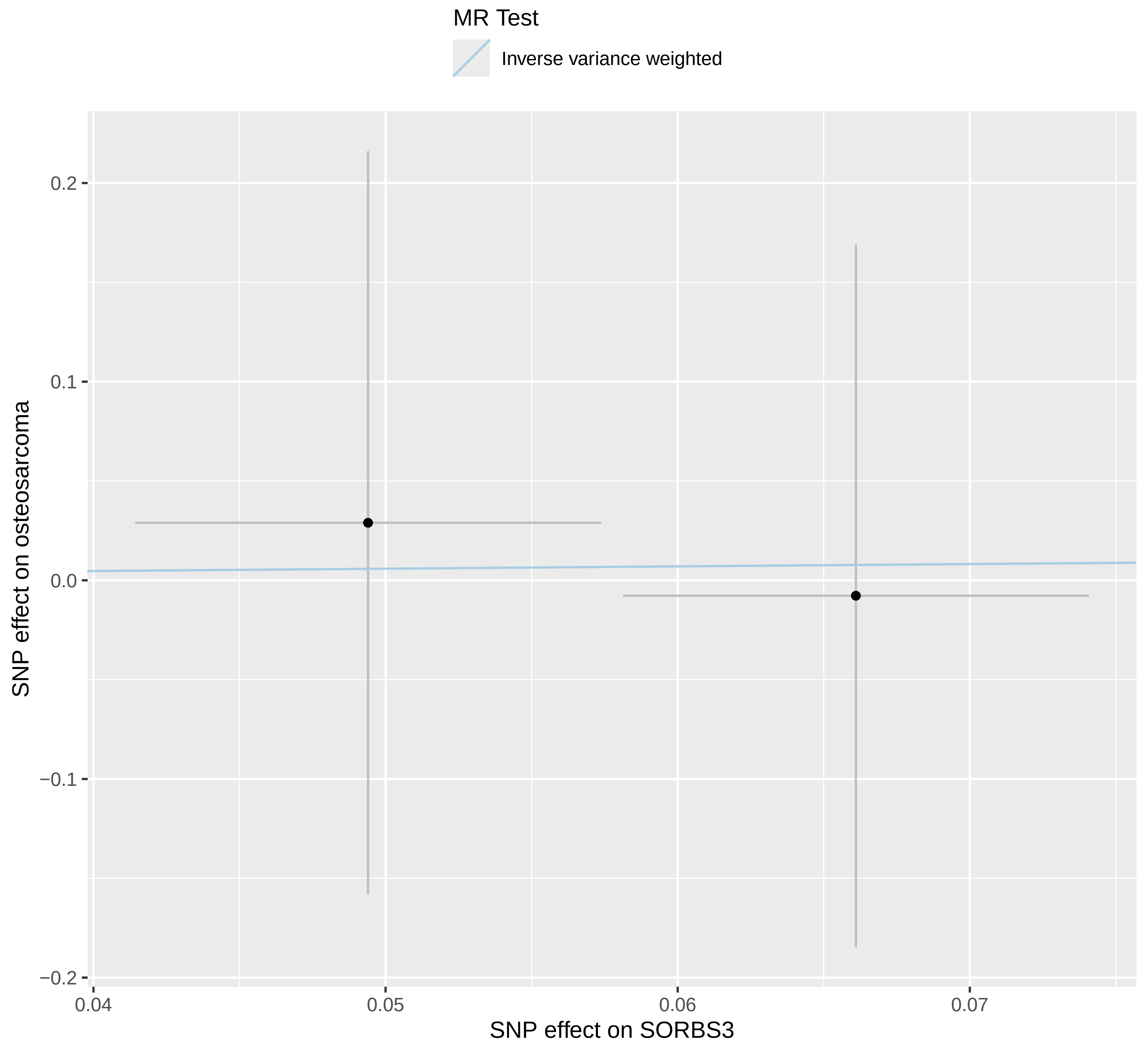

MR Test
Inverse variance weighted
0.2
0.1
0.0
−0.1
−0.2
SNP effect on osteosarcoma
0.04
0.05
0.06
0.07
SNP effect on SORBS3

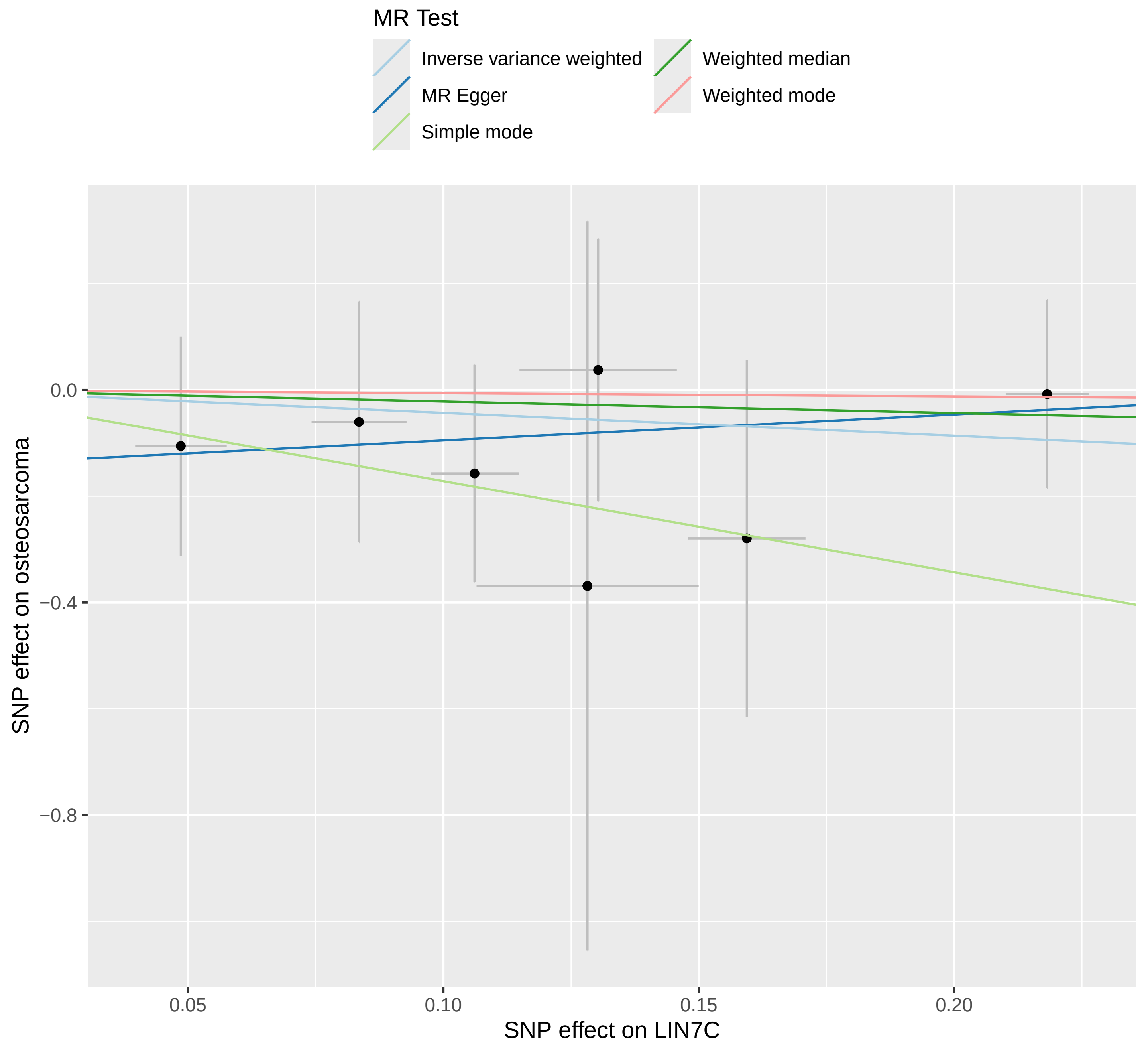

MR Test
Inverse variance weighted
MR Egger
Simple mode
Weighted median
Weighted mode
0.0
−0.4
−0.8
SNP effect on osteosarcoma
0.05
0.10
0.15
0.20
SNP effect on LIN7C

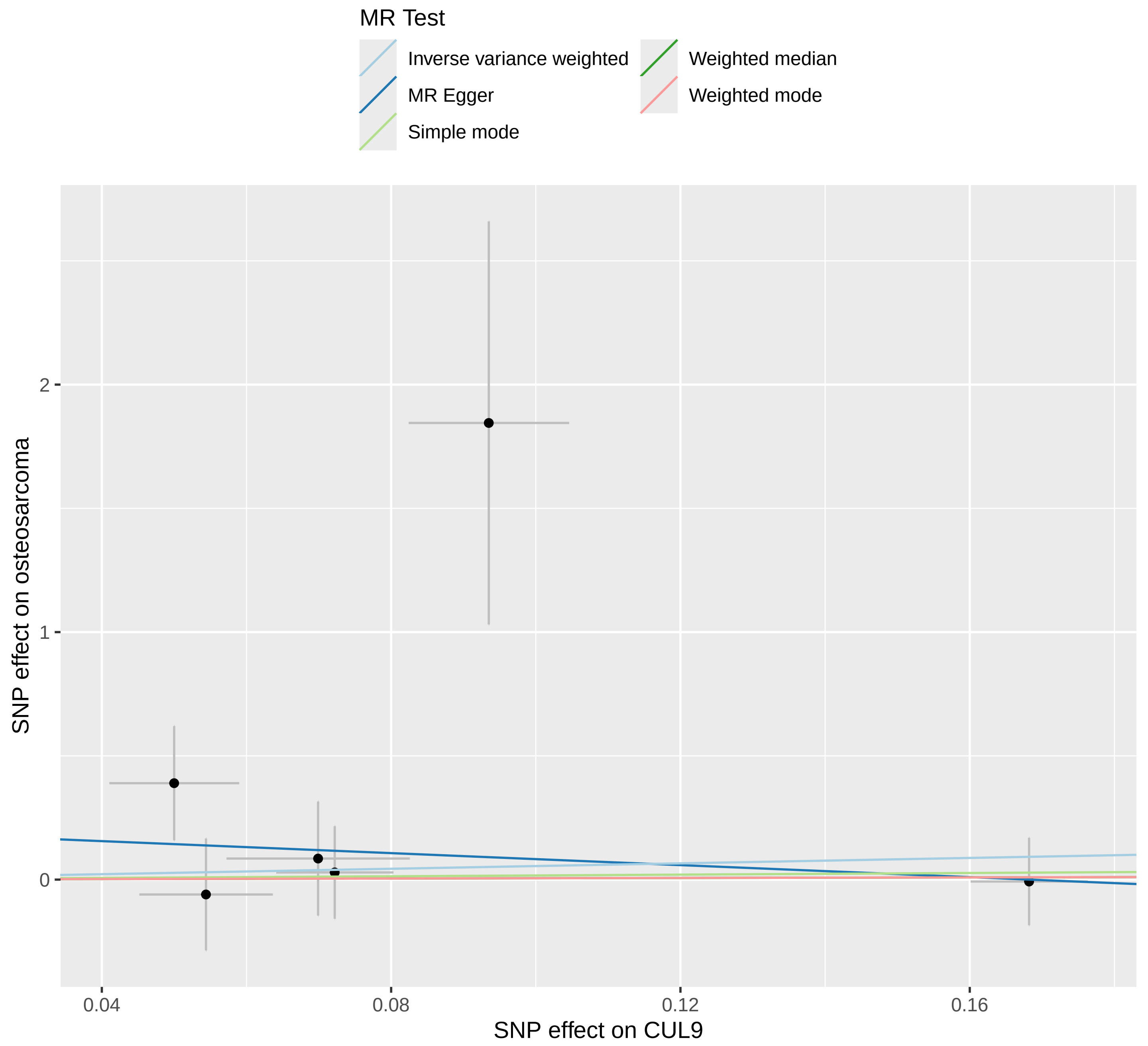
MR Test
Inverse variance weighted
MR Egger
Simple mode
Weighted median
Weighted mode
2
1
0
SNP effect on osteosarcoma
0.04
0.08
0.12
0.16
SNP effect on CUL9

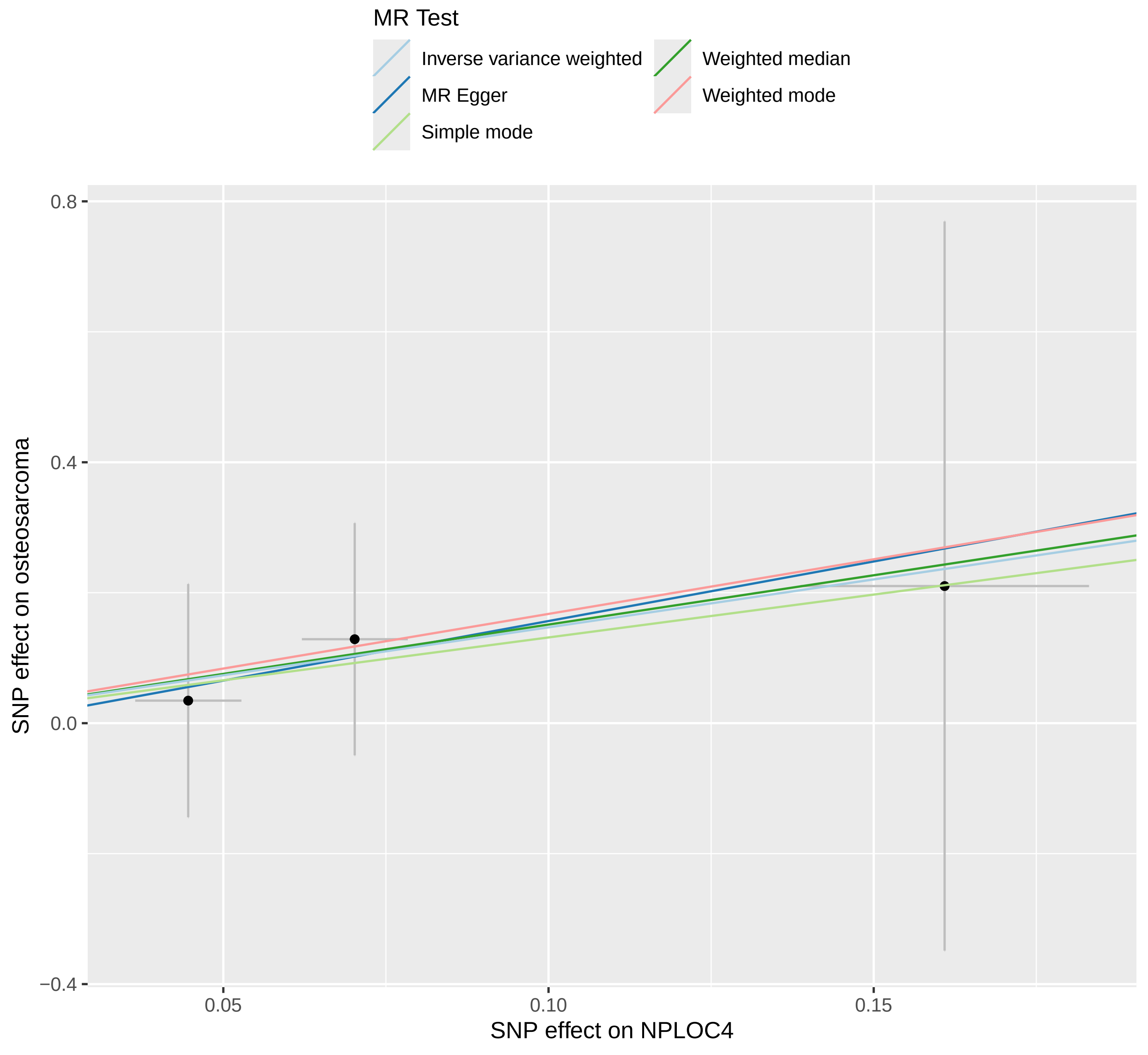
MR Test
Inverse variance weighted
MR Egger
Simple mode
Weighted median
Weighted mode
0.8
0.4
0.0
−0.4
0.05
0.10
0.15
SNP effect on osteosarcoma
SNP effect on NPLOC4

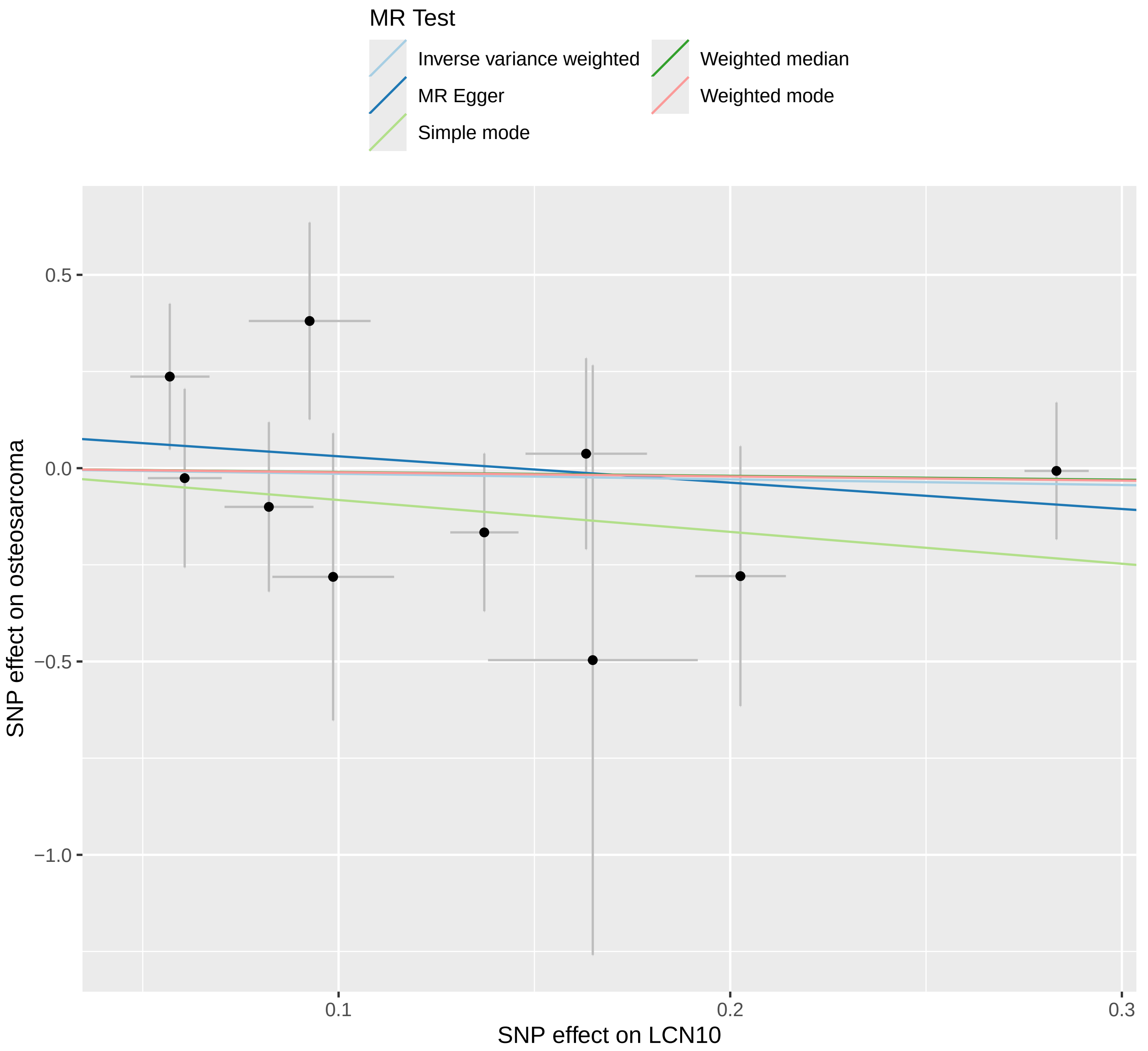

MR Test
Inverse variance weighted
MR Egger
Simple mode
Weighted median
Weighted mode
0.5
0.0
−0.5
−1.0
SNP effect on osteosarcoma
0.1
0.2
0.3
SNP effect on LCN10

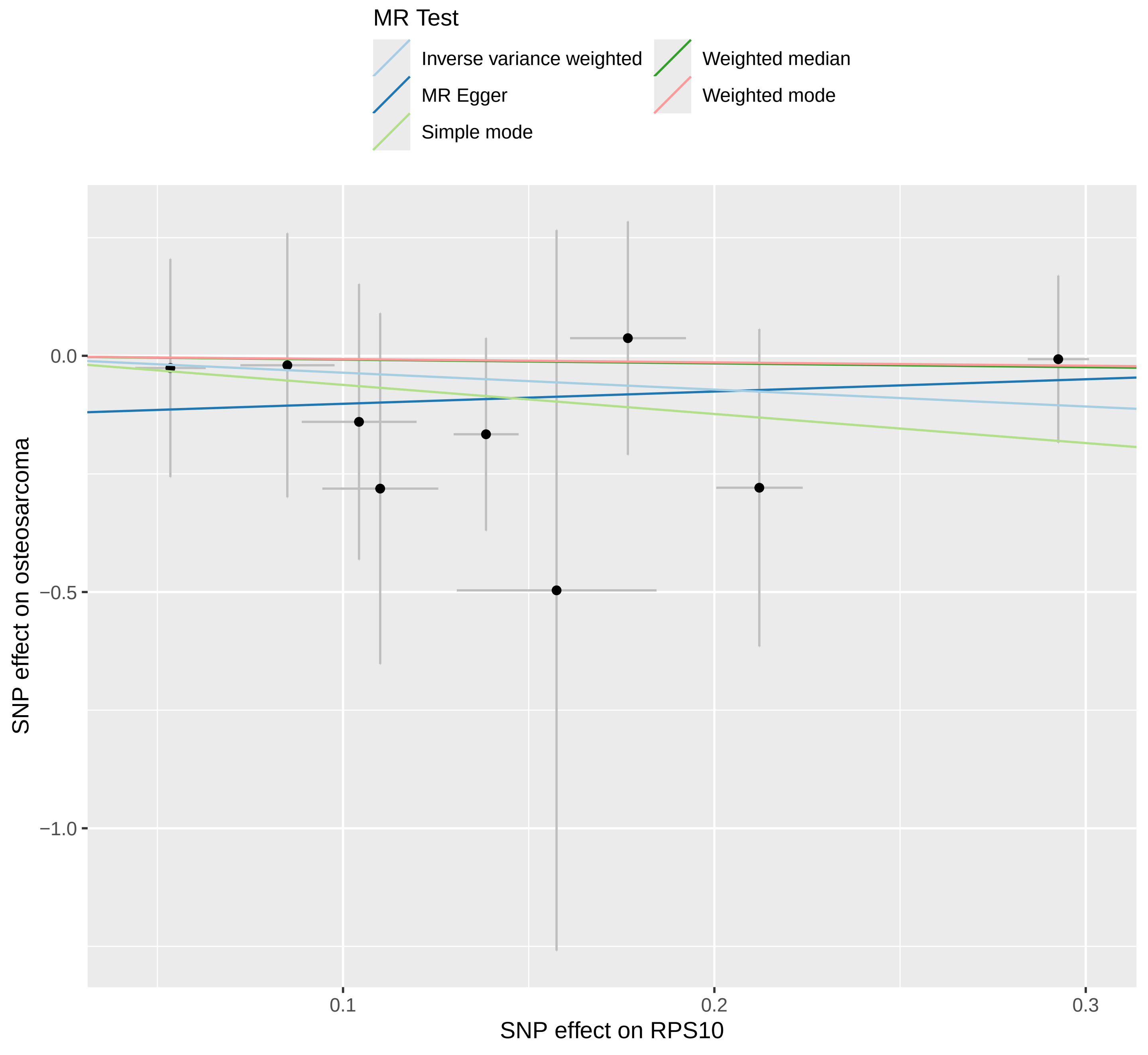
MR Test
Inverse variance weighted
MR Egger
Simple mode
Weighted median
Weighted mode
0.0
−0.5
−1.0
SNP effect on osteosarcoma
0.1
0.2
0.3
SNP effect on RPS10

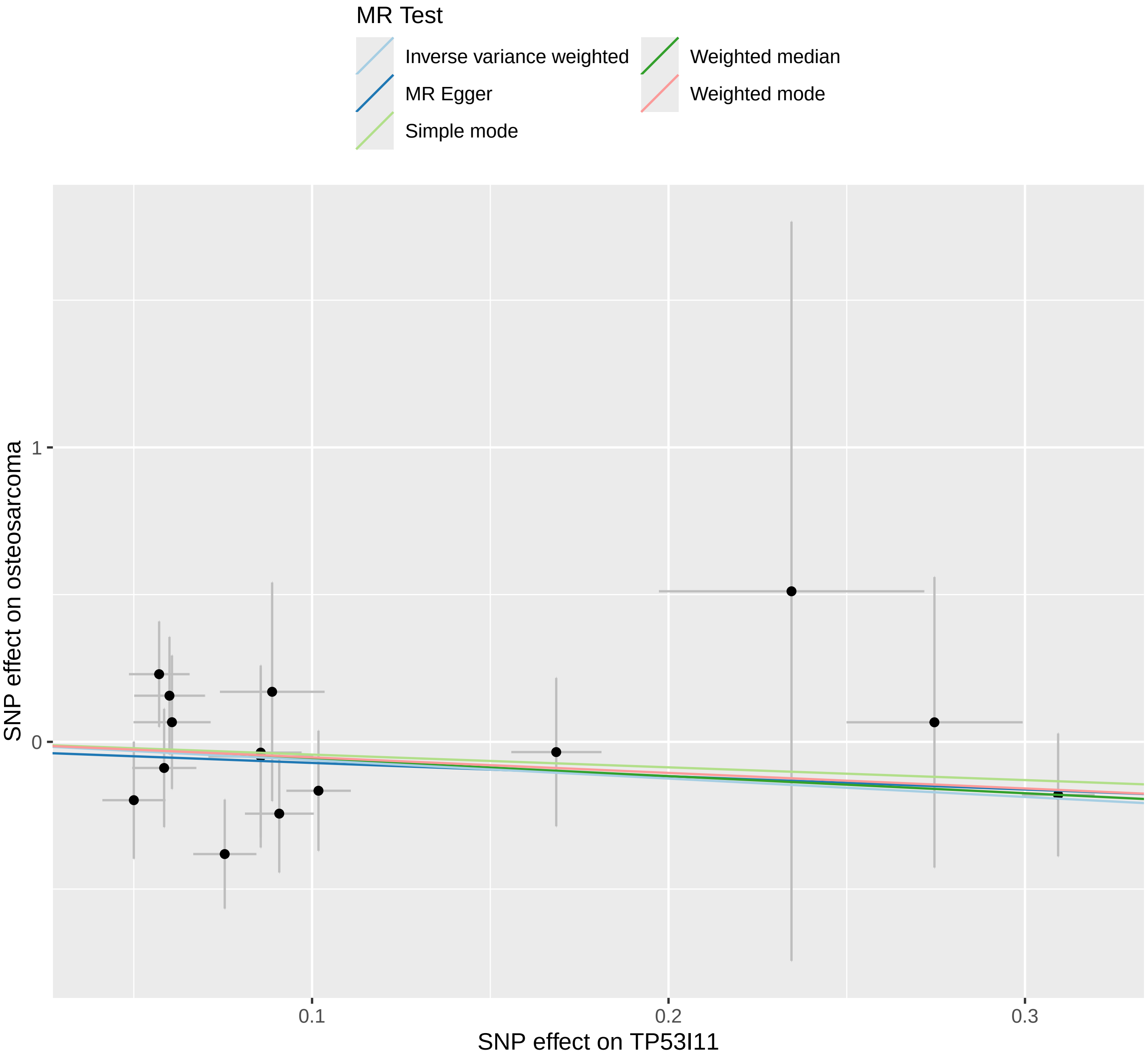
MR Test
Inverse variance weighted
MR Egger
Simple mode
Weighted median
Weighted mode
SNP effect on osteosarcoma
1
0
0.1
0.2
0.3
SNP effect on TP53I11

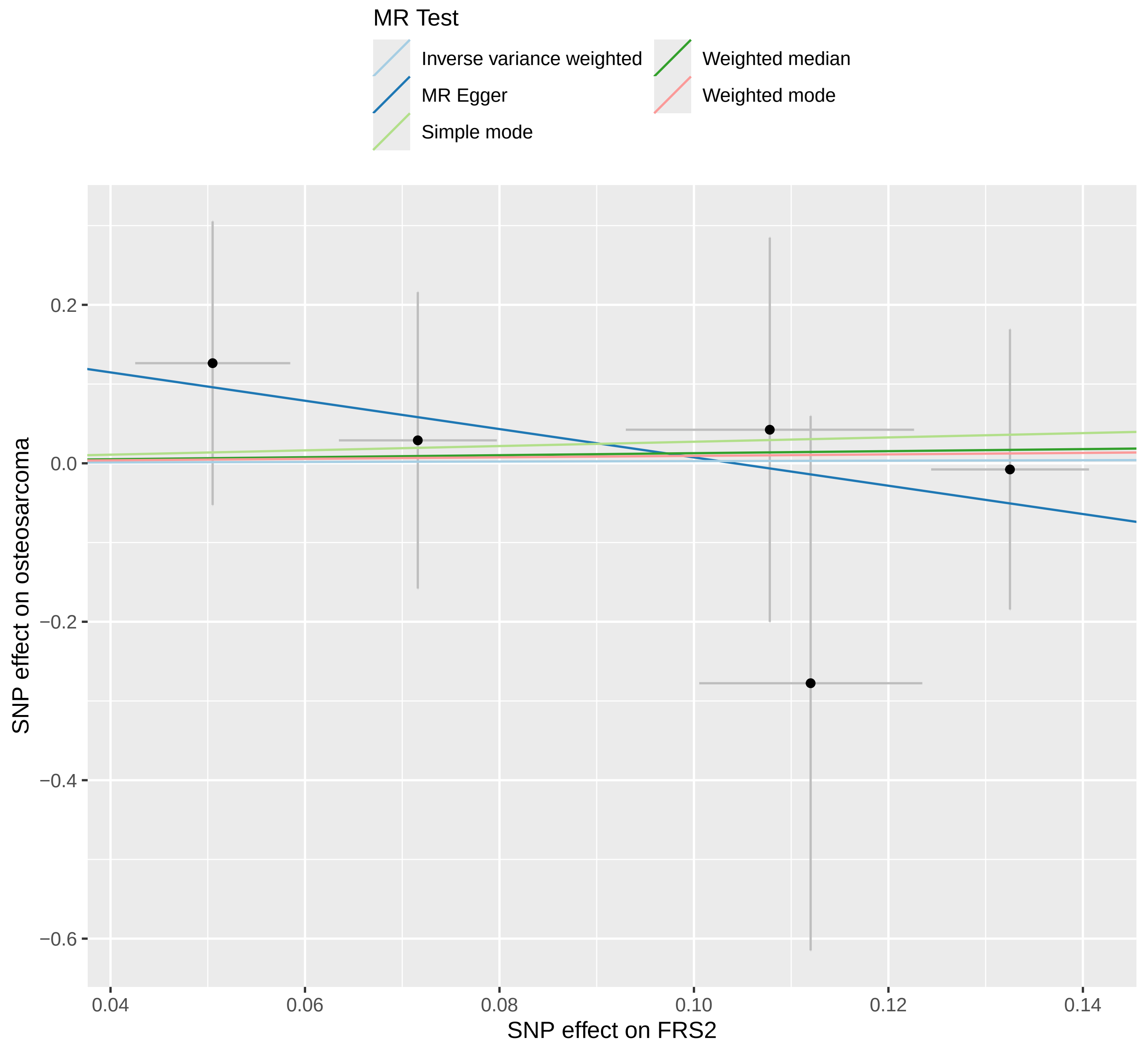

MR Test
Inverse variance weighted
MR Egger
Simple mode
Weighted median
Weighted mode
0.2
0.0
−0.2
−0.4
−0.6
0.04
0.06
0.08
0.10
0.12
0.14
SNP effect on FRS2
SNP effect on osteosarcoma

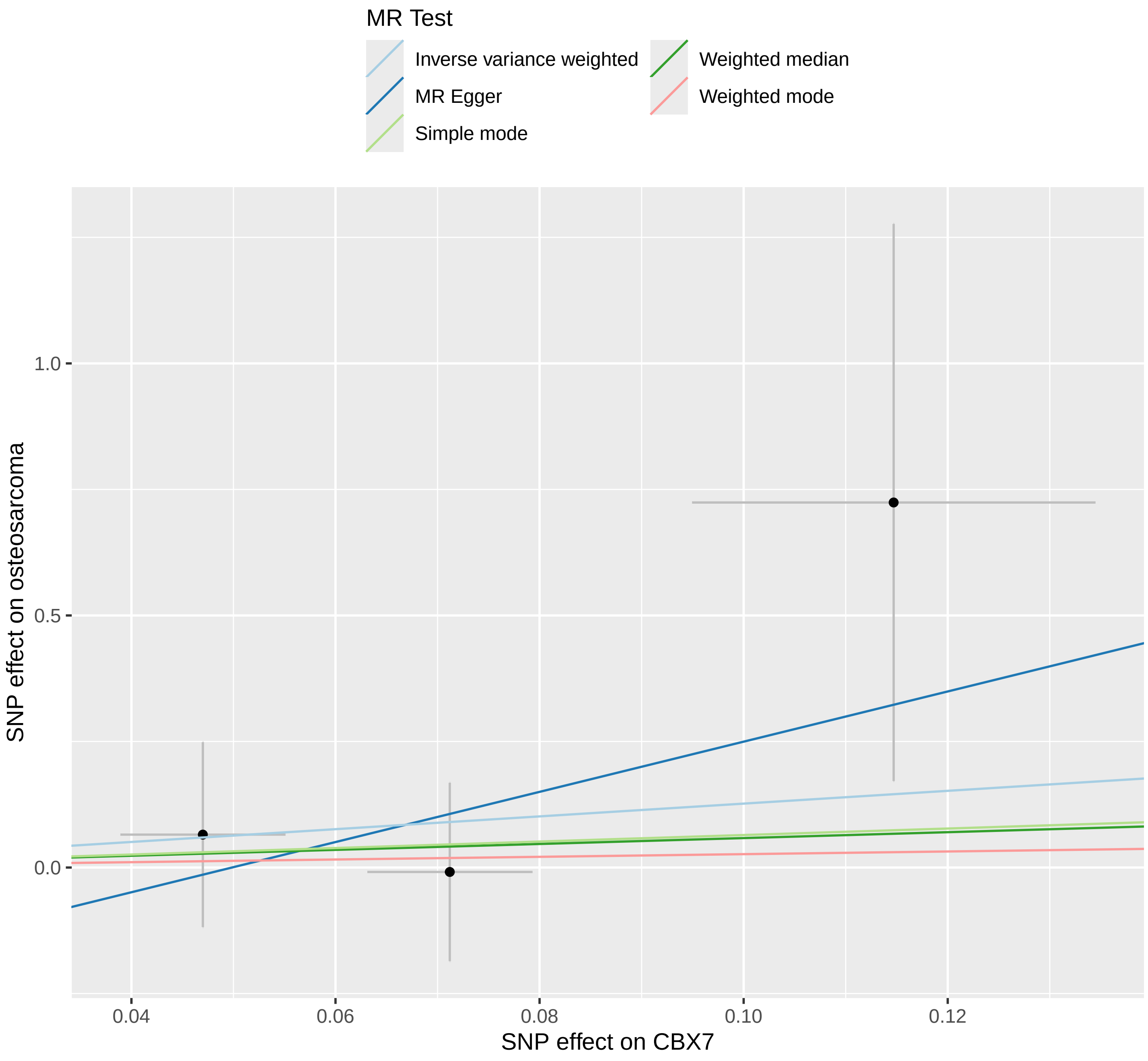

MR Test
Inverse variance weighted
MR Egger
Simple mode
Weighted median
Weighted mode
SNP effect on osteosarcoma
1.0
0.5
0.0
0.04
0.06
0.08
0.10
0.12
SNP effect on CBX7

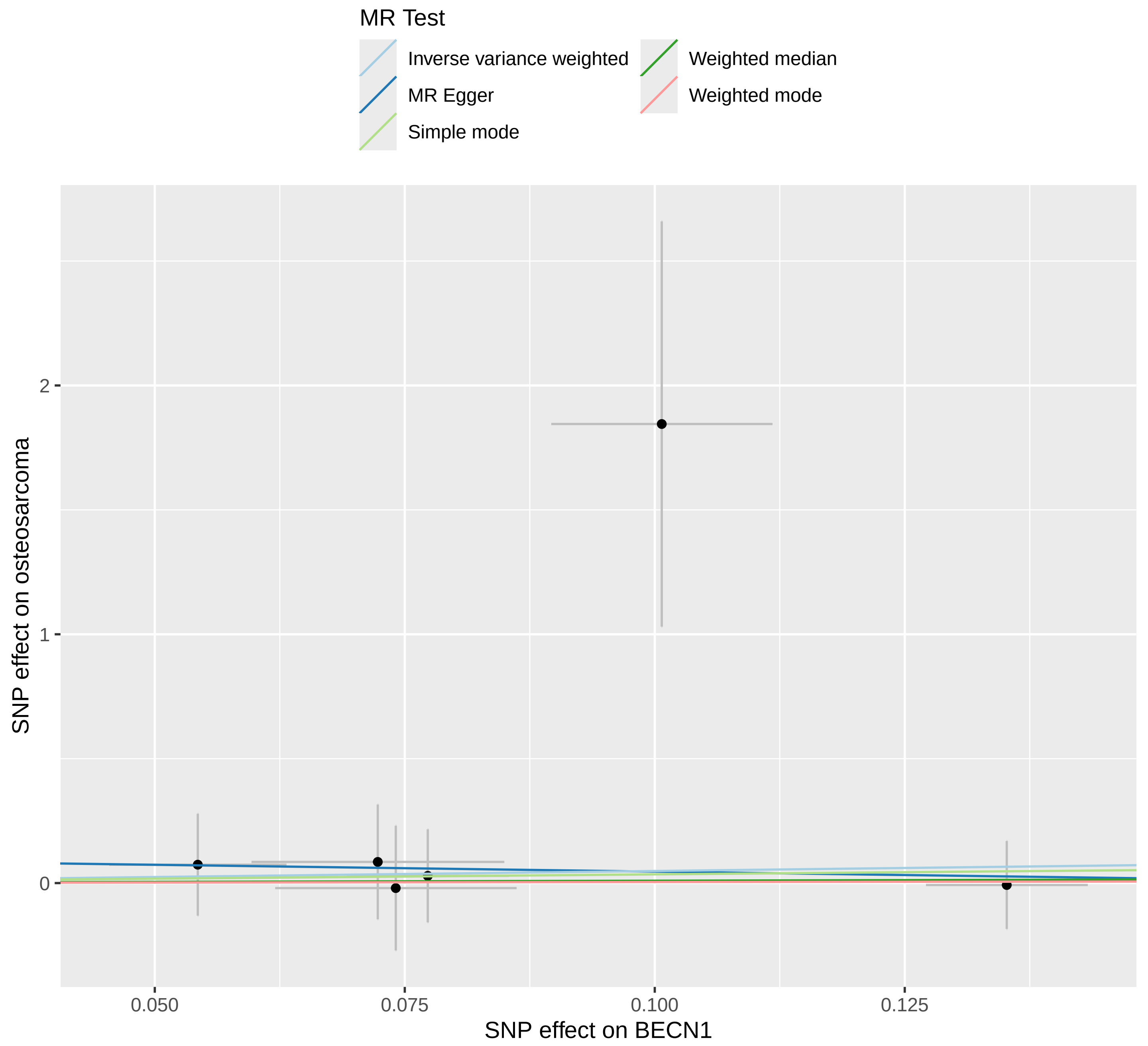

MR Test
Inverse variance weighted
MR Egger
Simple mode
Weighted median
Weighted mode
SNP effect on osteosarcoma
2
1
0
0.050
0.075
0.100
0.125
SNP effect on BECN1

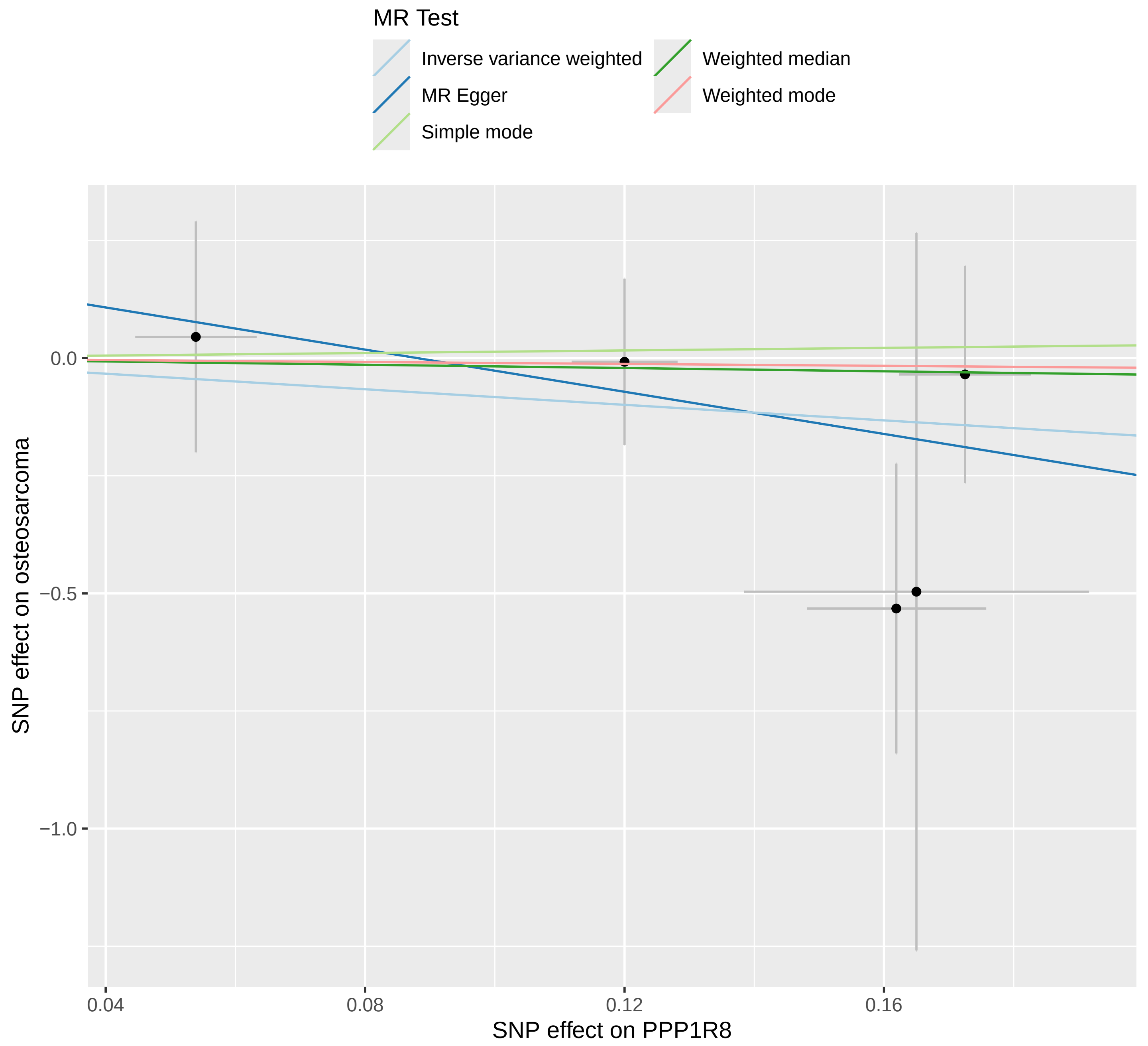

MR Test
Inverse variance weighted
MR Egger
Simple mode
Weighted median
Weighted mode
0.0
−0.5
−1.0
0.04
0.08
0.12
0.16
SNP effect on PPP1R8
SNP effect on osteosarcoma

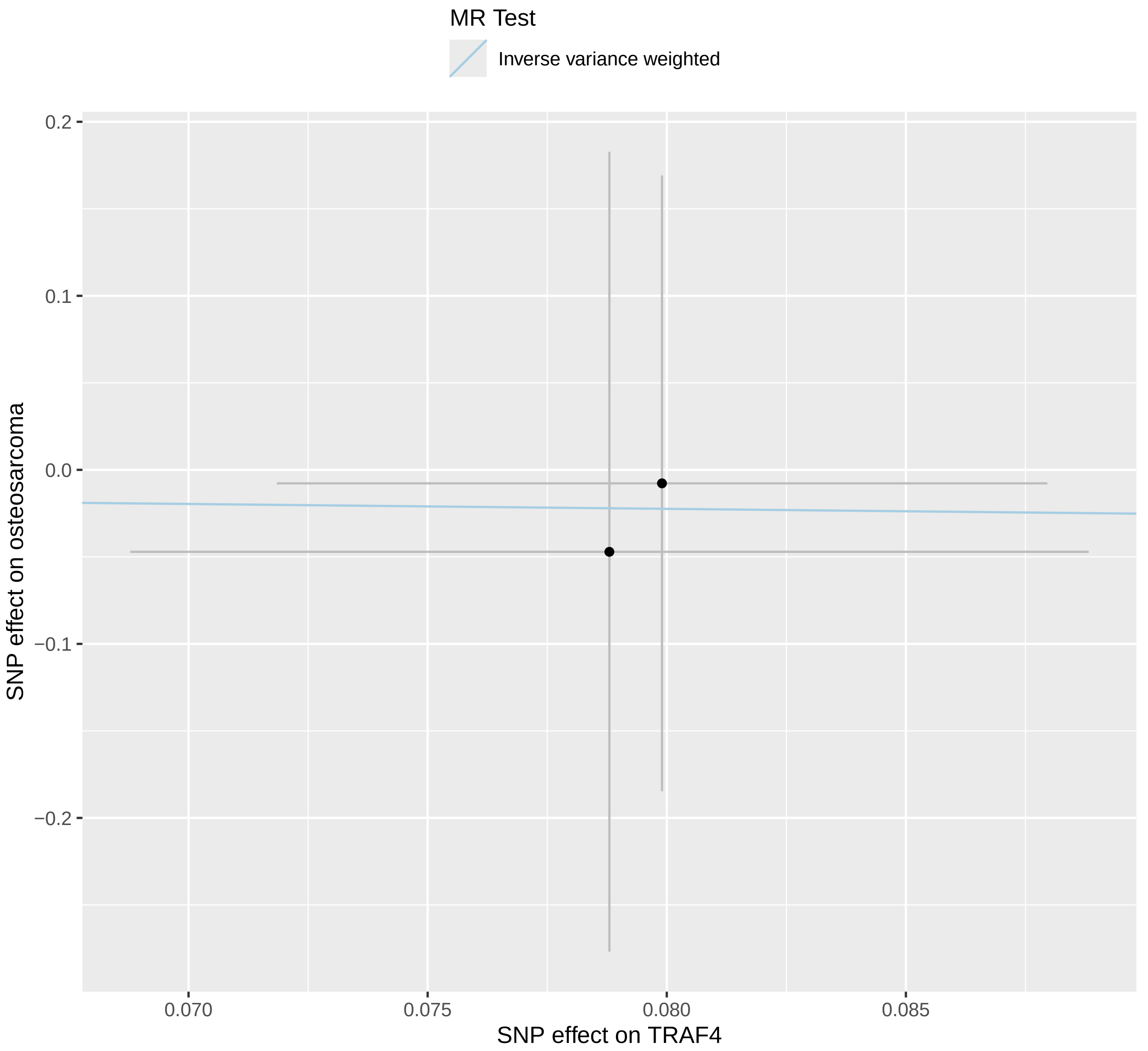
MR Test
Inverse variance weighted
0.2
0.1
0.0
−0.1
−0.2
SNP effect on osteosarcoma
0.070
0.075
0.080
0.085
SNP effect on TRAF4

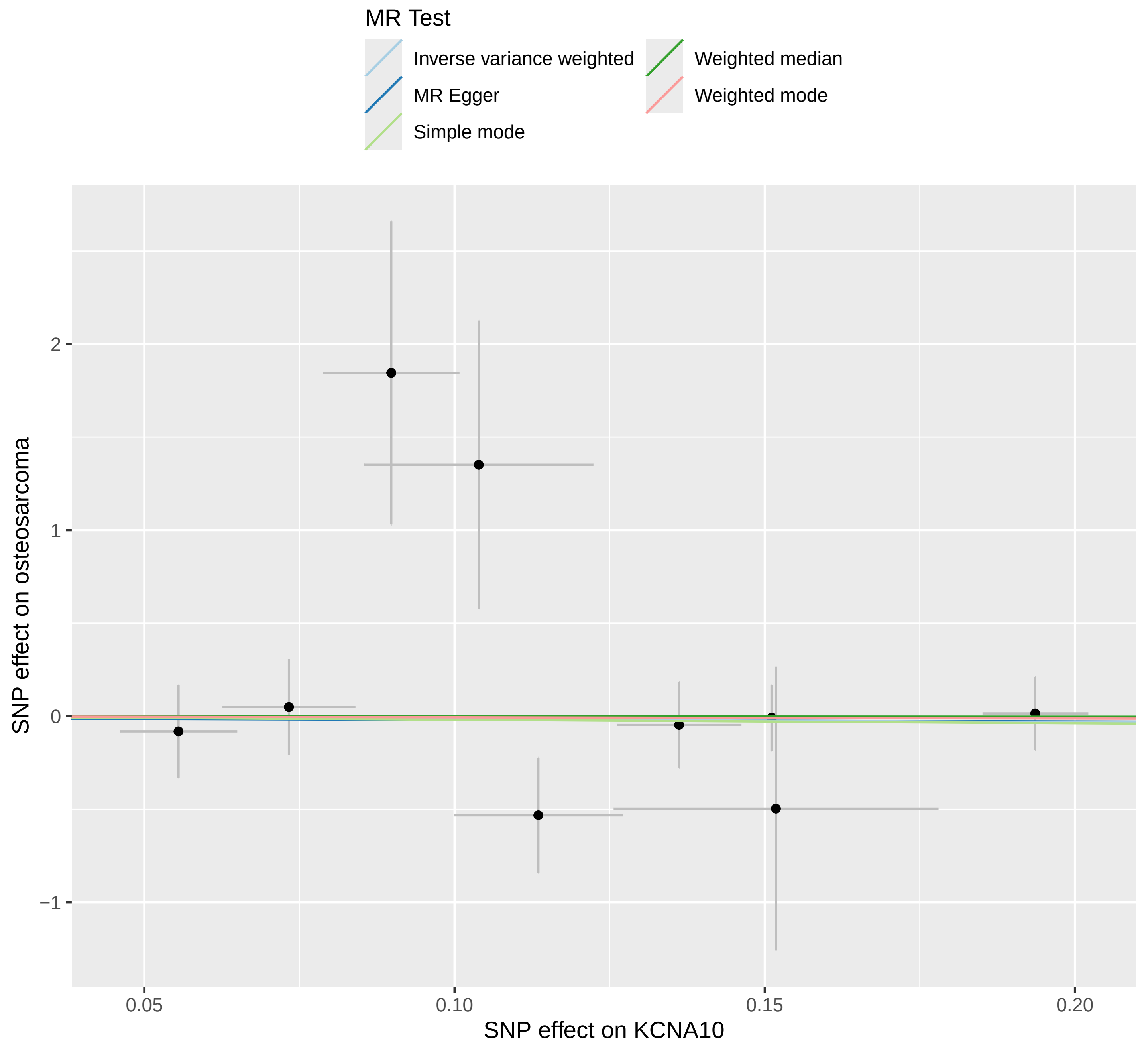
MR Test
Inverse variance weighted
MR Egger
Simple mode
Weighted median
Weighted mode
SNP effect on osteosarcoma
2
1
0
−1
0.05
0.10
0.15
0.20
SNP effect on KCNA10

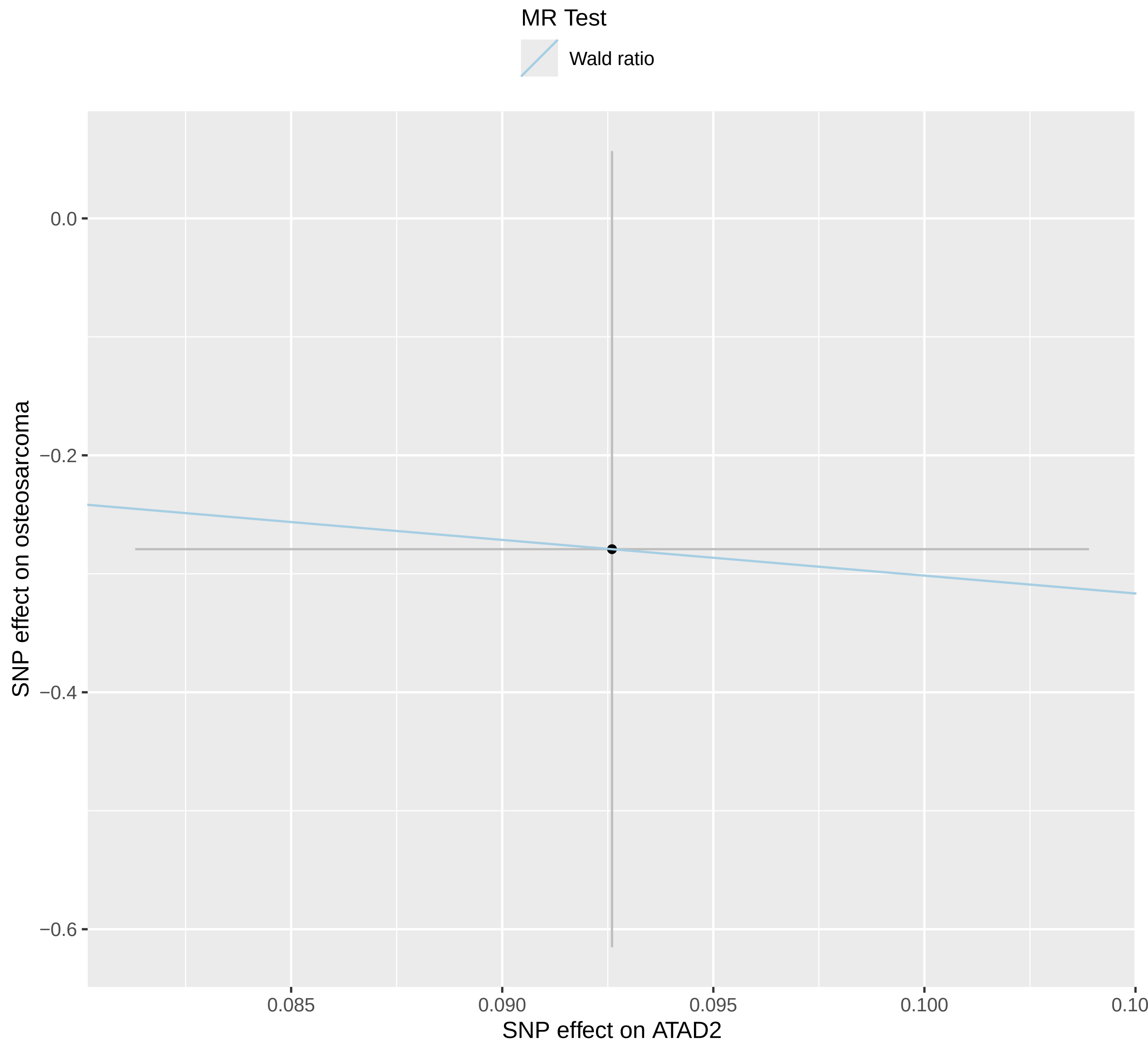

MR Test
Wald ratio
0.0
−0.2
−0.4
−0.6
SNP effect on osteosarcoma
0.085
0.090
0.095
0.100
SNP effect on ATAD2

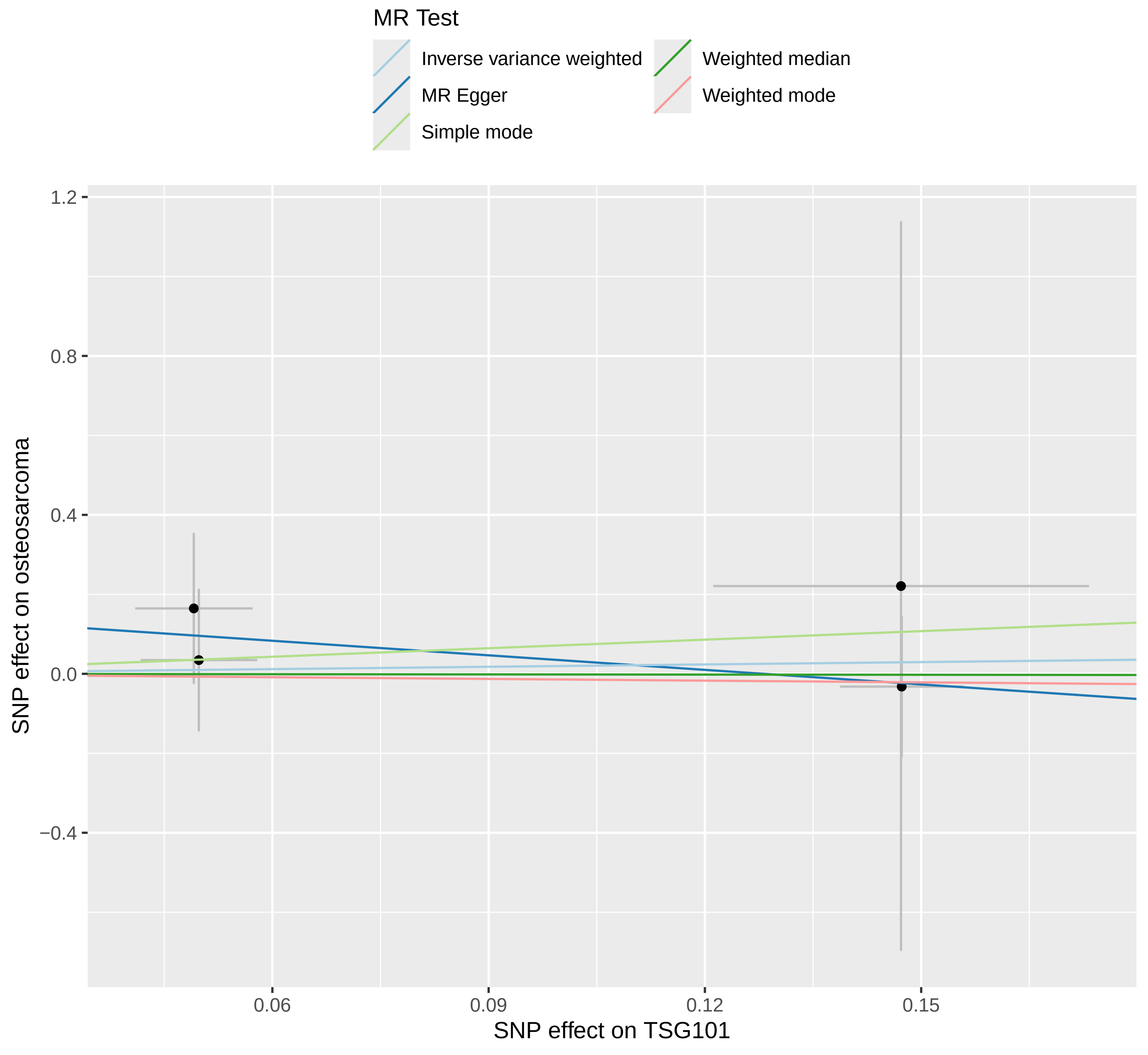
MR Test
Inverse variance weighted
MR Egger
Simple mode
Weighted median
Weighted mode
1.2
0.8
0.4
0.0
−0.4
0.06
0.09
0.12
0.15
SNP effect on osteosarcoma
SNP effect on TSG101

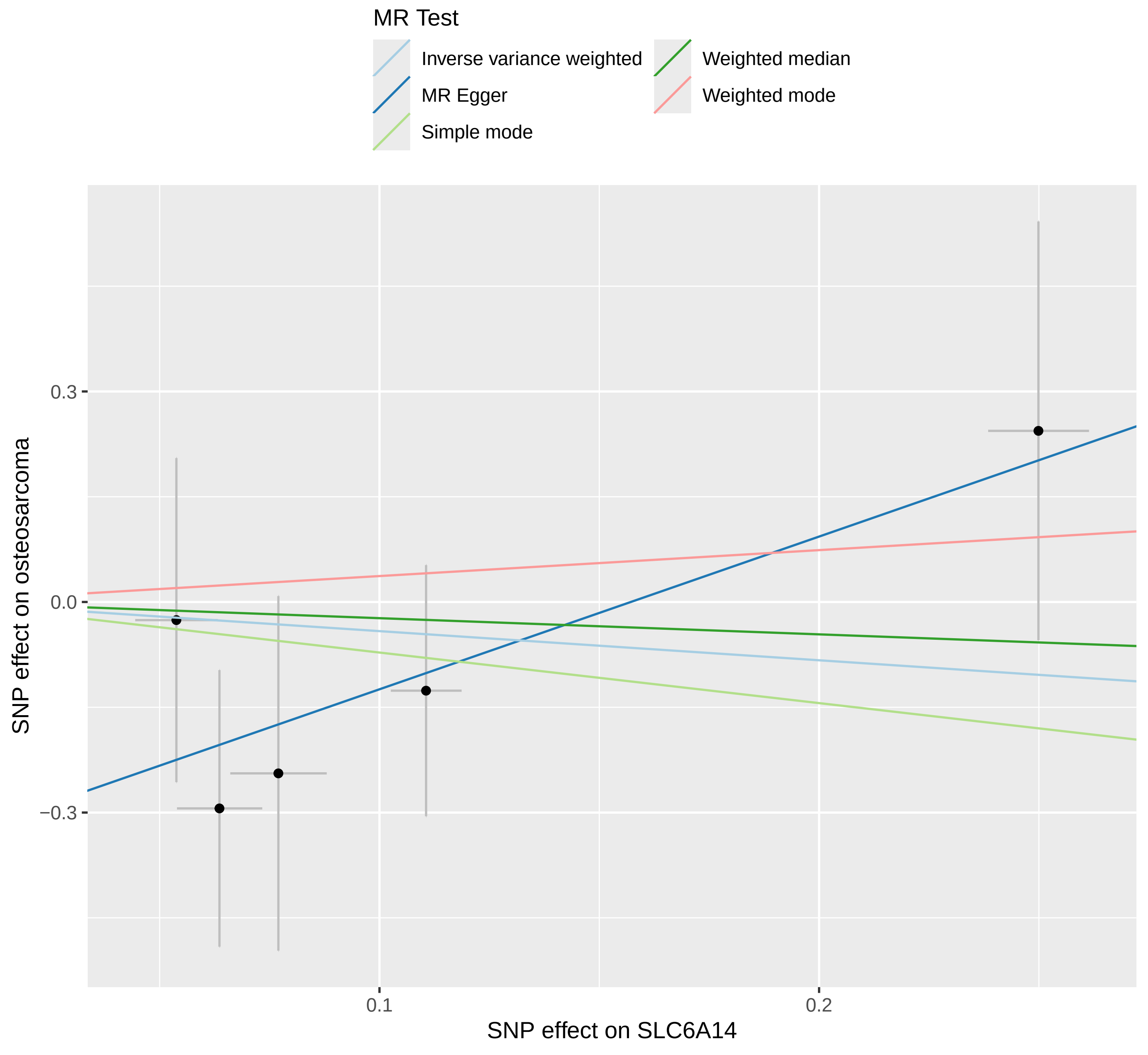
MR Test
Inverse variance weighted
Weighted median
MR Egger
Weighted mode
Simple mode
0.3
0.0
−0.3
0.1
0.2
SNP effect on osteosarcoma
SNP effect on SLC6A14

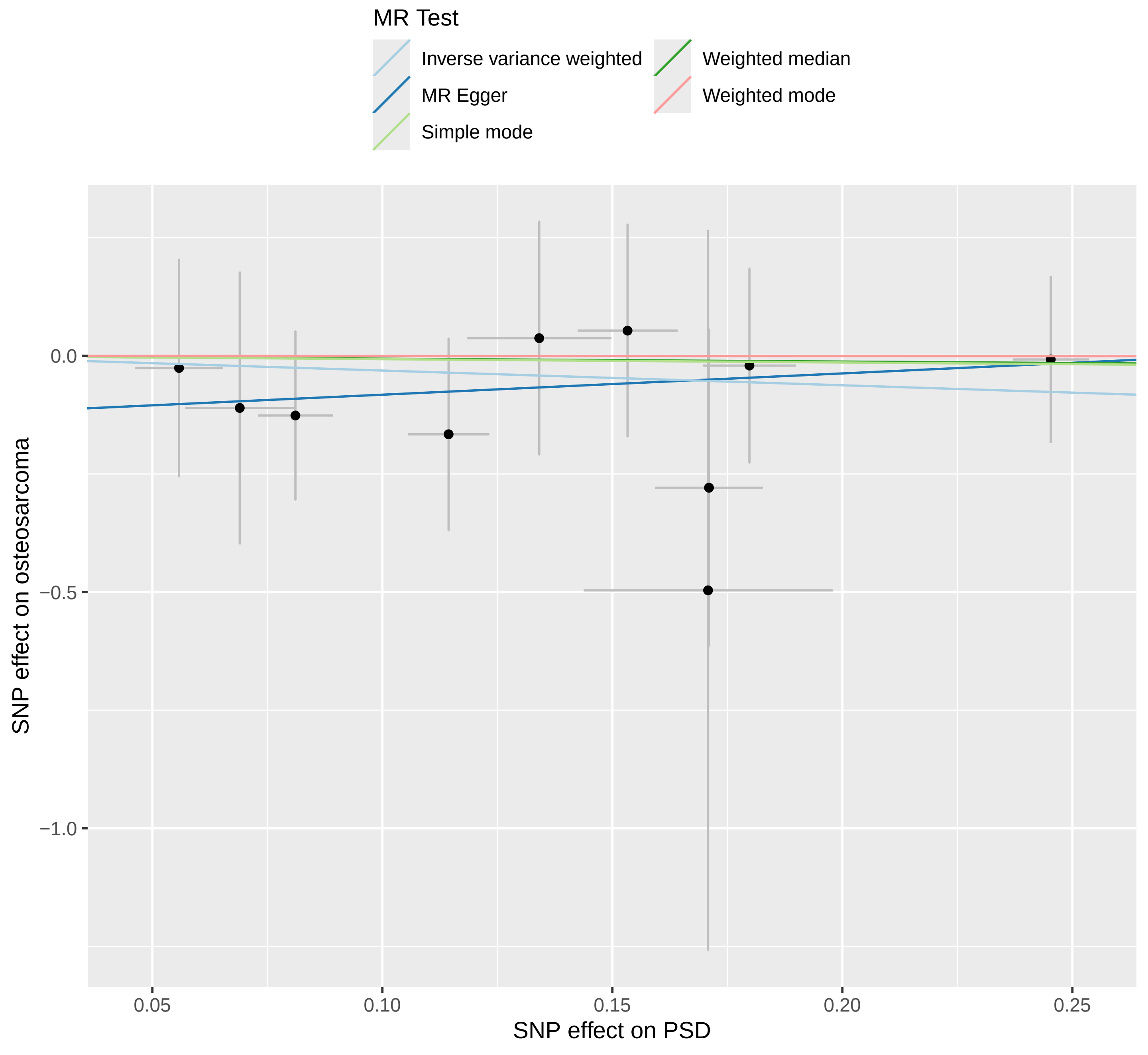

MR Test
Inverse variance weighted
MR Egger
Simple mode
Weighted median
Weighted mode
0.0
−0.5
−1.0
0.05
0.10
0.15
0.20
0.25
SNP effect on PSD
SNP effect on osteosarcoma

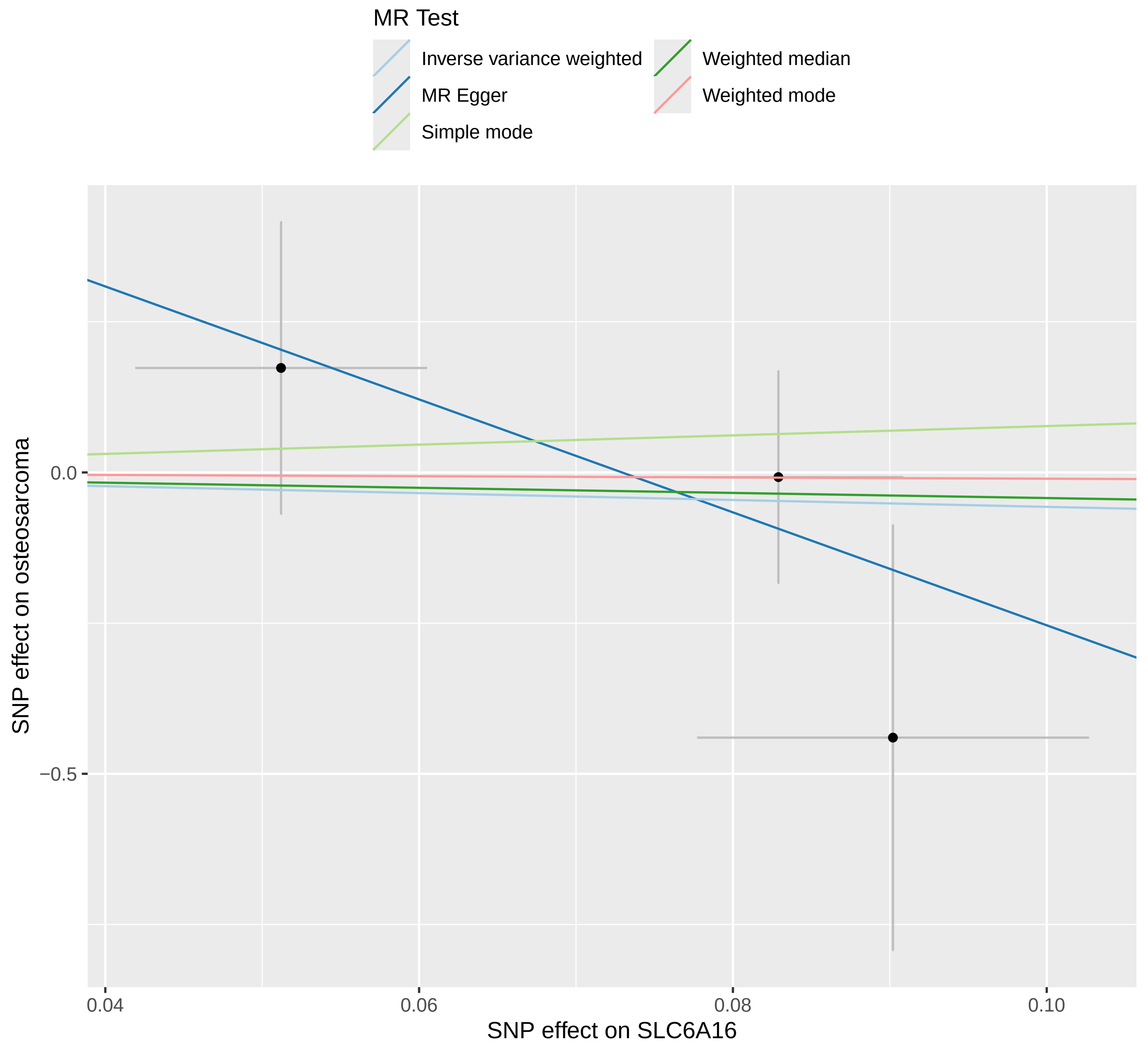
MR Test
Inverse variance weighted
MR Egger
Simple mode
Weighted median
Weighted mode
SNP effect on osteosarcoma
0.0
−0.5
0.04
0.06
0.08
0.10
SNP effect on SLC6A16

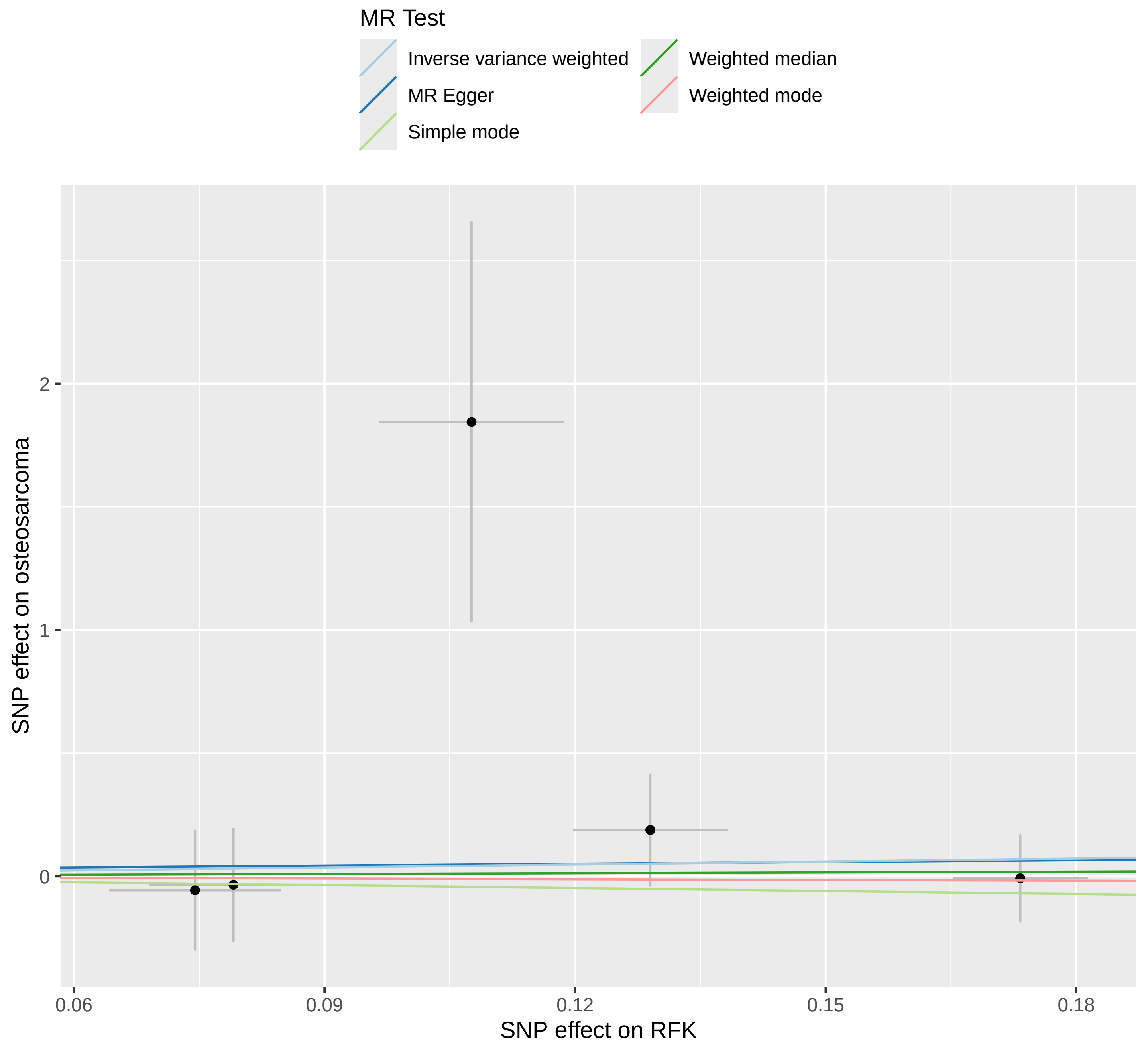
MR Test
Inverse variance weighted
MR Egger
Simple mode
Weighted median
Weighted mode
SNP effect on osteosarcoma
2
1
0
0.06
0.09
0.12
0.15
0.18
SNP effect on RFK

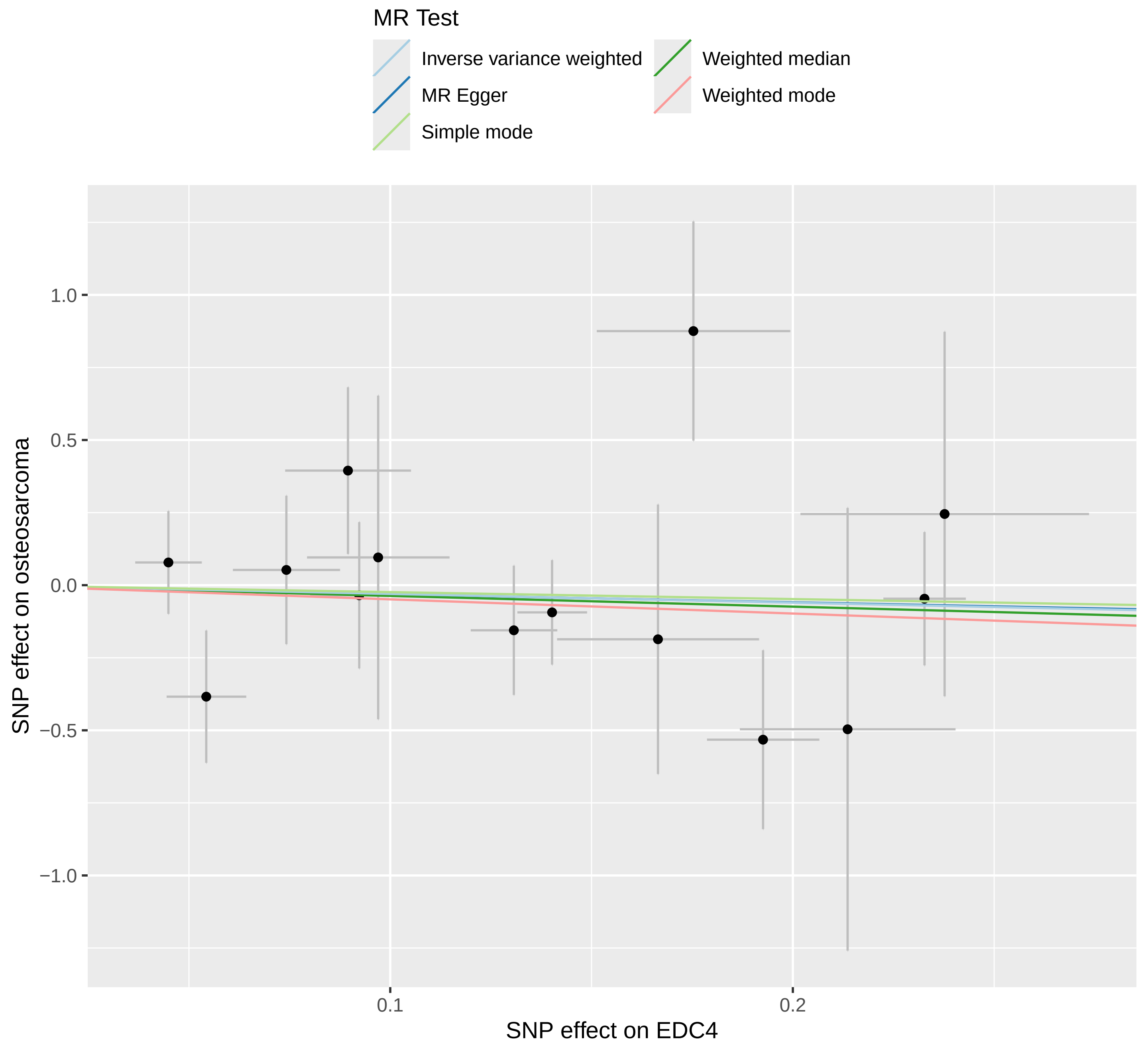
MR Test
Inverse variance weighted
MR Egger
Simple mode
Weighted median
Weighted mode
1.0
0.5
0.0
−0.5
−1.0
SNP effect on osteosarcoma
0.1
0.2
SNP effect on EDC4

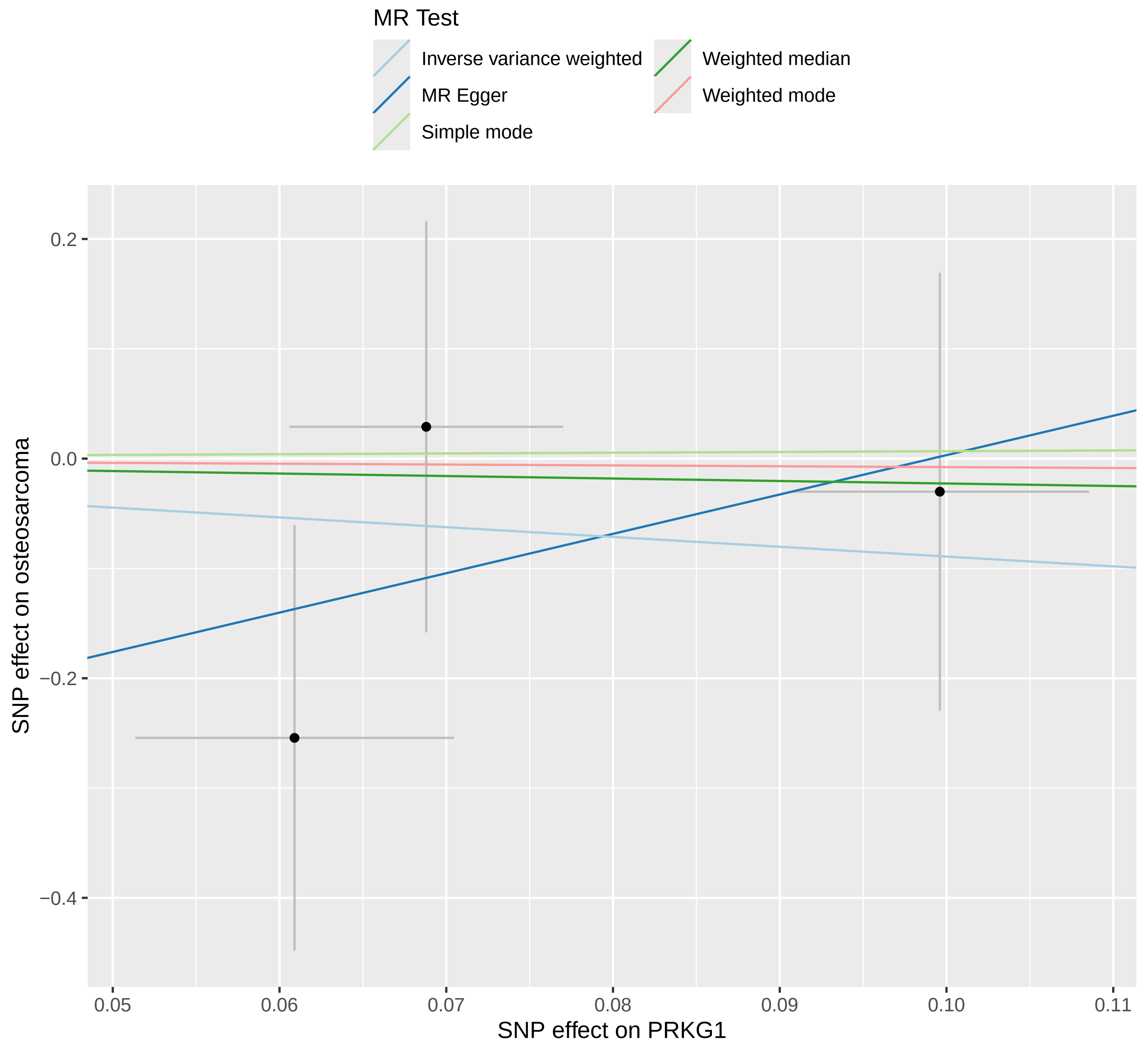
MR Test
Inverse variance weighted
MR Egger
Simple mode
Weighted median
Weighted mode
SNP effect on osteosarcoma
0.2
0.0
−0.2
−0.4
0.05
0.06
0.07
0.08
0.09
0.10
0.11
SNP effect on PRKG1

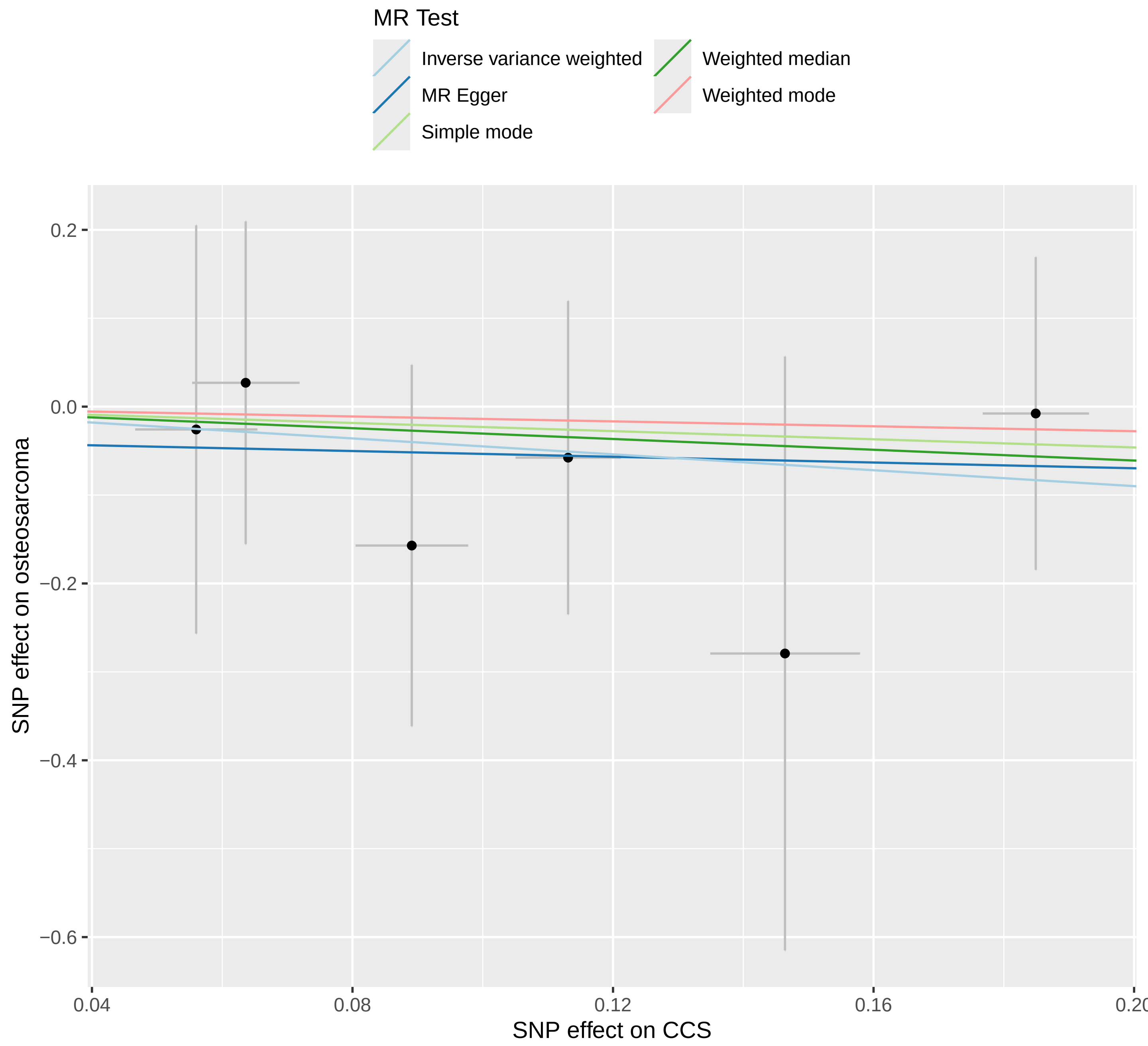

MR Test
Inverse variance weighted
MR Egger
Simple mode
Weighted median
Weighted mode
0.2
0.0
−0.2
−0.4
−0.6
SNP effect on osteosarcoma
0.04
0.08
0.12
0.16
0.20
SNP effect on CCS

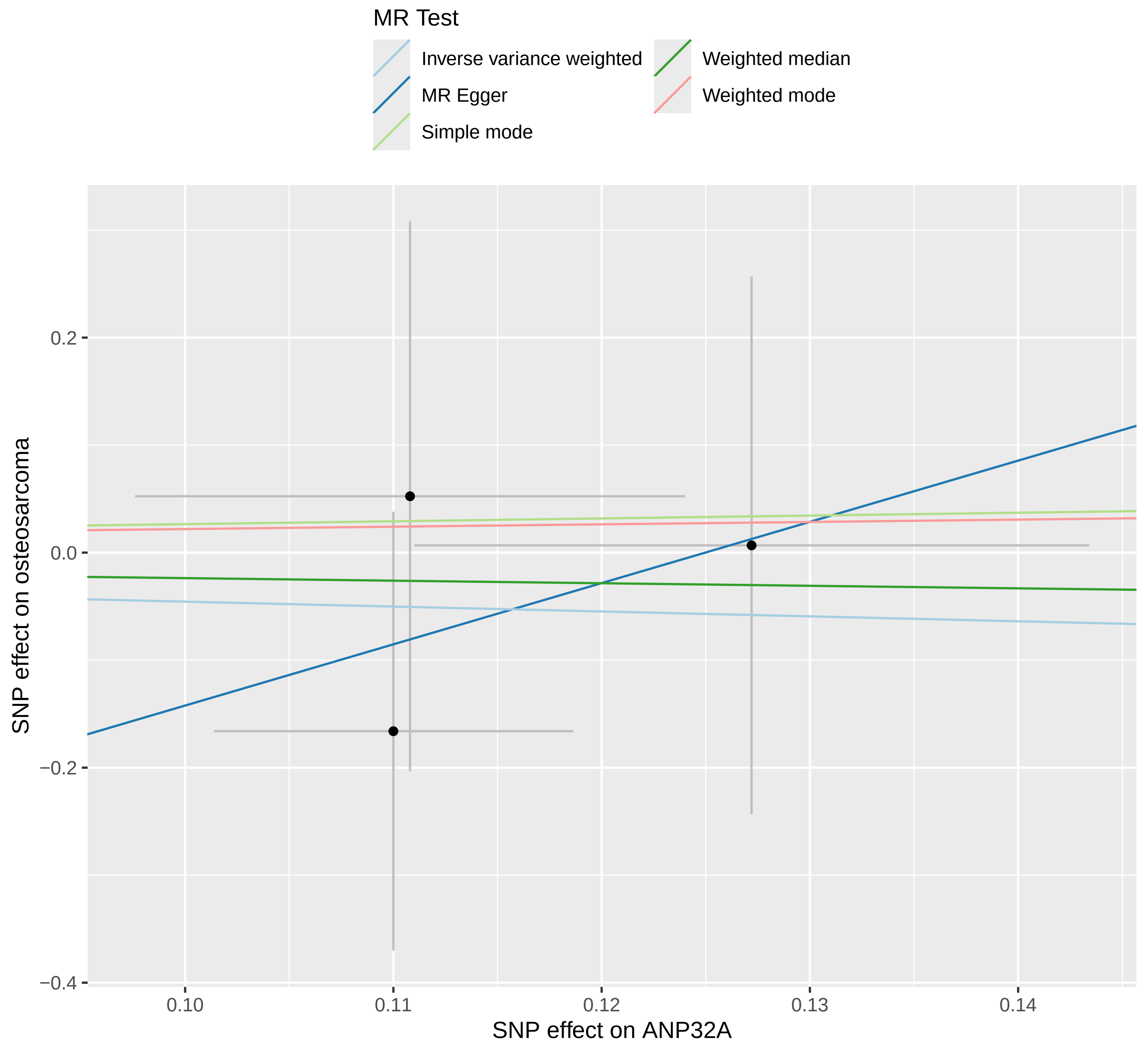
MR Test
Inverse variance weighted
MR Egger
Simple mode
Weighted median
Weighted mode
0.2
0.0
−0.2
−0.4
0.10
0.11
0.12
0.13
0.14
SNP effect on osteosarcoma
SNP effect on ANP32A

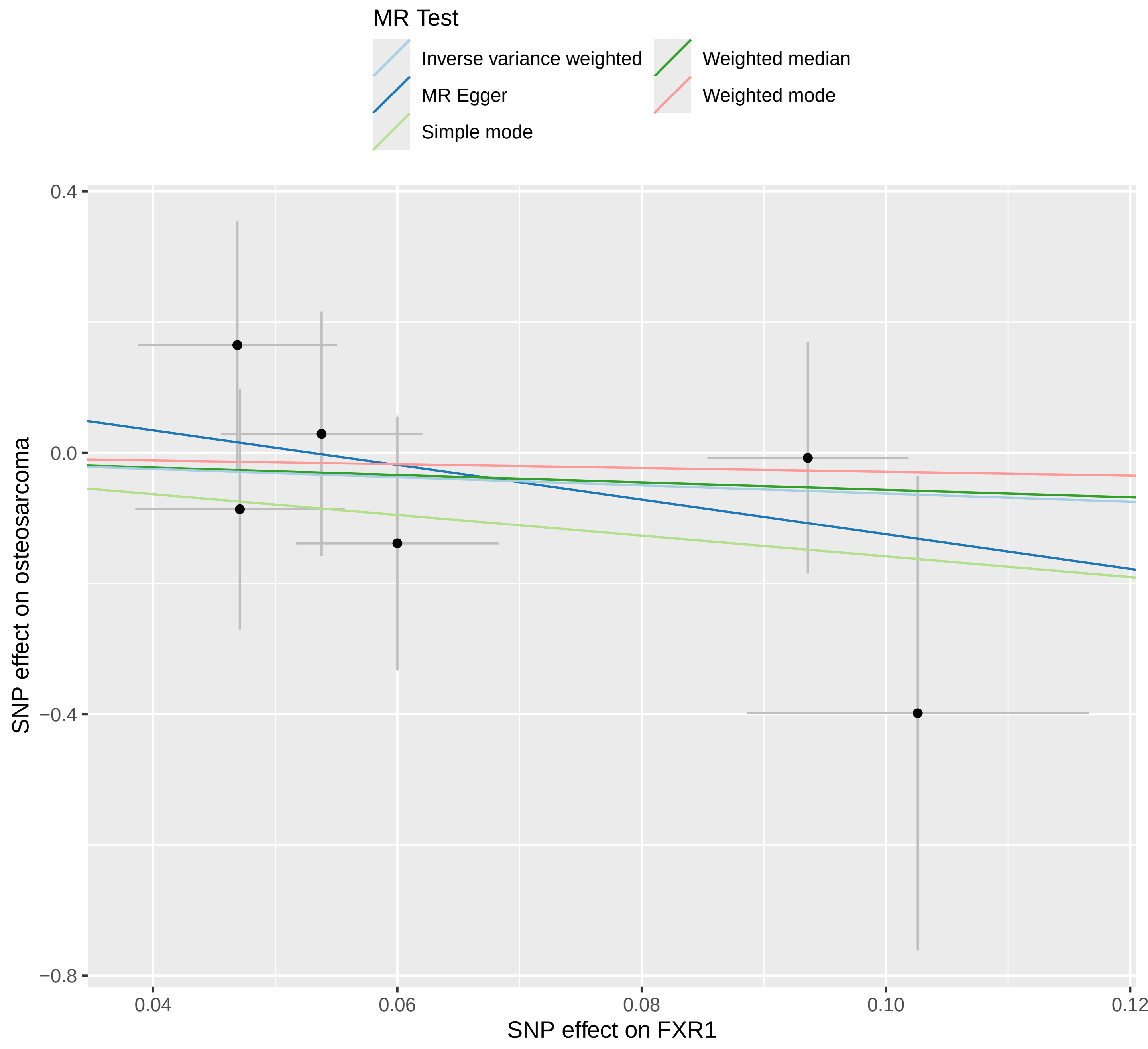
MR Test
Inverse variance weighted
MR Egger
Simple mode
Weighted median
Weighted mode
0.4
0.0
−0.4
−0.8
SNP effect on osteosarcoma
0.04
0.06
0.08
0.10
0.12
SNP effect on FXR1

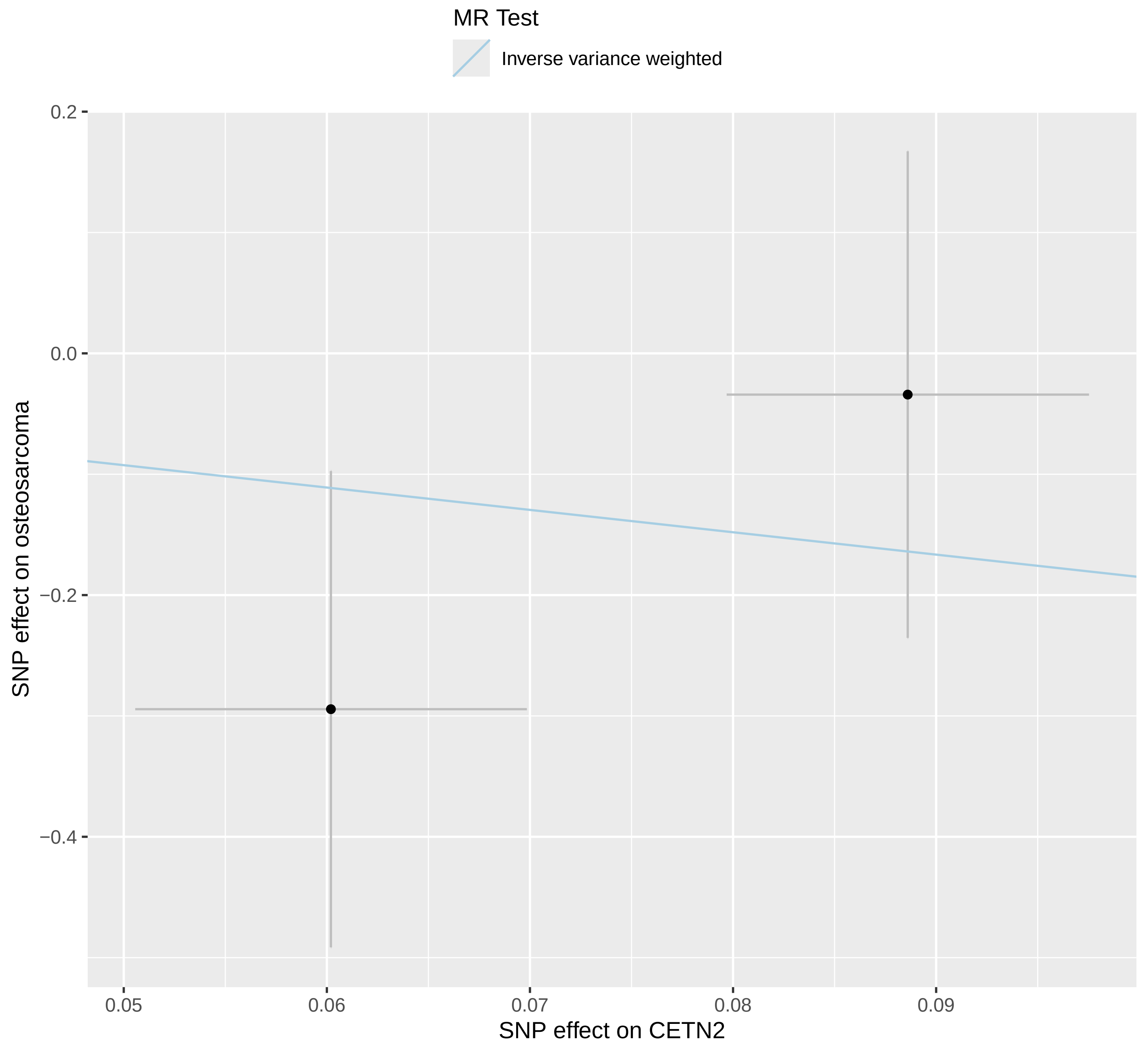
MR Test
Inverse variance weighted
0.2
0.0
−0.2
−0.4
0.05
0.06
0.07
0.08
0.09
SNP effect on CETN2
SNP effect on osteosarcoma

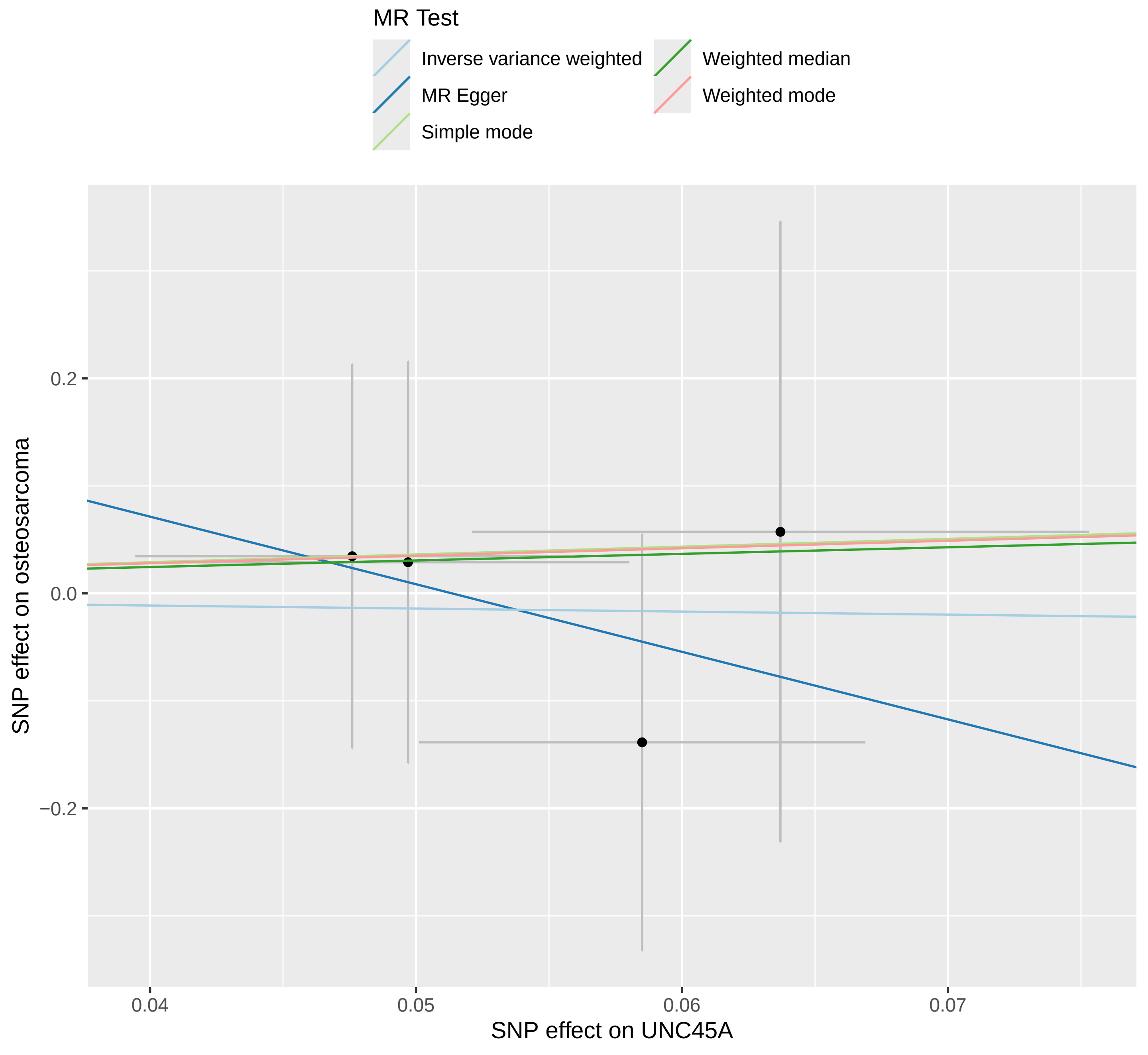

MR Test
Inverse variance weighted
MR Egger
Simple mode
Weighted median
Weighted mode
0.2
0.0
−0.2
0.04
0.05
0.06
0.07
SNP effect on osteosarcoma
SNP effect on UNC45A

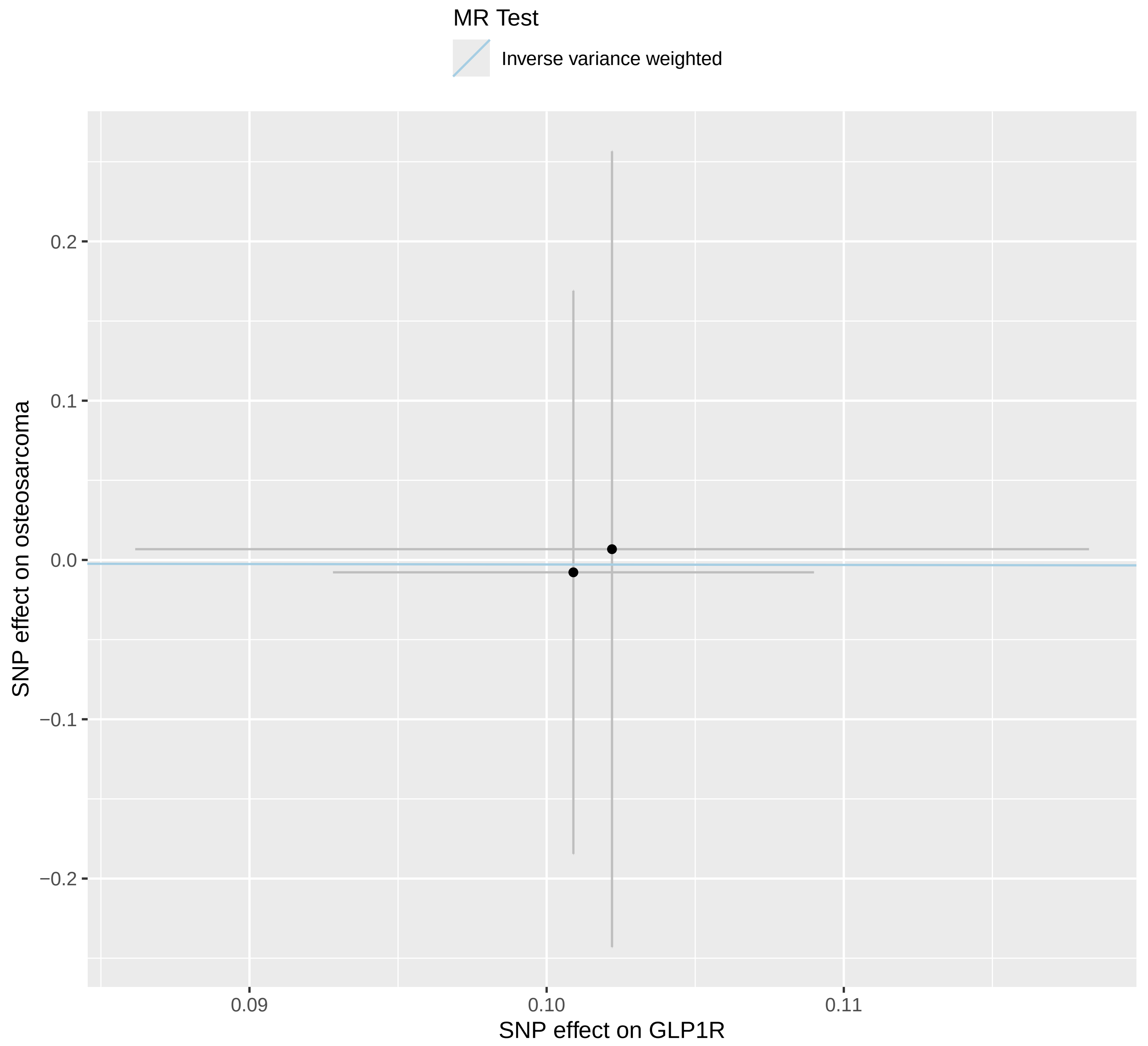
MR Test
Inverse variance weighted
0.2
0.1
0.0
−0.1
−0.2
SNP effect on osteosarcoma
0.09
0.10
0.11
SNP effect on GLP1R

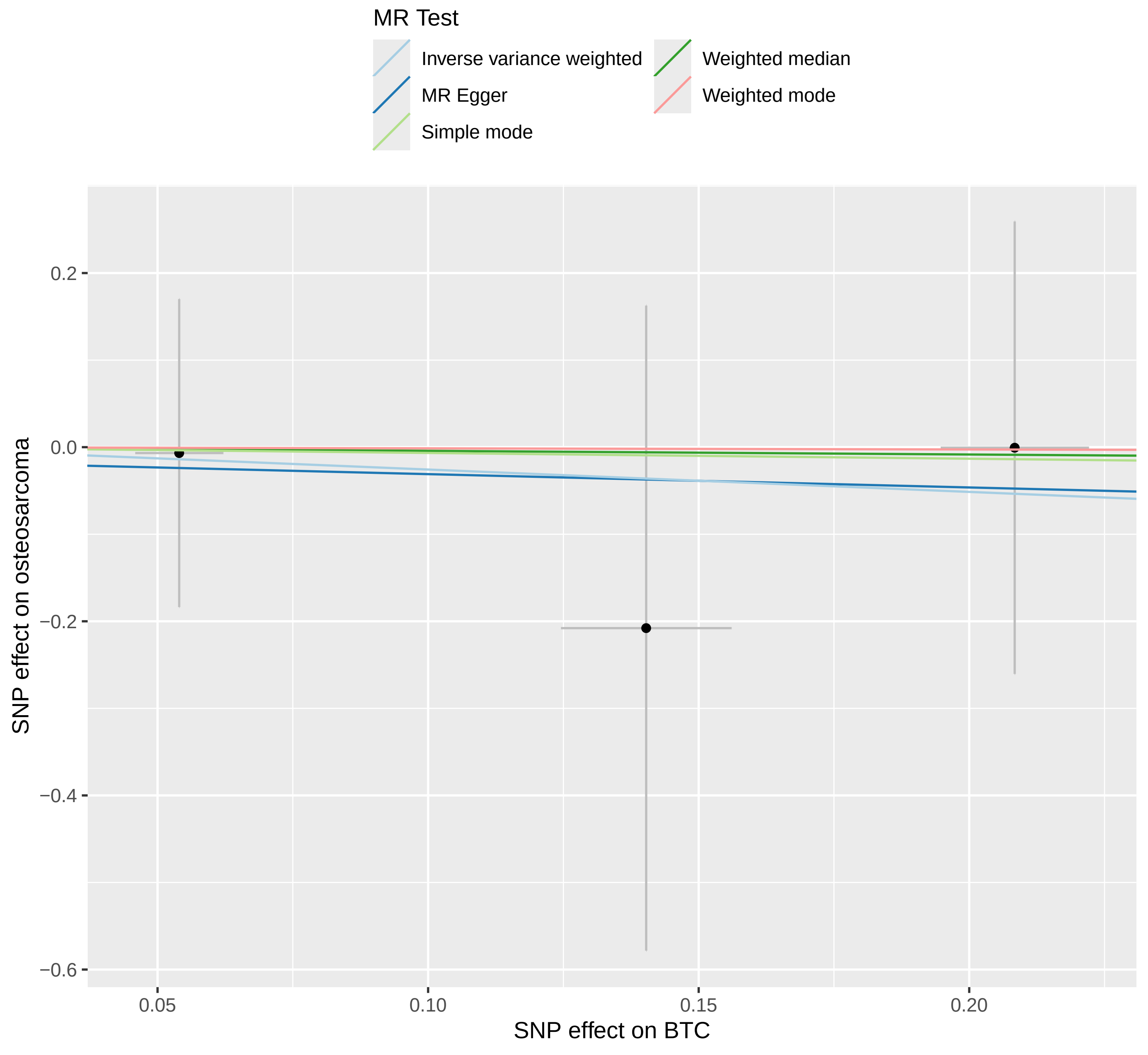
MR Test
Inverse variance weighted
MR Egger
Simple mode
Weighted median
Weighted mode
0.2
0.0
−0.2
−0.4
−0.6
SNP effect on osteosarcoma
0.05
0.10
0.15
0.20
SNP effect on BTC

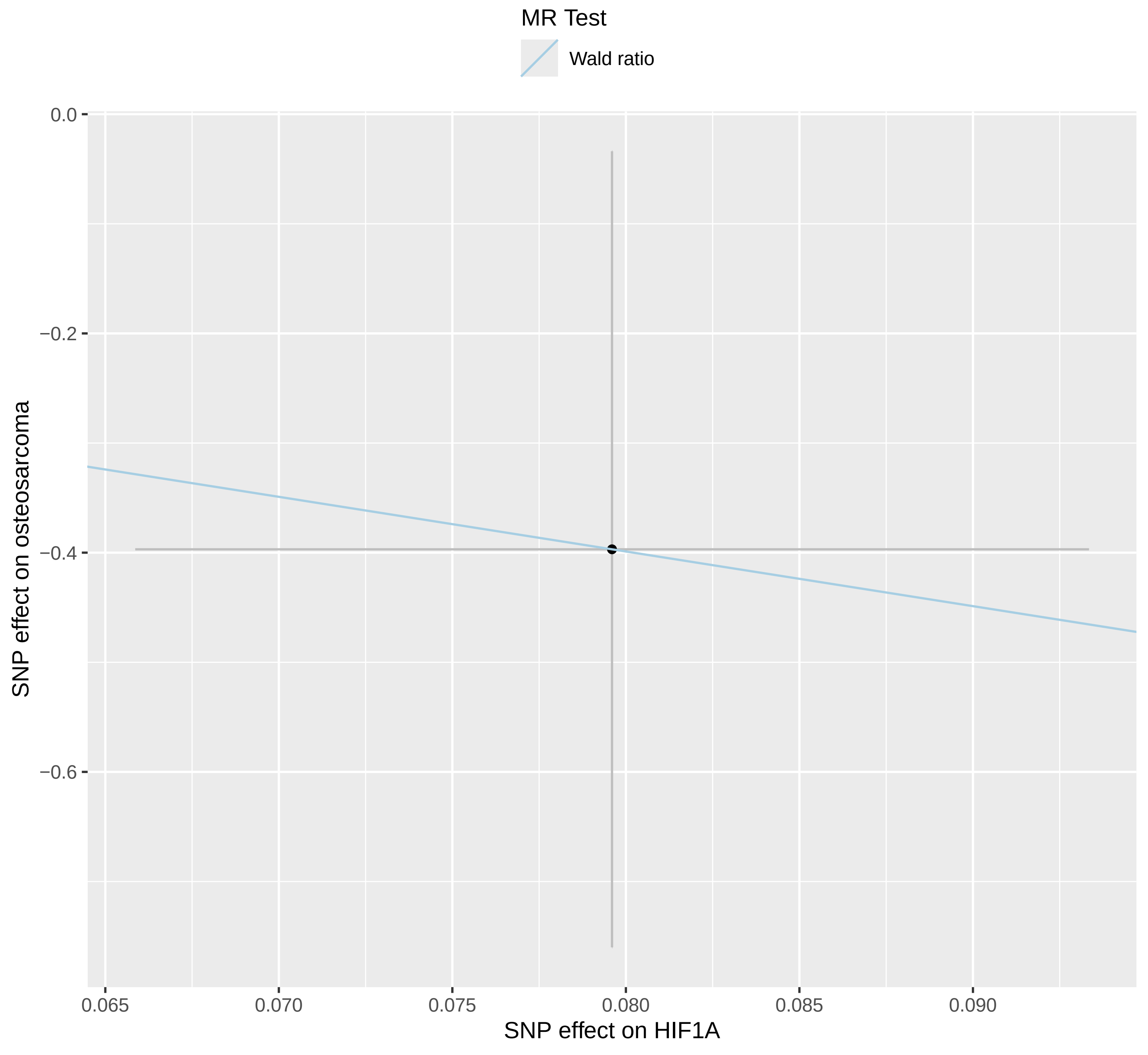

MR Test
Wald ratio
0.0
−0.2
−0.4
−0.6
SNP effect on osteosarcoma
0.065
0.070
0.075
0.080
0.085
0.090
SNP effect on HIF1A

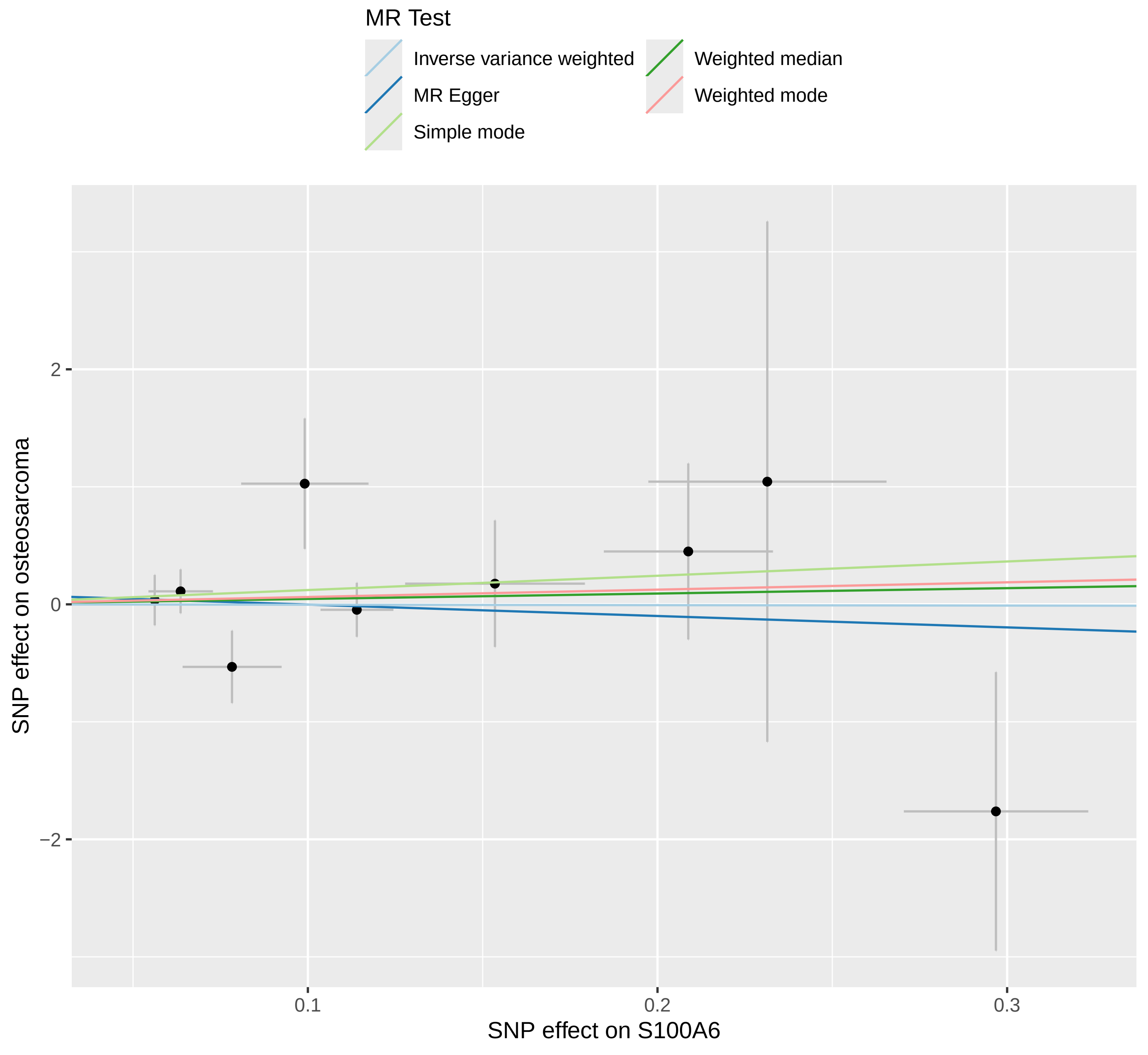
MR Test
Inverse variance weighted
MR Egger
Simple mode
Weighted median
Weighted mode
SNP effect on osteosarcoma
2
0
−2
0.1
0.2
0.3
SNP effect on S100A6

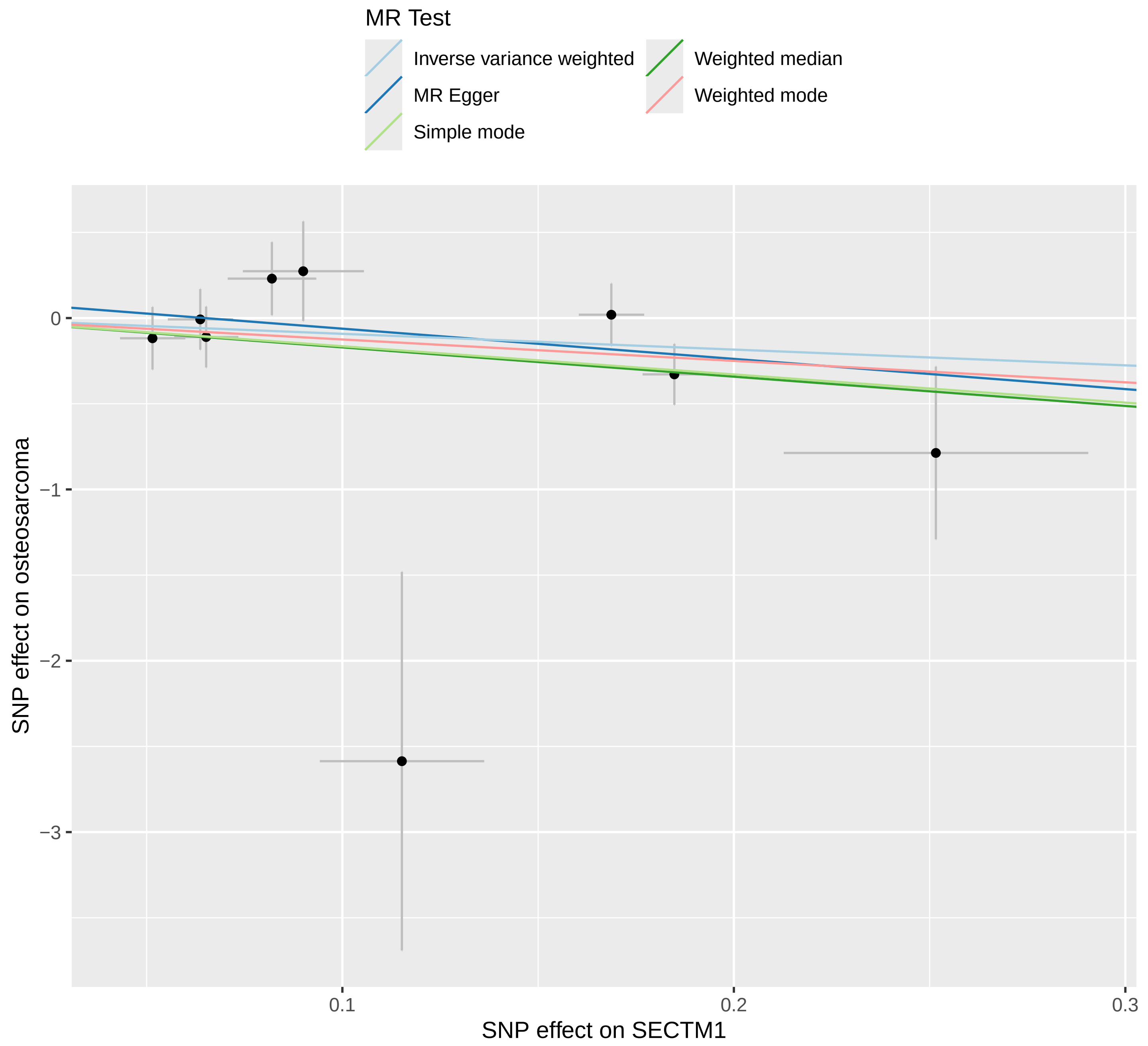
MR Test
Inverse variance weighted
MR Egger
Simple mode
Weighted median
Weighted mode
0
−1
−2
−3
0.1
0.2
0.3
SNP effect on SECTM1
SNP effect on osteosarcoma

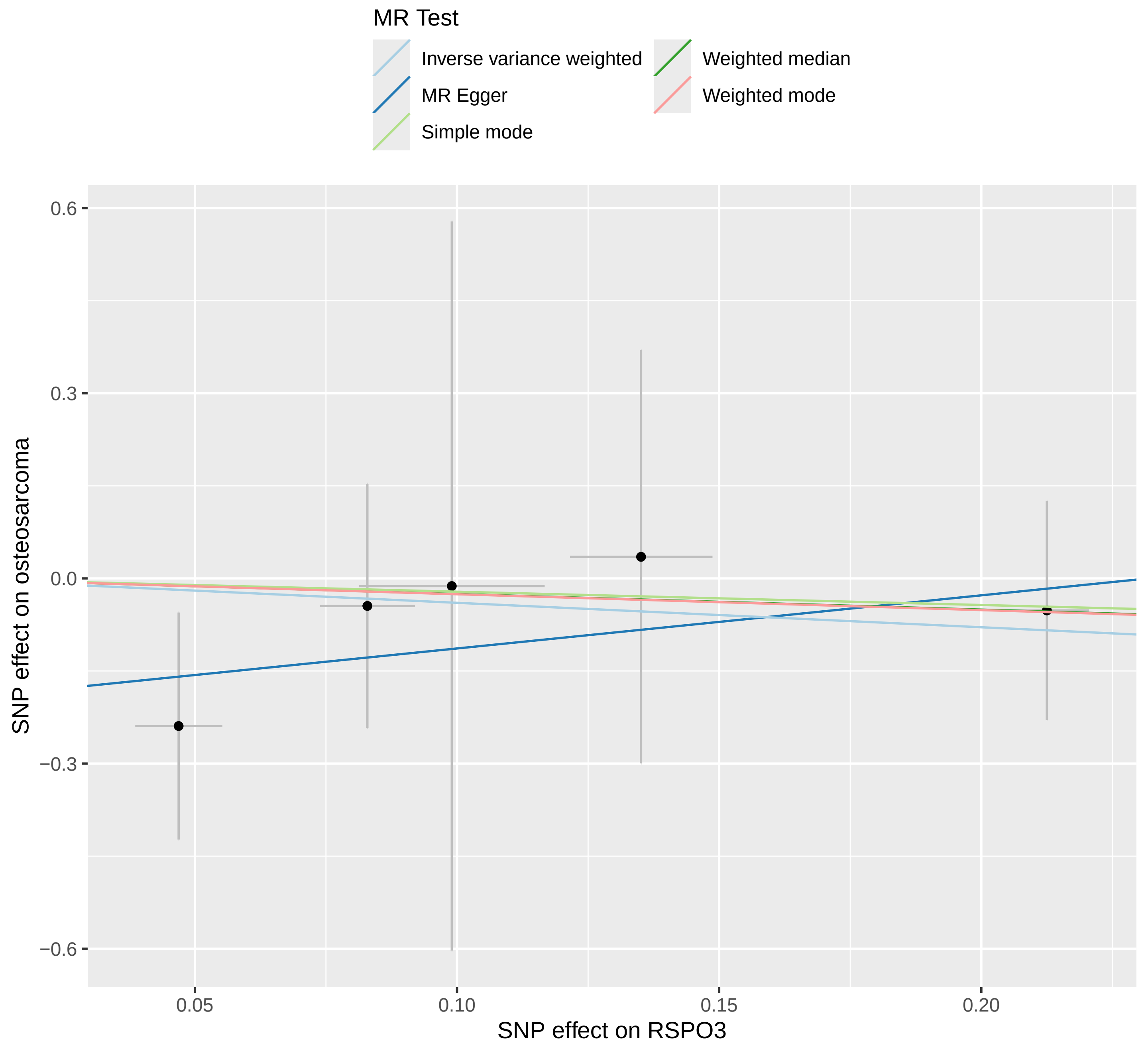
MR Test
Inverse variance weighted
MR Egger
Simple mode
Weighted median
Weighted mode
0.6
0.3
0.0
−0.3
−0.6
0.05
0.10
0.15
0.20
SNP effect on osteosarcoma
SNP effect on RSPO3

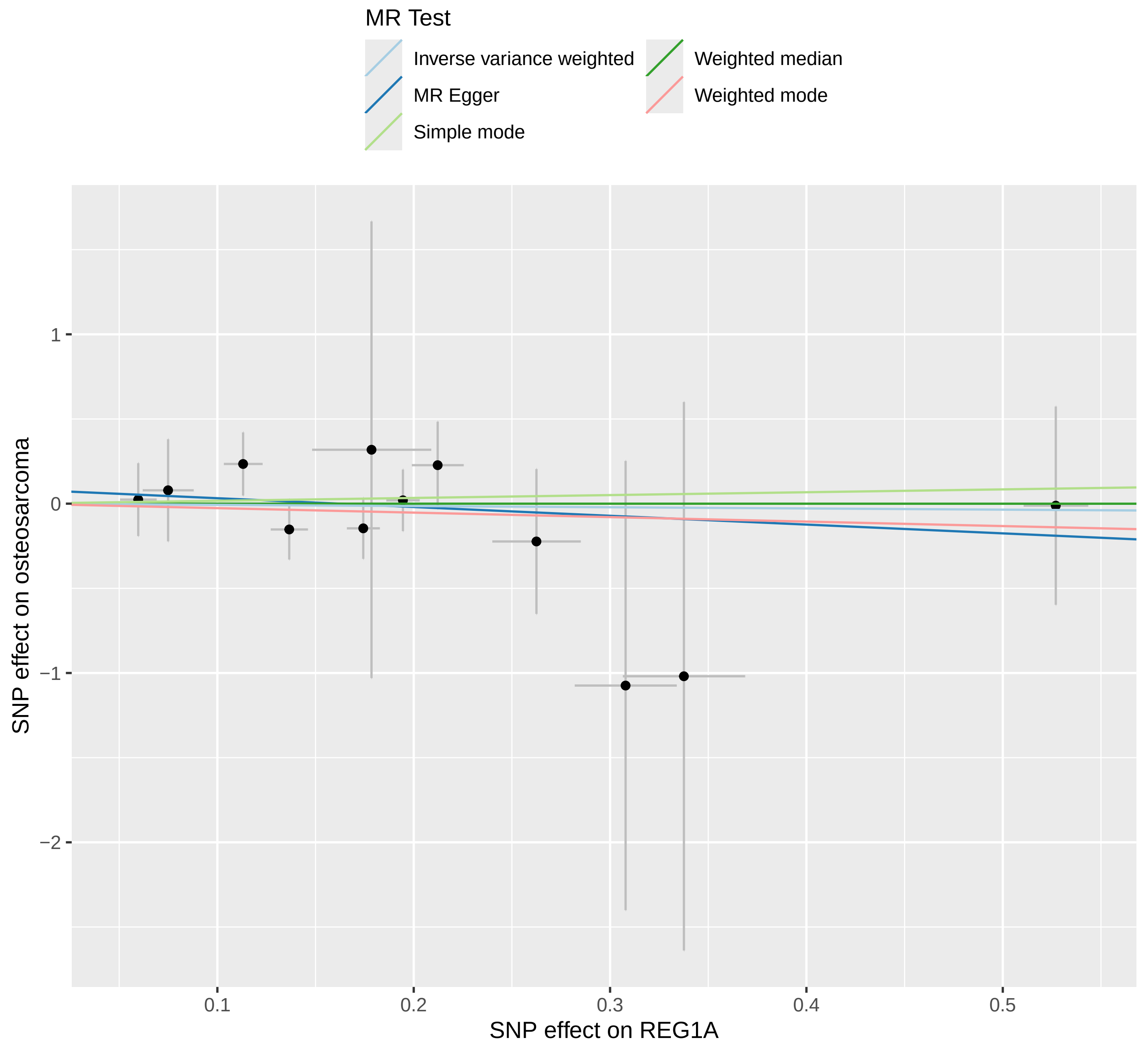
MR Test
Inverse variance weighted
MR Egger
Simple mode
Weighted median
Weighted mode
1
0
−1
−2
0.1
0.2
0.3
0.4
0.5
SNP effect on REG1A
SNP effect on osteosarcoma

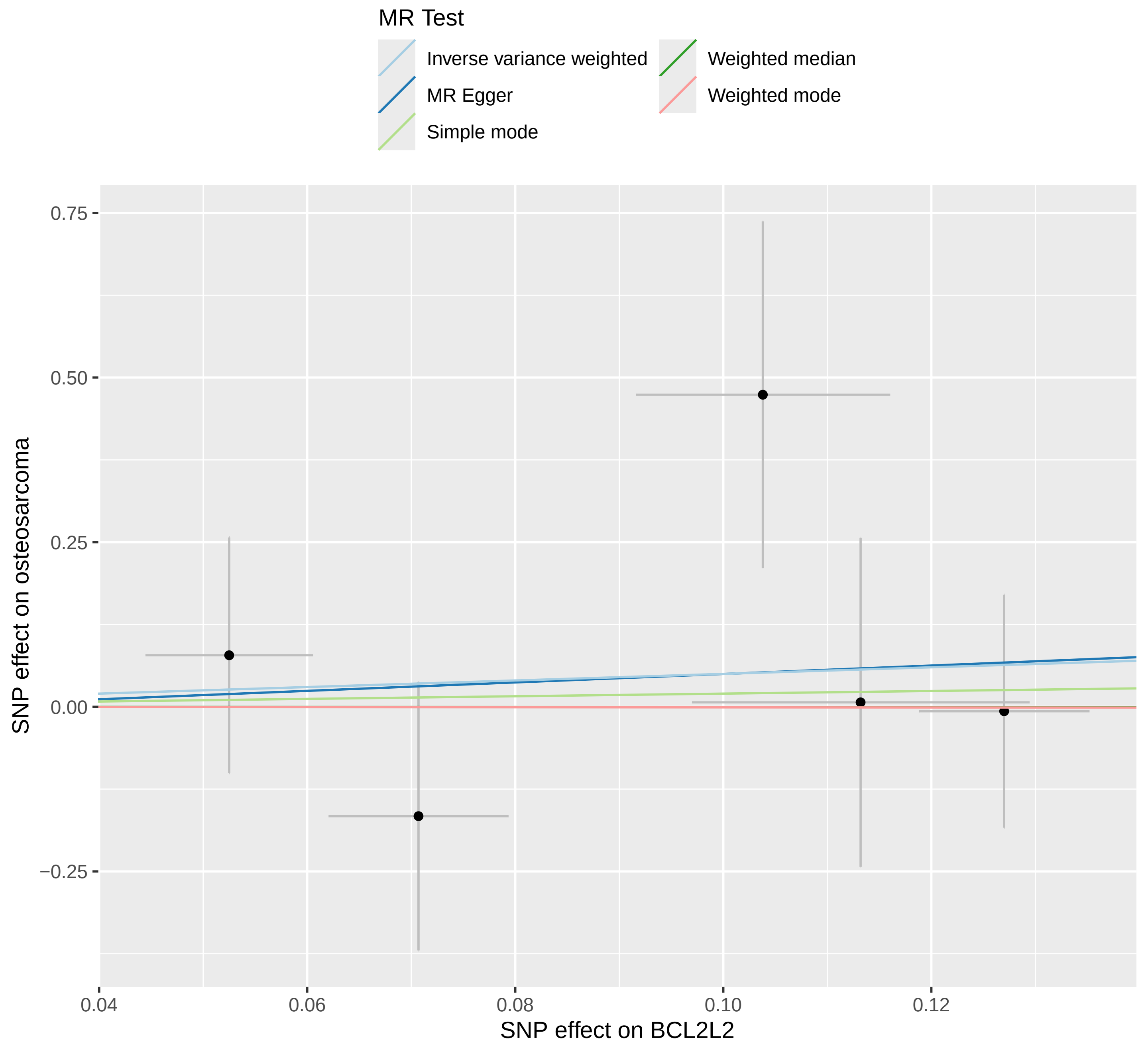
MR Test
Inverse variance weighted
MR Egger
Simple mode
Weighted median
Weighted mode
0.75
0.50
0.25
0.00
−0.25
SNP effect on osteosarcoma
0.04
0.06
0.08
0.10
0.12
SNP effect on BCL2L2

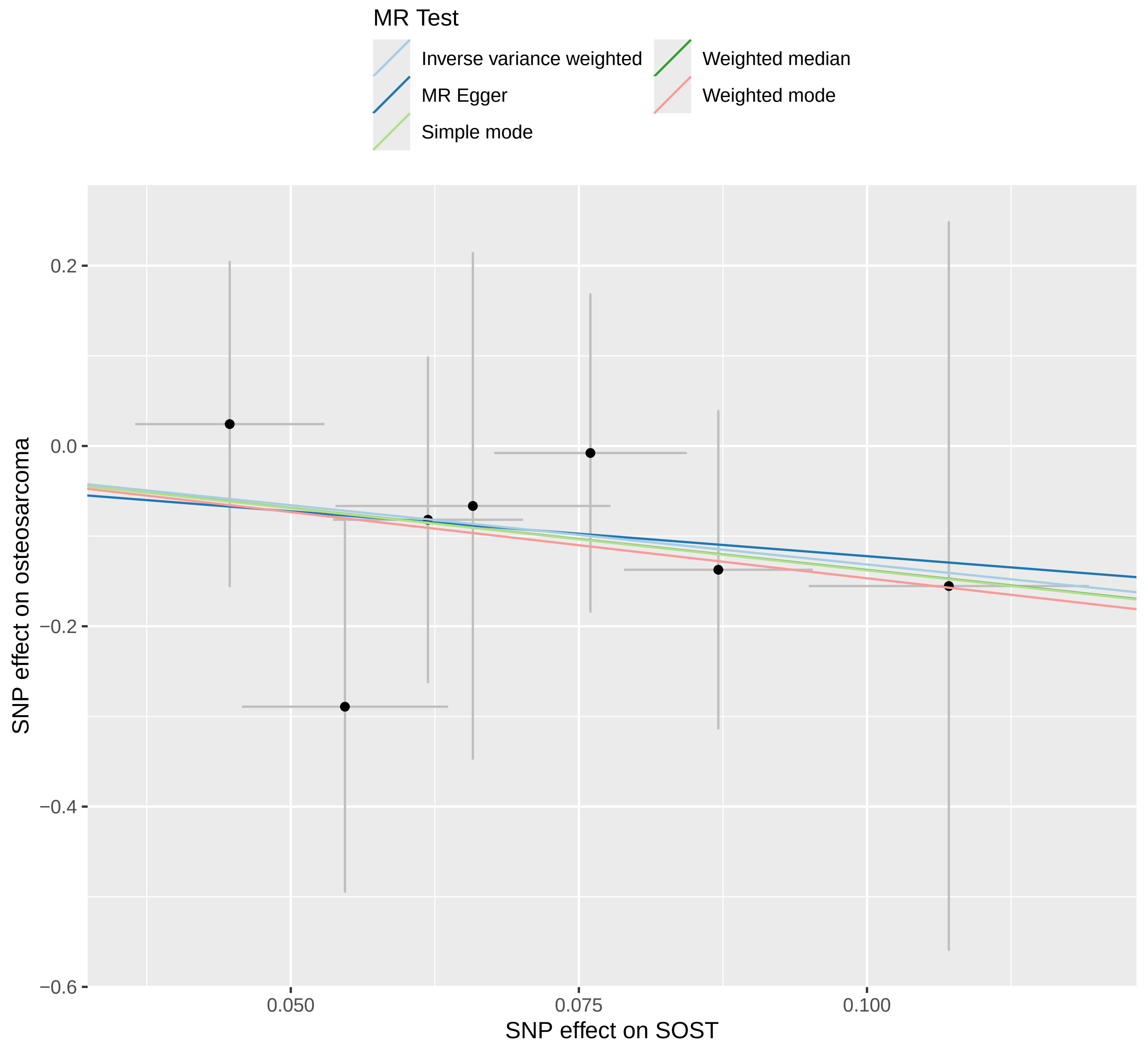

MR Test
Inverse variance weighted
MR Egger
Simple mode
Weighted median
Weighted mode
0.2
0.0
−0.2
−0.4
−0.6
SNP effect on osteosarcoma
0.050
0.075
0.100
SNP effect on SOST

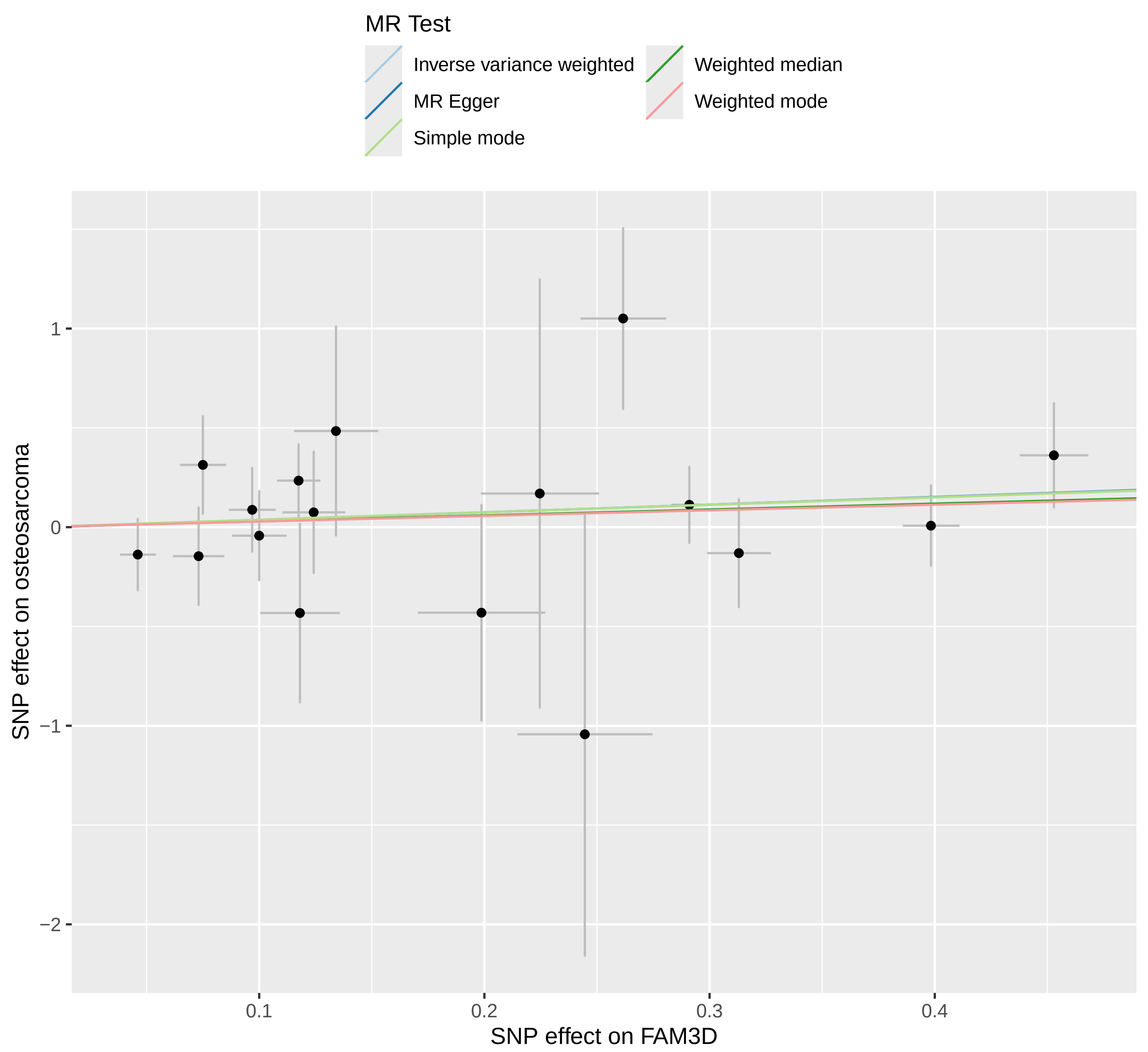
MR Test
Inverse variance weighted
MR Egger
Simple mode
Weighted median
Weighted mode
SNP effect on osteosarcoma
1
0
−1
−2
0.1
0.2
0.3
0.4
SNP effect on FAM3D

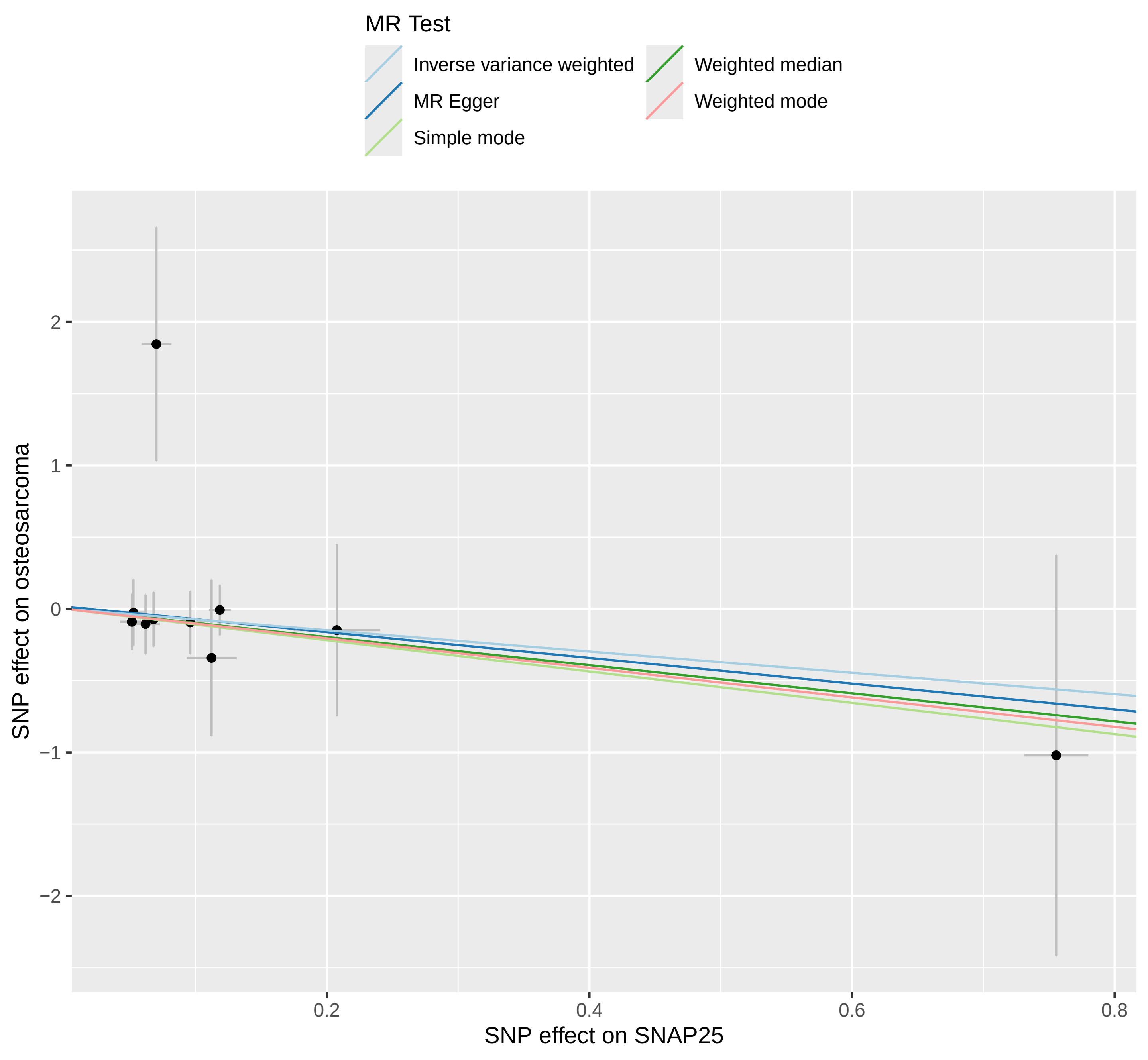
MR Test
Inverse variance weighted
MR Egger
Simple mode
Weighted median
Weighted mode
SNP effect on osteosarcoma
2
1
0
−1
−2
0.2
0.4
0.6
0.8
SNP effect on SNAP25

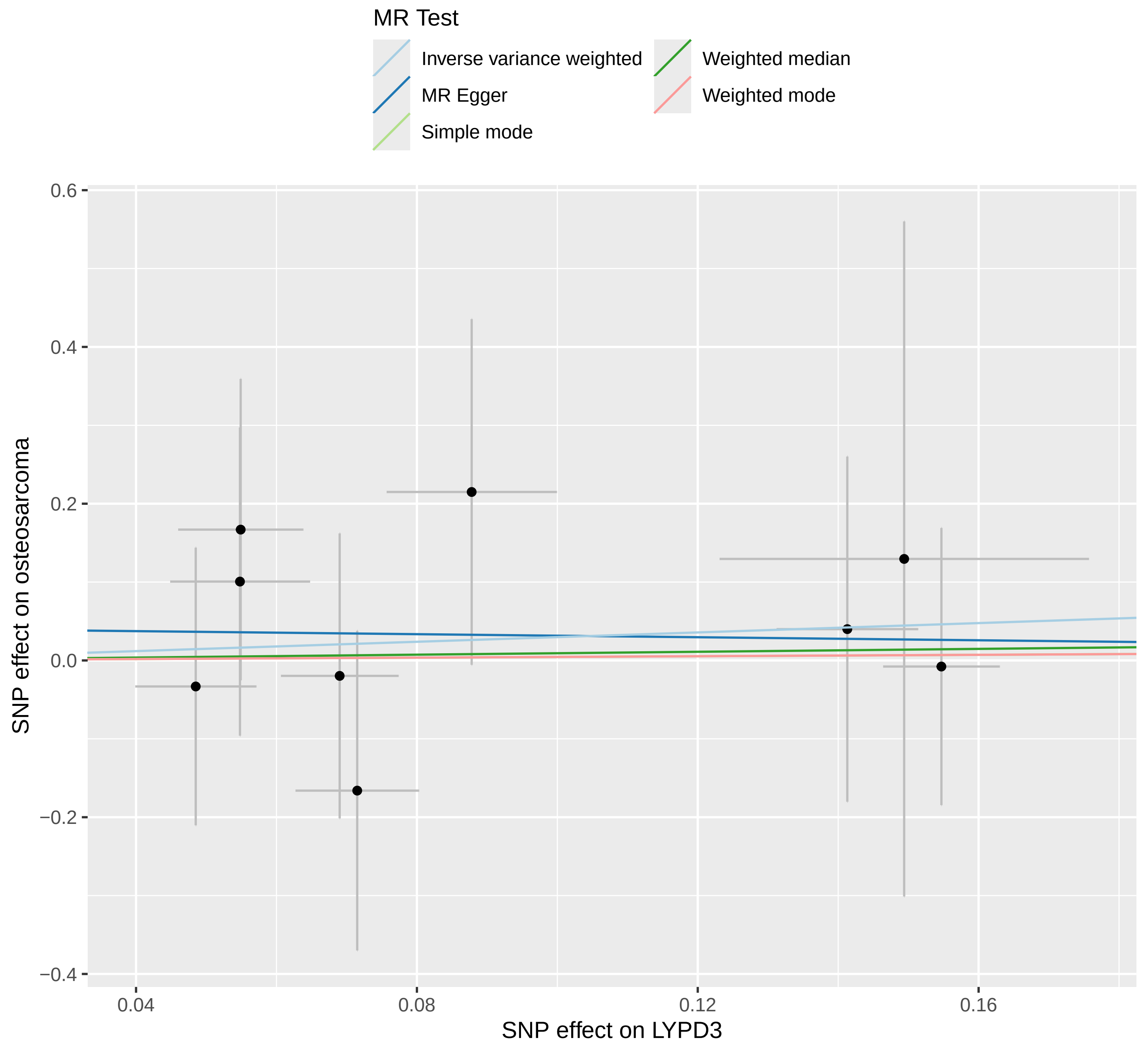
MR Test
Inverse variance weighted
MR Egger
Simple mode
Weighted median
Weighted mode
0.6
0.4
0.2
0.0
−0.2
−0.4
0.04
0.08
0.12
0.16
SNP effect on osteosarcoma
SNP effect on LYPD3

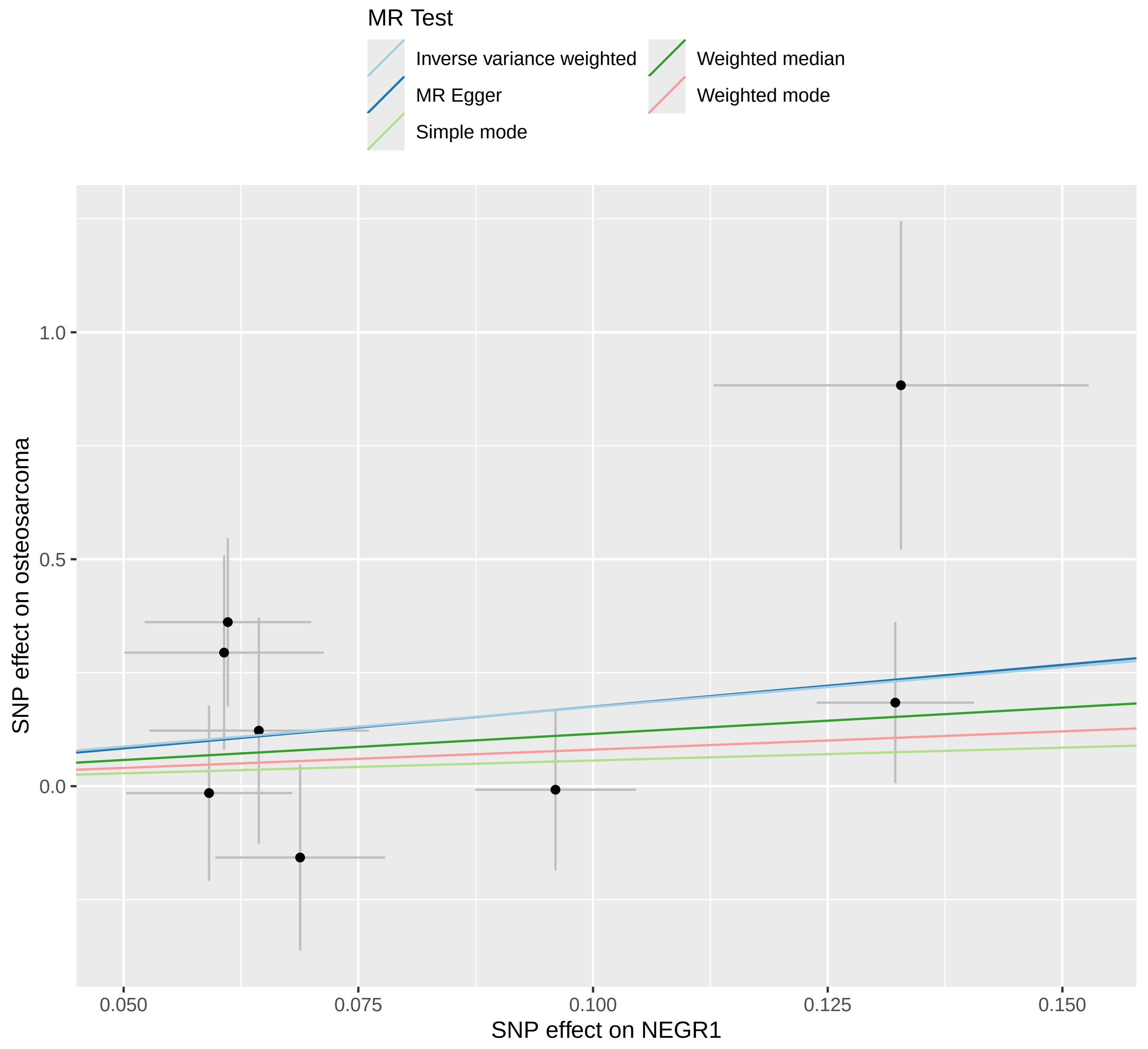
MR Test
Inverse variance weighted
MR Egger
Simple mode
Weighted median
Weighted mode
1.0
0.5
0.0
0.050
0.075
0.100
0.125
0.150
SNP effect on NEGR1
SNP effect on osteosarcoma

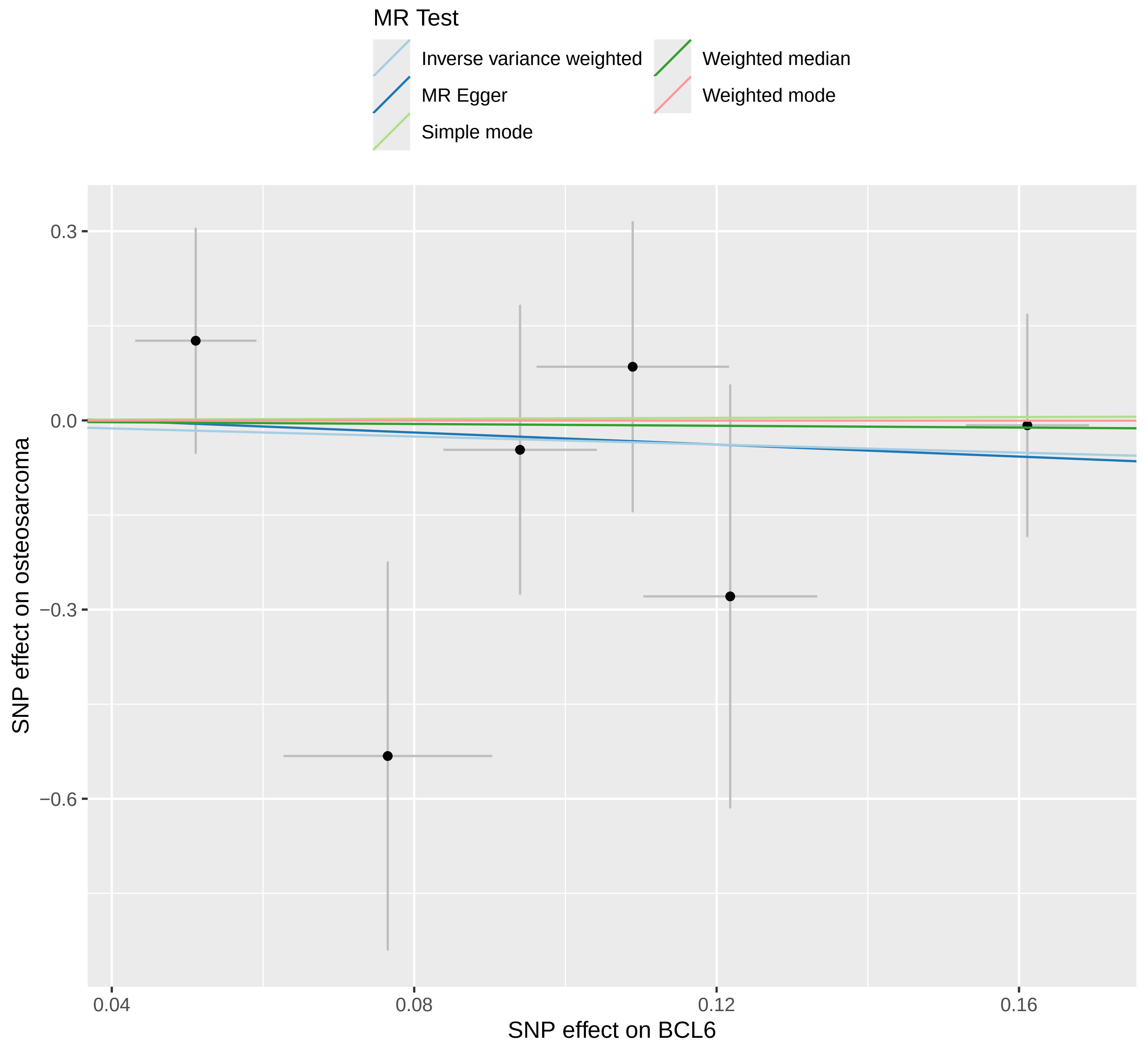
MR Test
Inverse variance weighted
MR Egger
Simple mode
Weighted median
Weighted mode
0.3
0.0
−0.3
−0.6
SNP effect on osteosarcoma
0.04
0.08
0.12
0.16
SNP effect on BCL6

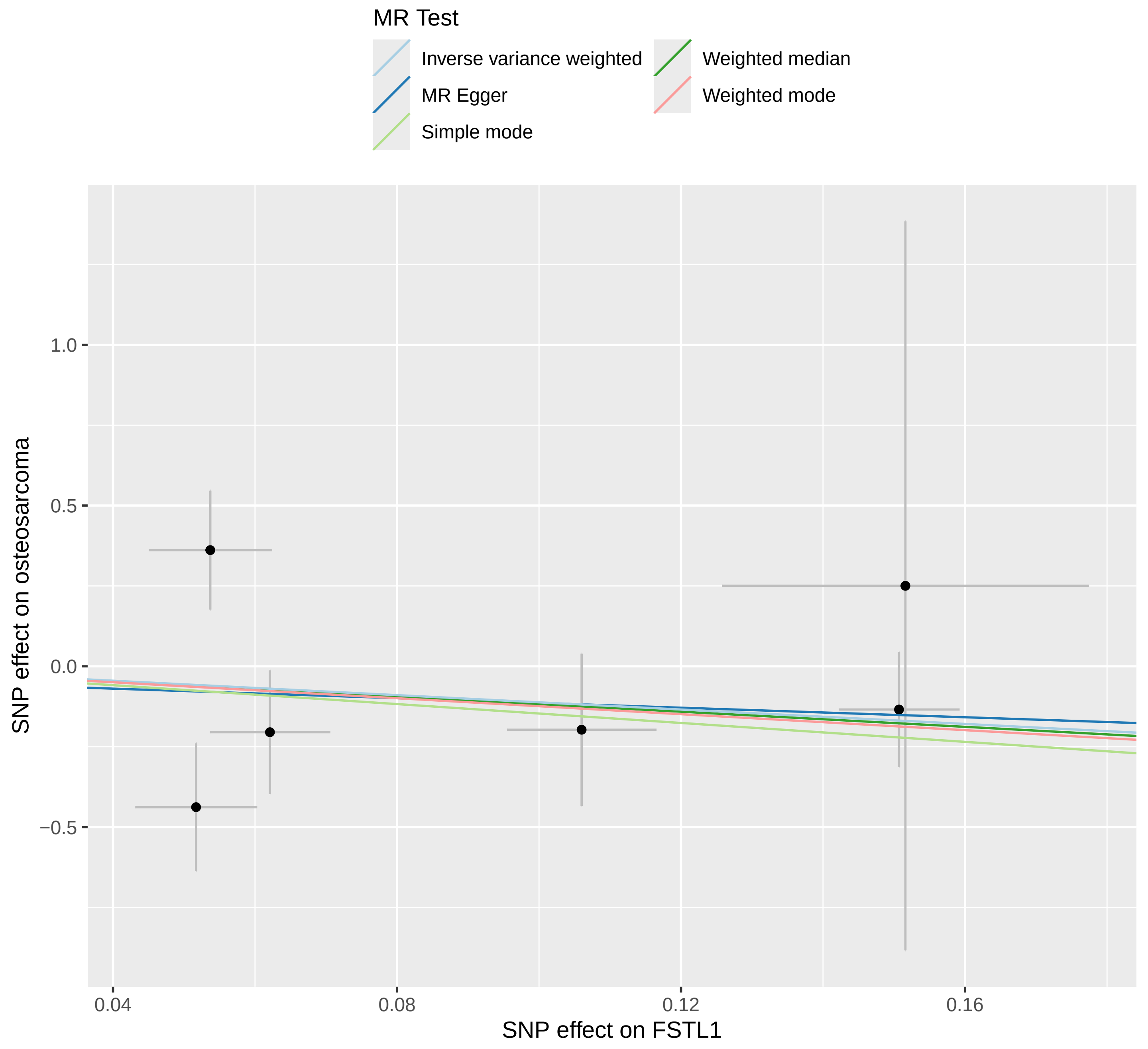
MR Test
Inverse variance weighted
MR Egger
Simple mode
Weighted median
Weighted mode
1.0
0.5
0.0
−0.5
SNP effect on osteosarcoma
0.04
0.08
0.12
0.16
SNP effect on FSTL1

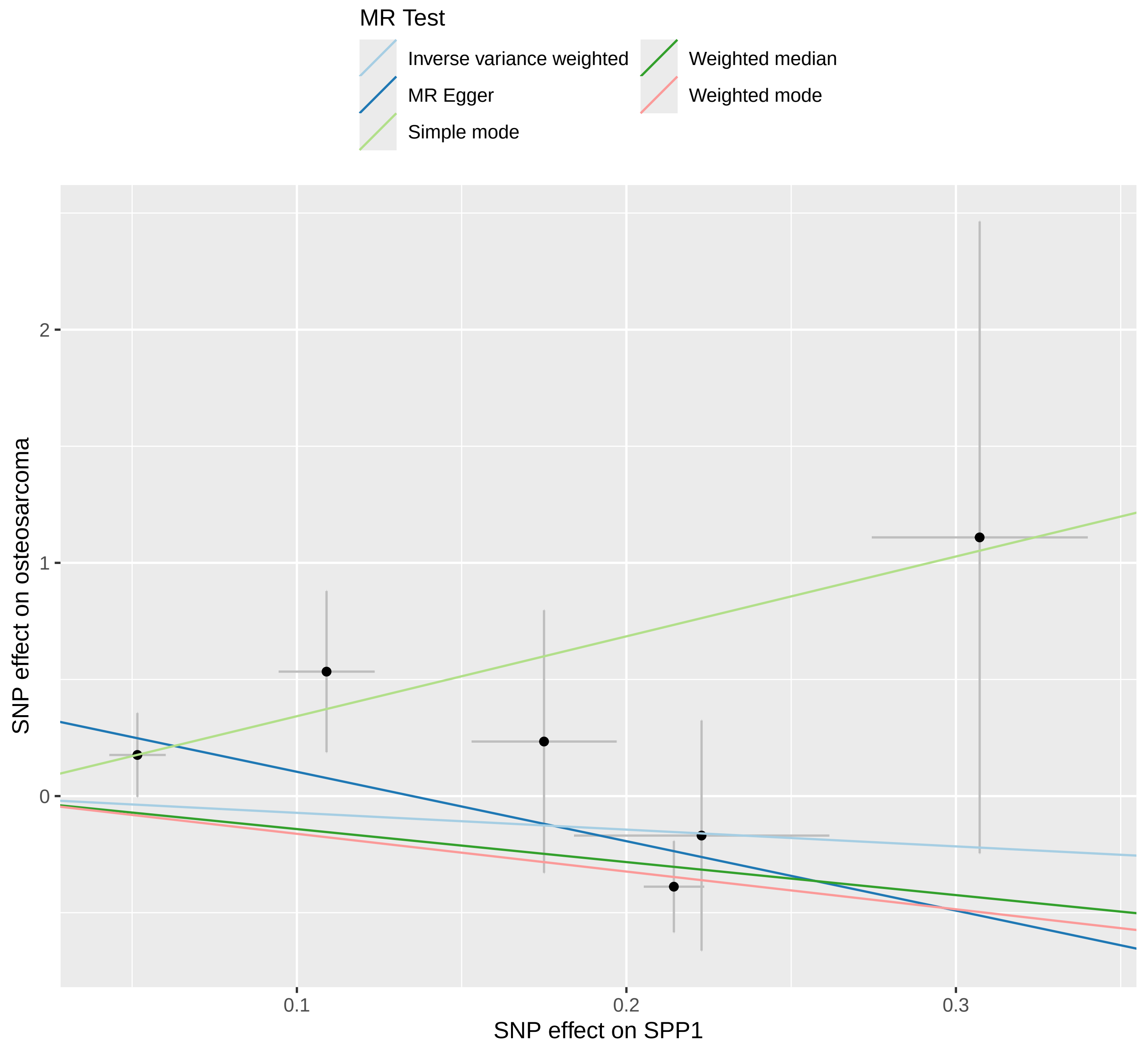
MR Test
Inverse variance weighted
MR Egger
Simple mode
Weighted median
Weighted mode
SNP effect on osteosarcoma
2
1
0
0.1
0.2
0.3
SNP effect on SPP1

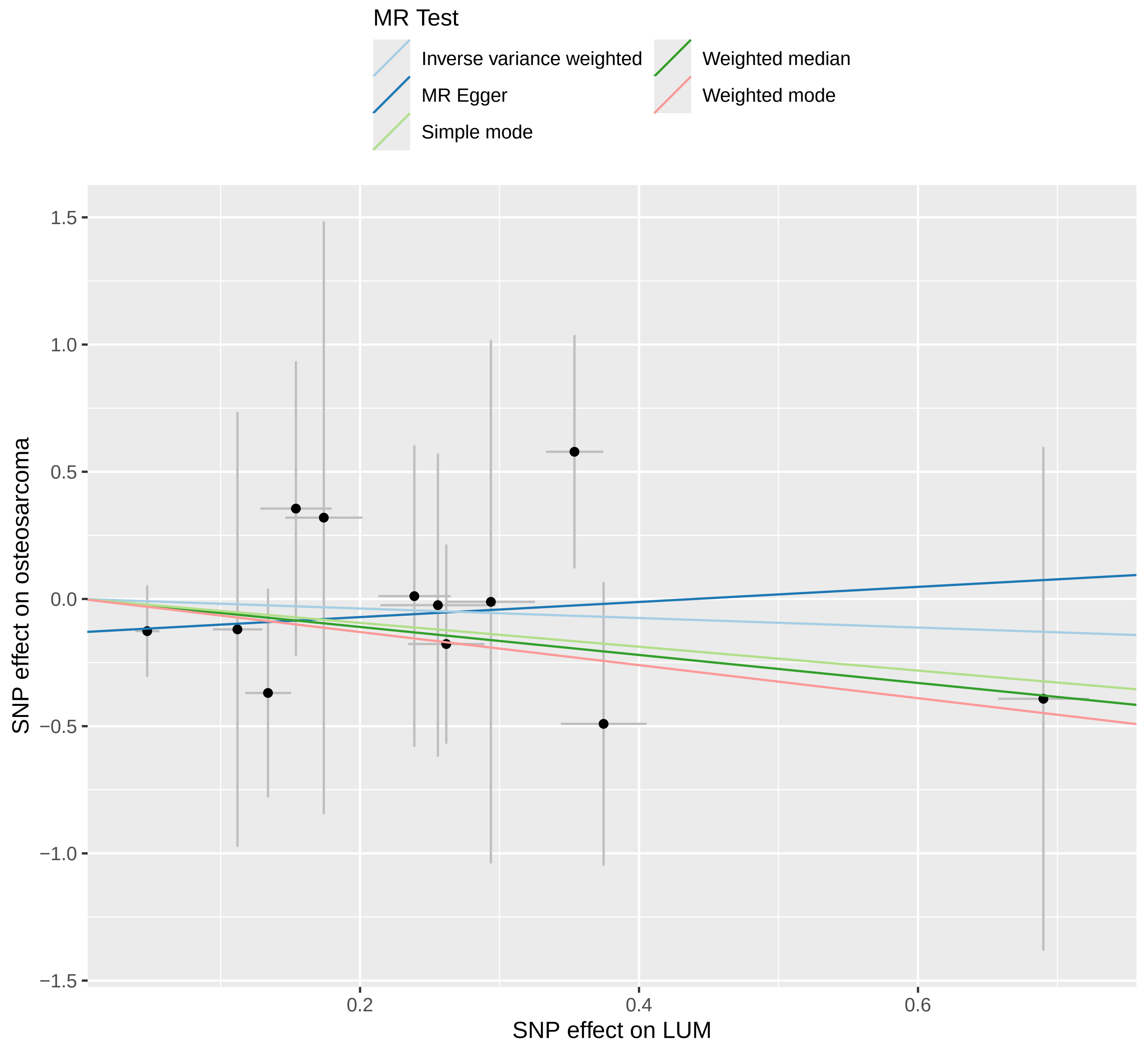
MR Test
Inverse variance weighted
MR Egger
Simple mode
Weighted median
Weighted mode
1.5
1.0
0.5
0.0
−0.5
−1.0
−1.5
SNP effect on osteosarcoma
0.2
0.4
0.6
SNP effect on LUM

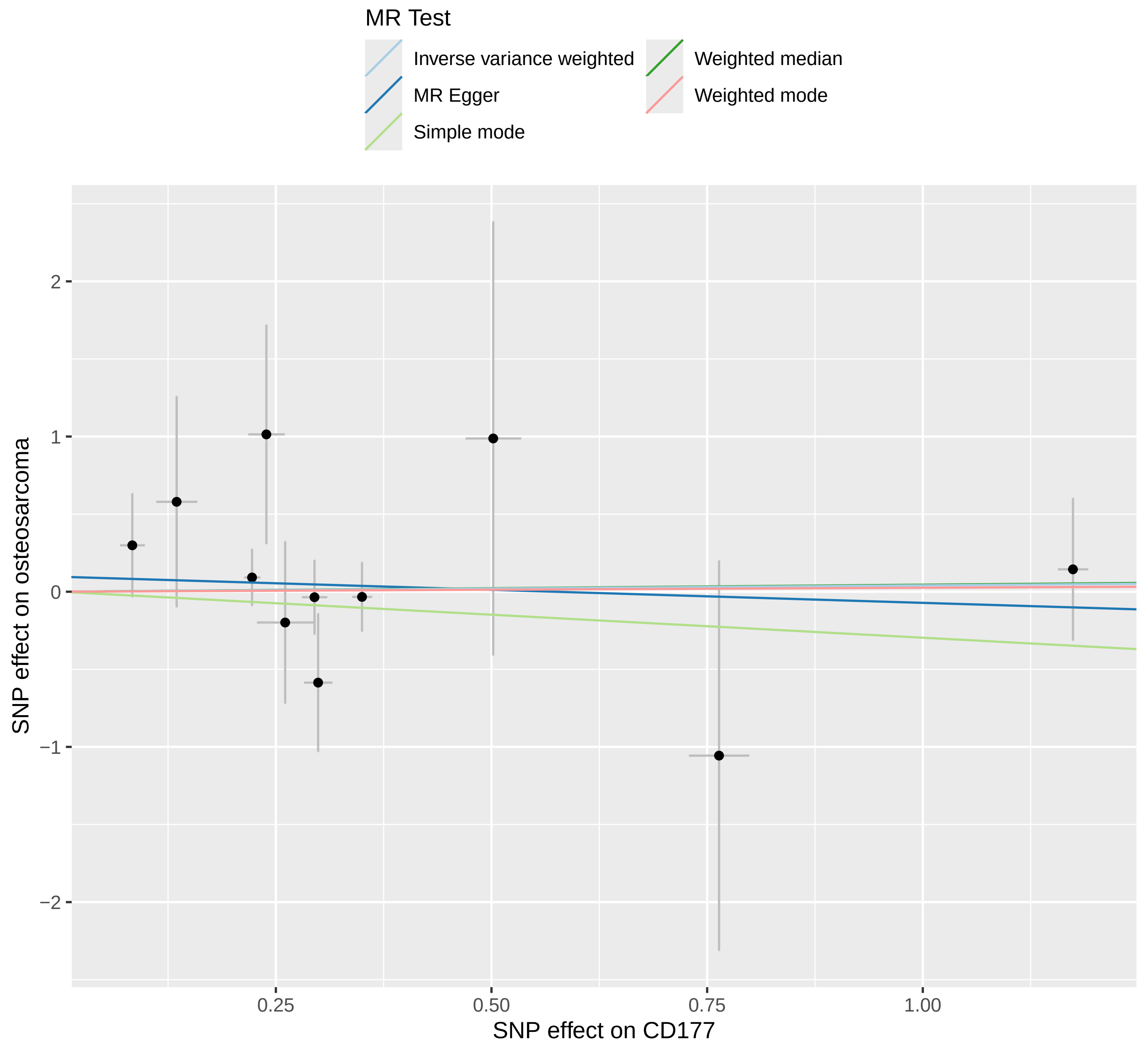
MR Test
Inverse variance weighted
MR Egger
Simple mode
Weighted median
Weighted mode
SNP effect on osteosarcoma
2
1
0
−1
−2
0.25
0.50
0.75
1.00
SNP effect on CD177

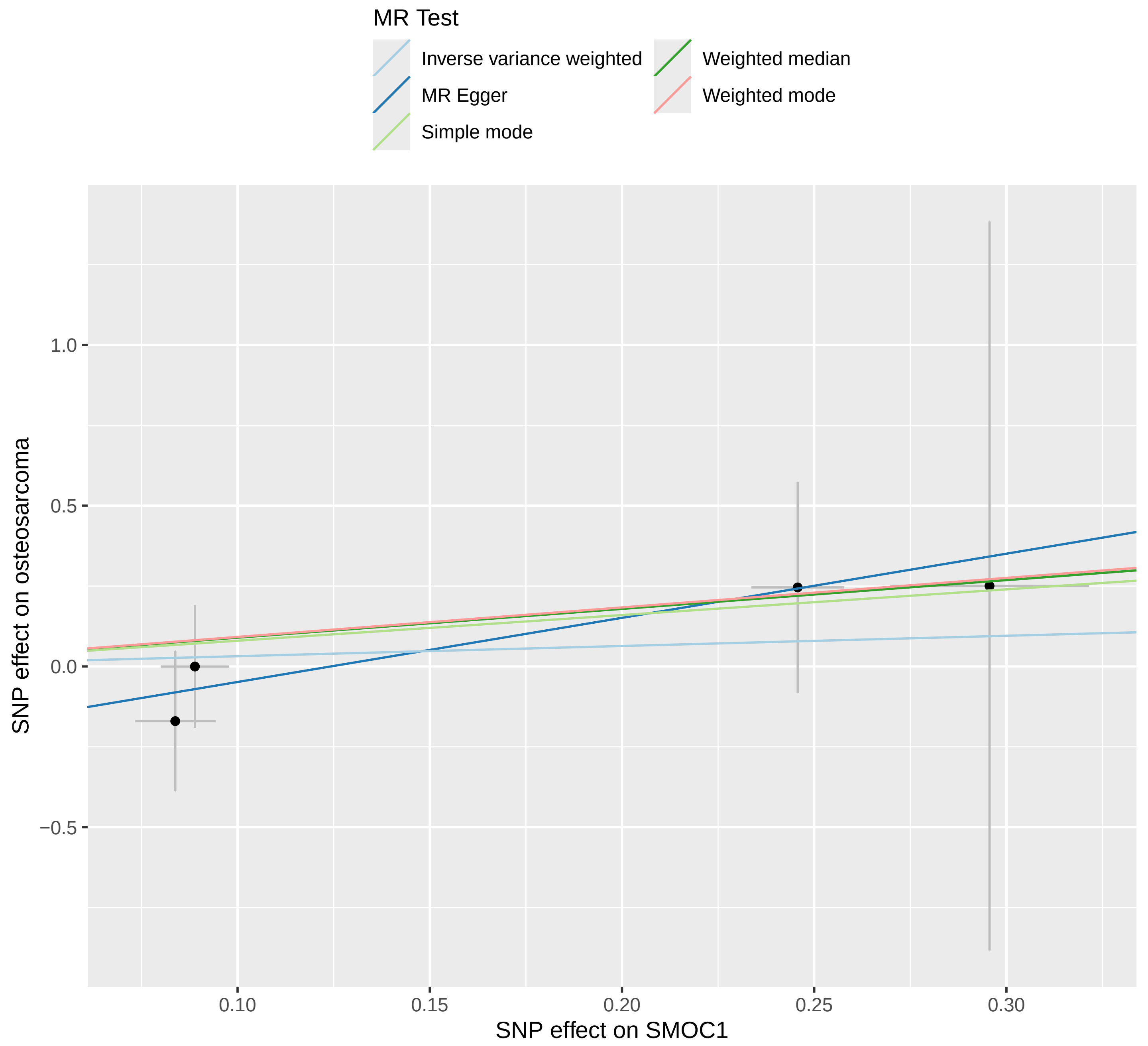

MR Test
Inverse variance weighted
MR Egger
Simple mode
Weighted median
Weighted mode
SNP effect on osteosarcoma
1.0
0.5
0.0
−0.5
0.10
0.15
0.20
0.25
0.30
SNP effect on SMOC1

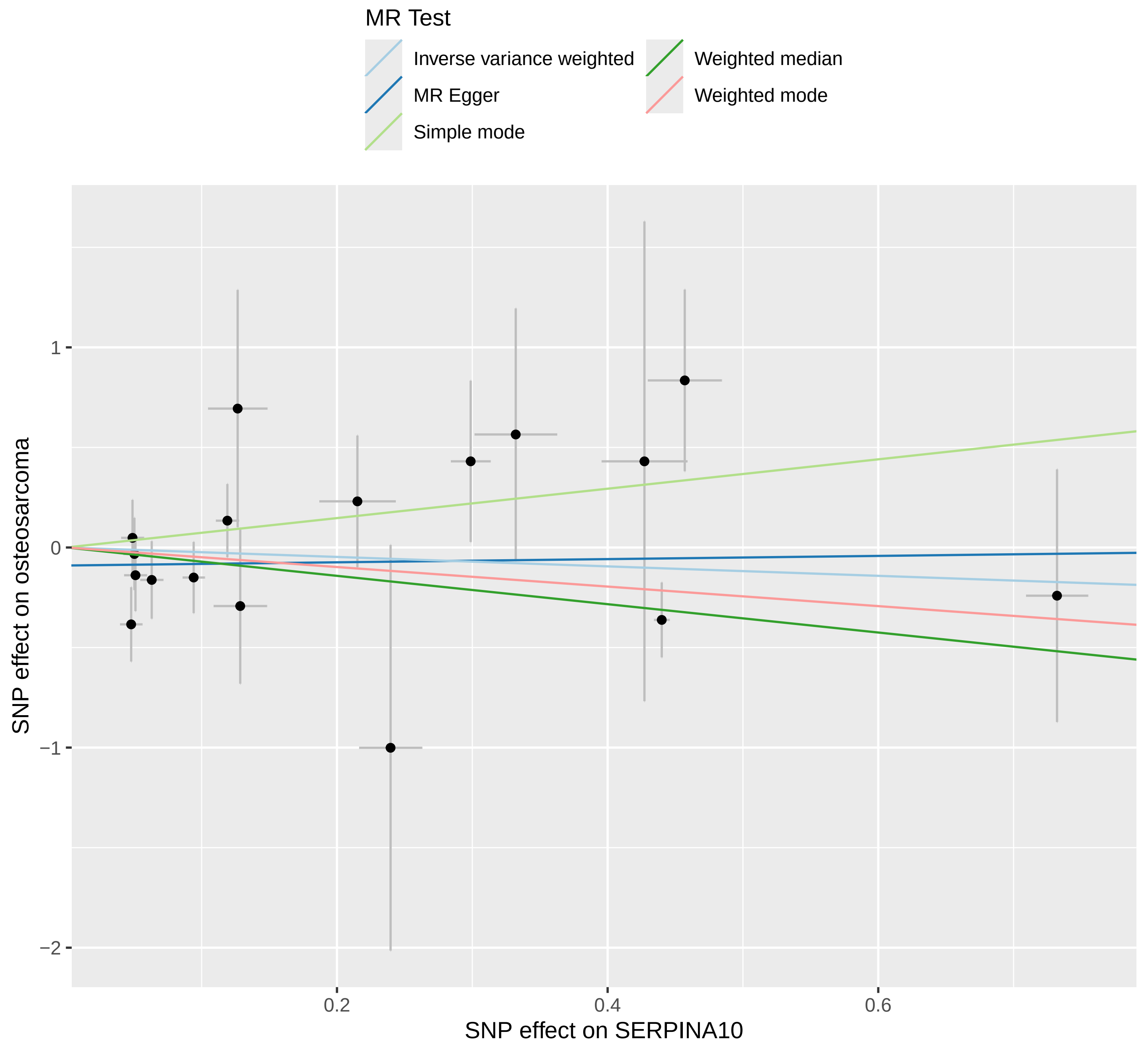
MR Test
Inverse variance weighted
MR Egger
Simple mode
Weighted median
Weighted mode
1
0
−1
−2
0.2
0.4
0.6
SNP effect on osteosarcoma
SNP effect on SERPINA10

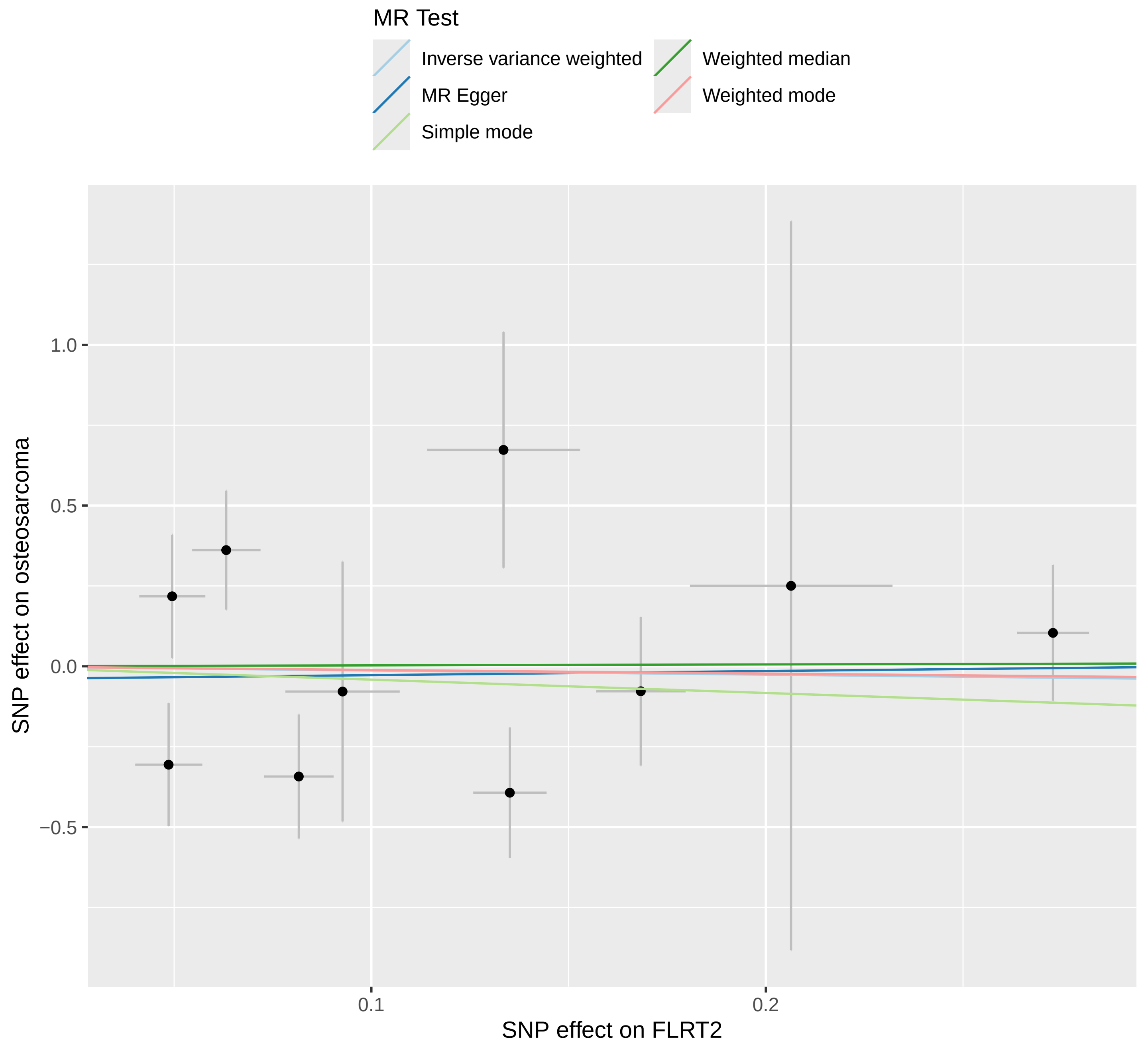
MR Test
Inverse variance weighted
MR Egger
Simple mode
Weighted median
Weighted mode
1.0
0.5
0.0
−0.5
0.1
0.2
SNP effect on osteosarcoma
SNP effect on FLRT2

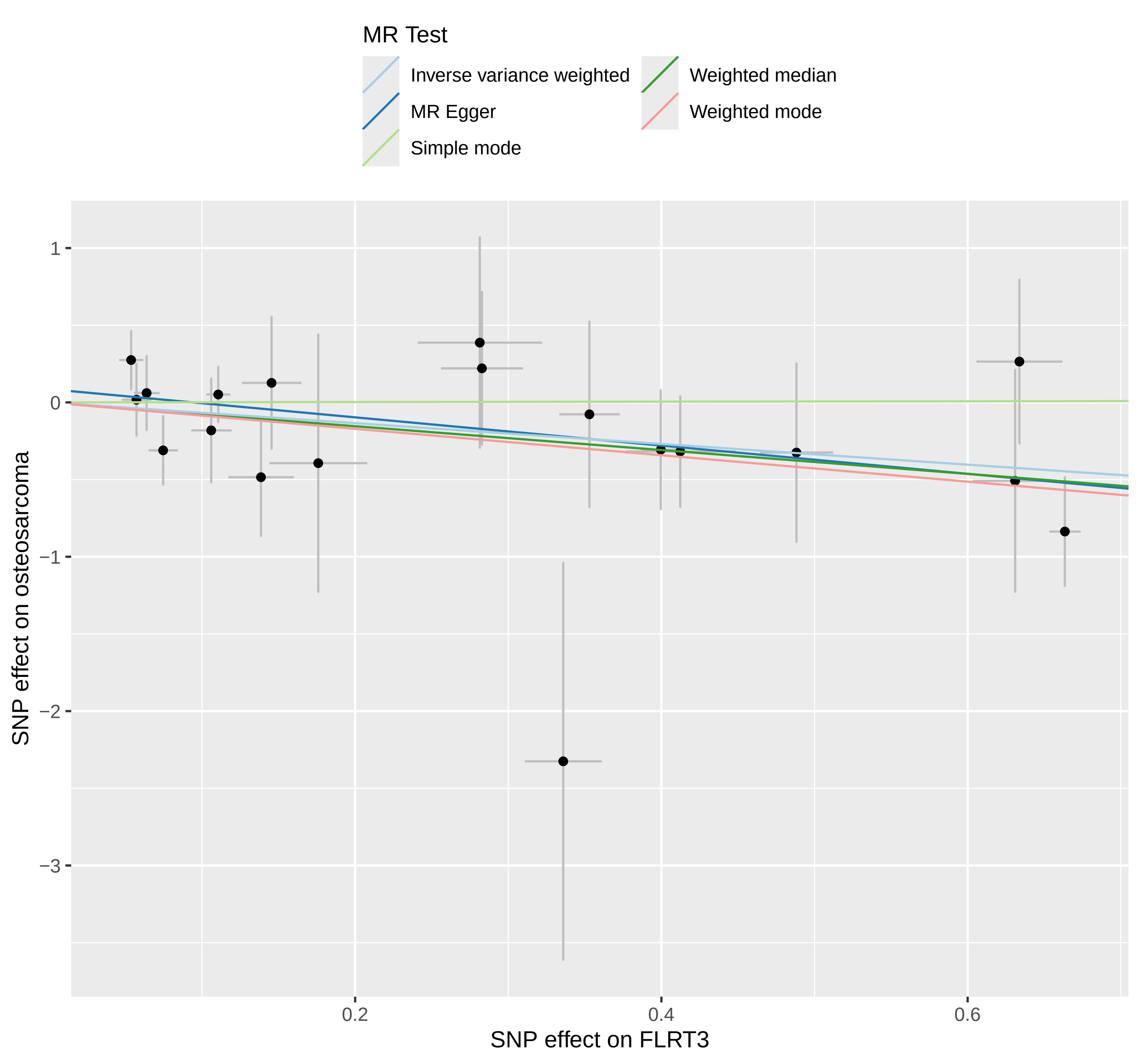
MR Test
Inverse variance weighted
MR Egger
Simple mode
Weighted median
Weighted mode
1
0
−1
−2
−3
0.2
0.4
0.6
SNP effect on osteosarcoma
SNP effect on FLRT3

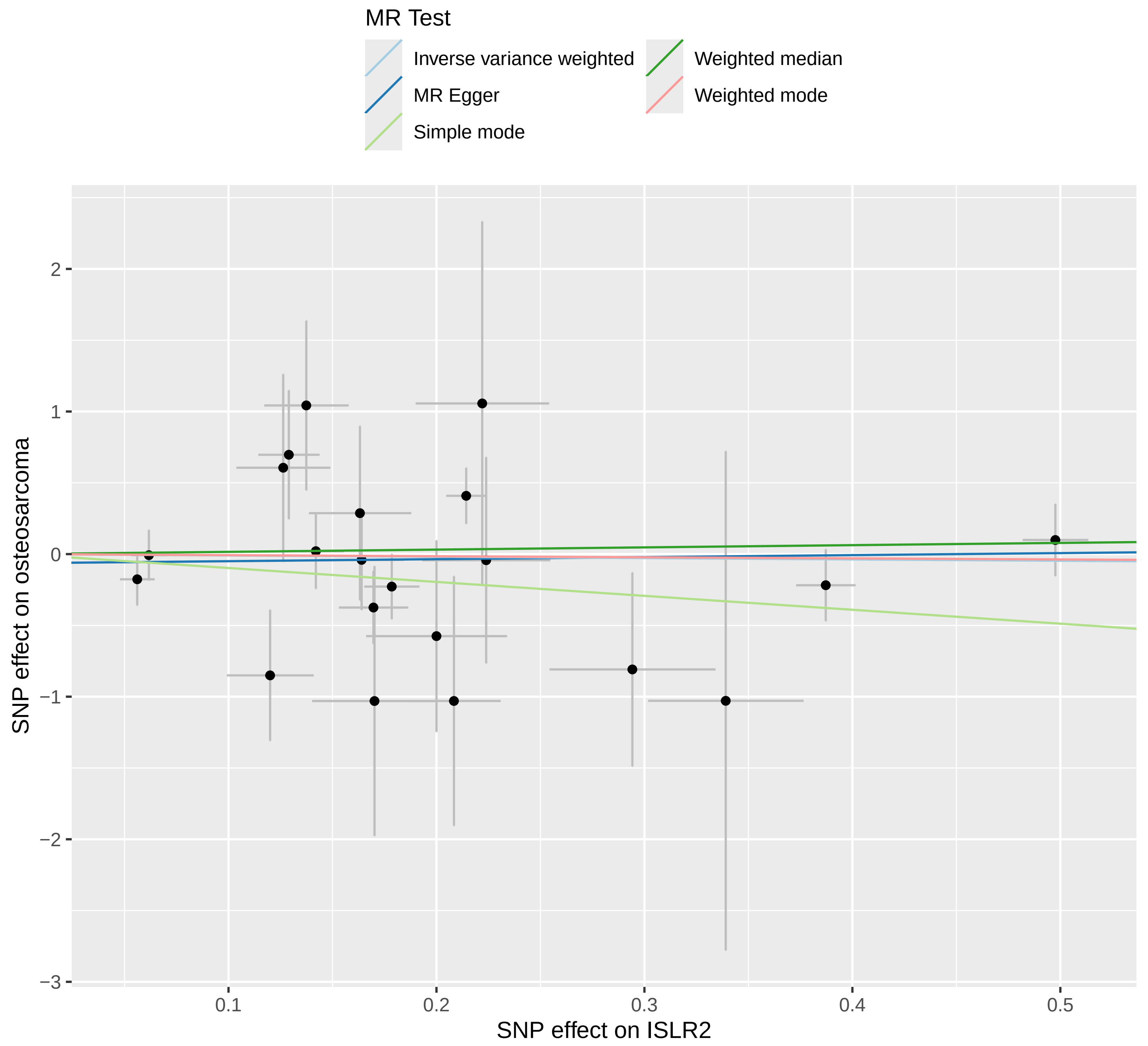

MR Test
Inverse variance weighted
MR Egger
Simple mode
Weighted median
Weighted mode
SNP effect on osteosarcoma
2
1
0
−1
−2
−3
0.1
0.2
0.3
0.4
0.5
SNP effect on ISLR2

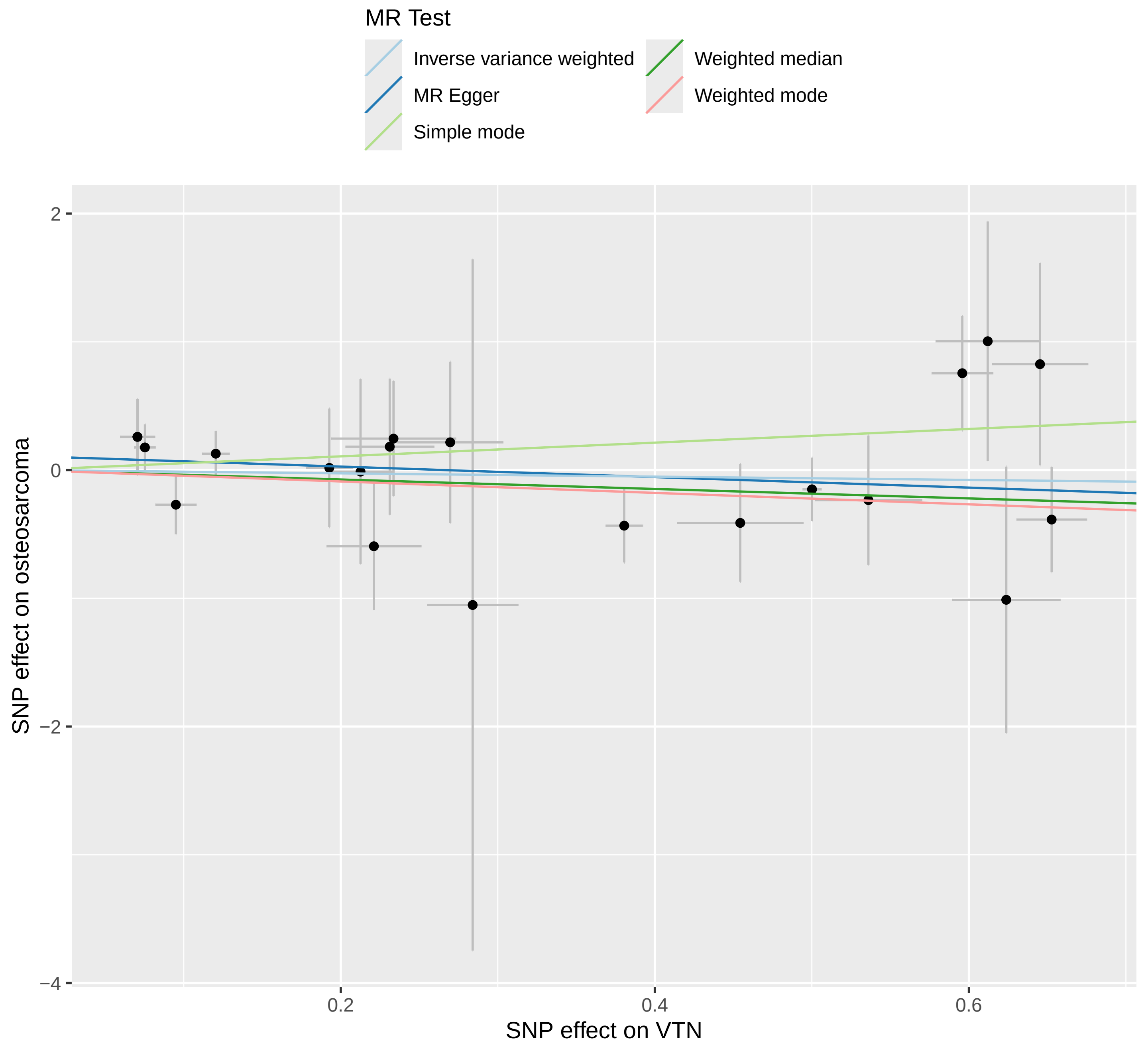
MR Test
Inverse variance weighted
MR Egger
Simple mode
Weighted median
Weighted mode
2
0
−2
−4
0.2
0.4
0.6
SNP effect on VTN
SNP effect on osteosarcoma

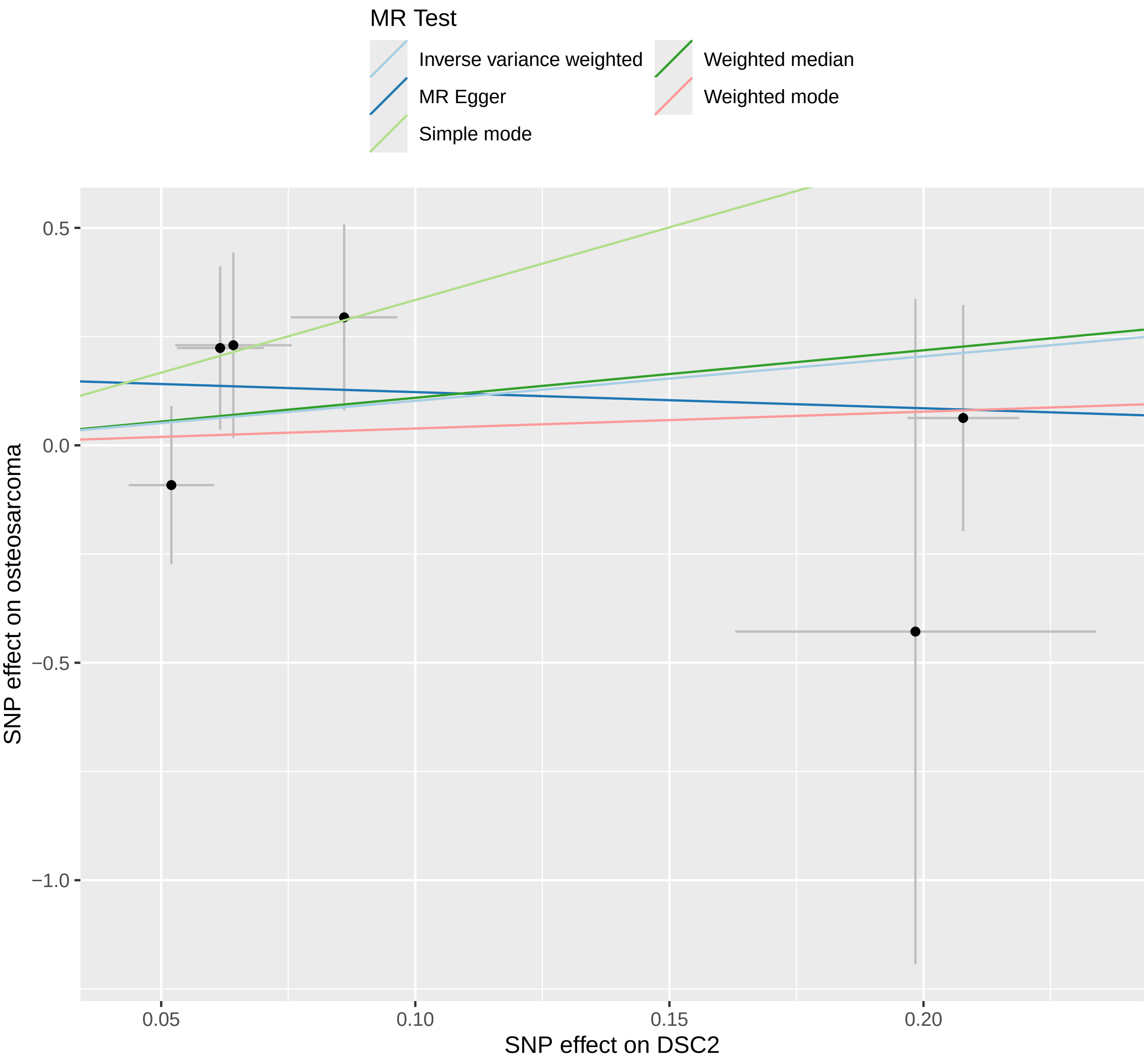
MR Test
Inverse variance weighted
MR Egger
Simple mode
Weighted median
Weighted mode
0.5
0.0
−0.5
−1.0
SNP effect on osteosarcoma
0.05
0.10
0.15
0.20
SNP effect on DSC2

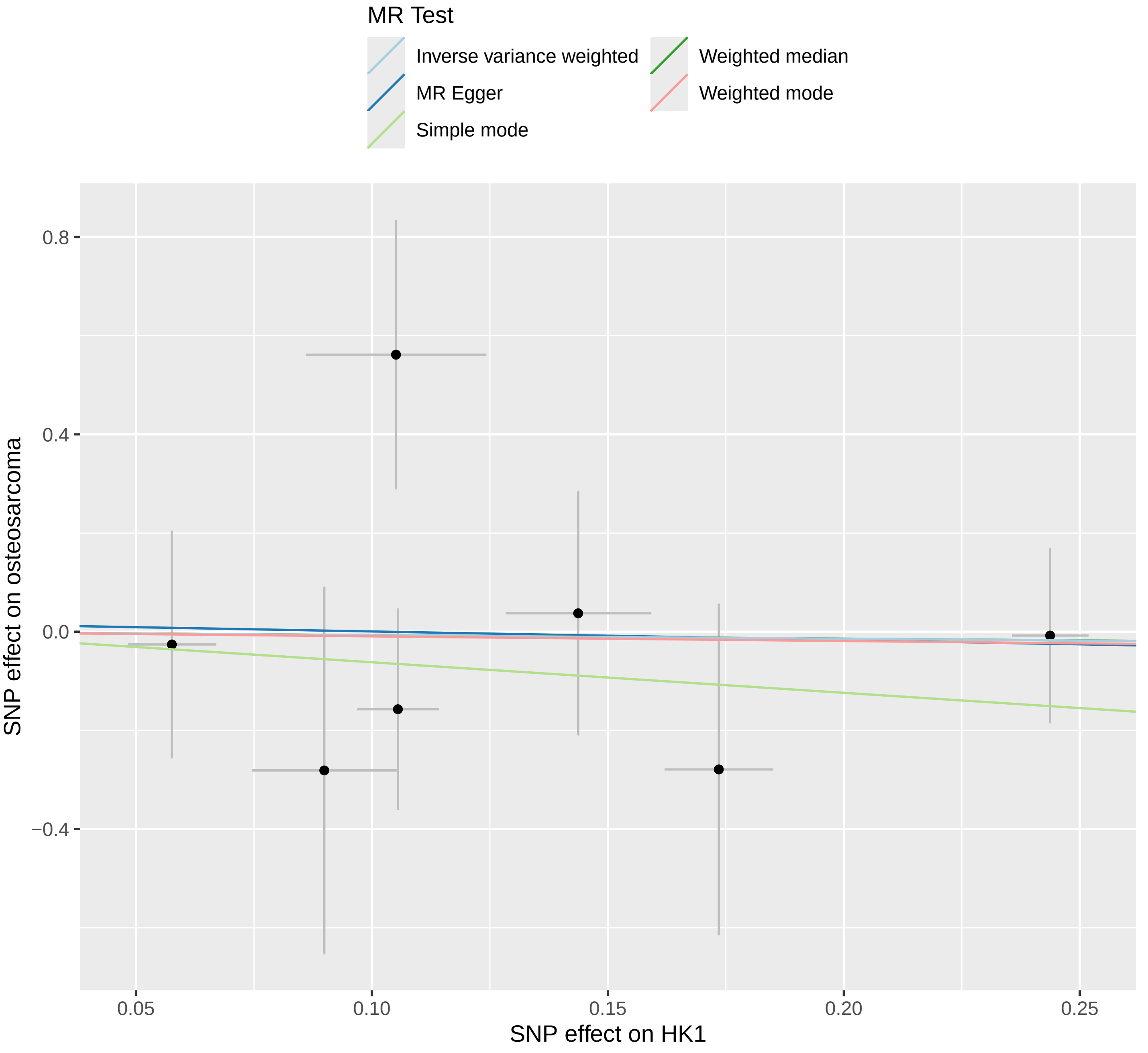

MR Test
Inverse variance weighted
MR Egger
Simple mode
Weighted median
Weighted mode
0.8
0.4
0.0
−0.4
SNP effect on osteosarcoma
0.05
0.10
0.15
0.20
0.25
SNP effect on HK1

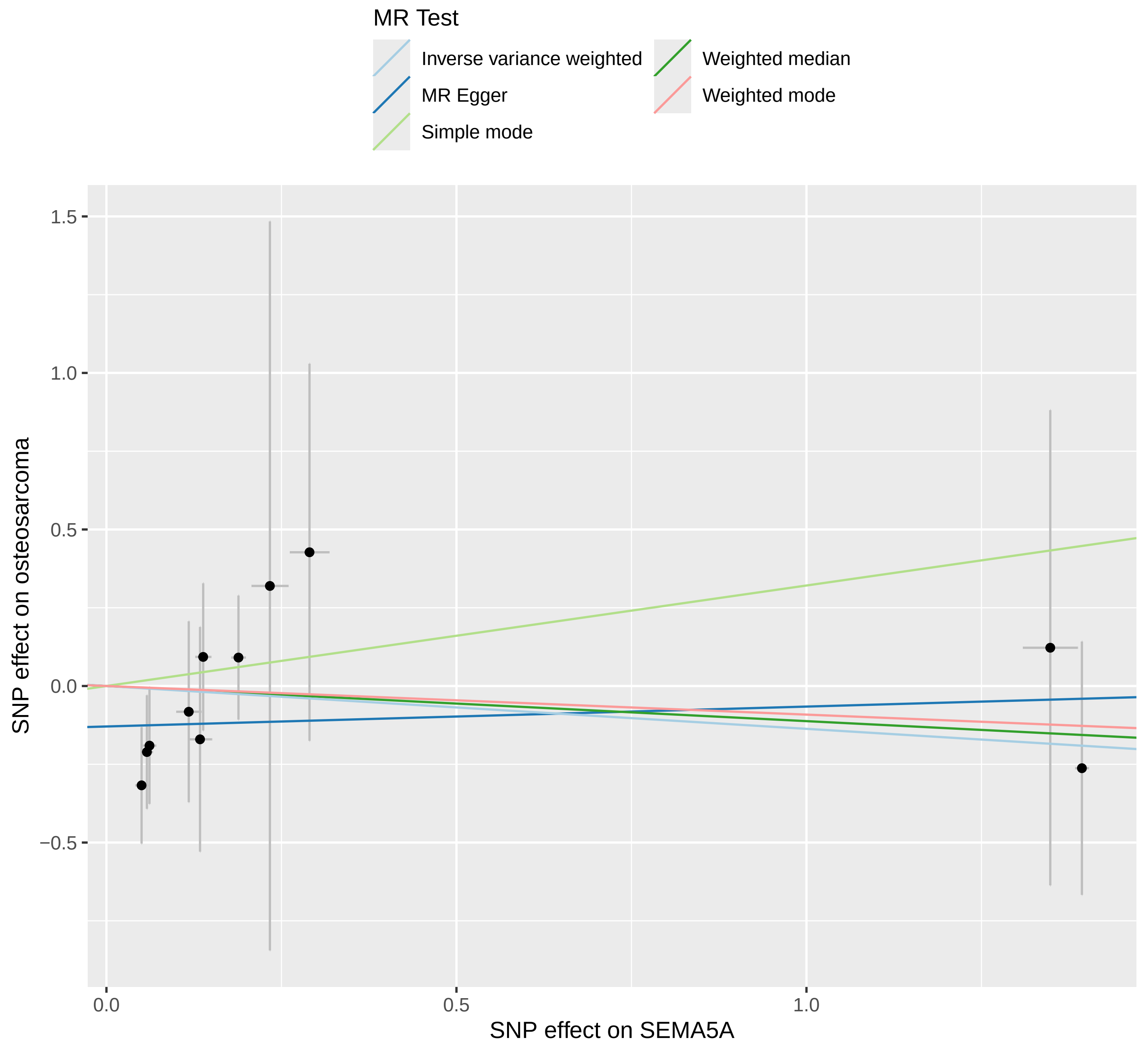
MR Test
Inverse variance weighted
MR Egger
Simple mode
Weighted median
Weighted mode
1.5
1.0
0.5
0.0
−0.5
SNP effect on osteosarcoma
0.0
0.5
1.0
SNP effect on SEMA5A

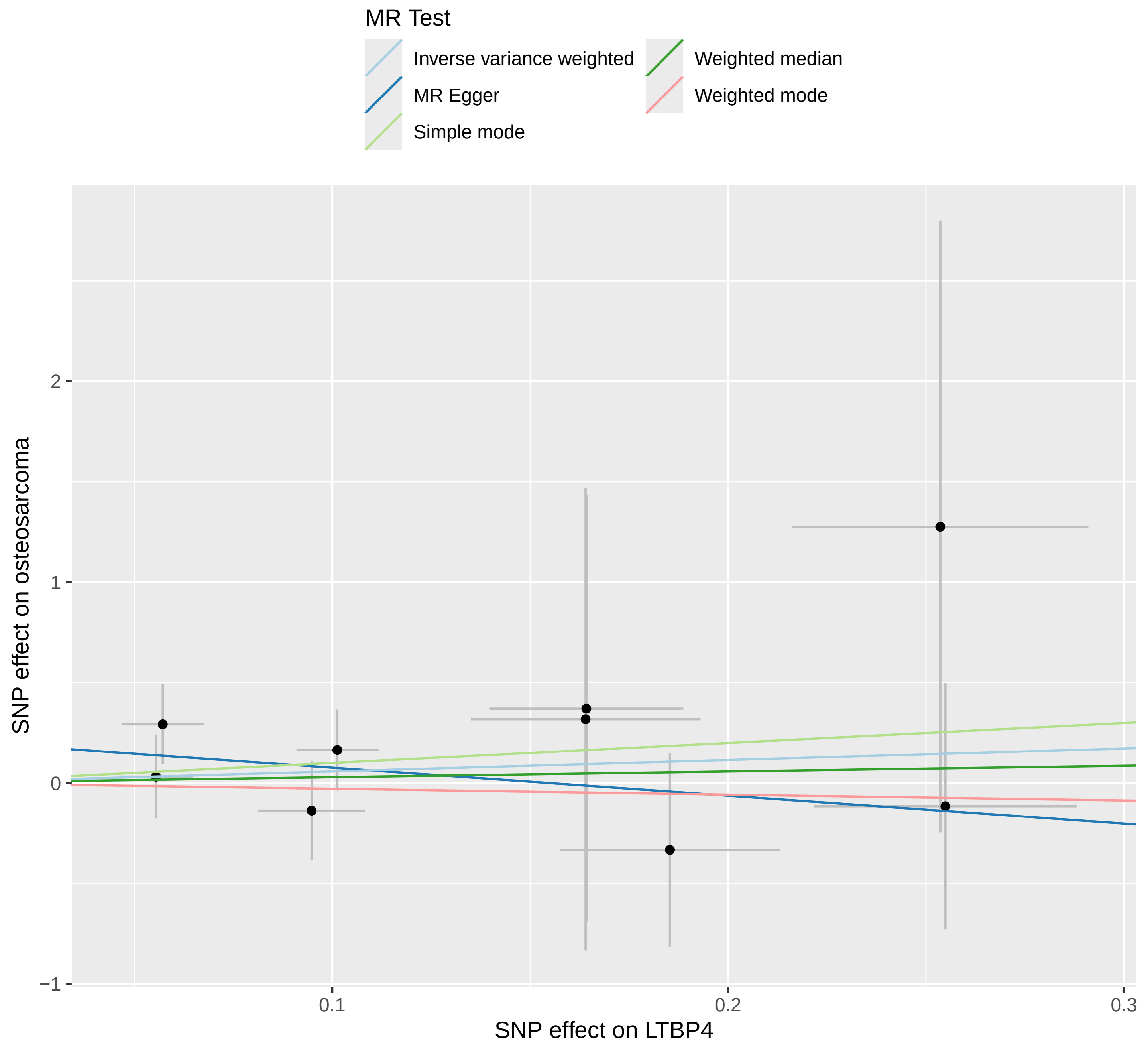
MR Test
Inverse variance weighted
MR Egger
Simple mode
Weighted median
Weighted mode
SNP effect on osteosarcoma
2
1
0
−1
0.1
0.2
0.3
SNP effect on LTBP4

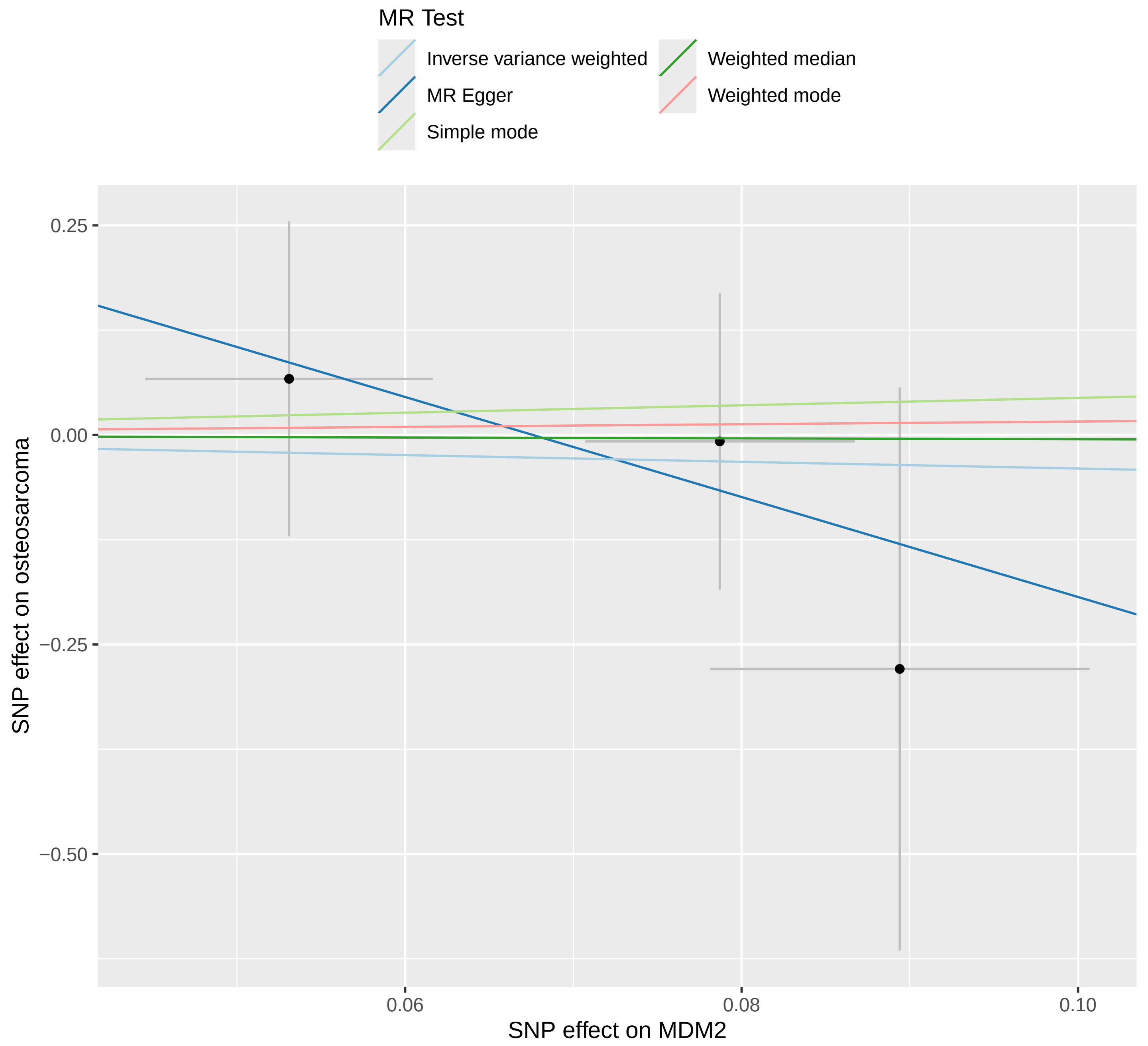

MR Test
Inverse variance weighted
MR Egger
Simple mode
Weighted median
Weighted mode
0.25
0.00
−0.25
−0.50
0.06
0.08
0.10
SNP effect on osteosarcoma
SNP effect on MDM2

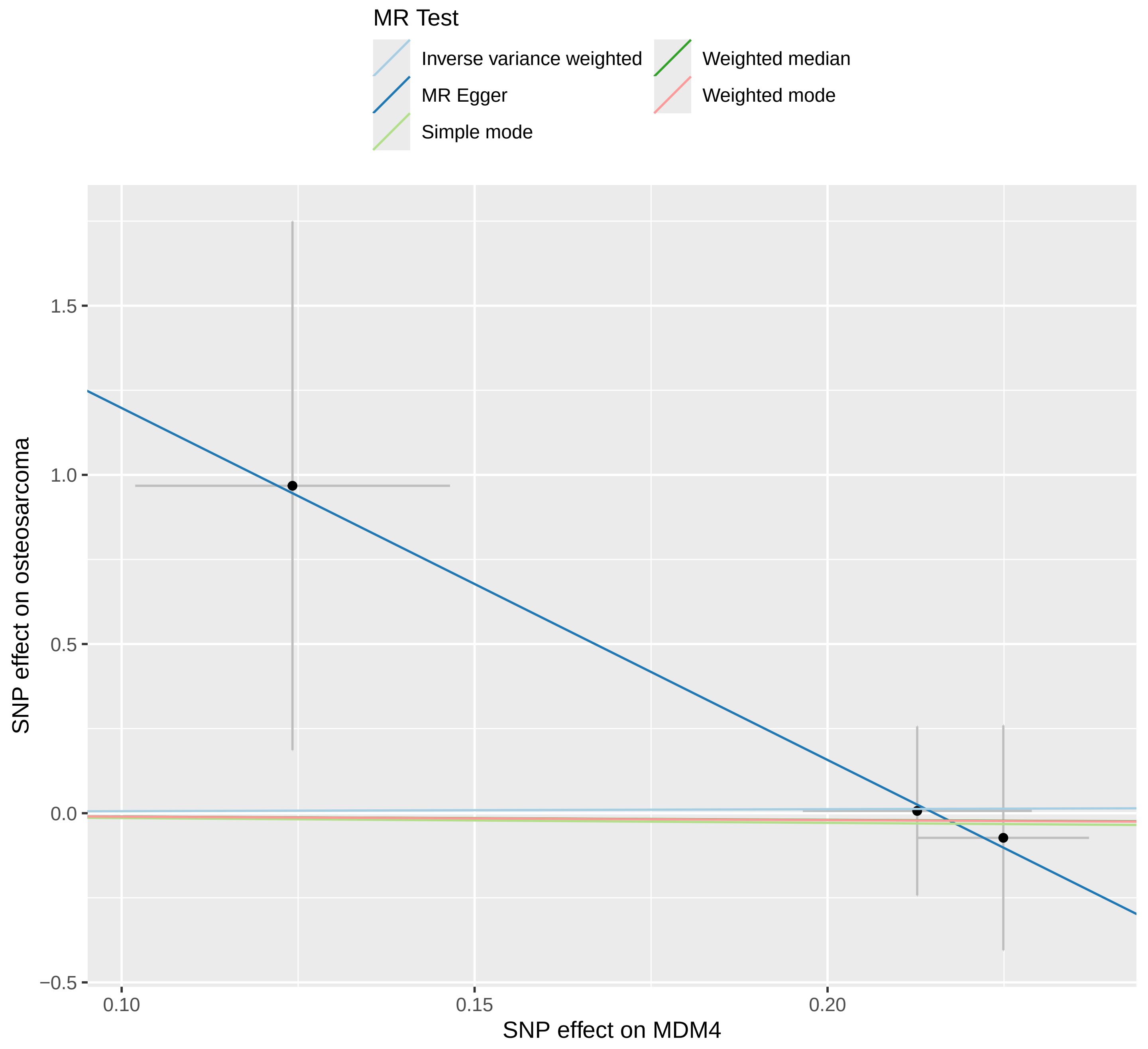
MR Test
Inverse variance weighted
MR Egger
Simple mode
Weighted median
Weighted mode
1.5
1.0
0.5
0.0
−0.5
0.10
0.15
0.20
SNP effect on osteosarcoma
SNP effect on MDM4

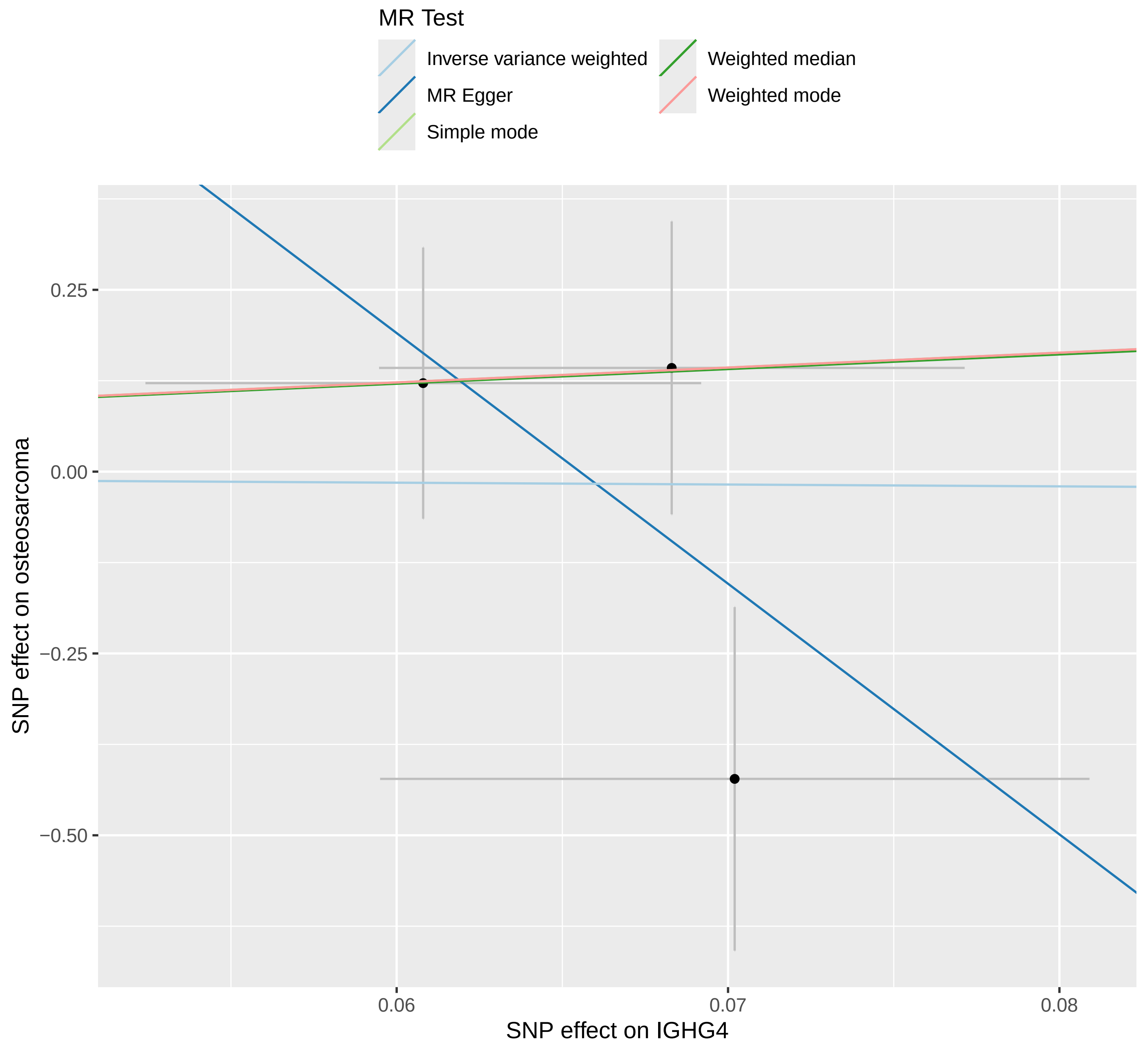

MR Test
Inverse variance weighted
MR Egger
Simple mode
Weighted median
Weighted mode
SNP effect on osteosarcoma
0.25
0.00
−0.25
−0.50
0.06
0.07
0.08
SNP effect on IGHG4

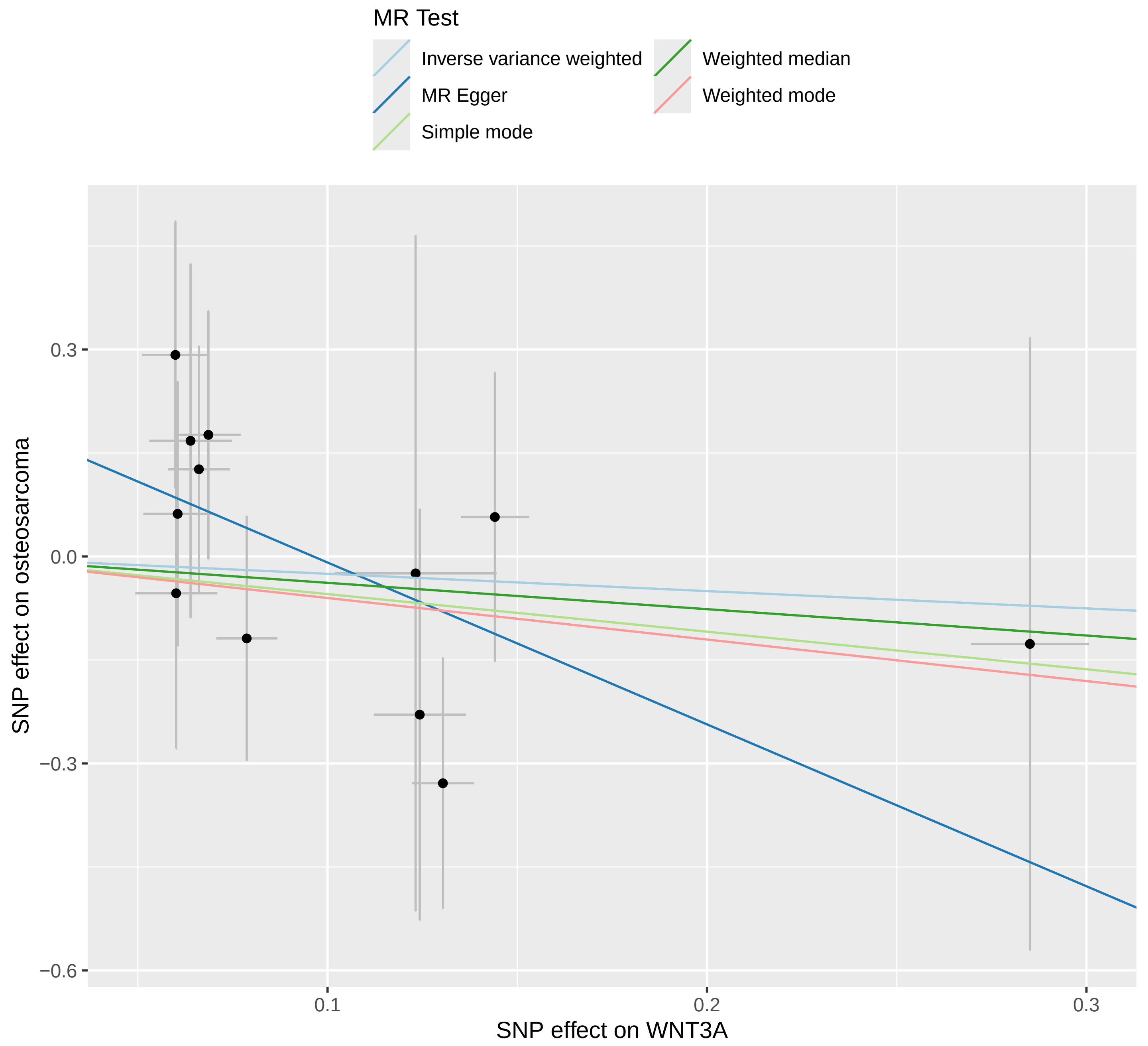
MR Test
Inverse variance weighted
MR Egger
Simple mode
Weighted median
Weighted mode
0.3
0.0
−0.3
−0.6
SNP effect on osteosarcoma
0.1
0.2
0.3
SNP effect on WNT3A

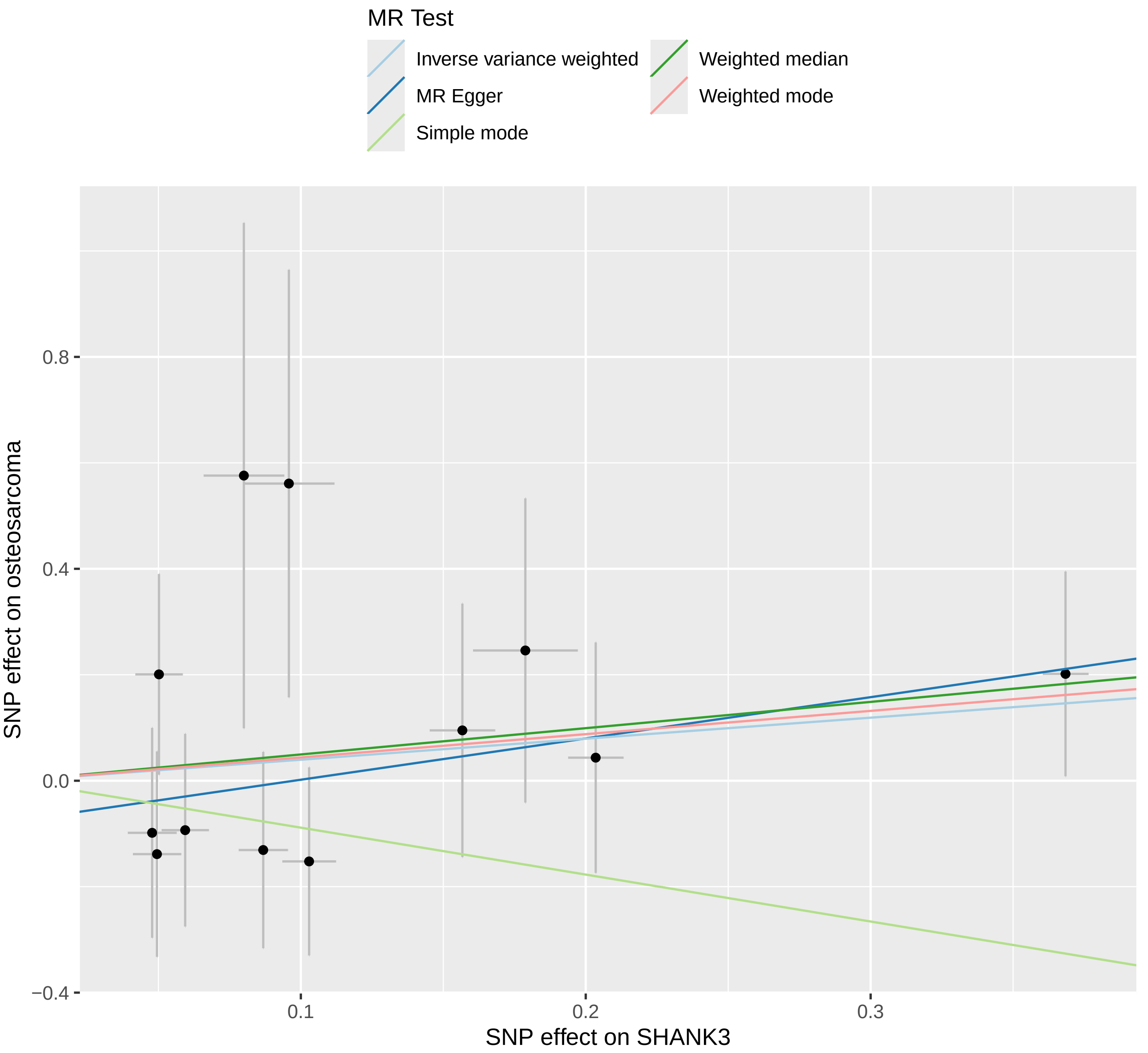
MR Test
Inverse variance weighted
MR Egger
Simple mode
Weighted median
Weighted mode
0.8
0.4
0.0
−0.4
0.1
0.2
0.3
SNP effect on SHANK3
SNP effect on osteosarcoma

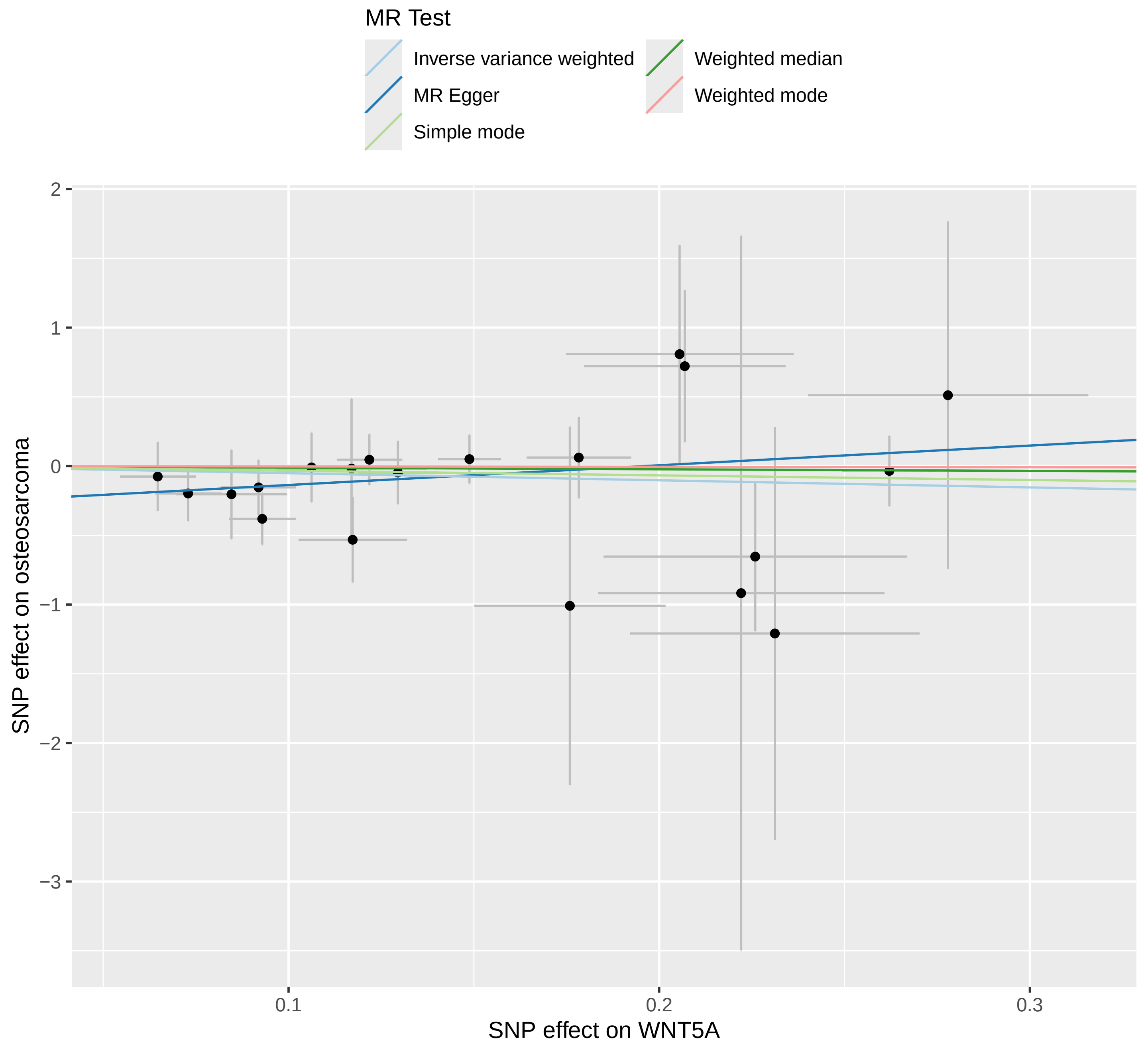

MR Test
Inverse variance weighted
MR Egger
Simple mode
Weighted median
Weighted mode
2
1
0
−1
−2
−3
0.1
0.2
0.3
SNP effect on osteosarcoma
SNP effect on WNT5A

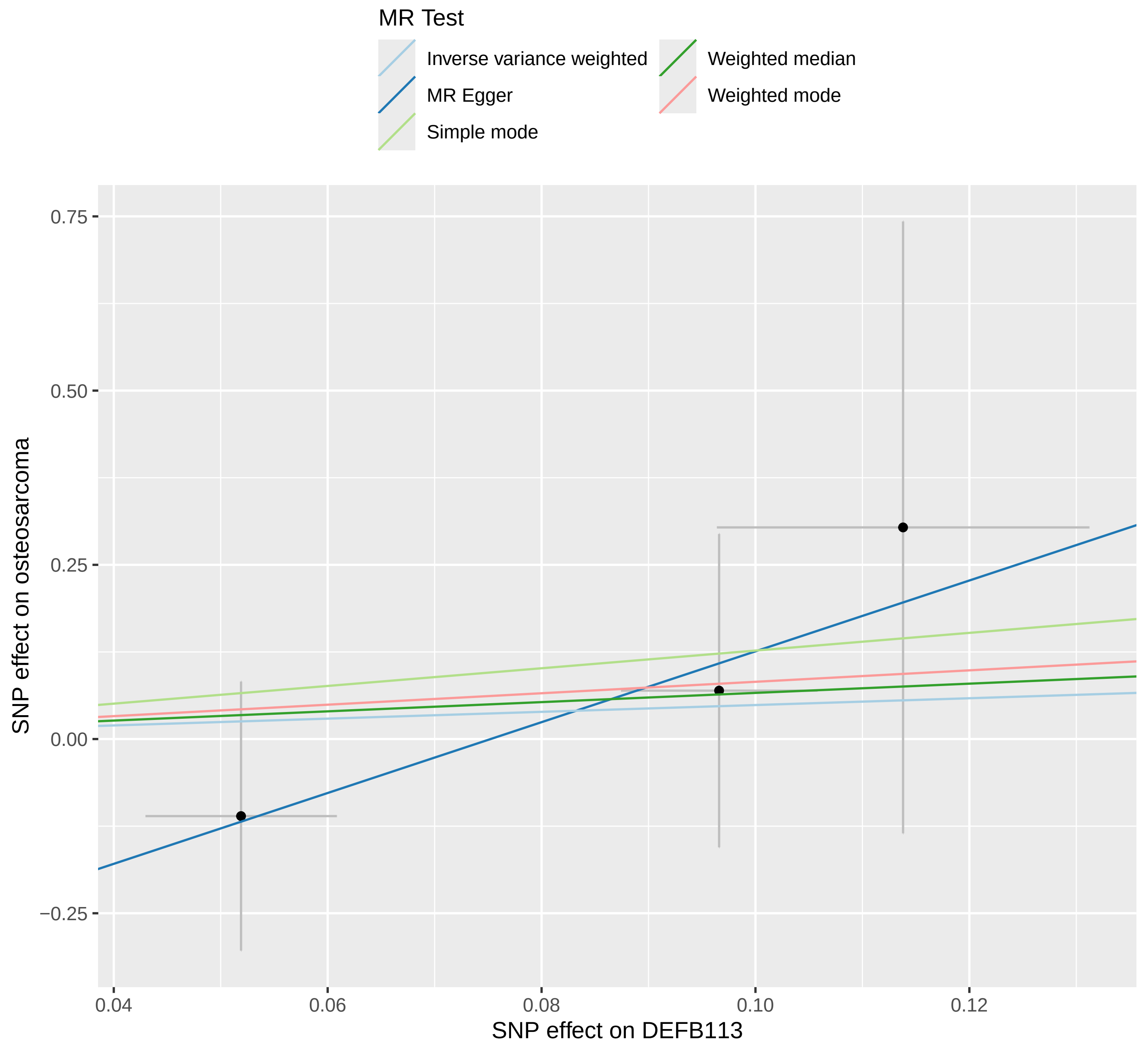
MR Test
Inverse variance weighted
MR Egger
Simple mode
Weighted median
Weighted mode
0.75
0.50
0.25
0.00
−0.25
SNP effect on osteosarcoma
0.04
0.06
0.08
0.10
0.12
SNP effect on DEFB113

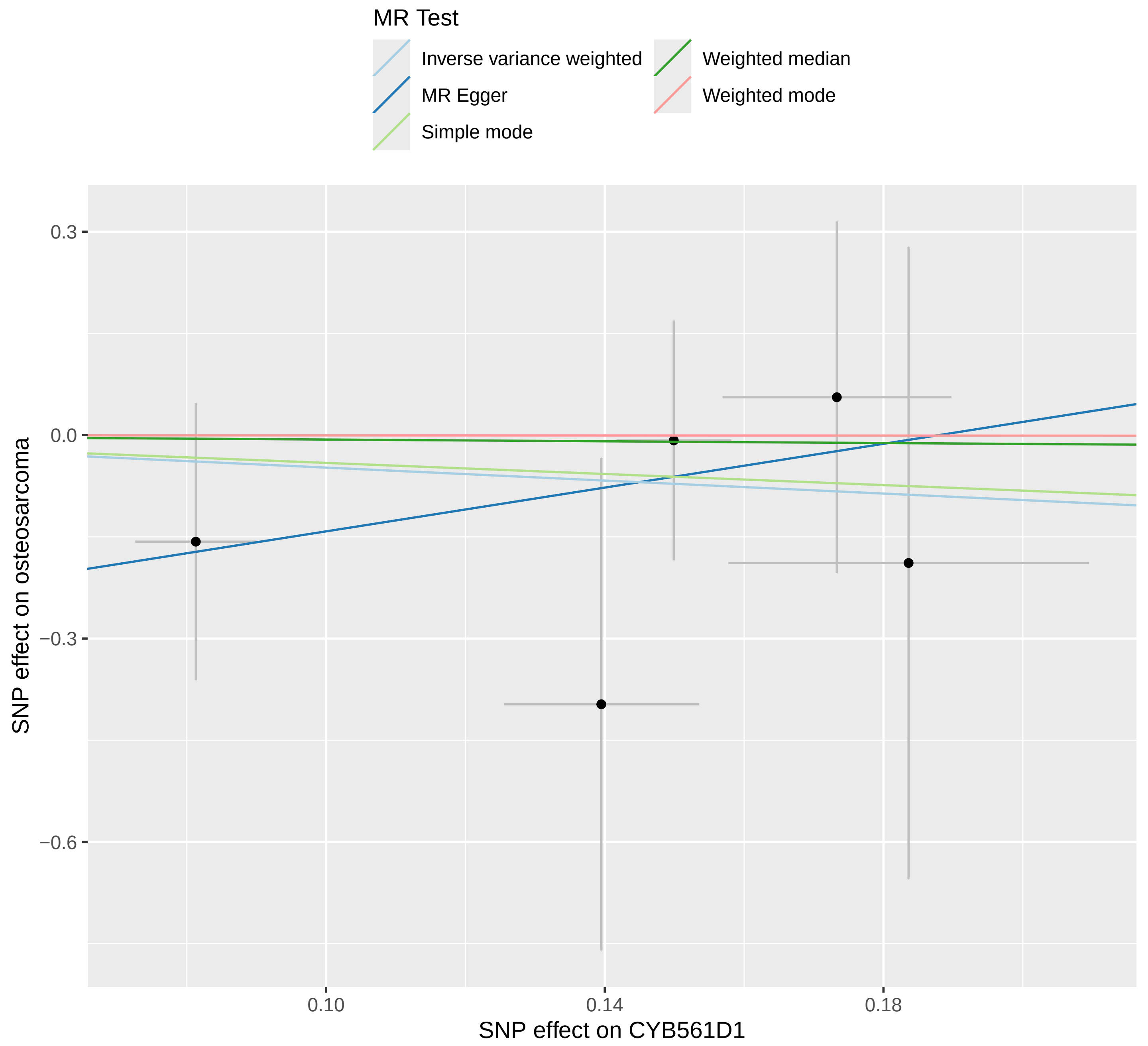
MR Test
Inverse variance weighted
MR Egger
Simple mode
Weighted median
Weighted mode
0.3
0.0
−0.3
−0.6
0.10
0.14
0.18
SNP effect on CYB561D1
SNP effect on osteosarcoma

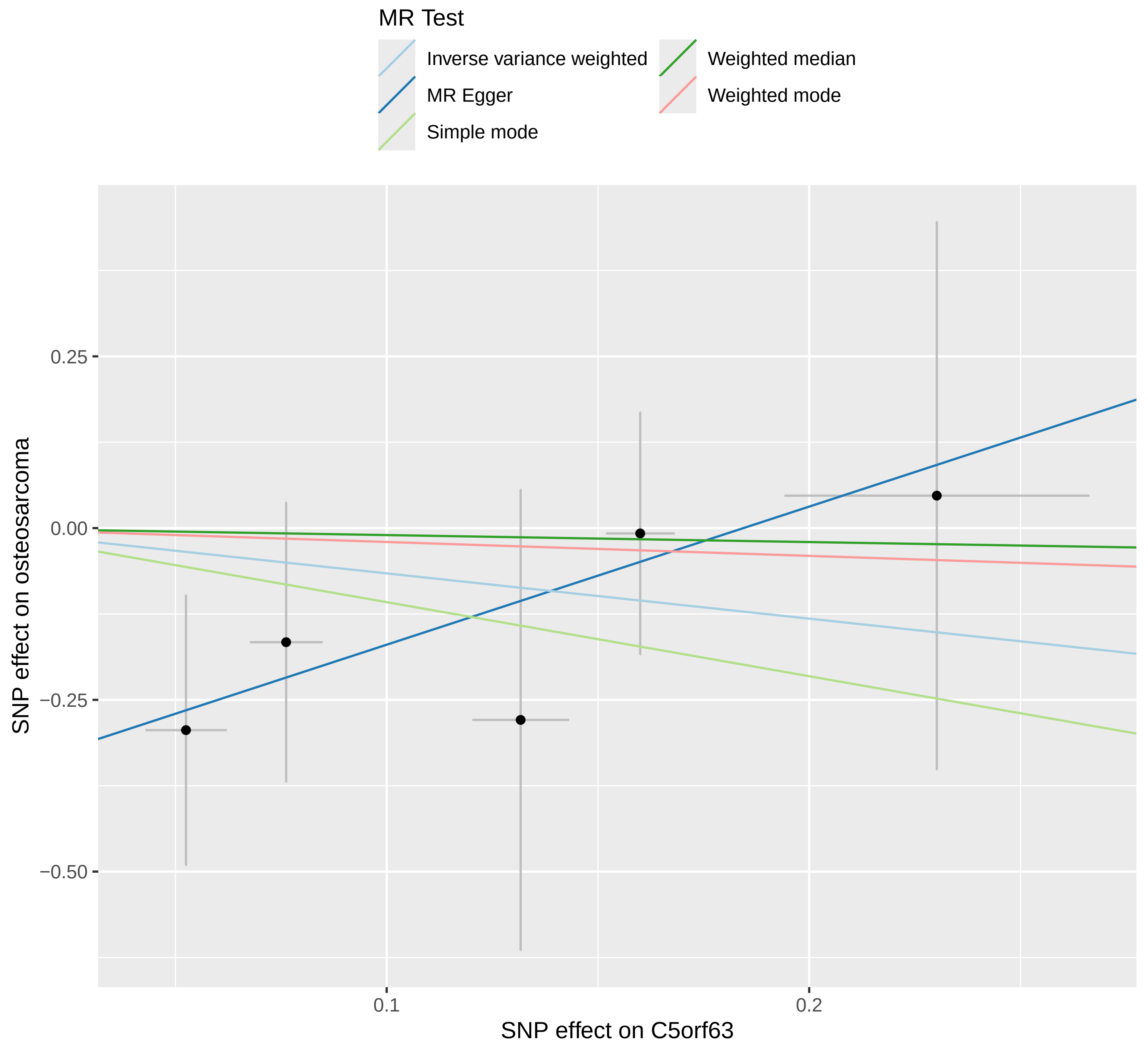
MR Test
Inverse variance weighted
MR Egger
Simple mode
Weighted median
Weighted mode
0.25
0.00
−0.25
−0.50
SNP effect on osteosarcoma
0.1
0.2
SNP effect on C5orf63

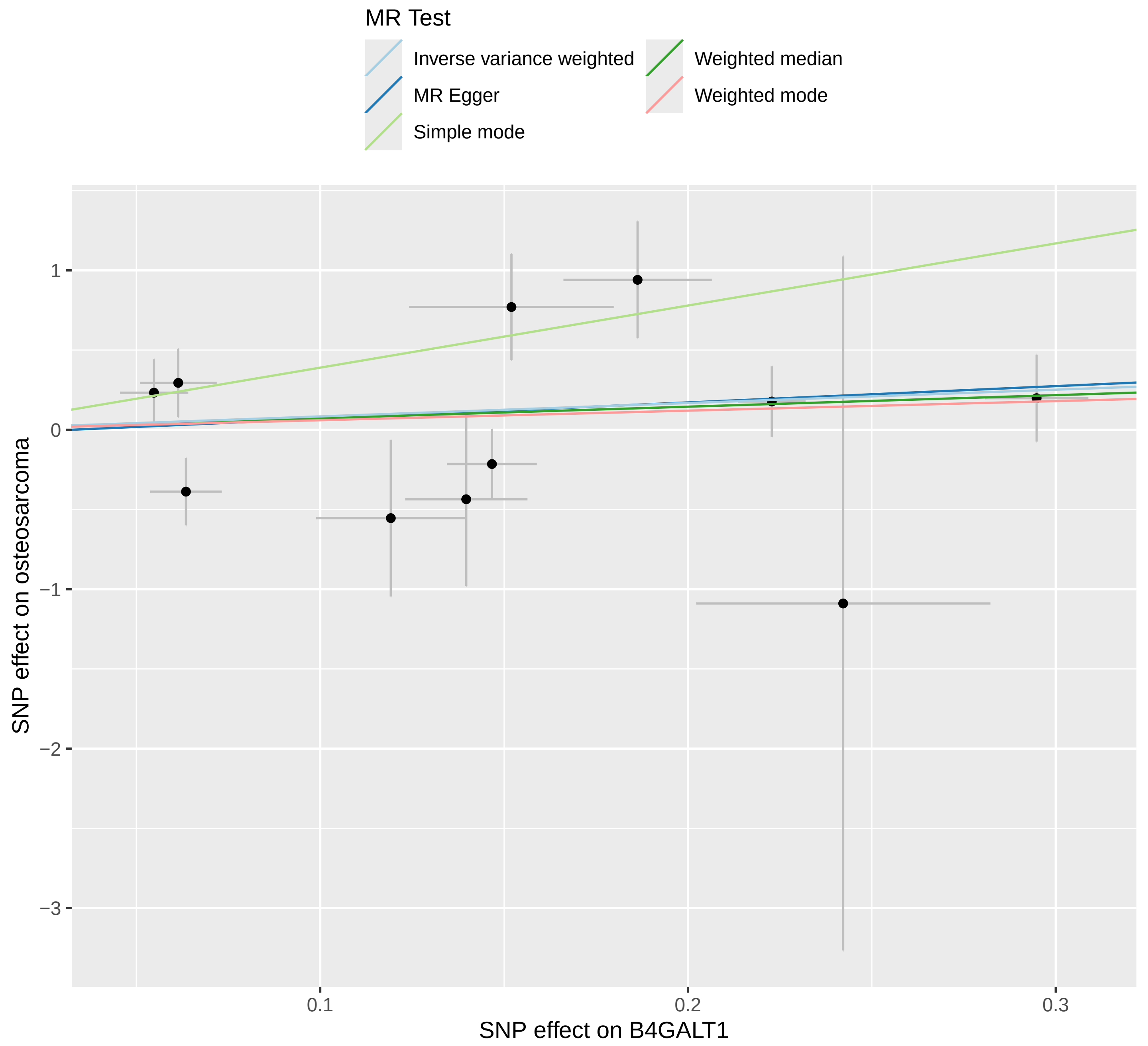
MR Test
Inverse variance weighted
MR Egger
Simple mode
Weighted median
Weighted mode
1
0
−1
−2
−3
0.1
0.2
0.3
SNP effect on osteosarcoma
SNP effect on B4GALT1

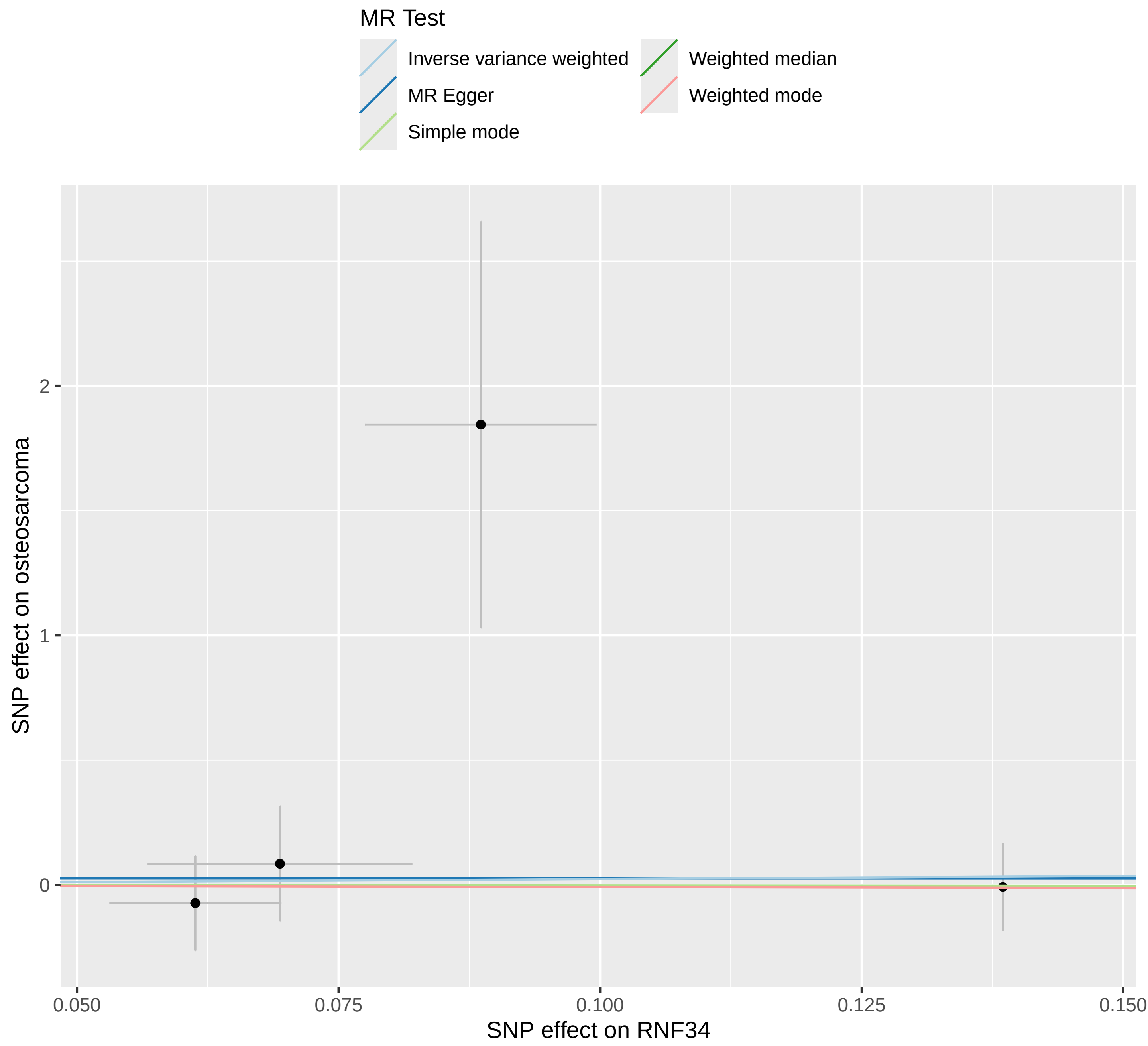
MR Test
Inverse variance weighted
MR Egger
Simple mode
Weighted median
Weighted mode
2
1
0
SNP effect on osteosarcoma
0.050
0.075
0.100
0.125
0.150
SNP effect on RNF34

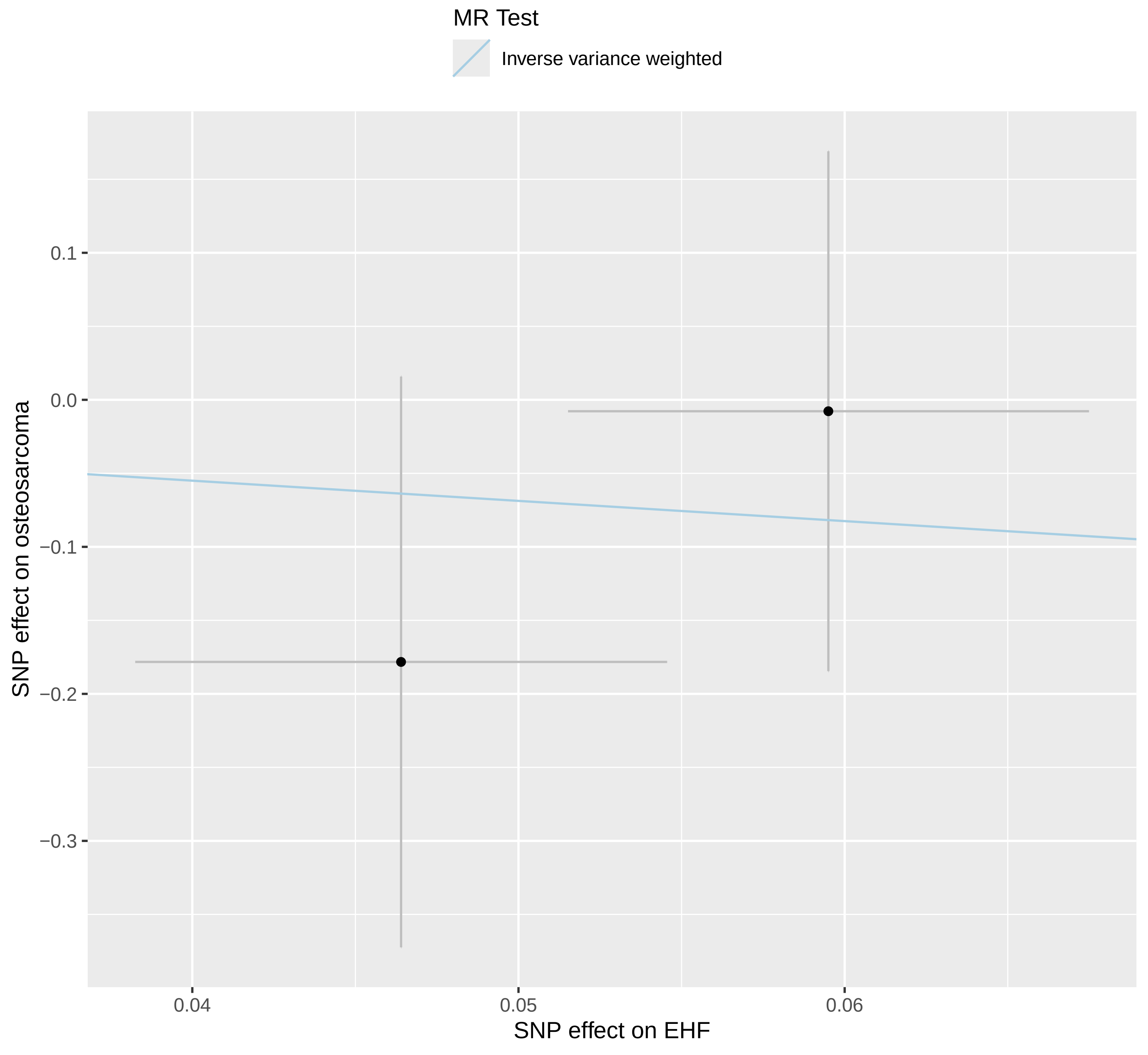

MR Test
Inverse variance weighted
0.1
0.0
−0.1
−0.2
−0.3
SNP effect on osteosarcoma
0.04
0.05
0.06
SNP effect on EHF

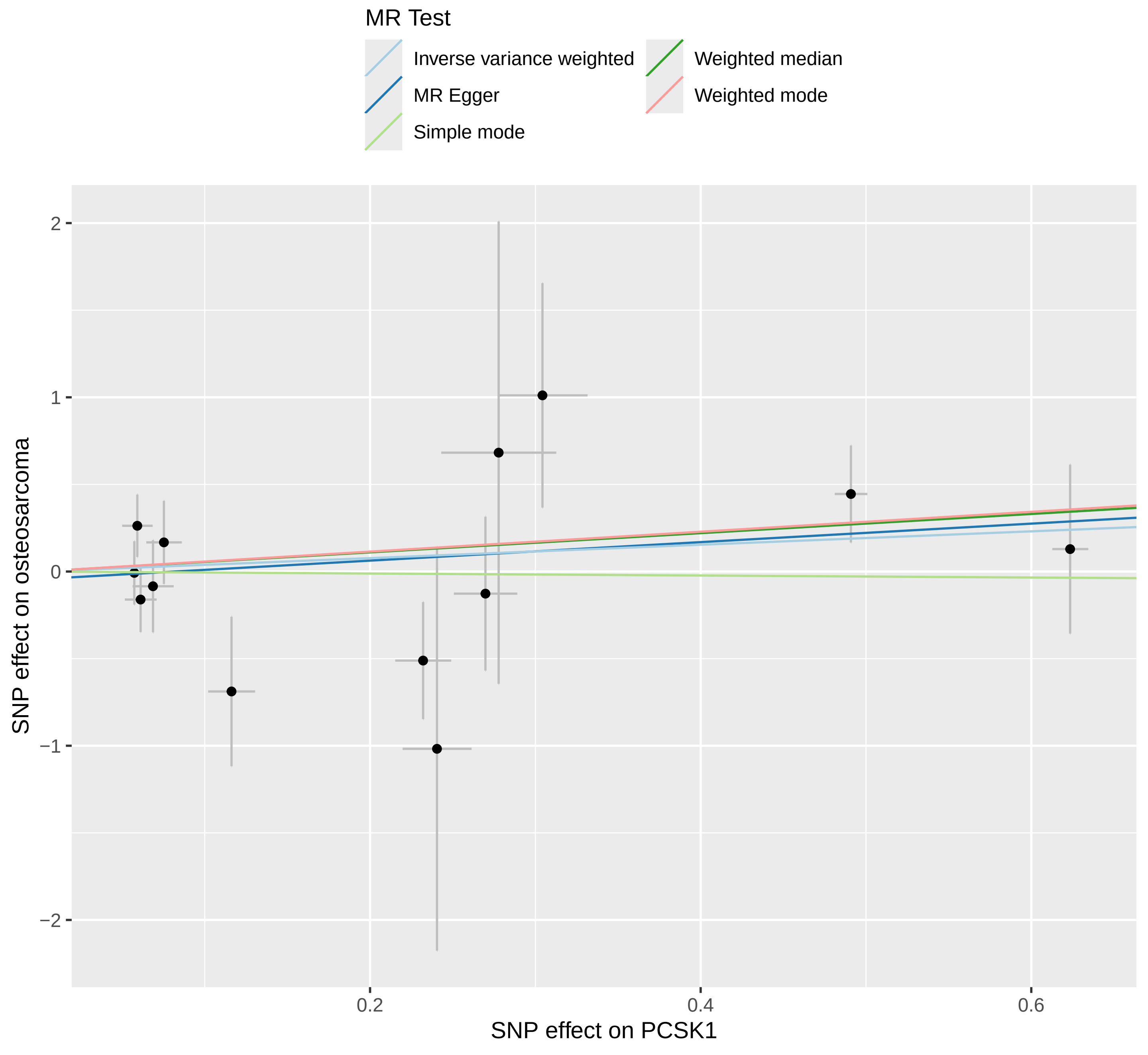
MR Test
Inverse variance weighted
MR Egger
Simple mode
Weighted median
Weighted mode
SNP effect on osteosarcoma
2
1
0
−1
−2
0.2
0.4
0.6
SNP effect on PCSK1

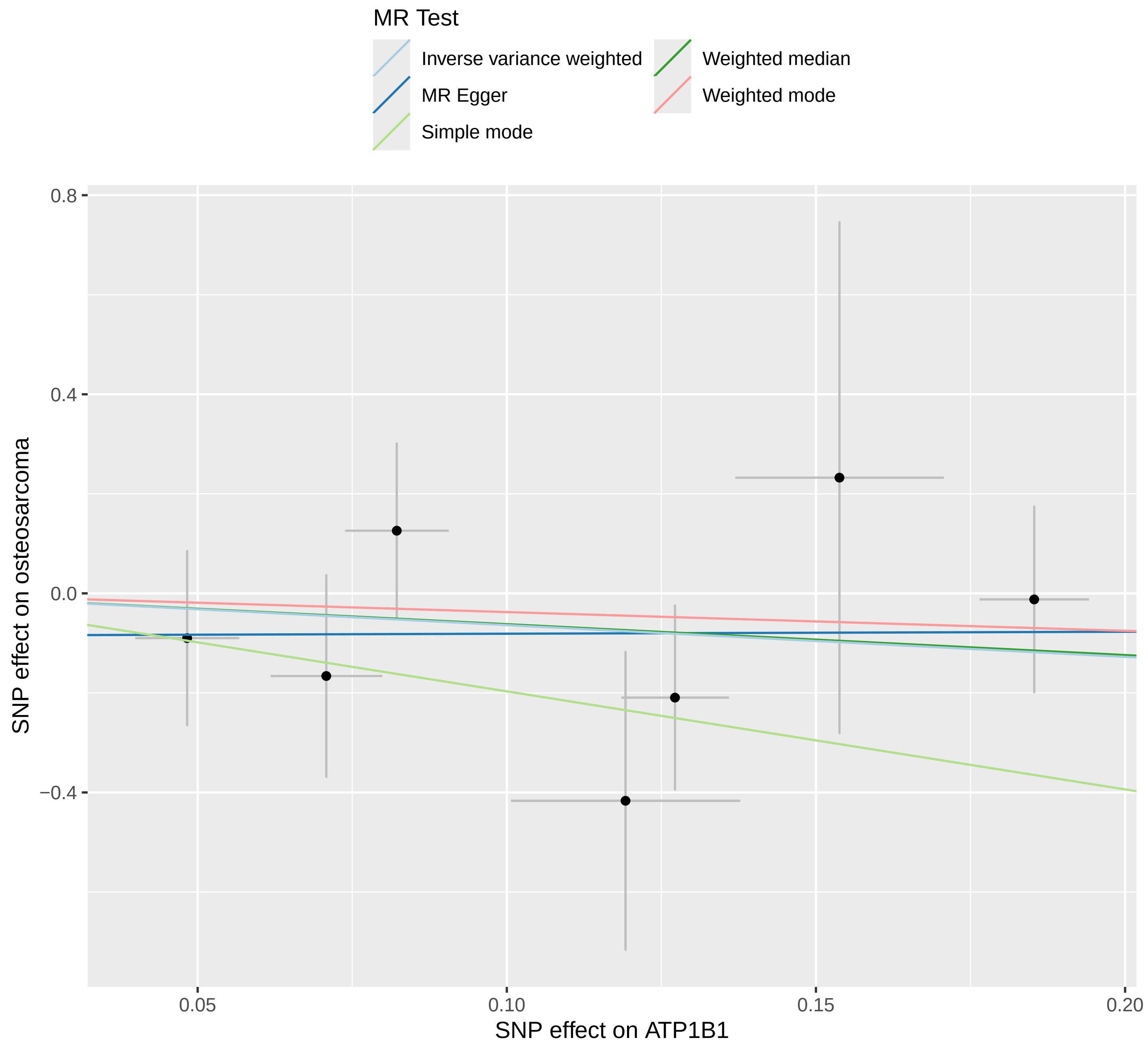
MR Test
Inverse variance weighted
MR Egger
Simple mode
Weighted median
Weighted mode
0.8
0.4
0.0
−0.4
SNP effect on osteosarcoma
0.05
0.10
0.15
0.20
SNP effect on ATP1B1

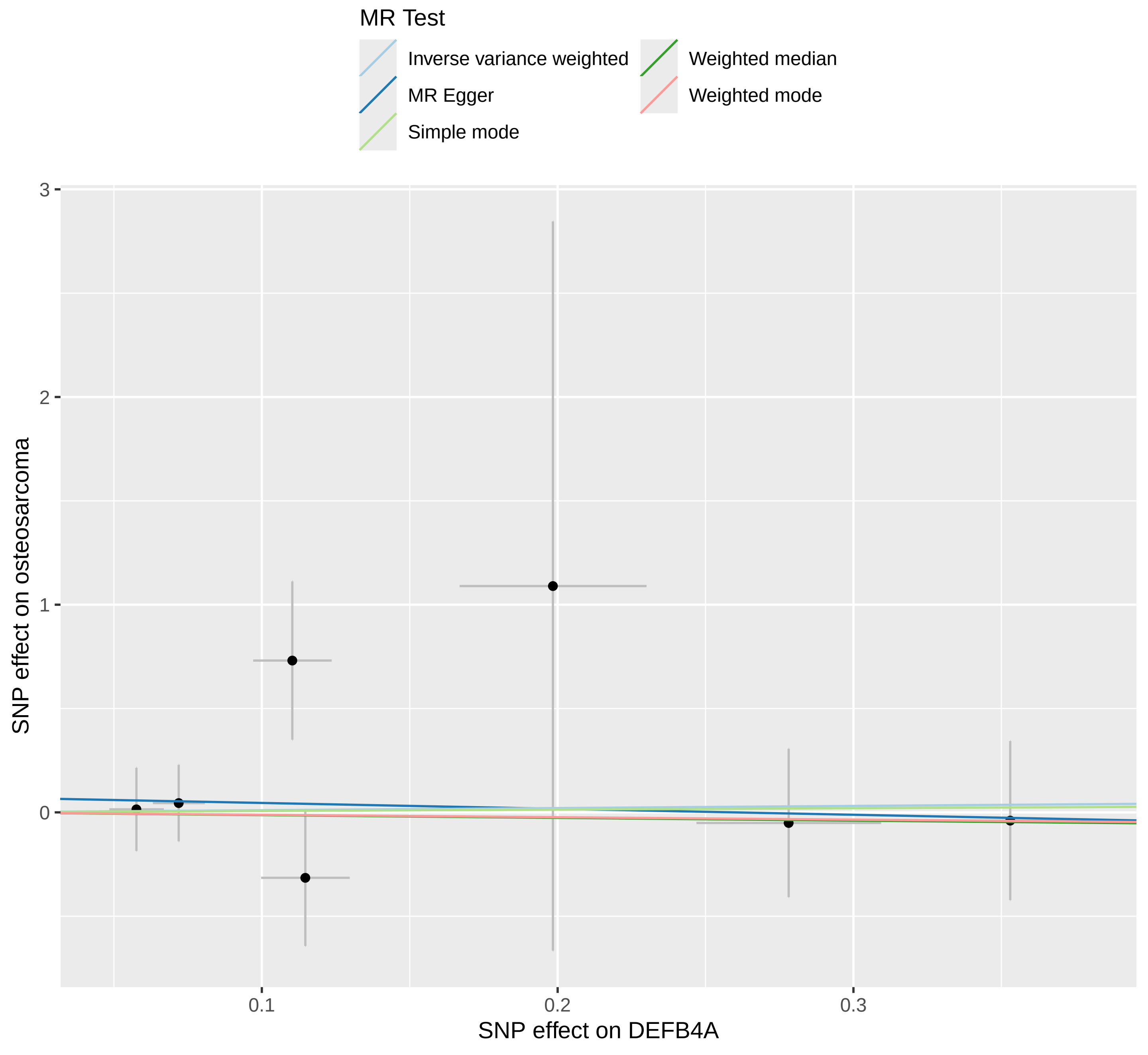
MR Test
Inverse variance weighted
MR Egger
Simple mode
Weighted median
Weighted mode
3
2
1
0
0.1
0.2
0.3
SNP effect on DEFB4A
SNP effect on osteosarcoma

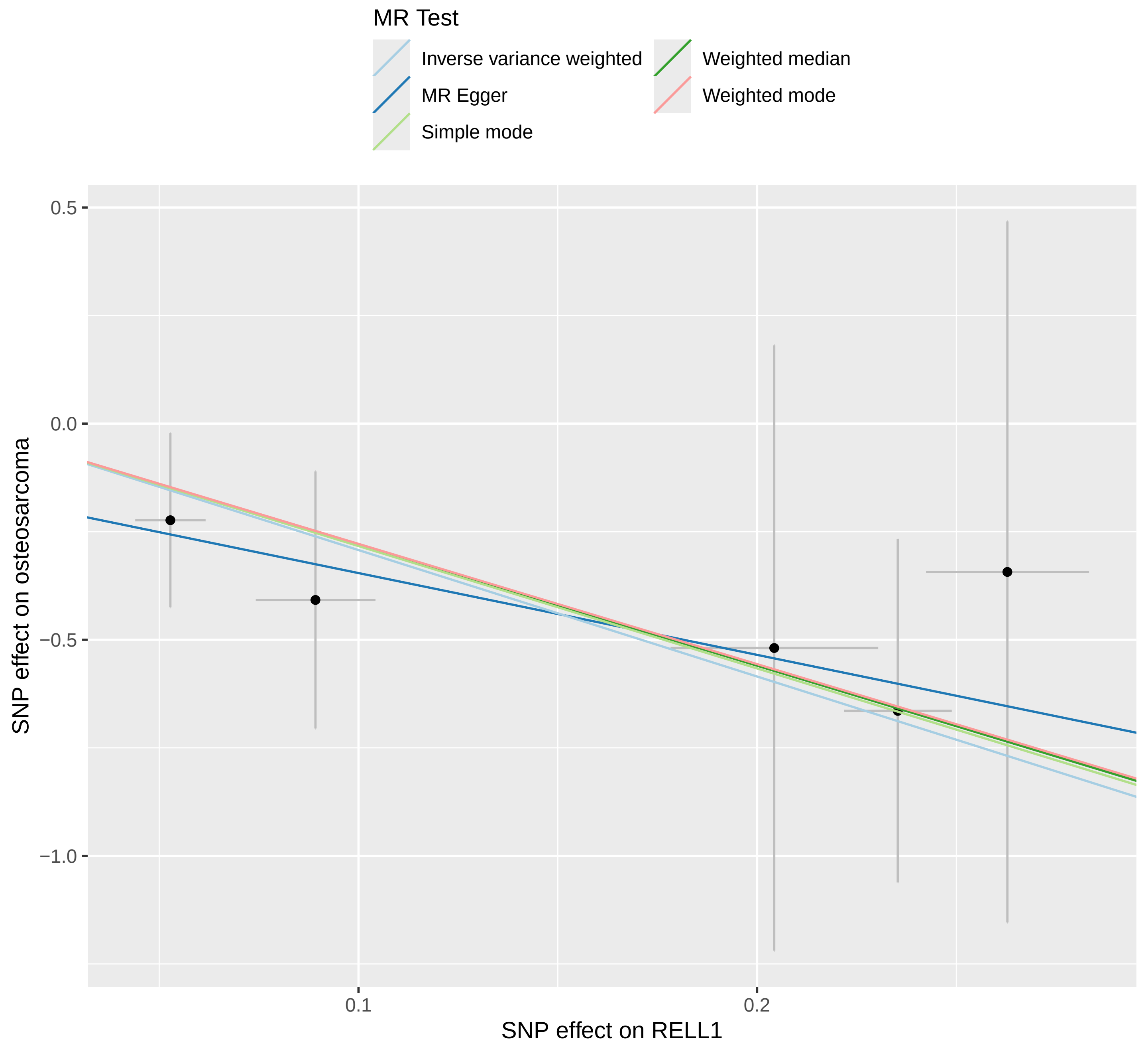

MR Test
Inverse variance weighted
MR Egger
Simple mode
Weighted median
Weighted mode
0.5
0.0
−0.5
−1.0
0.1
0.2
SNP effect on osteosarcoma
SNP effect on RELL1

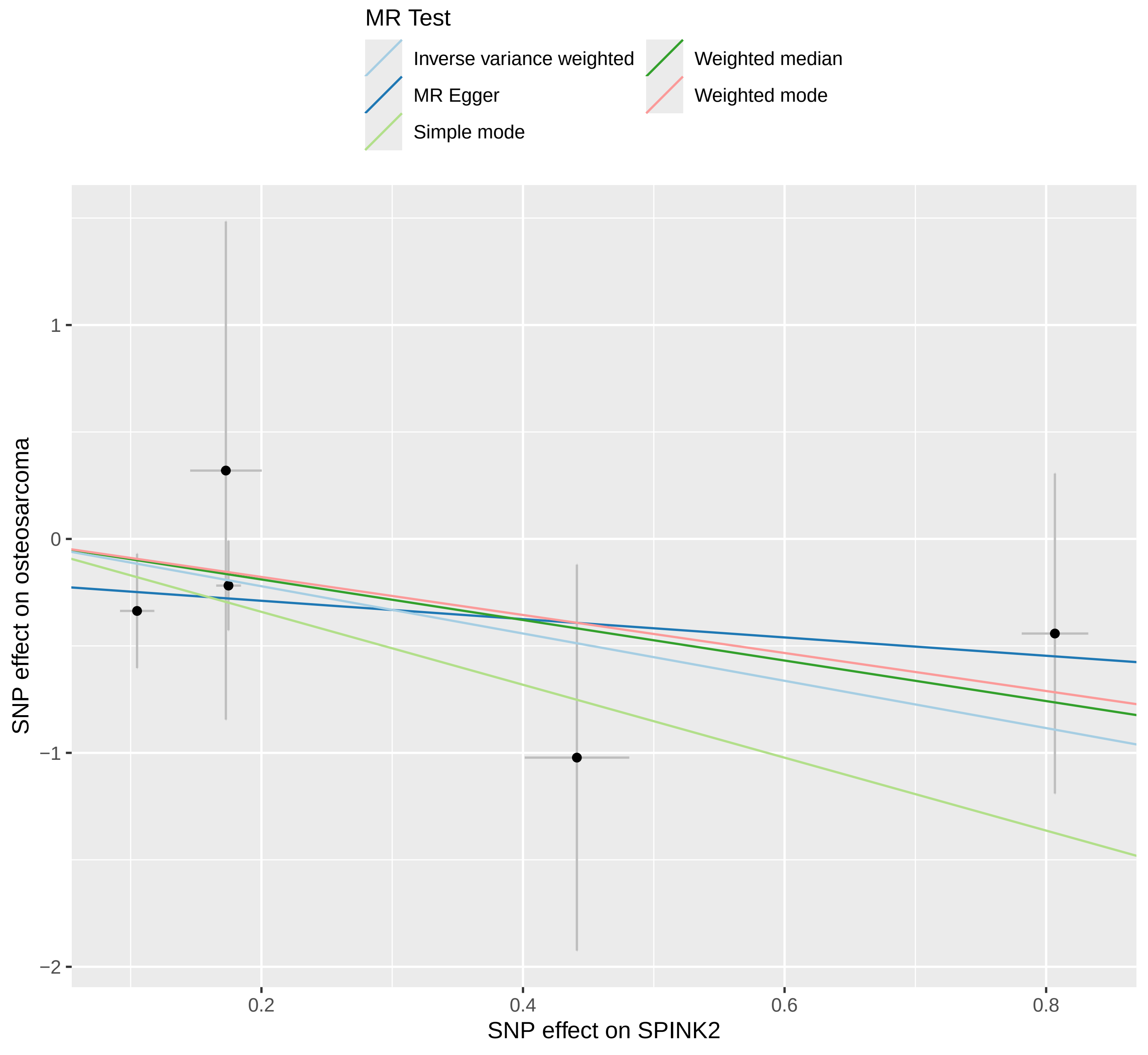

MR Test
Inverse variance weighted
MR Egger
Simple mode
Weighted median
Weighted mode
1
0
−1
−2
0.2
0.4
0.6
0.8
SNP effect on osteosarcoma
SNP effect on SPINK2

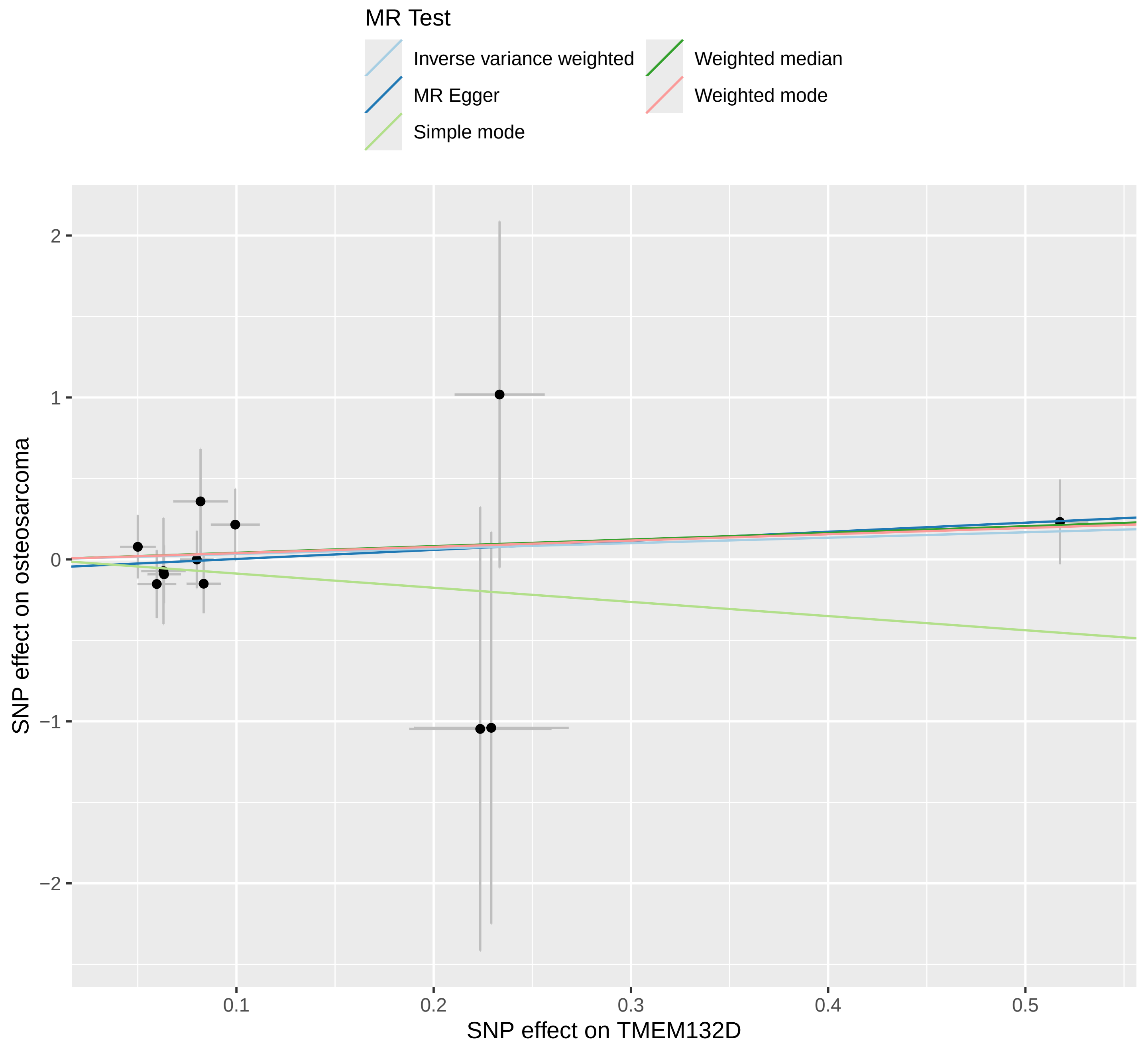
MR Test
Inverse variance weighted
MR Egger
Simple mode
Weighted median
Weighted mode
2
1
0
−1
−2
SNP effect on osteosarcoma
0.1
0.2
0.3
0.4
0.5
SNP effect on TMEM132D

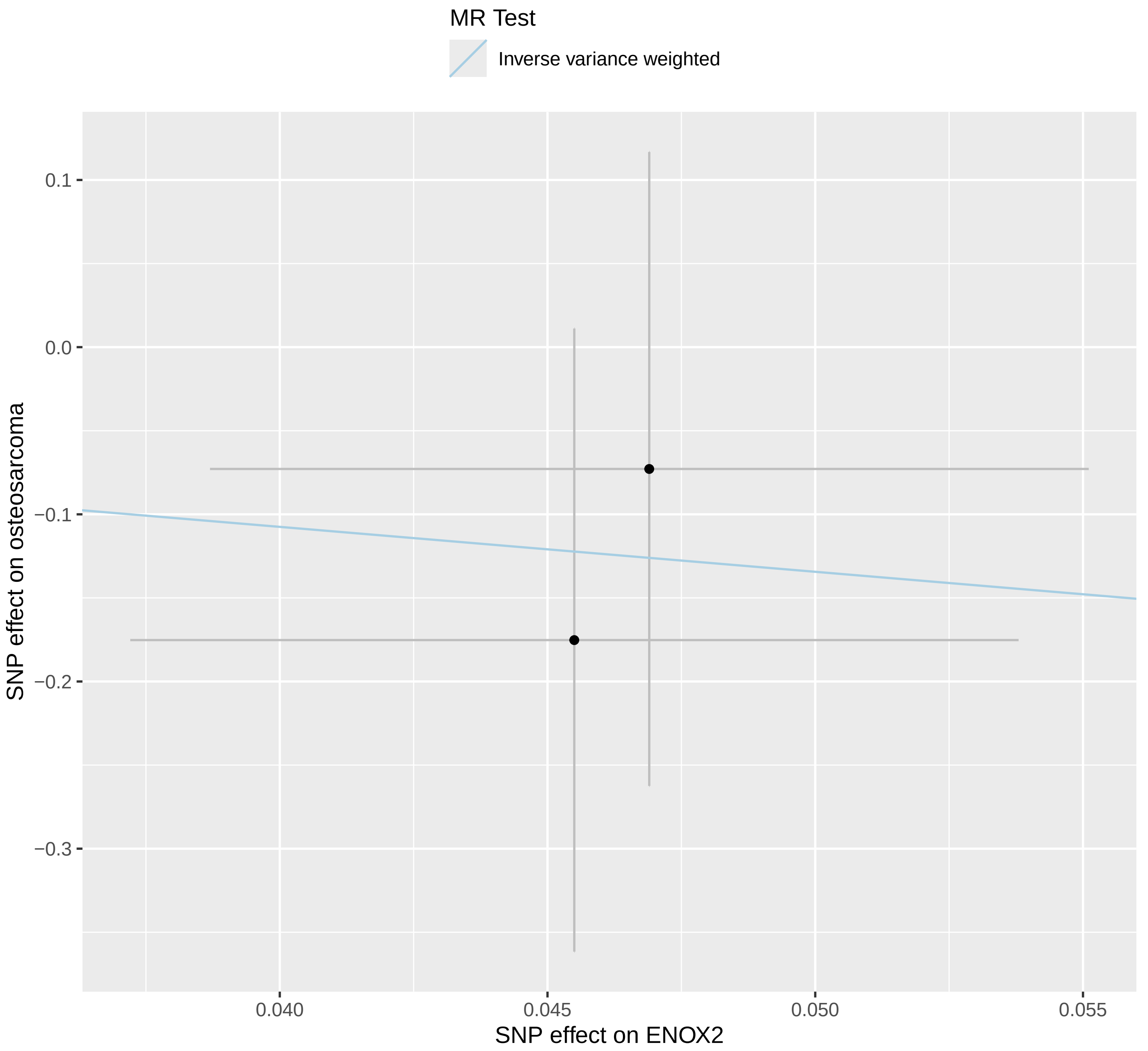
MR Test
Inverse variance weighted
0.1
0.0
−0.1
−0.2
−0.3
0.040
0.045
0.050
0.055
SNP effect on osteosarcoma
SNP effect on ENOX2

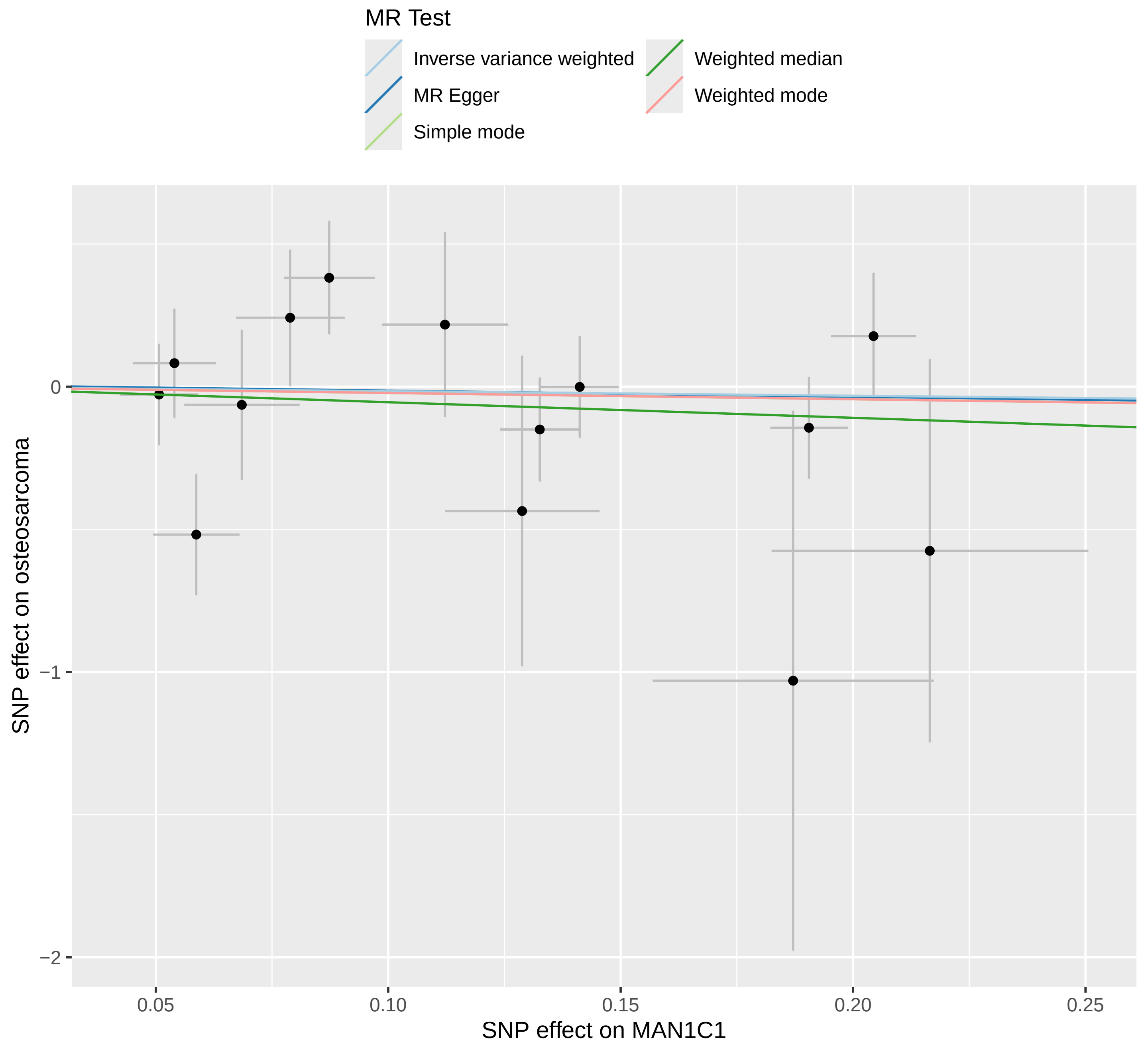
MR Test
Inverse variance weighted
MR Egger
Simple mode
Weighted median
Weighted mode
0
−1
−2
0.05
0.10
0.15
0.20
0.25
SNP effect on MAN1C1
SNP effect on osteosarcoma

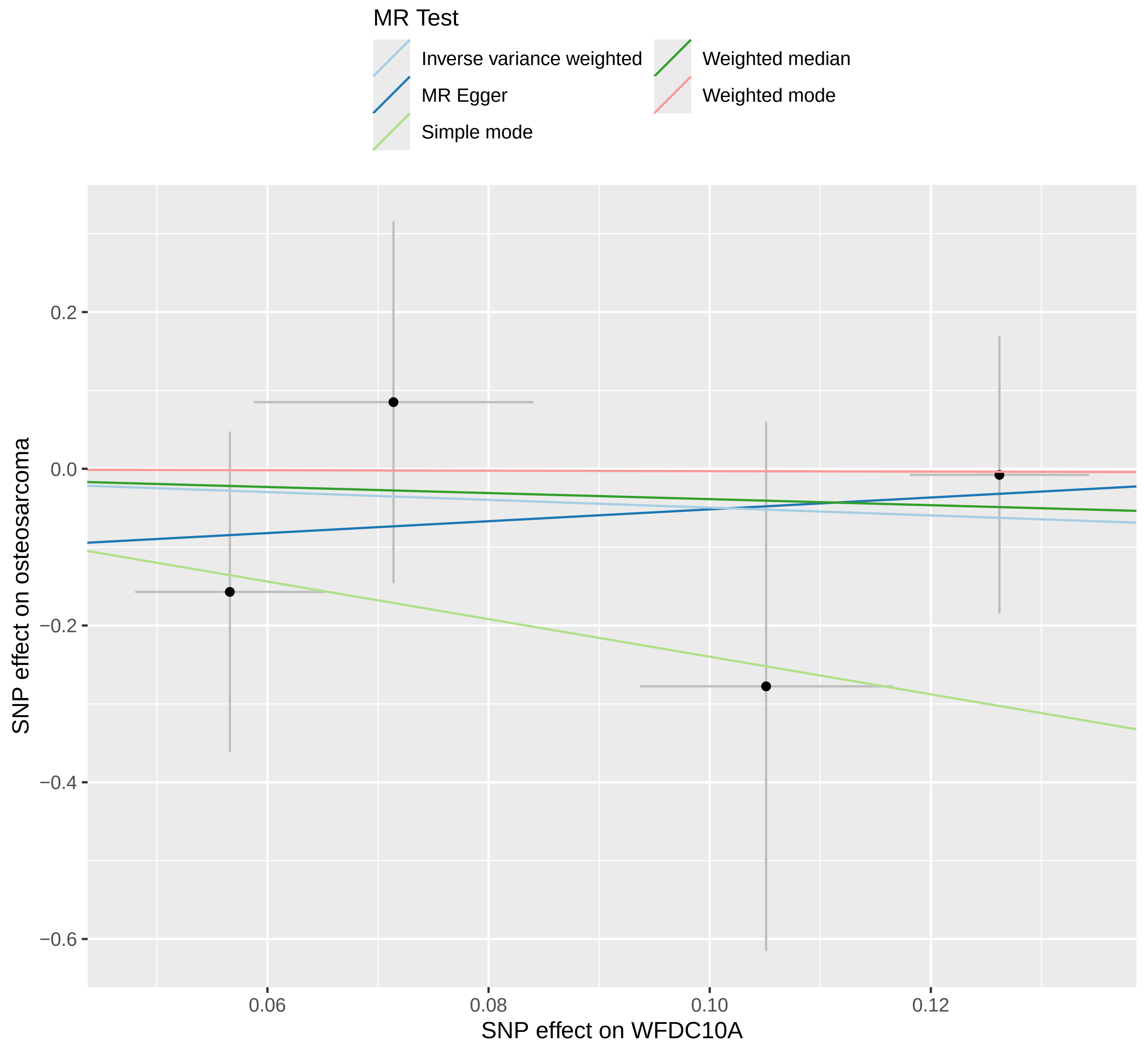

MR Test
Inverse variance weighted
MR Egger
Simple mode
Weighted median
Weighted mode
0.2
0.0
−0.2
−0.4
−0.6
0.06
0.08
0.10
0.12
SNP effect on WFDC10A
SNP effect on osteosarcoma

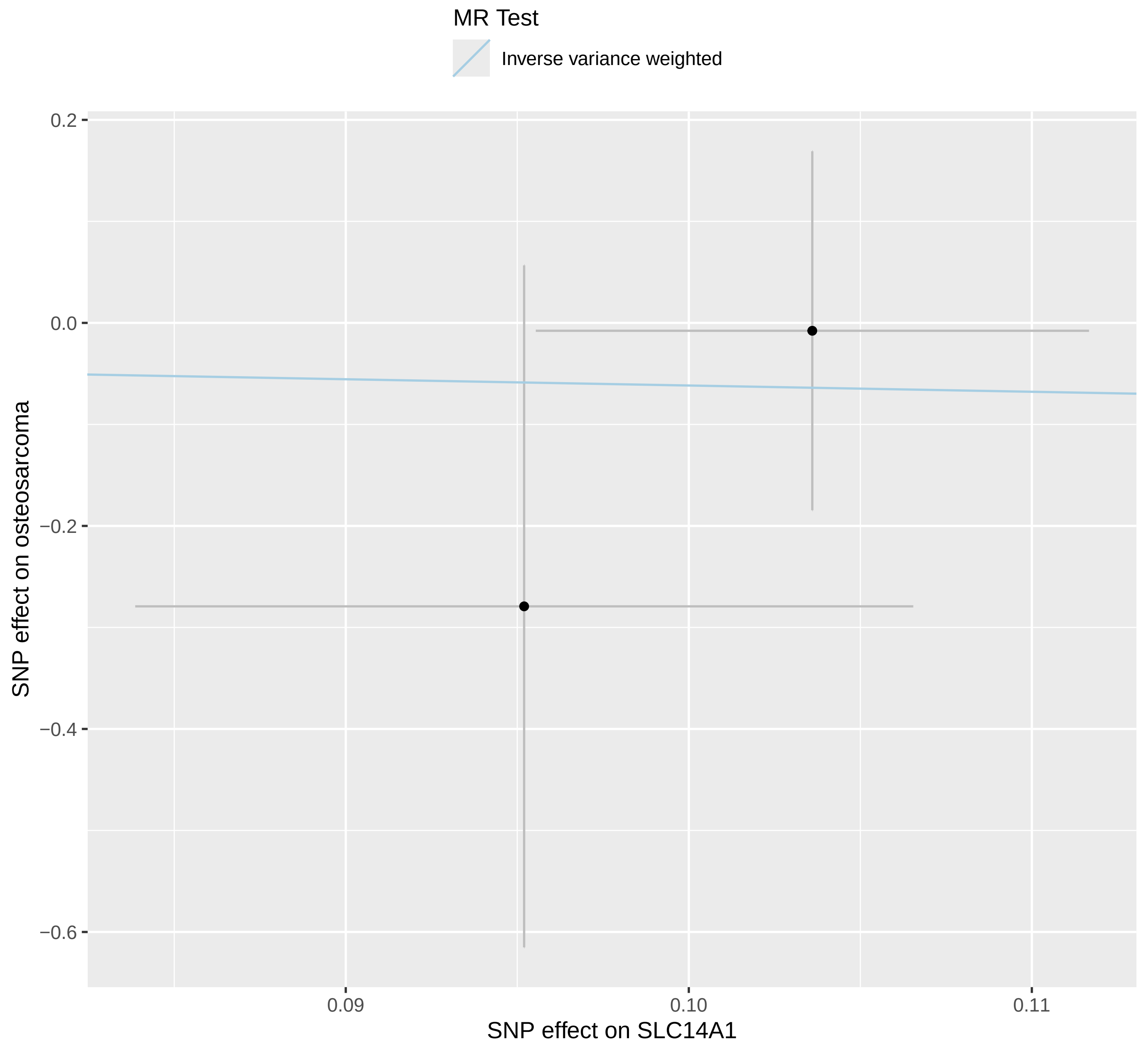
MR Test
Inverse variance weighted
0.2
0.0
−0.2
−0.4
−0.6
SNP effect on osteosarcoma
0.09
0.10
0.11
SNP effect on SLC14A1

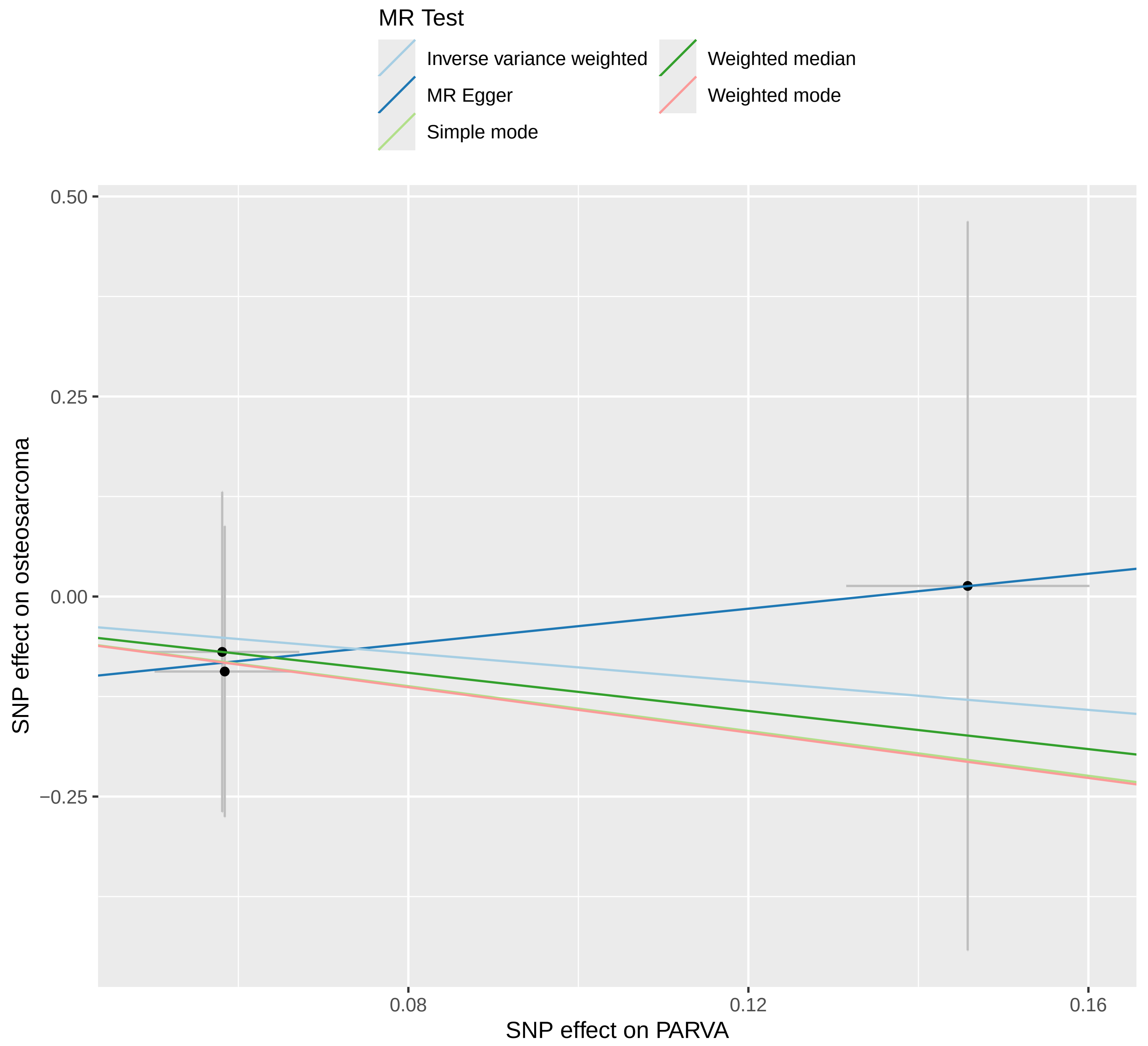
MR Test
Inverse variance weighted
MR Egger
Simple mode
Weighted median
Weighted mode
0.50
0.25
0.00
−0.25
SNP effect on osteosarcoma
0.08
0.12
0.16
SNP effect on PARVA

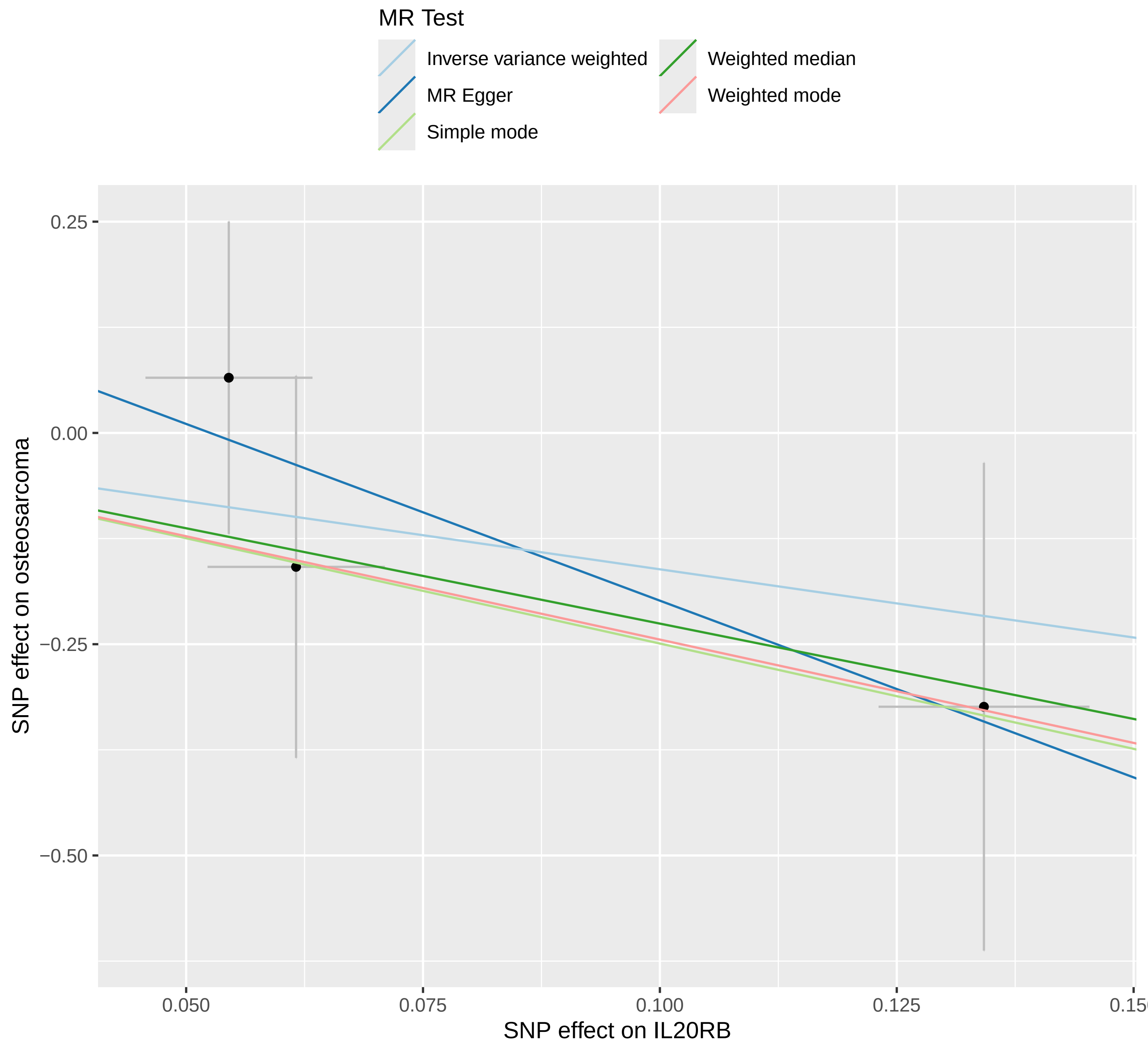
MR Test
Inverse variance weighted
MR Egger
Simple mode
Weighted median
Weighted mode
0.25
0.00
−0.25
−0.50
SNP effect on osteosarcoma
0.050
0.075
0.100
0.125
SNP effect on IL20RB

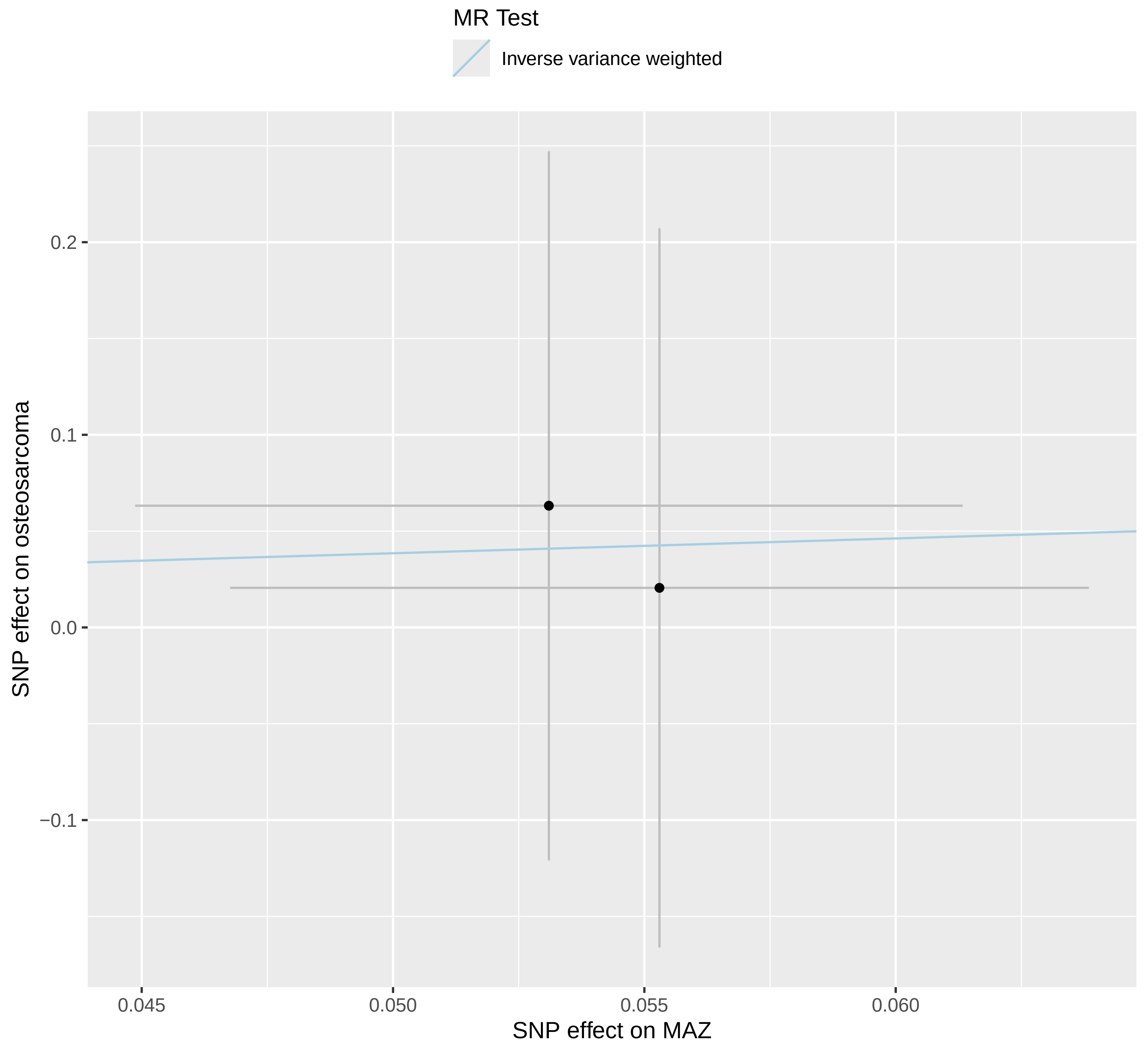

MR Test
Inverse variance weighted
SNP effect on osteosarcoma
0.2
0.1
0.0
−0.1
0.045
0.050
0.055
0.060
SNP effect on MAZ

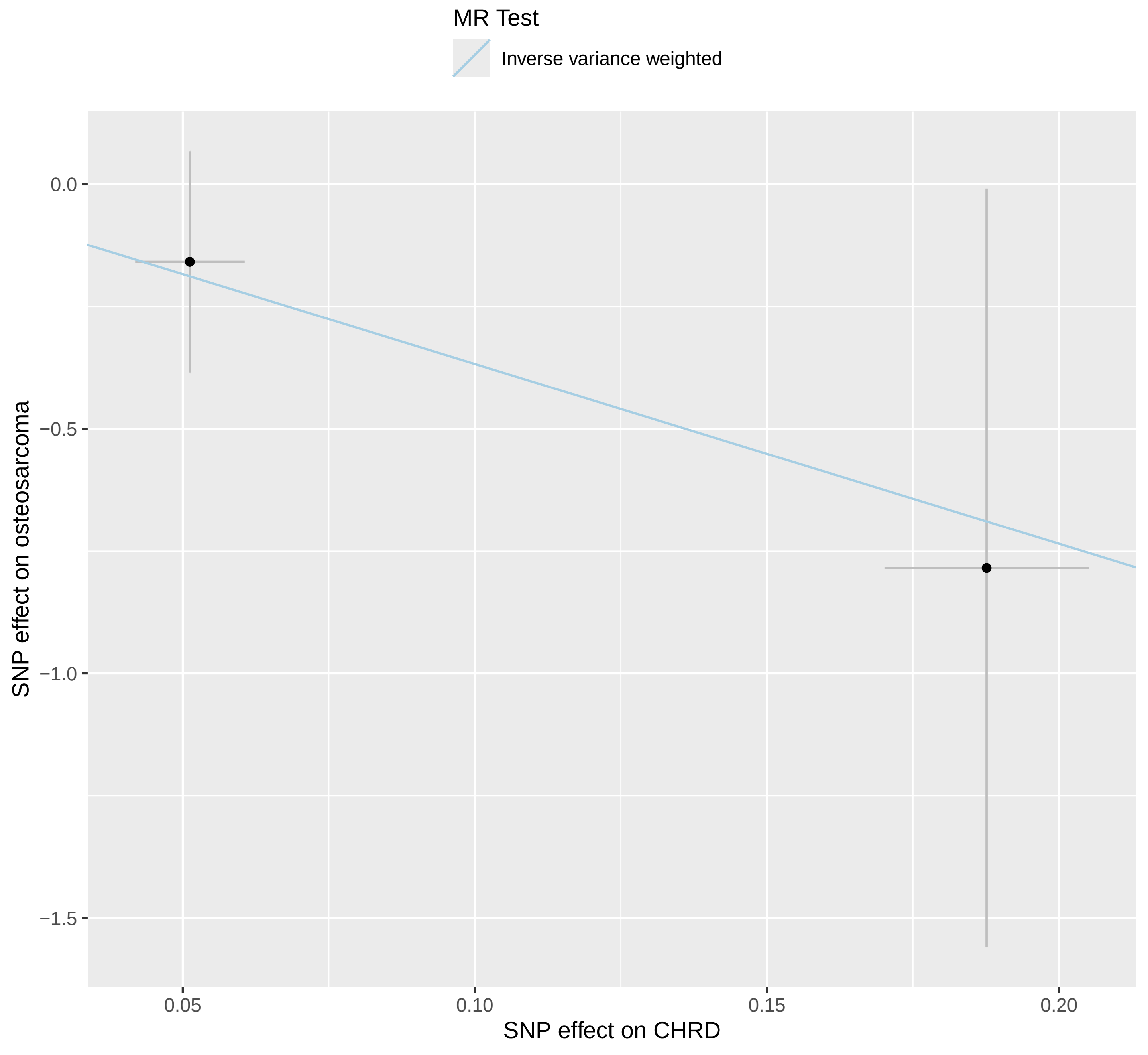

MR Test
Inverse variance weighted
0.0
−0.5
−1.0
−1.5
SNP effect on osteosarcoma
0.05
0.10
0.15
0.20
SNP effect on CHRD

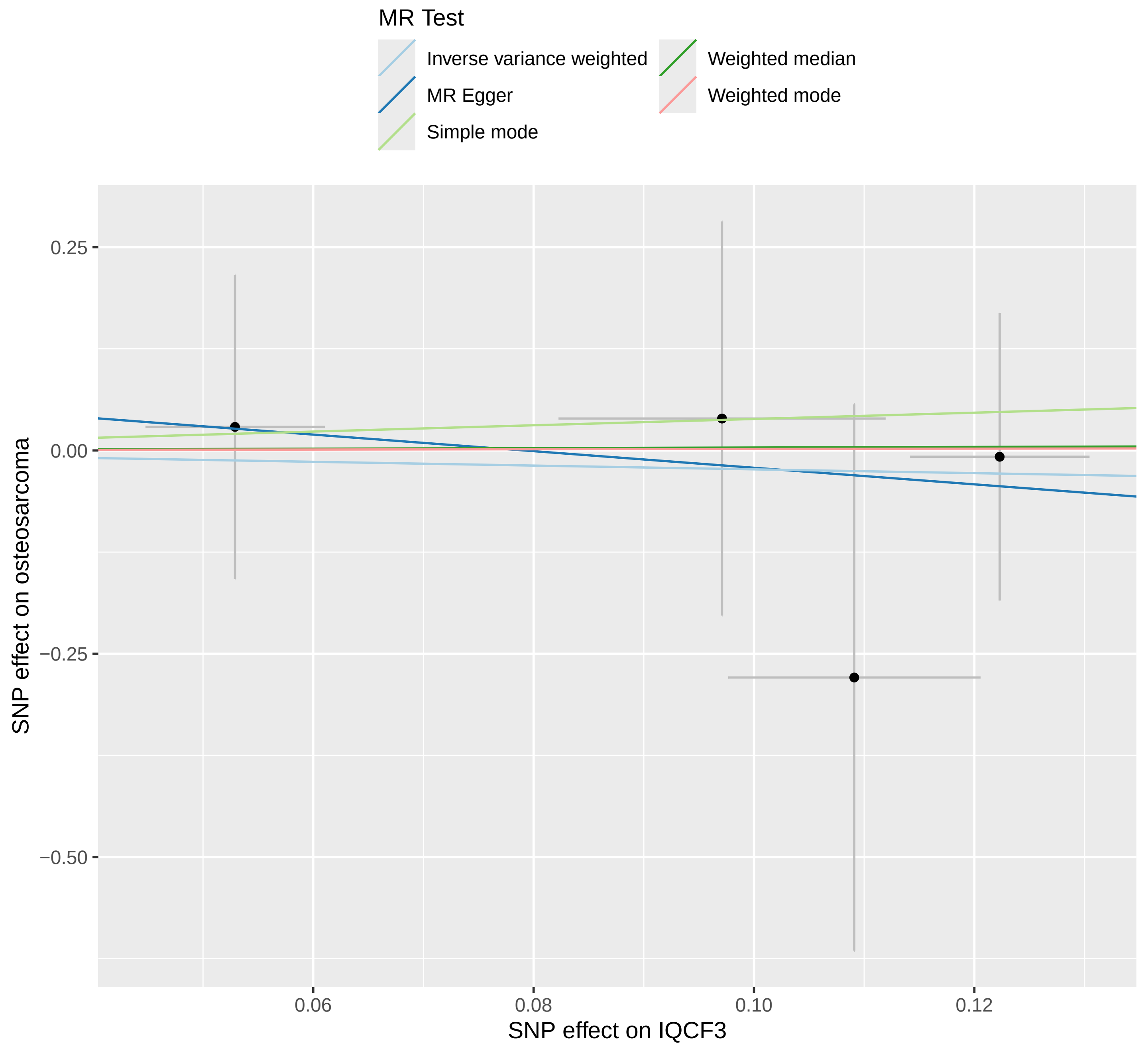
MR Test
Inverse variance weighted
MR Egger
Simple mode
Weighted median
Weighted mode
0.25
0.00
−0.25
−0.50
SNP effect on osteosarcoma
0.06
0.08
0.10
0.12
SNP effect on IQCF3

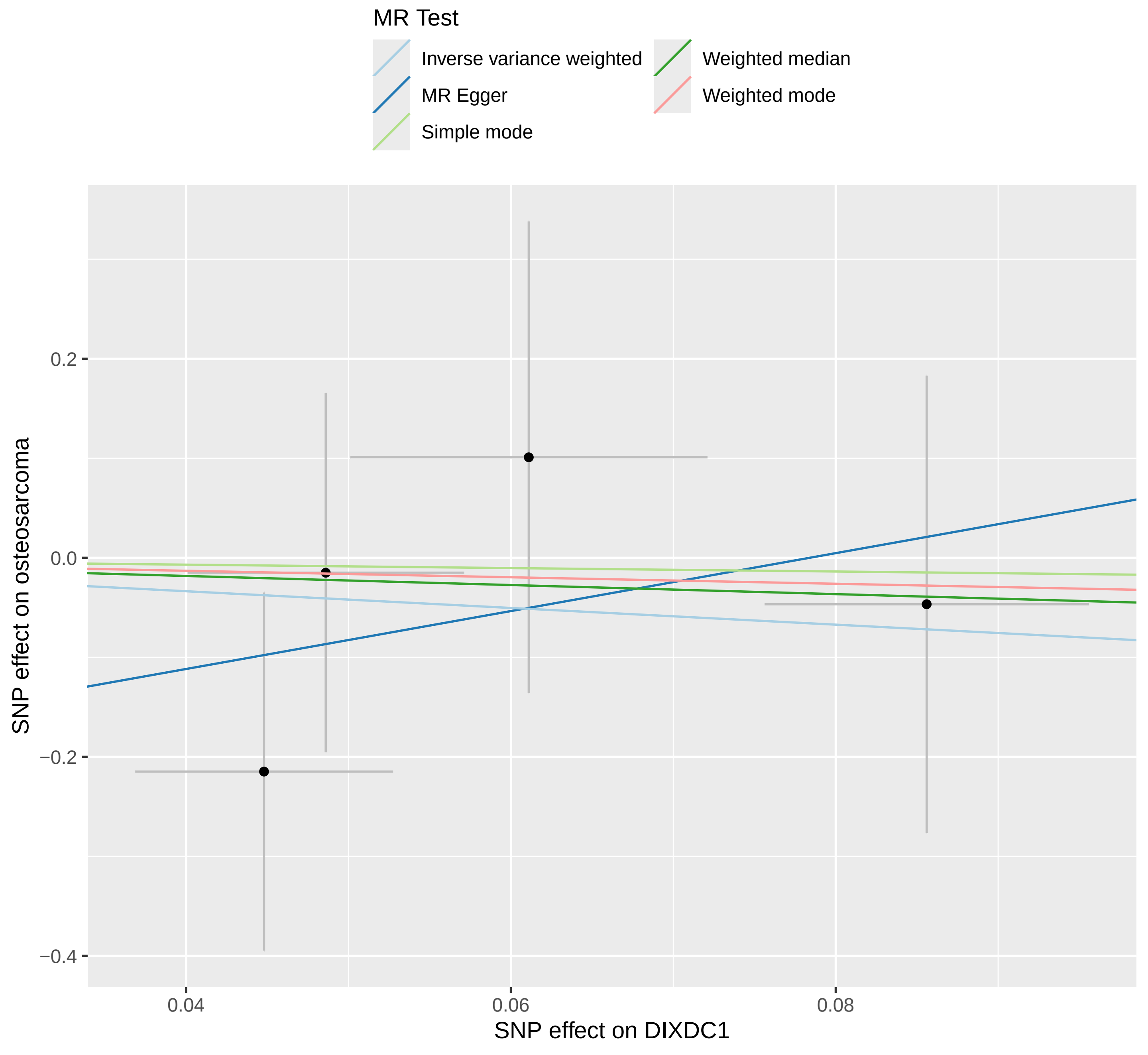
MR Test
Inverse variance weighted
MR Egger
Simple mode
Weighted median
Weighted mode
0.2
0.0
−0.2
−0.4
SNP effect on osteosarcoma
0.04
0.06
0.08
SNP effect on DIXDC1

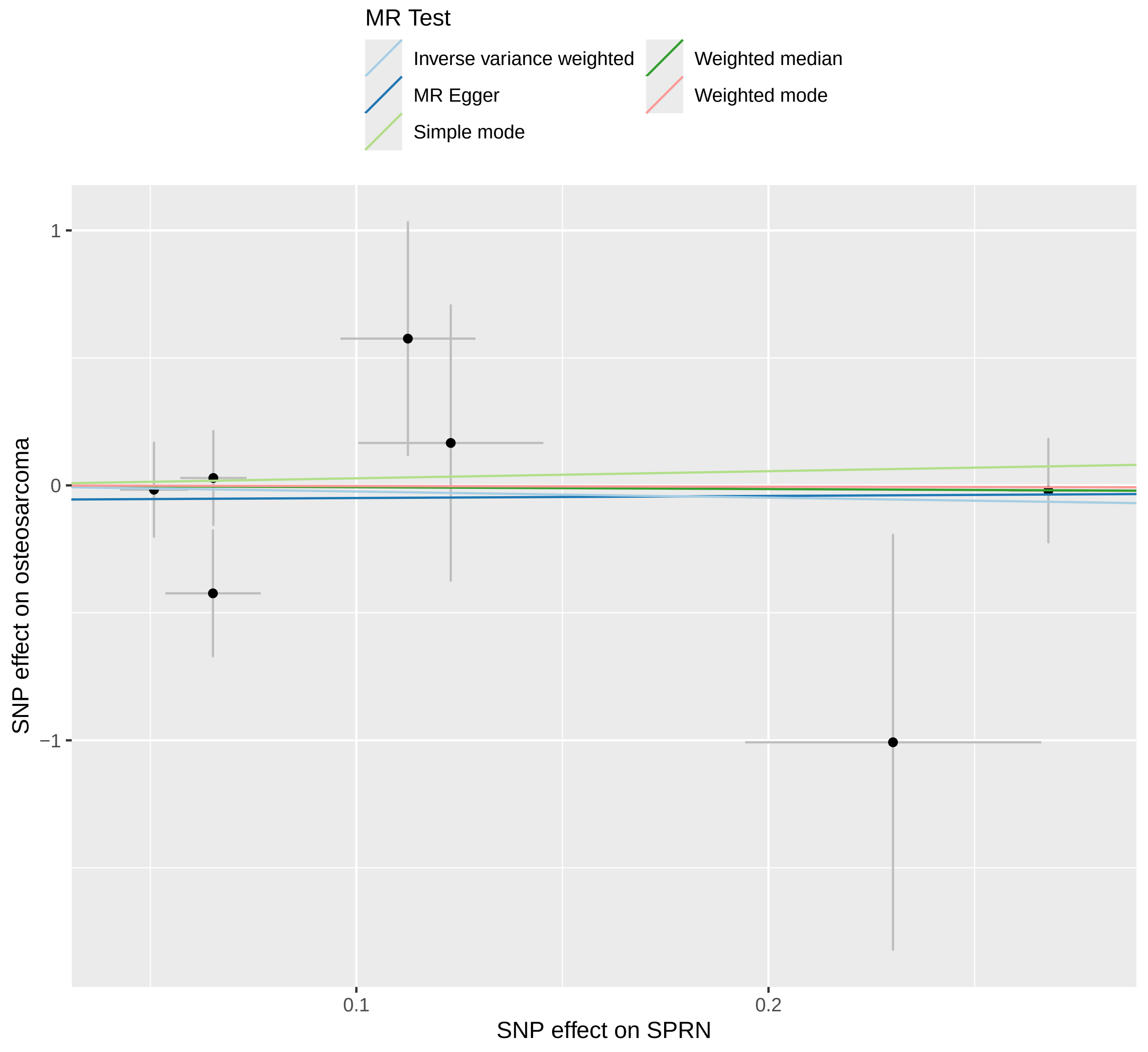

MR Test
Inverse variance weighted
MR Egger
Simple mode
Weighted median
Weighted mode
1
0
−1
0.1
0.2
SNP effect on SPRN
SNP effect on osteosarcoma

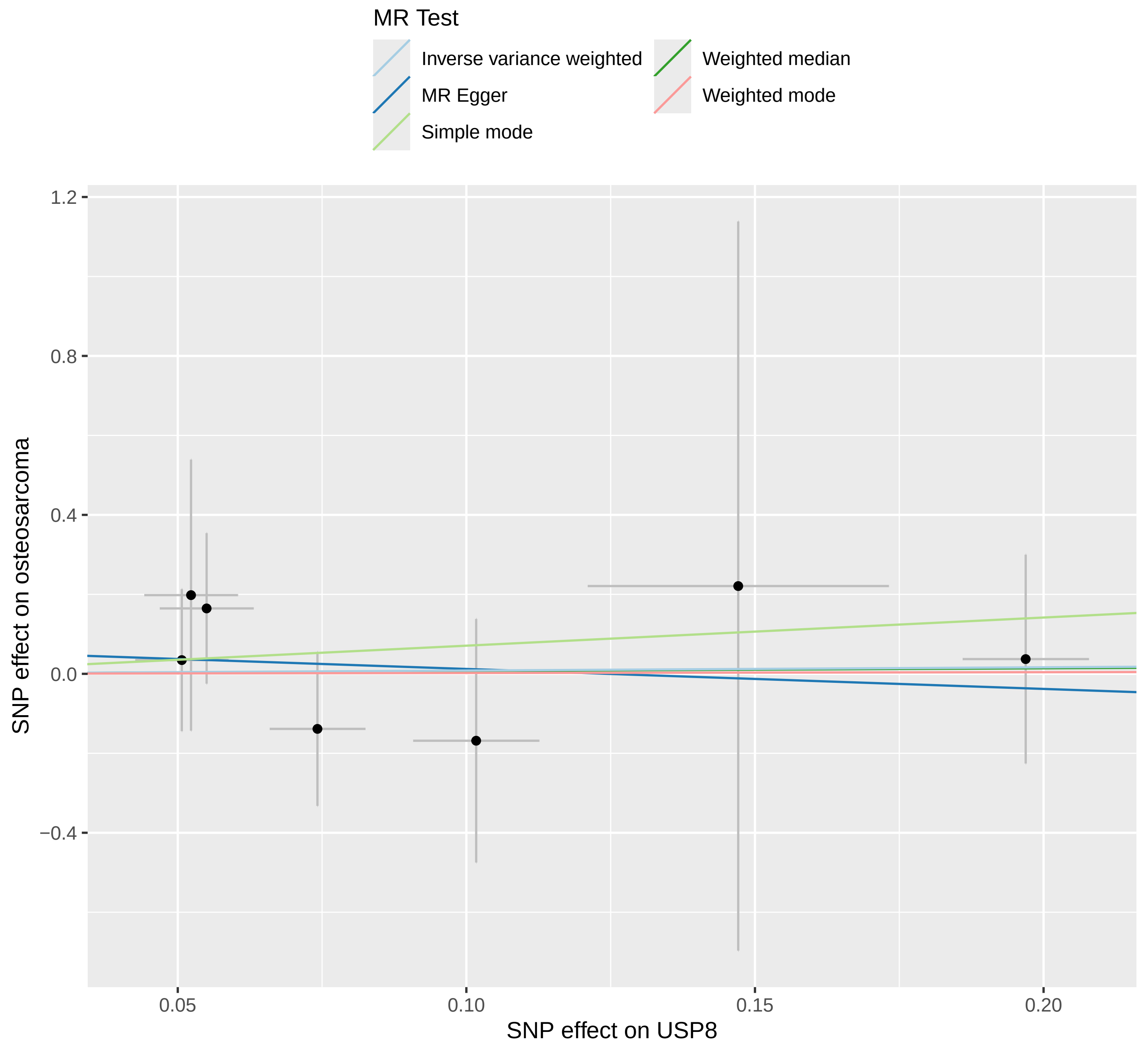
MR Test
Inverse variance weighted
MR Egger
Simple mode
Weighted median
Weighted mode
1.2
0.8
0.4
0.0
−0.4
SNP effect on osteosarcoma
0.05
0.10
0.15
0.20
SNP effect on USP8

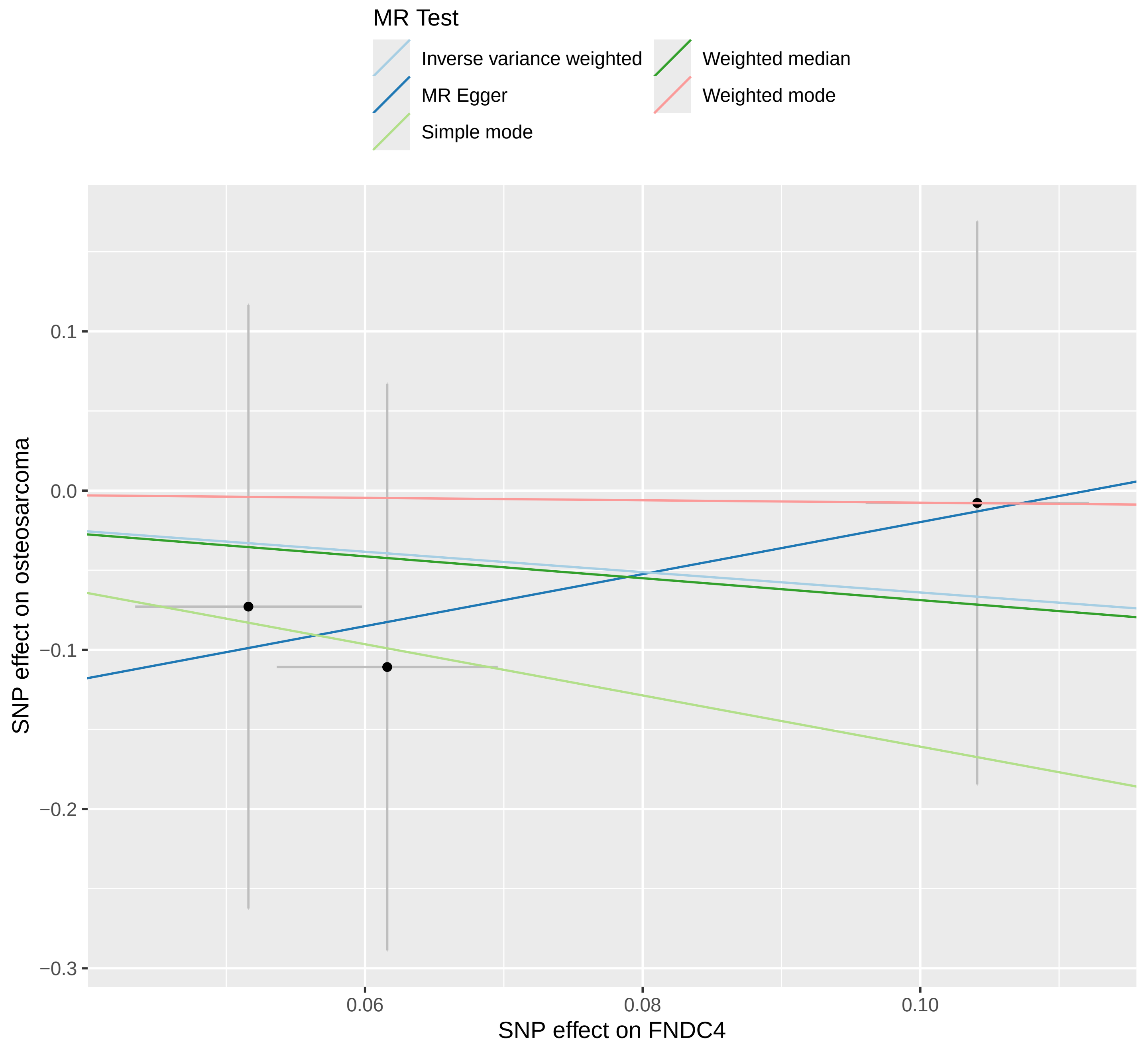

MR Test
Inverse variance weighted
MR Egger
Simple mode
Weighted median
Weighted mode
0.1
0.0
−0.1
−0.2
−0.3
0.06
0.08
0.10
SNP effect on osteosarcoma
SNP effect on FNDC4

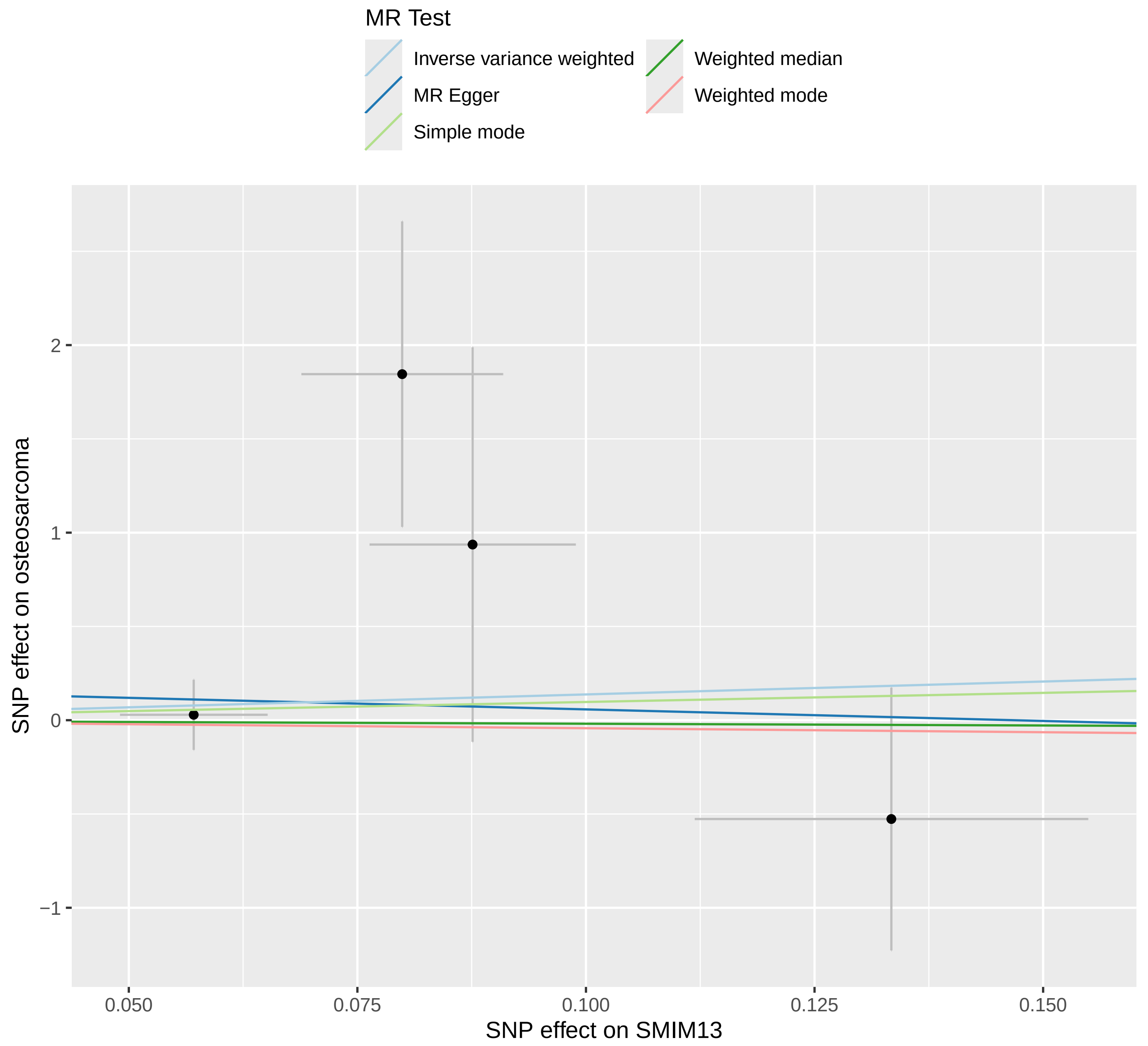
MR Test
Inverse variance weighted
MR Egger
Simple mode
Weighted median
Weighted mode
2
1
0
−1
SNP effect on osteosarcoma
0.050
0.075
0.100
0.125
0.150
SNP effect on SMIM13

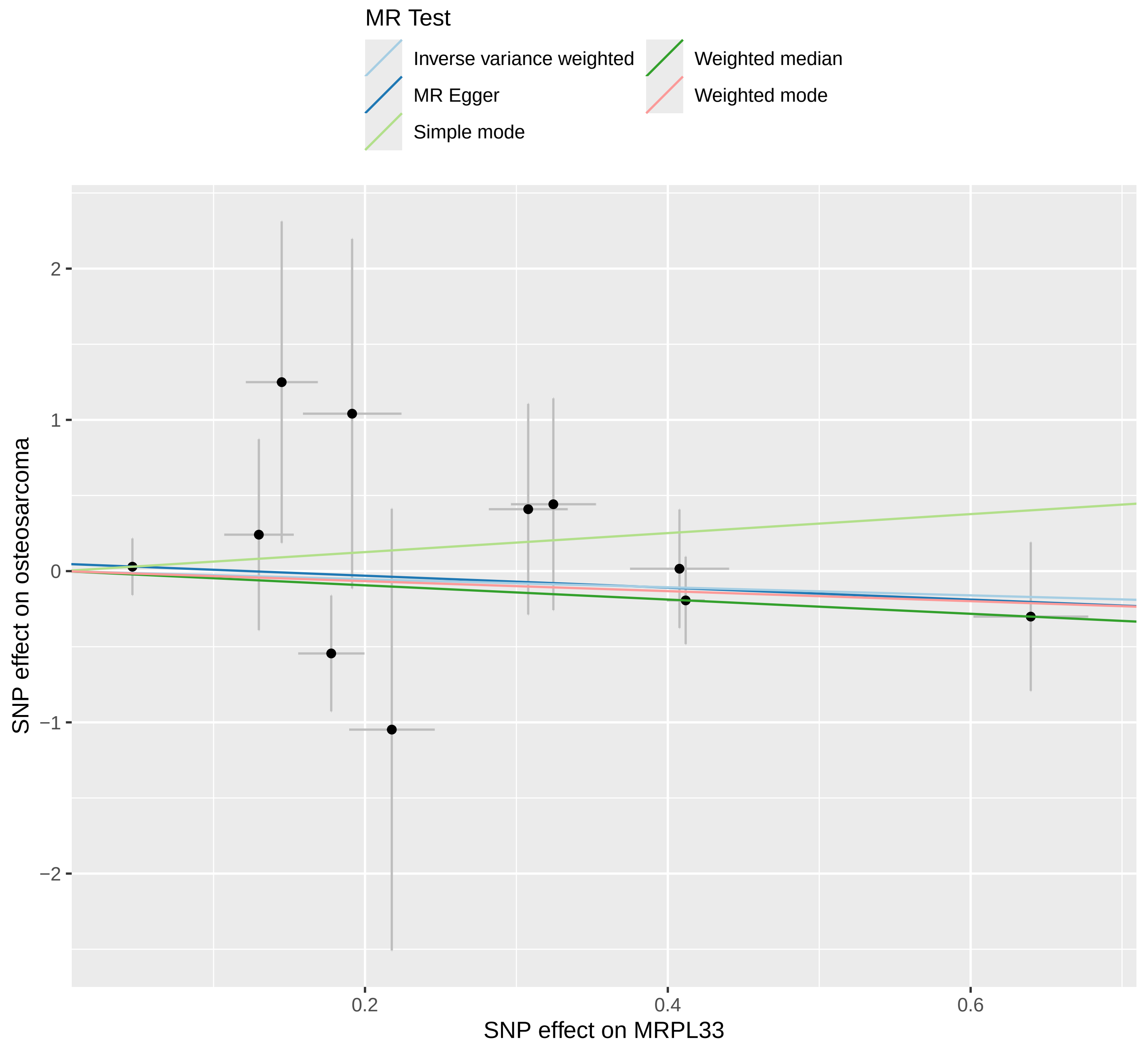

MR Test
Inverse variance weighted
MR Egger
Simple mode
Weighted median
Weighted mode
2
1
0
−1
−2
SNP effect on osteosarcoma
0.2
0.4
0.6
SNP effect on MRPL33

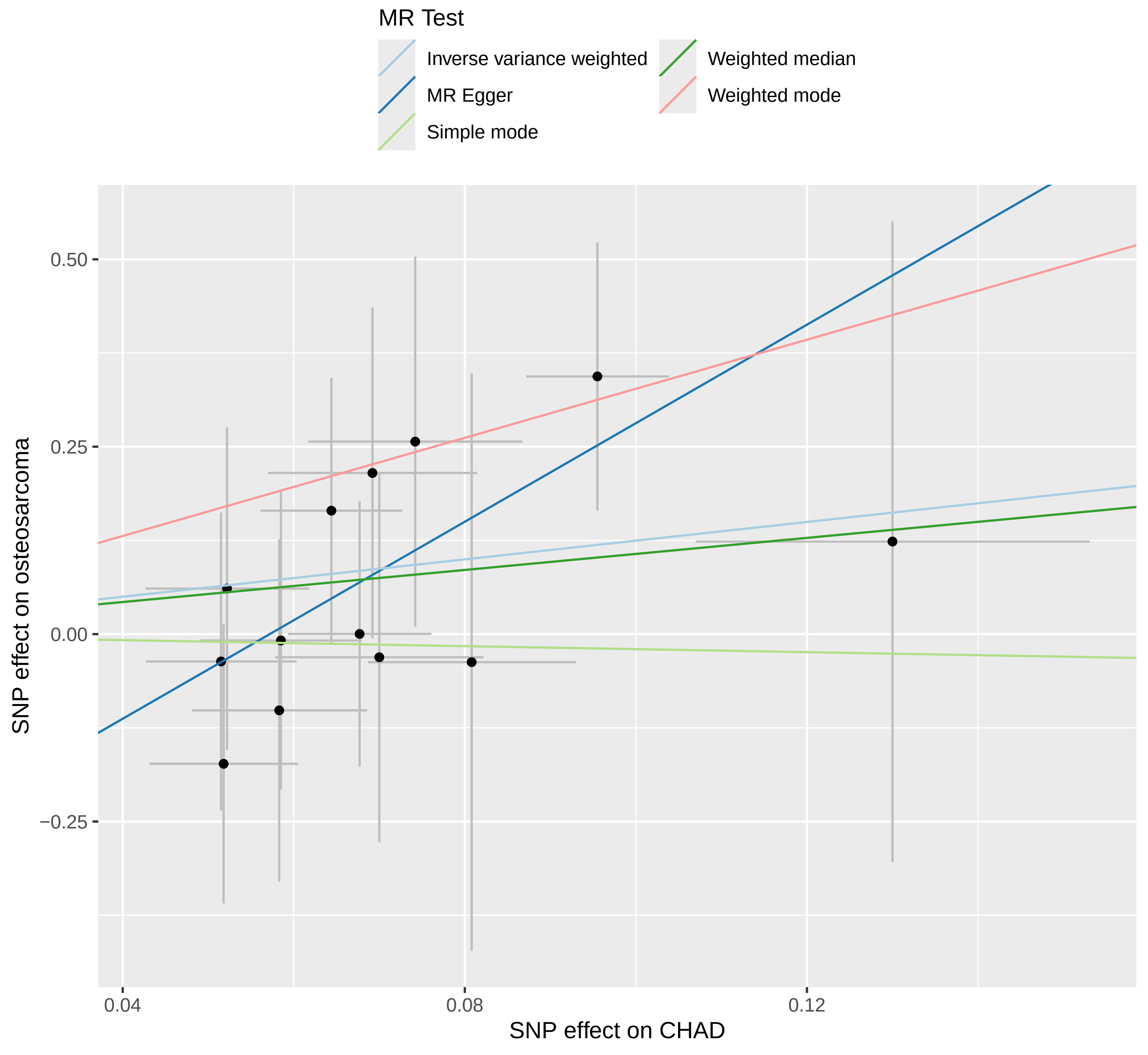

MR Test
Inverse variance weighted
MR Egger
Simple mode
Weighted median
Weighted mode
SNP effect on osteosarcoma
0.50
0.25
0.00
−0.25
0.04
0.08
0.12
SNP effect on CHAD

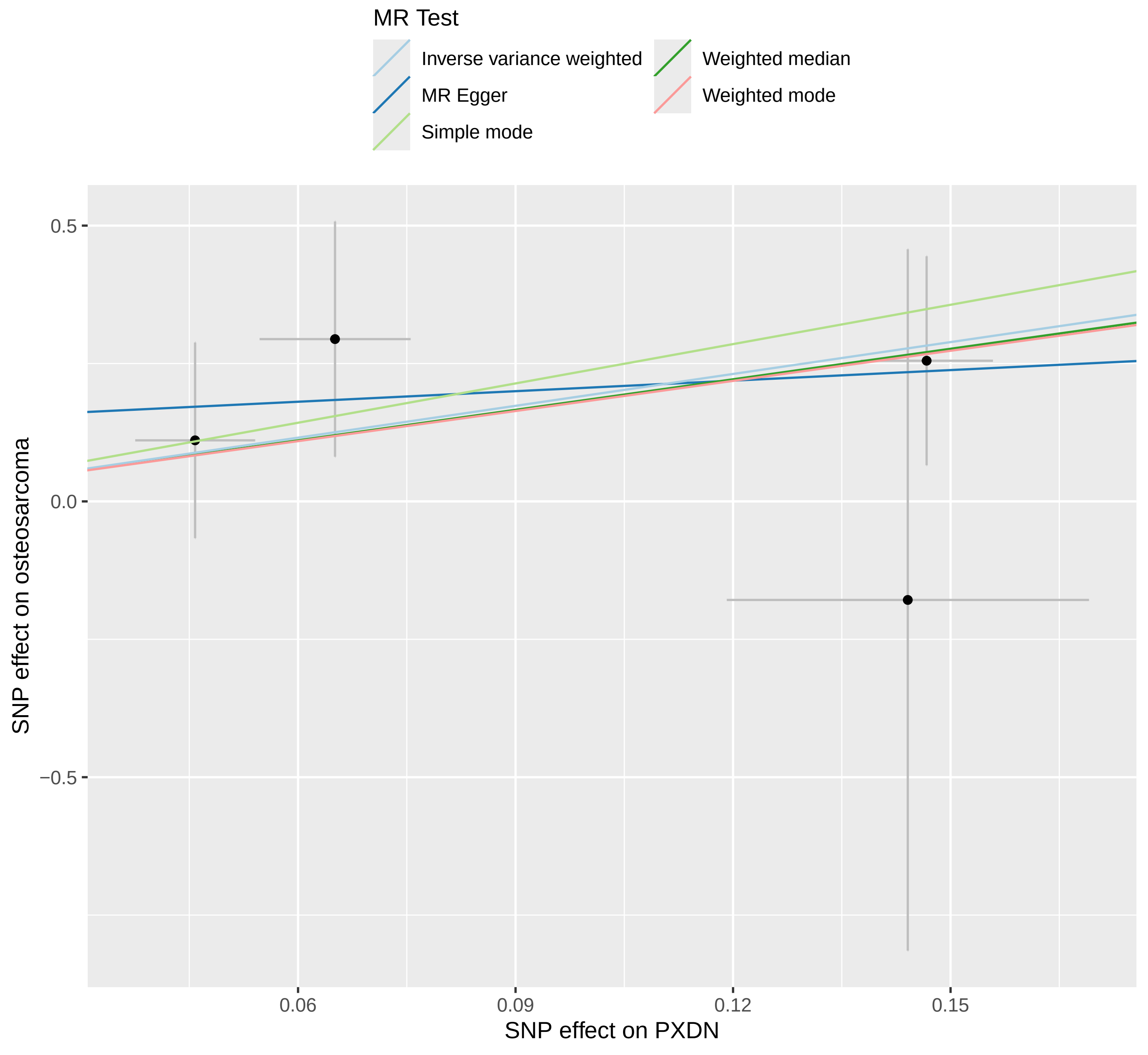
MR Test
Inverse variance weighted
MR Egger
Simple mode
Weighted median
Weighted mode
0.5
0.0
−0.5
SNP effect on osteosarcoma
0.06
0.09
0.12
0.15
SNP effect on PXDN

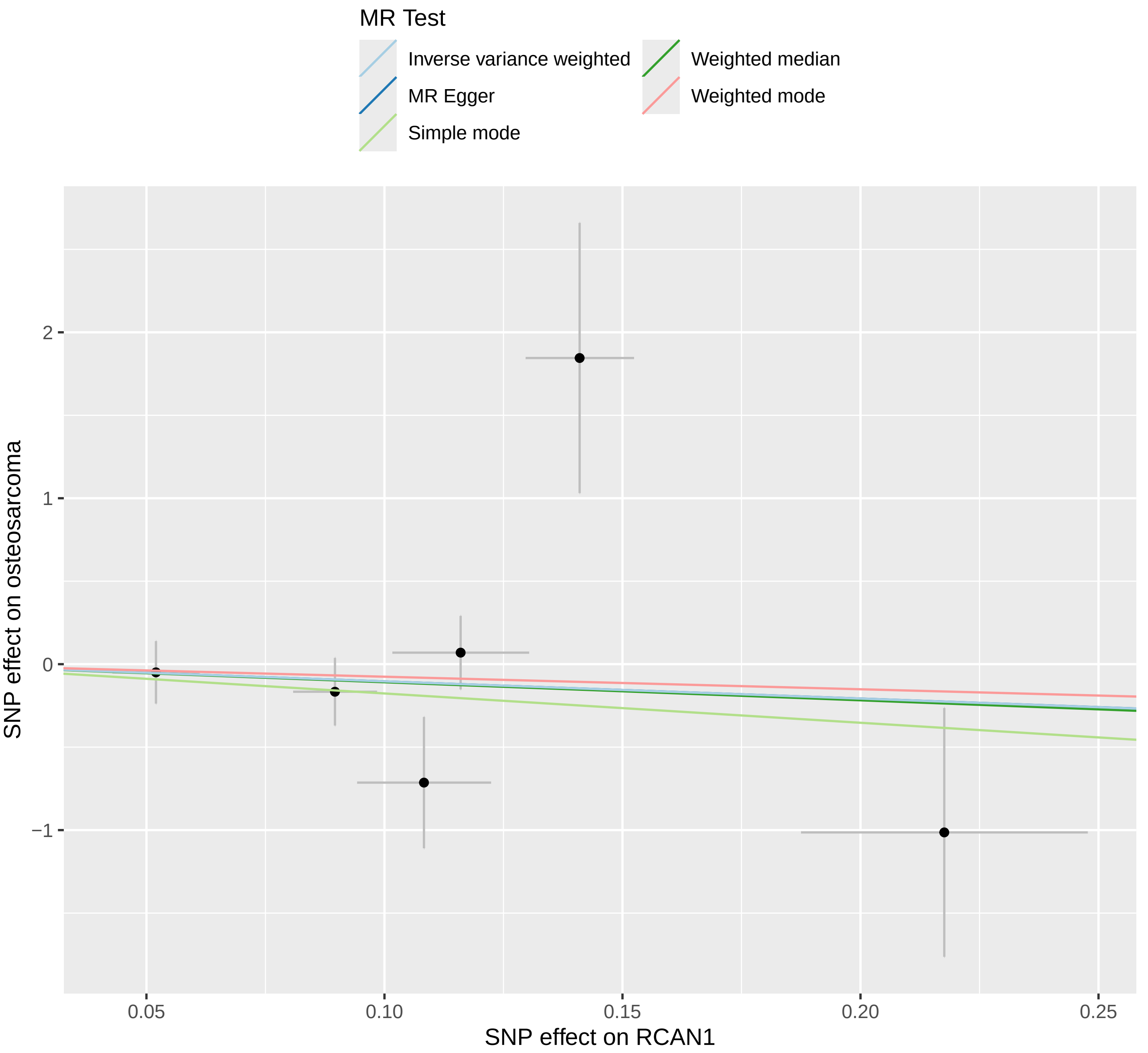
MR Test
Inverse variance weighted
MR Egger
Simple mode
Weighted median
Weighted mode
2
1
0
−1
0.05
0.10
0.15
0.20
0.25
SNP effect on osteosarcoma
SNP effect on RCAN1

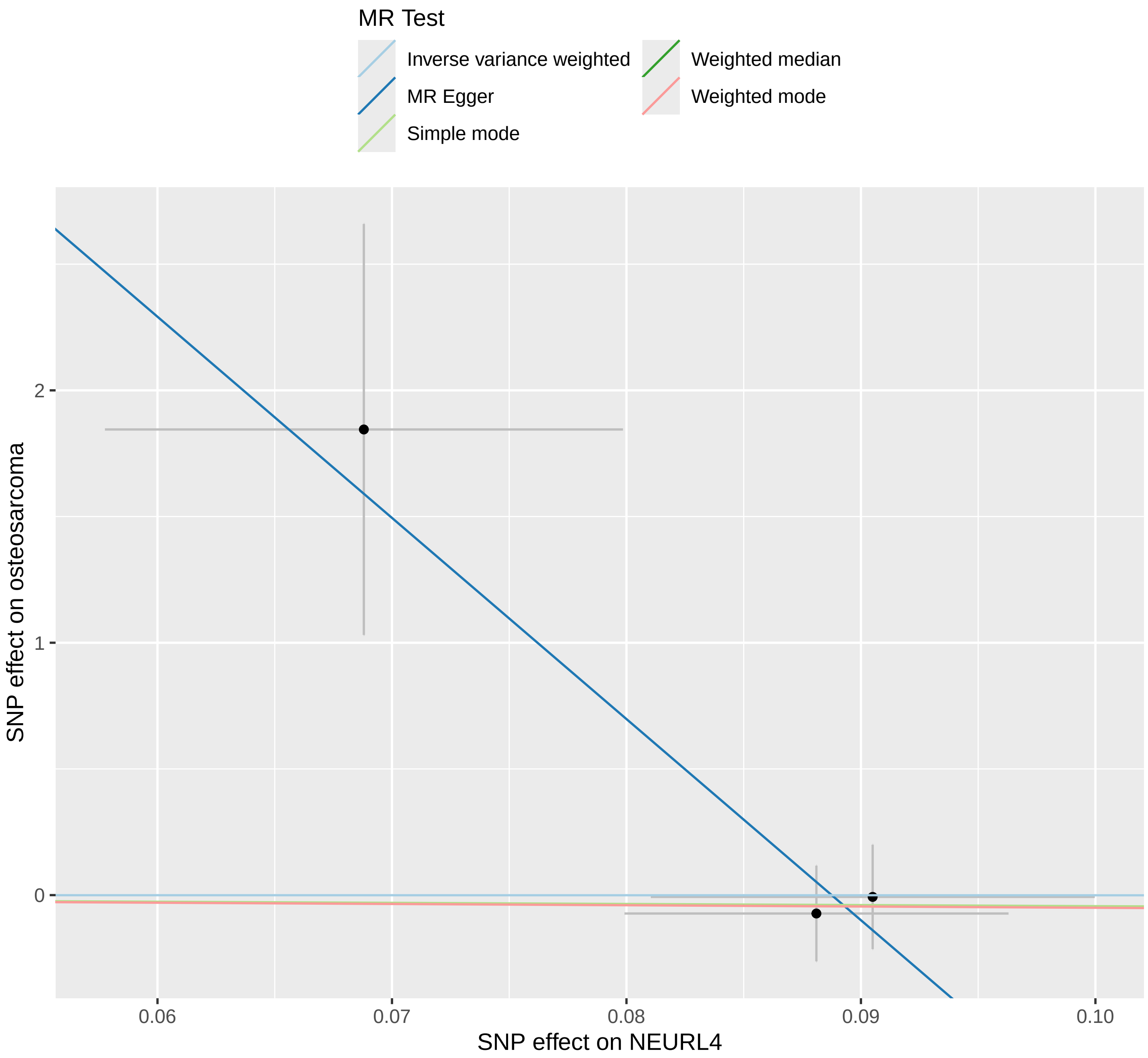
MR Test
Inverse variance weighted
MR Egger
Simple mode
Weighted median
Weighted mode
2
1
0
0.06
0.07
0.08
0.09
0.10
SNP effect on osteosarcoma
SNP effect on NEURL4

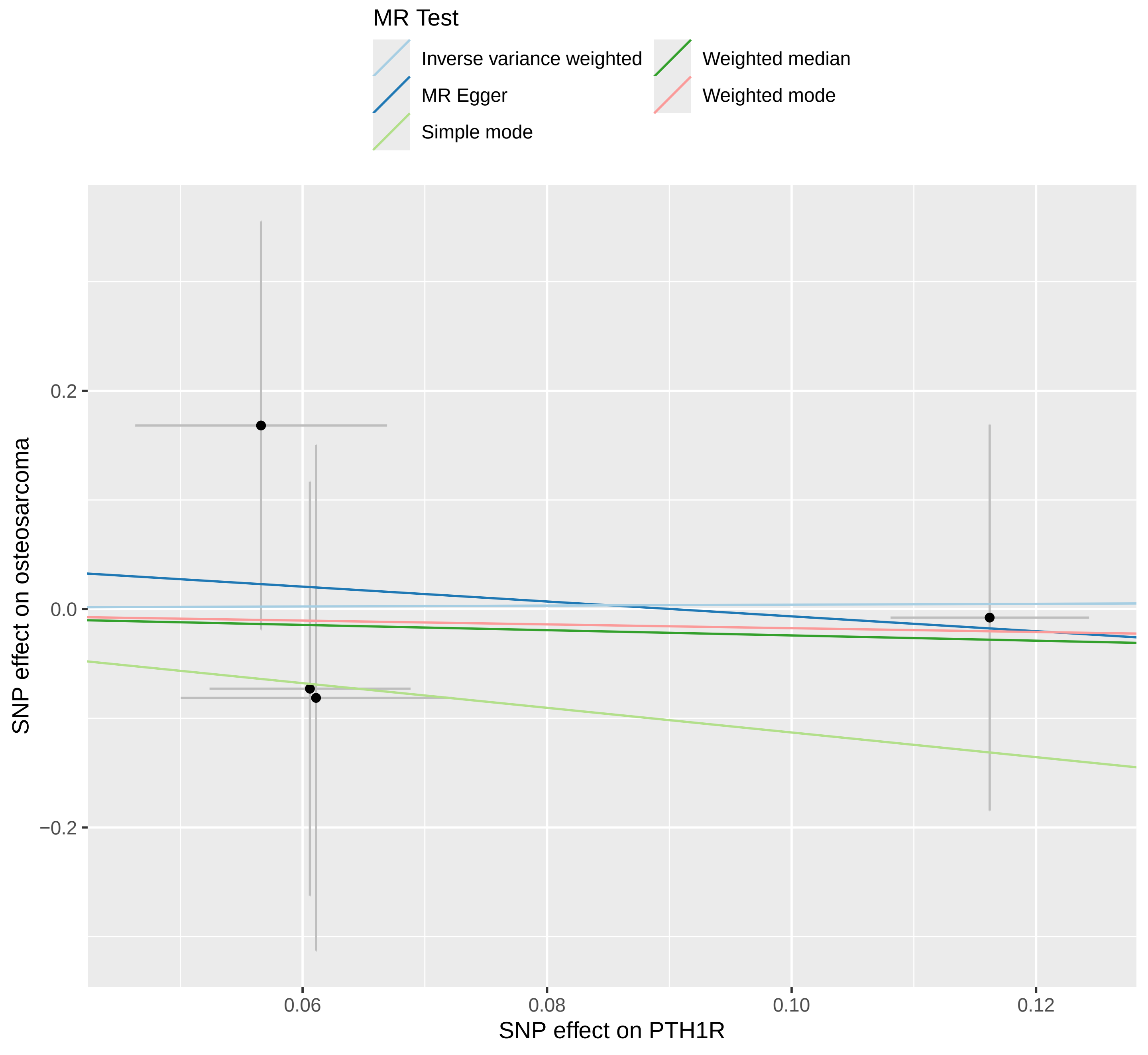

MR Test
Inverse variance weighted
MR Egger
Simple mode
Weighted median
Weighted mode
0.2
0.0
−0.2
SNP effect on osteosarcoma
0.06
0.08
0.10
0.12
SNP effect on PTH1R

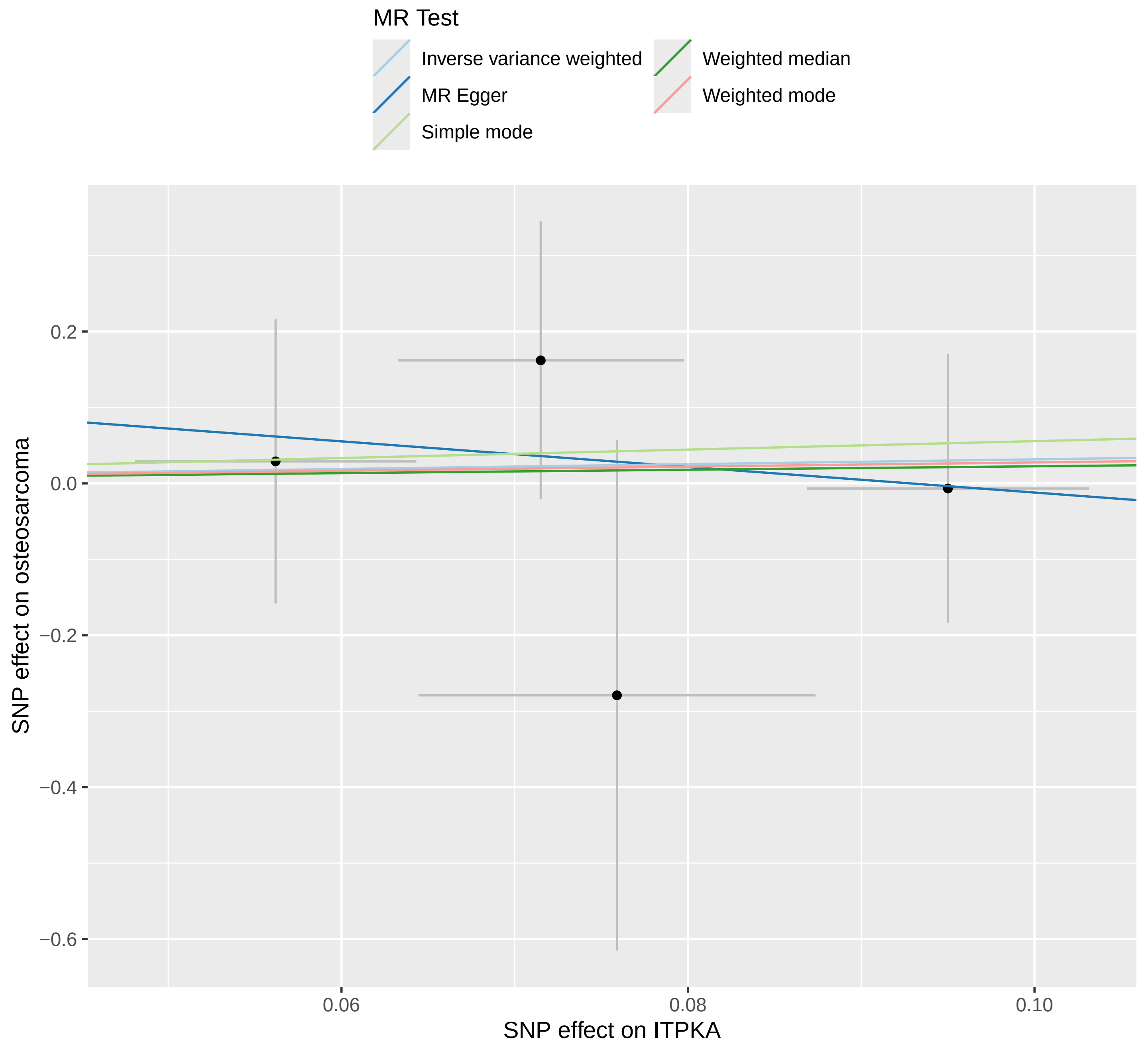
MR Test
Inverse variance weighted
MR Egger
Simple mode
Weighted median
Weighted mode
0.2
0.0
−0.2
−0.4
−0.6
SNP effect on osteosarcoma
0.06
0.08
0.10
SNP effect on ITPKA

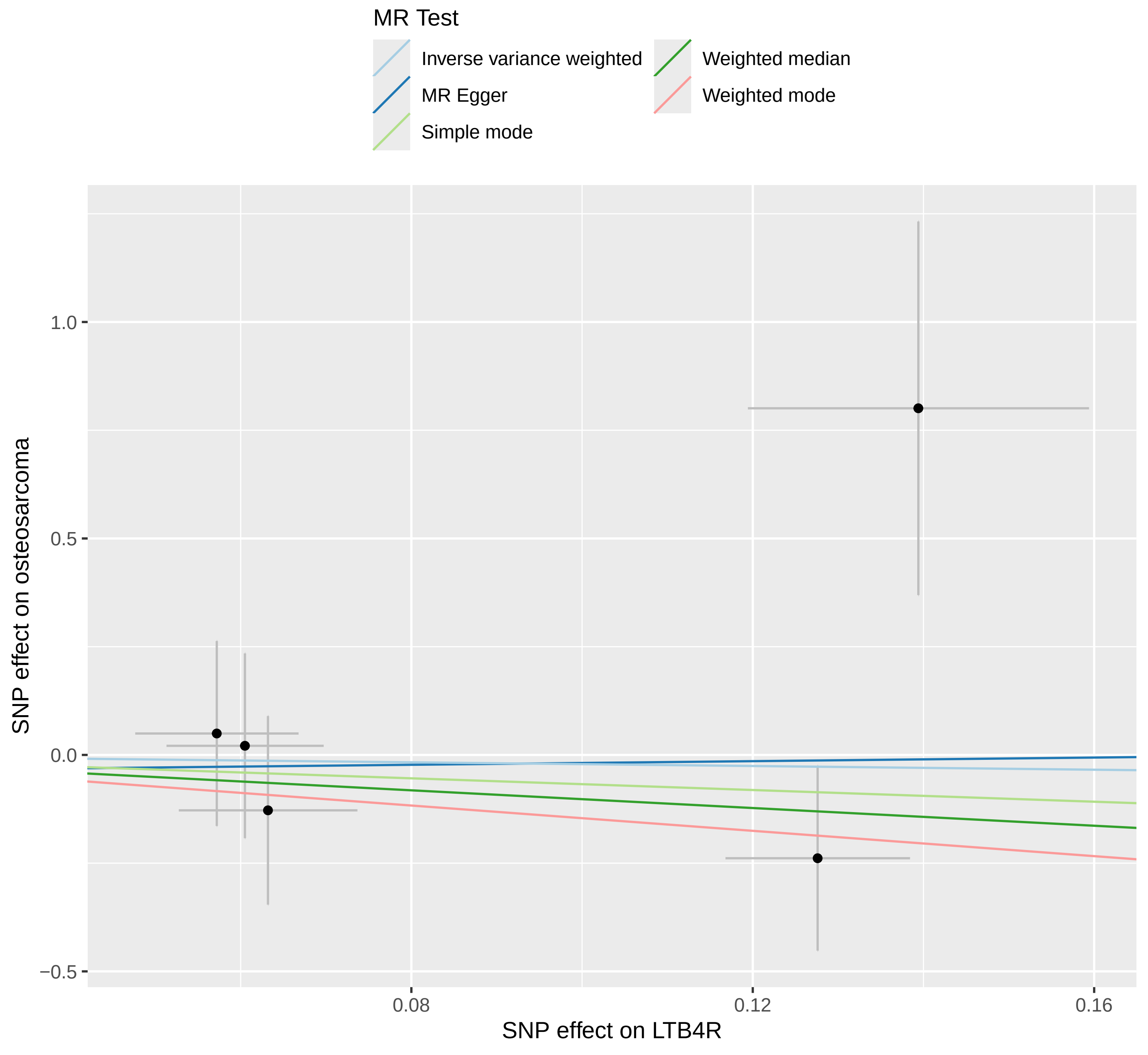

MR Test
Inverse variance weighted
MR Egger
Simple mode
Weighted median
Weighted mode
SNP effect on osteosarcoma
1.0
0.5
0.0
−0.5
0.08
0.12
0.16
SNP effect on LTB4R

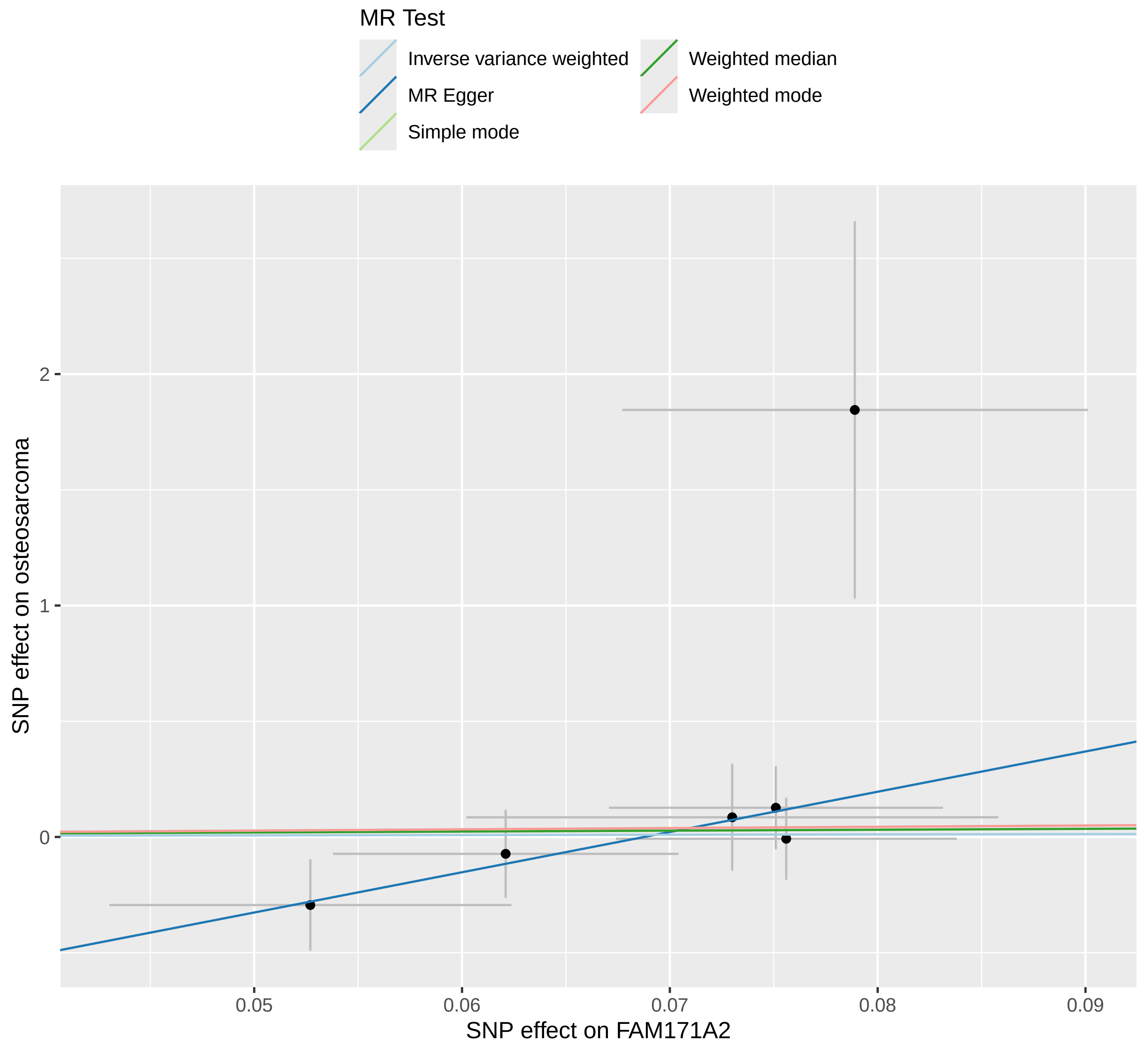

MR Test
Inverse variance weighted
MR Egger
Simple mode
Weighted median
Weighted mode
SNP effect on osteosarcoma
2
1
0
0.05
0.06
0.07
0.08
0.09
SNP effect on FAM171A2

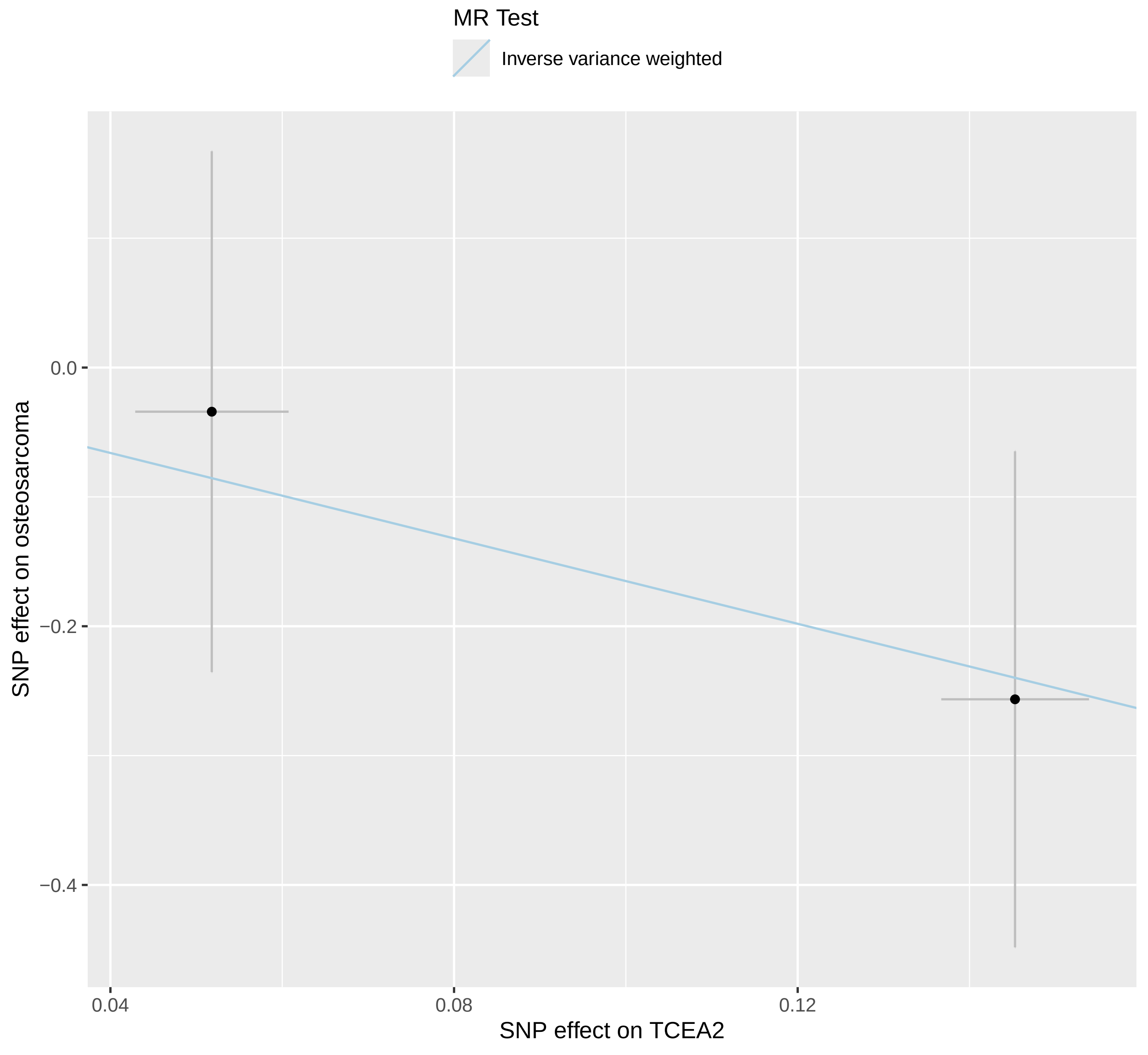

MR Test
Inverse variance weighted
0.0
−0.2
−0.4
SNP effect on osteosarcoma
0.04
0.08
0.12
SNP effect on TCEA2

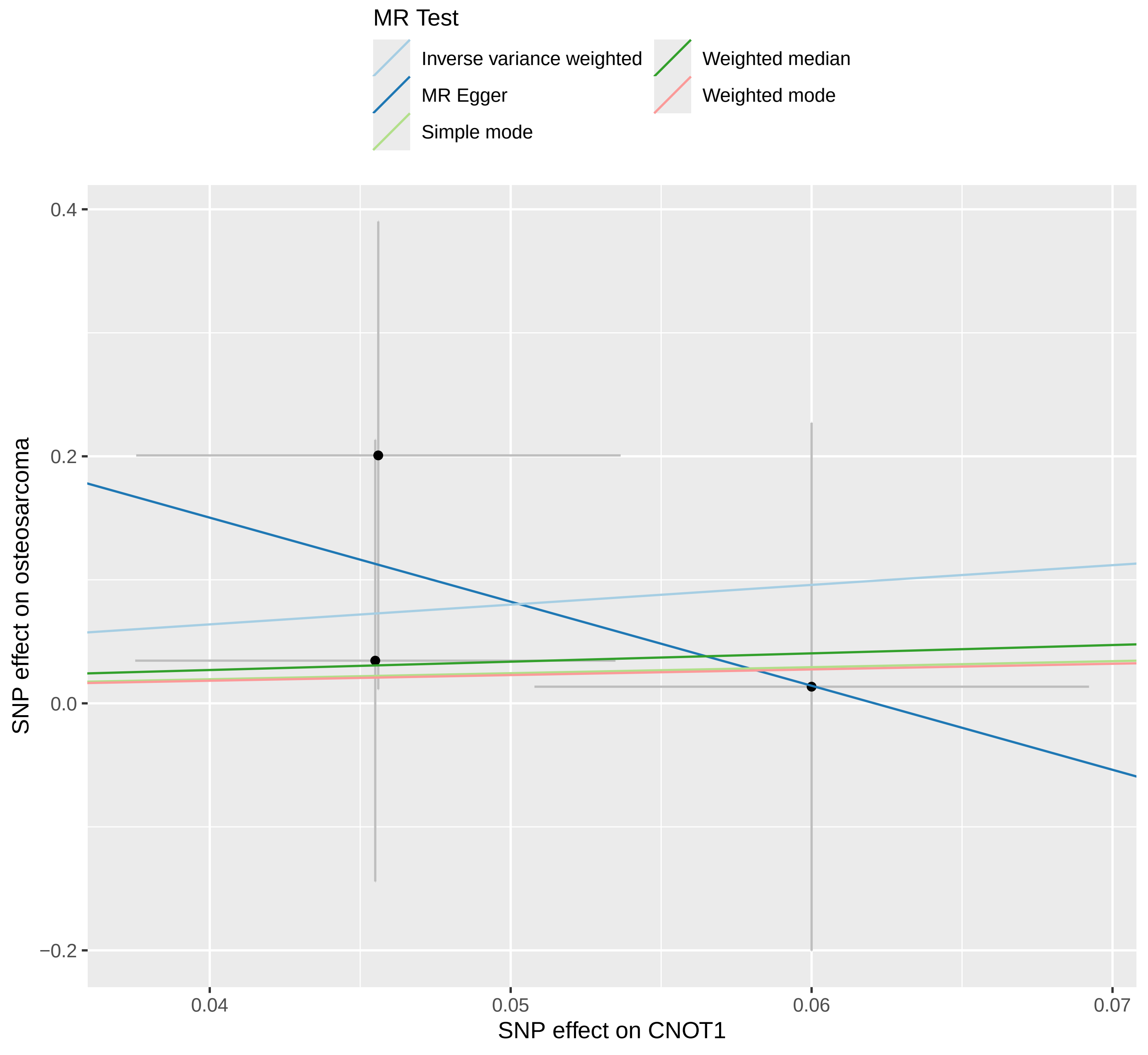

MR Test
Inverse variance weighted
MR Egger
Simple mode
Weighted median
Weighted mode
0.4
0.2
0.0
−0.2
0.04
0.05
0.06
0.07
SNP effect on CNOT1
SNP effect on osteosarcoma

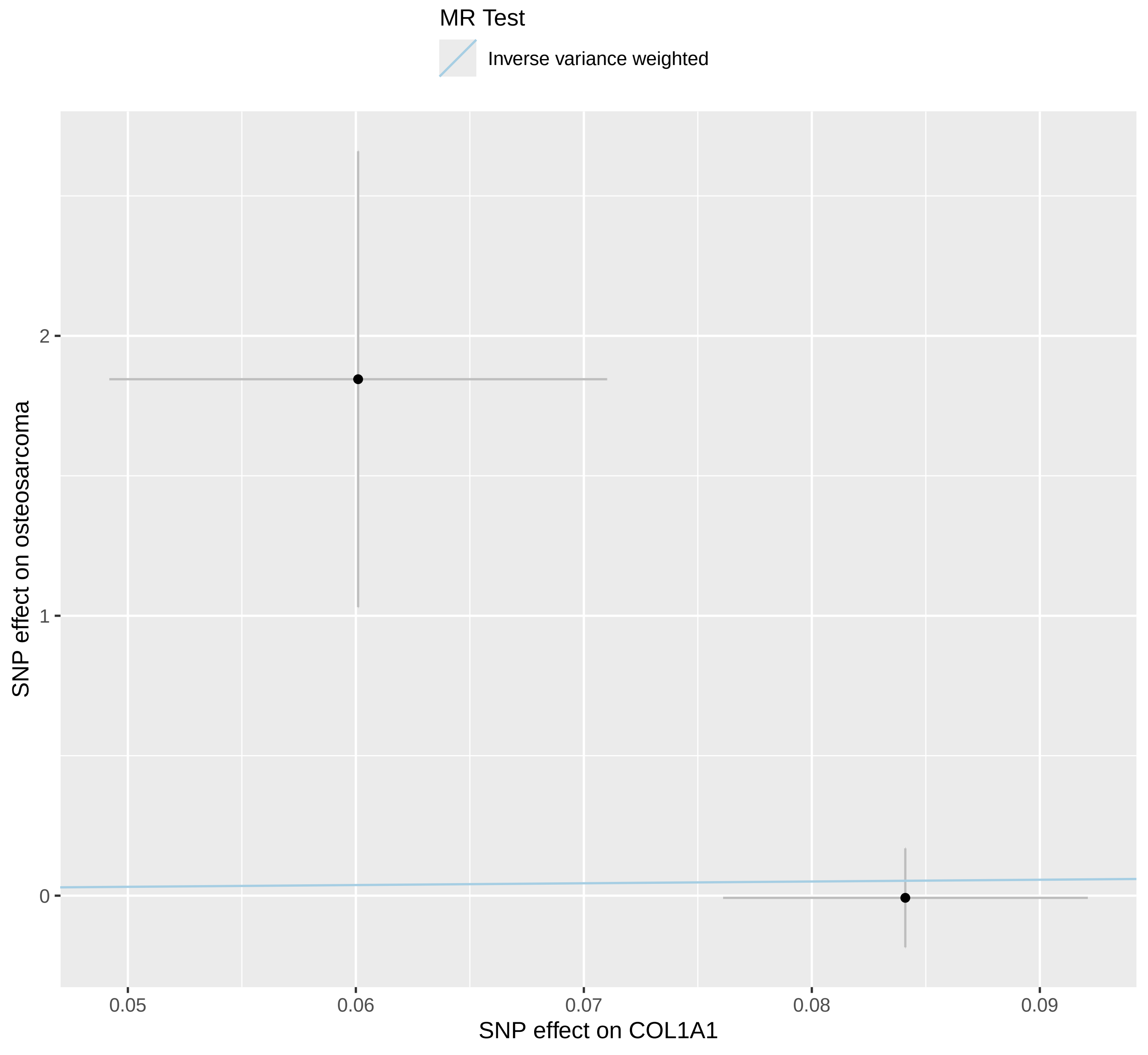
MR Test
Inverse variance weighted
SNP effect on osteosarcoma
2
1
0
0.05
0.06
0.07
0.08
0.09
SNP effect on COL1A1

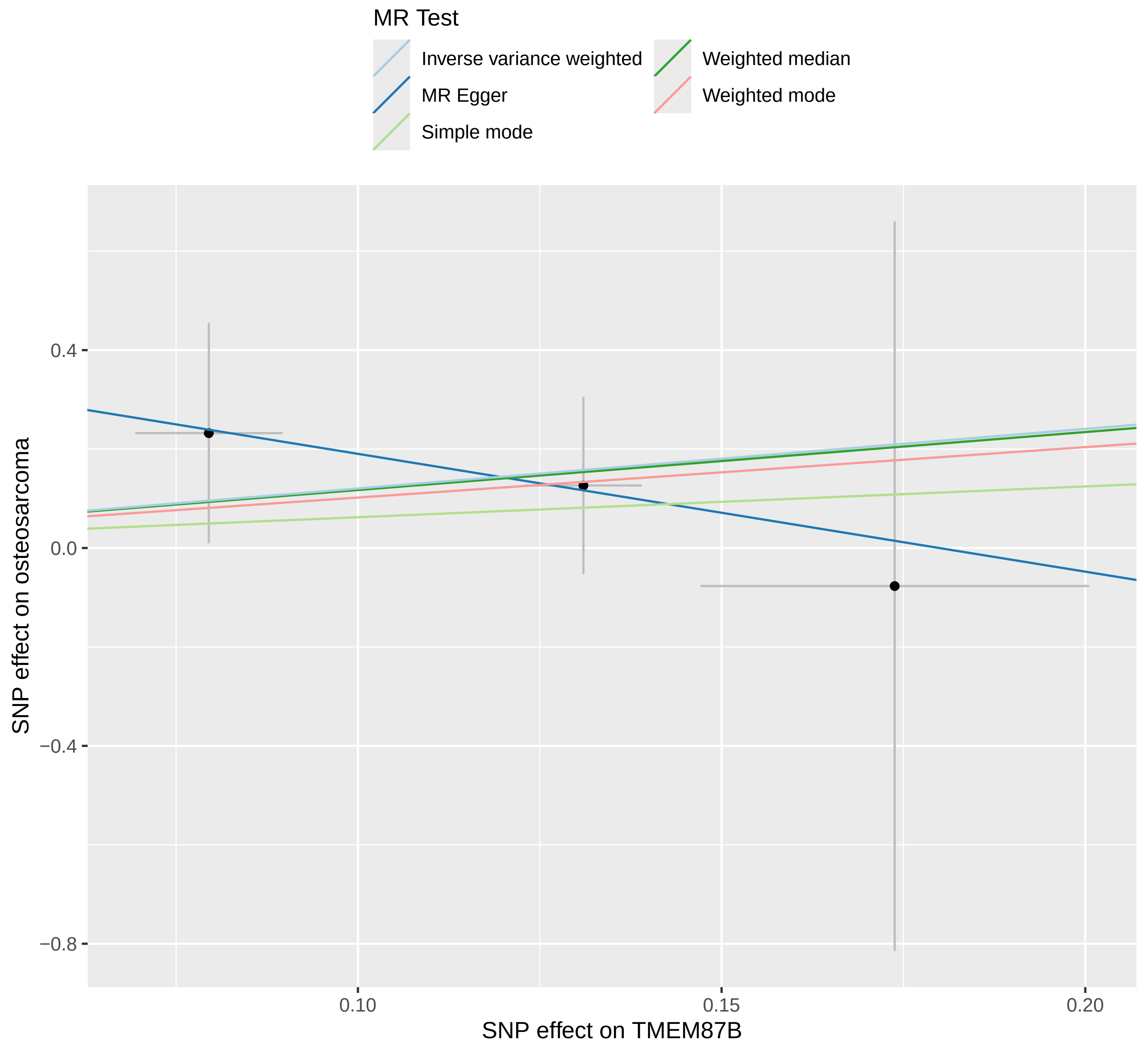
MR Test
Inverse variance weighted
MR Egger
Simple mode
Weighted median
Weighted mode
0.4
0.0
−0.4
−0.8
SNP effect on osteosarcoma
0.10
0.15
0.20
SNP effect on TMEM87B

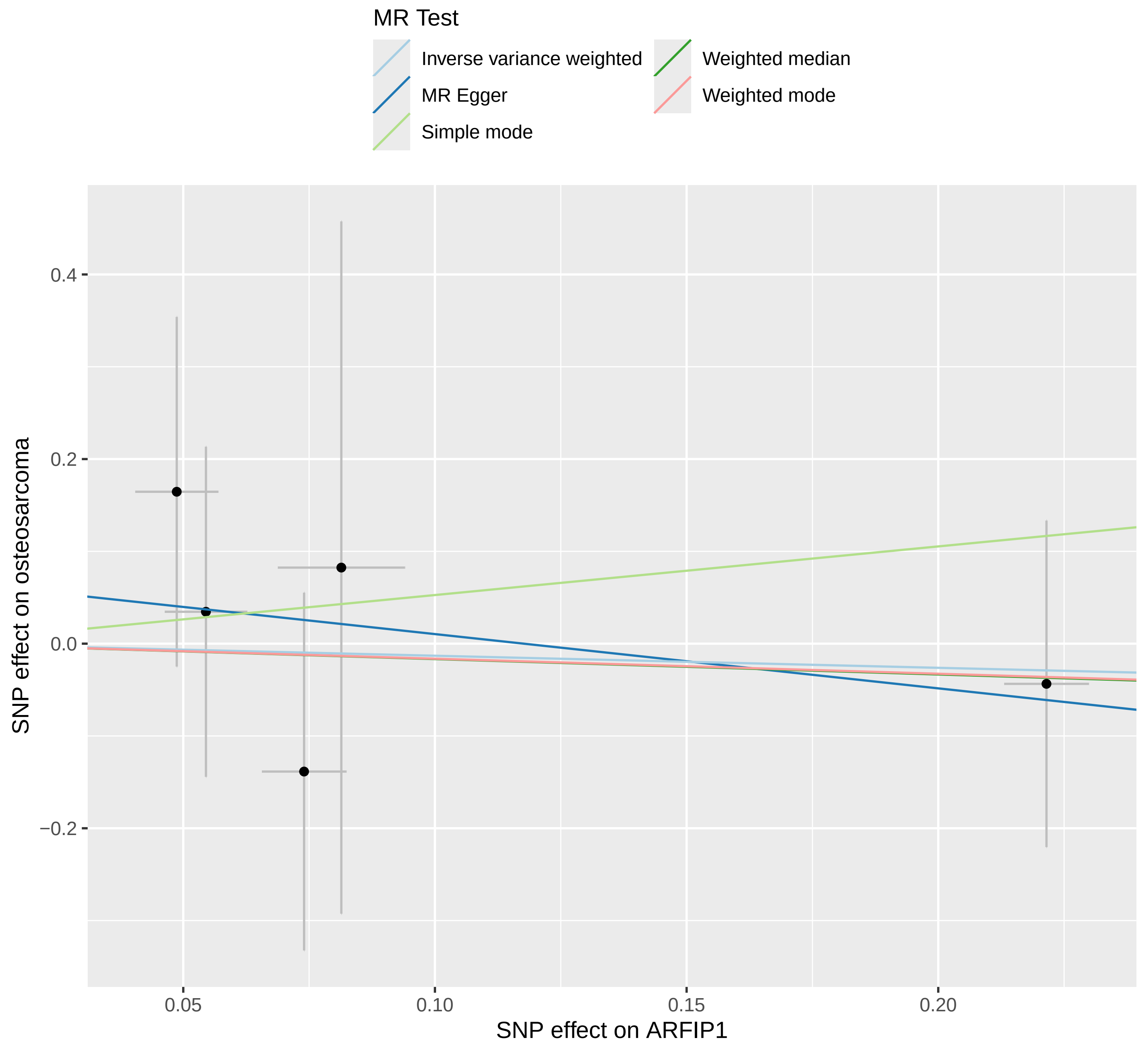
MR Test
Inverse variance weighted
MR Egger
Simple mode
Weighted median
Weighted mode
0.4
0.2
0.0
−0.2
0.05
0.10
0.15
0.20
SNP effect on osteosarcoma
SNP effect on ARFIP1

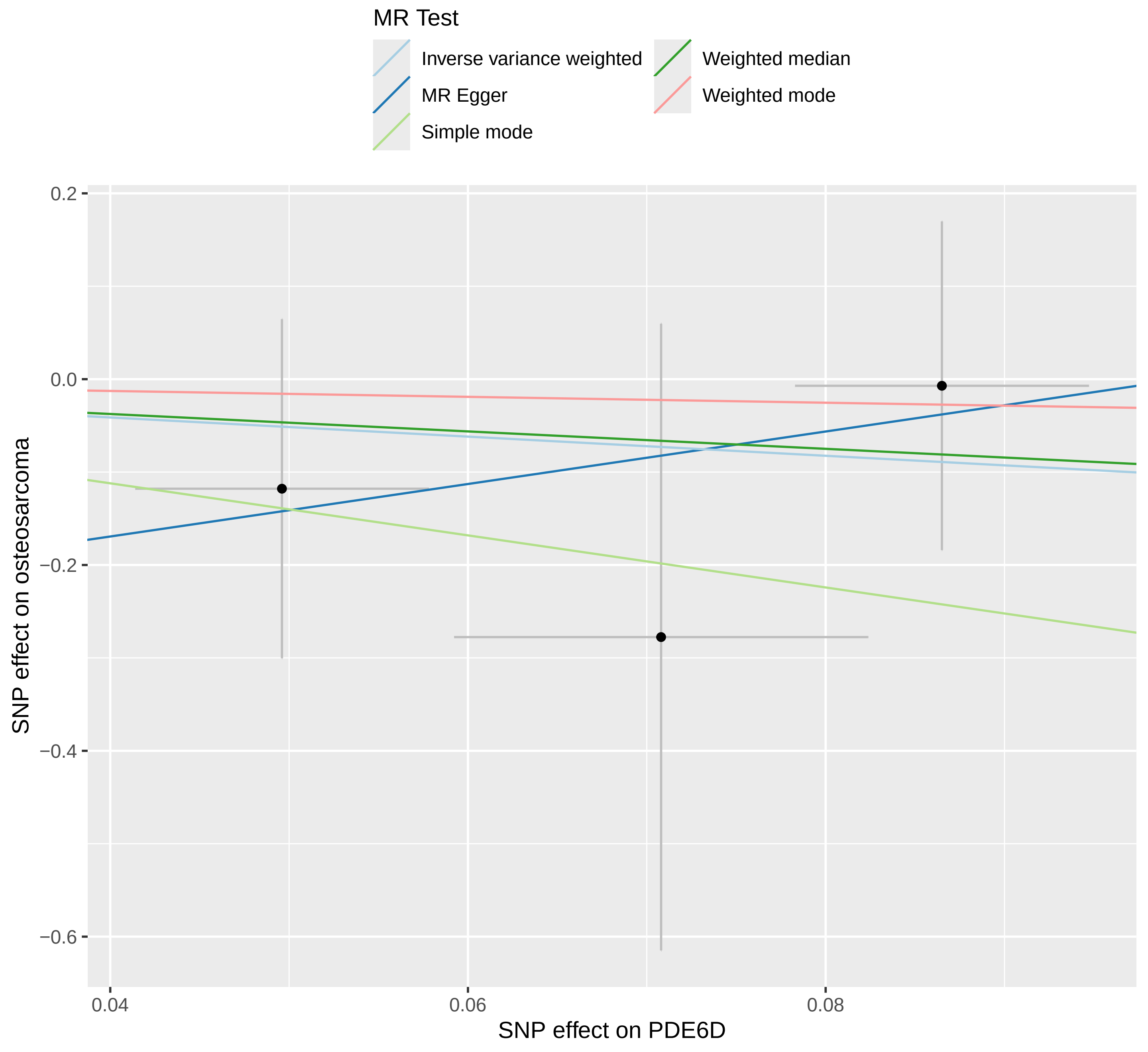
MR Test
Inverse variance weighted
MR Egger
Simple mode
Weighted median
Weighted mode
0.2
0.0
−0.2
−0.4
−0.6
SNP effect on osteosarcoma
0.04
0.06
0.08
SNP effect on PDE6D

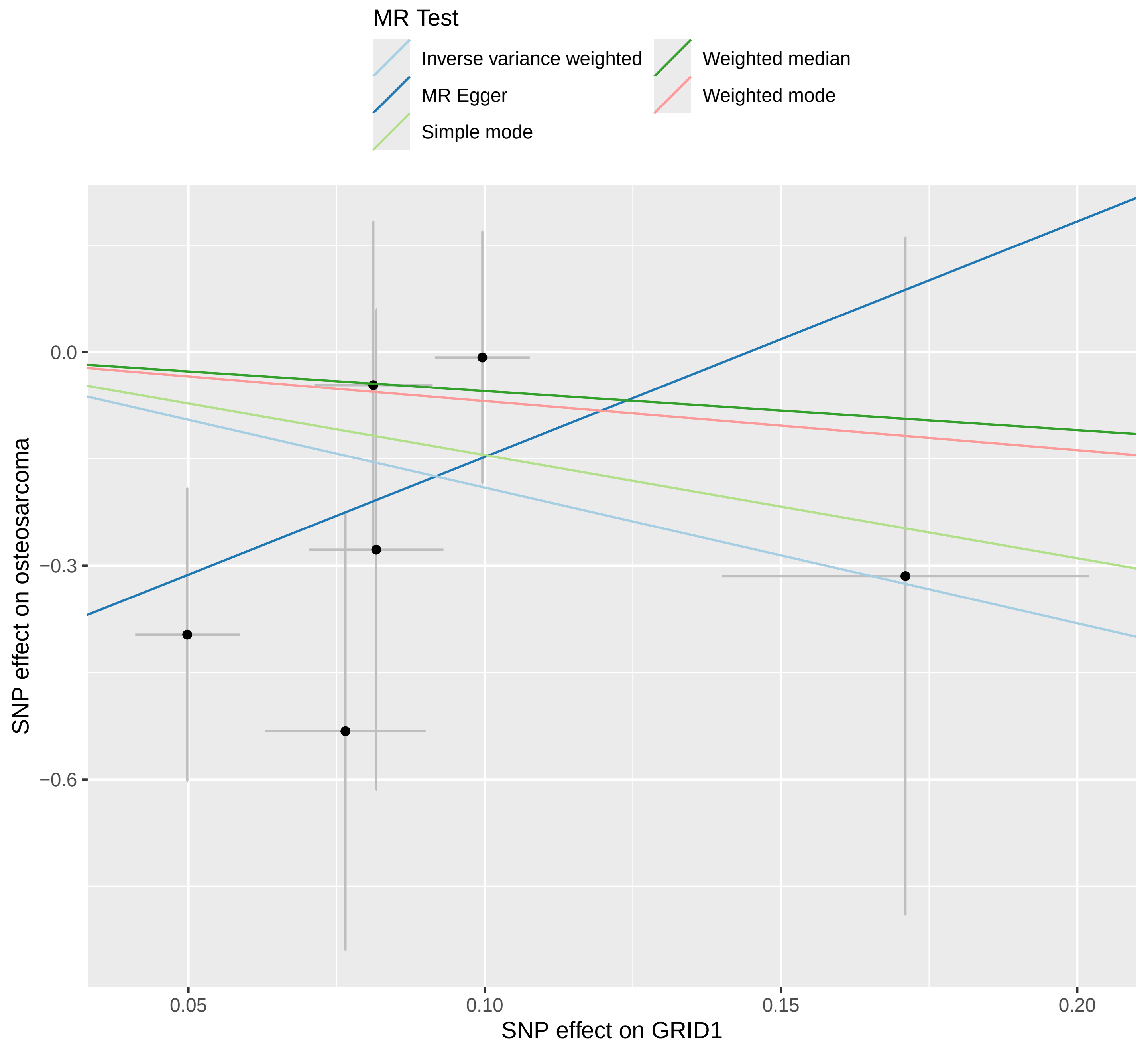
MR Test
Inverse variance weighted
MR Egger
Simple mode
Weighted median
Weighted mode
0.0
−0.3
−0.6
0.05
0.10
0.15
0.20
SNP effect on osteosarcoma
SNP effect on GRID1

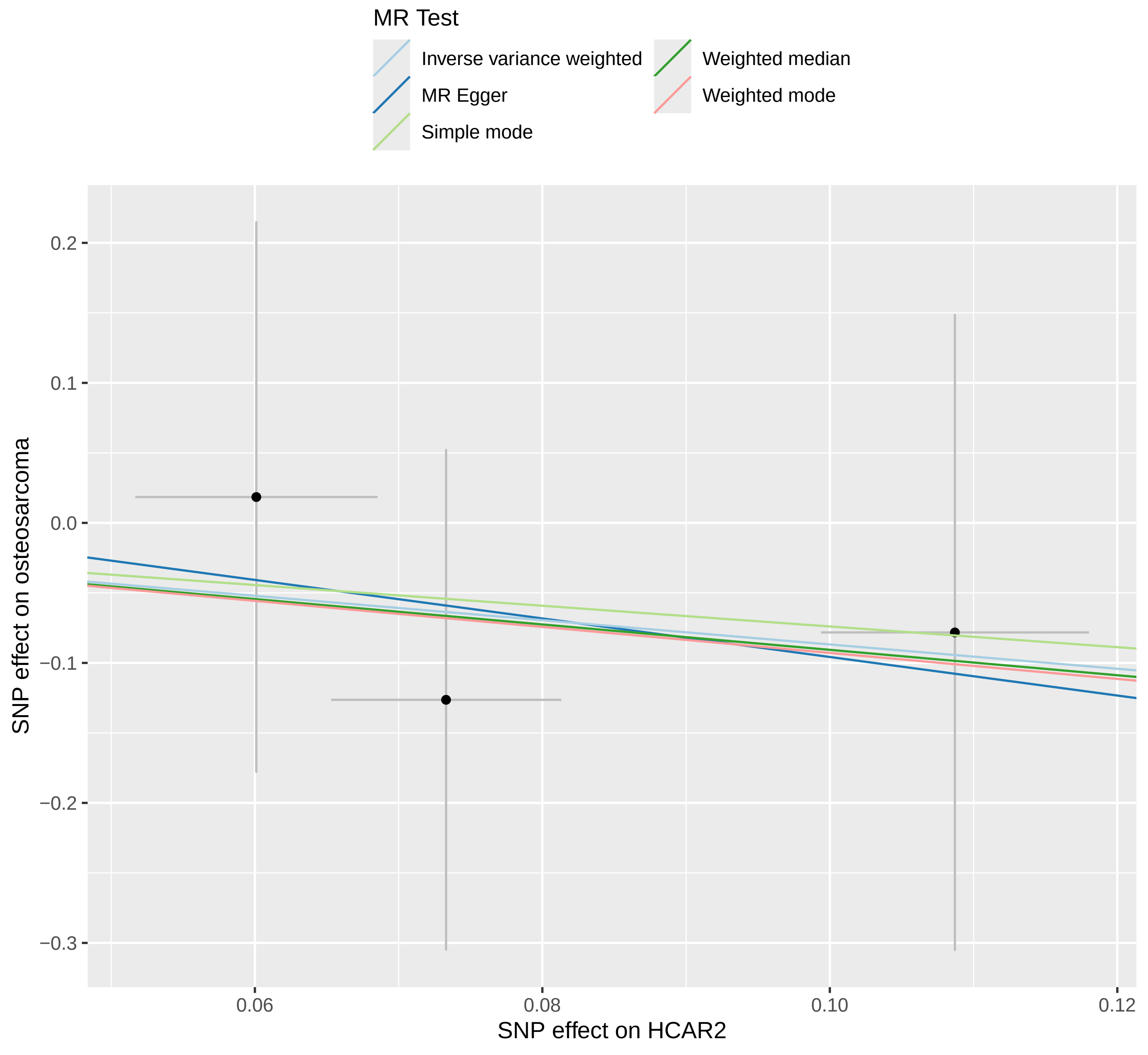

MR Test
Inverse variance weighted
MR Egger
Simple mode
Weighted median
Weighted mode
0.2
0.1
0.0
−0.1
−0.2
−0.3
0.06
0.08
0.10
0.12
SNP effect on HCAR2
SNP effect on osteosarcoma

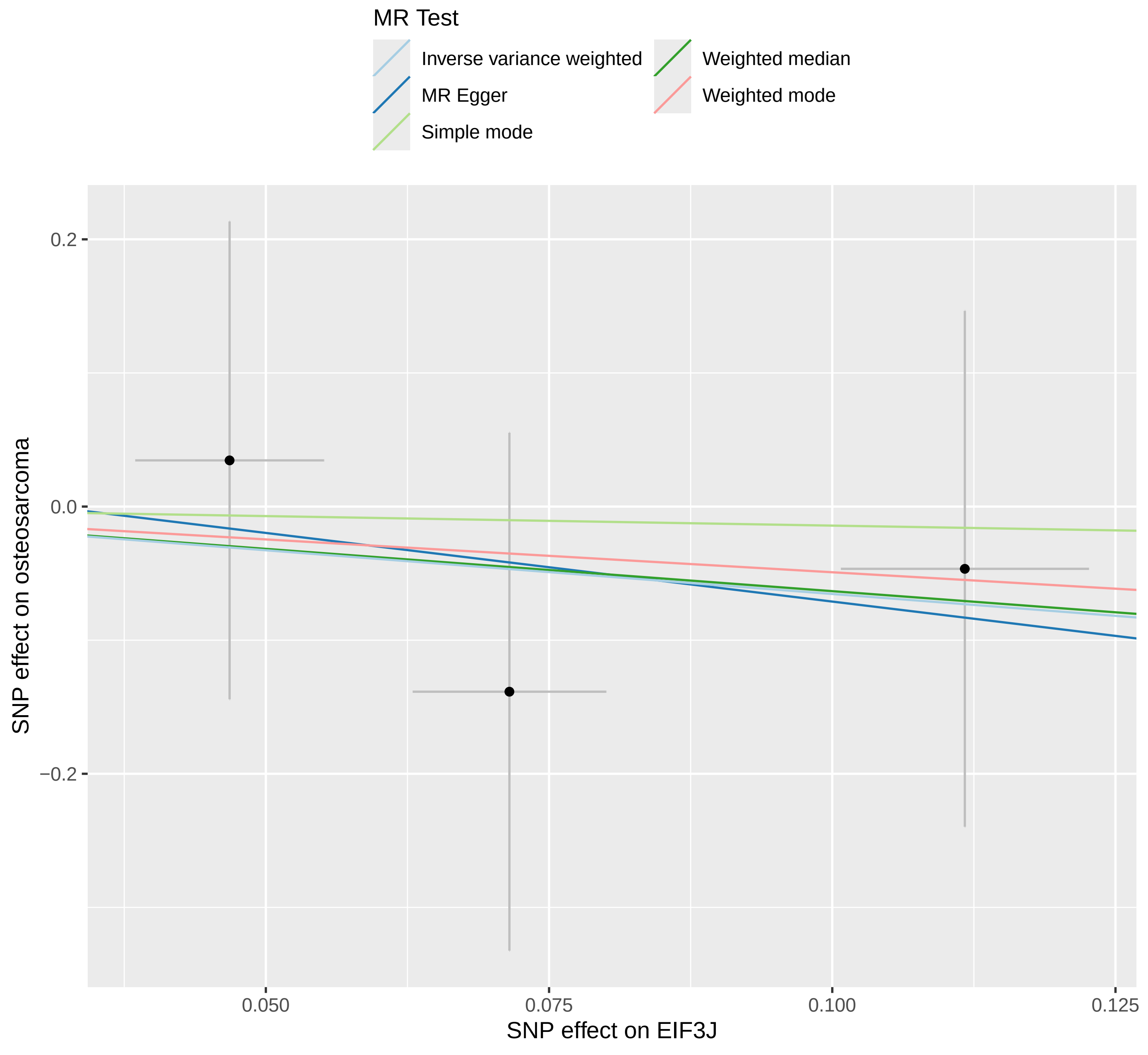

MR Test
Inverse variance weighted
MR Egger
Simple mode
Weighted median
Weighted mode
0.2
0.0
−0.2
0.050
0.075
0.100
0.125
SNP effect on EIF3J
SNP effect on osteosarcoma

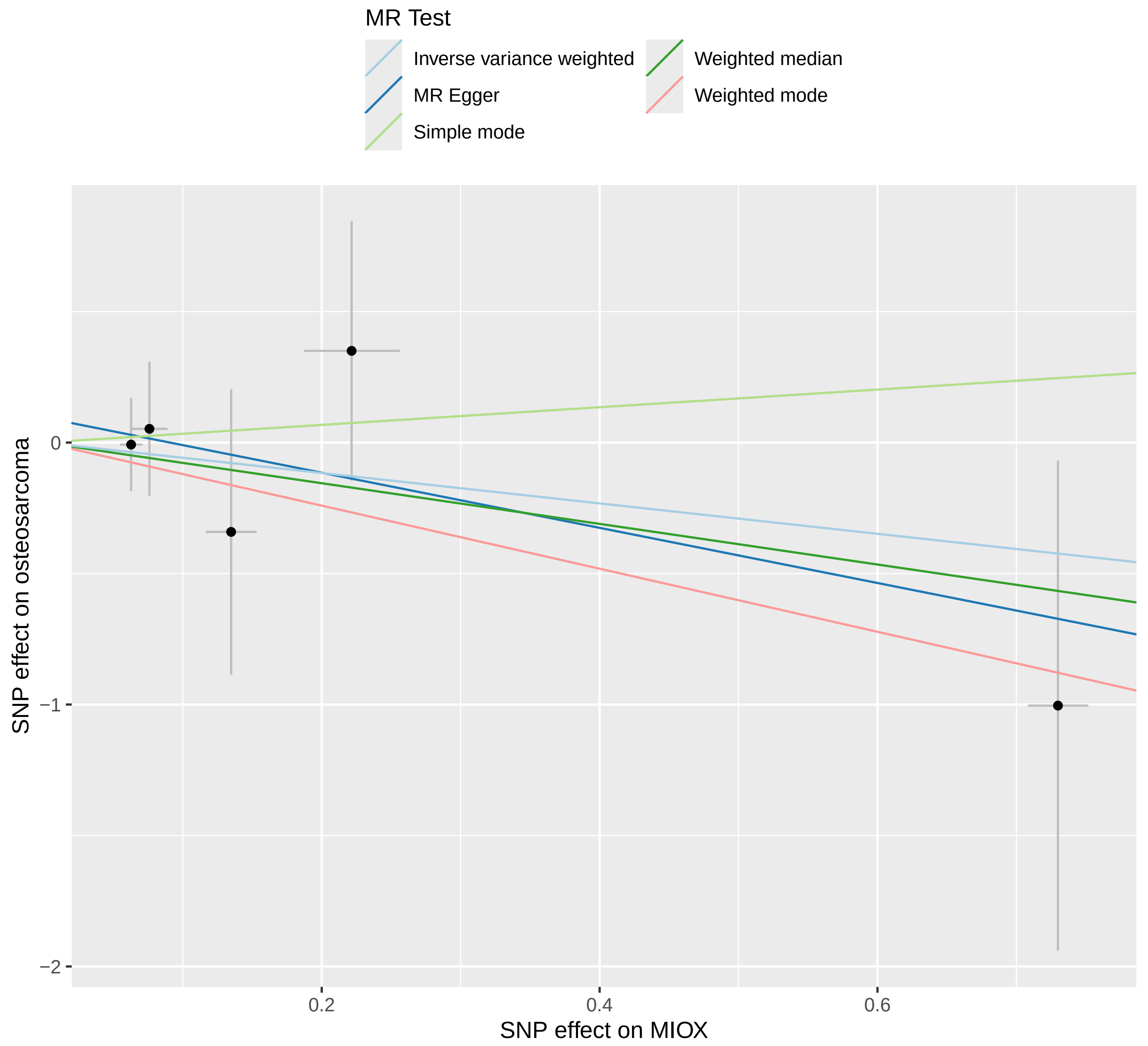
MR Test
Inverse variance weighted
MR Egger
Simple mode
Weighted median
Weighted mode
SNP effect on osteosarcoma
0
−1
−2
0.2
0.4
0.6
SNP effect on MIOX

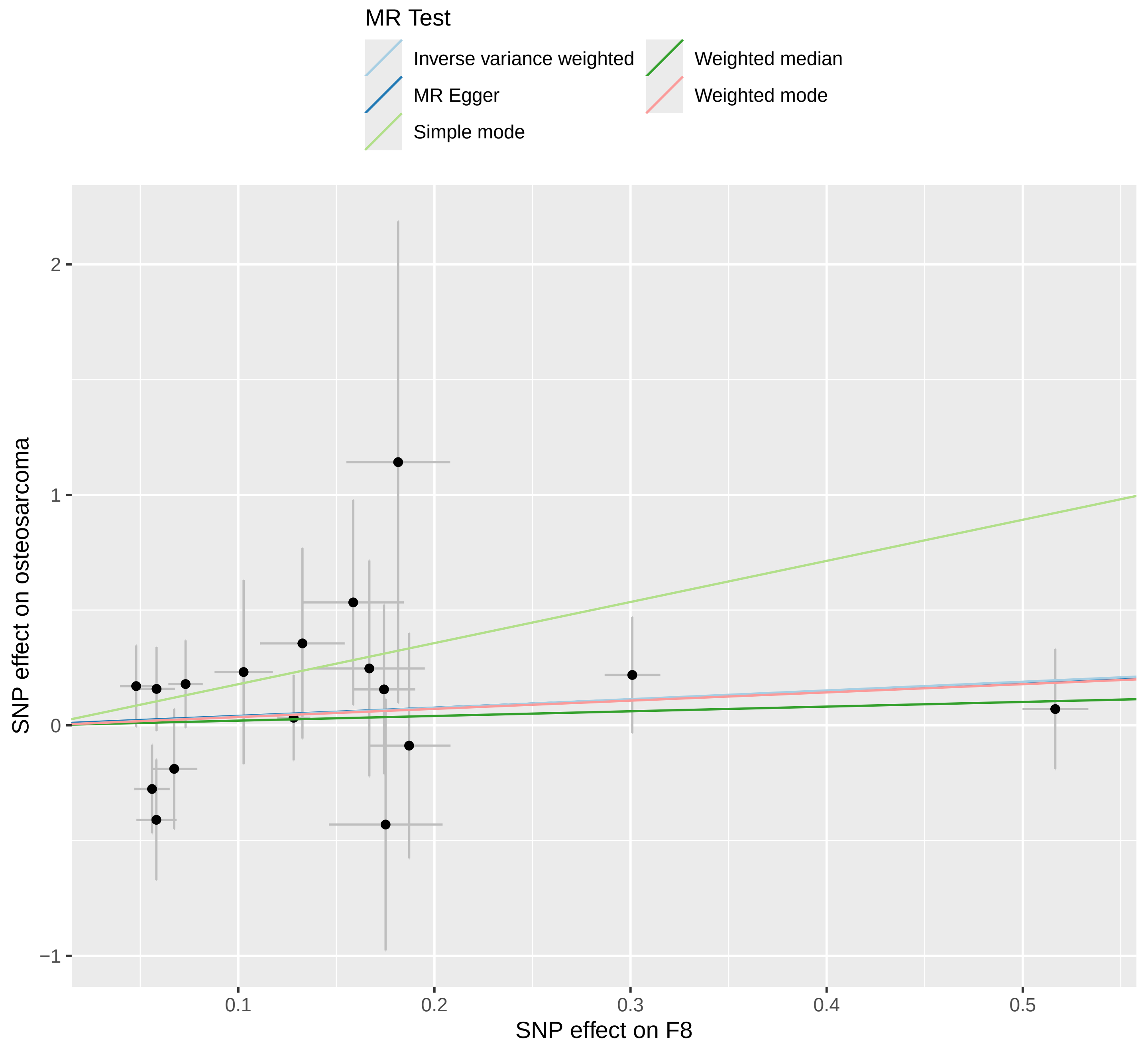

MR Test
Inverse variance weighted
MR Egger
Simple mode
Weighted median
Weighted mode
SNP effect on osteosarcoma
2
1
0
−1
0.1
0.2
0.3
0.4
0.5
SNP effect on F8

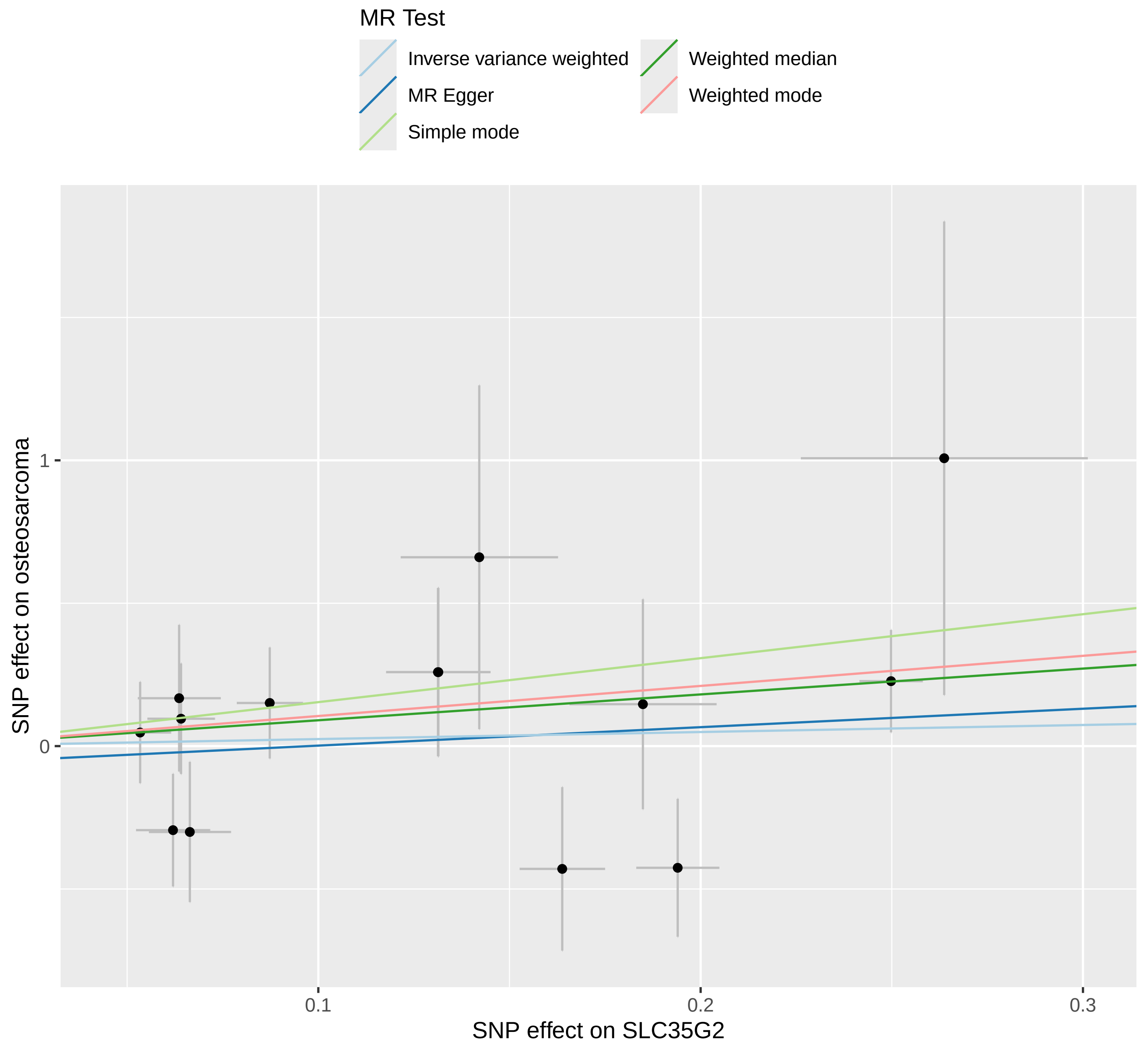
MR Test
Inverse variance weighted
MR Egger
Simple mode
Weighted median
Weighted mode
SNP effect on osteosarcoma
1
0
0.1
0.2
0.3
SNP effect on SLC35G2

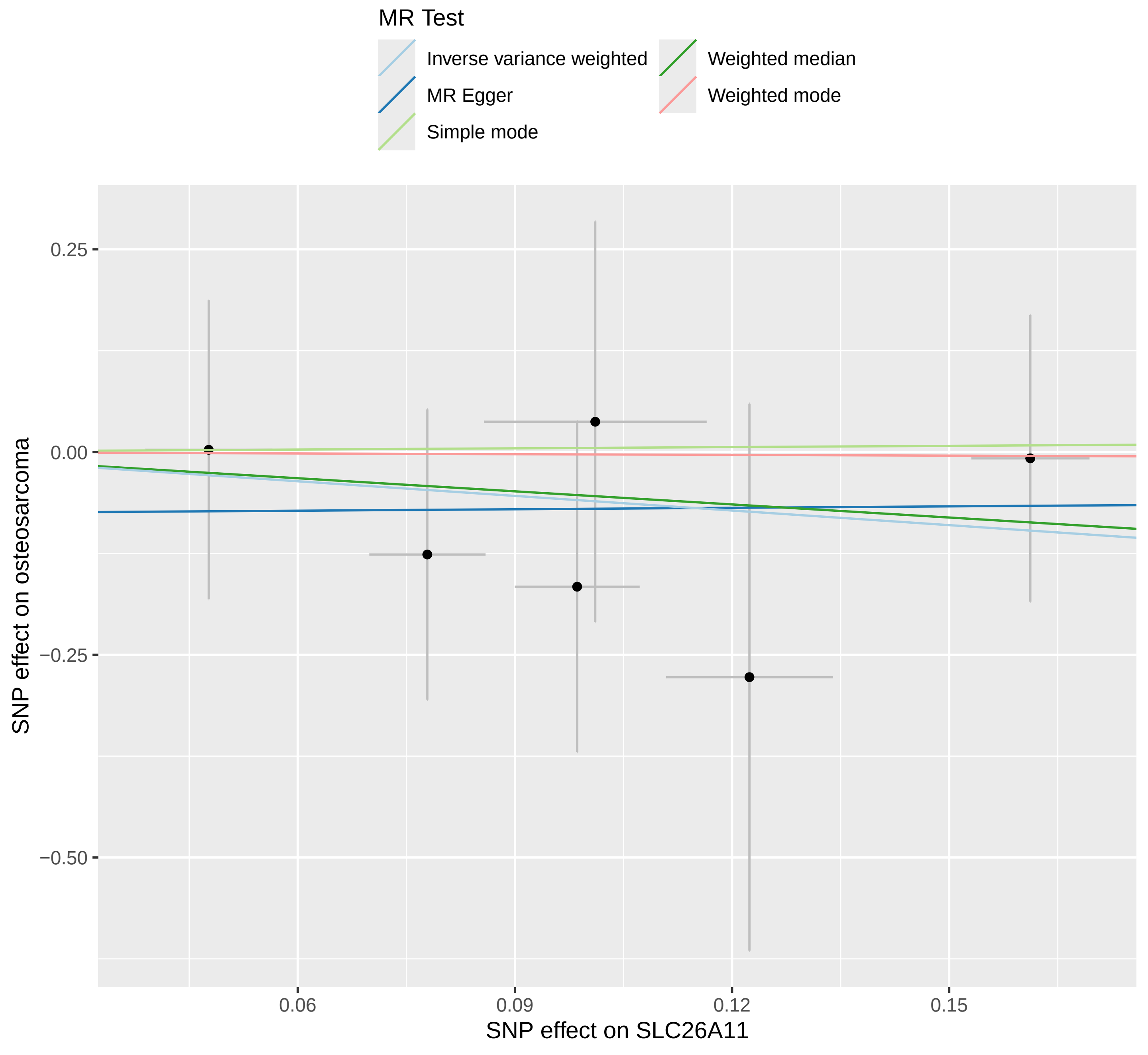

MR Test
Inverse variance weighted
MR Egger
Simple mode
Weighted median
Weighted mode
0.25
0.00
−0.25
−0.50
0.06
0.09
0.12
0.15
SNP effect on osteosarcoma
SNP effect on SLC26A11

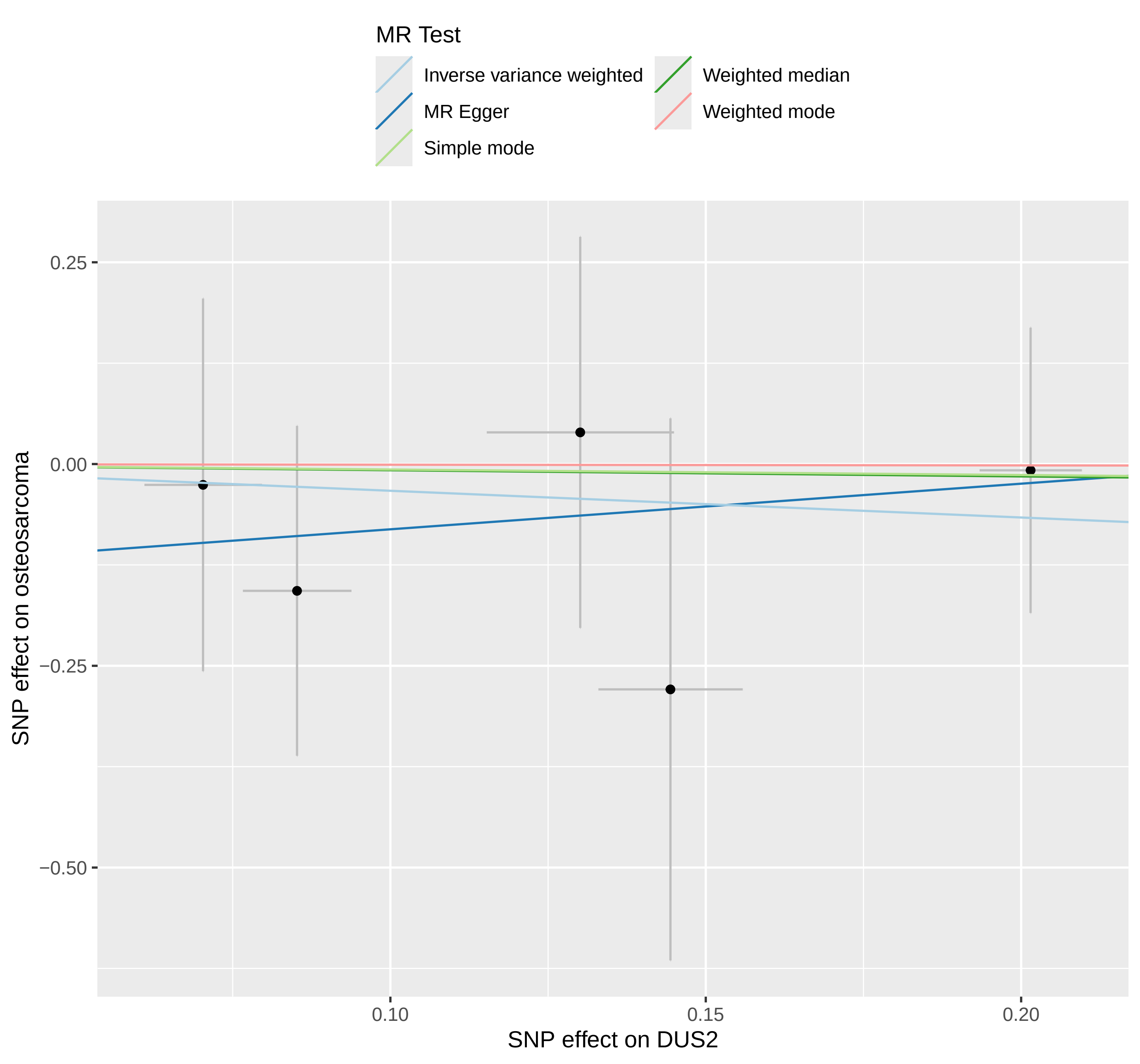

MR Test
Inverse variance weighted
MR Egger
Simple mode
Weighted median
Weighted mode
0.25
0.00
−0.25
−0.50
0.10
0.15
0.20
SNP effect on osteosarcoma
SNP effect on DUS2

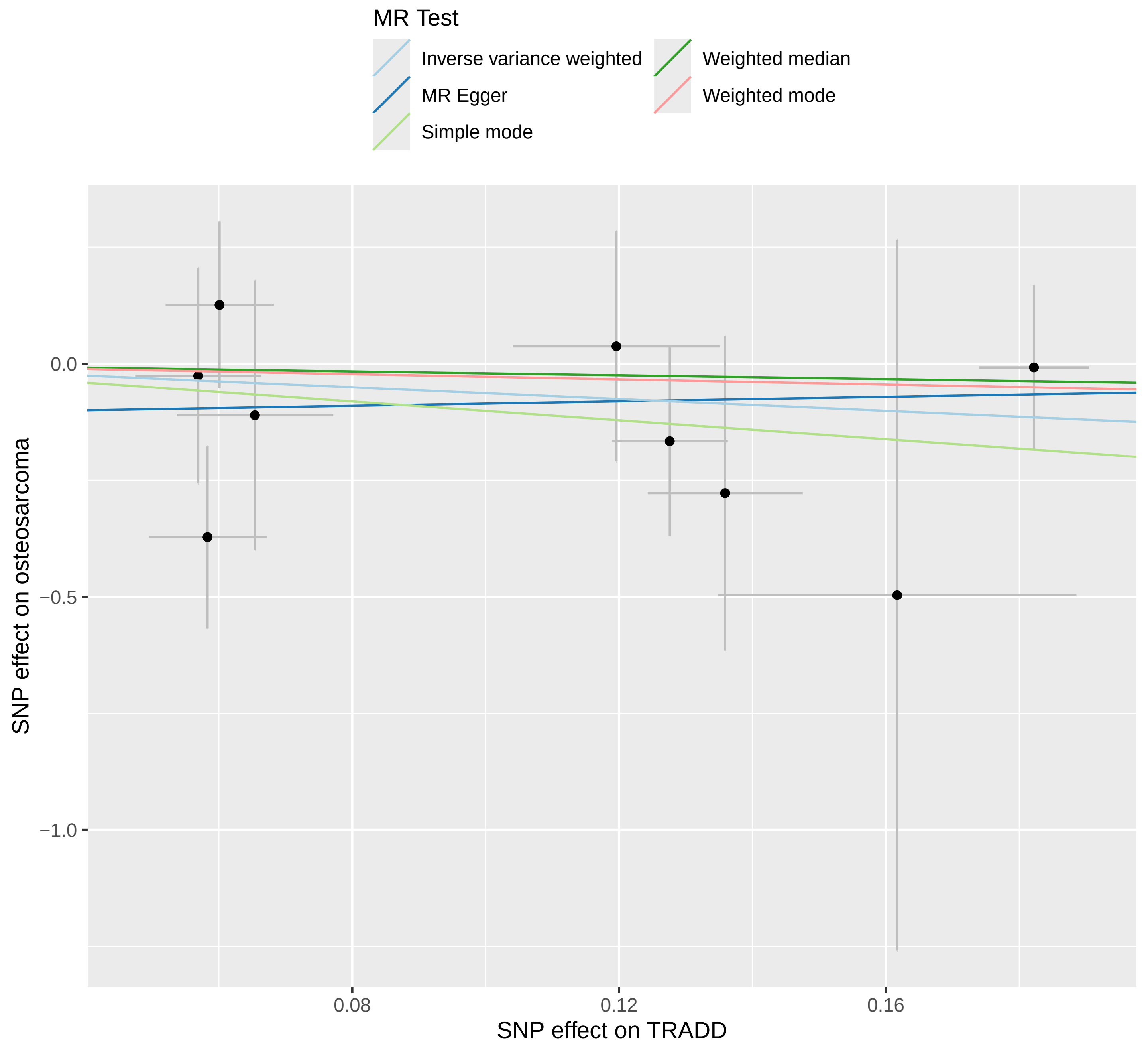

MR Test
Inverse variance weighted
MR Egger
Simple mode
Weighted median
Weighted mode
0.0
−0.5
−1.0
0.08
0.12
0.16
SNP effect on TRADD
SNP effect on osteosarcoma

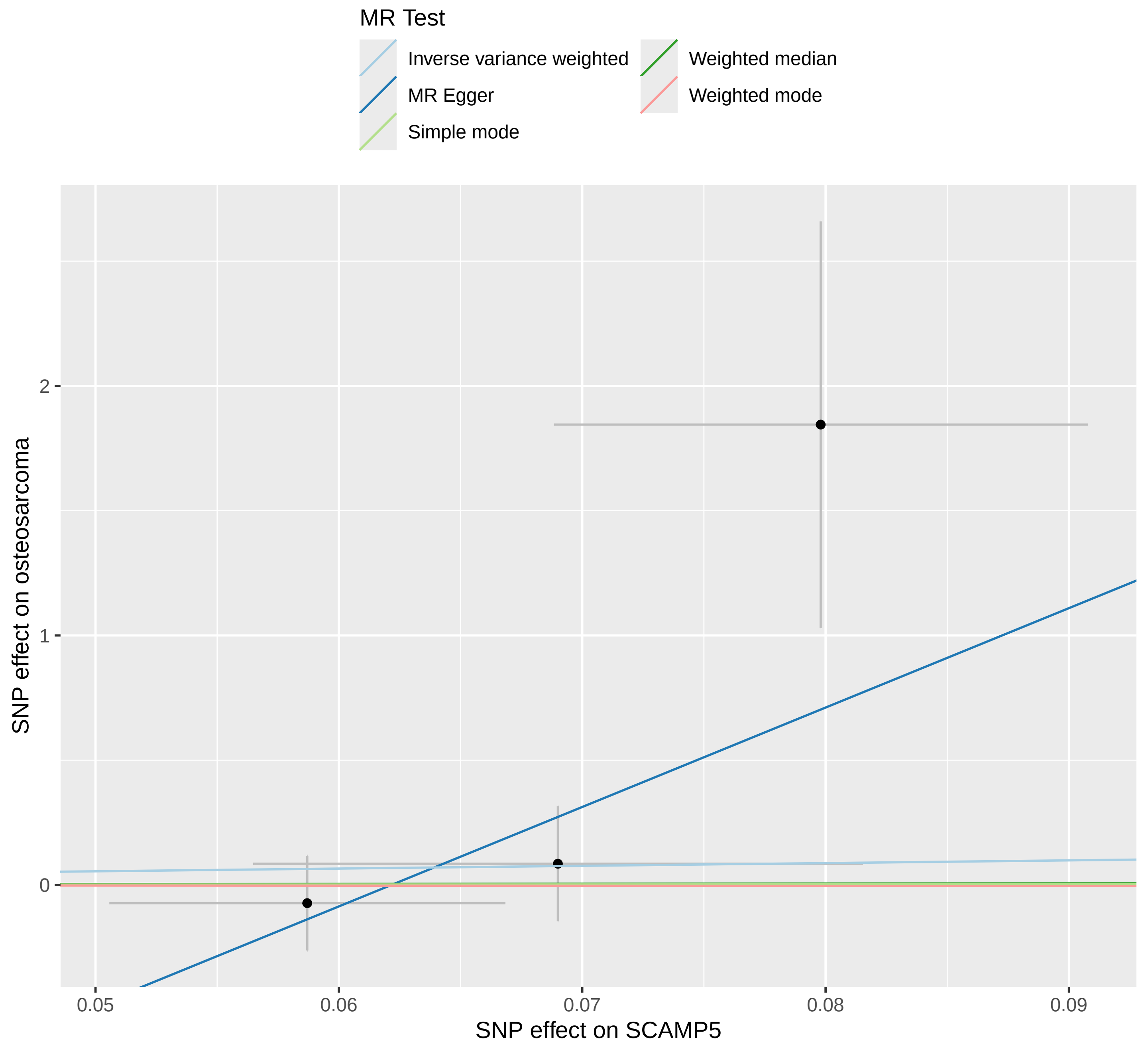
MR Test
Inverse variance weighted
MR Egger
Simple mode
Weighted median
Weighted mode
SNP effect on osteosarcoma
0
1
2
0.05
0.06
0.07
0.08
0.09
SNP effect on SCAMP5

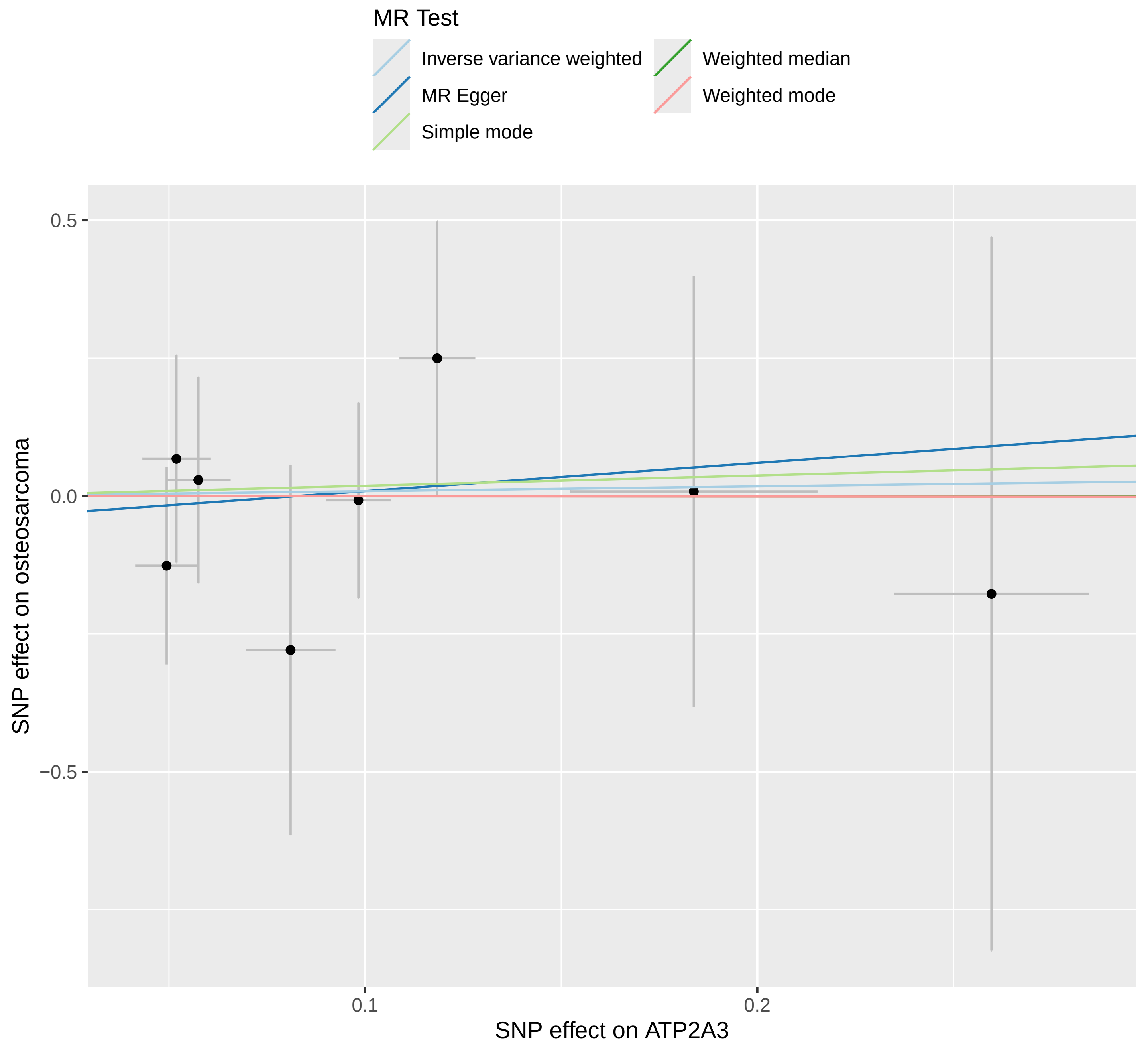
MR Test
Inverse variance weighted
MR Egger
Simple mode
Weighted median
Weighted mode
0.5
0.0
−0.5
0.1
0.2
SNP effect on osteosarcoma
SNP effect on ATP2A3

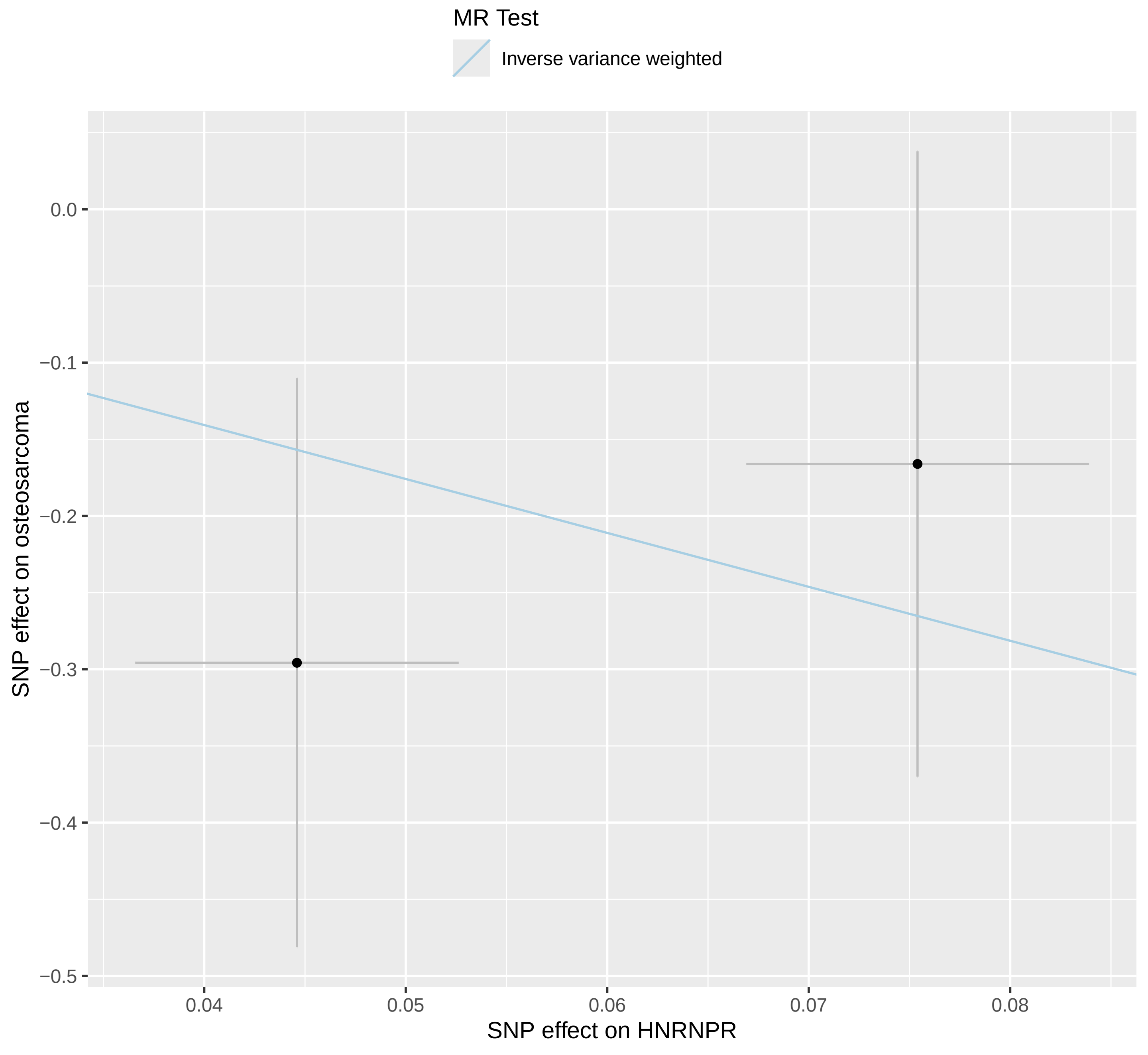
MR Test
Inverse variance weighted
0.0
−0.1
−0.2
−0.3
−0.4
−0.5
SNP effect on osteosarcoma
0.04
0.05
0.06
0.07
0.08
SNP effect on HNRNPR

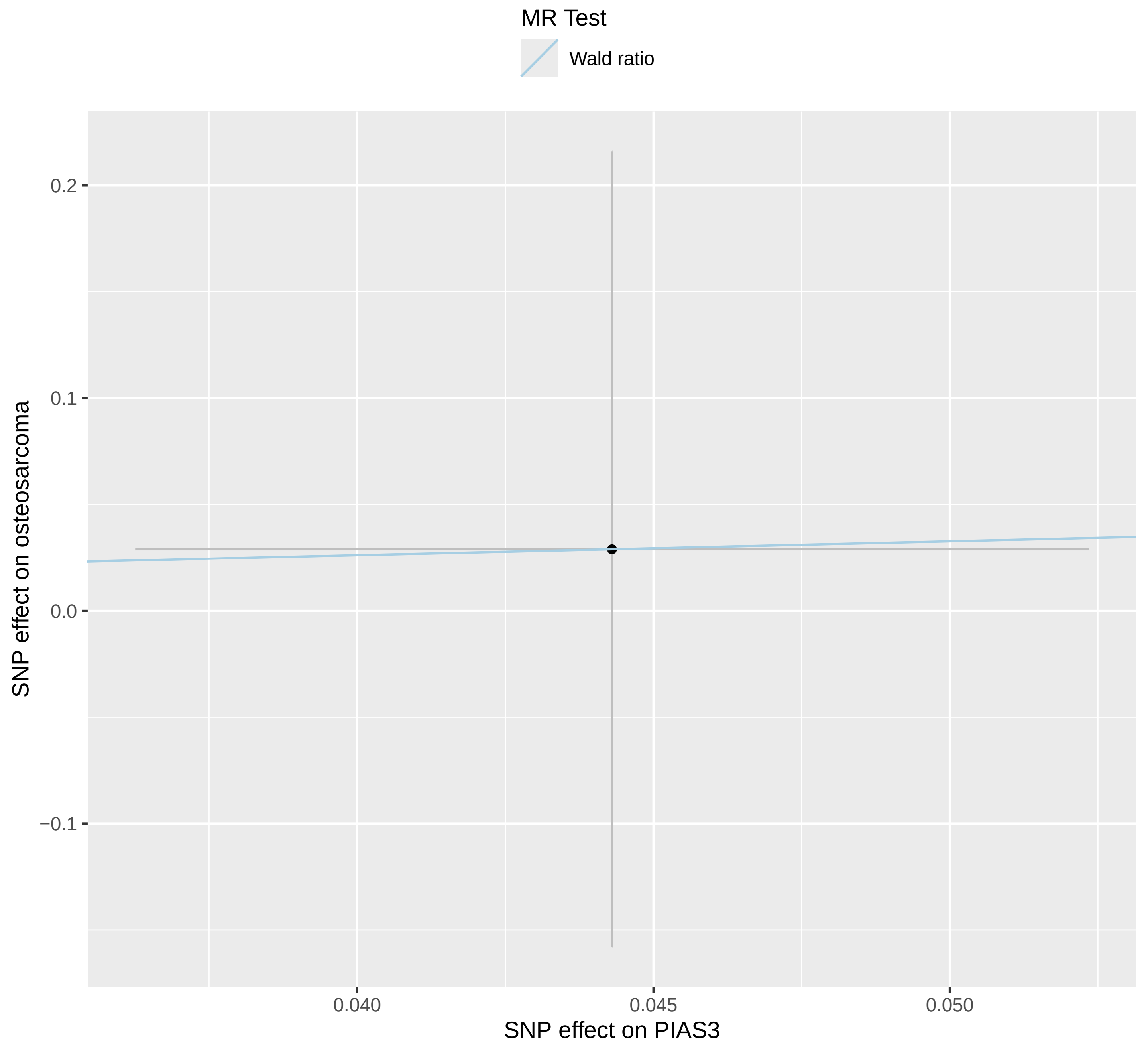

MR Test
Wald ratio
0.2
0.1
0.0
−0.1
0.040
0.045
0.050
SNP effect on PIAS3
SNP effect on osteosarcoma

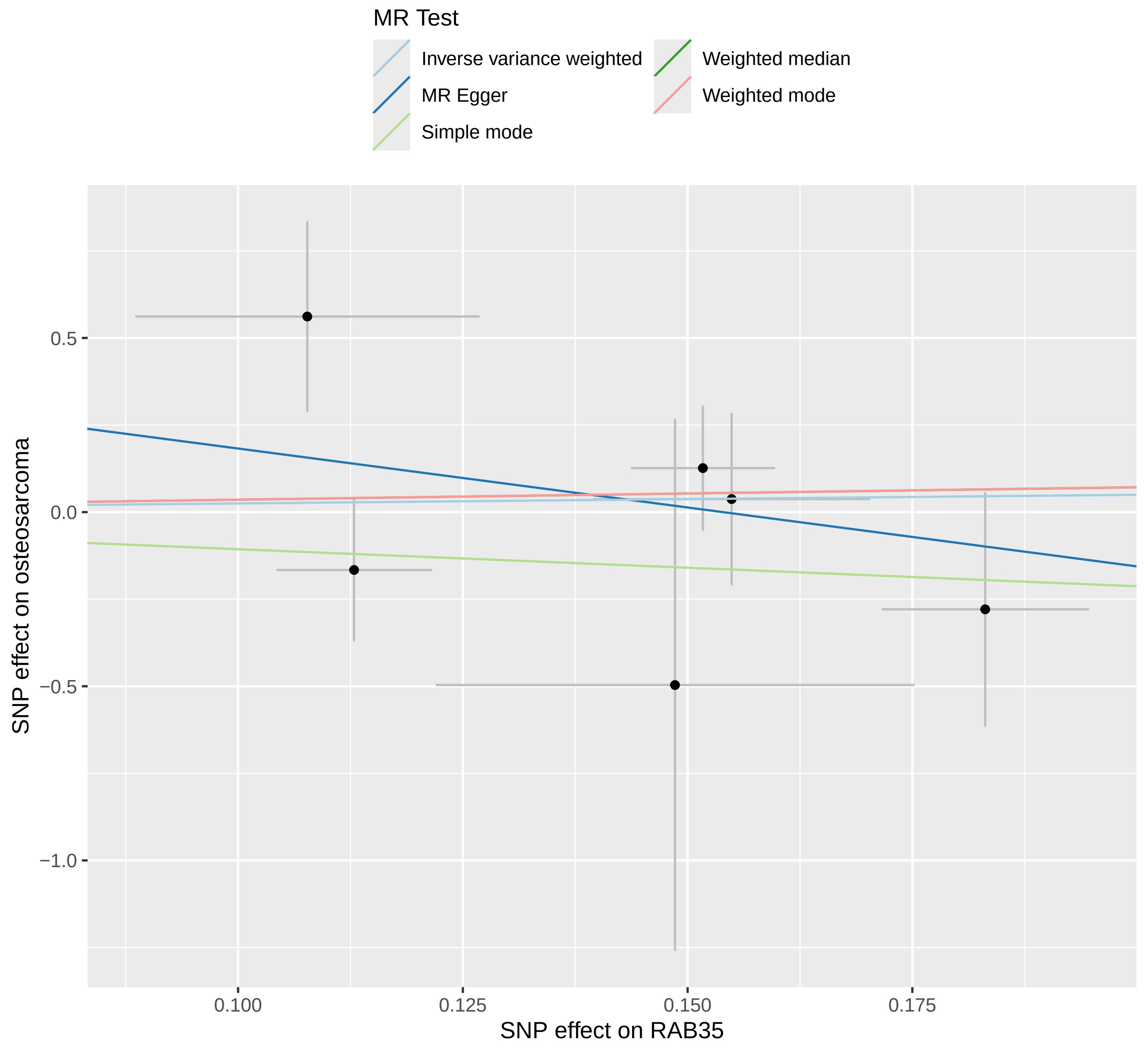
MR Test
Inverse variance weighted
MR Egger
Simple mode
Weighted median
Weighted mode
0.5
0.0
−0.5
−1.0
SNP effect on osteosarcoma
0.100
0.125
0.150
0.175
SNP effect on RAB35

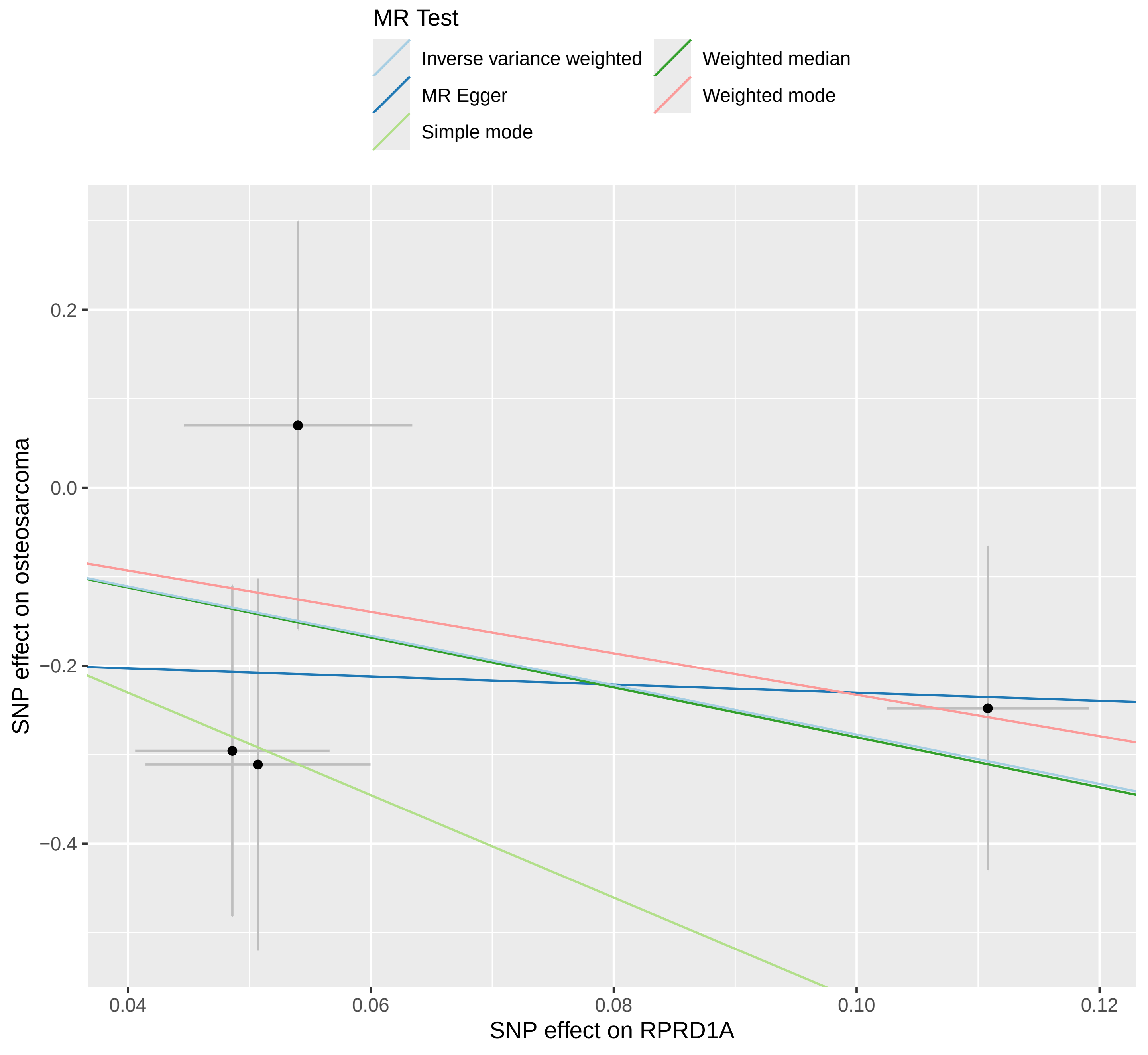

MR Test
Inverse variance weighted
MR Egger
Simple mode
Weighted median
Weighted mode
0.2
0.0
−0.2
−0.4
SNP effect on osteosarcoma
0.04
0.06
0.08
0.10
0.12
SNP effect on RPRD1A

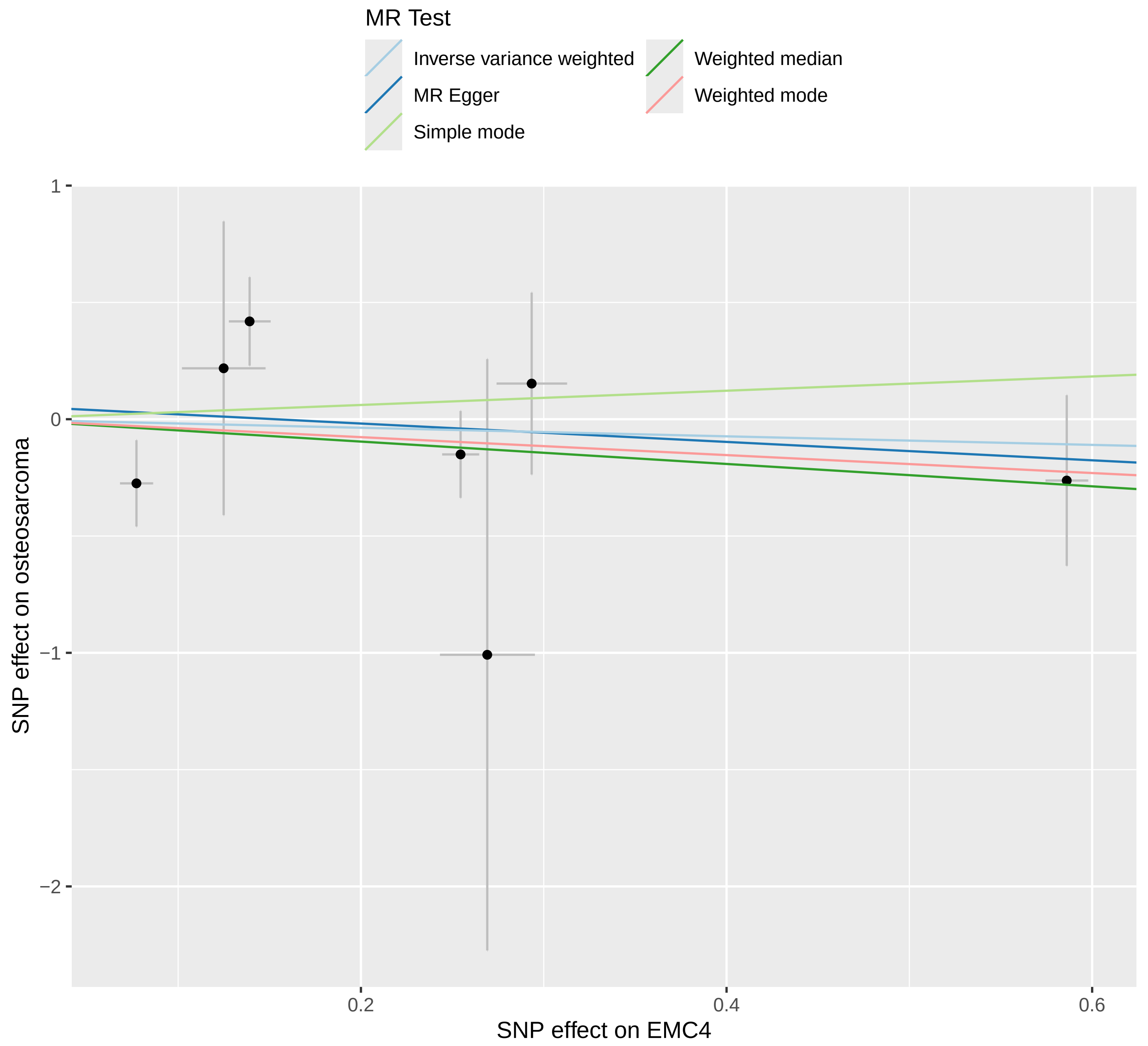
MR Test
Inverse variance weighted
MR Egger
Simple mode
Weighted median
Weighted mode
1
0
−1
−2
0.2
0.4
0.6
SNP effect on osteosarcoma
SNP effect on EMC4

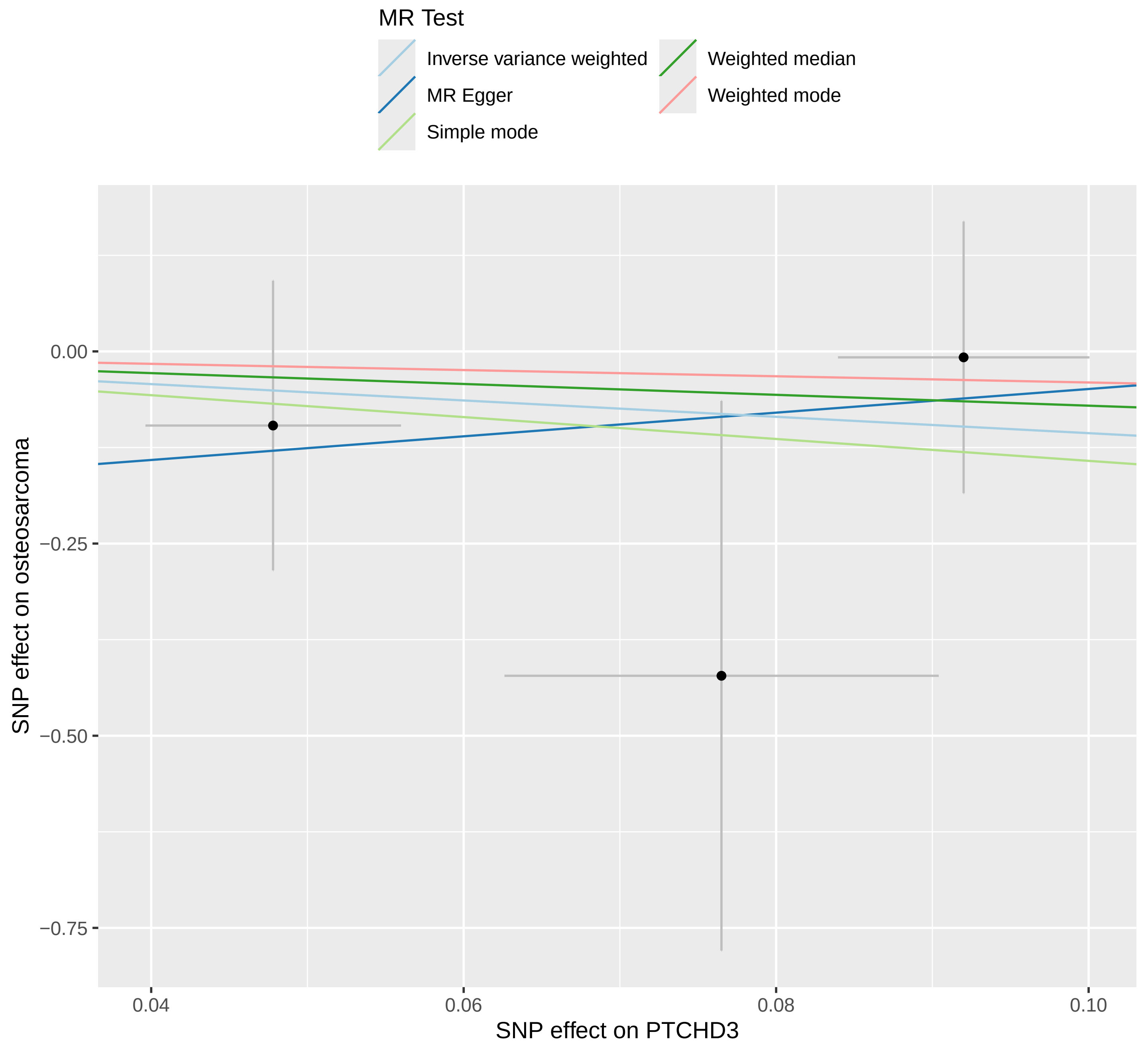

MR Test
Inverse variance weighted
MR Egger
Simple mode
Weighted median
Weighted mode
0.00
−0.25
−0.50
−0.75
SNP effect on osteosarcoma
0.04
0.06
0.08
0.10
SNP effect on PTCHD3

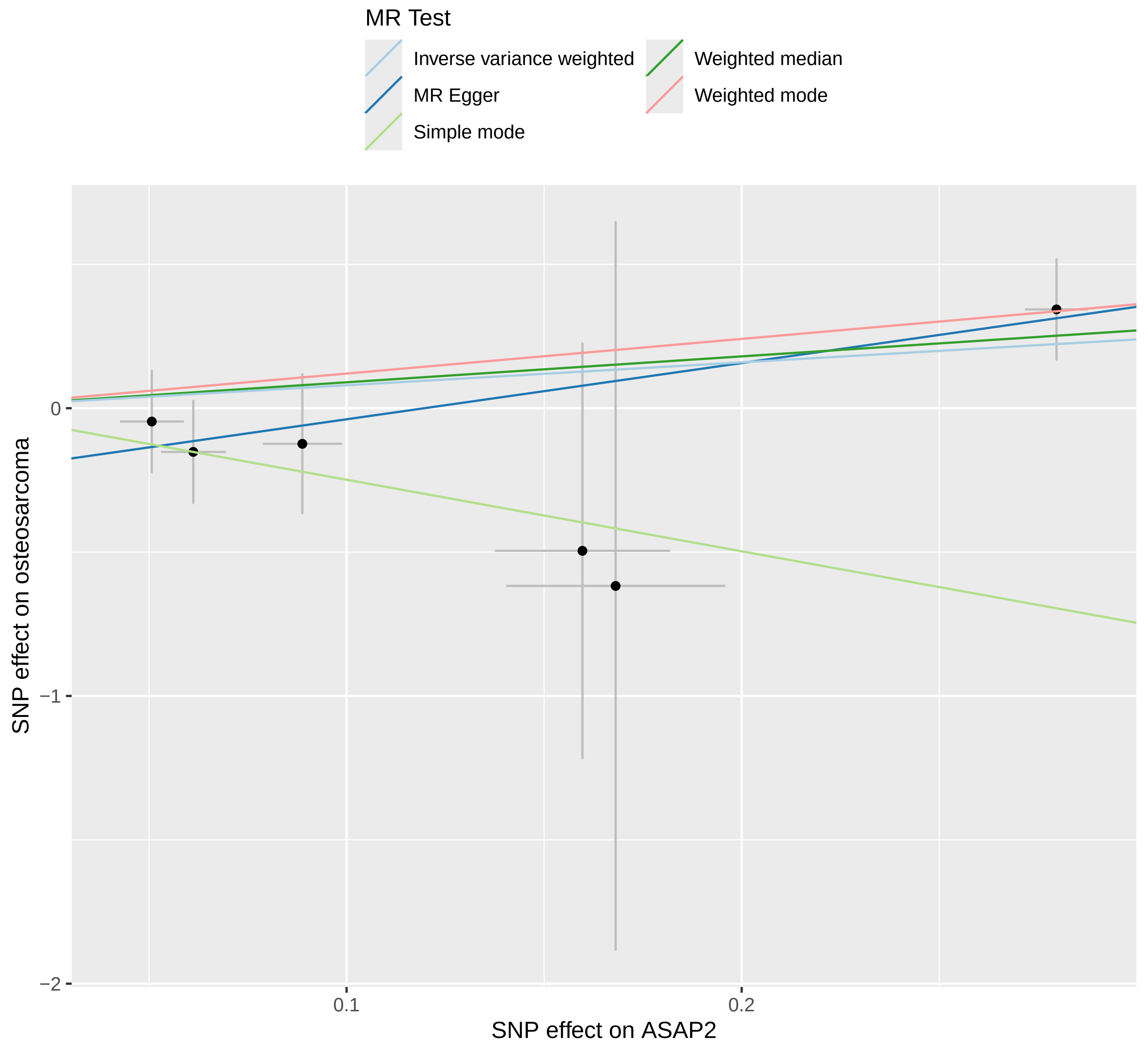

MR Test
Inverse variance weighted
MR Egger
Simple mode
Weighted median
Weighted mode
0
−1
−2
0.1
0.2
SNP effect on osteosarcoma
SNP effect on ASAP2

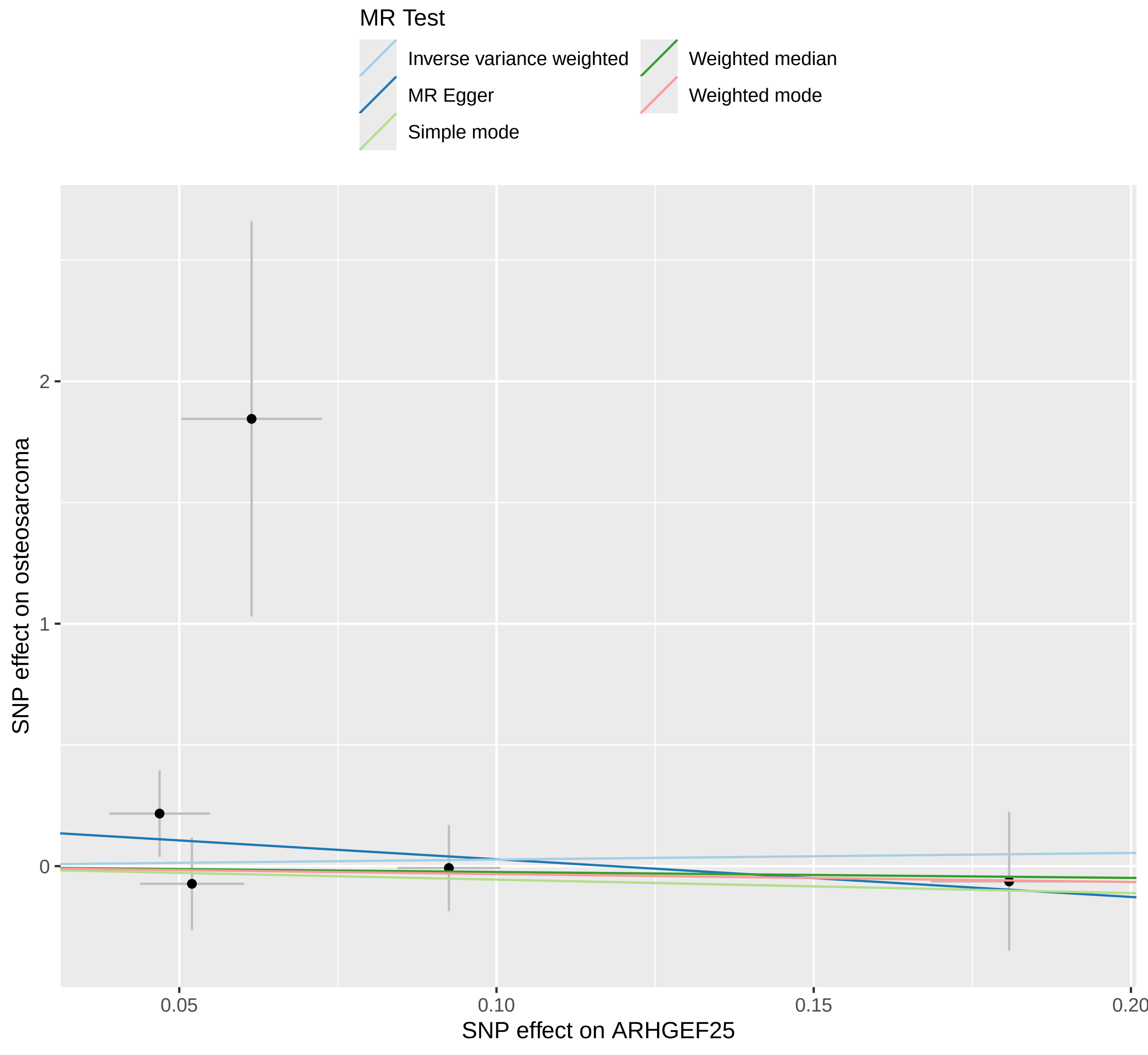

MR Test
Inverse variance weighted
MR Egger
Simple mode
Weighted median
Weighted mode
2
1
0
SNP effect on osteosarcoma
0.05
0.10
0.15
0.20
SNP effect on ARHGEF25

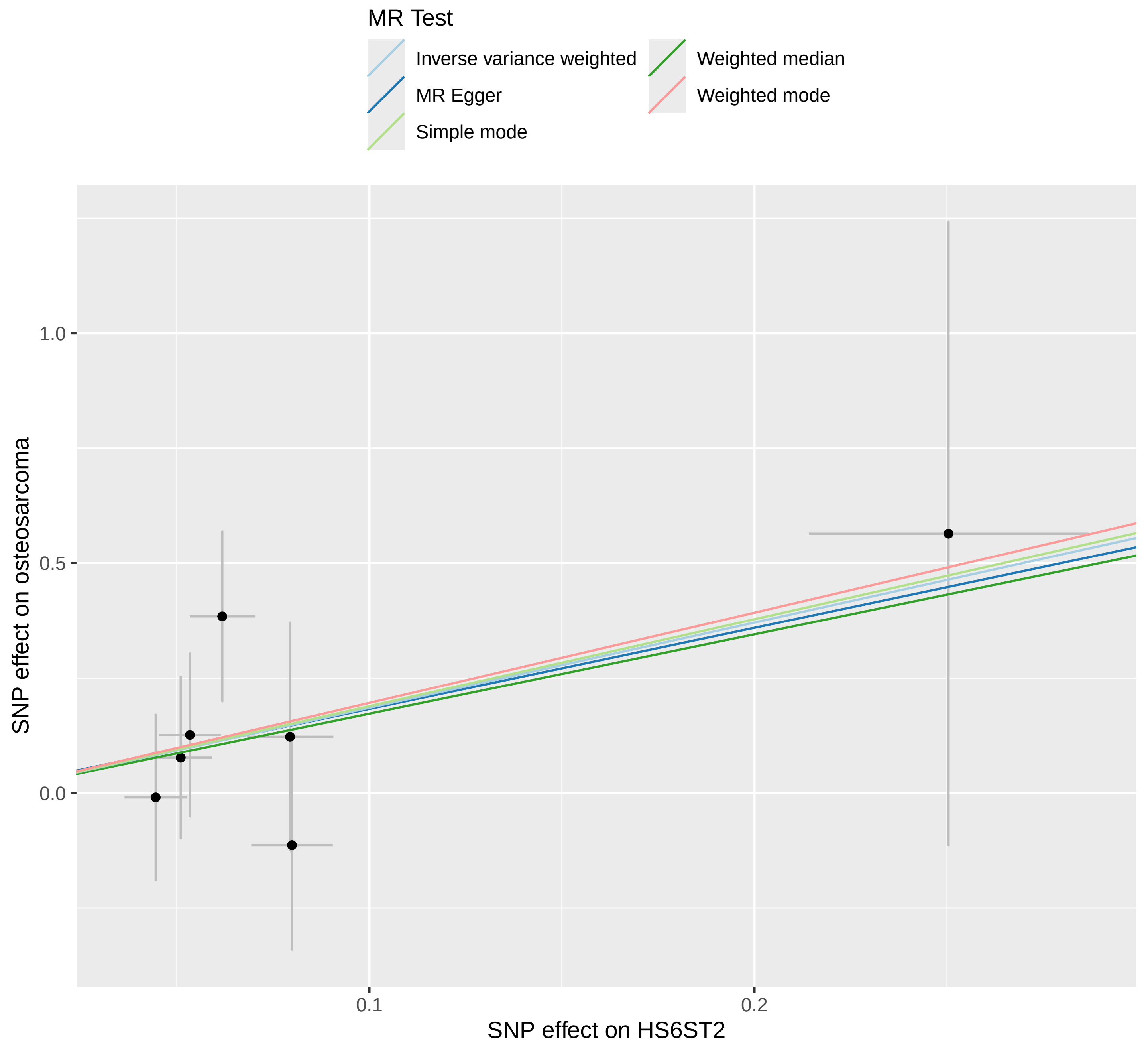
MR Test
Inverse variance weighted
MR Egger
Simple mode
Weighted median
Weighted mode
1.0
0.5
0.0
0.1
0.2
SNP effect on osteosarcoma
SNP effect on HS6ST2

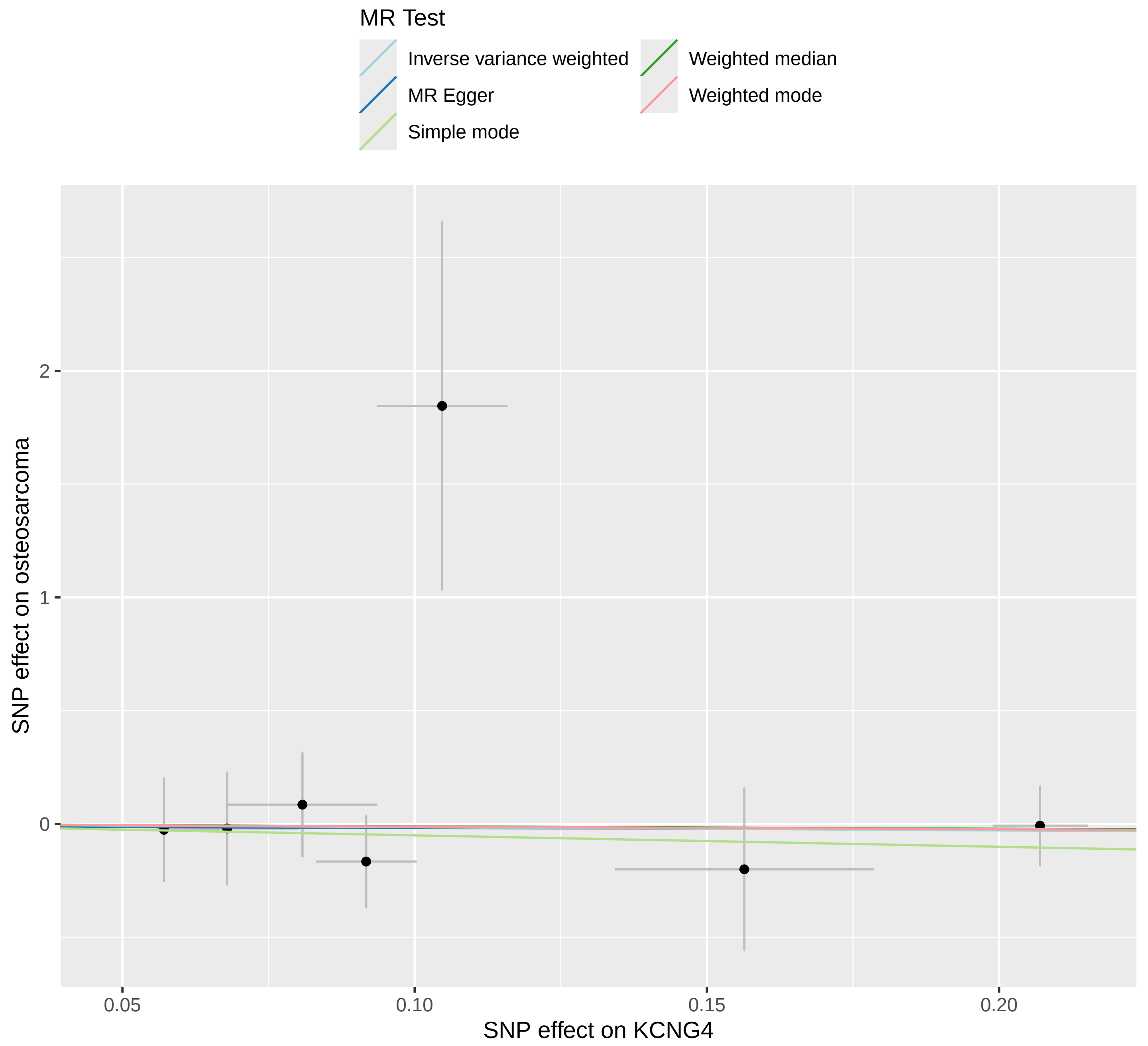
MR Test
Inverse variance weighted
MR Egger
Simple mode
Weighted median
Weighted mode
SNP effect on osteosarcoma
2
1
0
0.05
0.10
0.15
0.20
SNP effect on KCNG4

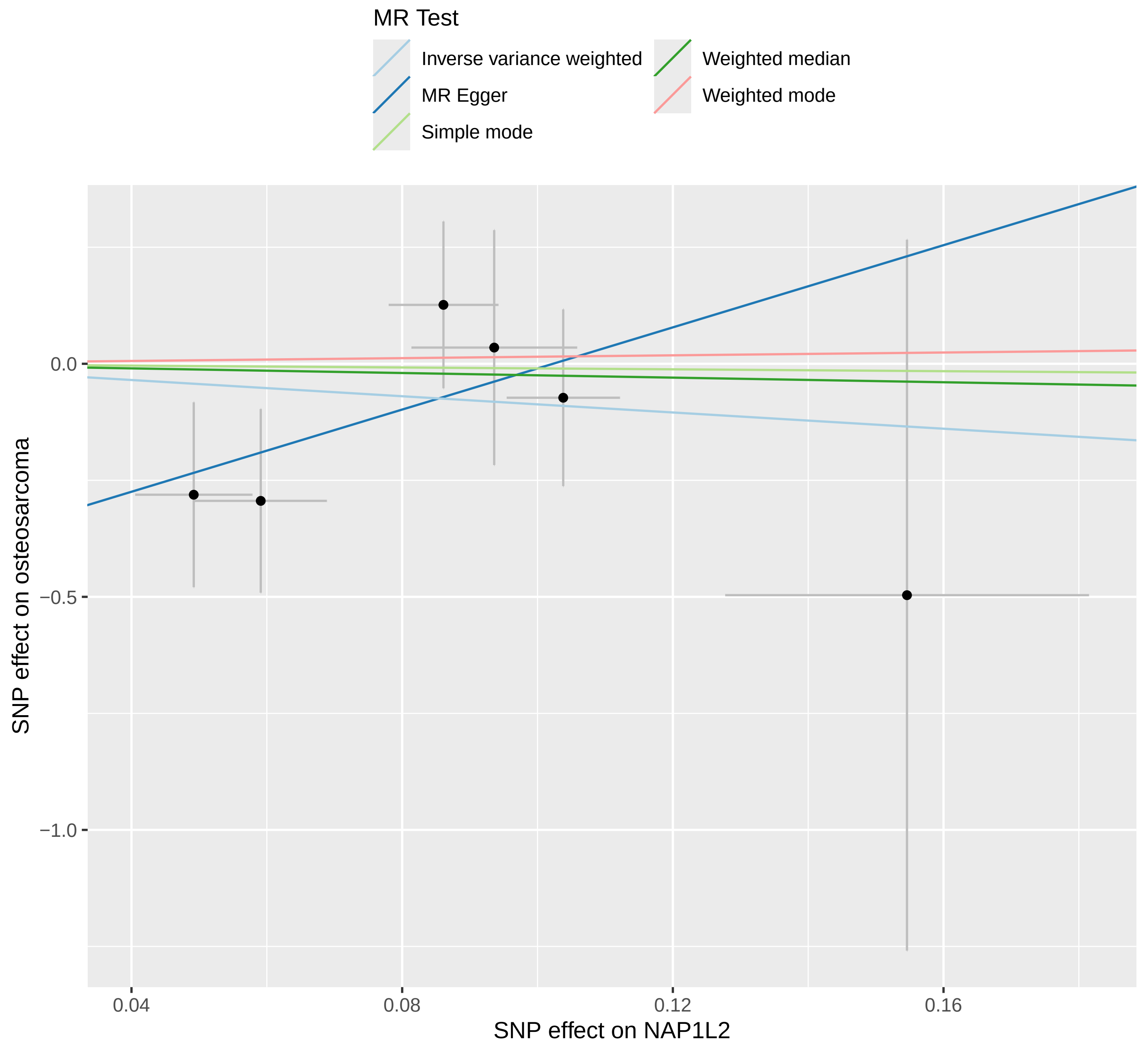

MR Test
Inverse variance weighted
MR Egger
Simple mode
Weighted median
Weighted mode
SNP effect on osteosarcoma
0.0
−0.5
−1.0
0.04
0.08
0.12
0.16
SNP effect on NAP1L2

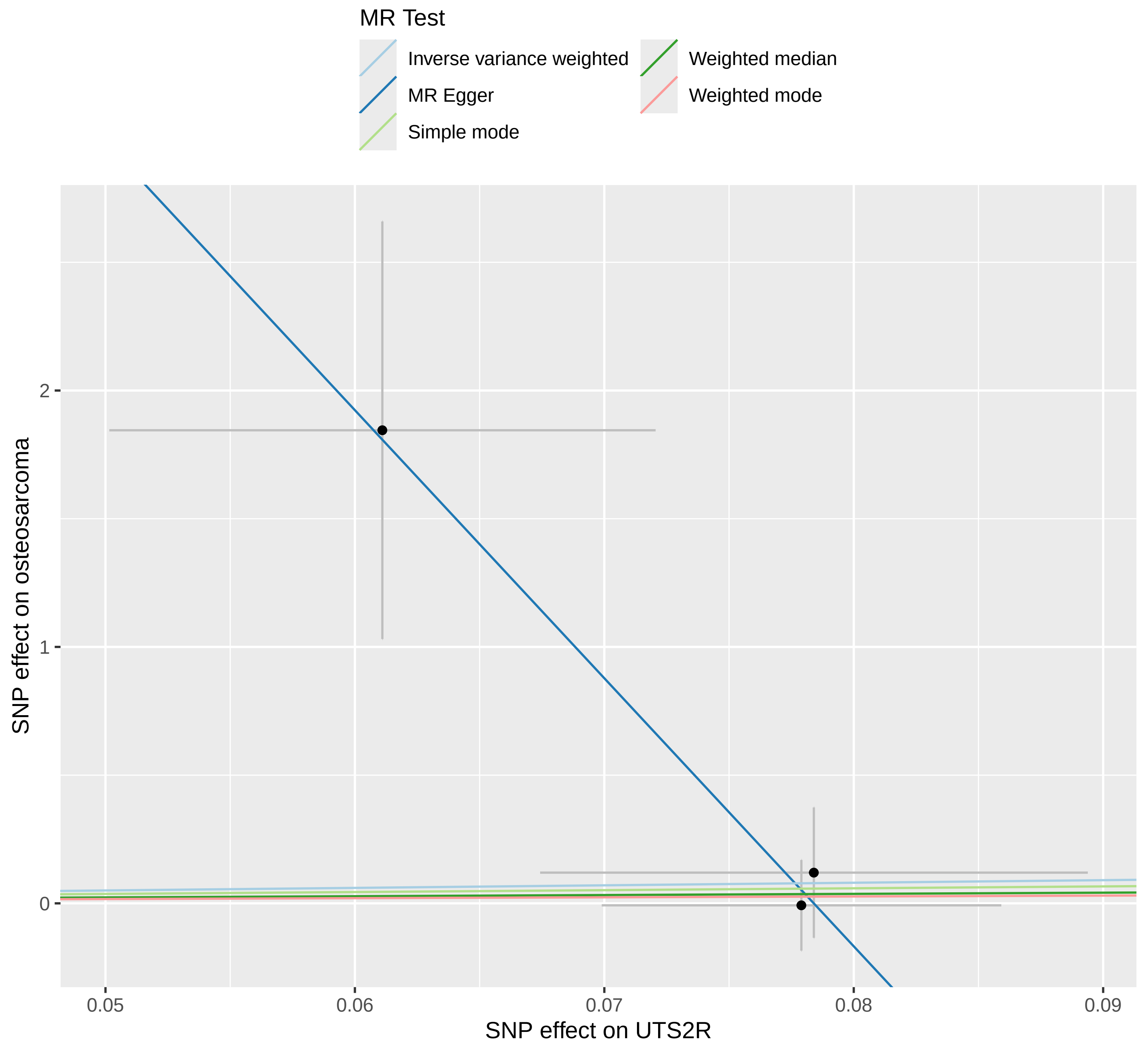
MR Test
Inverse variance weighted
MR Egger
Simple mode
Weighted median
Weighted mode
SNP effect on osteosarcoma
2
1
0
0.05
0.06
0.07
0.08
0.09
SNP effect on UTS2R

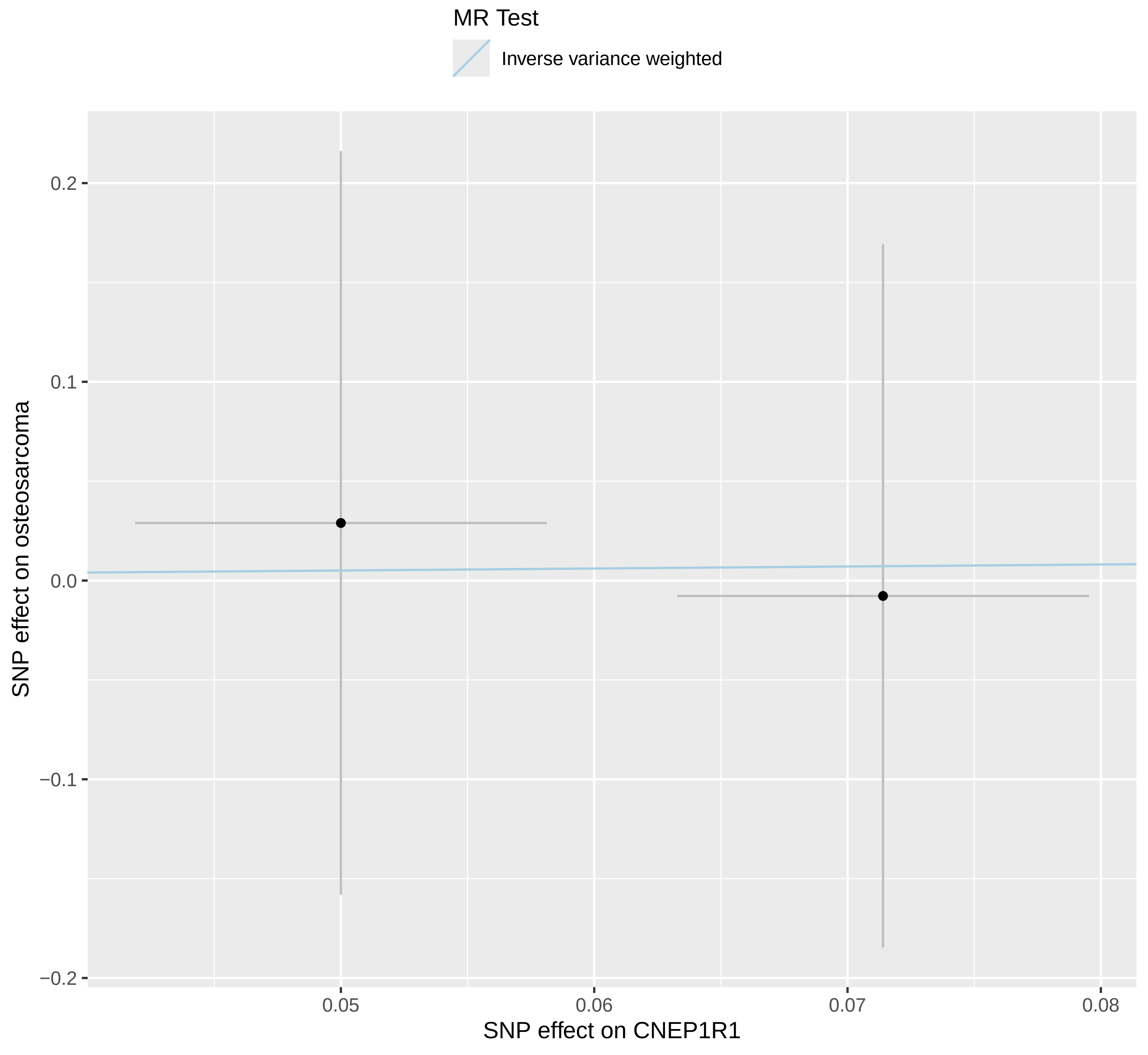
MR Test
Inverse variance weighted
0.2
0.1
0.0
−0.1
−0.2
0.05
0.06
0.07
0.08
SNP effect on osteosarcoma
SNP effect on CNEP1R1

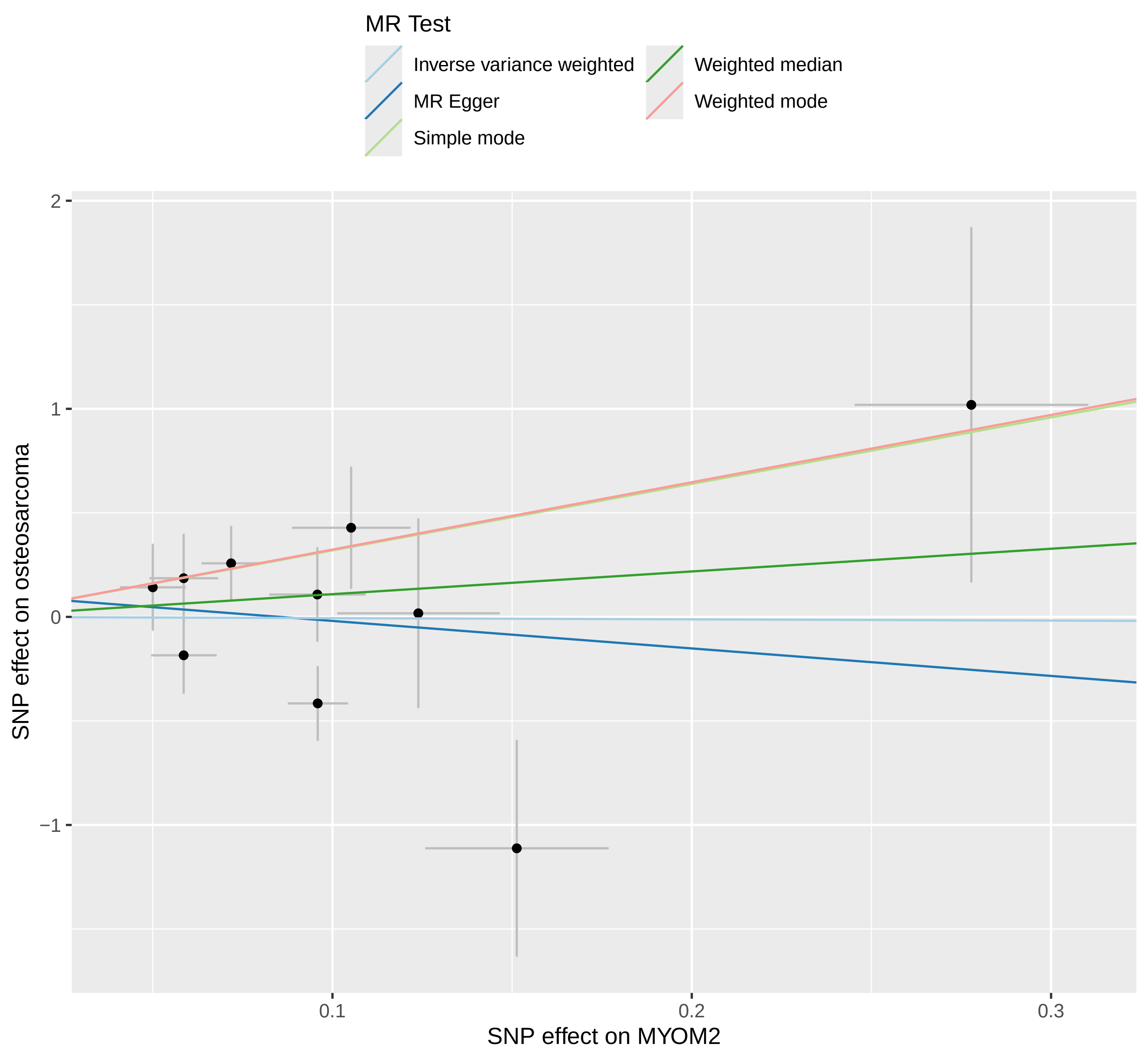

MR Test
Inverse variance weighted
MR Egger
Simple mode
Weighted median
Weighted mode
2
1
0
−1
0.1
0.2
0.3
SNP effect on osteosarcoma
SNP effect on MYOM2

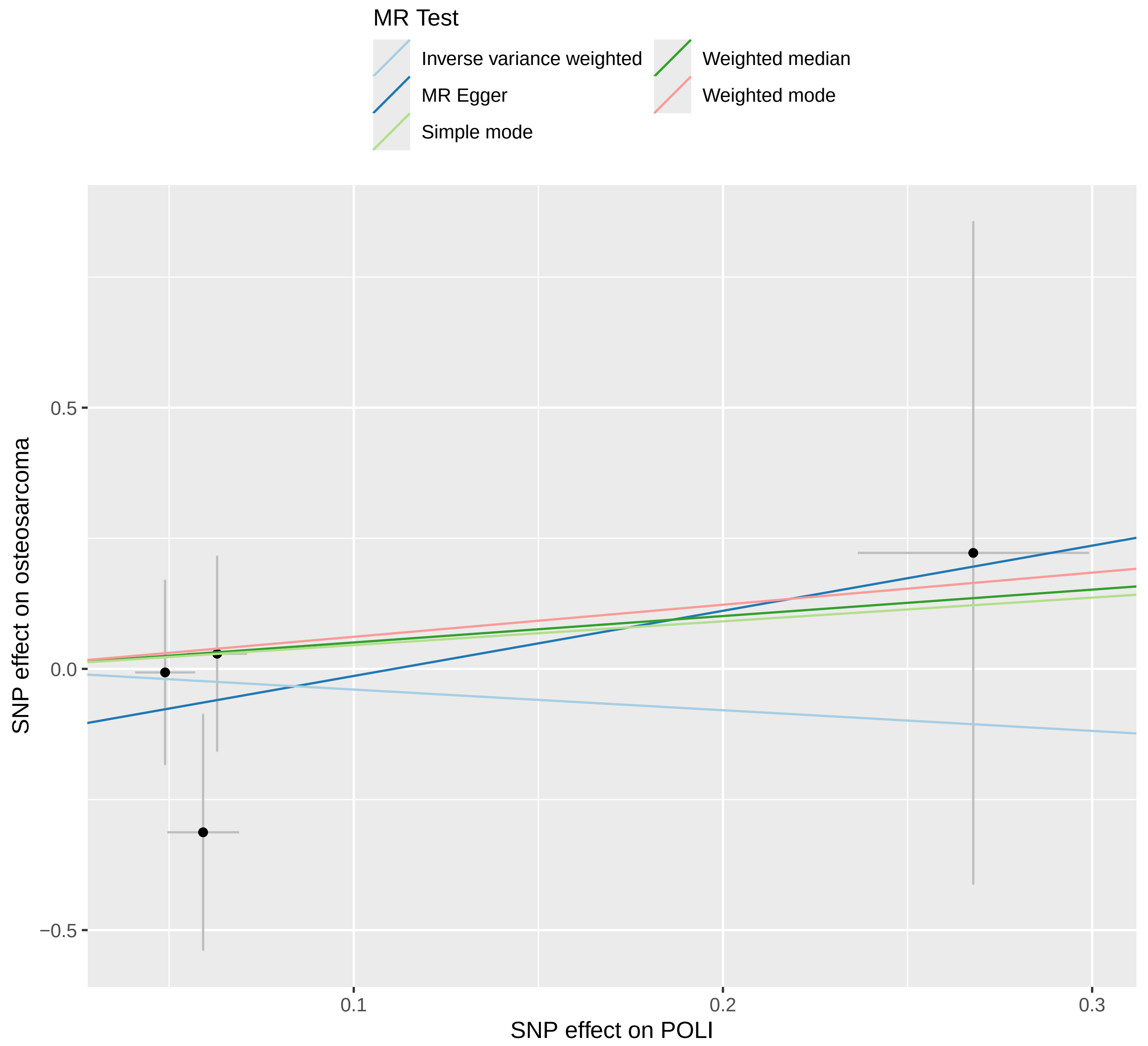
MR Test
Inverse variance weighted
MR Egger
Simple mode
Weighted median
Weighted mode
0.5
0.0
−0.5
0.1
0.2
0.3
SNP effect on osteosarcoma
SNP effect on POLI

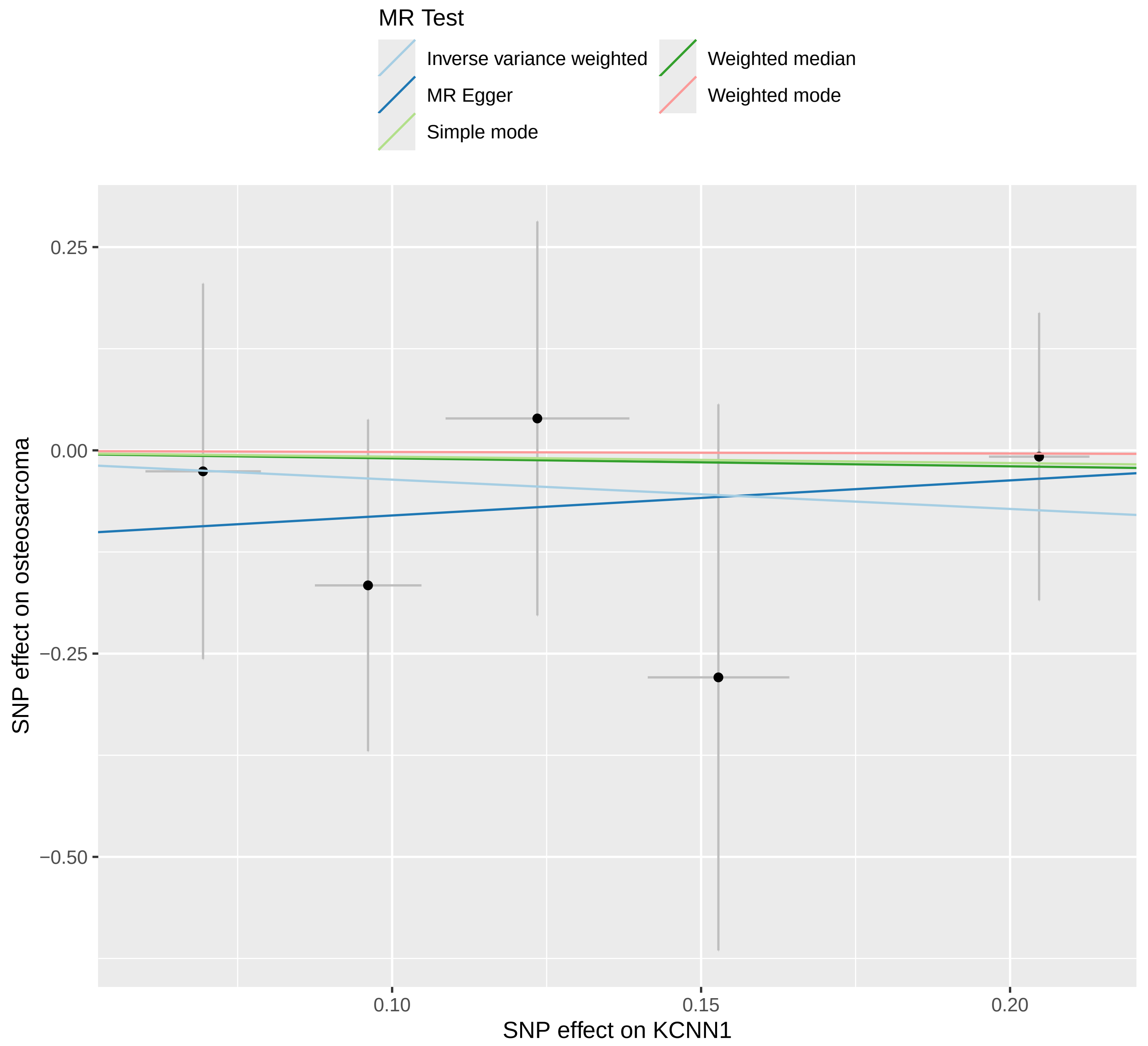
MR Test
Inverse variance weighted
MR Egger
Simple mode
Weighted median
Weighted mode
0.25
0.00
−0.25
−0.50
0.10
0.15
0.20
SNP effect on KCNN1
SNP effect on osteosarcoma

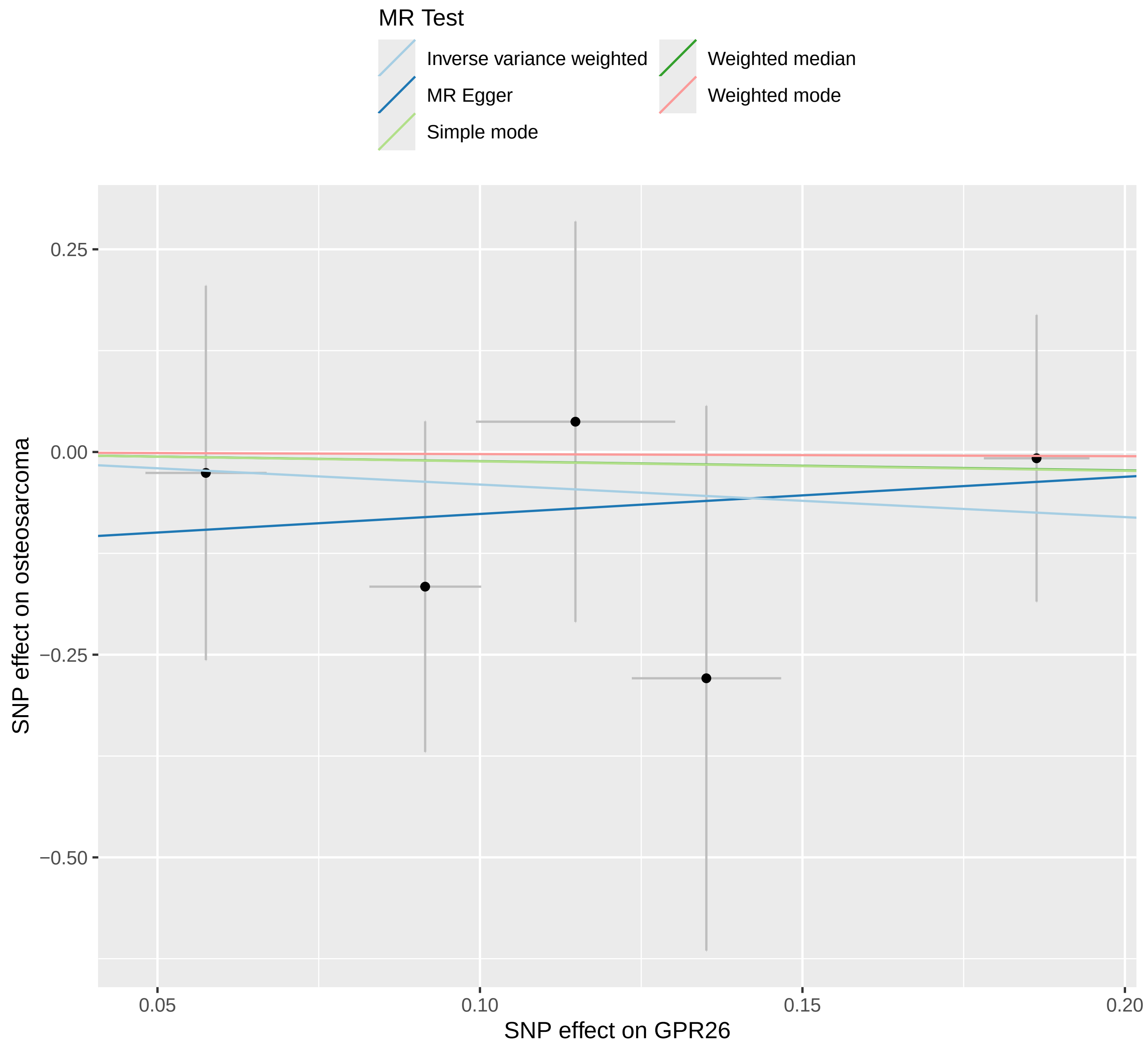
MR Test
Inverse variance weighted
MR Egger
Simple mode
Weighted median
Weighted mode
0.25
0.00
−0.25
−0.50
0.05
0.10
0.15
0.20
SNP effect on osteosarcoma
SNP effect on GPR26

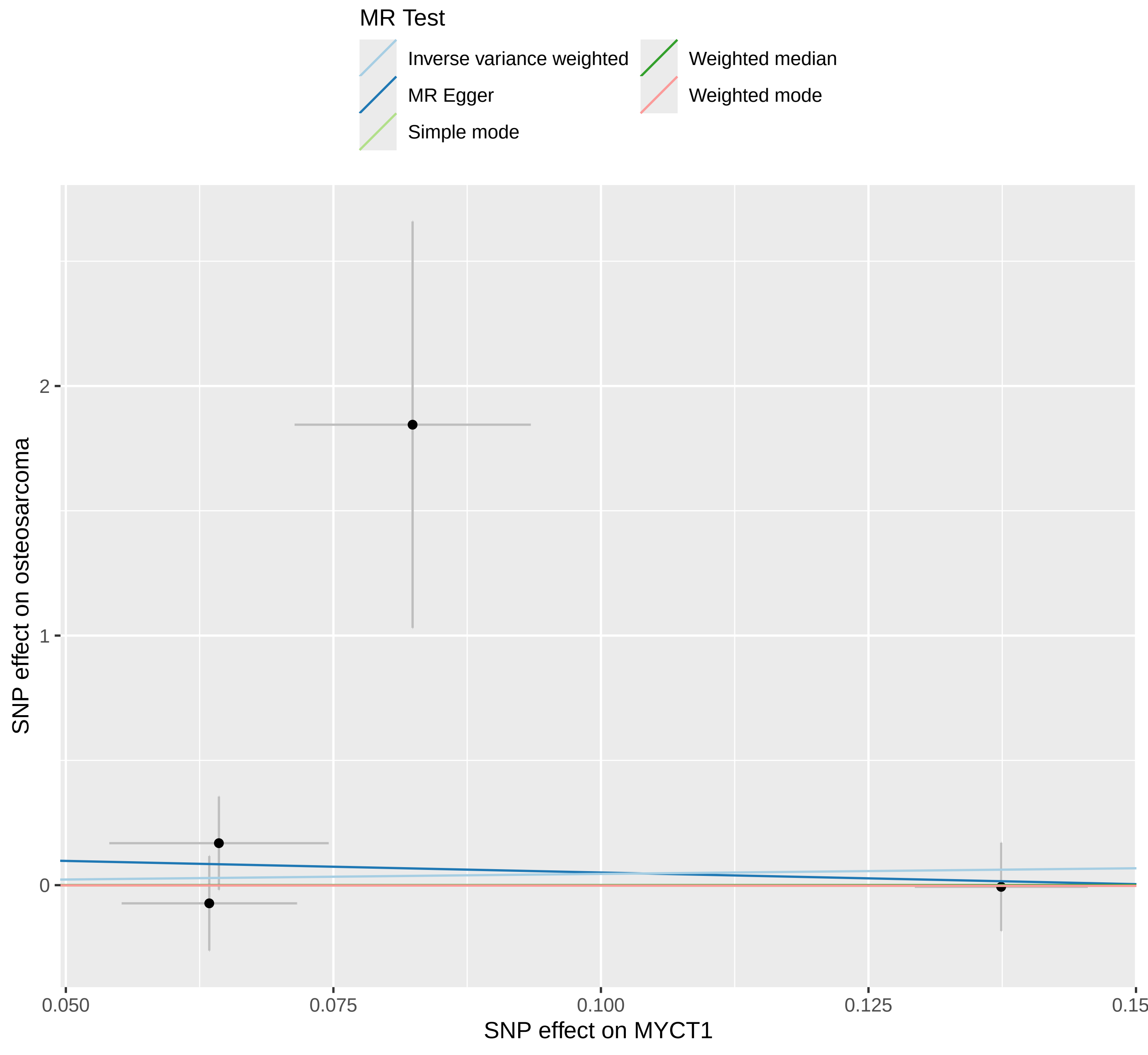

MR Test
Inverse variance weighted
MR Egger
Simple mode
Weighted median
Weighted mode
SNP effect on osteosarcoma
2
1
0
0.050
0.075
0.100
0.125
0.15
SNP effect on MYCT1

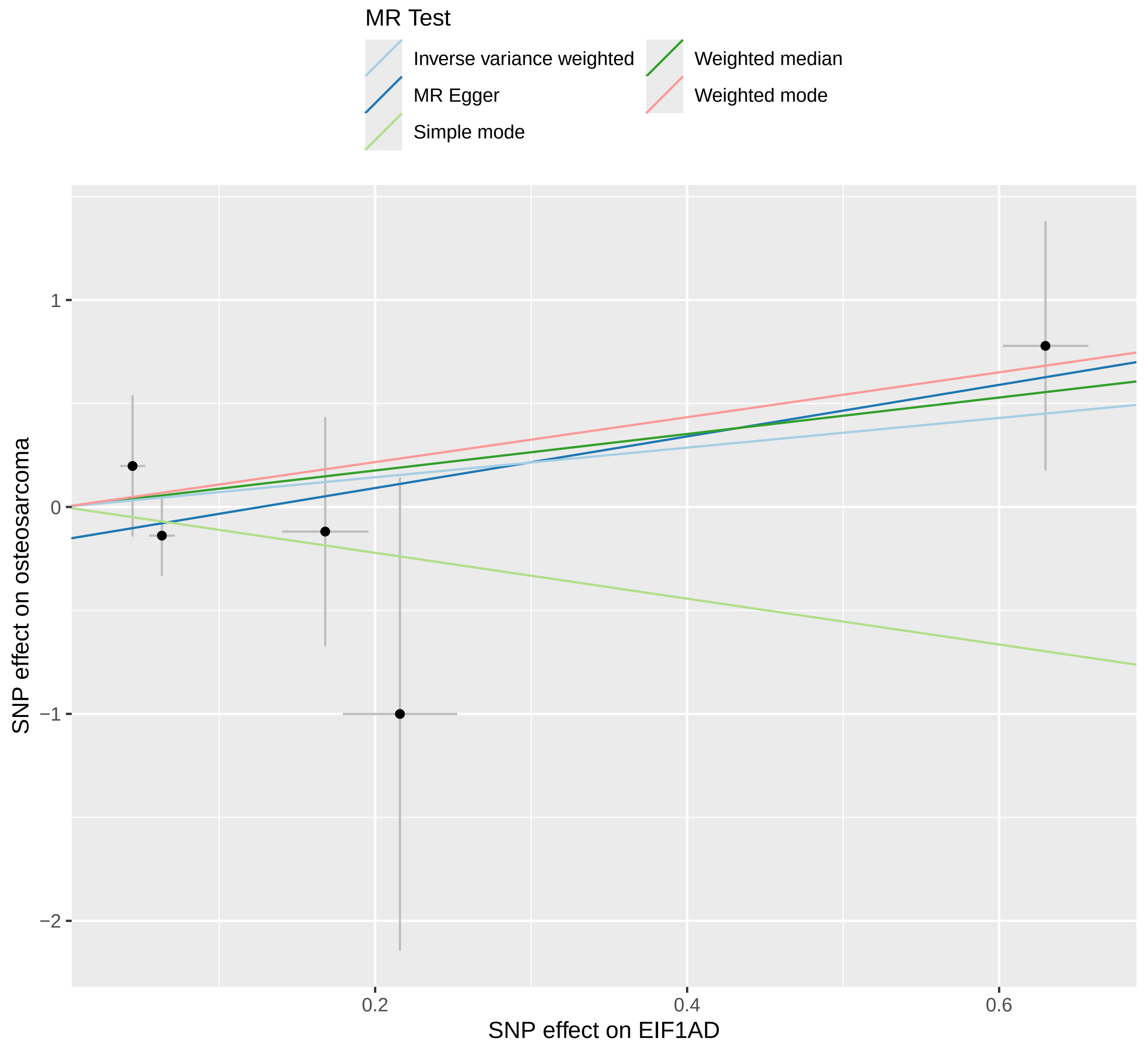
MR Test
Inverse variance weighted
MR Egger
Simple mode
Weighted median
Weighted mode
1
0
−1
−2
0.2
0.4
0.6
SNP effect on osteosarcoma
SNP effect on EIF1AD

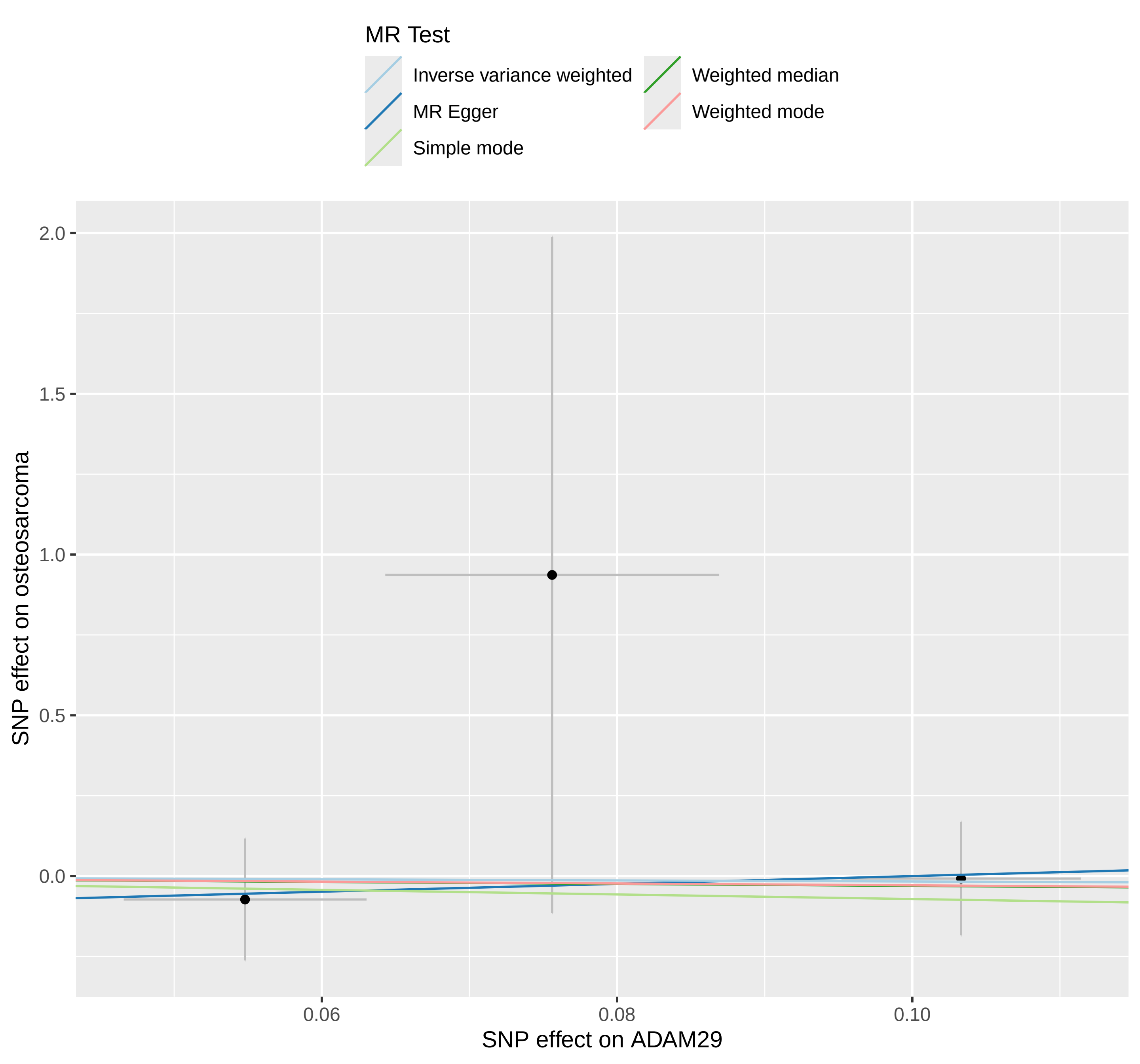

MR Test
Inverse variance weighted
MR Egger
Simple mode
Weighted median
Weighted mode
2.0
1.5
1.0
0.5
0.0
SNP effect on osteosarcoma
0.06
0.08
0.10
SNP effect on ADAM29

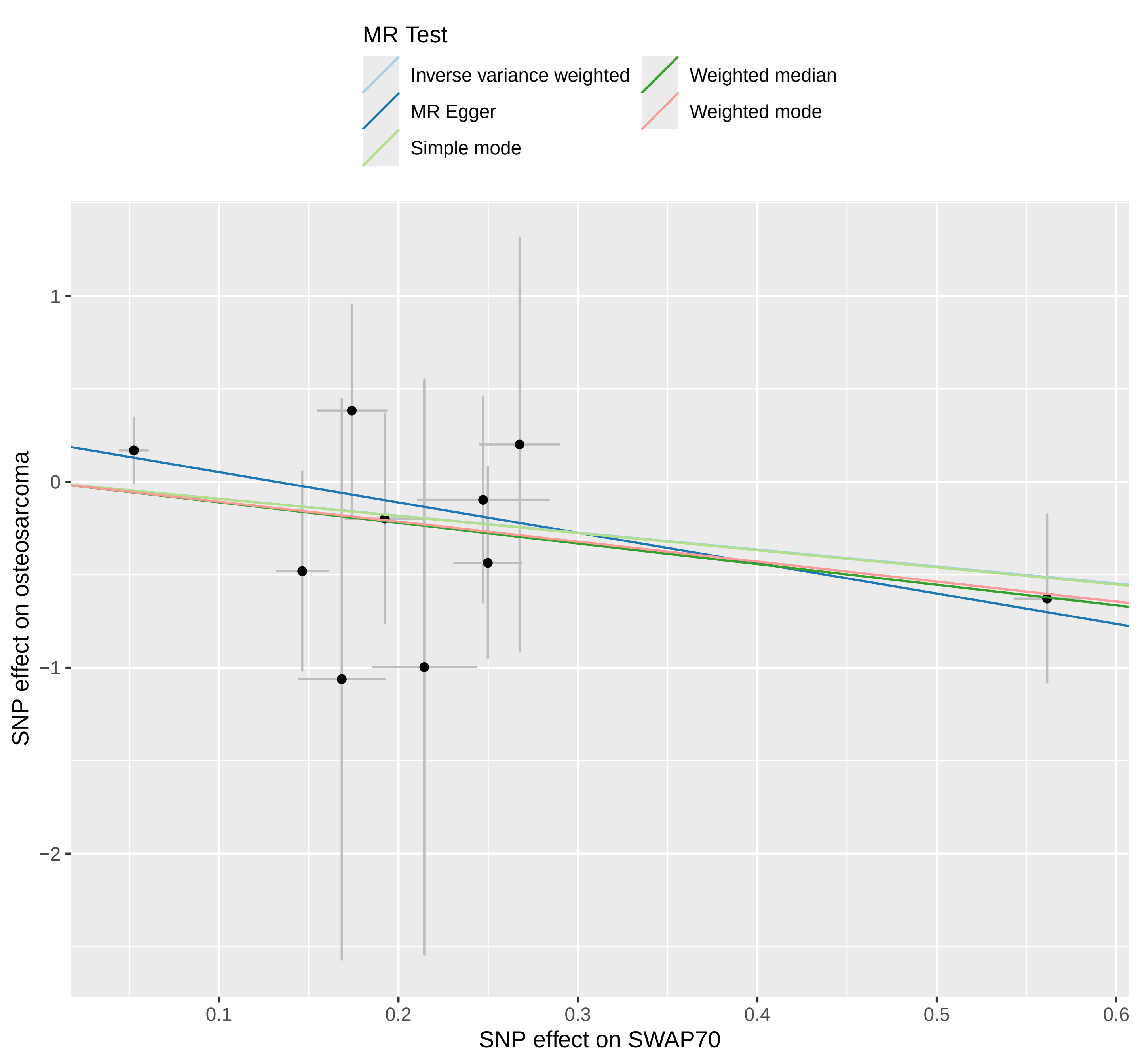

MR Test
Inverse variance weighted
MR Egger
Simple mode
Weighted median
Weighted mode
1
0
−1
−2
0.1
0.2
0.3
0.4
0.5
0.6
SNP effect on SWAP70
SNP effect on osteosarcoma

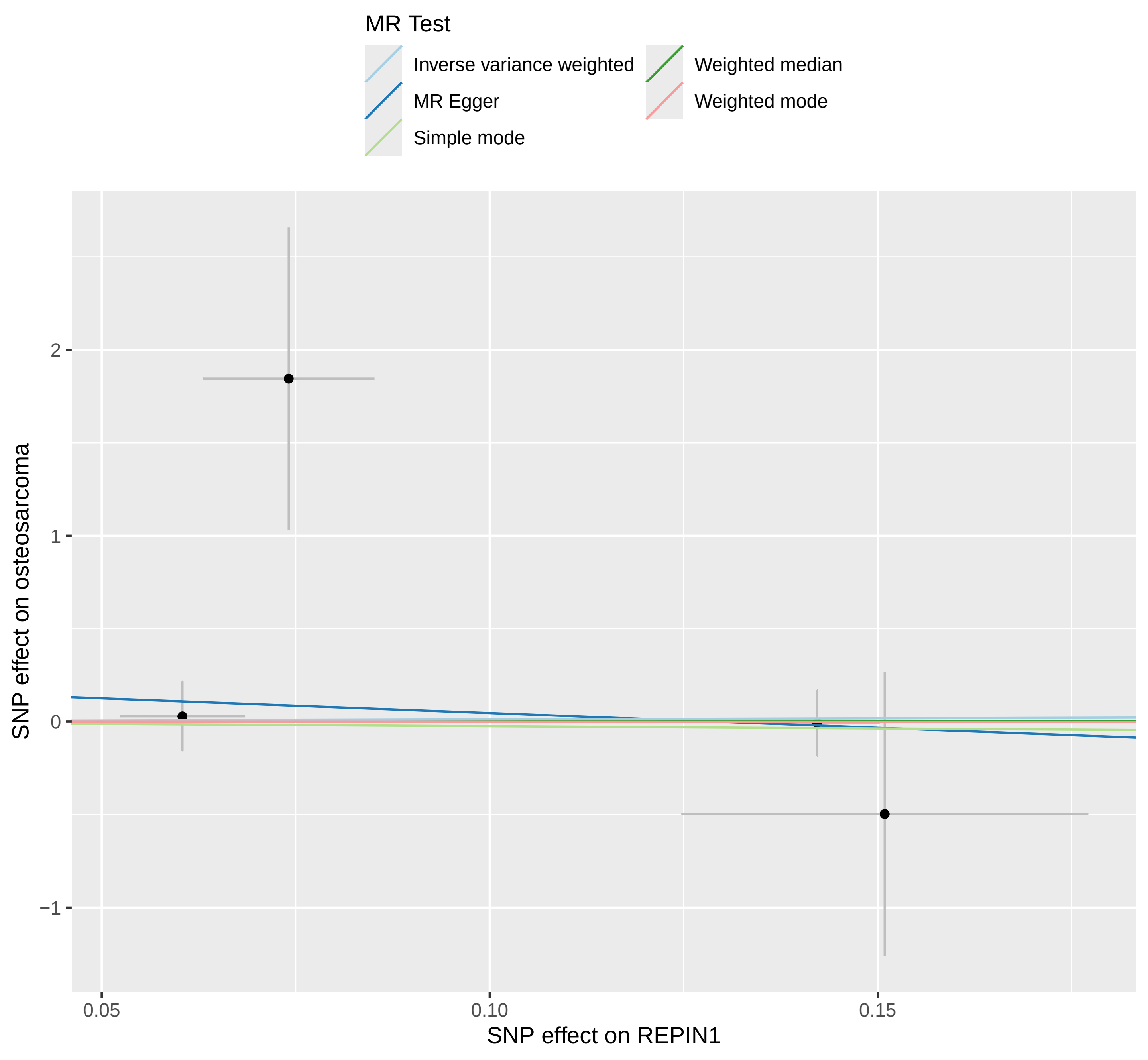

MR Test
Inverse variance weighted
MR Egger
Simple mode
Weighted median
Weighted mode
2
1
0
−1
0.05
0.10
0.15
SNP effect on REPIN1
SNP effect on osteosarcoma

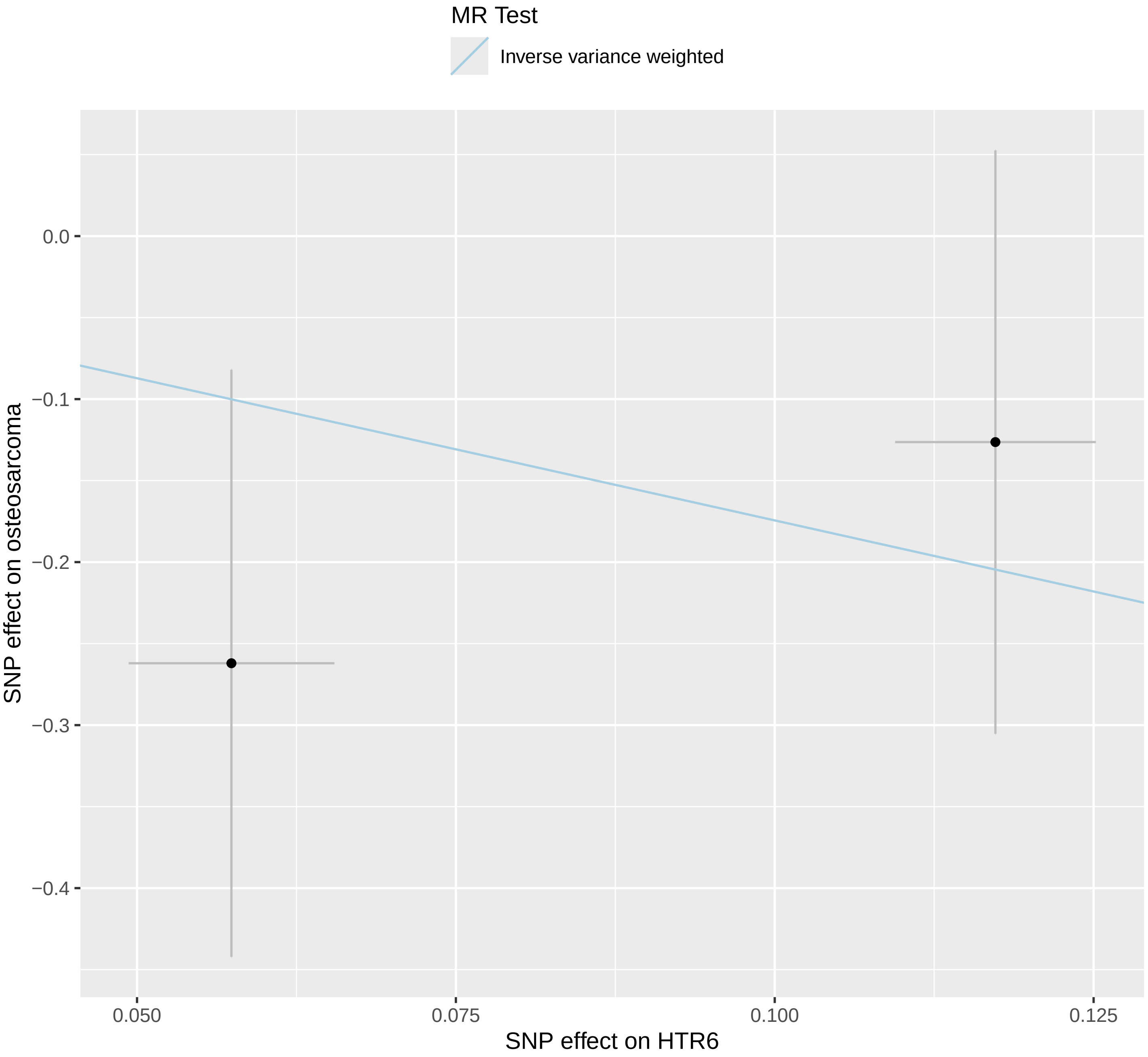
MR Test
Inverse variance weighted
0.0
−0.1
−0.2
−0.3
−0.4
SNP effect on osteosarcoma
0.050
0.075
0.100
0.125
SNP effect on HTR6

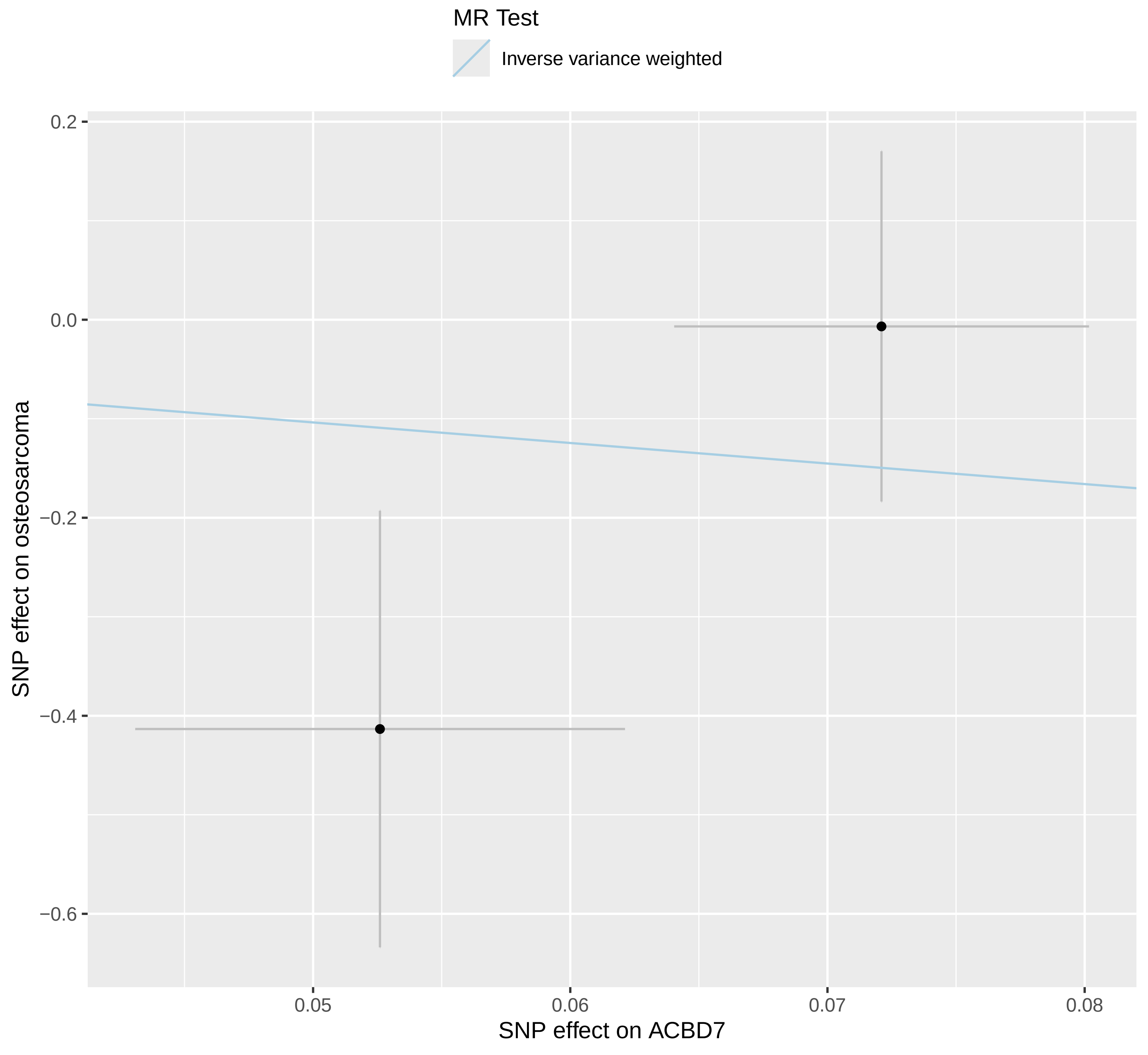

MR Test
Inverse variance weighted
0.2
0.0
−0.2
−0.4
−0.6
SNP effect on osteosarcoma
0.05
0.06
0.07
0.08
SNP effect on ACBD7

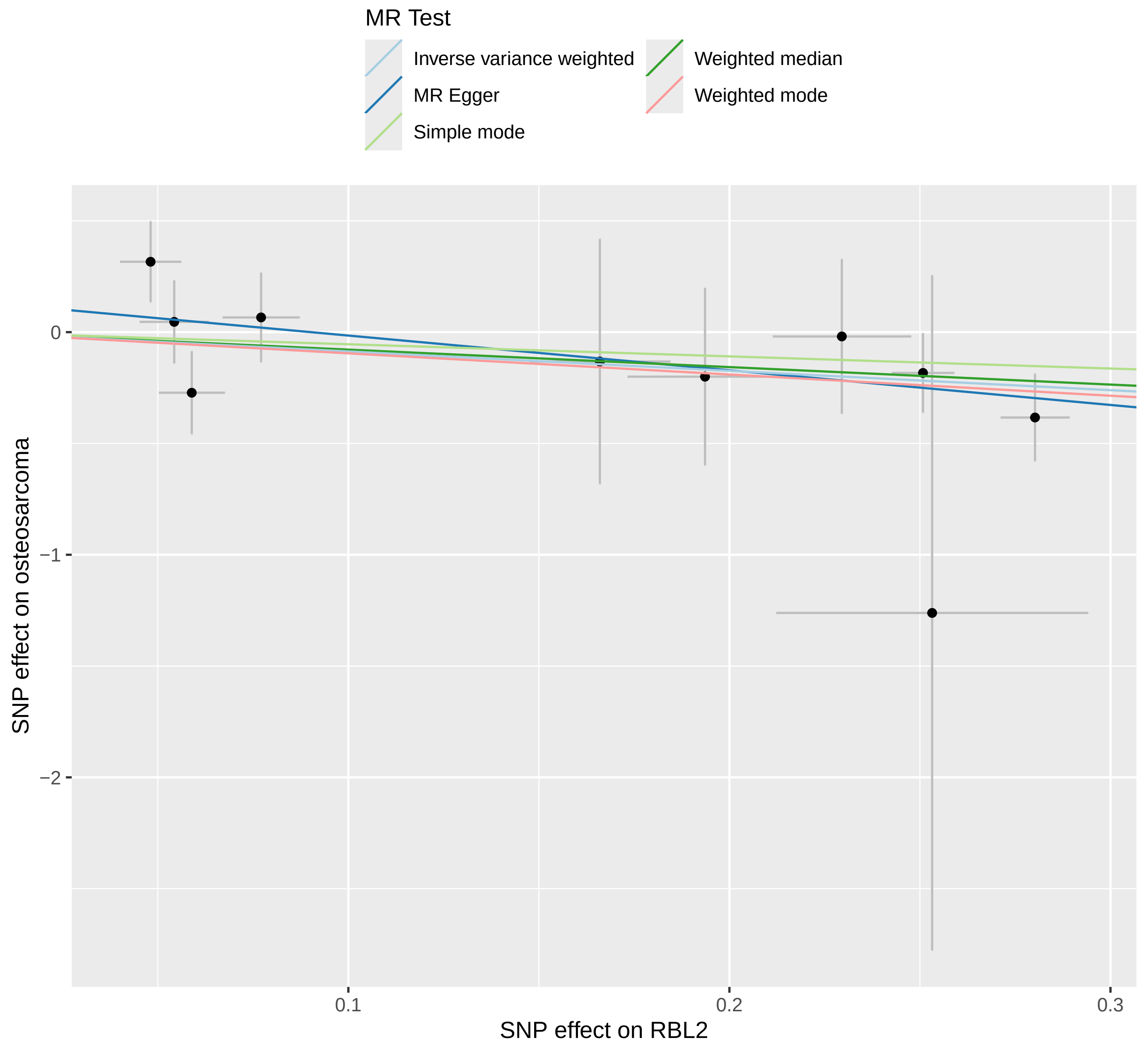
MR Test
Inverse variance weighted
MR Egger
Simple mode
Weighted median
Weighted mode
0
−1
−2
0.1
0.2
0.3
SNP effect on osteosarcoma
SNP effect on RBL2

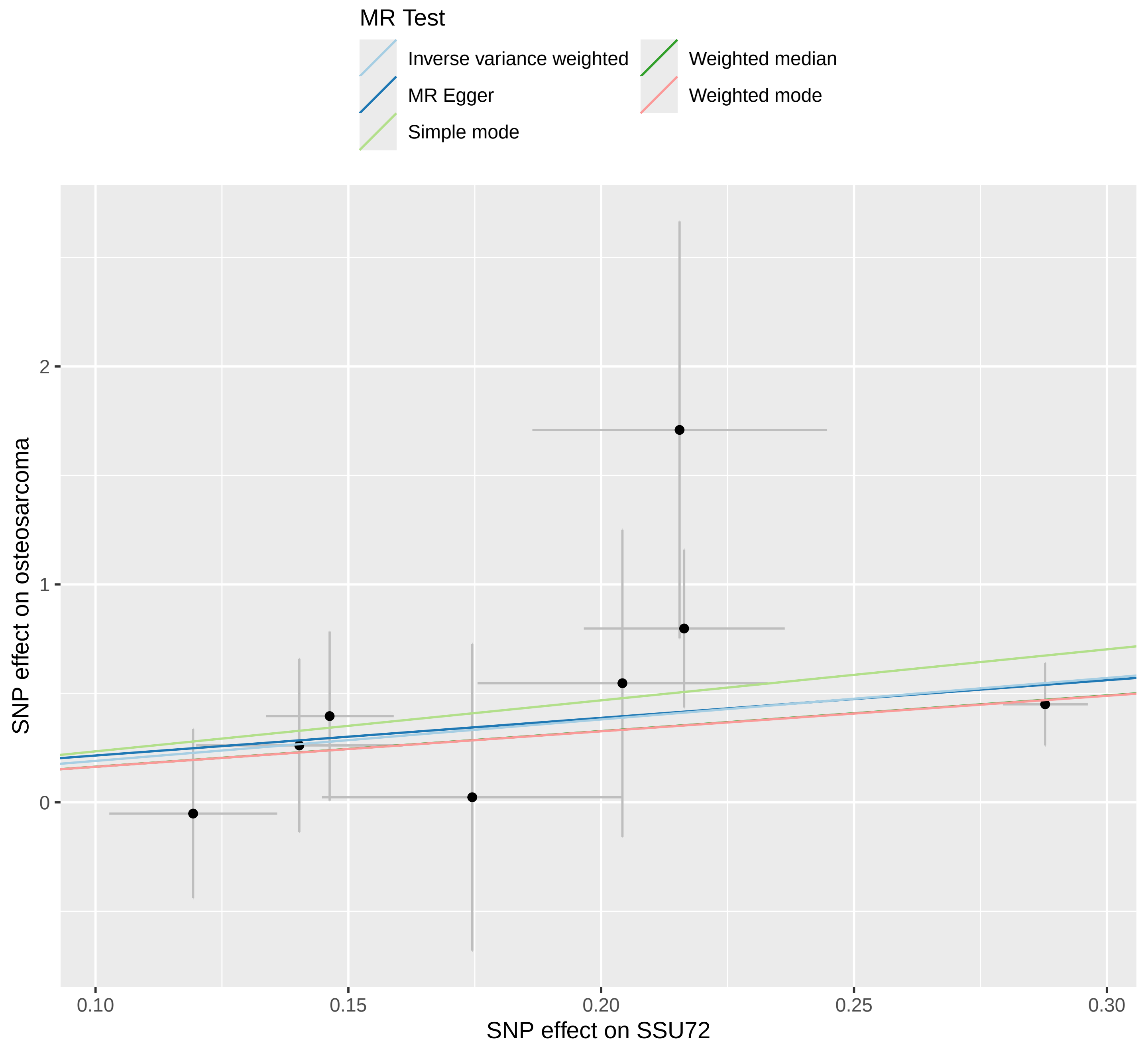
MR Test
Inverse variance weighted
MR Egger
Simple mode
Weighted median
Weighted mode
SNP effect on osteosarcoma
2
1
0
0.10
0.15
0.20
0.25
0.30
SNP effect on SSU72

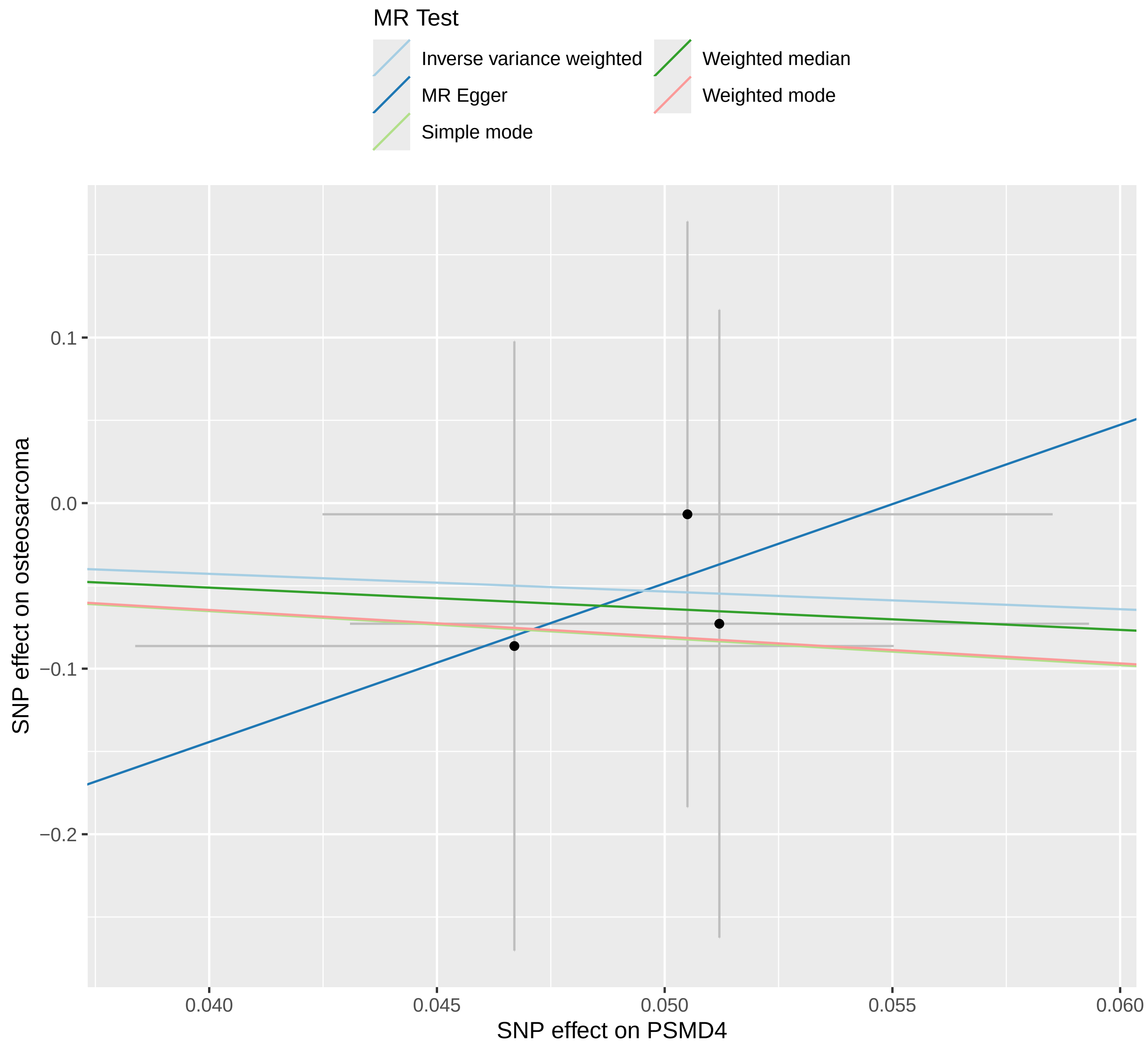

MR Test
Inverse variance weighted
MR Egger
Simple mode
Weighted median
Weighted mode
0.1
0.0
−0.1
−0.2
SNP effect on osteosarcoma
0.040
0.045
0.050
0.055
0.060
SNP effect on PSMD4

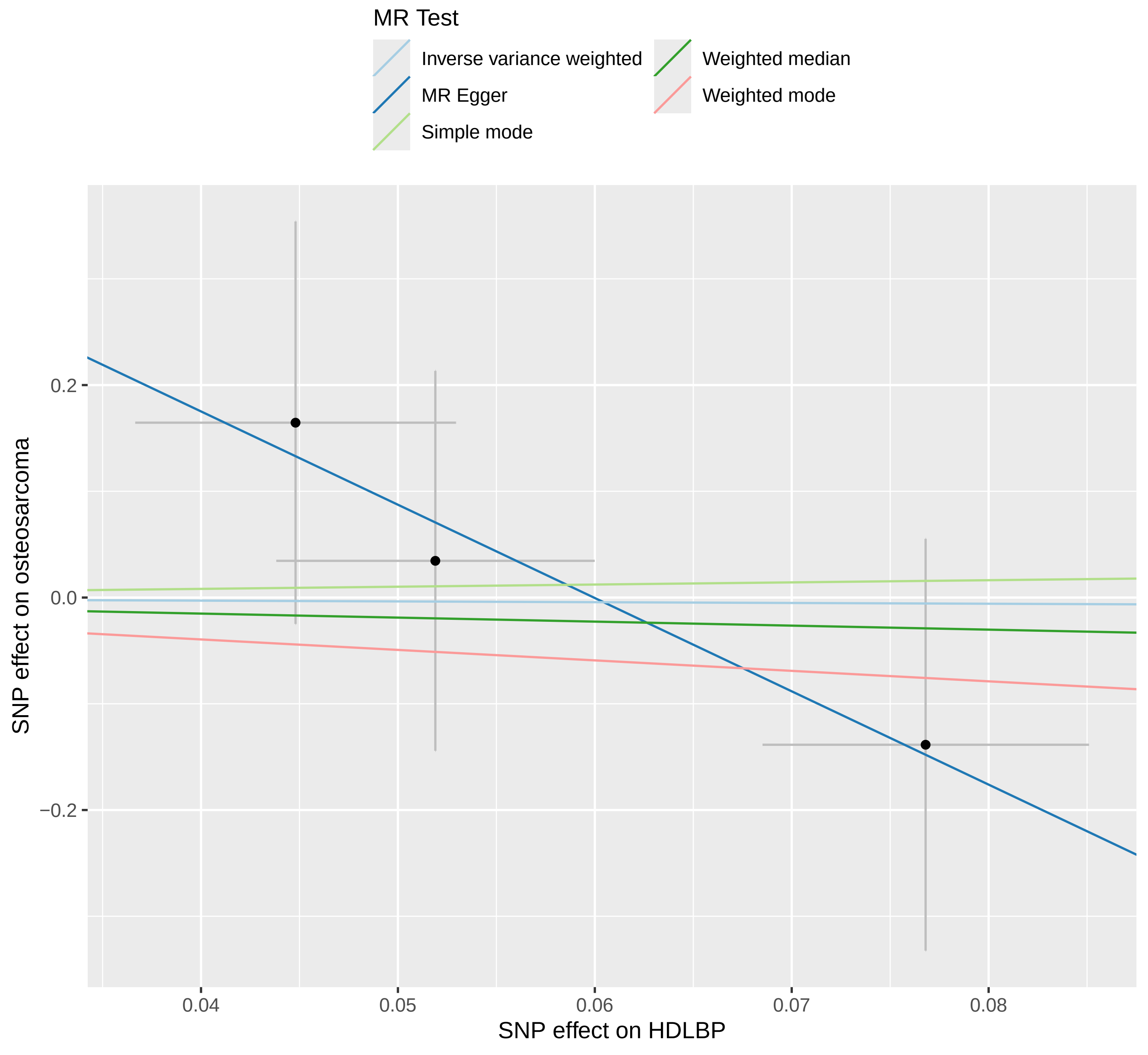
MR Test
Inverse variance weighted
MR Egger
Simple mode
Weighted median
Weighted mode
0.2
0.0
−0.2
SNP effect on osteosarcoma
0.04
0.05
0.06
0.07
0.08
SNP effect on HDLBP

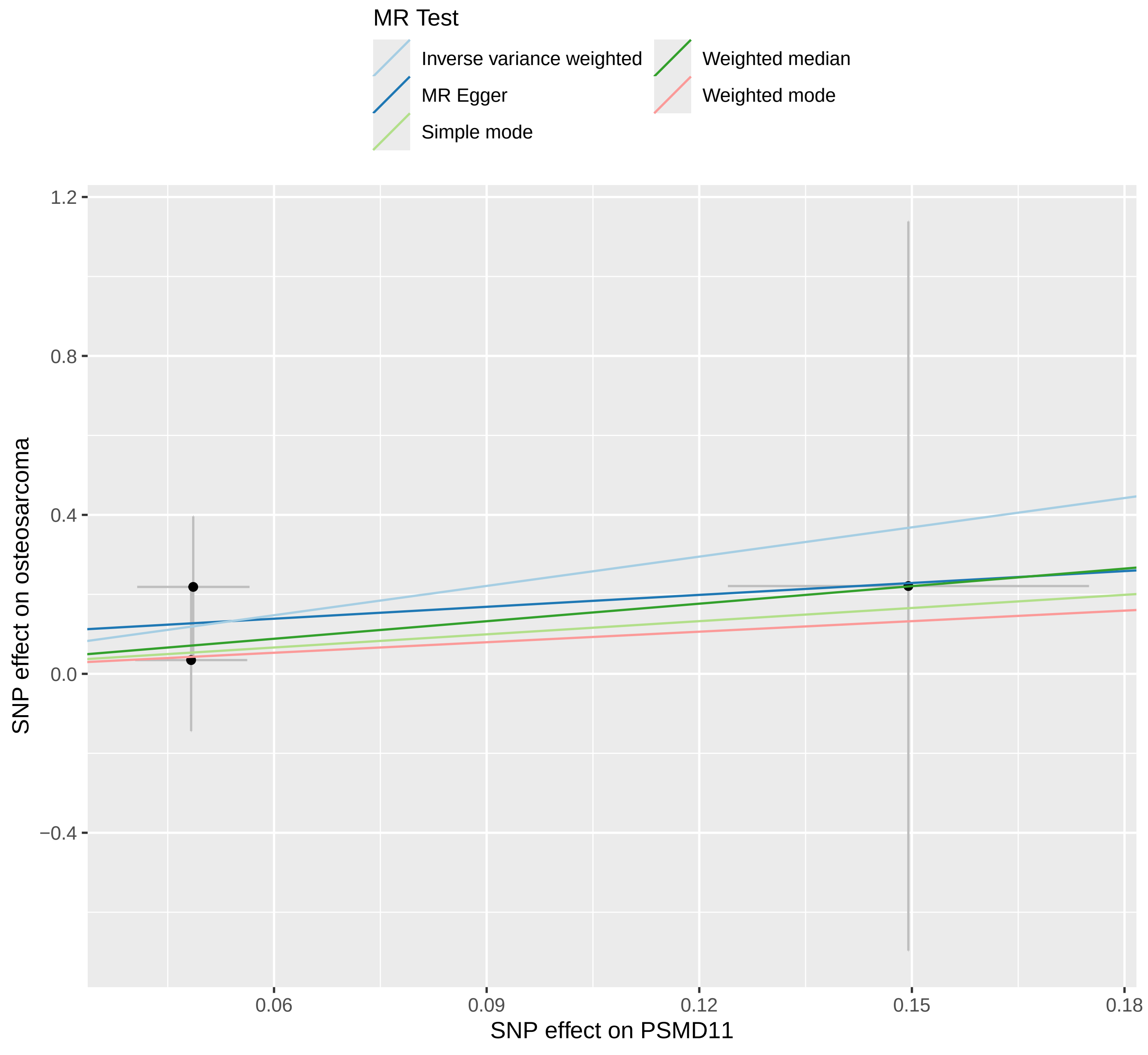

MR Test
Inverse variance weighted
MR Egger
Simple mode
Weighted median
Weighted mode
1.2
0.8
0.4
0.0
−0.4
0.06
0.09
0.12
0.15
0.18
SNP effect on PSMD11
SNP effect on osteosarcoma

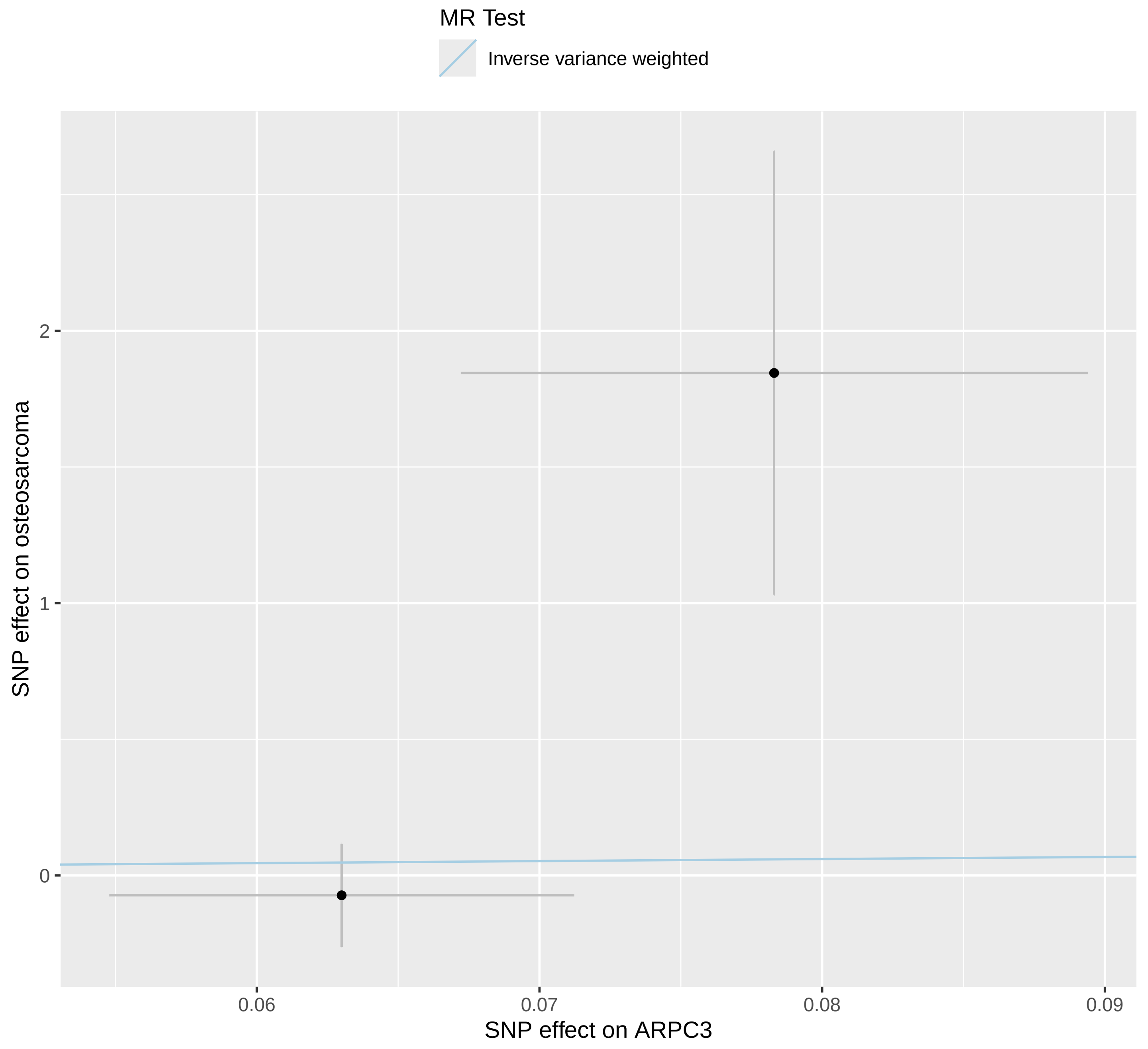

MR Test
Inverse variance weighted
SNP effect on osteosarcoma
SNP effect on ARPC3
0
1
2
0.06
0.07
0.08
0.09

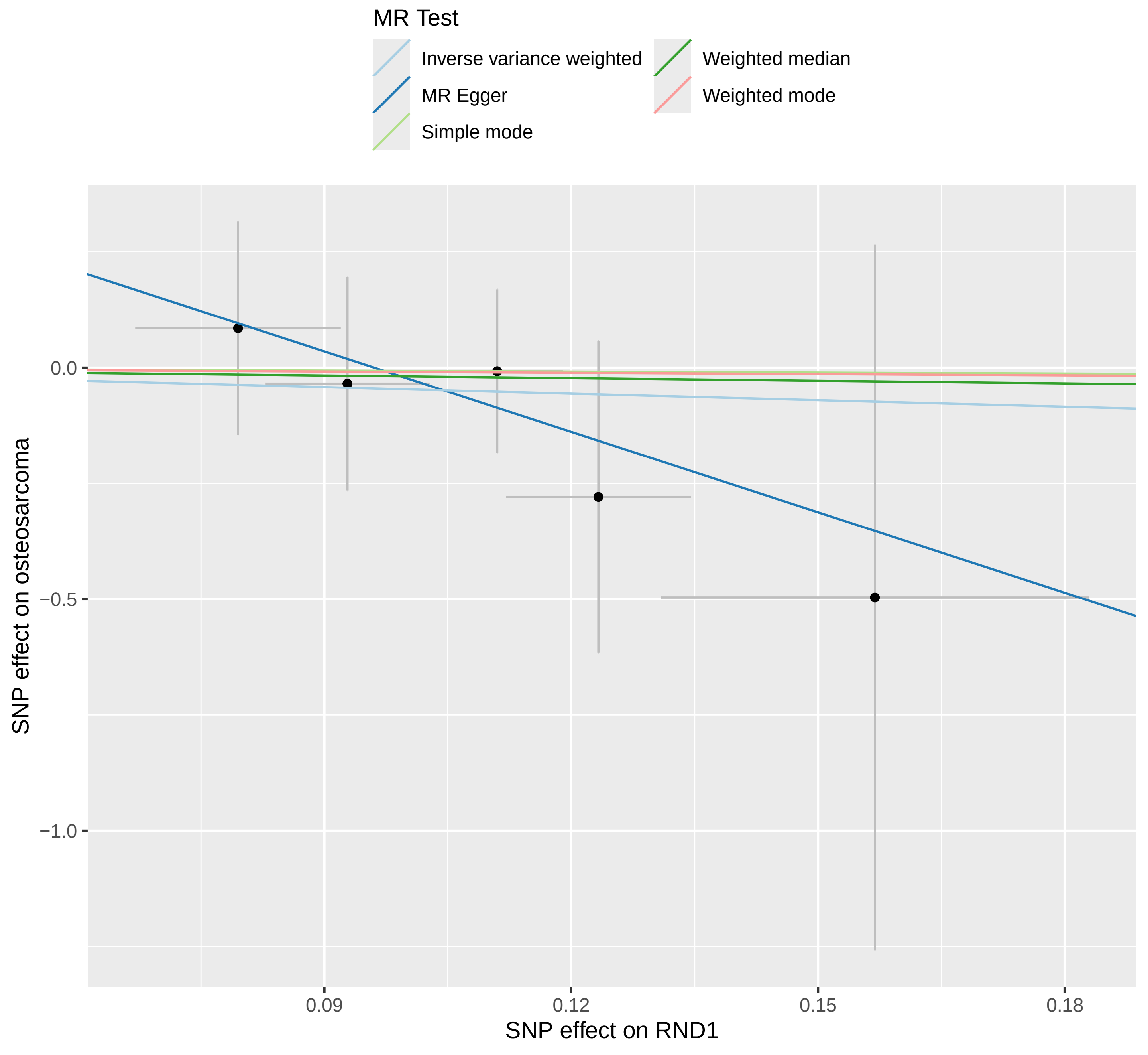
MR Test
Inverse variance weighted
MR Egger
Simple mode
Weighted median
Weighted mode
0.0
−0.5
−1.0
SNP effect on osteosarcoma
0.09
0.12
0.15
0.18
SNP effect on RND1

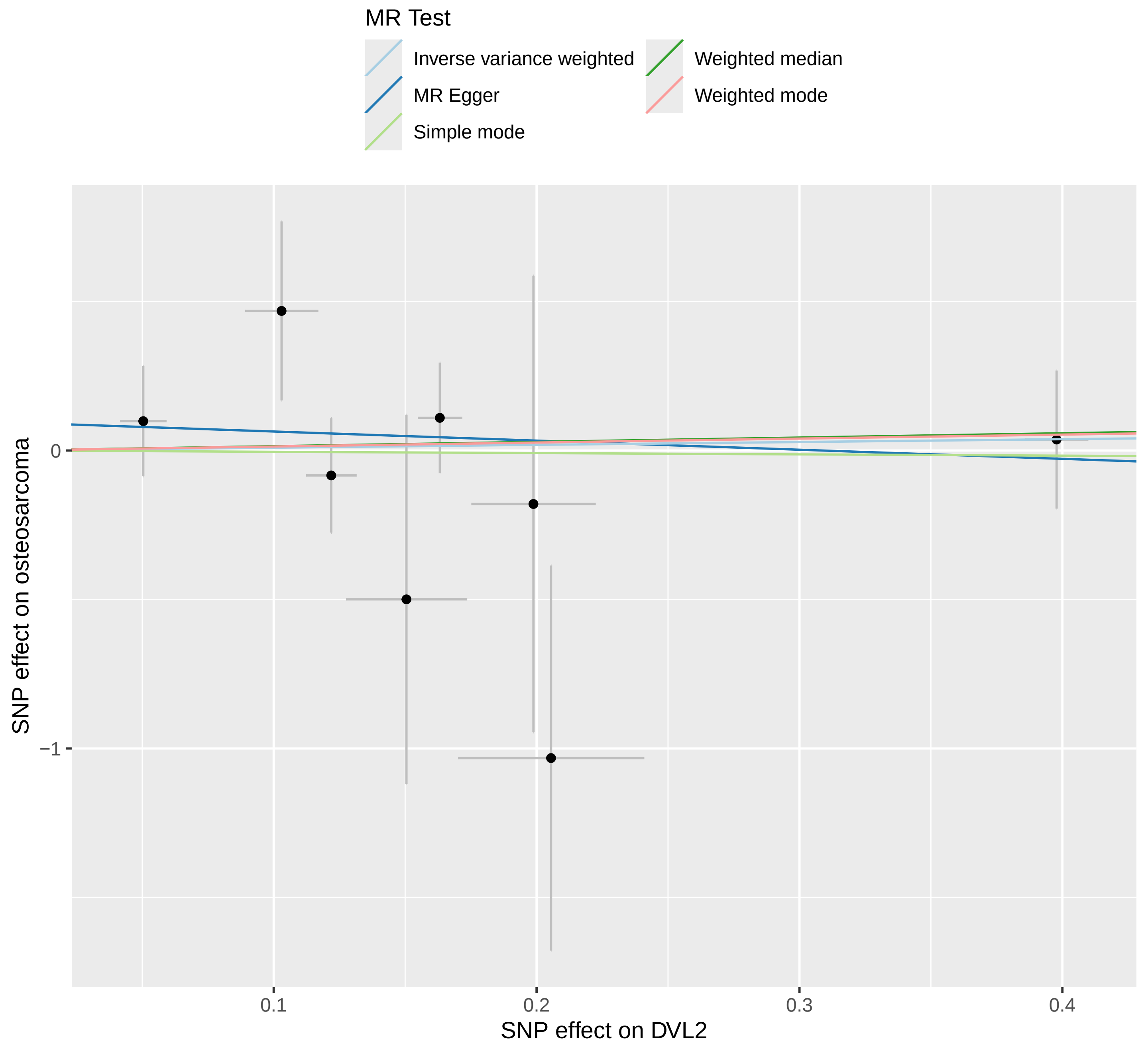
MR Test
Inverse variance weighted
MR Egger
Simple mode
Weighted median
Weighted mode
SNP effect on osteosarcoma
0
−1
0.1
0.2
0.3
0.4
SNP effect on DVL2

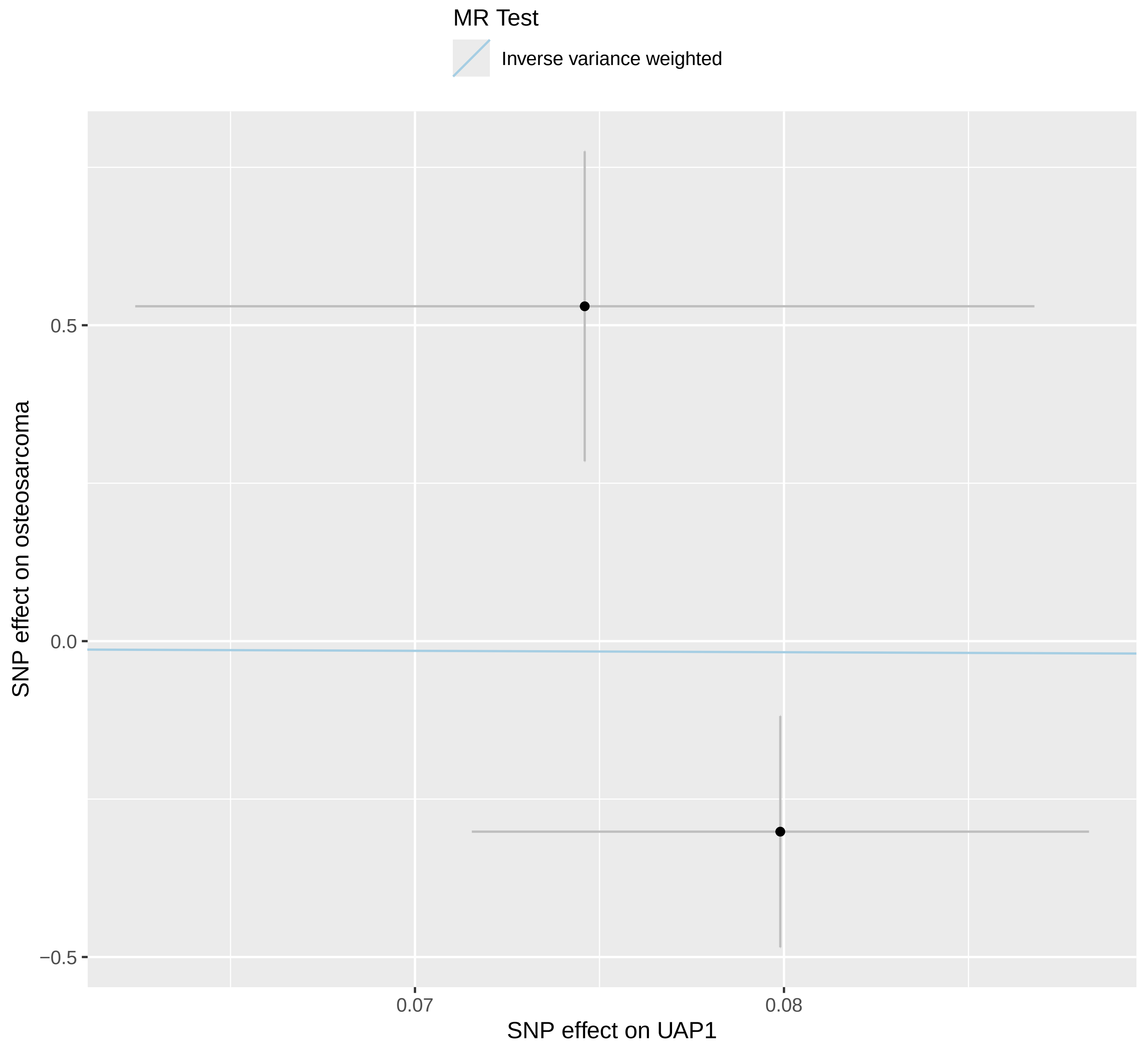
MR Test
Inverse variance weighted
0.5
0.0
−0.5
0.07
0.08
SNP effect on osteosarcoma
SNP effect on UAP1

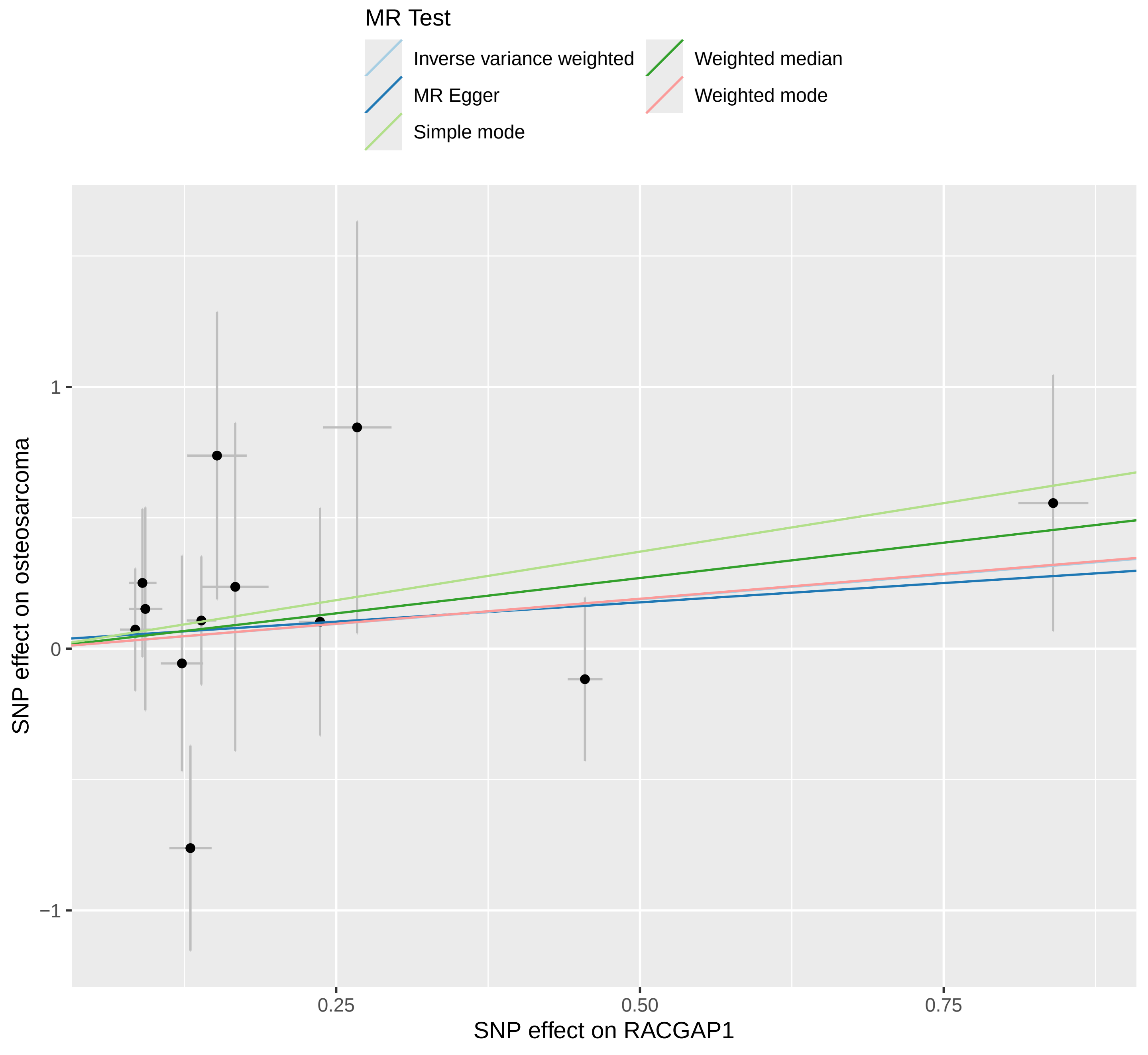

MR Test
Inverse variance weighted
MR Egger
Simple mode
Weighted median
Weighted mode
1
0
−1
SNP effect on osteosarcoma
0.25
0.50
0.75
SNP effect on RACGAP1

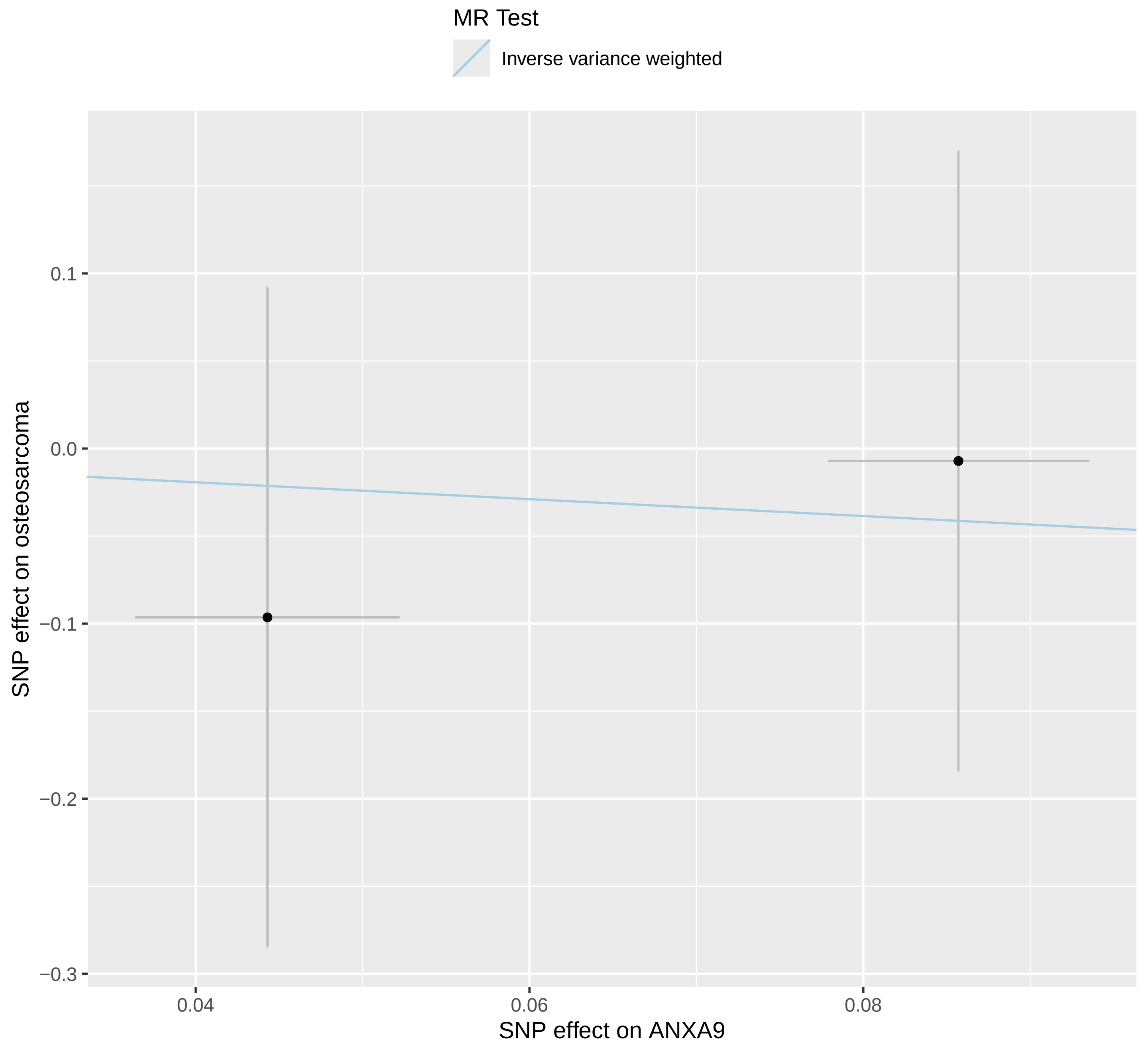
MR Test
Inverse variance weighted
SNP effect on osteosarcoma
0.1
0.0
−0.1
−0.2
−0.3
0.04
0.06
0.08
SNP effect on ANXA9

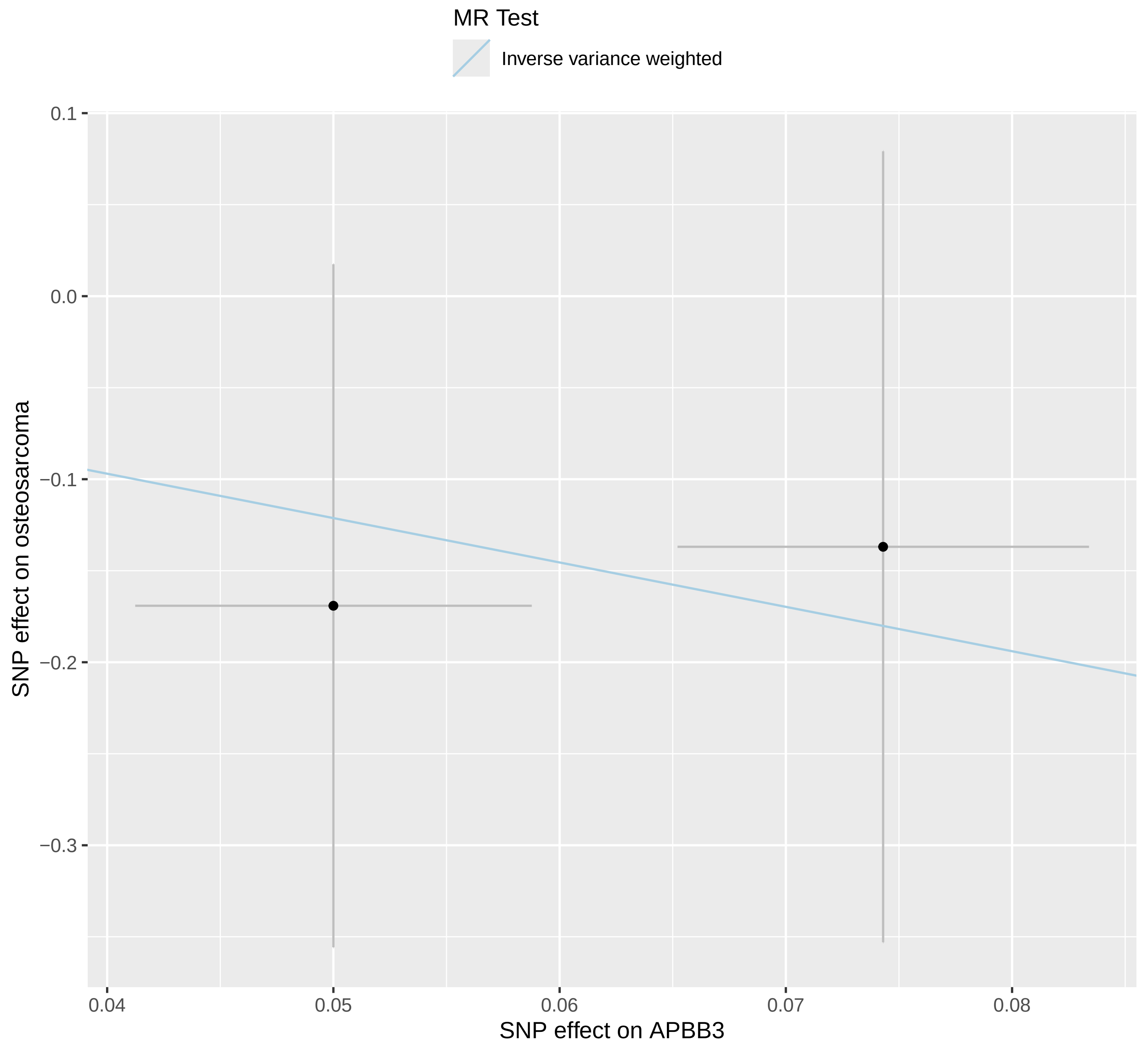
MR Test
Inverse variance weighted
0.1
0.0
−0.1
−0.2
−0.3
0.04
0.05
0.06
0.07
0.08
SNP effect on osteosarcoma
SNP effect on APBB3

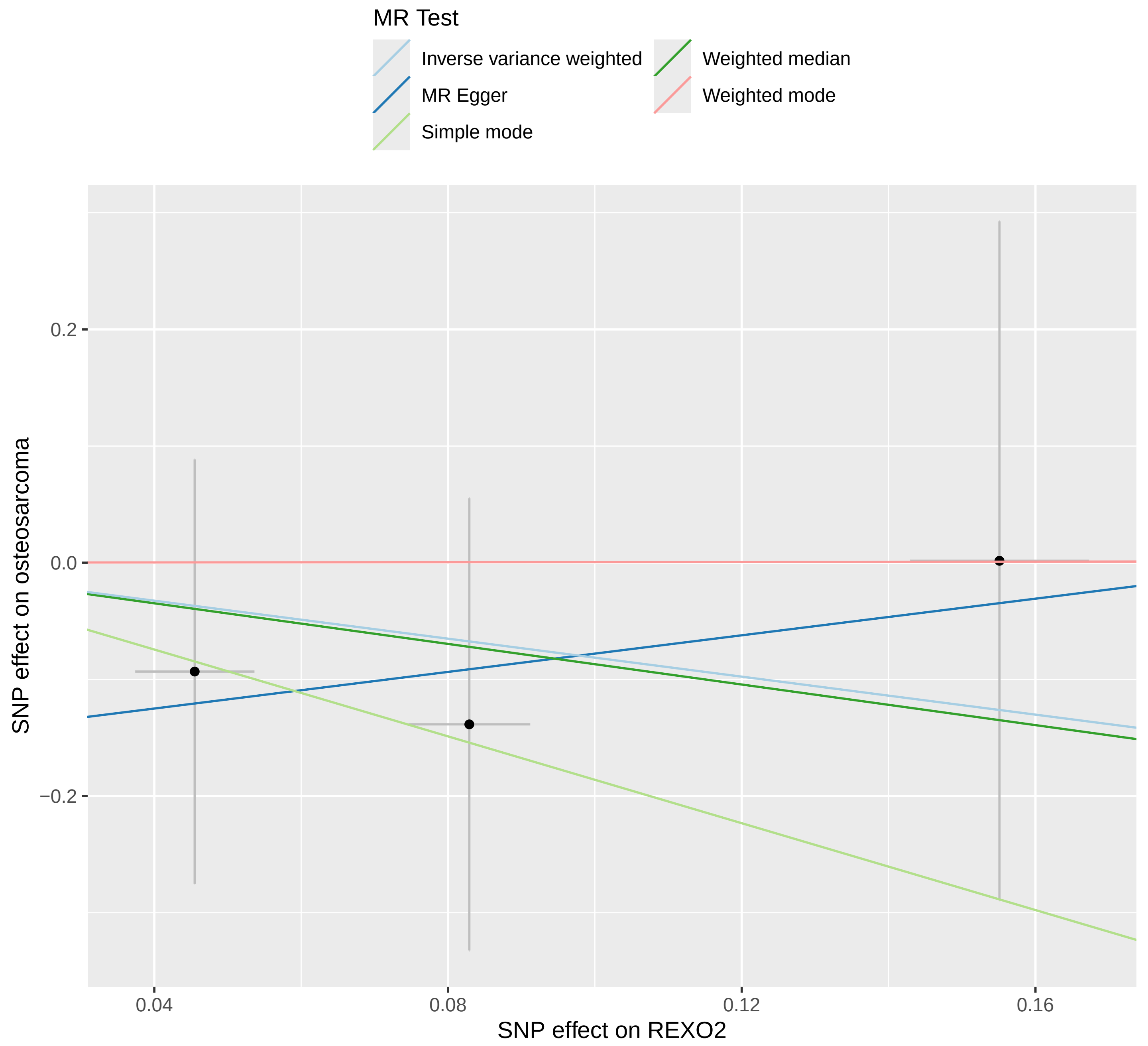

MR Test
Inverse variance weighted
MR Egger
Simple mode
Weighted median
Weighted mode
SNP effect on osteosarcoma
0.2
0.0
−0.2
0.04
0.08
0.12
0.16
SNP effect on REXO2

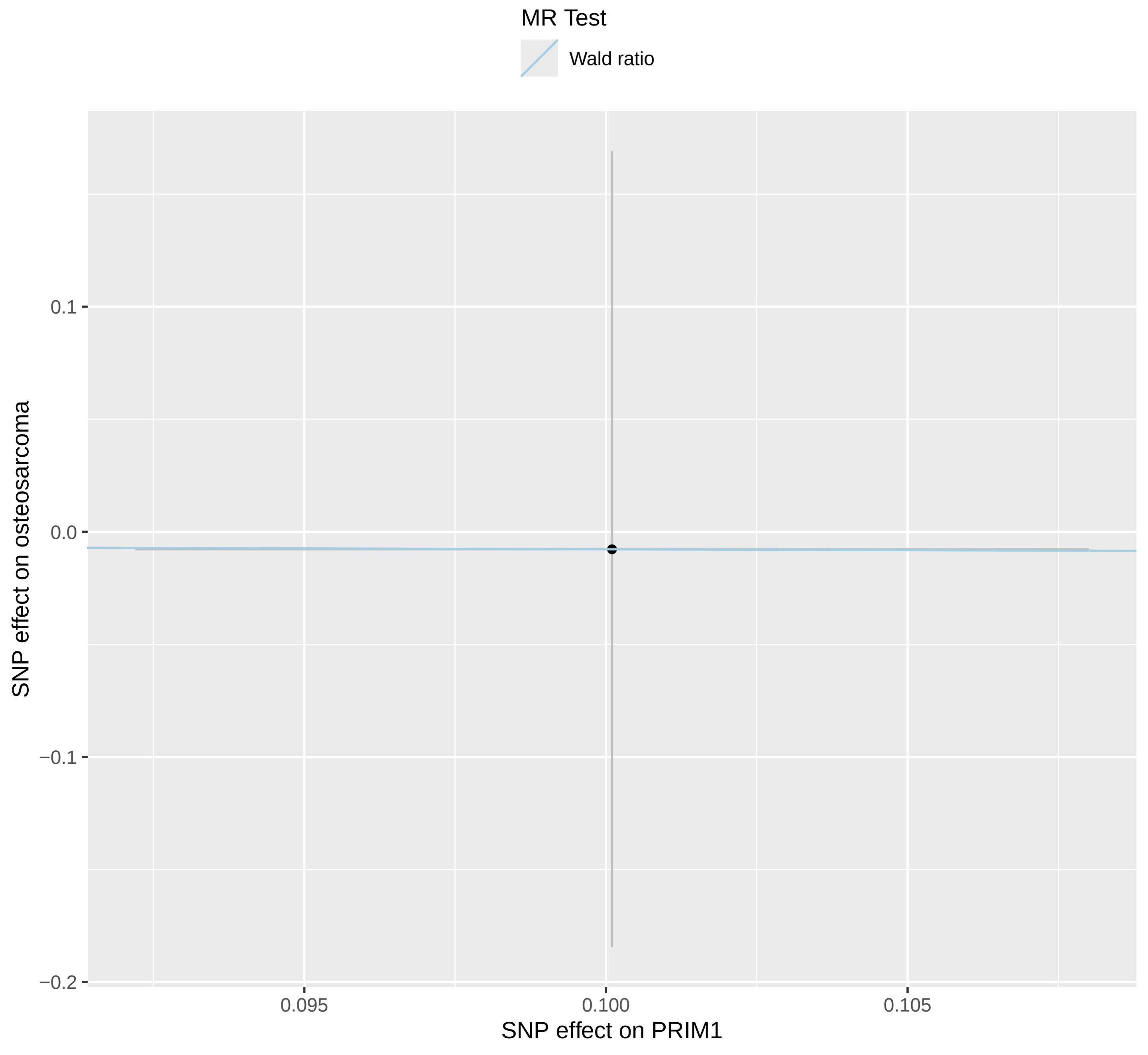

MR Test
Wald ratio
SNP effect on osteosarcoma
0.1
0.0
−0.1
−0.2
0.095
0.100
0.105
SNP effect on PRIM1

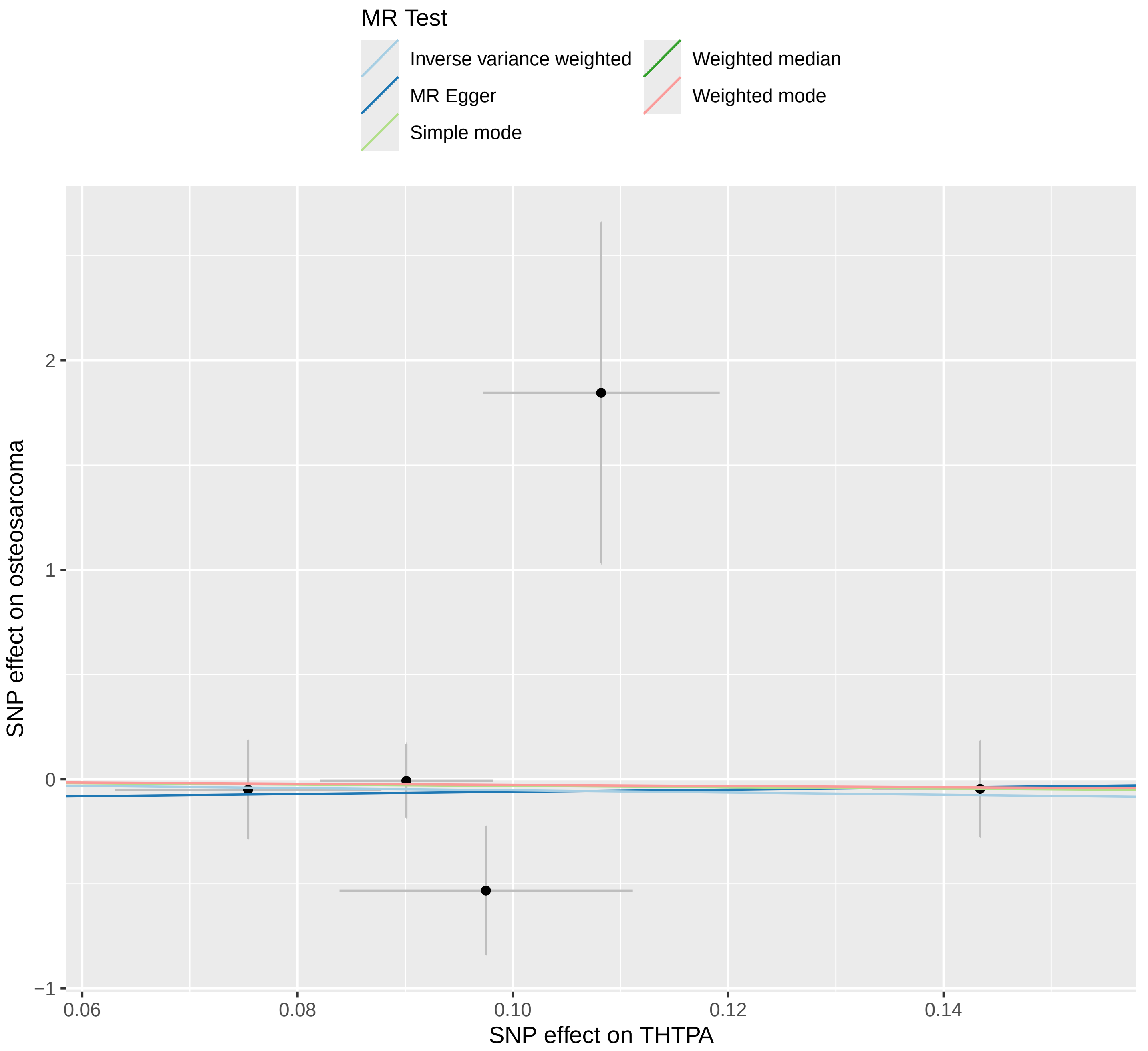
MR Test
Inverse variance weighted
MR Egger
Simple mode
Weighted median
Weighted mode
SNP effect on osteosarcoma
2
1
0
−1
0.06
0.08
0.10
0.12
0.14
SNP effect on THTPA

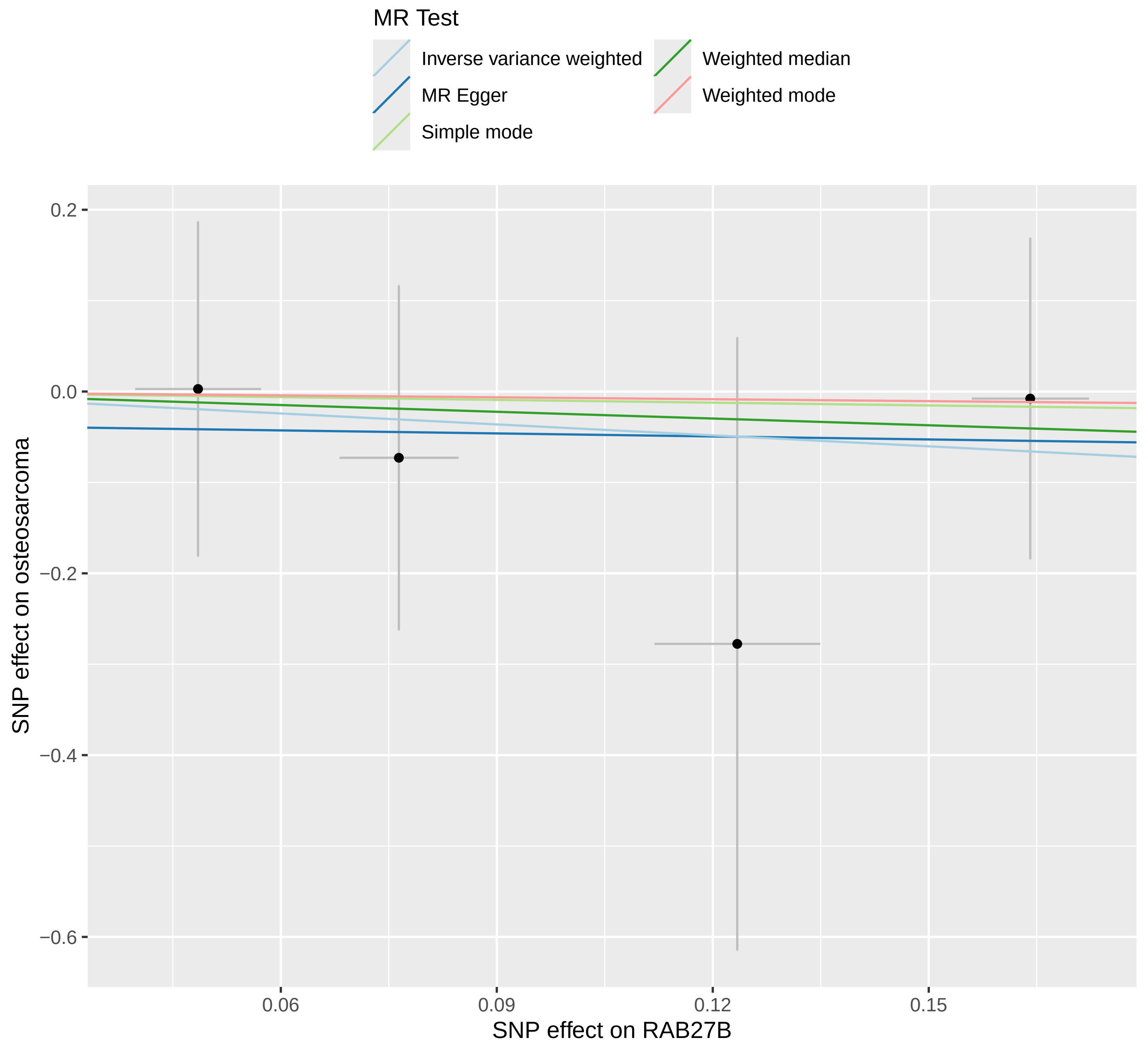
MR Test
Inverse variance weighted
MR Egger
Simple mode
Weighted median
Weighted mode
0.2
0.0
−0.2
−0.4
−0.6
SNP effect on osteosarcoma
0.06
0.09
0.12
0.15
SNP effect on RAB27B

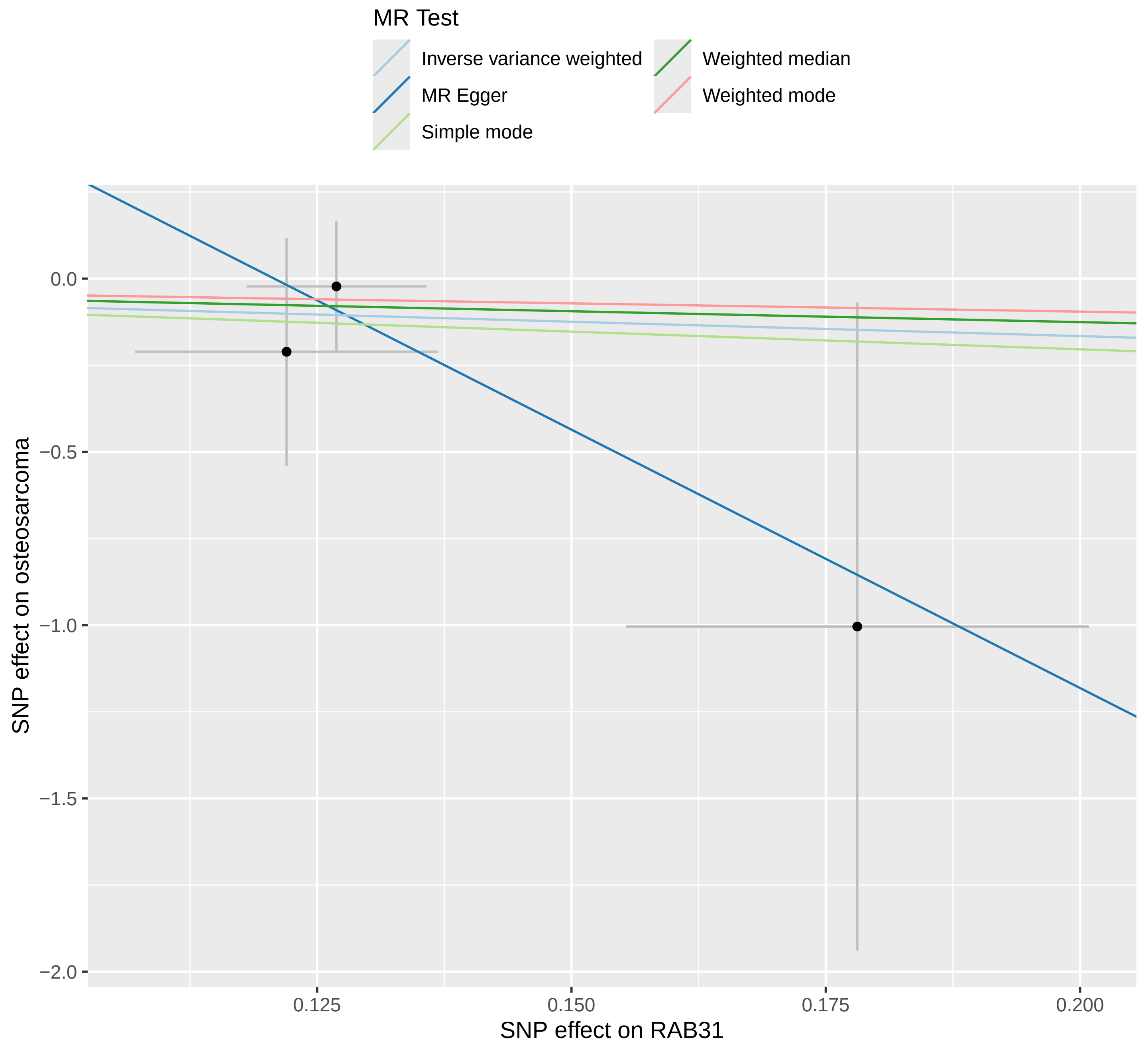
MR Test
Inverse variance weighted
MR Egger
Simple mode
Weighted median
Weighted mode
0.0
−0.5
−1.0
−1.5
−2.0
SNP effect on osteosarcoma
0.125
0.150
0.175
0.200
SNP effect on RAB31

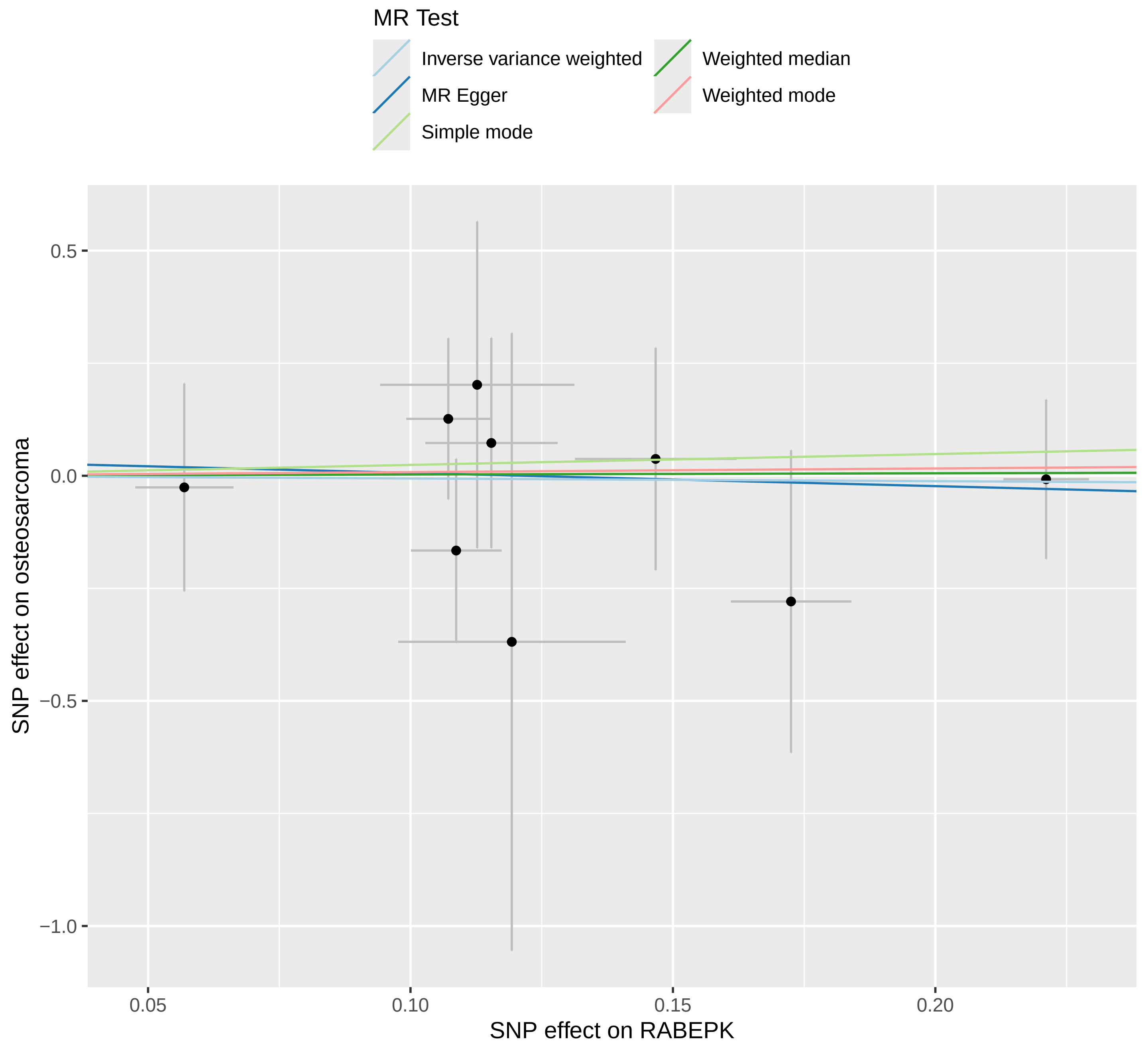

MR Test
Inverse variance weighted
MR Egger
Simple mode
Weighted median
Weighted mode
0.5
0.0
−0.5
−1.0
0.05
0.10
0.15
0.20
SNP effect on osteosarcoma
SNP effect on RABEPK

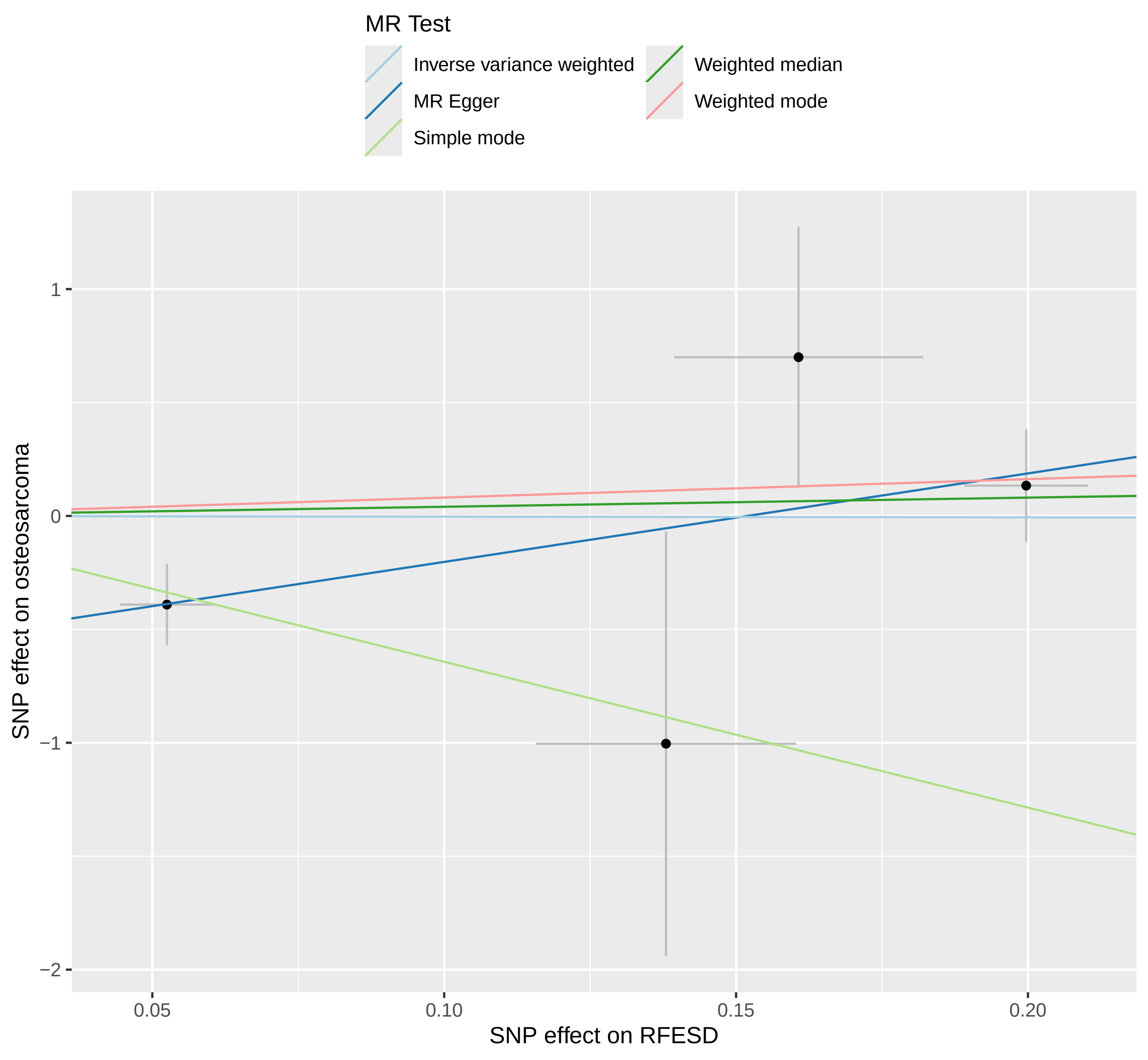
MR Test
Inverse variance weighted
MR Egger
Simple mode
Weighted median
Weighted mode
SNP effect on osteosarcoma
1
0
−1
−2
0.05
0.10
0.15
0.20
SNP effect on RFESD

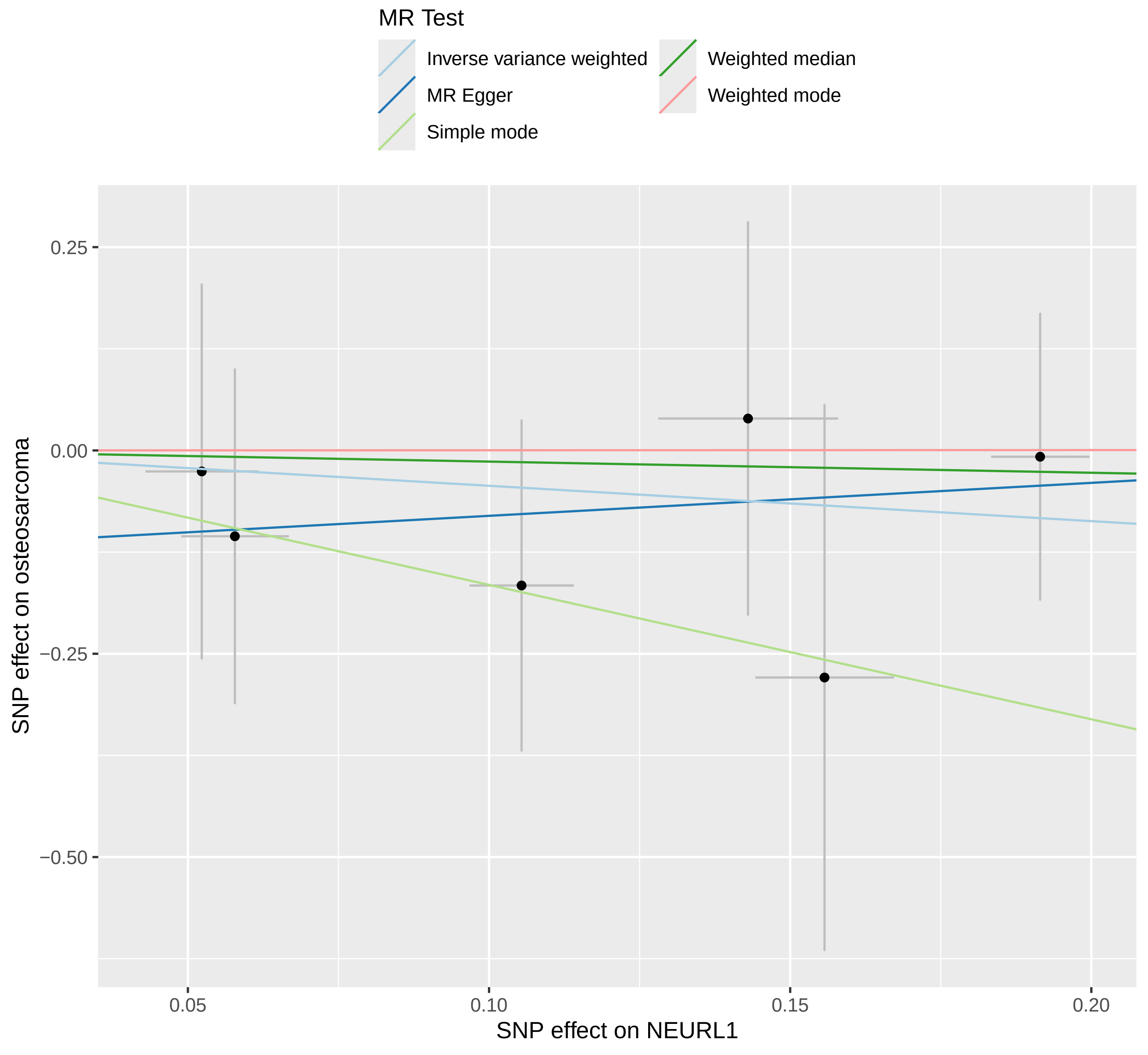

MR Test
Inverse variance weighted
MR Egger
Simple mode
Weighted median
Weighted mode
0.25
0.00
−0.25
−0.50
SNP effect on osteosarcoma
0.05
0.10
0.15
0.20
SNP effect on NEURL1

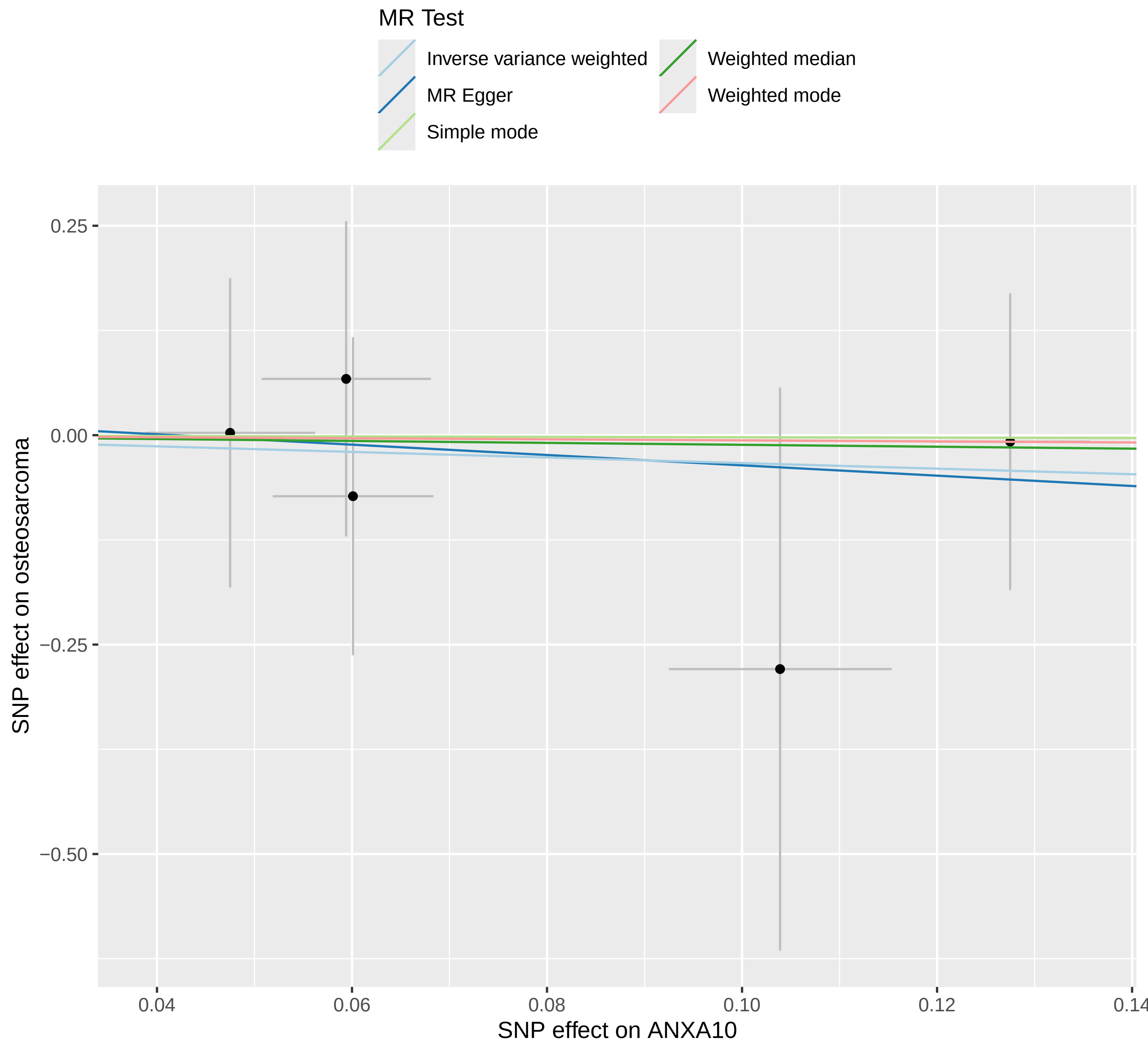

MR Test
Inverse variance weighted
MR Egger
Simple mode
Weighted median
Weighted mode
0.25
0.00
−0.25
−0.50
0.04
0.06
0.08
0.10
0.12
0.14
SNP effect on osteosarcoma
SNP effect on ANXA10

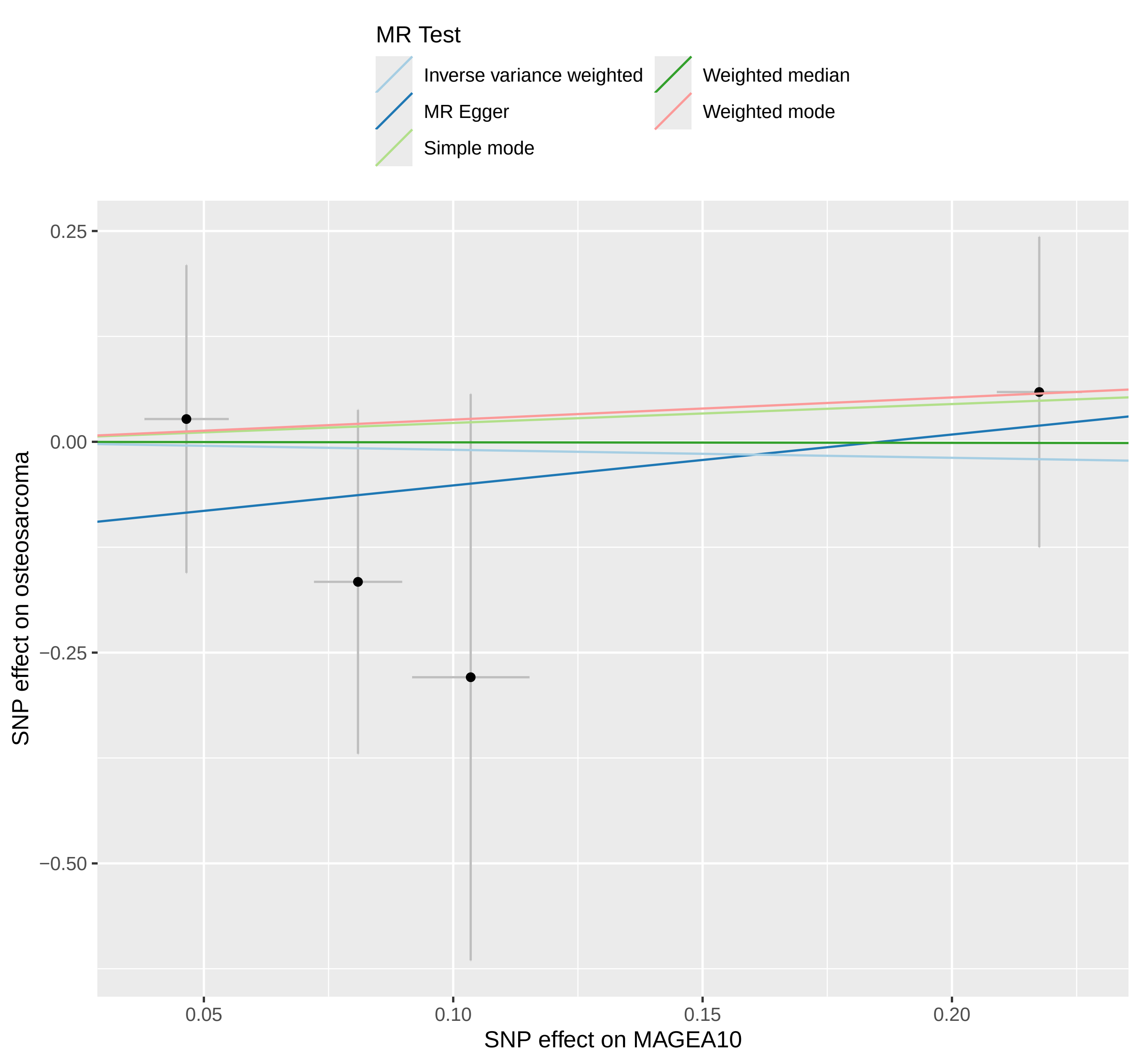

MR Test
Inverse variance weighted
MR Egger
Simple mode
Weighted median
Weighted mode
0.25
0.00
−0.25
−0.50
SNP effect on osteosarcoma
0.05
0.10
0.15
0.20
SNP effect on MAGEA10

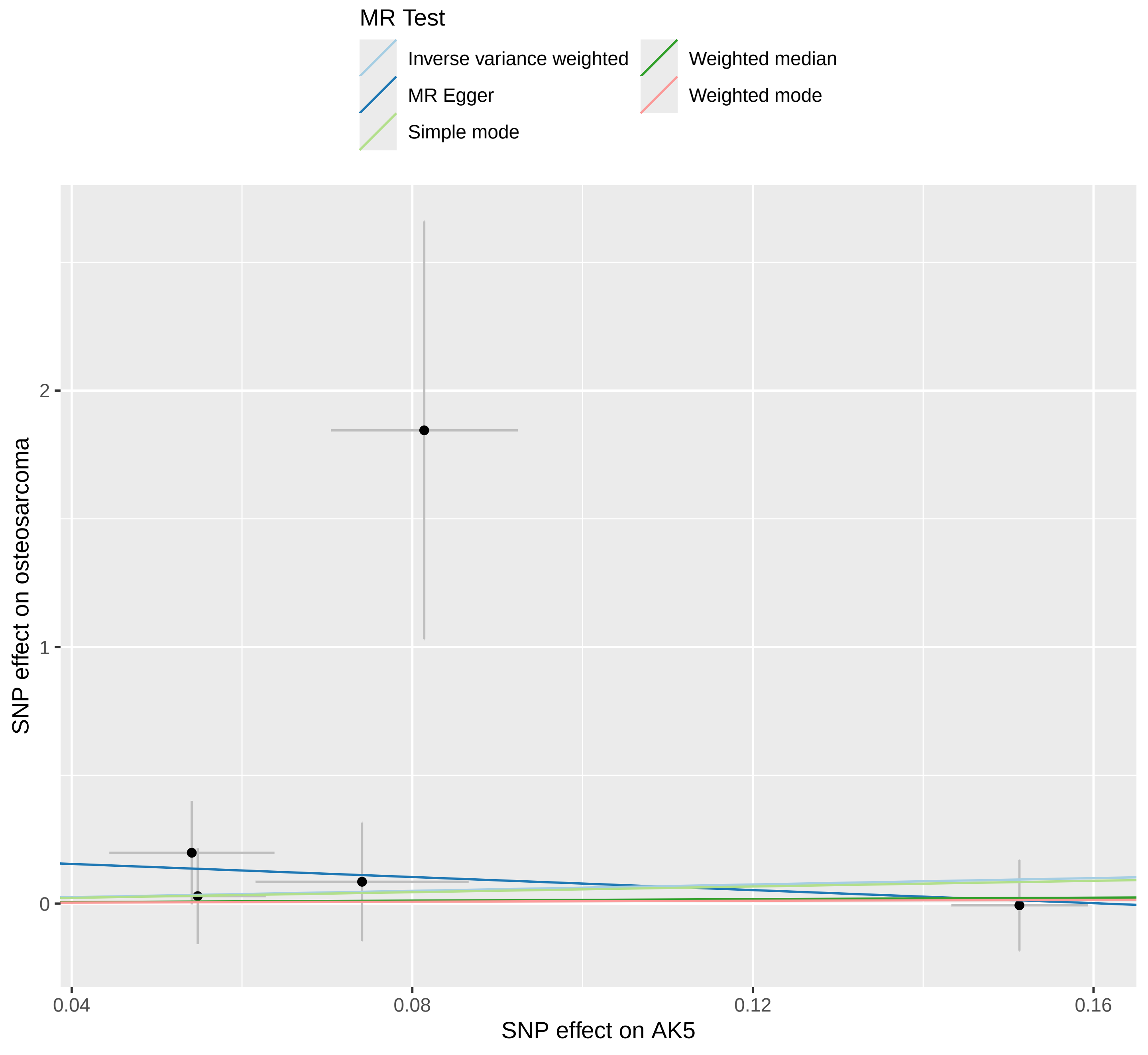
MR Test
Inverse variance weighted
MR Egger
Simple mode
Weighted median
Weighted mode
SNP effect on osteosarcoma
2
1
0
0.04
0.08
0.12
0.16
SNP effect on AK5

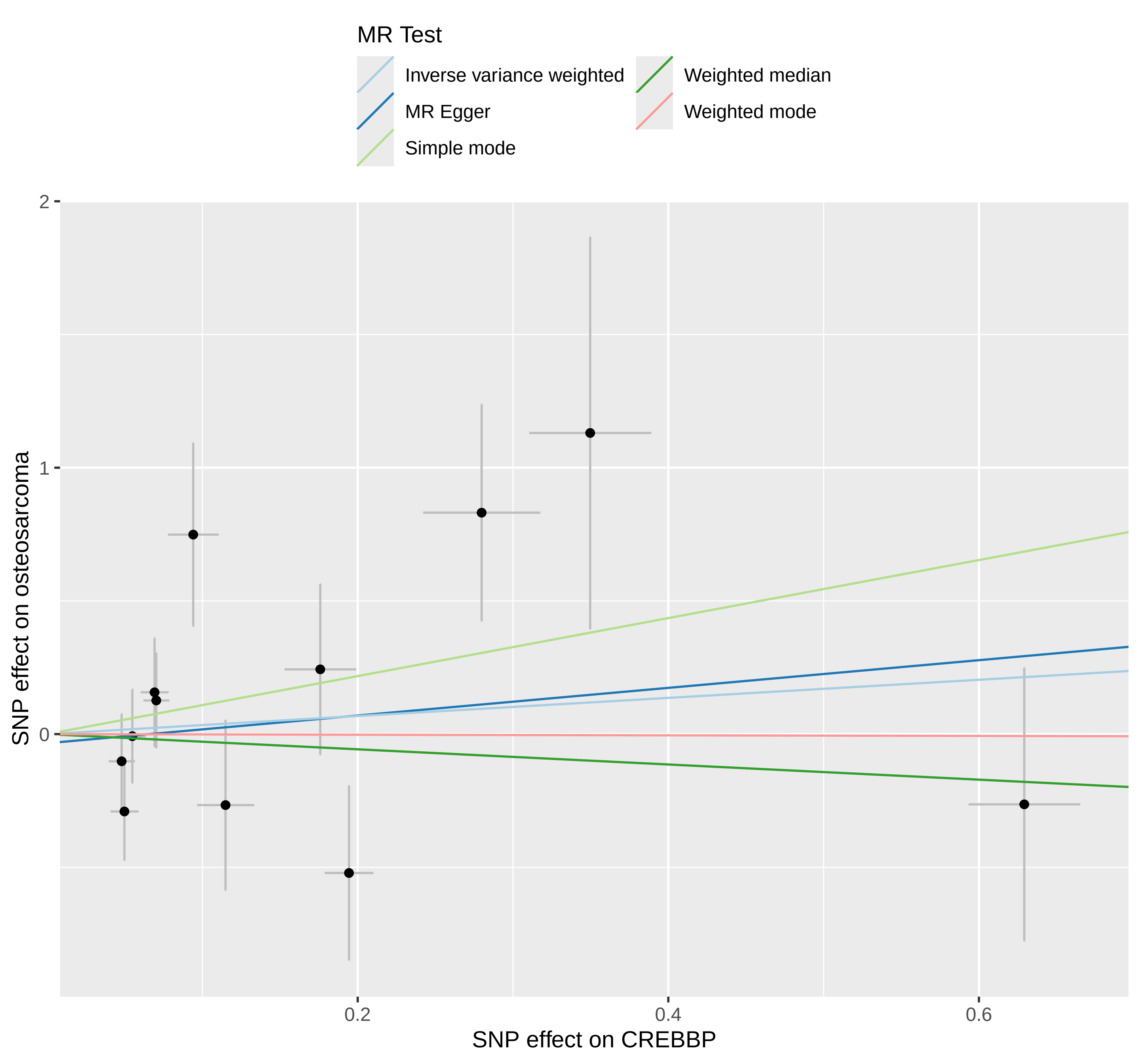
MR Test
Inverse variance weighted
MR Egger
Simple mode
Weighted median
Weighted mode
2
1
0
0.2
0.4
0.6
SNP effect on osteosarcoma
SNP effect on CREBBP

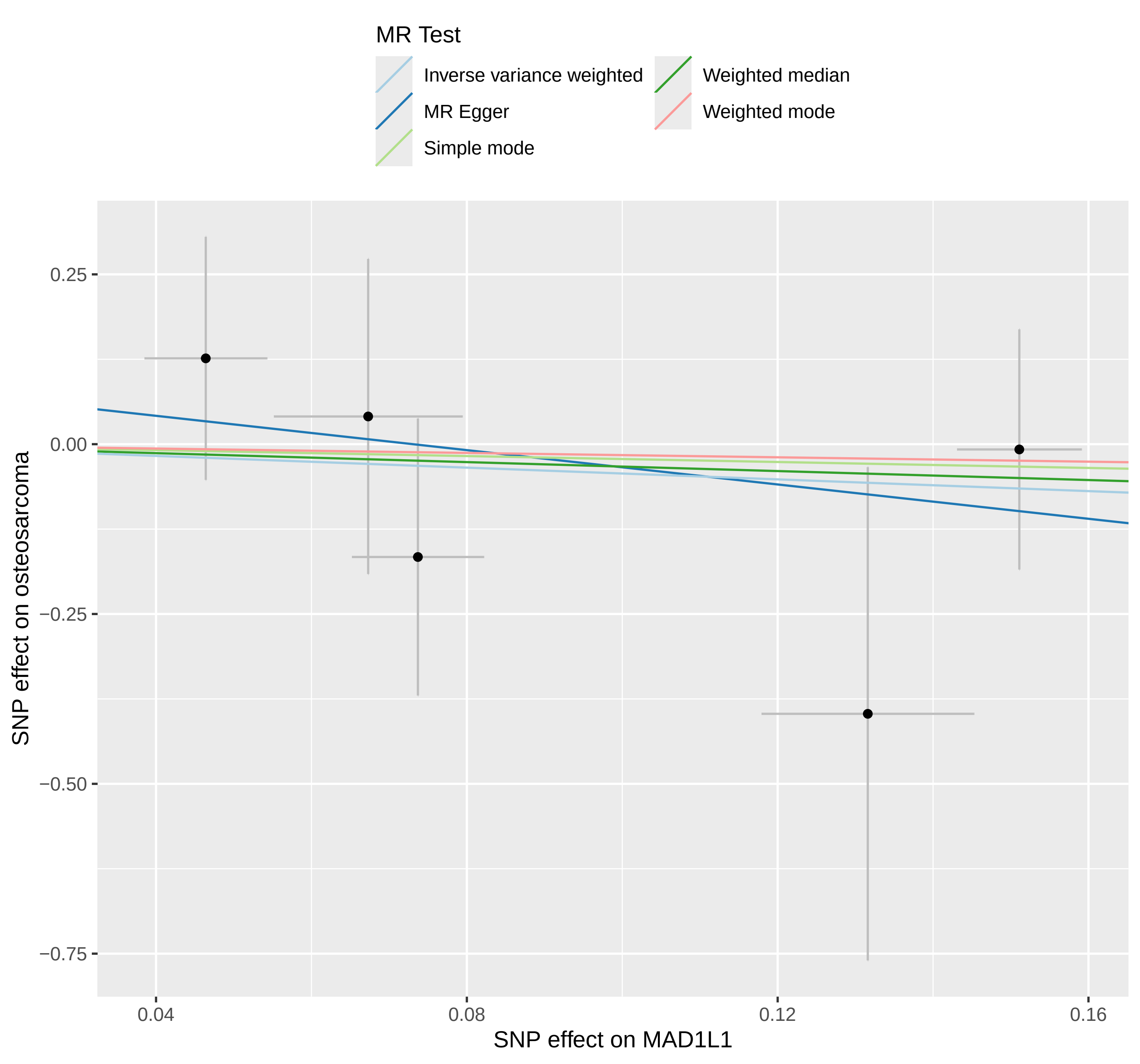
MR Test
Inverse variance weighted
MR Egger
Simple mode
Weighted median
Weighted mode
0.25
0.00
−0.25
−0.50
−0.75
SNP effect on osteosarcoma
0.04
0.08
0.12
0.16
SNP effect on MAD1L1

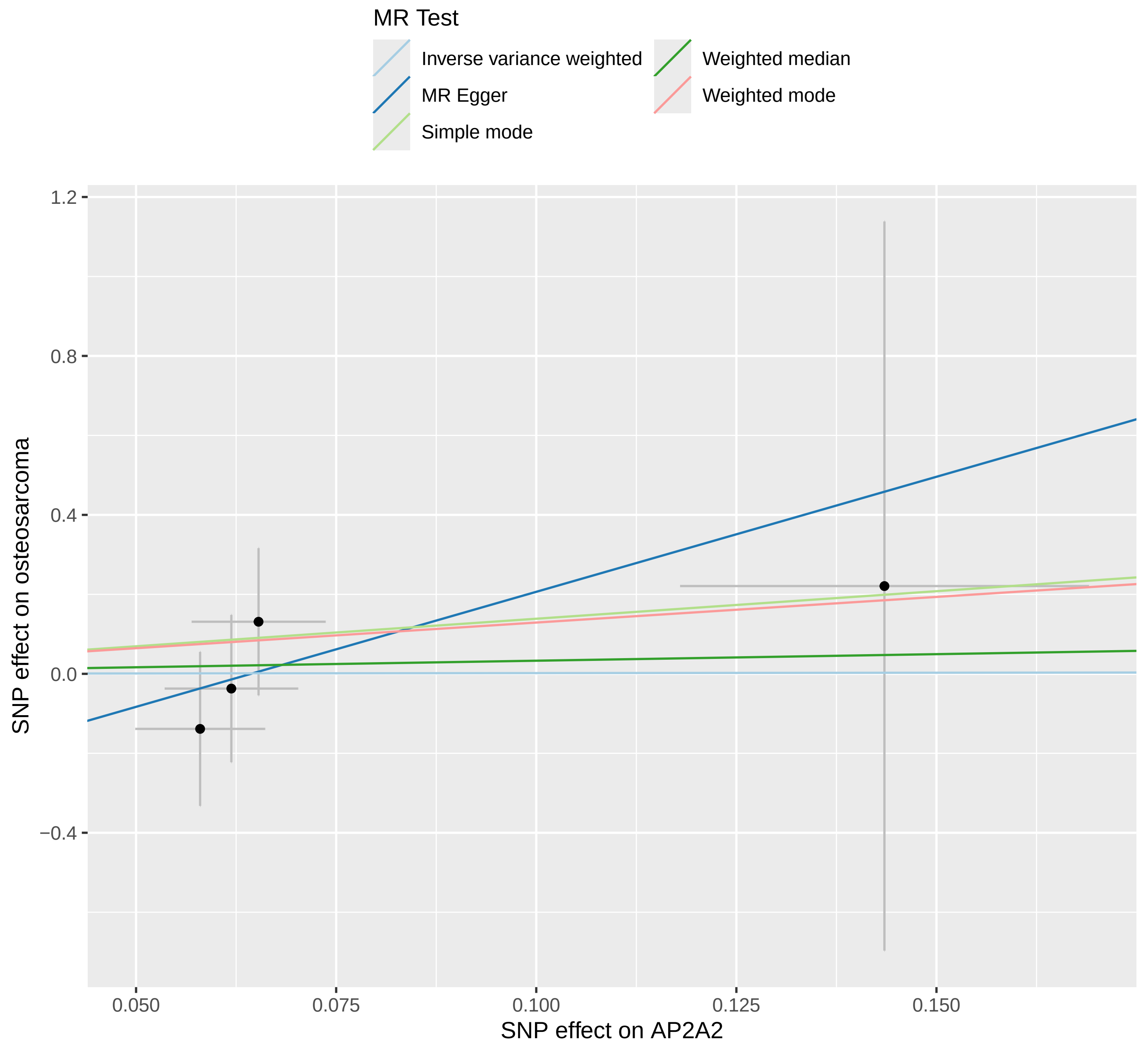

MR Test
Inverse variance weighted
MR Egger
Simple mode
Weighted median
Weighted mode
1.2
0.8
0.4
0.0
−0.4
SNP effect on osteosarcoma
0.050
0.075
0.100
0.125
0.150
SNP effect on AP2A2

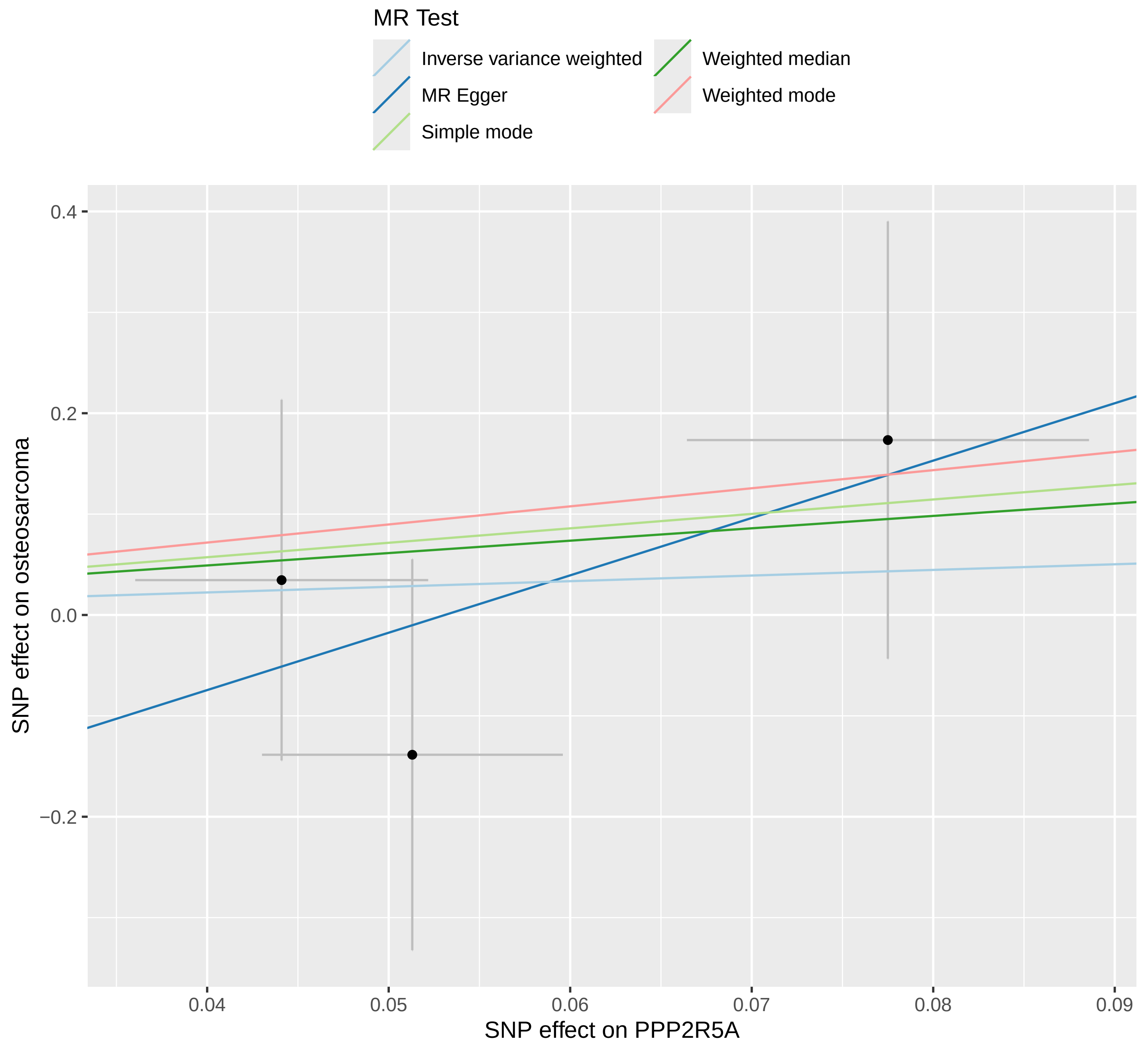
MR Test
Inverse variance weighted
MR Egger
Simple mode
Weighted median
Weighted mode
0.4
0.2
0.0
−0.2
SNP effect on osteosarcoma
0.04
0.05
0.06
0.07
0.08
0.09
SNP effect on PPP2R5A

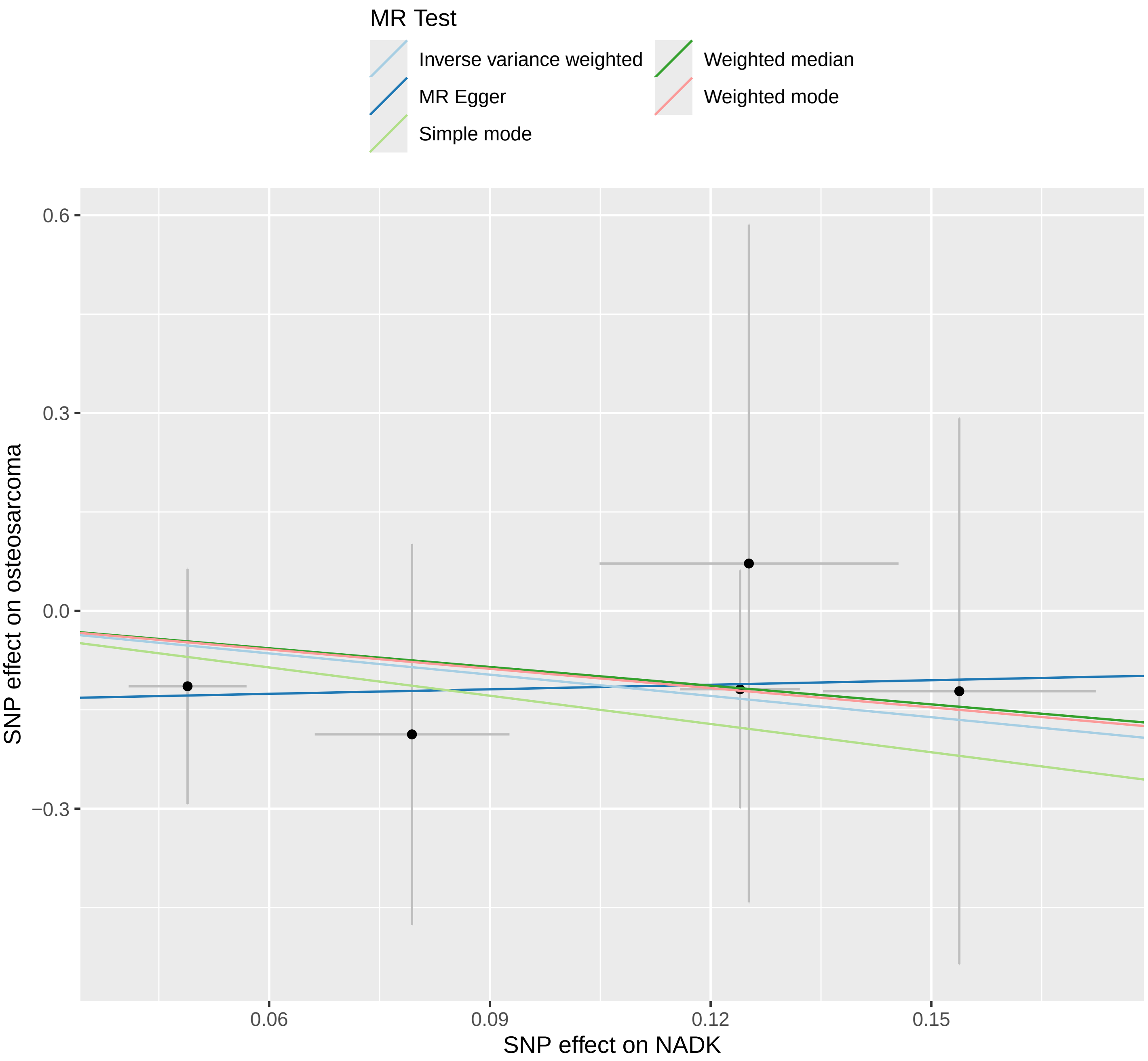
MR Test
Inverse variance weighted
MR Egger
Simple mode
Weighted median
Weighted mode
0.6
0.3
0.0
−0.3
SNP effect on osteosarcoma
0.06
0.09
0.12
0.15
SNP effect on NADK

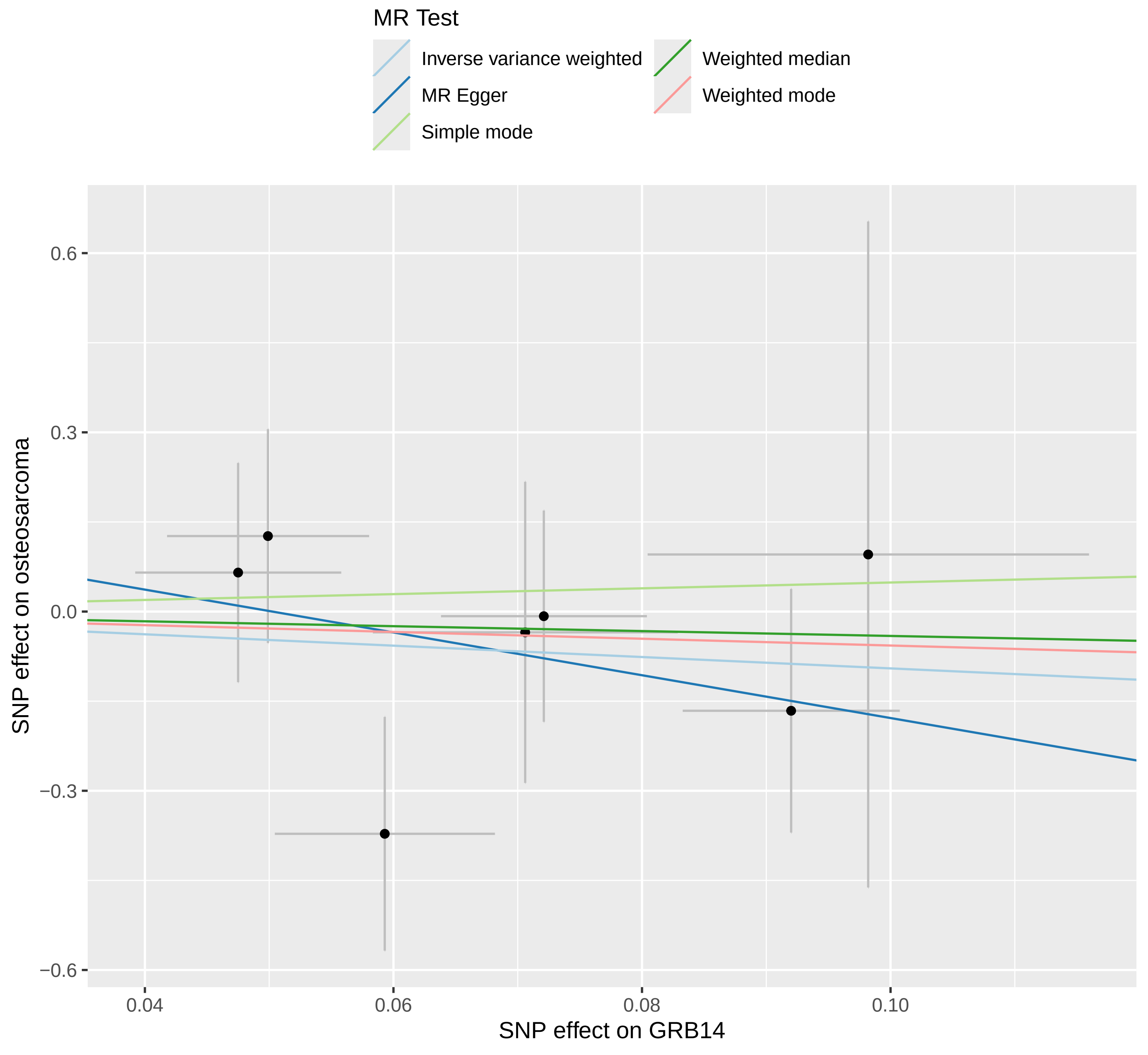
MR Test
Inverse variance weighted
MR Egger
Simple mode
Weighted median
Weighted mode
0.6
0.3
0.0
−0.3
−0.6
0.04
0.06
0.08
0.10
SNP effect on GRB14
SNP effect on osteosarcoma

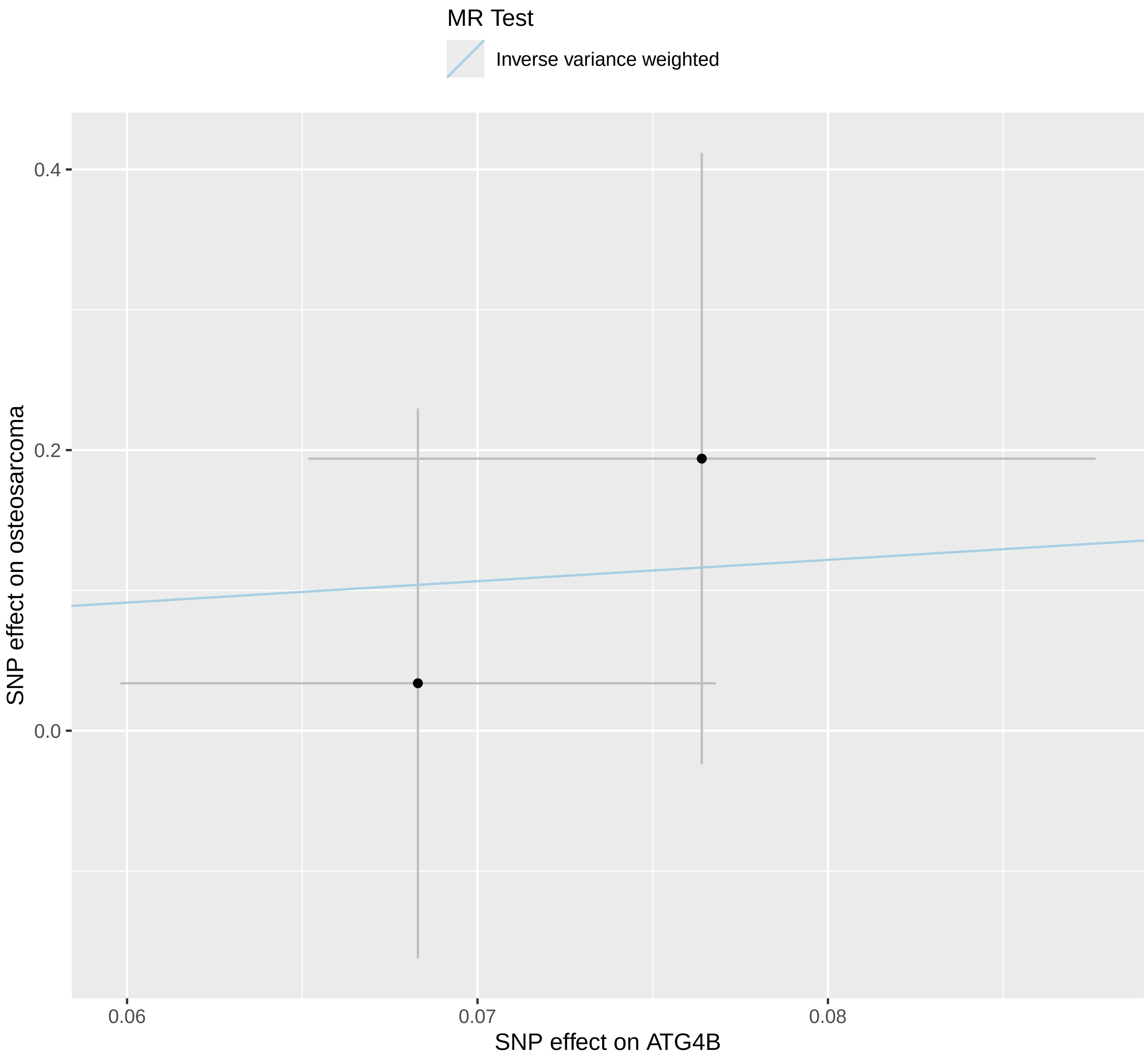
MR Test
Inverse variance weighted
0.4
0.2
0.0
SNP effect on osteosarcoma
0.06
0.07
0.08
SNP effect on ATG4B

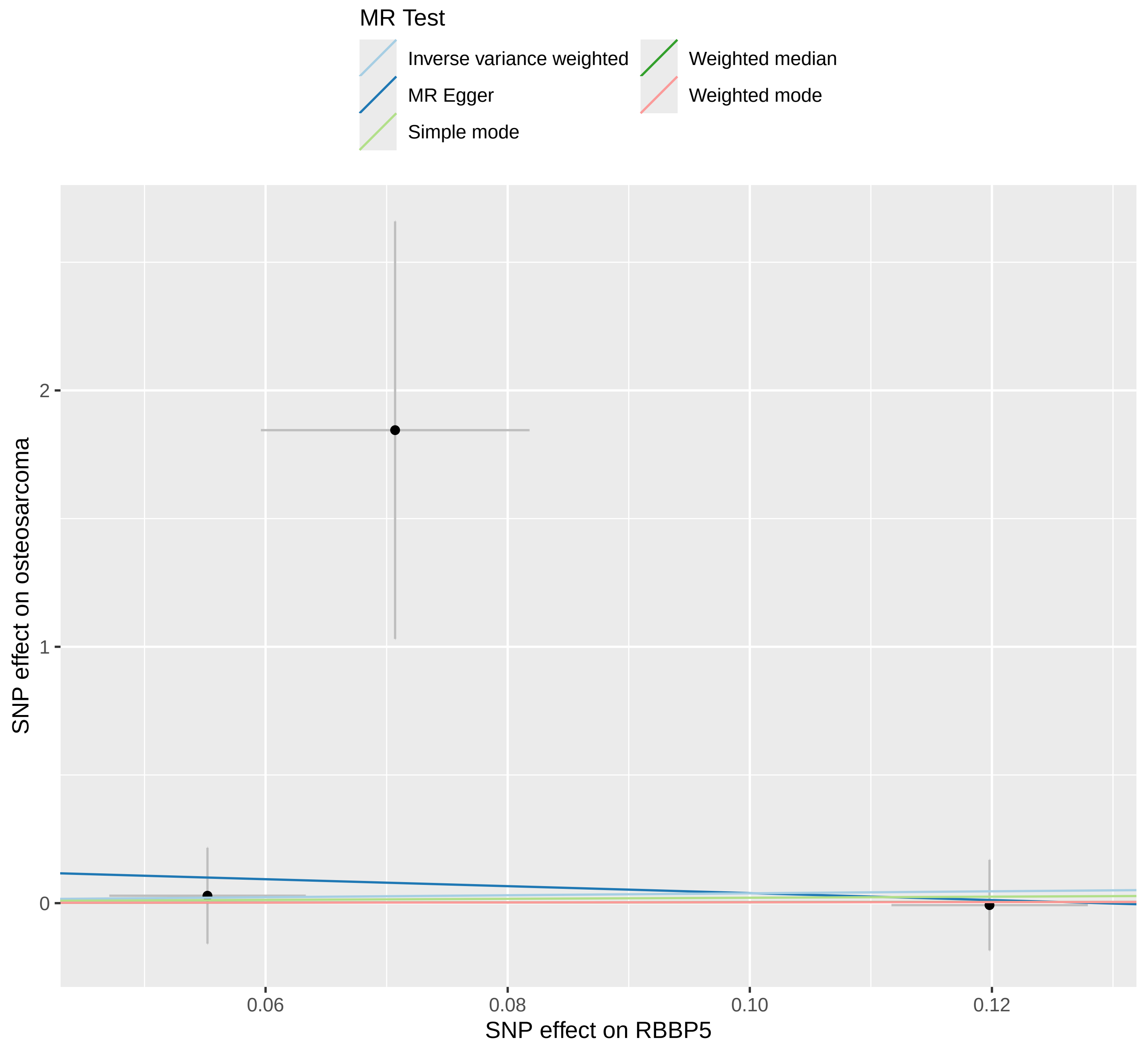

MR Test
Inverse variance weighted
MR Egger
Simple mode
Weighted median
Weighted mode
SNP effect on osteosarcoma
2
1
0
0.06
0.08
0.10
0.12
SNP effect on RBBP5

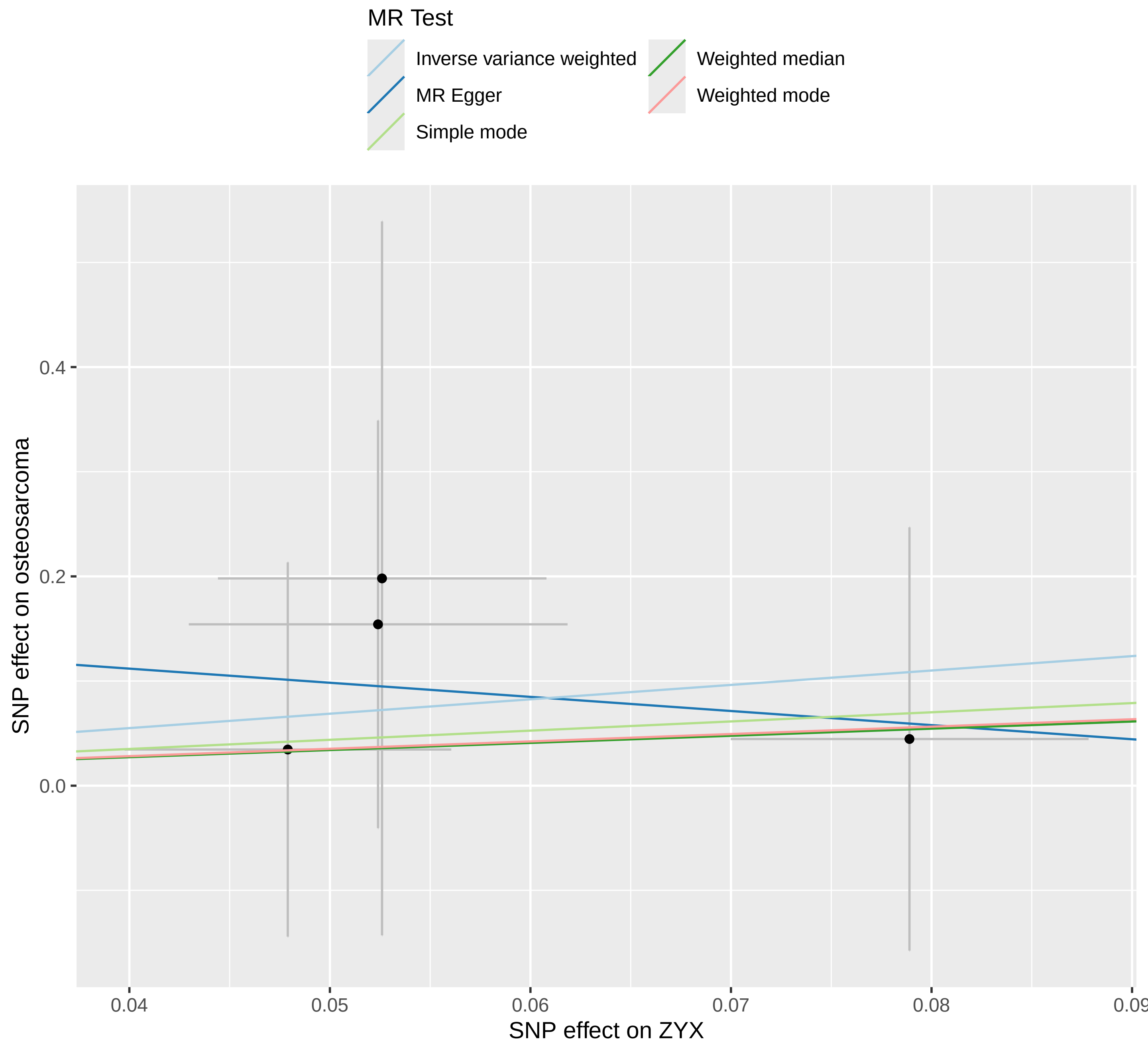

MR Test
Inverse variance weighted
MR Egger
Simple mode
Weighted median
Weighted mode
0.4
0.2
0.0
SNP effect on osteosarcoma
0.04
0.05
0.06
0.07
0.08
0.09
SNP effect on ZYX

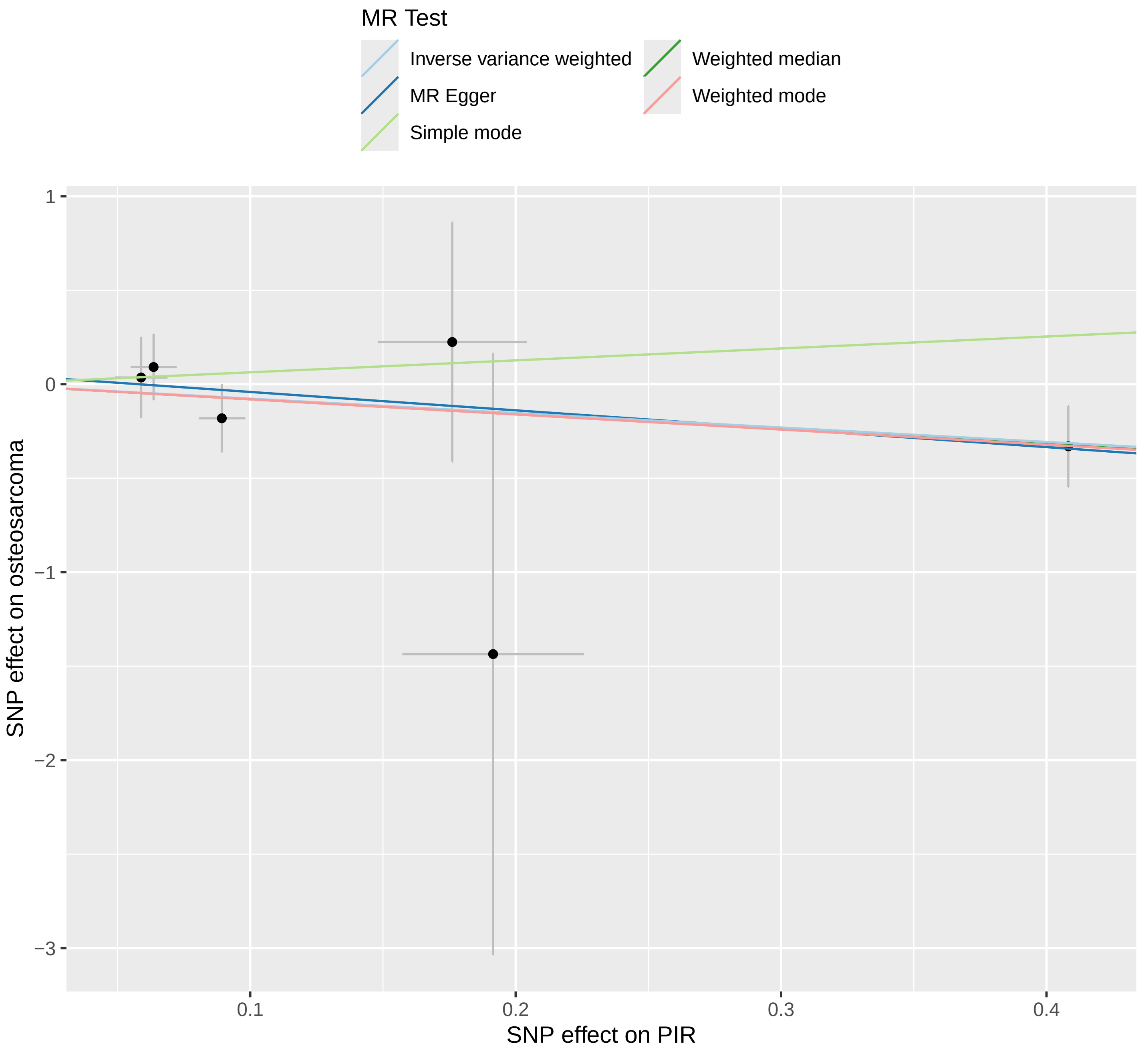
MR Test
Inverse variance weighted
MR Egger
Simple mode
Weighted median
Weighted mode
1
0
−1
−2
−3
0.1
0.2
0.3
0.4
SNP effect on osteosarcoma
SNP effect on PIR

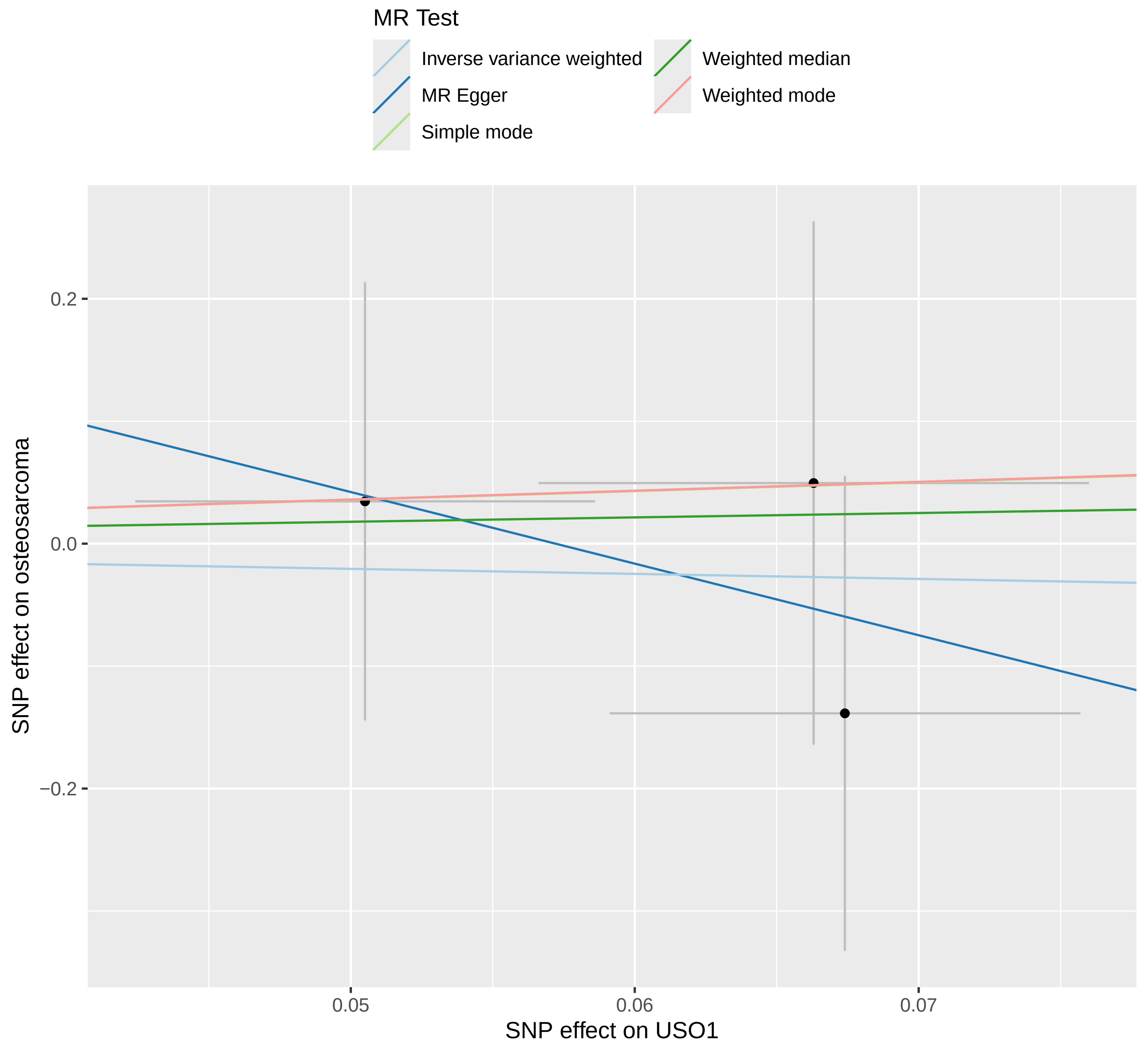
MR Test
Inverse variance weighted
MR Egger
Simple mode
Weighted median
Weighted mode
0.2
0.0
−0.2
SNP effect on osteosarcoma
0.05
0.06
0.07
SNP effect on USO1

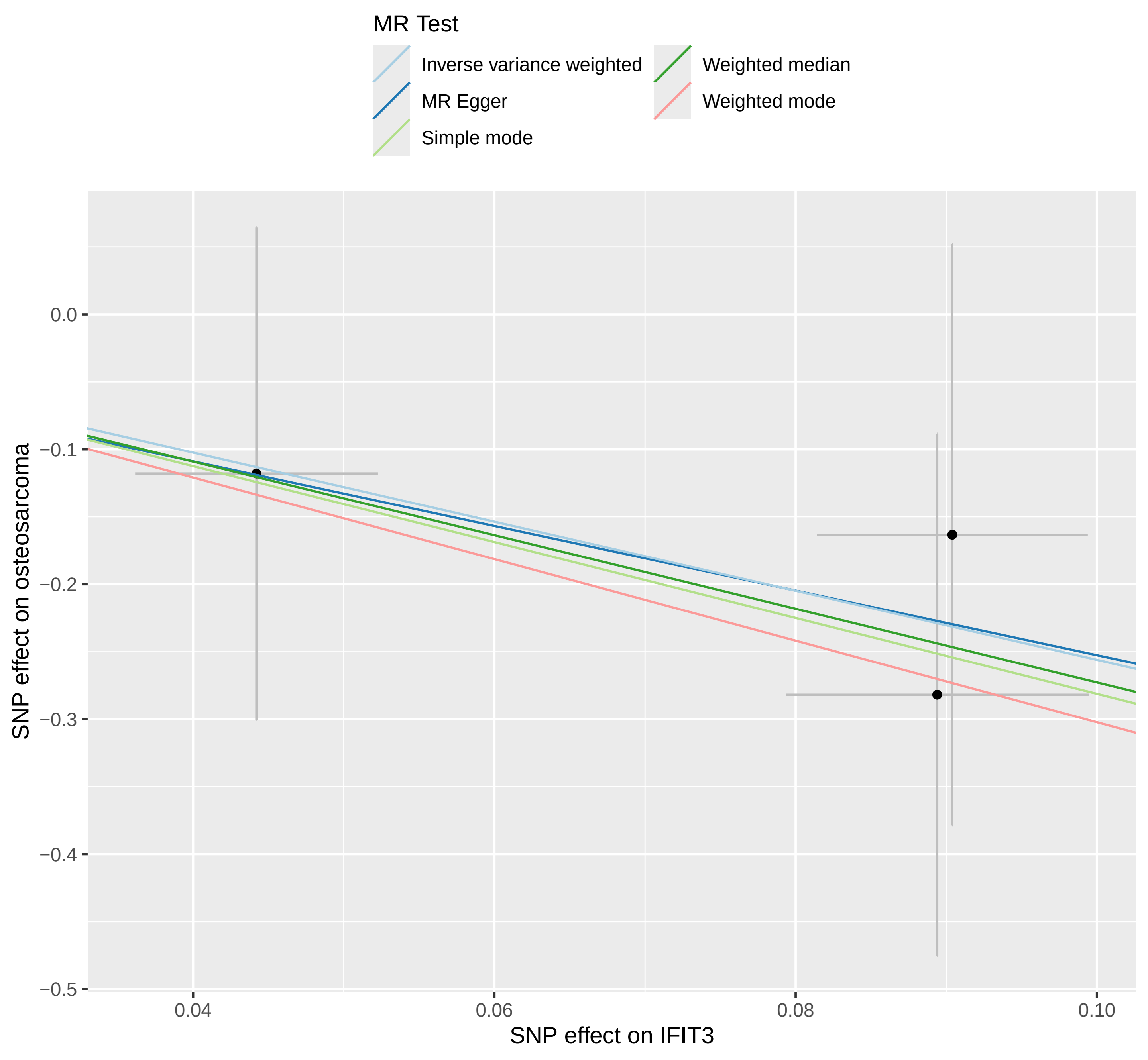
MR Test
Inverse variance weighted
MR Egger
Simple mode
Weighted median
Weighted mode
0.0
−0.1
−0.2
−0.3
−0.4
−0.5
SNP effect on osteosarcoma
0.04
0.06
0.08
0.10
SNP effect on IFIT3

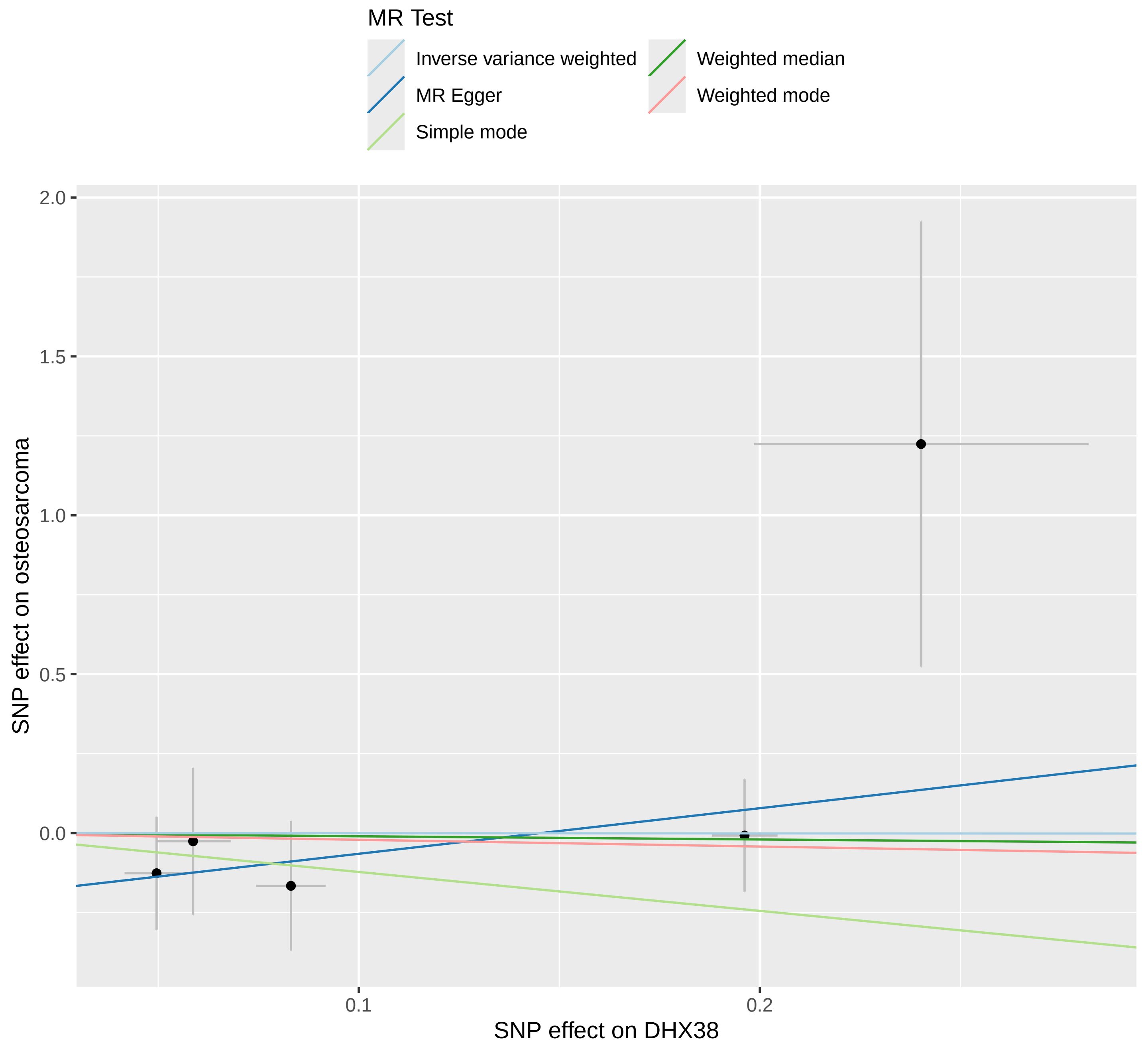
MR Test
Inverse variance weighted
MR Egger
Simple mode
Weighted median
Weighted mode
2.0
1.5
1.0
0.5
0.0
0.1
0.2
SNP effect on osteosarcoma
SNP effect on DHX38

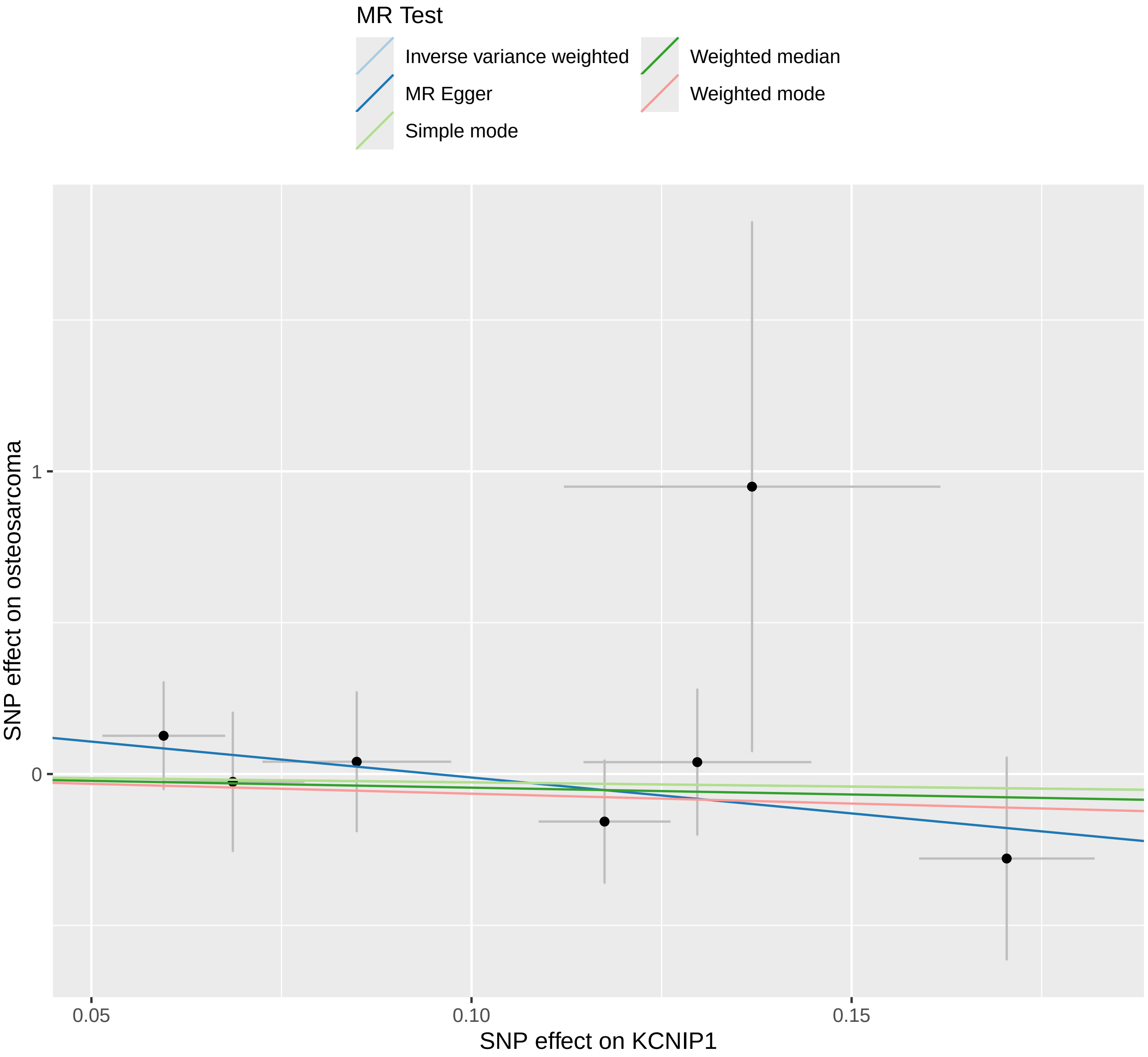
MR Test
Inverse variance weighted
MR Egger
Simple mode
Weighted median
Weighted mode
SNP effect on osteosarcoma
1
0
0.05
0.10
0.15
SNP effect on KCNIP1

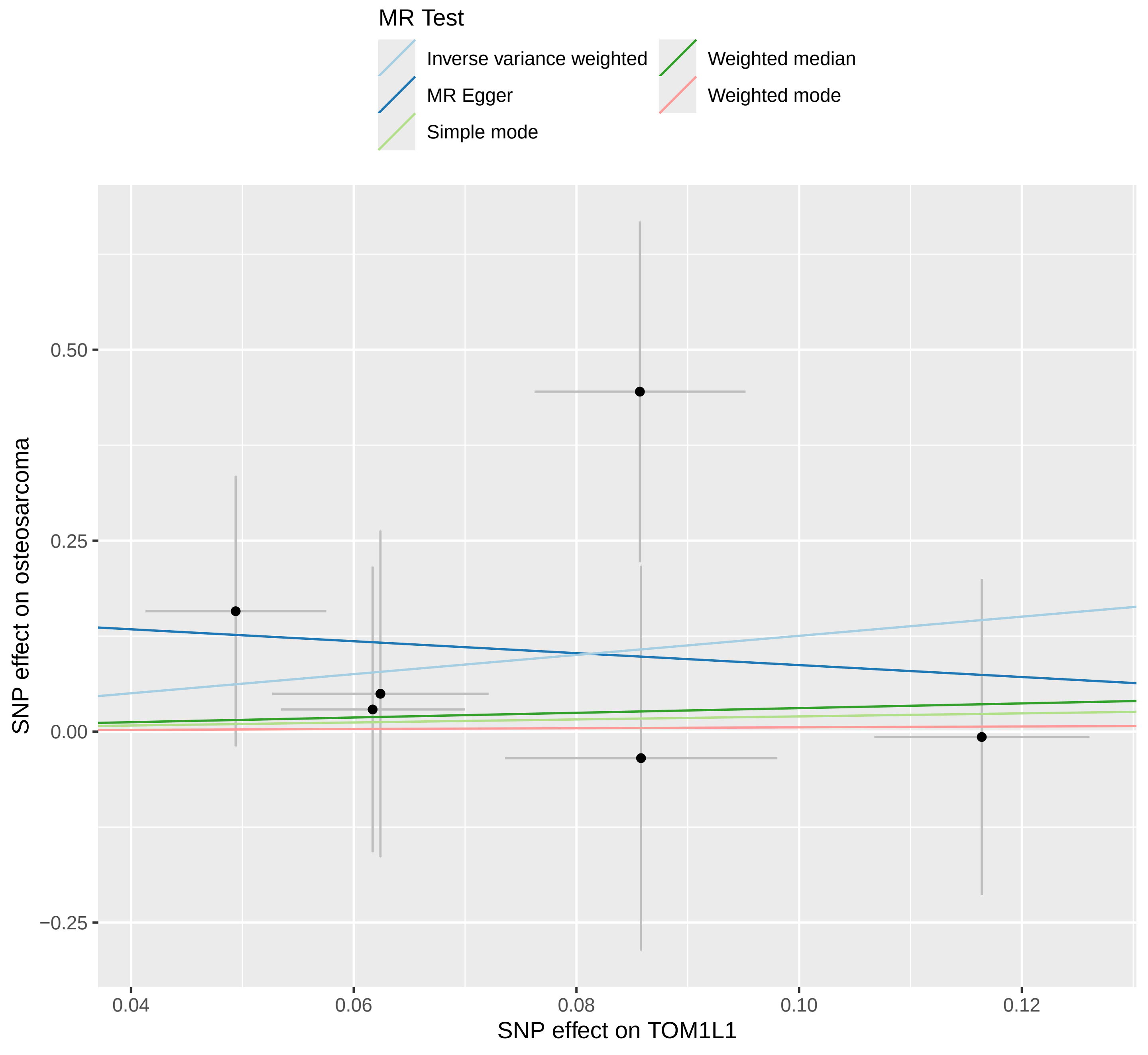
MR Test
Inverse variance weighted
MR Egger
Simple mode
Weighted median
Weighted mode
SNP effect on osteosarcoma
0.50
0.25
0.00
−0.25
0.04
0.06
0.08
0.10
0.12
SNP effect on TOM1L1

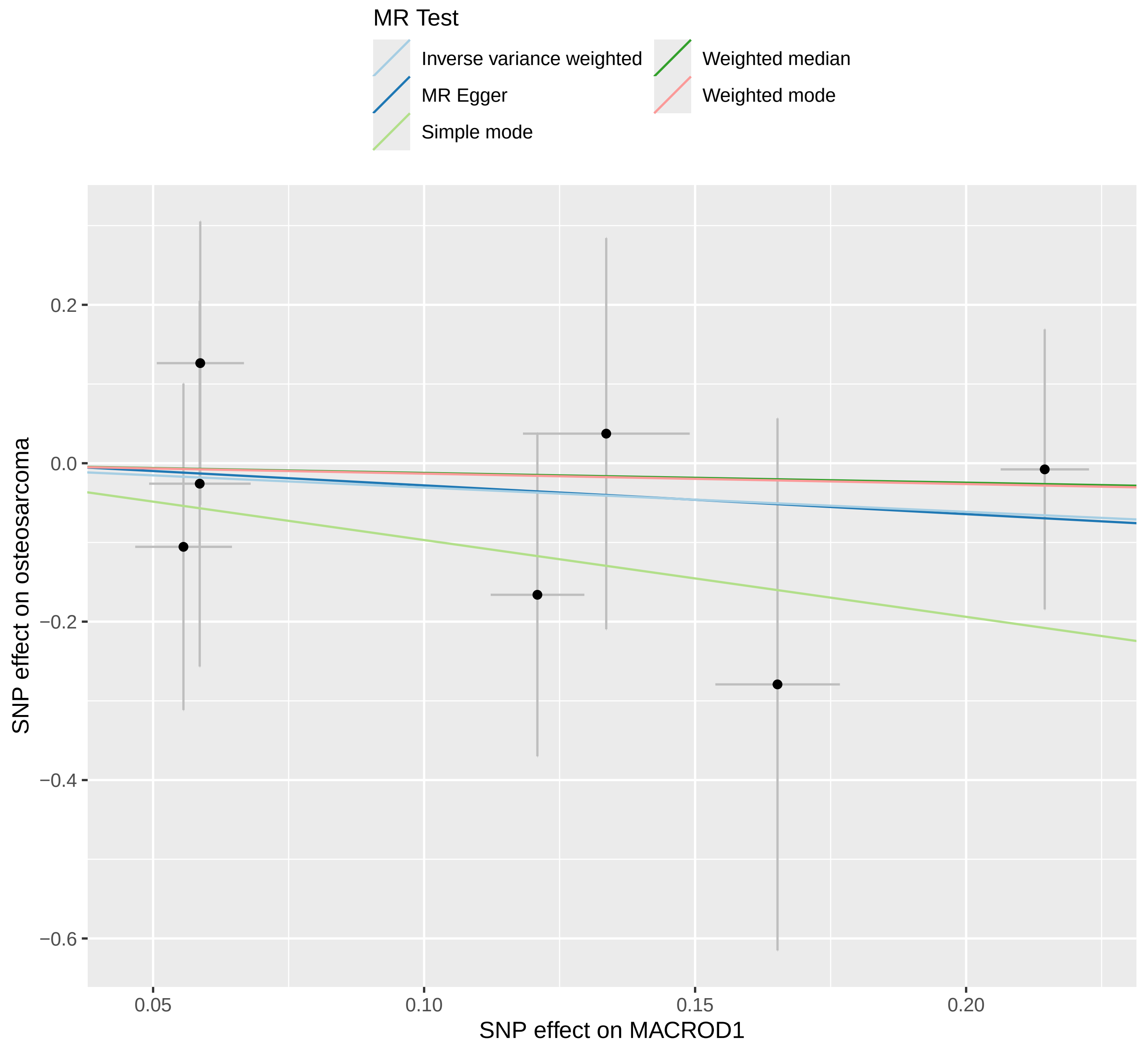
MR Test
Inverse variance weighted
MR Egger
Simple mode
Weighted median
Weighted mode
0.2
0.0
−0.2
−0.4
−0.6
SNP effect on osteosarcoma
0.05
0.10
0.15
0.20
SNP effect on MACROD1

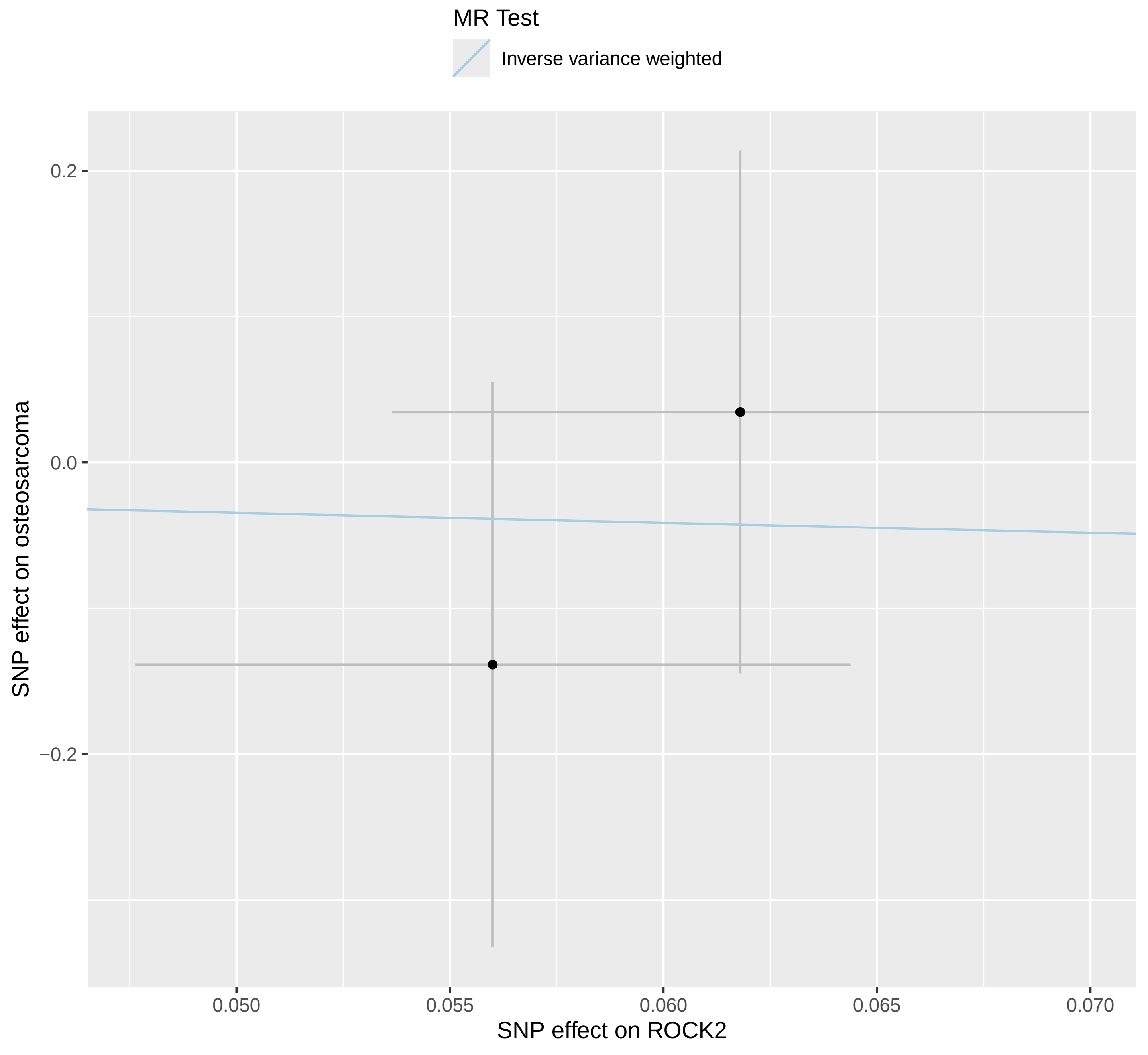

MR Test
Inverse variance weighted
0.2
0.0
−0.2
SNP effect on osteosarcoma
0.050
0.055
0.060
0.065
0.070
SNP effect on ROCK2

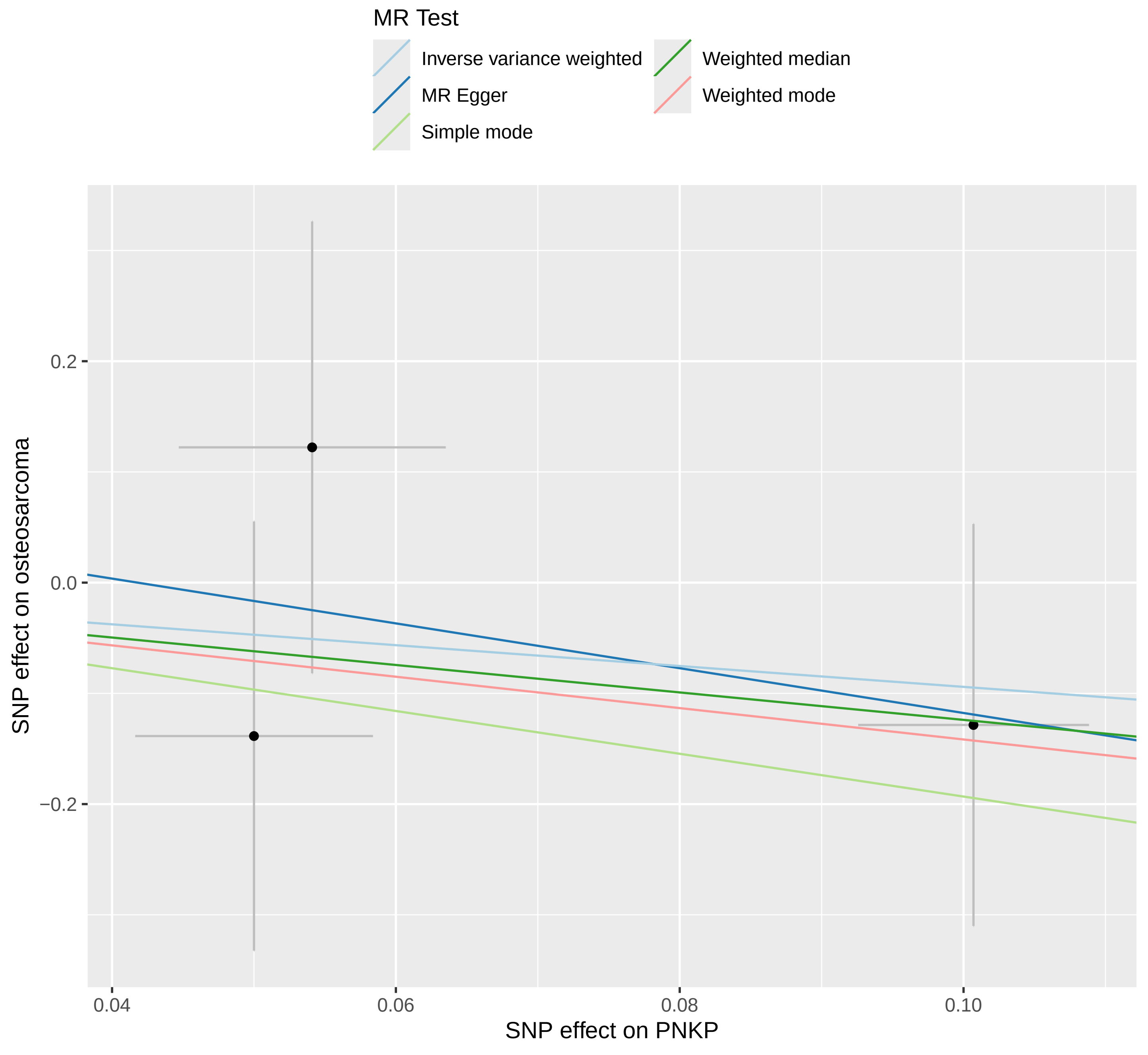
MR Test
Inverse variance weighted
MR Egger
Simple mode
Weighted median
Weighted mode
0.2
0.0
−0.2
SNP effect on osteosarcoma
0.04
0.06
0.08
0.10
SNP effect on PNKP

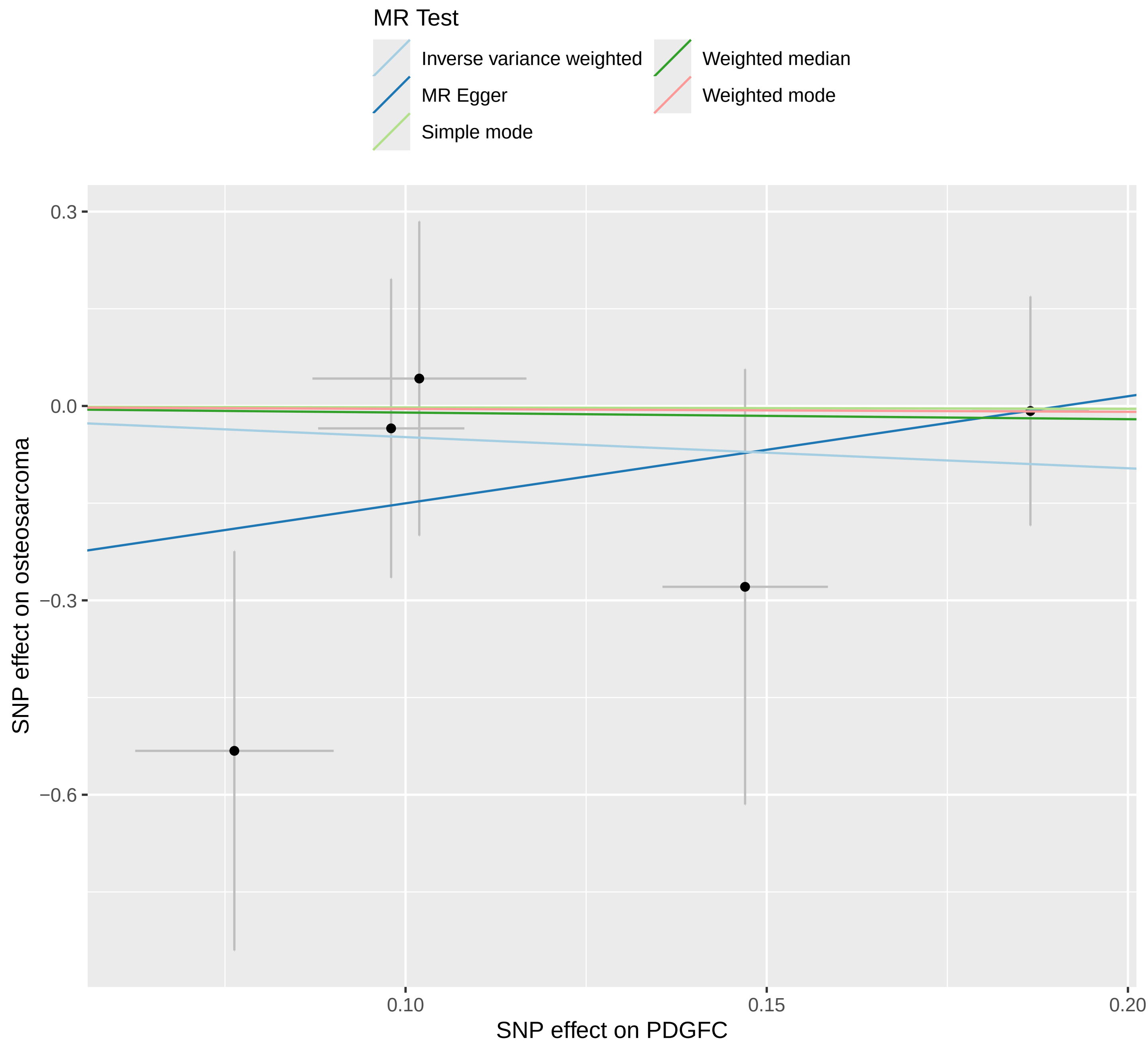
MR Test
Inverse variance weighted
MR Egger
Simple mode
Weighted median
Weighted mode
0.3
0.0
−0.3
−0.6
0.10
0.15
0.20
SNP effect on PDGFC
SNP effect on osteosarcoma

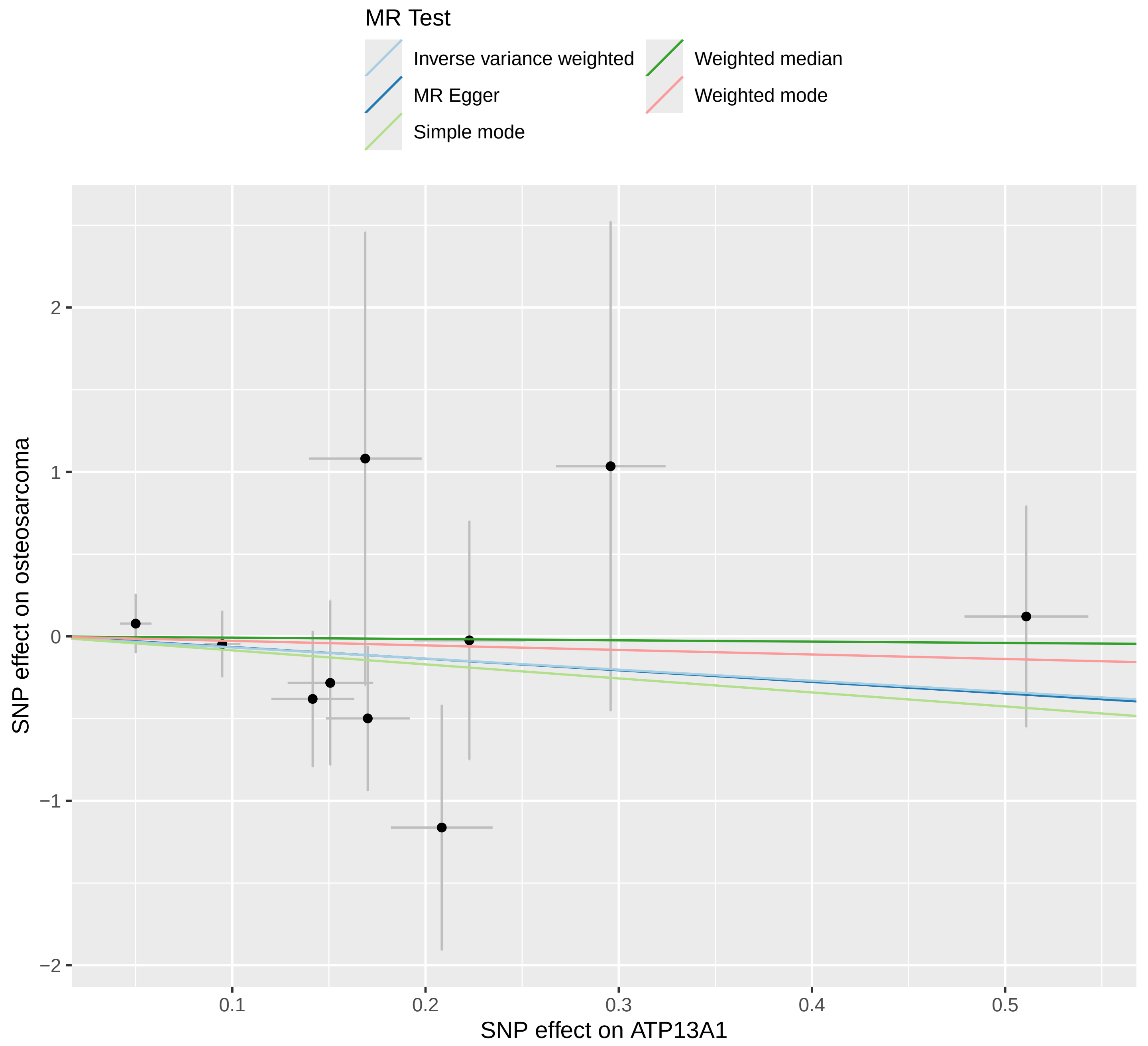
MR Test
Inverse variance weighted
MR Egger
Simple mode
Weighted median
Weighted mode
2
1
0
−1
−2
SNP effect on osteosarcoma
0.1
0.2
0.3
0.4
0.5
SNP effect on ATP13A1

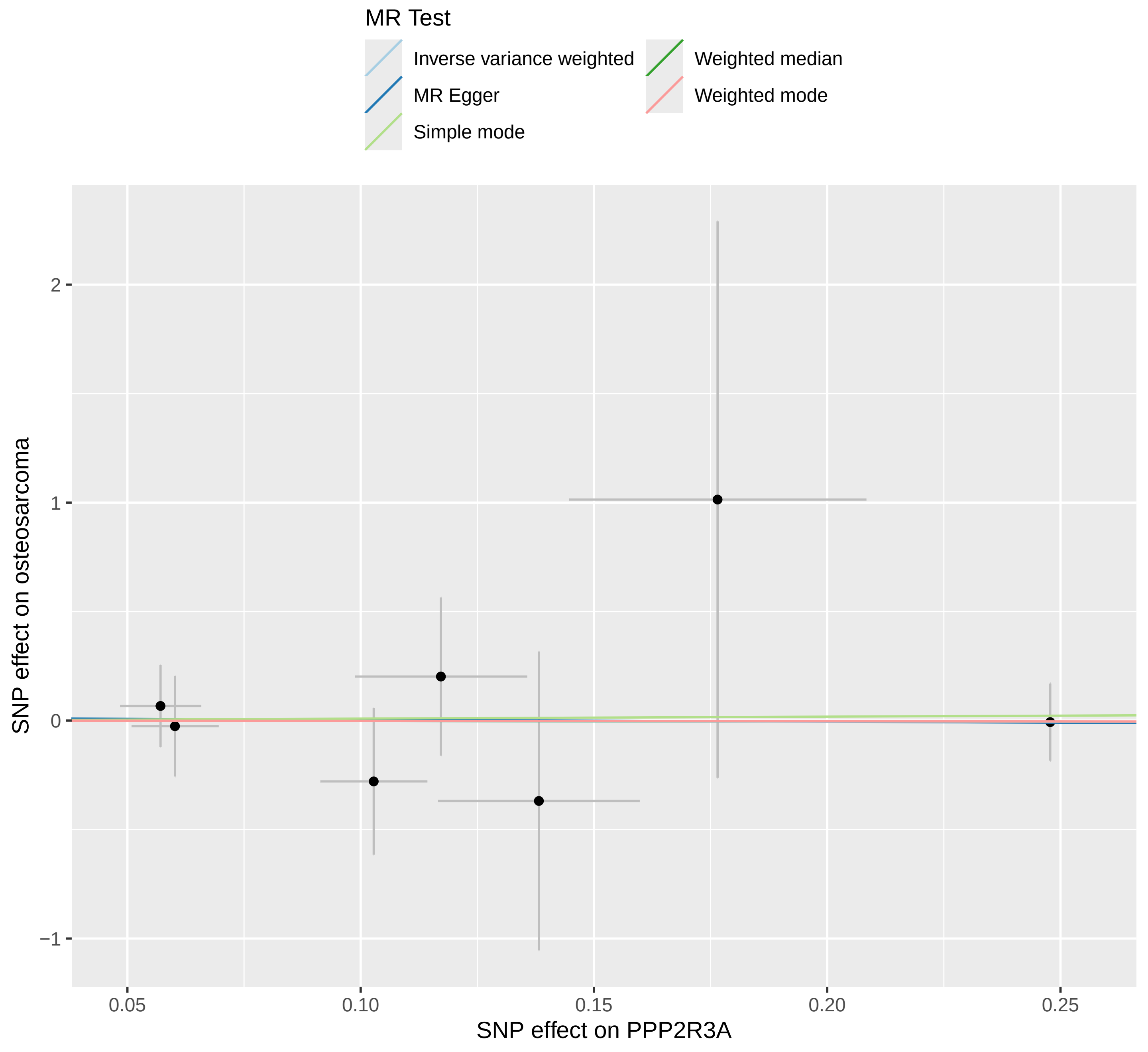

MR Test
Inverse variance weighted
MR Egger
Simple mode
Weighted median
Weighted mode
2
1
0
−1
0.05
0.10
0.15
0.20
0.25
SNP effect on osteosarcoma
SNP effect on PPP2R3A

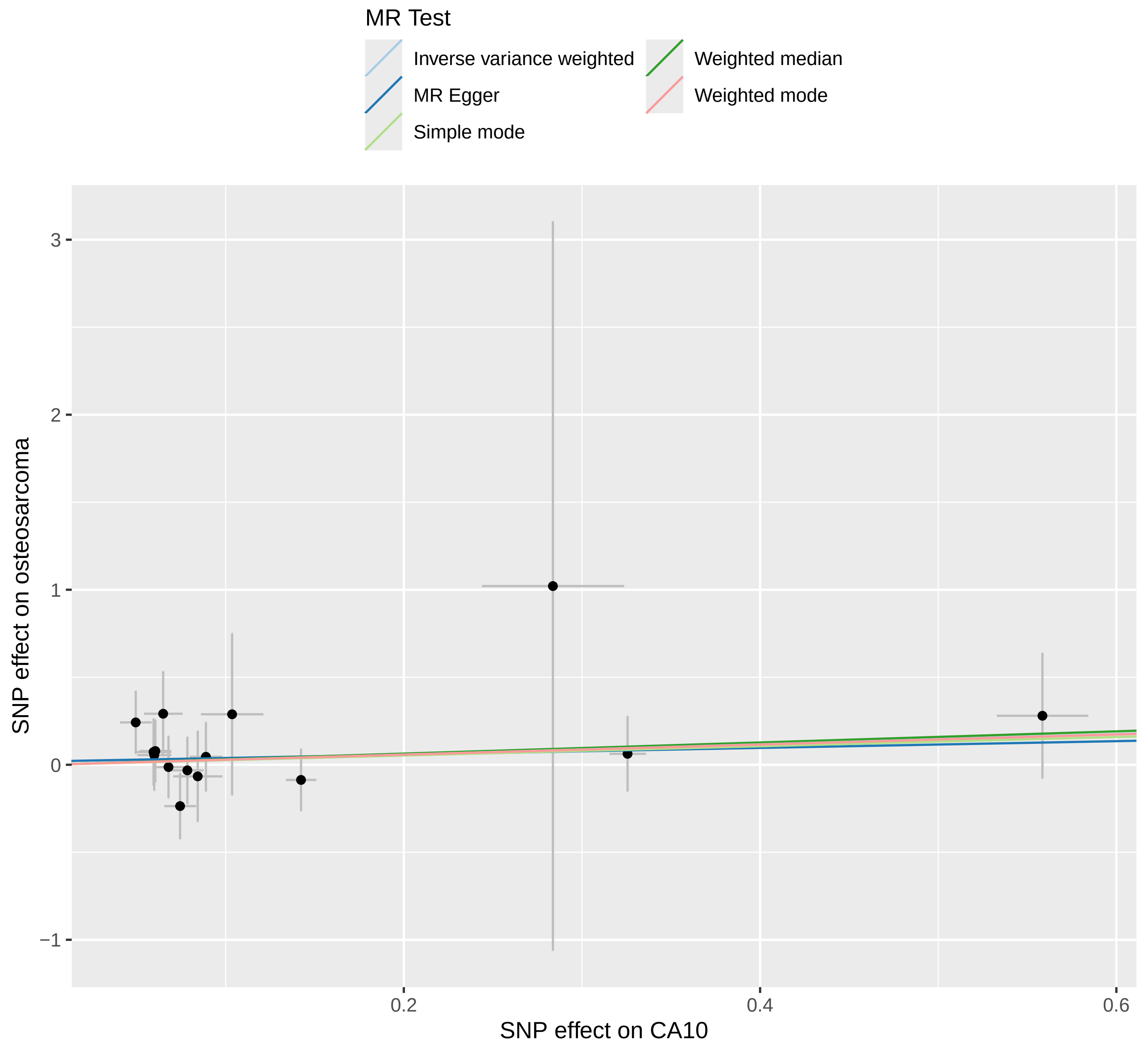
MR Test
Inverse variance weighted
MR Egger
Simple mode
Weighted median
Weighted mode
3
2
1
0
−1
0.2
0.4
0.6
SNP effect on osteosarcoma
SNP effect on CA10

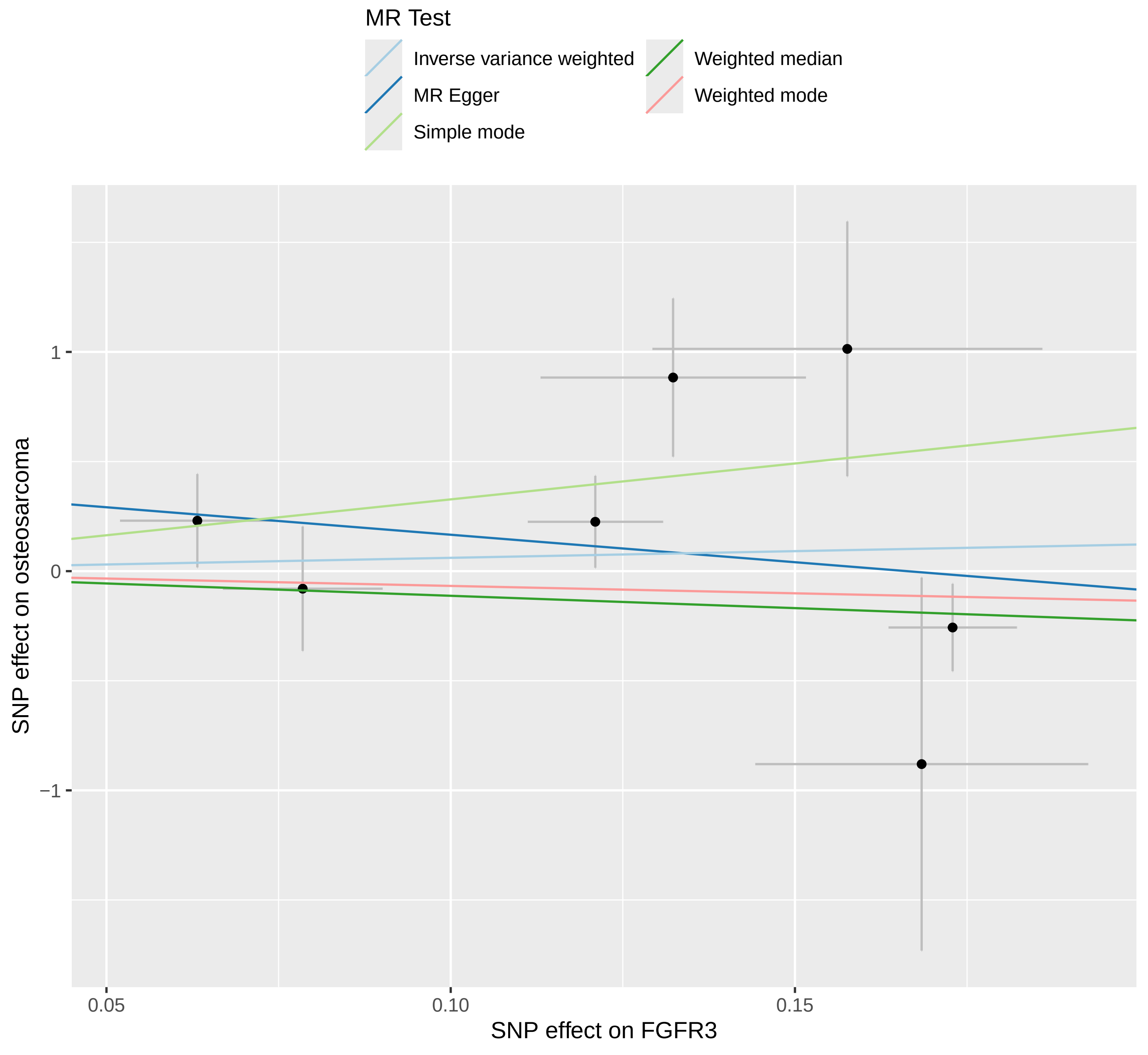
MR Test
Inverse variance weighted
MR Egger
Simple mode
Weighted median
Weighted mode
1
0
−1
0.05
0.10
0.15
SNP effect on FGFR3
SNP effect on osteosarcoma

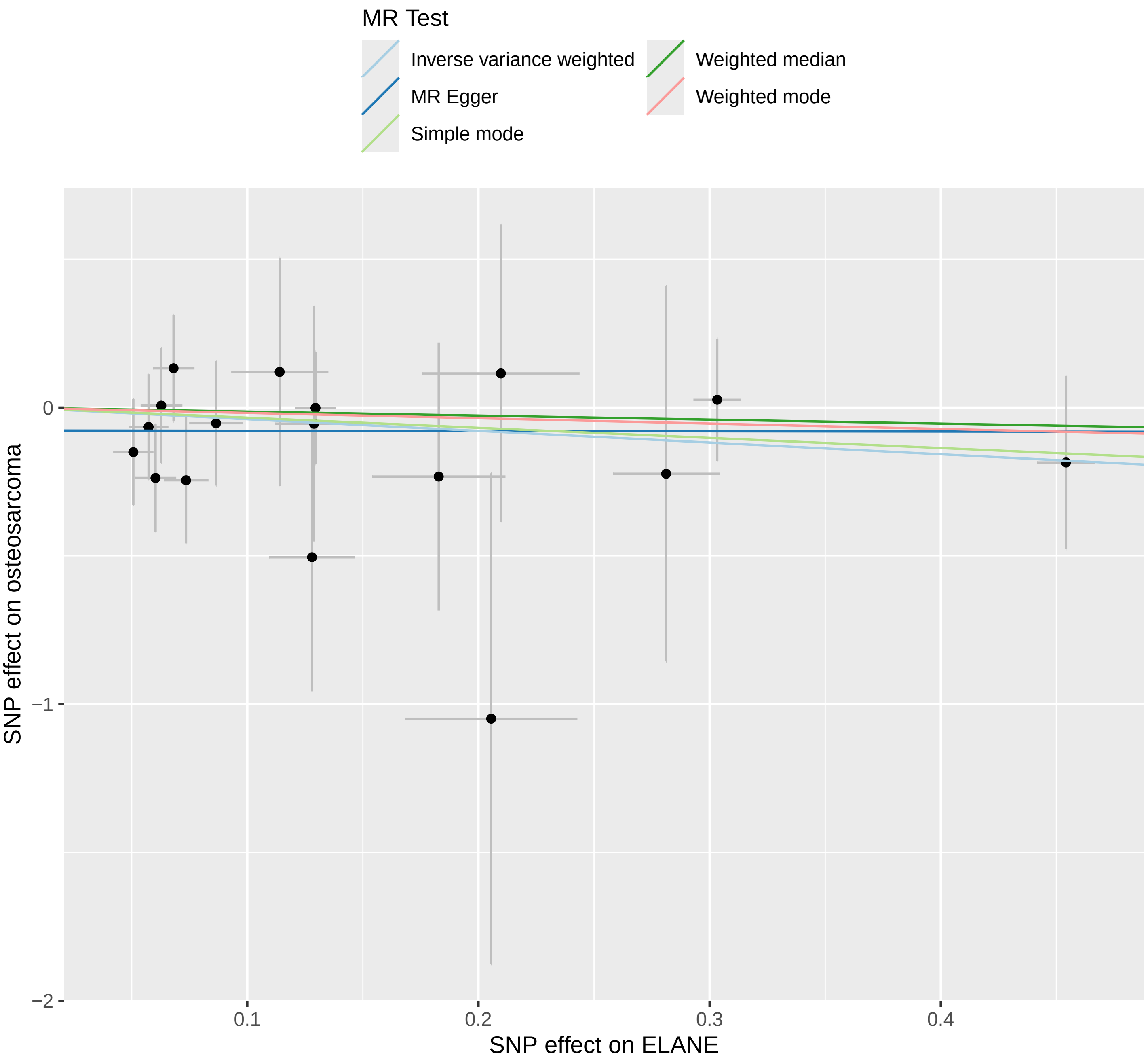
MR Test
Inverse variance weighted
MR Egger
Simple mode
Weighted median
Weighted mode
SNP effect on osteosarcoma
0
−1
−2
0.1
0.2
0.3
0.4
SNP effect on ELANE

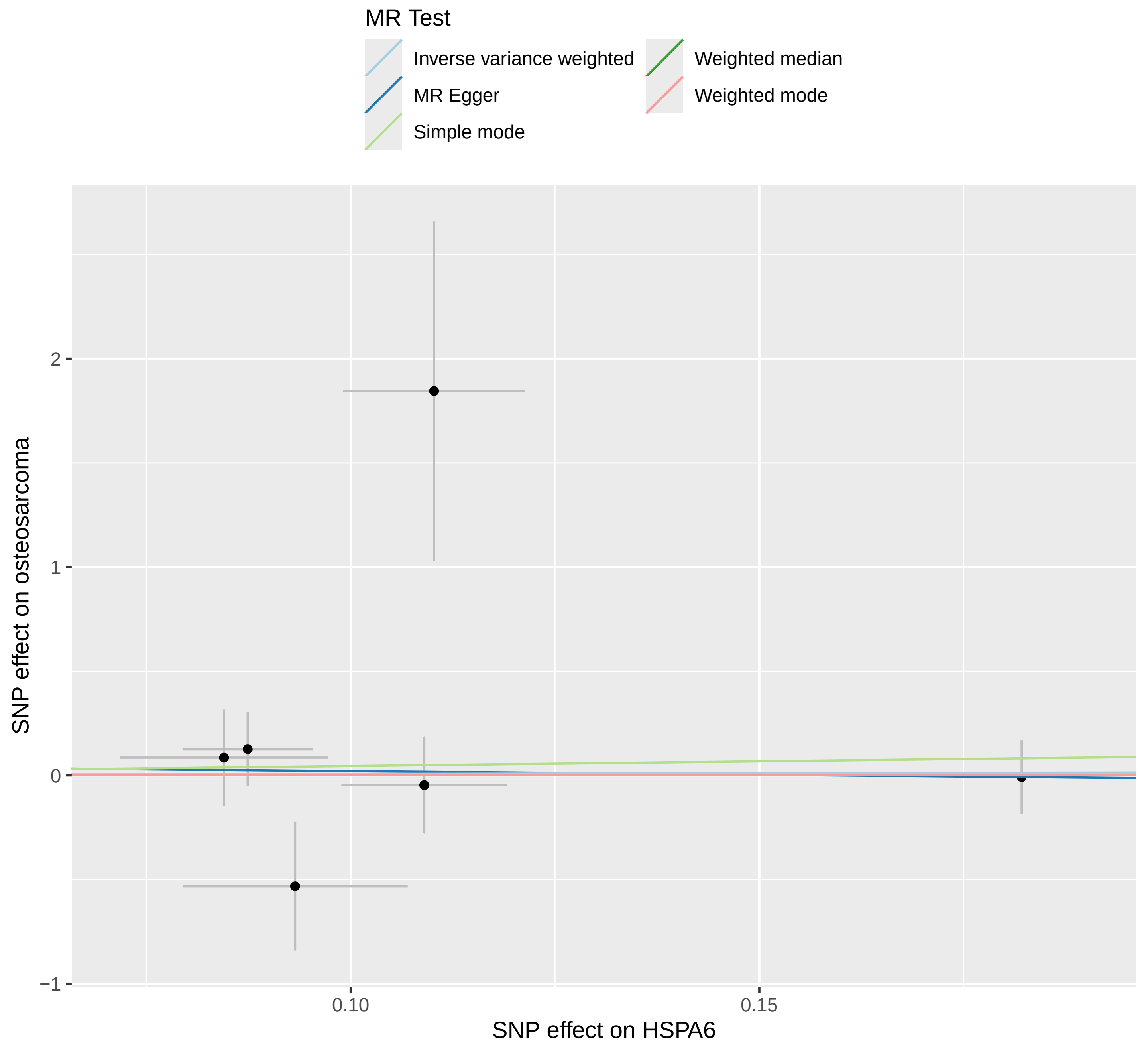
MR Test
Inverse variance weighted
MR Egger
Simple mode
Weighted median
Weighted mode
2
1
0
−1
0.10
0.15
SNP effect on HSPA6
SNP effect on osteosarcoma

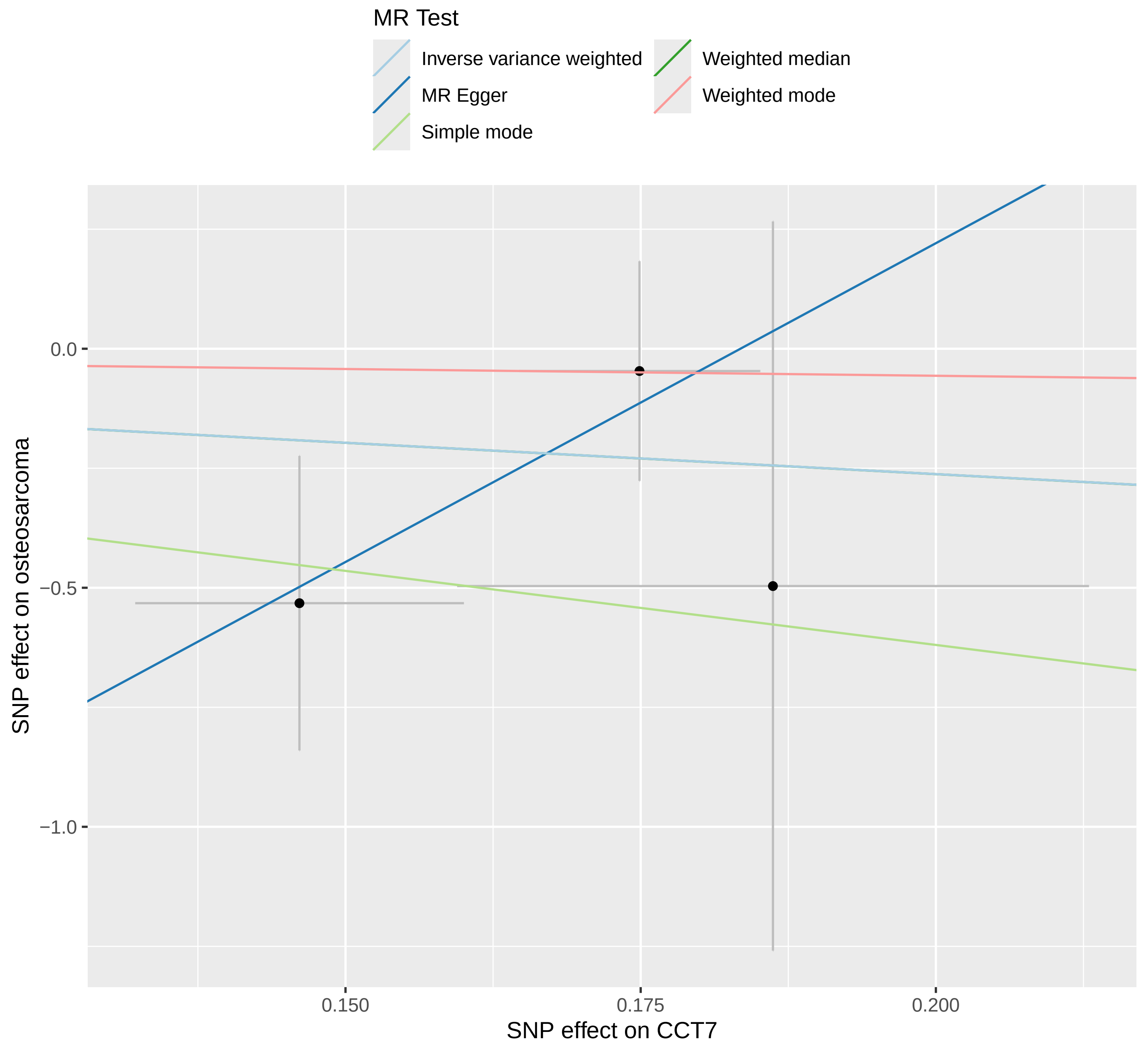

MR Test
Inverse variance weighted
MR Egger
Simple mode
Weighted median
Weighted mode
0.0
−0.5
−1.0
SNP effect on osteosarcoma
0.150
0.175
0.200
SNP effect on CCT7

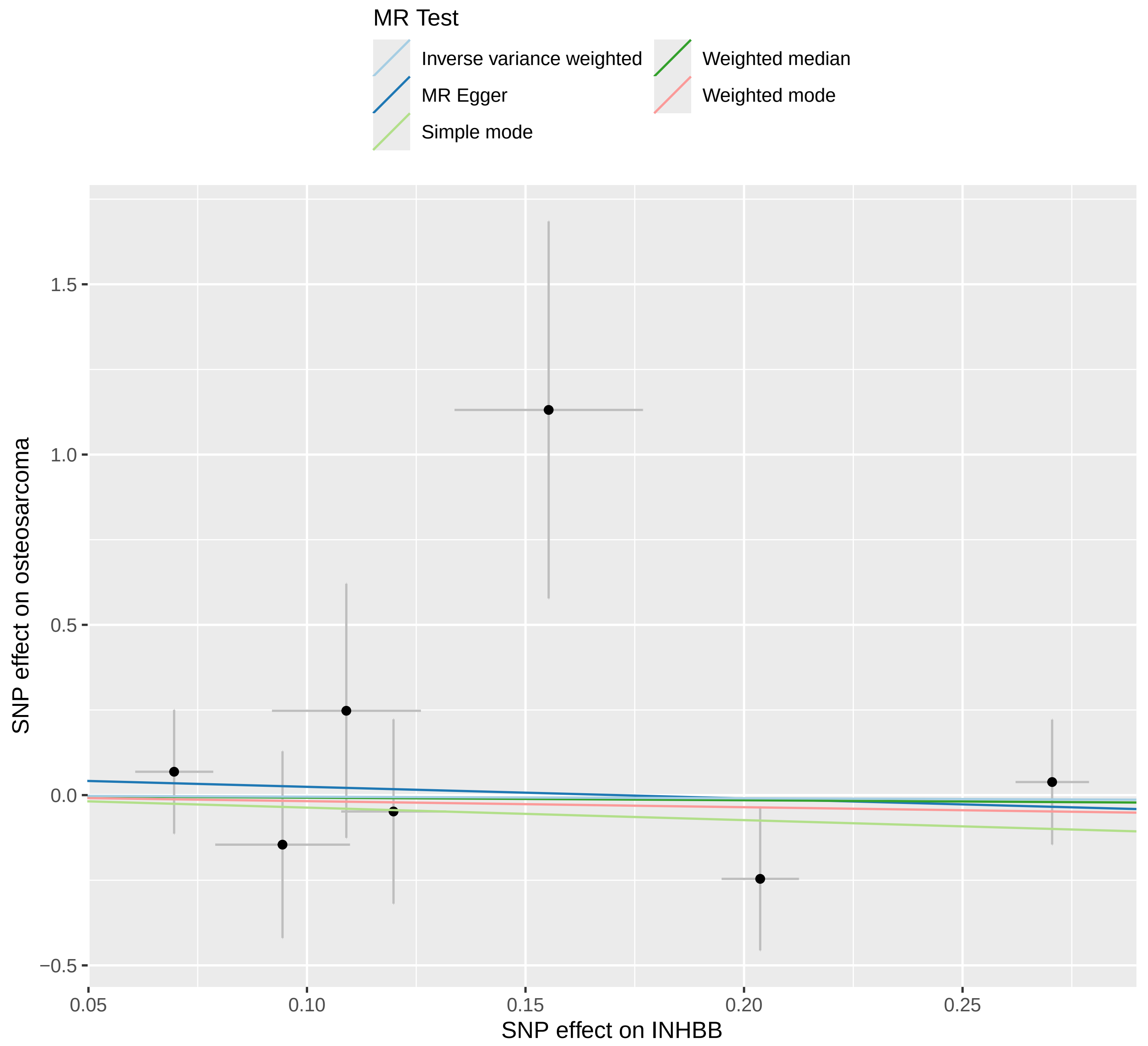
MR Test
Inverse variance weighted
MR Egger
Simple mode
Weighted median
Weighted mode
SNP effect on osteosarcoma
1.5
1.0
0.5
0.0
−0.5
0.05
0.10
0.15
0.20
0.25
SNP effect on INHBB

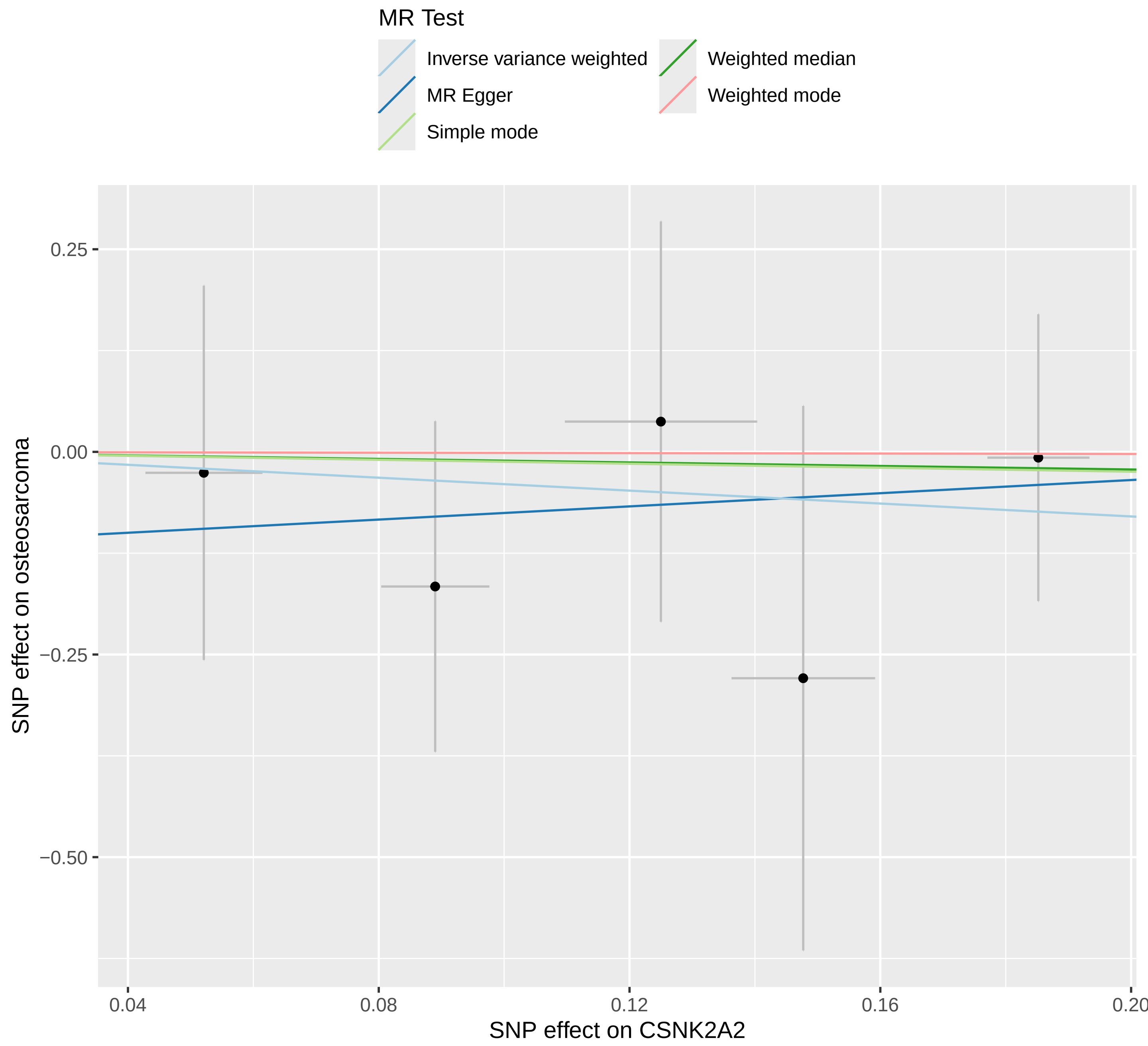
MR Test
Inverse variance weighted
MR Egger
Simple mode
Weighted median
Weighted mode
0.25
0.00
−0.25
−0.50
SNP effect on osteosarcoma
0.04
0.08
0.12
0.16
0.20
SNP effect on CSNK2A2

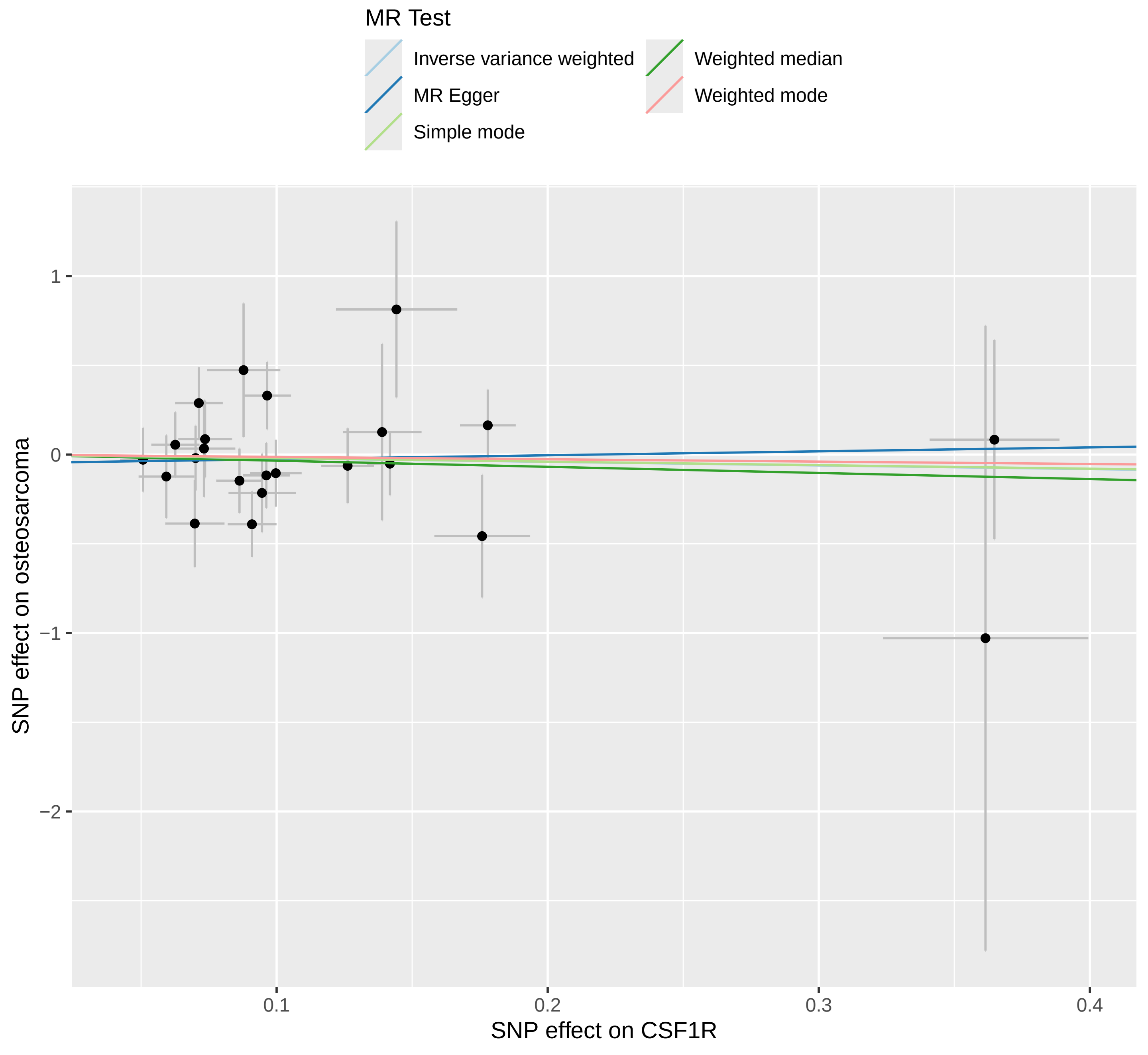

MR Test
Inverse variance weighted
MR Egger
Simple mode
Weighted median
Weighted mode
1
0
−1
−2
0.1
0.2
0.3
0.4
SNP effect on CSF1R
SNP effect on osteosarcoma

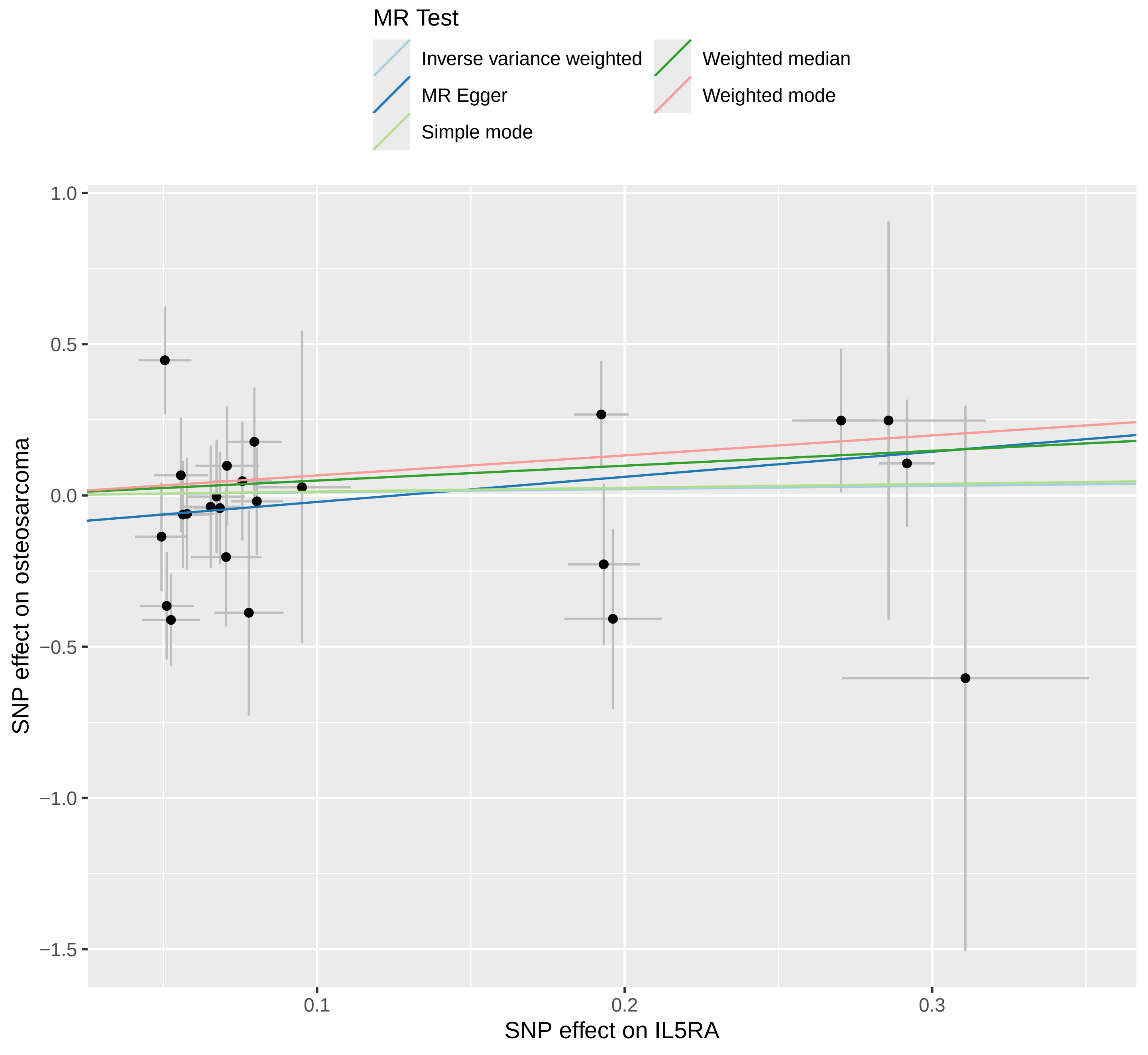

MR Test
Inverse variance weighted
MR Egger
Simple mode
Weighted median
Weighted mode
1.0
0.5
0.0
−0.5
−1.0
−1.5
0.1
0.2
0.3
SNP effect on osteosarcoma
SNP effect on IL5RA

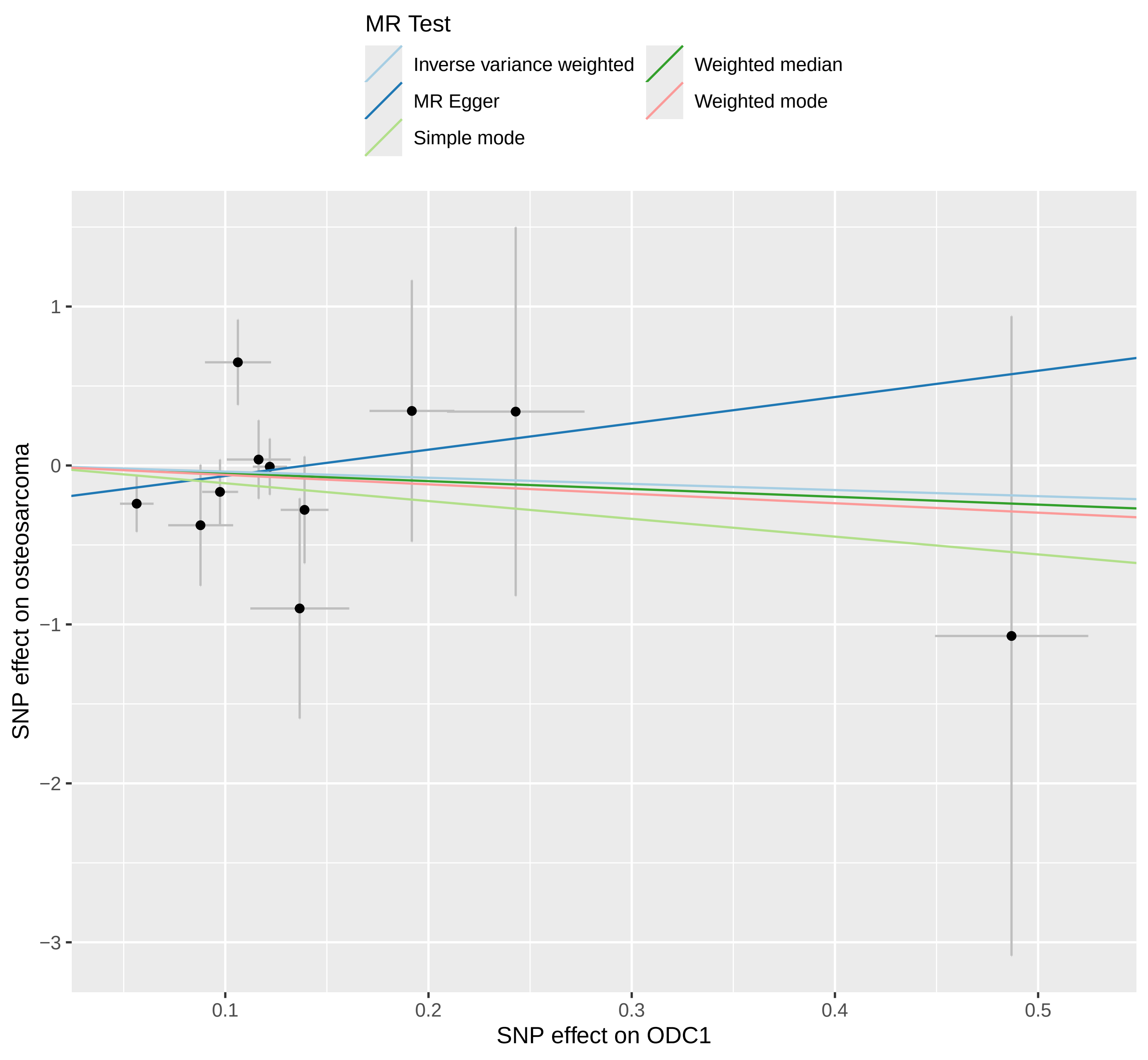

MR Test
Inverse variance weighted
MR Egger
Simple mode
Weighted median
Weighted mode
1
0
−1
−2
−3
0.1
0.2
0.3
0.4
0.5
SNP effect on osteosarcoma
SNP effect on ODC1

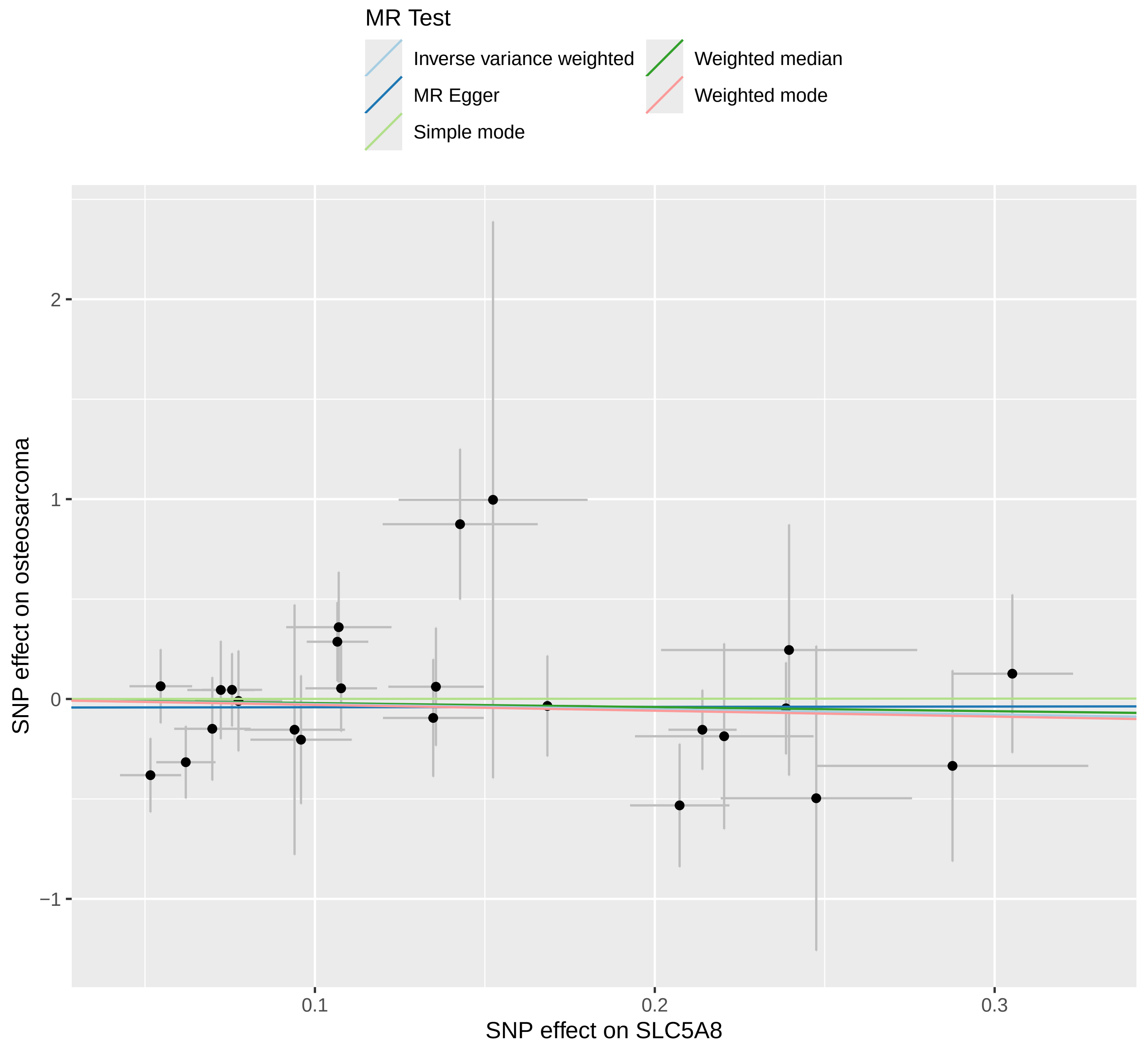
MR Test
Inverse variance weighted
MR Egger
Simple mode
Weighted median
Weighted mode
2
1
0
−1
0.1
0.2
0.3
SNP effect on osteosarcoma
SNP effect on SLC5A8

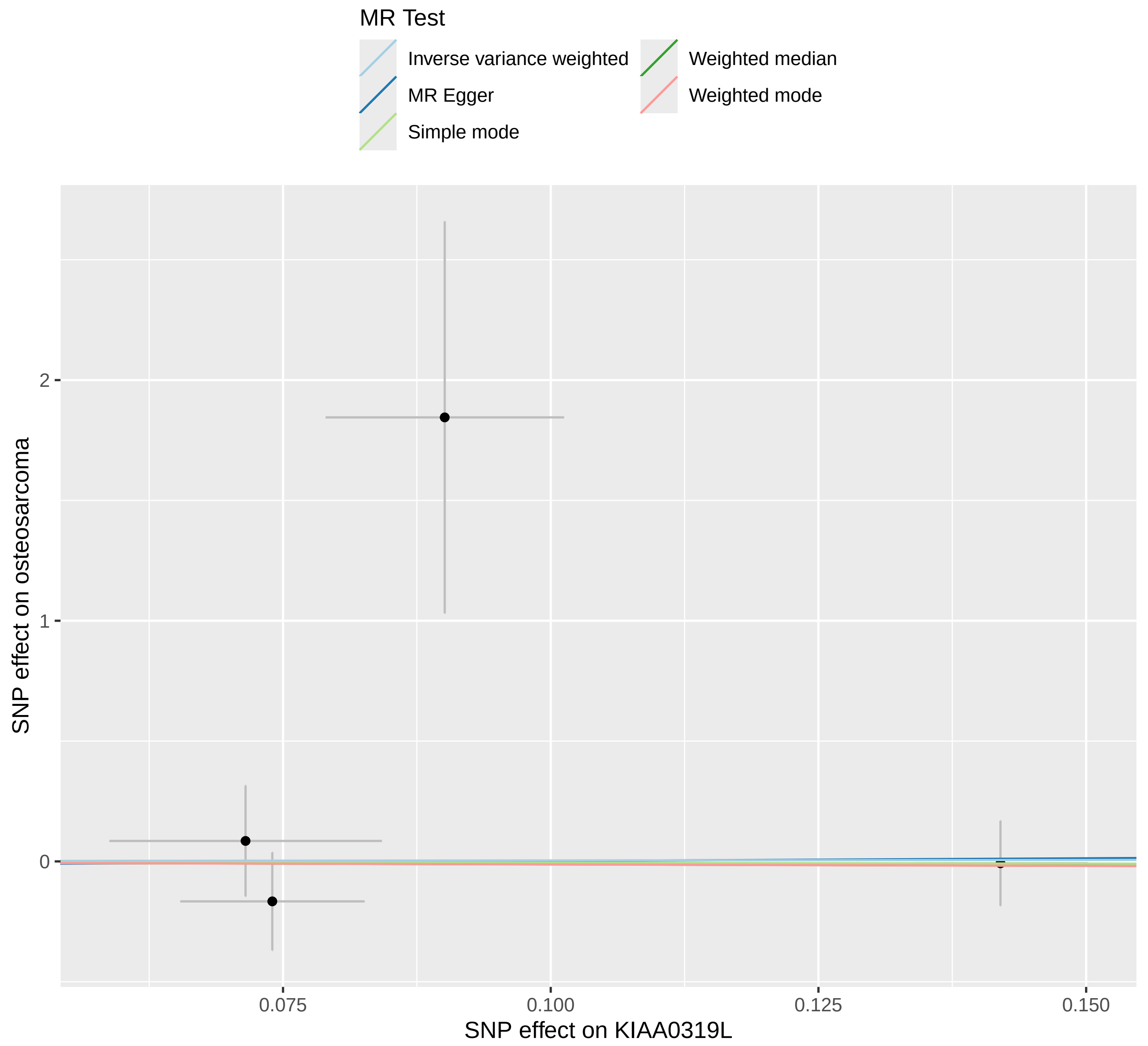
MR Test
Inverse variance weighted
MR Egger
Simple mode
Weighted median
Weighted mode
2
1
0
SNP effect on osteosarcoma
0.075
0.100
0.125
0.150
SNP effect on KIAA0319L

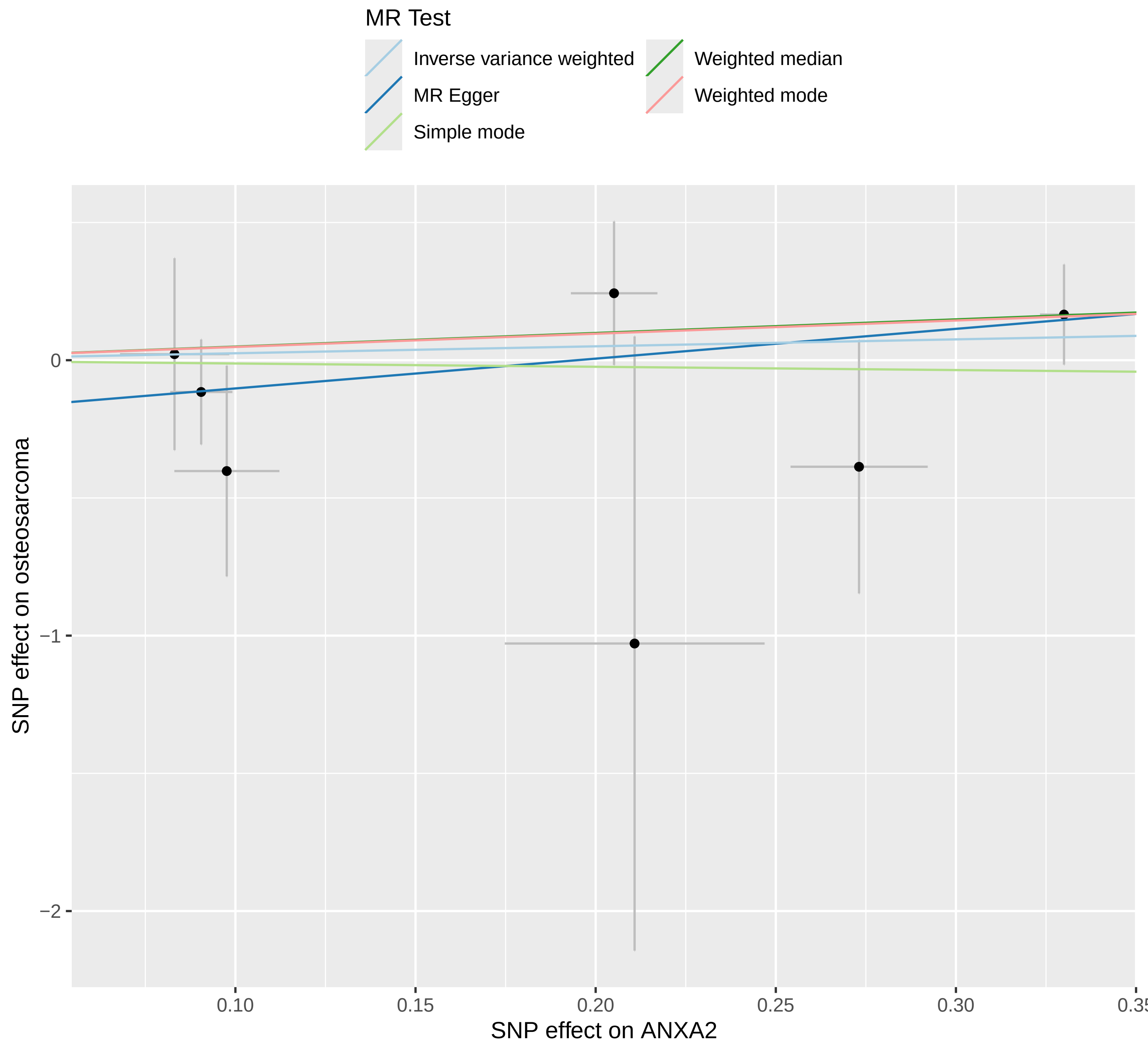
MR Test
Inverse variance weighted
MR Egger
Simple mode
Weighted median
Weighted mode
SNP effect on osteosarcoma
0
−1
−2
0.10
0.15
0.20
0.25
0.30
SNP effect on ANXA2

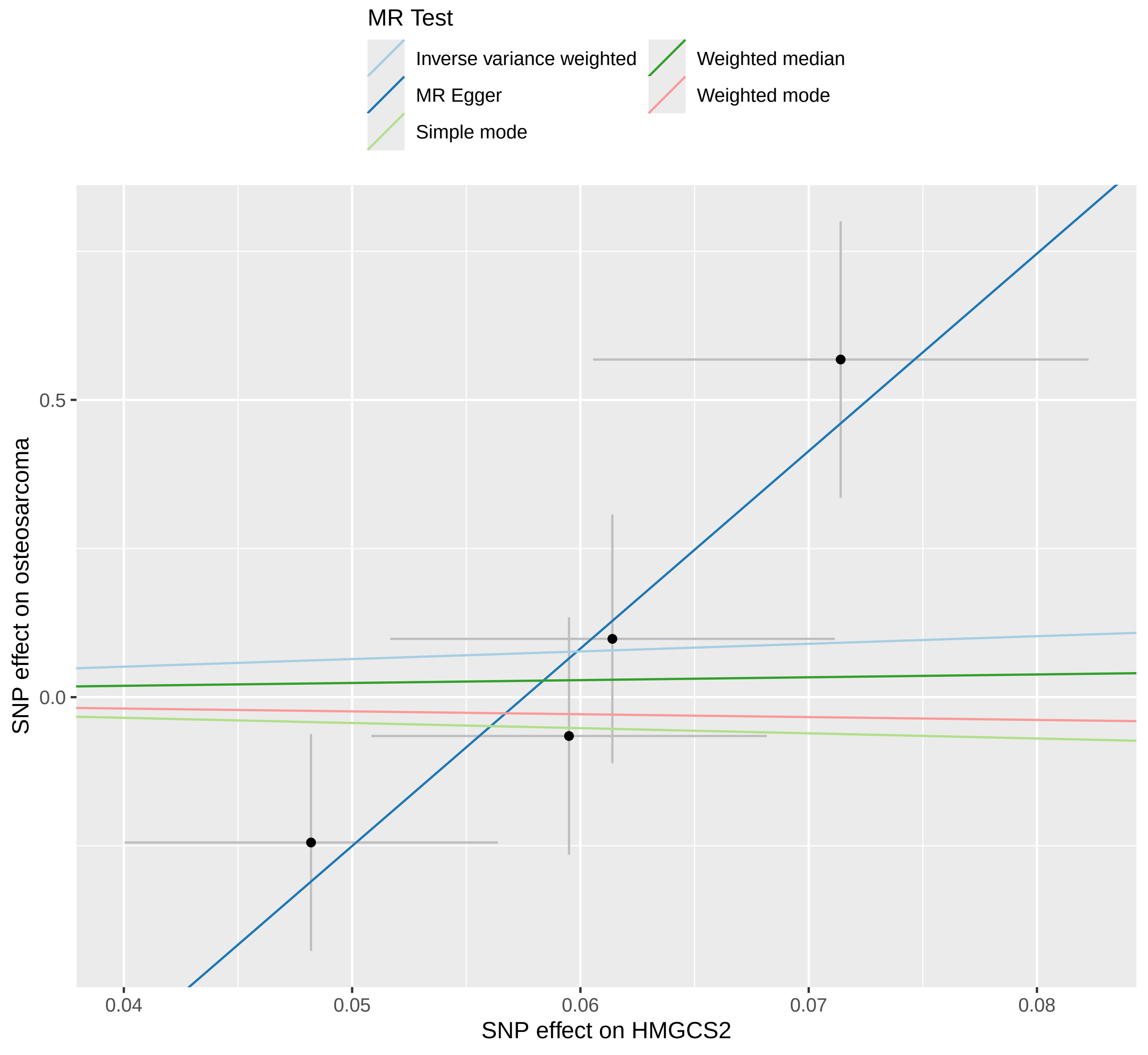
MR Test
Inverse variance weighted
MR Egger
Simple mode
Weighted median
Weighted mode
0.5
0.0
0.04
0.05
0.06
0.07
0.08
SNP effect on HMGCS2
SNP effect on osteosarcoma

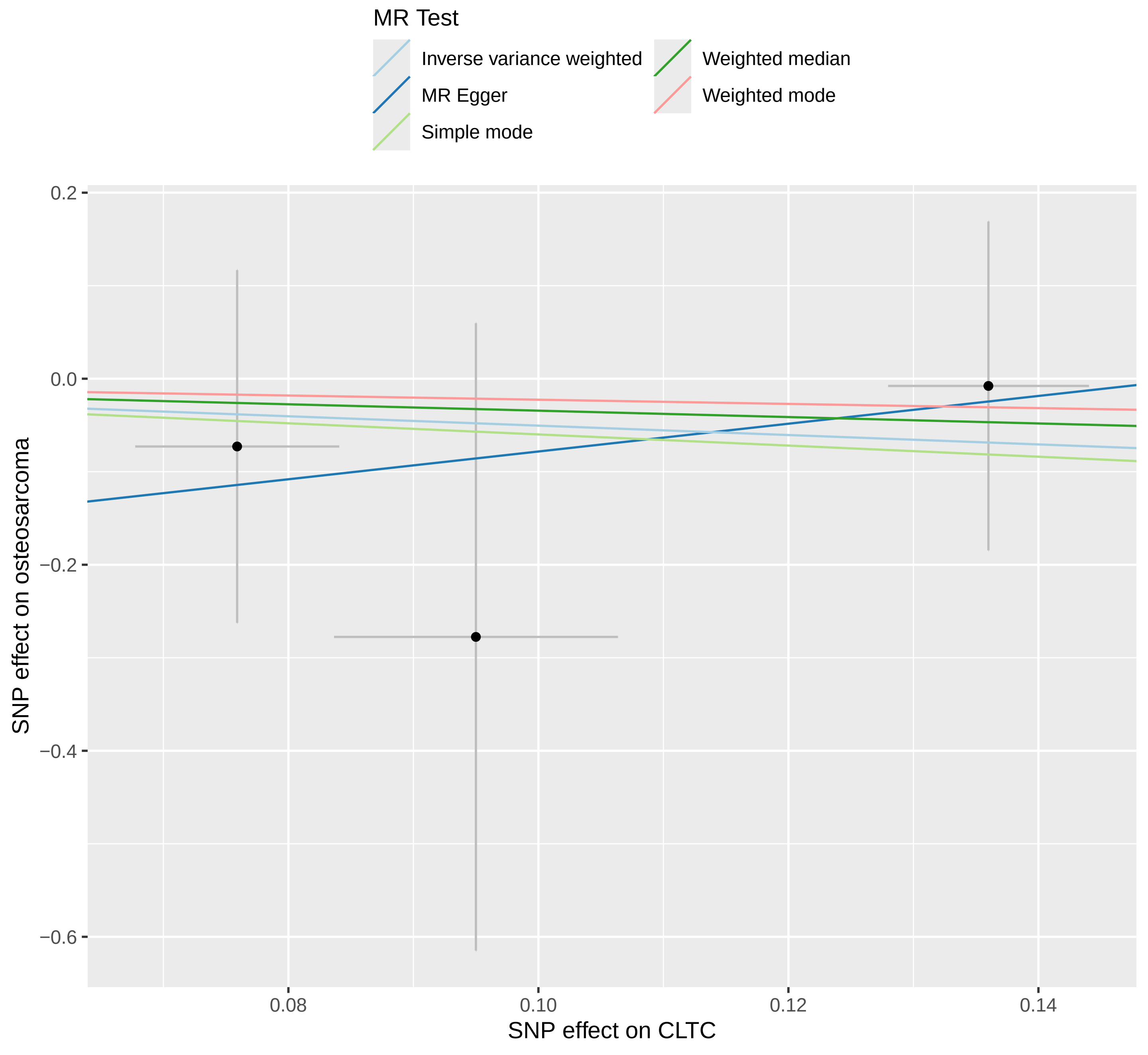
MR Test
Inverse variance weighted
MR Egger
Simple mode
Weighted median
Weighted mode
0.2
0.0
−0.2
−0.4
−0.6
SNP effect on osteosarcoma
0.08
0.10
0.12
0.14
SNP effect on CLTC

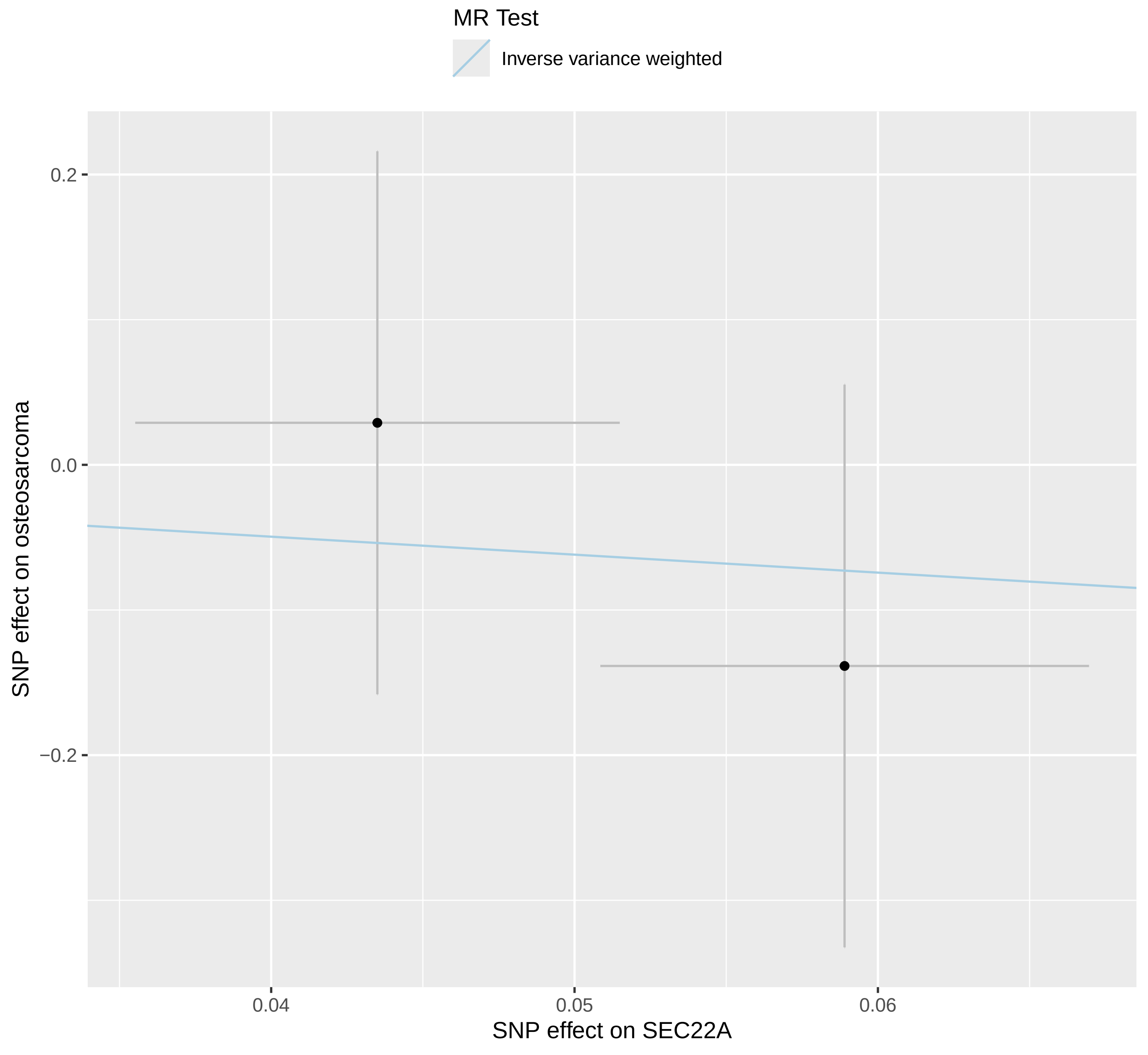
MR Test
Inverse variance weighted
0.2
0.0
−0.2
0.04
0.05
0.06
SNP effect on SEC22A
SNP effect on osteosarcoma

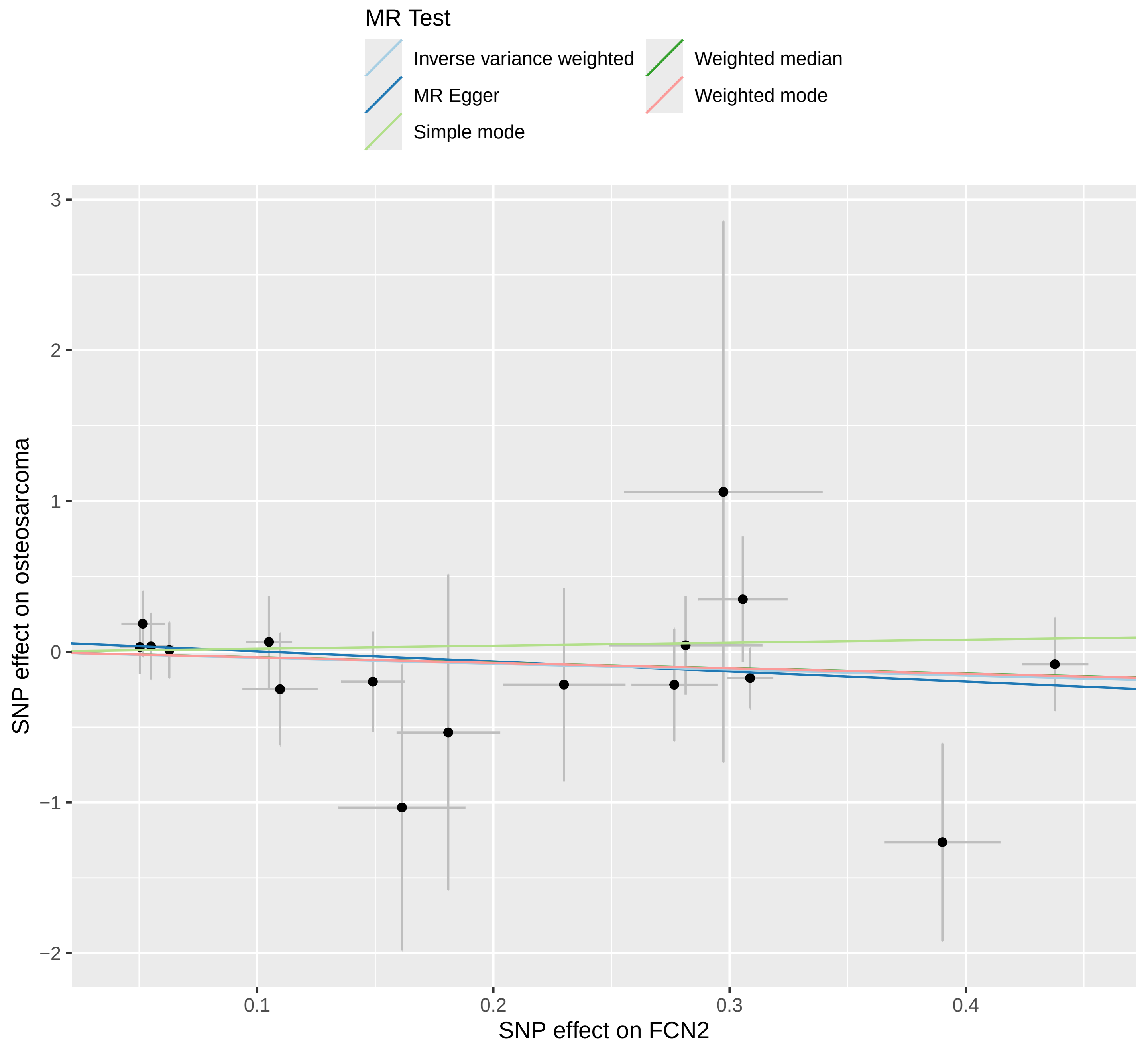
MR Test
Inverse variance weighted
MR Egger
Simple mode
Weighted median
Weighted mode
3
2
1
0
−1
−2
0.1
0.2
0.3
0.4
SNP effect on FCN2
SNP effect on osteosarcoma

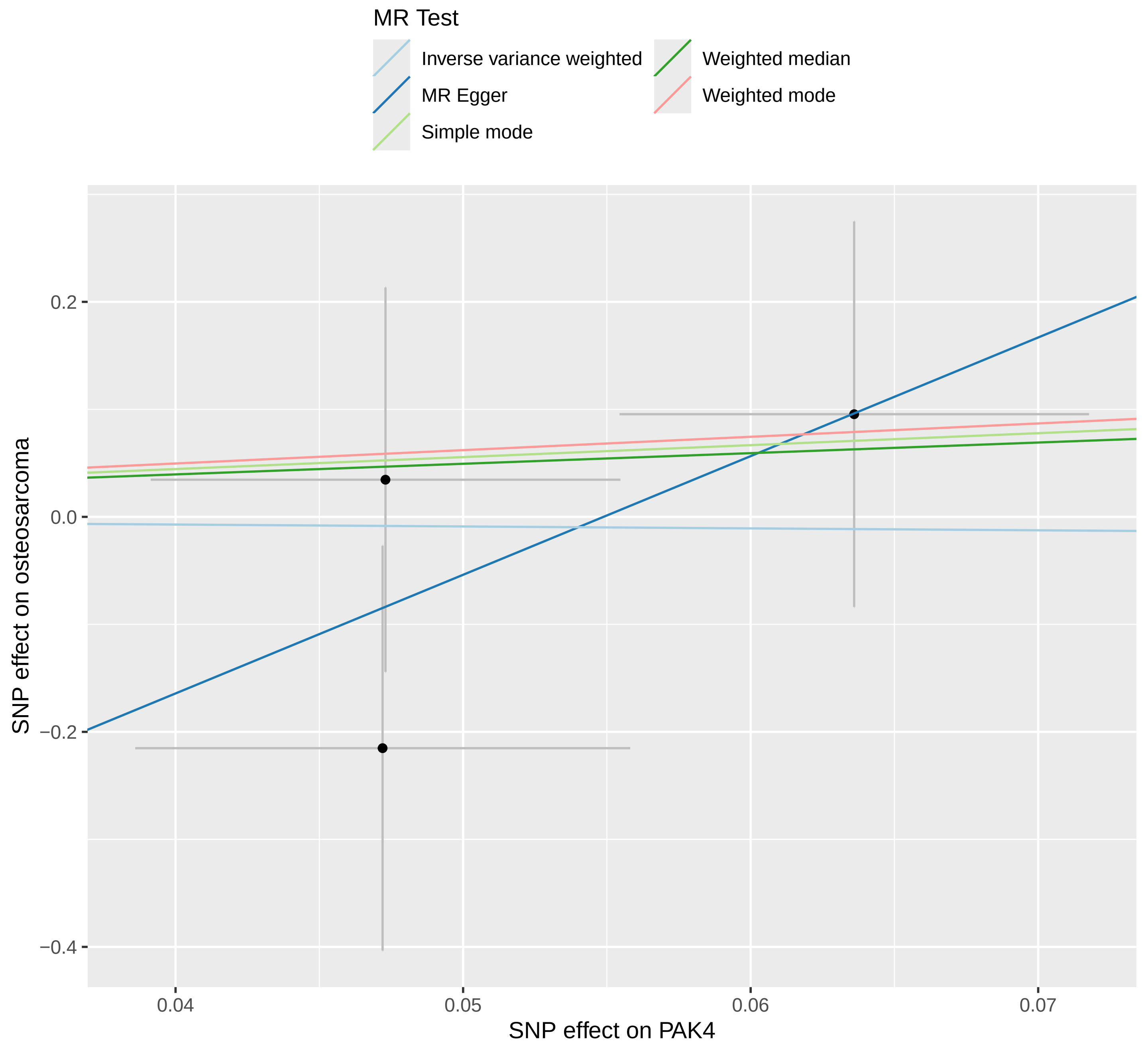
MR Test
Inverse variance weighted
MR Egger
Simple mode
Weighted median
Weighted mode
0.2
0.0
−0.2
−0.4
SNP effect on osteosarcoma
0.04
0.05
0.06
0.07
SNP effect on PAK4

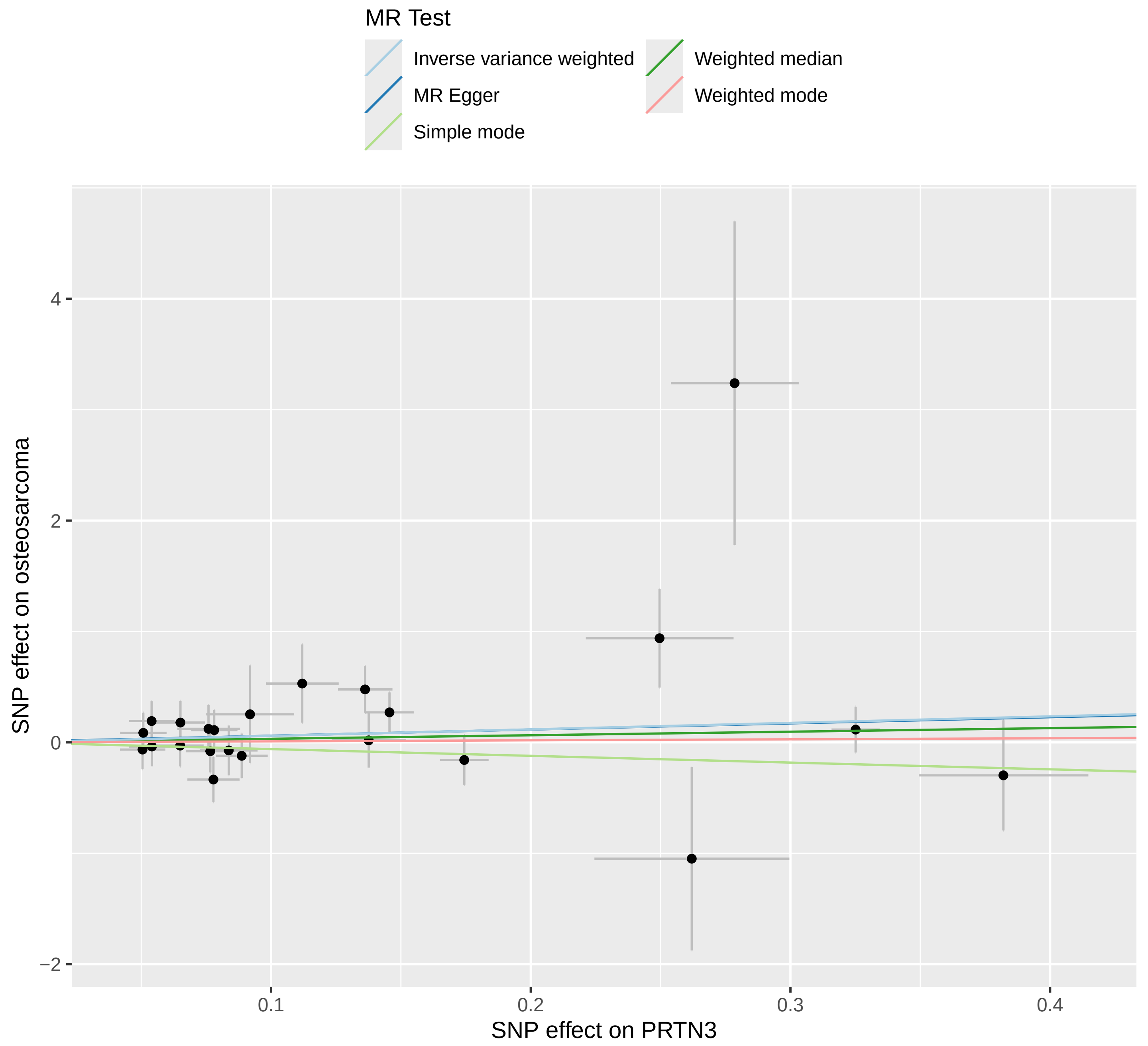
MR Test
Inverse variance weighted
MR Egger
Simple mode
Weighted median
Weighted mode
4
2
0
−2
0.1
0.2
0.3
0.4
SNP effect on osteosarcoma
SNP effect on PRTN3

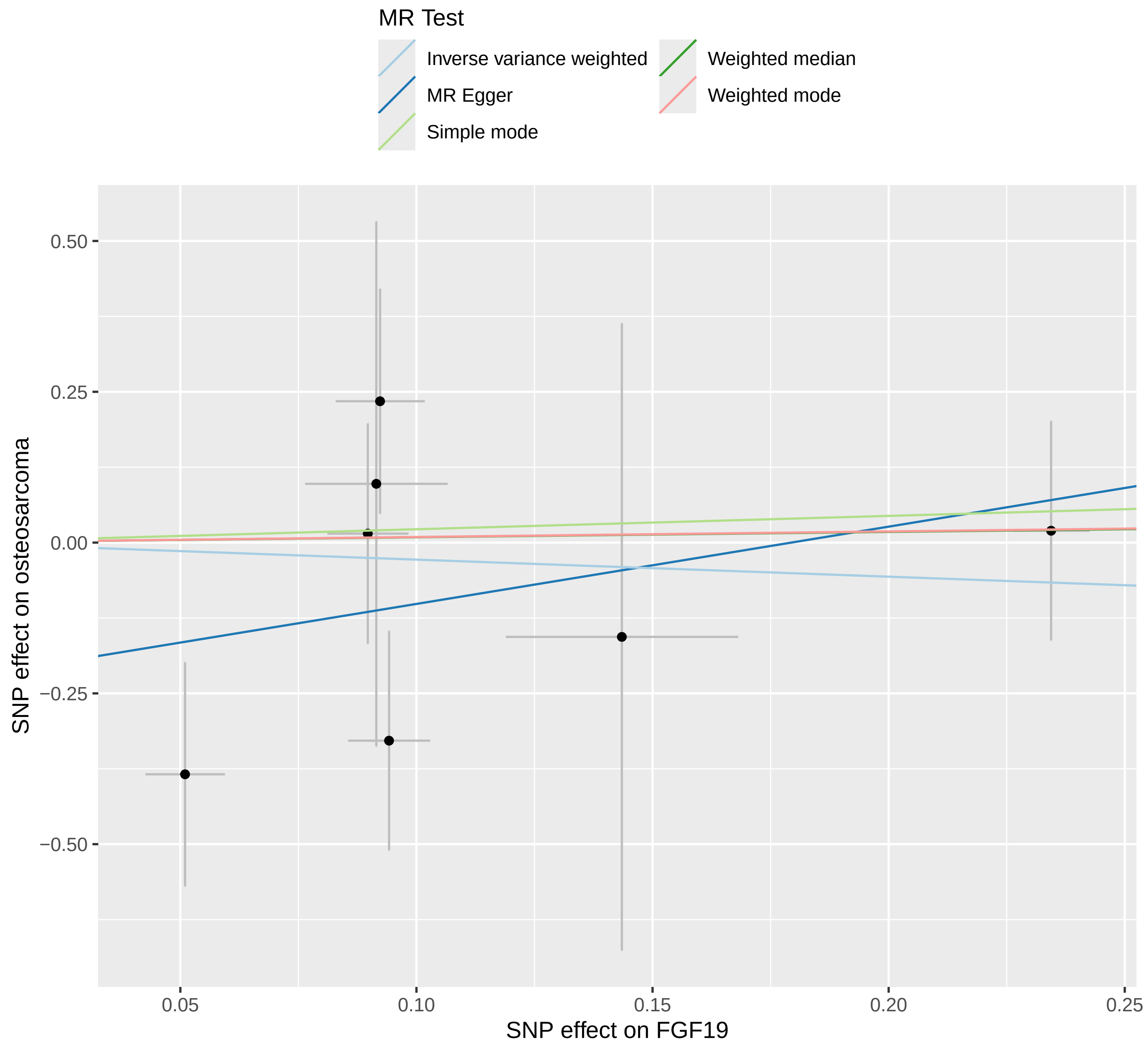
MR Test
Inverse variance weighted
MR Egger
Simple mode
Weighted median
Weighted mode
SNP effect on osteosarcoma
0.50
0.25
0.00
−0.25
−0.50
0.05
0.10
0.15
0.20
0.25
SNP effect on FGF19

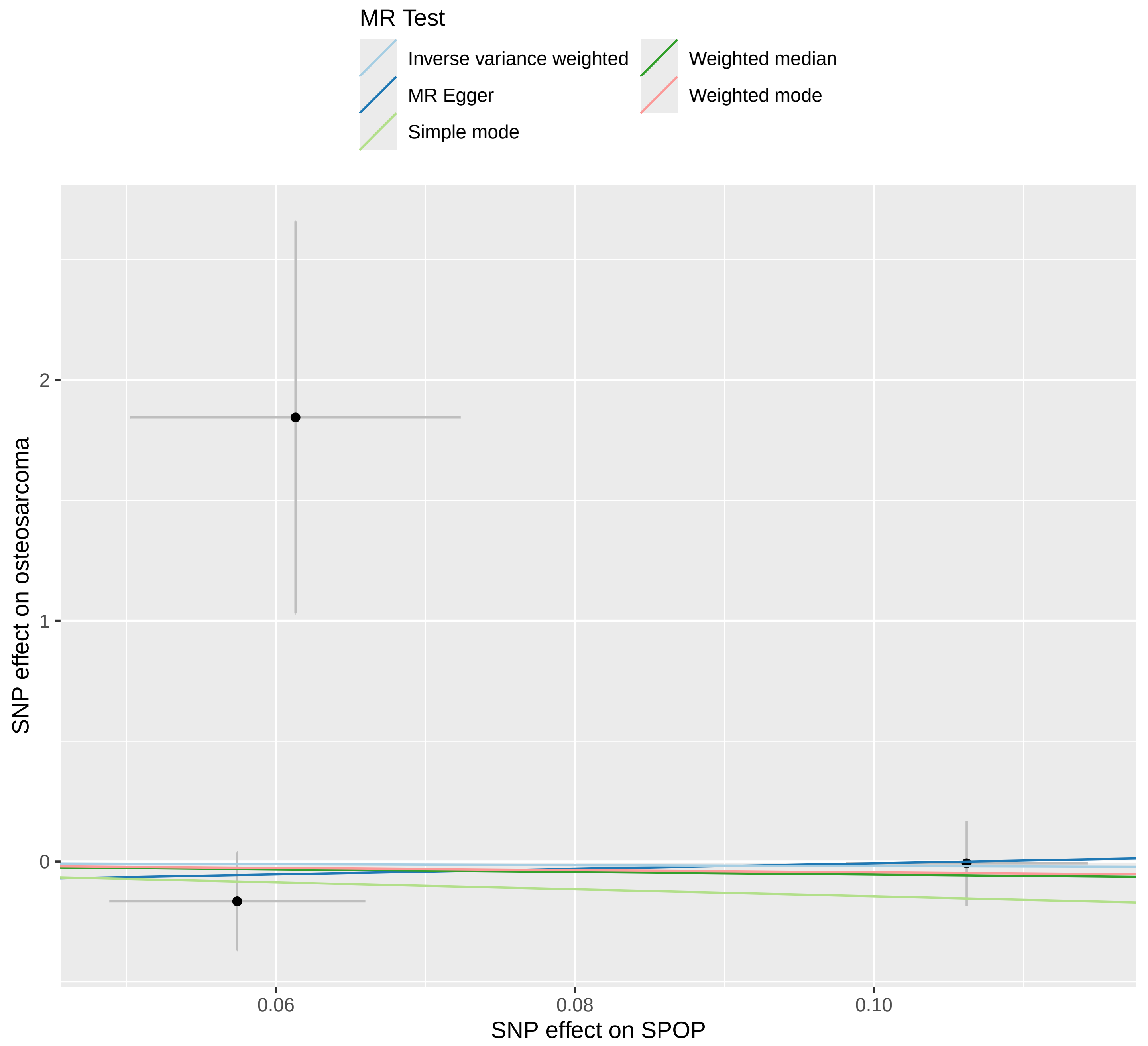

MR Test
Inverse variance weighted
MR Egger
Simple mode
Weighted median
Weighted mode
2
1
0
SNP effect on osteosarcoma
0.06
0.08
0.10
SNP effect on SPOP

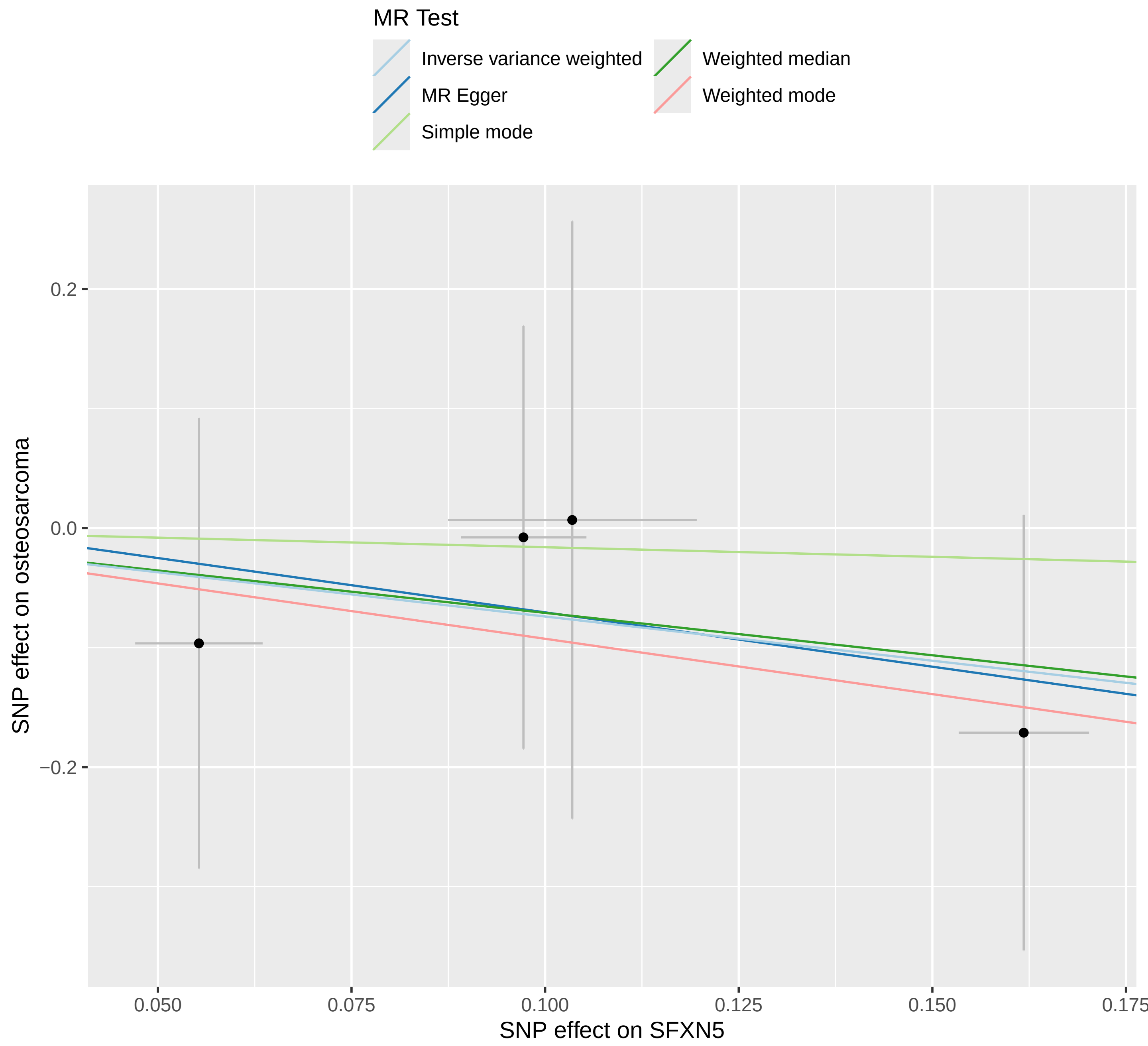
MR Test
Inverse variance weighted
MR Egger
Simple mode
Weighted median
Weighted mode
0.2
0.0
−0.2
SNP effect on osteosarcoma
0.050
0.075
0.100
0.125
0.150
0.175
SNP effect on SFXN5

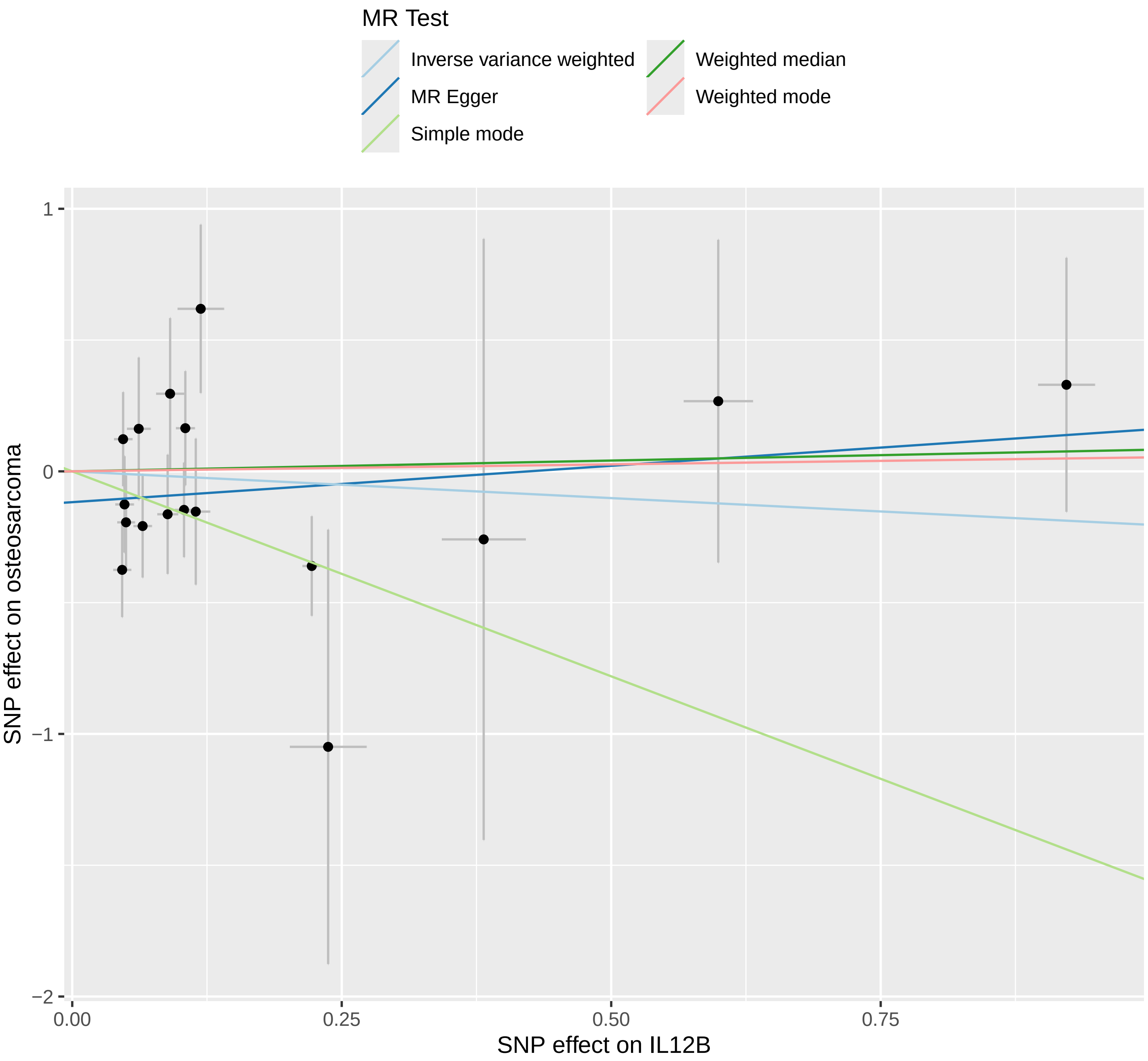

MR Test
Inverse variance weighted
MR Egger
Simple mode
Weighted median
Weighted mode
1
0
−1
−2
0.00
0.25
0.50
0.75
SNP effect on osteosarcoma
SNP effect on IL12B

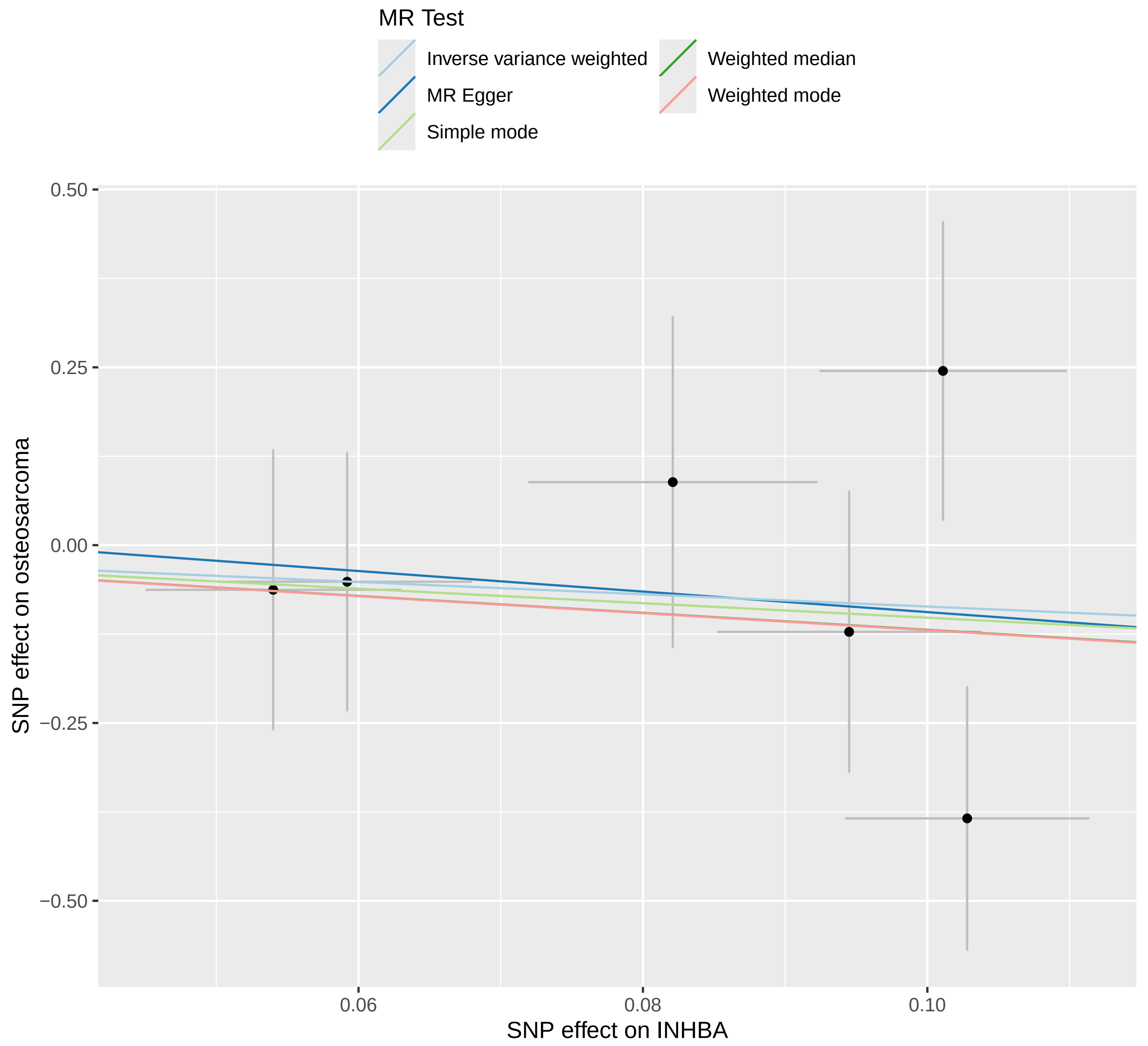

MR Test
Inverse variance weighted
MR Egger
Simple mode
Weighted median
Weighted mode
0.50
0.25
0.00
−0.25
−0.50
SNP effect on osteosarcoma
0.06
0.08
0.10
SNP effect on INHBA

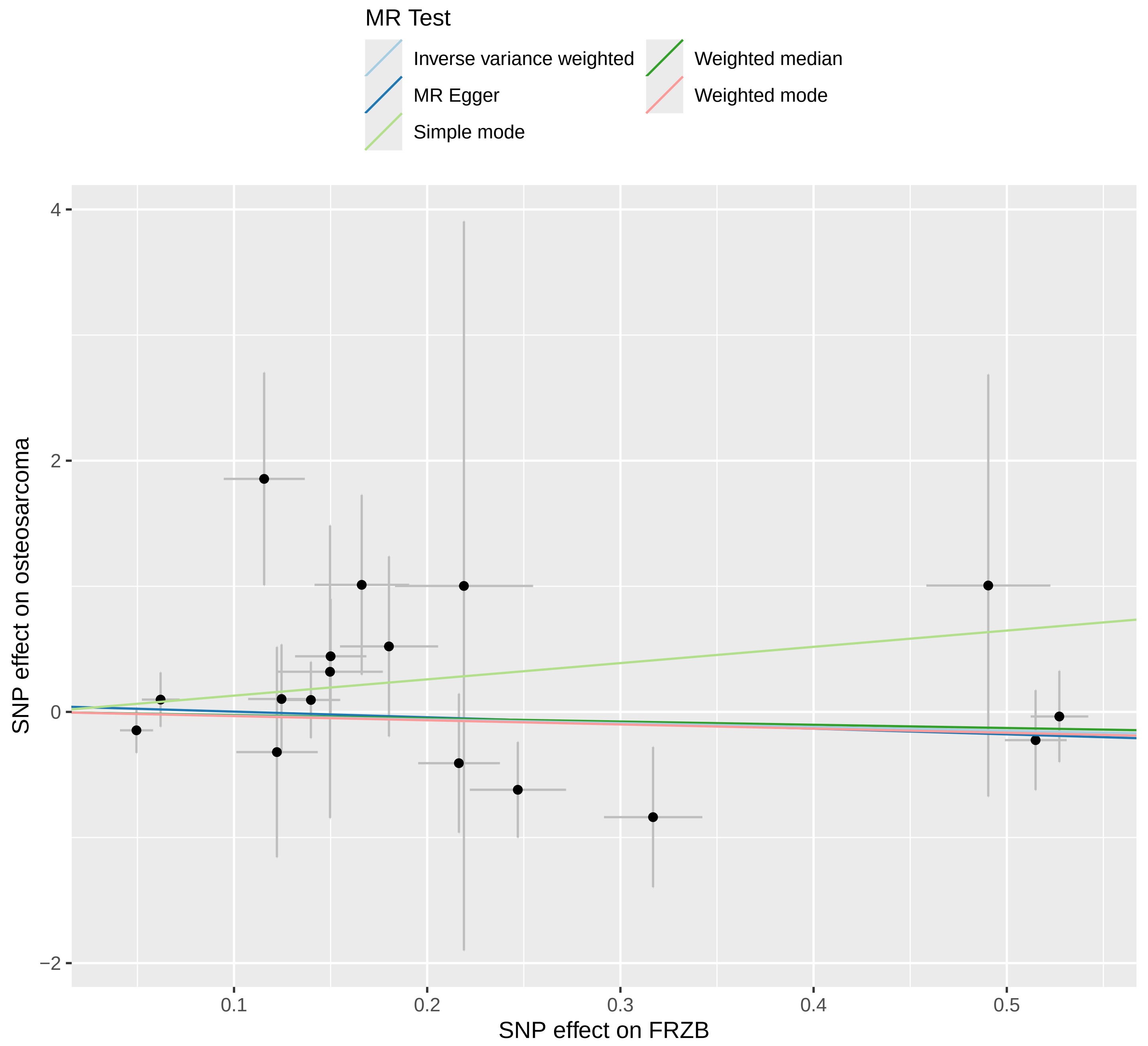

MR Test
Inverse variance weighted
MR Egger
Simple mode
Weighted median
Weighted mode
4
2
0
−2
SNP effect on osteosarcoma
0.1
0.2
0.3
0.4
0.5
SNP effect on FRZB

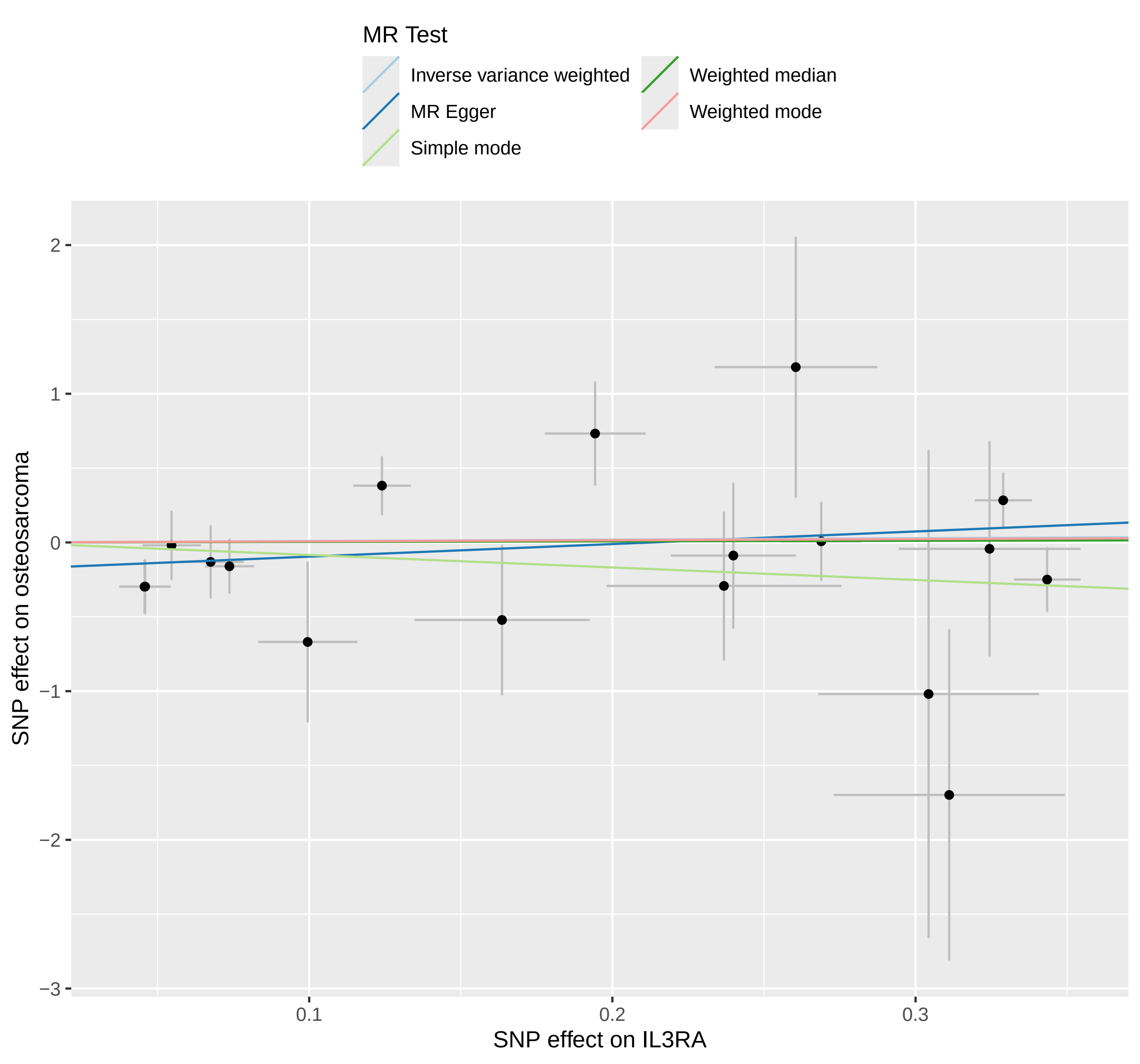
MR Test
Inverse variance weighted
MR Egger
Simple mode
Weighted median
Weighted mode
SNP effect on osteosarcoma
SNP effect on IL3RA
2
1
0
−1
−2
−3
0.1
0.2
0.3

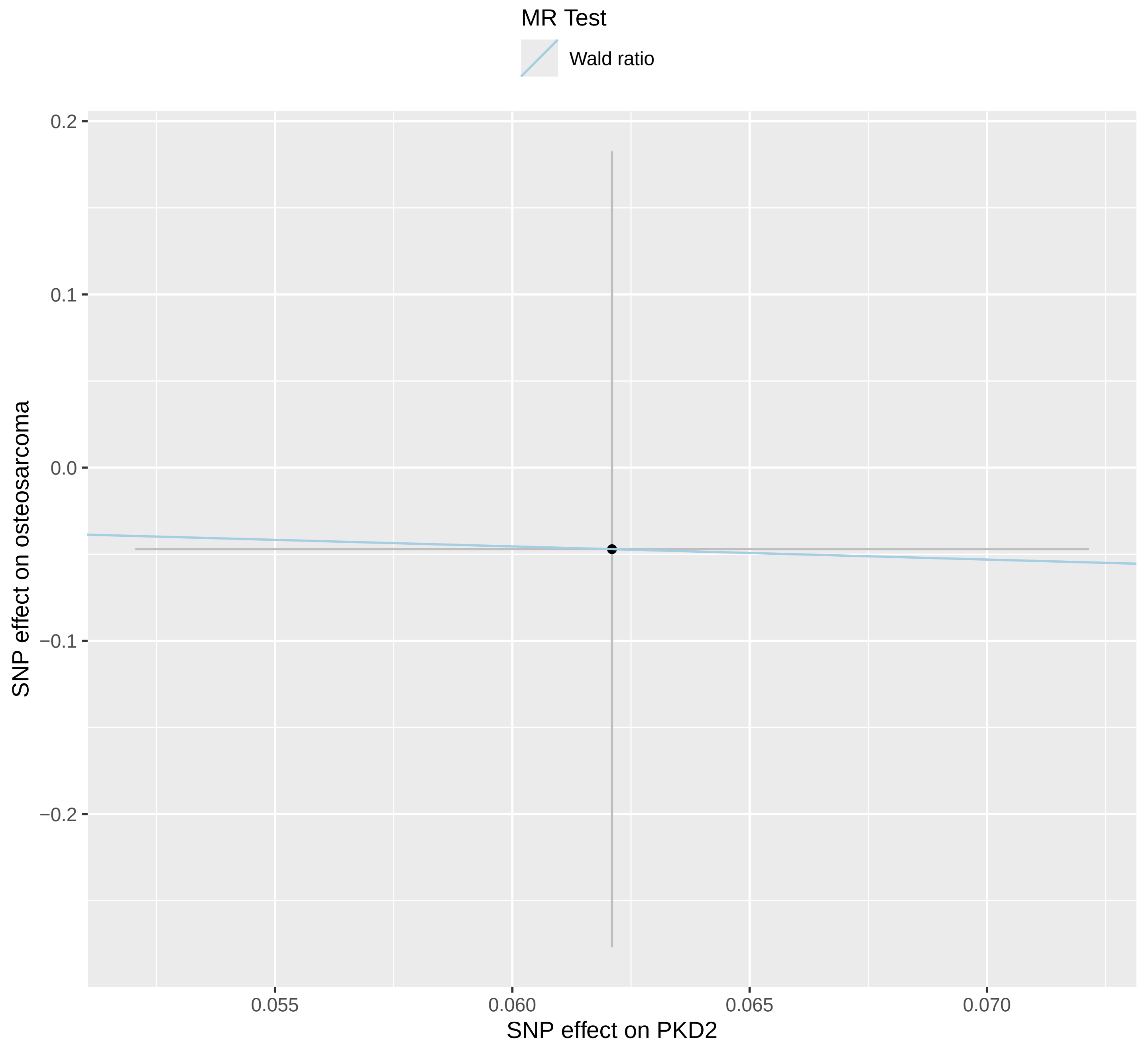
MR Test
Wald ratio
0.2
0.1
0.0
−0.1
−0.2
SNP effect on osteosarcoma
0.055
0.060
0.065
0.070
SNP effect on PKD2

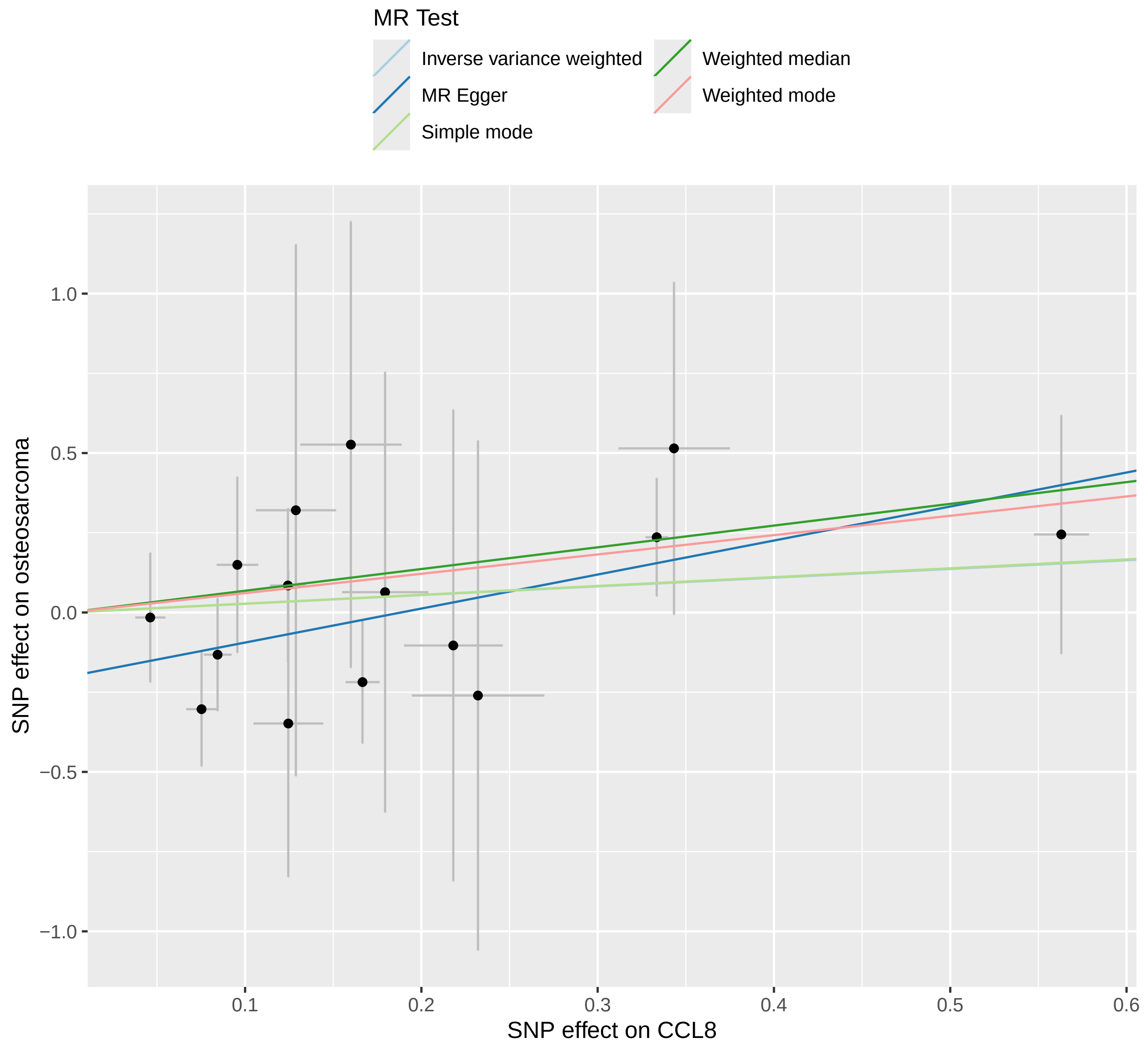

MR Test
Inverse variance weighted
MR Egger
Simple mode
Weighted median
Weighted mode
SNP effect on osteosarcoma
1.0
0.5
0.0
−0.5
−1.0
0.1
0.2
0.3
0.4
0.5
0.6
SNP effect on CCL8

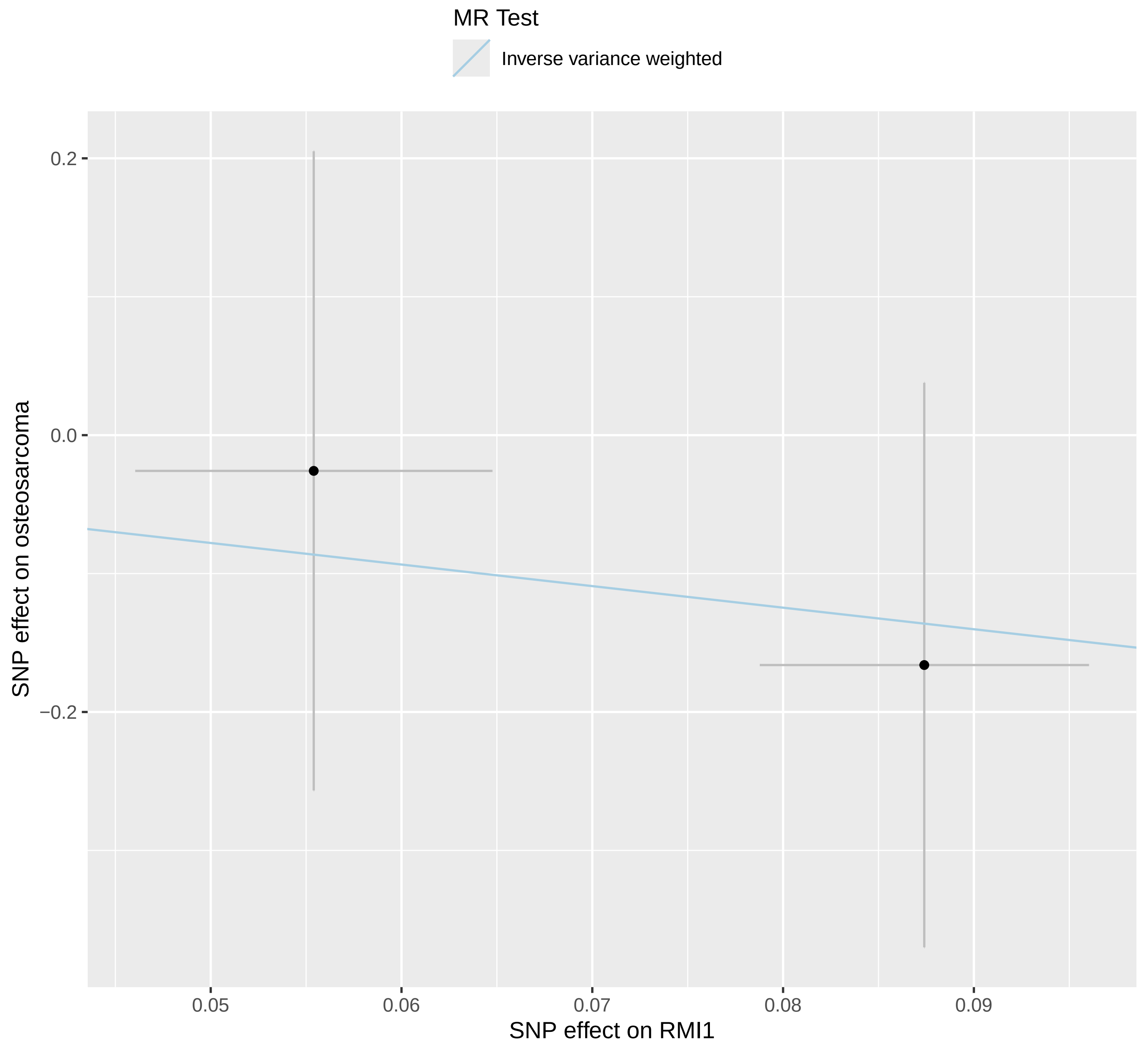
MR Test
Inverse variance weighted
0.2
0.0
−0.2
0.05
0.06
0.07
0.08
0.09
SNP effect on RMI1
SNP effect on osteosarcoma

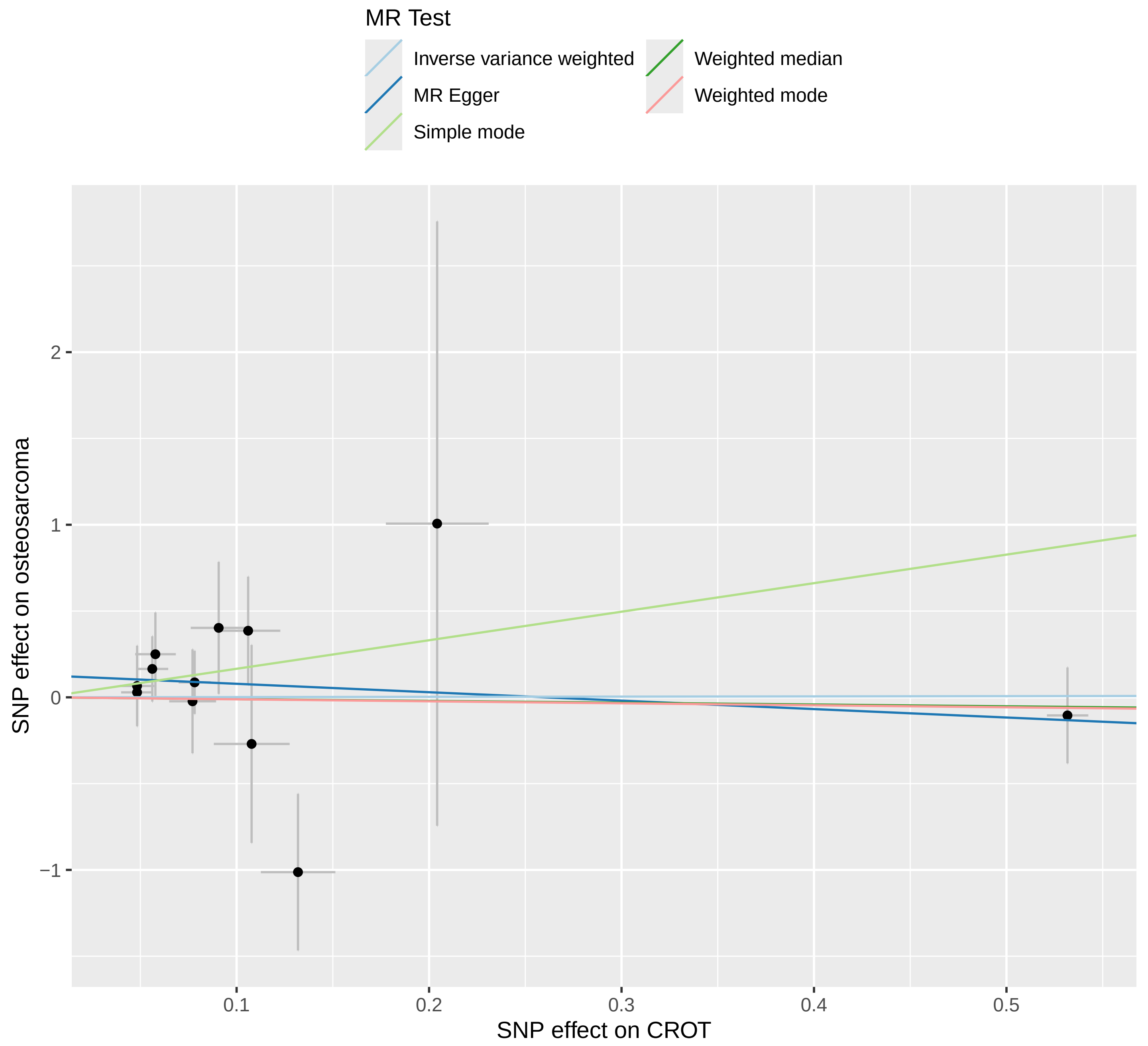
MR Test
Inverse variance weighted
MR Egger
Simple mode
Weighted median
Weighted mode
SNP effect on osteosarcoma
SNP effect on CROT
2
1
0
−1
0.1
0.2
0.3
0.4
0.5

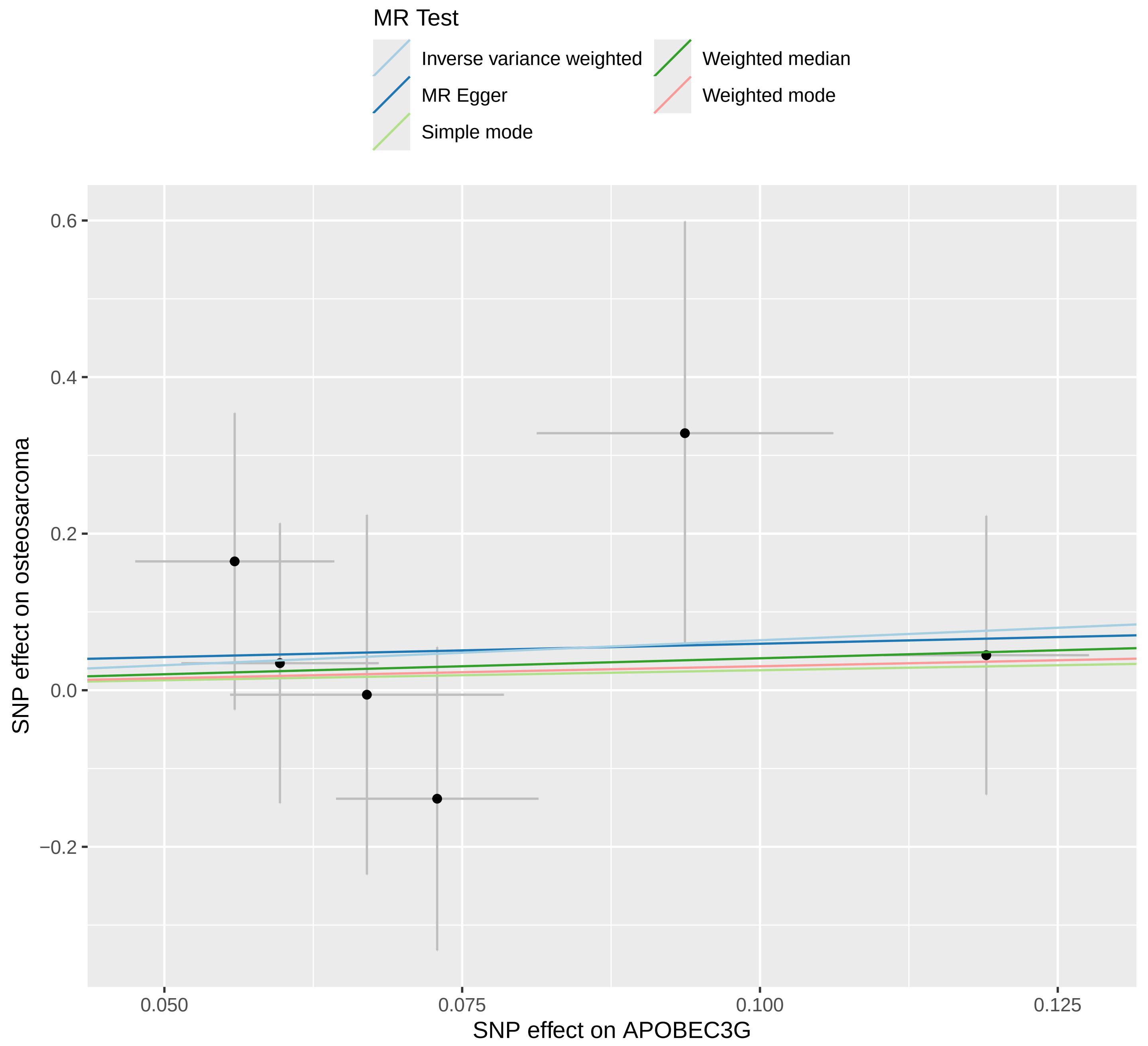

MR Test
Inverse variance weighted
MR Egger
Simple mode
Weighted median
Weighted mode
0.6
0.4
0.2
0.0
−0.2
0.050
0.075
0.100
0.125
SNP effect on osteosarcoma
SNP effect on APOBEC3G

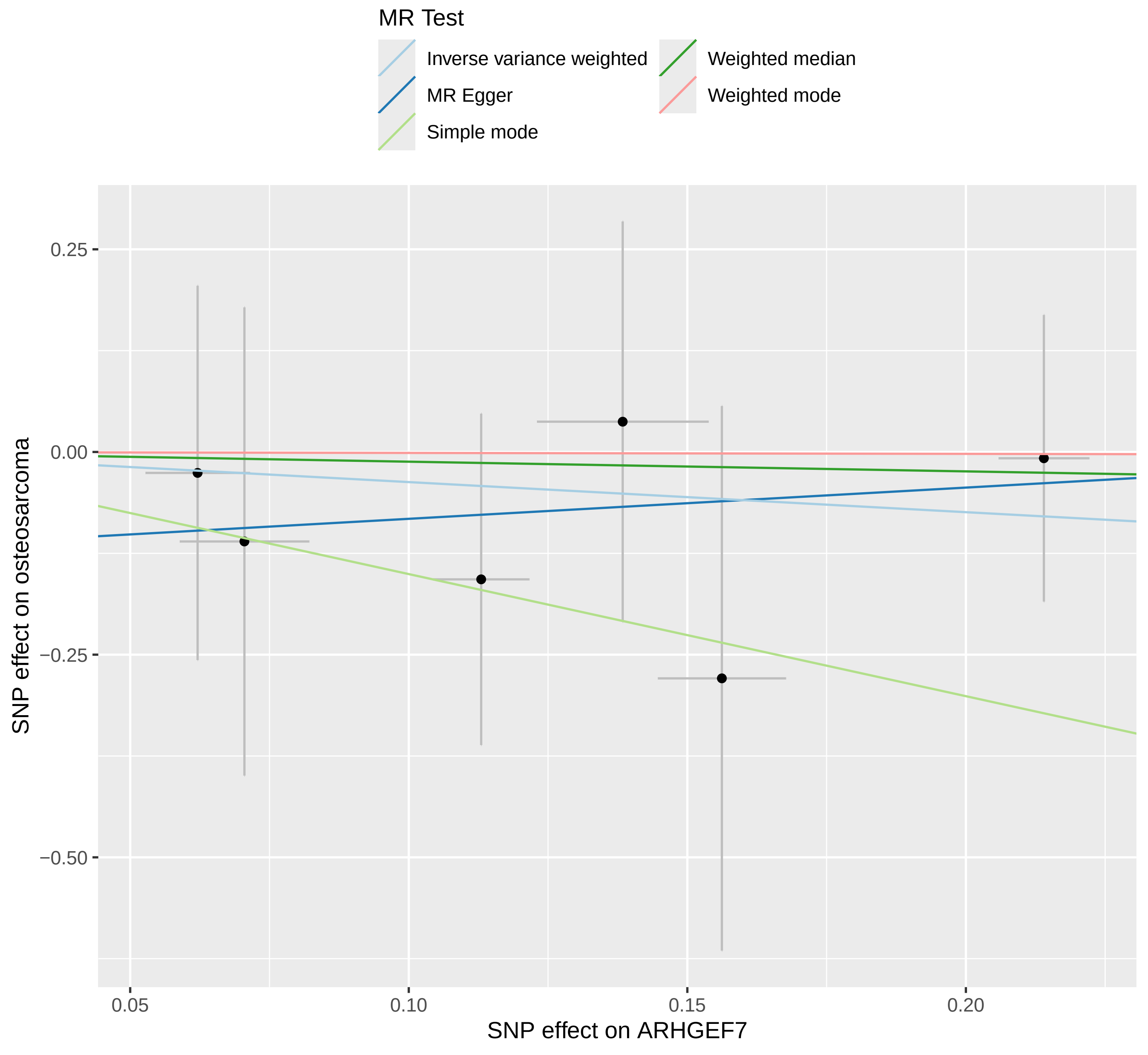
MR Test
Inverse variance weighted
MR Egger
Simple mode
Weighted median
Weighted mode
0.25
0.00
−0.25
−0.50
SNP effect on osteosarcoma
0.05
0.10
0.15
0.20
SNP effect on ARHGEF7

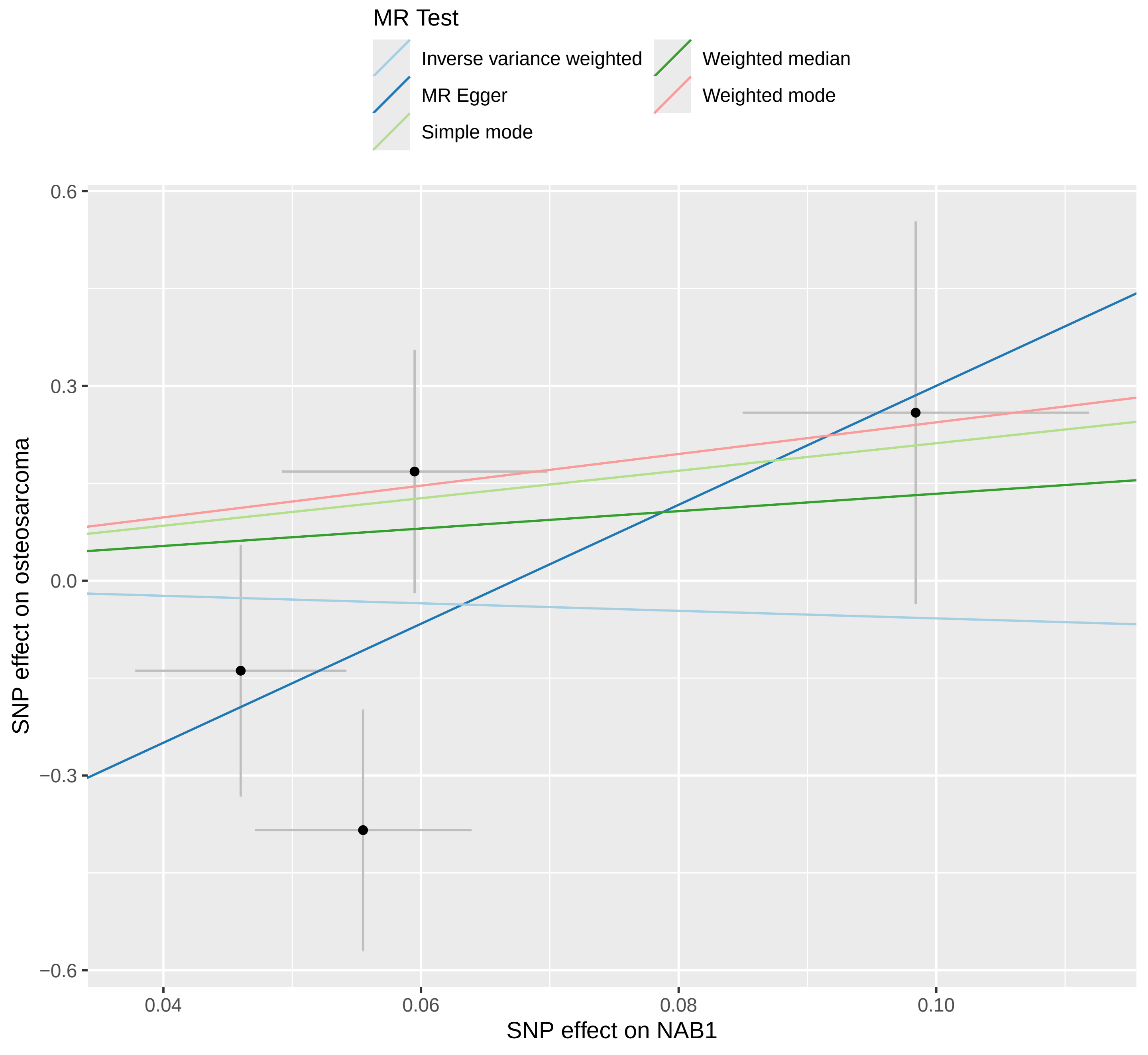
MR Test
Inverse variance weighted
MR Egger
Simple mode
Weighted median
Weighted mode
0.6
0.3
0.0
−0.3
−0.6
SNP effect on osteosarcoma
0.04
0.06
0.08
0.10
SNP effect on NAB1

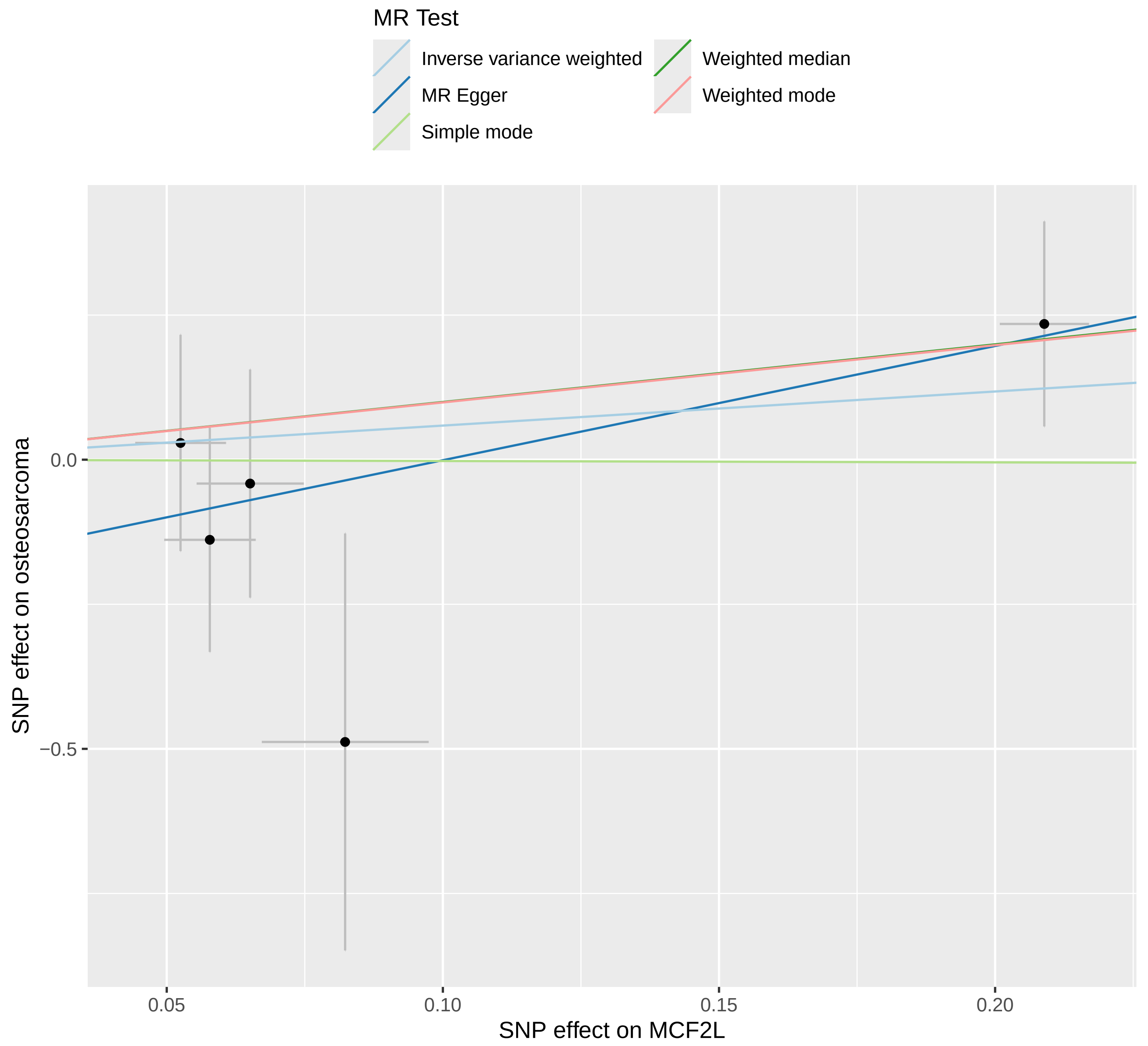
MR Test
Inverse variance weighted
MR Egger
Simple mode
Weighted median
Weighted mode
0.0
−0.5
0.05
0.10
0.15
0.20
SNP effect on osteosarcoma
SNP effect on MCF2L

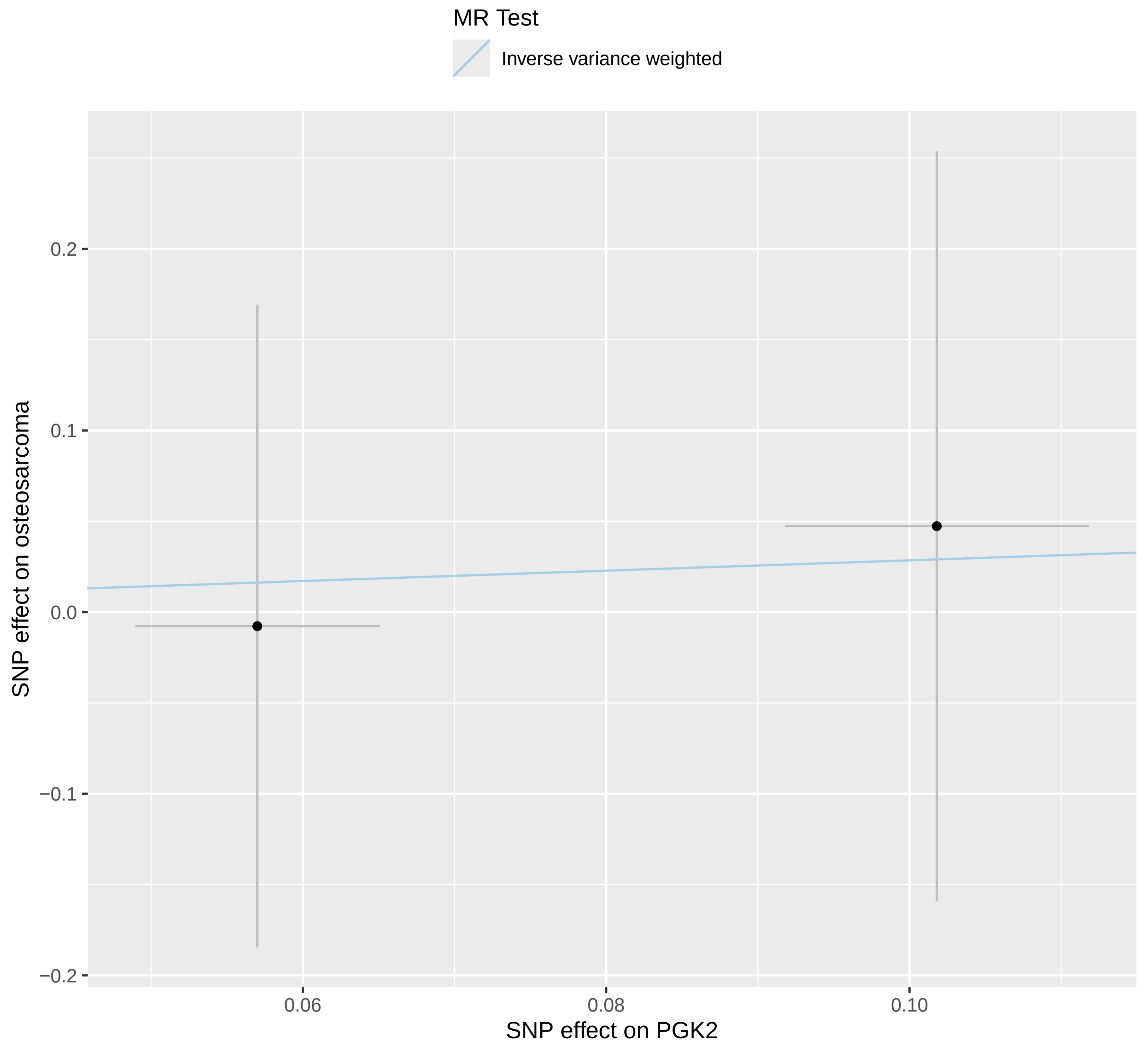
MR Test
Inverse variance weighted
0.2
0.1
0.0
−0.1
−0.2
SNP effect on osteosarcoma
0.06
0.08
0.10
SNP effect on PGK2

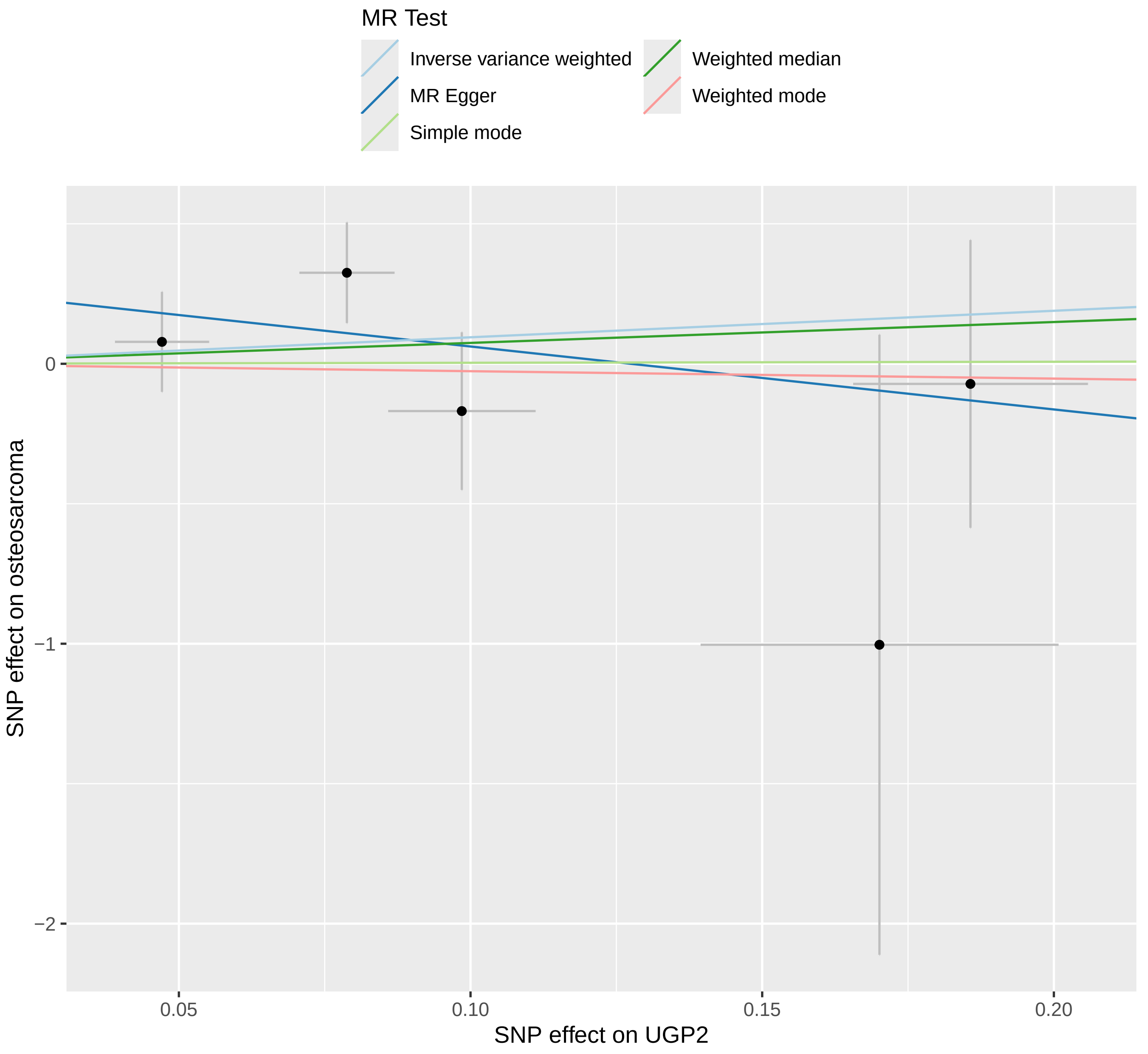
MR Test
Inverse variance weighted
MR Egger
Simple mode
Weighted median
Weighted mode
0
−1
−2
0.05
0.10
0.15
0.20
SNP effect on osteosarcoma
SNP effect on UGP2

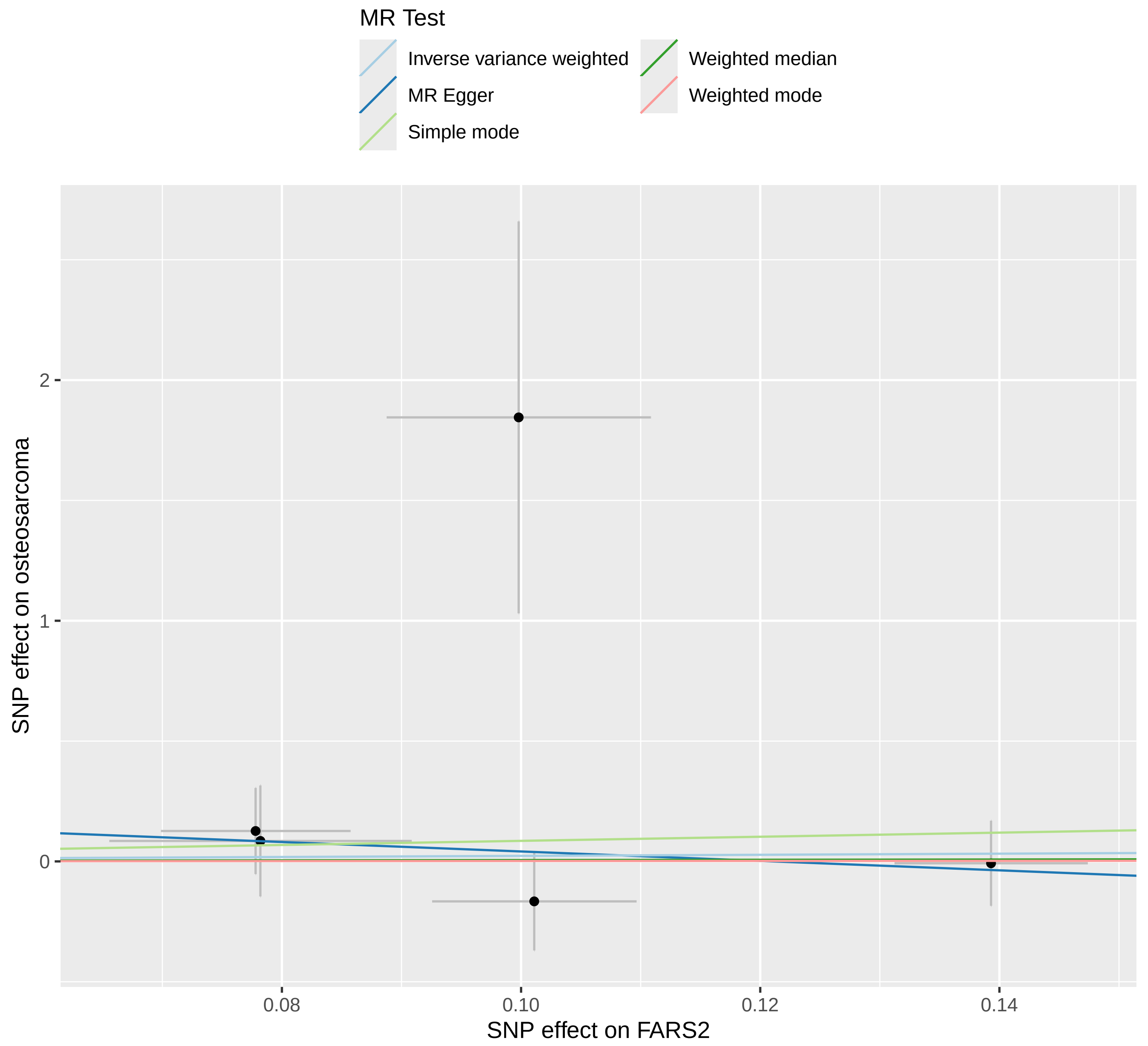
MR Test
Inverse variance weighted
MR Egger
Simple mode
Weighted median
Weighted mode
SNP effect on osteosarcoma
2
1
0
0.08
0.10
0.12
0.14
SNP effect on FARS2

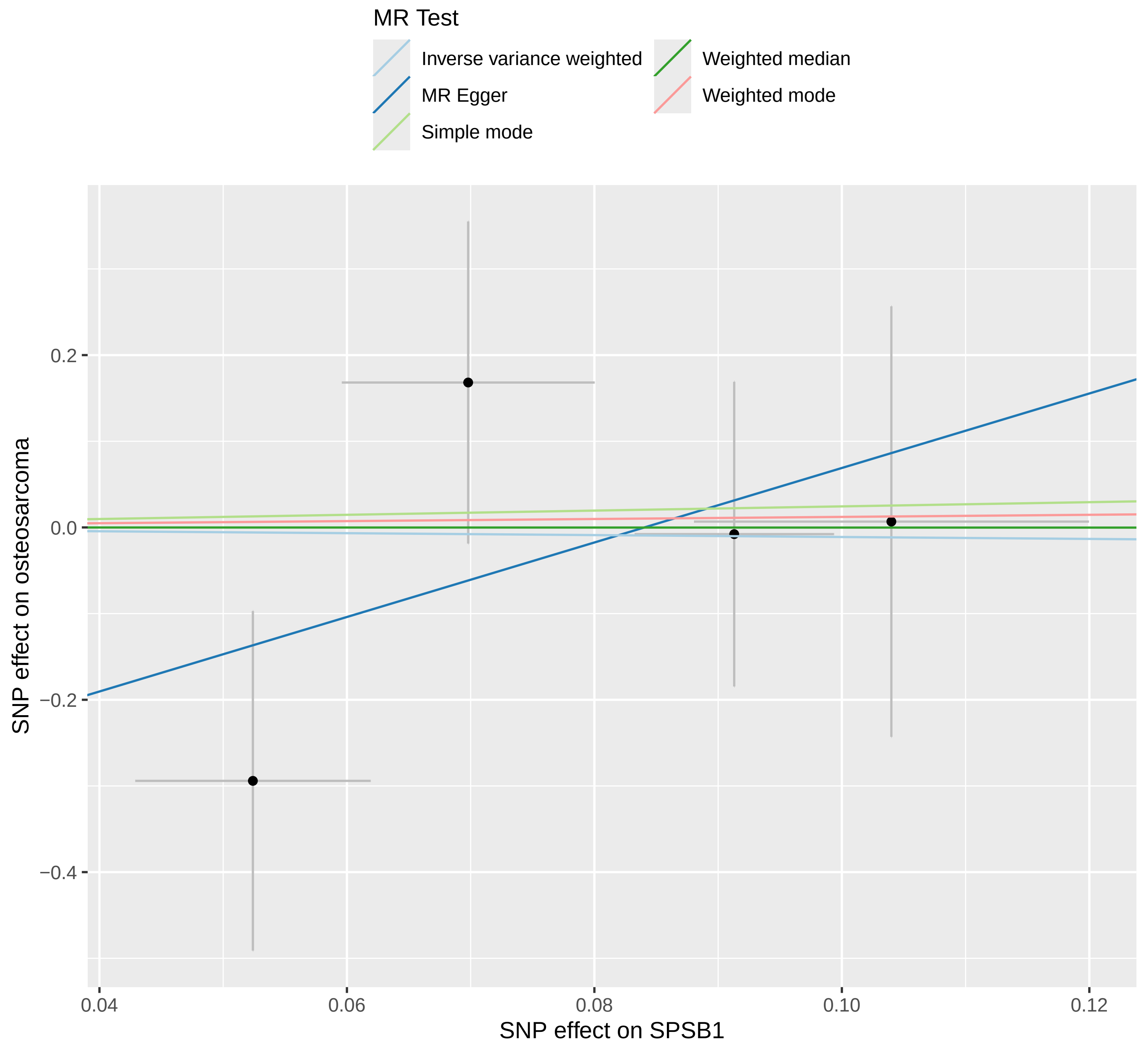

MR Test
Inverse variance weighted
MR Egger
Simple mode
Weighted median
Weighted mode
0.2
0.0
−0.2
−0.4
SNP effect on osteosarcoma
0.04
0.06
0.08
0.10
0.12
SNP effect on SPSB1

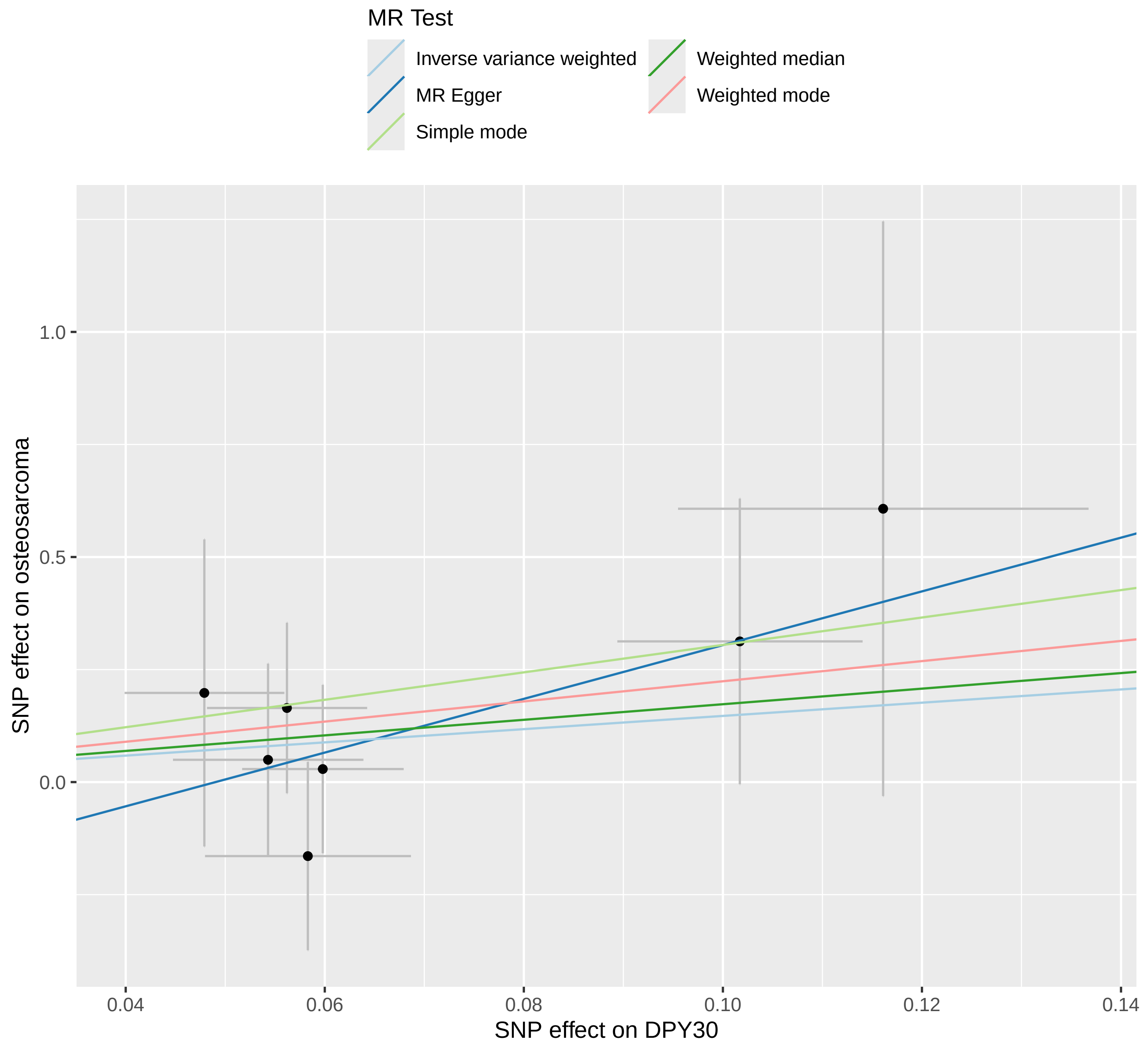
MR Test
Inverse variance weighted
MR Egger
Simple mode
Weighted median
Weighted mode
1.0
0.5
0.0
SNP effect on osteosarcoma
0.04
0.06
0.08
0.10
0.12
0.14
SNP effect on DPY30

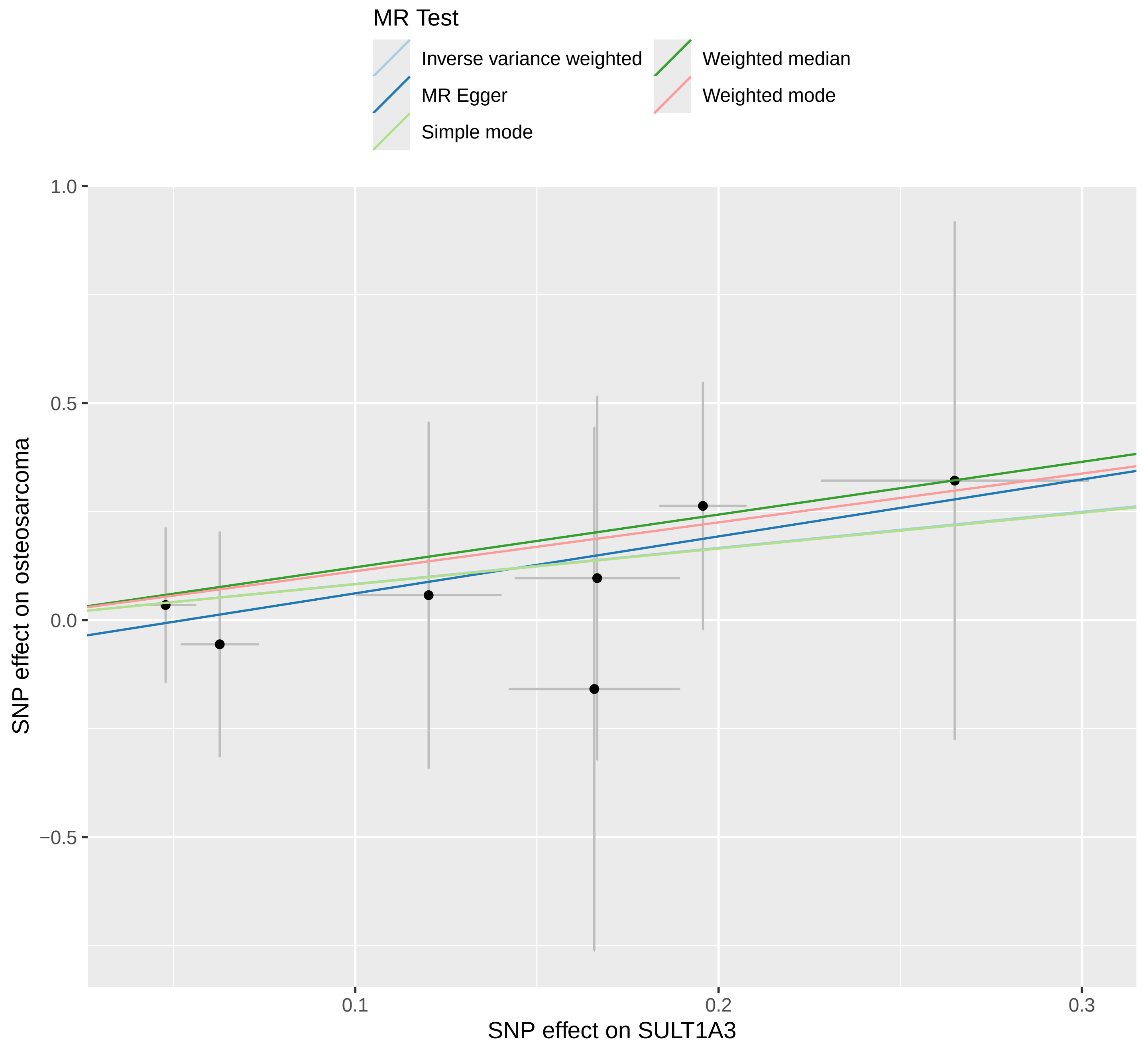
MR Test
Inverse variance weighted
MR Egger
Simple mode
Weighted median
Weighted mode
1.0
0.5
0.0
−0.5
0.1
0.2
0.3
SNP effect on osteosarcoma
SNP effect on SULT1A3

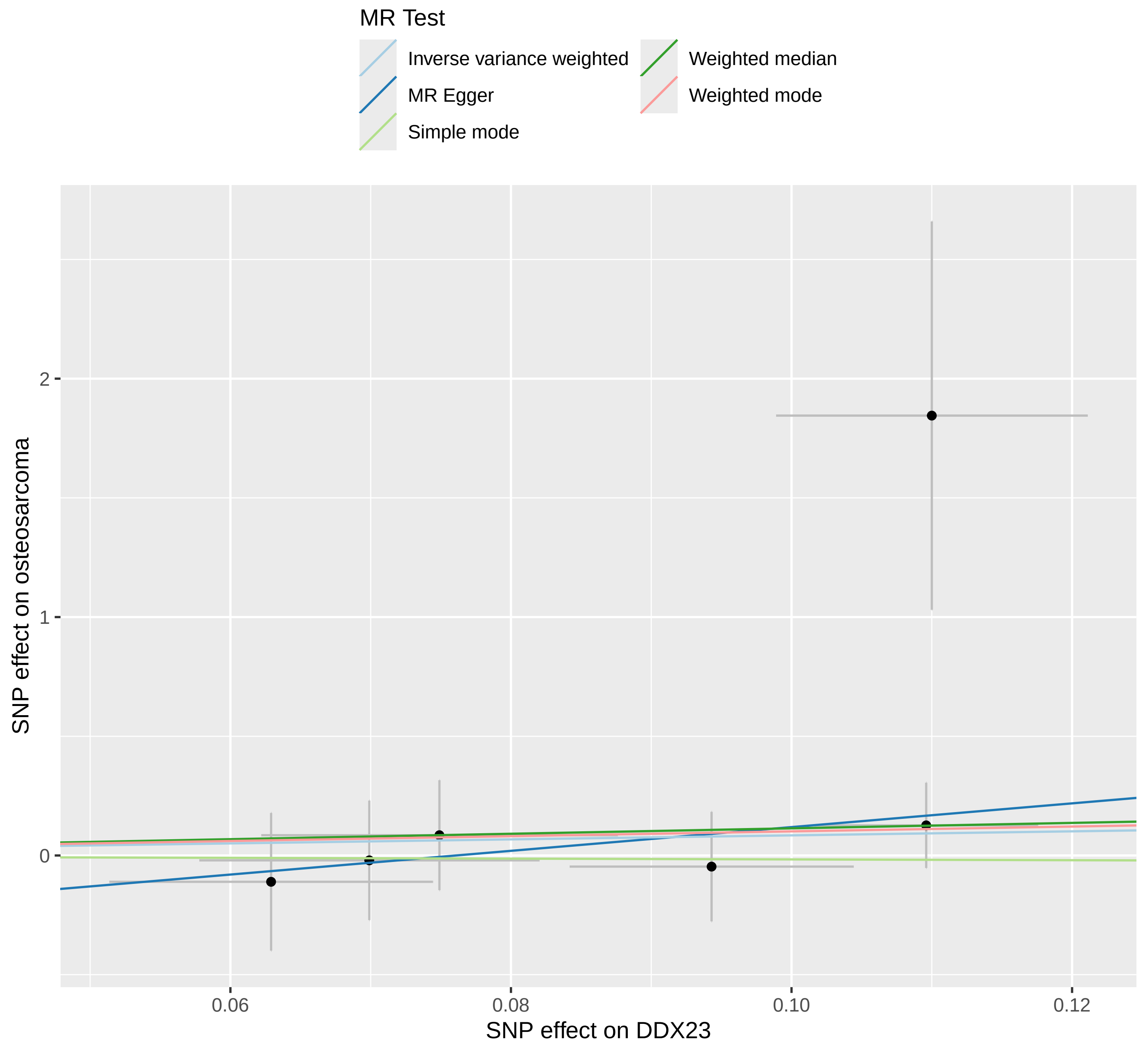
MR Test
Inverse variance weighted
MR Egger
Simple mode
Weighted median
Weighted mode
SNP effect on osteosarcoma
2
1
0
0.06
0.08
0.10
0.12
SNP effect on DDX23

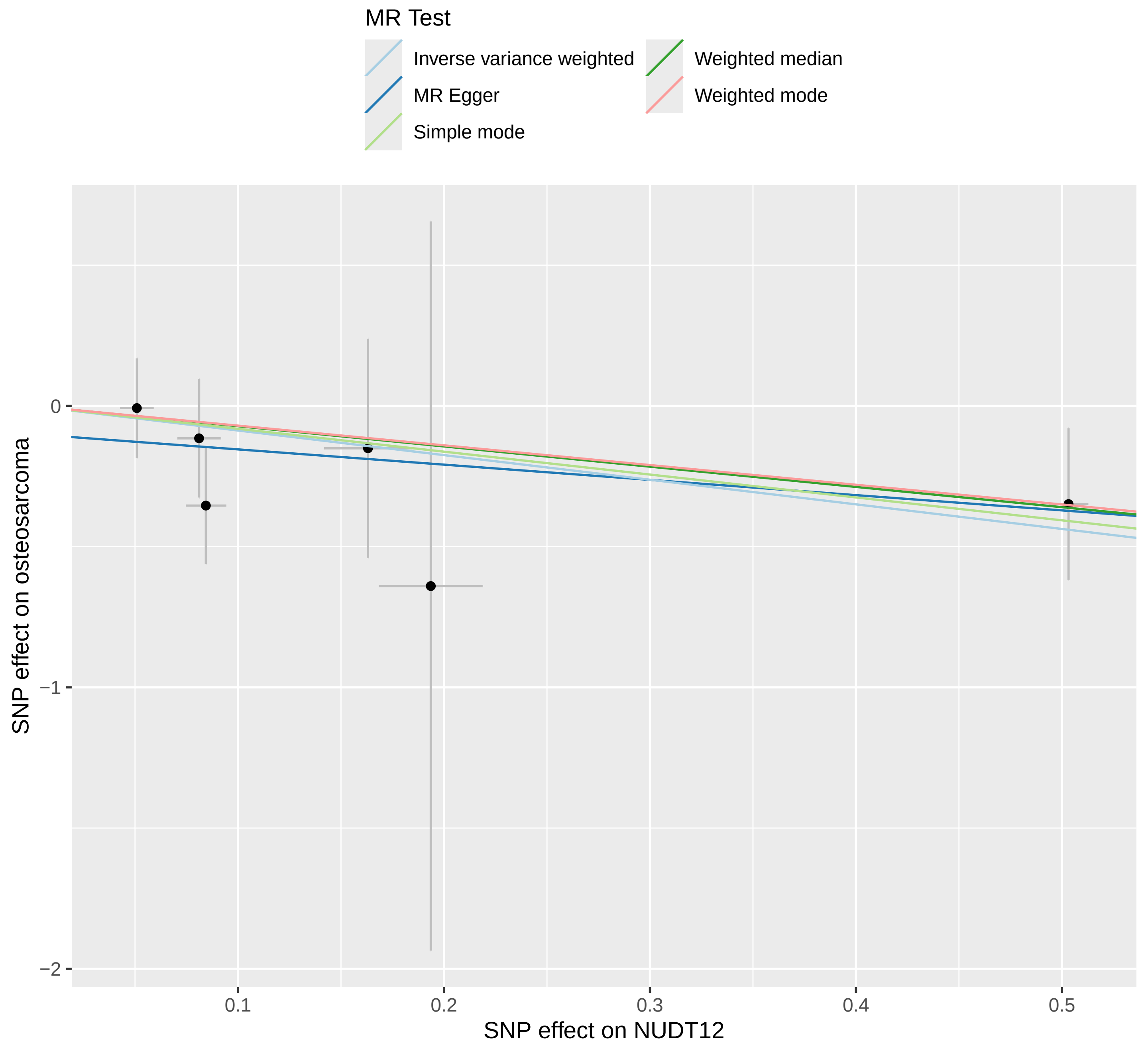
MR Test
Inverse variance weighted
MR Egger
Simple mode
Weighted median
Weighted mode
0
−1
−2
0.1
0.2
0.3
0.4
0.5
SNP effect on osteosarcoma
SNP effect on NUDT12

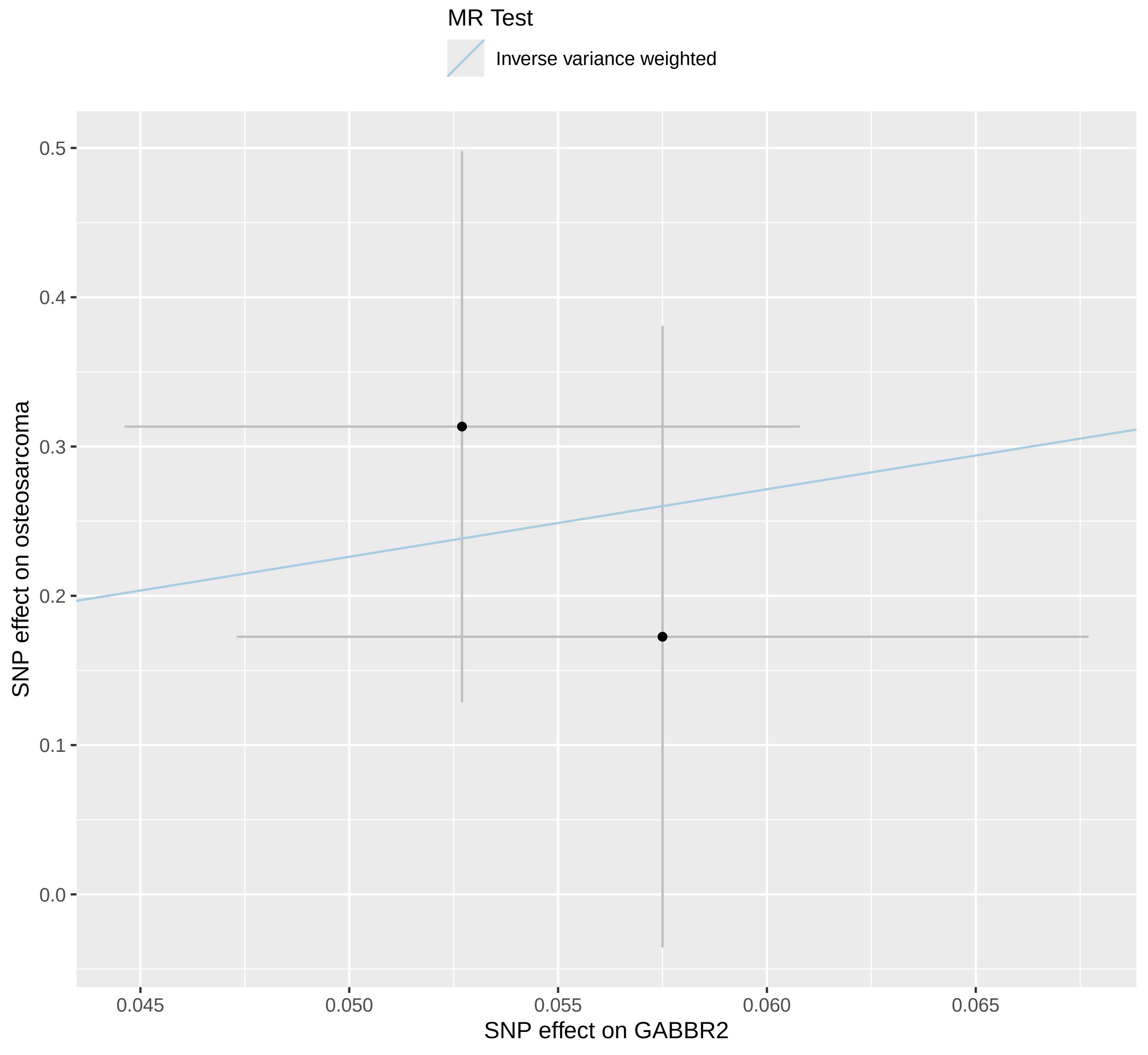

MR Test
Inverse variance weighted
SNP effect on osteosarcoma
0.5
0.4
0.3
0.2
0.1
0.0
0.045
0.050
0.055
0.060
0.065
SNP effect on GABBR2

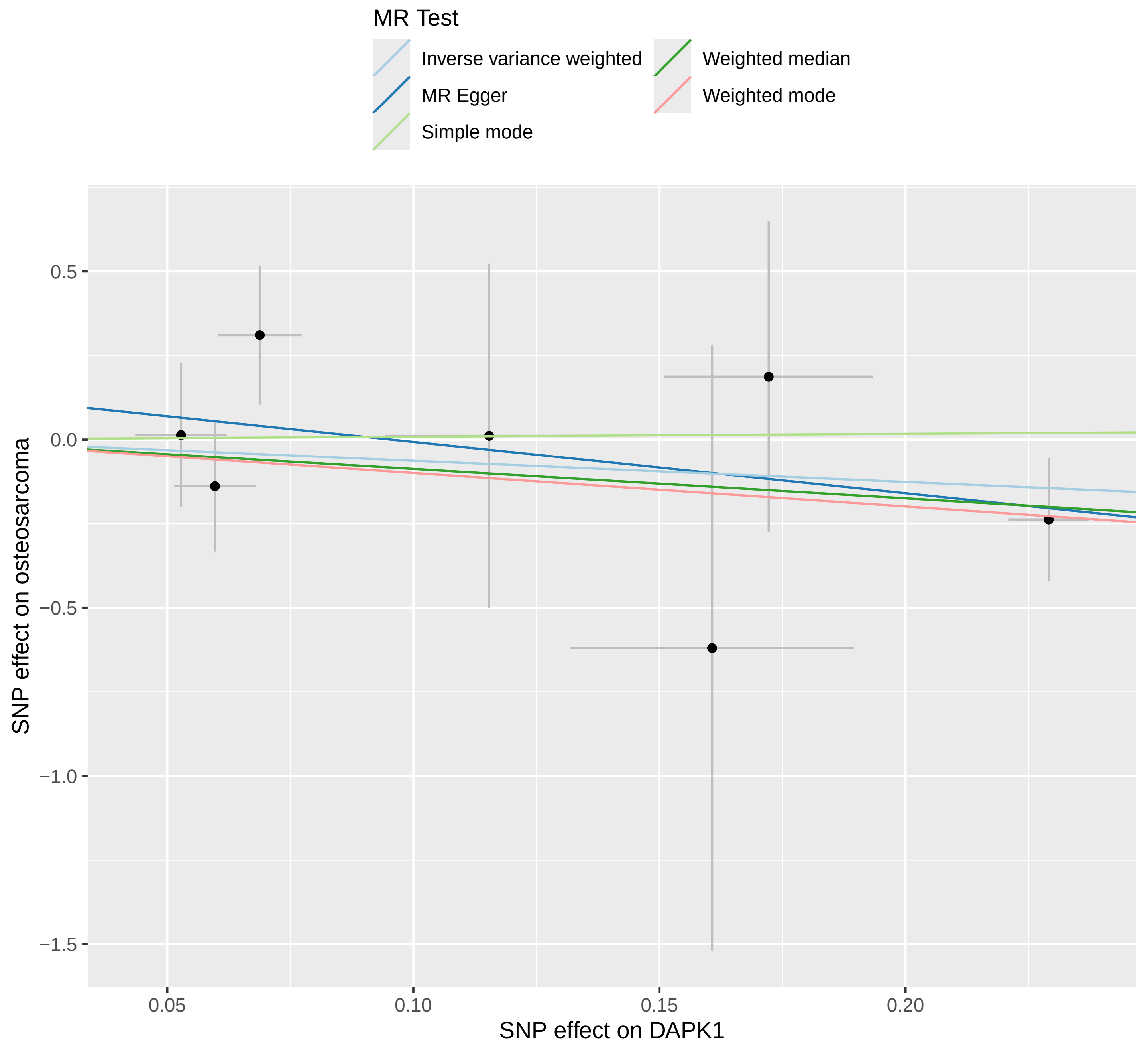
MR Test
Inverse variance weighted
MR Egger
Simple mode
Weighted median
Weighted mode
0.5
0.0
−0.5
−1.0
−1.5
0.05
0.10
0.15
0.20
SNP effect on osteosarcoma
SNP effect on DAPK1

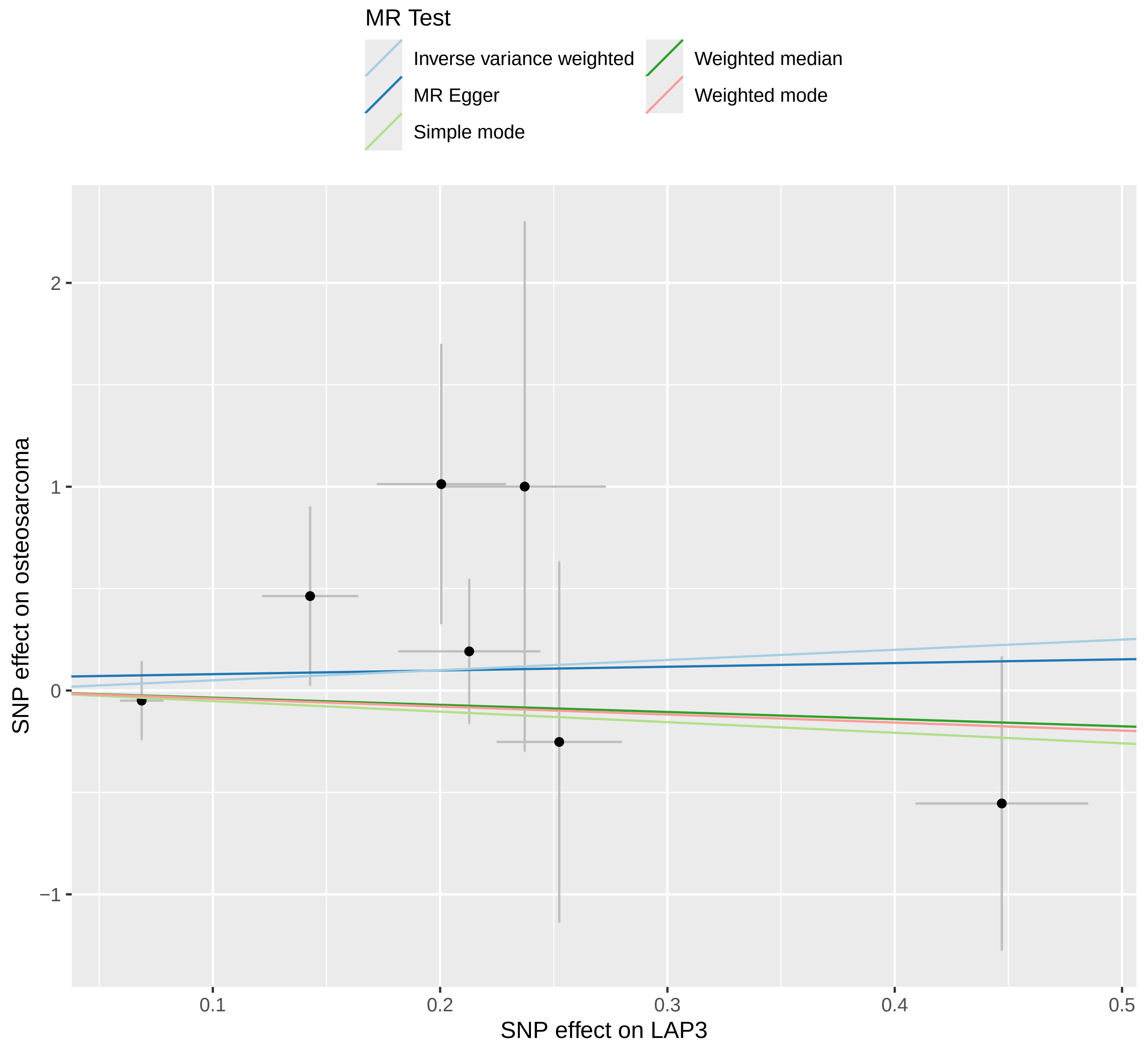
MR Test
Inverse variance weighted
MR Egger
Simple mode
Weighted median
Weighted mode
SNP effect on osteosarcoma
2
1
0
−1
0.1
0.2
0.3
0.4
0.5
SNP effect on LAP3

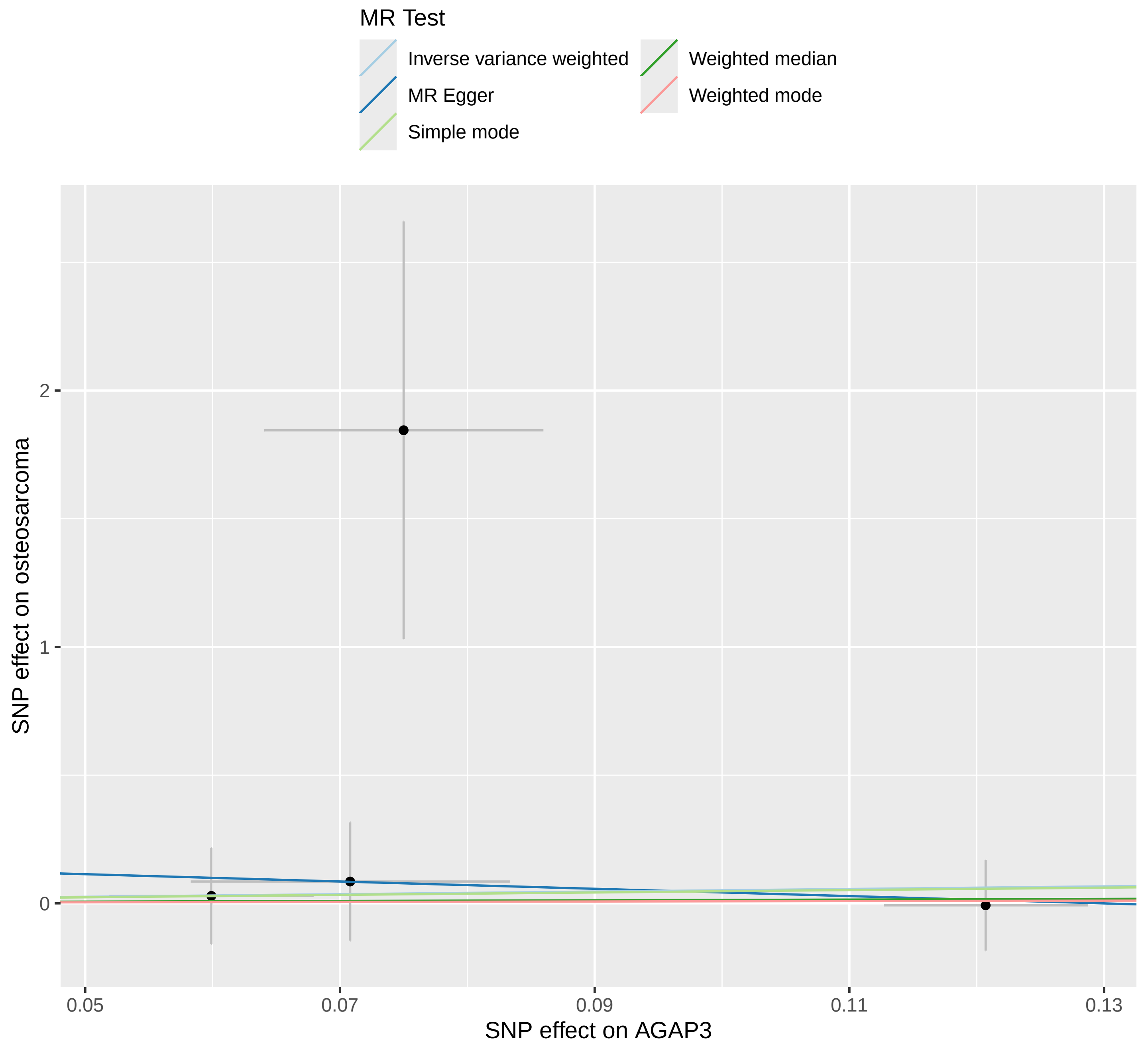
MR Test
Inverse variance weighted
MR Egger
Simple mode
Weighted median
Weighted mode
SNP effect on osteosarcoma
2
1
0
0.05
0.07
0.09
0.11
0.13
SNP effect on AGAP3

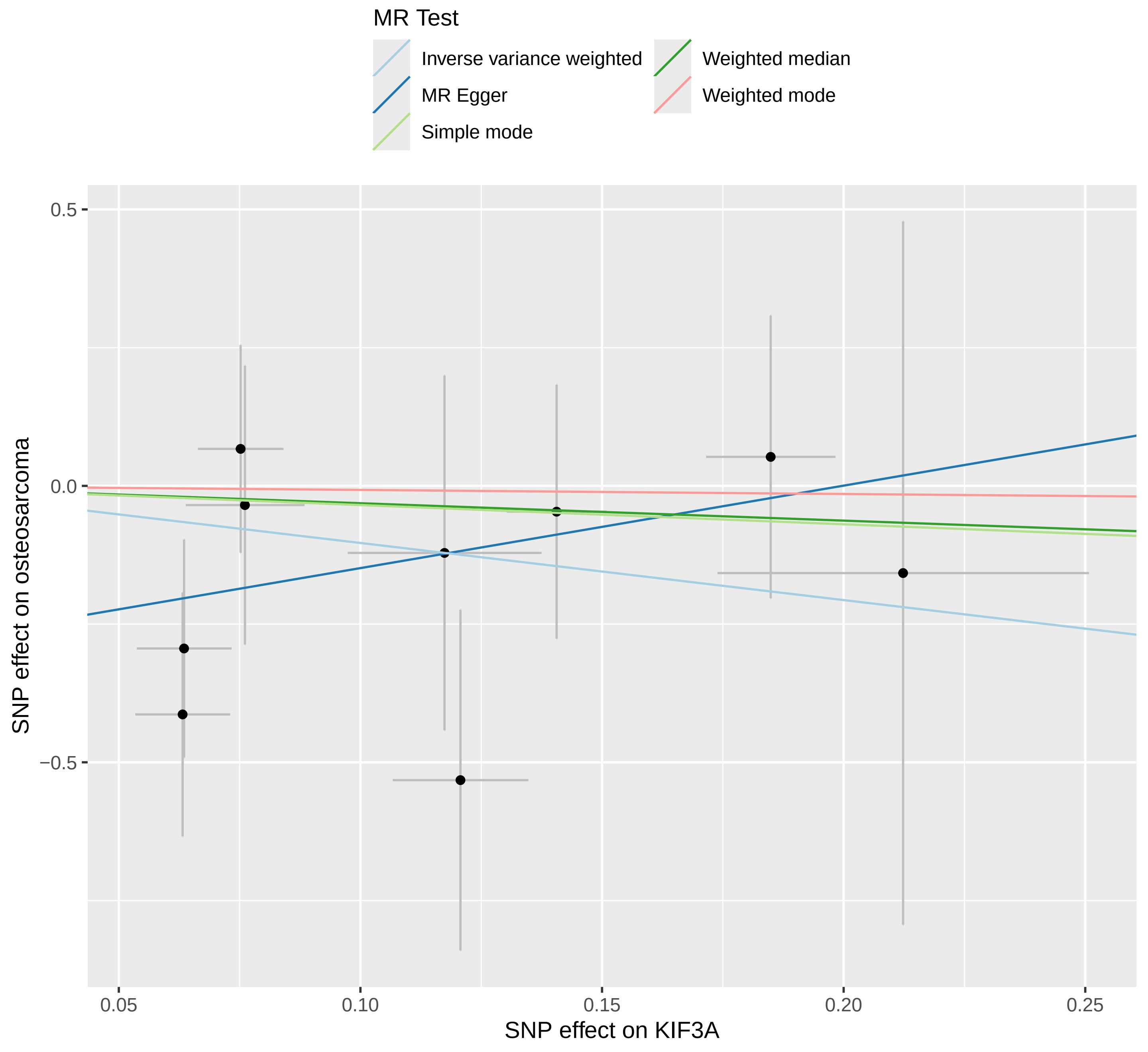
MR Test
Inverse variance weighted
MR Egger
Simple mode
Weighted median
Weighted mode
0.5
0.0
−0.5
0.05
0.10
0.15
0.20
0.25
SNP effect on osteosarcoma
SNP effect on KIF3A

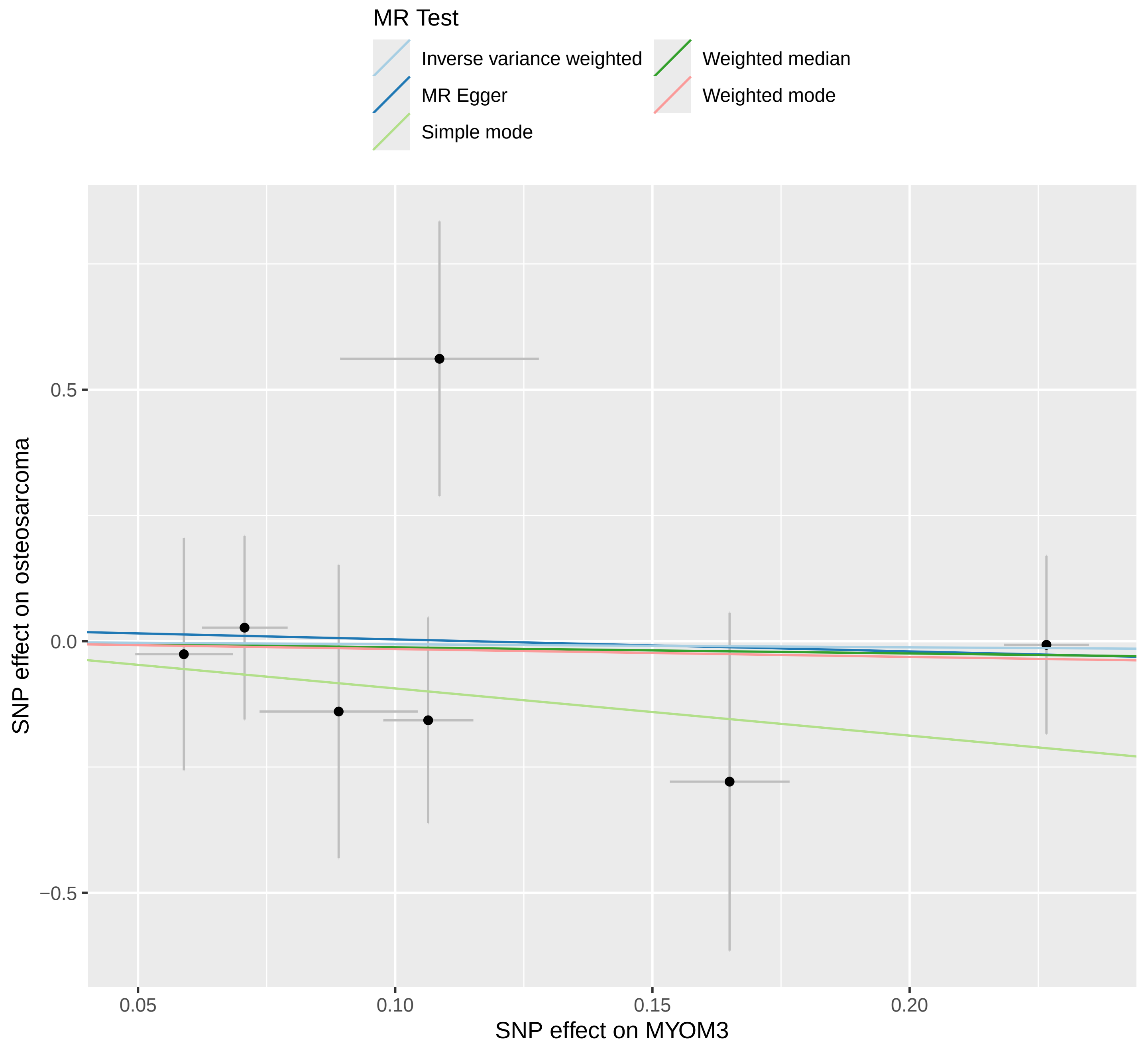
MR Test
Inverse variance weighted
MR Egger
Simple mode
Weighted median
Weighted mode
SNP effect on osteosarcoma
0.5
0.0
−0.5
0.05
0.10
0.15
0.20
SNP effect on MYOM3

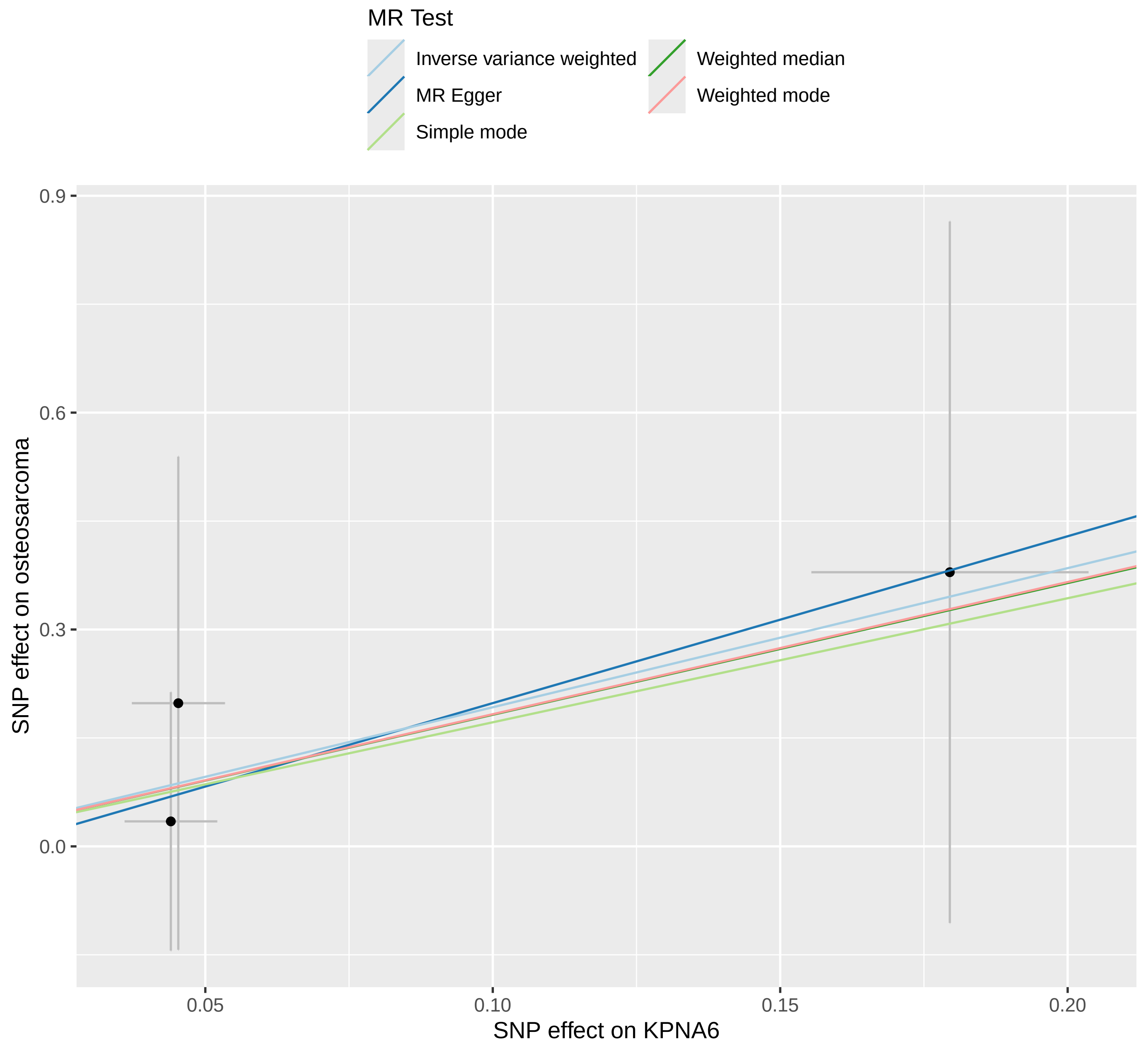
MR Test
Inverse variance weighted
MR Egger
Simple mode
Weighted median
Weighted mode
0.9
0.6
0.3
0.0
SNP effect on osteosarcoma
0.05
0.10
0.15
0.20
SNP effect on KPNA6

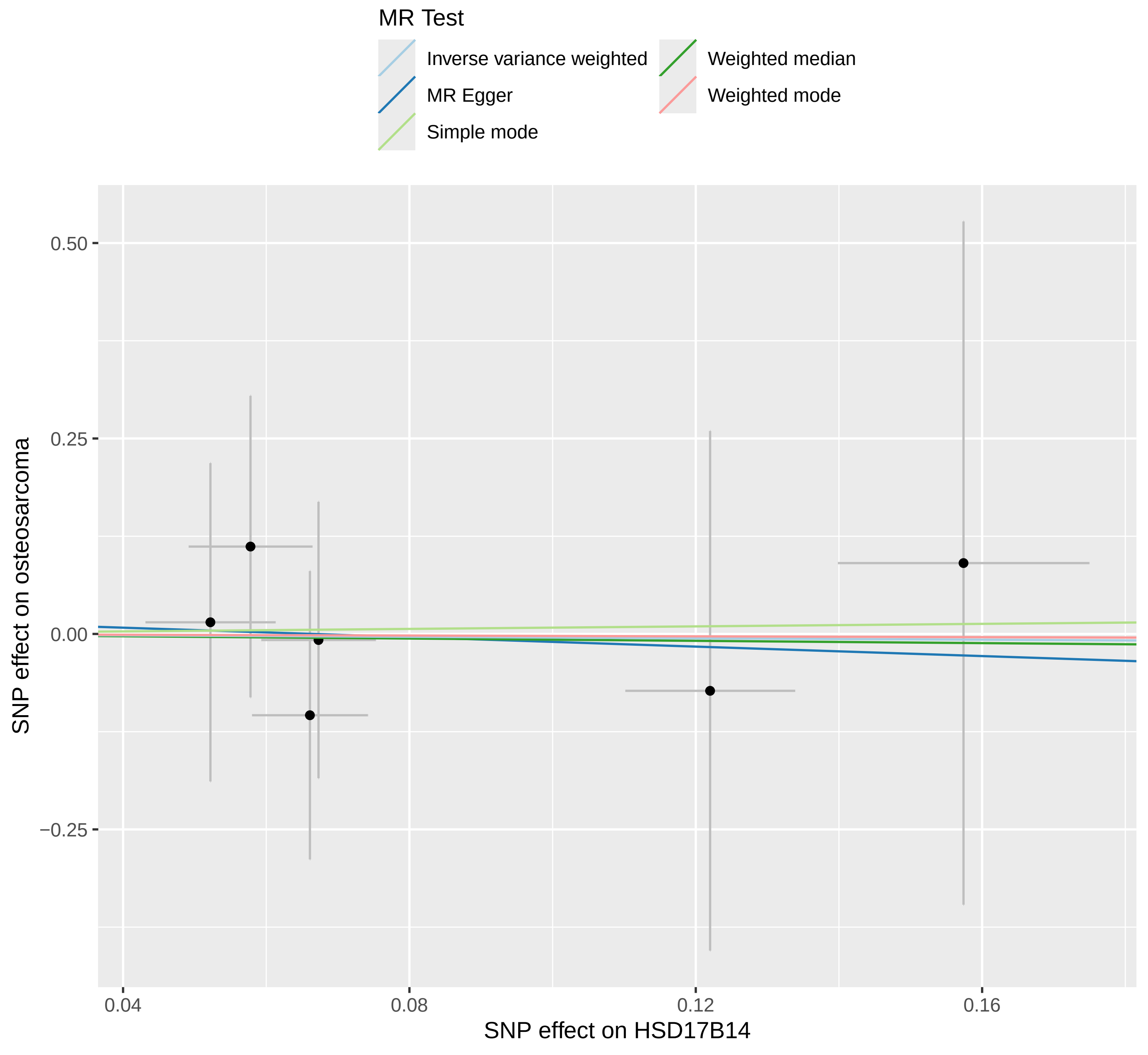
MR Test
Inverse variance weighted
MR Egger
Simple mode
Weighted median
Weighted mode
0.50
0.25
0.00
−0.25
0.04
0.08
0.12
0.16
SNP effect on HSD17B14
SNP effect on osteosarcoma

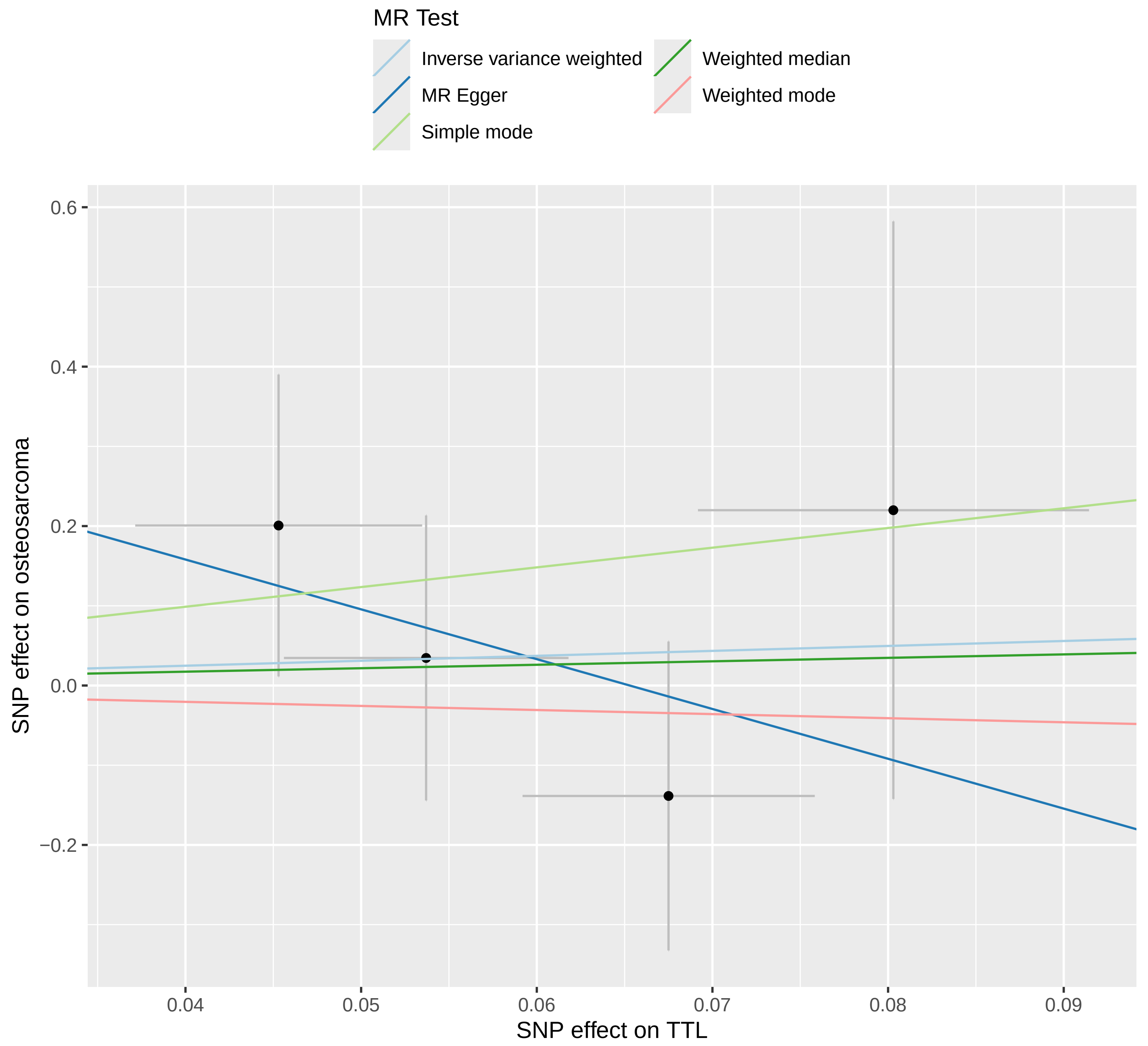
MR Test
Inverse variance weighted
MR Egger
Simple mode
Weighted median
Weighted mode
0.6
0.4
0.2
0.0
−0.2
SNP effect on osteosarcoma
0.04
0.05
0.06
0.07
0.08
0.09
SNP effect on TTL

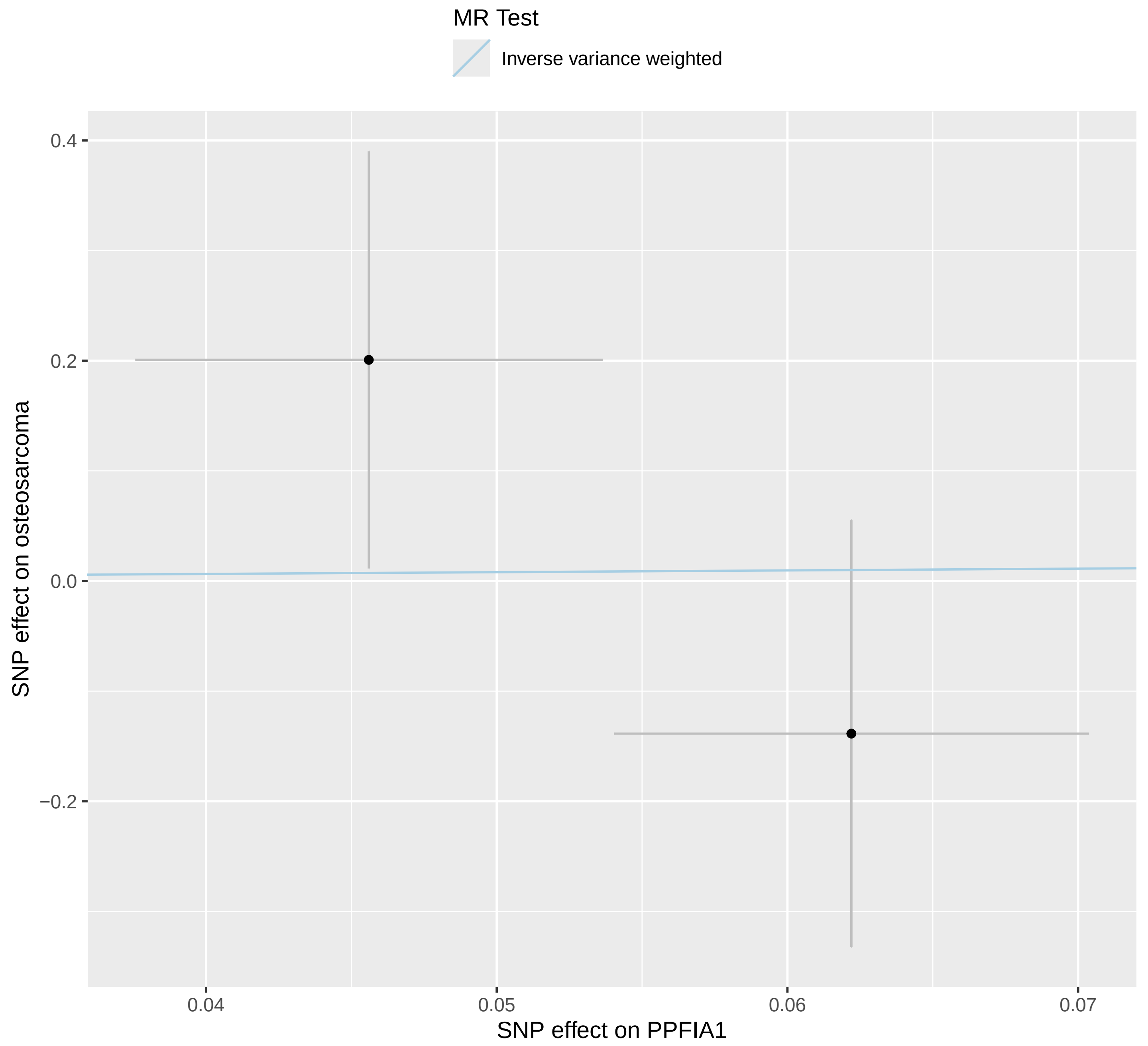

MR Test
Inverse variance weighted
0.4
0.2
0.0
−0.2
0.04
0.05
0.06
0.07
SNP effect on PPFIA1
SNP effect on osteosarcoma

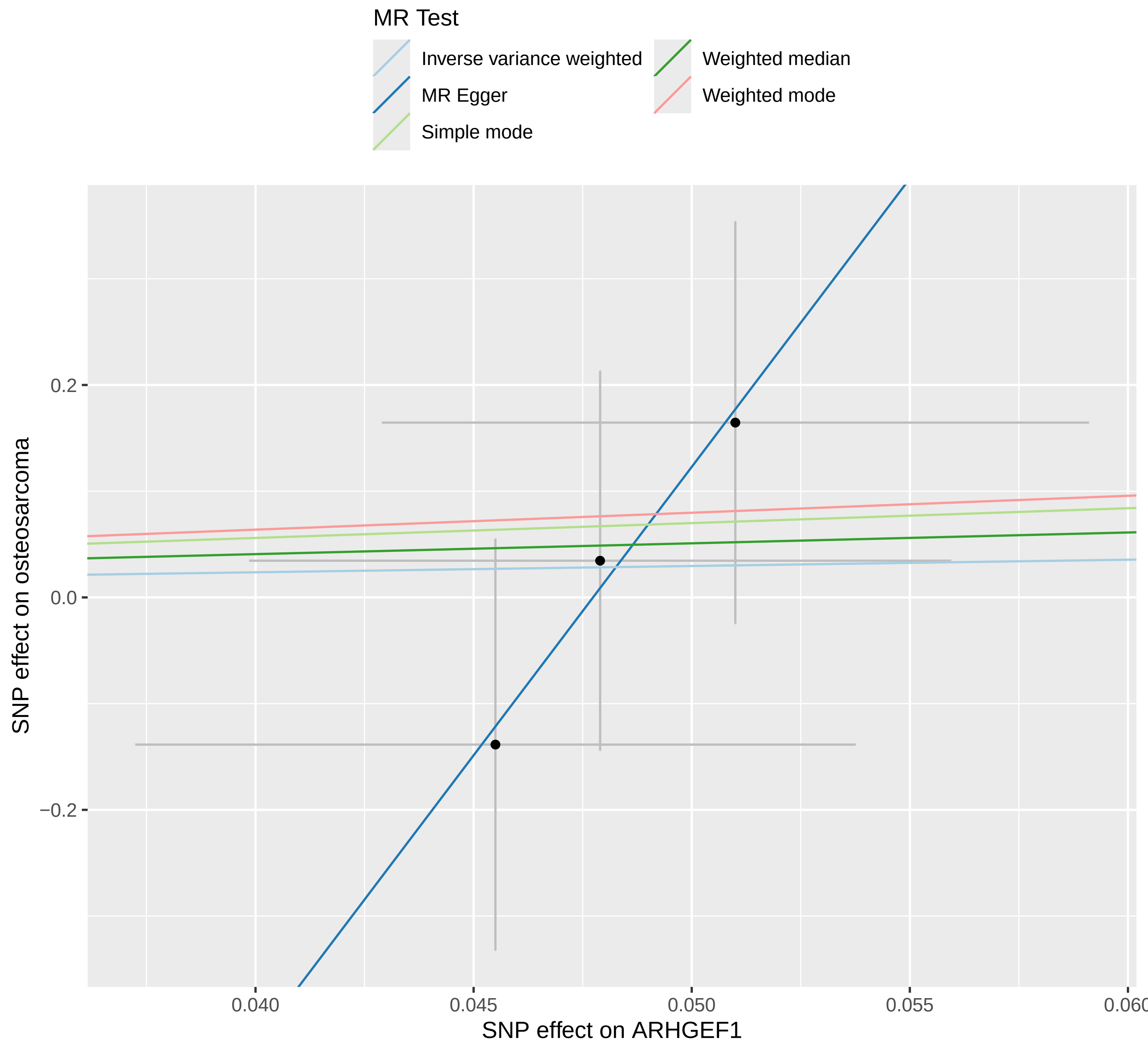
MR Test
Inverse variance weighted
MR Egger
Simple mode
Weighted median
Weighted mode
0.2
0.0
−0.2
0.040
0.045
0.050
0.055
0.060
SNP effect on osteosarcoma
SNP effect on ARHGEF1

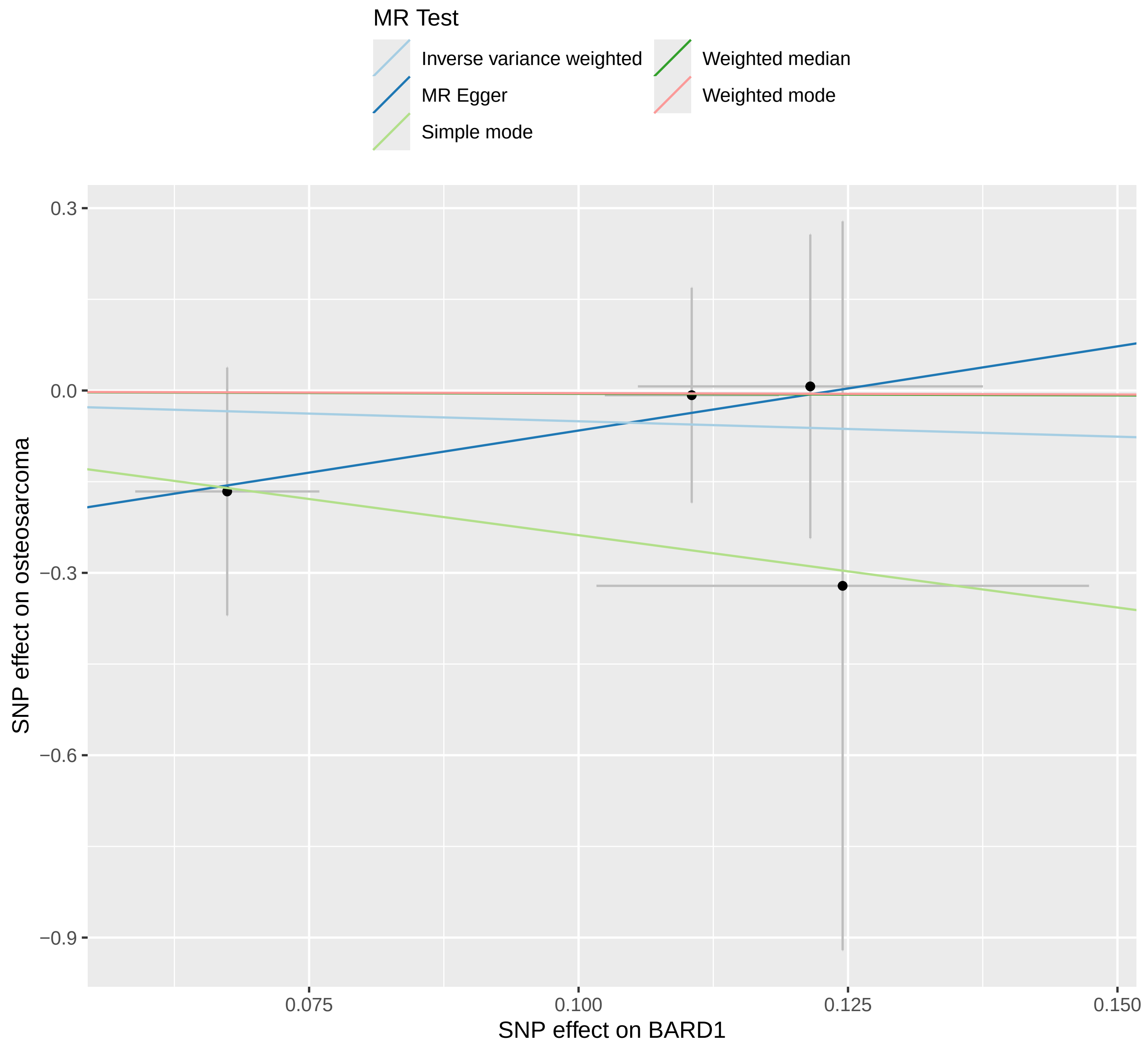
MR Test
Inverse variance weighted
MR Egger
Simple mode
Weighted median
Weighted mode
0.3
0.0
−0.3
−0.6
−0.9
0.075
0.100
0.125
0.150
SNP effect on BARD1
SNP effect on osteosarcoma

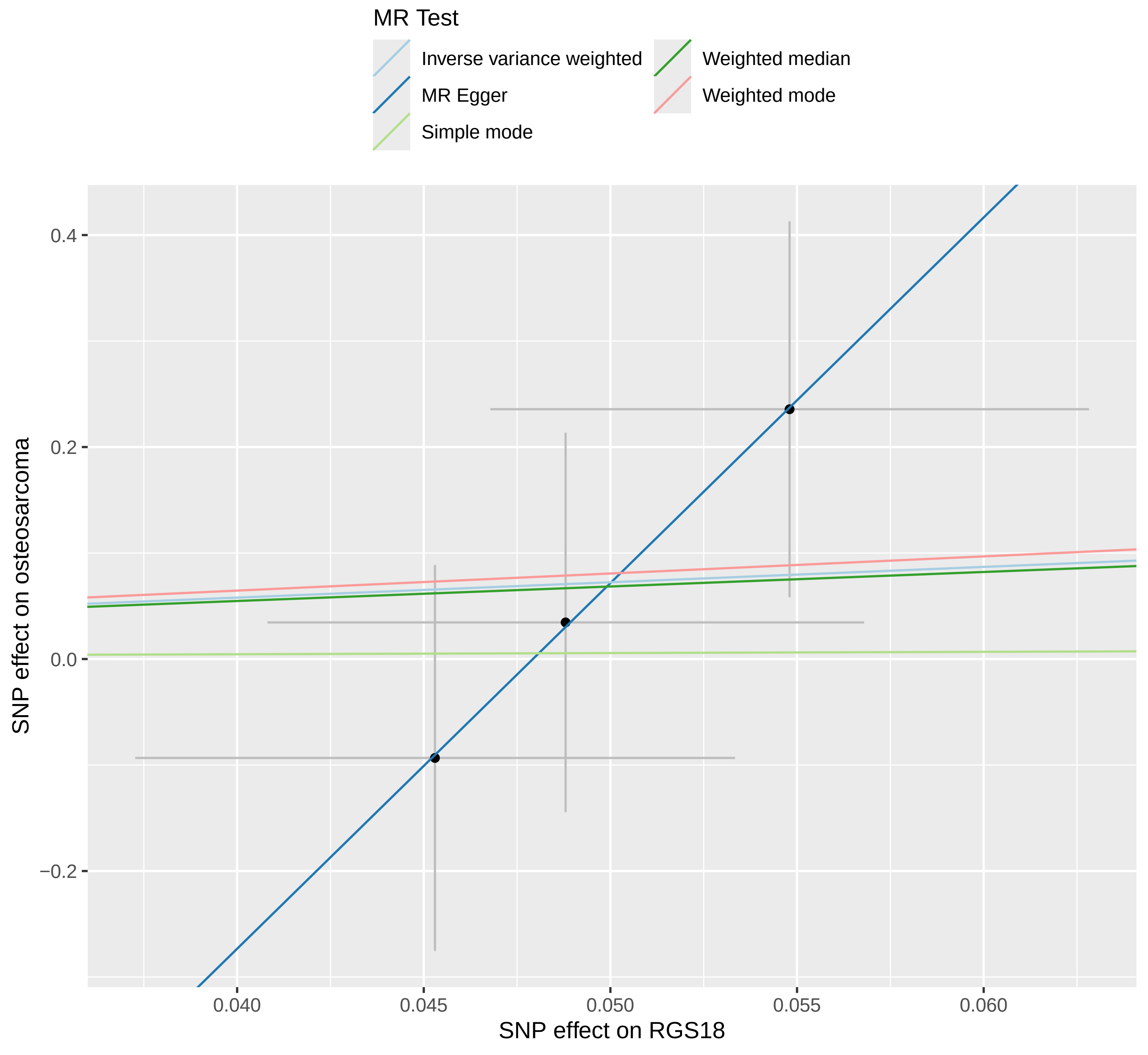
MR Test
Inverse variance weighted
MR Egger
Simple mode
Weighted median
Weighted mode
0.4
0.2
0.0
−0.2
SNP effect on osteosarcoma
0.040
0.045
0.050
0.055
0.060
SNP effect on RGS18

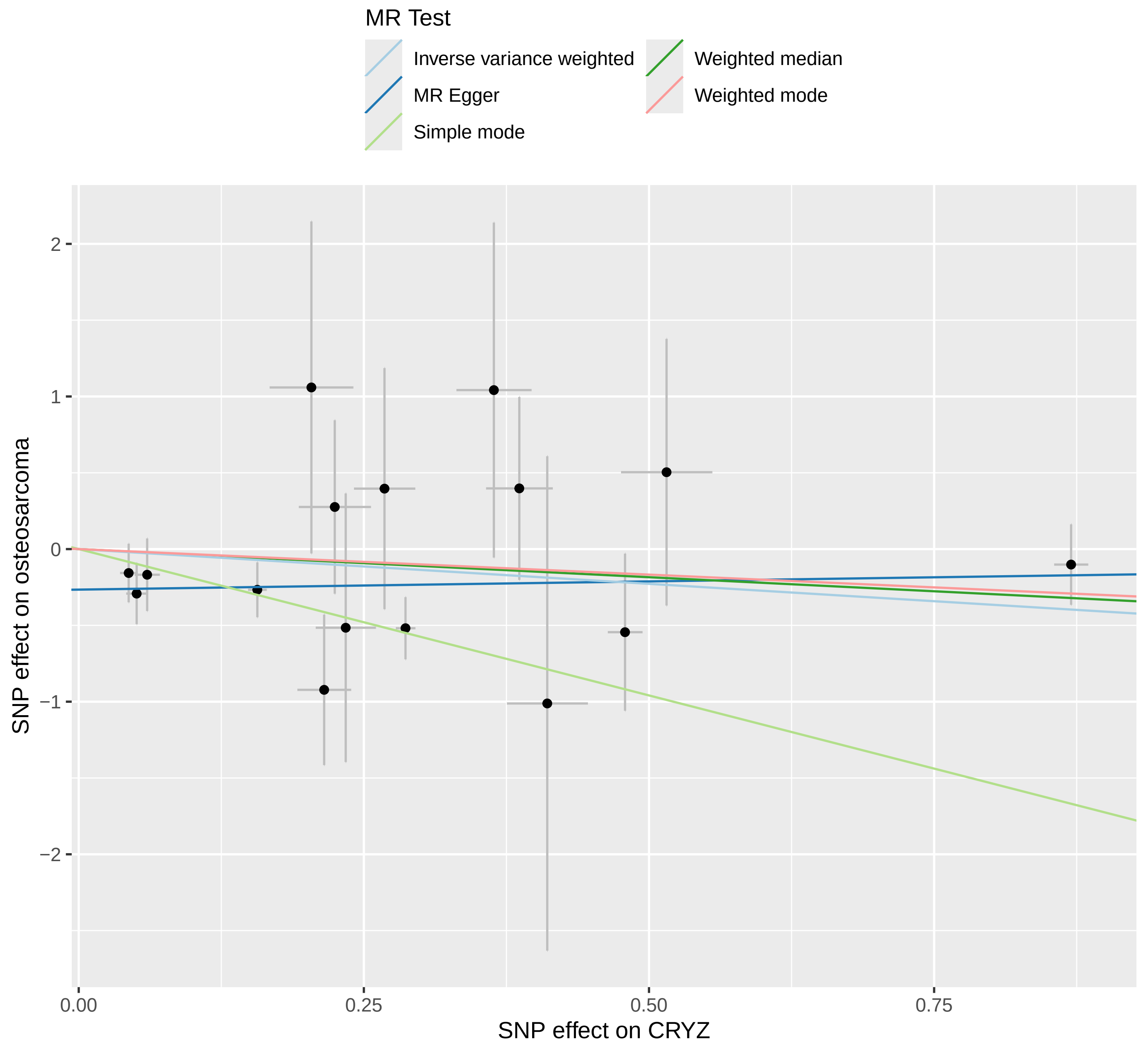
MR Test
Inverse variance weighted
MR Egger
Simple mode
Weighted median
Weighted mode
2
1
0
−1
−2
SNP effect on osteosarcoma
0.00
0.25
0.50
0.75
SNP effect on CRYZ

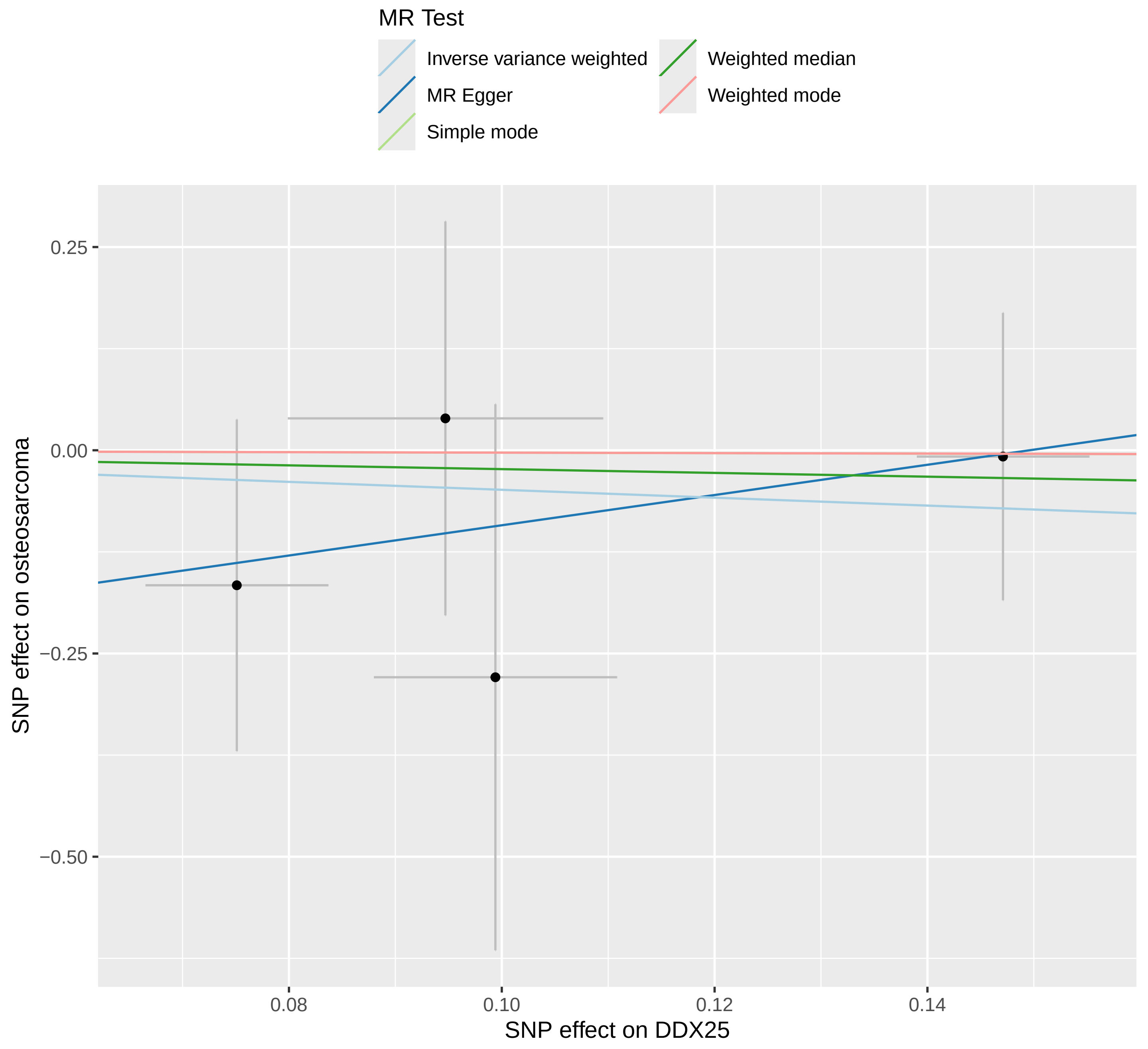
MR Test
Inverse variance weighted
MR Egger
Simple mode
Weighted median
Weighted mode
0.25
0.00
−0.25
−0.50
SNP effect on osteosarcoma
0.08
0.10
0.12
0.14
SNP effect on DDX25

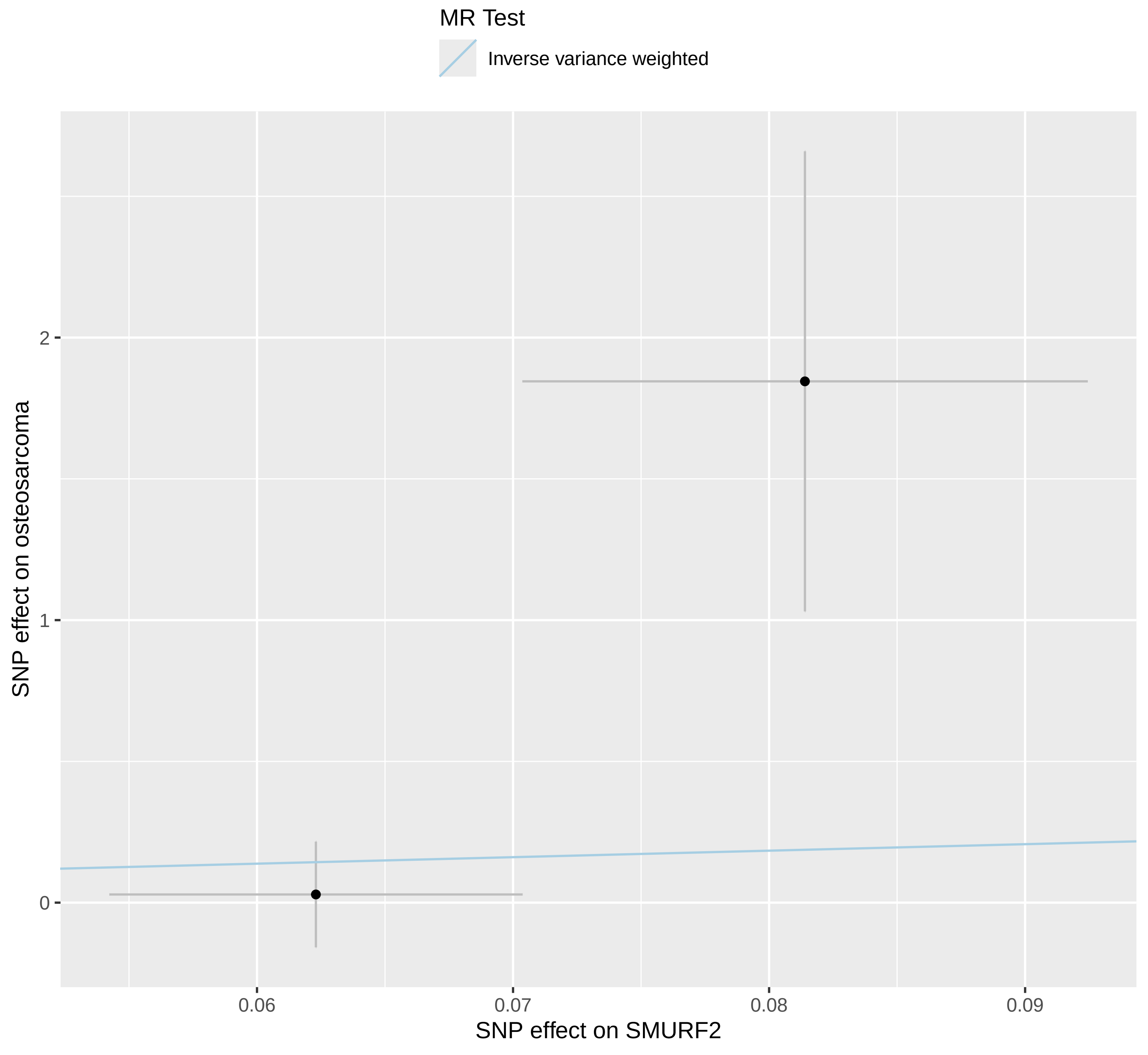
MR Test
Inverse variance weighted
SNP effect on osteosarcoma
2
1
0
0.06
0.07
0.08
0.09
SNP effect on SMURF2

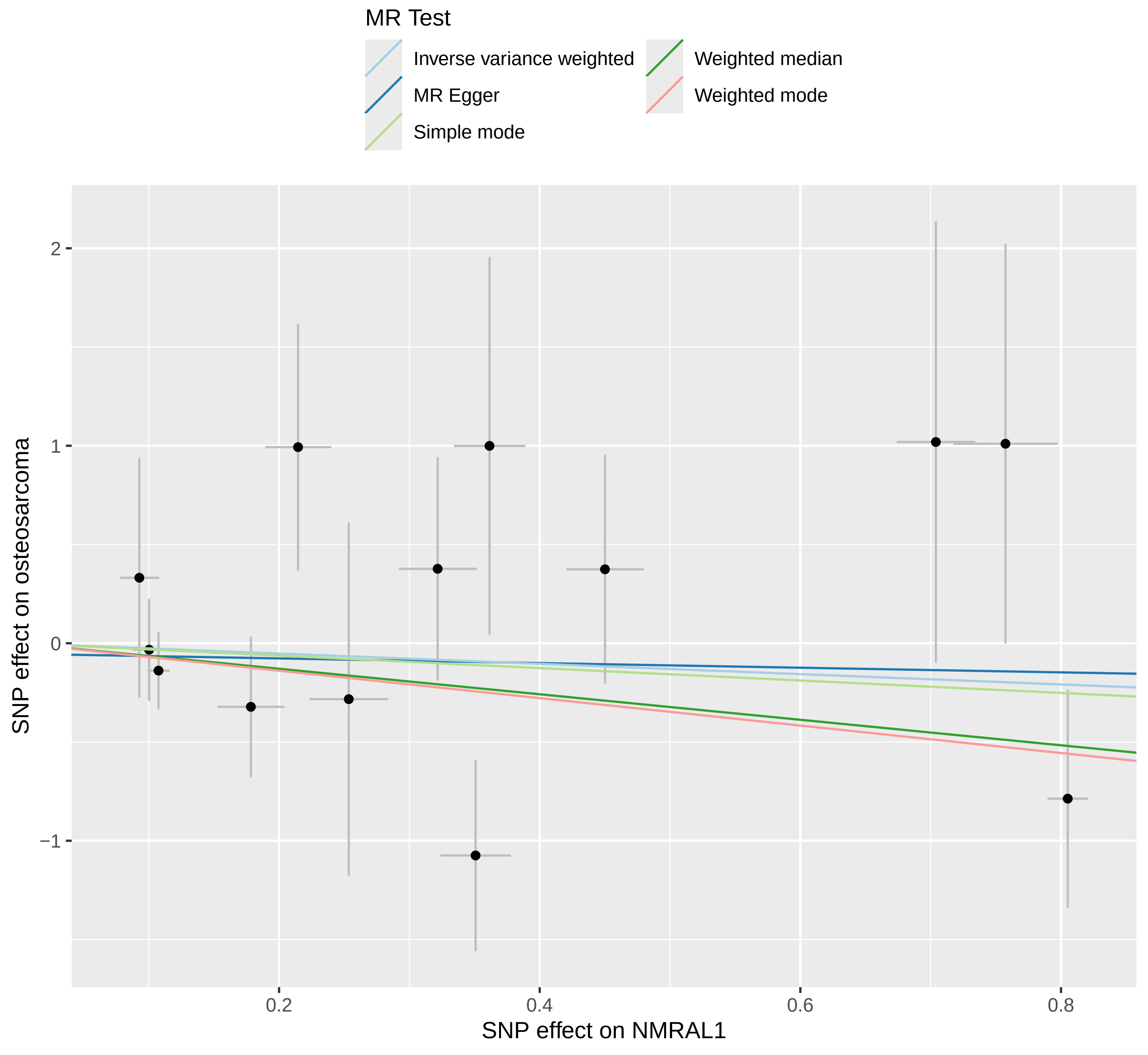
MR Test
Inverse variance weighted
MR Egger
Simple mode
Weighted median
Weighted mode
SNP effect on osteosarcoma
2
1
0
−1
0.2
0.4
0.6
0.8
SNP effect on NMRAL1

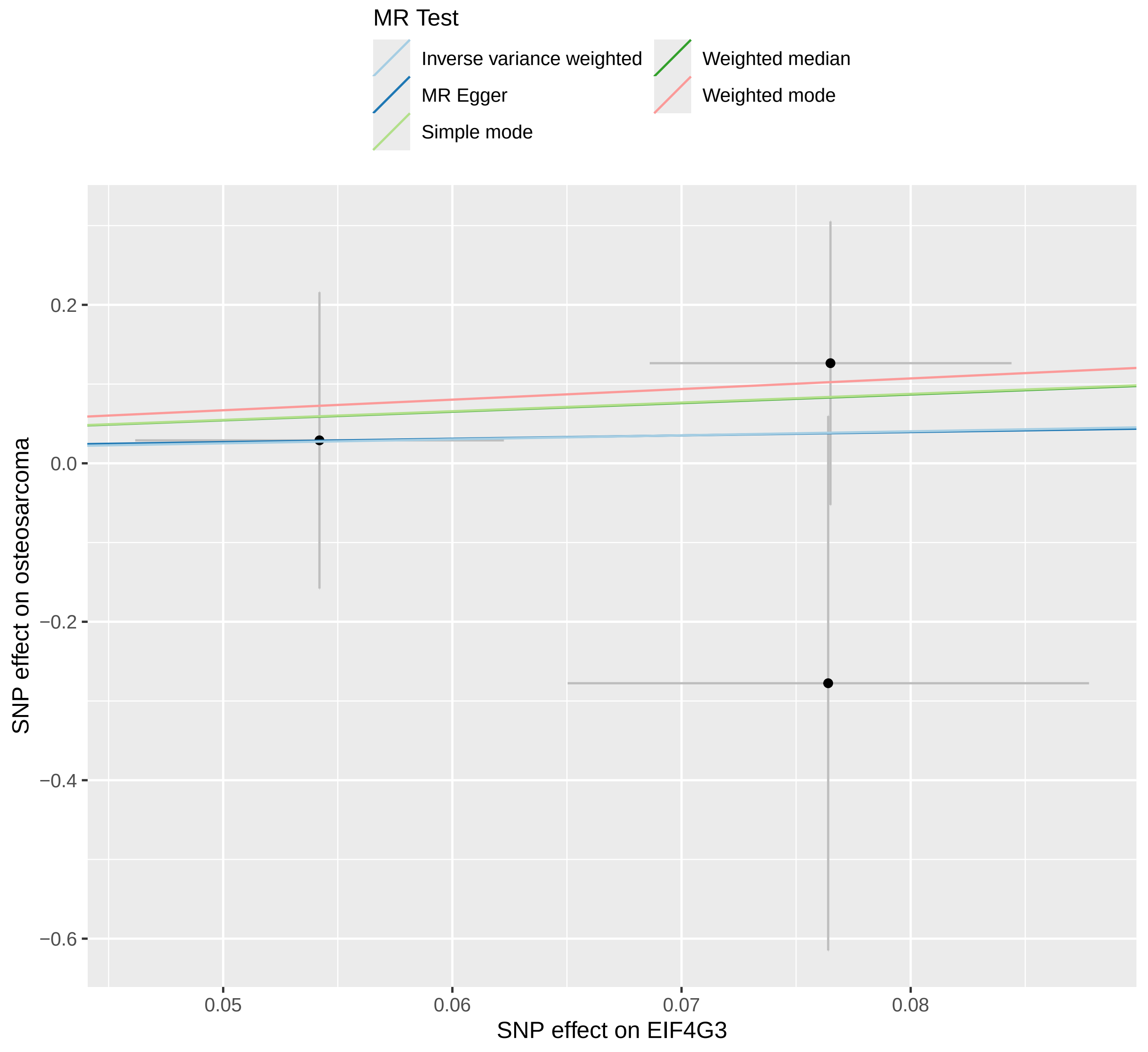

MR Test
Inverse variance weighted
MR Egger
Simple mode
Weighted median
Weighted mode
0.2
0.0
−0.2
−0.4
−0.6
SNP effect on osteosarcoma
0.05
0.06
0.07
0.08
SNP effect on EIF4G3

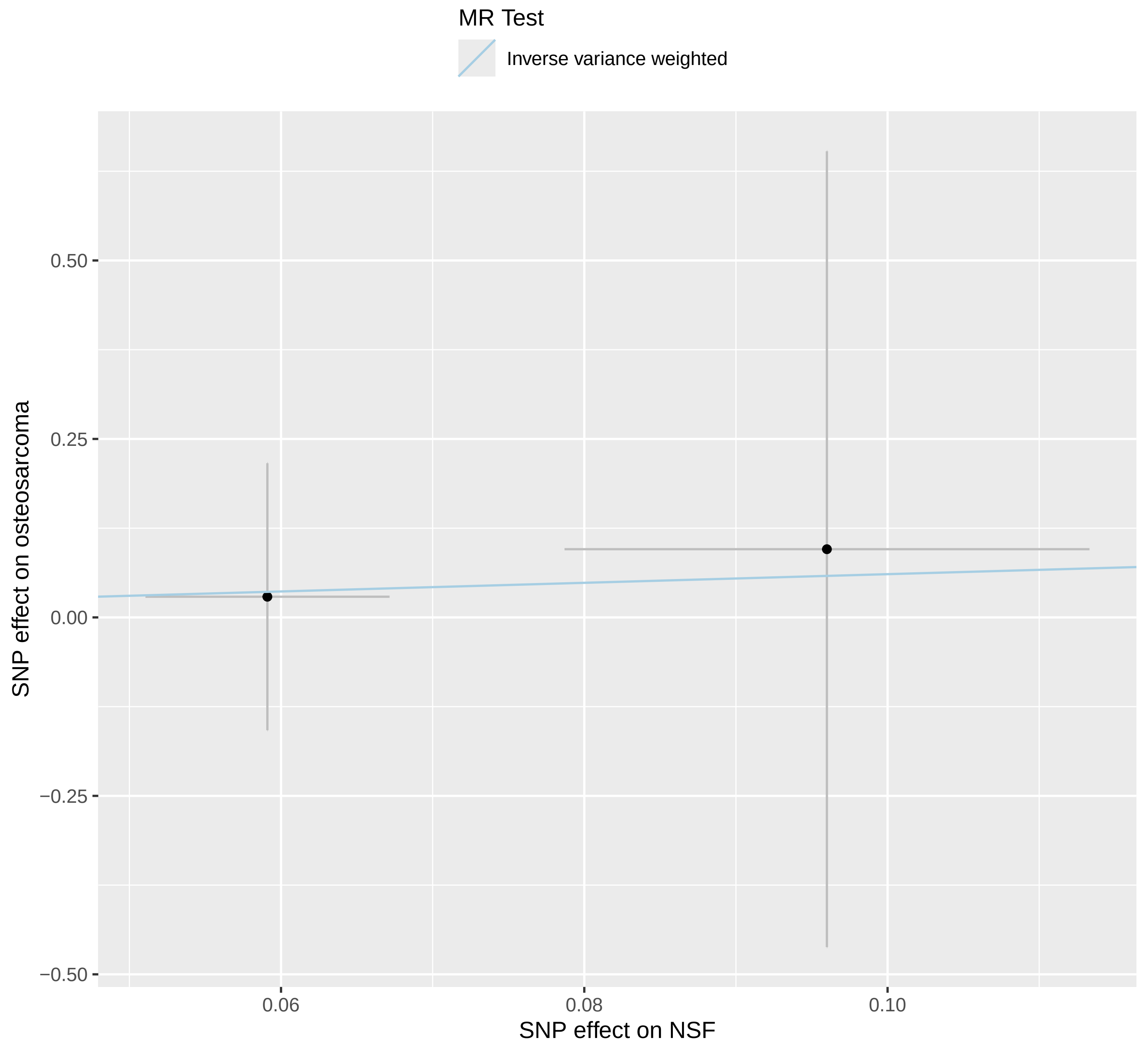
MR Test
Inverse variance weighted
0.50
0.25
0.00
−0.25
−0.50
0.06
0.08
0.10
SNP effect on osteosarcoma
SNP effect on NSF

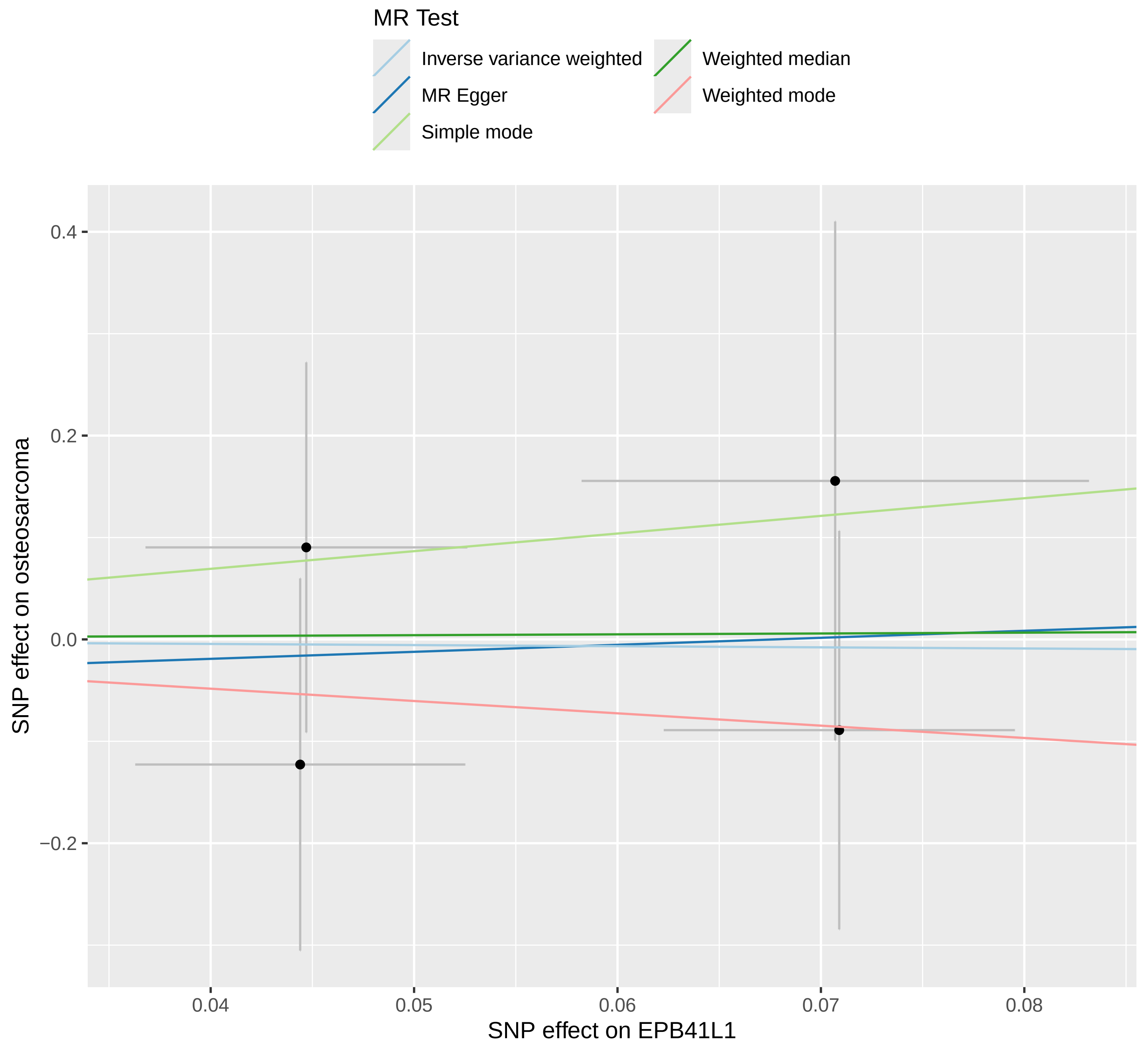
MR Test
Inverse variance weighted
MR Egger
Simple mode
Weighted median
Weighted mode
0.4
0.2
0.0
−0.2
SNP effect on osteosarcoma
0.04
0.05
0.06
0.07
0.08
SNP effect on EPB41L1

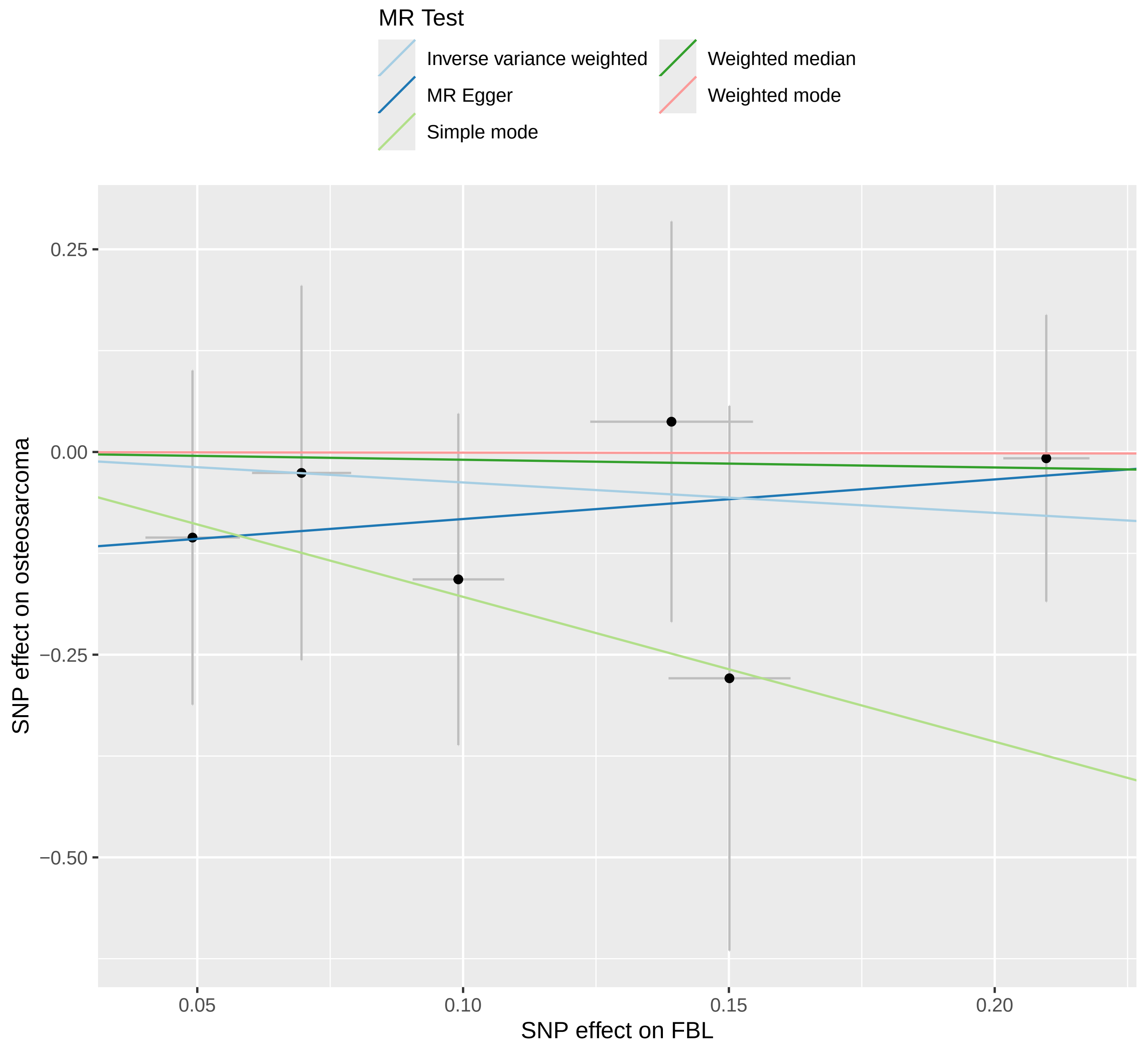

MR Test
Inverse variance weighted
MR Egger
Simple mode
Weighted median
Weighted mode
0.25
0.00
−0.25
−0.50
SNP effect on osteosarcoma
0.05
0.10
0.15
0.20
SNP effect on FBL

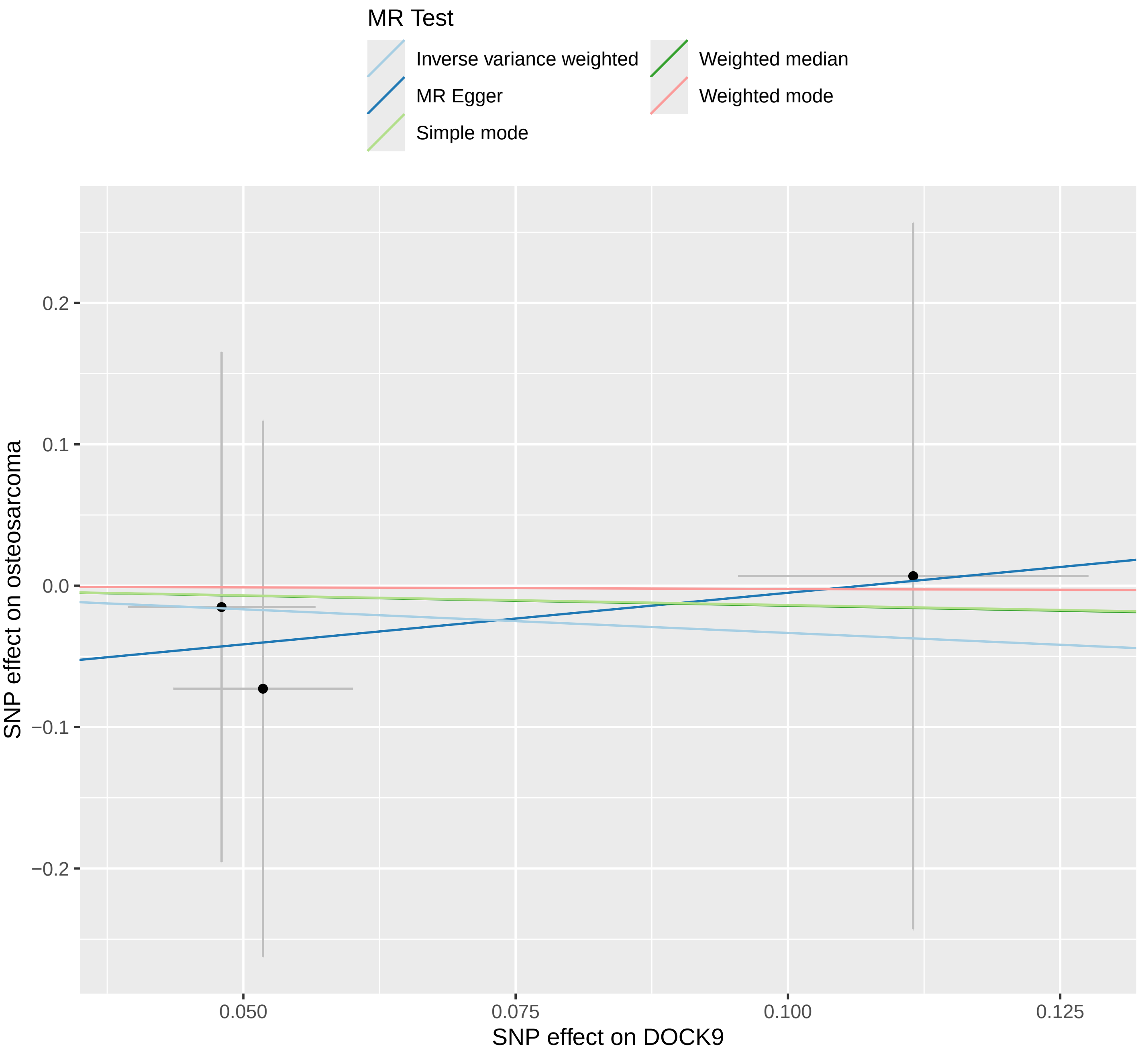
MR Test
Inverse variance weighted
MR Egger
Simple mode
Weighted median
Weighted mode
0.2
0.1
0.0
−0.1
−0.2
SNP effect on osteosarcoma
0.050
0.075
0.100
0.125
SNP effect on DOCK9

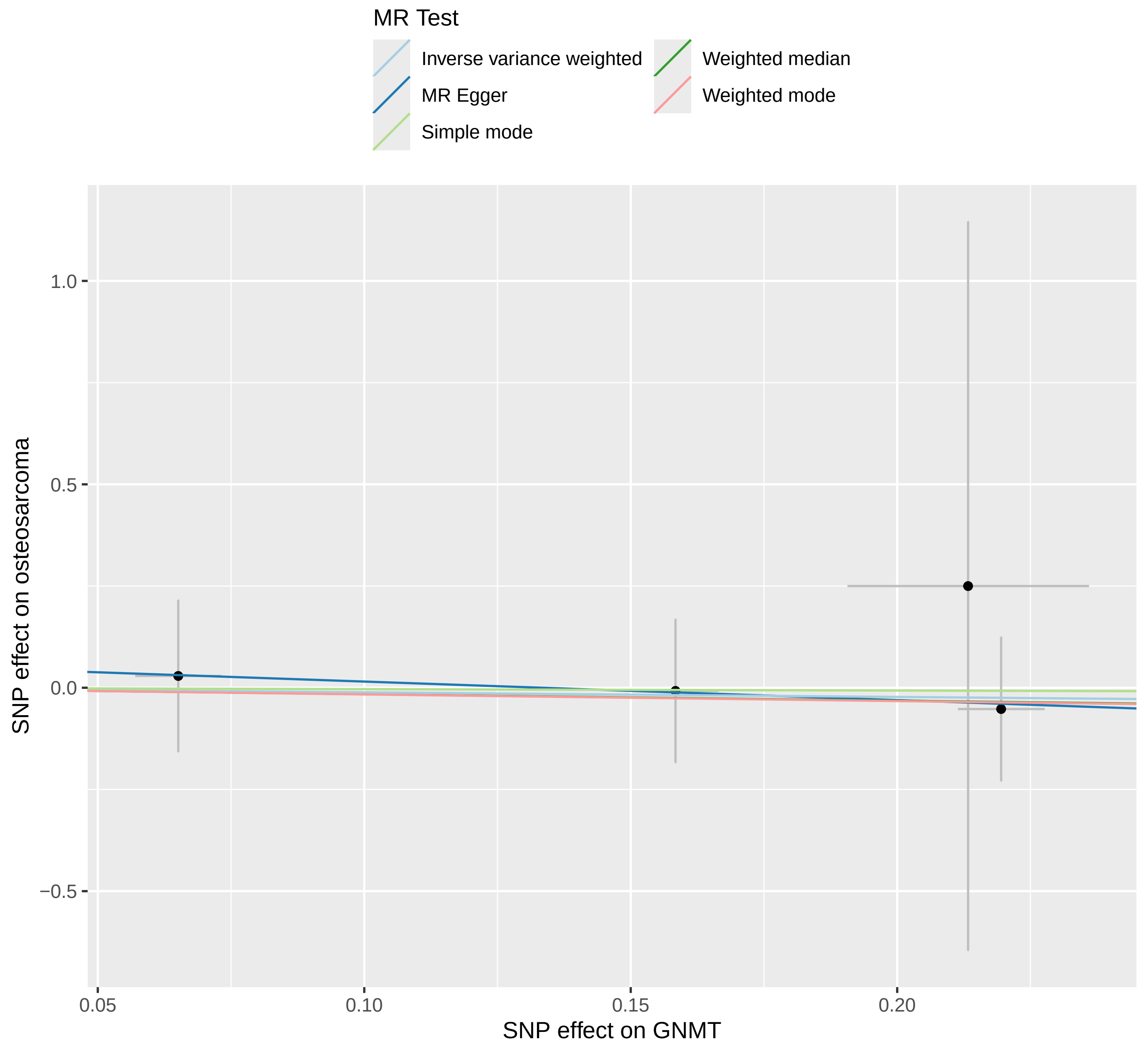
MR Test
Inverse variance weighted
MR Egger
Simple mode
Weighted median
Weighted mode
1.0
0.5
0.0
−0.5
SNP effect on osteosarcoma
0.05
0.10
0.15
0.20
SNP effect on GNMT

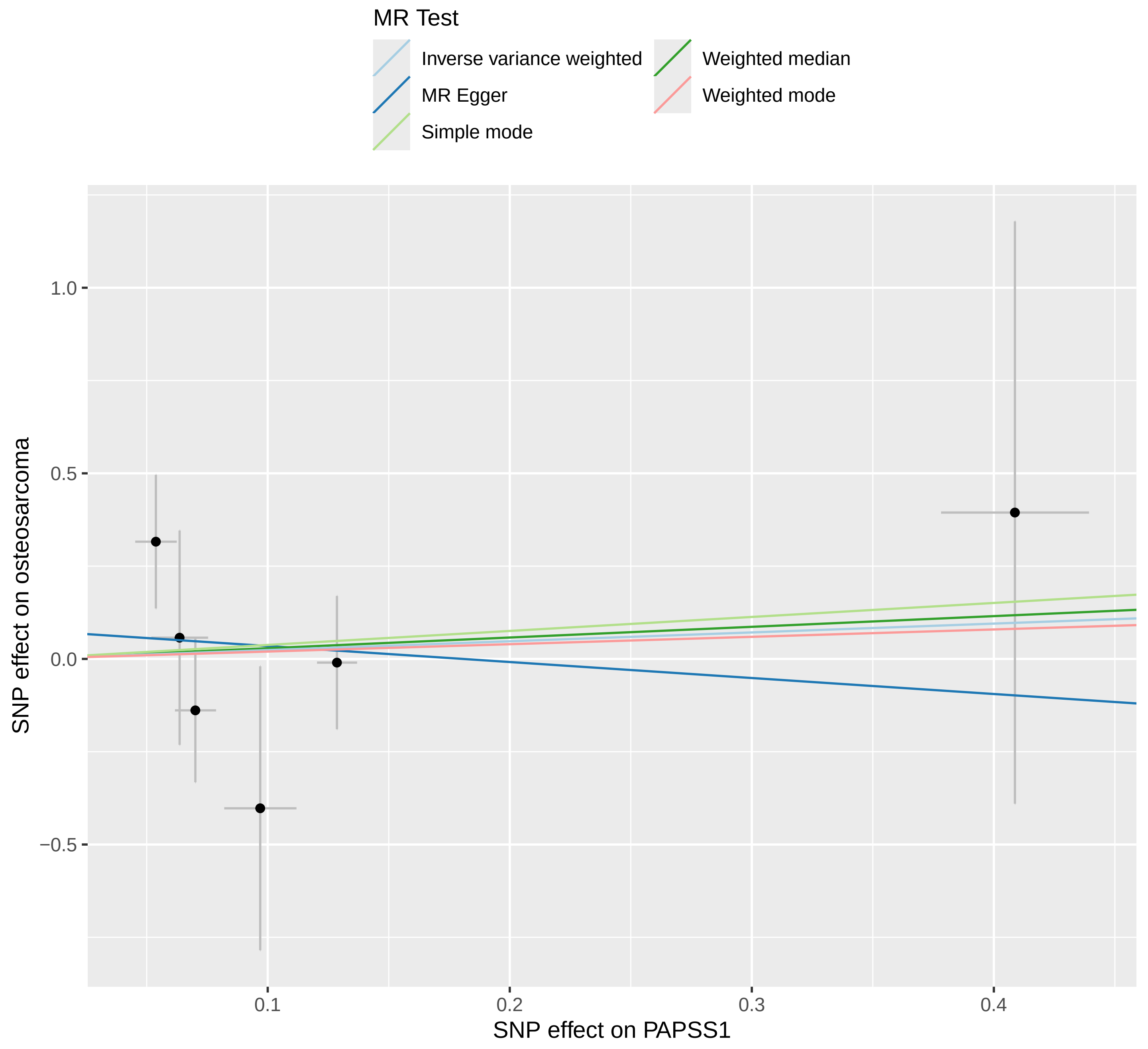
MR Test
Inverse variance weighted
MR Egger
Simple mode
Weighted median
Weighted mode
1.0
0.5
0.0
−0.5
0.1
0.2
0.3
0.4
SNP effect on osteosarcoma
SNP effect on PAPSS1

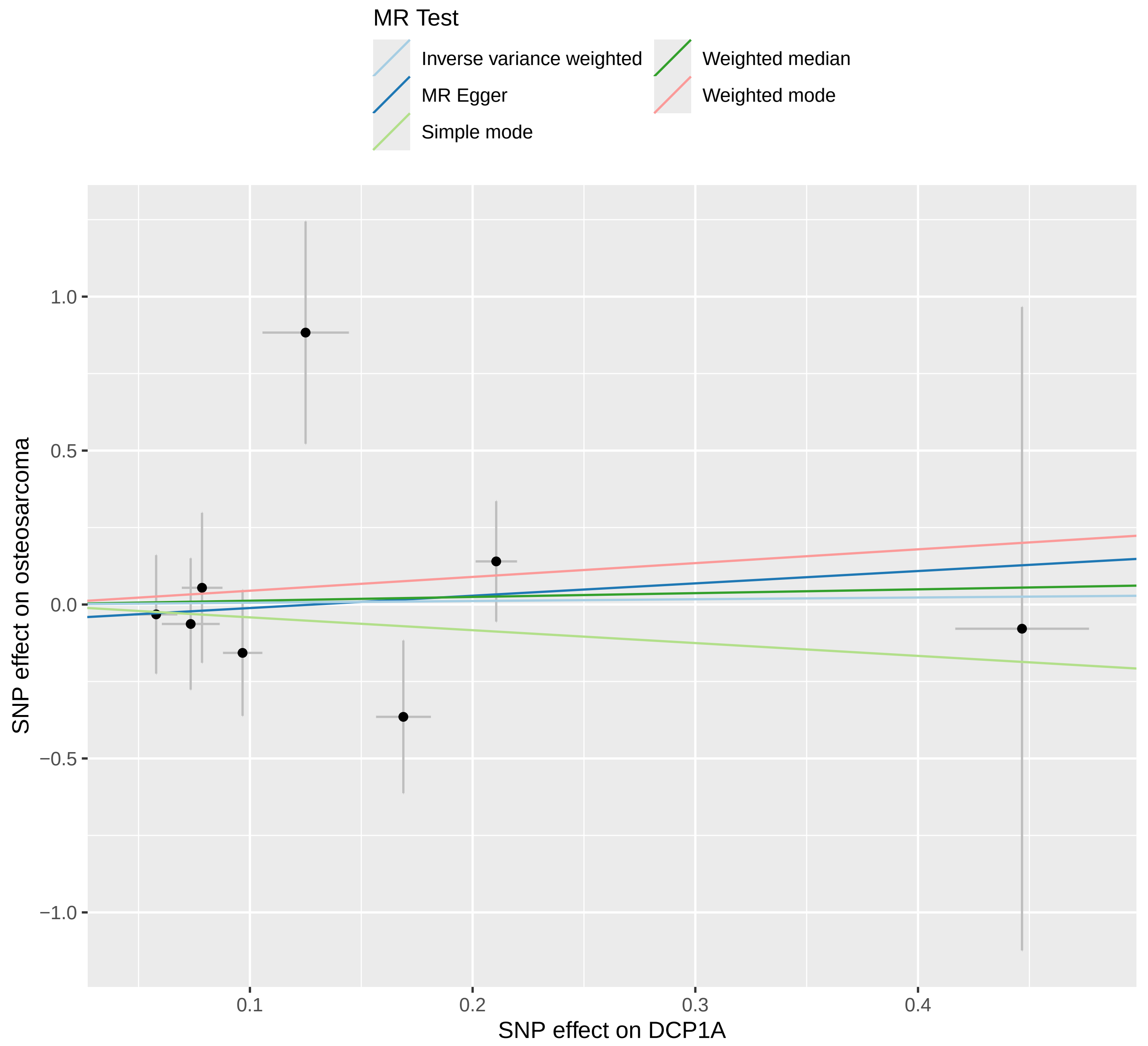

MR Test
Inverse variance weighted
MR Egger
Simple mode
Weighted median
Weighted mode
1.0
0.5
0.0
−0.5
−1.0
SNP effect on osteosarcoma
0.1
0.2
0.3
0.4
SNP effect on DCP1A

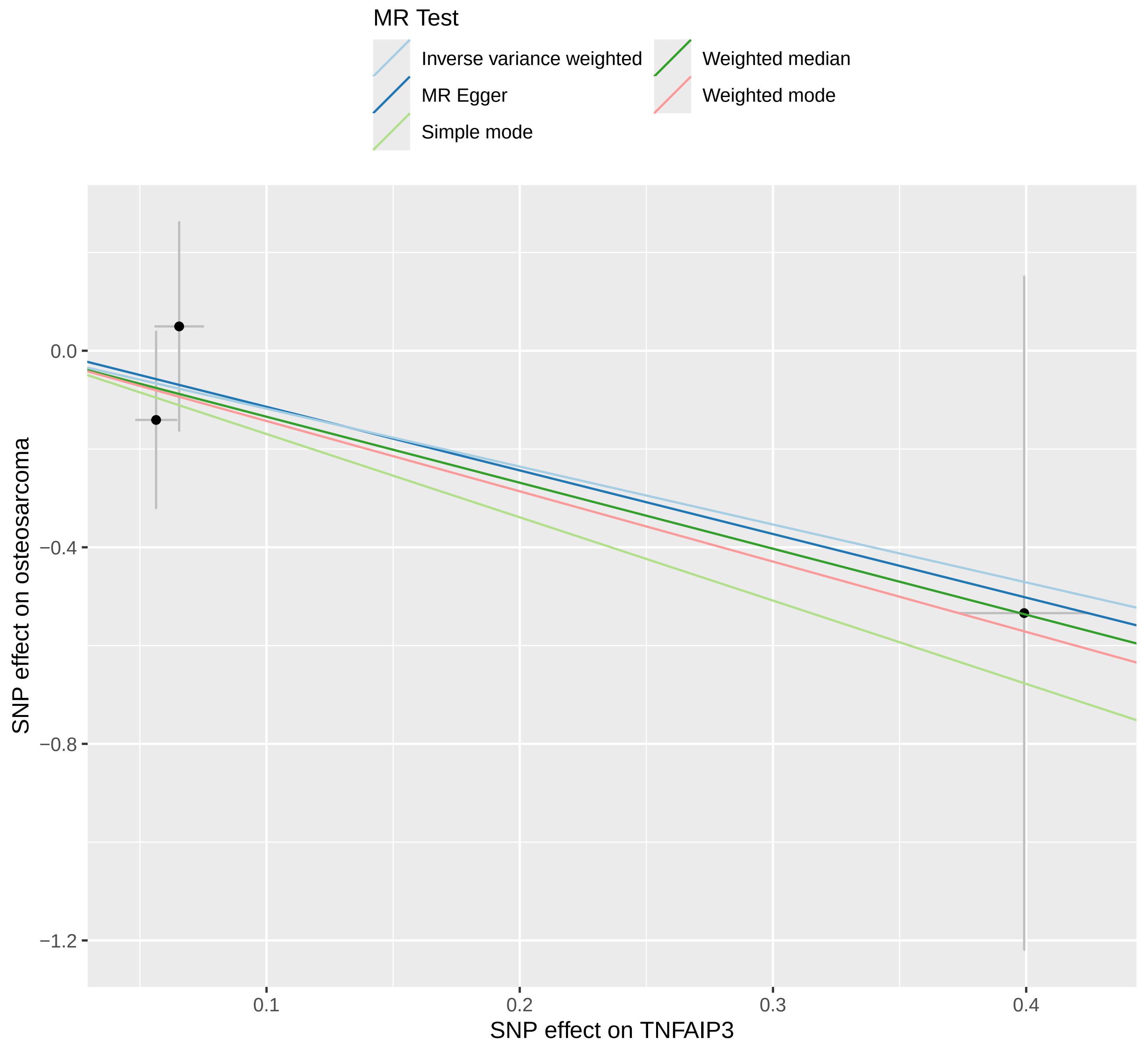
MR Test
Inverse variance weighted
MR Egger
Simple mode
Weighted median
Weighted mode
SNP effect on osteosarcoma
0.0
−0.4
−0.8
−1.2
0.1
0.2
0.3
0.4
SNP effect on TNFAIP3

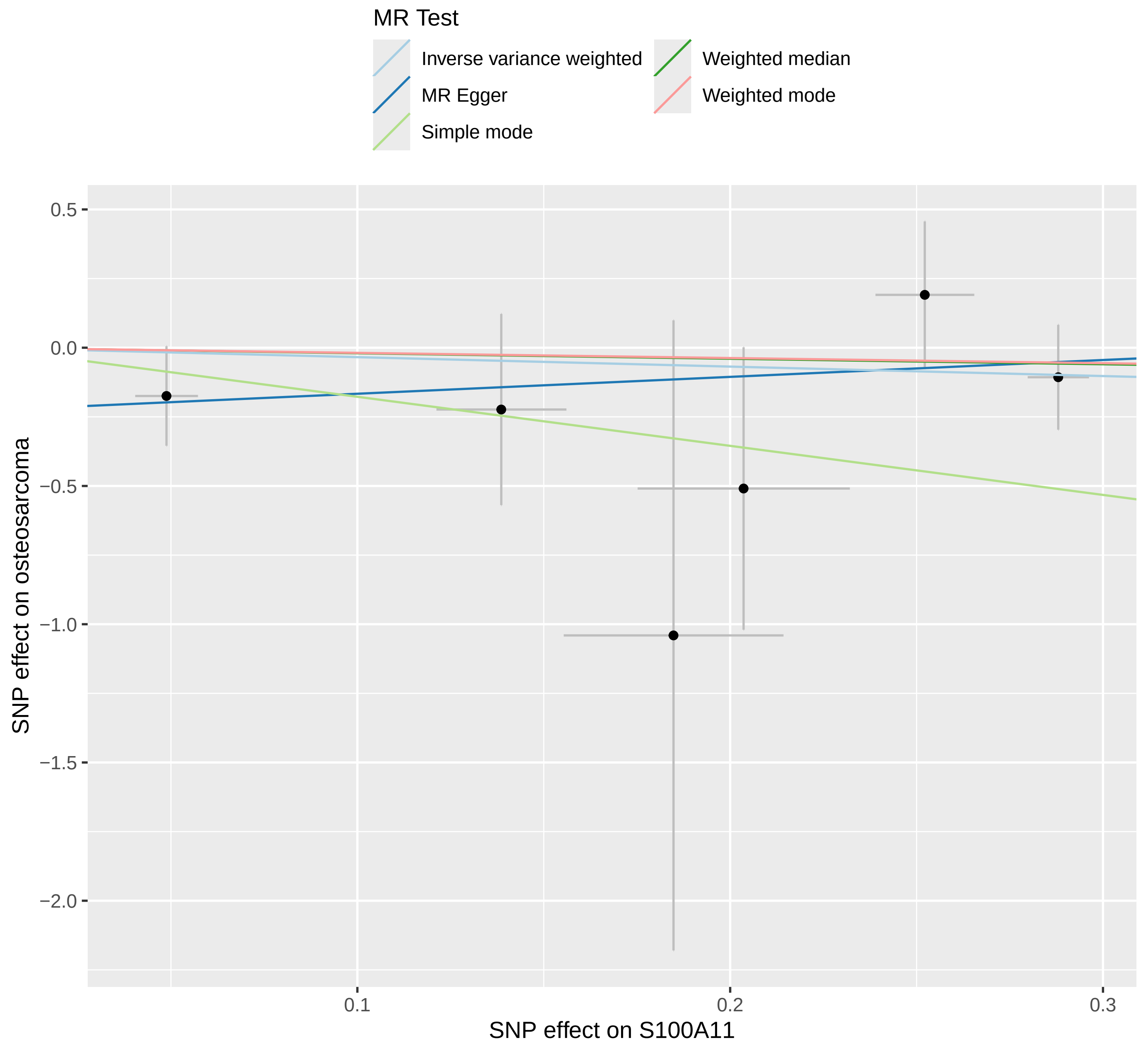

MR Test
Inverse variance weighted
MR Egger
Simple mode
Weighted median
Weighted mode
0.5
0.0
−0.5
−1.0
−1.5
−2.0
SNP effect on osteosarcoma
0.1
0.2
0.3
SNP effect on S100A11

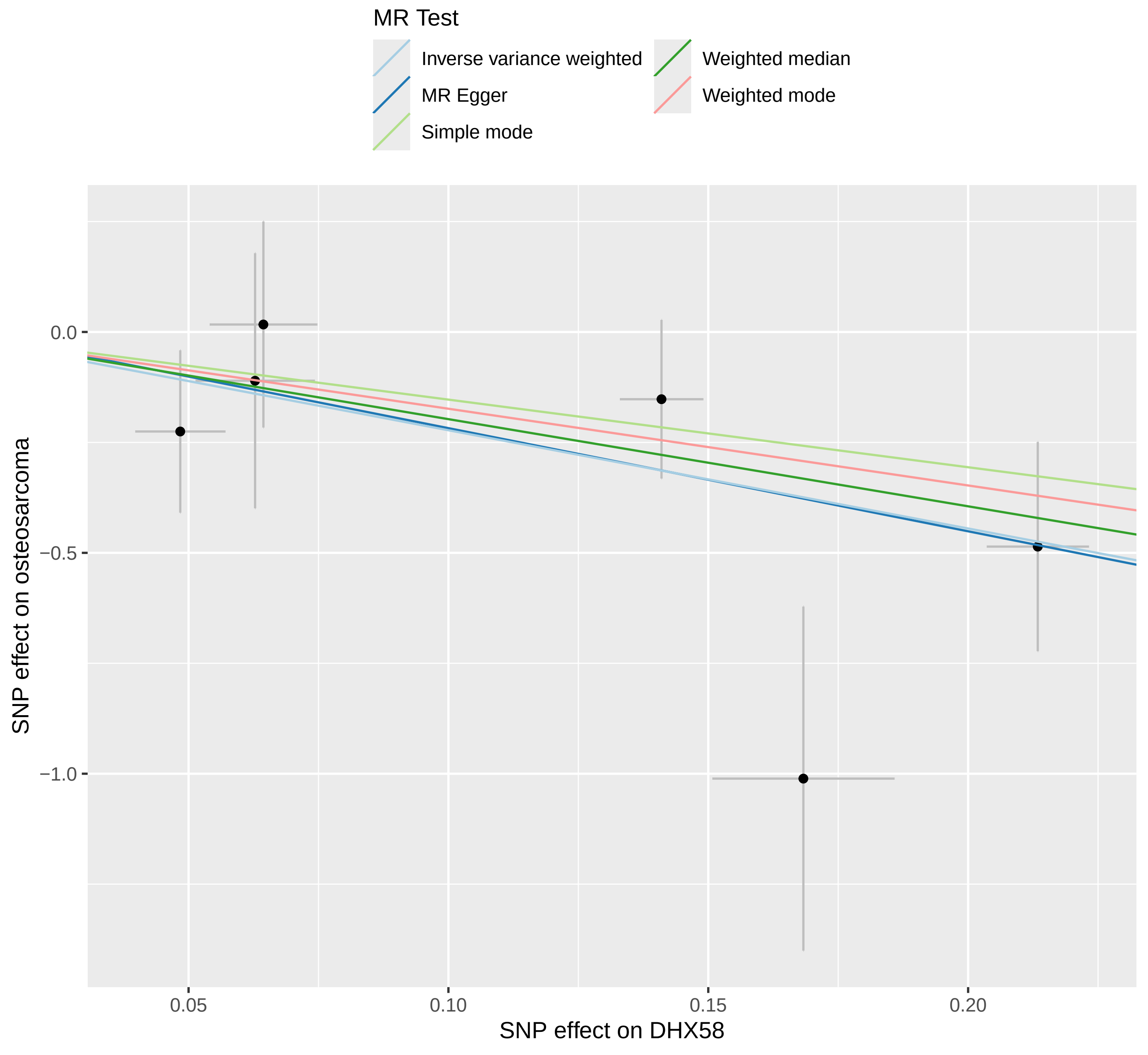

MR Test
Inverse variance weighted
MR Egger
Simple mode
Weighted median
Weighted mode
0.0
−0.5
−1.0
SNP effect on osteosarcoma
0.05
0.10
0.15
0.20
SNP effect on DHX58

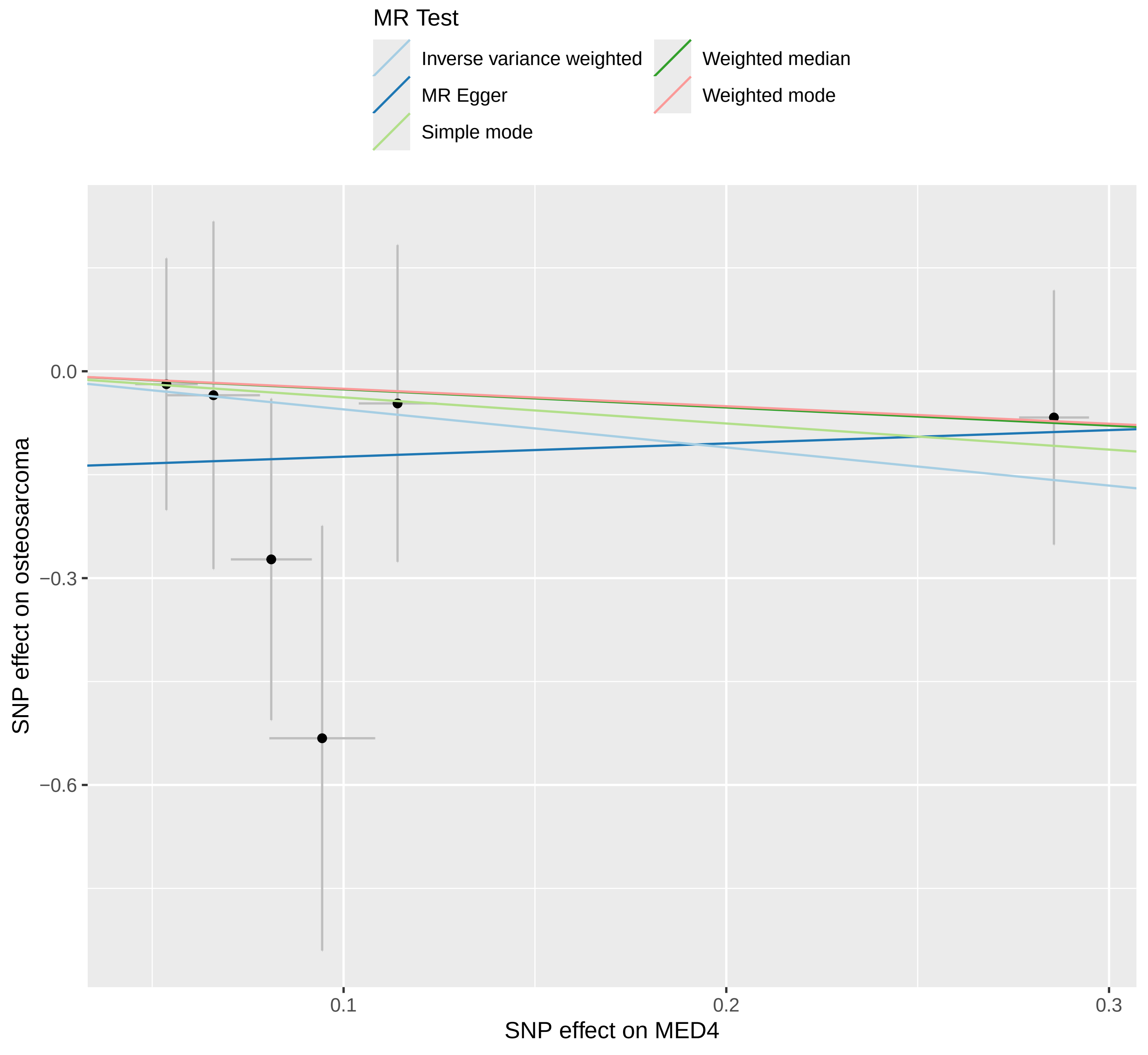
MR Test
Inverse variance weighted
MR Egger
Simple mode
Weighted median
Weighted mode
0.0
−0.3
−0.6
SNP effect on osteosarcoma
0.1
0.2
0.3
SNP effect on MED4

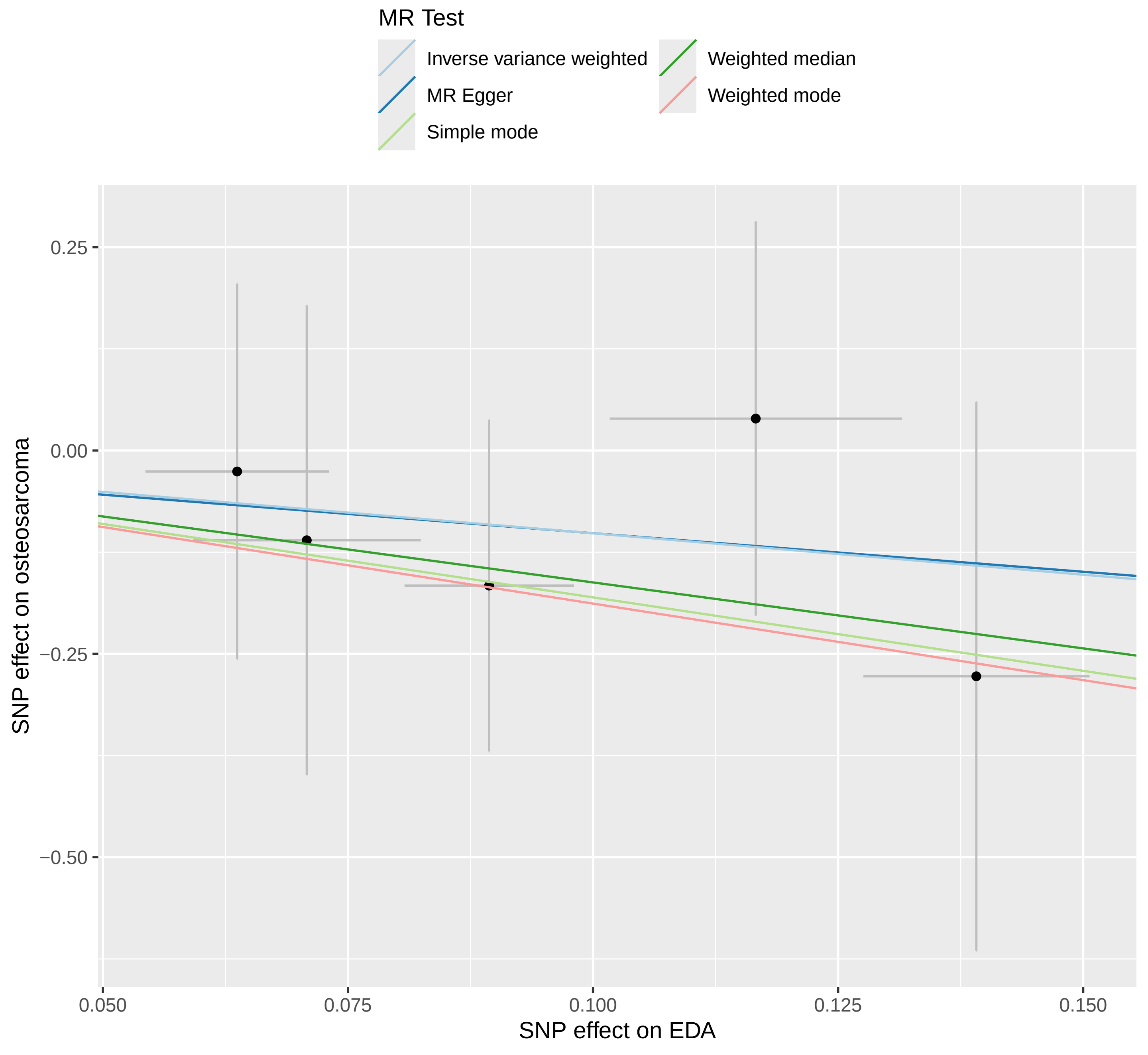
MR Test
Inverse variance weighted
MR Egger
Simple mode
Weighted median
Weighted mode
0.25
0.00
−0.25
−0.50
SNP effect on osteosarcoma
0.050
0.075
0.100
0.125
0.150
SNP effect on EDA

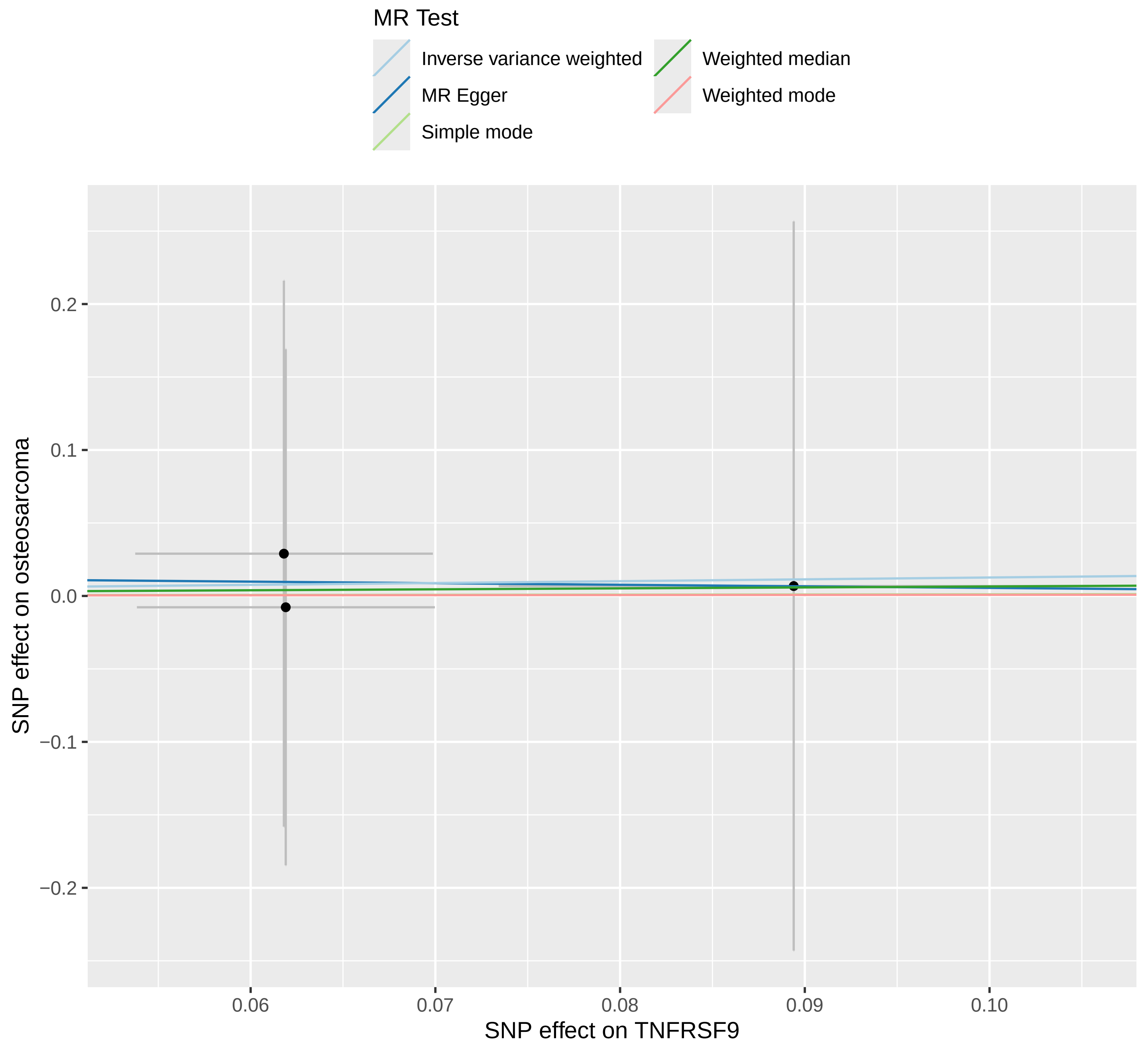

MR Test
Inverse variance weighted
MR Egger
Simple mode
Weighted median
Weighted mode
SNP effect on osteosarcoma
0.2
0.1
0.0
−0.1
−0.2
0.06
0.07
0.08
0.09
0.10
SNP effect on TNFRSF9

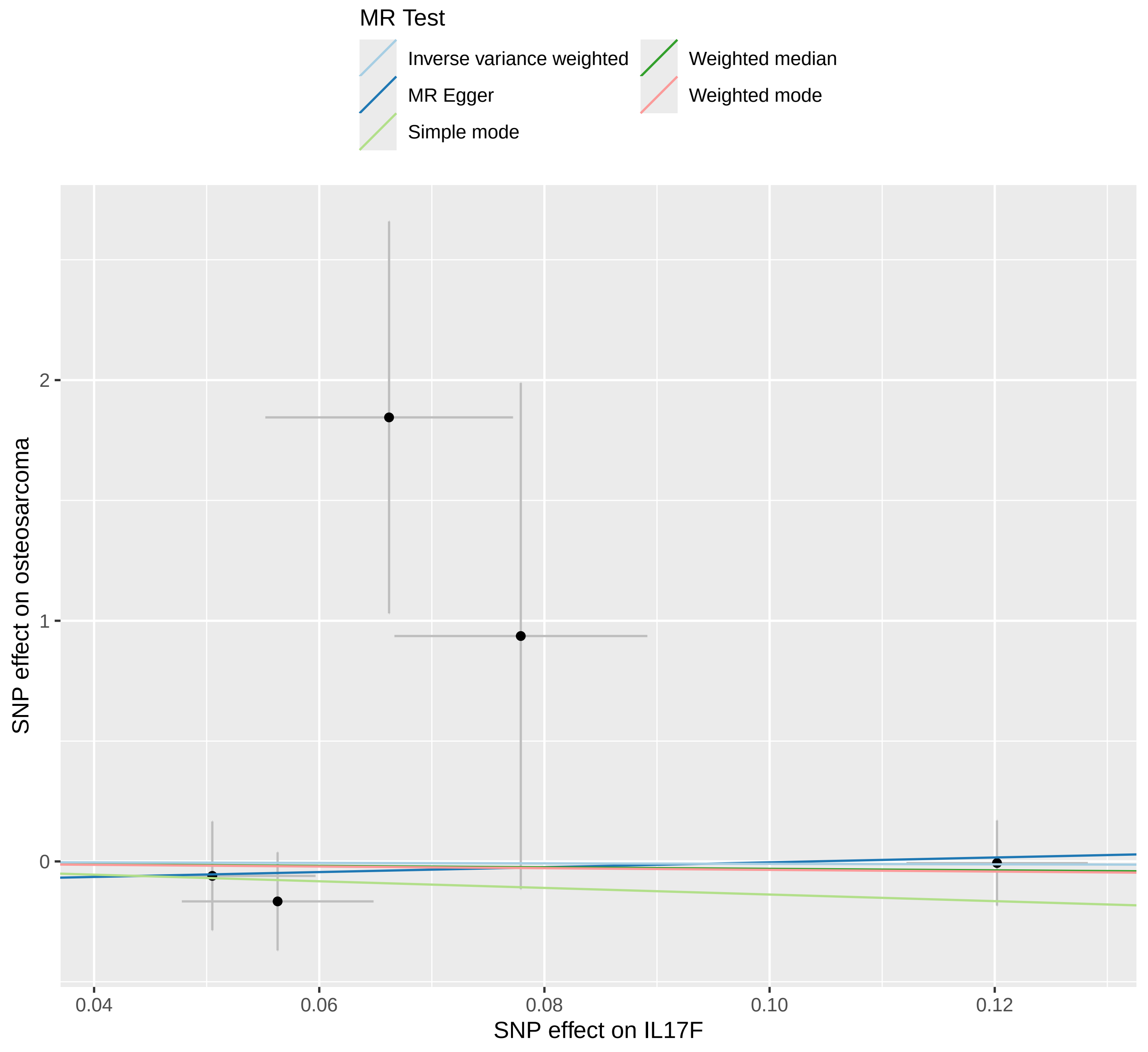
MR Test
Inverse variance weighted
MR Egger
Simple mode
Weighted median
Weighted mode
SNP effect on osteosarcoma
2
1
0
0.04
0.06
0.08
0.10
0.12
SNP effect on IL17F

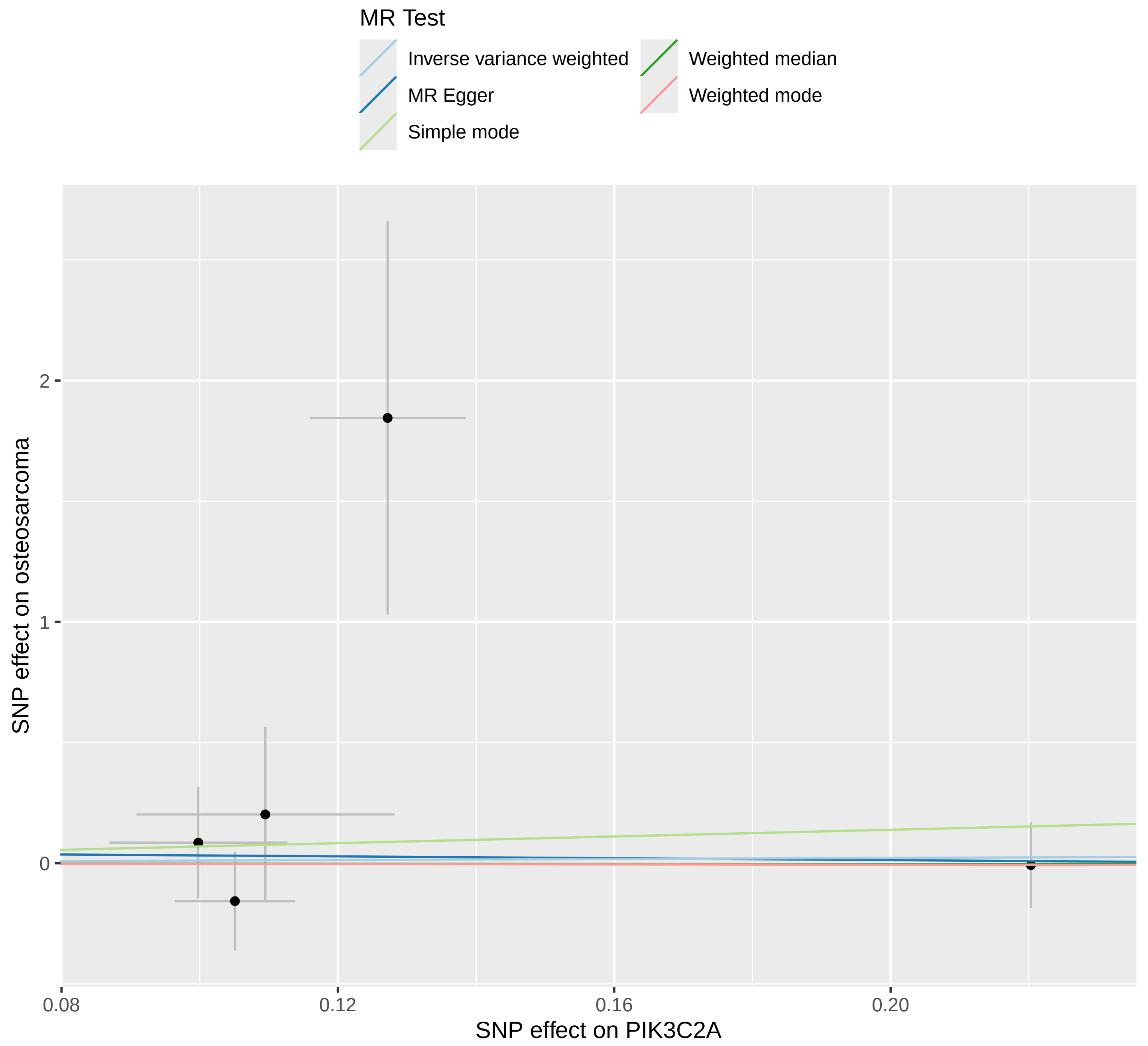
MR Test
Inverse variance weighted
MR Egger
Simple mode
Weighted median
Weighted mode
SNP effect on osteosarcoma
2
1
0
0.08
0.12
0.16
0.20
SNP effect on PIK3C2A

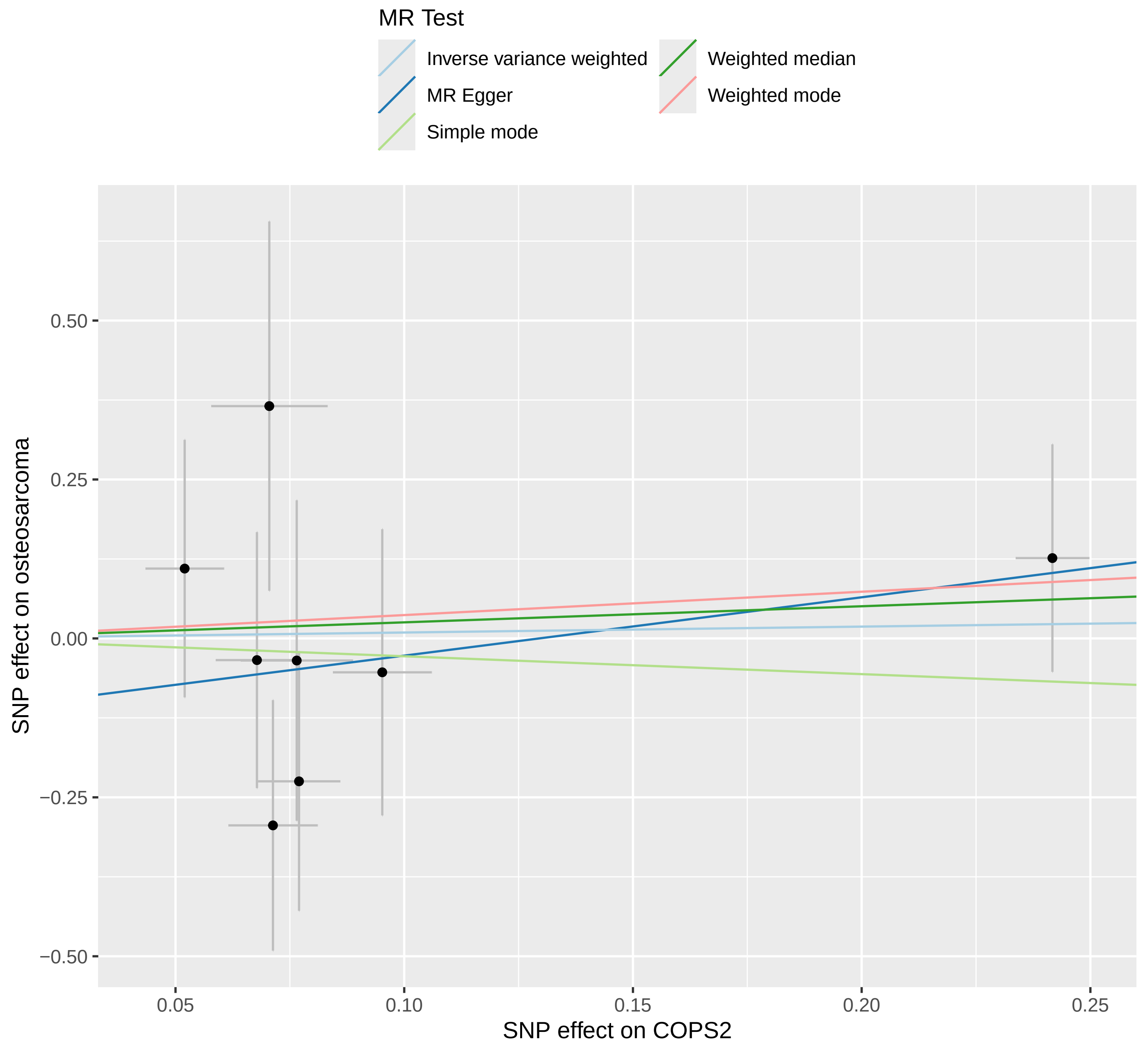

MR Test
Inverse variance weighted
MR Egger
Simple mode
Weighted median
Weighted mode
0.50
0.25
0.00
−0.25
−0.50
SNP effect on osteosarcoma
0.05
0.10
0.15
0.20
0.25
SNP effect on COPS2

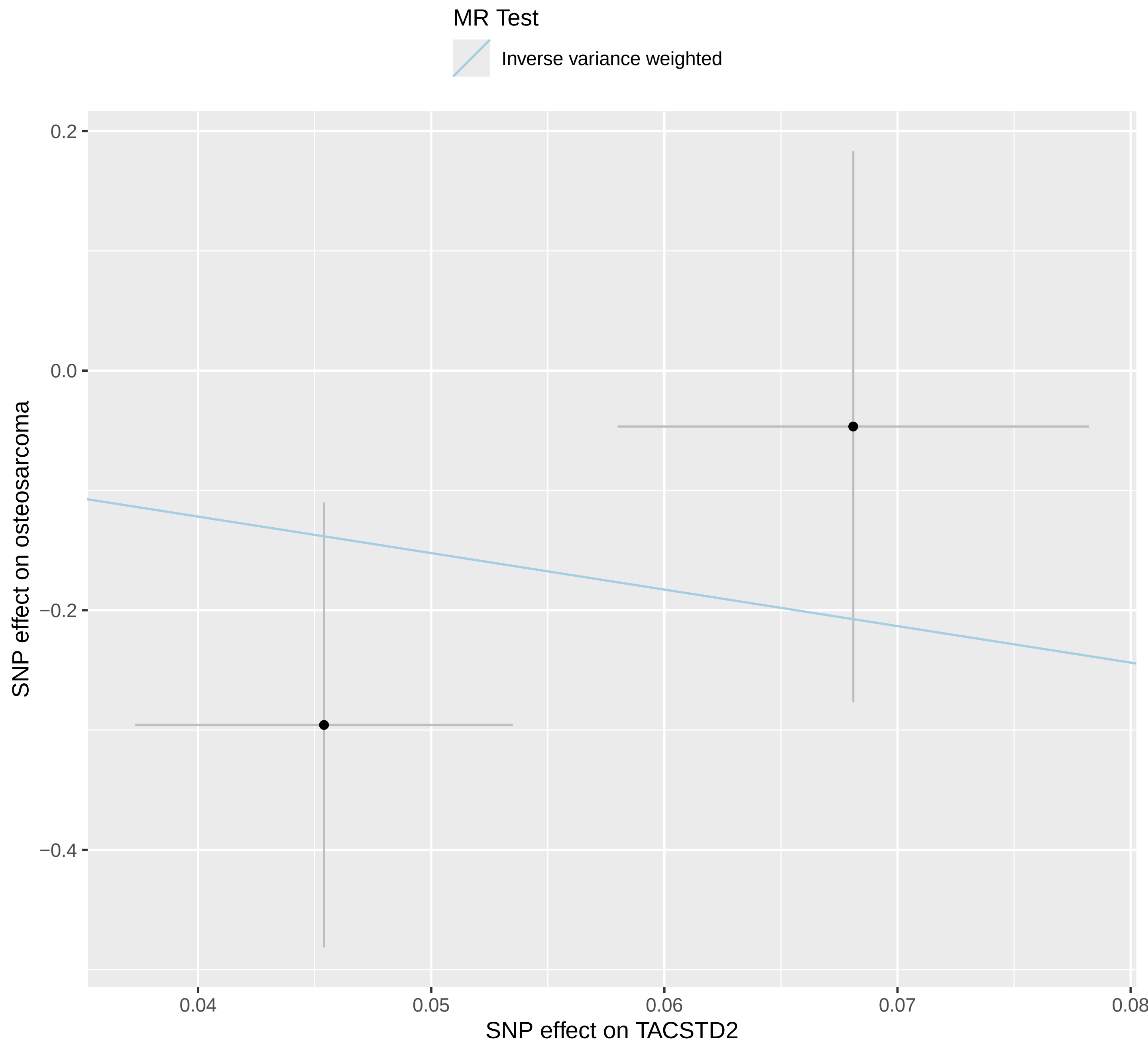

MR Test
Inverse variance weighted
0.2
0.0
−0.2
−0.4
0.04
0.05
0.06
0.07
0.08
SNP effect on TACSTD2
SNP effect on osteosarcoma

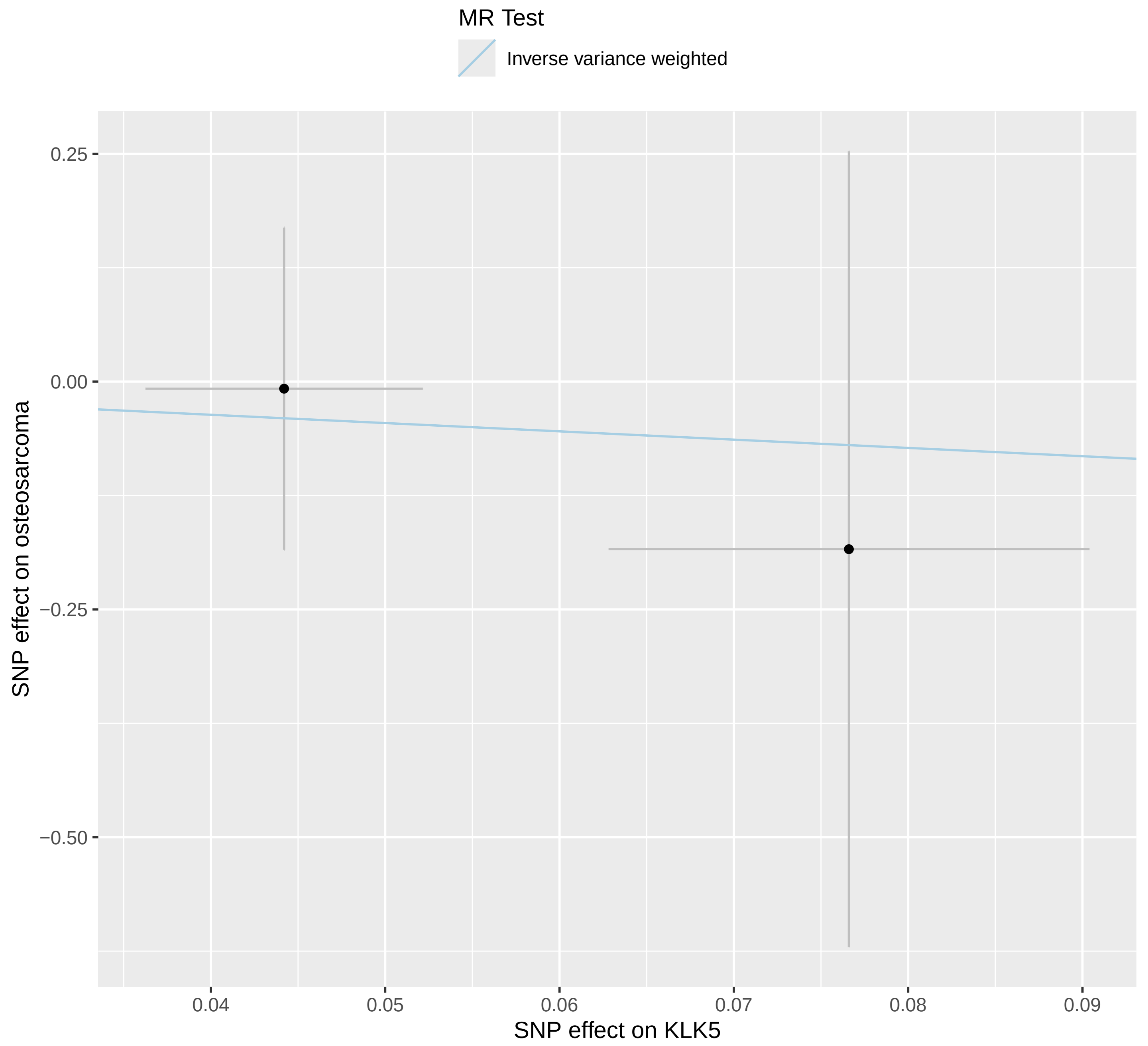
MR Test
Inverse variance weighted
0.25
0.00
−0.25
−0.50
SNP effect on osteosarcoma
0.04
0.05
0.06
0.07
0.08
0.09
SNP effect on KLK5

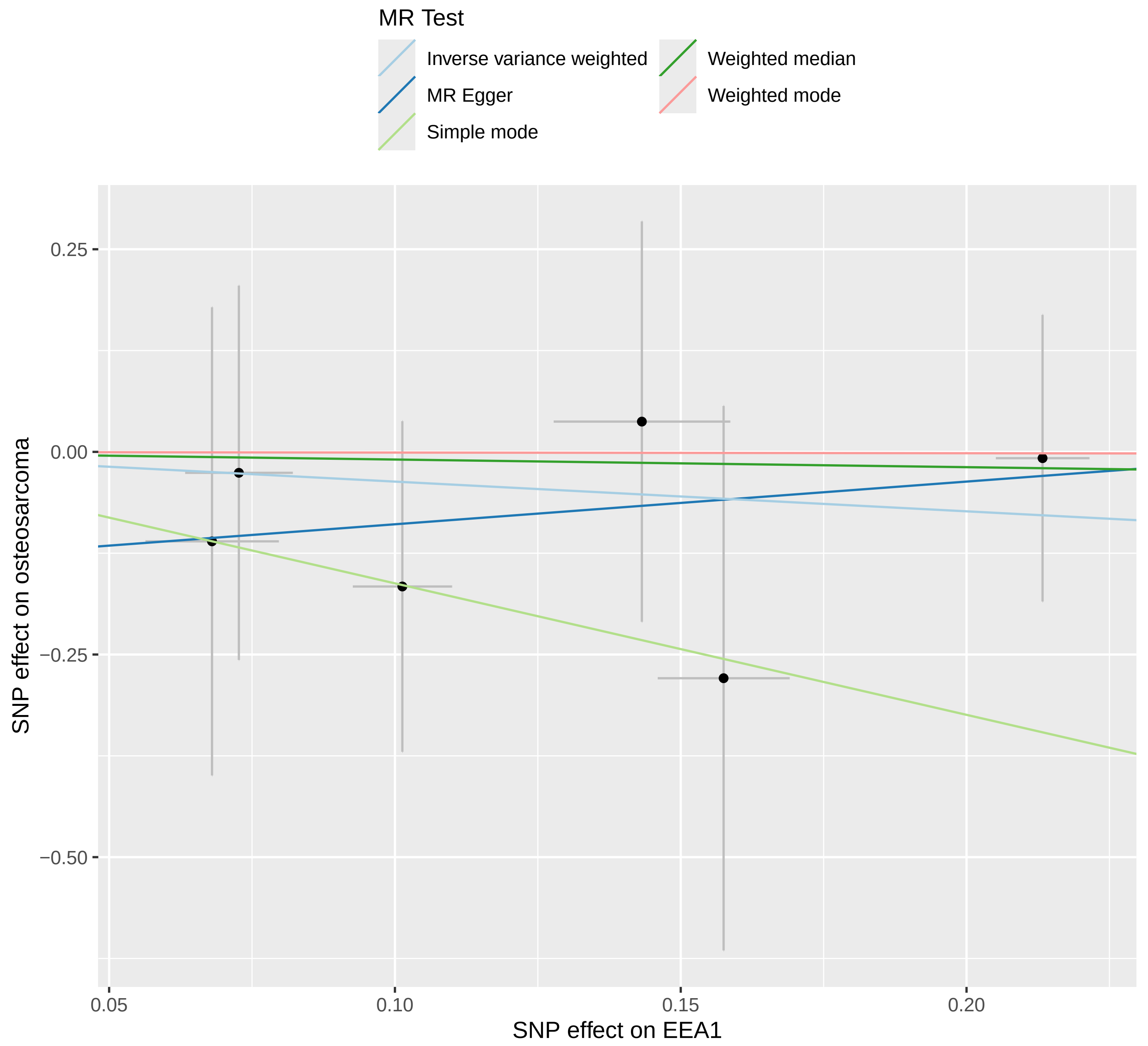
MR Test
Inverse variance weighted
MR Egger
Simple mode
Weighted median
Weighted mode
0.25
0.00
−0.25
−0.50
0.05
0.10
0.15
0.20
SNP effect on EEA1
SNP effect on osteosarcoma

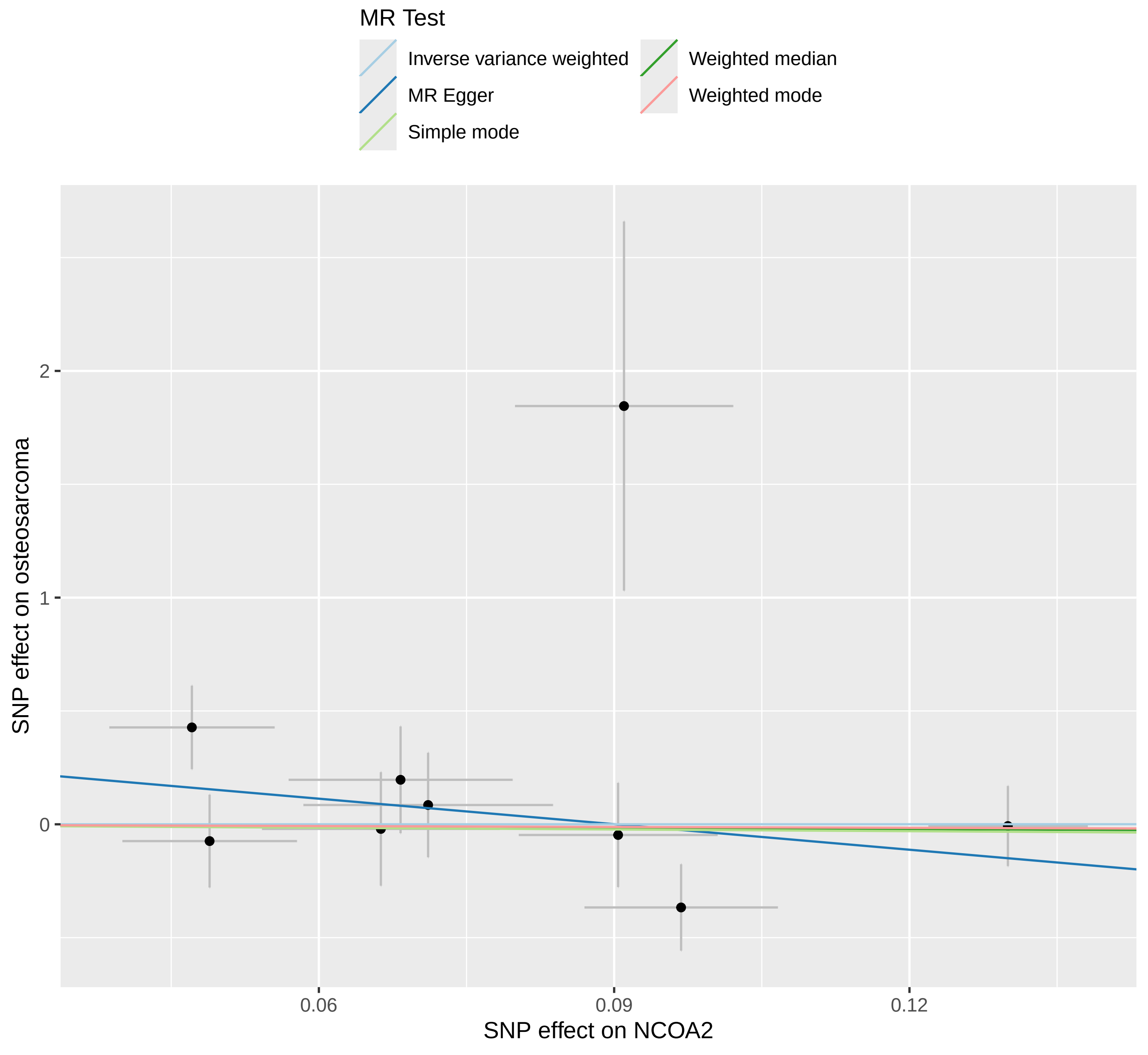
MR Test
Inverse variance weighted
MR Egger
Simple mode
Weighted median
Weighted mode
SNP effect on osteosarcoma
2
1
0
0.06
0.09
0.12
SNP effect on NCOA2

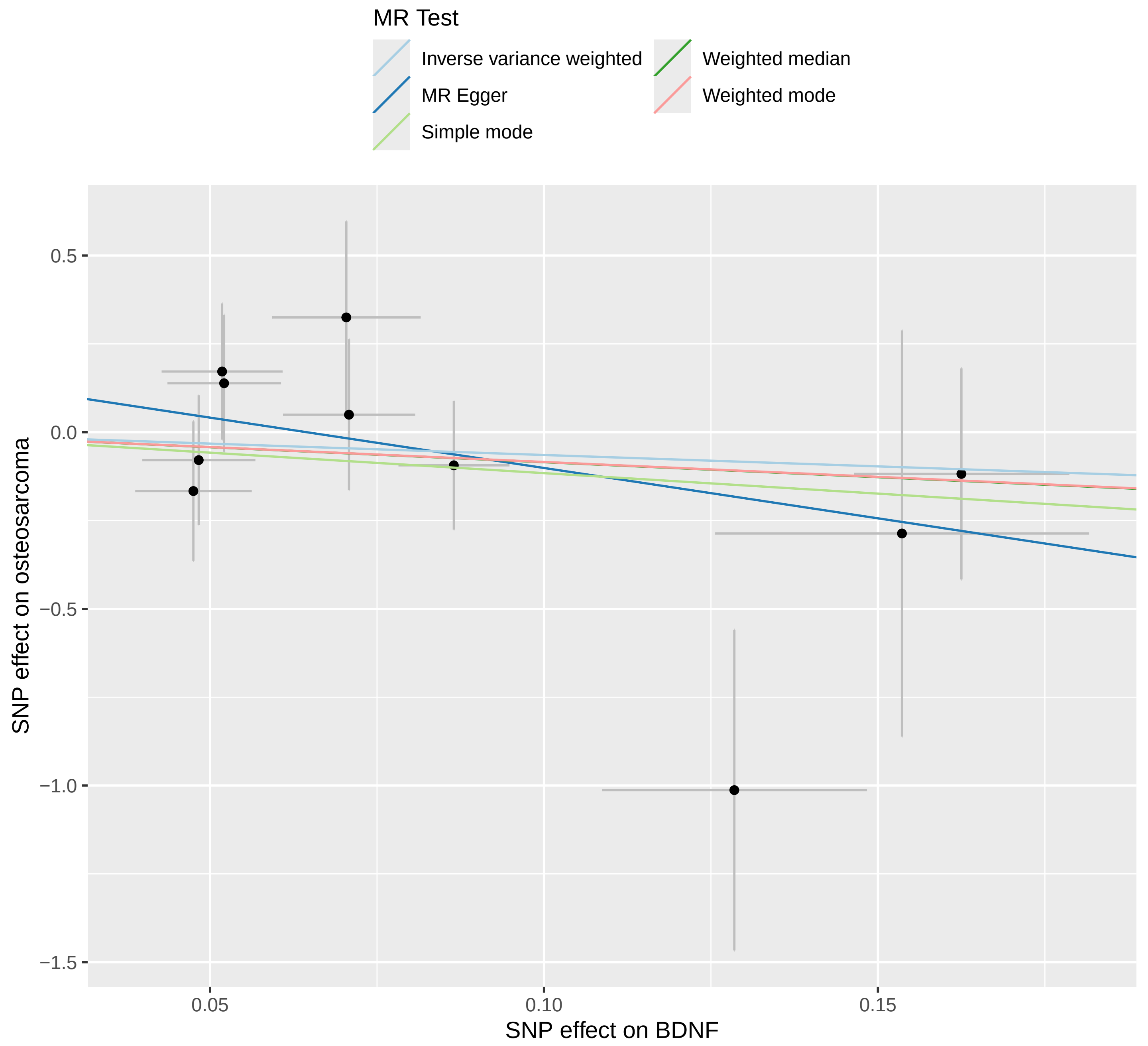
MR Test
Inverse variance weighted
MR Egger
Simple mode
Weighted median
Weighted mode
0.5
0.0
−0.5
−1.0
−1.5
SNP effect on osteosarcoma
0.05
0.10
0.15
SNP effect on BDNF

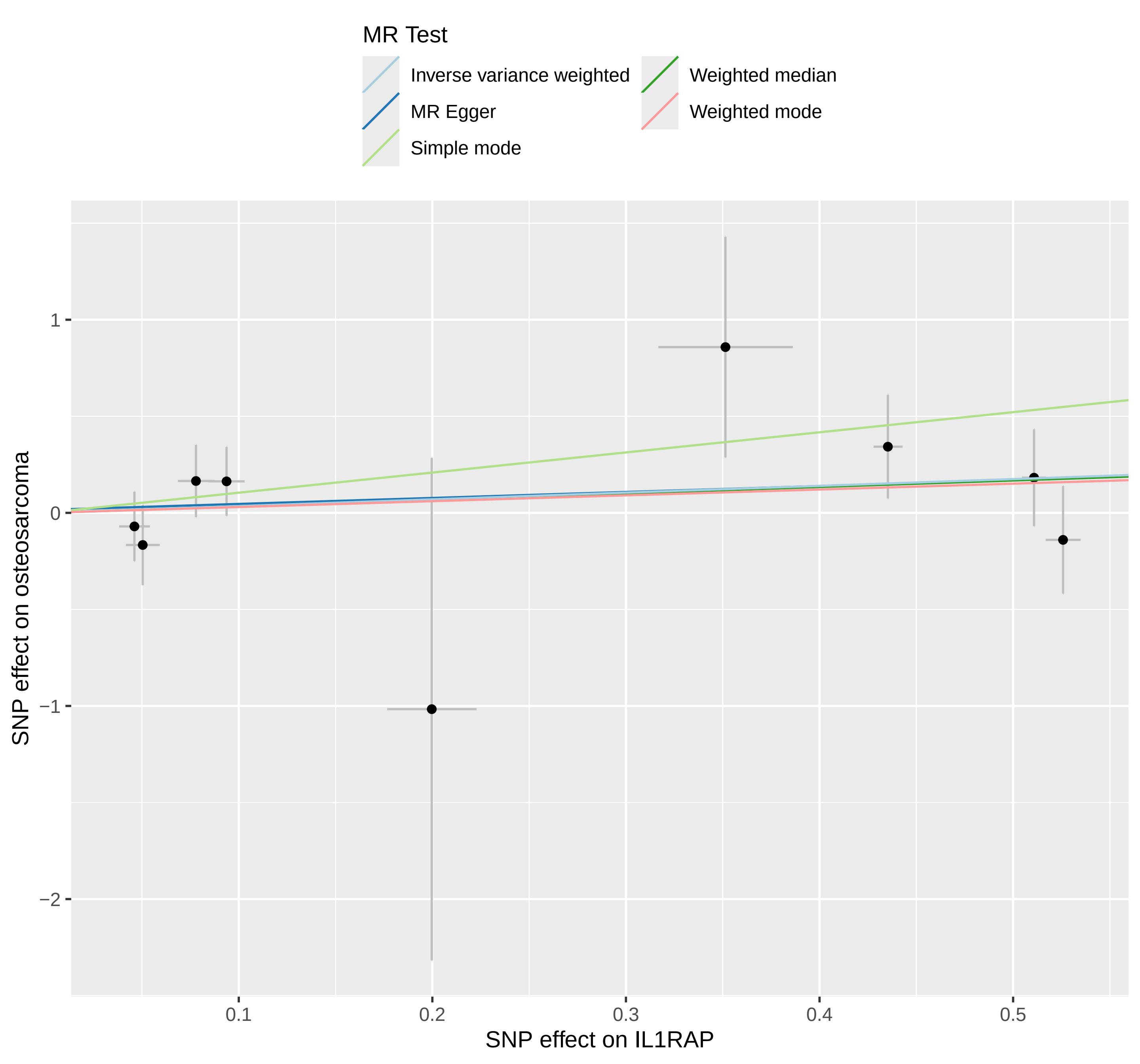
MR Test
Inverse variance weighted
MR Egger
Simple mode
Weighted median
Weighted mode
1
0
−1
−2
0.1
0.2
0.3
0.4
0.5
SNP effect on IL1RAP
SNP effect on osteosarcoma

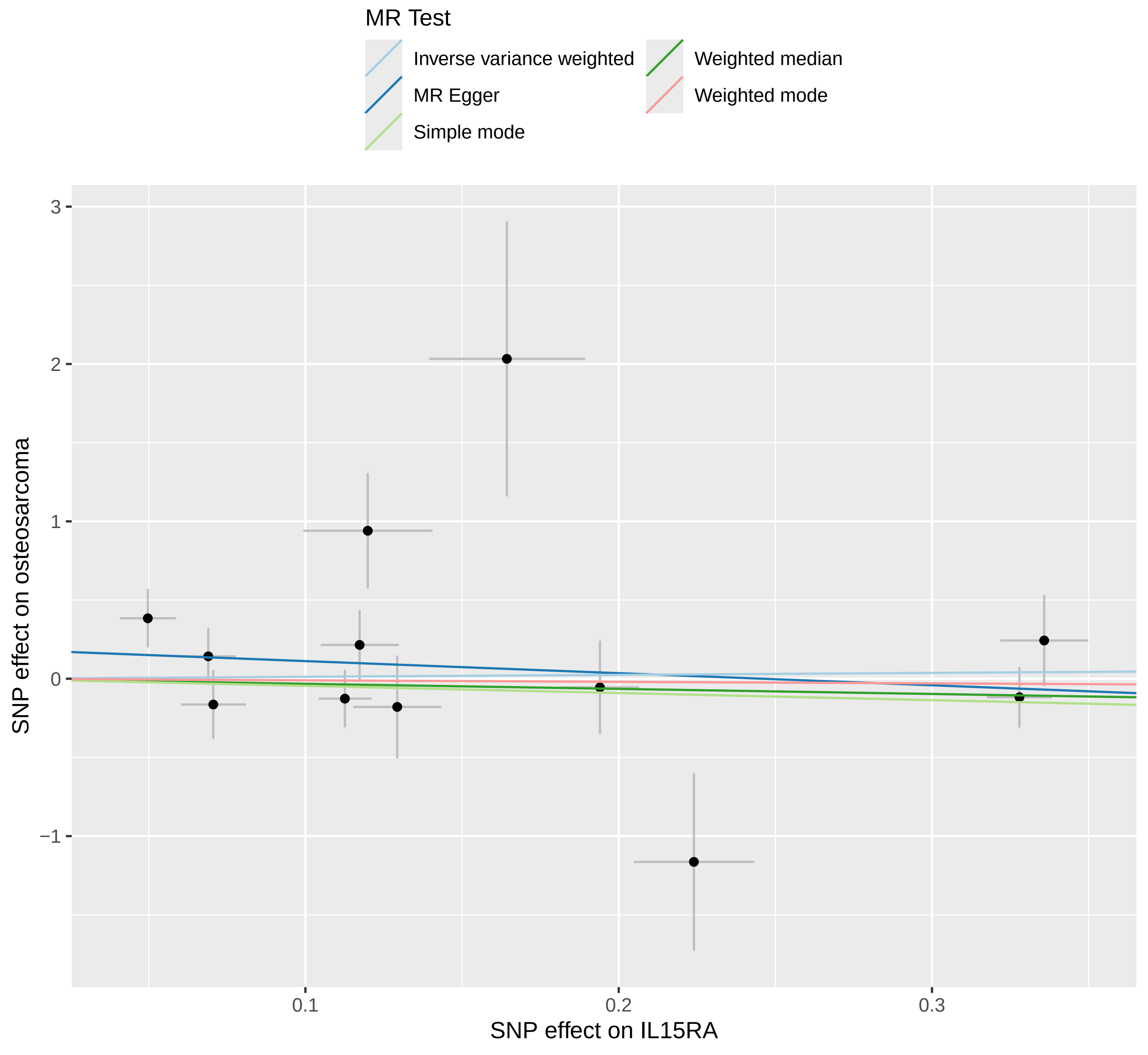
MR Test
Inverse variance weighted
MR Egger
Simple mode
Weighted median
Weighted mode
SNP effect on osteosarcoma
SNP effect on IL15RA
3
2
1
0
−1
0.1
0.2
0.3

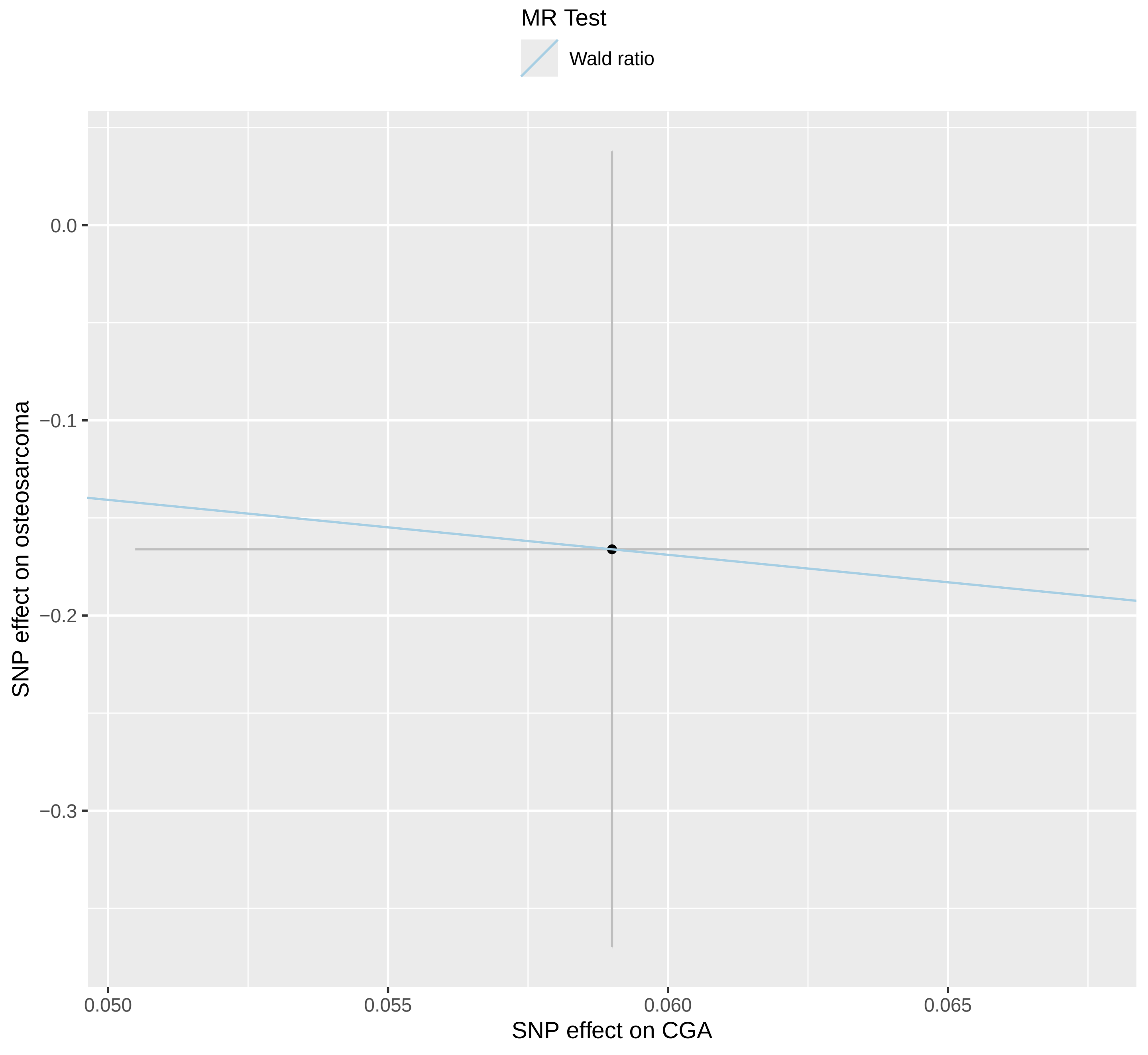
MR Test
Wald ratio
0.0
−0.1
−0.2
−0.3
0.050
0.055
0.060
0.065
SNP effect on osteosarcoma
SNP effect on CGA

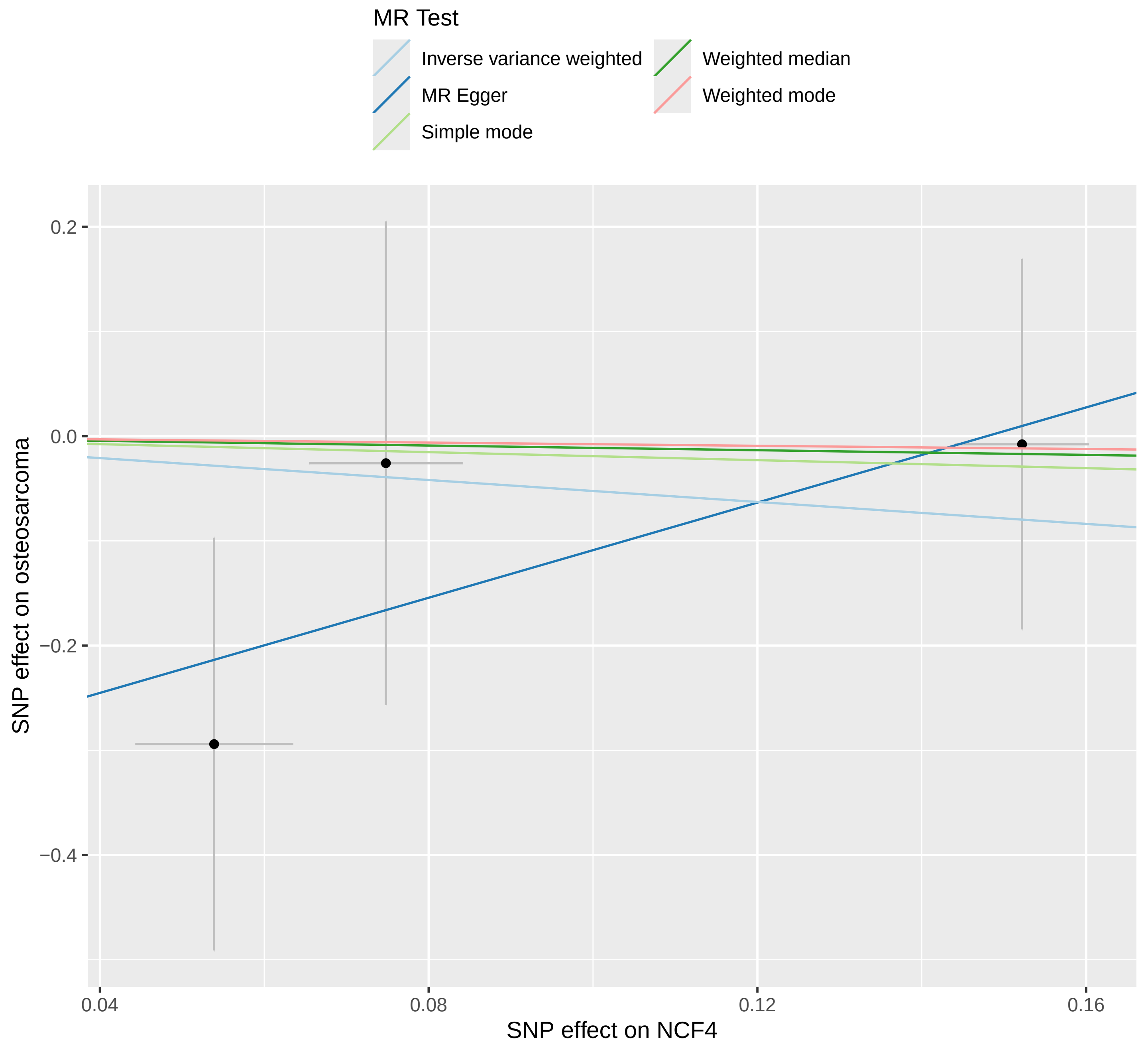
MR Test
Inverse variance weighted
MR Egger
Simple mode
Weighted median
Weighted mode
0.2
0.0
−0.2
−0.4
SNP effect on osteosarcoma
0.04
0.08
0.12
0.16
SNP effect on NCF4

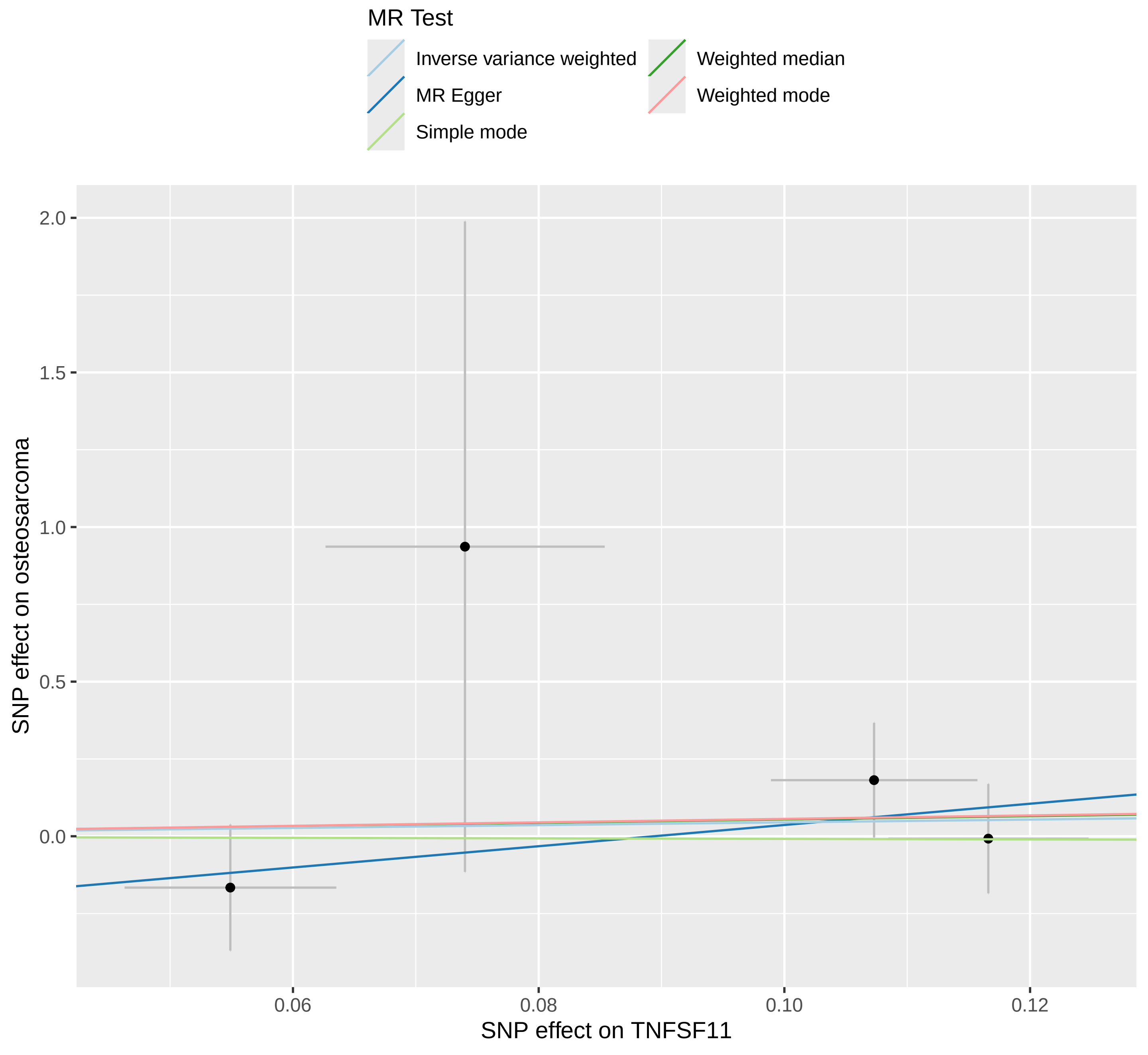
MR Test
Inverse variance weighted
MR Egger
Simple mode
Weighted median
Weighted mode
2.0
1.5
1.0
0.5
0.0
0.06
0.08
0.10
0.12
SNP effect on osteosarcoma
SNP effect on TNFSF11

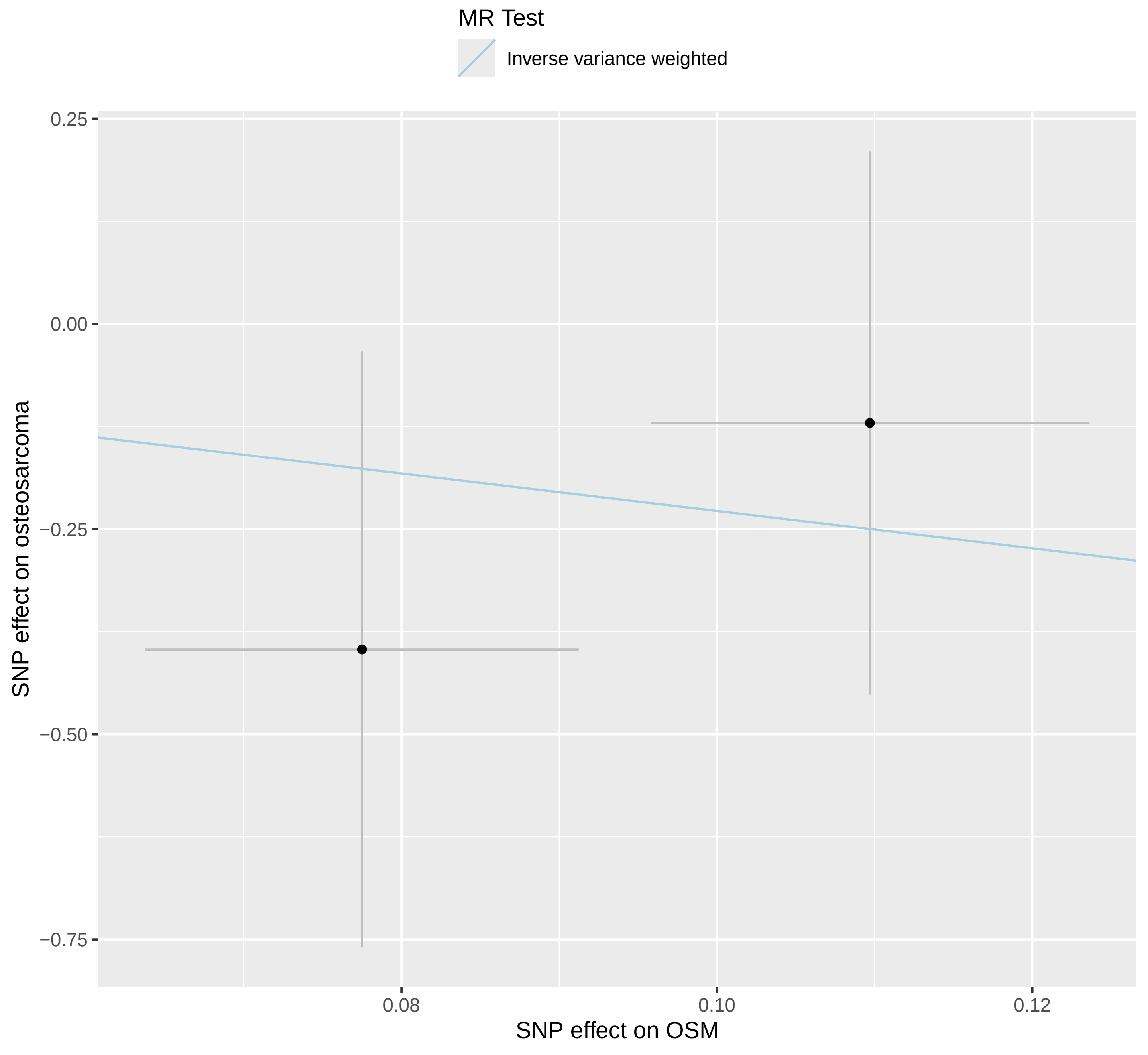
MR Test
Inverse variance weighted
0.25
0.00
−0.25
−0.50
−0.75
0.08
0.10
0.12
SNP effect on OSM
SNP effect on osteosarcoma

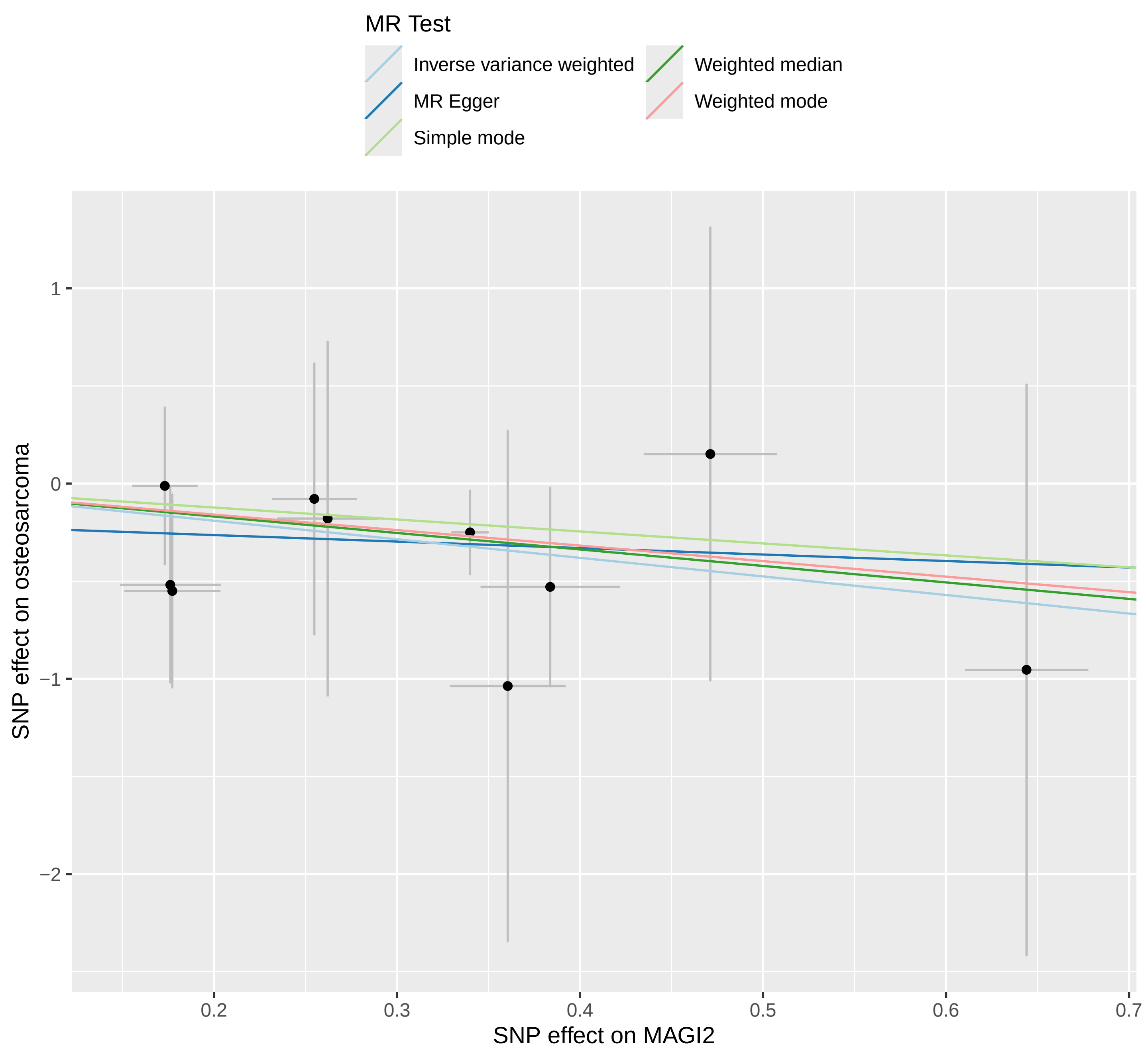
MR Test
Inverse variance weighted
MR Egger
Simple mode
Weighted median
Weighted mode
SNP effect on osteosarcoma
1
0
−1
−2
0.2
0.3
0.4
0.5
0.6
0.7
SNP effect on MAGI2

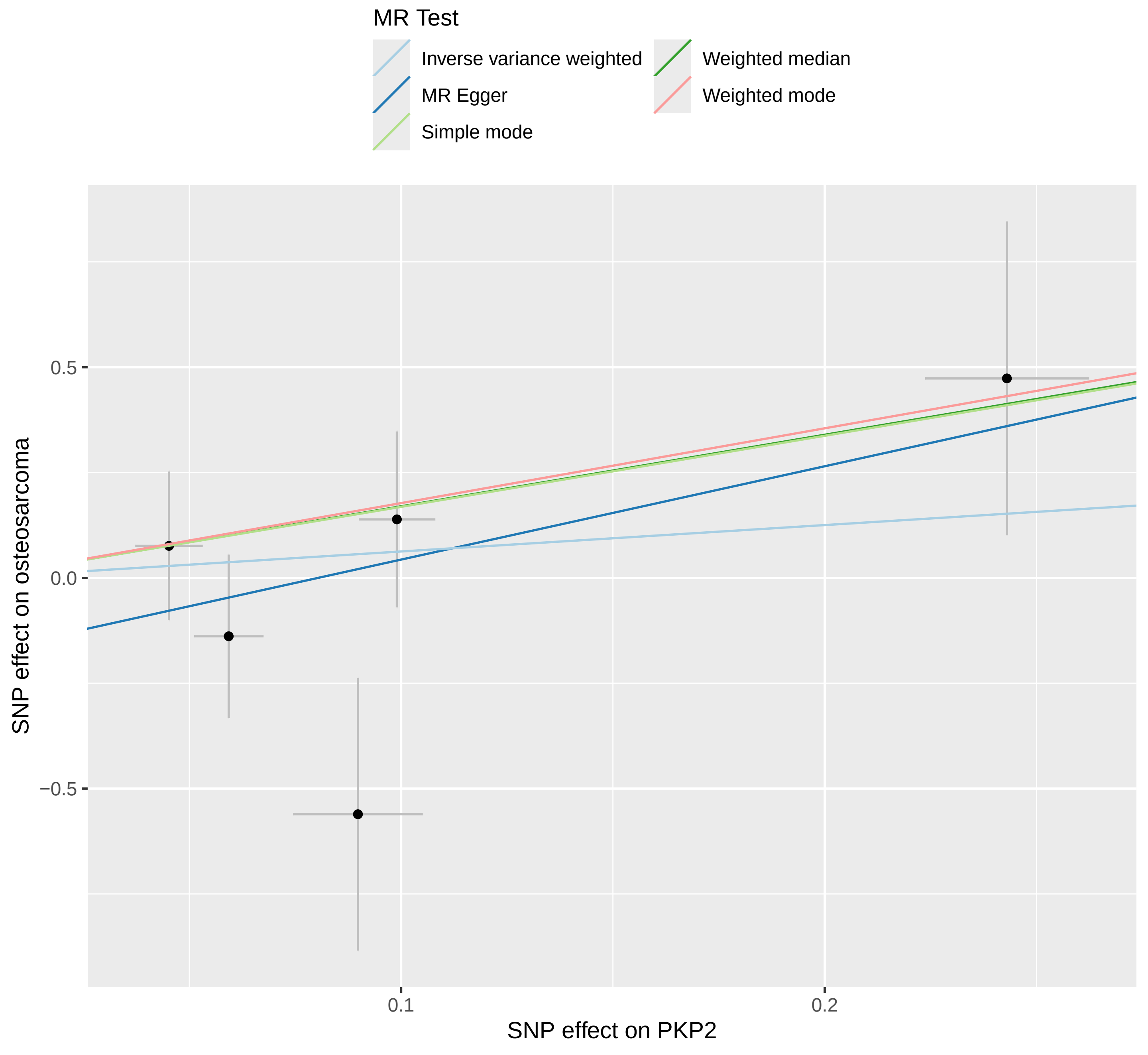
MR Test
Inverse variance weighted
MR Egger
Simple mode
Weighted median
Weighted mode
0.5
0.0
−0.5
0.1
0.2
SNP effect on osteosarcoma
SNP effect on PKP2

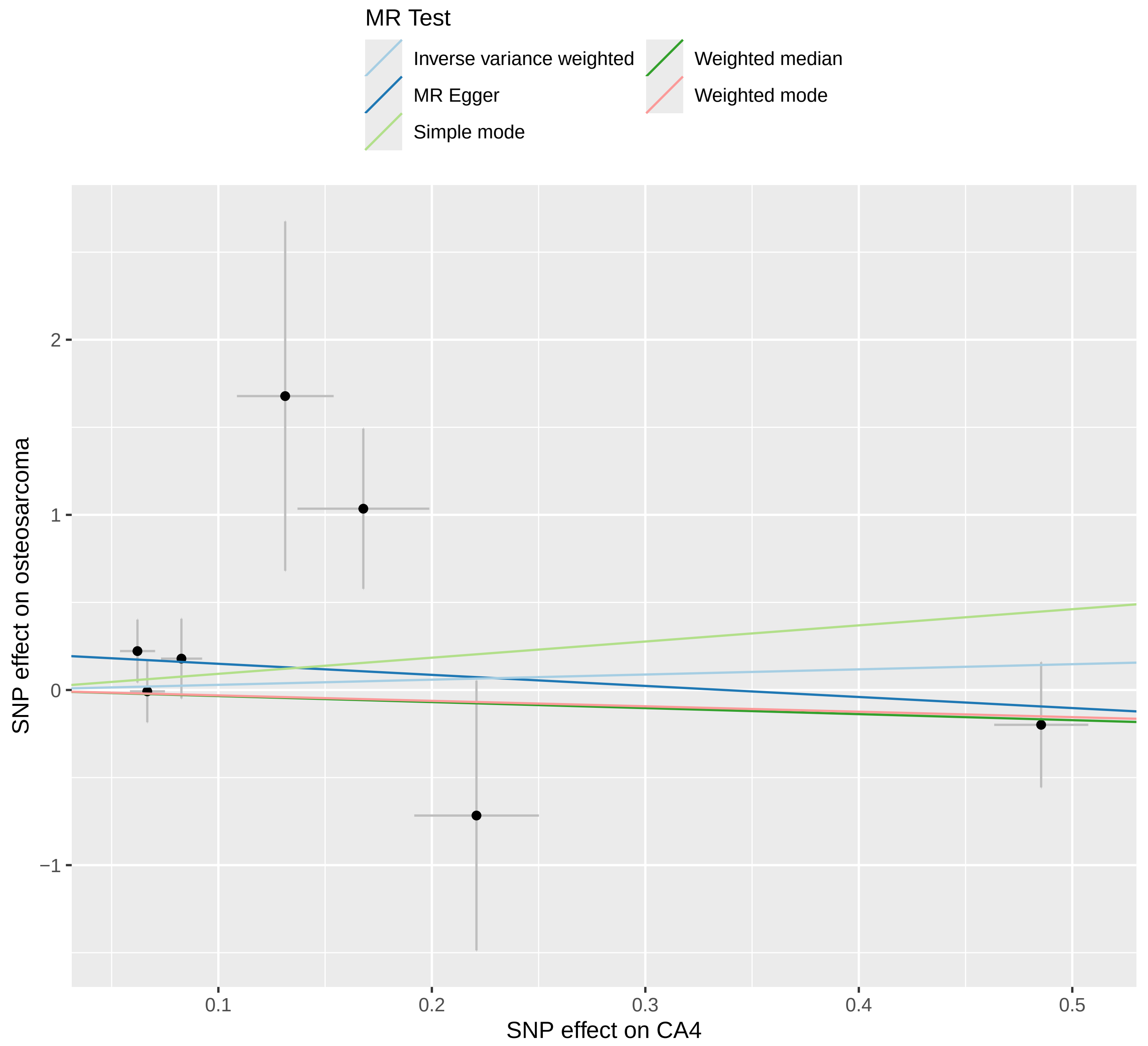
MR Test
Inverse variance weighted
MR Egger
Simple mode
Weighted median
Weighted mode
SNP effect on osteosarcoma
2
1
0
−1
0.1
0.2
0.3
0.4
0.5
SNP effect on CA4

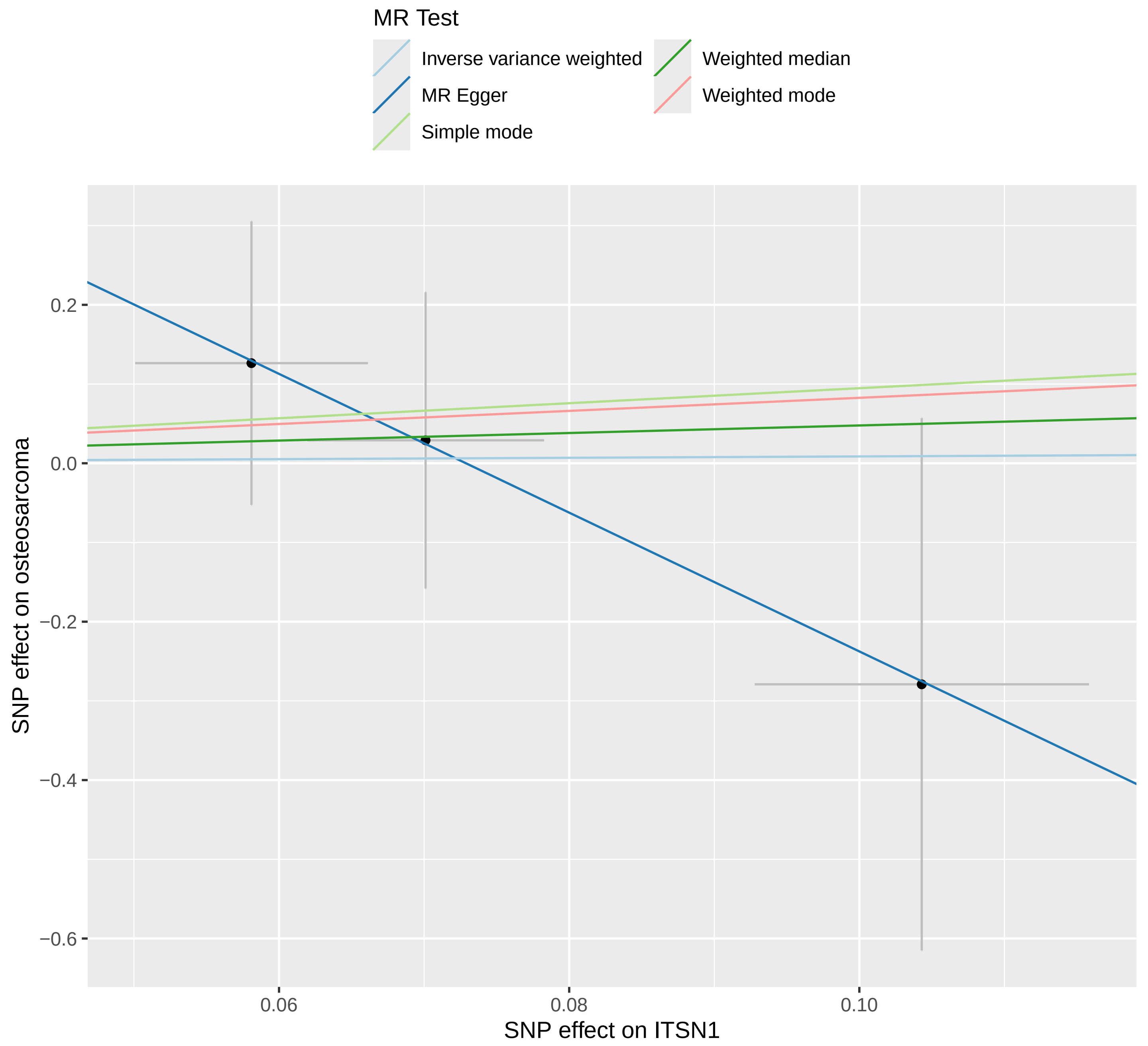
MR Test
Inverse variance weighted
MR Egger
Simple mode
Weighted median
Weighted mode
0.2
0.0
−0.2
−0.4
−0.6
SNP effect on osteosarcoma
0.06
0.08
0.10
SNP effect on ITSN1

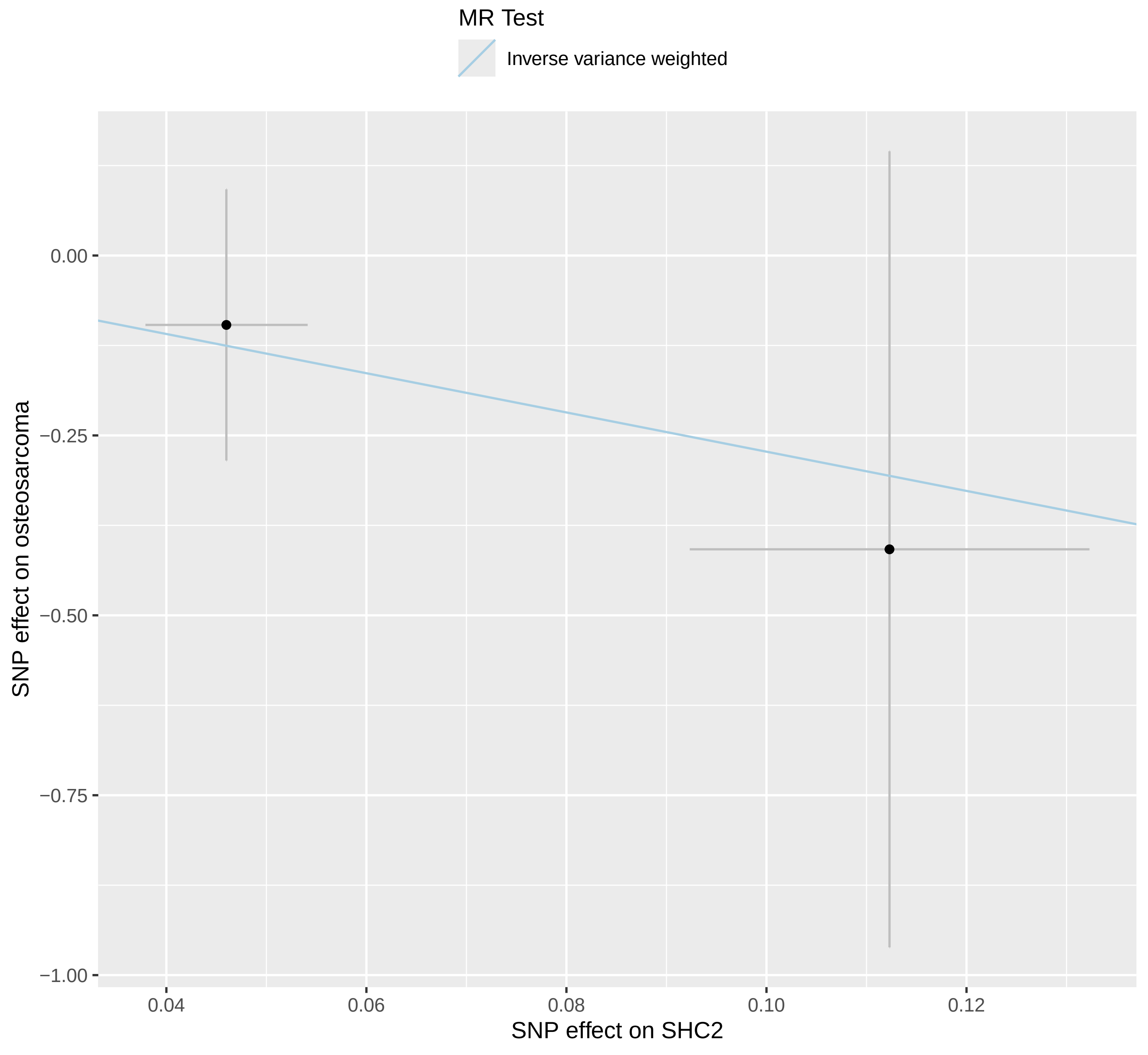
MR Test
Inverse variance weighted
0.00
−0.25
−0.50
−0.75
−1.00
SNP effect on osteosarcoma
0.04
0.06
0.08
0.10
0.12
SNP effect on SHC2

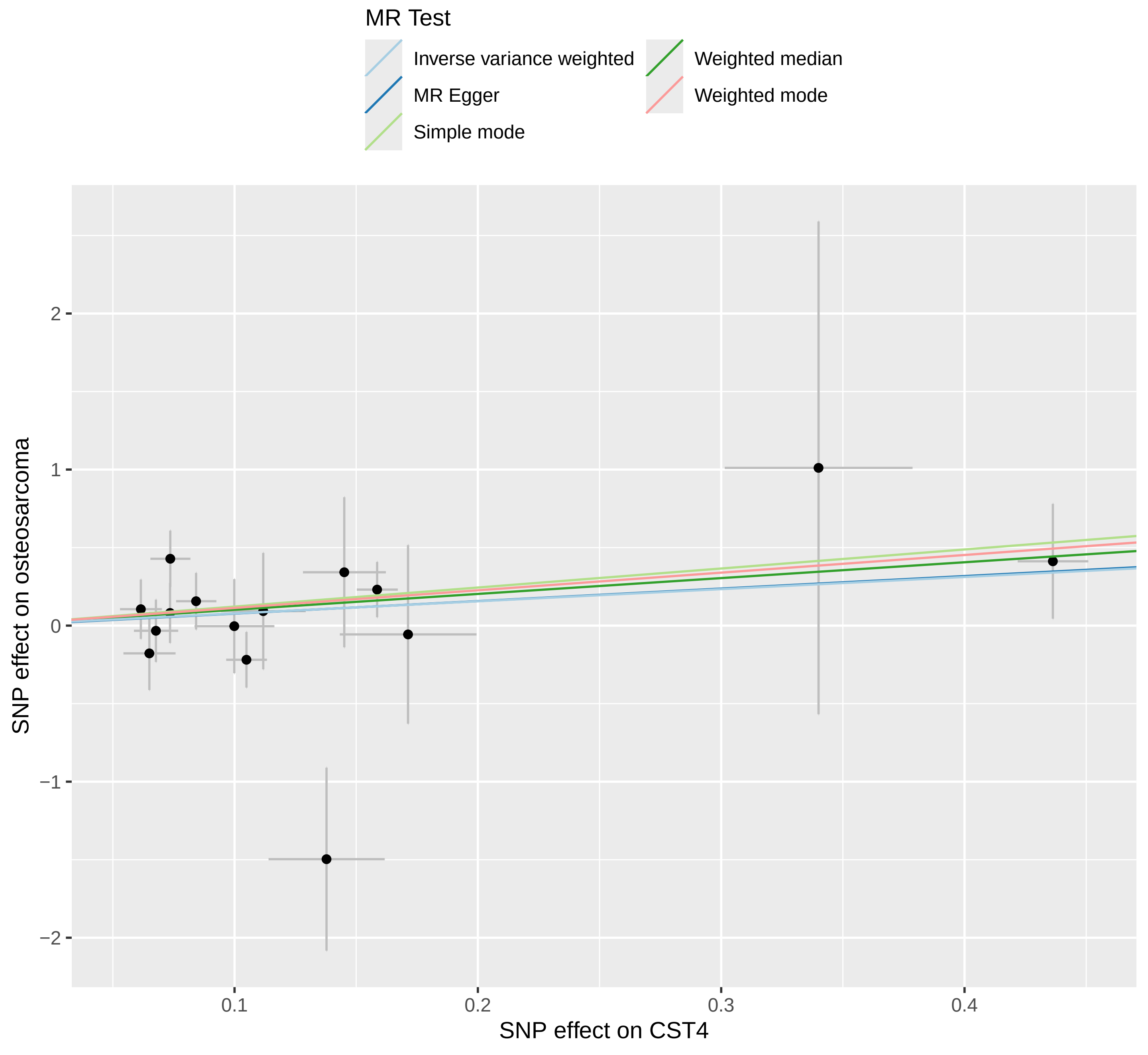
MR Test
Inverse variance weighted
MR Egger
Simple mode
Weighted median
Weighted mode
SNP effect on osteosarcoma
SNP effect on CST4
2
1
0
−1
−2
0.1
0.2
0.3
0.4

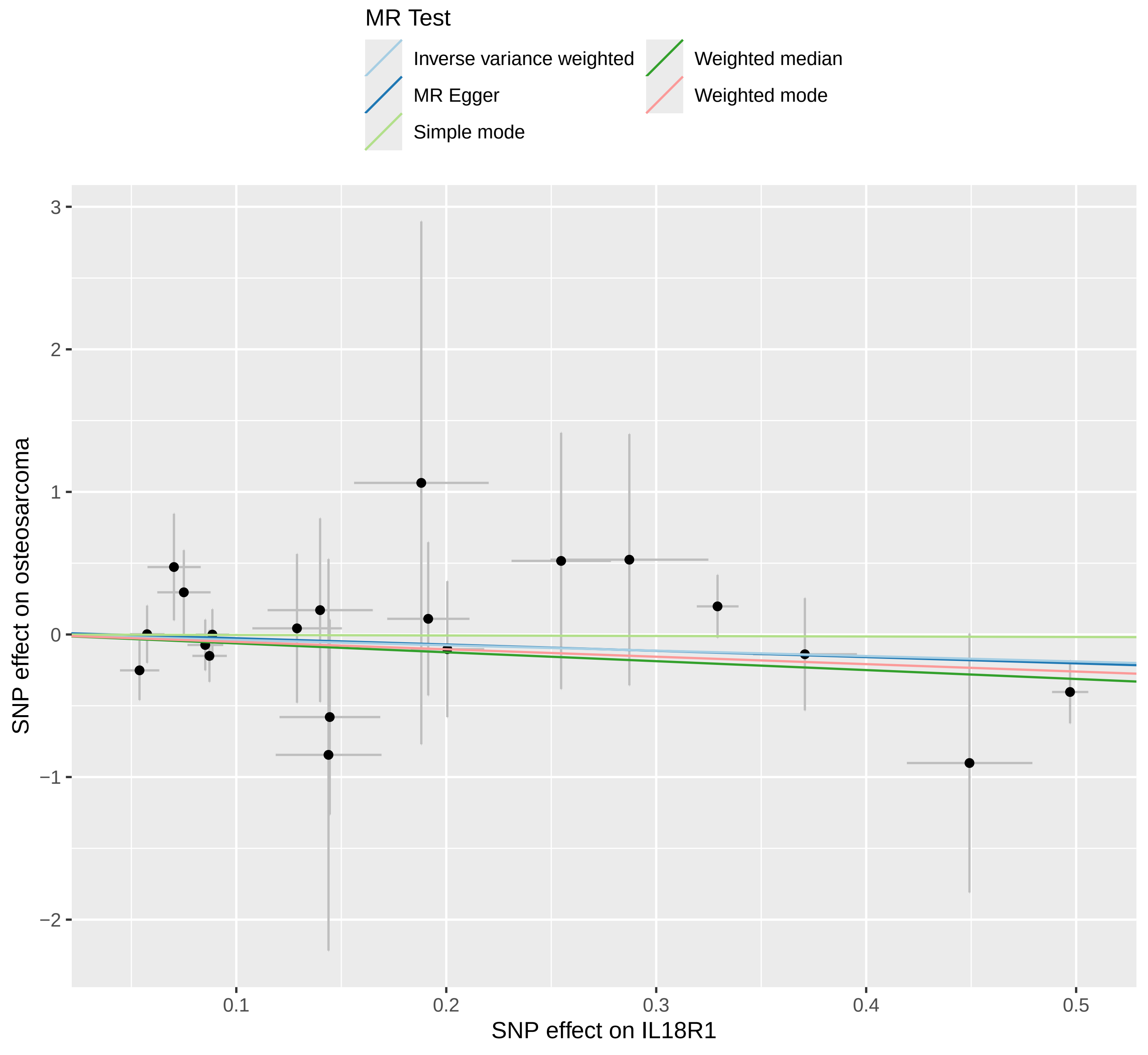
MR Test
Inverse variance weighted
MR Egger
Simple mode
Weighted median
Weighted mode
3
2
1
0
−1
−2
0.1
0.2
0.3
0.4
0.5
SNP effect on osteosarcoma
SNP effect on IL18R1

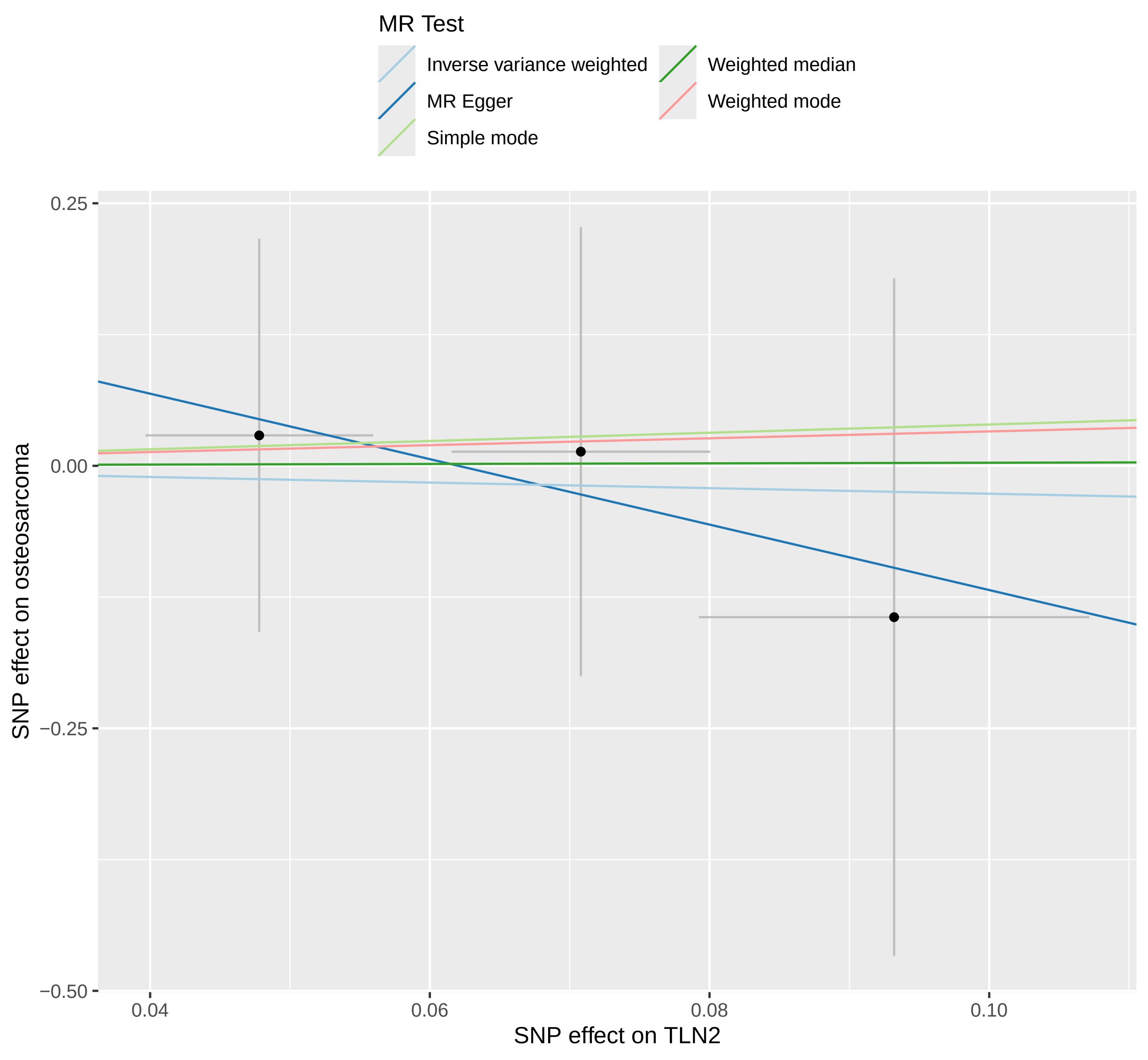

MR Test
Inverse variance weighted
MR Egger
Simple mode
Weighted median
Weighted mode
0.25
0.00
−0.25
−0.50
SNP effect on osteosarcoma
0.04
0.06
0.08
0.10
SNP effect on TLN2

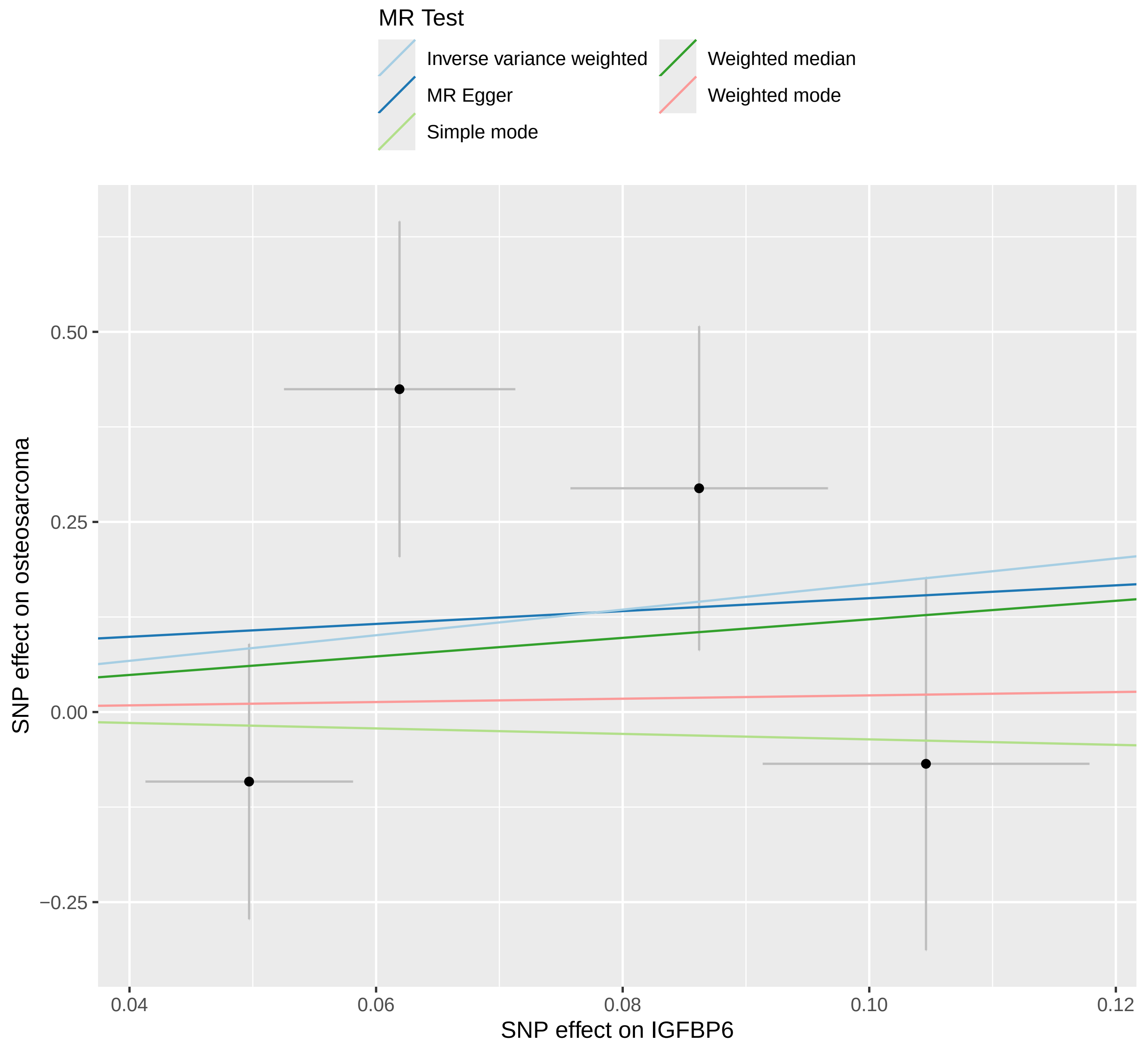
MR Test
Inverse variance weighted
MR Egger
Simple mode
Weighted median
Weighted mode
SNP effect on osteosarcoma
0.50
0.25
0.00
−0.25
0.04
0.06
0.08
0.10
0.12
SNP effect on IGFBP6

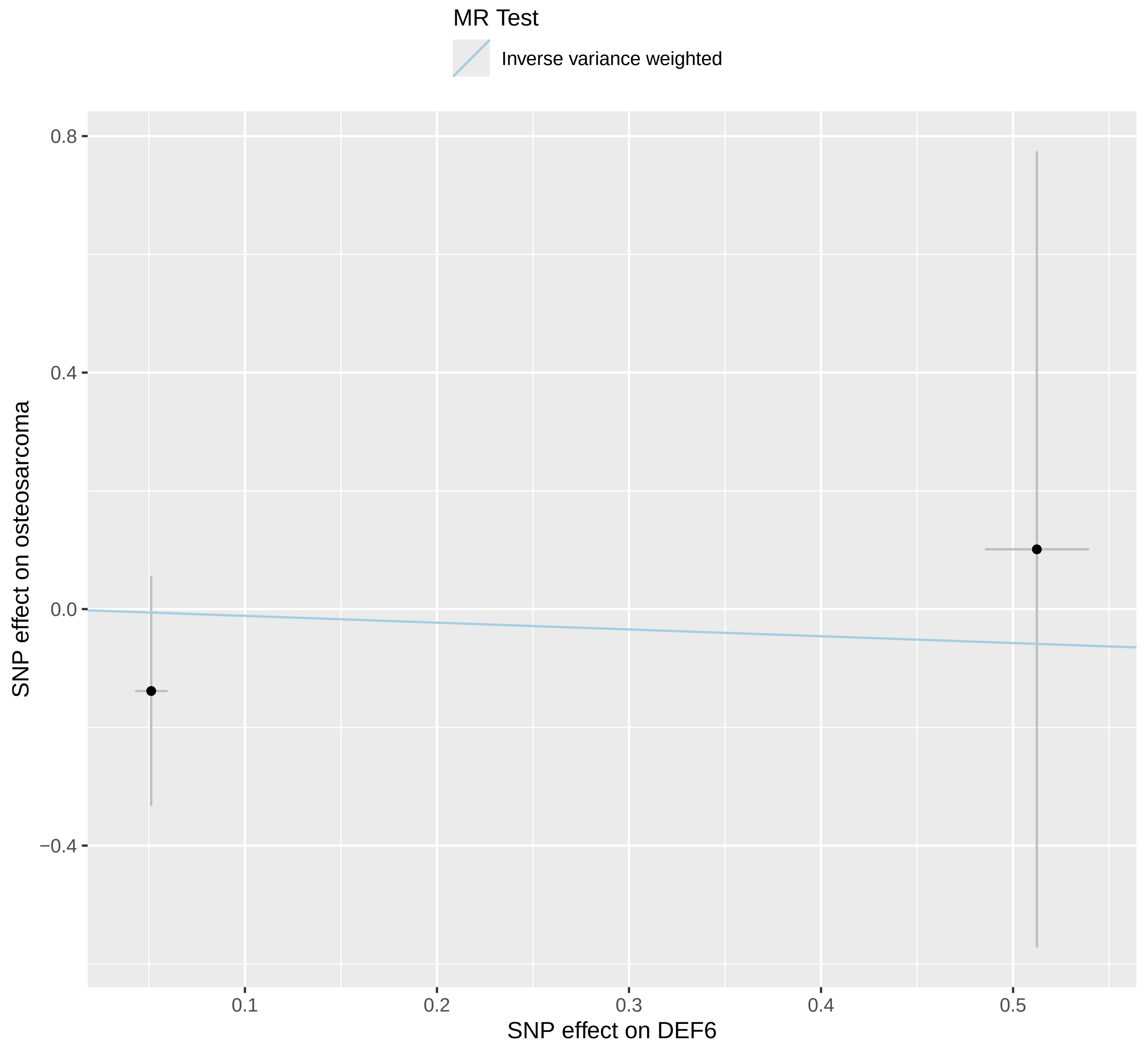

MR Test
Inverse variance weighted
0.8
0.4
0.0
−0.4
SNP effect on osteosarcoma
0.1
0.2
0.3
0.4
0.5
SNP effect on DEF6

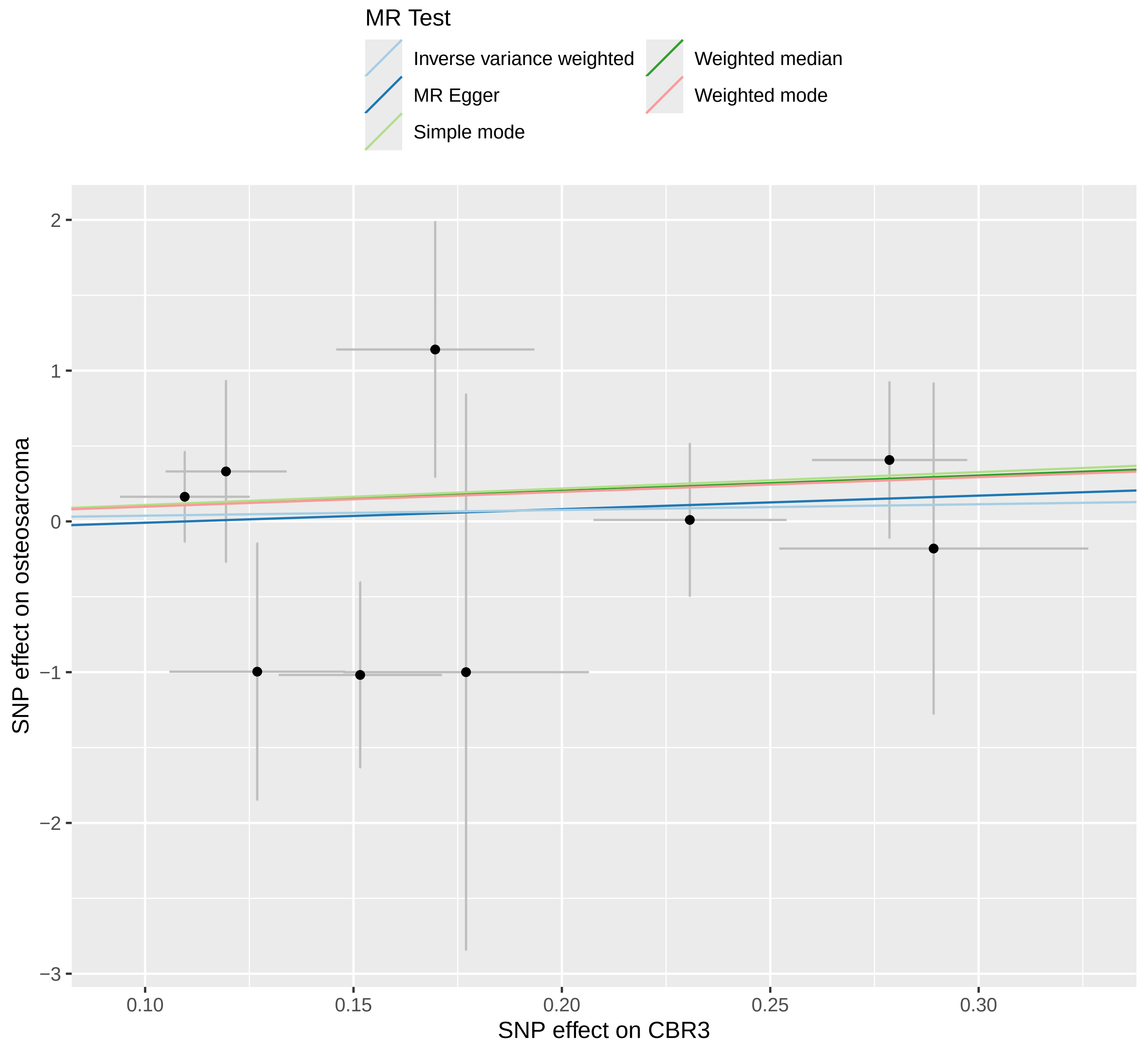

MR Test
Inverse variance weighted
MR Egger
Simple mode
Weighted median
Weighted mode
2
1
0
−1
−2
−3
SNP effect on osteosarcoma
0.10
0.15
0.20
0.25
0.30
SNP effect on CBR3

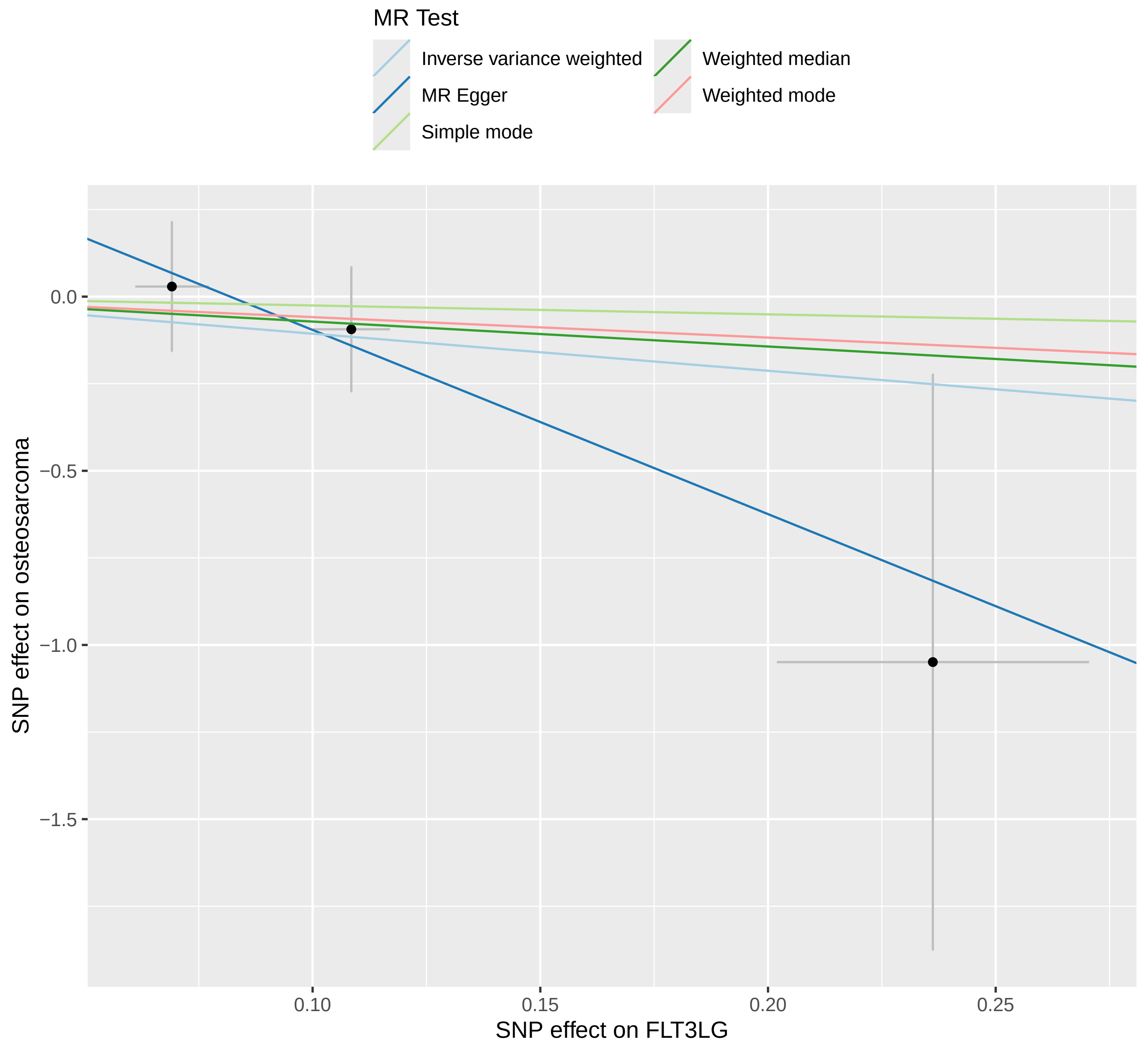
MR Test
Inverse variance weighted
MR Egger
Simple mode
Weighted median
Weighted mode
0.0
−0.5
−1.0
−1.5
0.10
0.15
0.20
0.25
SNP effect on FLT3LG
SNP effect on osteosarcoma

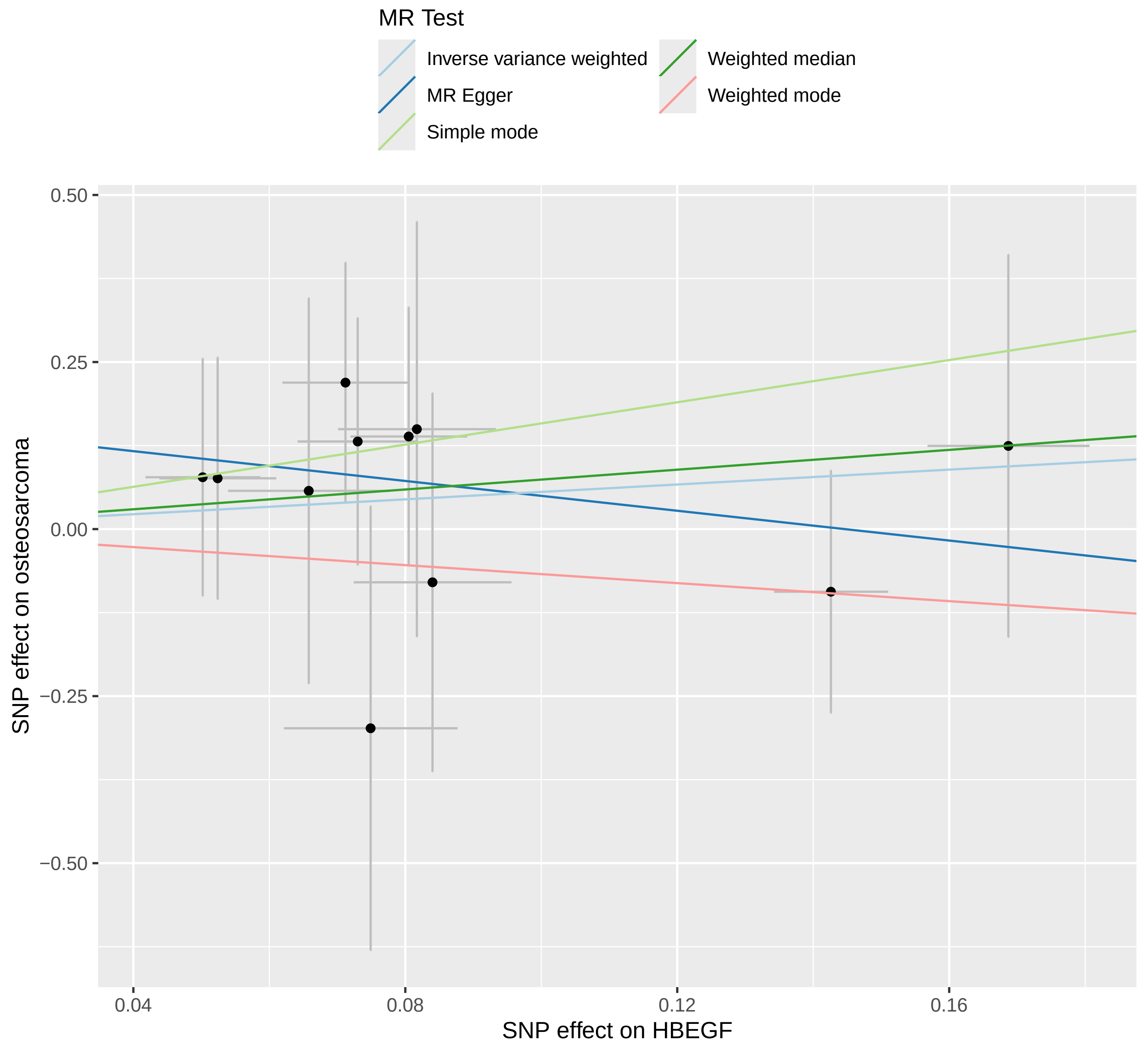
MR Test
Inverse variance weighted
MR Egger
Simple mode
Weighted median
Weighted mode
0.50
0.25
0.00
−0.25
−0.50
SNP effect on osteosarcoma
0.04
0.08
0.12
0.16
SNP effect on HBEGF

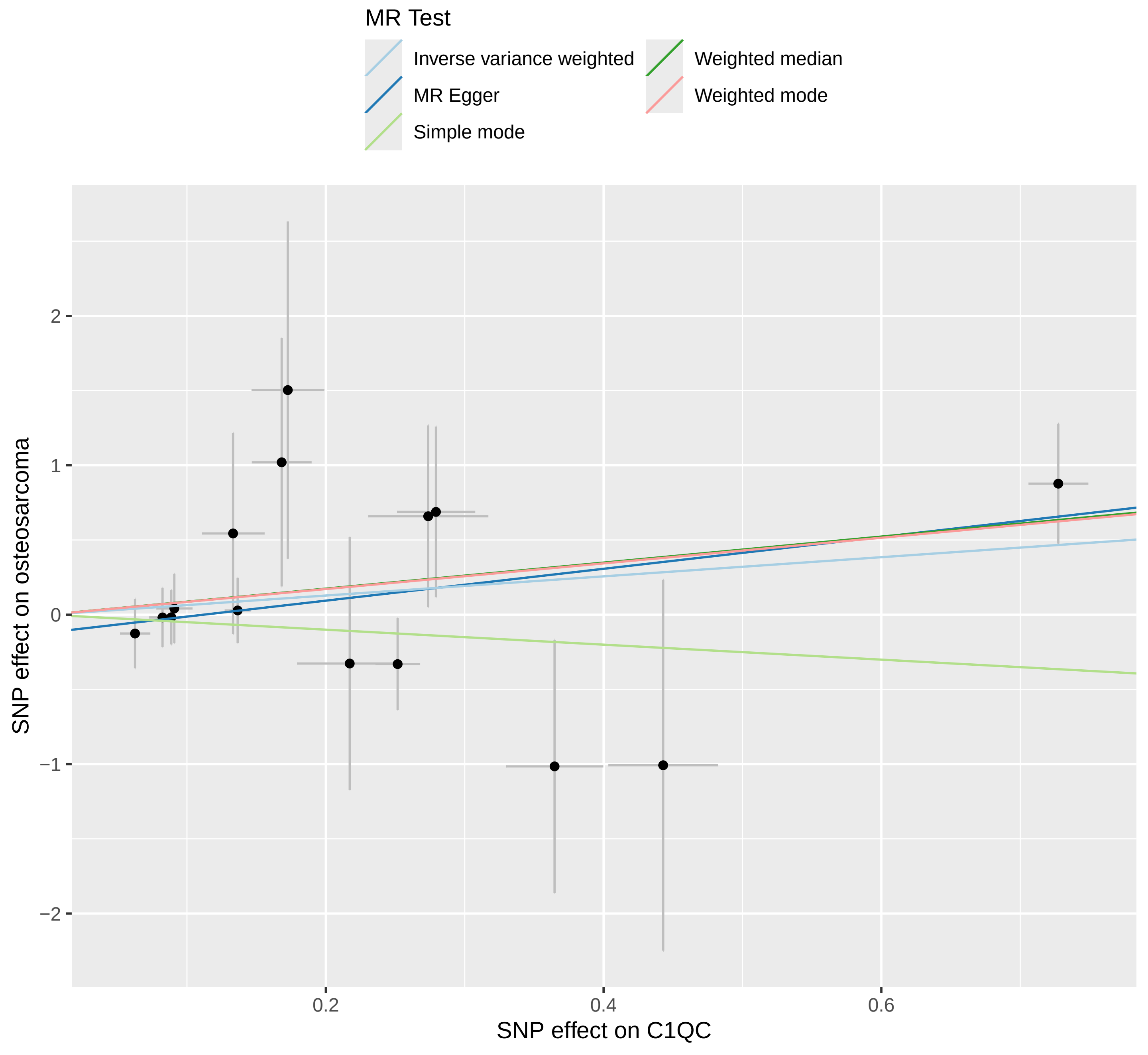

MR Test
Inverse variance weighted
MR Egger
Simple mode
Weighted median
Weighted mode
2
1
0
−1
−2
SNP effect on osteosarcoma
0.2
0.4
0.6
SNP effect on C1QC

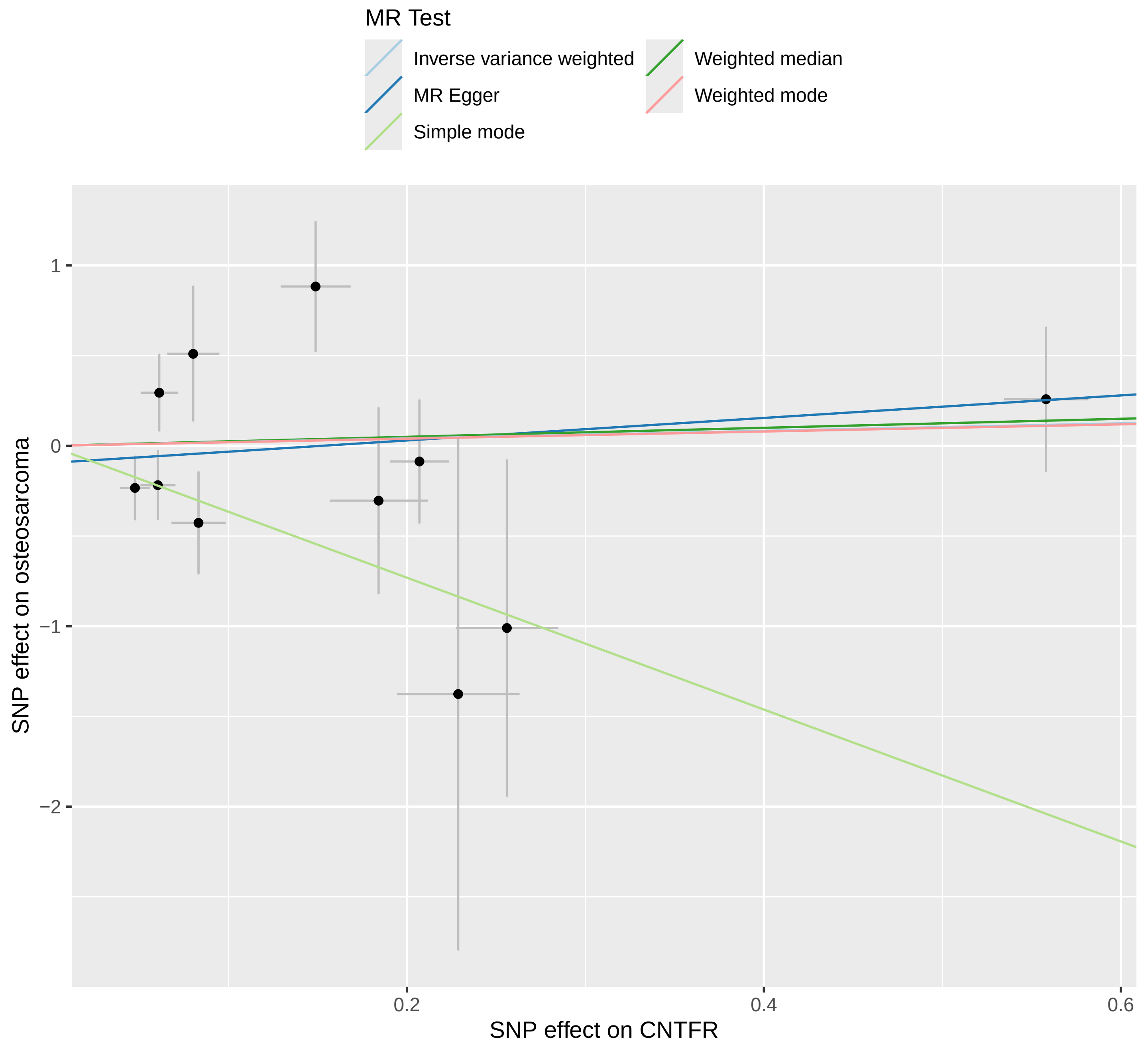
MR Test
Inverse variance weighted
MR Egger
Simple mode
Weighted median
Weighted mode
1
0
−1
−2
SNP effect on osteosarcoma
0.2
0.4
0.6
SNP effect on CNTFR

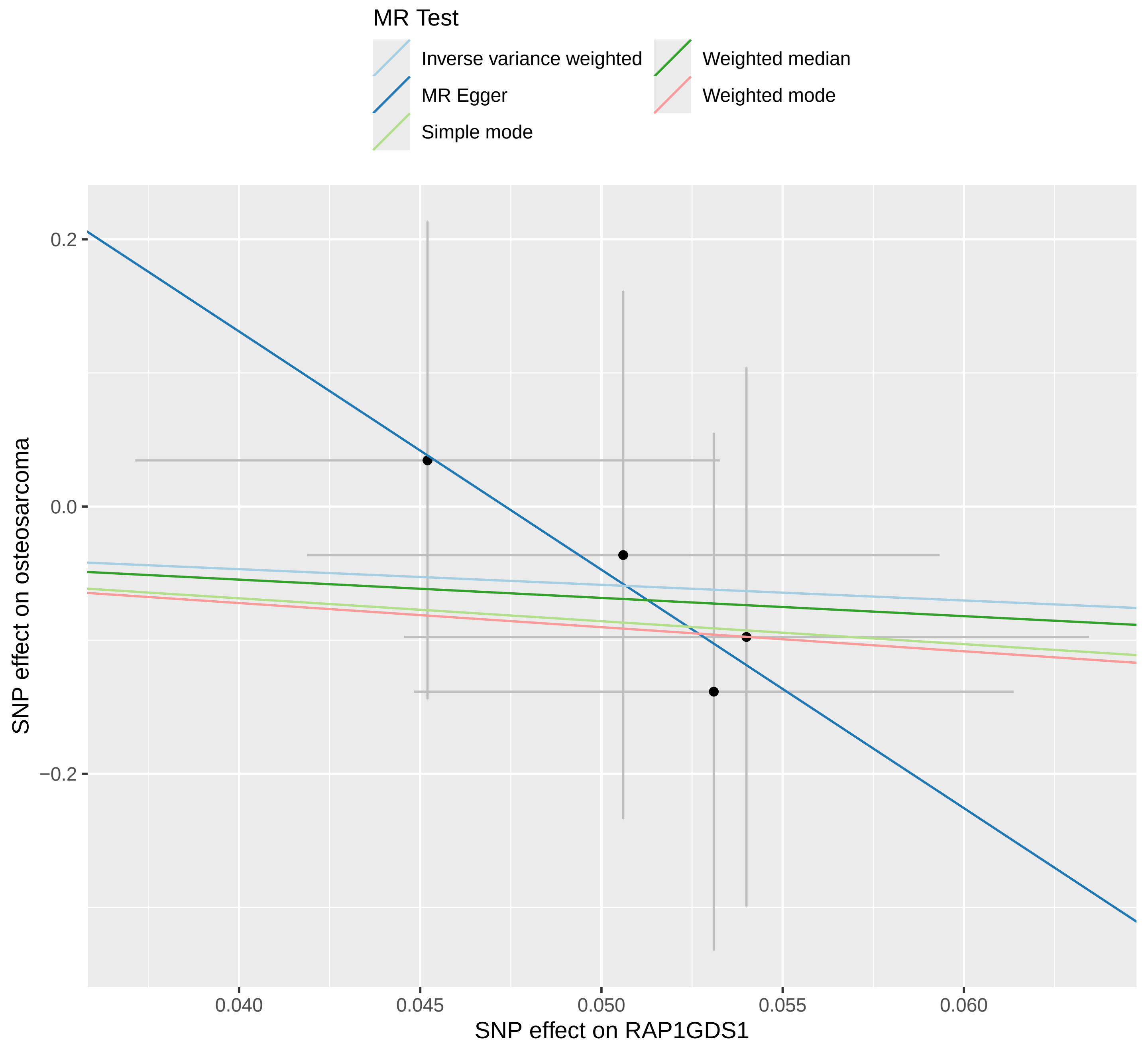
MR Test
Inverse variance weighted
MR Egger
Simple mode
Weighted median
Weighted mode
0.2
0.0
−0.2
0.040
0.045
0.050
0.055
0.060
SNP effect on RAP1GDS1
SNP effect on osteosarcoma

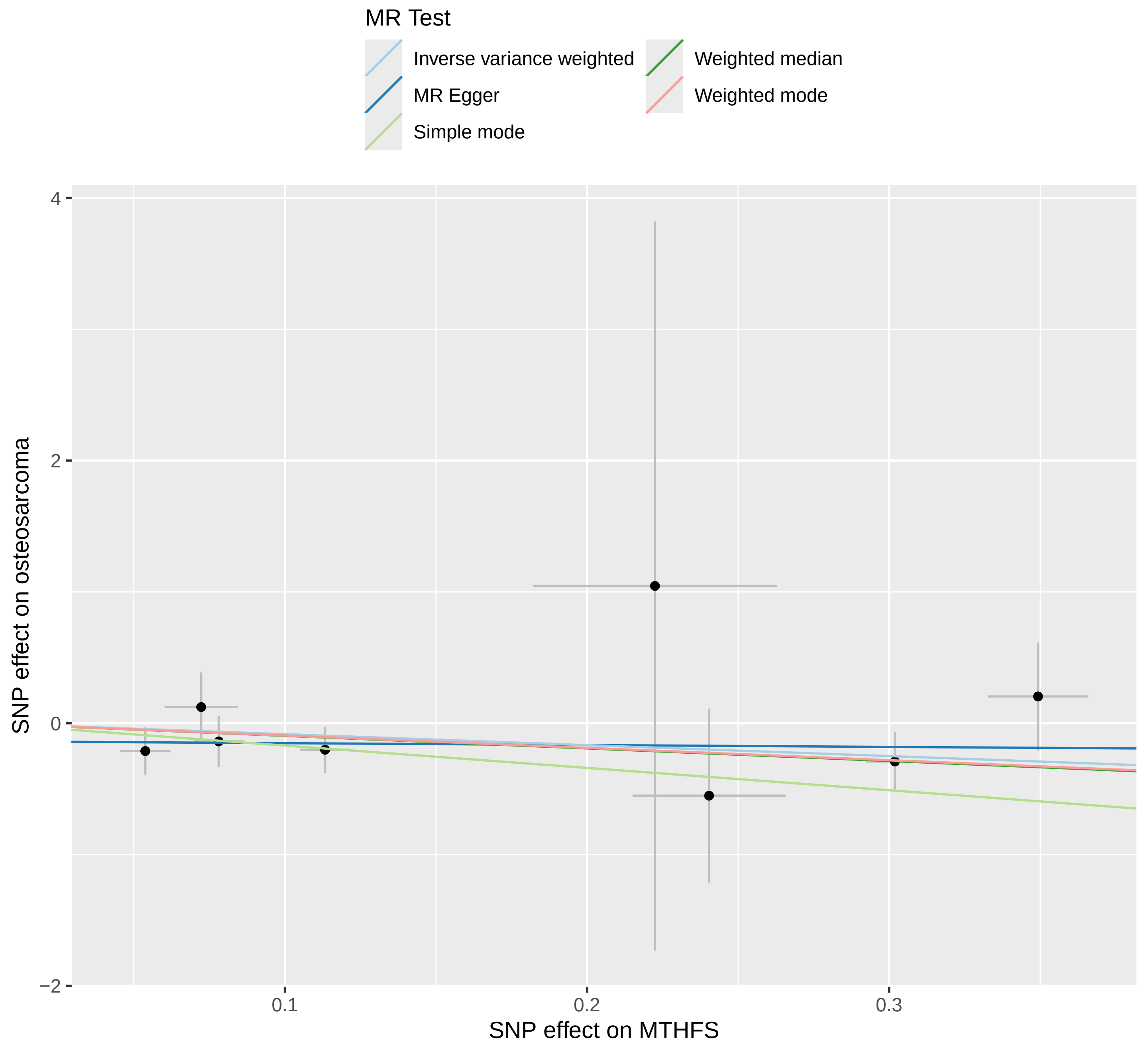
MR Test
Inverse variance weighted
MR Egger
Simple mode
Weighted median
Weighted mode
4
2
0
−2
0.1
0.2
0.3
SNP effect on osteosarcoma
SNP effect on MTHFS

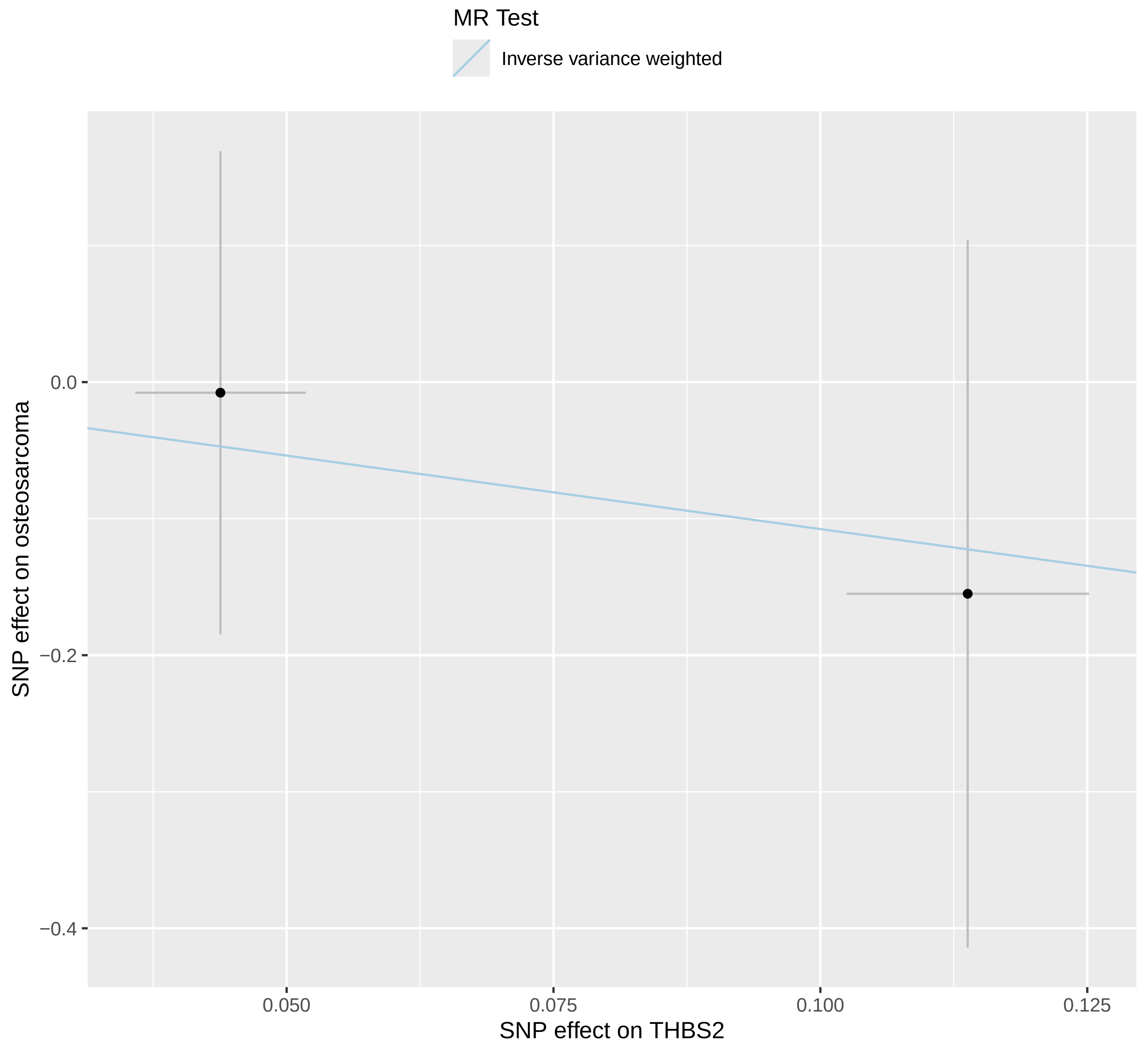

MR Test
Inverse variance weighted
0.0
−0.2
−0.4
SNP effect on osteosarcoma
0.050
0.075
0.100
0.125
SNP effect on THBS2

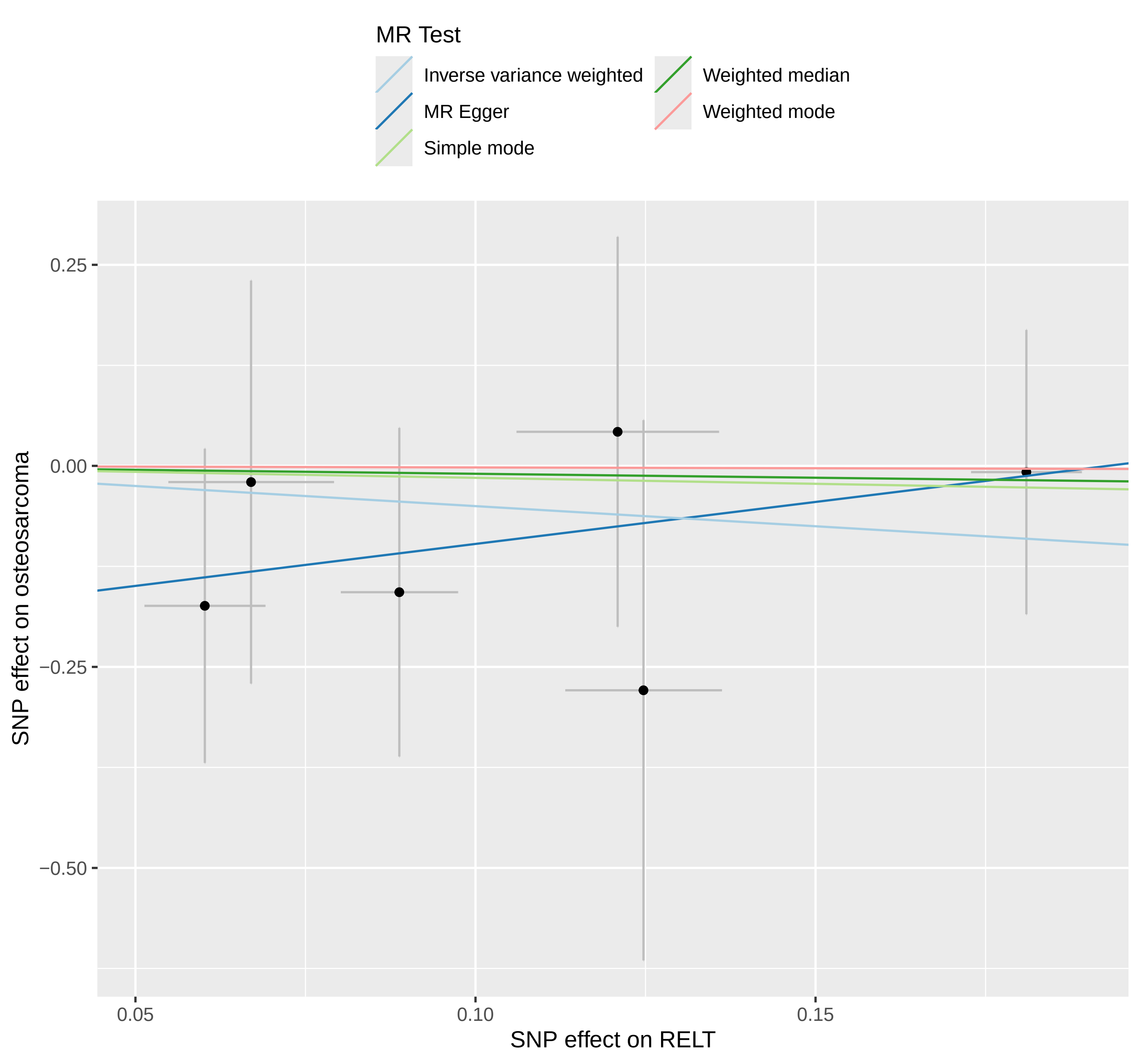
MR Test
Inverse variance weighted
MR Egger
Simple mode
Weighted median
Weighted mode
0.25
0.00
−0.25
−0.50
0.05
0.10
0.15
SNP effect on osteosarcoma
SNP effect on RELT

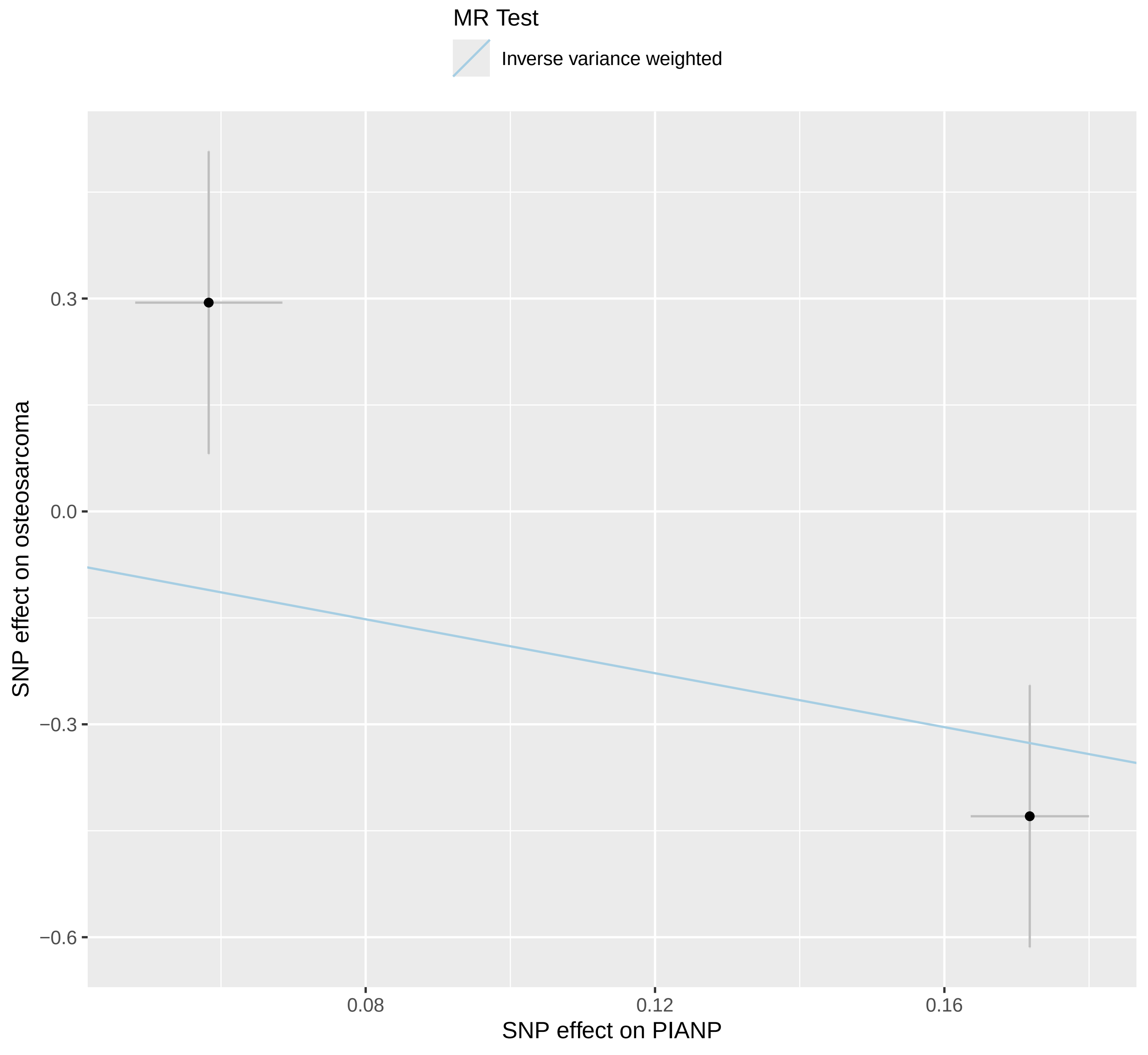
MR Test
Inverse variance weighted
0.3
0.0
−0.3
−0.6
SNP effect on osteosarcoma
0.08
0.12
0.16
SNP effect on PIANP

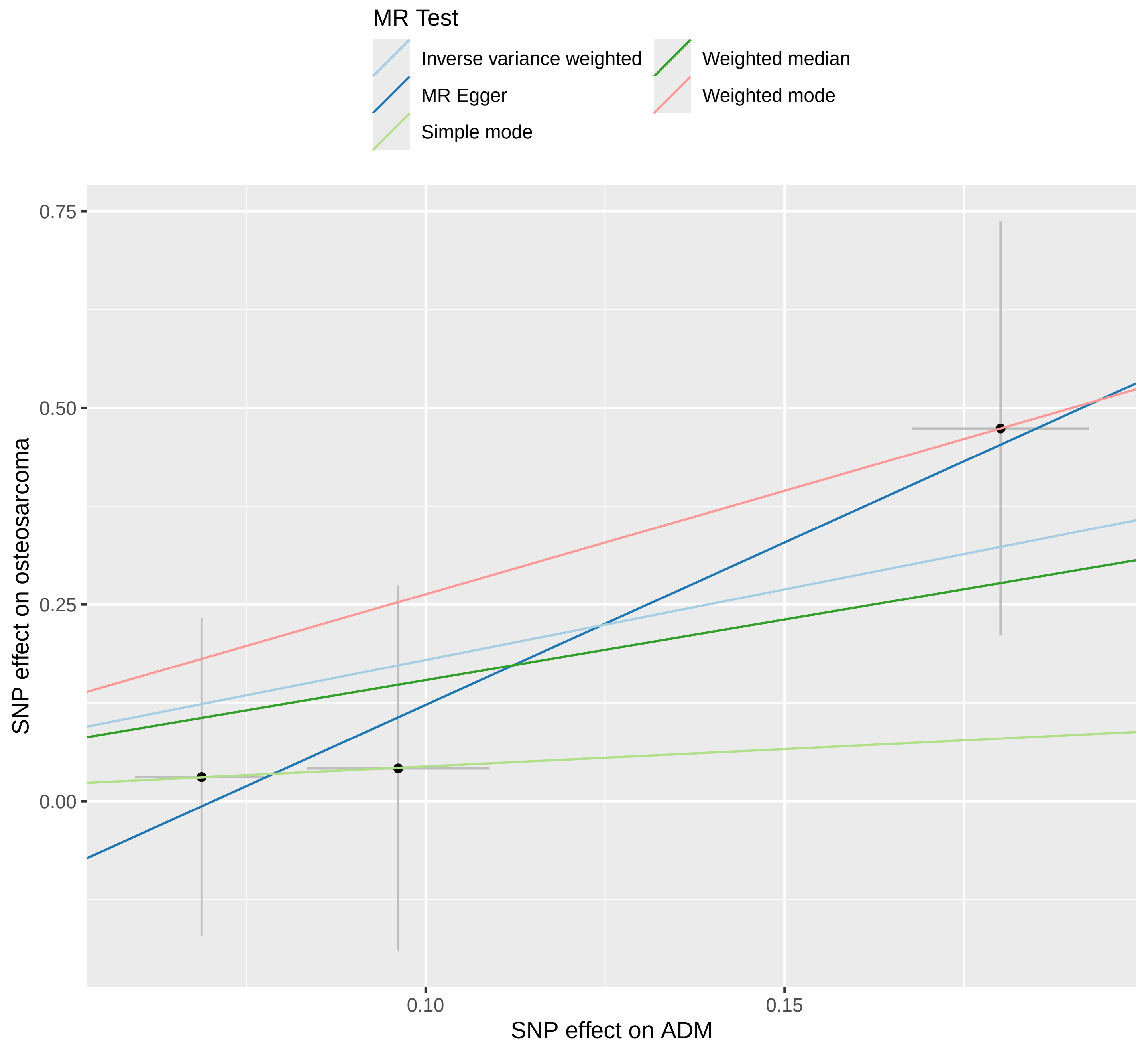
MR Test
Inverse variance weighted
MR Egger
Simple mode
Weighted median
Weighted mode
0.75
0.50
0.25
0.00
SNP effect on osteosarcoma
0.10
0.15
SNP effect on ADM

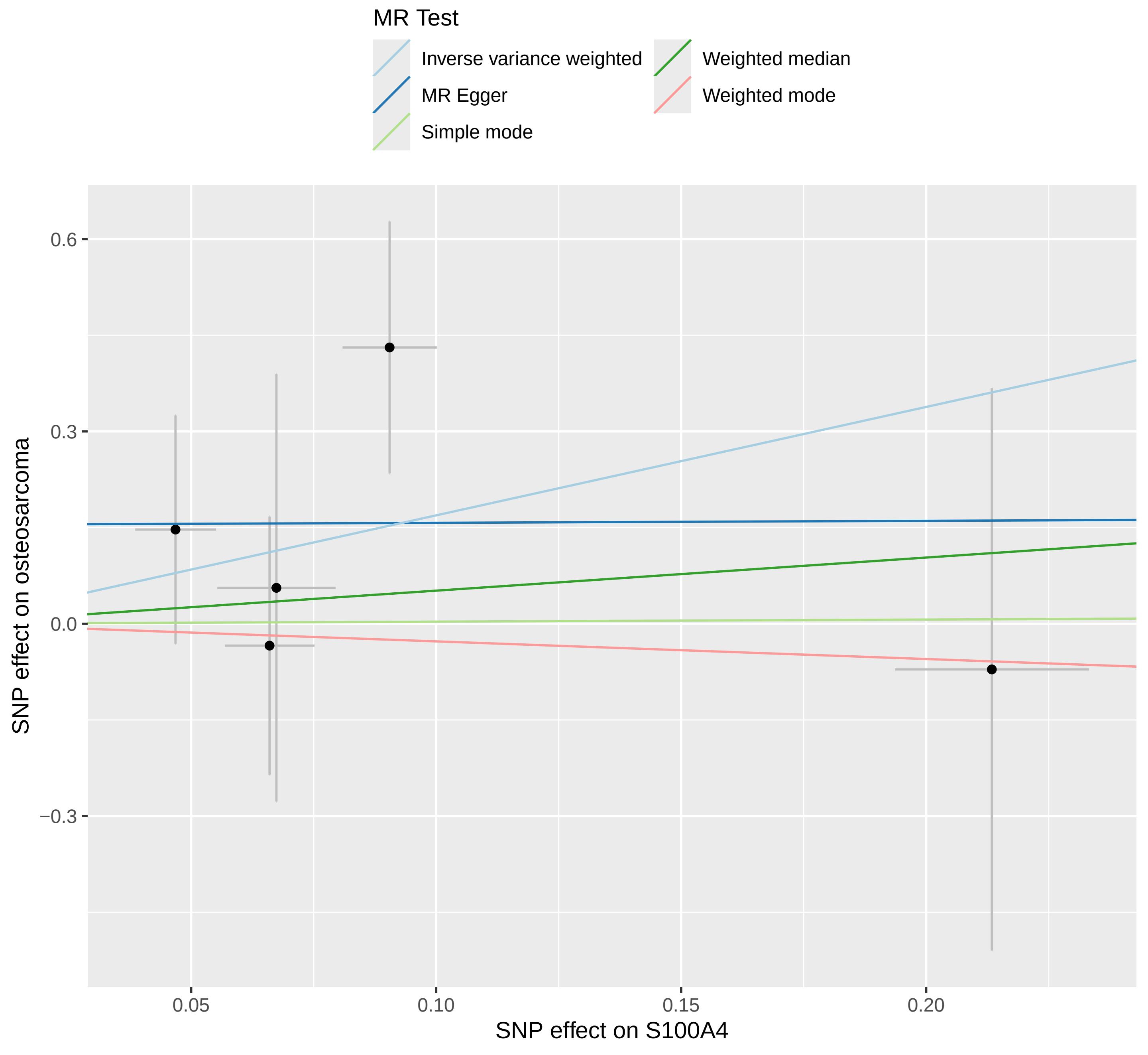
MR Test
Inverse variance weighted
MR Egger
Simple mode
Weighted median
Weighted mode
0.6
0.3
0.0
−0.3
0.05
0.10
0.15
0.20
SNP effect on S100A4
SNP effect on osteosarcoma

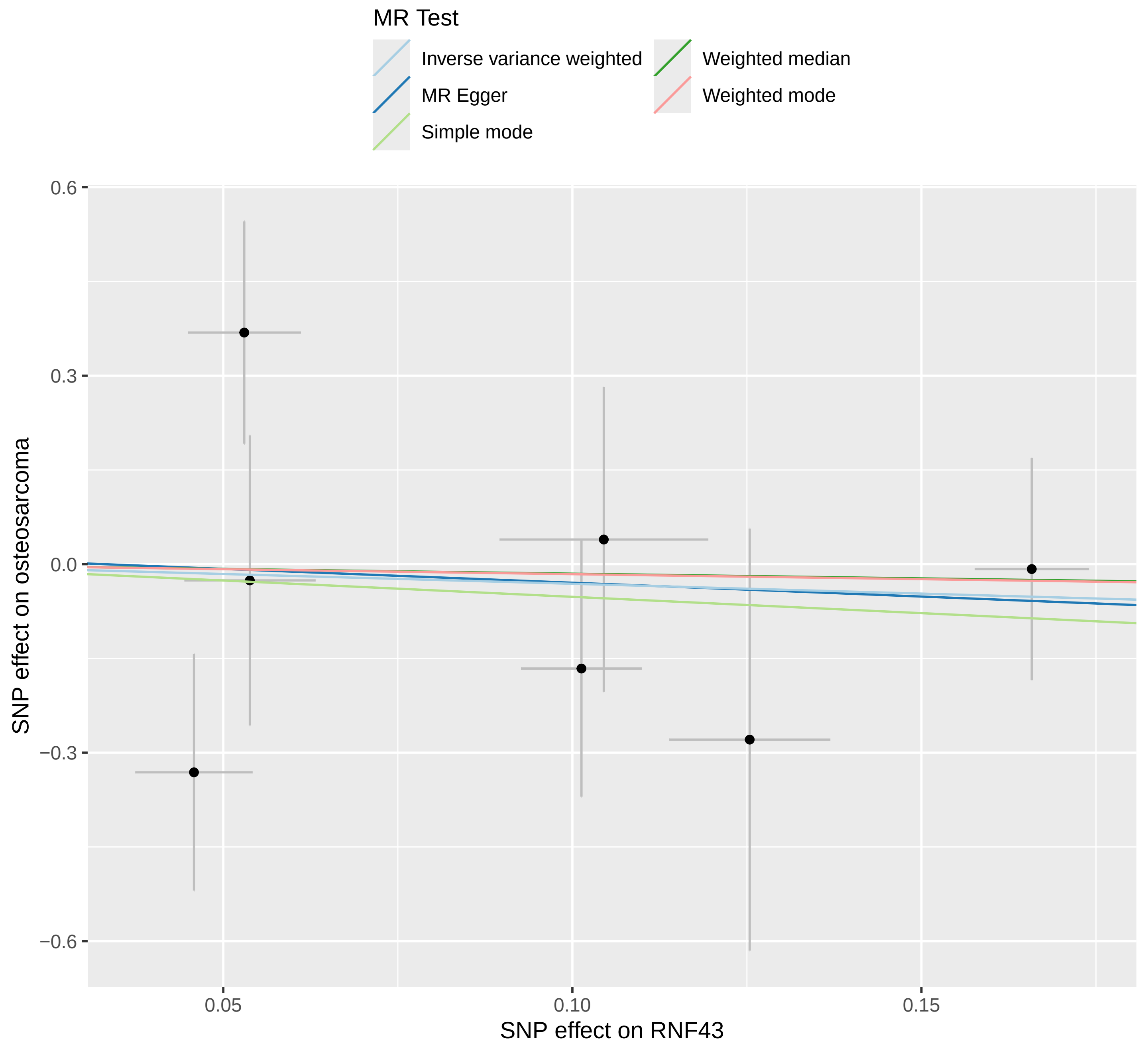

MR Test
Inverse variance weighted
MR Egger
Simple mode
Weighted median
Weighted mode
0.6
0.3
0.0
−0.3
−0.6
0.05
0.10
0.15
SNP effect on osteosarcoma
SNP effect on RNF43

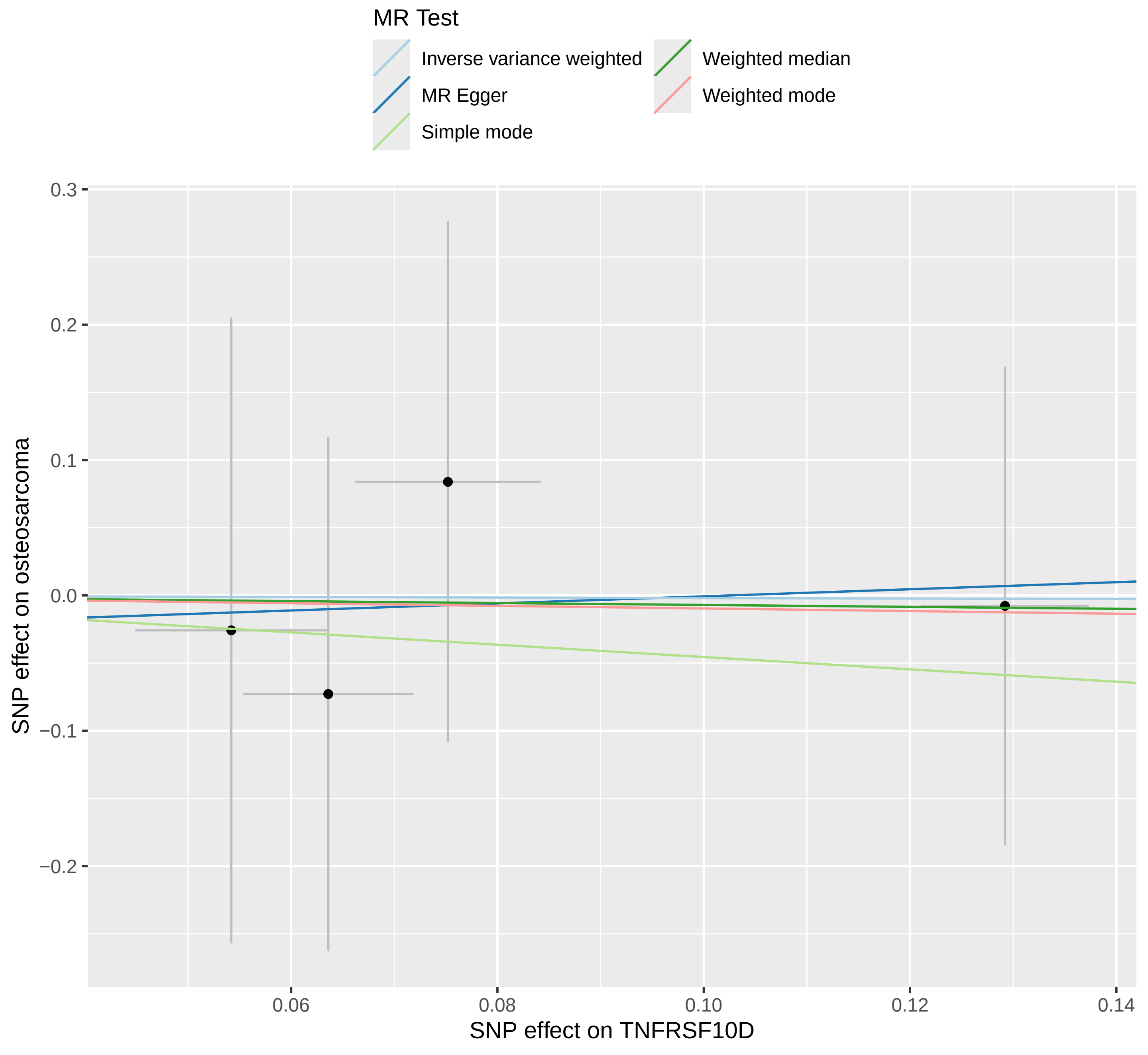

MR Test
Inverse variance weighted
MR Egger
Simple mode
Weighted median
Weighted mode
0.3
0.2
0.1
0.0
−0.1
−0.2
SNP effect on osteosarcoma
0.06
0.08
0.10
0.12
0.14
SNP effect on TNFRSF10D

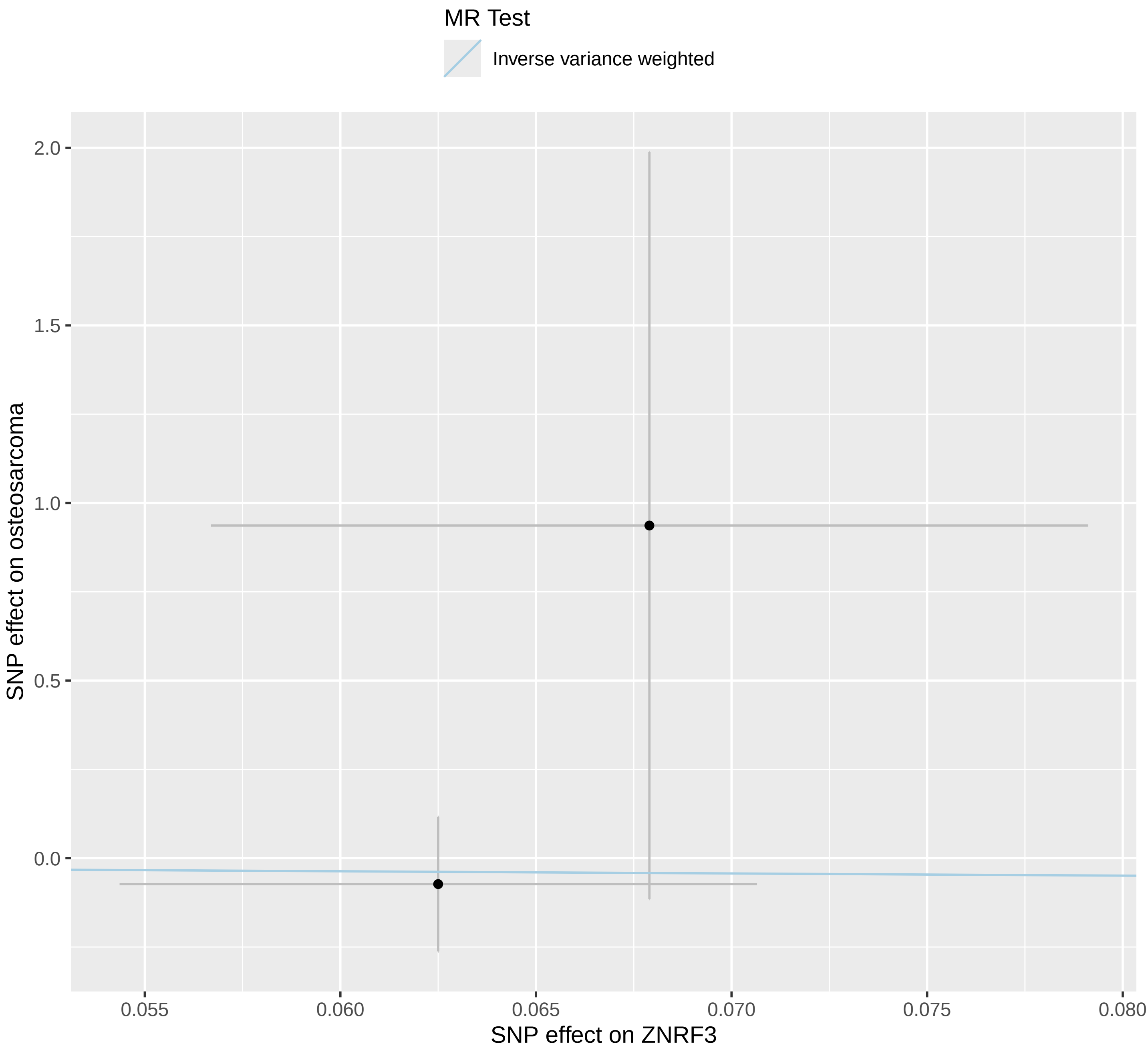

MR Test
Inverse variance weighted
2.0
1.5
1.0
0.5
0.0
SNP effect on osteosarcoma
0.055
0.060
0.065
0.070
0.075
0.080
SNP effect on ZNRF3

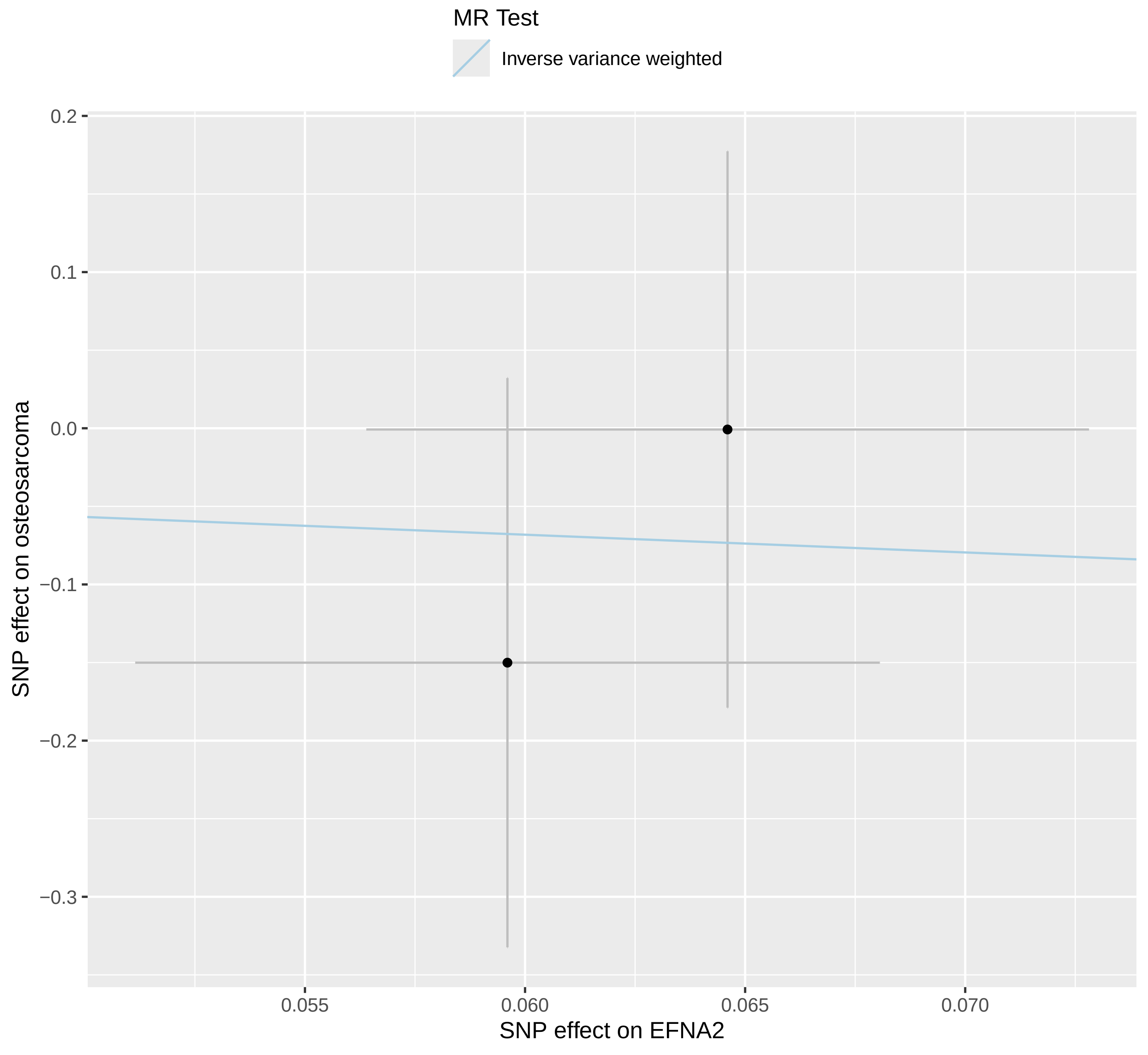
MR Test
Inverse variance weighted
0.2
0.1
0.0
−0.1
−0.2
−0.3
0.055
0.060
0.065
0.070
SNP effect on EFNA2
SNP effect on osteosarcoma

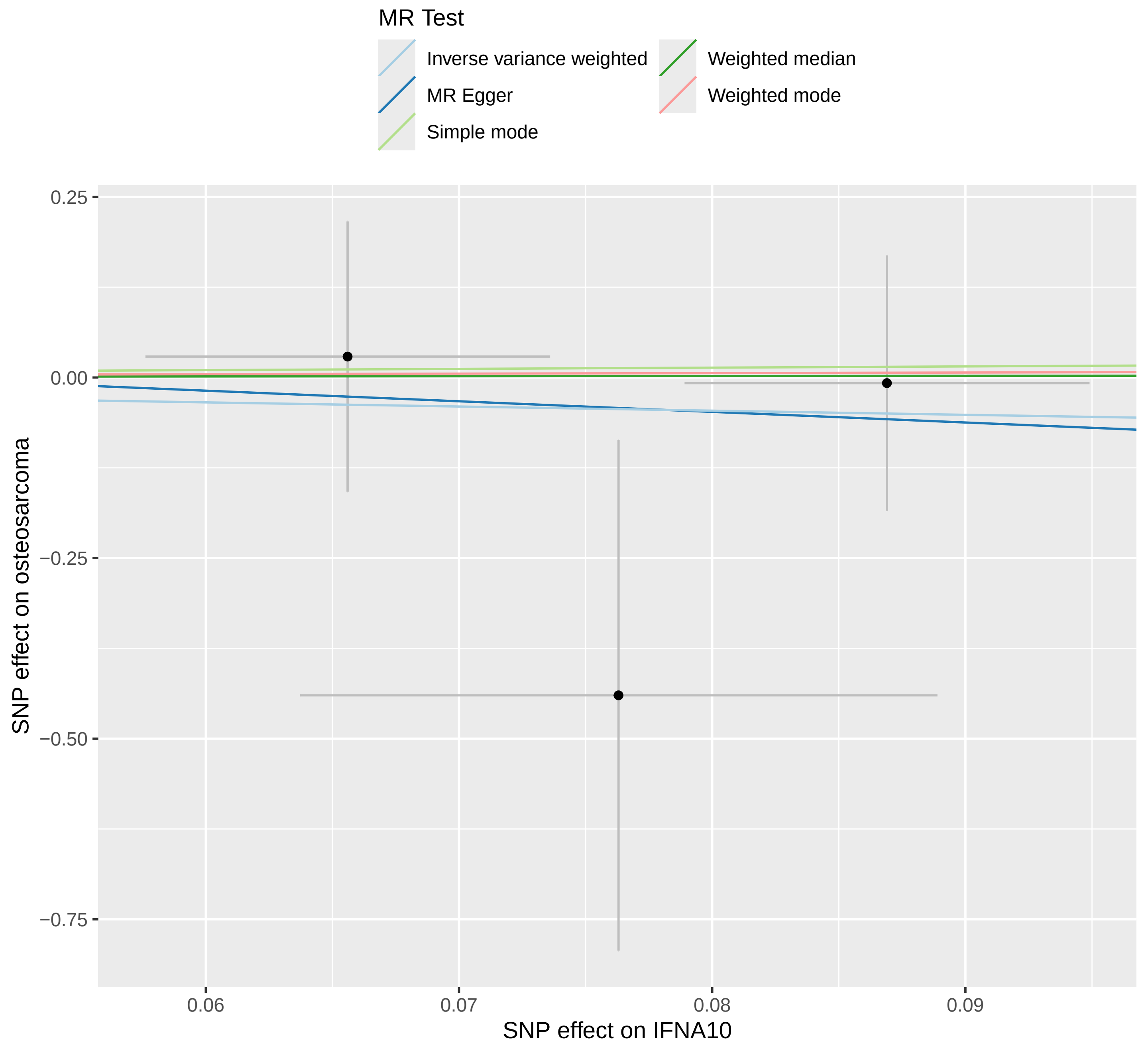
MR Test
Inverse variance weighted
MR Egger
Simple mode
Weighted median
Weighted mode
0.25
0.00
−0.25
−0.50
−0.75
SNP effect on osteosarcoma
0.06
0.07
0.08
0.09
SNP effect on IFNA10

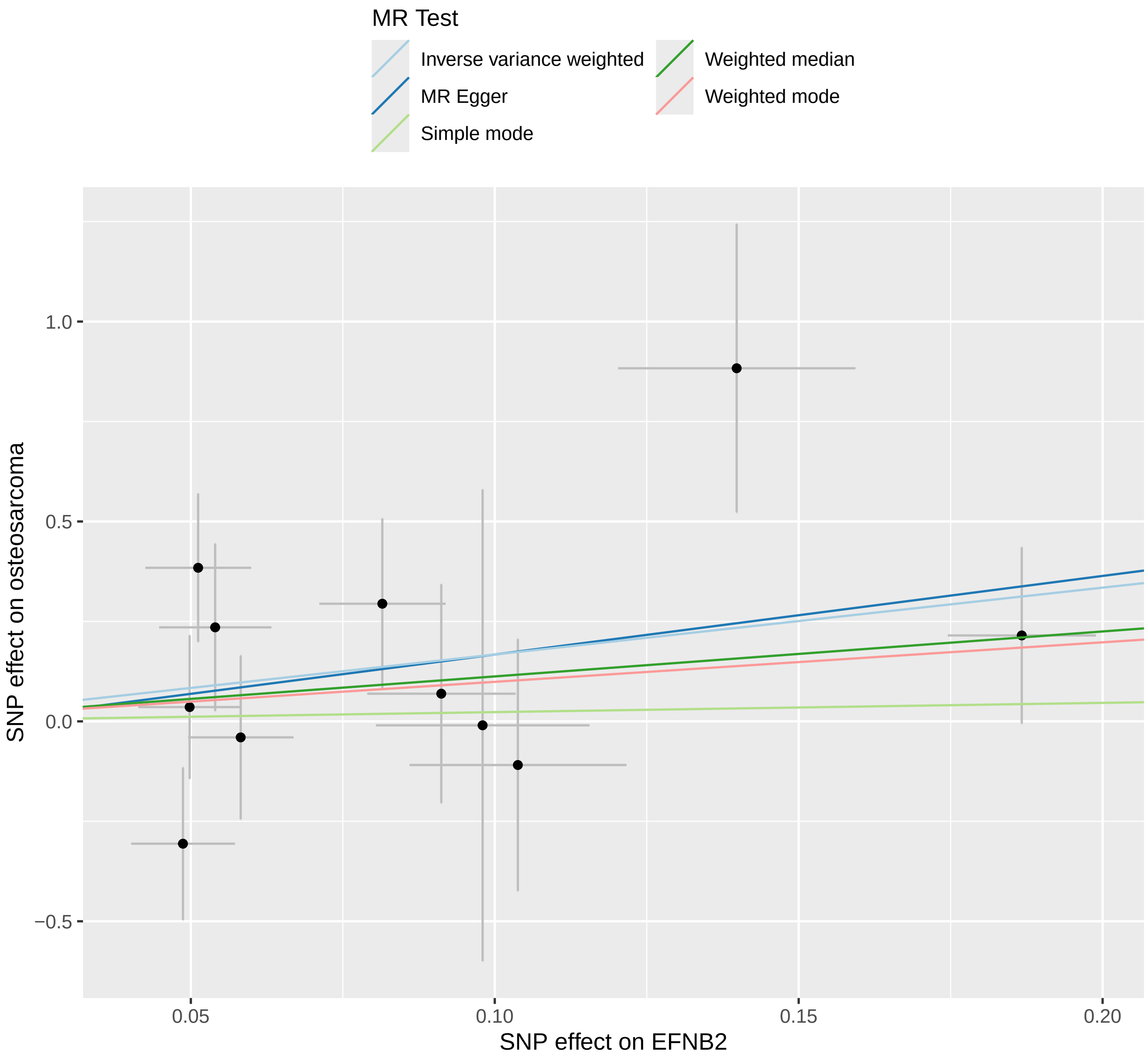

MR Test
Inverse variance weighted
MR Egger
Simple mode
Weighted median
Weighted mode
1.0
0.5
0.0
−0.5
0.05
0.10
0.15
0.20
SNP effect on osteosarcoma
SNP effect on EFNB2

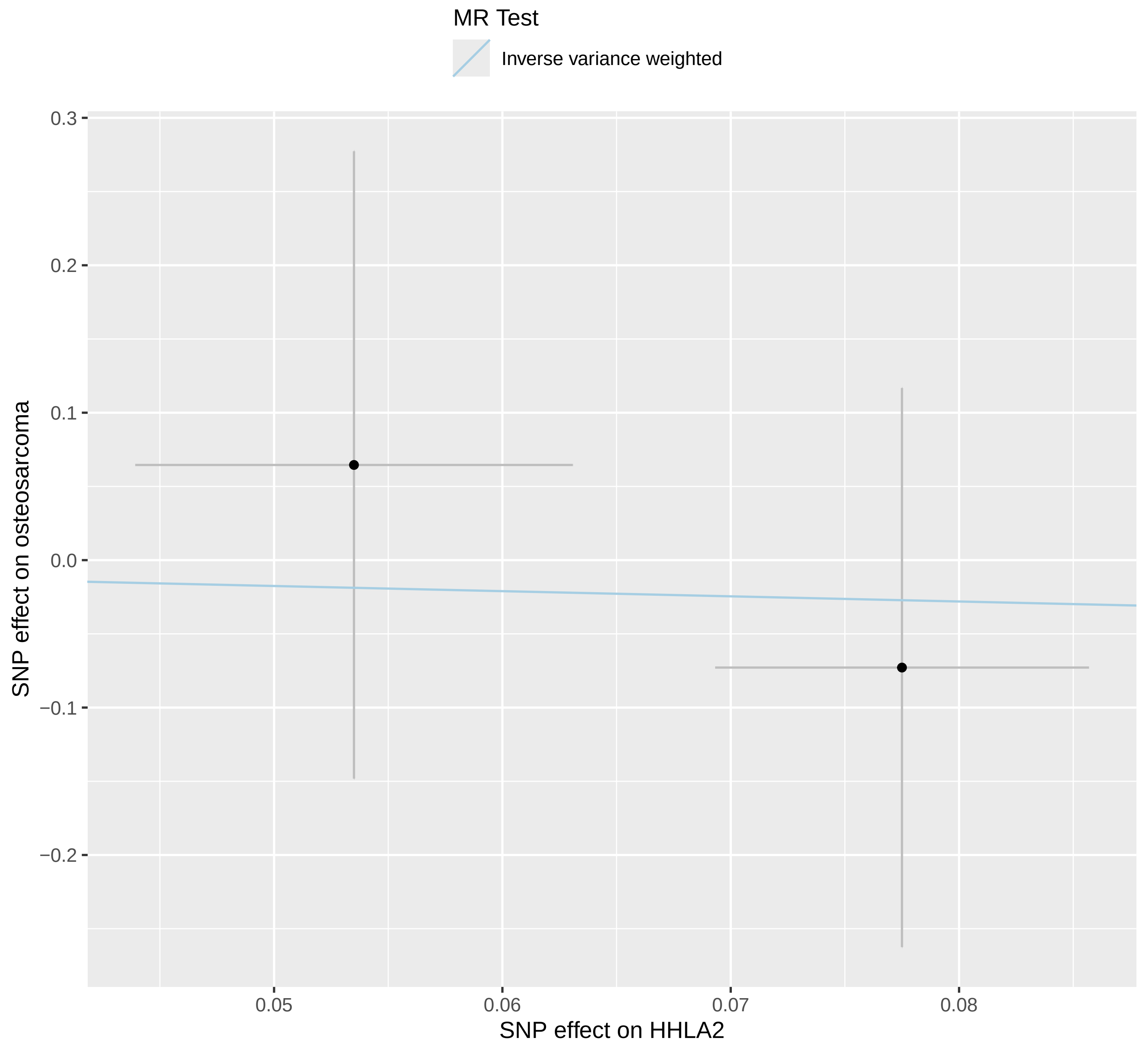
MR Test
Inverse variance weighted
0.3
0.2
0.1
0.0
−0.1
−0.2
SNP effect on osteosarcoma
0.05
0.06
0.07
0.08
SNP effect on HHLA2

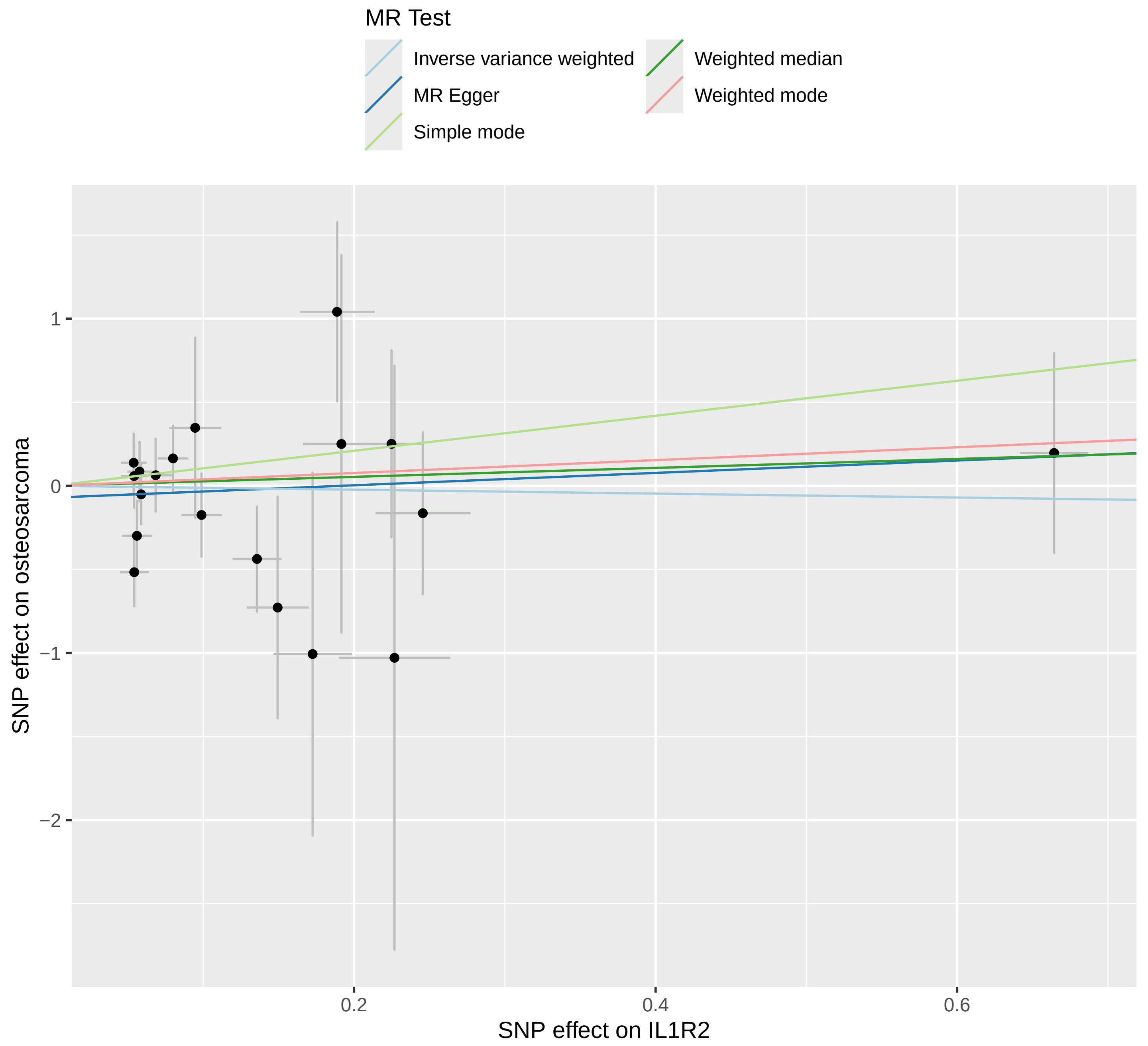

MR Test
Inverse variance weighted
MR Egger
Simple mode
Weighted median
Weighted mode
1
0
−1
−2
0.2
0.4
0.6
SNP effect on osteosarcoma
SNP effect on IL1R2

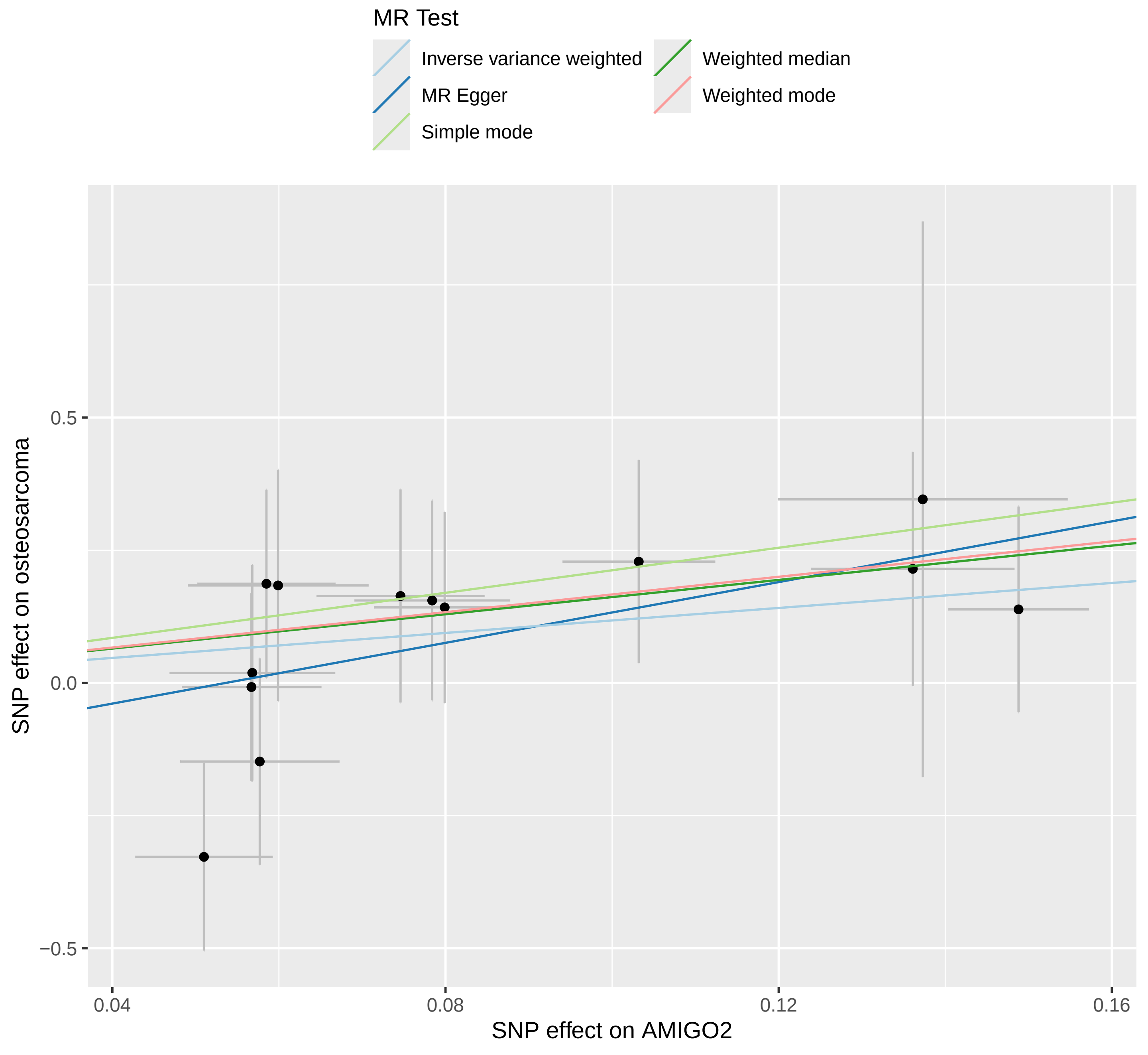
MR Test
Inverse variance weighted
MR Egger
Simple mode
Weighted median
Weighted mode
0.5
0.0
−0.5
0.04
0.08
0.12
0.16
SNP effect on osteosarcoma
SNP effect on AMIGO2

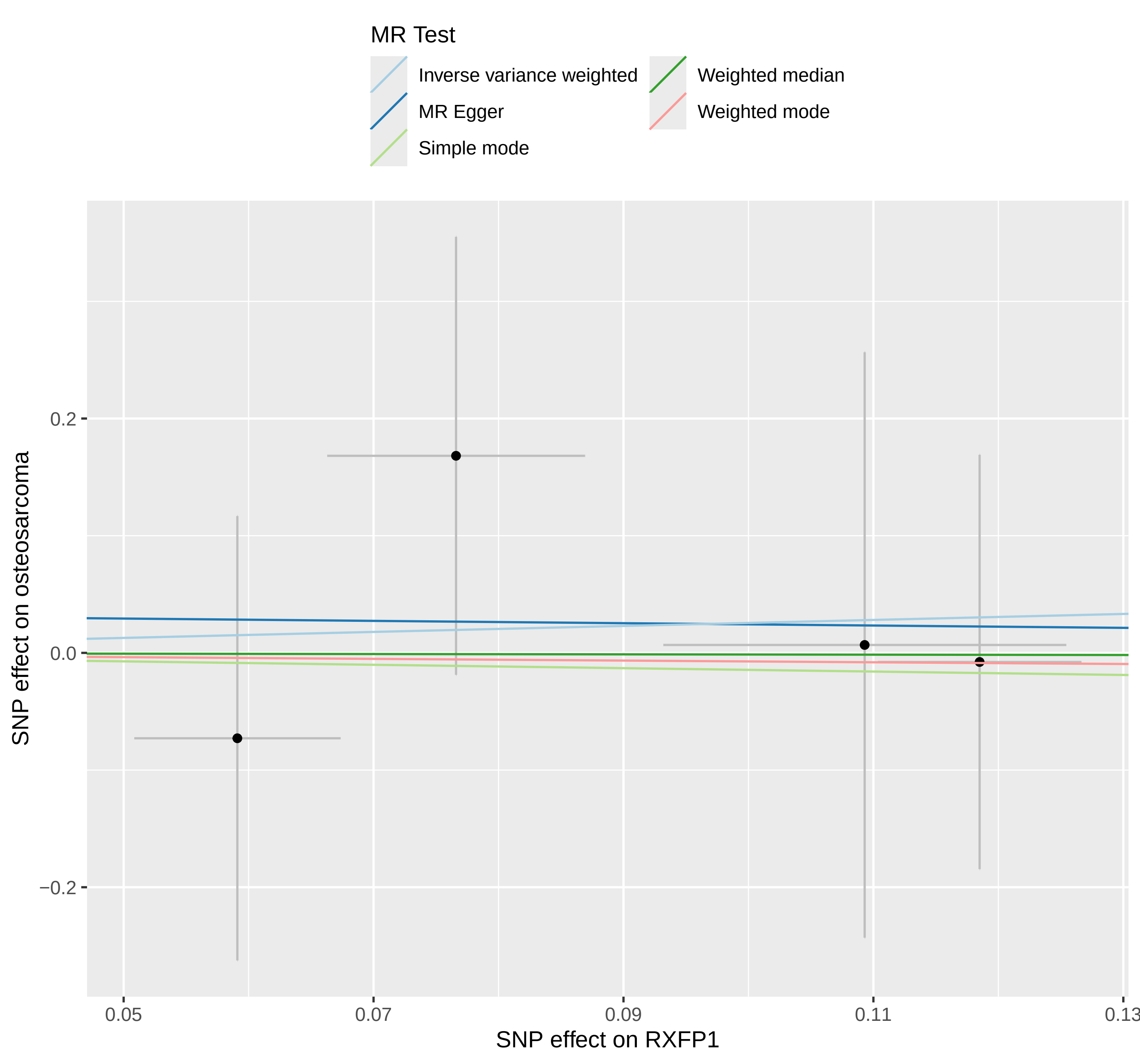
MR Test
Inverse variance weighted
MR Egger
Simple mode
Weighted median
Weighted mode
0.2
0.0
−0.2
0.05
0.07
0.09
0.11
0.13
SNP effect on osteosarcoma
SNP effect on RXFP1

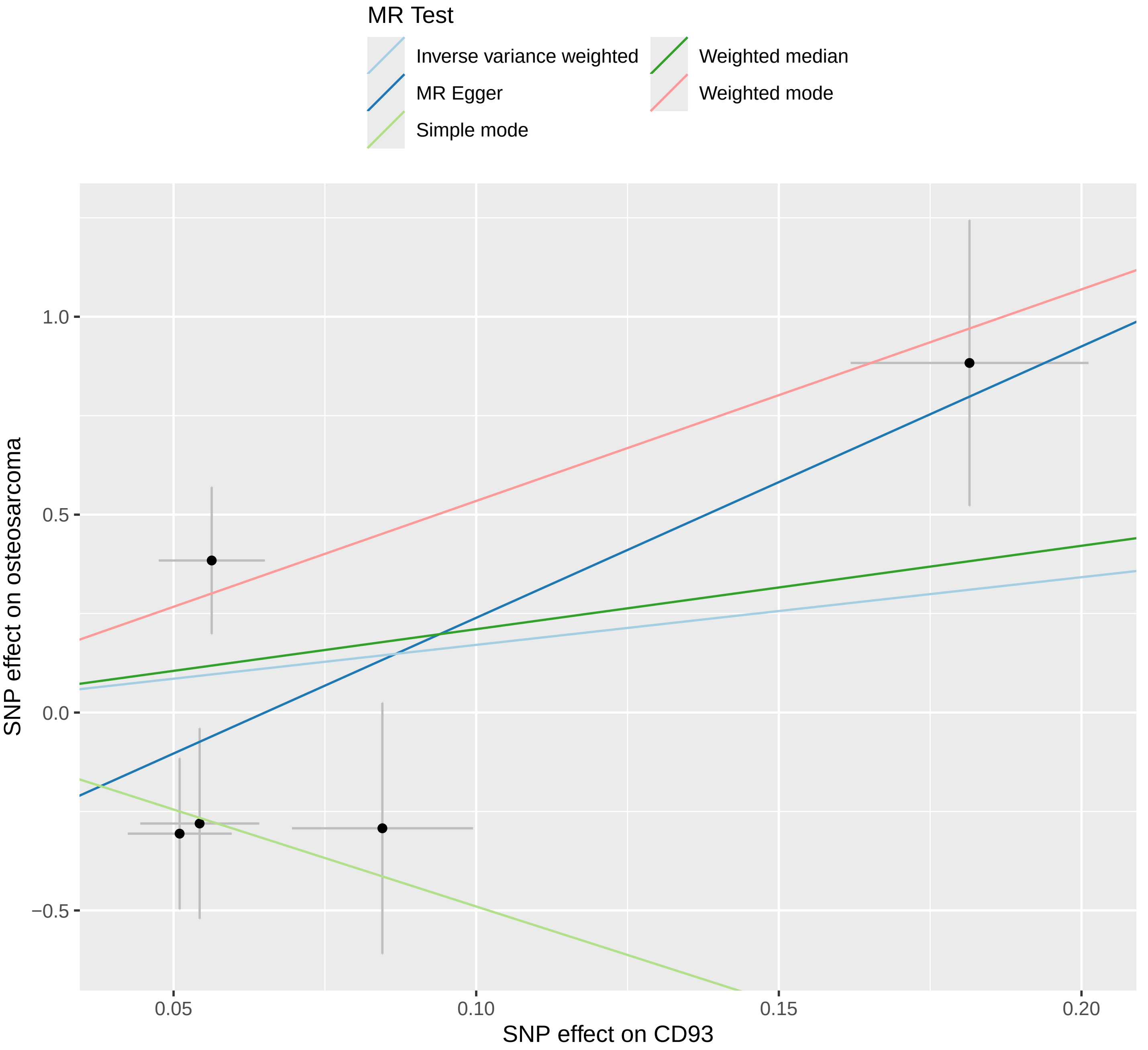
MR Test
Inverse variance weighted
MR Egger
Simple mode
Weighted median
Weighted mode
1.0
0.5
0.0
−0.5
SNP effect on osteosarcoma
0.05
0.10
0.15
0.20
SNP effect on CD93

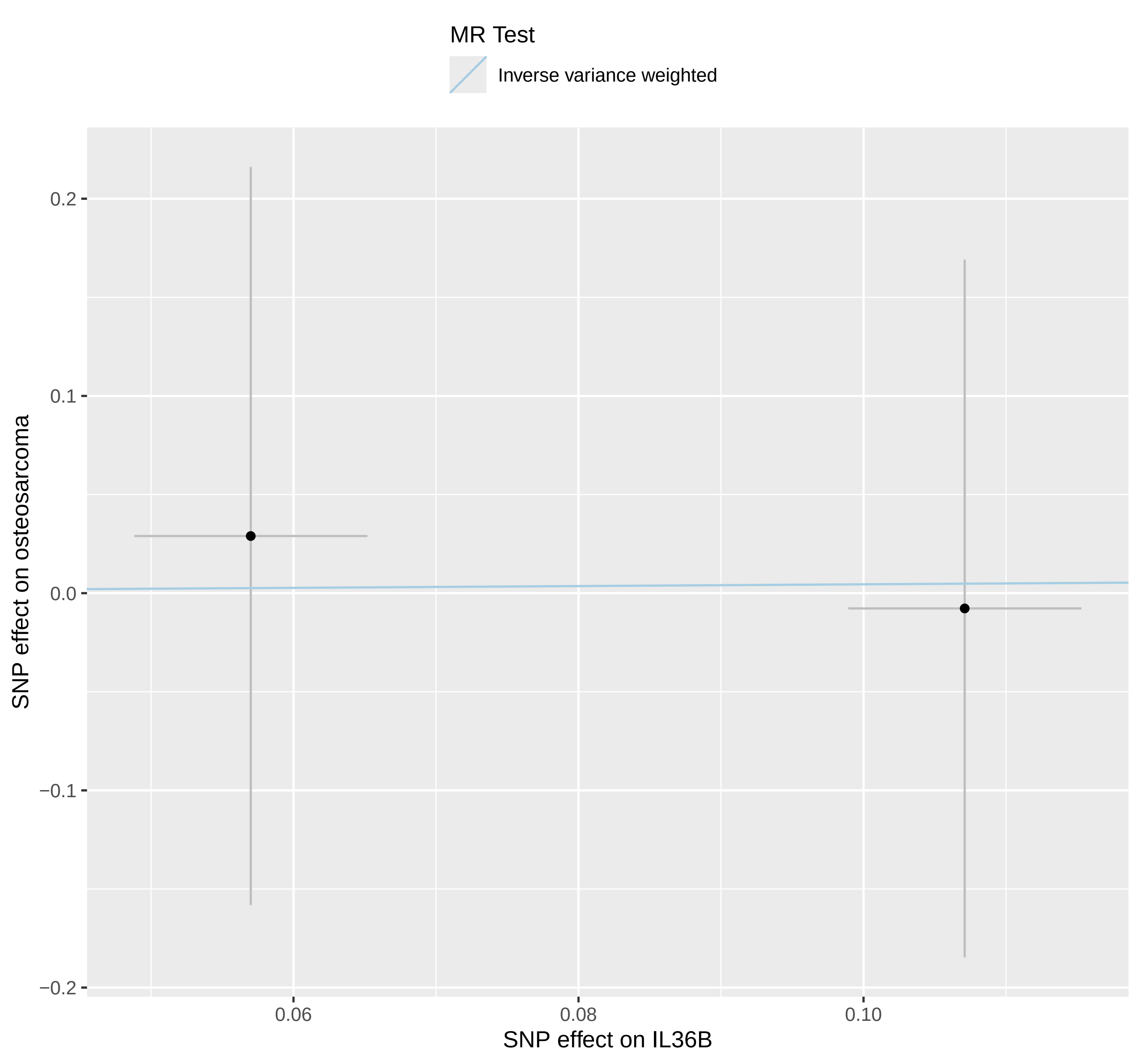
MR Test
Inverse variance weighted
SNP effect on osteosarcoma
0.2
0.1
0.0
−0.1
−0.2
0.06
0.08
0.10
SNP effect on IL36B

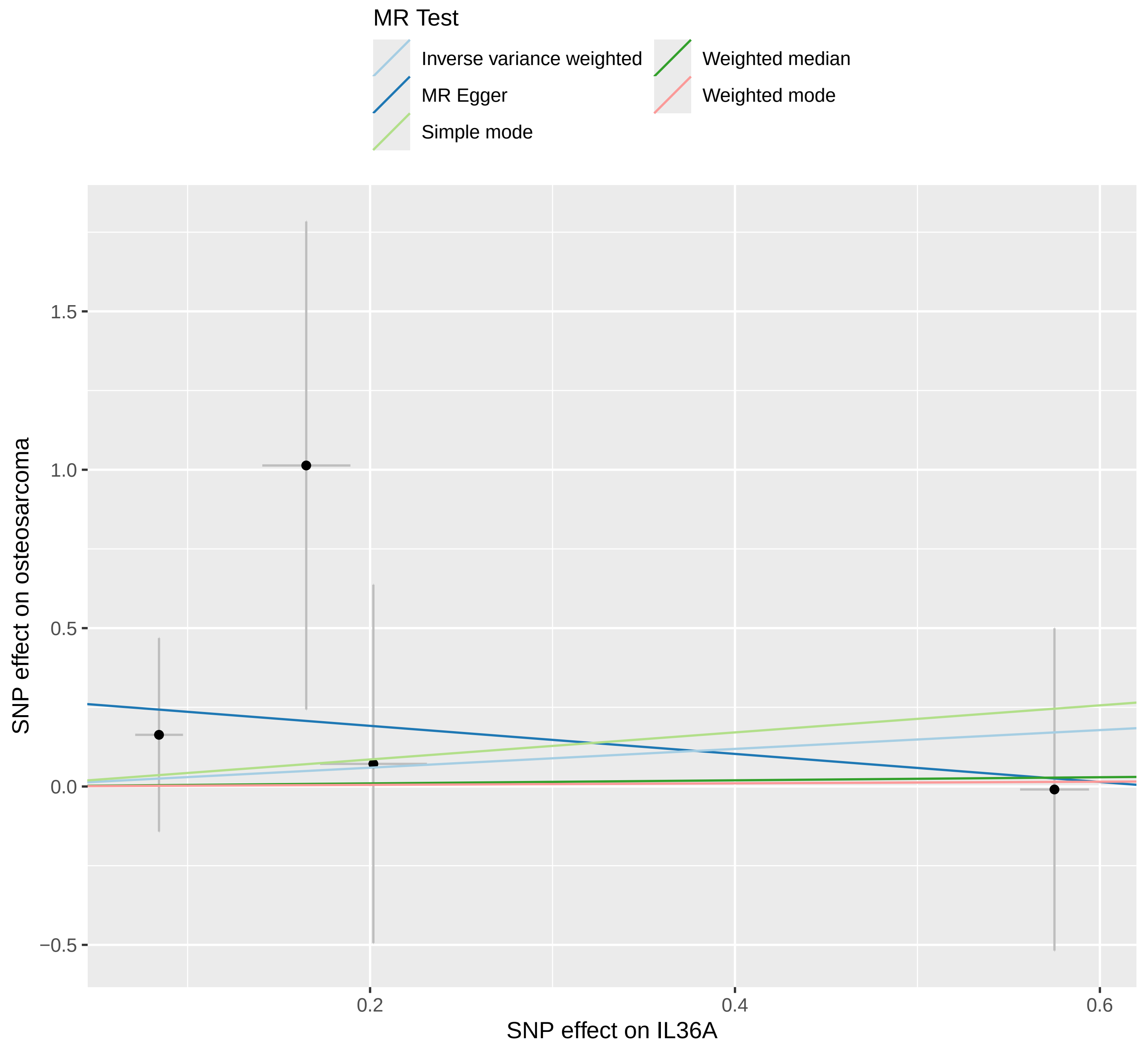

MR Test
Inverse variance weighted
MR Egger
Simple mode
Weighted median
Weighted mode
1.5
1.0
0.5
0.0
−0.5
SNP effect on osteosarcoma
0.2
0.4
0.6
SNP effect on IL36A

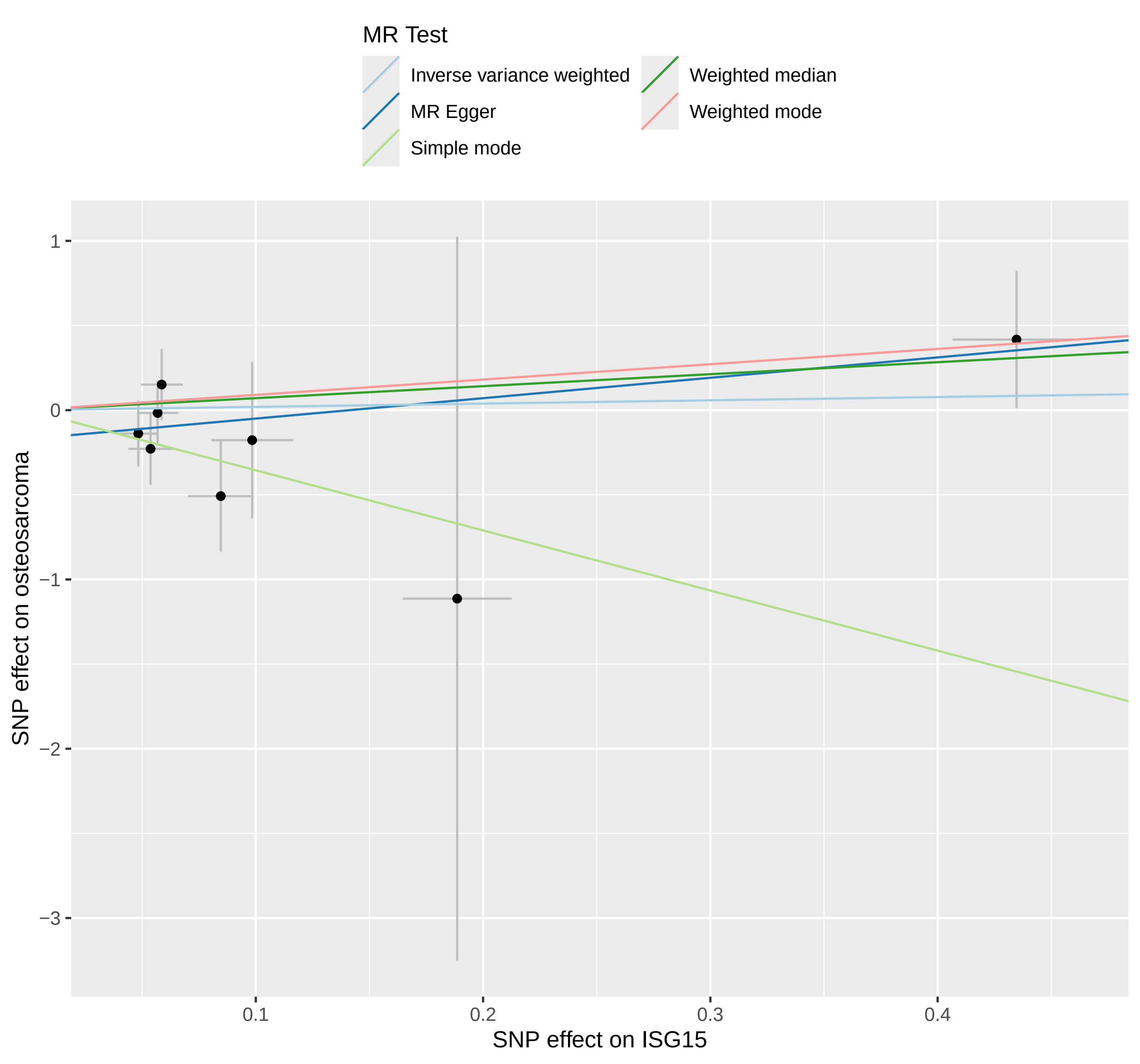
MR Test
Inverse variance weighted
MR Egger
Simple mode
Weighted median
Weighted mode
1
0
−1
−2
−3
0.1
0.2
0.3
0.4
SNP effect on osteosarcoma
SNP effect on ISG15

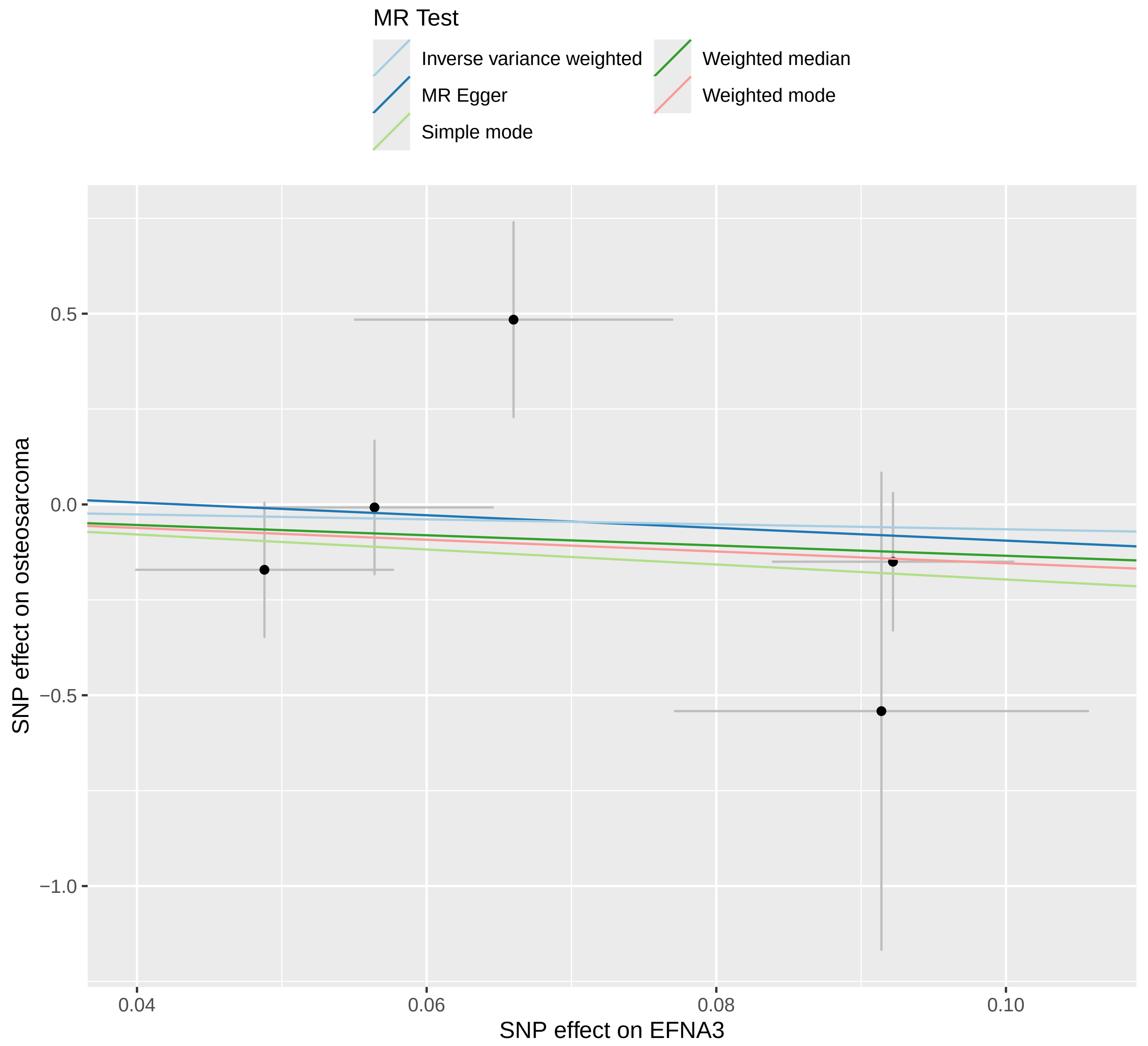
MR Test
Inverse variance weighted
MR Egger
Simple mode
Weighted median
Weighted mode
0.5
0.0
−0.5
−1.0
SNP effect on osteosarcoma
0.04
0.06
0.08
0.10
SNP effect on EFNA3

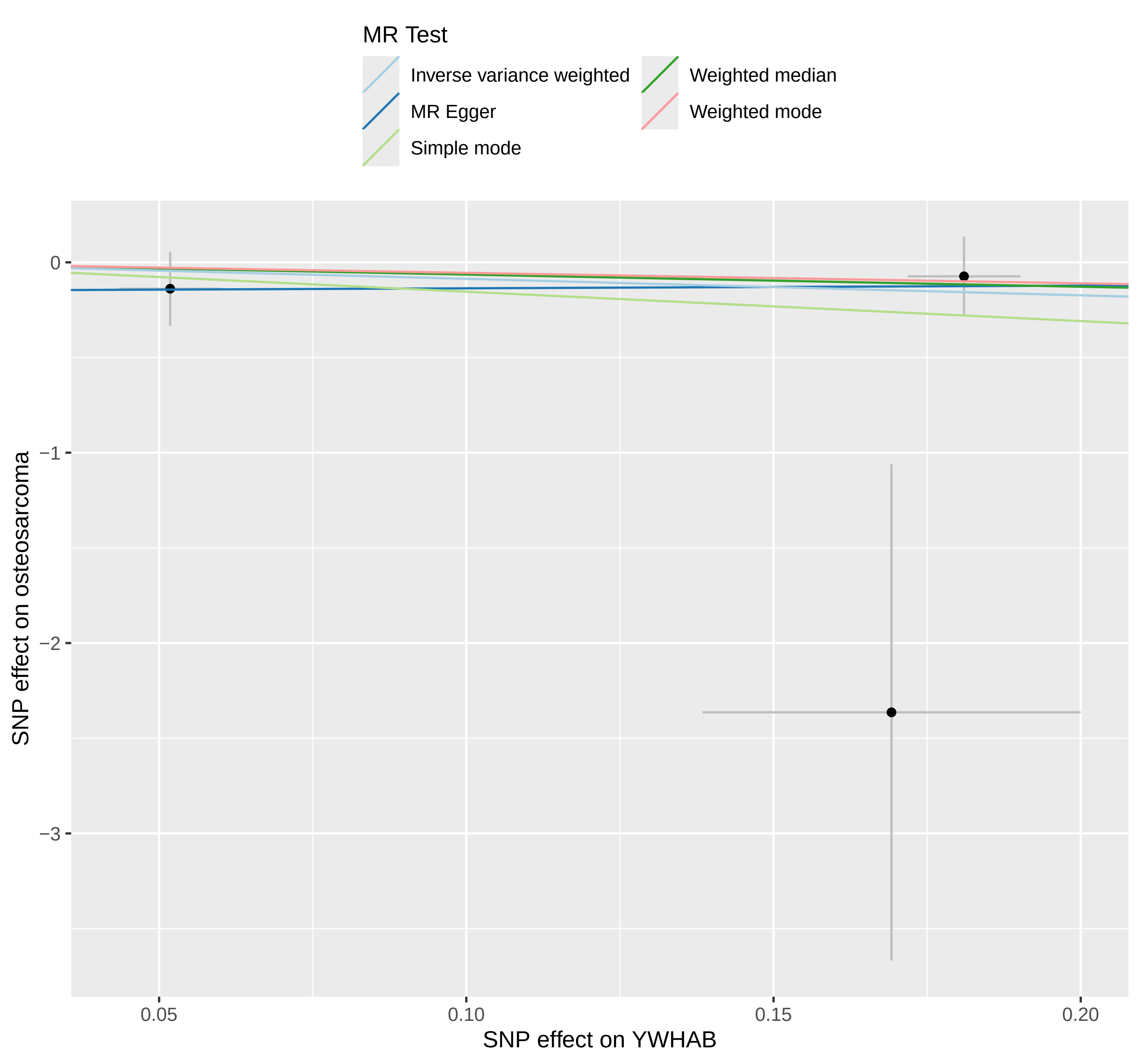

MR Test
Inverse variance weighted
MR Egger
Simple mode
Weighted median
Weighted mode
0
−1
−2
−3
SNP effect on osteosarcoma
0.05
0.10
0.15
0.20
SNP effect on YWHAB

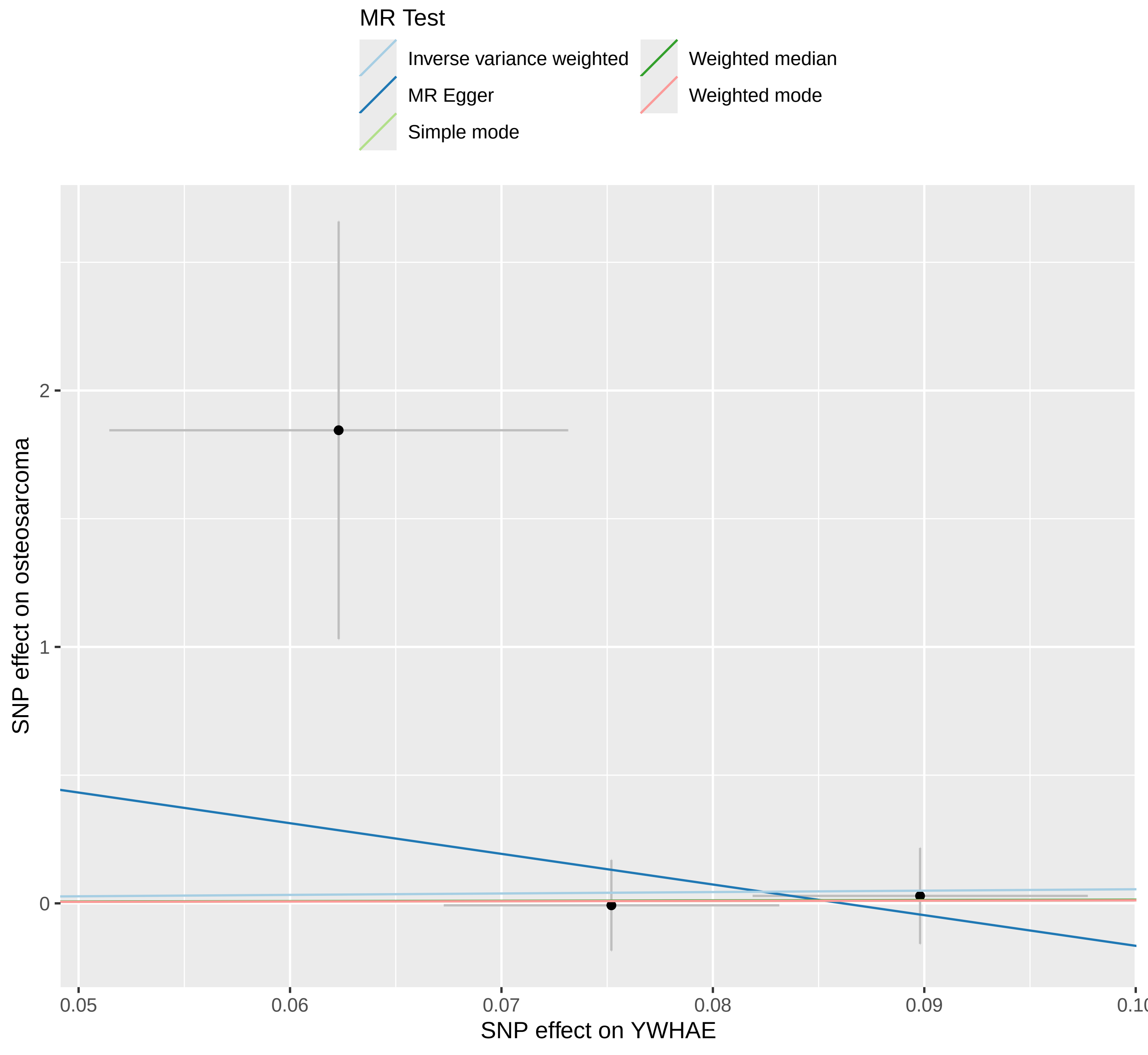

MR Test
Inverse variance weighted
MR Egger
Simple mode
Weighted median
Weighted mode
SNP effect on osteosarcoma
2
1
0
0.05
0.06
0.07
0.08
0.09
SNP effect on YWHAE

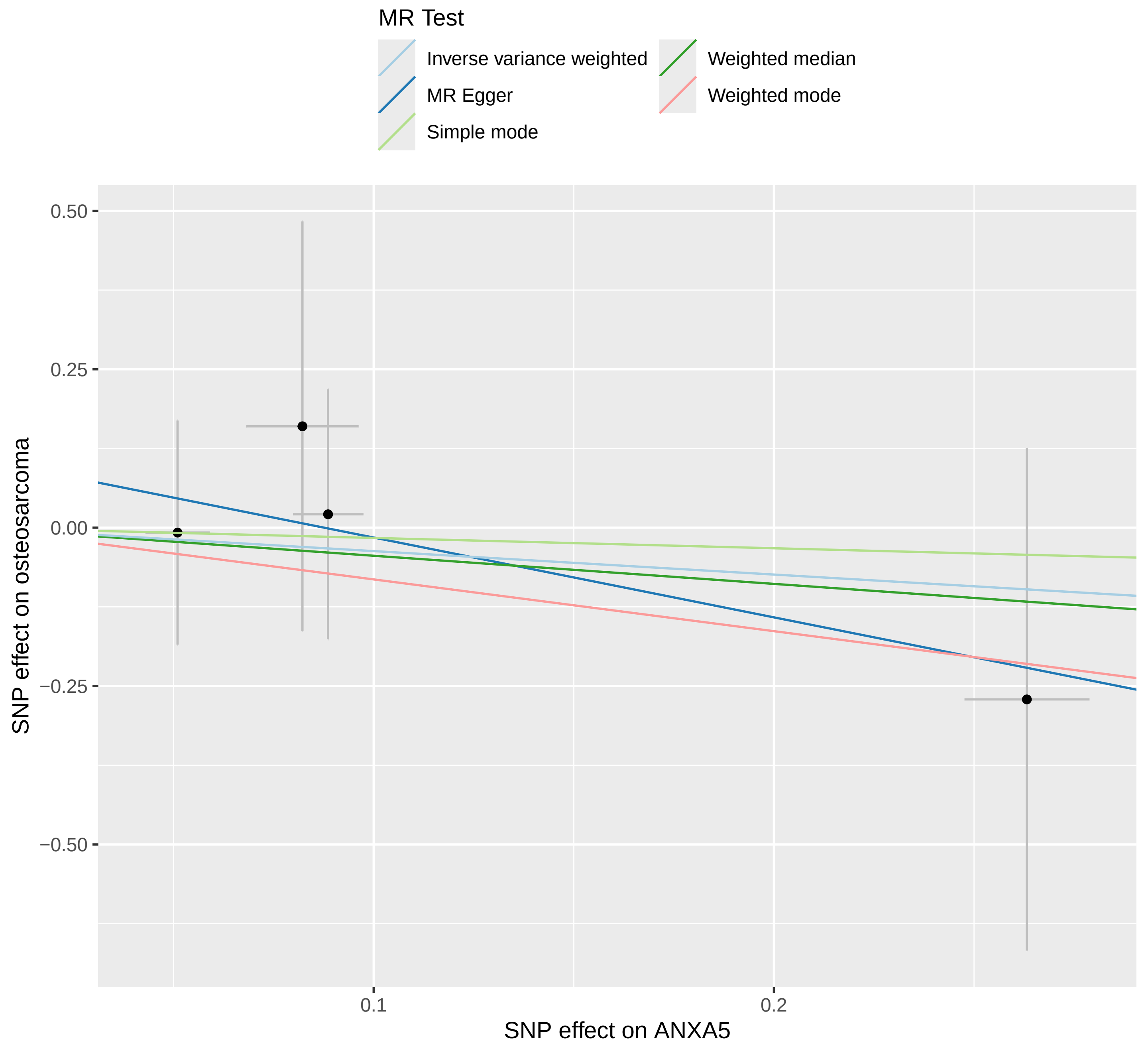
MR Test
Inverse variance weighted
MR Egger
Simple mode
Weighted median
Weighted mode
0.50
0.25
0.00
−0.25
−0.50
SNP effect on osteosarcoma
0.1
0.2
SNP effect on ANXA5

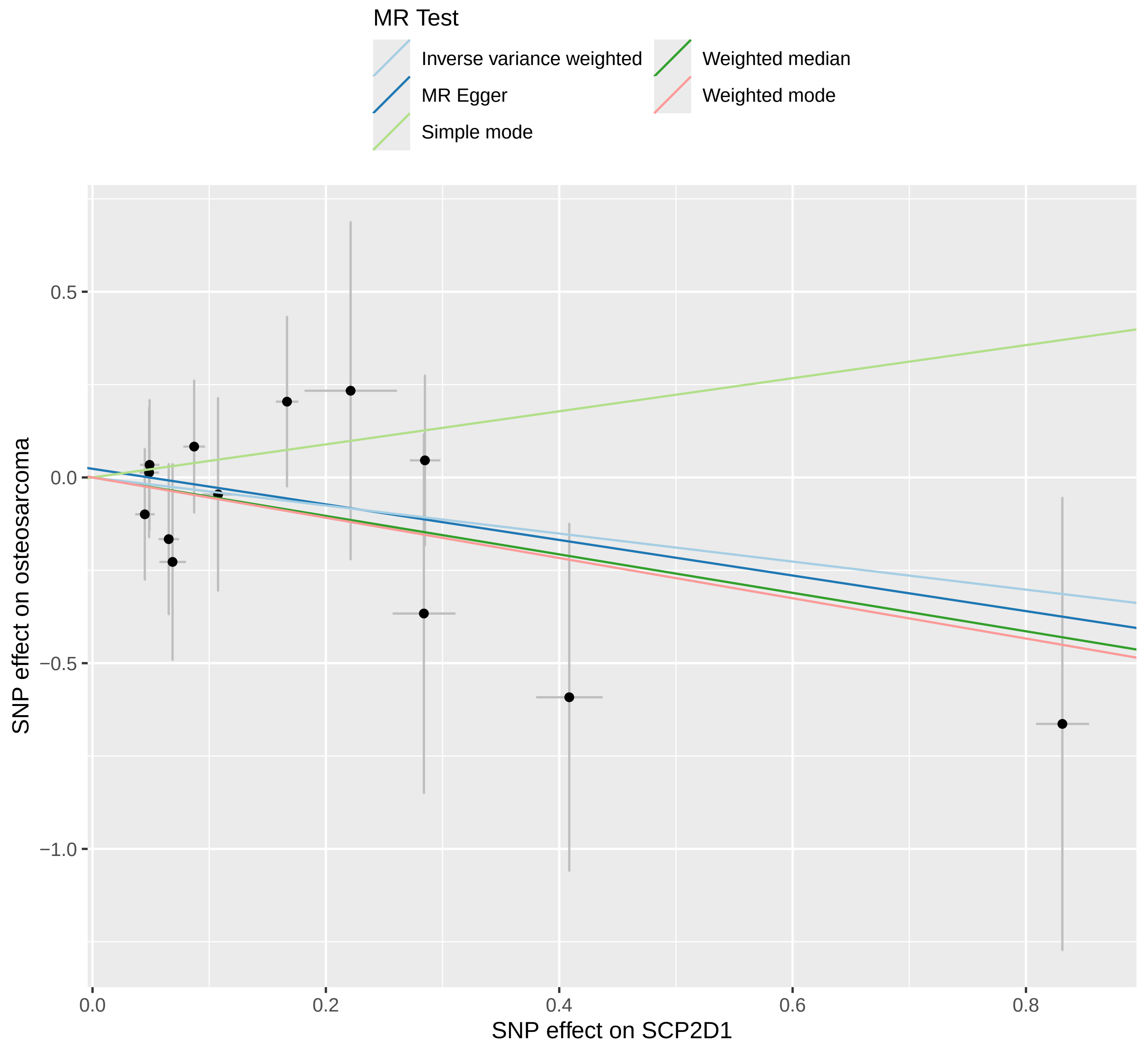
MR Test
Inverse variance weighted
MR Egger
Simple mode
Weighted median
Weighted mode
0.5
0.0
−0.5
−1.0
0.0
0.2
0.4
0.6
0.8
SNP effect on osteosarcoma
SNP effect on SCP2D1

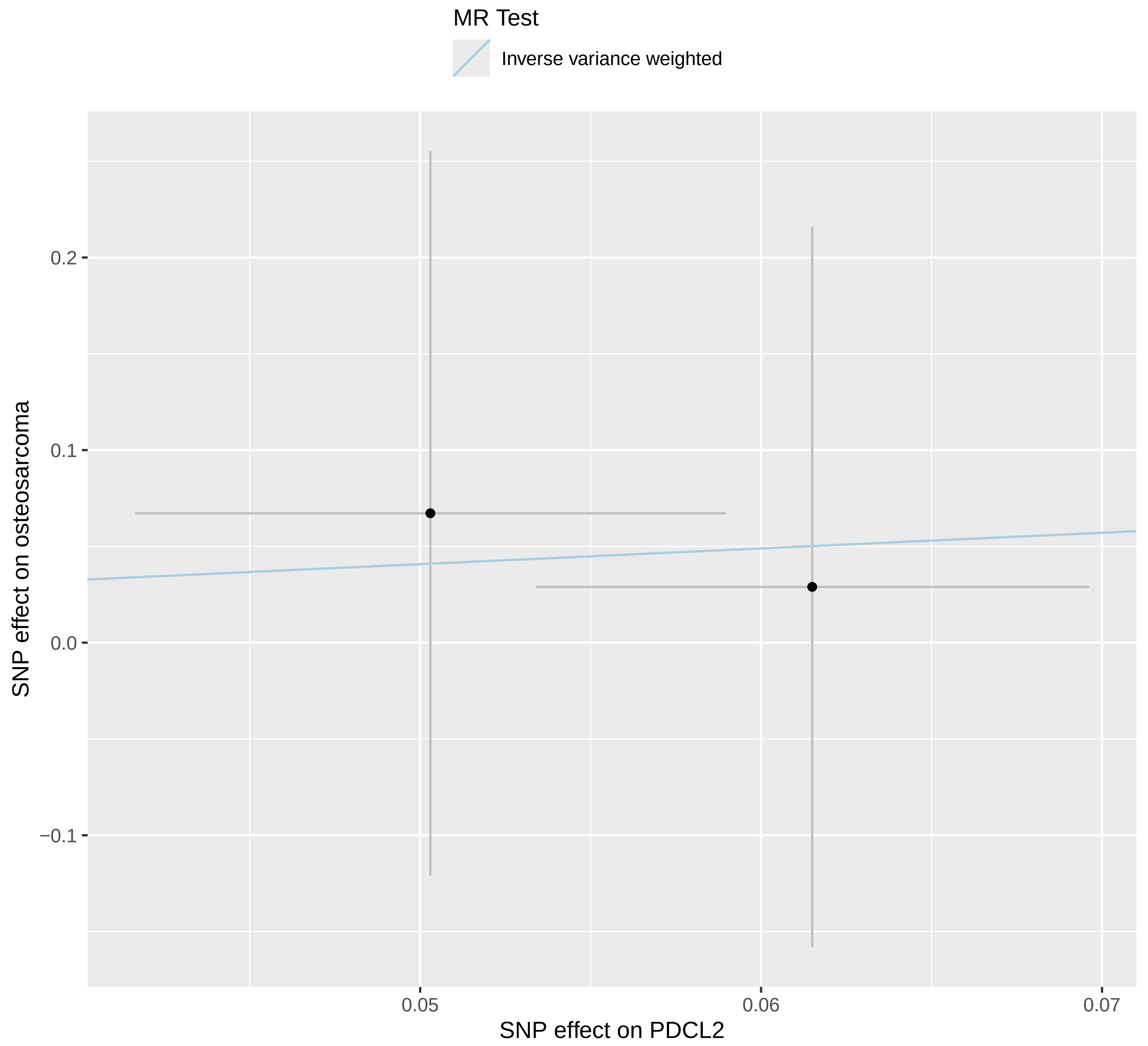
MR Test
Inverse variance weighted
0.2
0.1
0.0
−0.1
SNP effect on osteosarcoma
0.05
0.06
0.07
SNP effect on PDCL2

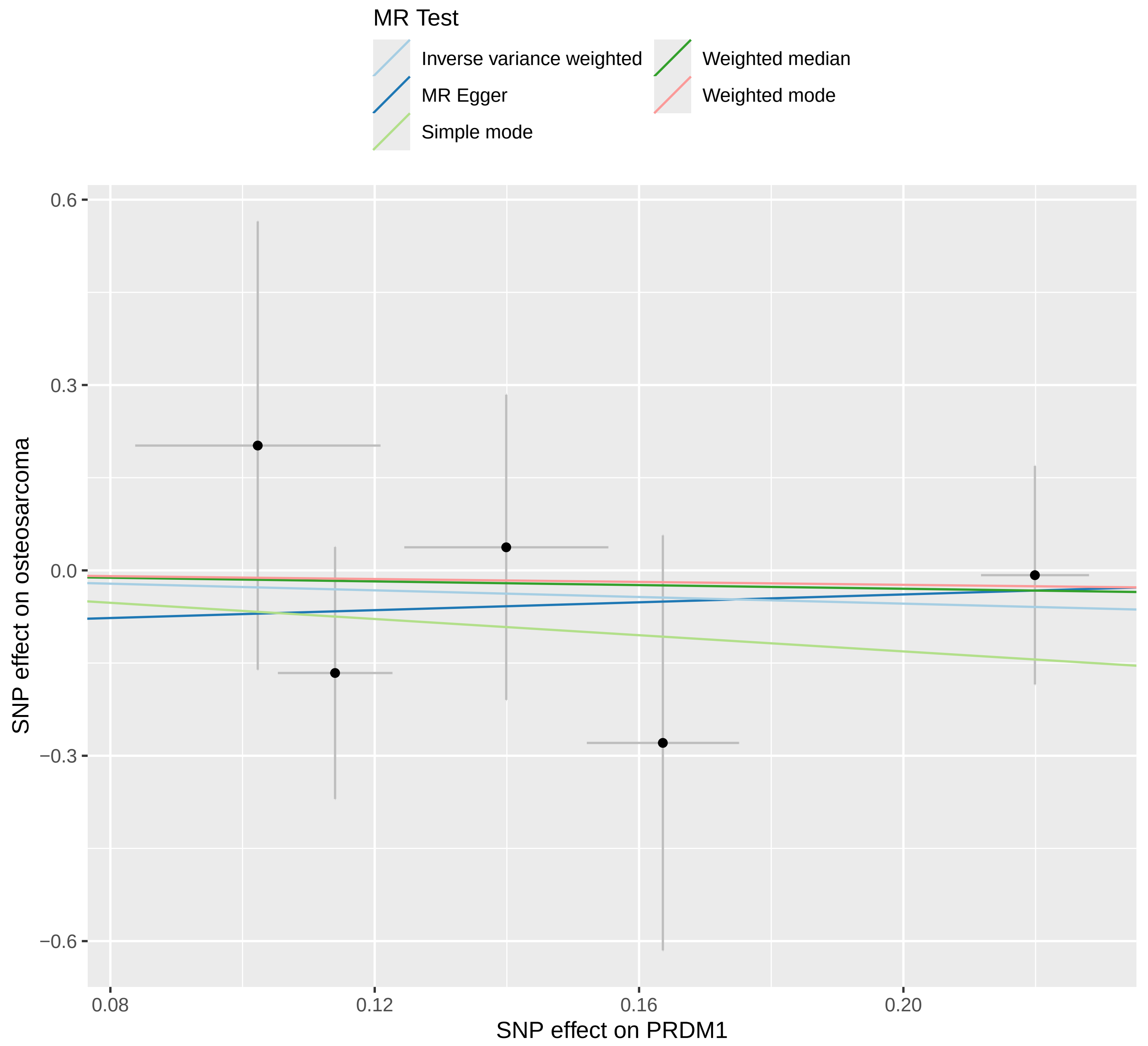
MR Test
Inverse variance weighted
MR Egger
Simple mode
Weighted median
Weighted mode
0.6
0.3
0.0
−0.3
−0.6
0.08
0.12
0.16
0.20
SNP effect on PRDM1
SNP effect on osteosarcoma

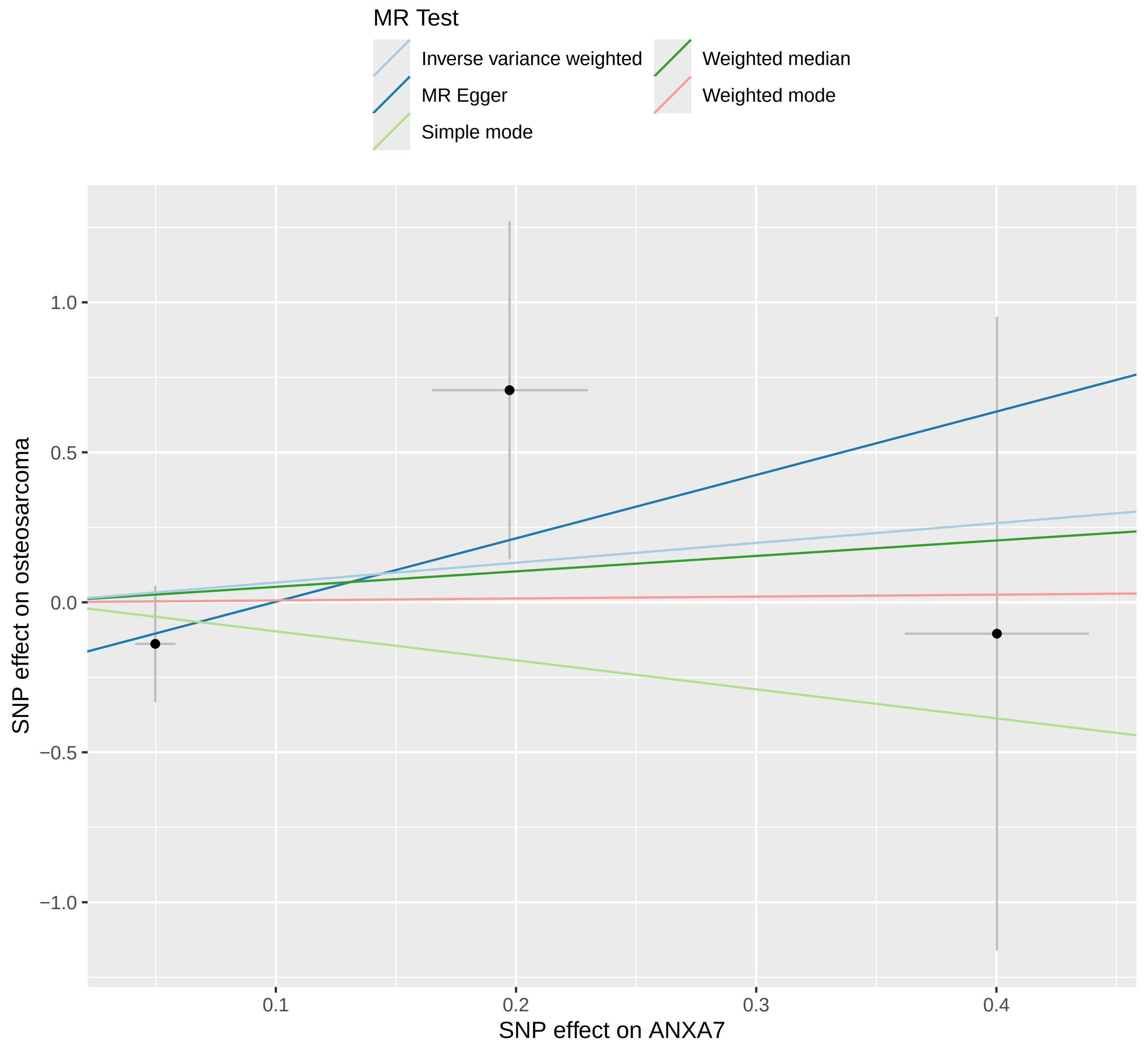

MR Test
Inverse variance weighted
MR Egger
Simple mode
Weighted median
Weighted mode
1.0
0.5
0.0
−0.5
−1.0
SNP effect on osteosarcoma
0.1
0.2
0.3
0.4
SNP effect on ANXA7

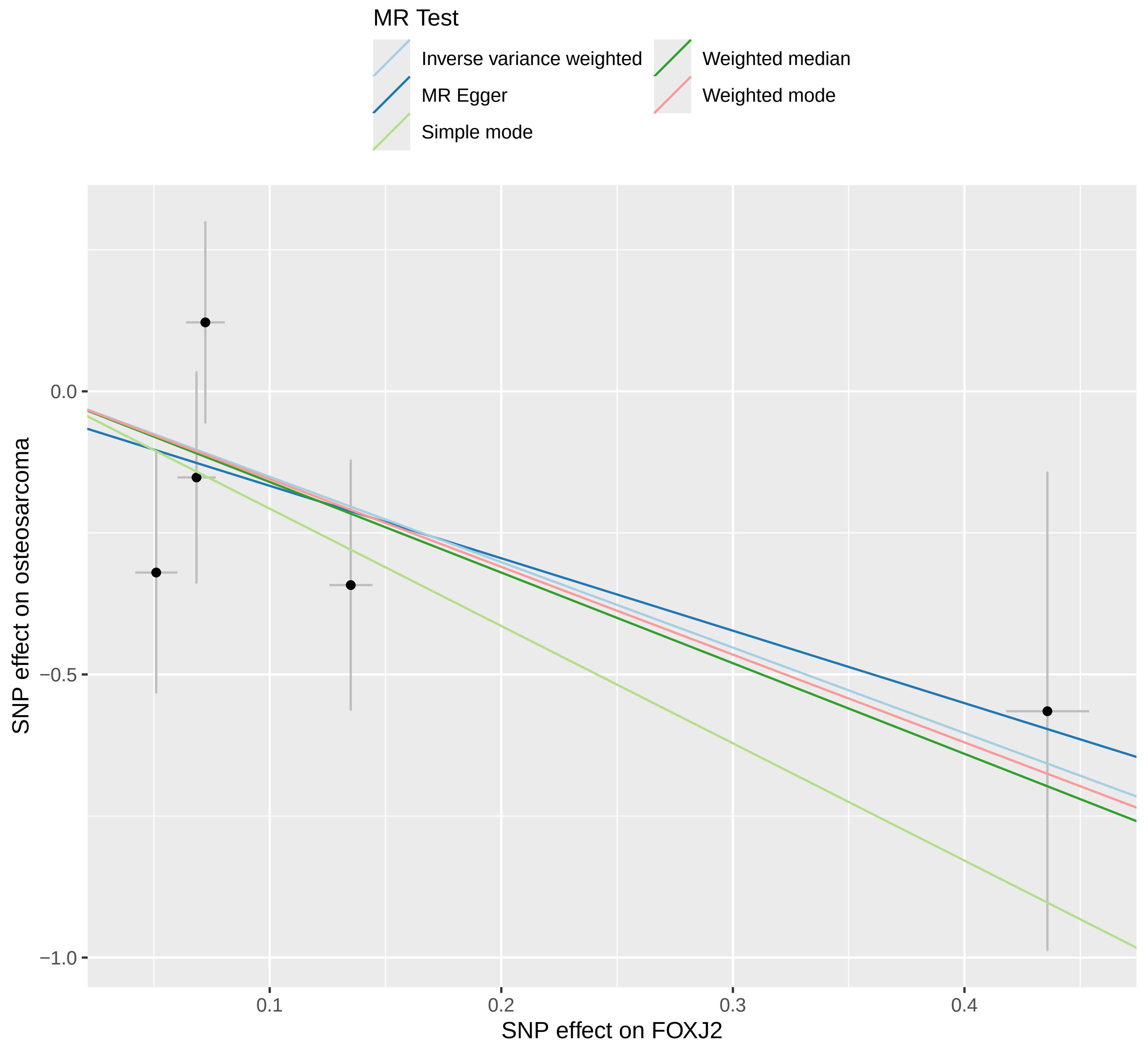
MR Test
Inverse variance weighted
MR Egger
Simple mode
Weighted median
Weighted mode
0.0
−0.5
−1.0
SNP effect on osteosarcoma
0.1
0.2
0.3
0.4
SNP effect on FOXJ2

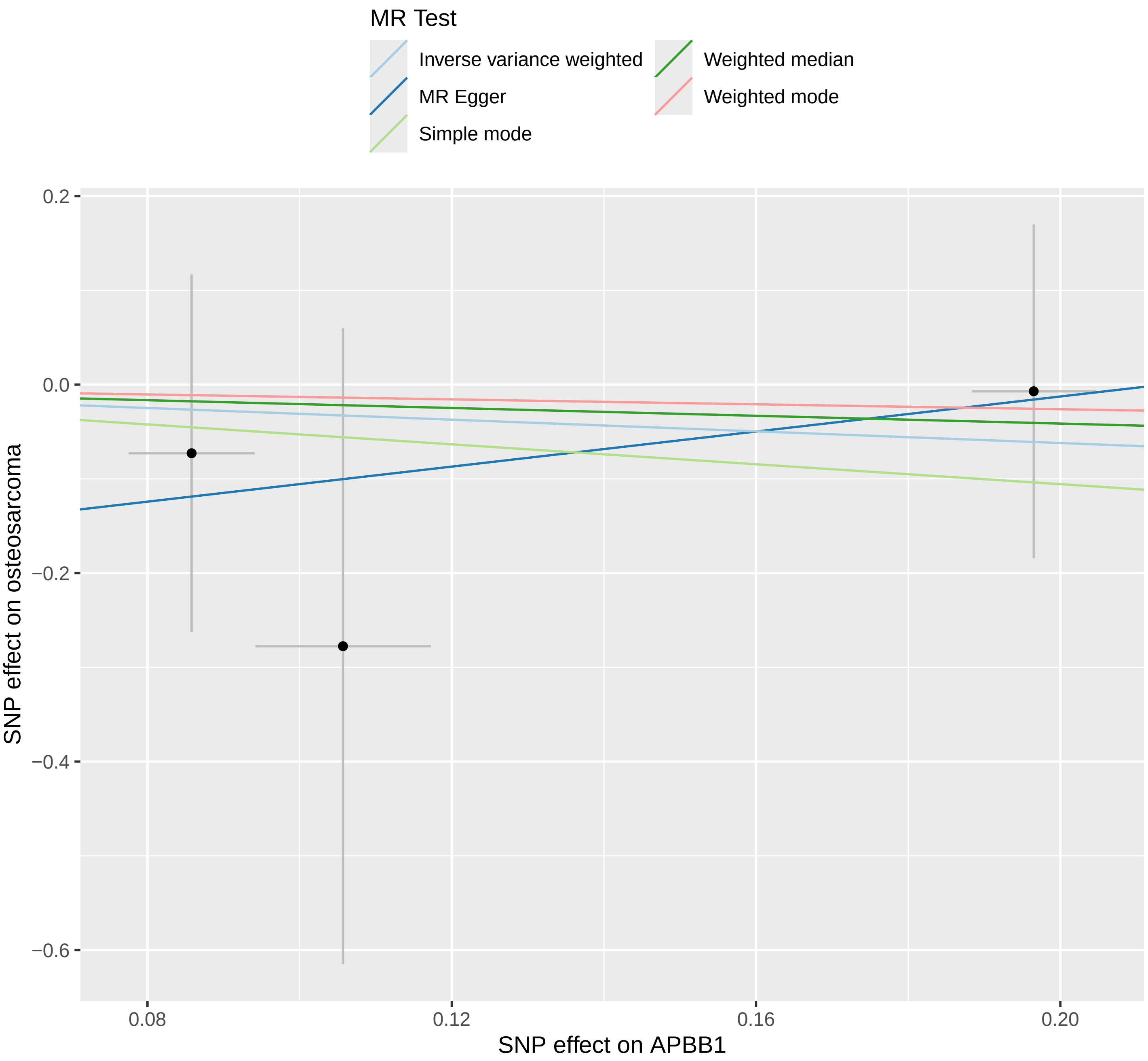

MR Test
Inverse variance weighted
MR Egger
Simple mode
Weighted median
Weighted mode
0.2
0.0
−0.2
−0.4
−0.6
SNP effect on osteosarcoma
0.08
0.12
0.16
0.20
SNP effect on APBB1

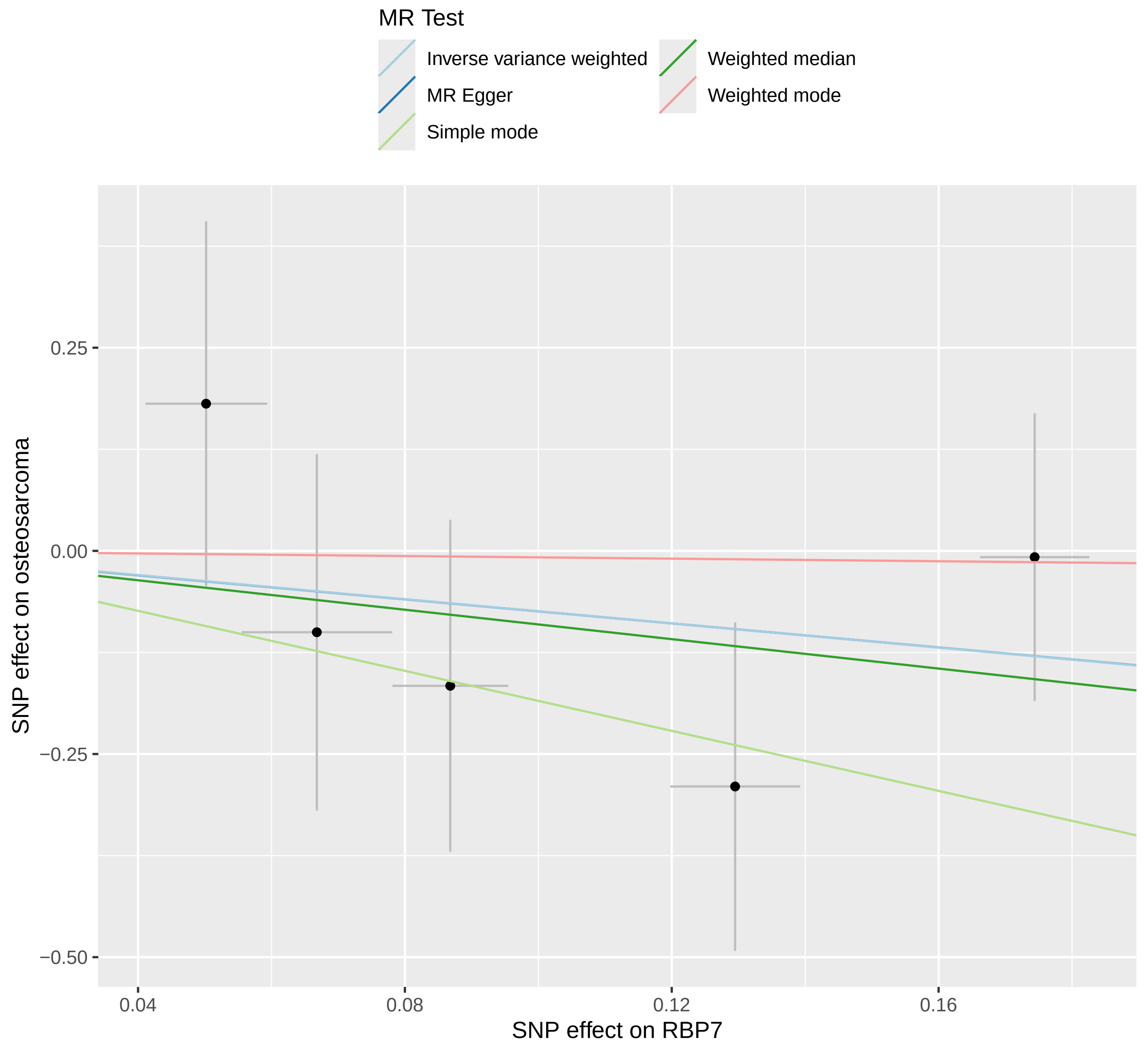
MR Test
Inverse variance weighted
MR Egger
Simple mode
Weighted median
Weighted mode
0.25
0.00
−0.25
−0.50
SNP effect on osteosarcoma
0.04
0.08
0.12
0.16
SNP effect on RBP7

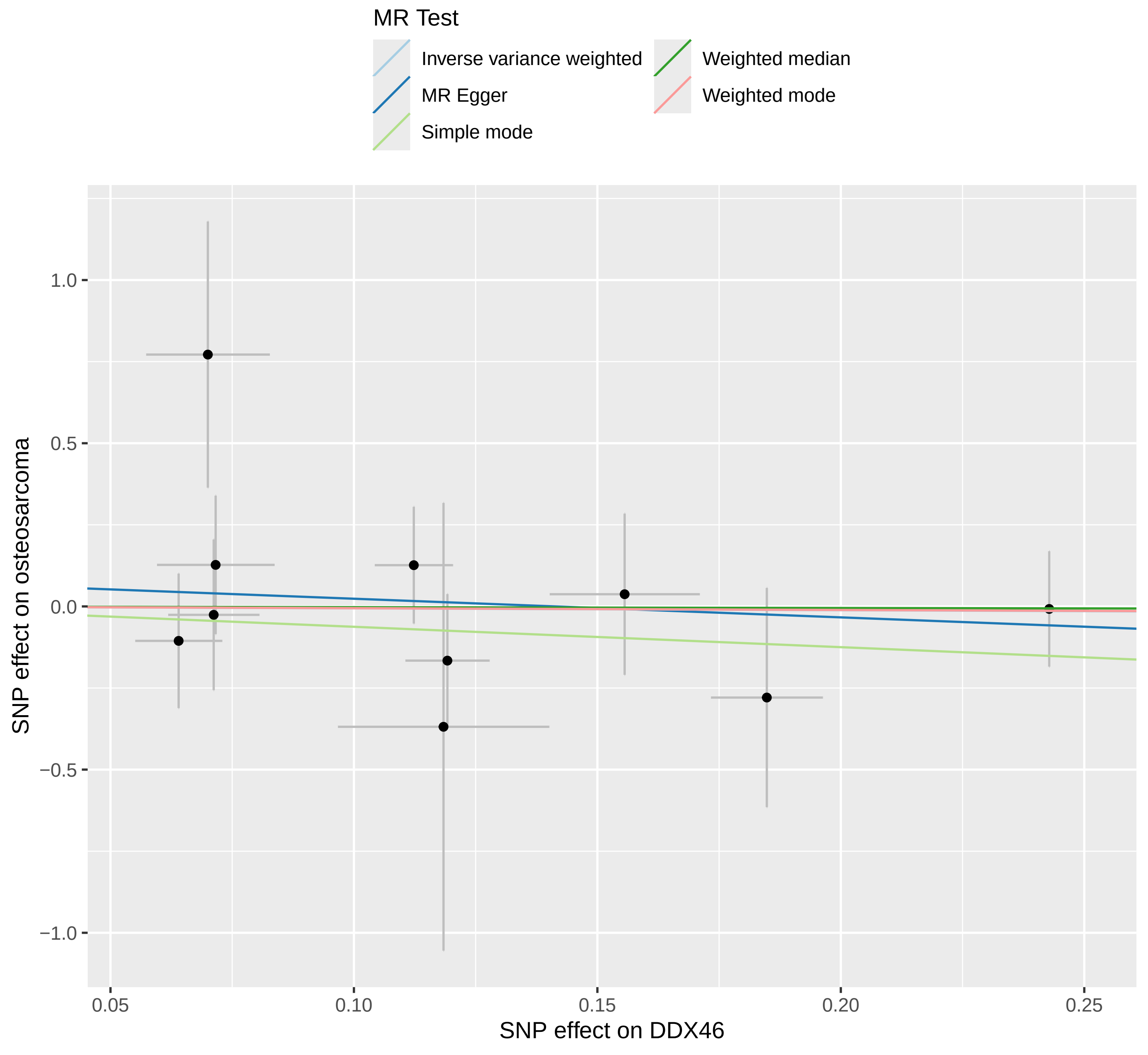

MR Test
Inverse variance weighted
MR Egger
Simple mode
Weighted median
Weighted mode
SNP effect on osteosarcoma
1.0
0.5
0.0
−0.5
−1.0
0.05
0.10
0.15
0.20
0.25
SNP effect on DDX46

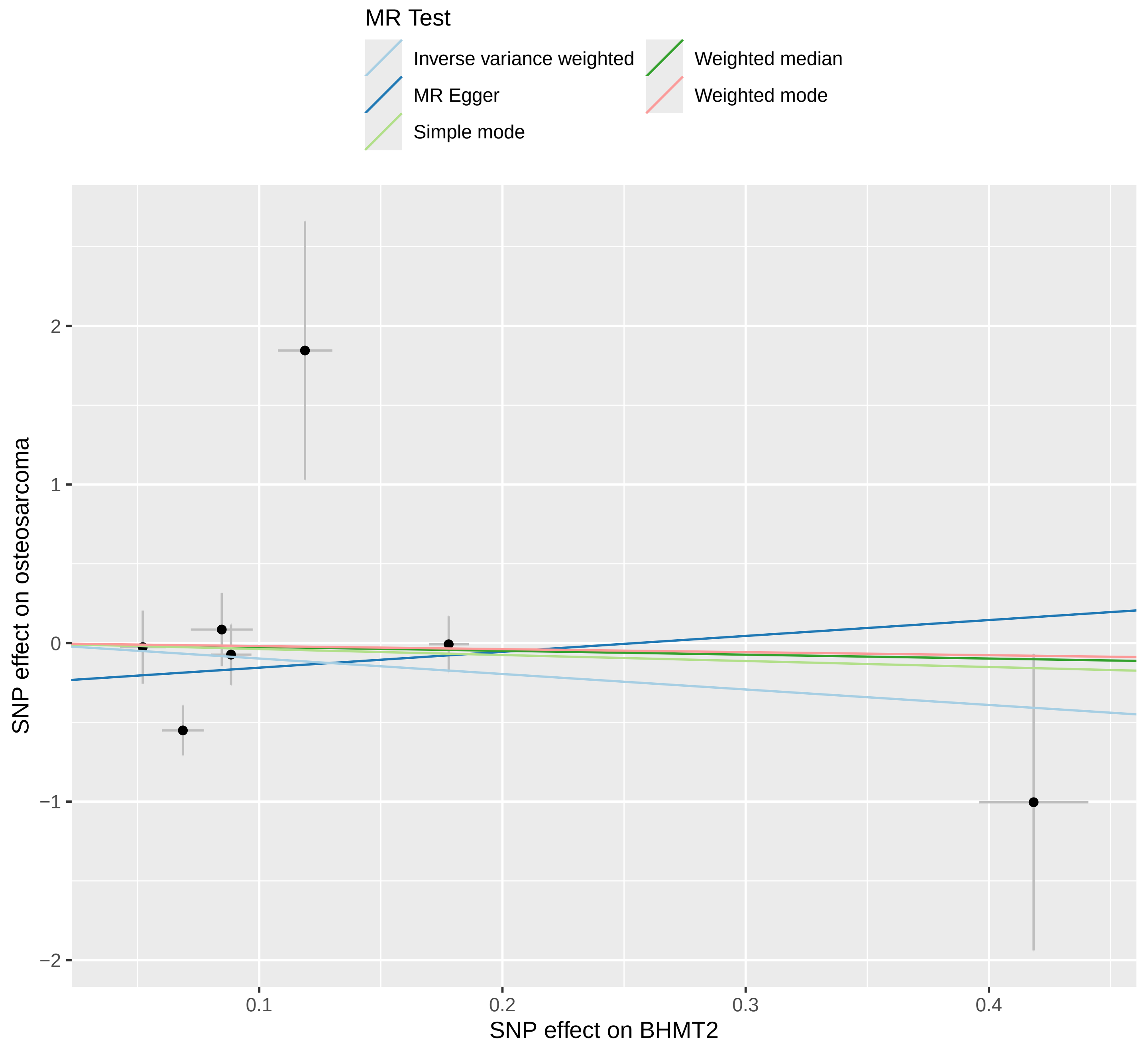
MR Test
Inverse variance weighted
MR Egger
Simple mode
Weighted median
Weighted mode
SNP effect on osteosarcoma
2
1
0
−1
−2
0.1
0.2
0.3
0.4
SNP effect on BHMT2

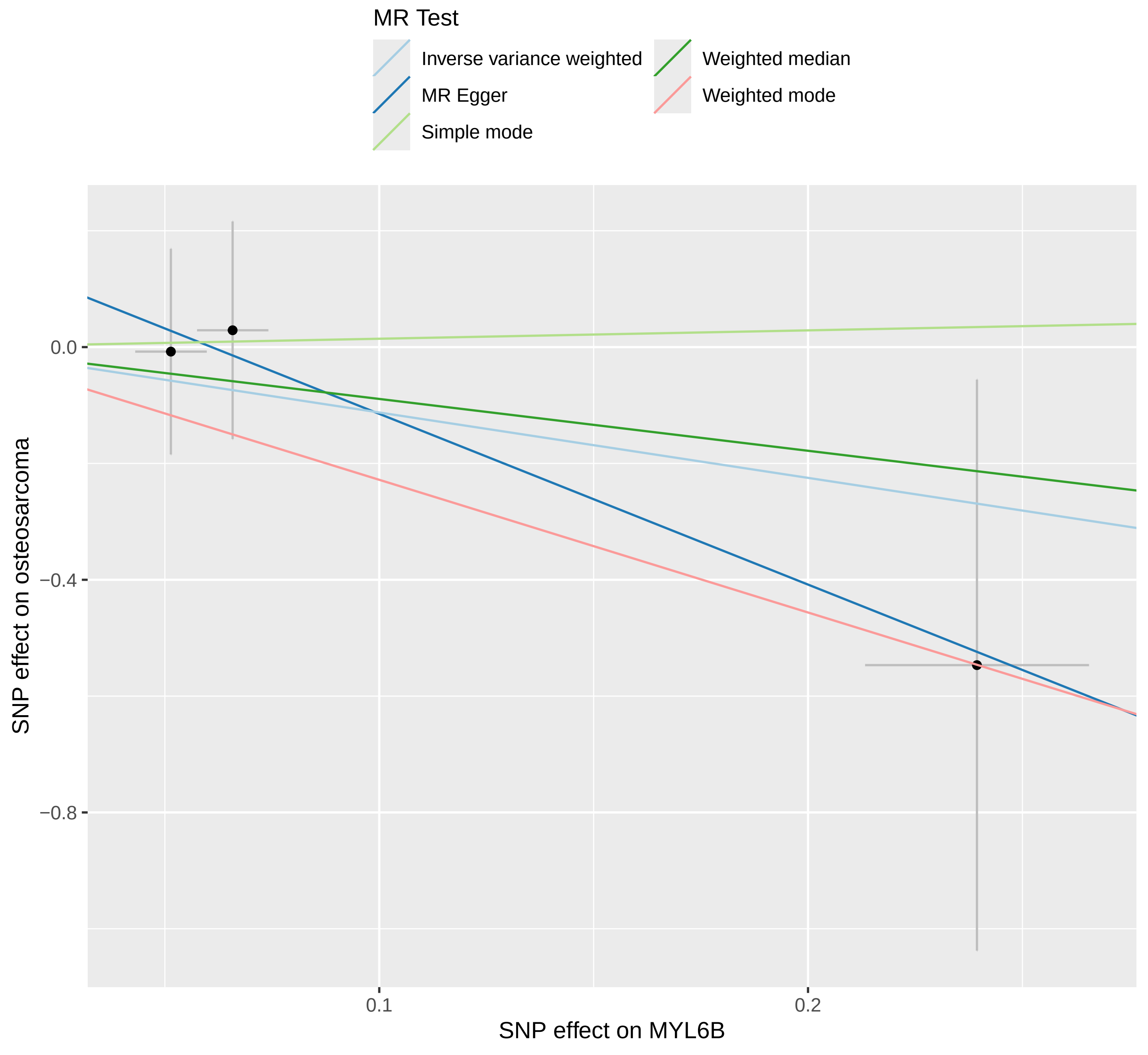
MR Test
Inverse variance weighted
MR Egger
Simple mode
Weighted median
Weighted mode
0.0
−0.4
−0.8
0.1
0.2
SNP effect on osteosarcoma
SNP effect on MYL6B

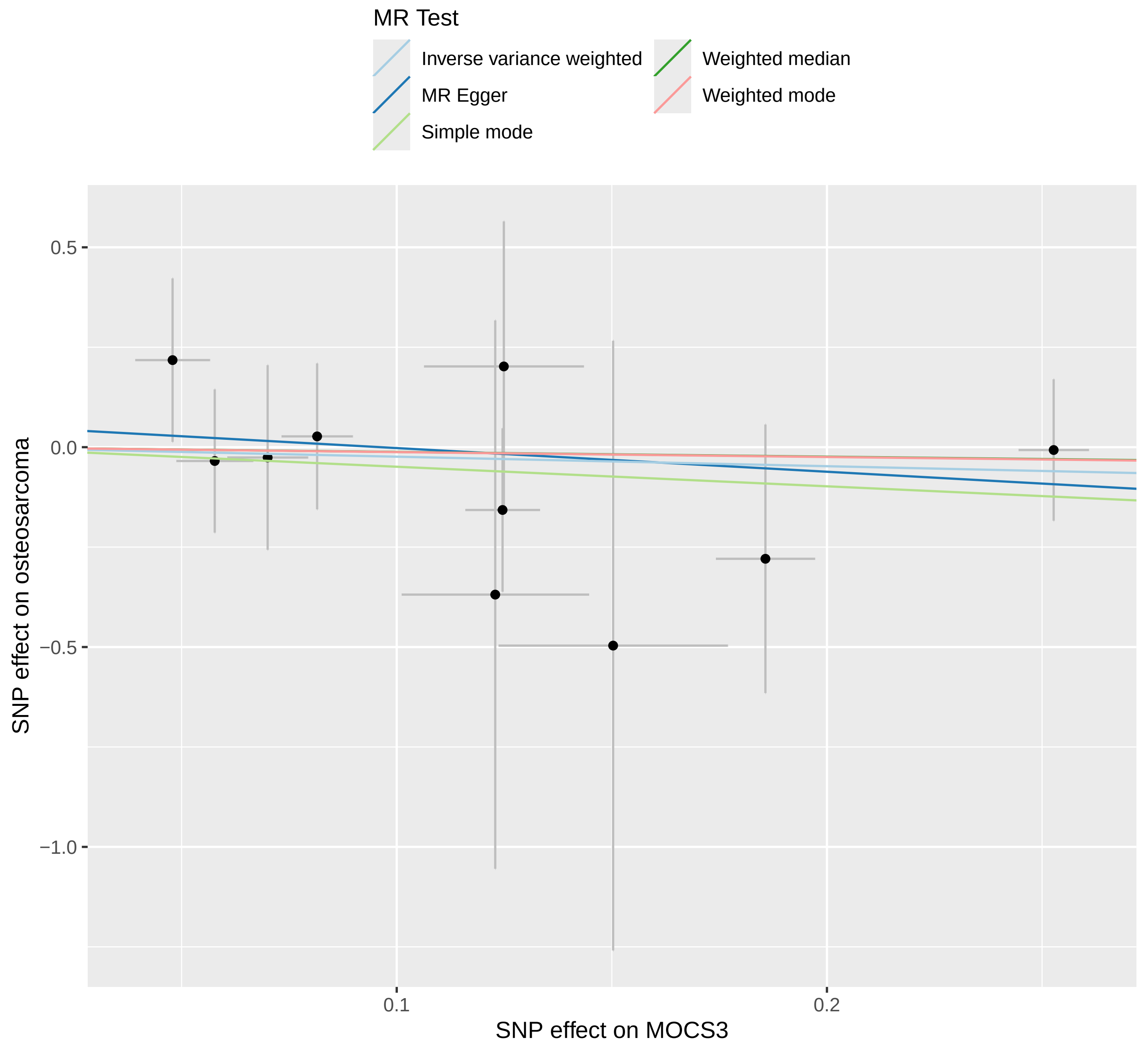
MR Test
Inverse variance weighted
MR Egger
Simple mode
Weighted median
Weighted mode
0.5
0.0
−0.5
−1.0
0.1
0.2
SNP effect on osteosarcoma
SNP effect on MOCS3

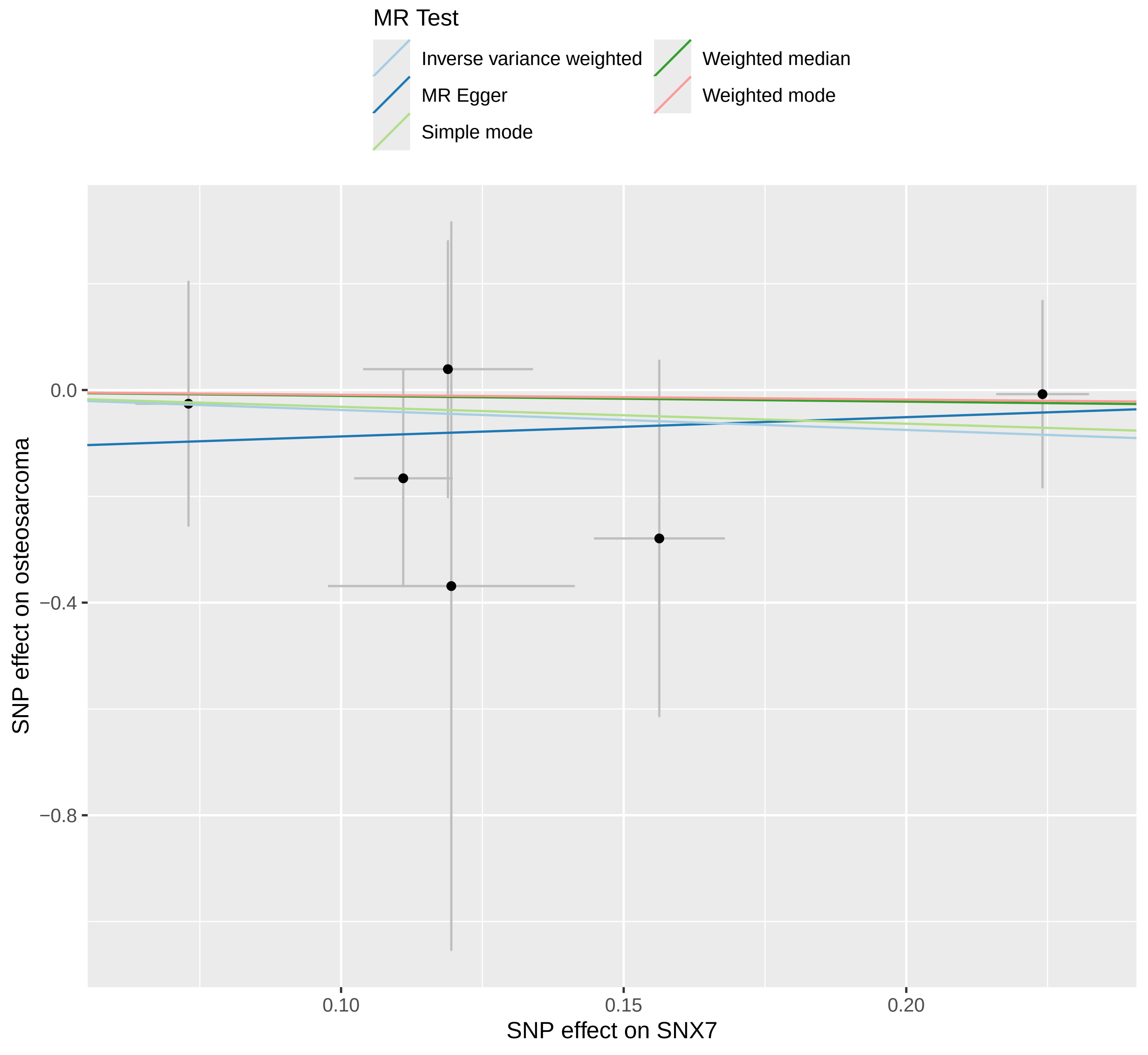
MR Test
Inverse variance weighted
MR Egger
Simple mode
Weighted median
Weighted mode
0.0
−0.4
−0.8
0.10
0.15
0.20
SNP effect on osteosarcoma
SNP effect on SNX7

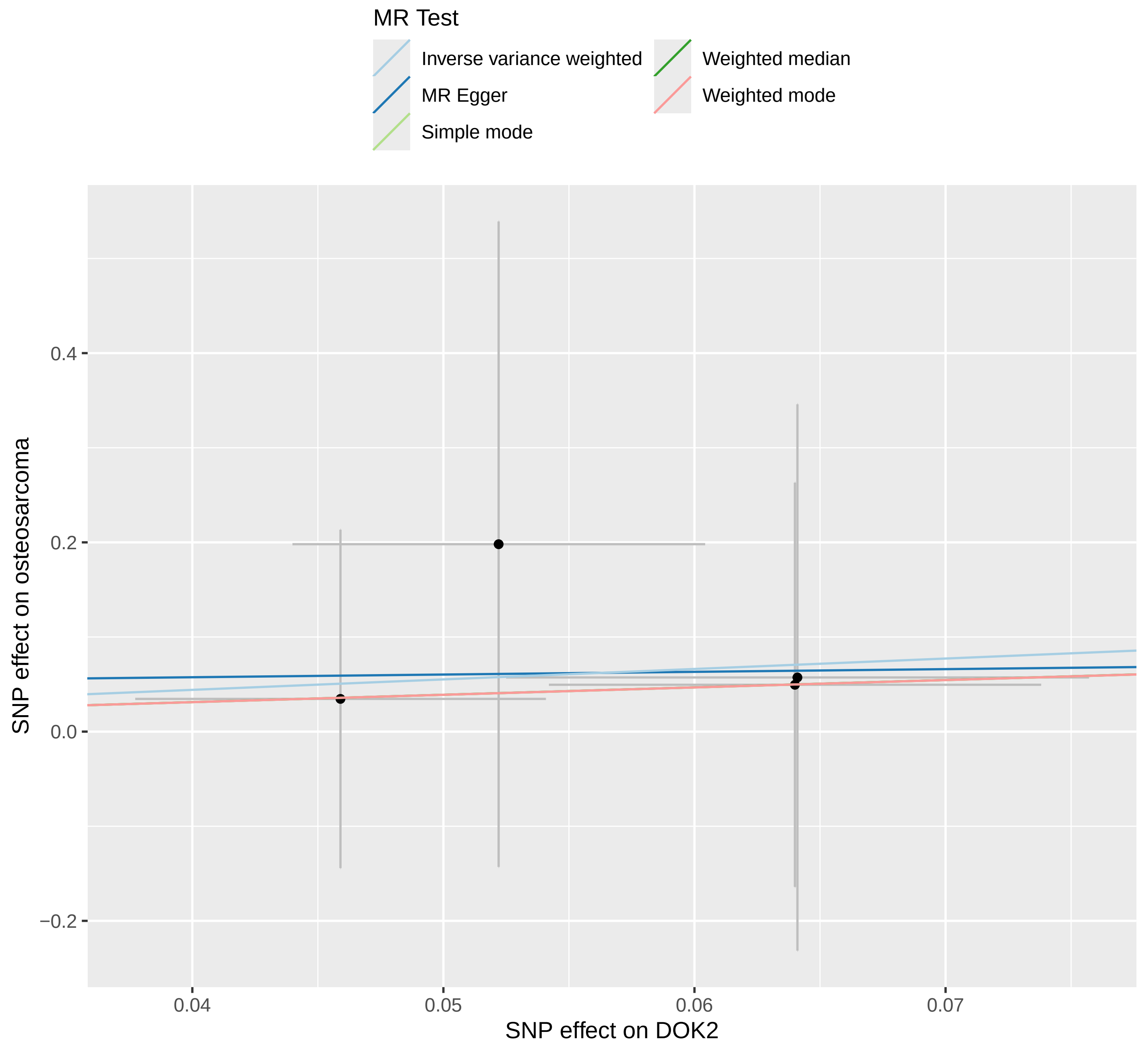

MR Test
Inverse variance weighted
MR Egger
Simple mode
Weighted median
Weighted mode
0.4
0.2
0.0
−0.2
SNP effect on osteosarcoma
0.04
0.05
0.06
0.07
SNP effect on DOK2

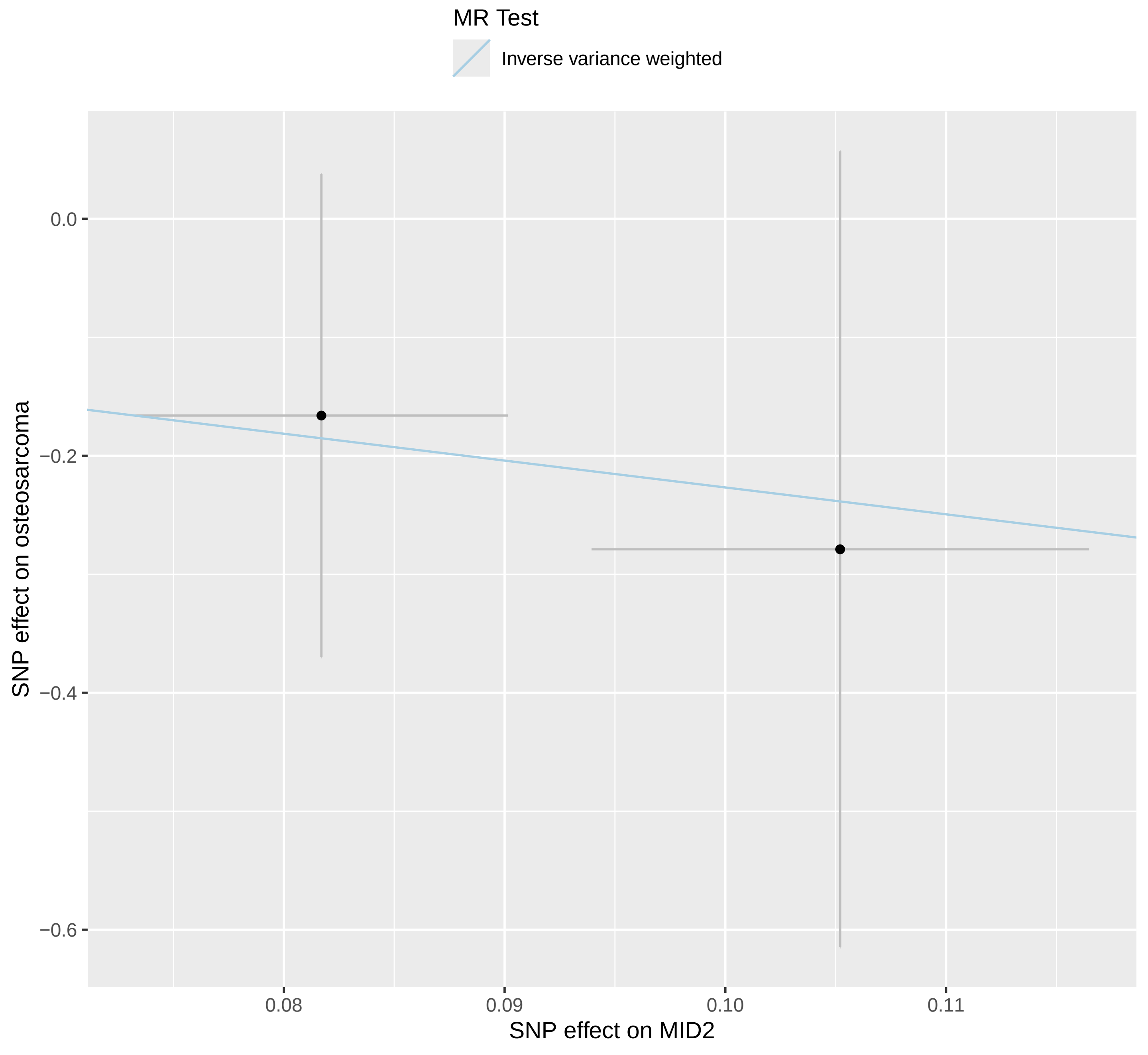
MR Test
Inverse variance weighted
0.0
−0.2
−0.4
−0.6
0.08
0.09
0.10
0.11
SNP effect on MID2
SNP effect on osteosarcoma

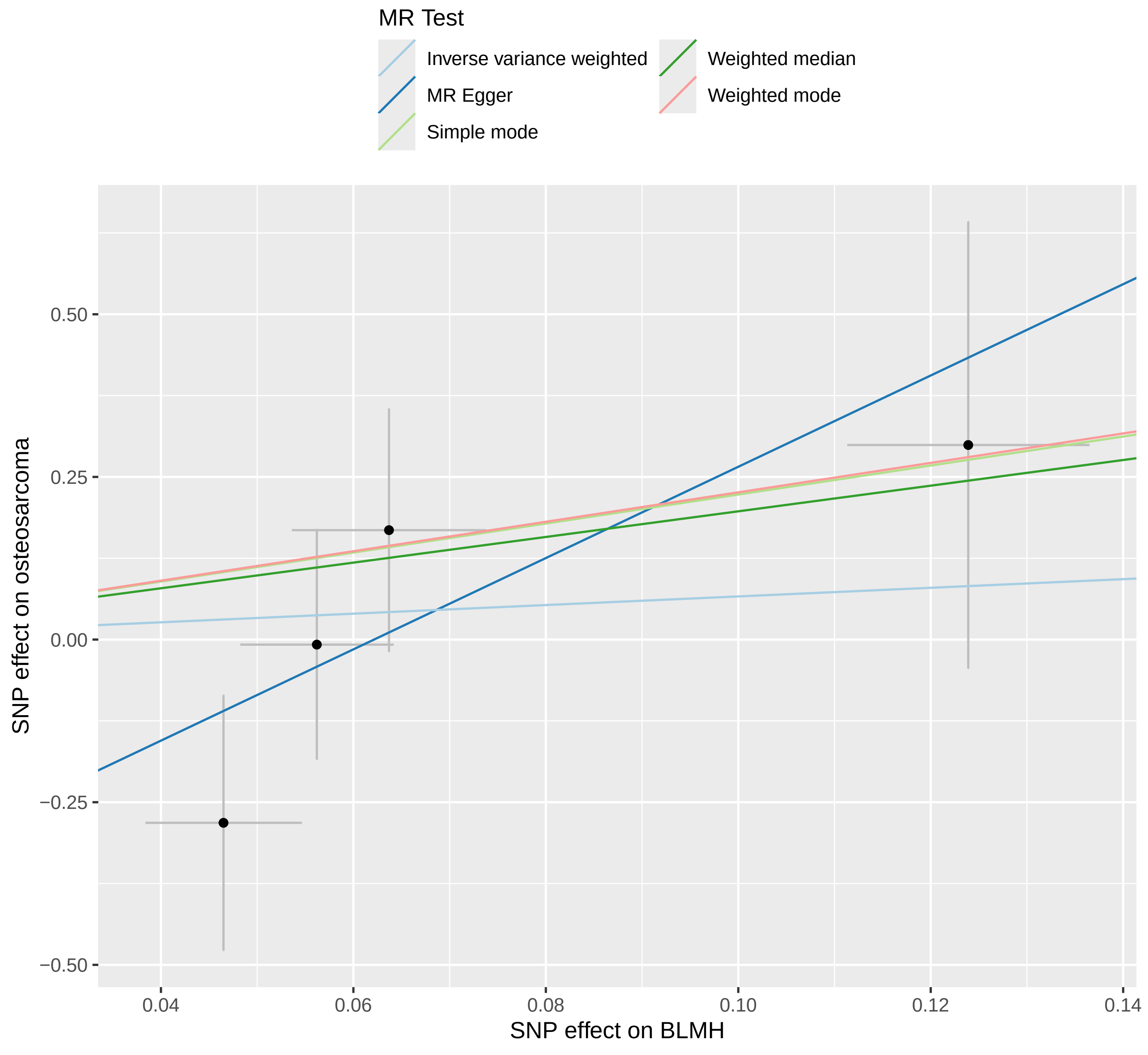
MR Test
Inverse variance weighted
MR Egger
Simple mode
Weighted median
Weighted mode
0.50
0.25
0.00
−0.25
−0.50
0.04
0.06
0.08
0.10
0.12
0.14
SNP effect on osteosarcoma
SNP effect on BLMH

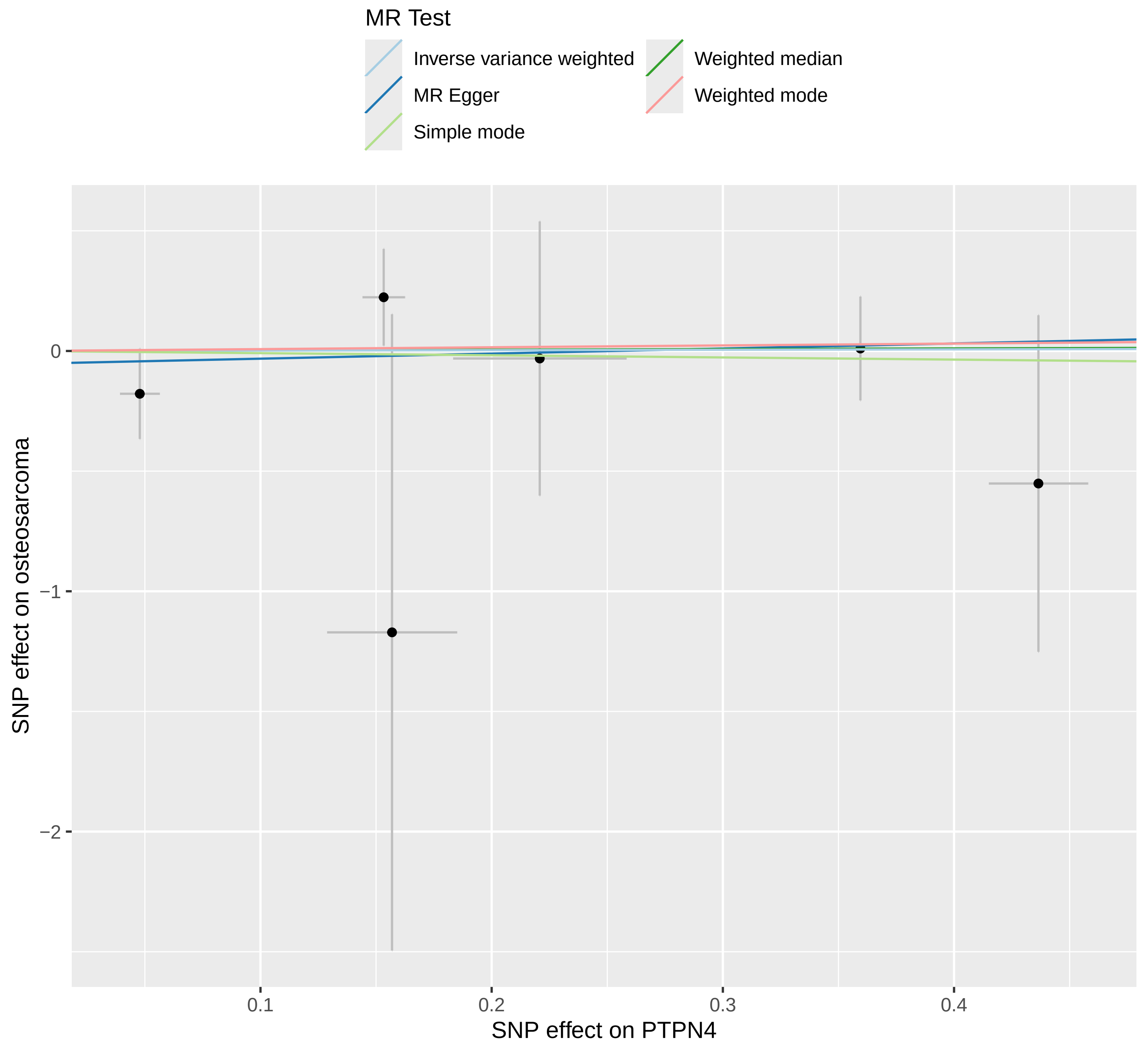
MR Test
Inverse variance weighted
MR Egger
Simple mode
Weighted median
Weighted mode
SNP effect on osteosarcoma
0
−1
−2
0.1
0.2
0.3
0.4
SNP effect on PTPN4

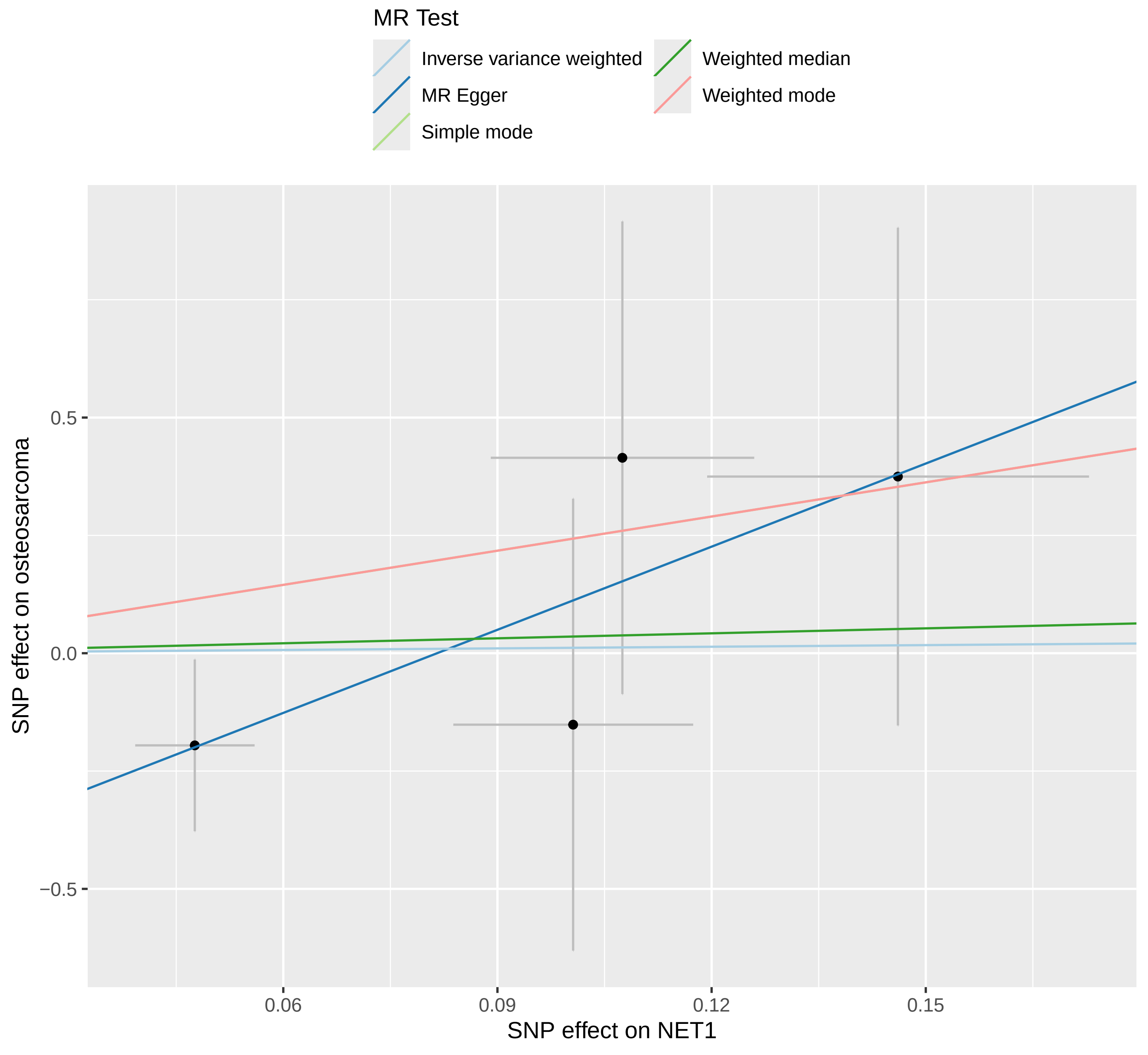
MR Test
Inverse variance weighted
MR Egger
Simple mode
Weighted median
Weighted mode
SNP effect on osteosarcoma
0.5
0.0
−0.5
0.06
0.09
0.12
0.15
SNP effect on NET1

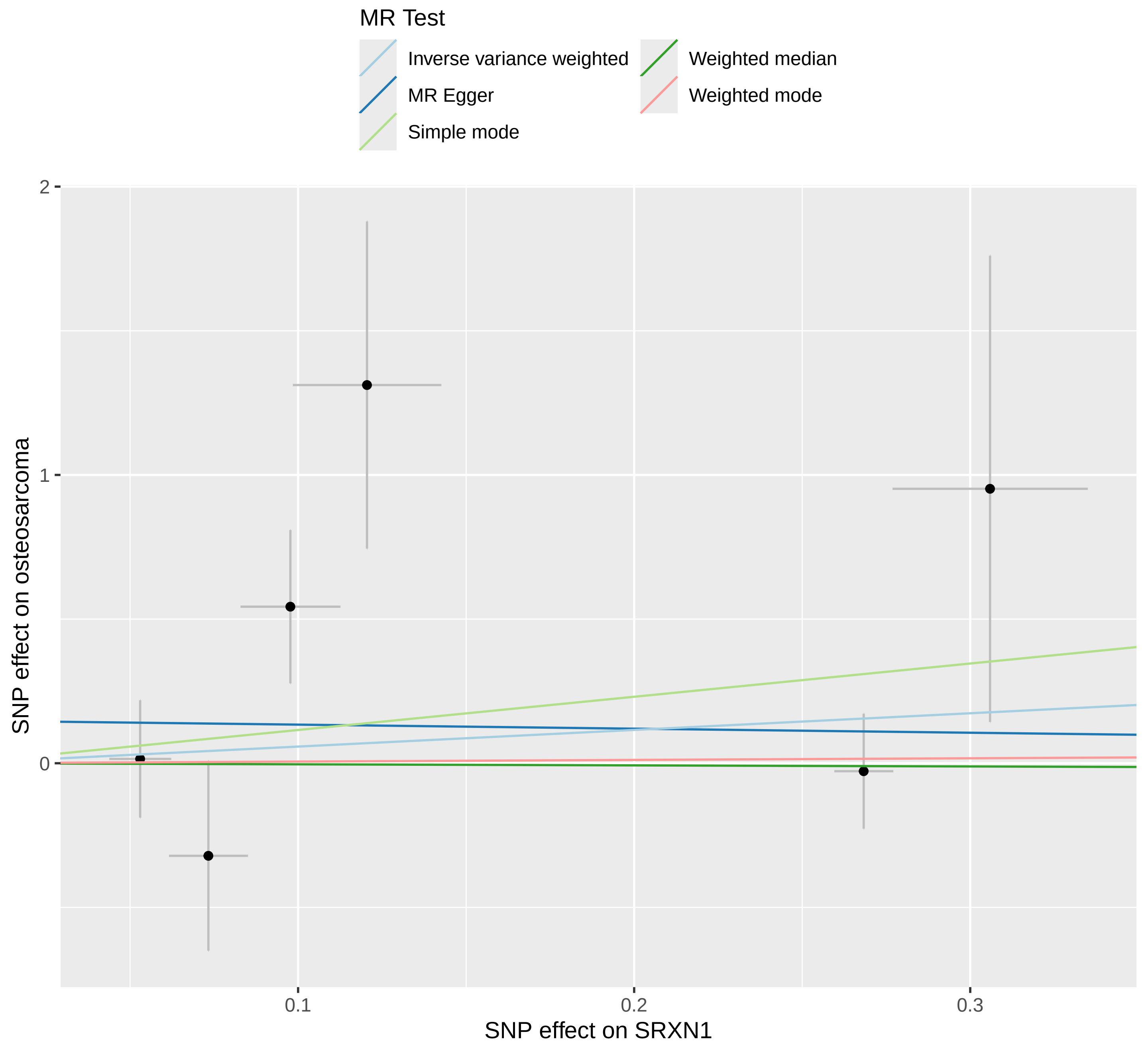
MR Test
Inverse variance weighted
MR Egger
Simple mode
Weighted median
Weighted mode
2
1
0
SNP effect on osteosarcoma
0.1
0.2
0.3
SNP effect on SRXN1

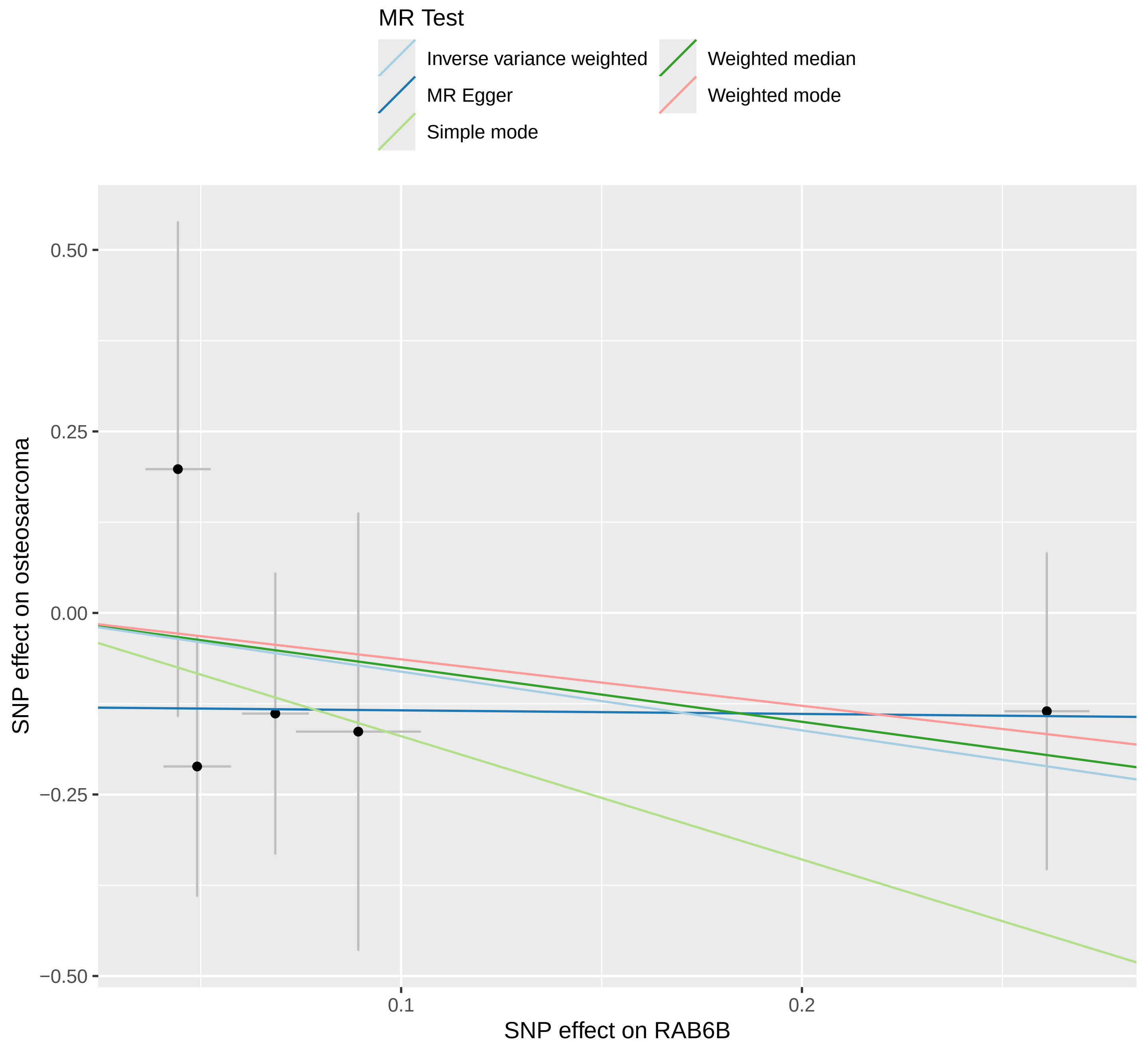
MR Test
Inverse variance weighted
MR Egger
Simple mode
Weighted median
Weighted mode
0.50
0.25
0.00
−0.25
−0.50
0.1
0.2
SNP effect on osteosarcoma
SNP effect on RAB6B

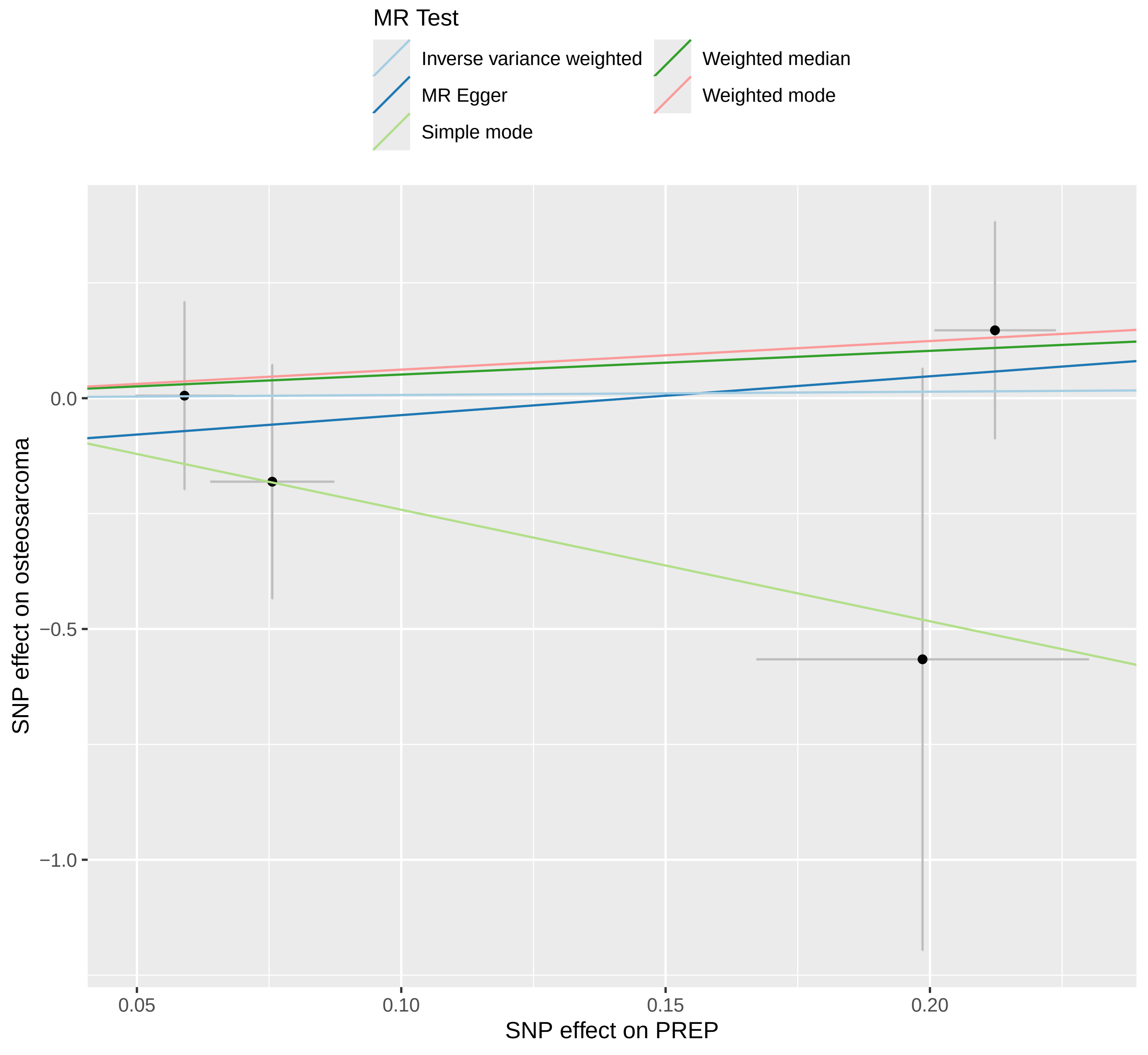
MR Test
Inverse variance weighted
MR Egger
Simple mode
Weighted median
Weighted mode
SNP effect on osteosarcoma
0.0
−0.5
−1.0
0.05
0.10
0.15
0.20
SNP effect on PREP

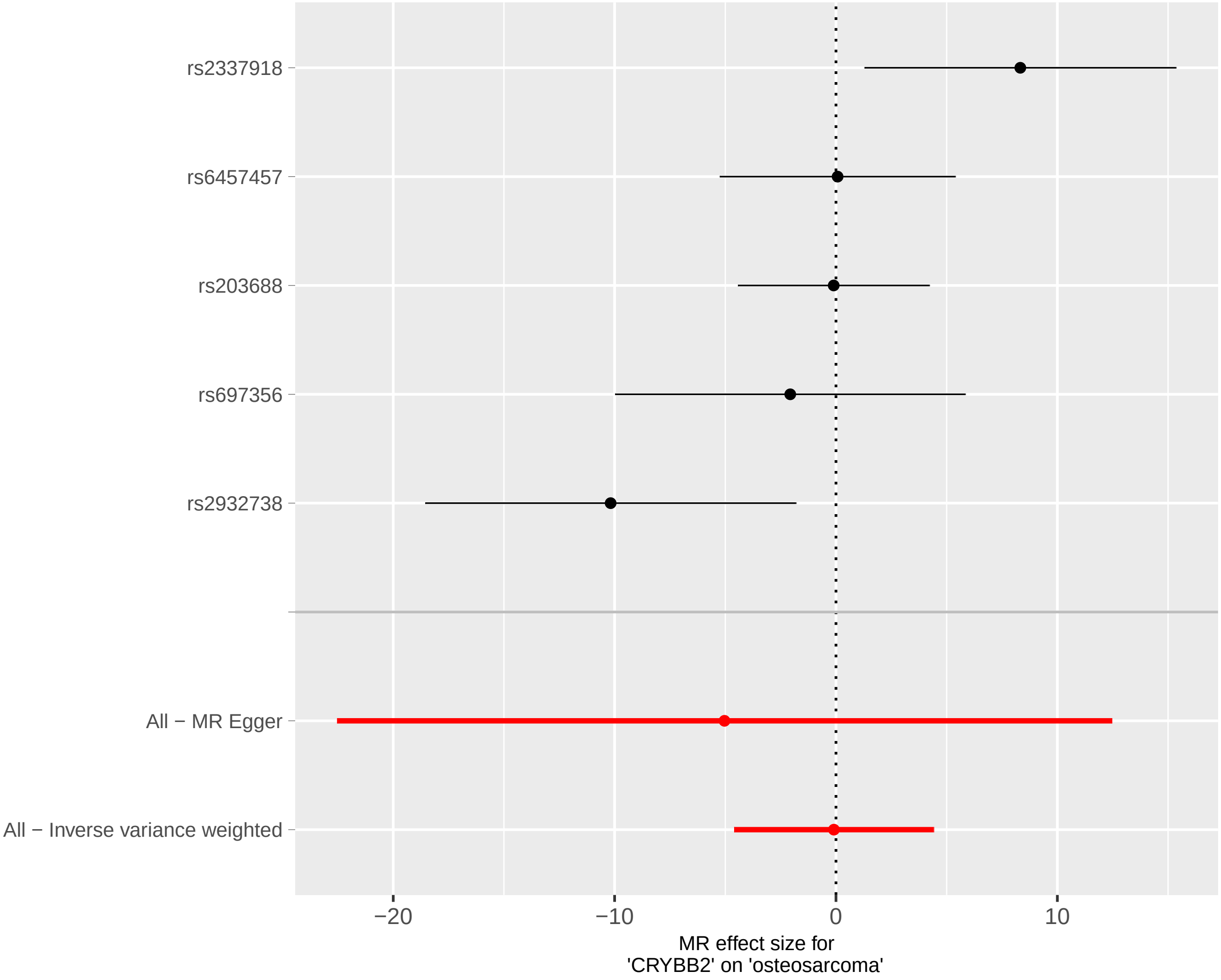
rs2337918
rs6457457
rs203688
rs697356
rs2932738
All − MR Egger
All − Inverse variance weighted
−20
−10
0
10
MR effect size for
'CRYBB2' on 'osteosarcoma'

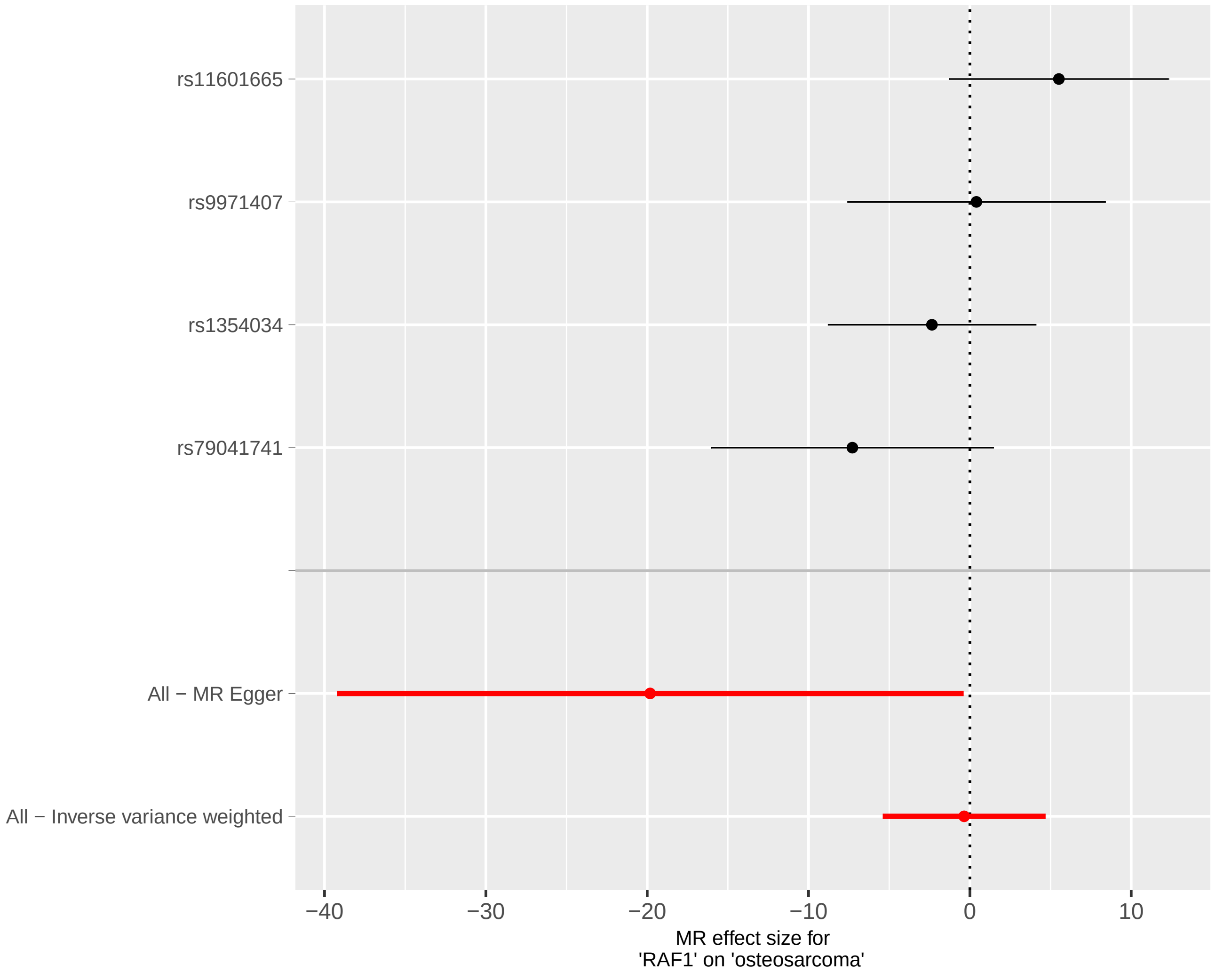

rs11601665
rs9971407
rs1354034
rs79041741
All − MR Egger
All − Inverse variance weighted
−40
−30
−20
−10
0
10
MR effect size for
'RAF1' on 'osteosarcoma'

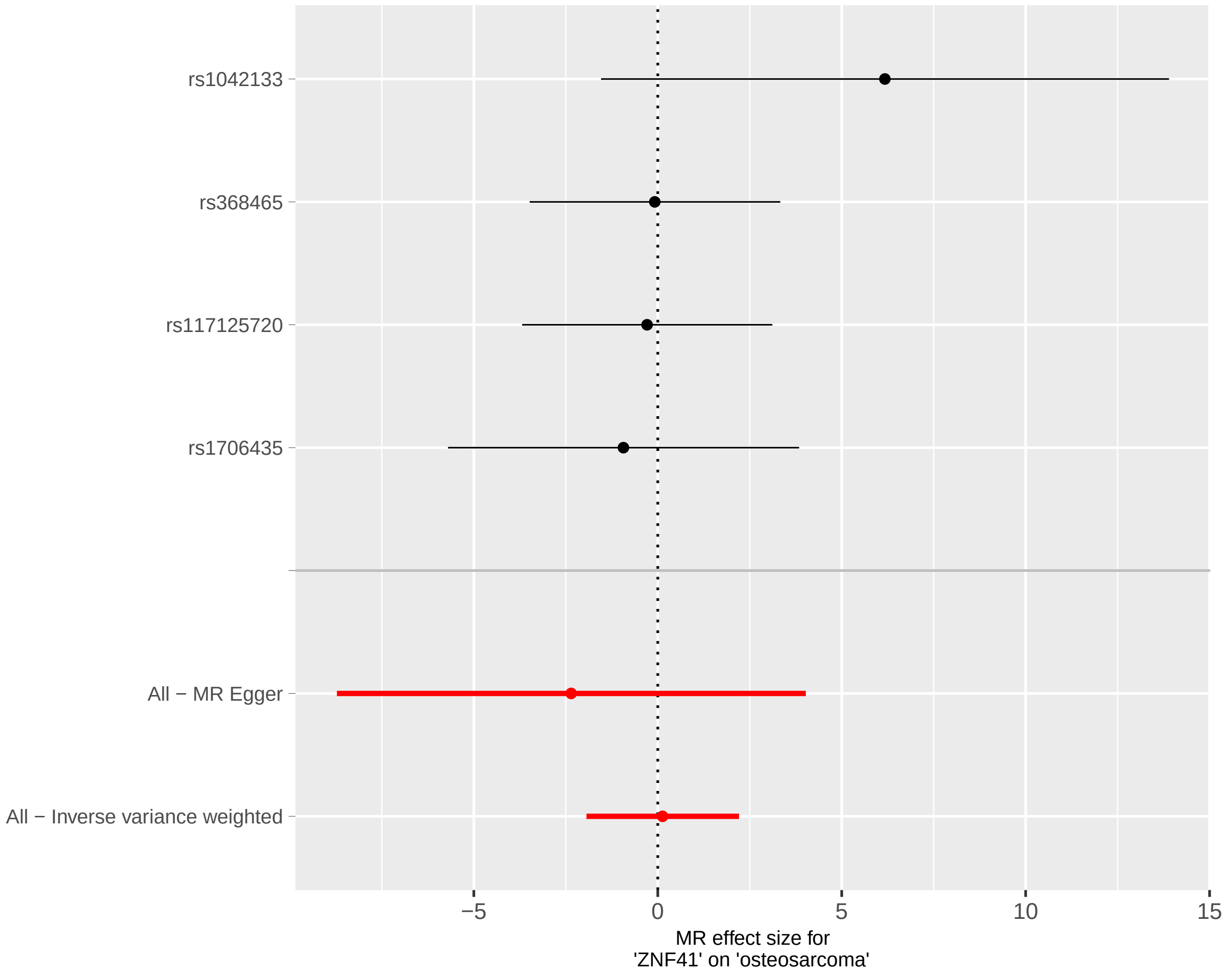
rs1042133
rs368465
rs117125720
rs1706435
All − MR Egger
All − Inverse variance weighted
−5
0
5
10
15
MR effect size for
'ZNF41' on 'osteosarcoma'

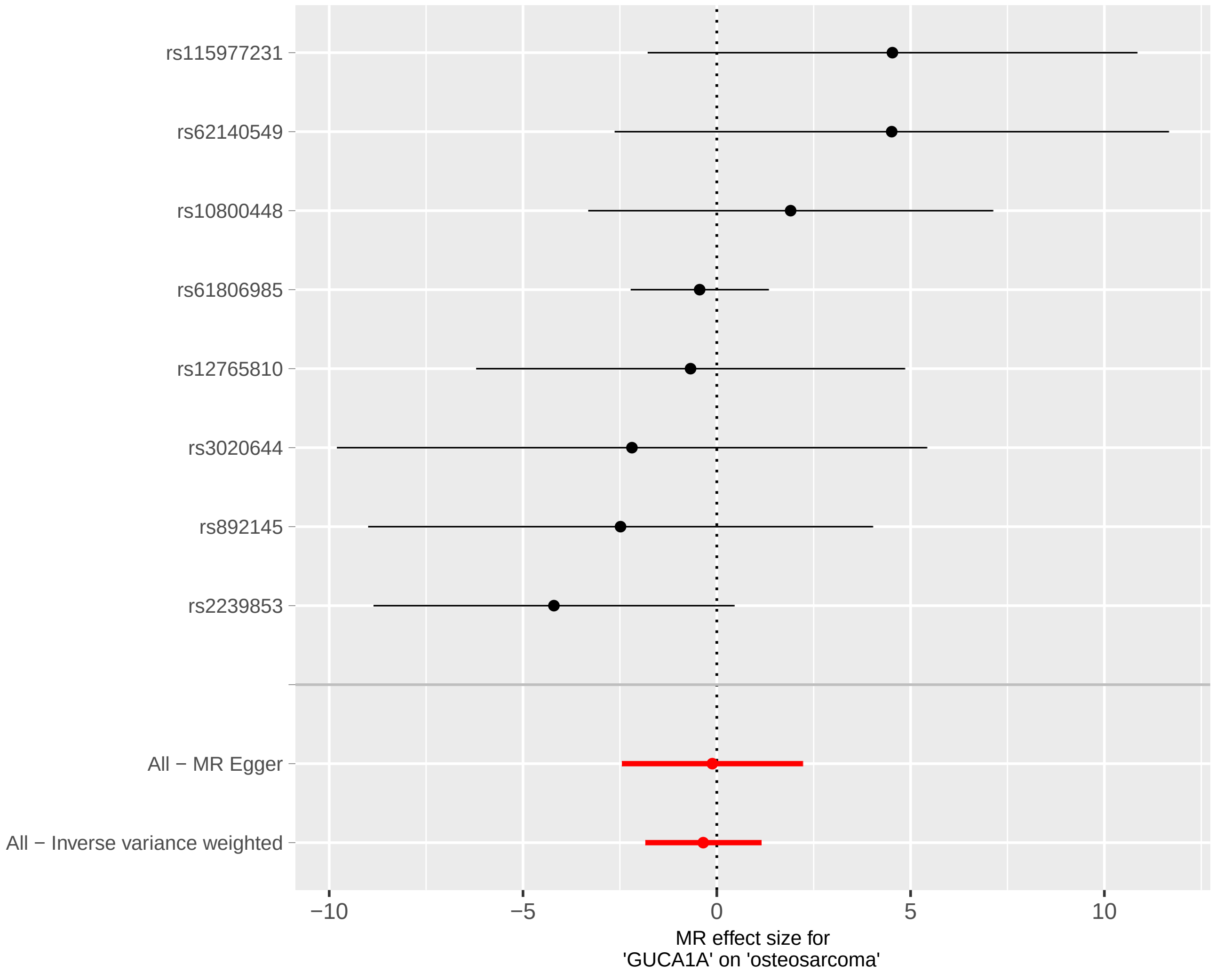
rs115977231
rs62140549
rs10800448
rs61806985
rs12765810
rs3020644
rs892145
rs2239853
All − MR Egger
All − Inverse variance weighted
−10
−5
0
5
10
MR effect size for
'GUCA1A' on 'osteosarcoma'

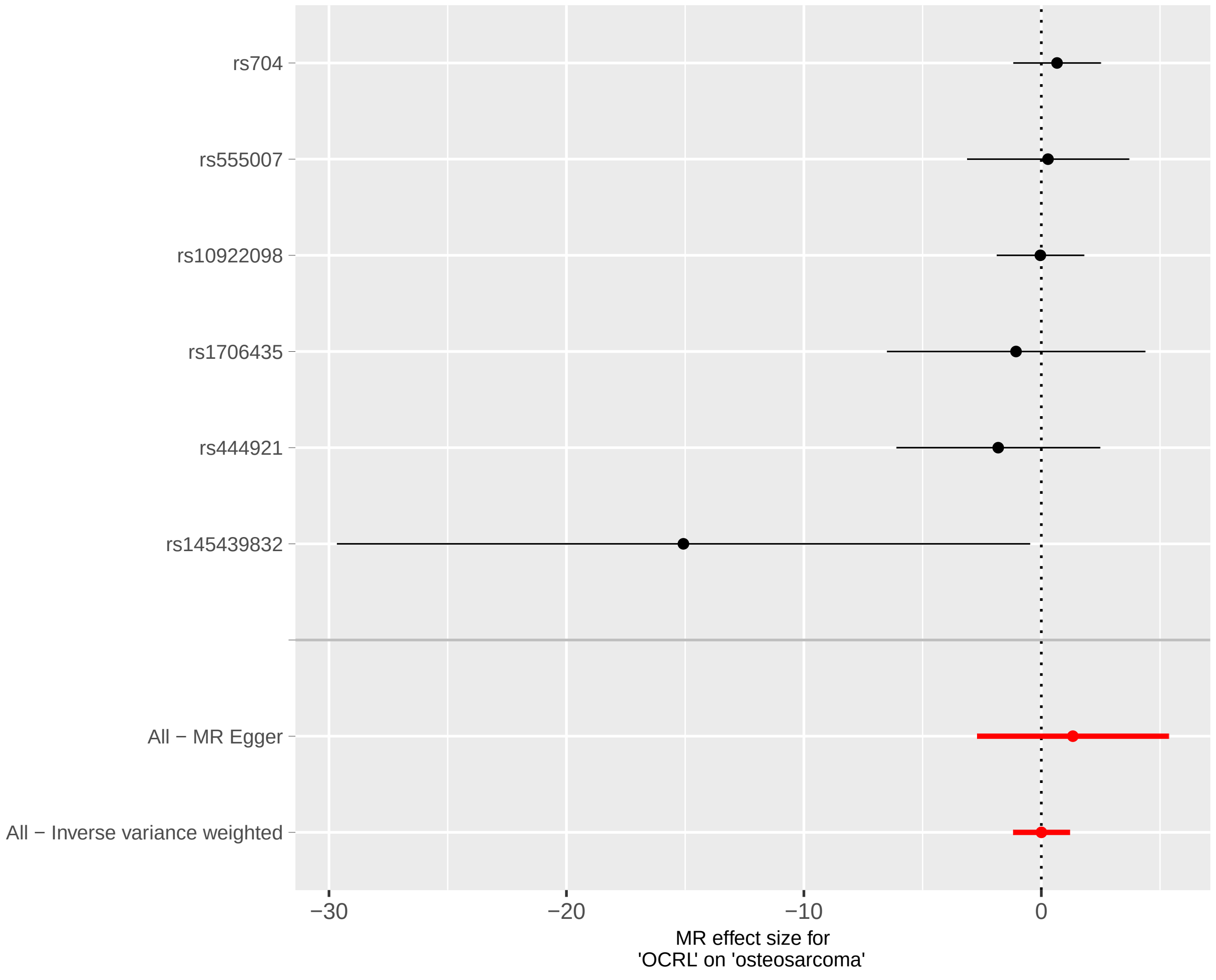

rs704
rs555007
rs10922098
rs1706435
rs444921
rs145439832
All − MR Egger
All − Inverse variance weighted
−30
−20
−10
0
MR effect size for
'OCRL' on 'osteosarcoma'

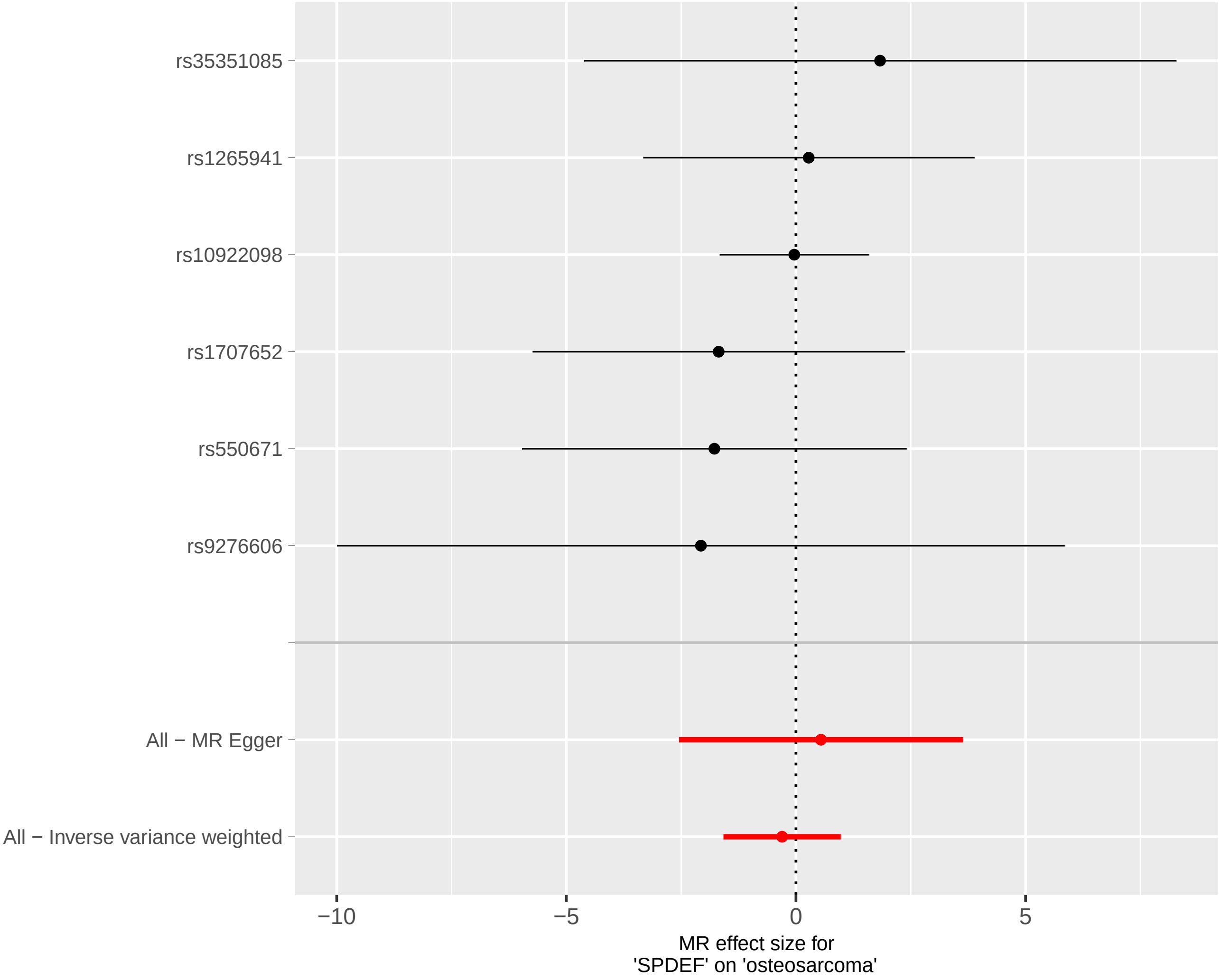

rs35351085
rs1265941
rs10922098
rs1707652
rs550671
rs9276606
All − MR Egger
All − Inverse variance weighted
−10
−5
0
5
MR effect size for
'SPDEF' on 'osteosarcoma'

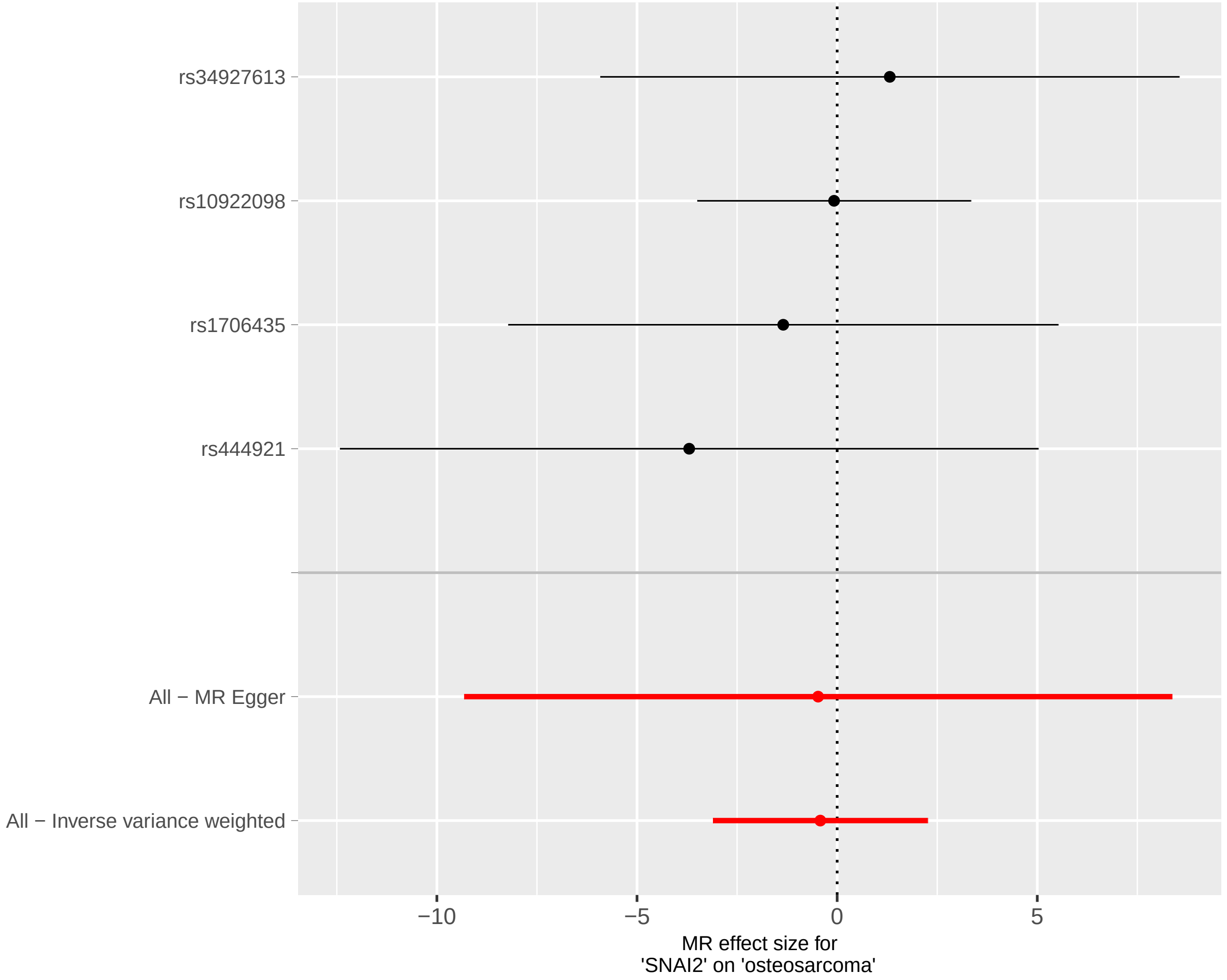
rs34927613
rs10922098
rs1706435
rs444921
All − MR Egger
All − Inverse variance weighted
−10
−5
0
5
MR effect size for
'SNAI2' on 'osteosarcoma'

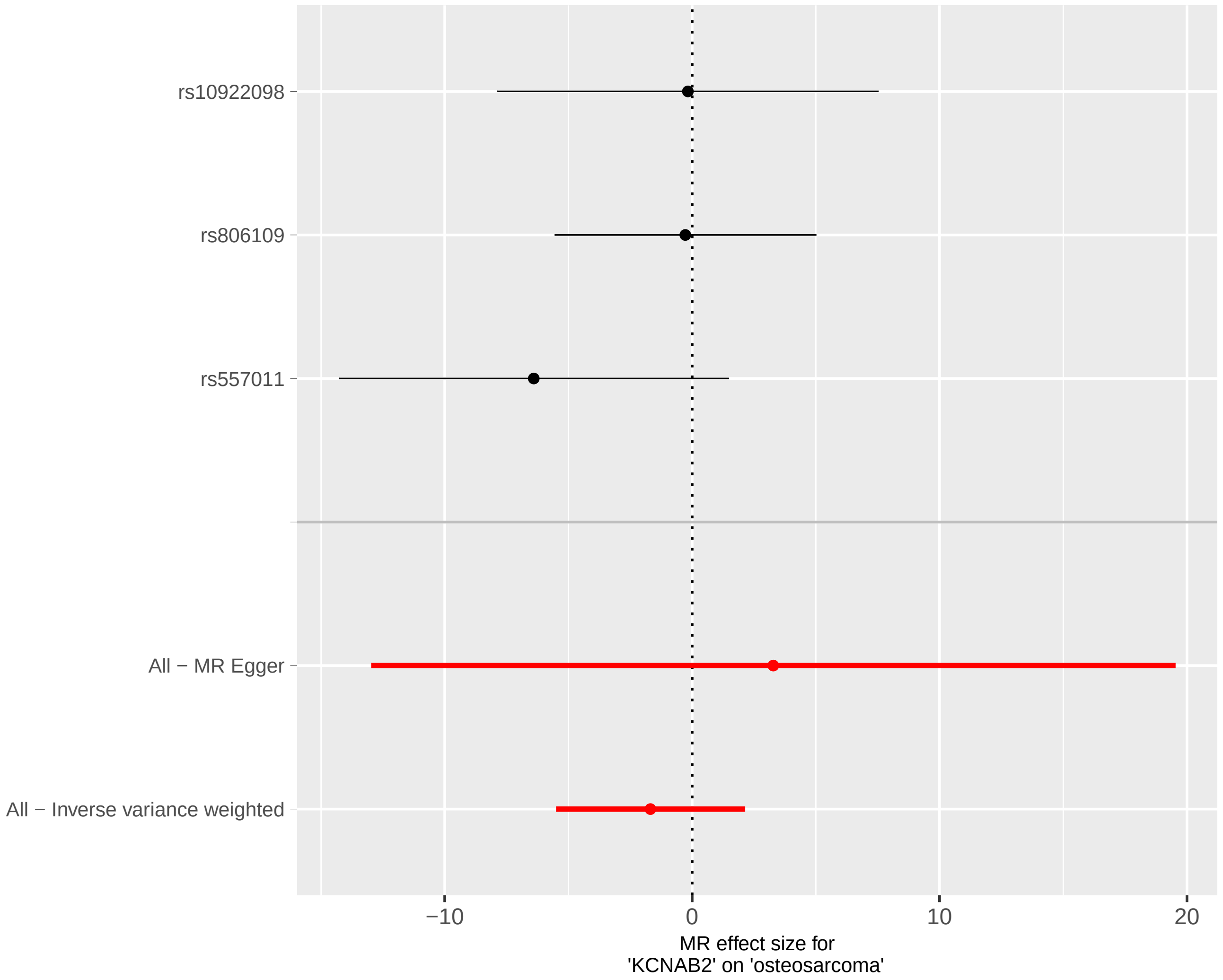

rs10922098
rs806109
rs557011
All − MR Egger
All − Inverse variance weighted
−10
0
10
20
MR effect size for
'KCNAB2' on 'osteosarcoma'

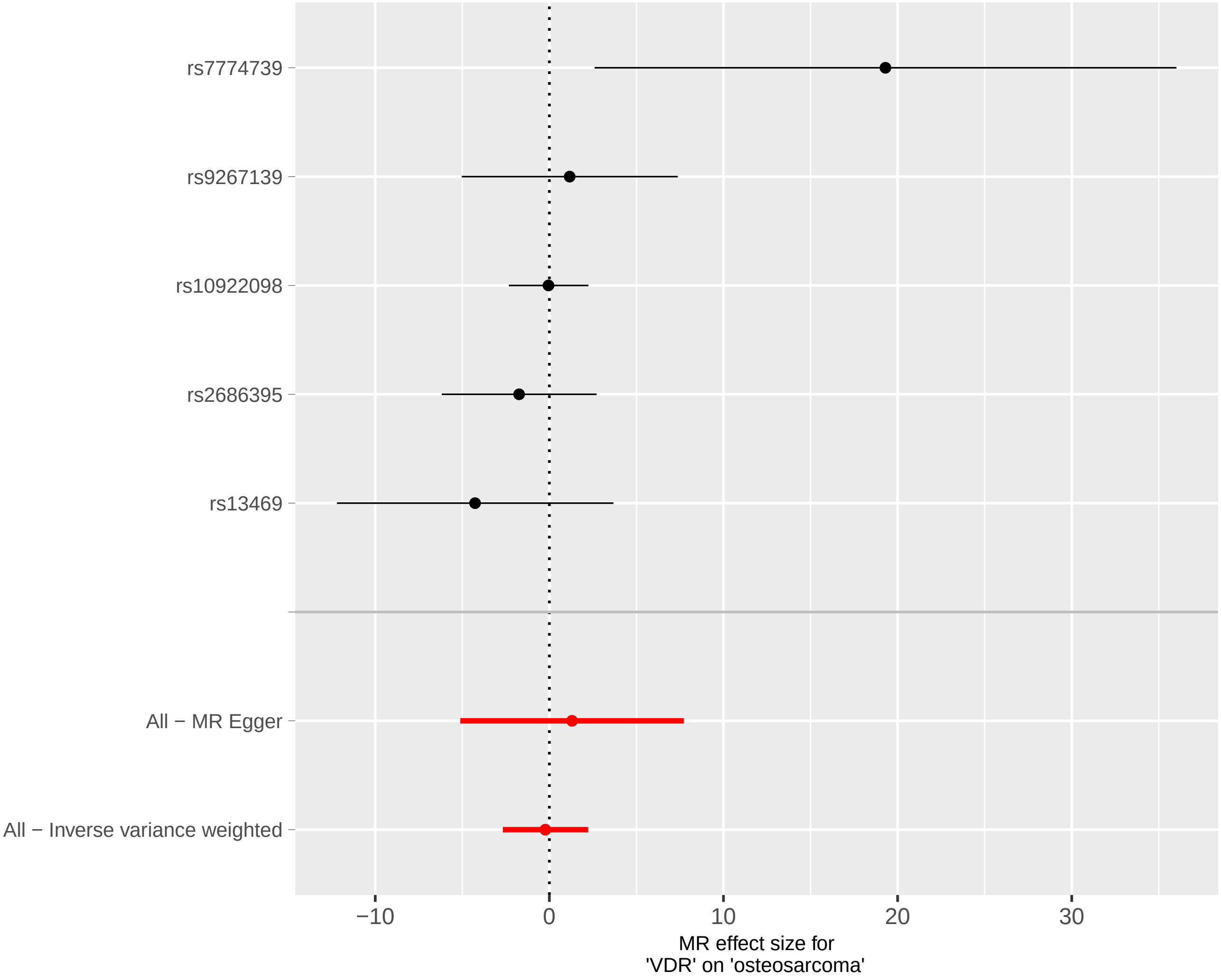
rs7774739
rs9267139
rs10922098
rs2686395
rs13469
All − MR Egger
All − Inverse variance weighted
−10
0
10
20
30
MR effect size for
'VDR' on 'osteosarcoma'

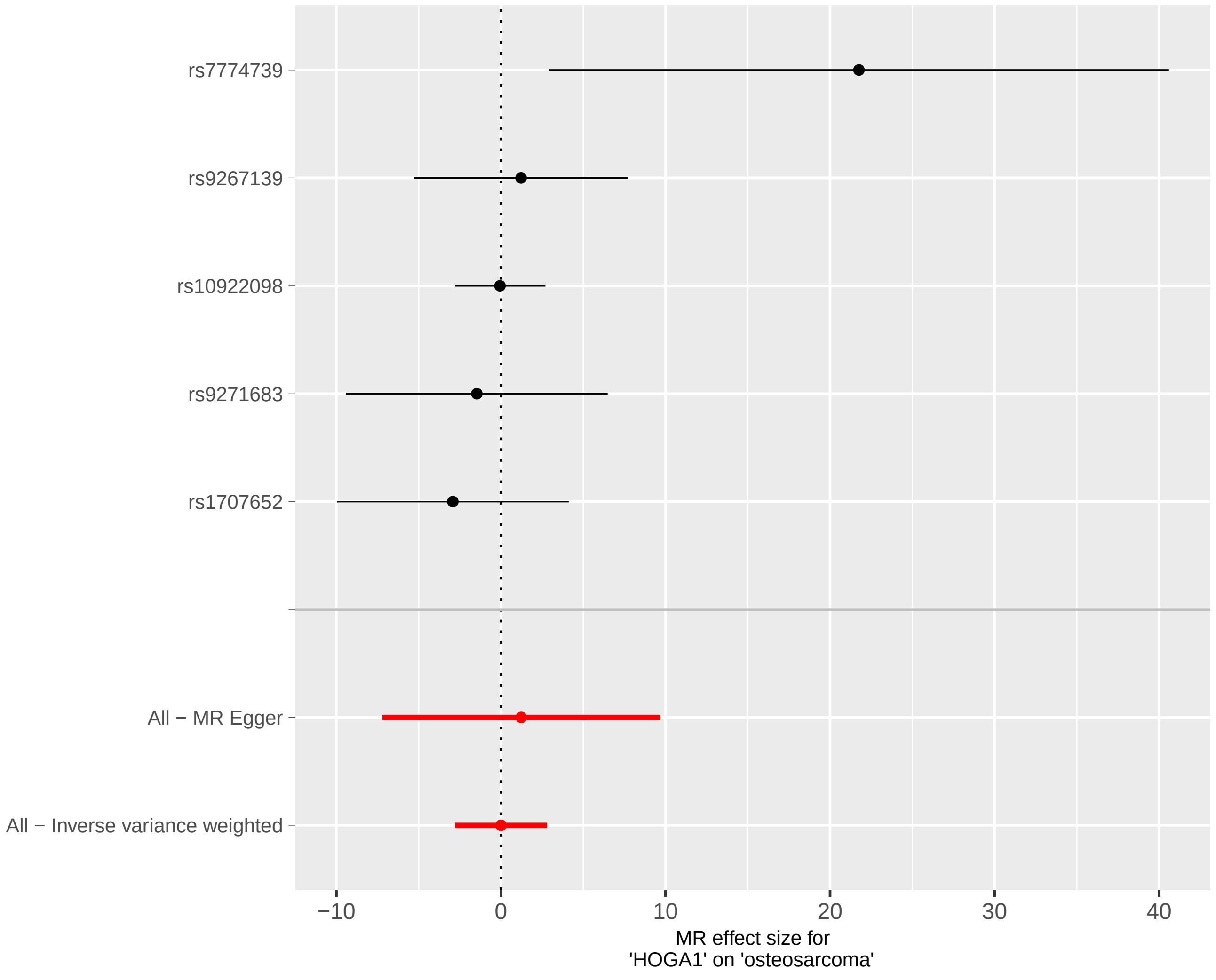

rs7774739
rs9267139
rs10922098
rs9271683
rs1707652
All − MR Egger
All − Inverse variance weighted
−10
0
10
20
30
40
MR effect size for
'HOGA1' on 'osteosarcoma'

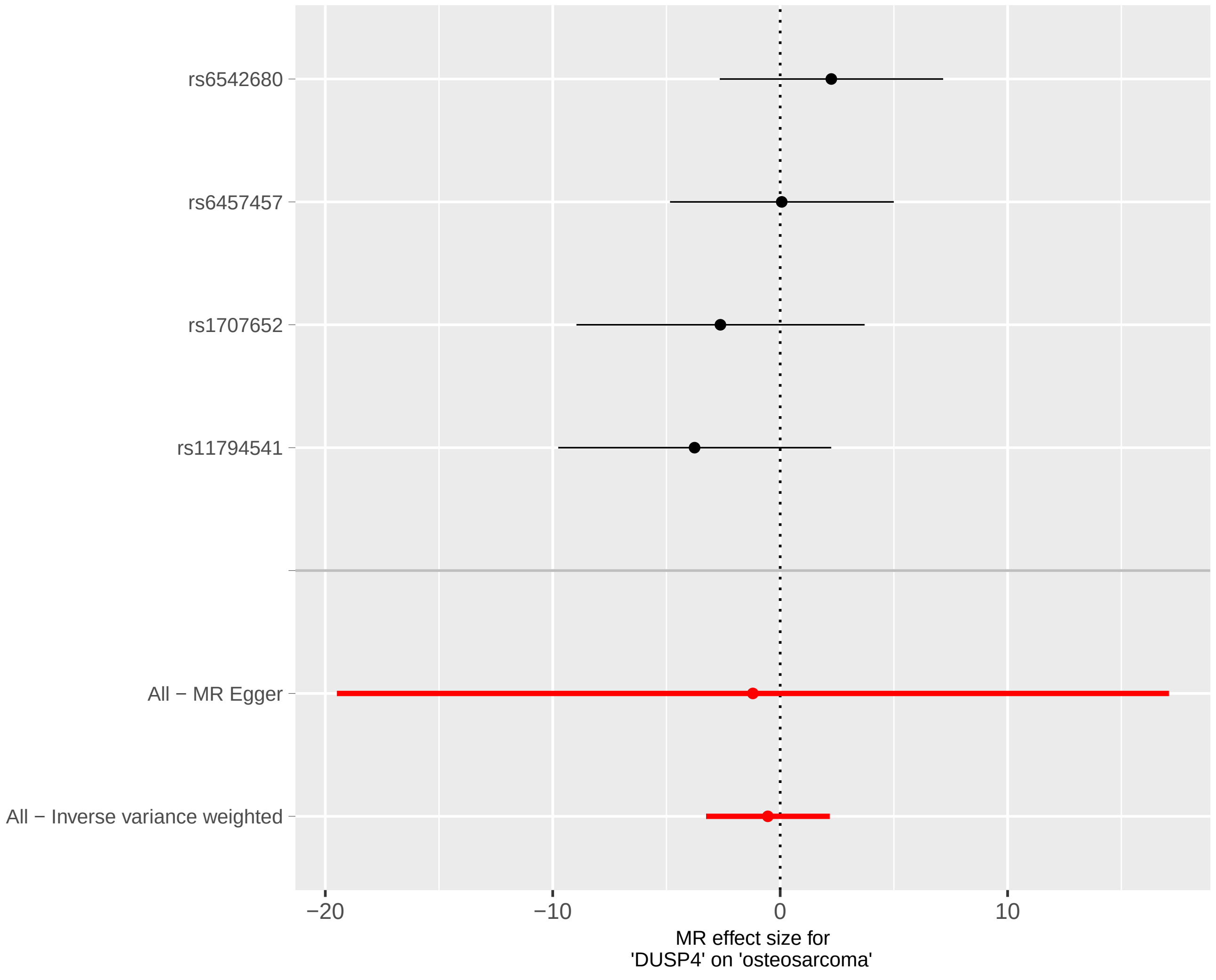
rs6542680
rs6457457
rs1707652
rs11794541
All − MR Egger
All − Inverse variance weighted
−20
−10
0
10
MR effect size for
'DUSP4' on 'osteosarcoma'

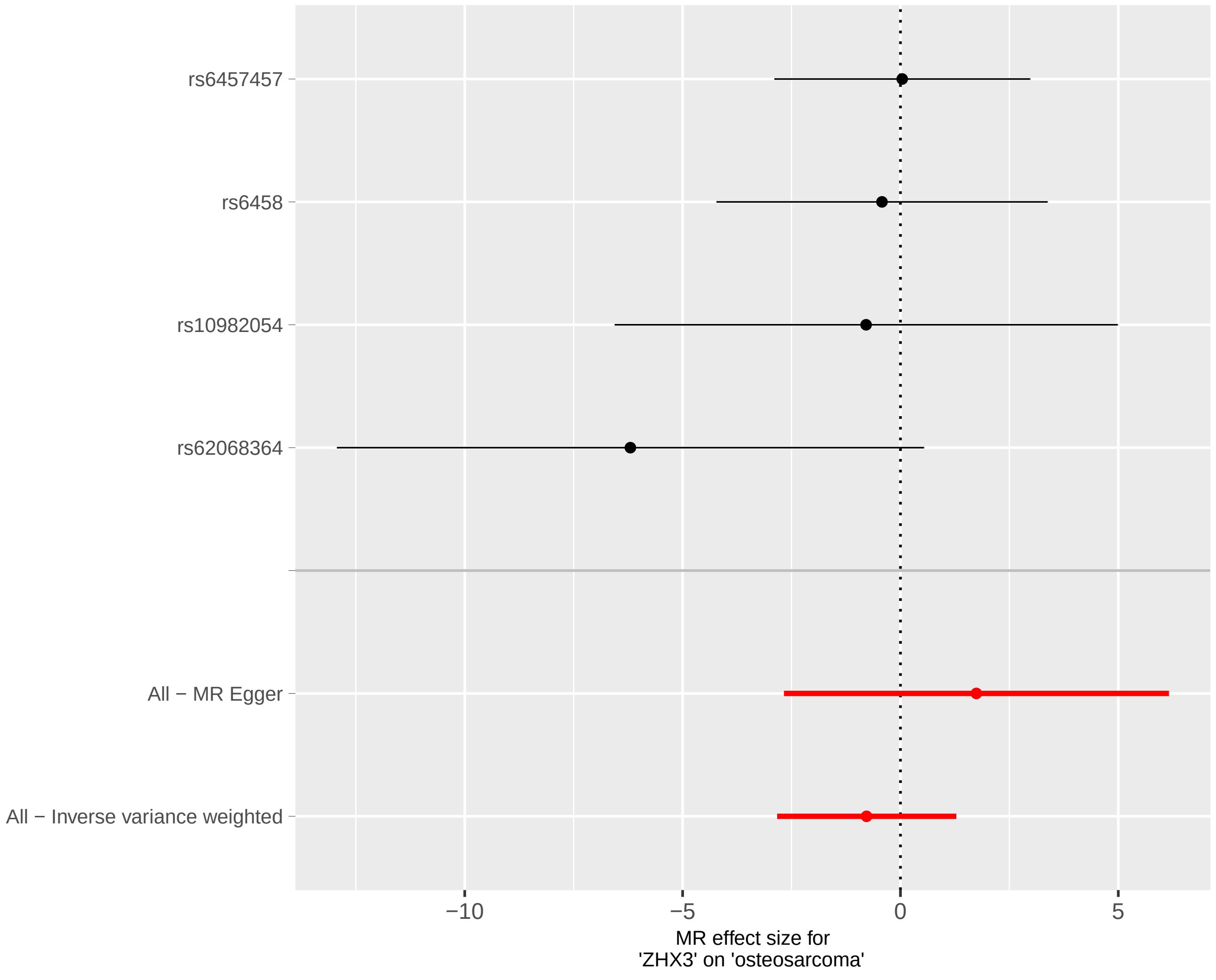

rs6457457
rs6458
rs10982054
rs62068364
All − MR Egger
All − Inverse variance weighted
−10
−5
0
5
MR effect size for
'ZHX3' on 'osteosarcoma'

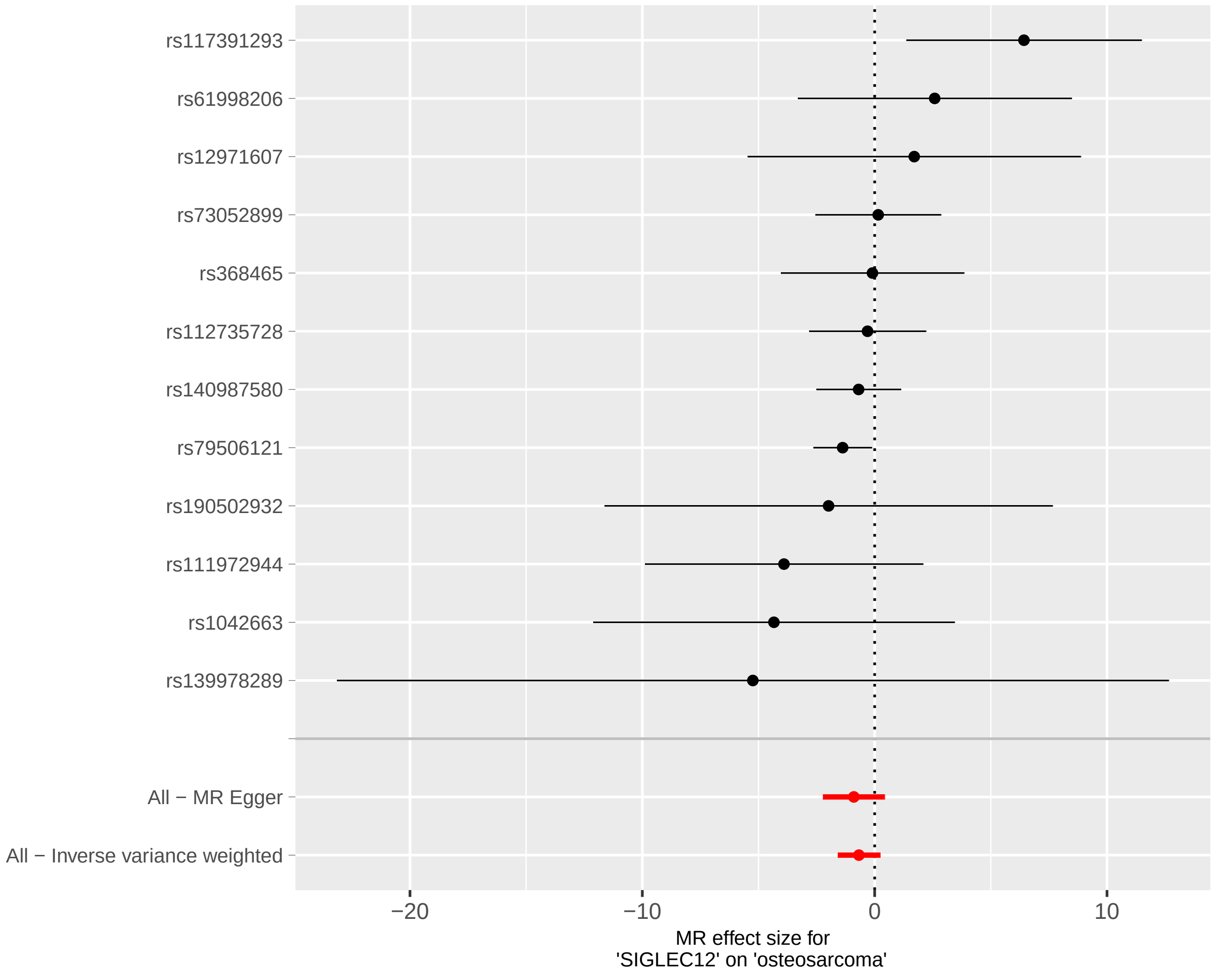
rs117391293
rs61998206
rs12971607
rs73052899
rs368465
rs112735728
rs140987580
rs79506121
rs190502932
rs111972944
rs1042663
rs139978289
All − MR Egger
All − Inverse variance weighted
−20
−10
0
10
MR effect size for
'SIGLEC12' on 'osteosarcoma'

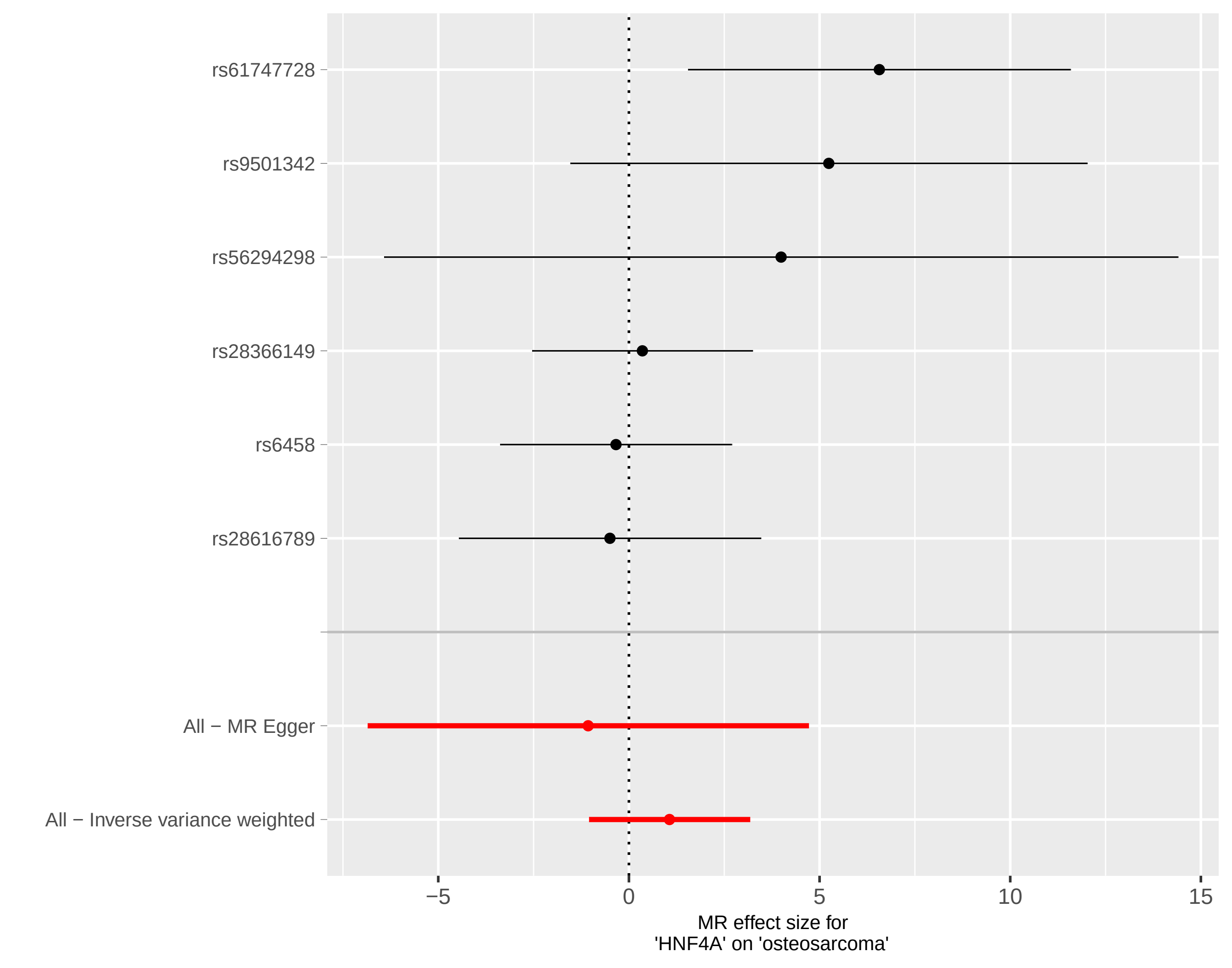
rs61747728
rs9501342
rs56294298
rs28366149
rs6458
rs28616789
All − MR Egger
All − Inverse variance weighted
−5
0
5
10
15
MR effect size for
'HNF4A' on 'osteosarcoma'

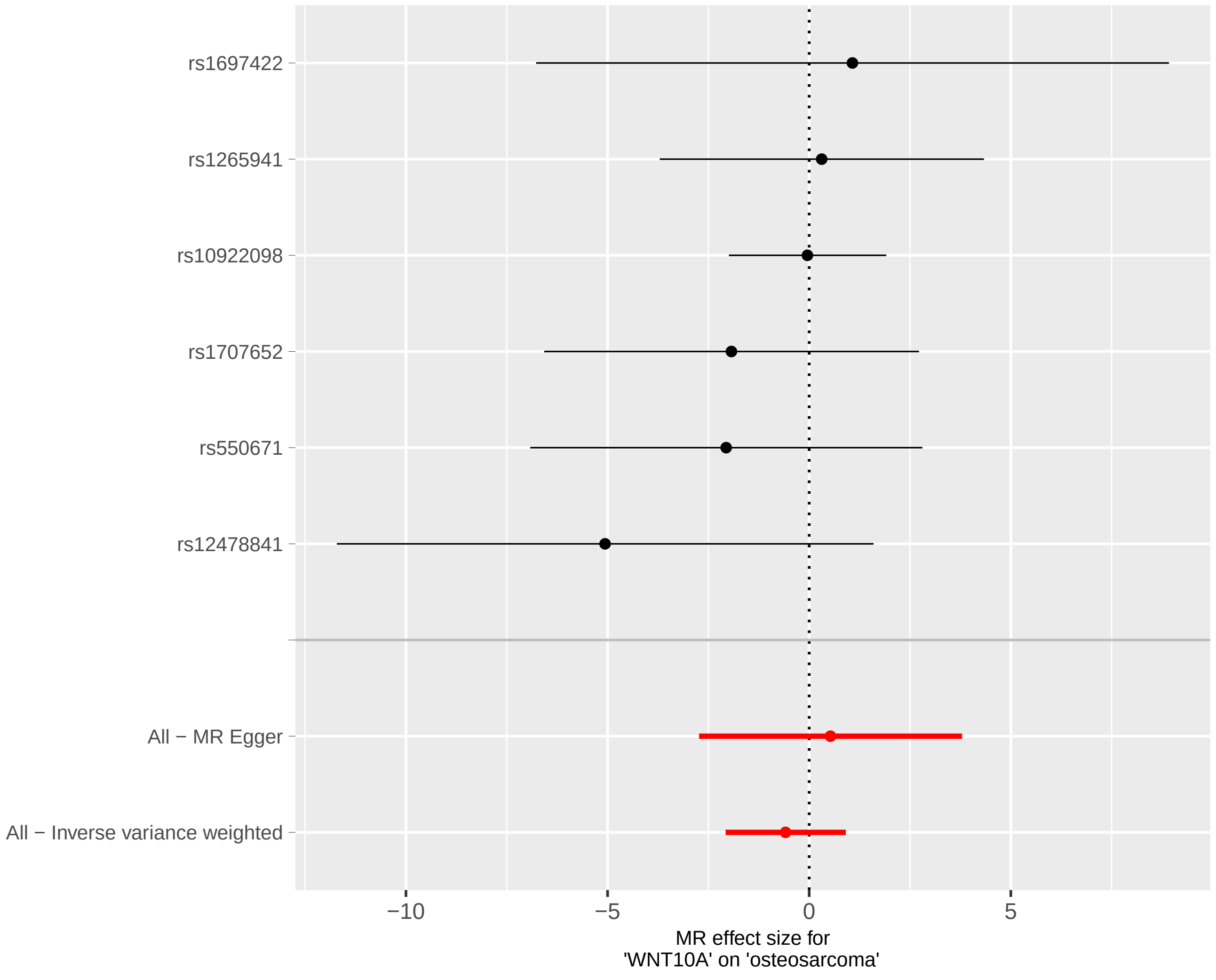

rs1697422
rs1265941
rs10922098
rs1707652
rs550671
rs12478841
All − MR Egger
All − Inverse variance weighted
−10
−5
0
5
MR effect size for
'WNT10A' on 'osteosarcoma'

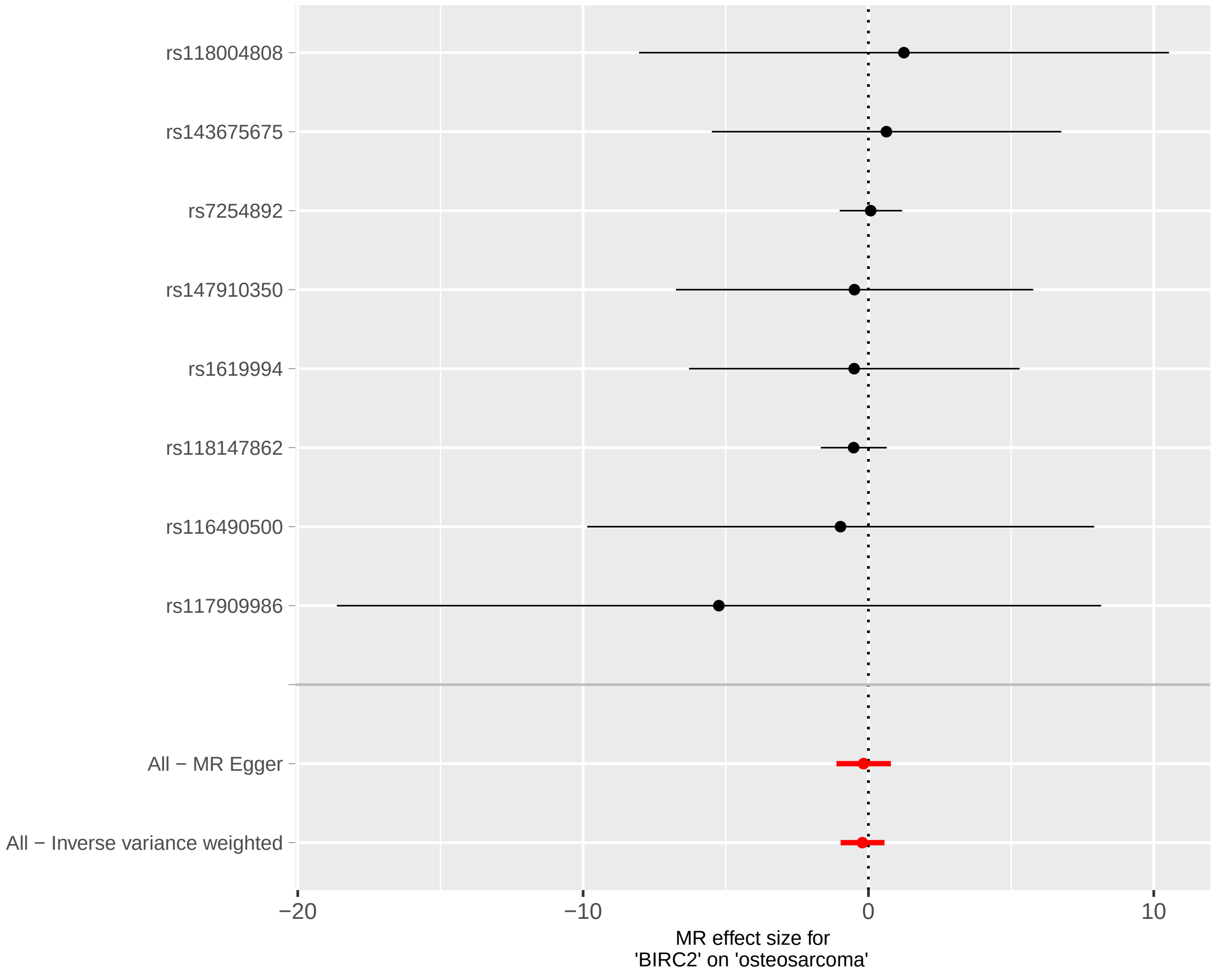
rs118004808
rs143675675
rs7254892
rs147910350
rs1619994
rs118147862
rs116490500
rs117909986
All − MR Egger
All − Inverse variance weighted
−20
−10
0
10
MR effect size for
'BIRC2' on 'osteosarcoma'

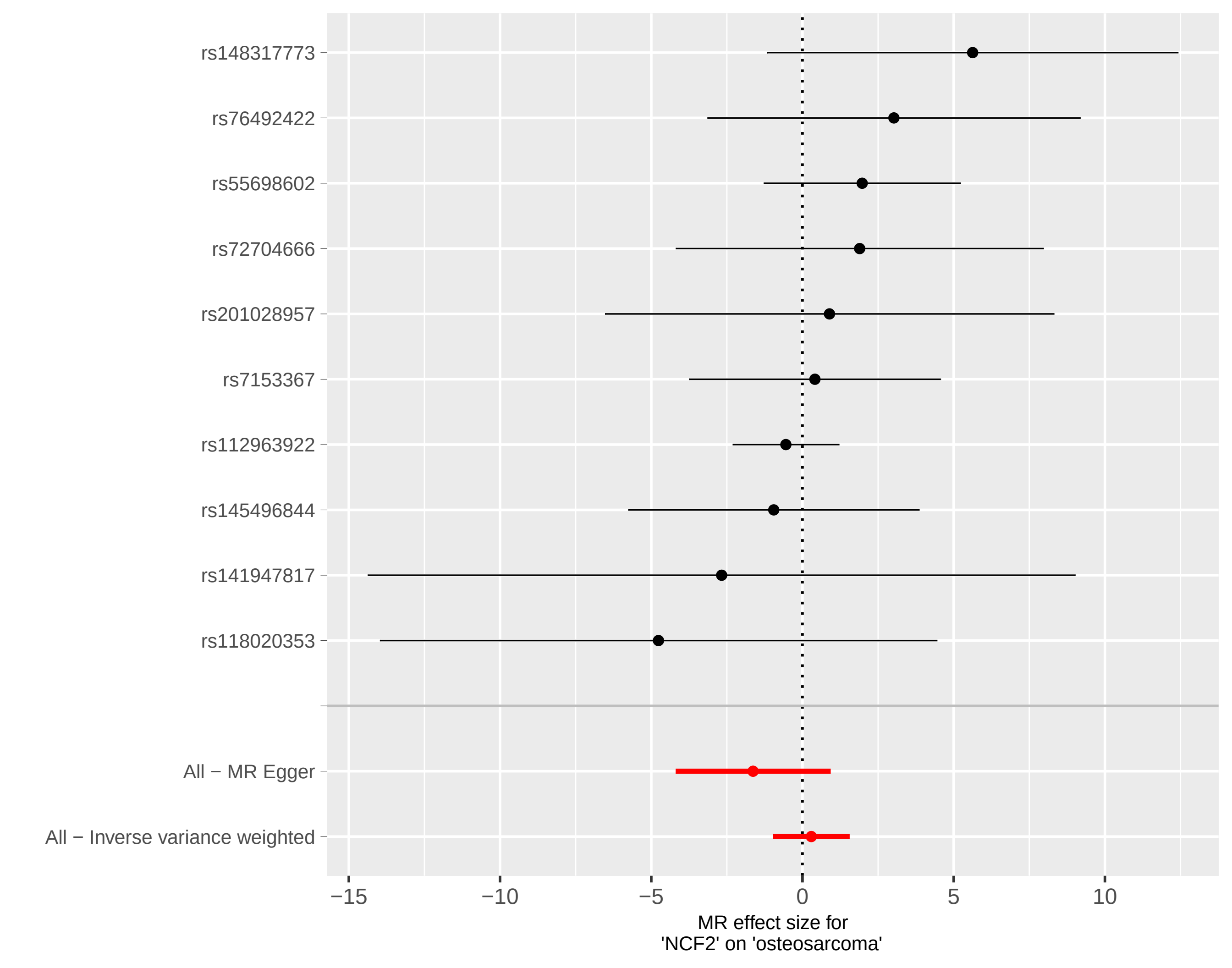

rs148317773
rs76492422
rs55698602
rs72704666
rs201028957
rs7153367
rs112963922
rs145496844
rs141947817
rs118020353
All − MR Egger
All − Inverse variance weighted
−15
−10
−5
0
5
10
MR effect size for
'NCF2' on 'osteosarcoma'

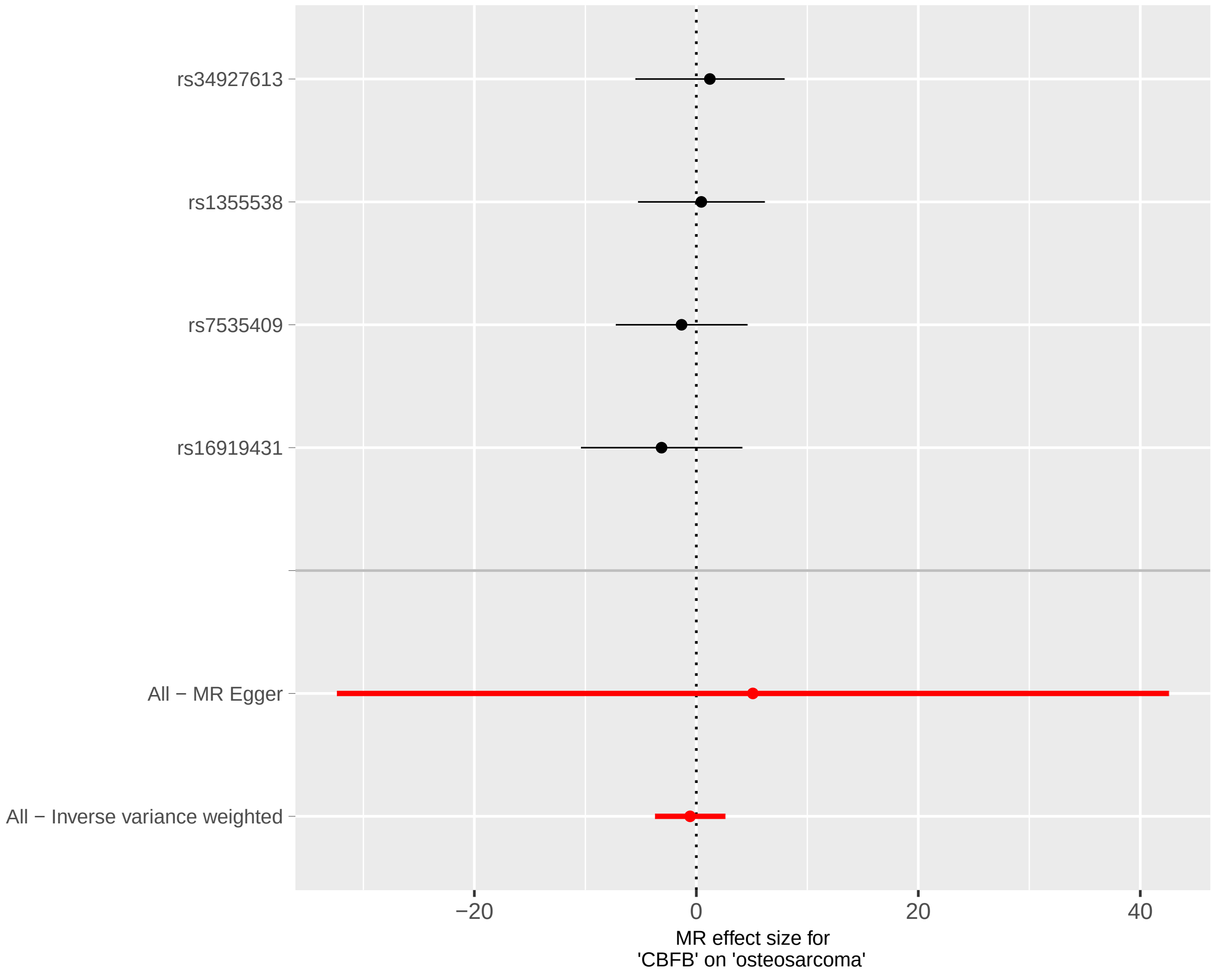
rs34927613
rs1355538
rs7535409
rs16919431
All − MR Egger
All − Inverse variance weighted
−20
0
20
40
MR effect size for
'CBFB' on 'osteosarcoma'

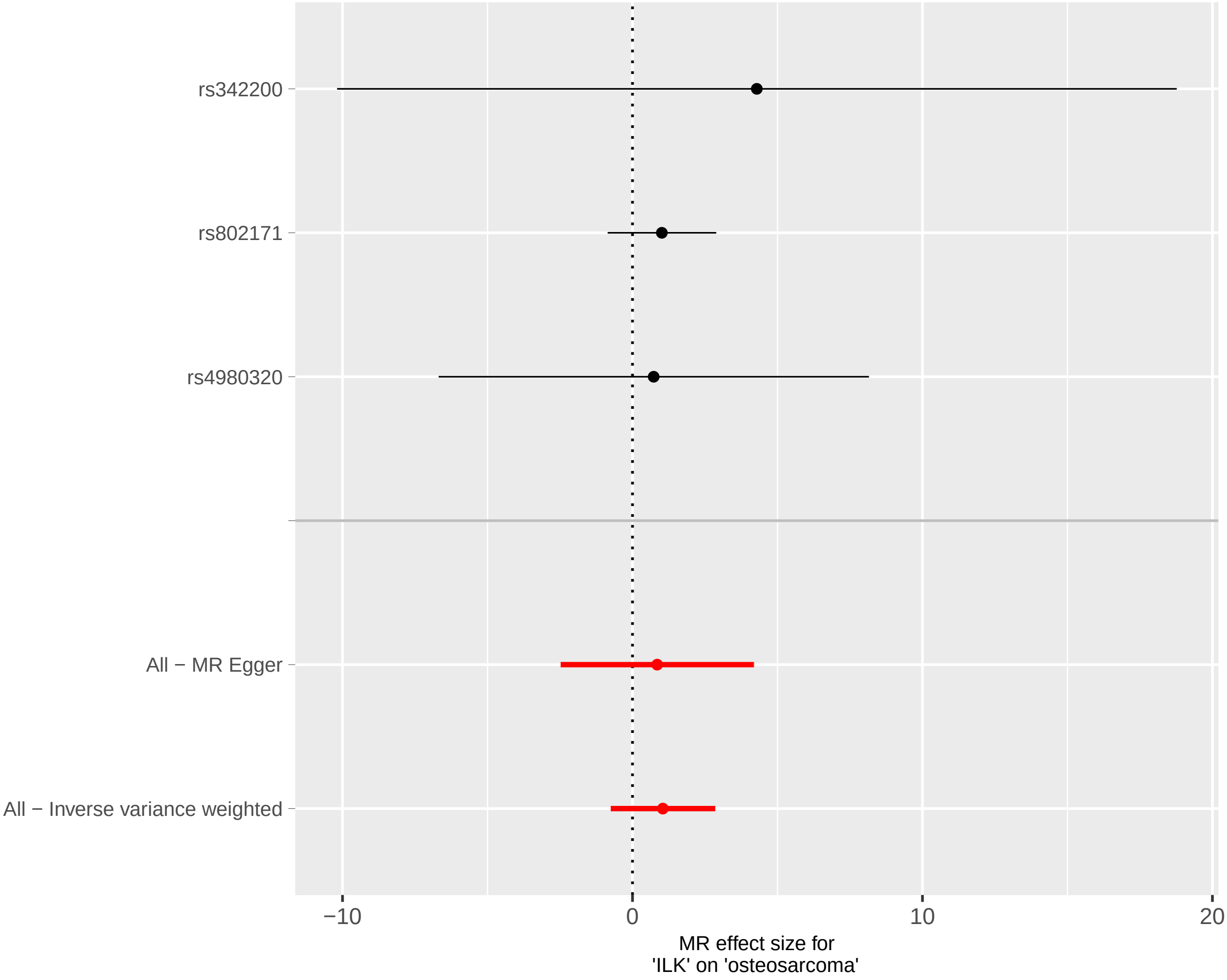

rs342200
rs802171
rs4980320
All − MR Egger
All − Inverse variance weighted
−10
0
10
20
MR effect size for
'ILK' on 'osteosarcoma'

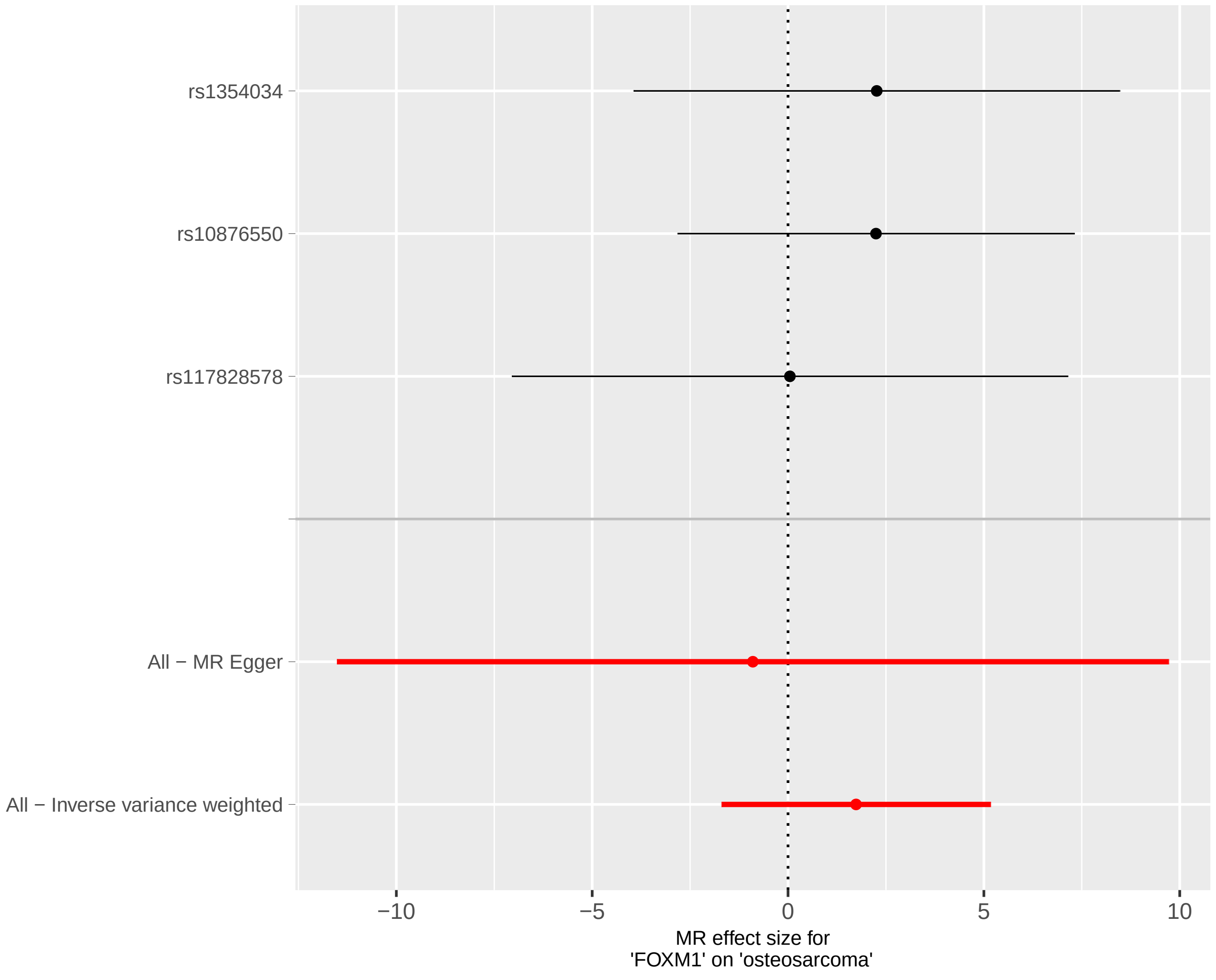

rs1354034
rs10876550
rs117828578
All − MR Egger
All − Inverse variance weighted
−10
−5
0
5
10
MR effect size for
'FOXM1' on 'osteosarcoma'

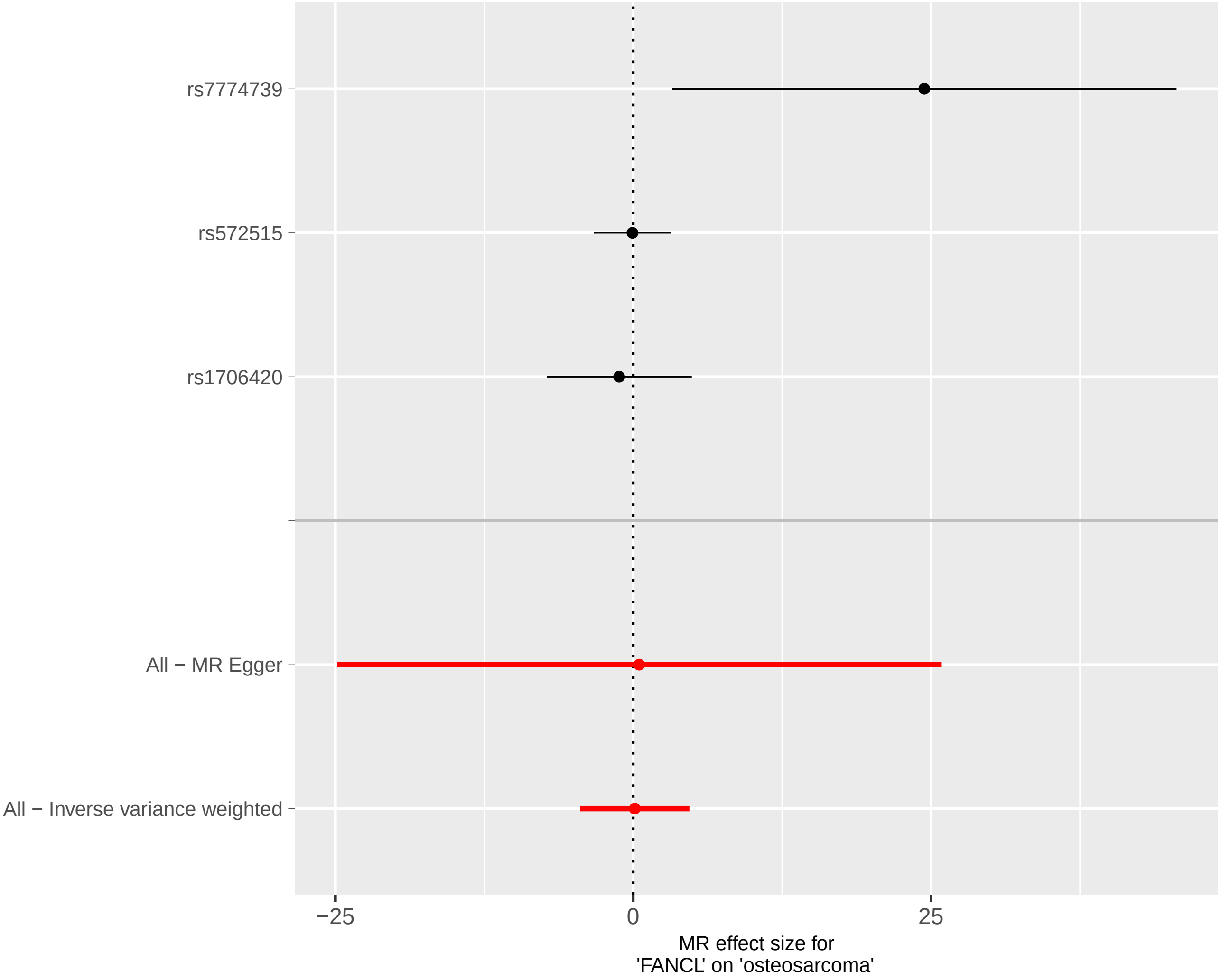
rs7774739
rs572515
rs1706420
All − MR Egger
All − Inverse variance weighted
−25
0
25
MR effect size for
'FANCL' on 'osteosarcoma'

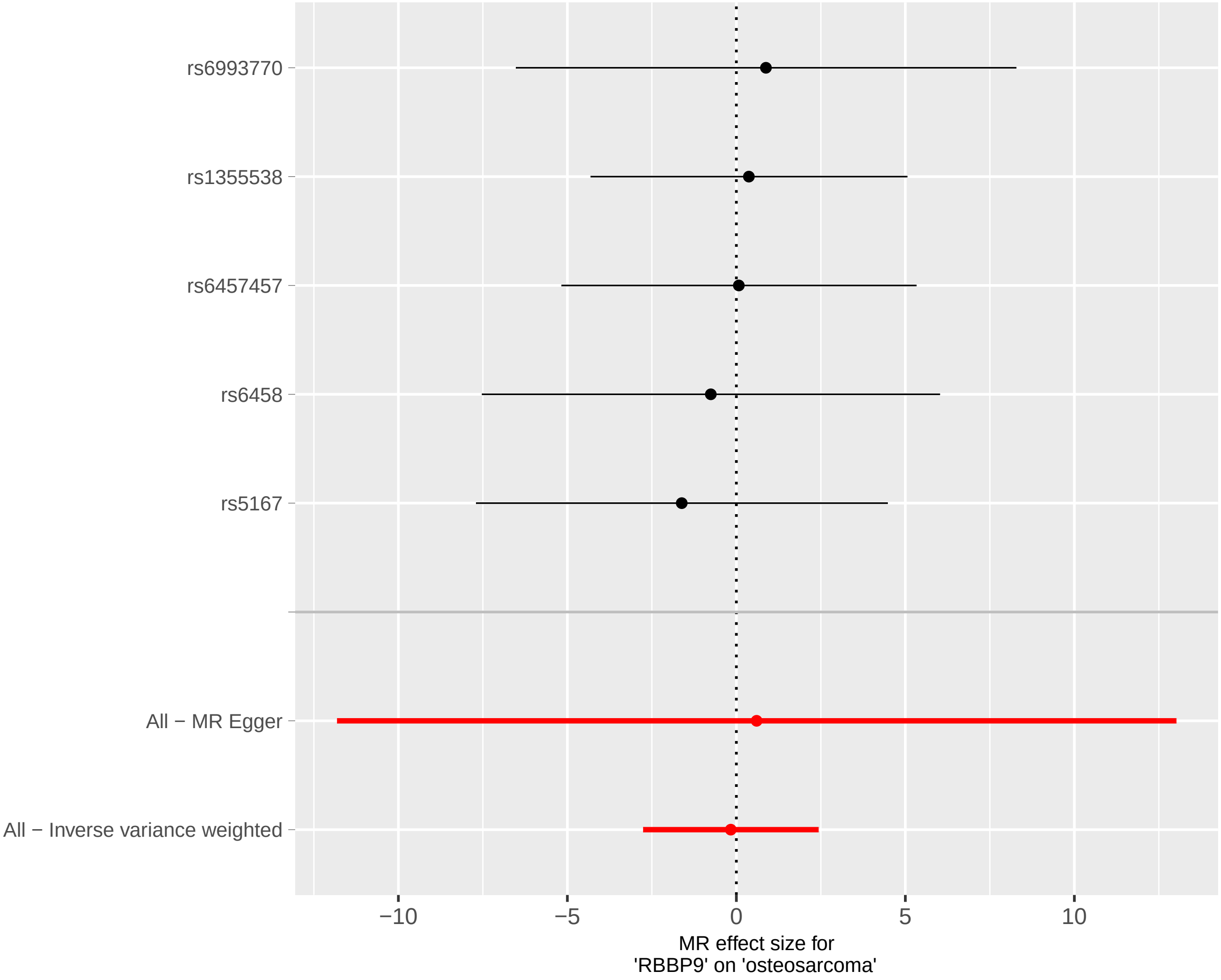

rs6993770
rs1355538
rs6457457
rs6458
rs5167
All − MR Egger
All − Inverse variance weighted
−10
−5
0
5
10
MR effect size for
'RBBP9' on 'osteosarcoma'

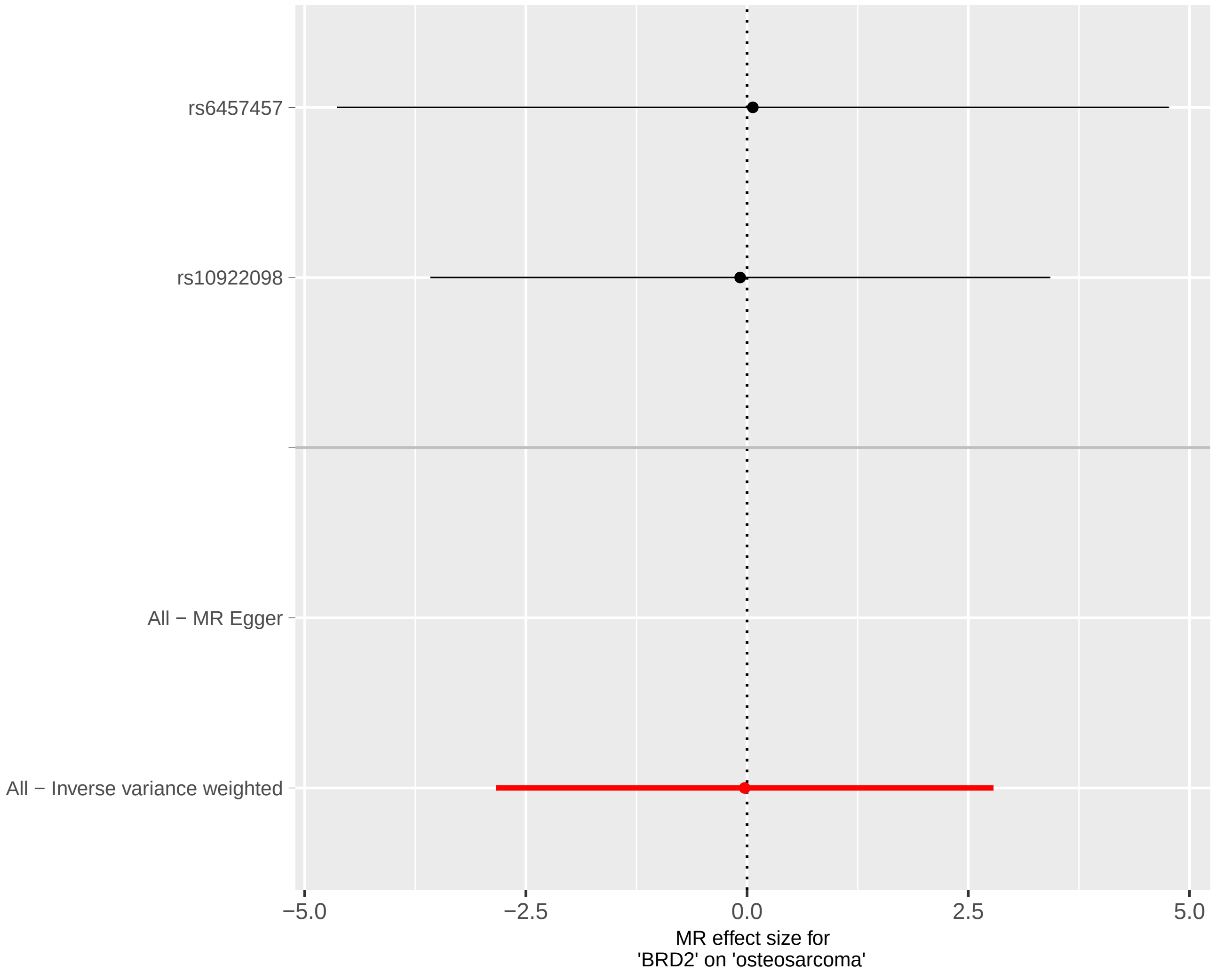
rs6457457
rs10922098
All − MR Egger
All − Inverse variance weighted
−5.0
−2.5
0.0
2.5
5.0
MR effect size for
'BRD2' on 'osteosarcoma'

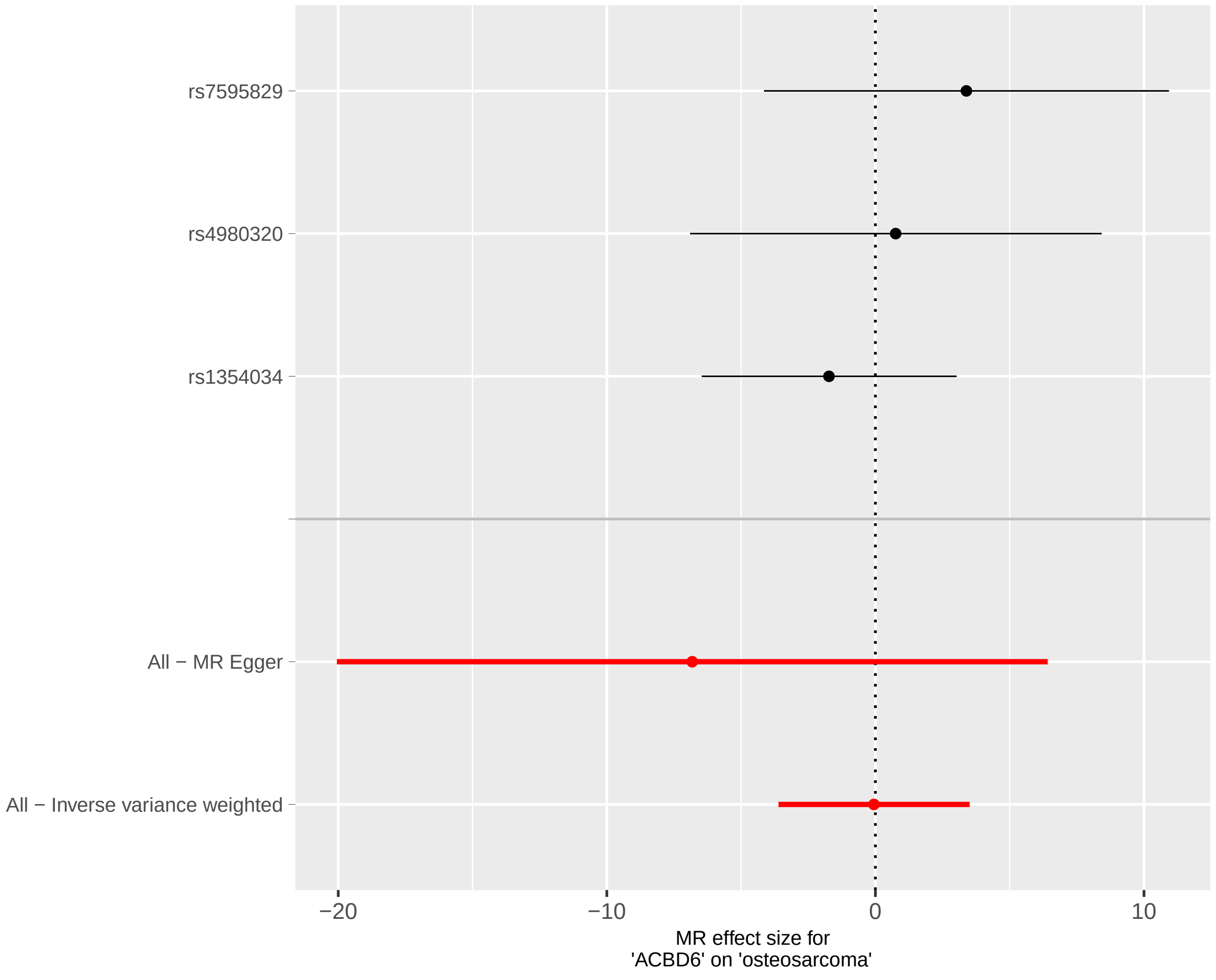
rs7595829
rs4980320
rs1354034
All − MR Egger
All − Inverse variance weighted
−20
−10
0
10
MR effect size for
'ACBD6' on 'osteosarcoma'

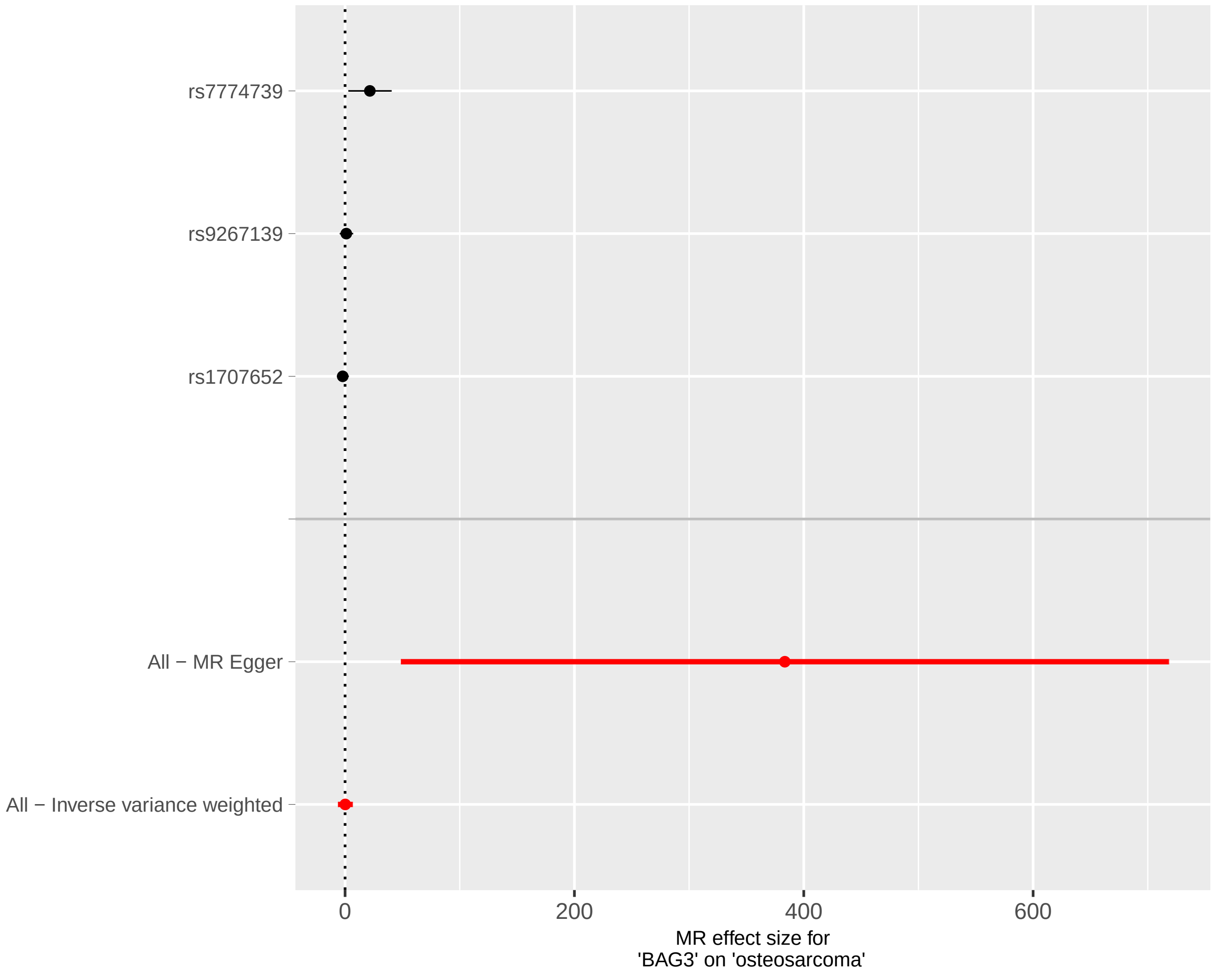

rs7774739
rs9267139
rs1707652
All − MR Egger
All − Inverse variance weighted
0
200
400
600
MR effect size for
'BAG3' on 'osteosarcoma'

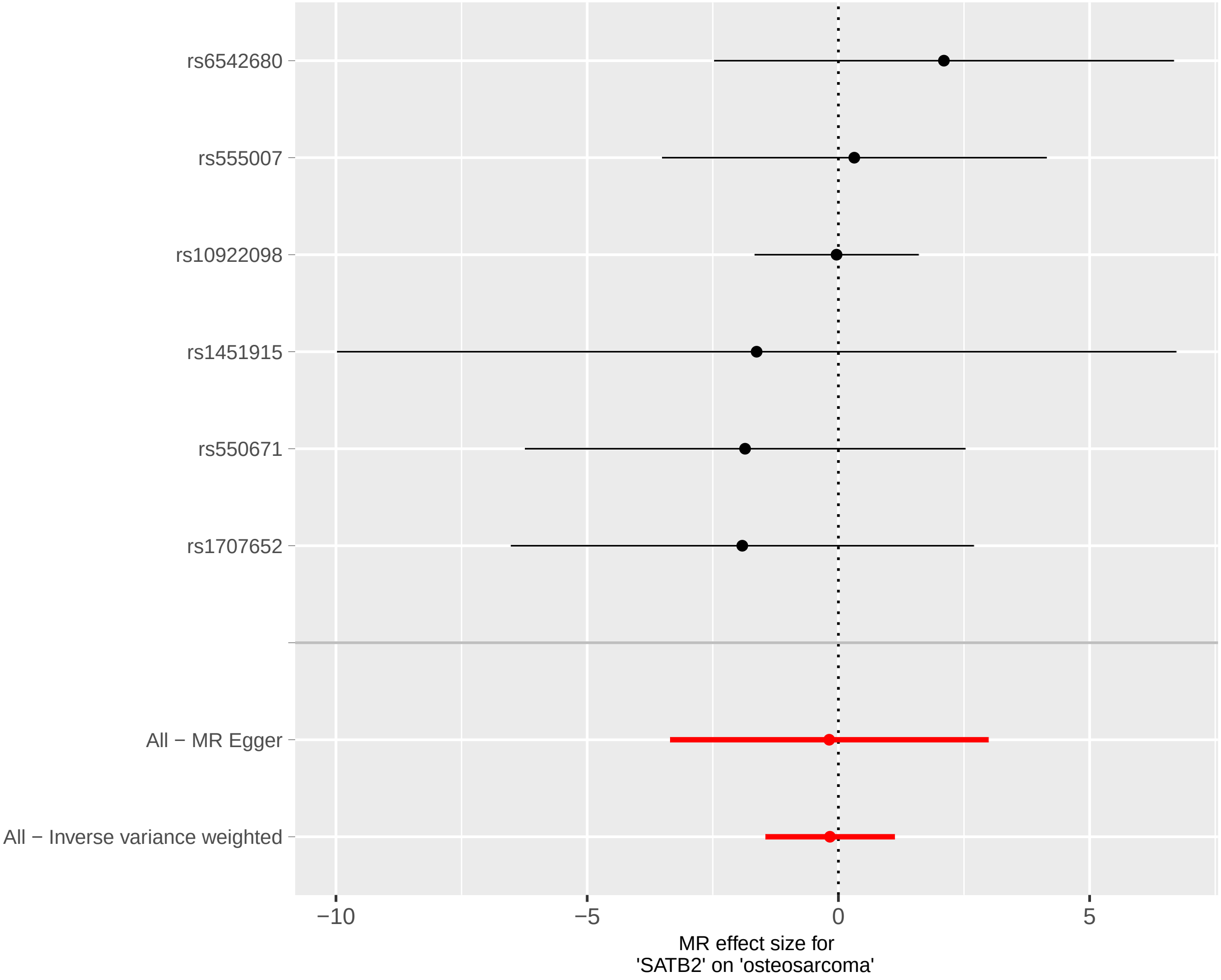

rs6542680
rs555007
rs10922098
rs1451915
rs550671
rs1707652
All − MR Egger
All − Inverse variance weighted
−10
−5
0
5
MR effect size for
'SATB2' on 'osteosarcoma'

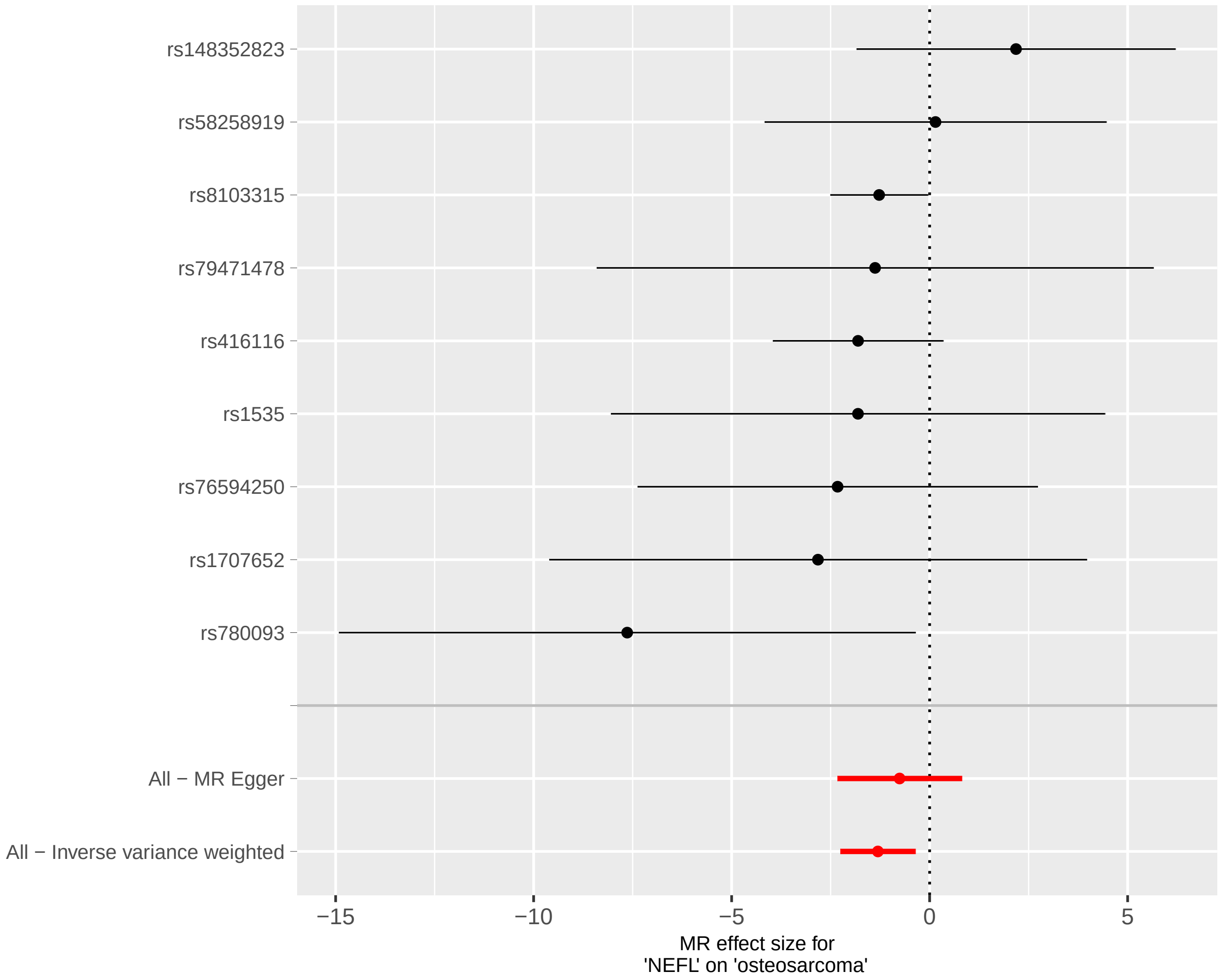
rs148352823
rs58258919
rs8103315
rs79471478
rs416116
rs1535
rs76594250
rs1707652
rs780093
All − MR Egger
All − Inverse variance weighted
−15
−10
−5
0
5
MR effect size for
'NEFL' on 'osteosarcoma'

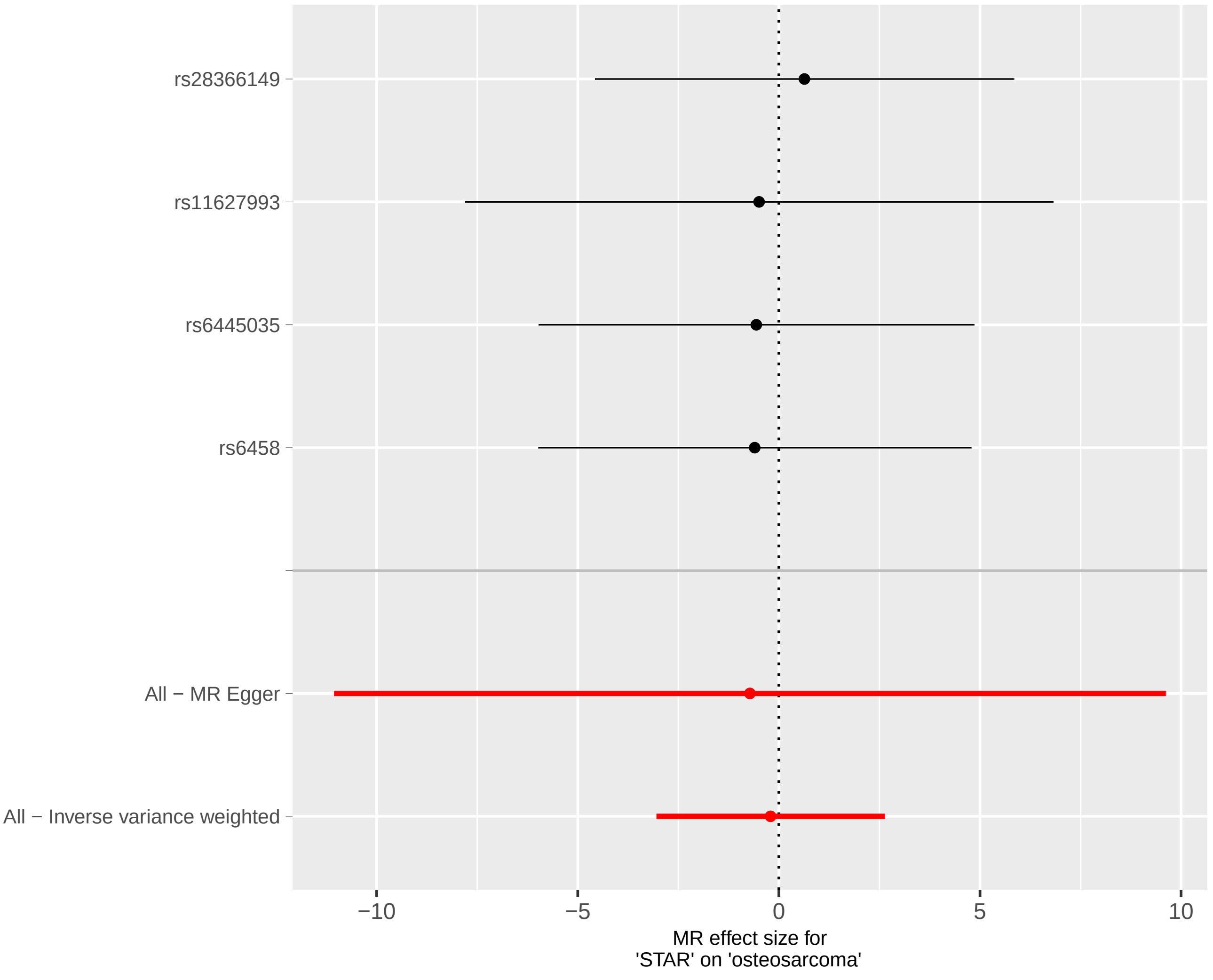
rs28366149
rs11627993
rs6445035
rs6458
All − MR Egger
All − Inverse variance weighted
−10
−5
0
5
10
MR effect size for
'STAR' on 'osteosarcoma'

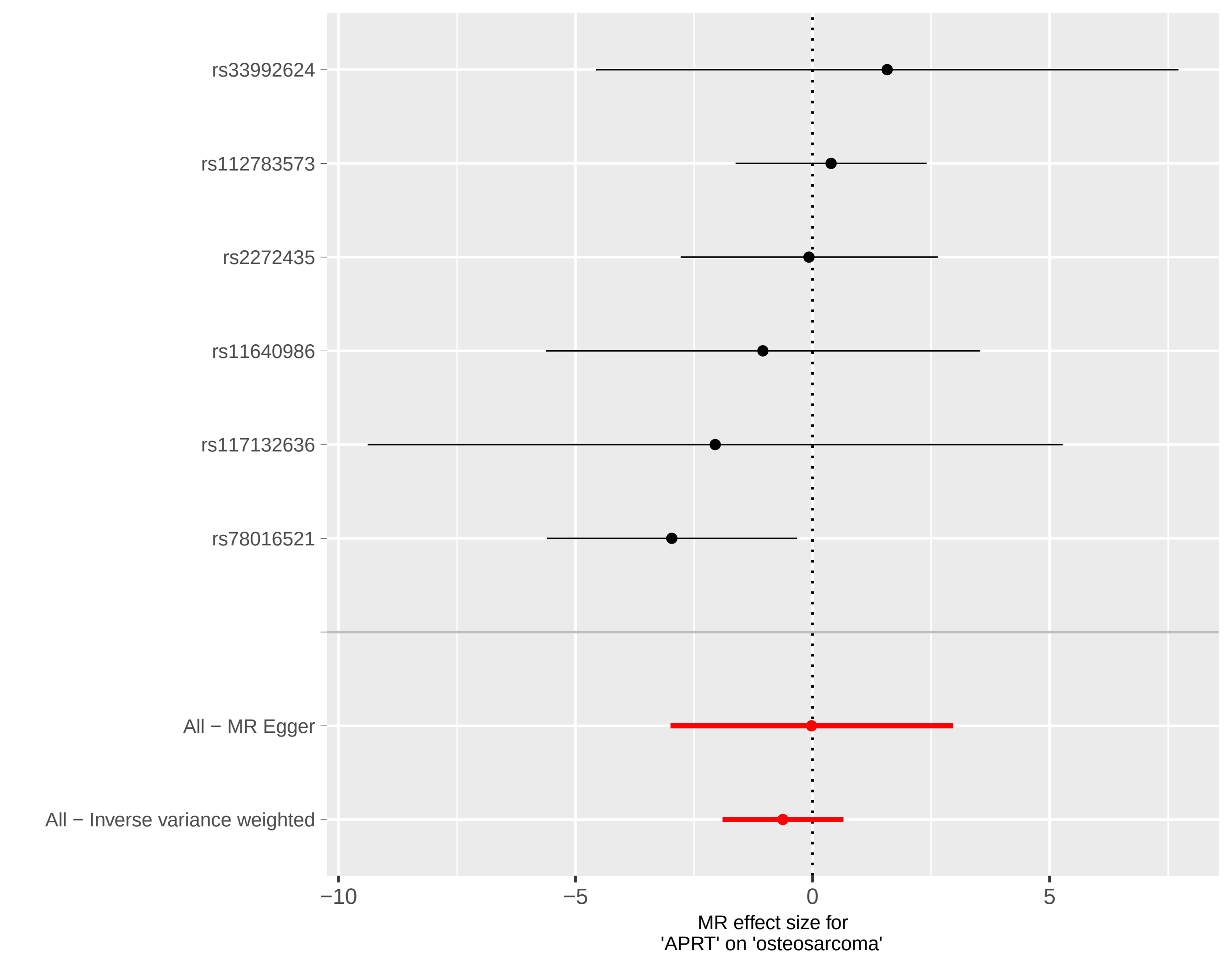
rs33992624
rs112783573
rs2272435
rs11640986
rs117132636
rs78016521
All − MR Egger
All − Inverse variance weighted
−10
−5
0
5
MR effect size for
'APRT' on 'osteosarcoma'

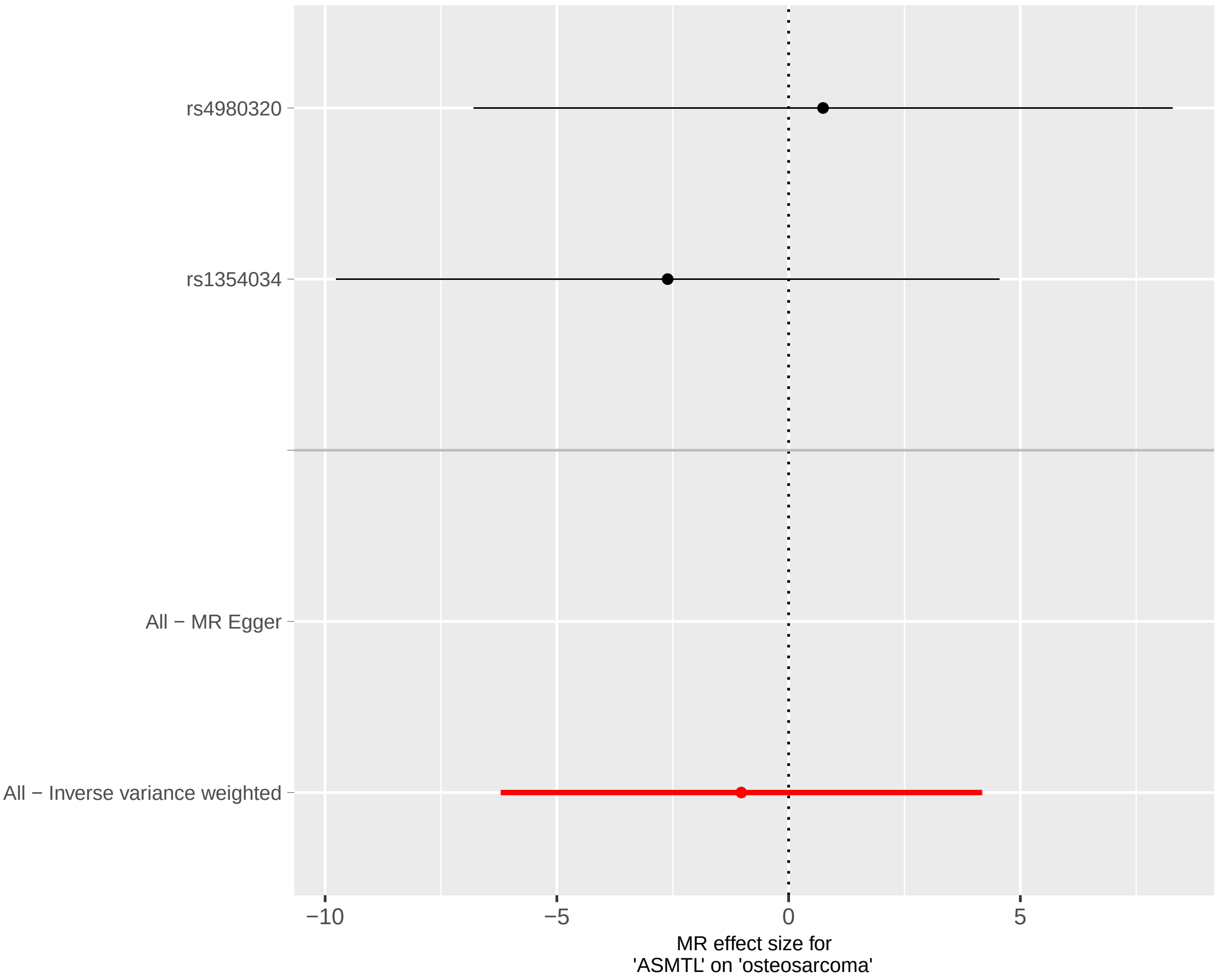
rs4980320
rs1354034
All − MR Egger
All − Inverse variance weighted
−10
−5
0
5
MR effect size for
'ASMTL' on 'osteosarcoma'

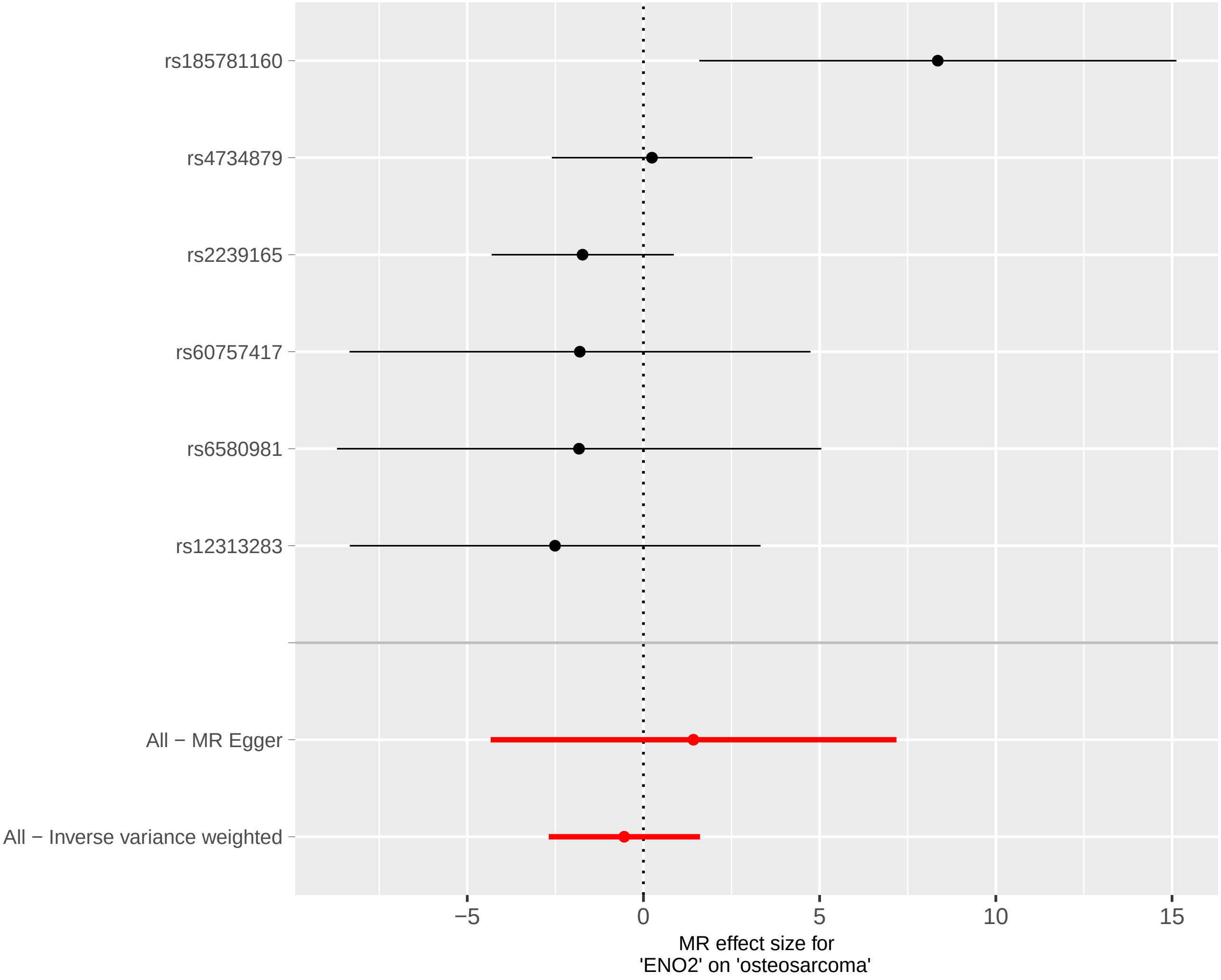
rs185781160
rs4734879
rs2239165
rs60757417
rs6580981
rs12313283
All − MR Egger
All − Inverse variance weighted
−5
0
5
10
15
MR effect size for
'ENO2' on 'osteosarcoma'

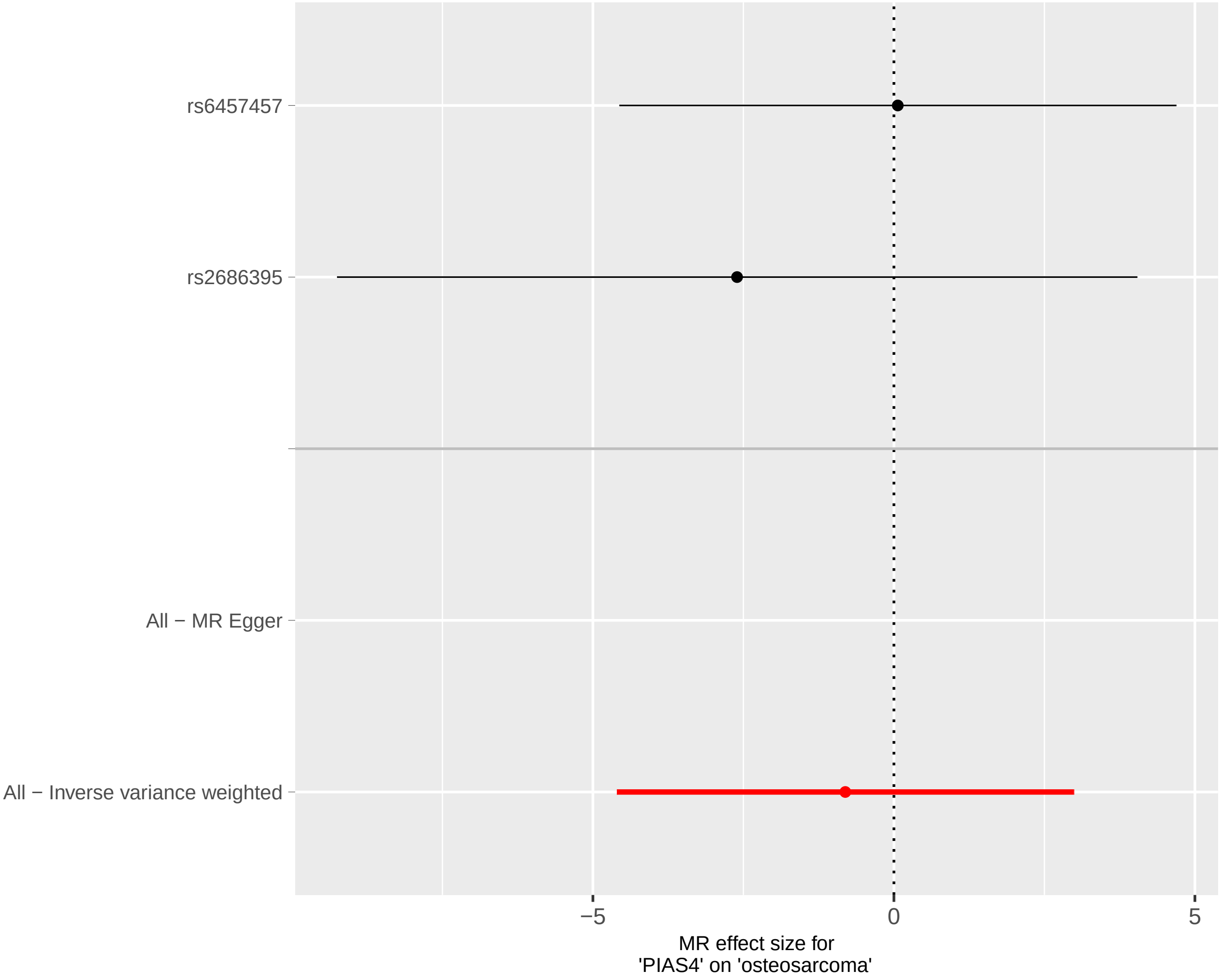
rs6457457
rs2686395
All − MR Egger
All − Inverse variance weighted
−5
0
5
MR effect size for
'PIAS4' on 'osteosarcoma'

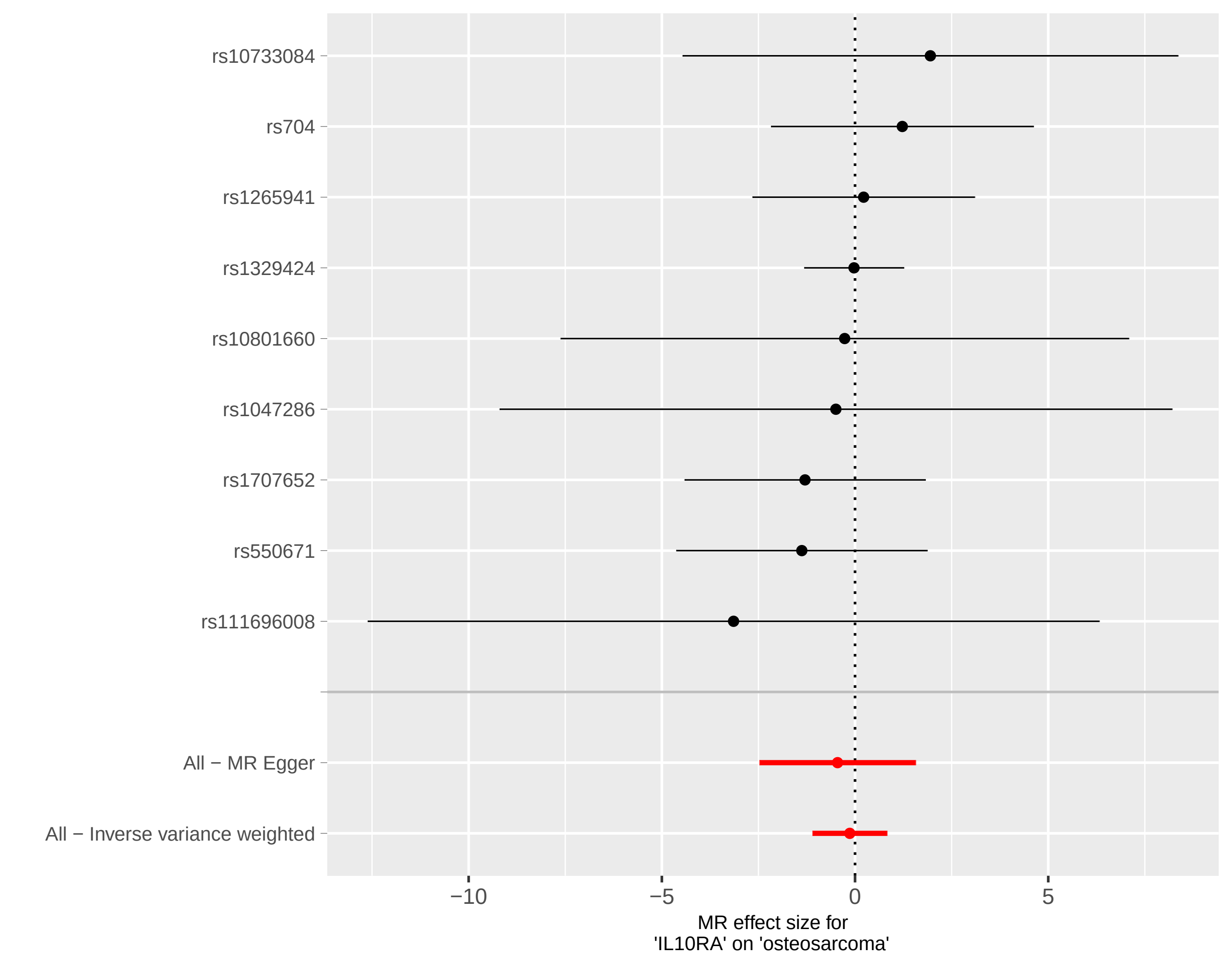
rs10733084
rs704
rs1265941
rs1329424
rs10801660
rs1047286
rs1707652
rs550671
rs111696008
All − MR Egger
All − Inverse variance weighted
−10
−5
0
5
MR effect size for
'IL10RA' on 'osteosarcoma'

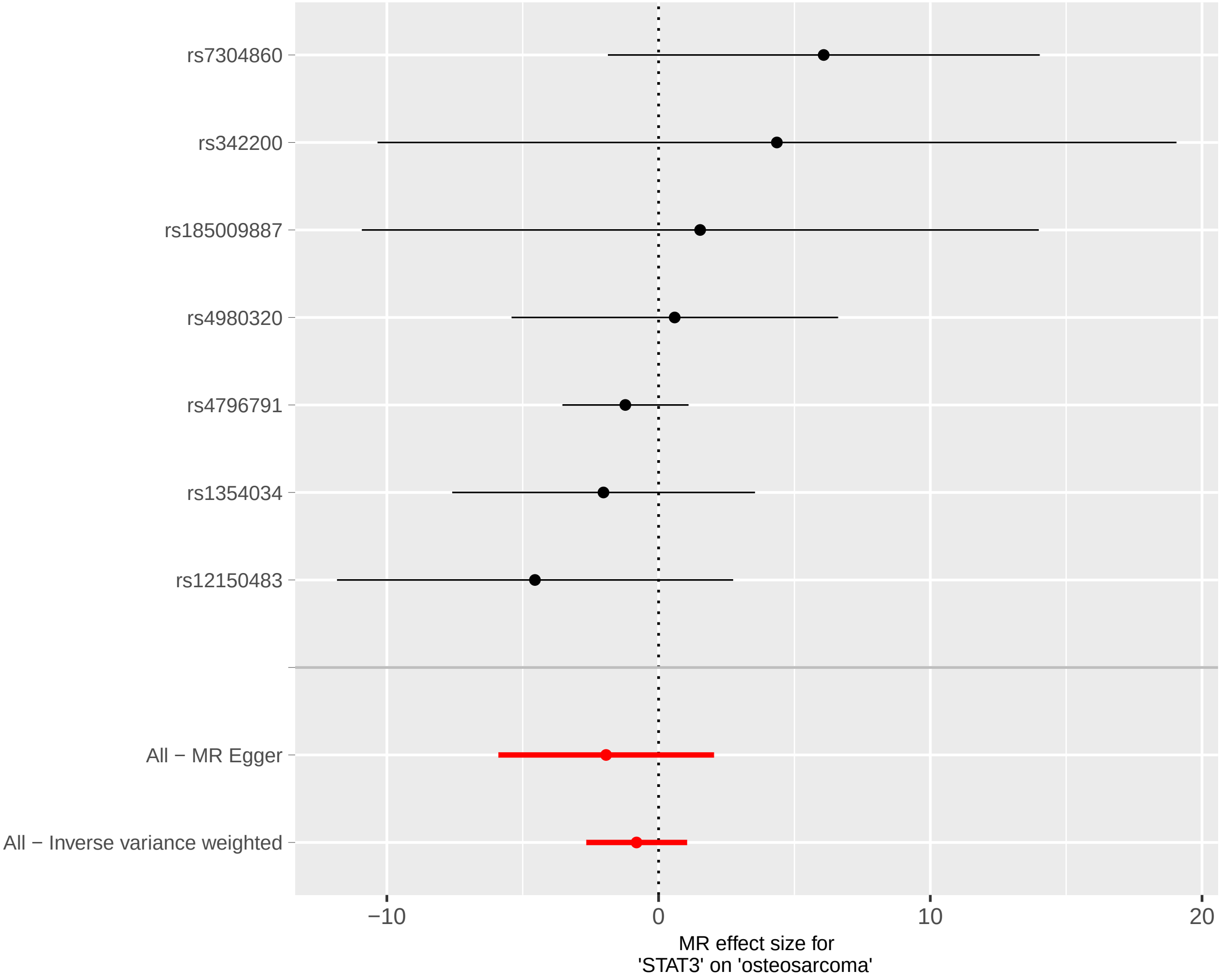

rs7304860
rs342200
rs185009887
rs4980320
rs4796791
rs1354034
rs12150483
All − MR Egger
All − Inverse variance weighted
−10
0
10
20
MR effect size for
'STAT3' on 'osteosarcoma'

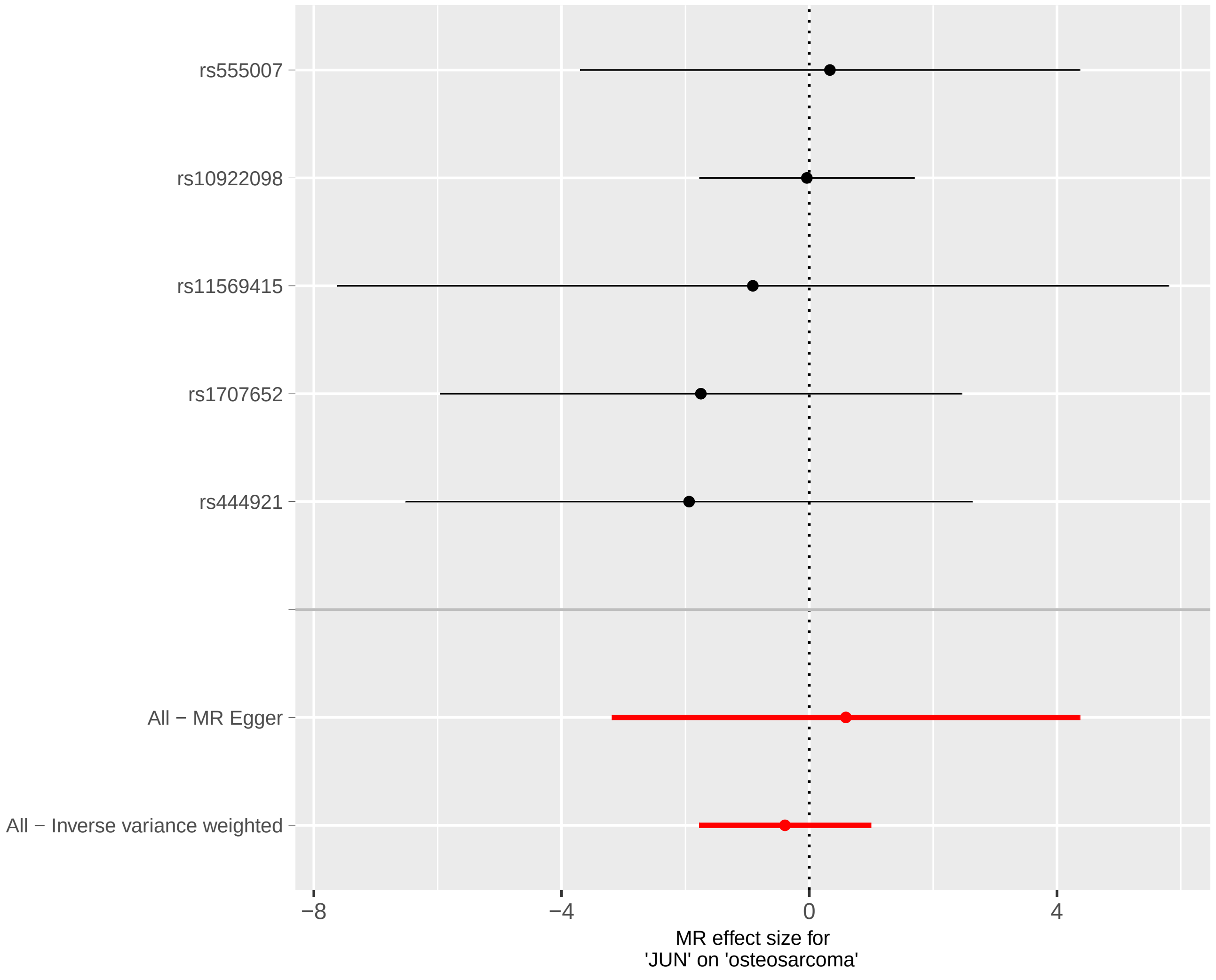

rs555007
rs10922098
rs11569415
rs1707652
rs444921
All − MR Egger
All − Inverse variance weighted
−8
−4
0
4
MR effect size for
'JUN' on 'osteosarcoma'

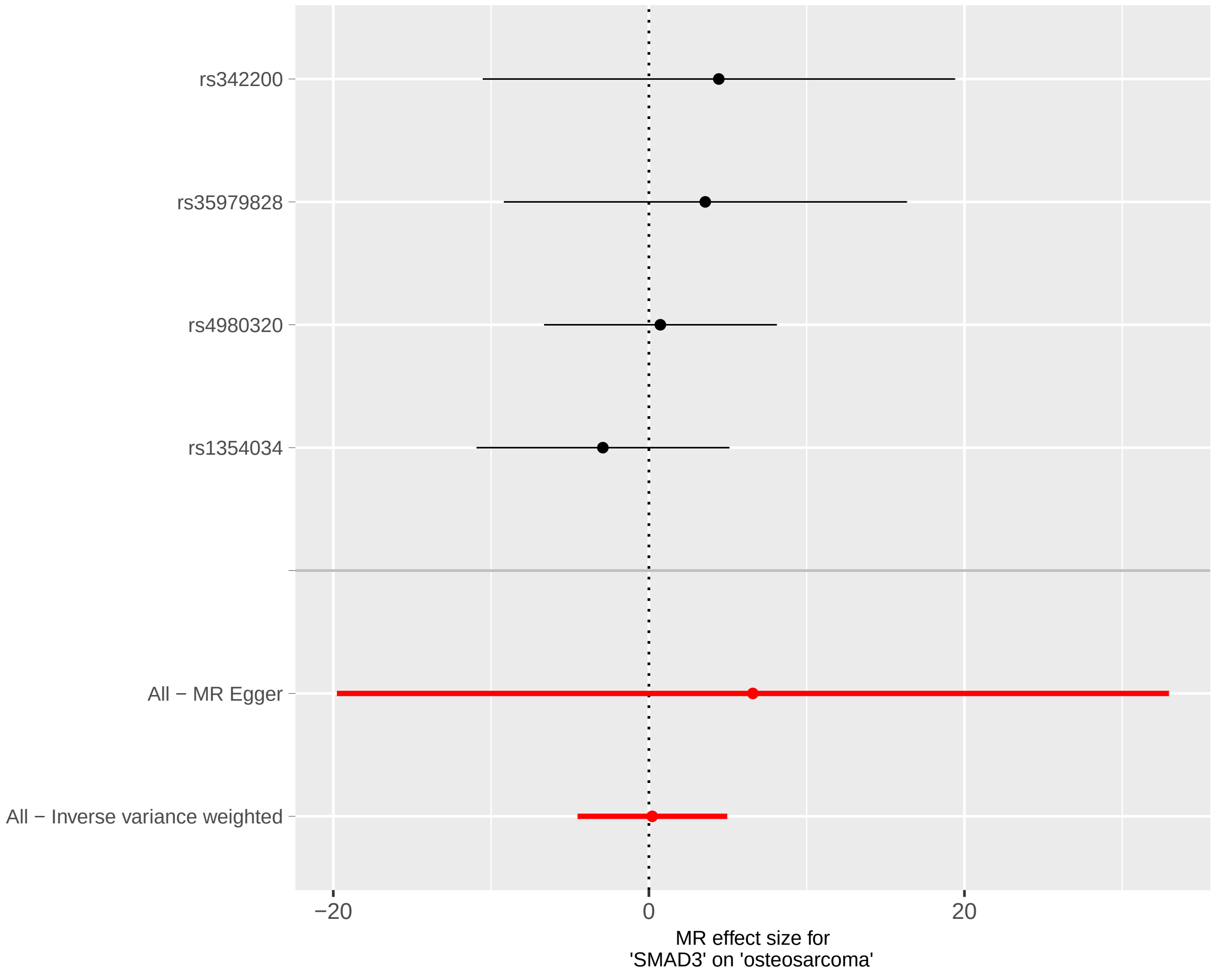
rs342200
rs35979828
rs4980320
rs1354034
All − MR Egger
All − Inverse variance weighted
−20
0
20
MR effect size for
'SMAD3' on 'osteosarcoma'

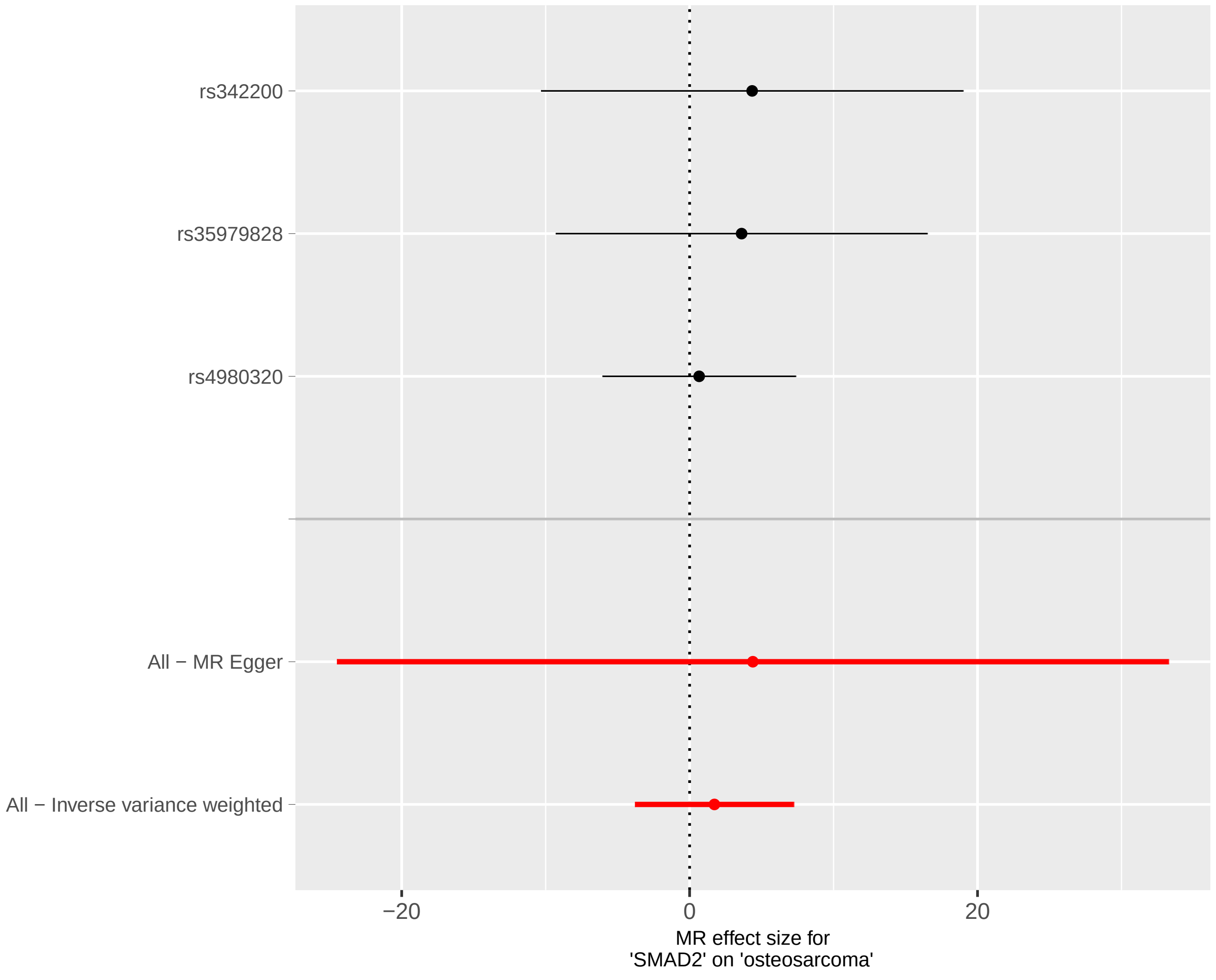

rs342200
rs35979828
rs4980320
All − MR Egger
All − Inverse variance weighted
−20
0
20
MR effect size for
'SMAD2' on 'osteosarcoma'

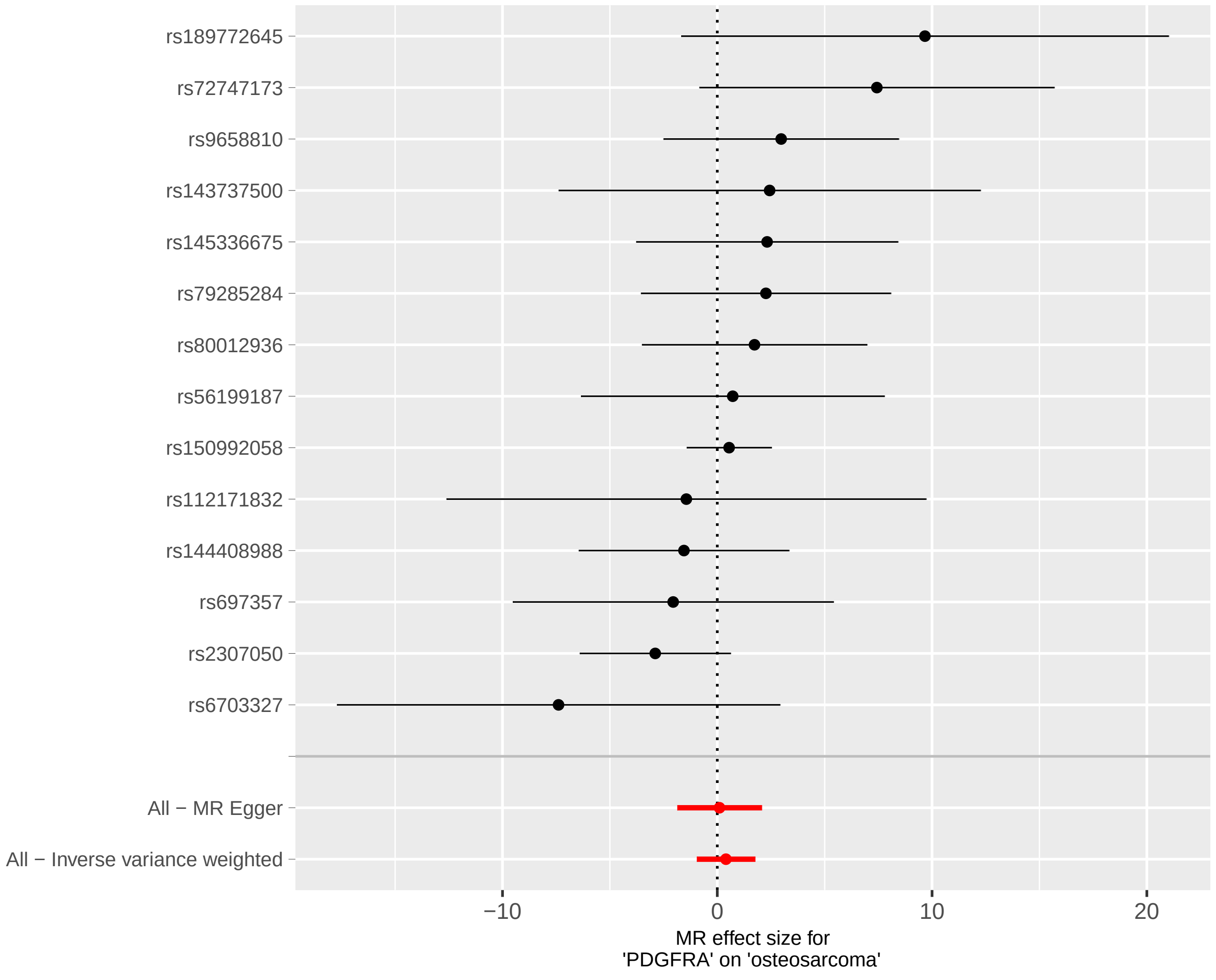
rs189772645
rs72747173
rs9658810
rs143737500
rs145336675
rs79285284
rs80012936
rs56199187
rs150992058
rs112171832
rs144408988
rs697357
rs2307050
rs6703327
All − MR Egger
All − Inverse variance weighted
−10
0
10
20
MR effect size for
'PDGFRA' on 'osteosarcoma'

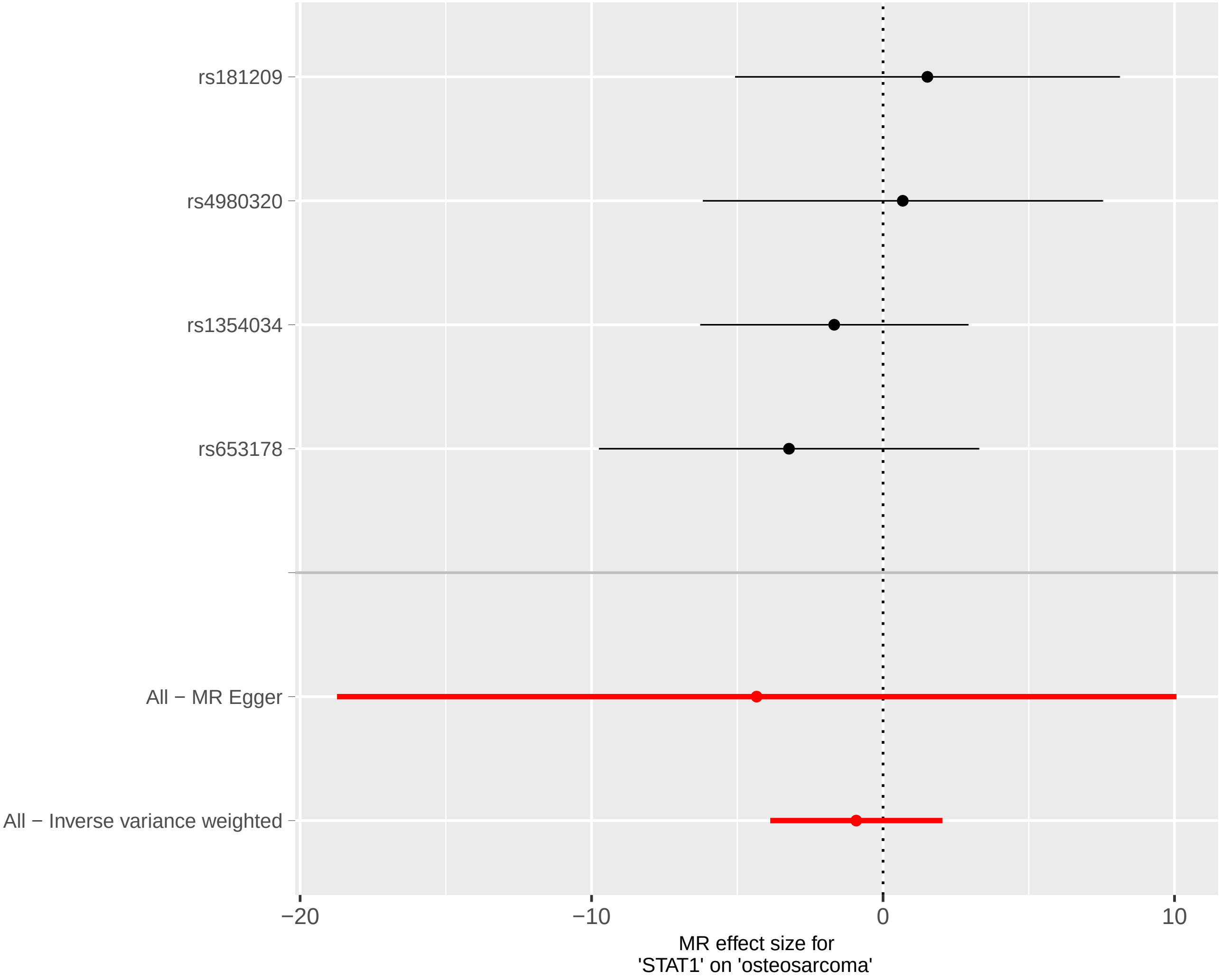

rs181209
rs4980320
rs1354034
rs653178
All − MR Egger
All − Inverse variance weighted
−20
−10
0
10
MR effect size for
'STAT1' on 'osteosarcoma'

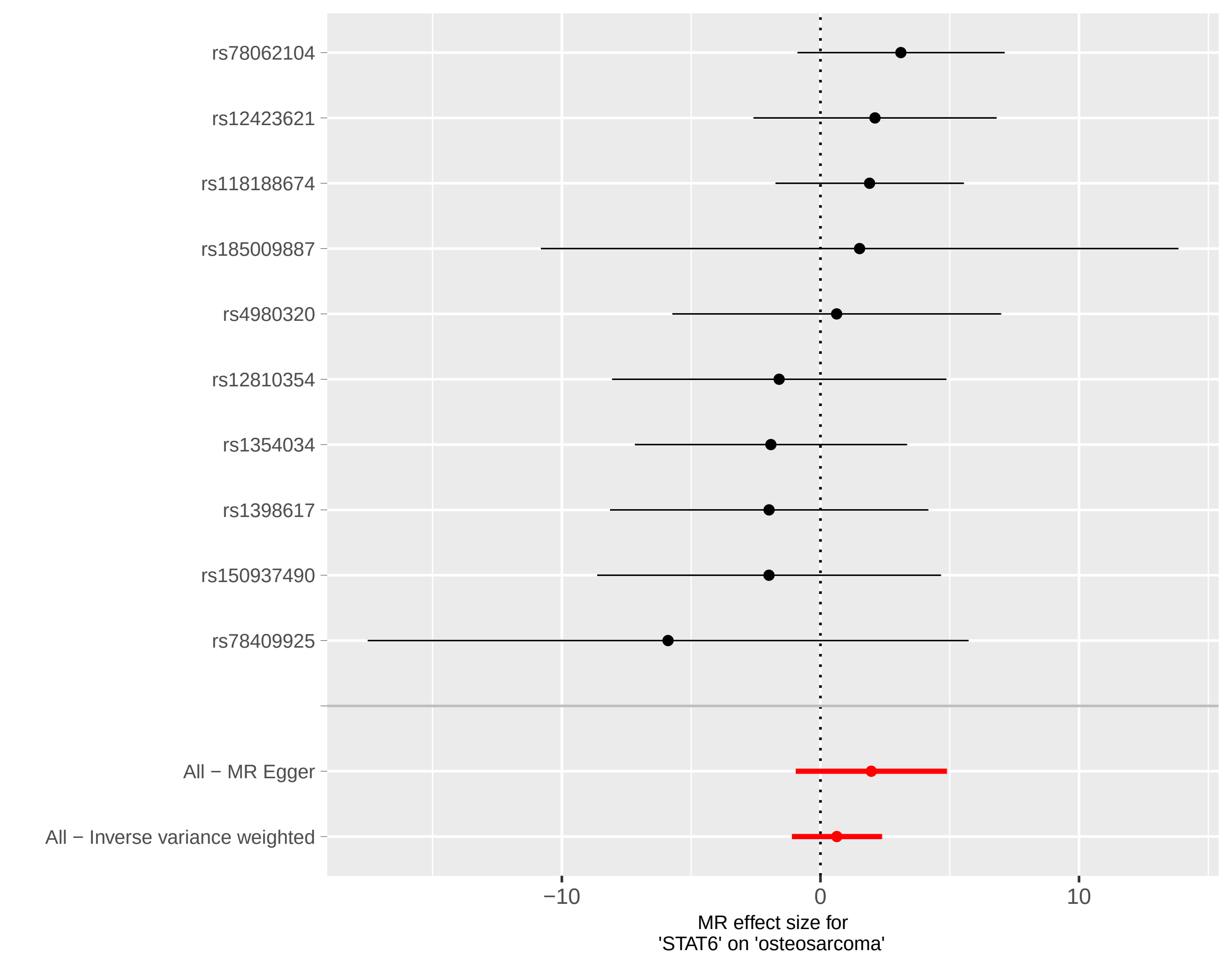

rs78062104
rs12423621
rs118188674
rs185009887
rs4980320
rs12810354
rs1354034
rs1398617
rs150937490
rs78409925
All − MR Egger
All − Inverse variance weighted
−10
0
10
MR effect size for
'STAT6' on 'osteosarcoma'

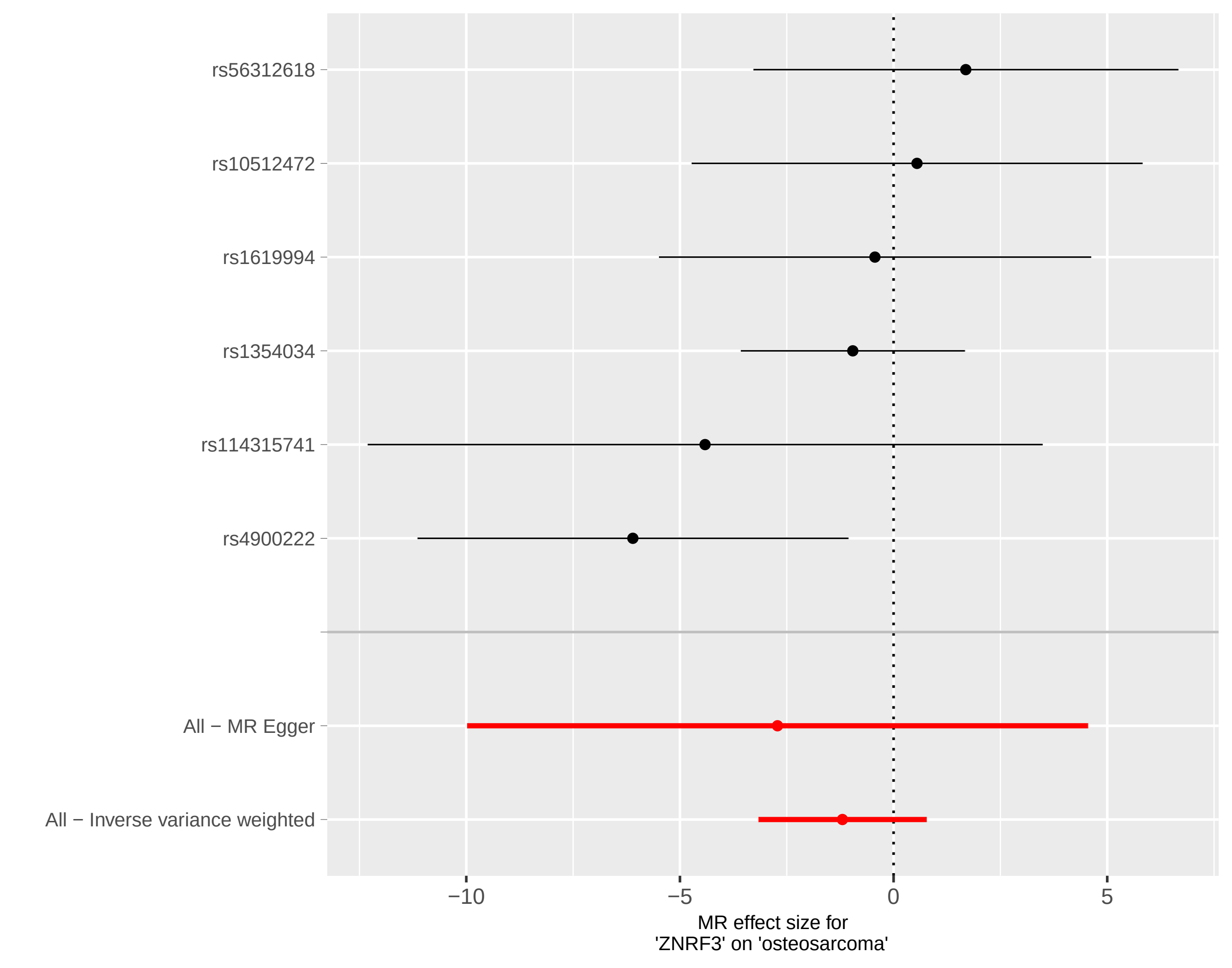

rs56312618
rs10512472
rs1619994
rs1354034
rs114315741
rs4900222
All − MR Egger
All − Inverse variance weighted
−10
−5
0
5
MR effect size for
'ZNRF3' on 'osteosarcoma'

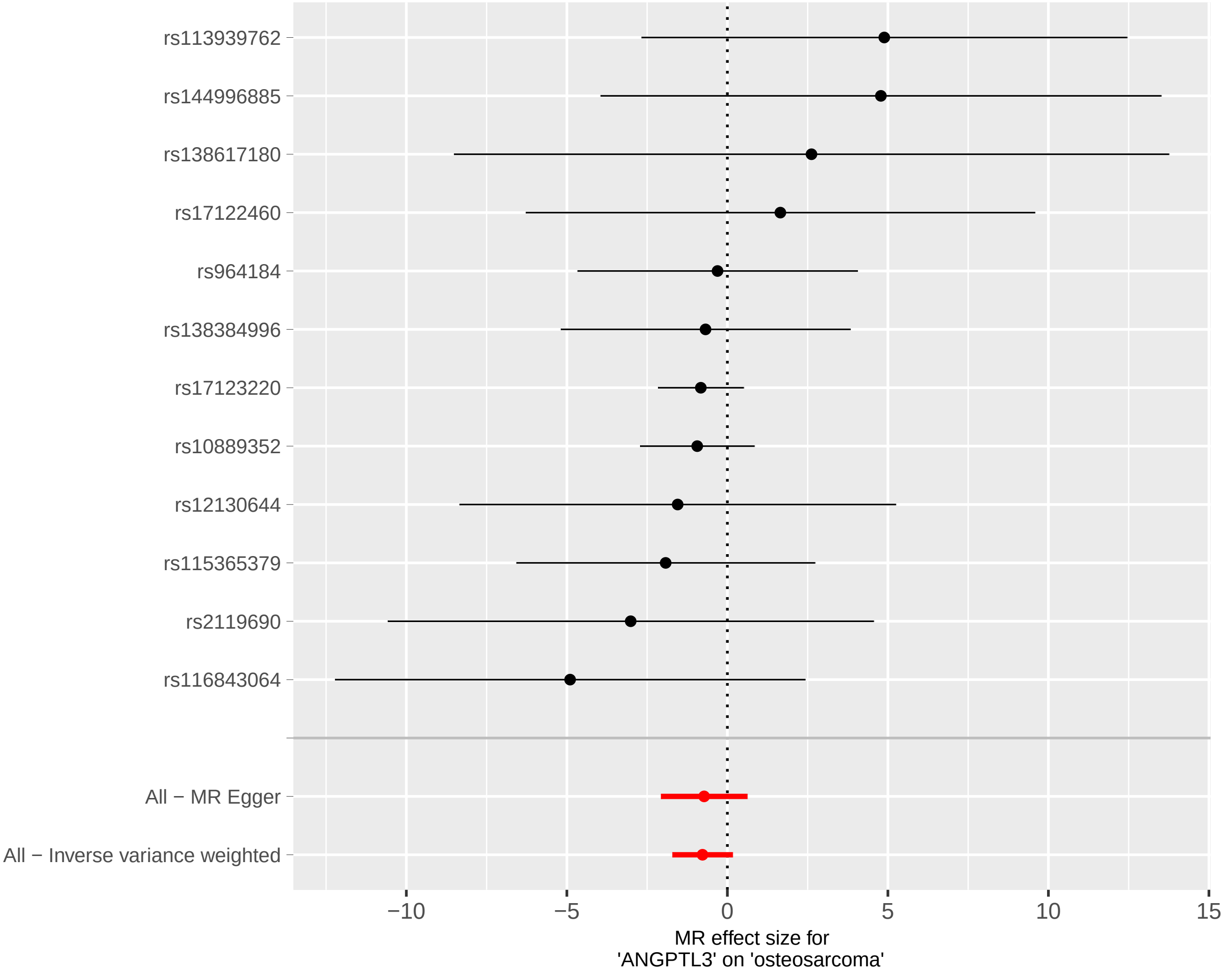

rs113939762
rs144996885
rs138617180
rs17122460
rs964184
rs138384996
rs17123220
rs10889352
rs12130644
rs115365379
rs2119690
rs116843064
All − MR Egger
All − Inverse variance weighted
−10
−5
0
5
10
15
MR effect size for
'ANGPTL3' on 'osteosarcoma'

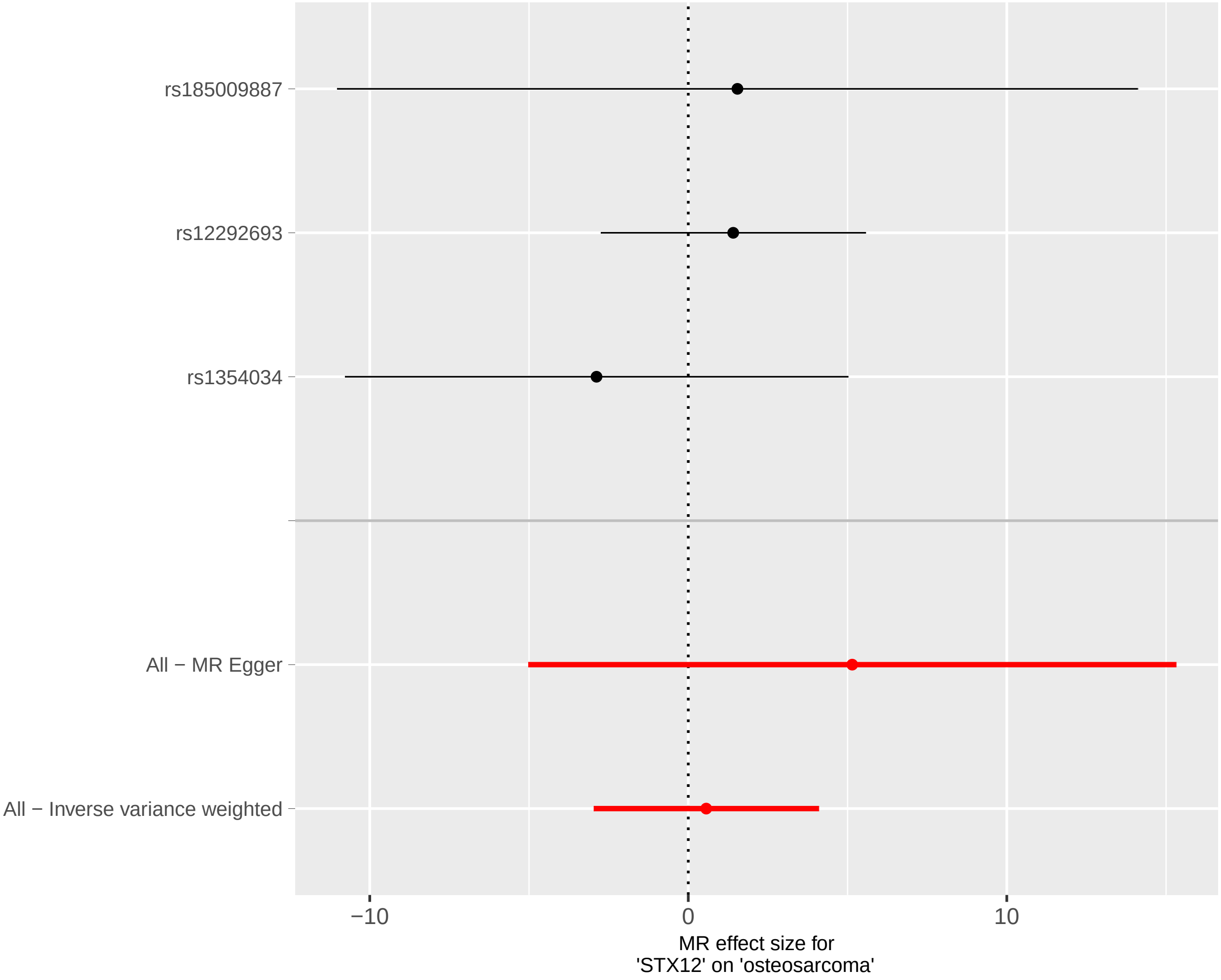

rs185009887
rs12292693
rs1354034
All − MR Egger
All − Inverse variance weighted
−10
0
10
MR effect size for
'STX12' on 'osteosarcoma'

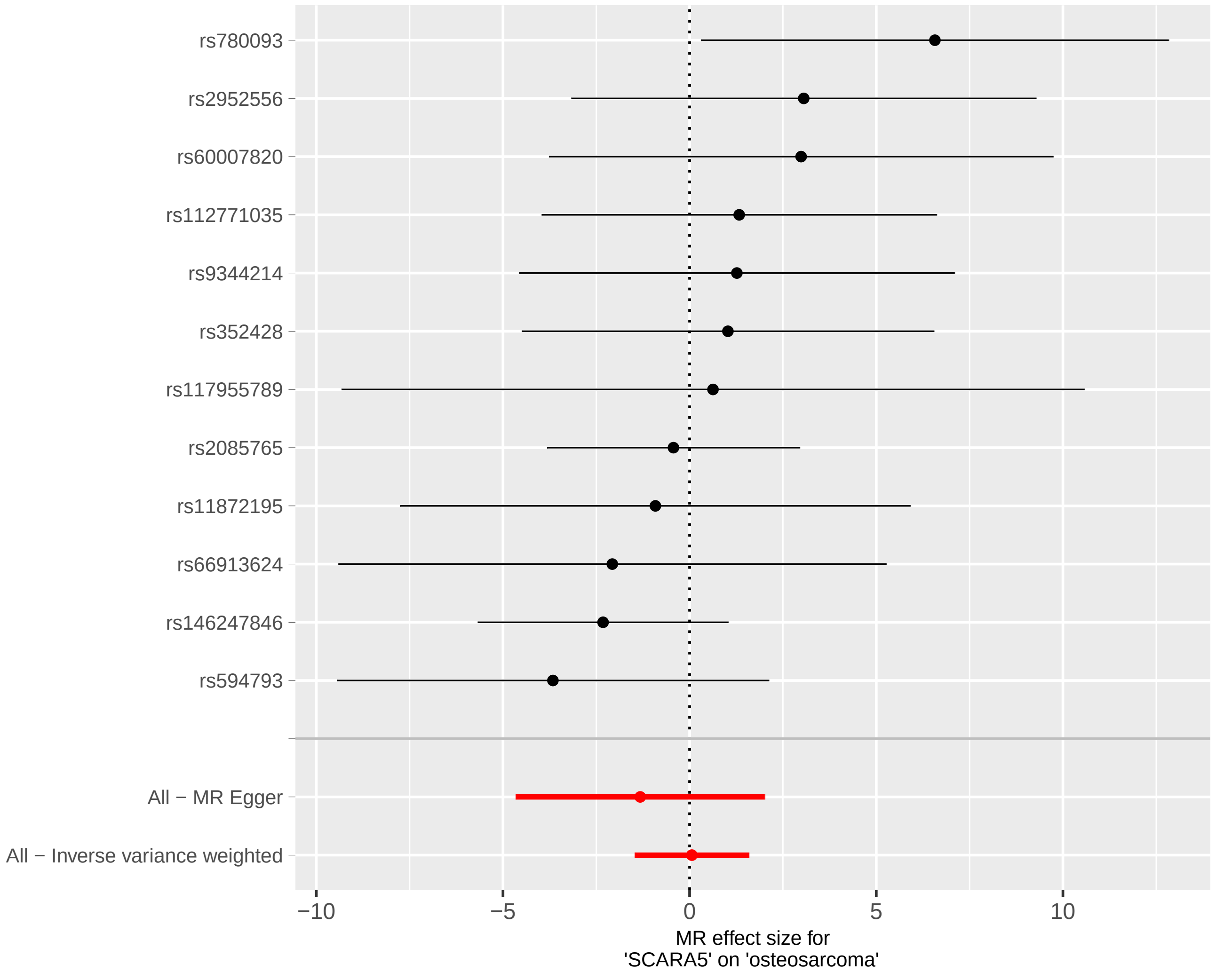

rs780093
rs2952556
rs60007820
rs112771035
rs9344214
rs352428
rs117955789
rs2085765
rs11872195
rs66913624
rs146247846
rs594793
All − MR Egger
All − Inverse variance weighted
−10
−5
0
5
10
MR effect size for
'SCARA5' on 'osteosarcoma'

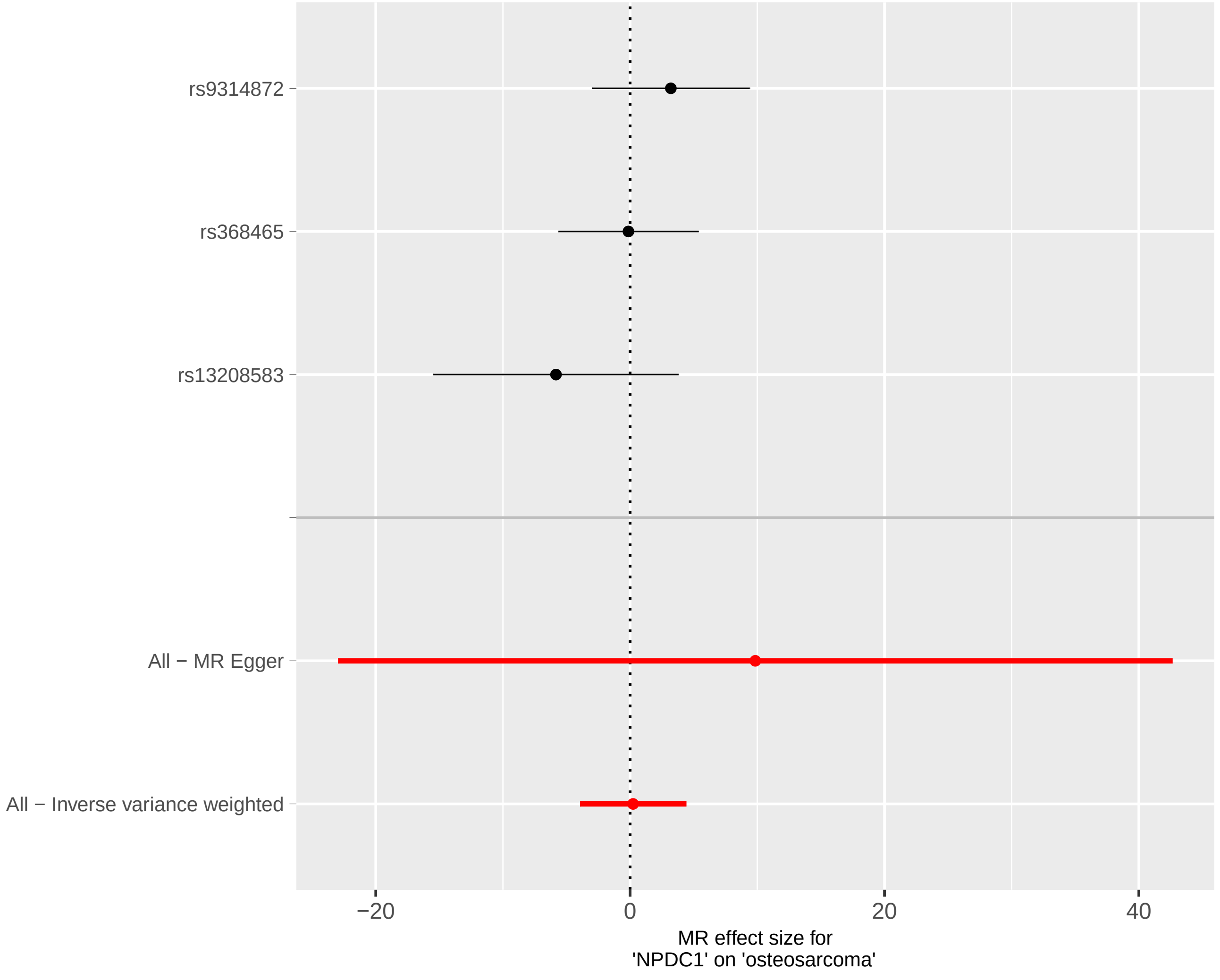
rs9314872
rs368465
rs13208583
All − MR Egger
All − Inverse variance weighted
−20
0
20
40
MR effect size for
'NPDC1' on 'osteosarcoma'

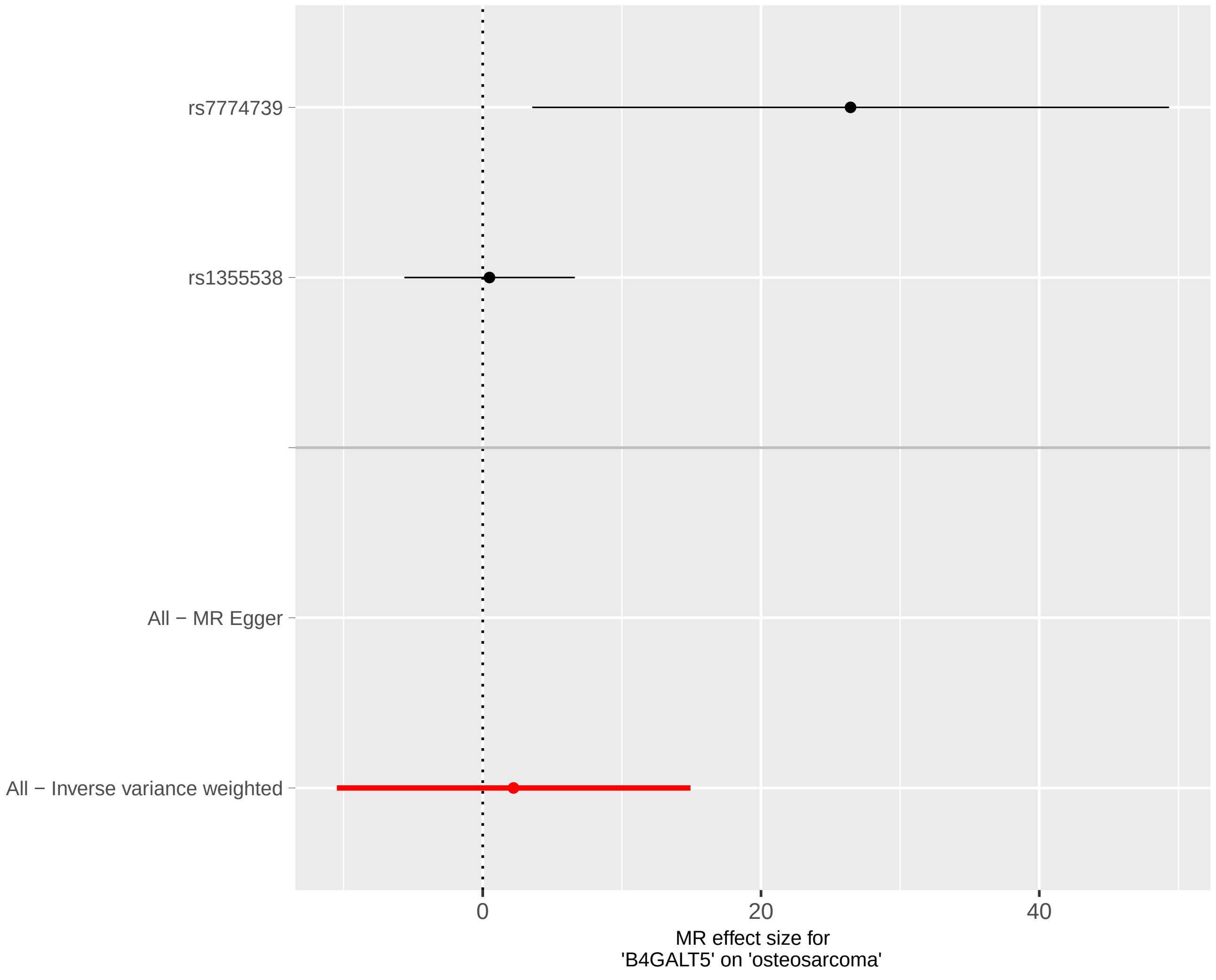

rs7774739
rs1355538
All − MR Egger
All − Inverse variance weighted
0
20
40
MR effect size for
'B4GALT5' on 'osteosarcoma'

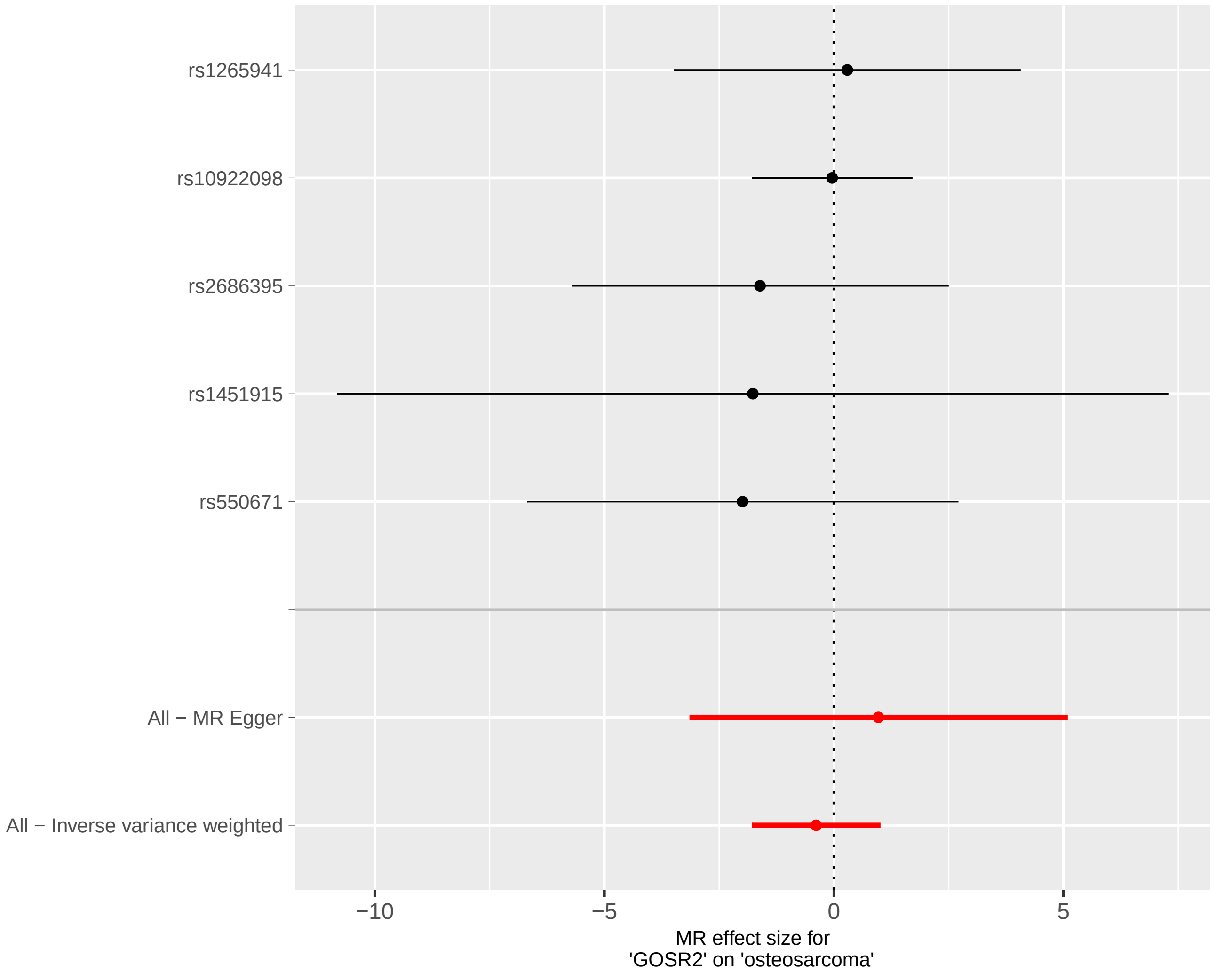
rs1265941
rs10922098
rs2686395
rs1451915
rs550671
All − MR Egger
All − Inverse variance weighted
−10
−5
0
5
MR effect size for
'GOSR2' on 'osteosarcoma'

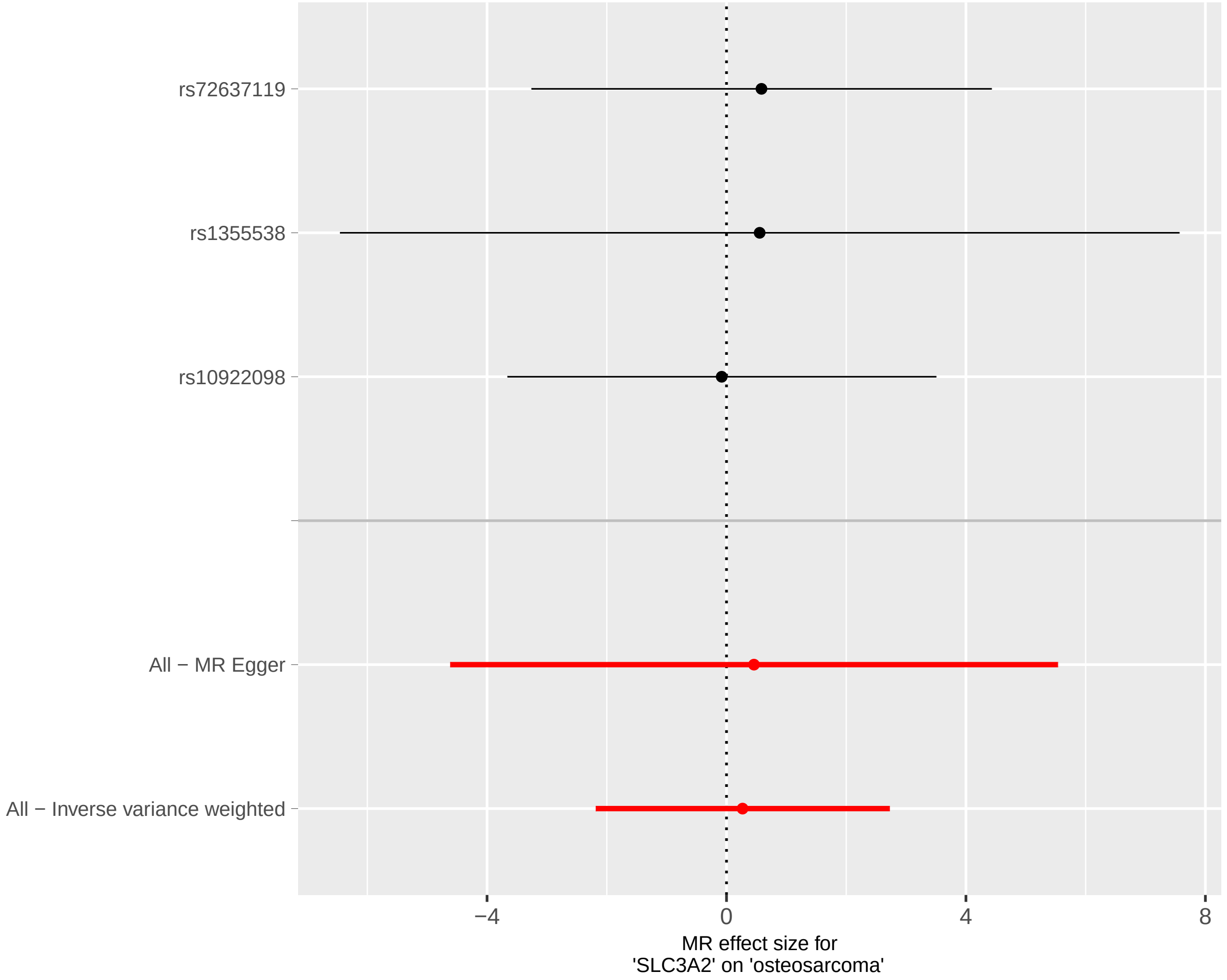

rs72637119
rs1355538
rs10922098
All − MR Egger
All − Inverse variance weighted
−4
0
4
8
MR effect size for
'SLC3A2' on 'osteosarcoma'

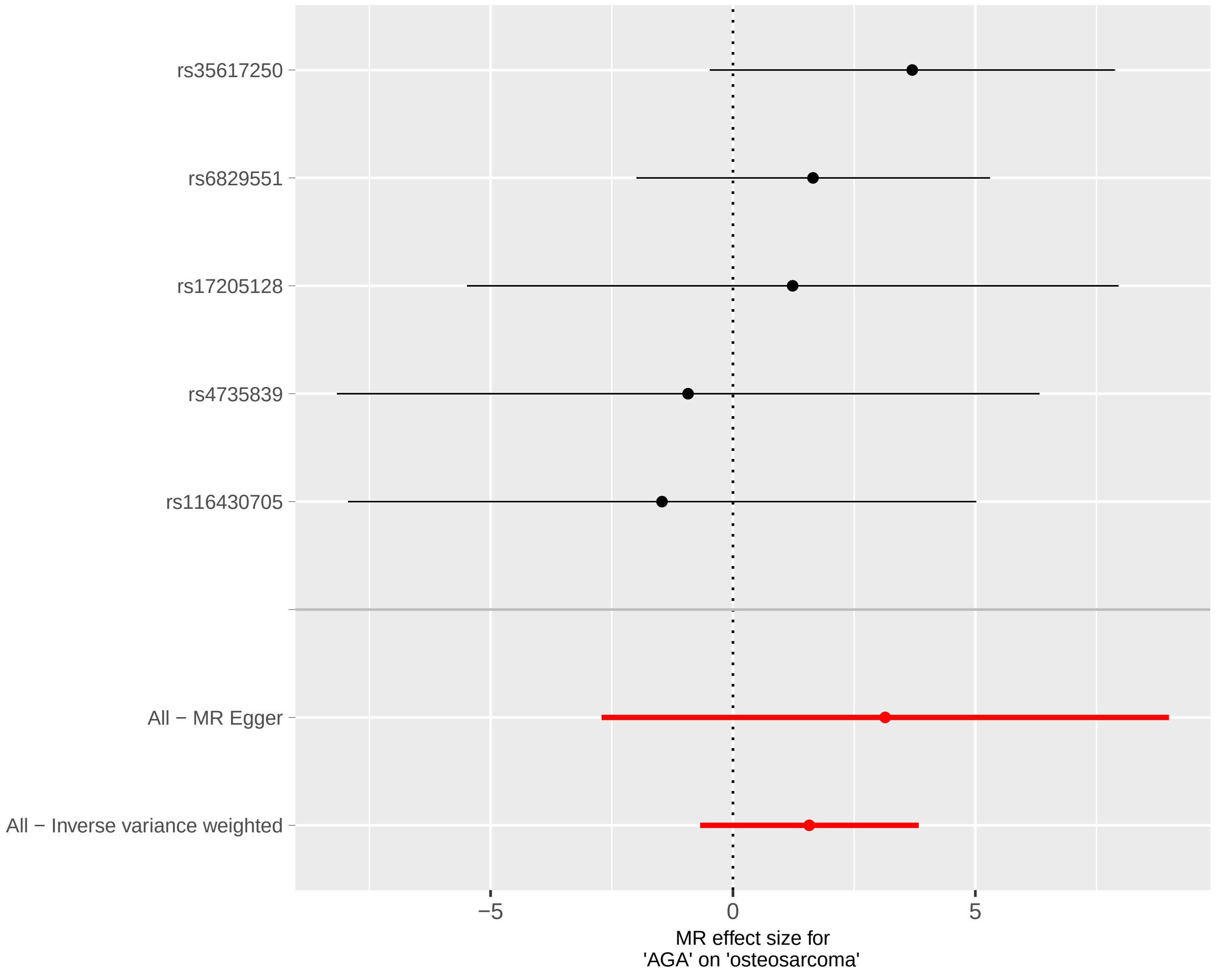

rs35617250
rs6829551
rs17205128
rs4735839
rs116430705
All − MR Egger
All − Inverse variance weighted
−5
0
5
MR effect size for
'AGA' on 'osteosarcoma'

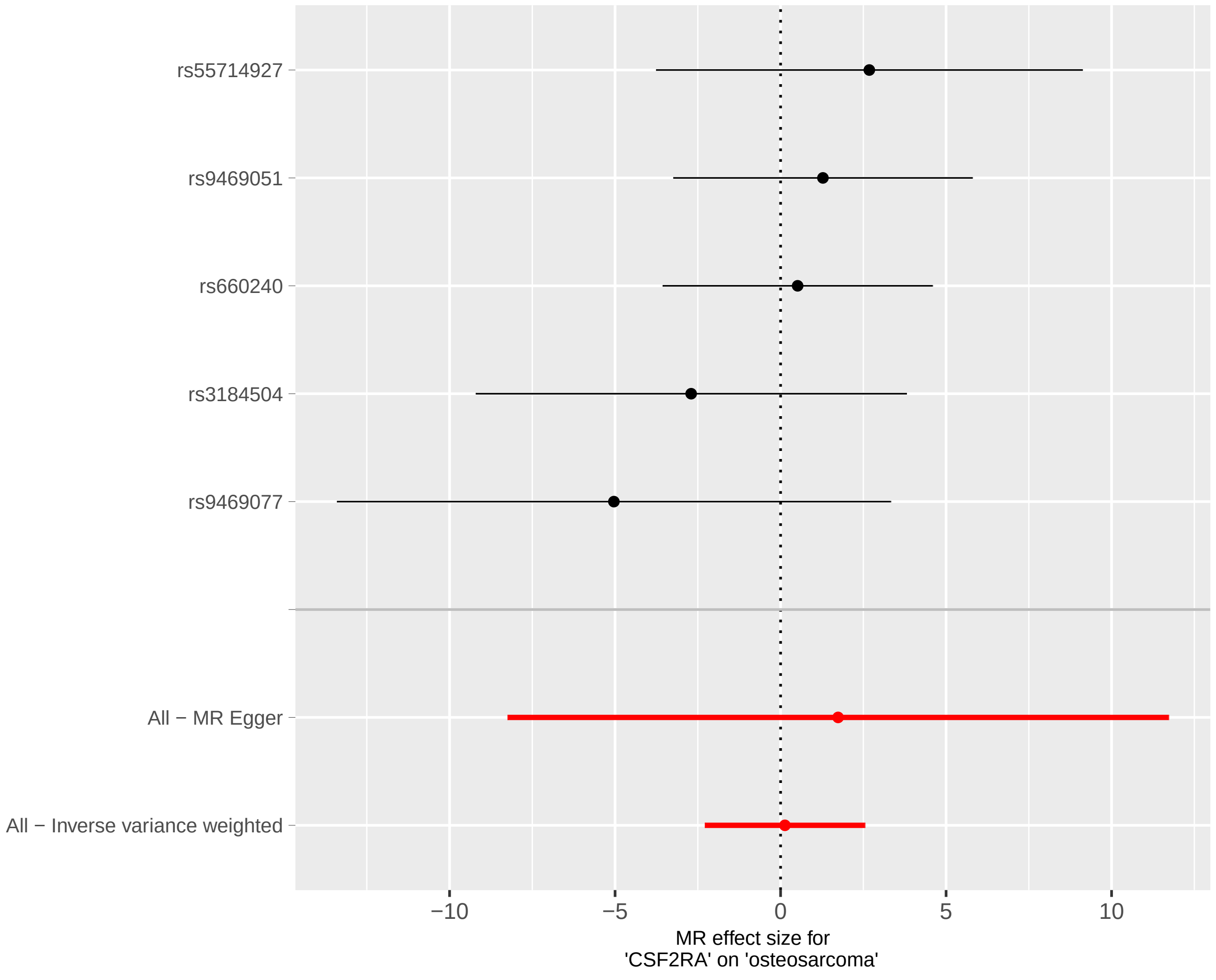
rs55714927
rs9469051
rs660240
rs3184504
rs9469077
All − MR Egger
All − Inverse variance weighted
−10
−5
0
5
10
MR effect size for
'CSF2RA' on 'osteosarcoma'

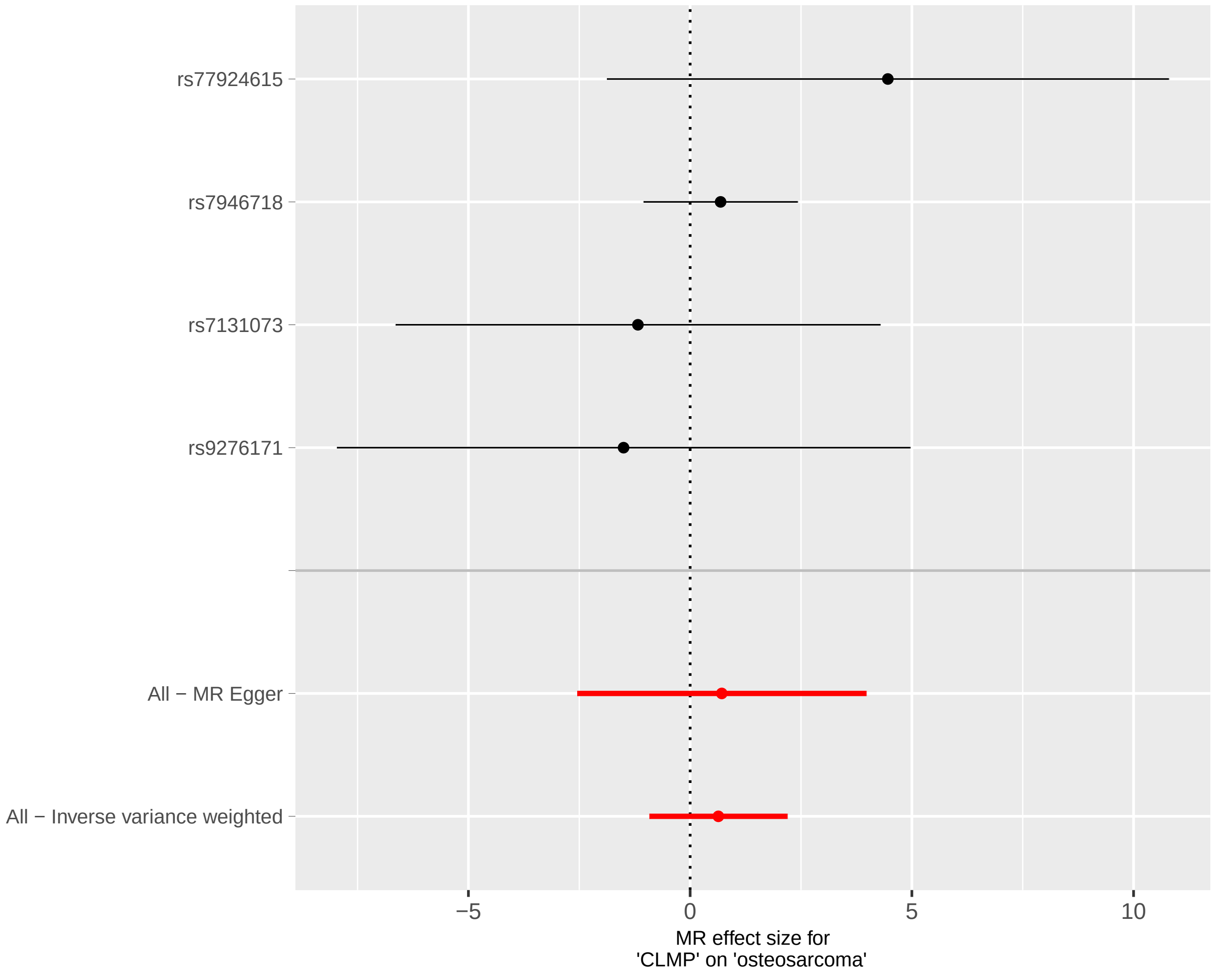
rs77924615
rs7946718
rs7131073
rs9276171
All − MR Egger
All − Inverse variance weighted
−5
0
5
10
MR effect size for
'CLMP' on 'osteosarcoma'

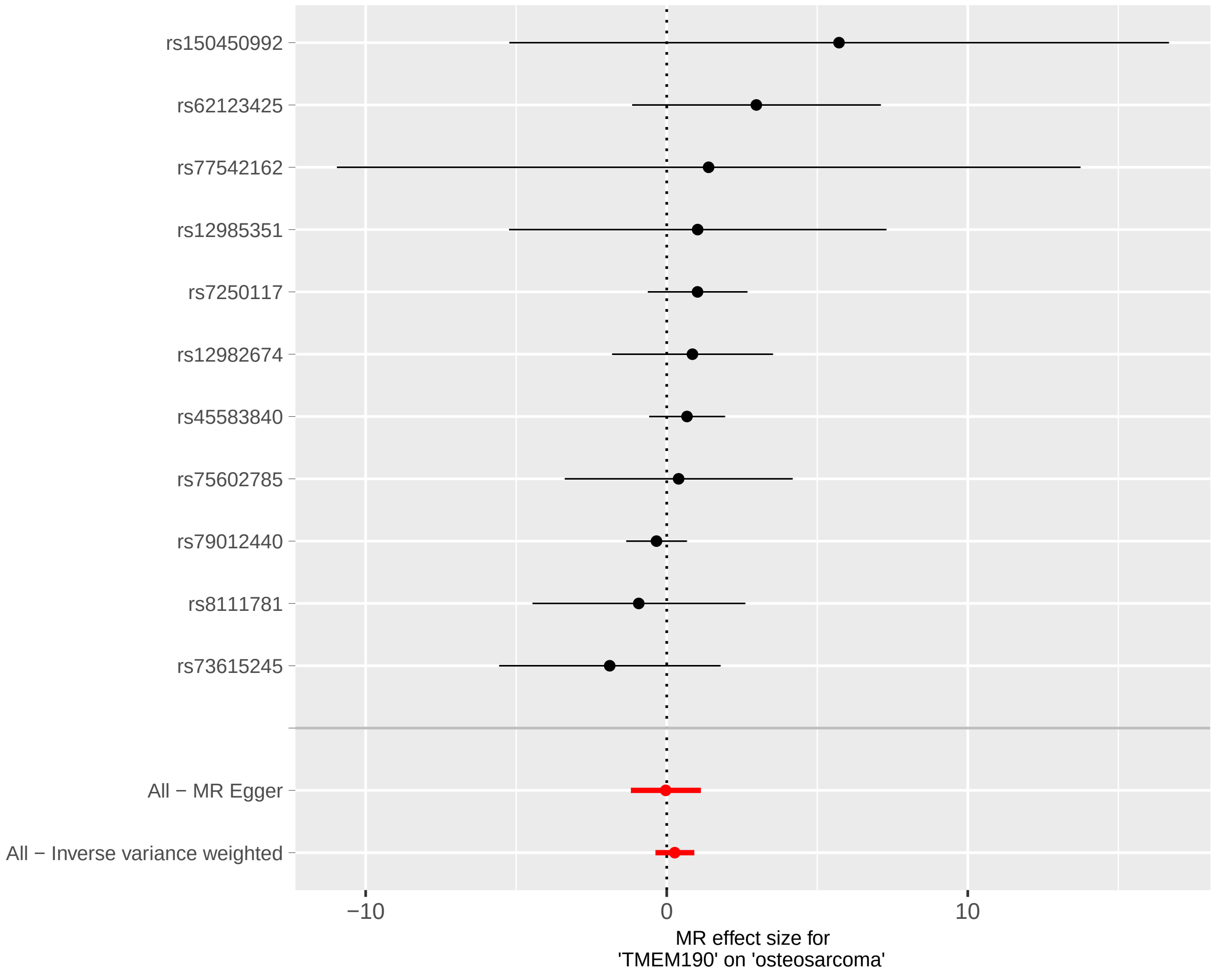
rs150450992
rs62123425
rs77542162
rs12985351
rs7250117
rs12982674
rs45583840
rs75602785
rs79012440
rs8111781
rs73615245
All − MR Egger
All − Inverse variance weighted
−10
0
10
MR effect size for
'TMEM190' on 'osteosarcoma'

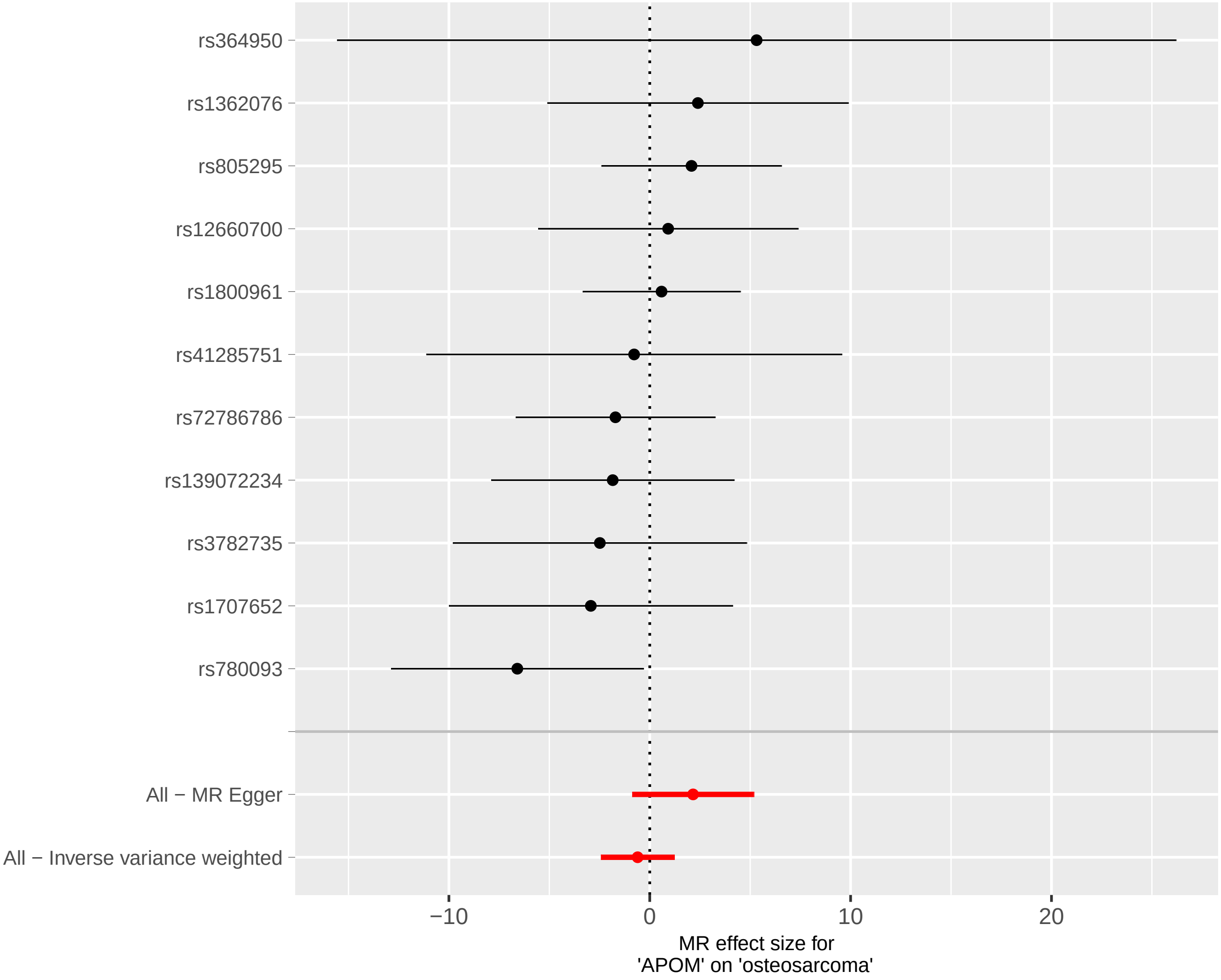

rs364950
rs1362076
rs805295
rs12660700
rs1800961
rs41285751
rs72786786
rs139072234
rs3782735
rs1707652
rs780093
All − MR Egger
All − Inverse variance weighted
−10
0
10
20
MR effect size for
'APOM' on 'osteosarcoma'

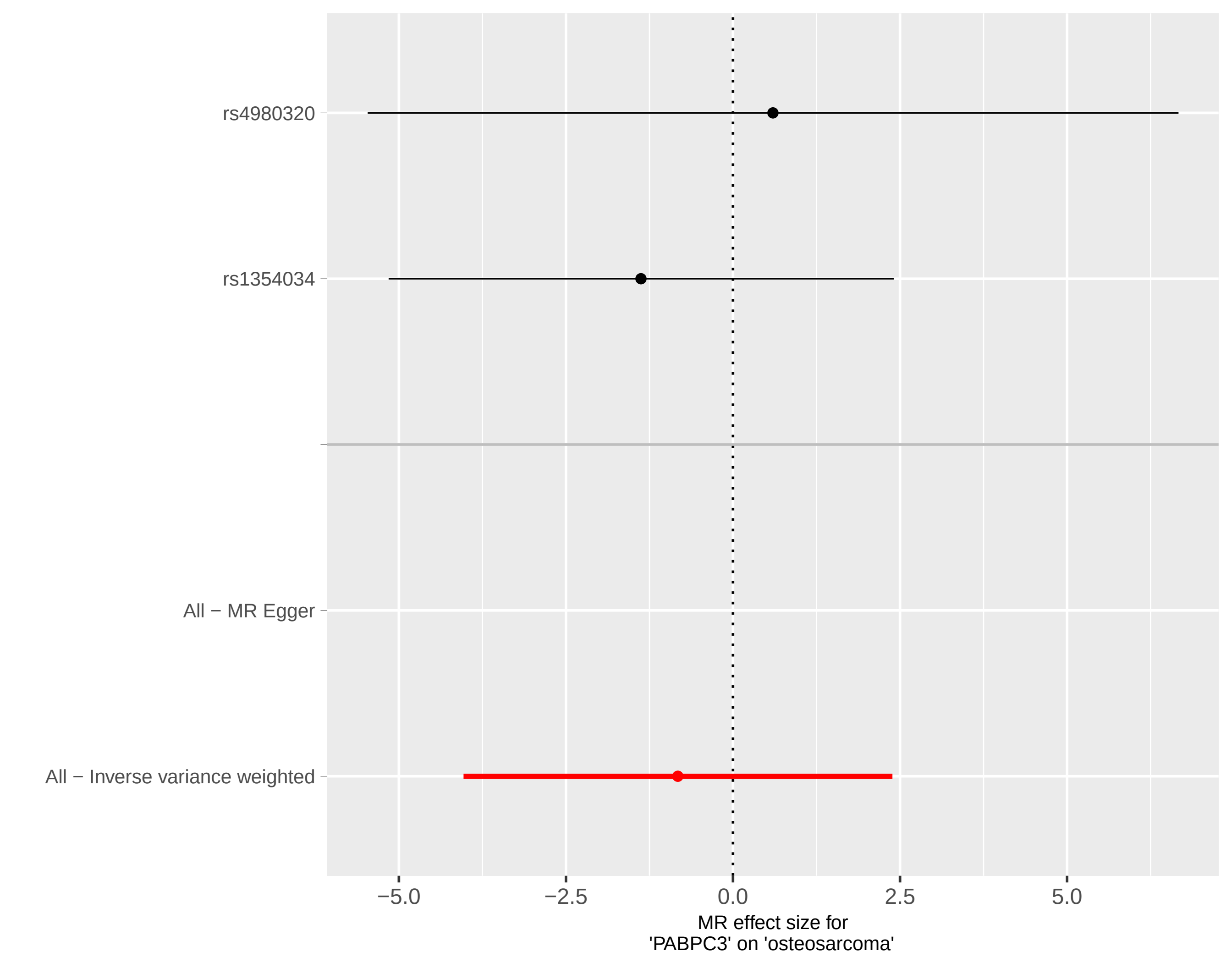

rs4980320
rs1354034
All − MR Egger
All − Inverse variance weighted
−5.0
−2.5
0.0
2.5
5.0
MR effect size for
'PABPC3' on 'osteosarcoma'

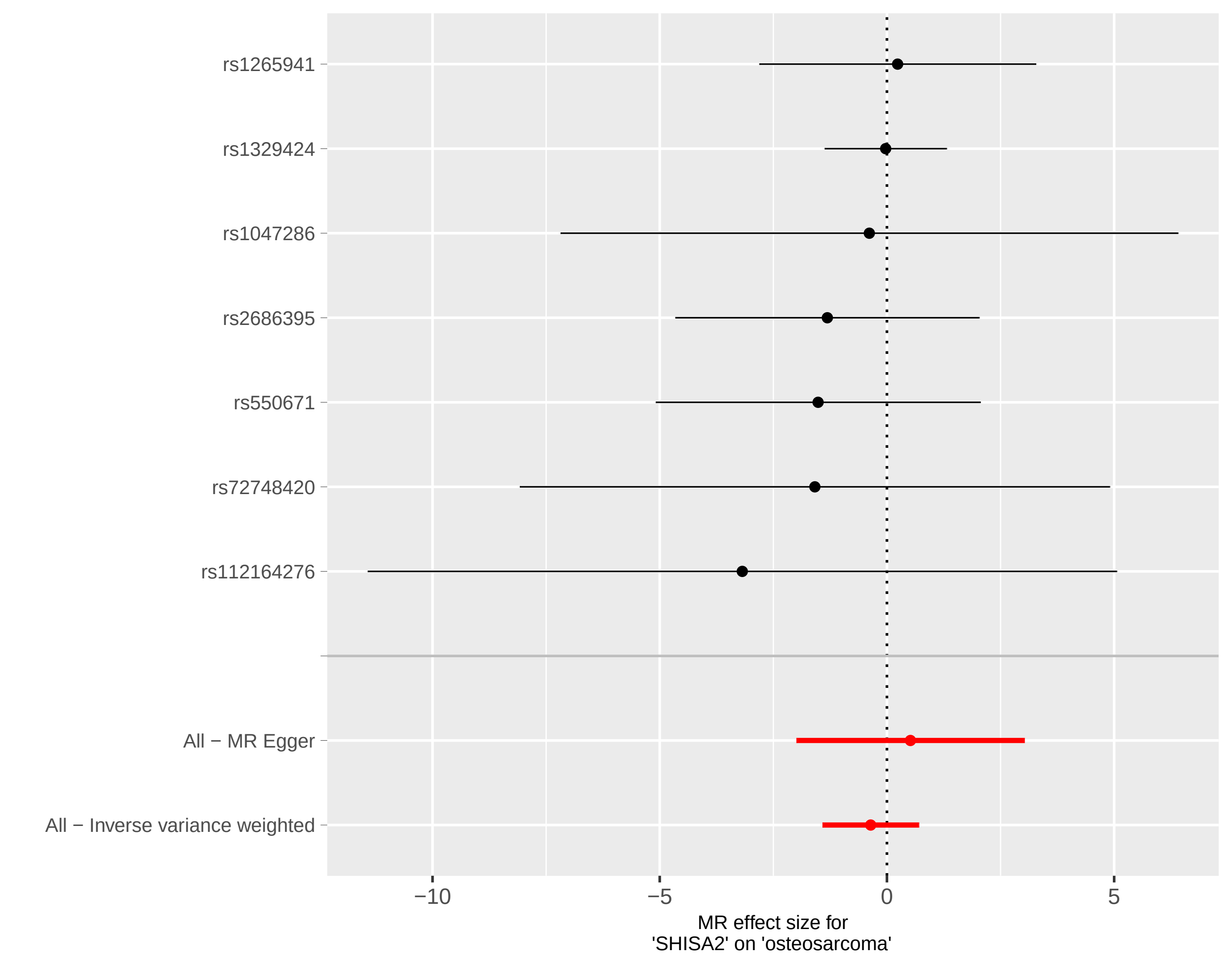
rs1265941
rs1329424
rs1047286
rs2686395
rs550671
rs72748420
rs112164276
All − MR Egger
All − Inverse variance weighted
−10
−5
0
5
MR effect size for
'SHISA2' on 'osteosarcoma'

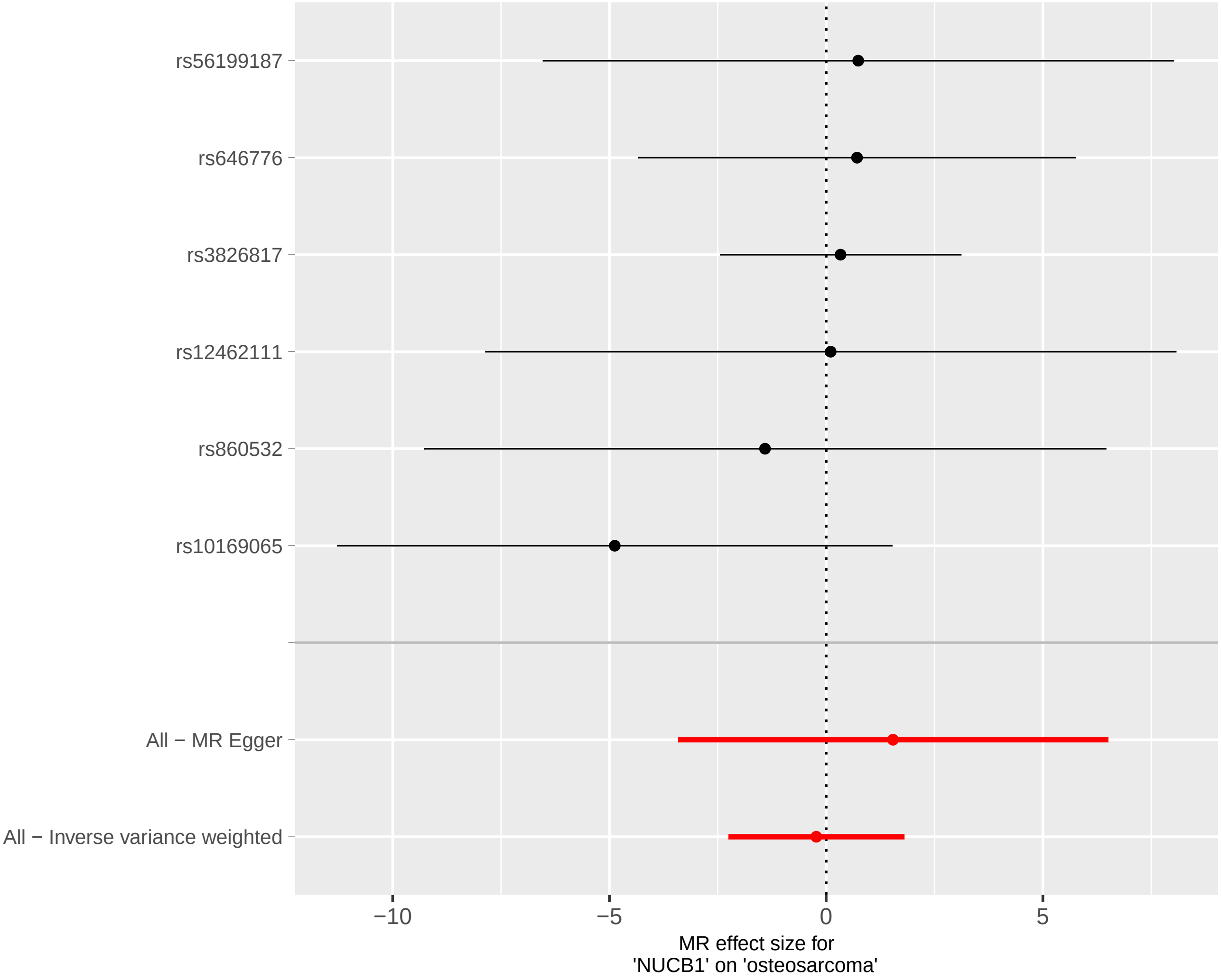

rs56199187
rs646776
rs3826817
rs12462111
rs860532
rs10169065
All − MR Egger
All − Inverse variance weighted
−10
−5
0
5
MR effect size for
'NUCB1' on 'osteosarcoma'

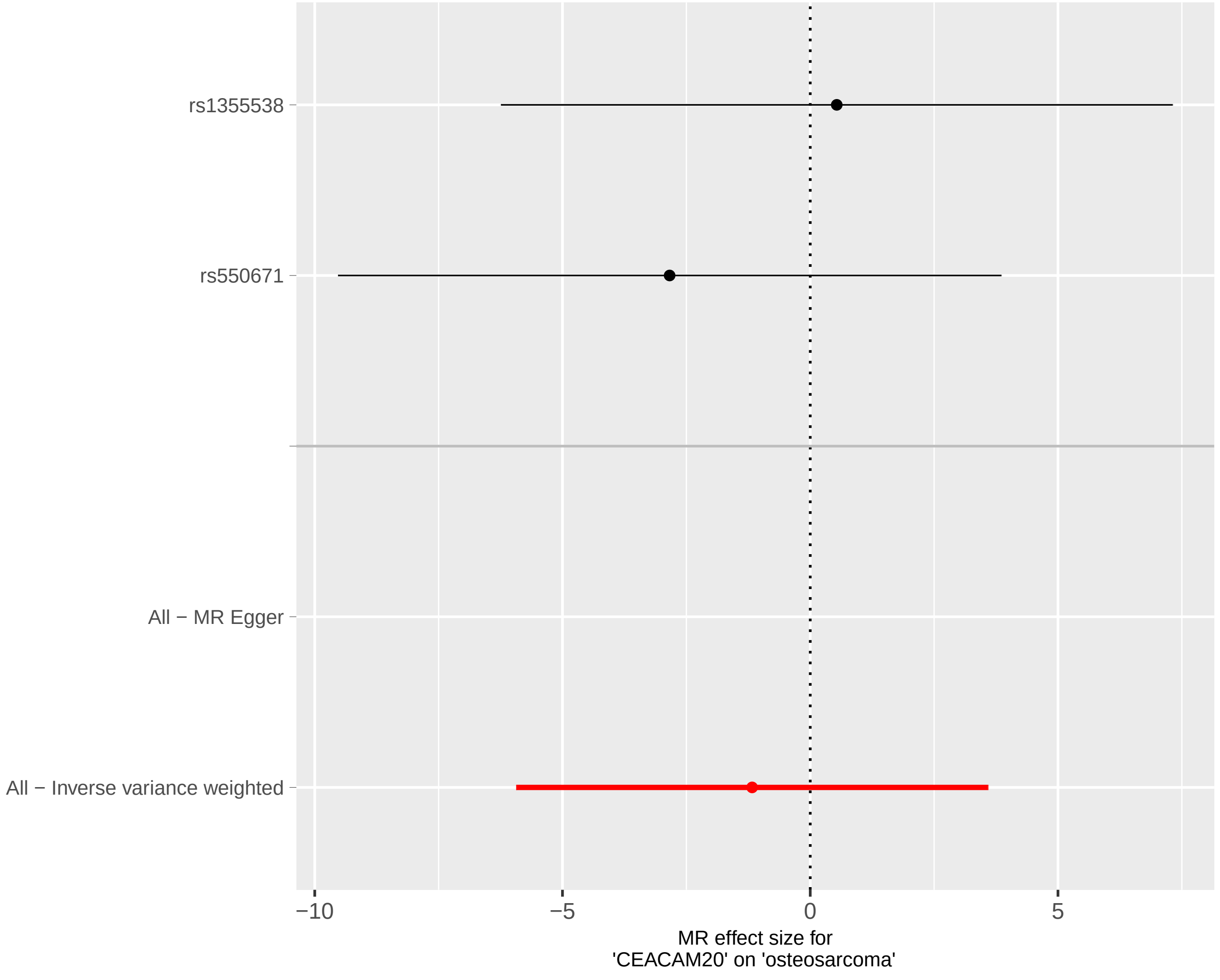
rs1355538
rs550671
All − MR Egger
All − Inverse variance weighted
−10
−5
0
5
MR effect size for
'CEACAM20' on 'osteosarcoma'

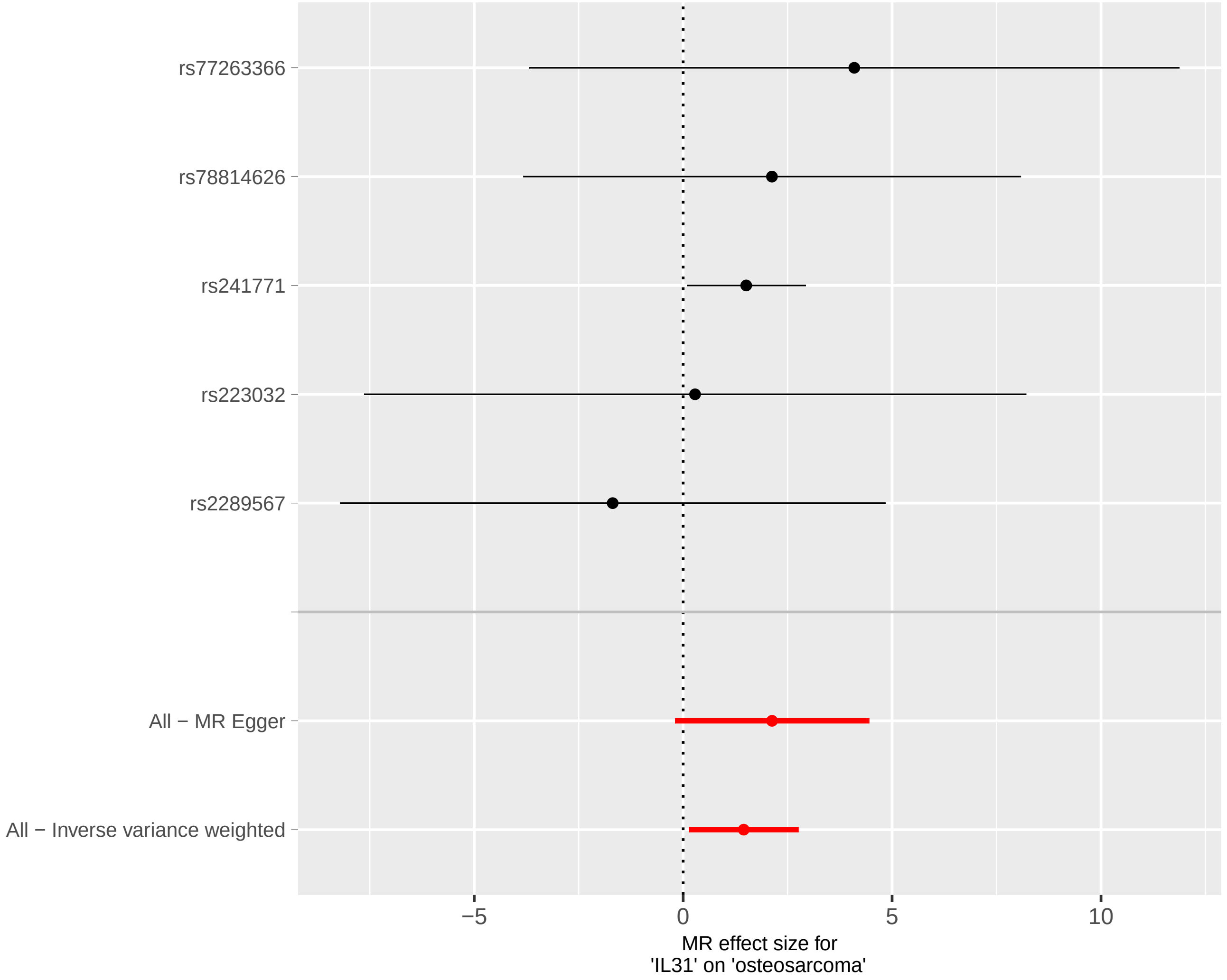
rs77263366
rs78814626
rs241771
rs223032
rs2289567
All − MR Egger
All − Inverse variance weighted
−5
0
5
10
MR effect size for
'IL31' on 'osteosarcoma'

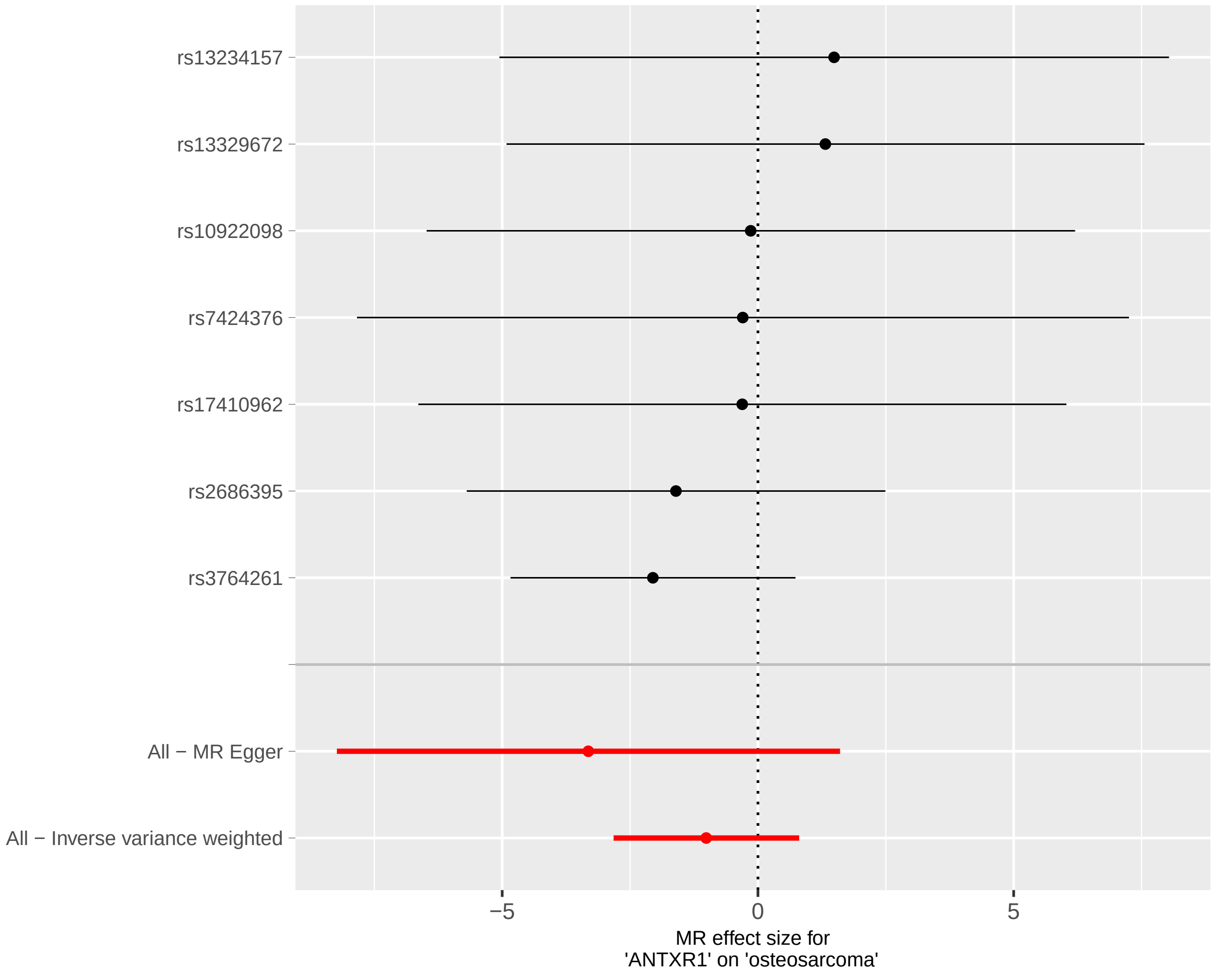

rs13234157
rs13329672
rs10922098
rs7424376
rs17410962
rs2686395
rs3764261
All − MR Egger
All − Inverse variance weighted
−5
0
5
MR effect size for
'ANTXR1' on 'osteosarcoma'

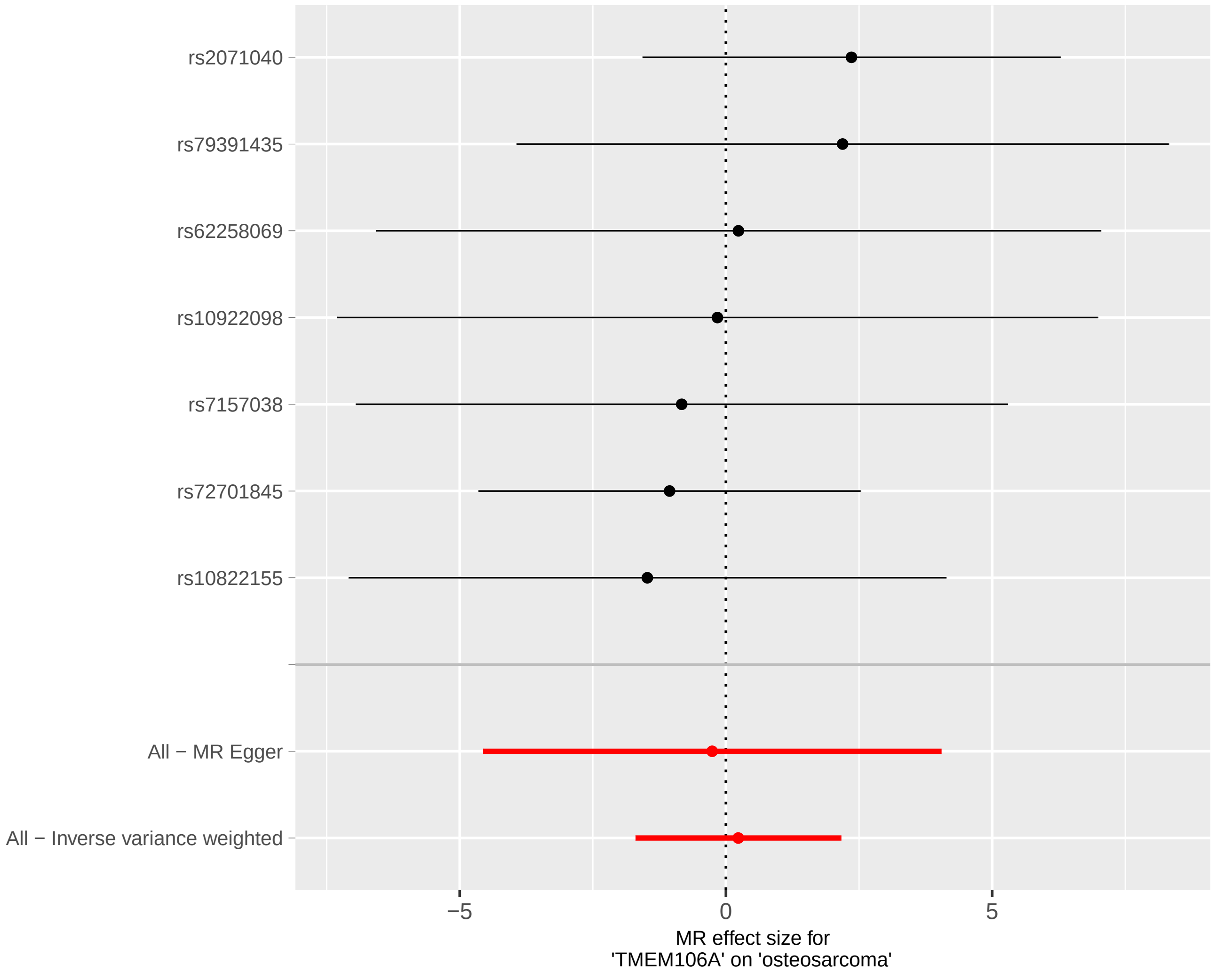
rs2071040
rs79391435
rs62258069
rs10922098
rs7157038
rs72701845
rs10822155
All − MR Egger
All − Inverse variance weighted
−5
0
5
MR effect size for
'TMEM106A' on 'osteosarcoma'

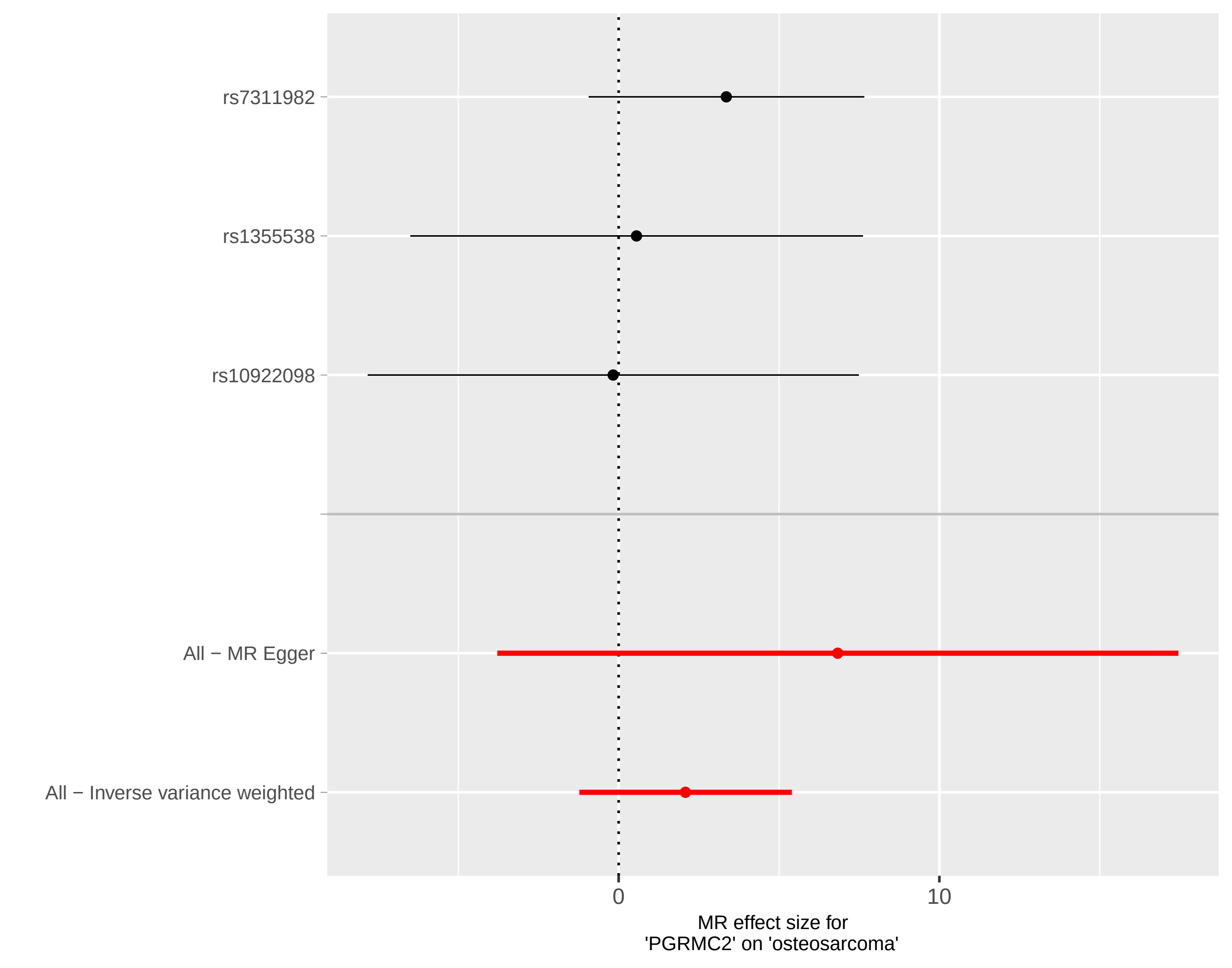
rs7311982
rs1355538
rs10922098
All − MR Egger
All − Inverse variance weighted
0
10
MR effect size for
'PGRMC2' on 'osteosarcoma'

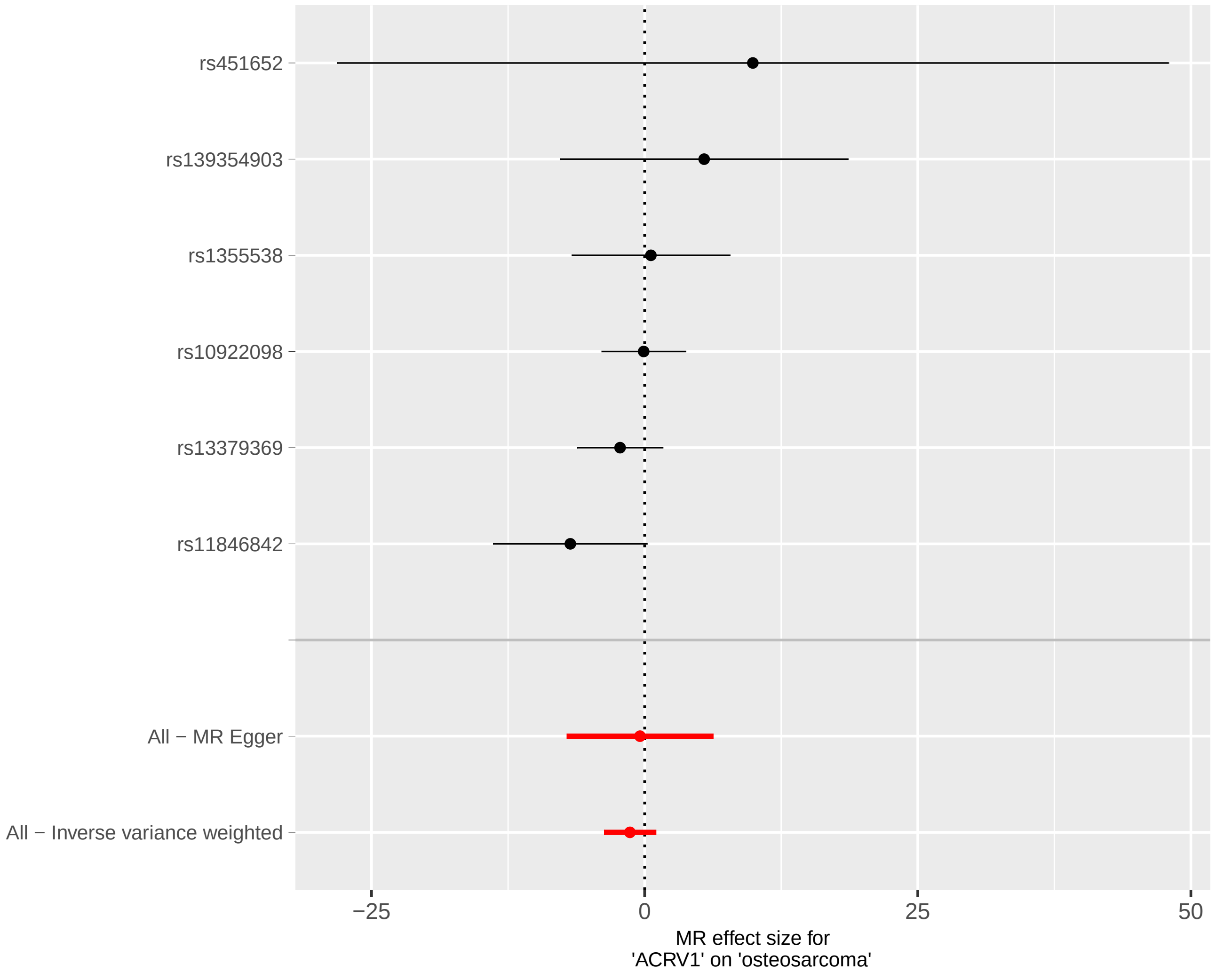
rs451652
rs139354903
rs1355538
rs10922098
rs13379369
rs11846842
All − MR Egger
All − Inverse variance weighted
−25
0
25
50
MR effect size for
'ACRV1' on 'osteosarcoma'

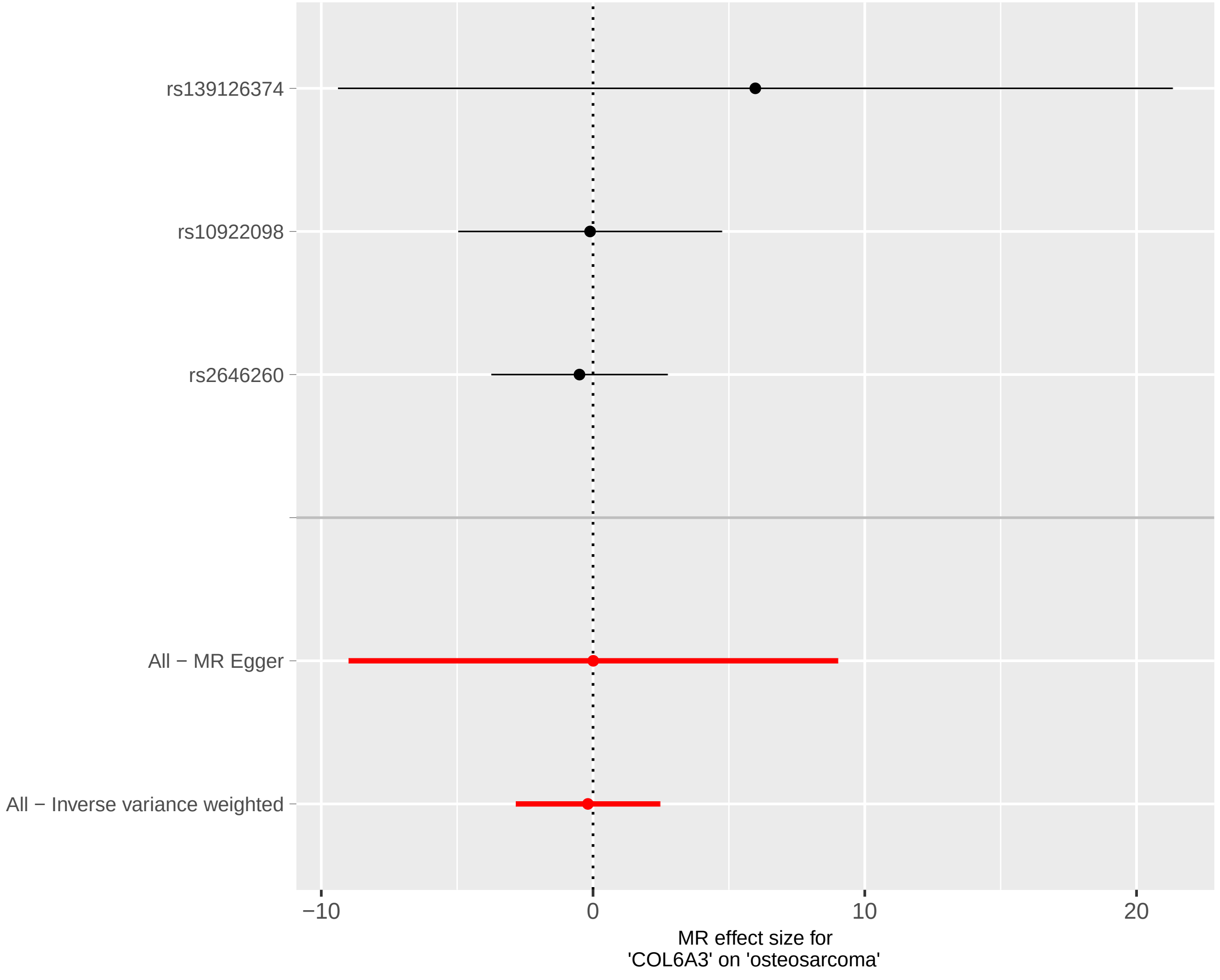

rs139126374
rs10922098
rs2646260
All − MR Egger
All − Inverse variance weighted
−10
0
10
20
MR effect size for
'COL6A3' on 'osteosarcoma'

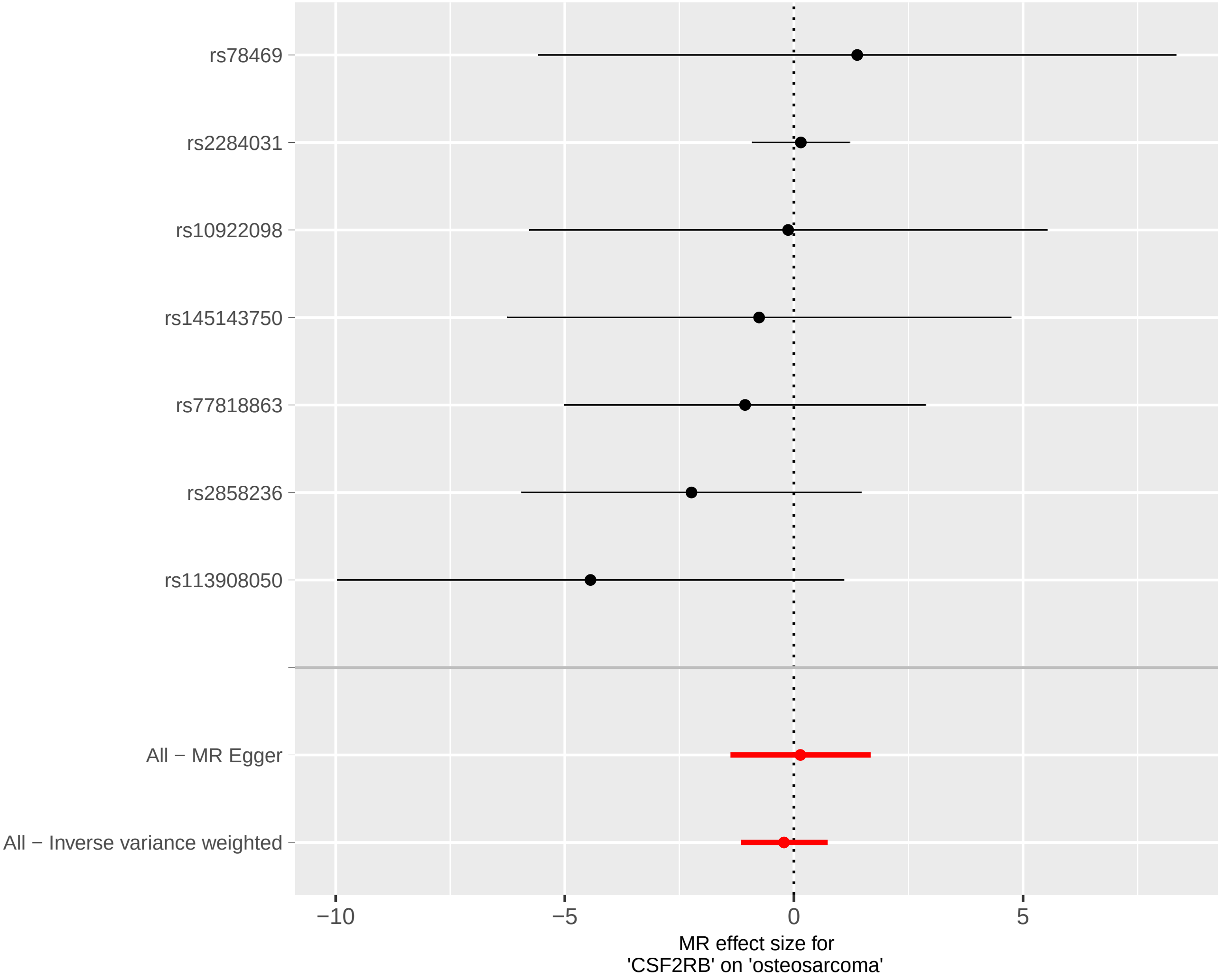

rs78469
rs2284031
rs10922098
rs145143750
rs77818863
rs2858236
rs113908050
All − MR Egger
All − Inverse variance weighted
−10
−5
0
5
MR effect size for
'CSF2RB' on 'osteosarcoma'

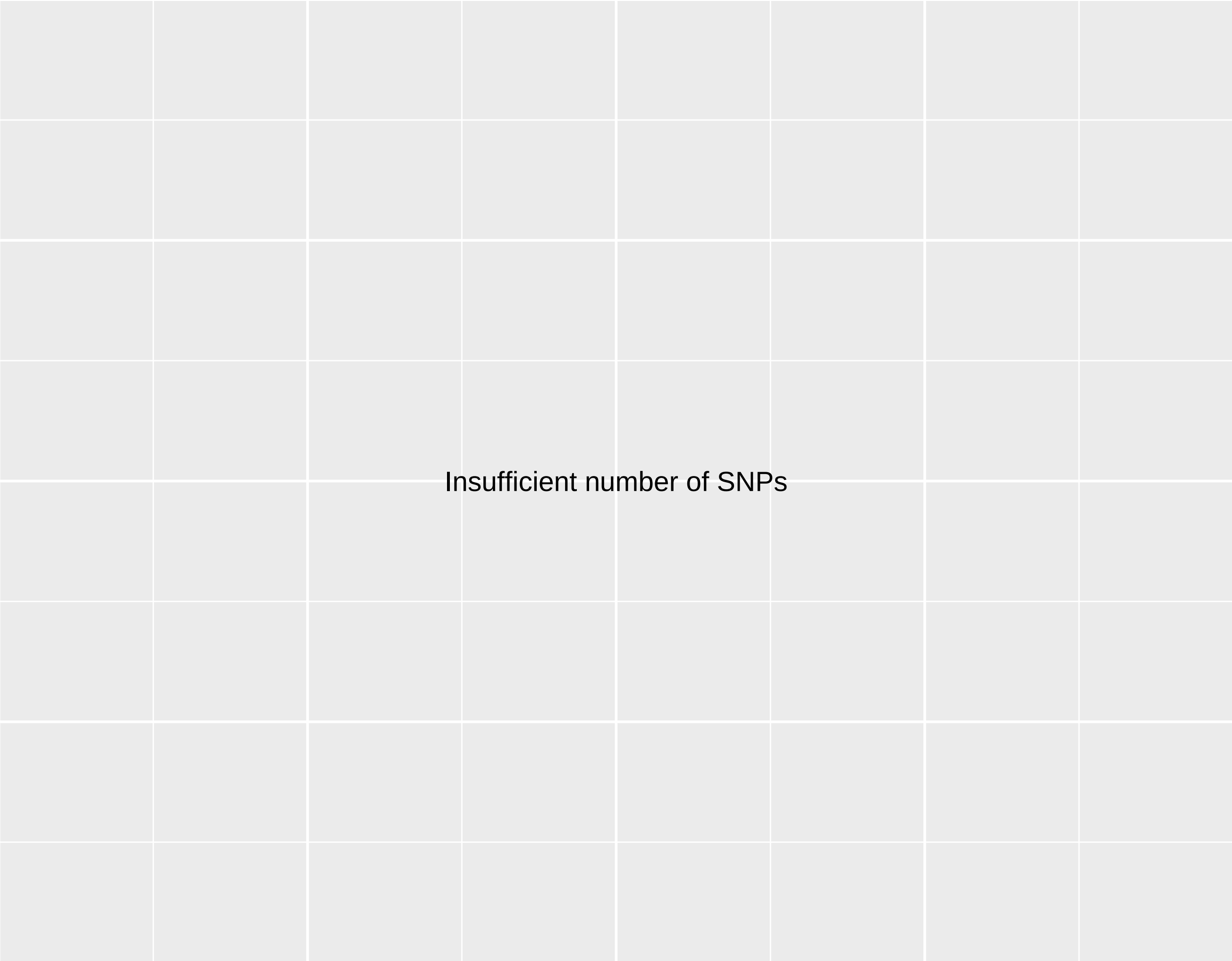
Insufficient number of SNPs

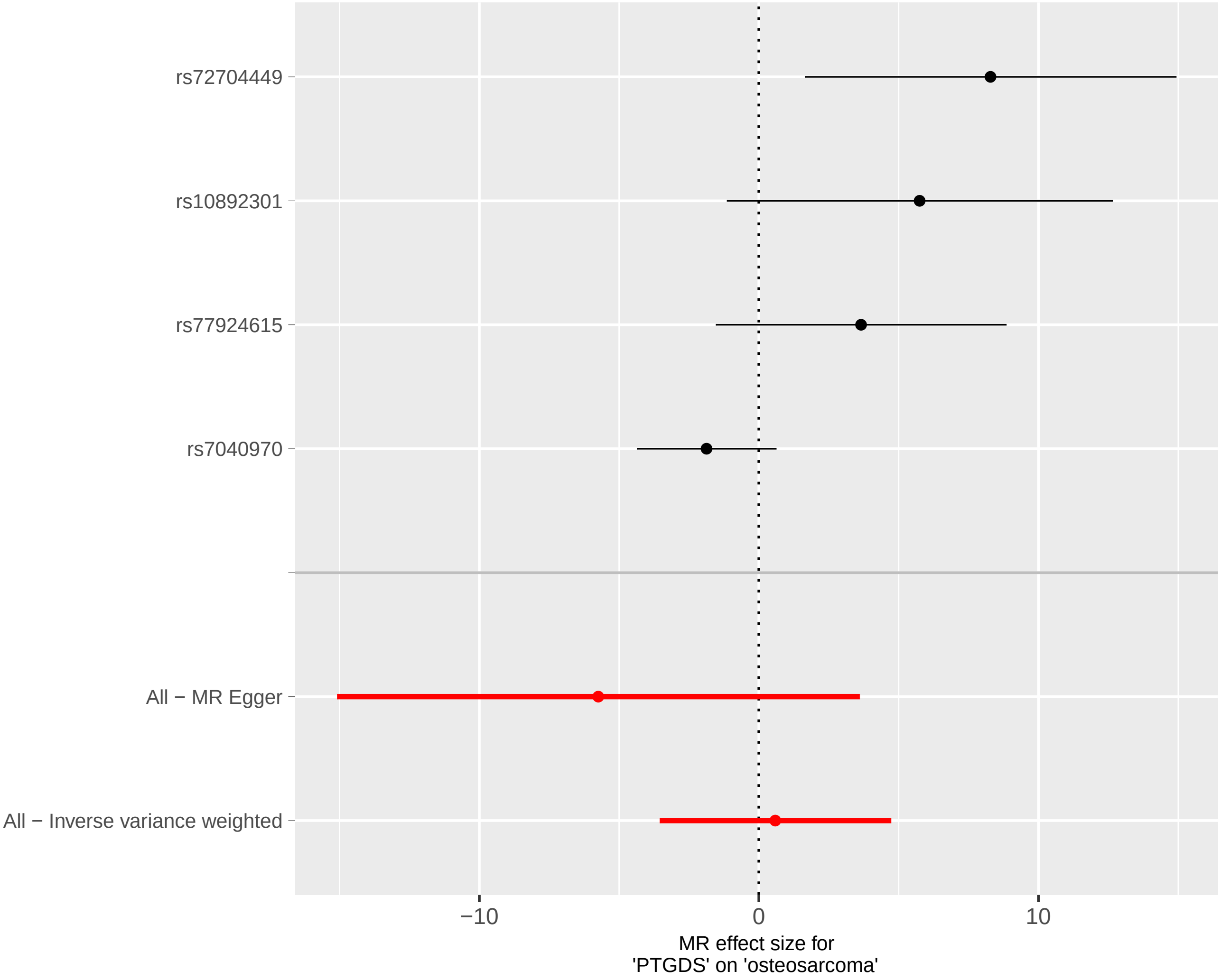

rs72704449
rs10892301
rs77924615
rs7040970
All − MR Egger
All − Inverse variance weighted
−10
0
10
MR effect size for
'PTGDS' on 'osteosarcoma'

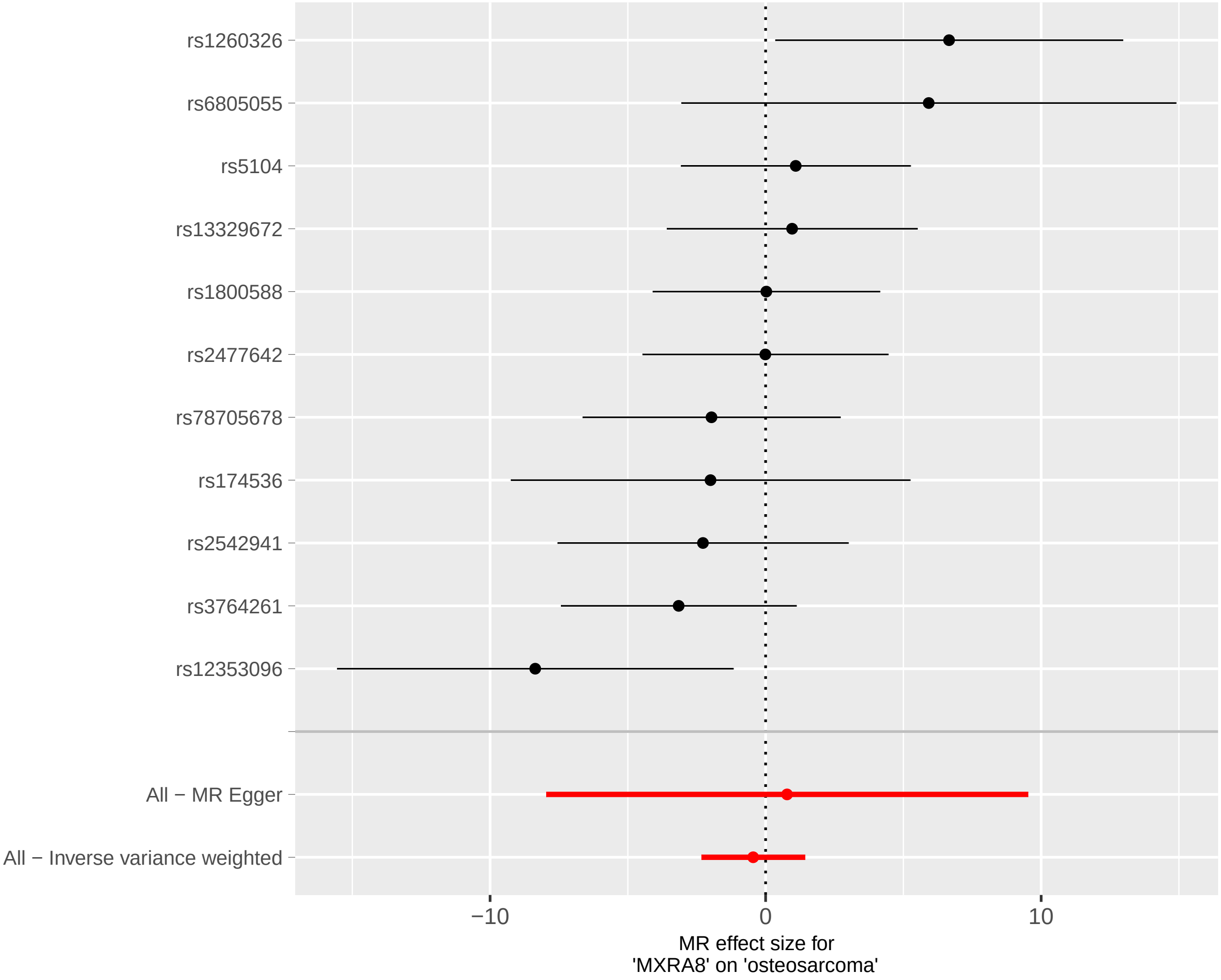

rs1260326
rs6805055
rs5104
rs13329672
rs1800588
rs2477642
rs78705678
rs174536
rs2542941
rs3764261
rs12353096
All − MR Egger
All − Inverse variance weighted
−10
0
10
MR effect size for
'MXRA8' on 'osteosarcoma'

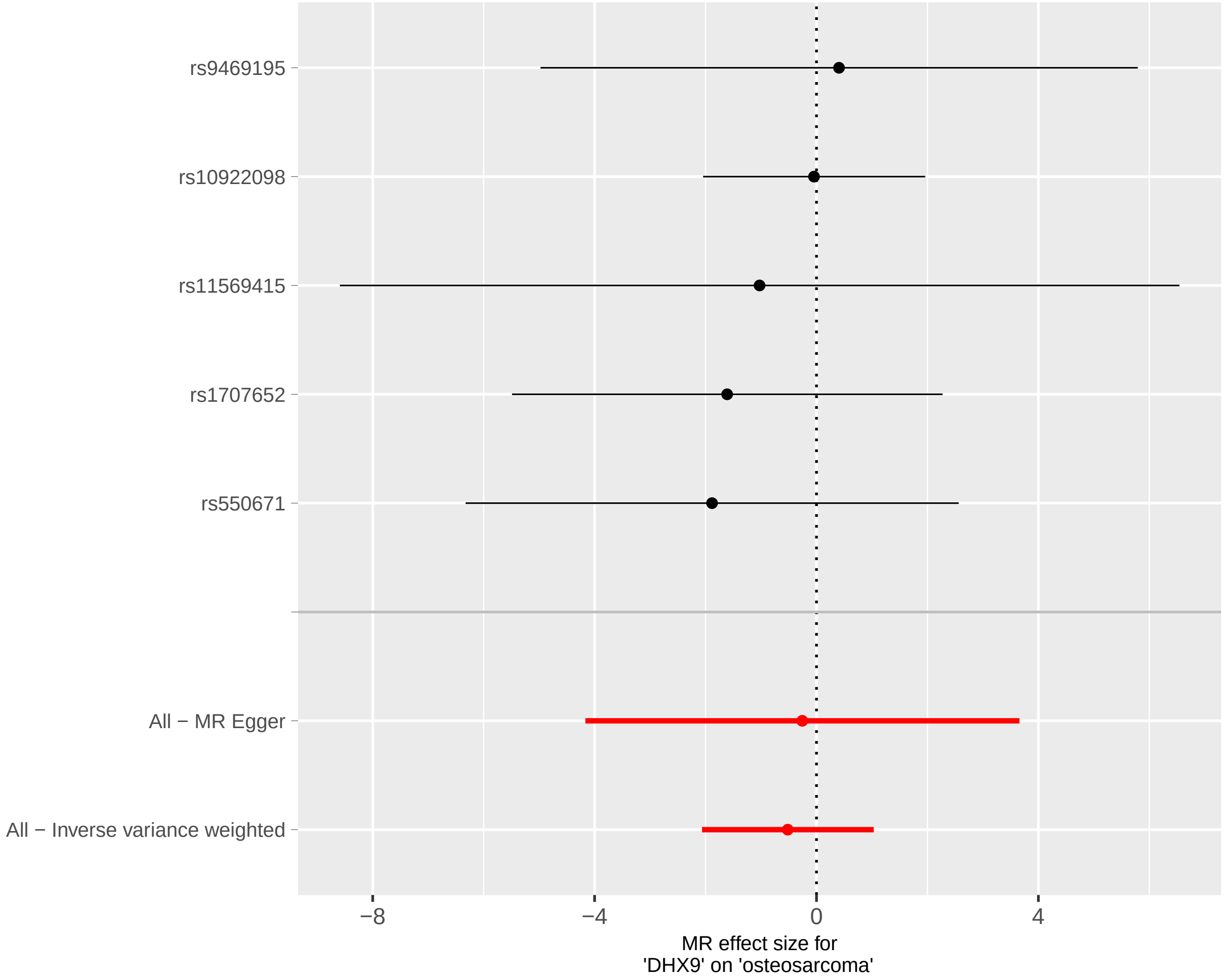

rs9469195
rs10922098
rs11569415
rs1707652
rs550671
All − MR Egger
All − Inverse variance weighted
−8
−4
0
4
MR effect size for
'DHX9' on 'osteosarcoma'

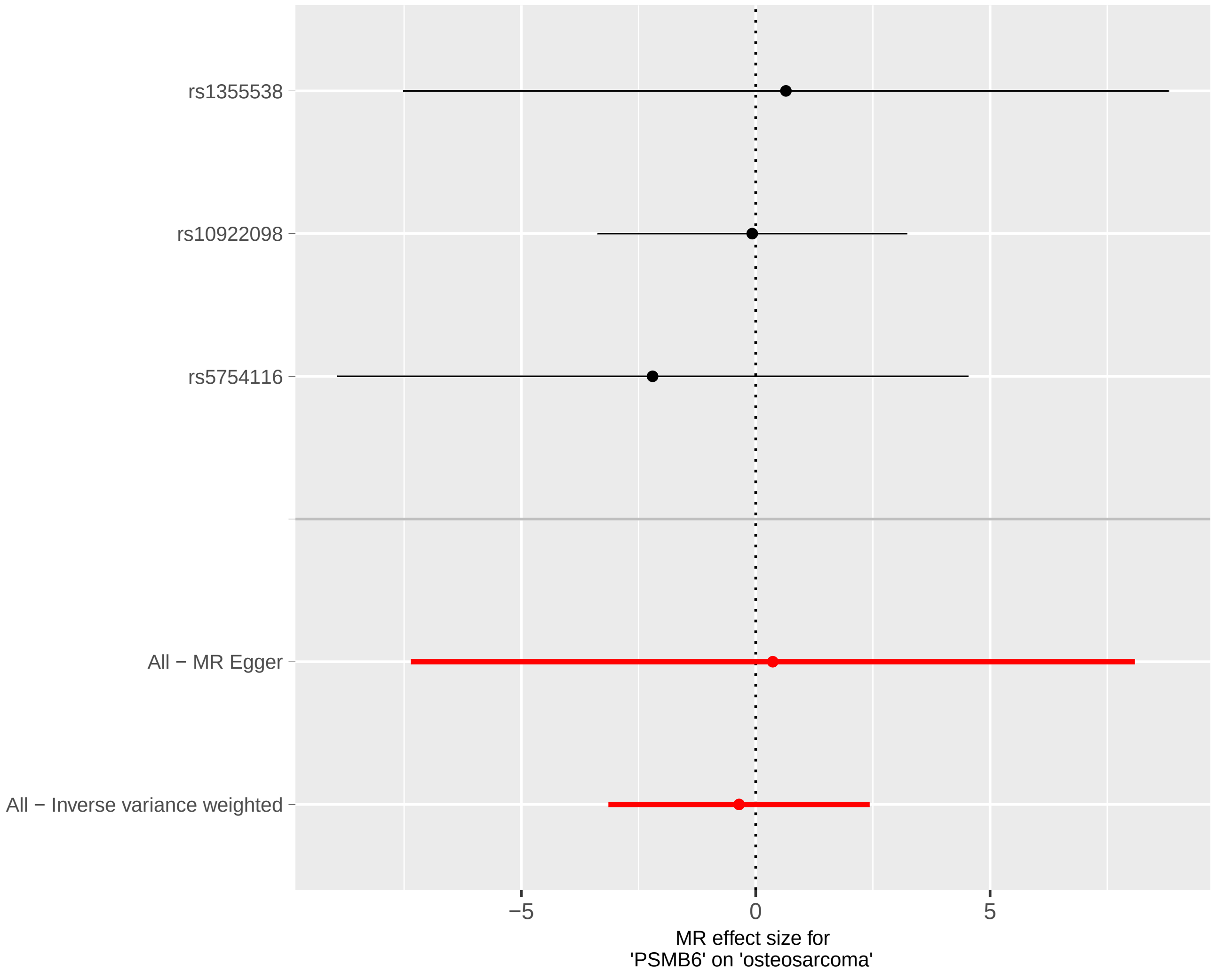
rs1355538
rs10922098
rs5754116
All − MR Egger
All − Inverse variance weighted
−5
0
5
MR effect size for
'PSMB6' on 'osteosarcoma'

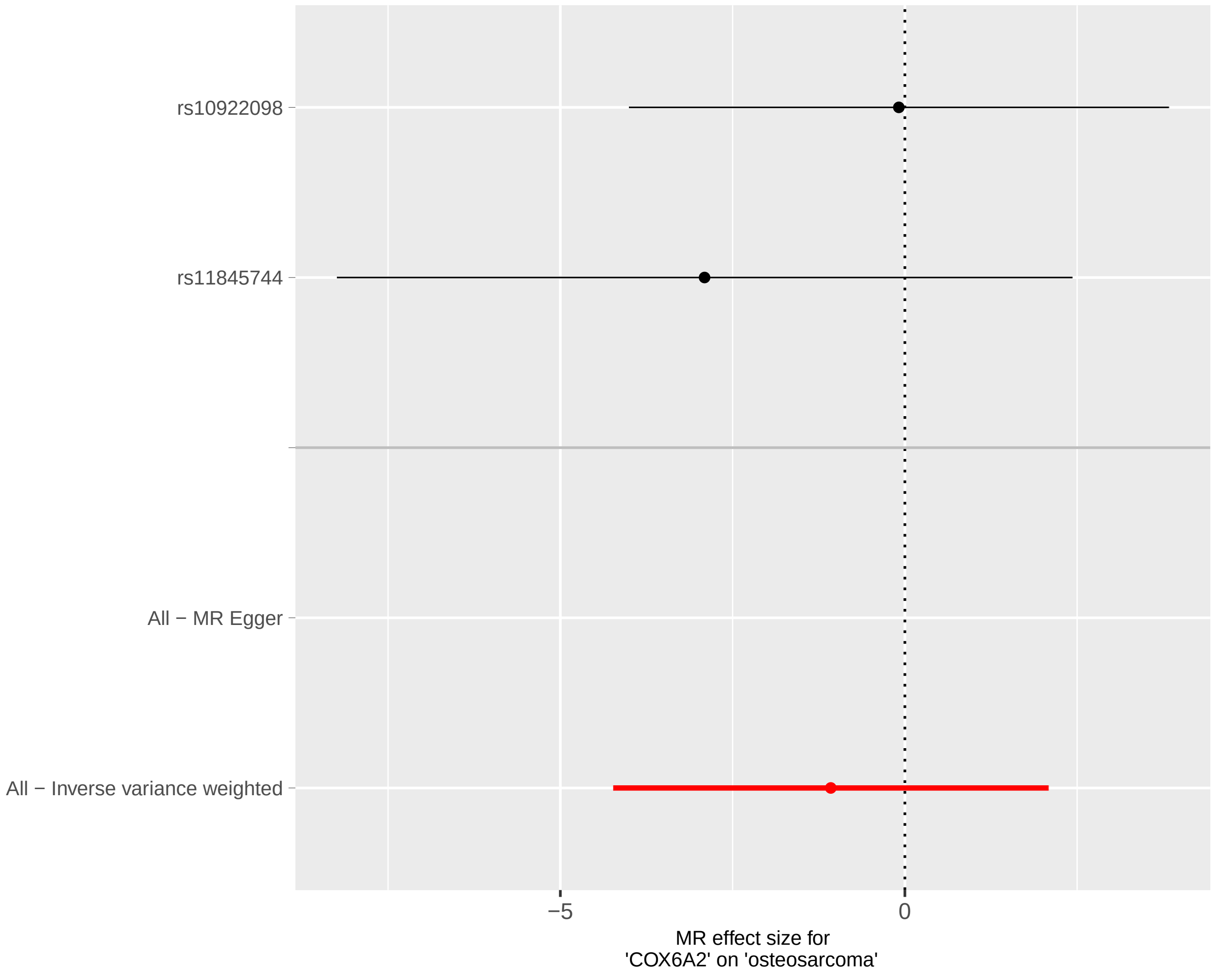

rs10922098
rs11845744
All − MR Egger
All − Inverse variance weighted
−5
0
MR effect size for
'COX6A2' on 'osteosarcoma'

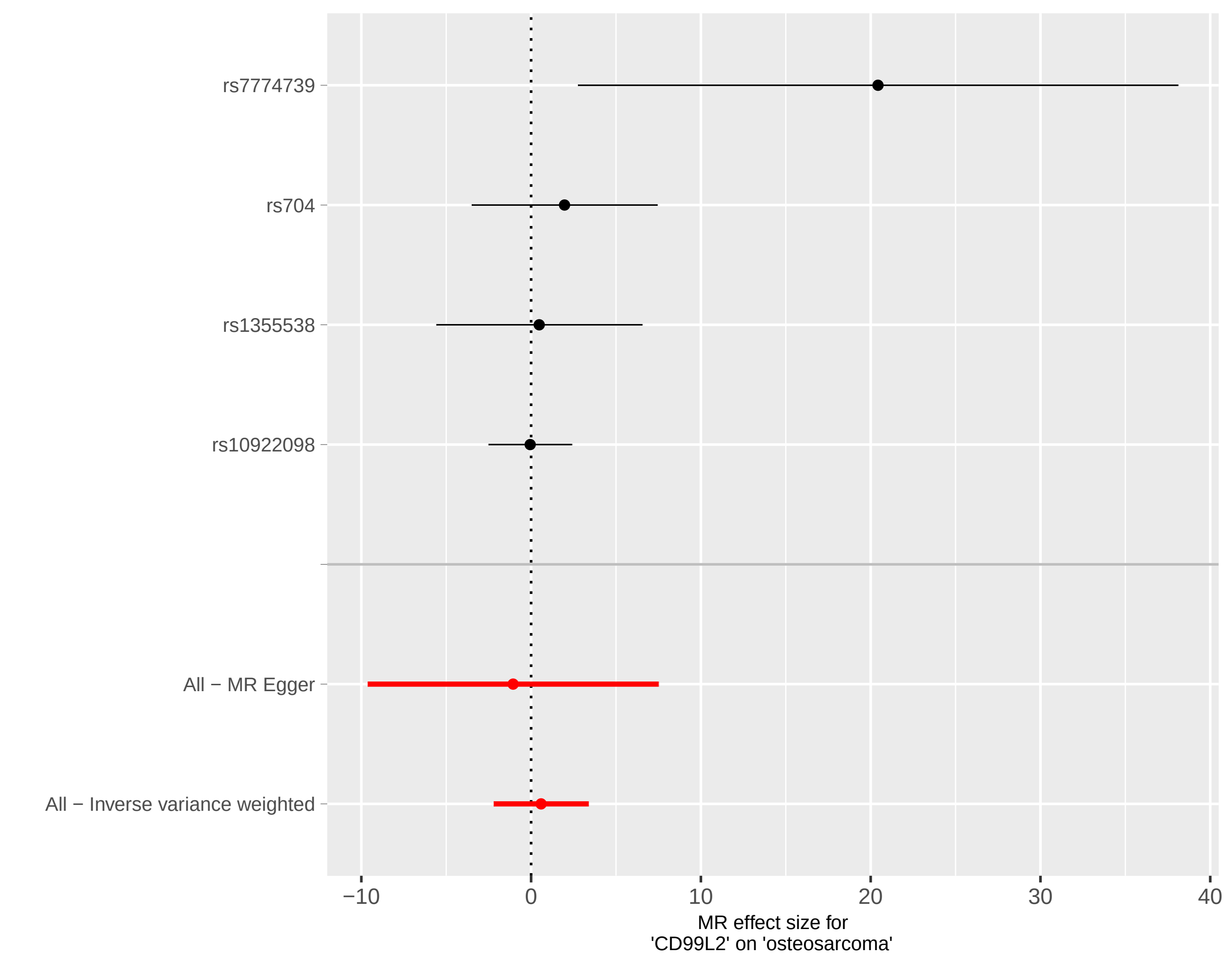

rs7774739
rs704
rs1355538
rs10922098
All − MR Egger
All − Inverse variance weighted
−10
0
10
20
30
40
MR effect size for
'CD99L2' on 'osteosarcoma'

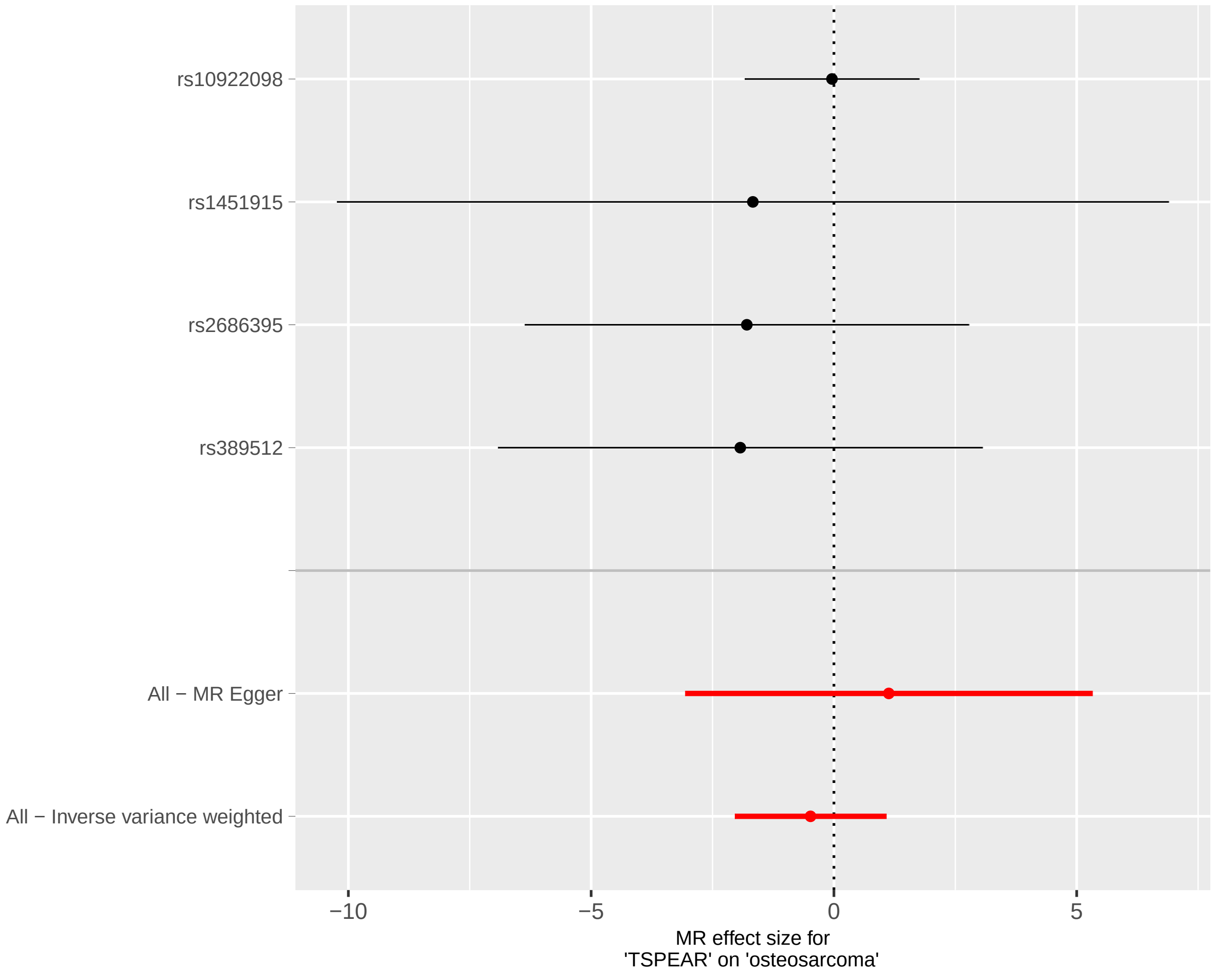

rs10922098
rs1451915
rs2686395
rs389512
All − MR Egger
All − Inverse variance weighted
−10
−5
0
5
MR effect size for
'TSPEAR' on 'osteosarcoma'

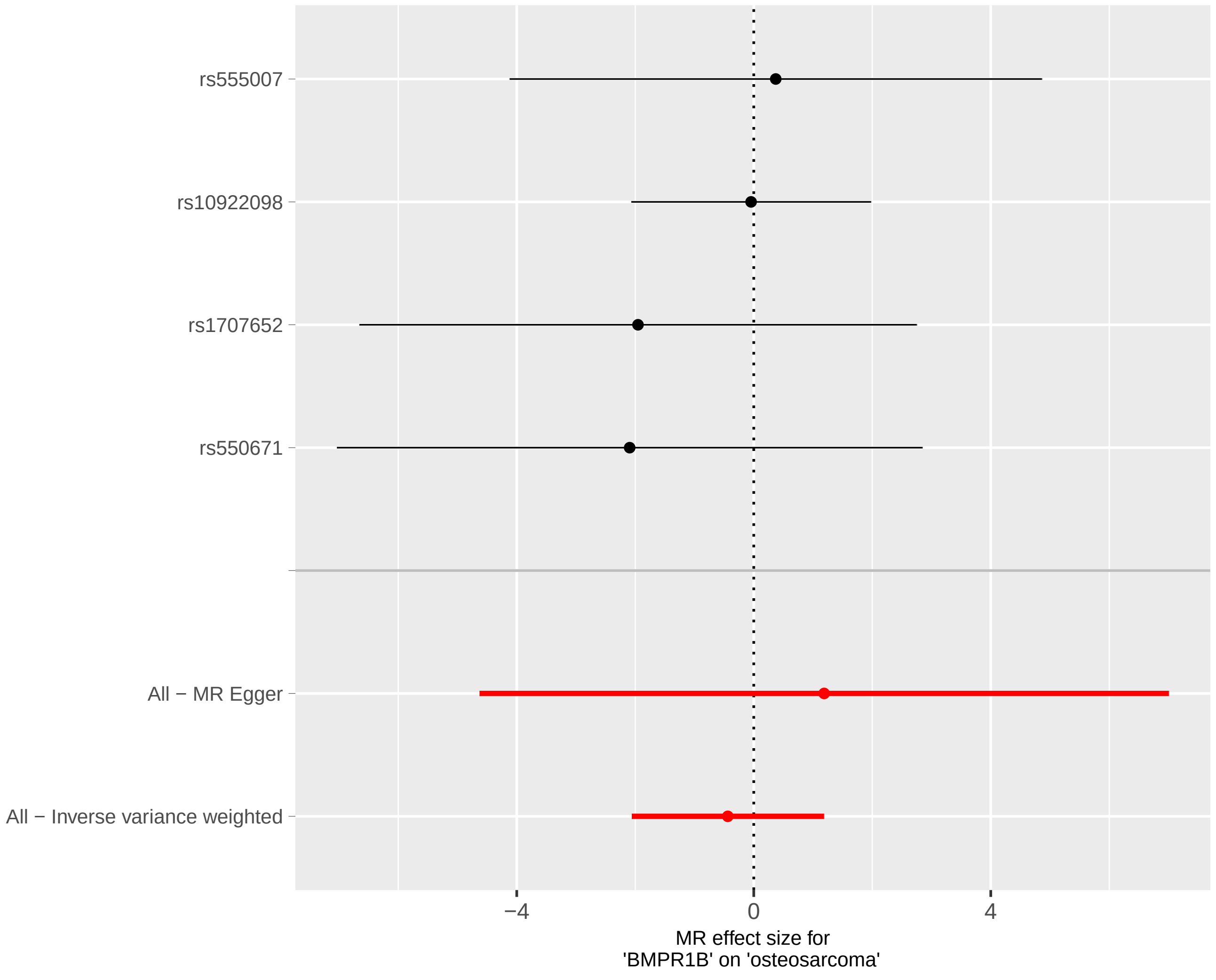
rs555007
rs10922098
rs1707652
rs550671
All − MR Egger
All − Inverse variance weighted
−4
0
4
MR effect size for
'BMPR1B' on 'osteosarcoma'

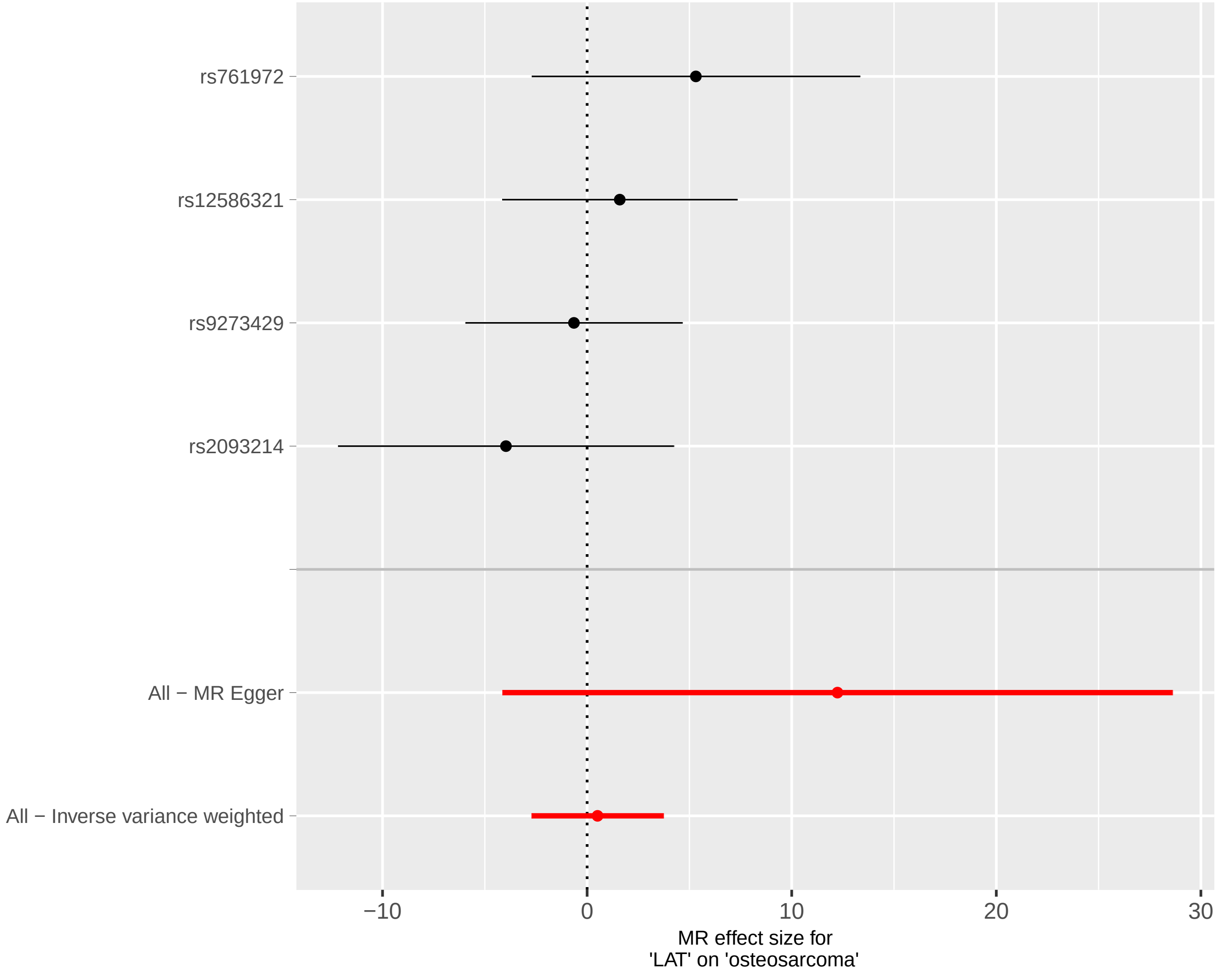
rs761972
rs12586321
rs9273429
rs2093214
All − MR Egger
All − Inverse variance weighted
−10
0
10
20
30
MR effect size for
'LAT' on 'osteosarcoma'

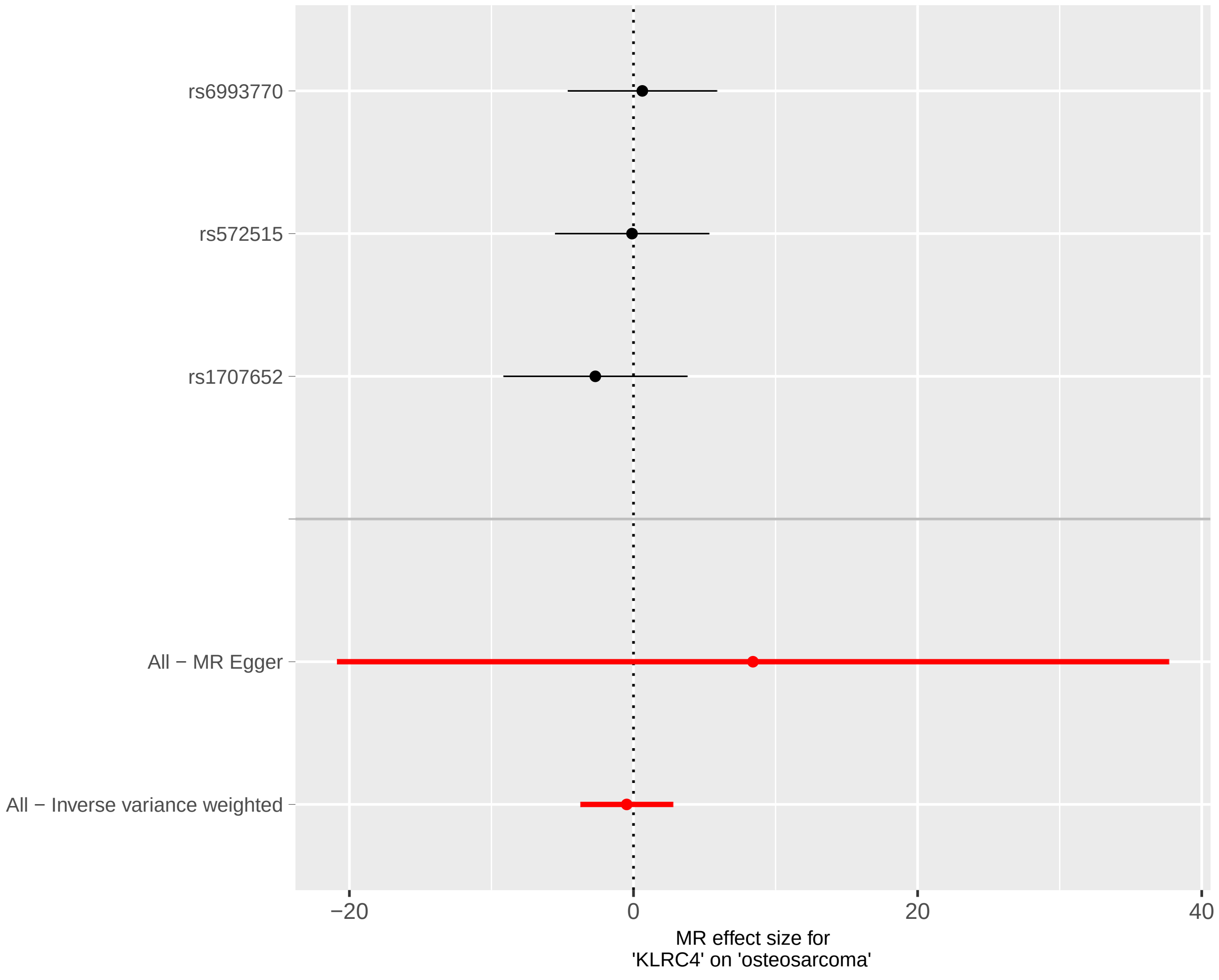
rs6993770
rs572515
rs1707652
All − MR Egger
All − Inverse variance weighted
−20
0
20
40
MR effect size for
'KLRC4' on 'osteosarcoma'

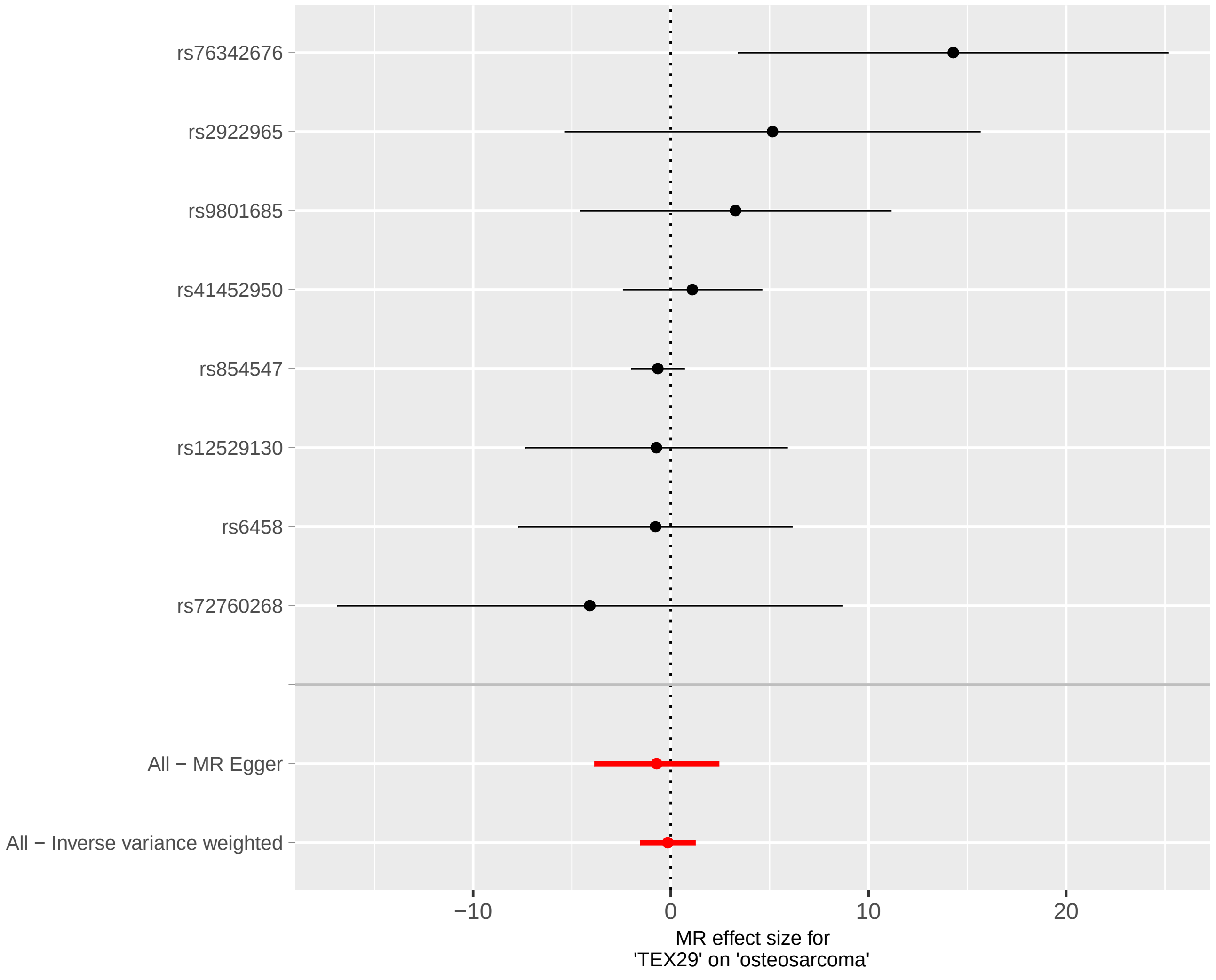
rs76342676
rs2922965
rs9801685
rs41452950
rs854547
rs12529130
rs6458
rs72760268
All − MR Egger
All − Inverse variance weighted
−10
0
10
20
MR effect size for
'TEX29' on 'osteosarcoma'

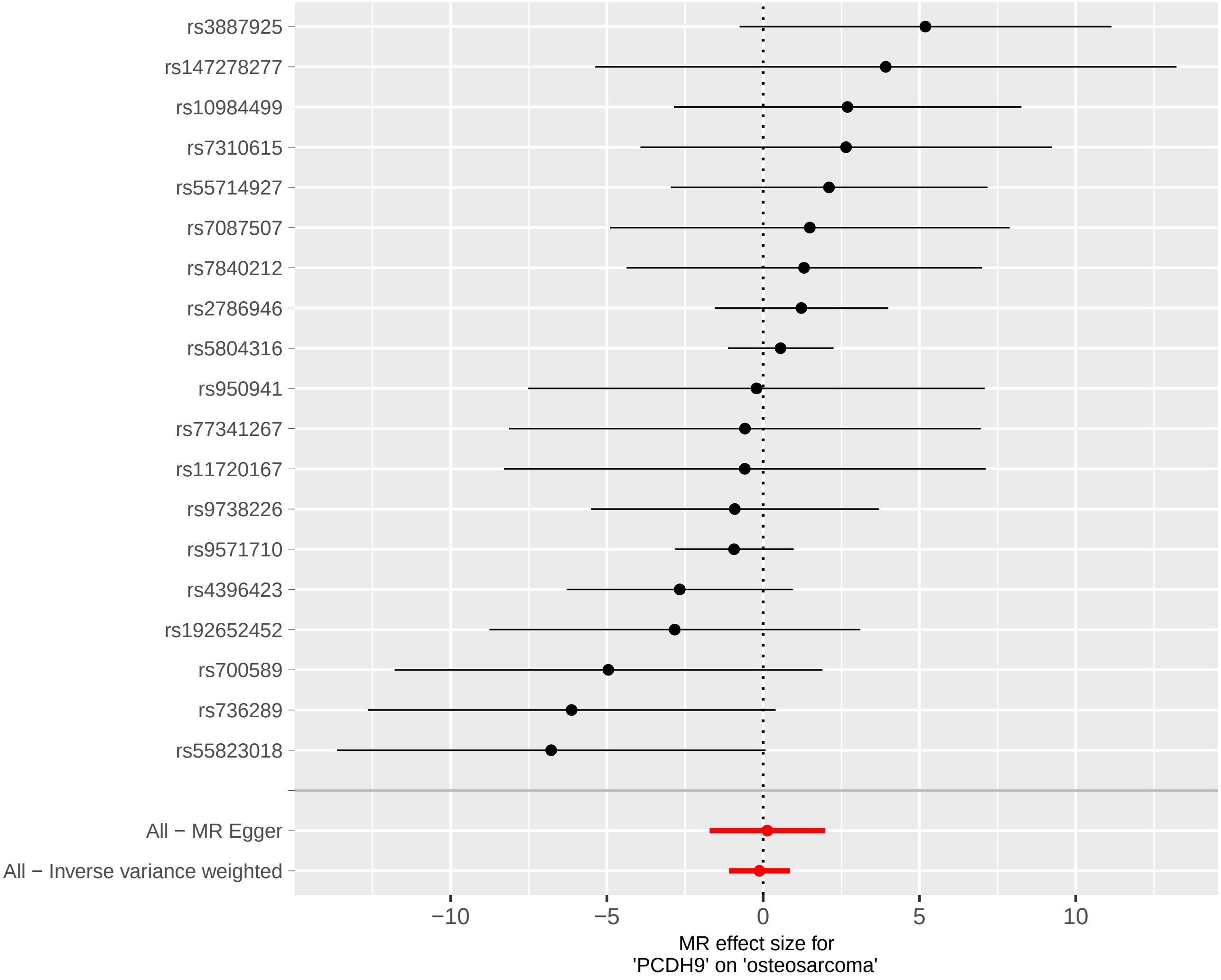

rs3887925
rs147278277
rs10984499
rs7310615
rs55714927
rs7087507
rs7840212
rs2786946
rs5804316
rs950941
rs77341267
rs11720167
rs9738226
rs9571710
rs4396423
rs192652452
rs700589
rs736289
rs55823018
All − MR Egger
All − Inverse variance weighted
−10
−5
0
5
10
MR effect size for
'PCDH9' on 'osteosarcoma'

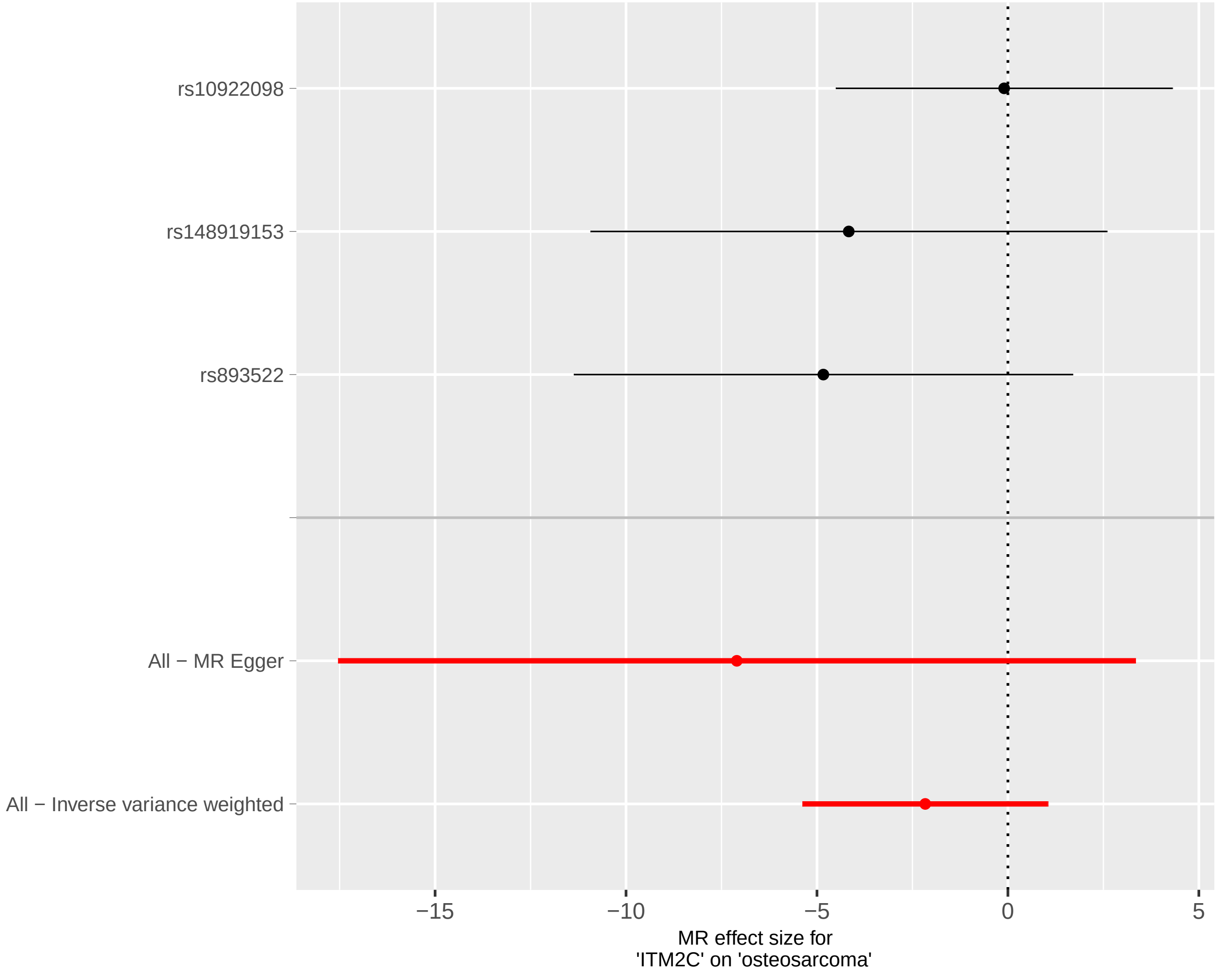

rs10922098
rs148919153
rs893522
All − MR Egger
All − Inverse variance weighted
−15
−10
−5
0
5
MR effect size for
'ITM2C' on 'osteosarcoma'

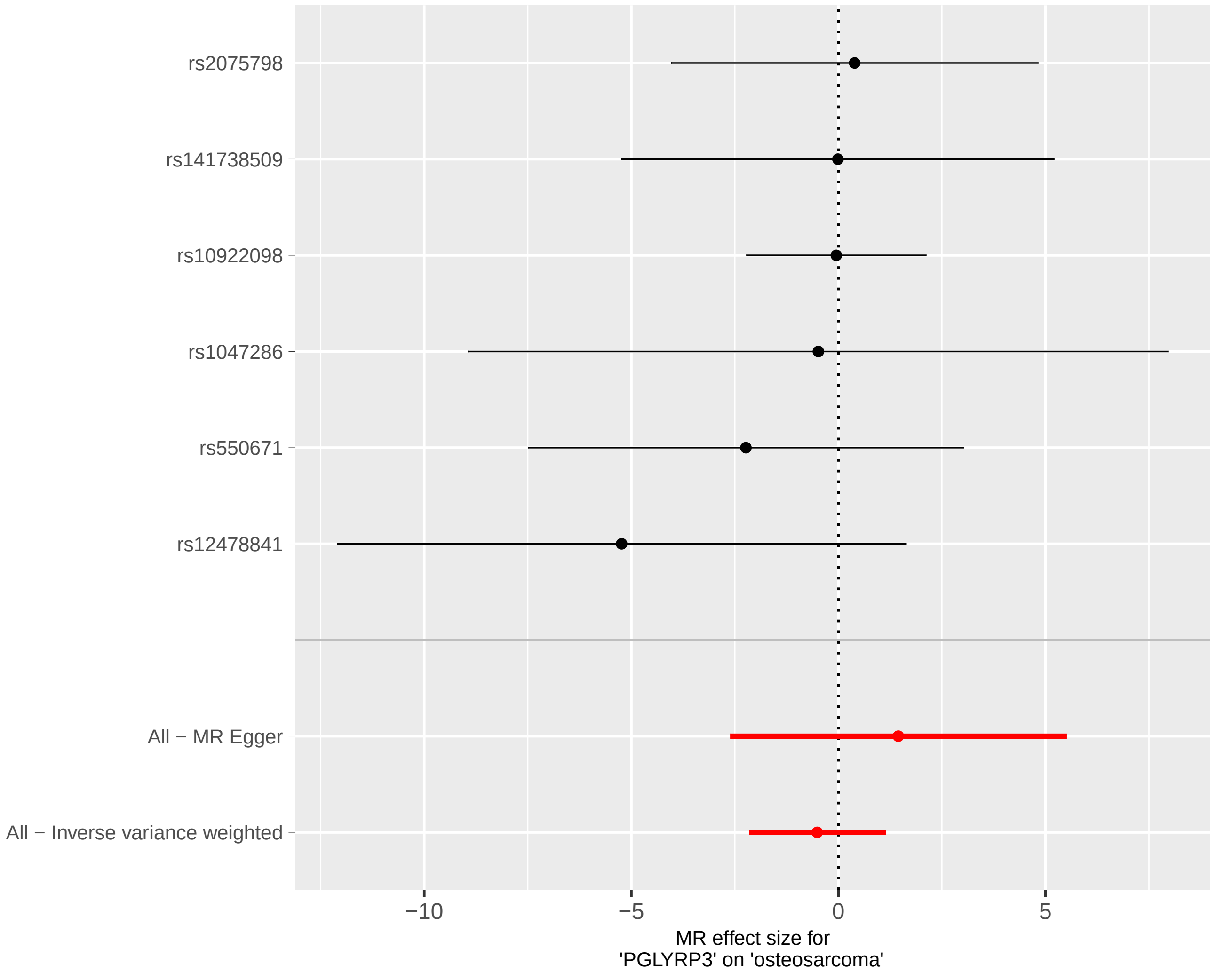
rs2075798
rs141738509
rs10922098
rs1047286
rs550671
rs12478841
All − MR Egger
All − Inverse variance weighted
−10
−5
0
5
MR effect size for
'PGLYRP3' on 'osteosarcoma'

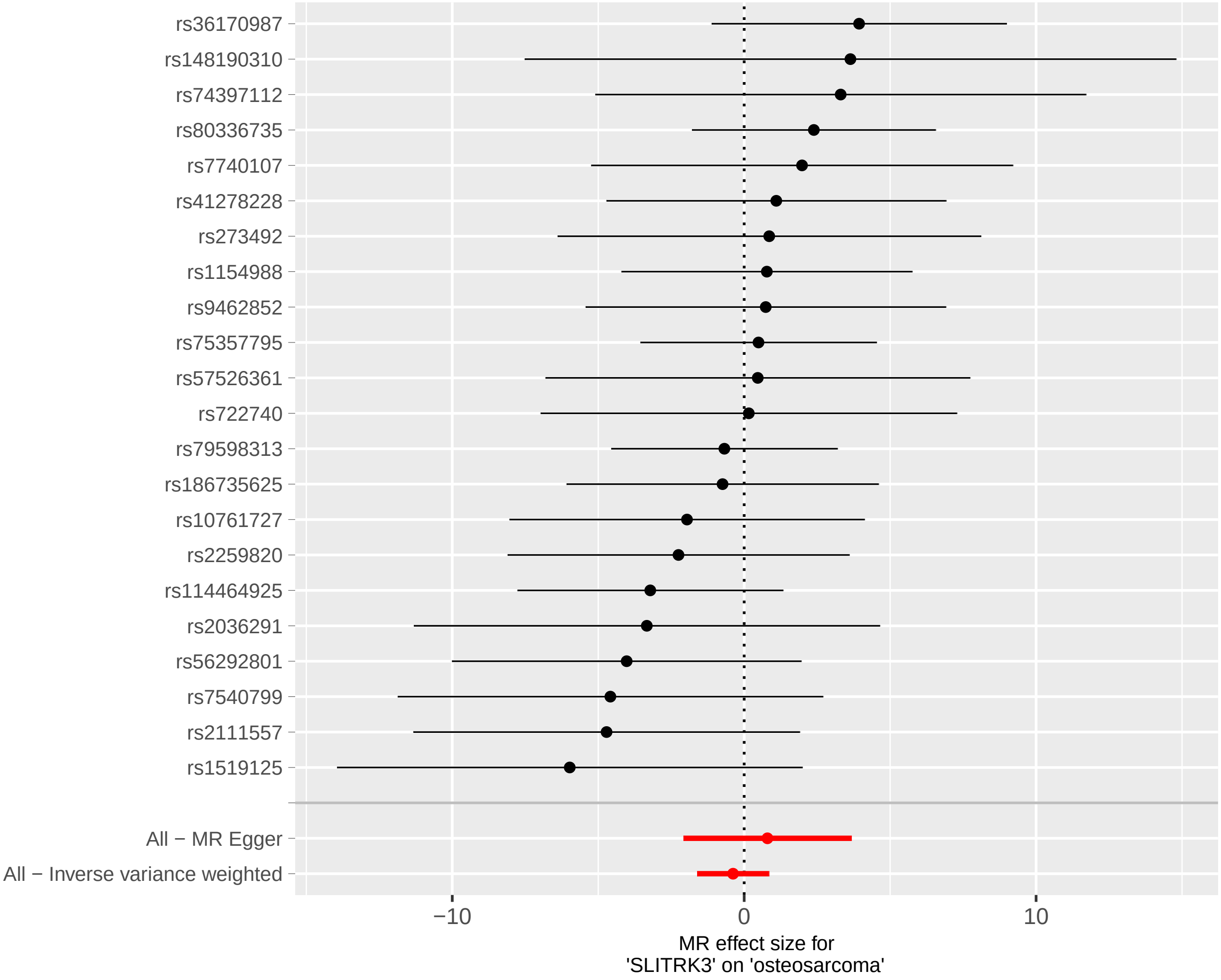

rs36170987
rs148190310
rs74397112
rs80336735
rs7740107
rs41278228
rs273492
rs1154988
rs9462852
rs75357795
rs57526361
rs722740
rs79598313
rs186735625
rs10761727
rs2259820
rs114464925
rs2036291
rs56292801
rs7540799
rs2111557
rs1519125
All − MR Egger
All − Inverse variance weighted
−10
0
10
MR effect size for
'SLITRK3' on 'osteosarcoma'

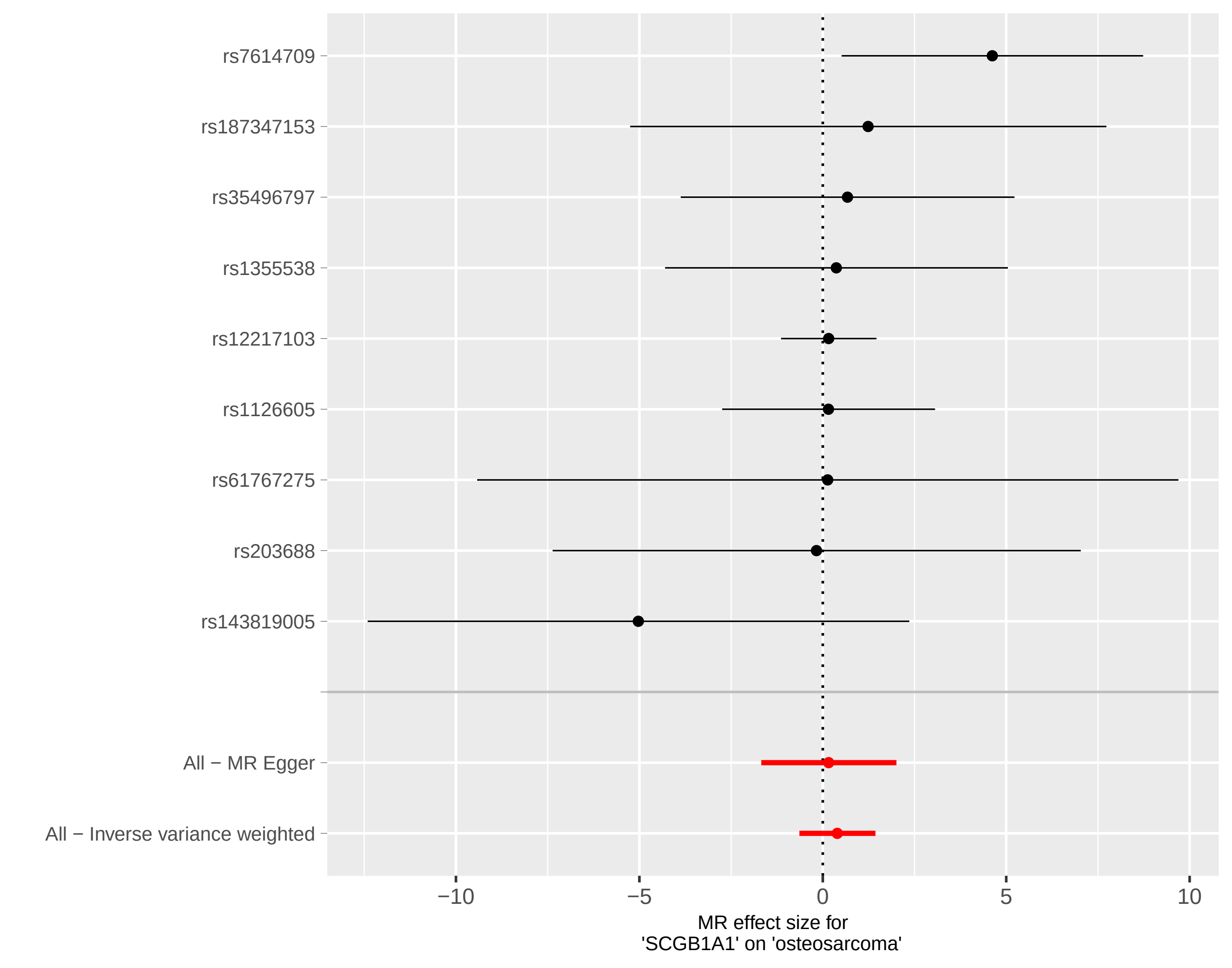

rs7614709
rs187347153
rs35496797
rs1355538
rs12217103
rs1126605
rs61767275
rs203688
rs143819005
All − MR Egger
All − Inverse variance weighted
−10
−5
0
5
10
MR effect size for
'SCGB1A1' on 'osteosarcoma'

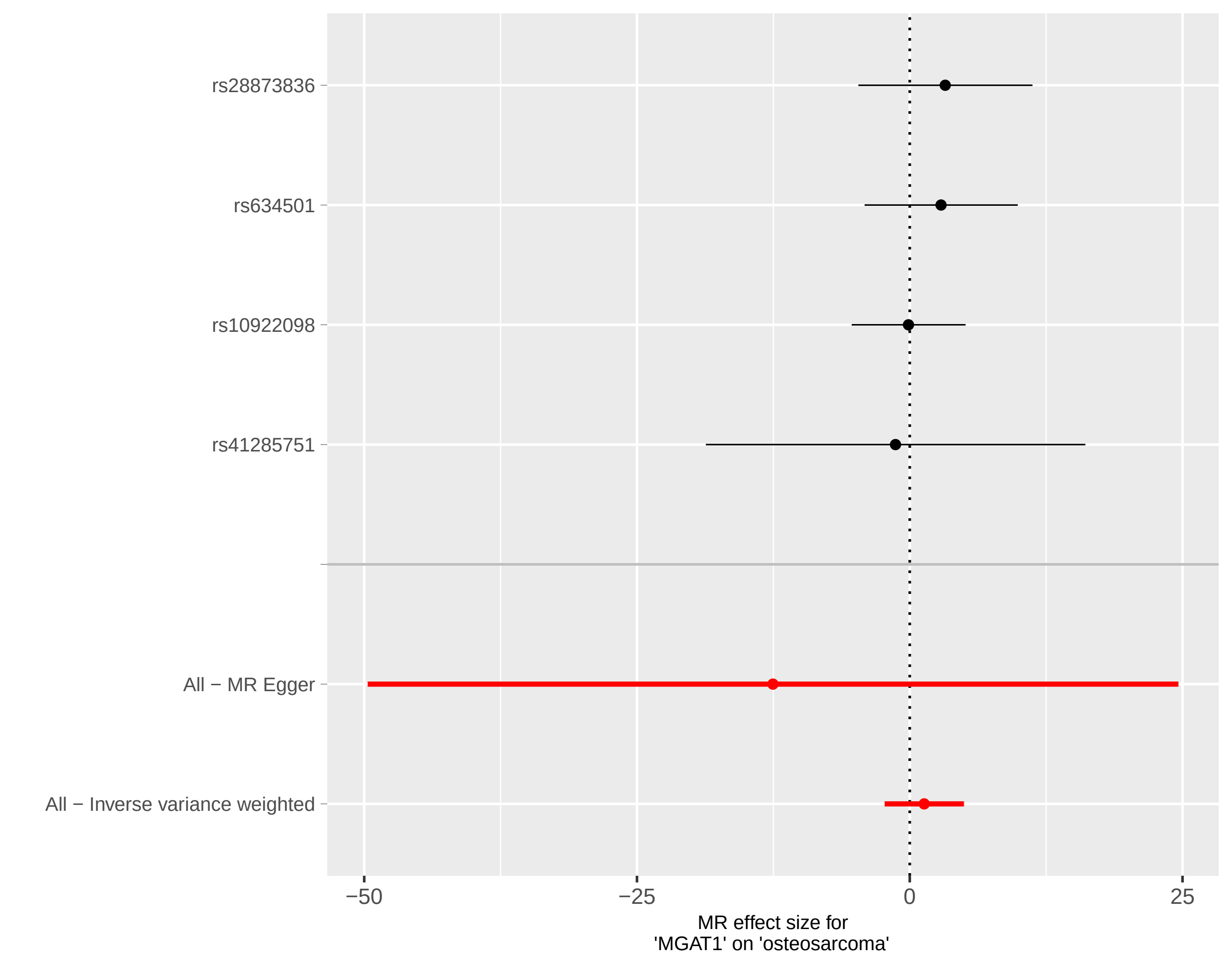
rs28873836
rs634501
rs10922098
rs41285751
All − MR Egger
All − Inverse variance weighted
−50
−25
0
25
MR effect size for
'MGAT1' on 'osteosarcoma'

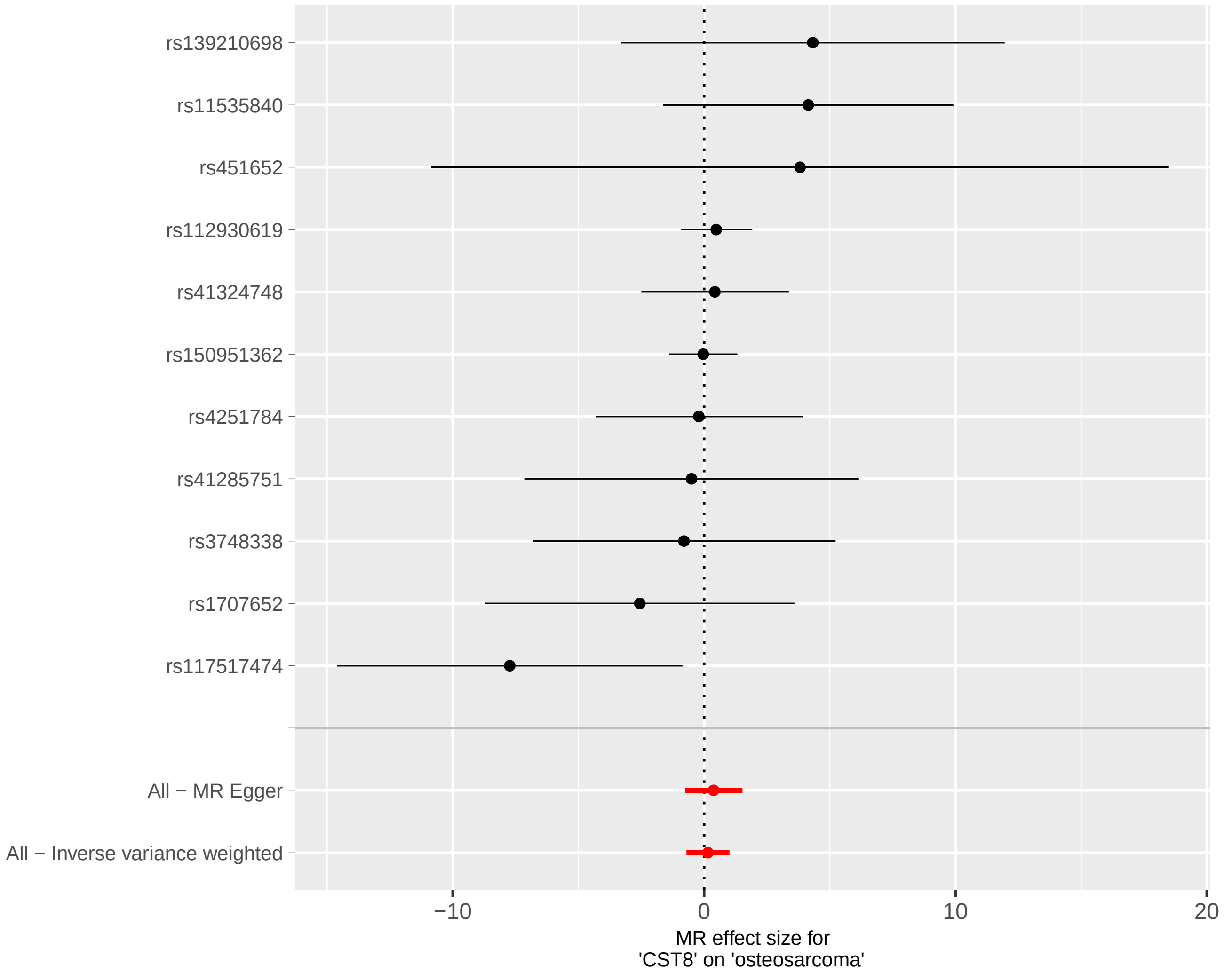

rs139210698
rs11535840
rs451652
rs112930619
rs41324748
rs150951362
rs4251784
rs41285751
rs3748338
rs1707652
rs117517474
All − MR Egger
All − Inverse variance weighted
−10
0
10
20
MR effect size for
'CST8' on 'osteosarcoma'

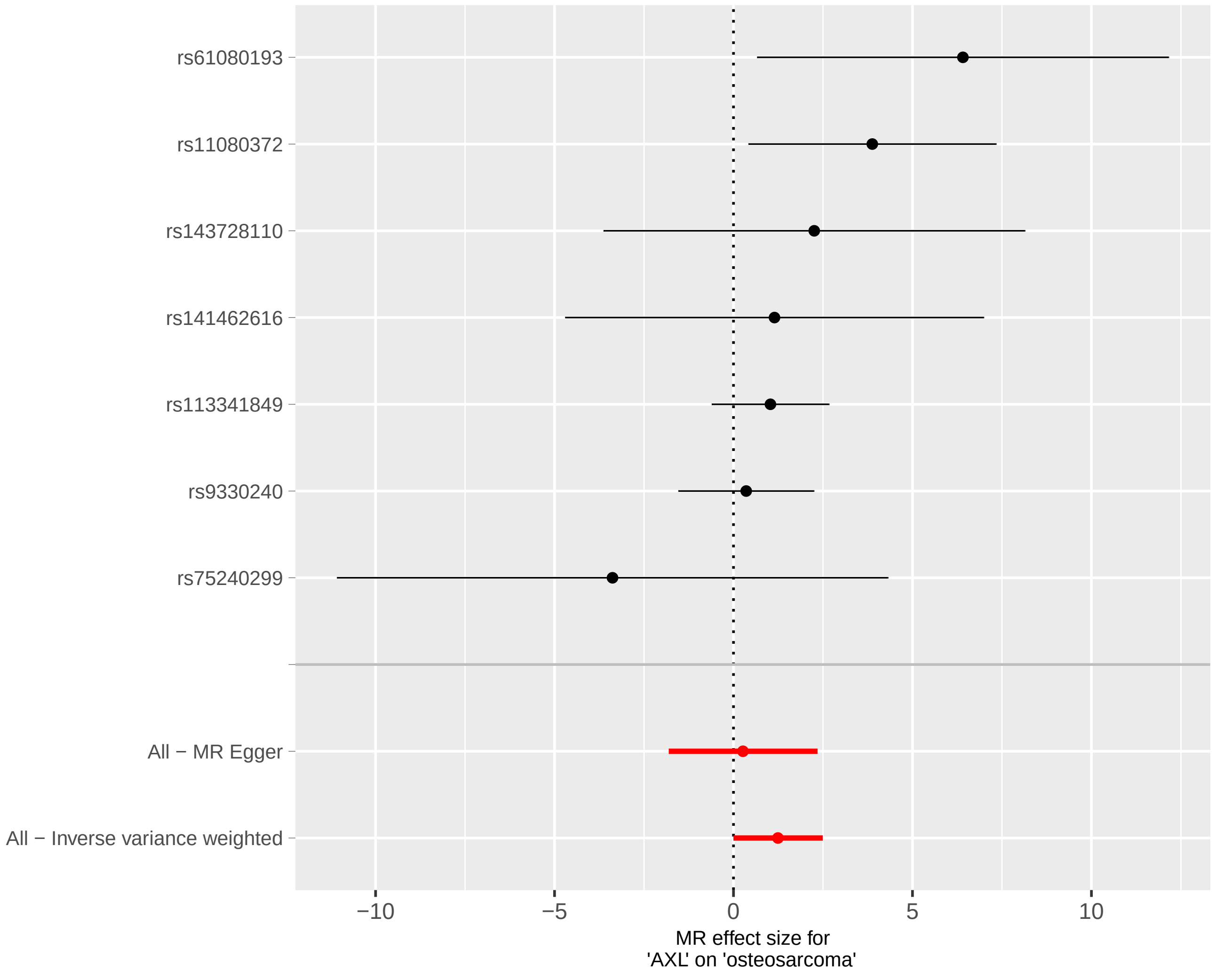

rs61080193
rs11080372
rs143728110
rs141462616
rs113341849
rs9330240
rs75240299
All − MR Egger
All − Inverse variance weighted
−10
−5
0
5
10
MR effect size for
'AXL' on 'osteosarcoma'

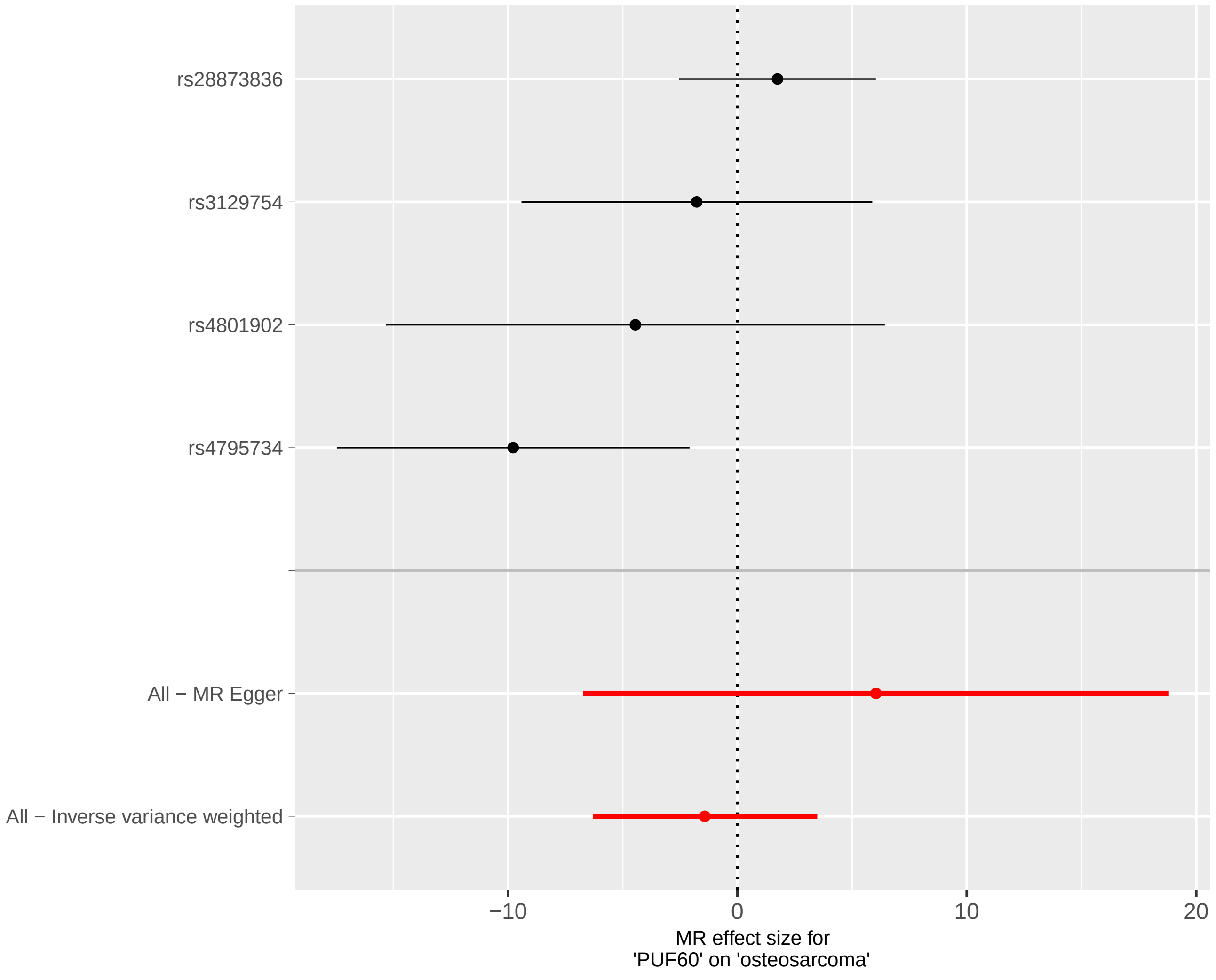
rs28873836
rs3129754
rs4801902
rs4795734
All − MR Egger
All − Inverse variance weighted
−10
0
10
20
MR effect size for
'PUF60' on 'osteosarcoma'

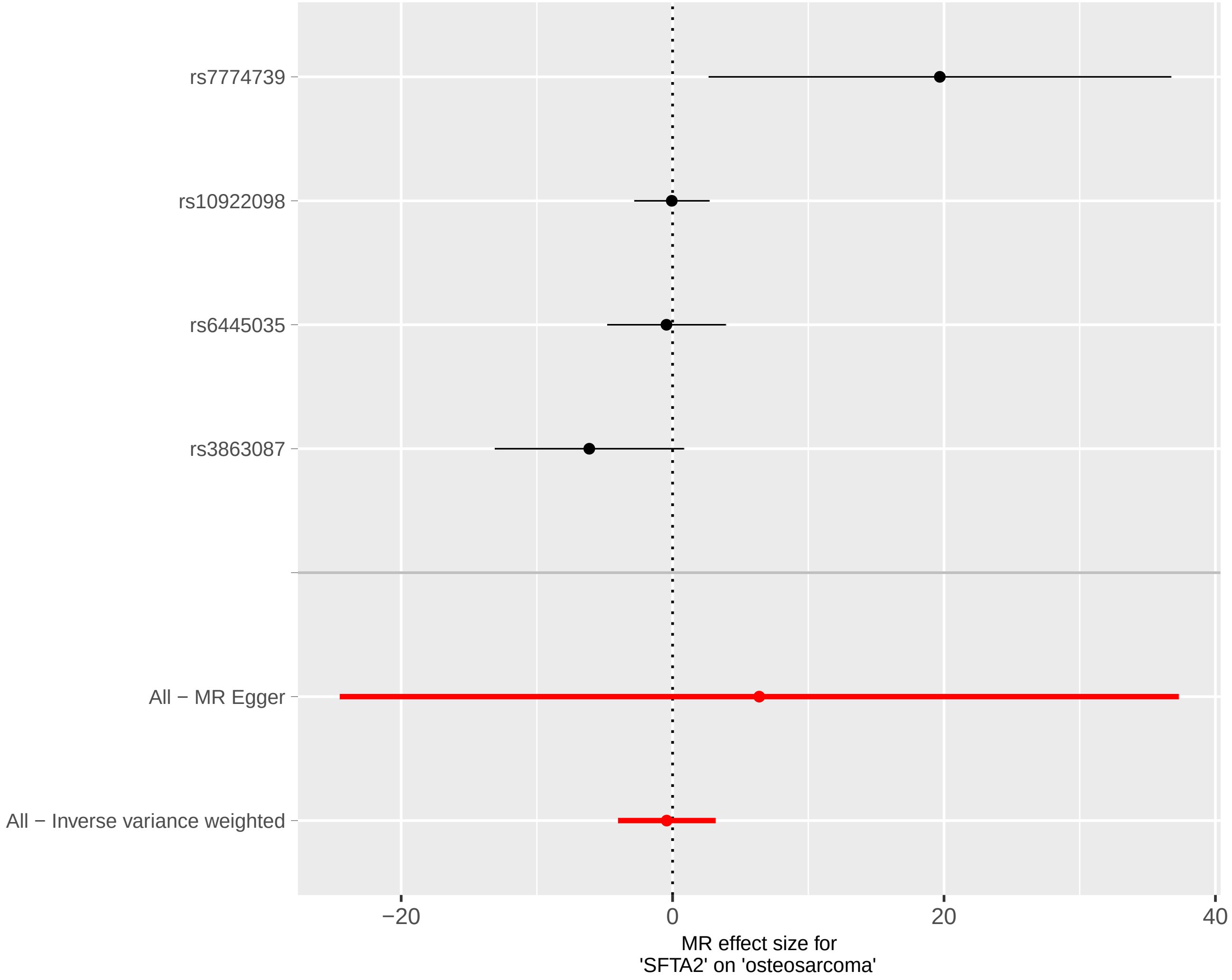

rs7774739
rs10922098
rs6445035
rs3863087
All − MR Egger
All − Inverse variance weighted
−20
0
20
40
MR effect size for
'SFTA2' on 'osteosarcoma'

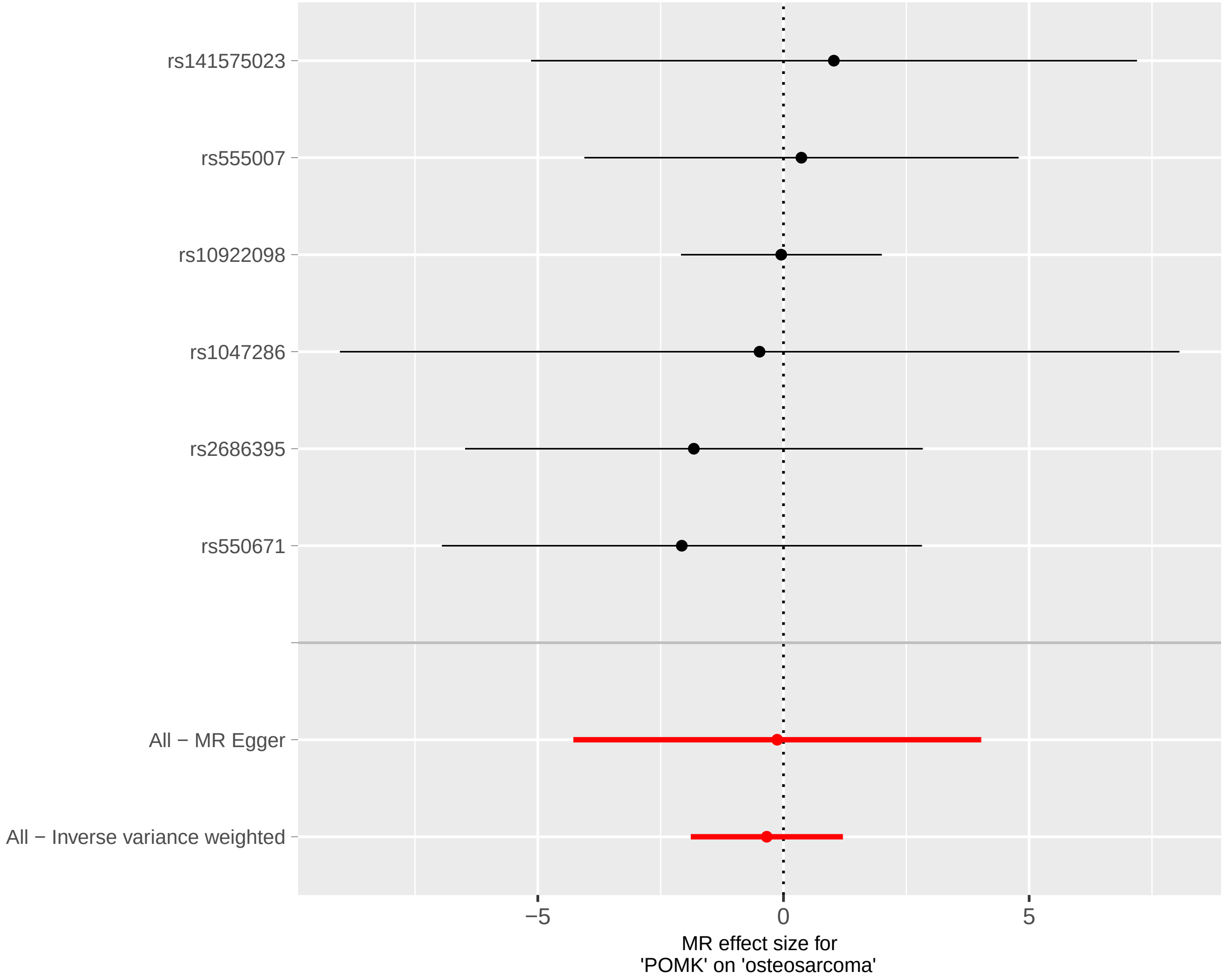

rs141575023
rs555007
rs10922098
rs1047286
rs2686395
rs550671
All − MR Egger
All − Inverse variance weighted
−5
0
5
MR effect size for
'POMK' on 'osteosarcoma'

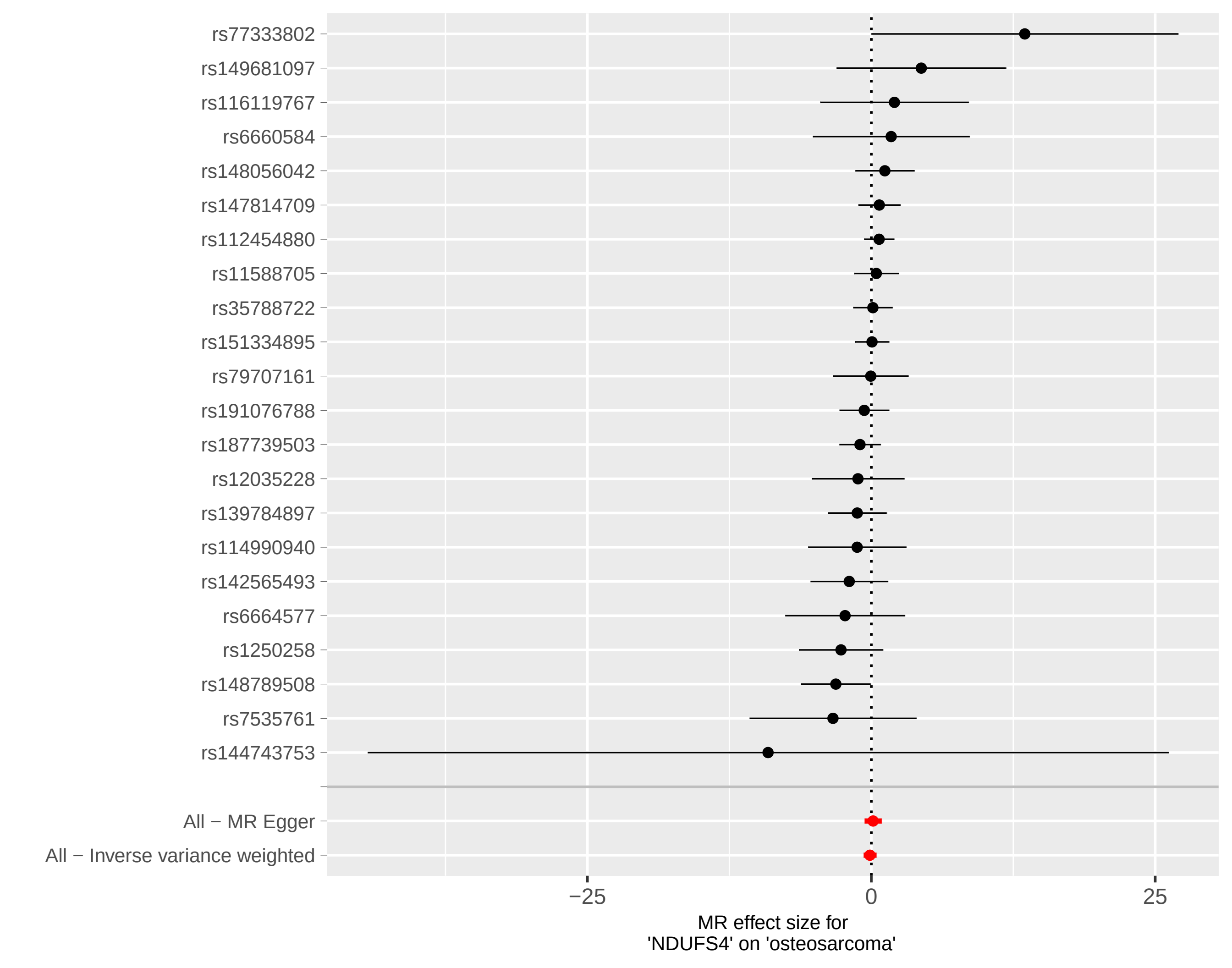
rs77333802
rs149681097
rs116119767
rs6660584
rs148056042
rs147814709
rs112454880
rs11588705
rs35788722
rs151334895
rs79707161
rs191076788
rs187739503
rs12035228
rs139784897
rs114990940
rs142565493
rs6664577
rs1250258
rs148789508
rs7535761
rs144743753
All − MR Egger
All − Inverse variance weighted
−25
0
25
MR effect size for
'NDUFS4' on 'osteosarcoma'

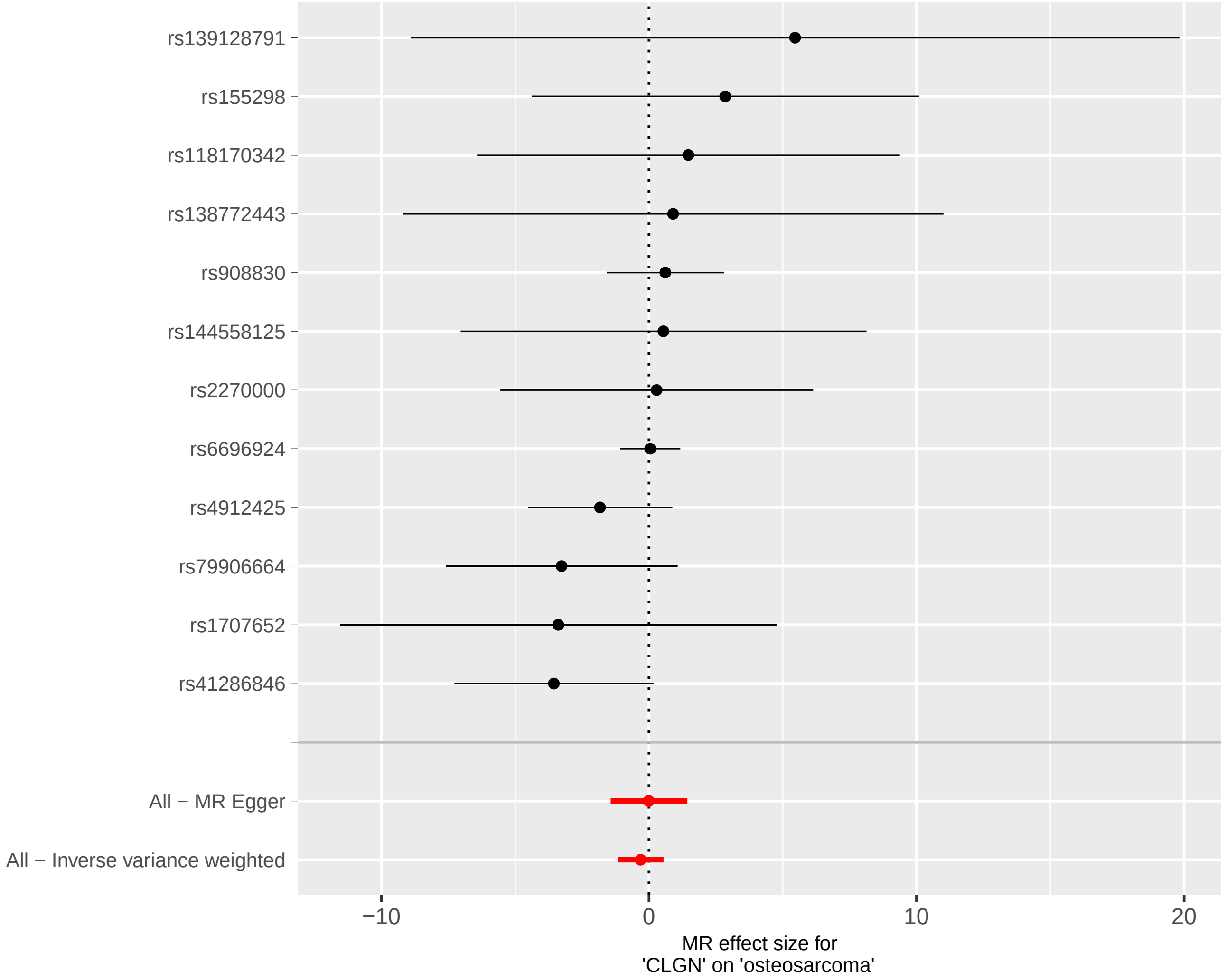
rs139128791
rs155298
rs118170342
rs138772443
rs908830
rs144558125
rs2270000
rs6696924
rs4912425
rs79906664
rs1707652
rs41286846
All − MR Egger
All − Inverse variance weighted
−10
0
10
20
MR effect size for
'CLGN' on 'osteosarcoma'

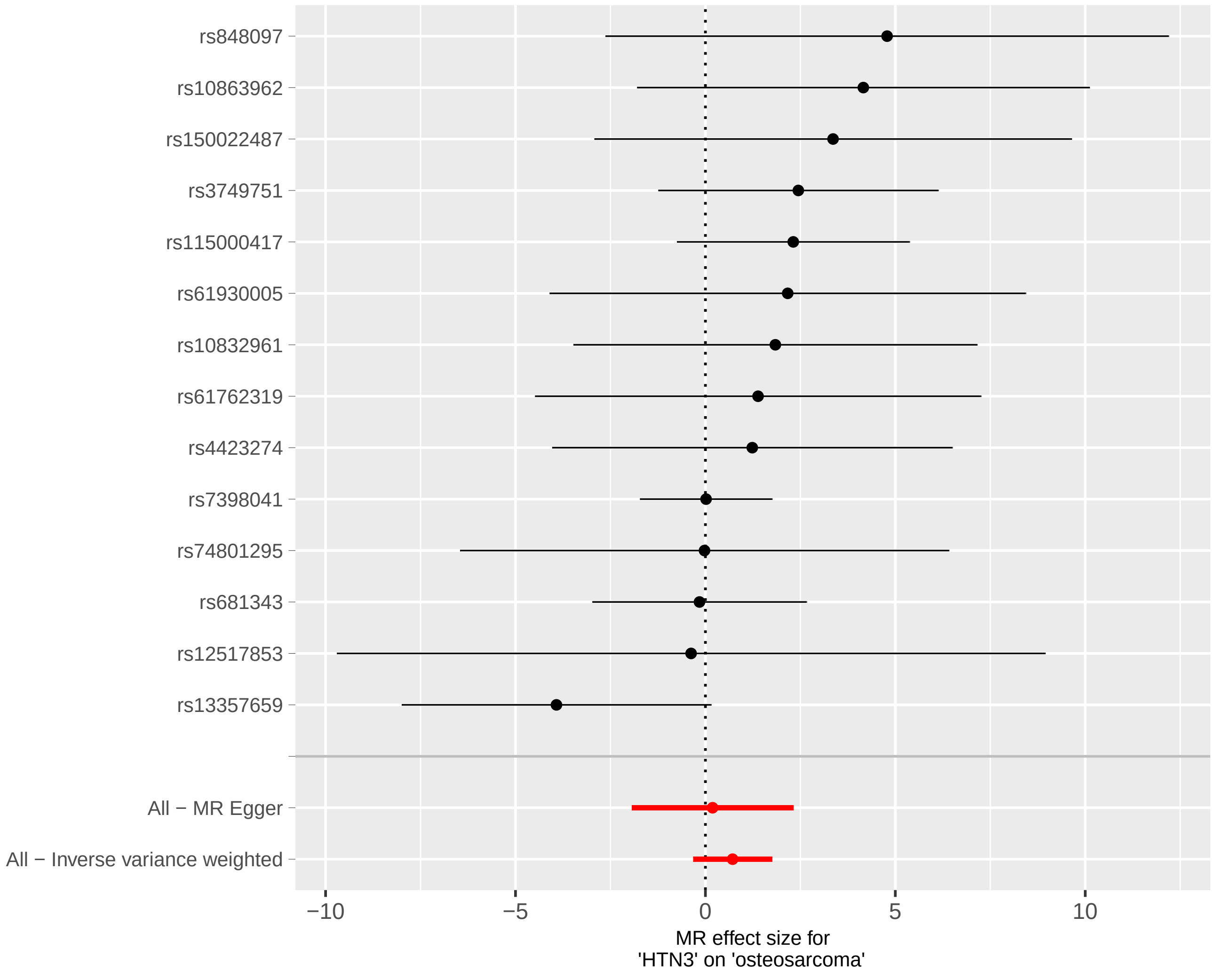
rs848097
rs10863962
rs150022487
rs3749751
rs115000417
rs61930005
rs10832961
rs61762319
rs4423274
rs7398041
rs74801295
rs681343
rs12517853
rs13357659
All − MR Egger
All − Inverse variance weighted
−10
−5
0
5
10
MR effect size for
'HTN3' on 'osteosarcoma'

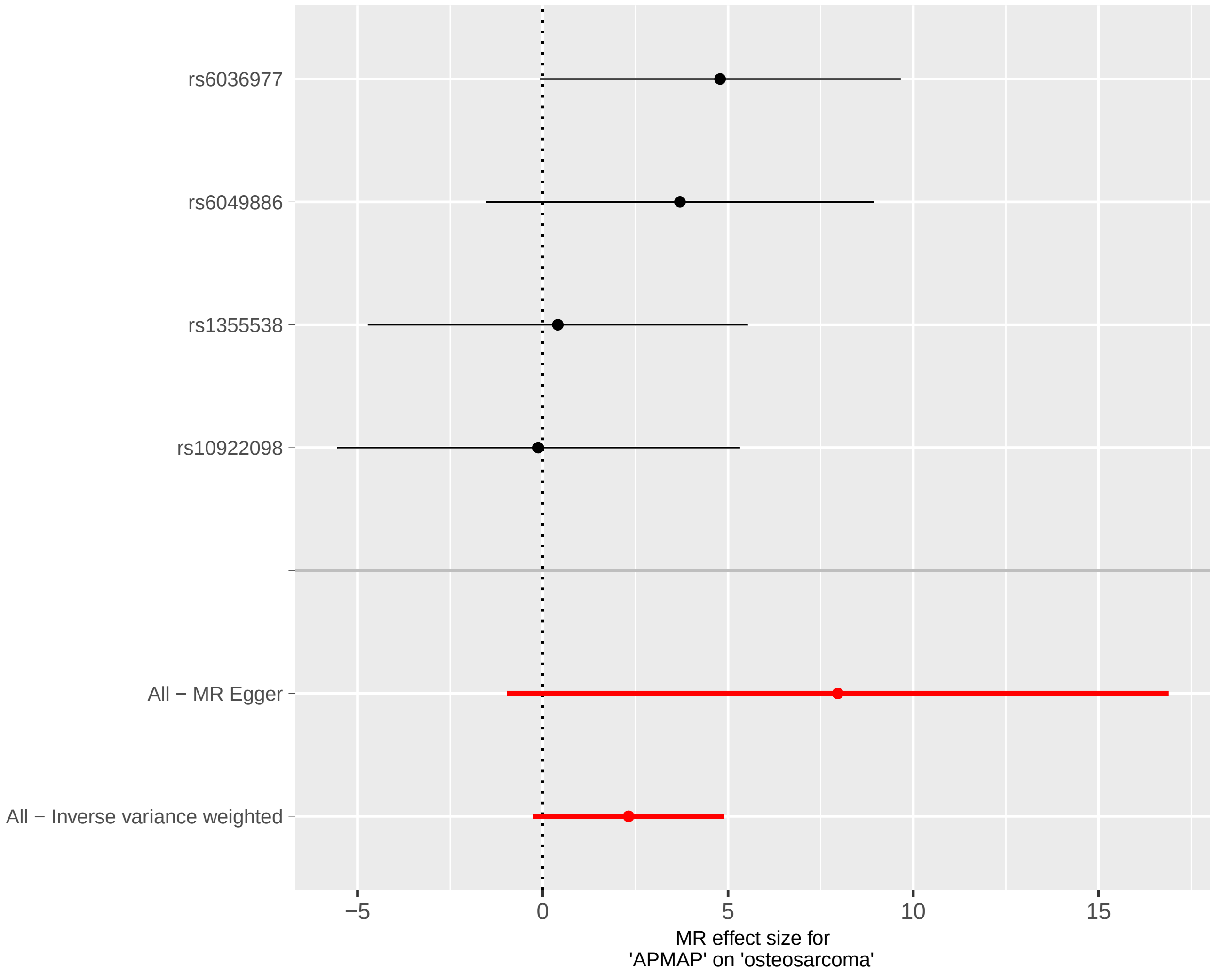
rs6036977
rs6049886
rs1355538
rs10922098
All − MR Egger
All − Inverse variance weighted
−5
0
5
10
15
MR effect size for
'APMAP' on 'osteosarcoma'

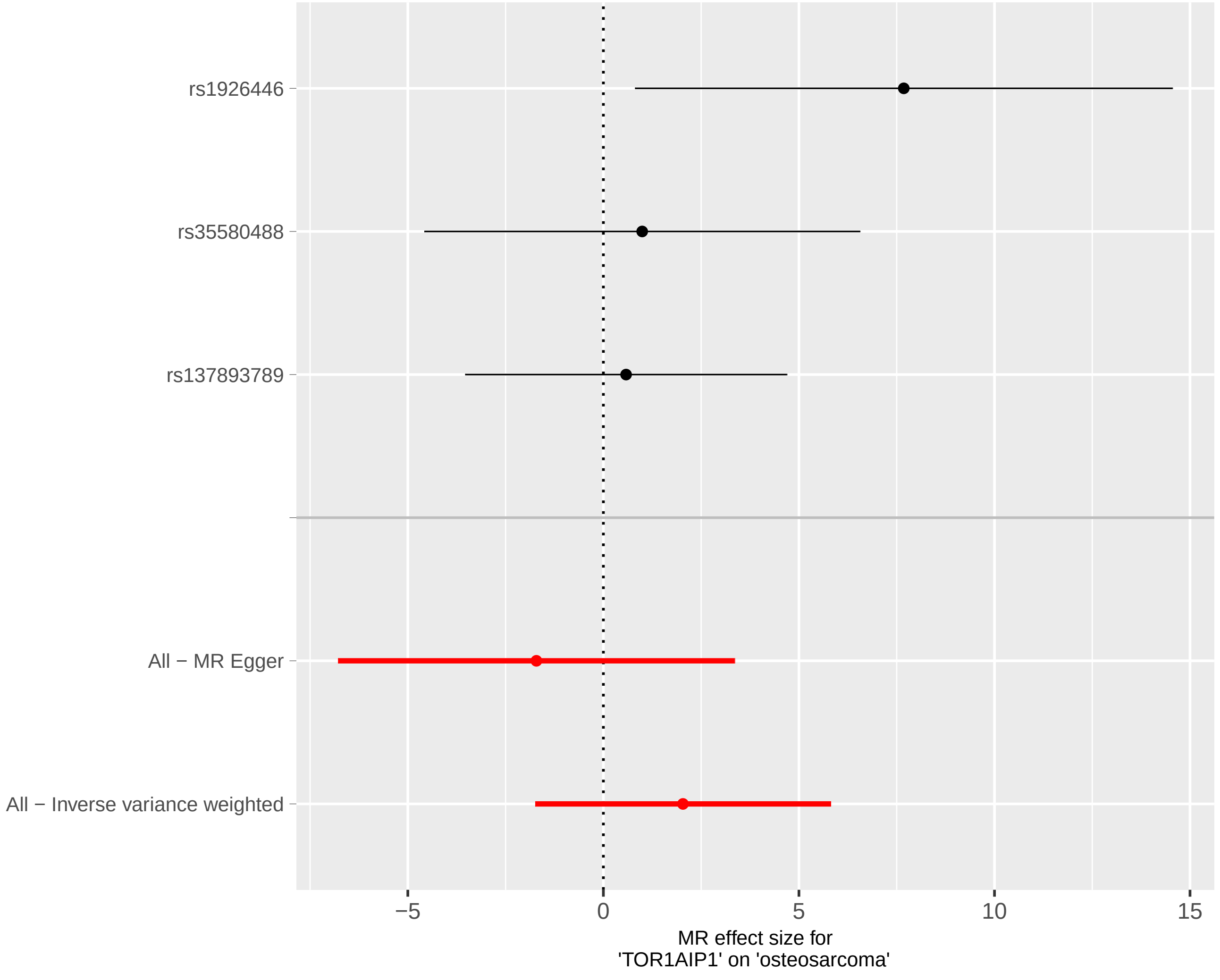

rs1926446
rs35580488
rs137893789
All − MR Egger
All − Inverse variance weighted
−5
0
5
10
15
MR effect size for
'TOR1AIP1' on 'osteosarcoma'

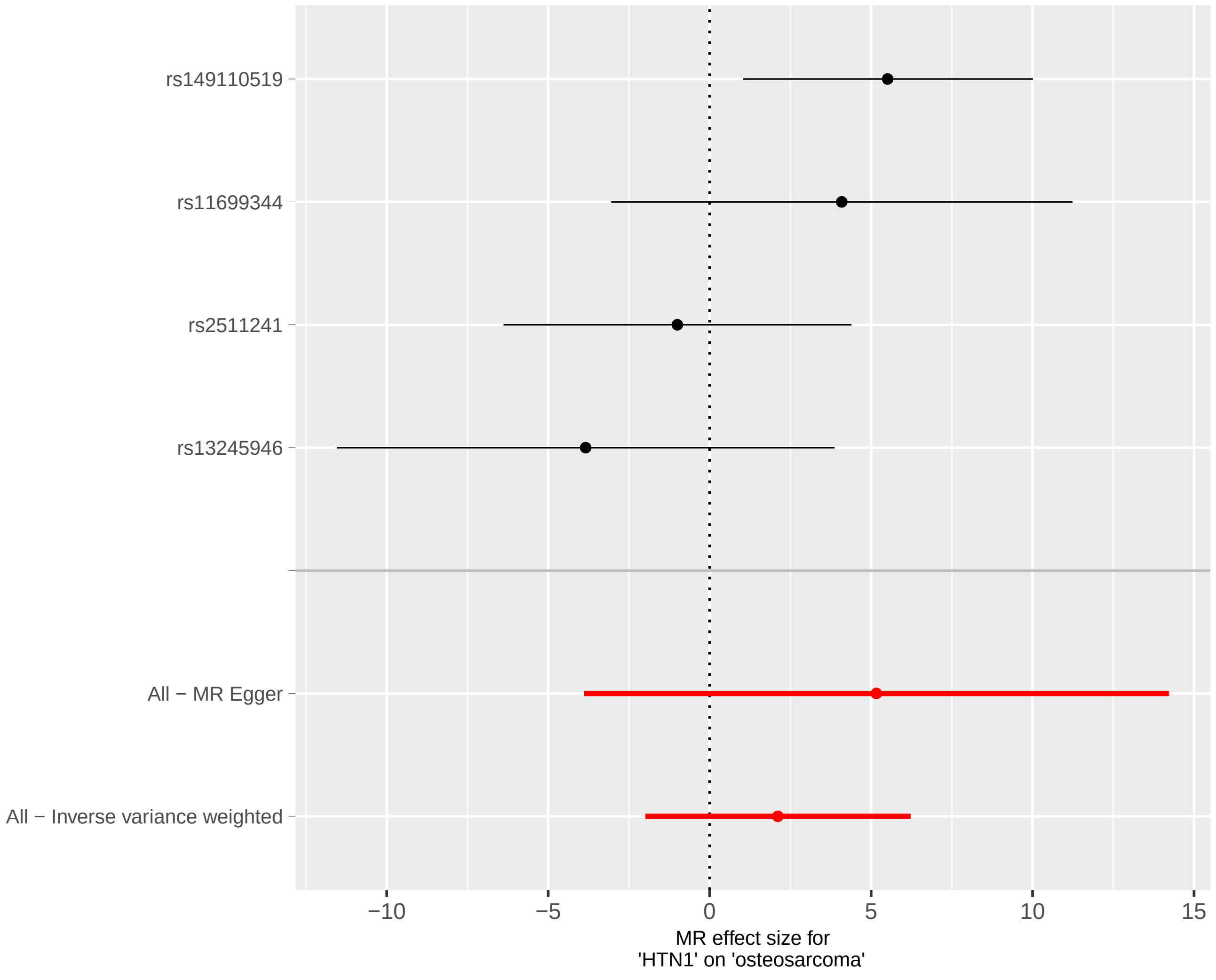

rs149110519
rs11699344
rs2511241
rs13245946
All − MR Egger
All − Inverse variance weighted
−10
−5
0
5
10
15
MR effect size for
'HTN1' on 'osteosarcoma'

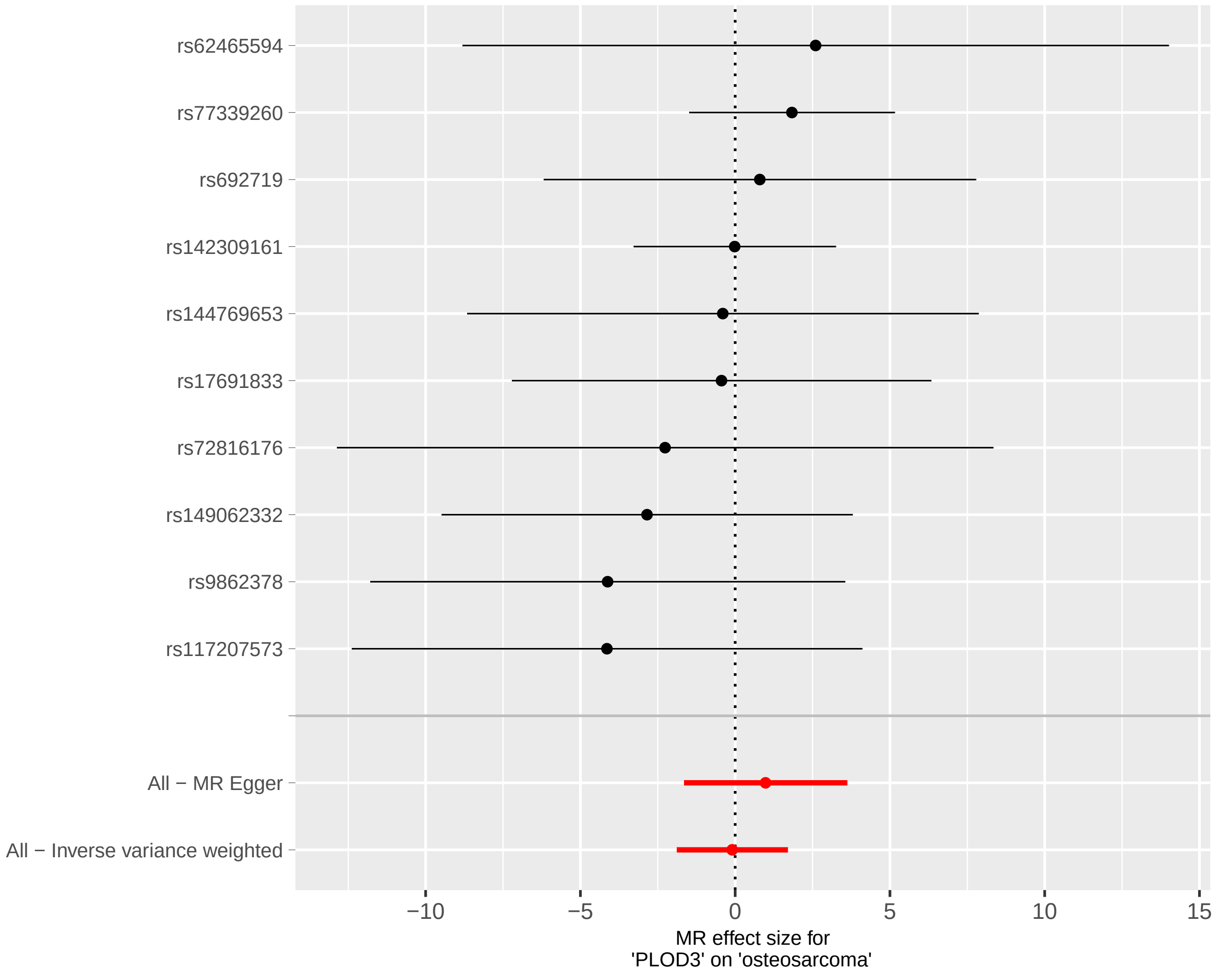
rs62465594
rs77339260
rs692719
rs142309161
rs144769653
rs17691833
rs72816176
rs149062332
rs9862378
rs117207573
All − MR Egger
All − Inverse variance weighted
−10
−5
0
5
10
15
MR effect size for
'PLOD3' on 'osteosarcoma'

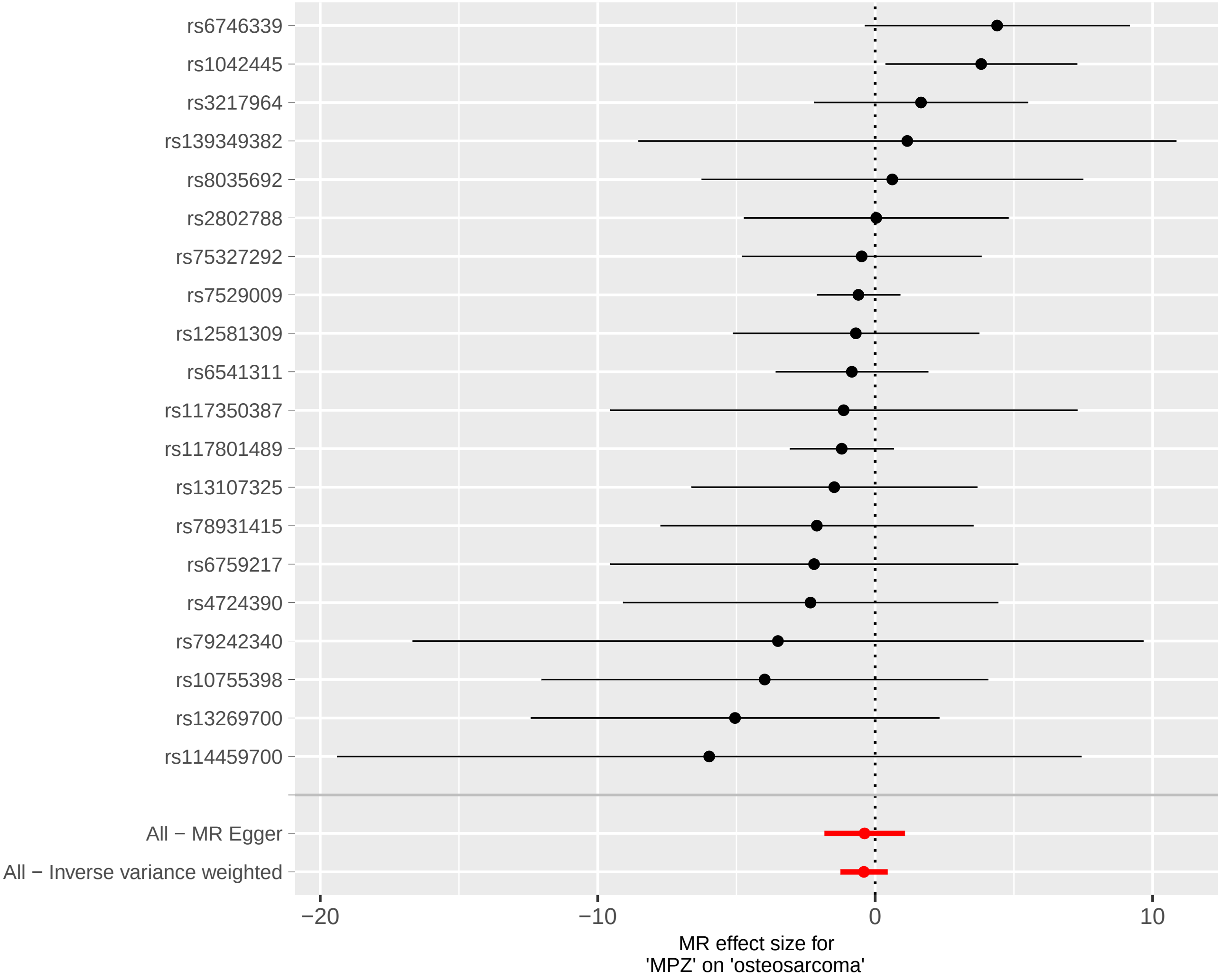

rs6746339
rs1042445
rs3217964
rs139349382
rs8035692
rs2802788
rs75327292
rs7529009
rs12581309
rs6541311
rs117350387
rs117801489
rs13107325
rs78931415
rs6759217
rs4724390
rs79242340
rs10755398
rs13269700
rs114459700
All − MR Egger
All − Inverse variance weighted
−20
−10
0
10
MR effect size for
'MPZ' on 'osteosarcoma'

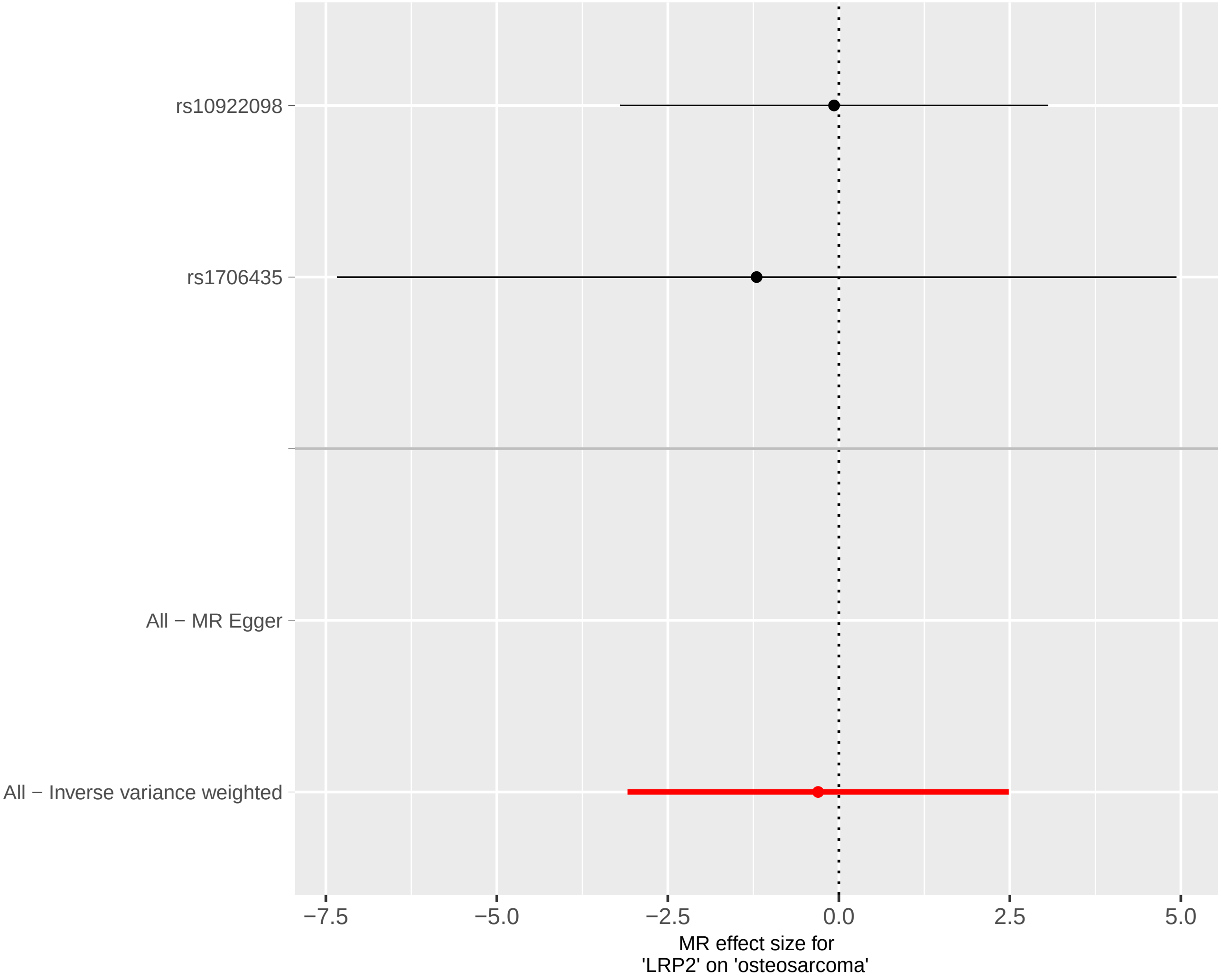
rs10922098
rs1706435
All − MR Egger
All − Inverse variance weighted
−7.5
−5.0
−2.5
0.0
2.5
5.0
MR effect size for
'LRP2' on 'osteosarcoma'

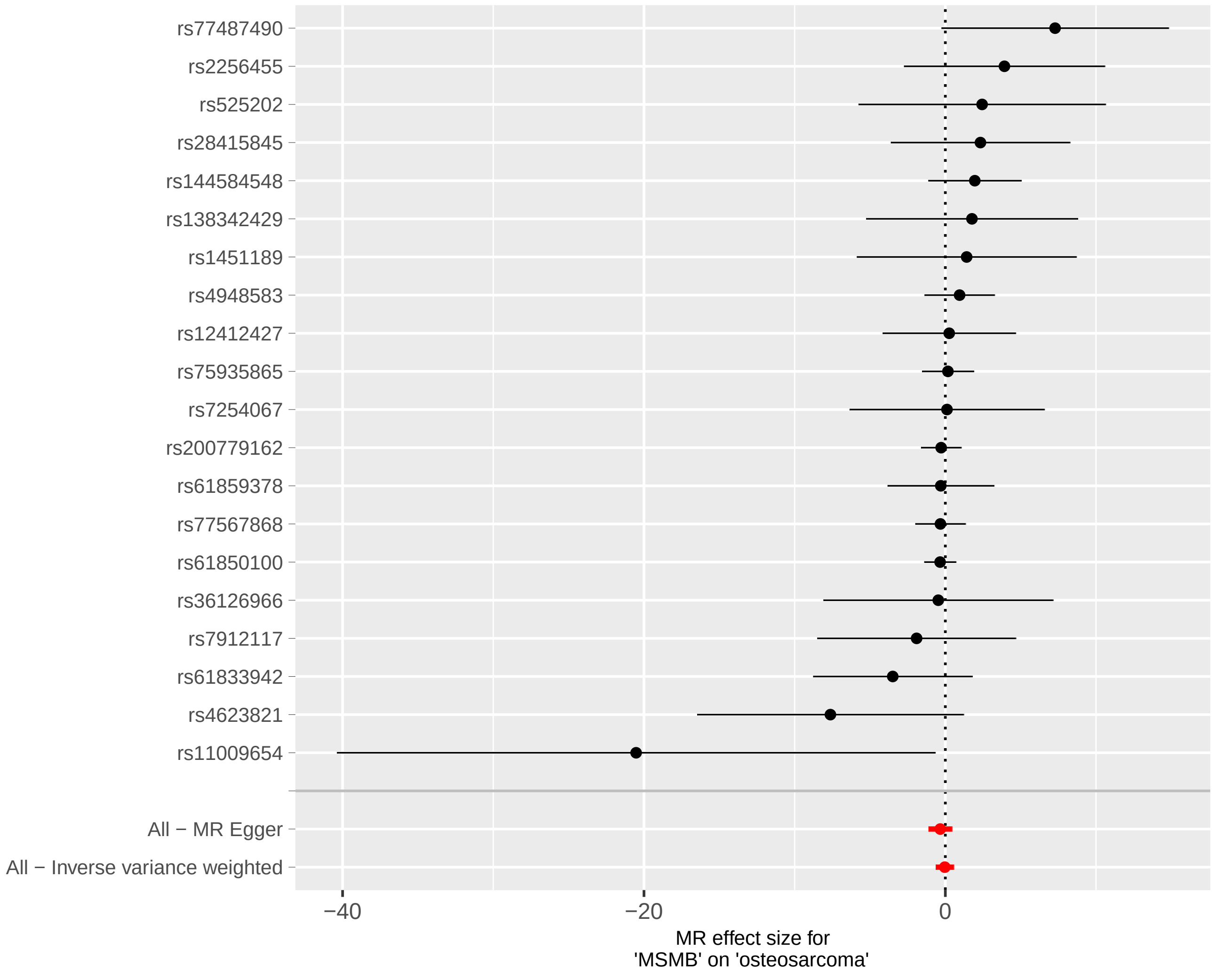
rs77487490
rs2256455
rs525202
rs28415845
rs144584548
rs138342429
rs1451189
rs4948583
rs12412427
rs75935865
rs7254067
rs200779162
rs61859378
rs77567868
rs61850100
rs36126966
rs7912117
rs61833942
rs4623821
rs11009654
All − MR Egger
All − Inverse variance weighted
−40
−20
0
MR effect size for
'MSMB' on 'osteosarcoma'

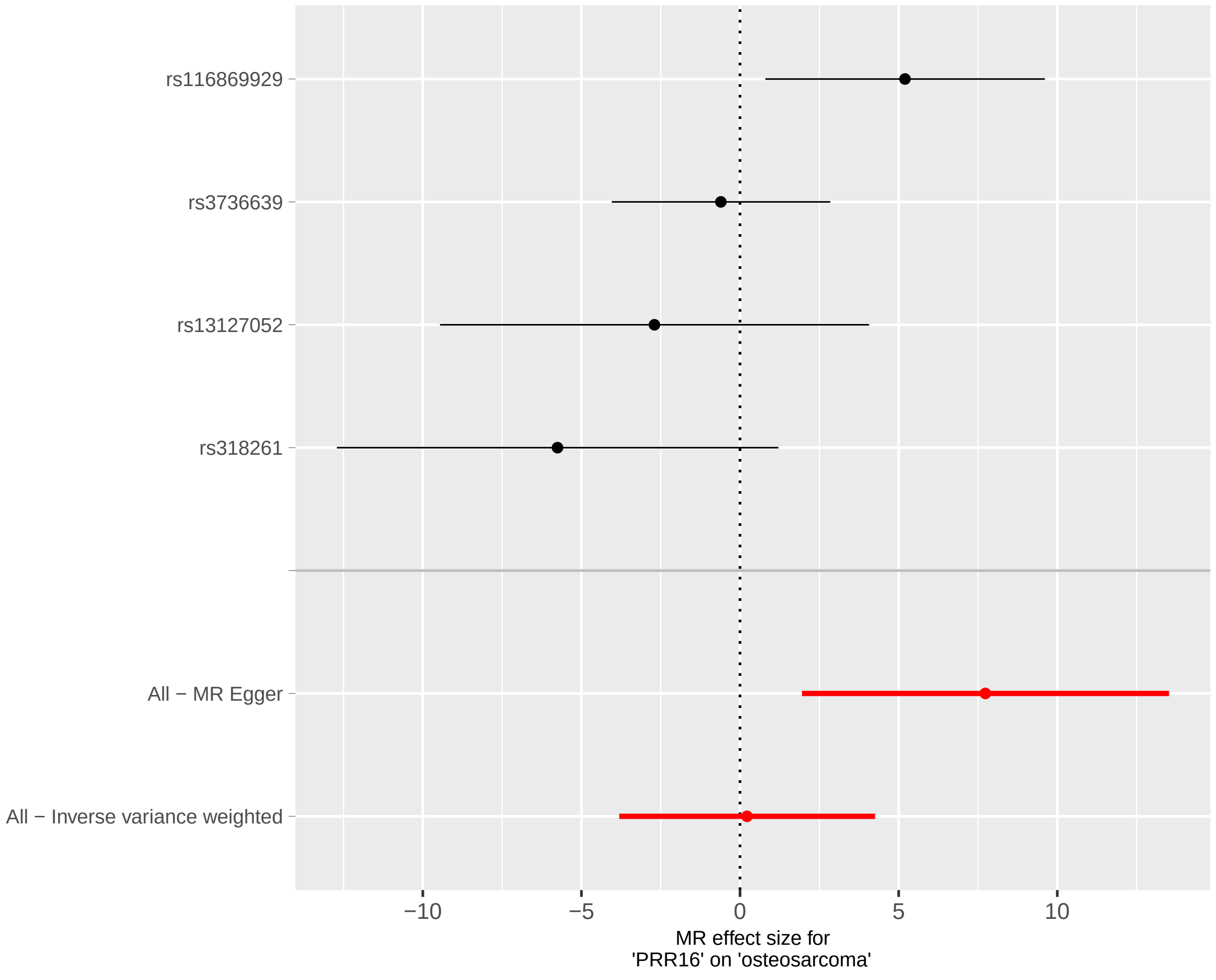
rs116869929
rs3736639
rs13127052
rs318261
All − MR Egger
All − Inverse variance weighted
−10
−5
0
5
10
MR effect size for
'PRR16' on 'osteosarcoma'

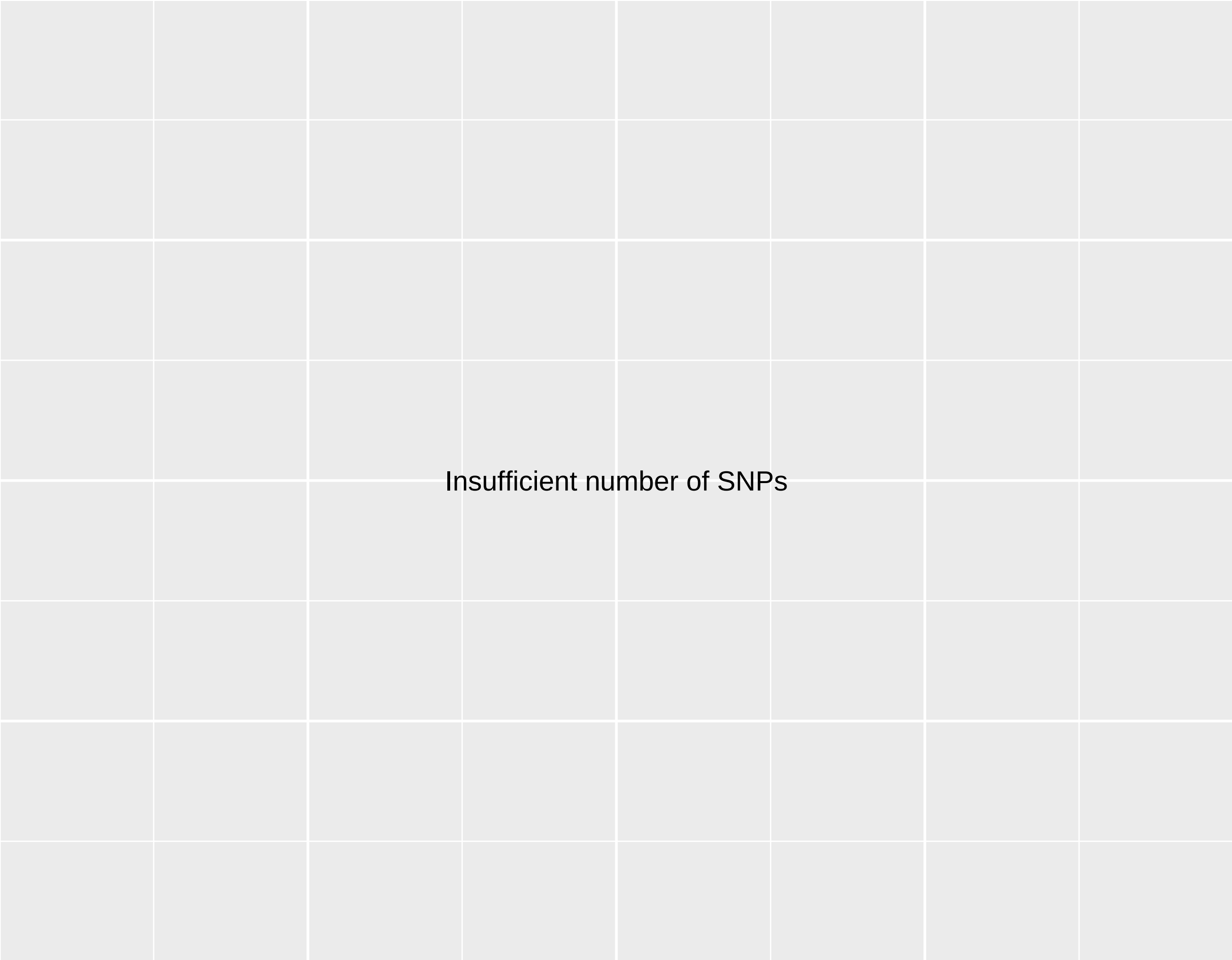
Insufficient number of SNPs

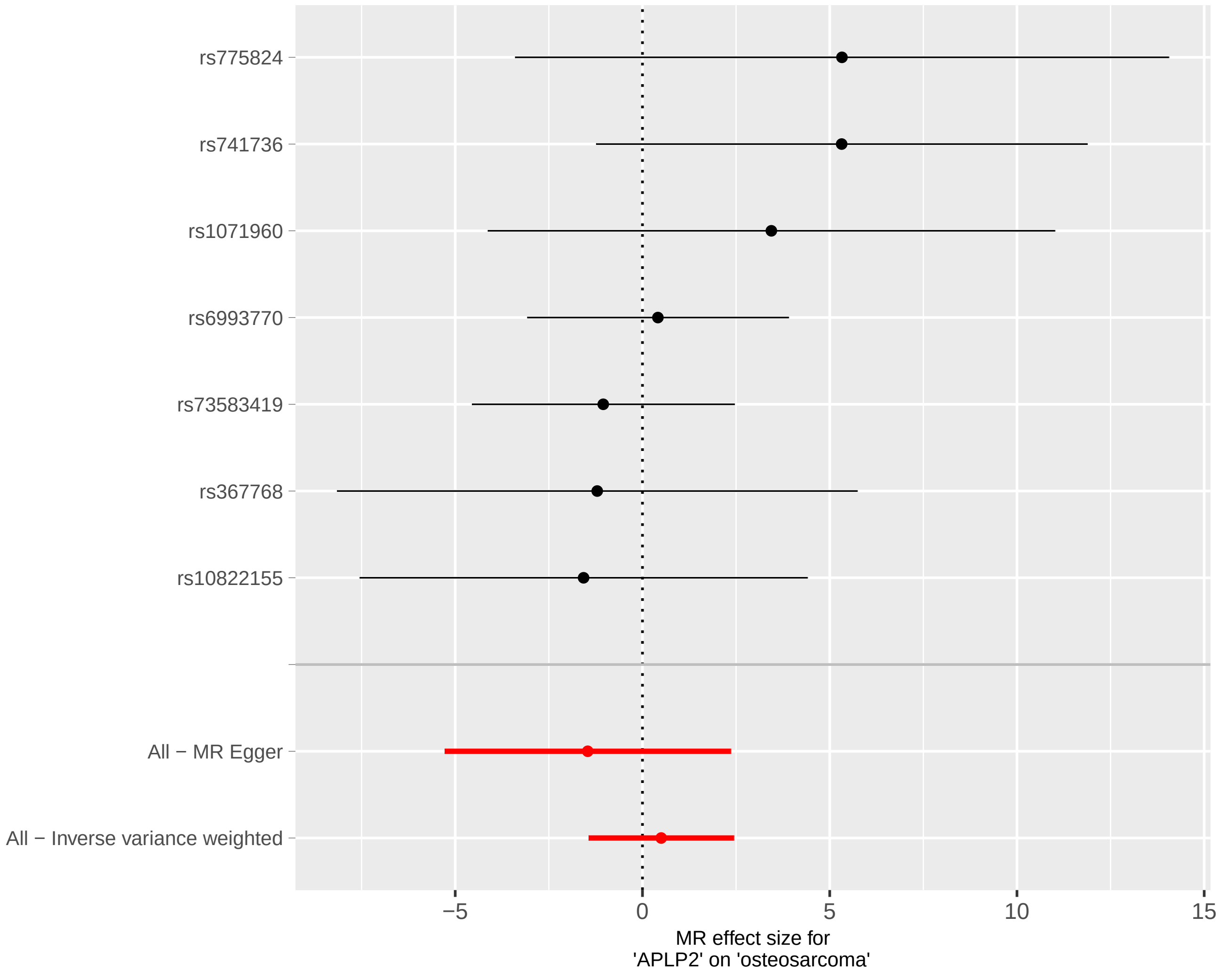
rs775824
rs741736
rs1071960
rs6993770
rs73583419
rs367768
rs10822155
All − MR Egger
All − Inverse variance weighted
−5
0
5
10
15
MR effect size for
'APLP2' on 'osteosarcoma'

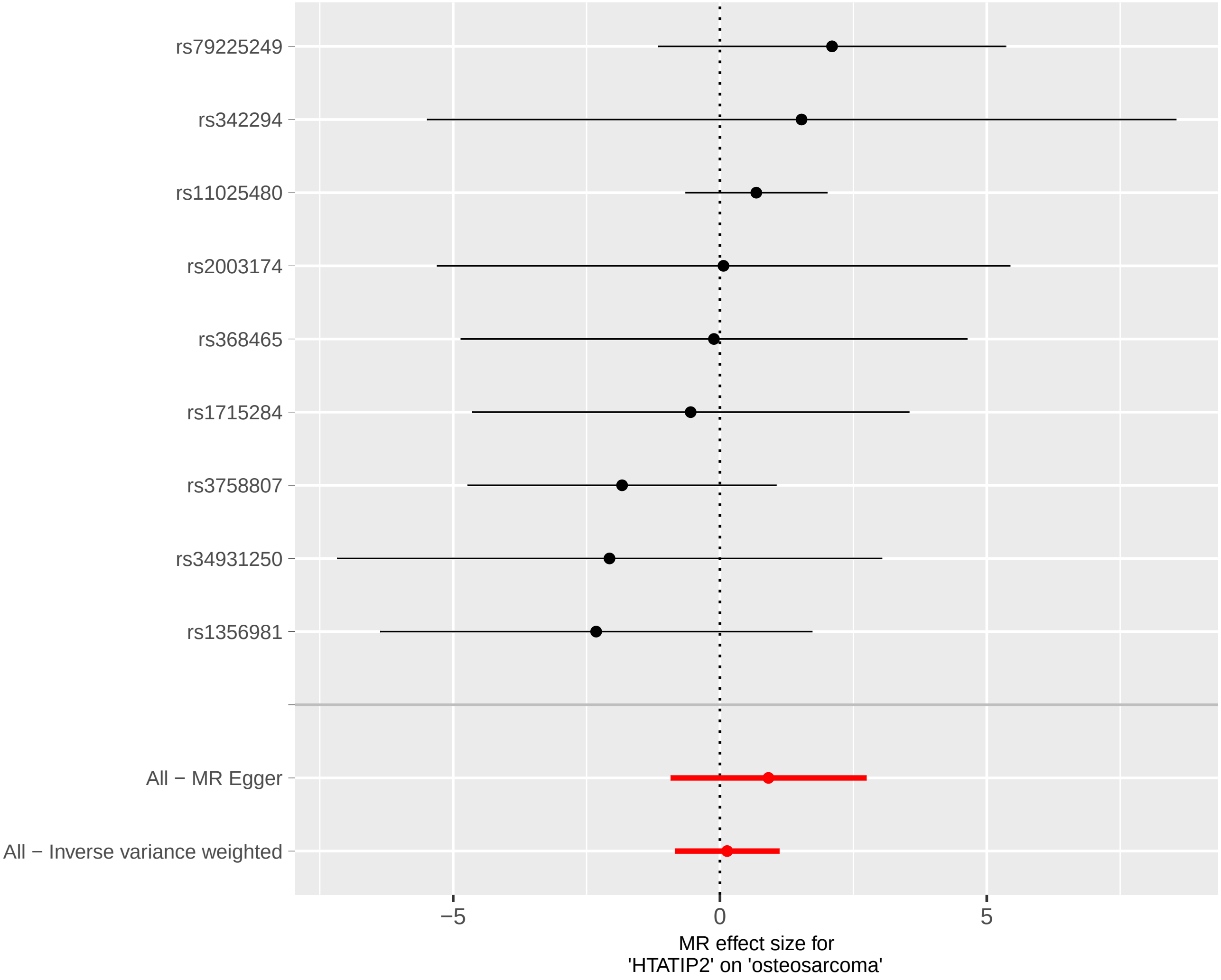
rs79225249
rs342294
rs11025480
rs2003174
rs368465
rs1715284
rs3758807
rs34931250
rs1356981
All − MR Egger
All − Inverse variance weighted
−5
0
5
MR effect size for
'HTATIP2' on 'osteosarcoma'

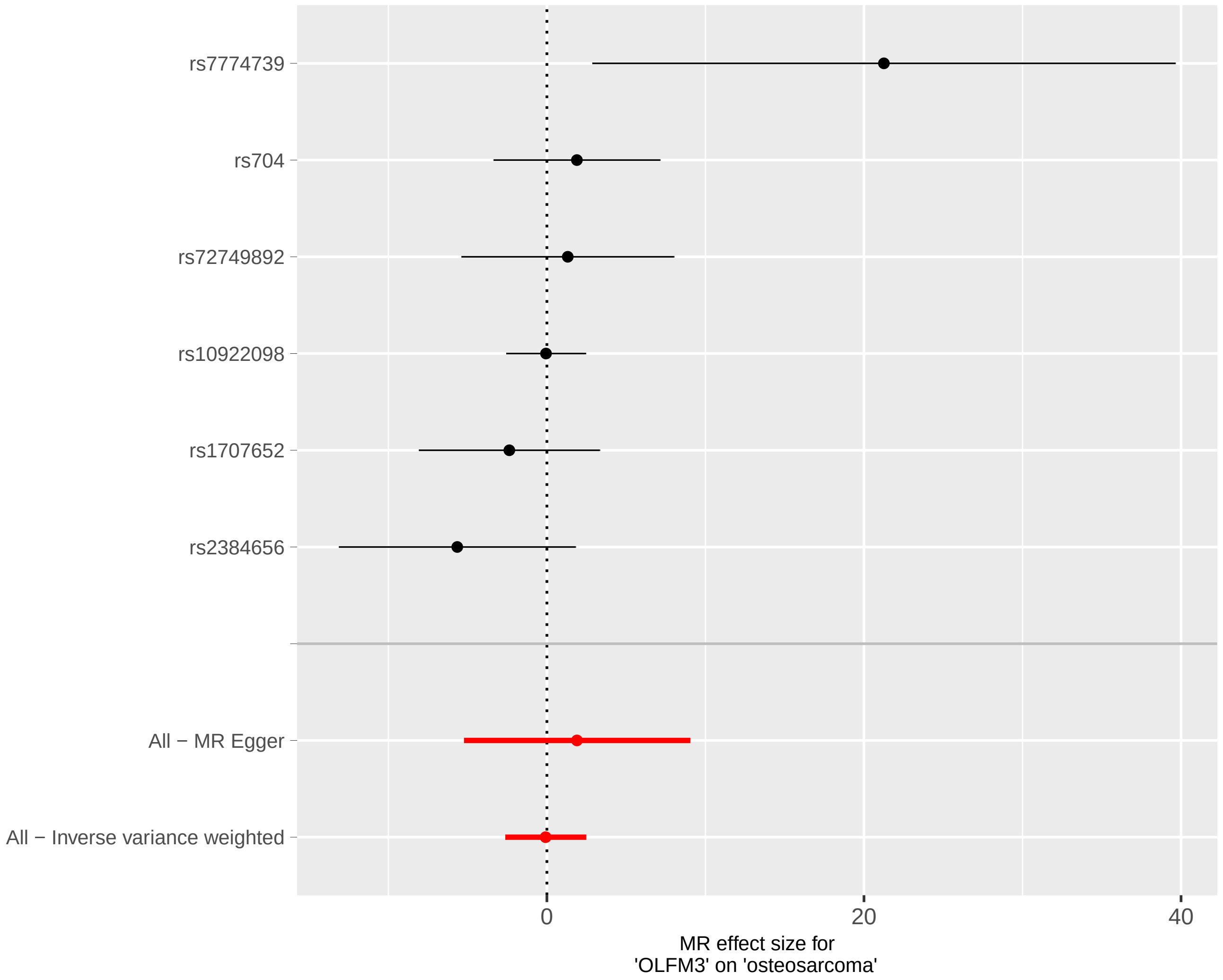

rs7774739
rs704
rs72749892
rs10922098
rs1707652
rs2384656
All − MR Egger
All − Inverse variance weighted
0
20
40
MR effect size for
'OLFM3' on 'osteosarcoma'

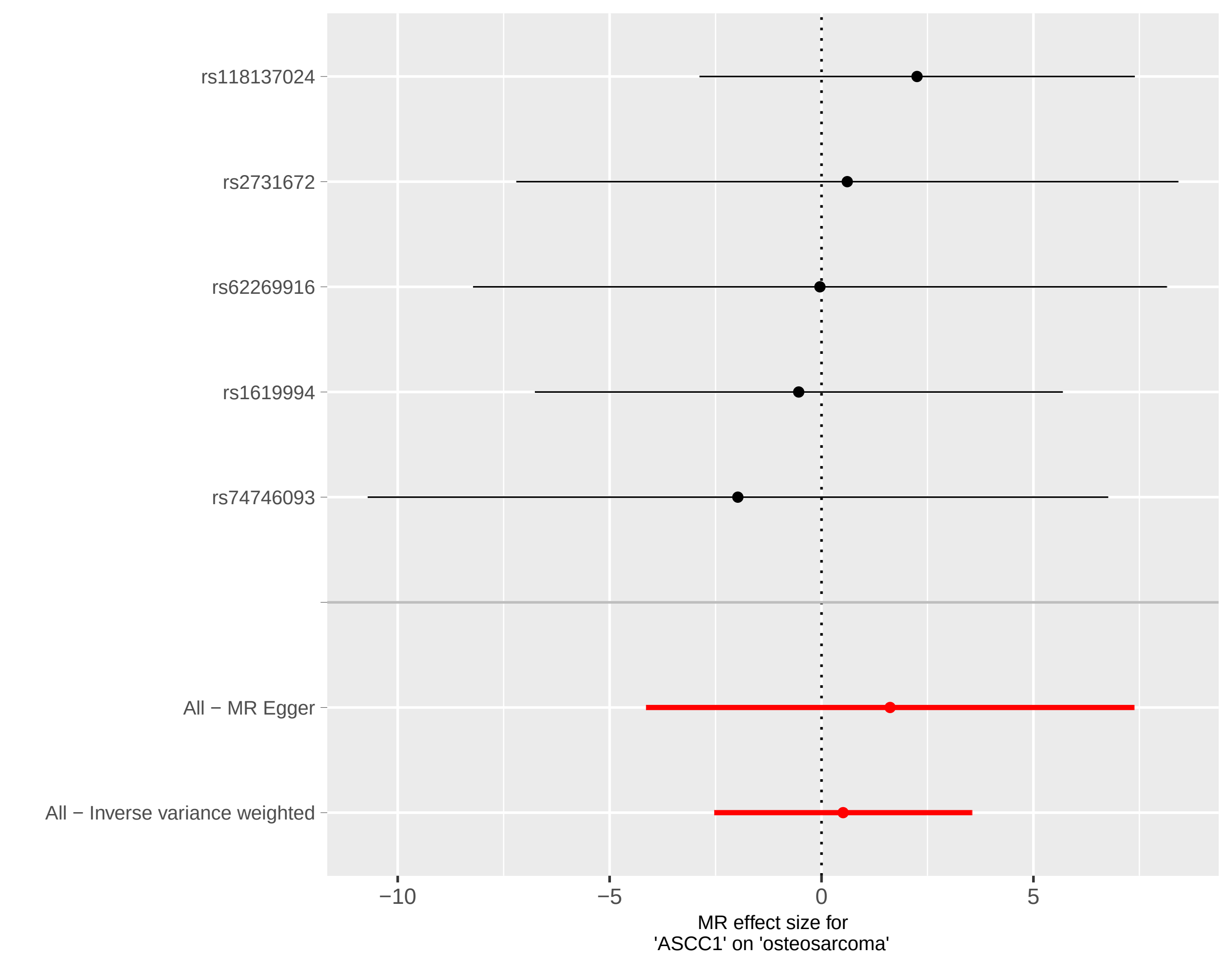
rs118137024
rs2731672
rs62269916
rs1619994
rs74746093
All − MR Egger
All − Inverse variance weighted
−10
−5
0
5
MR effect size for
'ASCC1' on 'osteosarcoma'

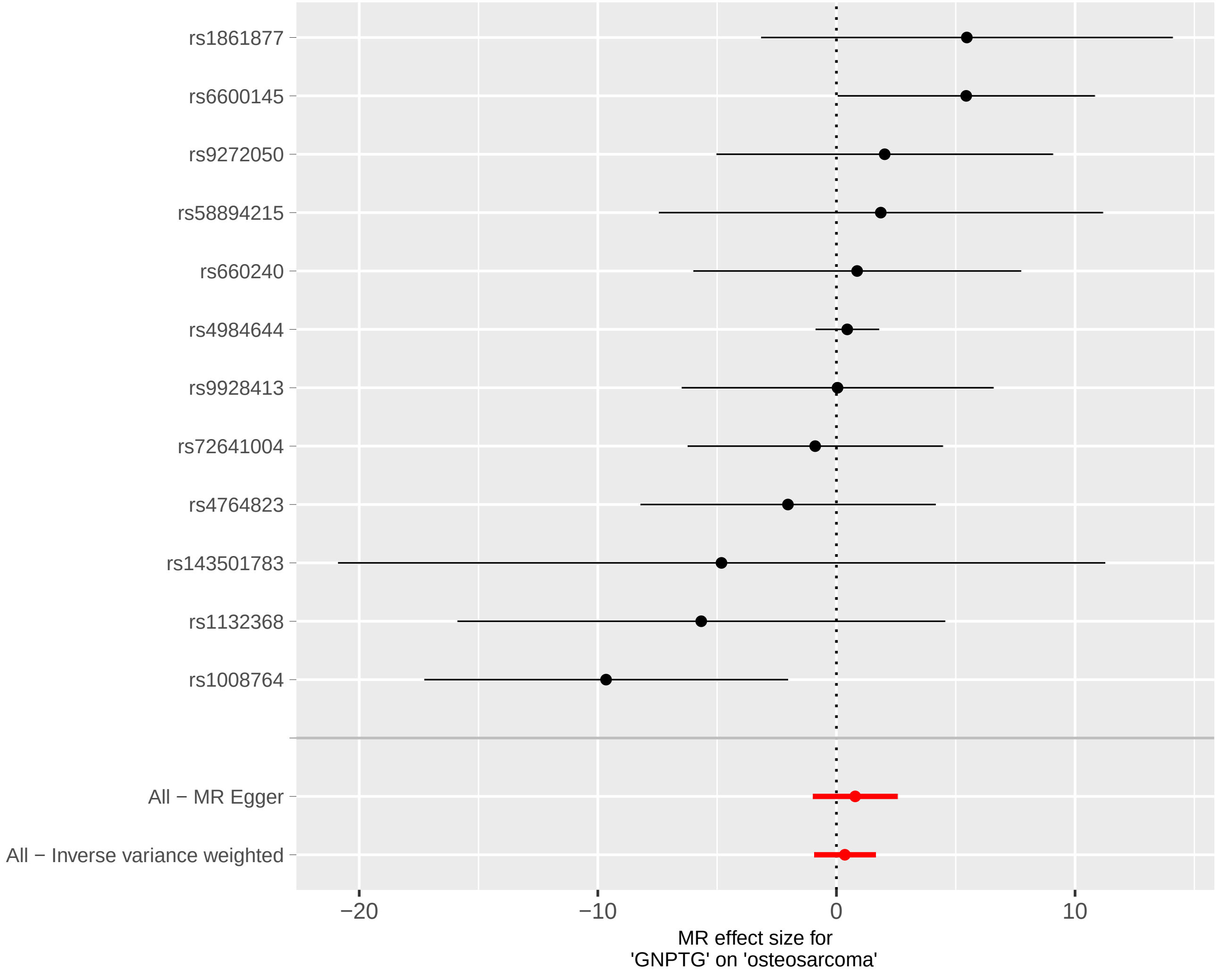
rs1861877
rs6600145
rs9272050
rs58894215
rs660240
rs4984644
rs9928413
rs72641004
rs4764823
rs143501783
rs1132368
rs1008764
All − MR Egger
All − Inverse variance weighted
−20
−10
0
10
MR effect size for
'GNPTG' on 'osteosarcoma'

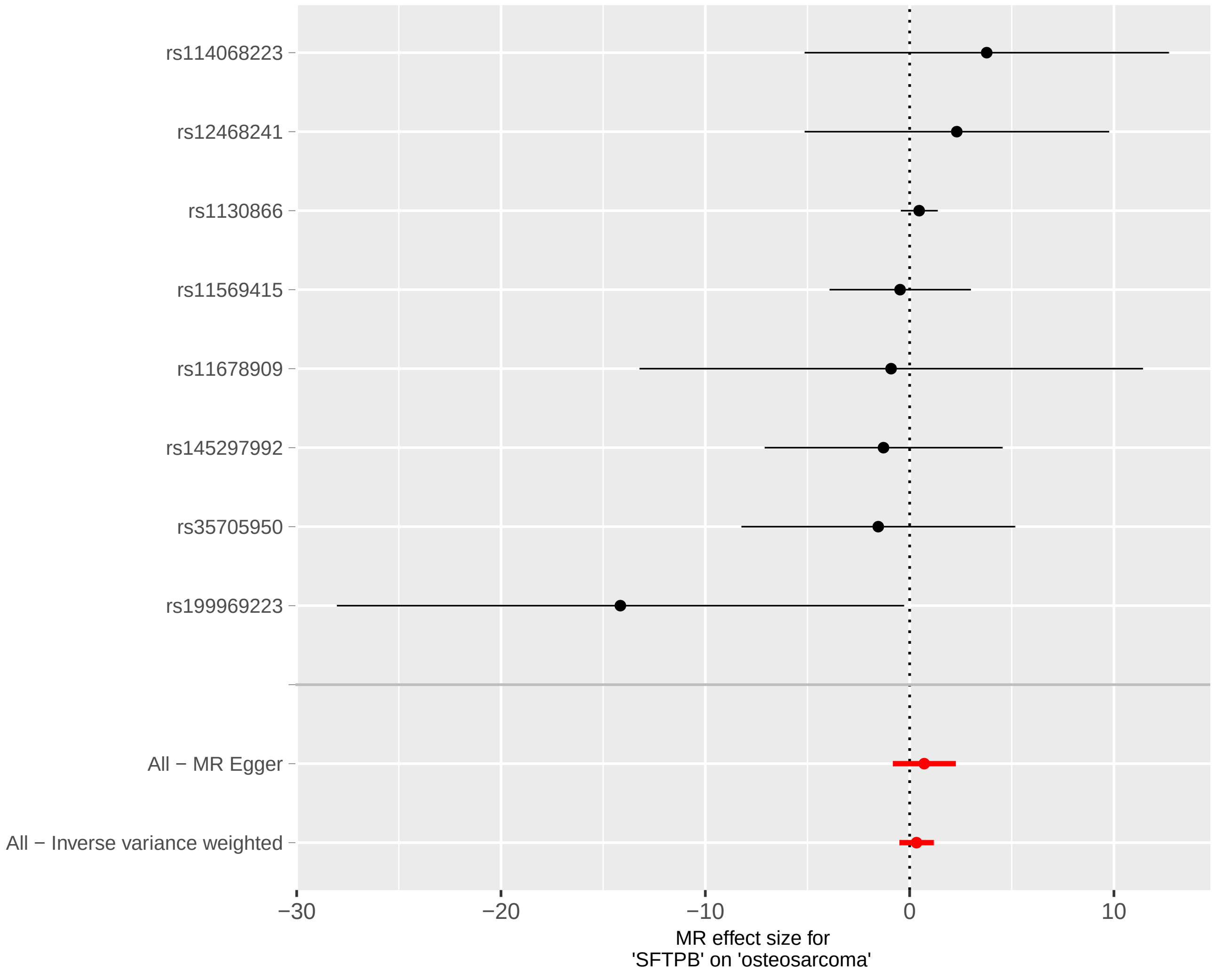
rs114068223
rs12468241
rs1130866
rs11569415
rs11678909
rs145297992
rs35705950
rs199969223
All − MR Egger
All − Inverse variance weighted
−30
−20
−10
0
10
MR effect size for
'SFTPB' on 'osteosarcoma'

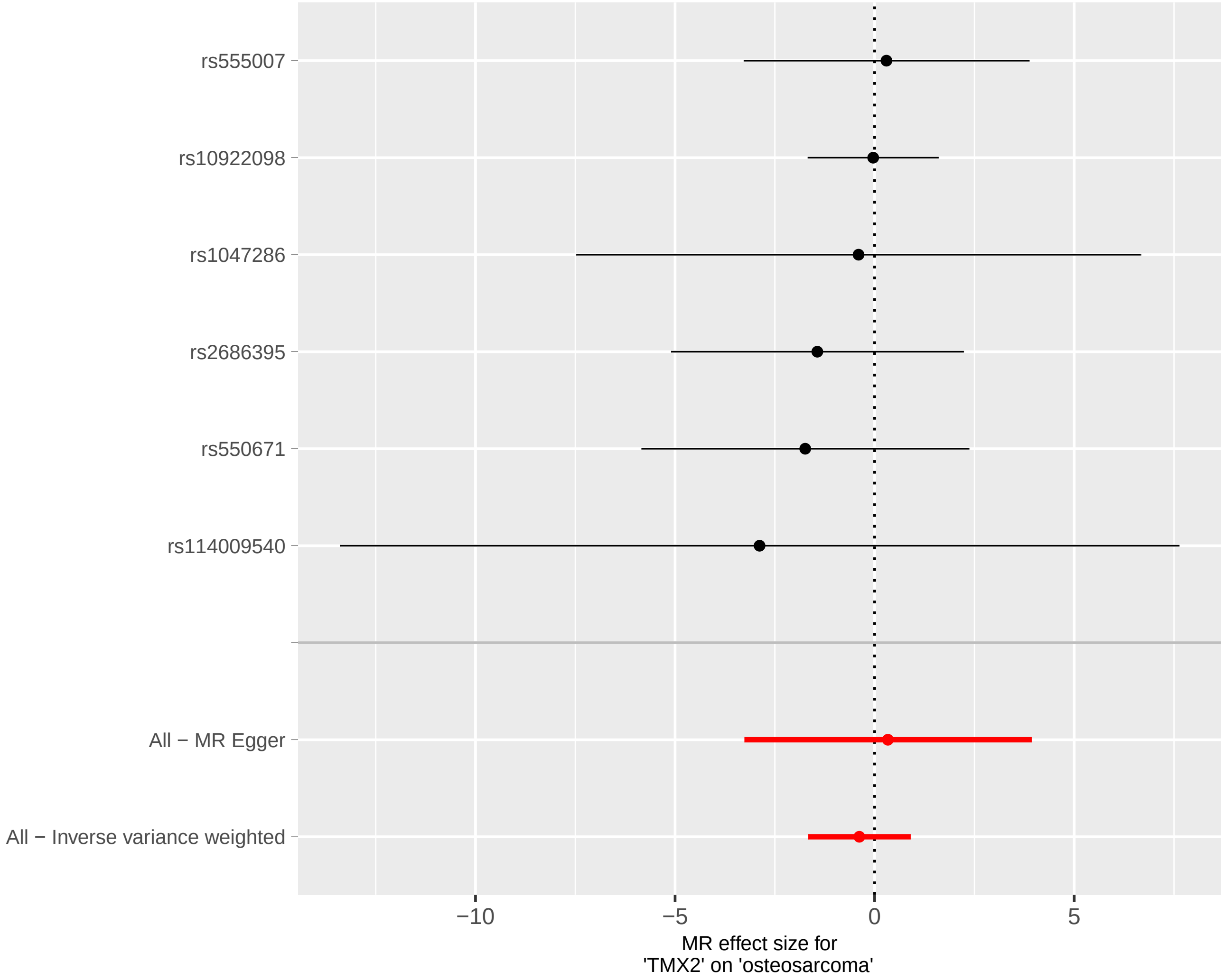

rs555007
rs10922098
rs1047286
rs2686395
rs550671
rs114009540
All − MR Egger
All − Inverse variance weighted
−10
−5
0
5
MR effect size for
'TMX2' on 'osteosarcoma'

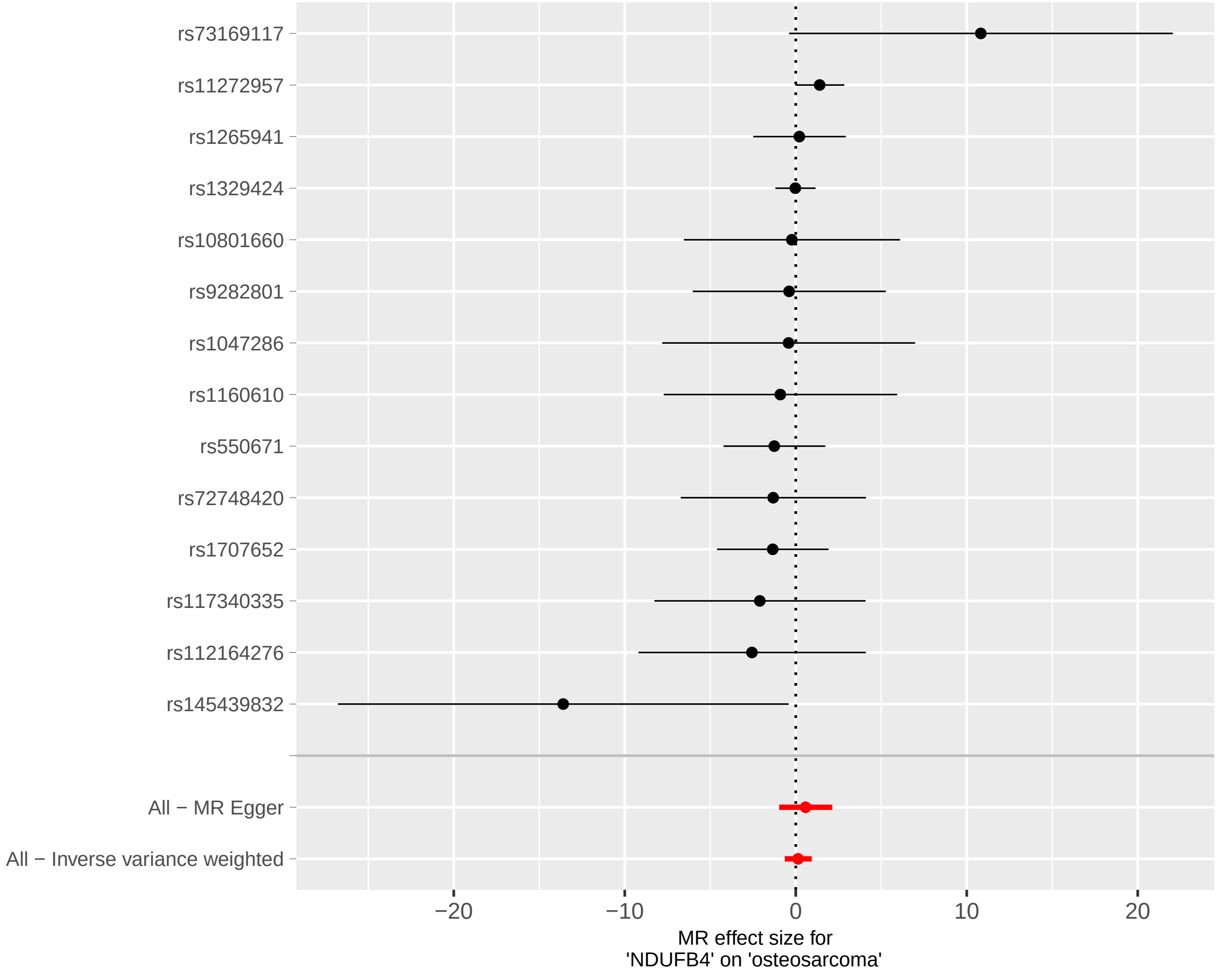

rs73169117
rs11272957
rs1265941
rs1329424
rs10801660
rs9282801
rs1047286
rs1160610
rs550671
rs72748420
rs1707652
rs117340335
rs112164276
rs145439832
All − MR Egger
All − Inverse variance weighted
−20
−10
0
10
20
MR effect size for
'NDUFB4' on 'osteosarcoma'

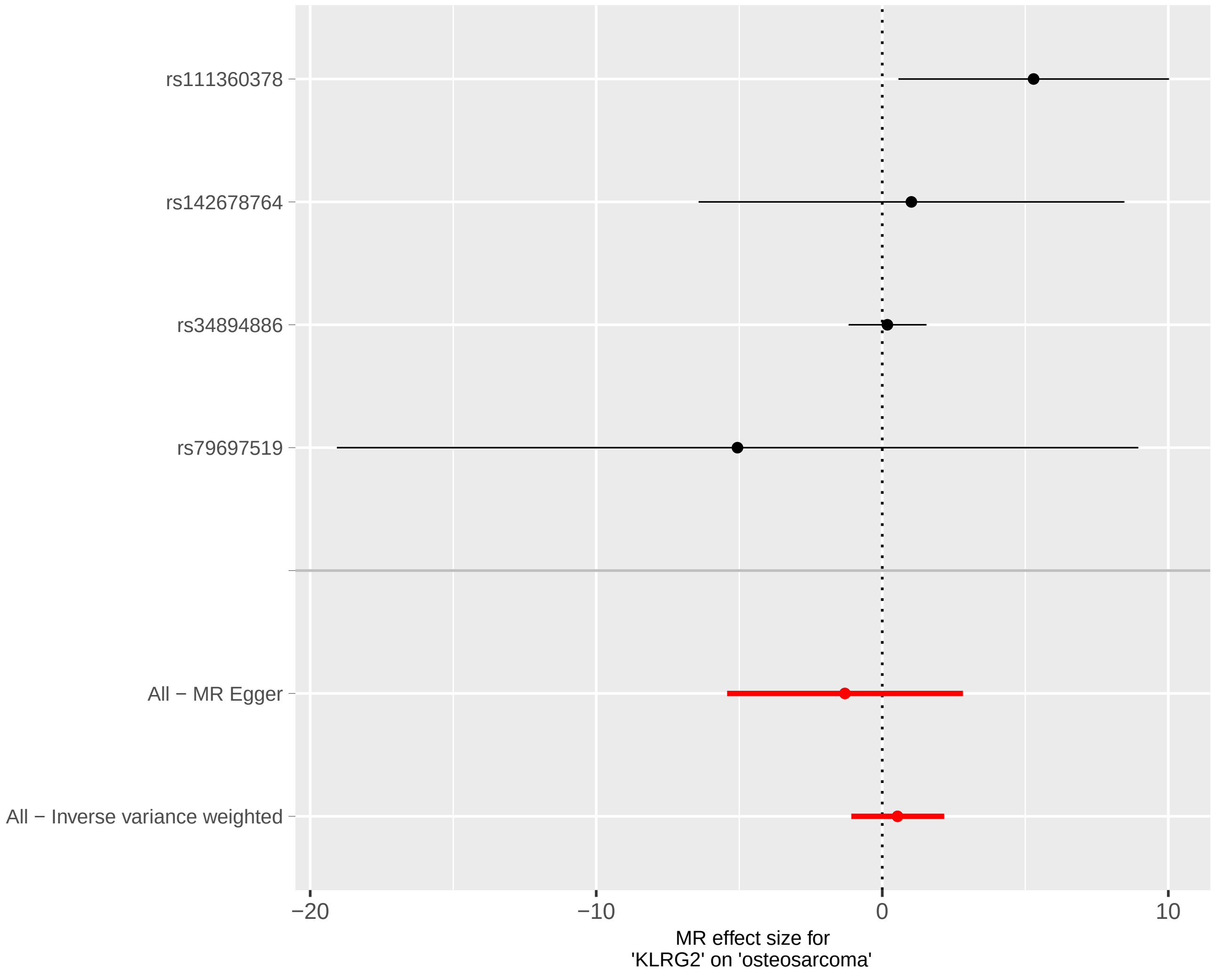

rs111360378
rs142678764
rs34894886
rs79697519
All − MR Egger
All − Inverse variance weighted
−20
−10
0
10
MR effect size for
'KLRG2' on 'osteosarcoma'

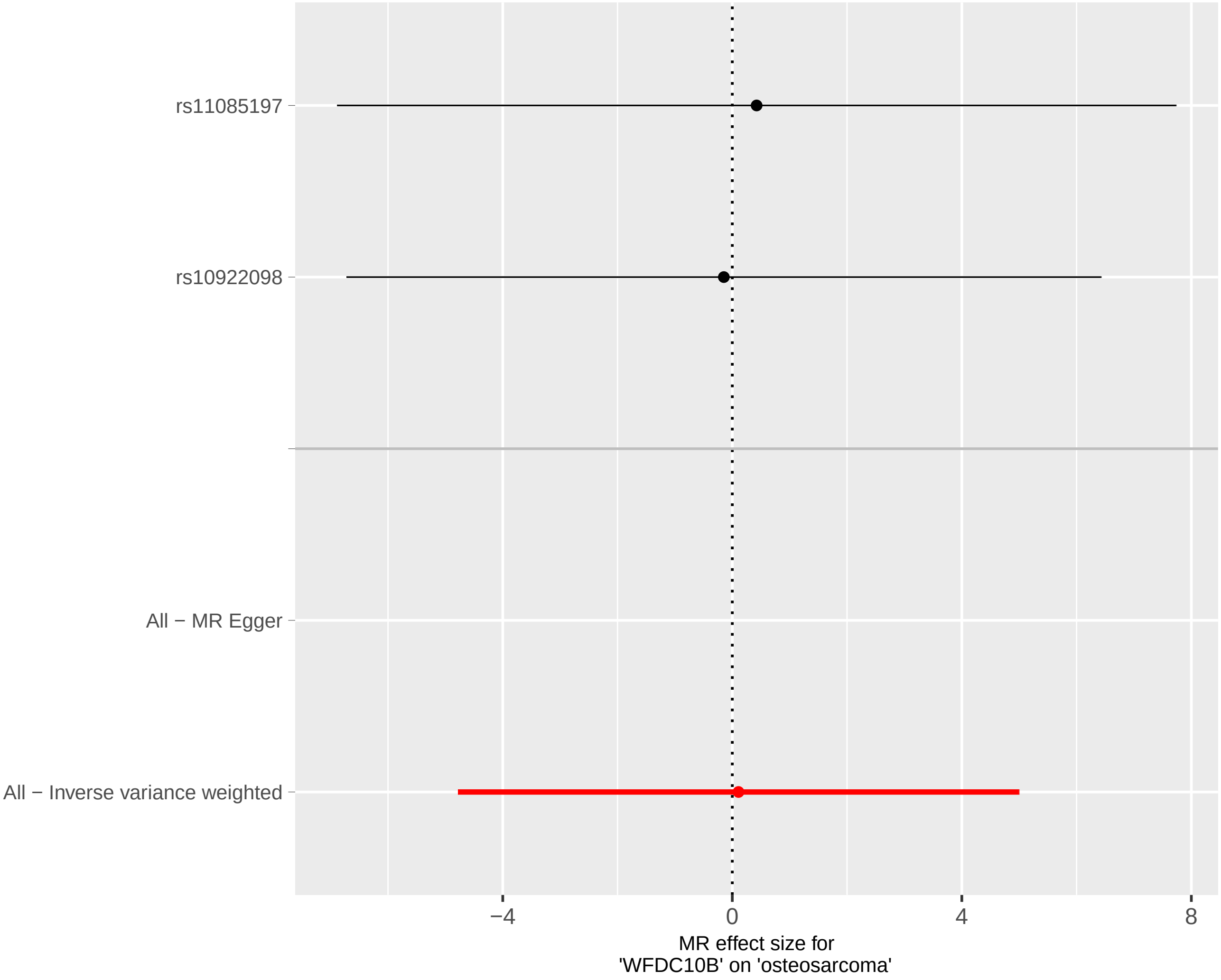
rs11085197
rs10922098
All − MR Egger
All − Inverse variance weighted
−4
0
4
8
MR effect size for
'WFDC10B' on 'osteosarcoma'

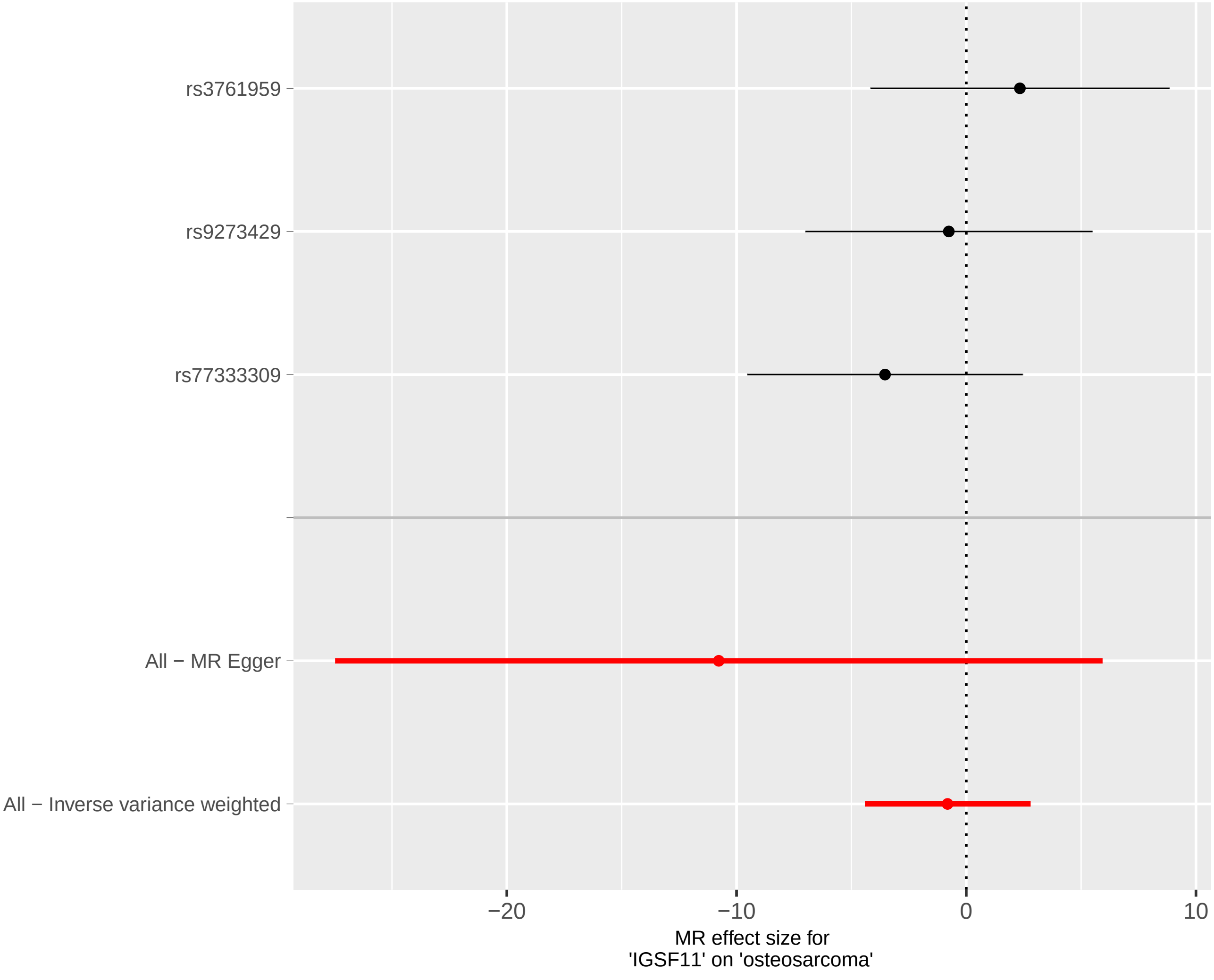

rs3761959
rs9273429
rs77333309
All − MR Egger
All − Inverse variance weighted
−20
−10
0
10
MR effect size for
'IGSF11' on 'osteosarcoma'

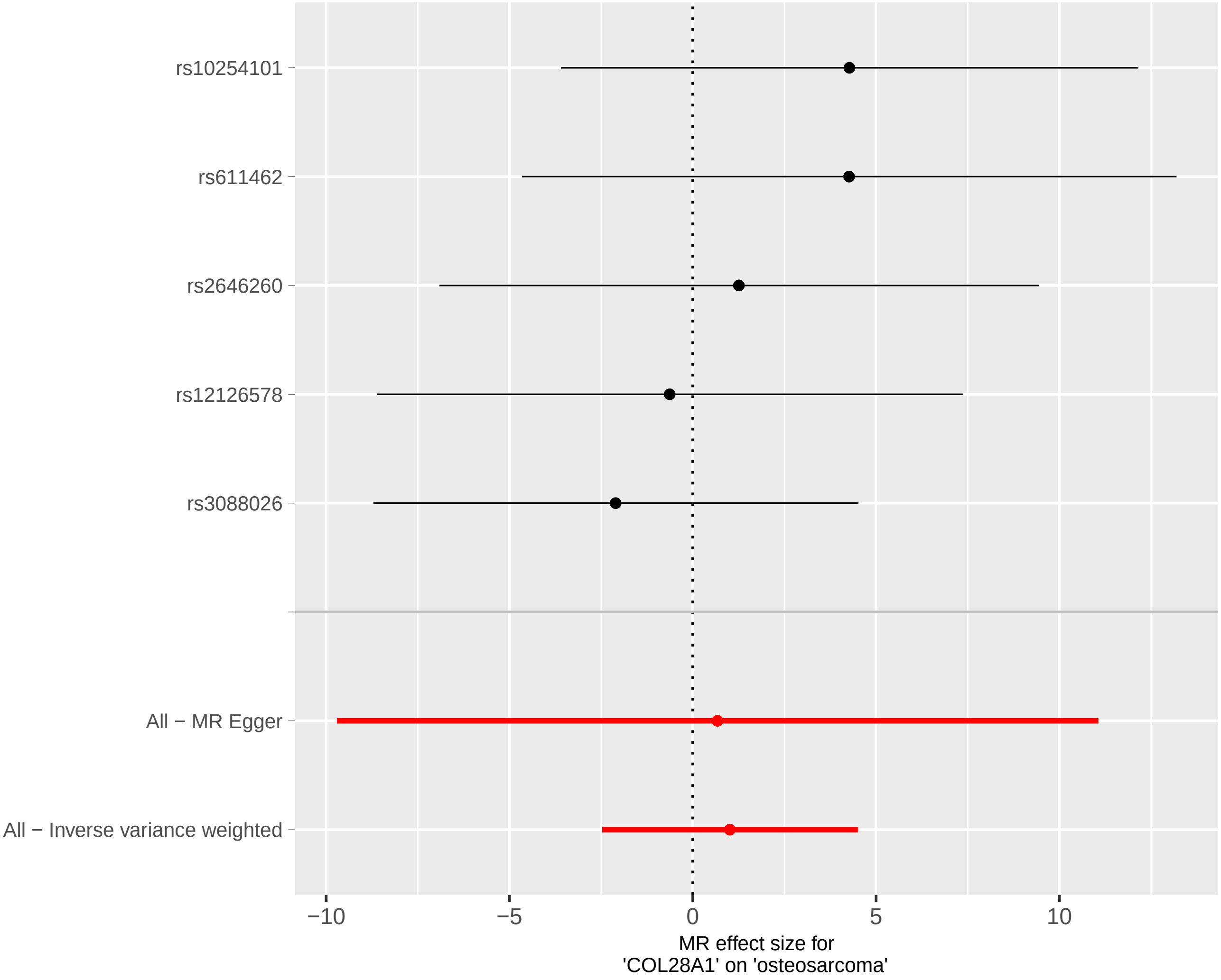

rs10254101
rs611462
rs2646260
rs12126578
rs3088026
All − MR Egger
All − Inverse variance weighted
−10
−5
0
5
10
MR effect size for
'COL28A1' on 'osteosarcoma'

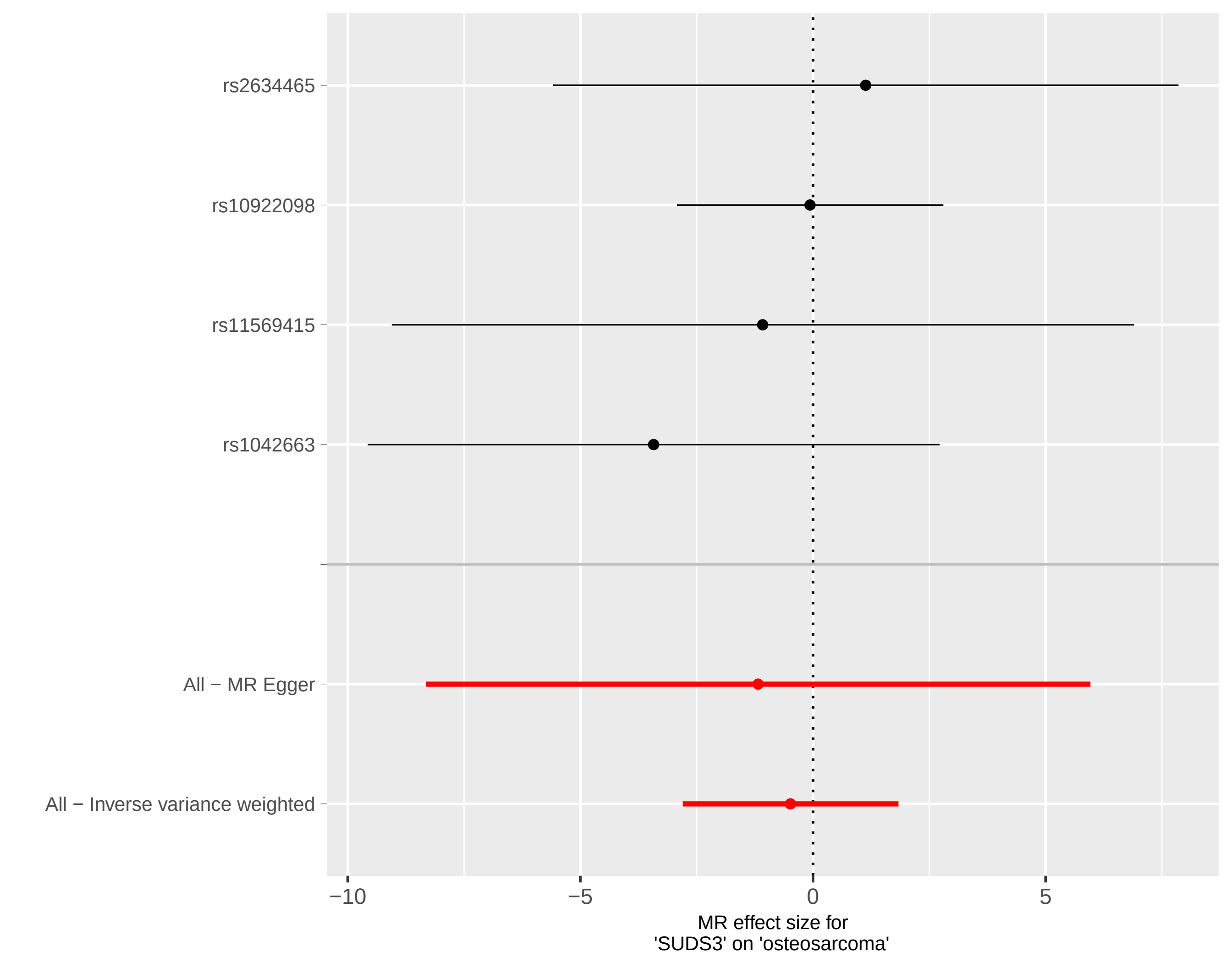

rs2634465
rs10922098
rs11569415
rs1042663
All − MR Egger
All − Inverse variance weighted
−10
−5
0
5
MR effect size for
'SUDS3' on 'osteosarcoma'

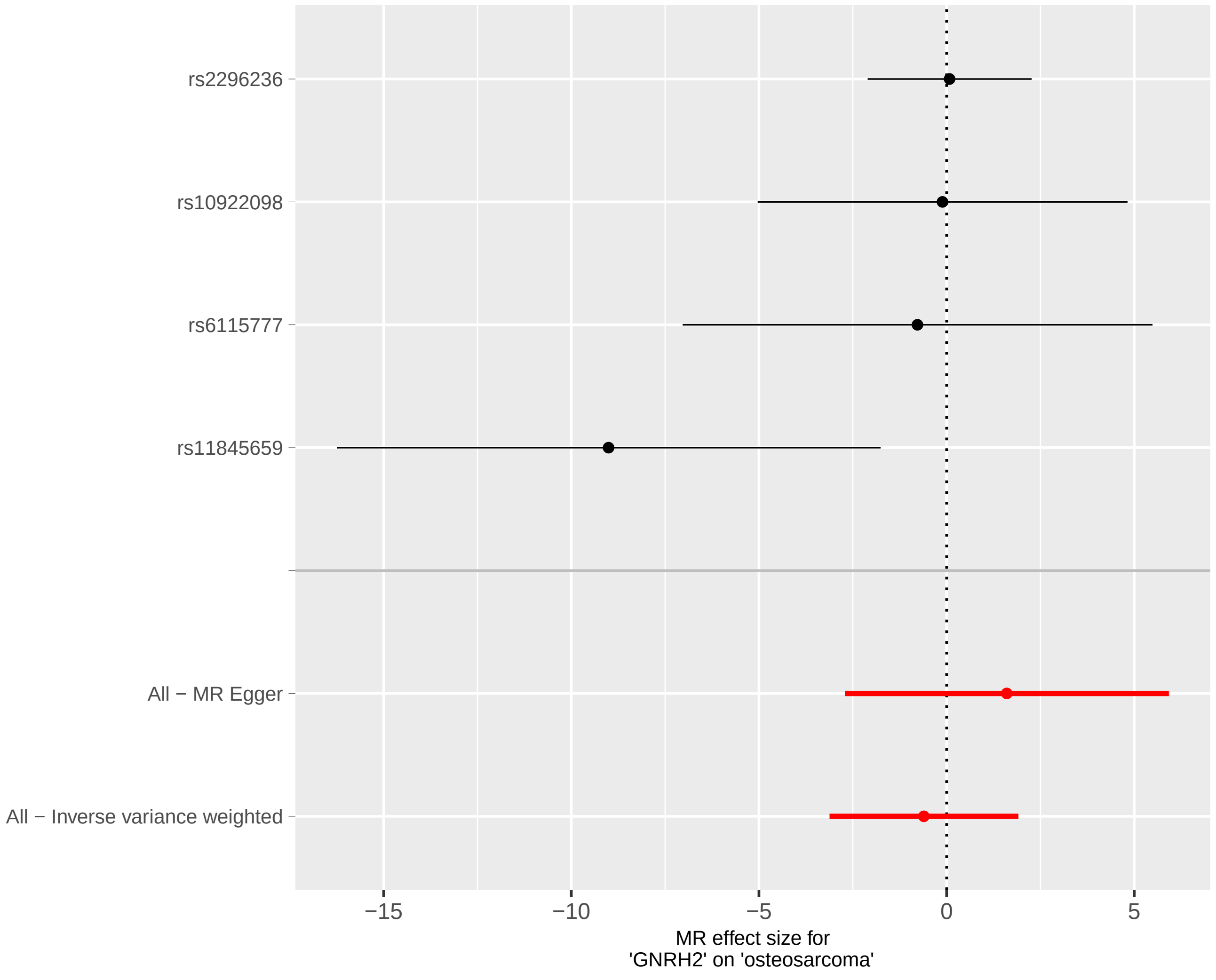
rs2296236
rs10922098
rs6115777
rs11845659
All − MR Egger
All − Inverse variance weighted
−15
−10
−5
0
5
MR effect size for
'GNRH2' on 'osteosarcoma'

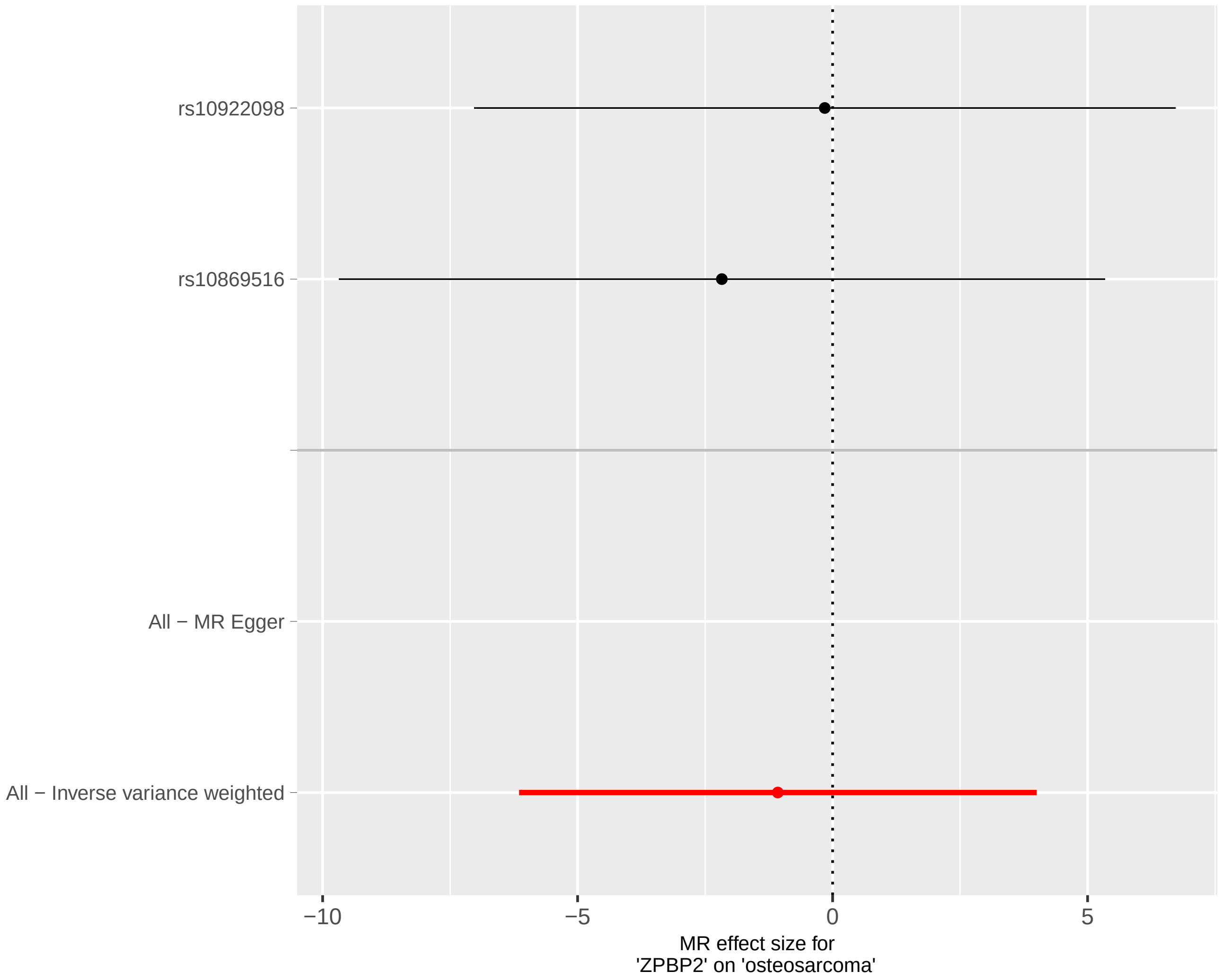

rs10922098
rs10869516
All − MR Egger
All − Inverse variance weighted
−10
−5
0
5
MR effect size for
'ZPBP2' on 'osteosarcoma'

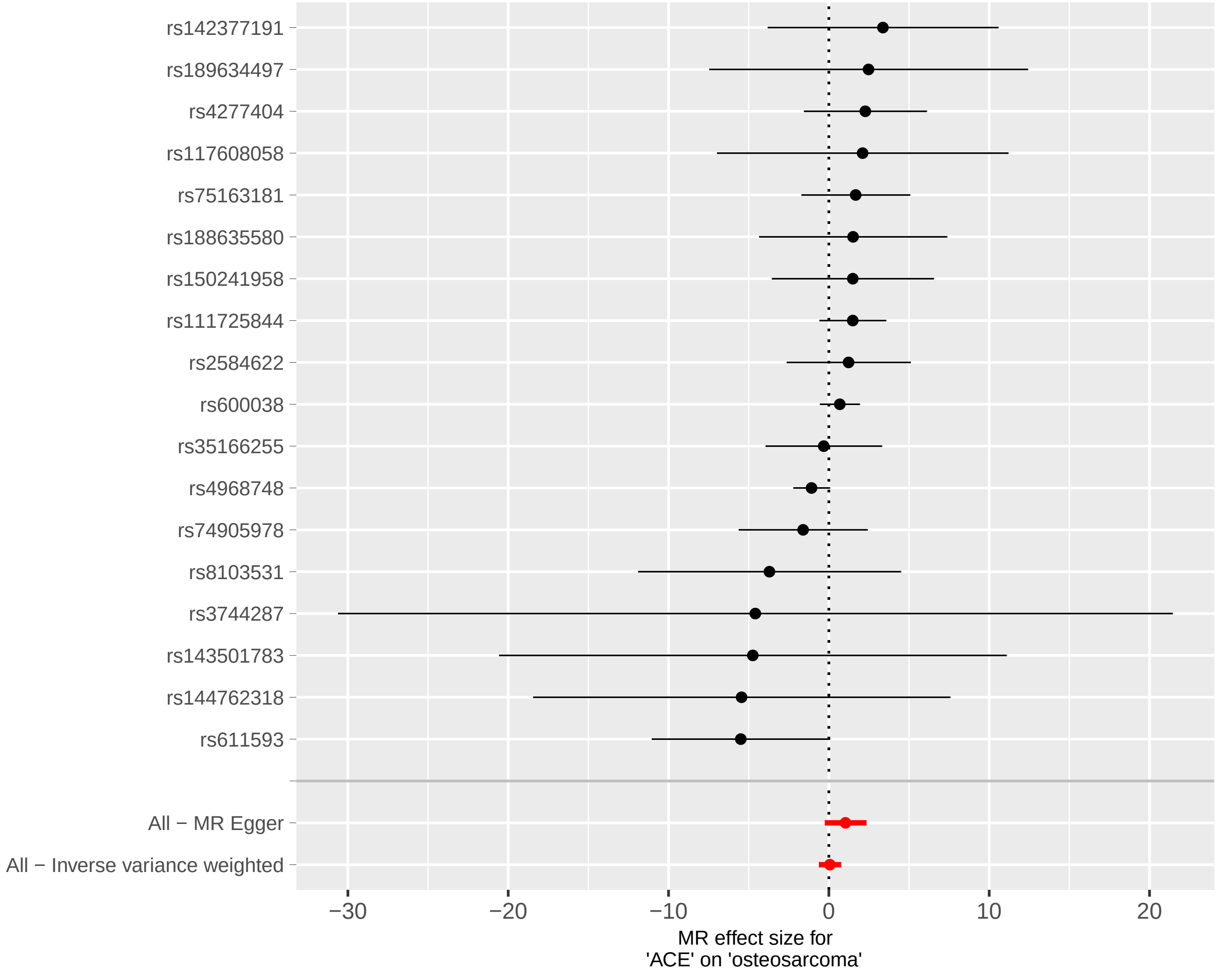
rs142377191
rs189634497
rs4277404
rs117608058
rs75163181
rs188635580
rs150241958
rs111725844
rs2584622
rs600038
rs35166255
rs4968748
rs74905978
rs8103531
rs3744287
rs143501783
rs144762318
rs611593
All − MR Egger
All − Inverse variance weighted
−30
−20
−10
0
10
20
MR effect size for
'ACE' on 'osteosarcoma'

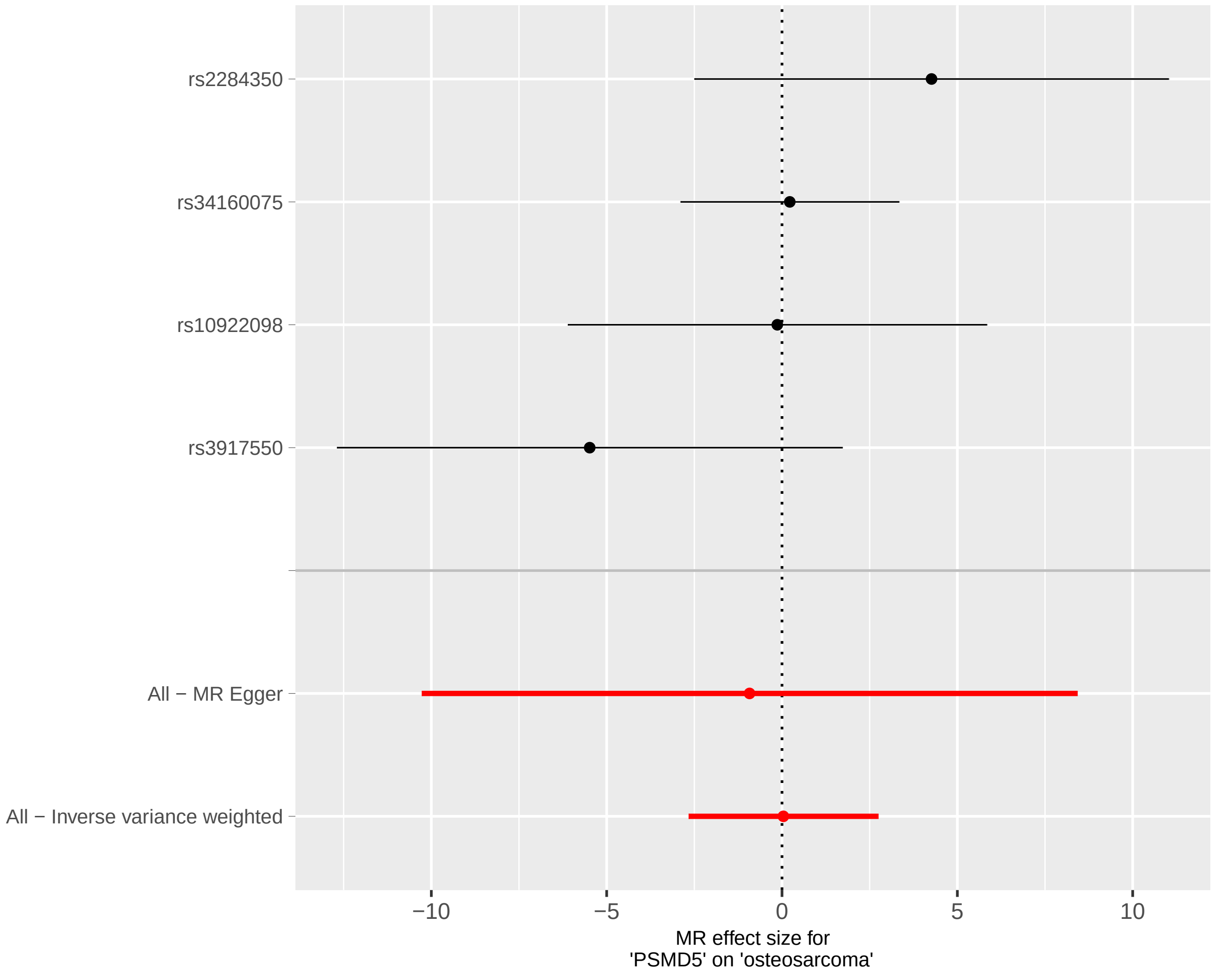

rs2284350
rs34160075
rs10922098
rs3917550
All − MR Egger
All − Inverse variance weighted
−10
−5
0
5
10
MR effect size for
'PSMD5' on 'osteosarcoma'

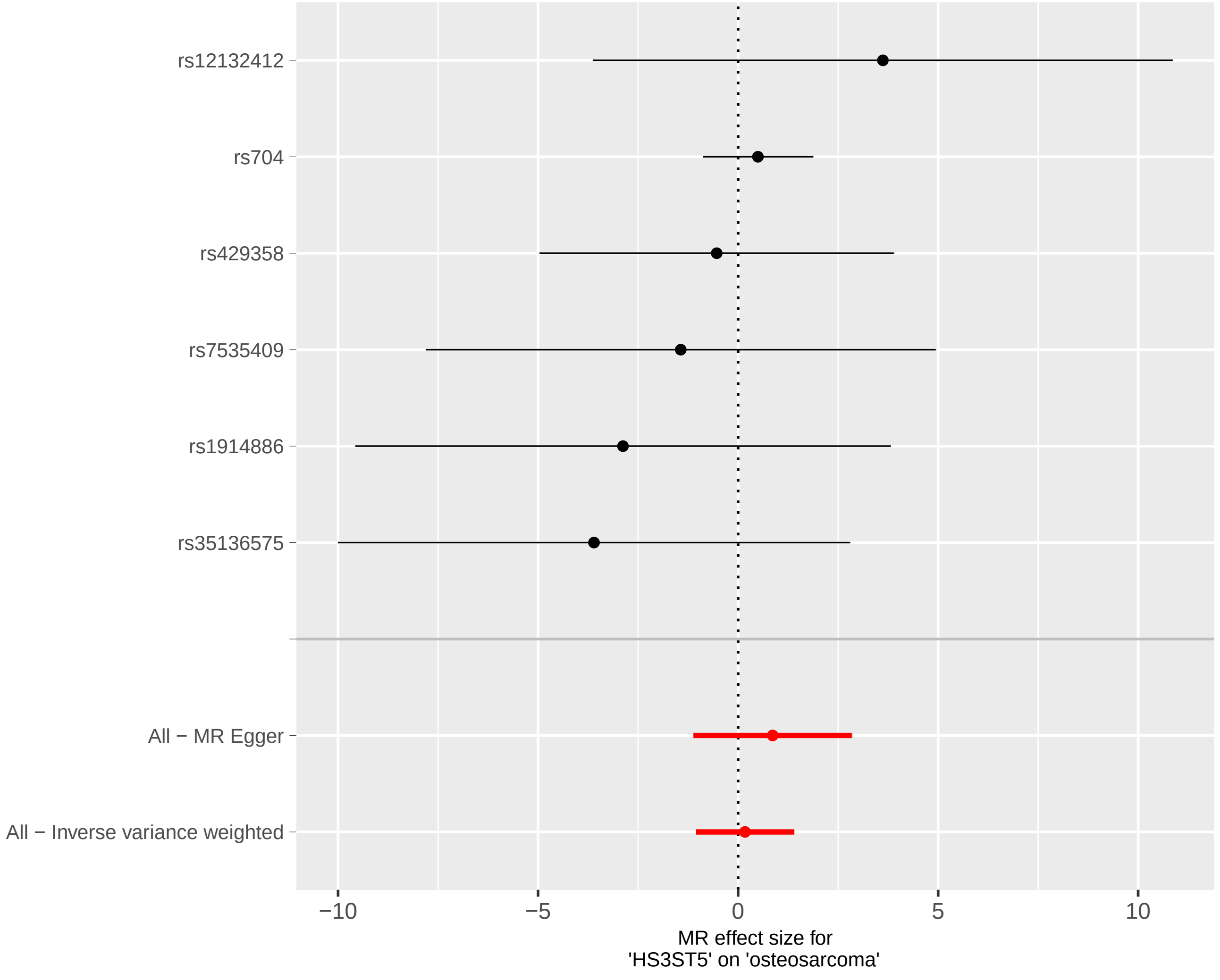

rs12132412
rs704
rs429358
rs7535409
rs1914886
rs35136575
All − MR Egger
All − Inverse variance weighted
−10
−5
0
5
10
MR effect size for
'HS3ST5' on 'osteosarcoma'

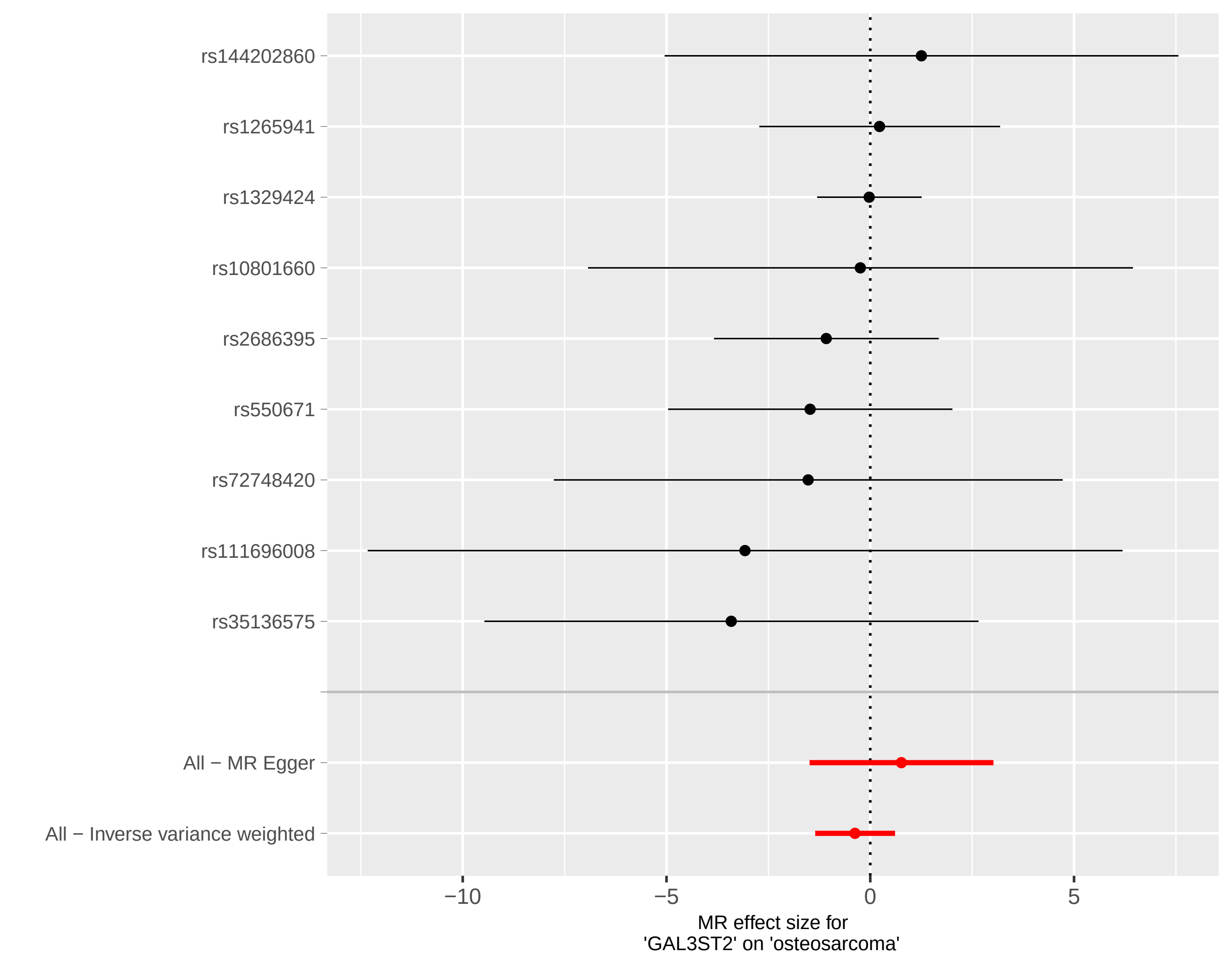
rs144202860
rs1265941
rs1329424
rs10801660
rs2686395
rs550671
rs72748420
rs111696008
rs35136575
All − MR Egger
All − Inverse variance weighted
−10
−5
0
5
MR effect size for
'GAL3ST2' on 'osteosarcoma'

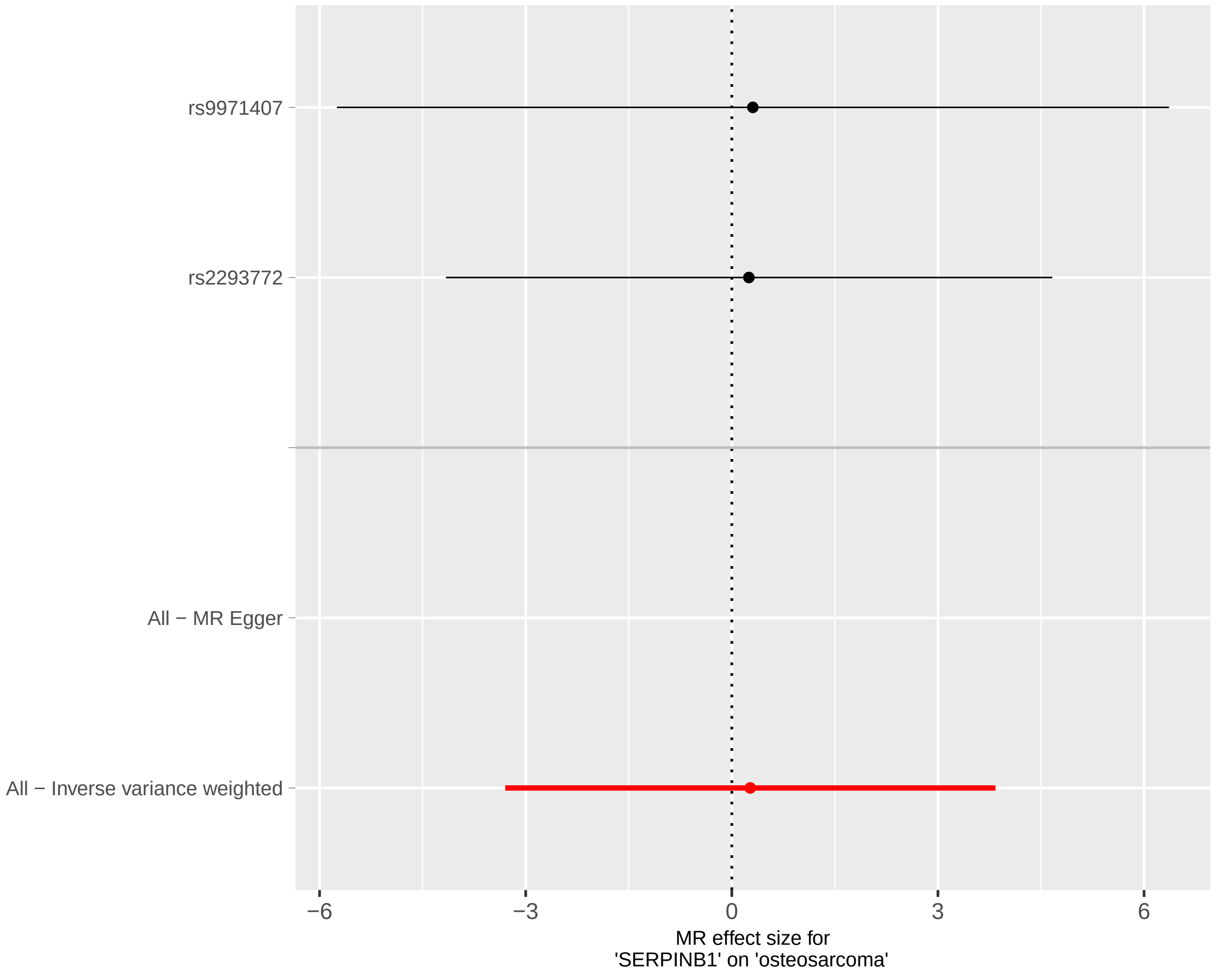
rs9971407
rs2293772
All − MR Egger
All − Inverse variance weighted
−6
−3
0
3
6
MR effect size for
'SERPINB1' on 'osteosarcoma'

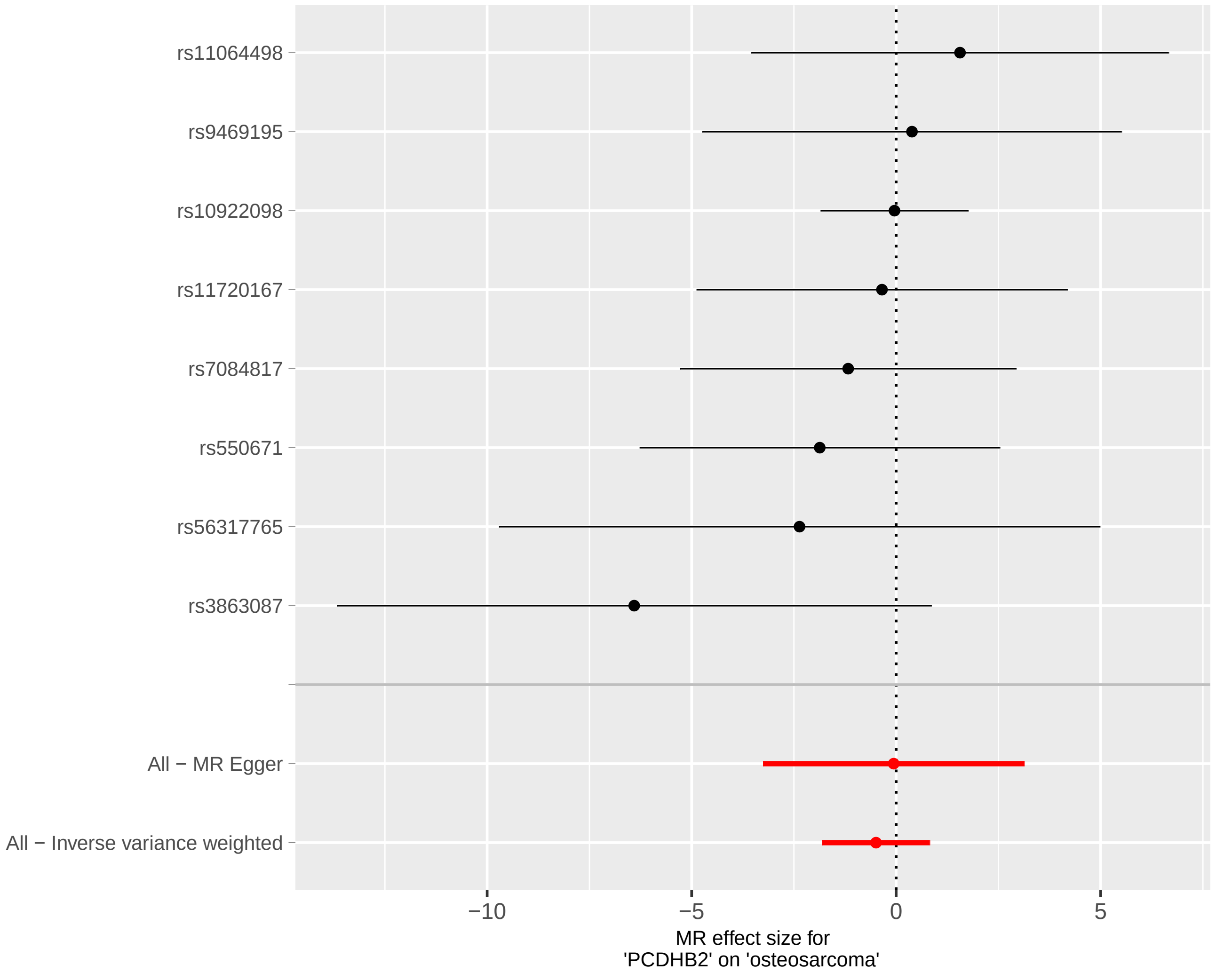

rs11064498
rs9469195
rs10922098
rs11720167
rs7084817
rs550671
rs56317765
rs3863087
All − MR Egger
All − Inverse variance weighted
−10
−5
0
5
MR effect size for
'PCDHB2' on 'osteosarcoma'

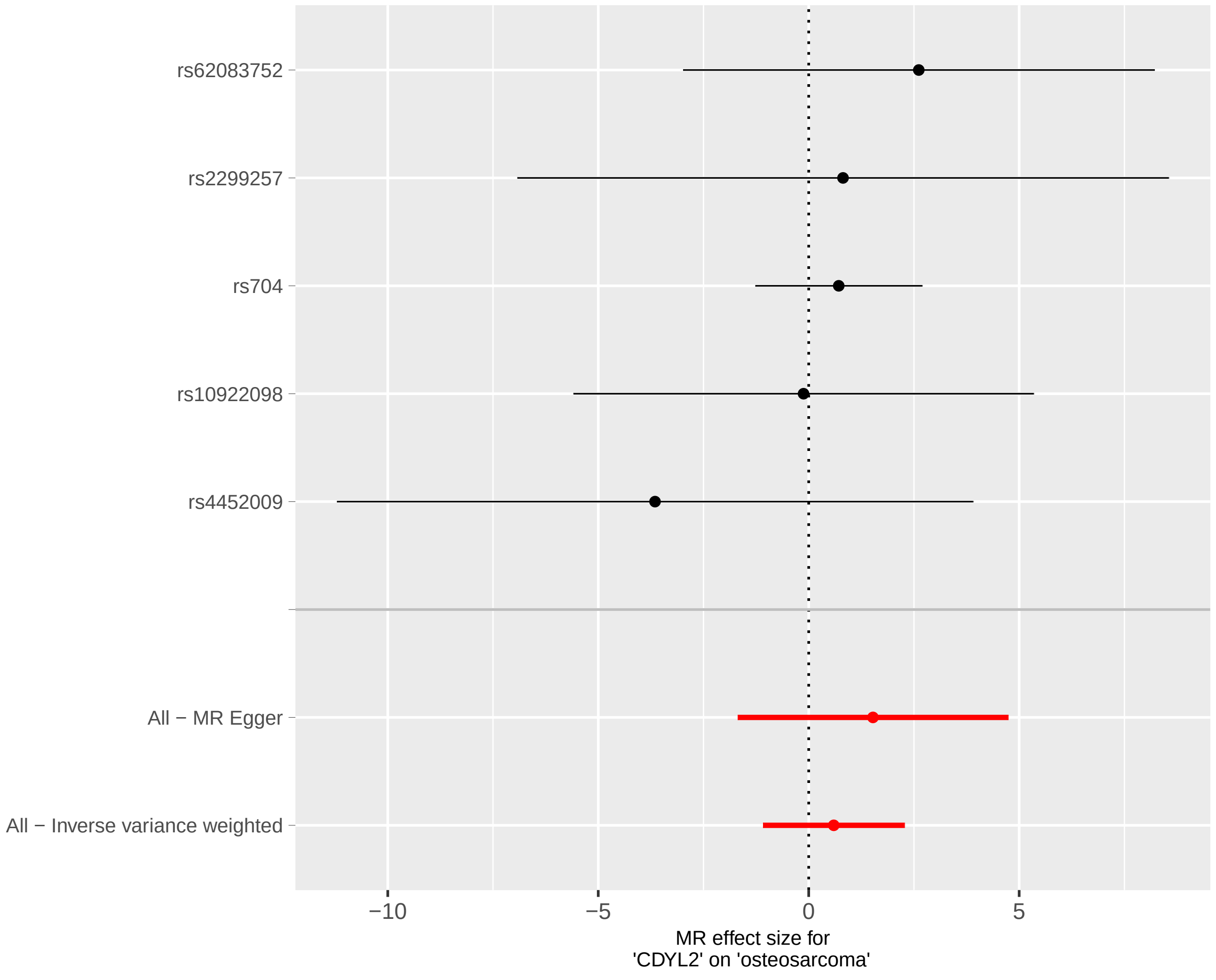
rs62083752
rs2299257
rs704
rs10922098
rs4452009
All − MR Egger
All − Inverse variance weighted
−10
−5
0
5
MR effect size for
'CDYL2' on 'osteosarcoma'

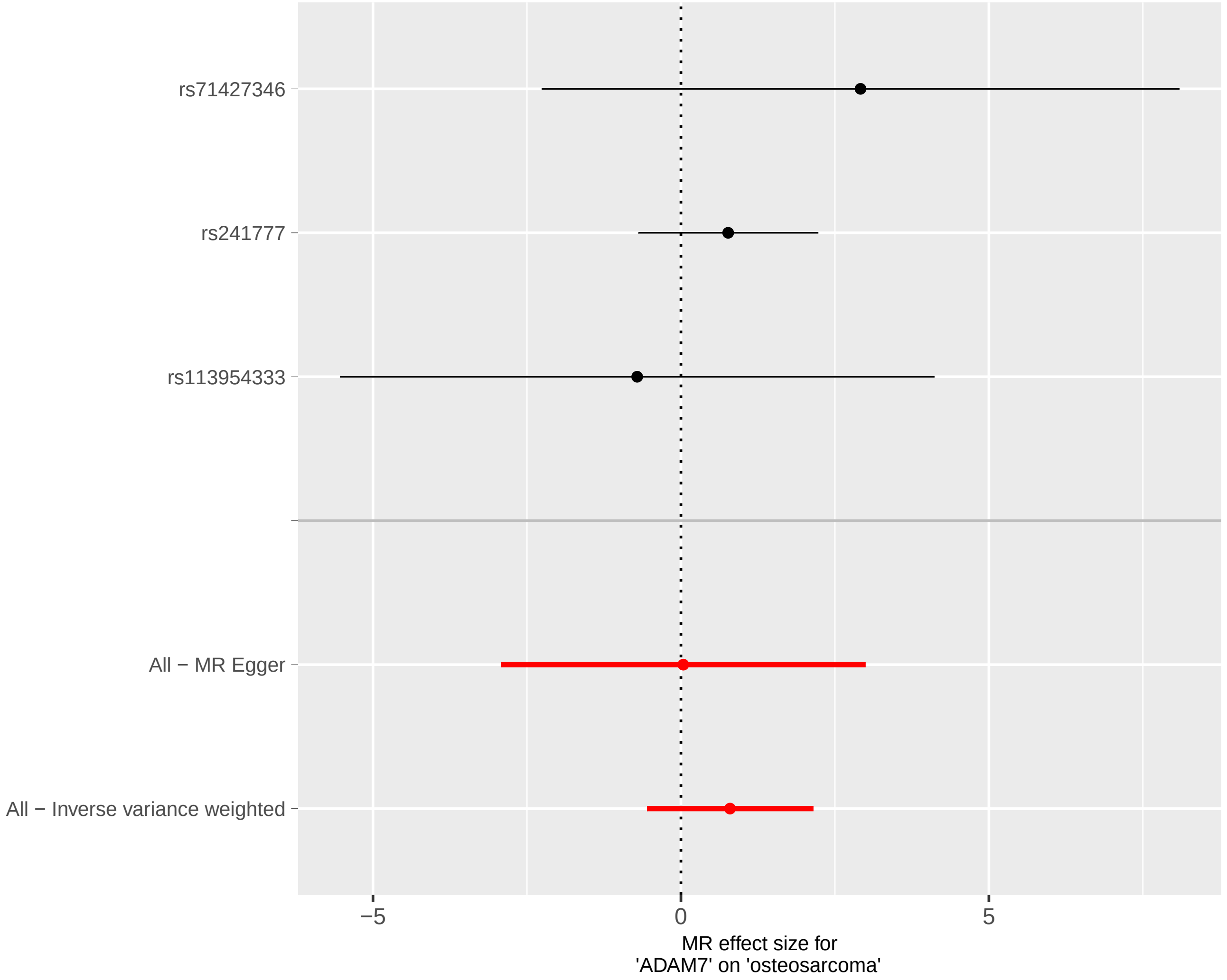

rs71427346
rs241777
rs113954333
All − MR Egger
All − Inverse variance weighted
−5
0
5
MR effect size for
'ADAM7' on 'osteosarcoma'

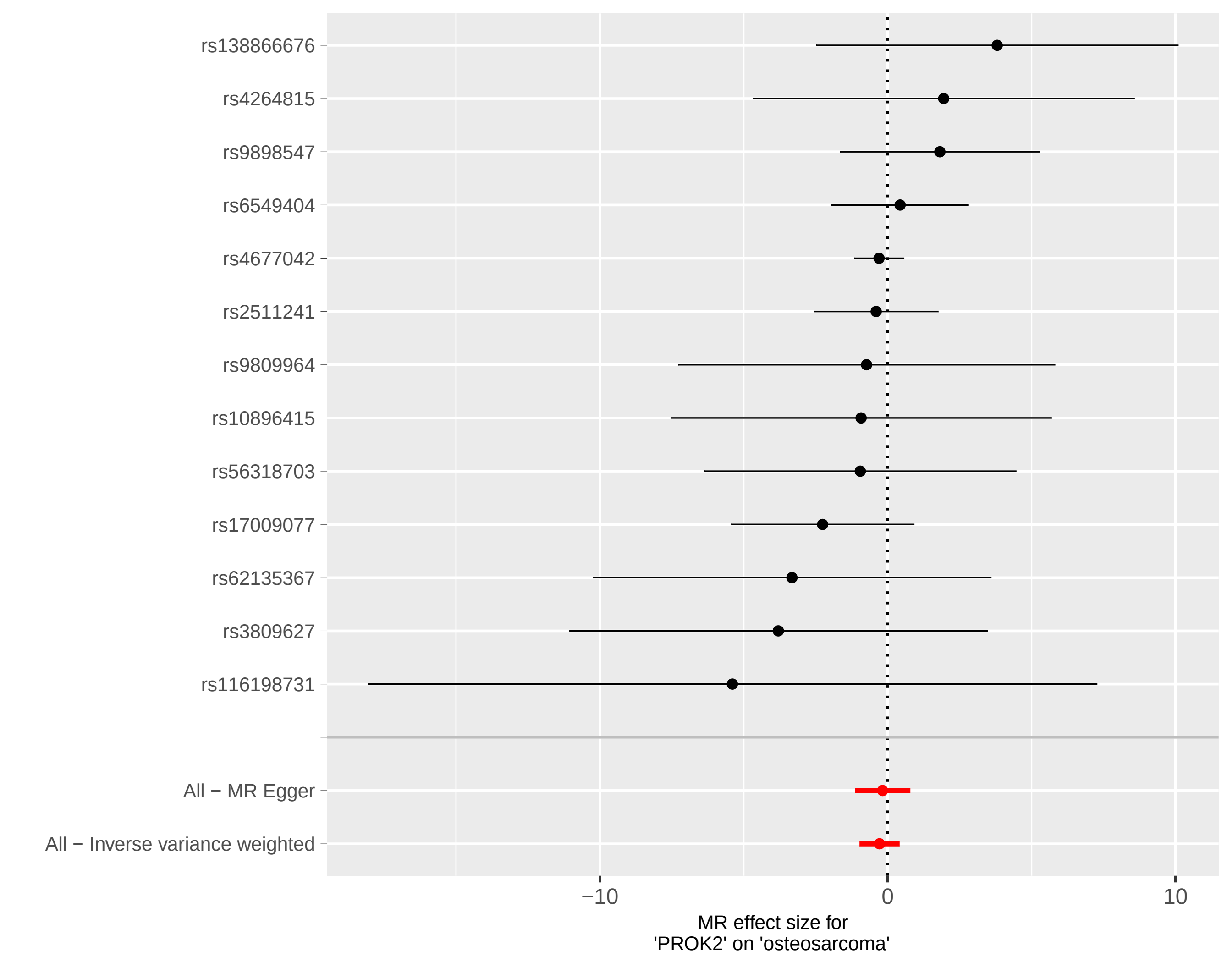
rs138866676
rs4264815
rs9898547
rs6549404
rs4677042
rs2511241
rs9809964
rs10896415
rs56318703
rs17009077
rs62135367
rs3809627
rs116198731
All − MR Egger
All − Inverse variance weighted
−10
0
10
MR effect size for
'PROK2' on 'osteosarcoma'

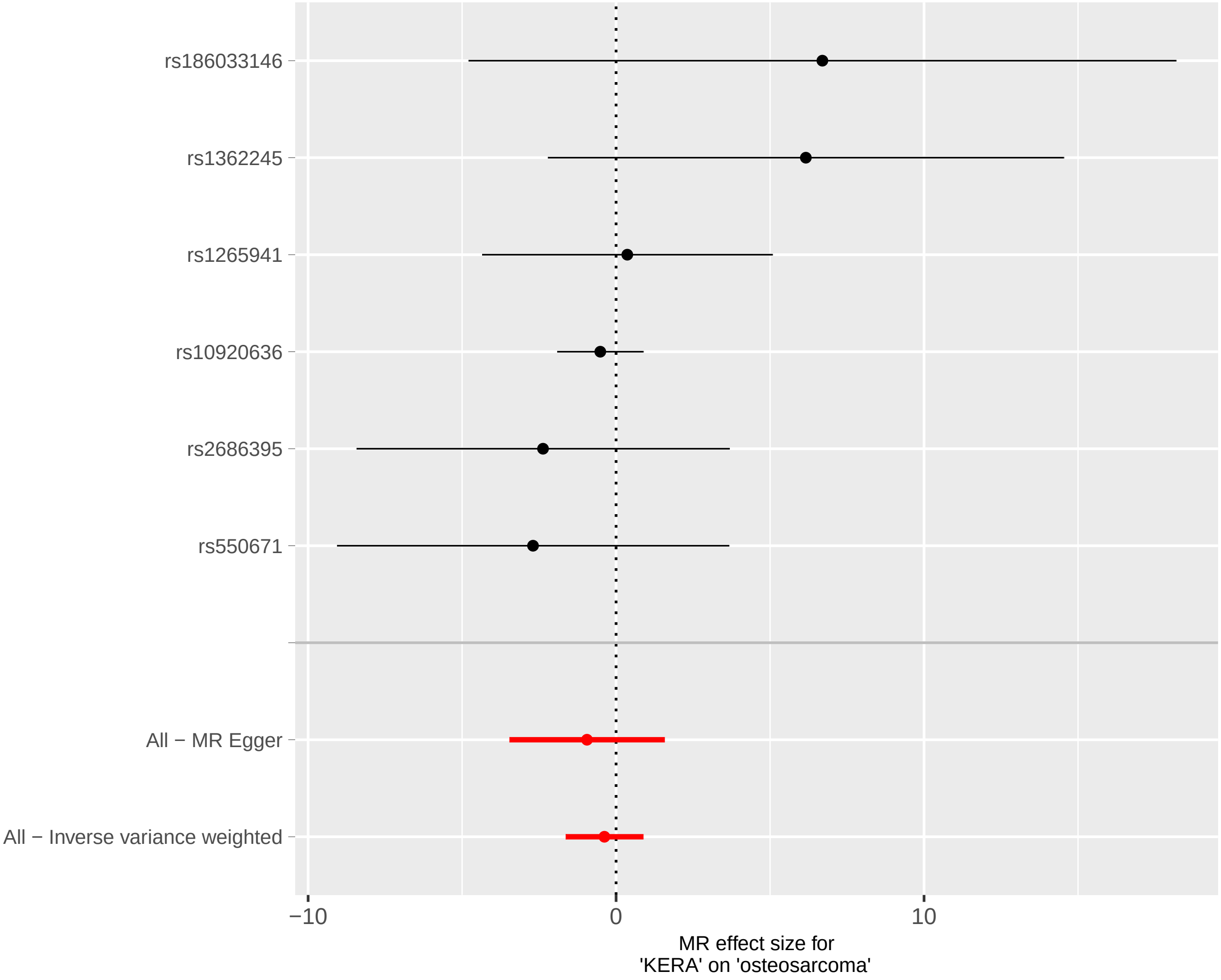

rs186033146
rs1362245
rs1265941
rs10920636
rs2686395
rs550671
All − MR Egger
All − Inverse variance weighted
−10
0
10
MR effect size for
'KERA' on 'osteosarcoma'

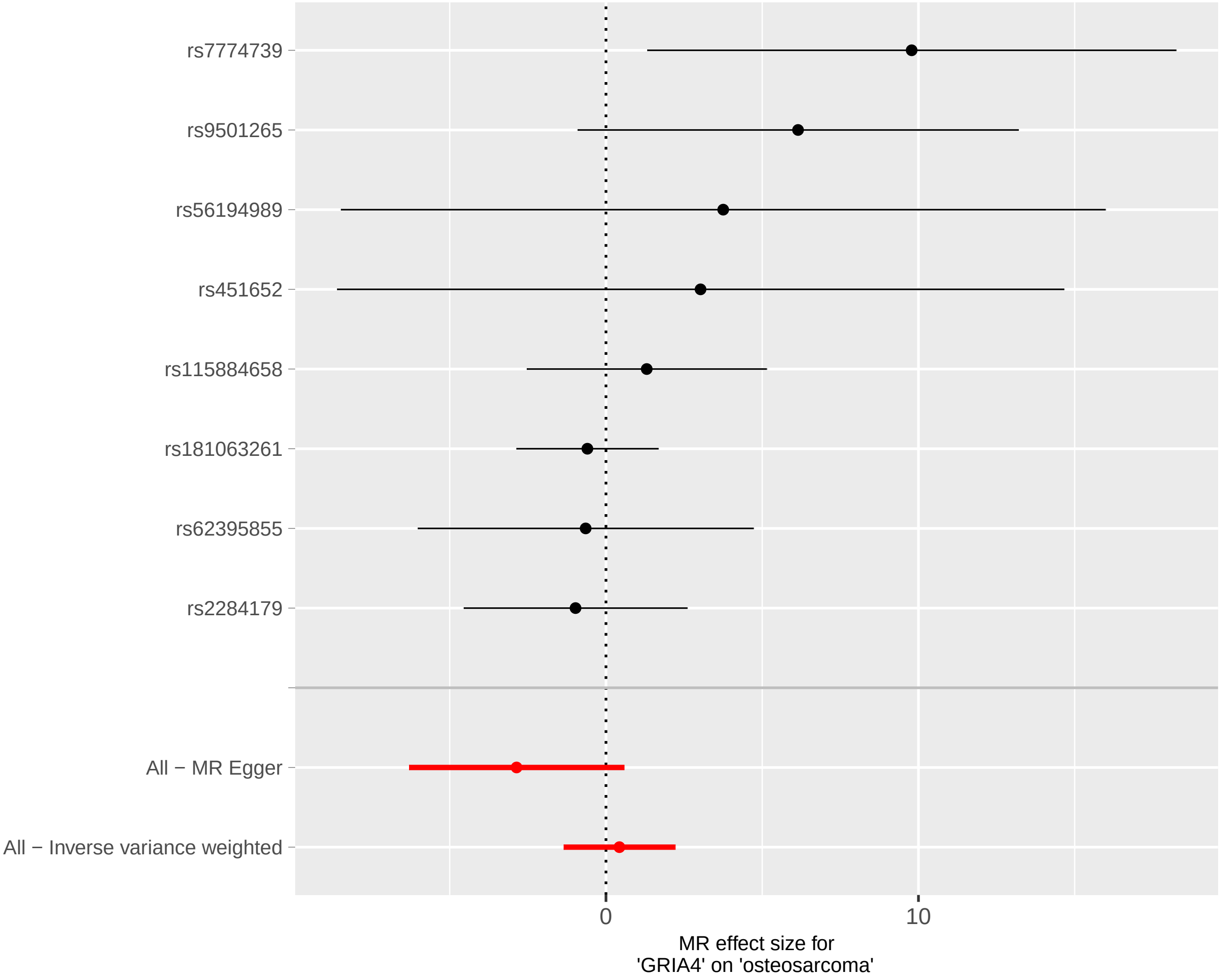
rs7774739
rs9501265
rs56194989
rs451652
rs115884658
rs181063261
rs62395855
rs2284179
All − MR Egger
All − Inverse variance weighted
0
10
MR effect size for
'GRIA4' on 'osteosarcoma'

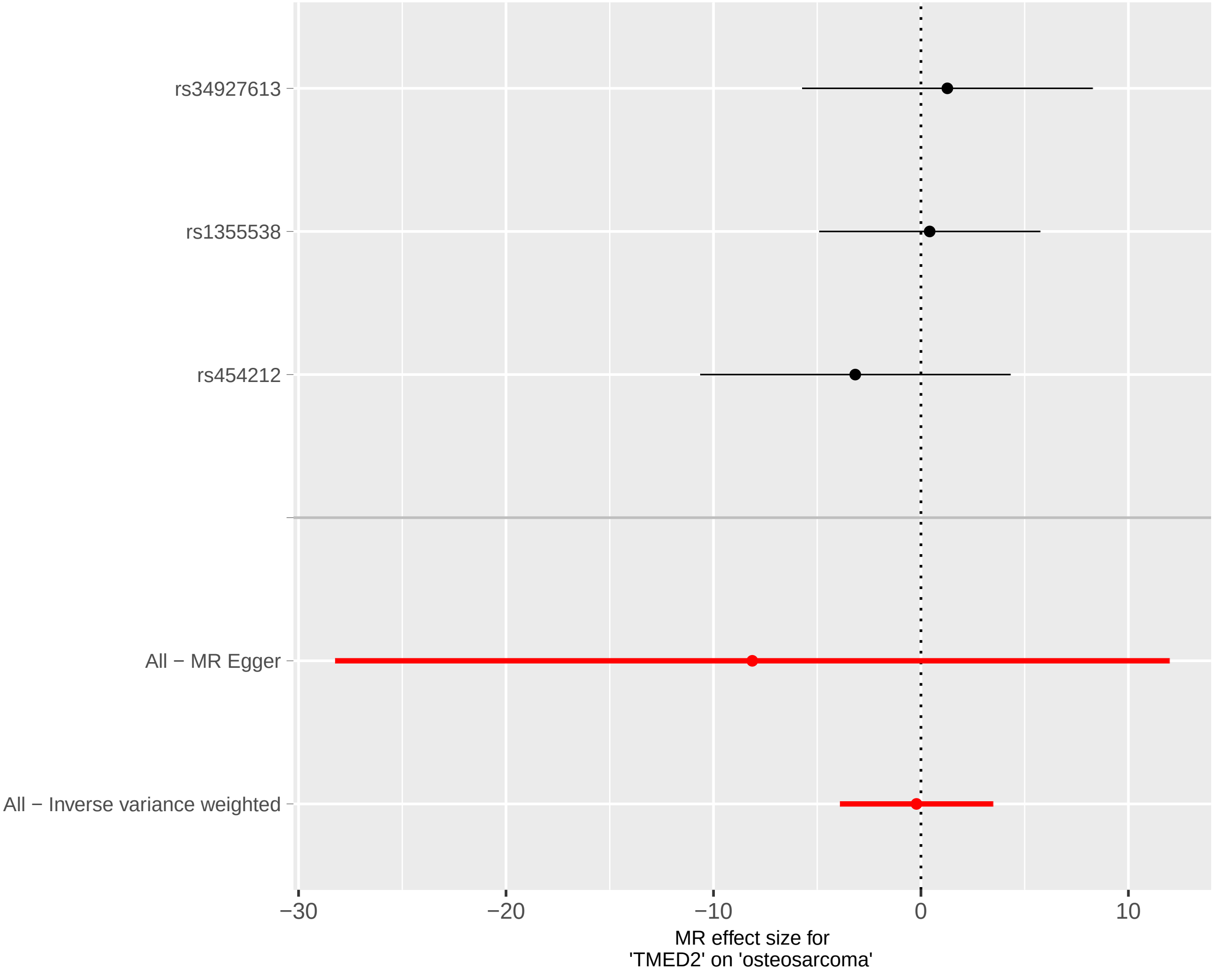

rs34927613
rs1355538
rs454212
All − MR Egger
All − Inverse variance weighted
−30
−20
−10
0
10
MR effect size for
'TMED2' on 'osteosarcoma'

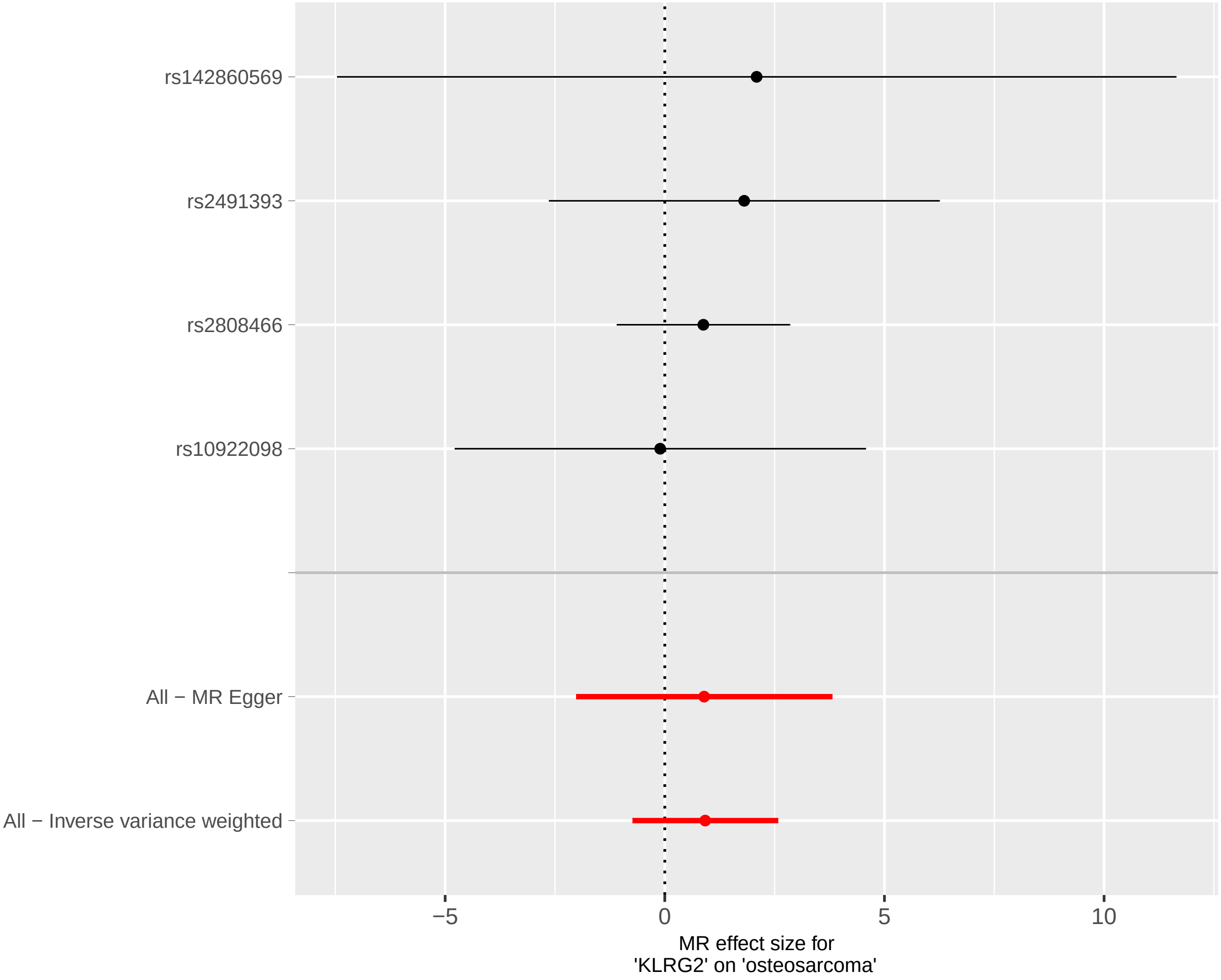
rs142860569
rs2491393
rs2808466
rs10922098
All − MR Egger
All − Inverse variance weighted
−5
0
5
10
MR effect size for
'KLRG2' on 'osteosarcoma'

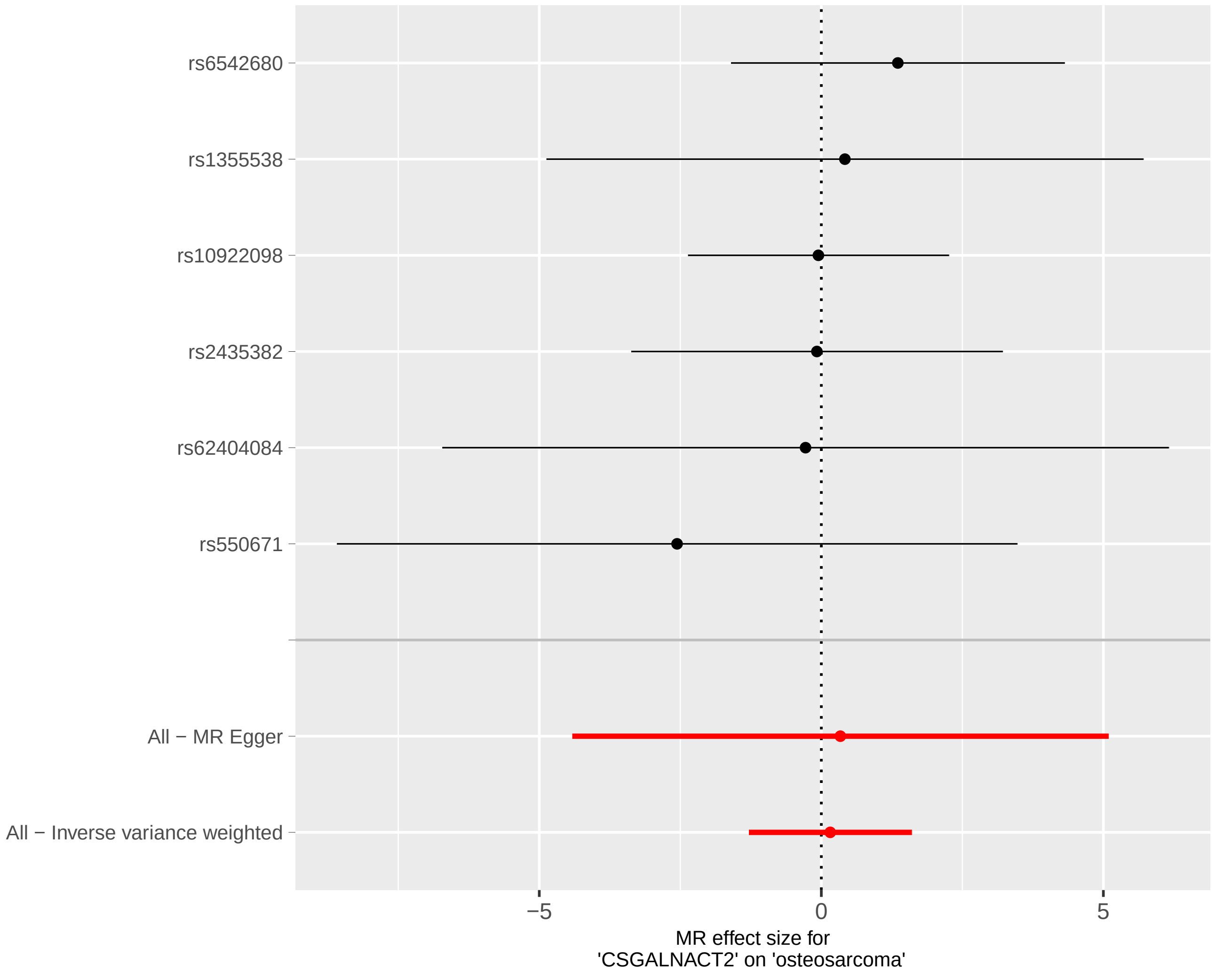

rs6542680
rs1355538
rs10922098
rs2435382
rs62404084
rs550671
All − MR Egger
All − Inverse variance weighted
−5
0
5
MR effect size for
'CSGALNACT2' on 'osteosarcoma'

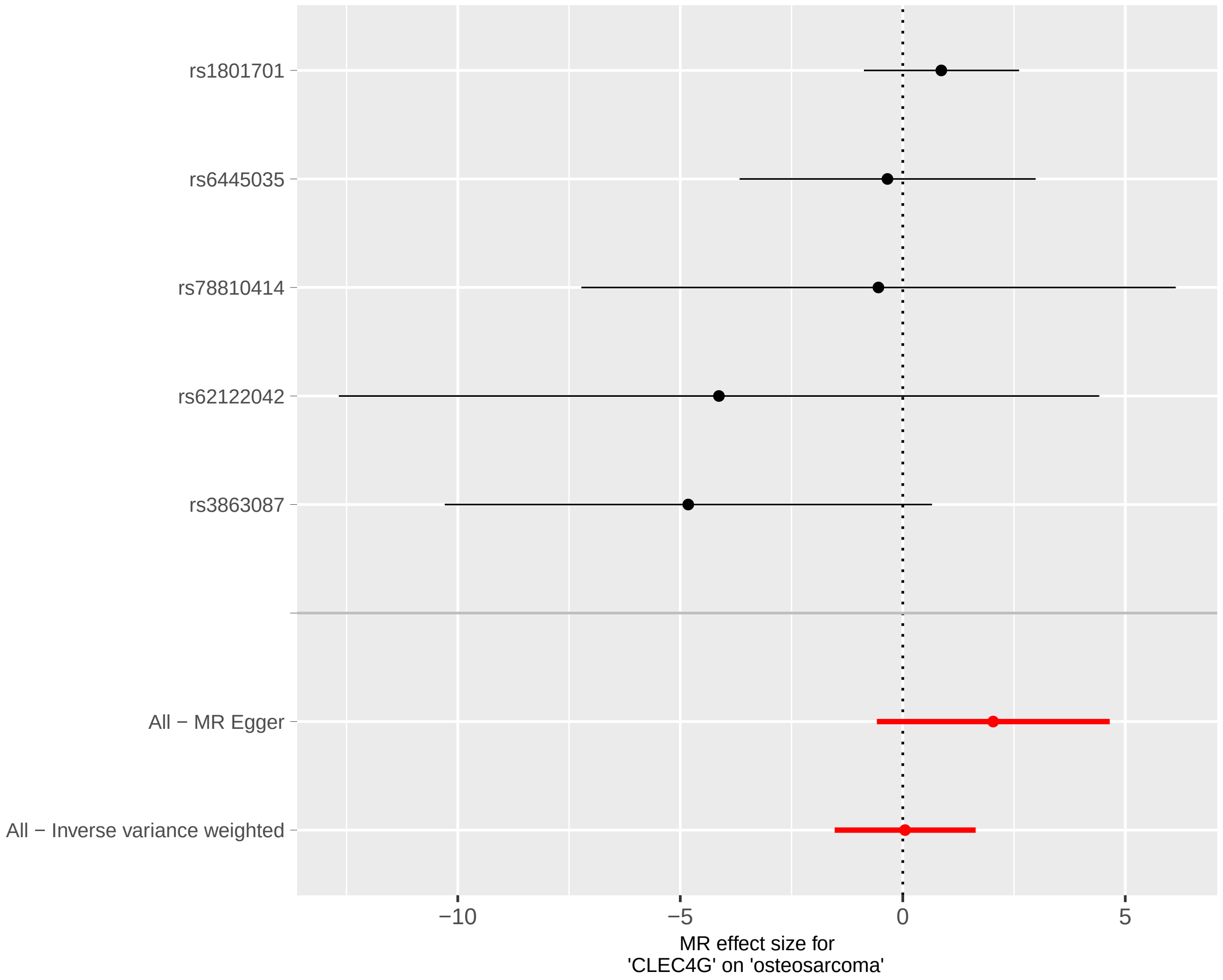
rs1801701
rs6445035
rs78810414
rs62122042
rs3863087
All − MR Egger
All − Inverse variance weighted
−10
−5
0
5
MR effect size for
'CLEC4G' on 'osteosarcoma'

Insufficient number of SNPs

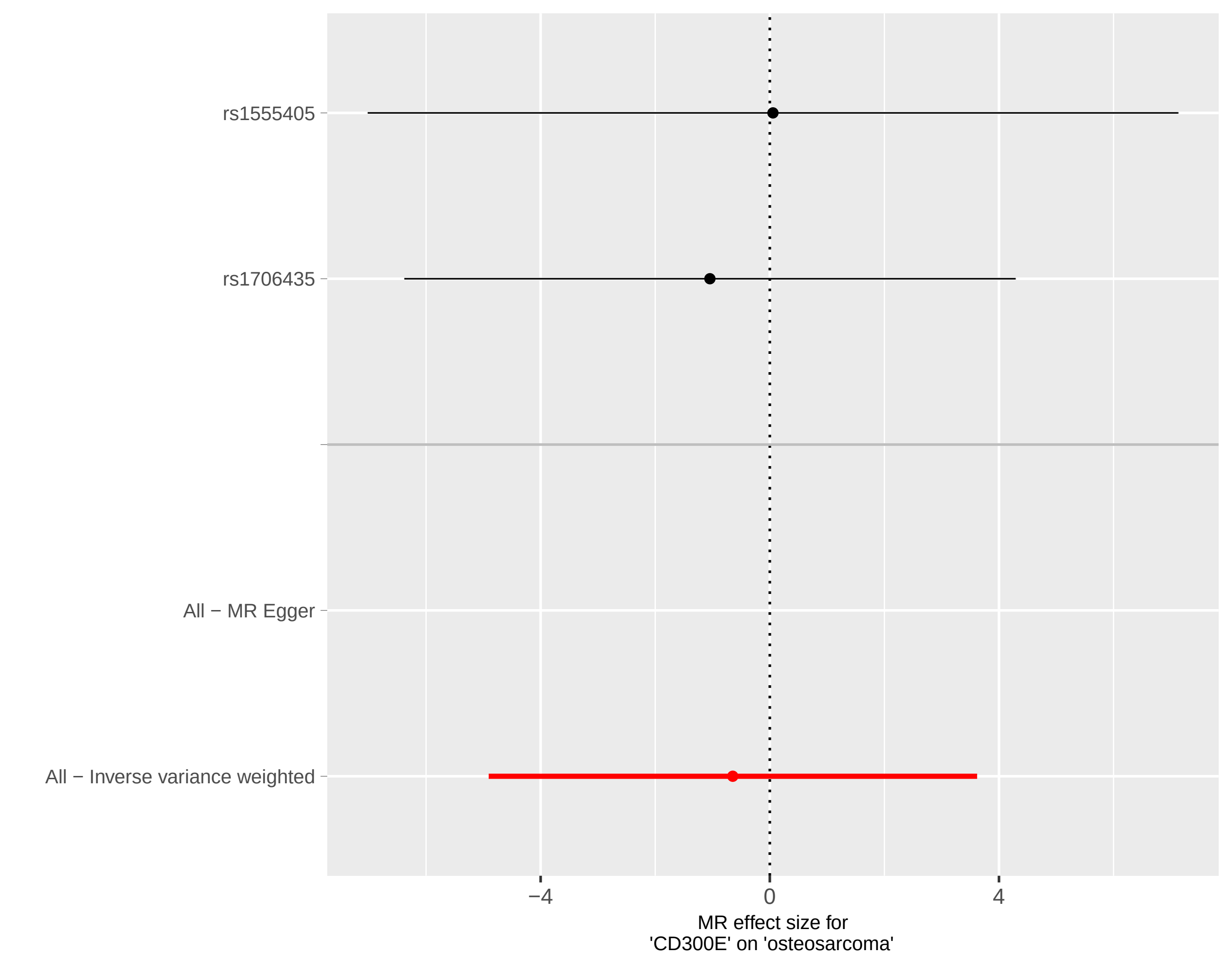
rs1555405
rs1706435
All − MR Egger
All − Inverse variance weighted
−4
0
4
MR effect size for
'CD300E' on 'osteosarcoma'

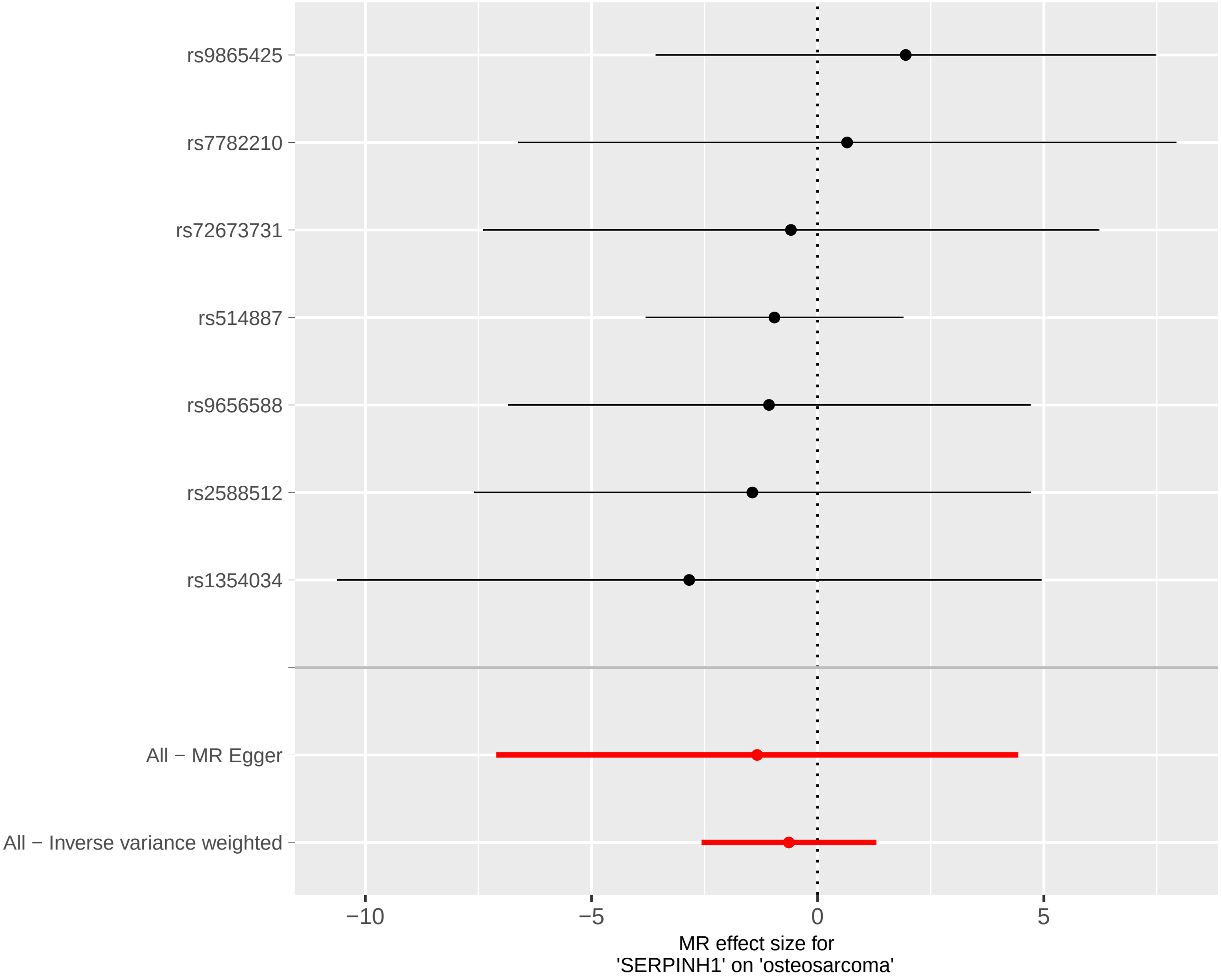

rs9865425
rs7782210
rs72673731
rs514887
rs9656588
rs2588512
rs1354034
All − MR Egger
All − Inverse variance weighted
−10
−5
0
5
MR effect size for
'SERPINH1' on 'osteosarcoma'

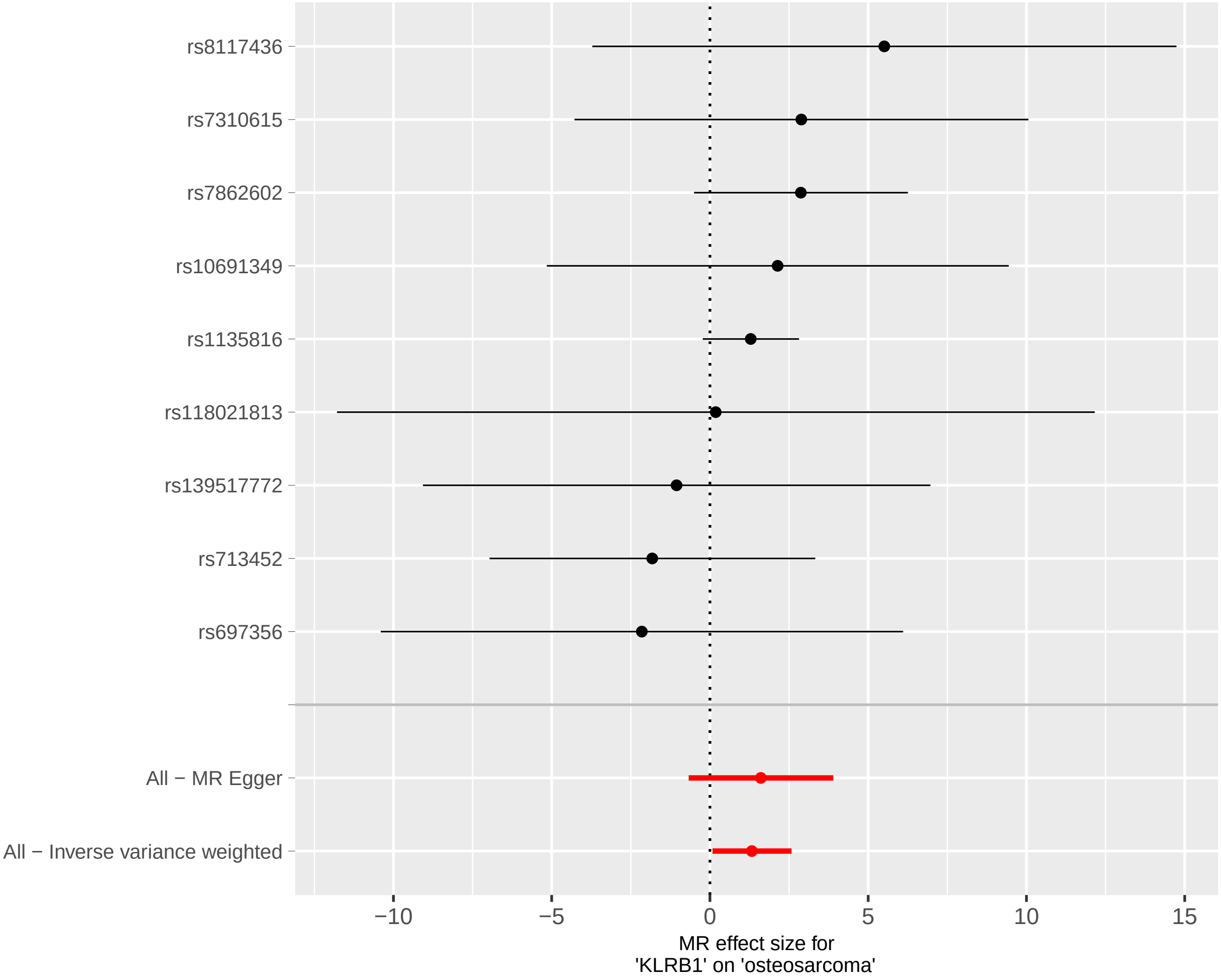
rs8117436
rs7310615
rs7862602
rs10691349
rs1135816
rs118021813
rs139517772
rs713452
rs697356
All − MR Egger
All − Inverse variance weighted
−10
−5
0
5
10
15
MR effect size for
'KLRB1' on 'osteosarcoma'

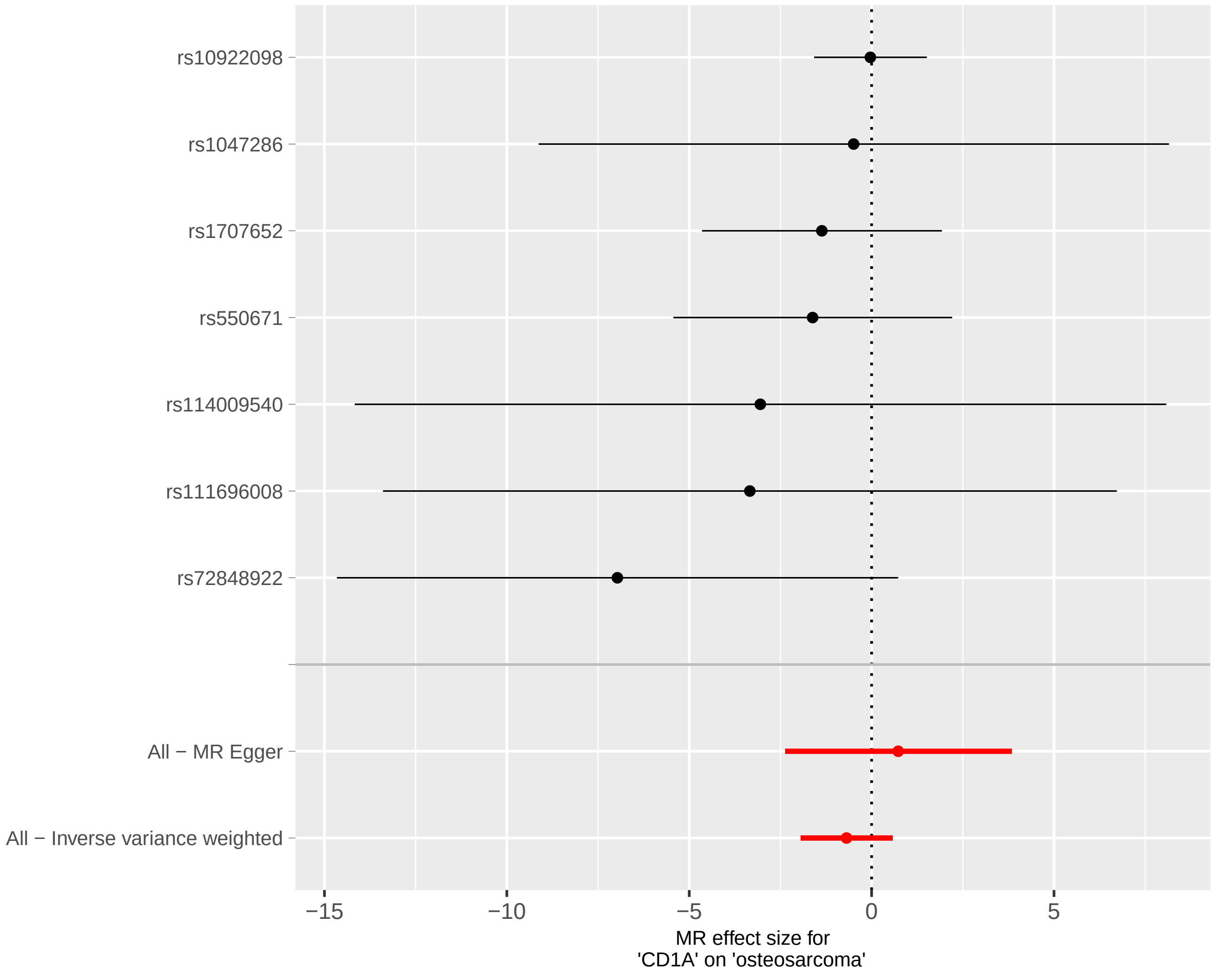
rs10922098
rs1047286
rs1707652
rs550671
rs114009540
rs111696008
rs72848922
All − MR Egger
All − Inverse variance weighted
−15
−10
−5
0
5
MR effect size for
'CD1A' on 'osteosarcoma'

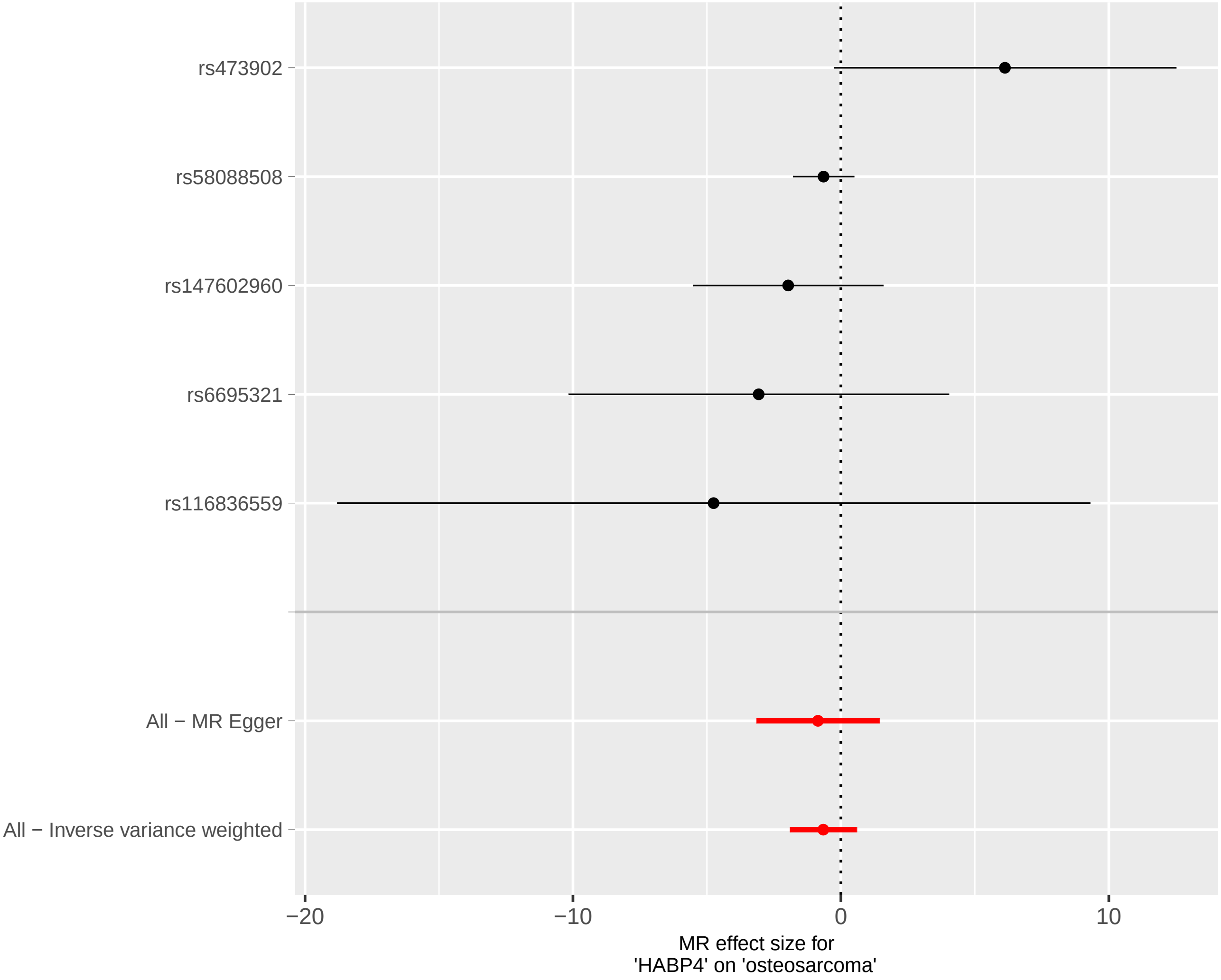
rs473902
rs58088508
rs147602960
rs6695321
rs116836559
All − MR Egger
All − Inverse variance weighted
−20
−10
0
10
MR effect size for
'HABP4' on 'osteosarcoma'

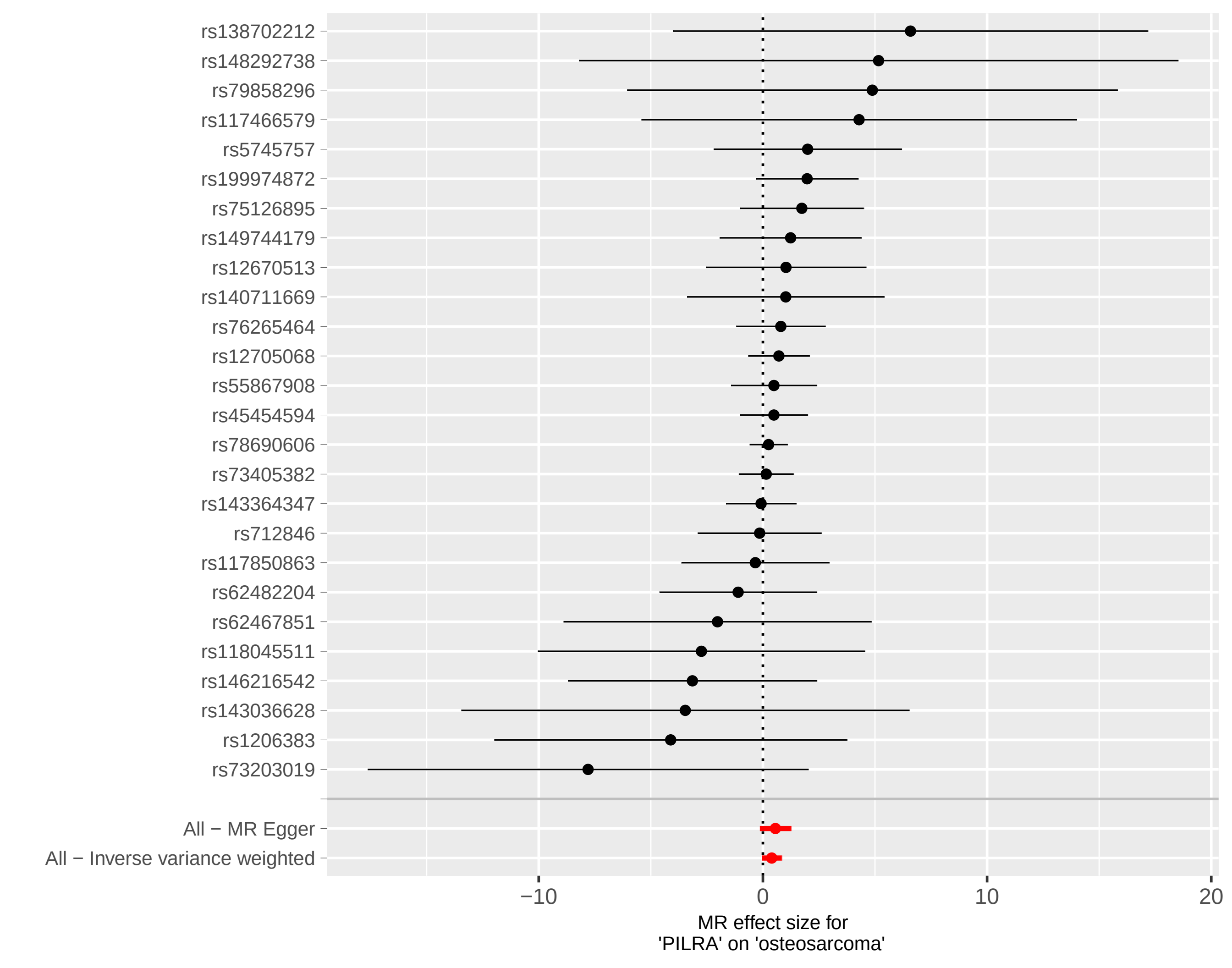

rs138702212
rs148292738
rs79858296
rs117466579
rs5745757
rs199974872
rs75126895
rs149744179
rs12670513
rs140711669
rs76265464
rs12705068
rs55867908
rs45454594
rs78690606
rs73405382
rs143364347
rs712846
rs117850863
rs62482204
rs62467851
rs118045511
rs146216542
rs143036628
rs1206383
rs73203019
All − MR Egger
All − Inverse variance weighted
−10
0
10
20
MR effect size for
'PILRA' on 'osteosarcoma'

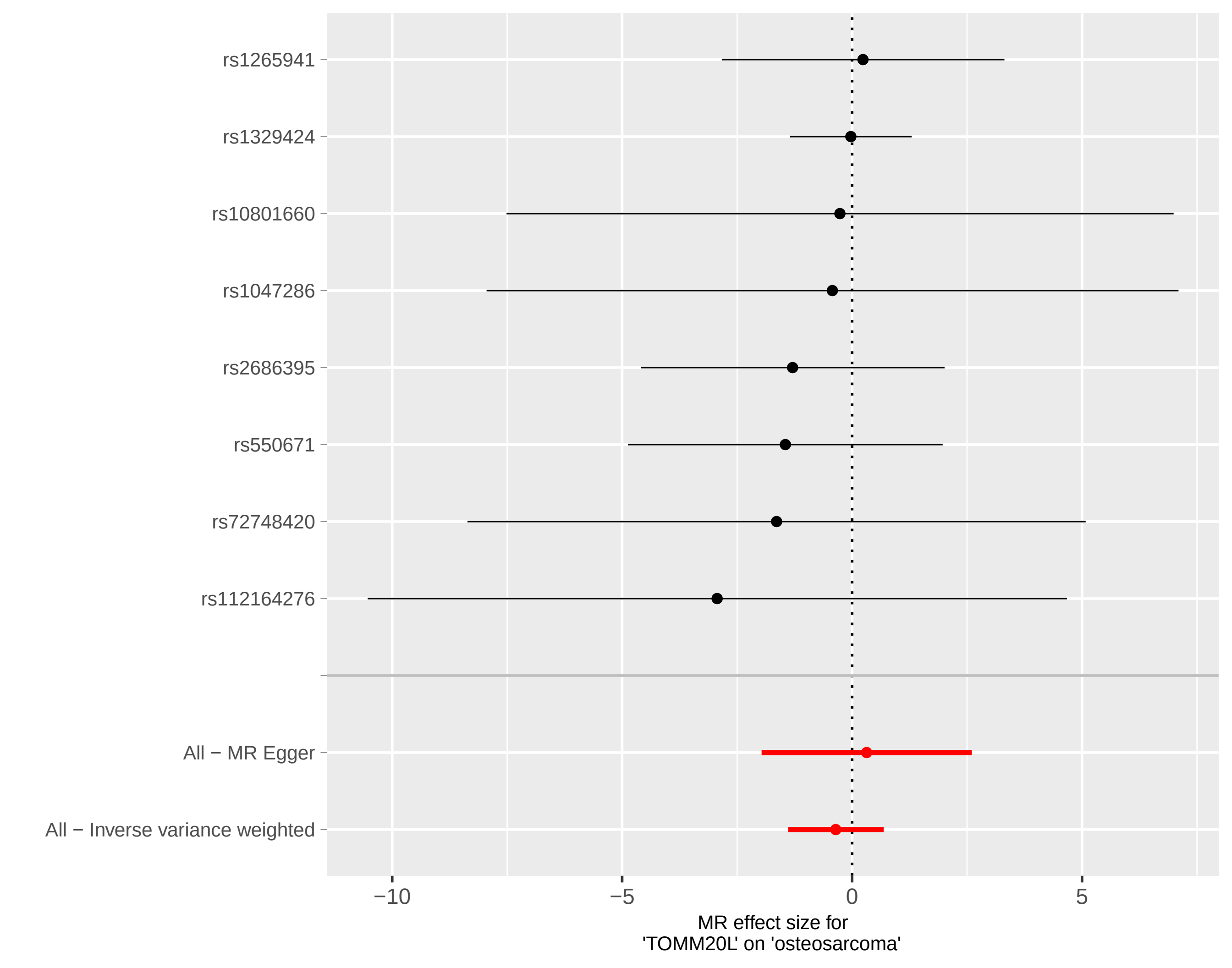
rs1265941
rs1329424
rs10801660
rs1047286
rs2686395
rs550671
rs72748420
rs112164276
All − MR Egger
All − Inverse variance weighted
−10
−5
0
5
MR effect size for
'TOMM20L' on 'osteosarcoma'

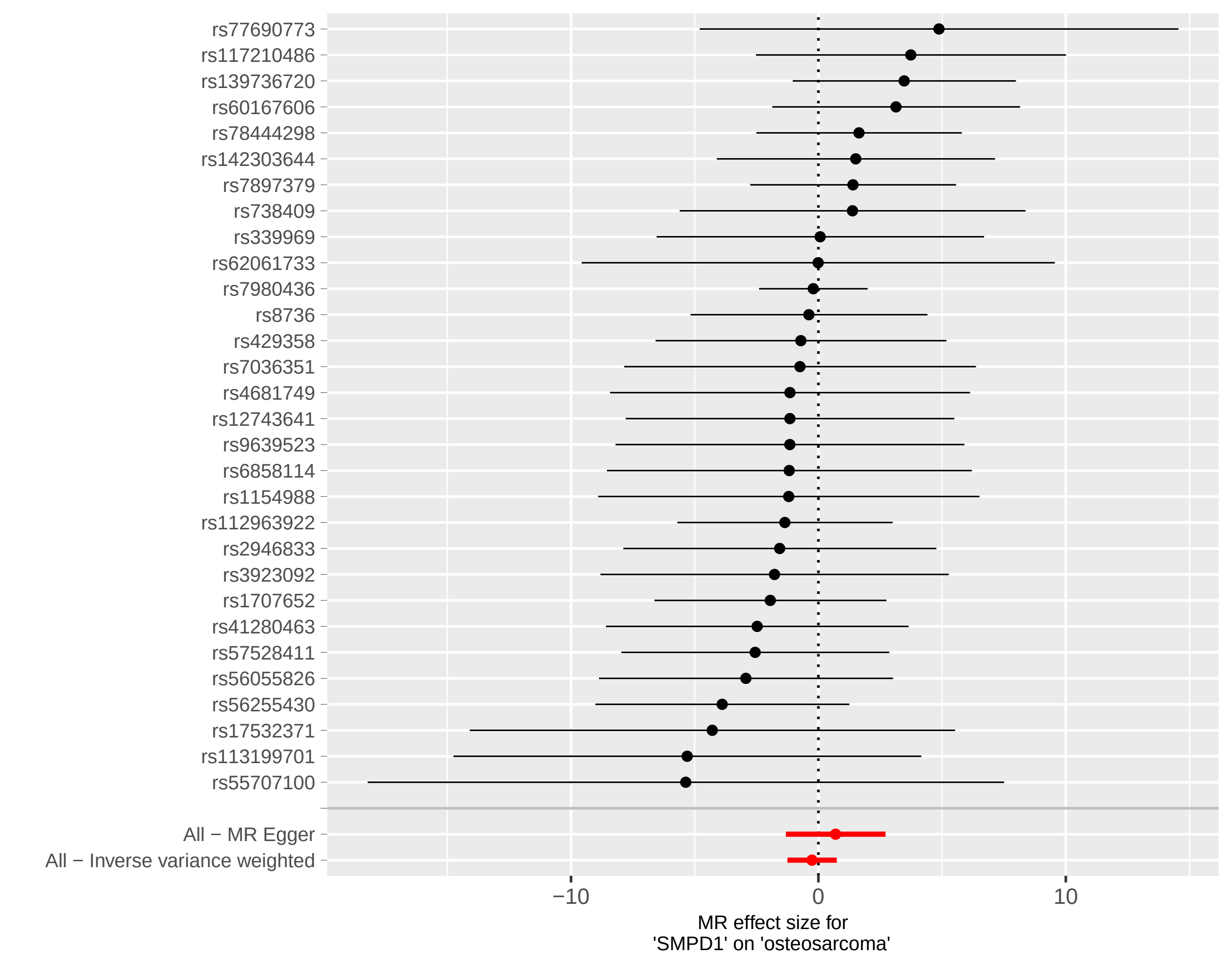
rs77690773
rs117210486
rs139736720
rs60167606
rs78444298
rs142303644
rs7897379
rs738409
rs339969
rs62061733
rs7980436
rs8736
rs429358
rs7036351
rs4681749
rs12743641
rs9639523
rs6858114
rs1154988
rs112963922
rs2946833
rs3923092
rs1707652
rs41280463
rs57528411
rs56055826
rs56255430
rs17532371
rs113199701
rs55707100
All − MR Egger
All − Inverse variance weighted
−10
0
10
MR effect size for
'SMPD1' on 'osteosarcoma'

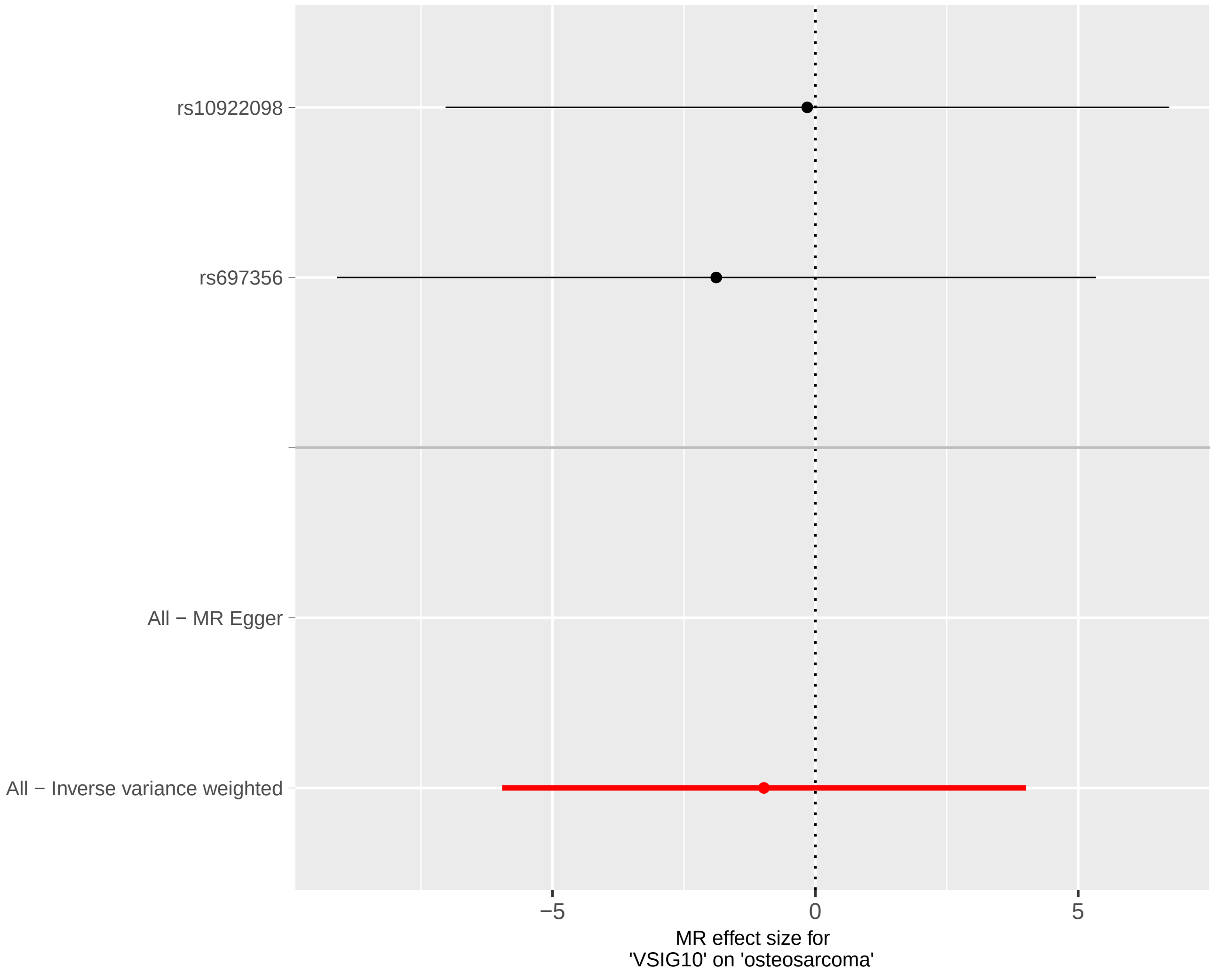

rs10922098
rs697356
All − MR Egger
All − Inverse variance weighted
−5
0
5
MR effect size for
'VSIG10' on 'osteosarcoma'

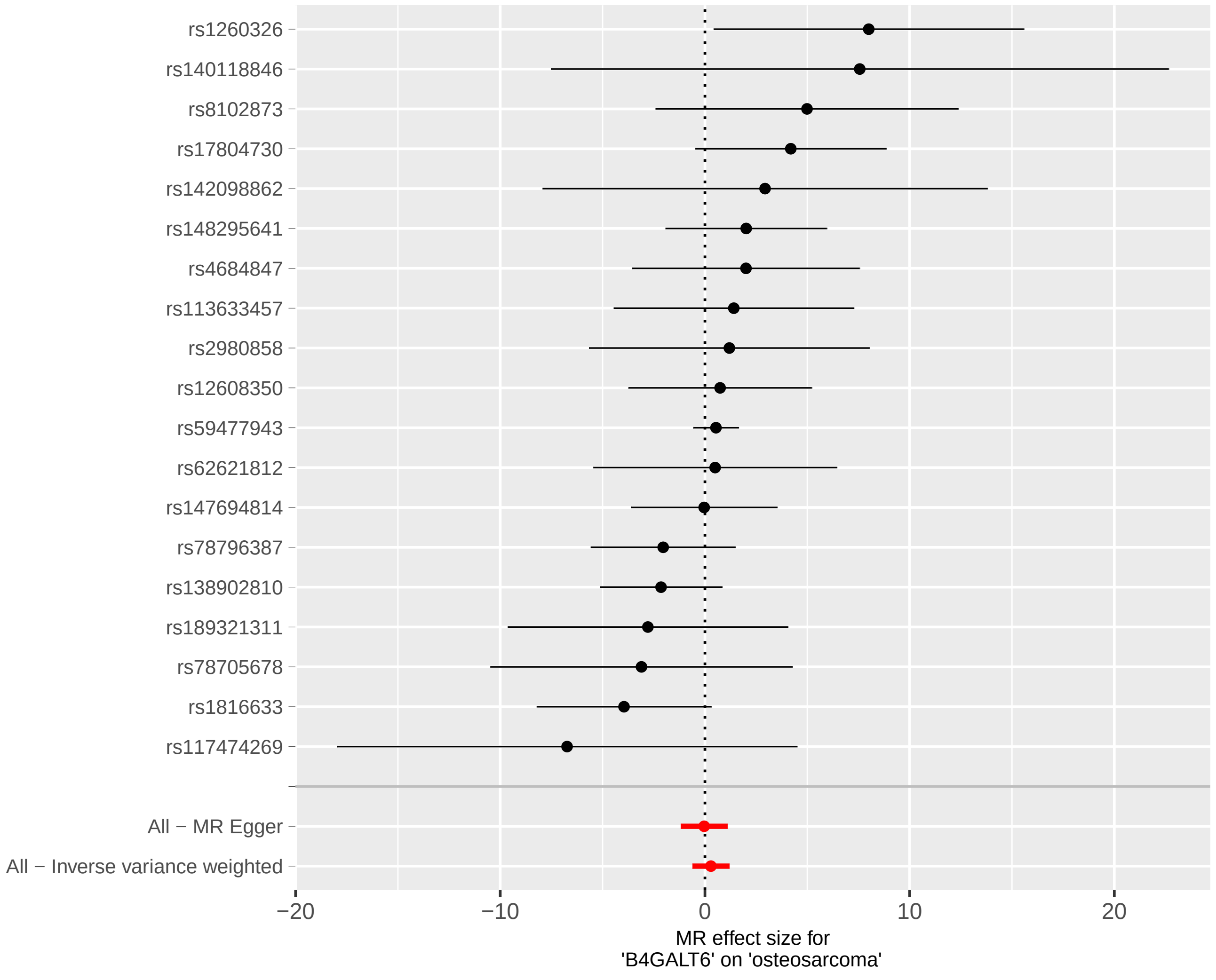

rs1260326
rs140118846
rs8102873
rs17804730
rs142098862
rs148295641
rs4684847
rs113633457
rs2980858
rs12608350
rs59477943
rs62621812
rs147694814
rs78796387
rs138902810
rs189321311
rs78705678
rs1816633
rs117474269
All − MR Egger
All − Inverse variance weighted
−20
−10
0
10
20
MR effect size for
'B4GALT6' on 'osteosarcoma'

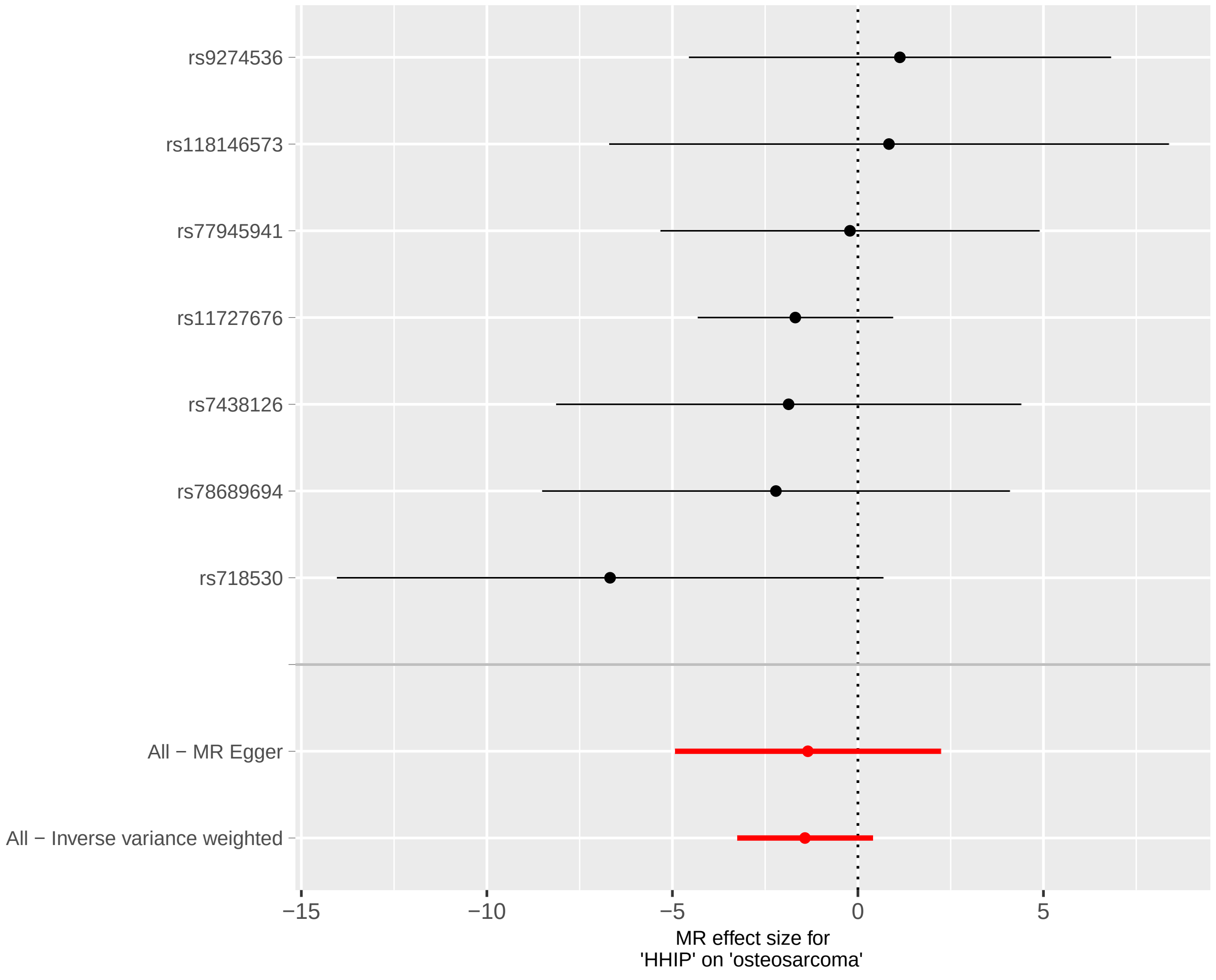

rs9274536
rs118146573
rs77945941
rs11727676
rs7438126
rs78689694
rs718530
All − MR Egger
All − Inverse variance weighted
−15
−10
−5
0
5
MR effect size for
'HHIP' on 'osteosarcoma'

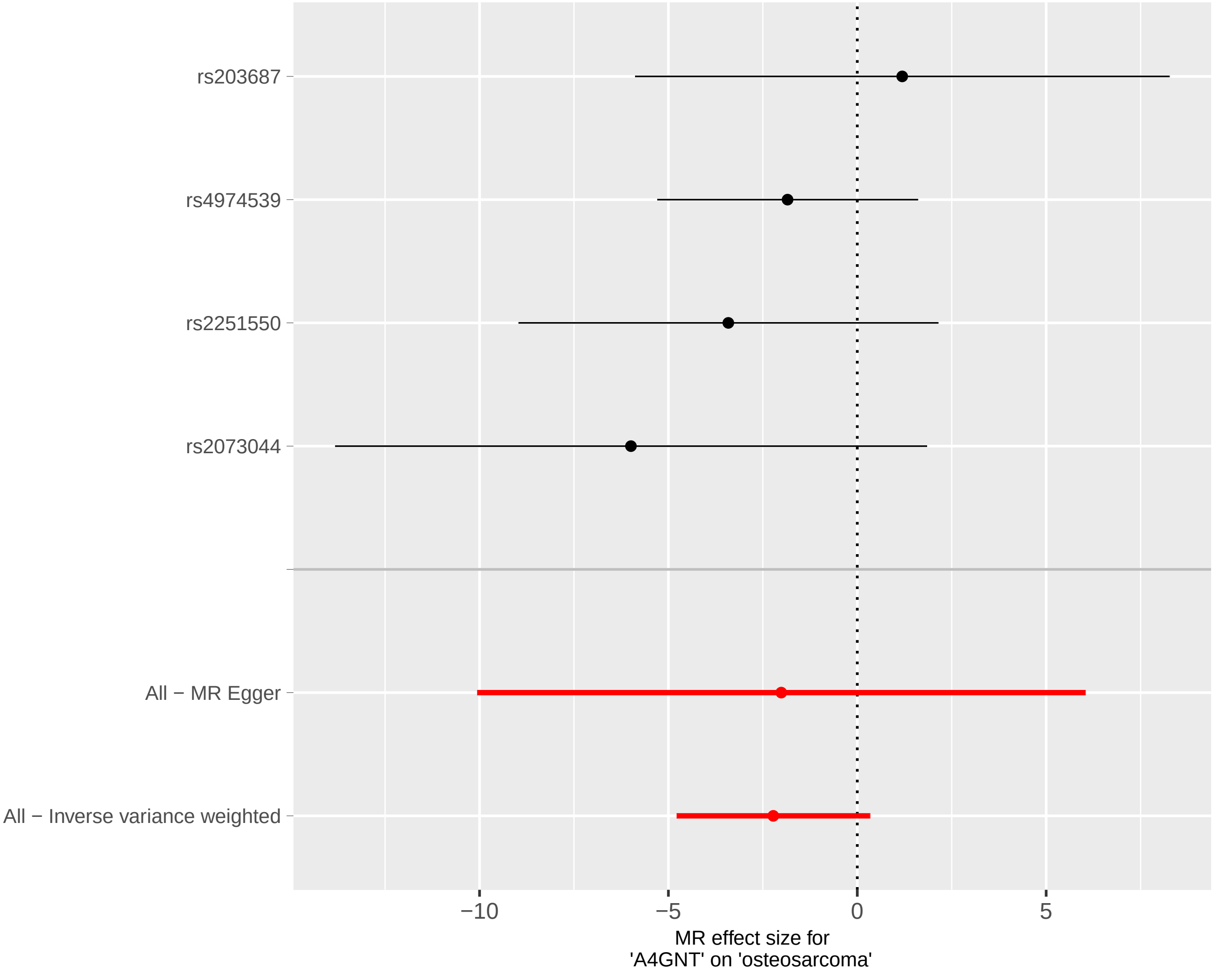

rs203687
rs4974539
rs2251550
rs2073044
All − MR Egger
All − Inverse variance weighted
−10
−5
0
5
MR effect size for
'A4GNT' on 'osteosarcoma'

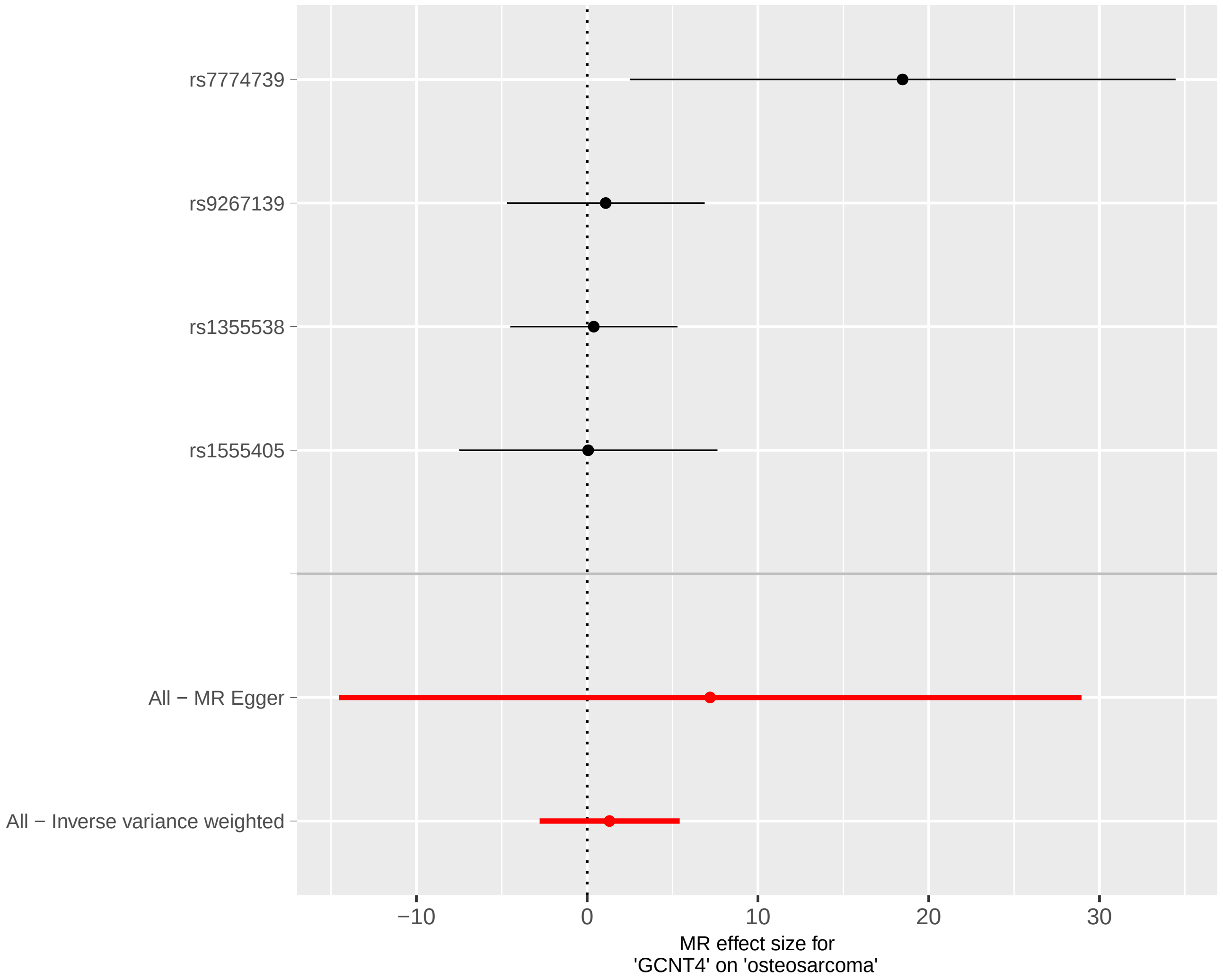
rs7774739
rs9267139
rs1355538
rs1555405
All − MR Egger
All − Inverse variance weighted
−10
0
10
20
30
MR effect size for
'GCNT4' on 'osteosarcoma'

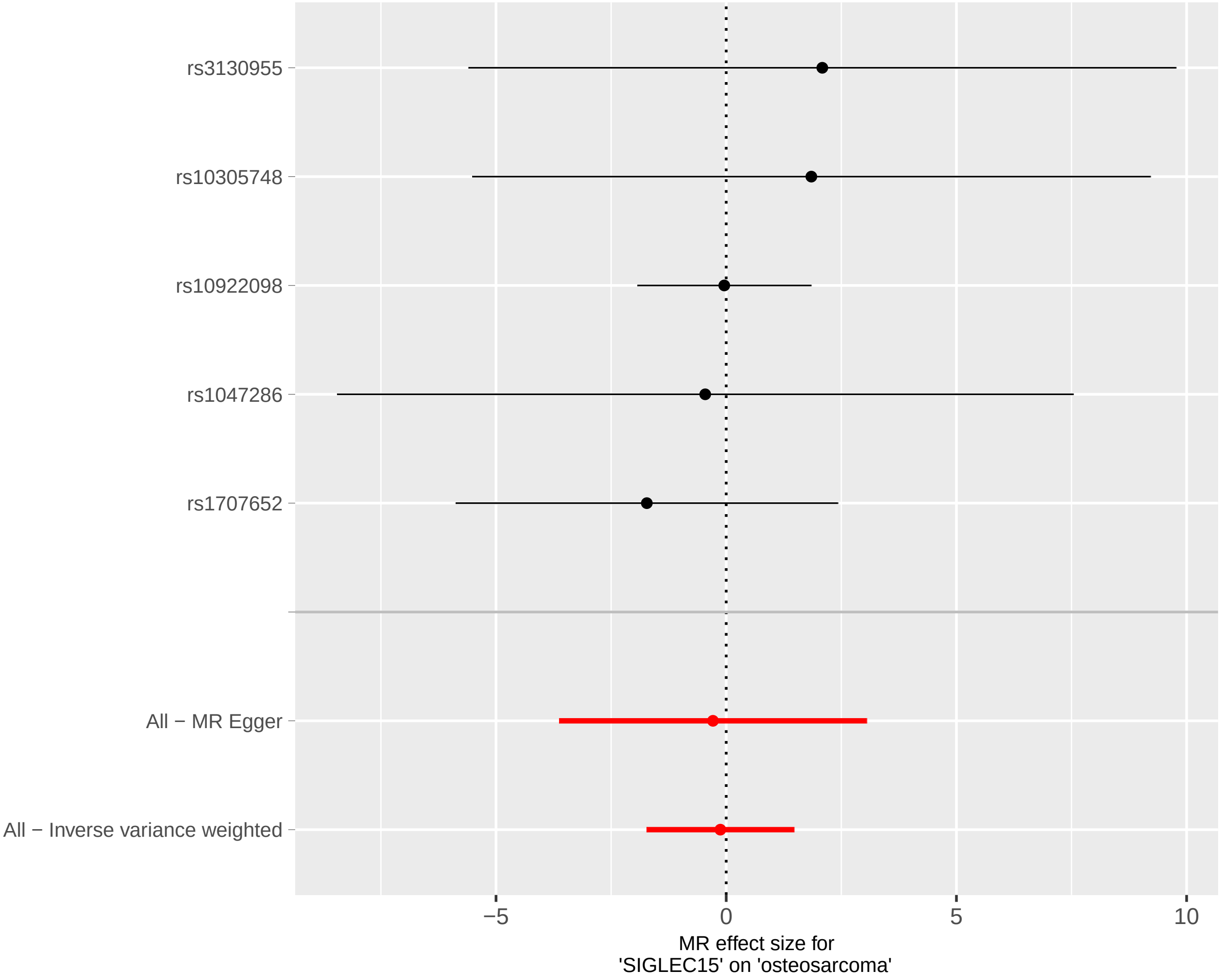
rs3130955
rs10305748
rs10922098
rs1047286
rs1707652
All − MR Egger
All − Inverse variance weighted
−5
0
5
10
MR effect size for
'SIGLEC15' on 'osteosarcoma'

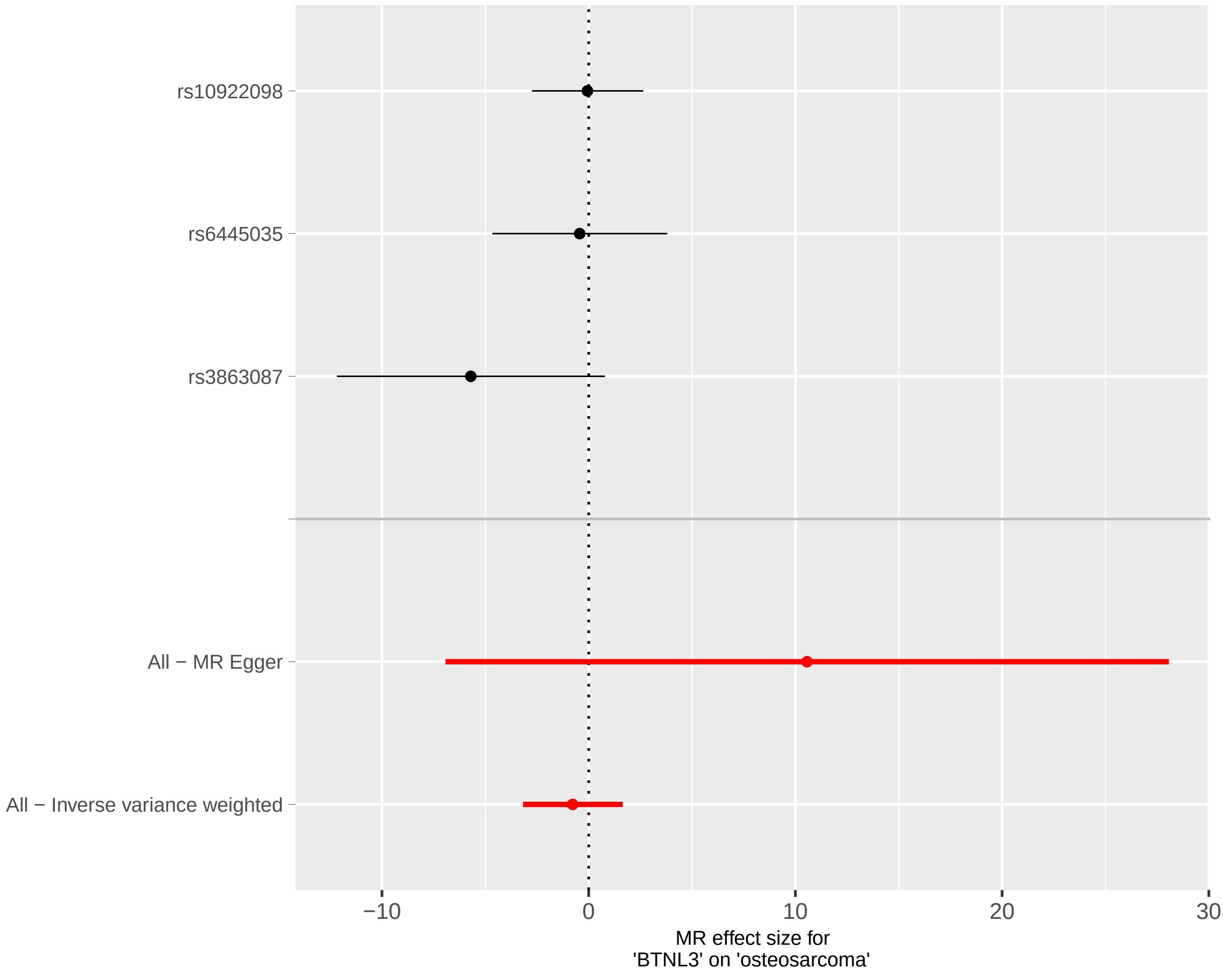

rs10922098
rs6445035
rs3863087
All − MR Egger
All − Inverse variance weighted
−10
0
10
20
30
MR effect size for
'BTNL3' on 'osteosarcoma'

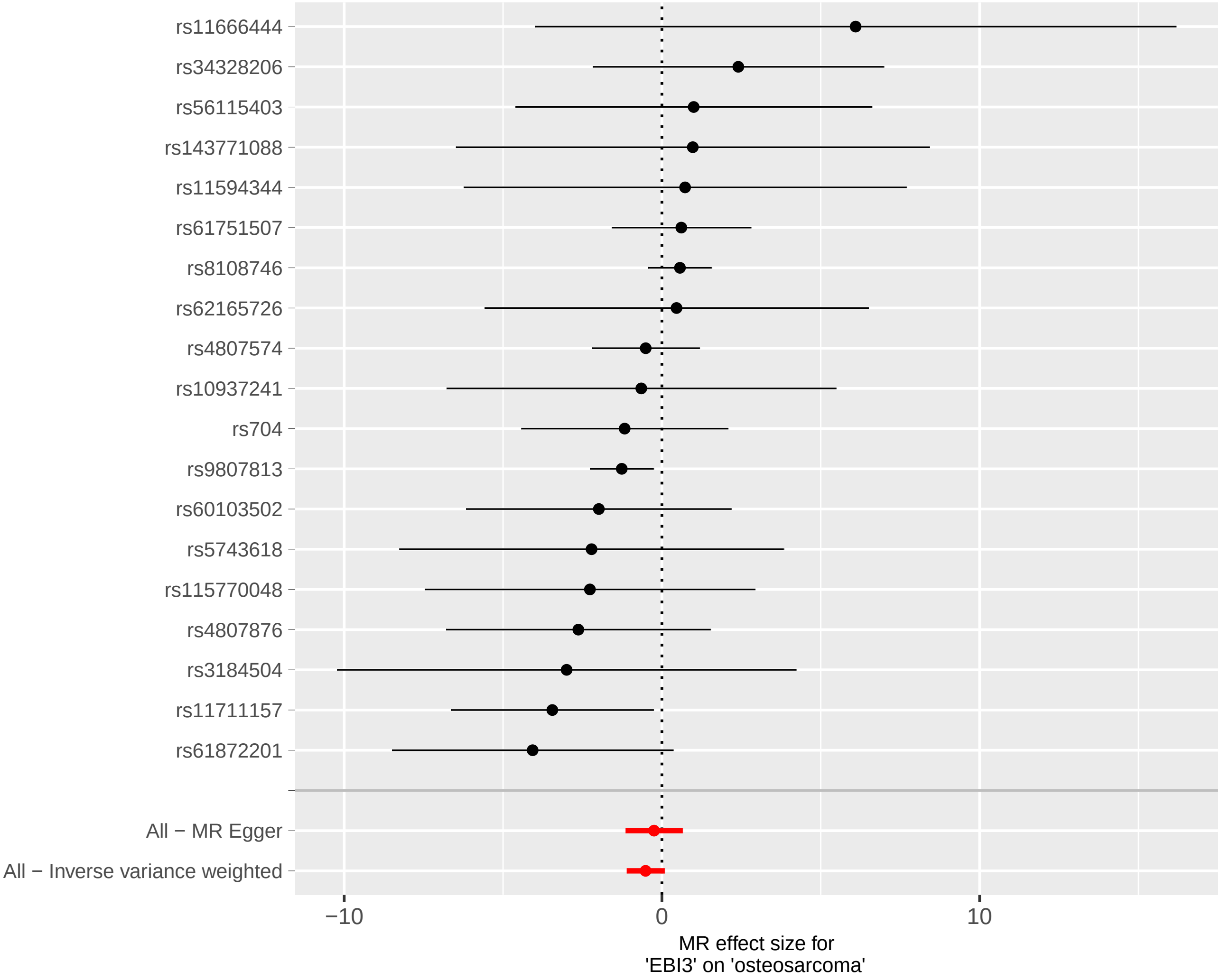

rs11666444
rs34328206
rs56115403
rs143771088
rs11594344
rs61751507
rs8108746
rs62165726
rs4807574
rs10937241
rs704
rs9807813
rs60103502
rs5743618
rs115770048
rs4807876
rs3184504
rs11711157
rs61872201
All − MR Egger
All − Inverse variance weighted
−10
0
10
MR effect size for
'EBI3' on 'osteosarcoma'

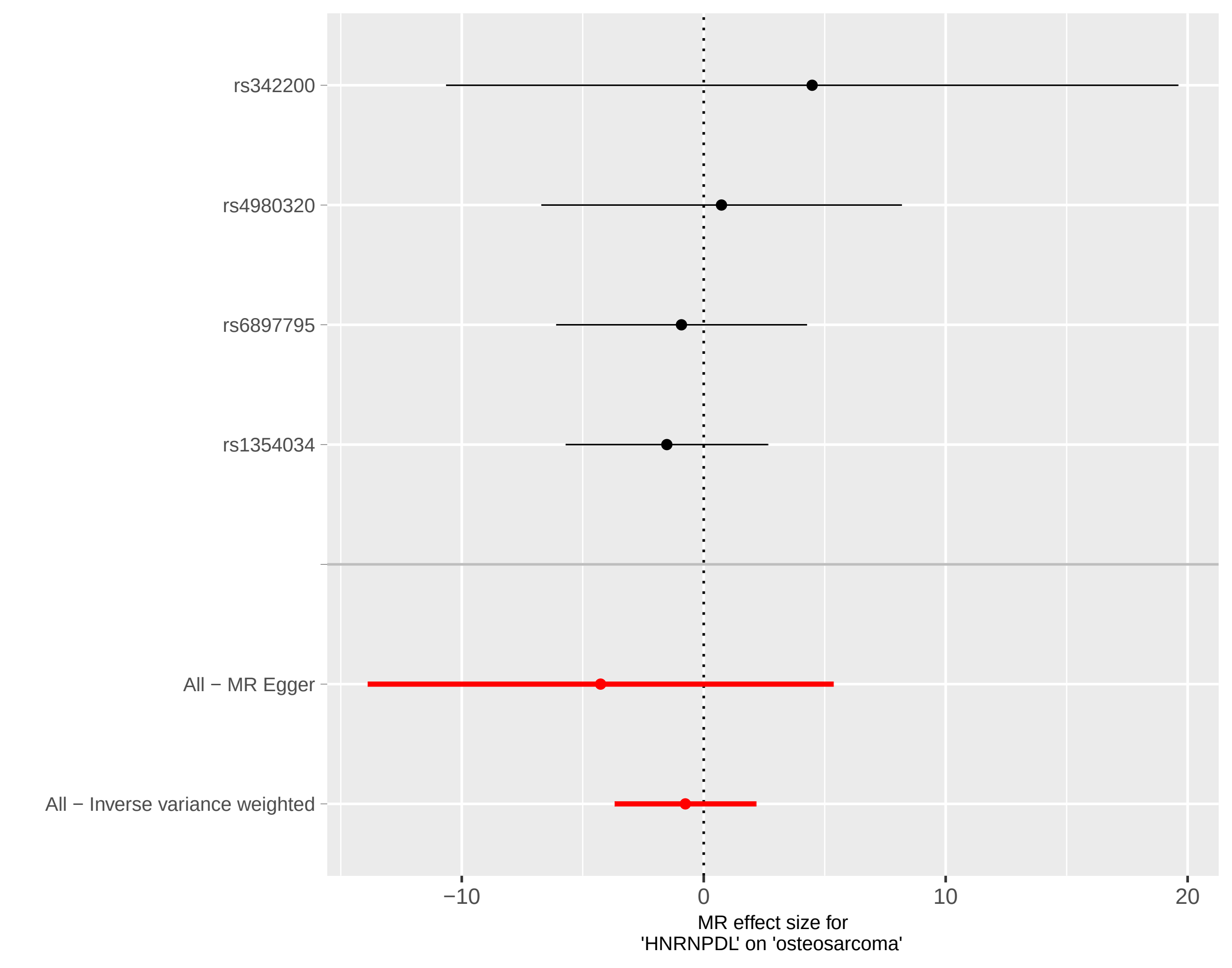
rs342200
rs4980320
rs6897795
rs1354034
All − MR Egger
All − Inverse variance weighted
−10
0
10
20
MR effect size for
'HNRNPDL' on 'osteosarcoma'

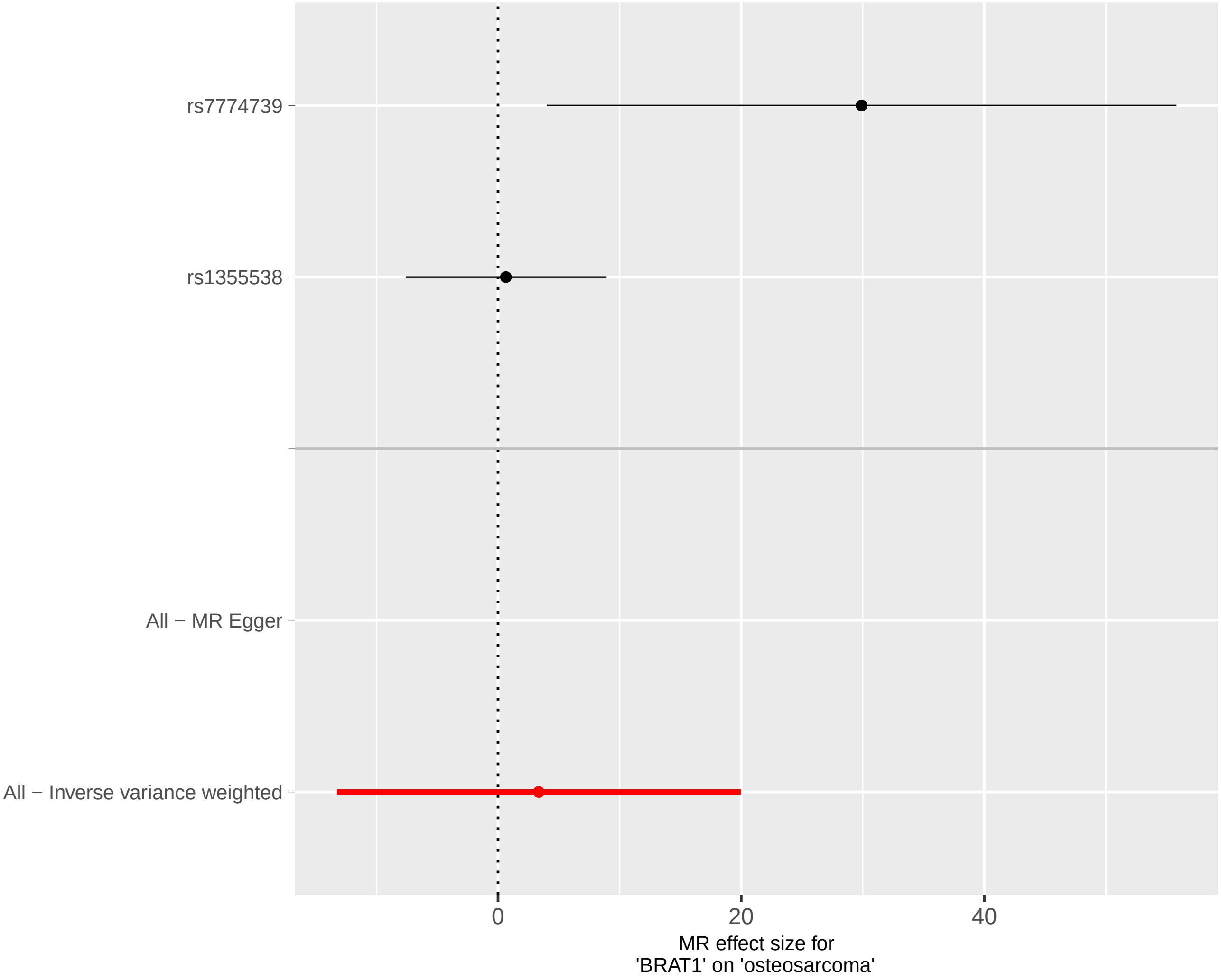

rs7774739
rs1355538
All − MR Egger
All − Inverse variance weighted
0
20
40
MR effect size for
'BRAT1' on 'osteosarcoma'

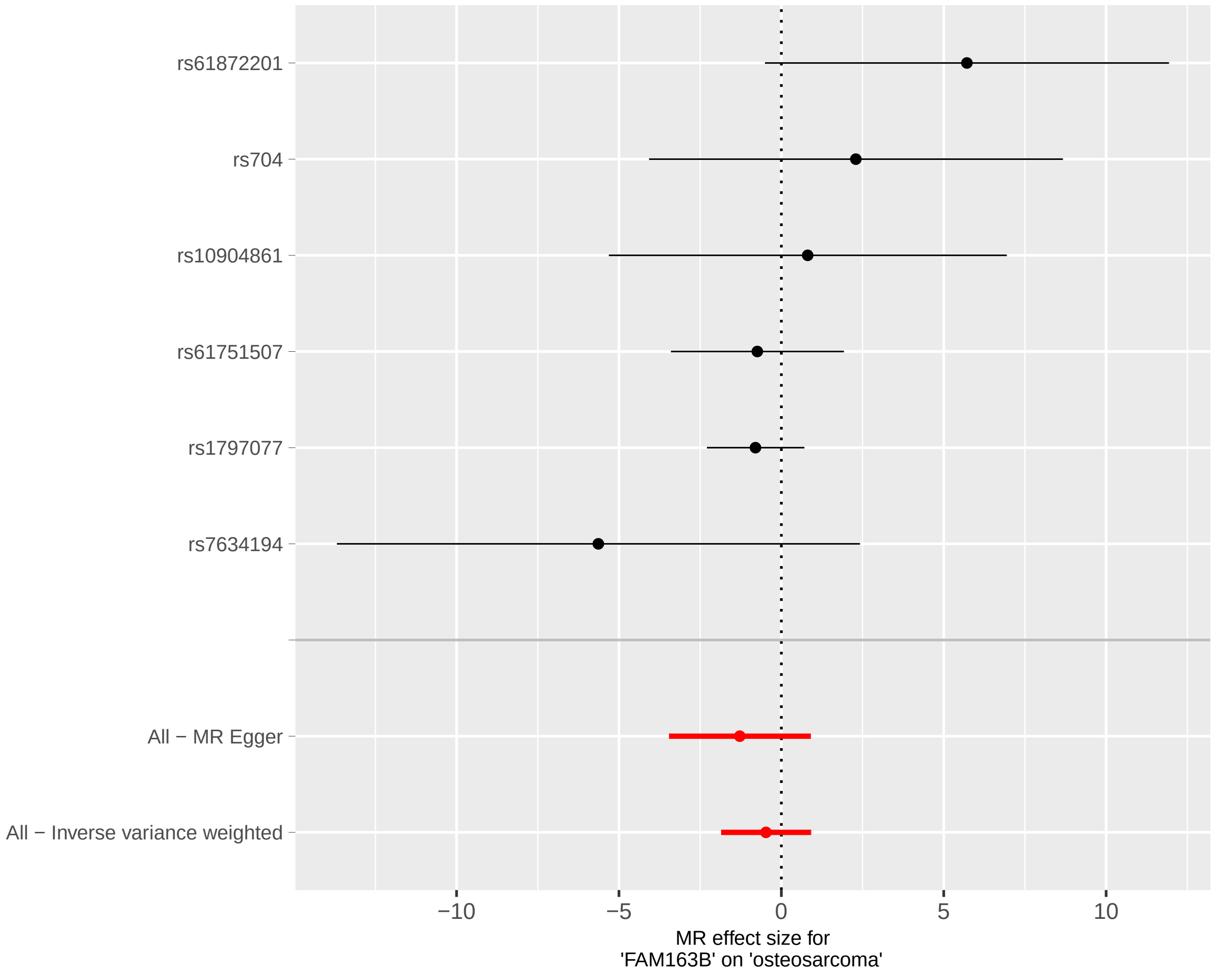

rs61872201
rs704
rs10904861
rs61751507
rs1797077
rs7634194
All − MR Egger
All − Inverse variance weighted
−10
−5
0
5
10
MR effect size for
'FAM163B' on 'osteosarcoma'

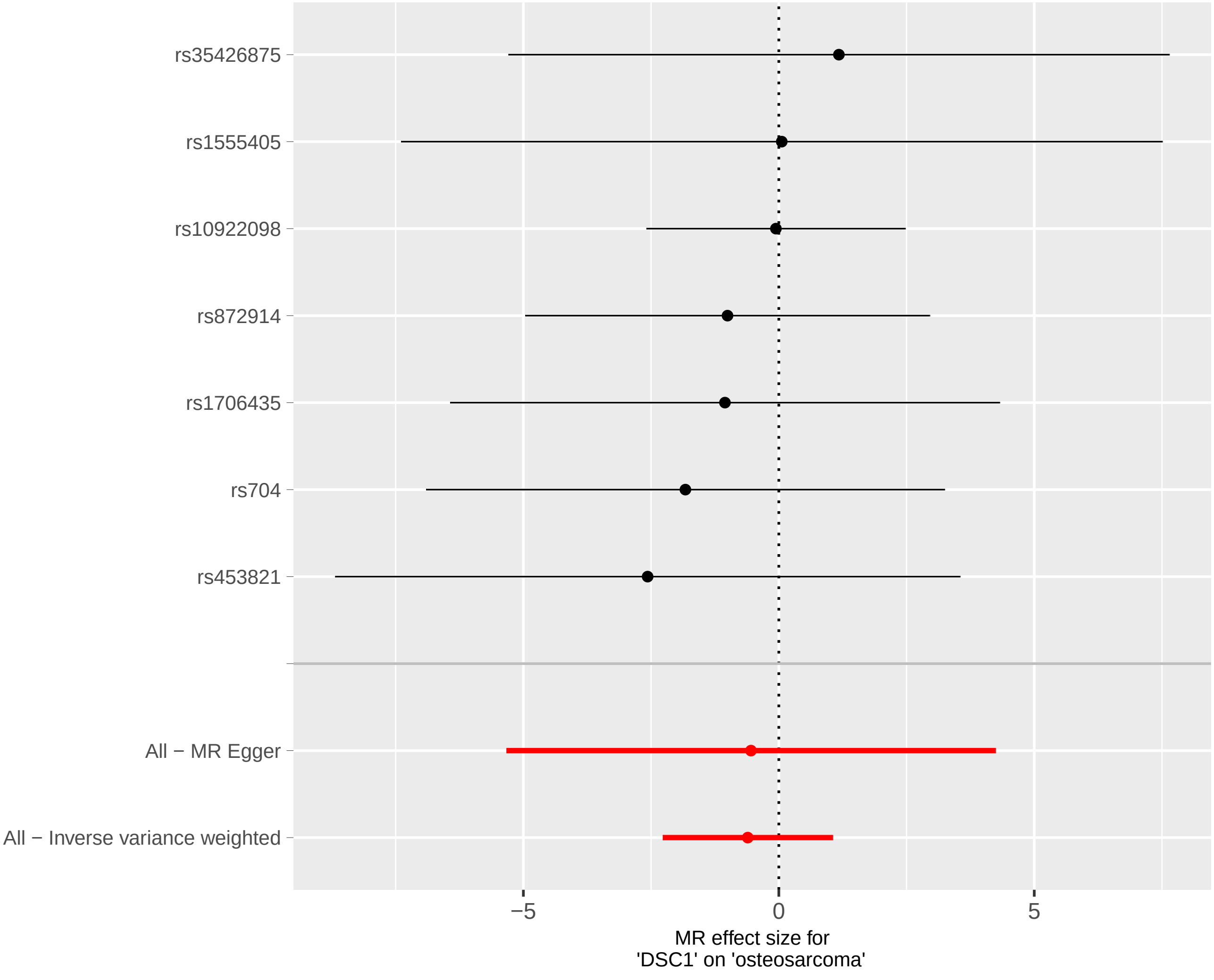

rs35426875
rs1555405
rs10922098
rs872914
rs1706435
rs704
rs453821
All − MR Egger
All − Inverse variance weighted
−5
0
5
MR effect size for
'DSC1' on 'osteosarcoma'

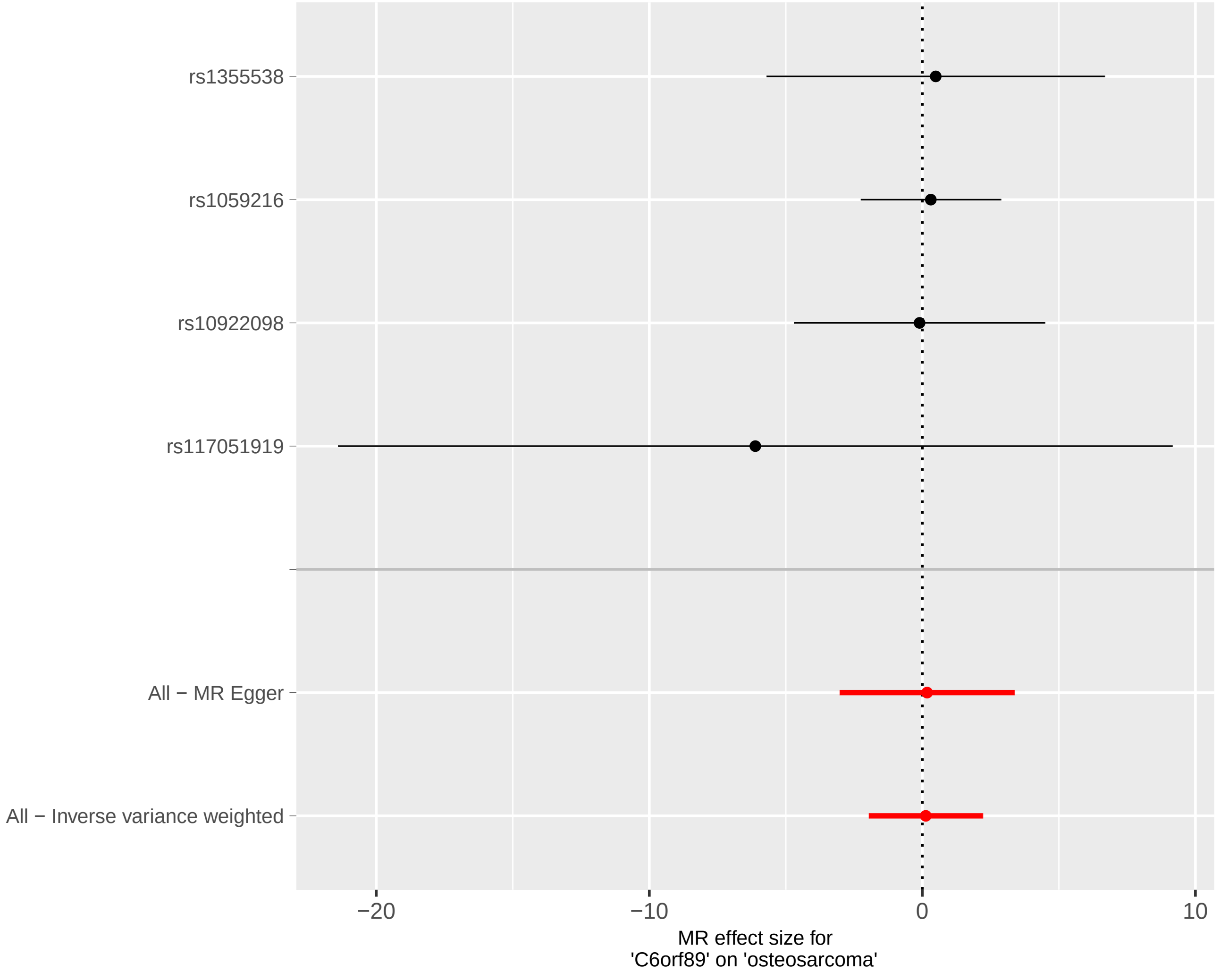

rs1355538
rs1059216
rs10922098
rs117051919
All − MR Egger
All − Inverse variance weighted
−20
−10
0
10
MR effect size for
'C6orf89' on 'osteosarcoma'

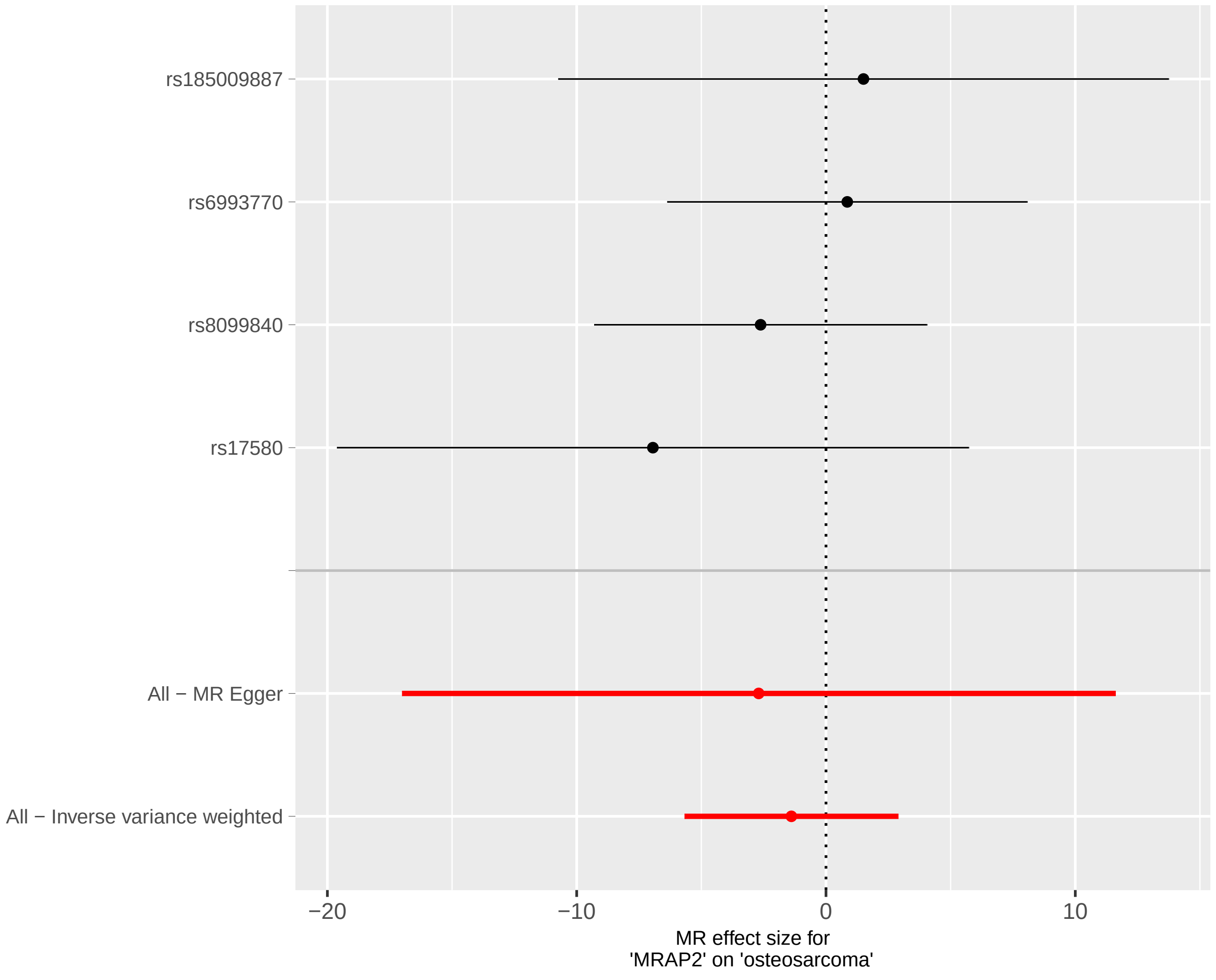

rs185009887
rs6993770
rs8099840
rs17580
All − MR Egger
All − Inverse variance weighted
−20
−10
0
10
MR effect size for
'MRAP2' on 'osteosarcoma'

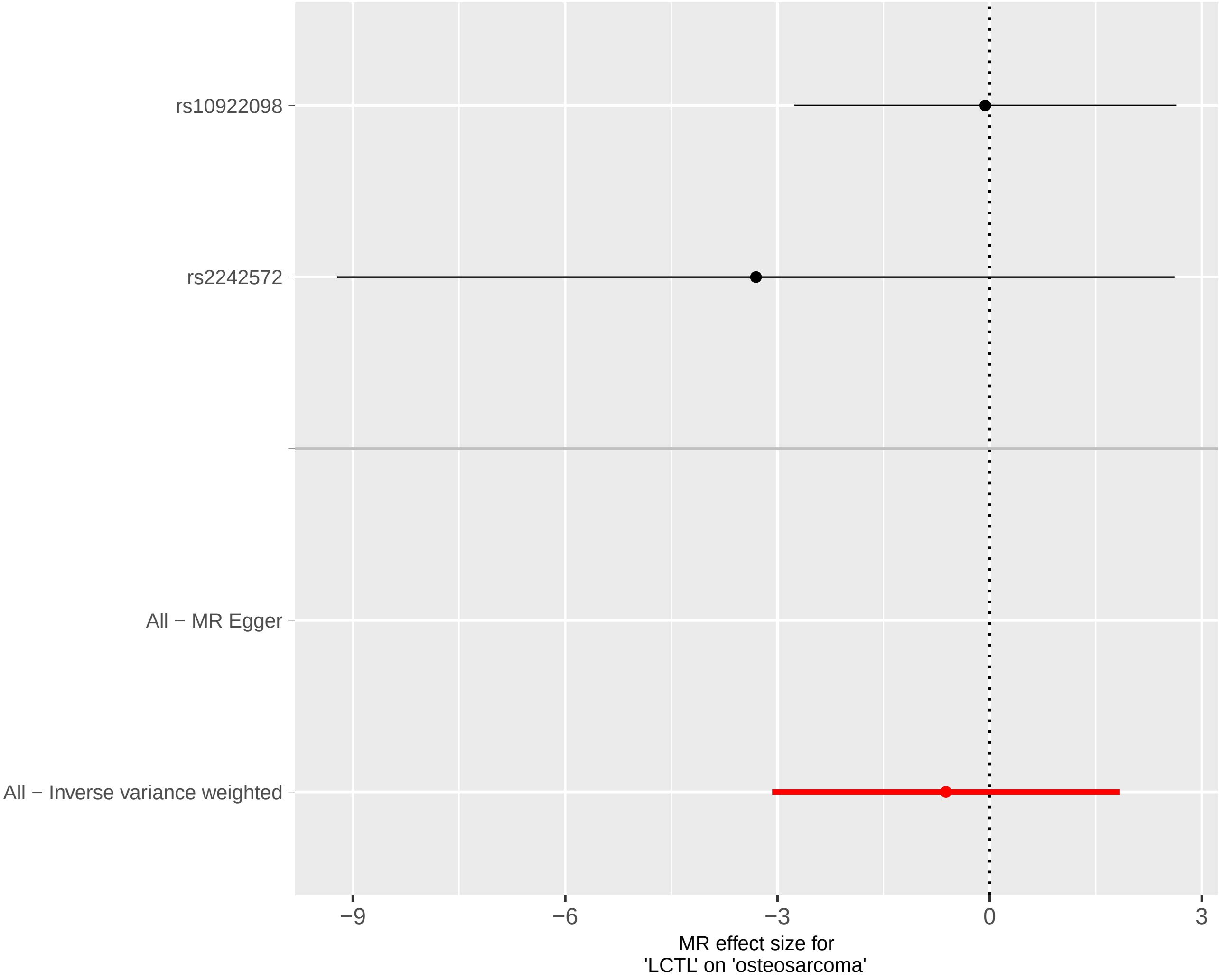
rs10922098
rs2242572
All − MR Egger
All − Inverse variance weighted
−9
−6
−3
0
3
MR effect size for
'LCTL' on 'osteosarcoma'

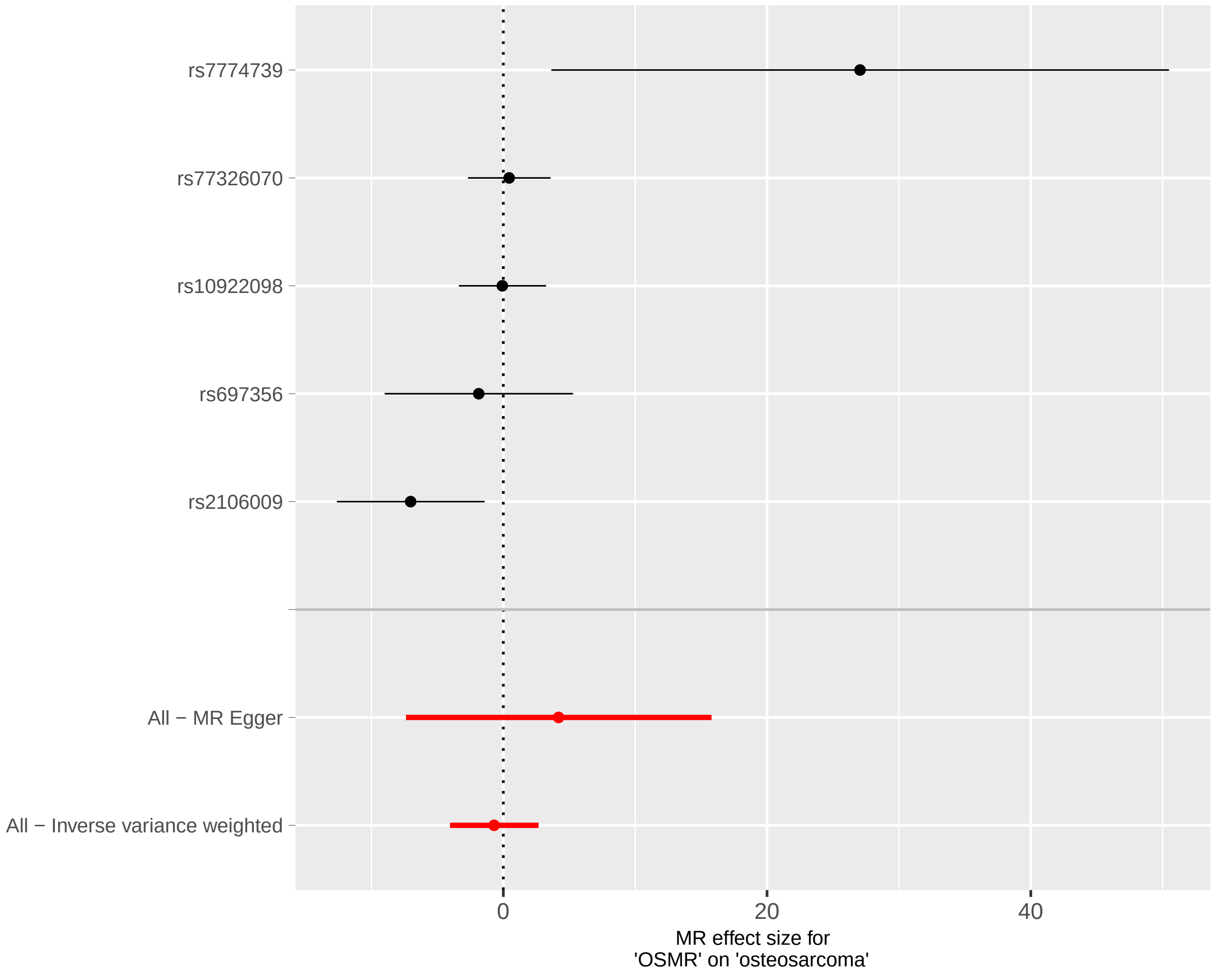

rs7774739
rs77326070
rs10922098
rs697356
rs2106009
All − MR Egger
All − Inverse variance weighted
0
20
40
MR effect size for
'OSMR' on 'osteosarcoma'

Insufficient number of SNPs

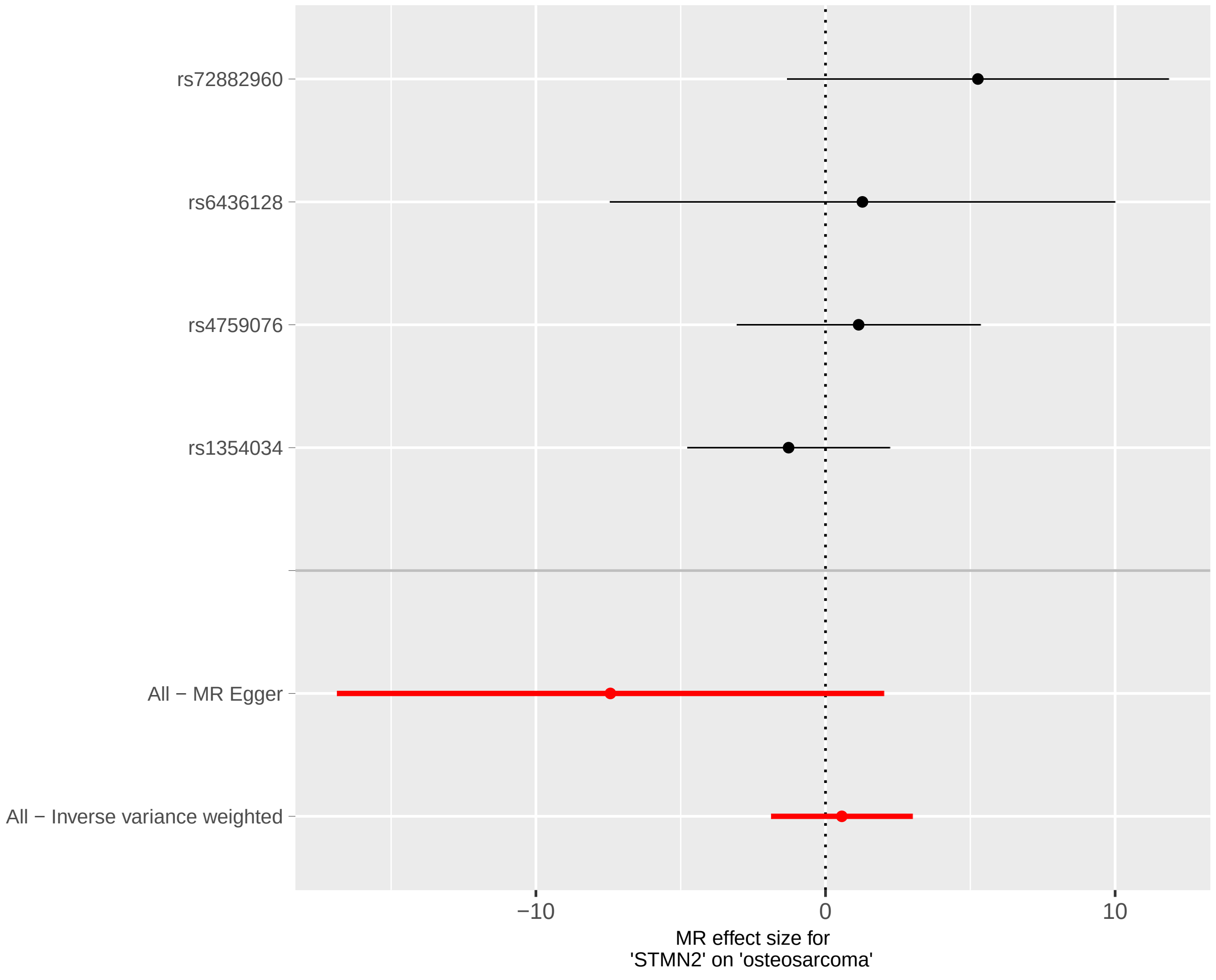
rs72882960
rs6436128
rs4759076
rs1354034
All − MR Egger
All − Inverse variance weighted
−10
0
10
MR effect size for
'STMN2' on 'osteosarcoma'

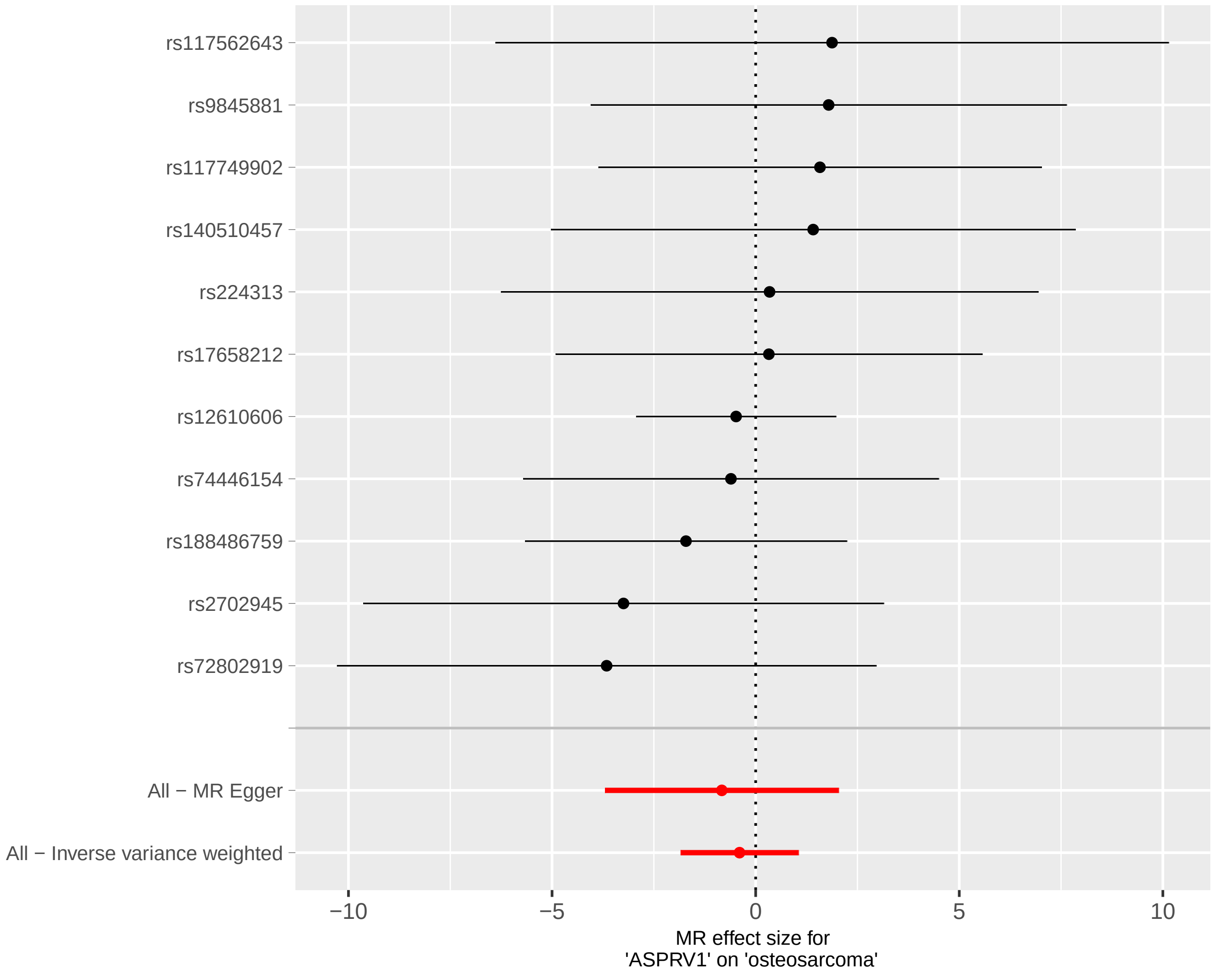
rs117562643
rs9845881
rs117749902
rs140510457
rs224313
rs17658212
rs12610606
rs74446154
rs188486759
rs2702945
rs72802919
All − MR Egger
All − Inverse variance weighted
−10
−5
0
5
10
MR effect size for
'ASPRV1' on 'osteosarcoma'

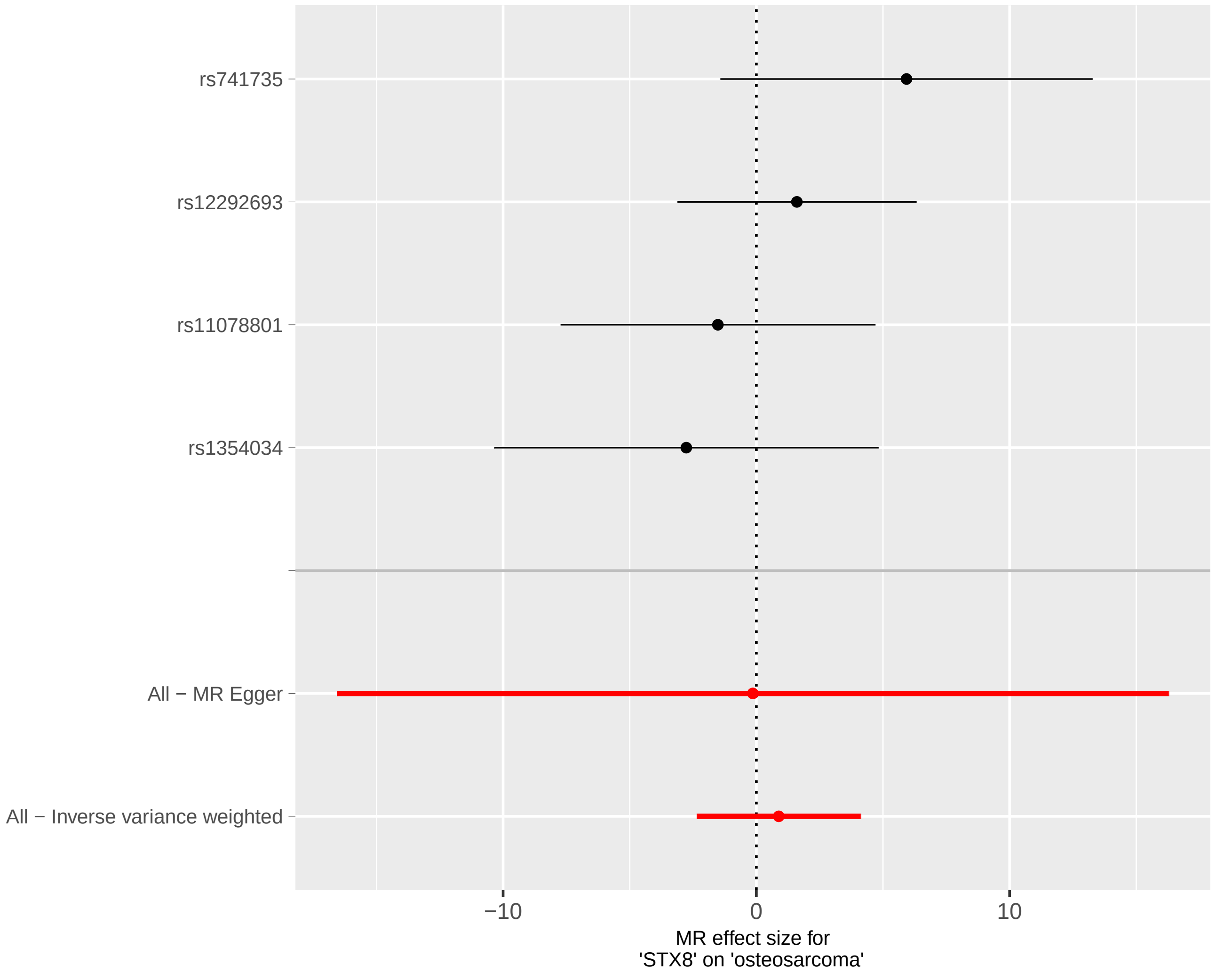
rs741735
rs12292693
rs11078801
rs1354034
All − MR Egger
All − Inverse variance weighted
−10
0
10
MR effect size for
'STX8' on 'osteosarcoma'

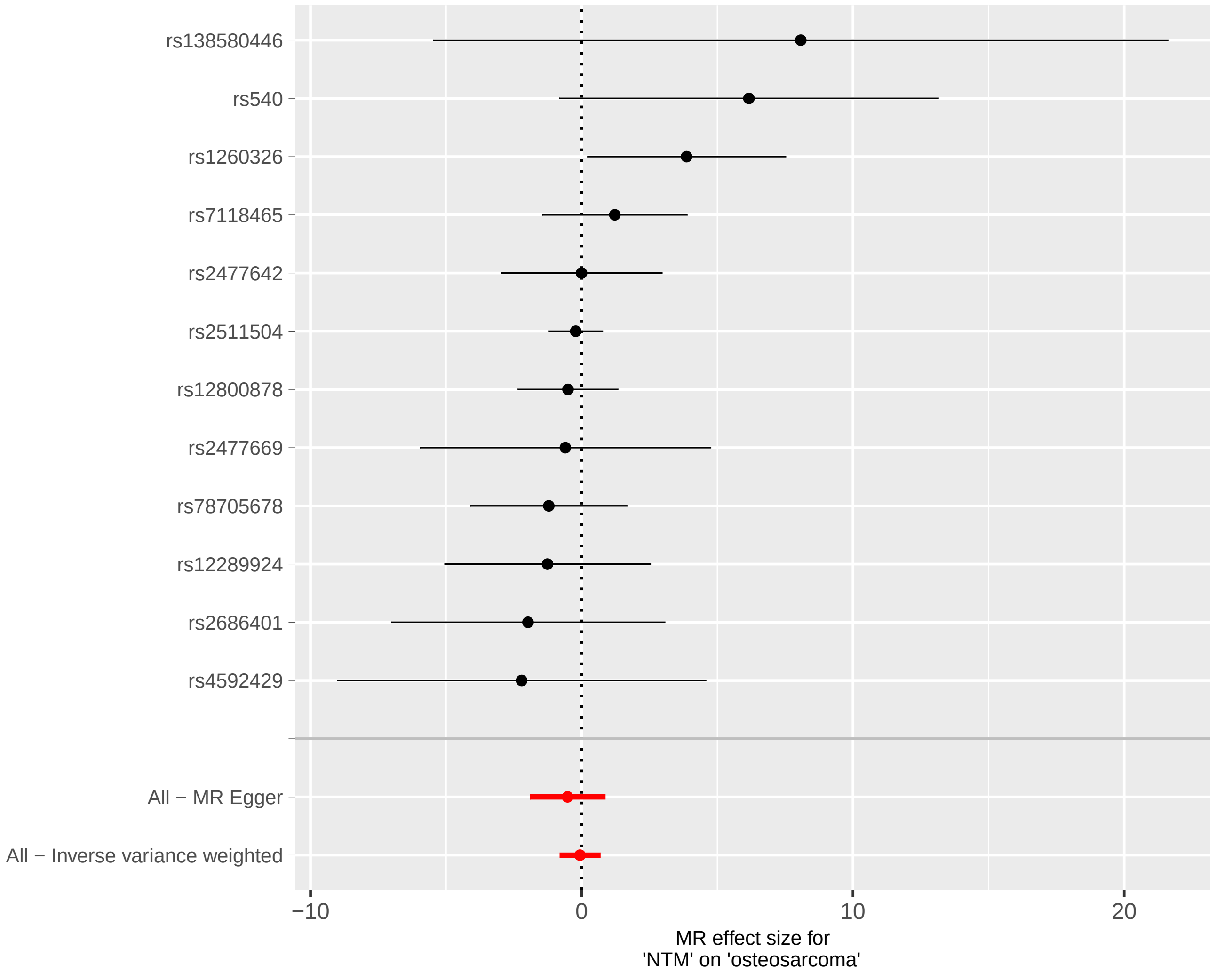
rs138580446
rs540
rs1260326
rs7118465
rs2477642
rs2511504
rs12800878
rs2477669
rs78705678
rs12289924
rs2686401
rs4592429
All − MR Egger
All − Inverse variance weighted
−10
0
10
20
MR effect size for
'NTM' on 'osteosarcoma'

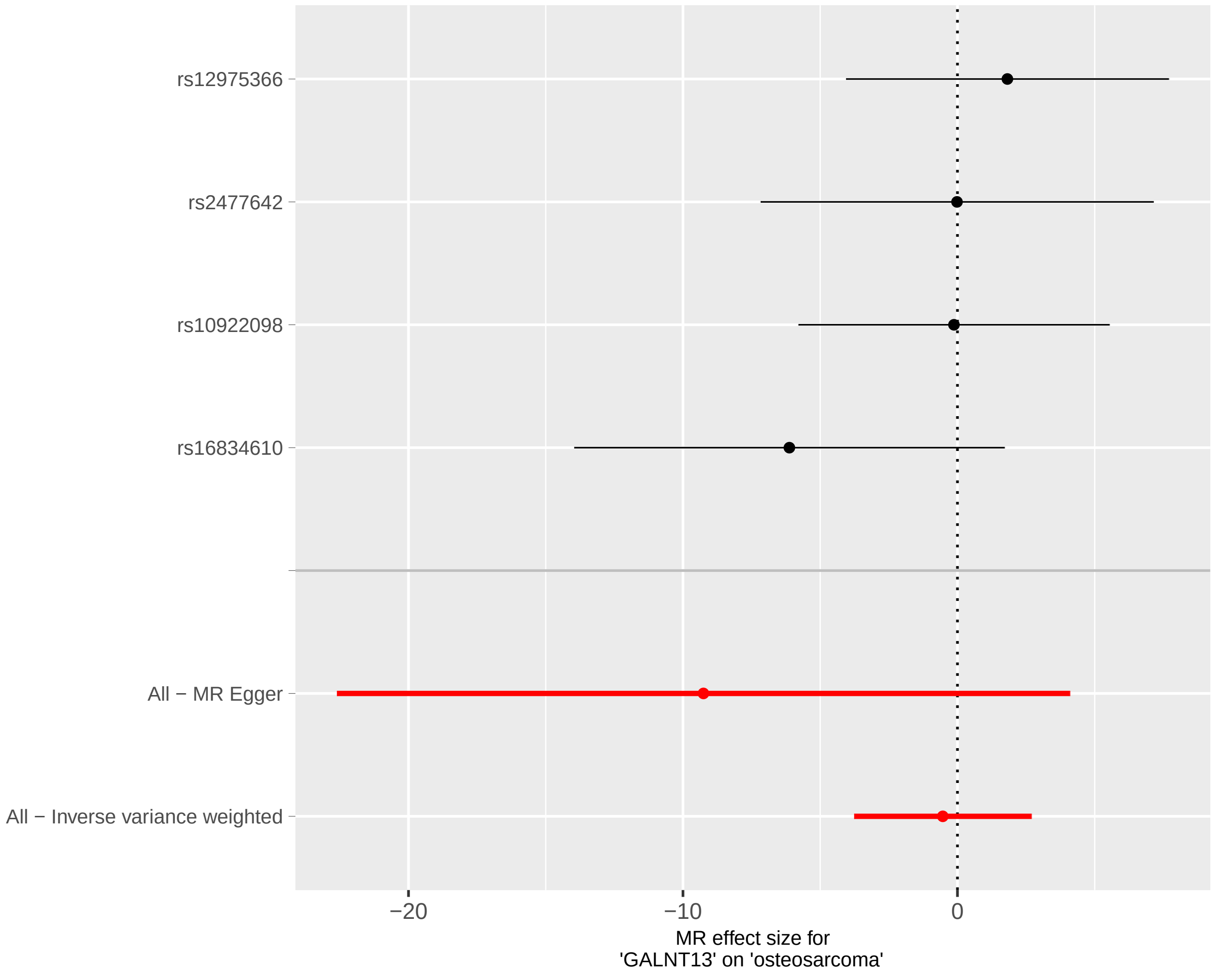

rs12975366
rs2477642
rs10922098
rs16834610
All − MR Egger
All − Inverse variance weighted
−20
−10
0
MR effect size for
'GALNT13' on 'osteosarcoma'

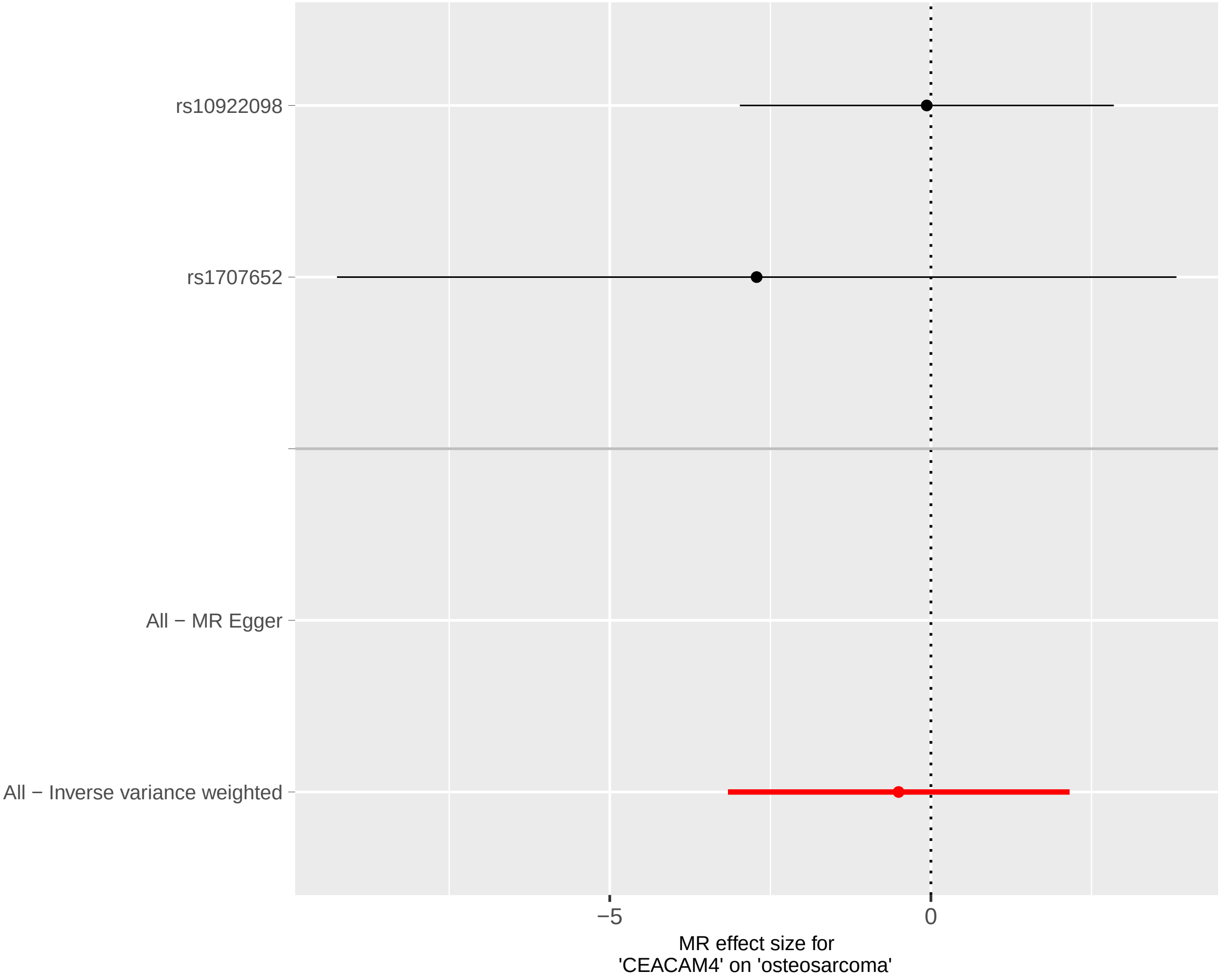
rs10922098
rs1707652
All − MR Egger
All − Inverse variance weighted
−5
0
MR effect size for
'CEACAM4' on 'osteosarcoma'

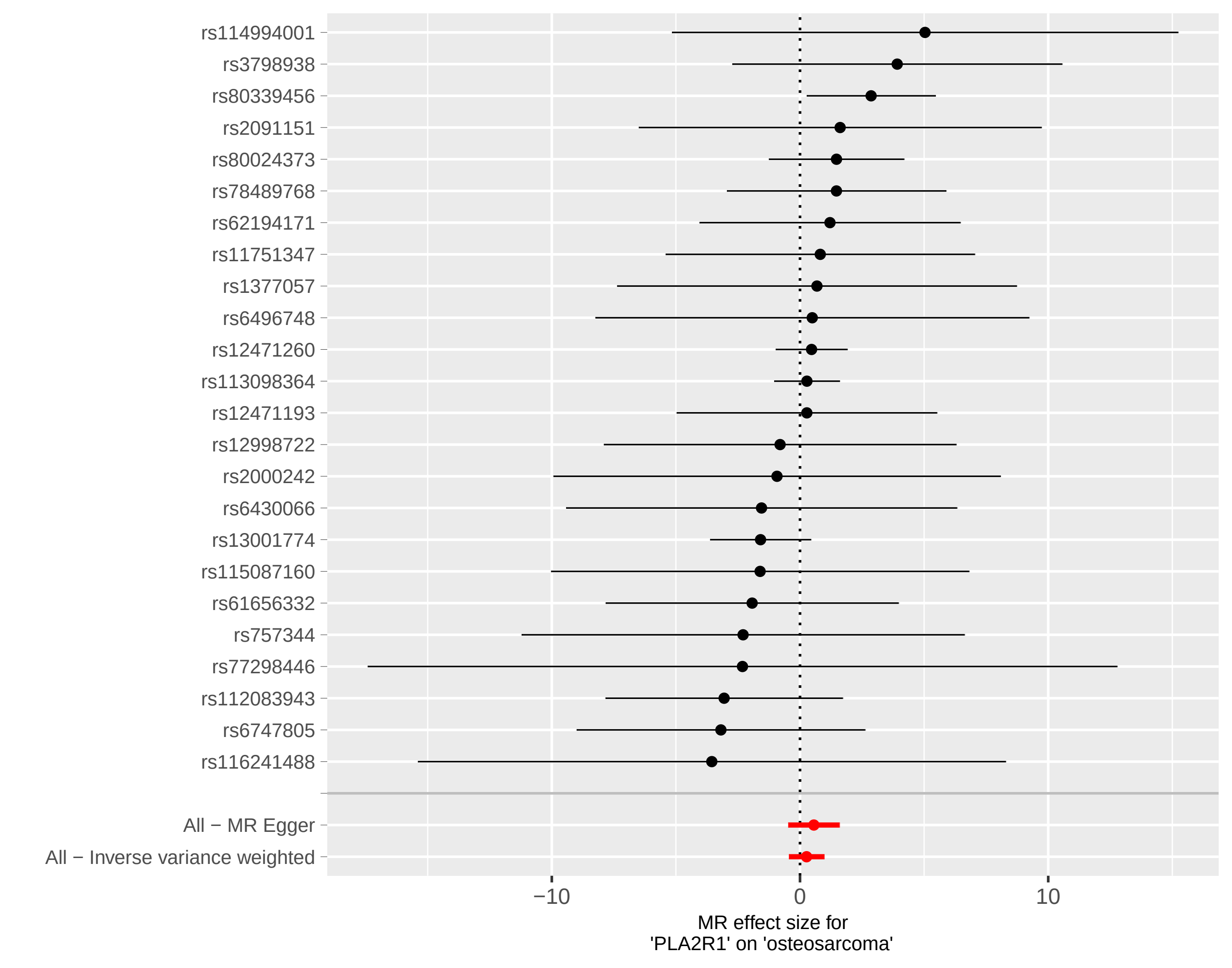
rs114994001
rs3798938
rs80339456
rs2091151
rs80024373
rs78489768
rs62194171
rs11751347
rs1377057
rs6496748
rs12471260
rs113098364
rs12471193
rs12998722
rs2000242
rs6430066
rs13001774
rs115087160
rs61656332
rs757344
rs77298446
rs112083943
rs6747805
rs116241488
All − MR Egger
All − Inverse variance weighted
−10
0
10
MR effect size for
'PLA2R1' on 'osteosarcoma'

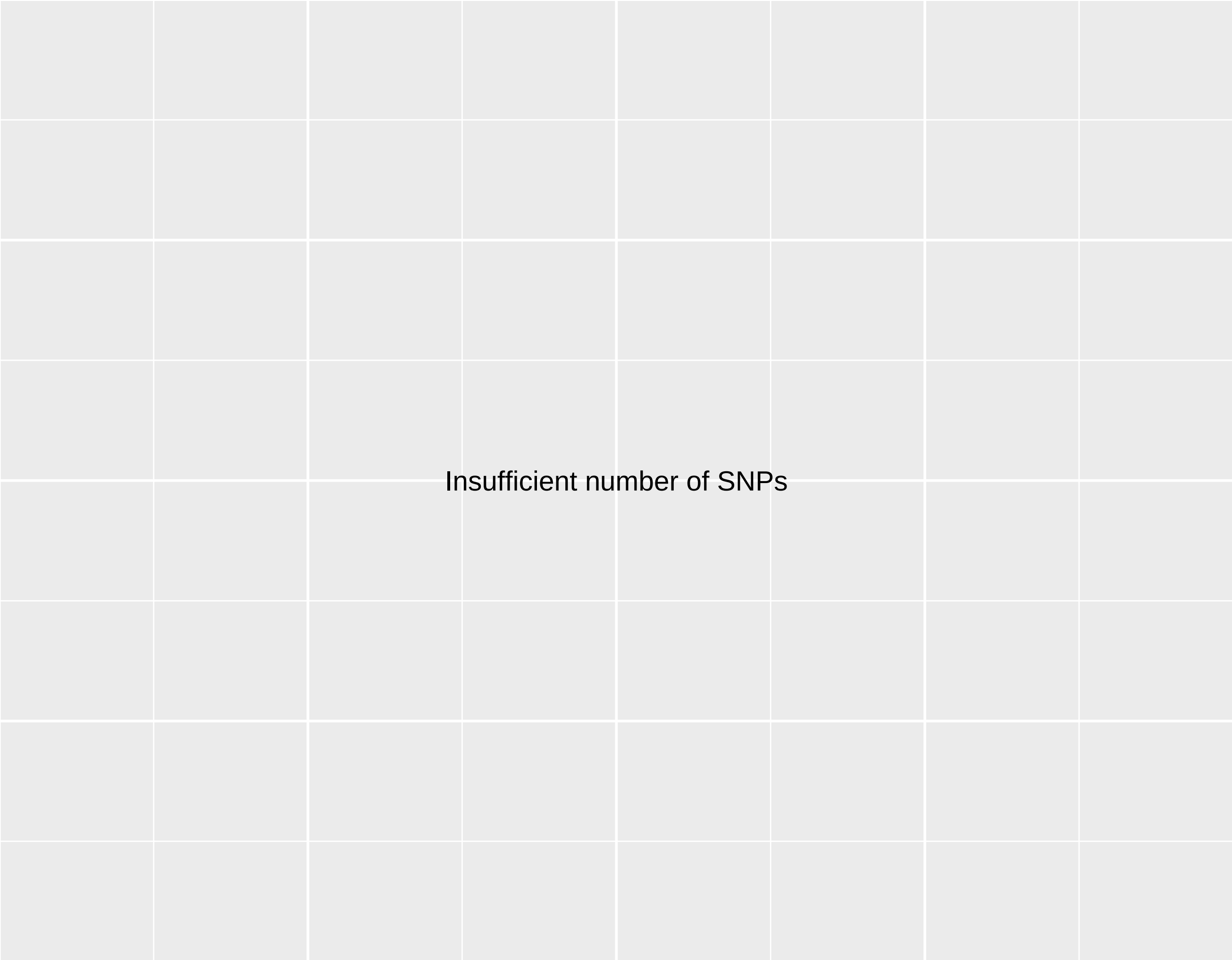
Insufficient number of SNPs

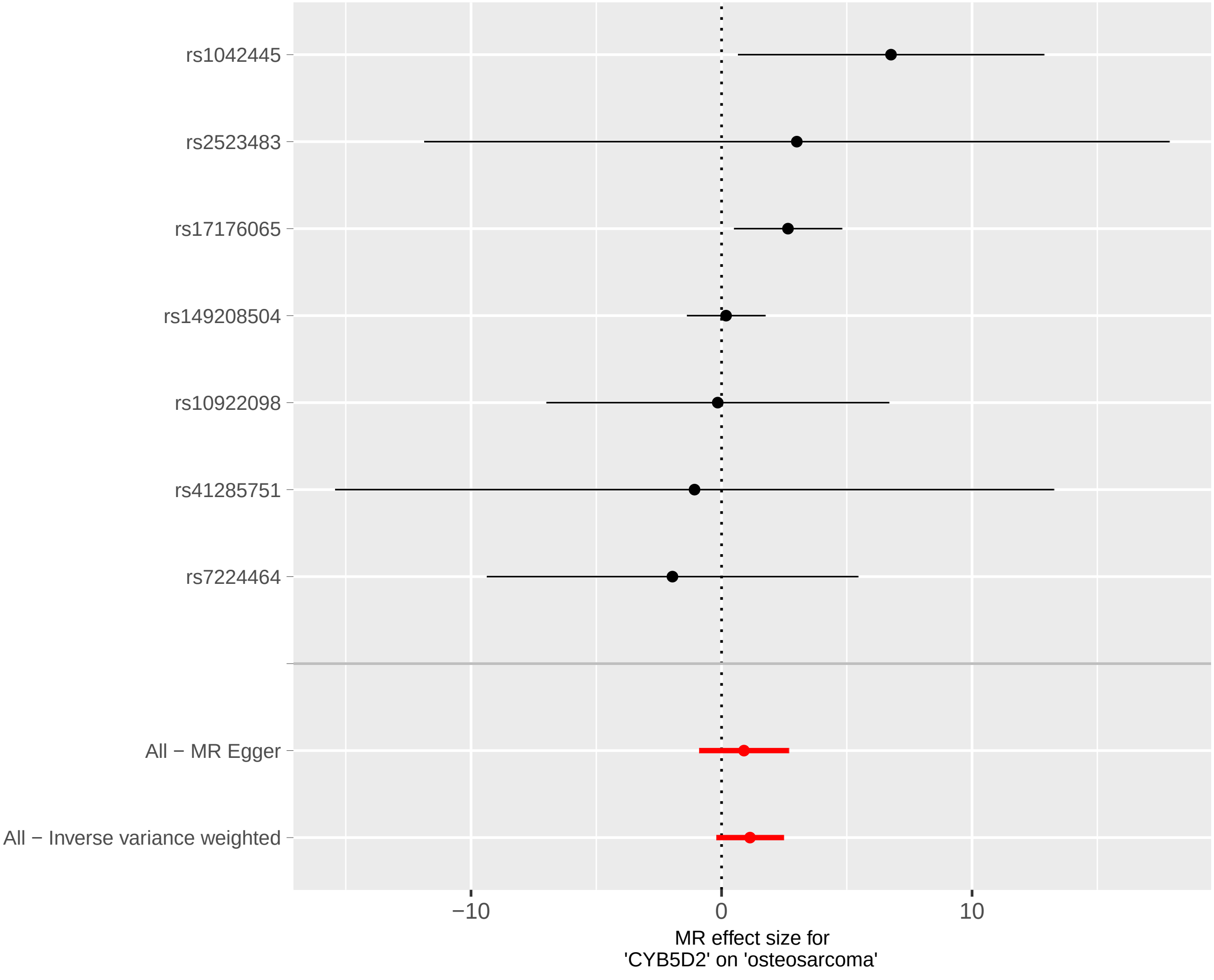
rs1042445
rs2523483
rs17176065
rs149208504
rs10922098
rs41285751
rs7224464
All − MR Egger
All − Inverse variance weighted
−10
0
10
MR effect size for
'CYB5D2' on 'osteosarcoma'

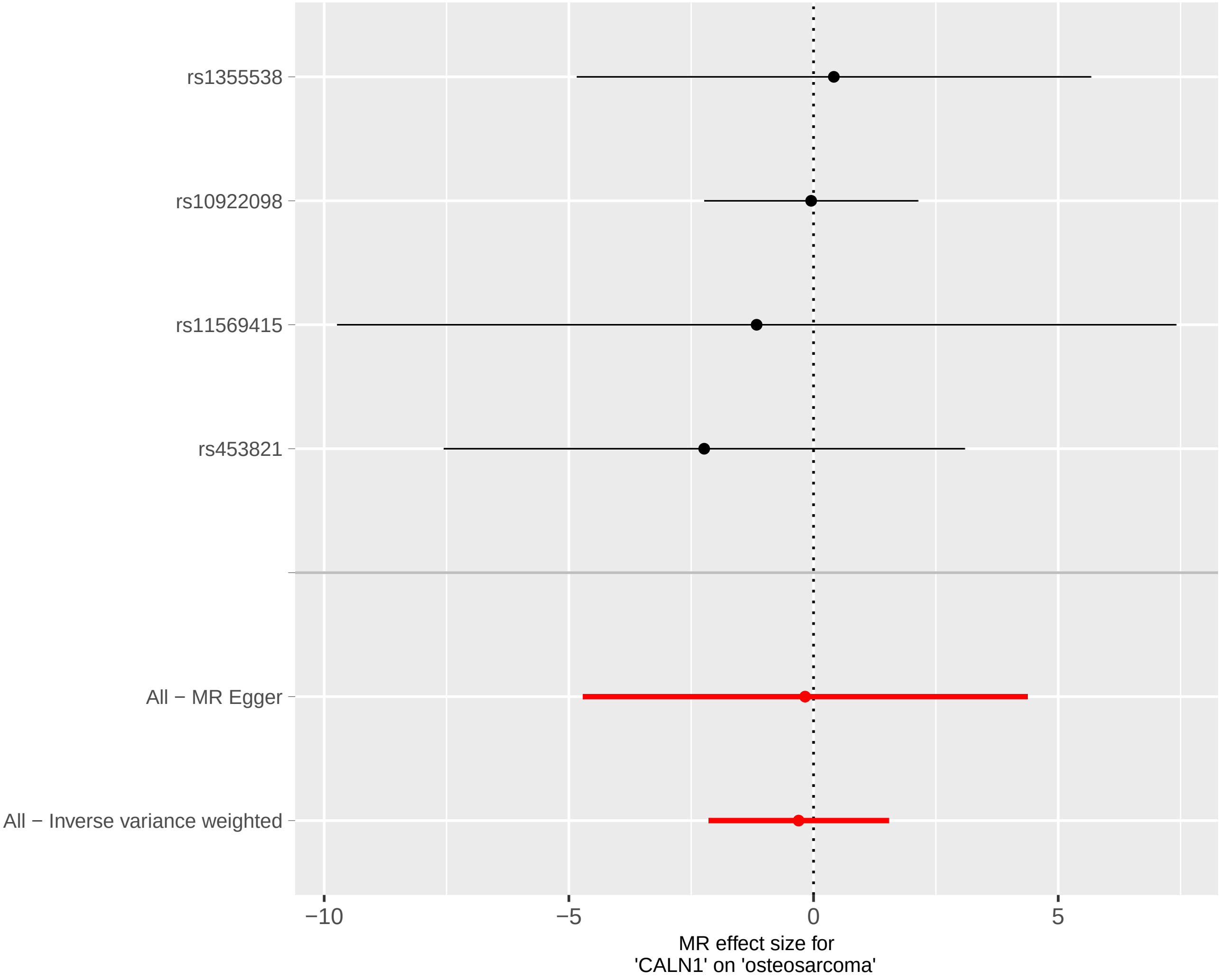

rs1355538
rs10922098
rs11569415
rs453821
All − MR Egger
All − Inverse variance weighted
−10
−5
0
5
MR effect size for
'CALN1' on 'osteosarcoma'

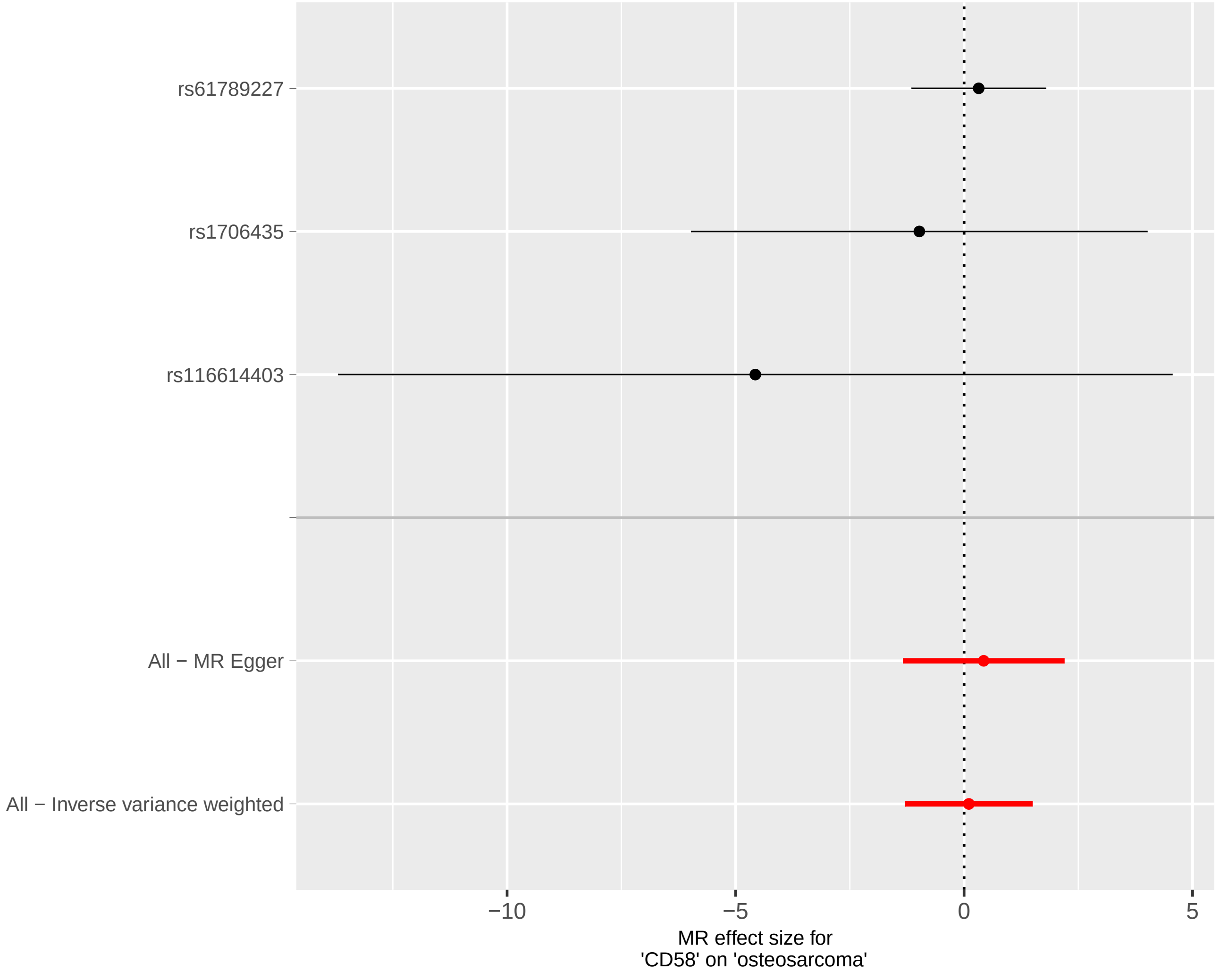

rs61789227
rs1706435
rs116614403
All − MR Egger
All − Inverse variance weighted
−10
−5
0
5
MR effect size for
'CD58' on 'osteosarcoma'

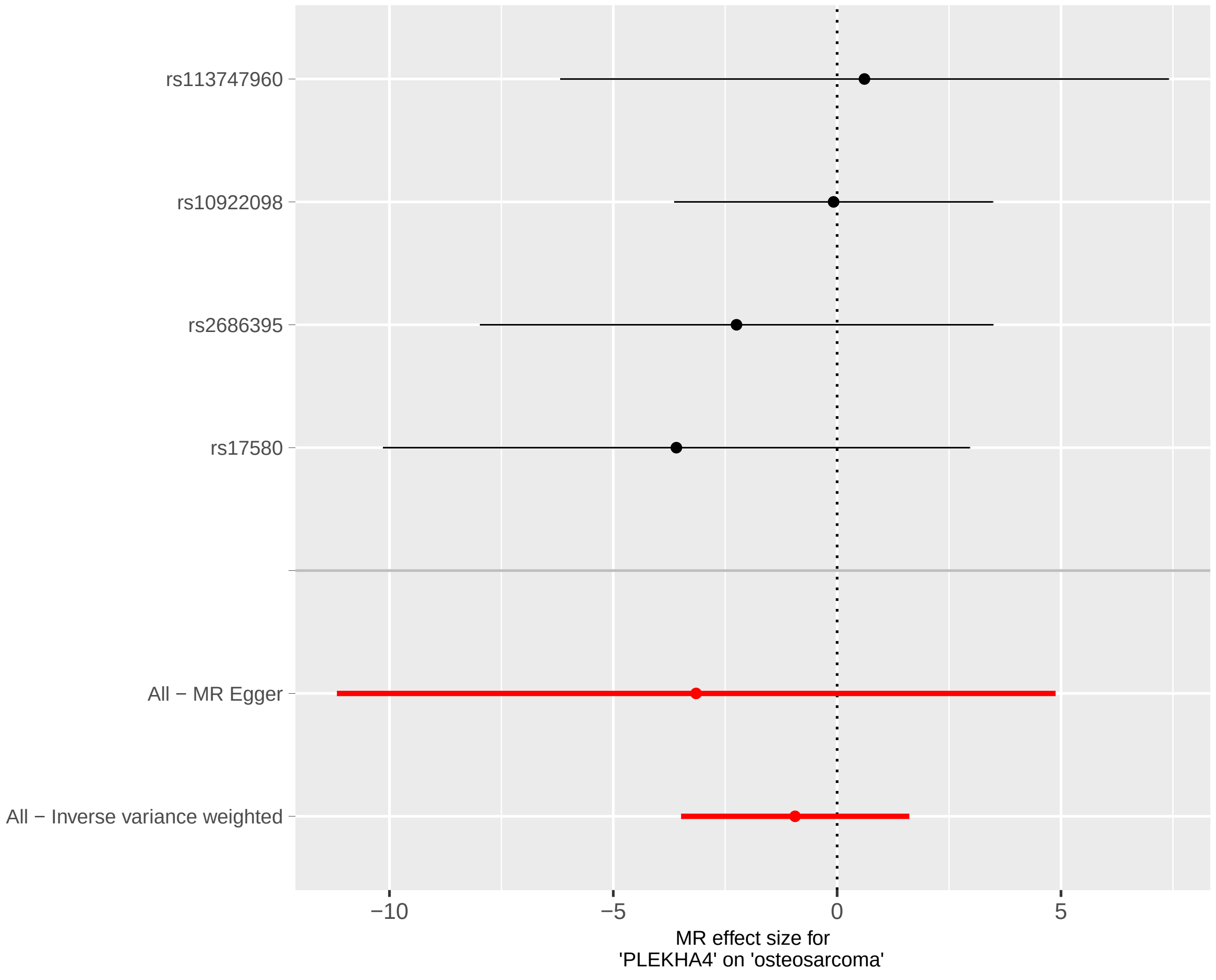

rs113747960
rs10922098
rs2686395
rs17580
All − MR Egger
All − Inverse variance weighted
−10
−5
0
5
MR effect size for
'PLEKHA4' on 'osteosarcoma'

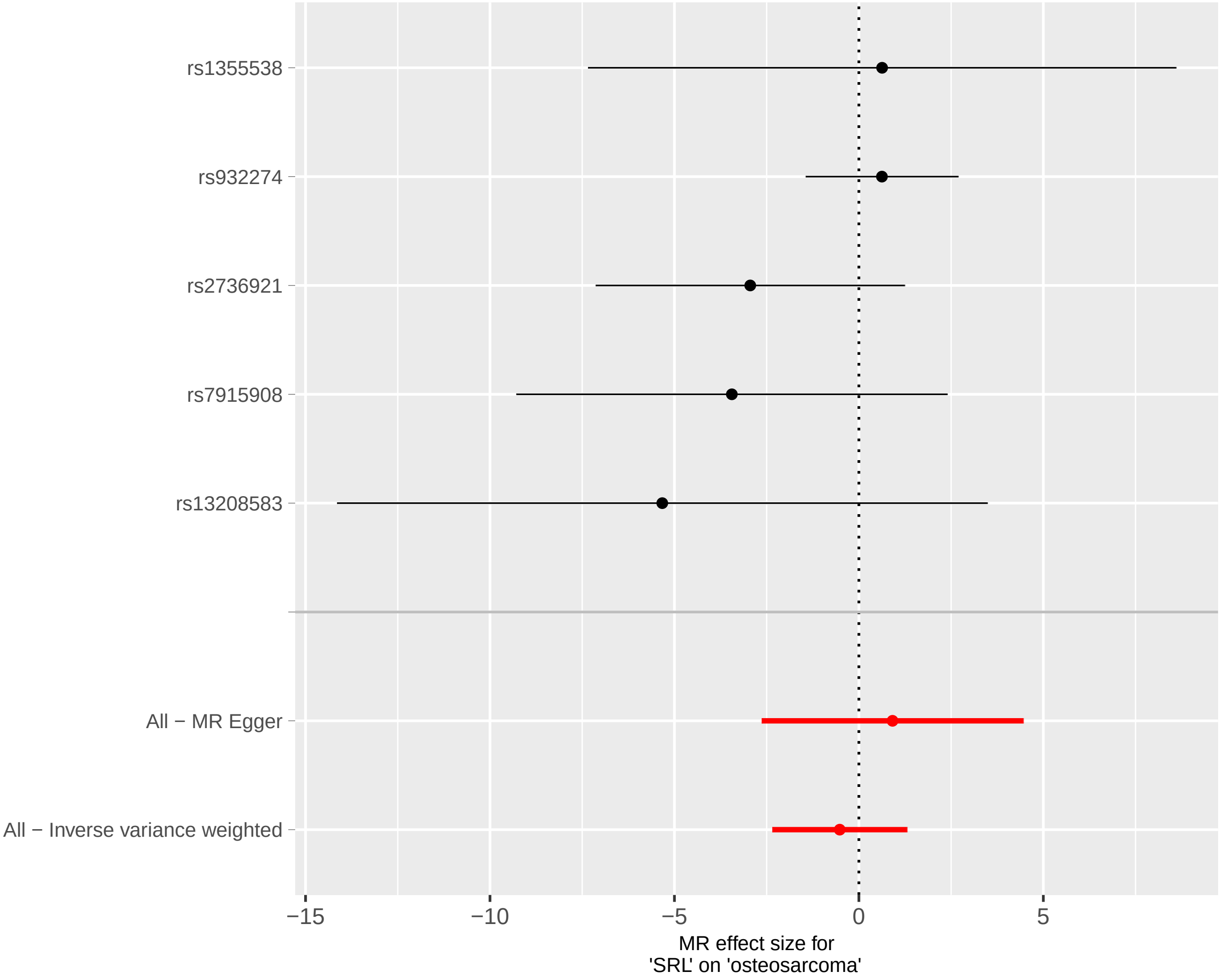
rs1355538
rs932274
rs2736921
rs7915908
rs13208583
All − MR Egger
All − Inverse variance weighted
−15
−10
−5
0
5
MR effect size for
'SRL' on 'osteosarcoma'

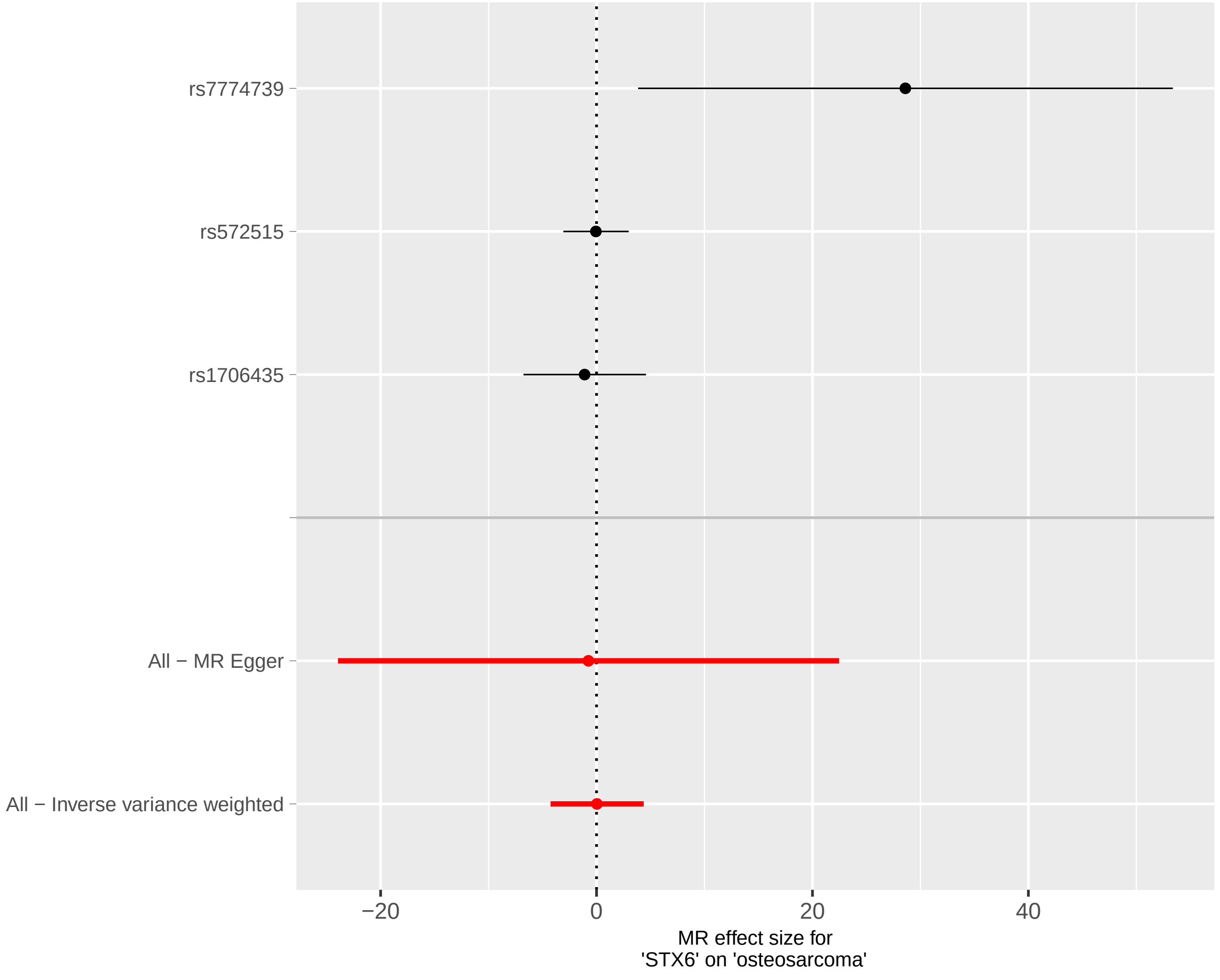

rs7774739
rs572515
rs1706435
All − MR Egger
All − Inverse variance weighted
−20
0
20
40
MR effect size for
'STX6' on 'osteosarcoma'

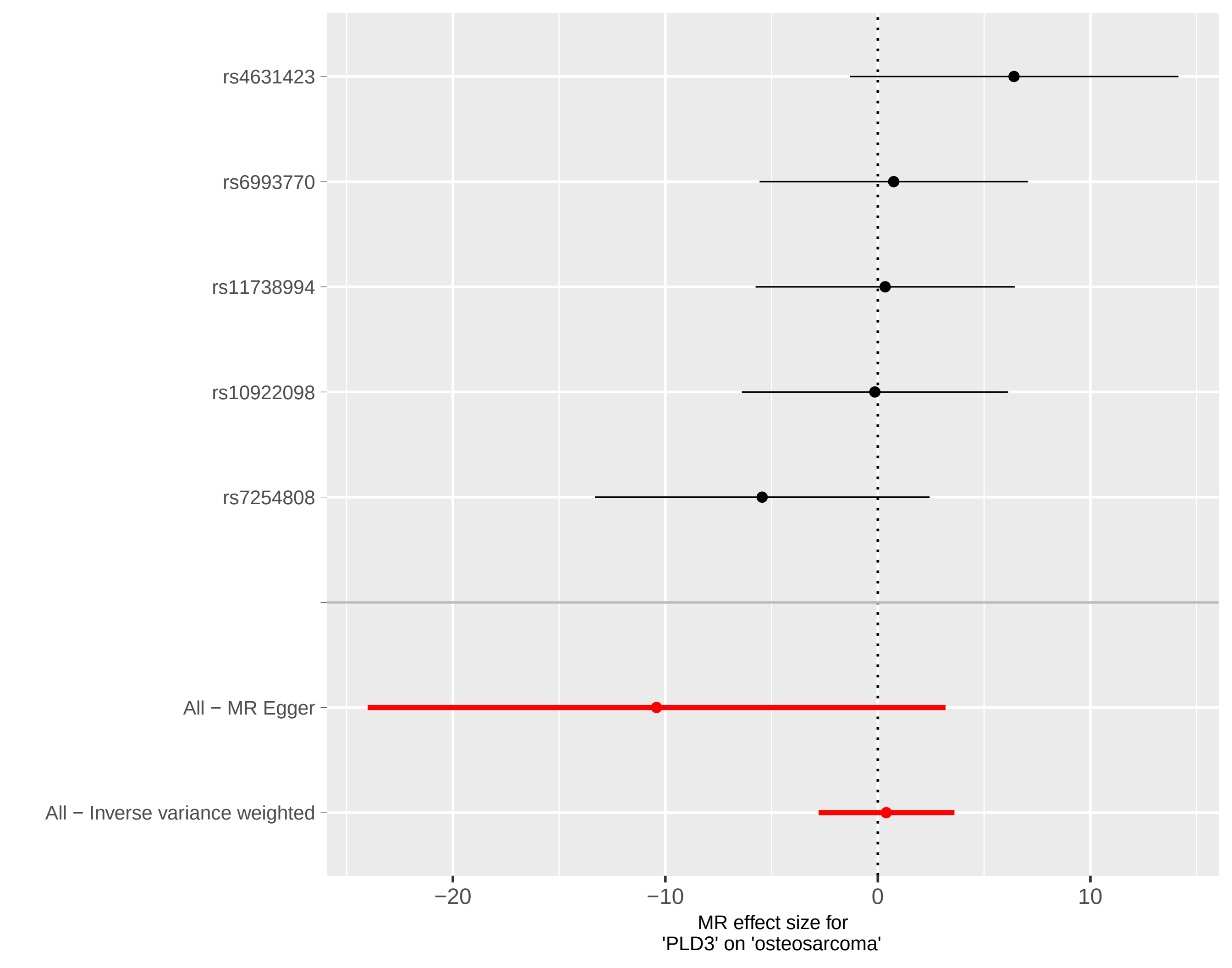

rs4631423
rs6993770
rs11738994
rs10922098
rs7254808
All − MR Egger
All − Inverse variance weighted
−20
−10
0
10
MR effect size for
'PLD3' on 'osteosarcoma'

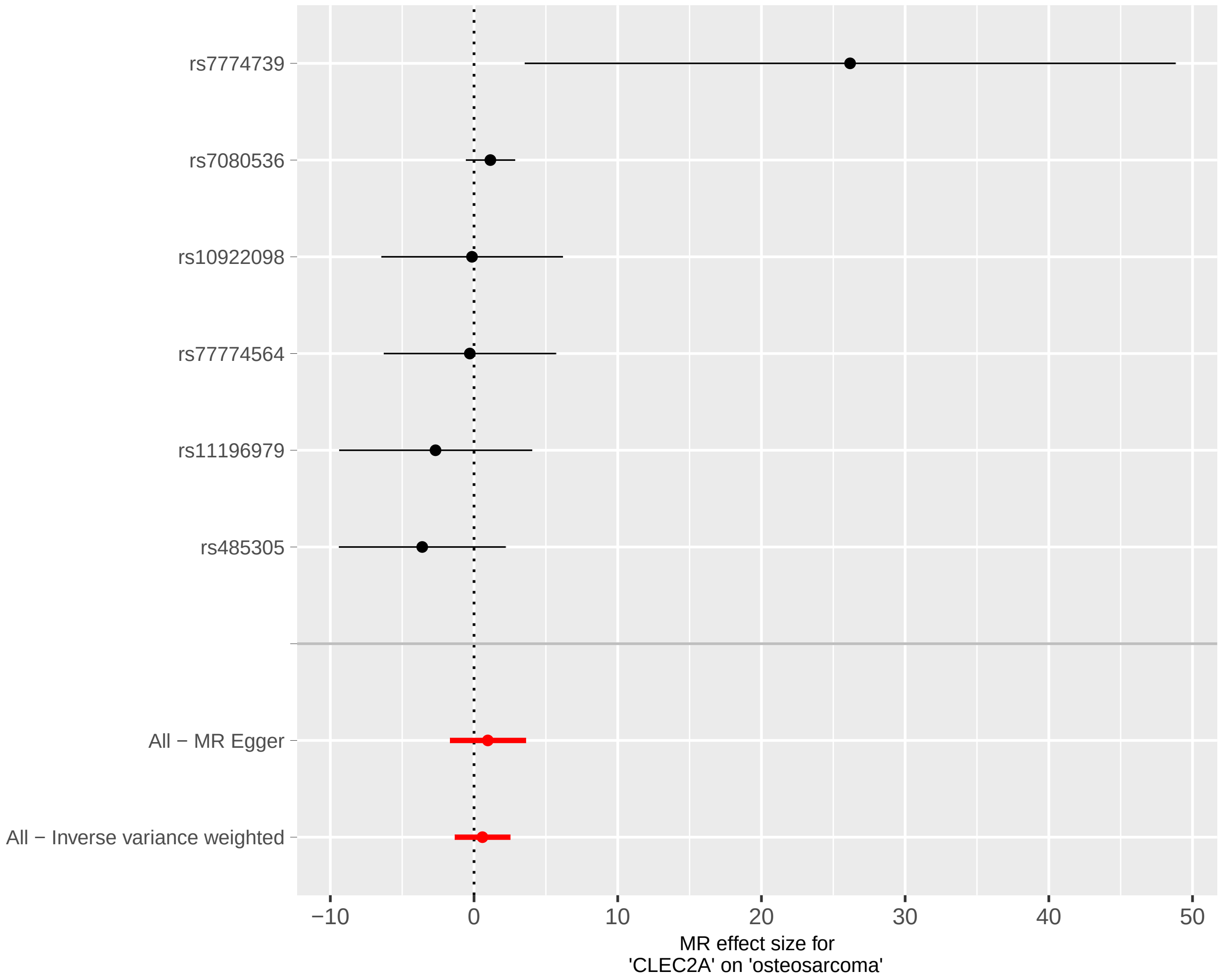

rs7774739
rs7080536
rs10922098
rs77774564
rs11196979
rs485305
All − MR Egger
All − Inverse variance weighted
−10
0
10
20
30
40
50
MR effect size for
'CLEC2A' on 'osteosarcoma'

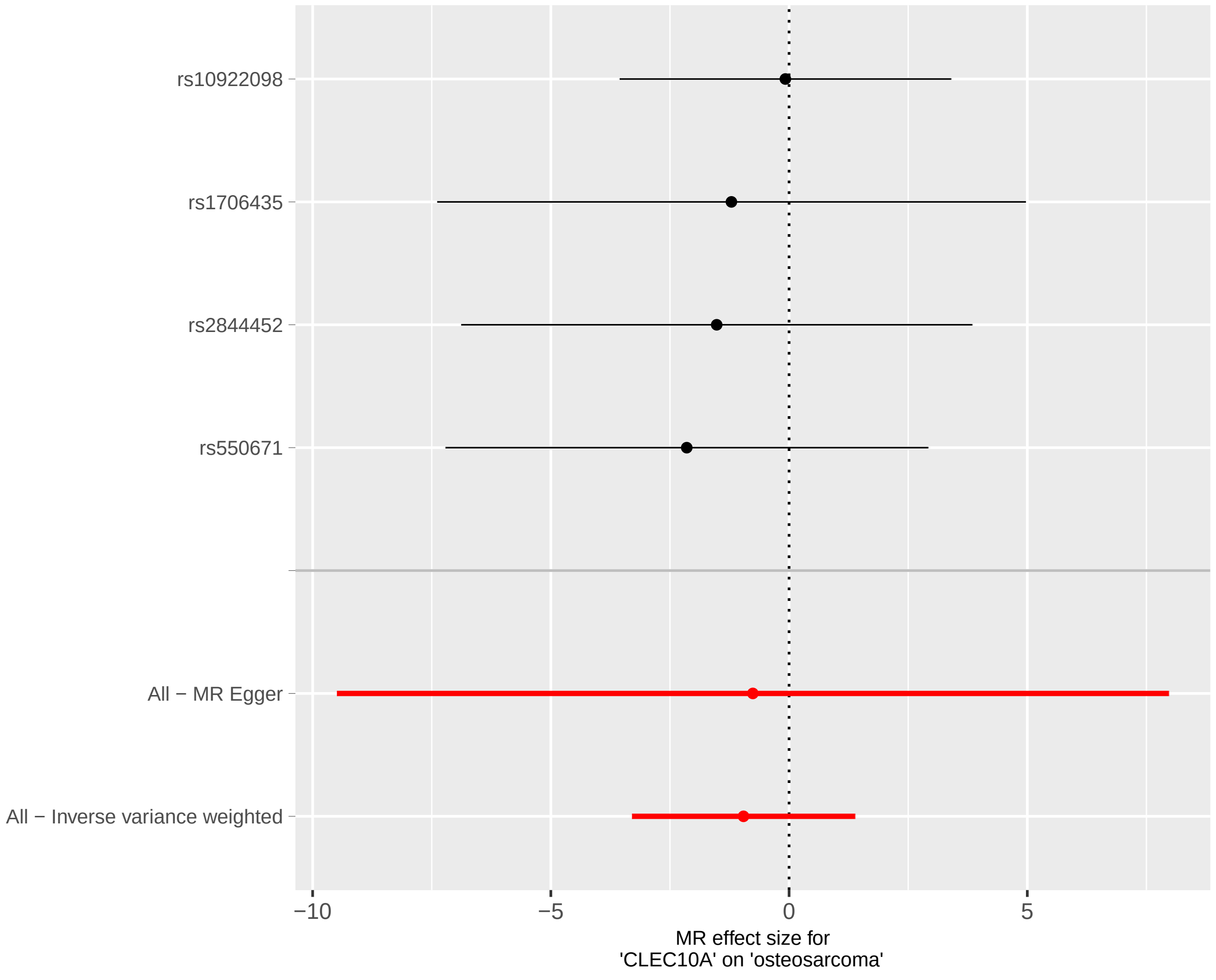

rs10922098
rs1706435
rs2844452
rs550671
All − MR Egger
All − Inverse variance weighted
−10
−5
0
5
MR effect size for
'CLEC10A' on 'osteosarcoma'

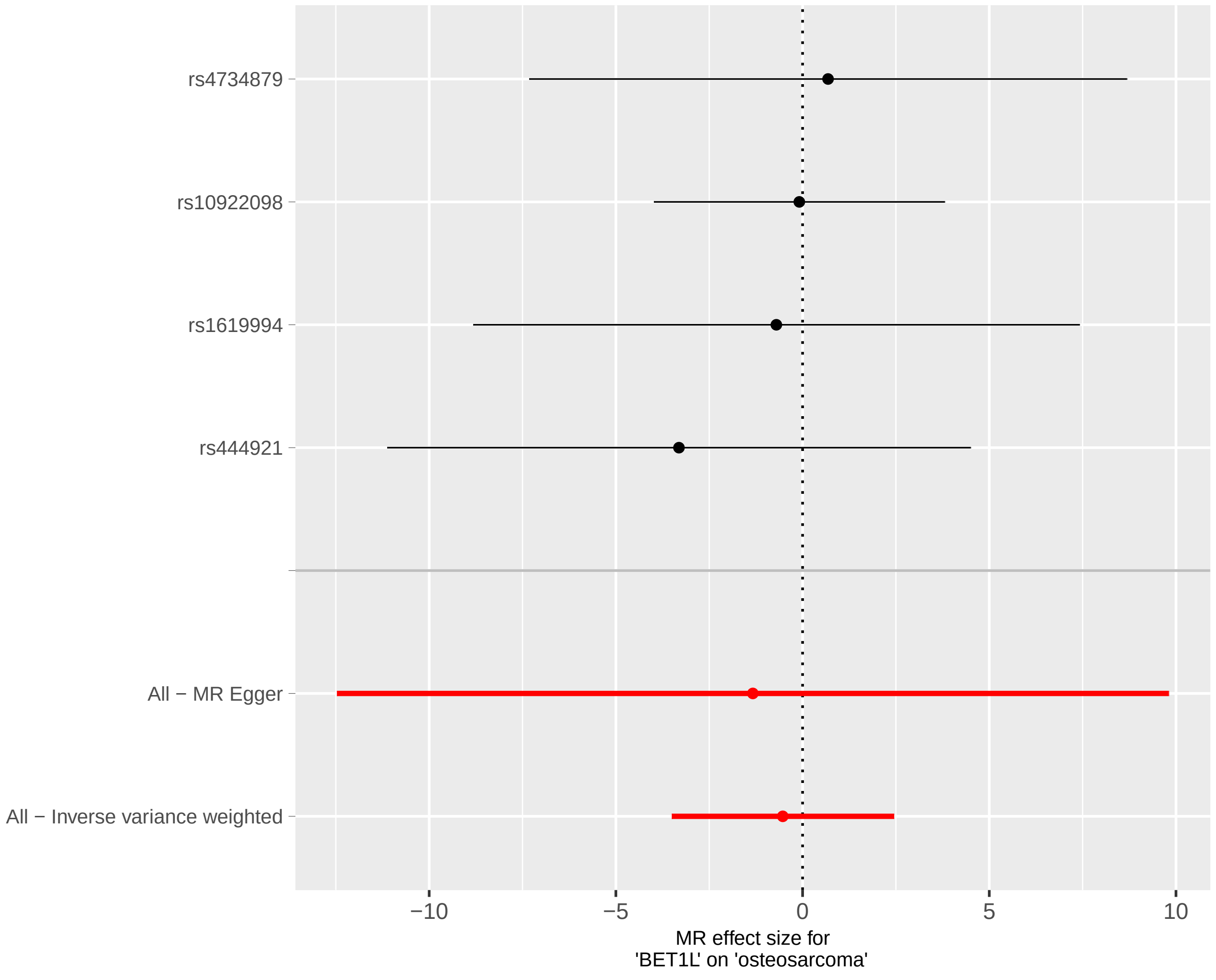

rs4734879
rs10922098
rs1619994
rs444921
All − MR Egger
All − Inverse variance weighted
−10
−5
0
5
10
MR effect size for
'BET1L' on 'osteosarcoma'

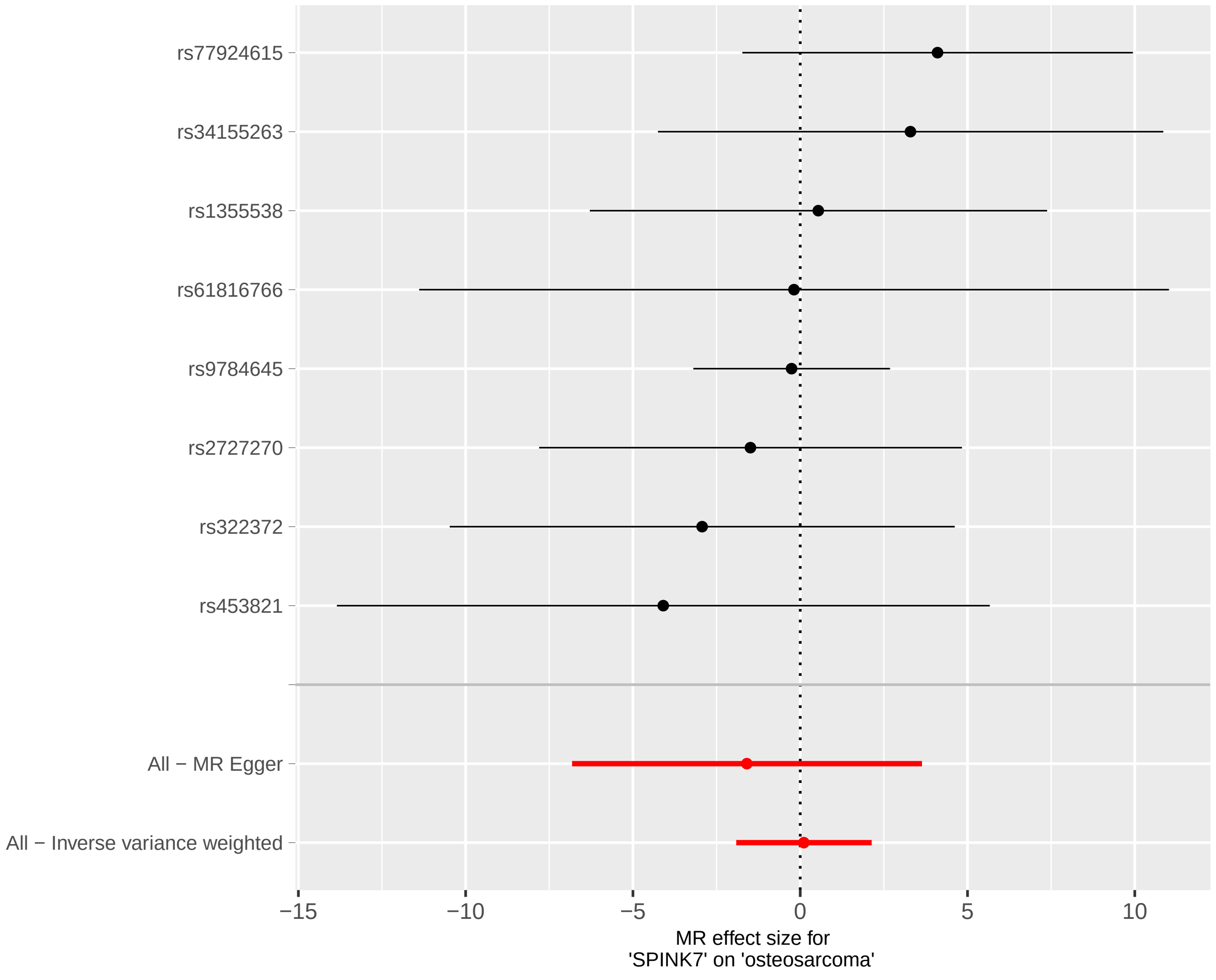
rs77924615
rs34155263
rs1355538
rs61816766
rs9784645
rs2727270
rs322372
rs453821
All − MR Egger
All − Inverse variance weighted
−15
−10
−5
0
5
10
MR effect size for
'SPINK7' on 'osteosarcoma'

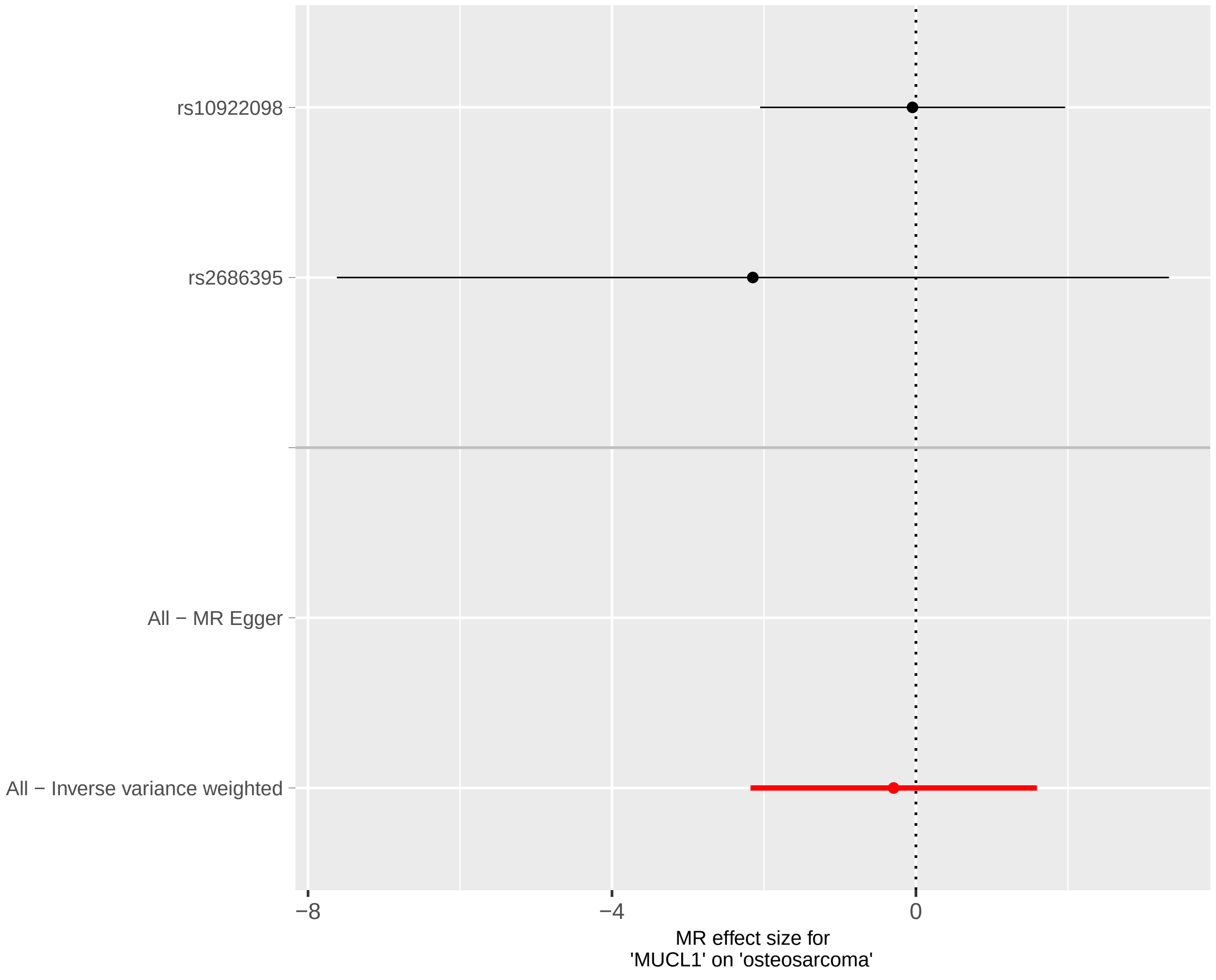

rs10922098
rs2686395
All − MR Egger
All − Inverse variance weighted
−8
−4
0
MR effect size for
'MUCL1' on 'osteosarcoma'

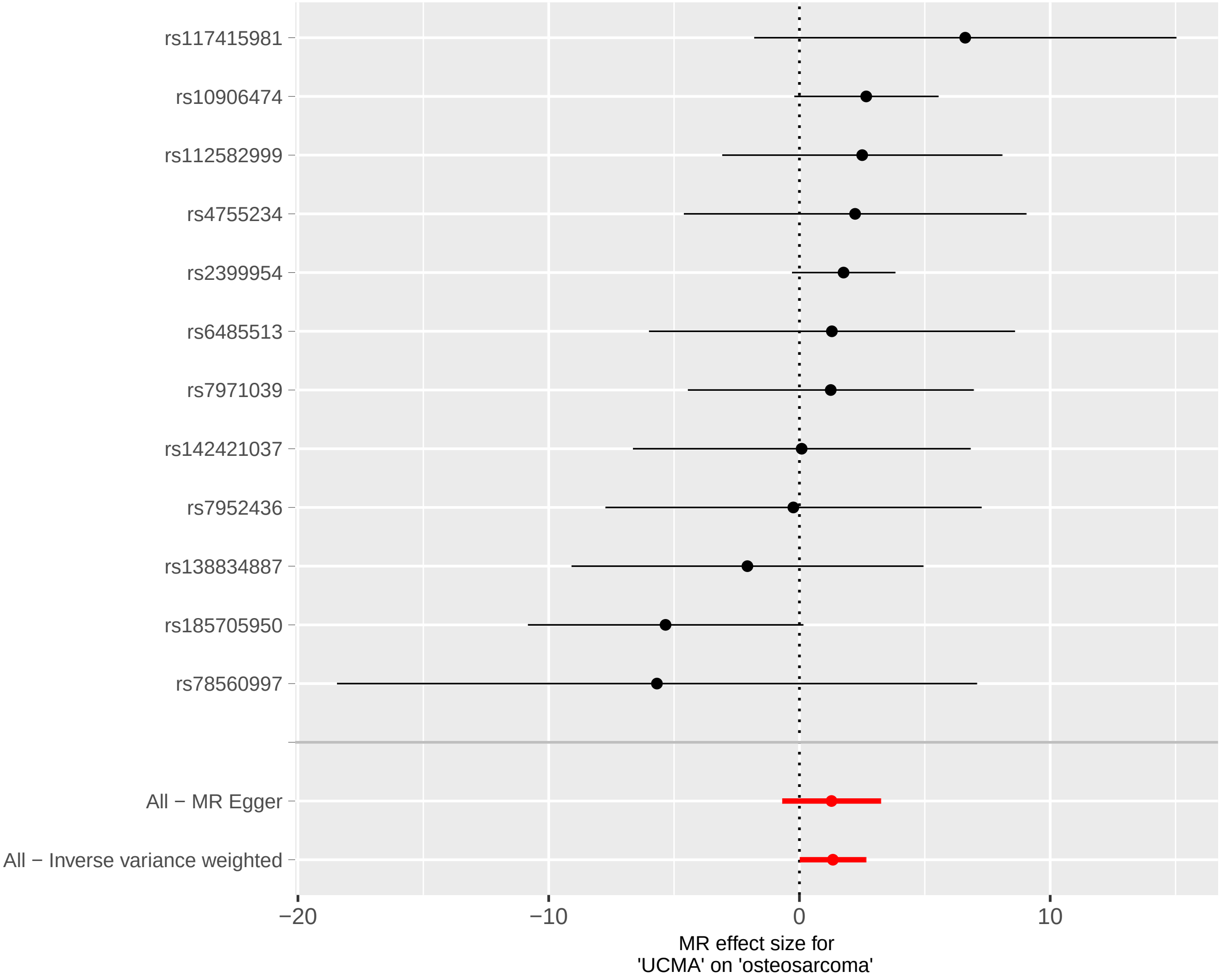

rs117415981
rs10906474
rs112582999
rs4755234
rs2399954
rs6485513
rs7971039
rs142421037
rs7952436
rs138834887
rs185705950
rs78560997
All − MR Egger
All − Inverse variance weighted
−20
−10
0
10
MR effect size for
'UCMA' on 'osteosarcoma'

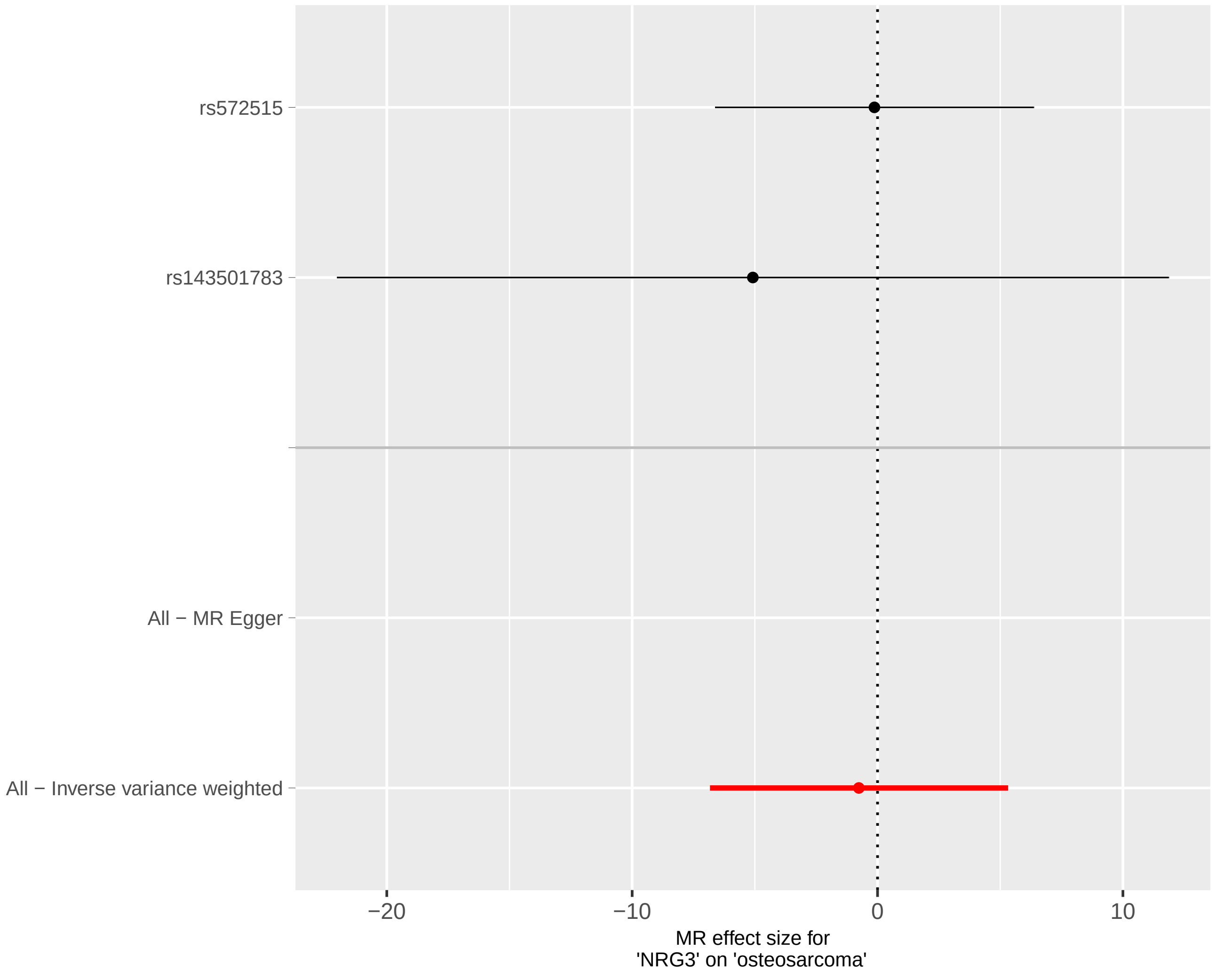

rs572515
rs143501783
All − MR Egger
All − Inverse variance weighted
−20
−10
0
10
MR effect size for
'NRG3' on 'osteosarcoma'

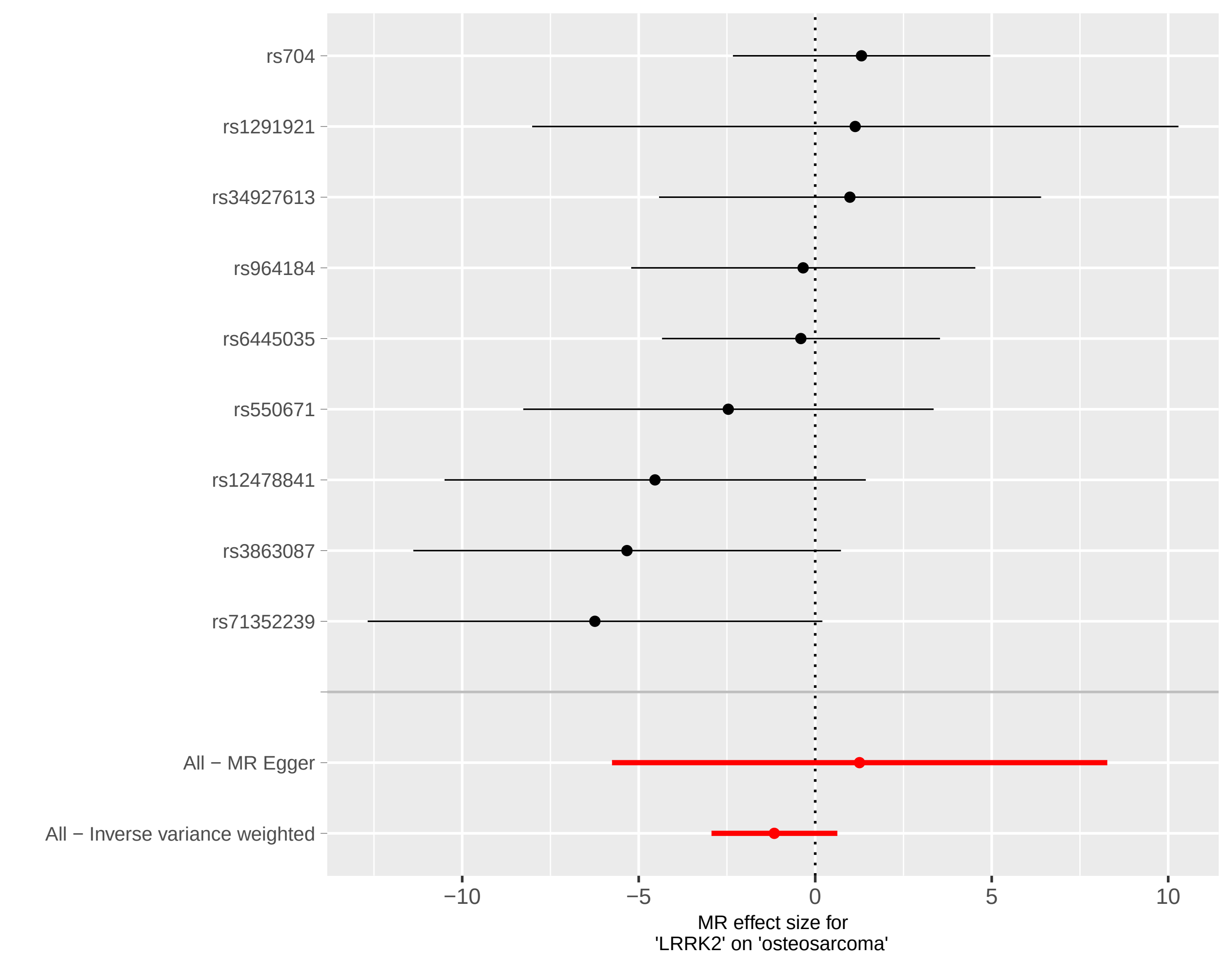
rs704
rs1291921
rs34927613
rs964184
rs6445035
rs550671
rs12478841
rs3863087
rs71352239
All − MR Egger
All − Inverse variance weighted
−10
−5
0
5
10
MR effect size for
'LRRK2' on 'osteosarcoma'

Insufficient number of SNPs

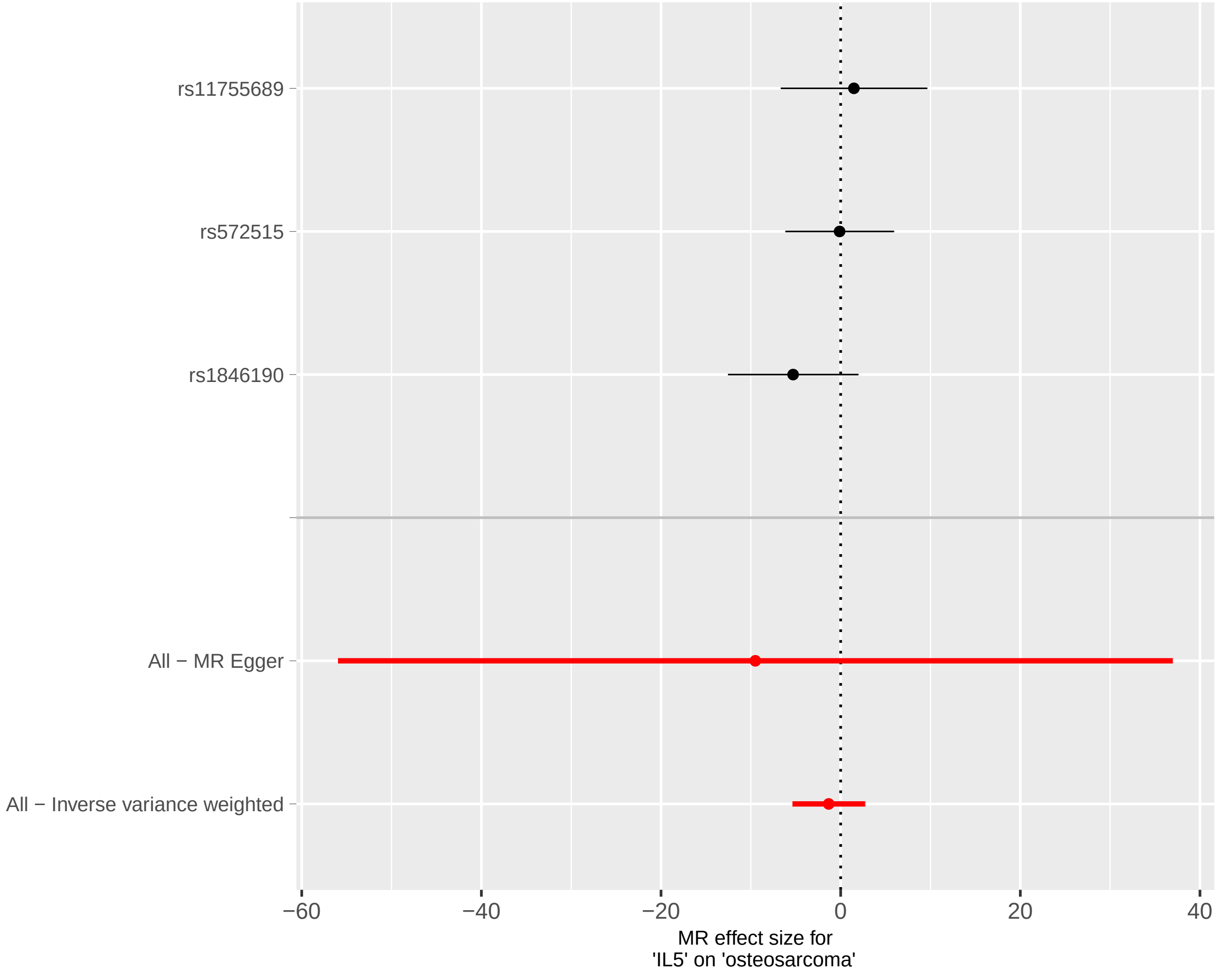
rs11755689
rs572515
rs1846190
All − MR Egger
All − Inverse variance weighted
−60
−40
−20
0
20
40
MR effect size for
'IL5' on 'osteosarcoma'

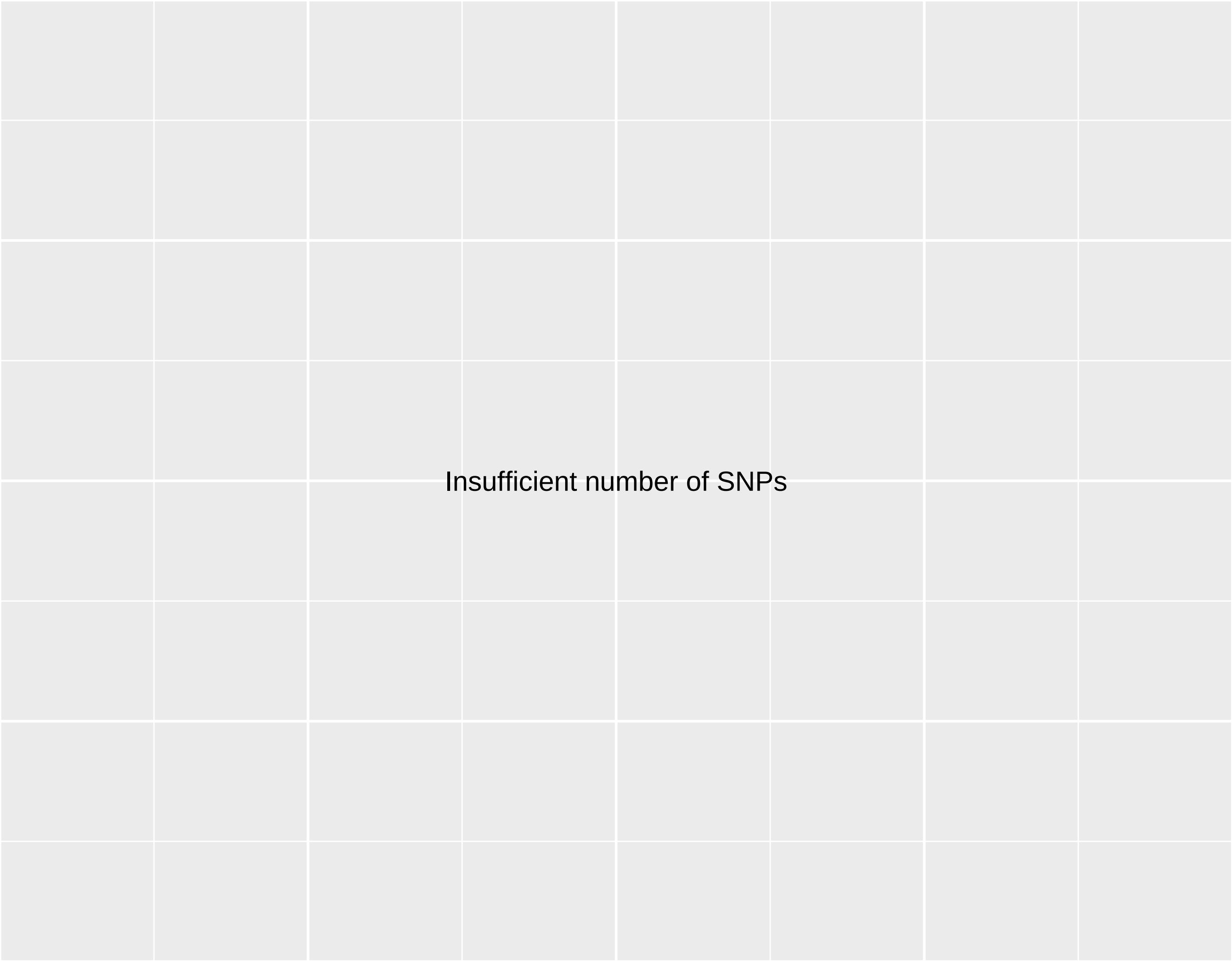
Insufficient number of SNPs

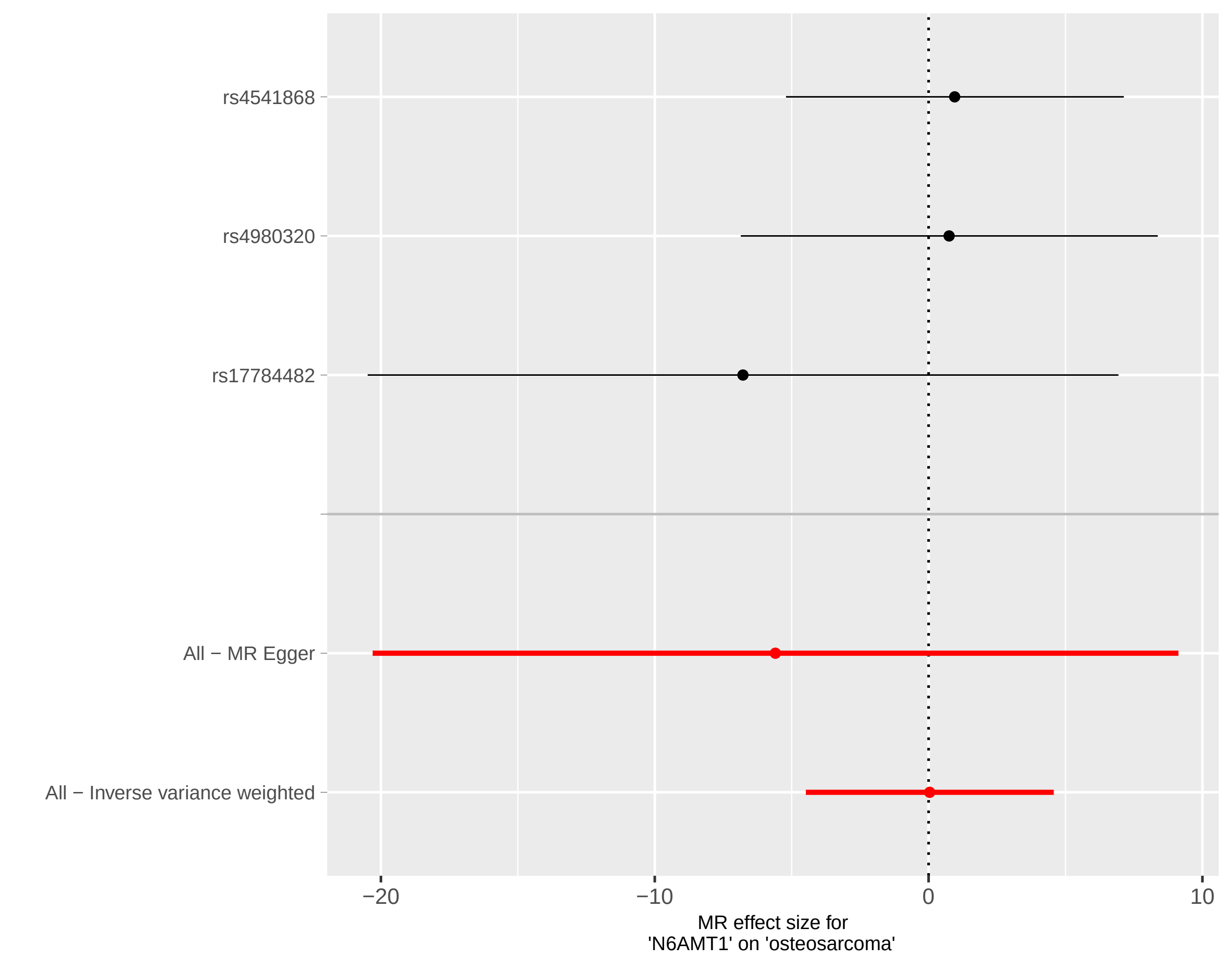
rs4541868
rs4980320
rs17784482
All − MR Egger
All − Inverse variance weighted
−20
−10
0
10
MR effect size for
'N6AMT1' on 'osteosarcoma'

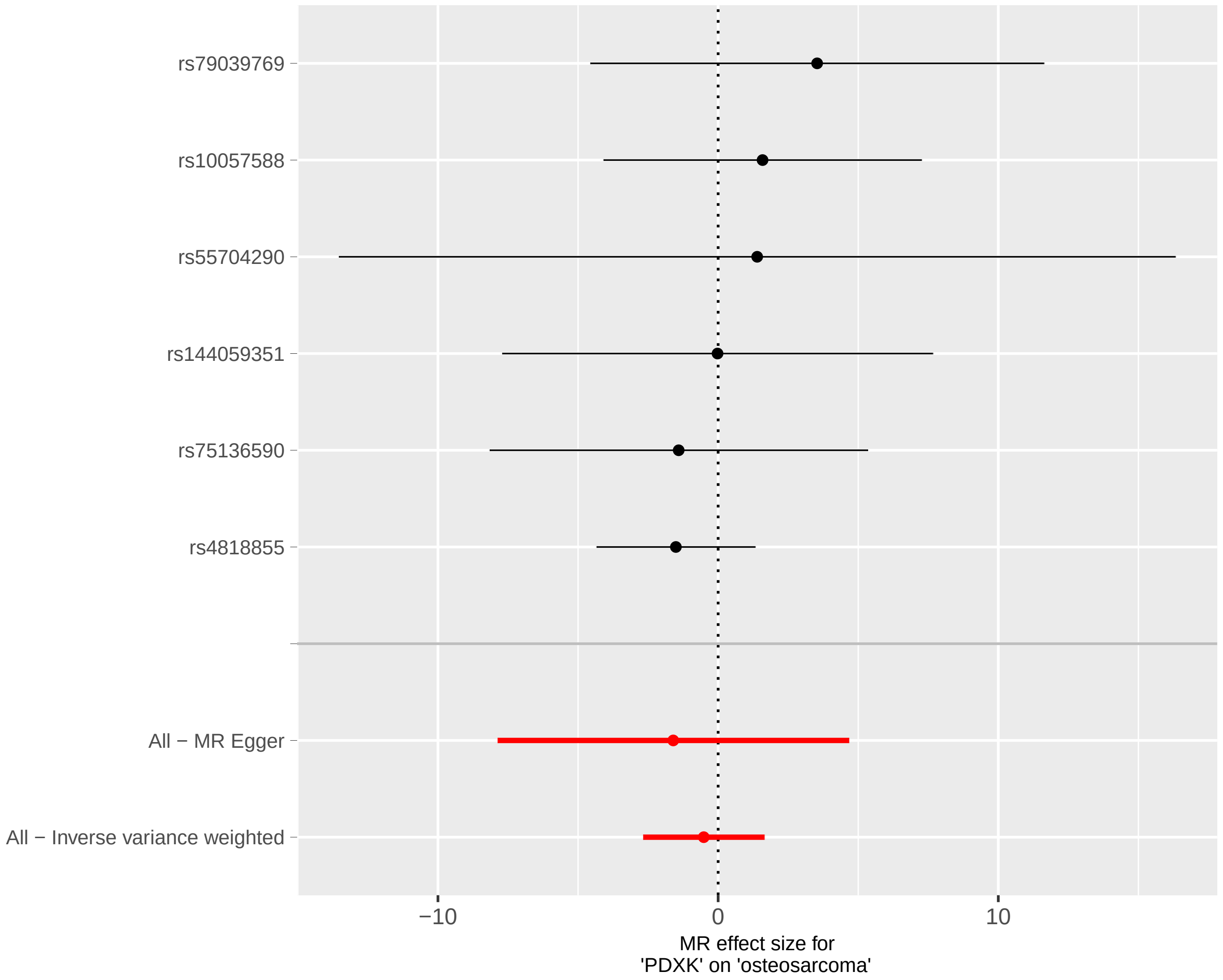

rs79039769
rs10057588
rs55704290
rs144059351
rs75136590
rs4818855
All − MR Egger
All − Inverse variance weighted
−10
0
10
MR effect size for
'PDXK' on 'osteosarcoma'

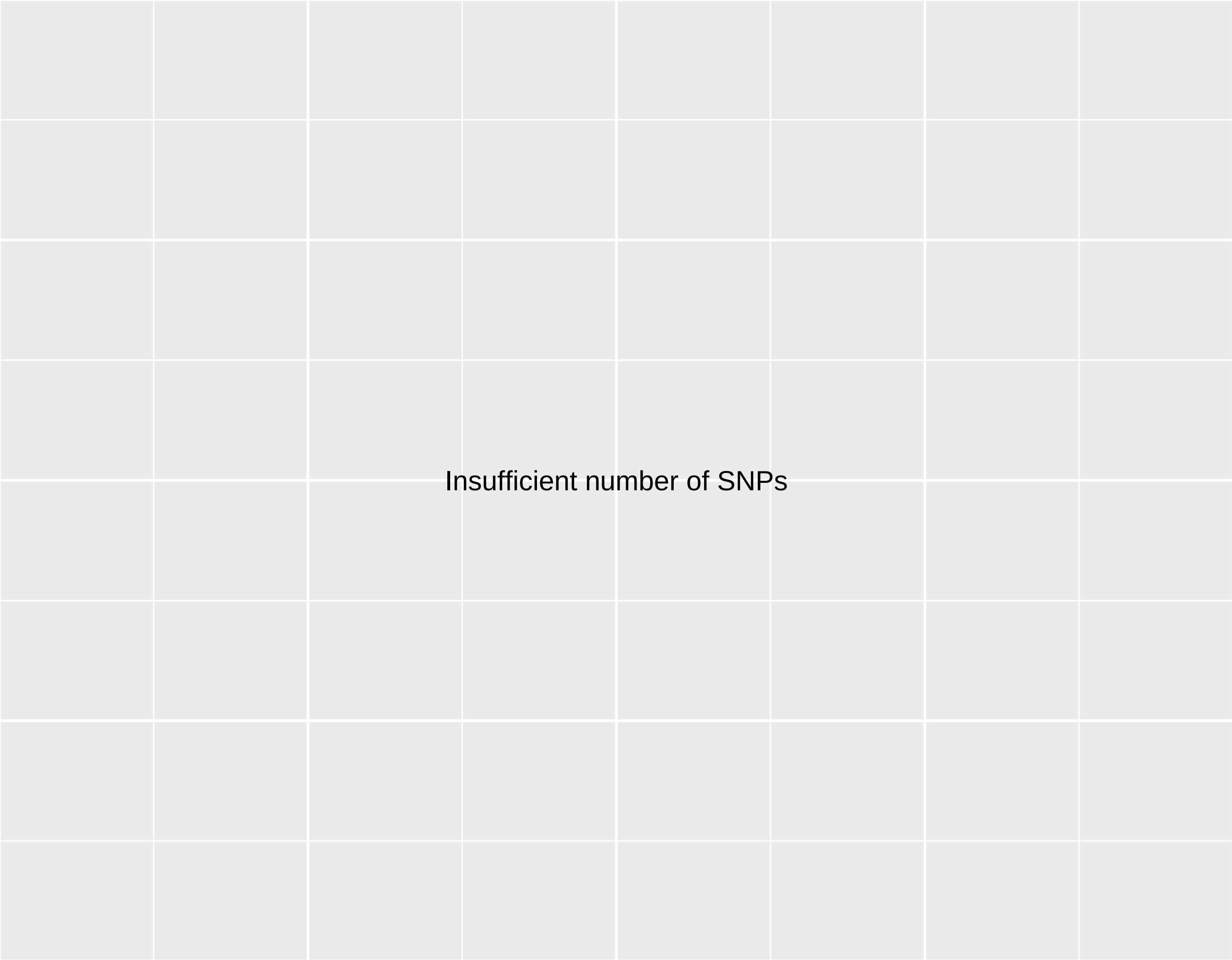
Insufficient number of SNPs

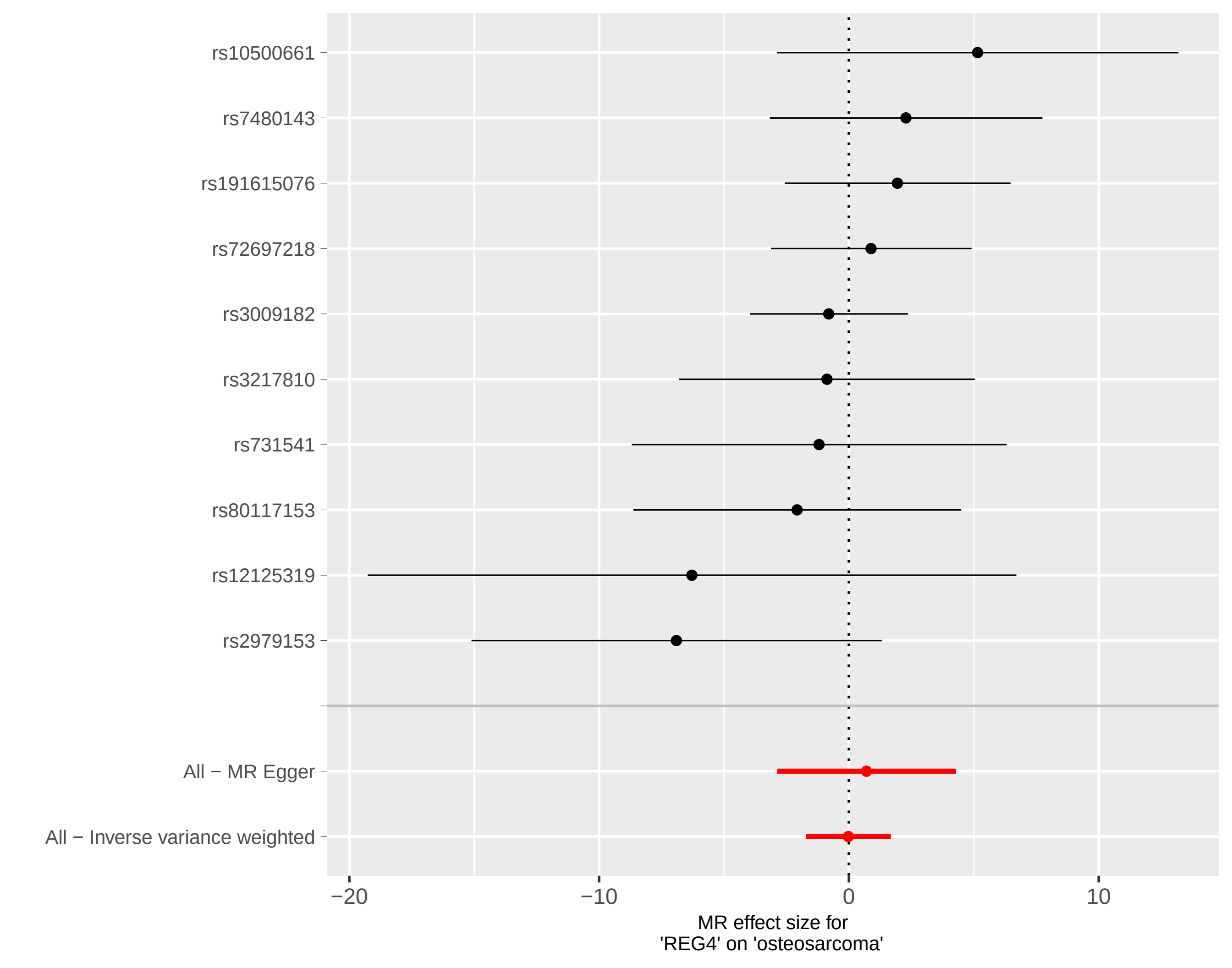

rs10500661
rs7480143
rs191615076
rs72697218
rs3009182
rs3217810
rs731541
rs80117153
rs12125319
rs2979153
All − MR Egger
All − Inverse variance weighted
−20
−10
0
10
MR effect size for
'REG4' on 'osteosarcoma'

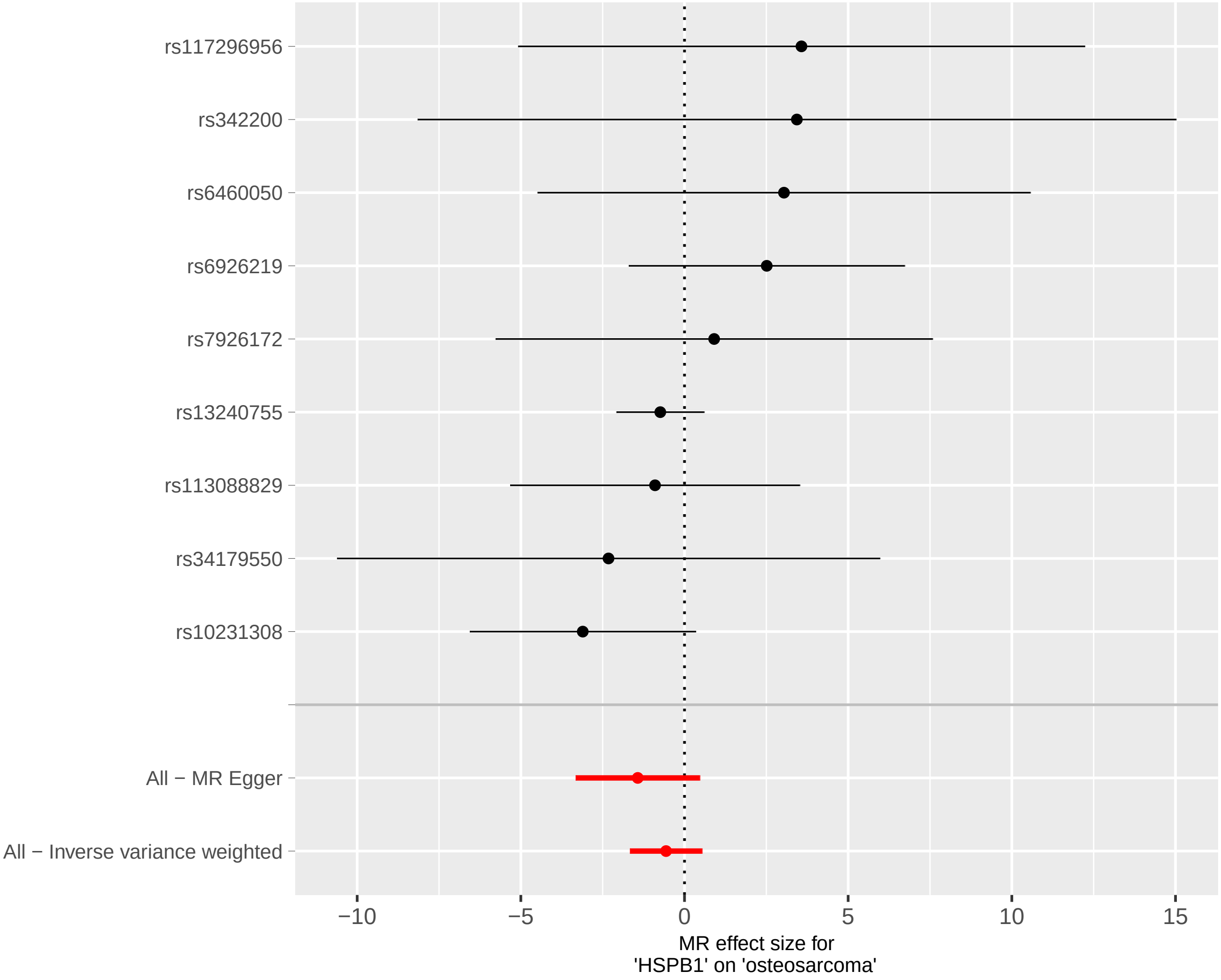
rs117296956
rs342200
rs6460050
rs6926219
rs7926172
rs13240755
rs113088829
rs34179550
rs10231308
All − MR Egger
All − Inverse variance weighted
−10
−5
0
5
10
15
MR effect size for
'HSPB1' on 'osteosarcoma'

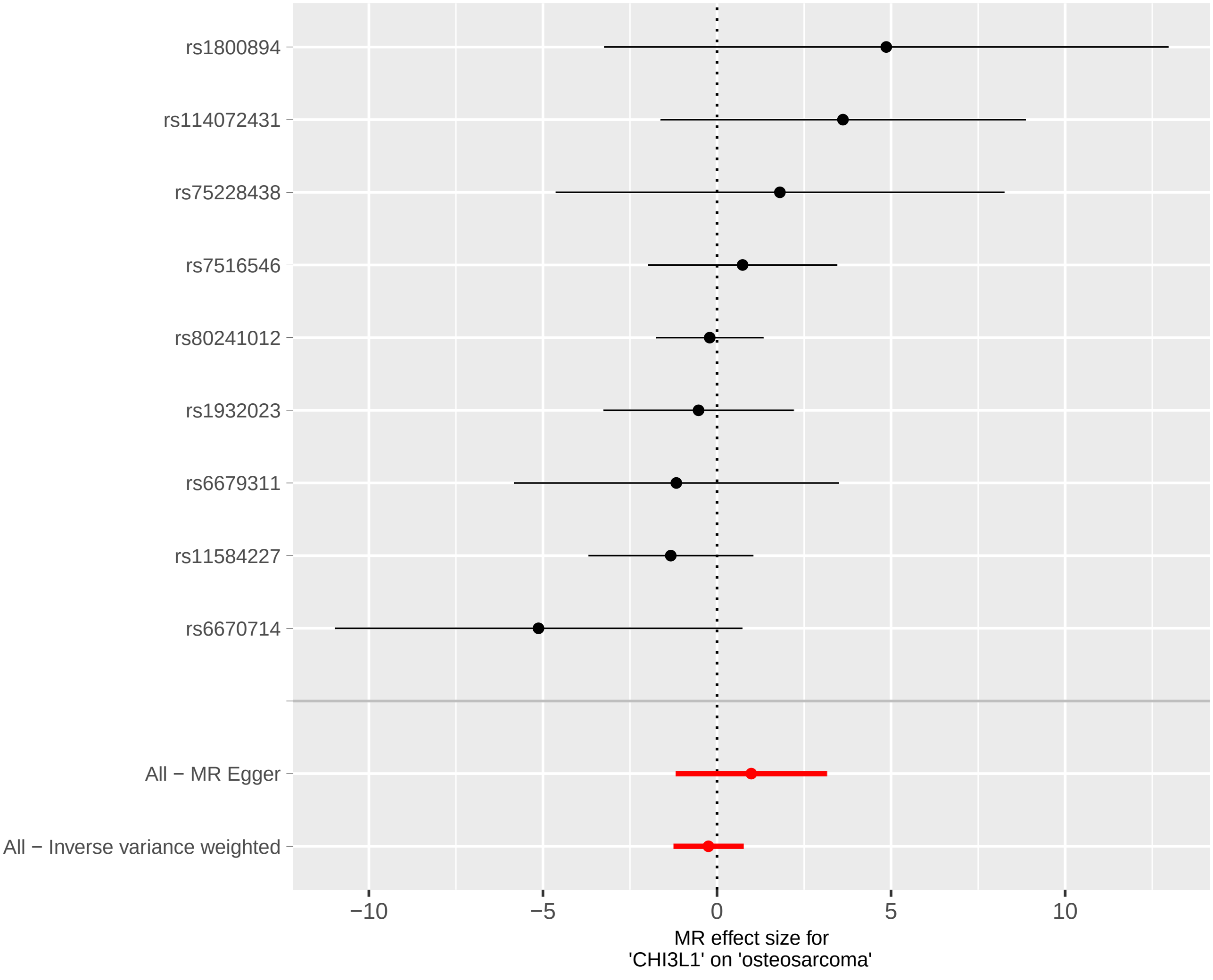
rs1800894
rs114072431
rs75228438
rs7516546
rs80241012
rs1932023
rs6679311
rs11584227
rs6670714
All − MR Egger
All − Inverse variance weighted
−10
−5
0
5
10
MR effect size for
'CHI3L1' on 'osteosarcoma'

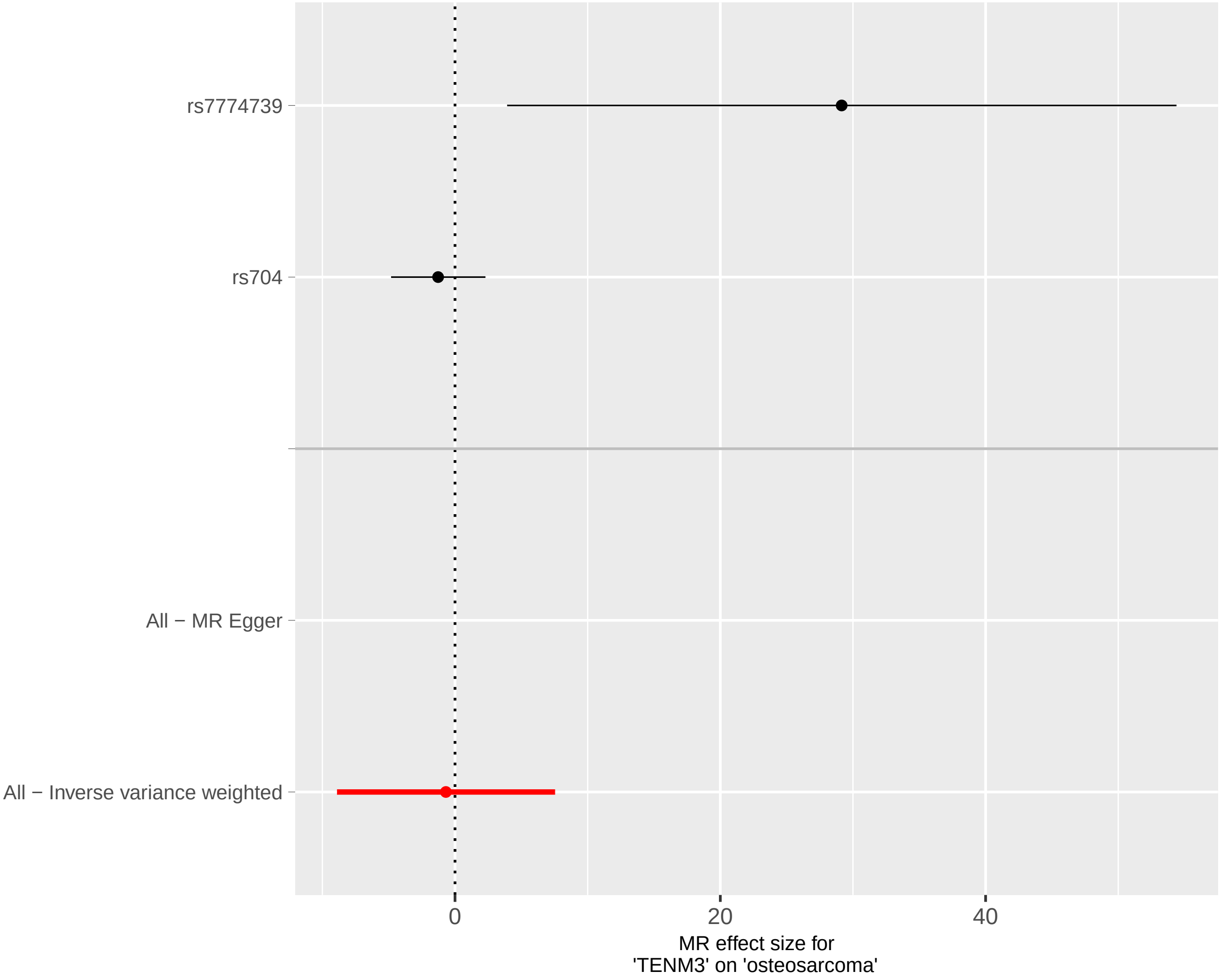
rs7774739
rs704
All − MR Egger
All − Inverse variance weighted
0
20
40
MR effect size for
'TENM3' on 'osteosarcoma'

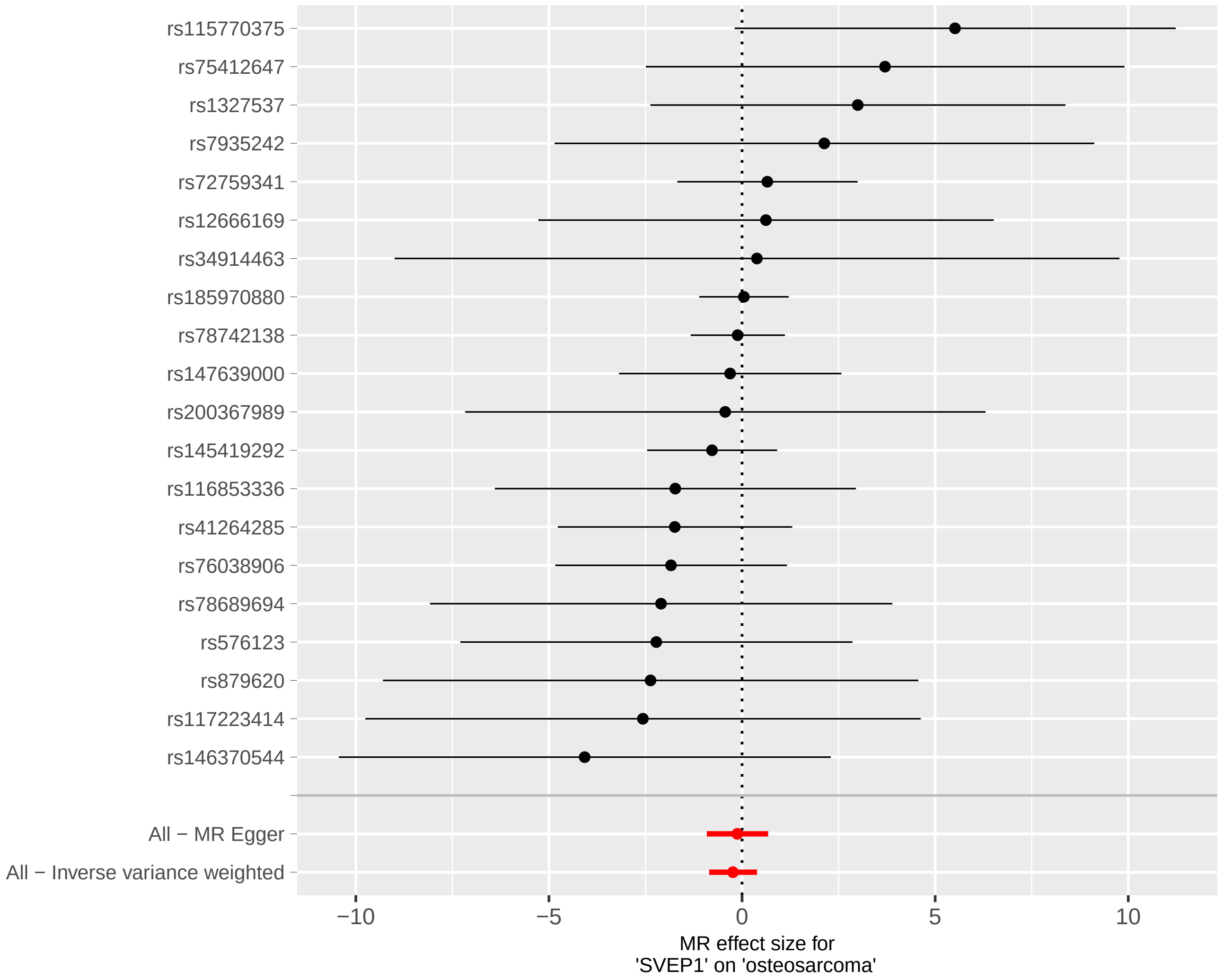
rs115770375
rs75412647
rs1327537
rs7935242
rs72759341
rs12666169
rs34914463
rs185970880
rs78742138
rs147639000
rs200367989
rs145419292
rs116853336
rs41264285
rs76038906
rs78689694
rs576123
rs879620
rs117223414
rs146370544
All − MR Egger
All − Inverse variance weighted
−10
−5
0
5
10
MR effect size for
'SVEP1' on 'osteosarcoma'

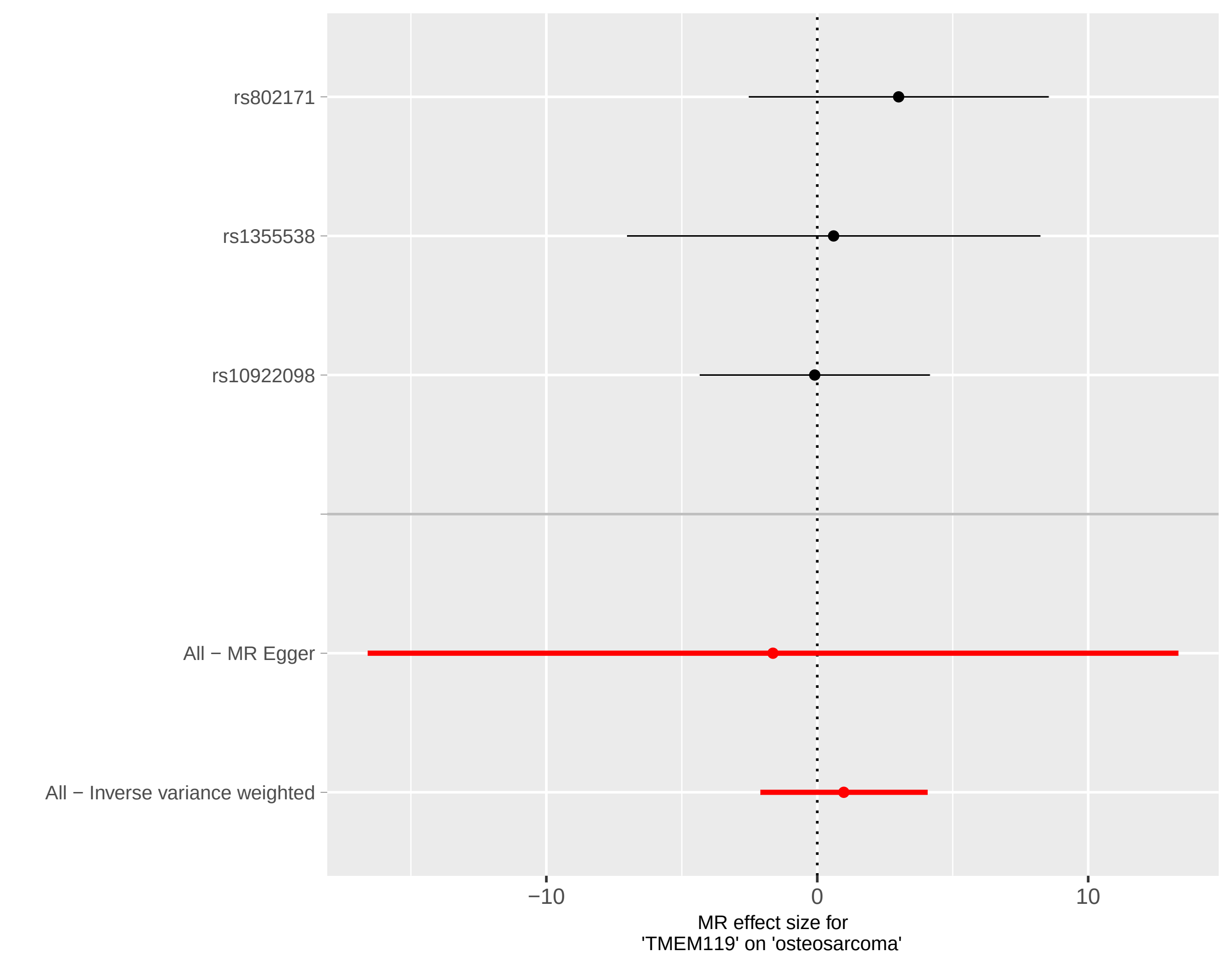
rs802171
rs1355538
rs10922098
All − MR Egger
All − Inverse variance weighted
−10
0
10
MR effect size for
'TMEM119' on 'osteosarcoma'

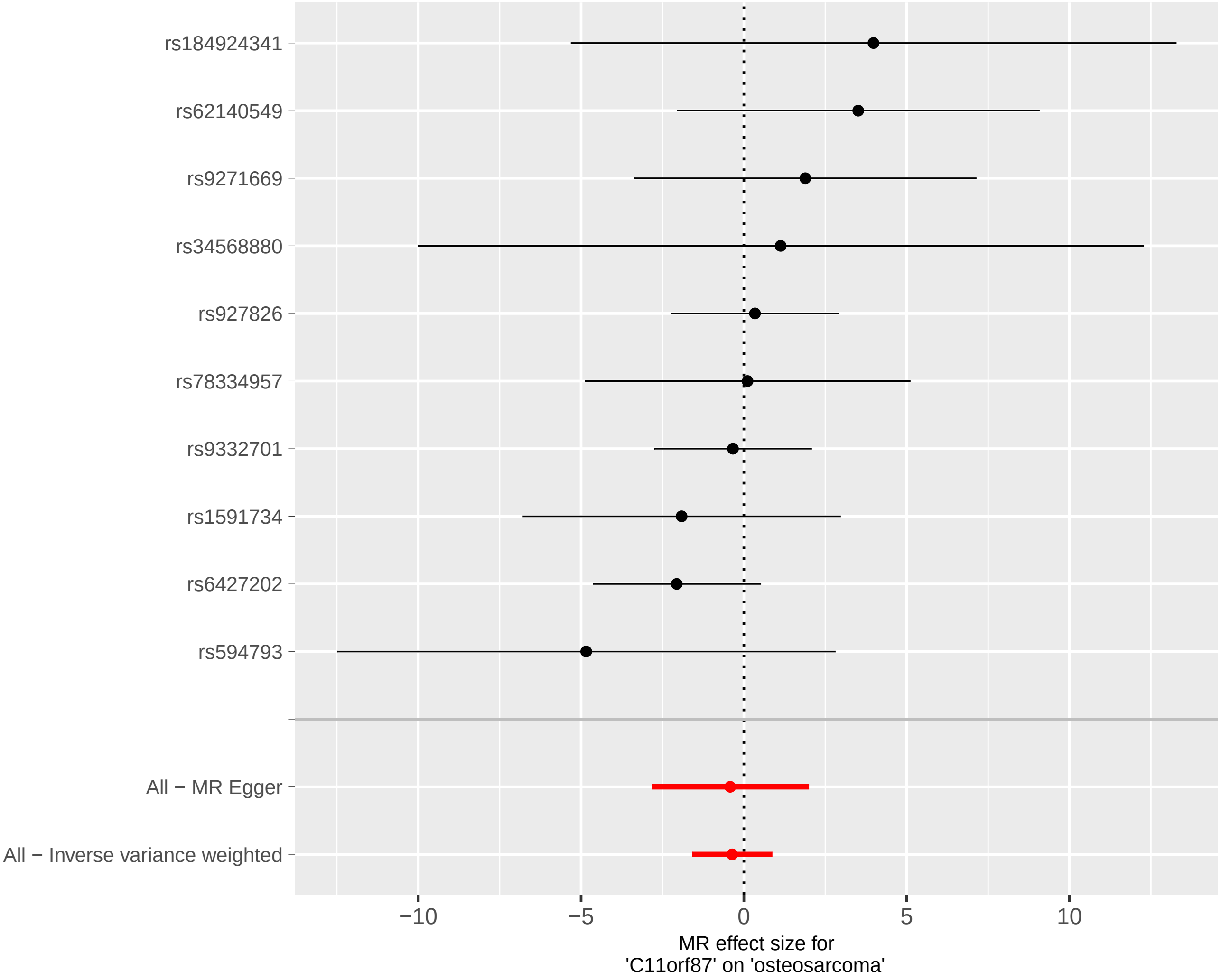
rs184924341
rs62140549
rs9271669
rs34568880
rs927826
rs78334957
rs9332701
rs1591734
rs6427202
rs594793
All − MR Egger
All − Inverse variance weighted
−10
−5
0
5
10
MR effect size for
'C11orf87' on 'osteosarcoma'

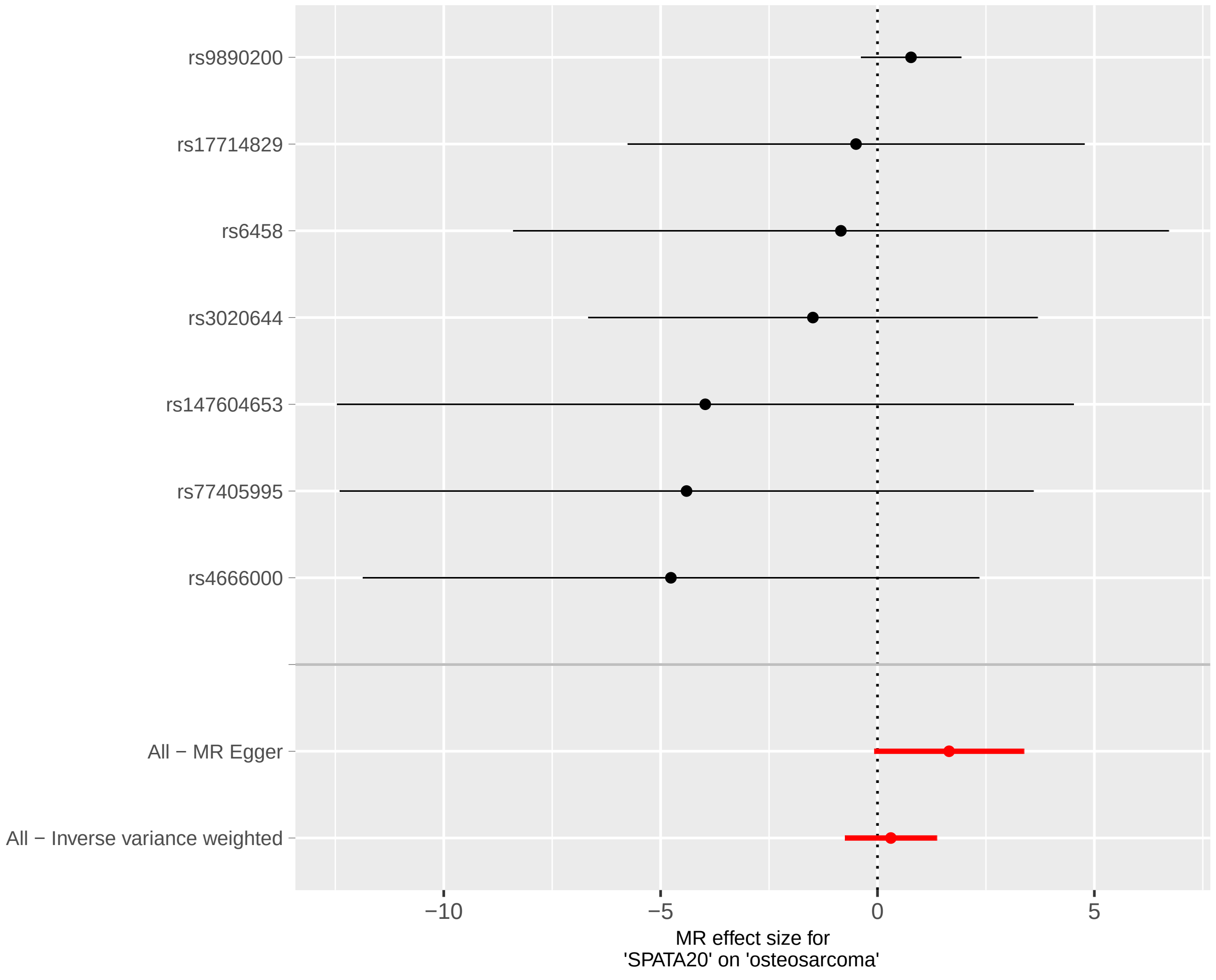

rs9890200
rs17714829
rs6458
rs3020644
rs147604653
rs77405995
rs4666000
All − MR Egger
All − Inverse variance weighted
−10
−5
0
5
MR effect size for
'SPATA20' on 'osteosarcoma'

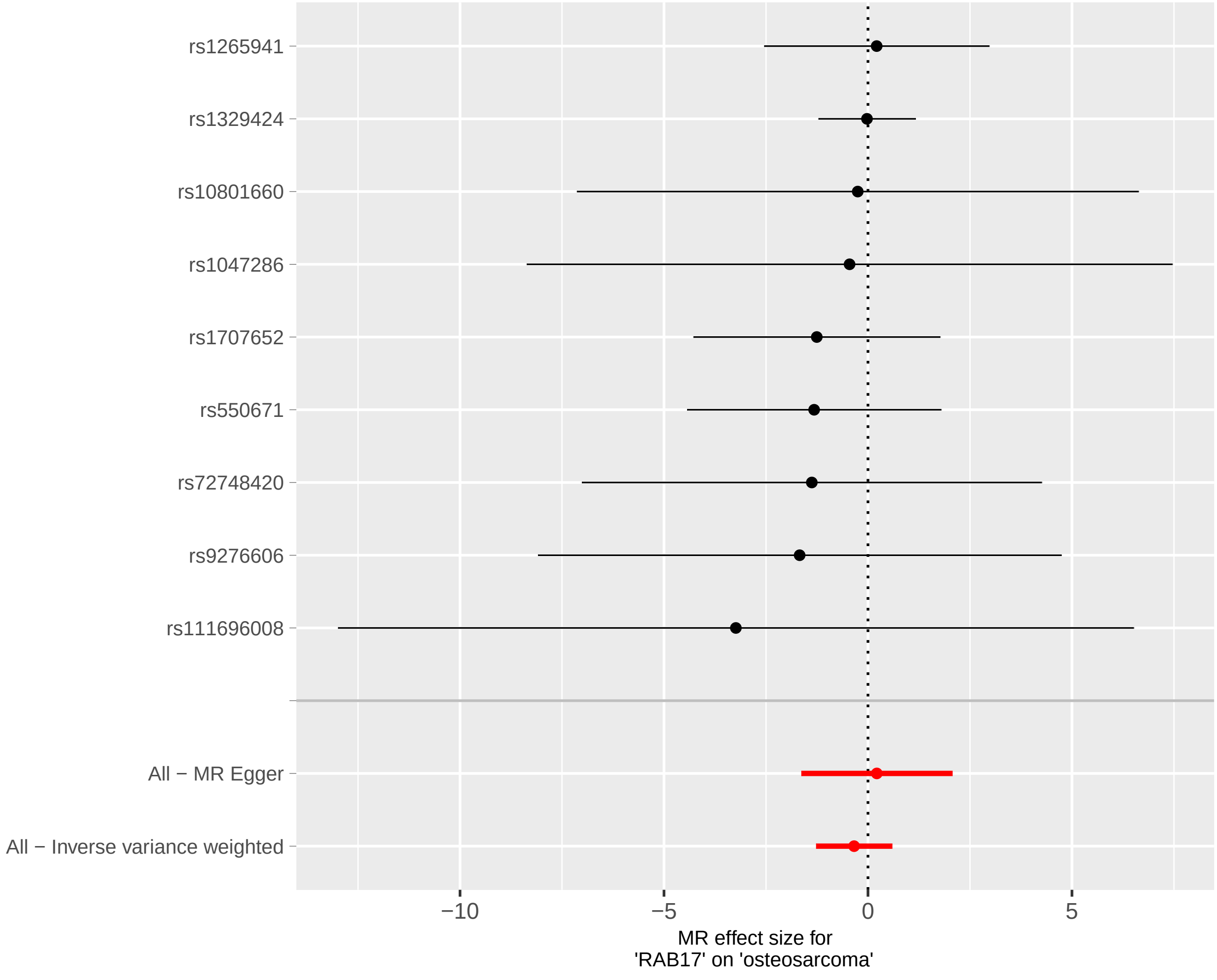
rs1265941
rs1329424
rs10801660
rs1047286
rs1707652
rs550671
rs72748420
rs9276606
rs111696008
All − MR Egger
All − Inverse variance weighted
−10
−5
0
5
MR effect size for
'RAB17' on 'osteosarcoma'

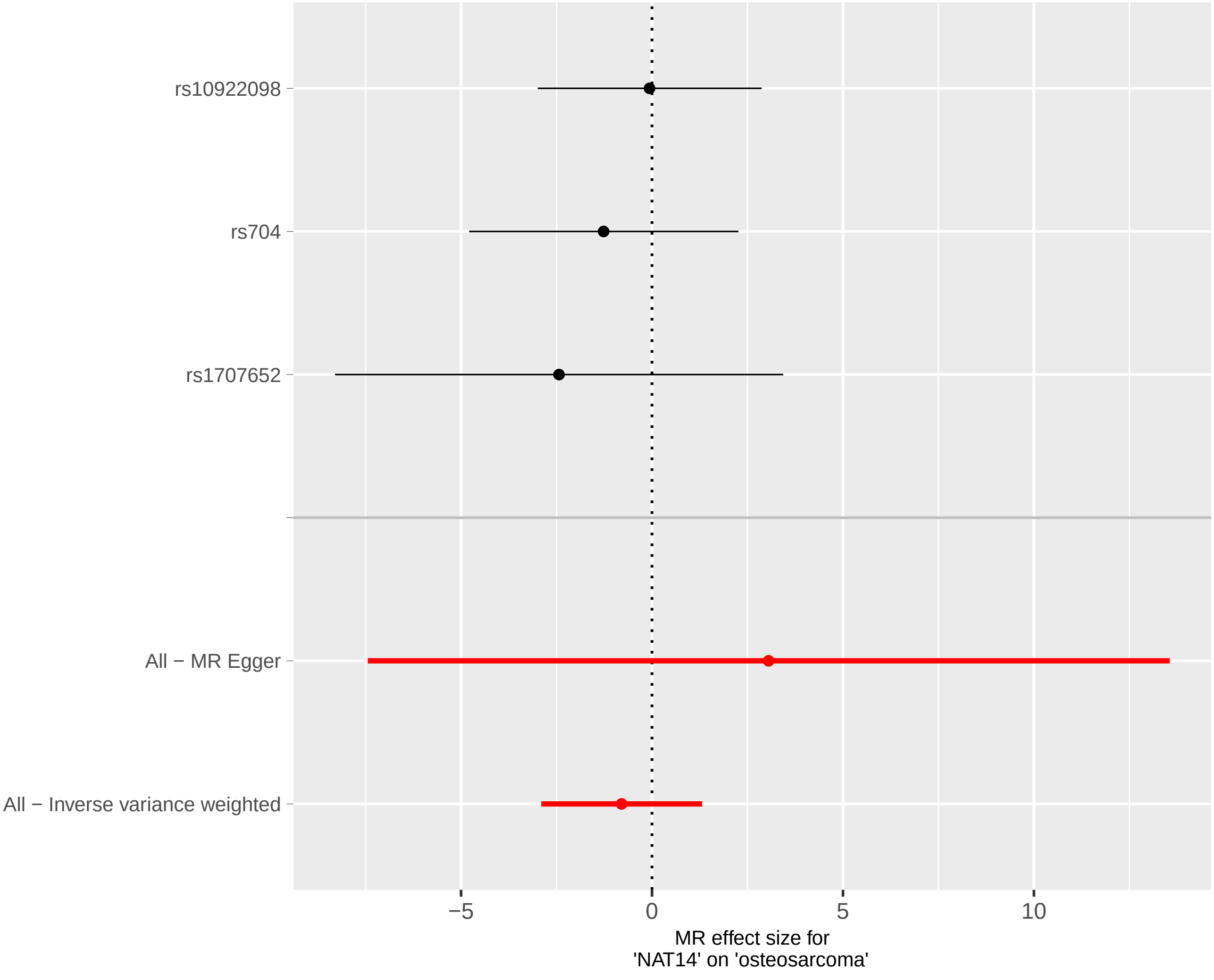
rs10922098
rs704
rs1707652
All − MR Egger
All − Inverse variance weighted
−5
0
5
10
MR effect size for
'NAT14' on 'osteosarcoma'

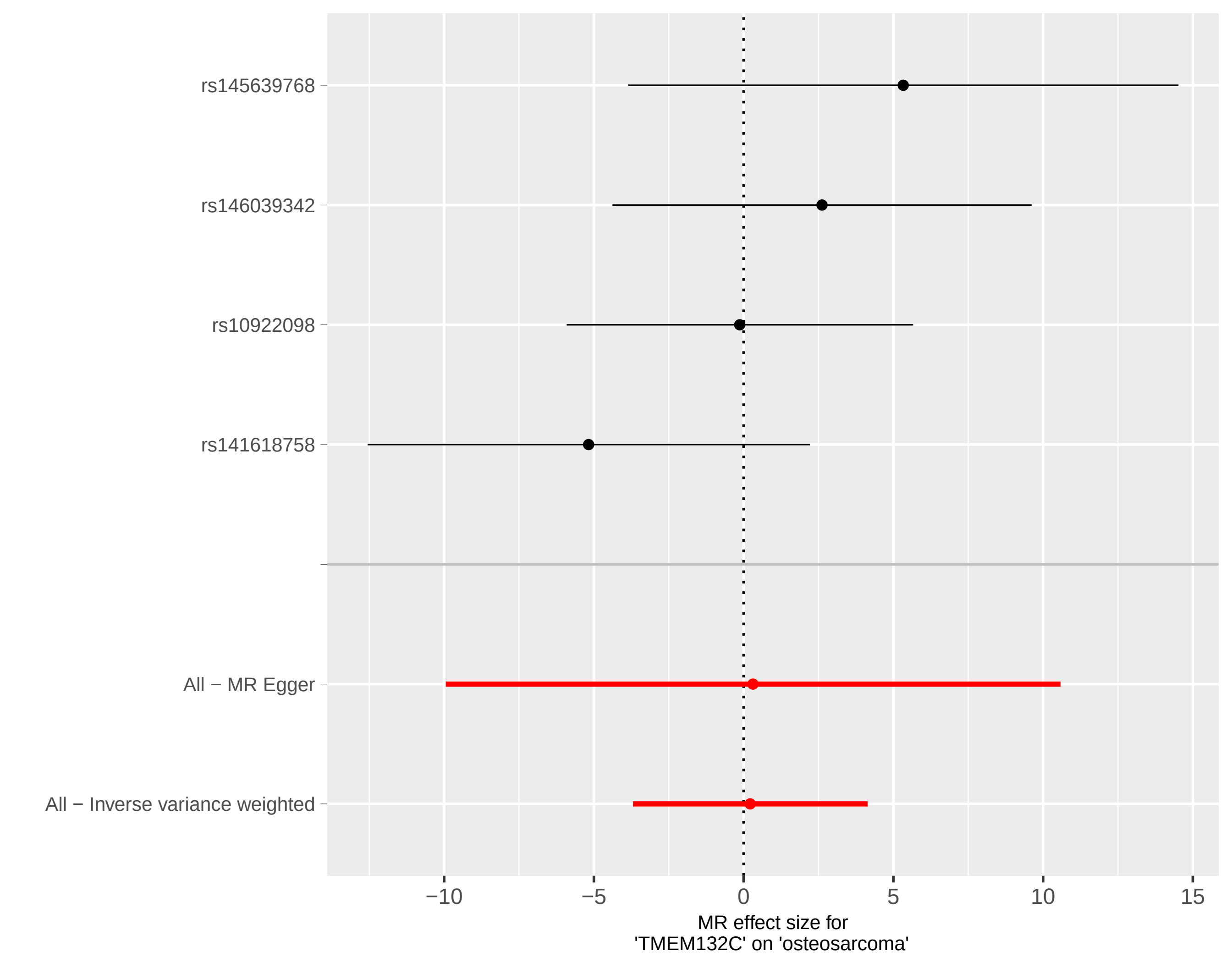

rs145639768
rs146039342
rs10922098
rs141618758
All − MR Egger
All − Inverse variance weighted
−10
−5
0
5
10
15
MR effect size for
'TMEM132C' on 'osteosarcoma'

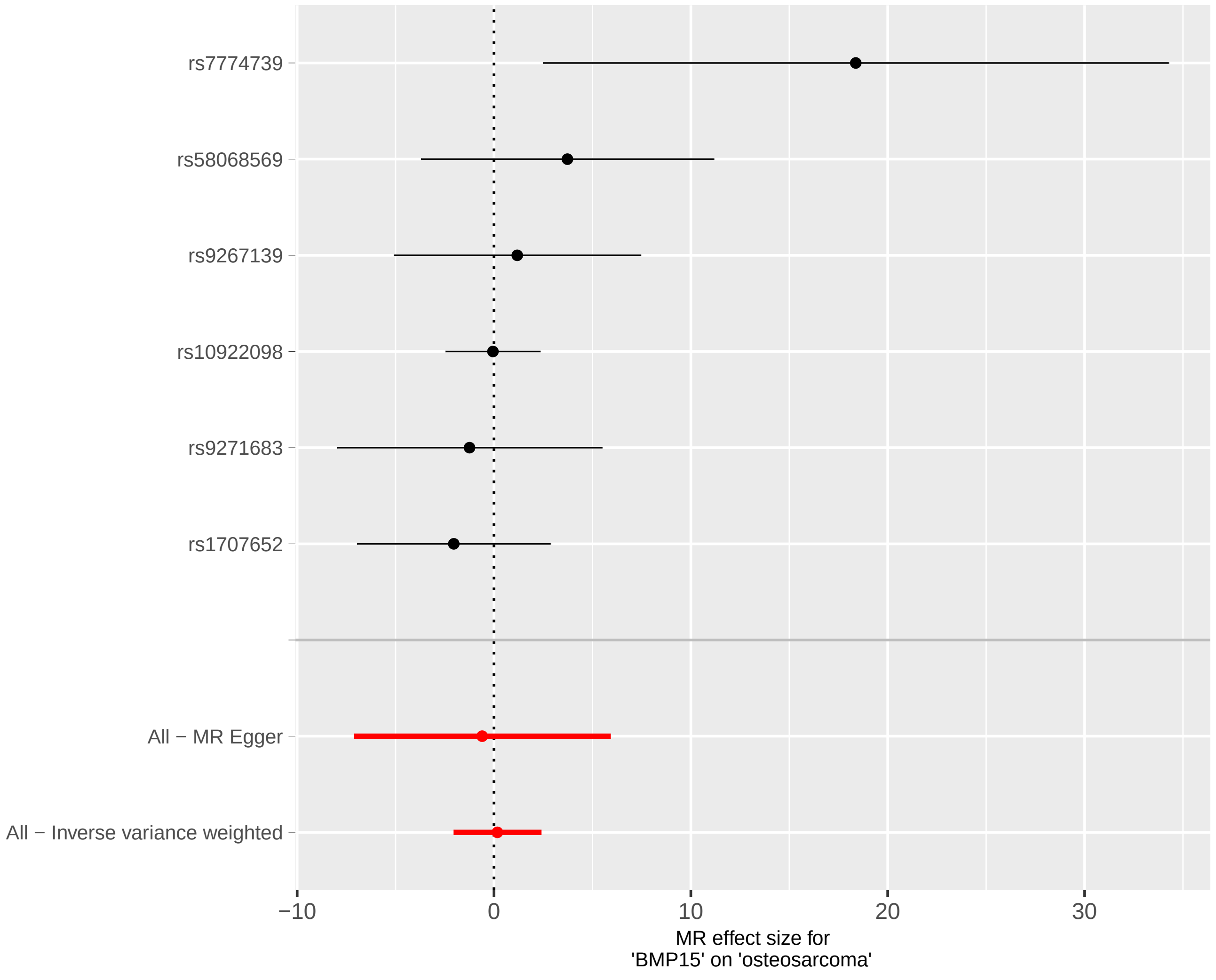

rs7774739
rs58068569
rs9267139
rs10922098
rs9271683
rs1707652
All − MR Egger
All − Inverse variance weighted
−10
0
10
20
30
MR effect size for
'BMP15' on 'osteosarcoma'

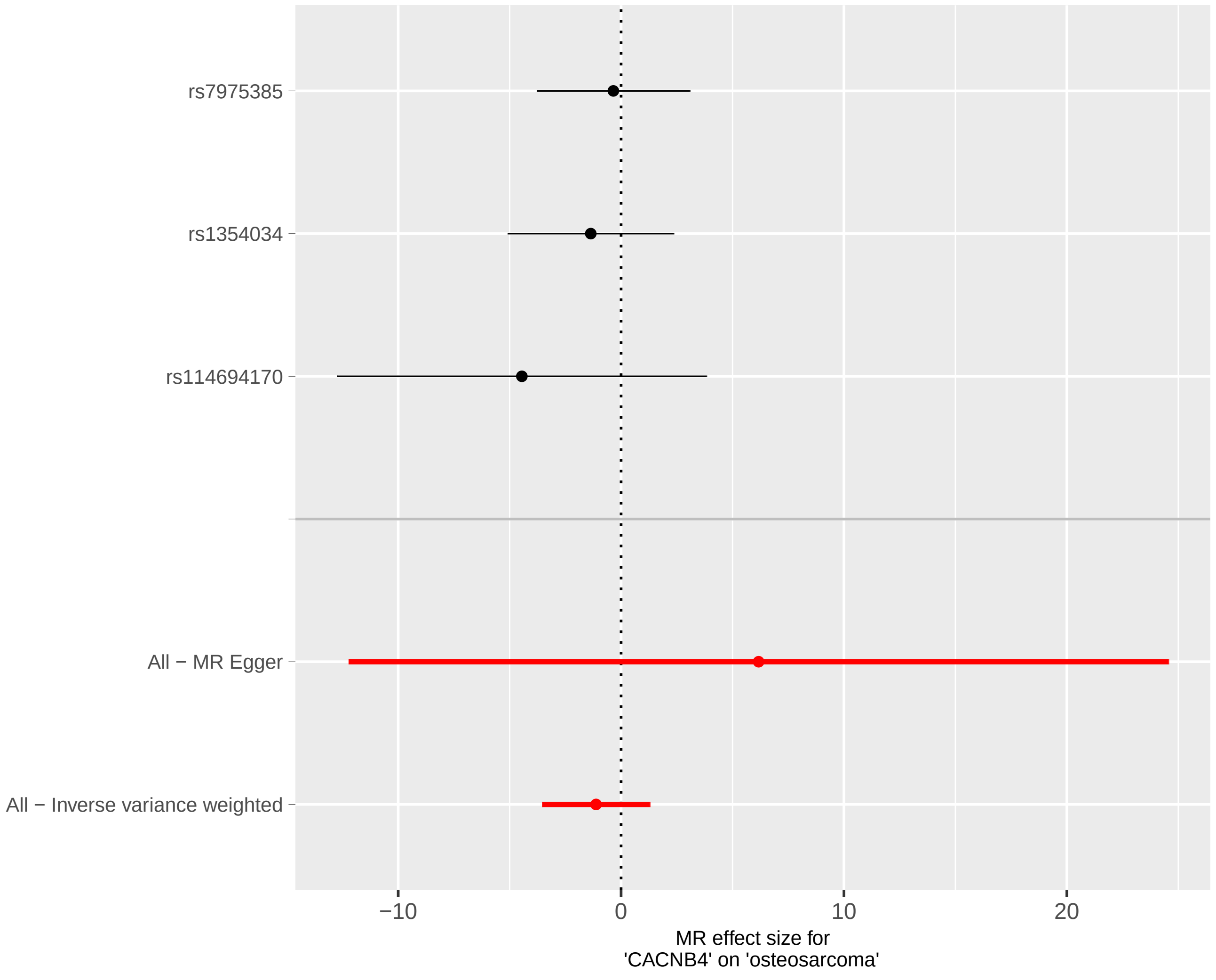
rs7975385
rs1354034
rs114694170
All − MR Egger
All − Inverse variance weighted
−10
0
10
20
MR effect size for
'CACNB4' on 'osteosarcoma'

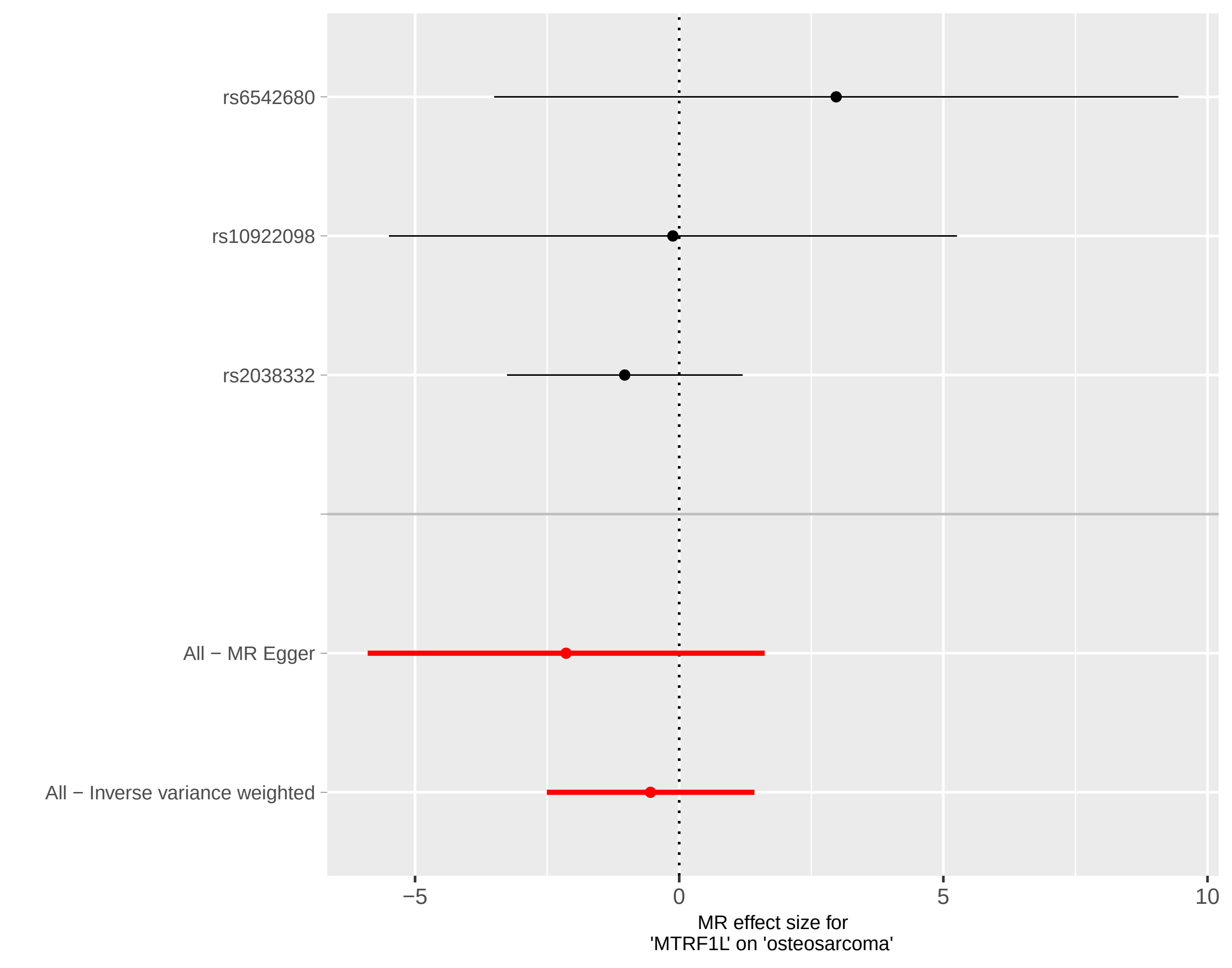

rs6542680
rs10922098
rs2038332
All − MR Egger
All − Inverse variance weighted
−5
0
5
10
MR effect size for
'MTRF1L' on 'osteosarcoma'

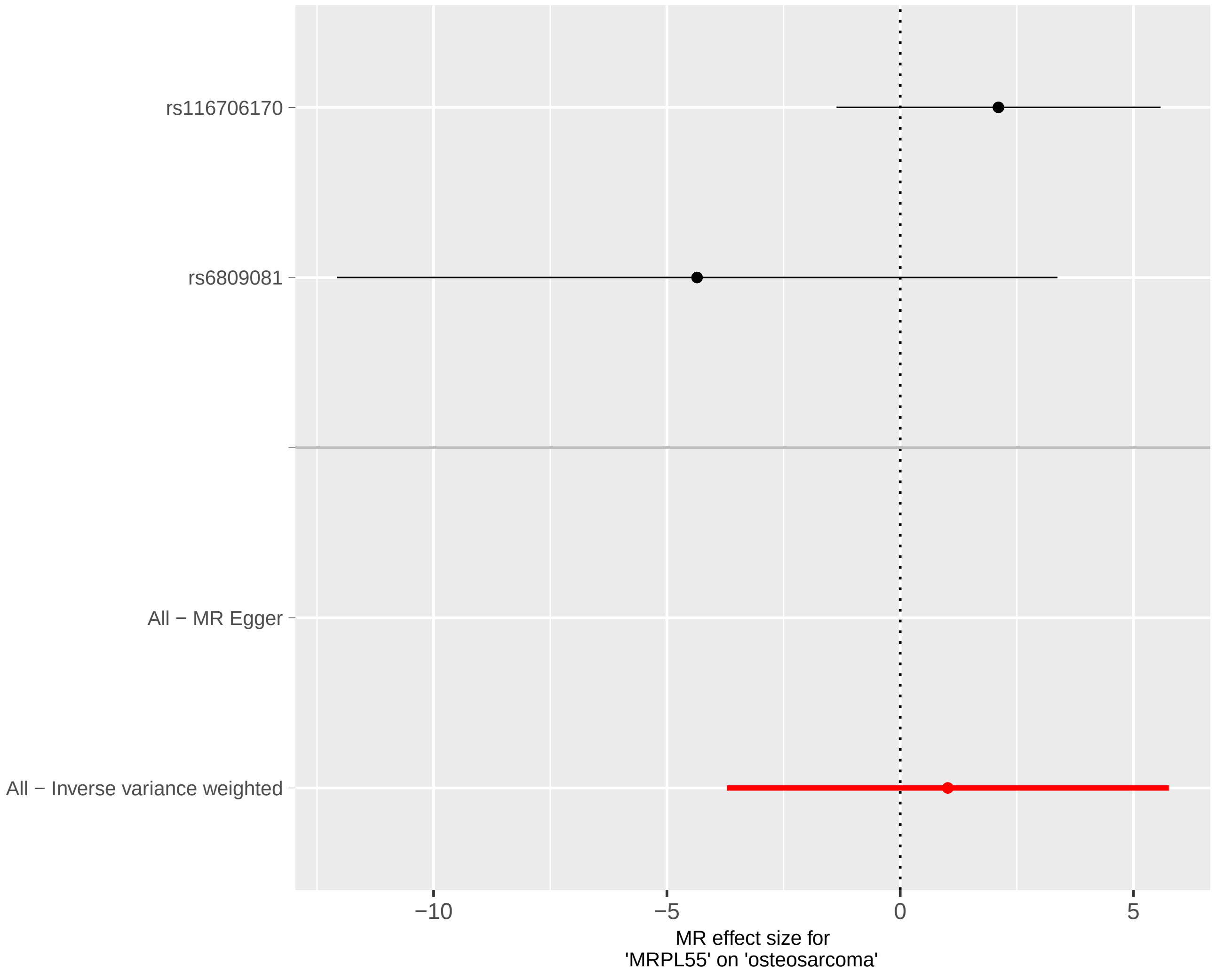

rs116706170
rs6809081
All − MR Egger
All − Inverse variance weighted
−10
−5
0
5
MR effect size for
'MRPL55' on 'osteosarcoma'

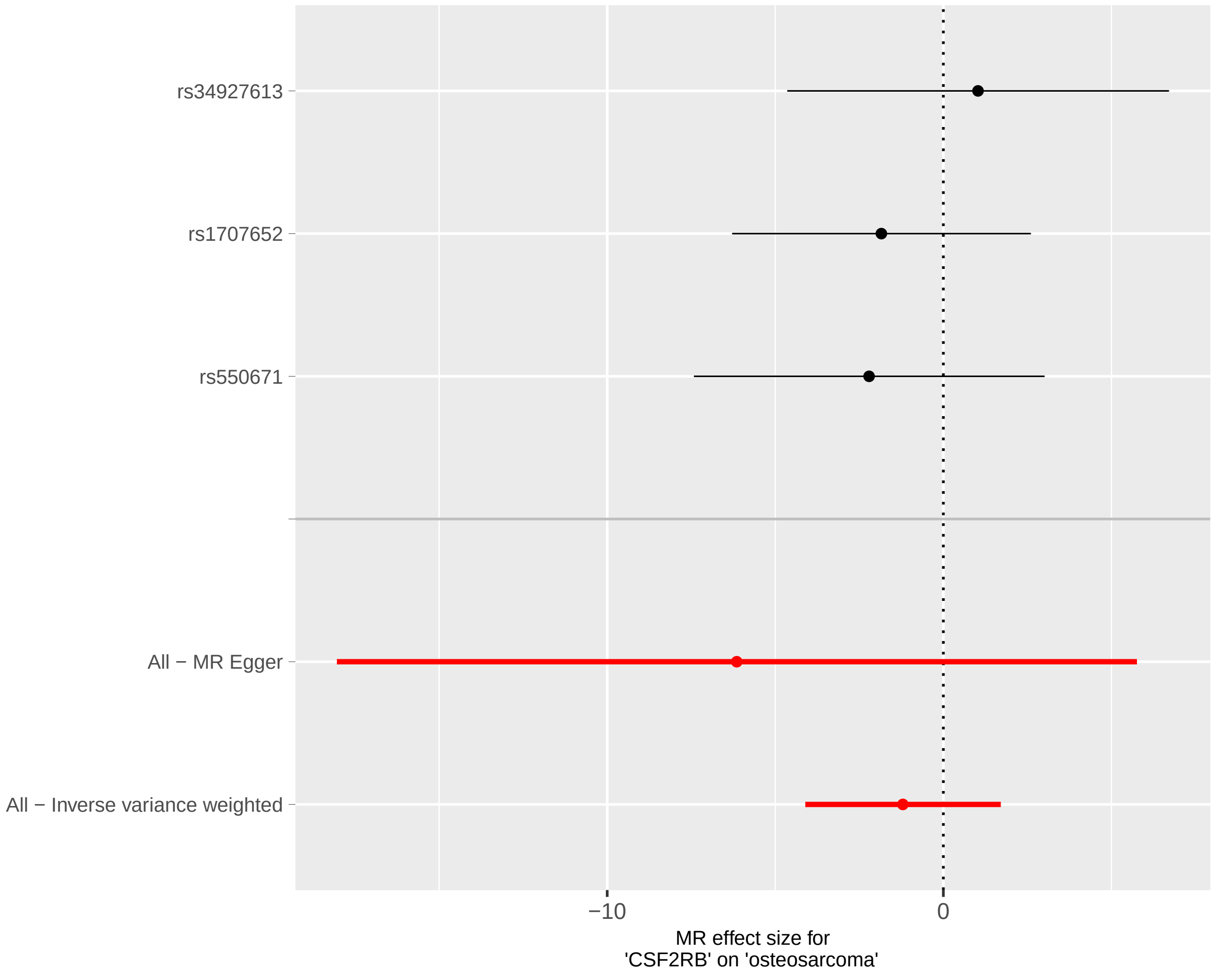

rs34927613
rs1707652
rs550671
All − MR Egger
All − Inverse variance weighted
−10
0
MR effect size for
'CSF2RB' on 'osteosarcoma'

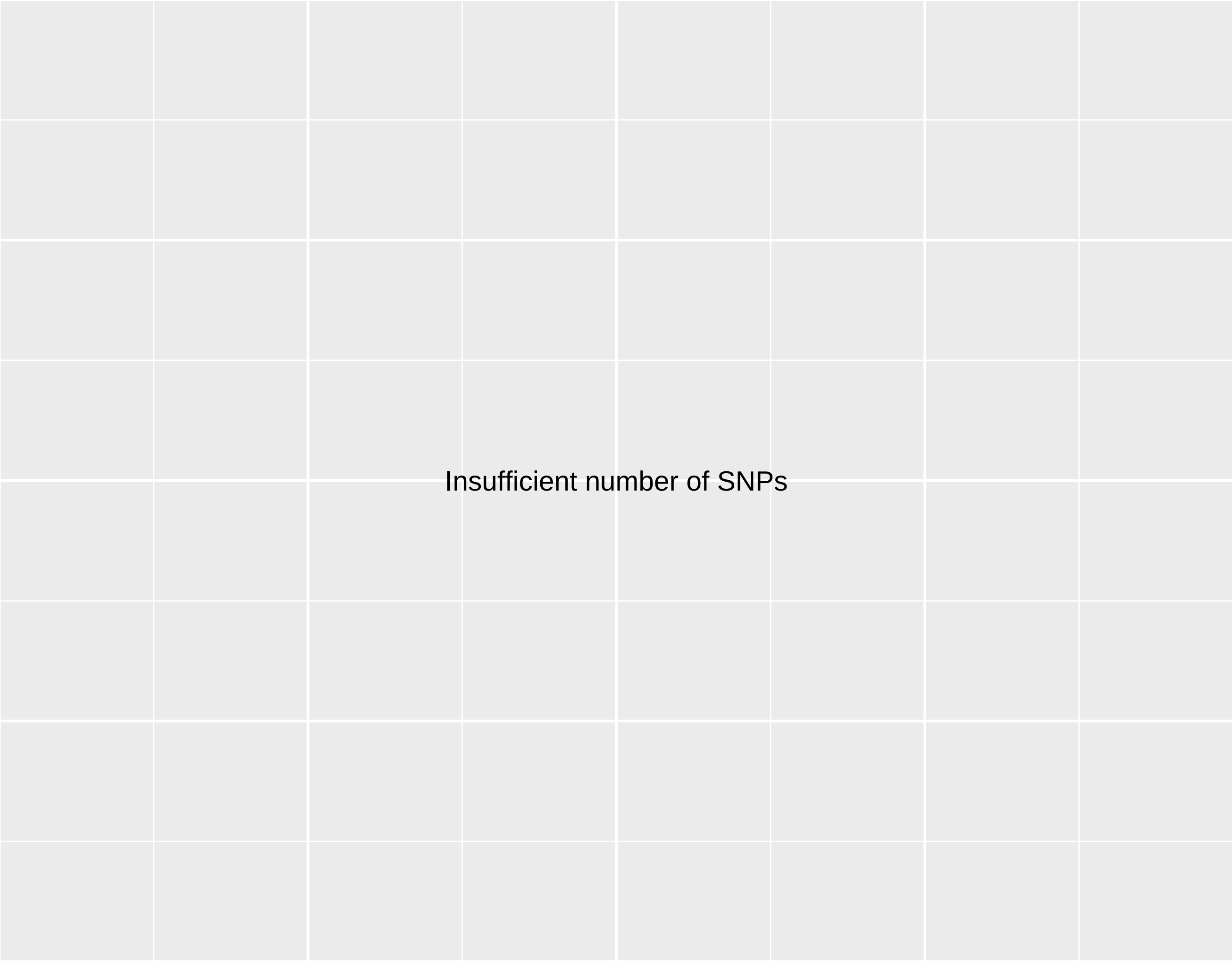
Insufficient number of SNPs

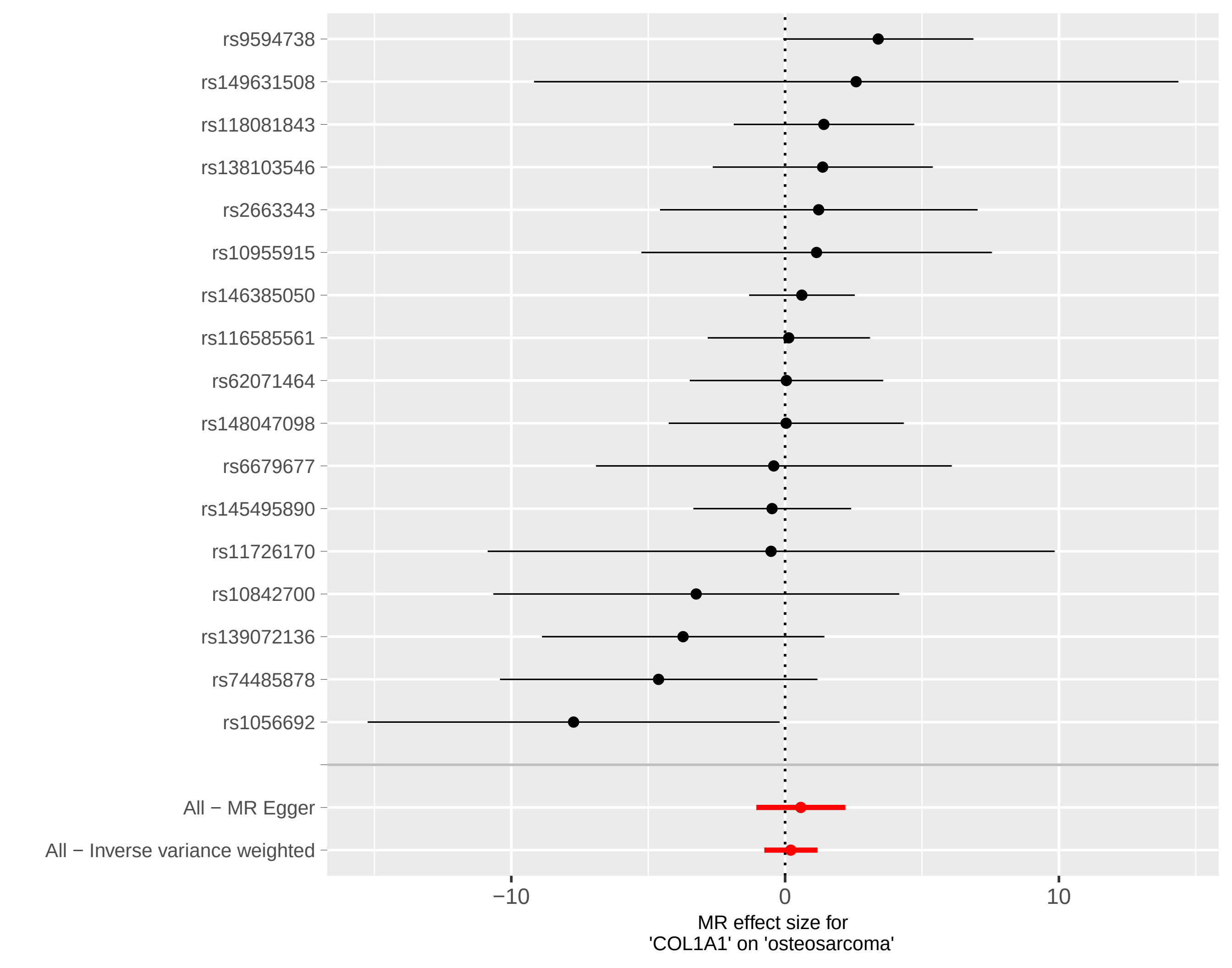

rs9594738
rs149631508
rs118081843
rs138103546
rs2663343
rs10955915
rs146385050
rs116585561
rs62071464
rs148047098
rs6679677
rs145495890
rs11726170
rs10842700
rs139072136
rs74485878
rs1056692
All − MR Egger
All − Inverse variance weighted
−10
0
10
MR effect size for
'COL1A1' on 'osteosarcoma'

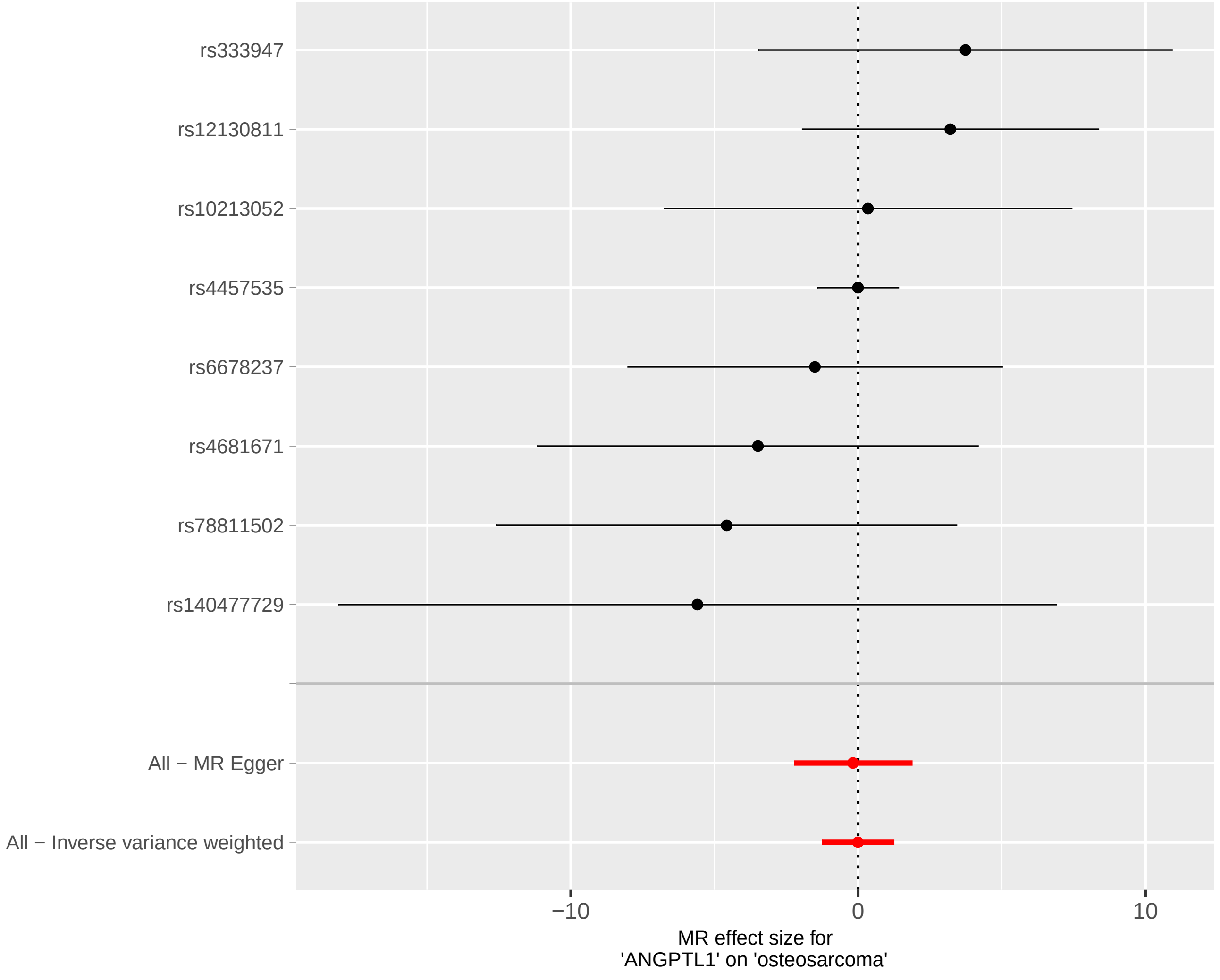

rs333947
rs12130811
rs10213052
rs4457535
rs6678237
rs4681671
rs78811502
rs140477729
All − MR Egger
All − Inverse variance weighted
−10
0
10
MR effect size for
'ANGPTL1' on 'osteosarcoma'

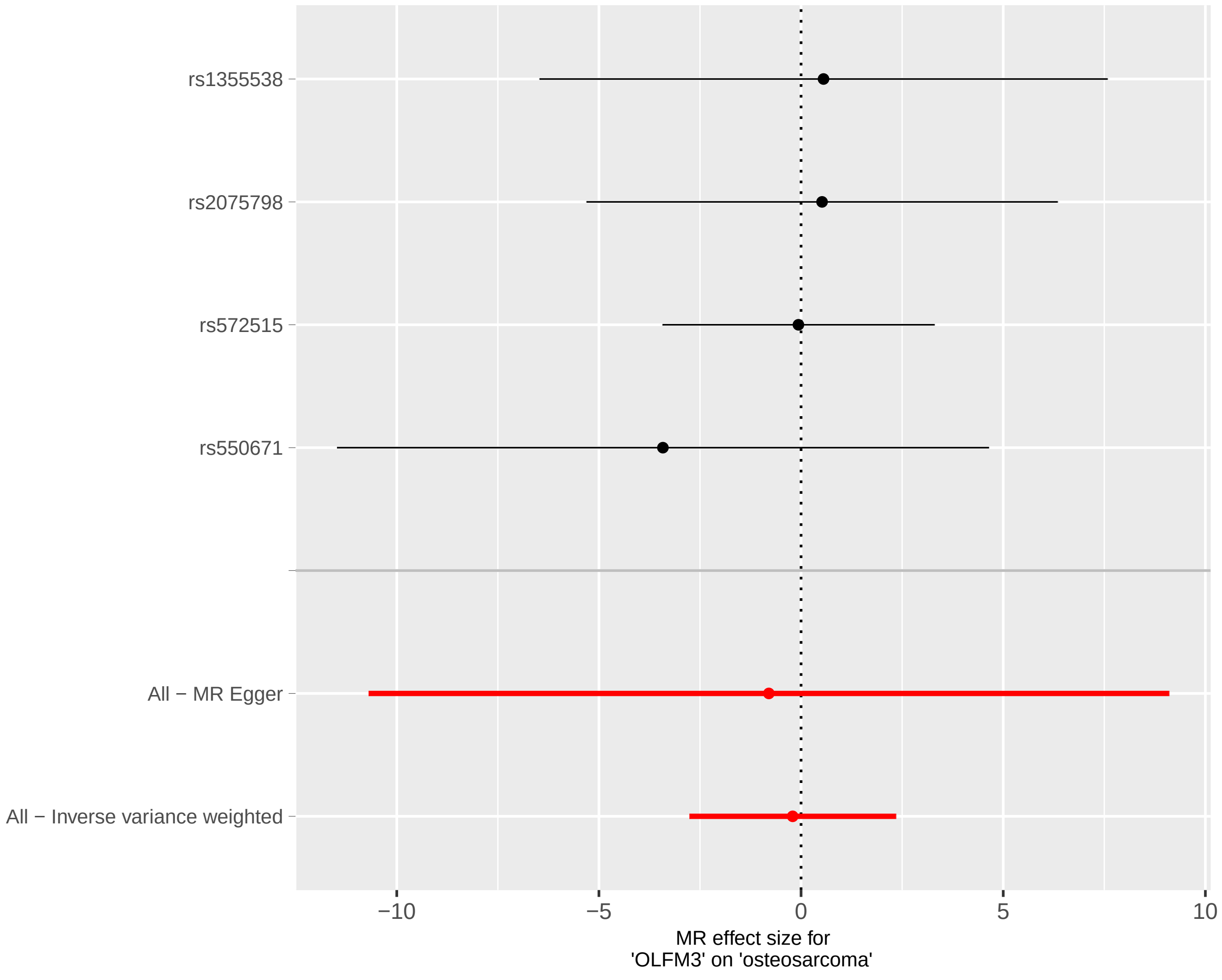

rs1355538
rs2075798
rs572515
rs550671
All − MR Egger
All − Inverse variance weighted
−10
−5
0
5
10
MR effect size for
'OLFM3' on 'osteosarcoma'

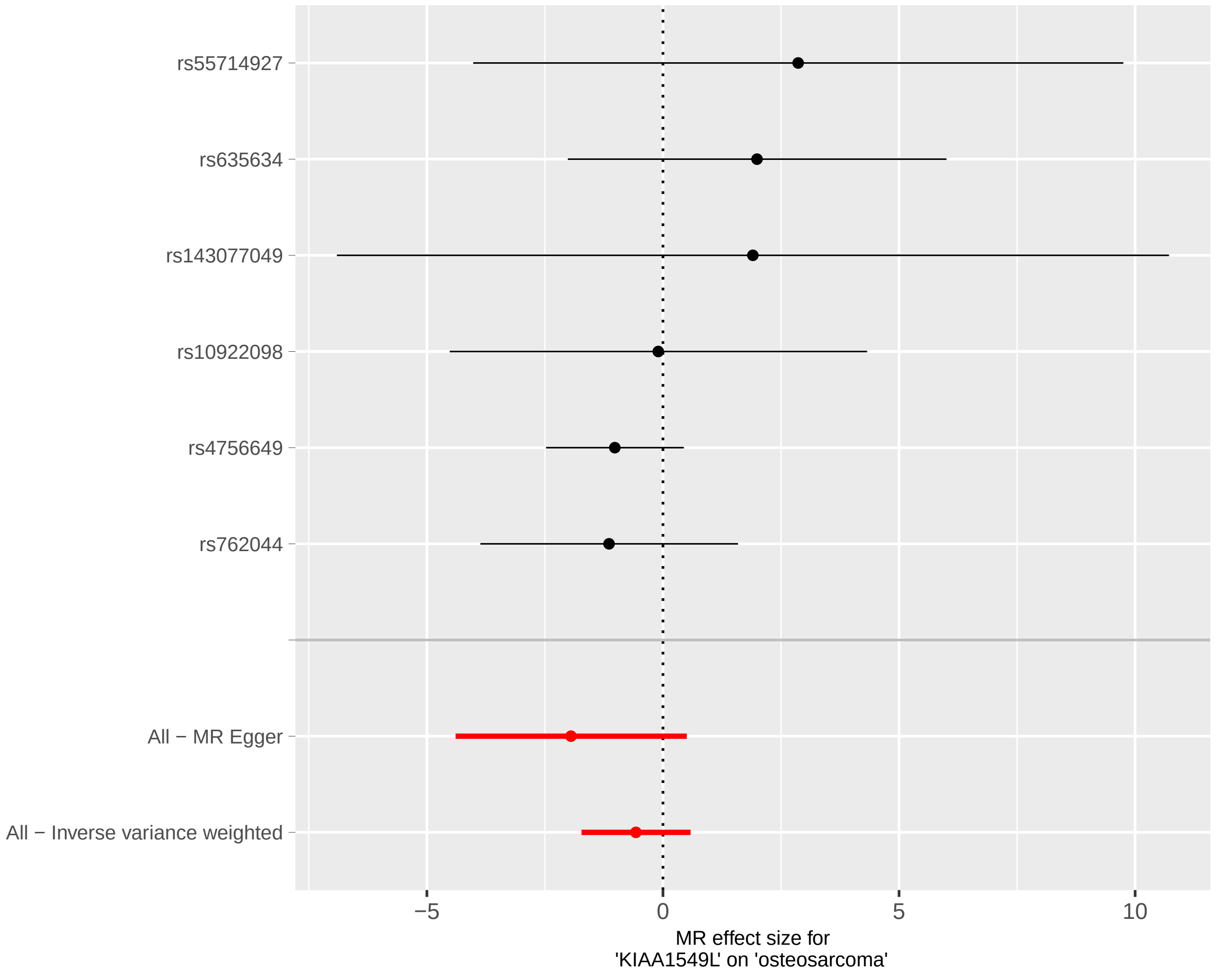
rs55714927
rs635634
rs143077049
rs10922098
rs4756649
rs762044
All − MR Egger
All − Inverse variance weighted
−5
0
5
10
MR effect size for
'KIAA1549L' on 'osteosarcoma'

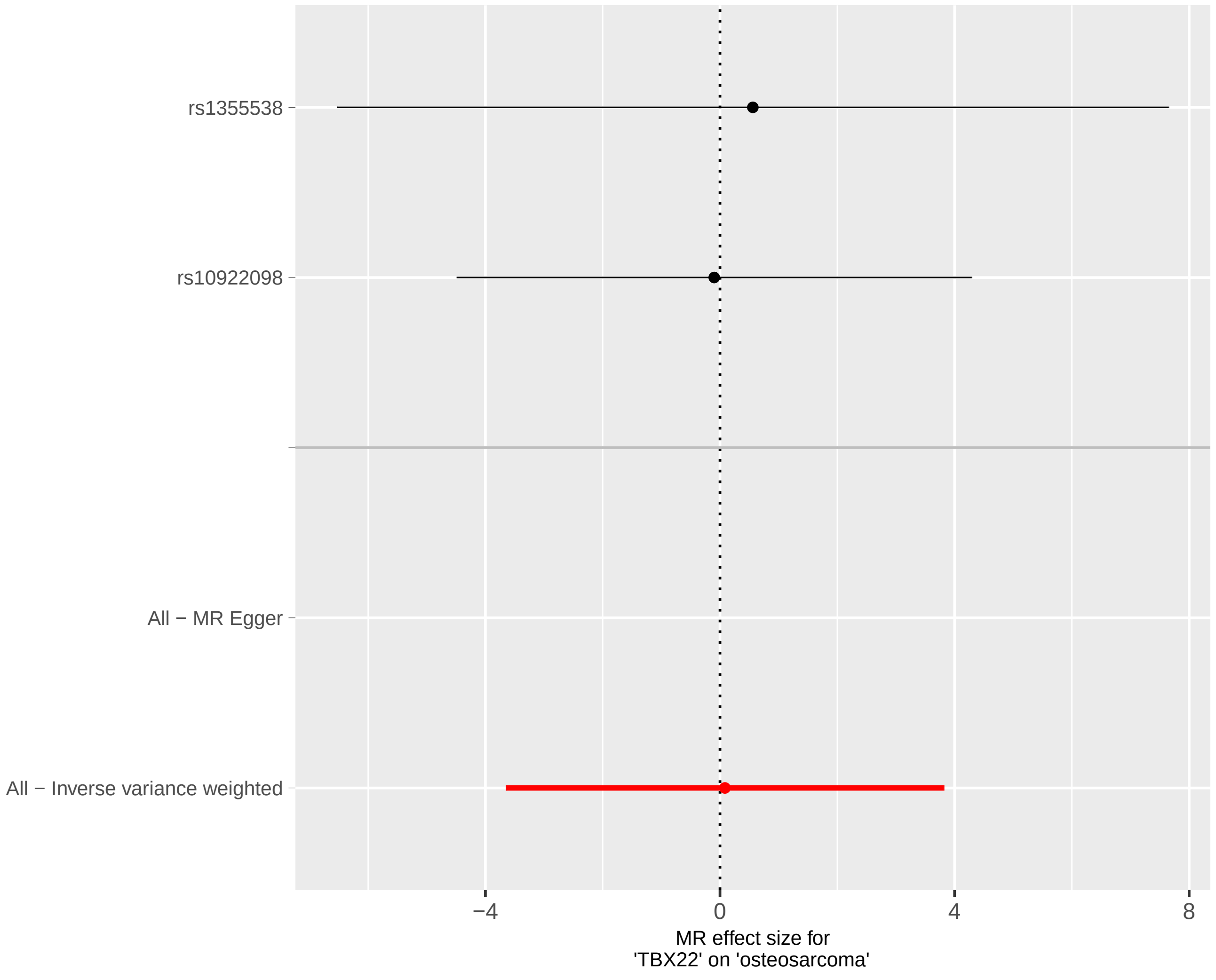
rs1355538
rs10922098
All − MR Egger
All − Inverse variance weighted
−4
0
4
8
MR effect size for
'TBX22' on 'osteosarcoma'

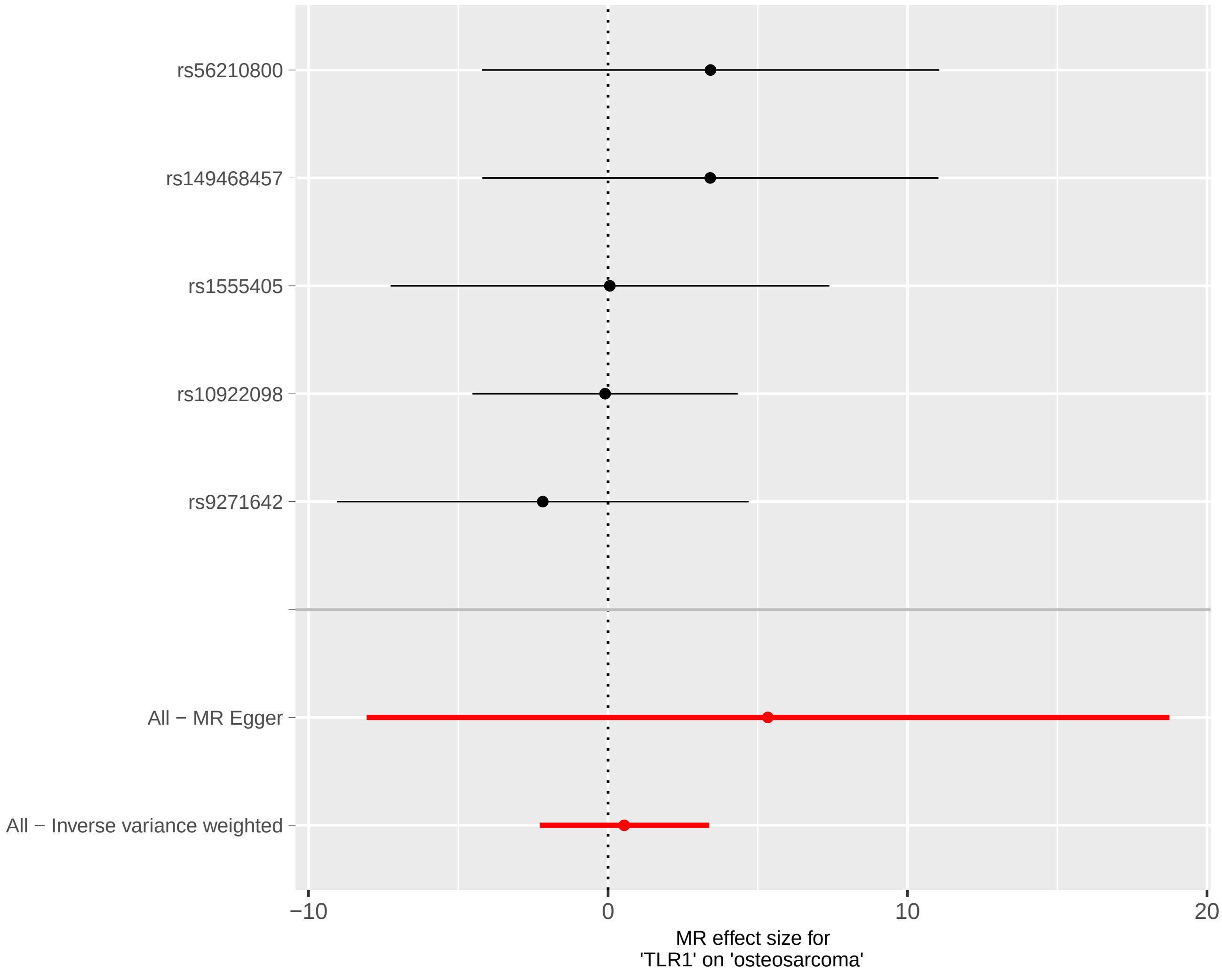

rs56210800
rs149468457
rs1555405
rs10922098
rs9271642
All − MR Egger
All − Inverse variance weighted
−10
0
10
20
MR effect size for
'TLR1' on 'osteosarcoma'

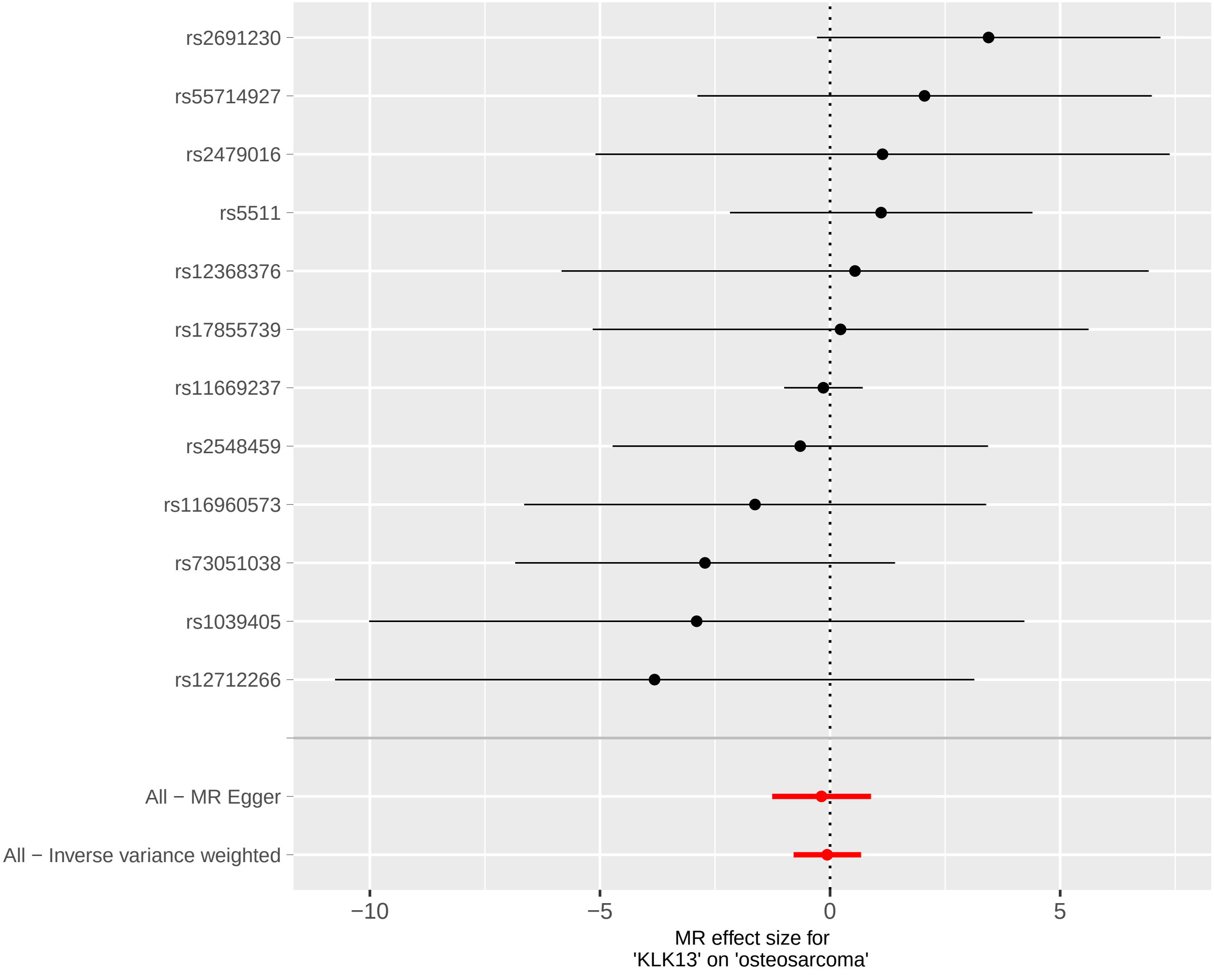

rs2691230
rs55714927
rs2479016
rs5511
rs12368376
rs17855739
rs11669237
rs2548459
rs116960573
rs73051038
rs1039405
rs12712266
All − MR Egger
All − Inverse variance weighted
−10
−5
0
5
MR effect size for
'KLK13' on 'osteosarcoma'

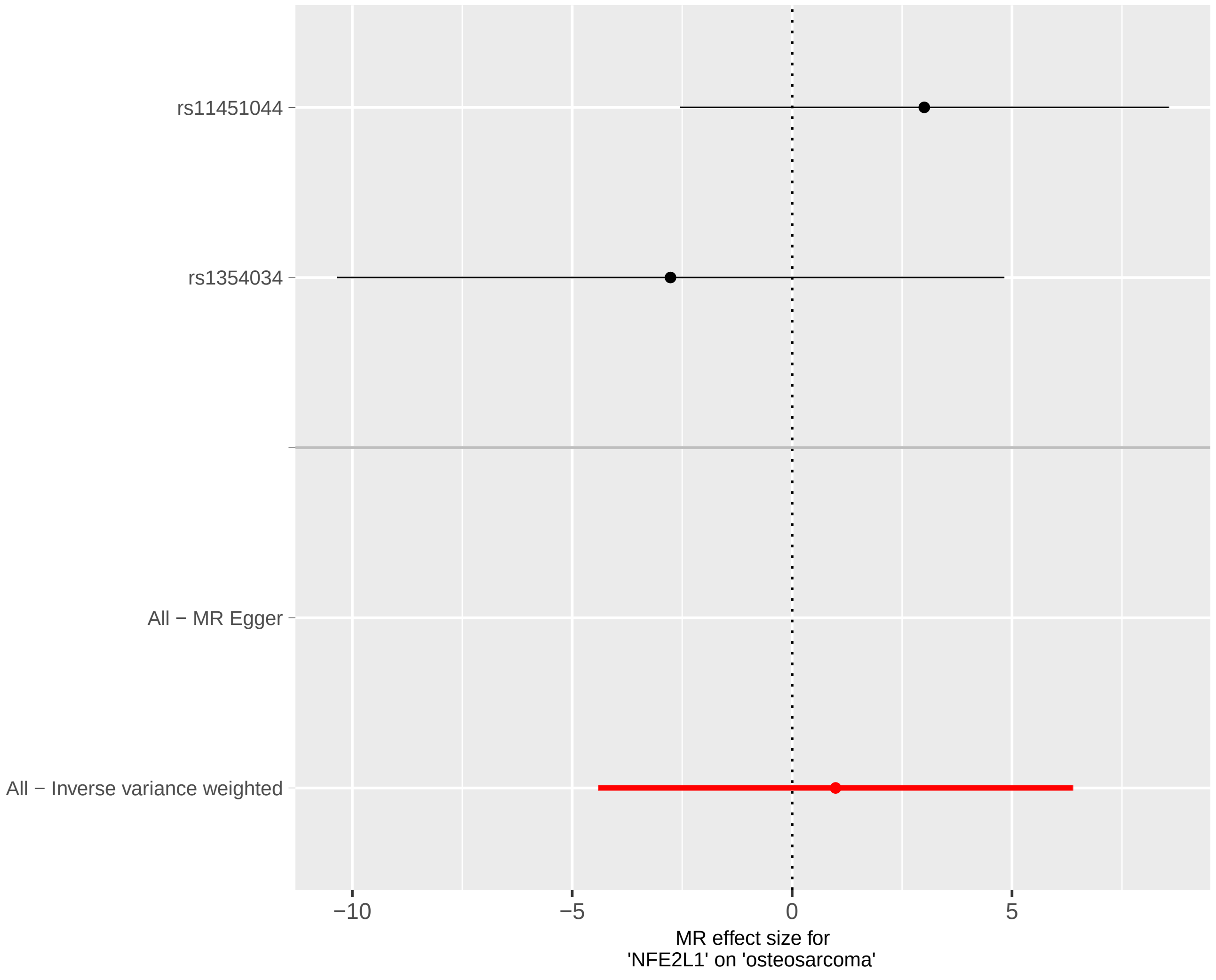

rs11451044
rs1354034
All − MR Egger
All − Inverse variance weighted
−10
−5
0
5
MR effect size for
'NFE2L1' on 'osteosarcoma'

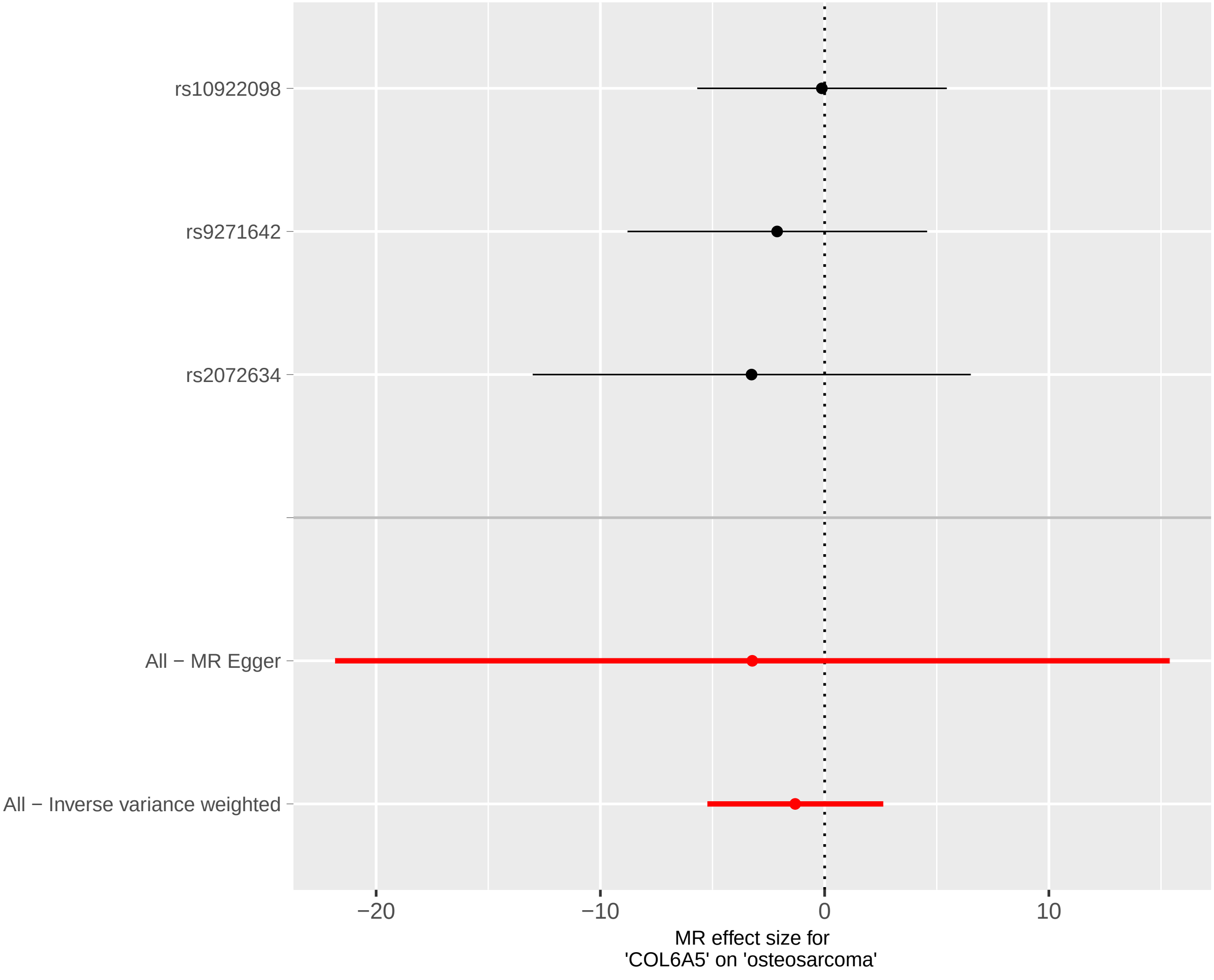

rs10922098
rs9271642
rs2072634
All − MR Egger
All − Inverse variance weighted
−20
−10
0
10
MR effect size for
'COL6A5' on 'osteosarcoma'

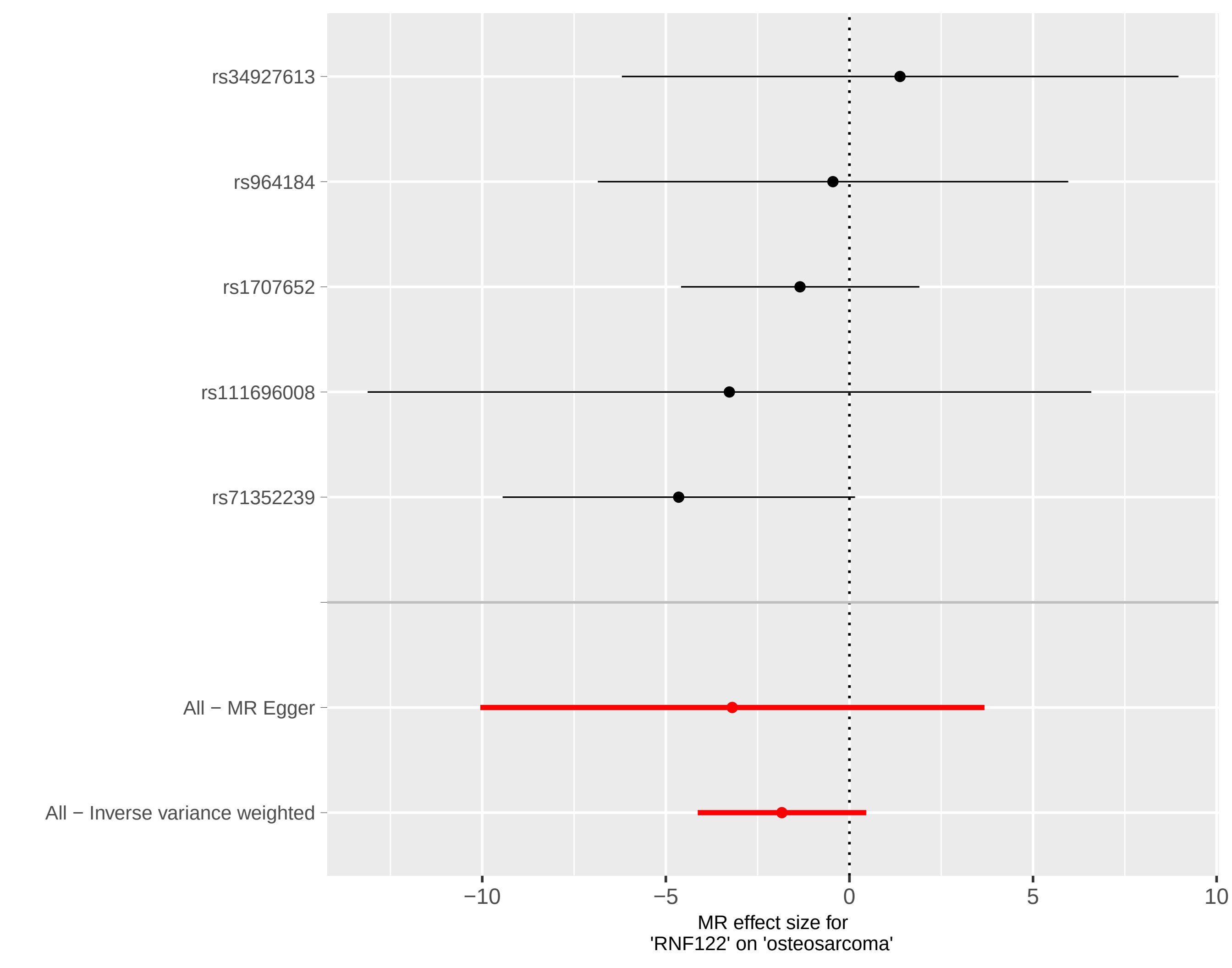

rs34927613
rs964184
rs1707652
rs111696008
rs71352239
All − MR Egger
All − Inverse variance weighted
−10
−5
0
5
10
MR effect size for
'RNF122' on 'osteosarcoma'

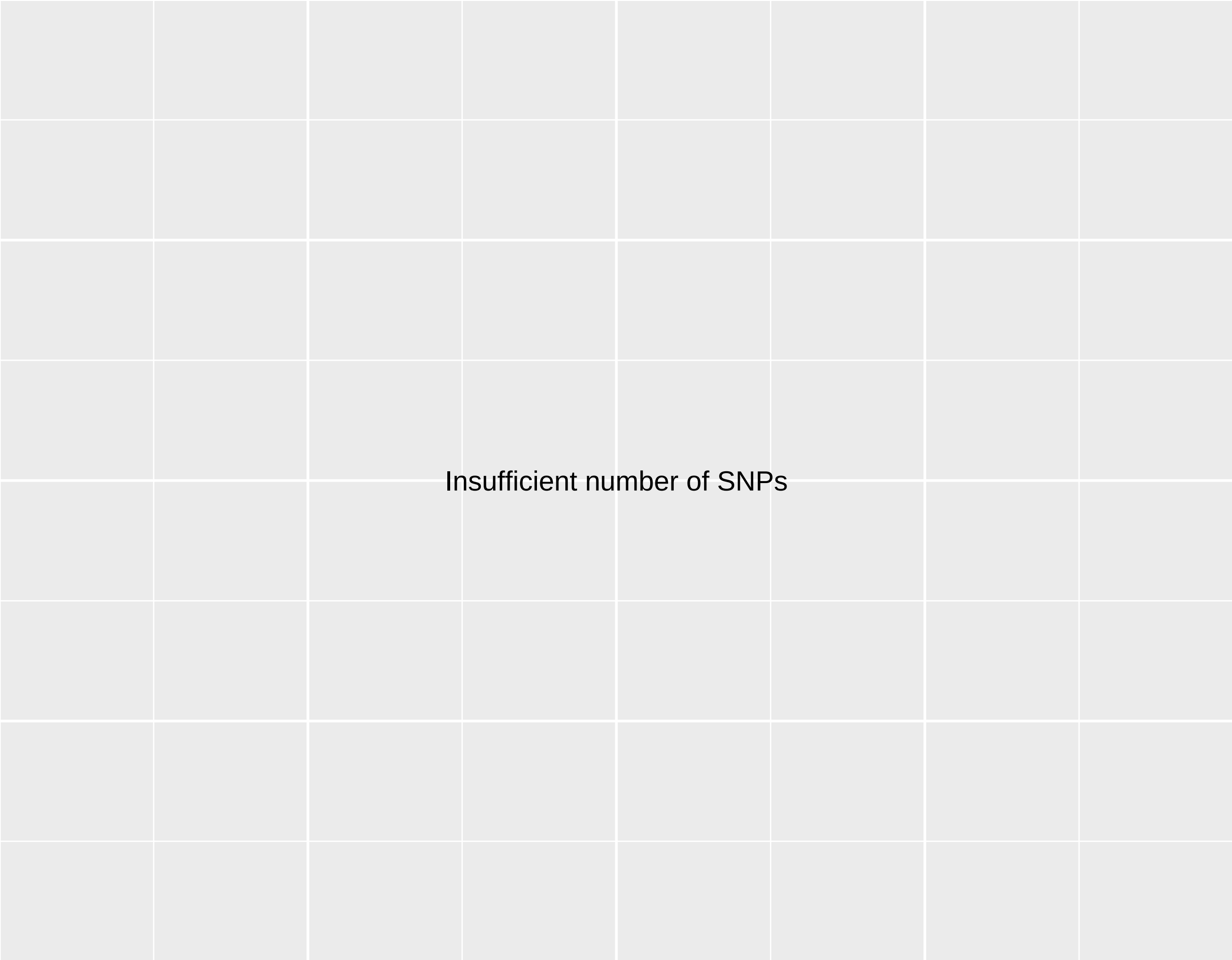
Insufficient number of SNPs

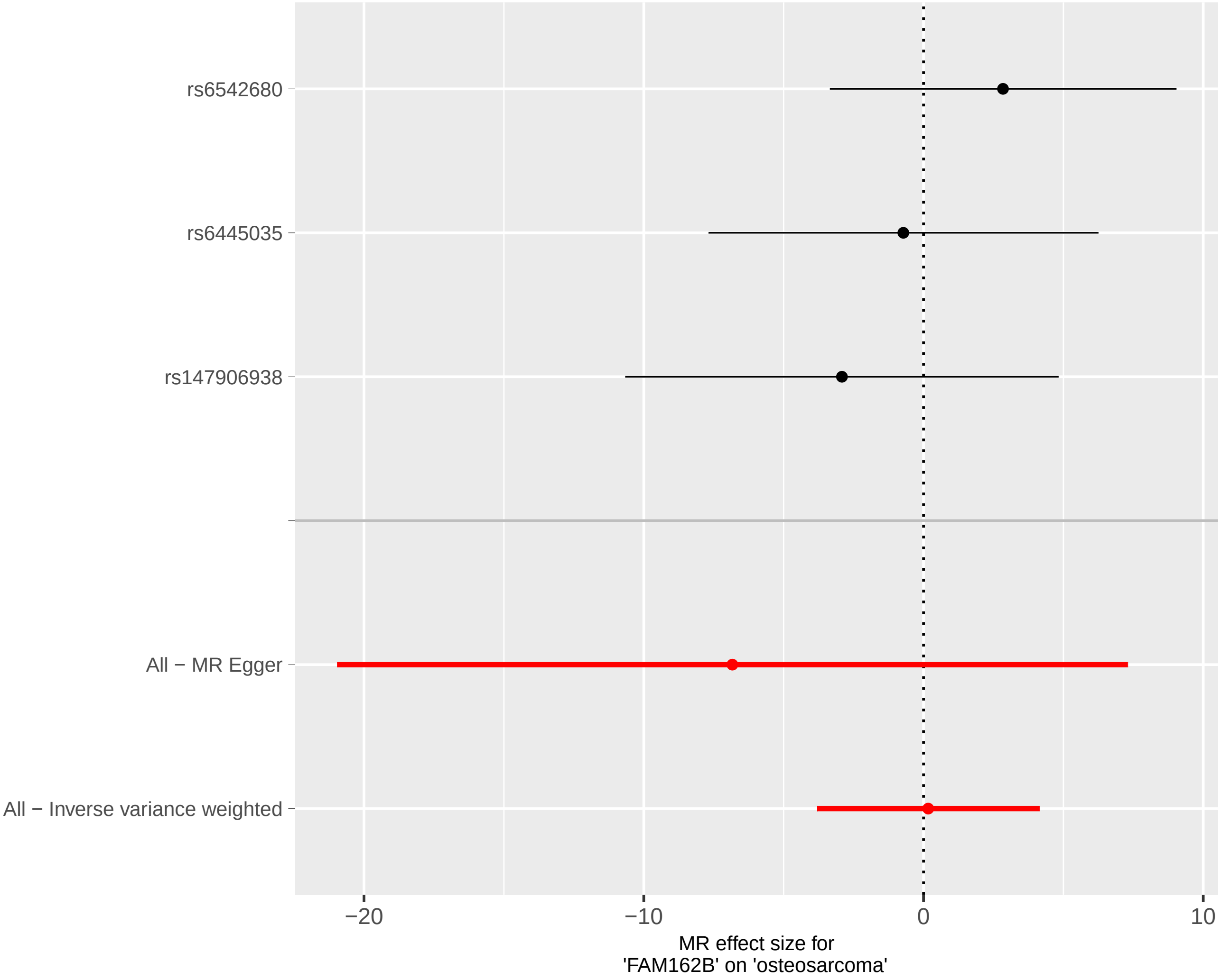
rs6542680
rs6445035
rs147906938
All − MR Egger
All − Inverse variance weighted
−20
−10
0
10
MR effect size for
'FAM162B' on 'osteosarcoma'

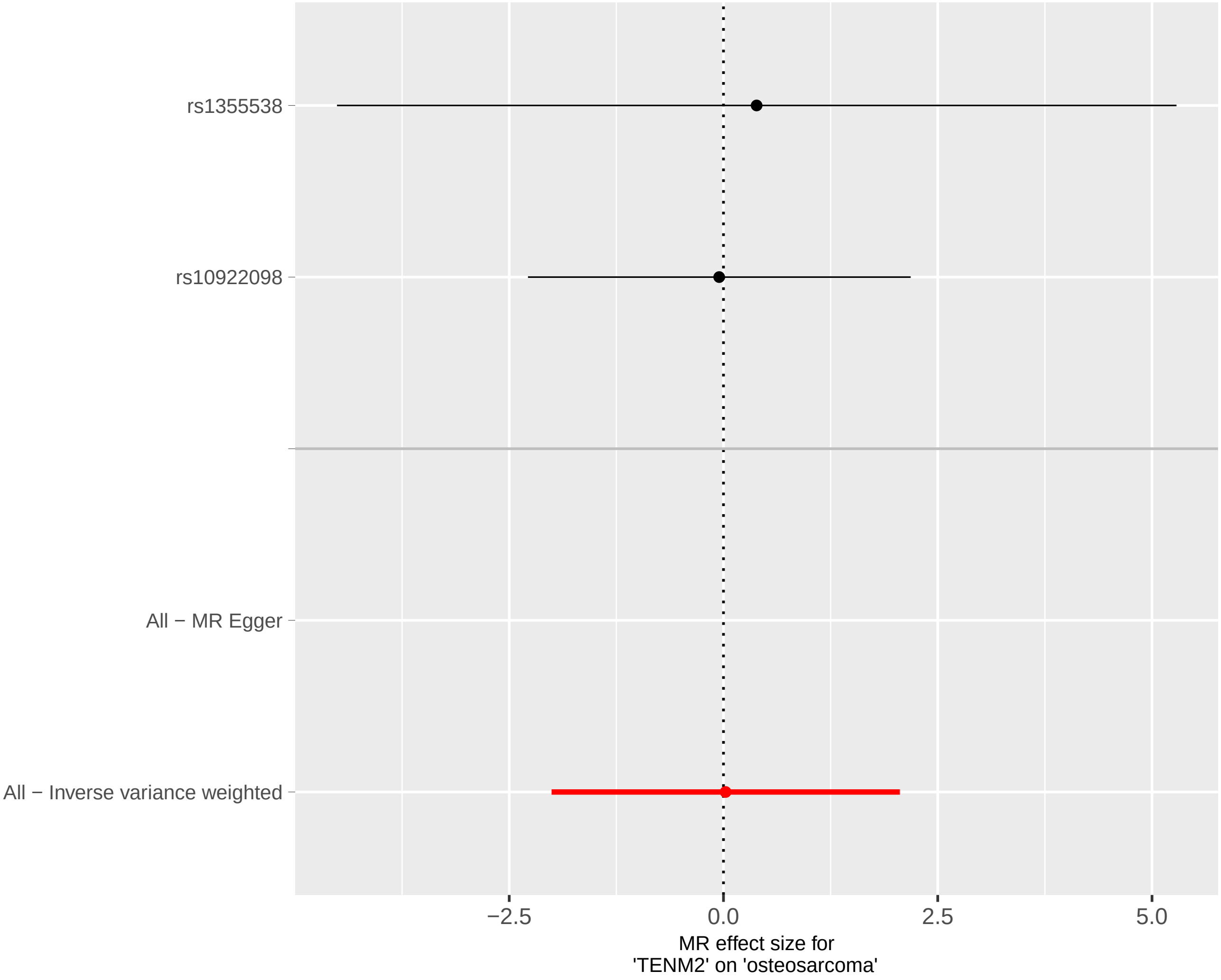
rs1355538
rs10922098
All − MR Egger
All − Inverse variance weighted
−2.5
0.0
2.5
5.0
MR effect size for
'TENM2' on 'osteosarcoma'

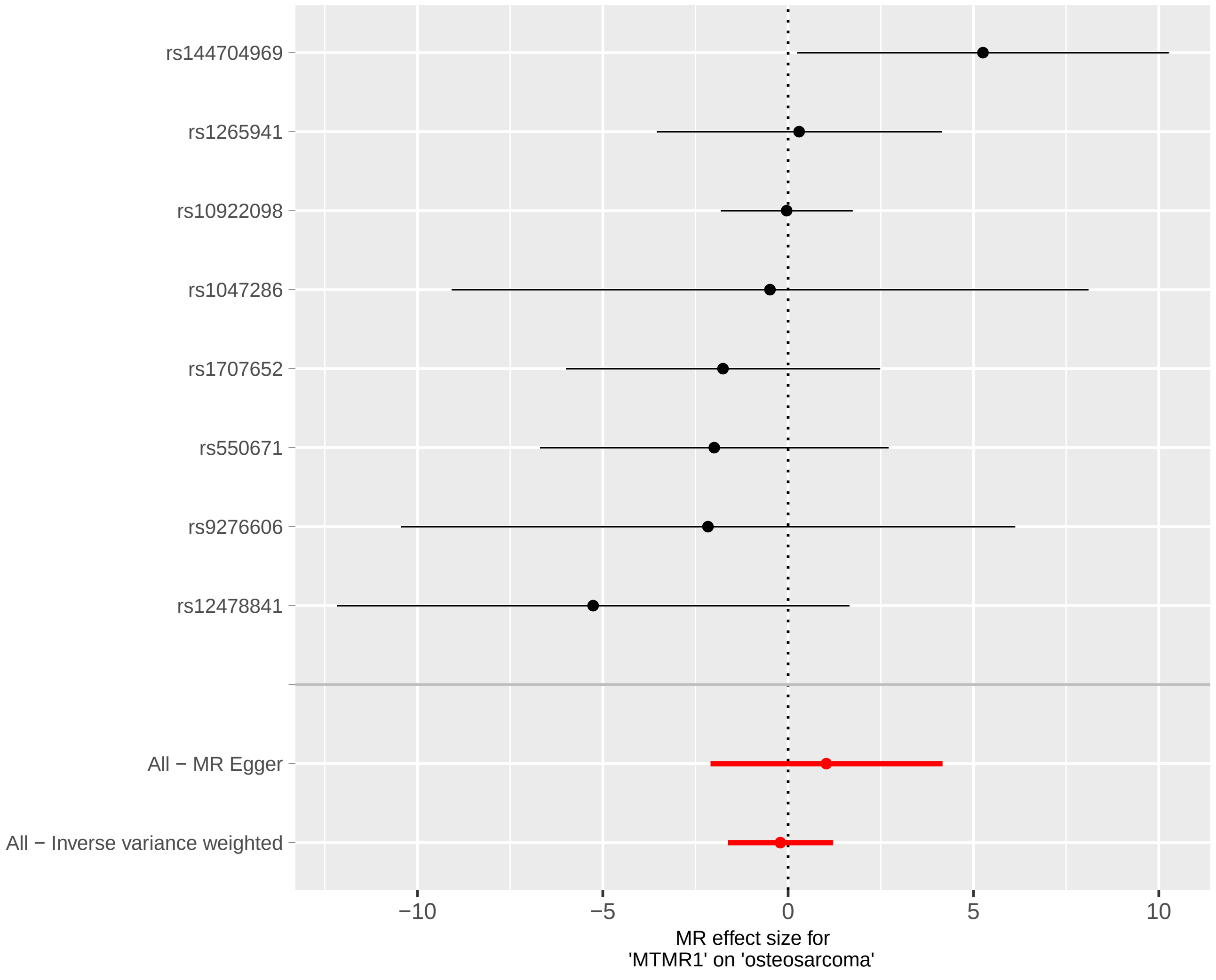

rs144704969
rs1265941
rs10922098
rs1047286
rs1707652
rs550671
rs9276606
rs12478841
All − MR Egger
All − Inverse variance weighted
−10
−5
0
5
10
MR effect size for
'MTMR1' on 'osteosarcoma'

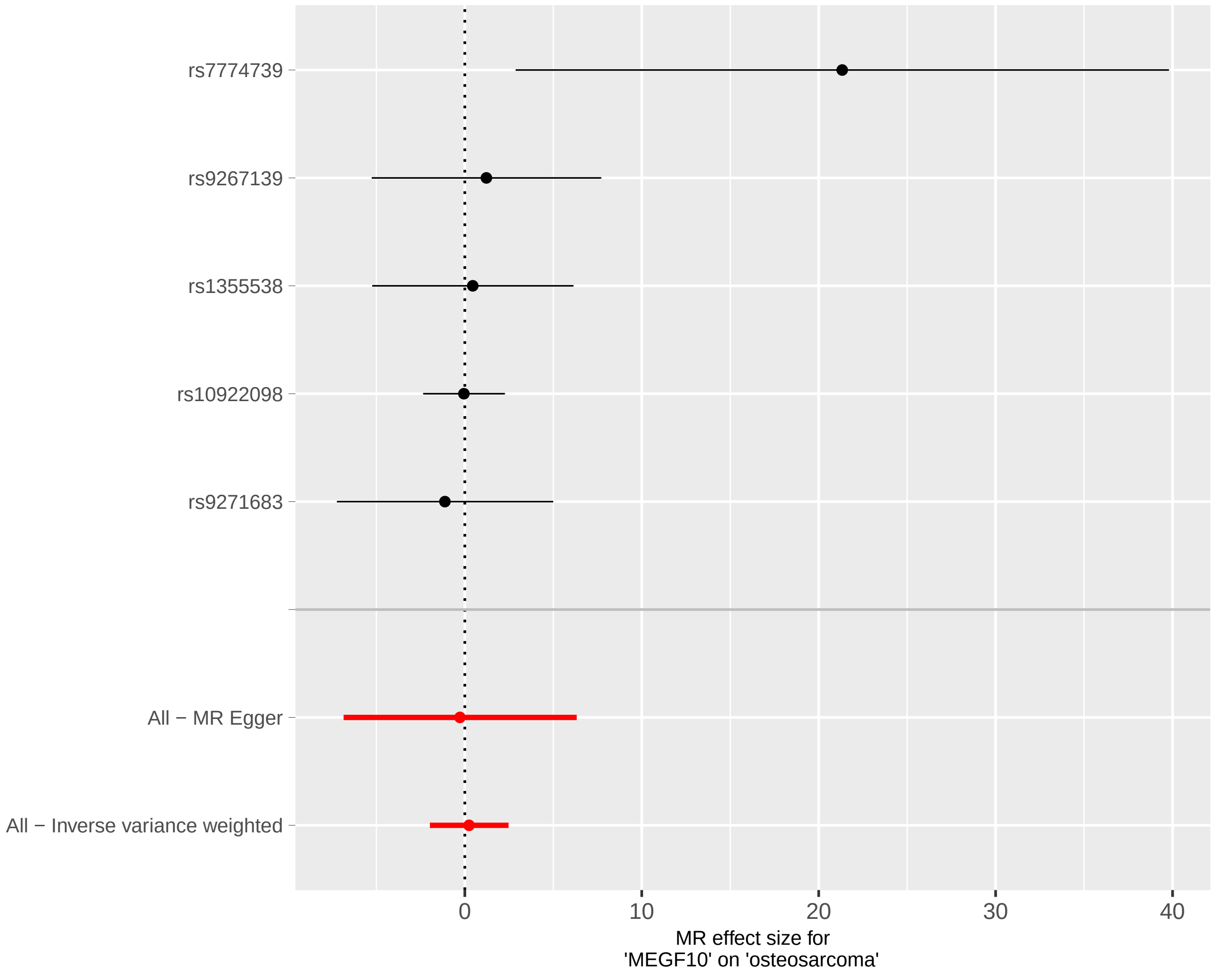

rs7774739
rs9267139
rs1355538
rs10922098
rs9271683
All − MR Egger
All − Inverse variance weighted
0
10
20
30
40
MR effect size for
'MEGF10' on 'osteosarcoma'

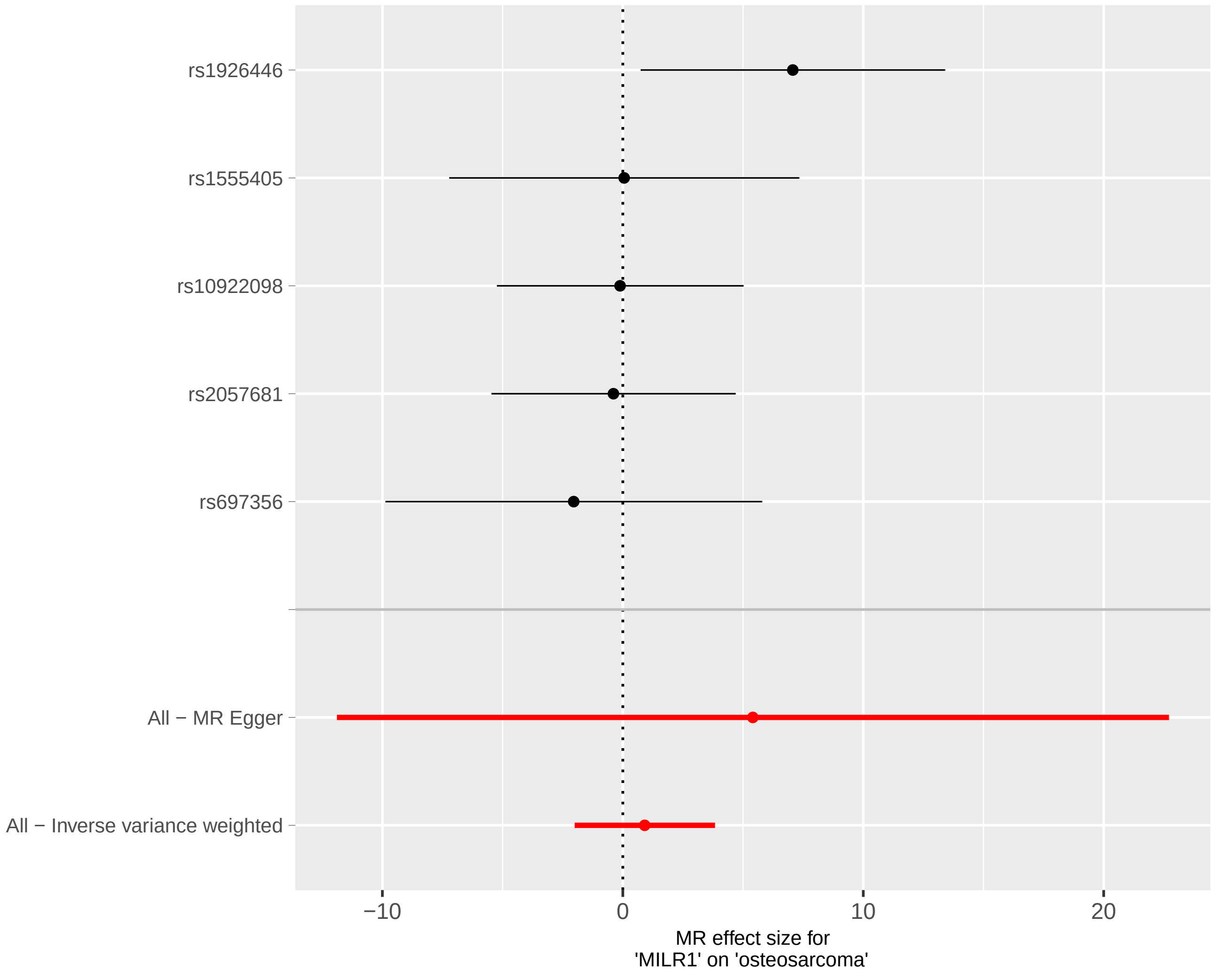

rs1926446
rs1555405
rs10922098
rs2057681
rs697356
All − MR Egger
All − Inverse variance weighted
−10
0
10
20
MR effect size for
'MILR1' on 'osteosarcoma'

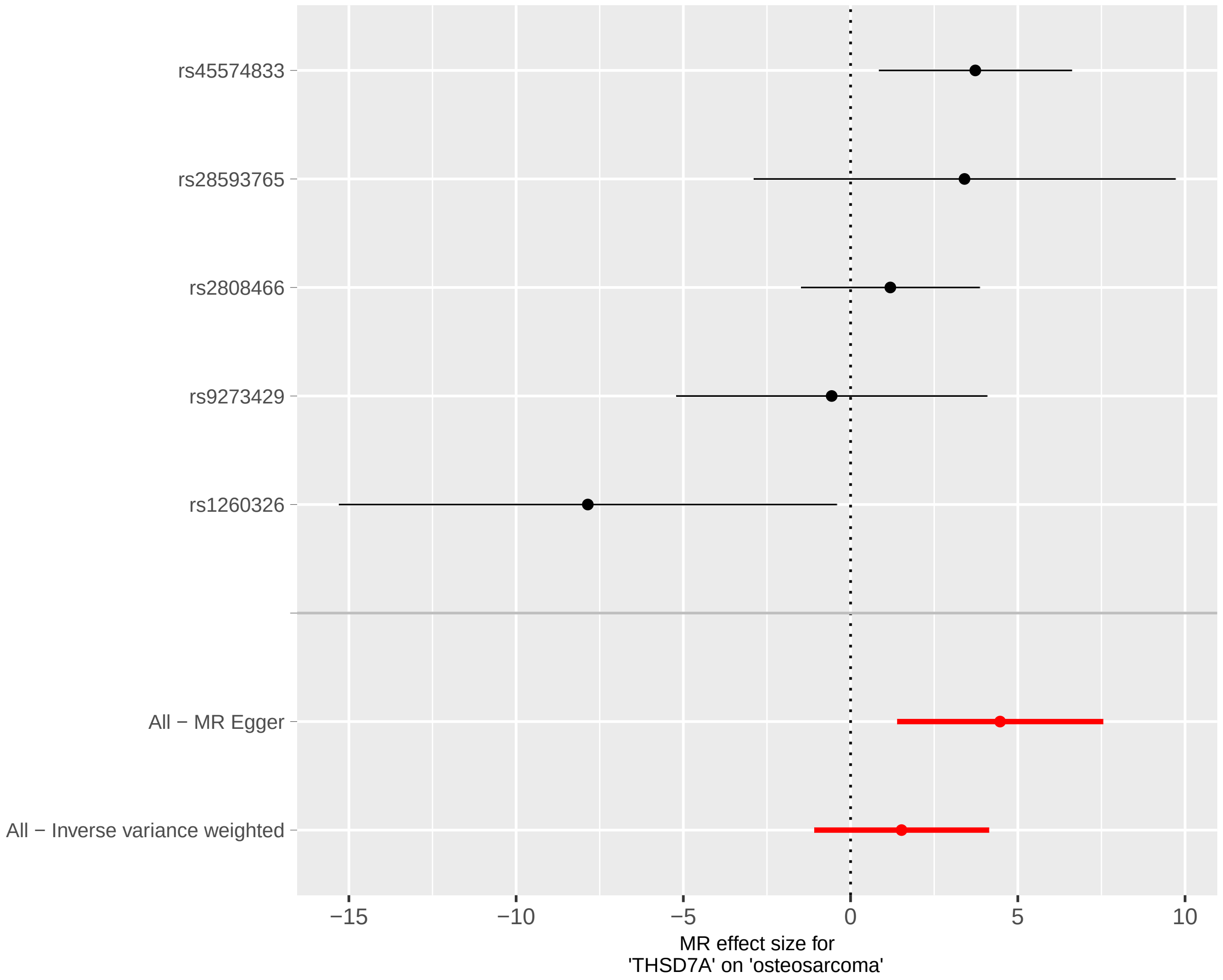
rs45574833
rs28593765
rs2808466
rs9273429
rs1260326
All − MR Egger
All − Inverse variance weighted
−15
−10
−5
0
5
10
MR effect size for
'THSD7A' on 'osteosarcoma'

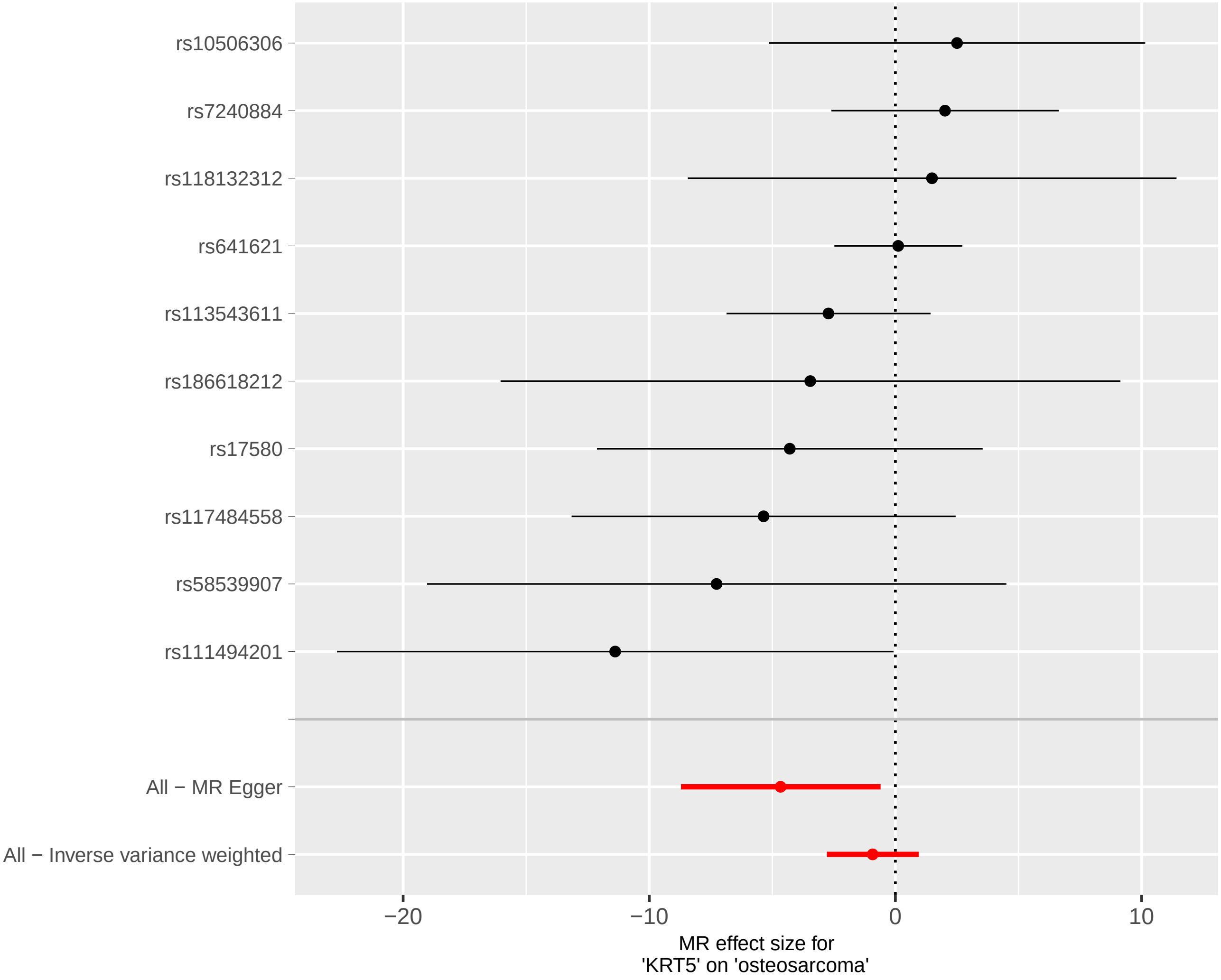

rs10506306
rs7240884
rs118132312
rs641621
rs113543611
rs186618212
rs17580
rs117484558
rs58539907
rs111494201
All − MR Egger
All − Inverse variance weighted
−20
−10
0
10
MR effect size for
'KRT5' on 'osteosarcoma'

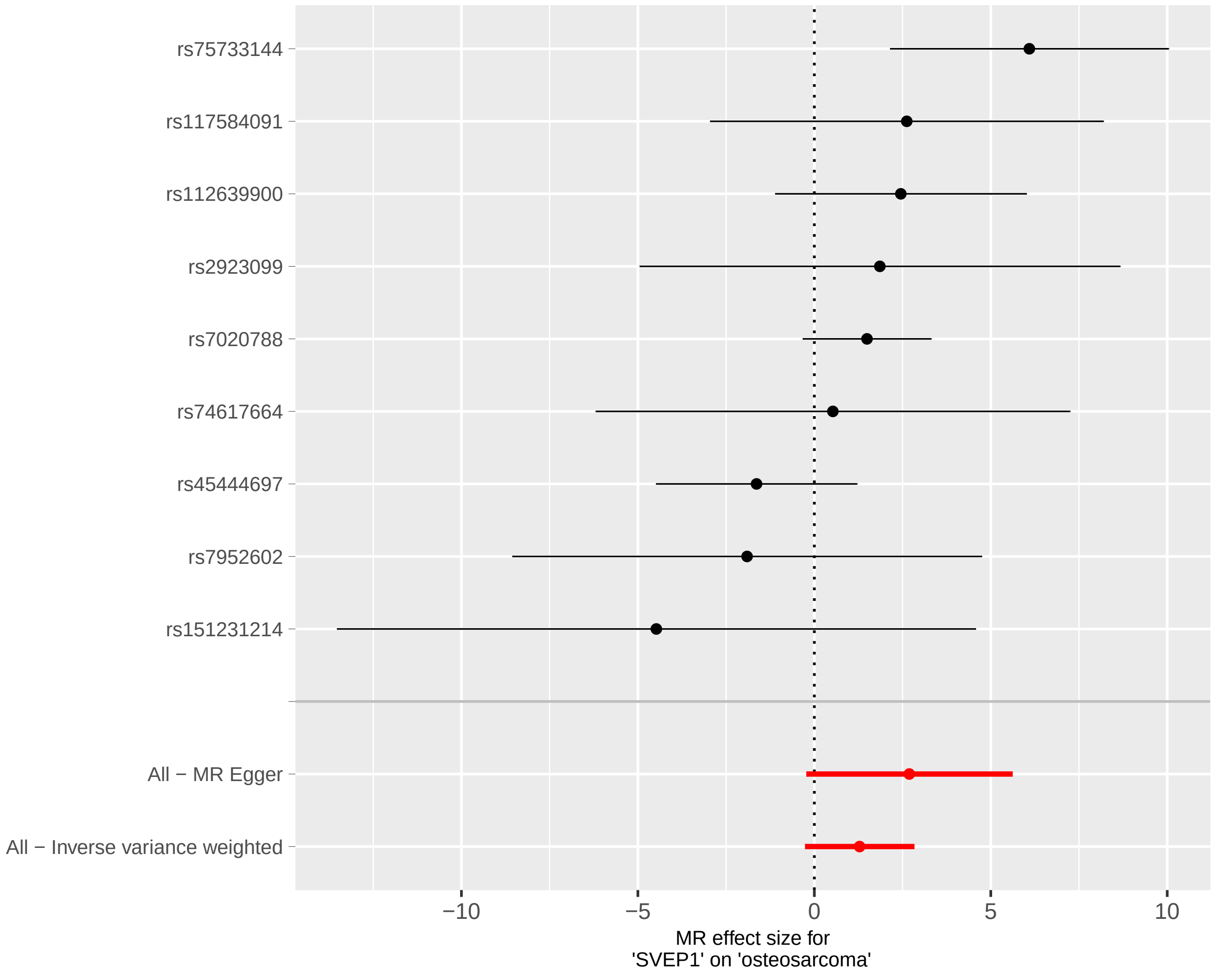

rs75733144
rs117584091
rs112639900
rs2923099
rs7020788
rs74617664
rs45444697
rs7952602
rs151231214
All − MR Egger
All − Inverse variance weighted
−10
−5
0
5
10
MR effect size for
'SVEP1' on 'osteosarcoma'

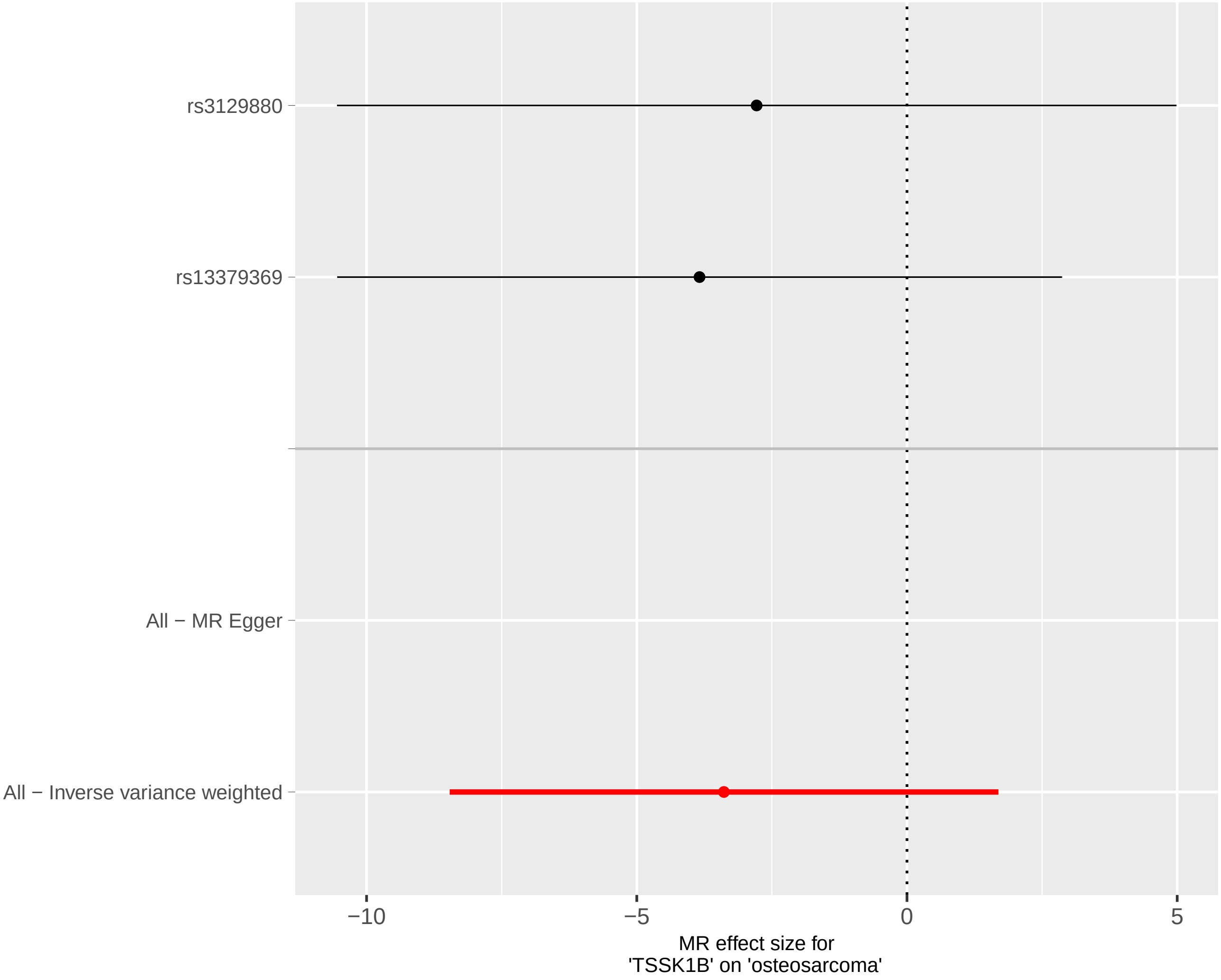

rs3129880
rs13379369
All − MR Egger
All − Inverse variance weighted
−10
−5
0
5
MR effect size for
'TSSK1B' on 'osteosarcoma'

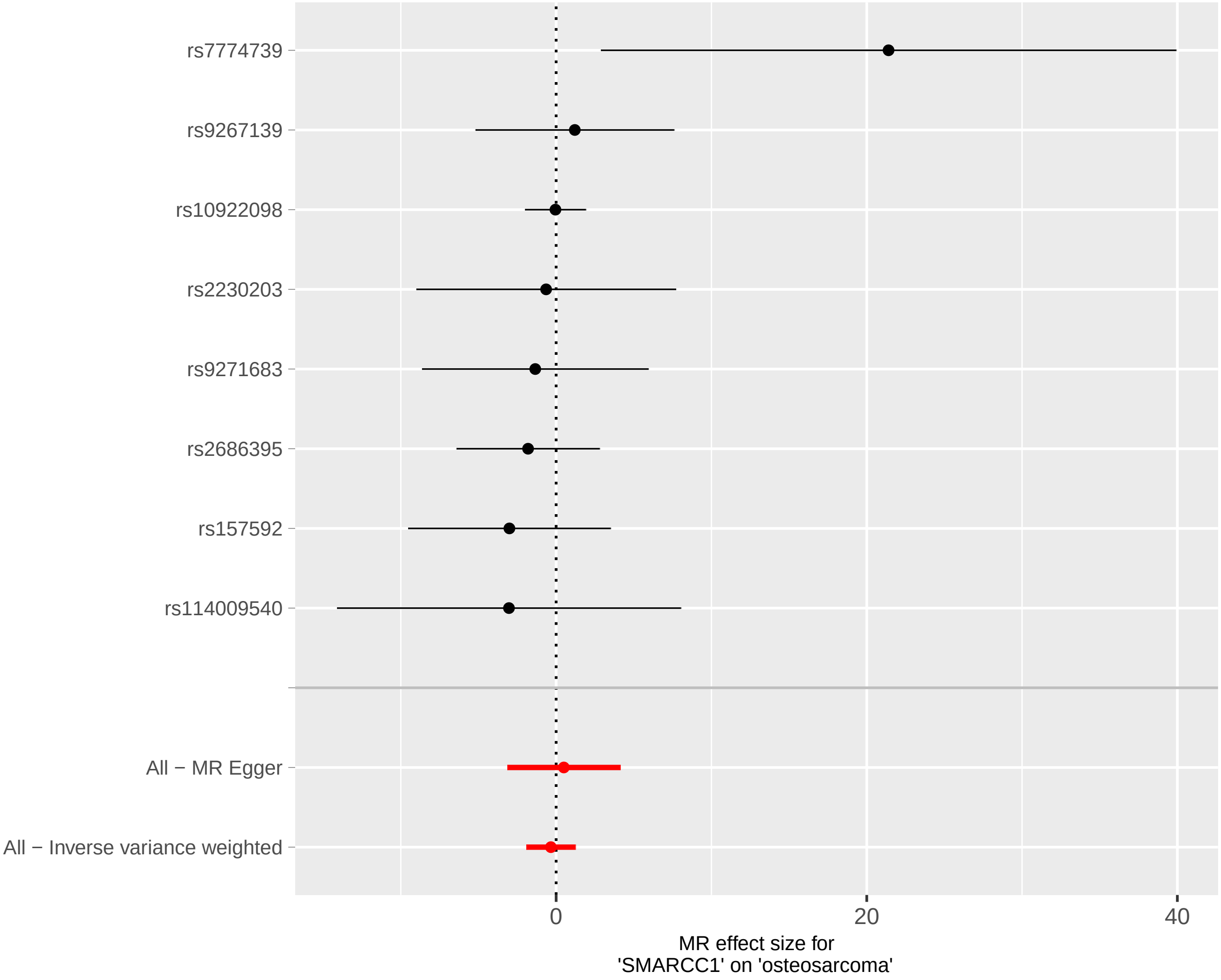
rs7774739
rs9267139
rs10922098
rs2230203
rs9271683
rs2686395
rs157592
rs114009540
All − MR Egger
All − Inverse variance weighted
0
20
40
MR effect size for
'SMARCC1' on 'osteosarcoma'

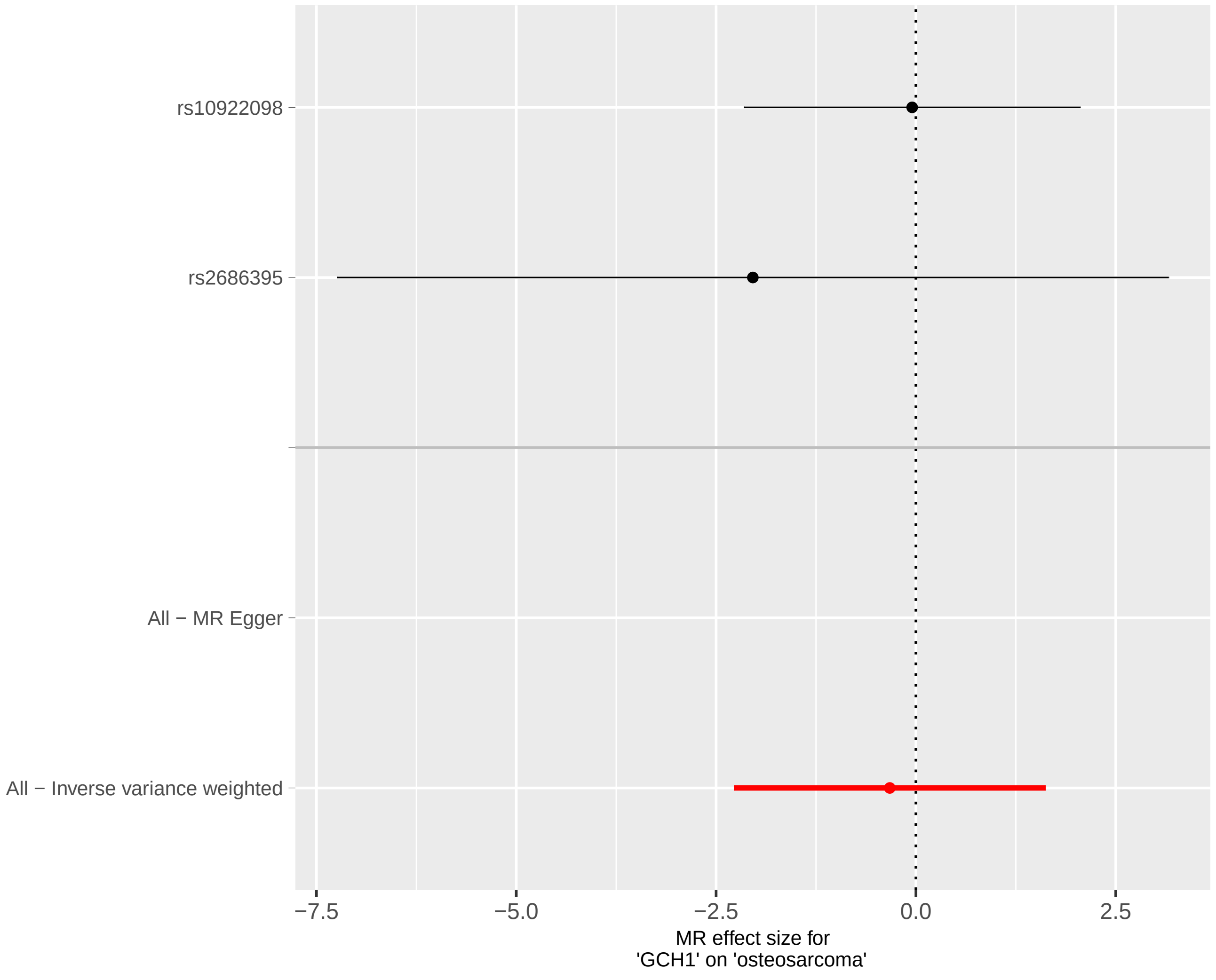

rs10922098
rs2686395
All − MR Egger
All − Inverse variance weighted
−7.5
−5.0
−2.5
0.0
2.5
MR effect size for
'GCH1' on 'osteosarcoma'

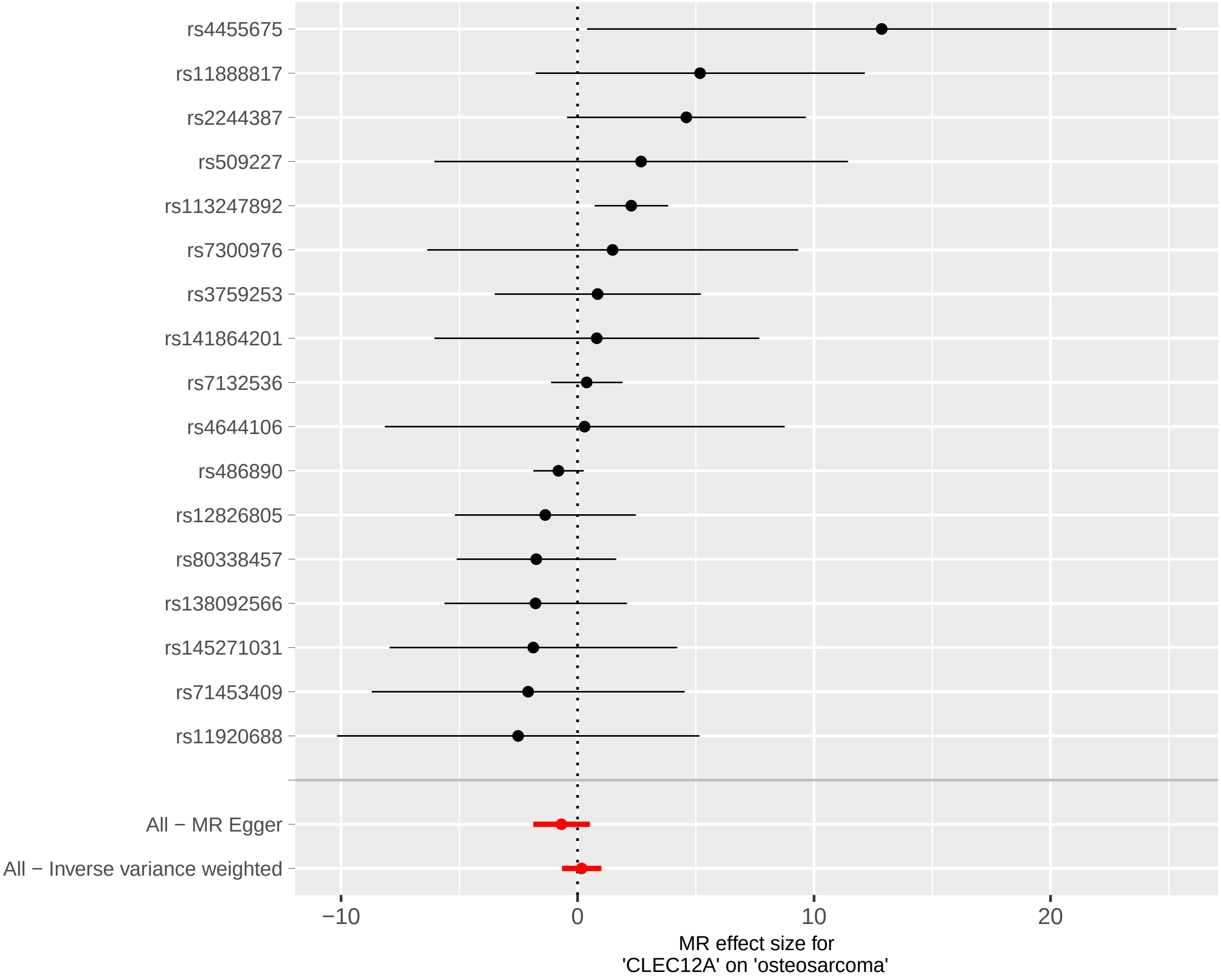

rs4455675
rs11888817
rs2244387
rs509227
rs113247892
rs7300976
rs3759253
rs141864201
rs7132536
rs4644106
rs486890
rs12826805
rs80338457
rs138092566
rs145271031
rs71453409
rs11920688
All − MR Egger
All − Inverse variance weighted
−10
0
10
20
MR effect size for
'CLEC12A' on 'osteosarcoma'

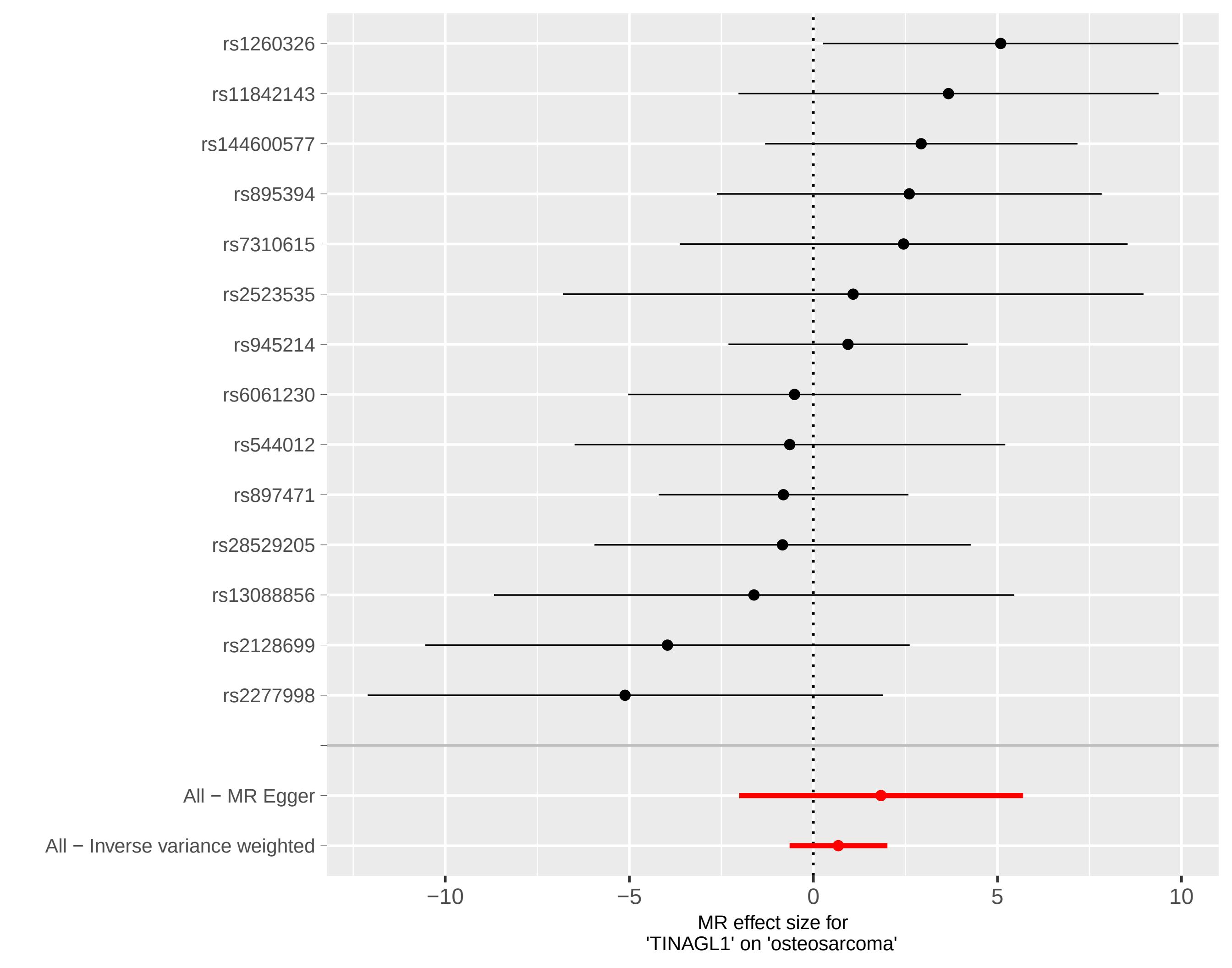

rs1260326
rs11842143
rs144600577
rs895394
rs7310615
rs2523535
rs945214
rs6061230
rs544012
rs897471
rs28529205
rs13088856
rs2128699
rs2277998
All − MR Egger
All − Inverse variance weighted
−10
−5
0
5
10
MR effect size for
'TINAGL1' on 'osteosarcoma'

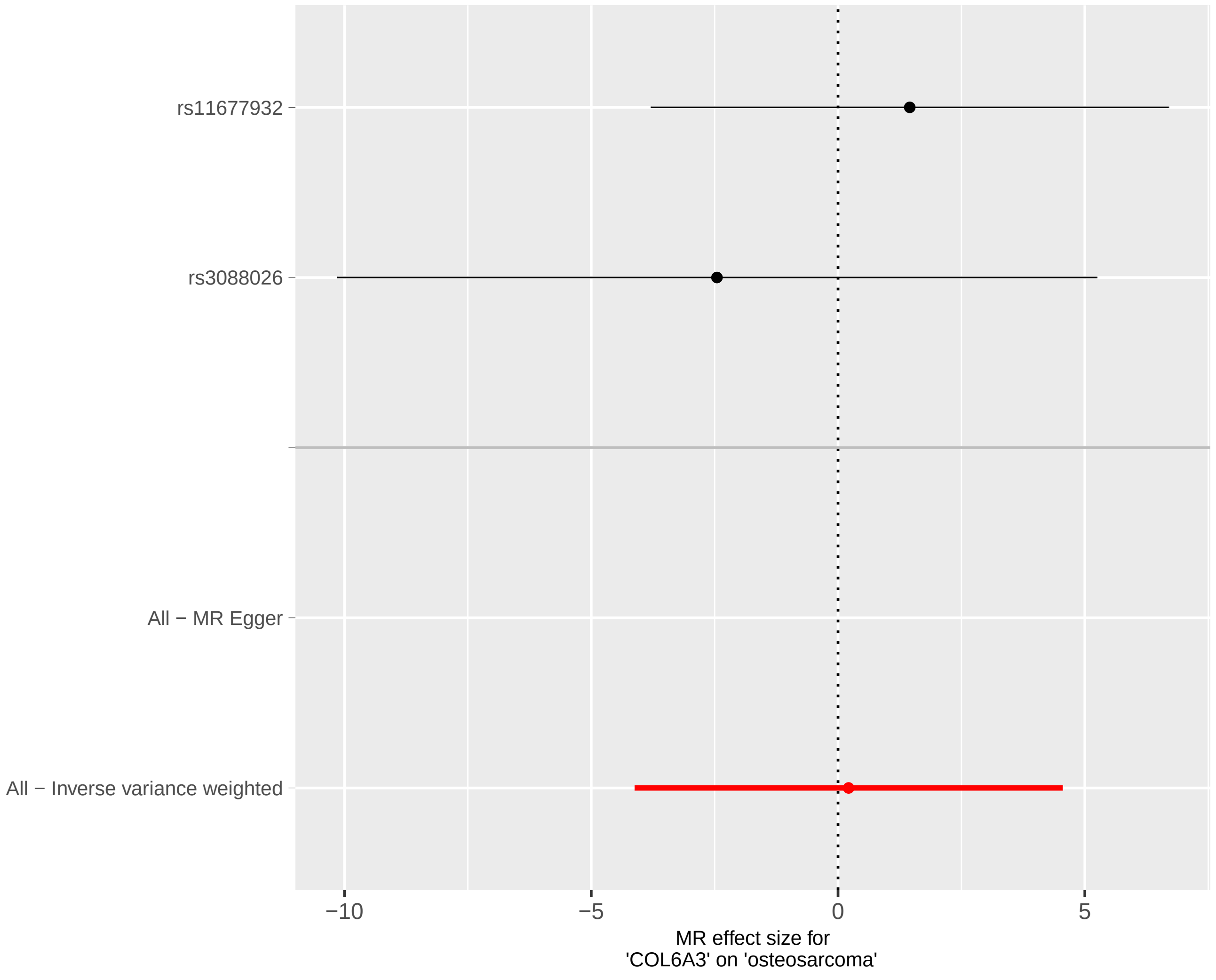
rs11677932
rs3088026
All − MR Egger
All − Inverse variance weighted
−10
−5
0
5
MR effect size for
'COL6A3' on 'osteosarcoma'

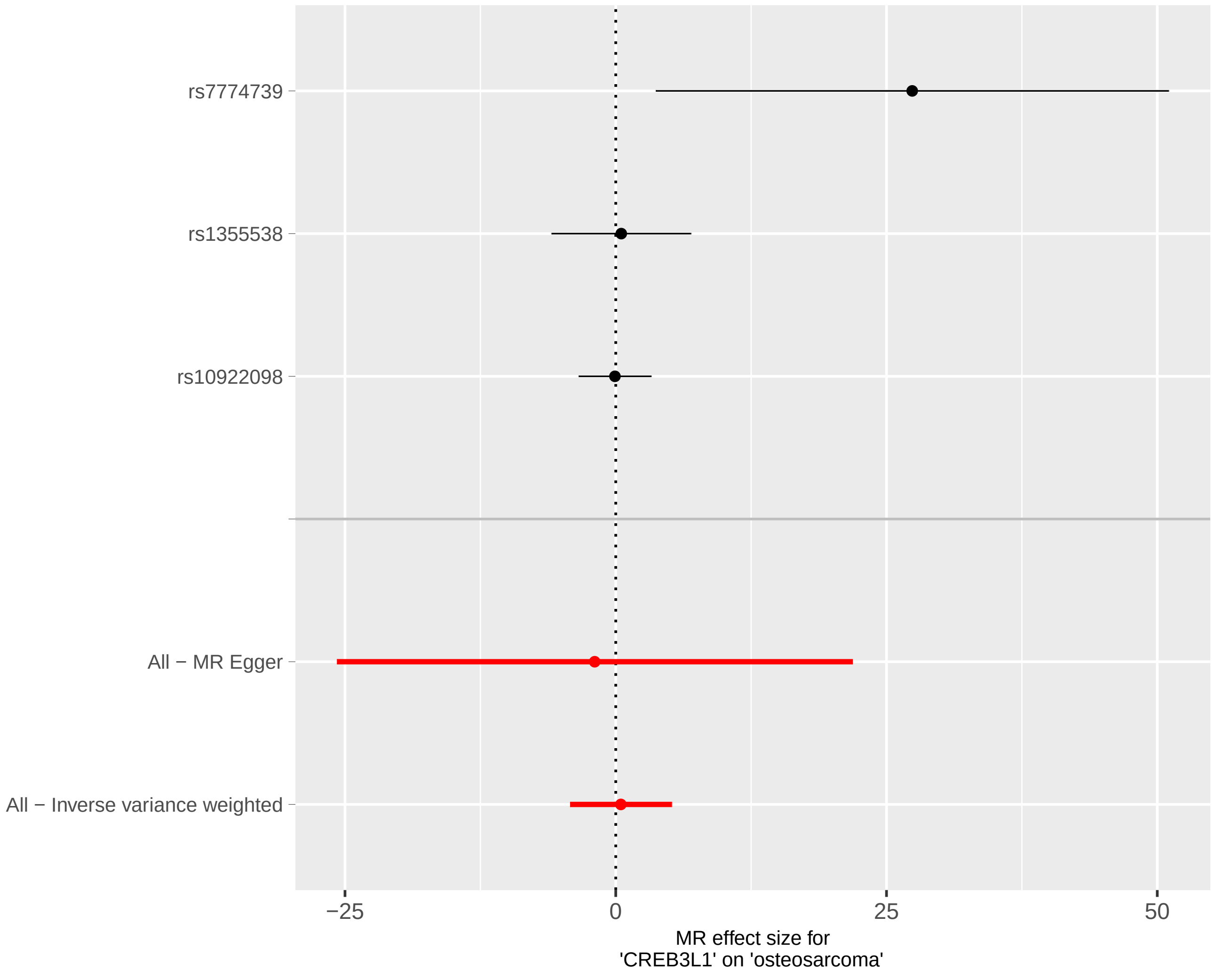

rs7774739
rs1355538
rs10922098
All − MR Egger
All − Inverse variance weighted
−25
0
25
50
MR effect size for
'CREB3L1' on 'osteosarcoma'

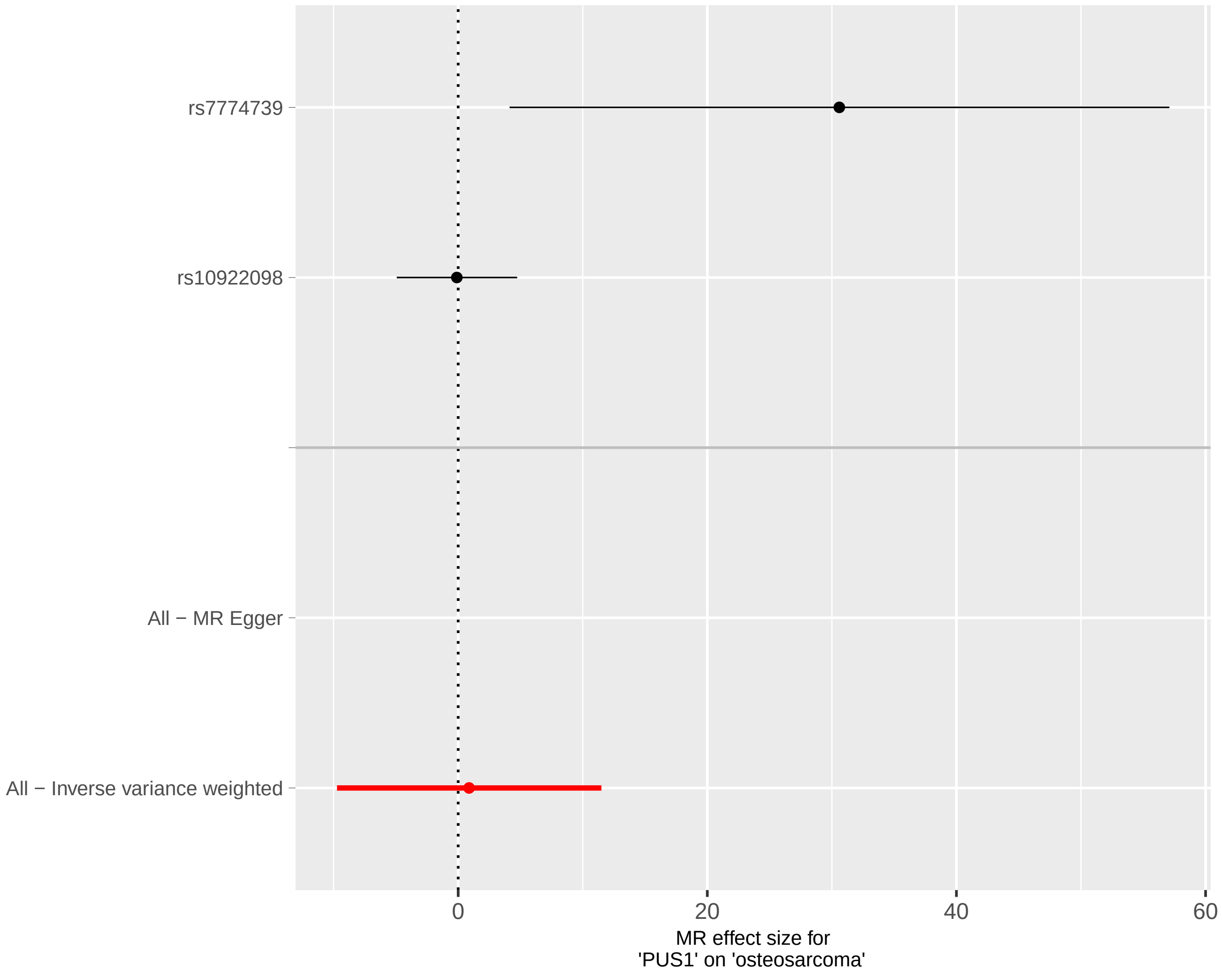
rs7774739
rs10922098
All − MR Egger
All − Inverse variance weighted
0
20
40
60
MR effect size for
'PUS1' on 'osteosarcoma'

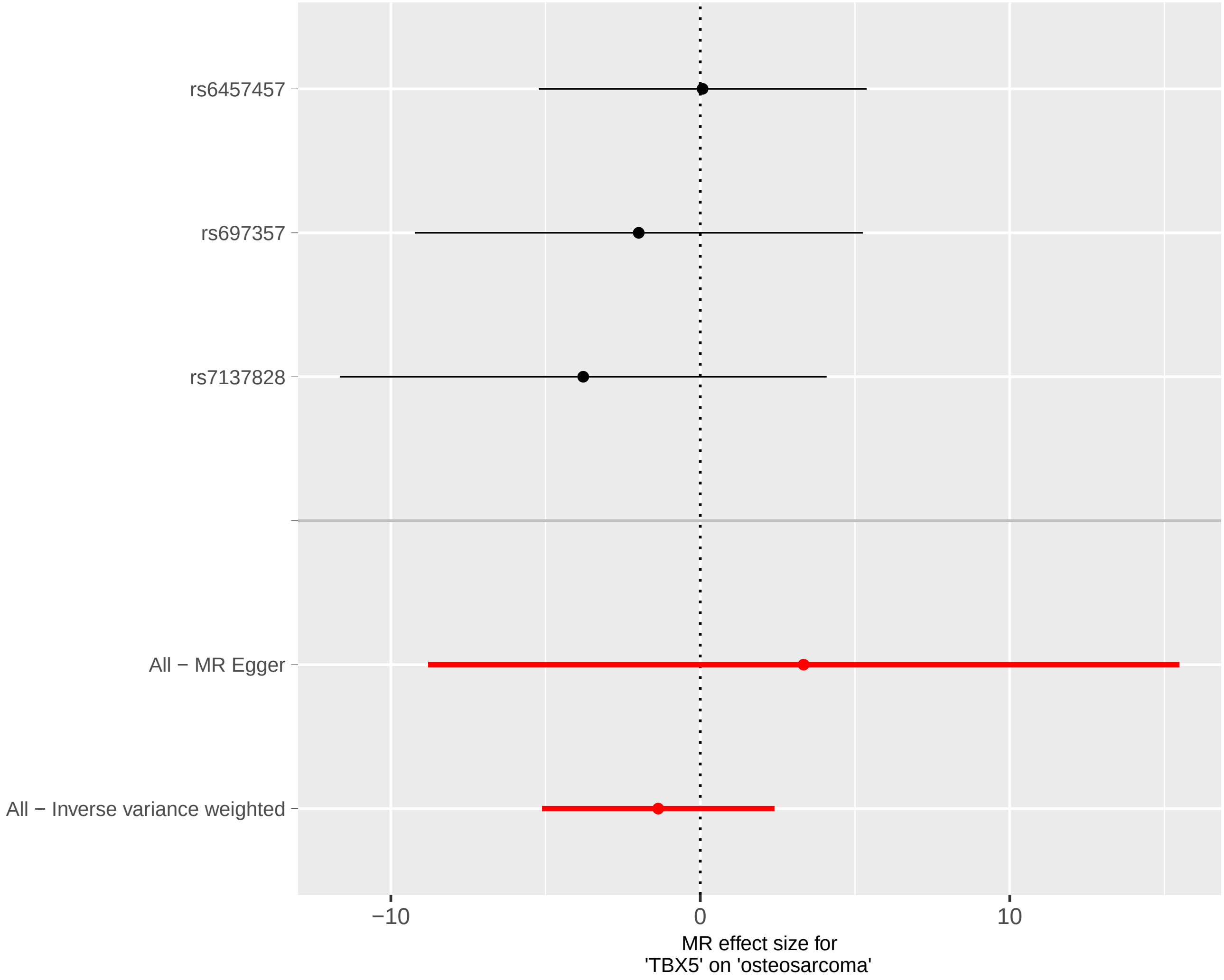
rs6457457
rs697357
rs7137828
All − MR Egger
All − Inverse variance weighted
−10
0
10
MR effect size for
'TBX5' on 'osteosarcoma'

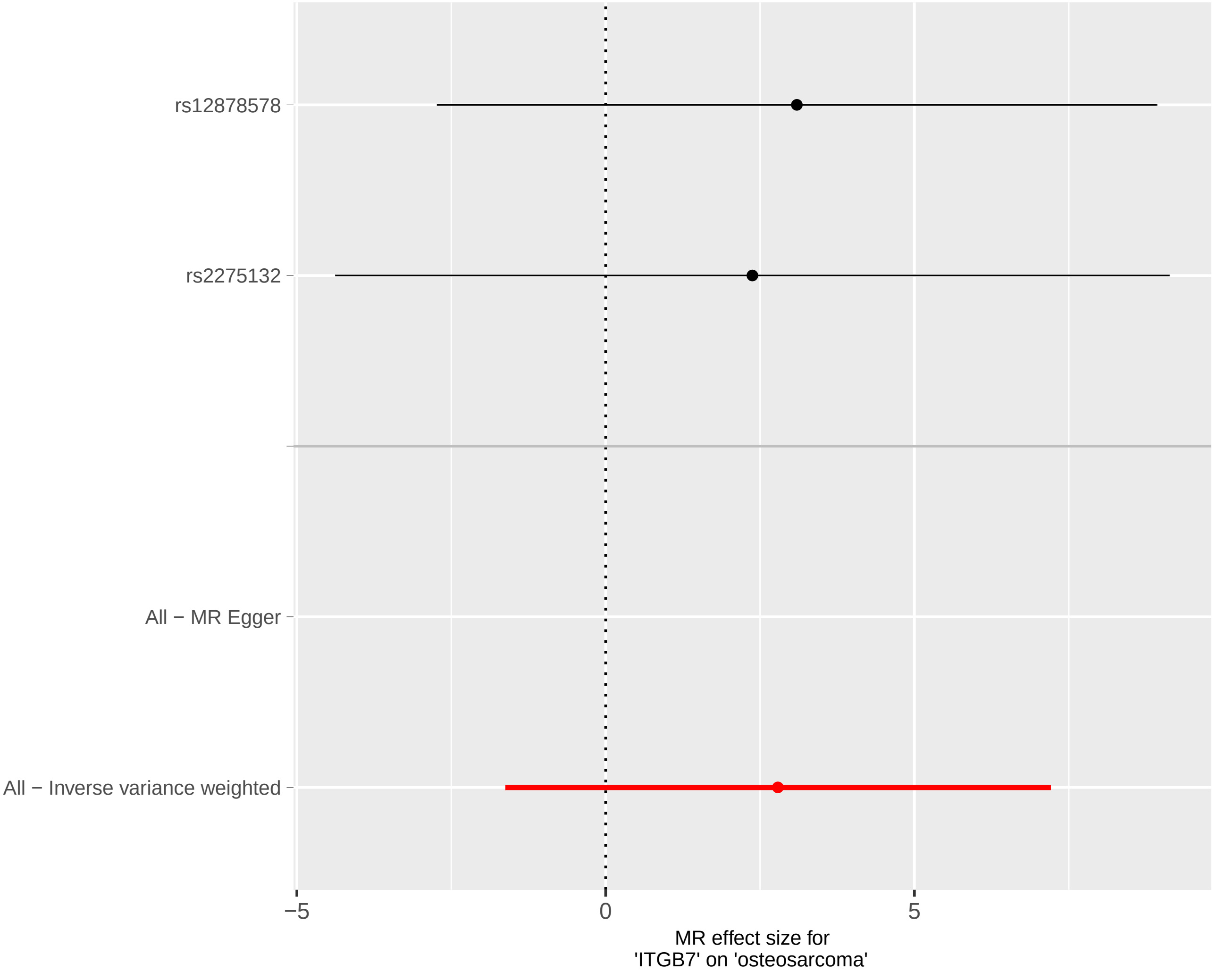

rs12878578
rs2275132
All − MR Egger
All − Inverse variance weighted
−5
0
5
MR effect size for
'ITGB7' on 'osteosarcoma'

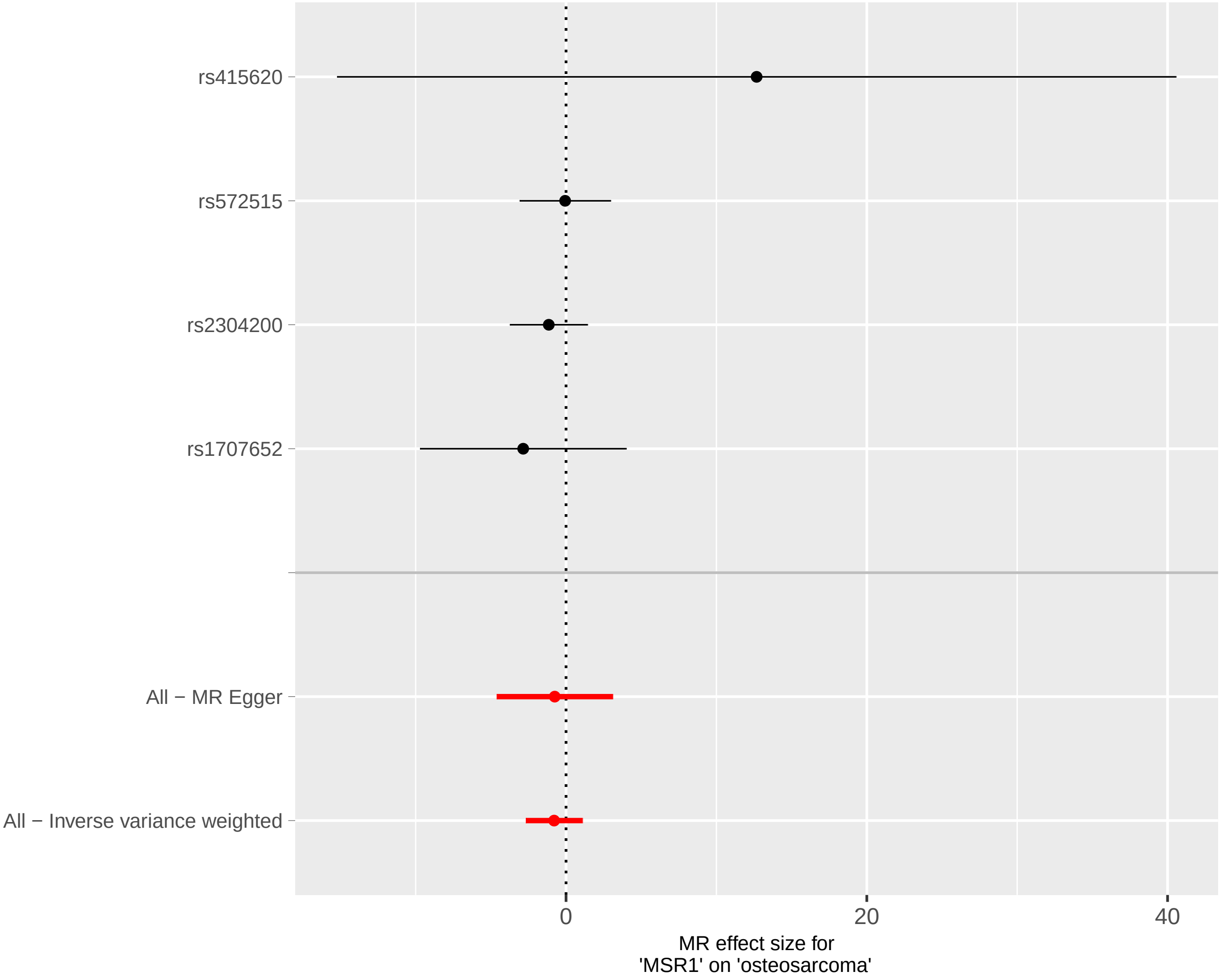

rs415620
rs572515
rs2304200
rs1707652
All − MR Egger
All − Inverse variance weighted
0
20
40
MR effect size for
'MSR1' on 'osteosarcoma'

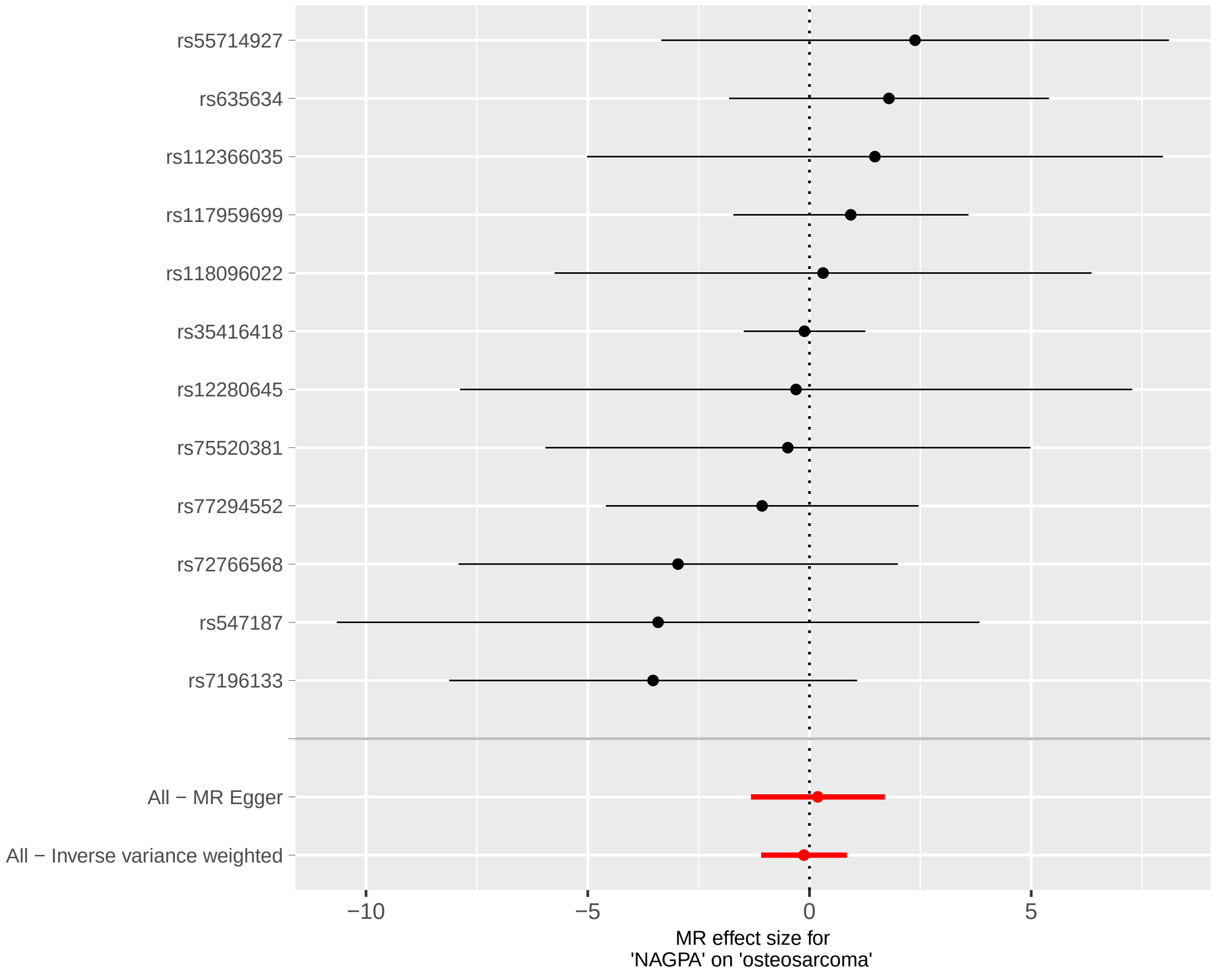

rs55714927
rs635634
rs112366035
rs117959699
rs118096022
rs35416418
rs12280645
rs75520381
rs77294552
rs72766568
rs547187
rs7196133
All − MR Egger
All − Inverse variance weighted
−10
−5
0
5
MR effect size for
'NAGPA' on 'osteosarcoma'

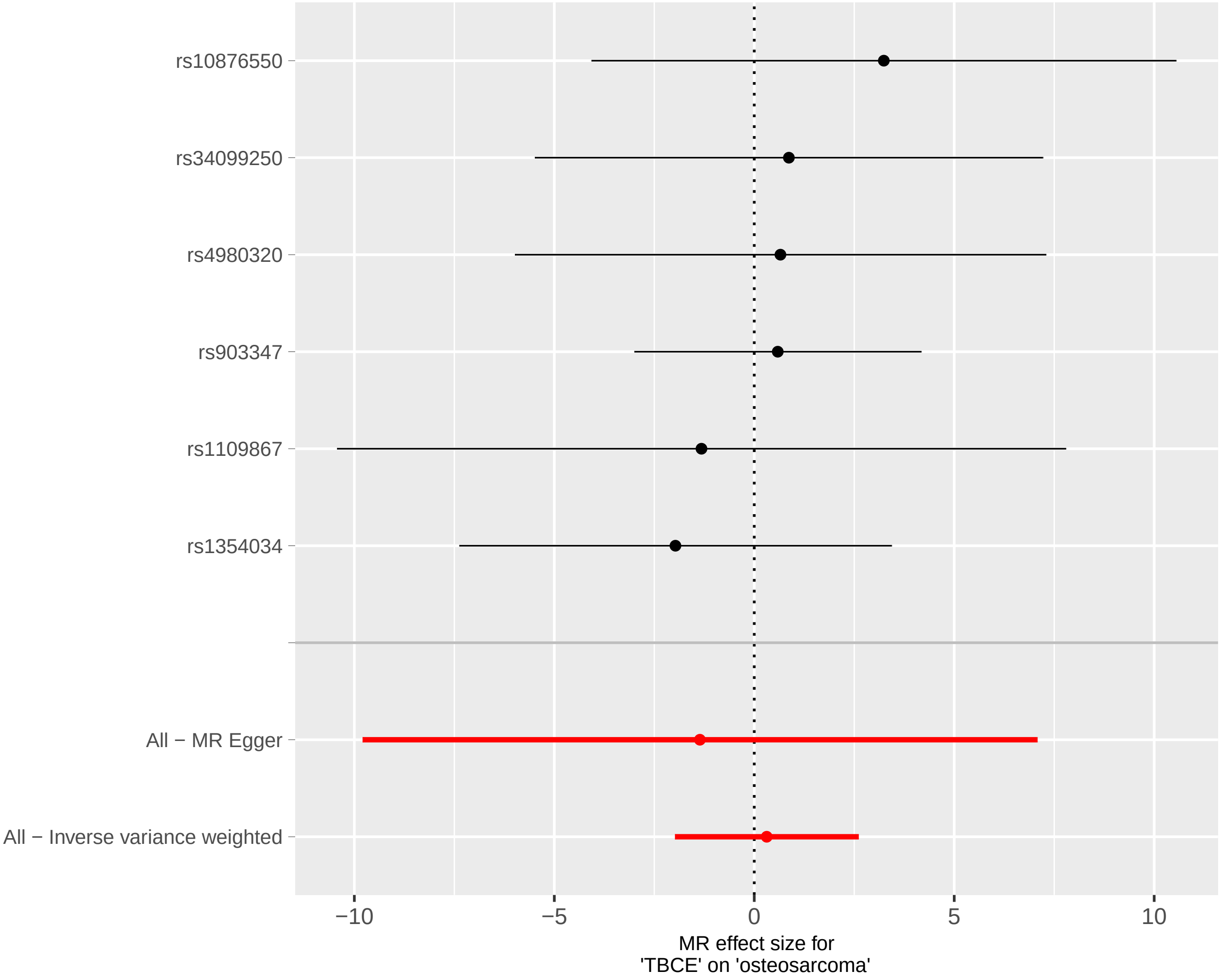
rs10876550
rs34099250
rs4980320
rs903347
rs1109867
rs1354034
All − MR Egger
All − Inverse variance weighted
−10
−5
0
5
10
MR effect size for
'TBCE' on 'osteosarcoma'

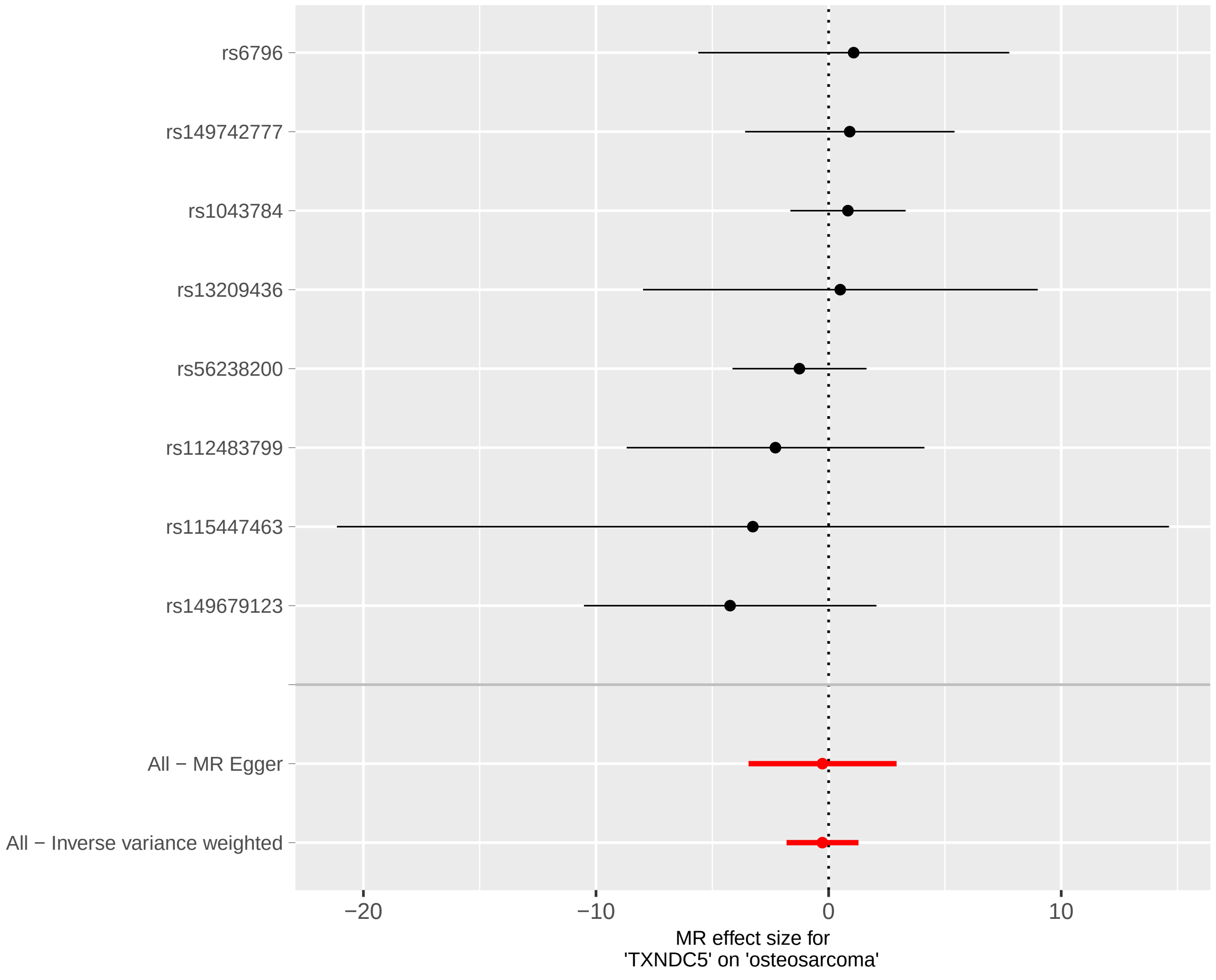
rs6796
rs149742777
rs1043784
rs13209436
rs56238200
rs112483799
rs115447463
rs149679123
All − MR Egger
All − Inverse variance weighted
−20
−10
0
10
MR effect size for
'TXNDC5' on 'osteosarcoma'

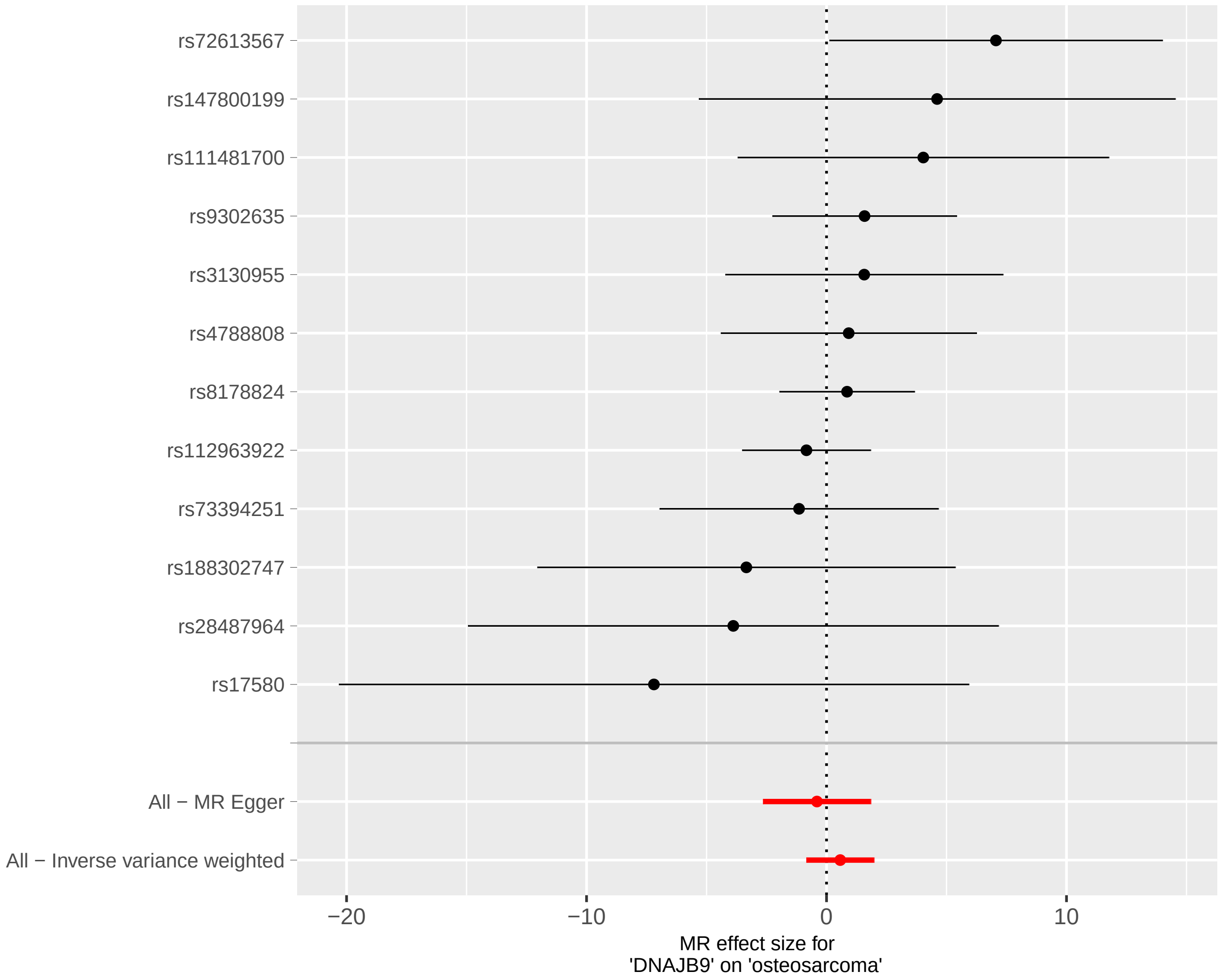

rs72613567
rs147800199
rs111481700
rs9302635
rs3130955
rs4788808
rs8178824
rs112963922
rs73394251
rs188302747
rs28487964
rs17580
All − MR Egger
All − Inverse variance weighted
−20
−10
0
10
MR effect size for
'DNAJB9' on 'osteosarcoma'

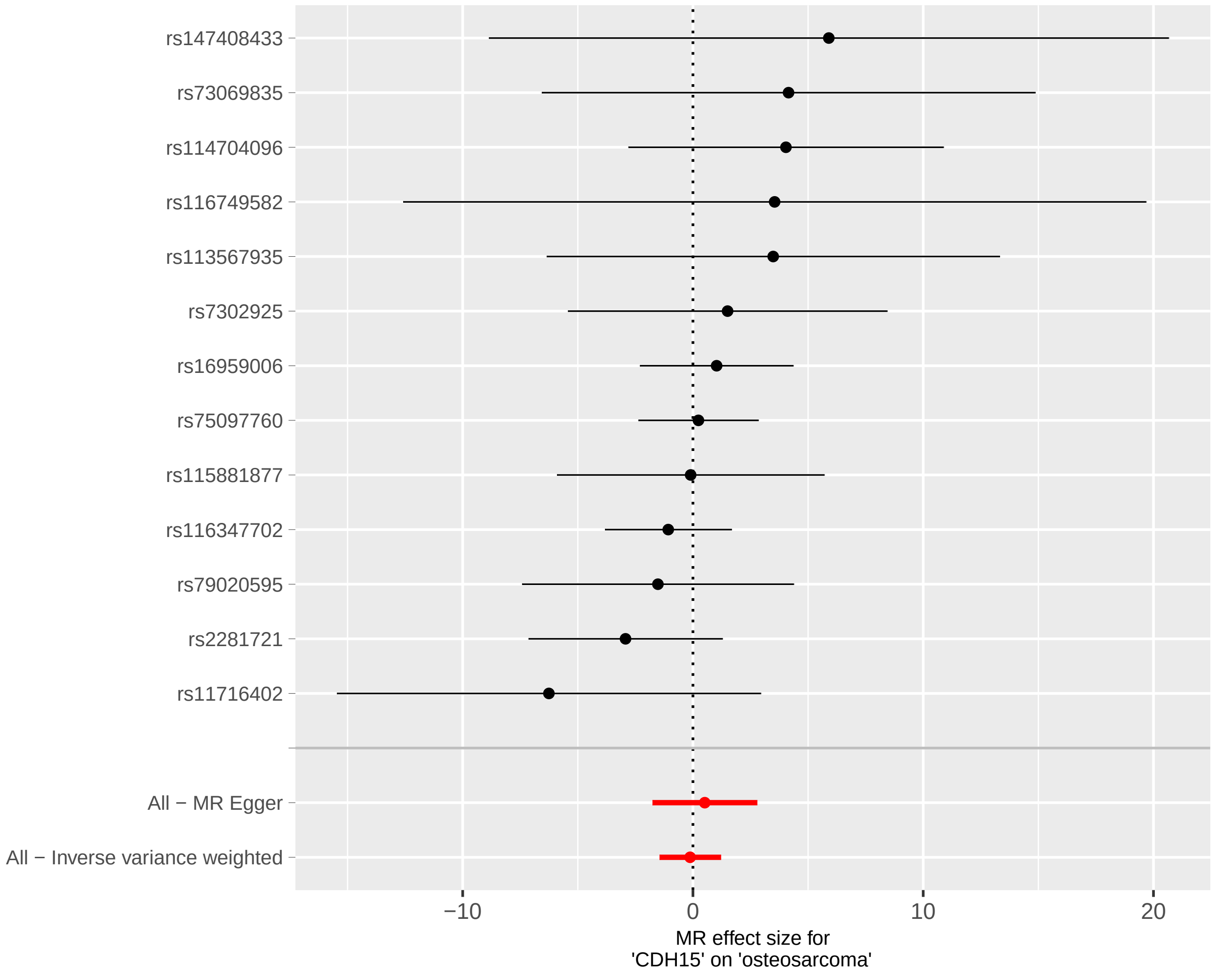
rs147408433
rs73069835
rs114704096
rs116749582
rs113567935
rs7302925
rs16959006
rs75097760
rs115881877
rs116347702
rs79020595
rs2281721
rs11716402
All − MR Egger
All − Inverse variance weighted
−10
0
10
20
MR effect size for
'CDH15' on 'osteosarcoma'

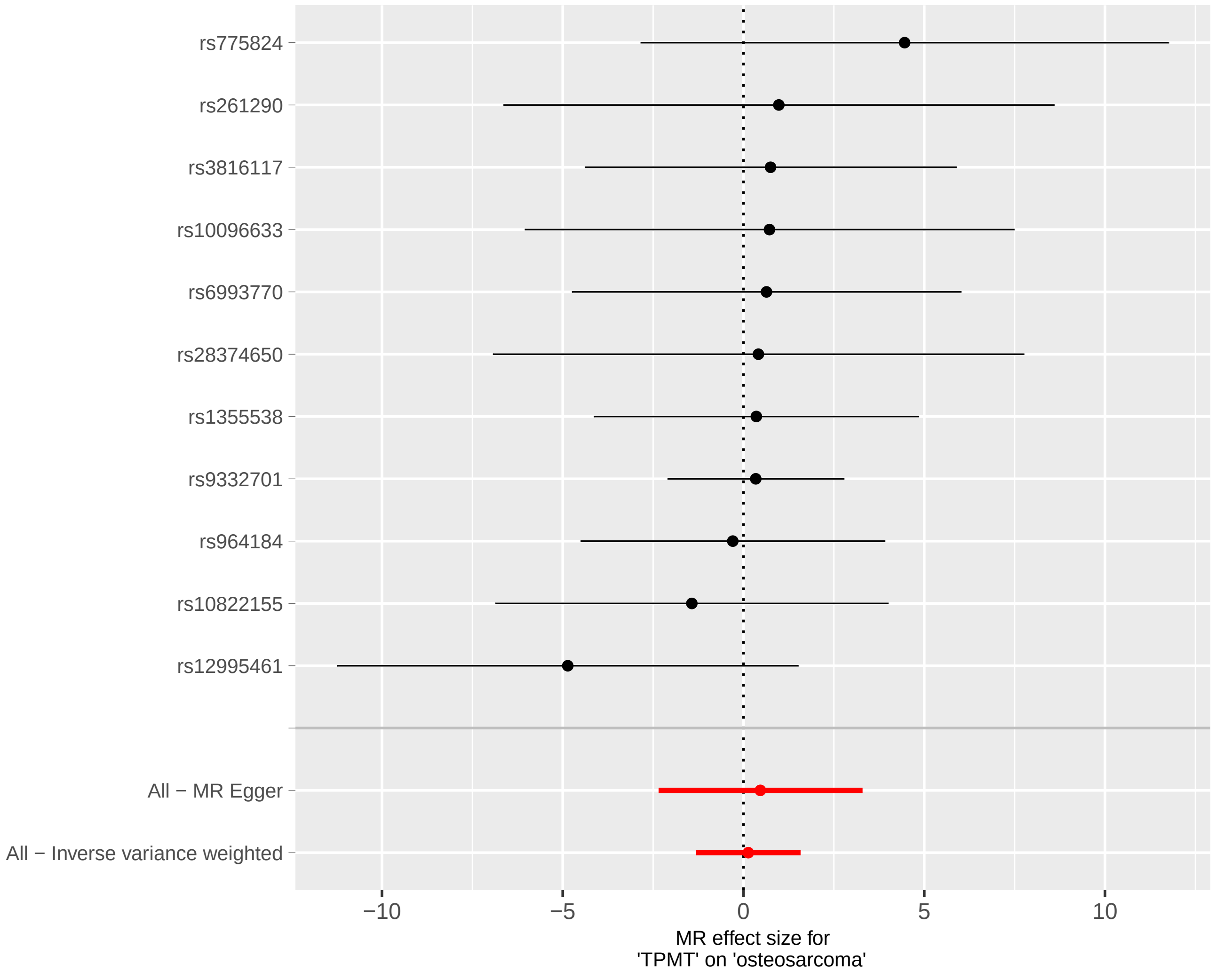

rs775824
rs261290
rs3816117
rs10096633
rs6993770
rs28374650
rs1355538
rs9332701
rs964184
rs10822155
rs12995461
All − MR Egger
All − Inverse variance weighted
−10
−5
0
5
10
MR effect size for
'TPMT' on 'osteosarcoma'

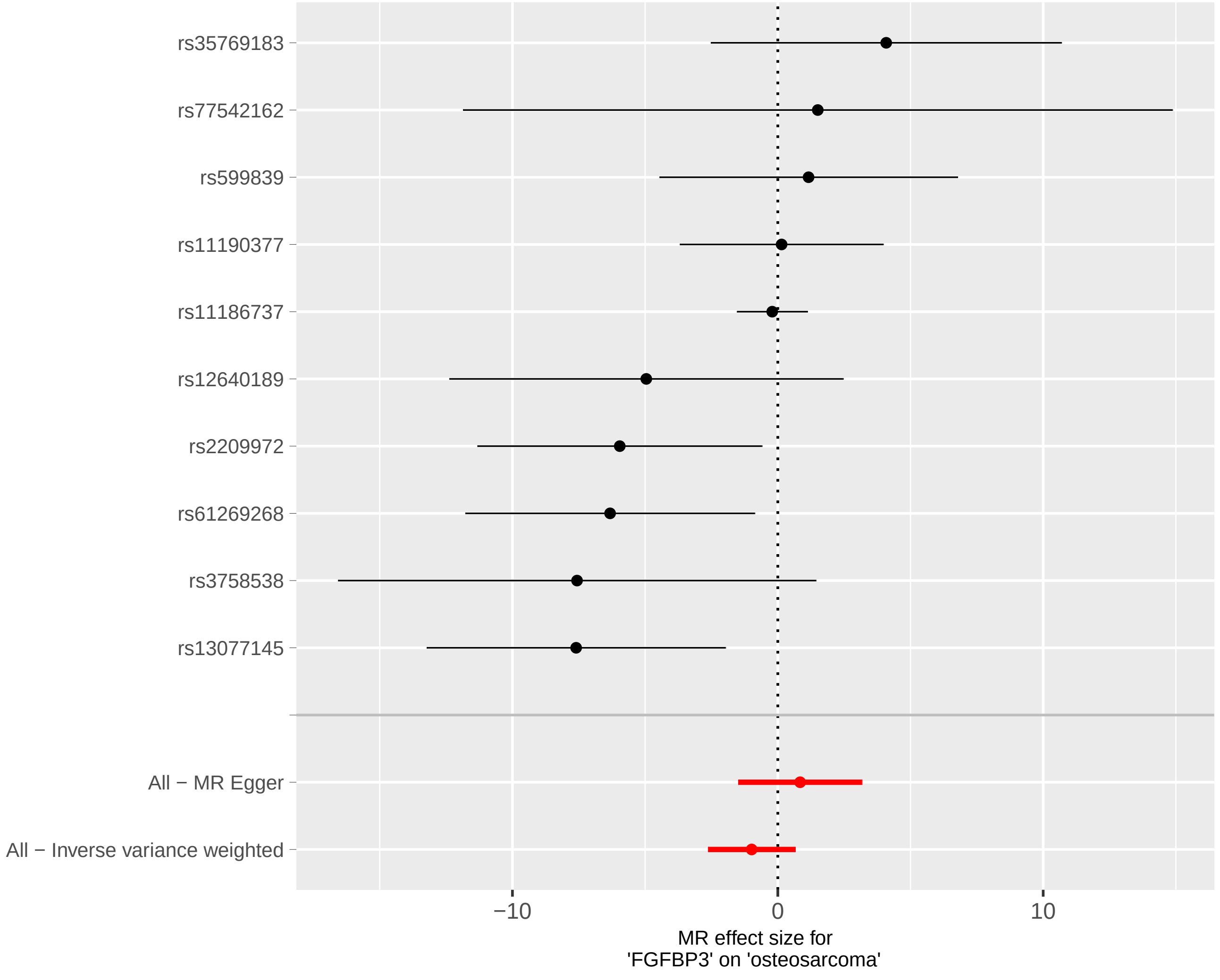

rs35769183
rs77542162
rs599839
rs11190377
rs11186737
rs12640189
rs2209972
rs61269268
rs3758538
rs13077145
All − MR Egger
All − Inverse variance weighted
−10
0
10
MR effect size for
'FGFBP3' on 'osteosarcoma'

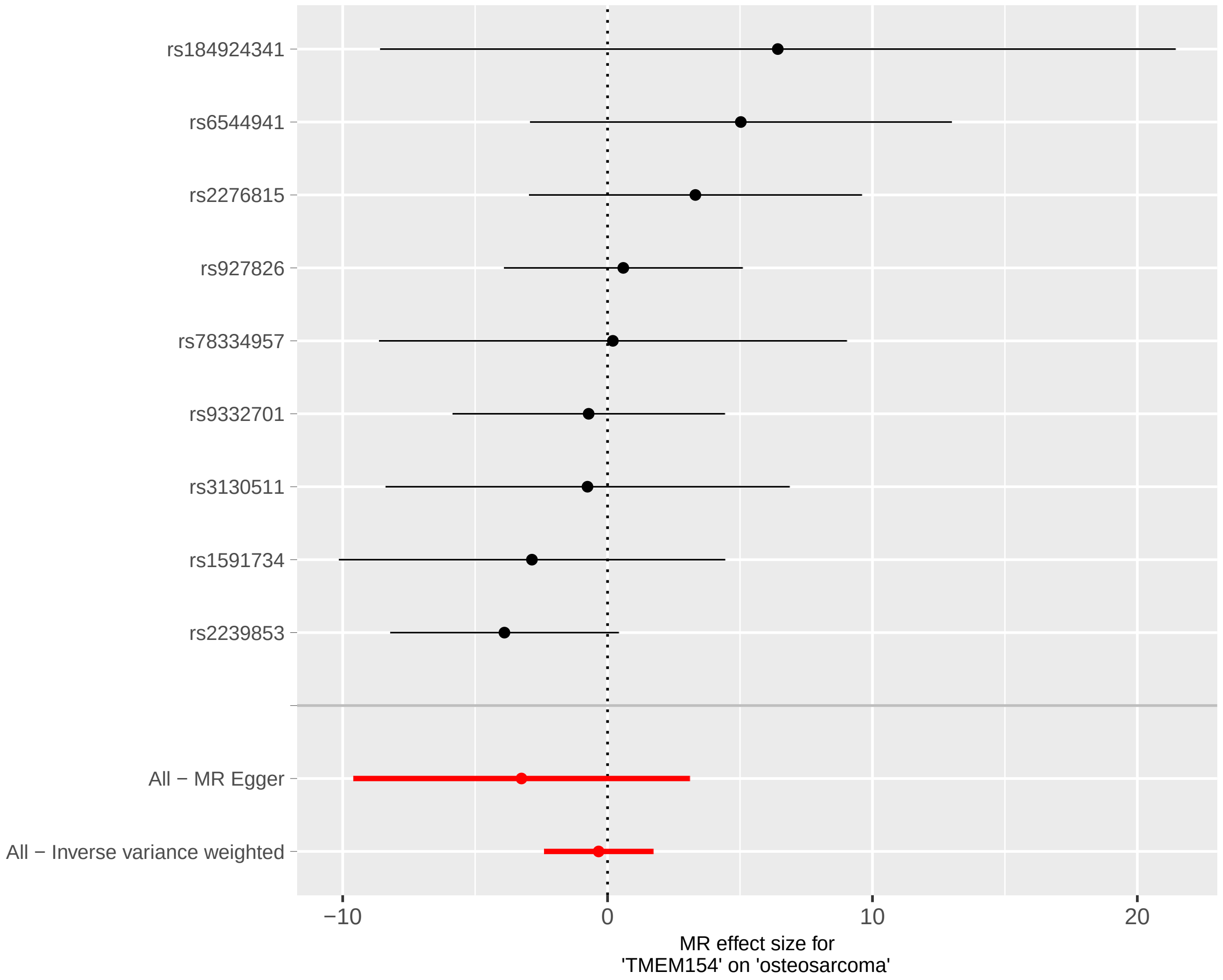

rs184924341
rs6544941
rs2276815
rs927826
rs78334957
rs9332701
rs3130511
rs1591734
rs2239853
All − MR Egger
All − Inverse variance weighted
−10
0
10
20
MR effect size for
'TMEM154' on 'osteosarcoma'

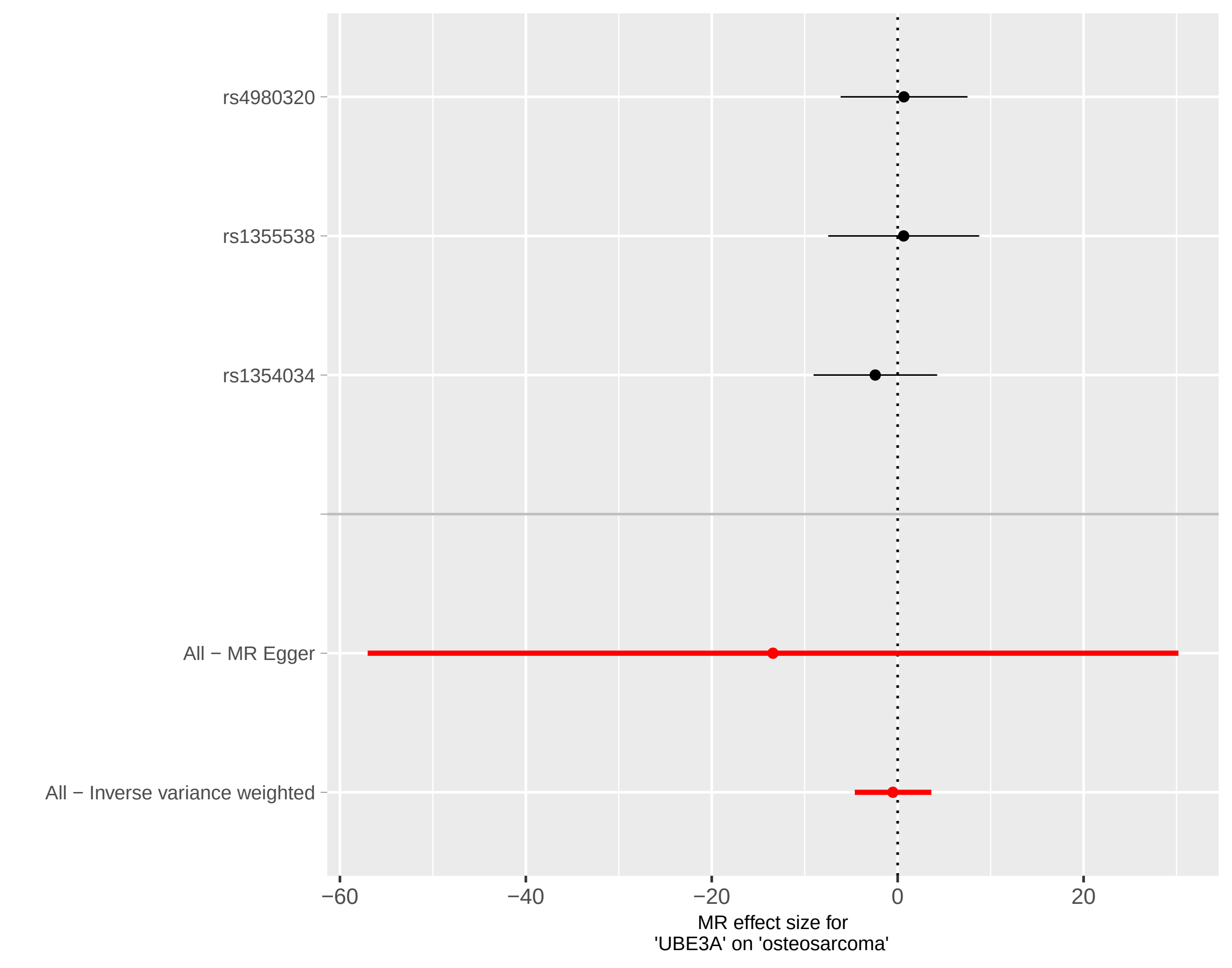
rs4980320
rs1355538
rs1354034
All − MR Egger
All − Inverse variance weighted
−60
−40
−20
0
20
MR effect size for
'UBE3A' on 'osteosarcoma'

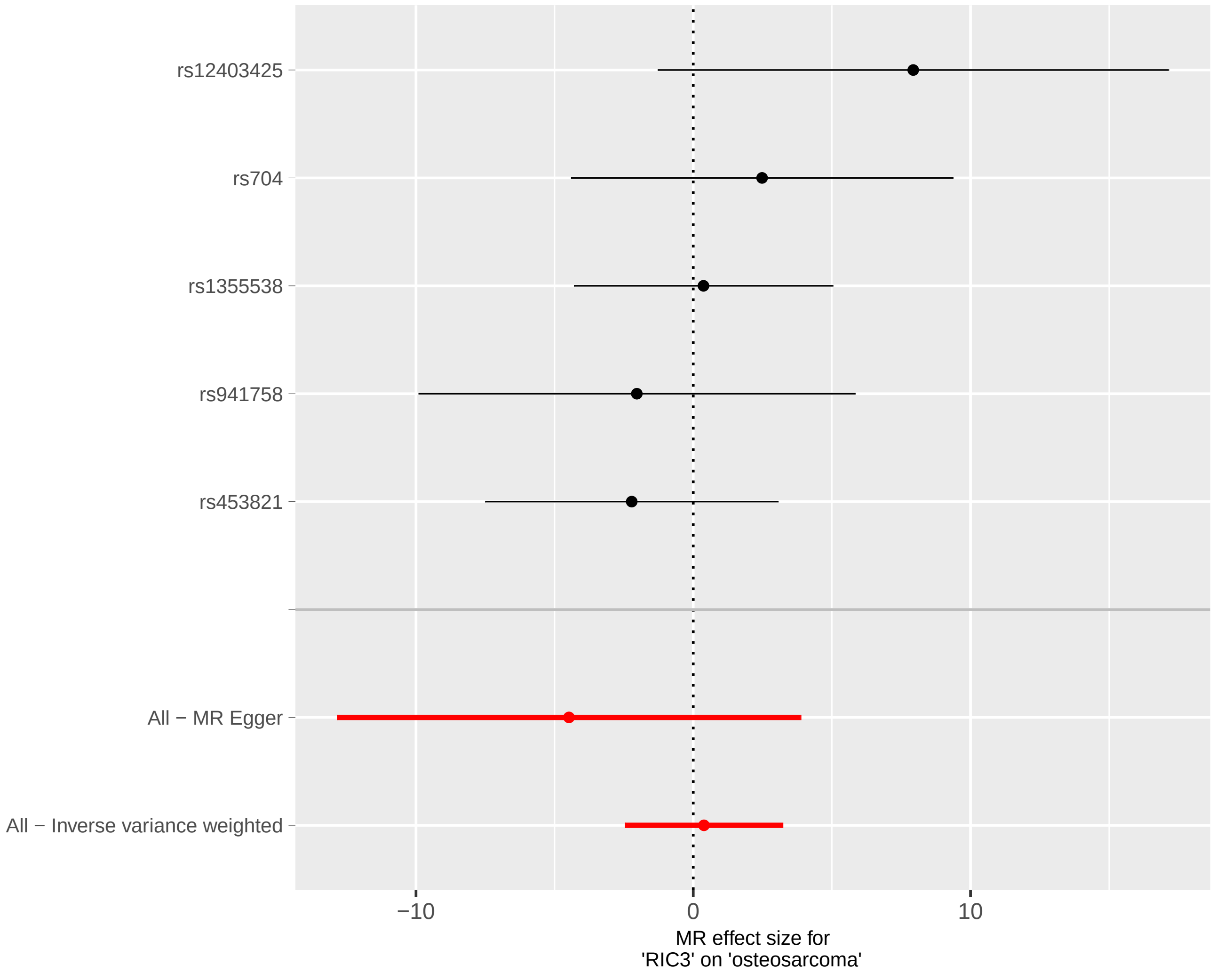

rs12403425
rs704
rs1355538
rs941758
rs453821
All − MR Egger
All − Inverse variance weighted
−10
0
10
MR effect size for
'RIC3' on 'osteosarcoma'

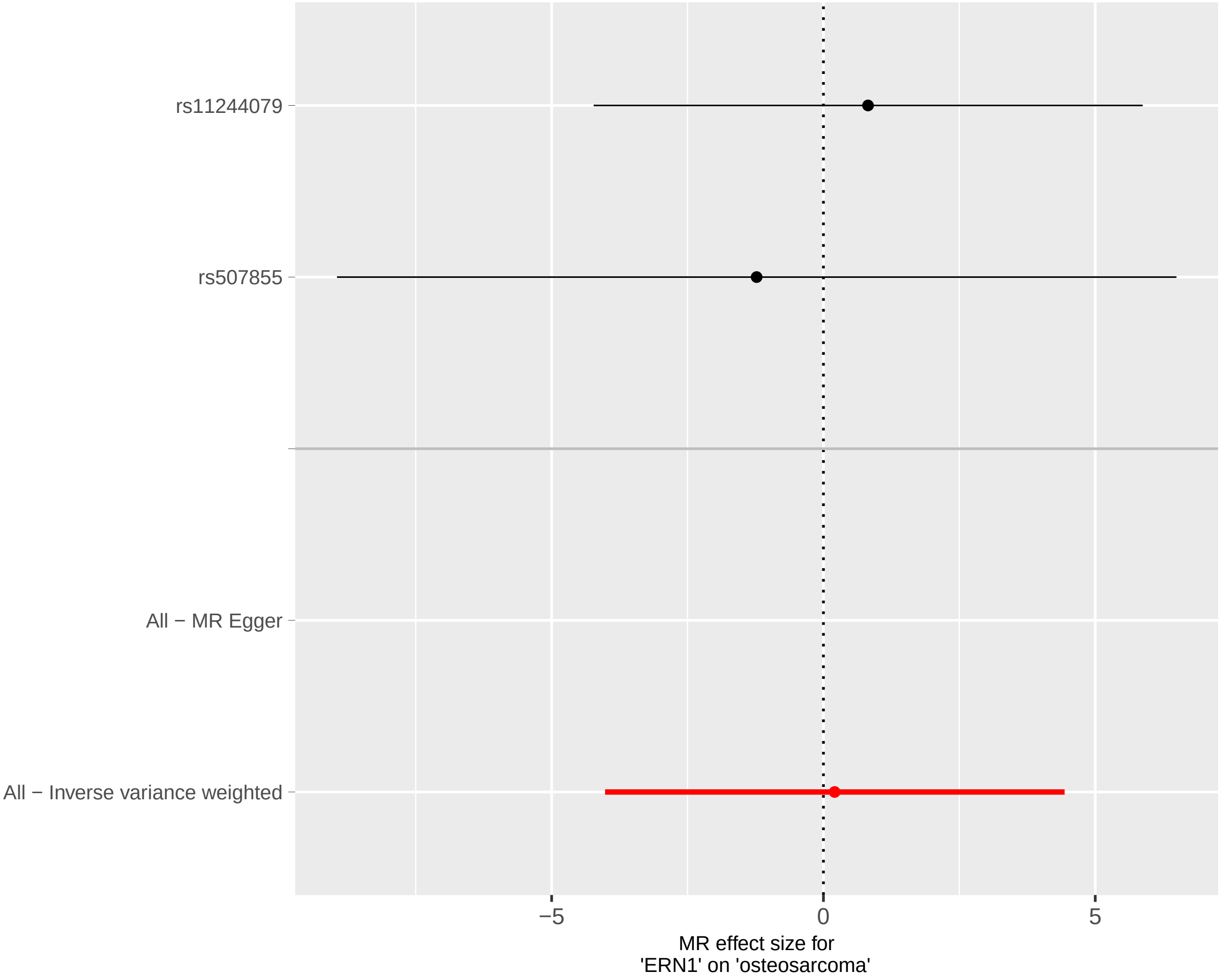
rs11244079
rs507855
All − MR Egger
All − Inverse variance weighted
−5
0
5
MR effect size for
'ERN1' on 'osteosarcoma'

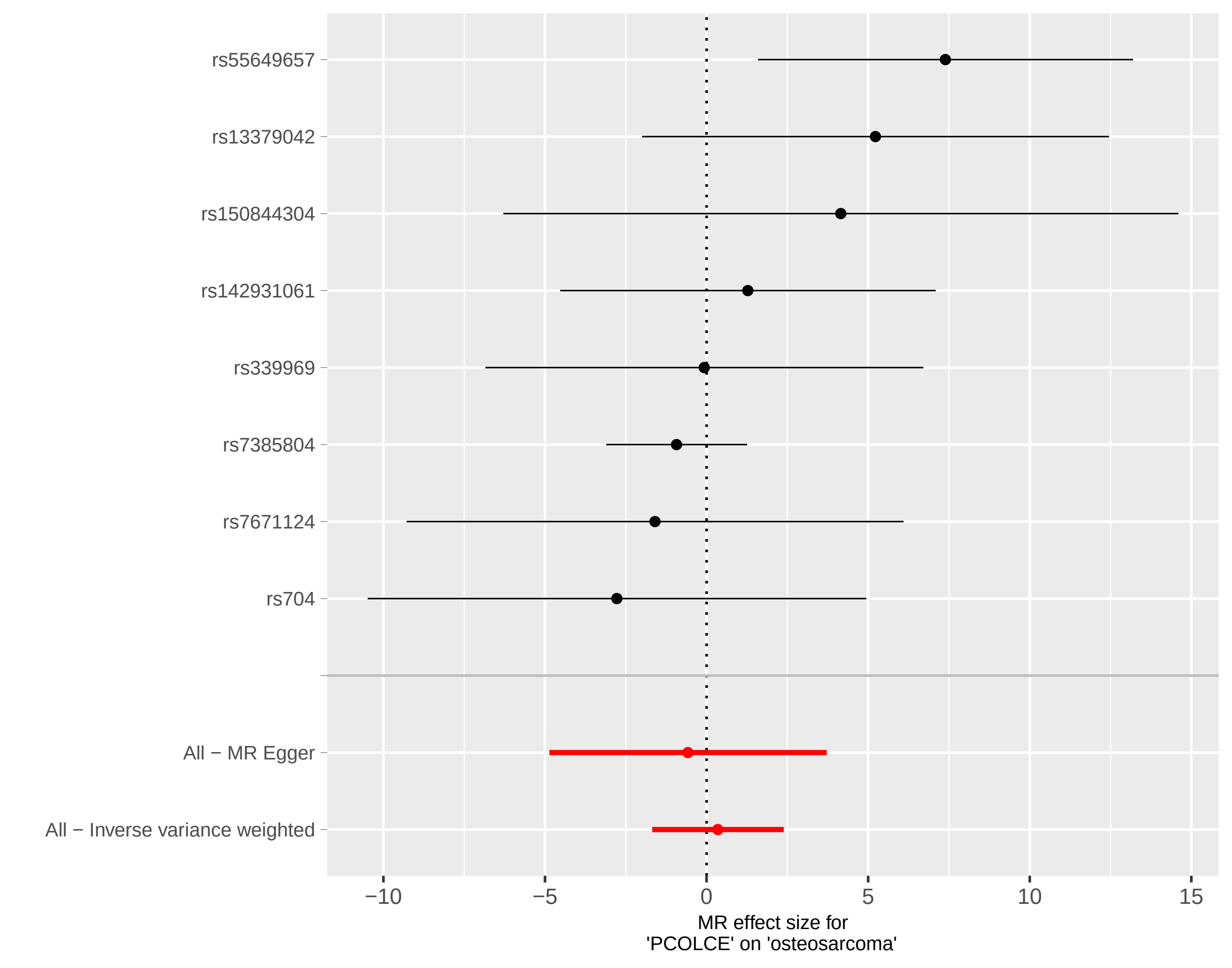

rs55649657
rs13379042
rs150844304
rs142931061
rs339969
rs7385804
rs7671124
rs704
All − MR Egger
All − Inverse variance weighted
−10
−5
0
5
10
15
MR effect size for
'PCOLCE' on 'osteosarcoma'

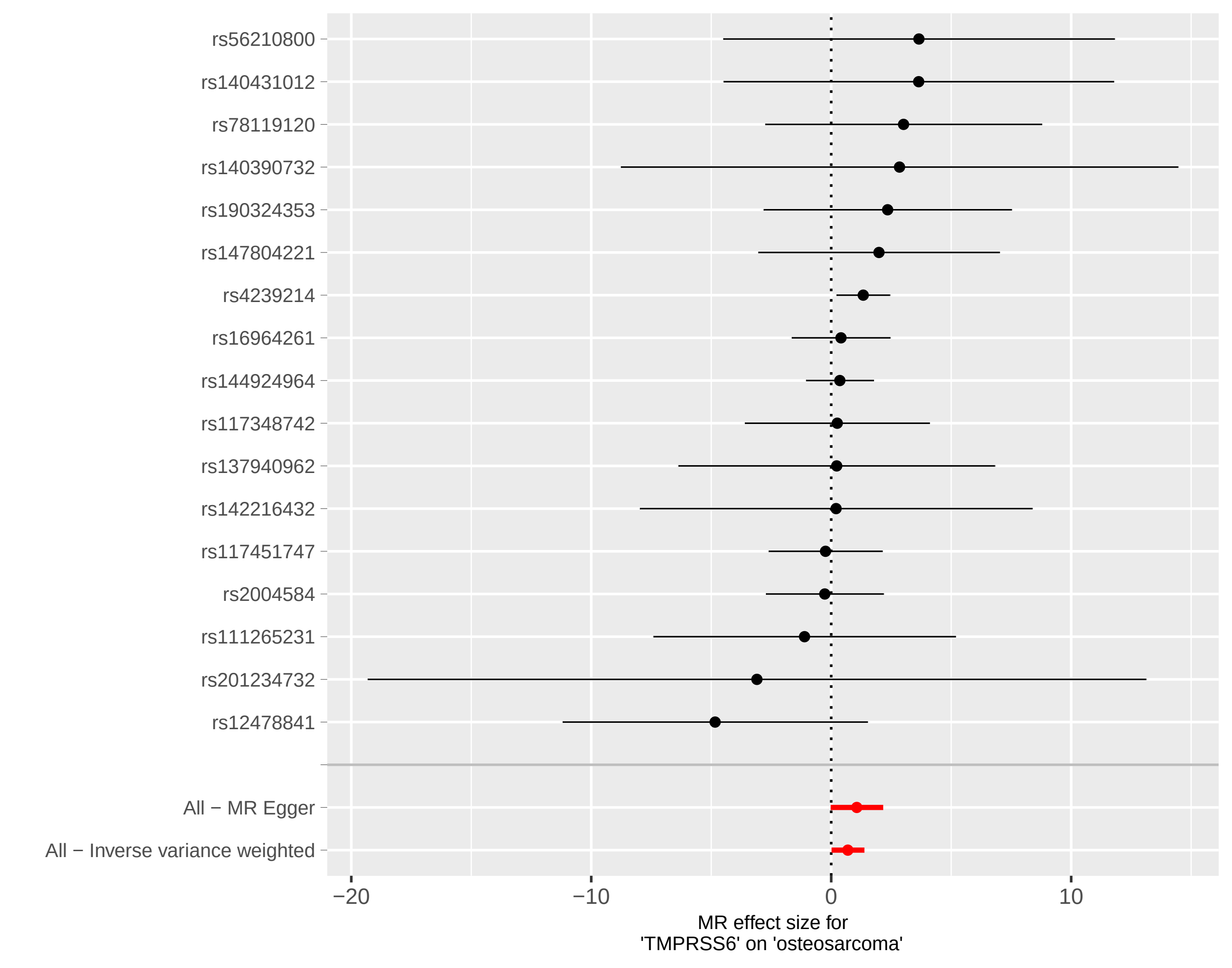
rs56210800
rs140431012
rs78119120
rs140390732
rs190324353
rs147804221
rs4239214
rs16964261
rs144924964
rs117348742
rs137940962
rs142216432
rs117451747
rs2004584
rs111265231
rs201234732
rs12478841
All − MR Egger
All − Inverse variance weighted
−20
−10
0
10
MR effect size for
'TMPRSS6' on 'osteosarcoma'

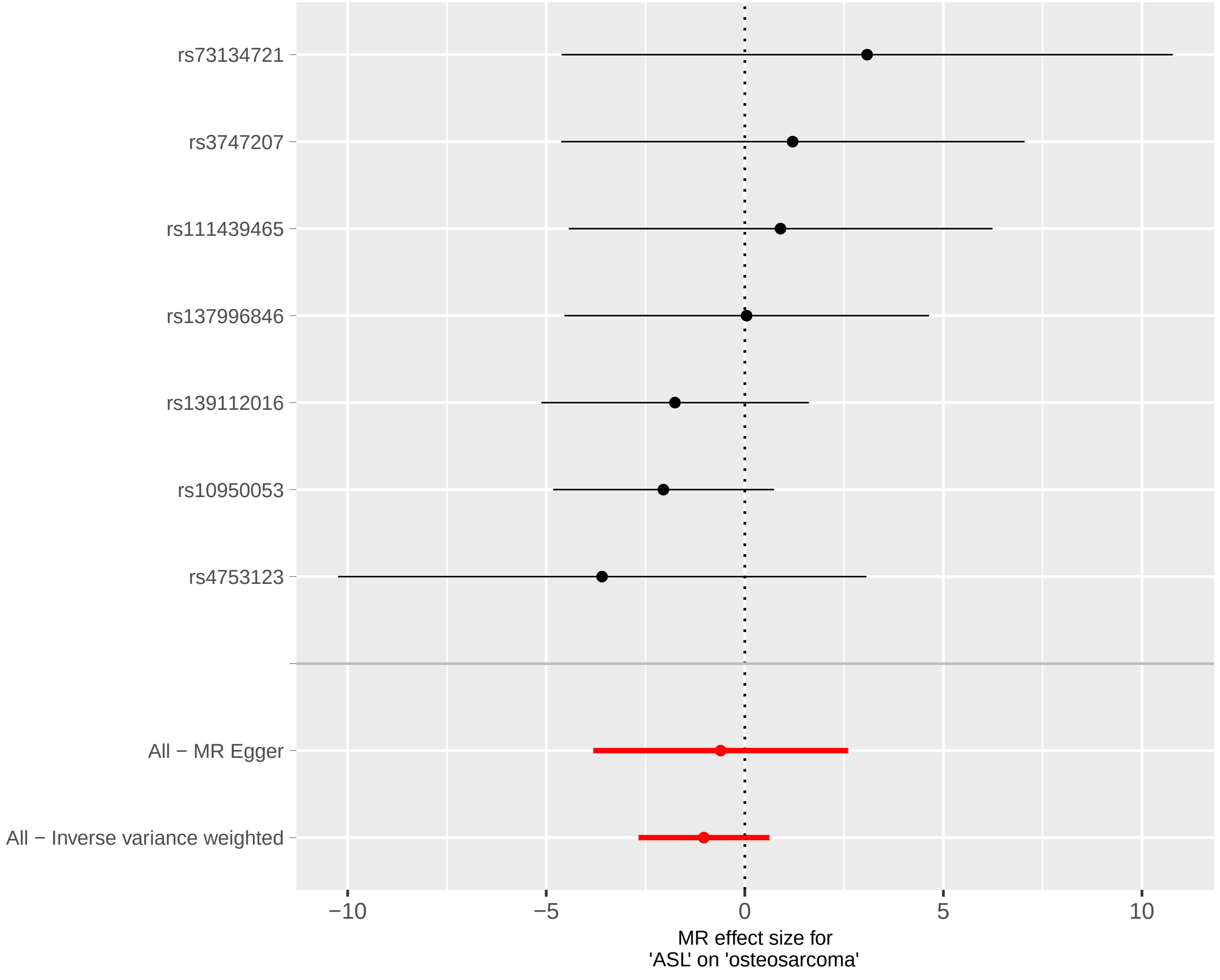
rs73134721
rs3747207
rs111439465
rs137996846
rs139112016
rs10950053
rs4753123
All − MR Egger
All − Inverse variance weighted
−10
−5
0
5
10
MR effect size for
'ASL' on 'osteosarcoma'

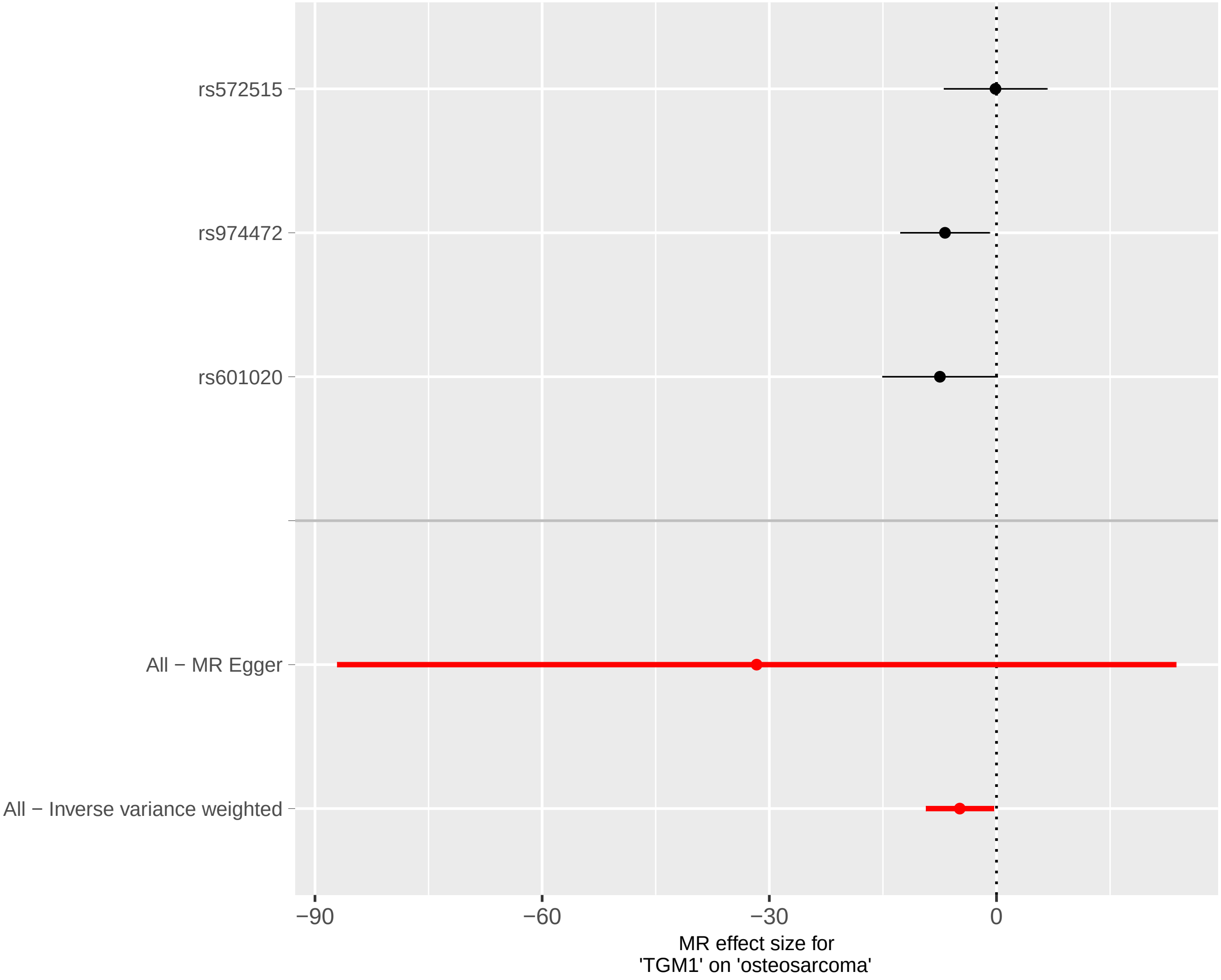

rs572515
rs974472
rs601020
All − MR Egger
All − Inverse variance weighted
−90
−60
−30
0
MR effect size for
'TGM1' on 'osteosarcoma'

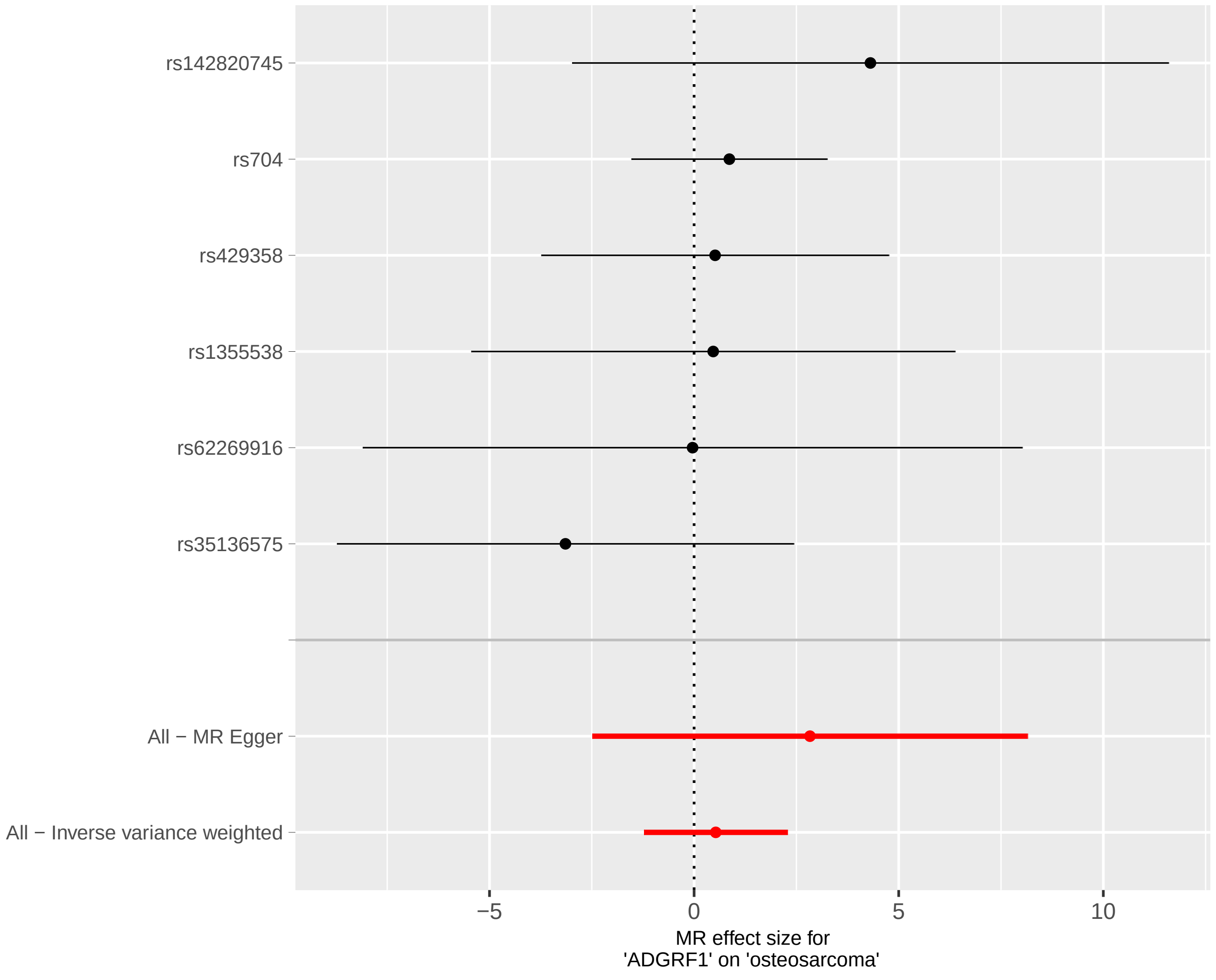

rs142820745
rs704
rs429358
rs1355538
rs62269916
rs35136575
All − MR Egger
All − Inverse variance weighted
−5
0
5
10
MR effect size for
'ADGRF1' on 'osteosarcoma'

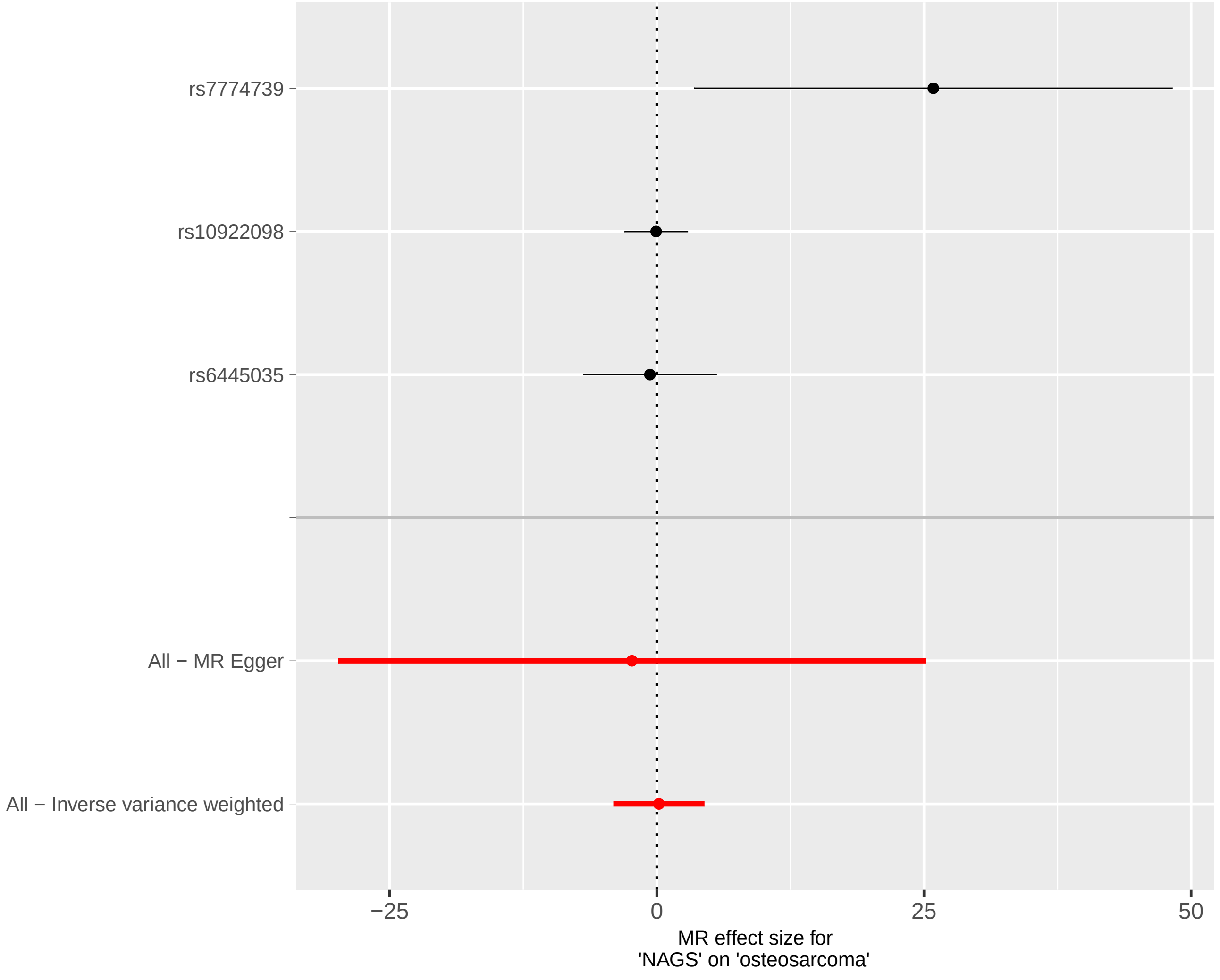

rs7774739
rs10922098
rs6445035
All − MR Egger
All − Inverse variance weighted
−25
0
25
50
MR effect size for
'NAGS' on 'osteosarcoma'

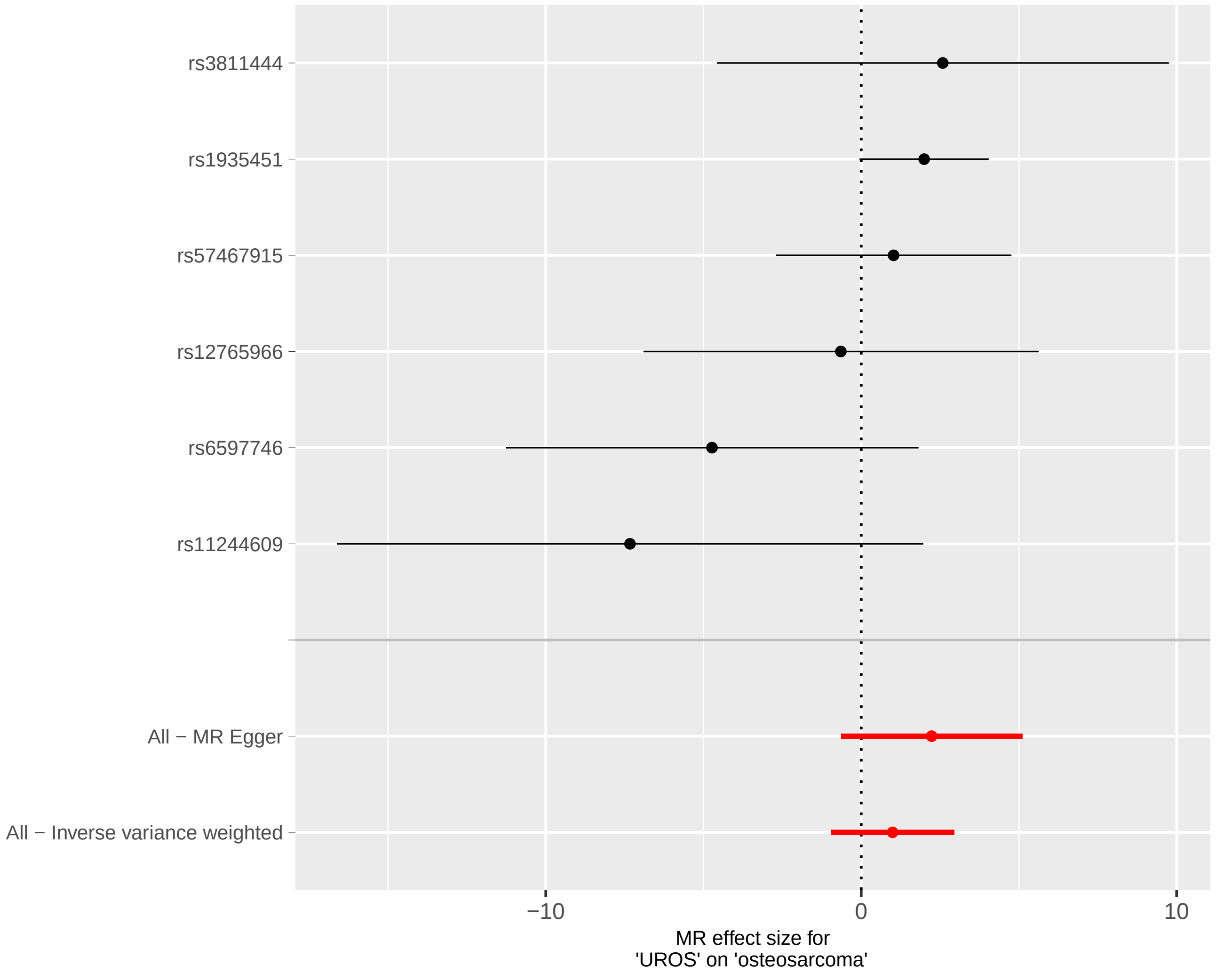

rs3811444
rs1935451
rs57467915
rs12765966
rs6597746
rs11244609
All − MR Egger
All − Inverse variance weighted
−10
0
10
MR effect size for
'UROS' on 'osteosarcoma'

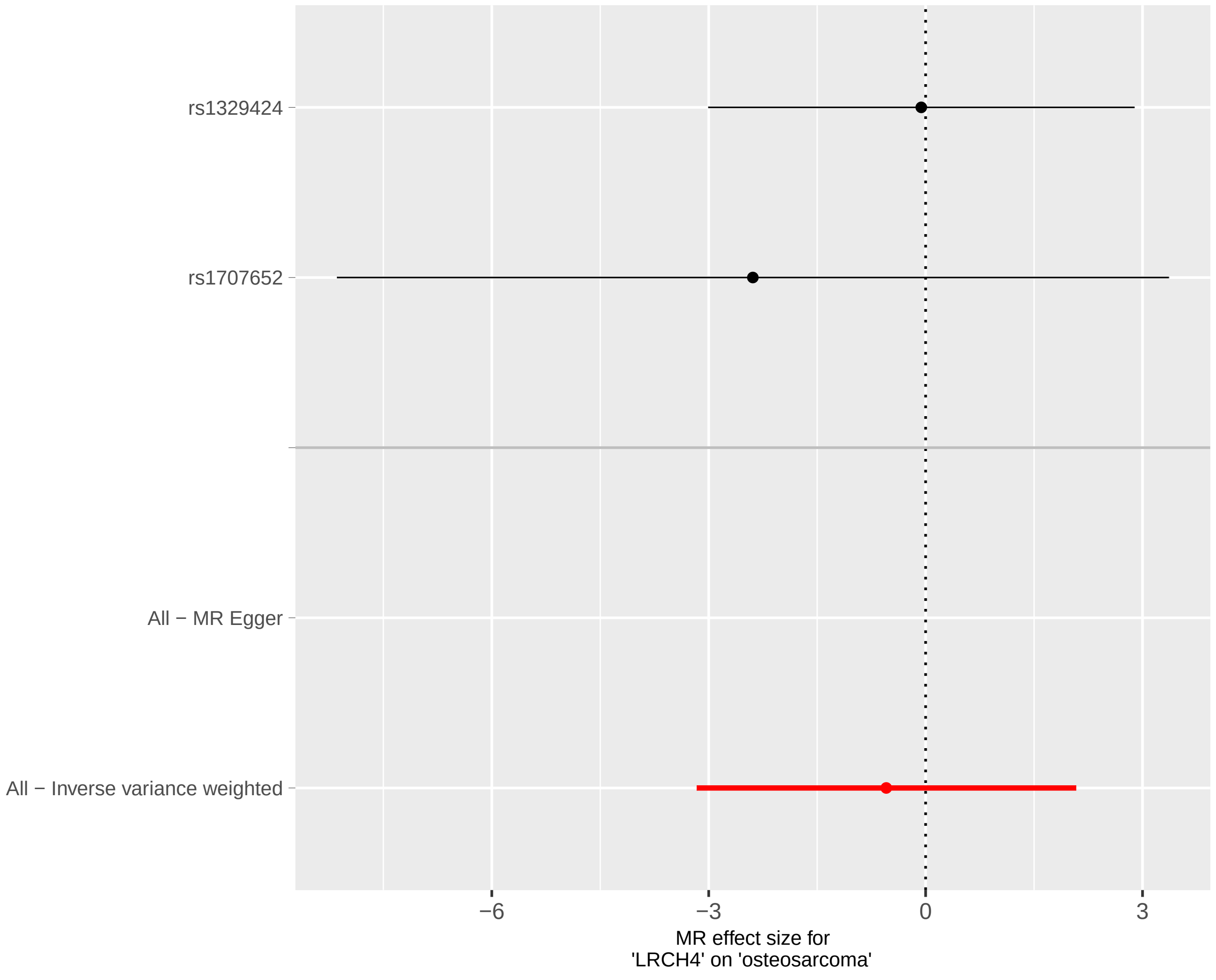

rs1329424
rs1707652
All − MR Egger
All − Inverse variance weighted
−6
−3
0
3
MR effect size for
'LRCH4' on 'osteosarcoma'

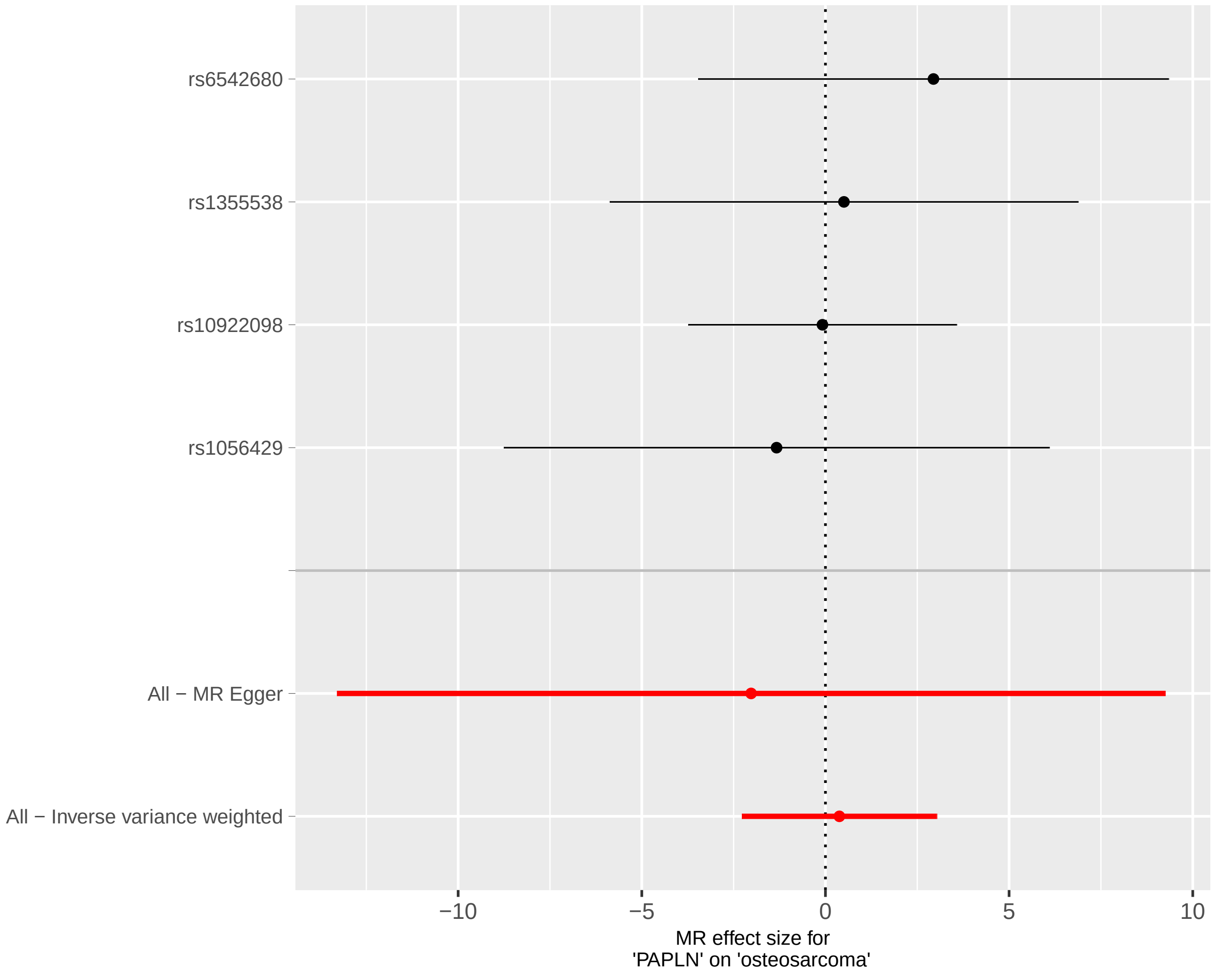
rs6542680
rs1355538
rs10922098
rs1056429
All − MR Egger
All − Inverse variance weighted
−10
−5
0
5
10
MR effect size for
'PAPLN' on 'osteosarcoma'

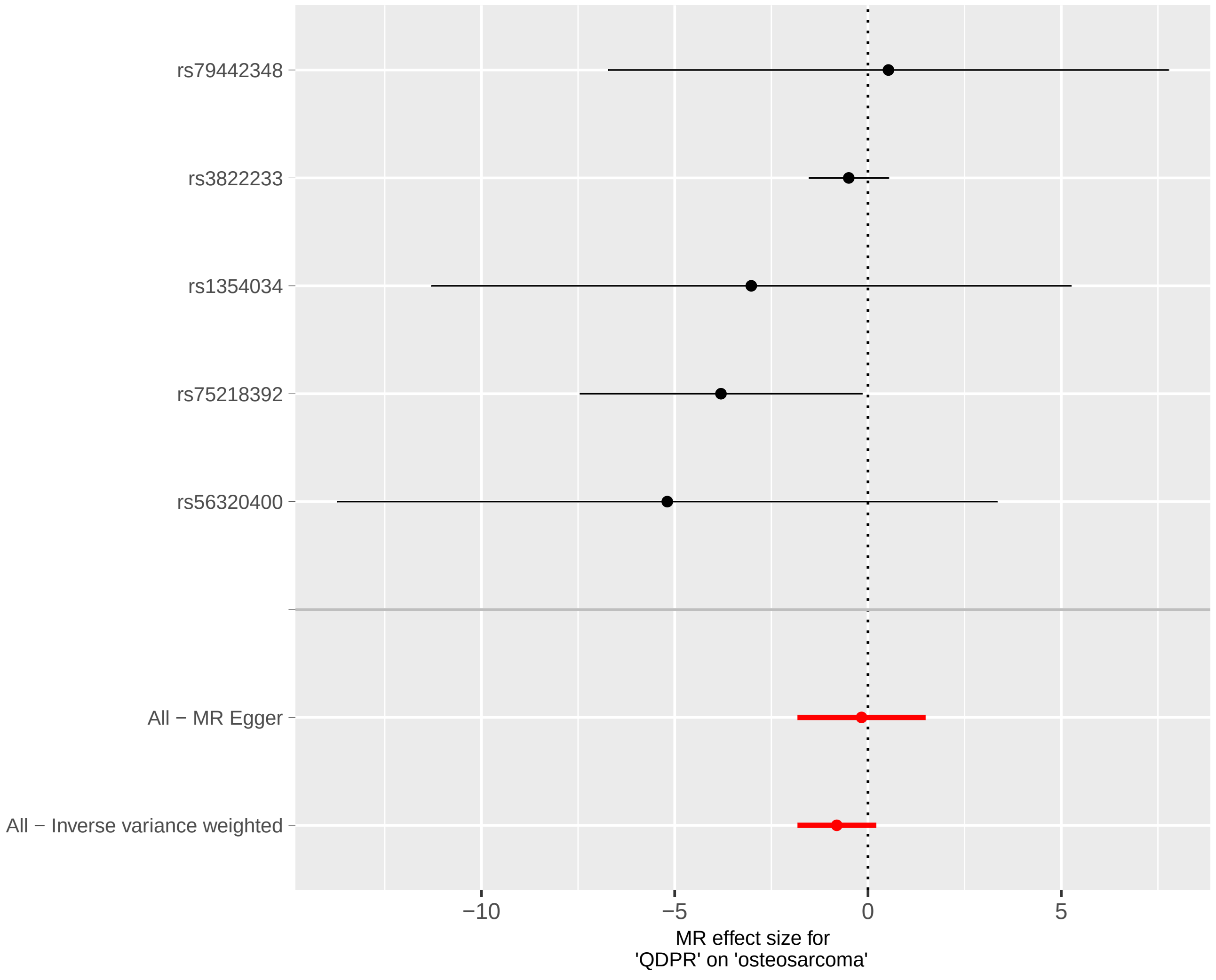

rs79442348
rs3822233
rs1354034
rs75218392
rs56320400
All − MR Egger
All − Inverse variance weighted
−10
−5
0
5
MR effect size for
'QDPR' on 'osteosarcoma'

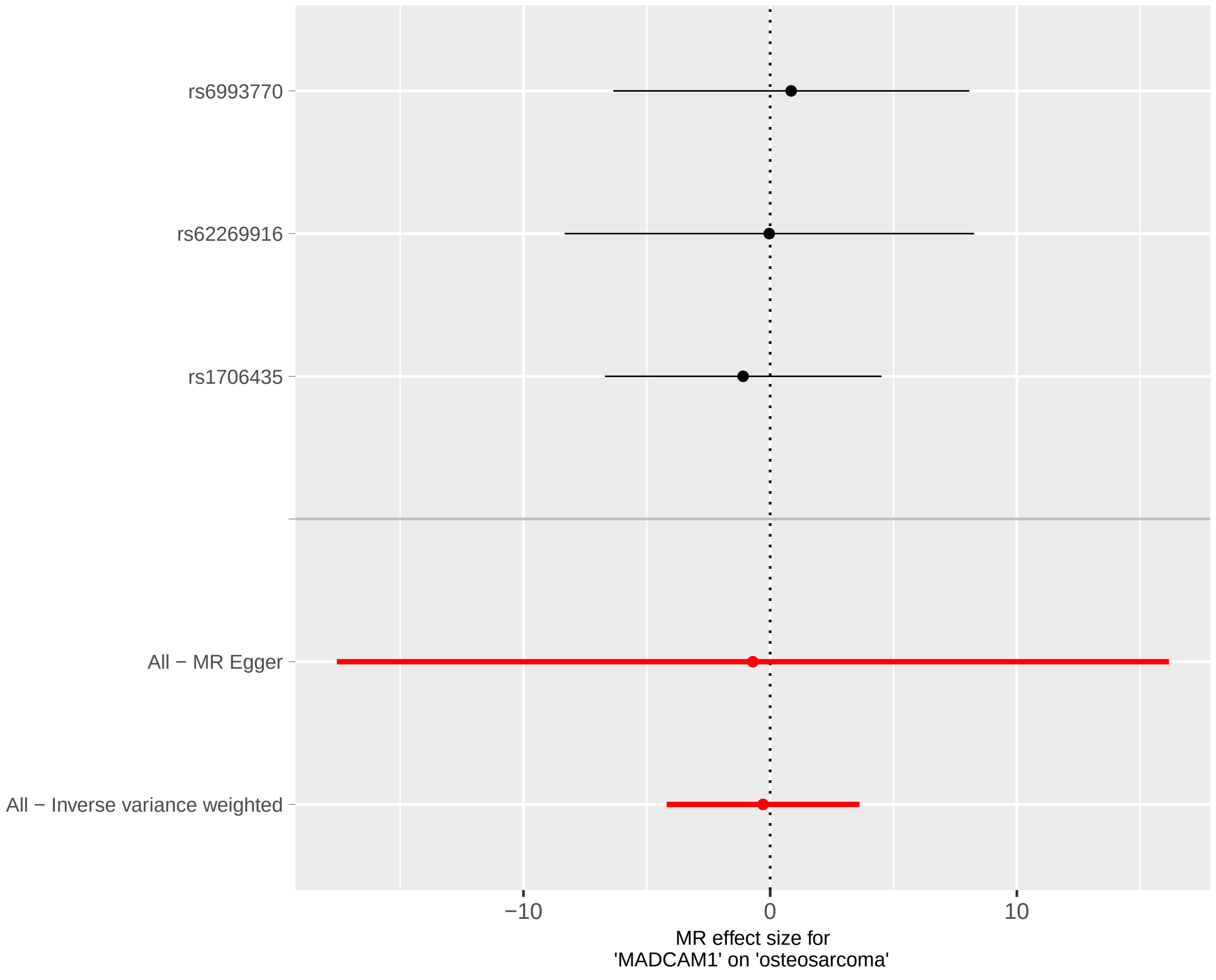
rs6993770
rs62269916
rs1706435
All − MR Egger
All − Inverse variance weighted
−10
0
10
MR effect size for
'MADCAM1' on 'osteosarcoma'

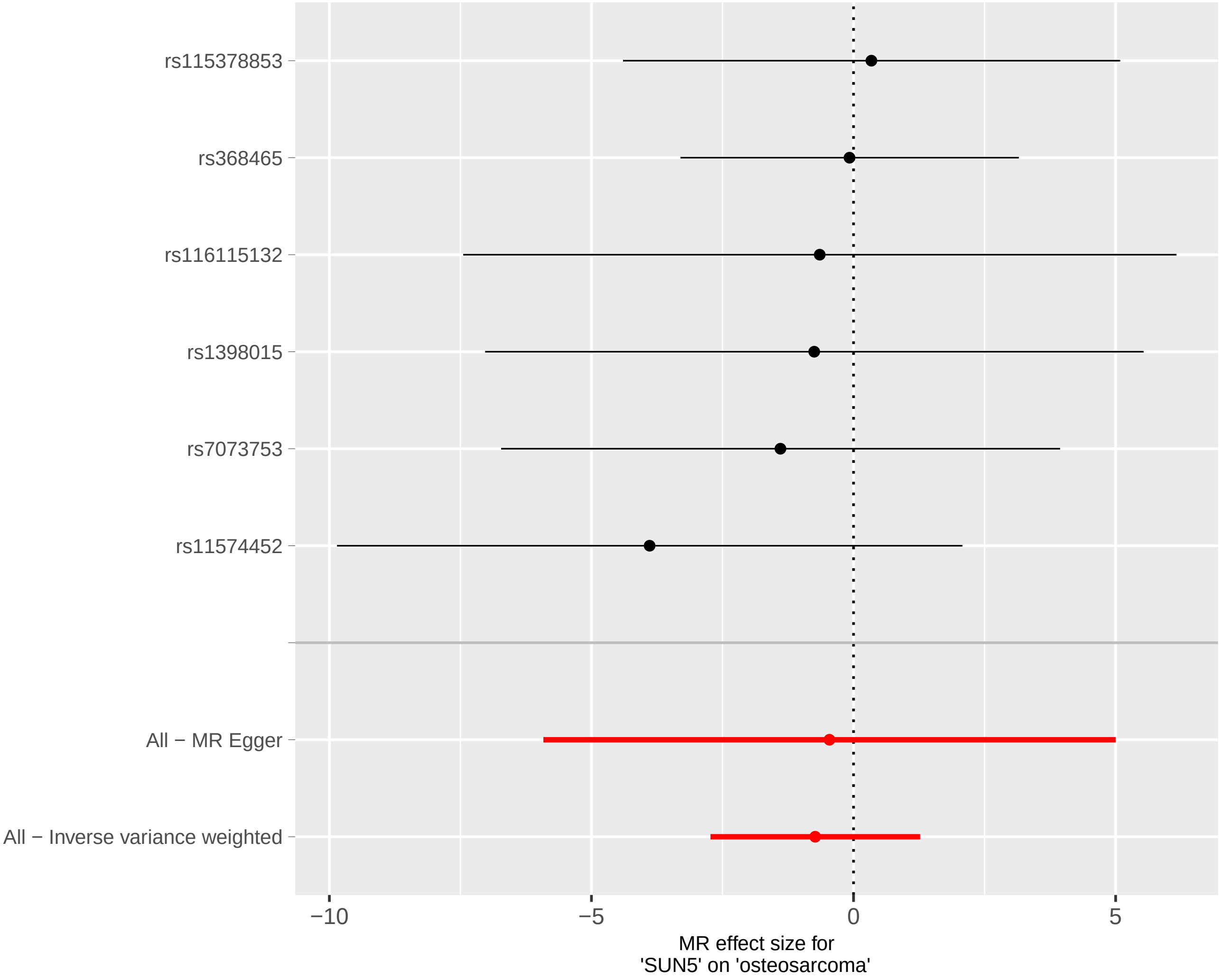
rs115378853
rs368465
rs116115132
rs1398015
rs7073753
rs11574452
All − MR Egger
All − Inverse variance weighted
−10
−5
0
5
MR effect size for
'SUN5' on 'osteosarcoma'

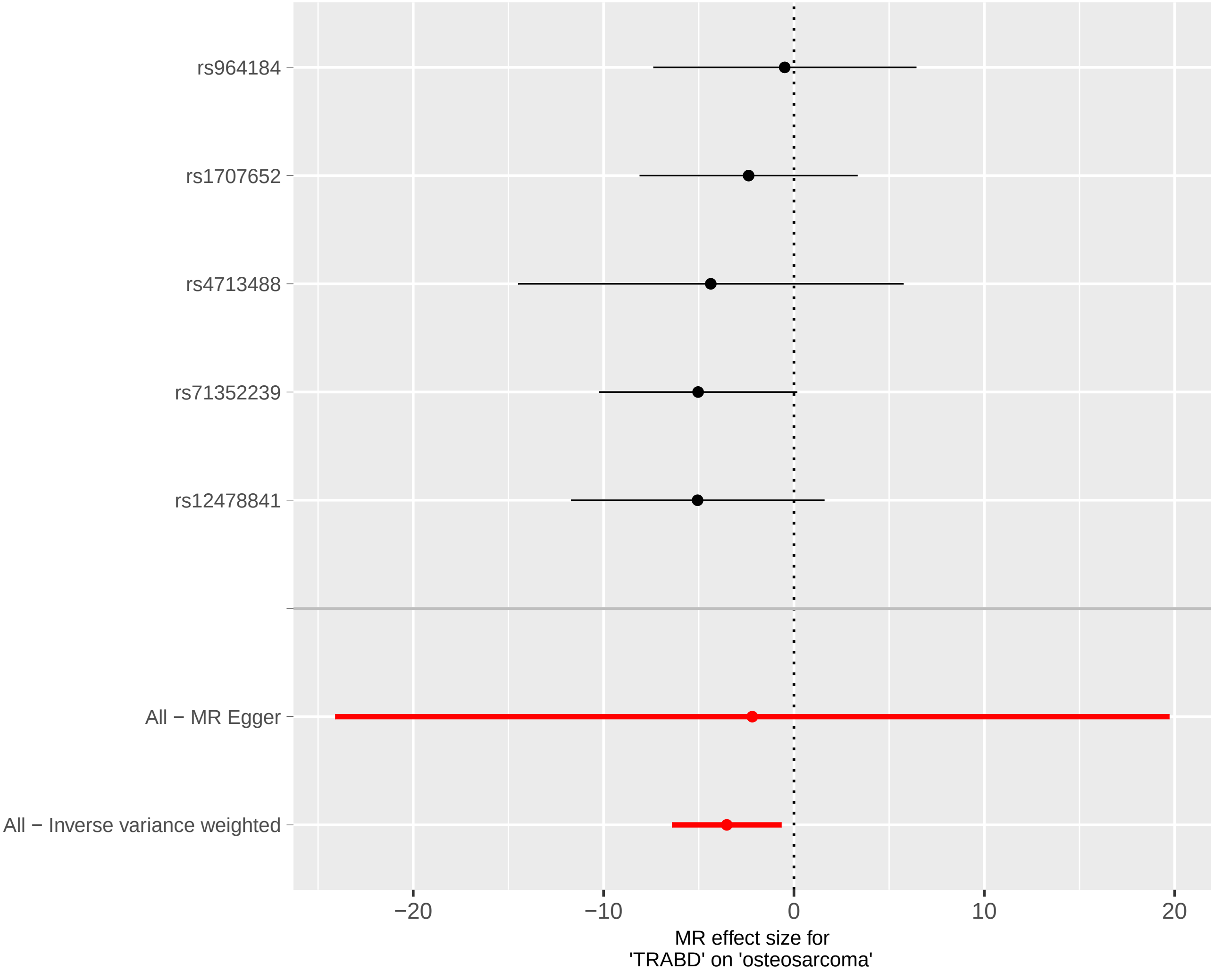

rs964184
rs1707652
rs4713488
rs71352239
rs12478841
All − MR Egger
All − Inverse variance weighted
−20
−10
0
10
20
MR effect size for
'TRABD' on 'osteosarcoma'

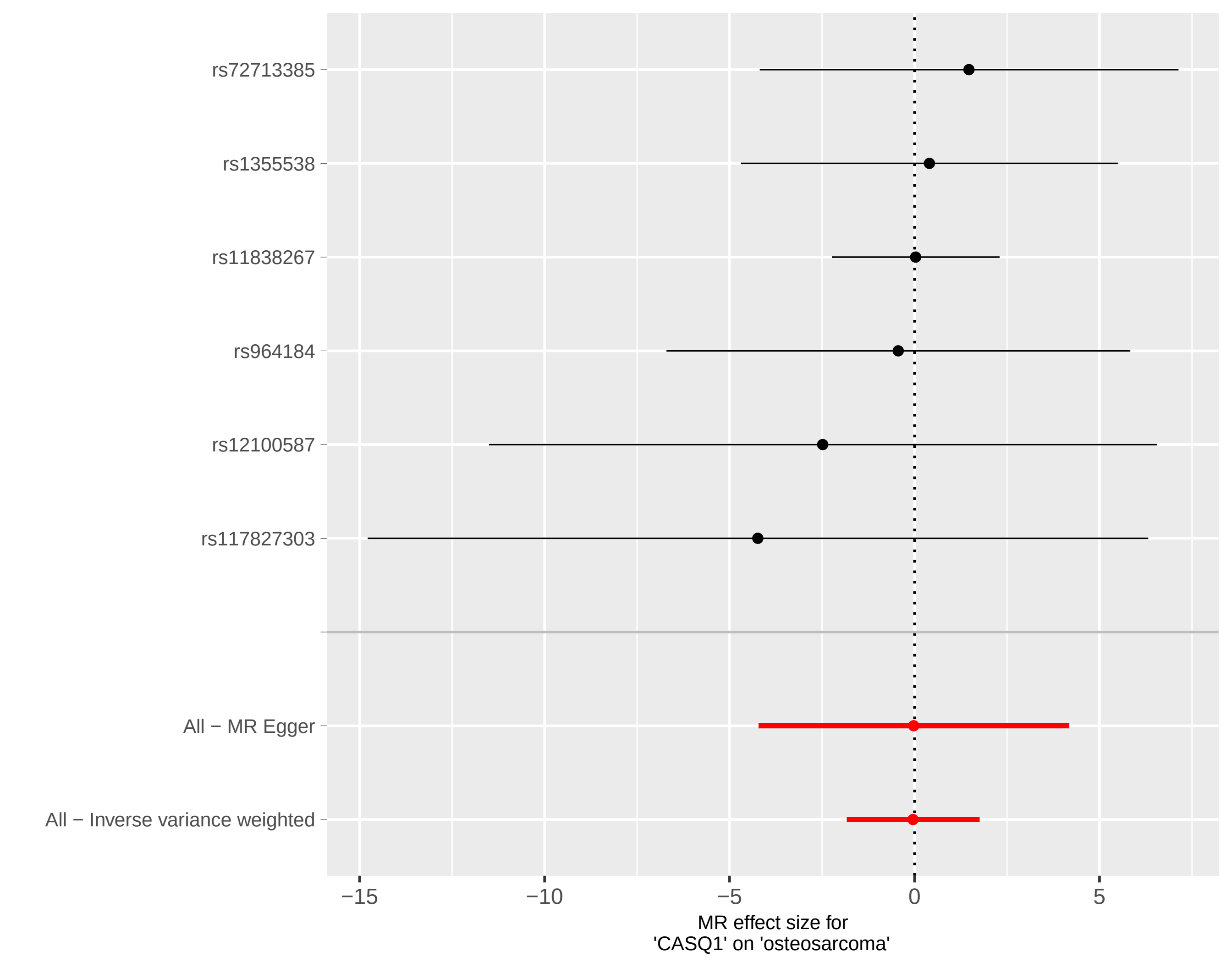
rs72713385
rs1355538
rs11838267
rs964184
rs12100587
rs117827303
All − MR Egger
All − Inverse variance weighted
−15
−10
−5
0
5
MR effect size for
'CASQ1' on 'osteosarcoma'

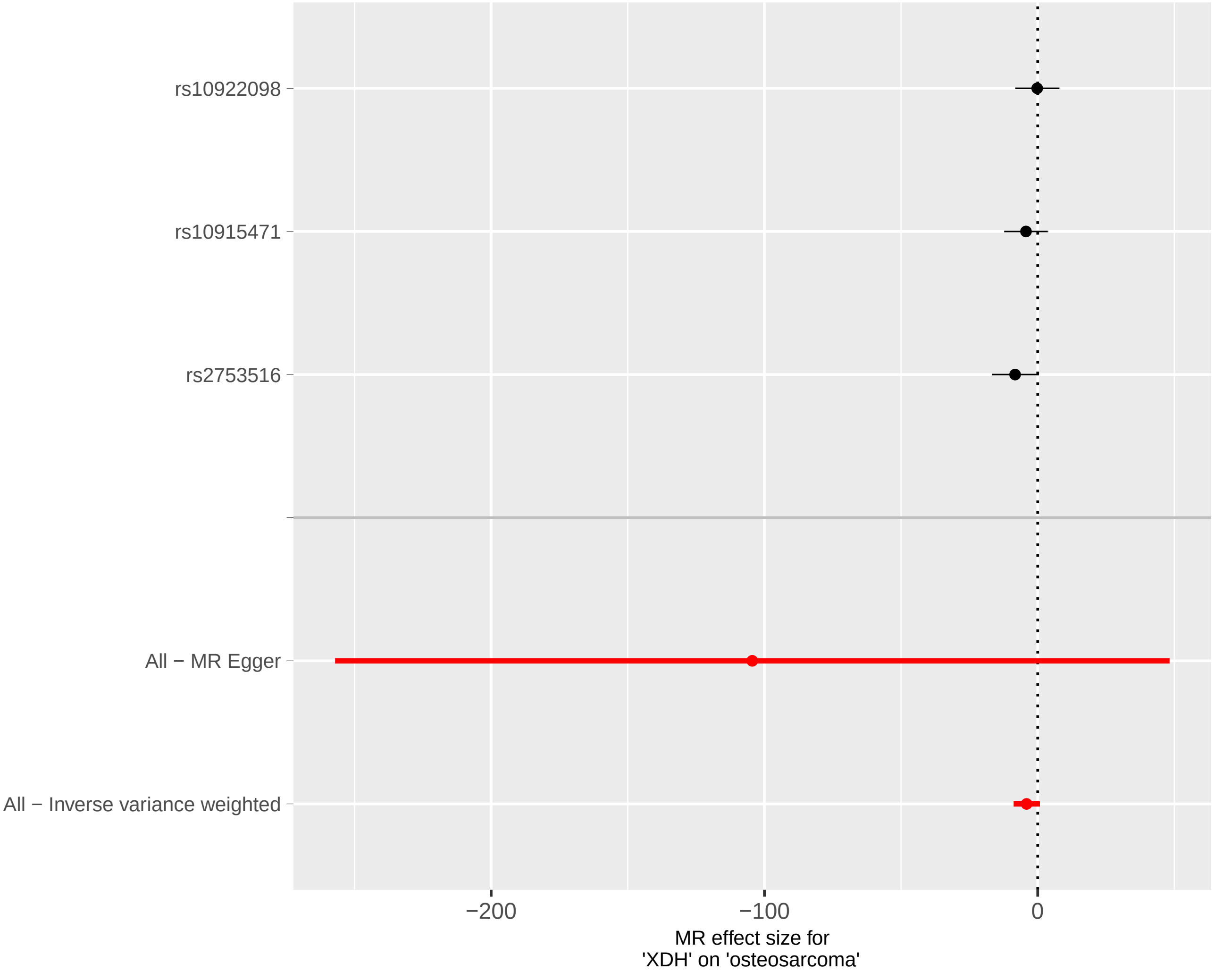
rs10922098
rs10915471
rs2753516
All − MR Egger
All − Inverse variance weighted
−200
−100
0
MR effect size for
'XDH' on 'osteosarcoma'

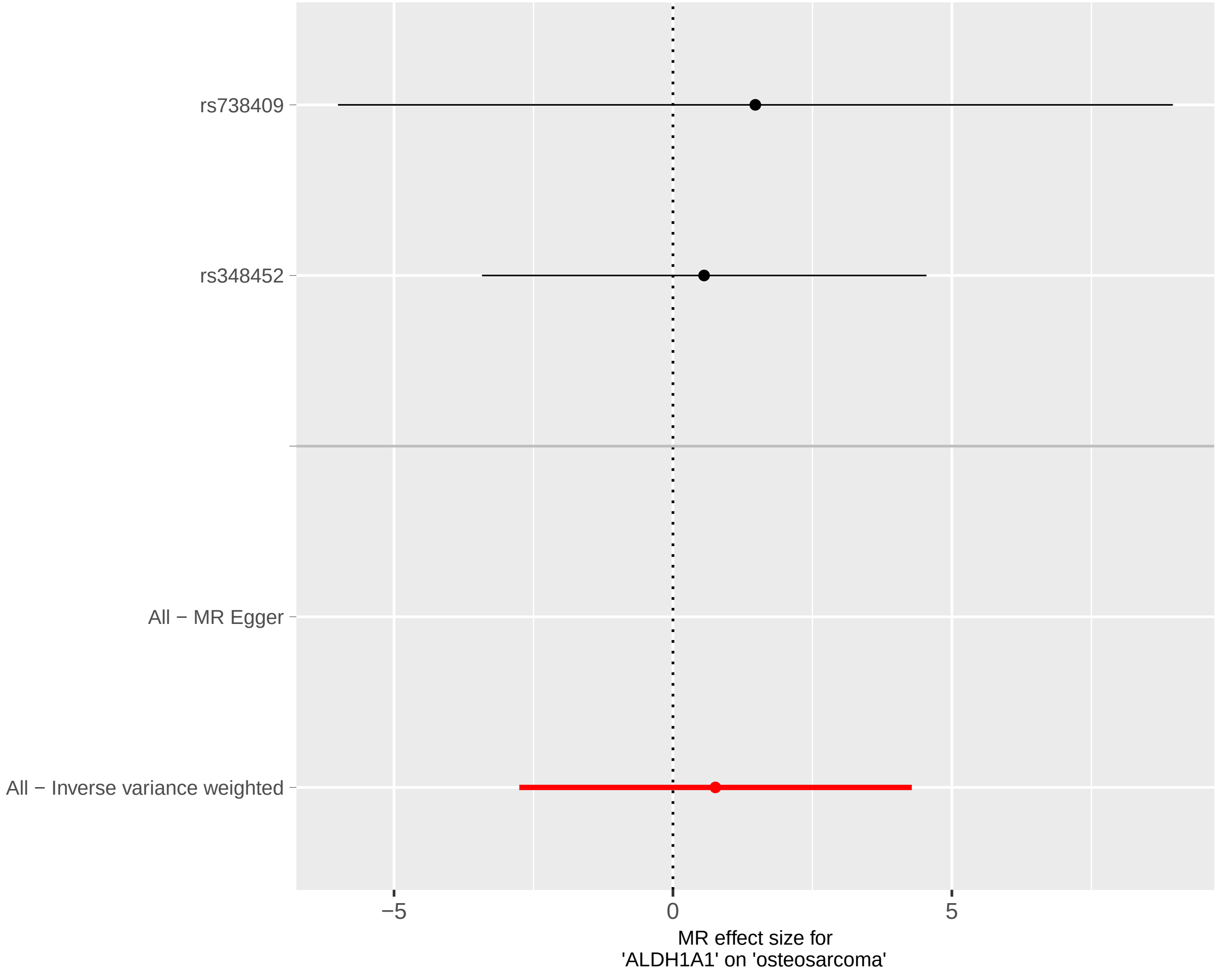

rs738409
rs348452
All − MR Egger
All − Inverse variance weighted
−5
0
5
MR effect size for
'ALDH1A1' on 'osteosarcoma'

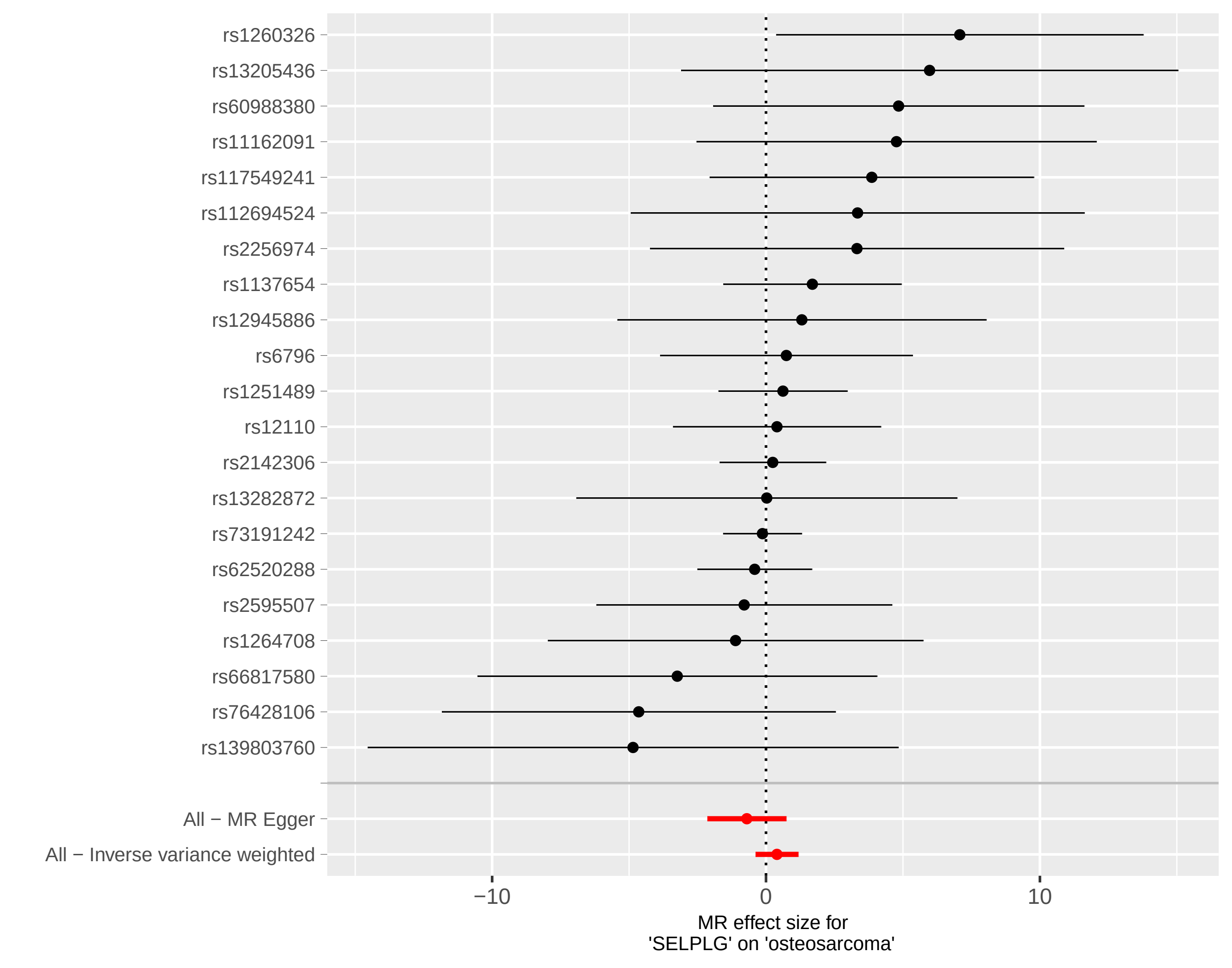

rs1260326
rs13205436
rs60988380
rs11162091
rs117549241
rs112694524
rs2256974
rs1137654
rs12945886
rs6796
rs1251489
rs12110
rs2142306
rs13282872
rs73191242
rs62520288
rs2595507
rs1264708
rs66817580
rs76428106
rs139803760
All − MR Egger
All − Inverse variance weighted
−10
0
10
MR effect size for
'SELPLG' on 'osteosarcoma'

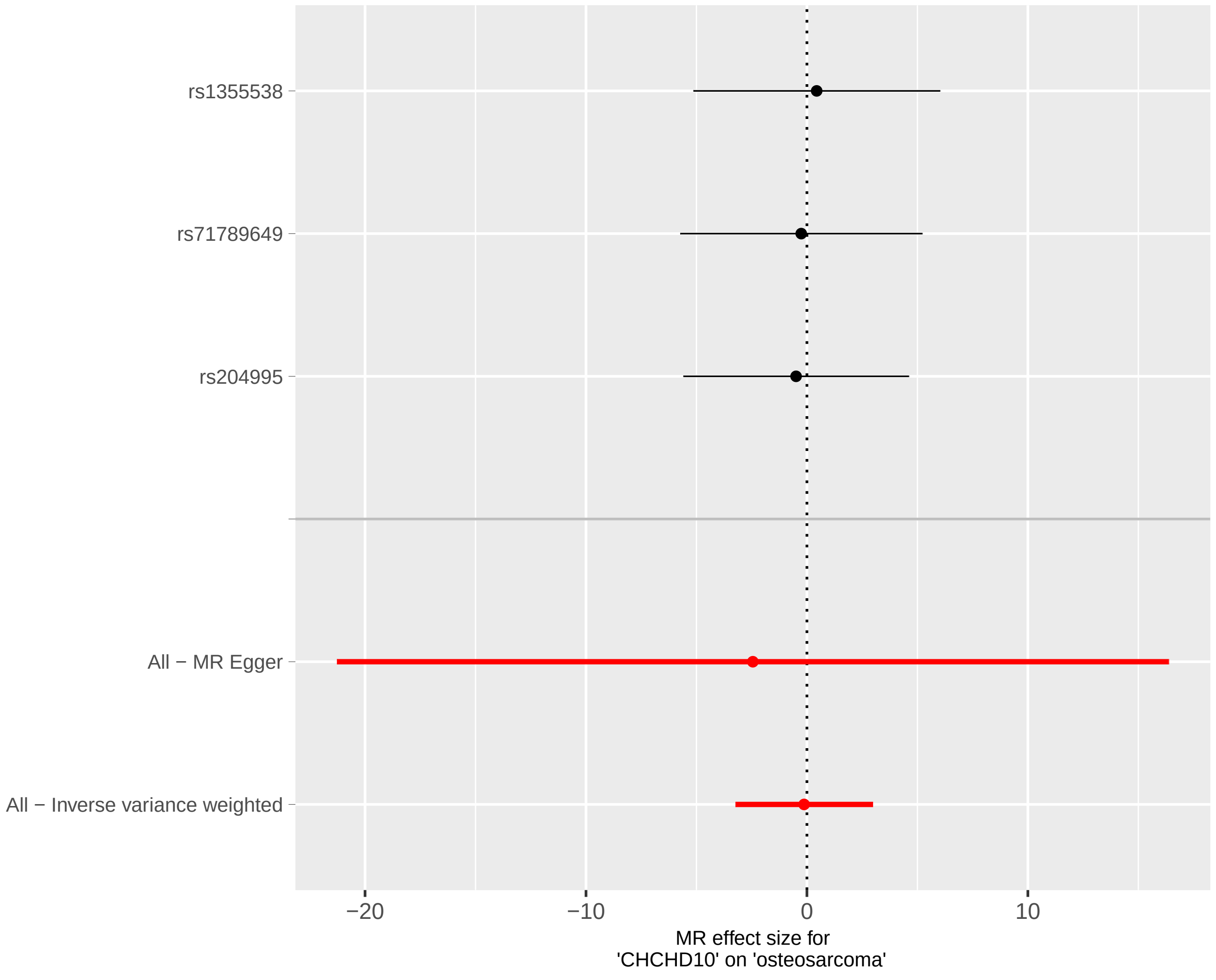

rs1355538
rs71789649
rs204995
All − MR Egger
All − Inverse variance weighted
−20
−10
0
10
MR effect size for
'CHCHD10' on 'osteosarcoma'

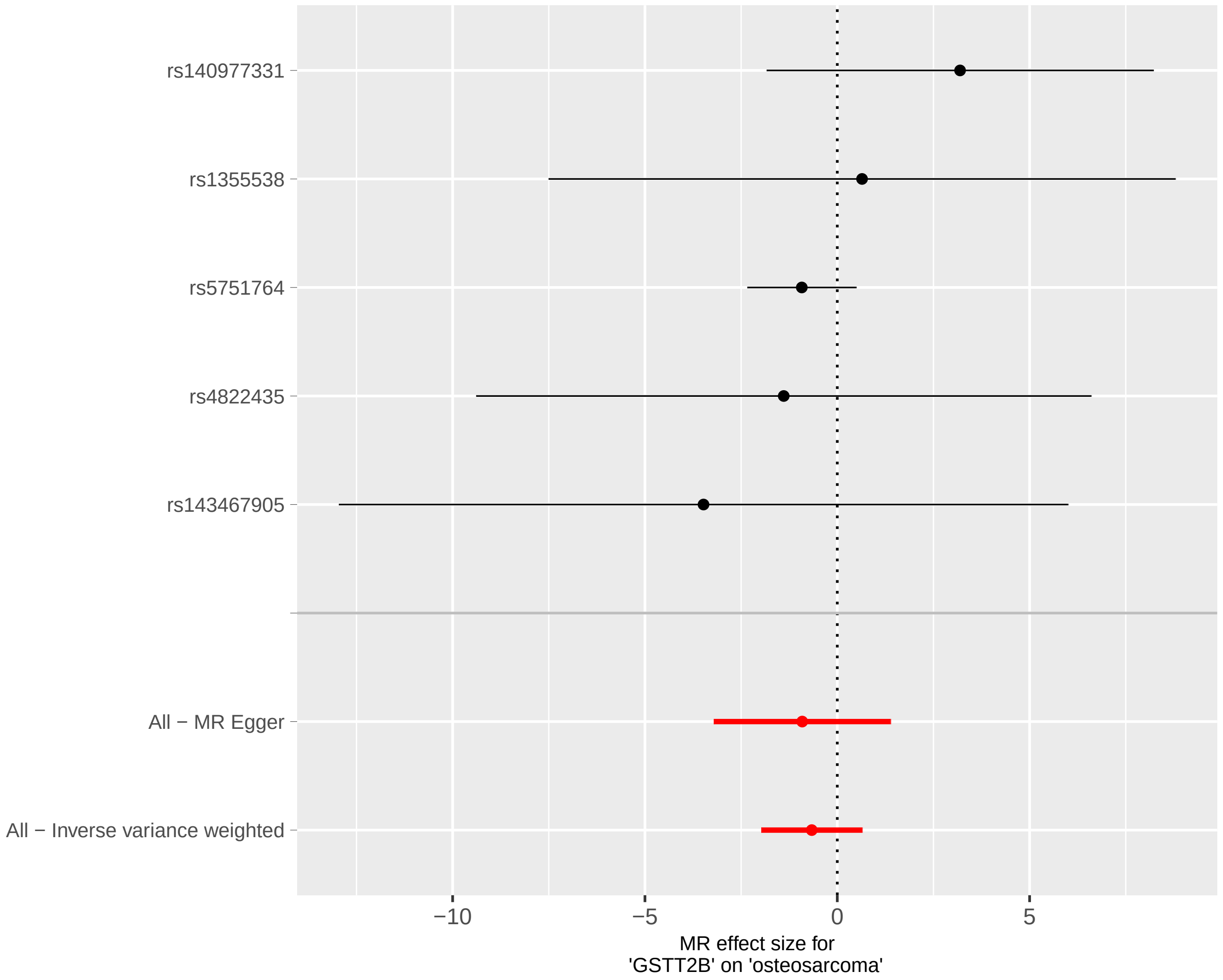

rs140977331
rs1355538
rs5751764
rs4822435
rs143467905
All − MR Egger
All − Inverse variance weighted
−10
−5
0
5
MR effect size for
'GSTT2B' on 'osteosarcoma'

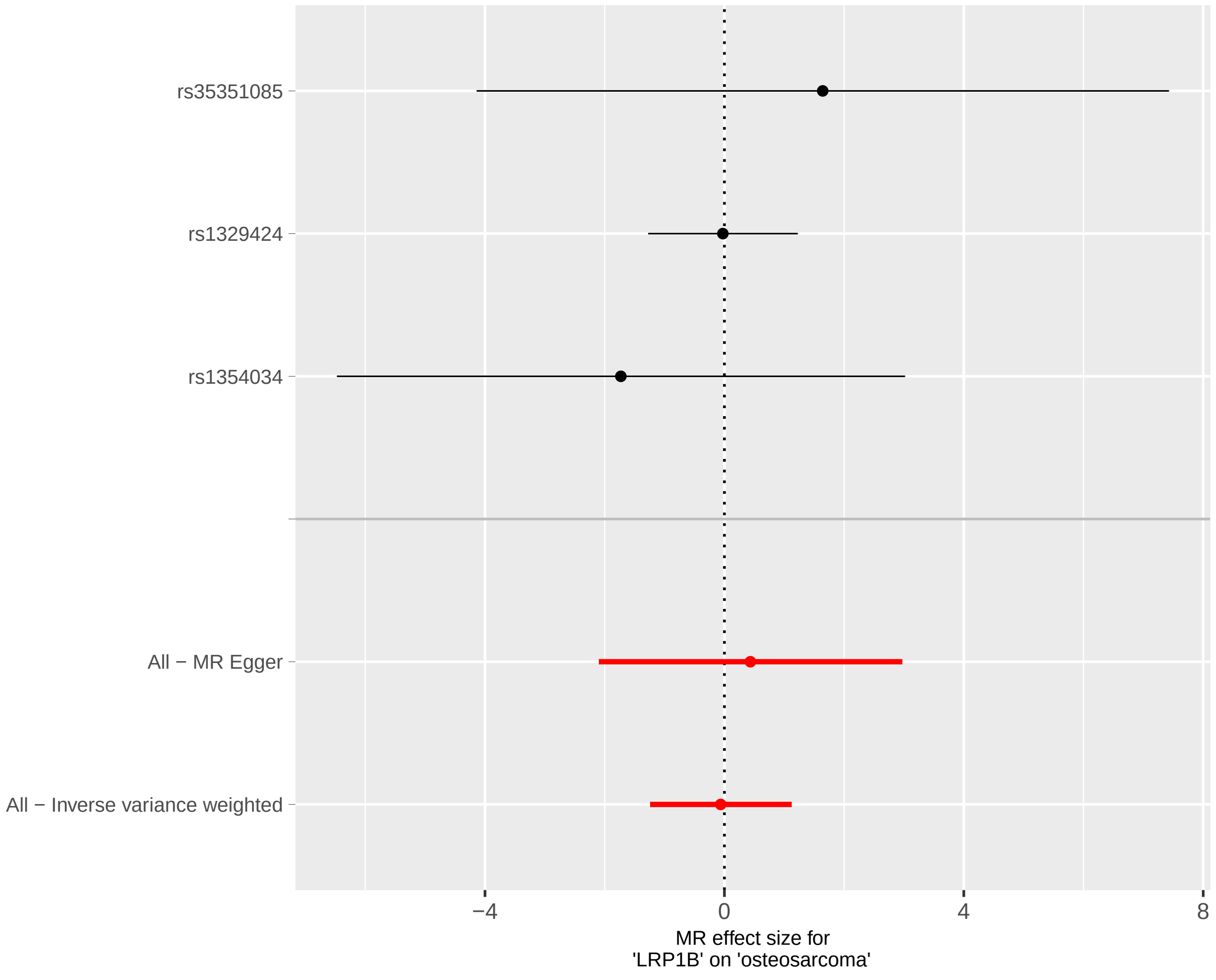
rs35351085
rs1329424
rs1354034
All − MR Egger
All − Inverse variance weighted
−4
0
4
8
MR effect size for
'LRP1B' on 'osteosarcoma'

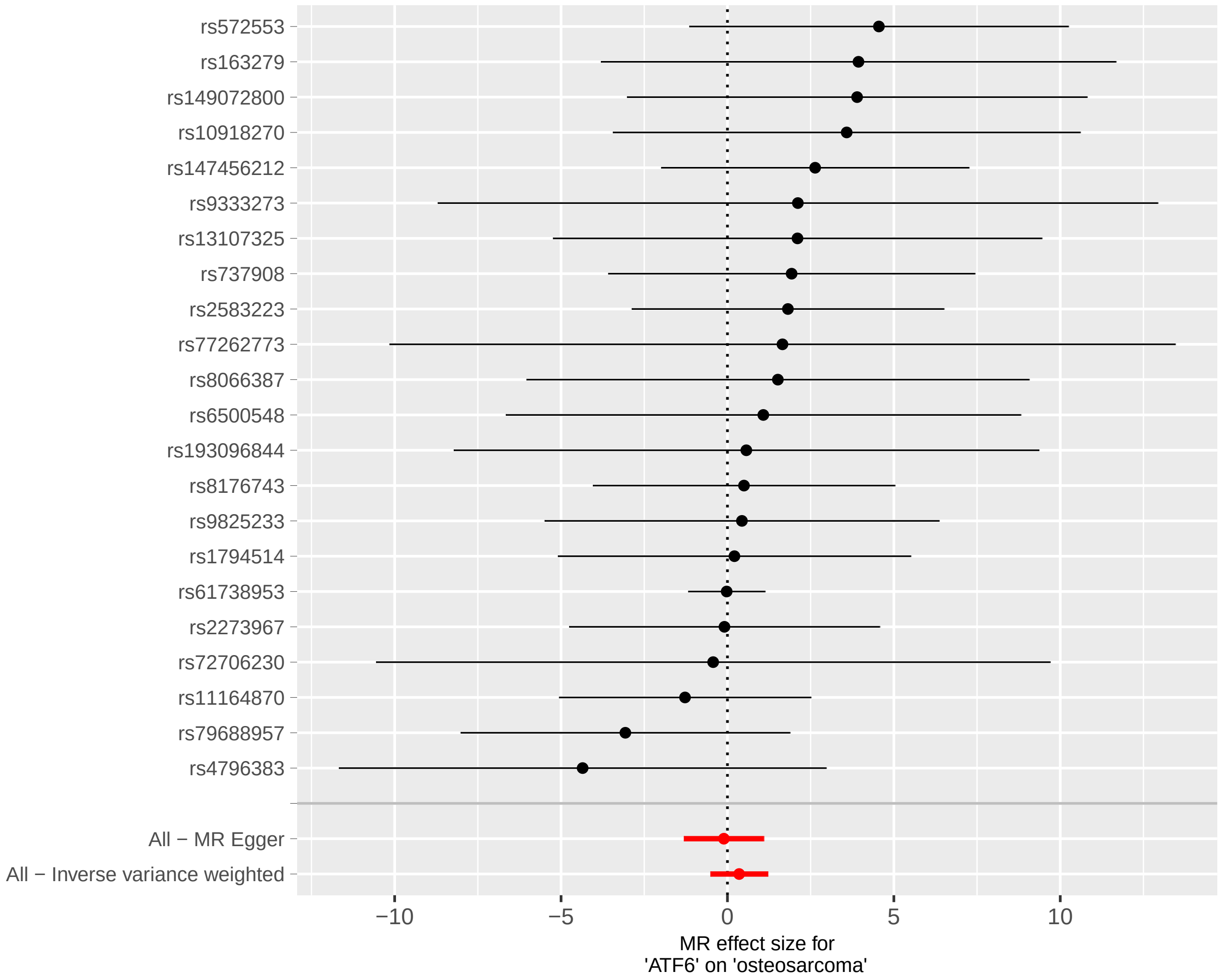

rs572553
rs163279
rs149072800
rs10918270
rs147456212
rs9333273
rs13107325
rs737908
rs2583223
rs77262773
rs8066387
rs6500548
rs193096844
rs8176743
rs9825233
rs1794514
rs61738953
rs2273967
rs72706230
rs11164870
rs79688957
rs4796383
All − MR Egger
All − Inverse variance weighted
−10
−5
0
5
10
MR effect size for
'ATF6' on 'osteosarcoma'

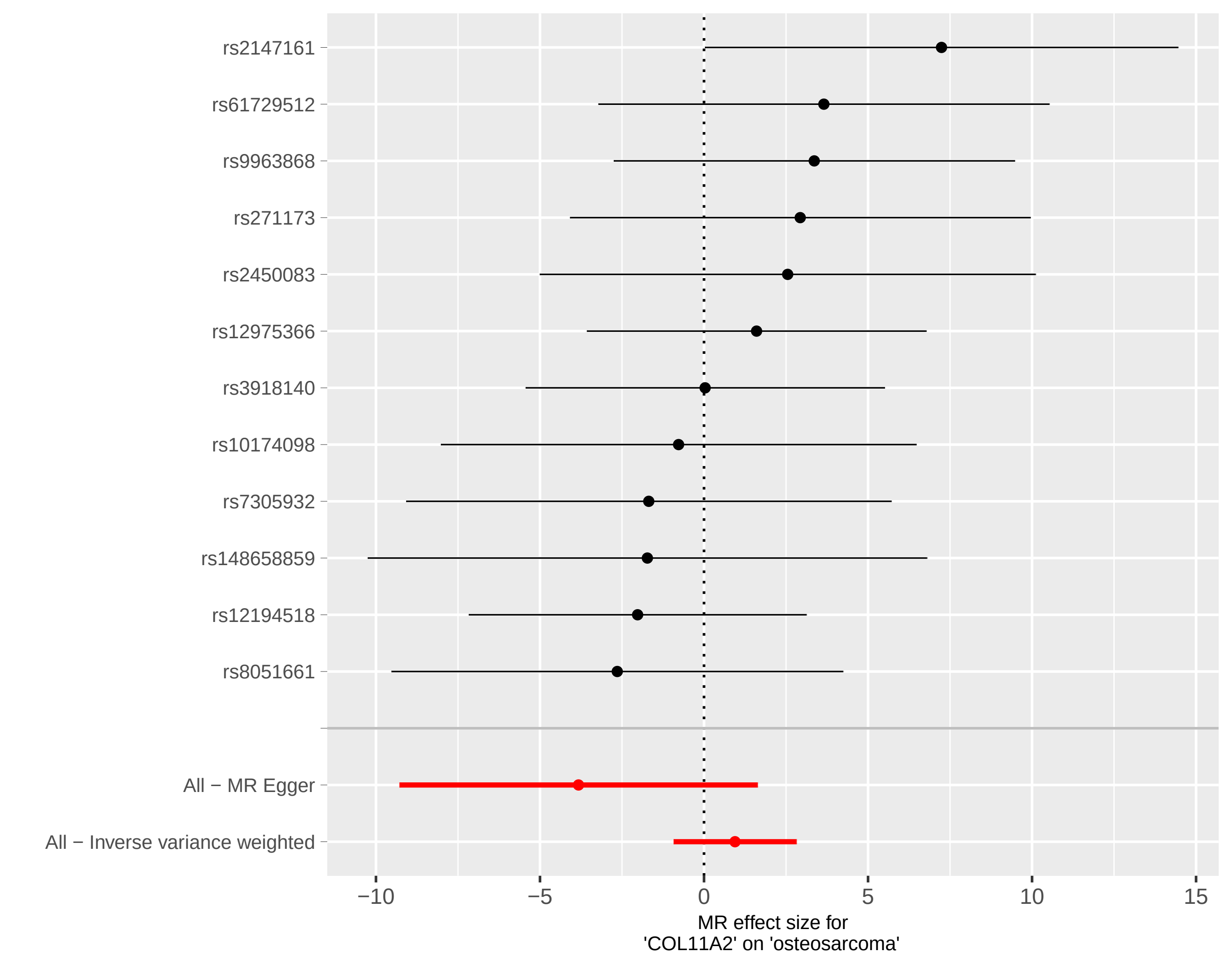
rs2147161
rs61729512
rs9963868
rs271173
rs2450083
rs12975366
rs3918140
rs10174098
rs7305932
rs148658859
rs12194518
rs8051661
All − MR Egger
All − Inverse variance weighted
−10
−5
0
5
10
15
MR effect size for
'COL11A2' on 'osteosarcoma'

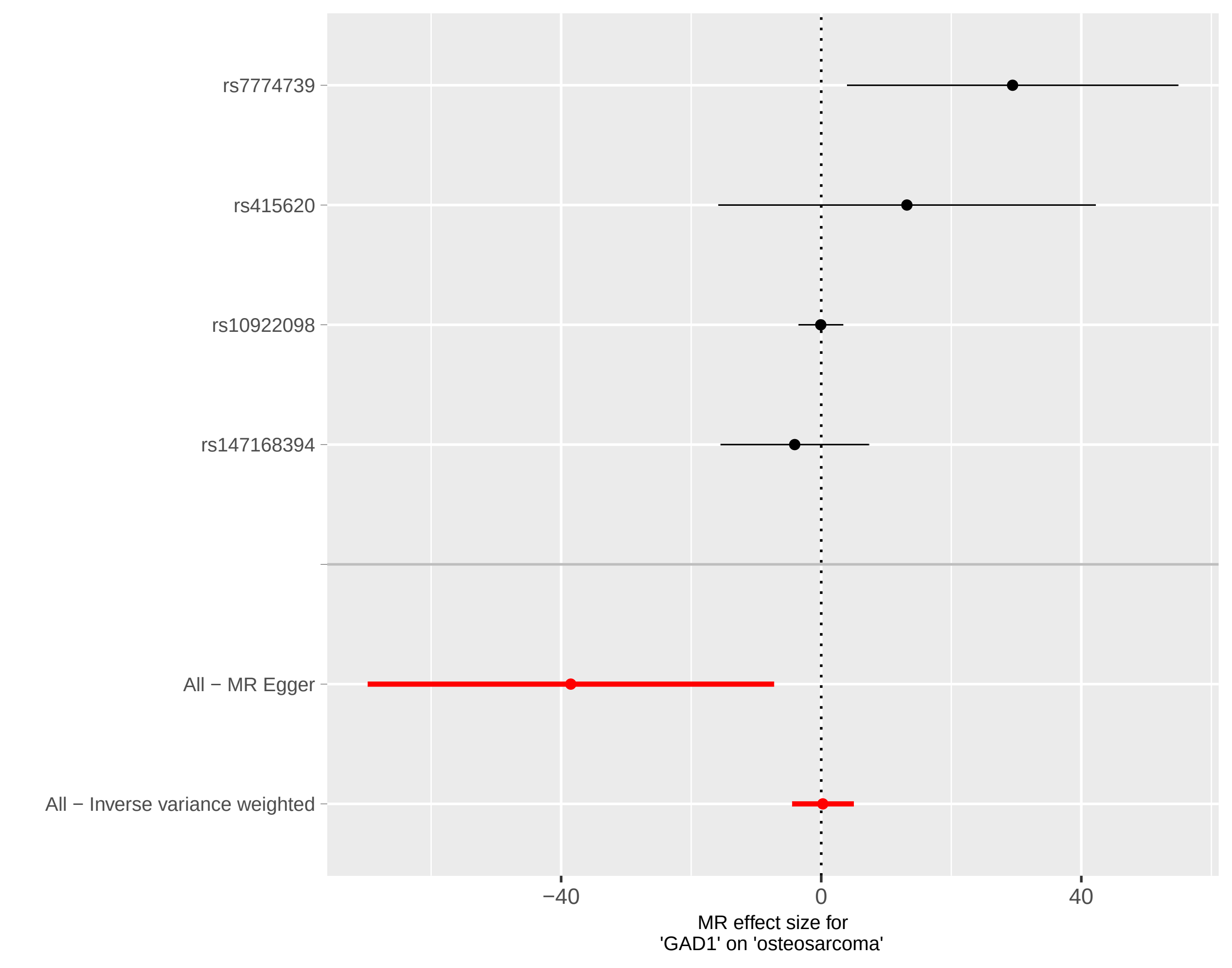

rs7774739
rs415620
rs10922098
rs147168394
All − MR Egger
All − Inverse variance weighted
−40
0
40
MR effect size for
'GAD1' on 'osteosarcoma'

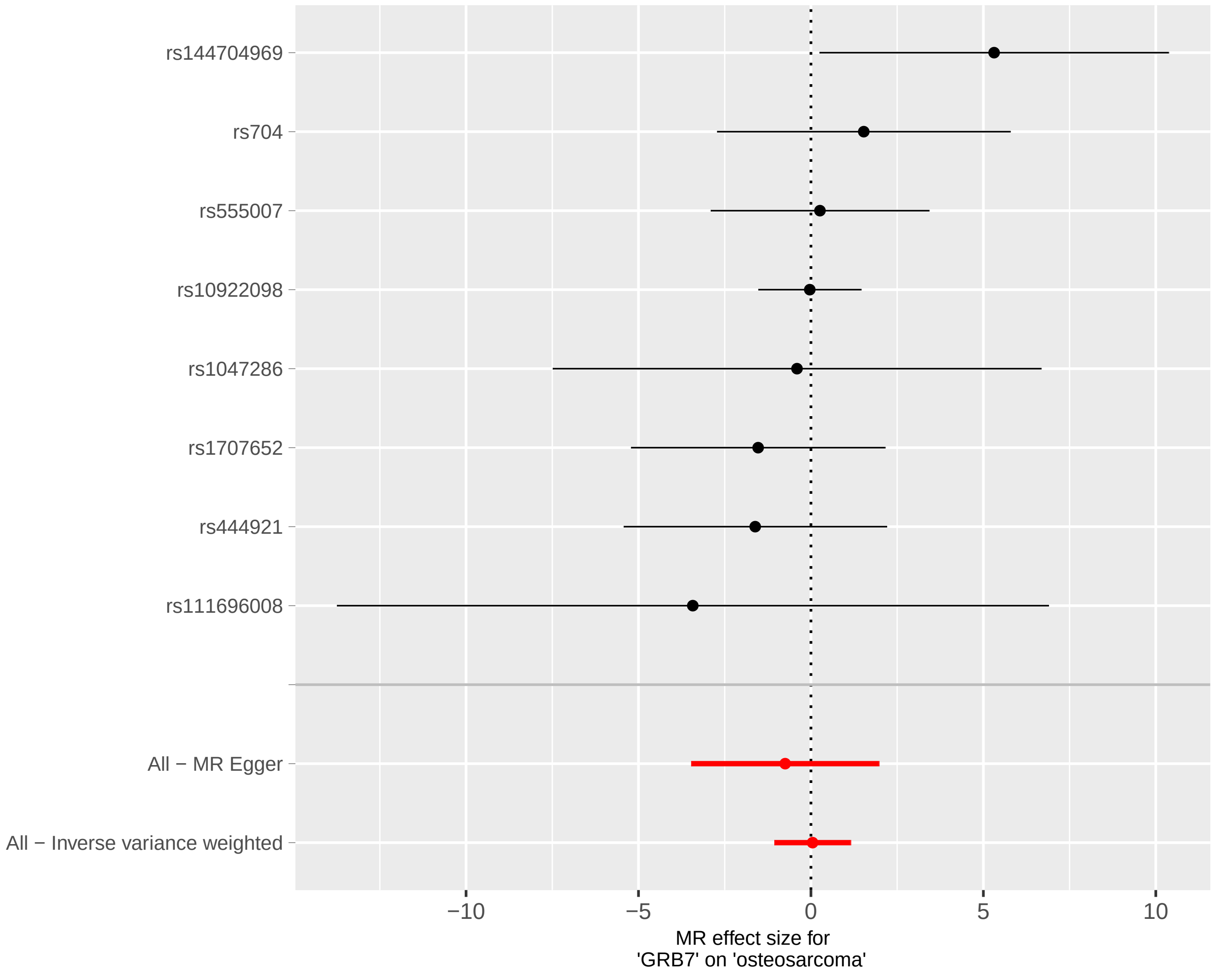

rs144704969
rs704
rs555007
rs10922098
rs1047286
rs1707652
rs444921
rs111696008
All − MR Egger
All − Inverse variance weighted
−10
−5
0
5
10
MR effect size for
'GRB7' on 'osteosarcoma'

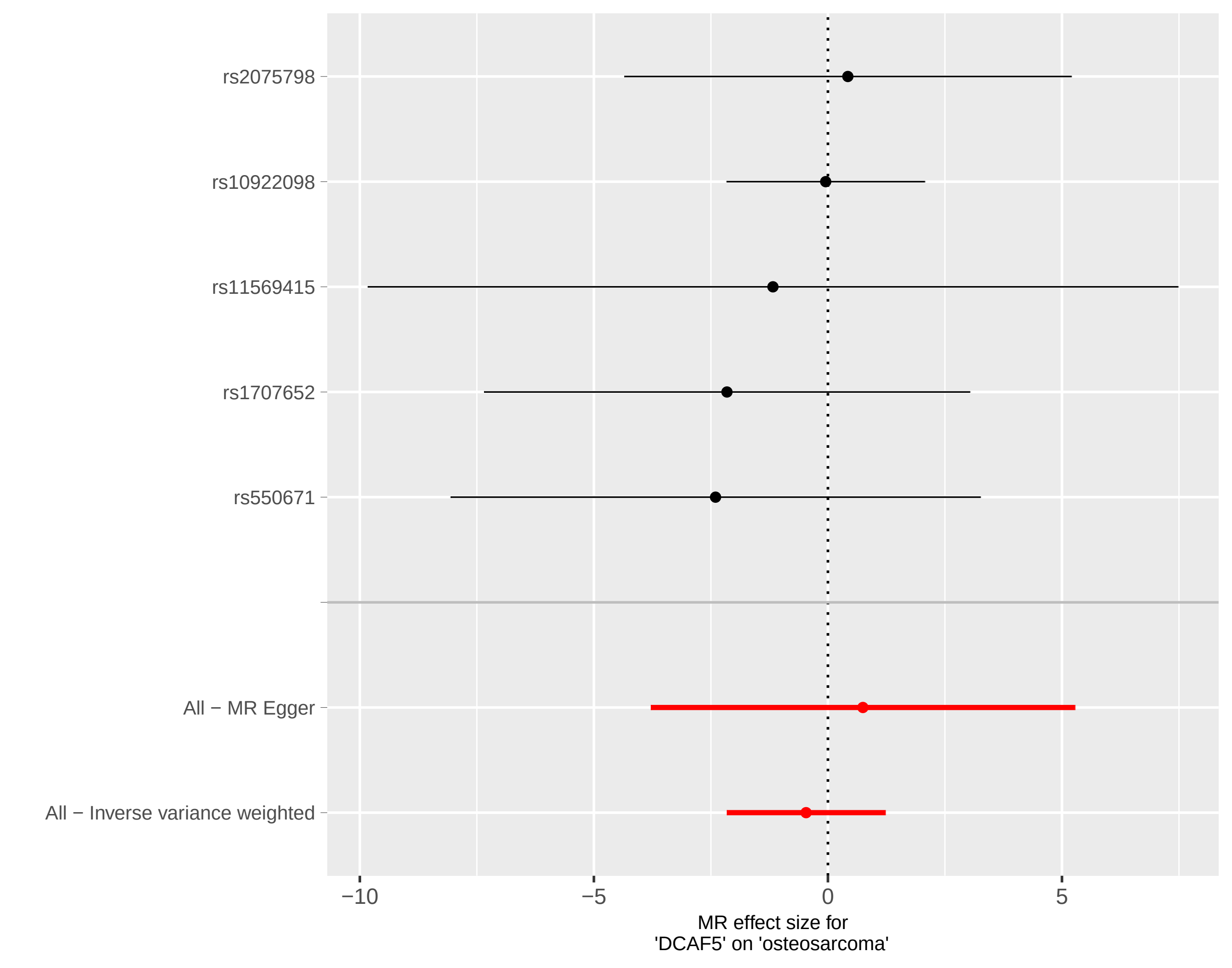
rs2075798
rs10922098
rs11569415
rs1707652
rs550671
All − MR Egger
All − Inverse variance weighted
−10
−5
0
5
MR effect size for
'DCAF5' on 'osteosarcoma'

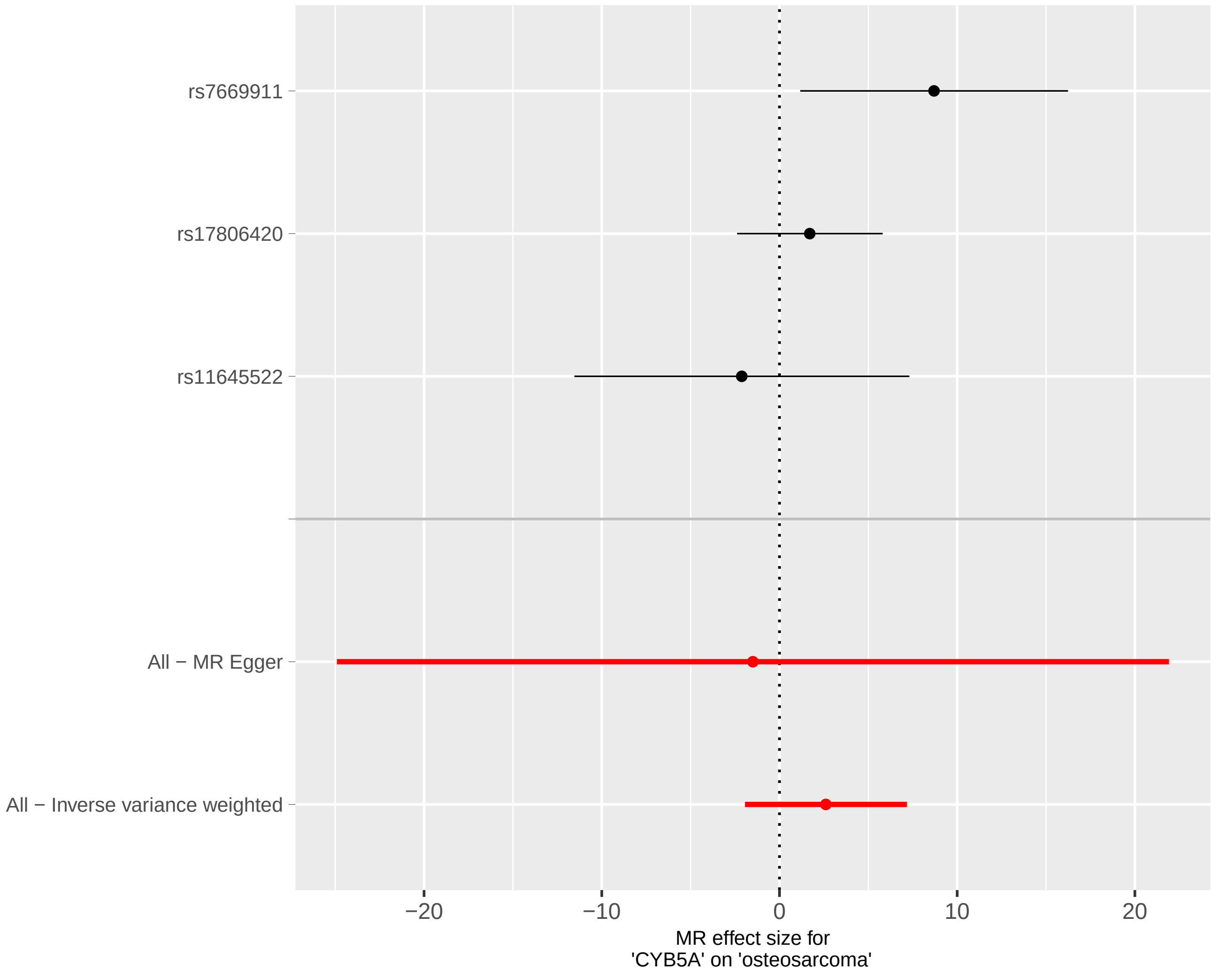
rs7669911
rs17806420
rs11645522
All − MR Egger
All − Inverse variance weighted
−20
−10
0
10
20
MR effect size for
'CYB5A' on 'osteosarcoma'

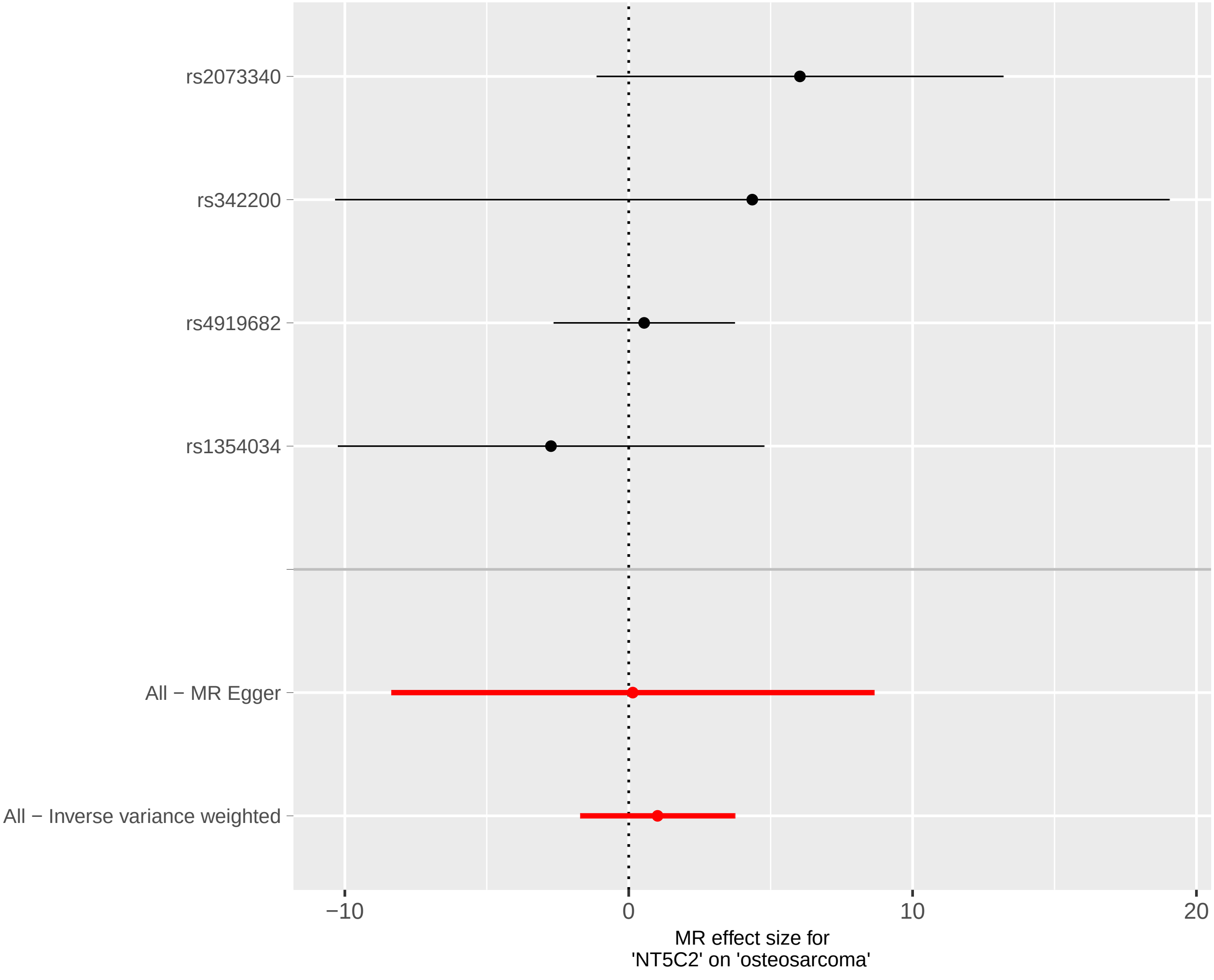

rs2073340
rs342200
rs4919682
rs1354034
All − MR Egger
All − Inverse variance weighted
−10
0
10
20
MR effect size for
'NT5C2' on 'osteosarcoma'

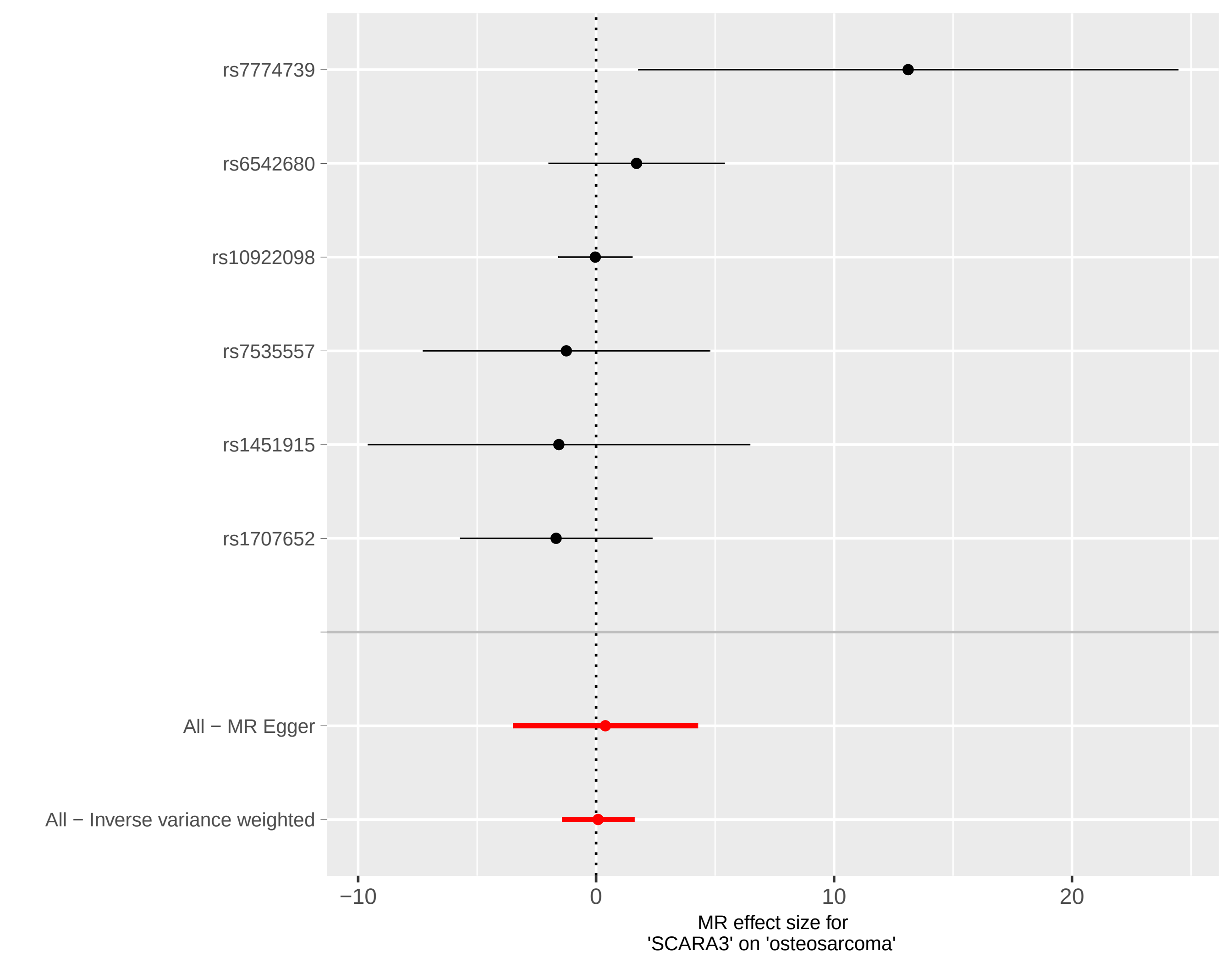

rs7774739
rs6542680
rs10922098
rs7535557
rs1451915
rs1707652
All − MR Egger
All − Inverse variance weighted
−10
0
10
20
MR effect size for
'SCARA3' on 'osteosarcoma'

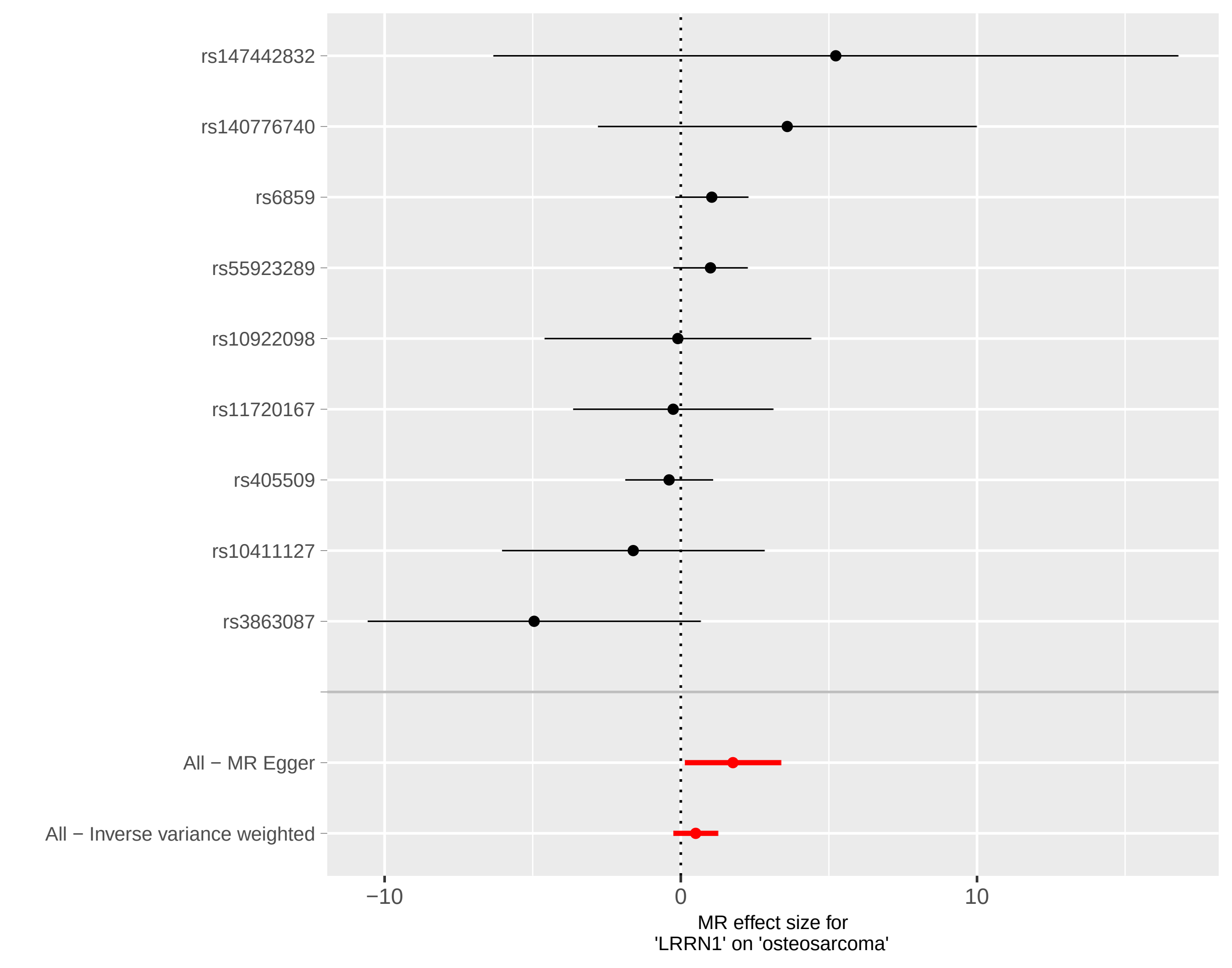

rs147442832
rs140776740
rs6859
rs55923289
rs10922098
rs11720167
rs405509
rs10411127
rs3863087
All − MR Egger
All − Inverse variance weighted
−10
0
10
MR effect size for
'LRRN1' on 'osteosarcoma'

Insufficient number of SNPs

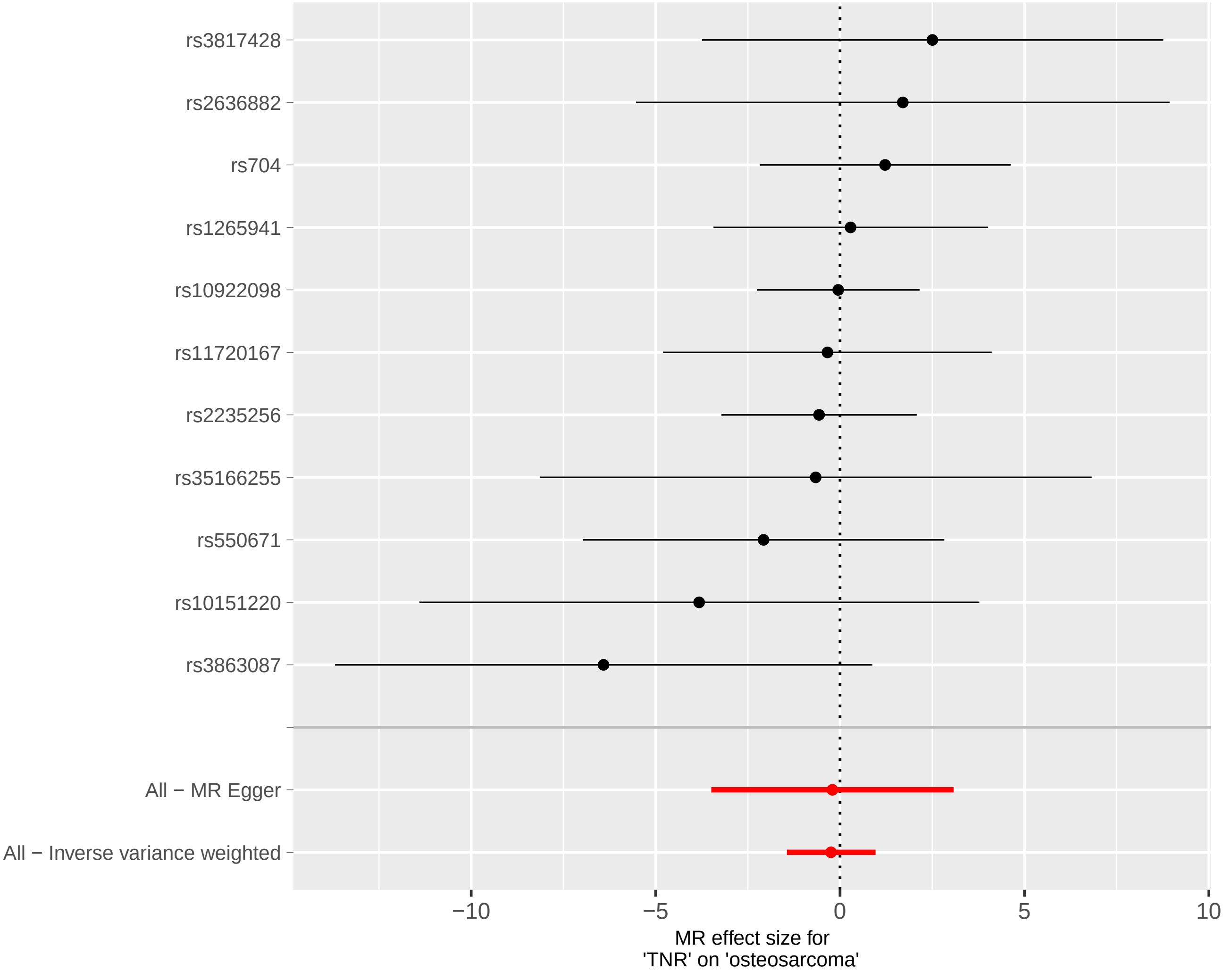

rs3817428
rs2636882
rs704
rs1265941
rs10922098
rs11720167
rs2235256
rs35166255
rs550671
rs10151220
rs3863087
All − MR Egger
All − Inverse variance weighted
−10
−5
0
5
10
MR effect size for
'TNR' on 'osteosarcoma'

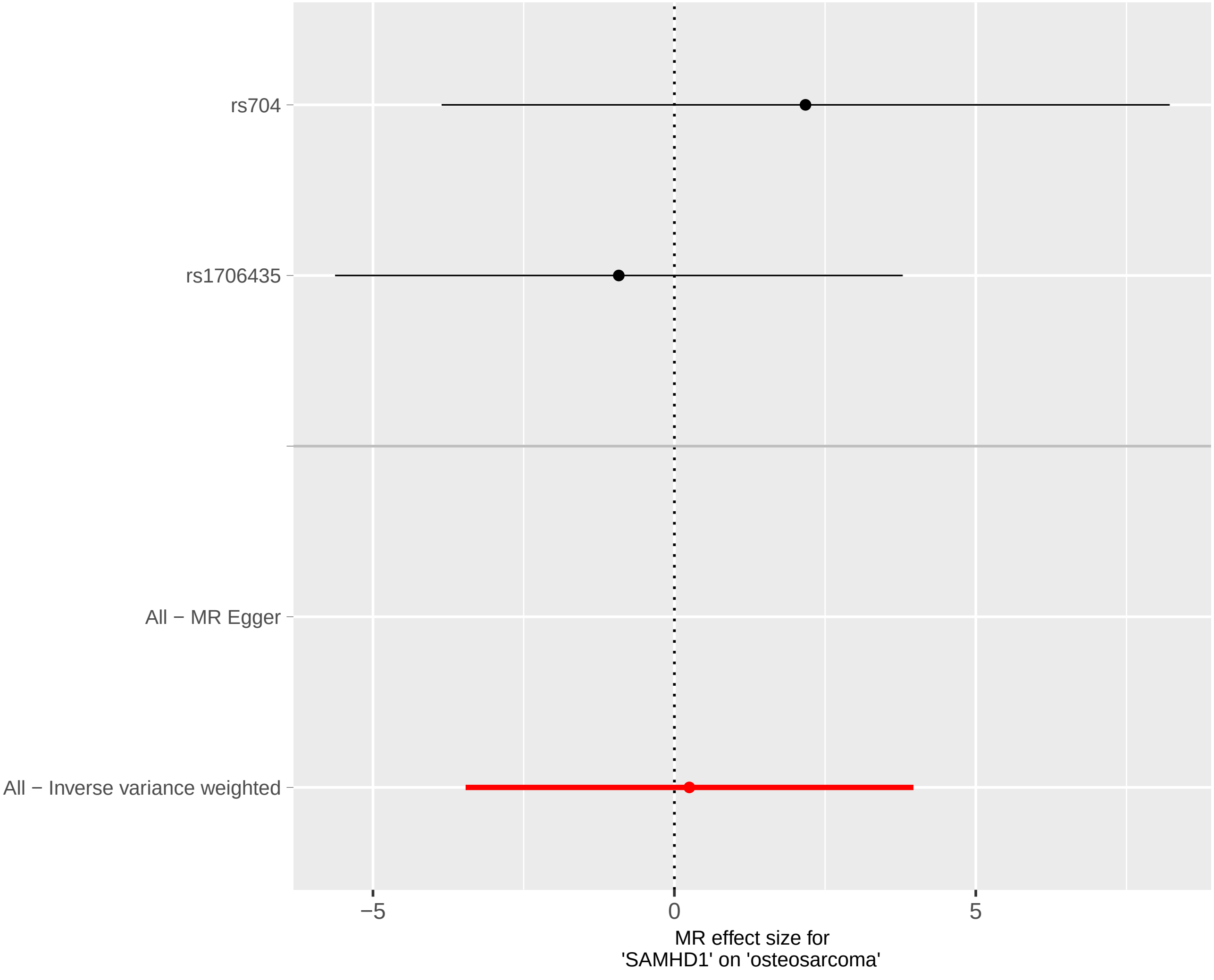
rs704
rs1706435
All − MR Egger
All − Inverse variance weighted
−5
0
5
MR effect size for
'SAMHD1' on 'osteosarcoma'

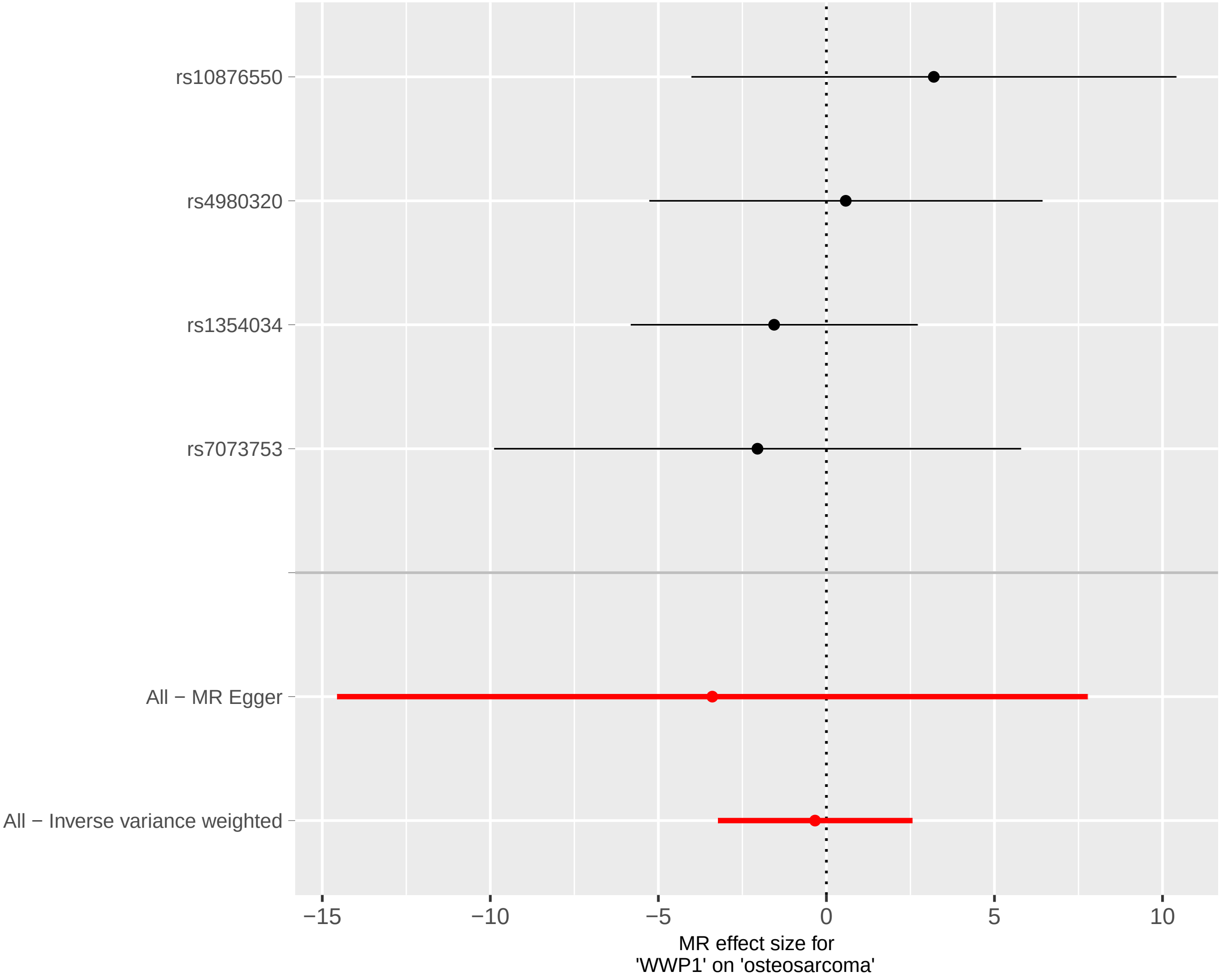

rs10876550
rs4980320
rs1354034
rs7073753
All − MR Egger
All − Inverse variance weighted
−15
−10
−5
0
5
10
MR effect size for
'WWP1' on 'osteosarcoma'

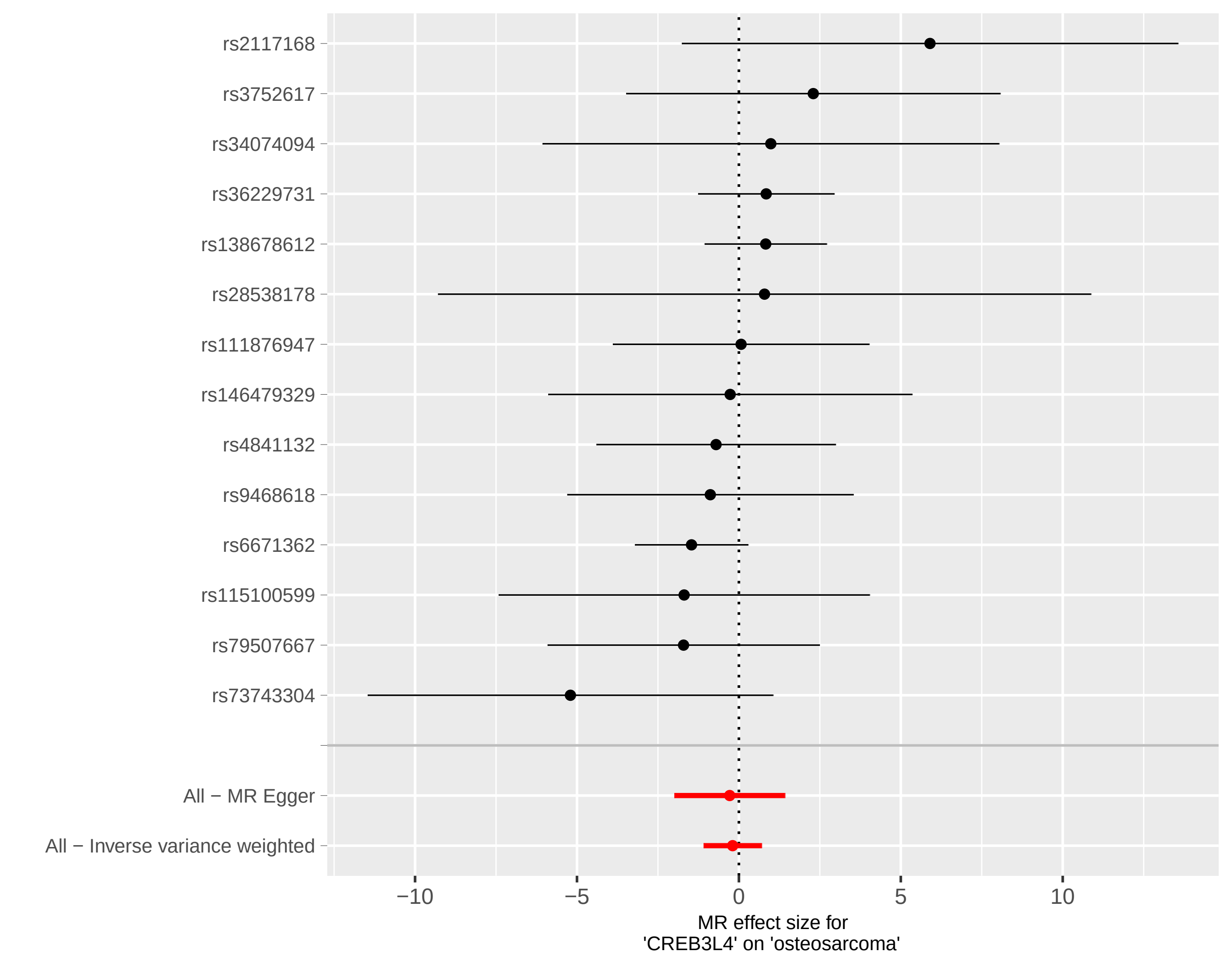

rs2117168
rs3752617
rs34074094
rs36229731
rs138678612
rs28538178
rs111876947
rs146479329
rs4841132
rs9468618
rs6671362
rs115100599
rs79507667
rs73743304
All − MR Egger
All − Inverse variance weighted
−10
−5
0
5
10
MR effect size for
'CREB3L4' on 'osteosarcoma'

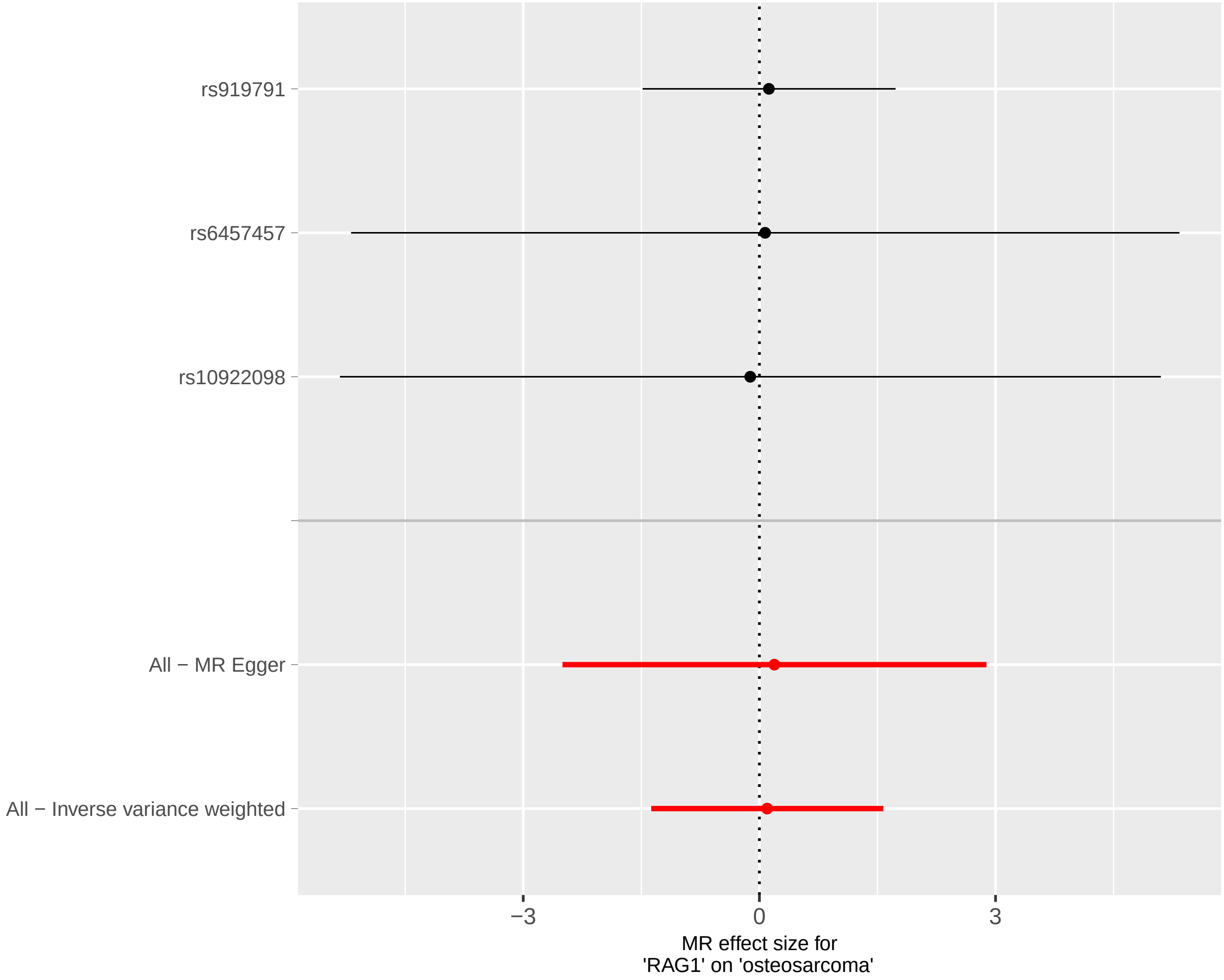
rs919791
rs6457457
rs10922098
All − MR Egger
All − Inverse variance weighted
−3
0
3
MR effect size for
'RAG1' on 'osteosarcoma'

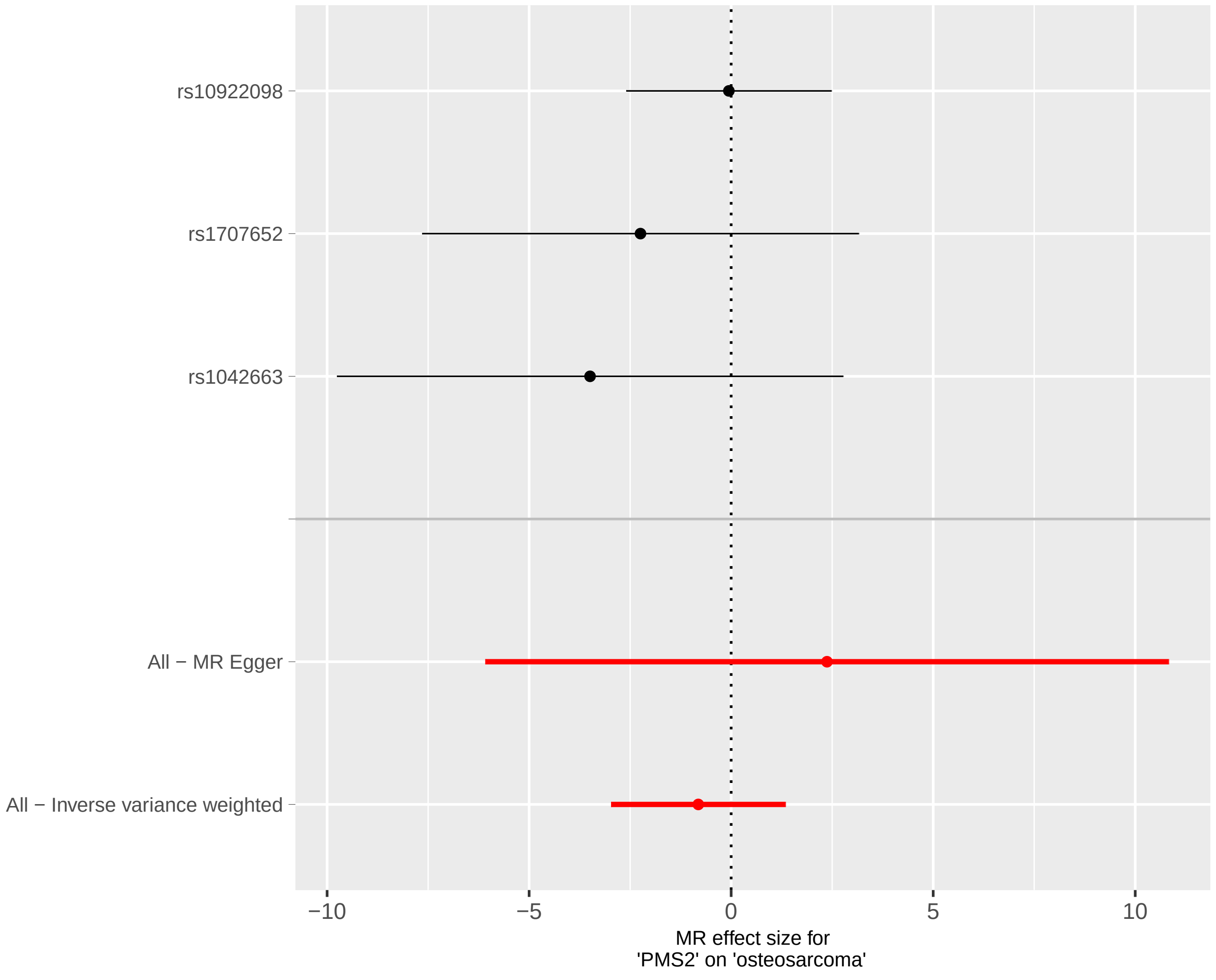
rs10922098
rs1707652
rs1042663
All − MR Egger
All − Inverse variance weighted
−10
−5
0
5
10
MR effect size for
'PMS2' on 'osteosarcoma'

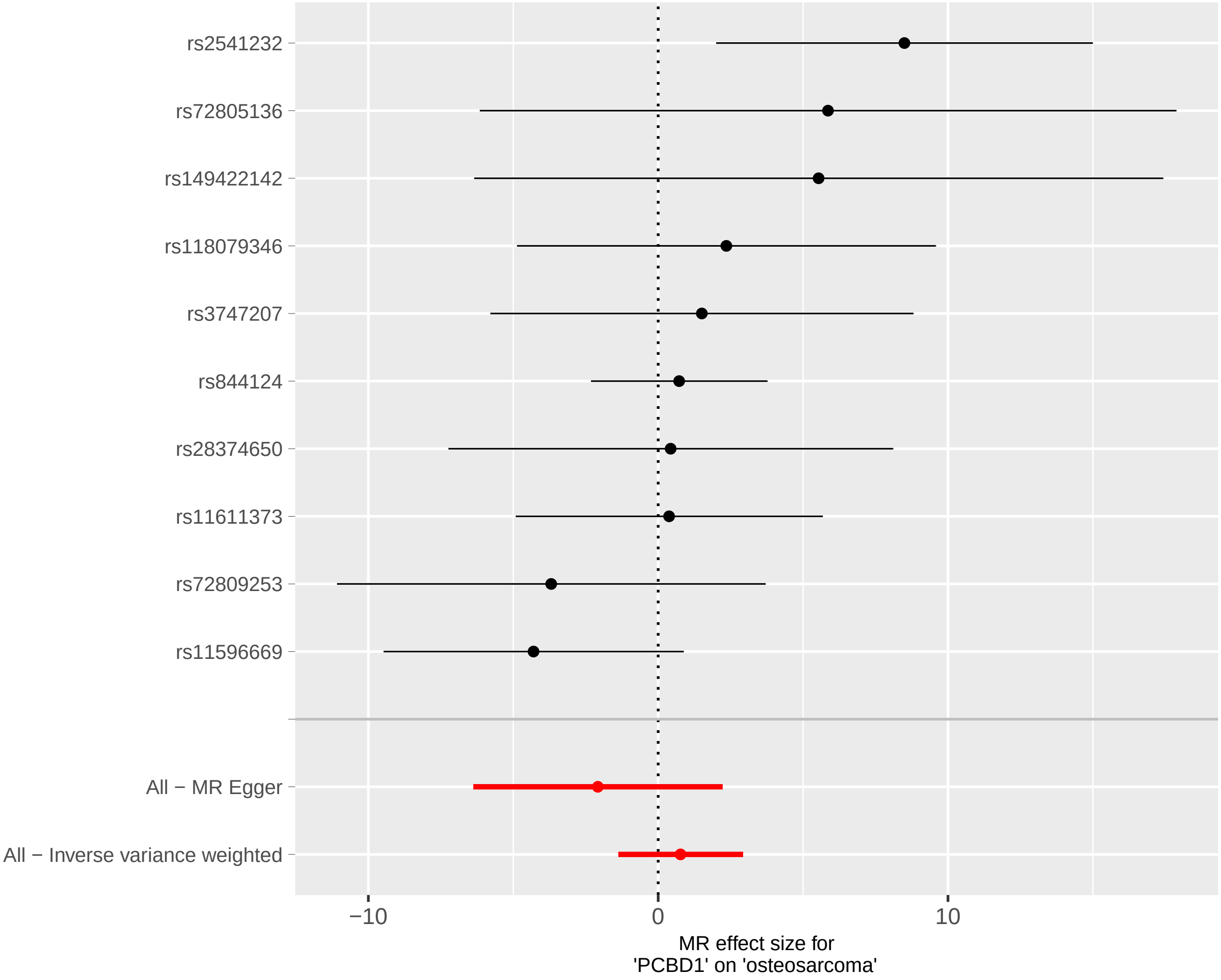

rs2541232
rs72805136
rs149422142
rs118079346
rs3747207
rs844124
rs28374650
rs11611373
rs72809253
rs11596669
All − MR Egger
All − Inverse variance weighted
−10
0
10
MR effect size for
'PCBD1' on 'osteosarcoma'

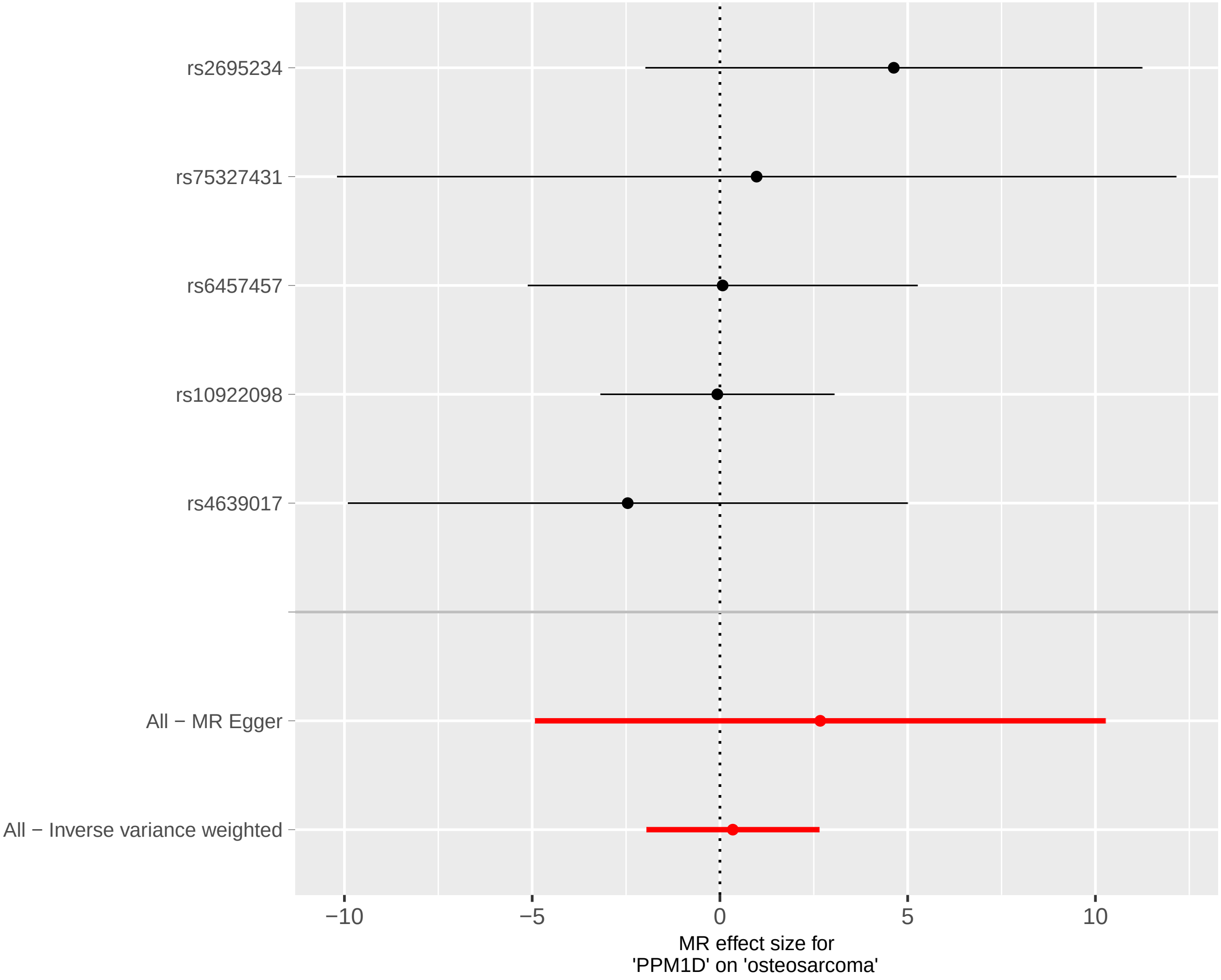

rs2695234
rs75327431
rs6457457
rs10922098
rs4639017
All − MR Egger
All − Inverse variance weighted
−10
−5
0
5
10
MR effect size for
'PPM1D' on 'osteosarcoma'

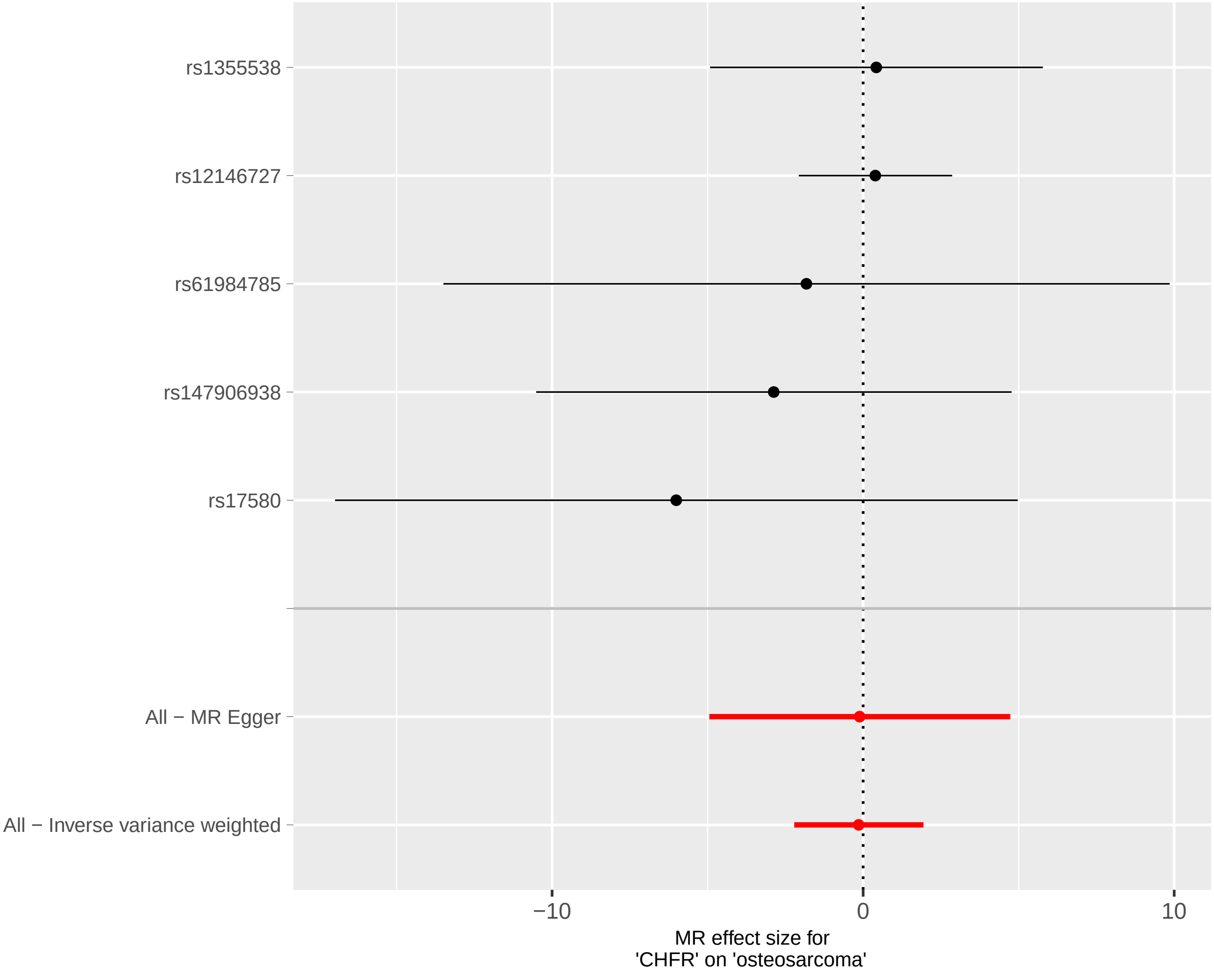
rs1355538
rs12146727
rs61984785
rs147906938
rs17580
All − MR Egger
All − Inverse variance weighted
−10
0
10
MR effect size for
'CHFR' on 'osteosarcoma'

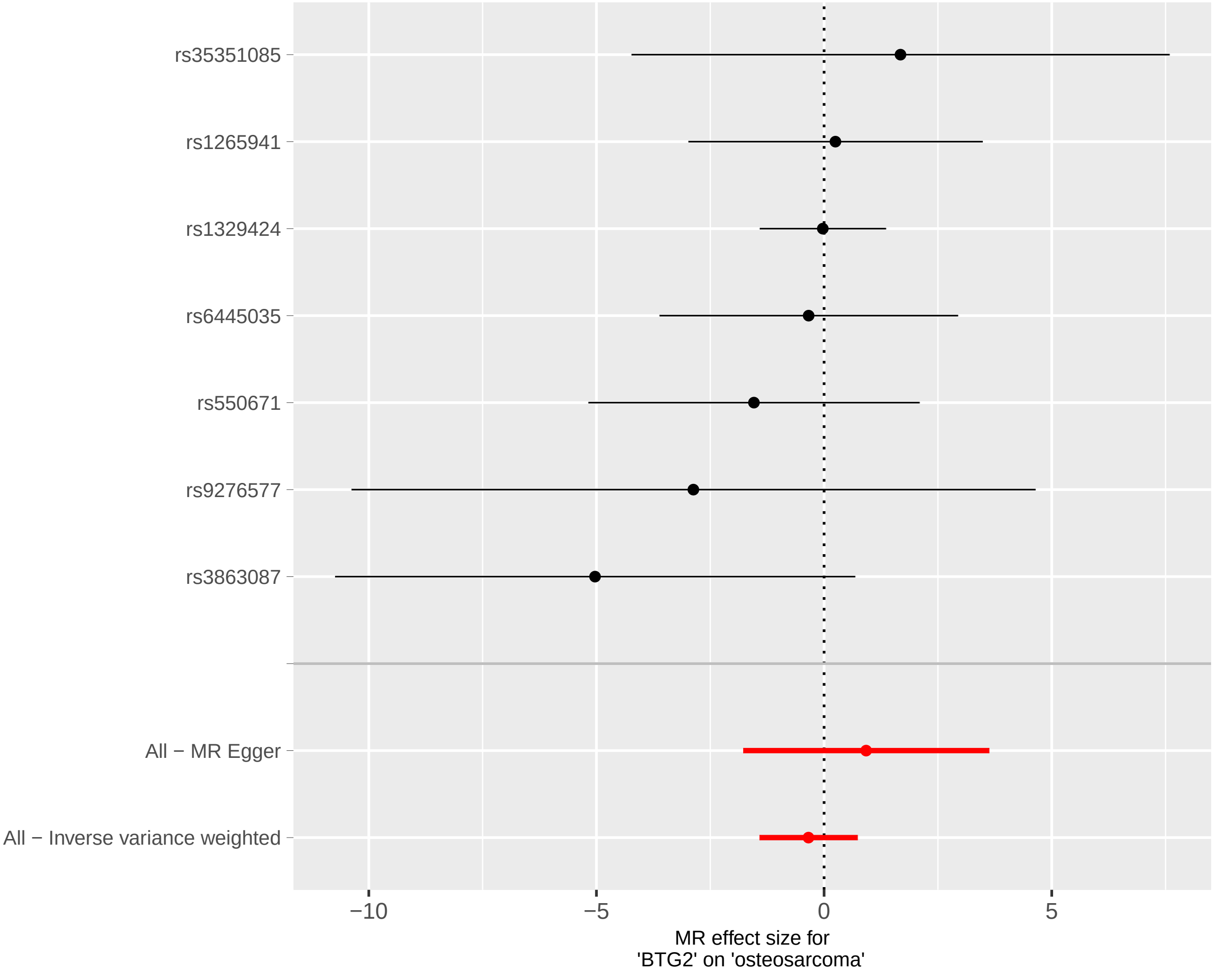

rs35351085
rs1265941
rs1329424
rs6445035
rs550671
rs9276577
rs3863087
All − MR Egger
All − Inverse variance weighted
−10
−5
0
5
MR effect size for
'BTG2' on 'osteosarcoma'

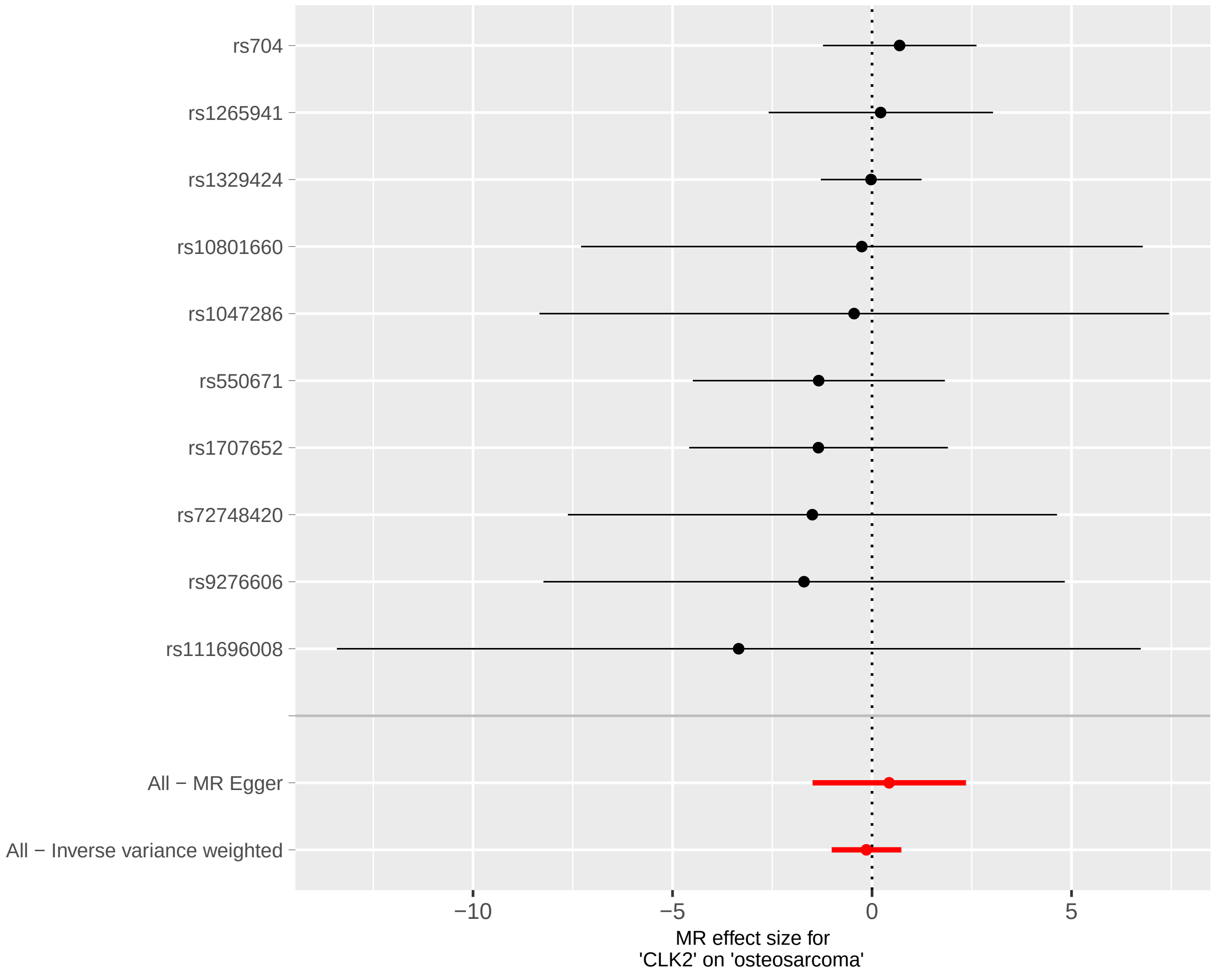

rs704
rs1265941
rs1329424
rs10801660
rs1047286
rs550671
rs1707652
rs72748420
rs9276606
rs111696008
All − MR Egger
All − Inverse variance weighted
−10
−5
0
5
MR effect size for
'CLK2' on 'osteosarcoma'

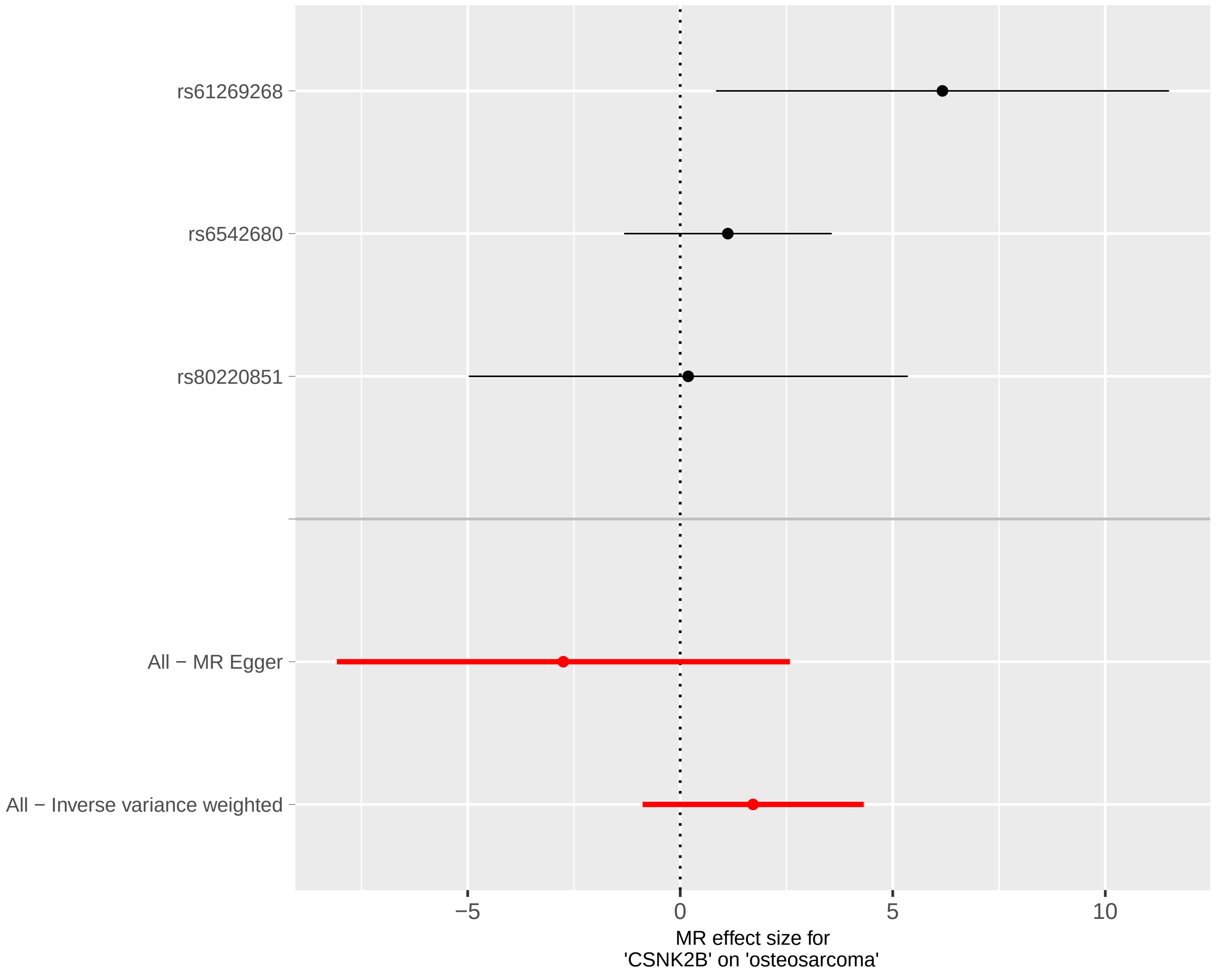

rs61269268
rs6542680
rs80220851
All − MR Egger
All − Inverse variance weighted
−5
0
5
10
MR effect size for
'CSNK2B' on 'osteosarcoma'

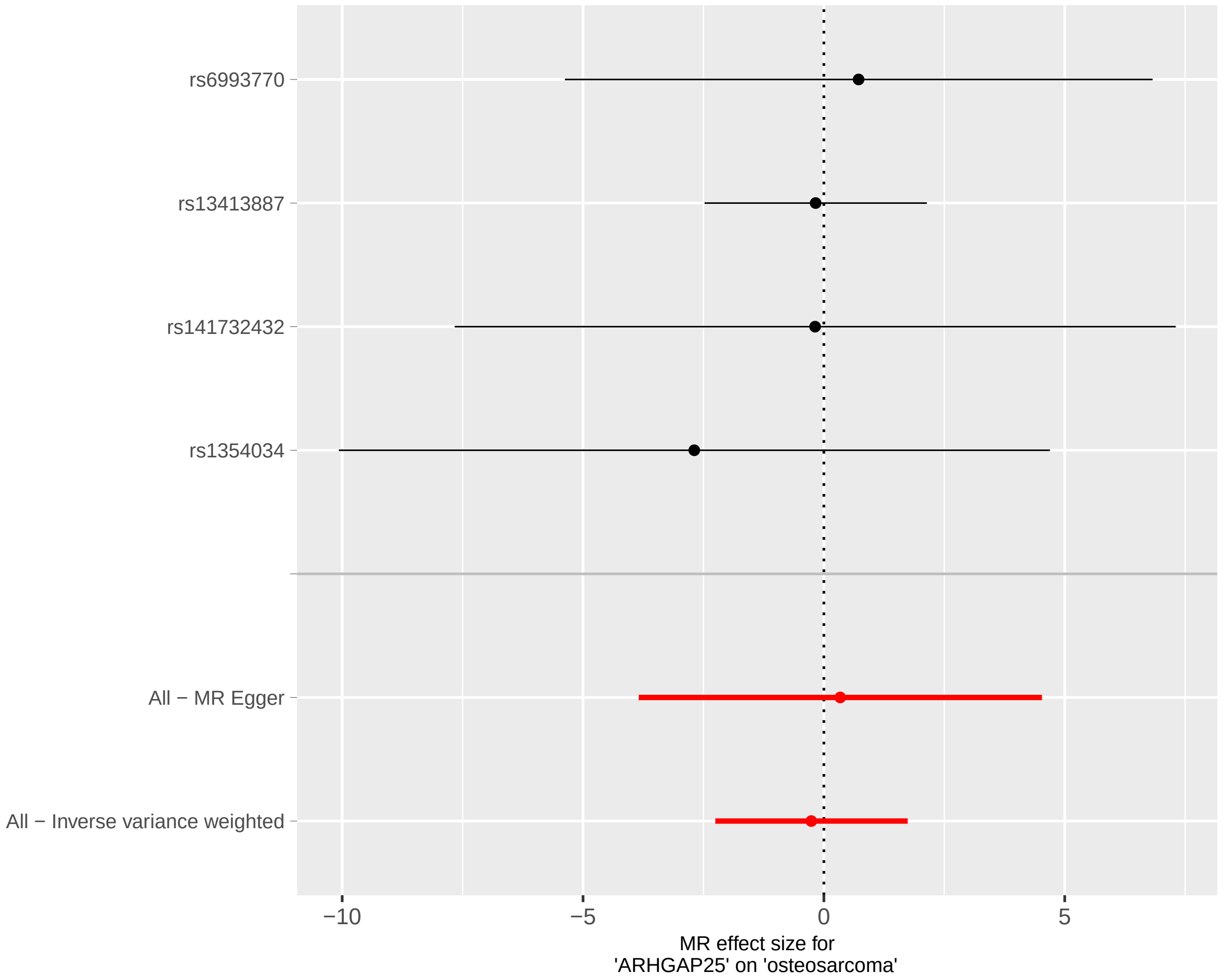
rs6993770
rs13413887
rs141732432
rs1354034
All − MR Egger
All − Inverse variance weighted
−10
−5
0
5
MR effect size for
'ARHGAP25' on 'osteosarcoma'

Insufficient number of SNPs

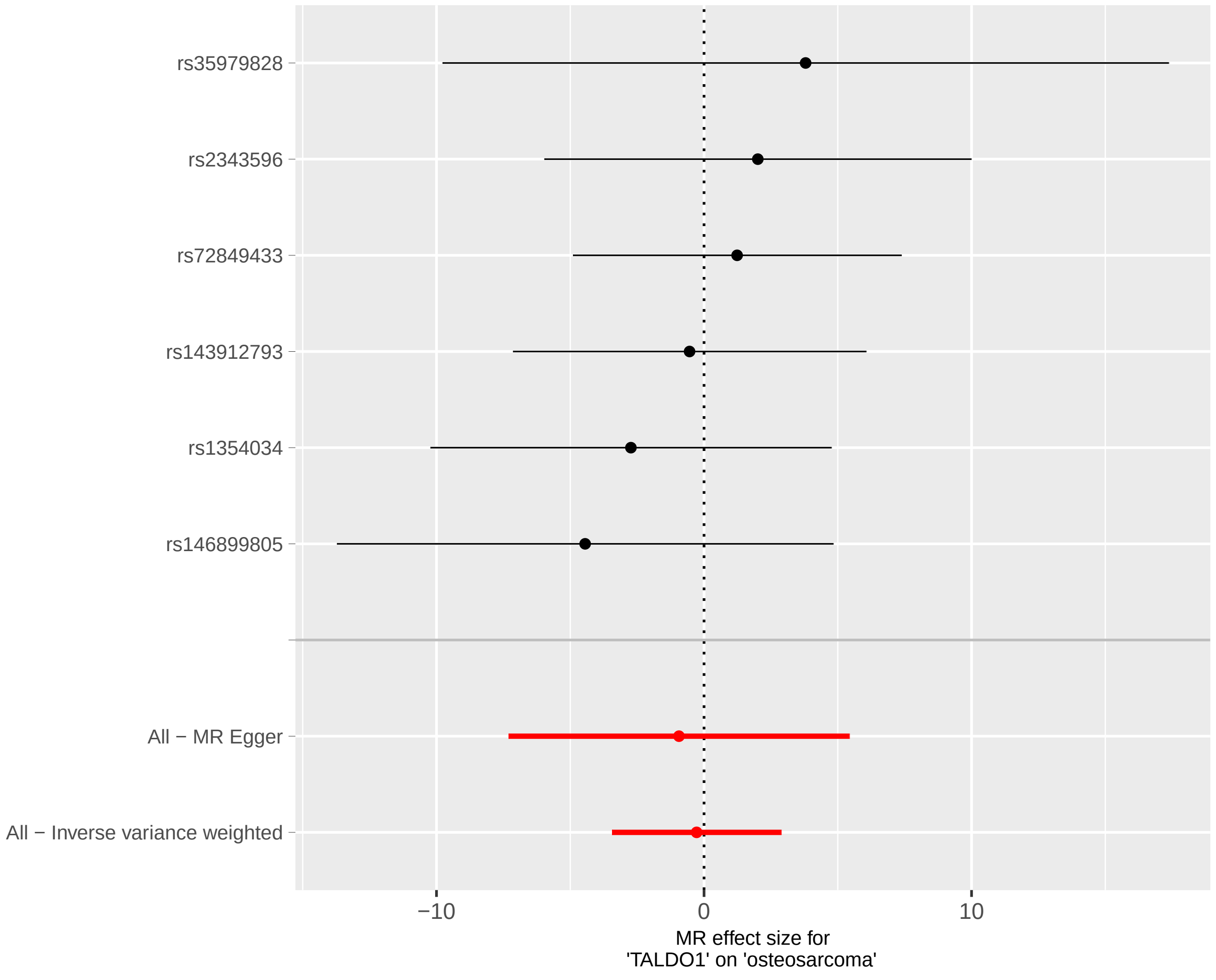

rs35979828
rs2343596
rs72849433
rs143912793
rs1354034
rs146899805
All − MR Egger
All − Inverse variance weighted
−10
0
10
MR effect size for
'TALDO1' on 'osteosarcoma'

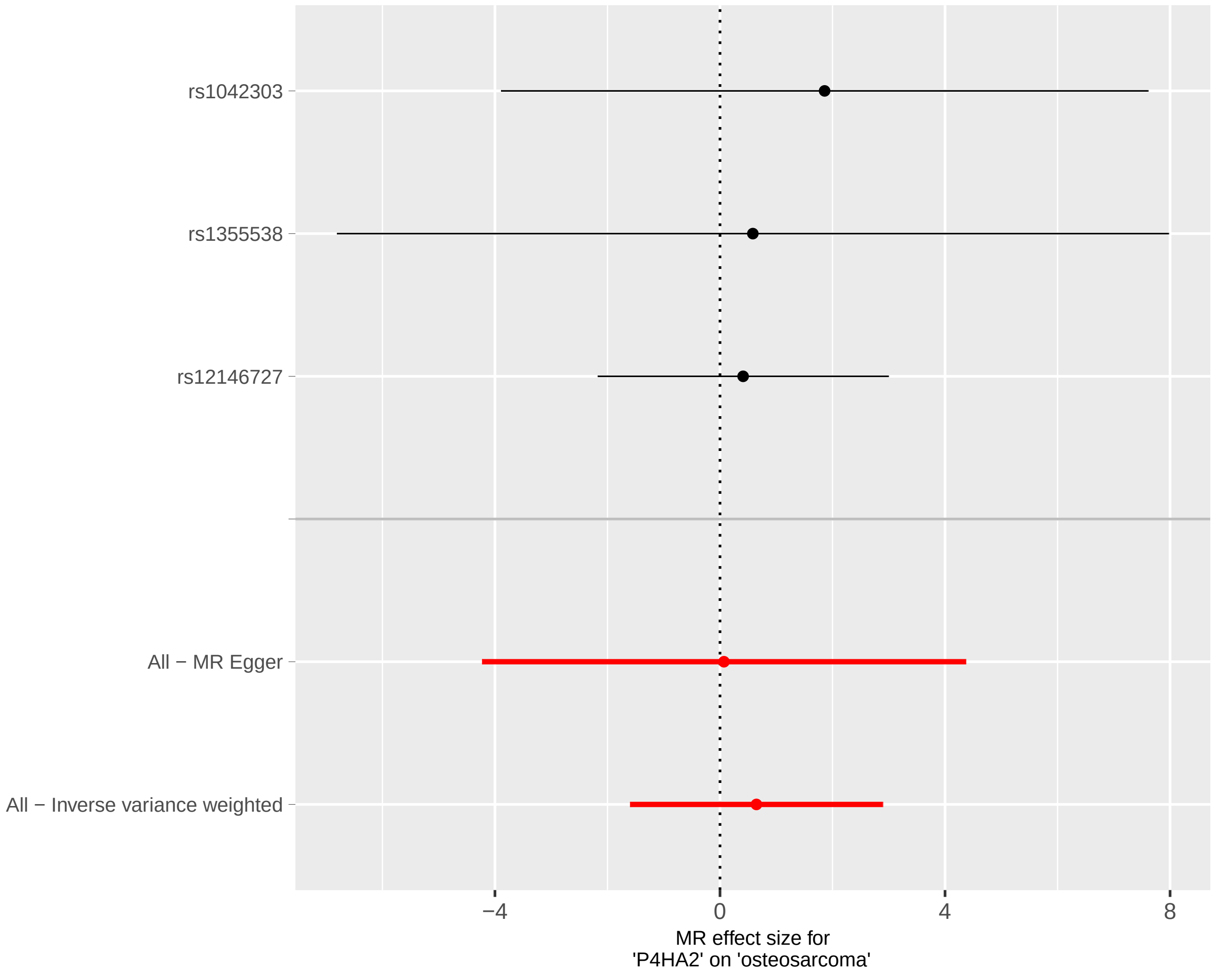

rs1042303
rs1355538
rs12146727
All − MR Egger
All − Inverse variance weighted
−4
0
4
8
MR effect size for
'P4HA2' on 'osteosarcoma'

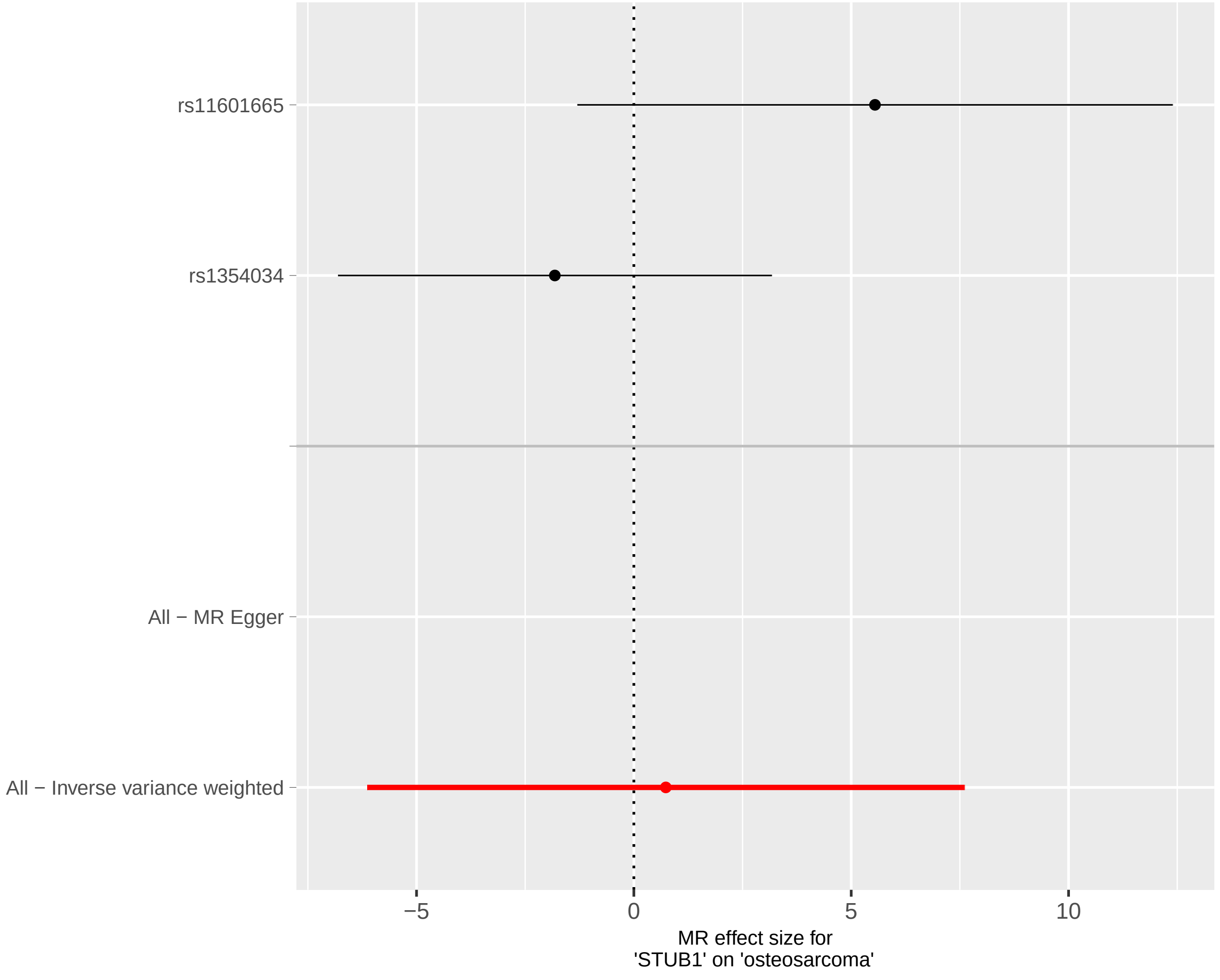
rs11601665
rs1354034
All − MR Egger
All − Inverse variance weighted
−5
0
5
10
MR effect size for
'STUB1' on 'osteosarcoma'

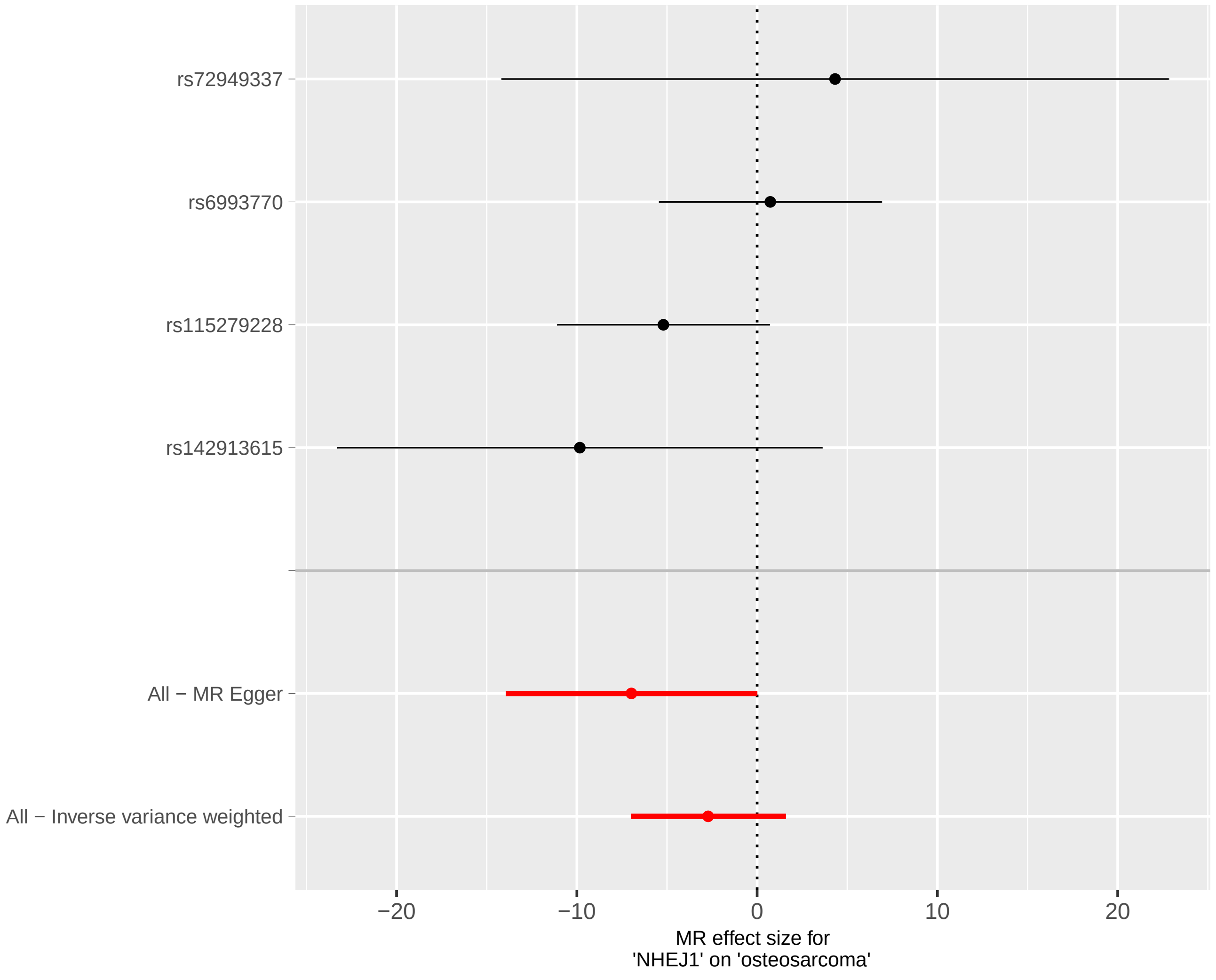
rs72949337
rs6993770
rs115279228
rs142913615
All − MR Egger
All − Inverse variance weighted
−20
−10
0
10
20
MR effect size for
'NHEJ1' on 'osteosarcoma'

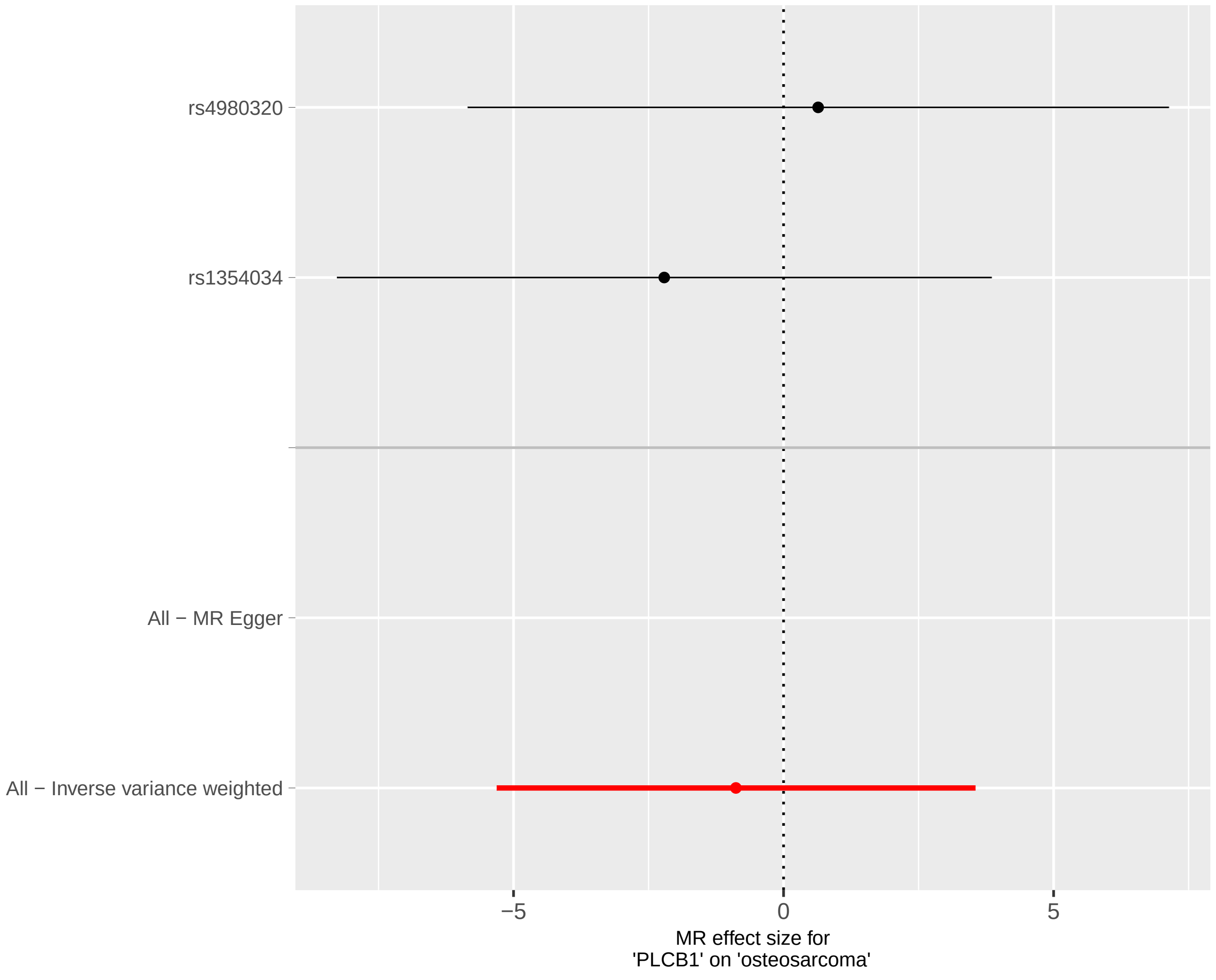
rs4980320
rs1354034
All − MR Egger
All − Inverse variance weighted
−5
0
5
MR effect size for
'PLCB1' on 'osteosarcoma'

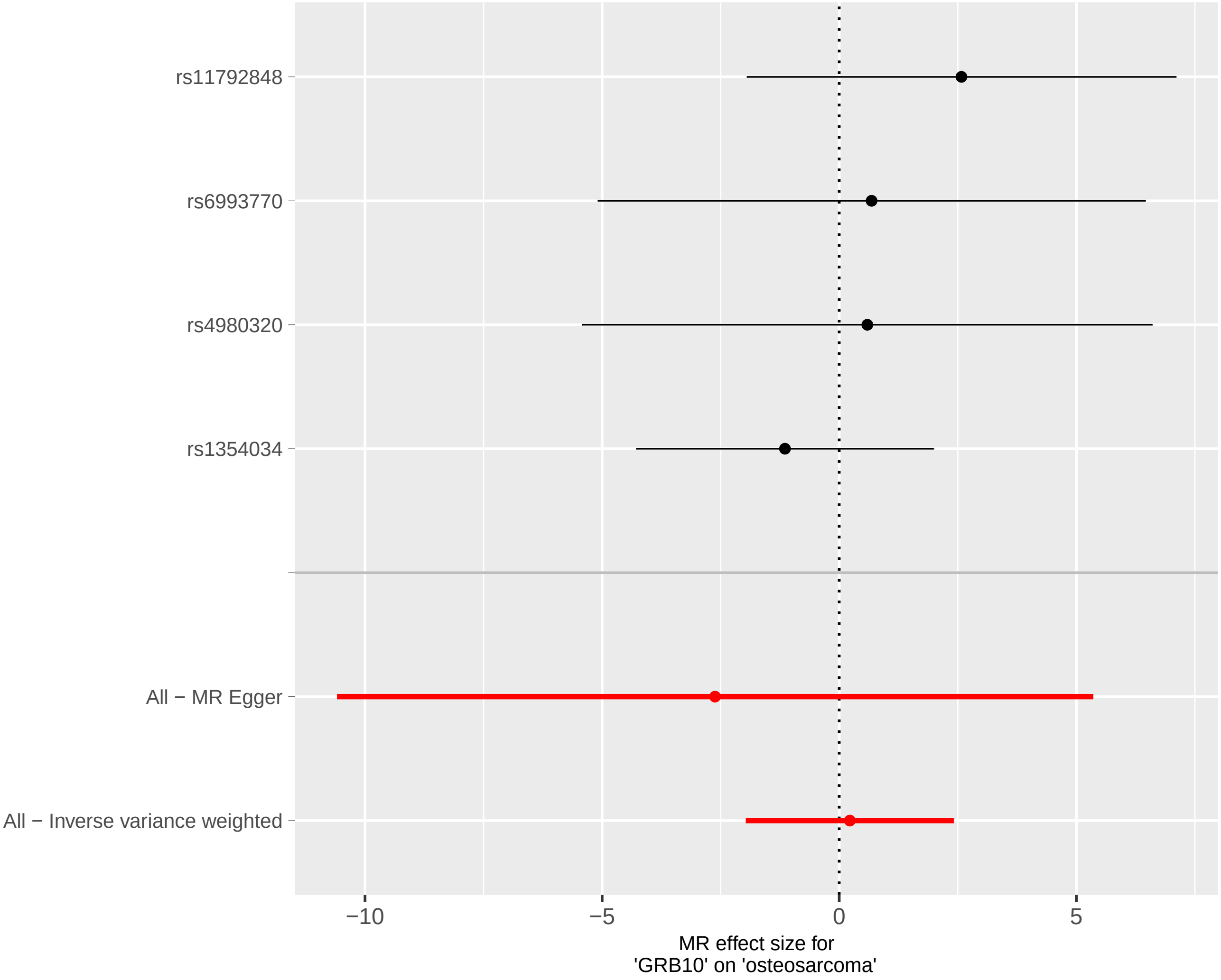

rs11792848
rs6993770
rs4980320
rs1354034
All − MR Egger
All − Inverse variance weighted
−10
−5
0
5
MR effect size for
'GRB10' on 'osteosarcoma'

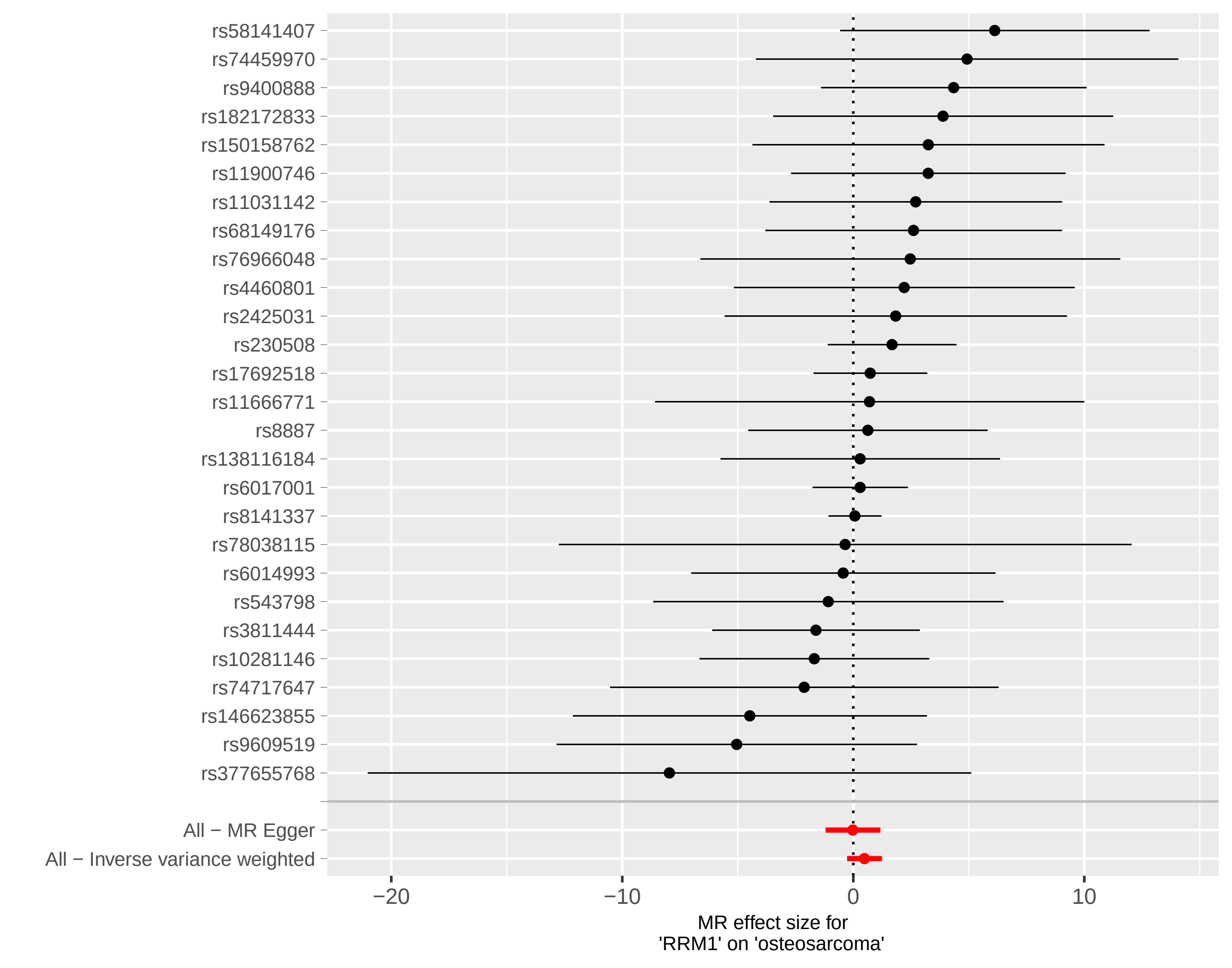

rs58141407
rs74459970
rs9400888
rs182172833
rs150158762
rs11900746
rs11031142
rs68149176
rs76966048
rs4460801
rs2425031
rs230508
rs17692518
rs11666771
rs8887
rs138116184
rs6017001
rs8141337
rs78038115
rs6014993
rs543798
rs3811444
rs10281146
rs74717647
rs146623855
rs9609519
rs377655768
All − MR Egger
All − Inverse variance weighted
−20
−10
0
10
MR effect size for
'RRM1' on 'osteosarcoma'

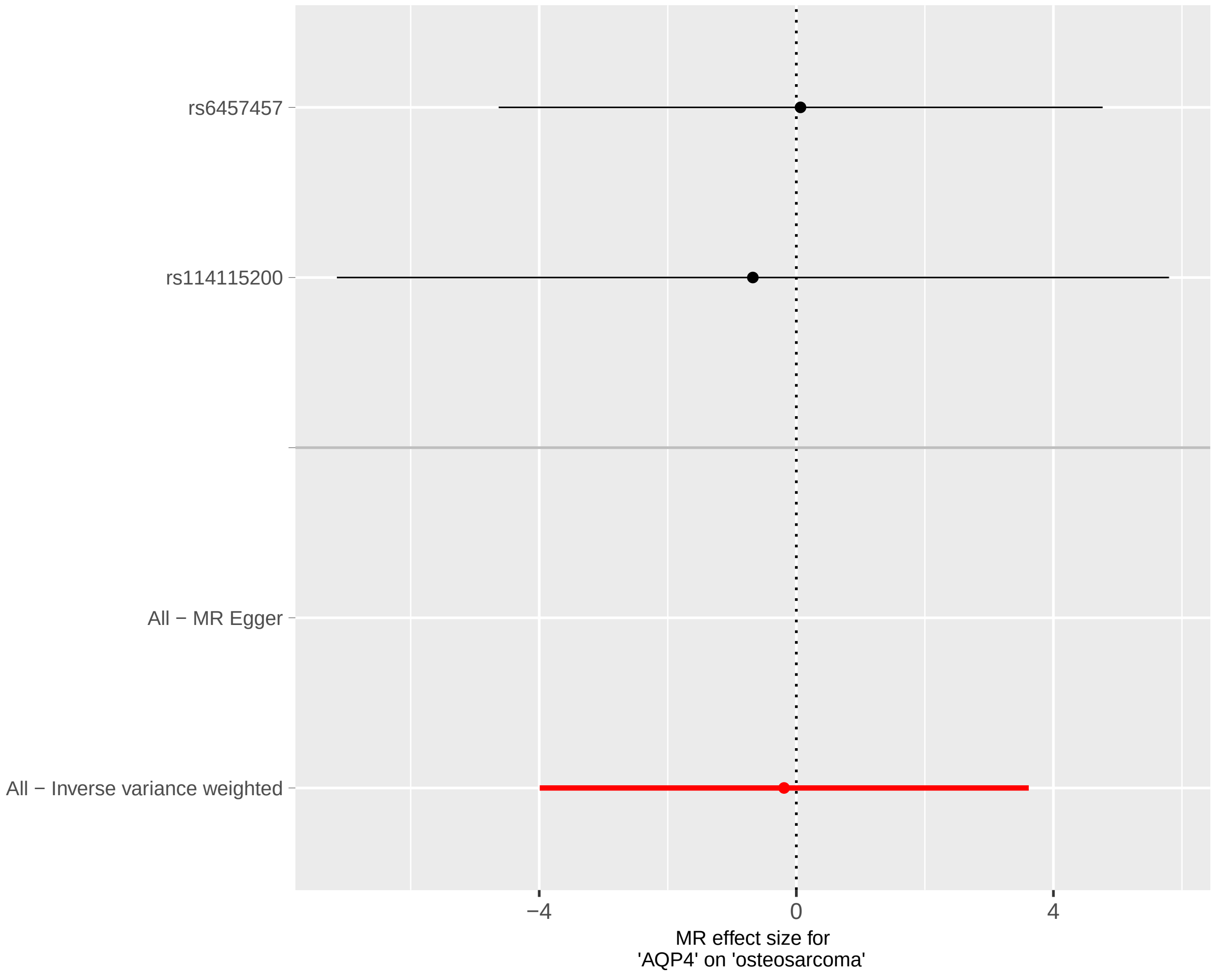
rs6457457
rs114115200
All − MR Egger
All − Inverse variance weighted
−4
0
4
MR effect size for
'AQP4' on 'osteosarcoma'

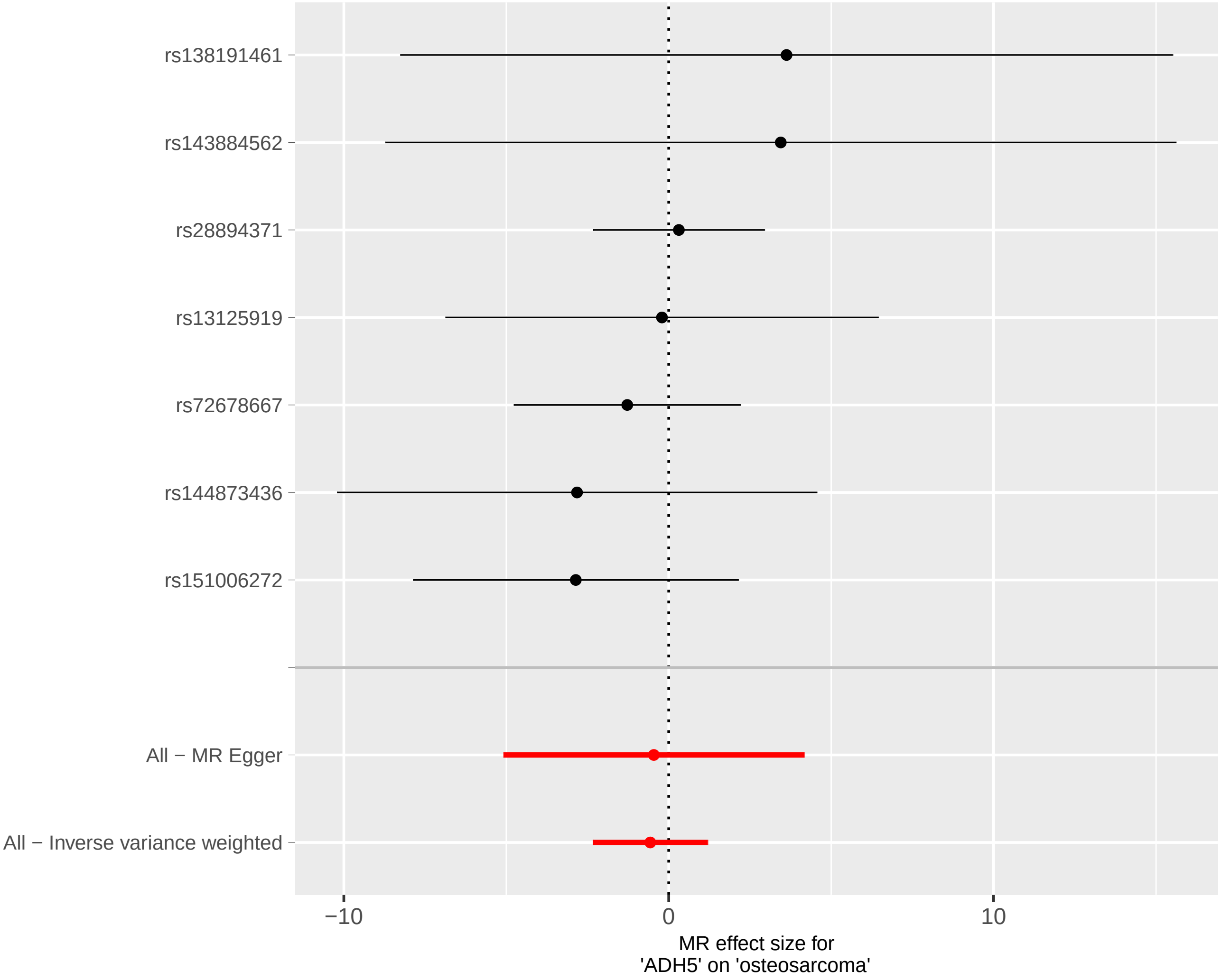

rs138191461
rs143884562
rs28894371
rs13125919
rs72678667
rs144873436
rs151006272
All − MR Egger
All − Inverse variance weighted
−10
0
10
MR effect size for
'ADH5' on 'osteosarcoma'

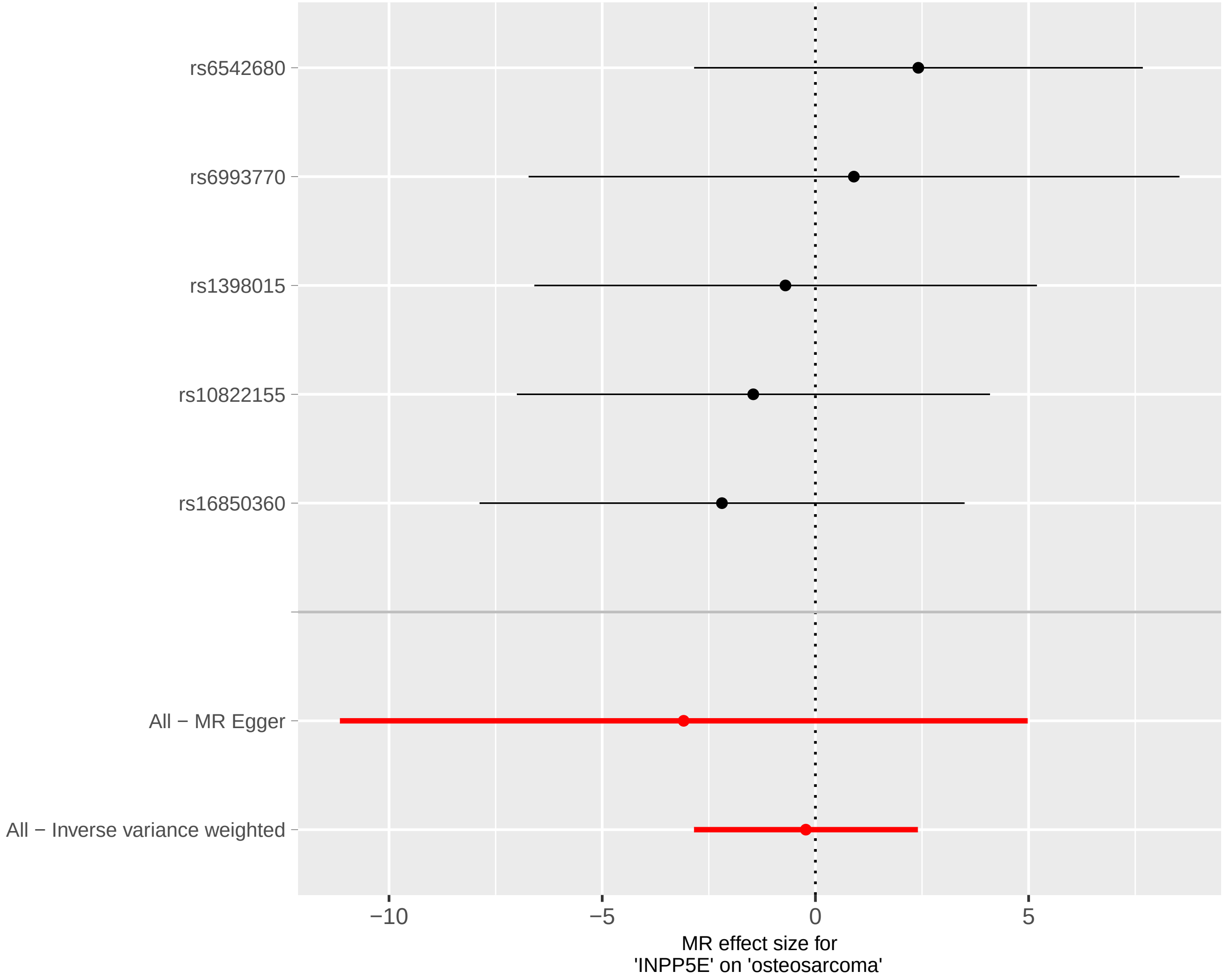

rs6542680
rs6993770
rs1398015
rs10822155
rs16850360
All − MR Egger
All − Inverse variance weighted
−10
−5
0
5
MR effect size for
'INPP5E' on 'osteosarcoma'

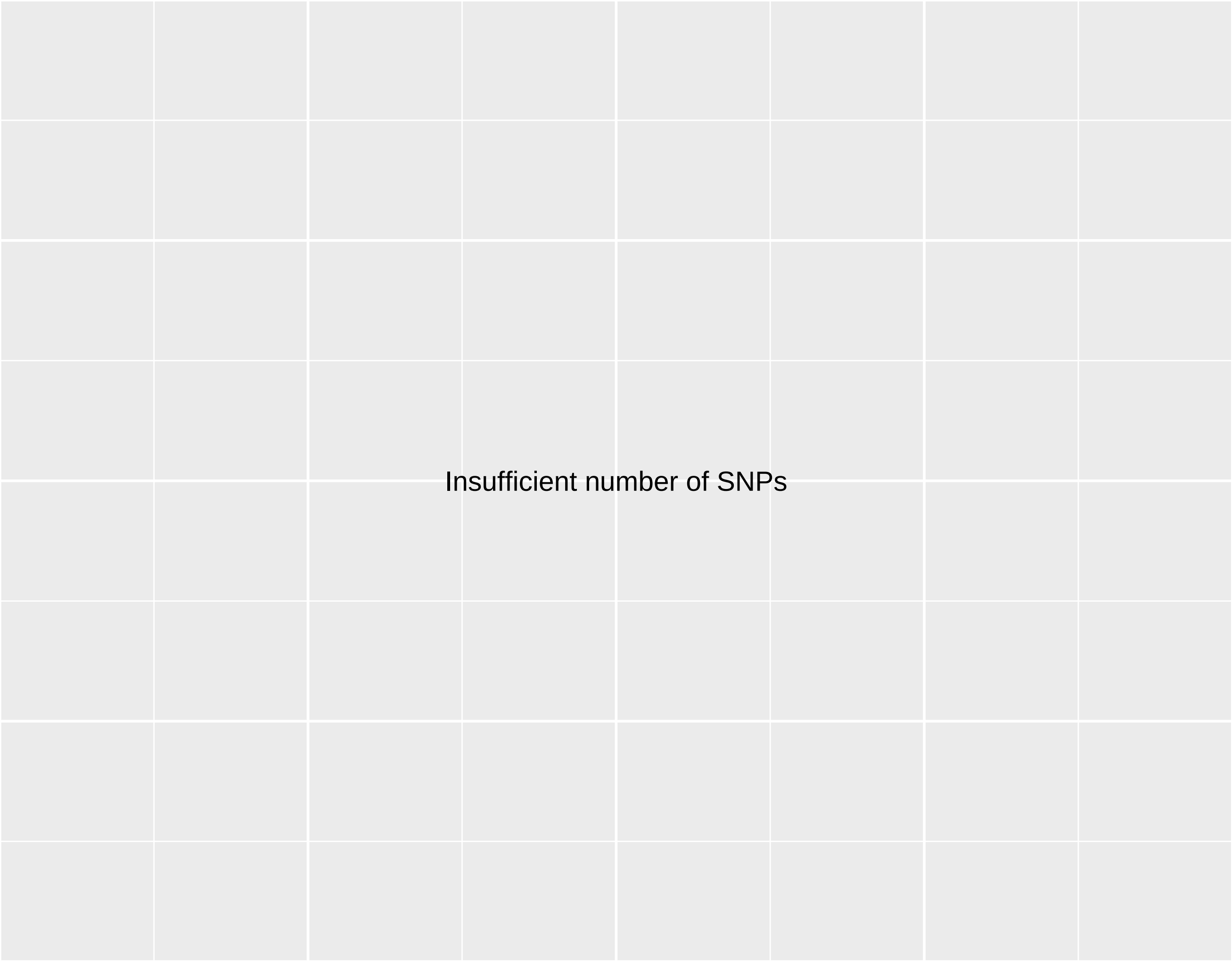
Insufficient number of SNPs

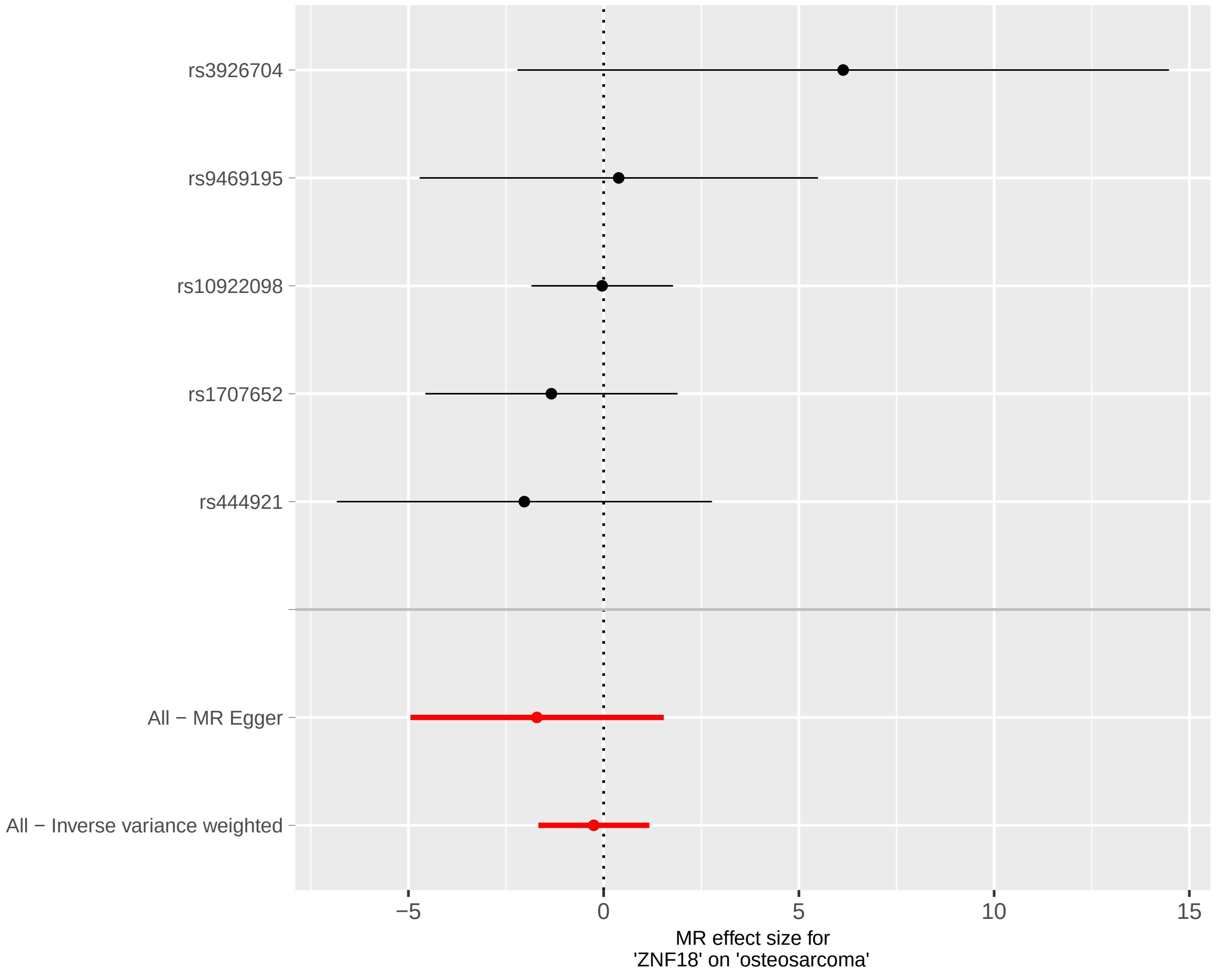

rs3926704
rs9469195
rs10922098
rs1707652
rs444921
All − MR Egger
All − Inverse variance weighted
−5
0
5
10
15
MR effect size for
'ZNF18' on 'osteosarcoma'

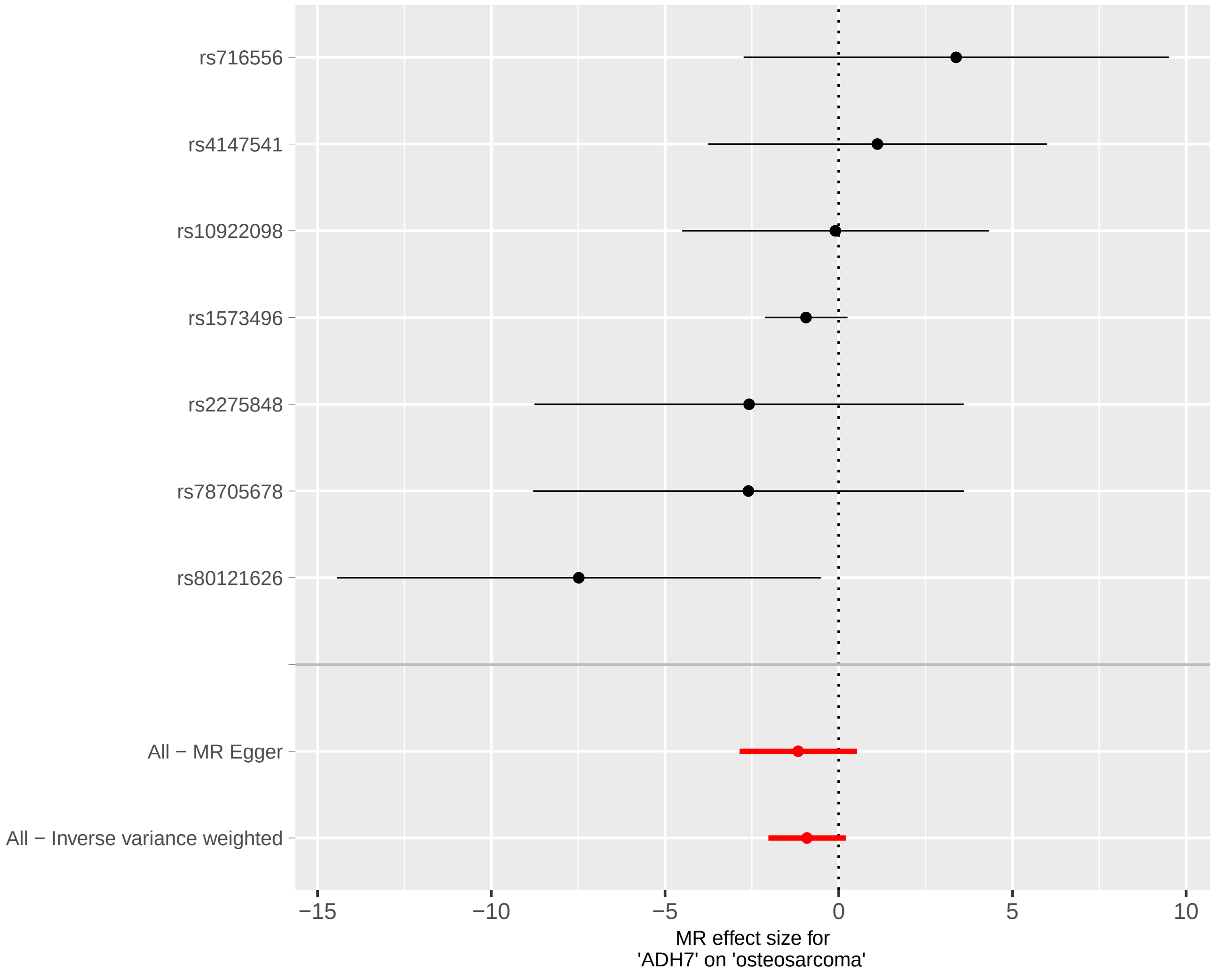

rs716556
rs4147541
rs10922098
rs1573496
rs2275848
rs78705678
rs80121626
All − MR Egger
All − Inverse variance weighted
−15
−10
−5
0
5
10
MR effect size for
'ADH7' on 'osteosarcoma'

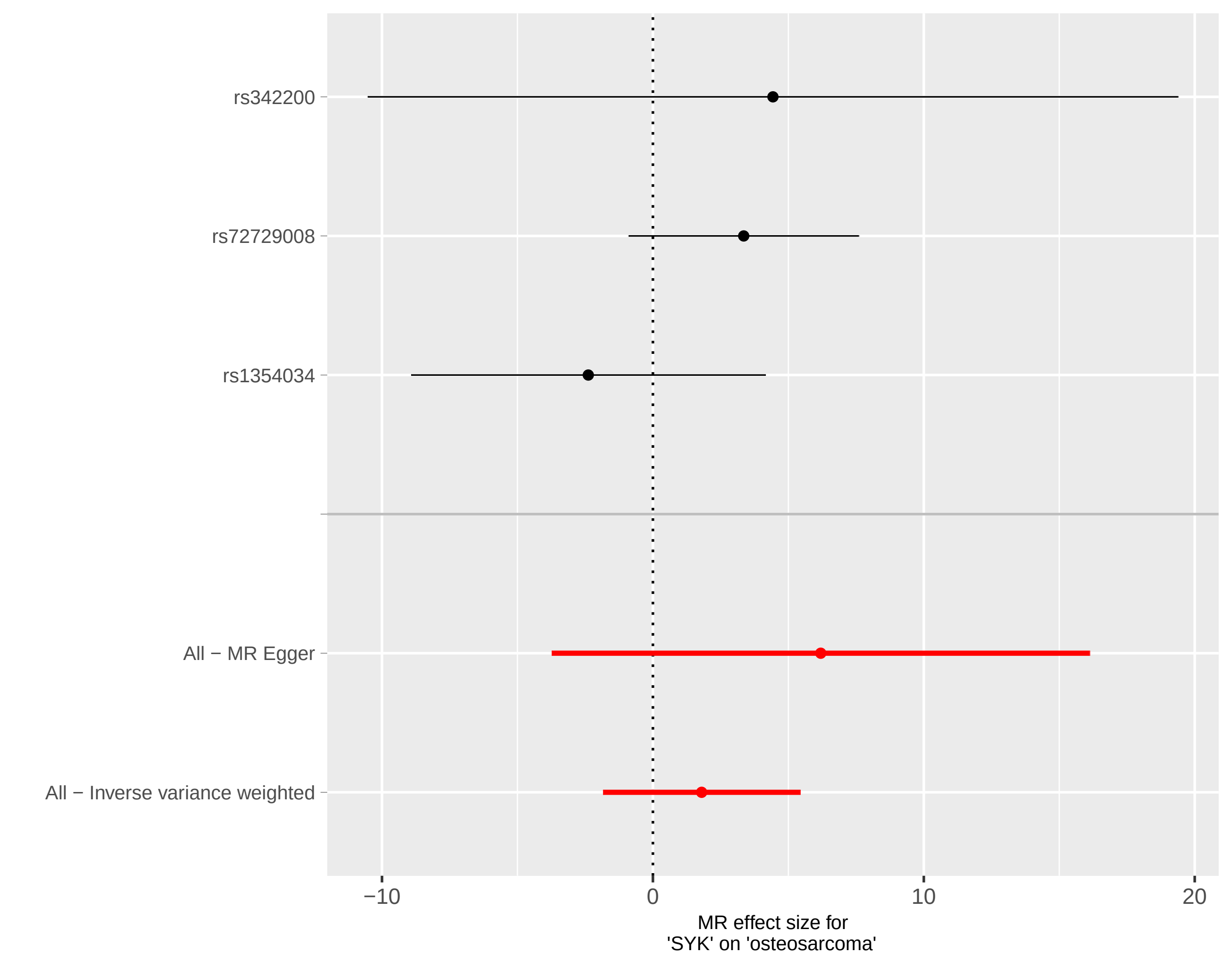
rs342200
rs72729008
rs1354034
All − MR Egger
All − Inverse variance weighted
−10
0
10
20
MR effect size for
'SYK' on 'osteosarcoma'

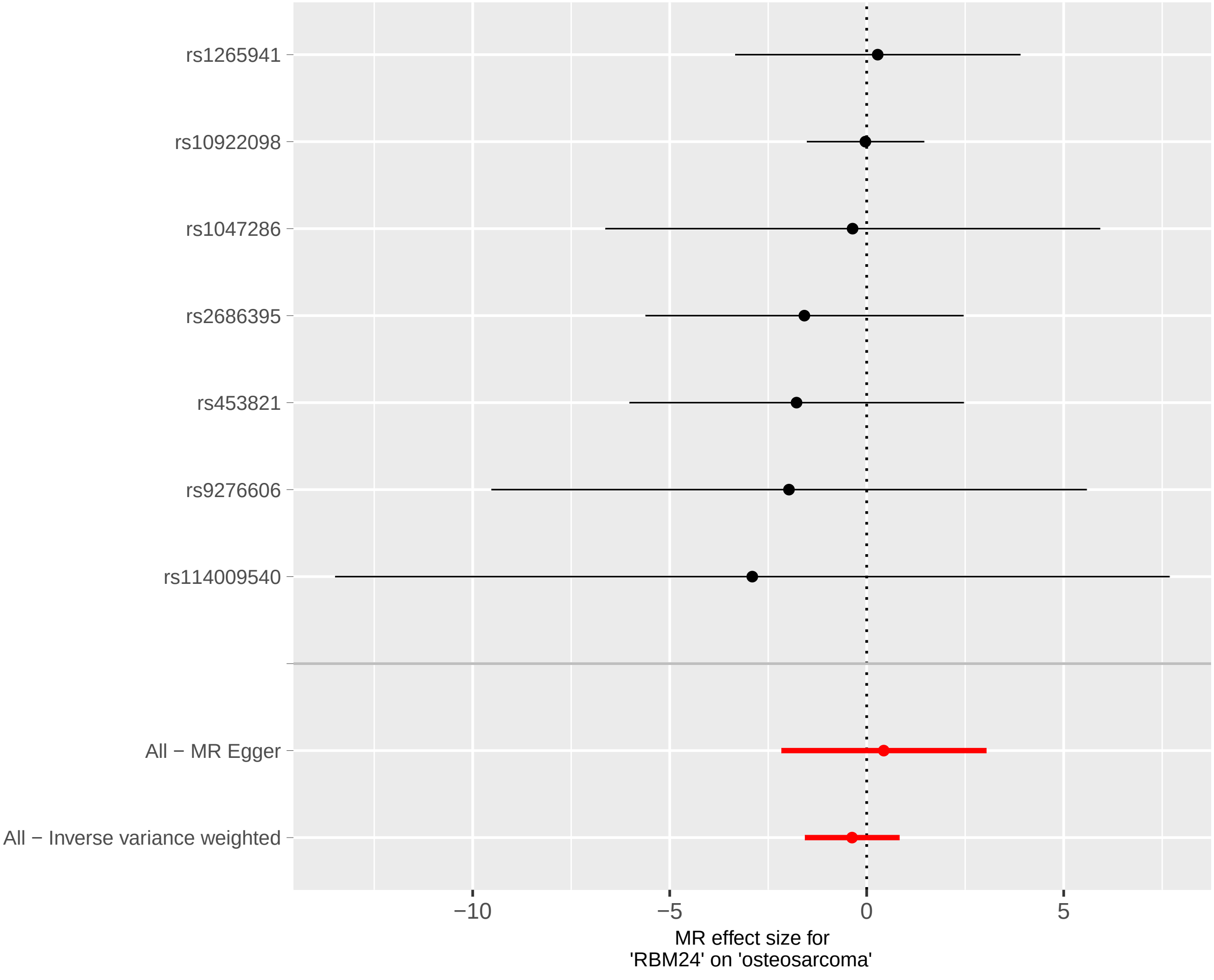

rs1265941
rs10922098
rs1047286
rs2686395
rs453821
rs9276606
rs114009540
All − MR Egger
All − Inverse variance weighted
−10
−5
0
5
MR effect size for
'RBM24' on 'osteosarcoma'

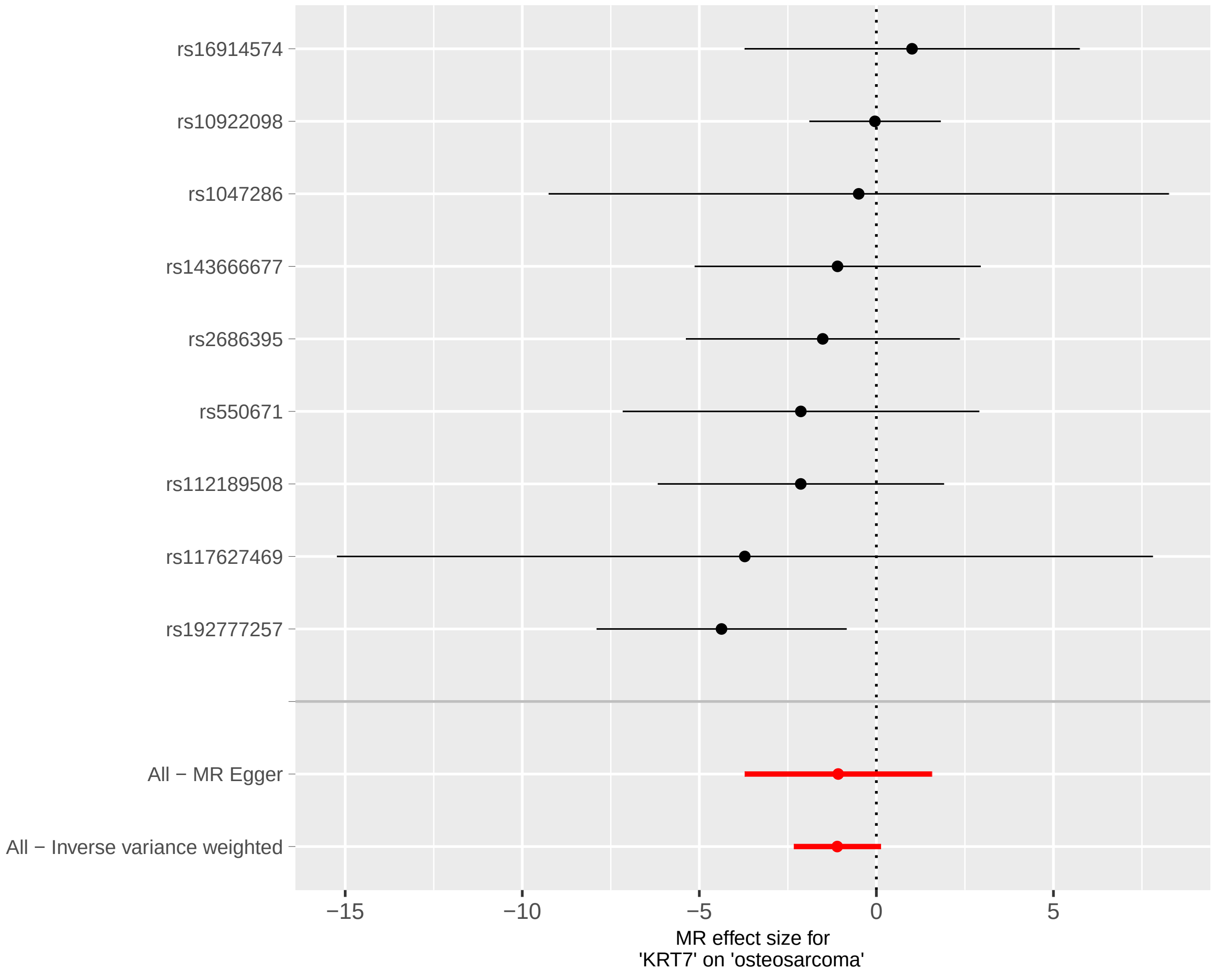
rs16914574
rs10922098
rs1047286
rs143666677
rs2686395
rs550671
rs112189508
rs117627469
rs192777257
All − MR Egger
All − Inverse variance weighted
−15
−10
−5
0
5
MR effect size for
'KRT7' on 'osteosarcoma'

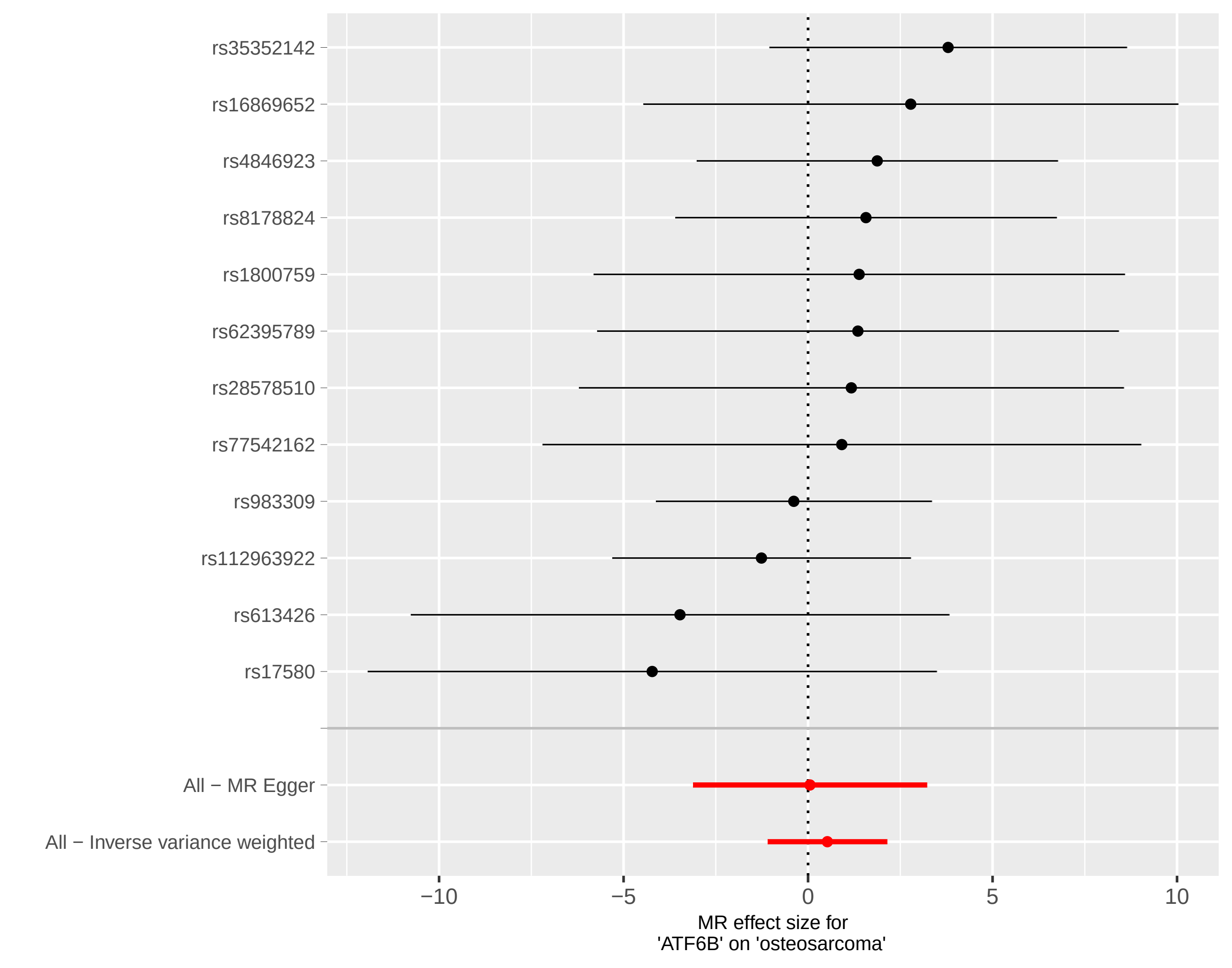

rs35352142
rs16869652
rs4846923
rs8178824
rs1800759
rs62395789
rs28578510
rs77542162
rs983309
rs112963922
rs613426
rs17580
All − MR Egger
All − Inverse variance weighted
−10
−5
0
5
10
MR effect size for
'ATF6B' on 'osteosarcoma'

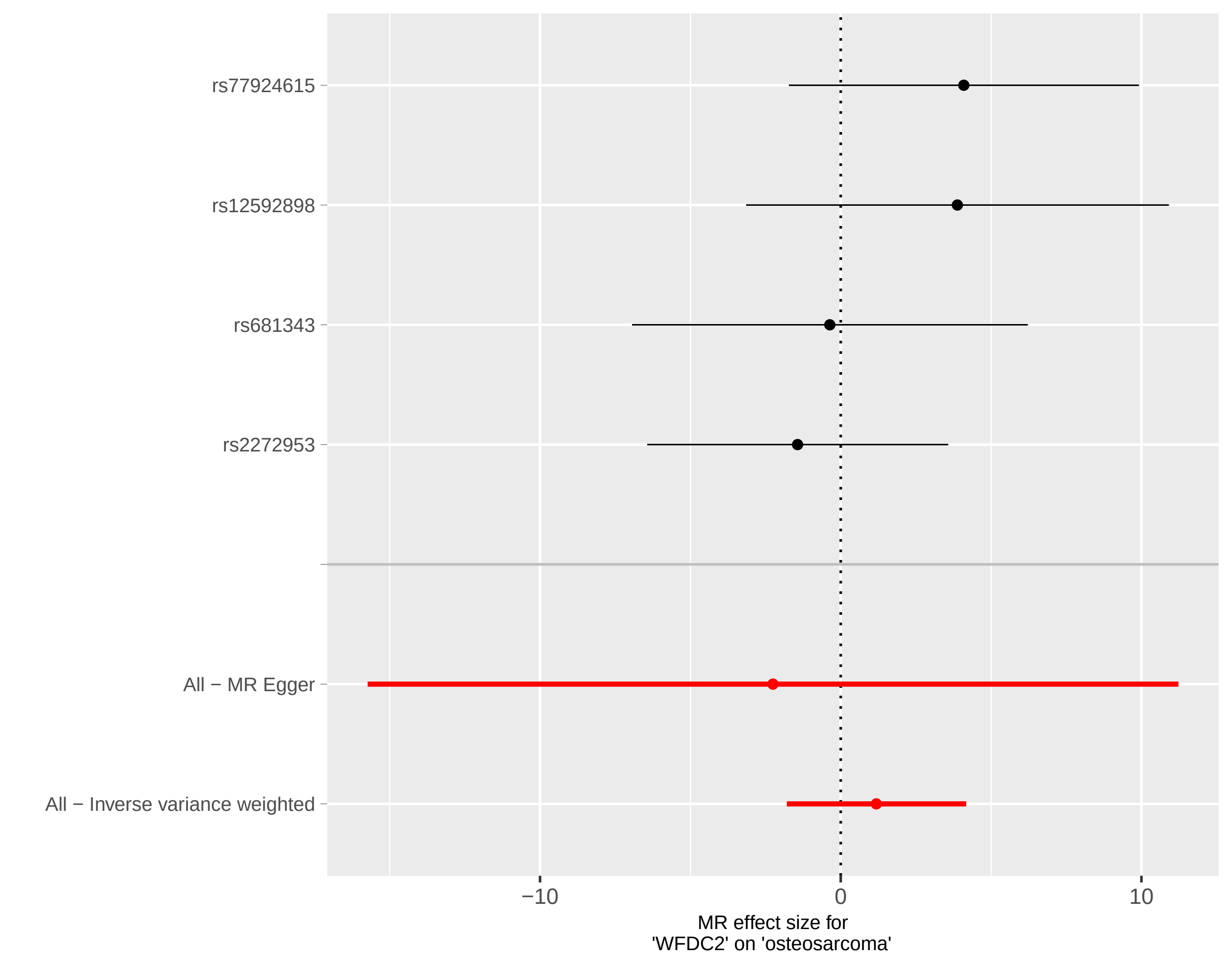

rs77924615
rs12592898
rs681343
rs2272953
All − MR Egger
All − Inverse variance weighted
−10
0
10
MR effect size for
'WFDC2' on 'osteosarcoma'

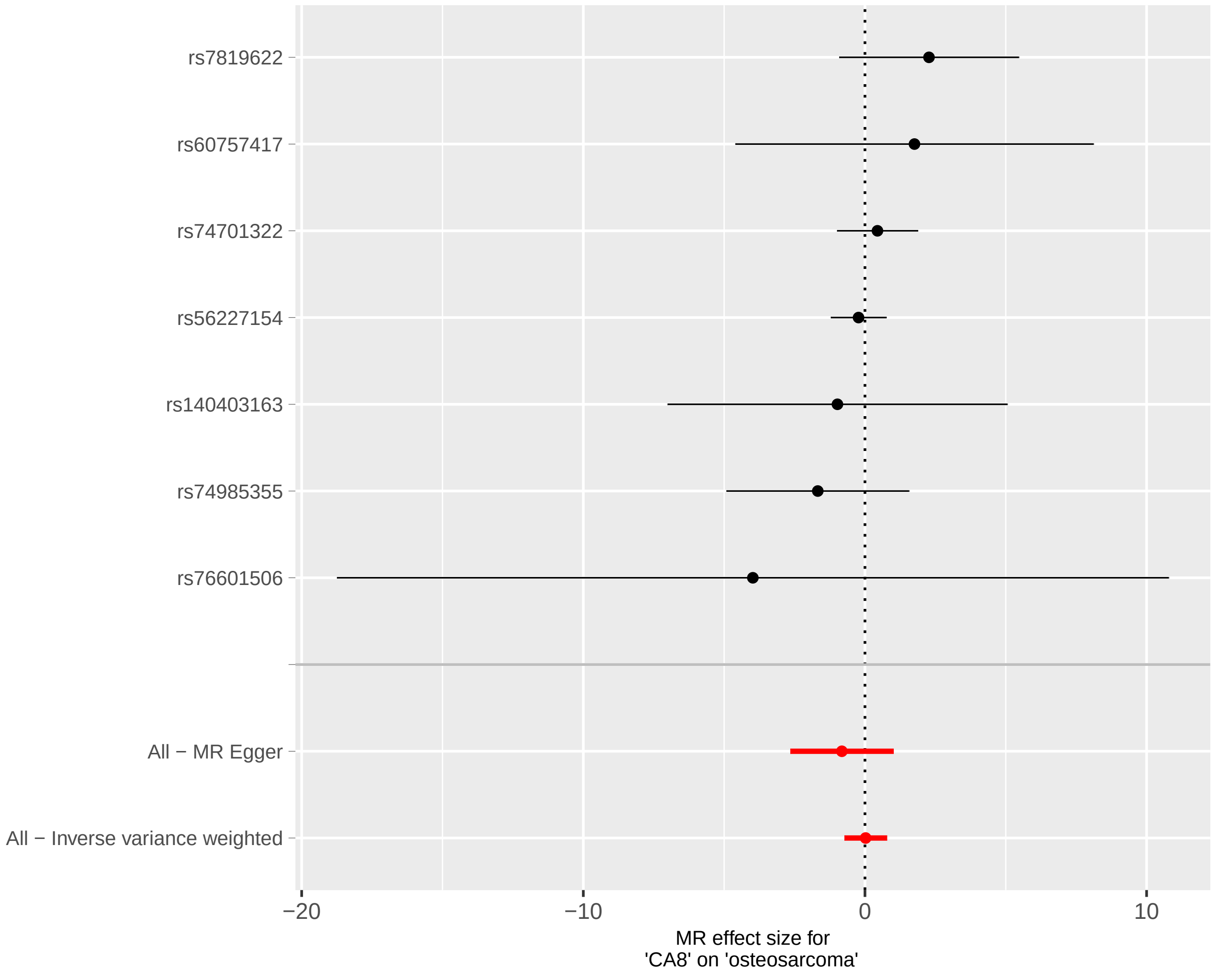
rs7819622
rs60757417
rs74701322
rs56227154
rs140403163
rs74985355
rs76601506
All − MR Egger
All − Inverse variance weighted
−20
−10
0
10
MR effect size for
'CA8' on 'osteosarcoma'

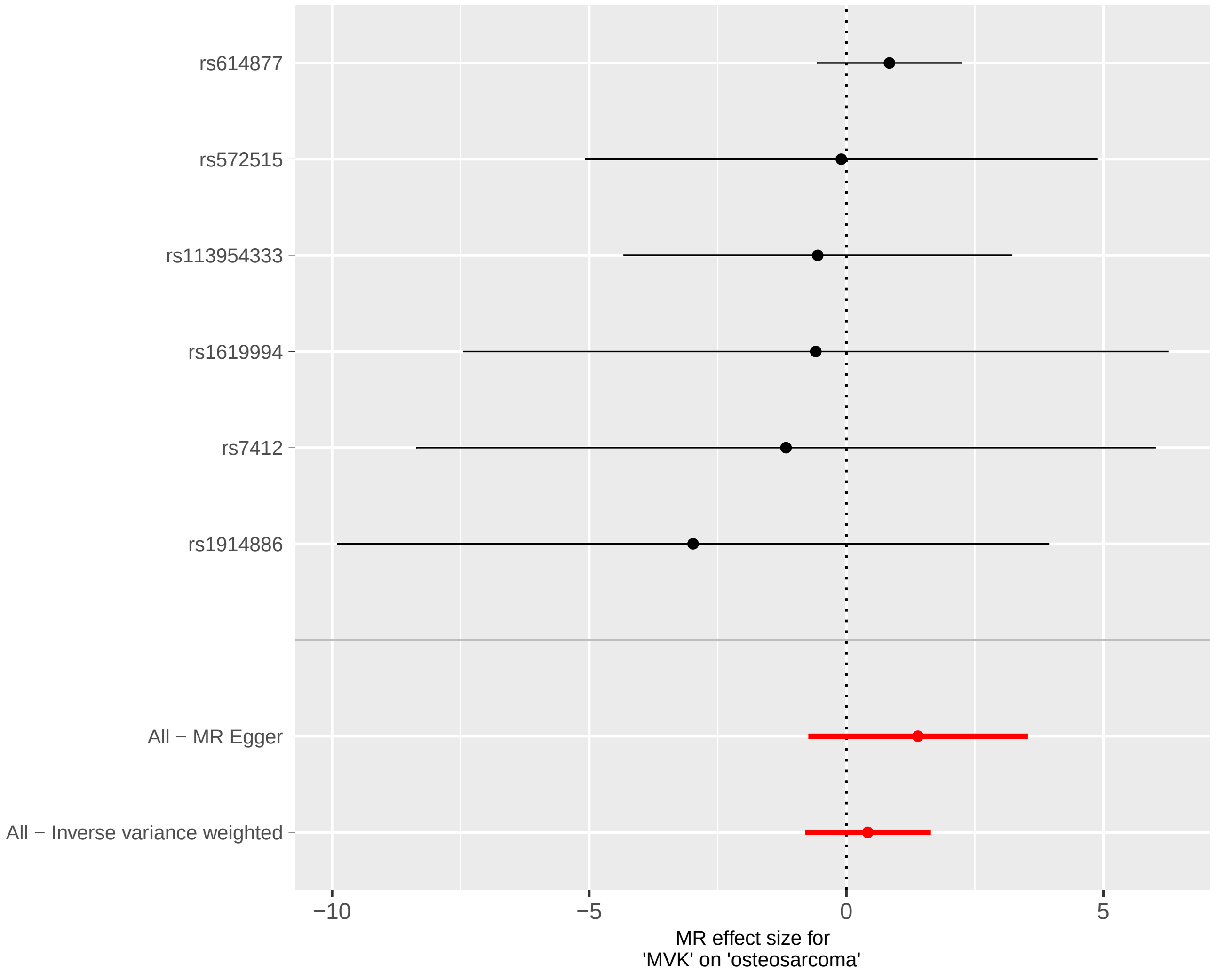
rs614877
rs572515
rs113954333
rs1619994
rs7412
rs1914886
All − MR Egger
All − Inverse variance weighted
−10
−5
0
5
MR effect size for
'MVK' on 'osteosarcoma'

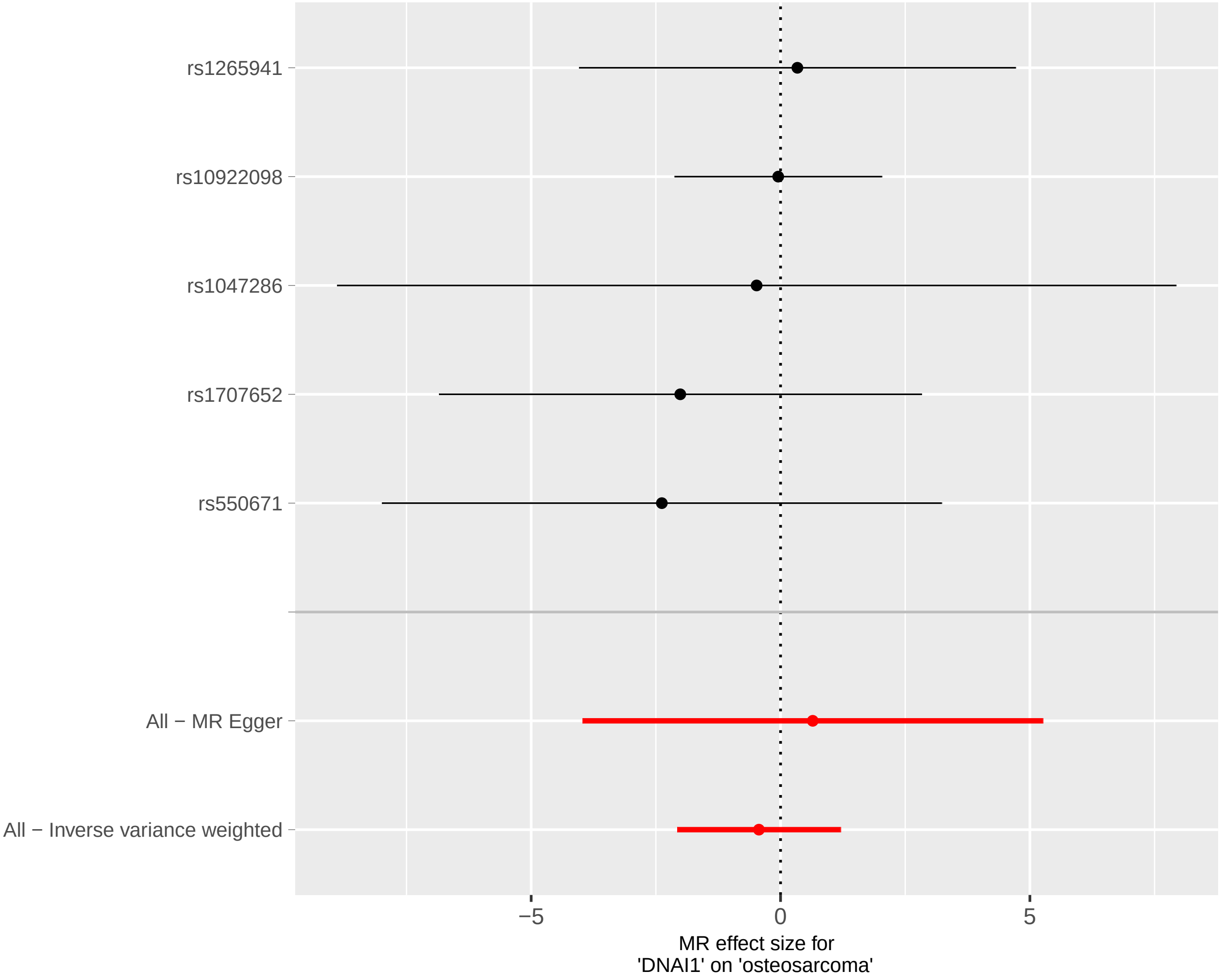
rs1265941
rs10922098
rs1047286
rs1707652
rs550671
All − MR Egger
All − Inverse variance weighted
−5
0
5
MR effect size for
'DNAI1' on 'osteosarcoma'

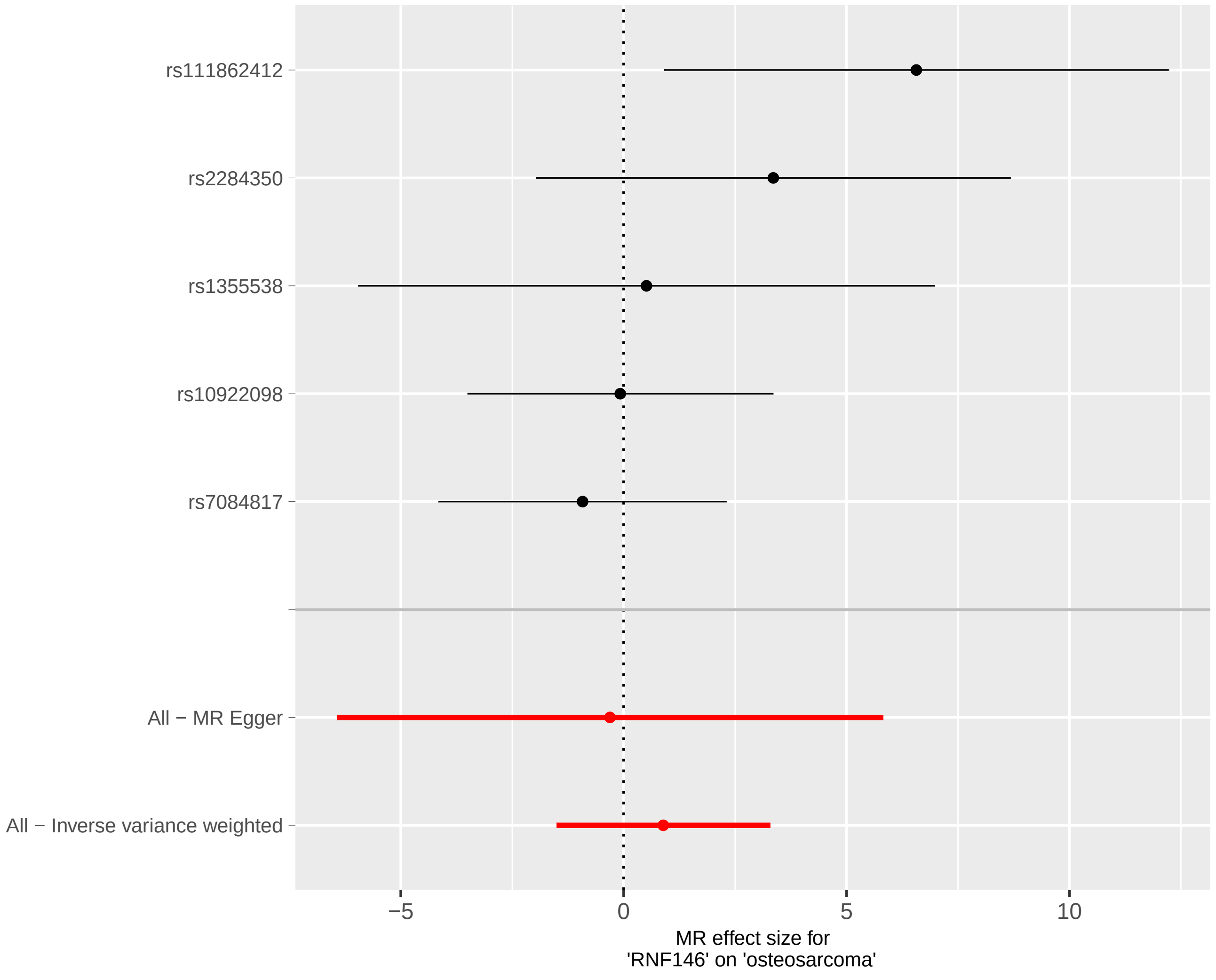

rs111862412
rs2284350
rs1355538
rs10922098
rs7084817
All − MR Egger
All − Inverse variance weighted
−5
0
5
10
MR effect size for
'RNF146' on 'osteosarcoma'

Insufficient number of SNPs

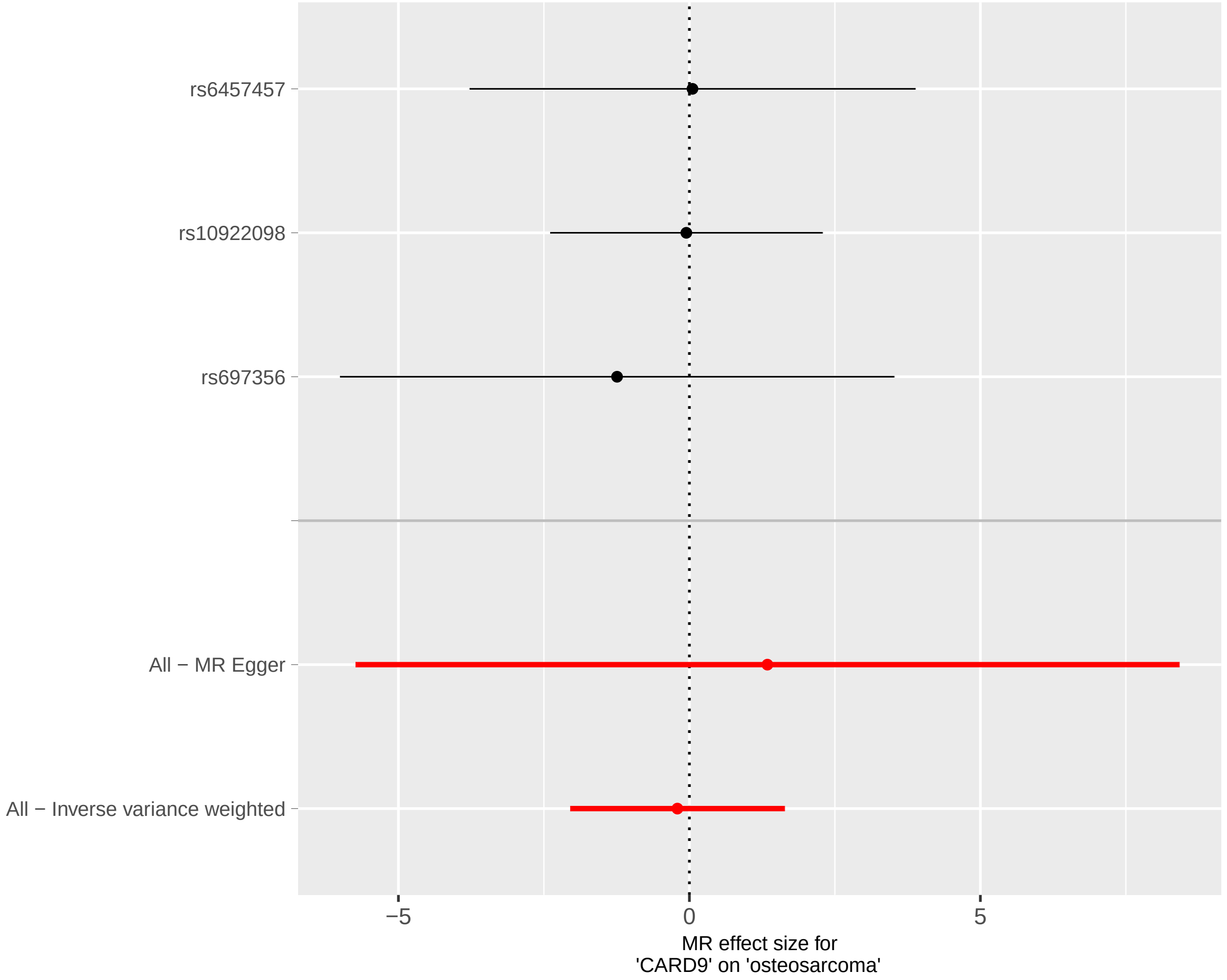
rs6457457
rs10922098
rs697356
All − MR Egger
All − Inverse variance weighted
−5
0
5
MR effect size for
'CARD9' on 'osteosarcoma'

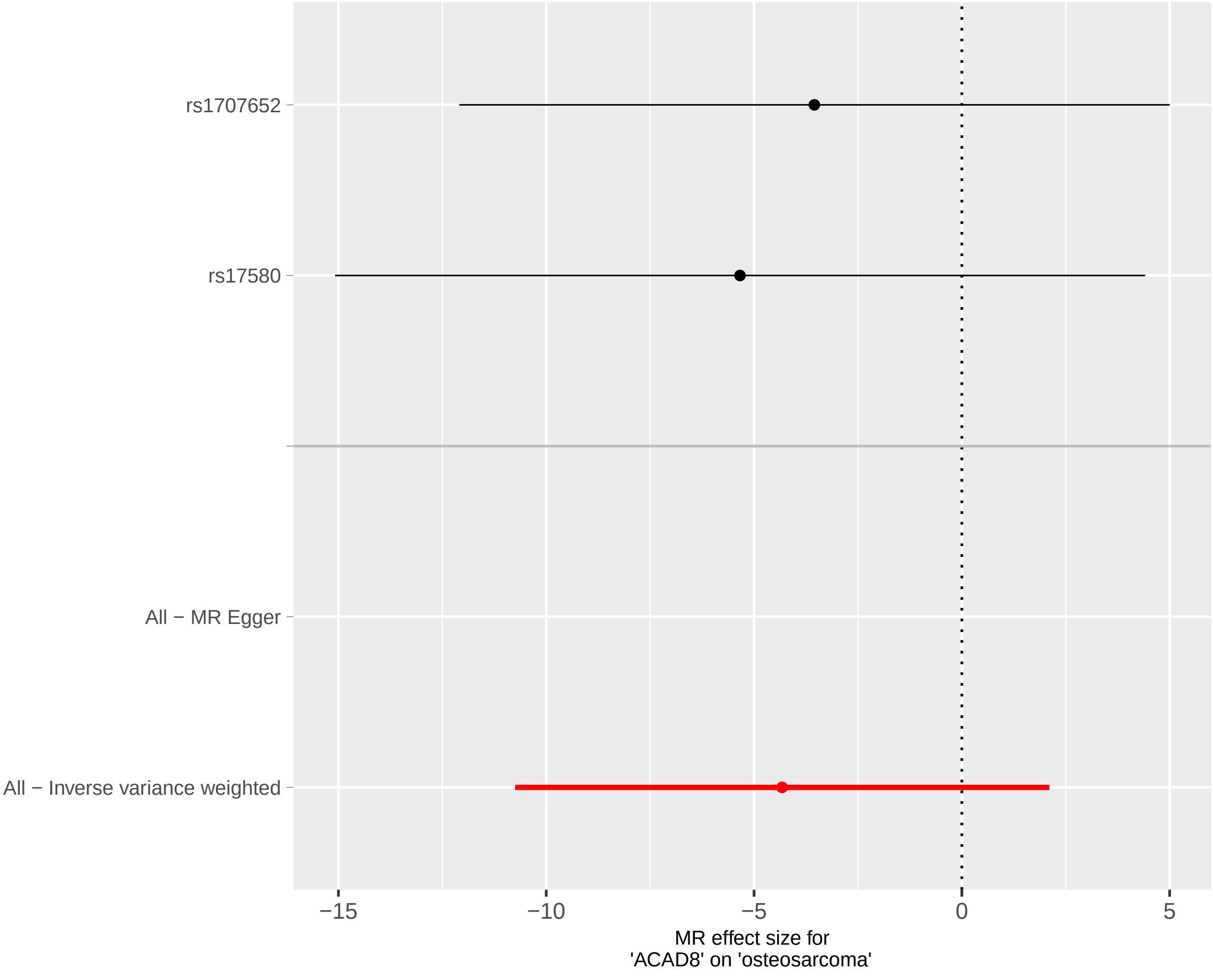

rs1707652
rs17580
All − MR Egger
All − Inverse variance weighted
−15
−10
−5
0
5
MR effect size for
'ACAD8' on 'osteosarcoma'

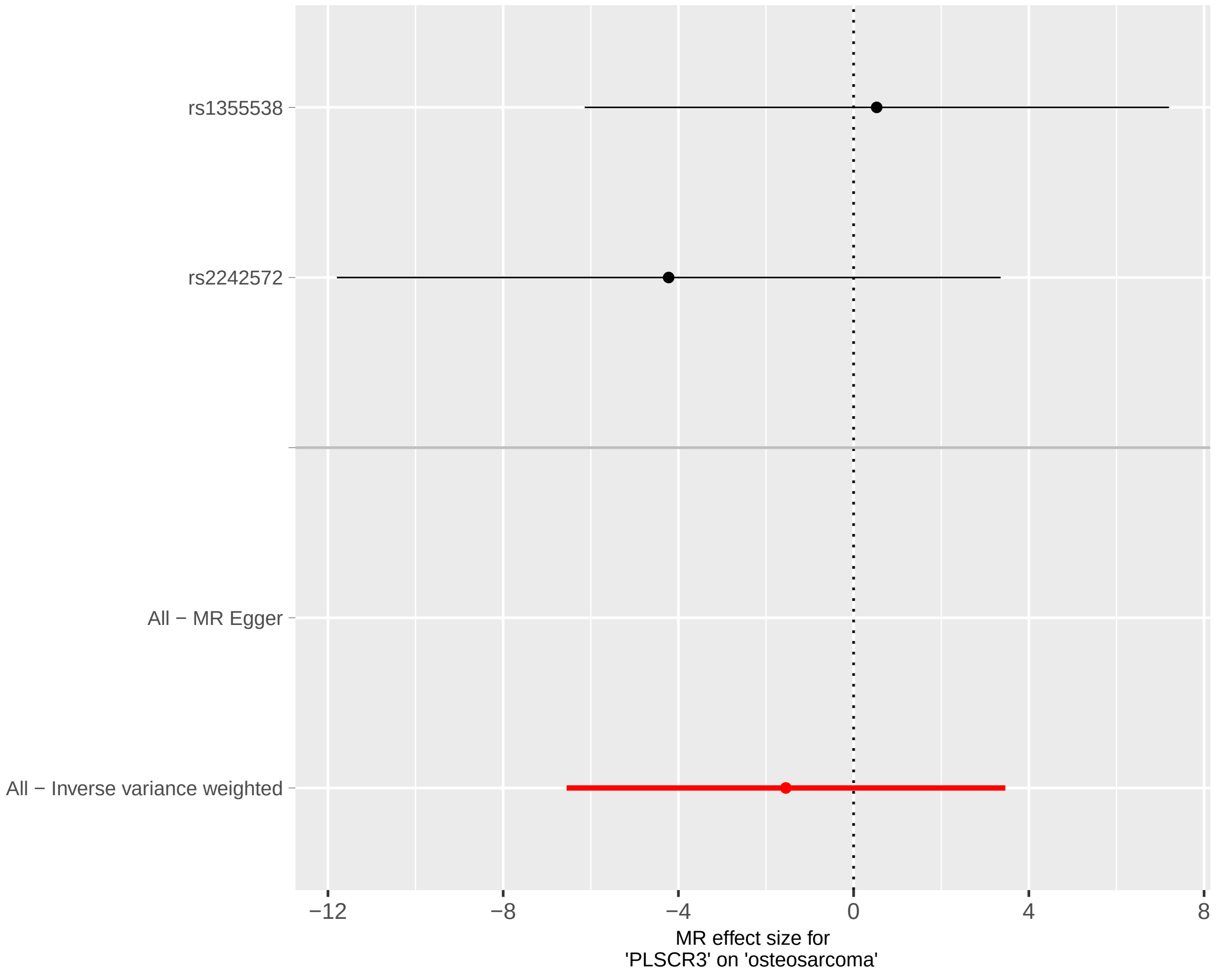
rs1355538
rs2242572
All − MR Egger
All − Inverse variance weighted
−12
−8
−4
0
4
8
MR effect size for
'PLSCR3' on 'osteosarcoma'

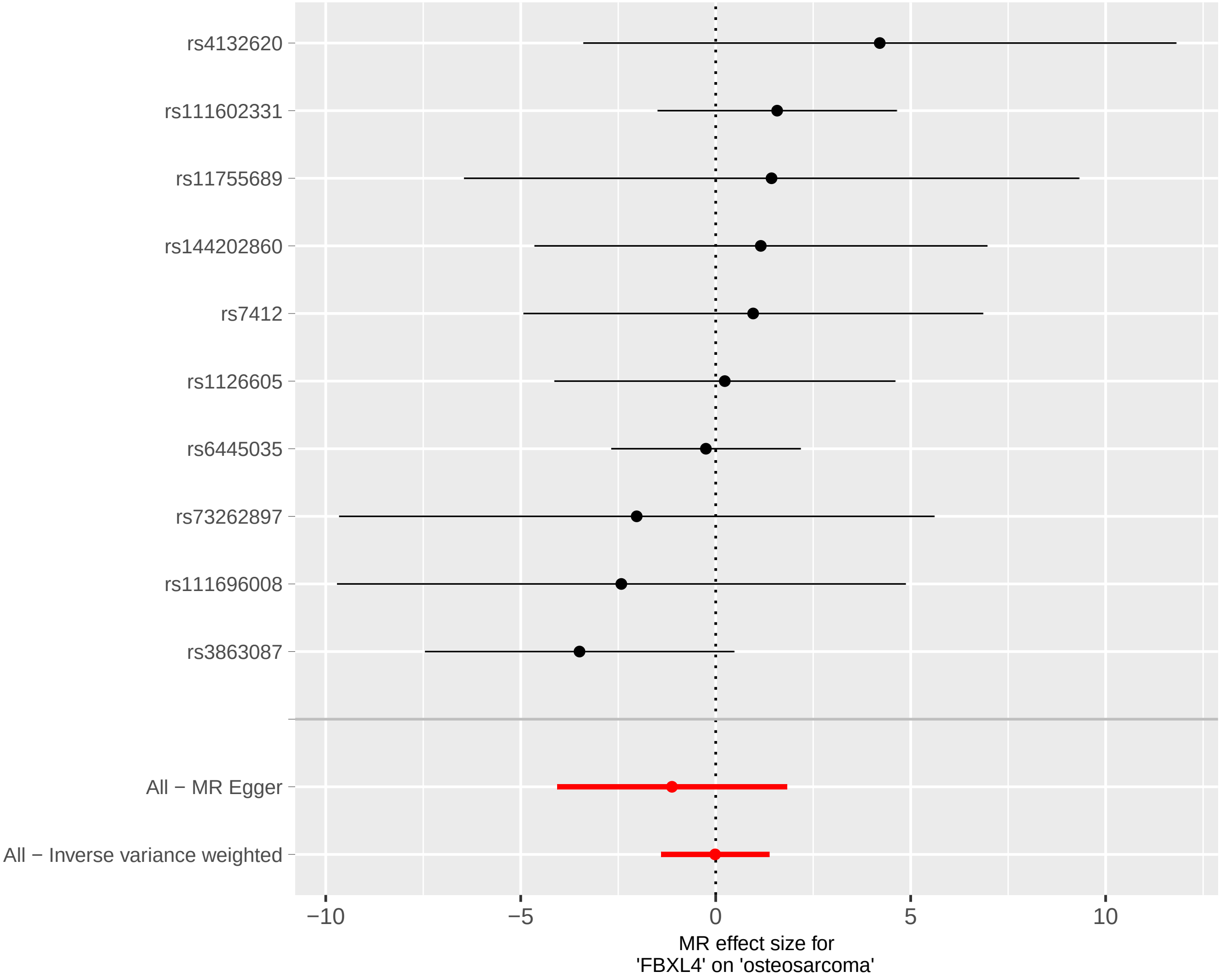
rs4132620
rs111602331
rs11755689
rs144202860
rs7412
rs1126605
rs6445035
rs73262897
rs111696008
rs3863087
All − MR Egger
All − Inverse variance weighted
−10
−5
0
5
10
MR effect size for
'FBXL4' on 'osteosarcoma'

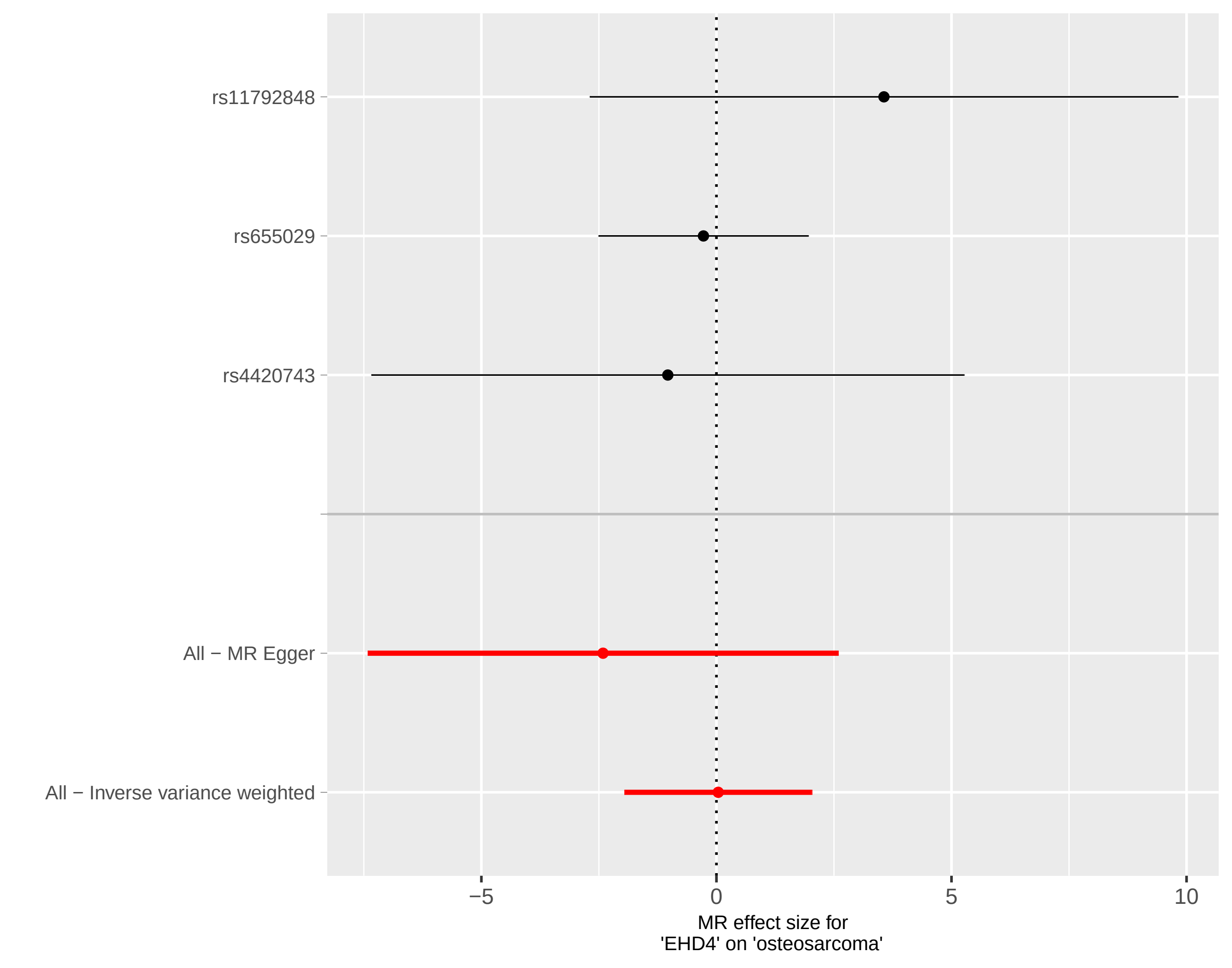
rs11792848
rs655029
rs4420743
All − MR Egger
All − Inverse variance weighted
−5
0
5
10
MR effect size for
'EHD4' on 'osteosarcoma'

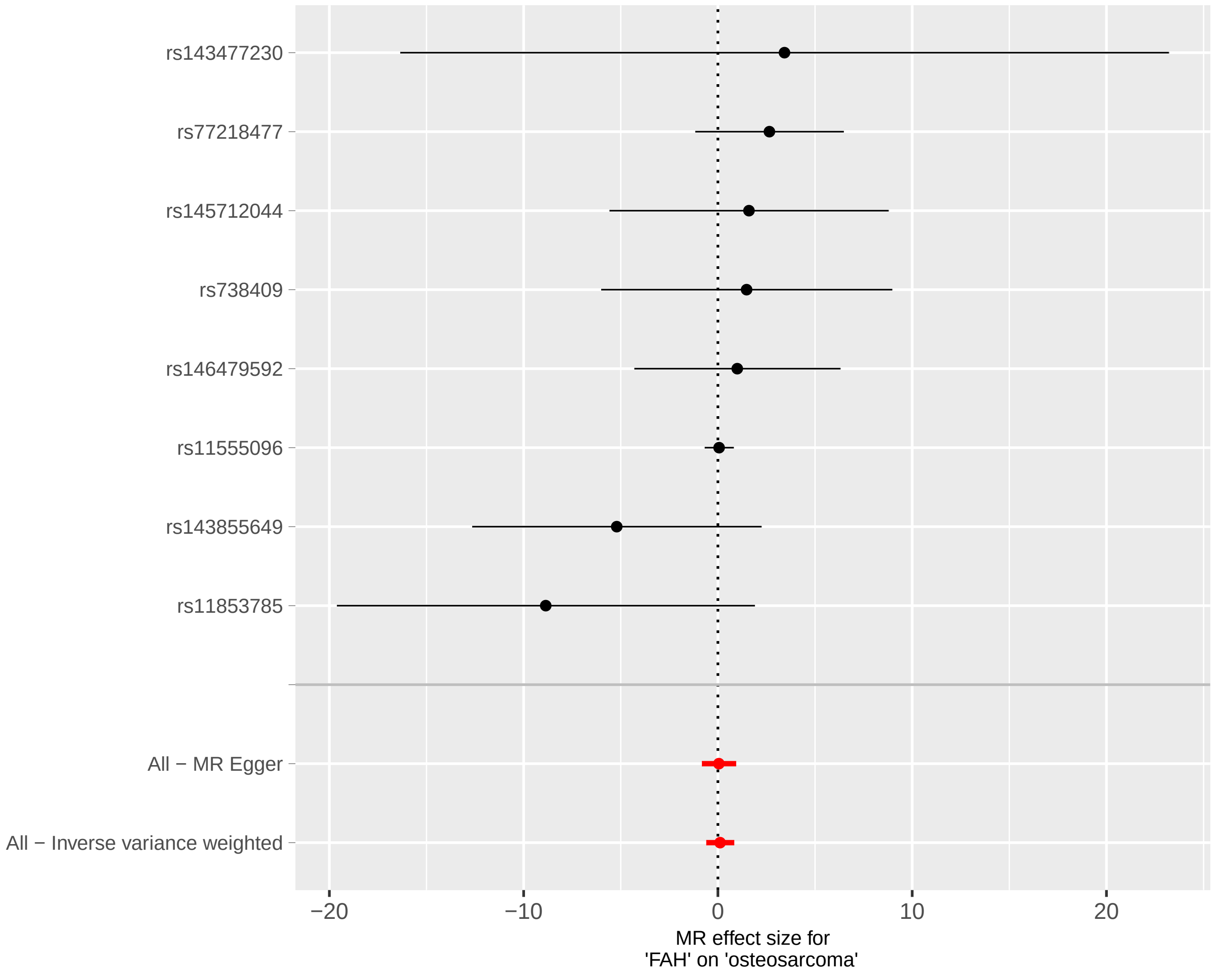

rs143477230
rs77218477
rs145712044
rs738409
rs146479592
rs11555096
rs143855649
rs11853785
All − MR Egger
All − Inverse variance weighted
−20
−10
0
10
20
MR effect size for
'FAH' on 'osteosarcoma'

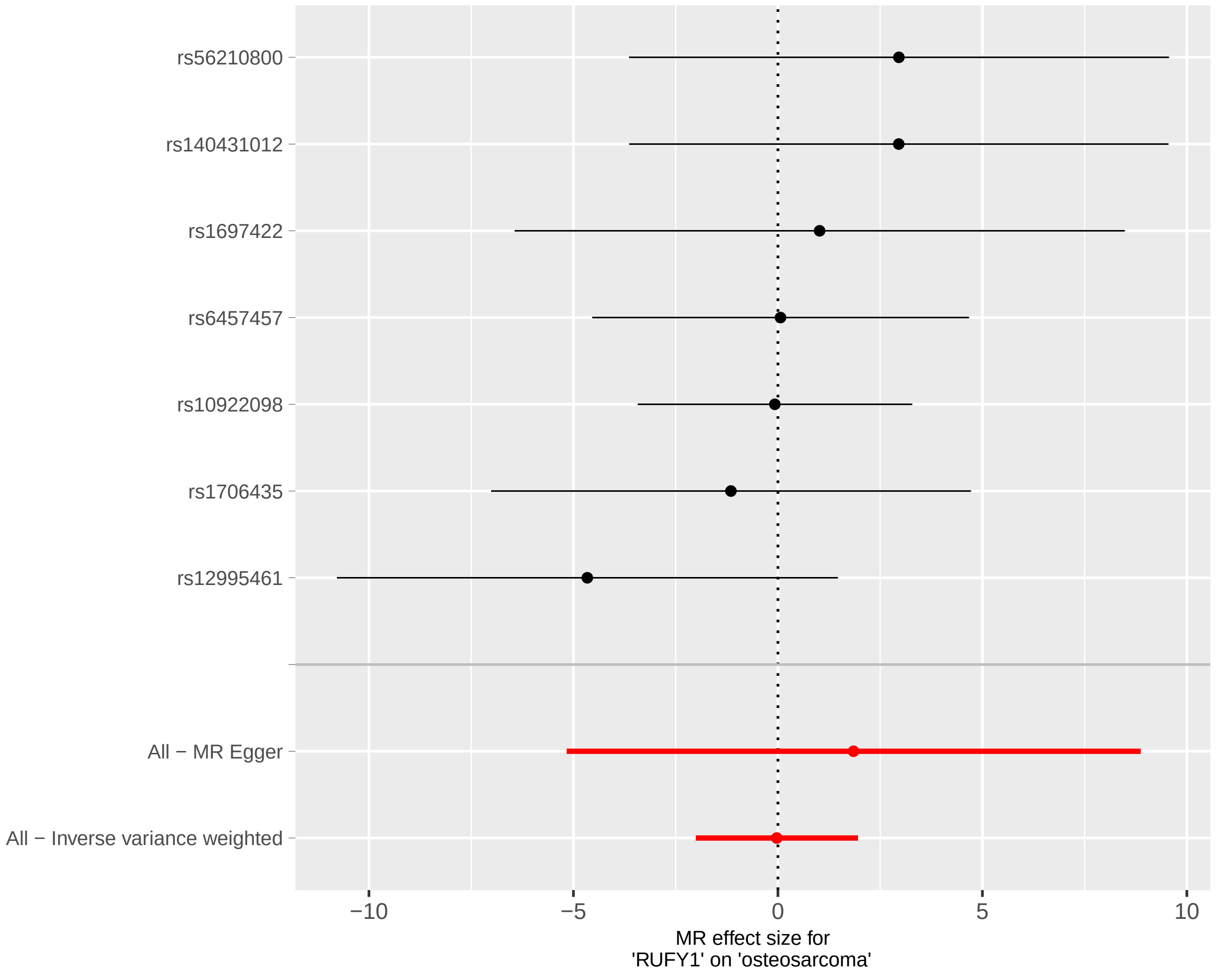
rs56210800
rs140431012
rs1697422
rs6457457
rs10922098
rs1706435
rs12995461
All − MR Egger
All − Inverse variance weighted
−10
−5
0
5
10
MR effect size for
'RUFY1' on 'osteosarcoma'

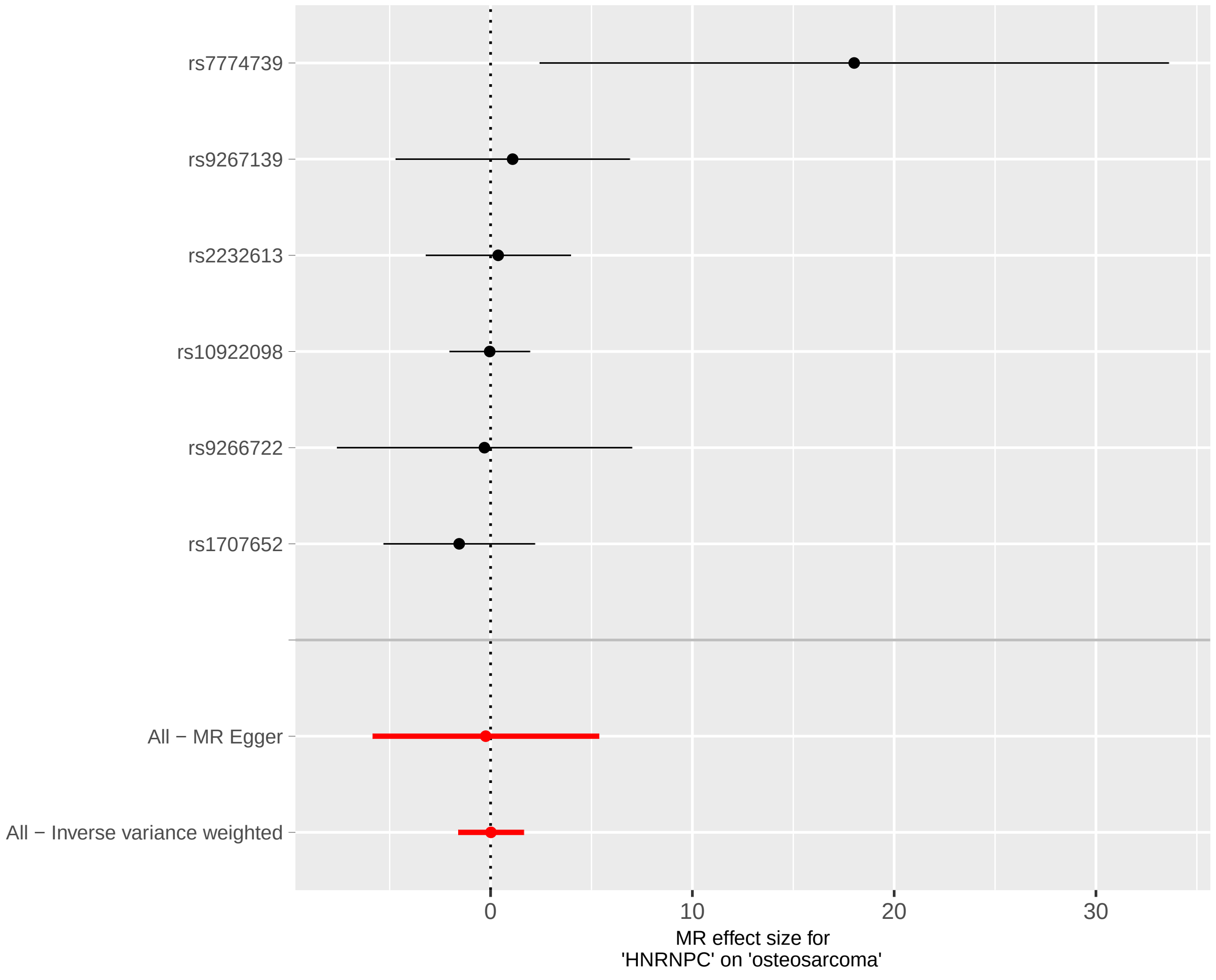

rs7774739
rs9267139
rs2232613
rs10922098
rs9266722
rs1707652
All − MR Egger
All − Inverse variance weighted
0
10
20
30
MR effect size for
'HNRNPC' on 'osteosarcoma'

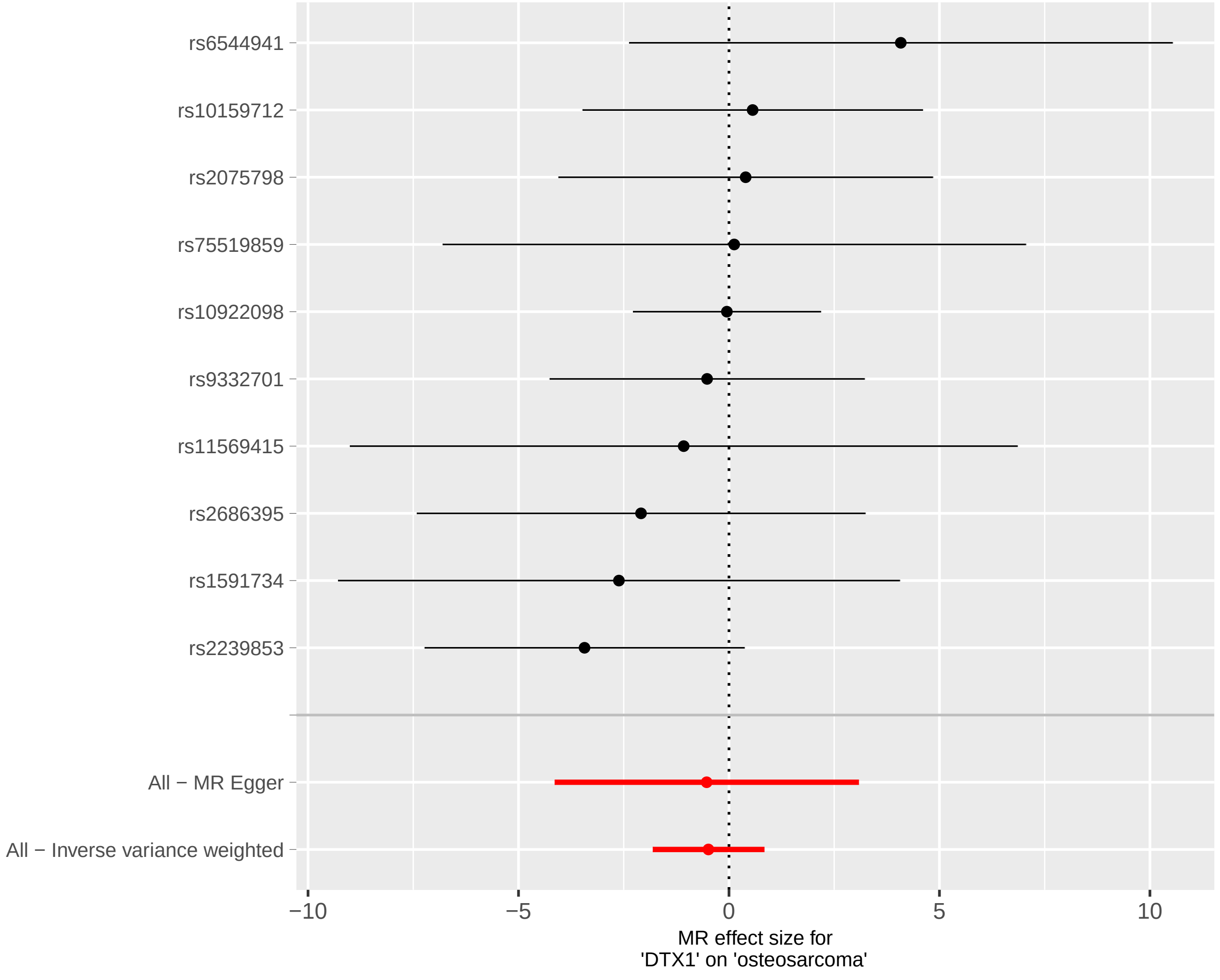
rs6544941
rs10159712
rs2075798
rs75519859
rs10922098
rs9332701
rs11569415
rs2686395
rs1591734
rs2239853
All − MR Egger
All − Inverse variance weighted
−10
−5
0
5
10
MR effect size for
'DTX1' on 'osteosarcoma'

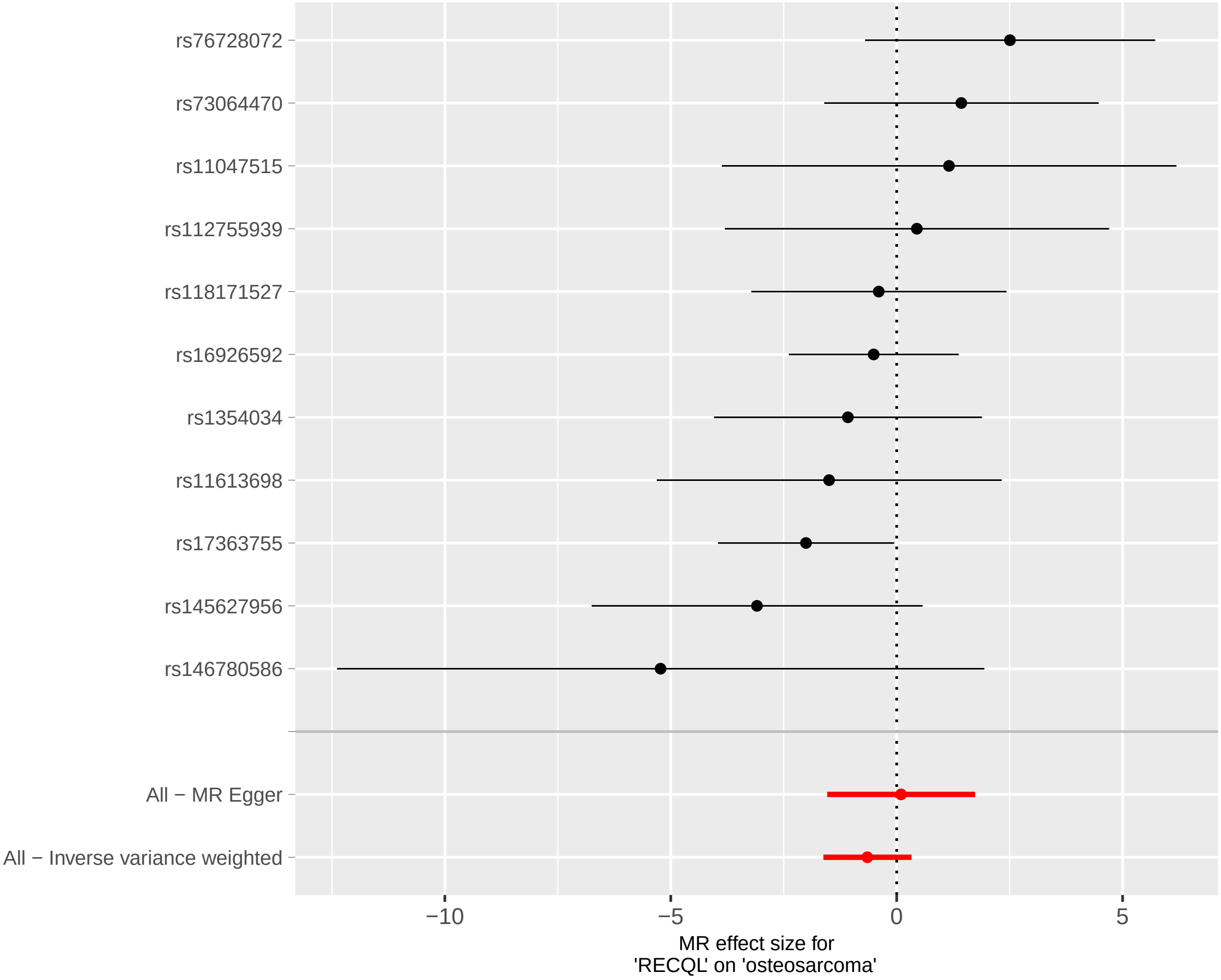

rs76728072
rs73064470
rs11047515
rs112755939
rs118171527
rs16926592
rs1354034
rs11613698
rs17363755
rs145627956
rs146780586
All − MR Egger
All − Inverse variance weighted
−10
−5
0
5
MR effect size for
'RECQL' on 'osteosarcoma'

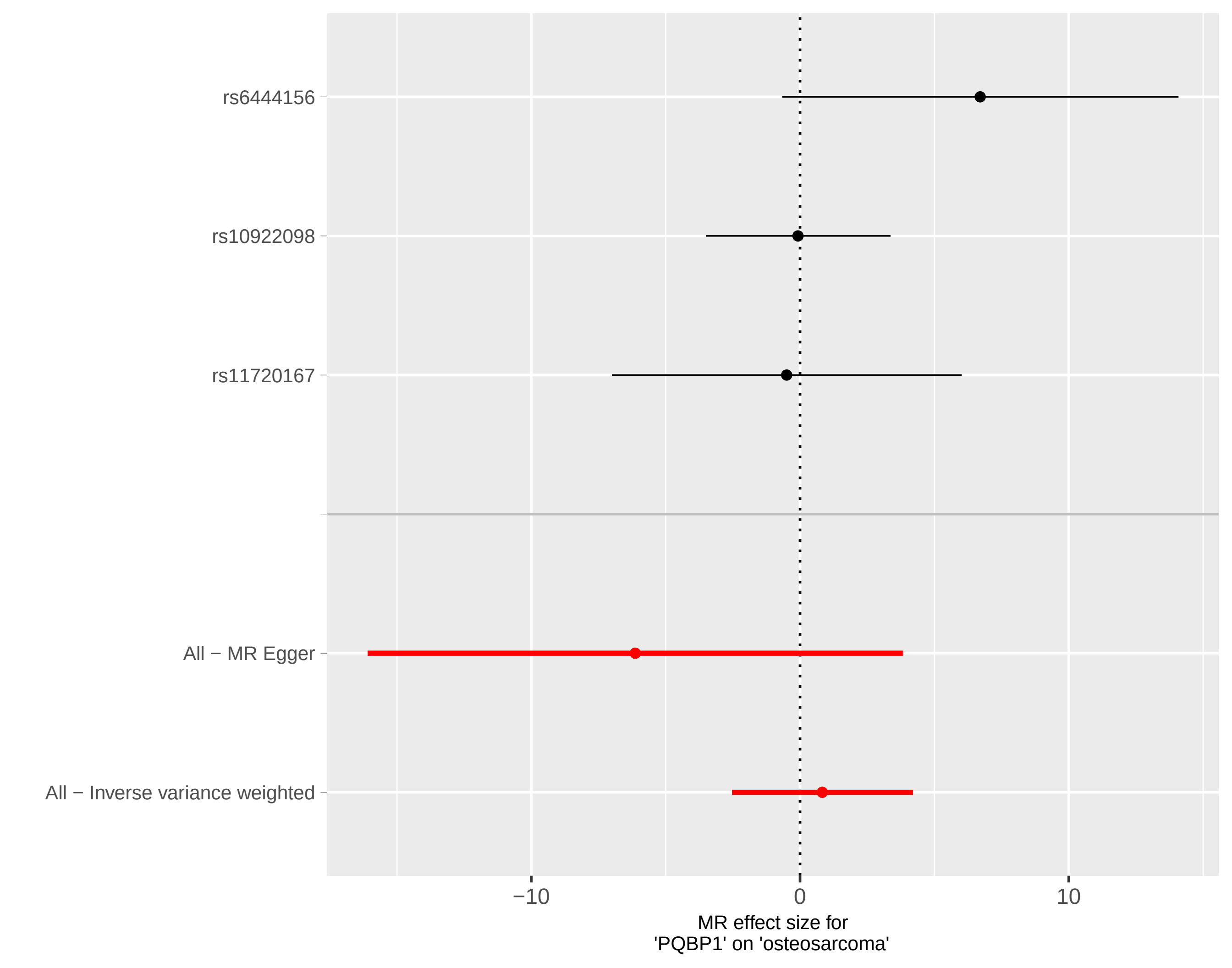

rs6444156
rs10922098
rs11720167
All − MR Egger
All − Inverse variance weighted
−10
0
10
MR effect size for
'PQBP1' on 'osteosarcoma'

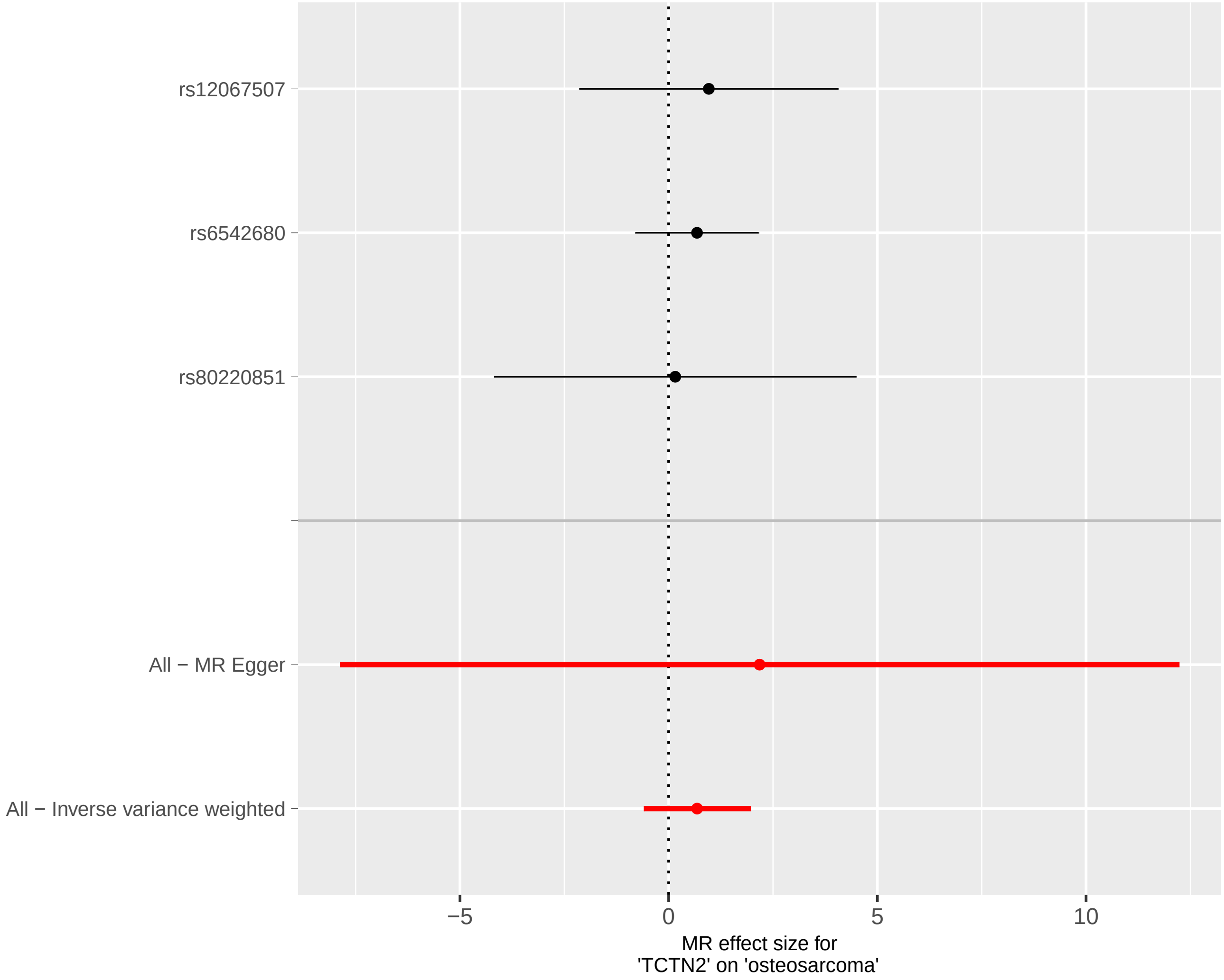

rs12067507
rs6542680
rs80220851
All − MR Egger
All − Inverse variance weighted
−5
0
5
10
MR effect size for
'TCTN2' on 'osteosarcoma'

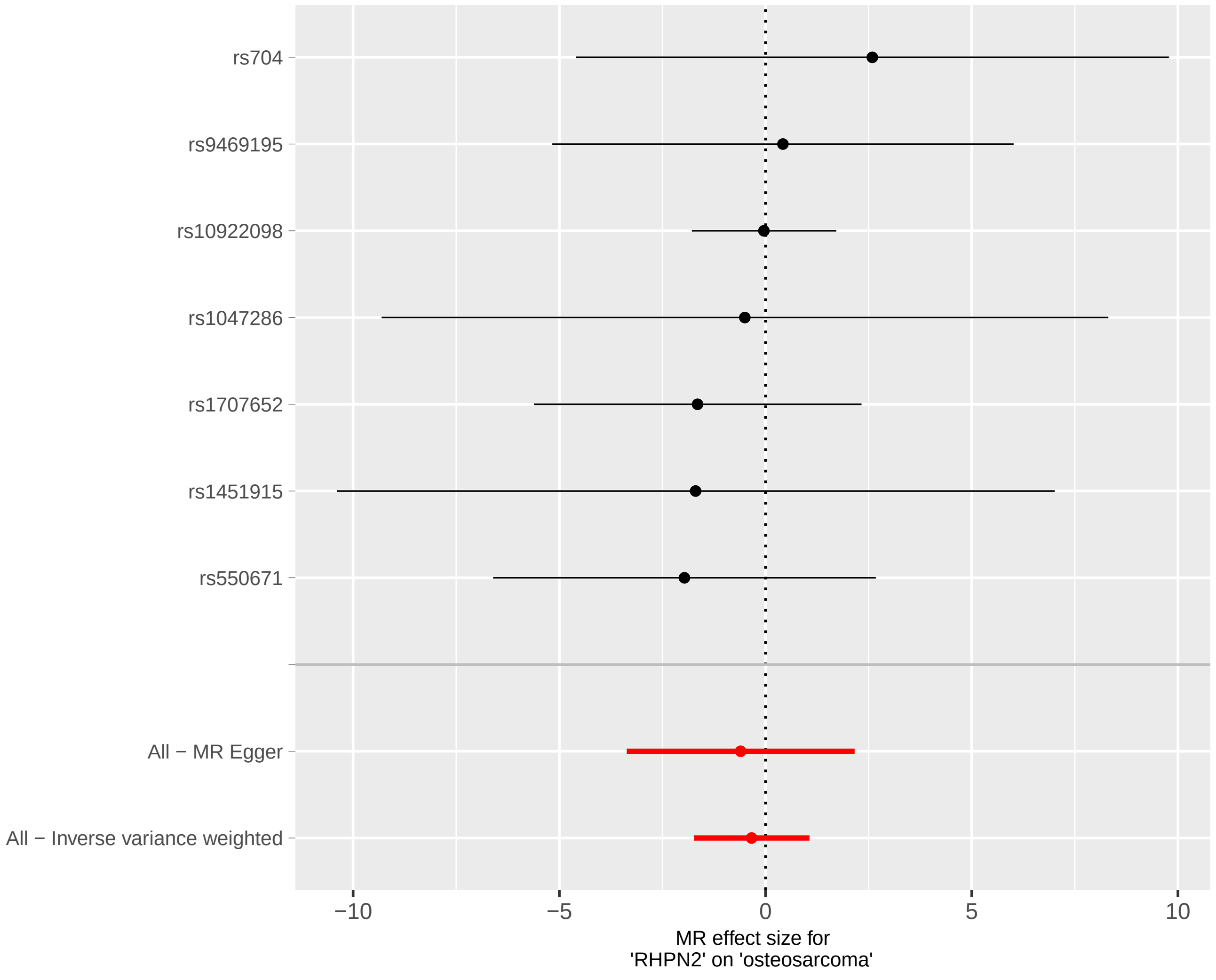
rs704
rs9469195
rs10922098
rs1047286
rs1707652
rs1451915
rs550671
All − MR Egger
All − Inverse variance weighted
−10
−5
0
5
10
MR effect size for
'RHPN2' on 'osteosarcoma'

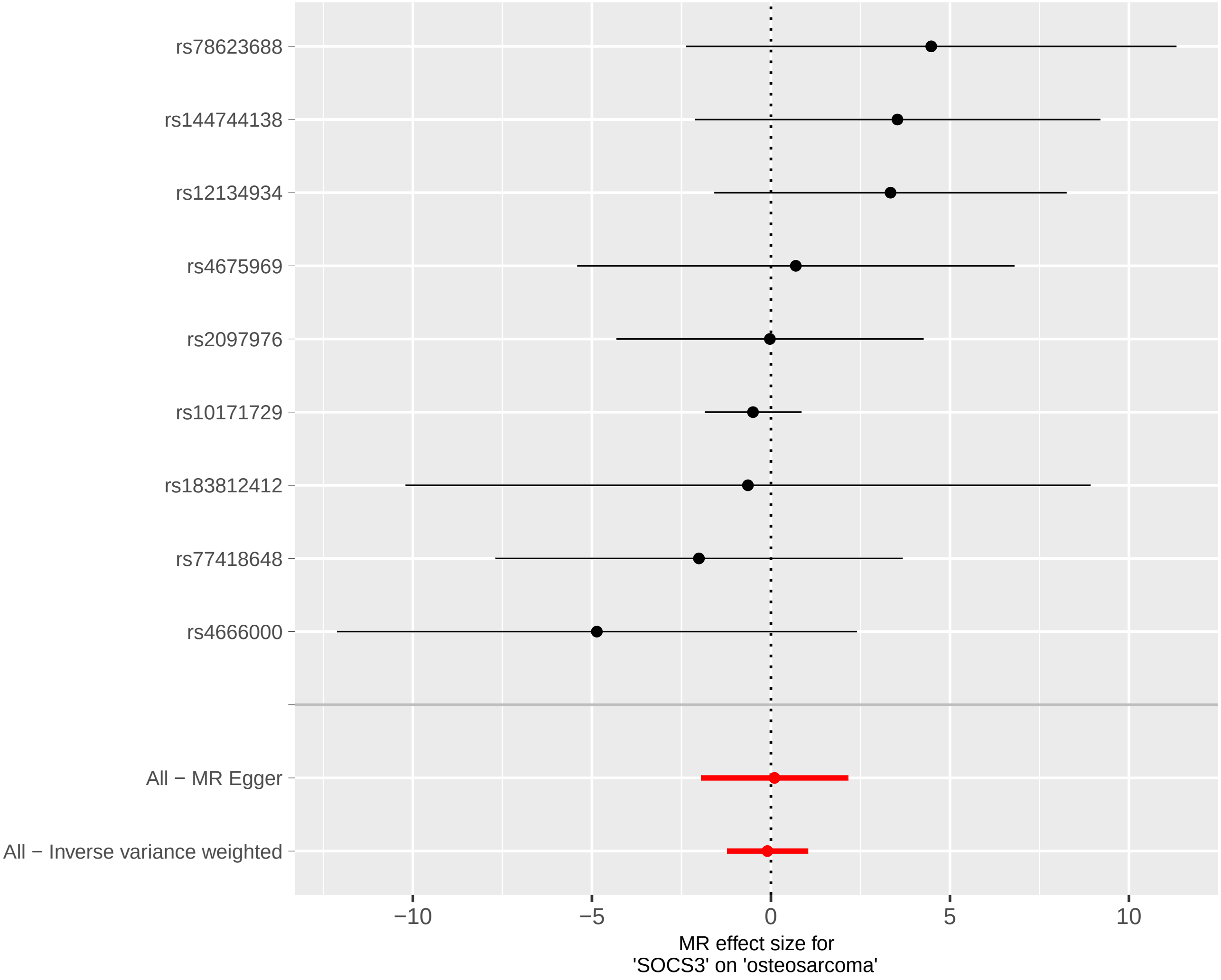

rs78623688
rs144744138
rs12134934
rs4675969
rs2097976
rs10171729
rs183812412
rs77418648
rs4666000
All − MR Egger
All − Inverse variance weighted
−10
−5
0
5
10
MR effect size for
'SOCS3' on 'osteosarcoma'

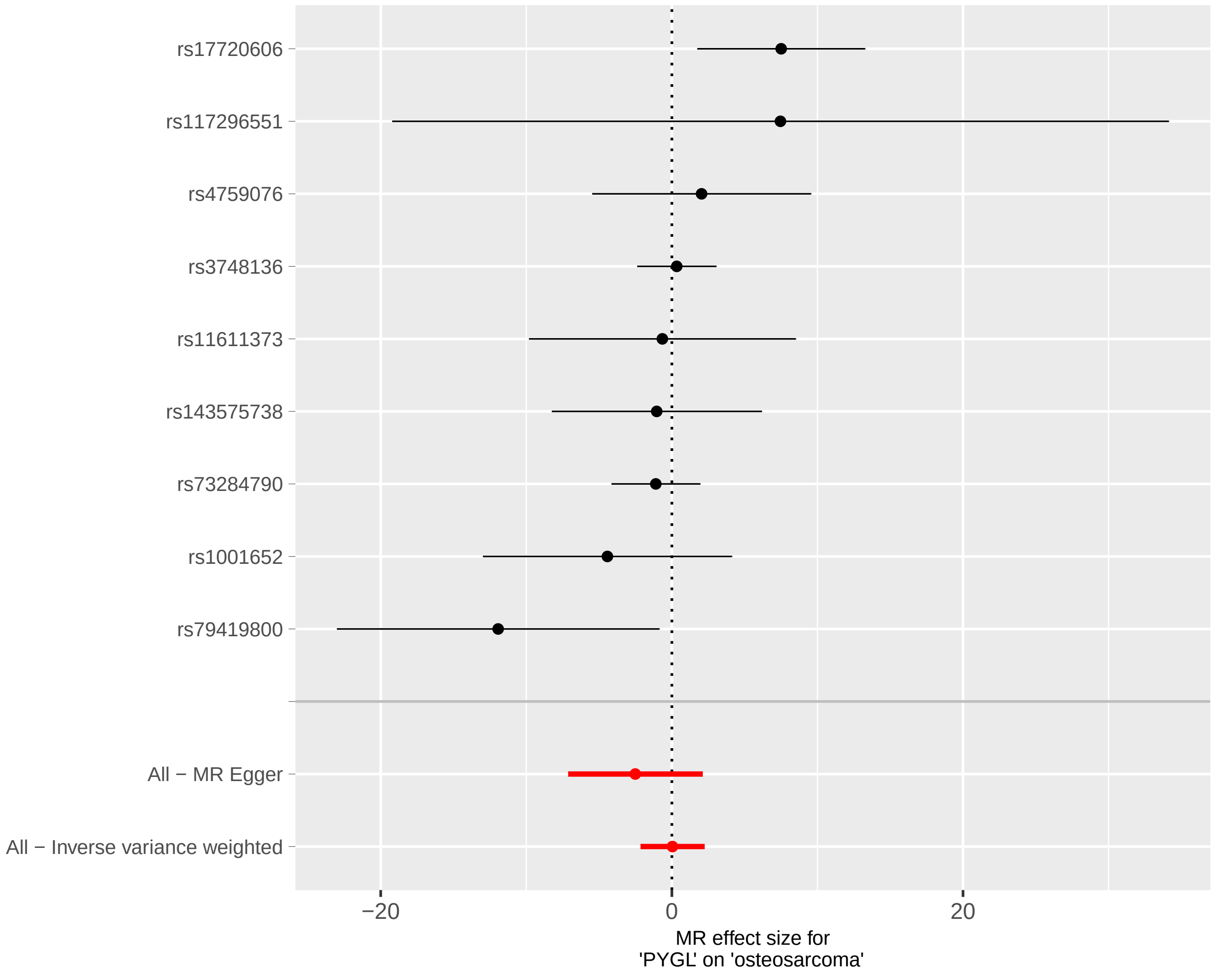
rs17720606
rs117296551
rs4759076
rs3748136
rs11611373
rs143575738
rs73284790
rs1001652
rs79419800
All − MR Egger
All − Inverse variance weighted
−20
0
20
MR effect size for
'PYGL' on 'osteosarcoma'

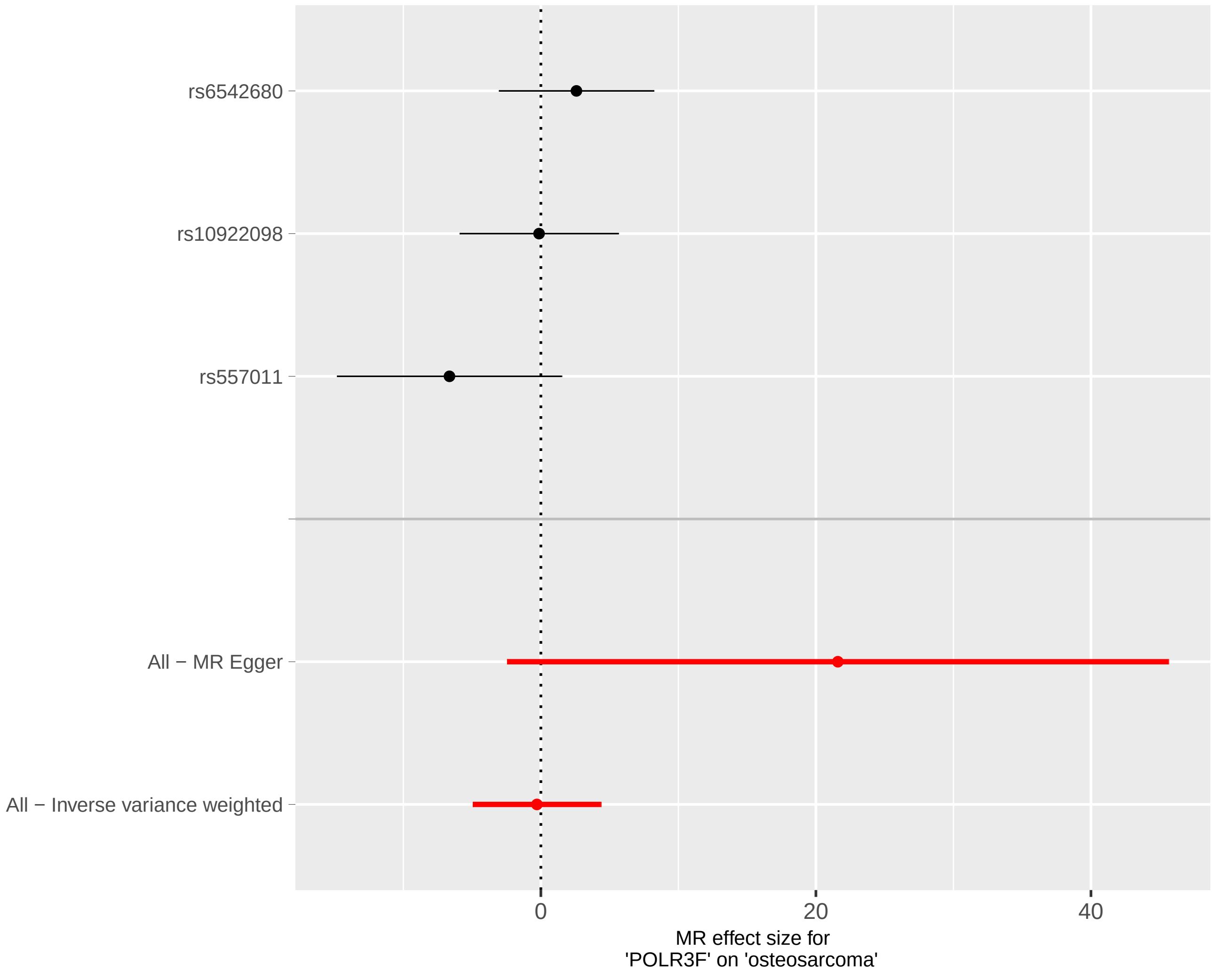
rs6542680
rs10922098
rs557011
All − MR Egger
All − Inverse variance weighted
0
20
40
MR effect size for
'POLR3F' on 'osteosarcoma'

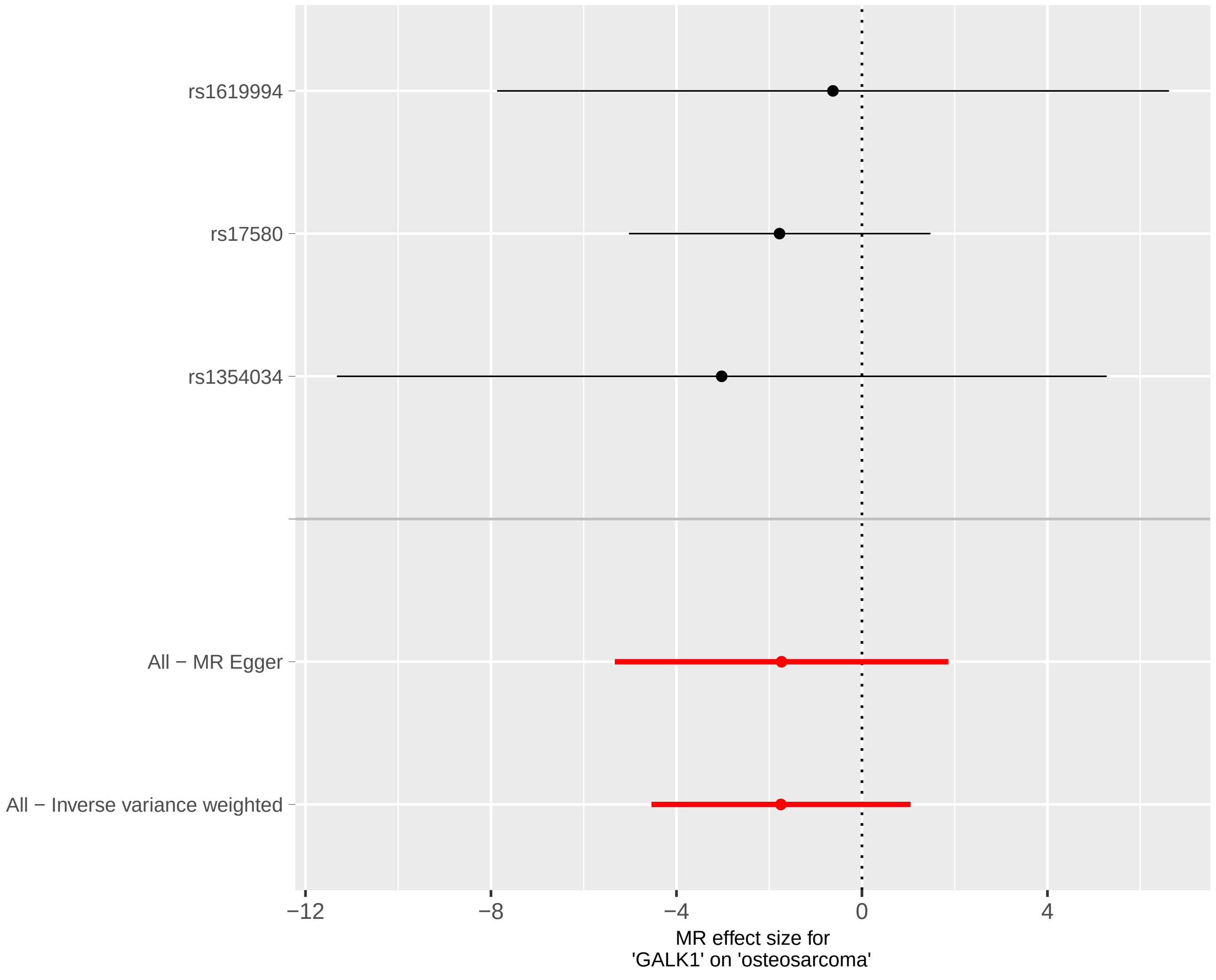

rs1619994
rs17580
rs1354034
All − MR Egger
All − Inverse variance weighted
−12
−8
−4
0
4
MR effect size for
'GALK1' on 'osteosarcoma'

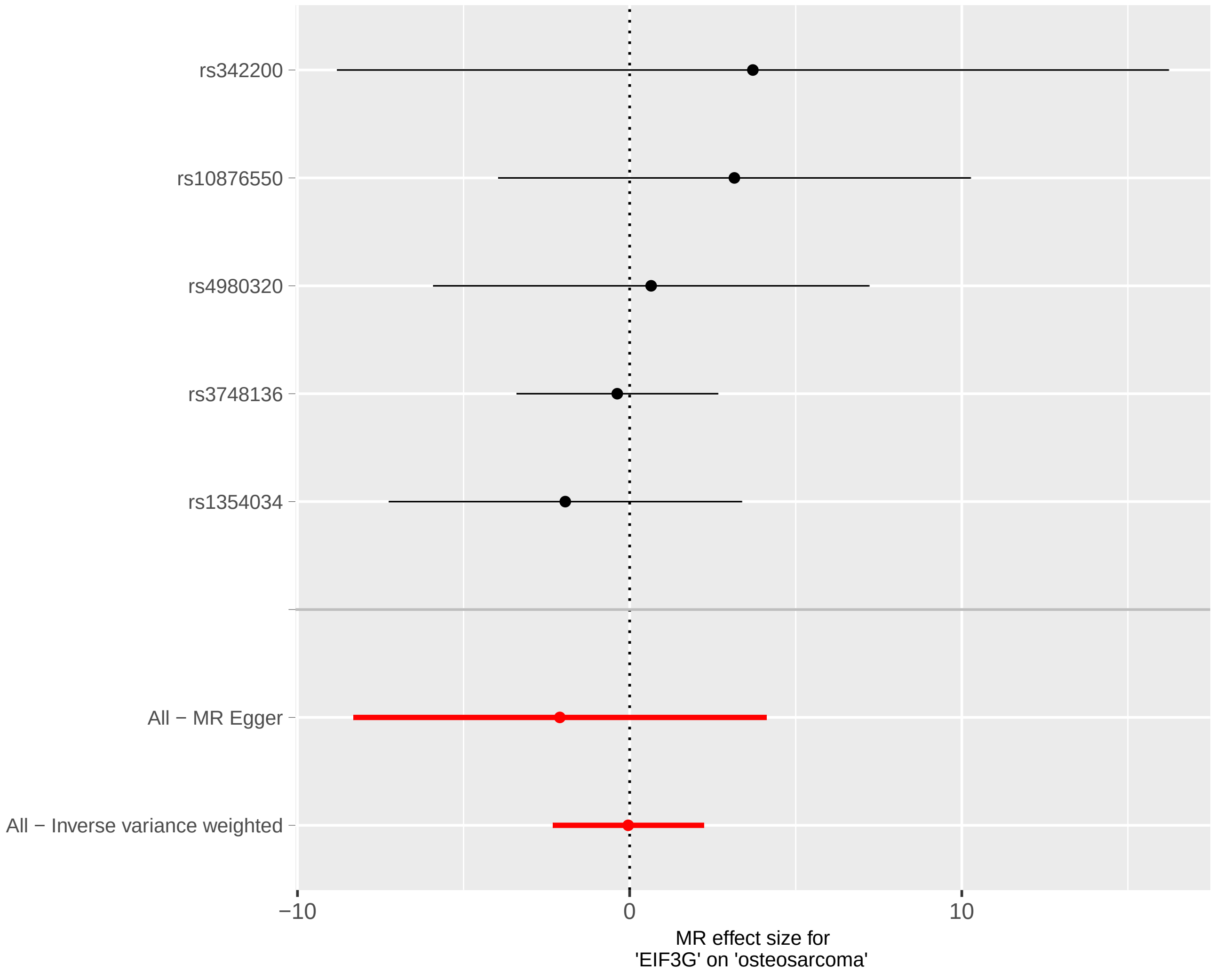

rs342200
rs10876550
rs4980320
rs3748136
rs1354034
All − MR Egger
All − Inverse variance weighted
−10
0
10
MR effect size for
'EIF3G' on 'osteosarcoma'

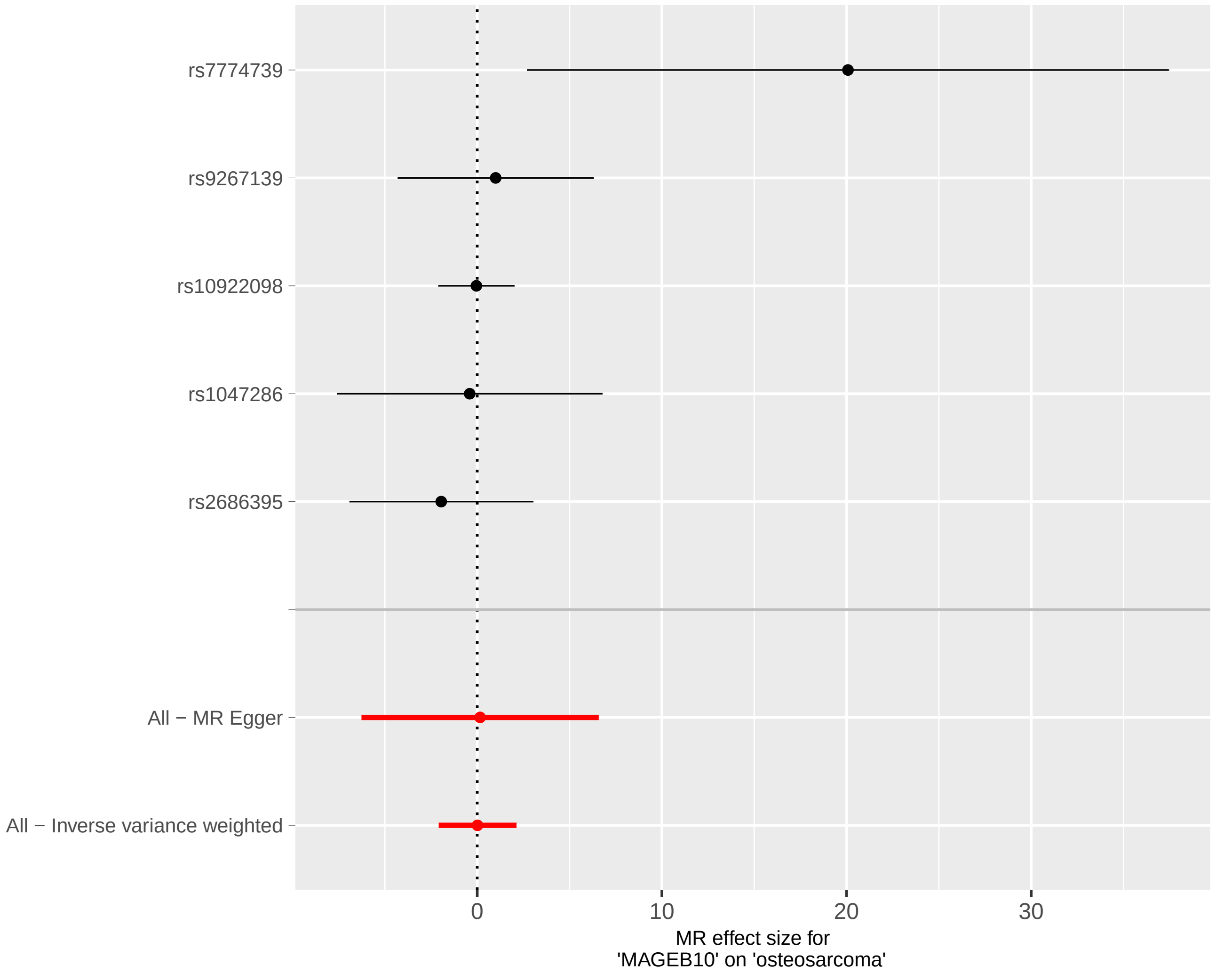

rs7774739
rs9267139
rs10922098
rs1047286
rs2686395
All − MR Egger
All − Inverse variance weighted
0
10
20
30
MR effect size for
'MAGEB10' on 'osteosarcoma'

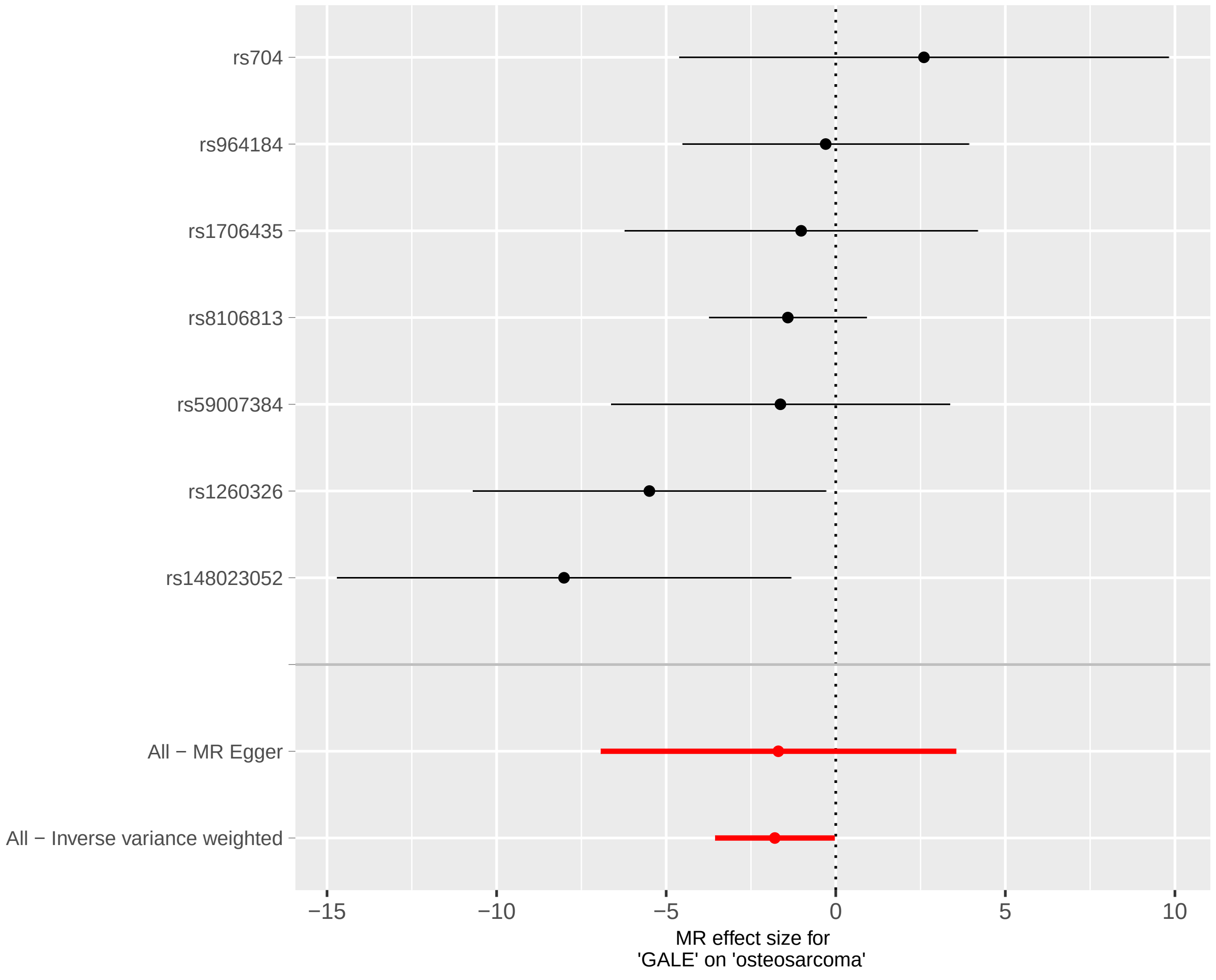
rs704
rs964184
rs1706435
rs8106813
rs59007384
rs1260326
rs148023052
All − MR Egger
All − Inverse variance weighted
−15
−10
−5
0
5
10
MR effect size for
'GALE' on 'osteosarcoma'

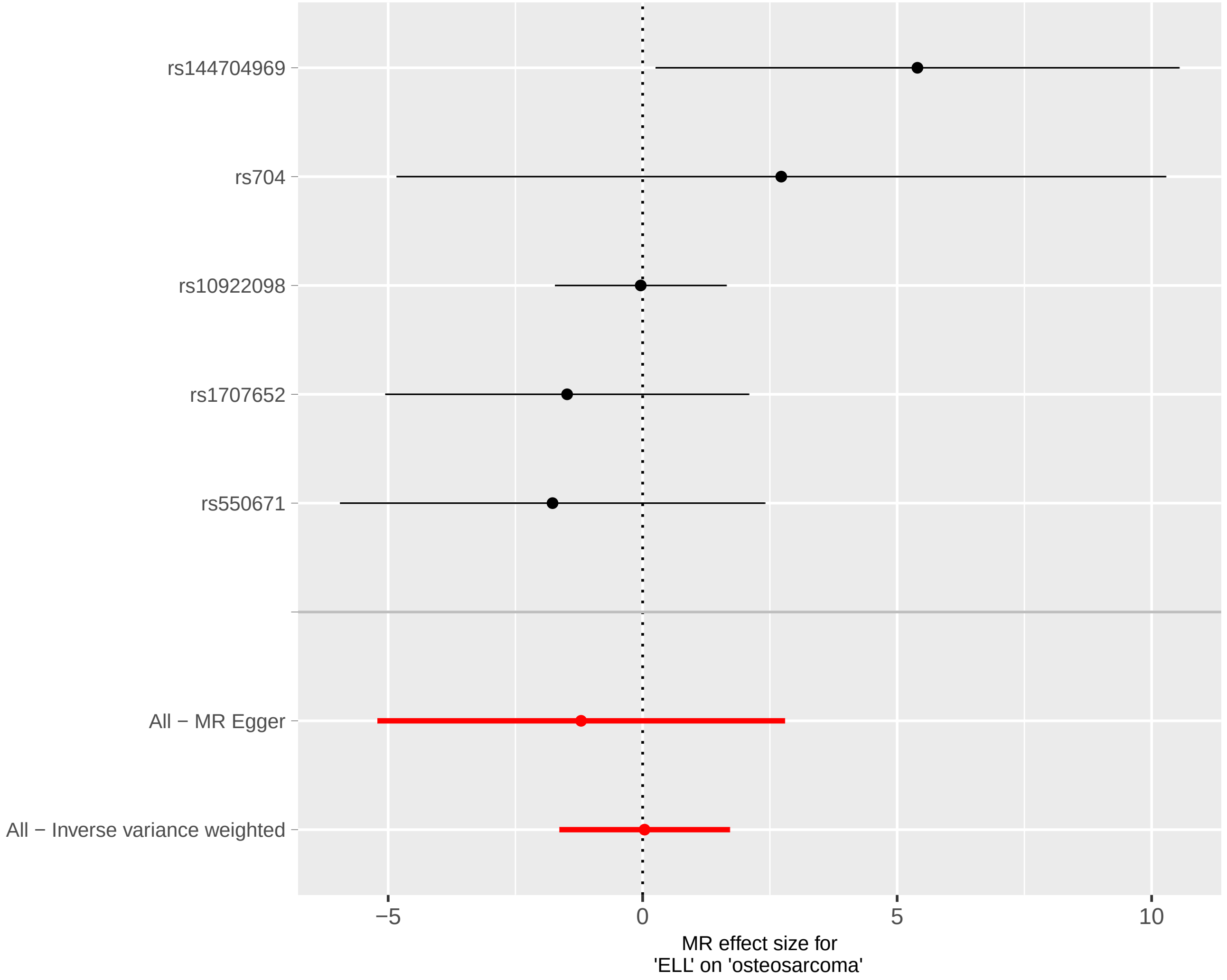
rs144704969
rs704
rs10922098
rs1707652
rs550671
All − MR Egger
All − Inverse variance weighted
−5
0
5
10
MR effect size for
'ELL' on 'osteosarcoma'

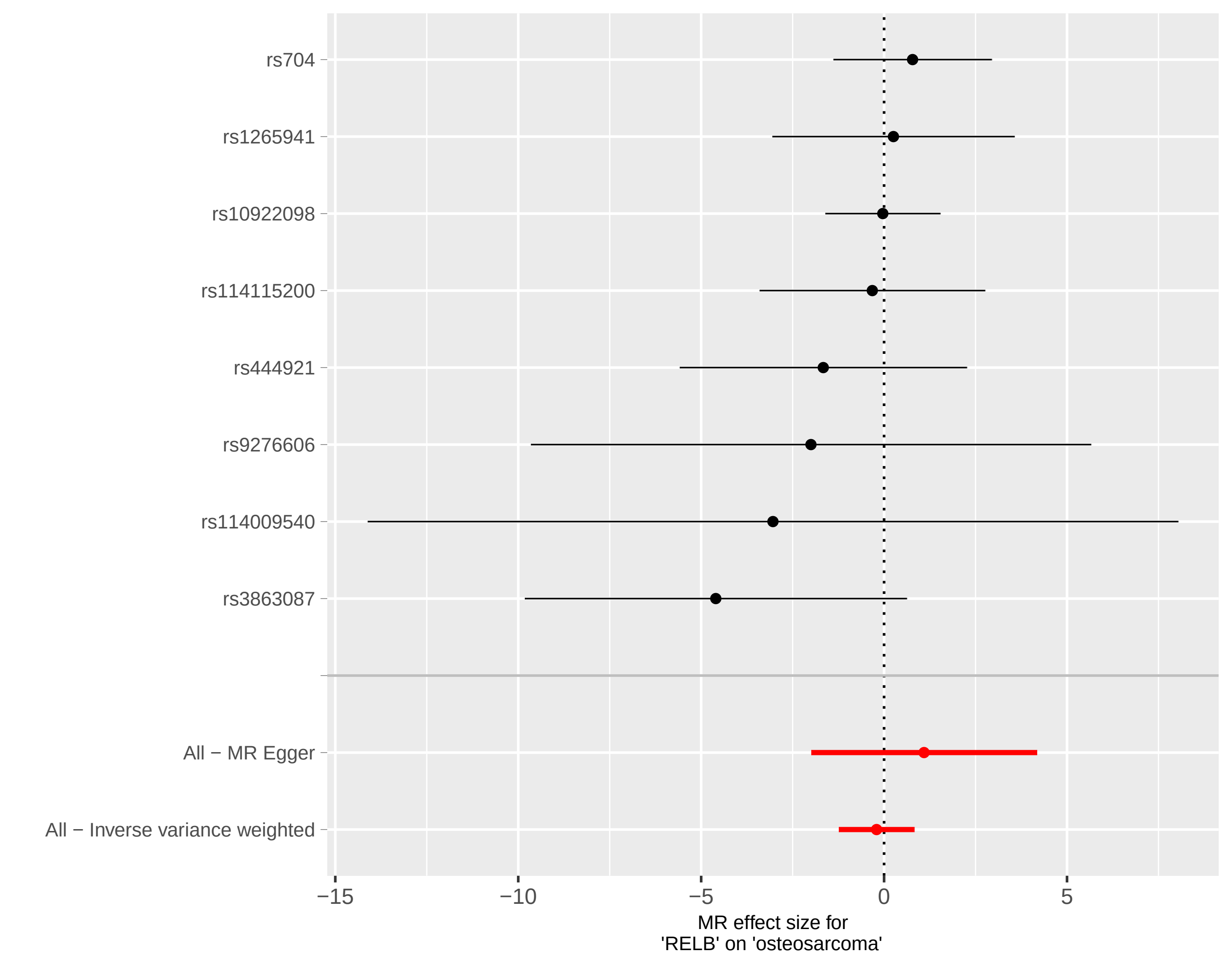
rs704
rs1265941
rs10922098
rs114115200
rs444921
rs9276606
rs114009540
rs3863087
All − MR Egger
All − Inverse variance weighted
−15
−10
−5
0
5
MR effect size for
'RELB' on 'osteosarcoma'

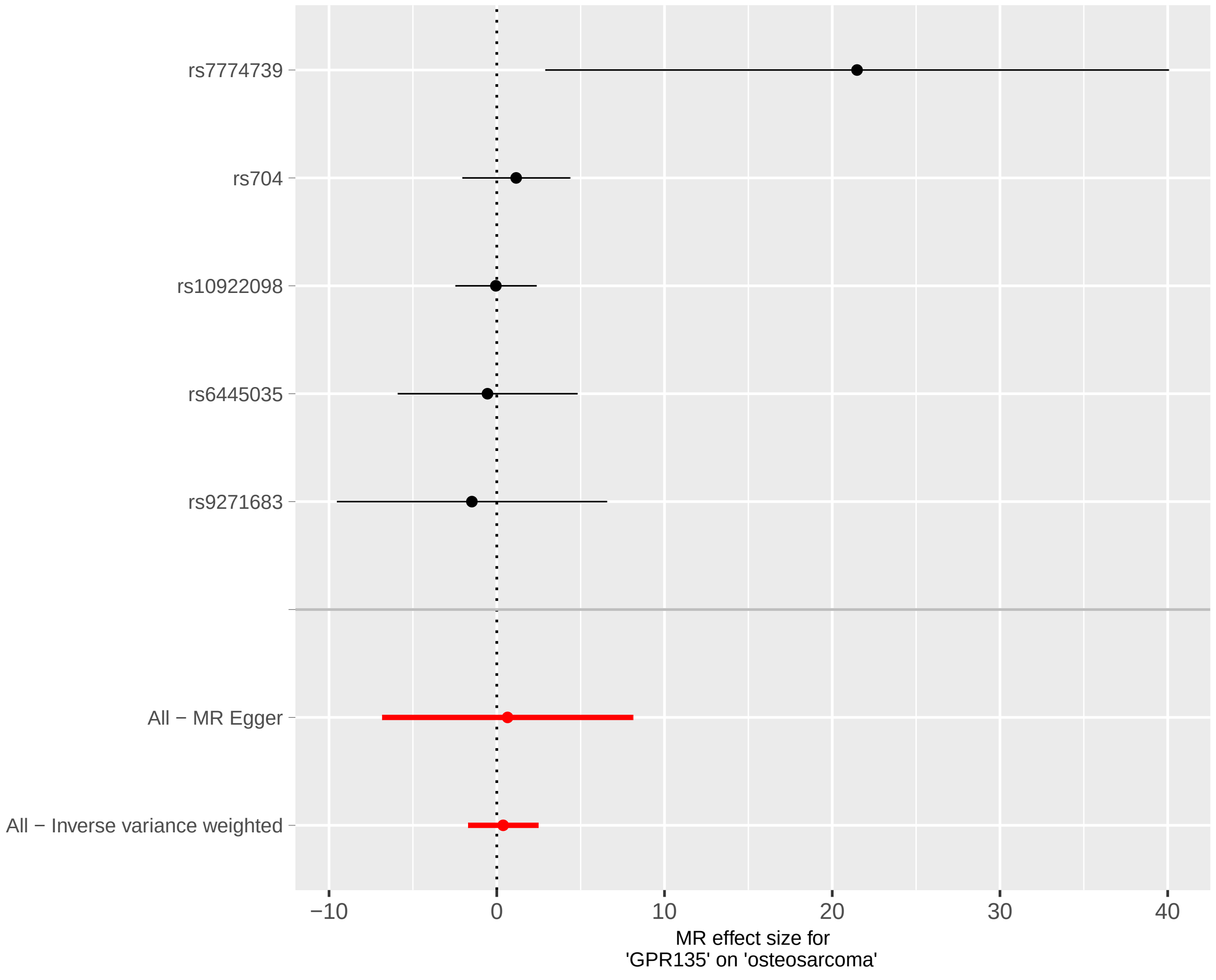

rs7774739
rs704
rs10922098
rs6445035
rs9271683
All − MR Egger
All − Inverse variance weighted
−10
0
10
20
30
40
MR effect size for
'GPR135' on 'osteosarcoma'

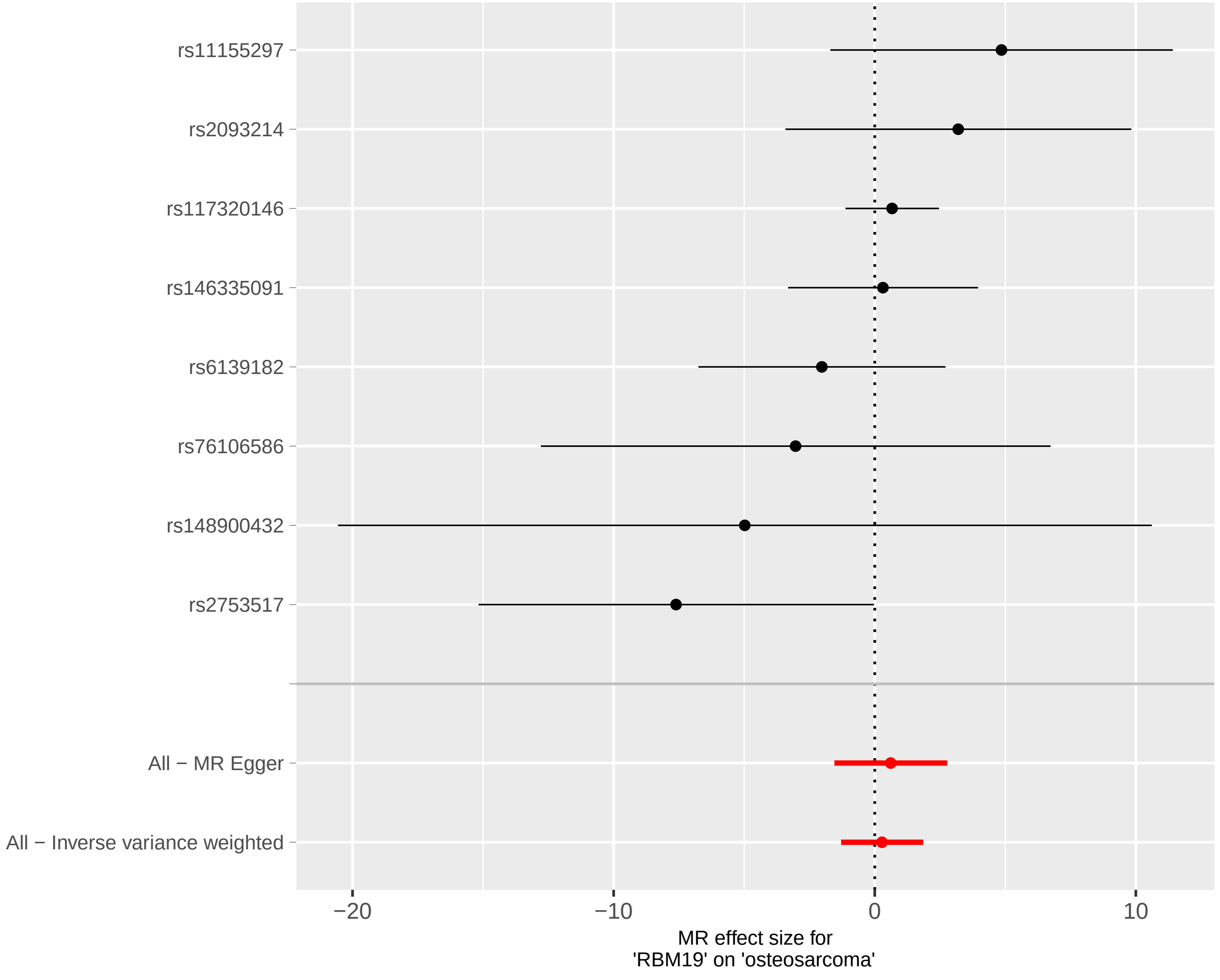

rs11155297
rs2093214
rs117320146
rs146335091
rs6139182
rs76106586
rs148900432
rs2753517
All − MR Egger
All − Inverse variance weighted
−20
−10
0
10
MR effect size for
'RBM19' on 'osteosarcoma'

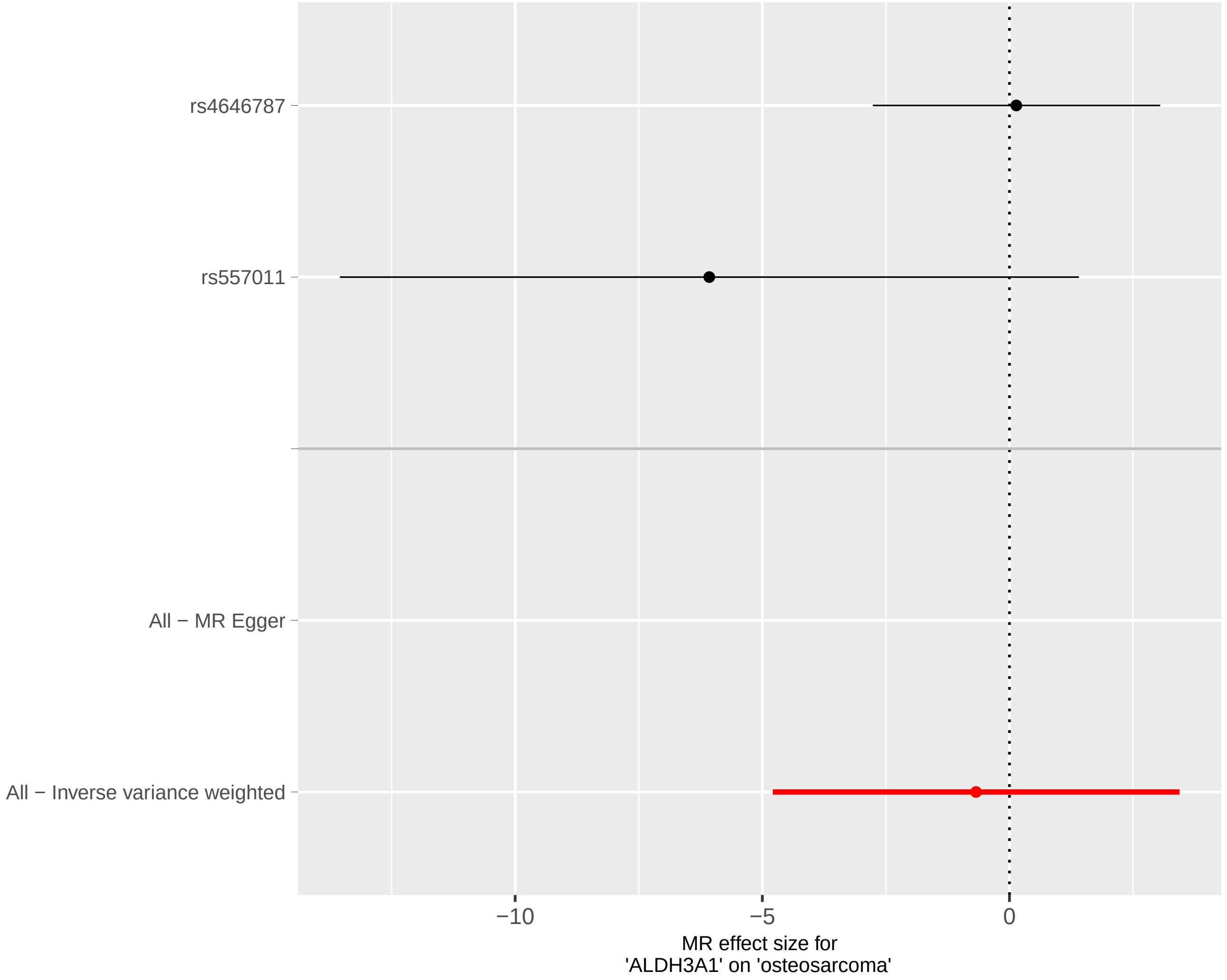

rs4646787
rs557011
All − MR Egger
All − Inverse variance weighted
−10
−5
0
MR effect size for
'ALDH3A1' on 'osteosarcoma'

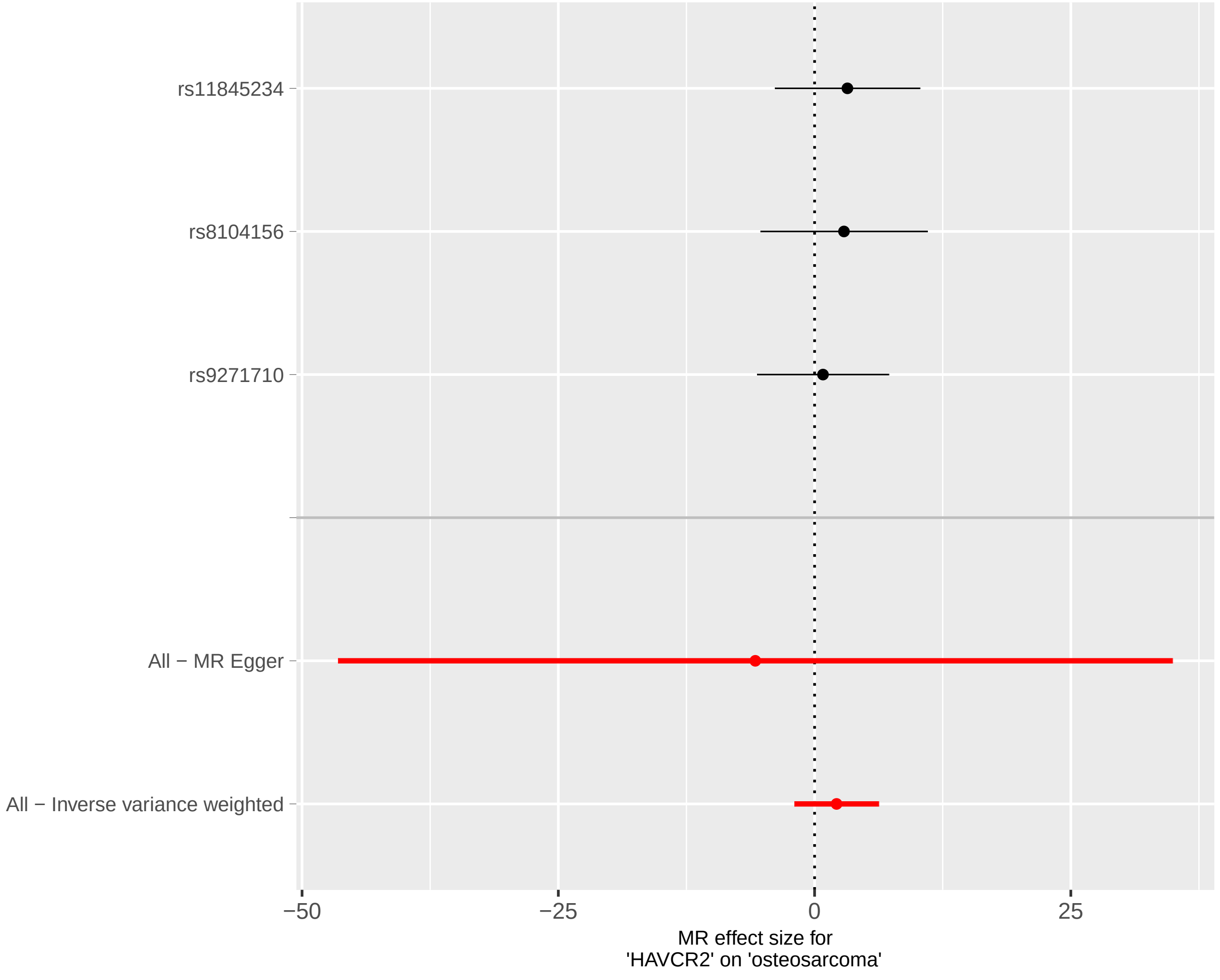
rs11845234
rs8104156
rs9271710
All − MR Egger
All − Inverse variance weighted
−50
−25
0
25
MR effect size for
'HAVCR2' on 'osteosarcoma'

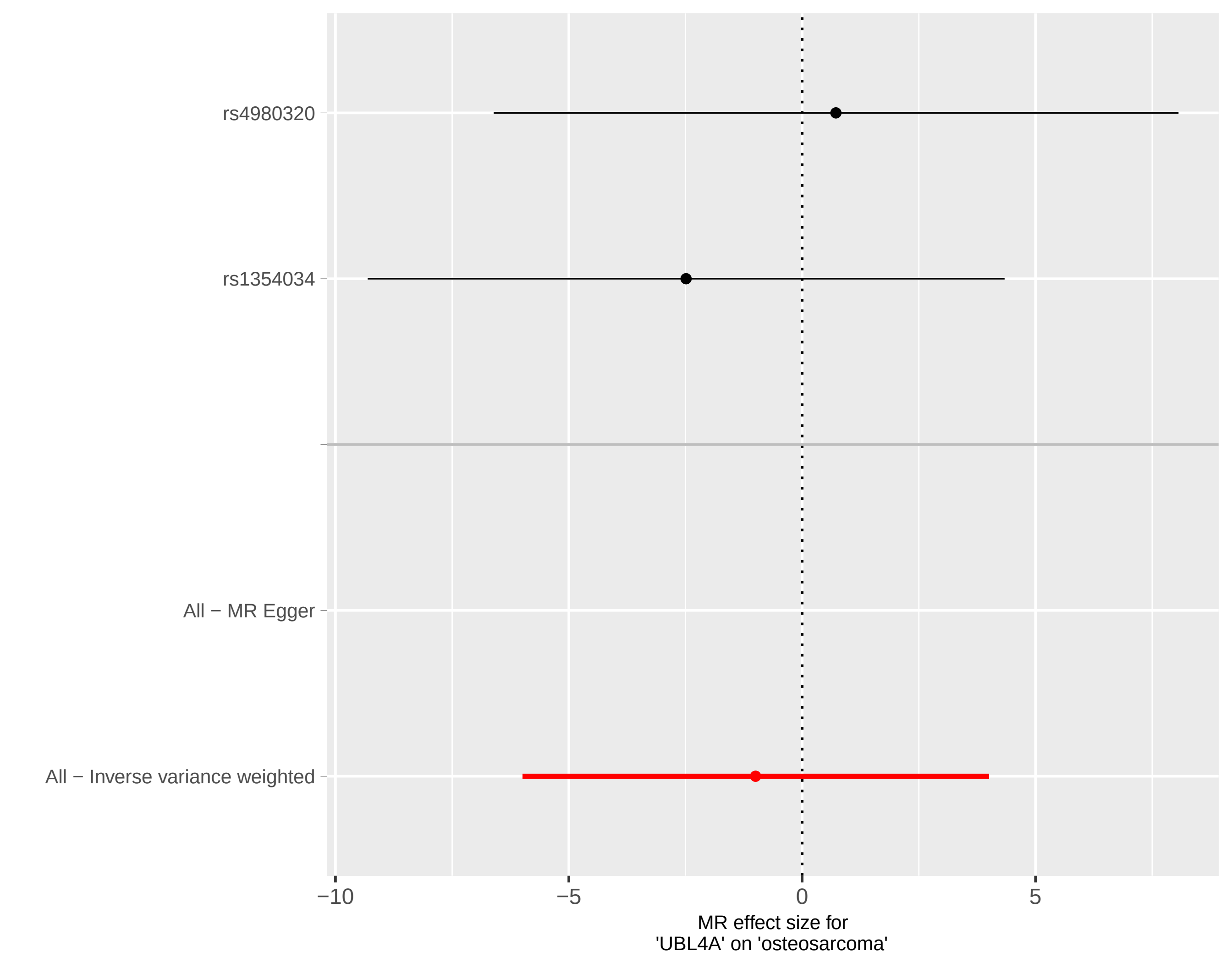
rs4980320
rs1354034
All − MR Egger
All − Inverse variance weighted
−10
−5
0
5
MR effect size for
'UBL4A' on 'osteosarcoma'

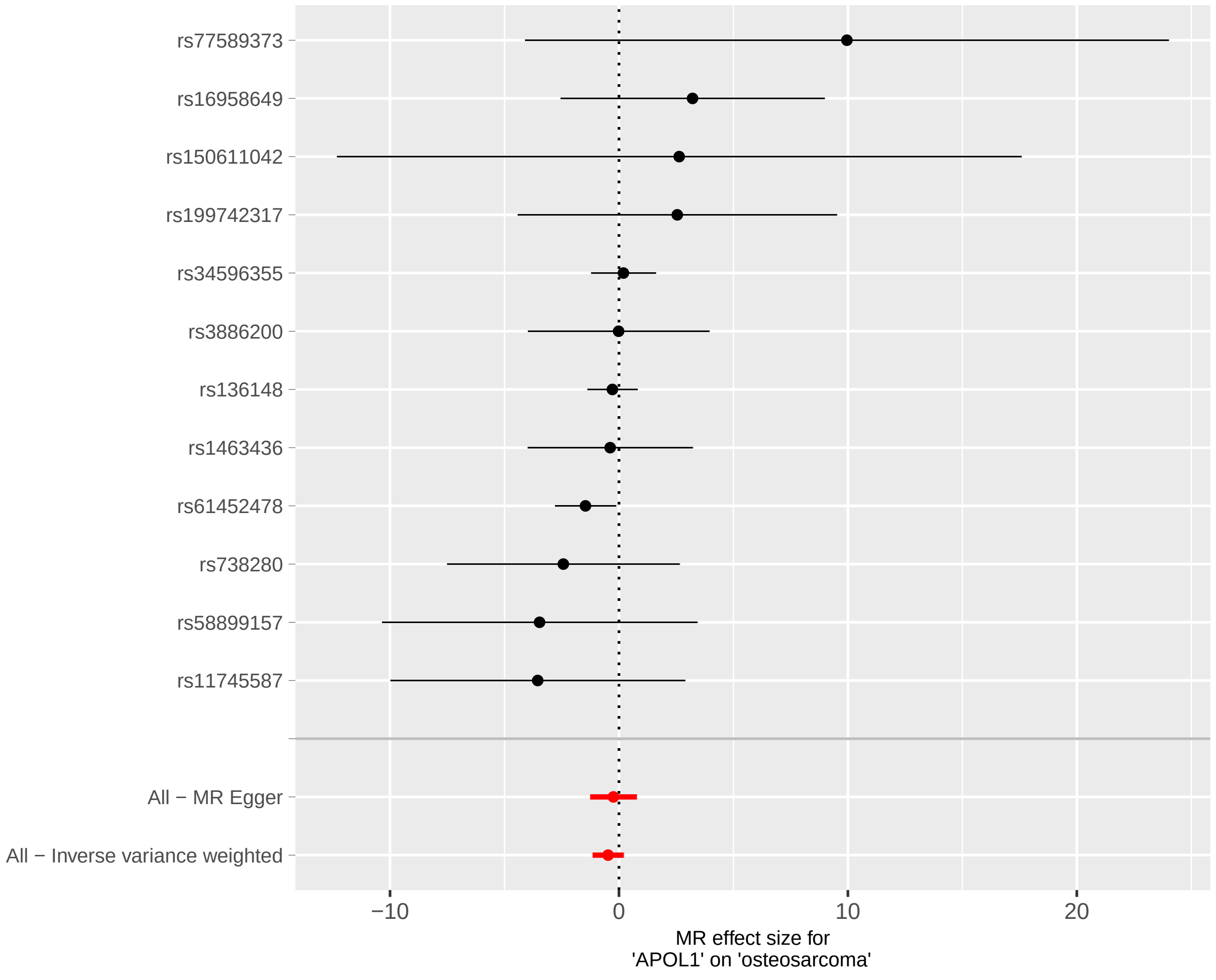

rs77589373
rs16958649
rs150611042
rs199742317
rs34596355
rs3886200
rs136148
rs1463436
rs61452478
rs738280
rs58899157
rs11745587
All − MR Egger
All − Inverse variance weighted
−10
0
10
20
MR effect size for
'APOL1' on 'osteosarcoma'

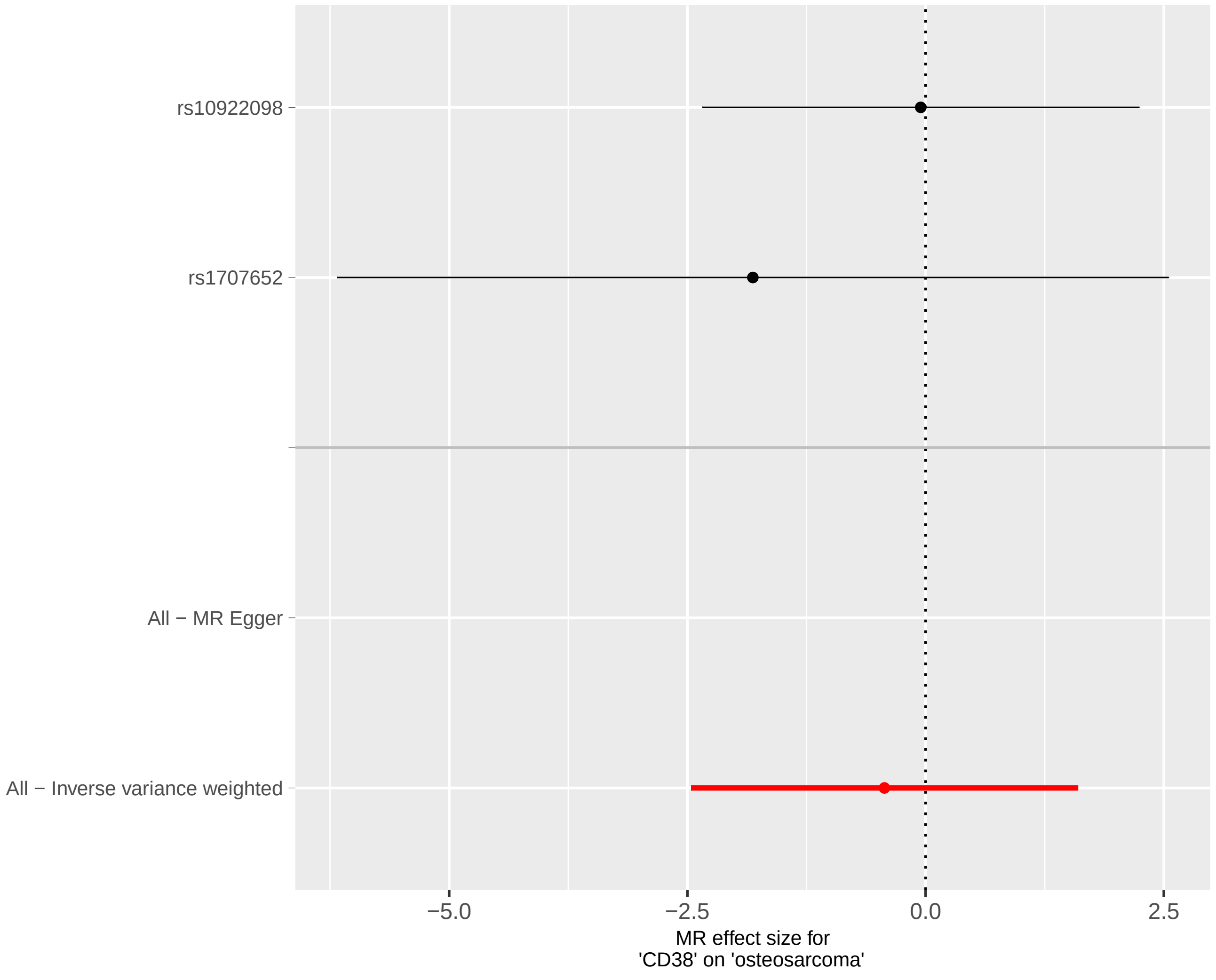
rs10922098
rs1707652
All − MR Egger
All − Inverse variance weighted
−5.0
−2.5
0.0
2.5
MR effect size for
'CD38' on 'osteosarcoma'

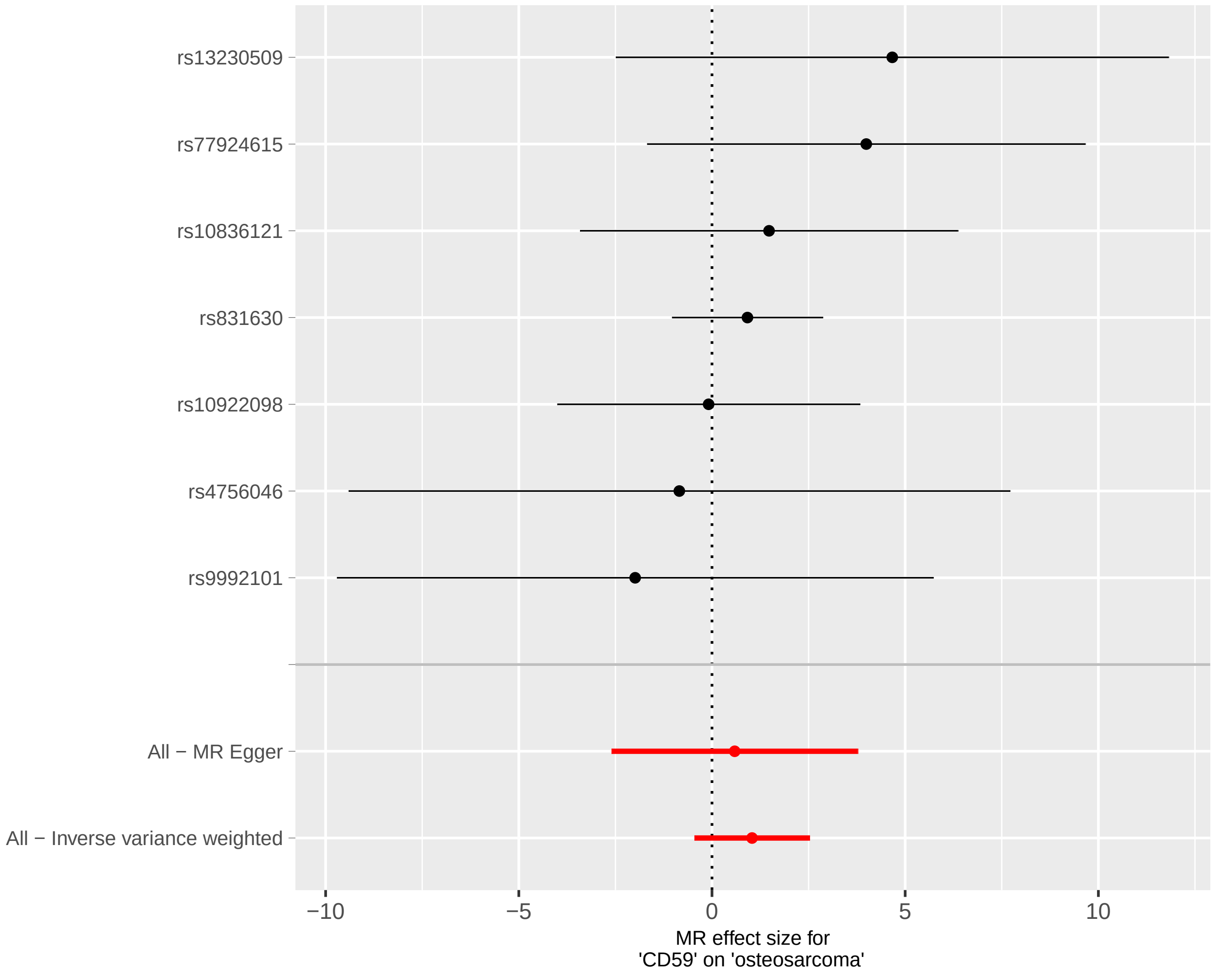

rs13230509
rs77924615
rs10836121
rs831630
rs10922098
rs4756046
rs9992101
All − MR Egger
All − Inverse variance weighted
−10
−5
0
5
10
MR effect size for
'CD59' on 'osteosarcoma'

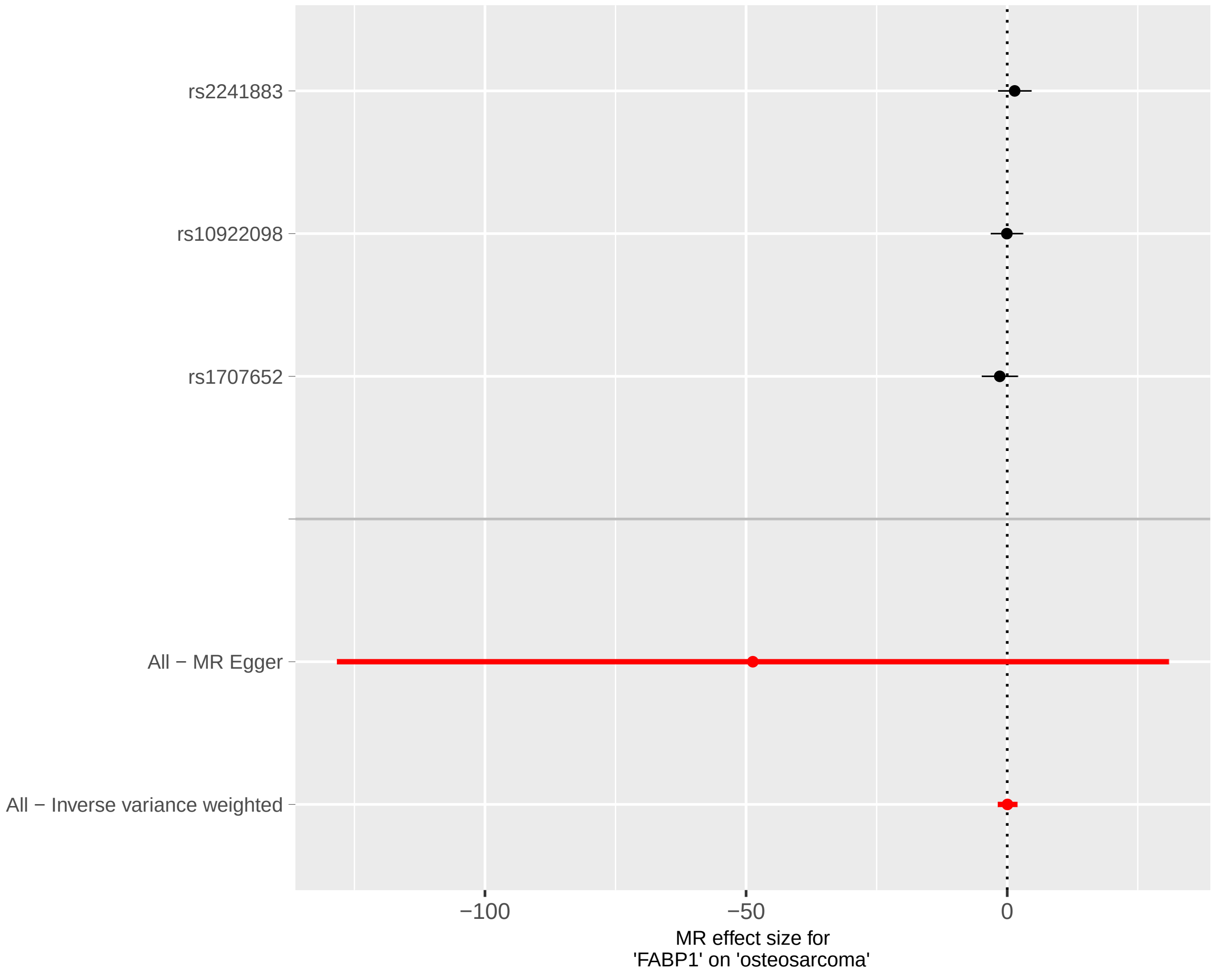
rs2241883
rs10922098
rs1707652
All − MR Egger
All − Inverse variance weighted
−100
−50
0
MR effect size for
'FABP1' on 'osteosarcoma'

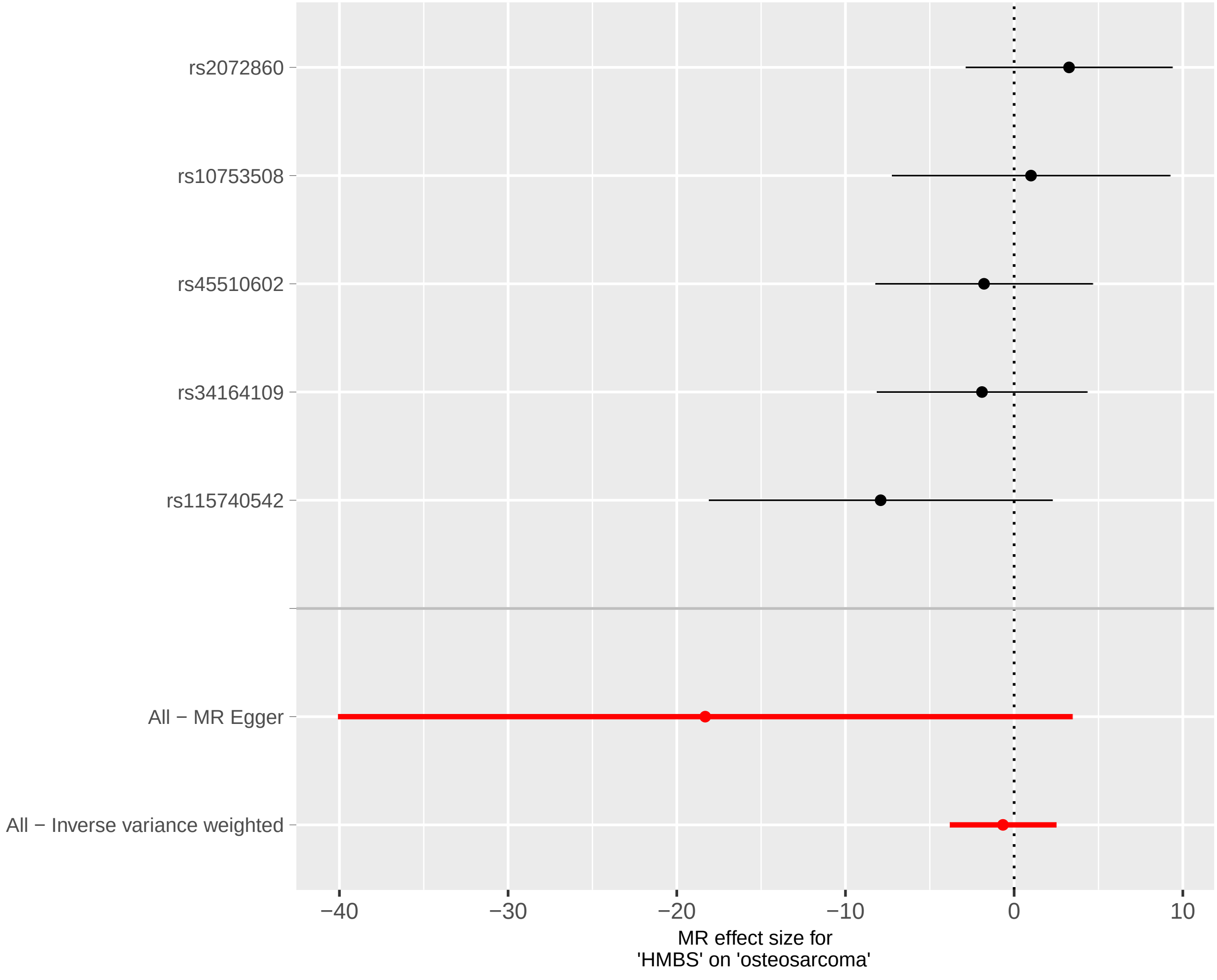
rs2072860
rs10753508
rs45510602
rs34164109
rs115740542
All − MR Egger
All − Inverse variance weighted
−40
−30
−20
−10
0
10
MR effect size for
'HMBS' on 'osteosarcoma'

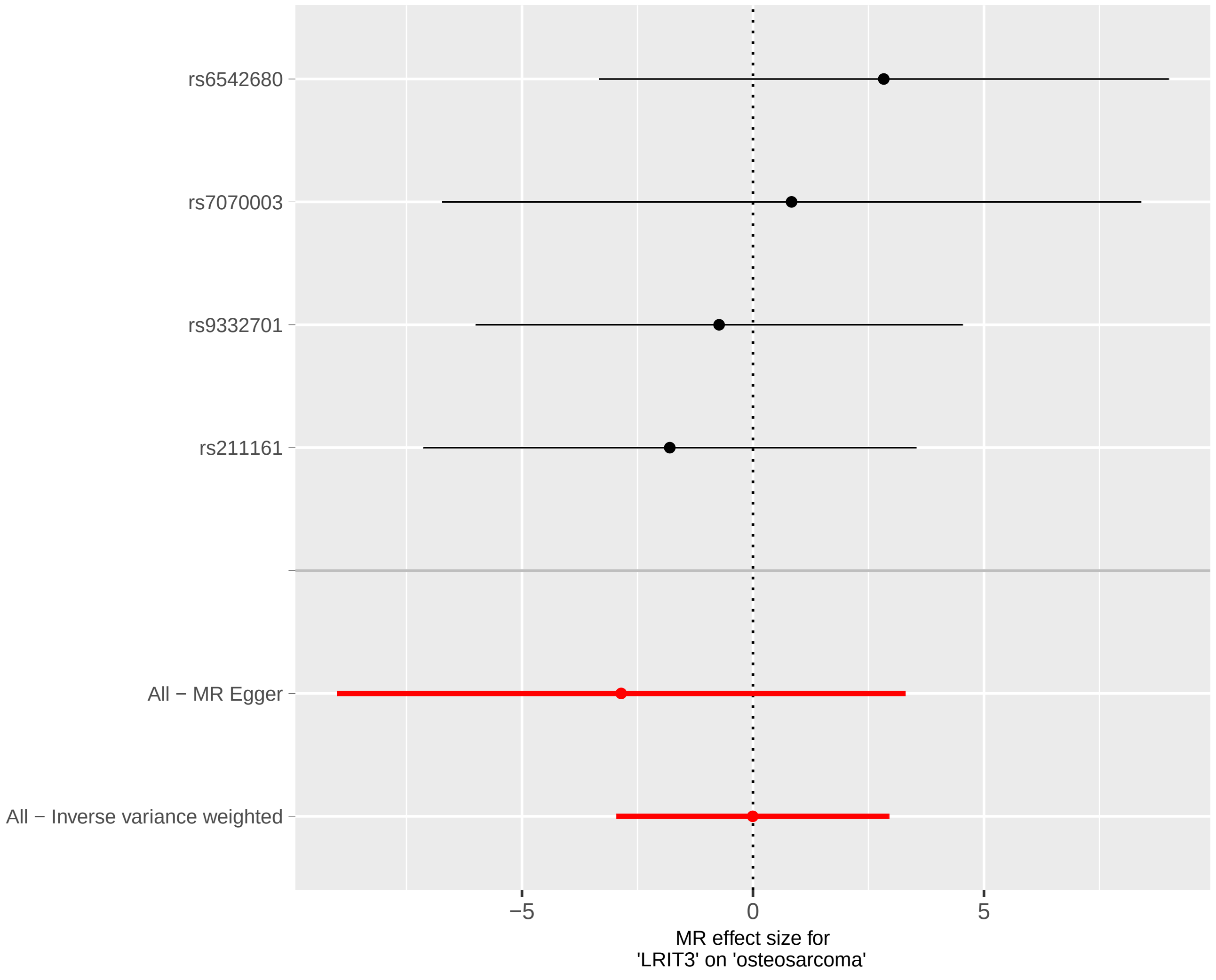
rs6542680
rs7070003
rs9332701
rs211161
All − MR Egger
All − Inverse variance weighted
−5
0
5
MR effect size for
'LRIT3' on 'osteosarcoma'

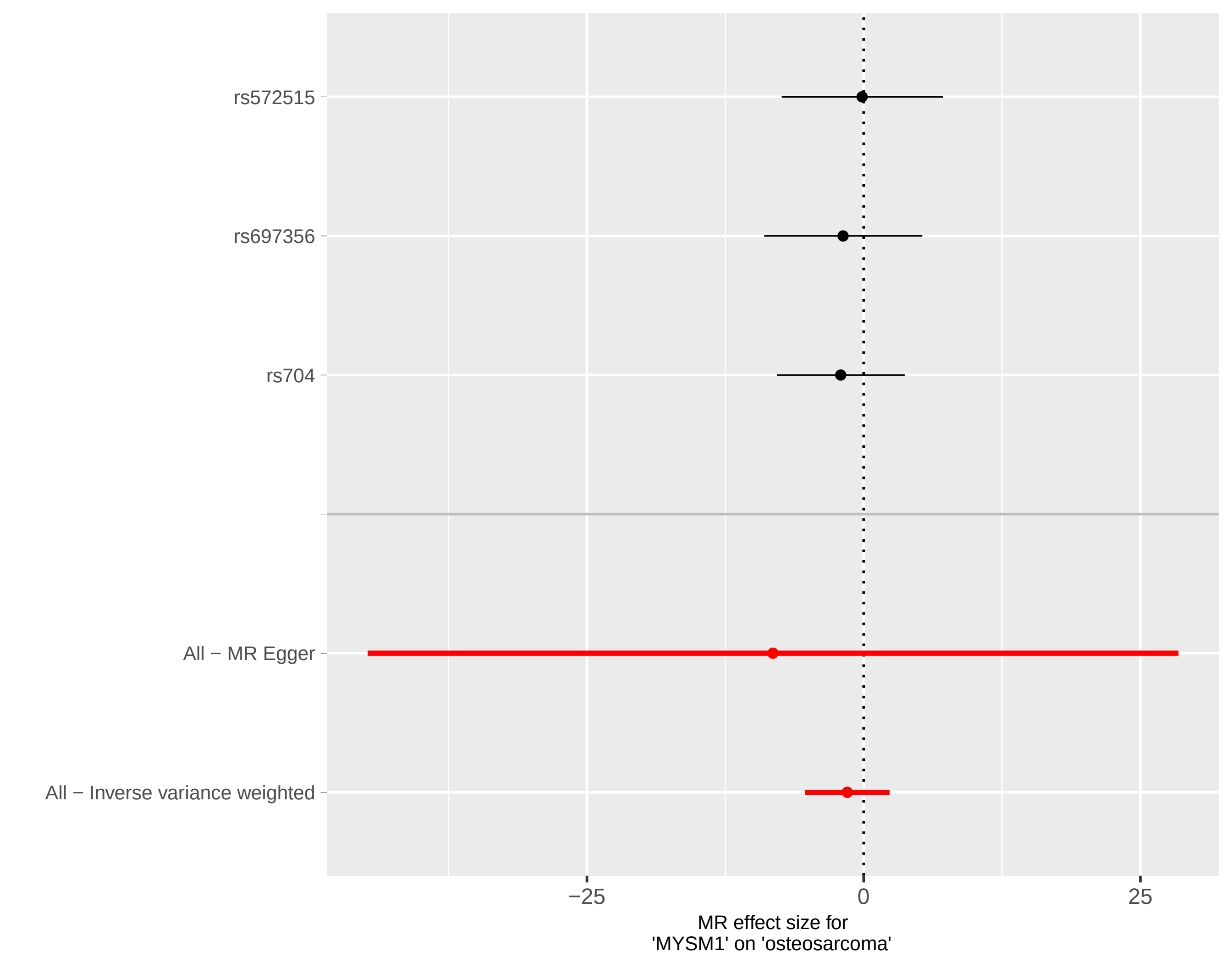

rs572515
rs697356
rs704
All − MR Egger
All − Inverse variance weighted
−25
0
25
MR effect size for
'MYSM1' on 'osteosarcoma'

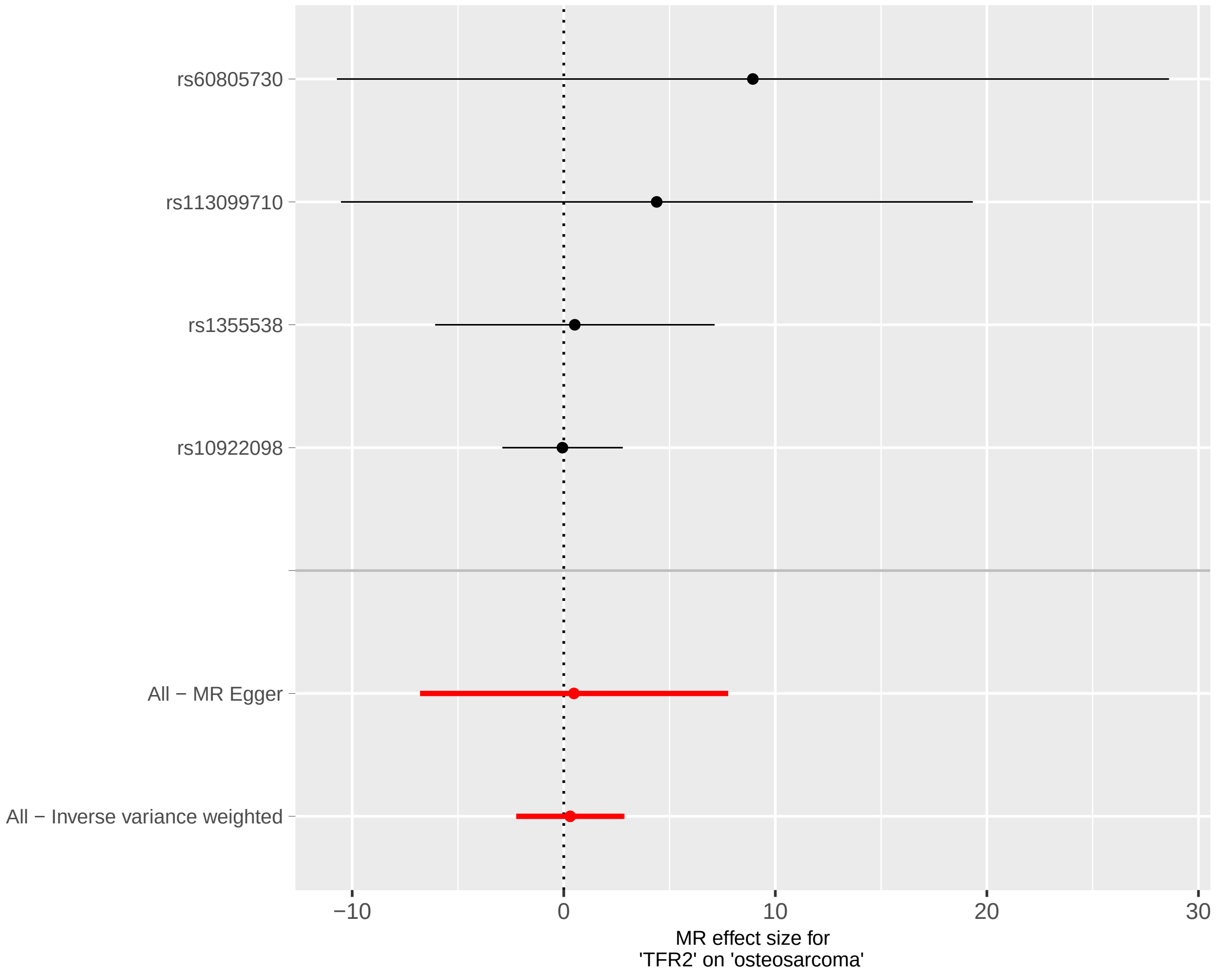
rs60805730
rs113099710
rs1355538
rs10922098
All − MR Egger
All − Inverse variance weighted
−10
0
10
20
30
MR effect size for
'TFR2' on 'osteosarcoma'

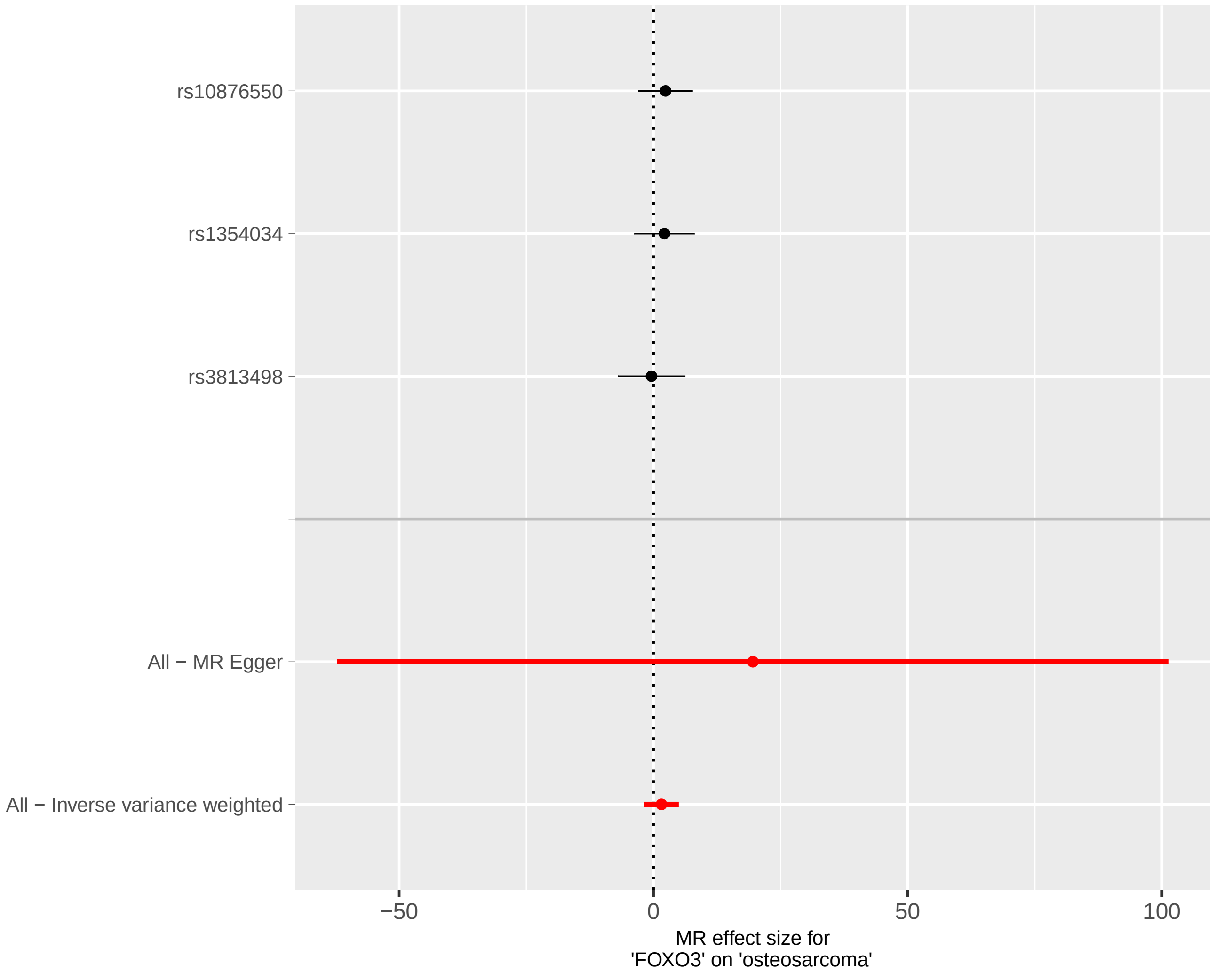

rs10876550
rs1354034
rs3813498
All − MR Egger
All − Inverse variance weighted
−50
0
50
100
MR effect size for
'FOXO3' on 'osteosarcoma'

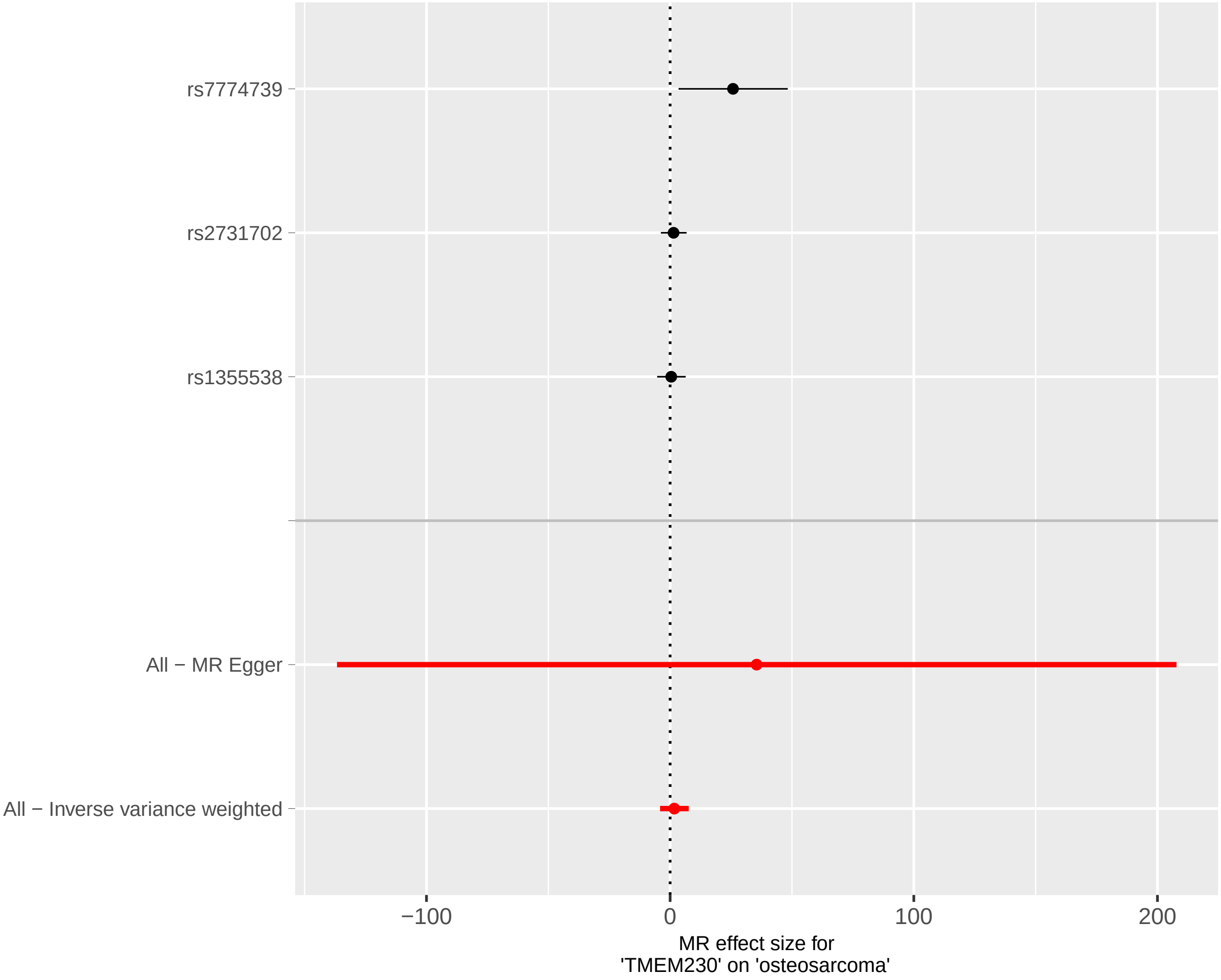

rs7774739
rs2731702
rs1355538
All − MR Egger
All − Inverse variance weighted
−100
0
100
200
MR effect size for
'TMEM230' on 'osteosarcoma'

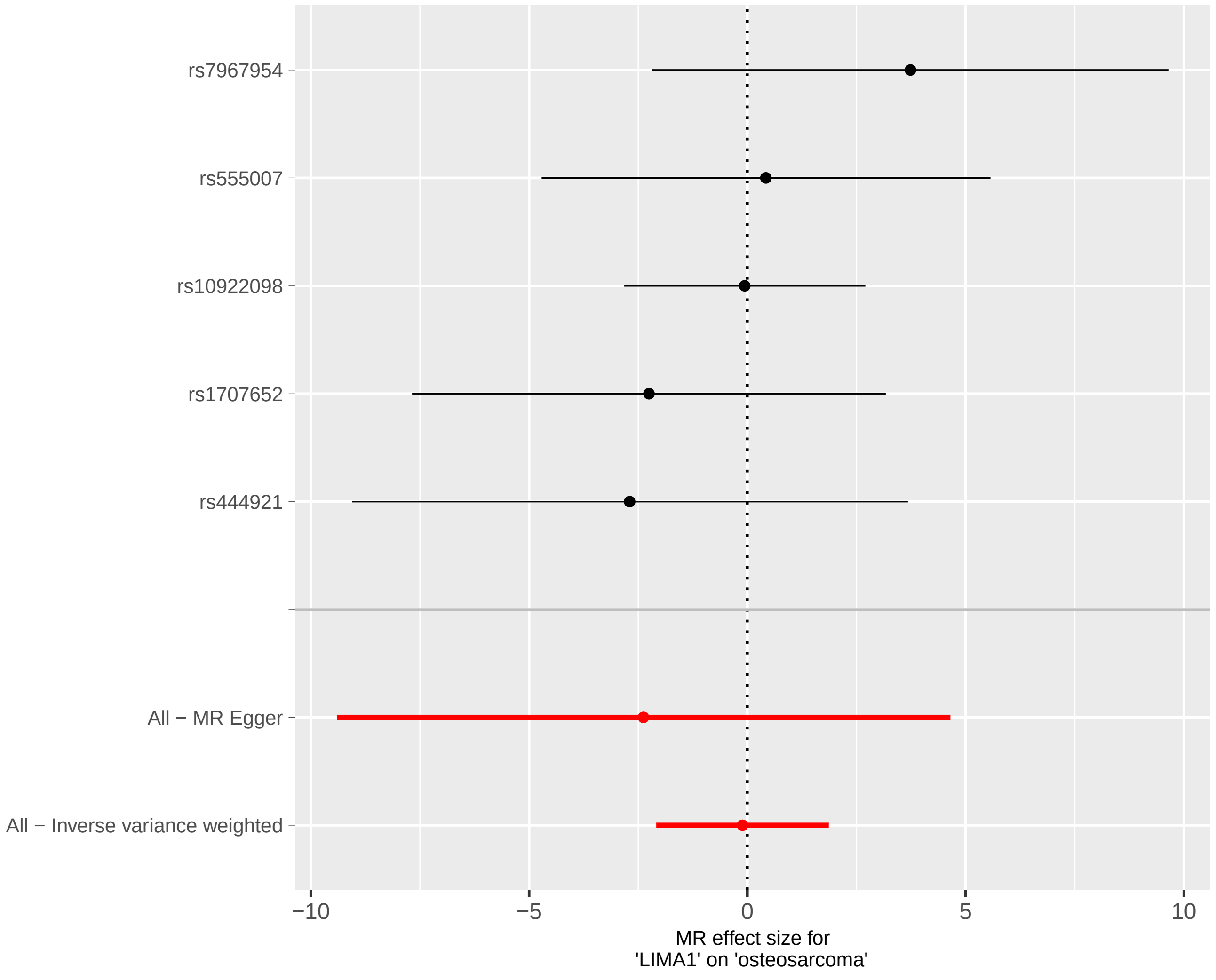

rs7967954
rs555007
rs10922098
rs1707652
rs444921
All − MR Egger
All − Inverse variance weighted
−10
−5
0
5
10
MR effect size for
'LIMA1' on 'osteosarcoma'

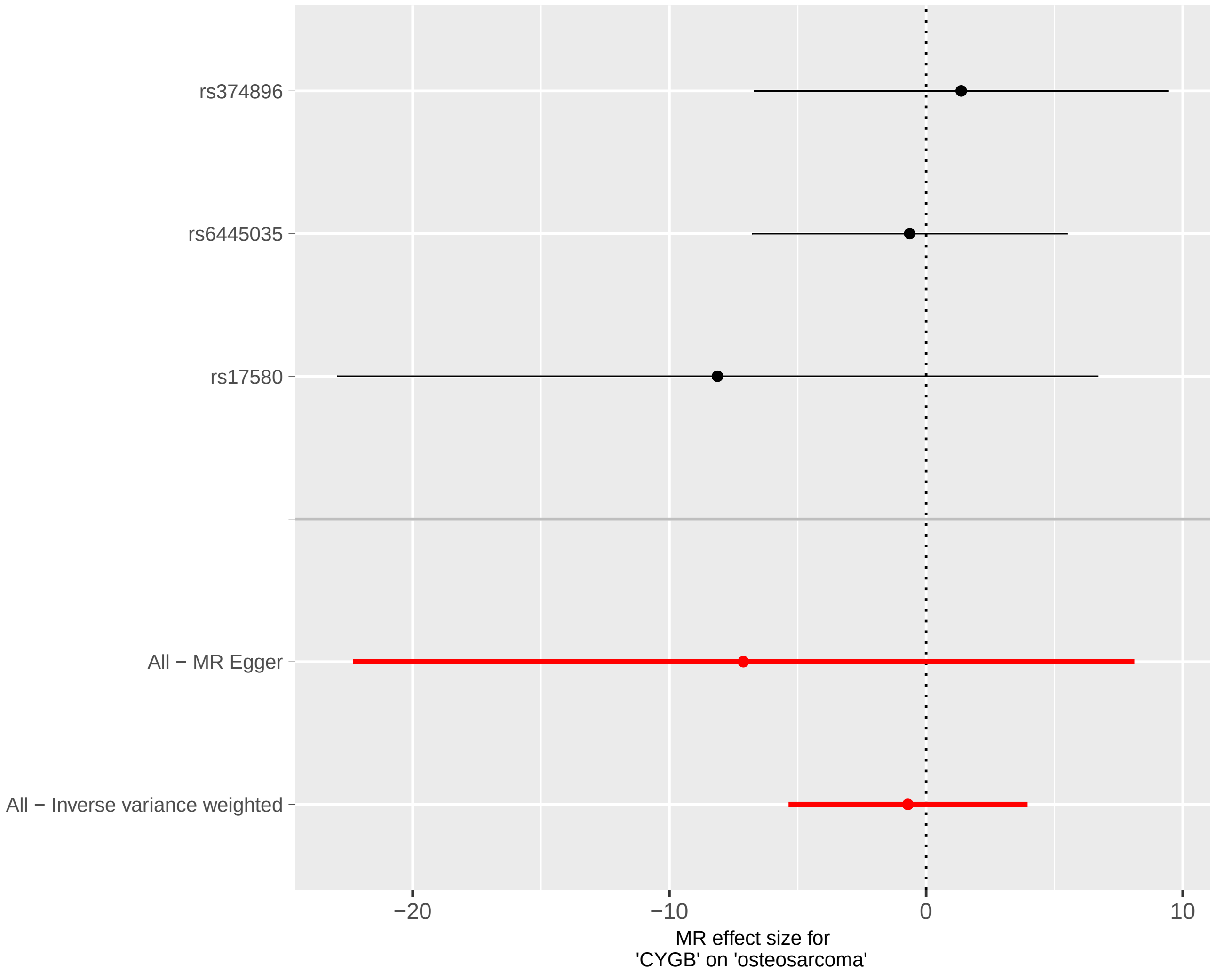

rs374896
rs6445035
rs17580
All − MR Egger
All − Inverse variance weighted
−20
−10
0
10
MR effect size for
'CYGB' on 'osteosarcoma'

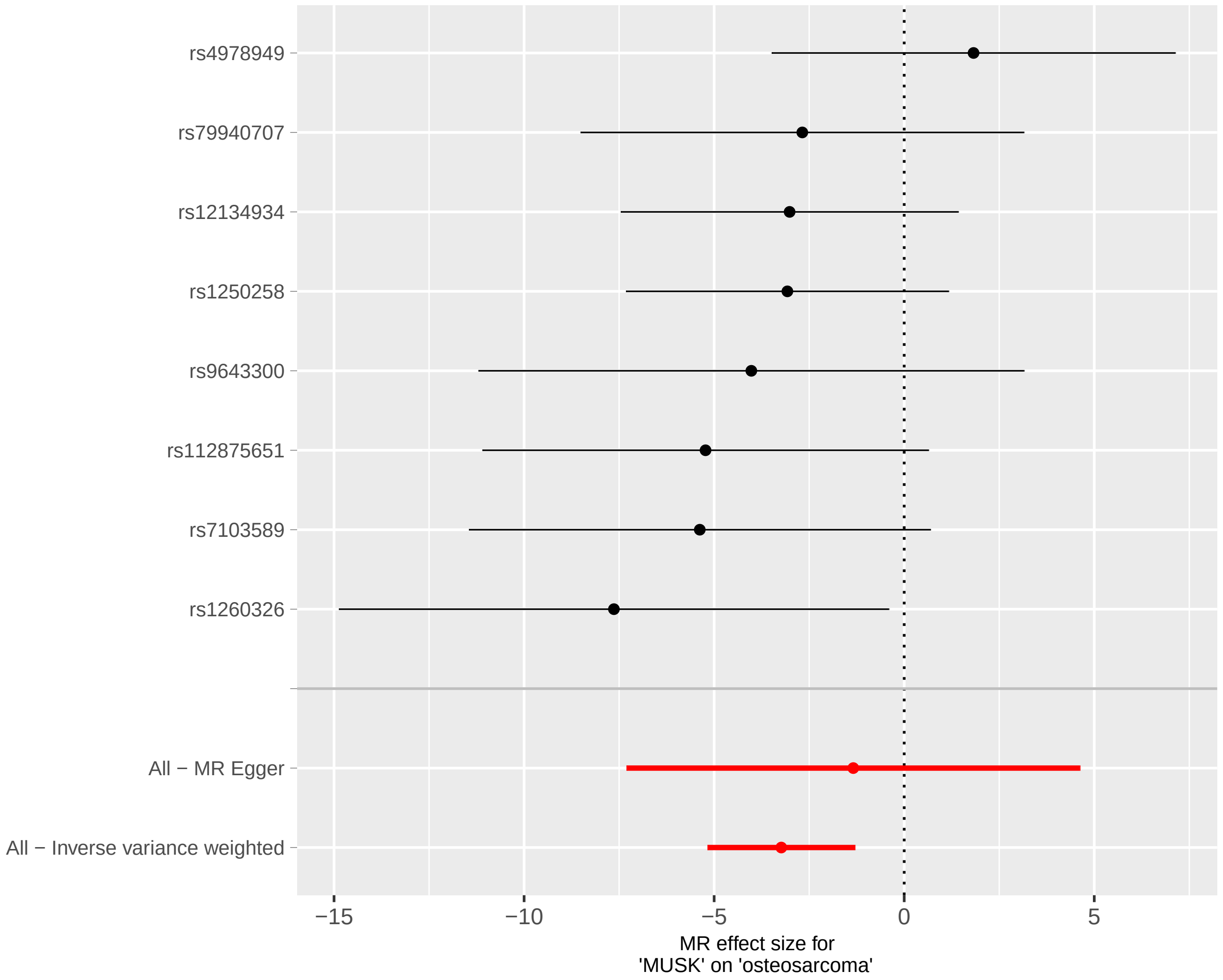
rs4978949
rs79940707
rs12134934
rs1250258
rs9643300
rs112875651
rs7103589
rs1260326
All − MR Egger
All − Inverse variance weighted
−15
−10
−5
0
5
MR effect size for
'MUSK' on 'osteosarcoma'

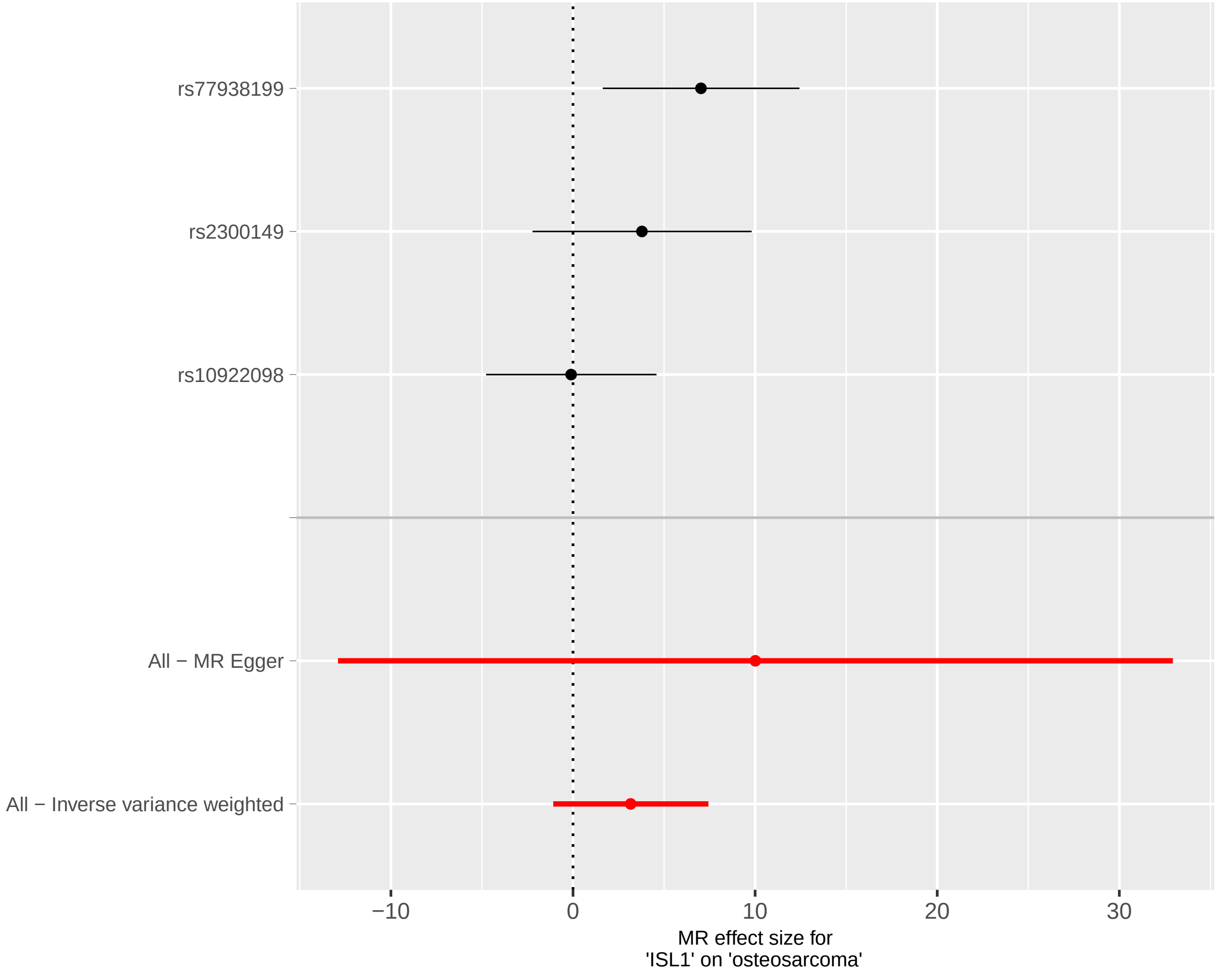
rs77938199
rs2300149
rs10922098
All − MR Egger
All − Inverse variance weighted
−10
0
10
20
30
MR effect size for
'ISL1' on 'osteosarcoma'

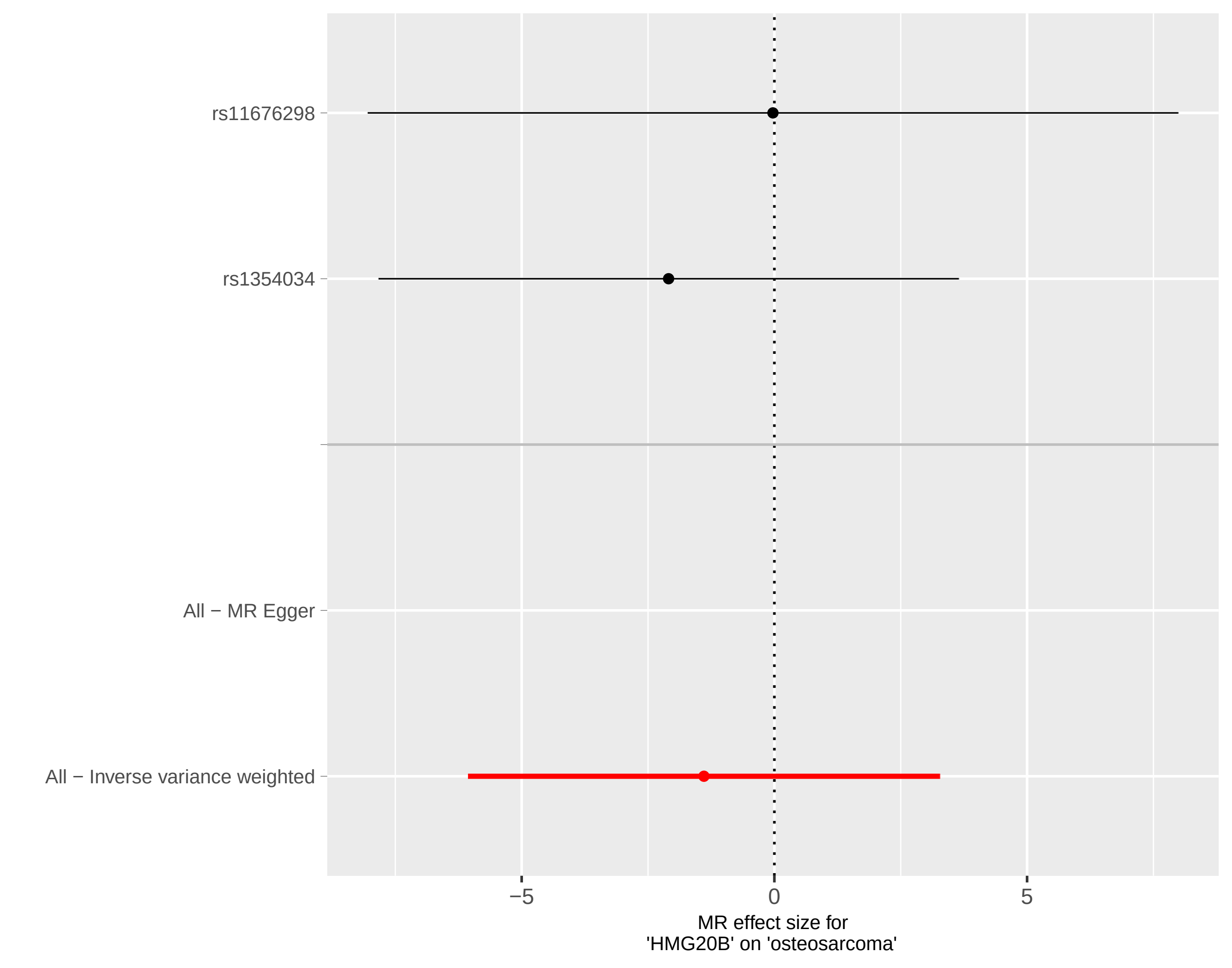

rs11676298
rs1354034
All − MR Egger
All − Inverse variance weighted
−5
0
5
MR effect size for
'HMG20B' on 'osteosarcoma'

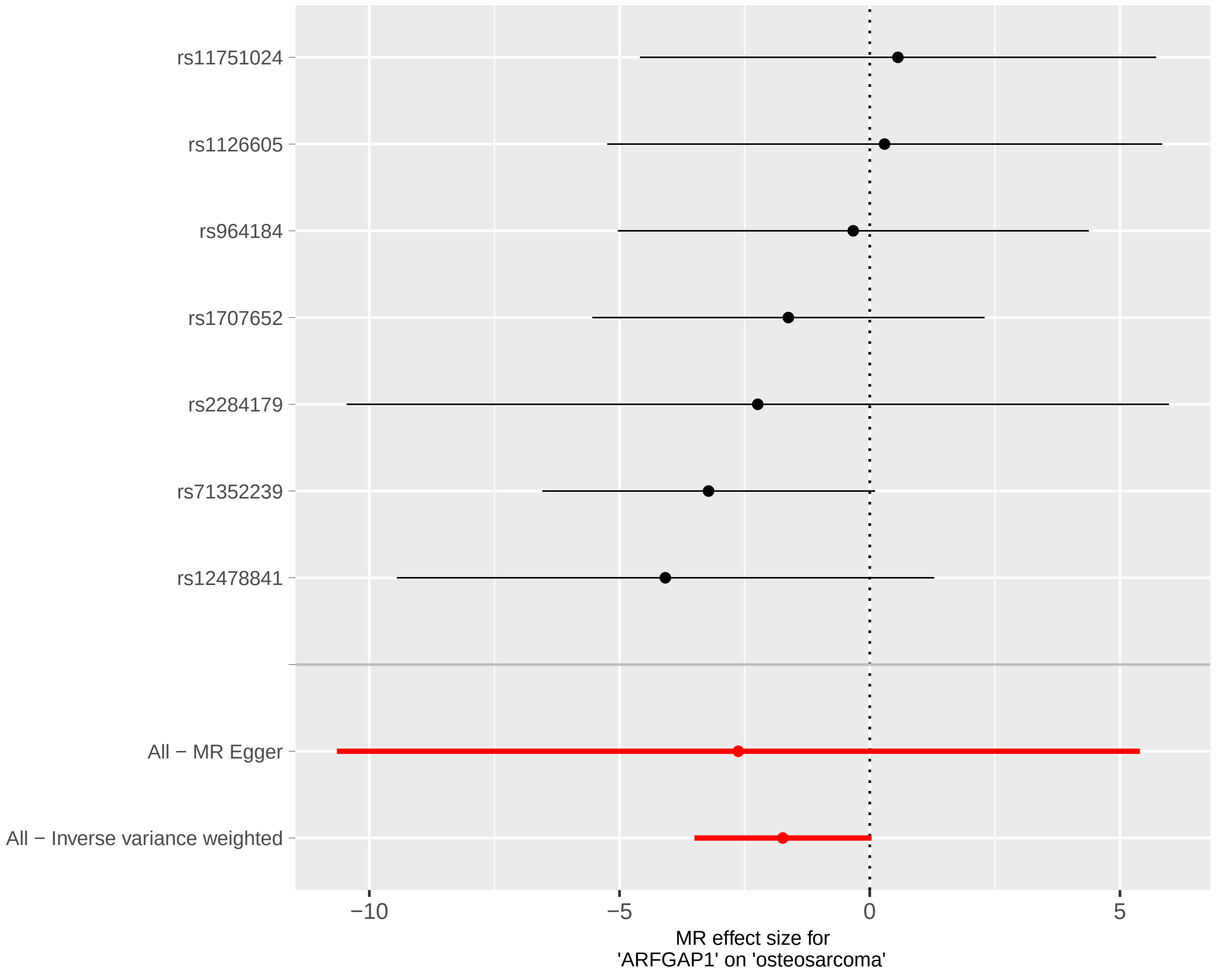
rs11751024
rs1126605
rs964184
rs1707652
rs2284179
rs71352239
rs12478841
All − MR Egger
All − Inverse variance weighted
−10
−5
0
5
MR effect size for
'ARFGAP1' on 'osteosarcoma'

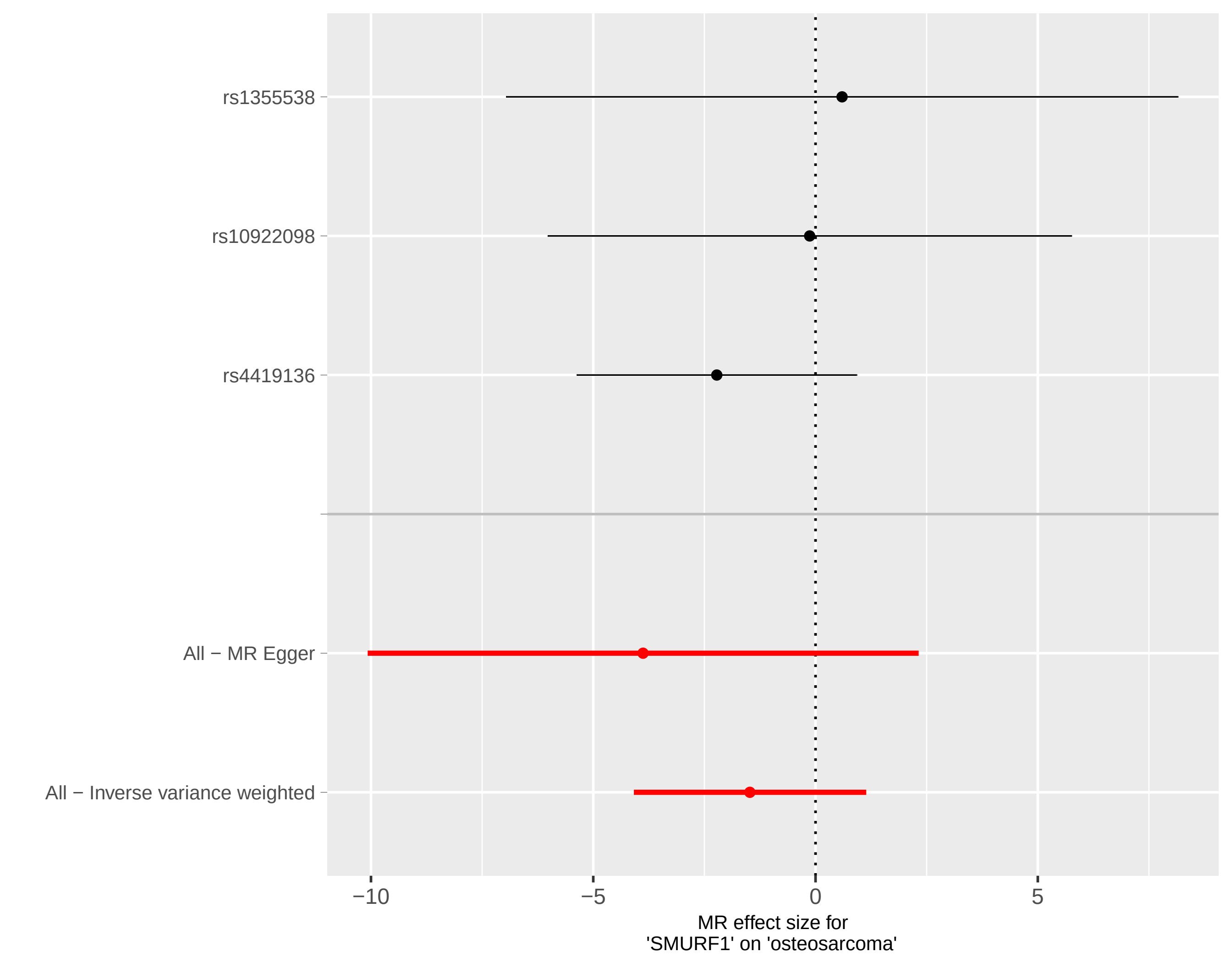

rs1355538
rs10922098
rs4419136
All − MR Egger
All − Inverse variance weighted
−10
−5
0
5
MR effect size for
'SMURF1' on 'osteosarcoma'

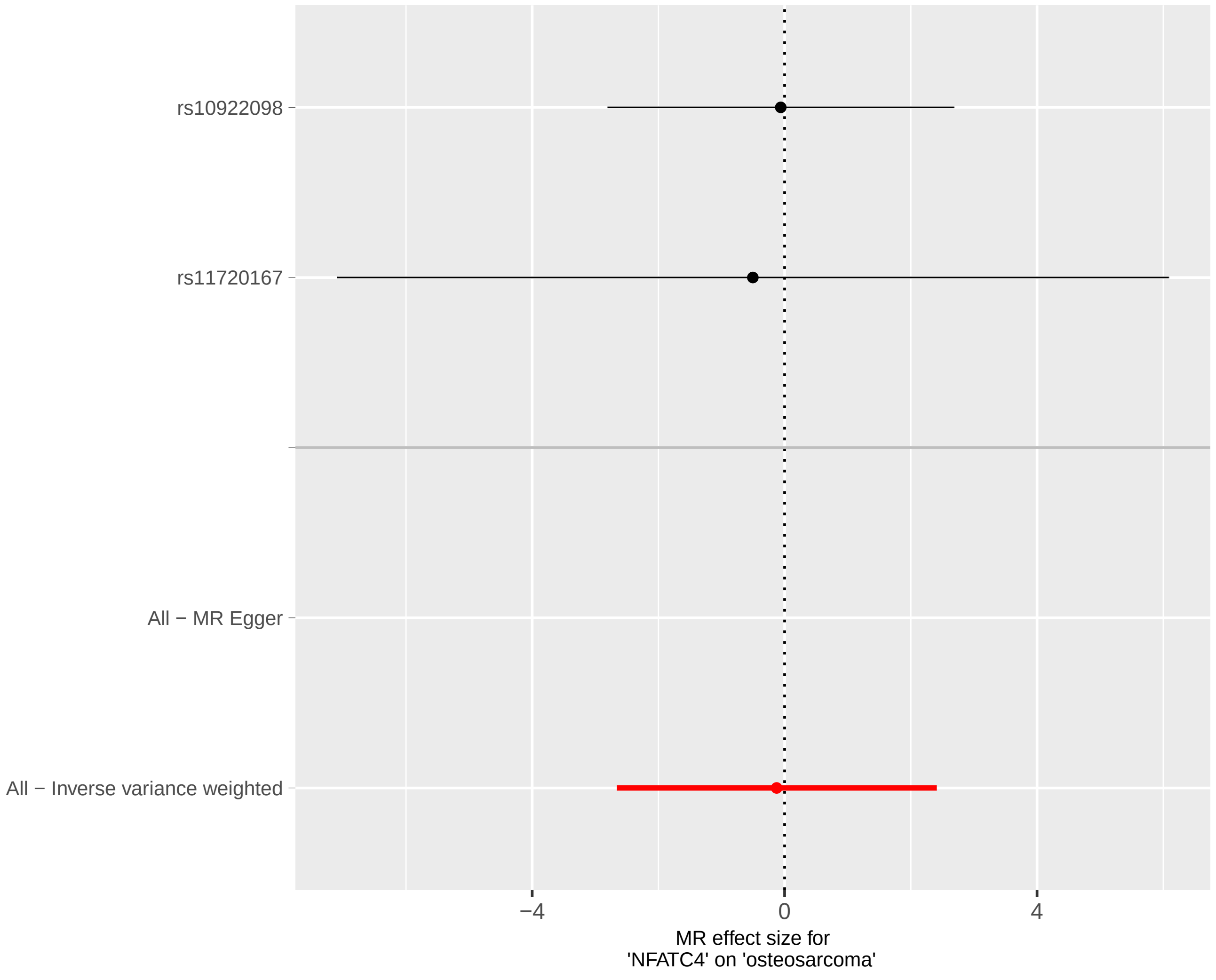
rs10922098
rs11720167
All − MR Egger
All − Inverse variance weighted
−4
0
4
MR effect size for
'NFATC4' on 'osteosarcoma'

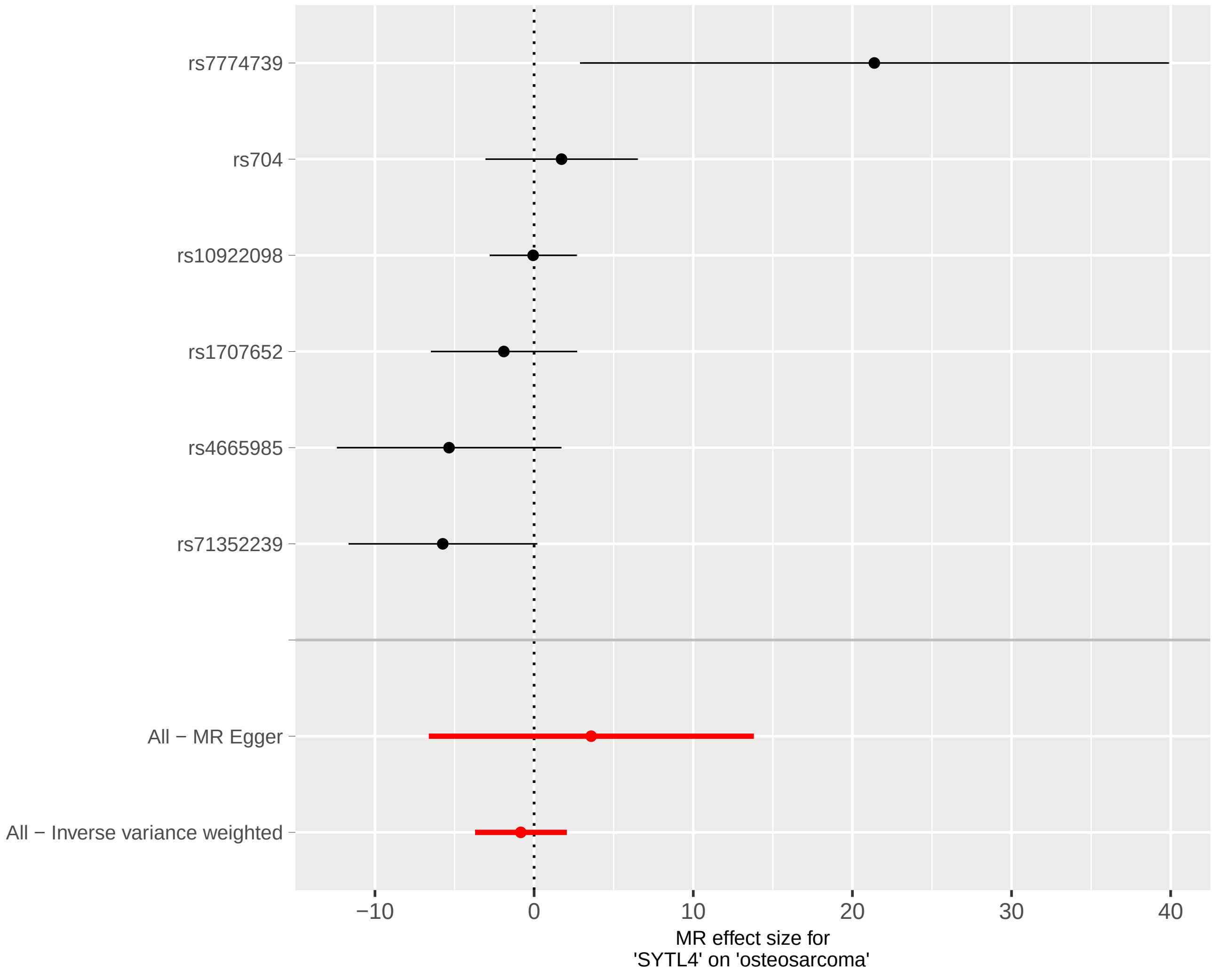

rs7774739
rs704
rs10922098
rs1707652
rs4665985
rs71352239
All − MR Egger
All − Inverse variance weighted
−10
0
10
20
30
40
MR effect size for
'SYTL4' on 'osteosarcoma'

Insufficient number of SNPs

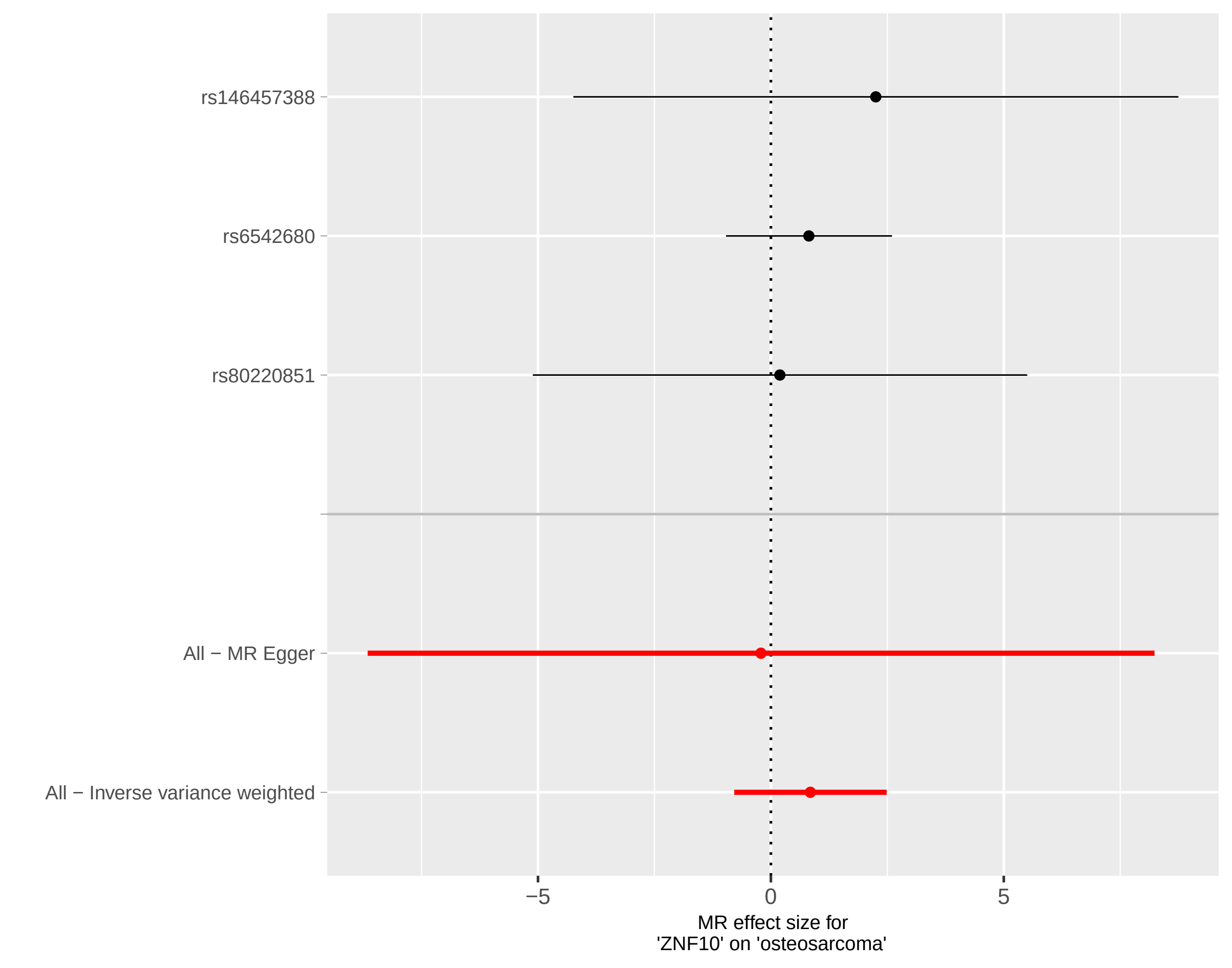

rs146457388
rs6542680
rs80220851
All − MR Egger
All − Inverse variance weighted
−5
0
5
MR effect size for
'ZNF10' on 'osteosarcoma'

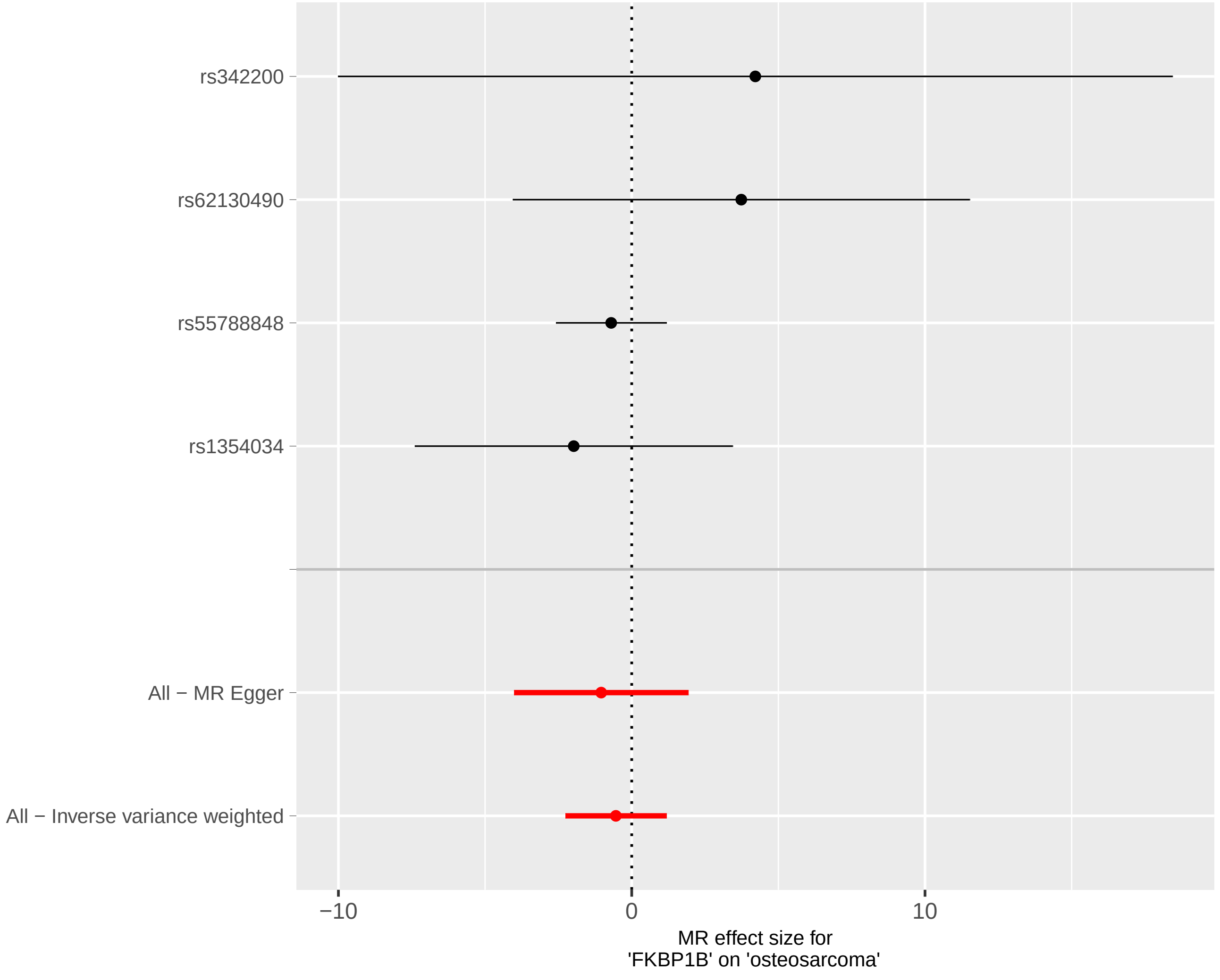
rs342200
rs62130490
rs55788848
rs1354034
All − MR Egger
All − Inverse variance weighted
−10
0
10
MR effect size for
'FKBP1B' on 'osteosarcoma'

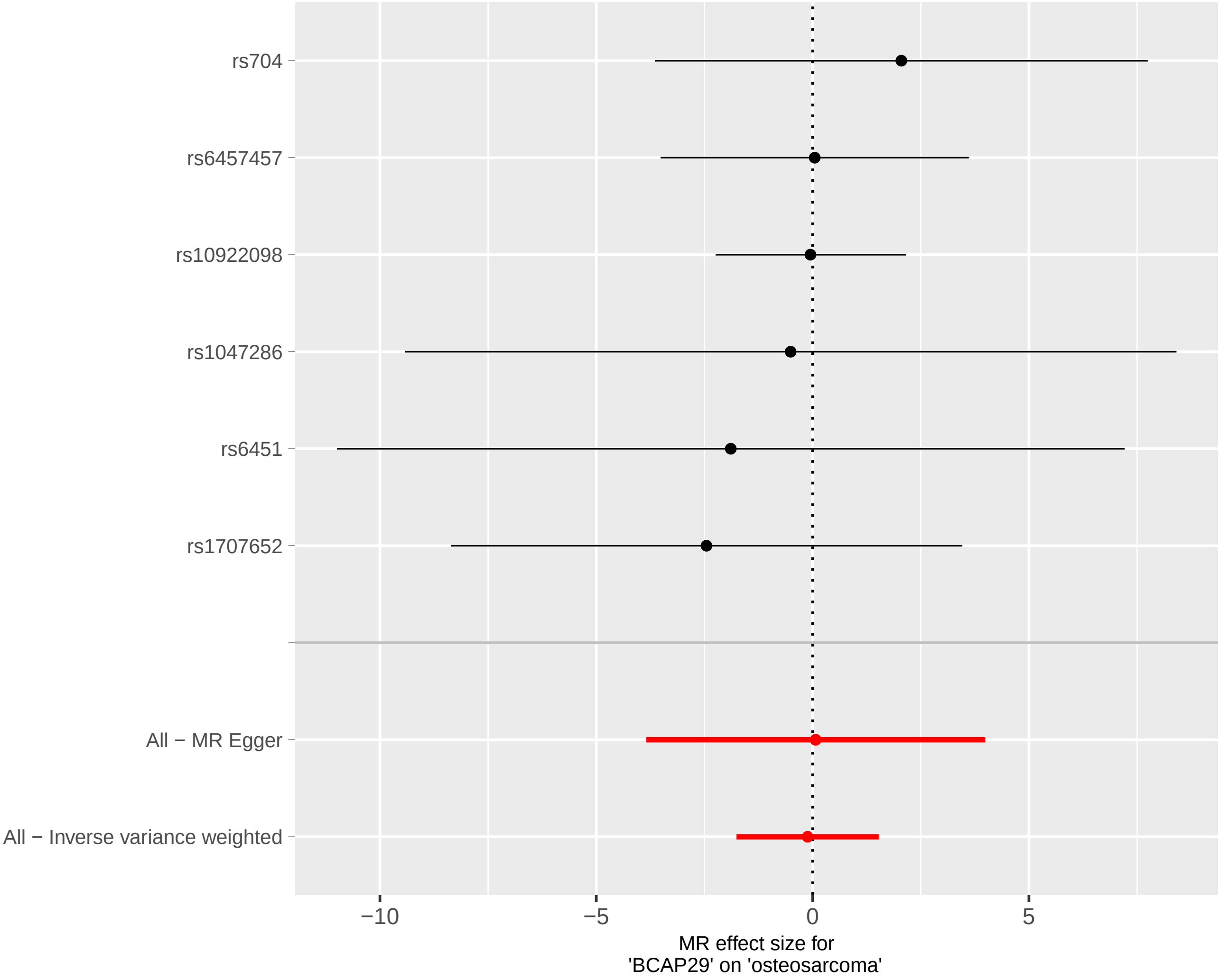

rs704
rs6457457
rs10922098
rs1047286
rs6451
rs1707652
All − MR Egger
All − Inverse variance weighted
−10
−5
0
5
MR effect size for
'BCAP29' on 'osteosarcoma'

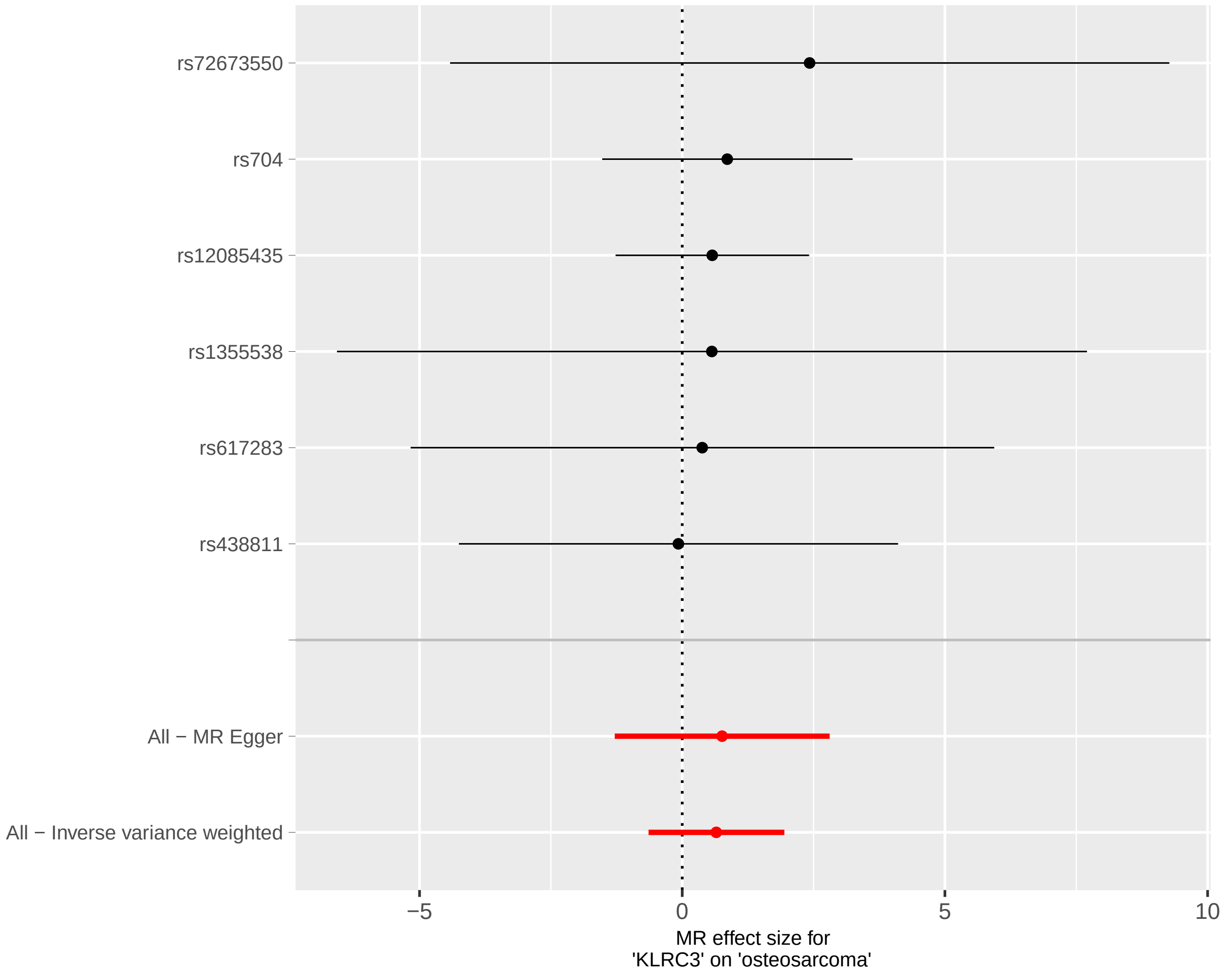

rs72673550
rs704
rs12085435
rs1355538
rs617283
rs438811
All − MR Egger
All − Inverse variance weighted
−5
0
5
10
MR effect size for
'KLRC3' on 'osteosarcoma'

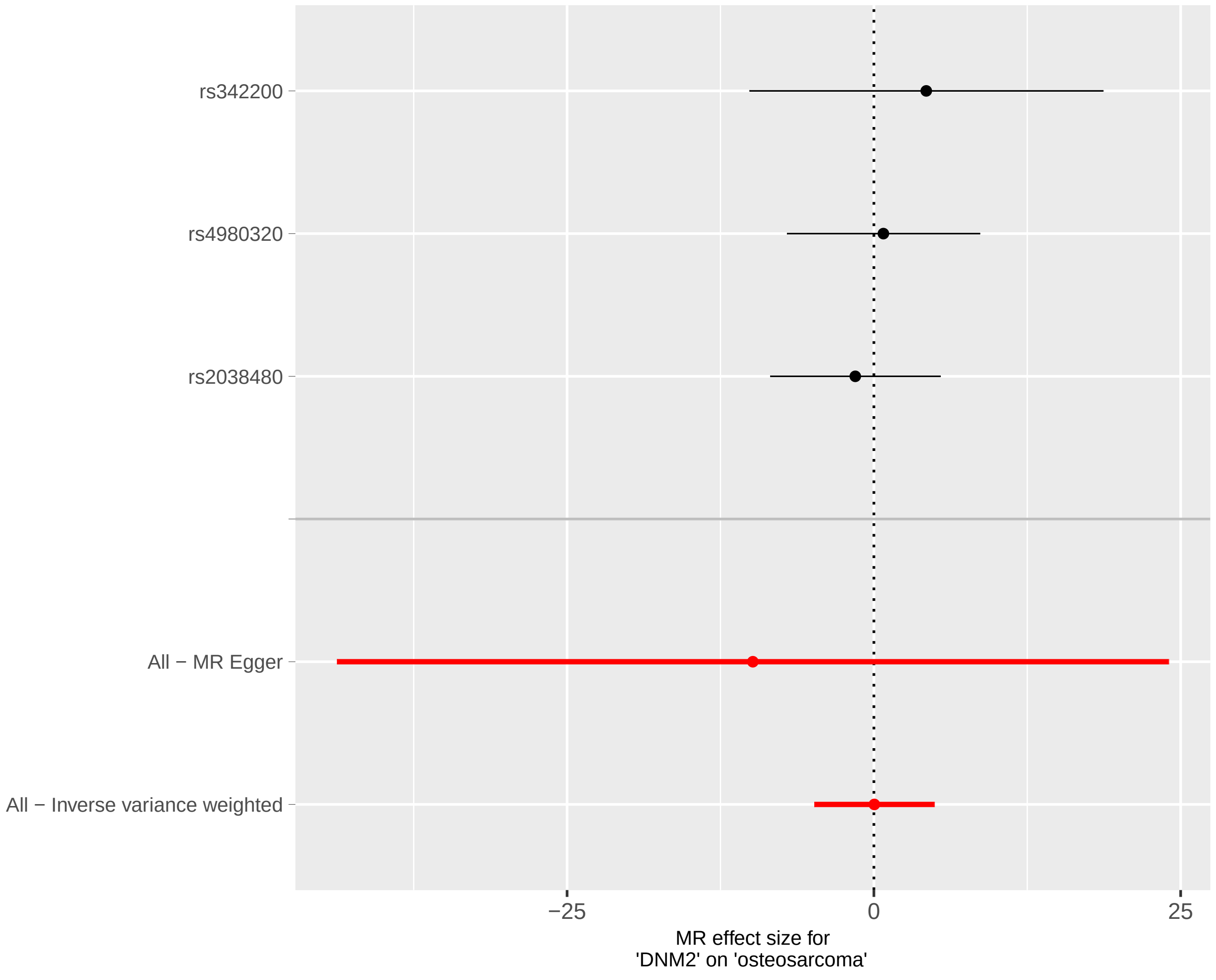
rs342200
rs4980320
rs2038480
All − MR Egger
All − Inverse variance weighted
−25
0
25
MR effect size for
'DNM2' on 'osteosarcoma'

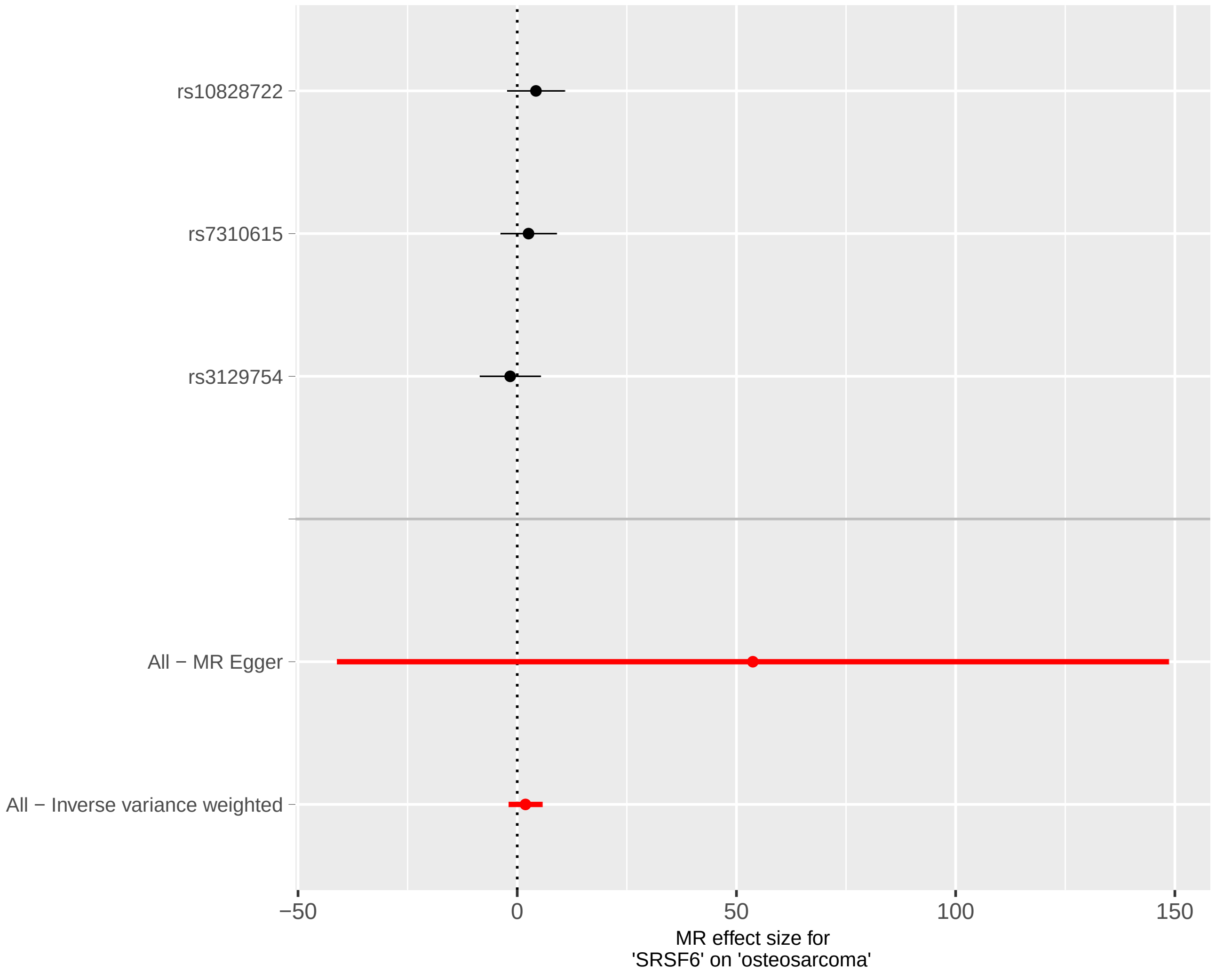

rs10828722
rs7310615
rs3129754
All − MR Egger
All − Inverse variance weighted
−50
0
50
100
150
MR effect size for
'SRSF6' on 'osteosarcoma'

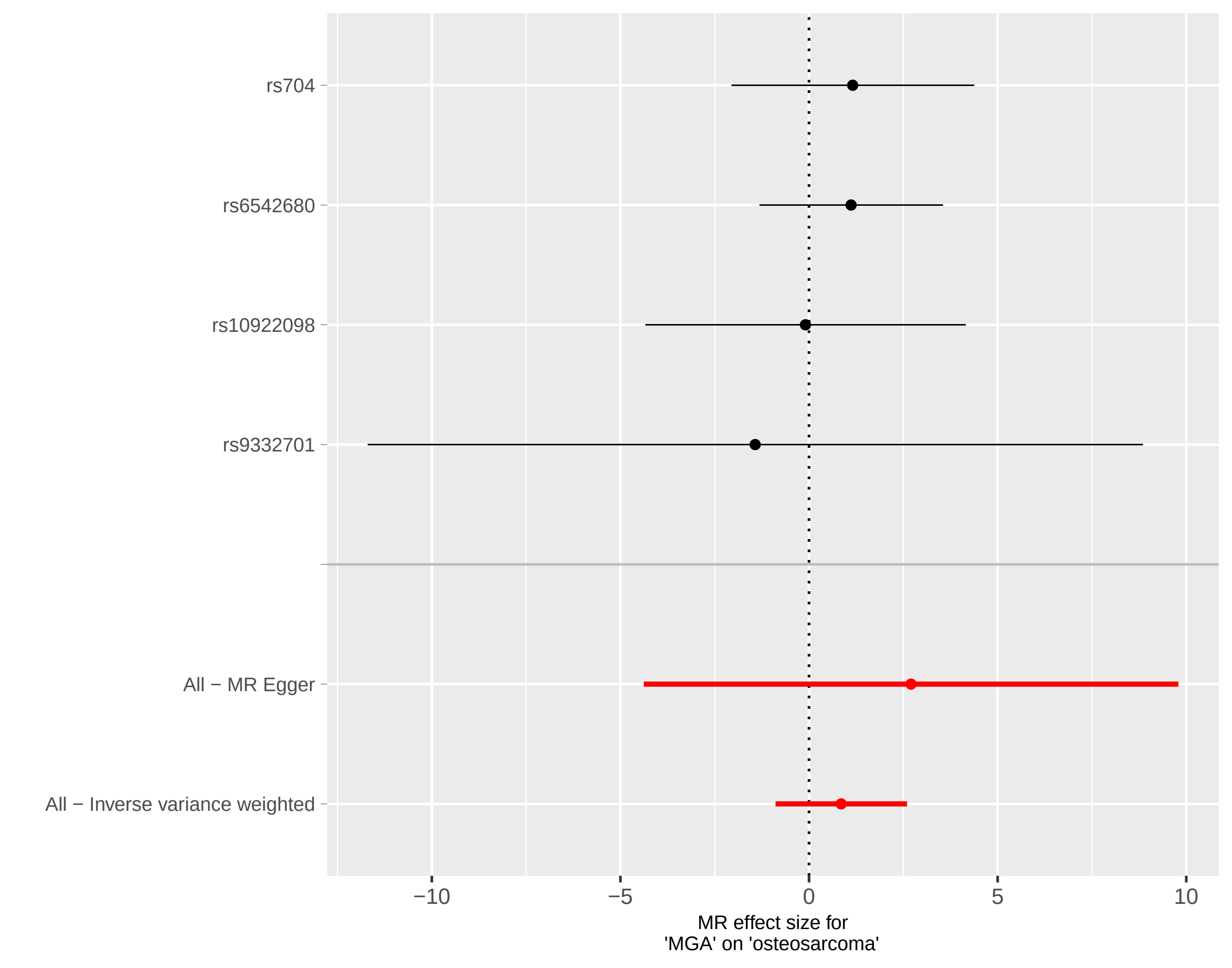
rs704
rs6542680
rs10922098
rs9332701
All − MR Egger
All − Inverse variance weighted
−10
−5
0
5
10
MR effect size for
'MGA' on 'osteosarcoma'

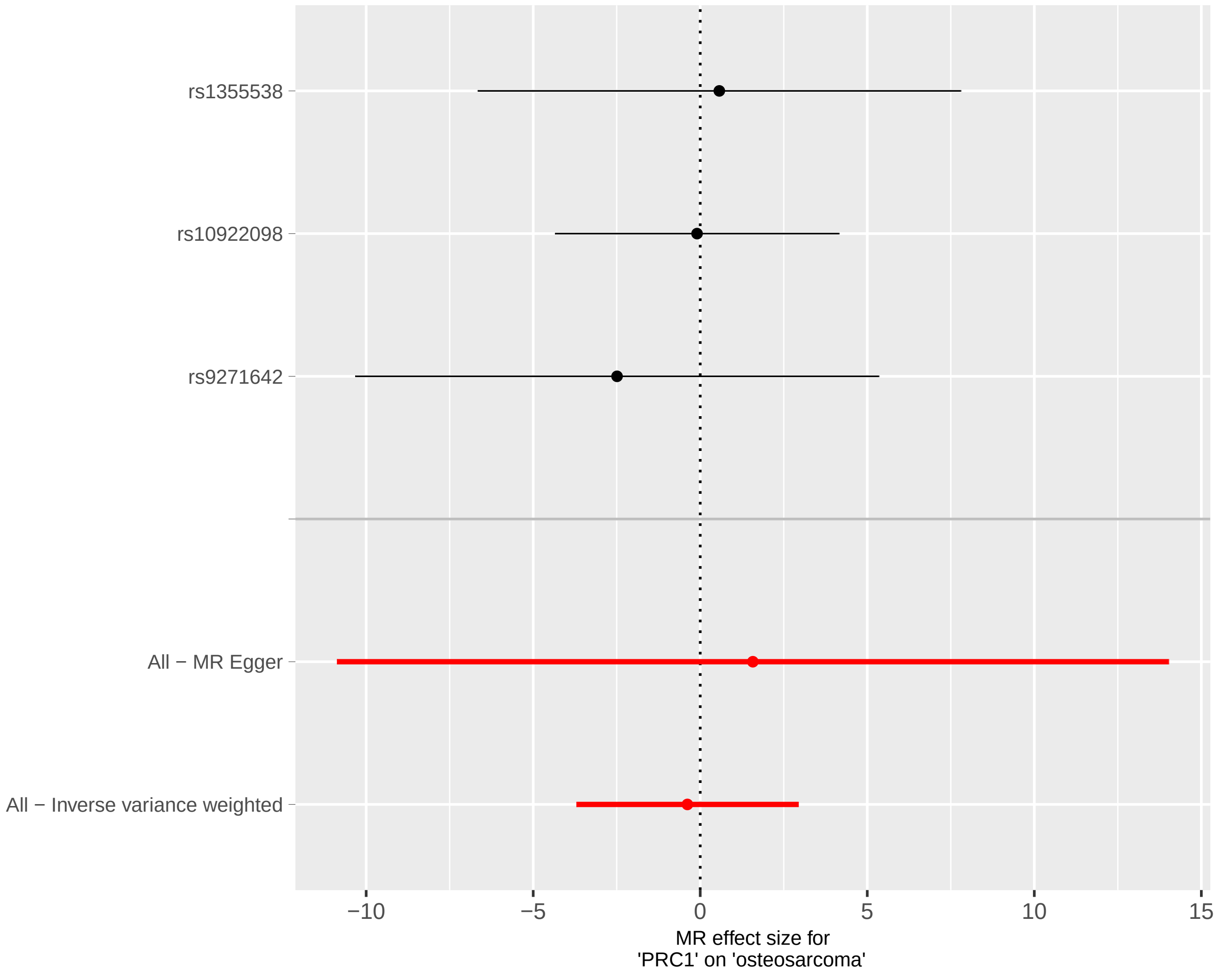

rs1355538
rs10922098
rs9271642
All − MR Egger
All − Inverse variance weighted
−10
−5
0
5
10
15
MR effect size for
'PRC1' on 'osteosarcoma'

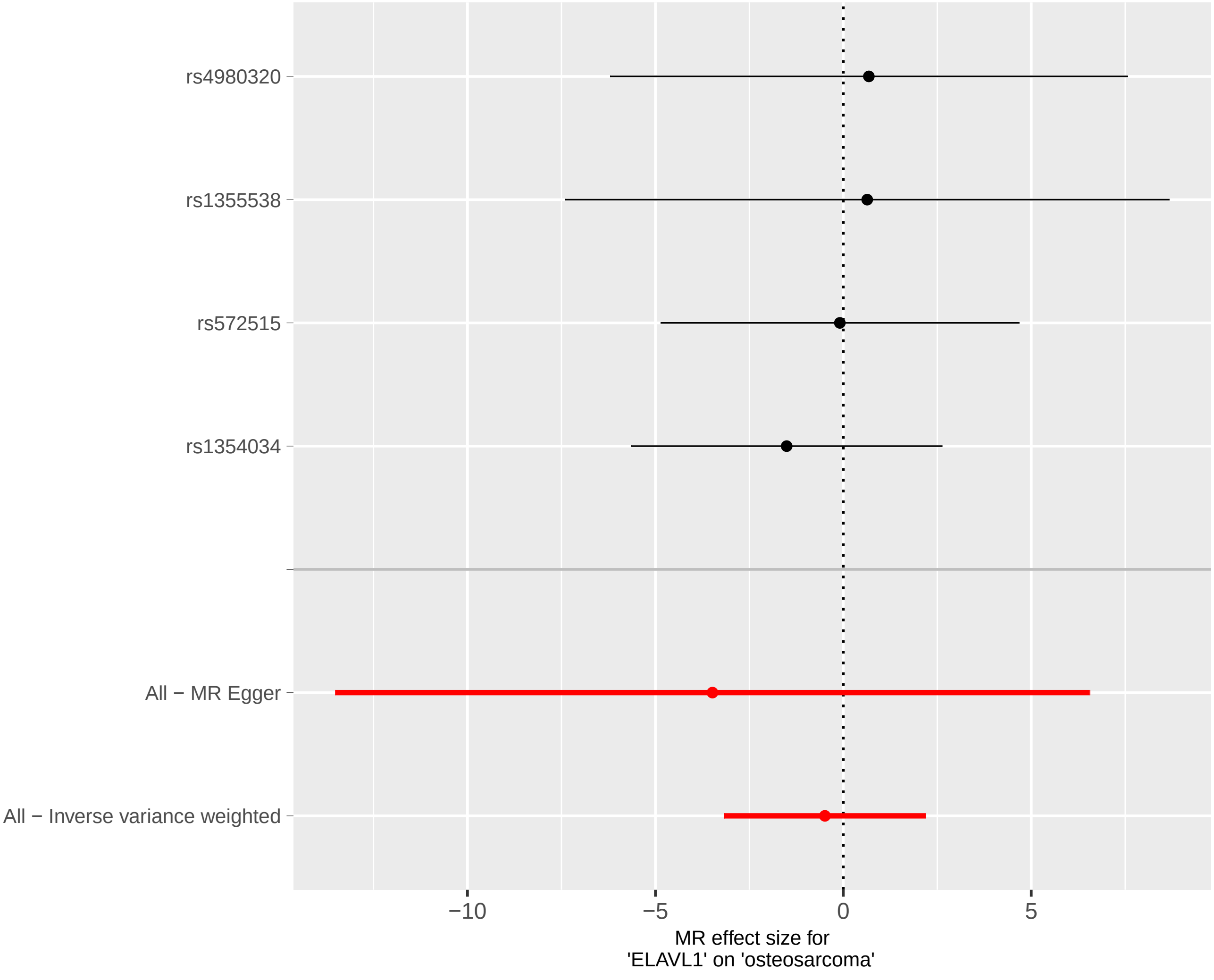
rs4980320
rs1355538
rs572515
rs1354034
All − MR Egger
All − Inverse variance weighted
−10
−5
0
5
MR effect size for
'ELAVL1' on 'osteosarcoma'

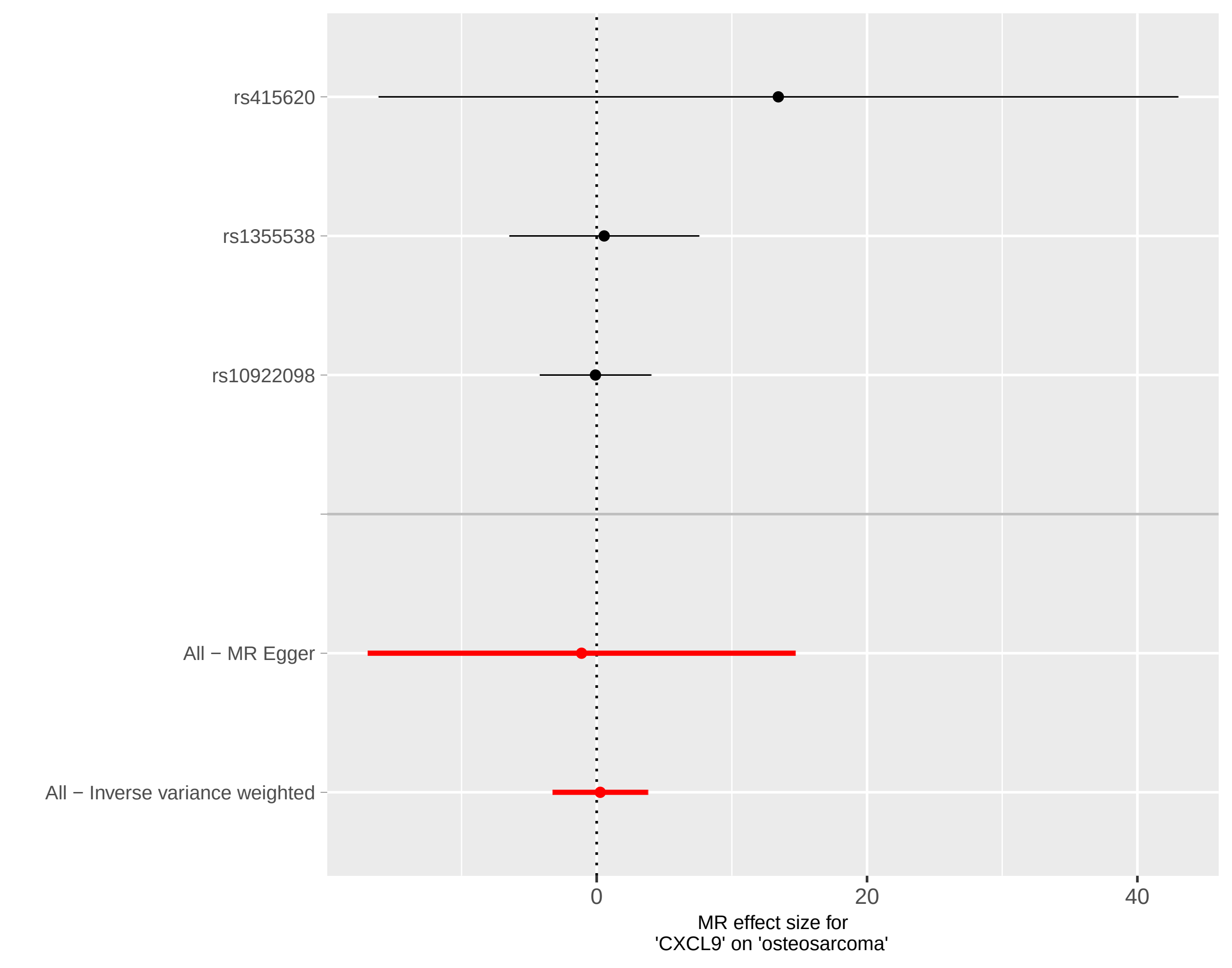
rs415620
rs1355538
rs10922098
All − MR Egger
All − Inverse variance weighted
0
20
40
MR effect size for
'CXCL9' on 'osteosarcoma'

Insufficient number of SNPs

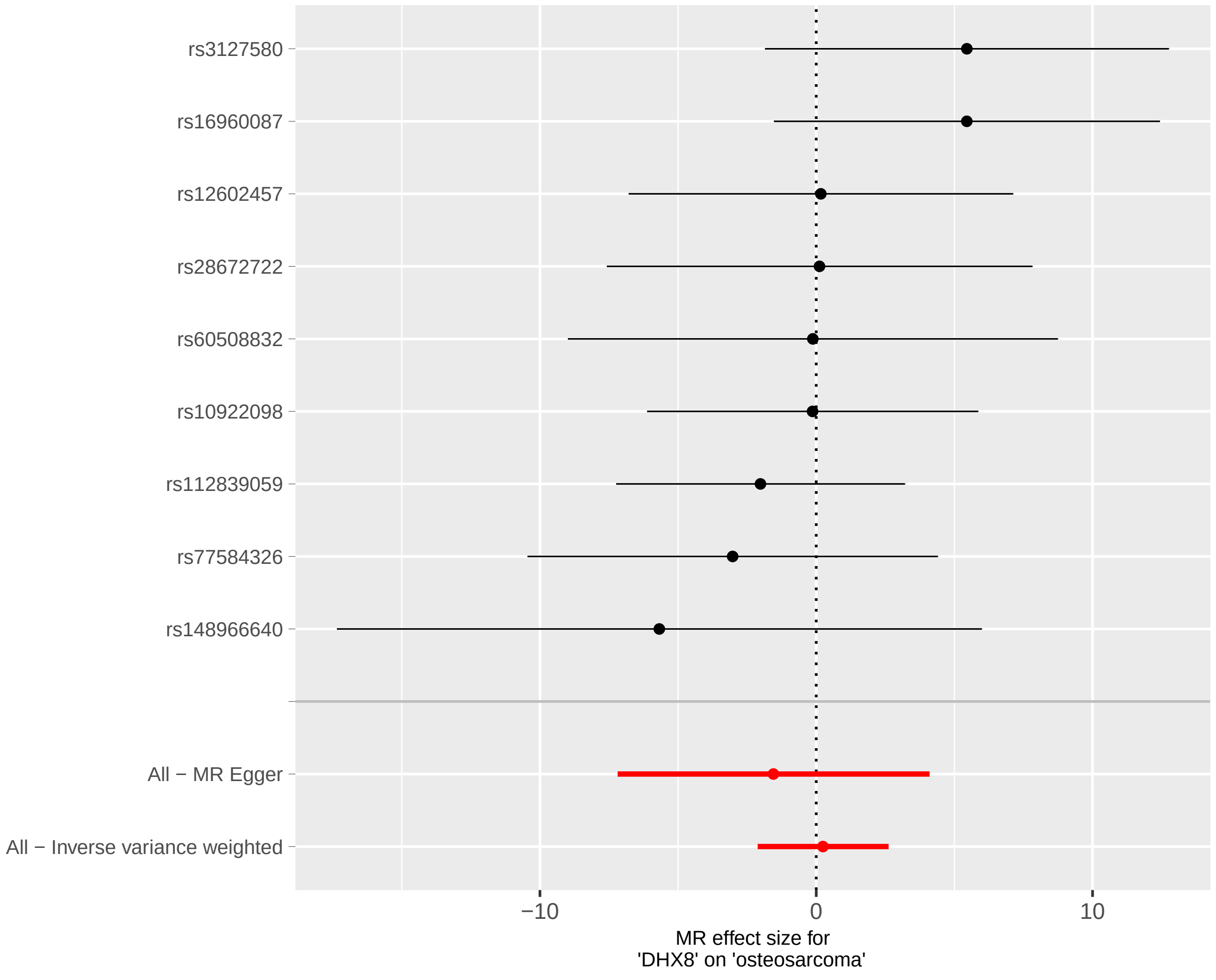

rs3127580
rs16960087
rs12602457
rs28672722
rs60508832
rs10922098
rs112839059
rs77584326
rs148966640
All − MR Egger
All − Inverse variance weighted
−10
0
10
MR effect size for
'DHX8' on 'osteosarcoma'

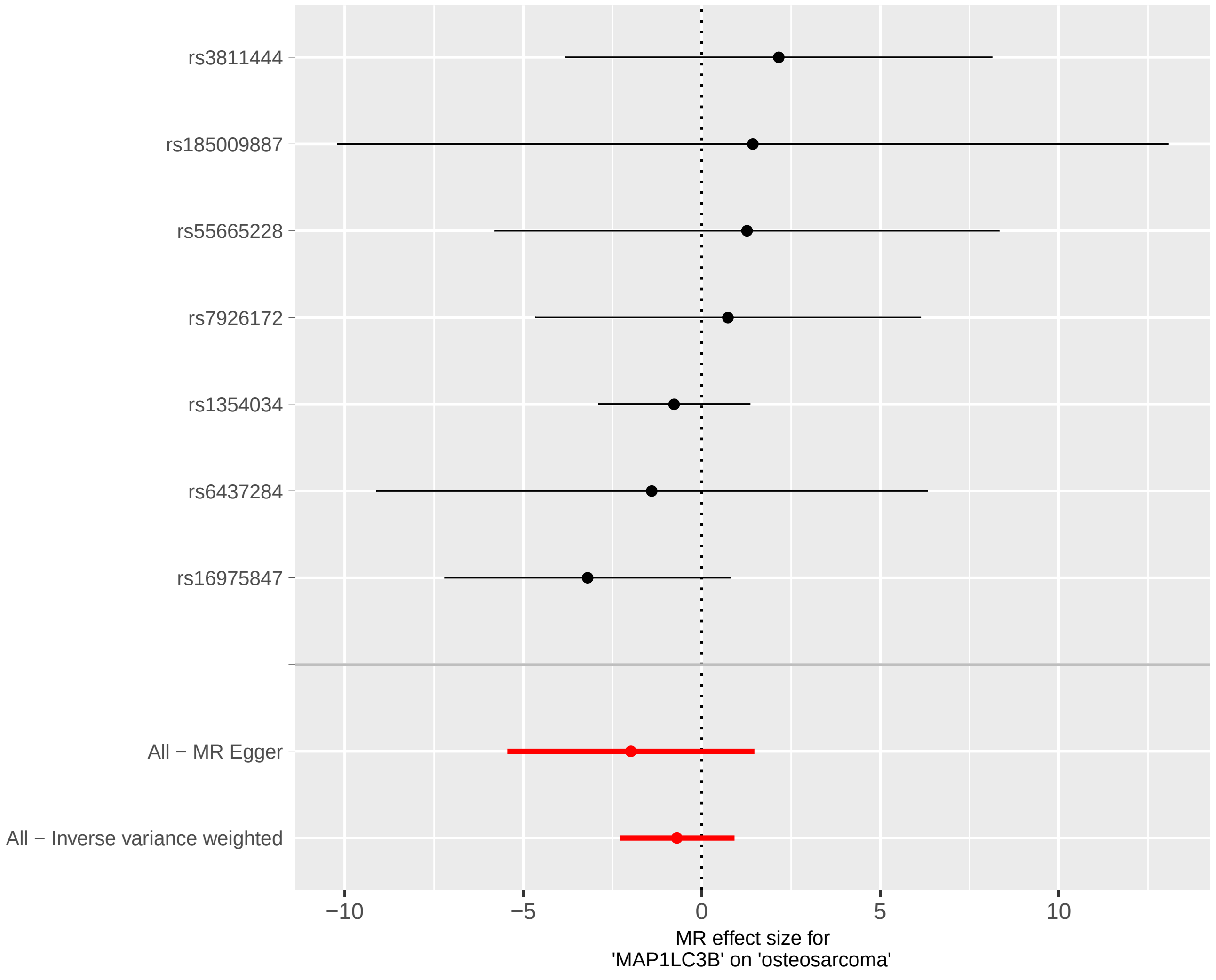
rs3811444
rs185009887
rs55665228
rs7926172
rs1354034
rs6437284
rs16975847
All − MR Egger
All − Inverse variance weighted
−10
−5
0
5
10
MR effect size for
'MAP1LC3B' on 'osteosarcoma'

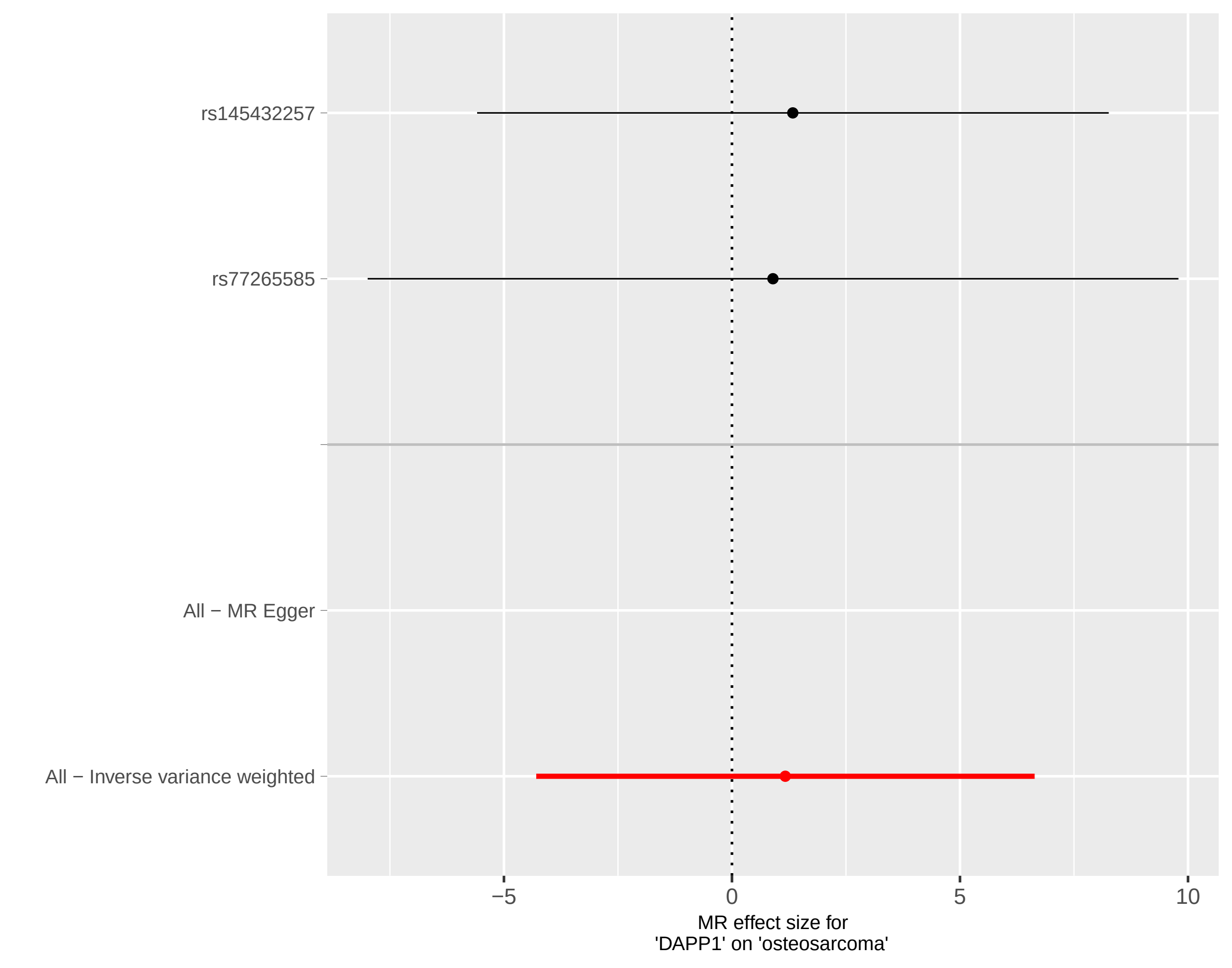

rs145432257
rs77265585
All − MR Egger
All − Inverse variance weighted
−5
0
5
10
MR effect size for
'DAPP1' on 'osteosarcoma'

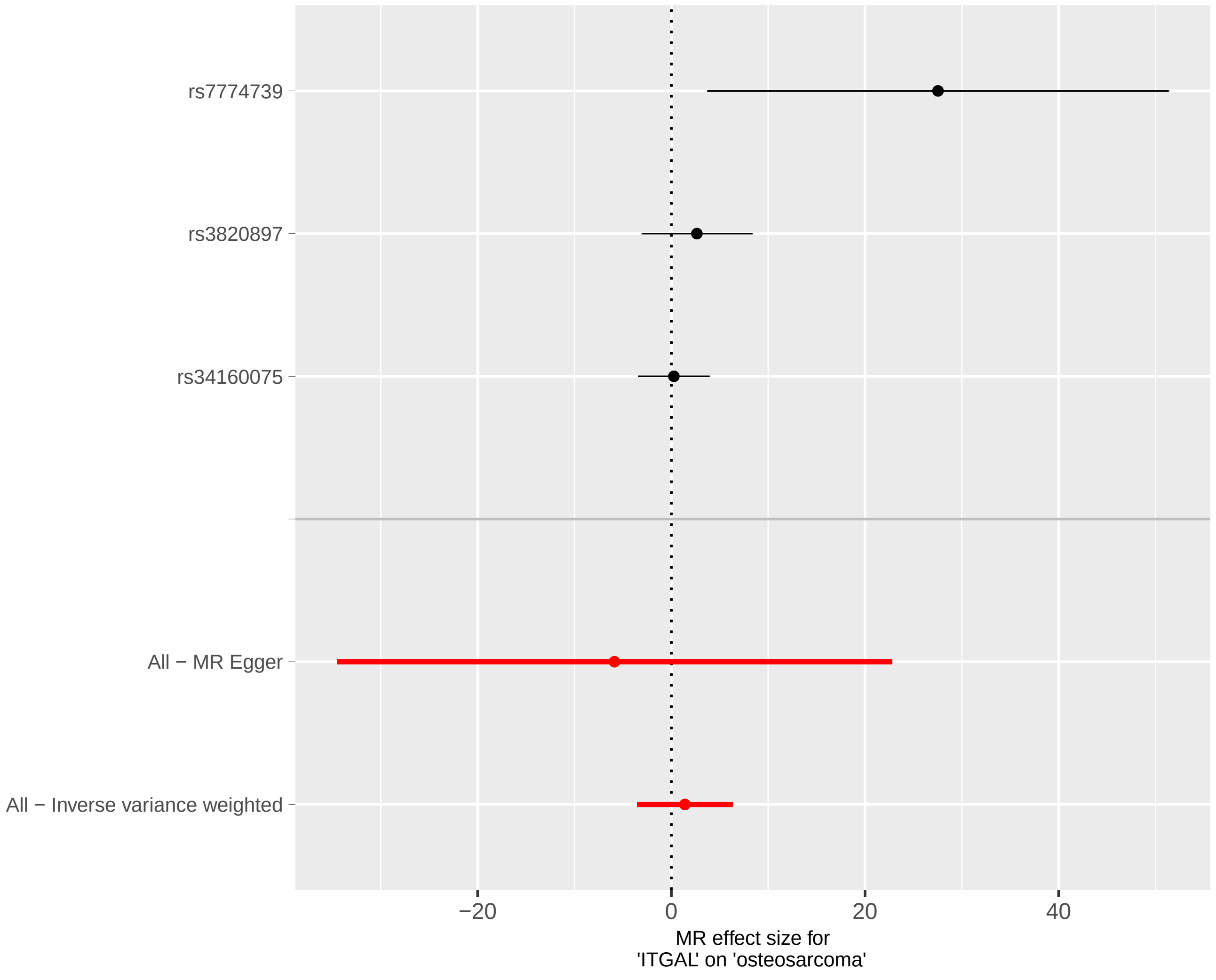
rs7774739
rs3820897
rs34160075
All − MR Egger
All − Inverse variance weighted
−20
0
20
40
MR effect size for
'ITGAL' on 'osteosarcoma'

Insufficient number of SNPs

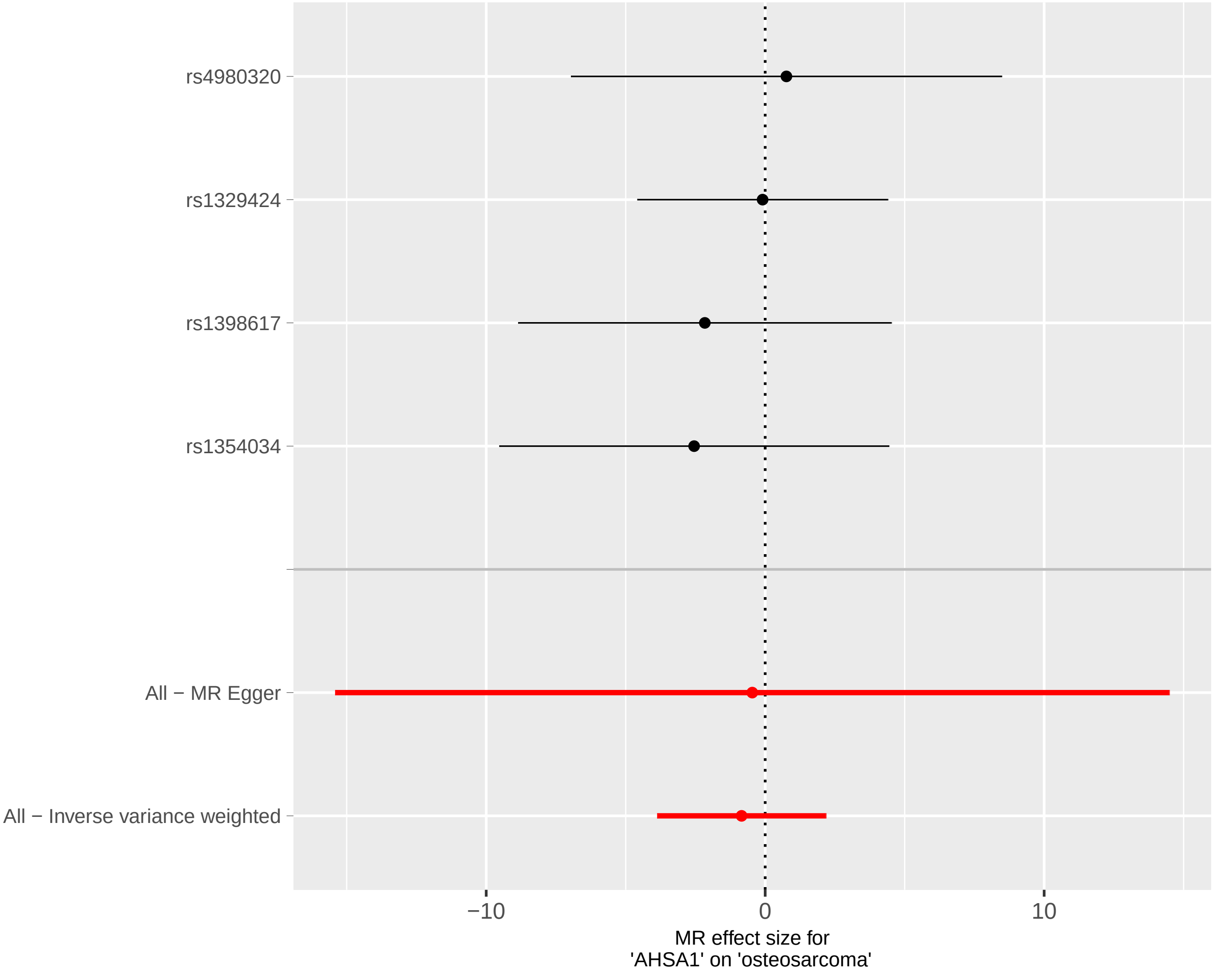
rs4980320
rs1329424
rs1398617
rs1354034
All − MR Egger
All − Inverse variance weighted
−10
0
10
MR effect size for
'AHSA1' on 'osteosarcoma'

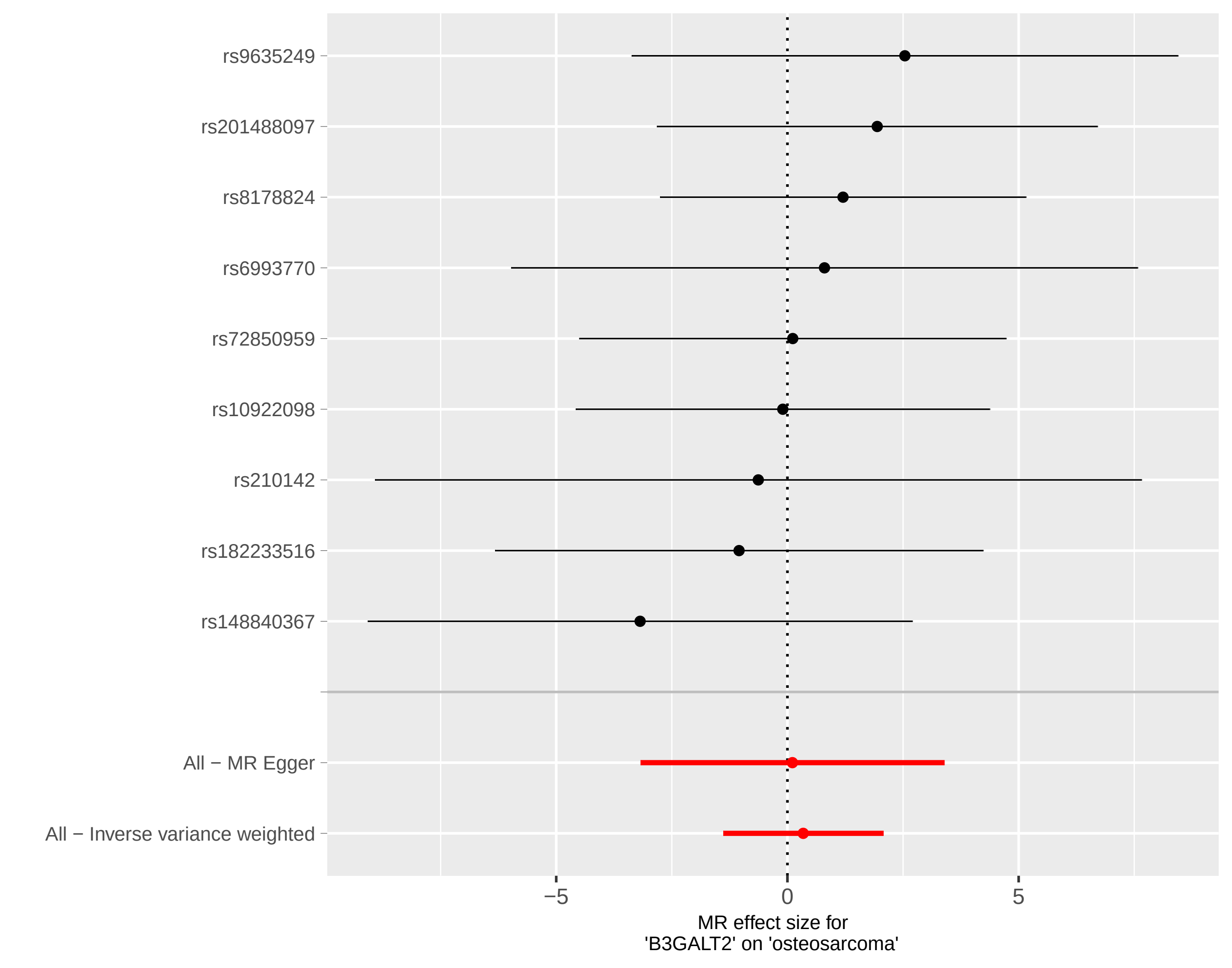

rs9635249
rs201488097
rs8178824
rs6993770
rs72850959
rs10922098
rs210142
rs182233516
rs148840367
All − MR Egger
All − Inverse variance weighted
−5
0
5
MR effect size for
'B3GALT2' on 'osteosarcoma'

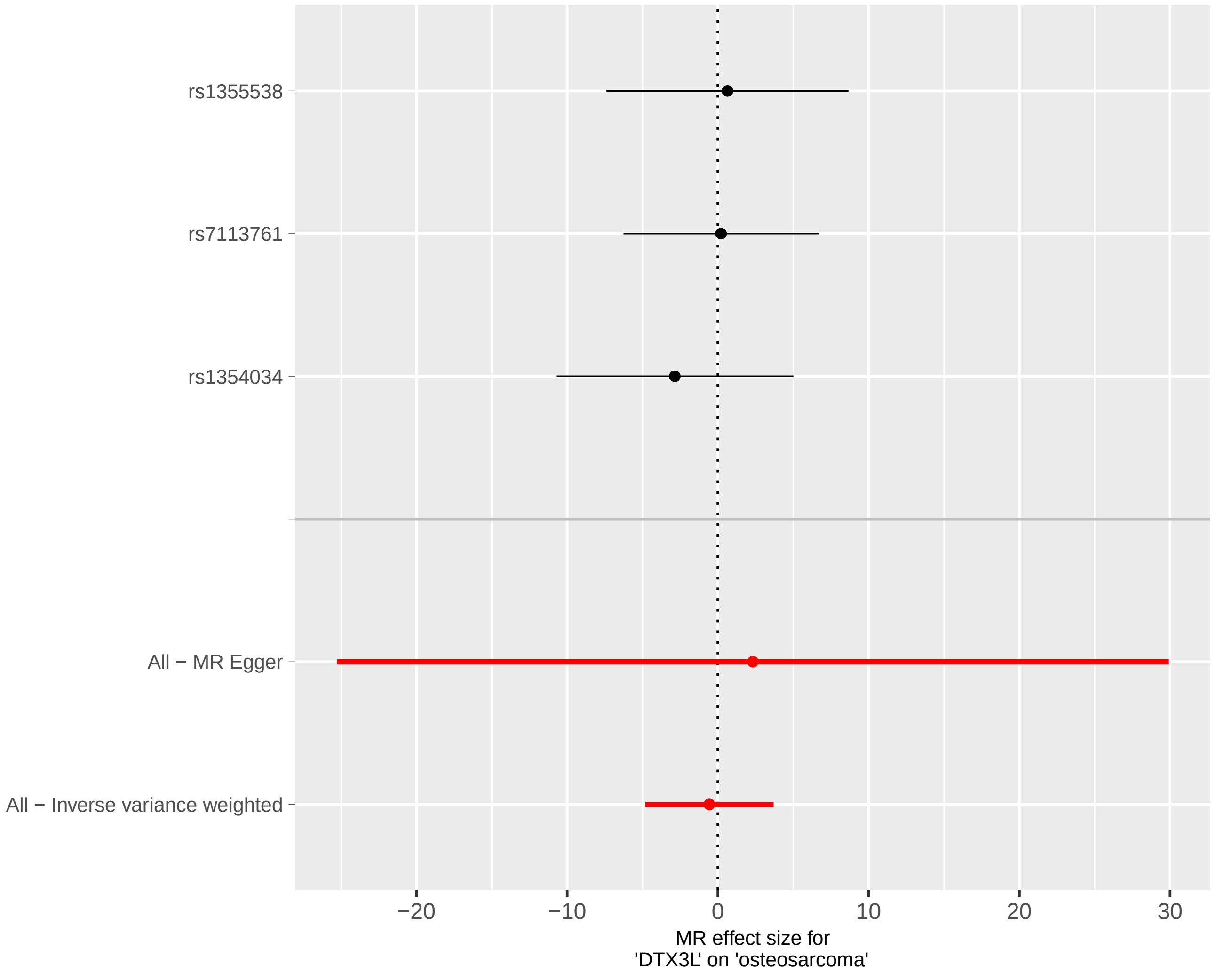

rs1355538
rs7113761
rs1354034
All − MR Egger
All − Inverse variance weighted
−20
−10
0
10
20
30
MR effect size for
'DTX3L' on 'osteosarcoma'

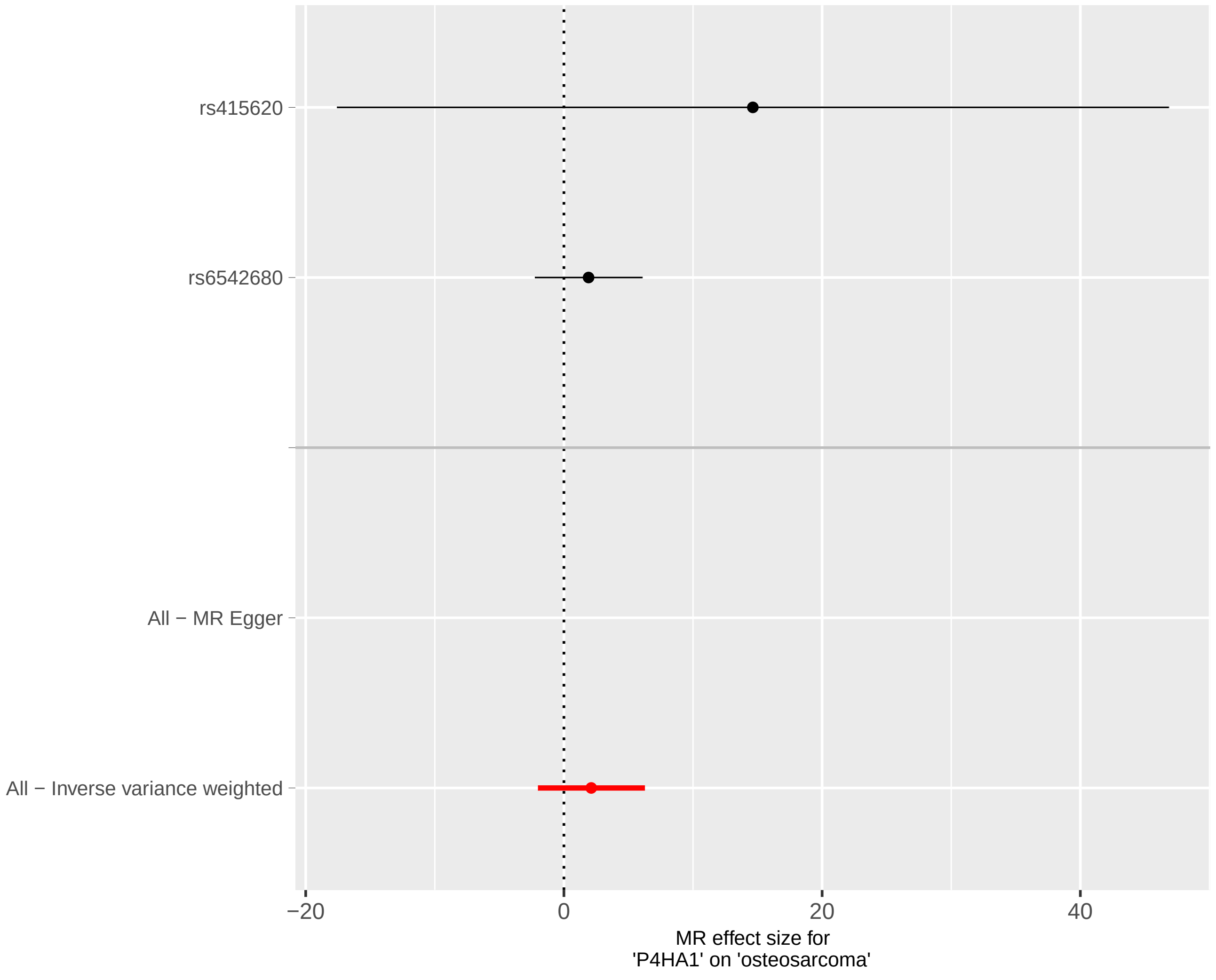

rs415620
rs6542680
All − MR Egger
All − Inverse variance weighted
−20
0
20
40
MR effect size for
'P4HA1' on 'osteosarcoma'

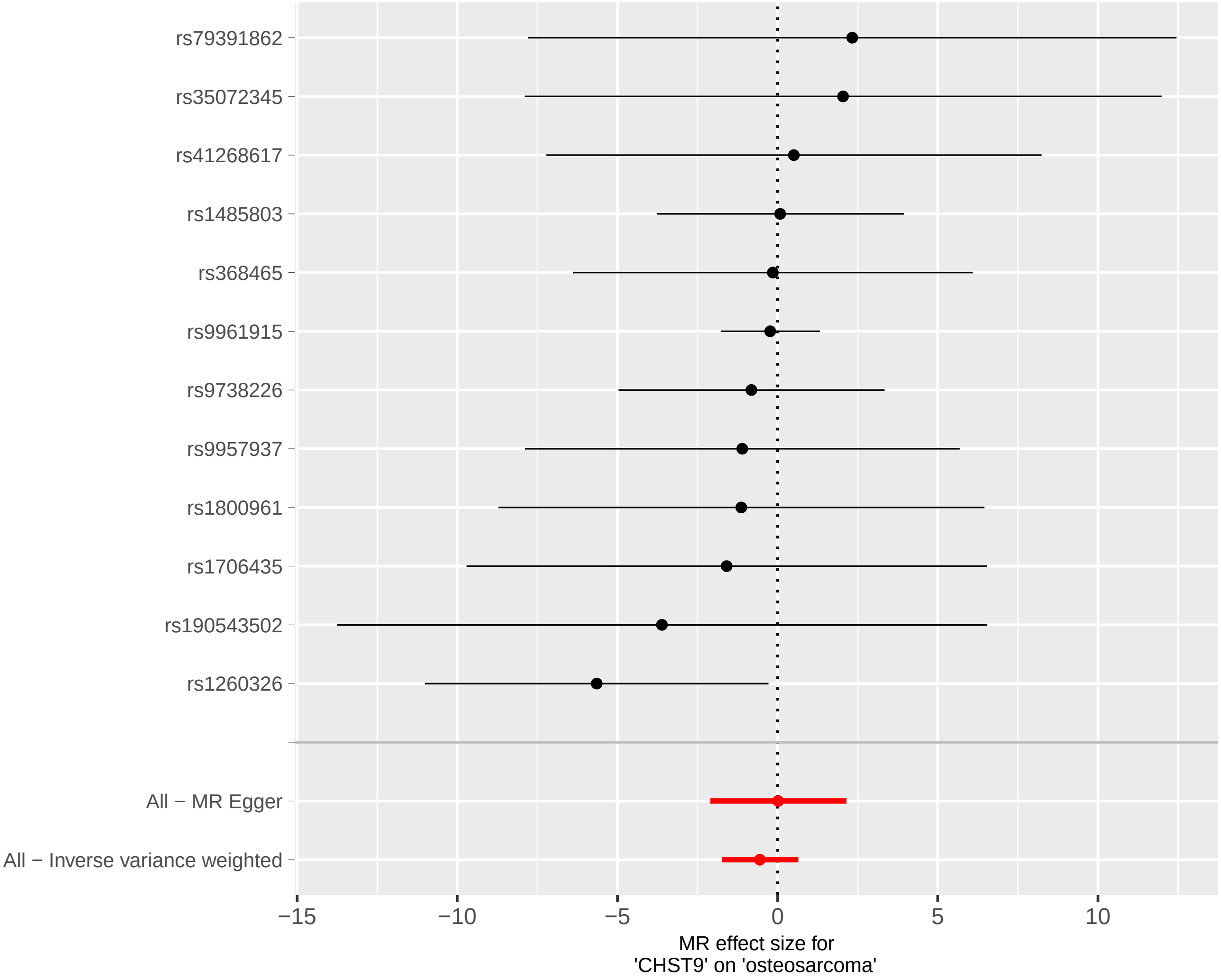

rs79391862
rs35072345
rs41268617
rs1485803
rs368465
rs9961915
rs9738226
rs9957937
rs1800961
rs1706435
rs190543502
rs1260326
All − MR Egger
All − Inverse variance weighted
−15
−10
−5
0
5
10
MR effect size for
'CHST9' on 'osteosarcoma'

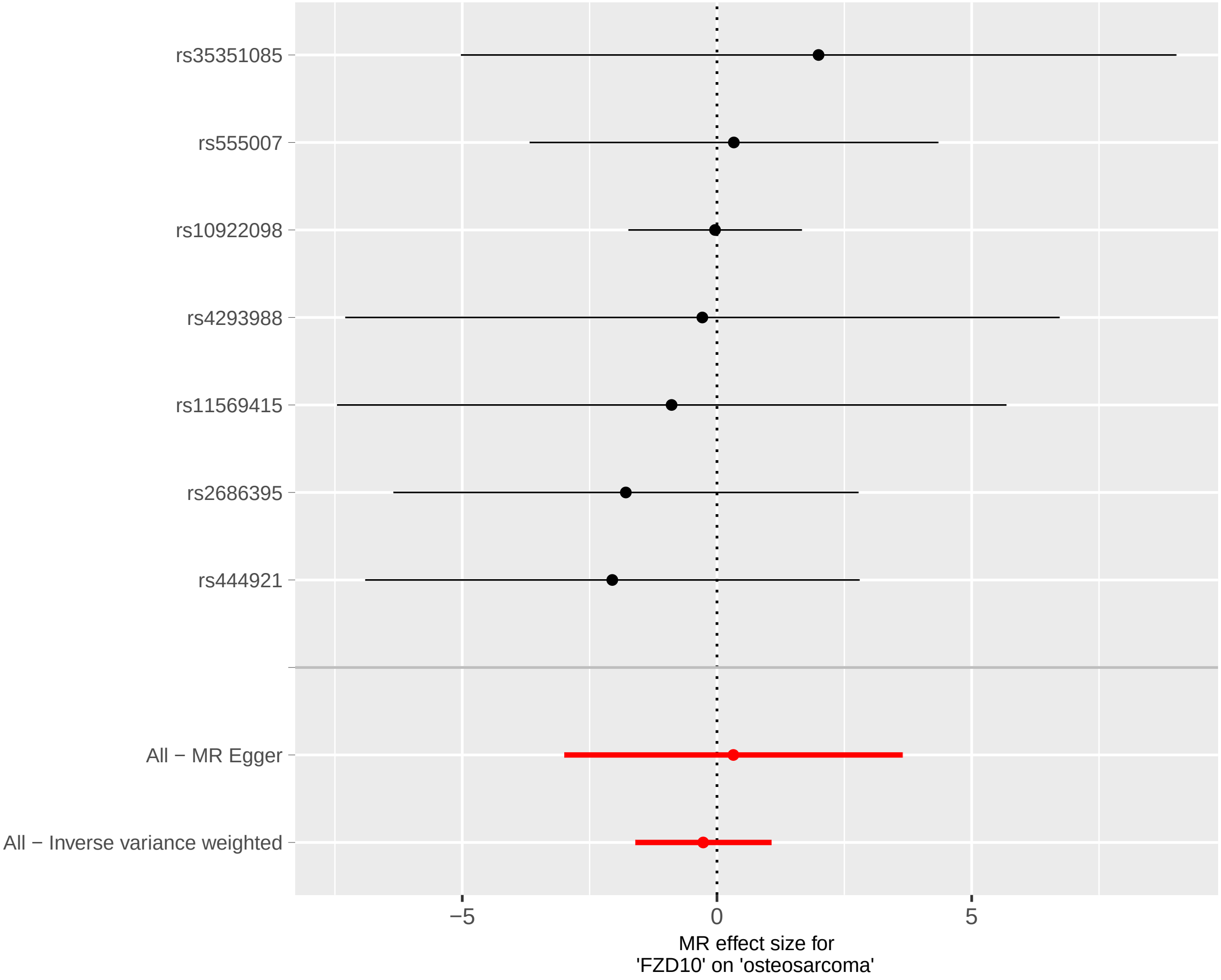

rs35351085
rs555007
rs10922098
rs4293988
rs11569415
rs2686395
rs444921
All − MR Egger
All − Inverse variance weighted
−5
0
5
MR effect size for
'FZD10' on 'osteosarcoma'

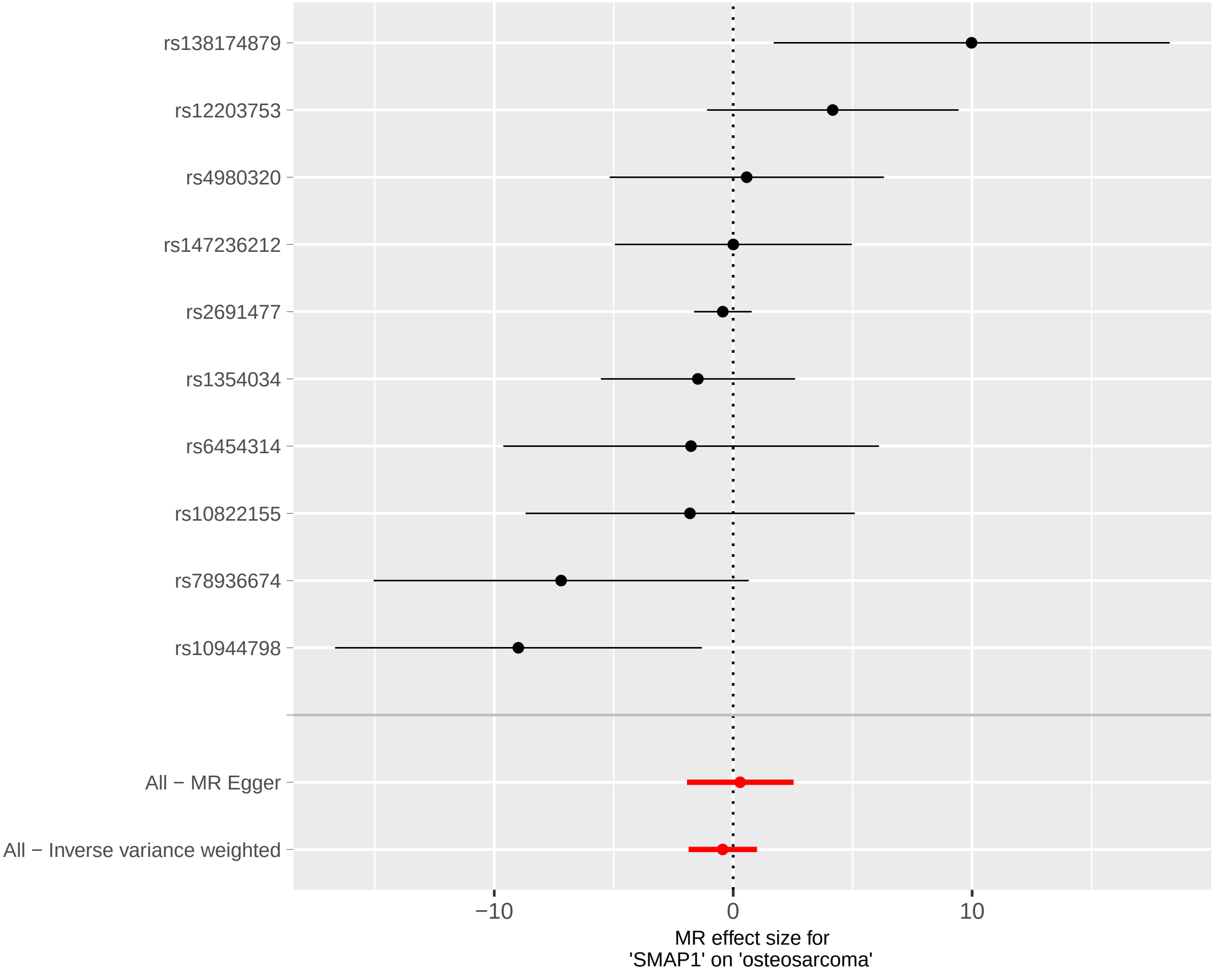
rs138174879
rs12203753
rs4980320
rs147236212
rs2691477
rs1354034
rs6454314
rs10822155
rs78936674
rs10944798
All − MR Egger
All − Inverse variance weighted
−10
0
10
MR effect size for
'SMAP1' on 'osteosarcoma'

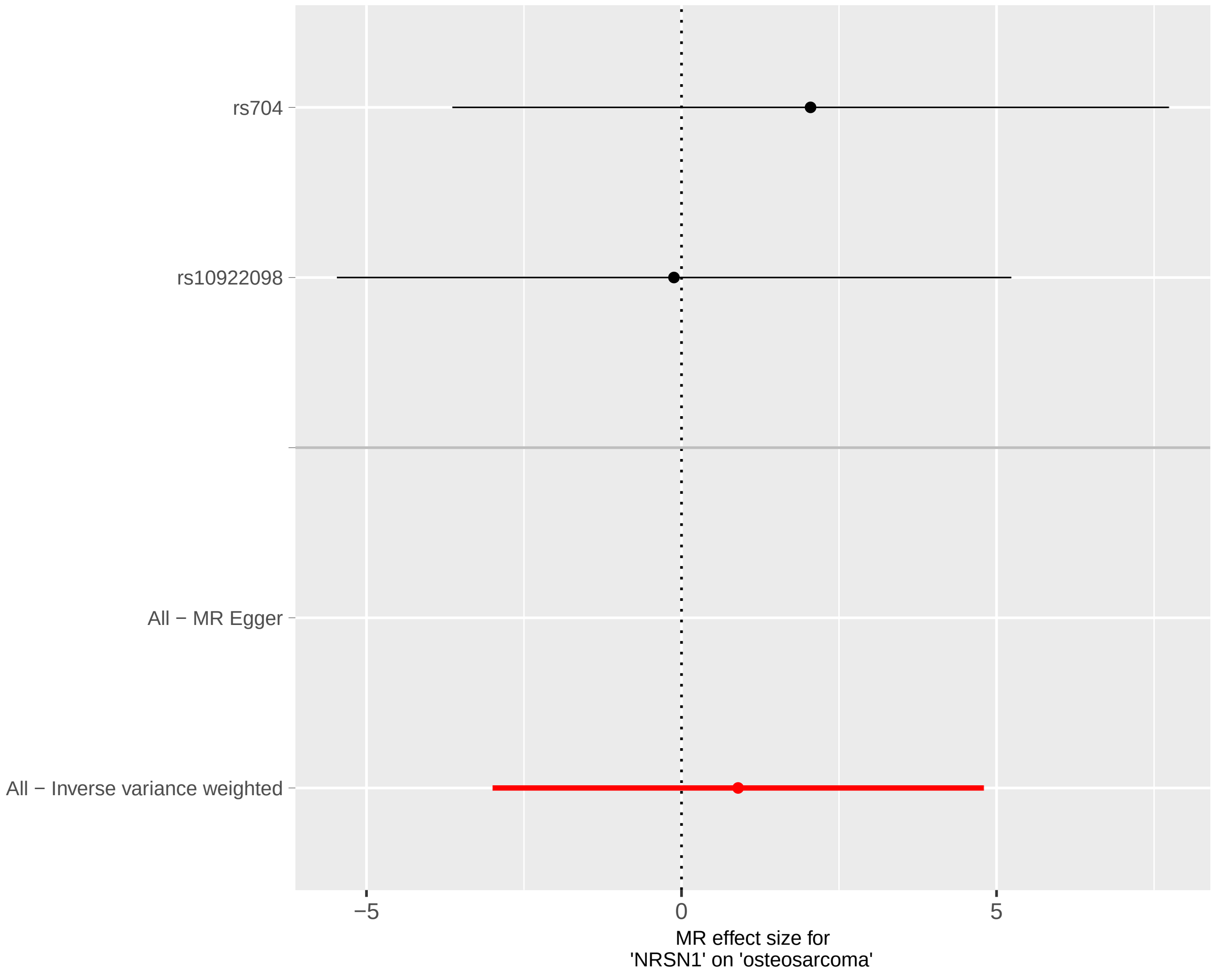

rs704
rs10922098
All − MR Egger
All − Inverse variance weighted
−5
0
5
MR effect size for
'NRSN1' on 'osteosarcoma'

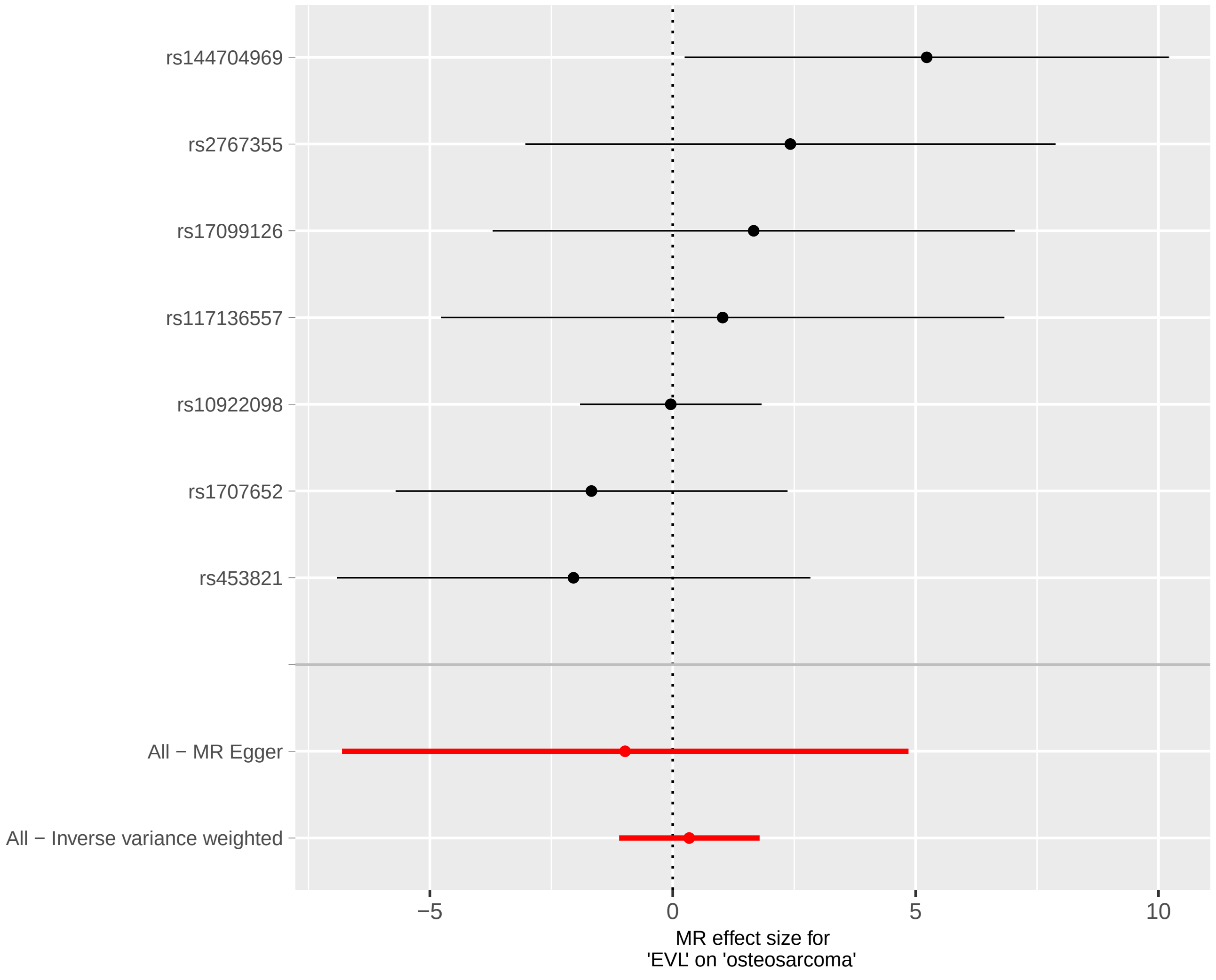
rs144704969
rs2767355
rs17099126
rs117136557
rs10922098
rs1707652
rs453821
All − MR Egger
All − Inverse variance weighted
−5
0
5
10
MR effect size for
'EVL' on 'osteosarcoma'

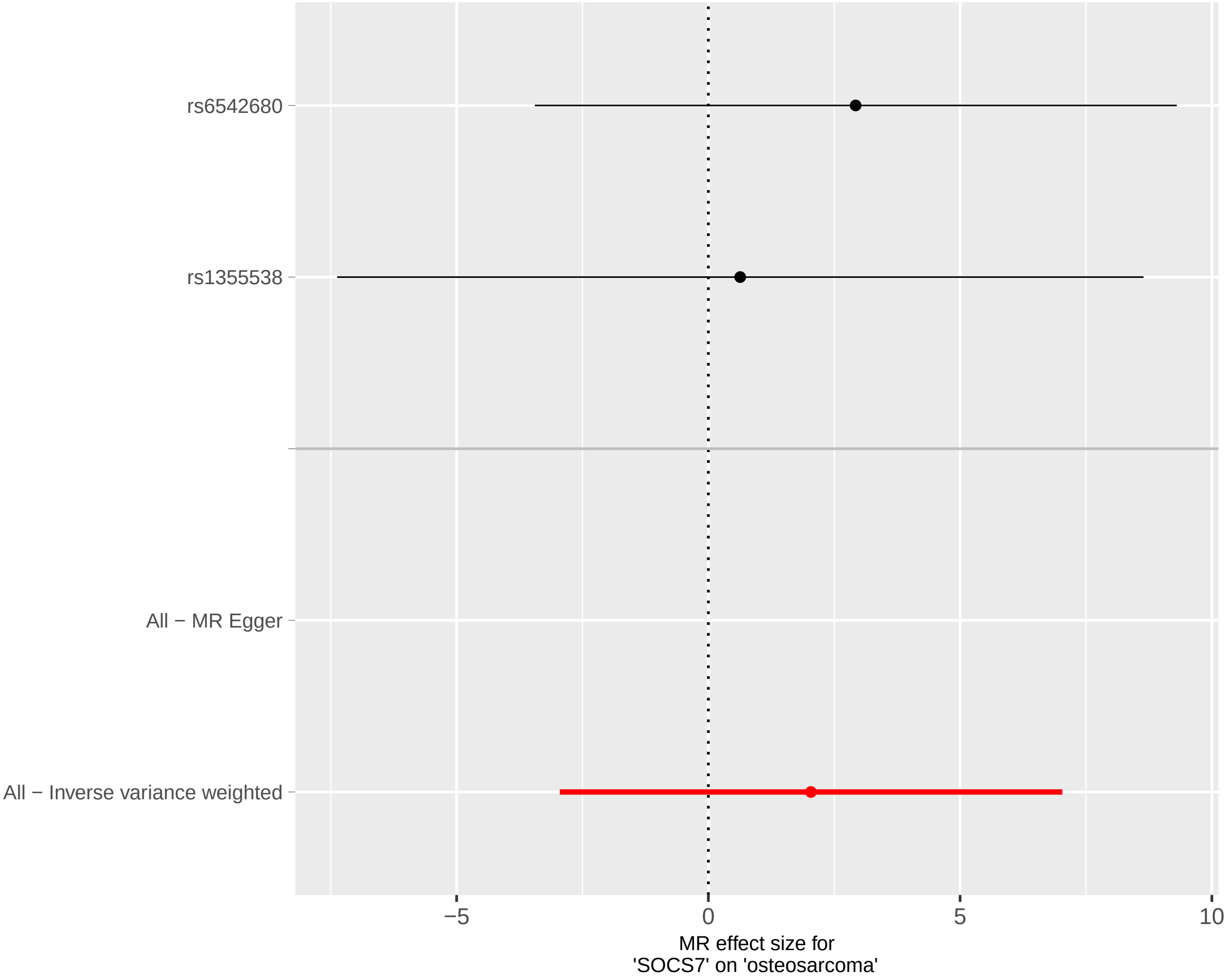

rs6542680
rs1355538
All − MR Egger
All − Inverse variance weighted
−5
0
5
10
MR effect size for
'SOCS7' on 'osteosarcoma'

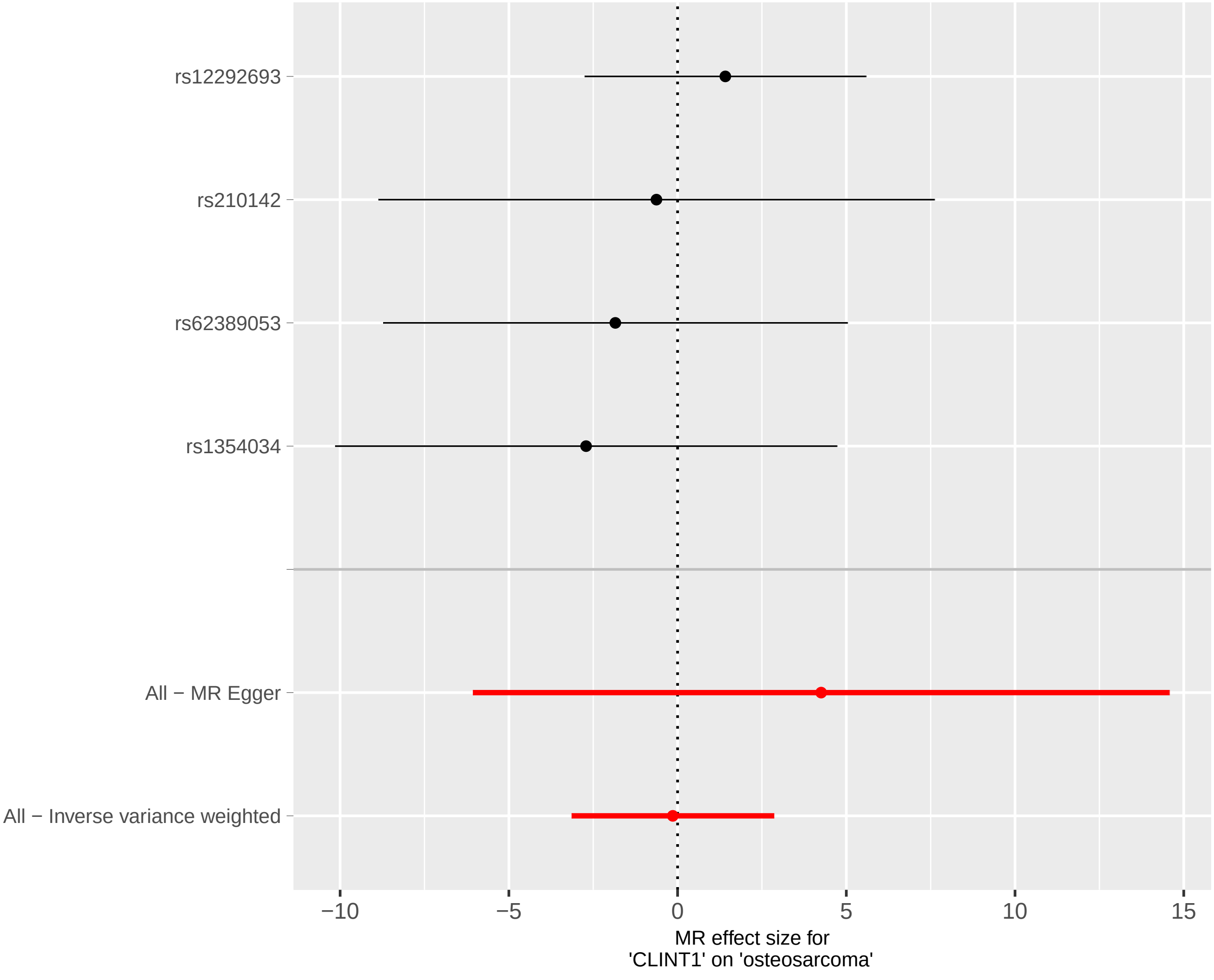
rs12292693
rs210142
rs62389053
rs1354034
All − MR Egger
All − Inverse variance weighted
−10
−5
0
5
10
15
MR effect size for
'CLINT1' on 'osteosarcoma'

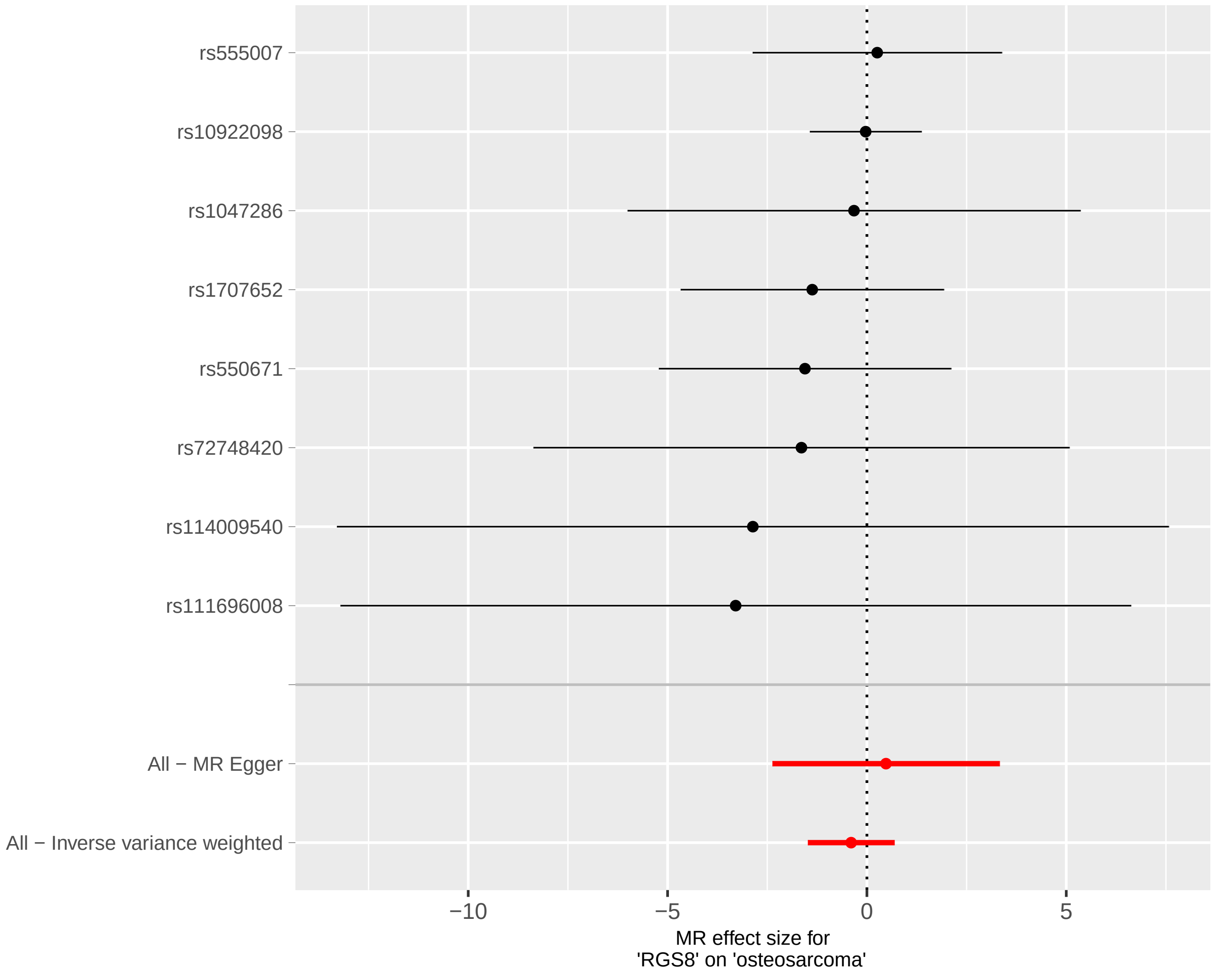

rs555007
rs10922098
rs1047286
rs1707652
rs550671
rs72748420
rs114009540
rs111696008
All − MR Egger
All − Inverse variance weighted
−10
−5
0
5
MR effect size for
'RGS8' on 'osteosarcoma'

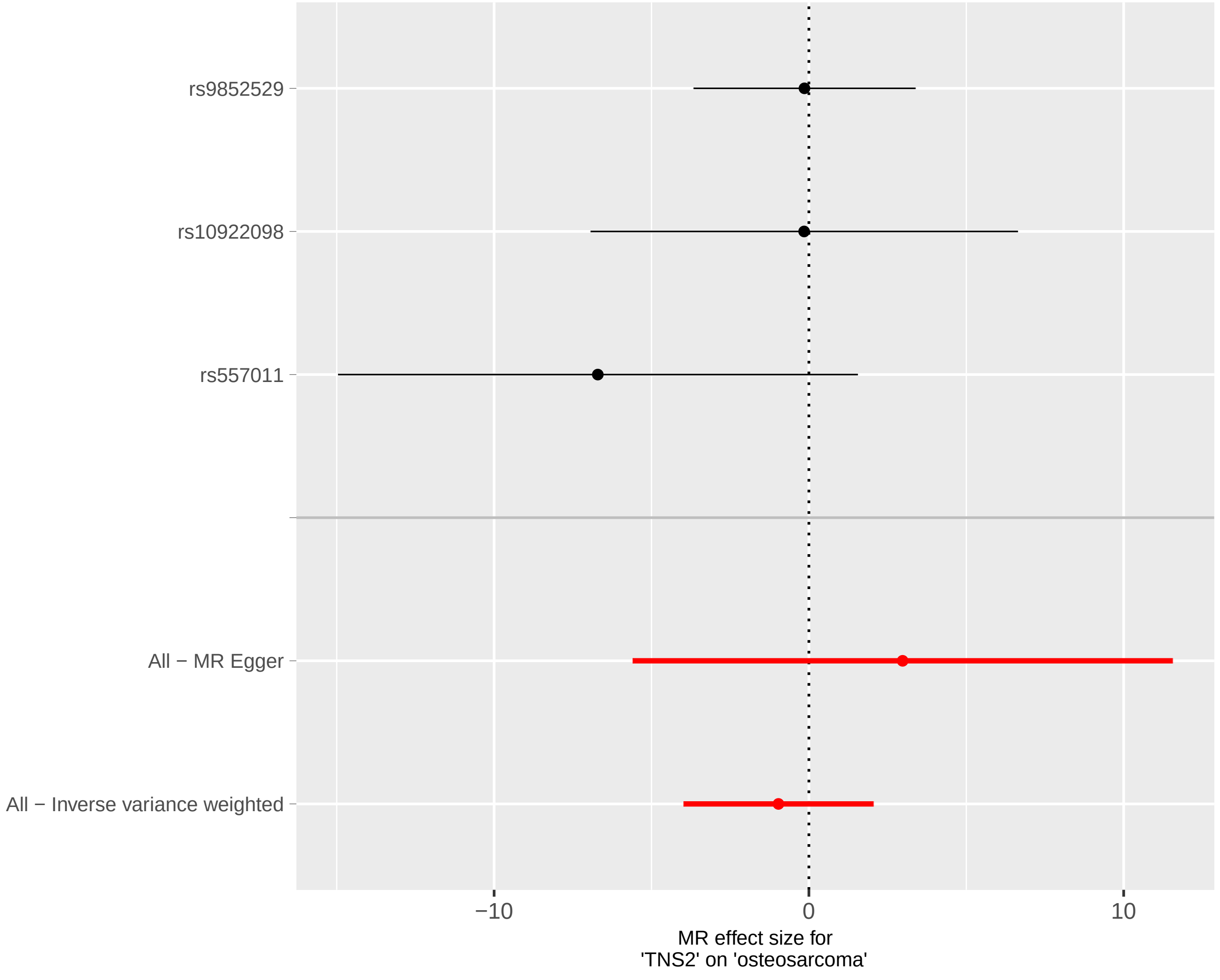
rs9852529
rs10922098
rs557011
All − MR Egger
All − Inverse variance weighted
−10
0
10
MR effect size for
'TNS2' on 'osteosarcoma'

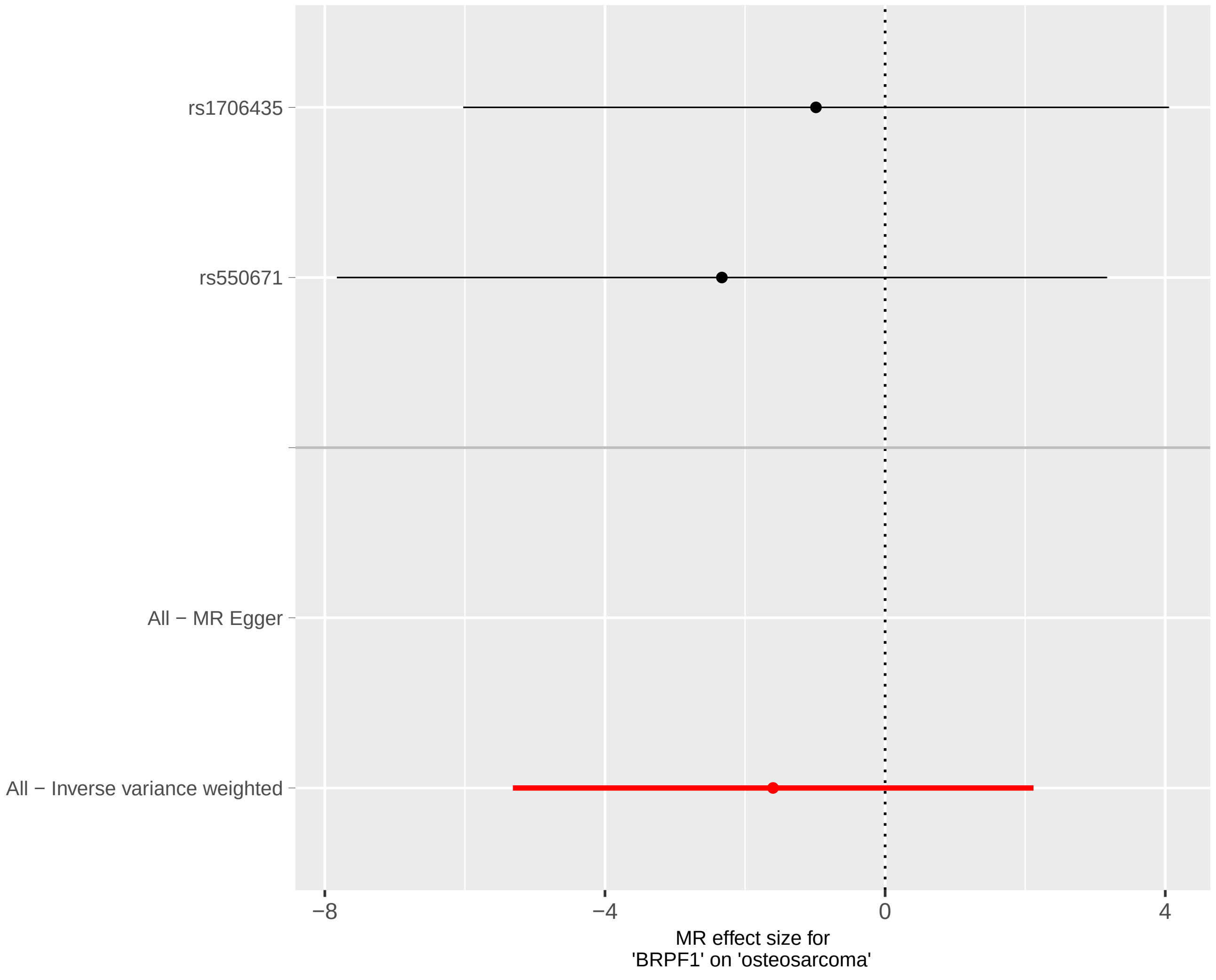
rs1706435
rs550671
All − MR Egger
All − Inverse variance weighted
−8
−4
0
4
MR effect size for
'BRPF1' on 'osteosarcoma'

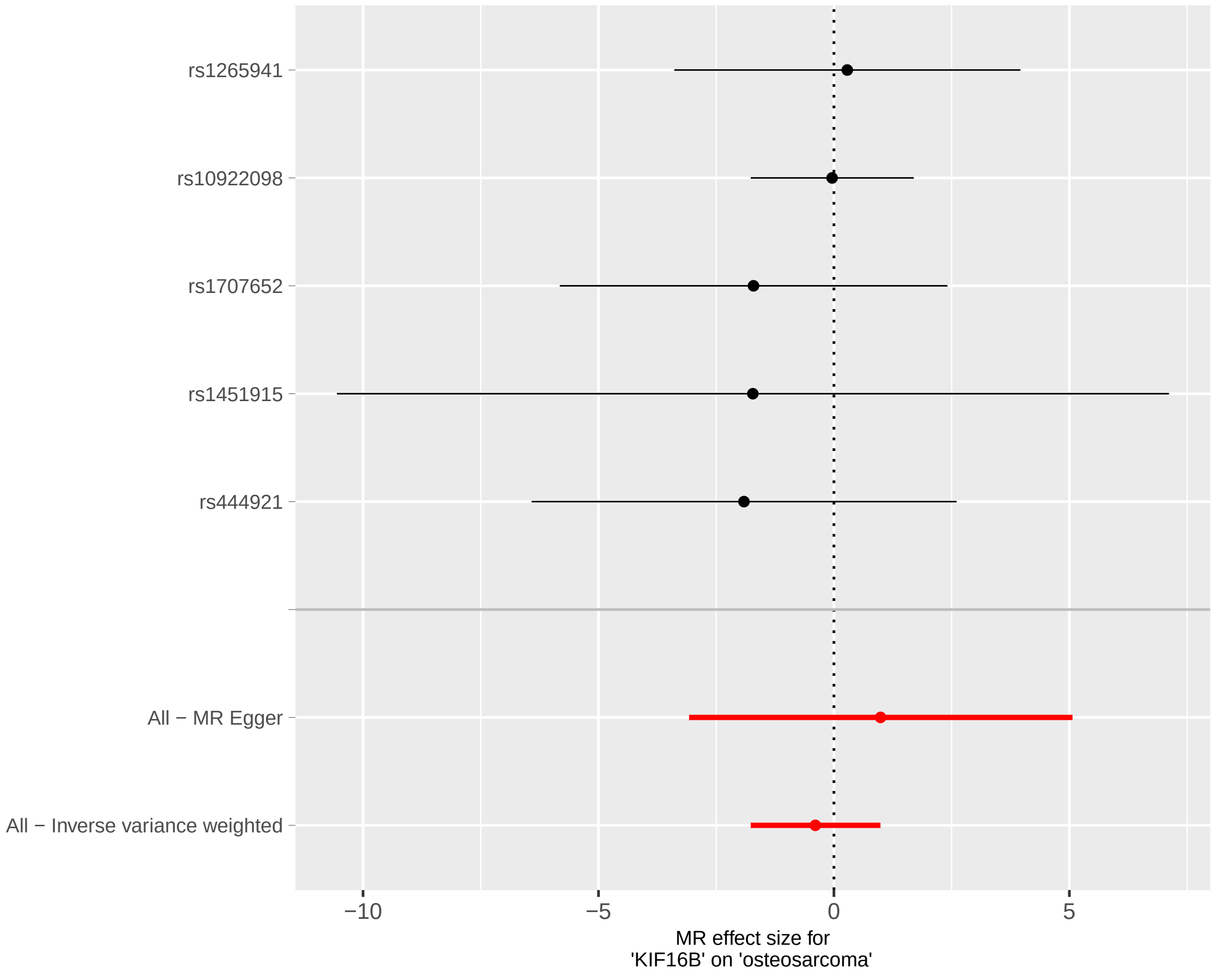
rs1265941
rs10922098
rs1707652
rs1451915
rs444921
All − MR Egger
All − Inverse variance weighted
−10
−5
0
5
MR effect size for
'KIF16B' on 'osteosarcoma'

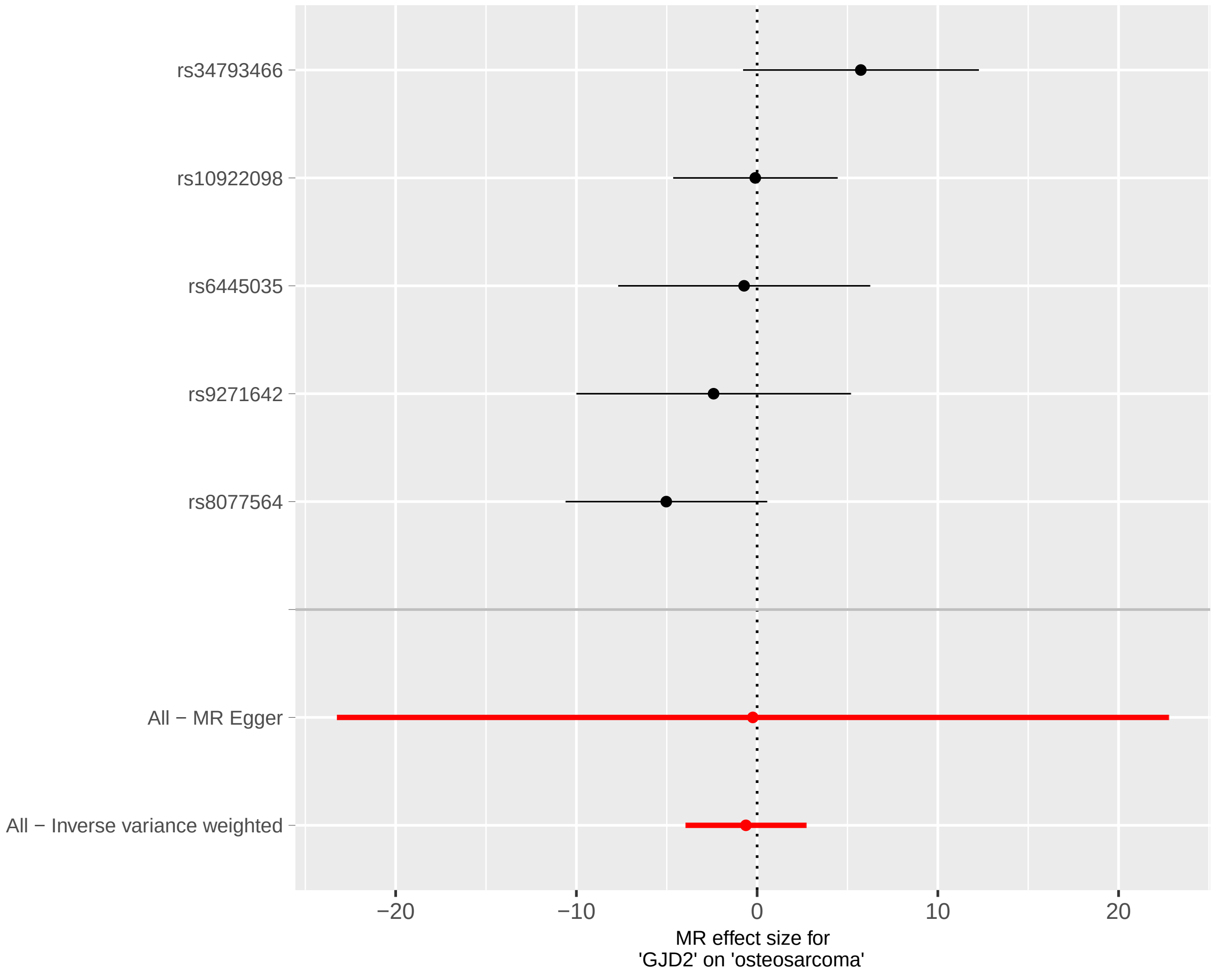

rs34793466
rs10922098
rs6445035
rs9271642
rs8077564
All − MR Egger
All − Inverse variance weighted
−20
−10
0
10
20
MR effect size for
'GJD2' on 'osteosarcoma'

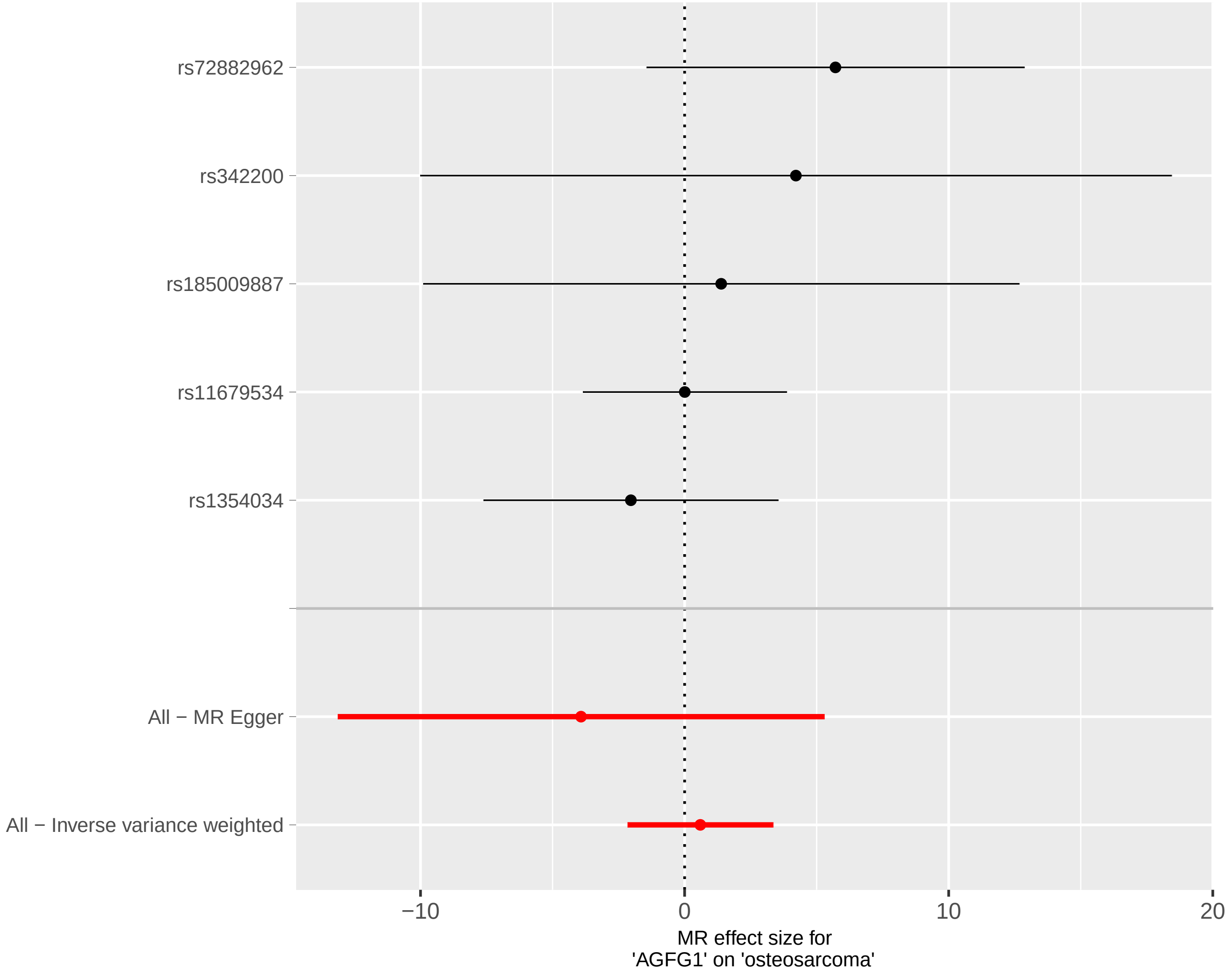

rs72882962
rs342200
rs185009887
rs11679534
rs1354034
All − MR Egger
All − Inverse variance weighted
−10
0
10
20
MR effect size for
'AGFG1' on 'osteosarcoma'

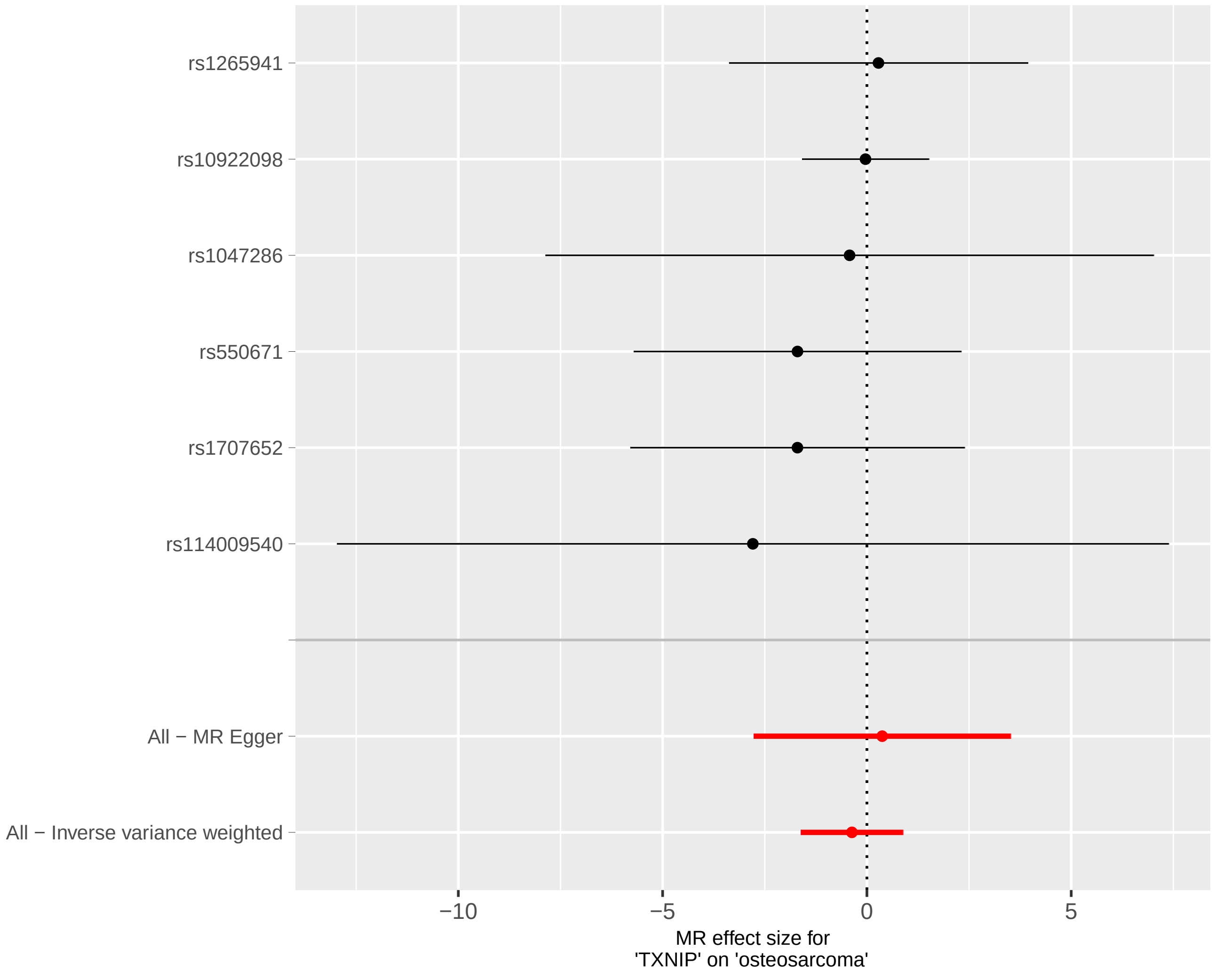

rs1265941
rs10922098
rs1047286
rs550671
rs1707652
rs114009540
All − MR Egger
All − Inverse variance weighted
−10
−5
0
5
MR effect size for
'TXNIP' on 'osteosarcoma'

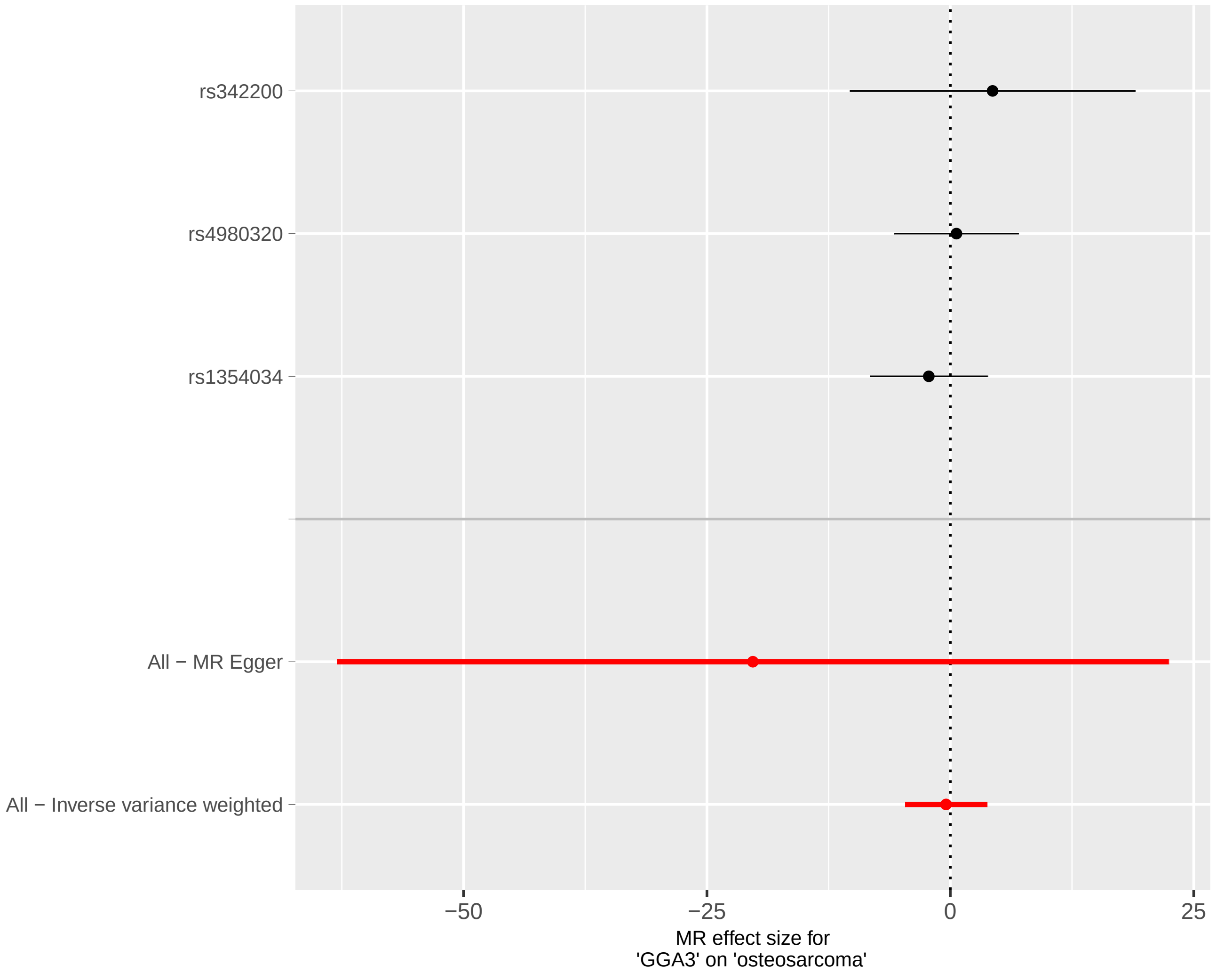
rs342200
rs4980320
rs1354034
All − MR Egger
All − Inverse variance weighted
−50
−25
0
25
MR effect size for
'GGA3' on 'osteosarcoma'

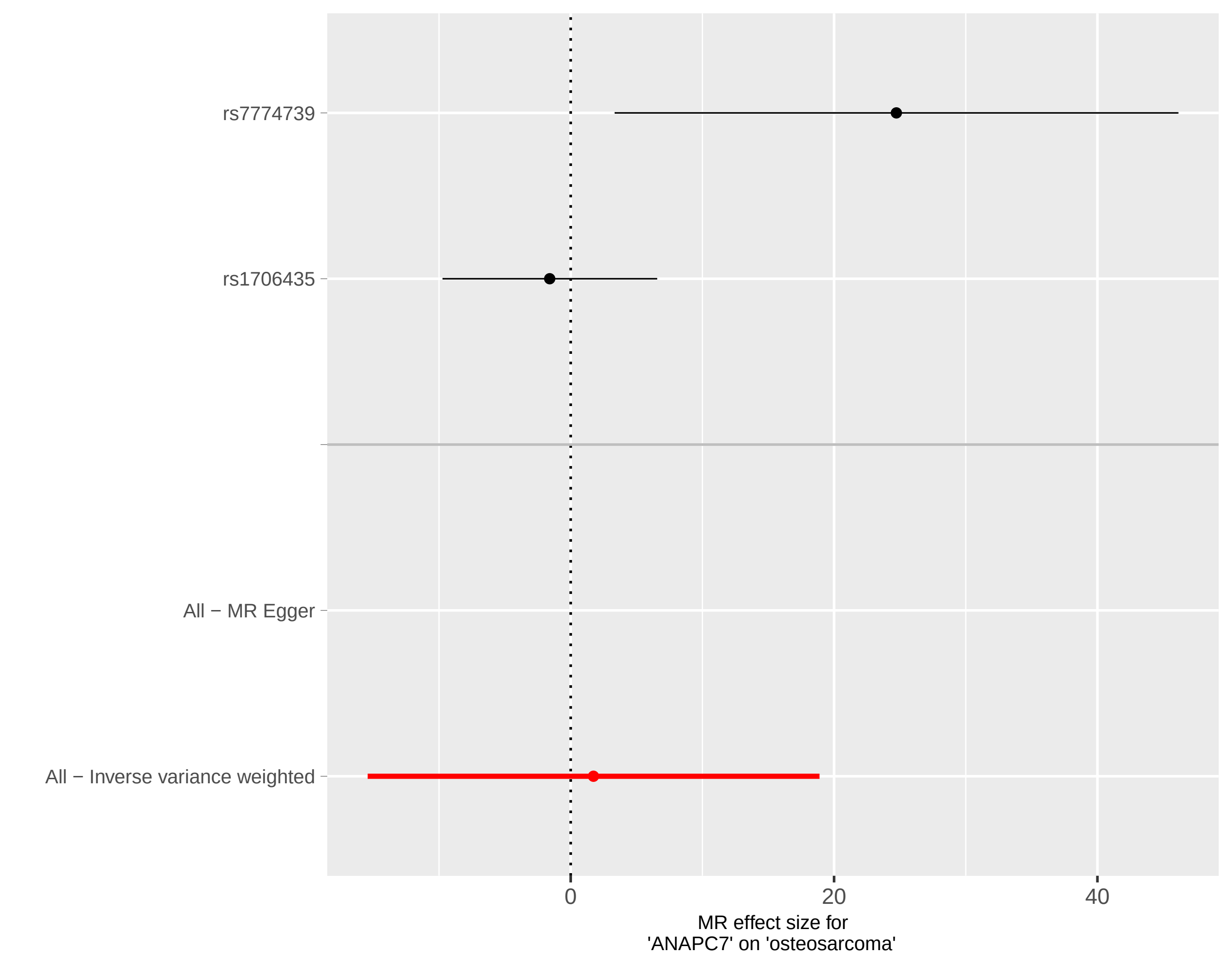

rs7774739
rs1706435
All − MR Egger
All − Inverse variance weighted
0
20
40
MR effect size for
'ANAPC7' on 'osteosarcoma'

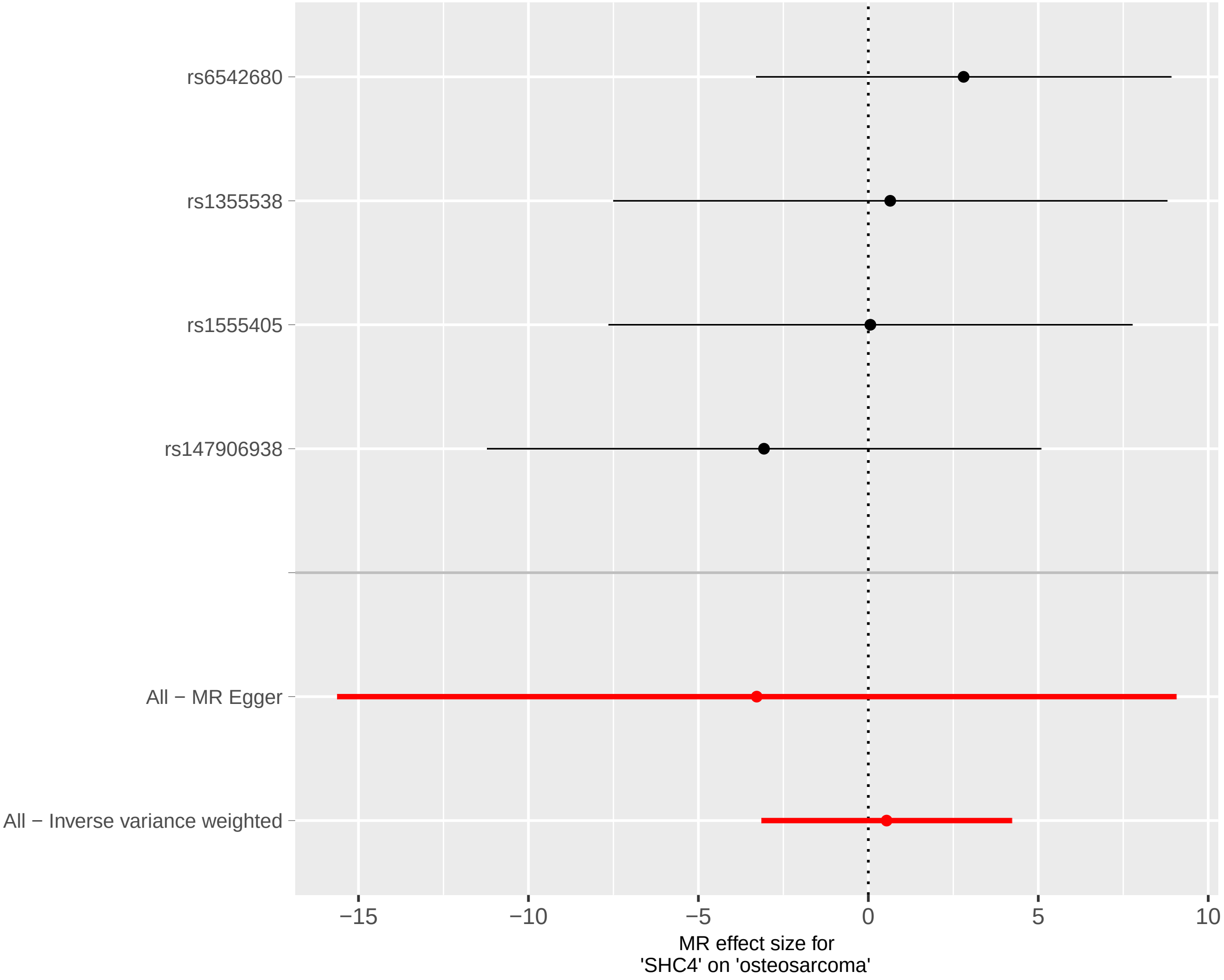
rs6542680
rs1355538
rs1555405
rs147906938
All − MR Egger
All − Inverse variance weighted
−15
−10
−5
0
5
10
MR effect size for
'SHC4' on 'osteosarcoma'

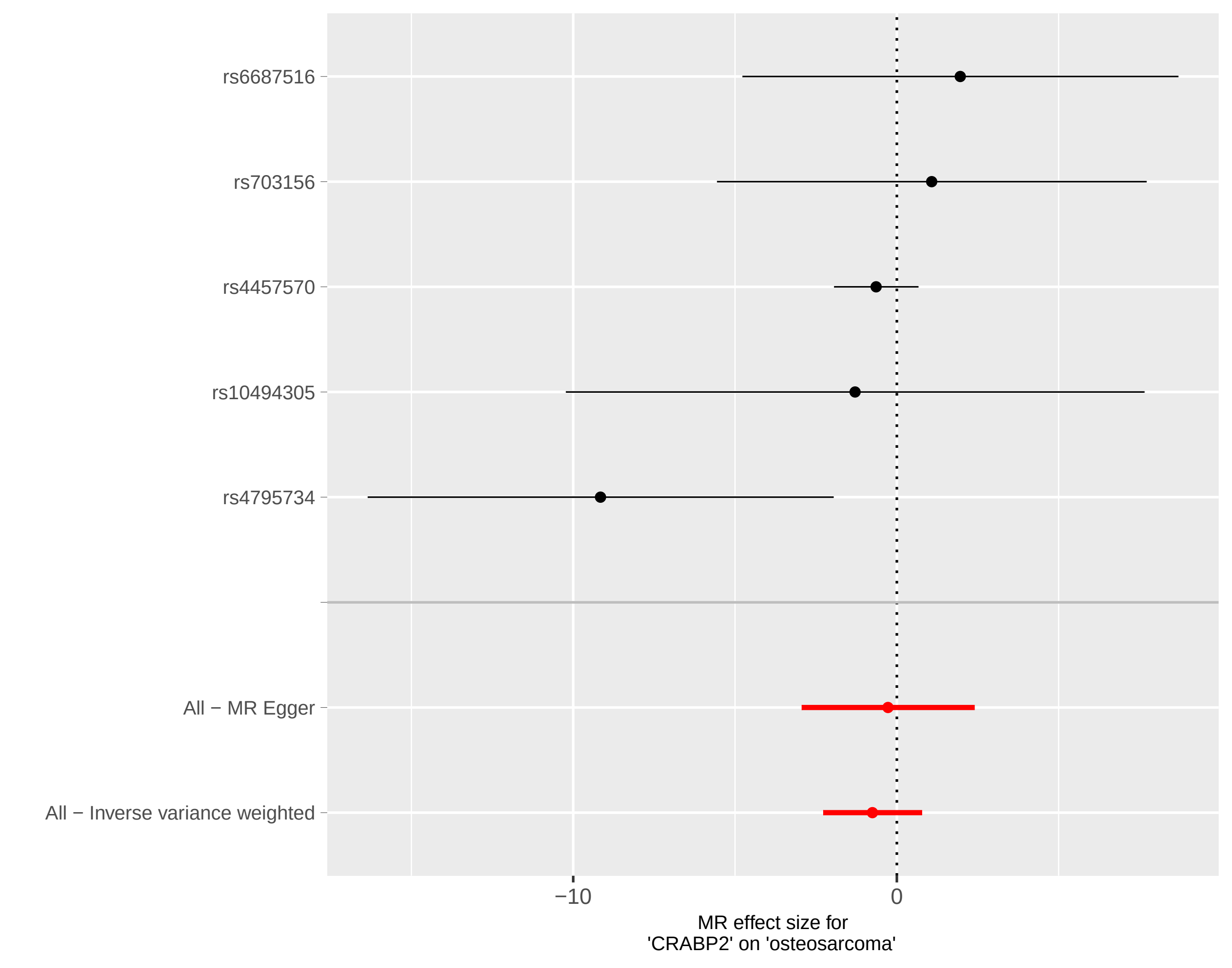
rs6687516
rs703156
rs4457570
rs10494305
rs4795734
All − MR Egger
All − Inverse variance weighted
−10
0
MR effect size for
'CRABP2' on 'osteosarcoma'

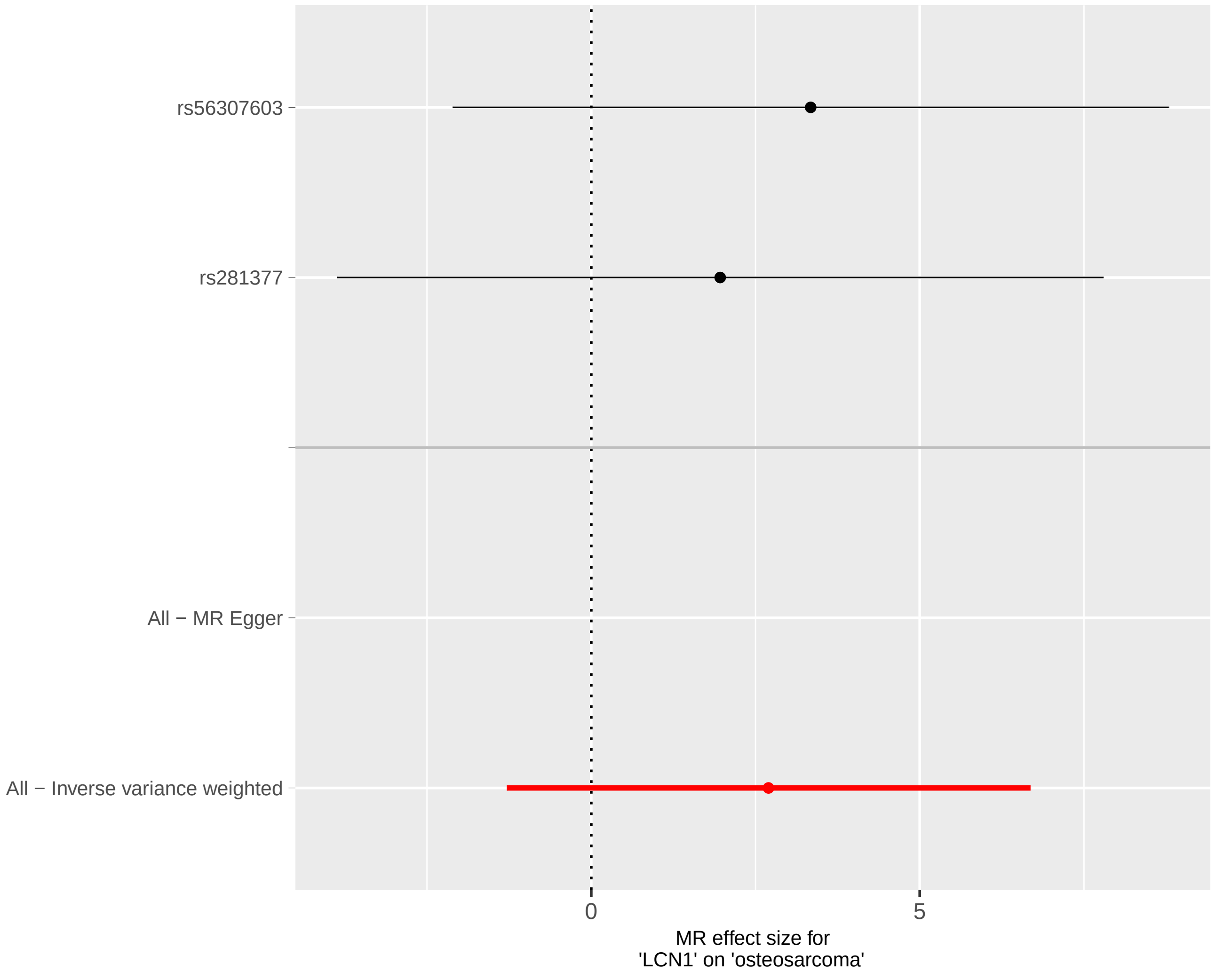
rs56307603
rs281377
All − MR Egger
All − Inverse variance weighted
0
5
MR effect size for
'LCN1' on 'osteosarcoma'

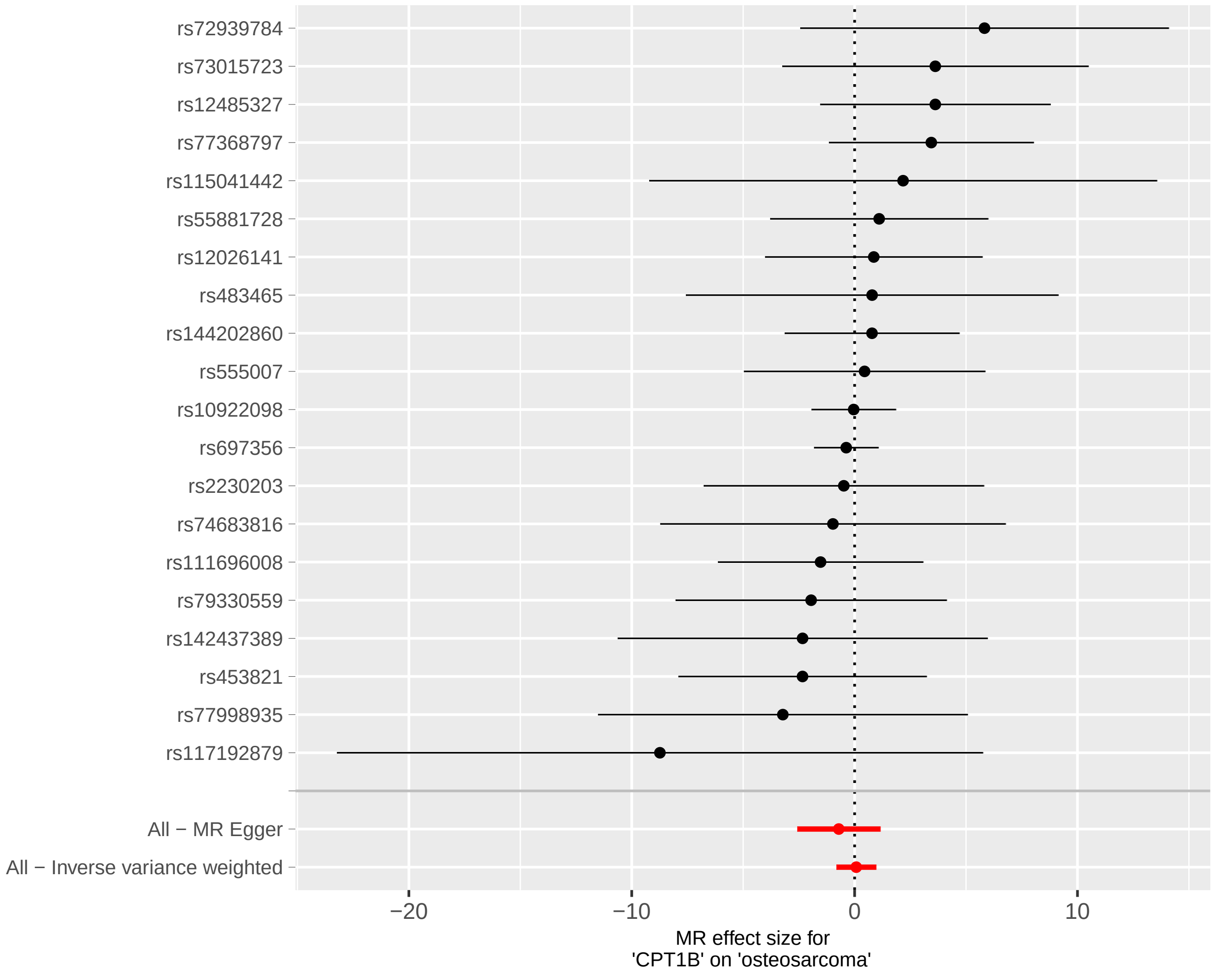

rs72939784
rs73015723
rs12485327
rs77368797
rs115041442
rs55881728
rs12026141
rs483465
rs144202860
rs555007
rs10922098
rs697356
rs2230203
rs74683816
rs111696008
rs79330559
rs142437389
rs453821
rs77998935
rs117192879
All − MR Egger
All − Inverse variance weighted
−20
−10
0
10
MR effect size for
'CPT1B' on 'osteosarcoma'

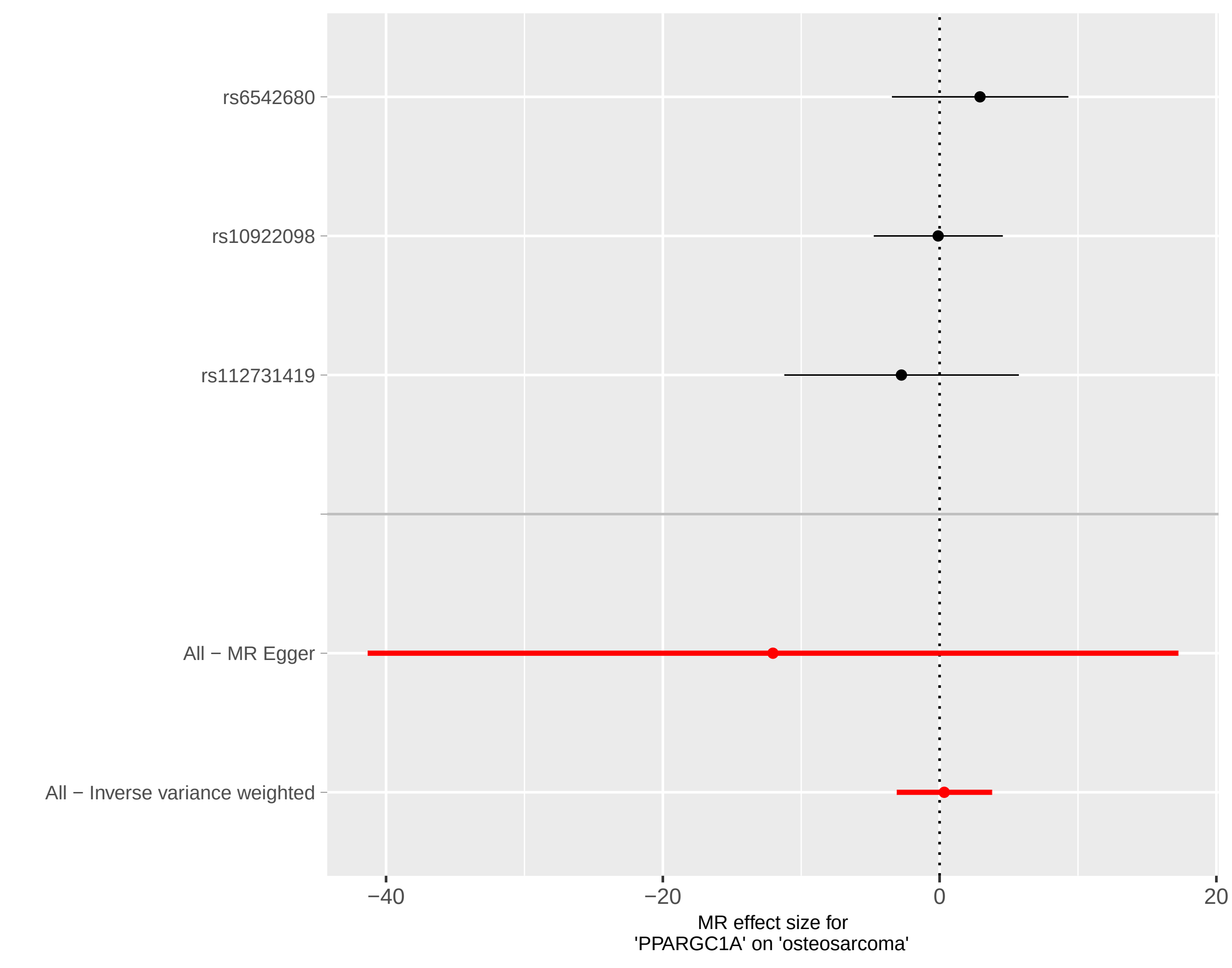
rs6542680
rs10922098
rs112731419
All − MR Egger
All − Inverse variance weighted
−40
−20
0
20
MR effect size for
'PPARGC1A' on 'osteosarcoma'

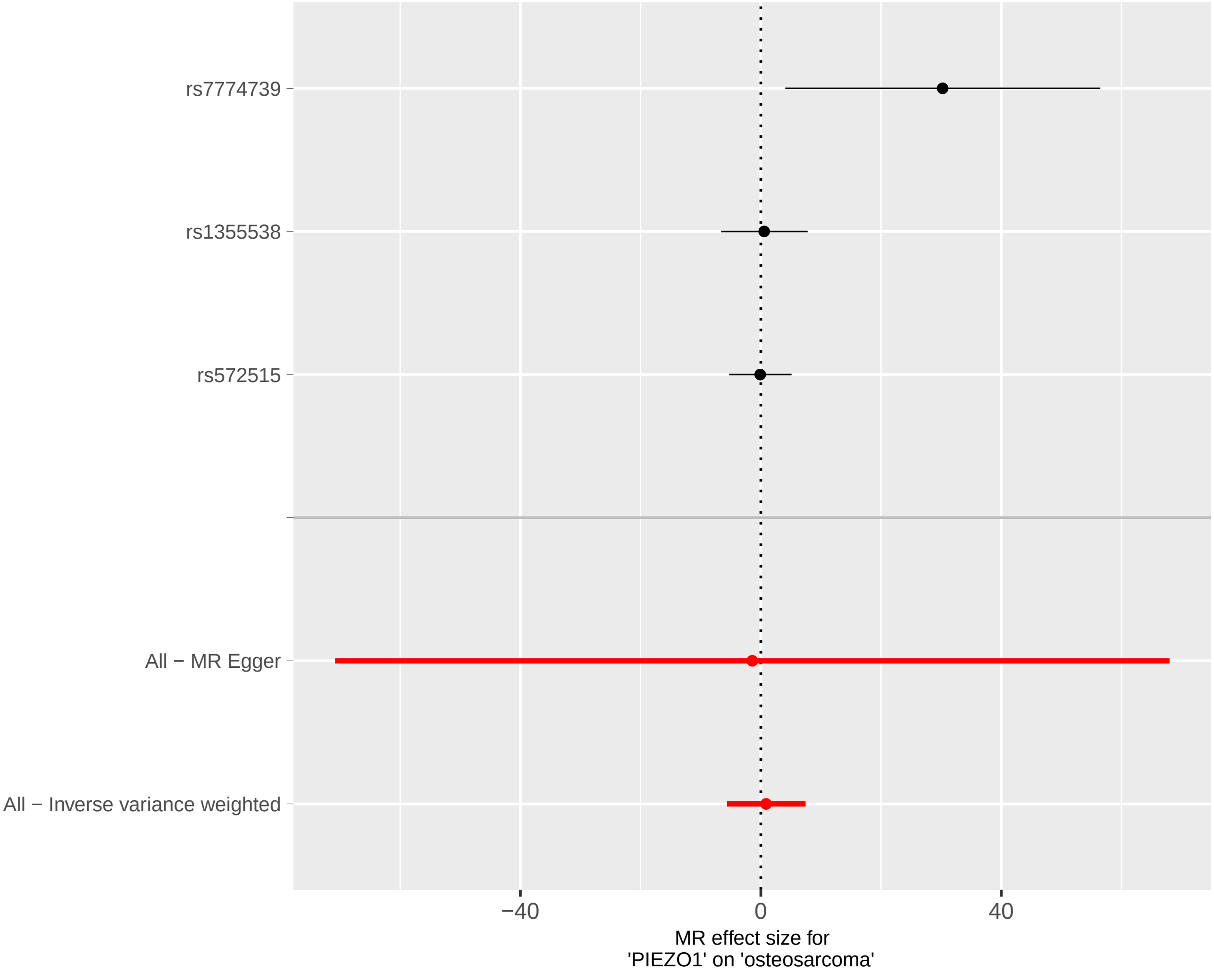

rs7774739
rs1355538
rs572515
All − MR Egger
All − Inverse variance weighted
−40
0
40
MR effect size for
'PIEZO1' on 'osteosarcoma'

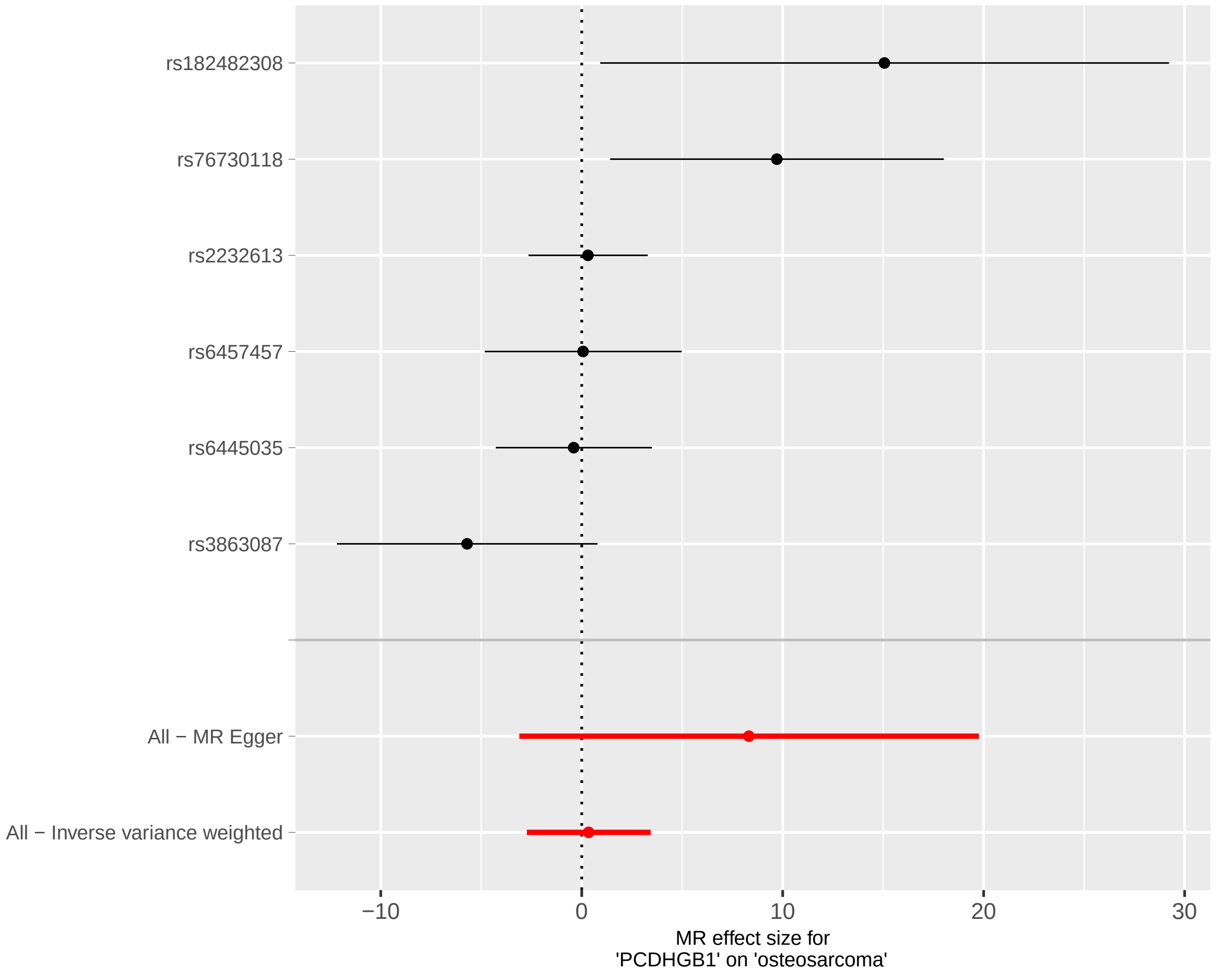
rs182482308
rs76730118
rs2232613
rs6457457
rs6445035
rs3863087
All − MR Egger
All − Inverse variance weighted
−10
0
10
20
30
MR effect size for
'PCDHGB1' on 'osteosarcoma'

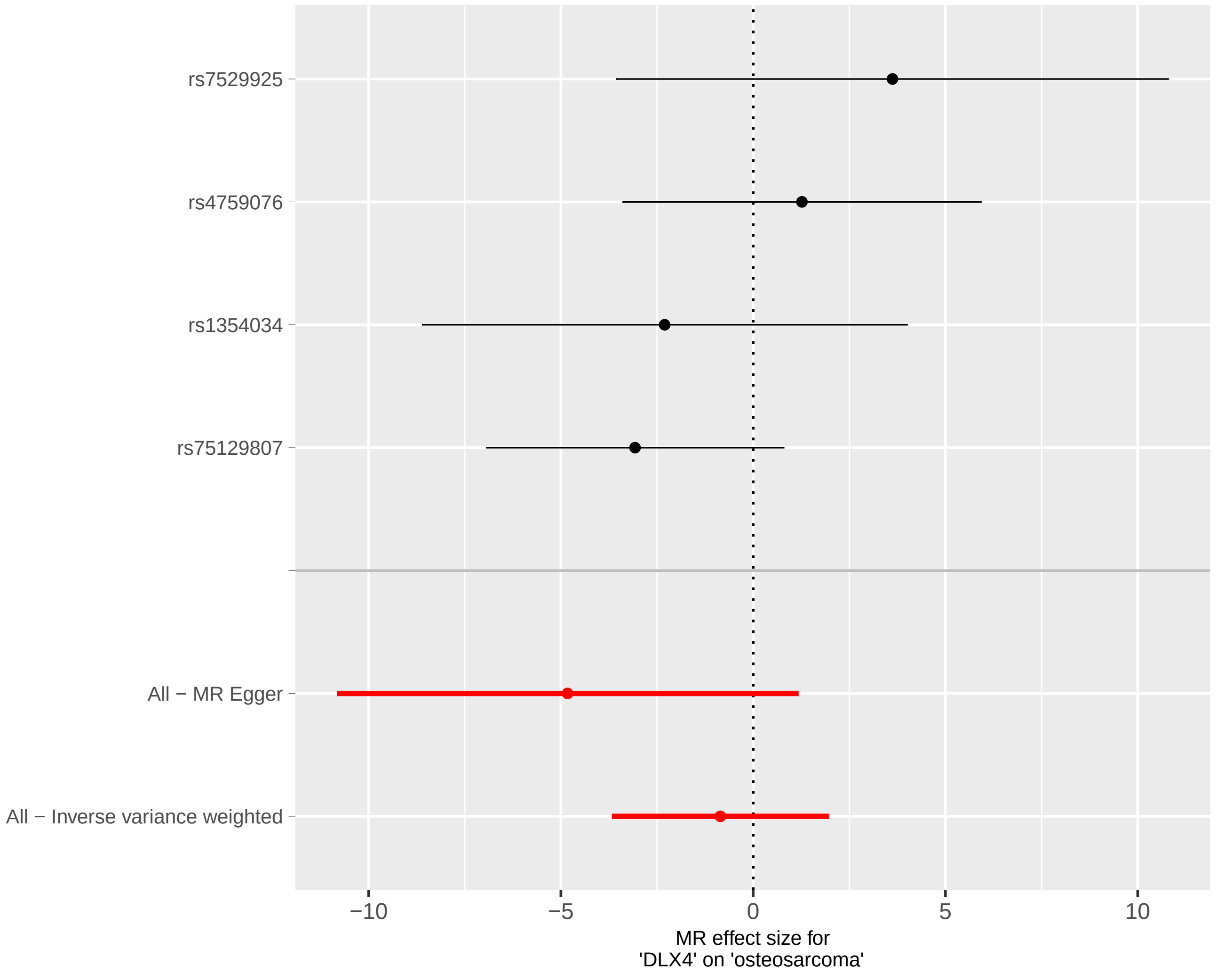

rs7529925
rs4759076
rs1354034
rs75129807
All − MR Egger
All − Inverse variance weighted
−10
−5
0
5
10
MR effect size for
'DLX4' on 'osteosarcoma'

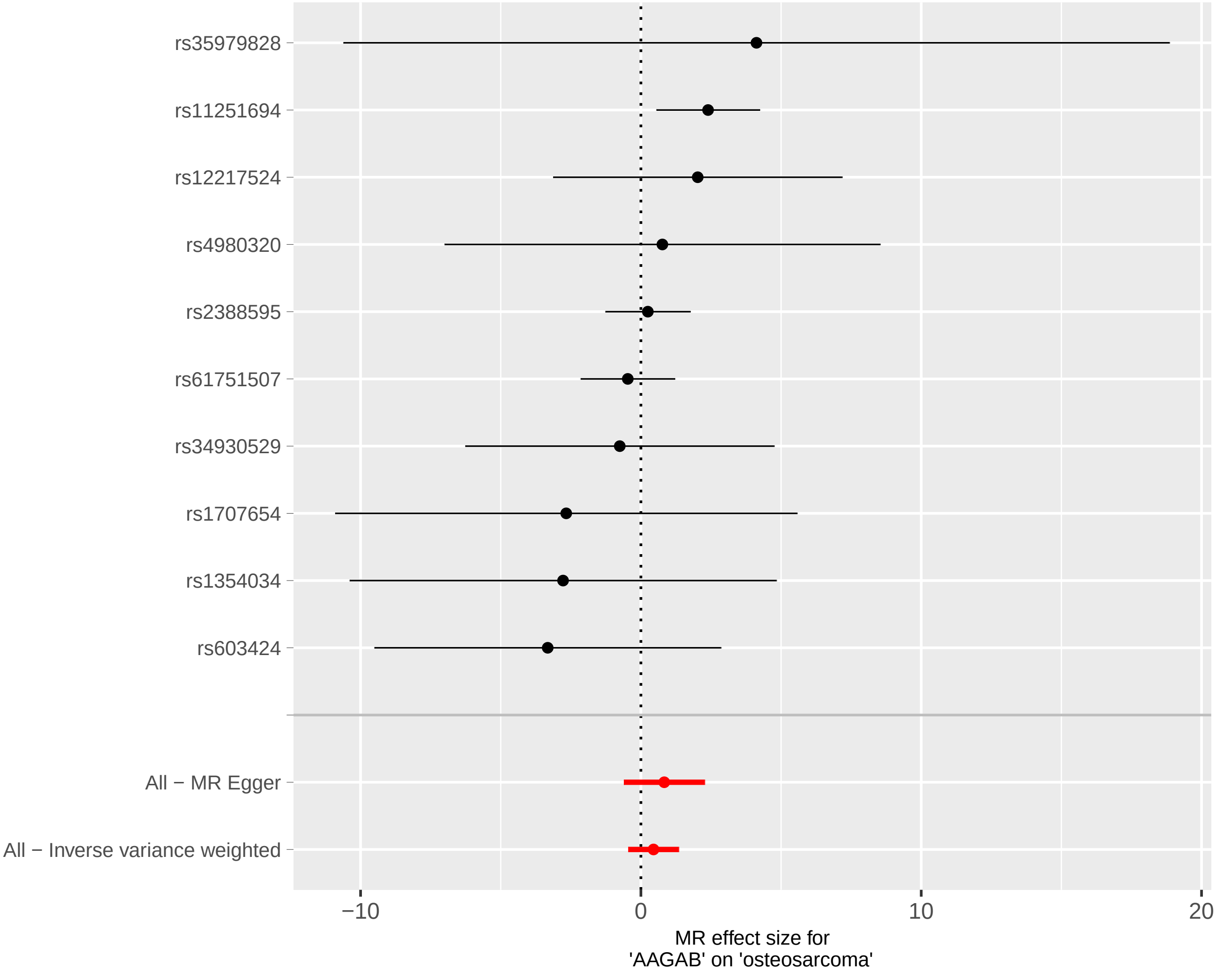
rs35979828
rs11251694
rs12217524
rs4980320
rs2388595
rs61751507
rs34930529
rs1707654
rs1354034
rs603424
All − MR Egger
All − Inverse variance weighted
−10
0
10
20
MR effect size for
'AAGAB' on 'osteosarcoma'

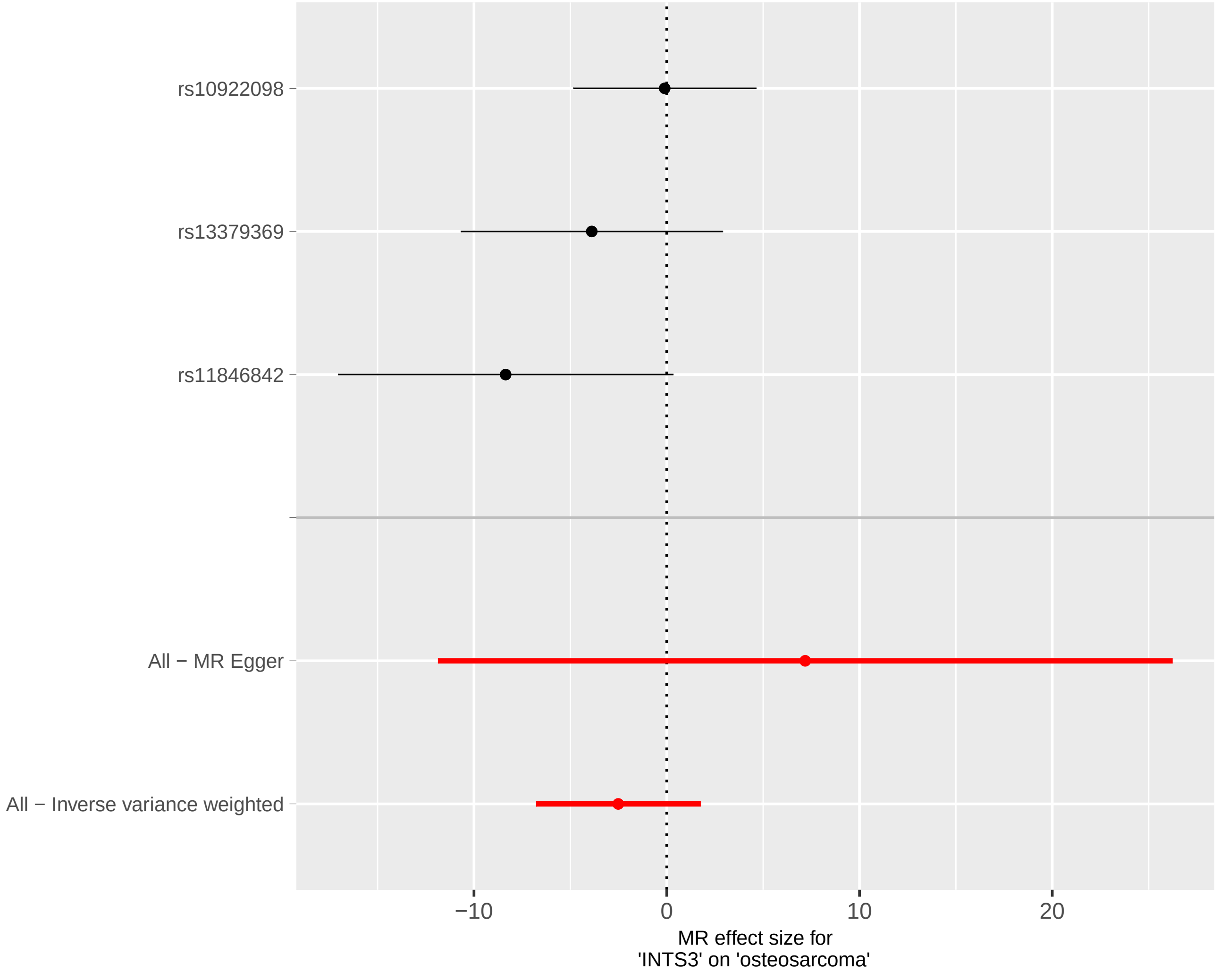

rs10922098
rs13379369
rs11846842
All − MR Egger
All − Inverse variance weighted
−10
0
10
20
MR effect size for
'INTS3' on 'osteosarcoma'

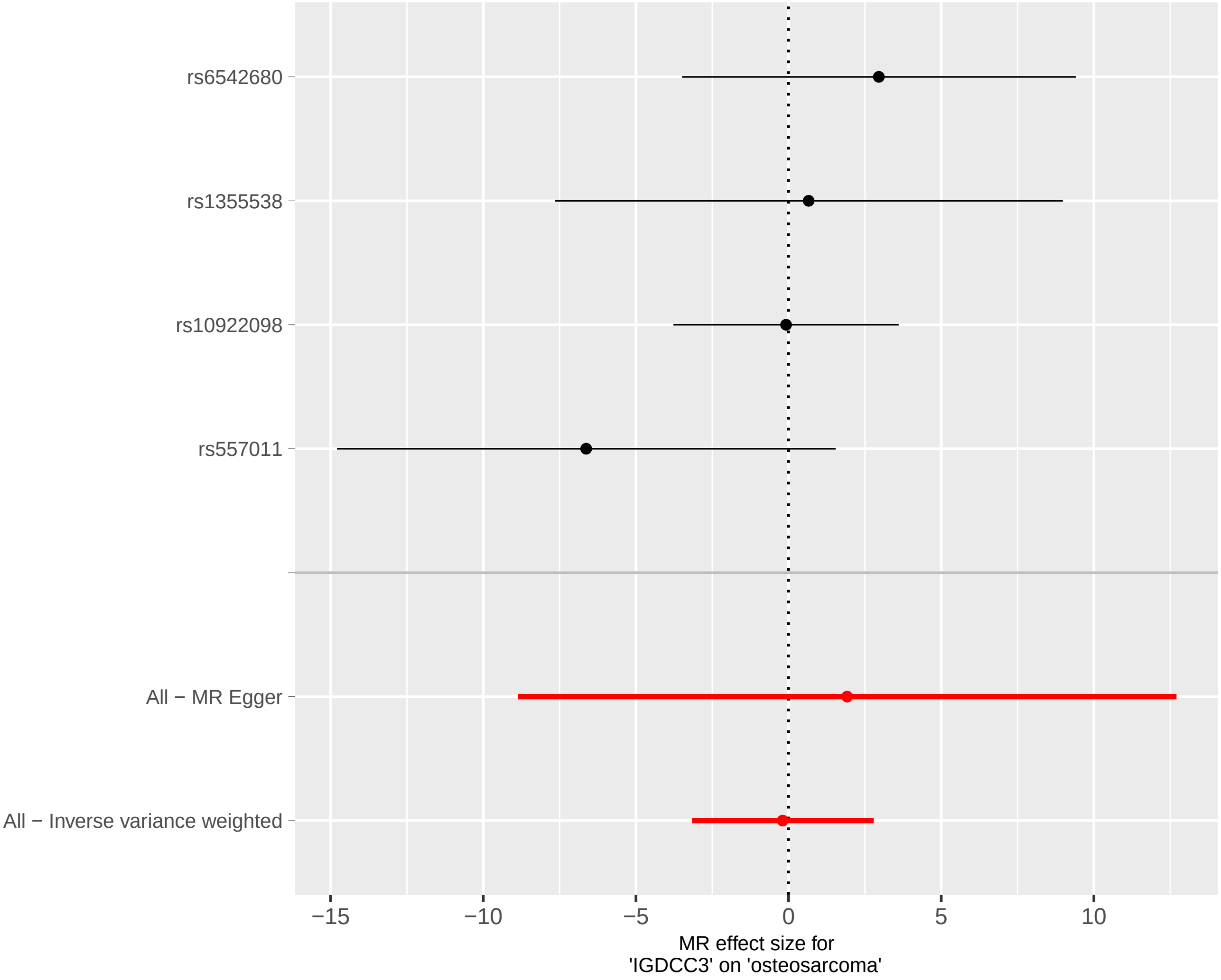
rs6542680
rs1355538
rs10922098
rs557011
All − MR Egger
All − Inverse variance weighted
−15
−10
−5
0
5
10
MR effect size for
'IGDCC3' on 'osteosarcoma'

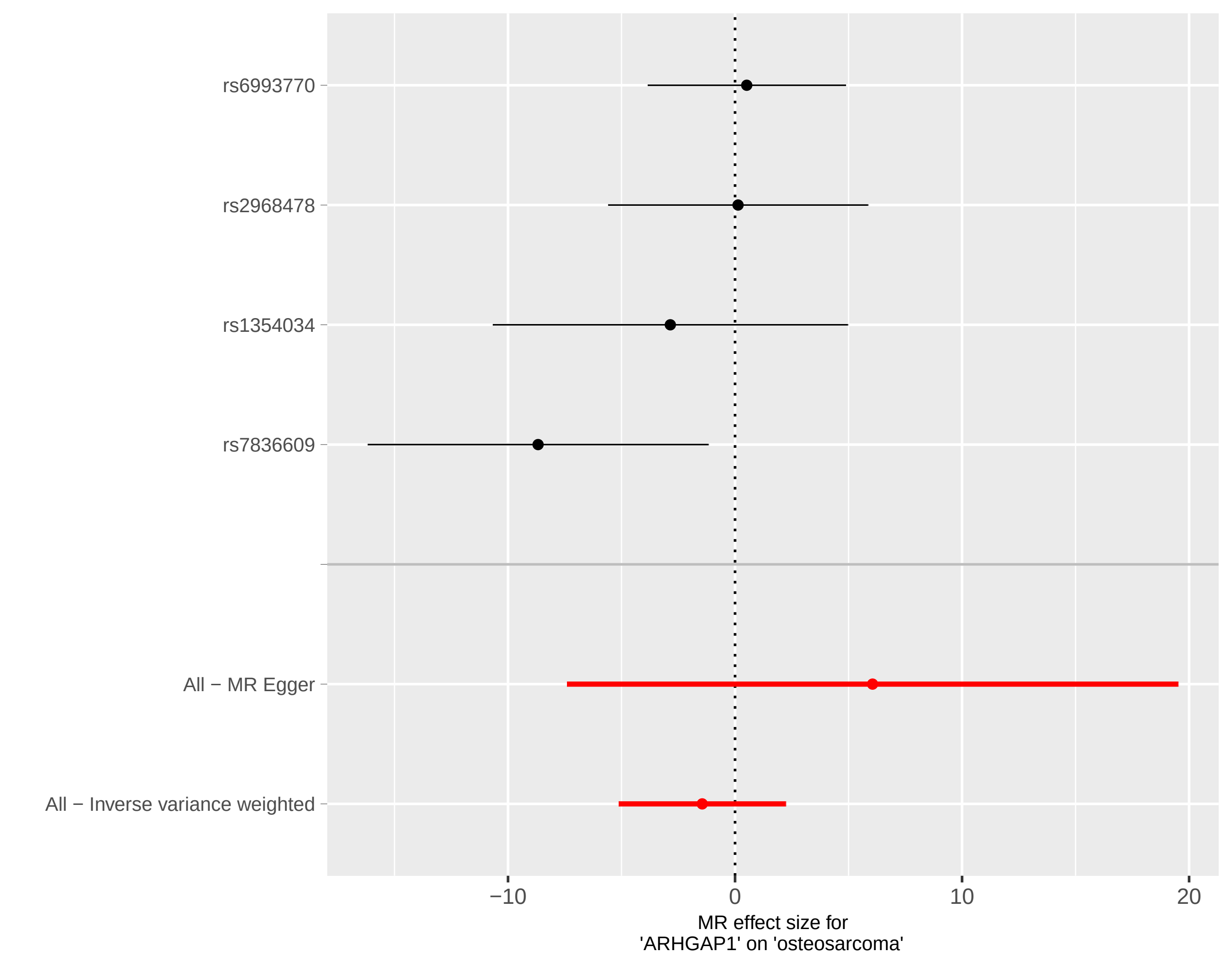
rs6993770
rs2968478
rs1354034
rs7836609
All − MR Egger
All − Inverse variance weighted
−10
0
10
20
MR effect size for
'ARHGAP1' on 'osteosarcoma'

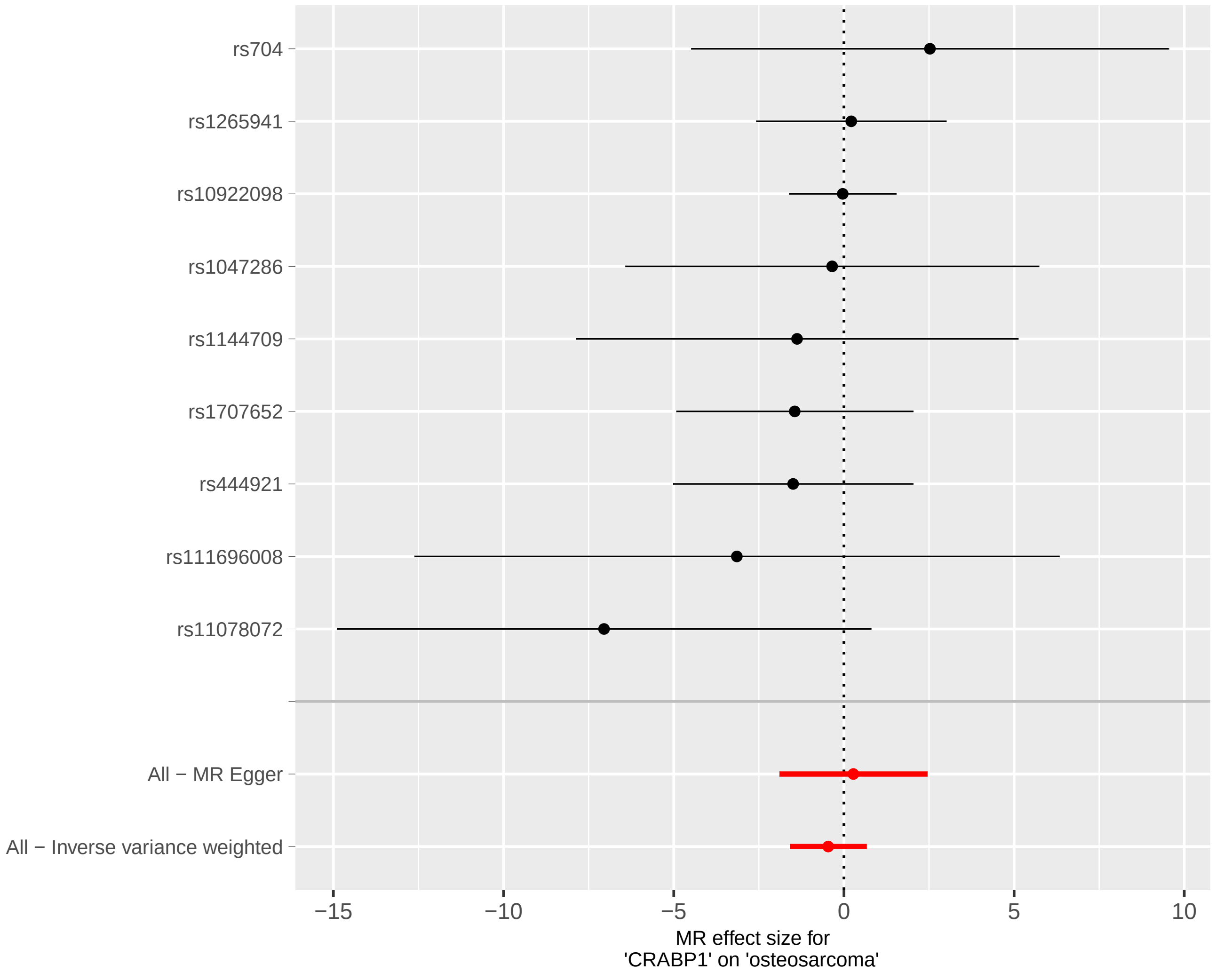

rs704
rs1265941
rs10922098
rs1047286
rs1144709
rs1707652
rs444921
rs111696008
rs11078072
All − MR Egger
All − Inverse variance weighted
−15
−10
−5
0
5
10
MR effect size for
'CRABP1' on 'osteosarcoma'

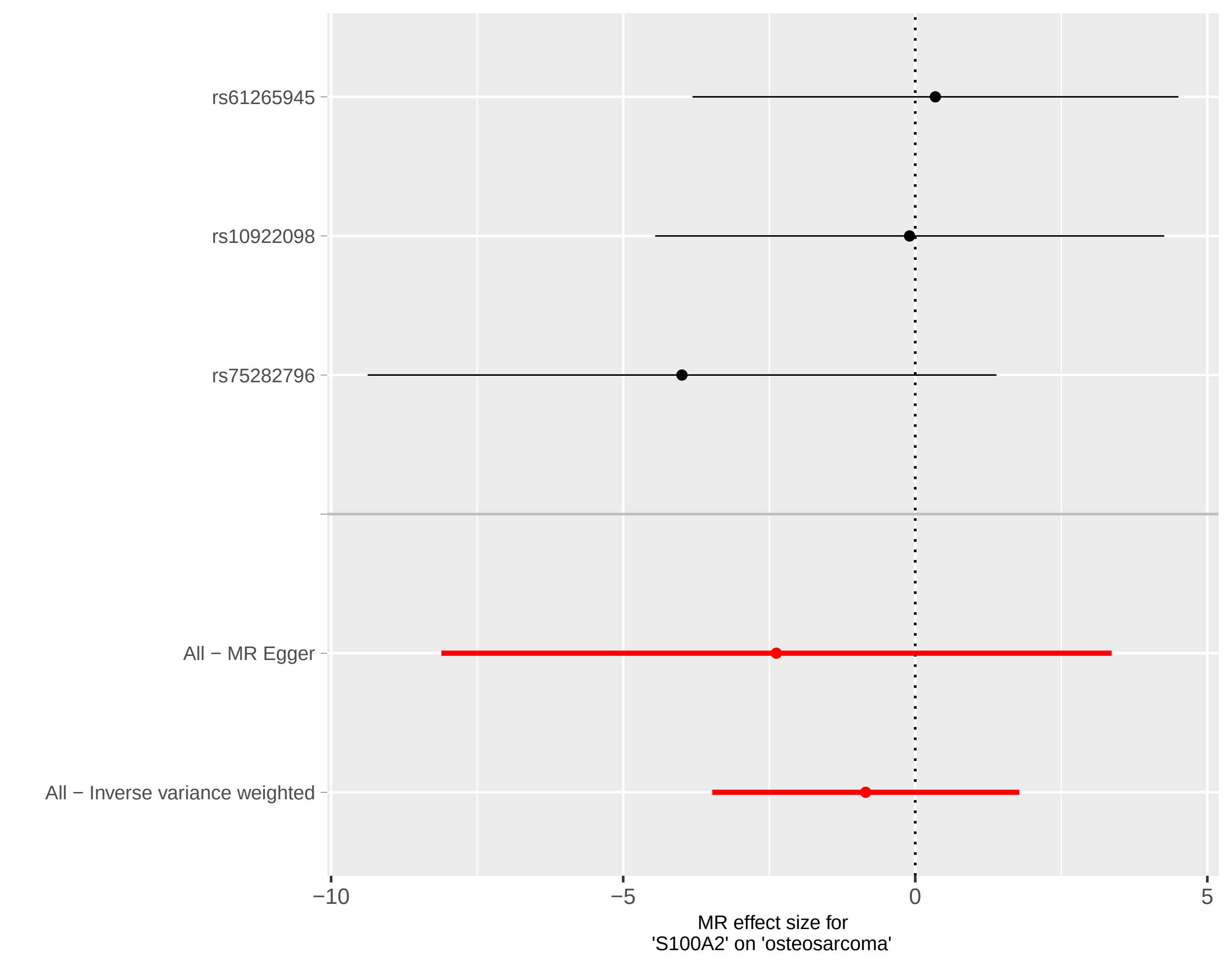

rs61265945
rs10922098
rs75282796
All − MR Egger
All − Inverse variance weighted
−10
−5
0
5
MR effect size for
'S100A2' on 'osteosarcoma'

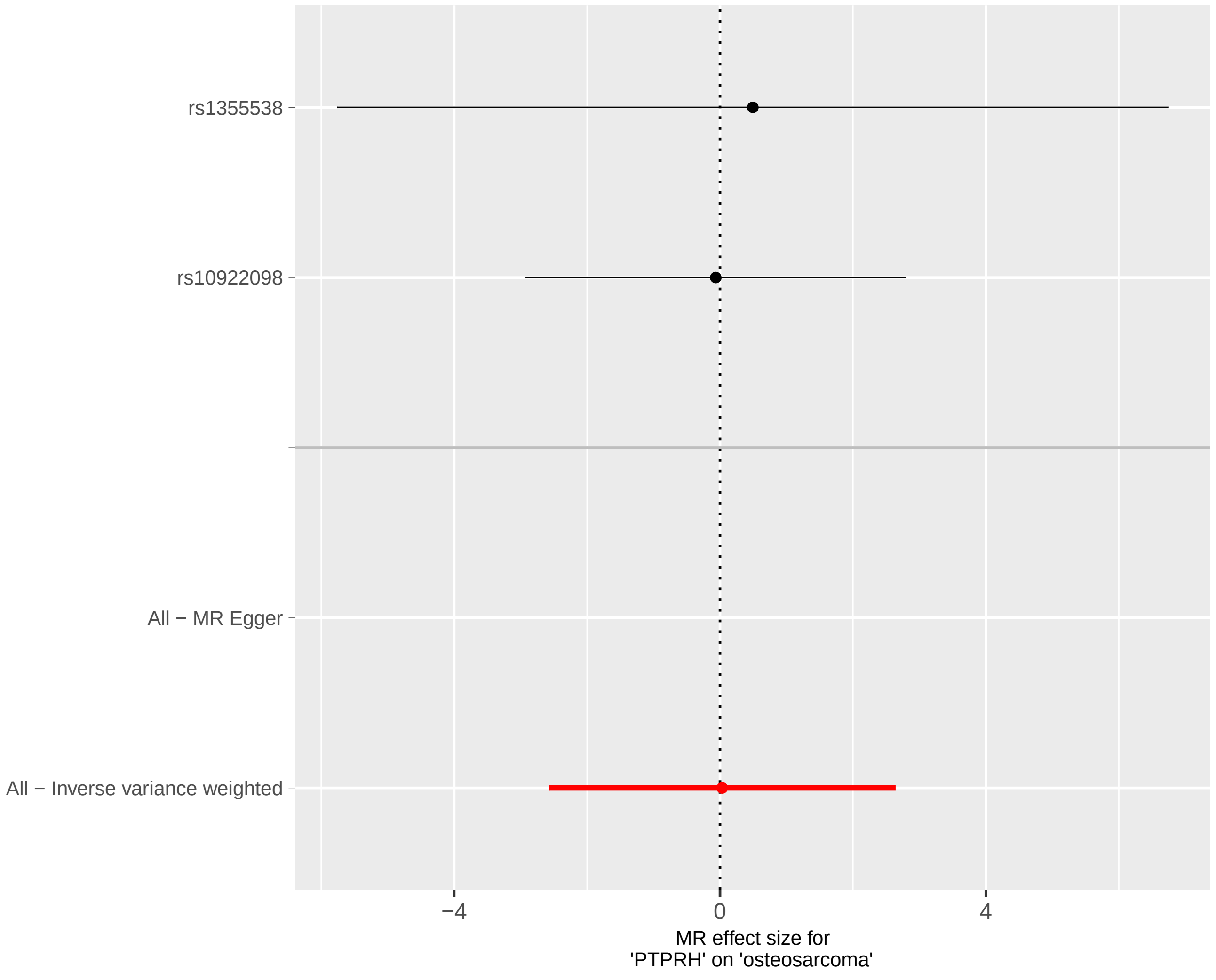

rs1355538
rs10922098
All − MR Egger
All − Inverse variance weighted
−4
0
4
MR effect size for
'PTPRH' on 'osteosarcoma'

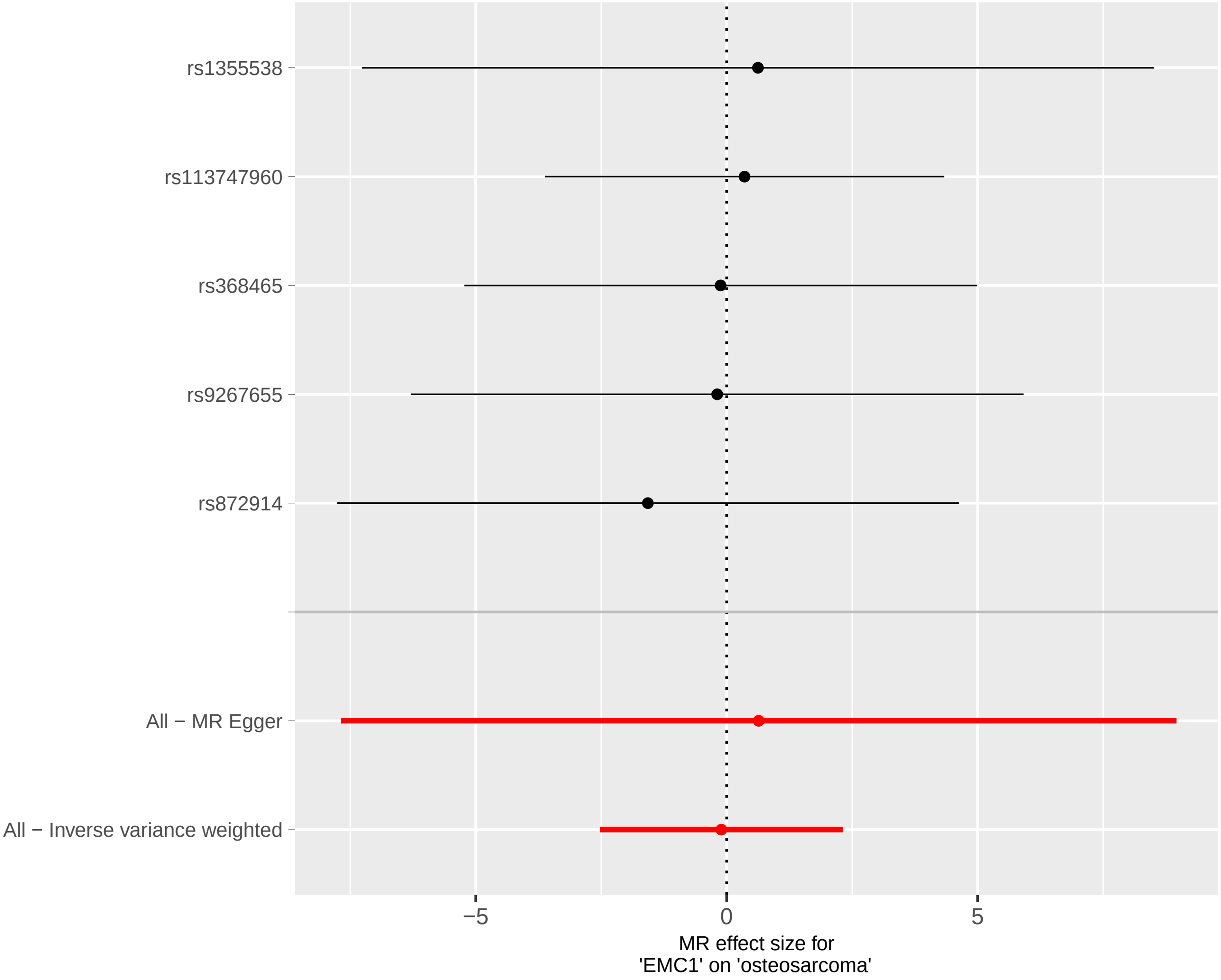

rs1355538
rs113747960
rs368465
rs9267655
rs872914
All − MR Egger
All − Inverse variance weighted
−5
0
5
MR effect size for
'EMC1' on 'osteosarcoma'

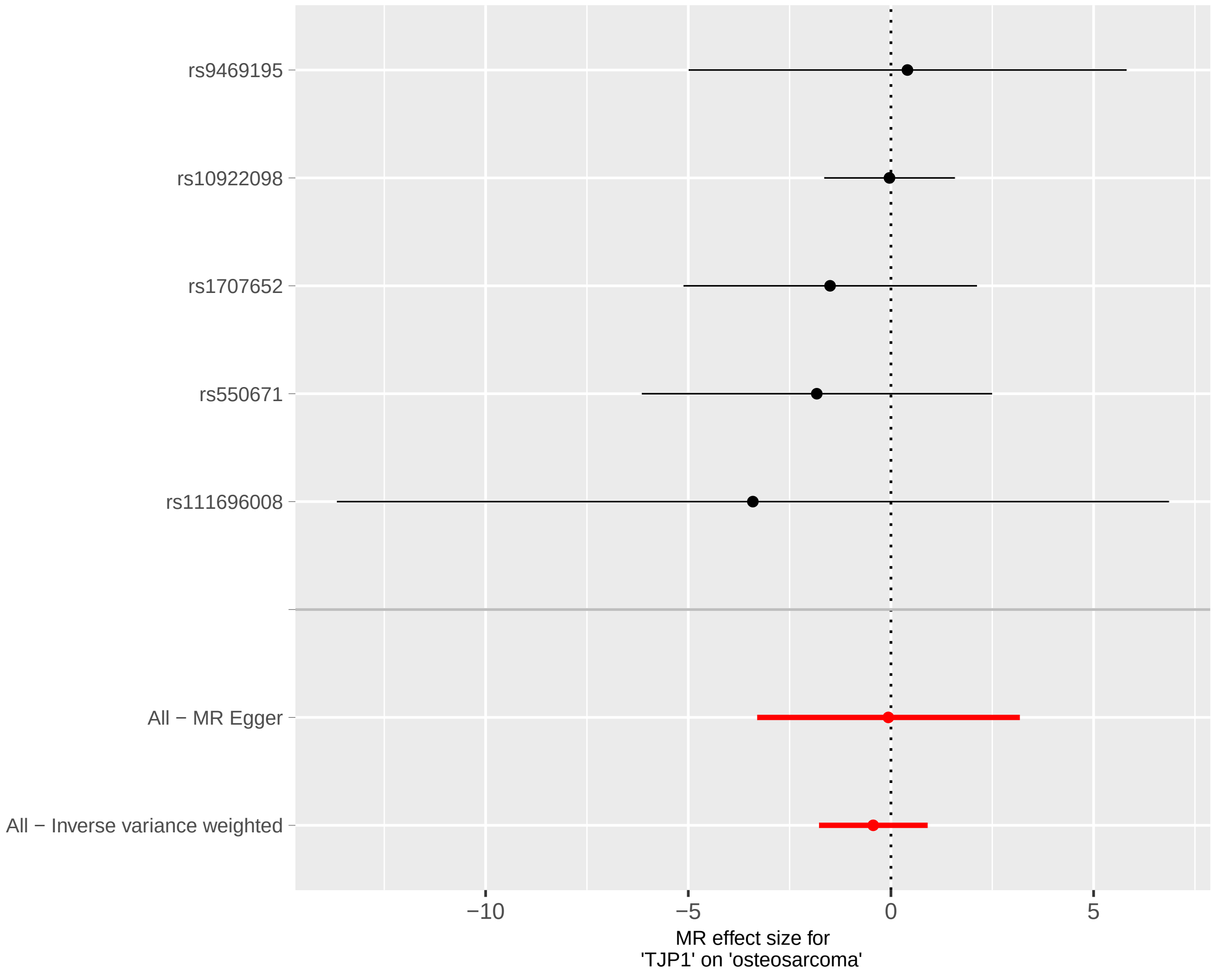

rs9469195
rs10922098
rs1707652
rs550671
rs111696008
All − MR Egger
All − Inverse variance weighted
−10
−5
0
5
MR effect size for
'TJP1' on 'osteosarcoma'

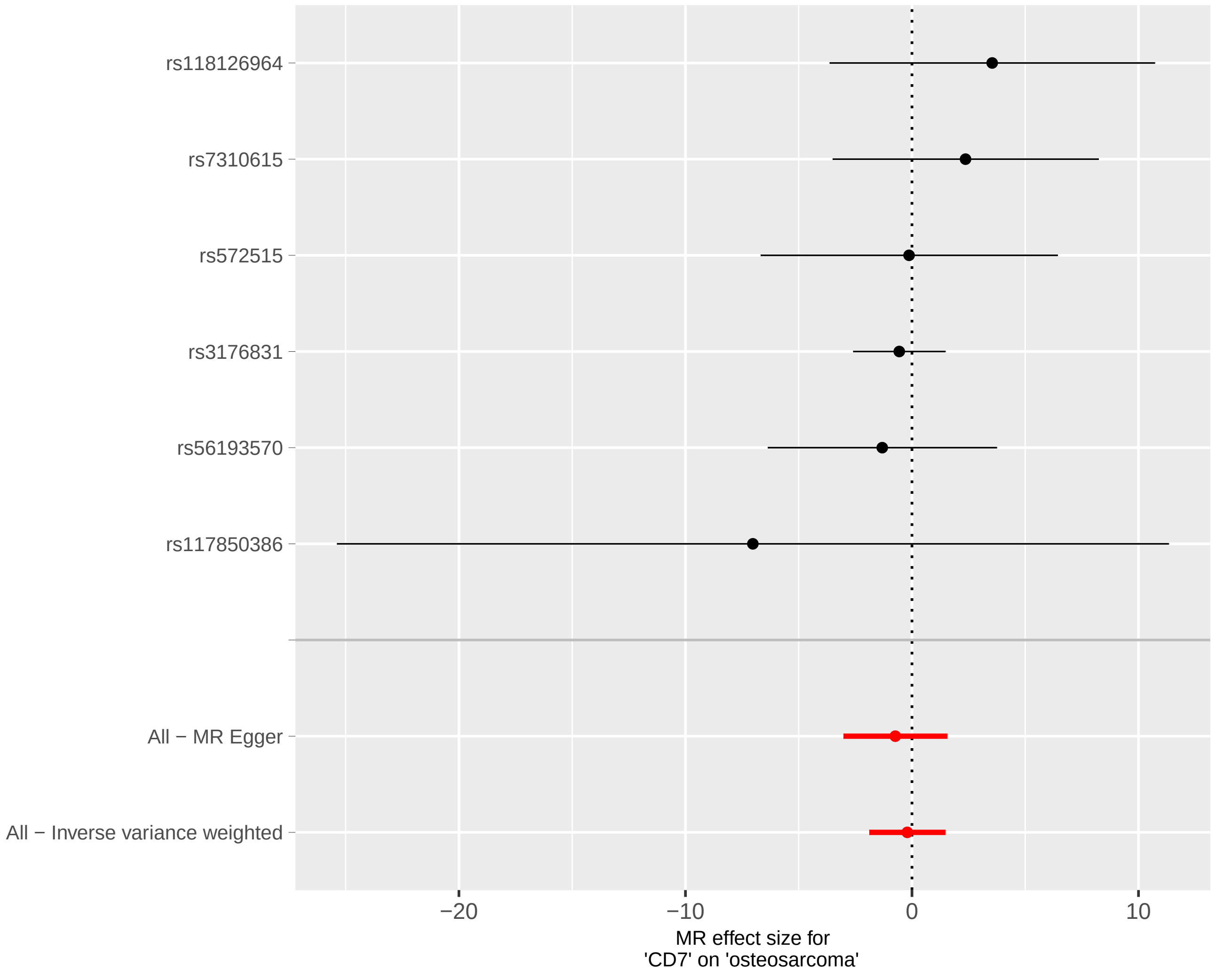
rs118126964
rs7310615
rs572515
rs3176831
rs56193570
rs117850386
All − MR Egger
All − Inverse variance weighted
−20
−10
0
10
MR effect size for
'CD7' on 'osteosarcoma'

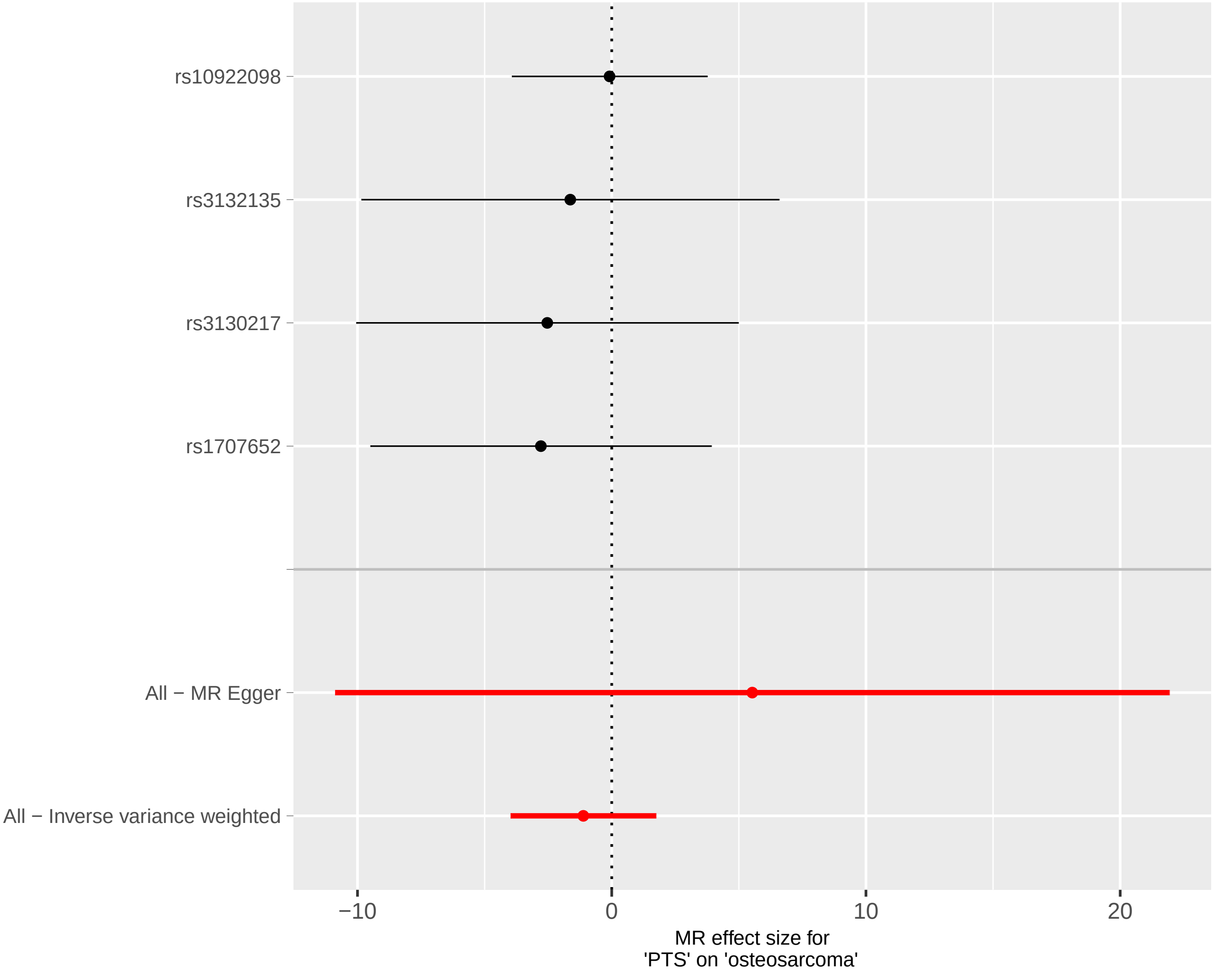
rs10922098
rs3132135
rs3130217
rs1707652
All − MR Egger
All − Inverse variance weighted
−10
0
10
20
MR effect size for
'PTS' on 'osteosarcoma'

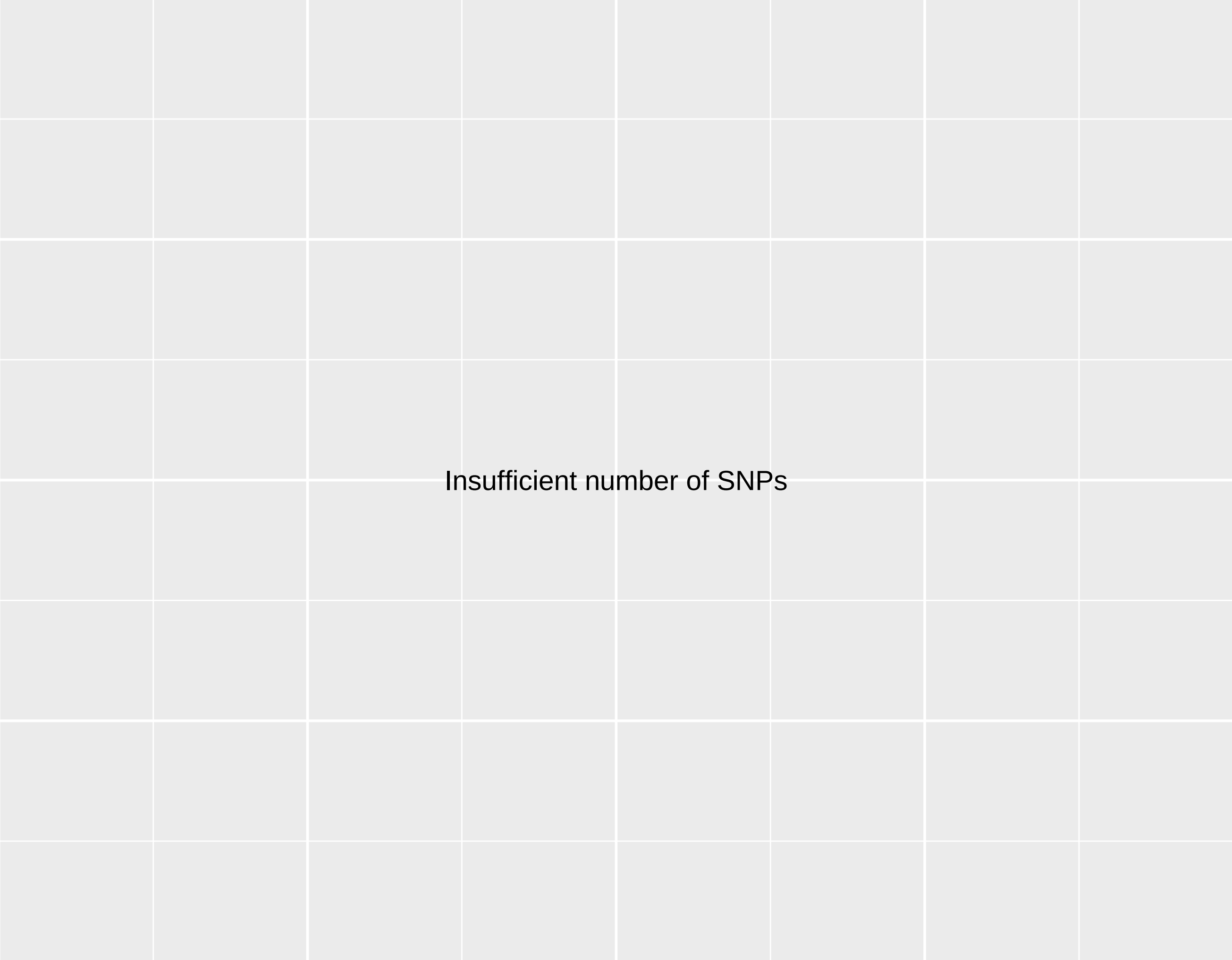
Insufficient number of SNPs

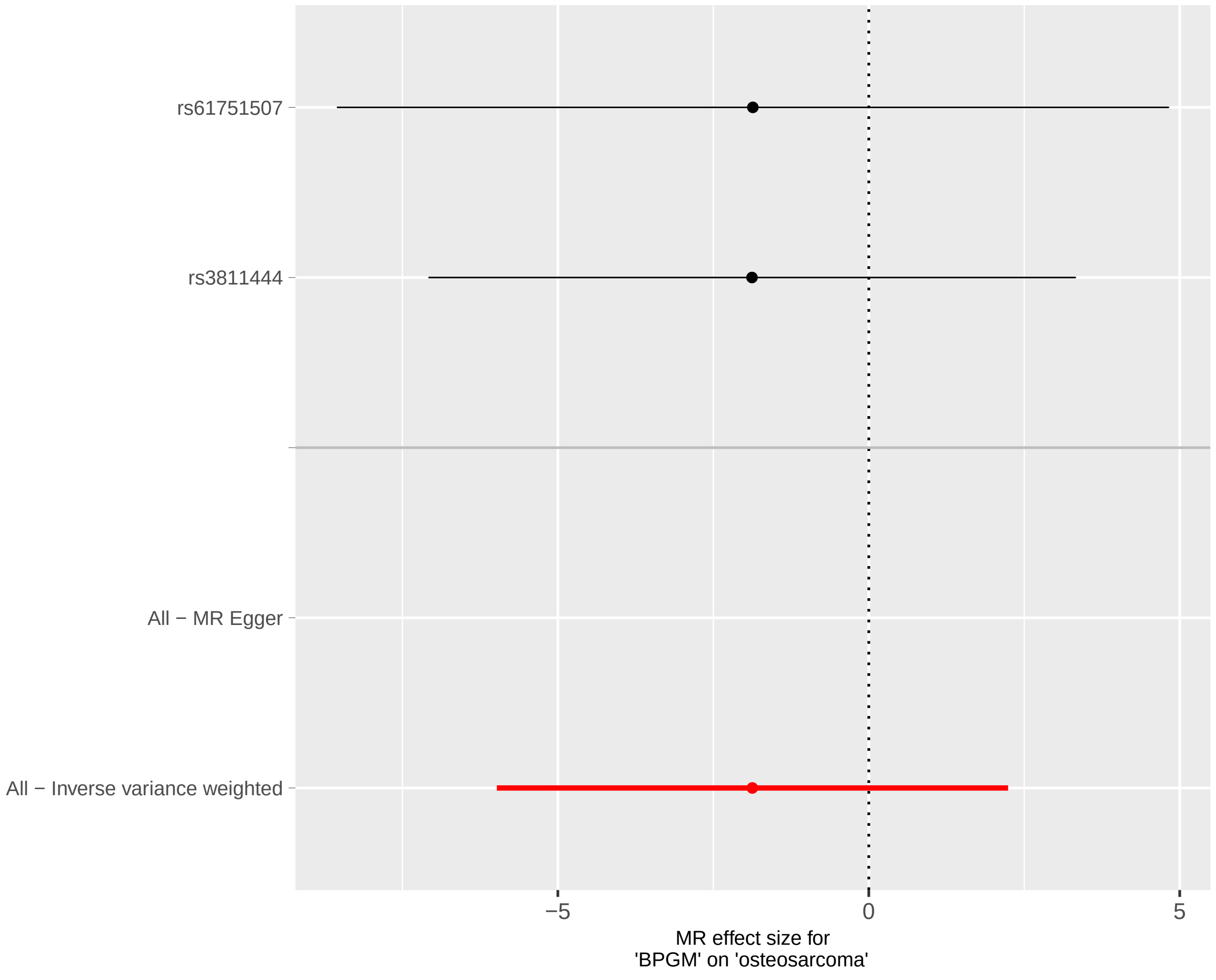

rs61751507
rs3811444
All − MR Egger
All − Inverse variance weighted
−5
0
5
MR effect size for
'BPGM' on 'osteosarcoma'

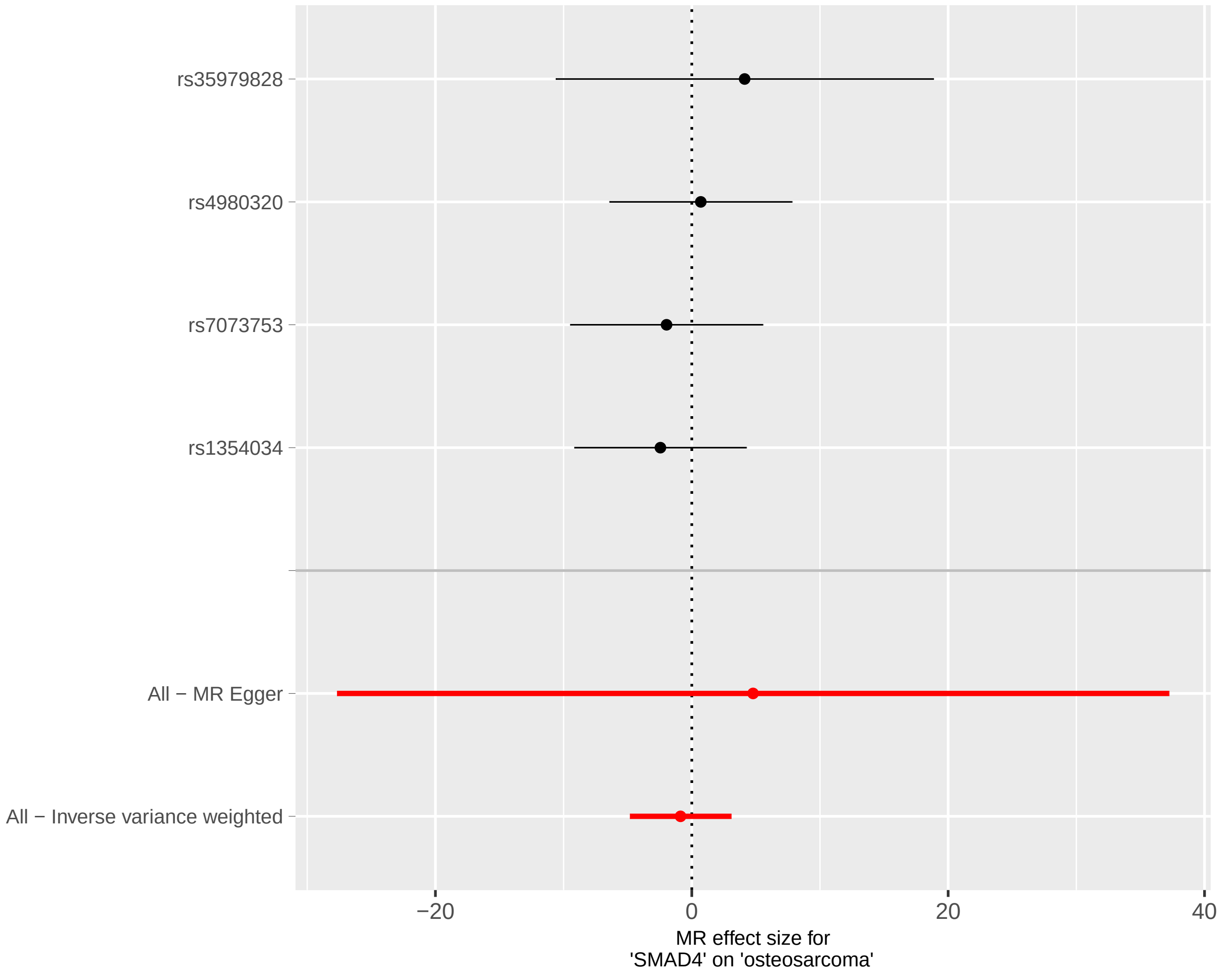
rs35979828
rs4980320
rs7073753
rs1354034
All − MR Egger
All − Inverse variance weighted
−20
0
20
40
MR effect size for
'SMAD4' on 'osteosarcoma'

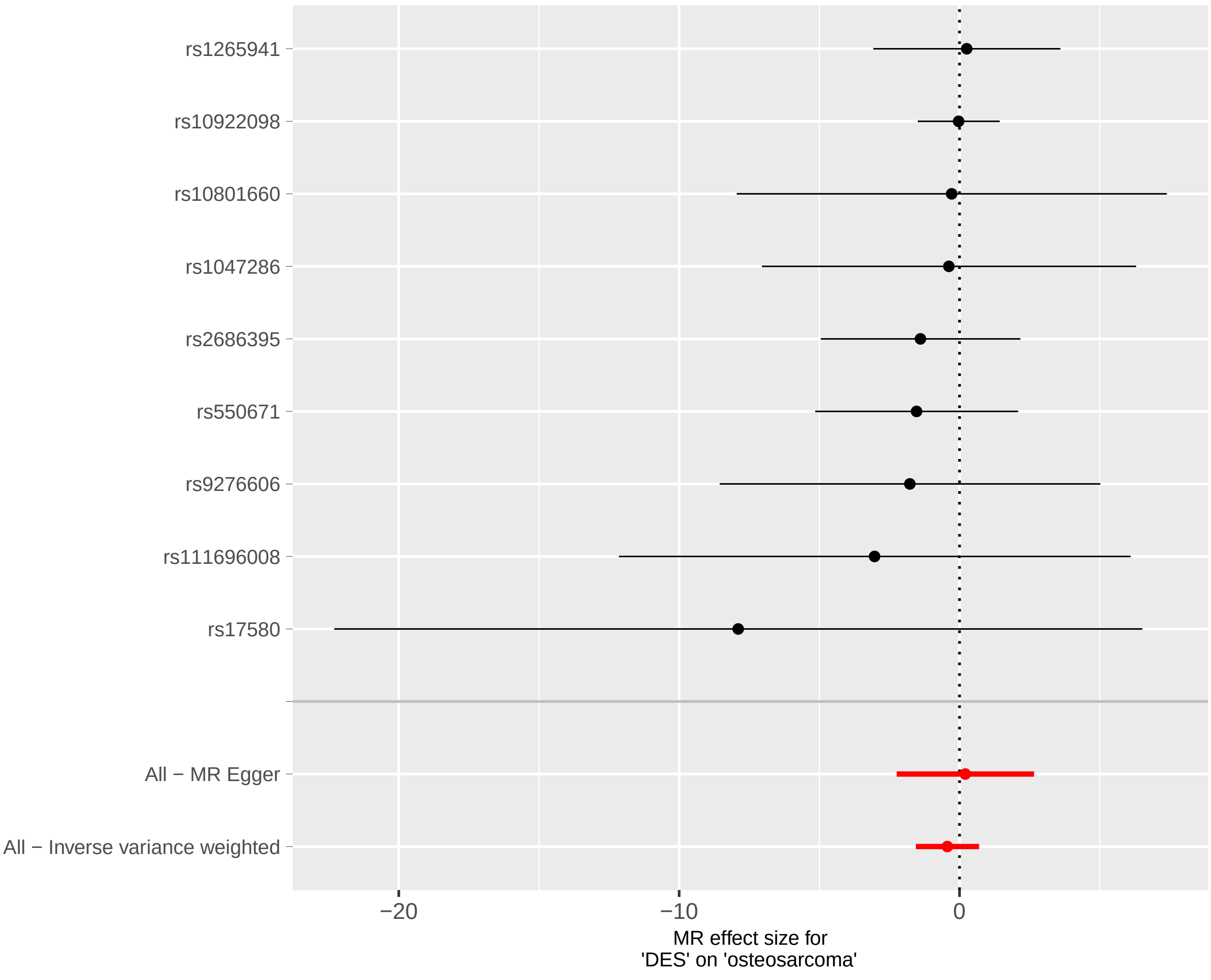
rs1265941
rs10922098
rs10801660
rs1047286
rs2686395
rs550671
rs9276606
rs111696008
rs17580
All − MR Egger
All − Inverse variance weighted
−20
−10
0
MR effect size for
'DES' on 'osteosarcoma'

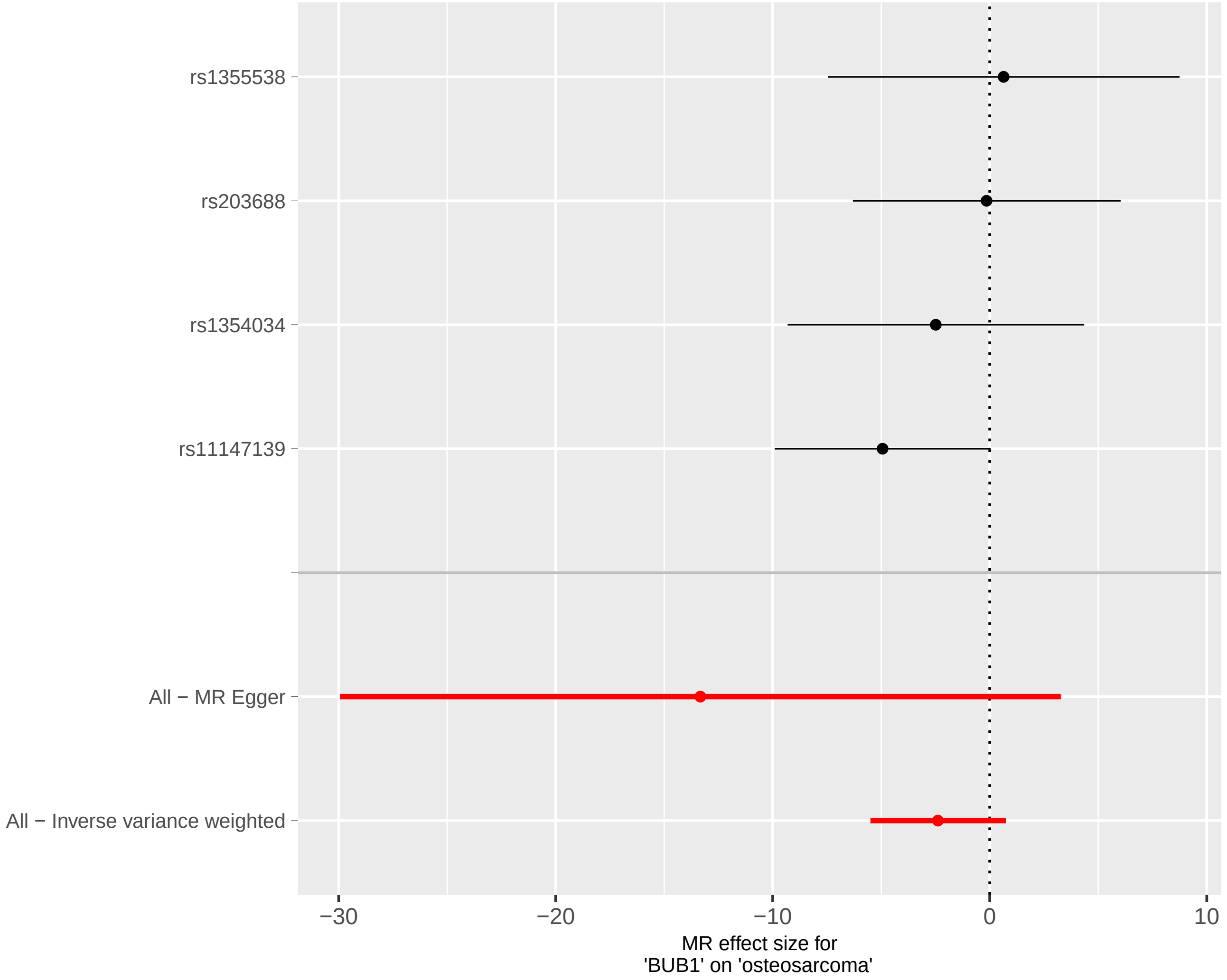

rs1355538
rs203688
rs1354034
rs11147139
All − MR Egger
All − Inverse variance weighted
−30
−20
−10
0
10
MR effect size for
'BUB1' on 'osteosarcoma'

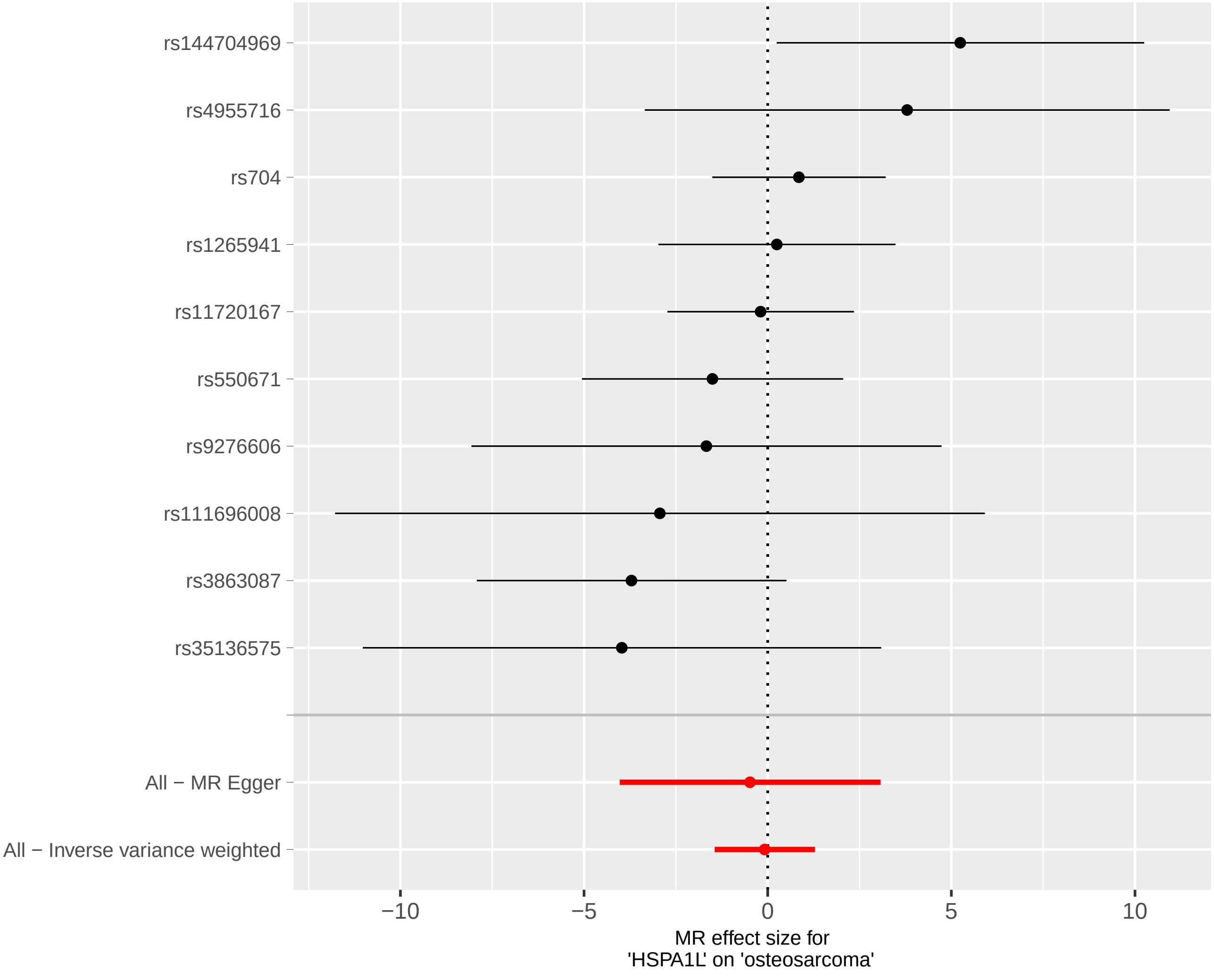
rs144704969
rs4955716
rs704
rs1265941
rs11720167
rs550671
rs9276606
rs111696008
rs3863087
rs35136575
All − MR Egger
All − Inverse variance weighted
−10
−5
0
5
10
MR effect size for
'HSPA1L' on 'osteosarcoma'

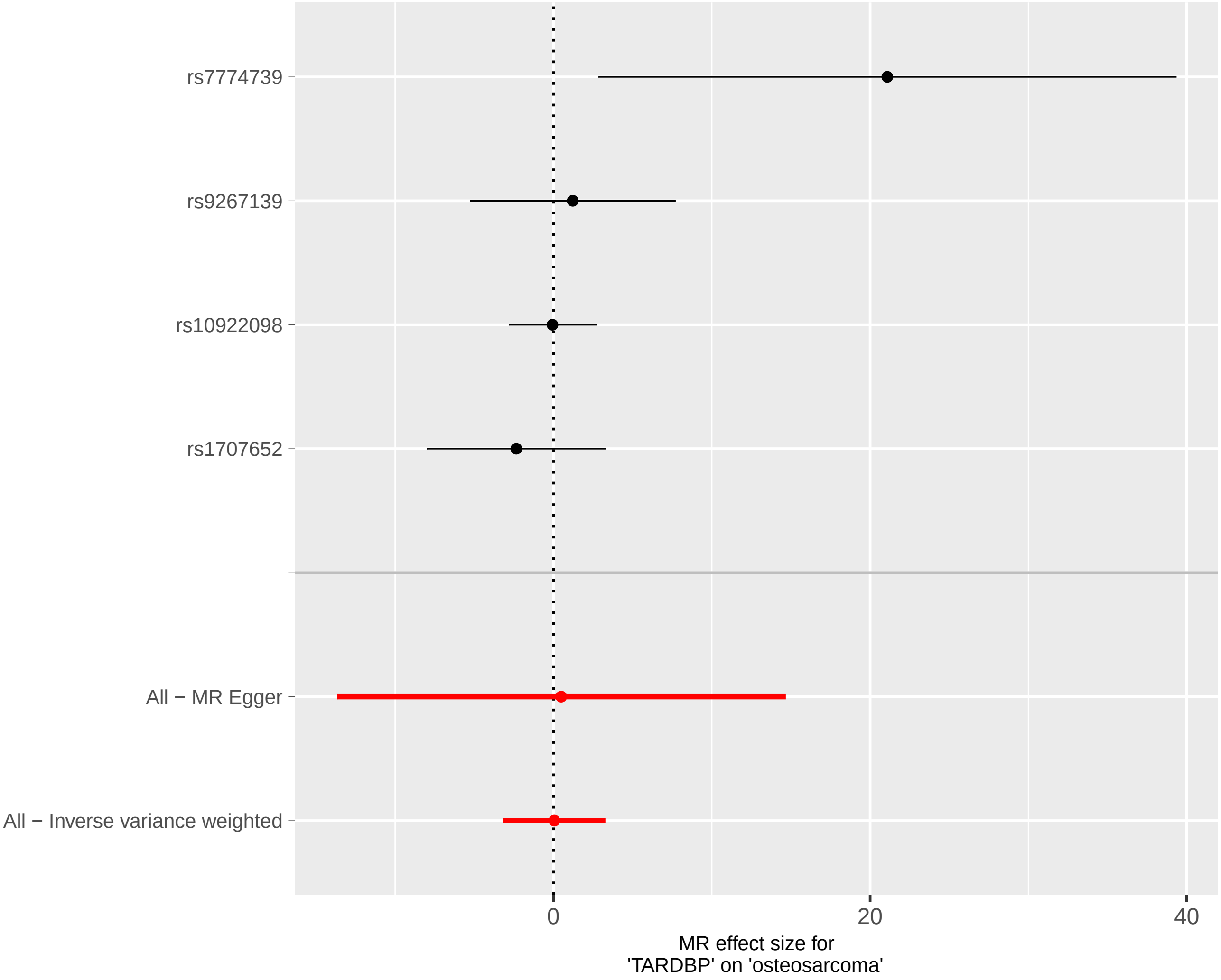
rs7774739
rs9267139
rs10922098
rs1707652
All − MR Egger
All − Inverse variance weighted
0
20
40
MR effect size for
'TARDBP' on 'osteosarcoma'

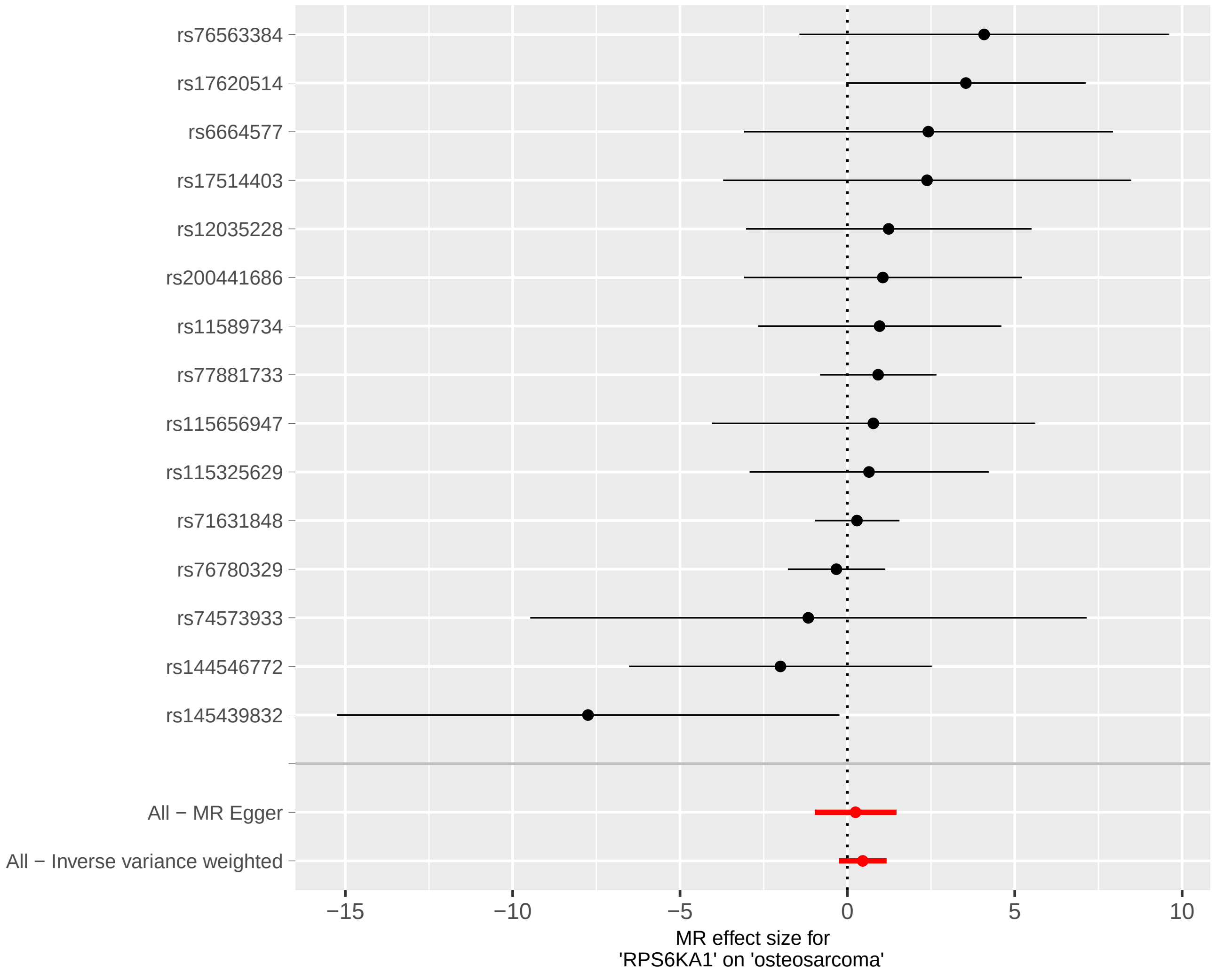
rs76563384
rs17620514
rs6664577
rs17514403
rs12035228
rs200441686
rs11589734
rs77881733
rs115656947
rs115325629
rs71631848
rs76780329
rs74573933
rs144546772
rs145439832
All − MR Egger
All − Inverse variance weighted
−15
−10
−5
0
5
10
MR effect size for
'RPS6KA1' on 'osteosarcoma'

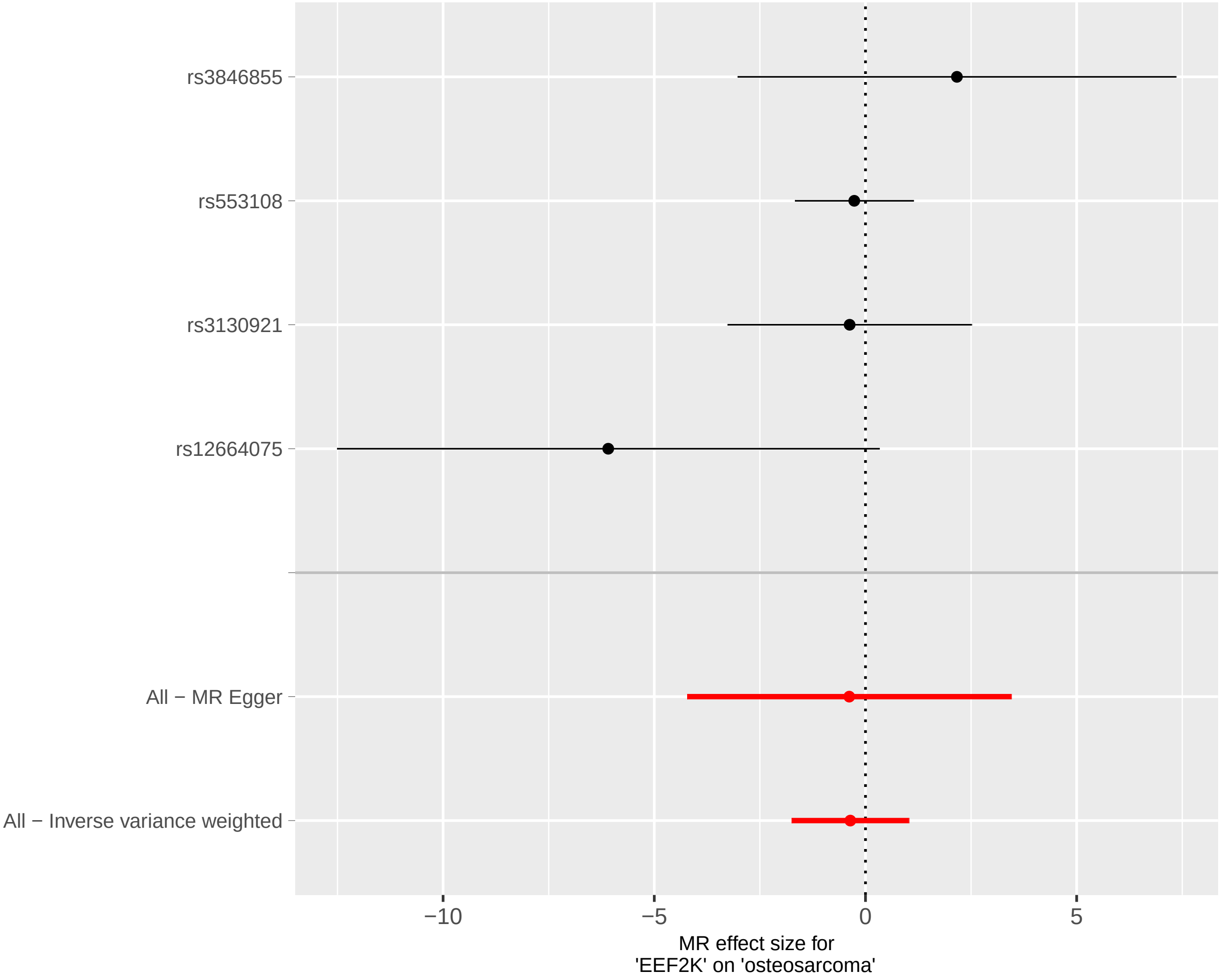

rs3846855
rs553108
rs3130921
rs12664075
All − MR Egger
All − Inverse variance weighted
−10
−5
0
5
MR effect size for
'EEF2K' on 'osteosarcoma'

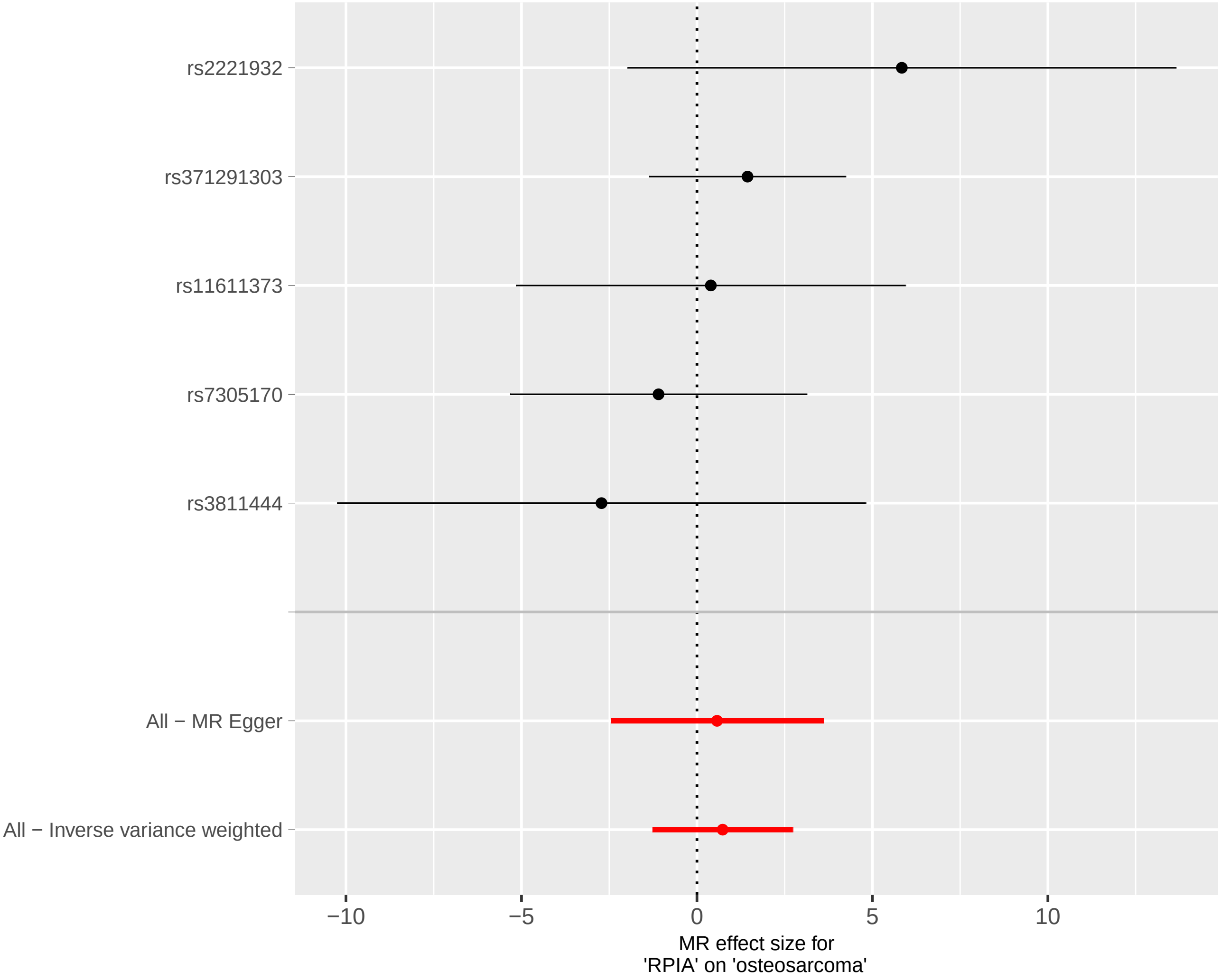
rs2221932
rs371291303
rs11611373
rs7305170
rs3811444
All − MR Egger
All − Inverse variance weighted
−10
−5
0
5
10
MR effect size for
'RPIA' on 'osteosarcoma'

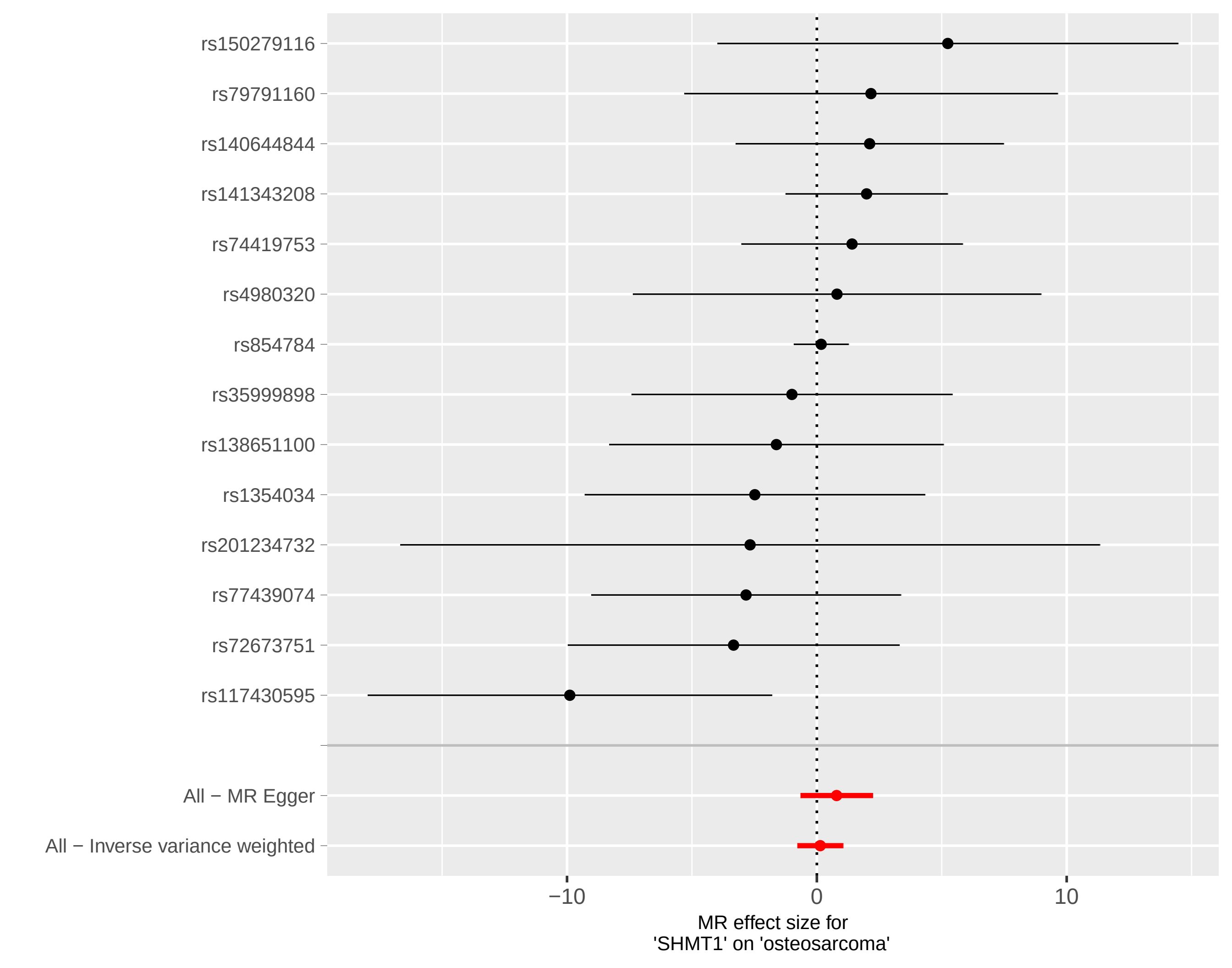

rs150279116
rs79791160
rs140644844
rs141343208
rs74419753
rs4980320
rs854784
rs35999898
rs138651100
rs1354034
rs201234732
rs77439074
rs72673751
rs117430595
All − MR Egger
All − Inverse variance weighted
−10
0
10
MR effect size for
'SHMT1' on 'osteosarcoma'

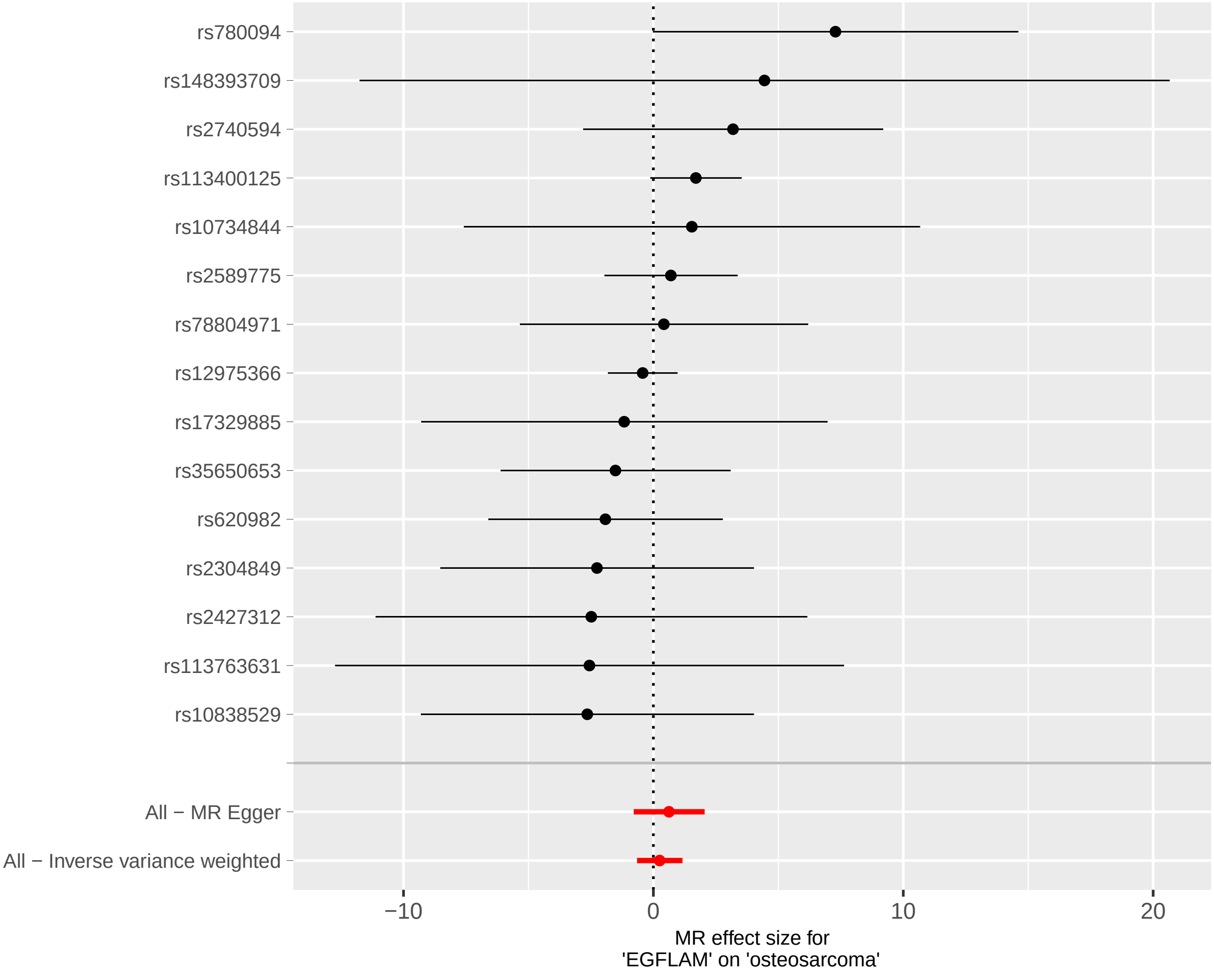
rs780094
rs148393709
rs2740594
rs113400125
rs10734844
rs2589775
rs78804971
rs12975366
rs17329885
rs35650653
rs620982
rs2304849
rs2427312
rs113763631
rs10838529
All − MR Egger
All − Inverse variance weighted
−10
0
10
20
MR effect size for
'EGFLAM' on 'osteosarcoma'

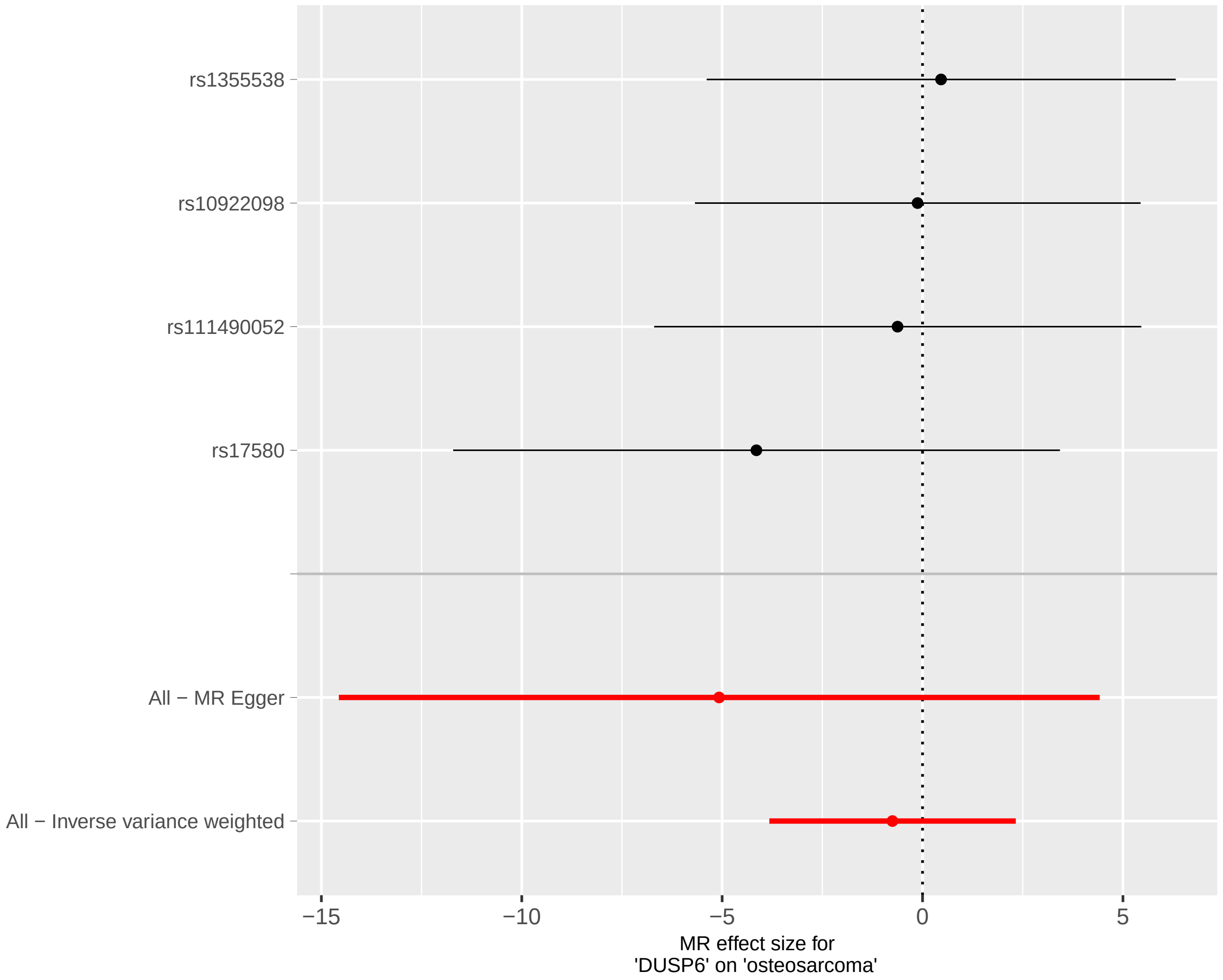

rs1355538
rs10922098
rs111490052
rs17580
All − MR Egger
All − Inverse variance weighted
−15
−10
−5
0
5
MR effect size for
'DUSP6' on 'osteosarcoma'

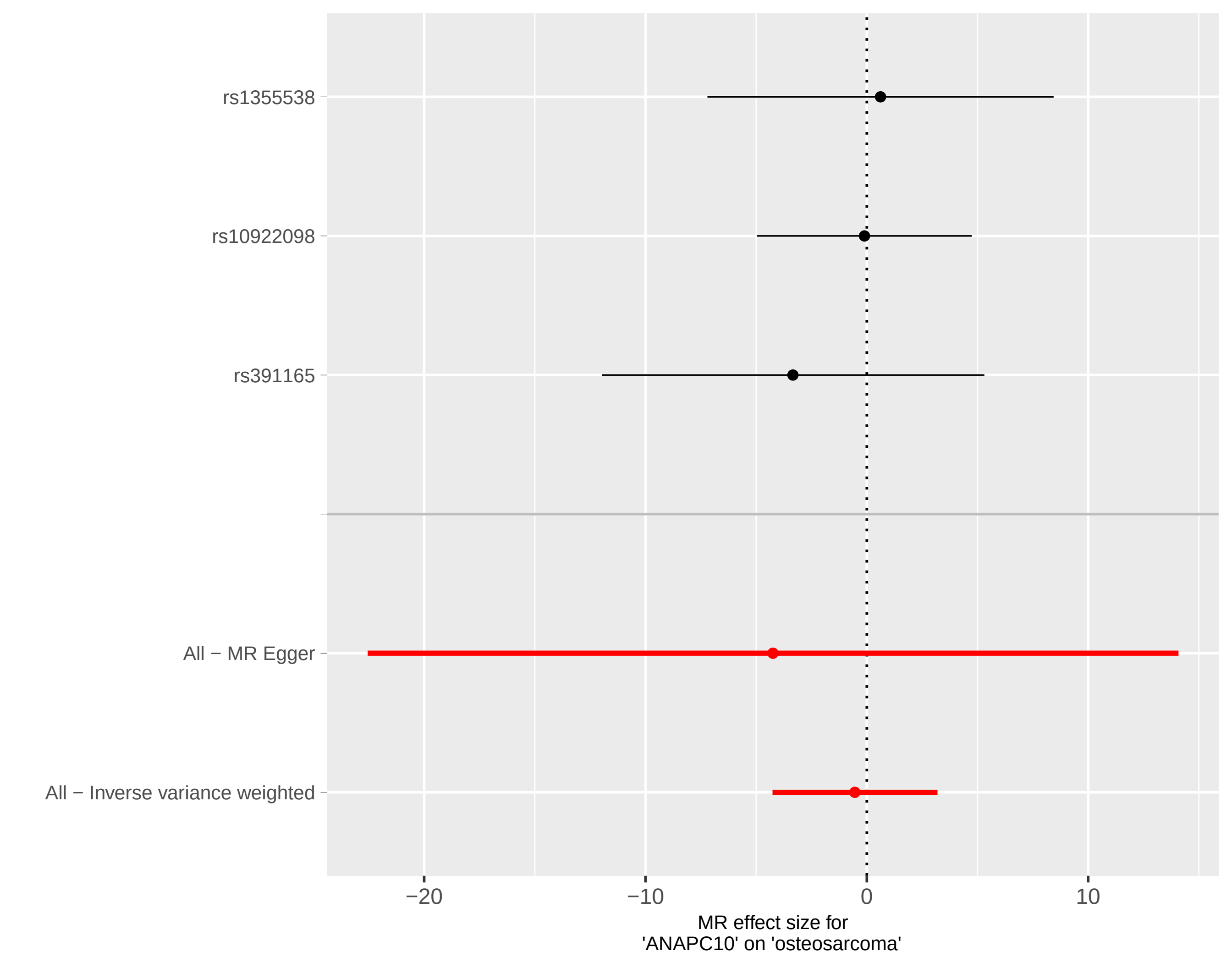

rs1355538
rs10922098
rs391165
All − MR Egger
All − Inverse variance weighted
−20
−10
0
10
MR effect size for
'ANAPC10' on 'osteosarcoma'

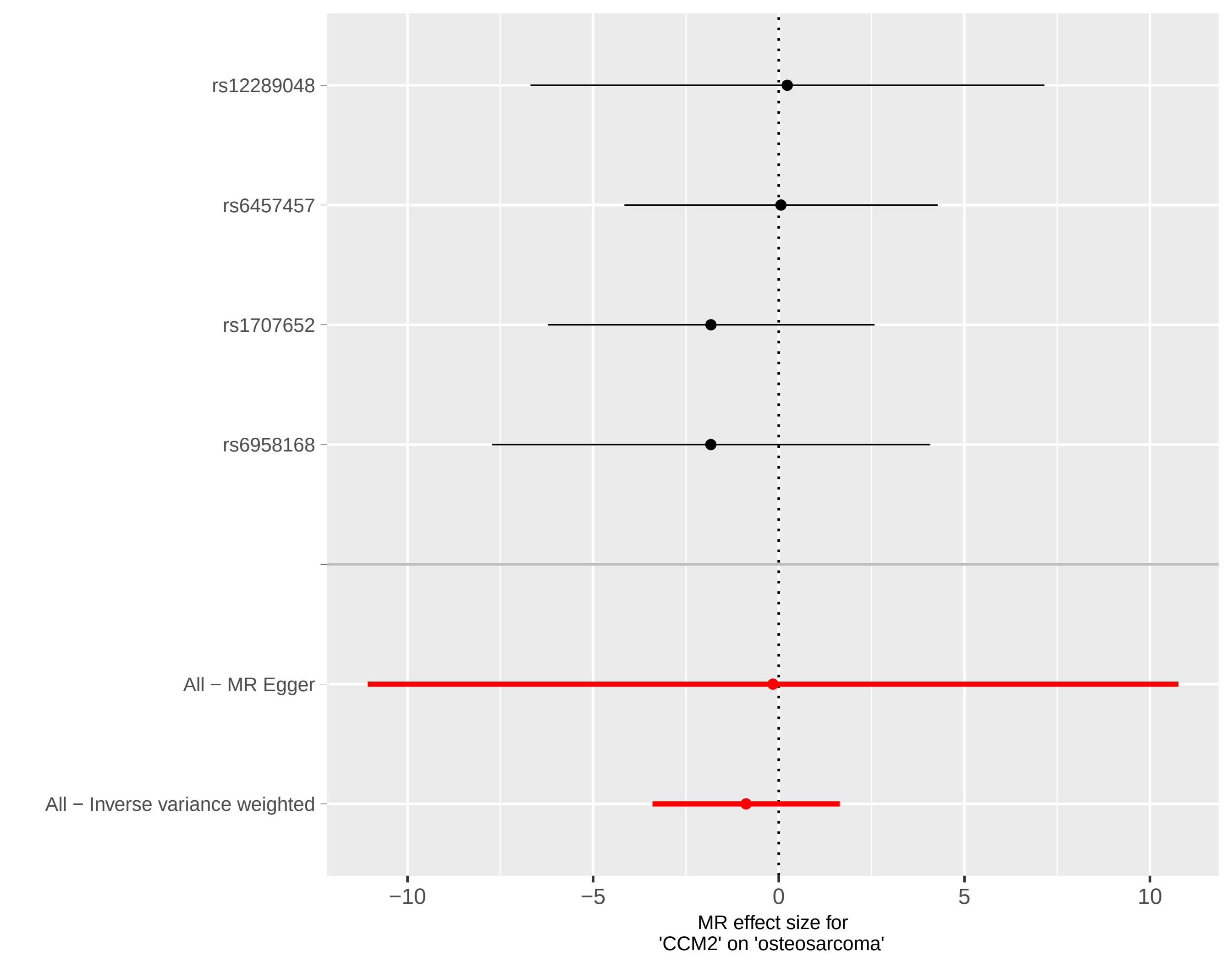
rs12289048
rs6457457
rs1707652
rs6958168
All − MR Egger
All − Inverse variance weighted
−10
−5
0
5
10
MR effect size for
'CCM2' on 'osteosarcoma'

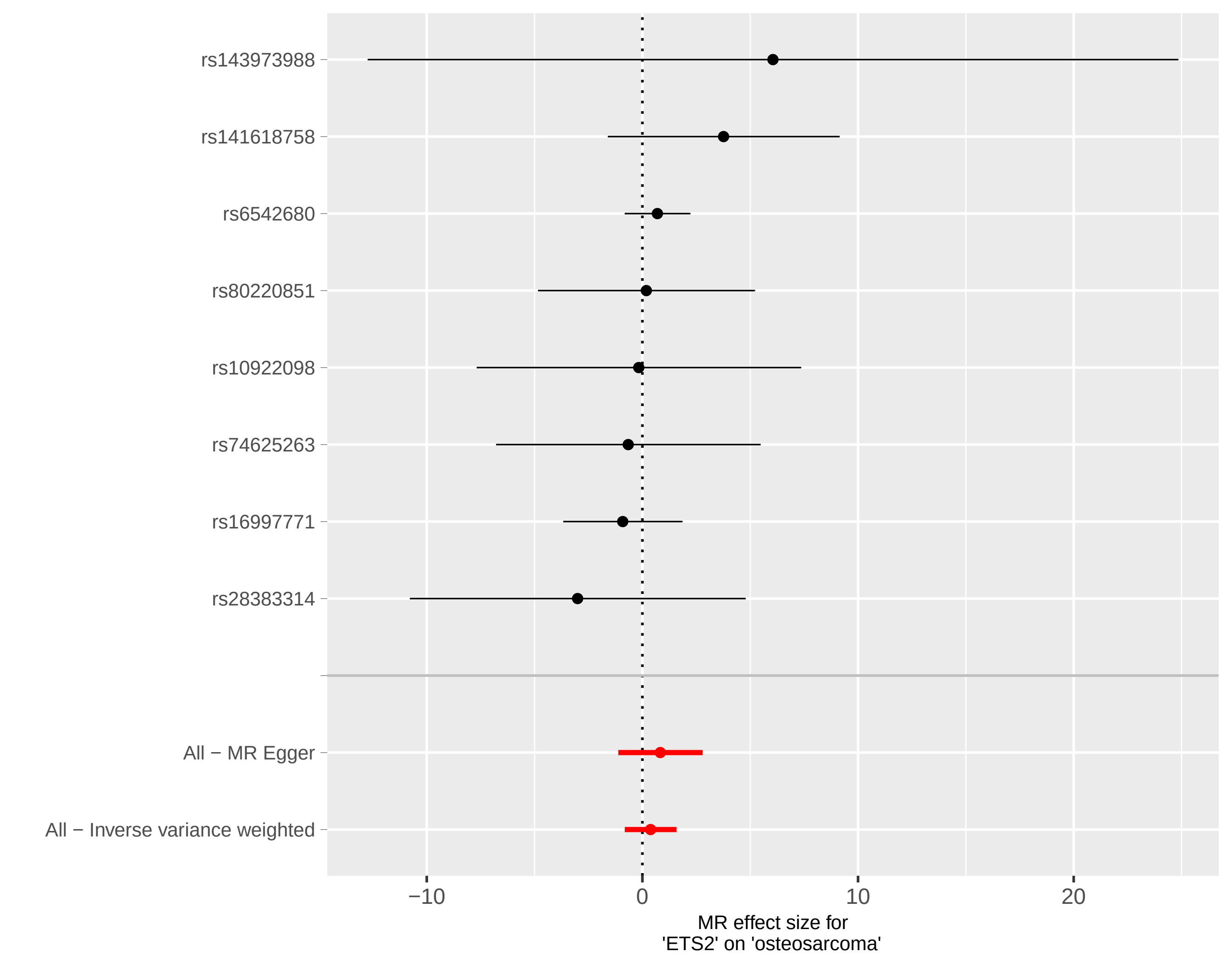

rs143973988
rs141618758
rs6542680
rs80220851
rs10922098
rs74625263
rs16997771
rs28383314
All − MR Egger
All − Inverse variance weighted
−10
0
10
20
MR effect size for
'ETS2' on 'osteosarcoma'

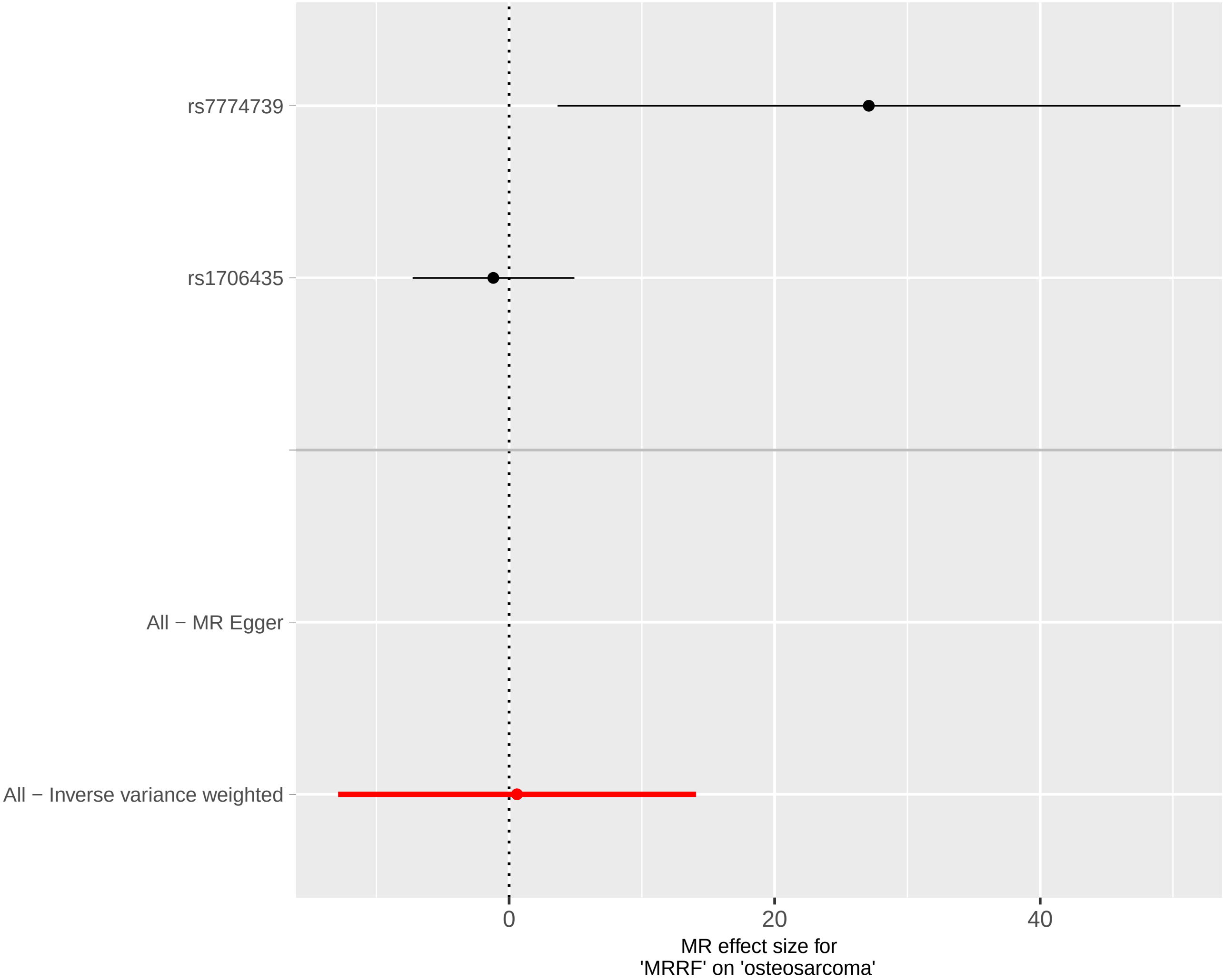

rs7774739
rs1706435
All − MR Egger
All − Inverse variance weighted
0
20
40
MR effect size for
'MRRF' on 'osteosarcoma'

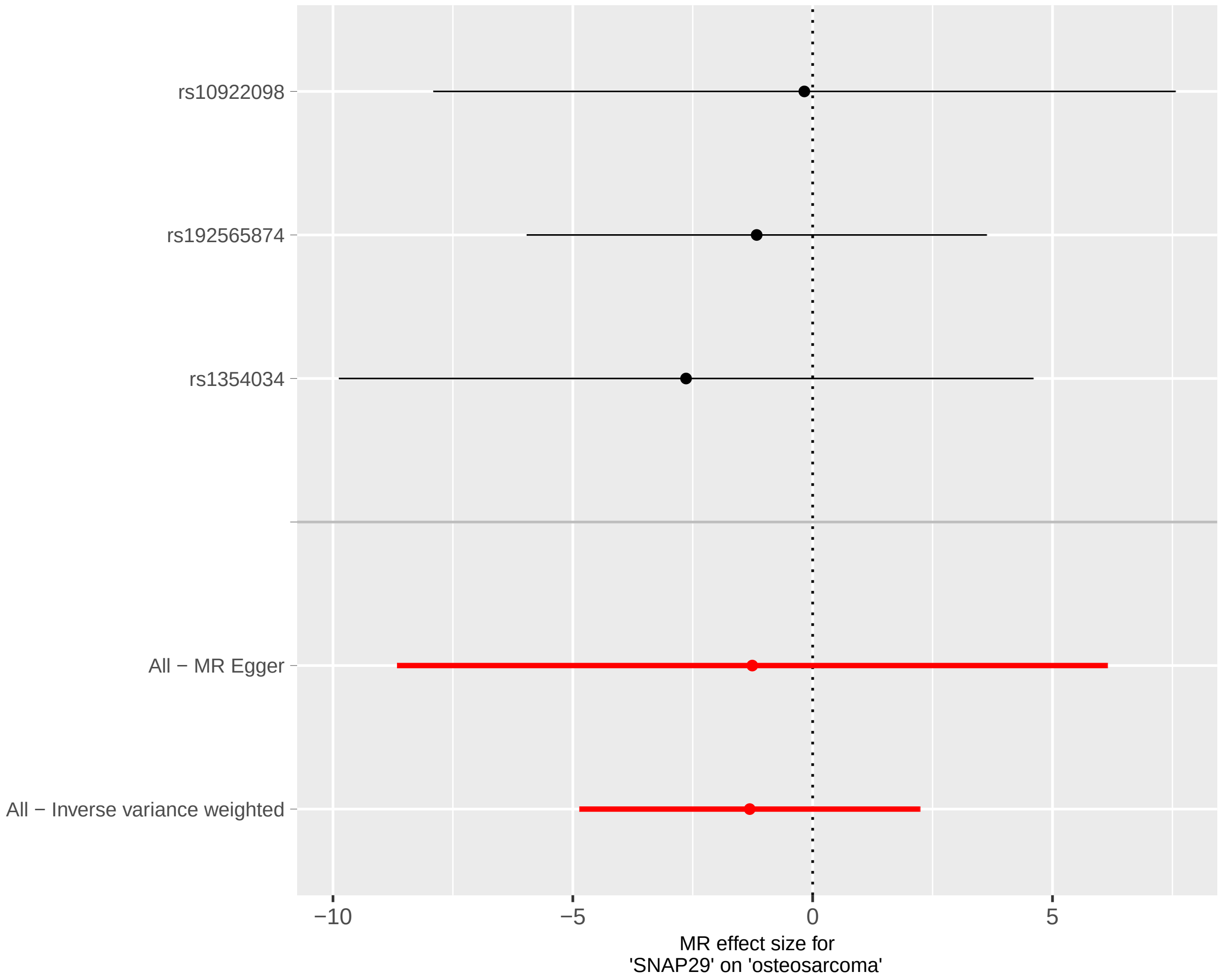

rs10922098
rs192565874
rs1354034
All − MR Egger
All − Inverse variance weighted
−10
−5
0
5
MR effect size for
'SNAP29' on 'osteosarcoma'

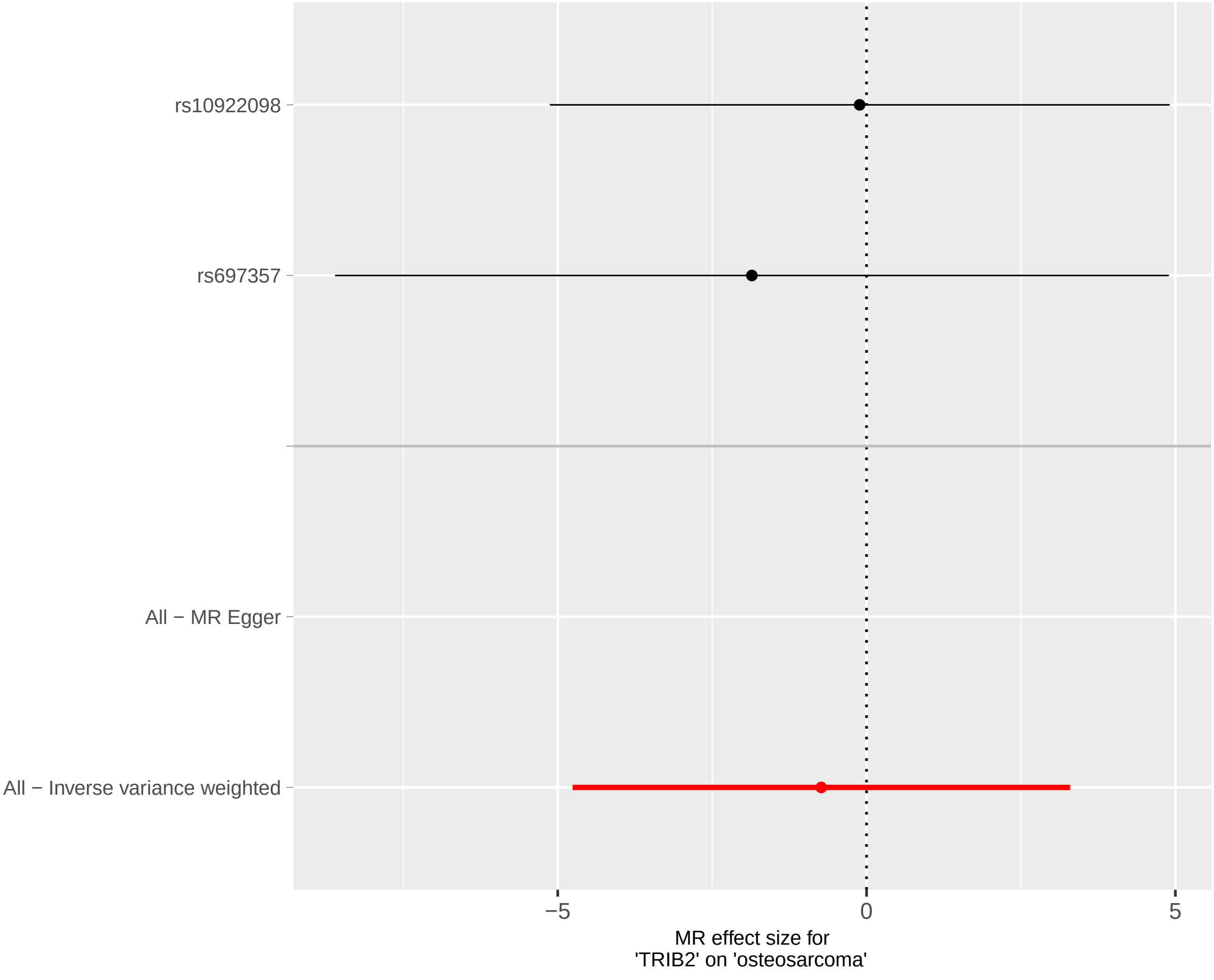
rs10922098
rs697357
All − MR Egger
All − Inverse variance weighted
−5
0
5
MR effect size for
'TRIB2' on 'osteosarcoma'

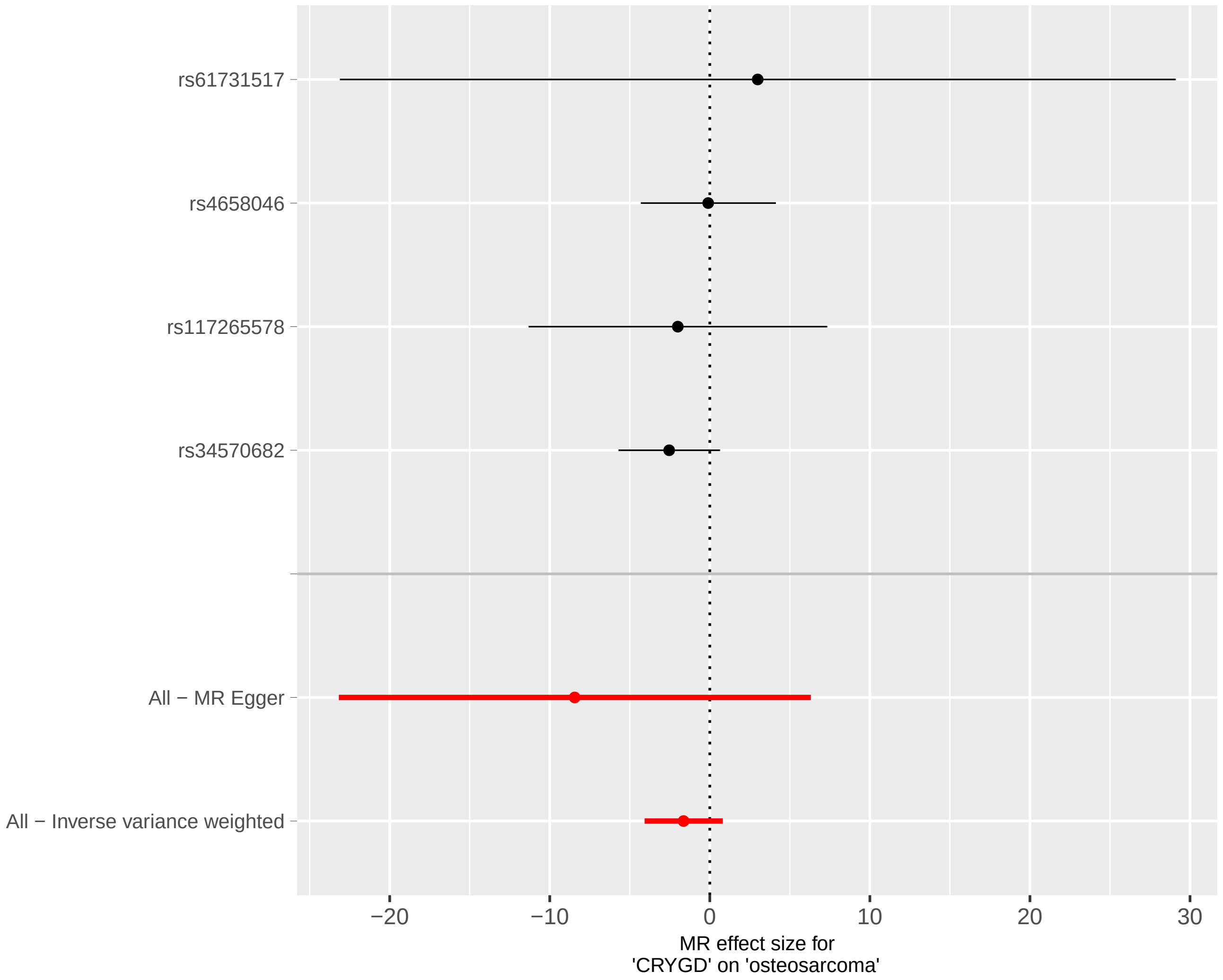

rs61731517
rs4658046
rs117265578
rs34570682
All − MR Egger
All − Inverse variance weighted
−20
−10
0
10
20
30
MR effect size for
'CRYGD' on 'osteosarcoma'

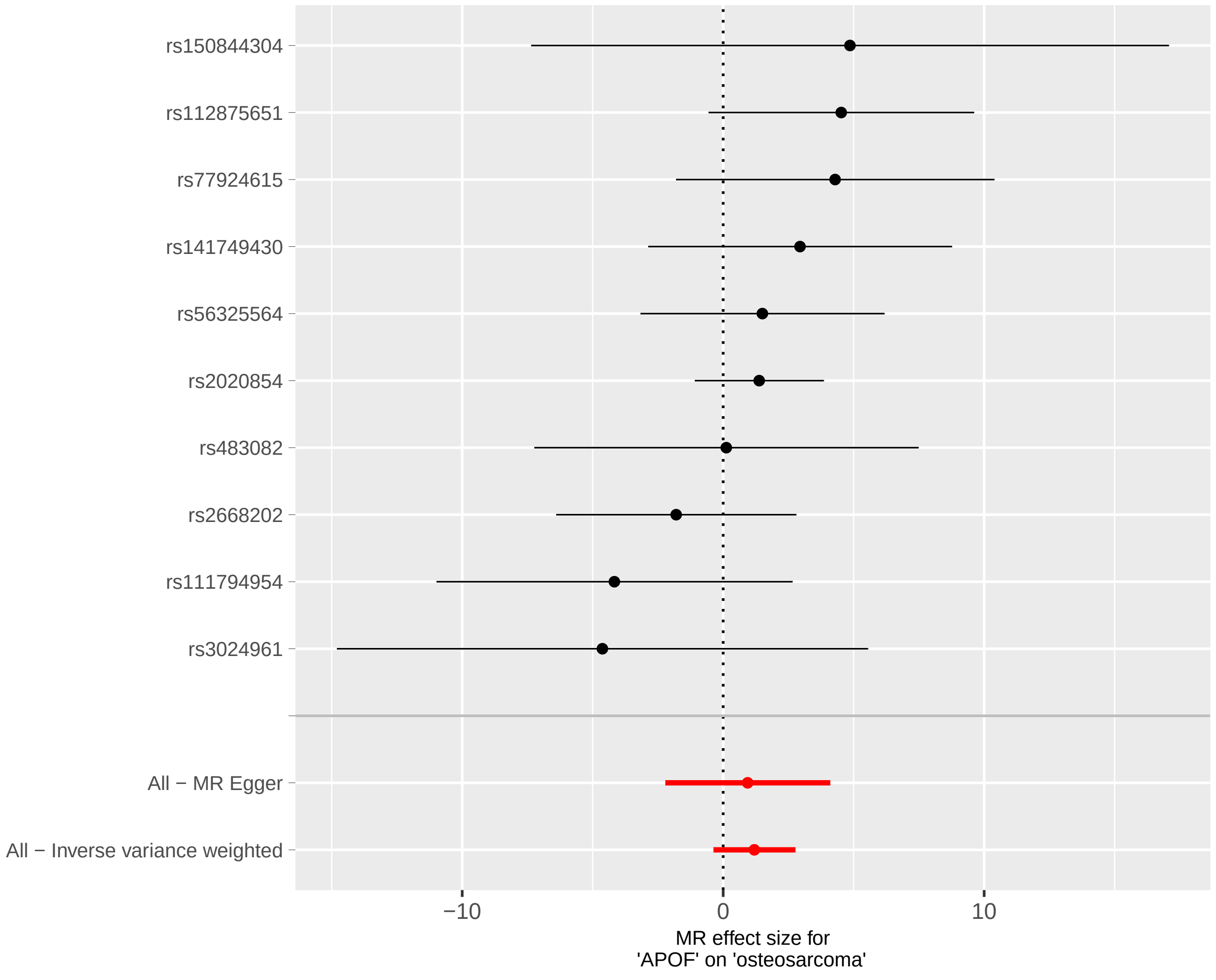

rs150844304
rs112875651
rs77924615
rs141749430
rs56325564
rs2020854
rs483082
rs2668202
rs111794954
rs3024961
All − MR Egger
All − Inverse variance weighted
−10
0
10
MR effect size for
'APOF' on 'osteosarcoma'

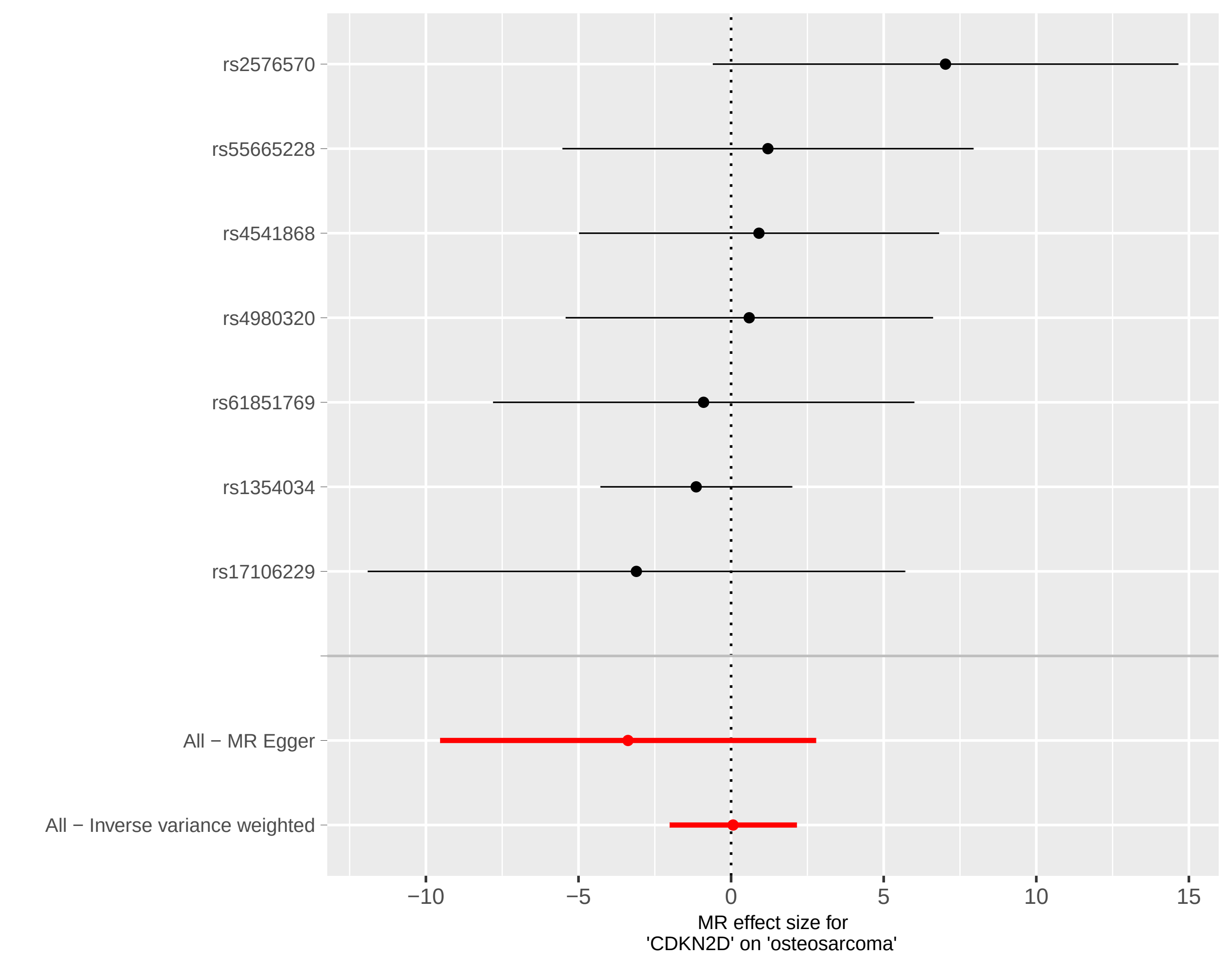

rs2576570
rs55665228
rs4541868
rs4980320
rs61851769
rs1354034
rs17106229
All − MR Egger
All − Inverse variance weighted
−10
−5
0
5
10
15
MR effect size for
'CDKN2D' on 'osteosarcoma'

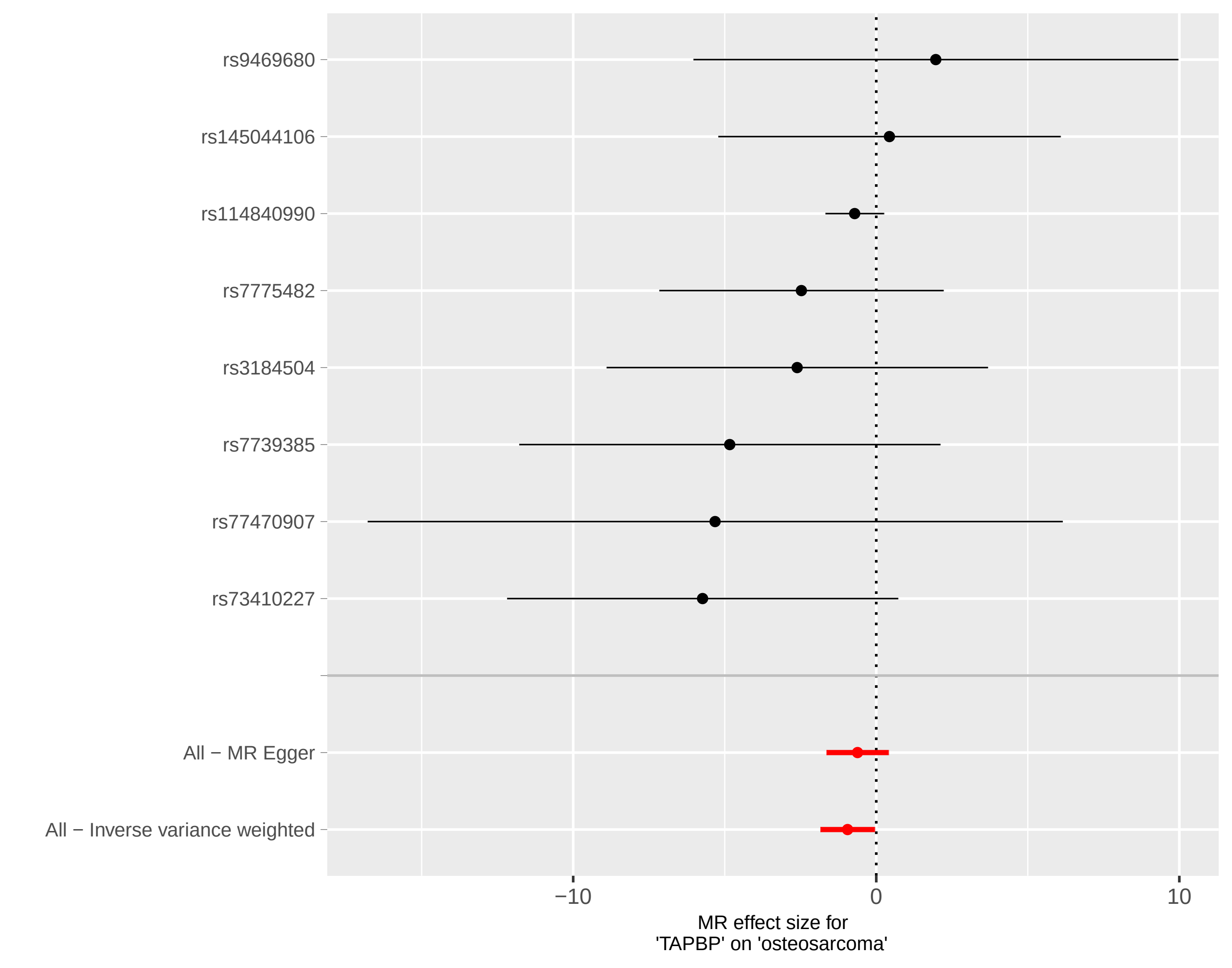

rs9469680
rs145044106
rs114840990
rs7775482
rs3184504
rs7739385
rs77470907
rs73410227
All − MR Egger
All − Inverse variance weighted
−10
0
10
MR effect size for
'TAPBP' on 'osteosarcoma'

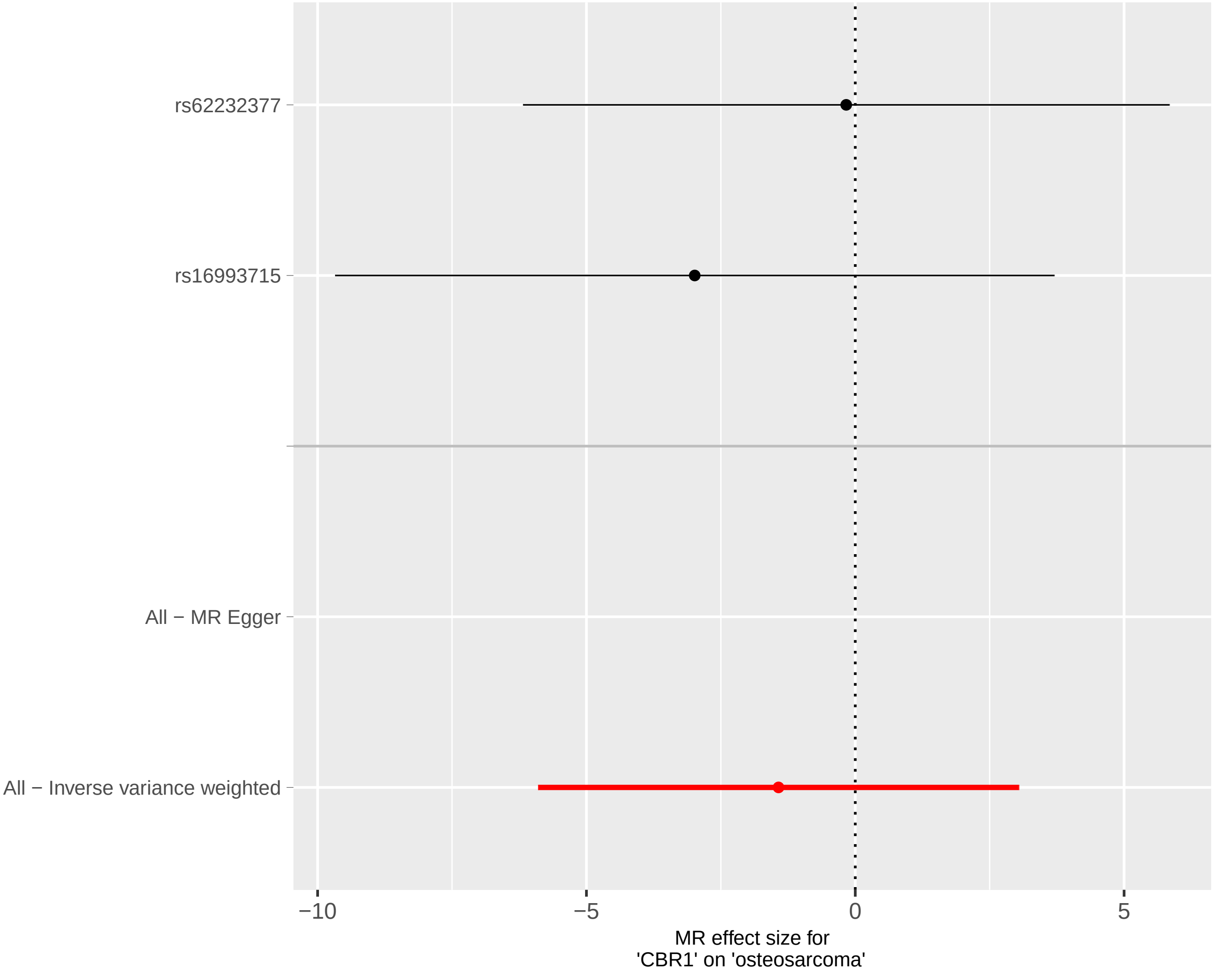

rs62232377
rs16993715
All − MR Egger
All − Inverse variance weighted
−10
−5
0
5
MR effect size for
'CBR1' on 'osteosarcoma'

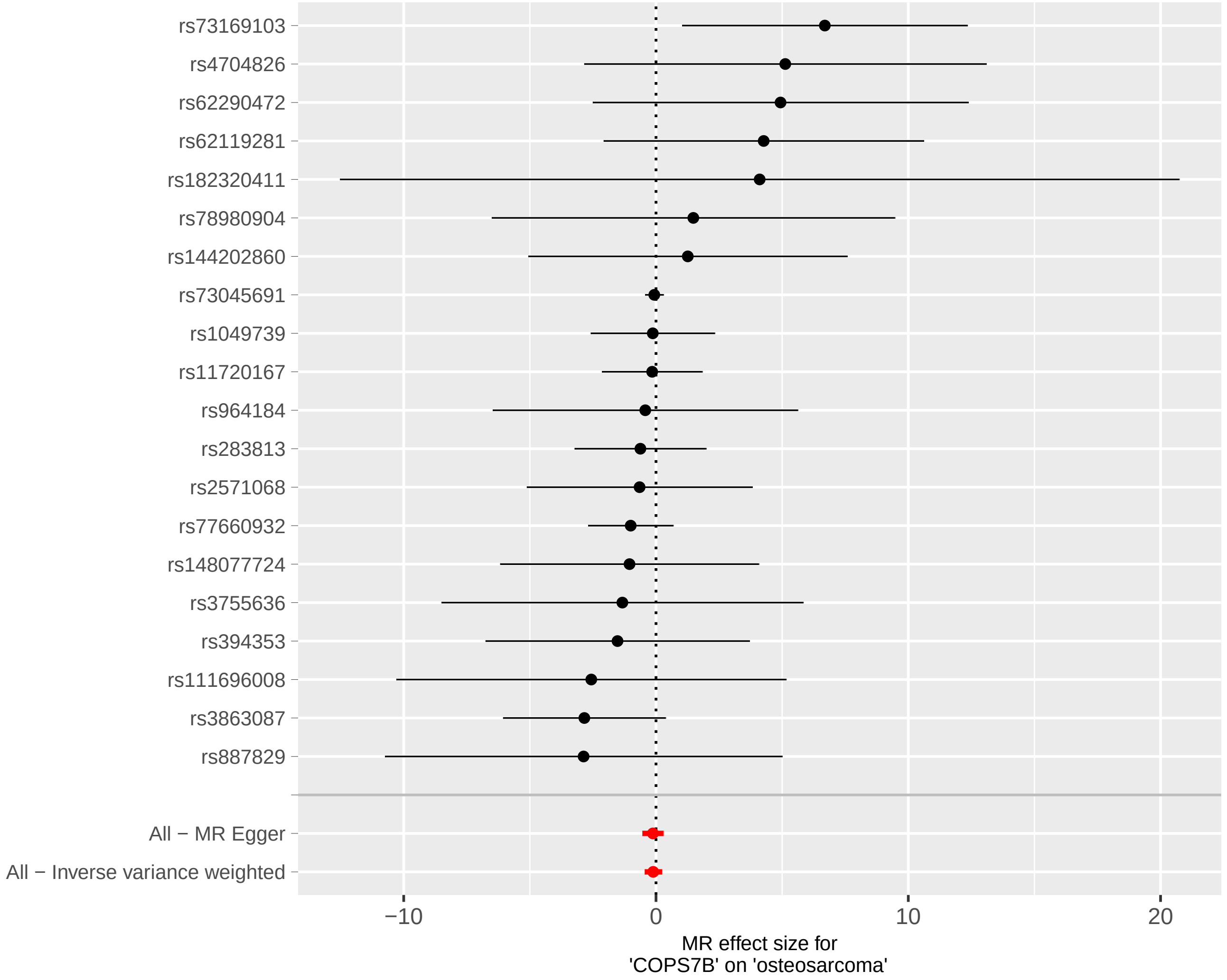

rs73169103
rs4704826
rs62290472
rs62119281
rs182320411
rs78980904
rs144202860
rs73045691
rs1049739
rs11720167
rs964184
rs283813
rs2571068
rs77660932
rs148077724
rs3755636
rs394353
rs111696008
rs3863087
rs887829
All − MR Egger
All − Inverse variance weighted
−10
0
10
20
MR effect size for
'COPS7B' on 'osteosarcoma'

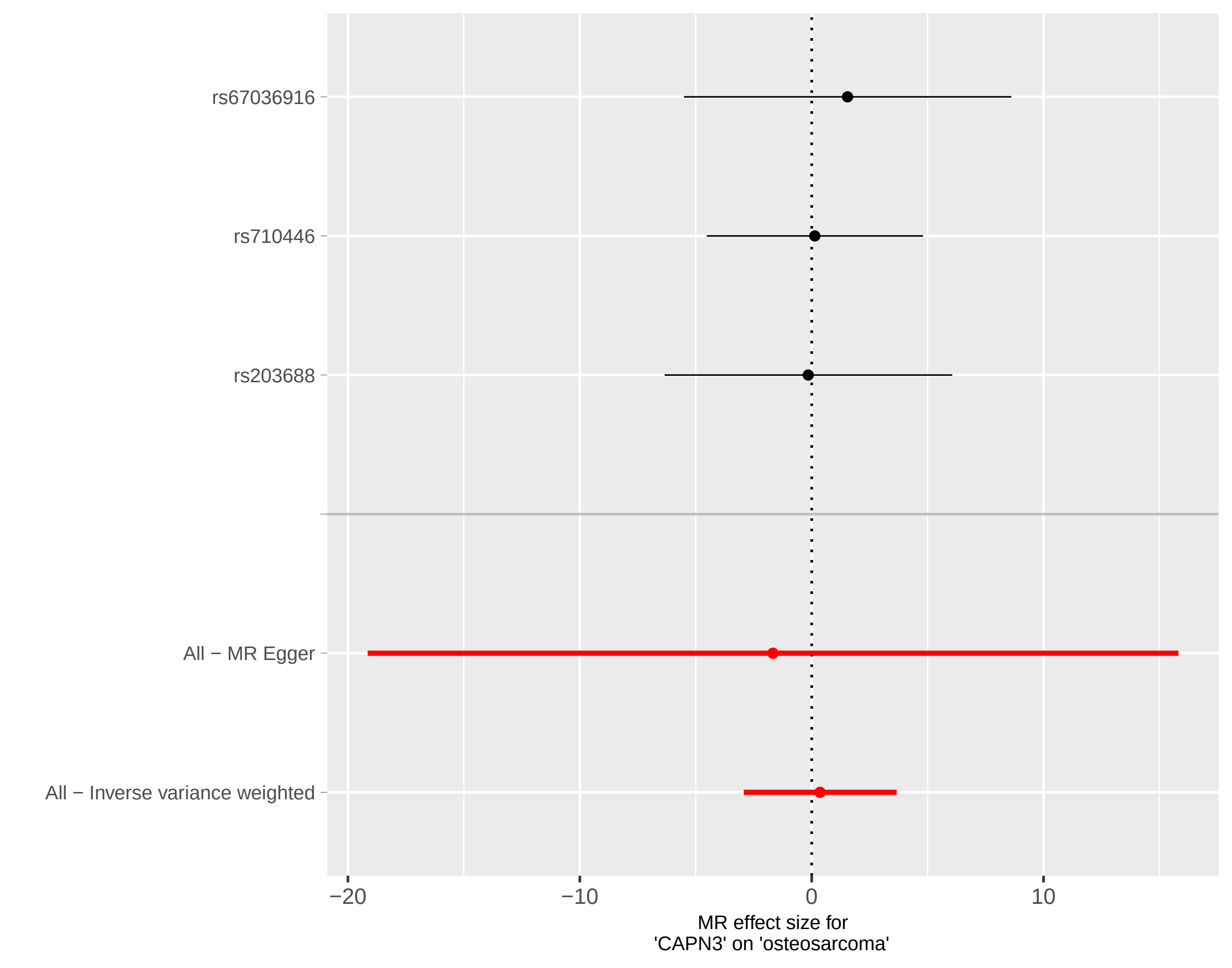
rs67036916
rs710446
rs203688
All − MR Egger
All − Inverse variance weighted
−20
−10
0
10
MR effect size for
'CAPN3' on 'osteosarcoma'

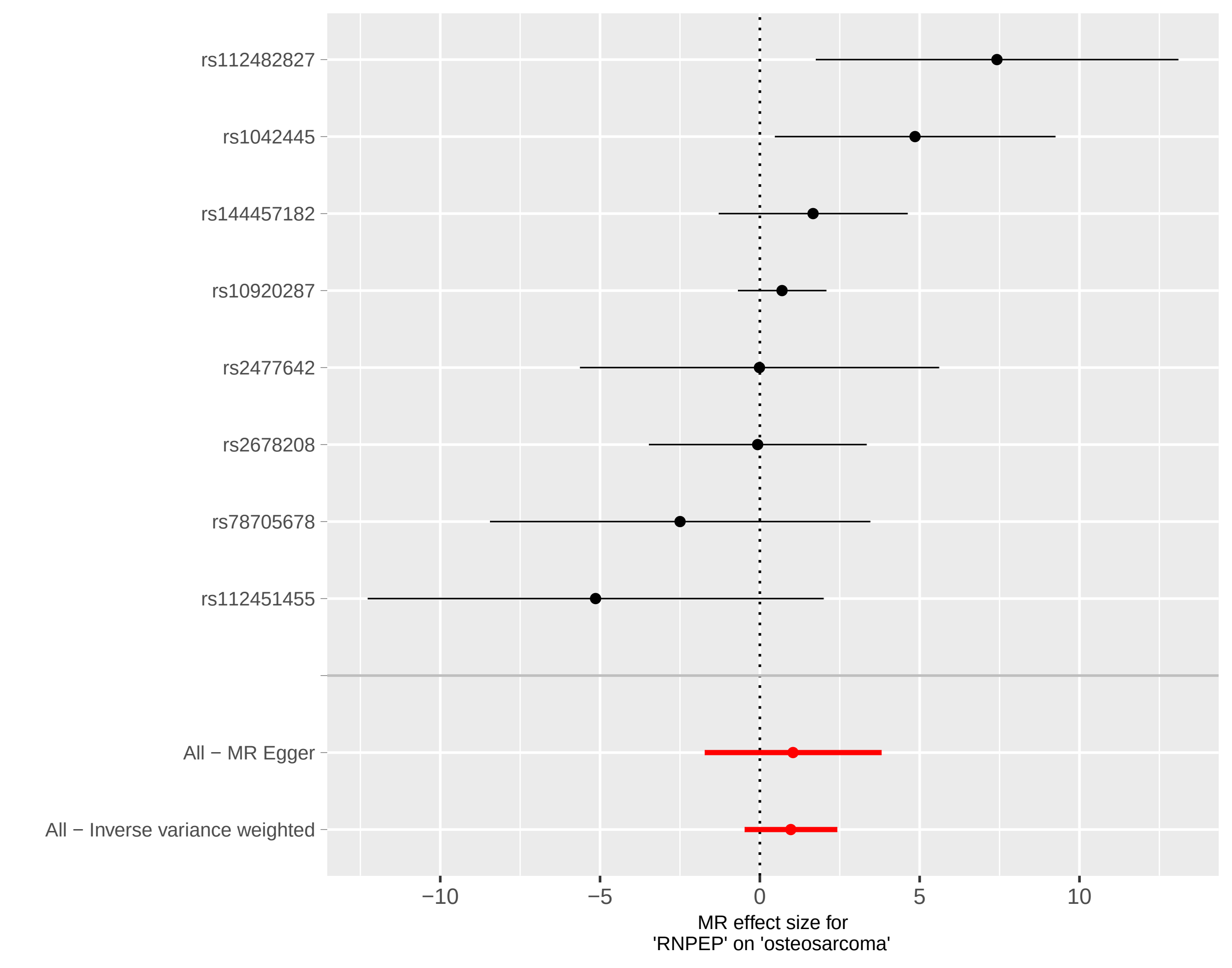

rs112482827
rs1042445
rs144457182
rs10920287
rs2477642
rs2678208
rs78705678
rs112451455
All − MR Egger
All − Inverse variance weighted
−10
−5
0
5
10
MR effect size for
'RNPEP' on 'osteosarcoma'

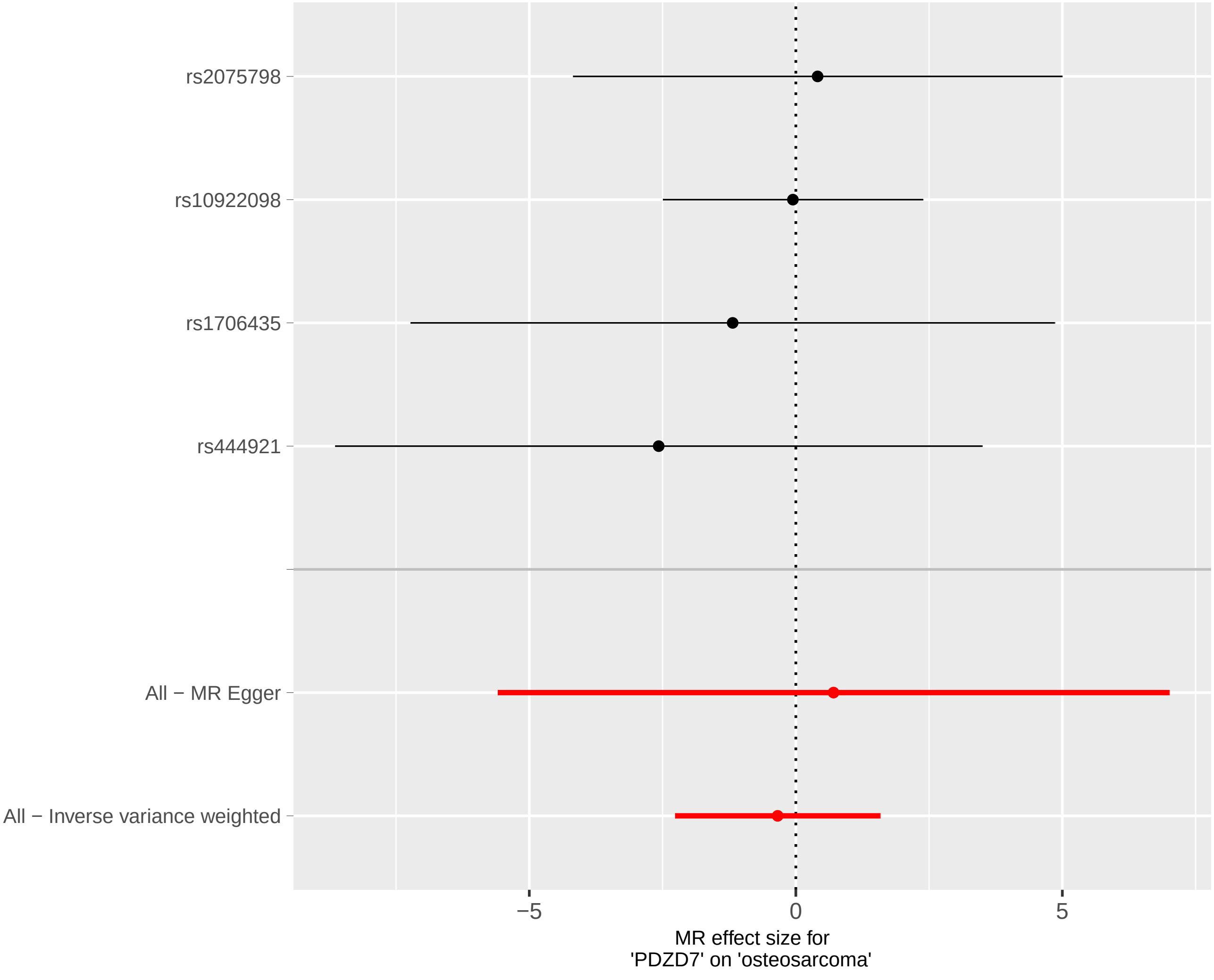

rs2075798
rs10922098
rs1706435
rs444921
All − MR Egger
All − Inverse variance weighted
−5
0
5
MR effect size for
'PDZD7' on 'osteosarcoma'

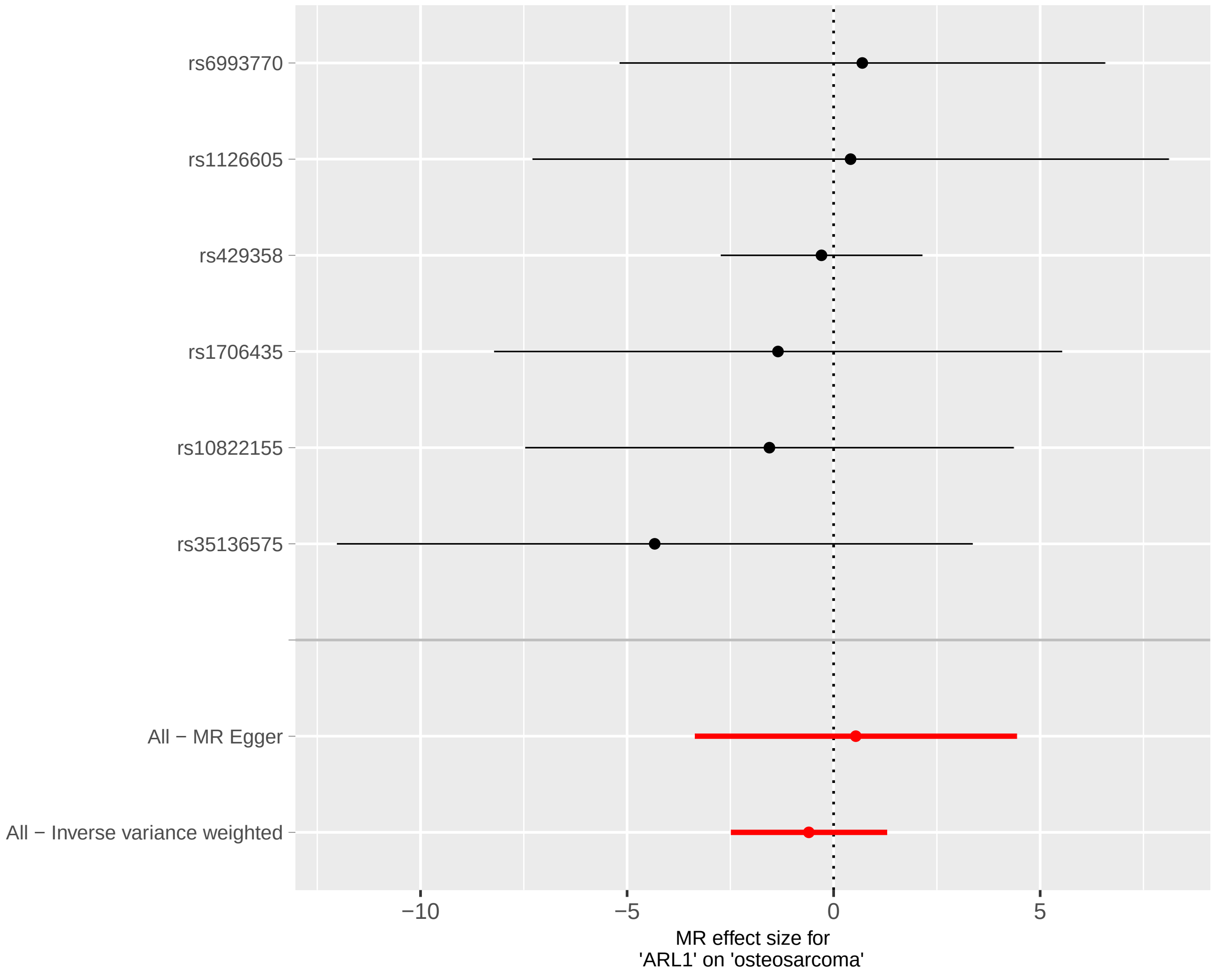

rs6993770
rs1126605
rs429358
rs1706435
rs10822155
rs35136575
All − MR Egger
All − Inverse variance weighted
−10
−5
0
5
MR effect size for
'ARL1' on 'osteosarcoma'

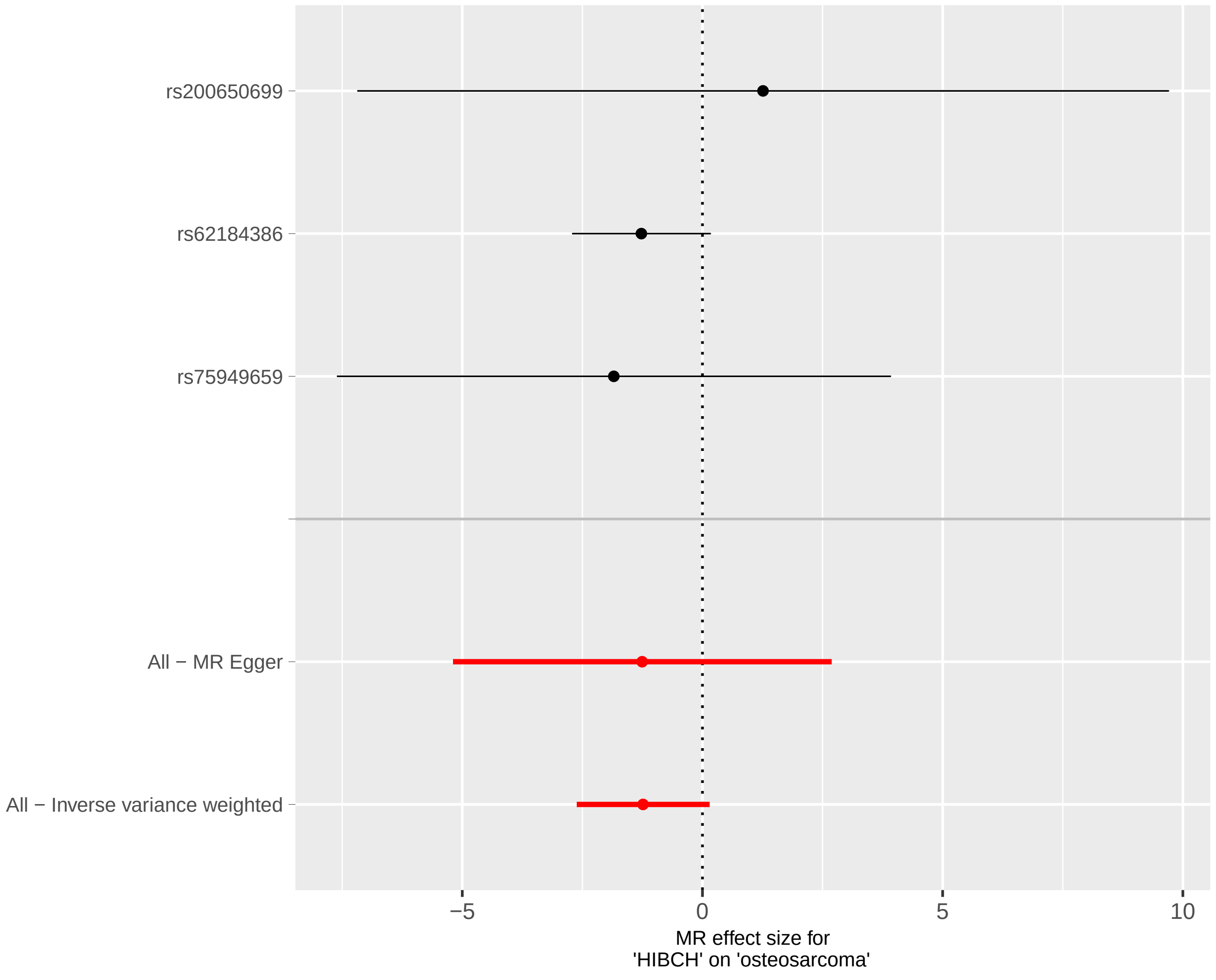
rs200650699
rs62184386
rs75949659
All − MR Egger
All − Inverse variance weighted
−5
0
5
10
MR effect size for
'HIBCH' on 'osteosarcoma'

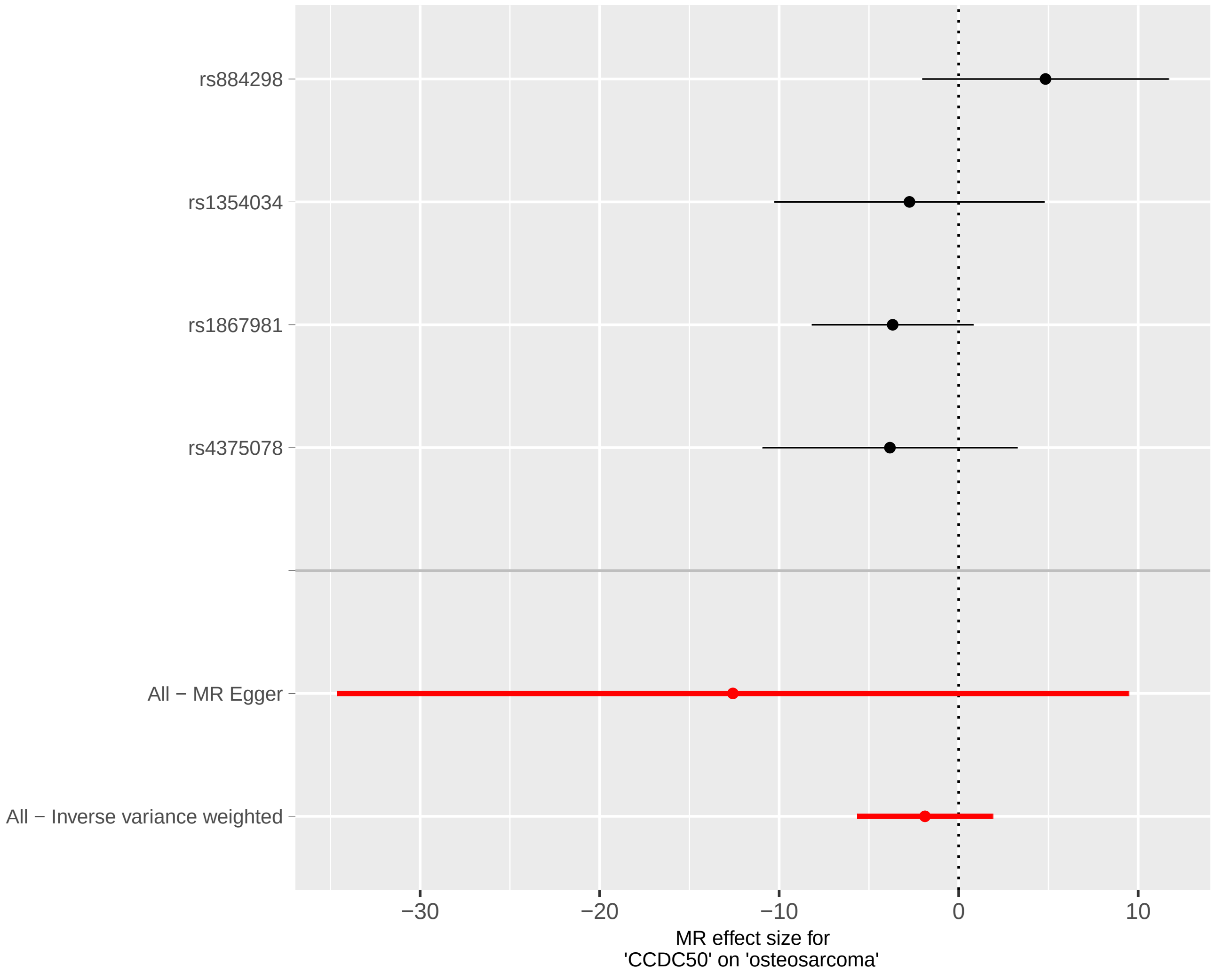
rs884298
rs1354034
rs1867981
rs4375078
All − MR Egger
All − Inverse variance weighted
−30
−20
−10
0
10
MR effect size for
'CCDC50' on 'osteosarcoma'

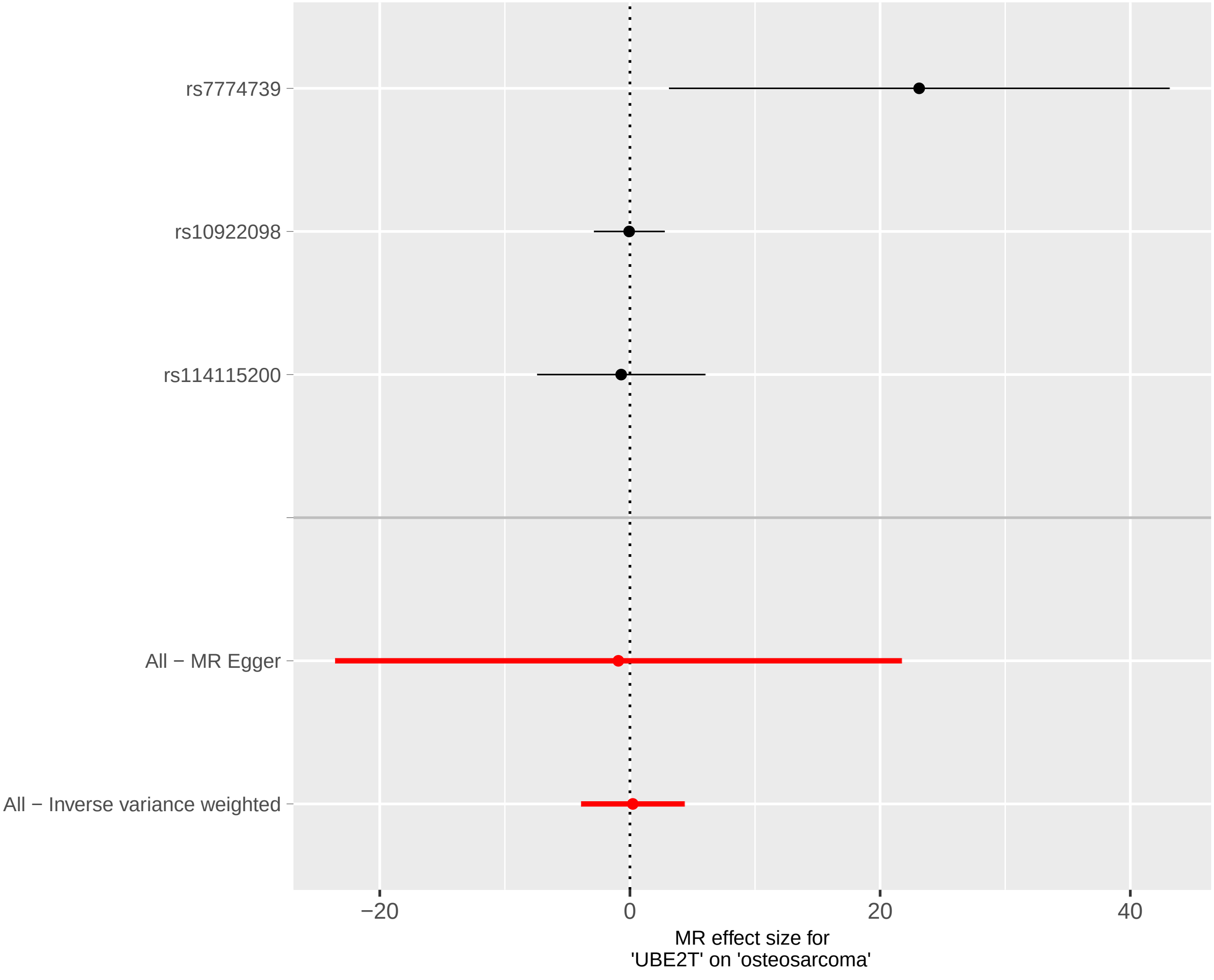

rs7774739
rs10922098
rs114115200
All − MR Egger
All − Inverse variance weighted
−20
0
20
40
MR effect size for
'UBE2T' on 'osteosarcoma'

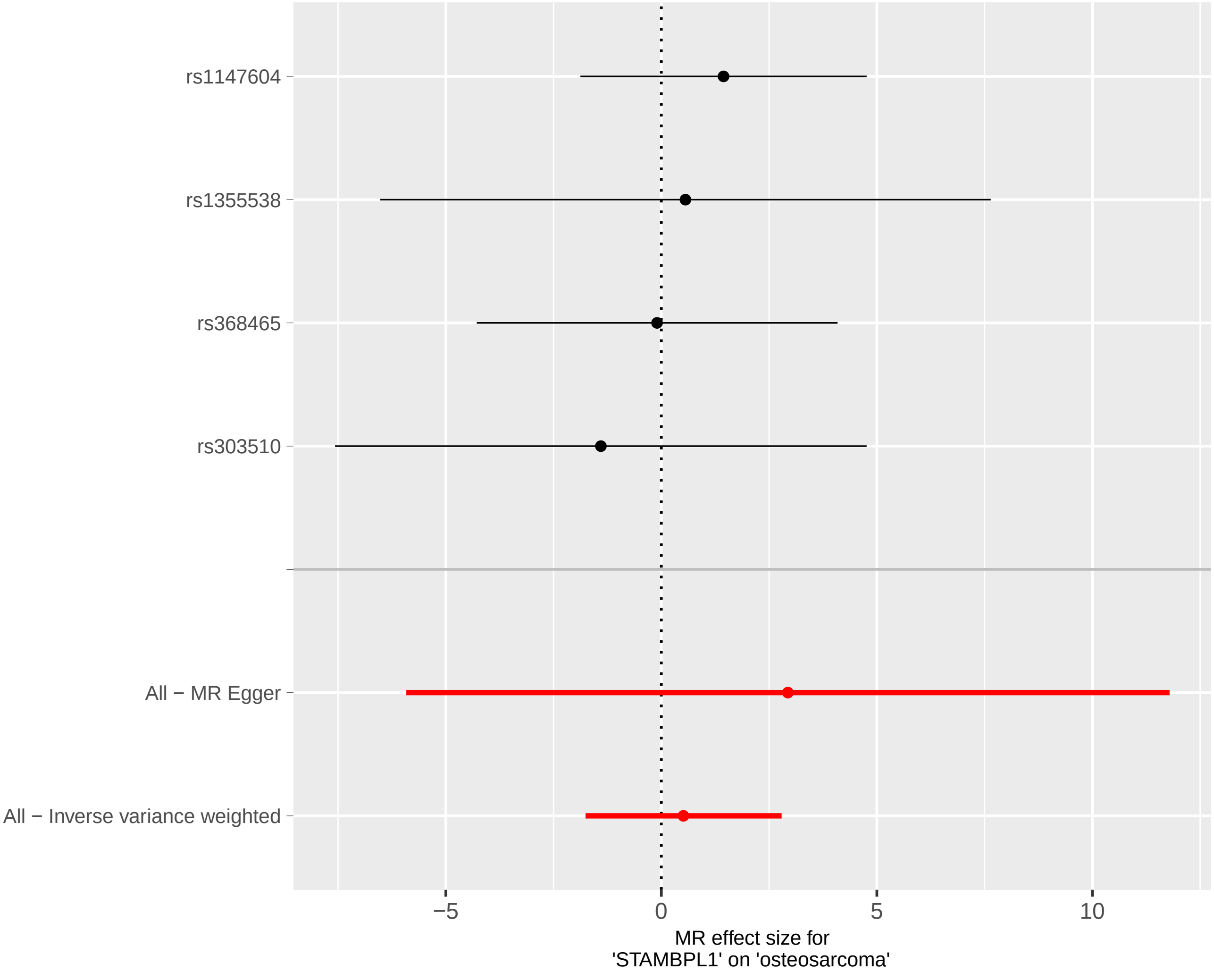

rs1147604
rs1355538
rs368465
rs303510
All − MR Egger
All − Inverse variance weighted
−5
0
5
10
MR effect size for
'STAMBPL1' on 'osteosarcoma'

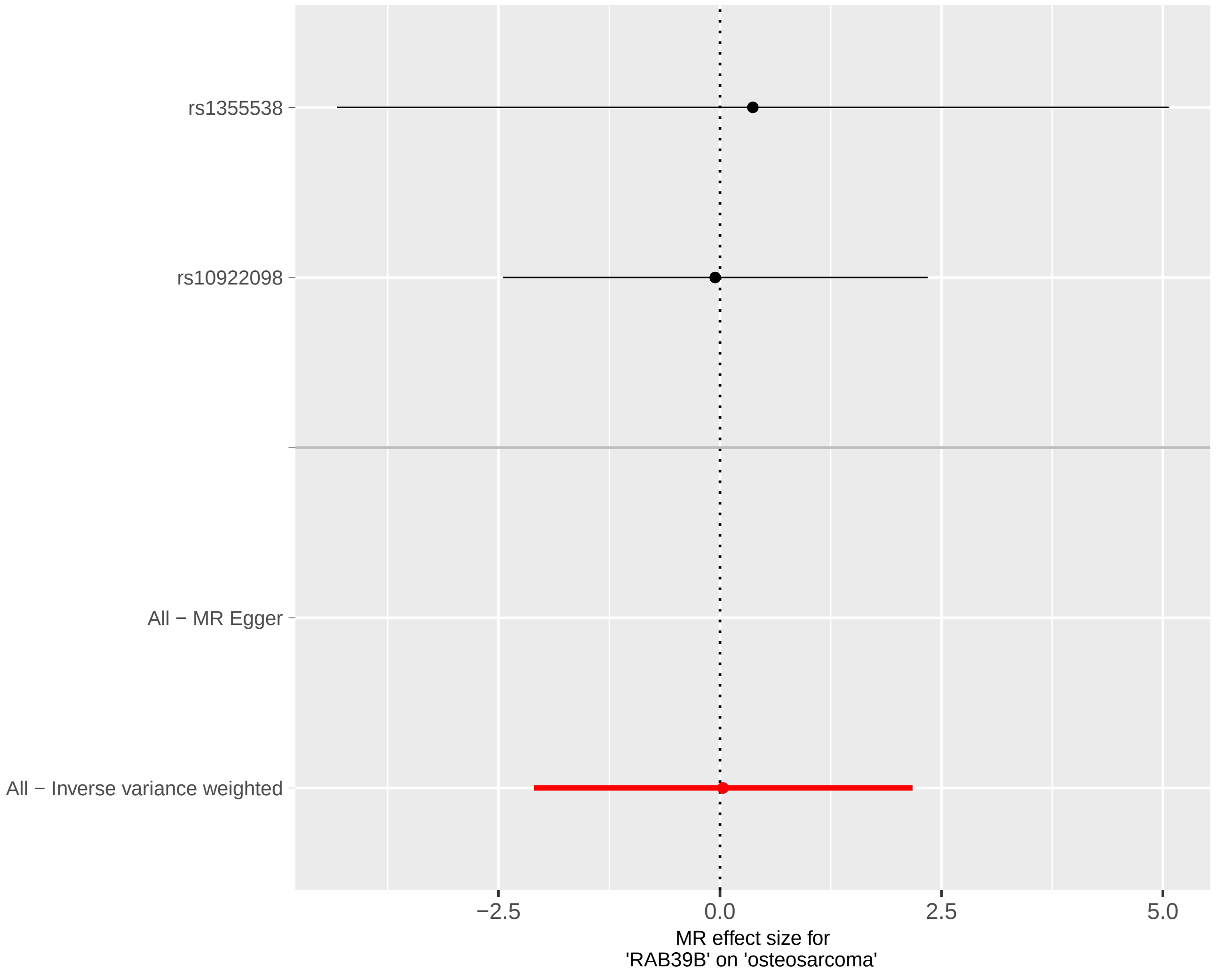

rs1355538
rs10922098
All − MR Egger
All − Inverse variance weighted
−2.5
0.0
2.5
5.0
MR effect size for
'RAB39B' on 'osteosarcoma'

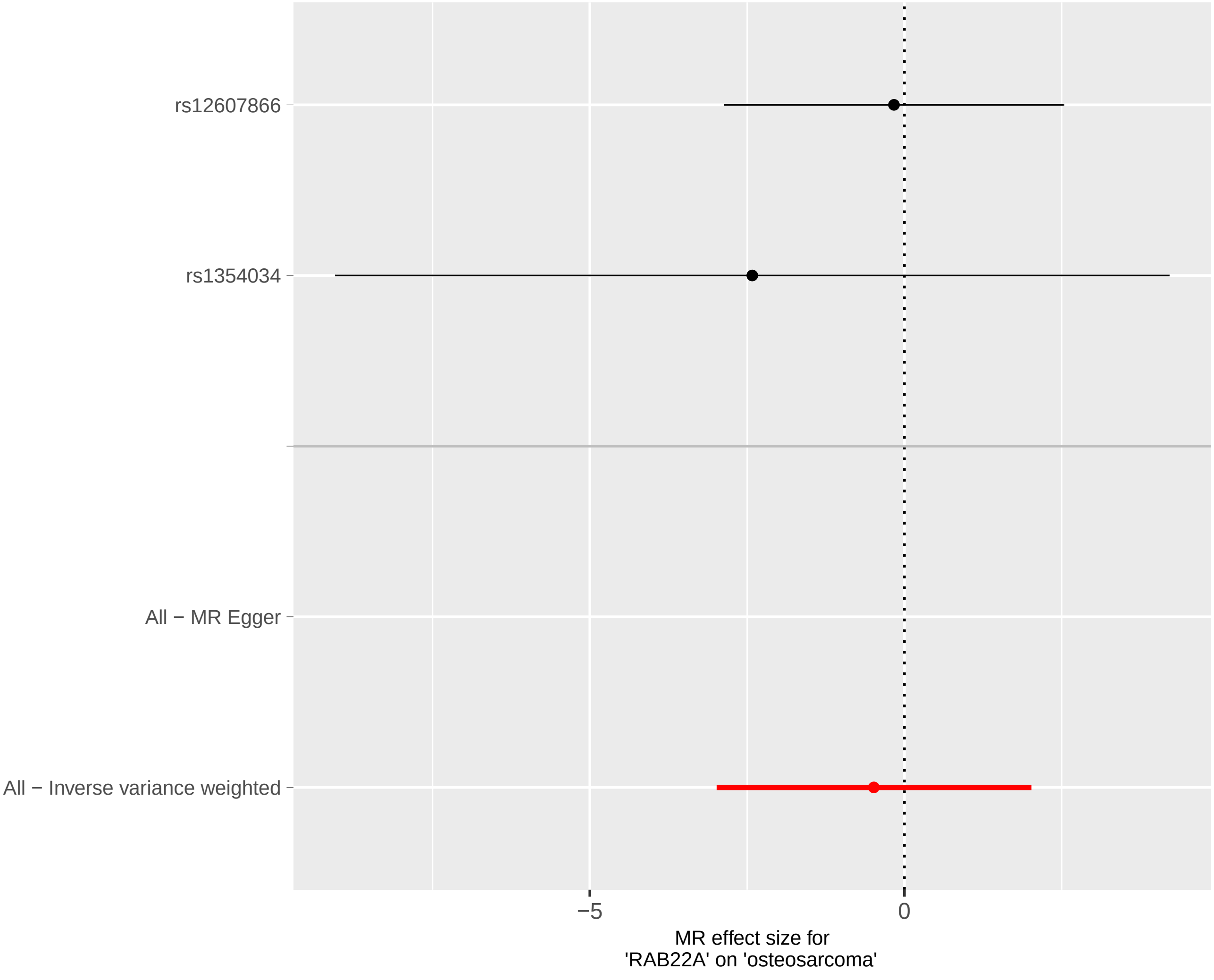
rs12607866
rs1354034
All − MR Egger
All − Inverse variance weighted
−5
0
MR effect size for
'RAB22A' on 'osteosarcoma'

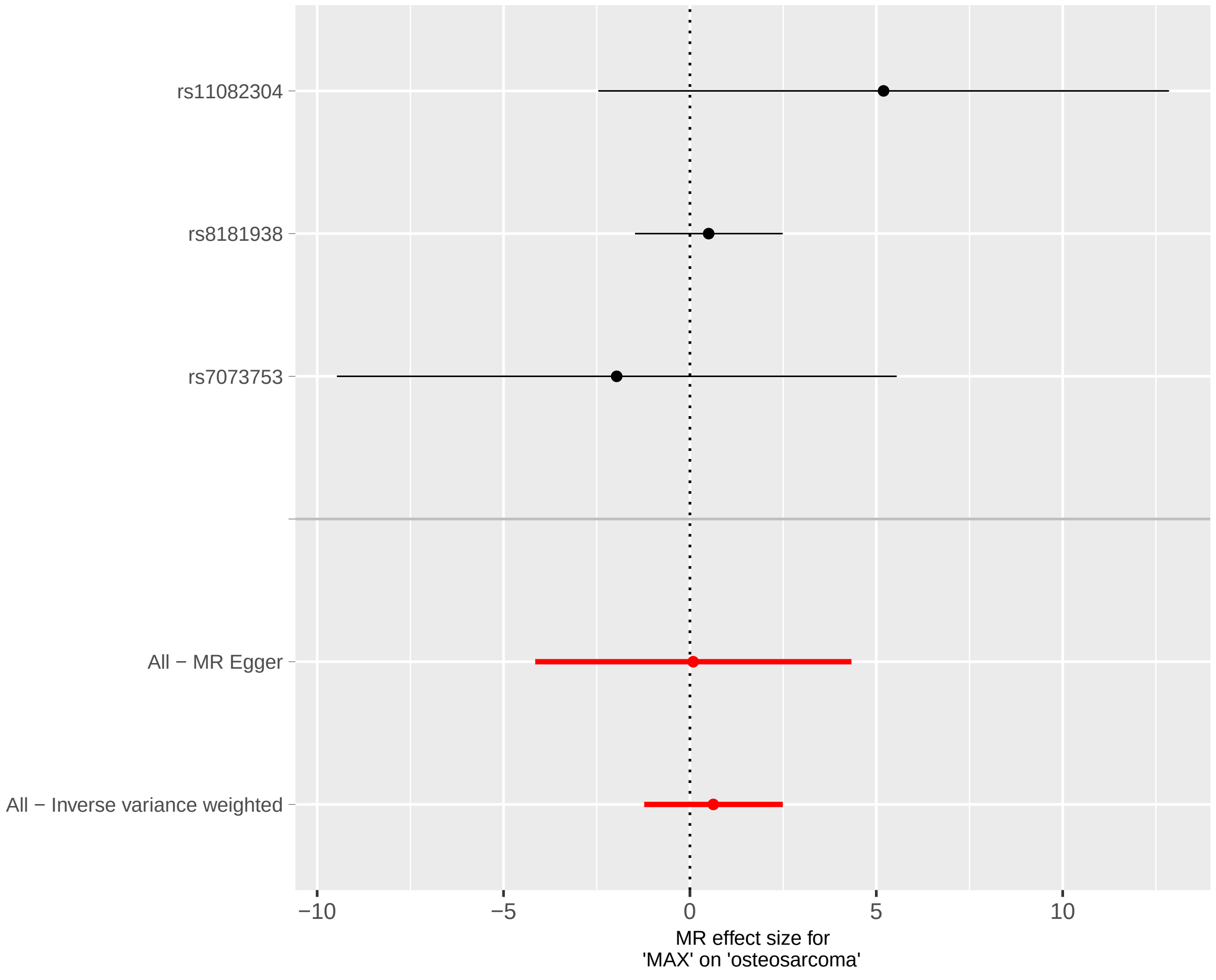

rs11082304
rs8181938
rs7073753
All − MR Egger
All − Inverse variance weighted
−10
−5
0
5
10
MR effect size for
'MAX' on 'osteosarcoma'

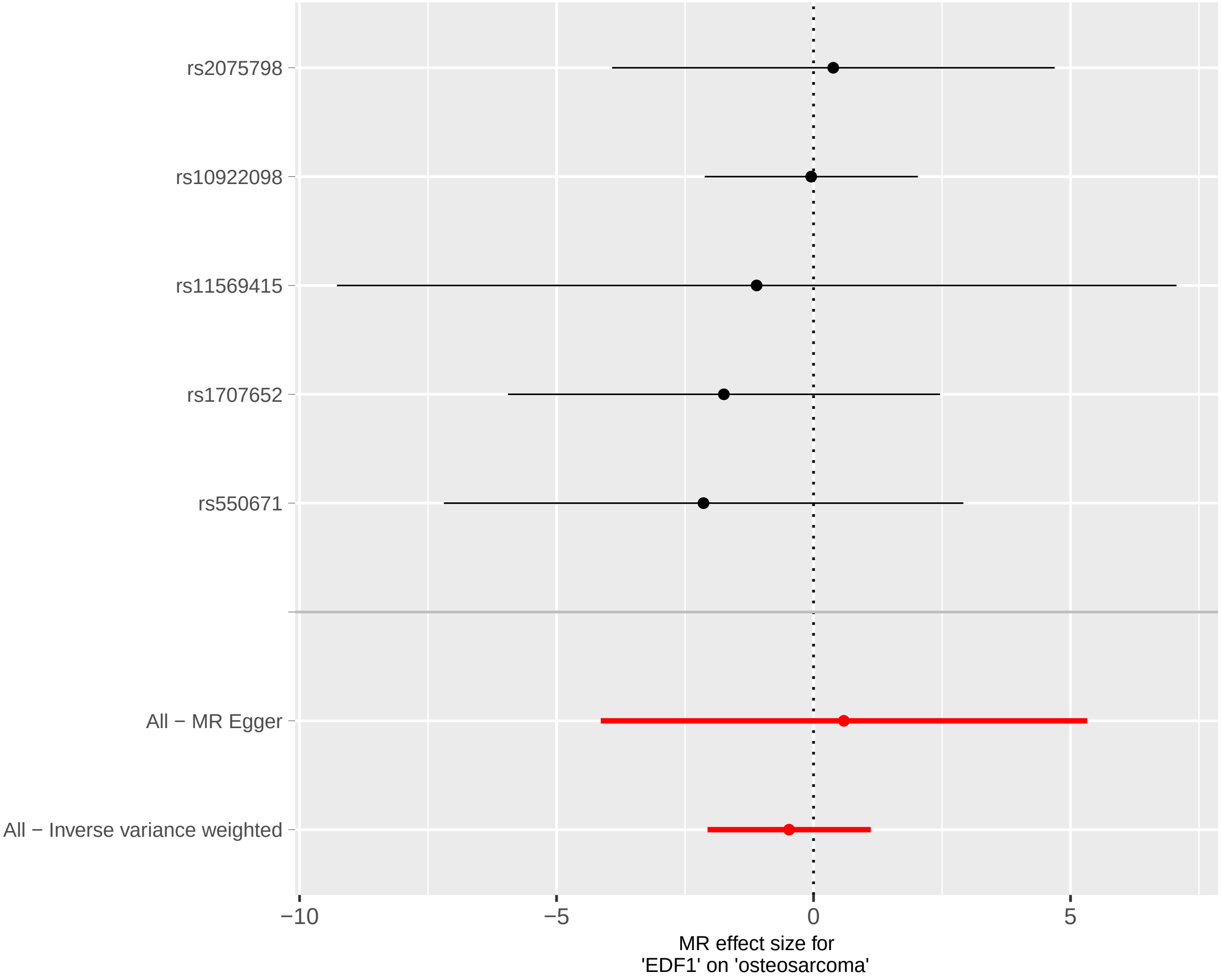

rs2075798
rs10922098
rs11569415
rs1707652
rs550671
All − MR Egger
All − Inverse variance weighted
−10
−5
0
5
MR effect size for
'EDF1' on 'osteosarcoma'

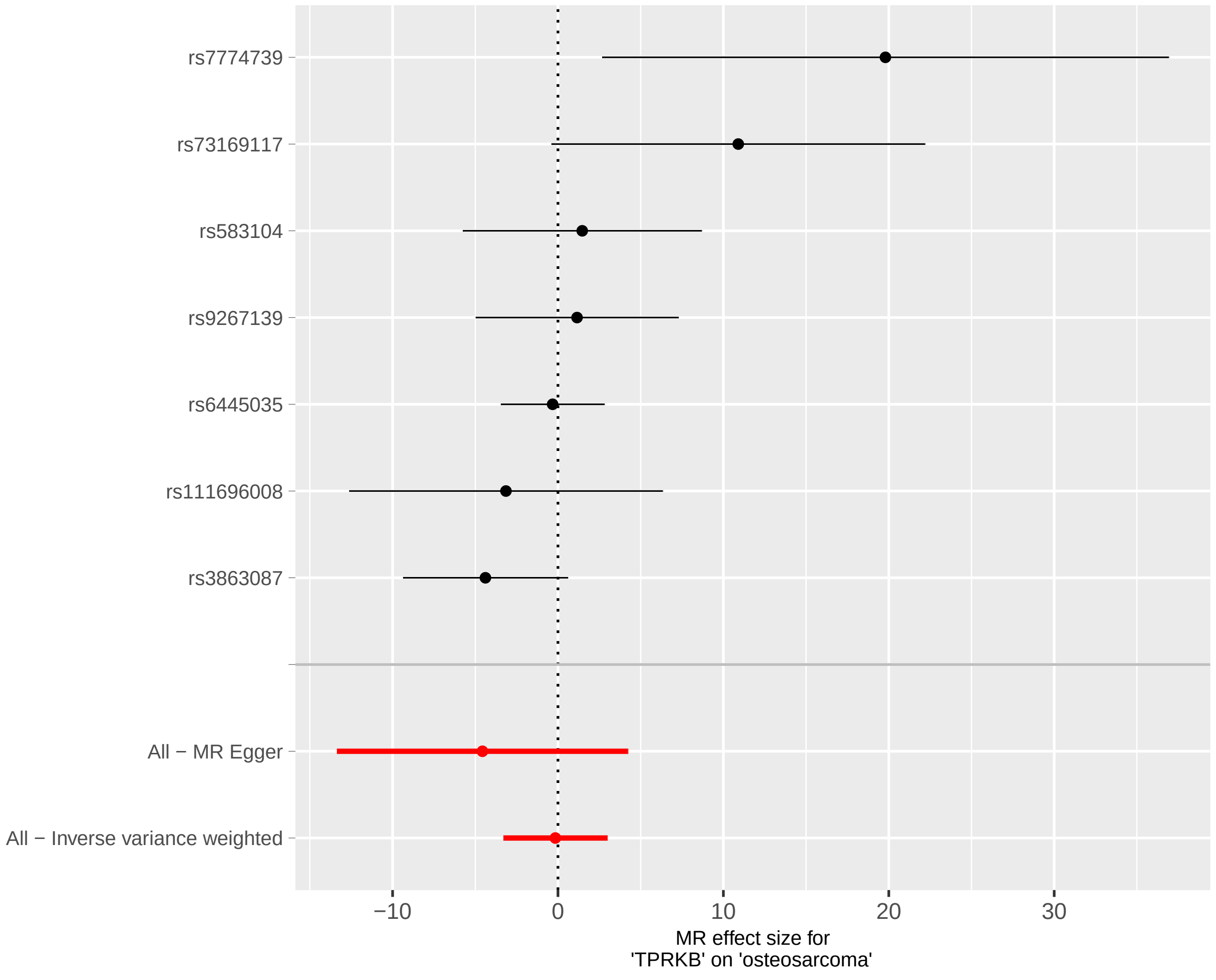

rs7774739
rs73169117
rs583104
rs9267139
rs6445035
rs111696008
rs3863087
All − MR Egger
All − Inverse variance weighted
−10
0
10
20
30
MR effect size for
'TPRKB' on 'osteosarcoma'

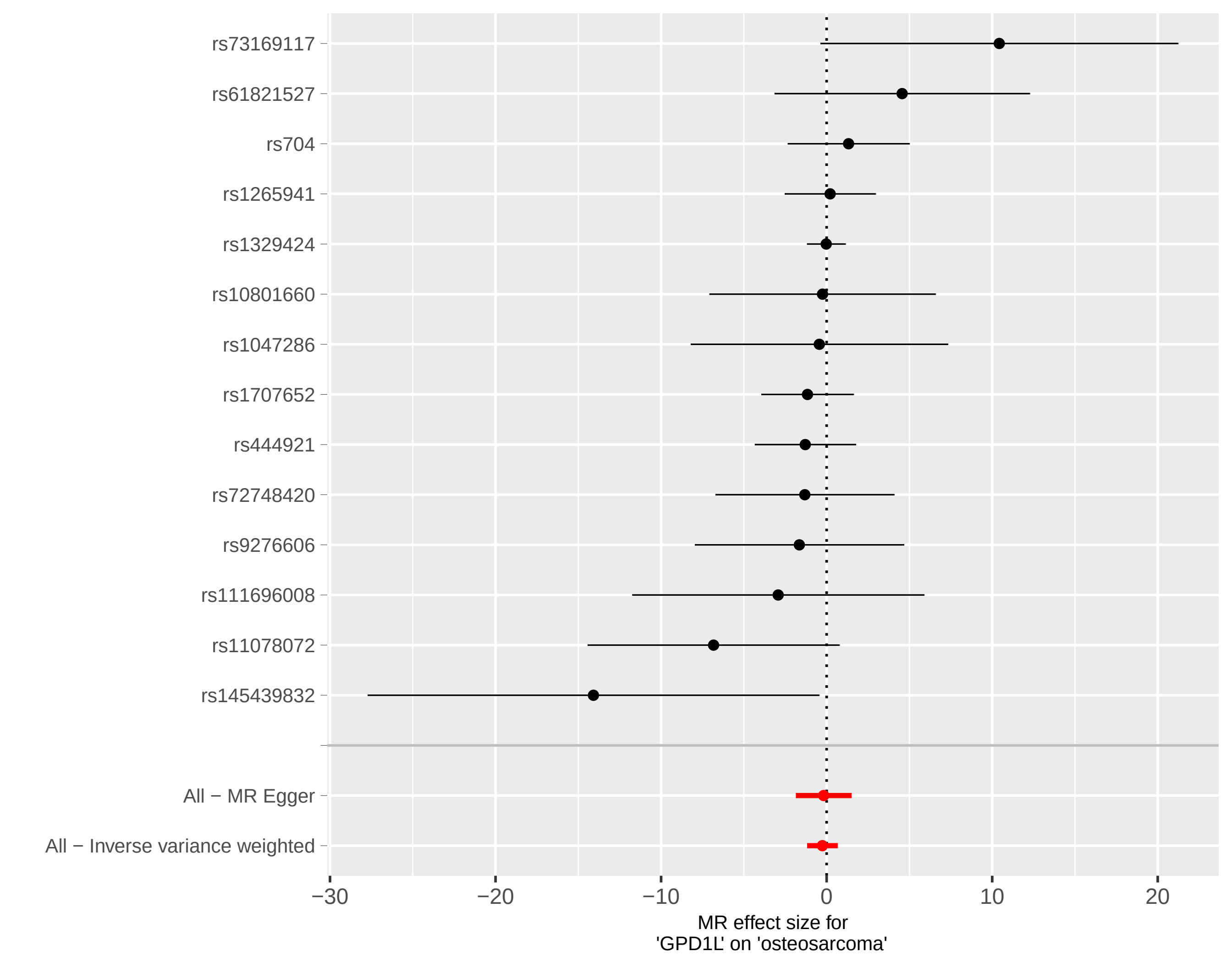

rs73169117
rs61821527
rs704
rs1265941
rs1329424
rs10801660
rs1047286
rs1707652
rs444921
rs72748420
rs9276606
rs111696008
rs11078072
rs145439832
All − MR Egger
All − Inverse variance weighted
−30
−20
−10
0
10
20
MR effect size for
'GPD1L' on 'osteosarcoma'

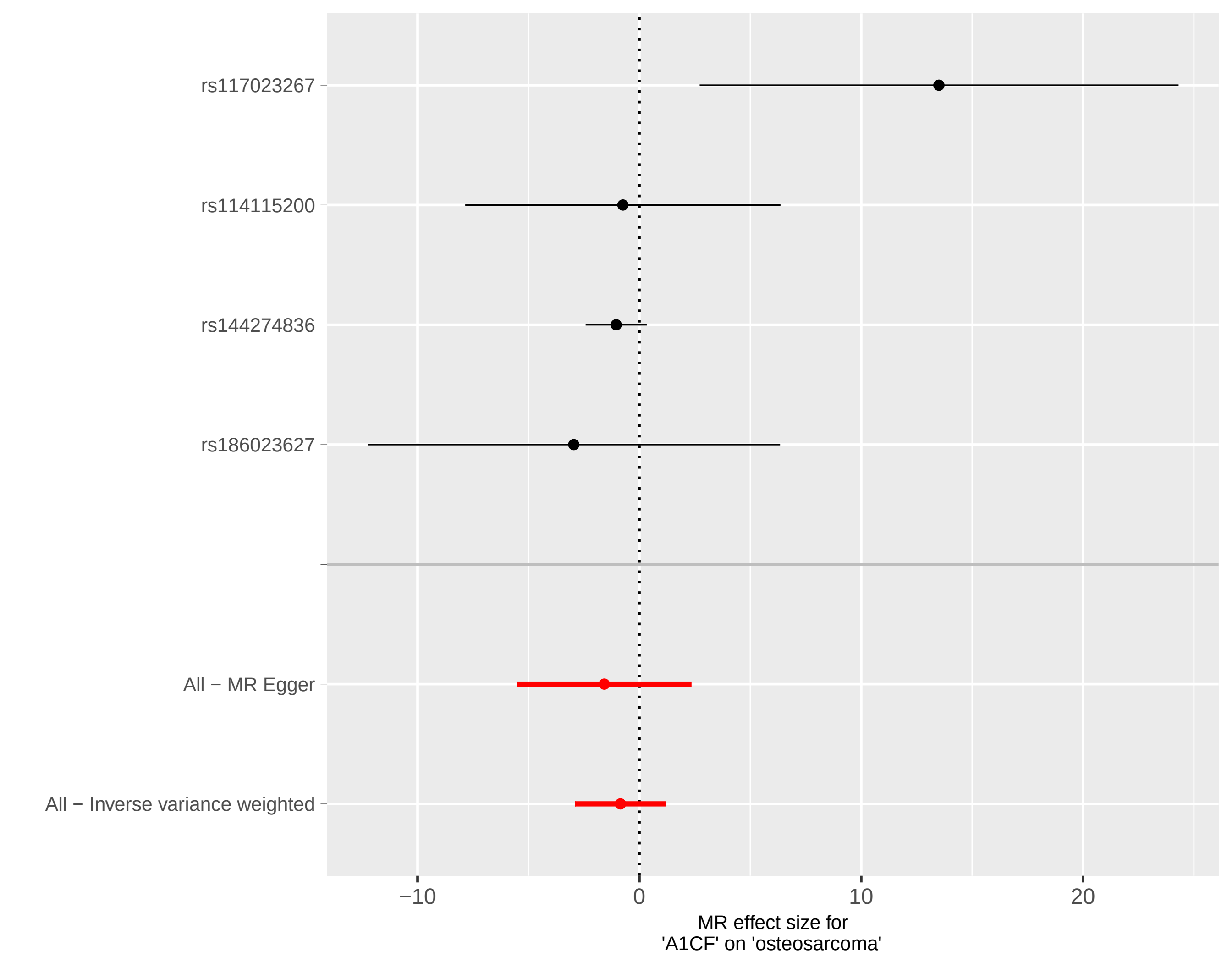

rs117023267
rs114115200
rs144274836
rs186023627
All − MR Egger
All − Inverse variance weighted
−10
0
10
20
MR effect size for
'A1CF' on 'osteosarcoma'

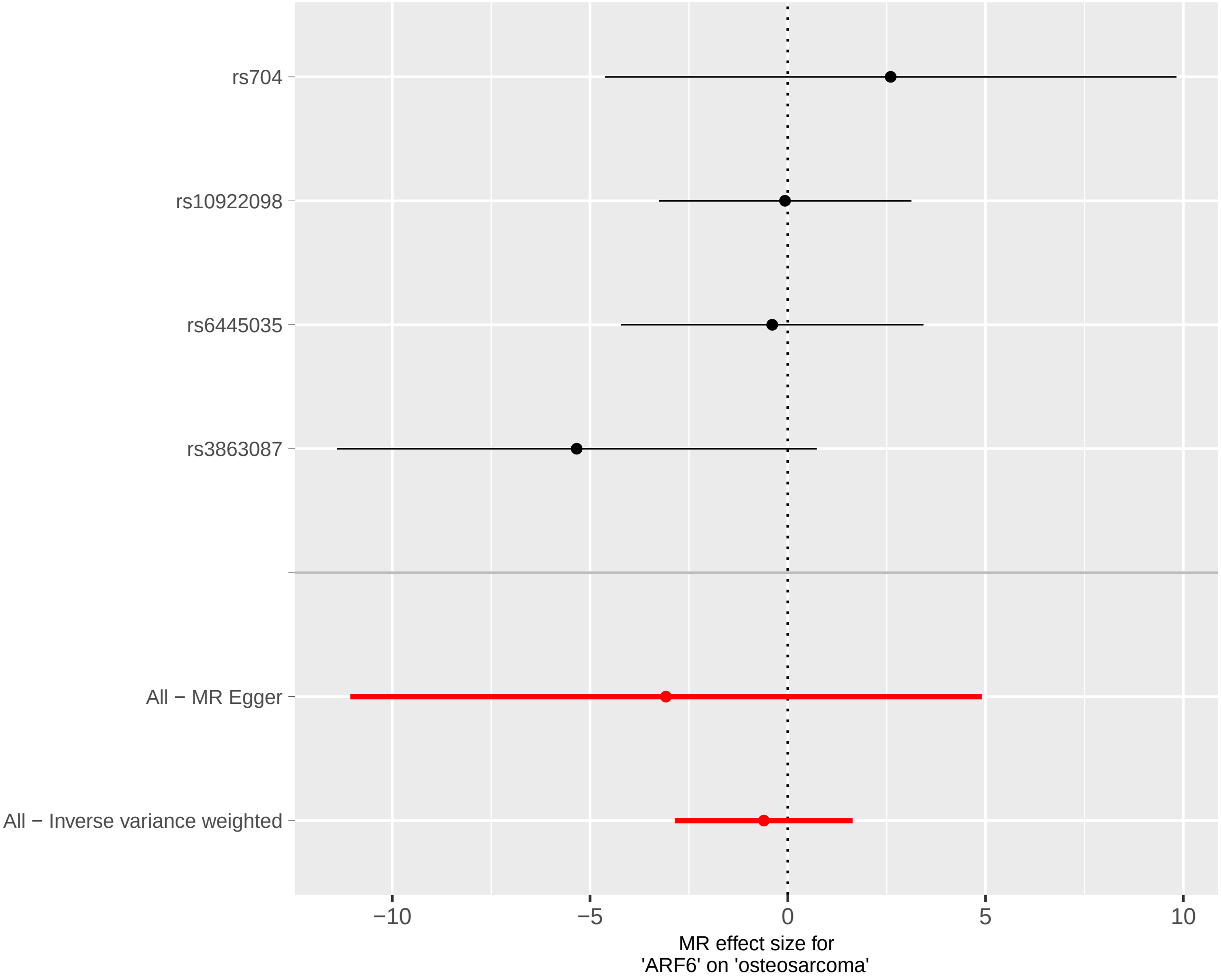

rs704
rs10922098
rs6445035
rs3863087
All − MR Egger
All − Inverse variance weighted
−10
−5
0
5
10
MR effect size for
'ARF6' on 'osteosarcoma'

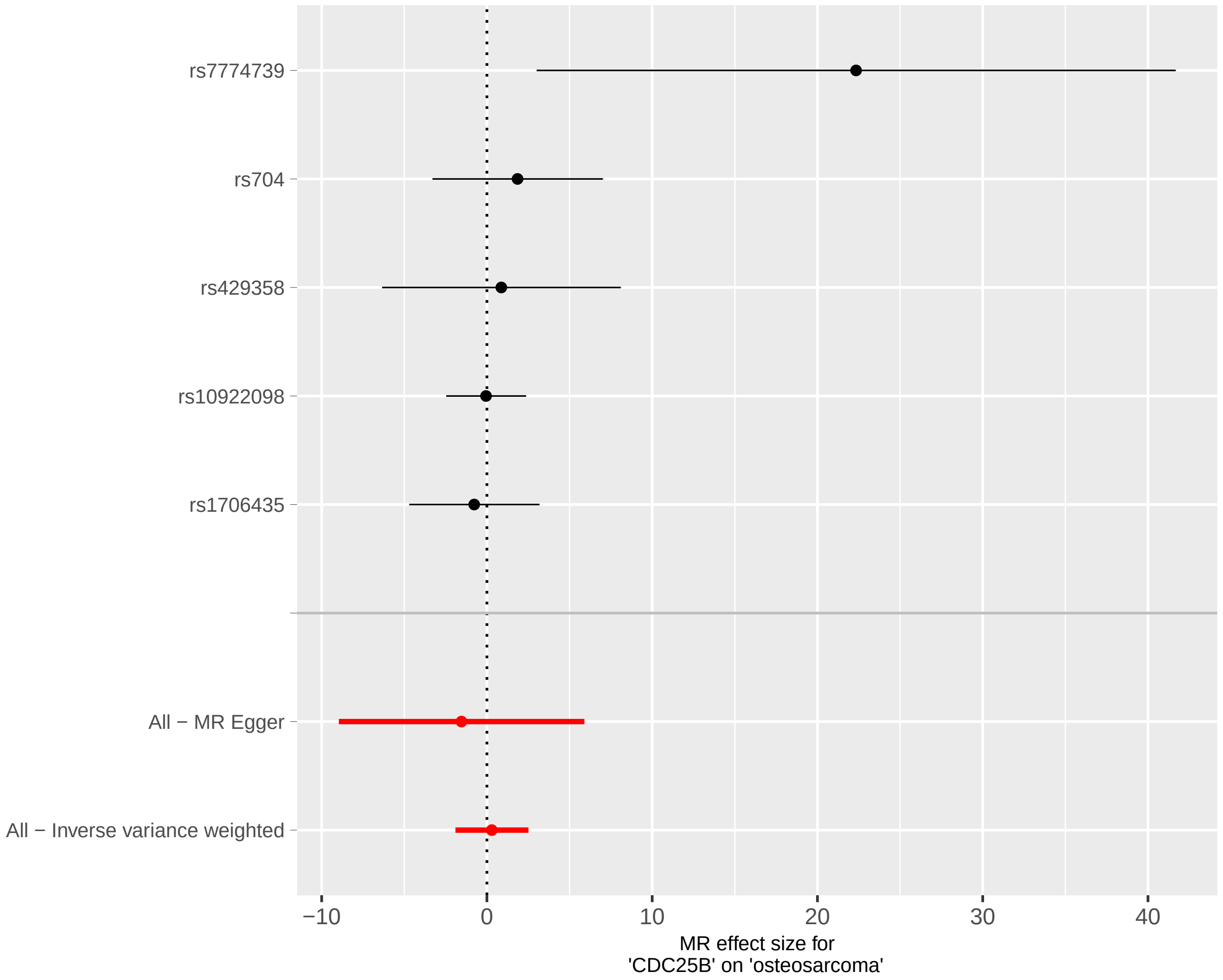

rs7774739
rs704
rs429358
rs10922098
rs1706435
All − MR Egger
All − Inverse variance weighted
−10
0
10
20
30
40
MR effect size for
'CDC25B' on 'osteosarcoma'

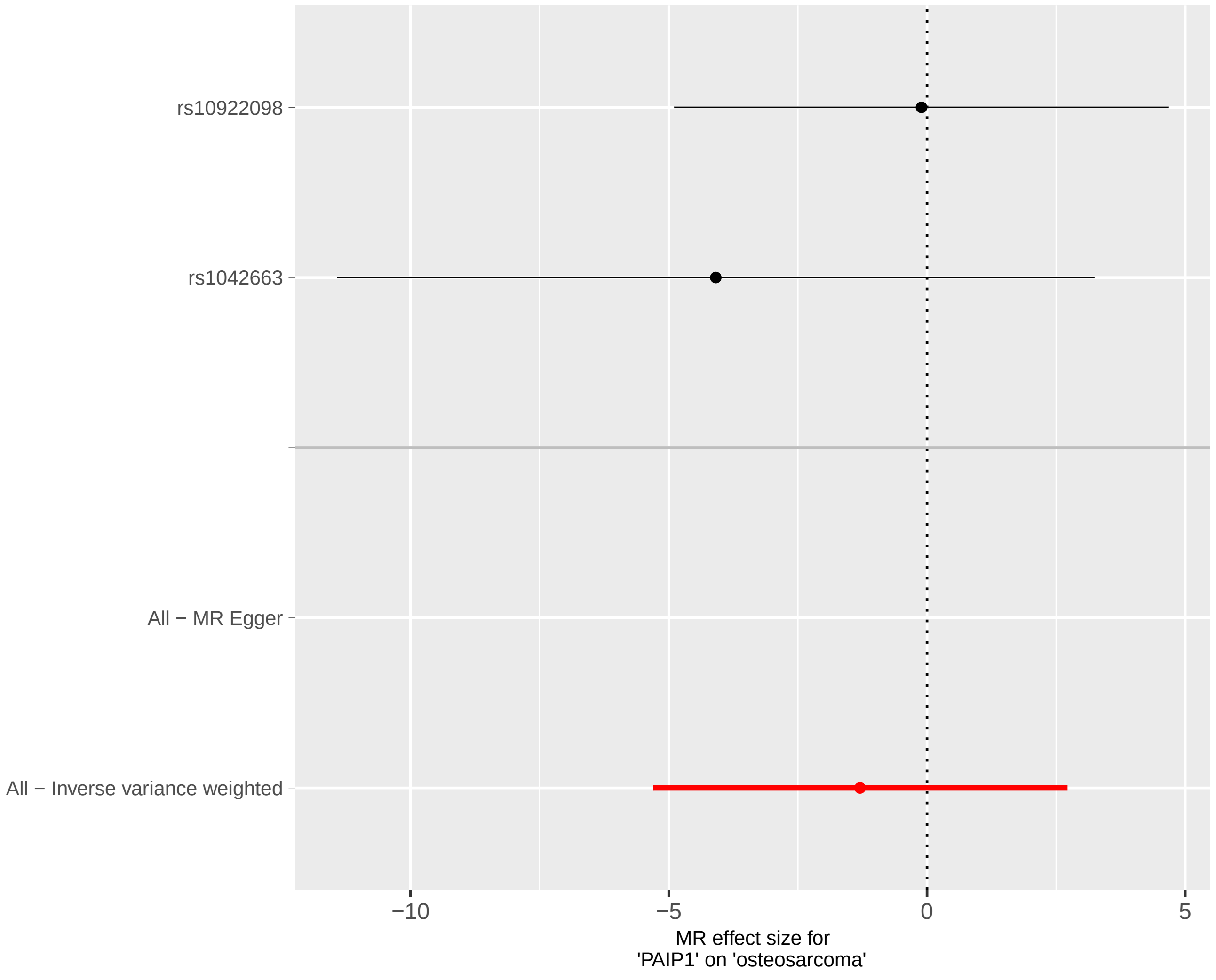
rs10922098
rs1042663
All − MR Egger
All − Inverse variance weighted
−10
−5
0
5
MR effect size for
'PAIP1' on 'osteosarcoma'

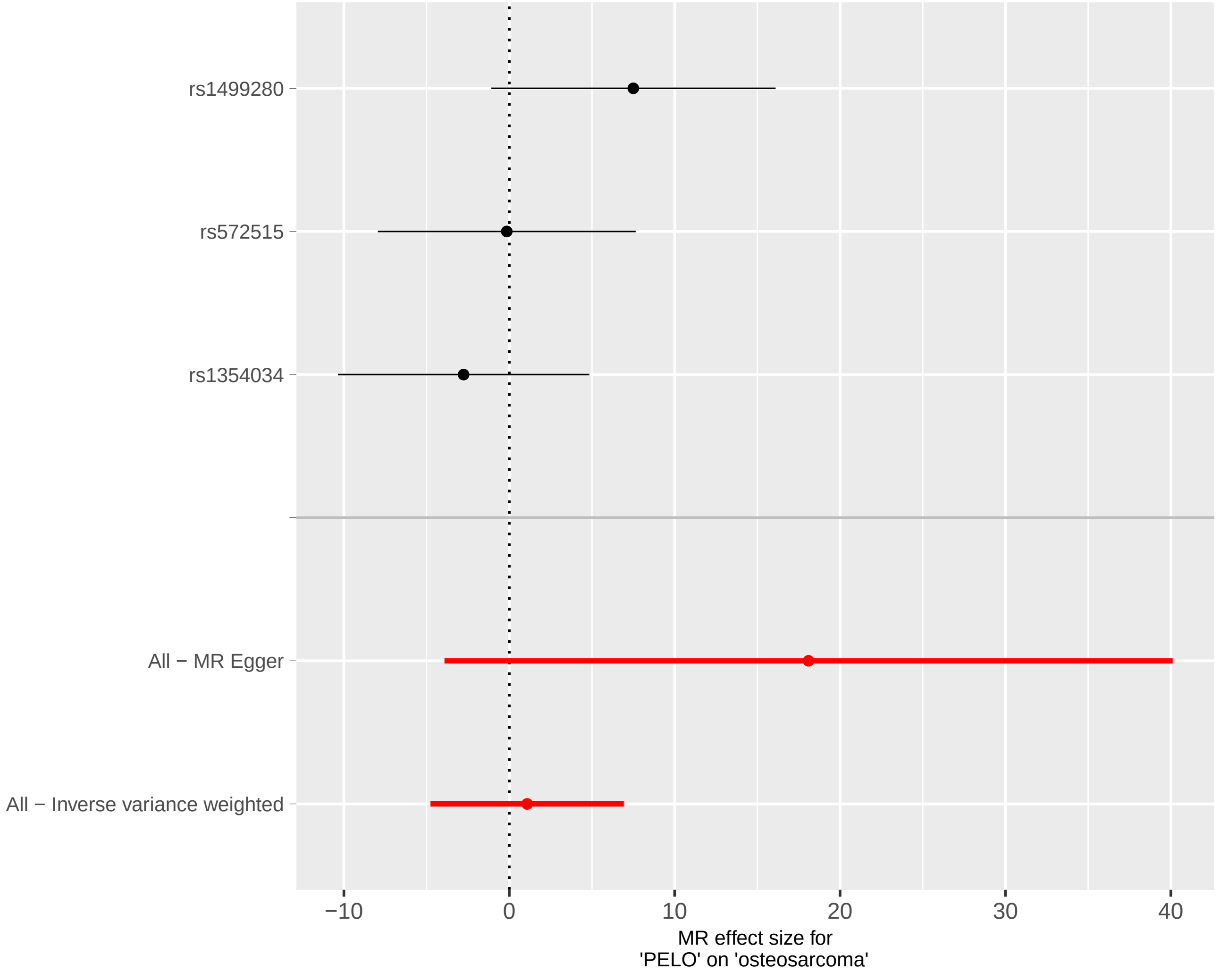

rs1499280
rs572515
rs1354034
All − MR Egger
All − Inverse variance weighted
−10
0
10
20
30
40
MR effect size for
'PELO' on 'osteosarcoma'

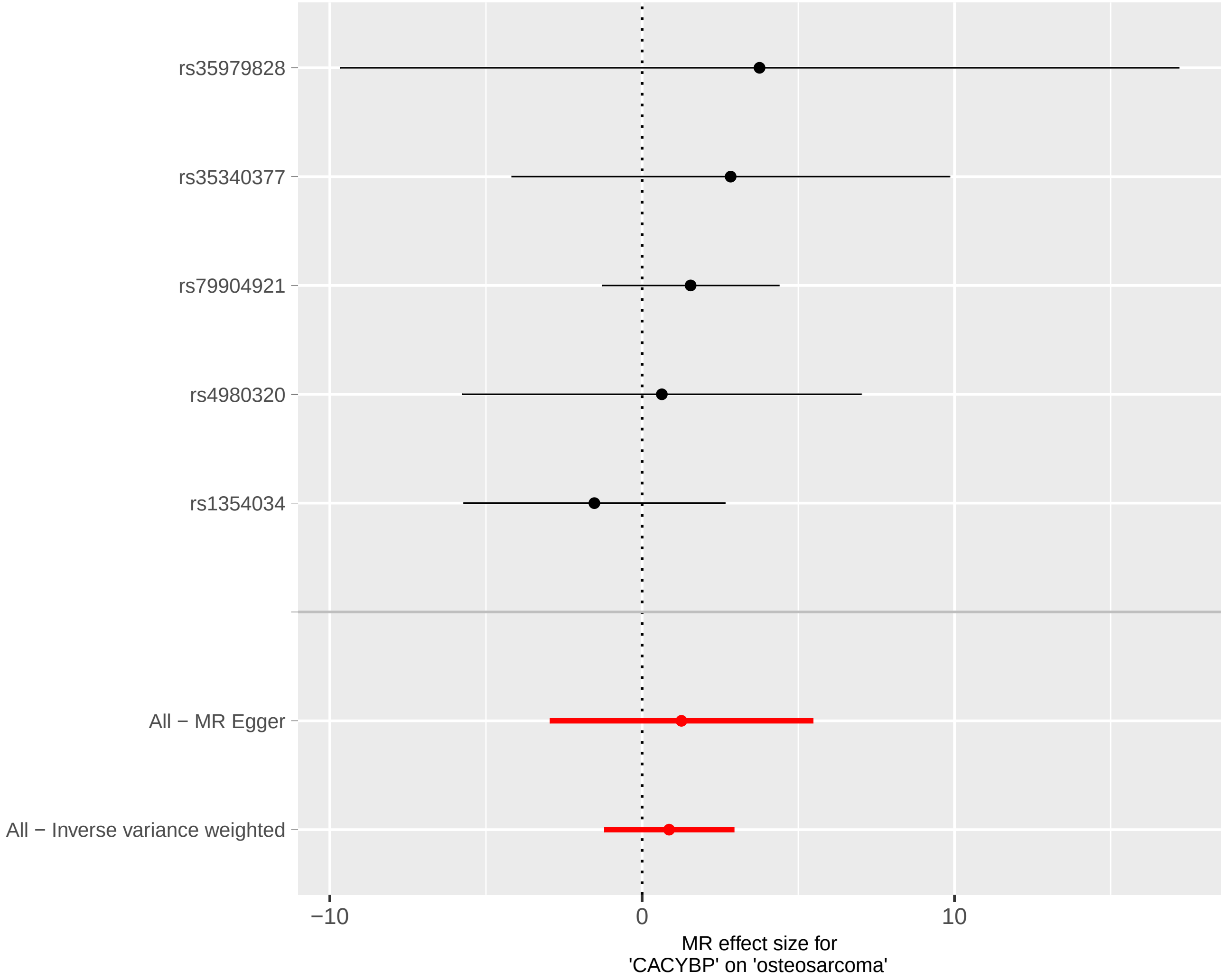

rs35979828
rs35340377
rs79904921
rs4980320
rs1354034
All − MR Egger
All − Inverse variance weighted
−10
0
10
MR effect size for
'CACYBP' on 'osteosarcoma'

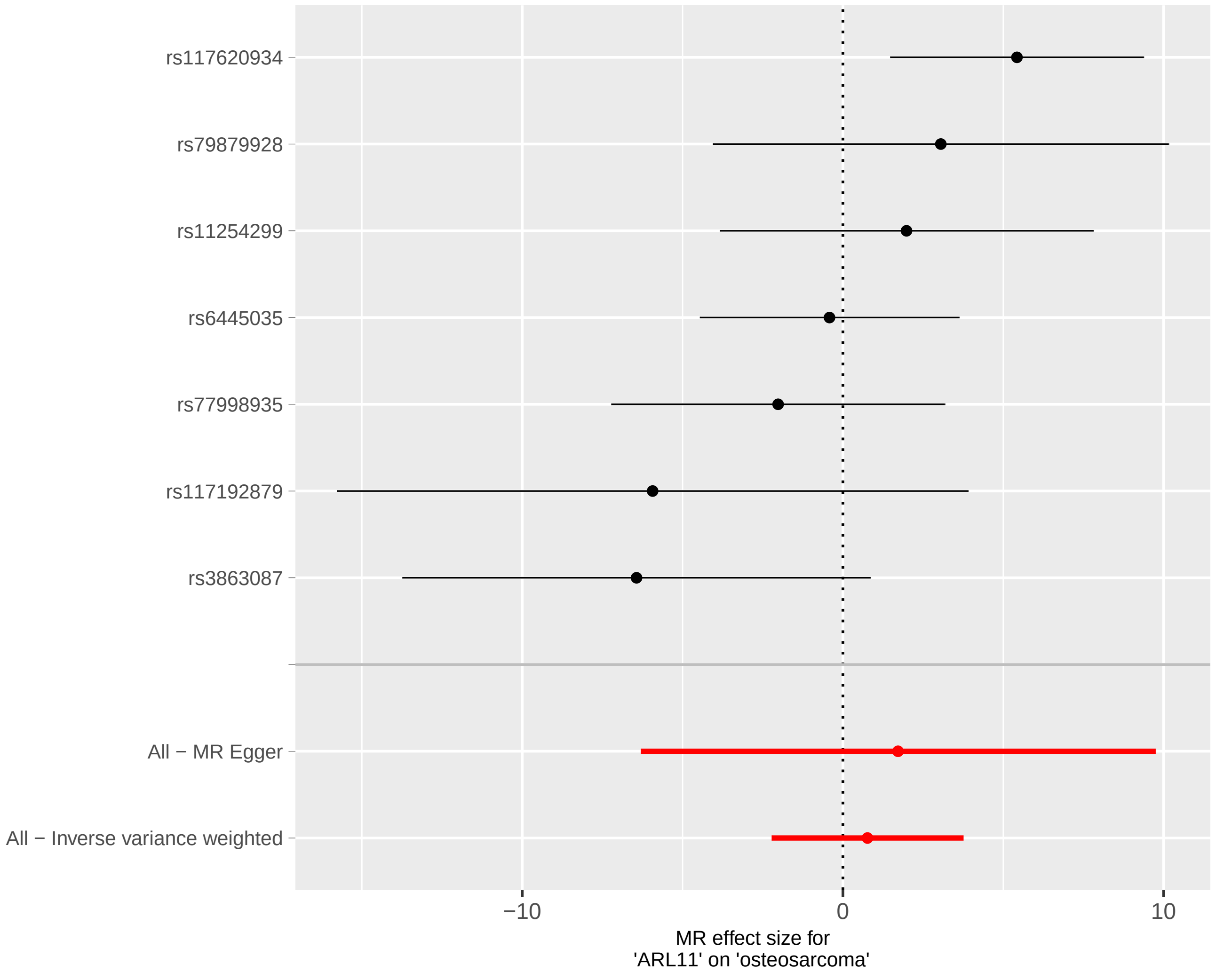
rs117620934
rs79879928
rs11254299
rs6445035
rs77998935
rs117192879
rs3863087
All − MR Egger
All − Inverse variance weighted
−10
0
10
MR effect size for
'ARL11' on 'osteosarcoma'

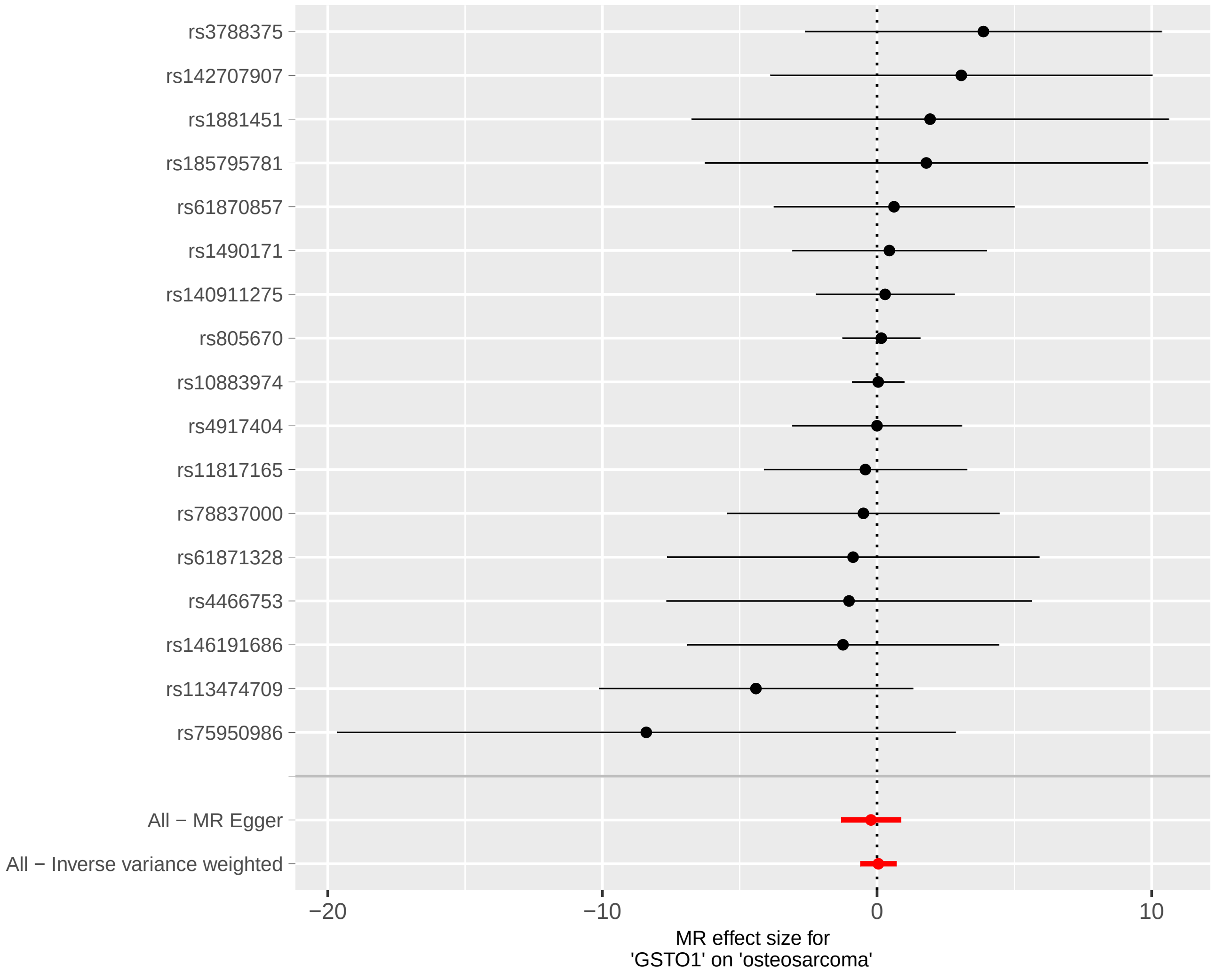

rs3788375
rs142707907
rs1881451
rs185795781
rs61870857
rs1490171
rs140911275
rs805670
rs10883974
rs4917404
rs11817165
rs78837000
rs61871328
rs4466753
rs146191686
rs113474709
rs75950986
All − MR Egger
All − Inverse variance weighted
−20
−10
0
10
MR effect size for
'GSTO1' on 'osteosarcoma'

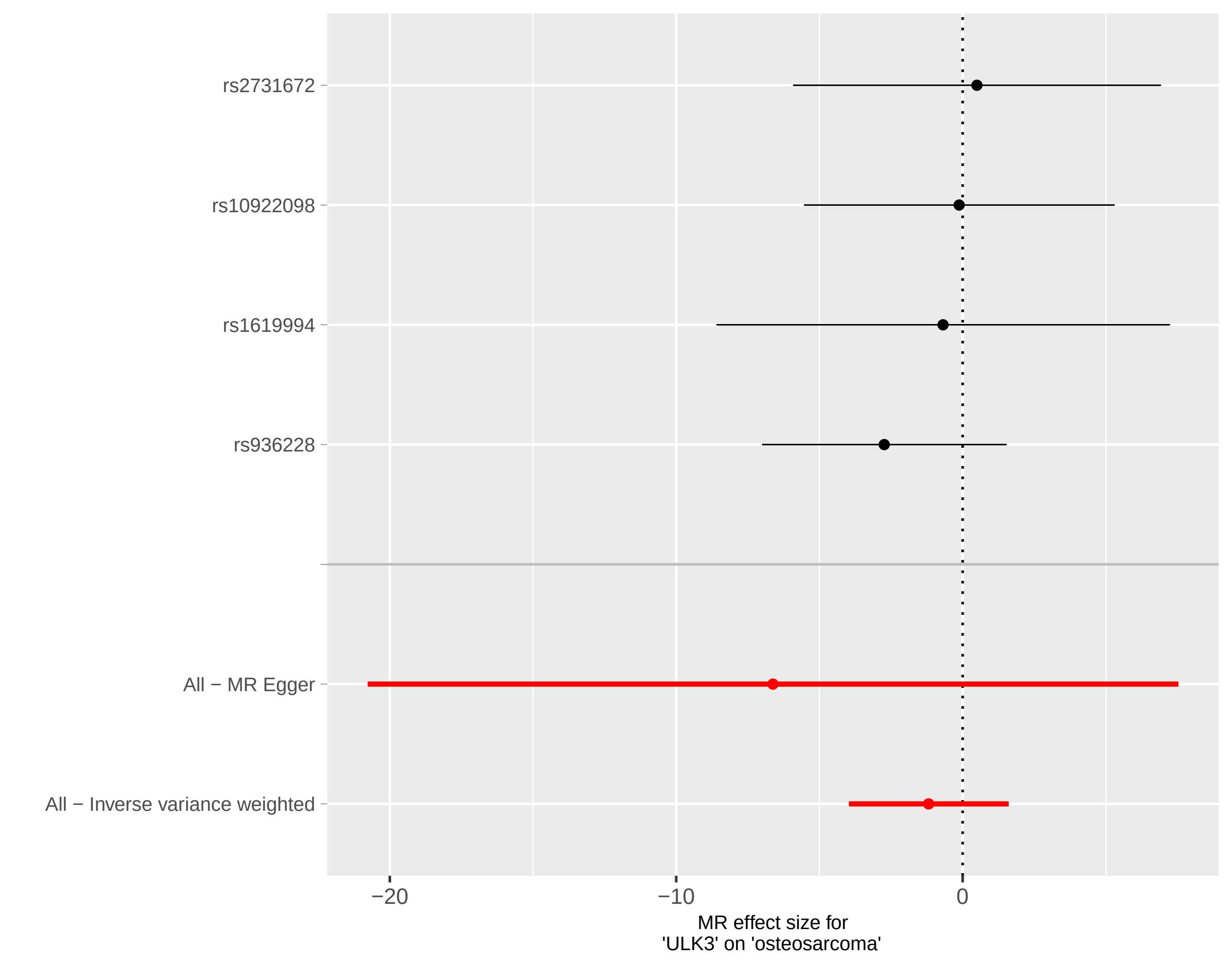
rs2731672
rs10922098
rs1619994
rs936228
All − MR Egger
All − Inverse variance weighted
−20
−10
0
MR effect size for
'ULK3' on 'osteosarcoma'

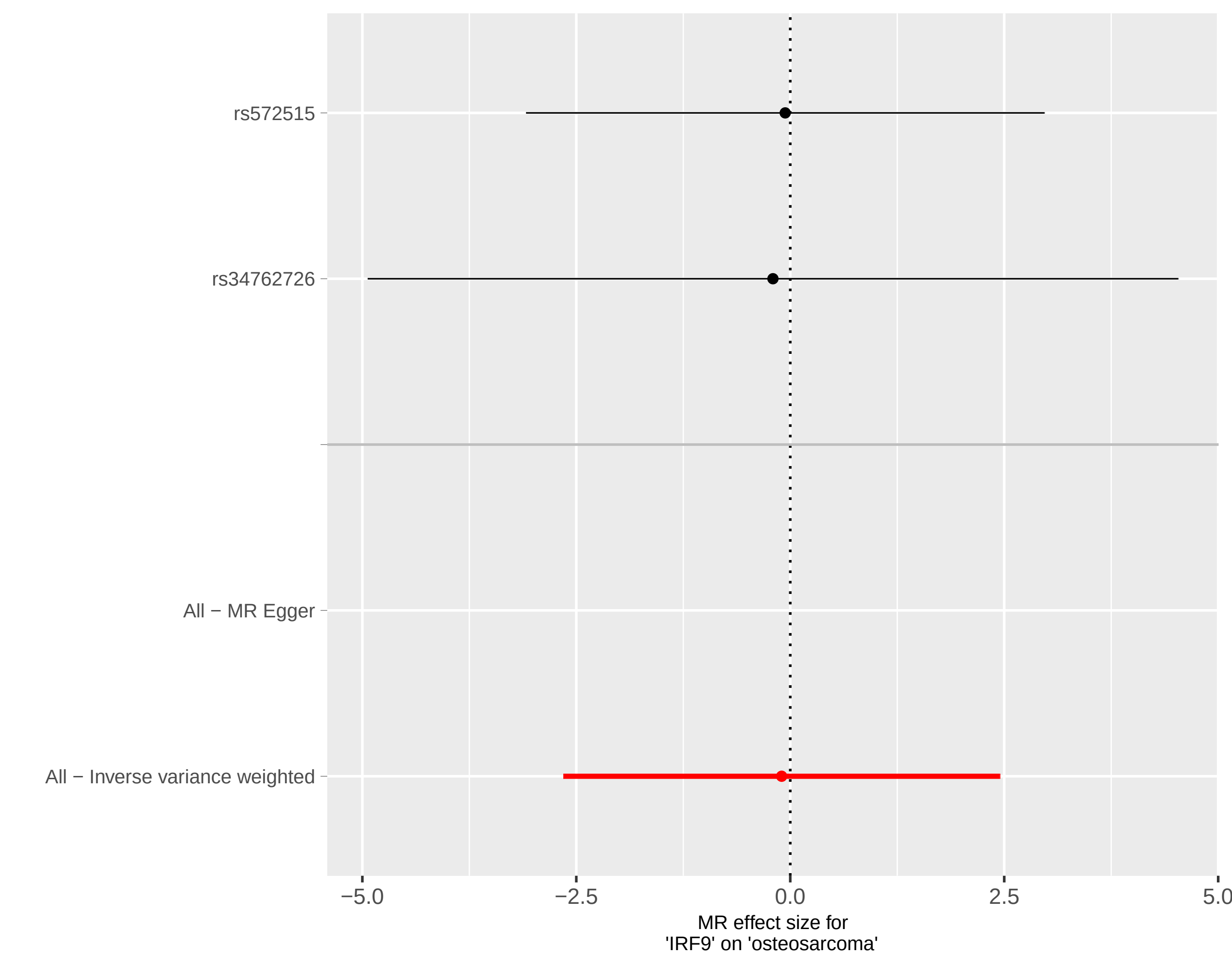

rs572515
rs34762726
All − MR Egger
All − Inverse variance weighted
−5.0
−2.5
0.0
2.5
5.0
MR effect size for
'IRF9' on 'osteosarcoma'

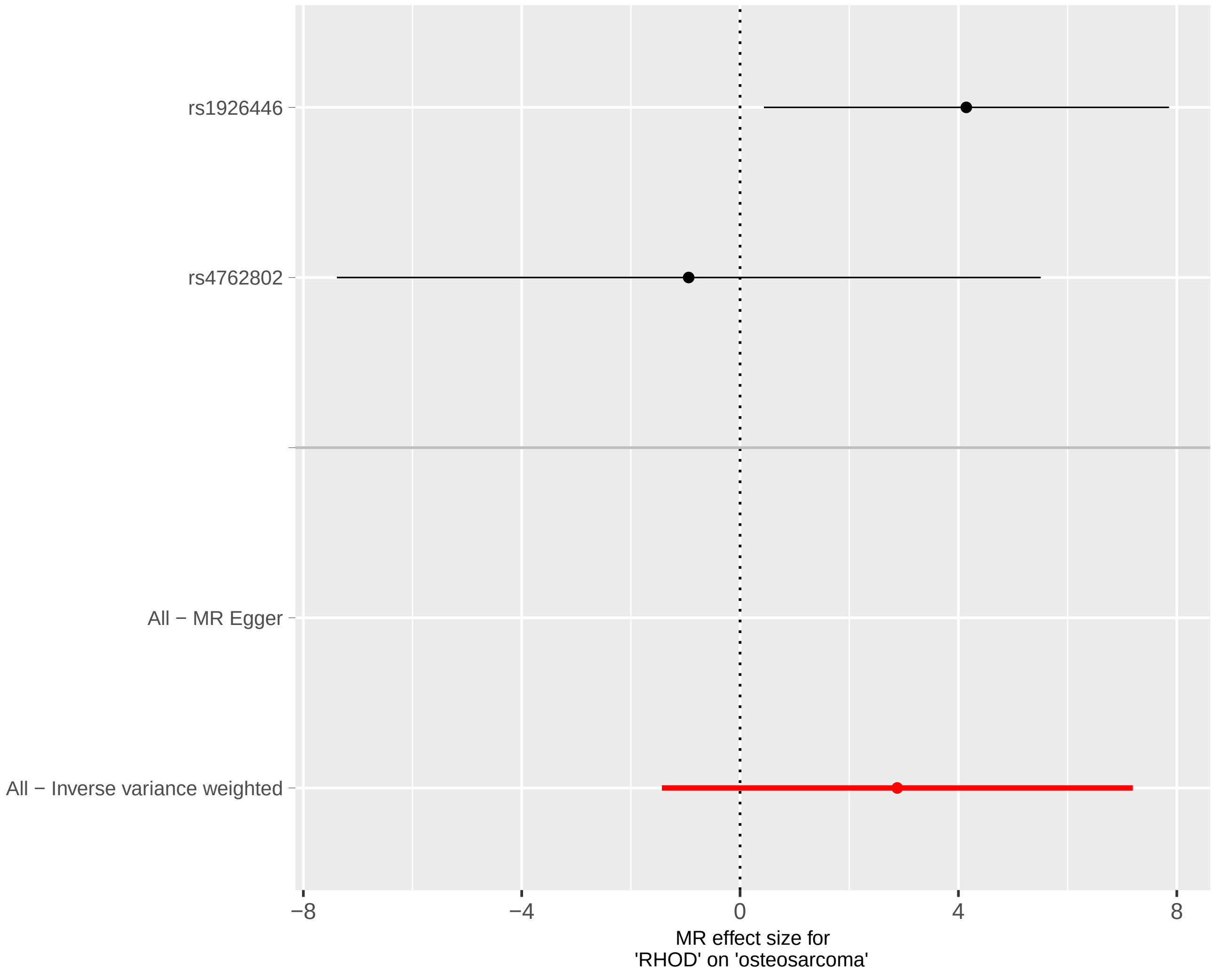
rs1926446
rs4762802
All − MR Egger
All − Inverse variance weighted
−8
−4
0
4
8
MR effect size for
'RHOD' on 'osteosarcoma'

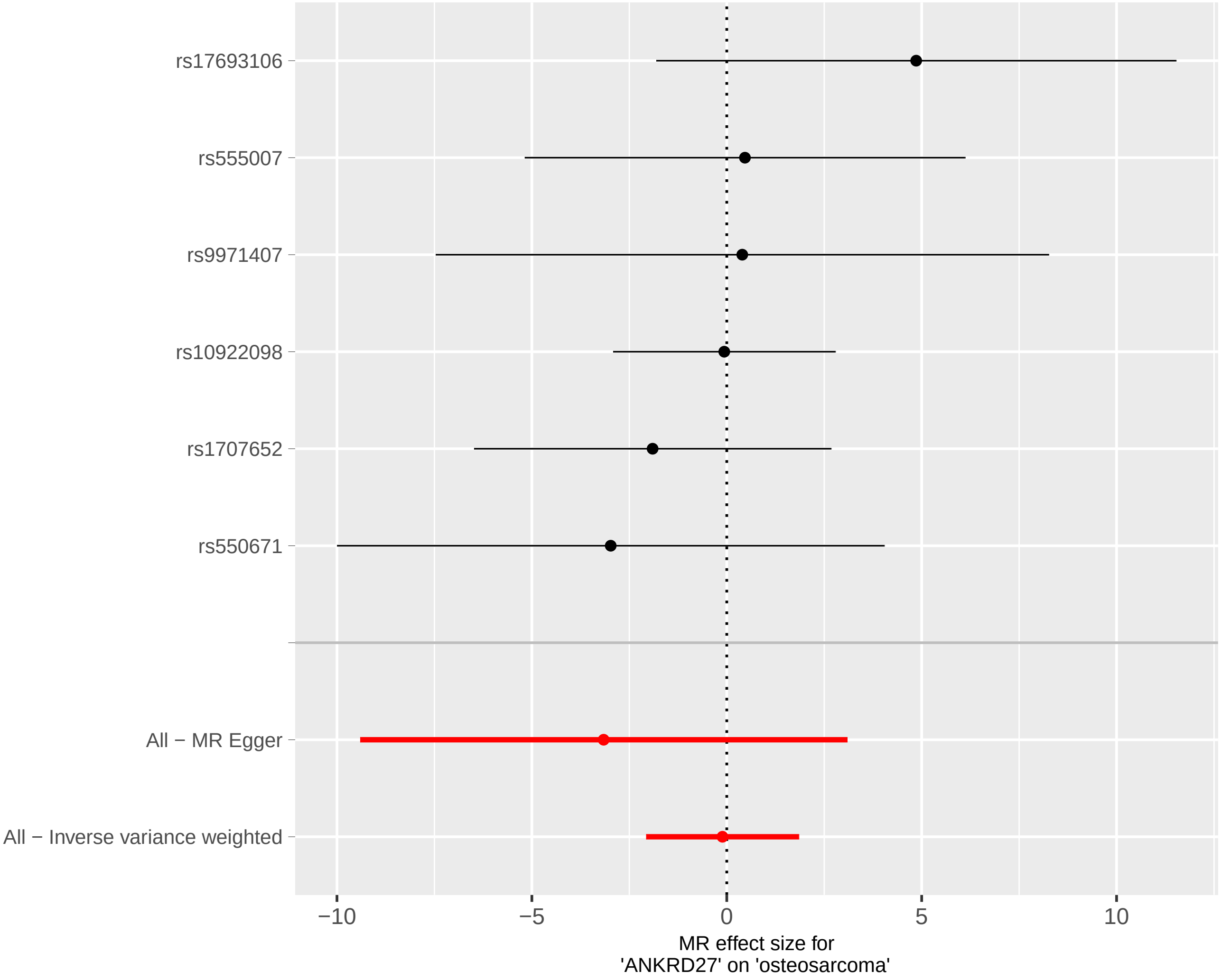
rs17693106
rs555007
rs9971407
rs10922098
rs1707652
rs550671
All − MR Egger
All − Inverse variance weighted
−10
−5
0
5
10
MR effect size for
'ANKRD27' on 'osteosarcoma'

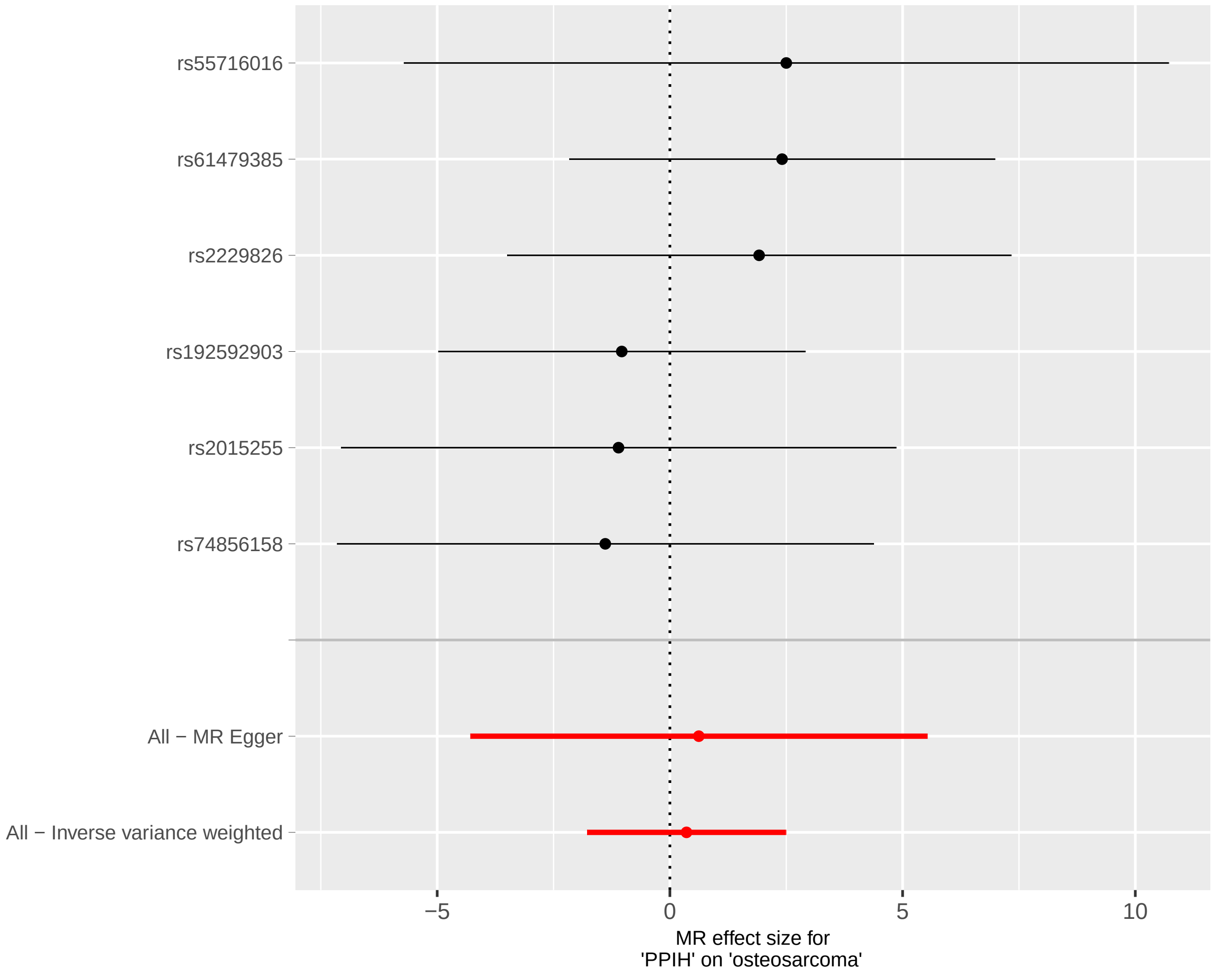

rs55716016
rs61479385
rs2229826
rs192592903
rs2015255
rs74856158
All − MR Egger
All − Inverse variance weighted
−5
0
5
10
MR effect size for
'PPIH' on 'osteosarcoma'

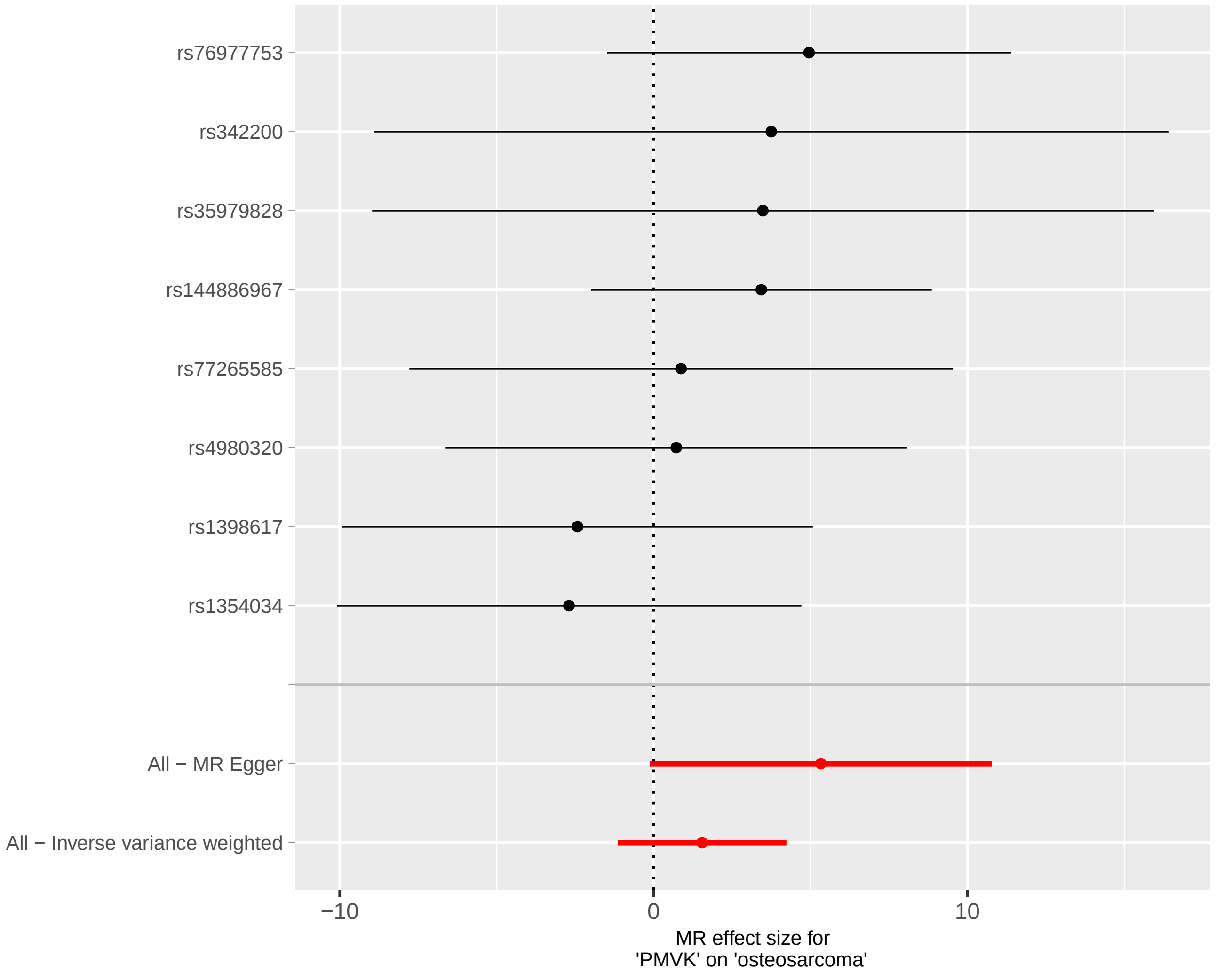
rs76977753
rs342200
rs35979828
rs144886967
rs77265585
rs4980320
rs1398617
rs1354034
All − MR Egger
All − Inverse variance weighted
−10
0
10
MR effect size for
'PMVK' on 'osteosarcoma'

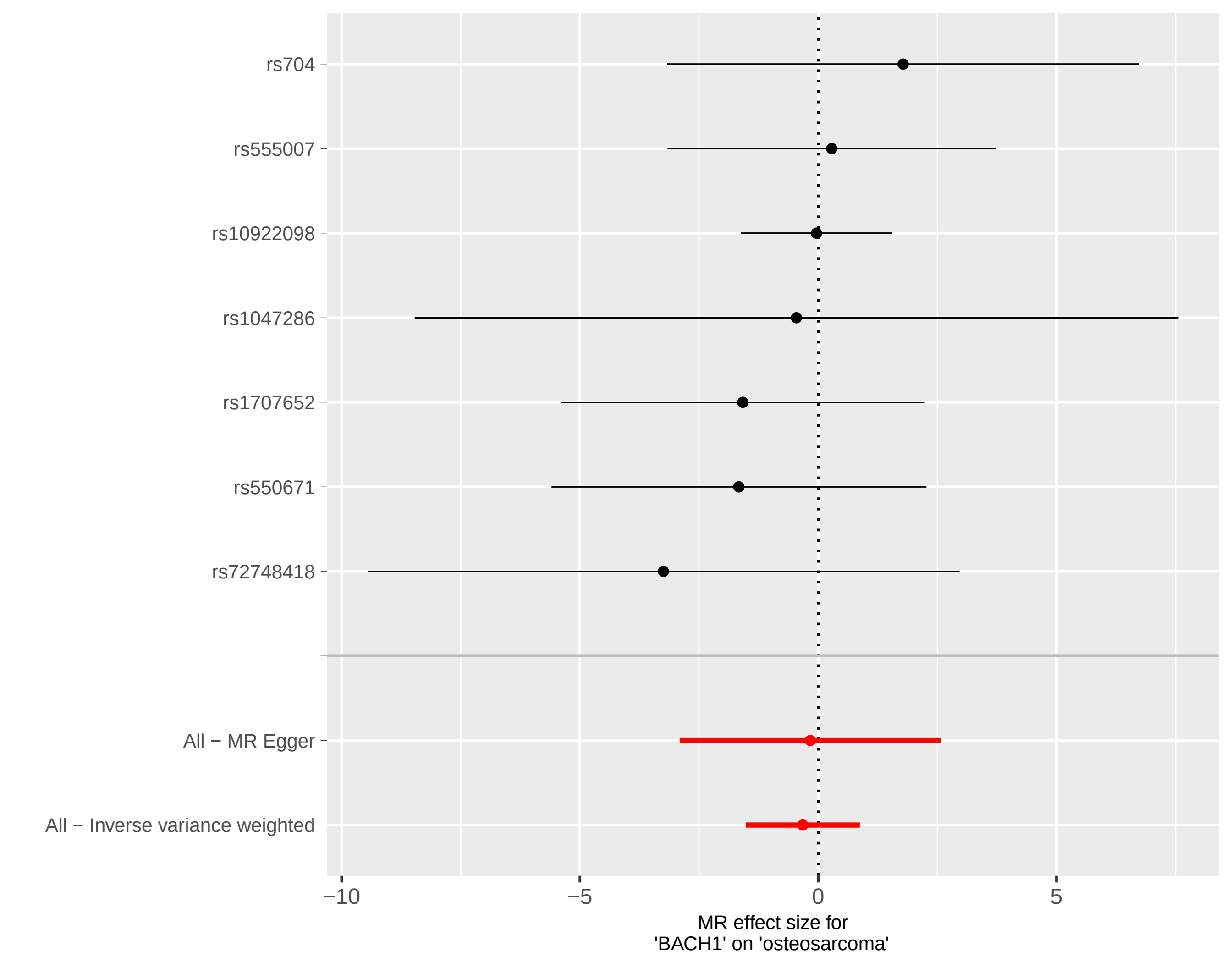
rs704
rs555007
rs10922098
rs1047286
rs1707652
rs550671
rs72748418
All − MR Egger
All − Inverse variance weighted
−10
−5
0
5
MR effect size for
'BACH1' on 'osteosarcoma'

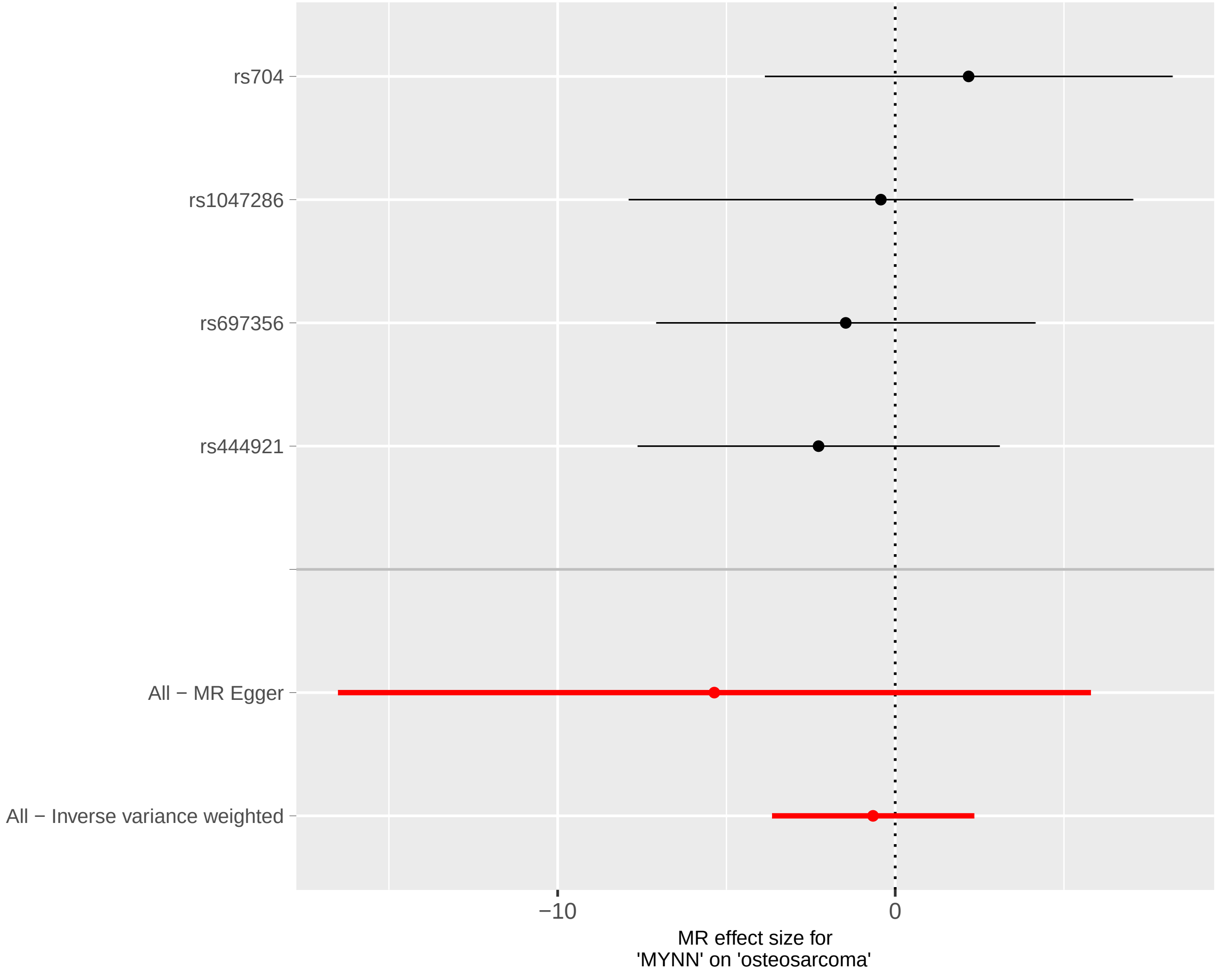

rs704
rs1047286
rs697356
rs444921
All − MR Egger
All − Inverse variance weighted
−10
0
MR effect size for
'MYNN' on 'osteosarcoma'

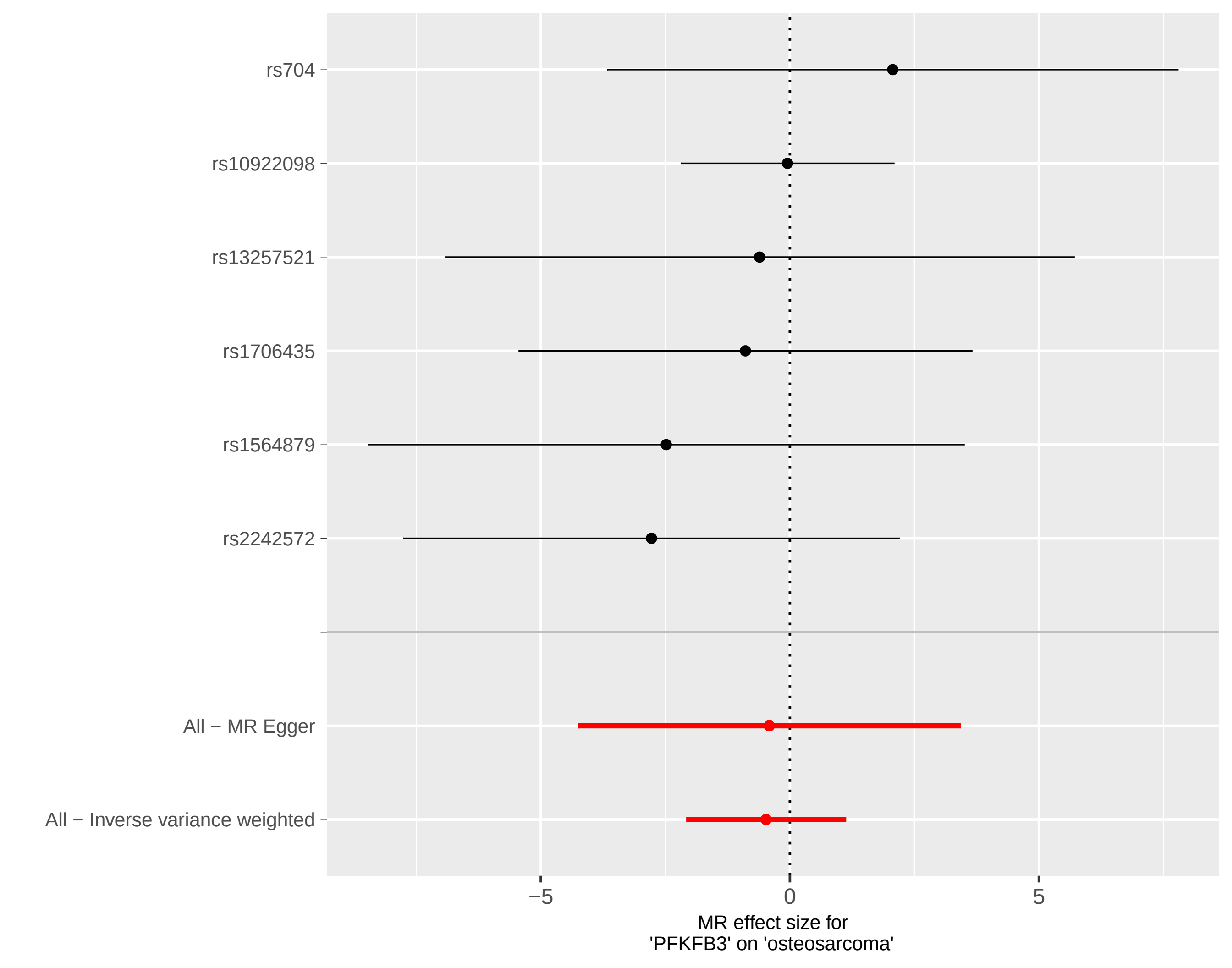
rs704
rs10922098
rs13257521
rs1706435
rs1564879
rs2242572
All − MR Egger
All − Inverse variance weighted
−5
0
5
MR effect size for
'PFKFB3' on 'osteosarcoma'

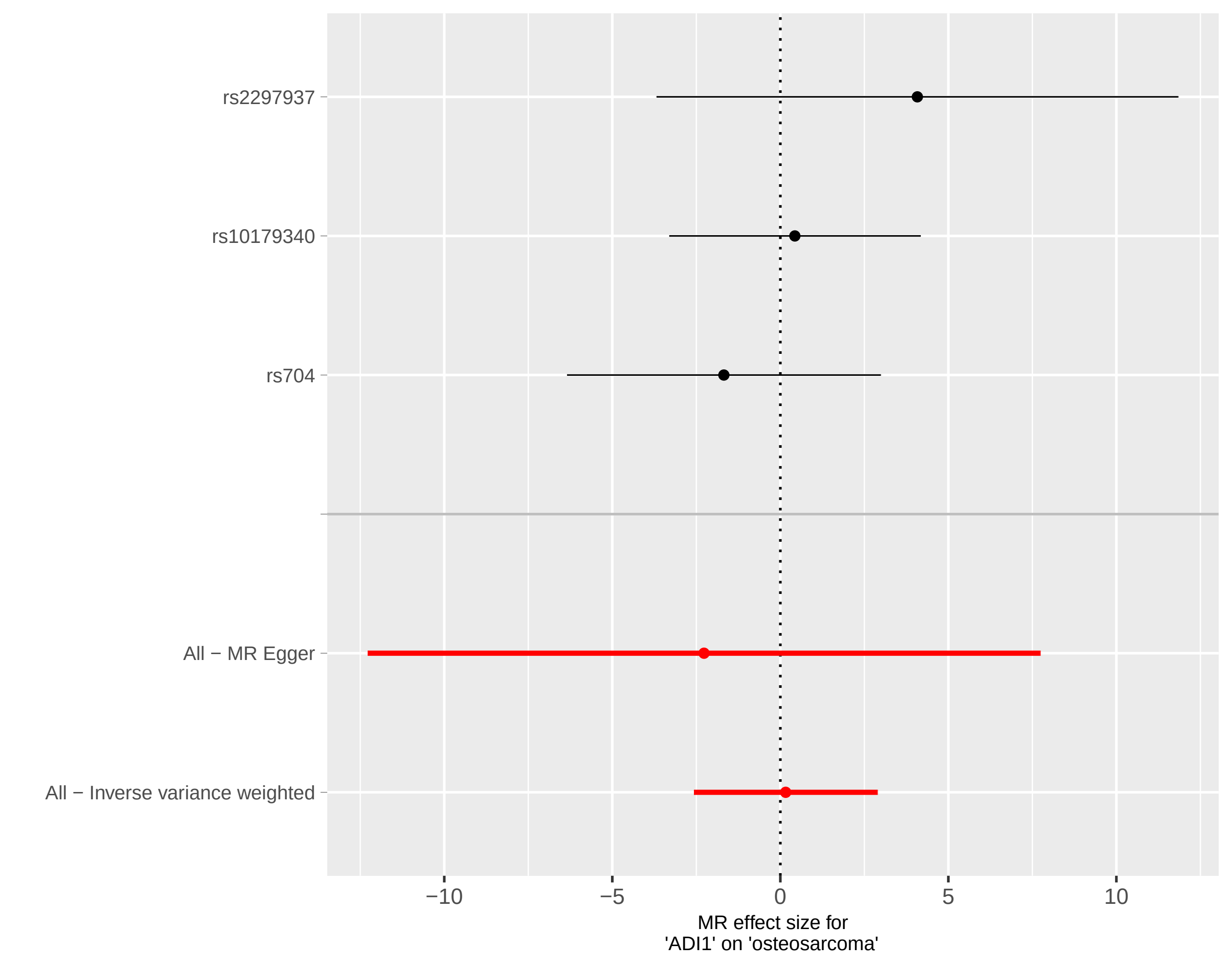

rs2297937
rs10179340
rs704
All − MR Egger
All − Inverse variance weighted
−10
−5
0
5
10
MR effect size for
'ADI1' on 'osteosarcoma'

Insufficient number of SNPs

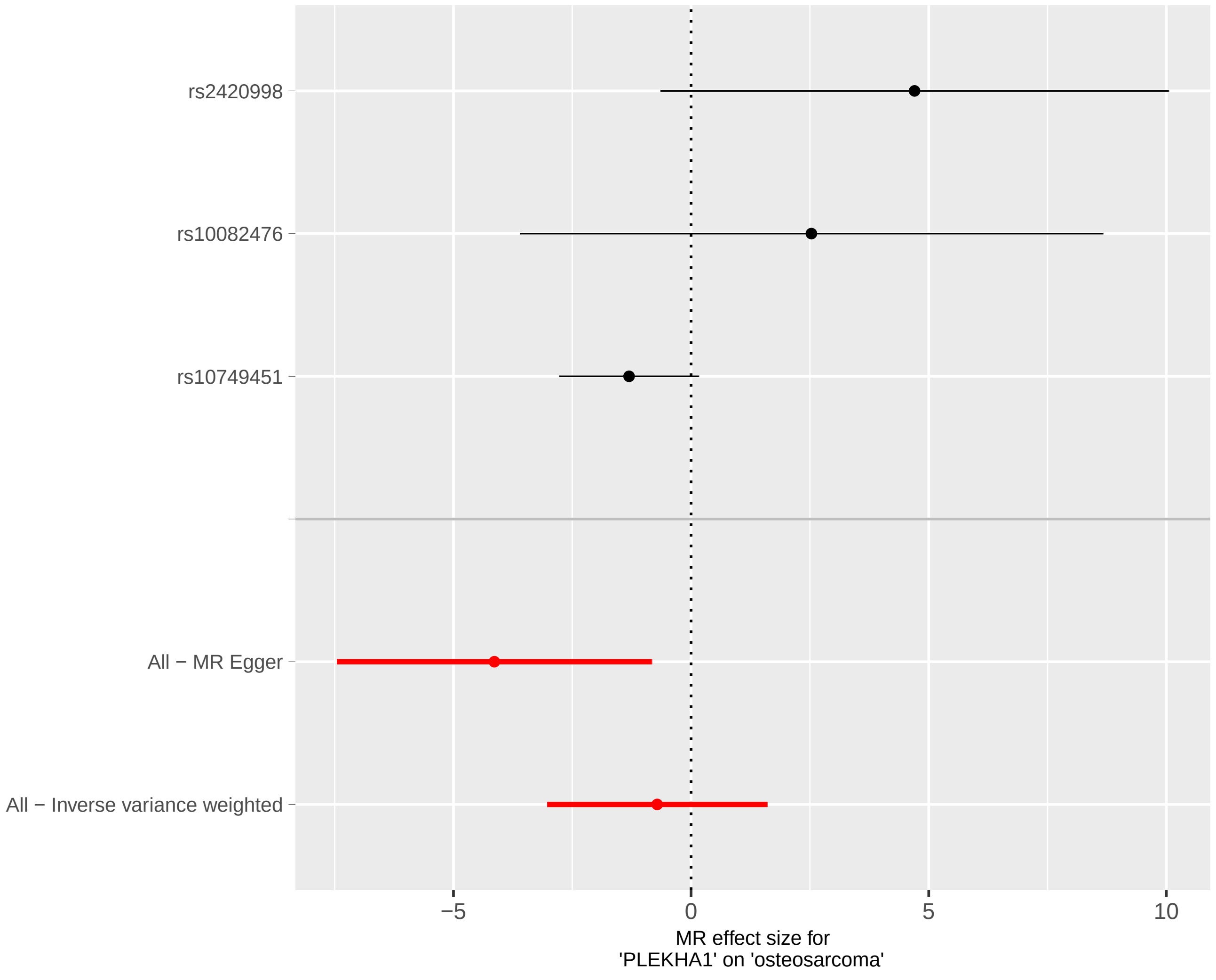

rs2420998
rs10082476
rs10749451
All − MR Egger
All − Inverse variance weighted
−5
0
5
10
MR effect size for
'PLEKHA1' on 'osteosarcoma'

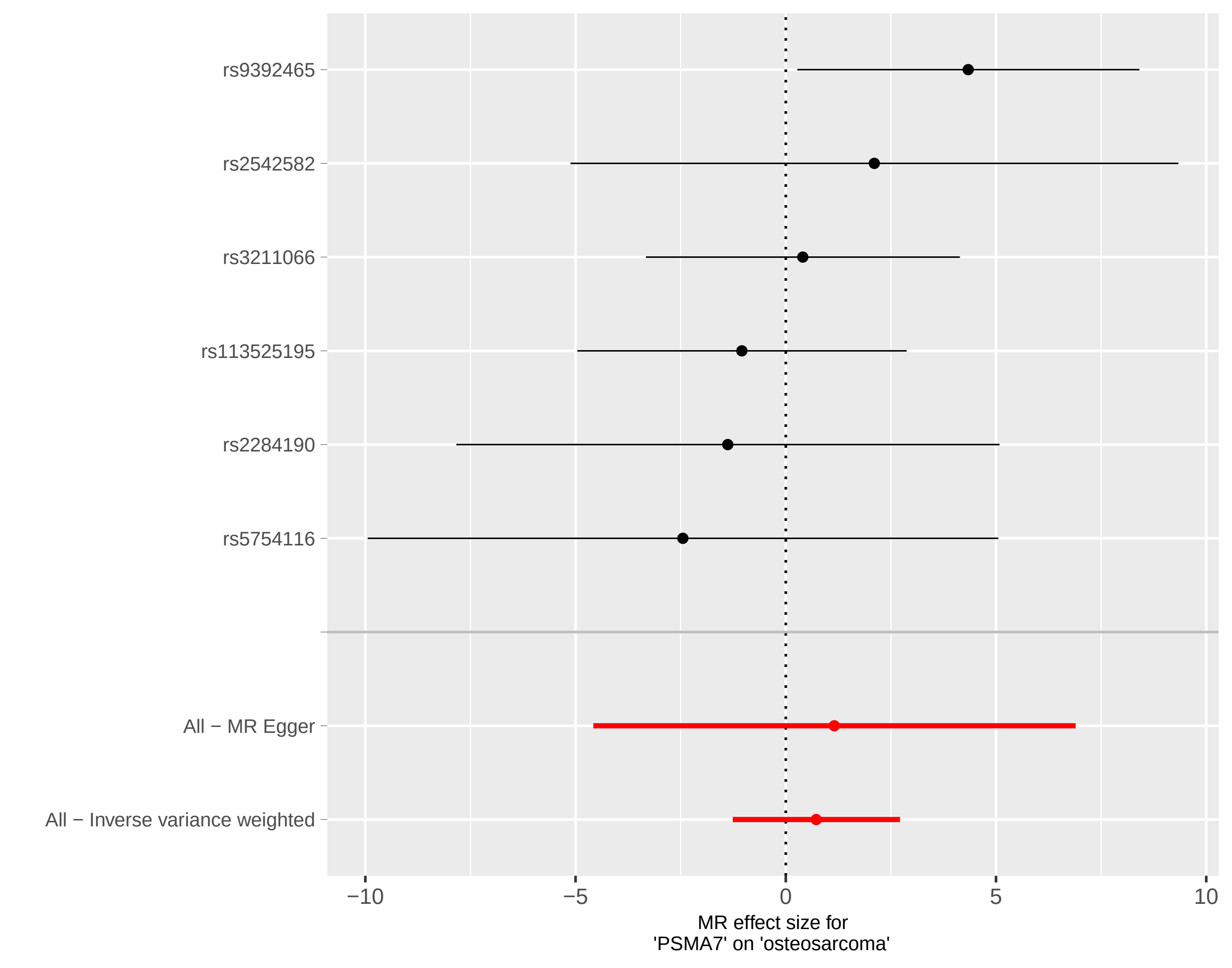

rs9392465
rs2542582
rs3211066
rs113525195
rs2284190
rs5754116
All − MR Egger
All − Inverse variance weighted
−10
−5
0
5
10
MR effect size for
'PSMA7' on 'osteosarcoma'

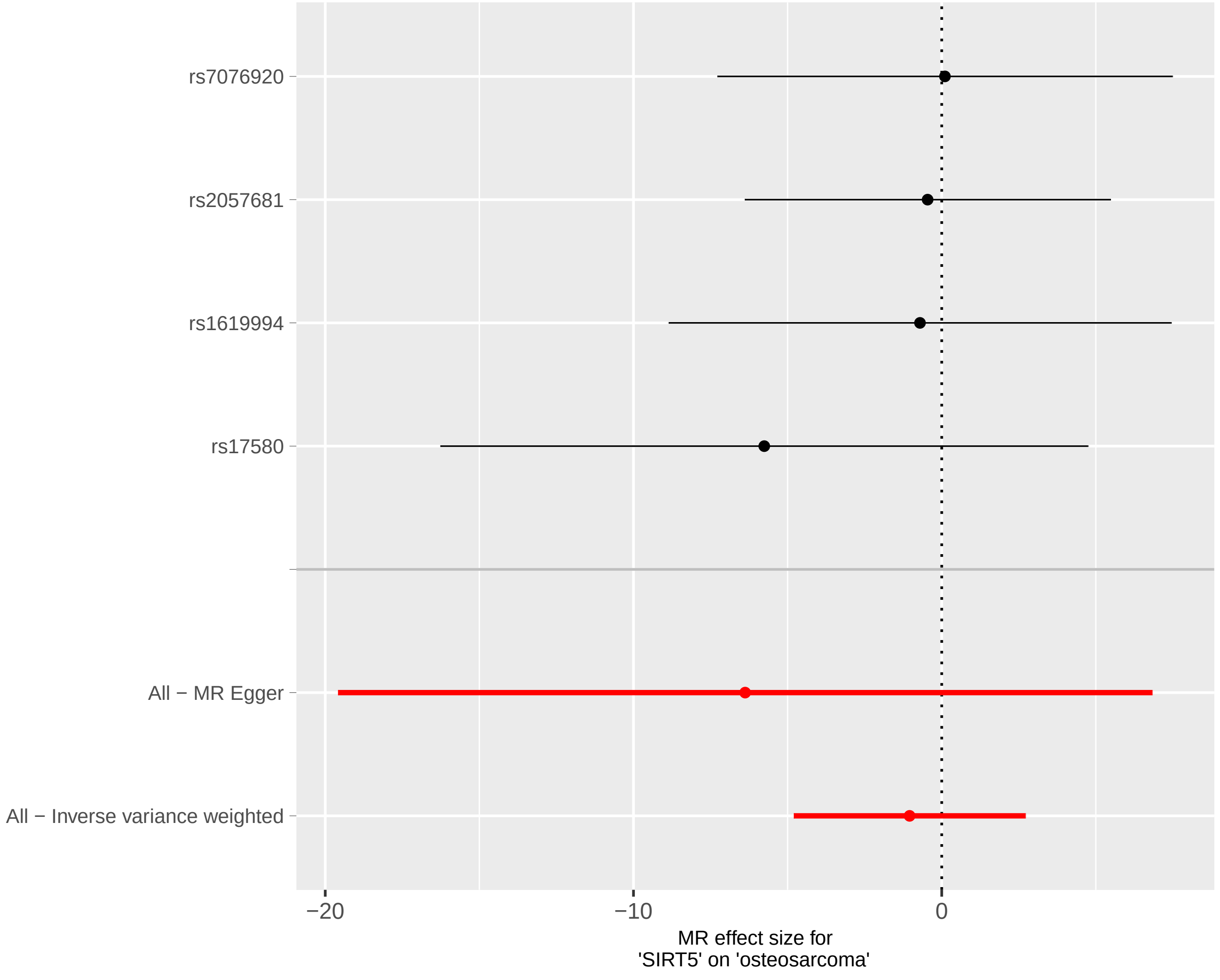
rs7076920
rs2057681
rs1619994
rs17580
All − MR Egger
All − Inverse variance weighted
−20
−10
0
MR effect size for
'SIRT5' on 'osteosarcoma'

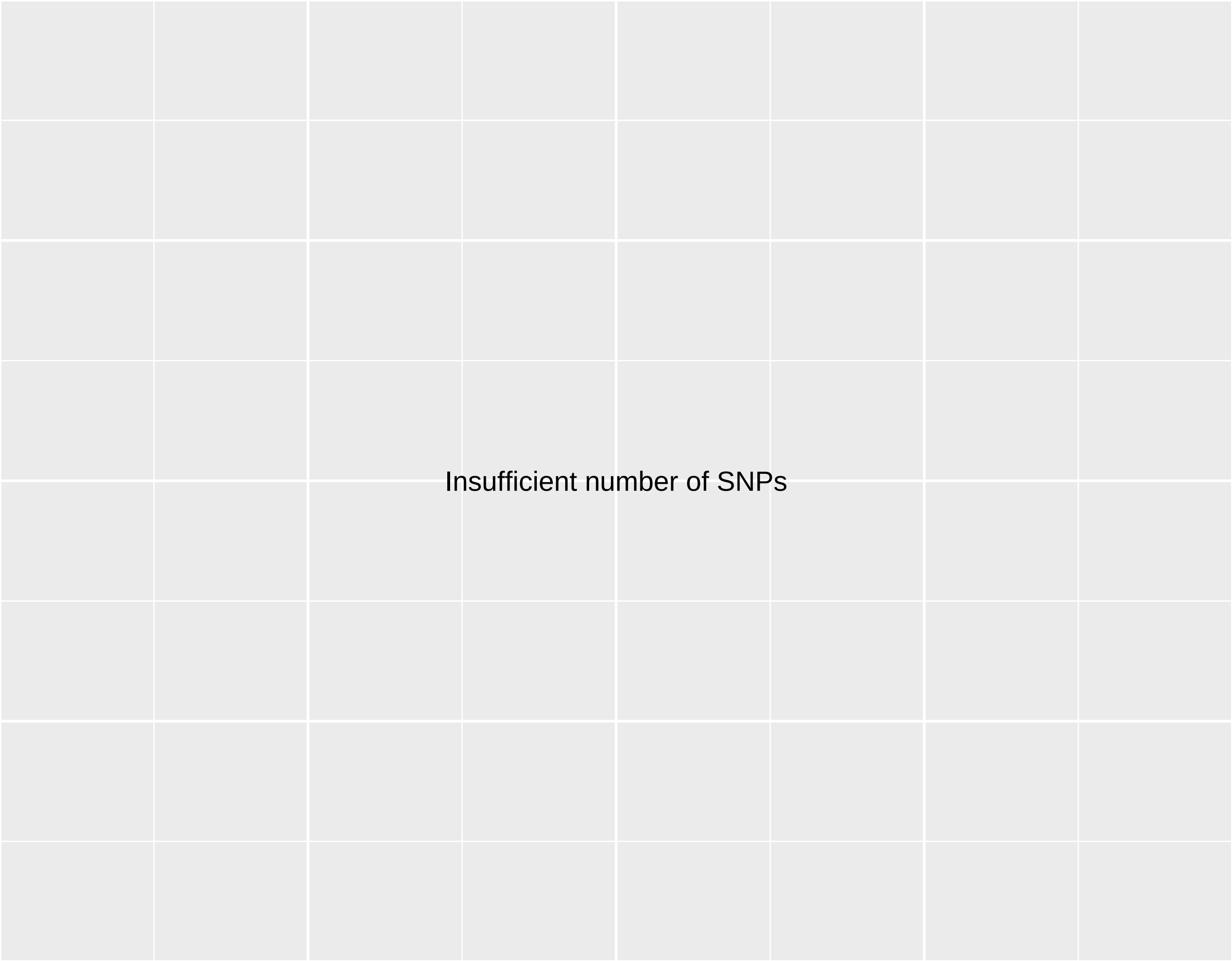
Insufficient number of SNPs

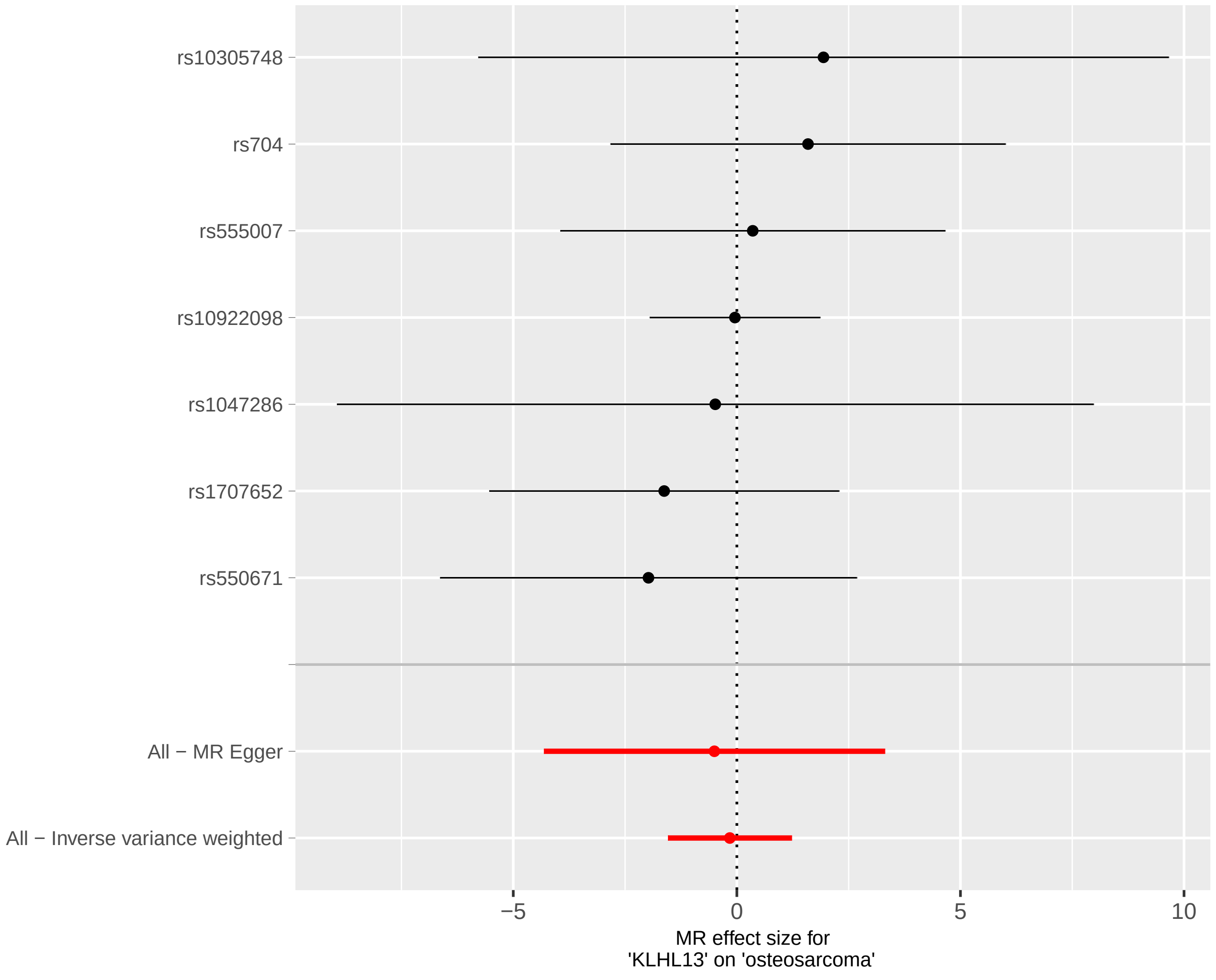

rs10305748
rs704
rs555007
rs10922098
rs1047286
rs1707652
rs550671
All − MR Egger
All − Inverse variance weighted
−5
0
5
10
MR effect size for
'KLHL13' on 'osteosarcoma'

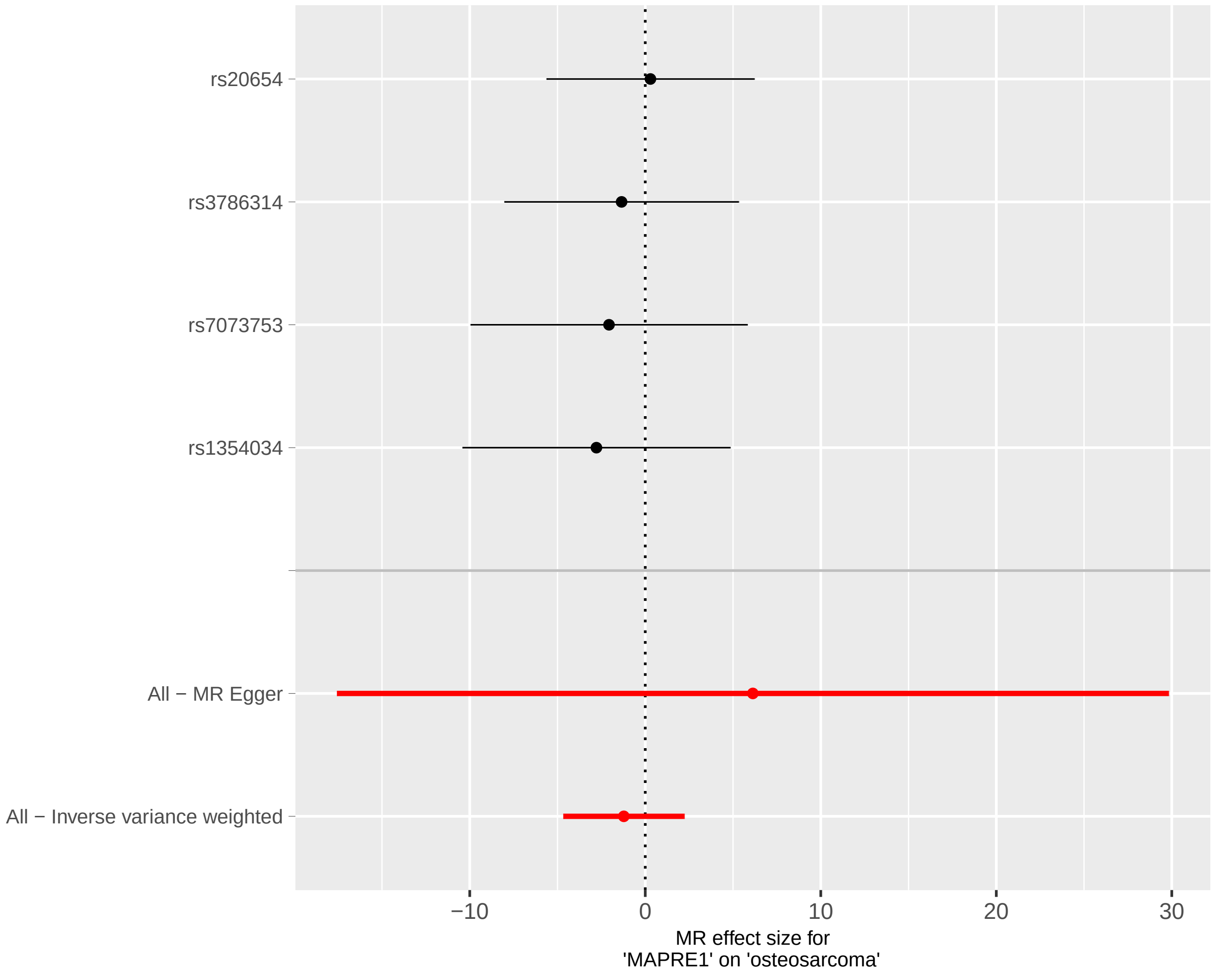

rs20654
rs3786314
rs7073753
rs1354034
All − MR Egger
All − Inverse variance weighted
−10
0
10
20
30
MR effect size for
'MAPRE1' on 'osteosarcoma'

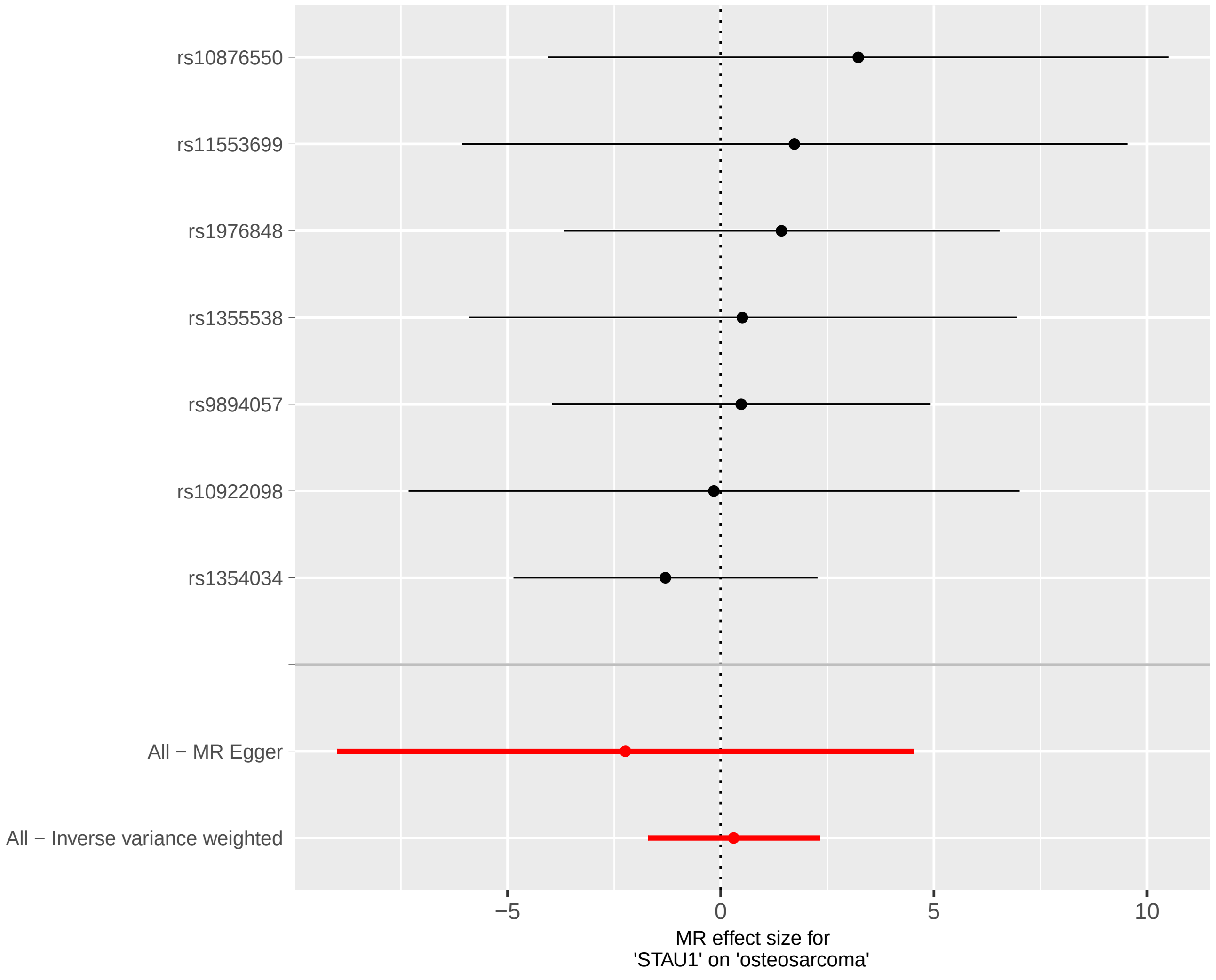

rs10876550
rs11553699
rs1976848
rs1355538
rs9894057
rs10922098
rs1354034
All − MR Egger
All − Inverse variance weighted
−5
0
5
10
MR effect size for
'STAU1' on 'osteosarcoma'

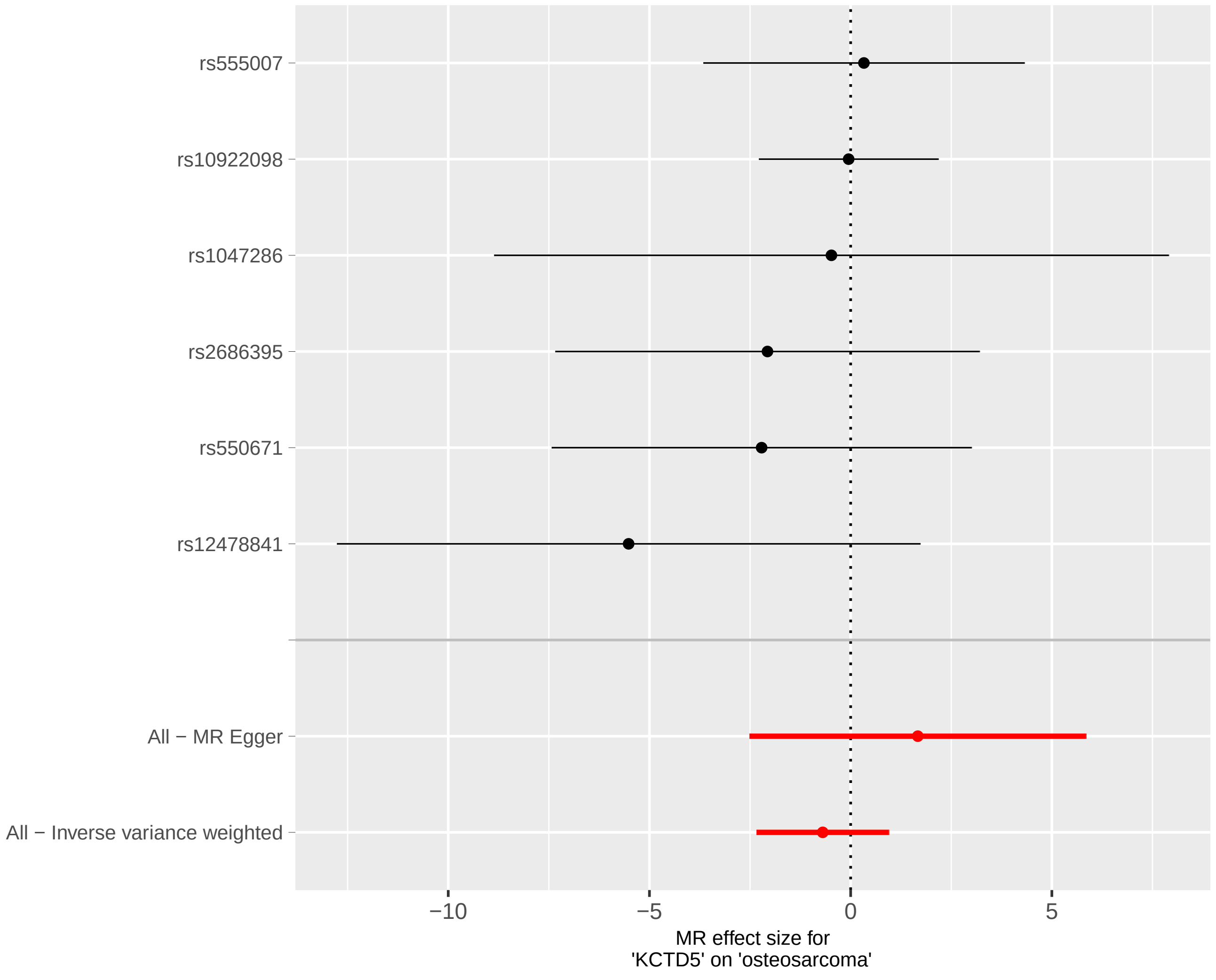
rs555007
rs10922098
rs1047286
rs2686395
rs550671
rs12478841
All − MR Egger
All − Inverse variance weighted
−10
−5
0
5
MR effect size for
'KCTD5' on 'osteosarcoma'

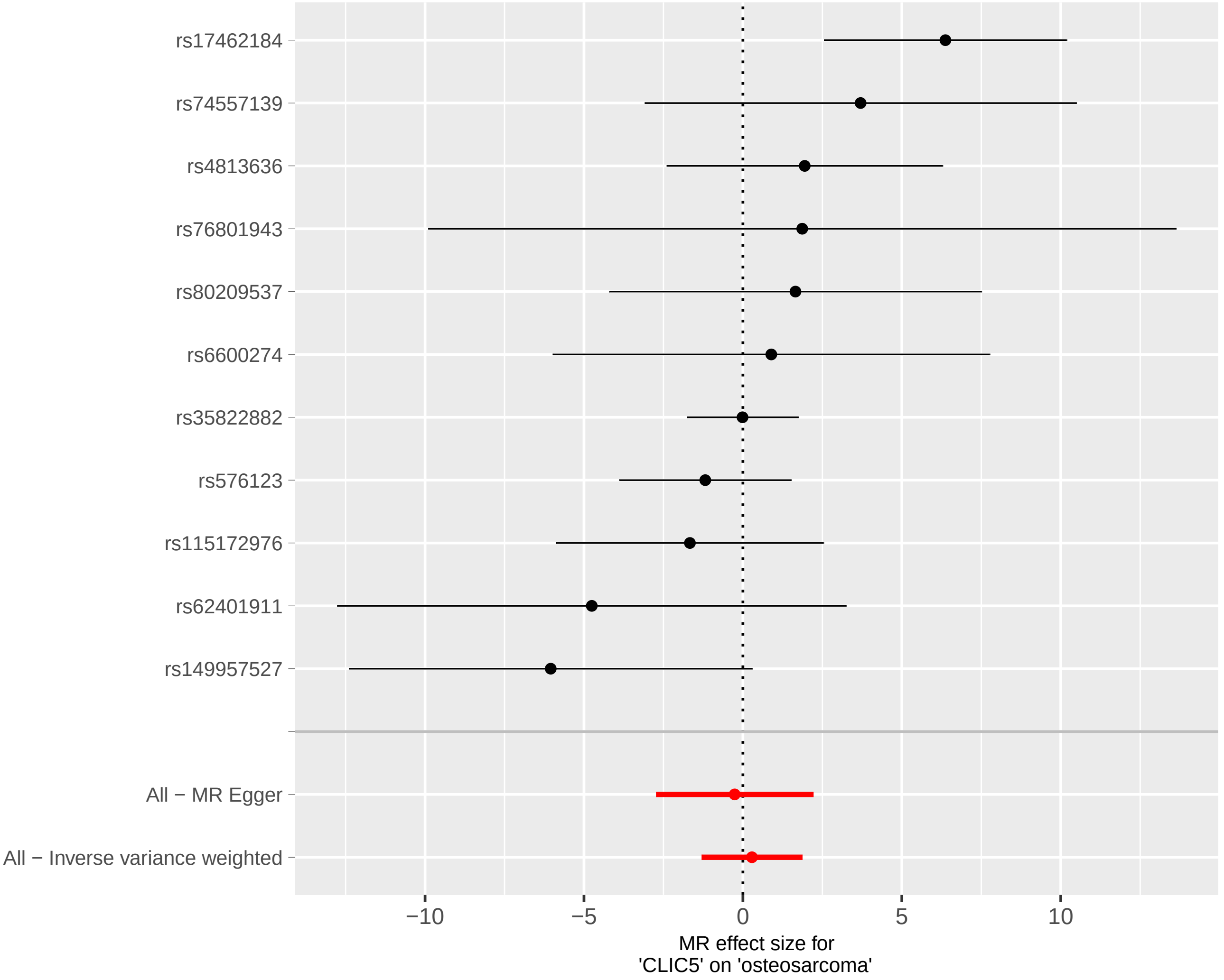
rs17462184
rs74557139
rs4813636
rs76801943
rs80209537
rs6600274
rs35822882
rs576123
rs115172976
rs62401911
rs149957527
All − MR Egger
All − Inverse variance weighted
−10
−5
0
5
10
MR effect size for
'CLIC5' on 'osteosarcoma'

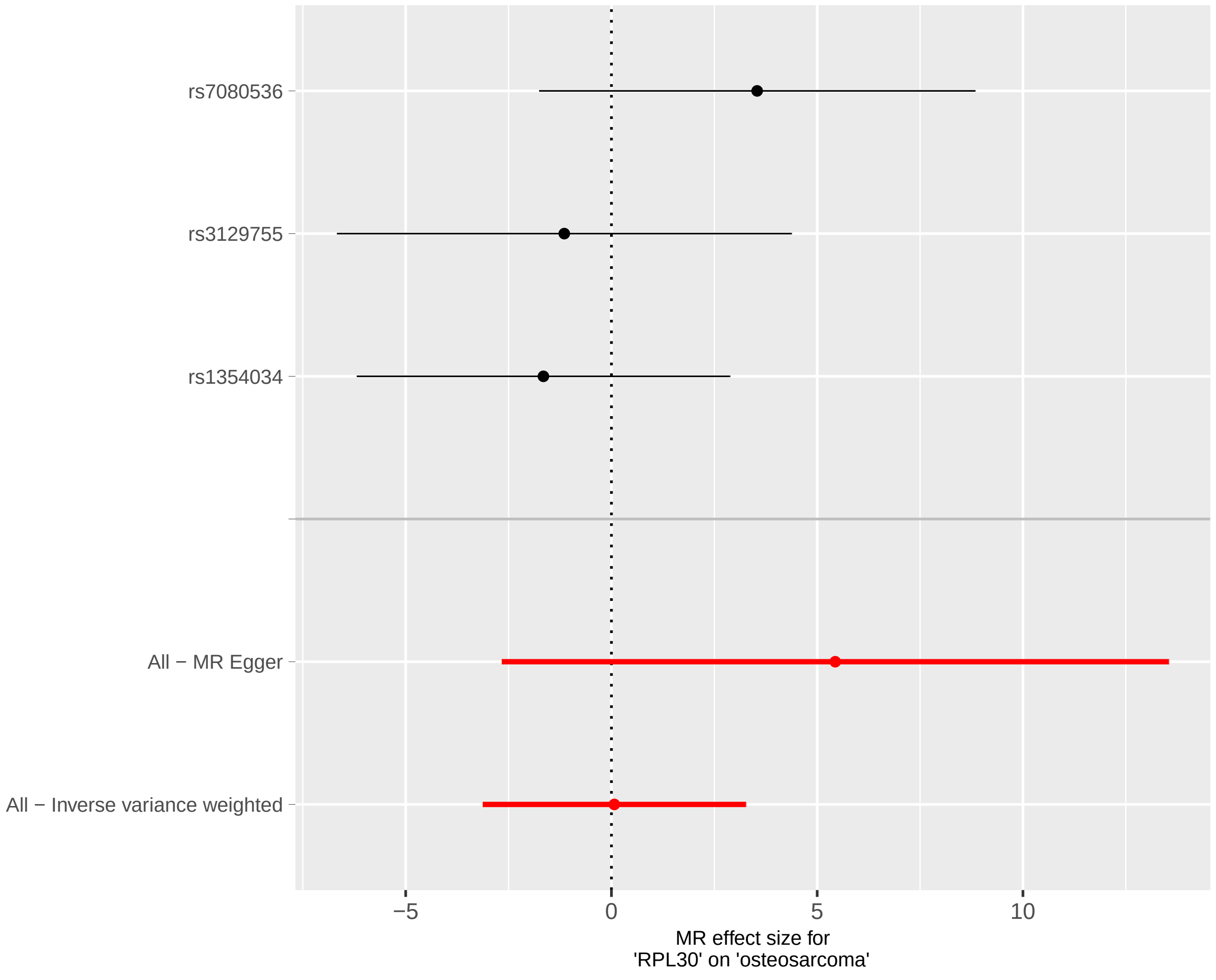

rs7080536
rs3129755
rs1354034
All − MR Egger
All − Inverse variance weighted
−5
0
5
10
MR effect size for
'RPL30' on 'osteosarcoma'

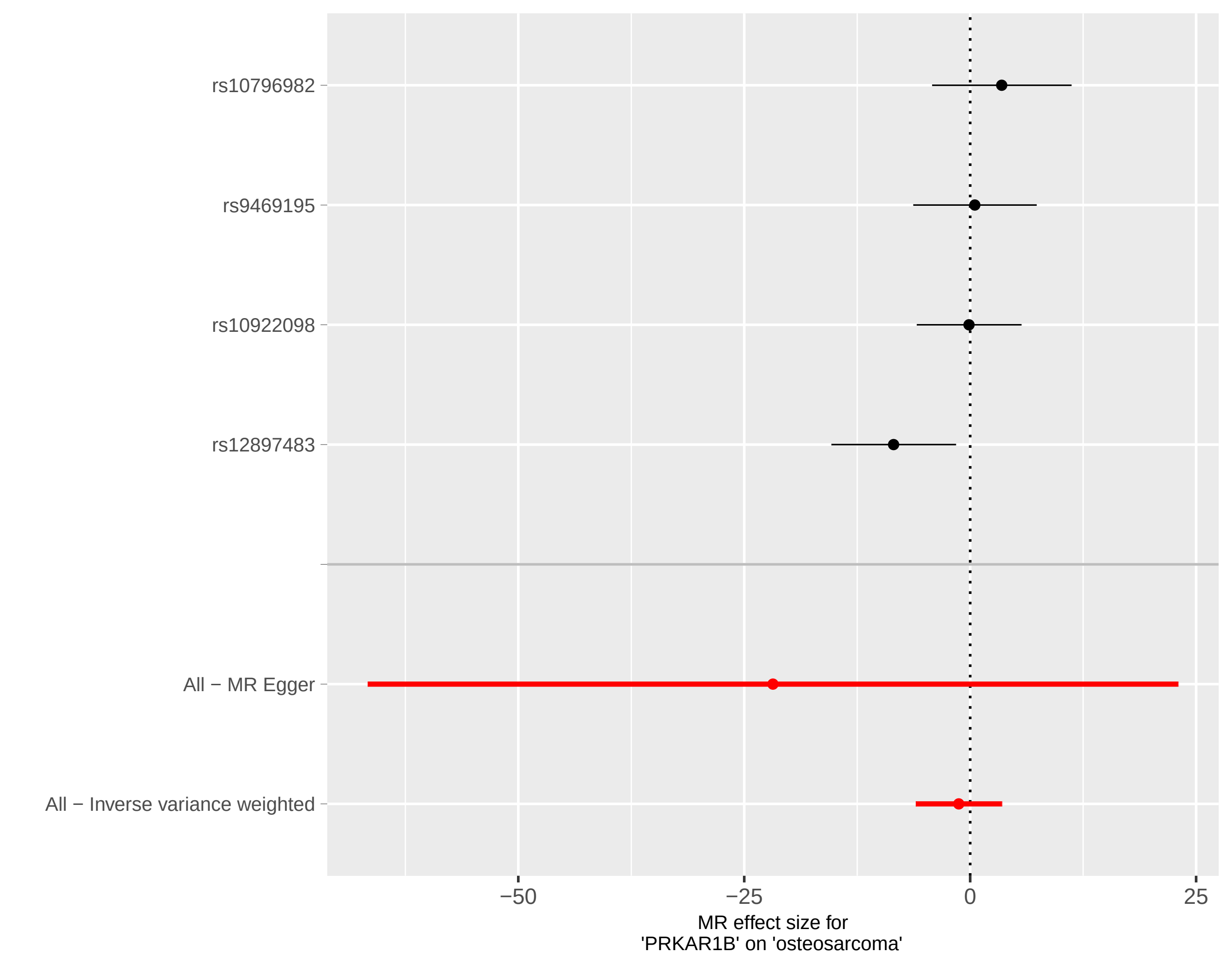
rs10796982
rs9469195
rs10922098
rs12897483
All − MR Egger
All − Inverse variance weighted
−50
−25
0
25
MR effect size for
'PRKAR1B' on 'osteosarcoma'

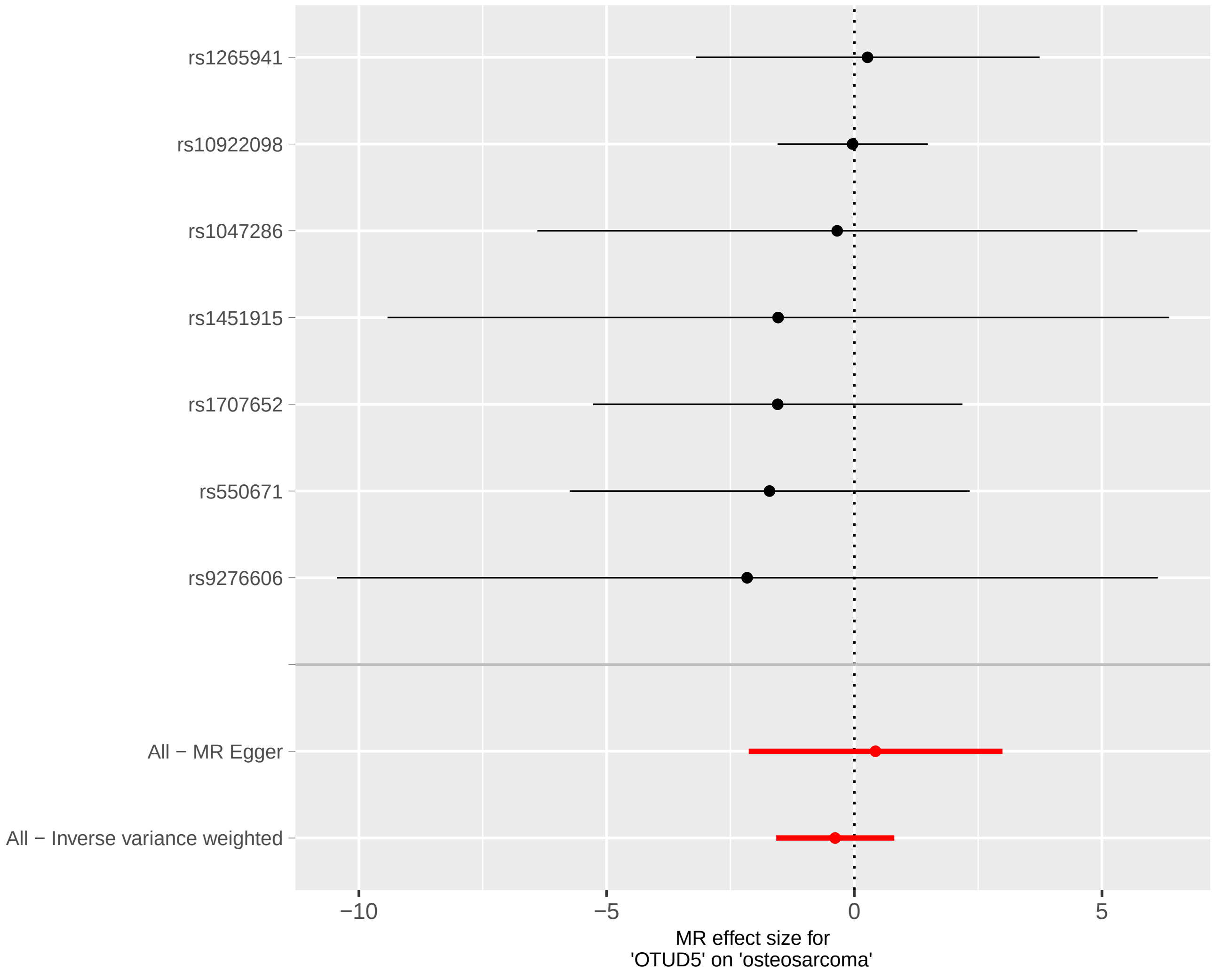
rs1265941
rs10922098
rs1047286
rs1451915
rs1707652
rs550671
rs9276606
All − MR Egger
All − Inverse variance weighted
−10
−5
0
5
MR effect size for
'OTUD5' on 'osteosarcoma'

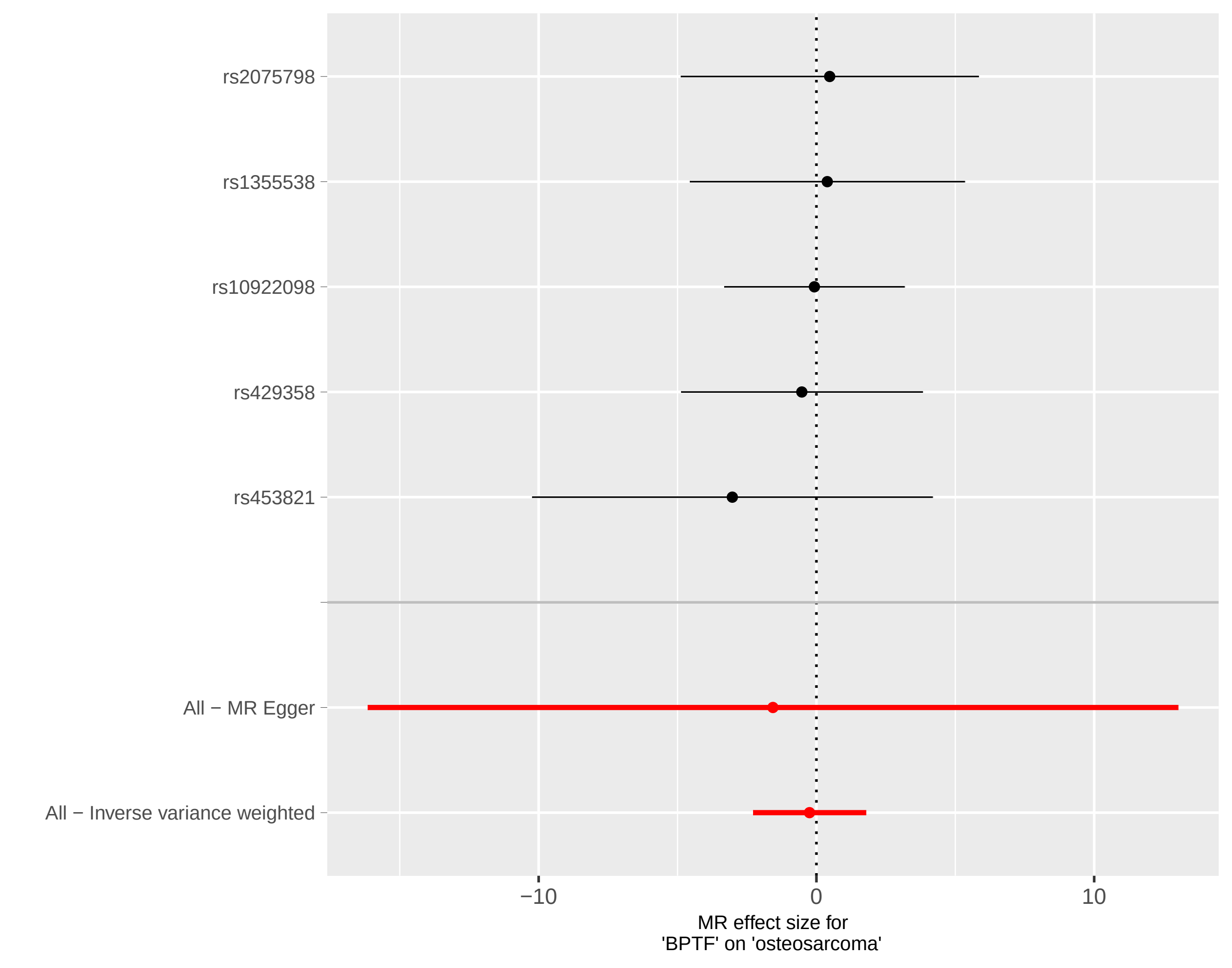
rs2075798
rs1355538
rs10922098
rs429358
rs453821
All − MR Egger
All − Inverse variance weighted
−10
0
10
MR effect size for
'BPTF' on 'osteosarcoma'

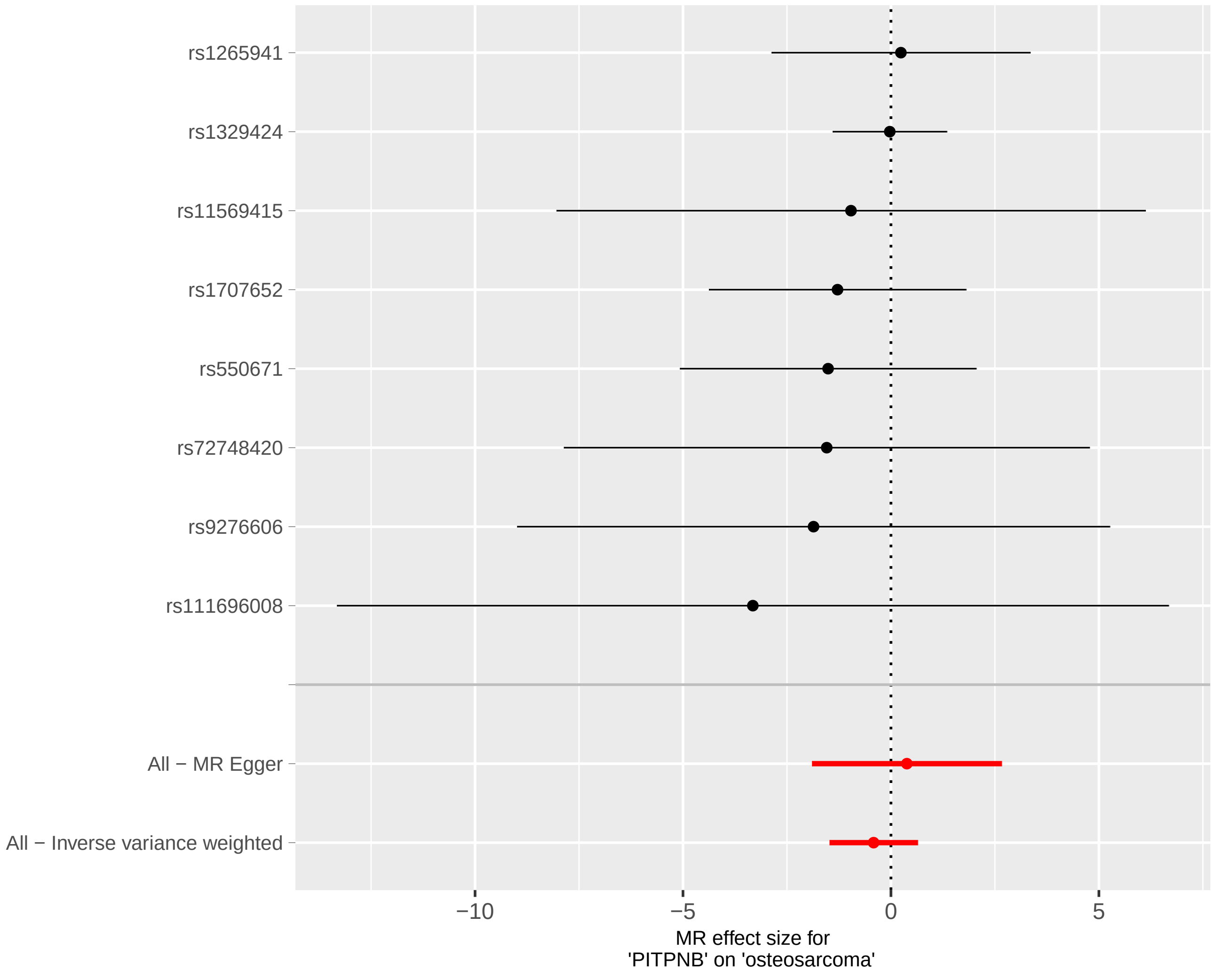
rs1265941
rs1329424
rs11569415
rs1707652
rs550671
rs72748420
rs9276606
rs111696008
All − MR Egger
All − Inverse variance weighted
−10
−5
0
5
MR effect size for
'PITPNB' on 'osteosarcoma'

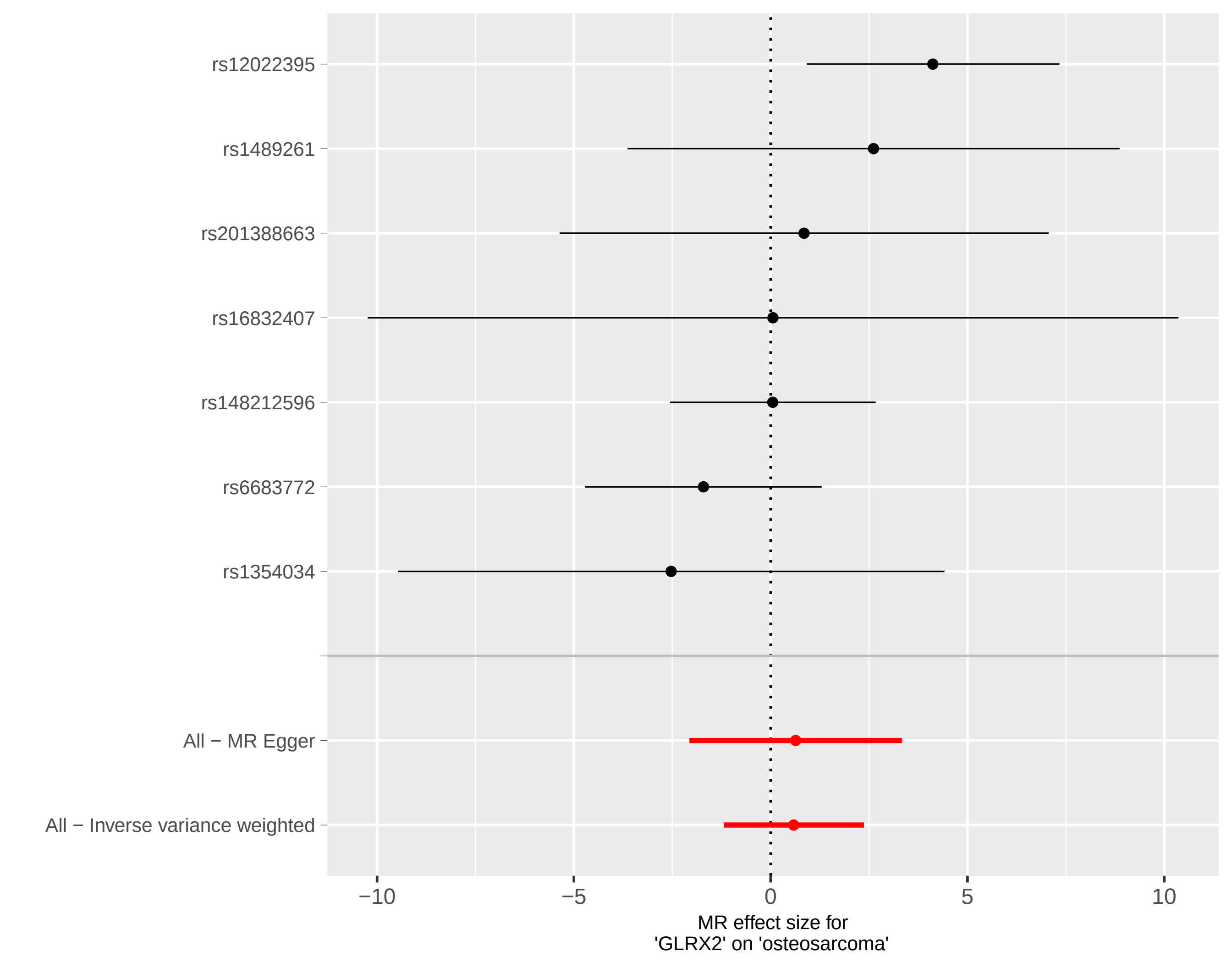

rs12022395
rs1489261
rs201388663
rs16832407
rs148212596
rs6683772
rs1354034
All − MR Egger
All − Inverse variance weighted
−10
−5
0
5
10
MR effect size for
'GLRX2' on 'osteosarcoma'

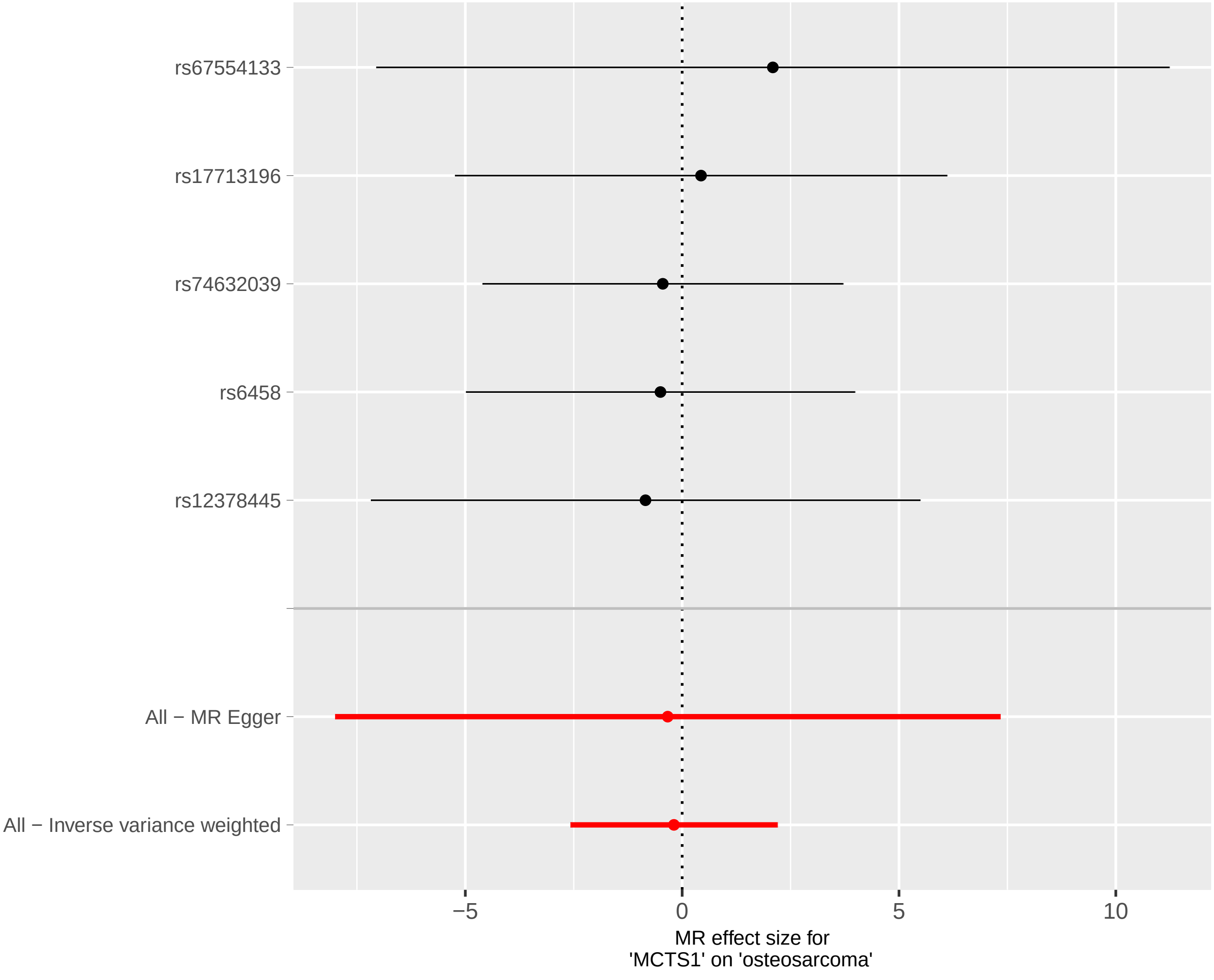

rs67554133
rs17713196
rs74632039
rs6458
rs12378445
All − MR Egger
All − Inverse variance weighted
−5
0
5
10
MR effect size for
'MCTS1' on 'osteosarcoma'

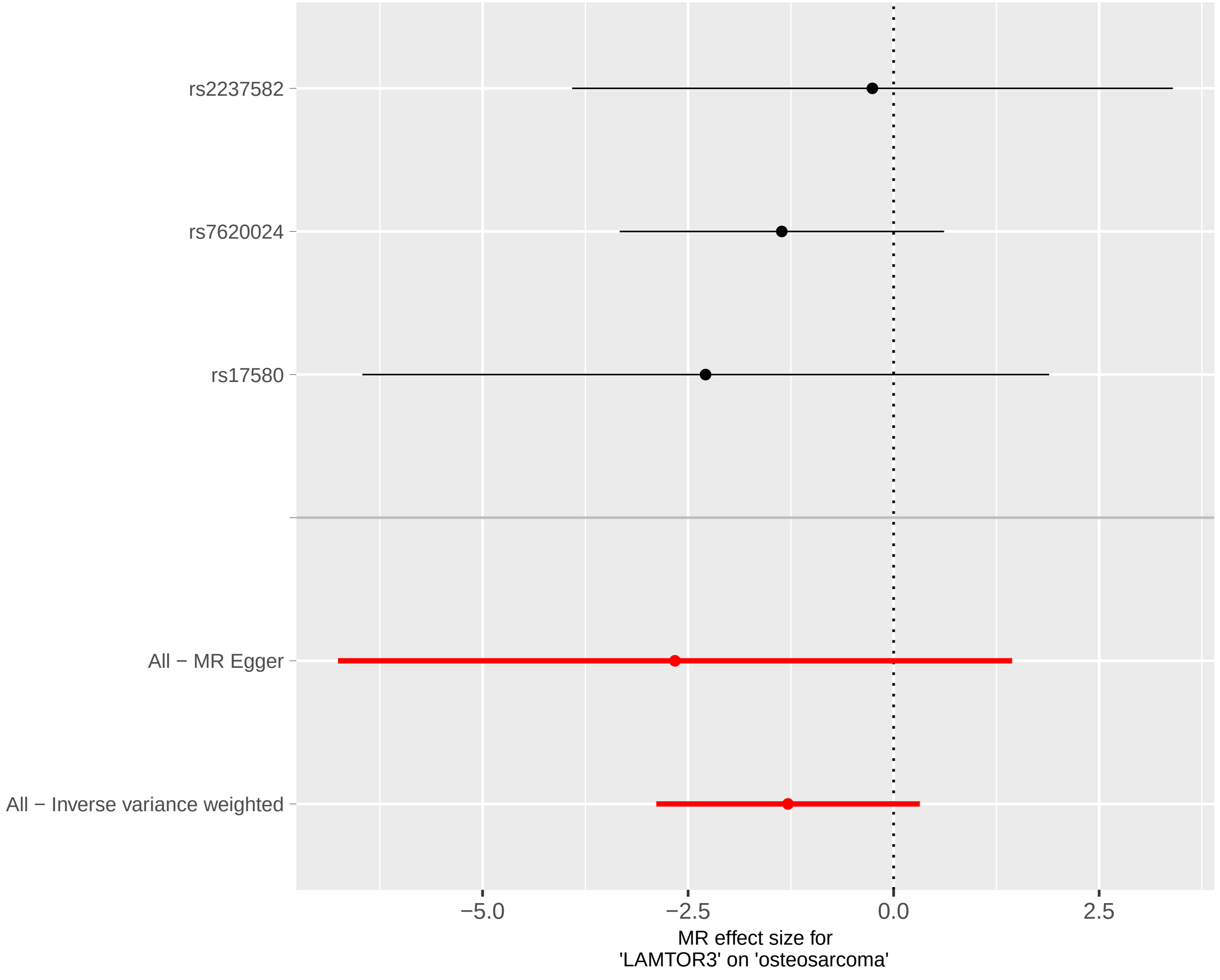

rs2237582
rs7620024
rs17580
All − MR Egger
All − Inverse variance weighted
−5.0
−2.5
0.0
2.5
MR effect size for
'LAMTOR3' on 'osteosarcoma'

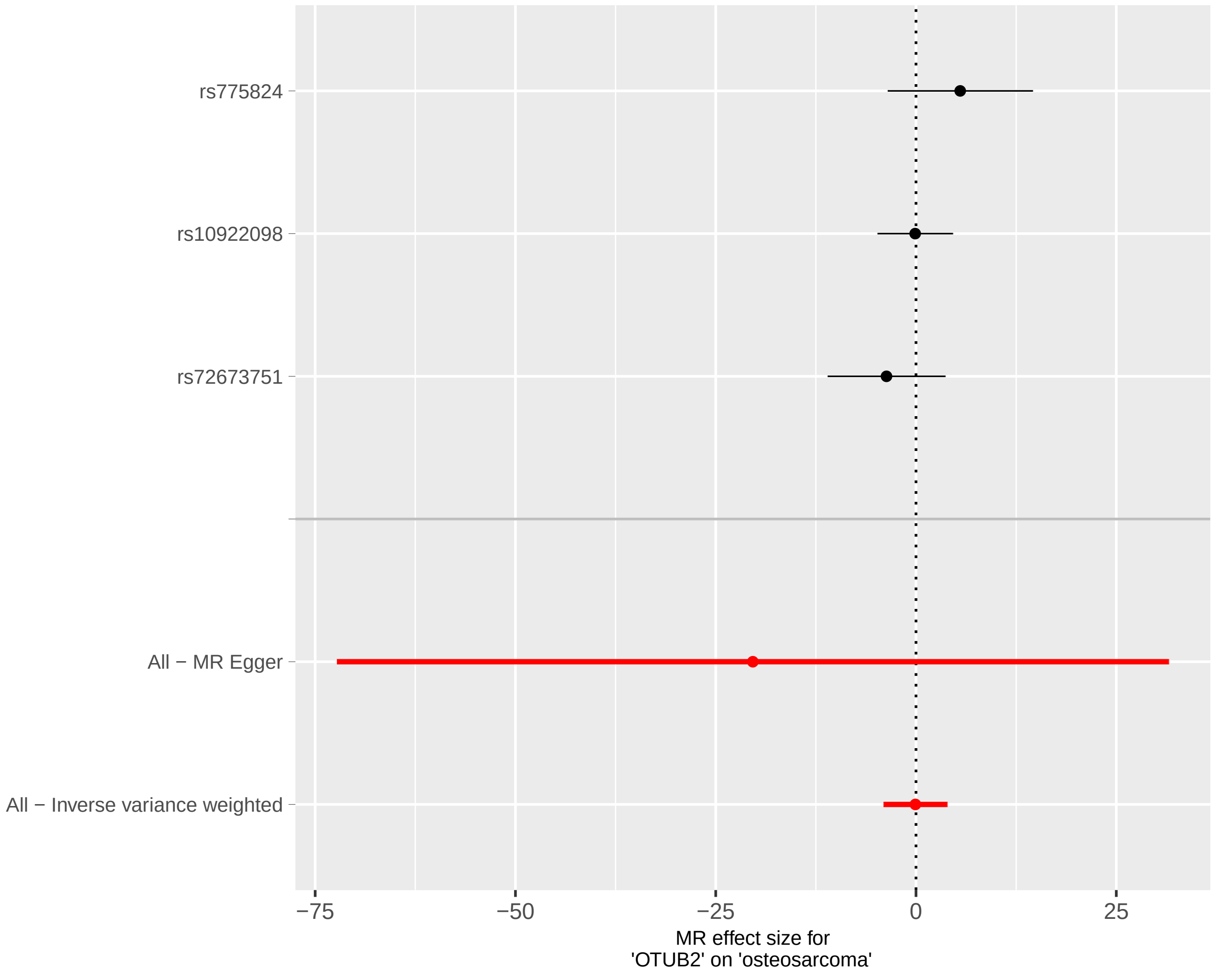

rs775824
rs10922098
rs72673751
All − MR Egger
All − Inverse variance weighted
−75
−50
−25
0
25
MR effect size for
'OTUB2' on 'osteosarcoma'

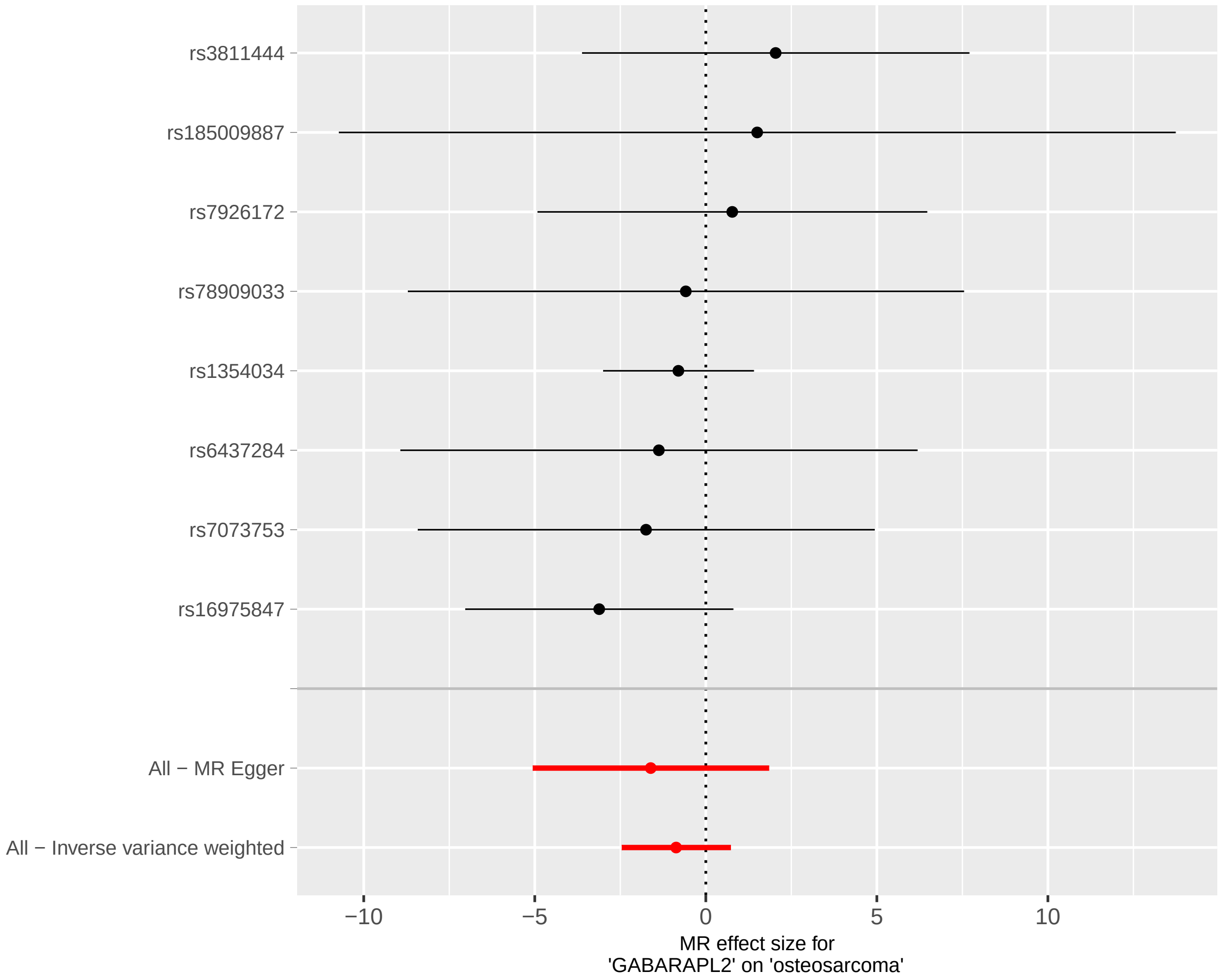

rs3811444
rs185009887
rs7926172
rs78909033
rs1354034
rs6437284
rs7073753
rs16975847
All − MR Egger
All − Inverse variance weighted
−10
−5
0
5
10
MR effect size for
'GABARAPL2' on 'osteosarcoma'

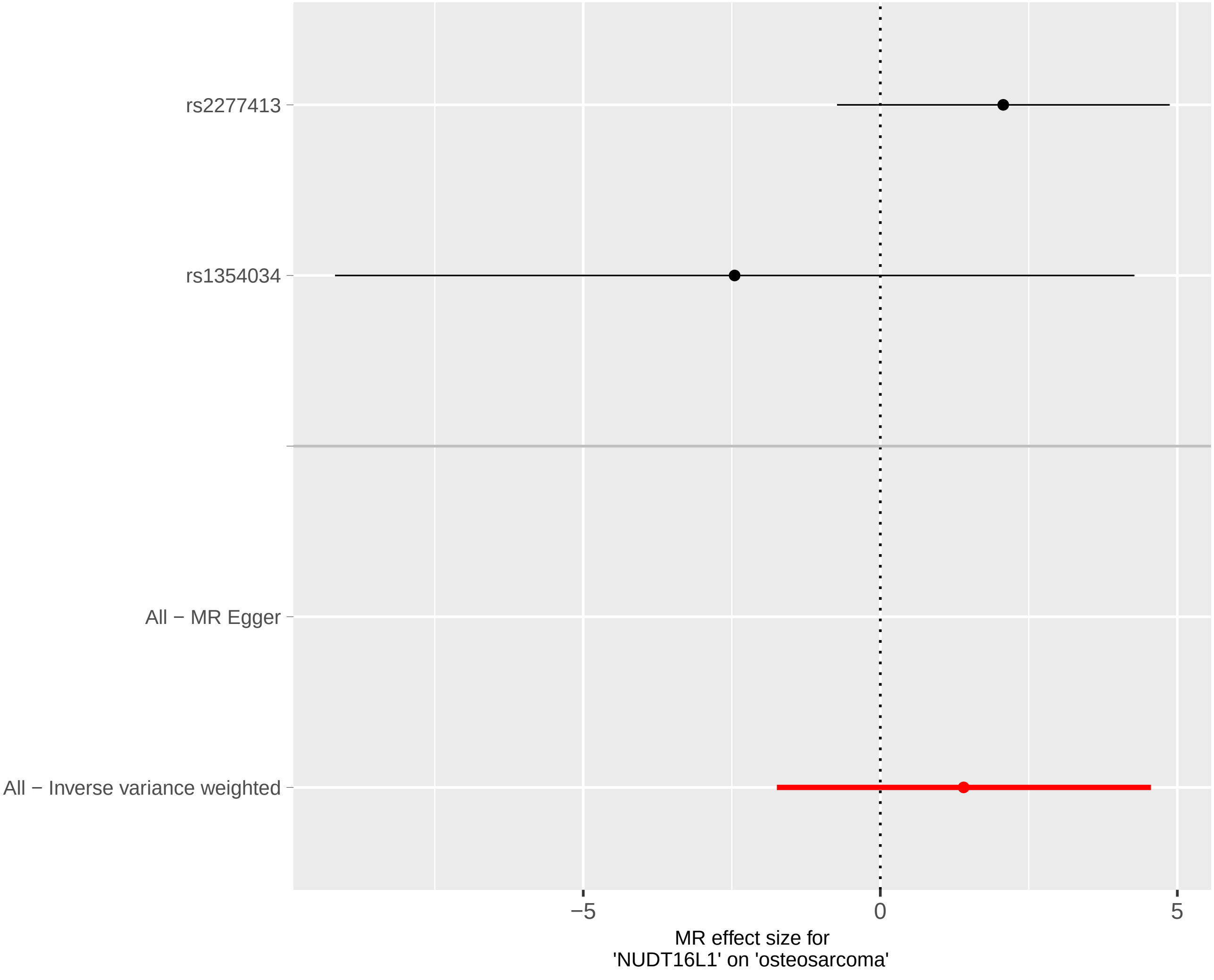

rs2277413
rs1354034
All − MR Egger
All − Inverse variance weighted
−5
0
5
MR effect size for
'NUDT16L1' on 'osteosarcoma'

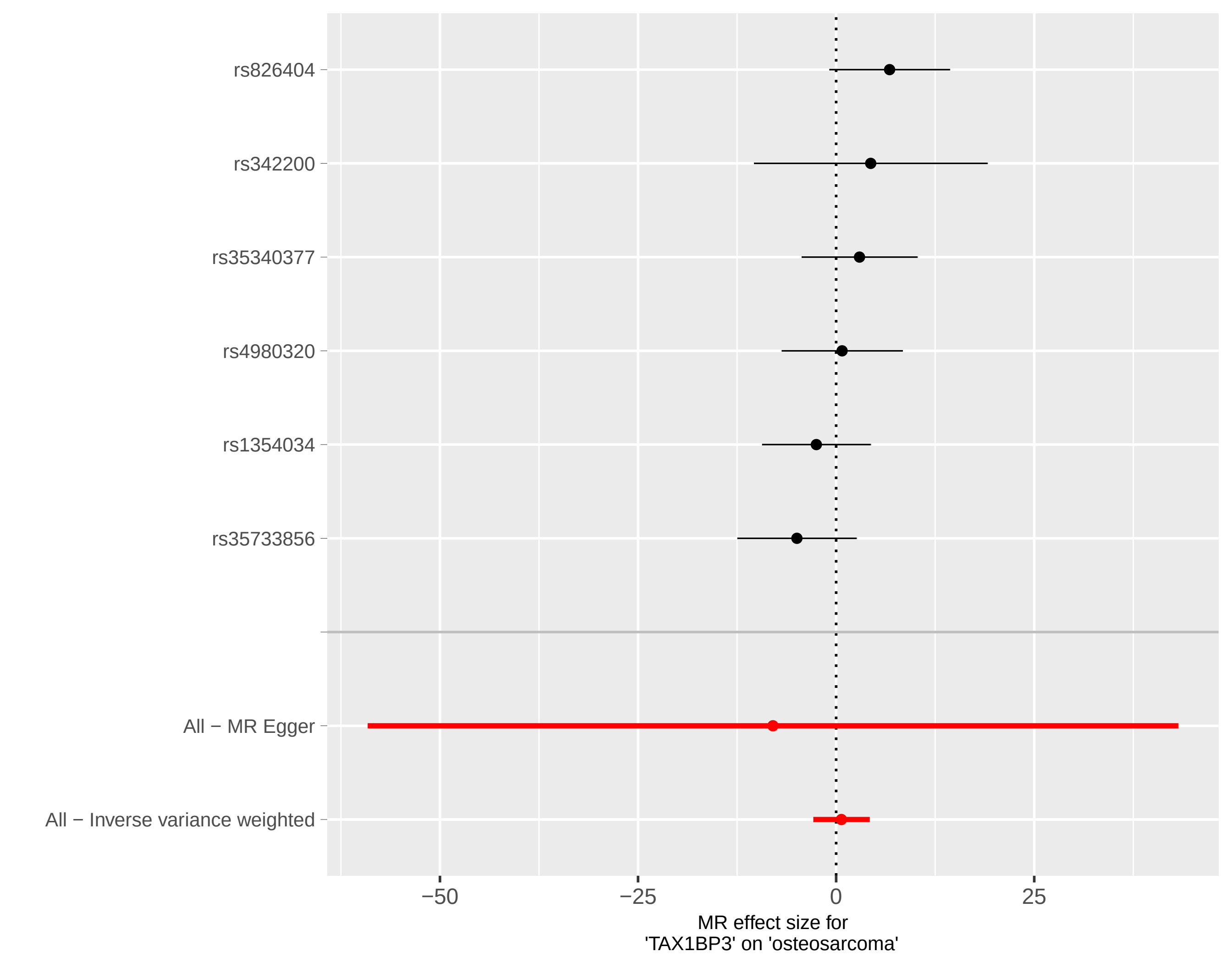

rs826404
rs342200
rs35340377
rs4980320
rs1354034
rs35733856
All − MR Egger
All − Inverse variance weighted
−50
−25
0
25
MR effect size for
'TAX1BP3' on 'osteosarcoma'

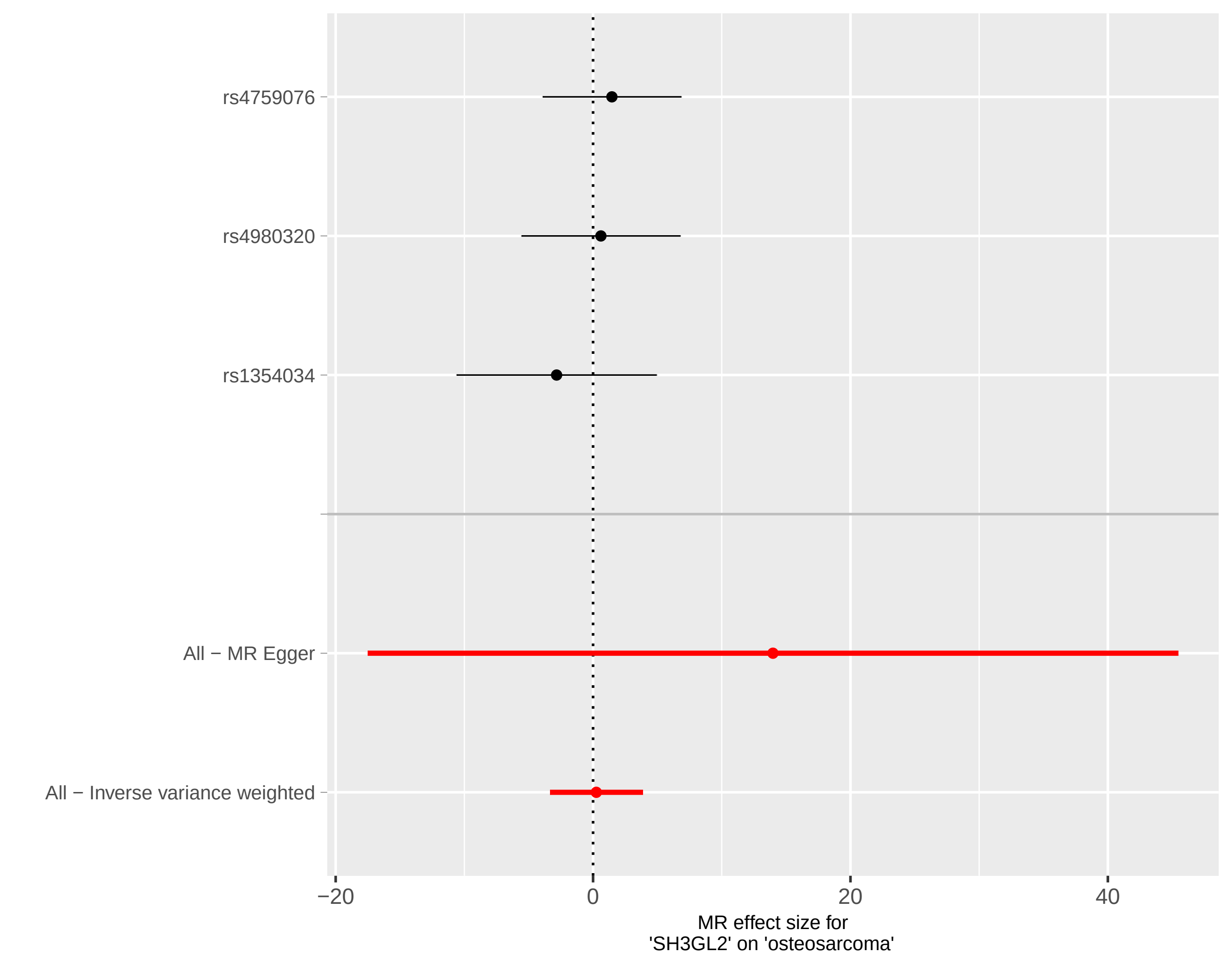

rs4759076
rs4980320
rs1354034
All − MR Egger
All − Inverse variance weighted
−20
0
20
40
MR effect size for
'SH3GL2' on 'osteosarcoma'

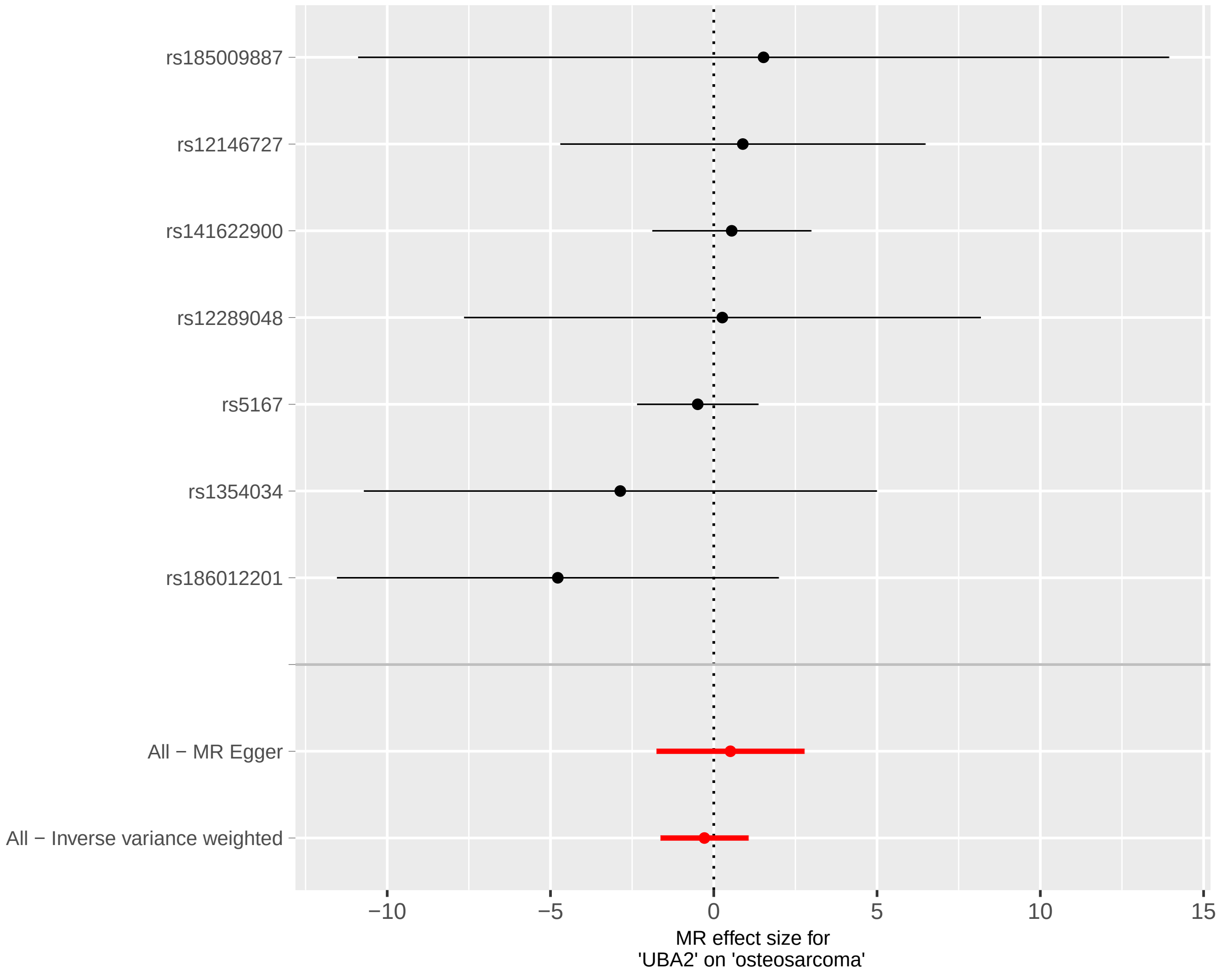
rs185009887
rs12146727
rs141622900
rs12289048
rs5167
rs1354034
rs186012201
All − MR Egger
All − Inverse variance weighted
−10
−5
0
5
10
15
MR effect size for
'UBA2' on 'osteosarcoma'

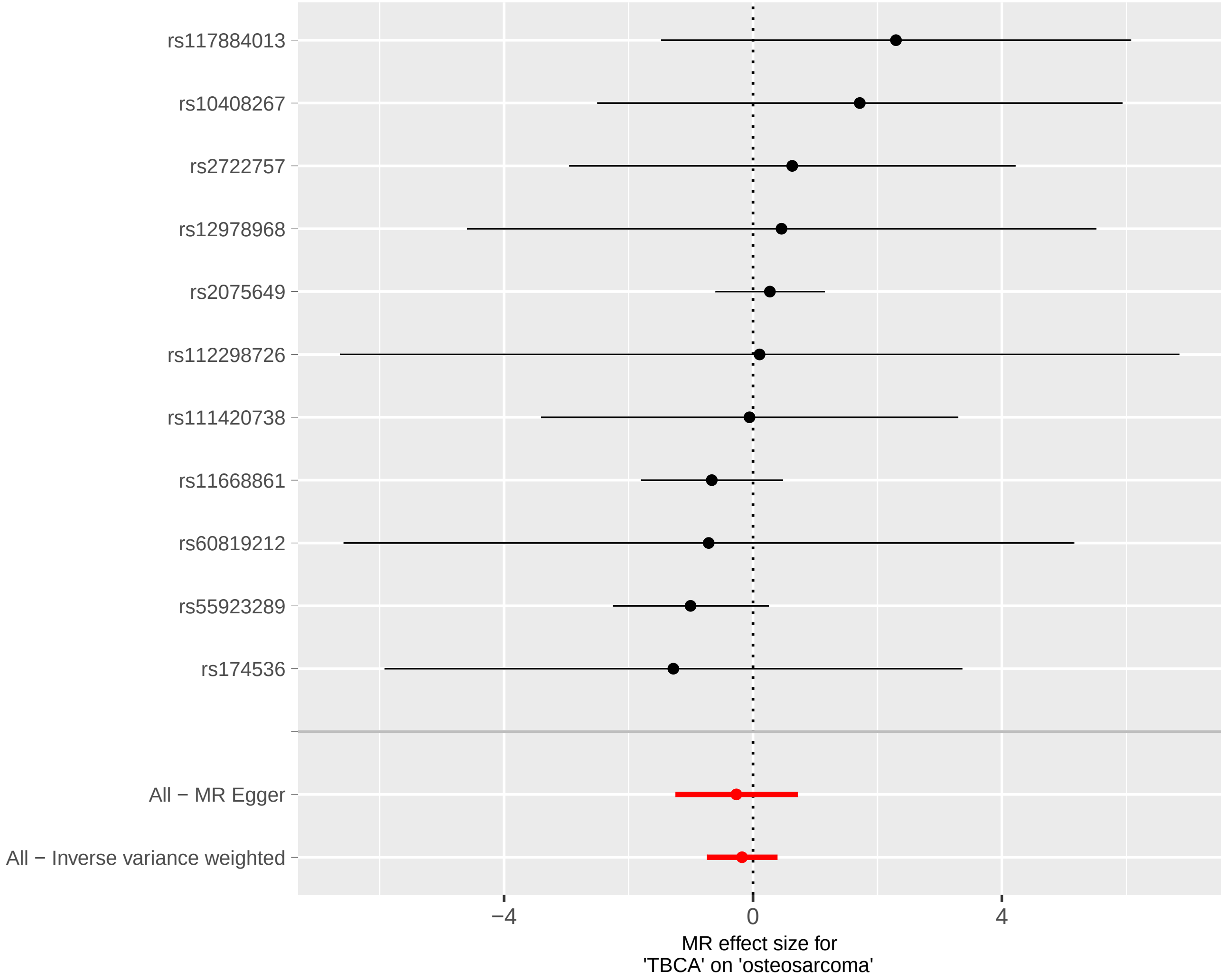

rs117884013
rs10408267
rs2722757
rs12978968
rs2075649
rs112298726
rs111420738
rs11668861
rs60819212
rs55923289
rs174536
All − MR Egger
All − Inverse variance weighted
−4
0
4
MR effect size for
'TBCA' on 'osteosarcoma'

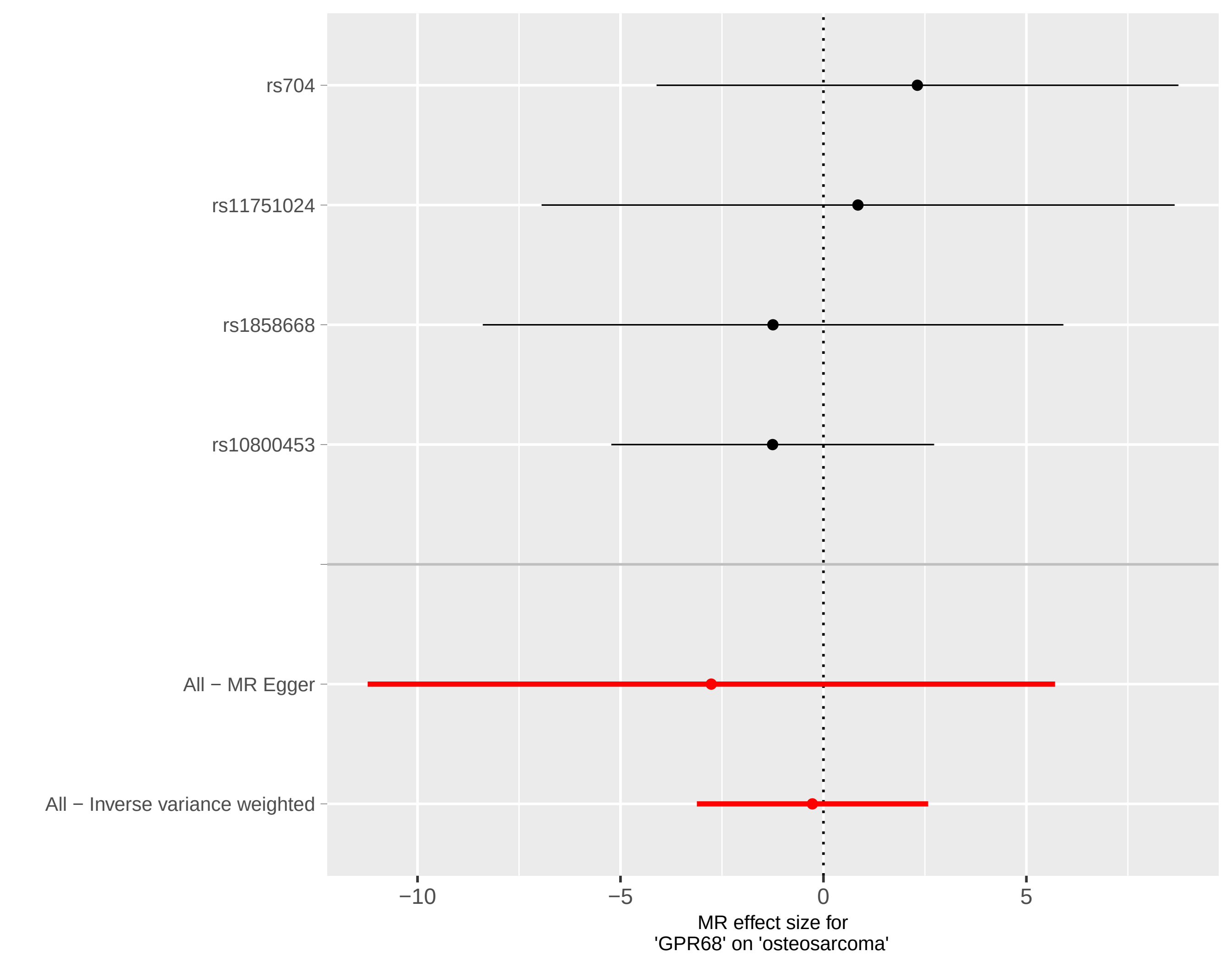
rs704
rs11751024
rs1858668
rs10800453
All − MR Egger
All − Inverse variance weighted
−10
−5
0
5
MR effect size for
'GPR68' on 'osteosarcoma'

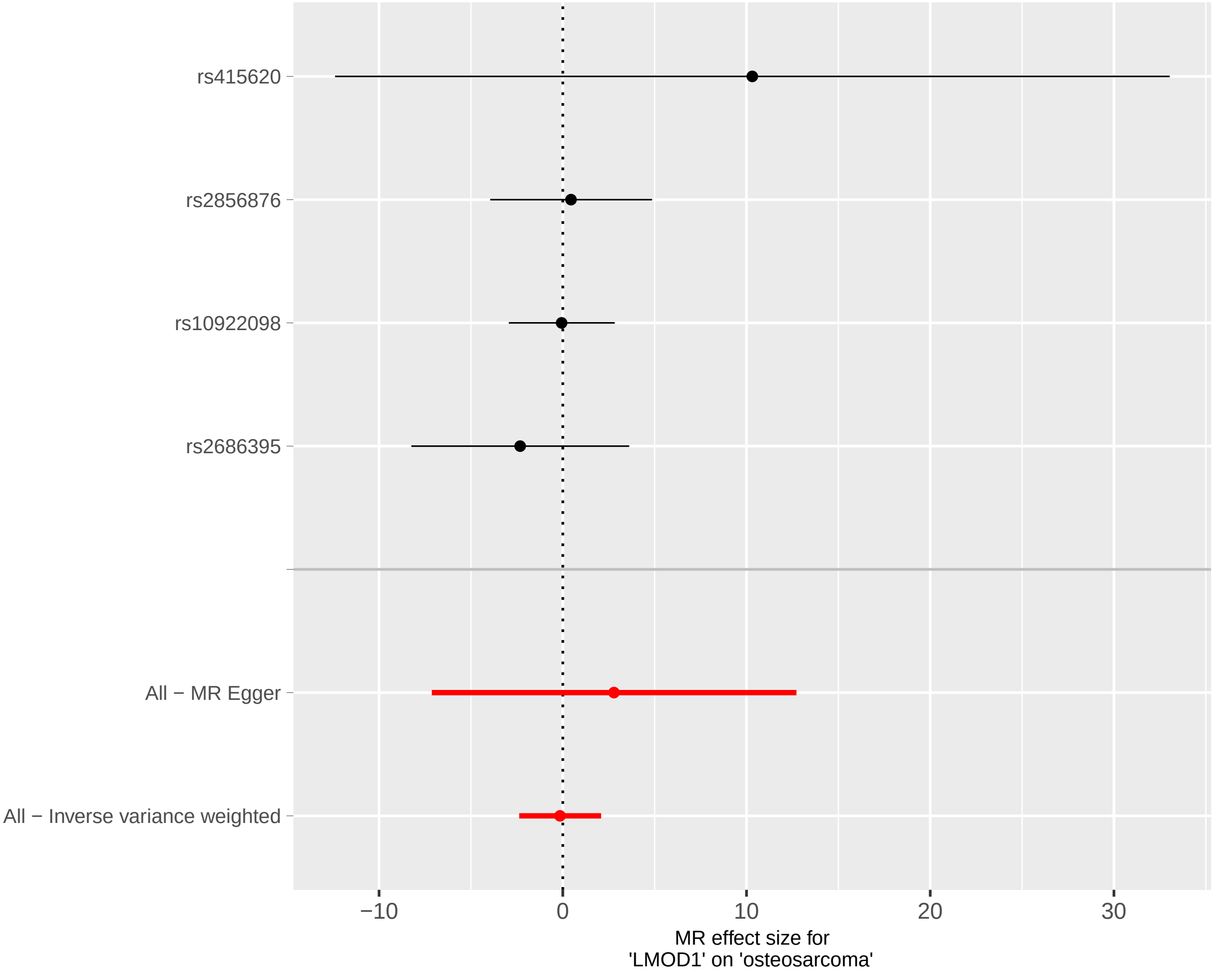

rs415620
rs2856876
rs10922098
rs2686395
All − MR Egger
All − Inverse variance weighted
−10
0
10
20
30
MR effect size for
'LMOD1' on 'osteosarcoma'

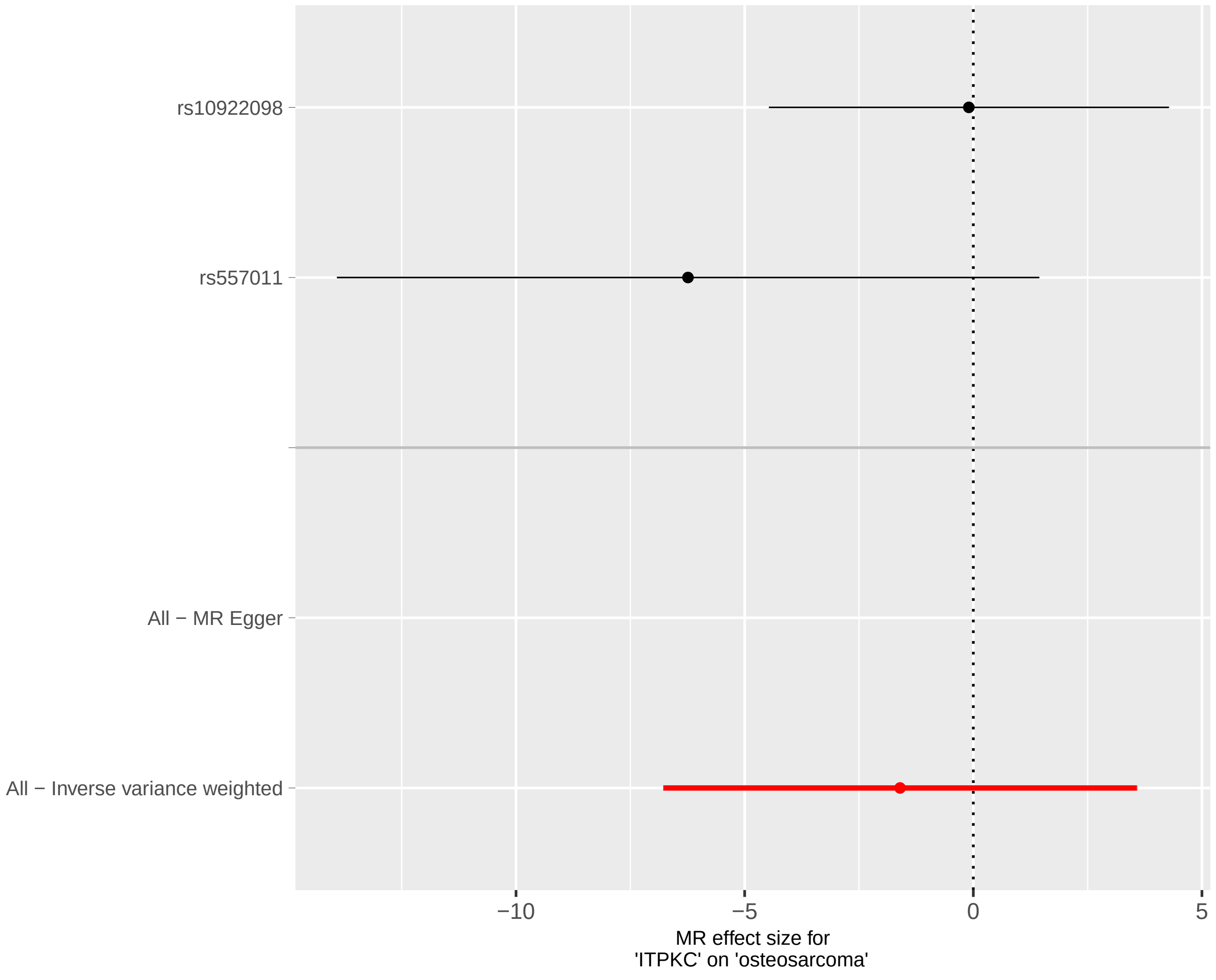

rs10922098
rs557011
All − MR Egger
All − Inverse variance weighted
−10
−5
0
5
MR effect size for
'ITPKC' on 'osteosarcoma'

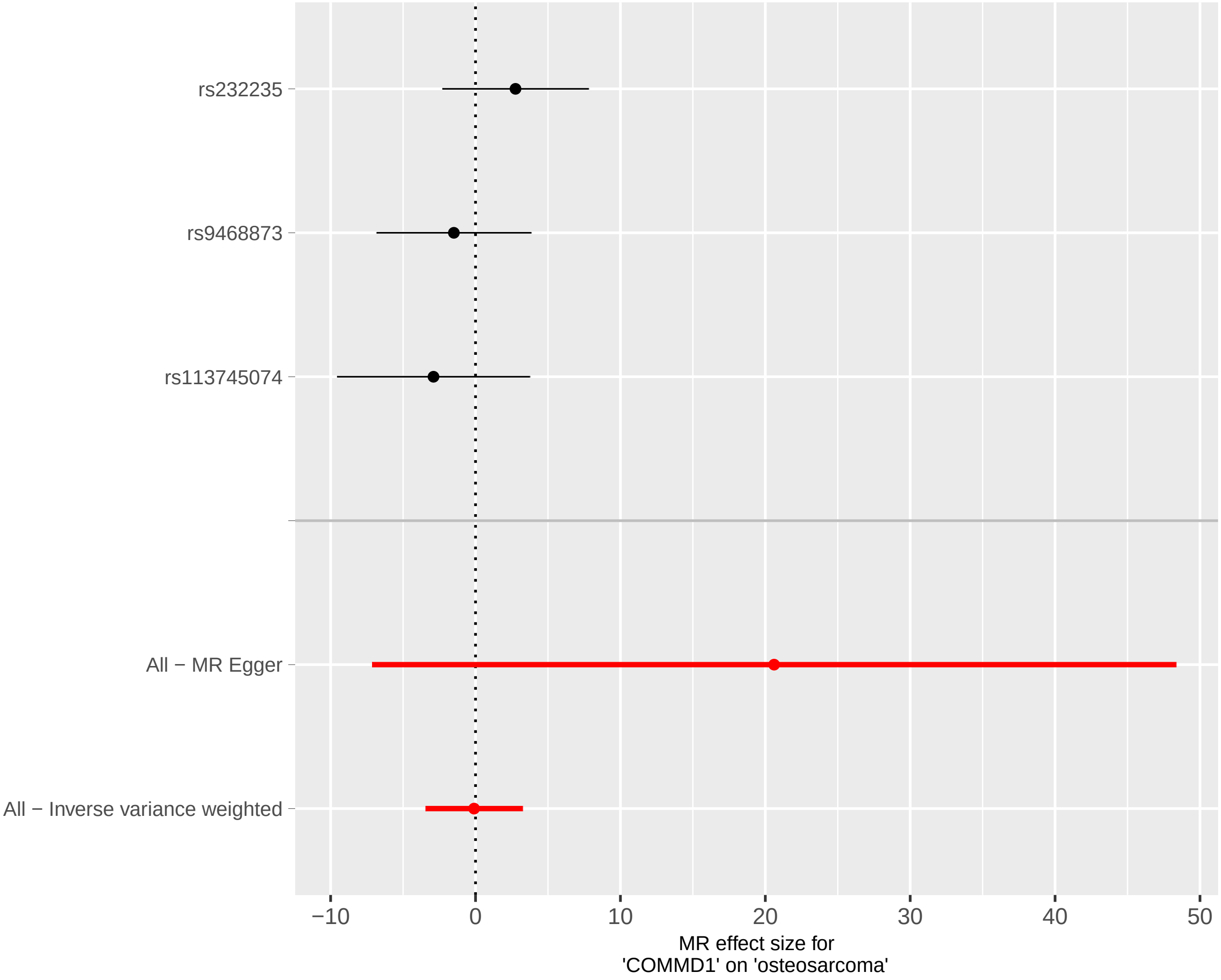
rs232235
rs9468873
rs113745074
All − MR Egger
All − Inverse variance weighted
−10
0
10
20
30
40
50
MR effect size for
'COMMD1' on 'osteosarcoma'

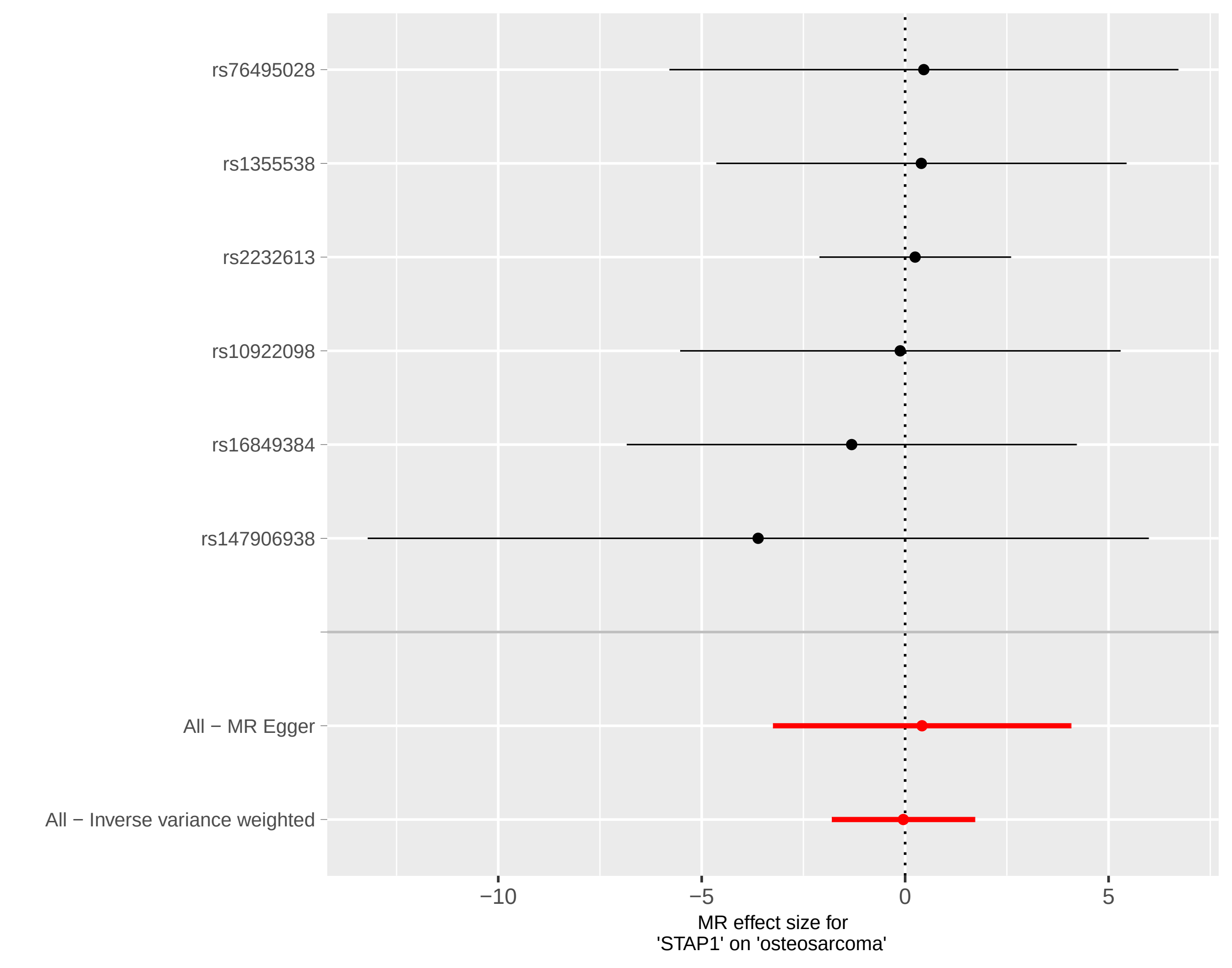
rs76495028
rs1355538
rs2232613
rs10922098
rs16849384
rs147906938
All − MR Egger
All − Inverse variance weighted
−10
−5
0
5
MR effect size for
'STAP1' on 'osteosarcoma'

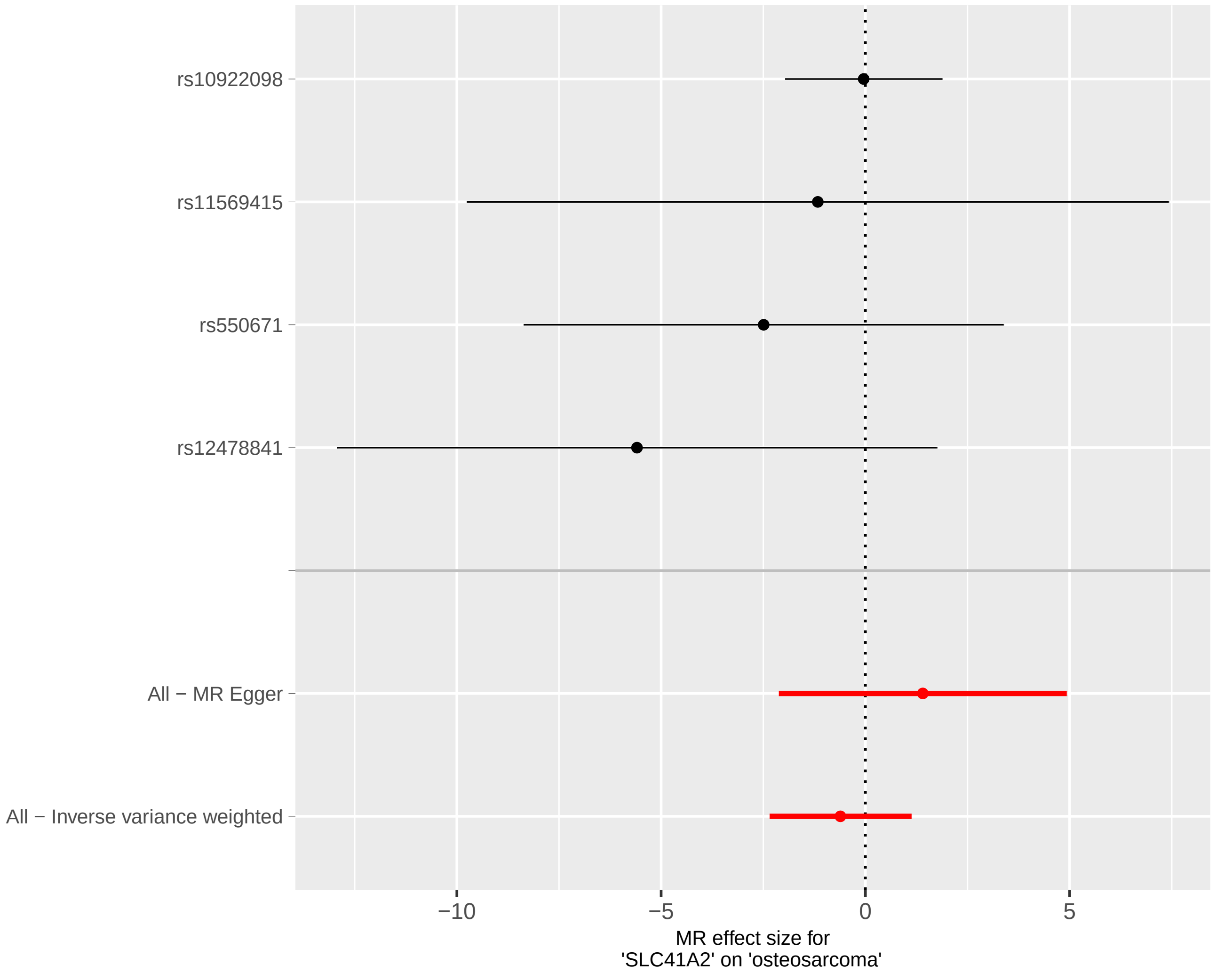
rs10922098
rs11569415
rs550671
rs12478841
All − MR Egger
All − Inverse variance weighted
−10
−5
0
5
MR effect size for
'SLC41A2' on 'osteosarcoma'

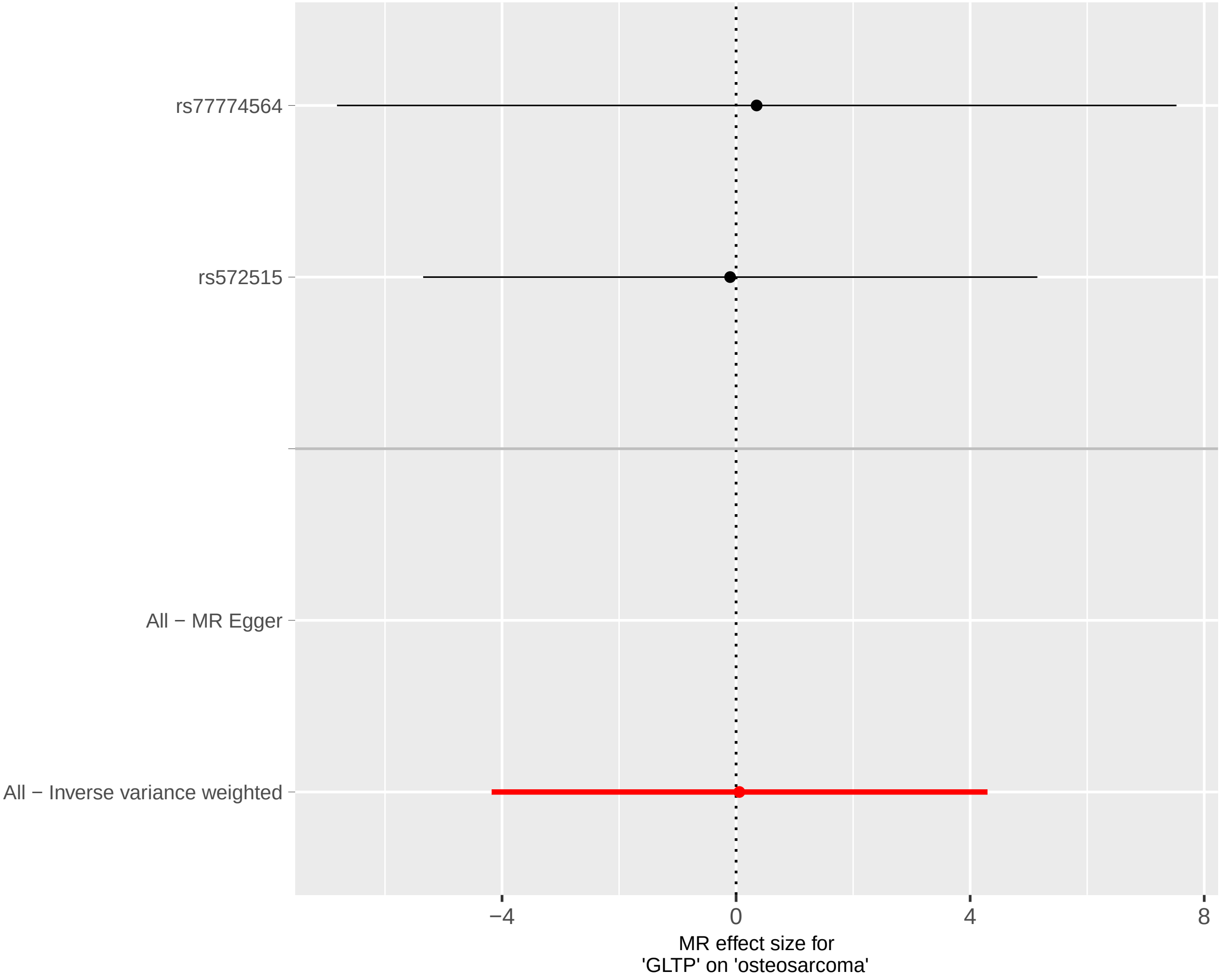

rs77774564
rs572515
All − MR Egger
All − Inverse variance weighted
−4
0
4
8
MR effect size for
'GLTP' on 'osteosarcoma'

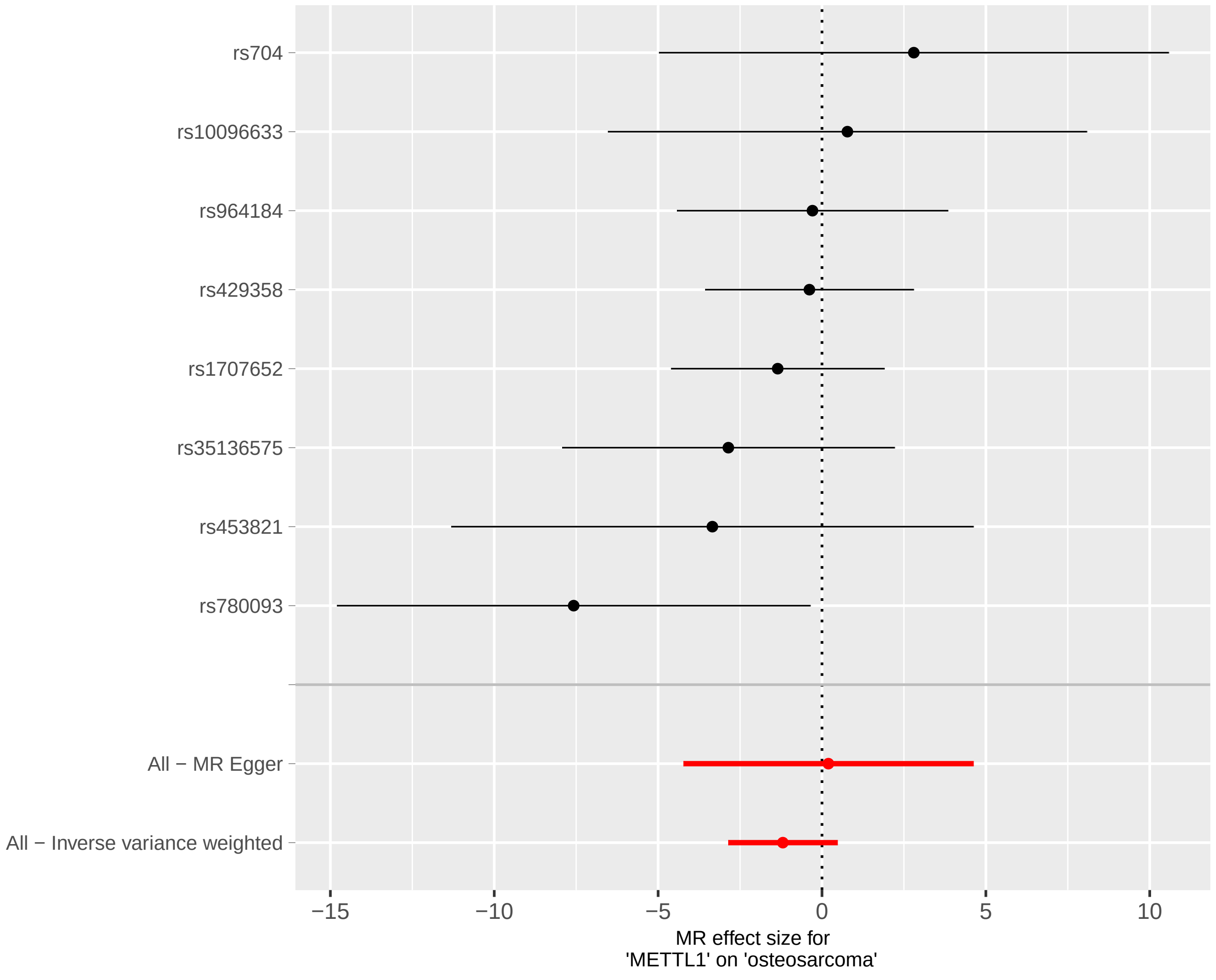

rs704
rs10096633
rs964184
rs429358
rs1707652
rs35136575
rs453821
rs780093
All − MR Egger
All − Inverse variance weighted
−15
−10
−5
0
5
10
MR effect size for
'METTL1' on 'osteosarcoma'

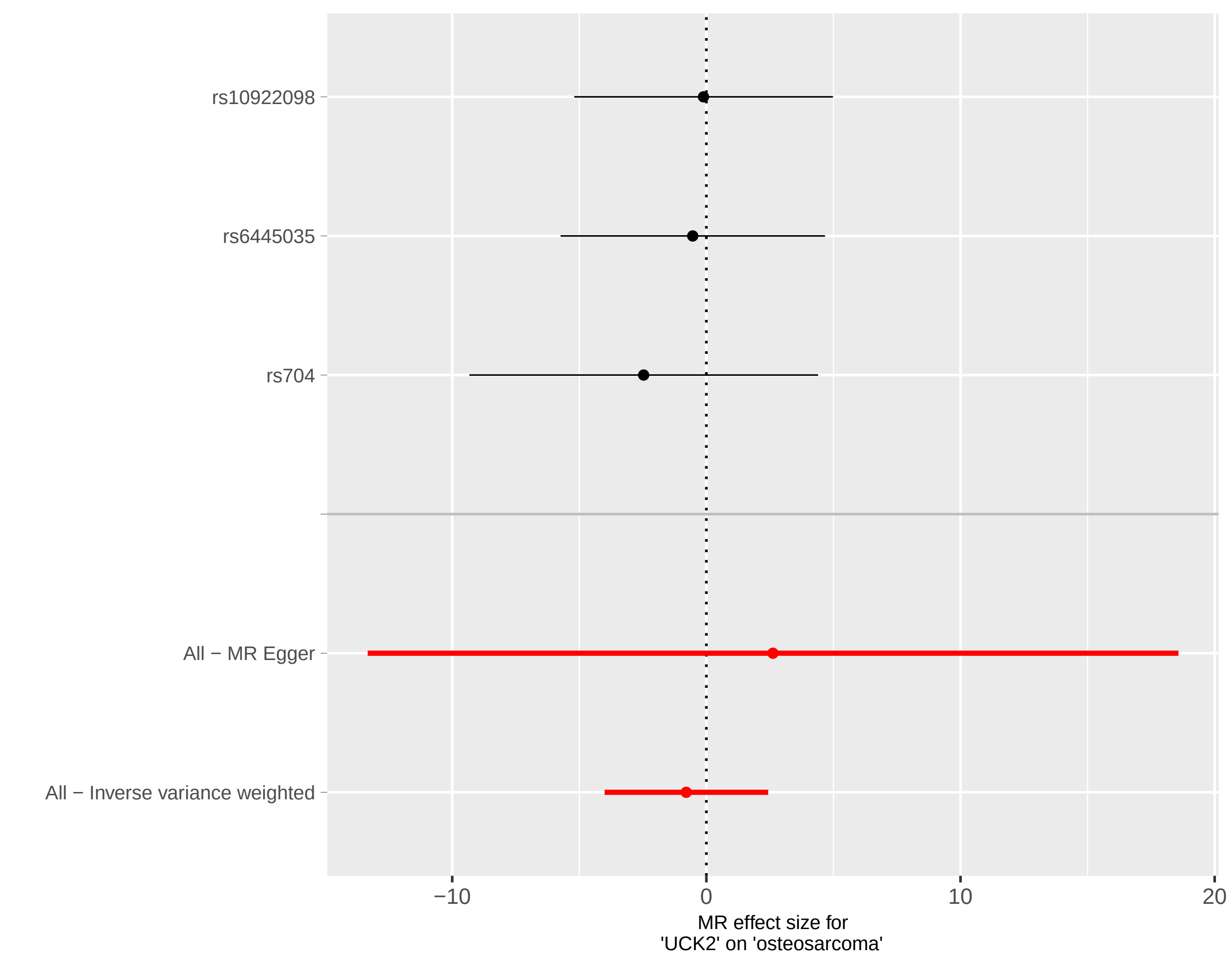
rs10922098
rs6445035
rs704
All − MR Egger
All − Inverse variance weighted
−10
0
10
20
MR effect size for
'UCK2' on 'osteosarcoma'

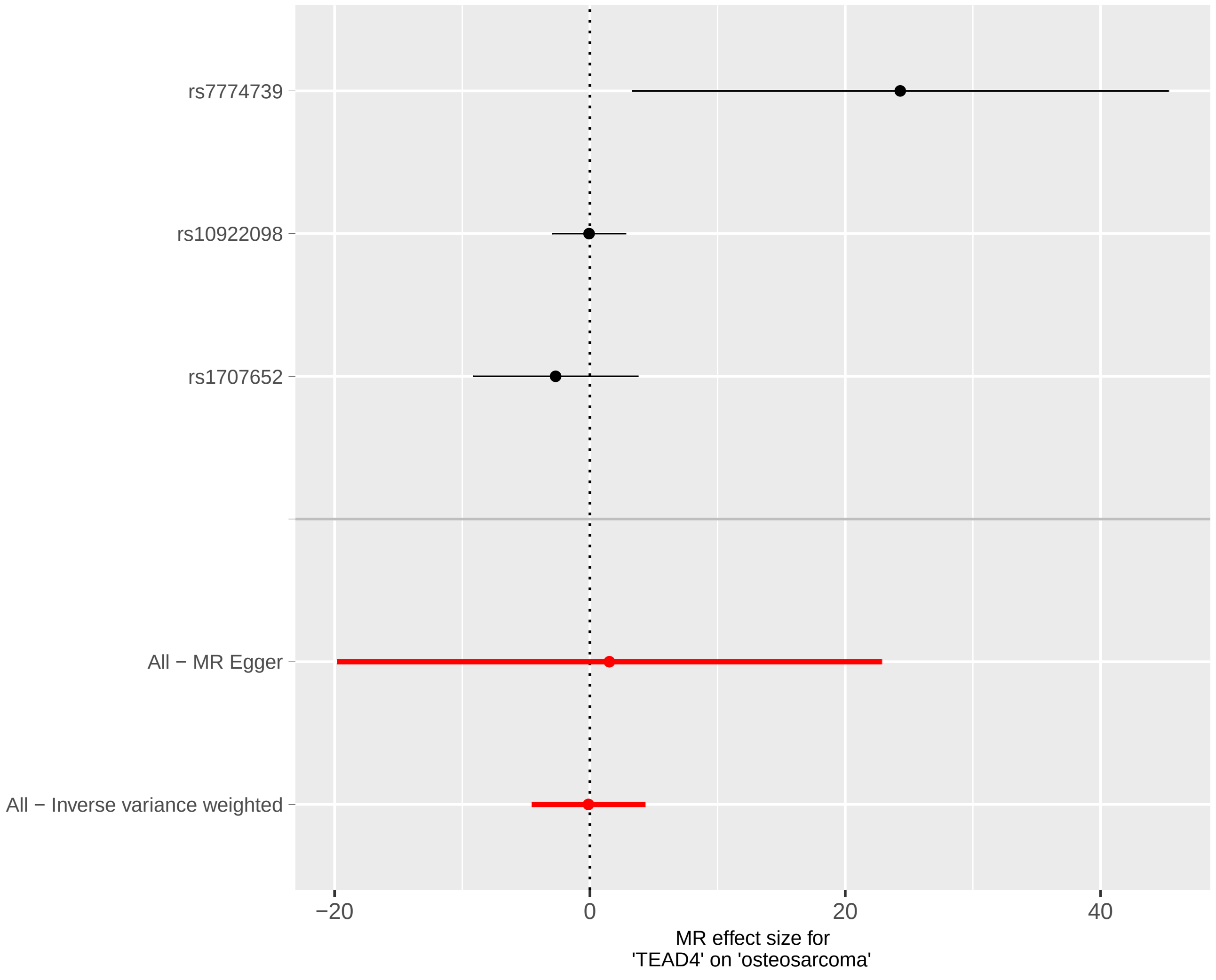

rs7774739
rs10922098
rs1707652
All − MR Egger
All − Inverse variance weighted
−20
0
20
40
MR effect size for
'TEAD4' on 'osteosarcoma'

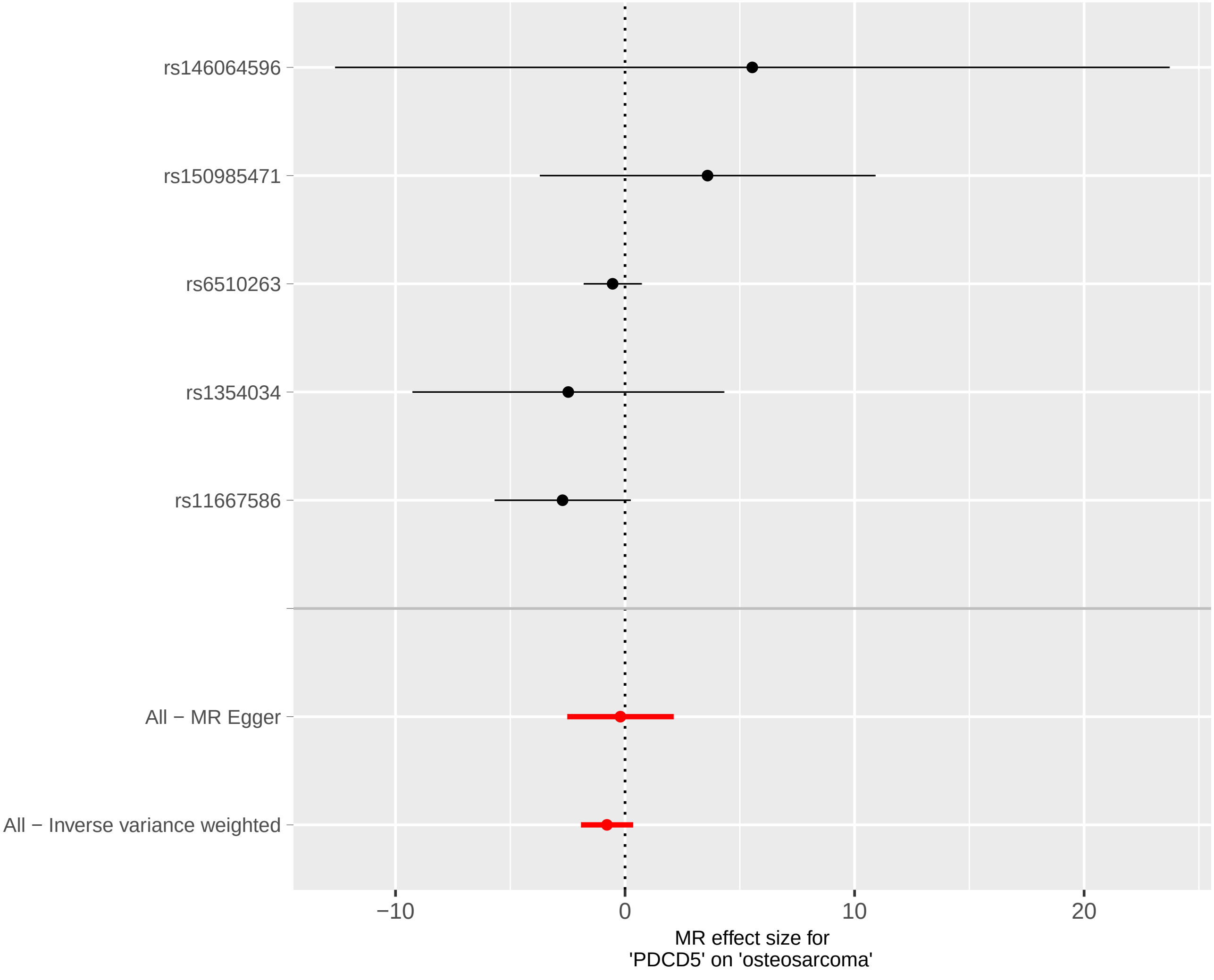
rs146064596
rs150985471
rs6510263
rs1354034
rs11667586
All − MR Egger
All − Inverse variance weighted
−10
0
10
20
MR effect size for
'PDCD5' on 'osteosarcoma'

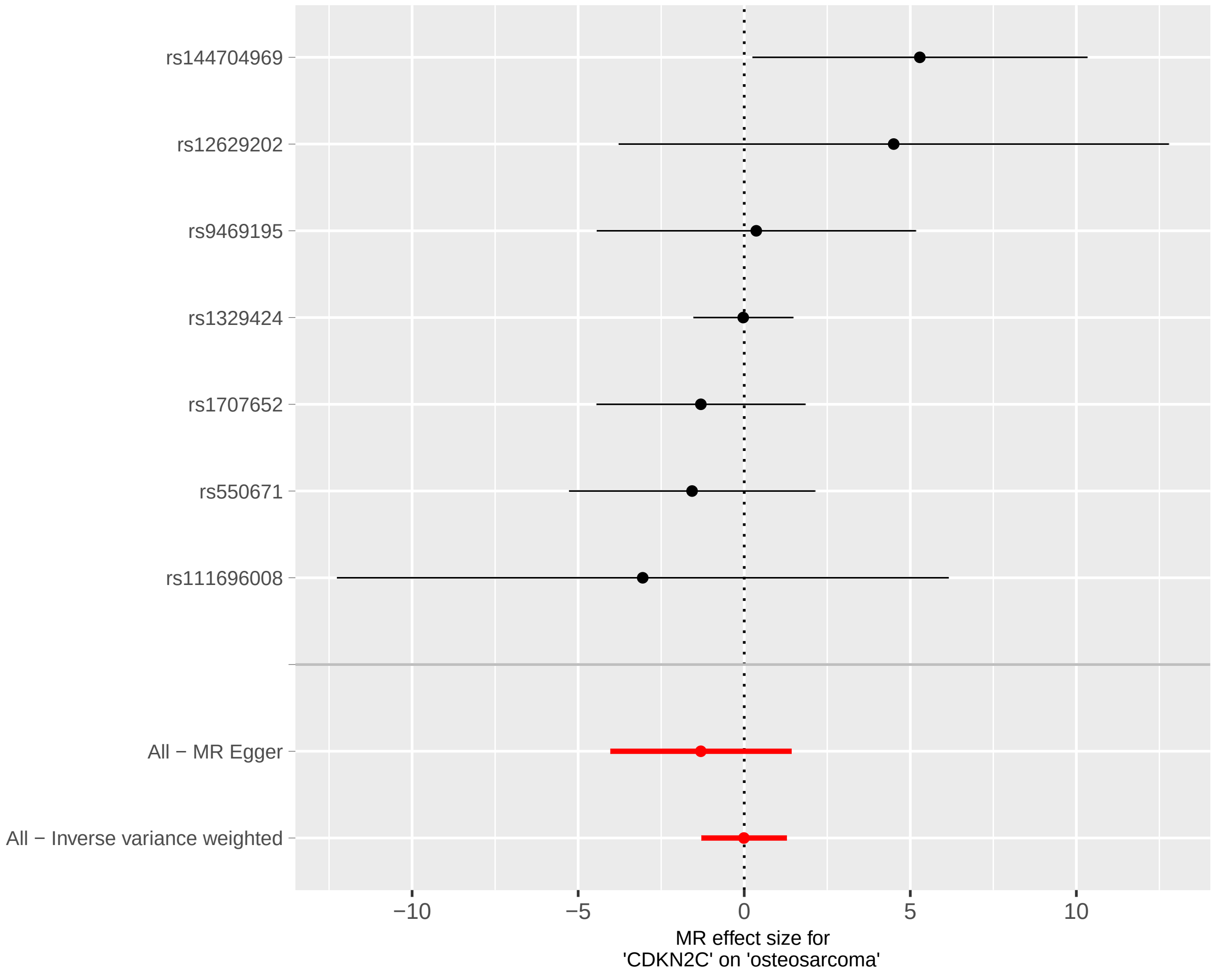
rs144704969
rs12629202
rs9469195
rs1329424
rs1707652
rs550671
rs11696008
All − MR Egger
All − Inverse variance weighted
−10
−5
0
5
10
MR effect size for
'CDKN2C' on 'osteosarcoma'

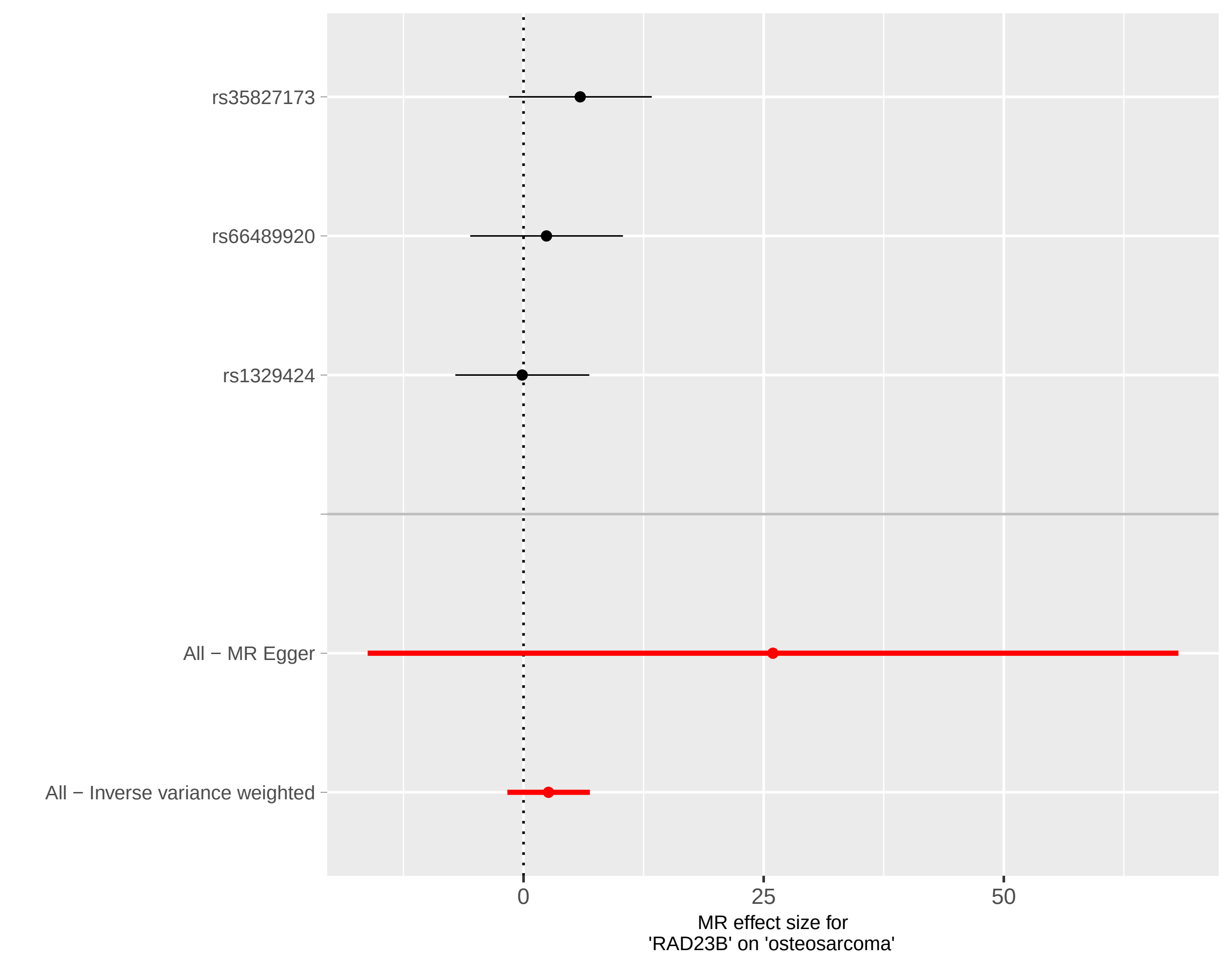
rs35827173
rs66489920
rs1329424
All − MR Egger
All − Inverse variance weighted
0
25
50
MR effect size for
'RAD23B' on 'osteosarcoma'

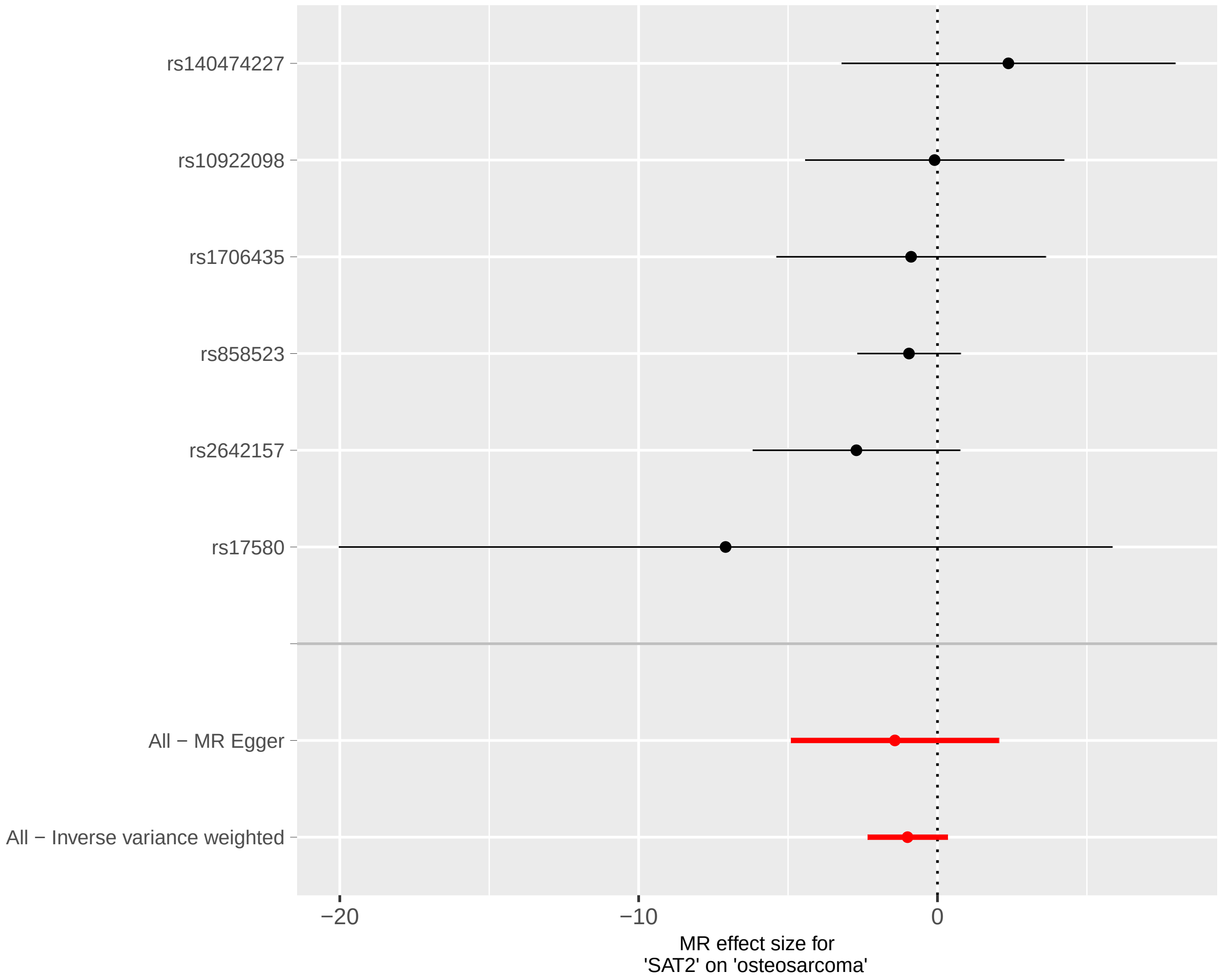
rs140474227
rs10922098
rs1706435
rs858523
rs2642157
rs17580
All − MR Egger
All − Inverse variance weighted
−20
−10
0
MR effect size for
'SAT2' on 'osteosarcoma'

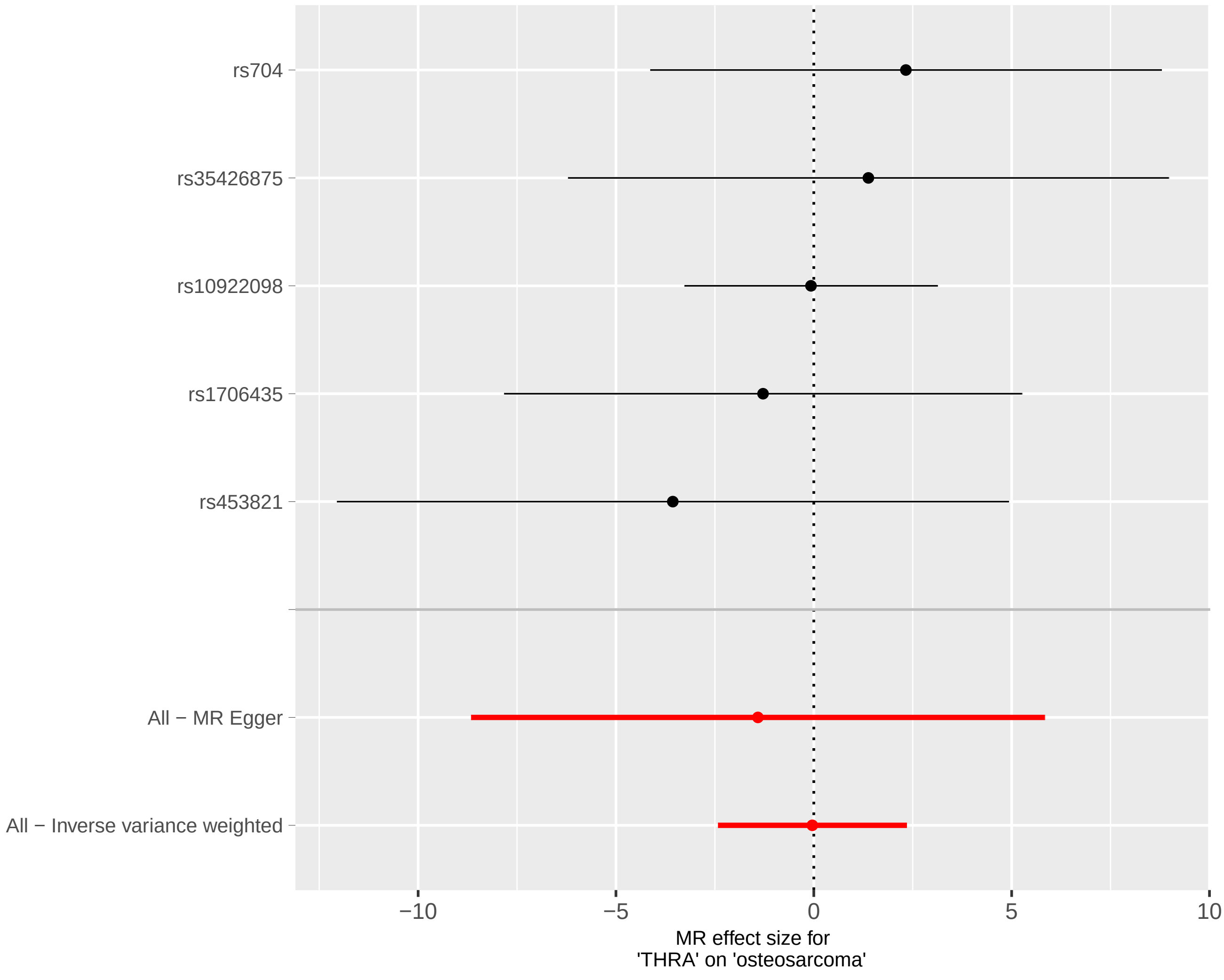

rs704
rs35426875
rs10922098
rs1706435
rs453821
All − MR Egger
All − Inverse variance weighted
−10
−5
0
5
10
MR effect size for
'THRA' on 'osteosarcoma'

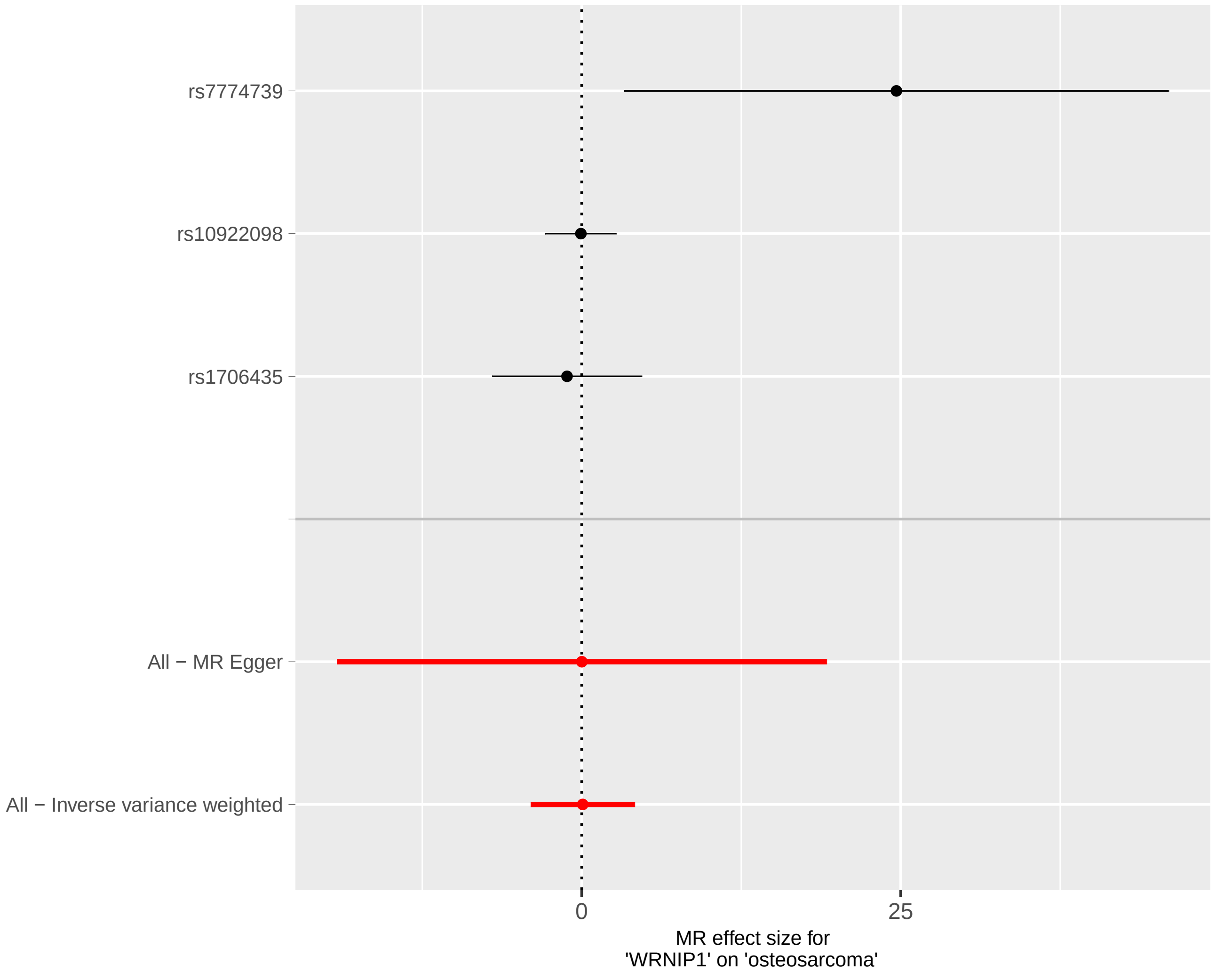
rs7774739
rs10922098
rs1706435
All − MR Egger
All − Inverse variance weighted
0
25
MR effect size for
'WRNIP1' on 'osteosarcoma'

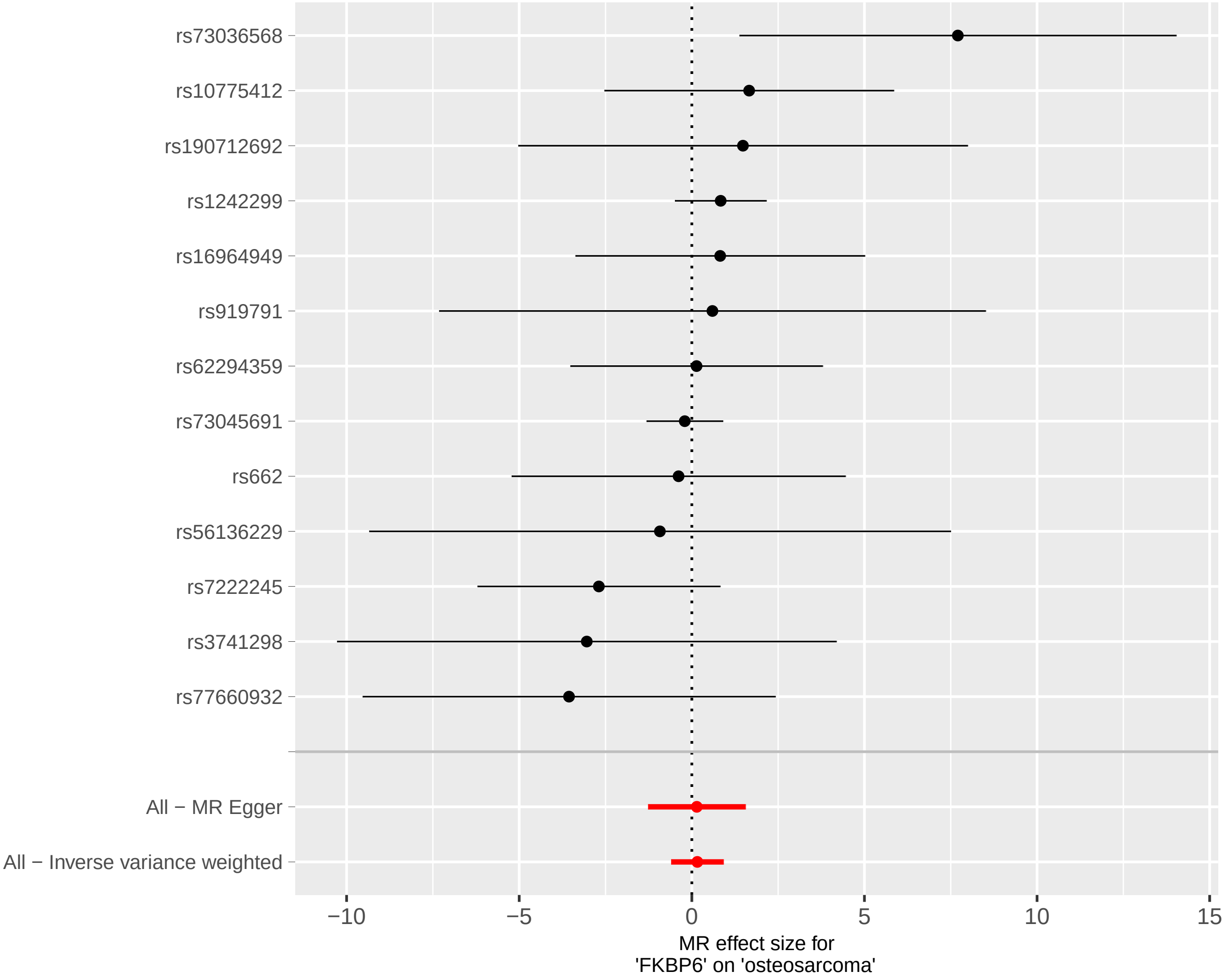

rs73036568
rs10775412
rs190712692
rs1242299
rs16964949
rs919791
rs62294359
rs73045691
rs662
rs56136229
rs7222245
rs3741298
rs77660932
All − MR Egger
All − Inverse variance weighted
−10
−5
0
5
10
15
MR effect size for
'FKBP6' on 'osteosarcoma'

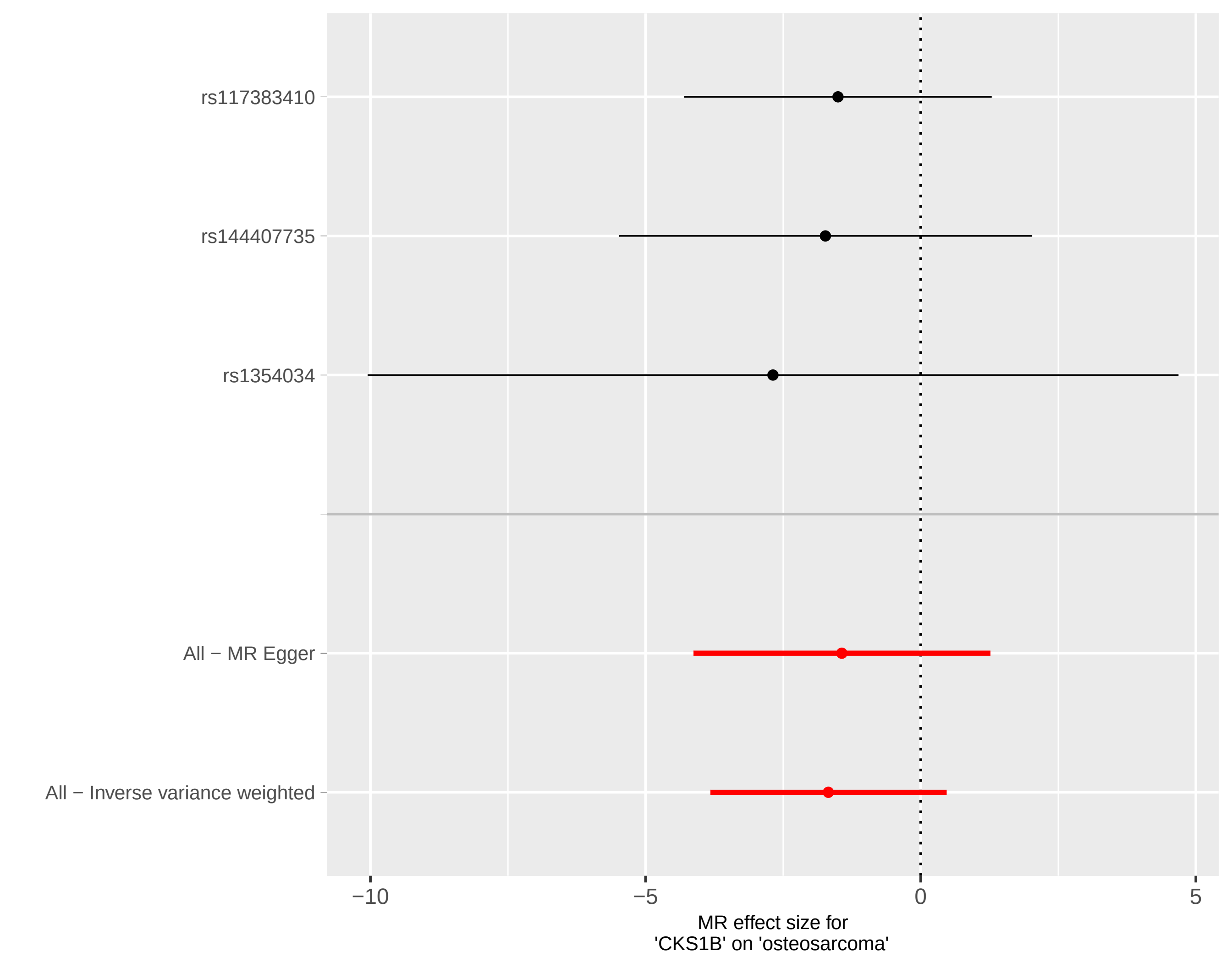

rs117383410
rs144407735
rs1354034
All − MR Egger
All − Inverse variance weighted
−10
−5
0
5
MR effect size for
'CKS1B' on 'osteosarcoma'

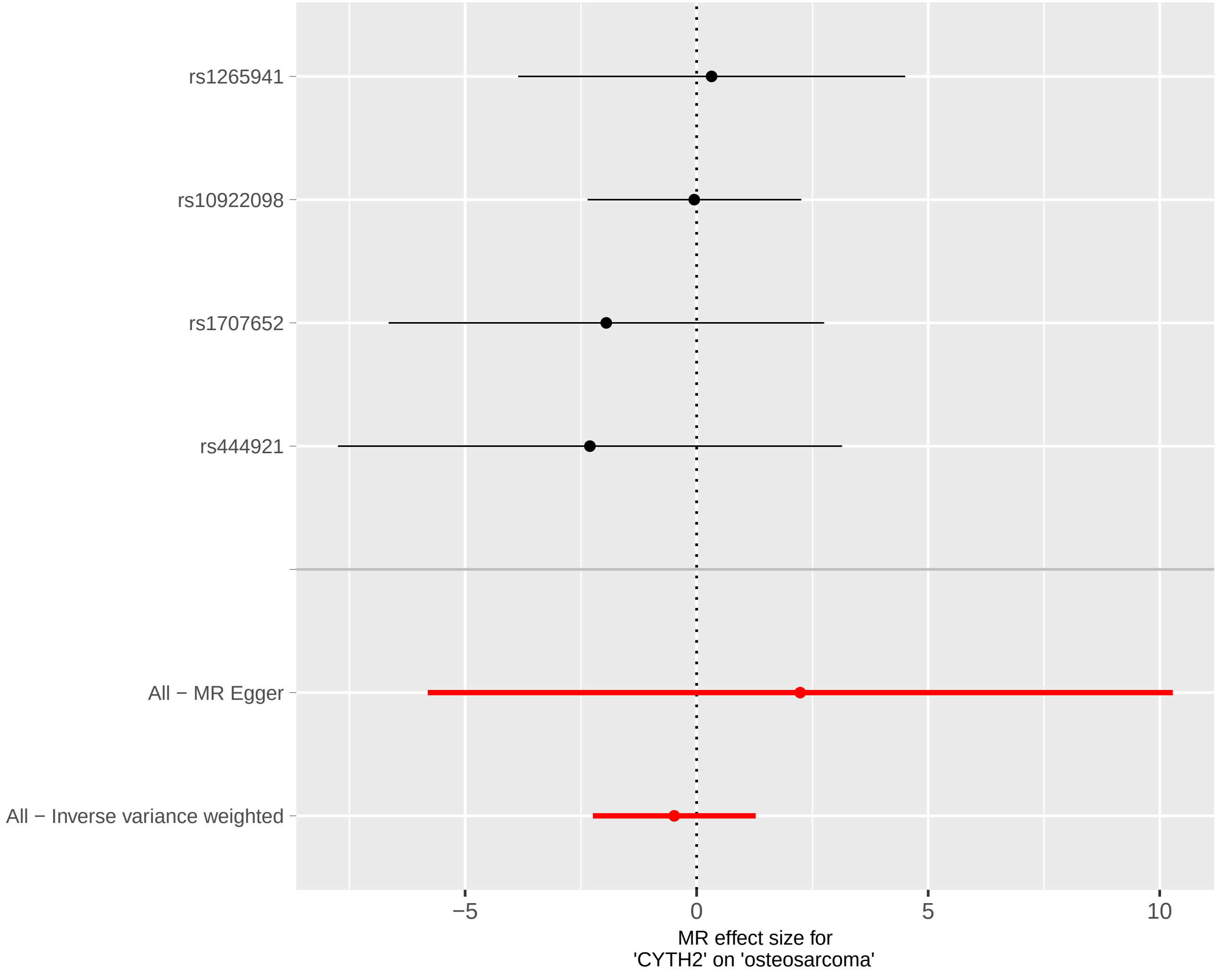
rs1265941
rs10922098
rs1707652
rs444921
All − MR Egger
All − Inverse variance weighted
−5
0
5
10
MR effect size for
'CYTH2' on 'osteosarcoma'

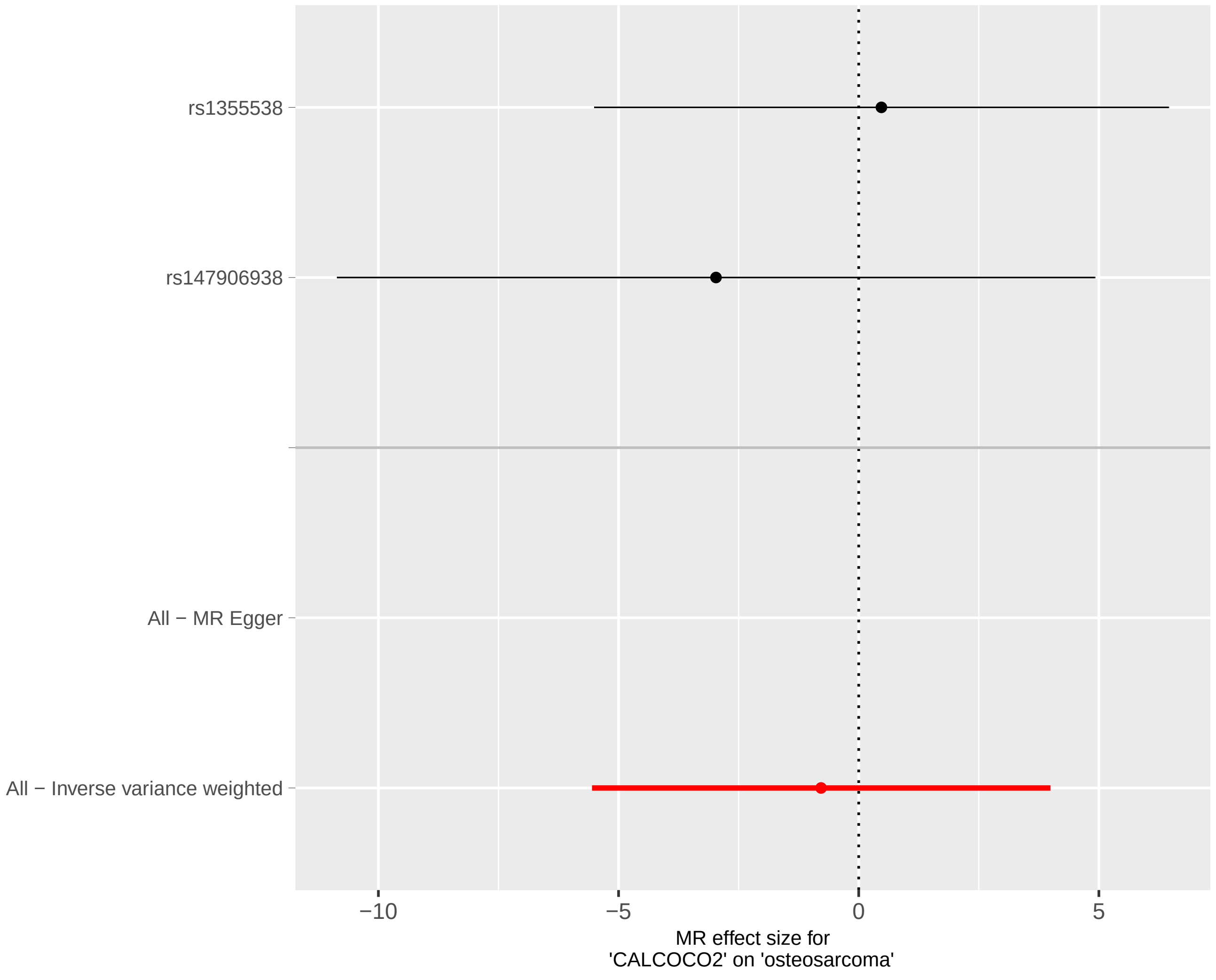

rs1355538
rs147906938
All − MR Egger
All − Inverse variance weighted
−10
−5
0
5
MR effect size for
'CALCOCO2' on 'osteosarcoma'

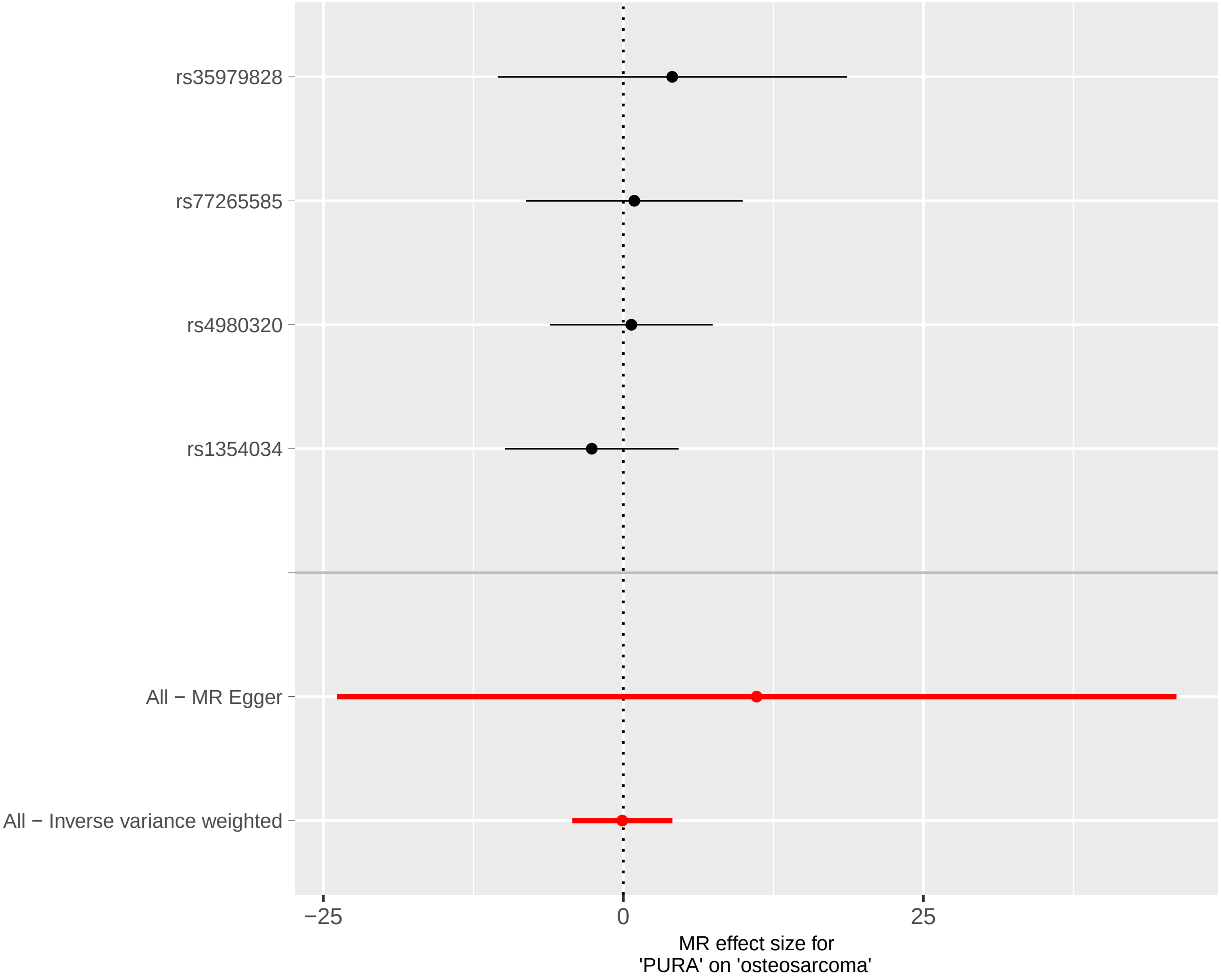
rs35979828
rs77265585
rs4980320
rs1354034
All − MR Egger
All − Inverse variance weighted
−25
0
25
MR effect size for
'PURA' on 'osteosarcoma'

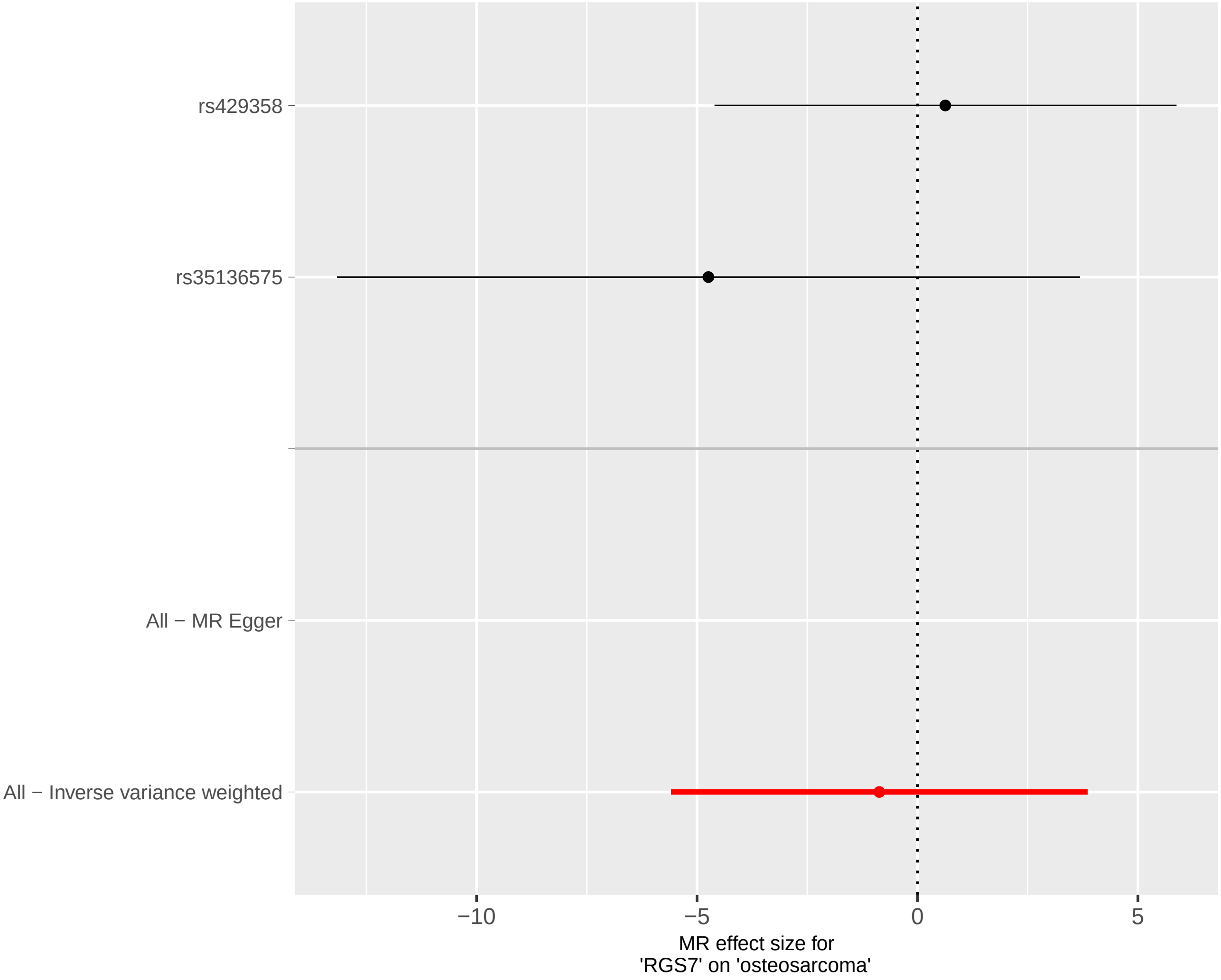

rs429358
rs35136575
All − MR Egger
All − Inverse variance weighted
−10
−5
0
5
MR effect size for
'RGS7' on 'osteosarcoma'

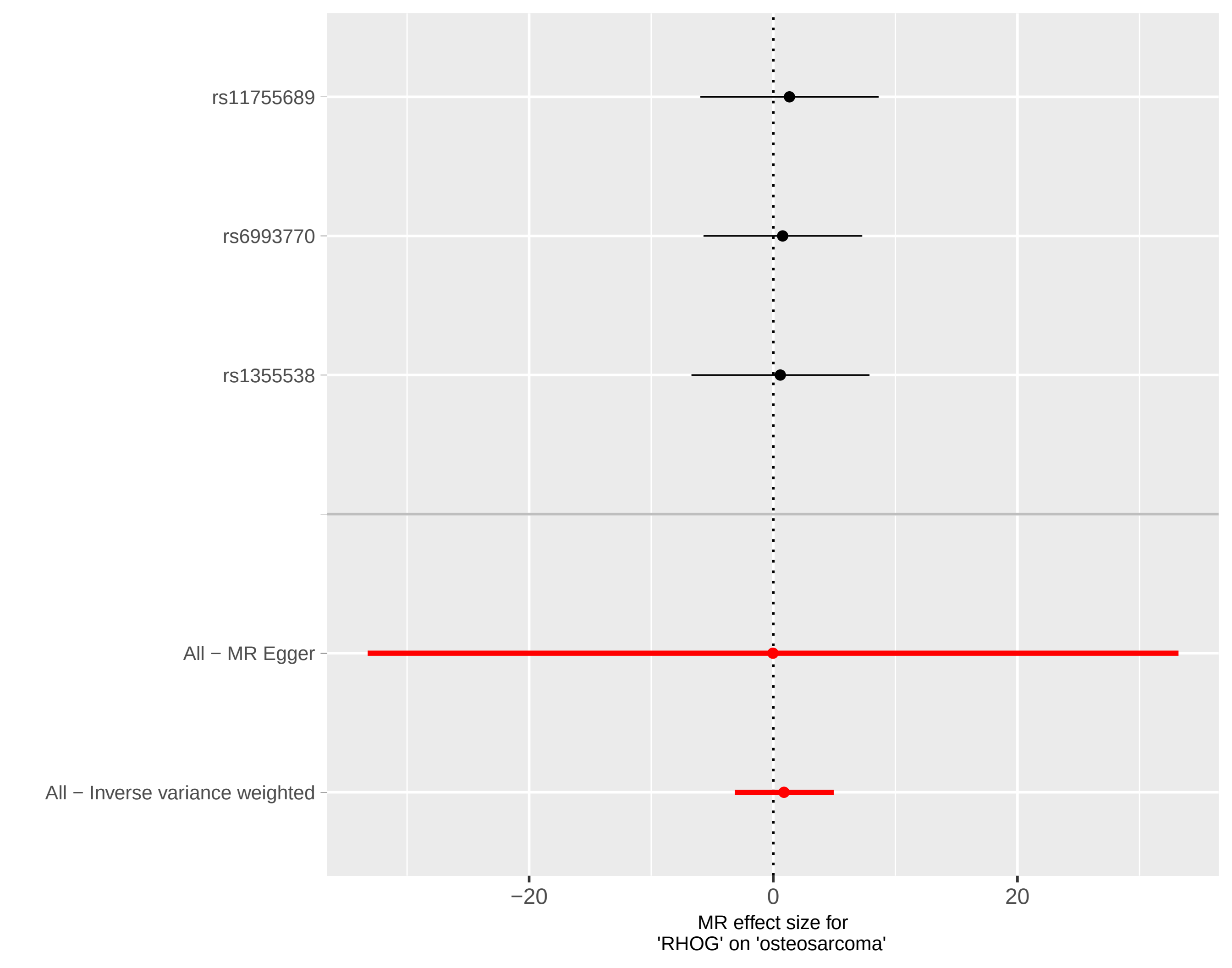
rs11755689
rs6993770
rs1355538
All − MR Egger
All − Inverse variance weighted
−20
0
20
MR effect size for
'RHOG' on 'osteosarcoma'

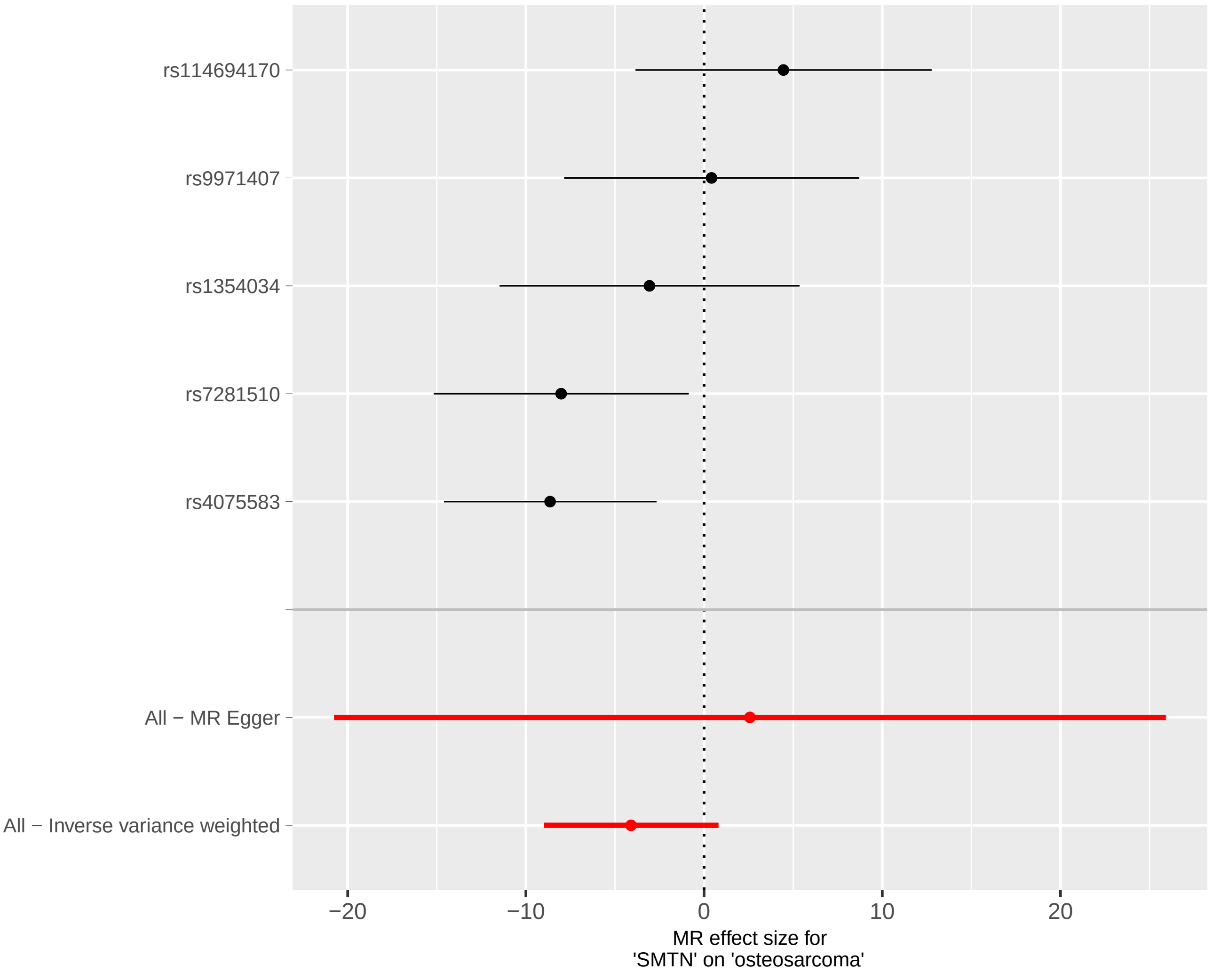
rs114694170
rs9971407
rs1354034
rs7281510
rs4075583
All − MR Egger
All − Inverse variance weighted
−20
−10
0
10
20
MR effect size for
'SMTN' on 'osteosarcoma'

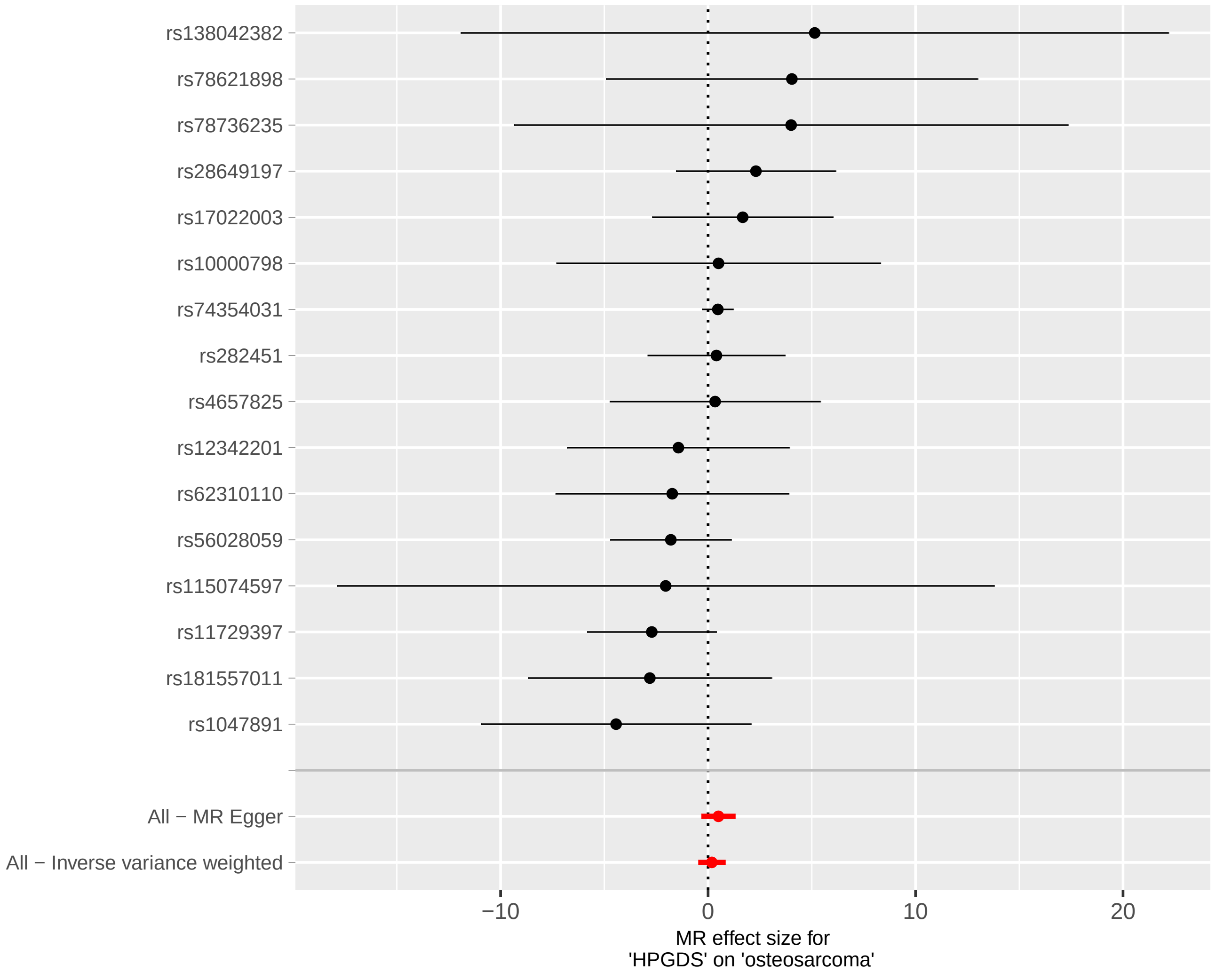

rs138042382
rs78621898
rs78736235
rs28649197
rs17022003
rs10000798
rs74354031
rs282451
rs4657825
rs12342201
rs62310110
rs56028059
rs115074597
rs11729397
rs181557011
rs1047891
All − MR Egger
All − Inverse variance weighted
−10
0
10
20
MR effect size for
'HPGDS' on 'osteosarcoma'

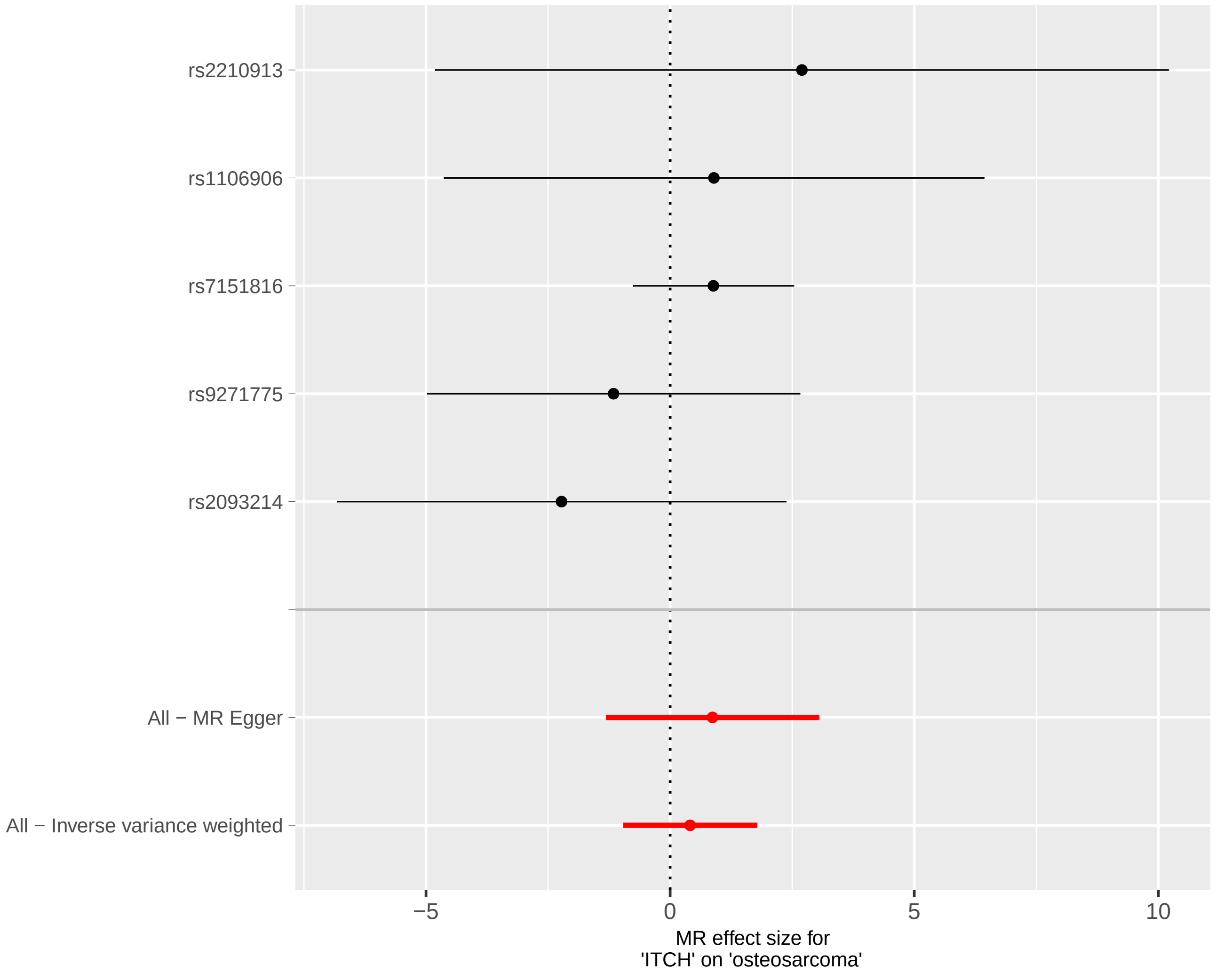
rs2210913
rs1106906
rs7151816
rs9271775
rs2093214
All − MR Egger
All − Inverse variance weighted
−5
0
5
10
MR effect size for
'ITCH' on 'osteosarcoma'

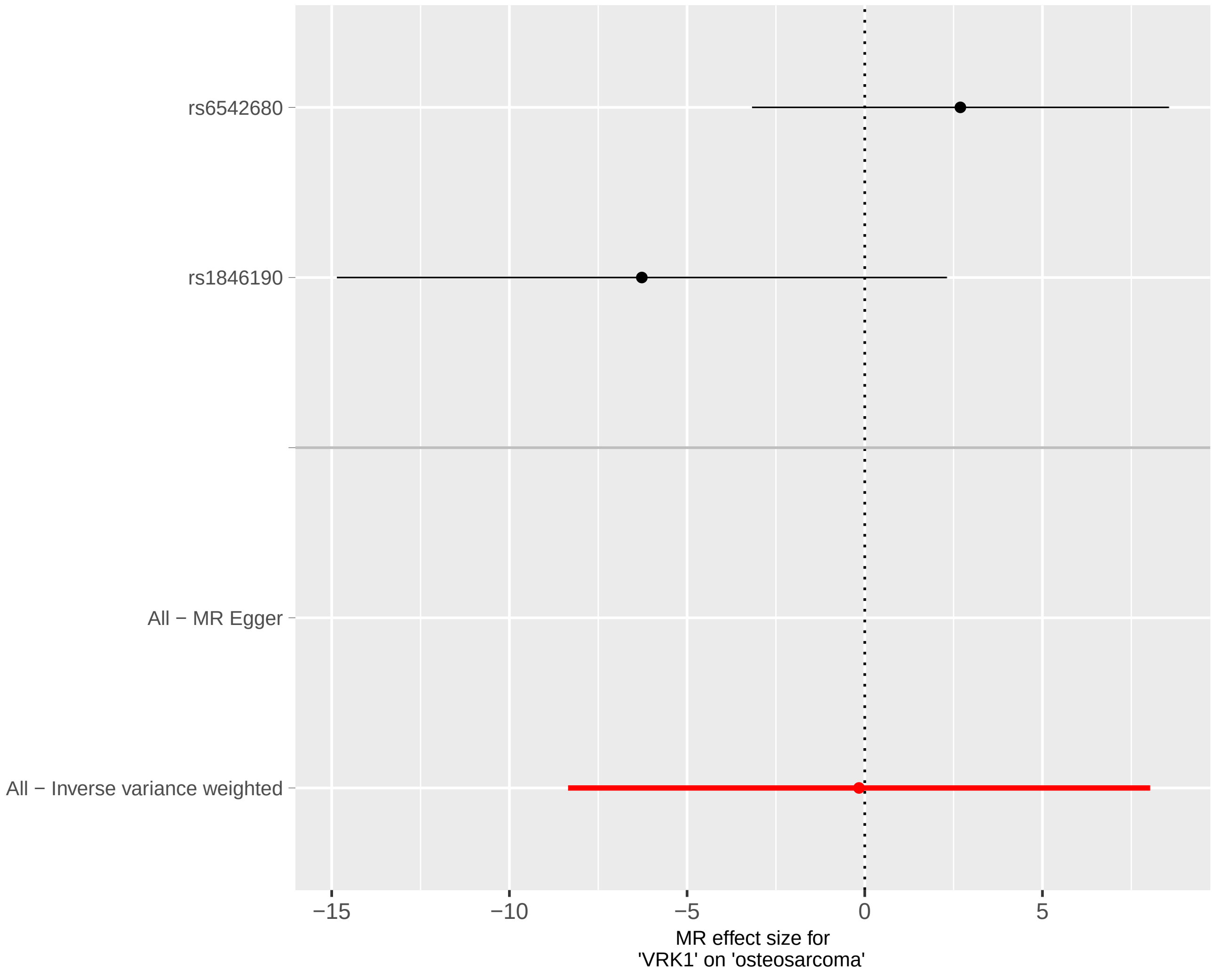

rs6542680
rs1846190
All − MR Egger
All − Inverse variance weighted
−15
−10
−5
0
5
MR effect size for
'VRK1' on 'osteosarcoma'

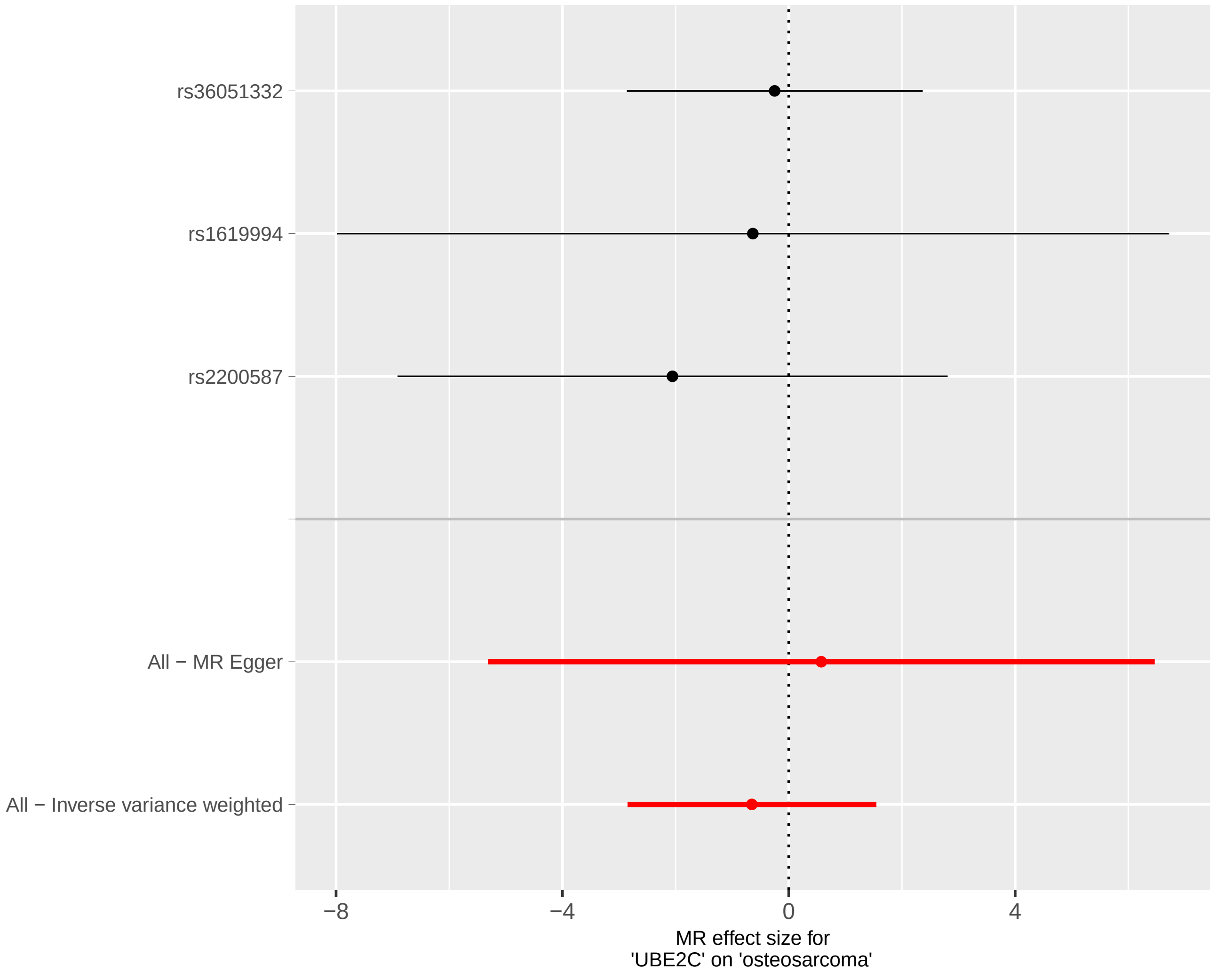
rs36051332
rs1619994
rs2200587
All − MR Egger
All − Inverse variance weighted
−8
−4
0
4
MR effect size for
'UBE2C' on 'osteosarcoma'

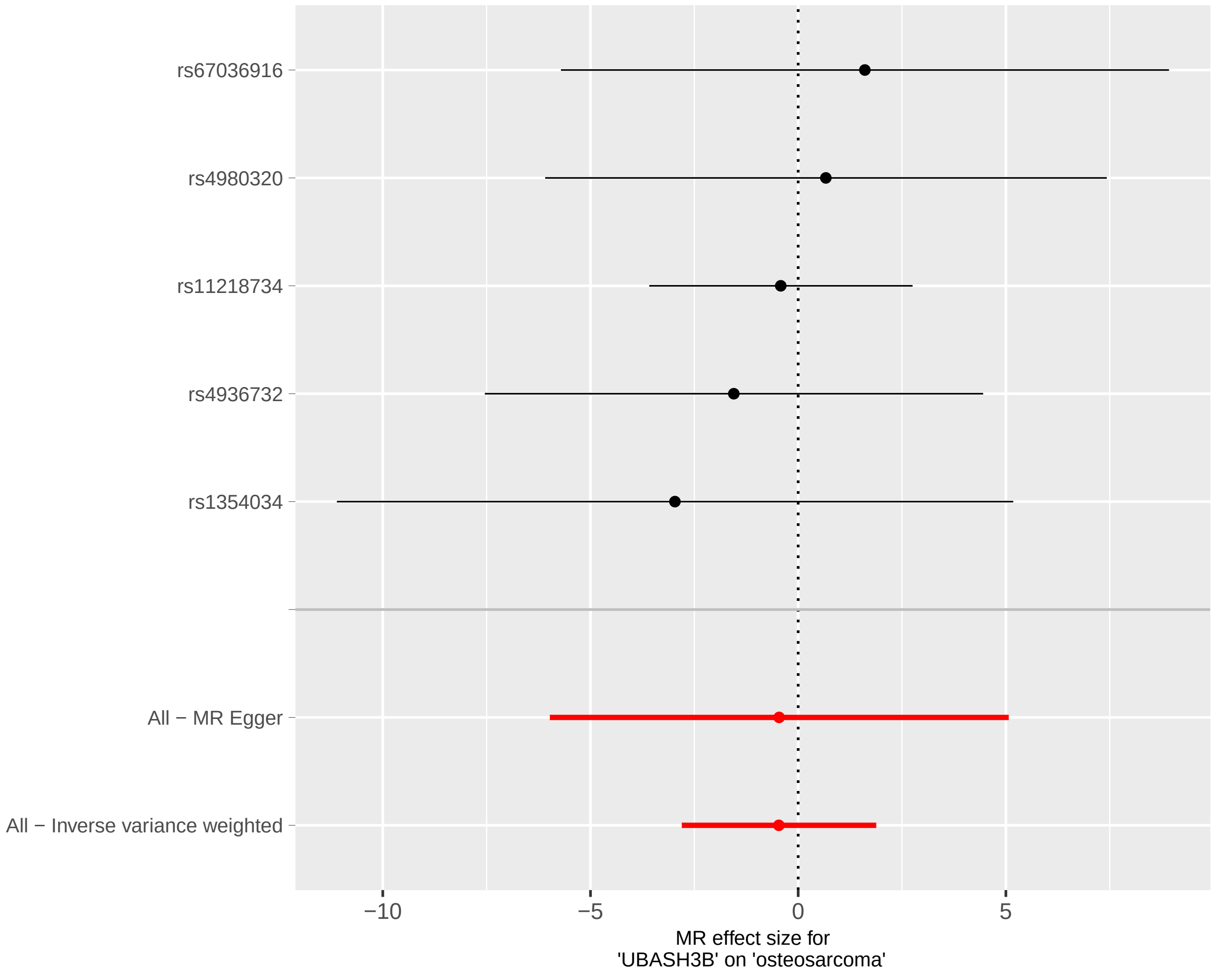
rs67036916
rs4980320
rs11218734
rs4936732
rs1354034
All − MR Egger
All − Inverse variance weighted
−10
−5
0
5
MR effect size for
'UBASH3B' on 'osteosarcoma'

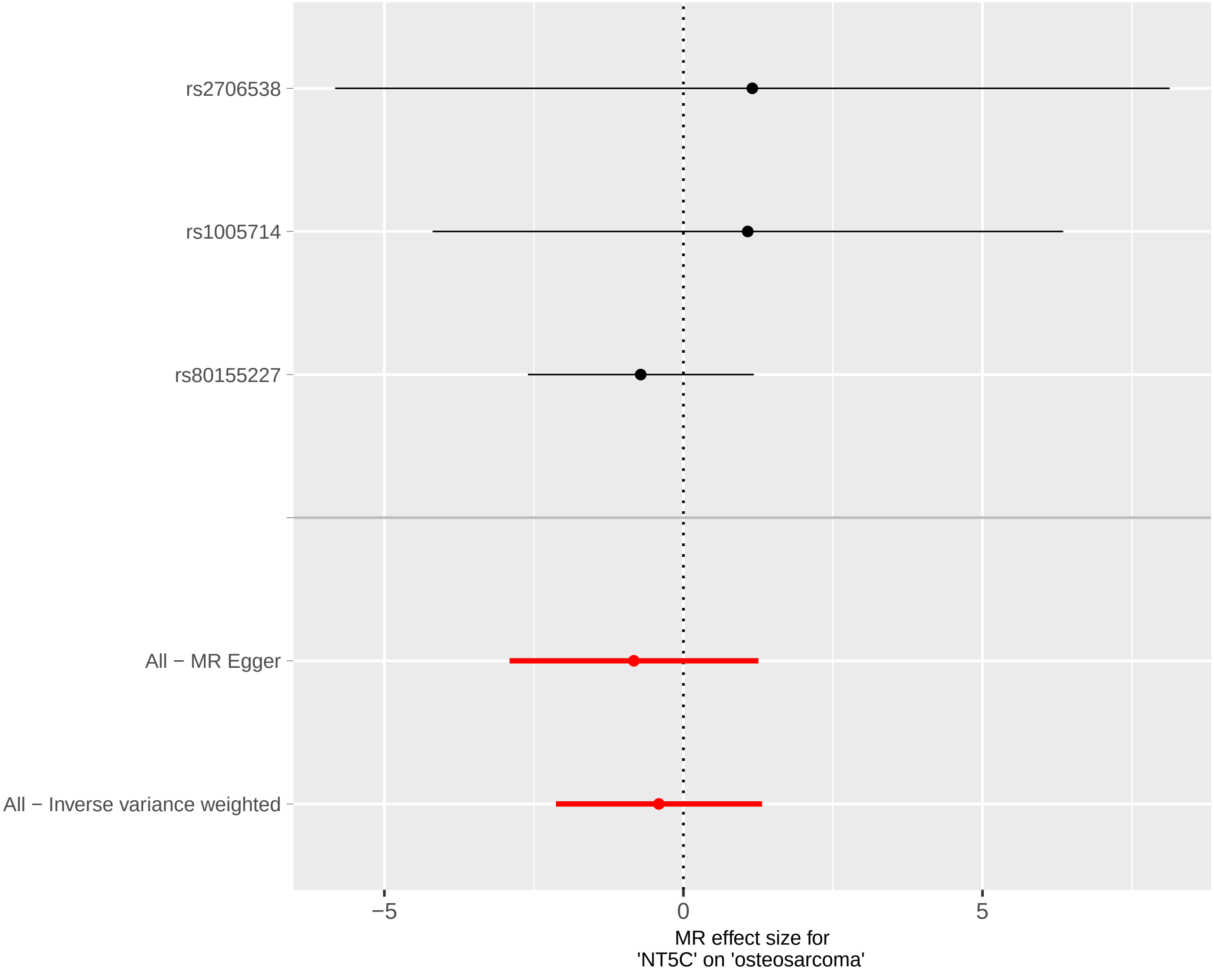
rs2706538
rs1005714
rs80155227
All − MR Egger
All − Inverse variance weighted
−5
0
5
MR effect size for
'NT5C' on 'osteosarcoma'

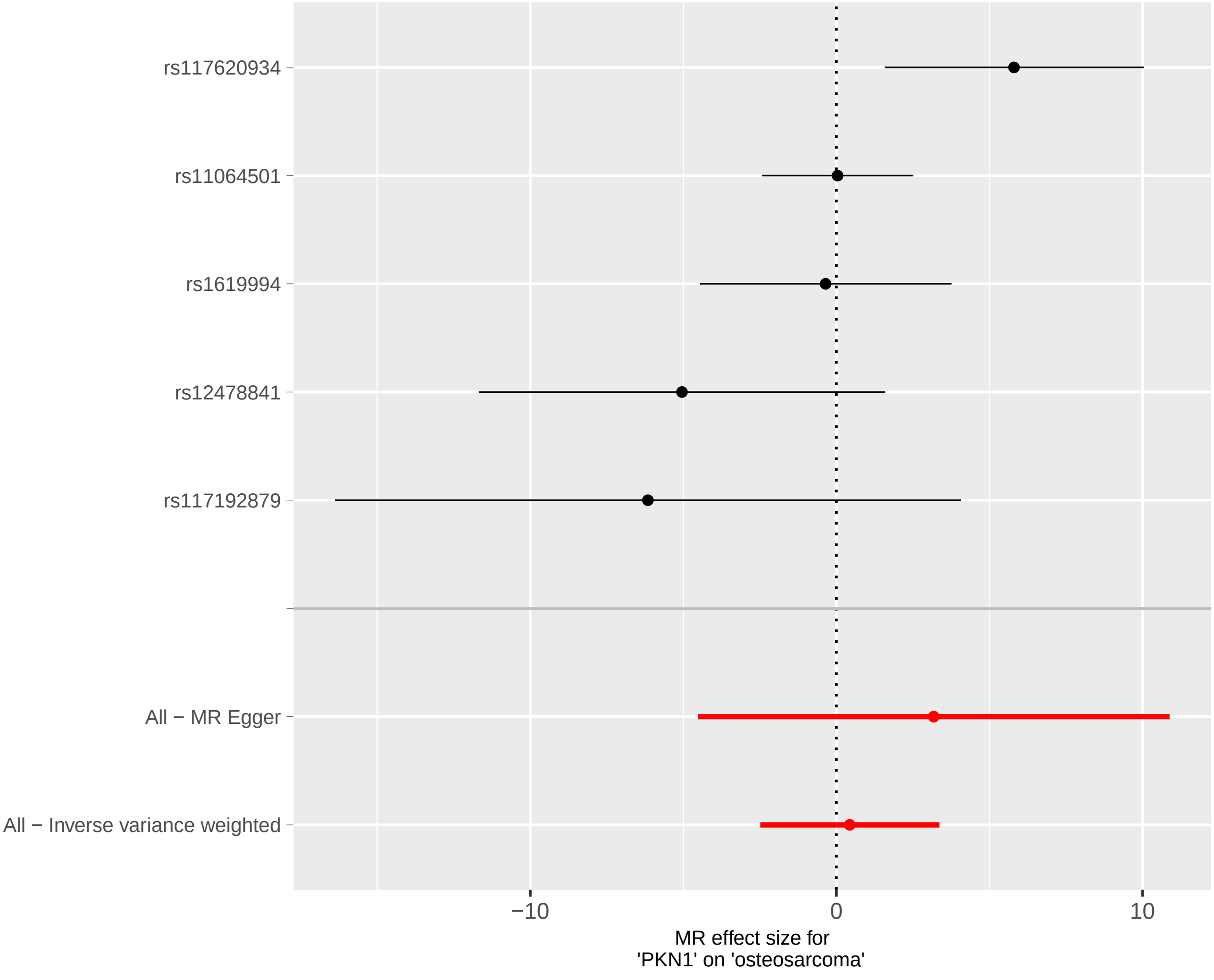
rs117620934
rs11064501
rs1619994
rs12478841
rs117192879
All − MR Egger
All − Inverse variance weighted
−10
0
10
MR effect size for
'PKN1' on 'osteosarcoma'

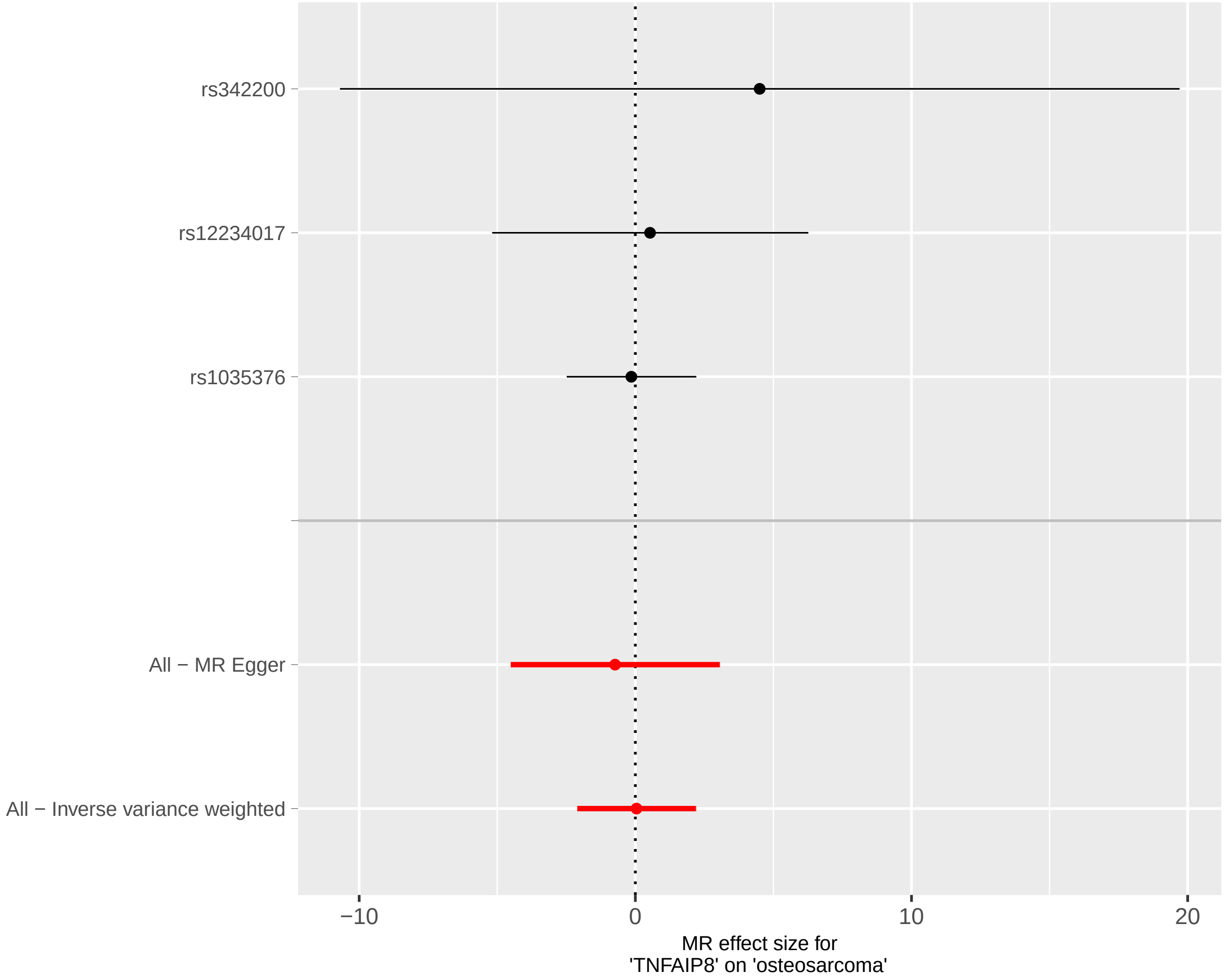

rs342200
rs12234017
rs1035376
All − MR Egger
All − Inverse variance weighted
−10
0
10
20
MR effect size for
'TNFAIP8' on 'osteosarcoma'

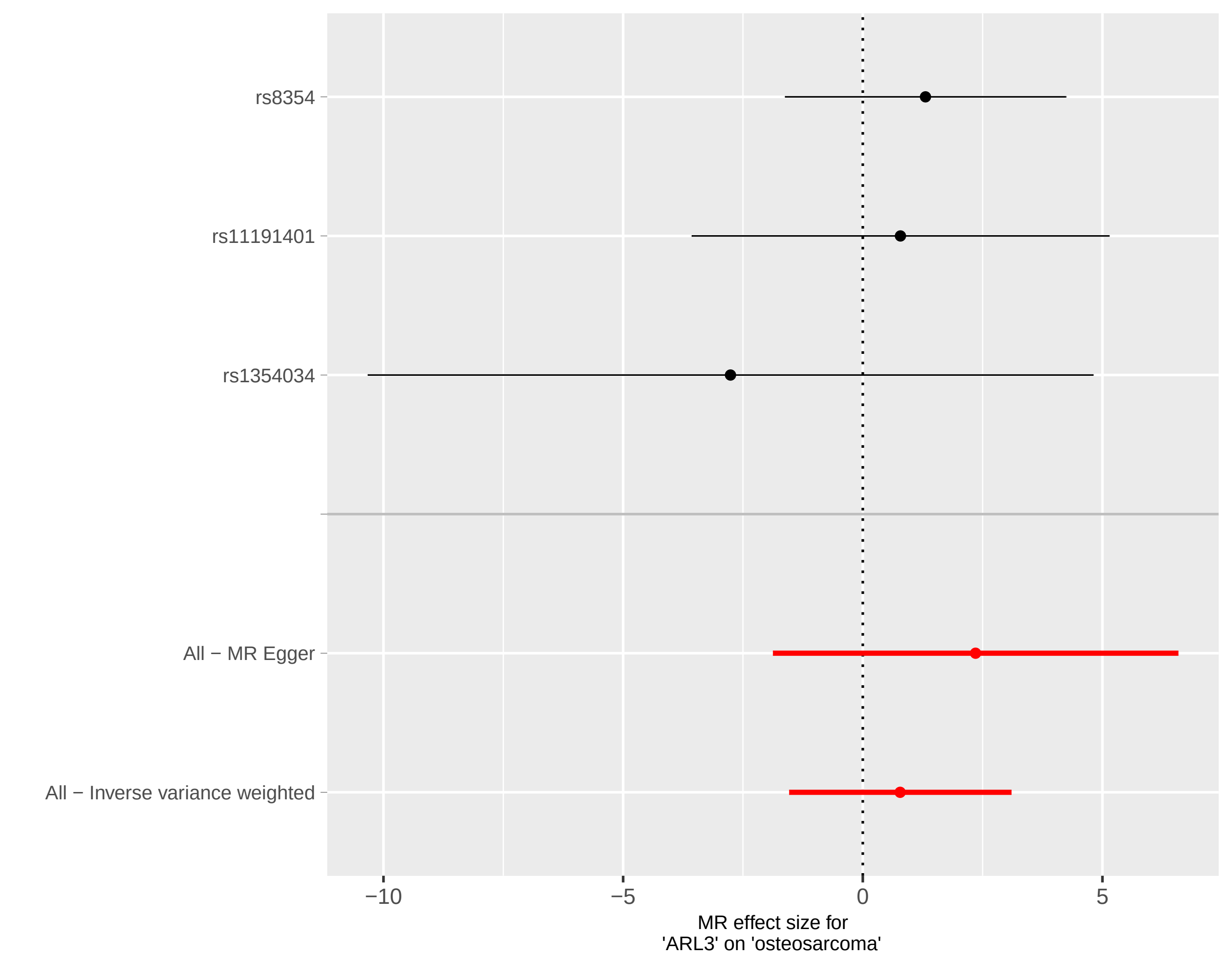
rs8354
rs11191401
rs1354034
All − MR Egger
All − Inverse variance weighted
−10
−5
0
5
MR effect size for
'ARL3' on 'osteosarcoma'

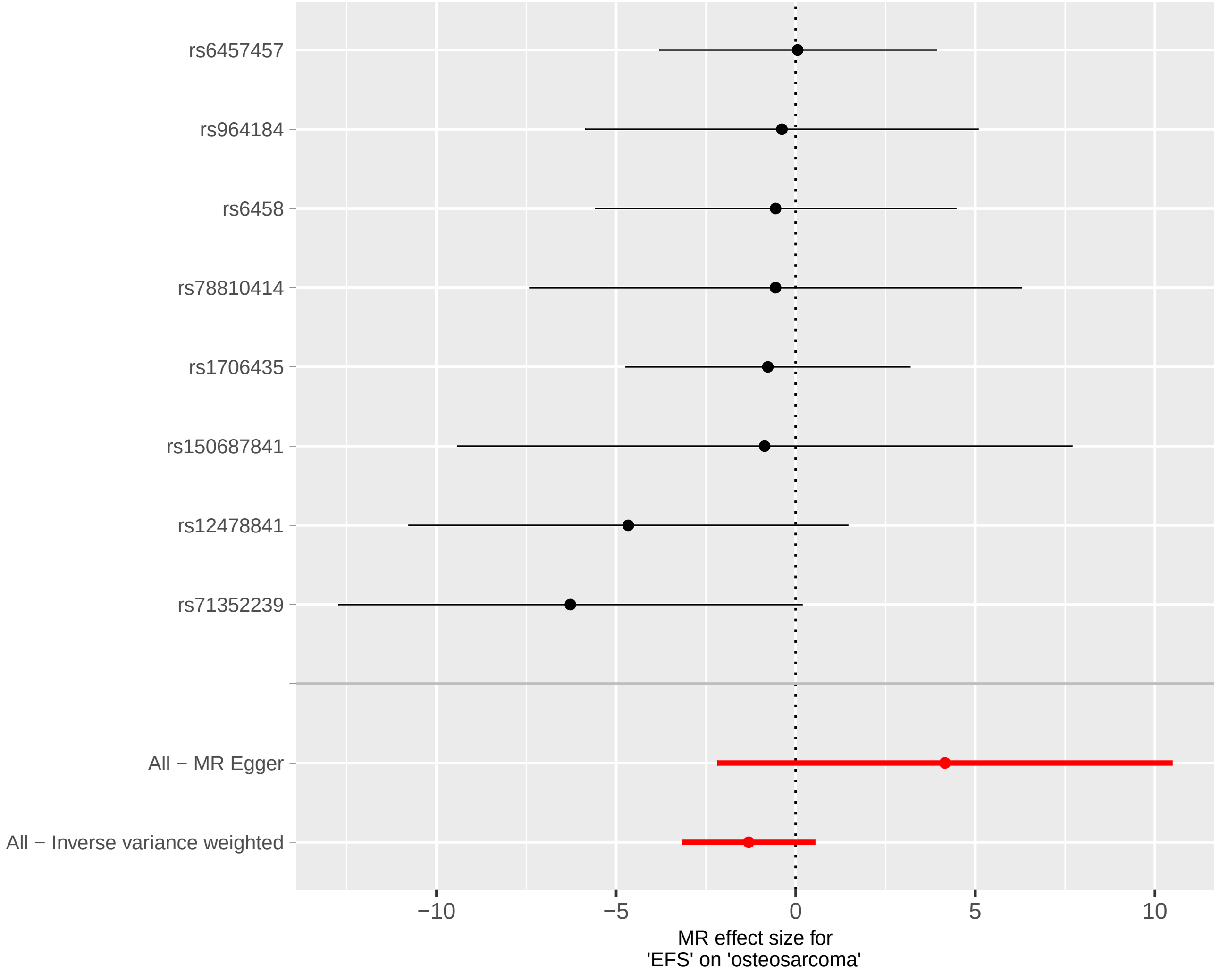
rs6457457
rs964184
rs6458
rs78810414
rs1706435
rs150687841
rs12478841
rs71352239
All − MR Egger
All − Inverse variance weighted
−10
−5
0
5
10
MR effect size for
'EFS' on 'osteosarcoma'

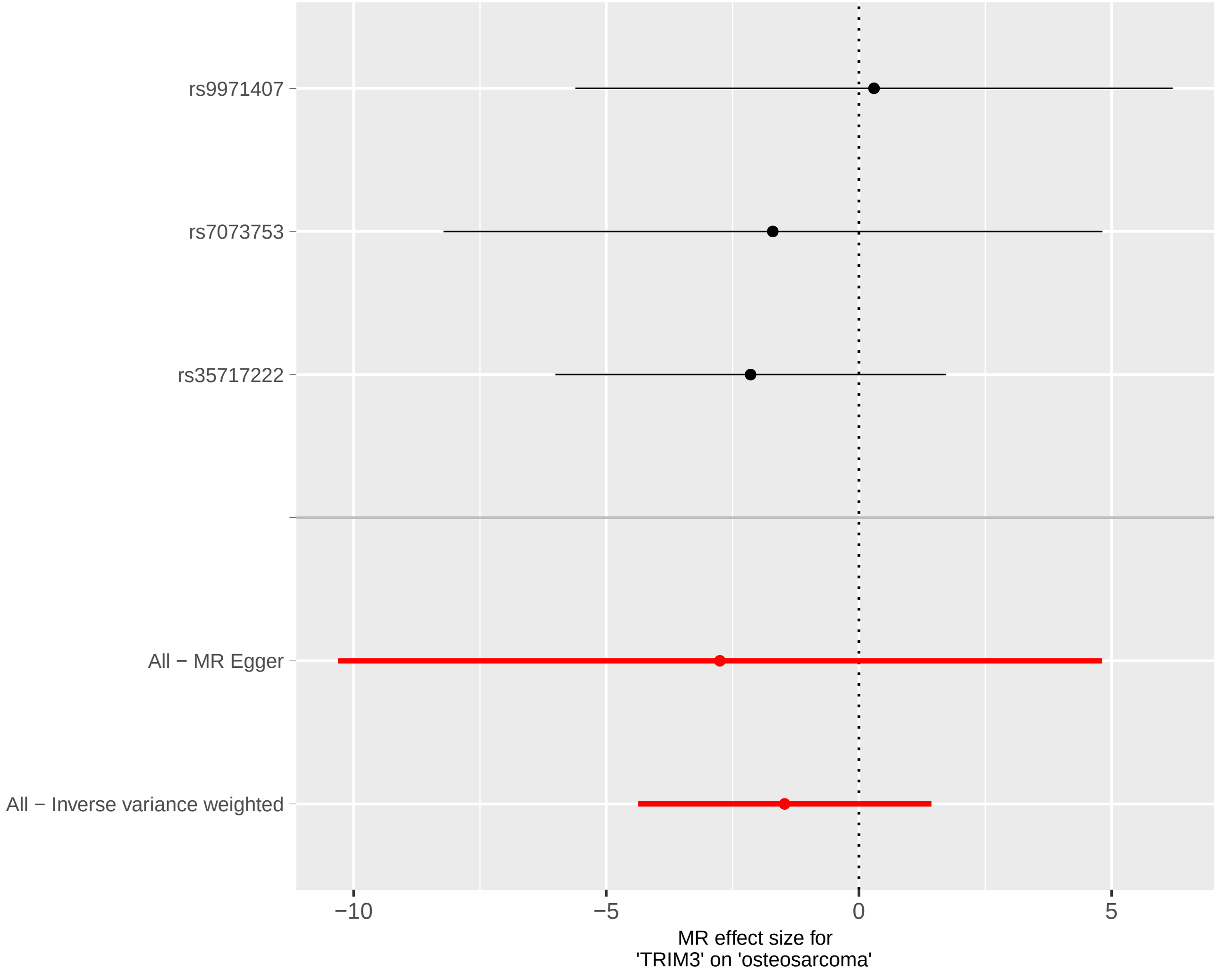

rs9971407
rs7073753
rs35717222
All − MR Egger
All − Inverse variance weighted
−10
−5
0
5
MR effect size for
'TRIM3' on 'osteosarcoma'

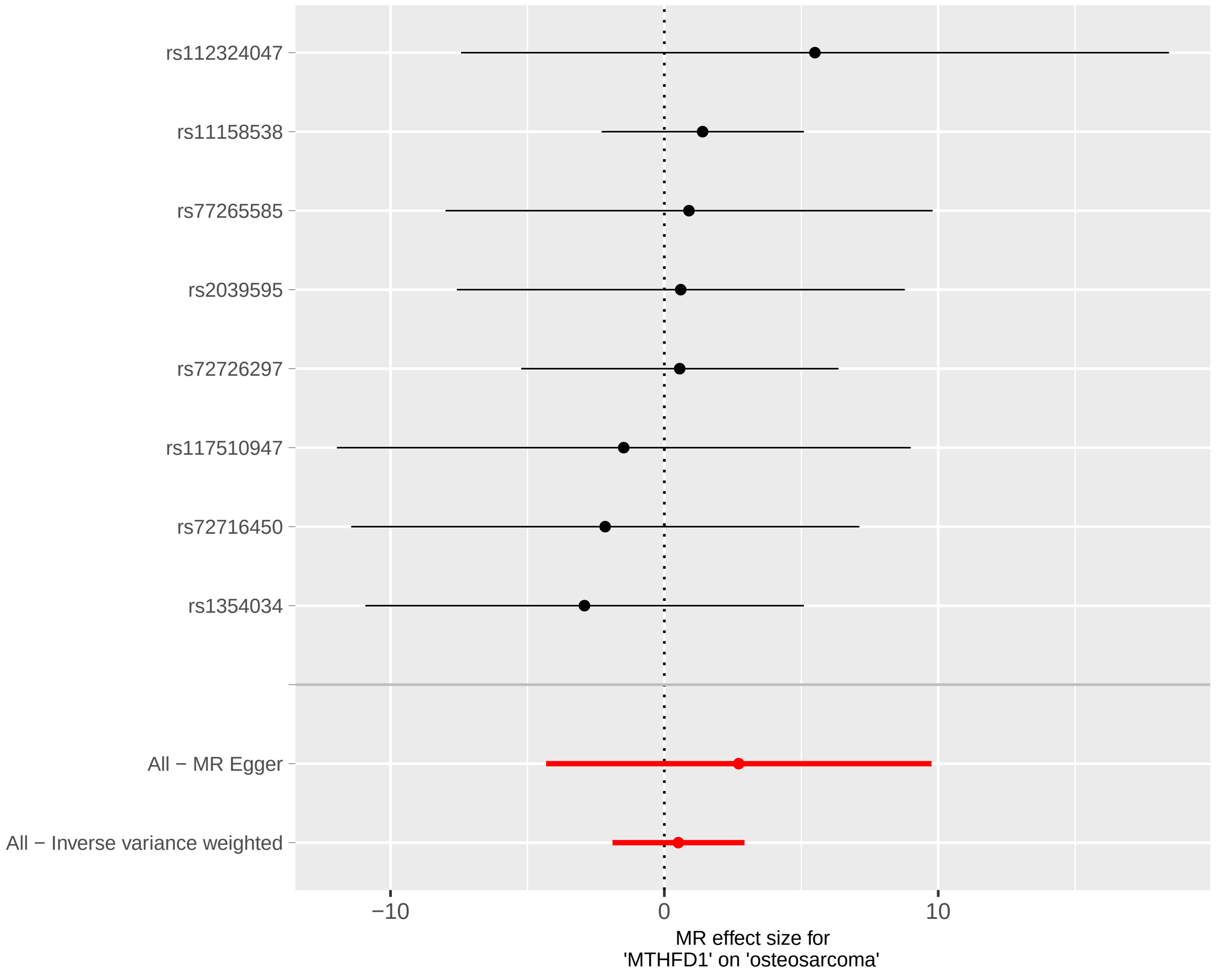

rs112324047
rs11158538
rs77265585
rs2039595
rs72726297
rs117510947
rs72716450
rs1354034
All − MR Egger
All − Inverse variance weighted
−10
0
10
MR effect size for
'MTHFD1' on 'osteosarcoma'

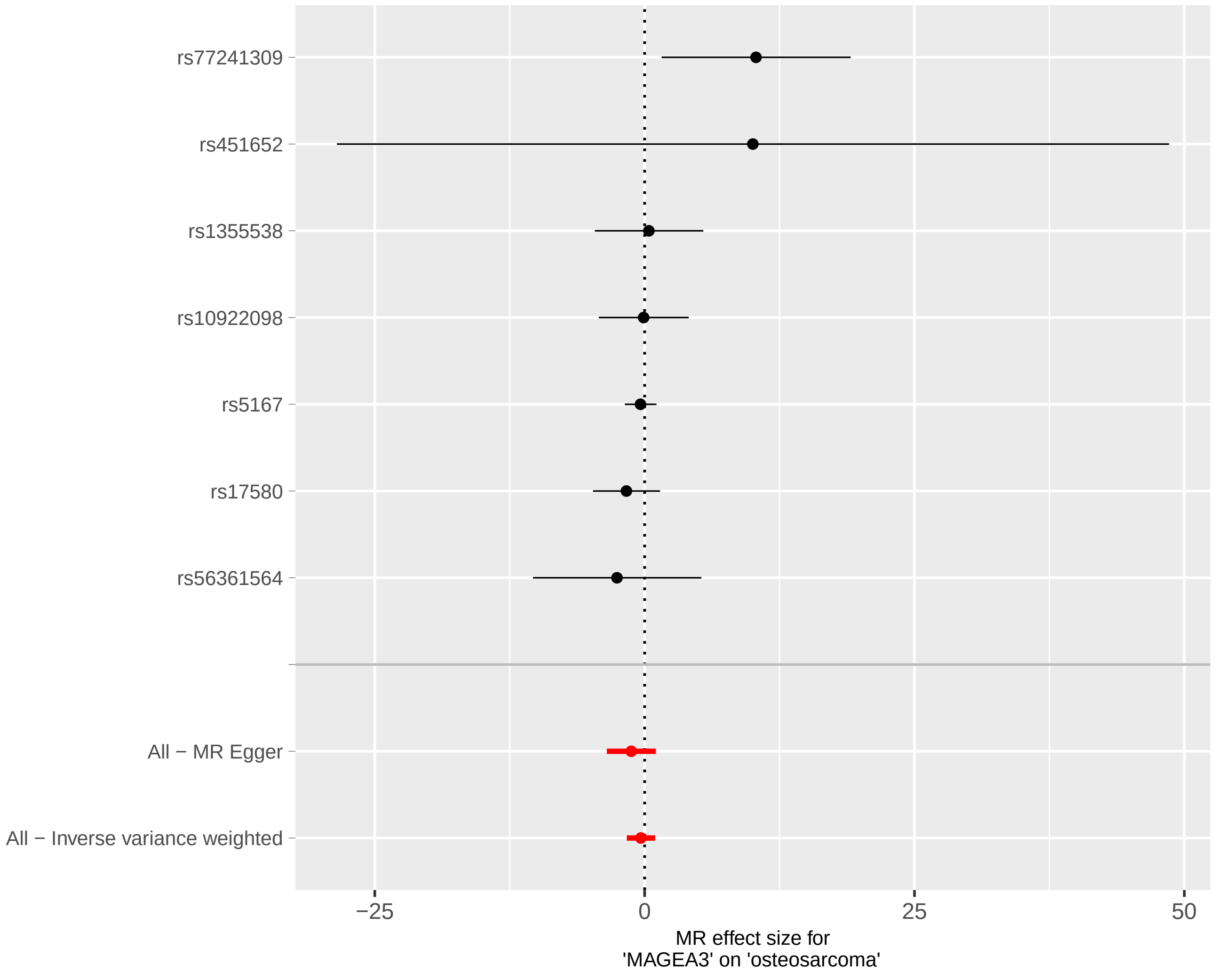
rs77241309
rs451652
rs1355538
rs10922098
rs5167
rs17580
rs56361564
All − MR Egger
All − Inverse variance weighted
−25
0
25
50
MR effect size for
'MAGEA3' on 'osteosarcoma'

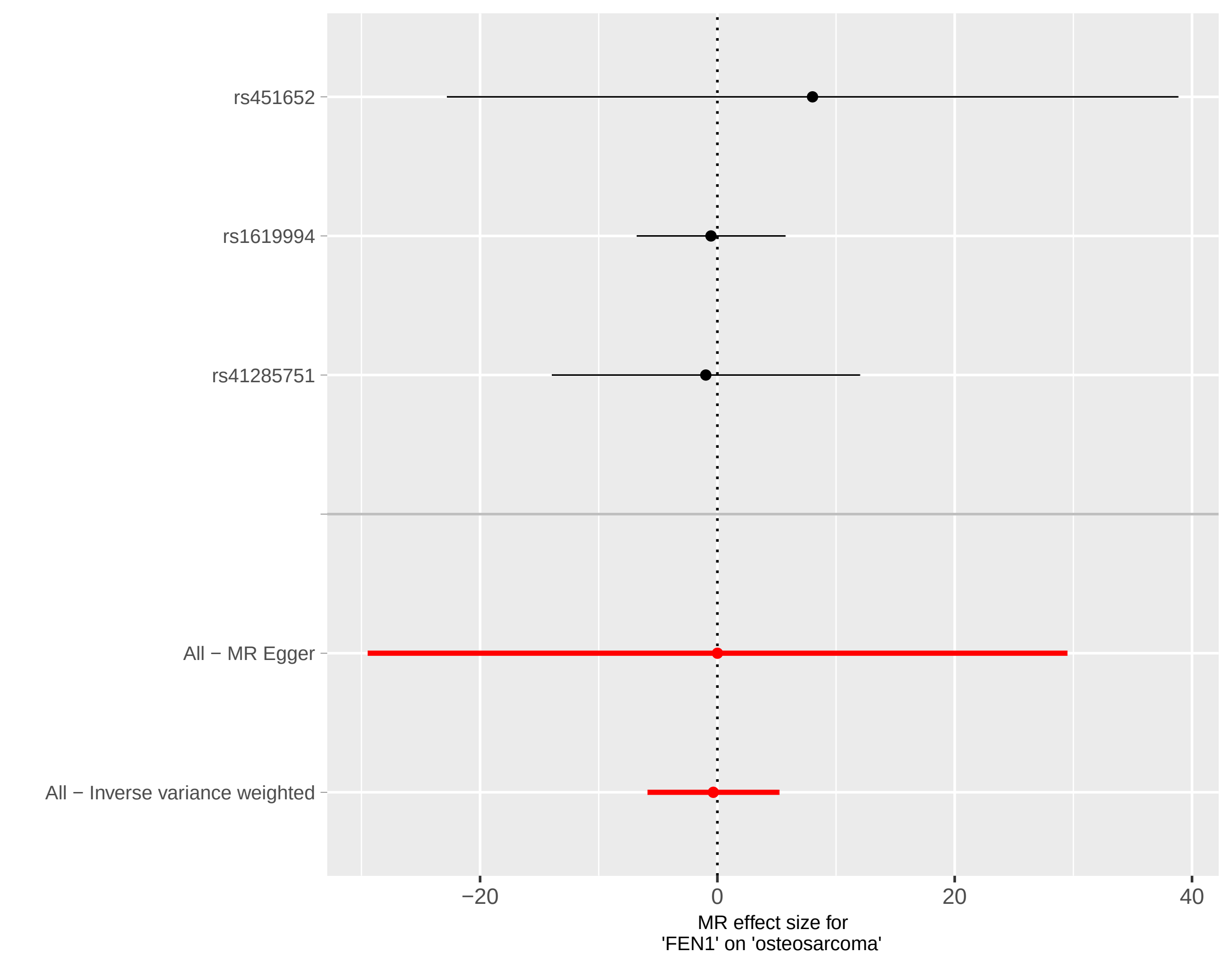
rs451652
rs1619994
rs41285751
All − MR Egger
All − Inverse variance weighted
−20
0
20
40
MR effect size for
'FEN1' on 'osteosarcoma'

Insufficient number of SNPs

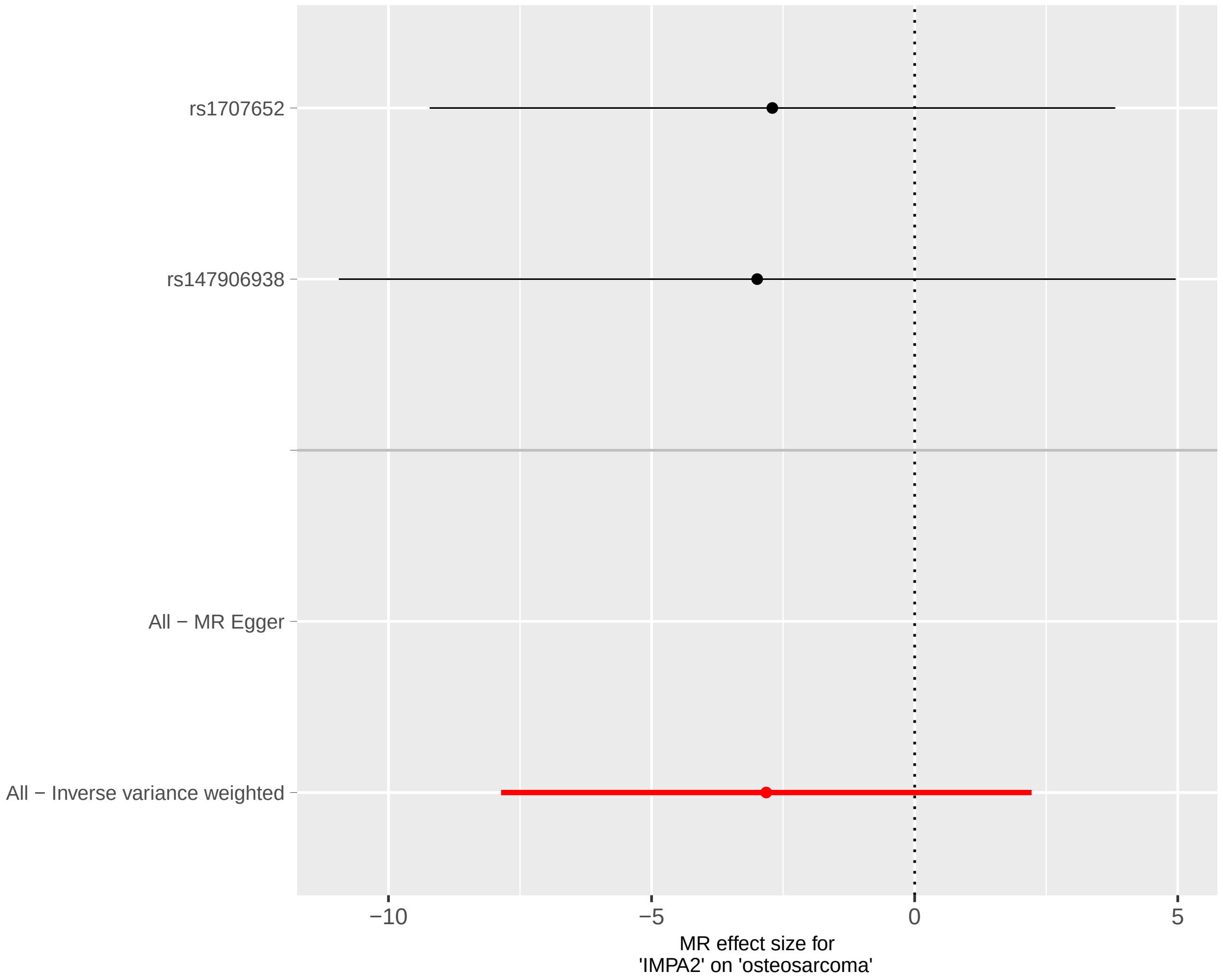

rs1707652
rs147906938
All − MR Egger
All − Inverse variance weighted
−10
−5
0
5
MR effect size for
'IMPA2' on 'osteosarcoma'

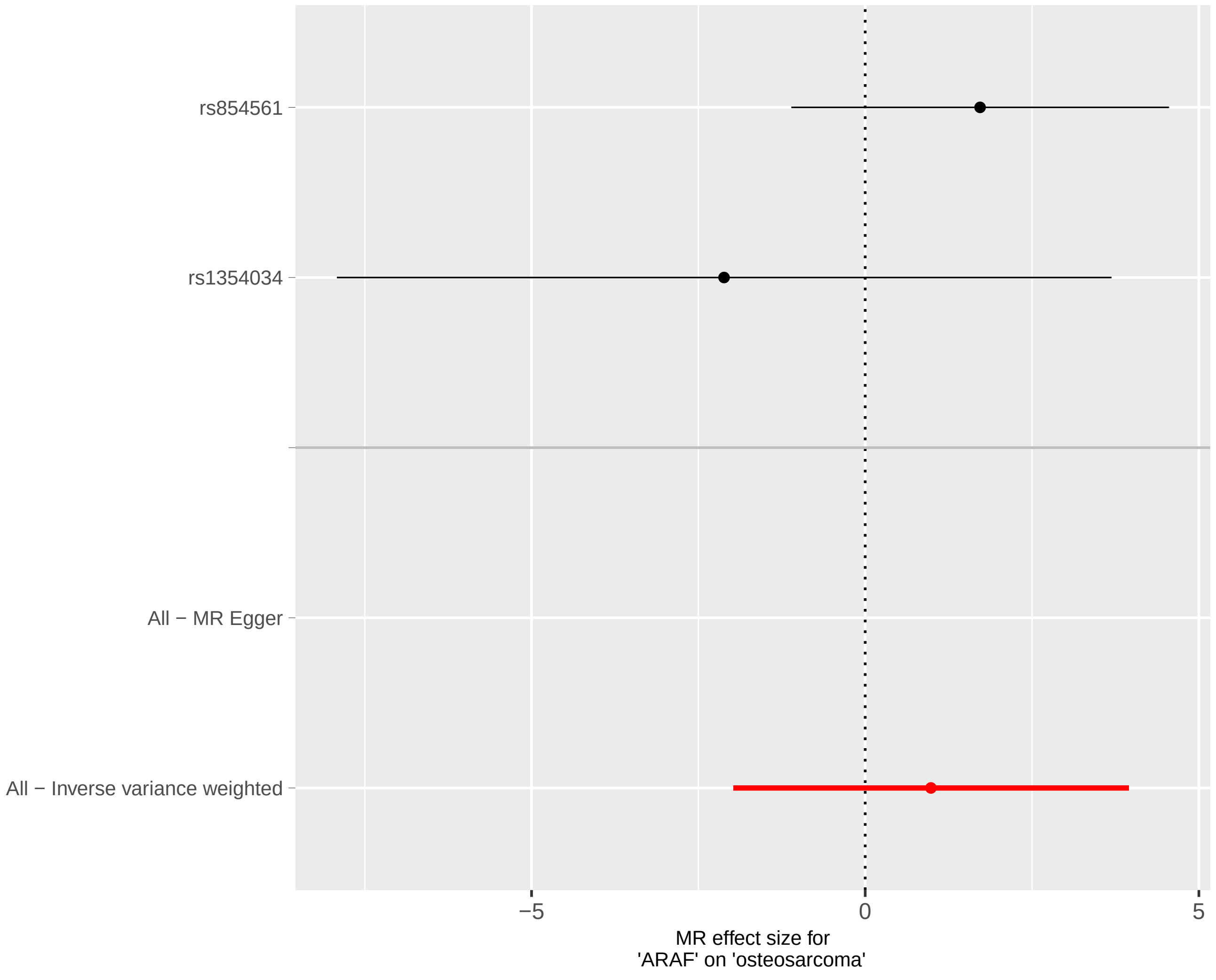

rs854561
rs1354034
All − MR Egger
All − Inverse variance weighted
−5
0
5
MR effect size for
'ARAF' on 'osteosarcoma'

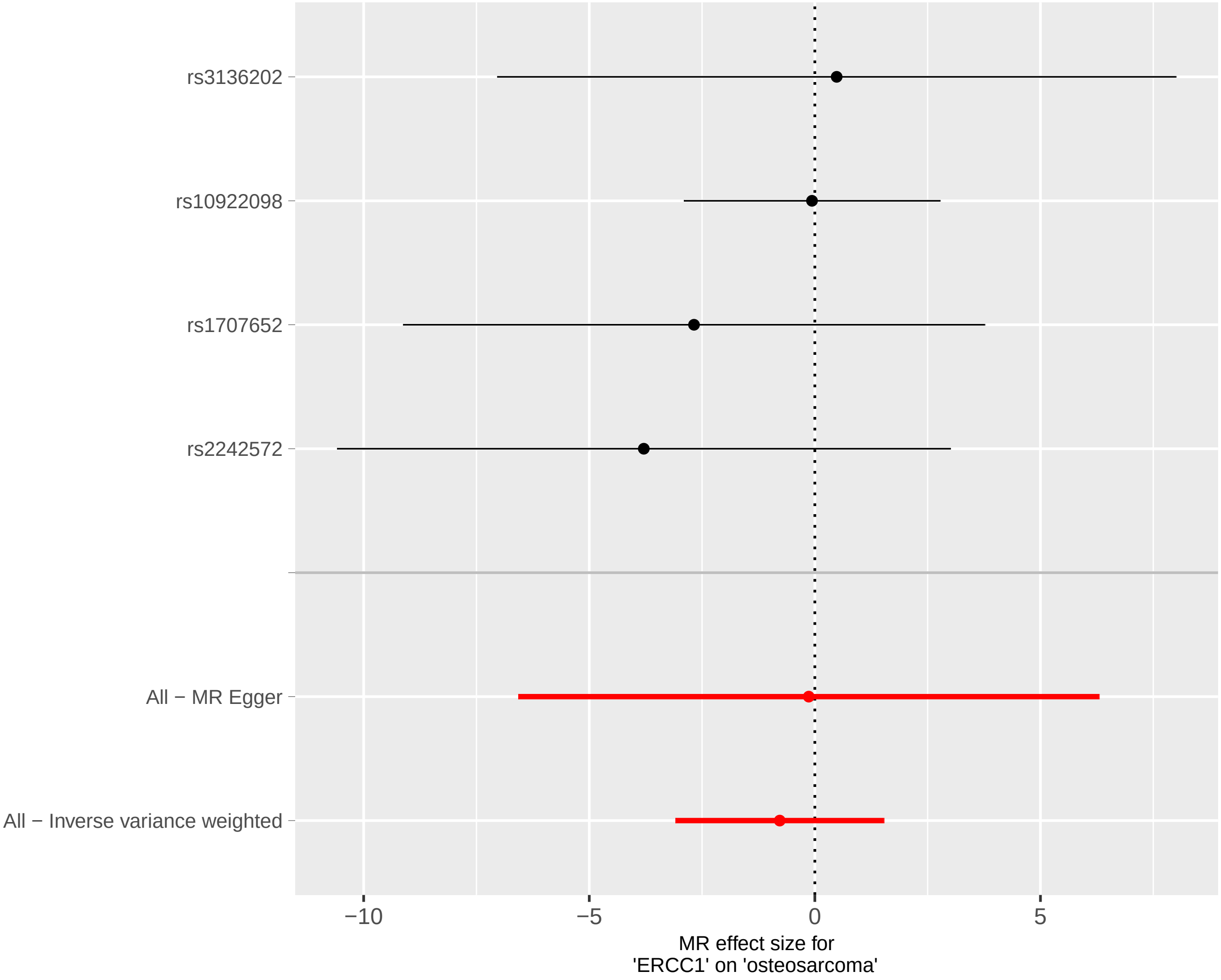

rs3136202
rs10922098
rs1707652
rs2242572
All − MR Egger
All − Inverse variance weighted
−10
−5
0
5
MR effect size for
'ERCC1' on 'osteosarcoma'

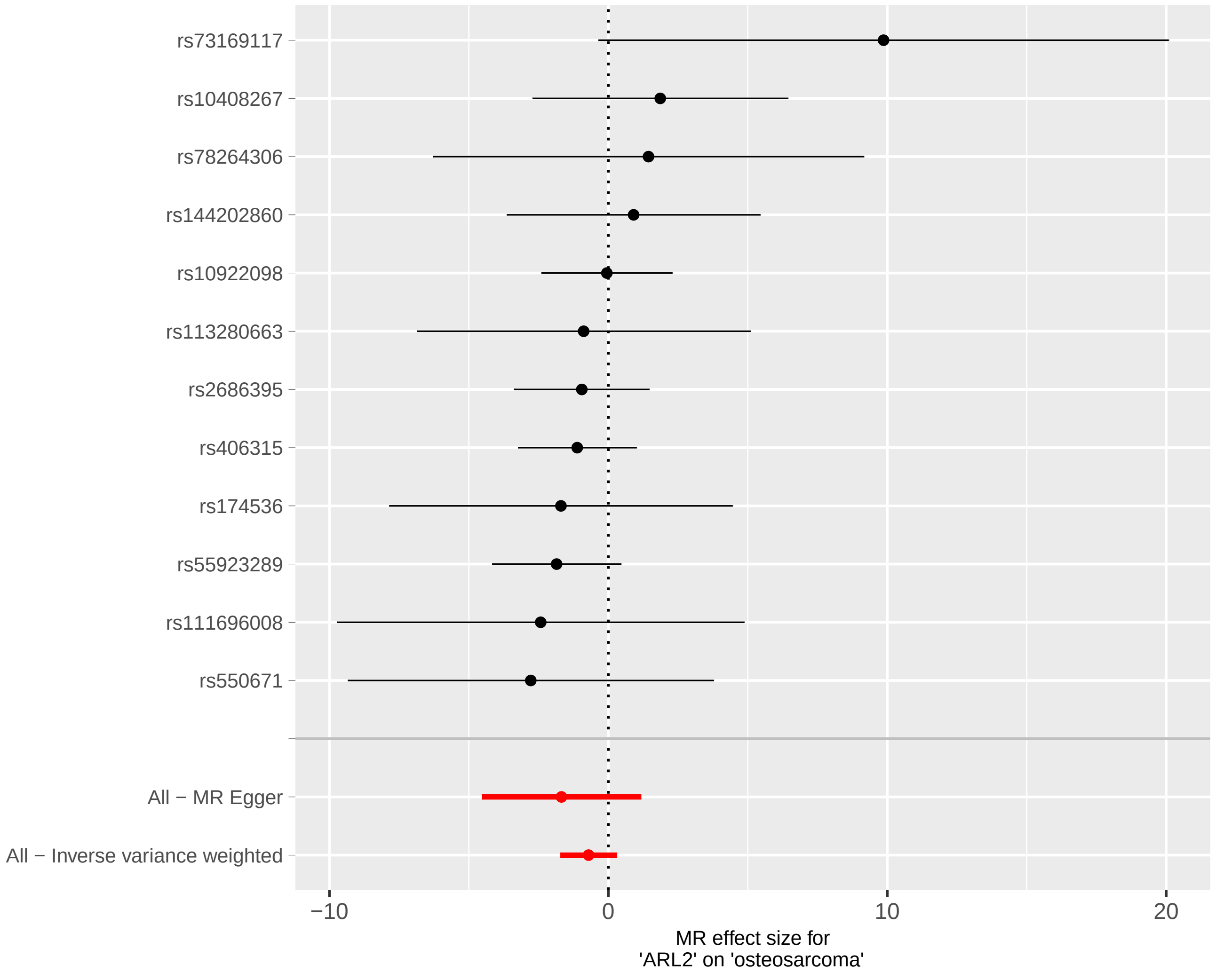
rs73169117
rs10408267
rs78264306
rs144202860
rs10922098
rs113280663
rs2686395
rs406315
rs174536
rs55923289
rs111696008
rs550671
All − MR Egger
All − Inverse variance weighted
−10
0
10
20
MR effect size for
'ARL2' on 'osteosarcoma'

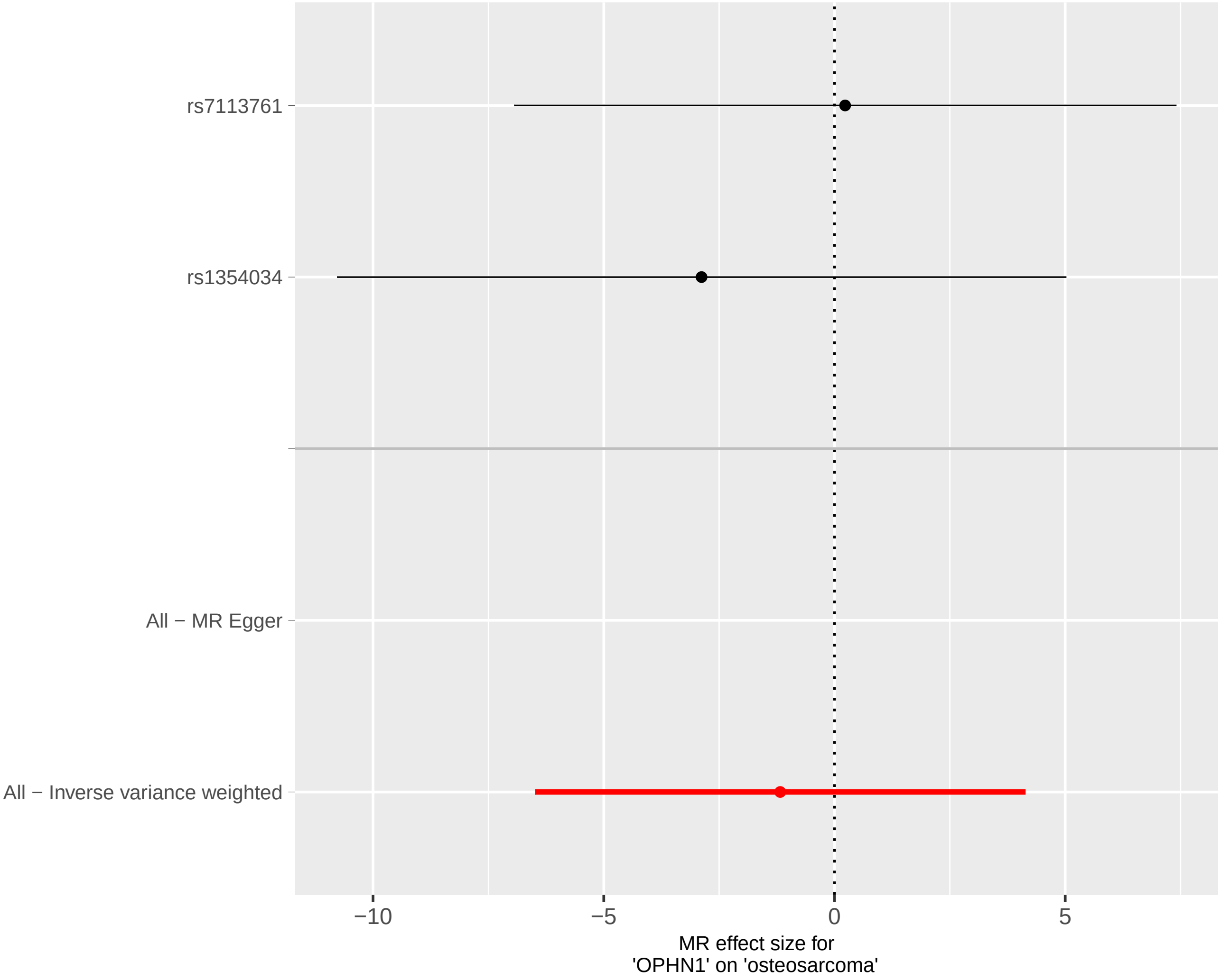

rs7113761
rs1354034
All − MR Egger
All − Inverse variance weighted
−10
−5
0
5
MR effect size for
'OPHN1' on 'osteosarcoma'

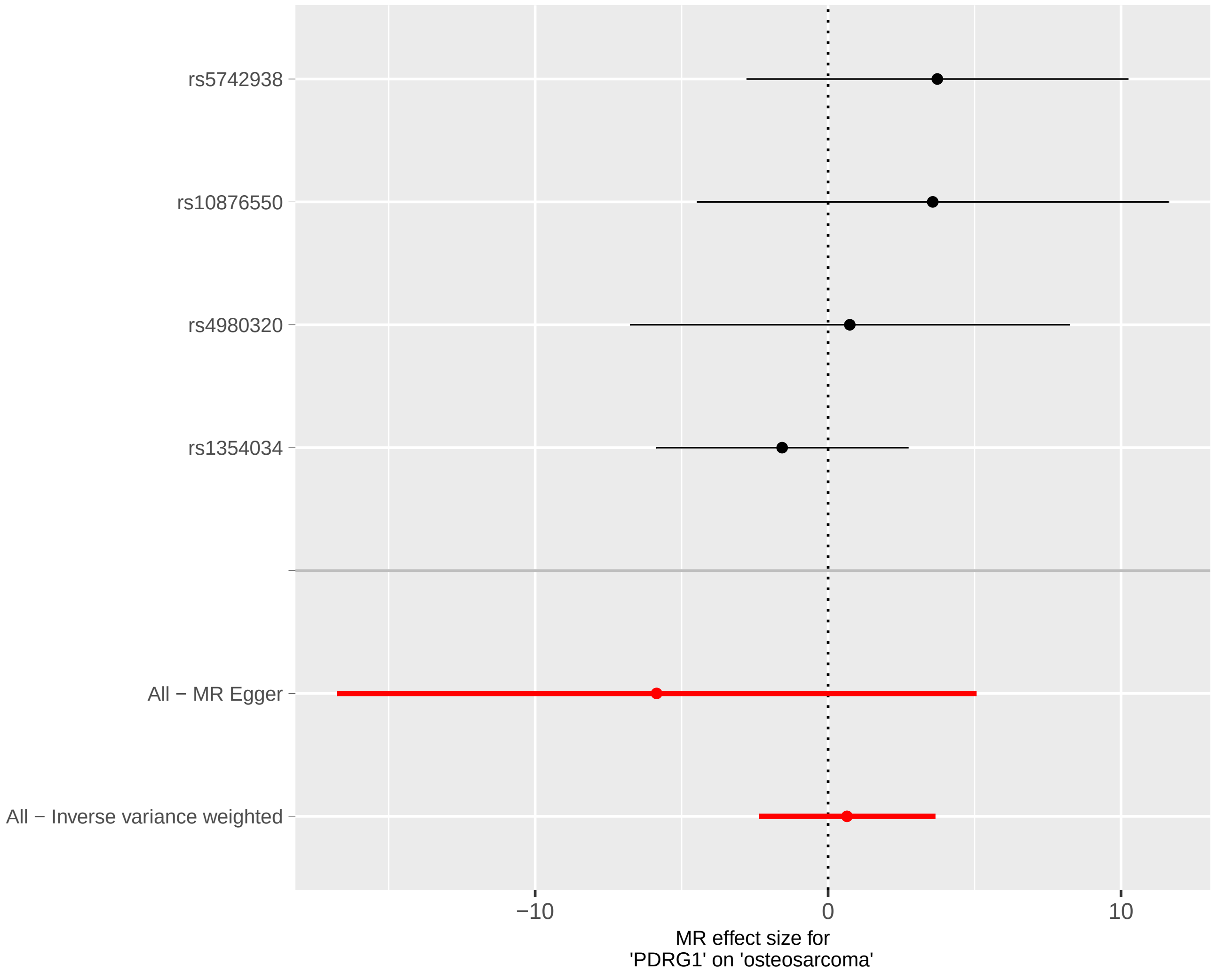
rs5742938
rs10876550
rs4980320
rs1354034
All − MR Egger
All − Inverse variance weighted
−10
0
10
MR effect size for
'PDRG1' on 'osteosarcoma'

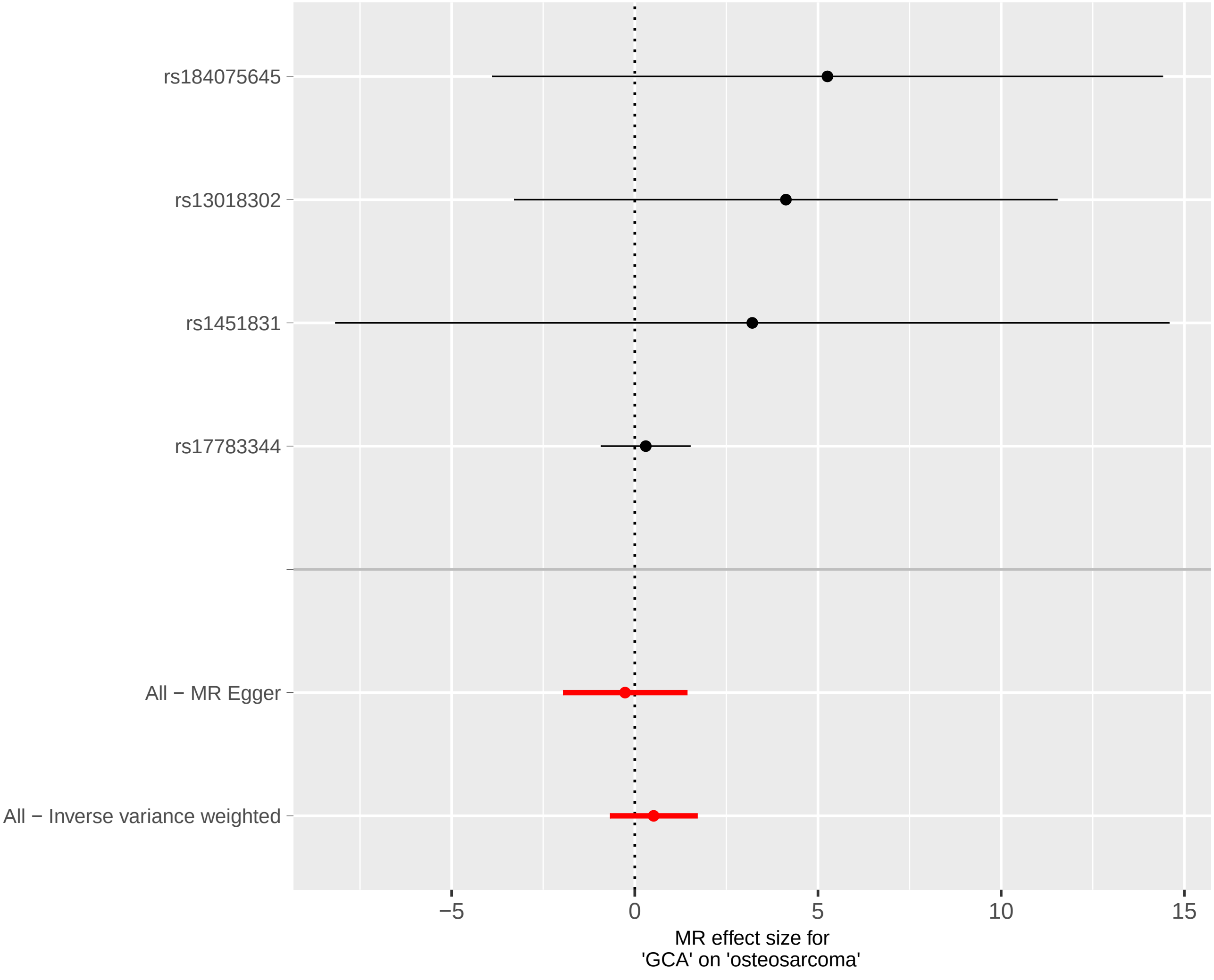
rs184075645
rs13018302
rs1451831
rs17783344
All − MR Egger
All − Inverse variance weighted
−5
0
5
10
15
MR effect size for
'GCA' on 'osteosarcoma'

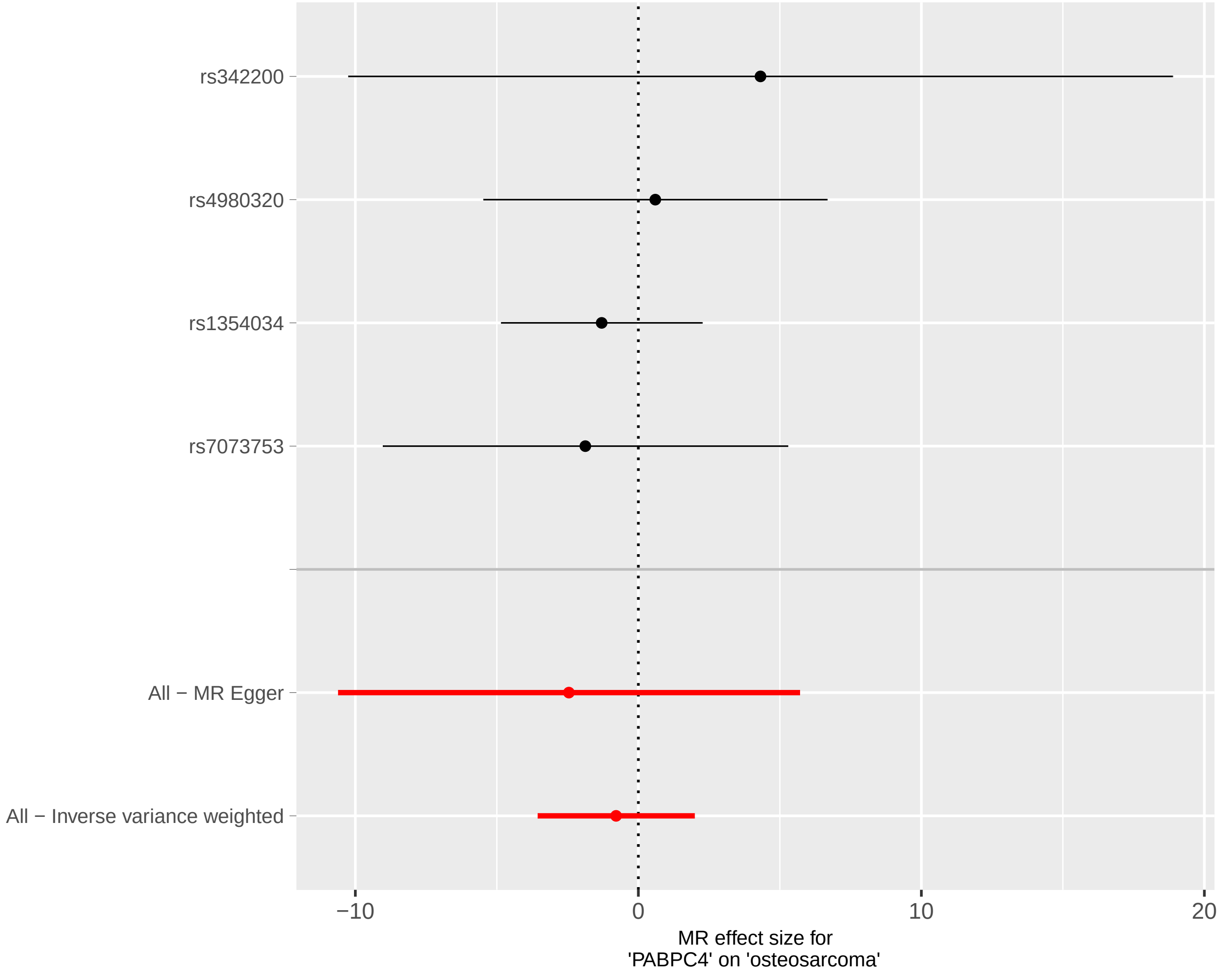
rs342200
rs4980320
rs1354034
rs7073753
All − MR Egger
All − Inverse variance weighted
−10
0
10
20
MR effect size for
'PABPC4' on 'osteosarcoma'

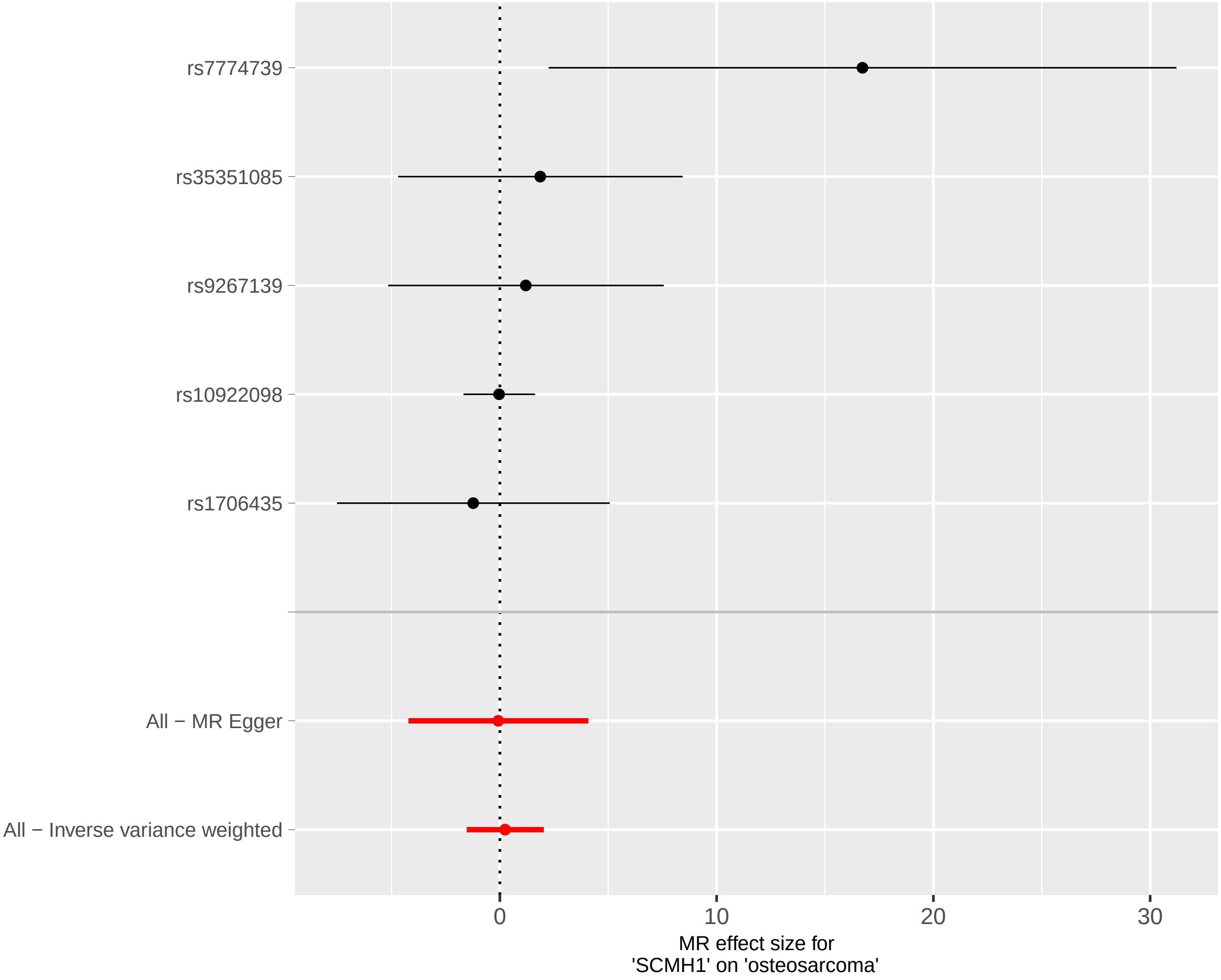

rs7774739
rs35351085
rs9267139
rs10922098
rs1706435
All − MR Egger
All − Inverse variance weighted
0
10
20
30
MR effect size for
'SCMH1' on 'osteosarcoma'

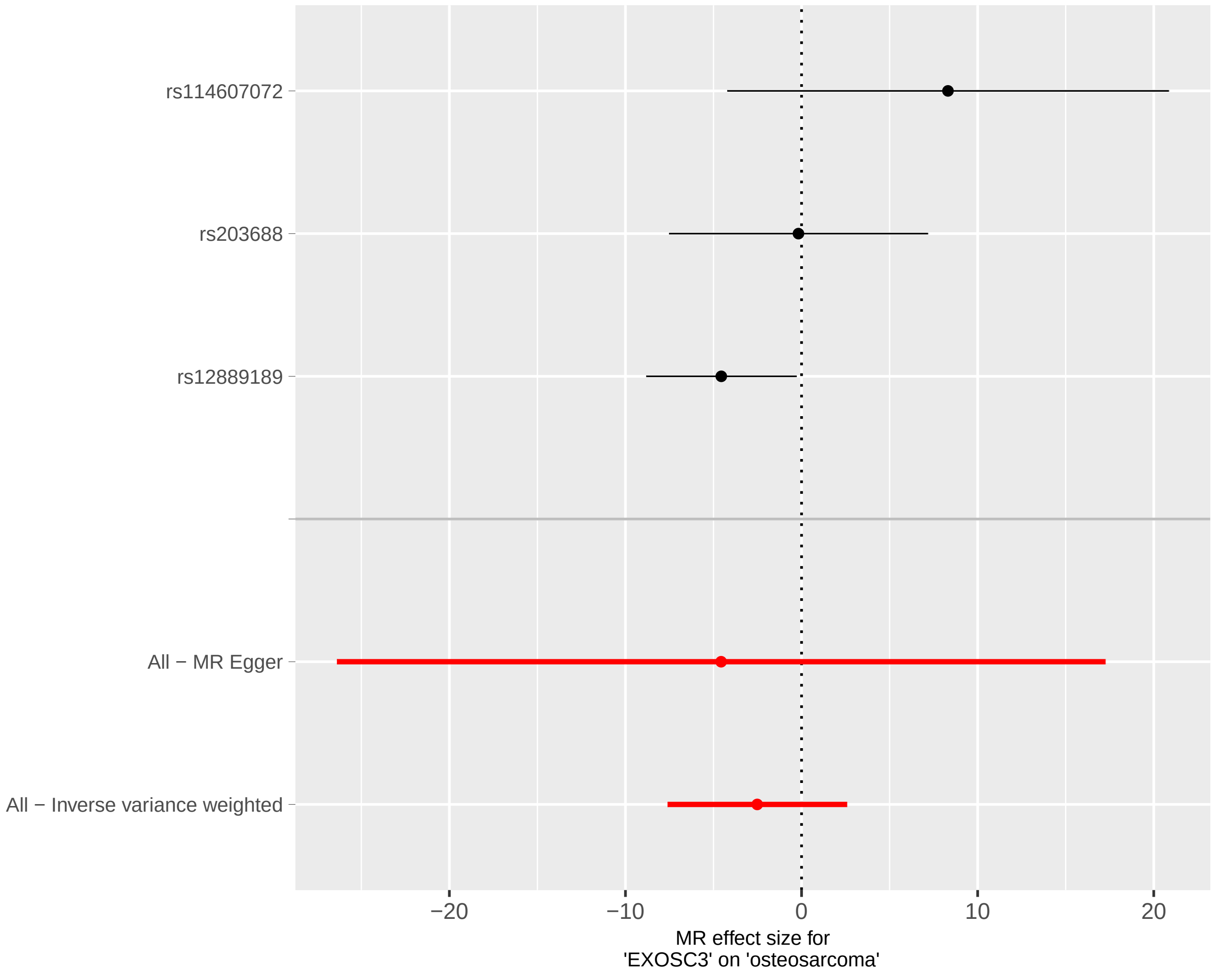
rs114607072
rs203688
rs12889189
All − MR Egger
All − Inverse variance weighted
−20
−10
0
10
20
MR effect size for
'EXOSC3' on 'osteosarcoma'

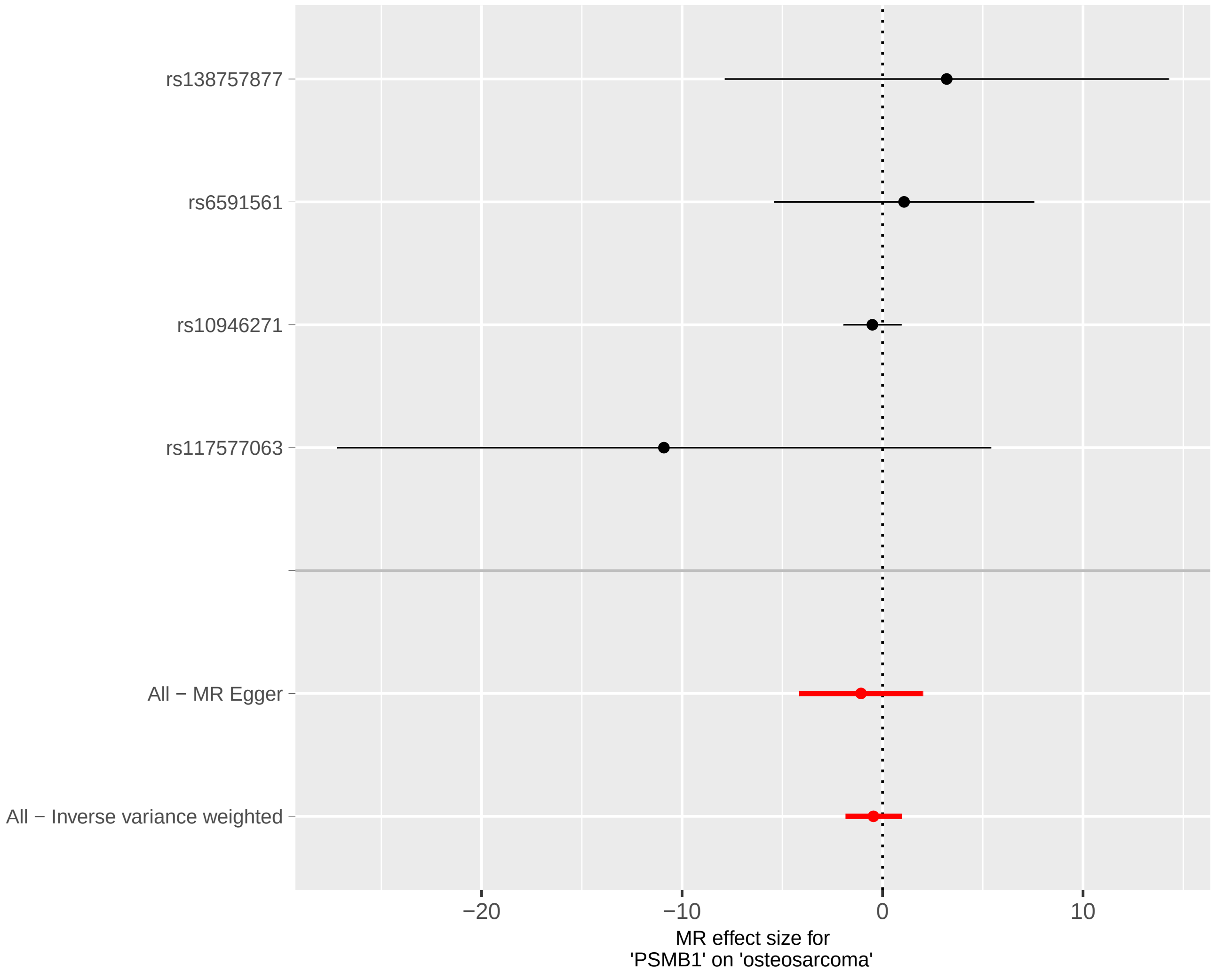
rs138757877
rs6591561
rs10946271
rs117577063
All − MR Egger
All − Inverse variance weighted
−20
−10
0
10
MR effect size for
'PSMB1' on 'osteosarcoma'

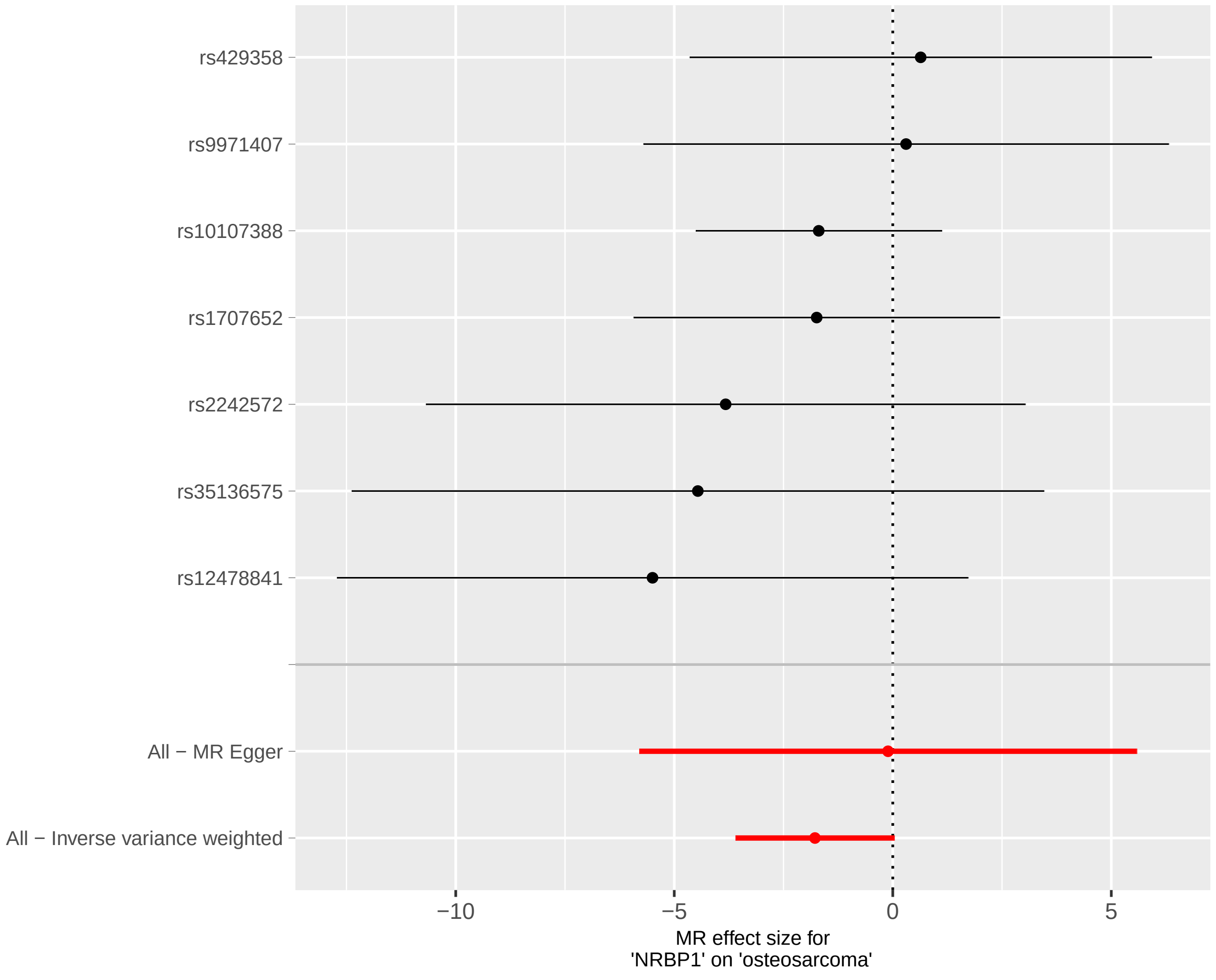

rs429358
rs9971407
rs10107388
rs1707652
rs2242572
rs35136575
rs12478841
All − MR Egger
All − Inverse variance weighted
−10
−5
0
5
MR effect size for
'NRBP1' on 'osteosarcoma'

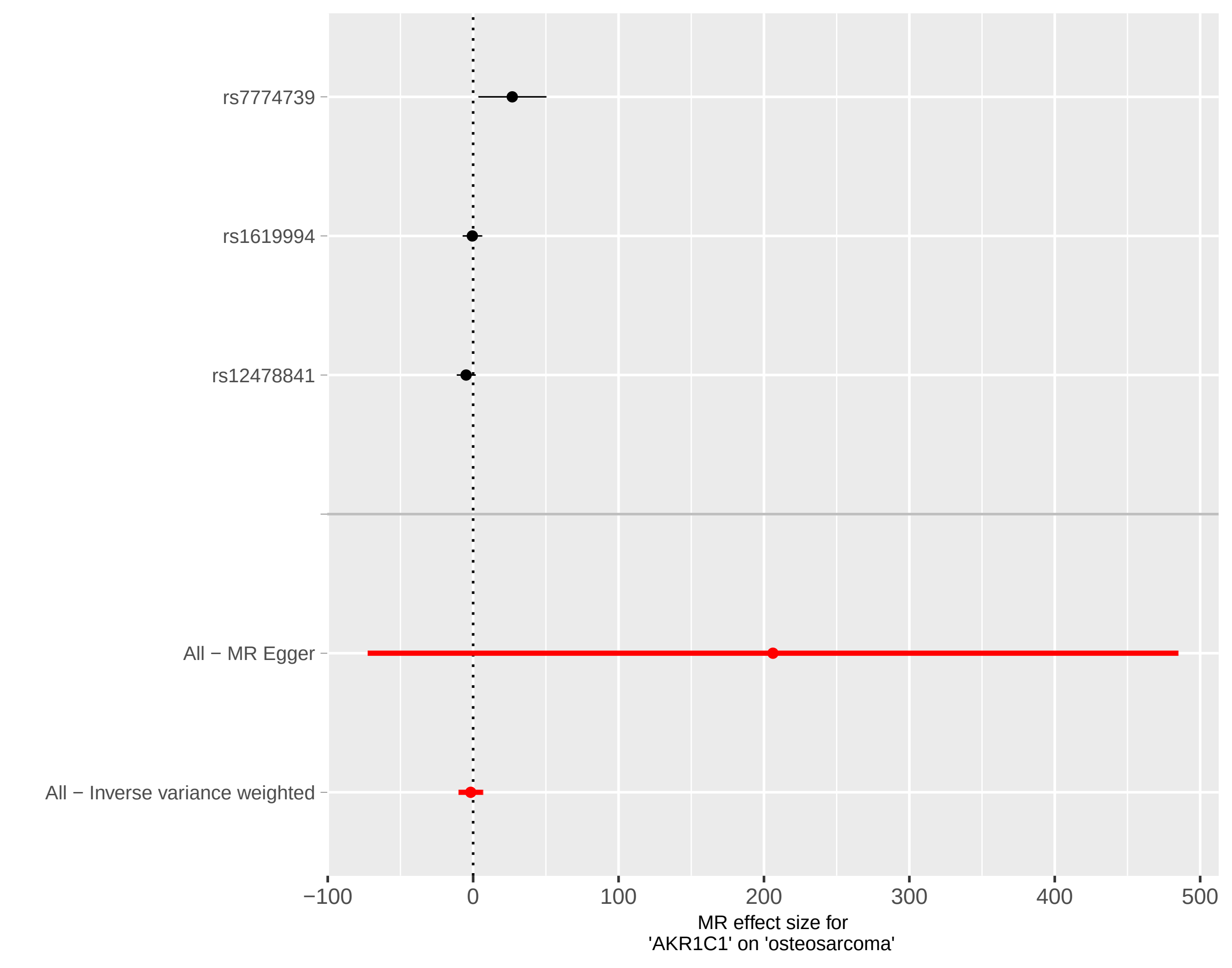
rs7774739
rs1619994
rs12478841
All − MR Egger
All − Inverse variance weighted
−100
0
100
200
300
400
500
MR effect size for
'AKR1C1' on 'osteosarcoma'

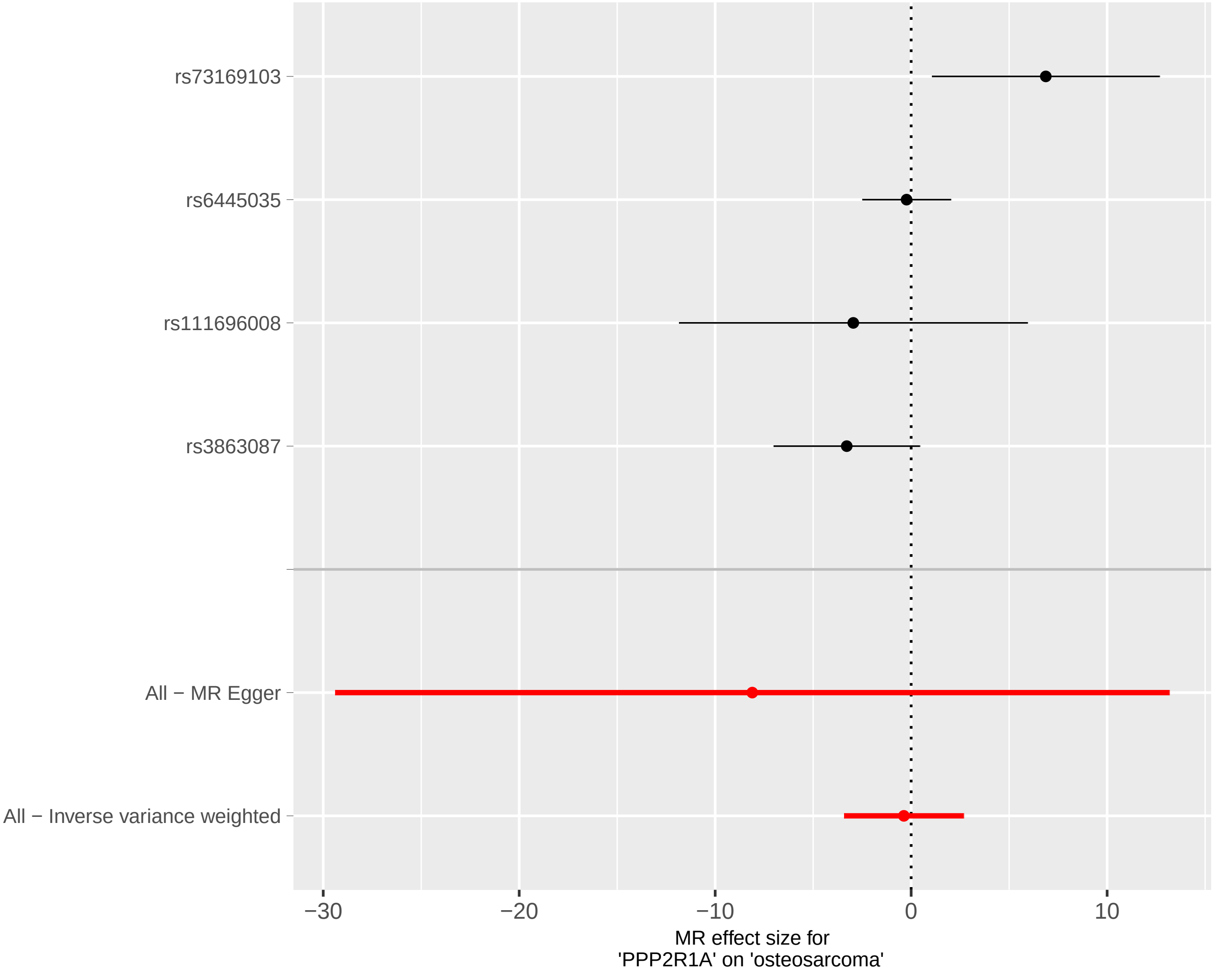
rs73169103
rs6445035
rs111696008
rs3863087
All − MR Egger
All − Inverse variance weighted
−30
−20
−10
0
10
MR effect size for
'PPP2R1A' on 'osteosarcoma'

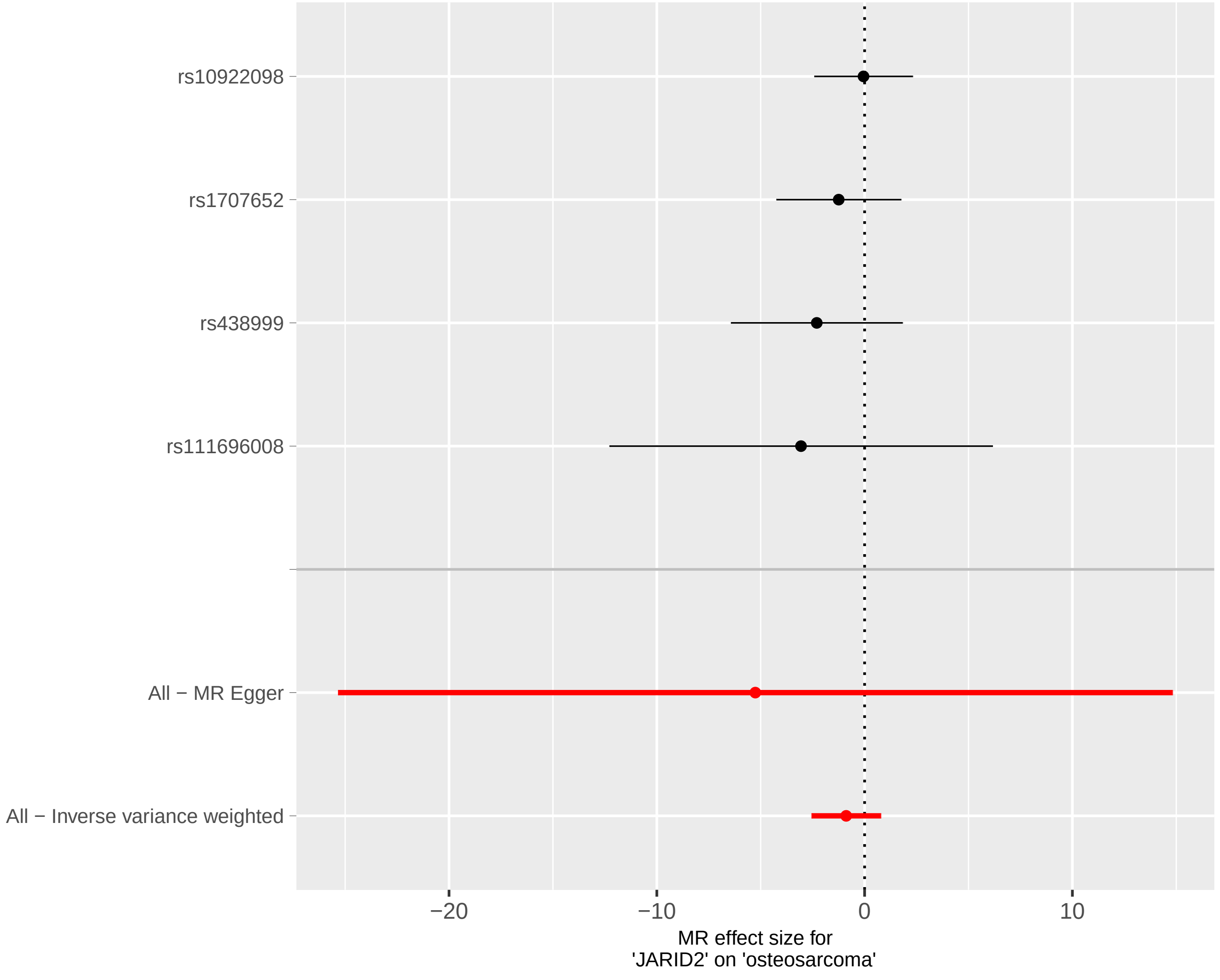
rs10922098
rs1707652
rs438999
rs111696008
All − MR Egger
All − Inverse variance weighted
−20
−10
0
10
MR effect size for
'JARID2' on 'osteosarcoma'

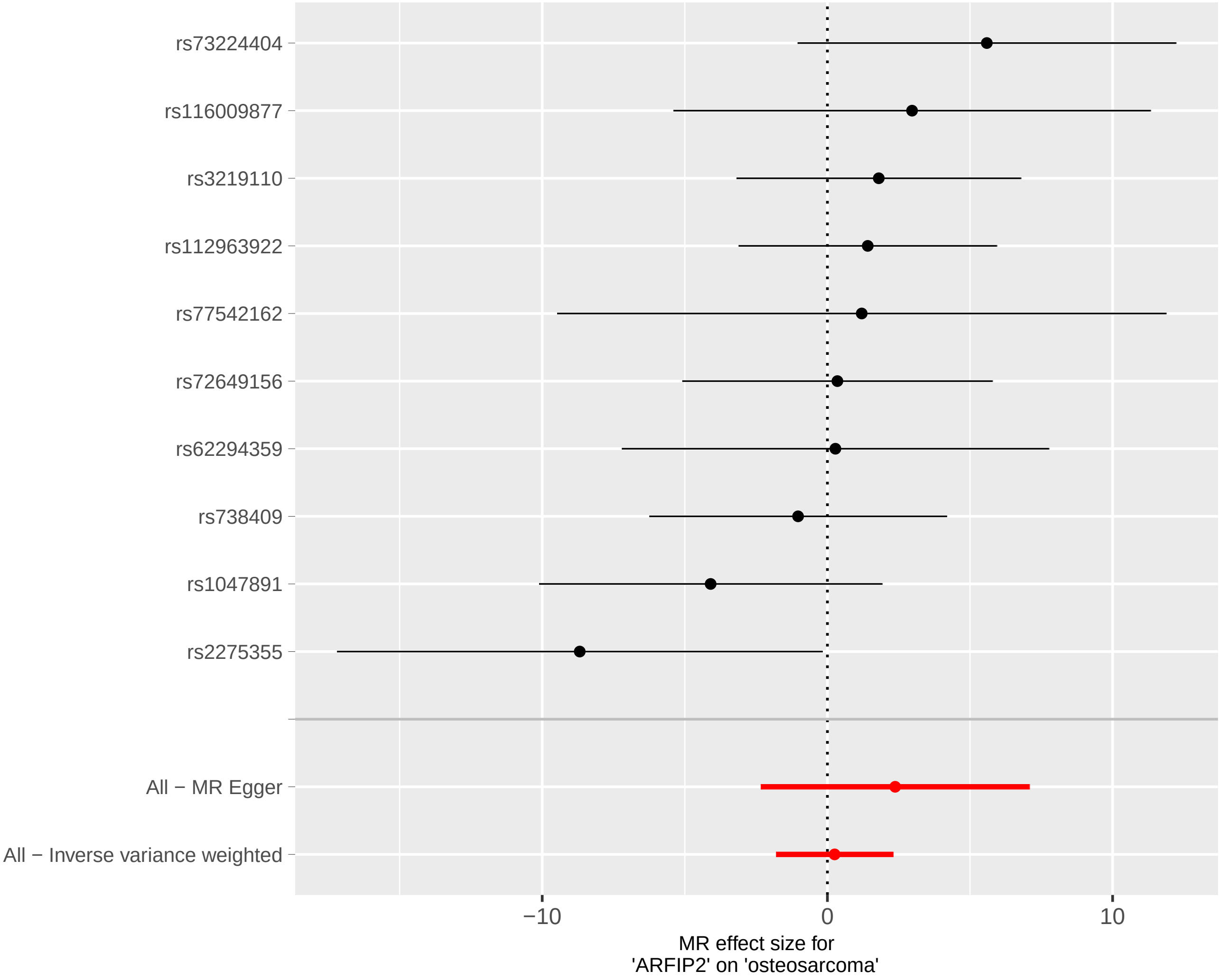

rs73224404
rs116009877
rs3219110
rs112963922
rs77542162
rs72649156
rs62294359
rs738409
rs1047891
rs2275355
All − MR Egger
All − Inverse variance weighted
−10
0
10
MR effect size for
'ARFIP2' on 'osteosarcoma'

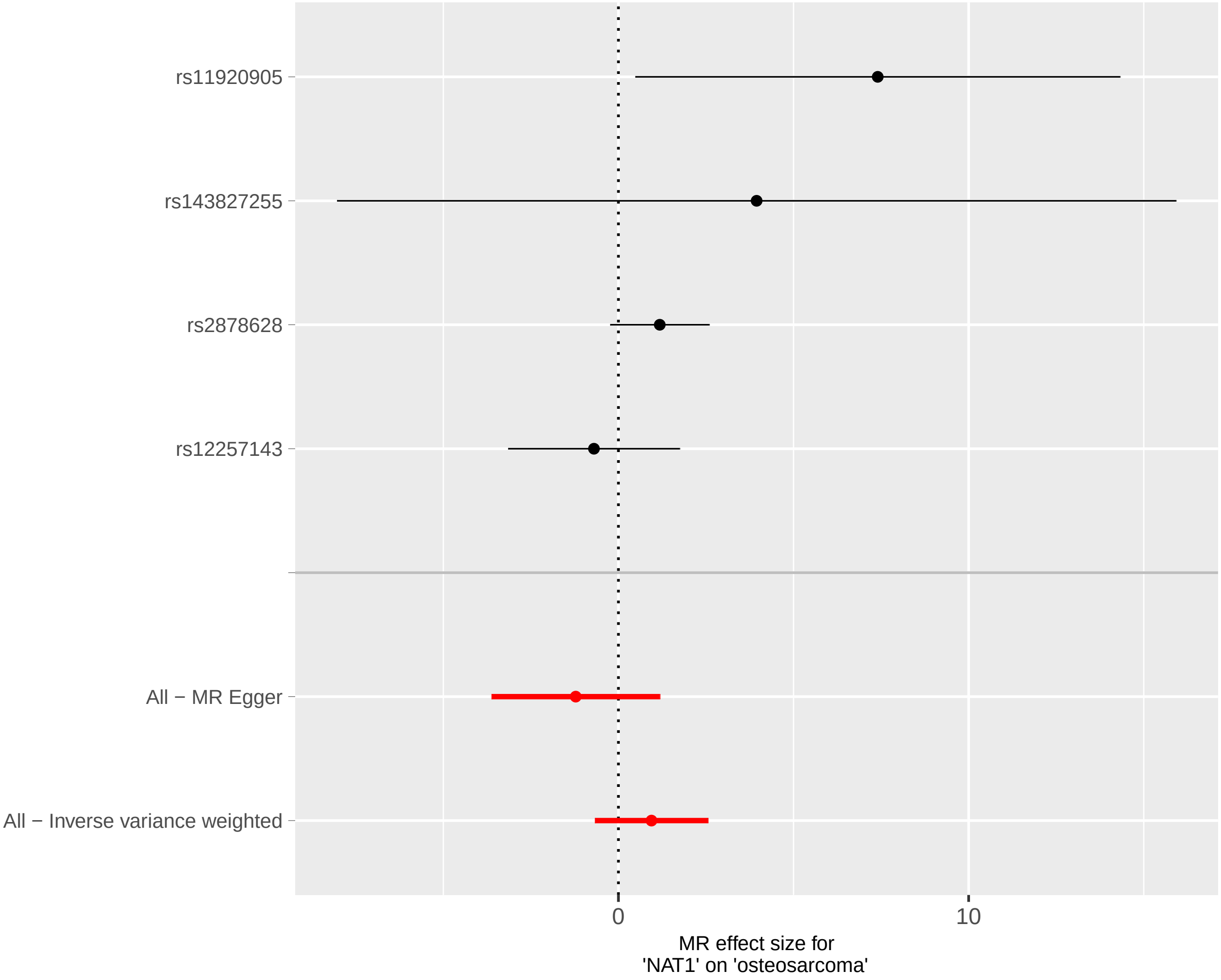
rs11920905
rs143827255
rs2878628
rs12257143
All − MR Egger
All − Inverse variance weighted
0
10
MR effect size for
'NAT1' on 'osteosarcoma'

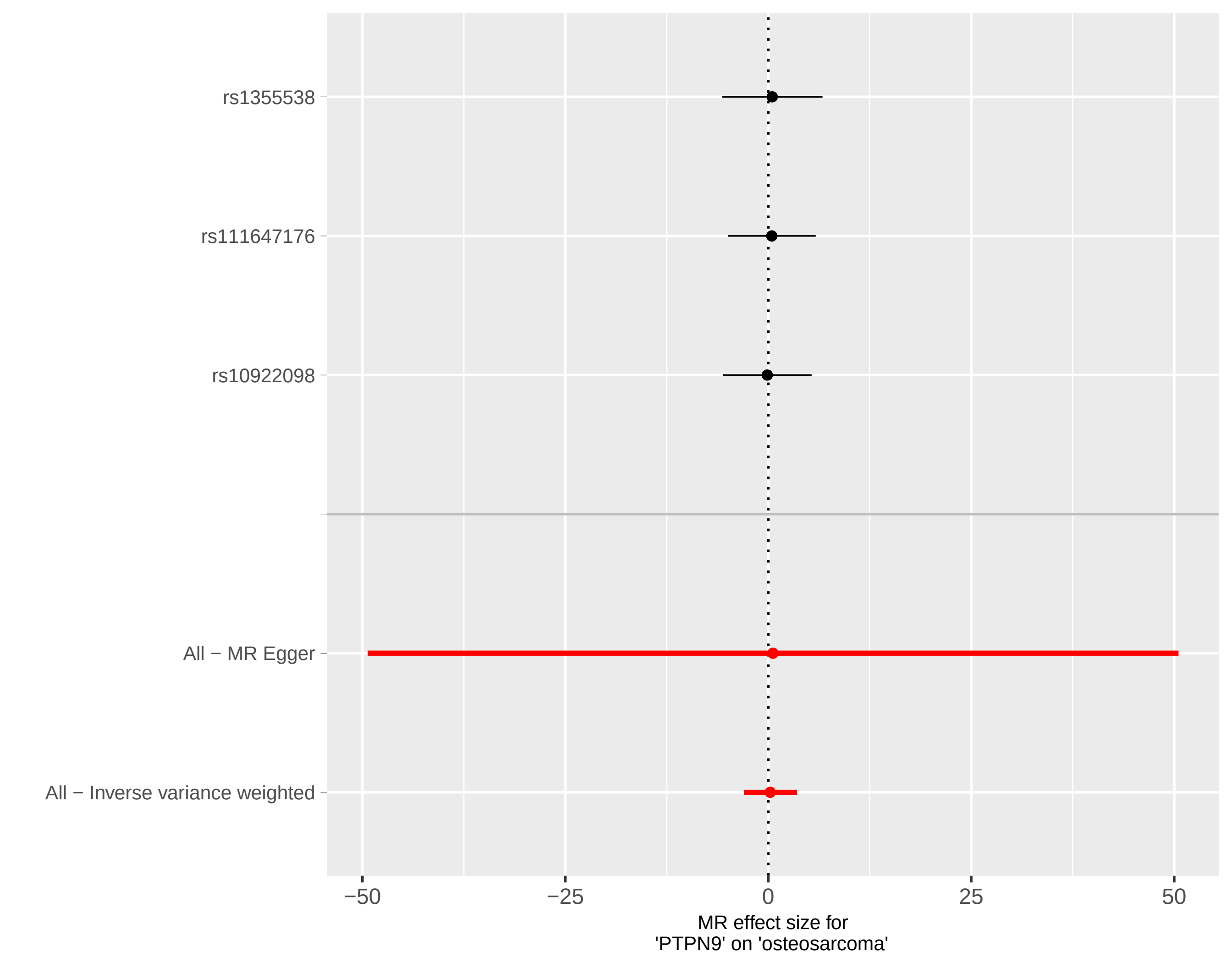

rs1355538
rs111647176
rs10922098
All − MR Egger
All − Inverse variance weighted
−50
−25
0
25
50
MR effect size for
'PTPN9' on 'osteosarcoma'

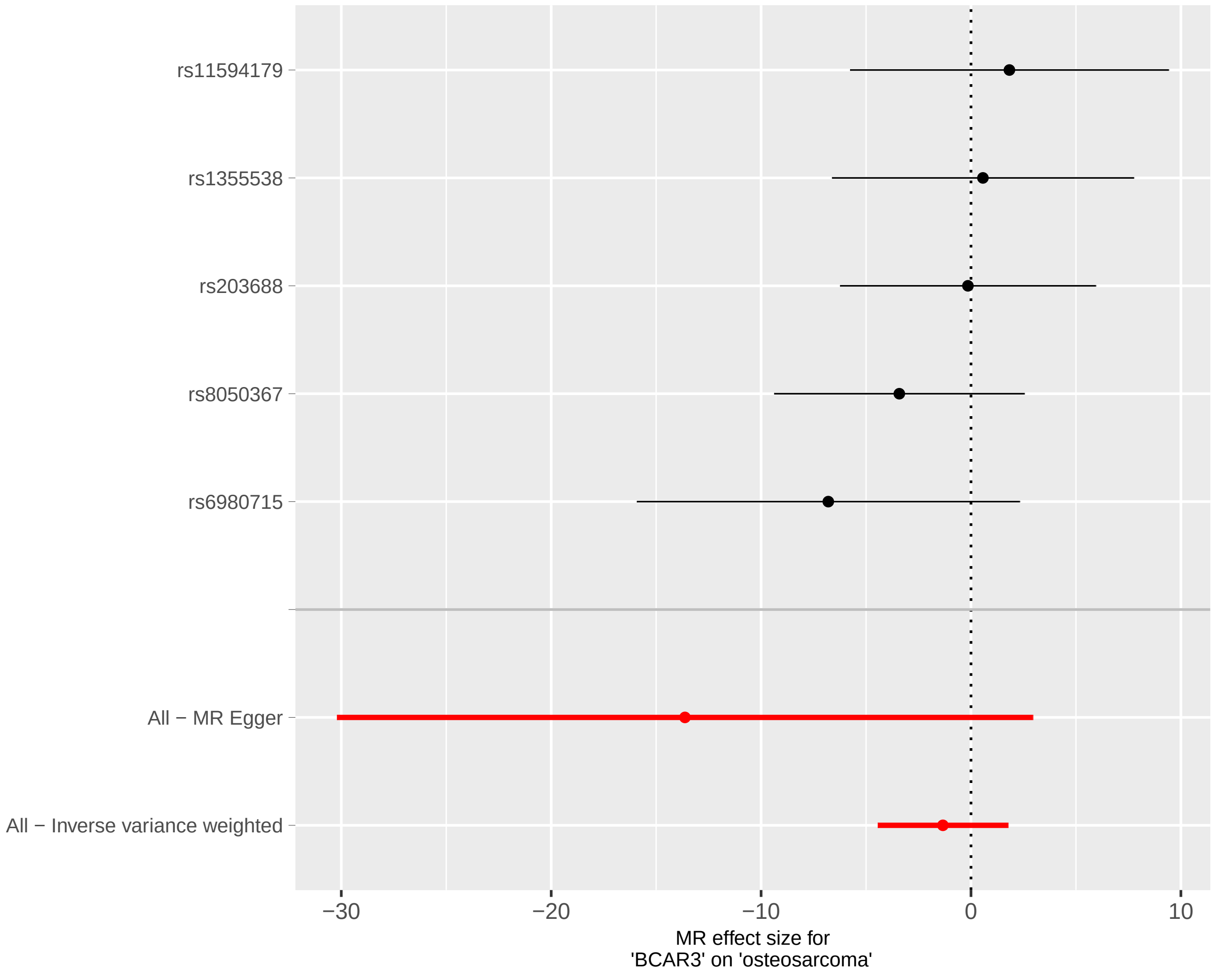
rs11594179
rs1355538
rs203688
rs8050367
rs6980715
All − MR Egger
All − Inverse variance weighted
−30
−20
−10
0
10
MR effect size for
'BCAR3' on 'osteosarcoma'

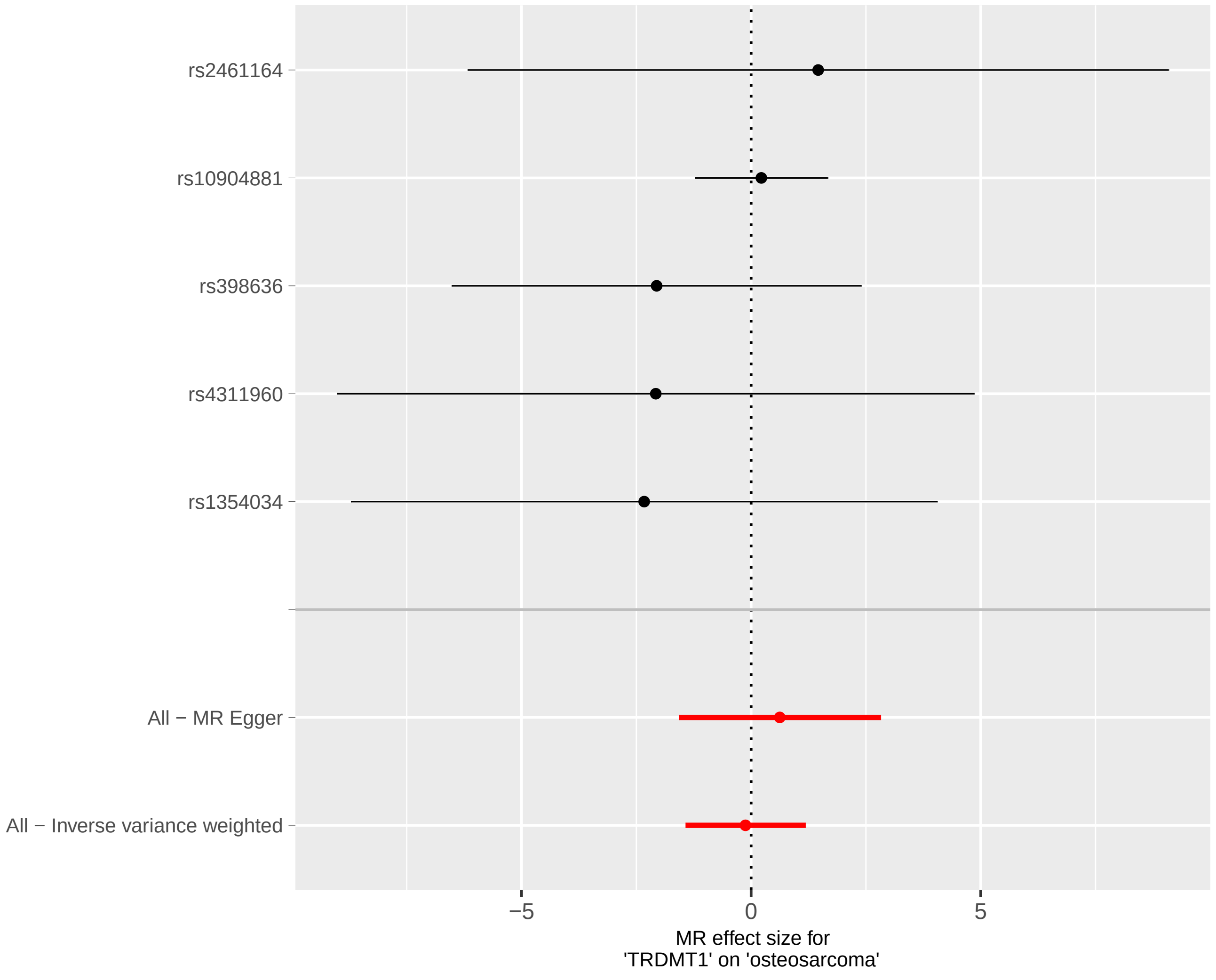

rs2461164
rs10904881
rs398636
rs4311960
rs1354034
All − MR Egger
All − Inverse variance weighted
−5
0
5
MR effect size for
'TRDMT1' on 'osteosarcoma'

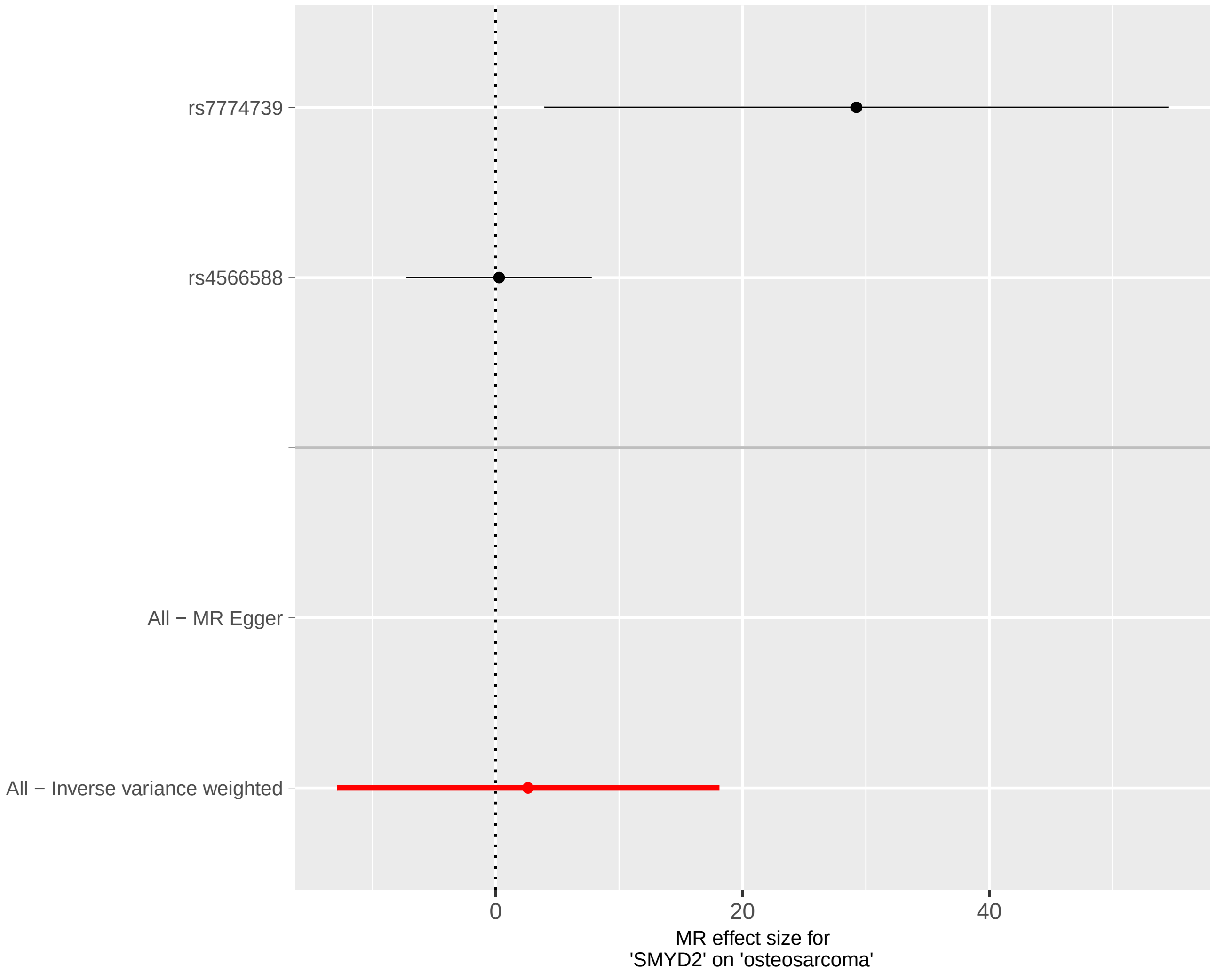
rs7774739
rs4566588
All − MR Egger
All − Inverse variance weighted
0
20
40
MR effect size for
'SMYD2' on 'osteosarcoma'

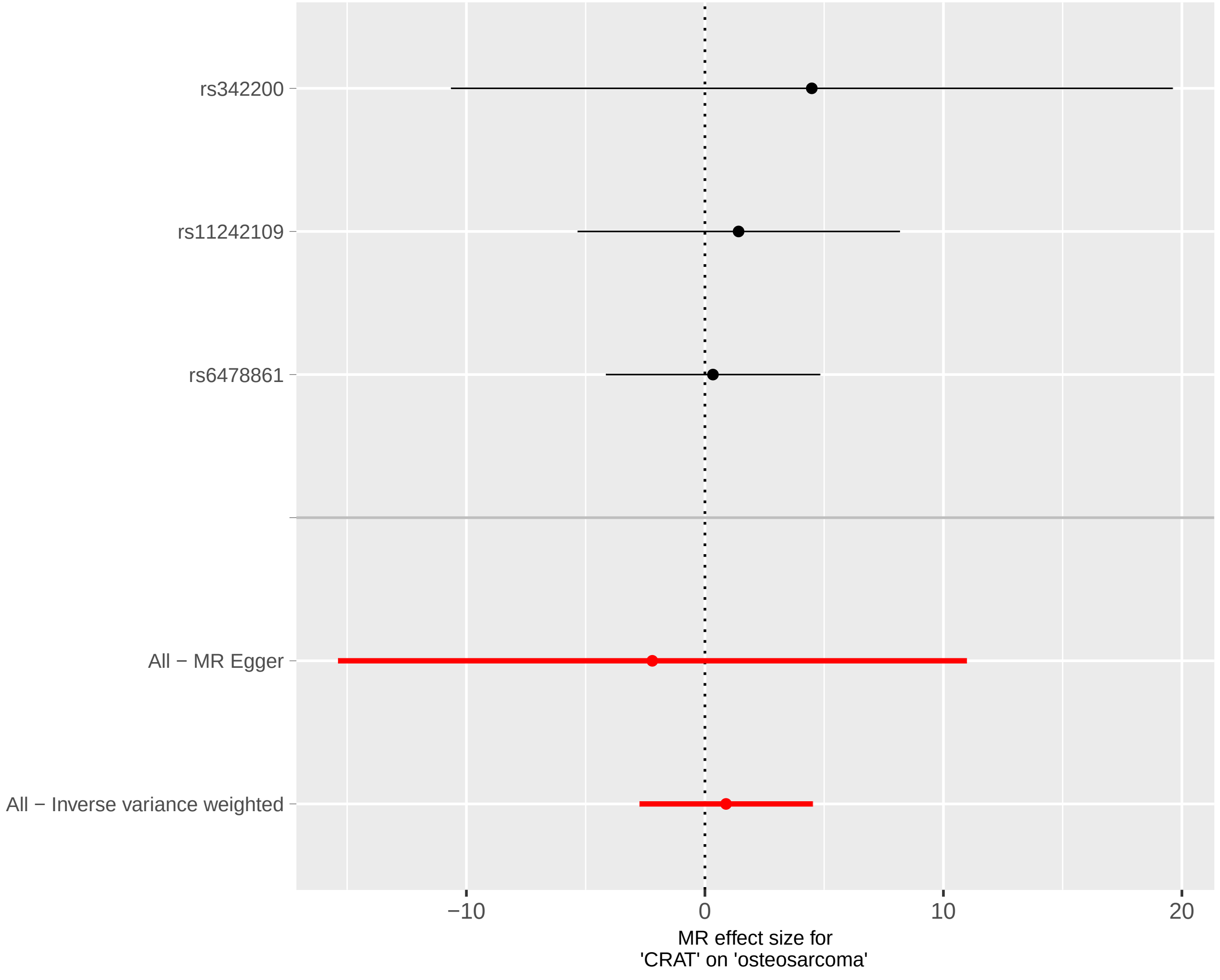
rs342200
rs11242109
rs6478861
All − MR Egger
All − Inverse variance weighted
−10
0
10
20
MR effect size for
'CRAT' on 'osteosarcoma'

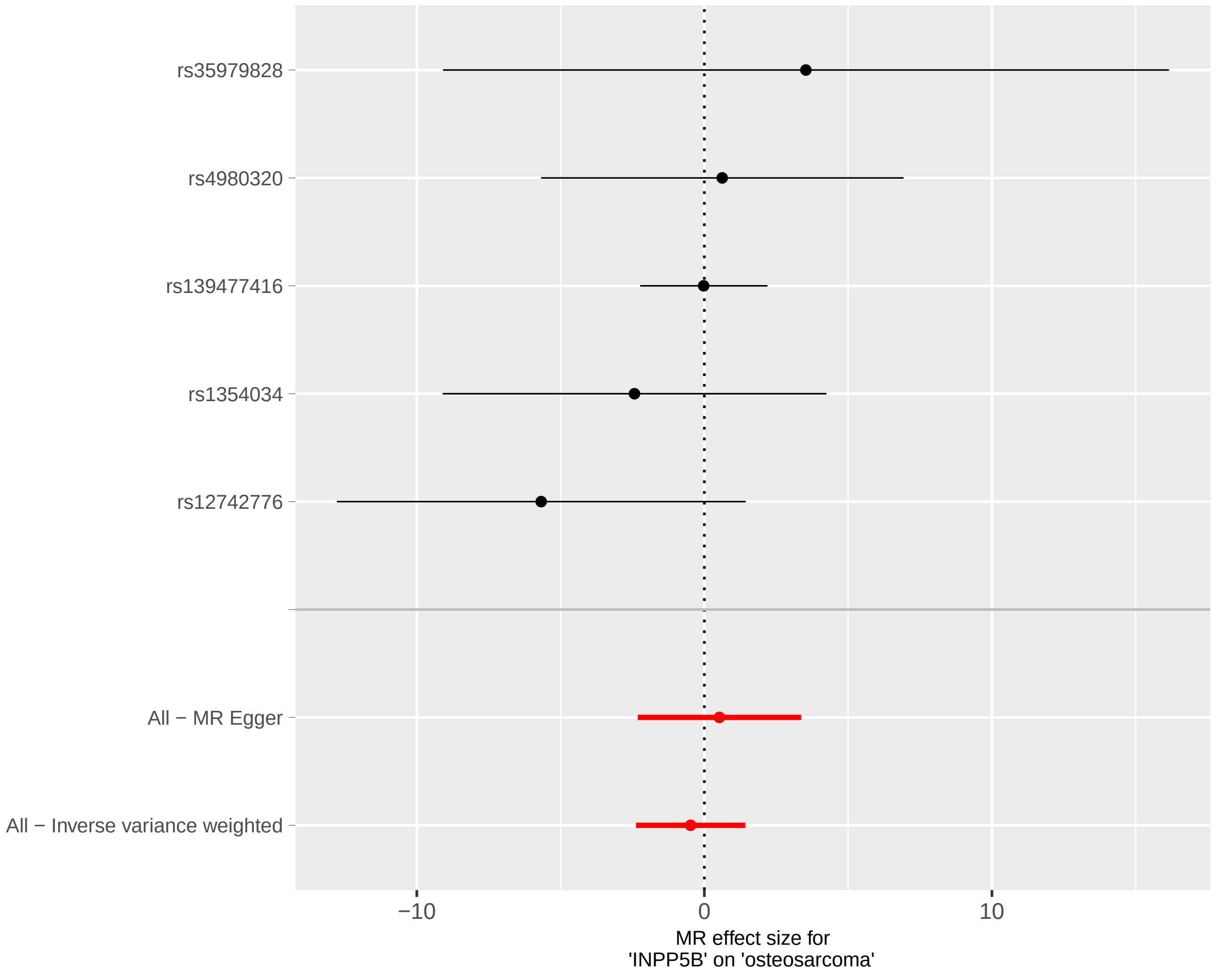
rs35979828
rs4980320
rs139477416
rs1354034
rs12742776
All − MR Egger
All − Inverse variance weighted
−10
0
10
MR effect size for
'INPP5B' on 'osteosarcoma'

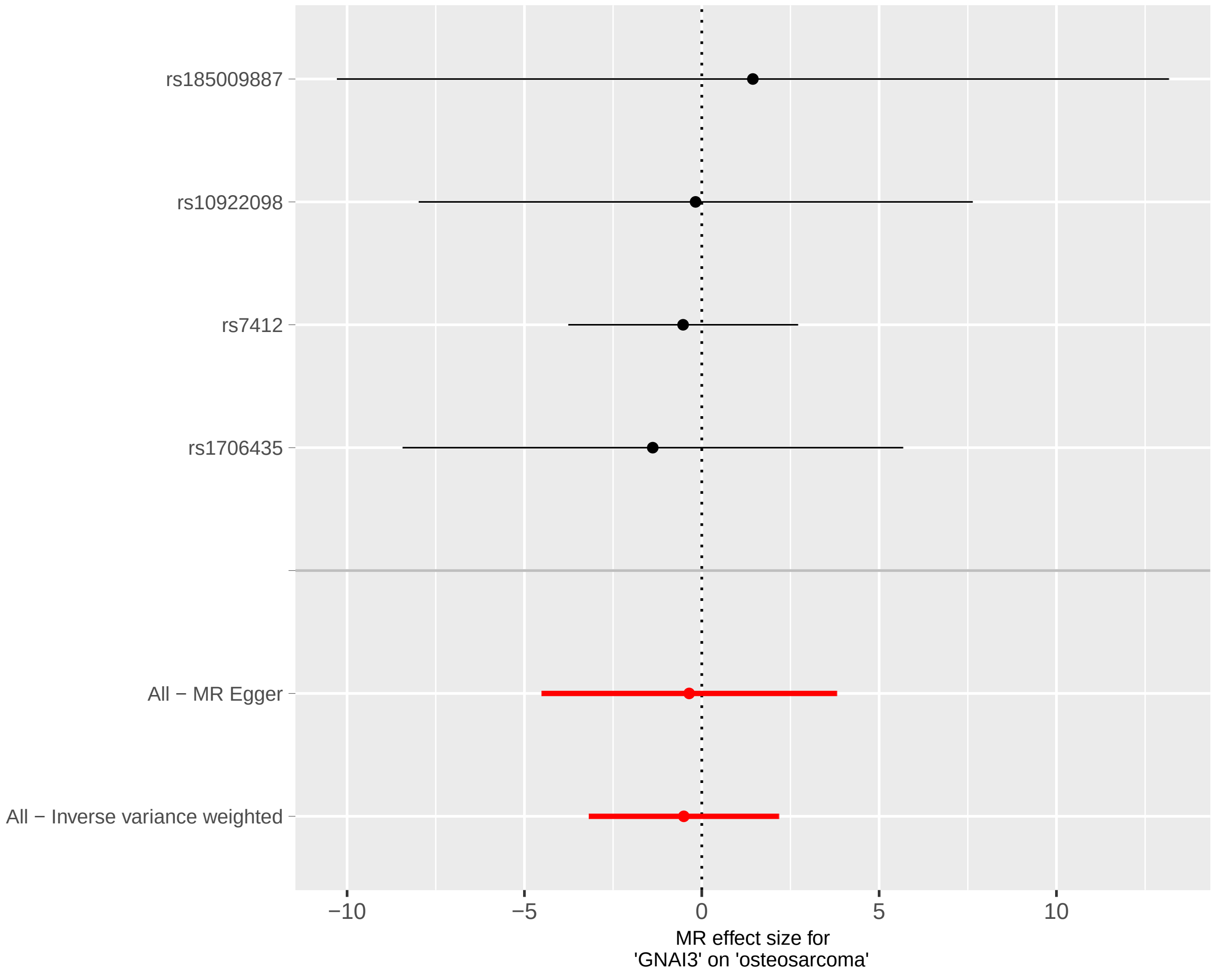
rs185009887
rs10922098
rs7412
rs1706435
All − MR Egger
All − Inverse variance weighted
−10
−5
0
5
10
MR effect size for
'GNAI3' on 'osteosarcoma'

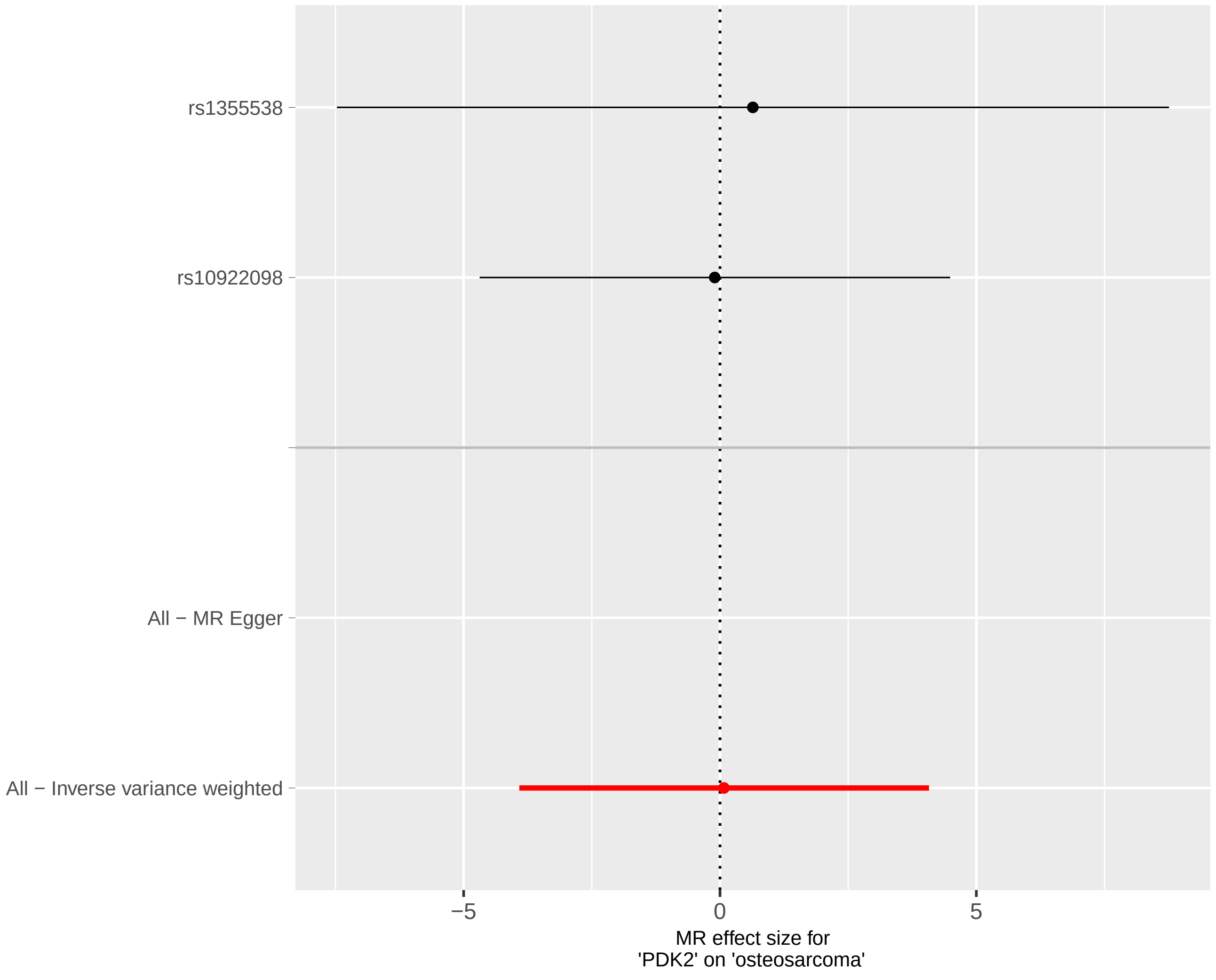

rs1355538
rs10922098
All − MR Egger
All − Inverse variance weighted
−5
0
5
MR effect size for
'PDK2' on 'osteosarcoma'

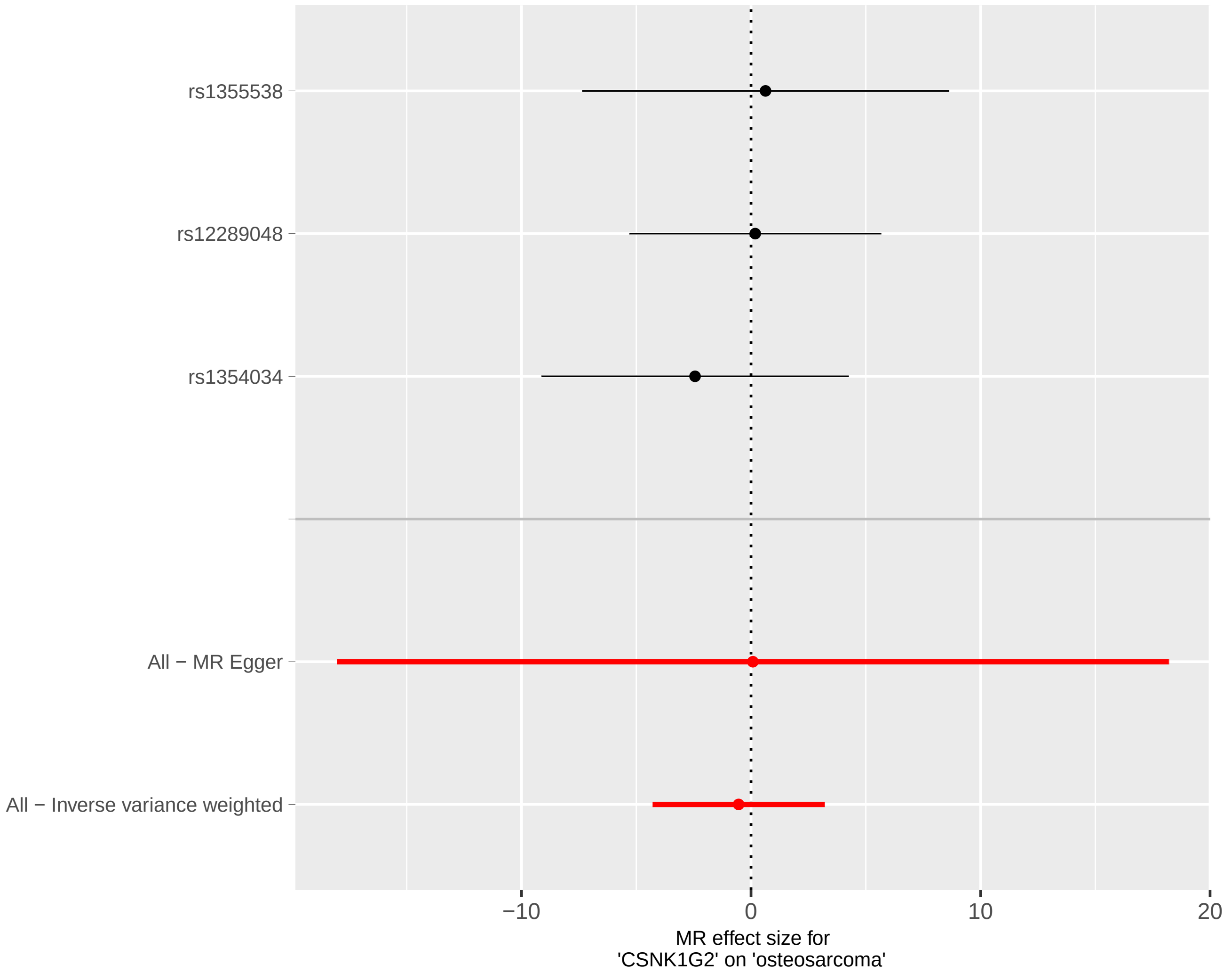
rs1355538
rs12289048
rs1354034
All − MR Egger
All − Inverse variance weighted
−10
0
10
20
MR effect size for
'CSNK1G2' on 'osteosarcoma'

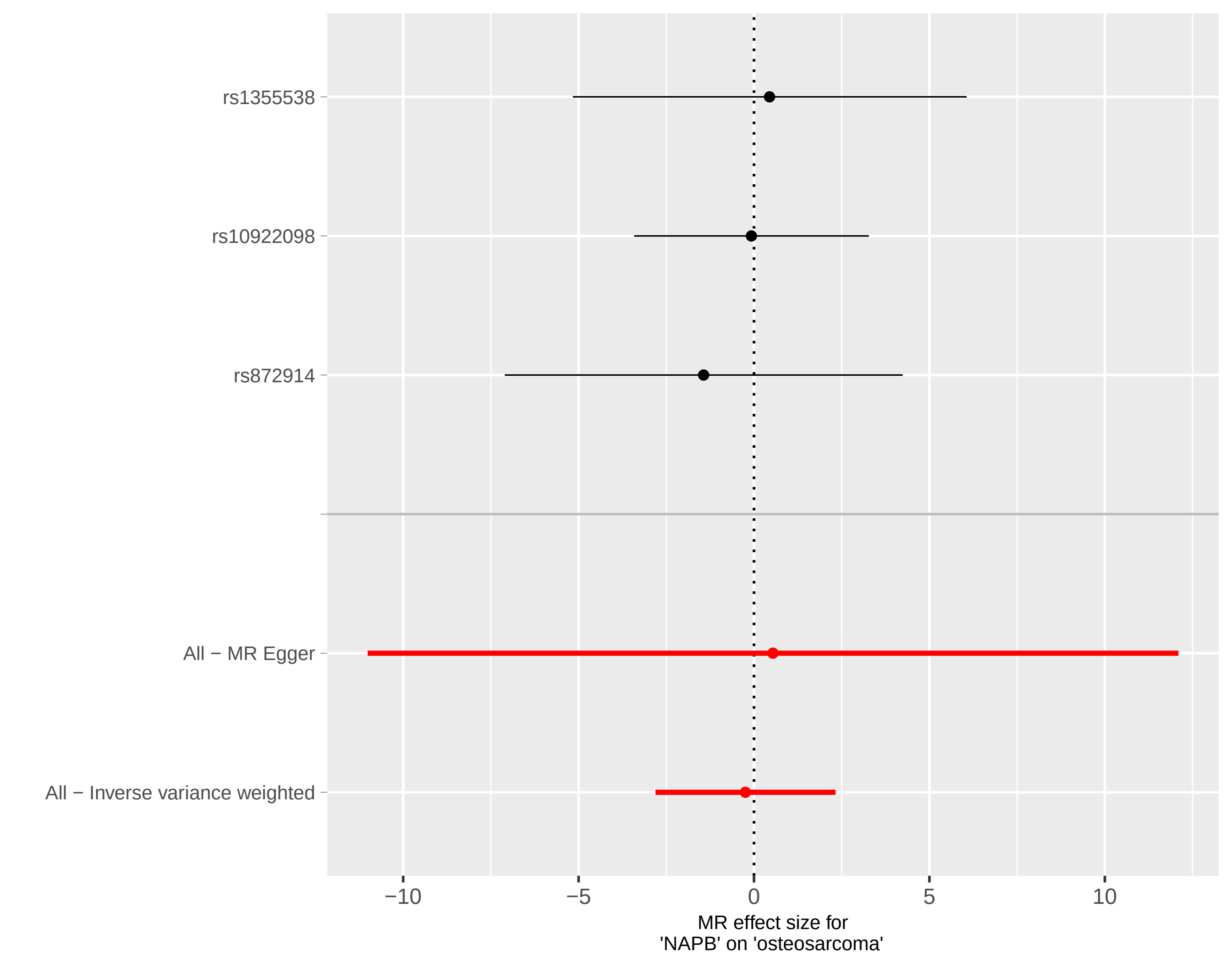
rs1355538
rs10922098
rs872914
All − MR Egger
All − Inverse variance weighted
−10
−5
0
5
10
MR effect size for
'NAPB' on 'osteosarcoma'

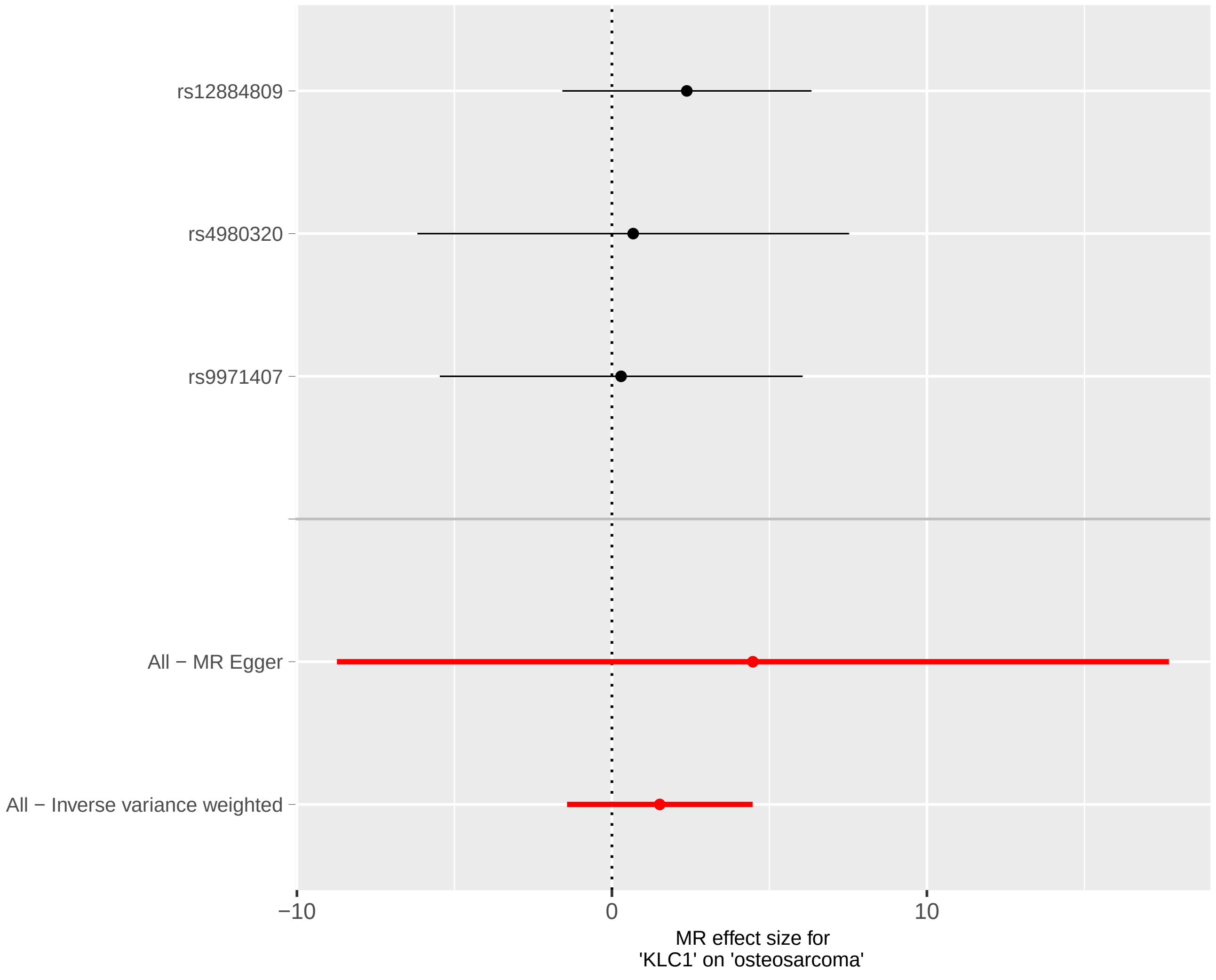

rs12884809
rs4980320
rs9971407
All − MR Egger
All − Inverse variance weighted
−10
0
10
MR effect size for
'KLC1' on 'osteosarcoma'

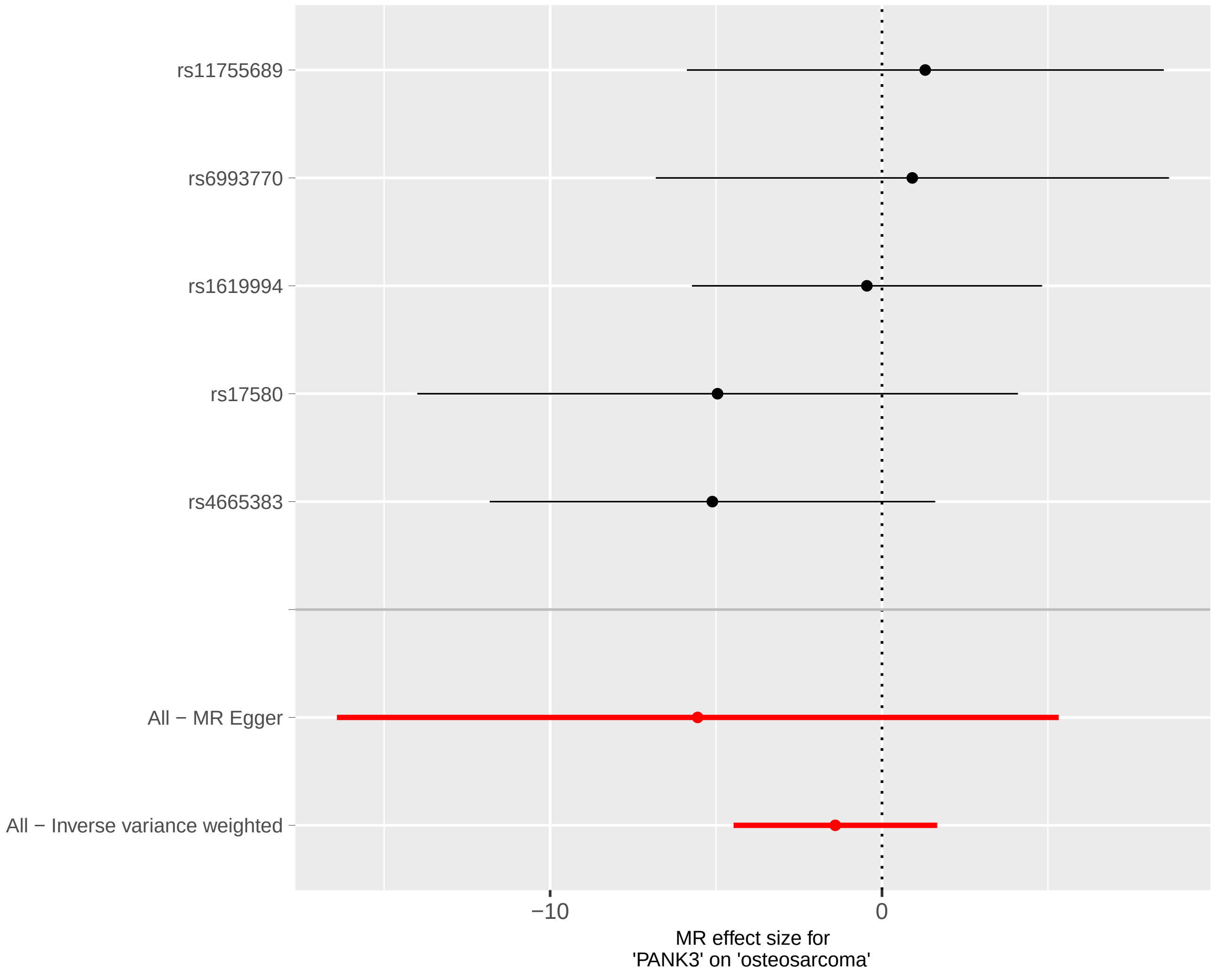

rs11755689
rs6993770
rs1619994
rs17580
rs4665383
All − MR Egger
All − Inverse variance weighted
−10
0
MR effect size for
'PANK3' on 'osteosarcoma'

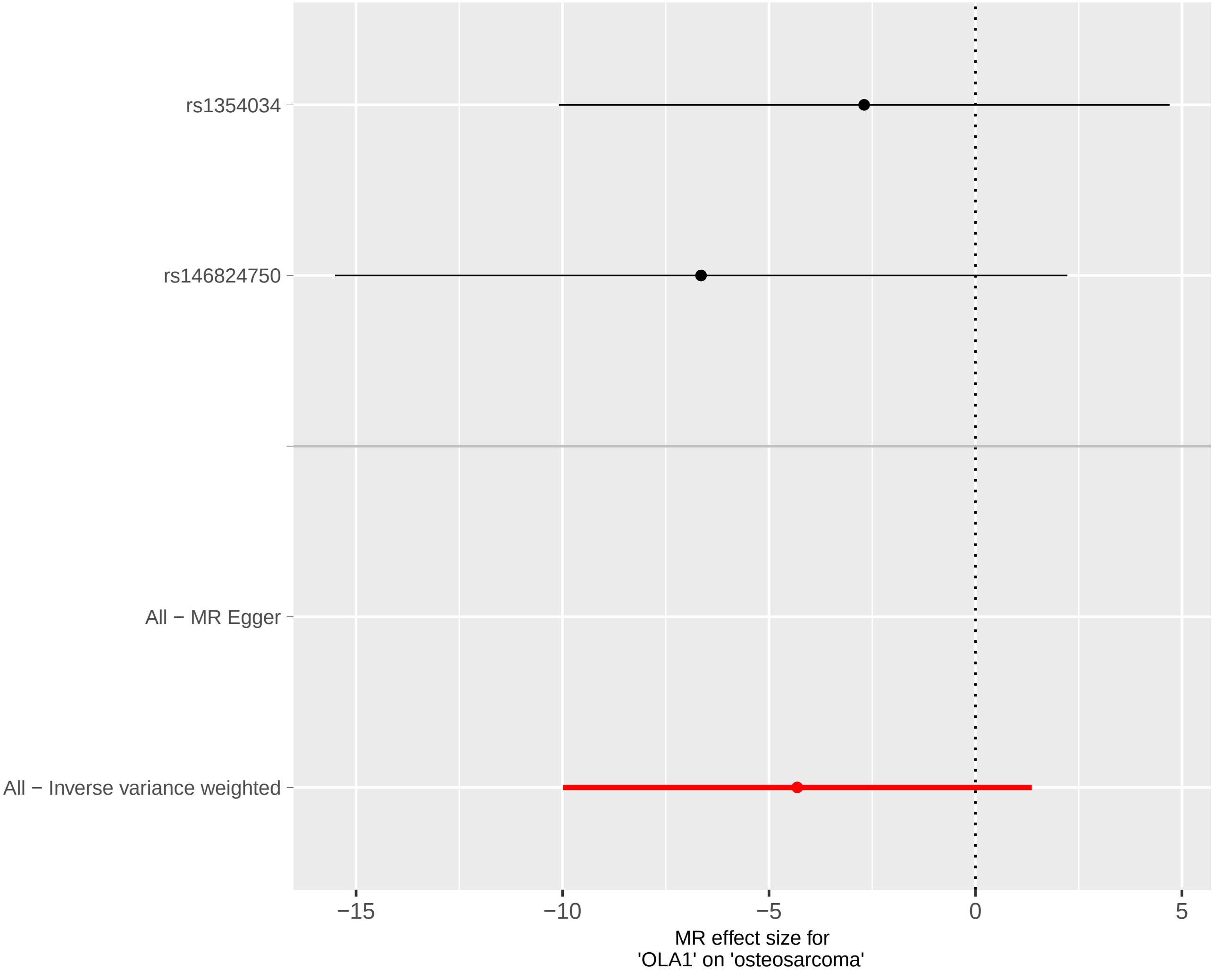

rs1354034
rs146824750
All − MR Egger
All − Inverse variance weighted
−15
−10
−5
0
5
MR effect size for
'OLA1' on 'osteosarcoma'

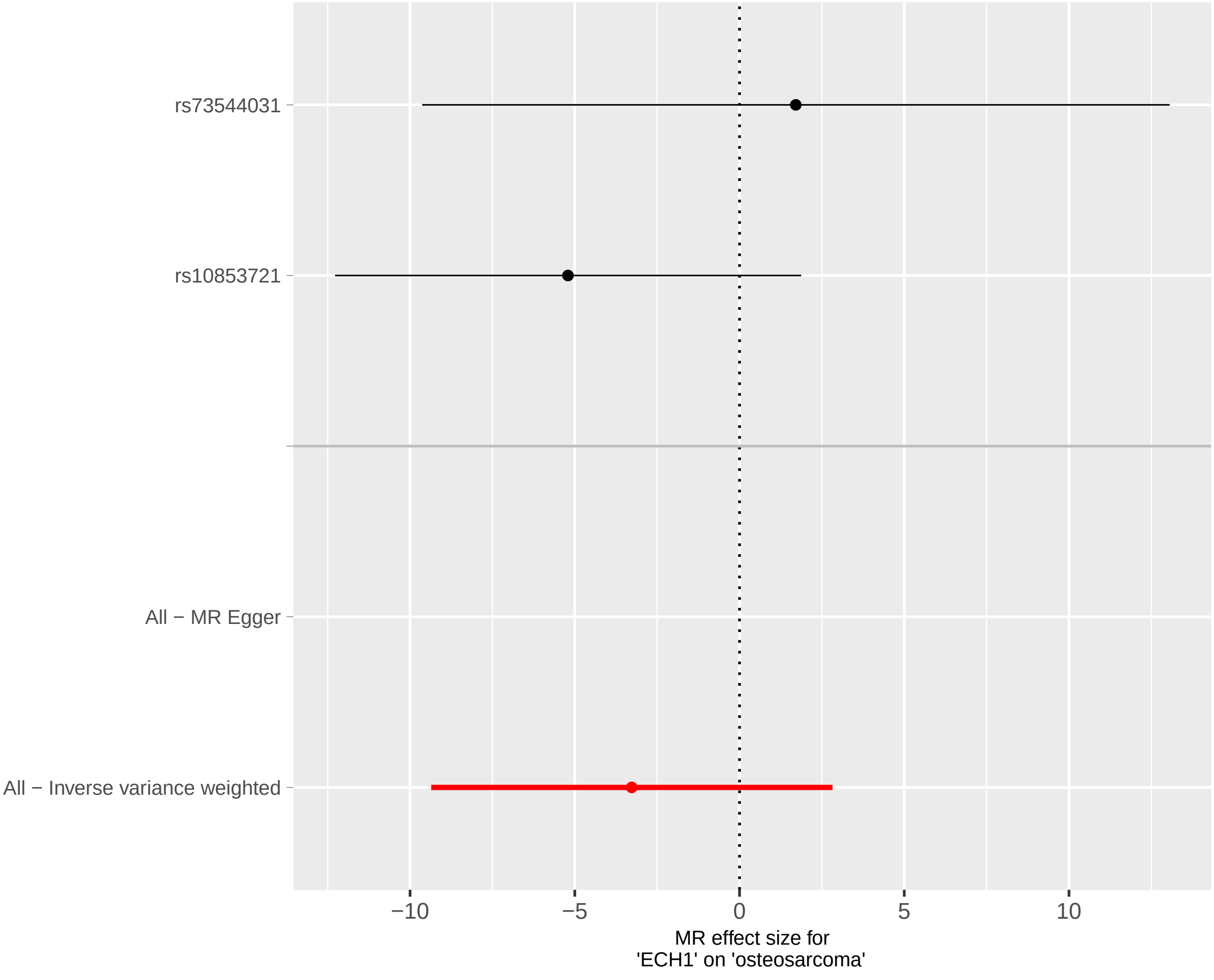

rs73544031
rs10853721
All − MR Egger
All − Inverse variance weighted
−10
−5
0
5
10
MR effect size for
'ECH1' on 'osteosarcoma'

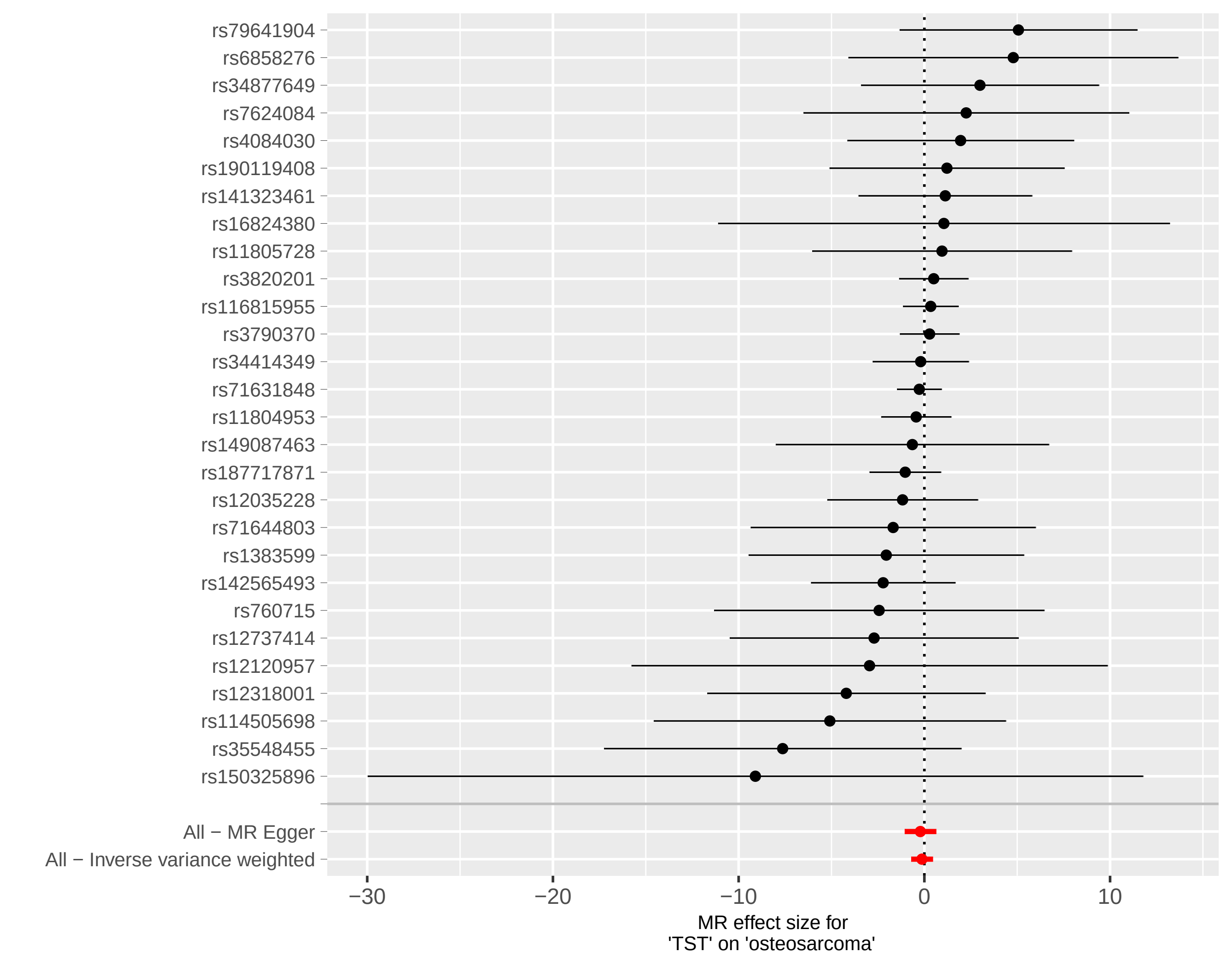

rs79641904
rs6858276
rs34877649
rs7624084
rs4084030
rs190119408
rs141323461
rs16824380
rs11805728
rs3820201
rs116815955
rs3790370
rs34414349
rs71631848
rs11804953
rs149087463
rs187717871
rs12035228
rs71644803
rs1383599
rs142565493
rs760715
rs12737414
rs12120957
rs12318001
rs114505698
rs35548455
rs150325896
All − MR Egger
All − Inverse variance weighted
−30
−20
−10
0
10
MR effect size for
'TST' on 'osteosarcoma'

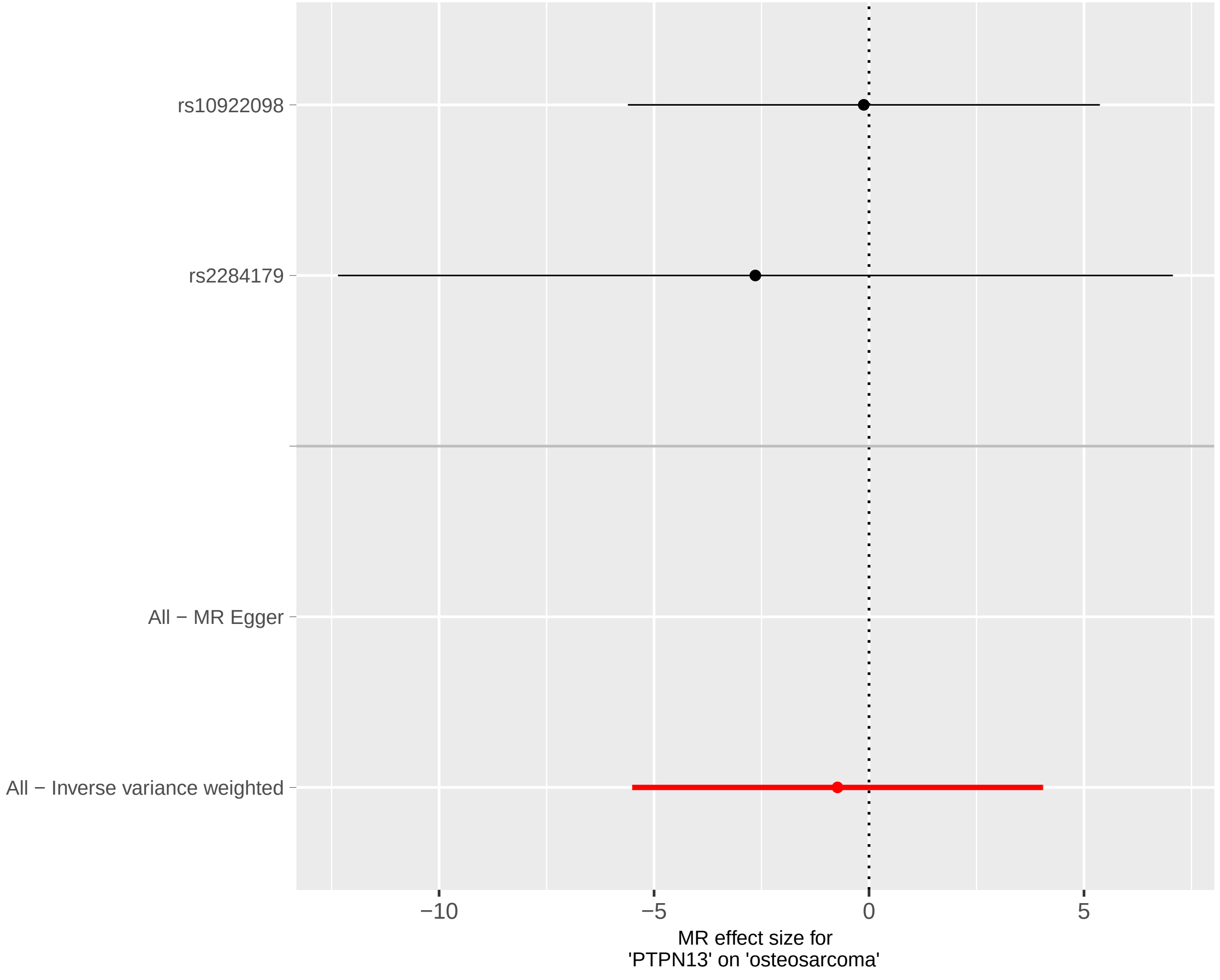
rs10922098
rs2284179
All − MR Egger
All − Inverse variance weighted
−10
−5
0
5
MR effect size for
'PTPN13' on 'osteosarcoma'

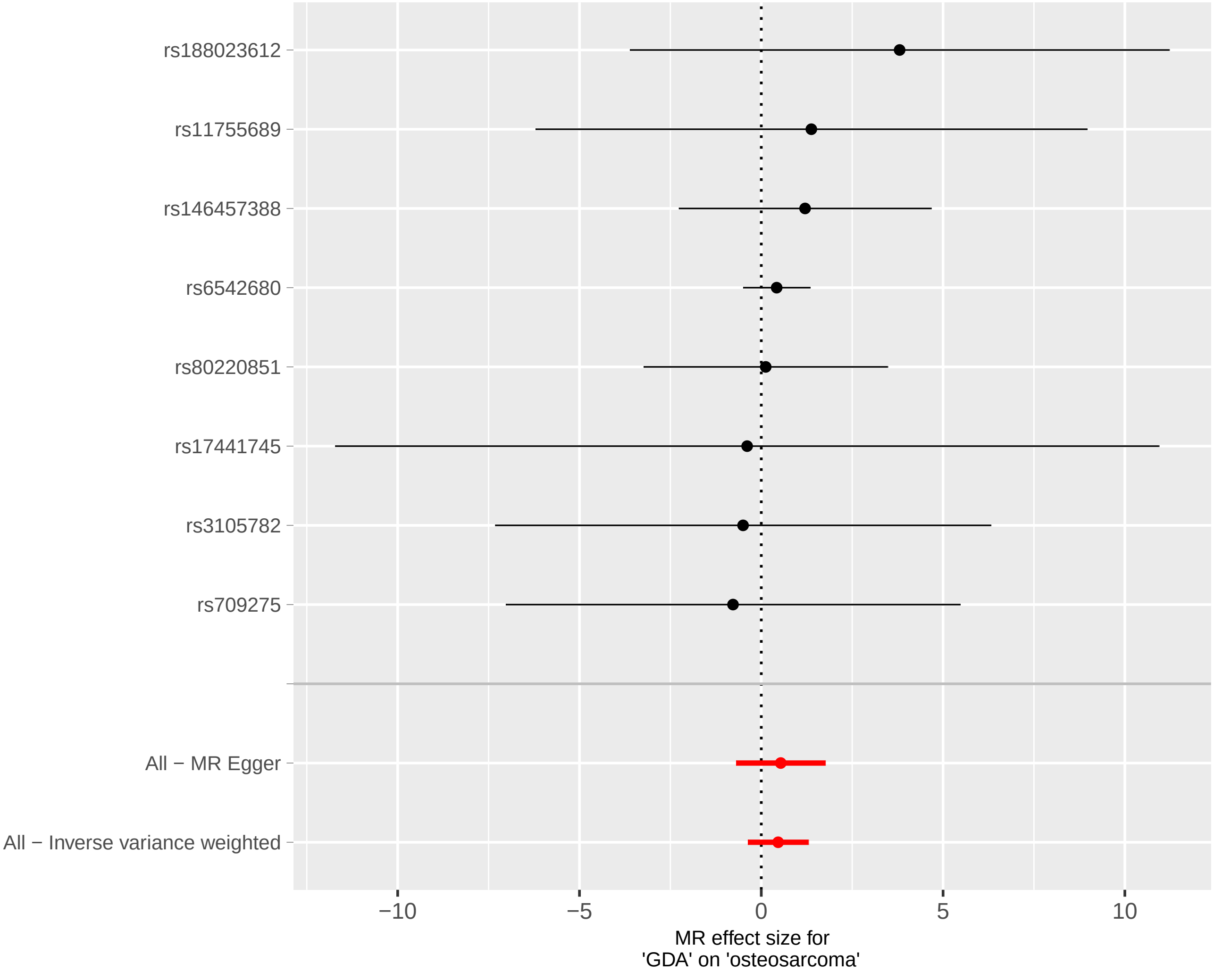

rs188023612
rs11755689
rs146457388
rs6542680
rs80220851
rs17441745
rs3105782
rs709275
All − MR Egger
All − Inverse variance weighted
−10
−5
0
5
10
MR effect size for
'GDA' on 'osteosarcoma'

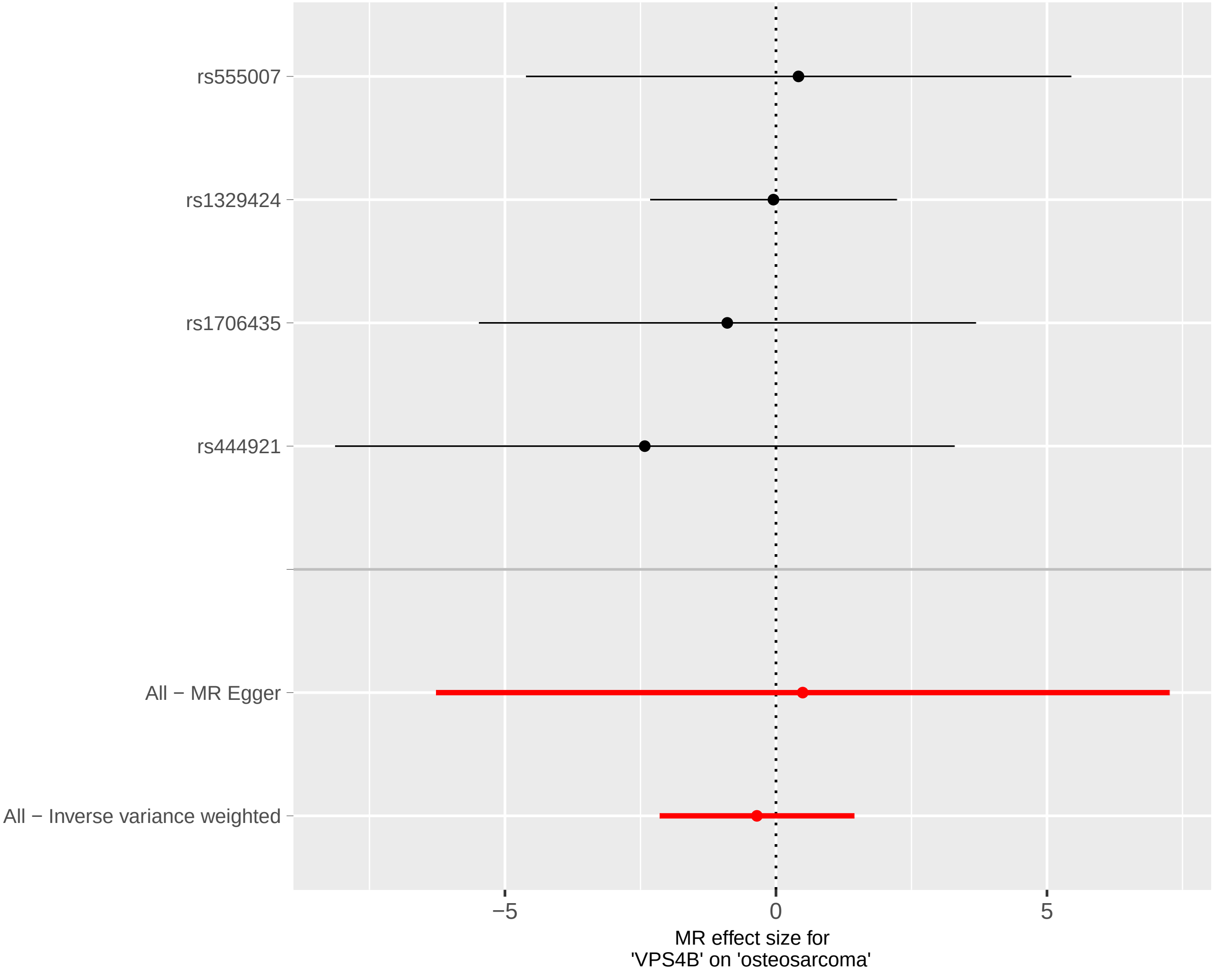
rs555007
rs1329424
rs1706435
rs444921
All − MR Egger
All − Inverse variance weighted
−5
0
5
MR effect size for
'VPS4B' on 'osteosarcoma'

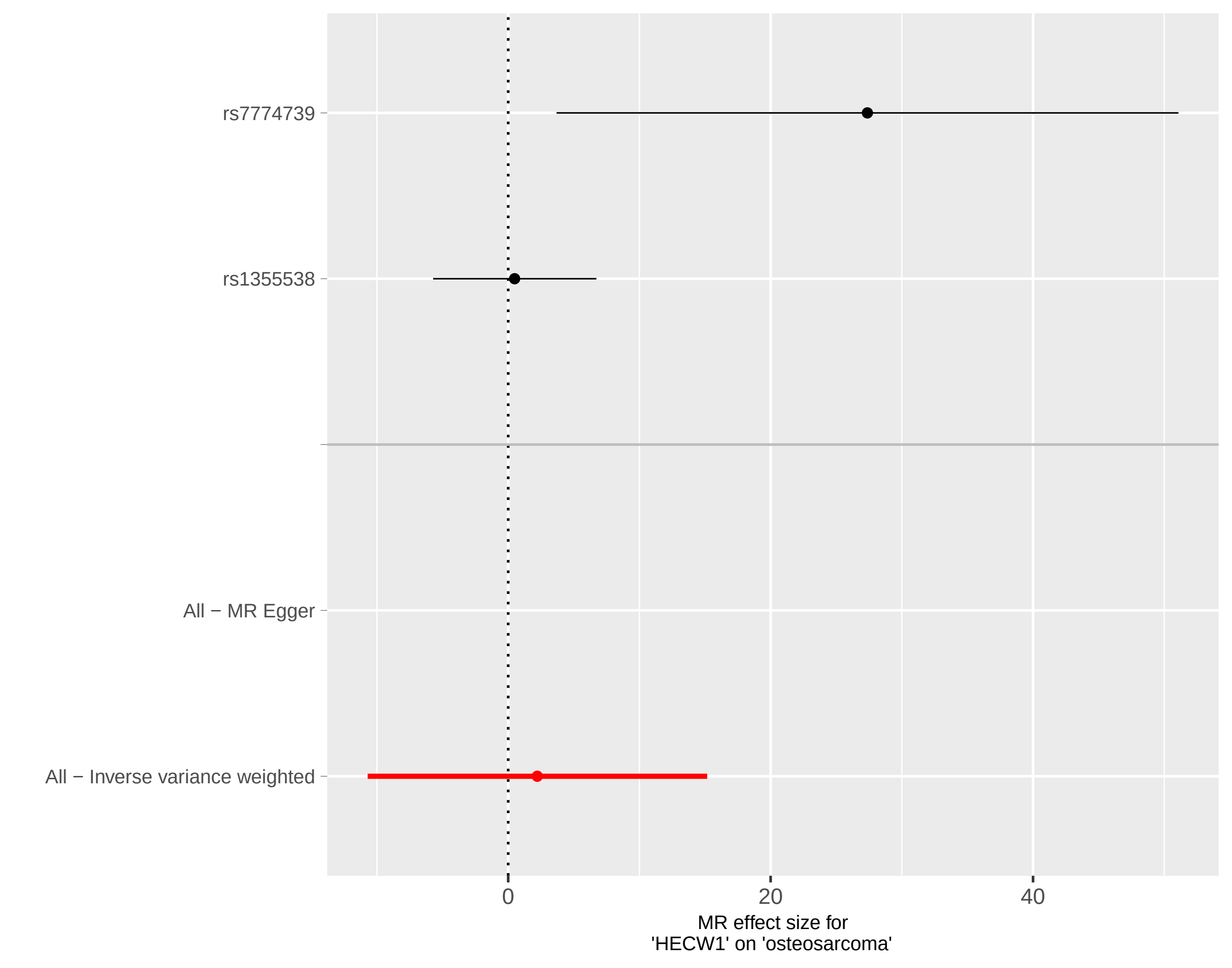
rs7774739
rs1355538
All − MR Egger
All − Inverse variance weighted
0
20
40
MR effect size for
'HECW1' on 'osteosarcoma'

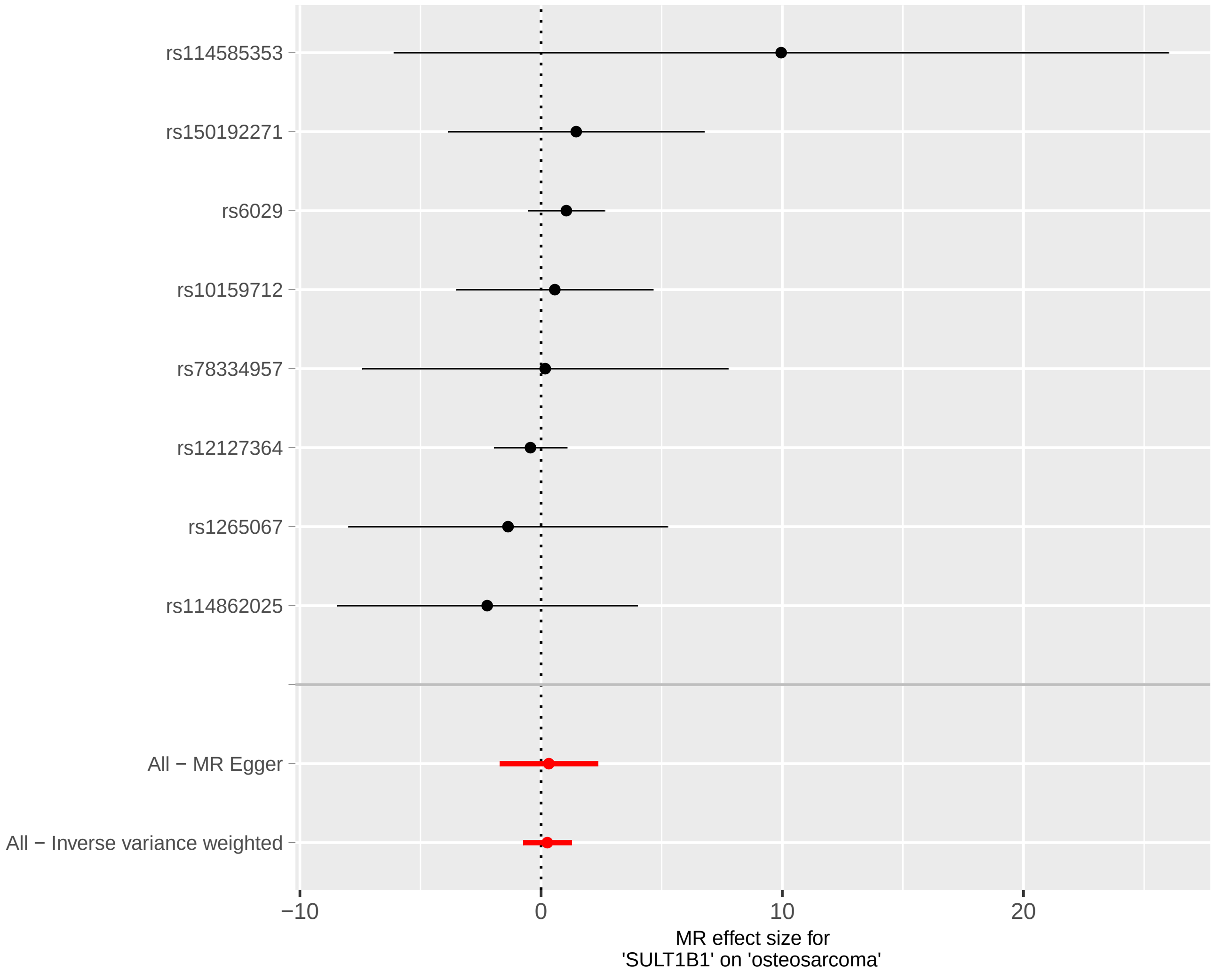
rs114585353
rs150192271
rs6029
rs10159712
rs78334957
rs12127364
rs1265067
rs114862025
All − MR Egger
All − Inverse variance weighted
−10
0
10
20
MR effect size for
'SULT1B1' on 'osteosarcoma'

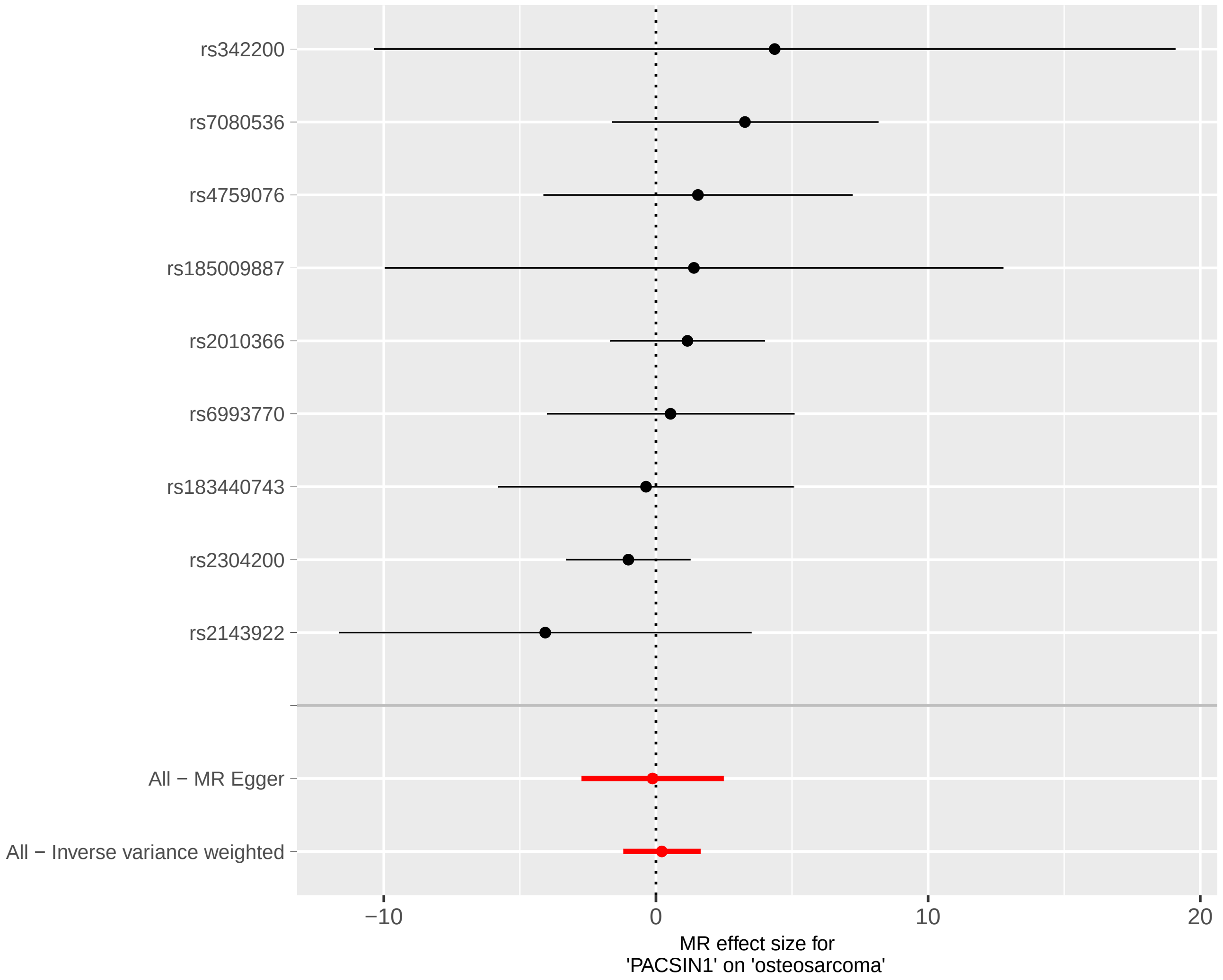
rs342200
rs7080536
rs4759076
rs185009887
rs2010366
rs6993770
rs183440743
rs2304200
rs2143922
All − MR Egger
All − Inverse variance weighted
−10
0
10
20
MR effect size for
'PACSIN1' on 'osteosarcoma'

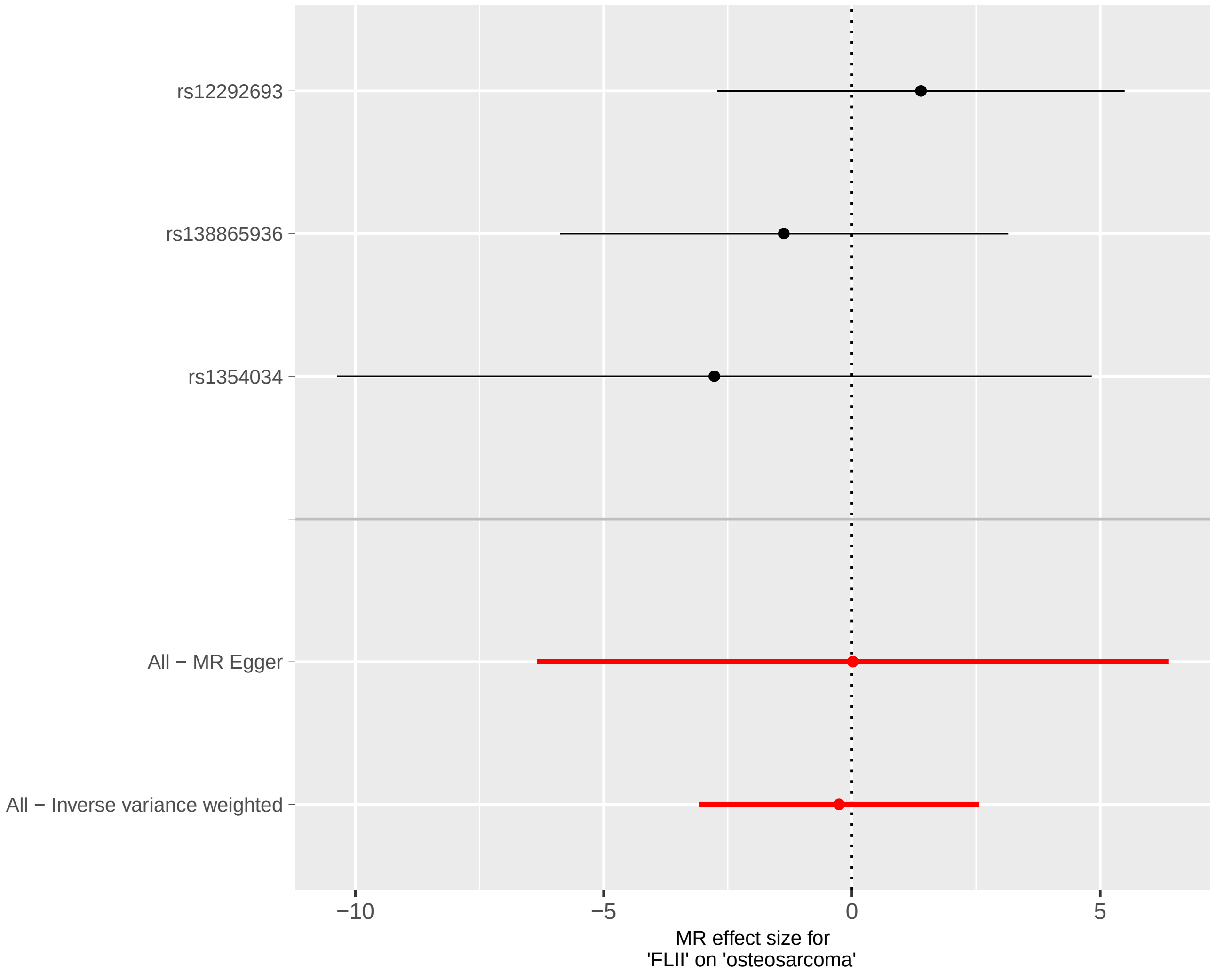
rs12292693
rs138865936
rs1354034
All − MR Egger
All − Inverse variance weighted
−10
−5
0
5
MR effect size for
'FLII' on 'osteosarcoma'

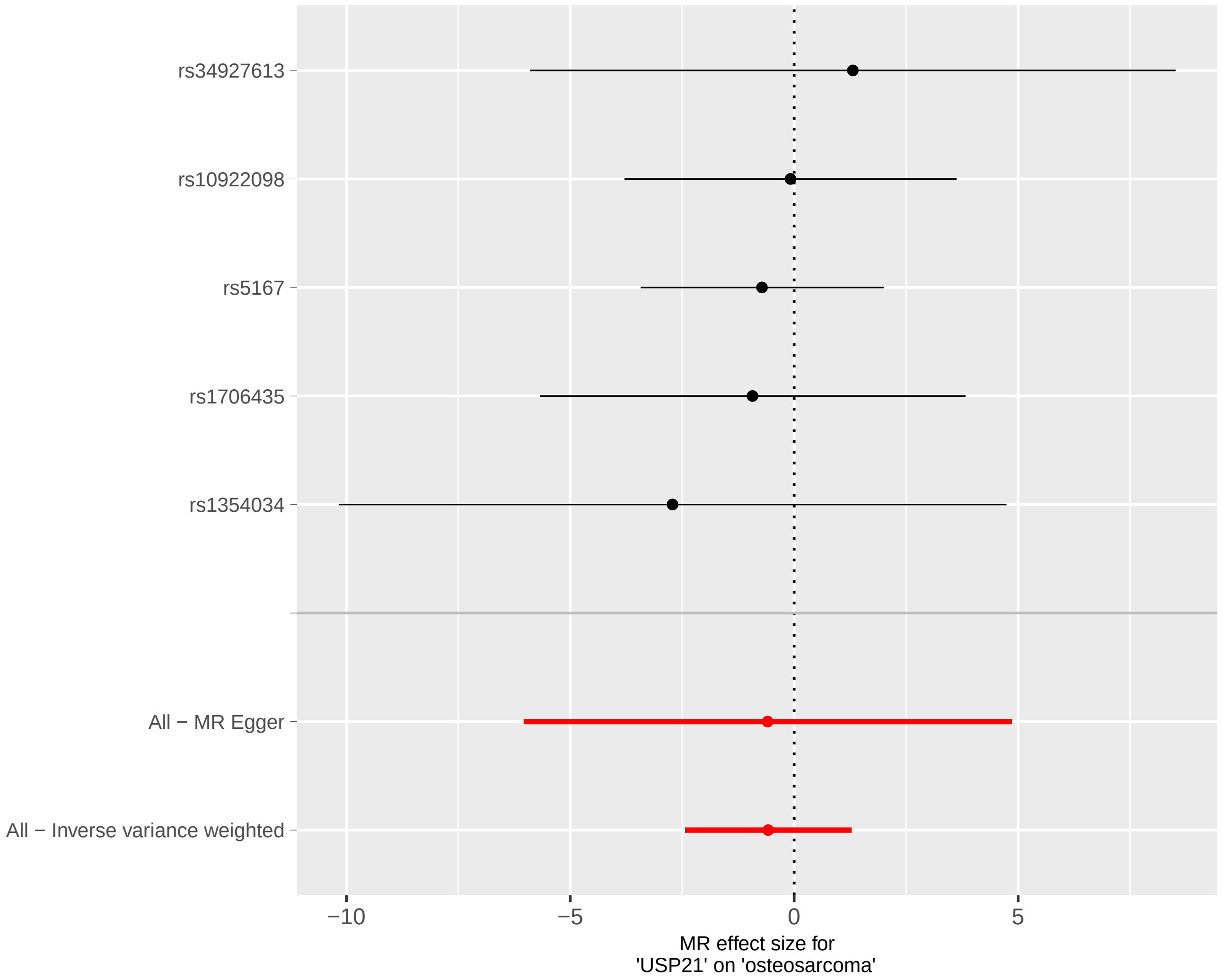

rs34927613
rs10922098
rs5167
rs1706435
rs1354034
All − MR Egger
All − Inverse variance weighted
−10
−5
0
5
MR effect size for
'USP21' on 'osteosarcoma'

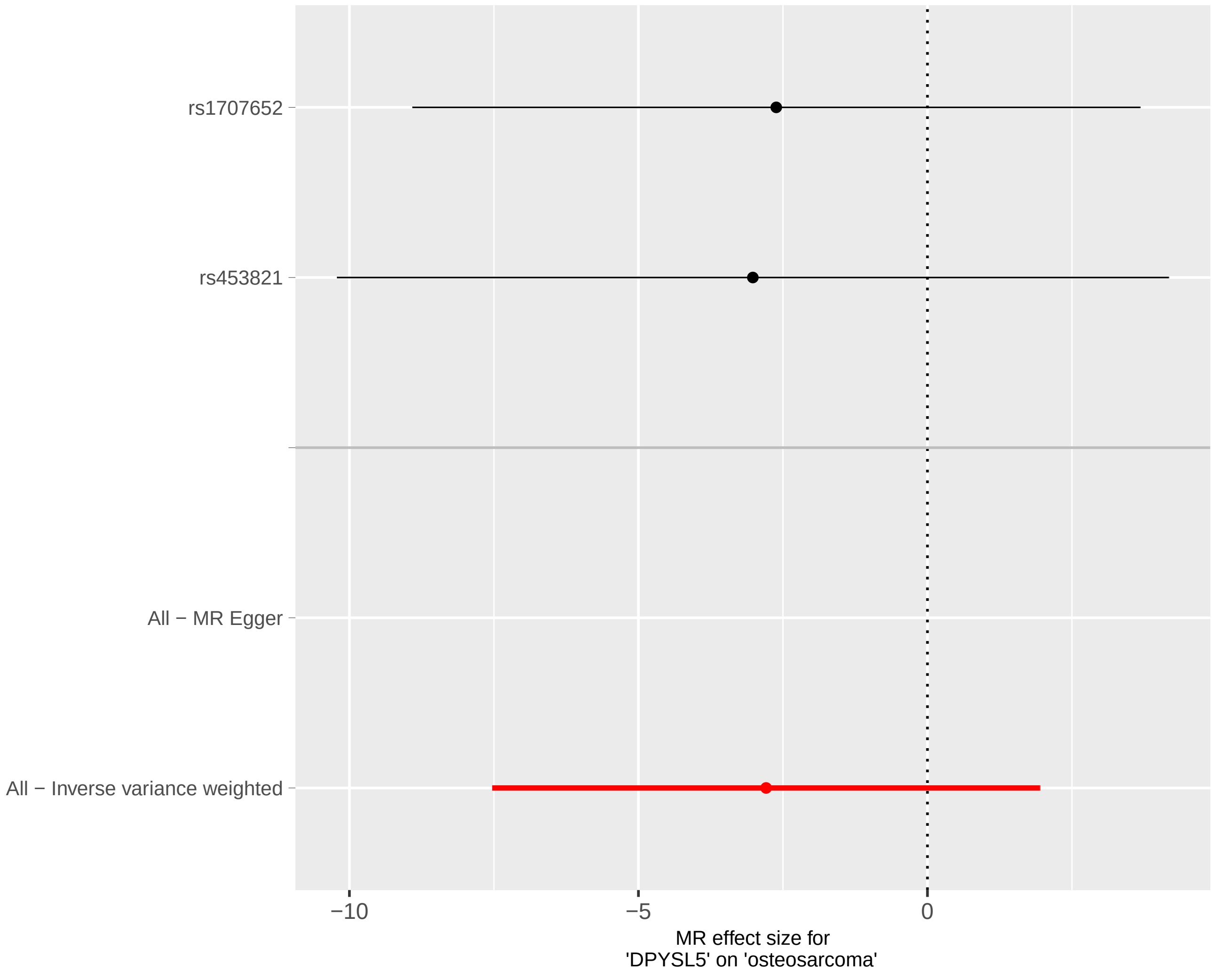

rs1707652
rs453821
All − MR Egger
All − Inverse variance weighted
−10
−5
0
MR effect size for
'DPYSL5' on 'osteosarcoma'

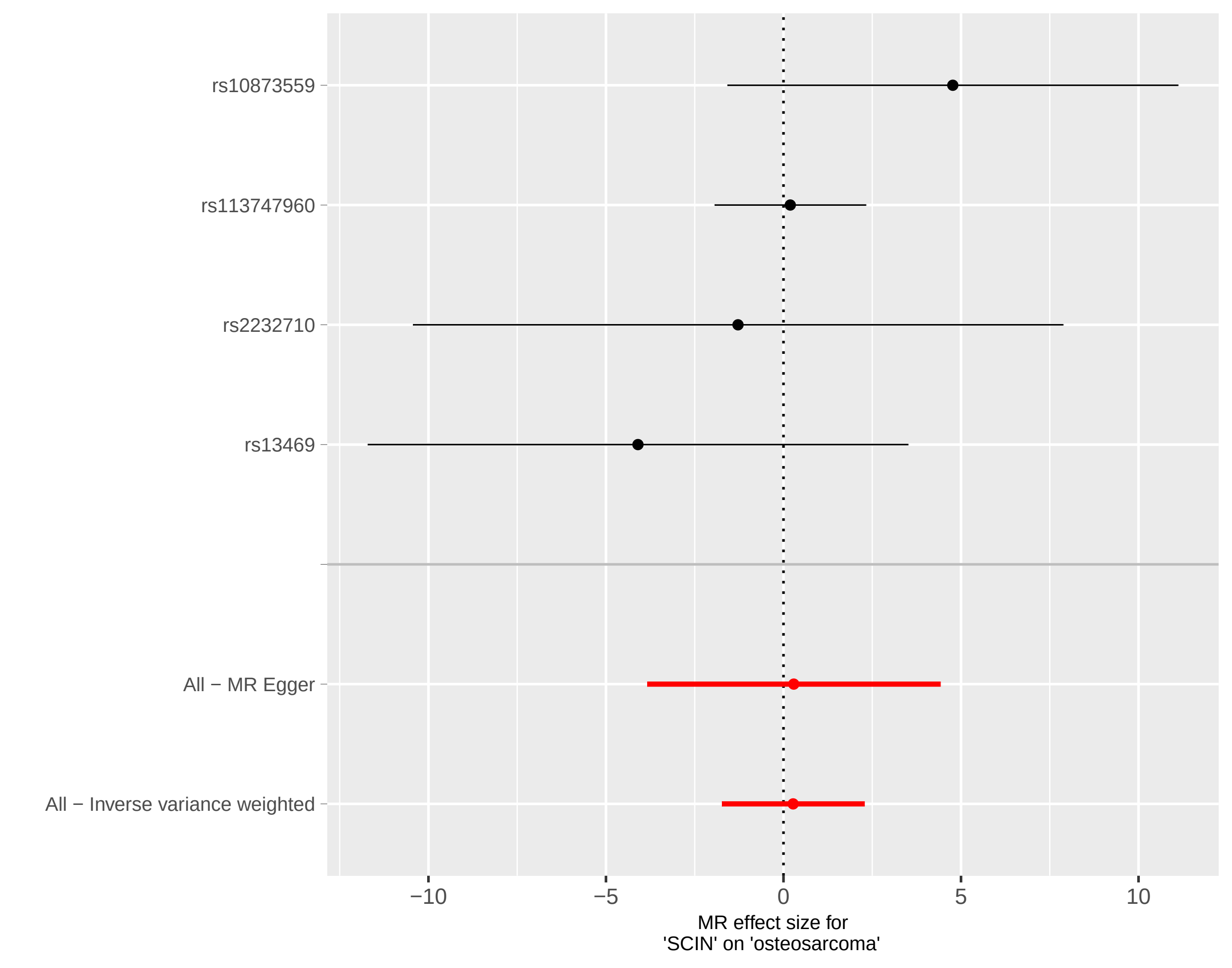

rs10873559
rs113747960
rs2232710
rs13469
All − MR Egger
All − Inverse variance weighted
−10
−5
0
5
10
MR effect size for
'SCIN' on 'osteosarcoma'

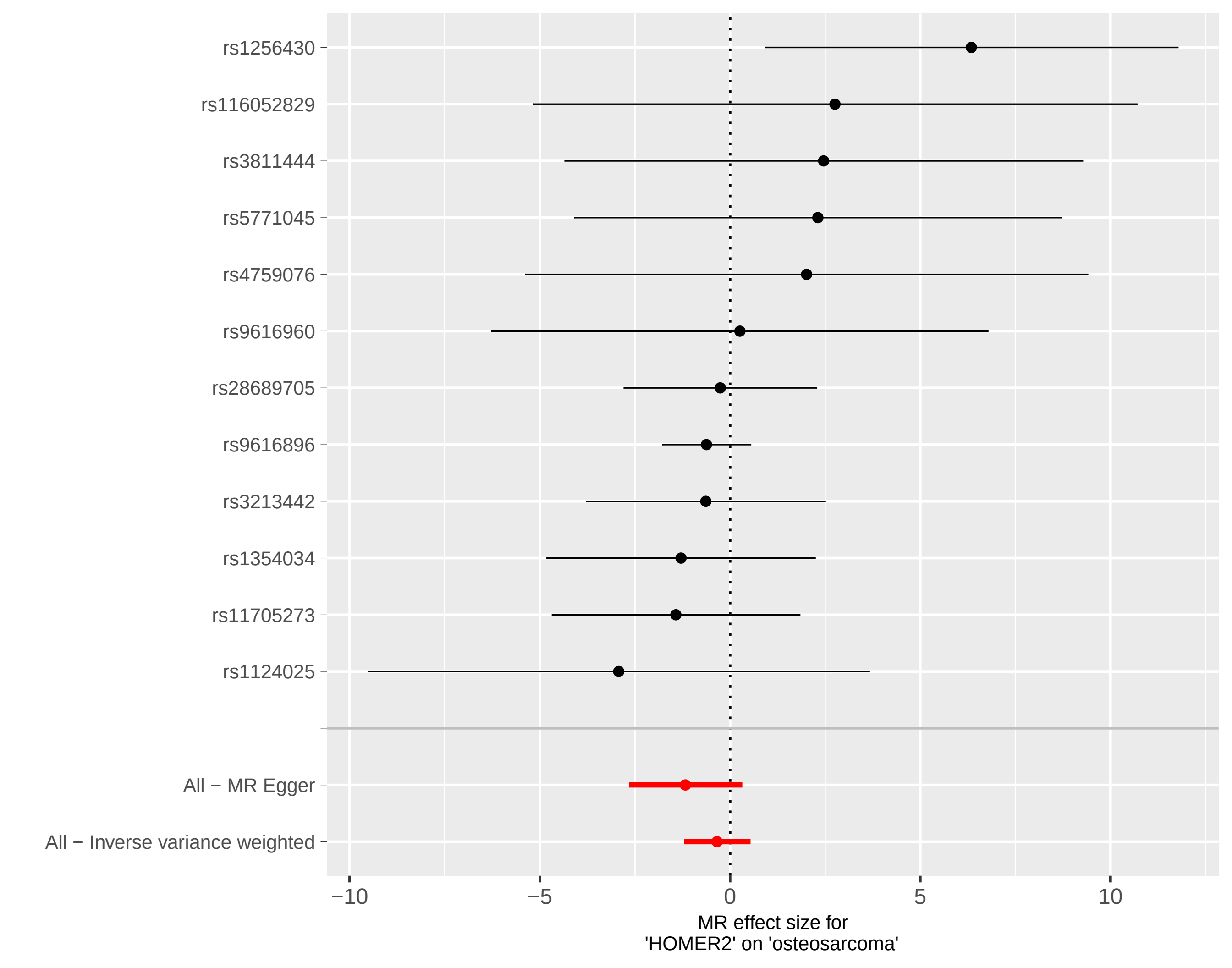
rs1256430
rs116052829
rs3811444
rs5771045
rs4759076
rs9616960
rs28689705
rs9616896
rs3213442
rs1354034
rs11705273
rs1124025
All − MR Egger
All − Inverse variance weighted
−10
−5
0
5
10
MR effect size for
'HOMER2' on 'osteosarcoma'

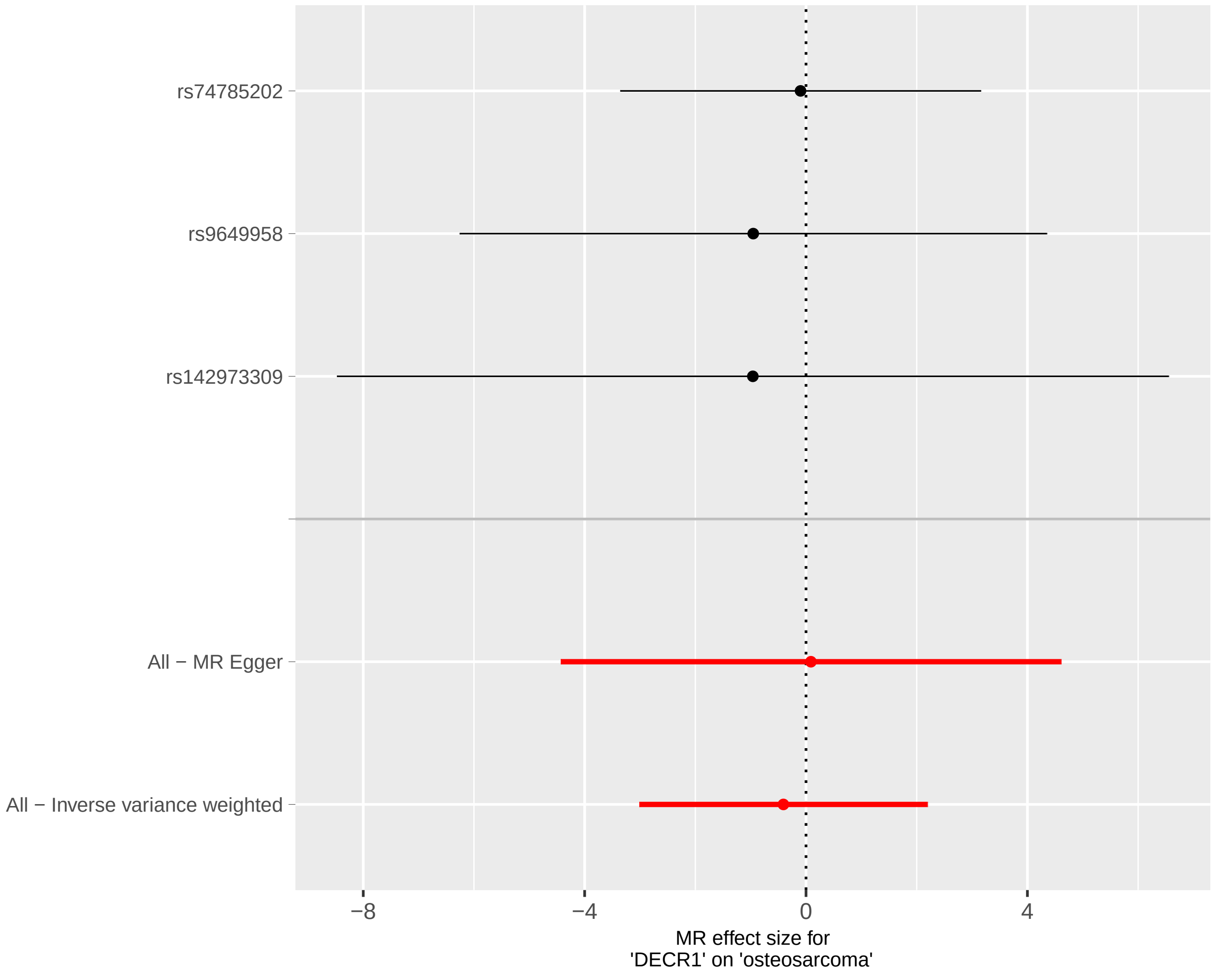

rs74785202
rs9649958
rs142973309
All − MR Egger
All − Inverse variance weighted
−8
−4
0
4
MR effect size for
'DECR1' on 'osteosarcoma'

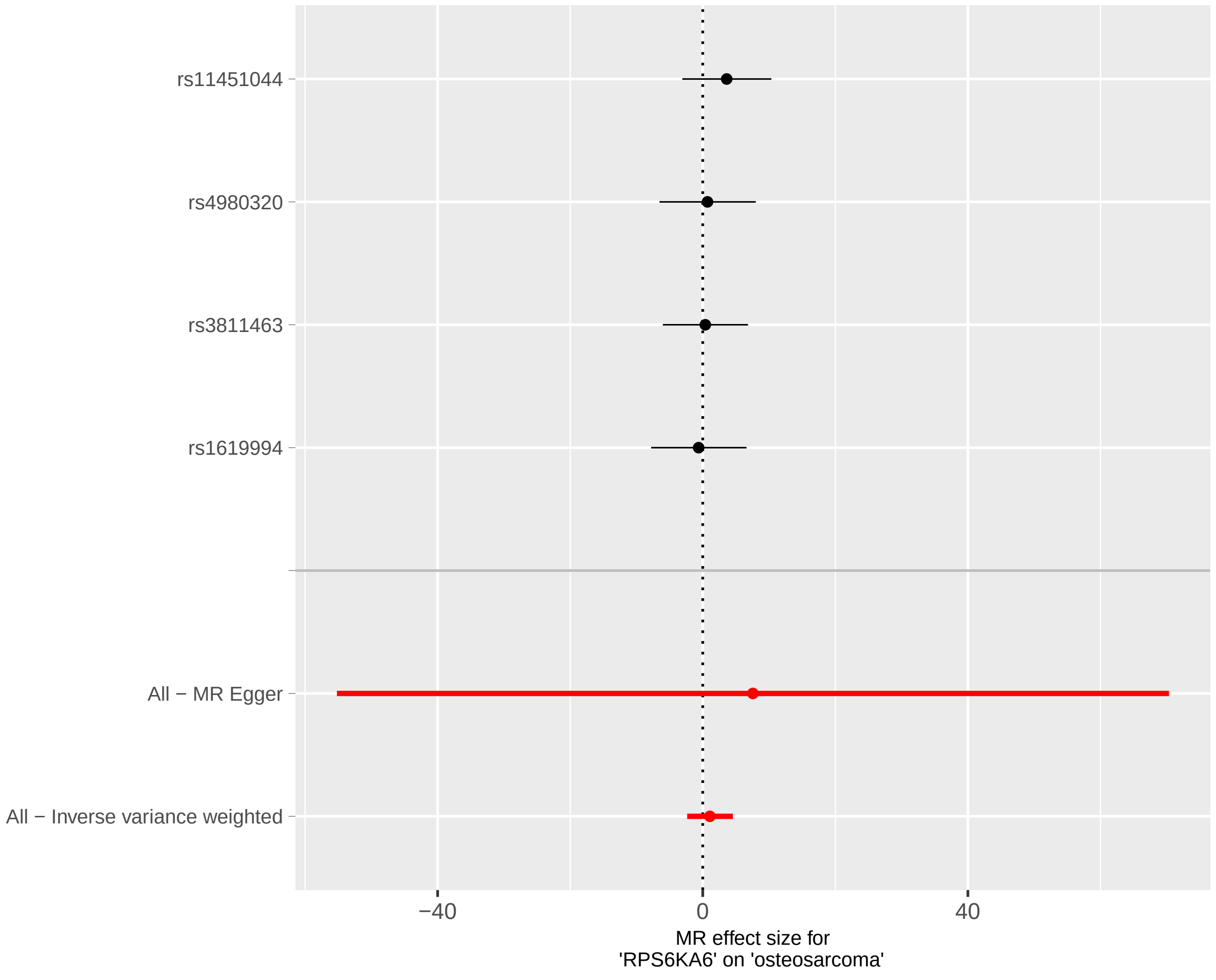

rs11451044
rs4980320
rs3811463
rs1619994
All − MR Egger
All − Inverse variance weighted
−40
0
40
MR effect size for
'RPS6KA6' on 'osteosarcoma'

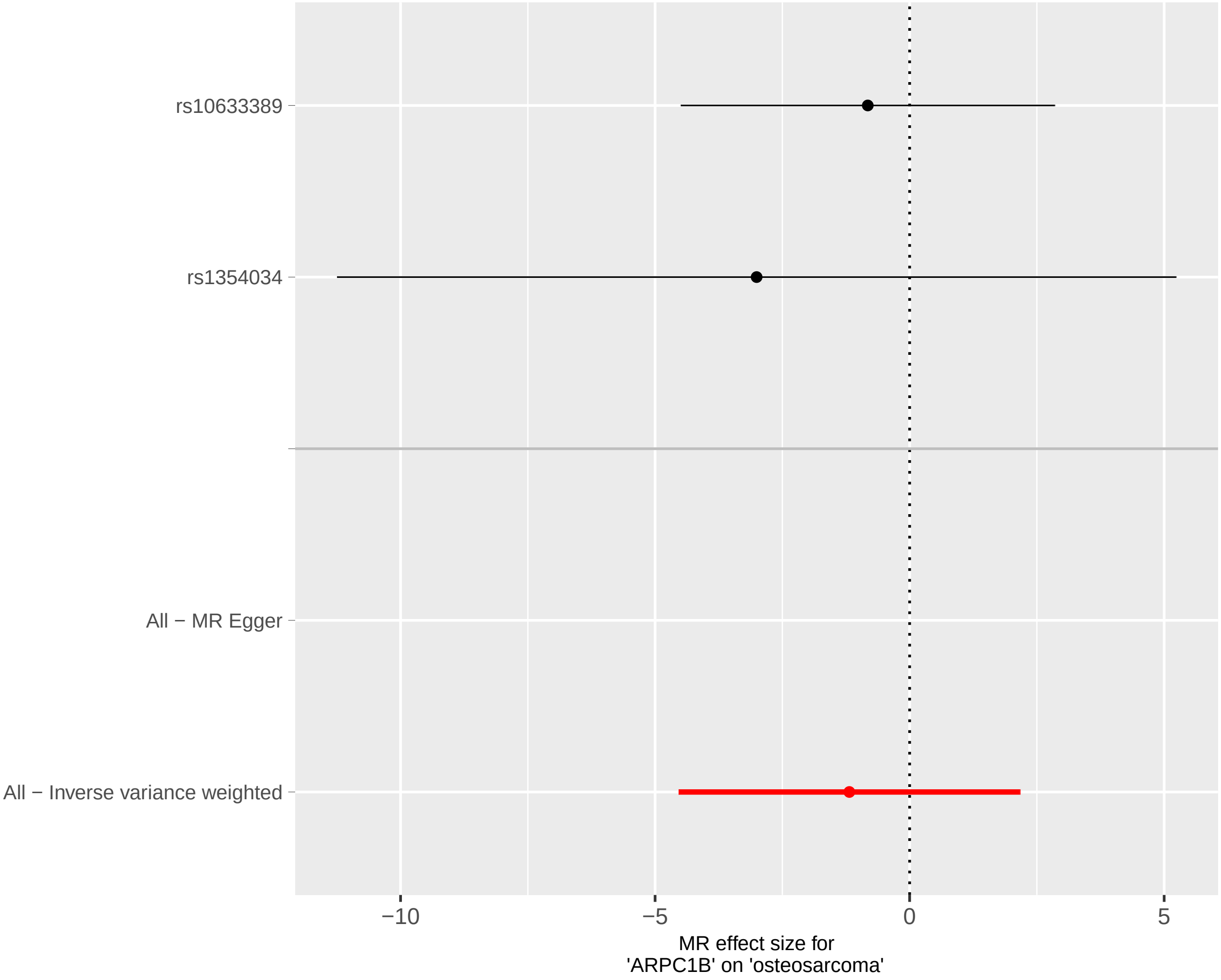

rs10633389
rs1354034
All − MR Egger
All − Inverse variance weighted
−10
−5
0
5
MR effect size for
'ARPC1B' on 'osteosarcoma'

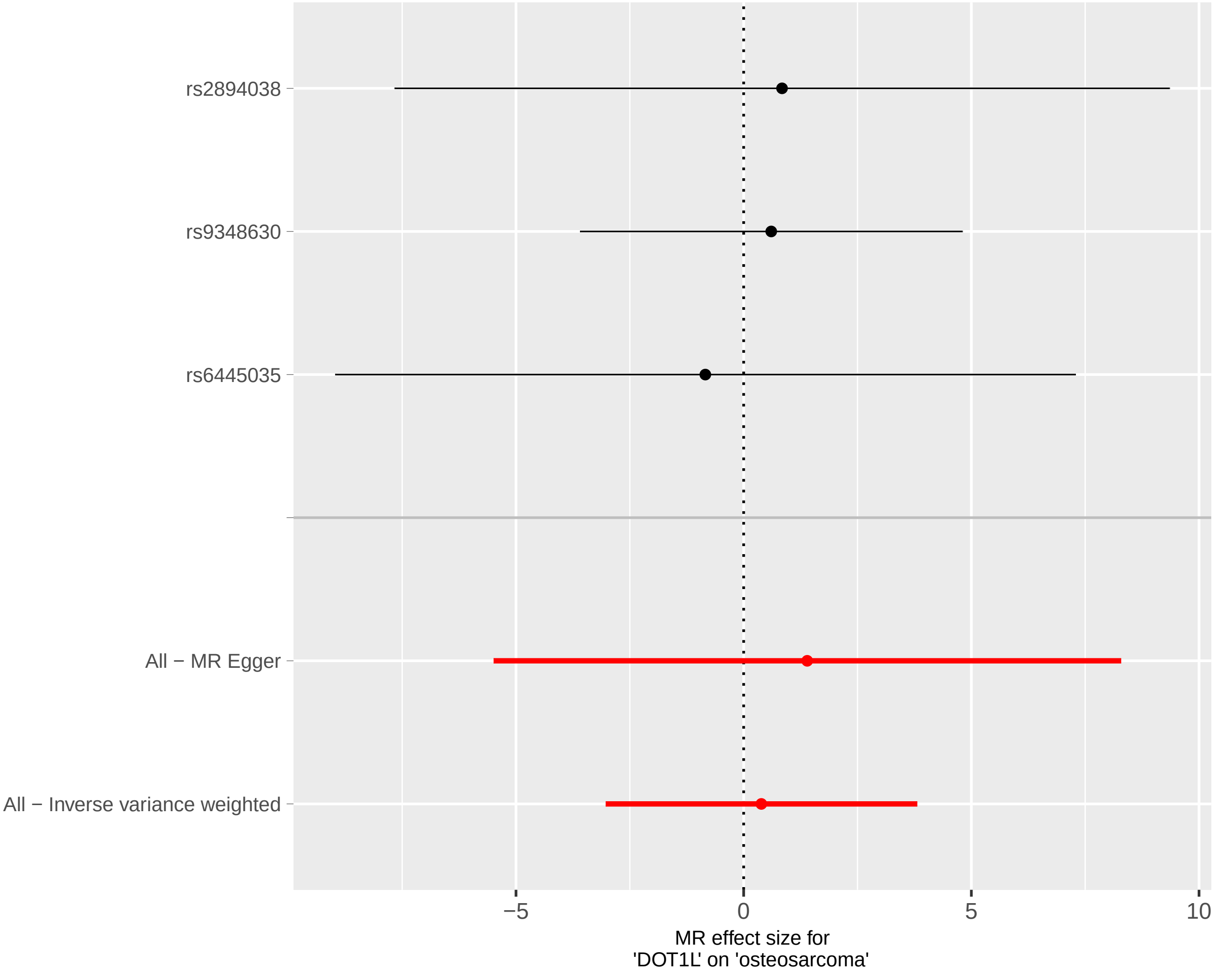

rs2894038
rs9348630
rs6445035
All − MR Egger
All − Inverse variance weighted
−5
0
5
10
MR effect size for
'DOT1L' on 'osteosarcoma'

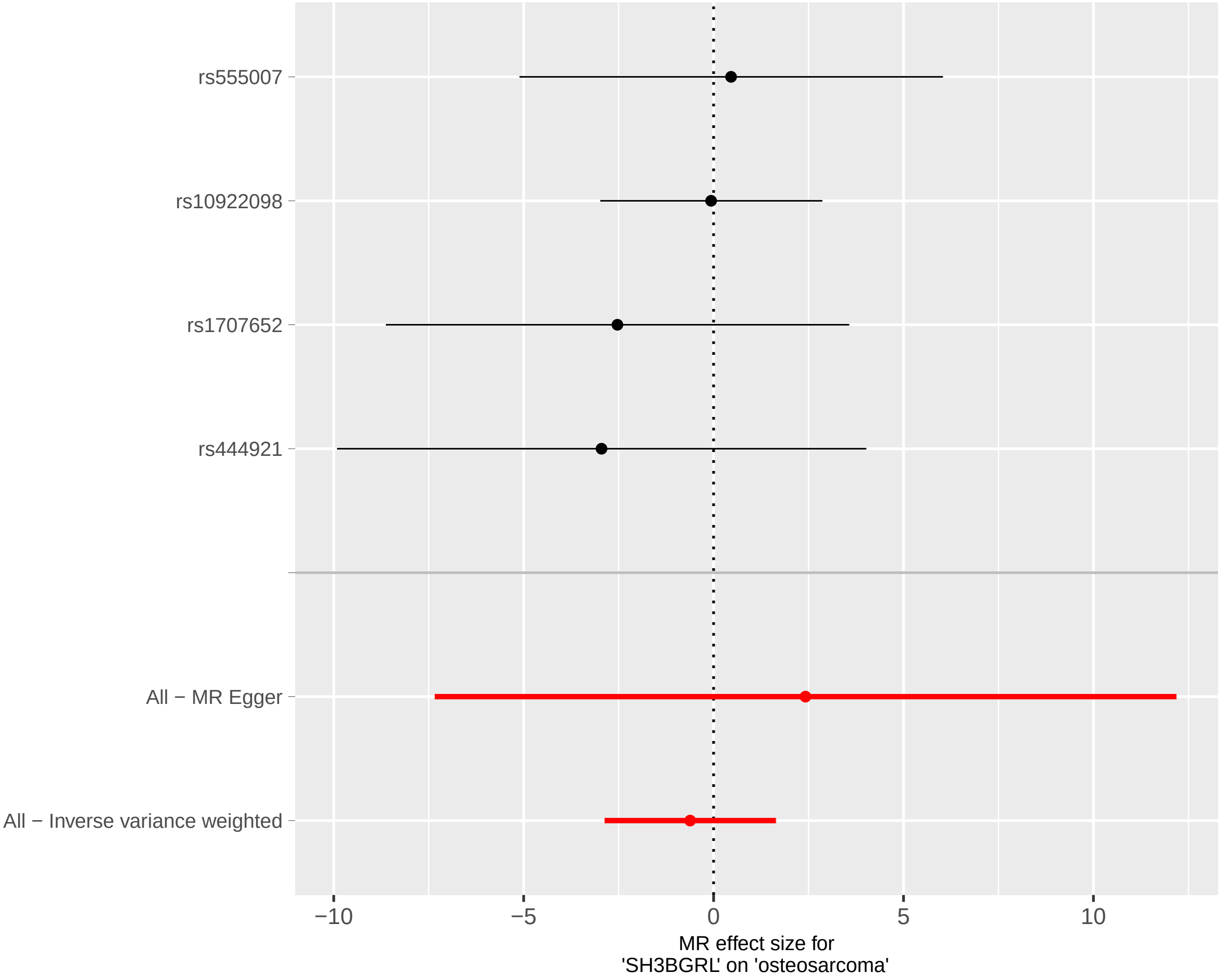

rs555007
rs10922098
rs1707652
rs444921
All − MR Egger
All − Inverse variance weighted
−10
−5
0
5
10
MR effect size for
'SH3BGRL' on 'osteosarcoma'

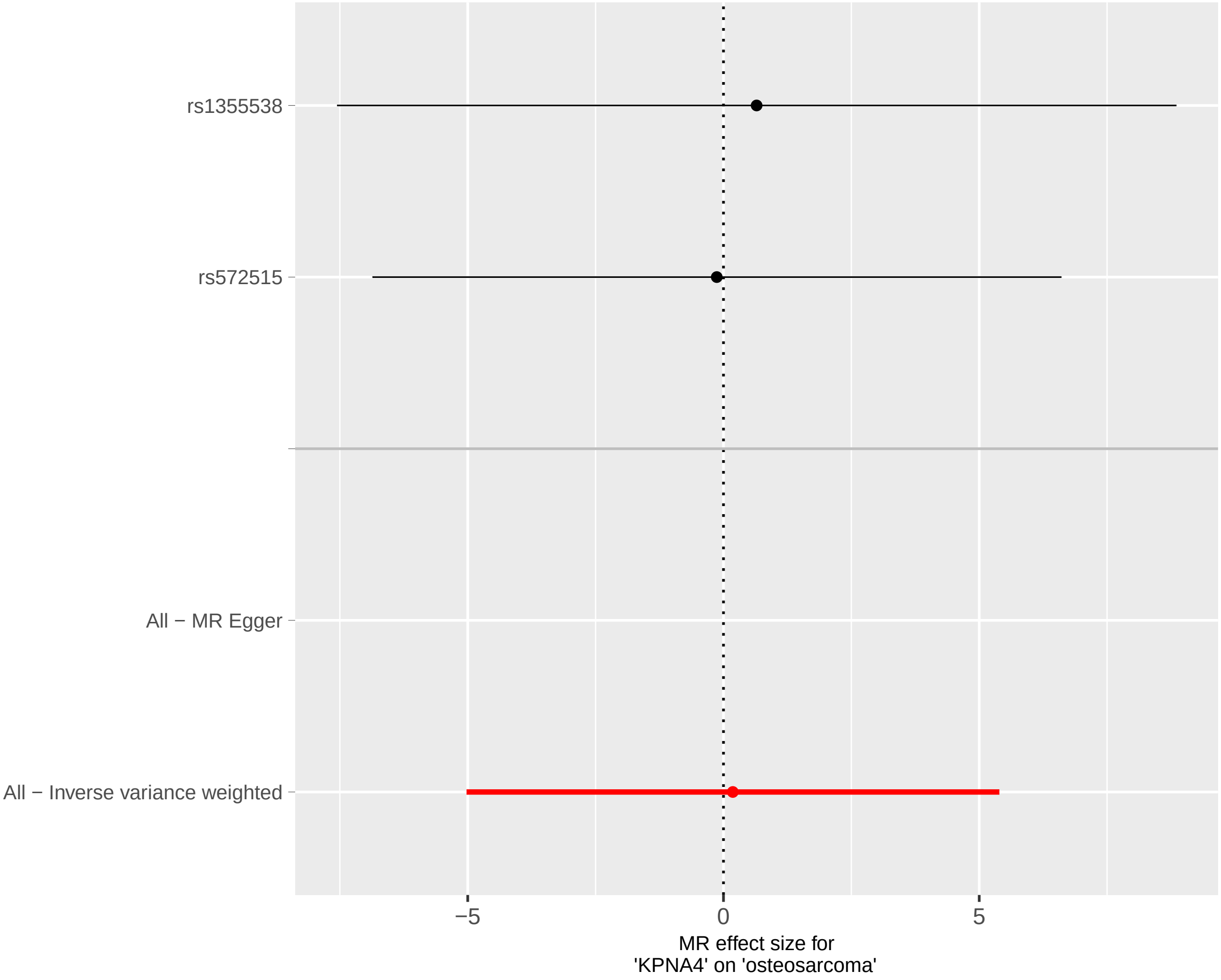

rs1355538
rs572515
All − MR Egger
All − Inverse variance weighted
−5
0
5
MR effect size for
'KPNA4' on 'osteosarcoma'

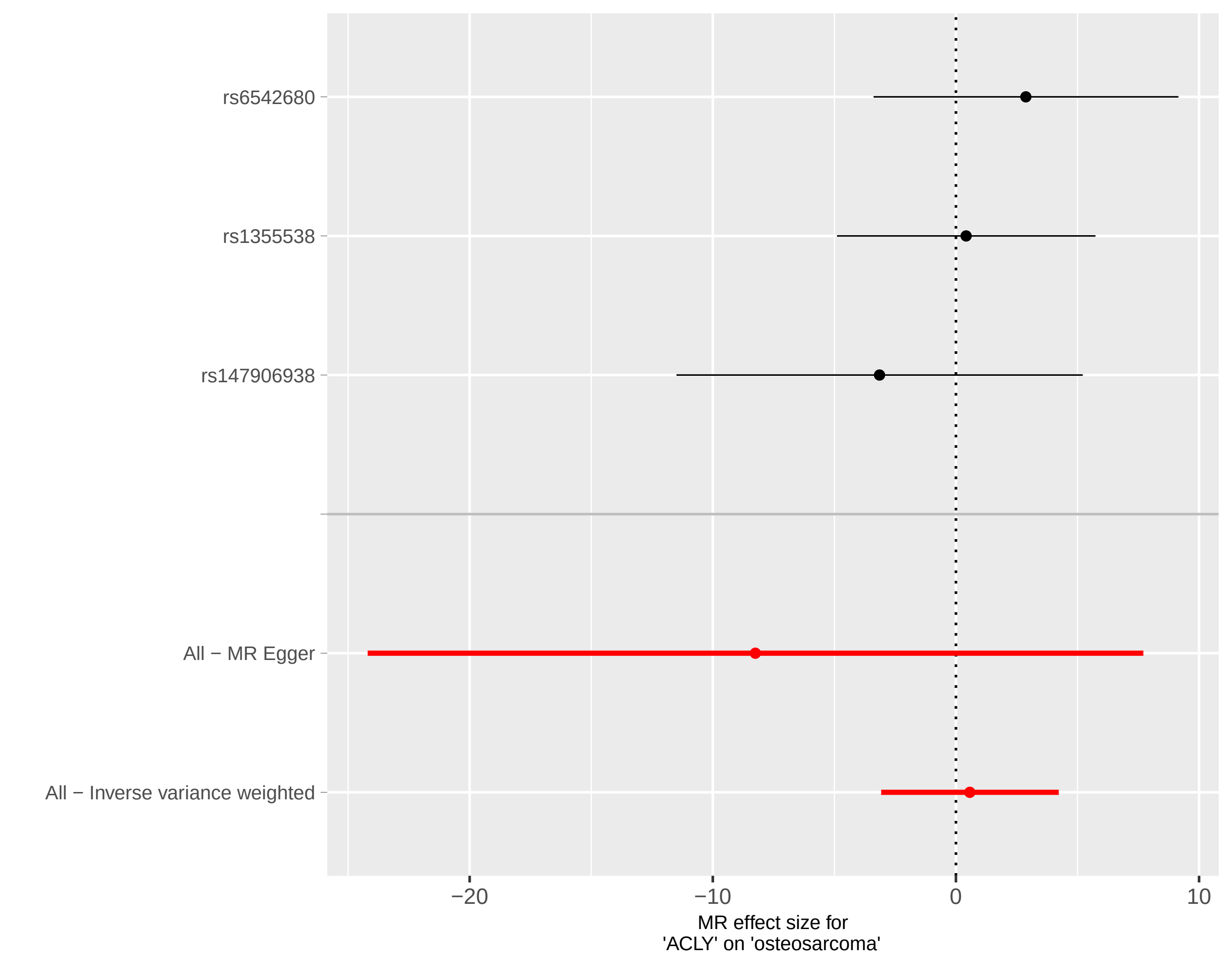
rs6542680
rs1355538
rs147906938
All − MR Egger
All − Inverse variance weighted
−20
−10
0
10
MR effect size for
'ACLY' on 'osteosarcoma'

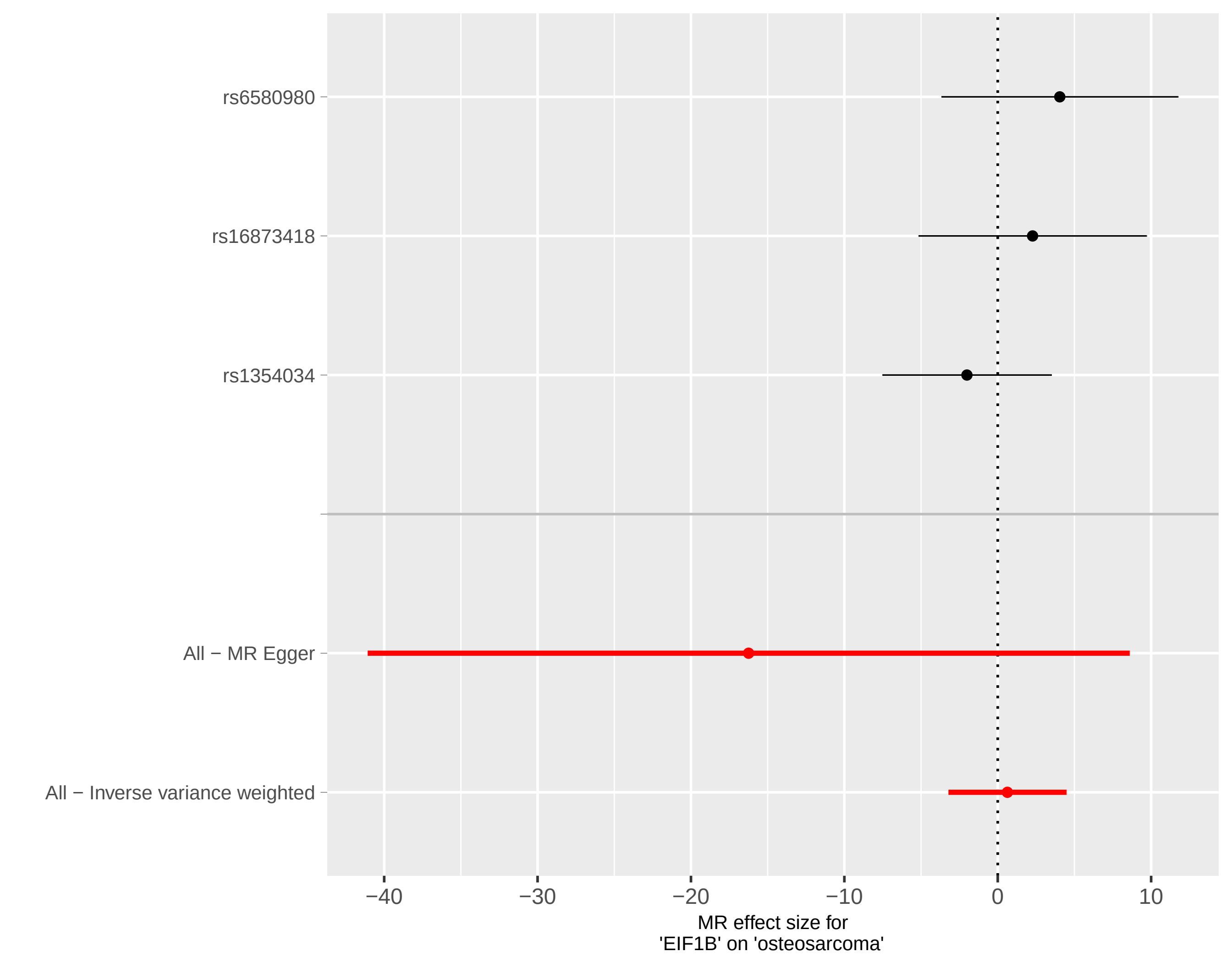

rs6580980
rs16873418
rs1354034
All − MR Egger
All − Inverse variance weighted
−40
−30
−20
−10
0
10
MR effect size for
'EIF1B' on 'osteosarcoma'

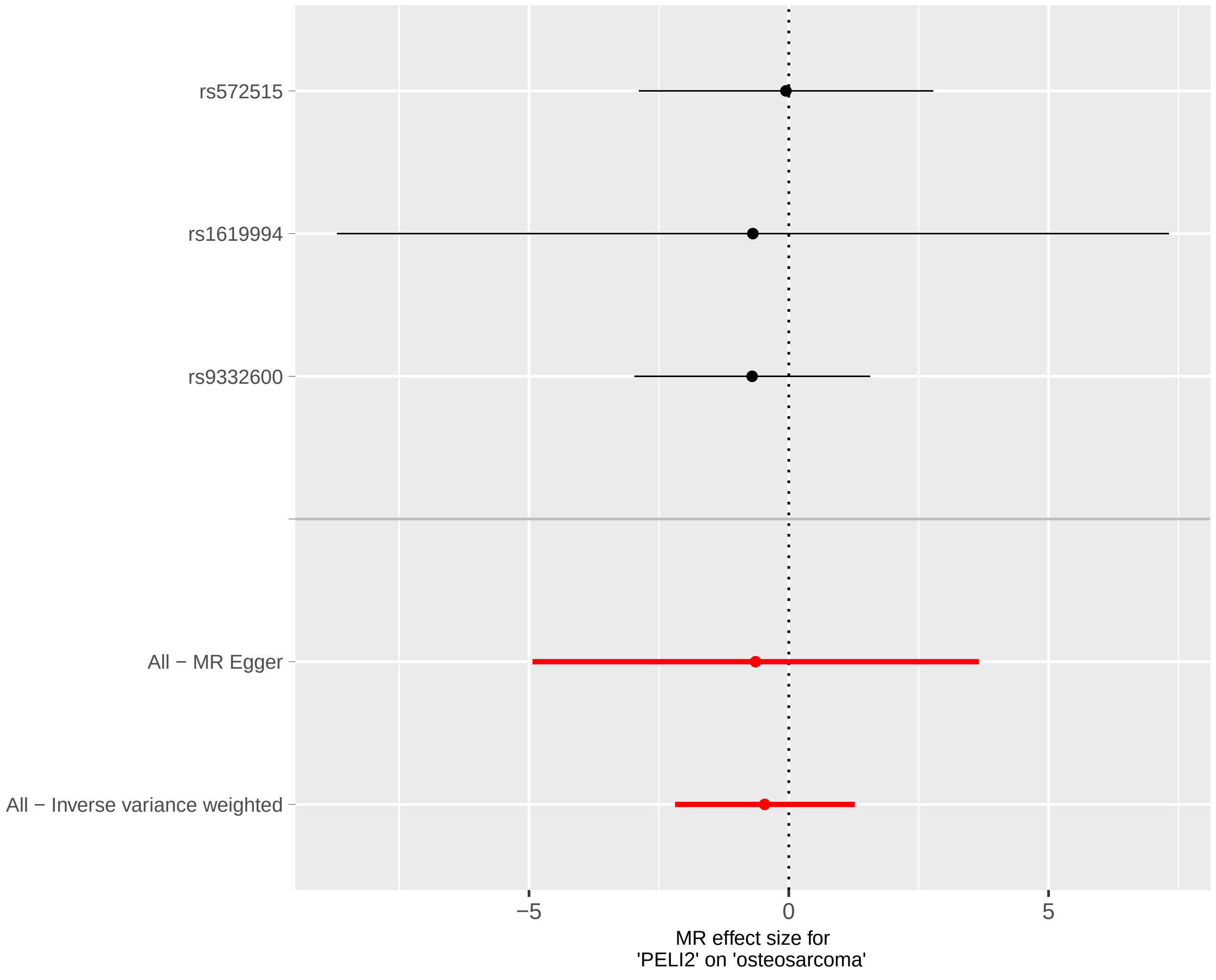
rs572515
rs1619994
rs9332600
All − MR Egger
All − Inverse variance weighted
−5
0
5
MR effect size for
'PELI2' on 'osteosarcoma'

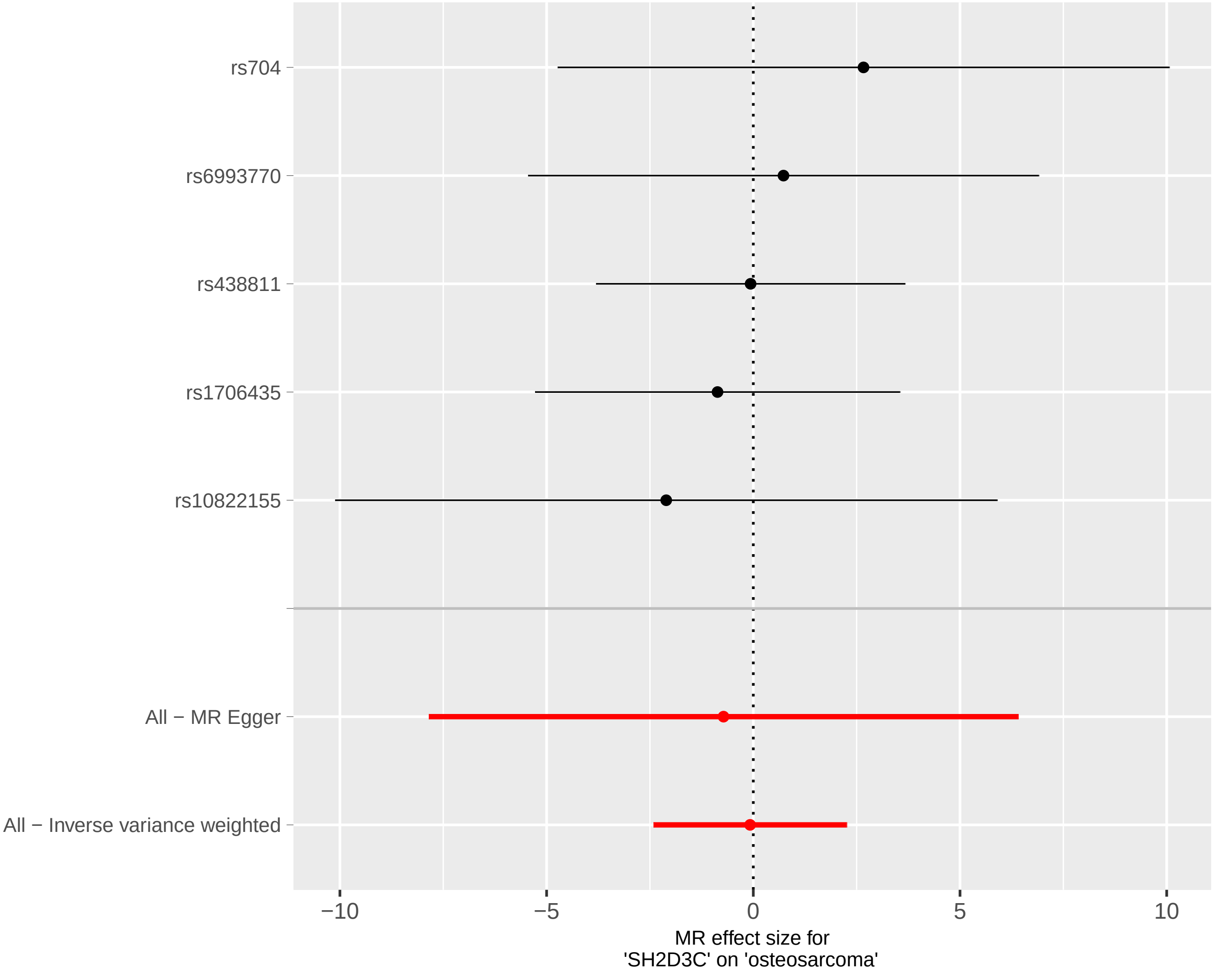
rs704
rs6993770
rs438811
rs1706435
rs10822155
All − MR Egger
All − Inverse variance weighted
−10
−5
0
5
10
MR effect size for
'SH2D3C' on 'osteosarcoma'

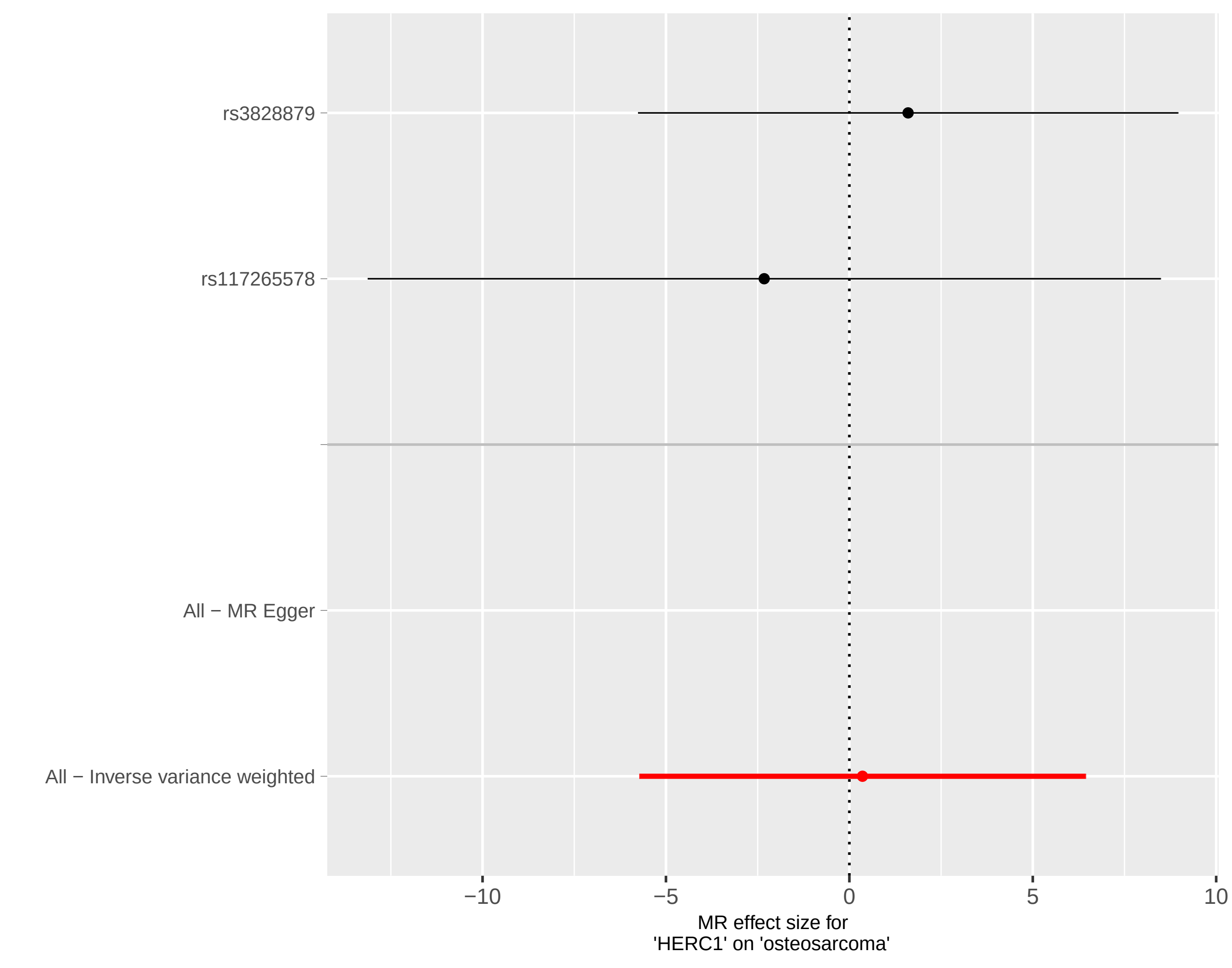
rs3828879
rs117265578
All − MR Egger
All − Inverse variance weighted
−10
−5
0
5
10
MR effect size for
'HERC1' on 'osteosarcoma'

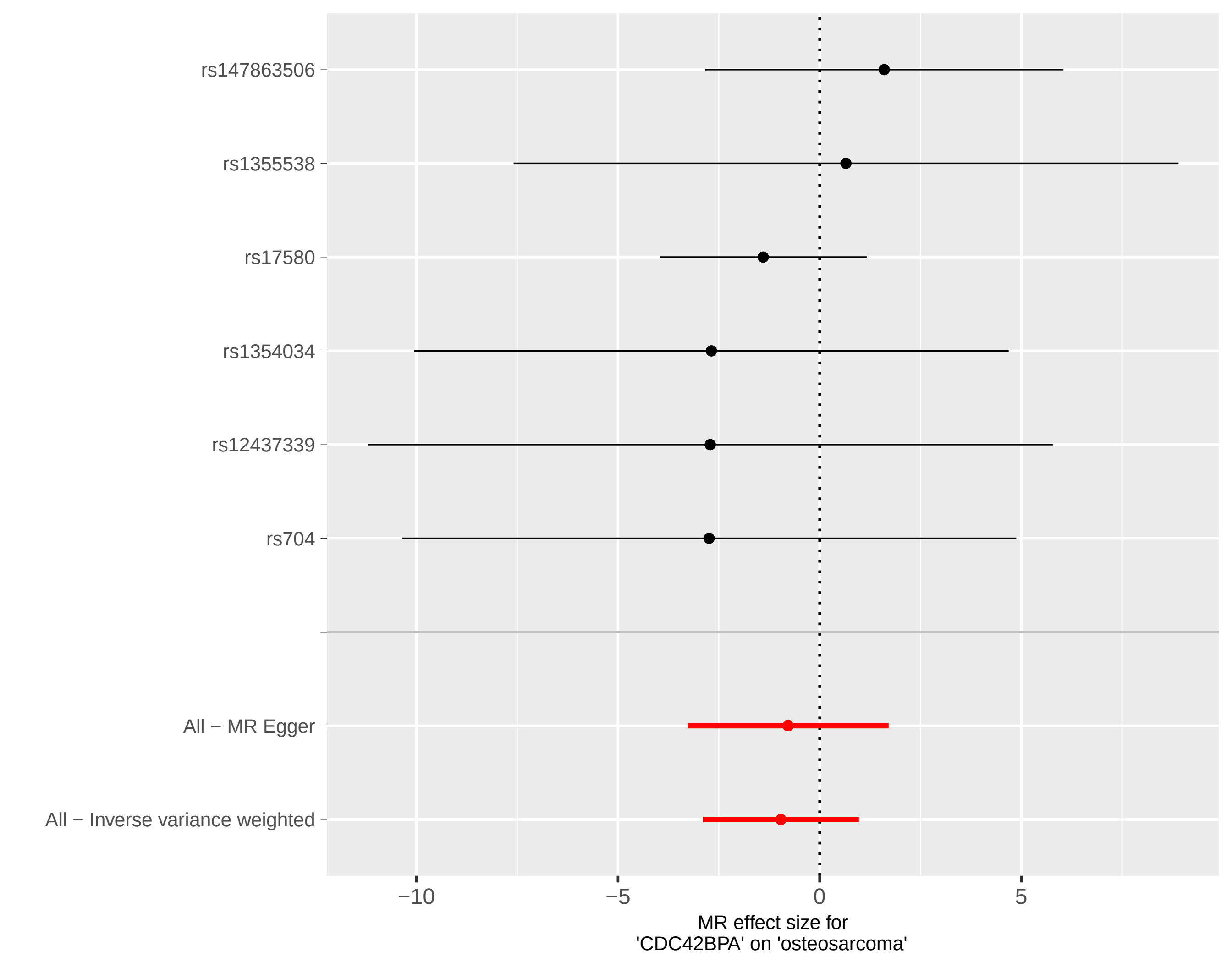

rs147863506
rs1355538
rs17580
rs1354034
rs12437339
rs704
All − MR Egger
All − Inverse variance weighted
−10
−5
0
5
MR effect size for
'CDC42BPA' on 'osteosarcoma'

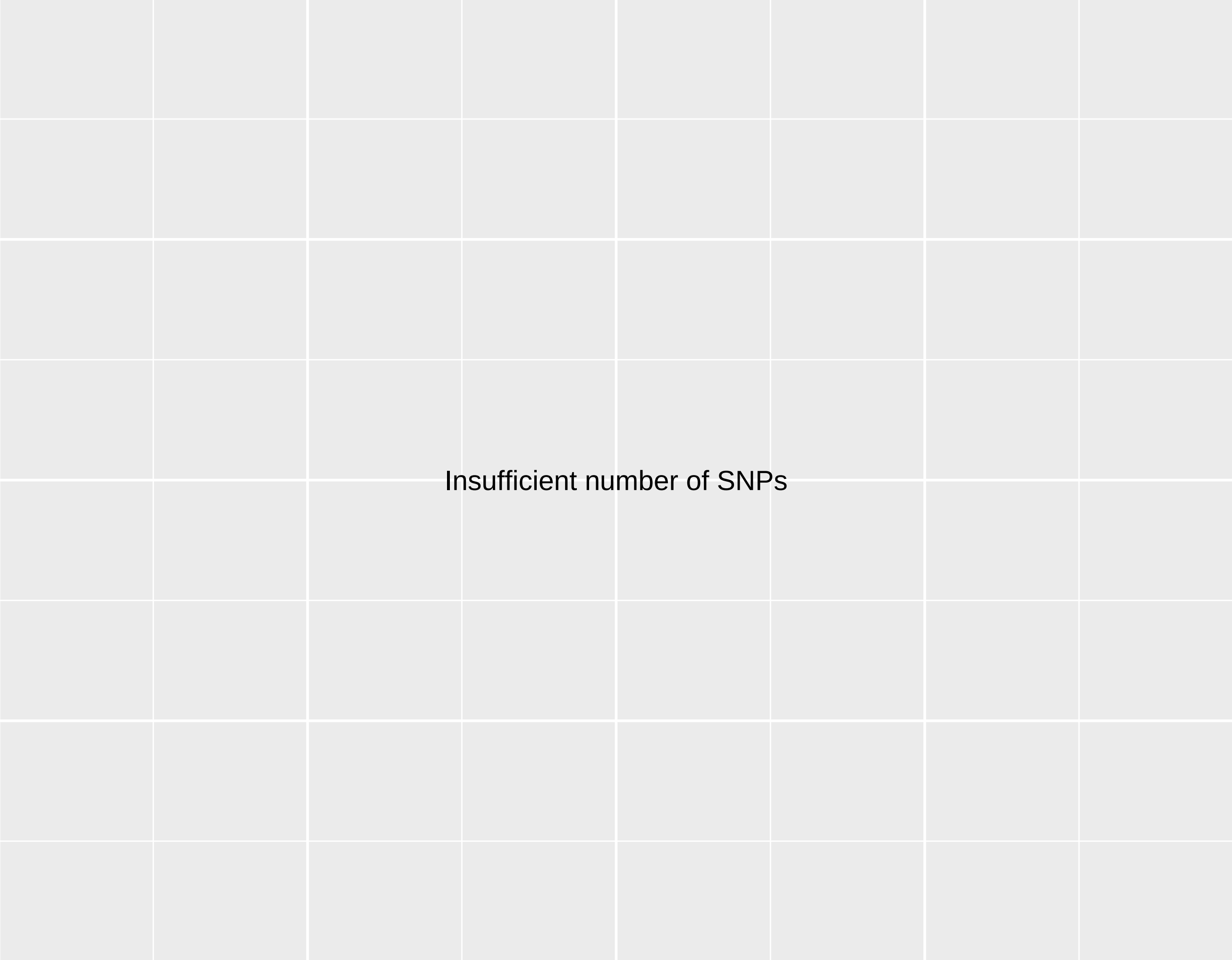
Insufficient number of SNPs

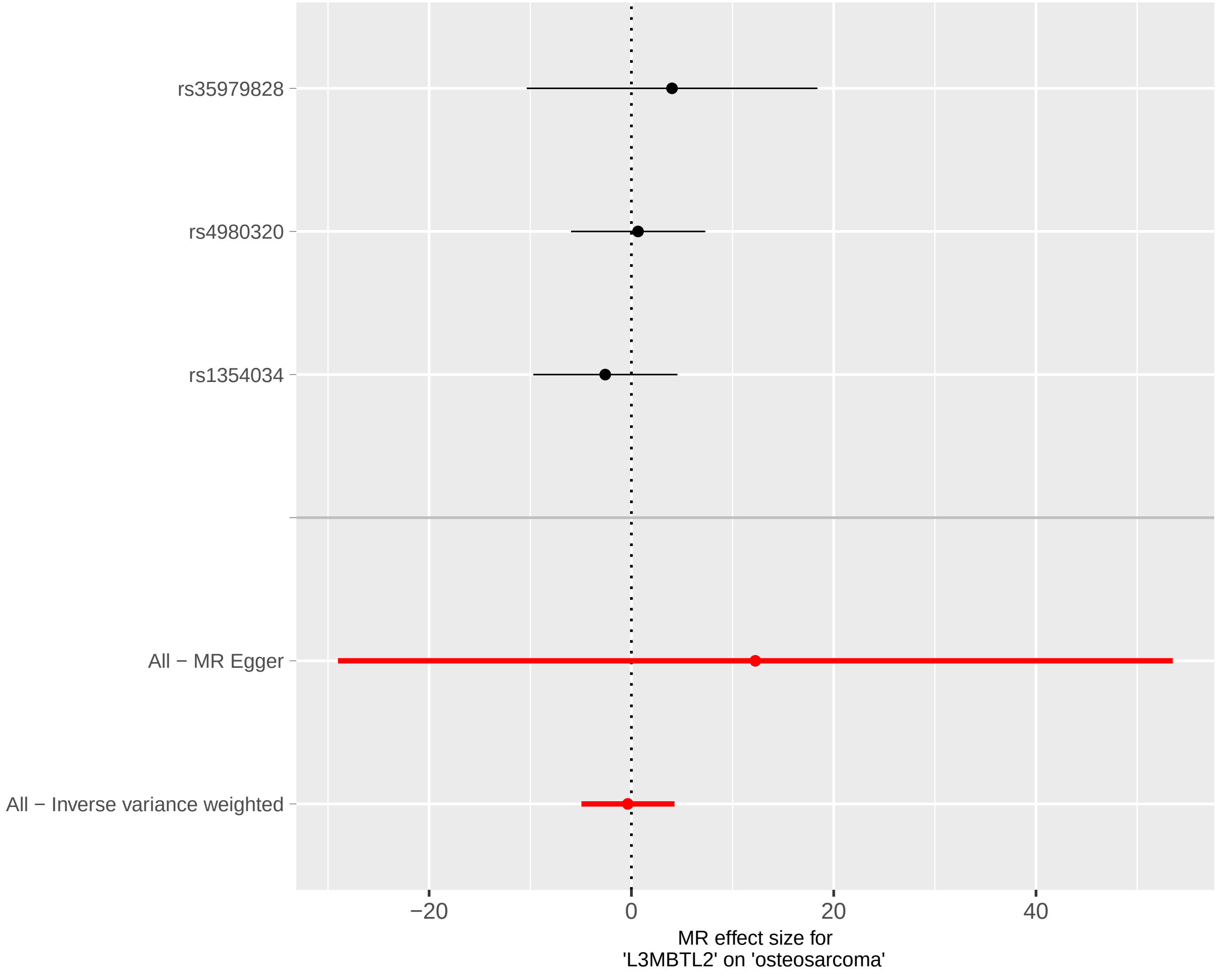

rs35979828
rs4980320
rs1354034
All − MR Egger
All − Inverse variance weighted
−20
0
20
40
MR effect size for
'L3MBTL2' on 'osteosarcoma'

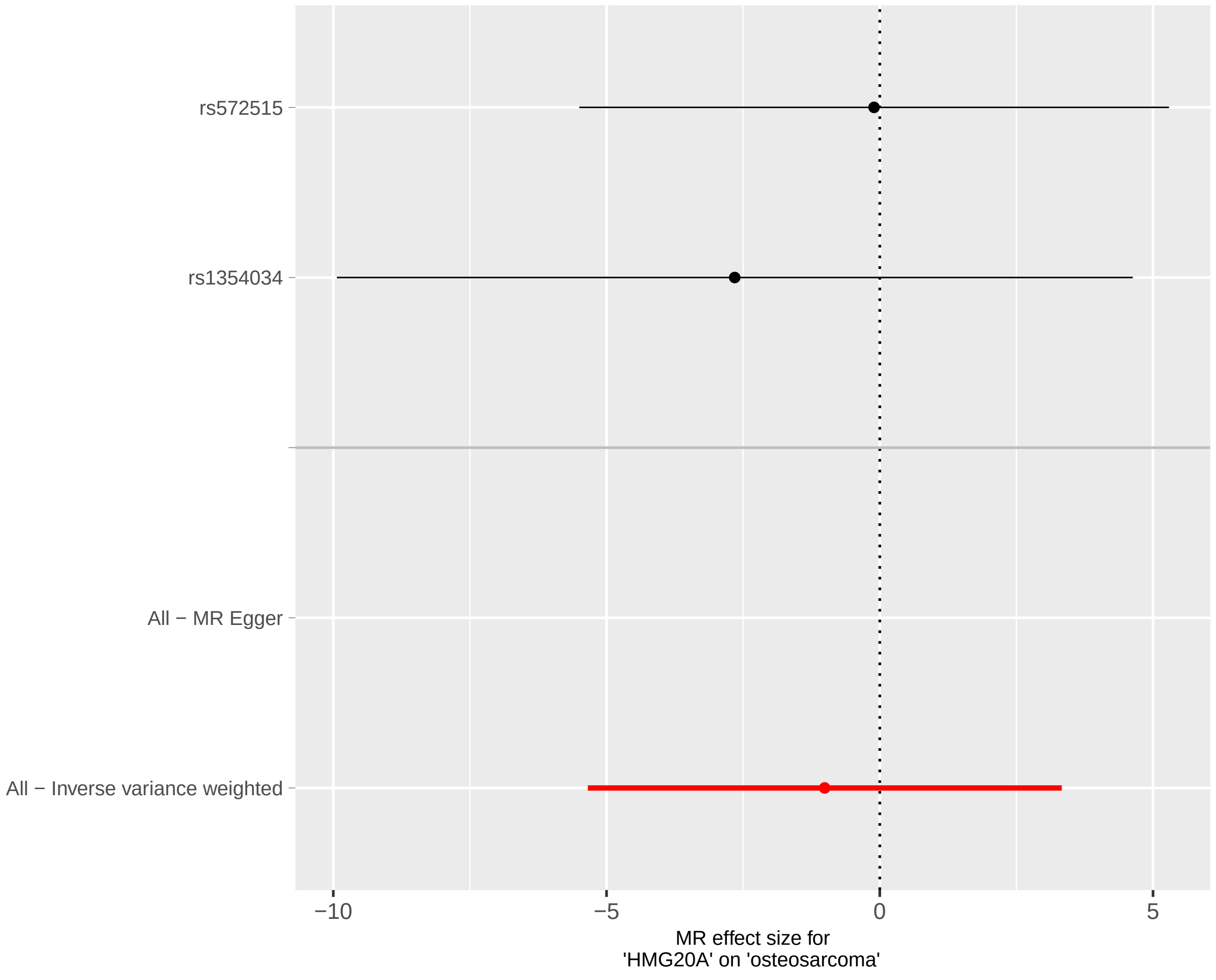

rs572515
rs1354034
All − MR Egger
All − Inverse variance weighted
−10
−5
0
5
MR effect size for
'HMG20A' on 'osteosarcoma'

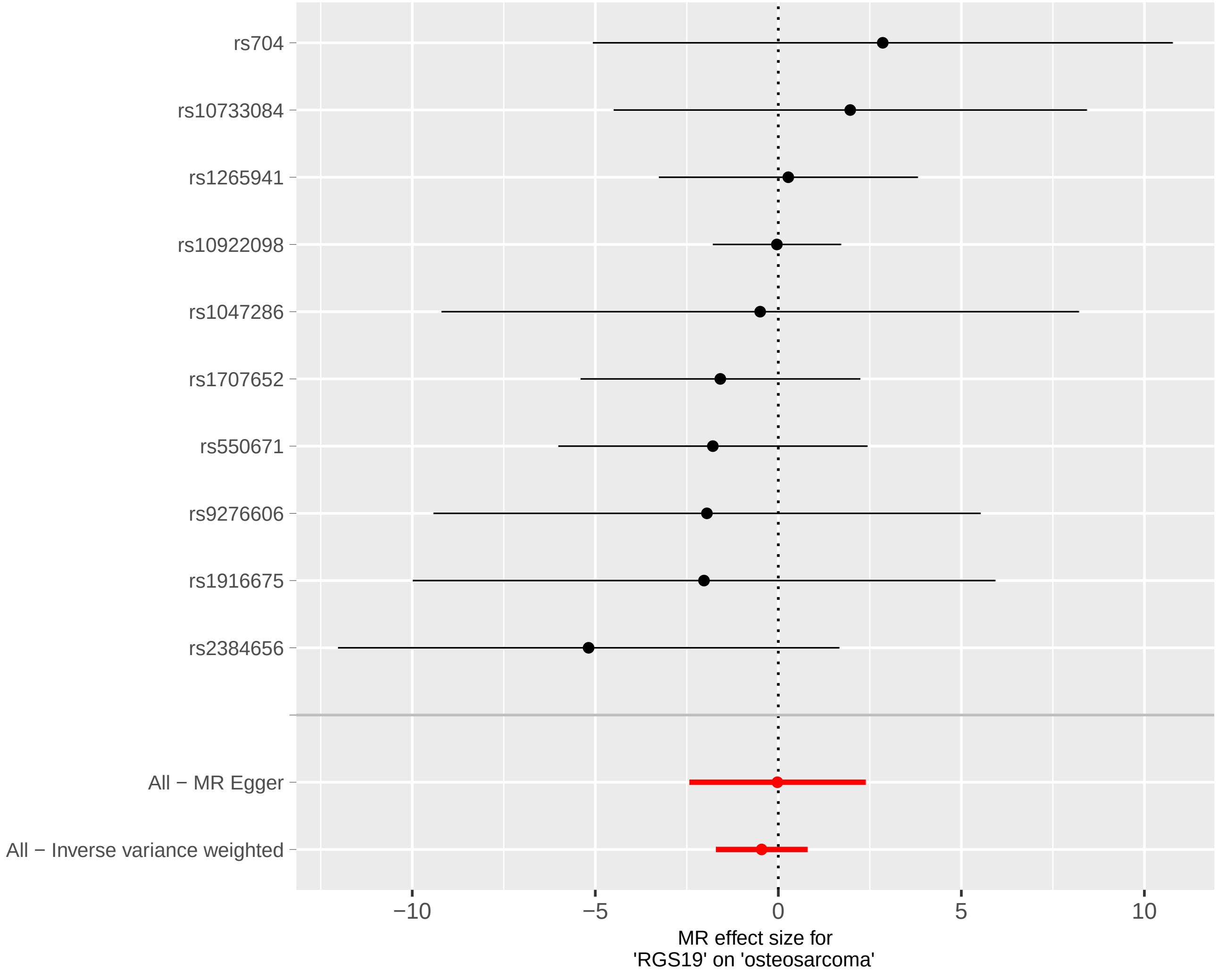

rs704
rs10733084
rs1265941
rs10922098
rs1047286
rs1707652
rs550671
rs9276606
rs1916675
rs2384656
All − MR Egger
All − Inverse variance weighted
−10
−5
0
5
10
MR effect size for
'RGS19' on 'osteosarcoma'

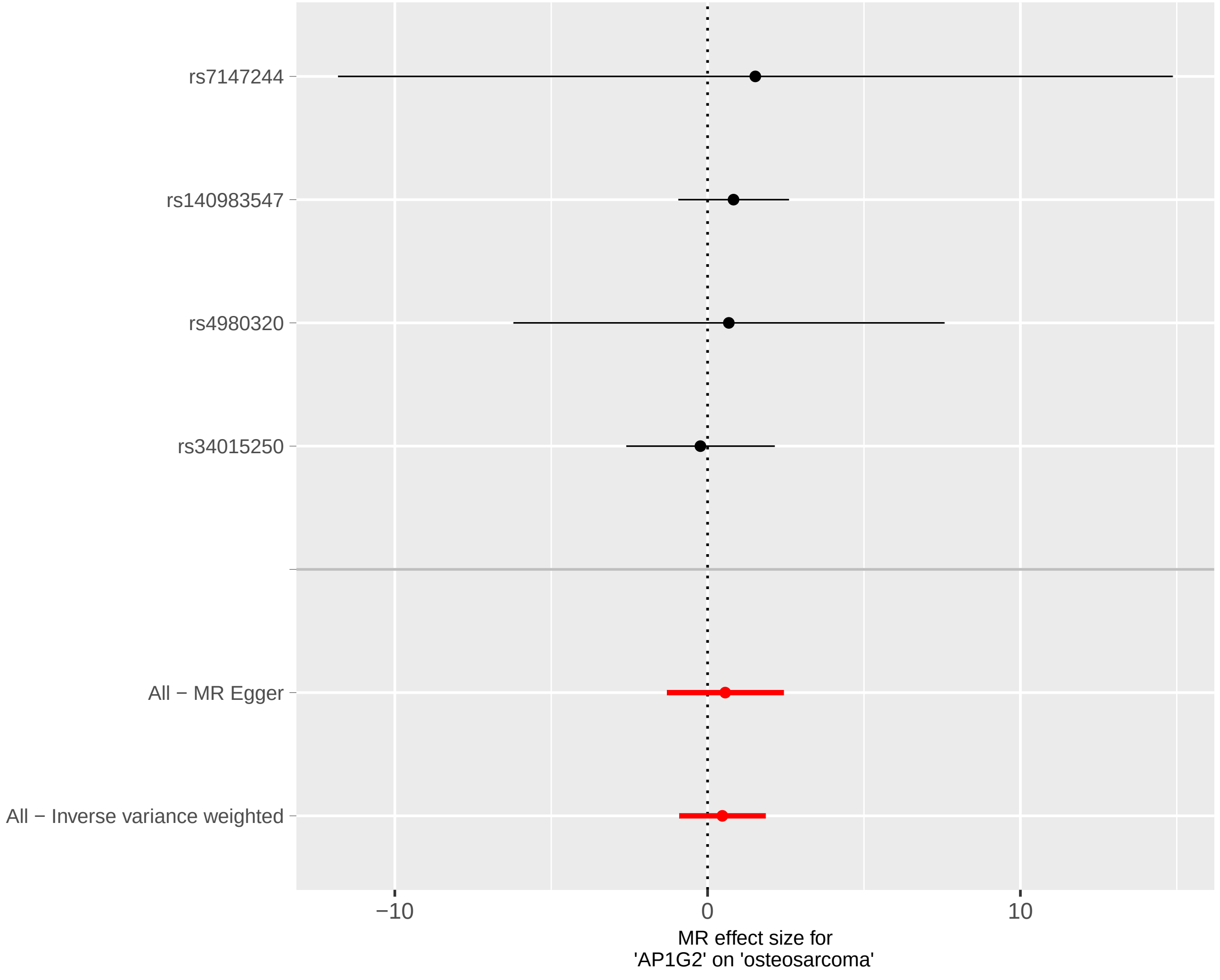
rs7147244
rs140983547
rs4980320
rs34015250
All − MR Egger
All − Inverse variance weighted
−10
0
10
MR effect size for
'AP1G2' on 'osteosarcoma'

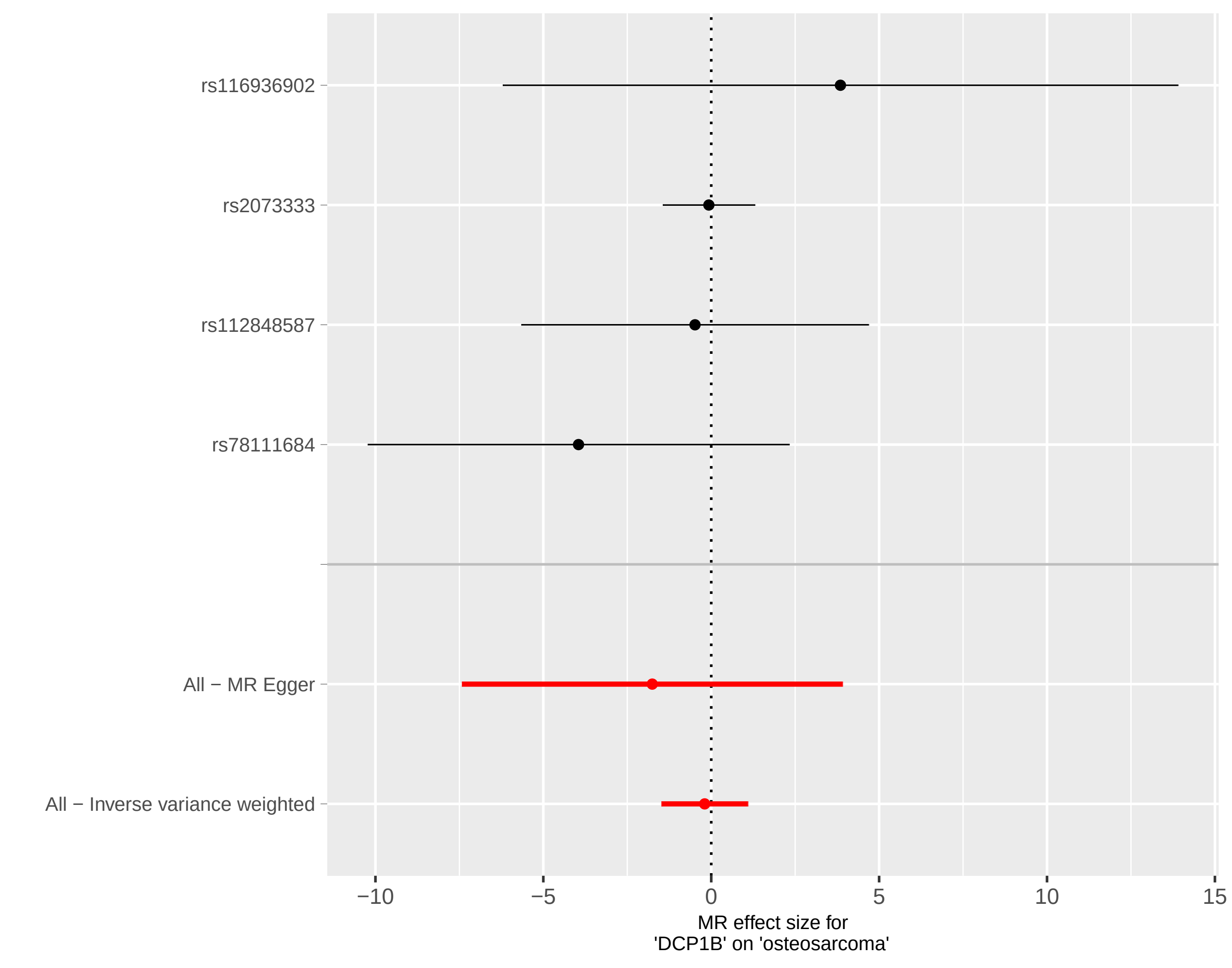
rs116936902
rs2073333
rs112848587
rs78111684
All − MR Egger
All − Inverse variance weighted
−10
−5
0
5
10
15
MR effect size for
'DCP1B' on 'osteosarcoma'

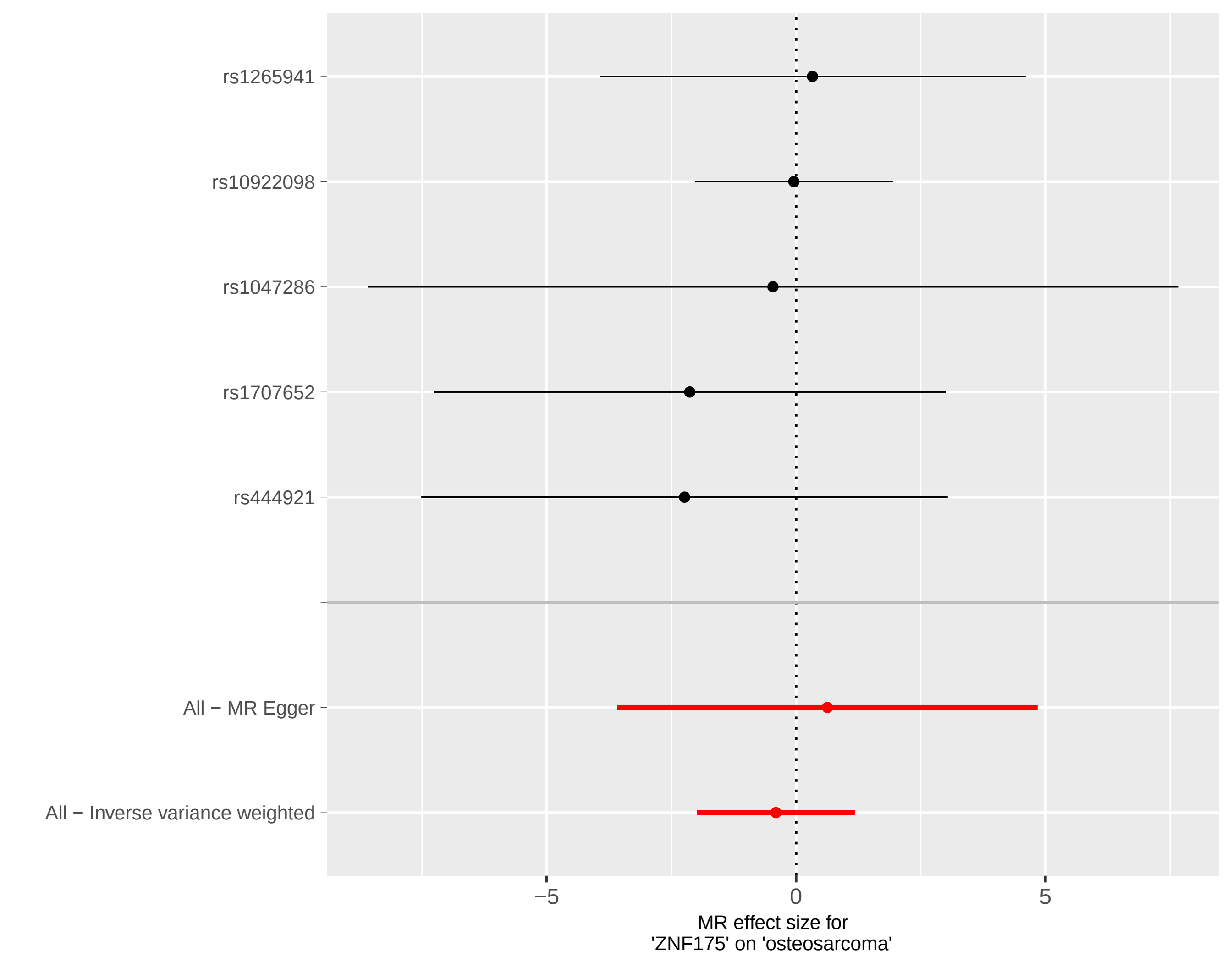
rs1265941
rs10922098
rs1047286
rs1707652
rs444921
All − MR Egger
All − Inverse variance weighted
−5
0
5
MR effect size for
'ZNF175' on 'osteosarcoma'

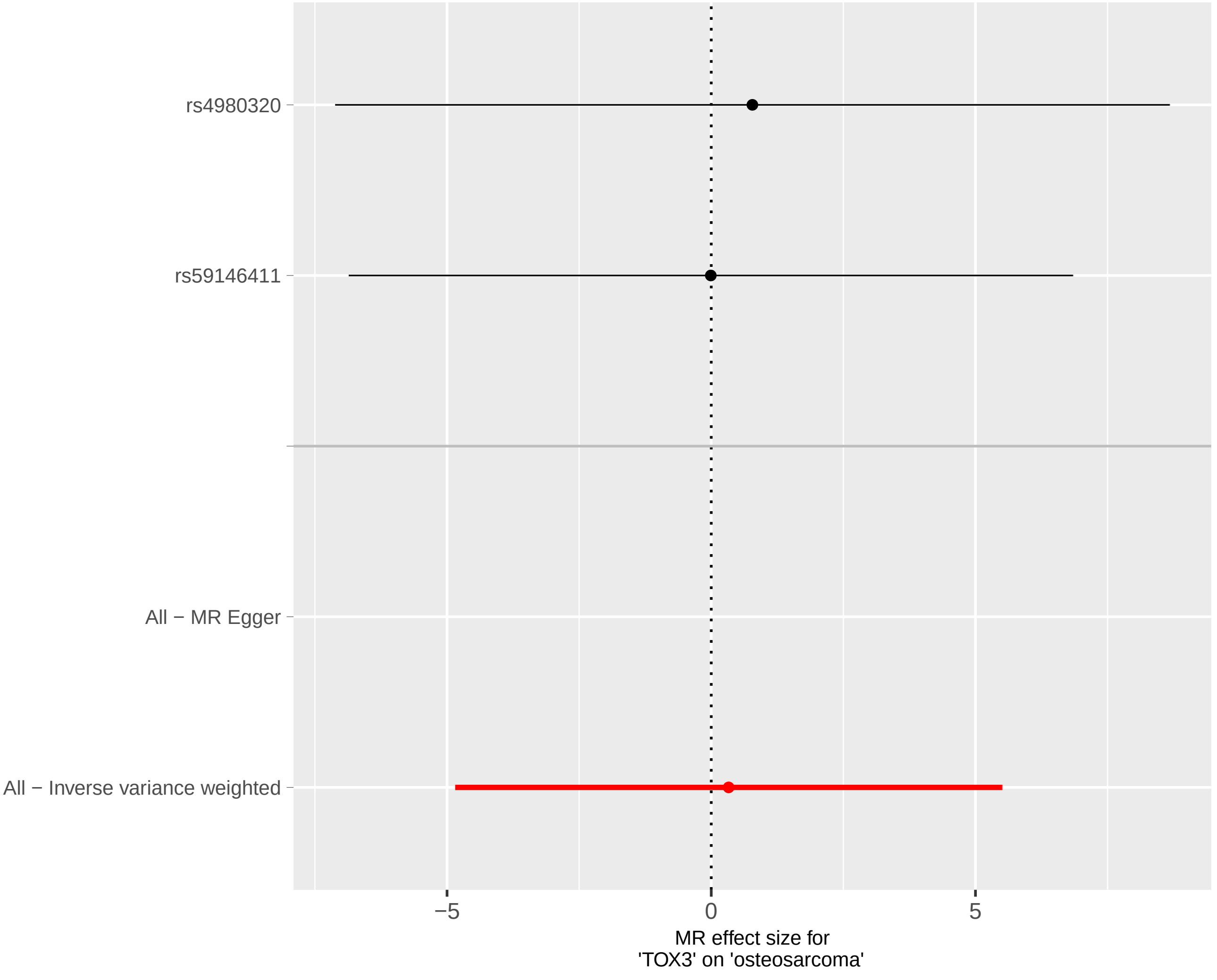

rs4980320
rs59146411
All − MR Egger
All − Inverse variance weighted
−5
0
5
MR effect size for
'TOX3' on 'osteosarcoma'

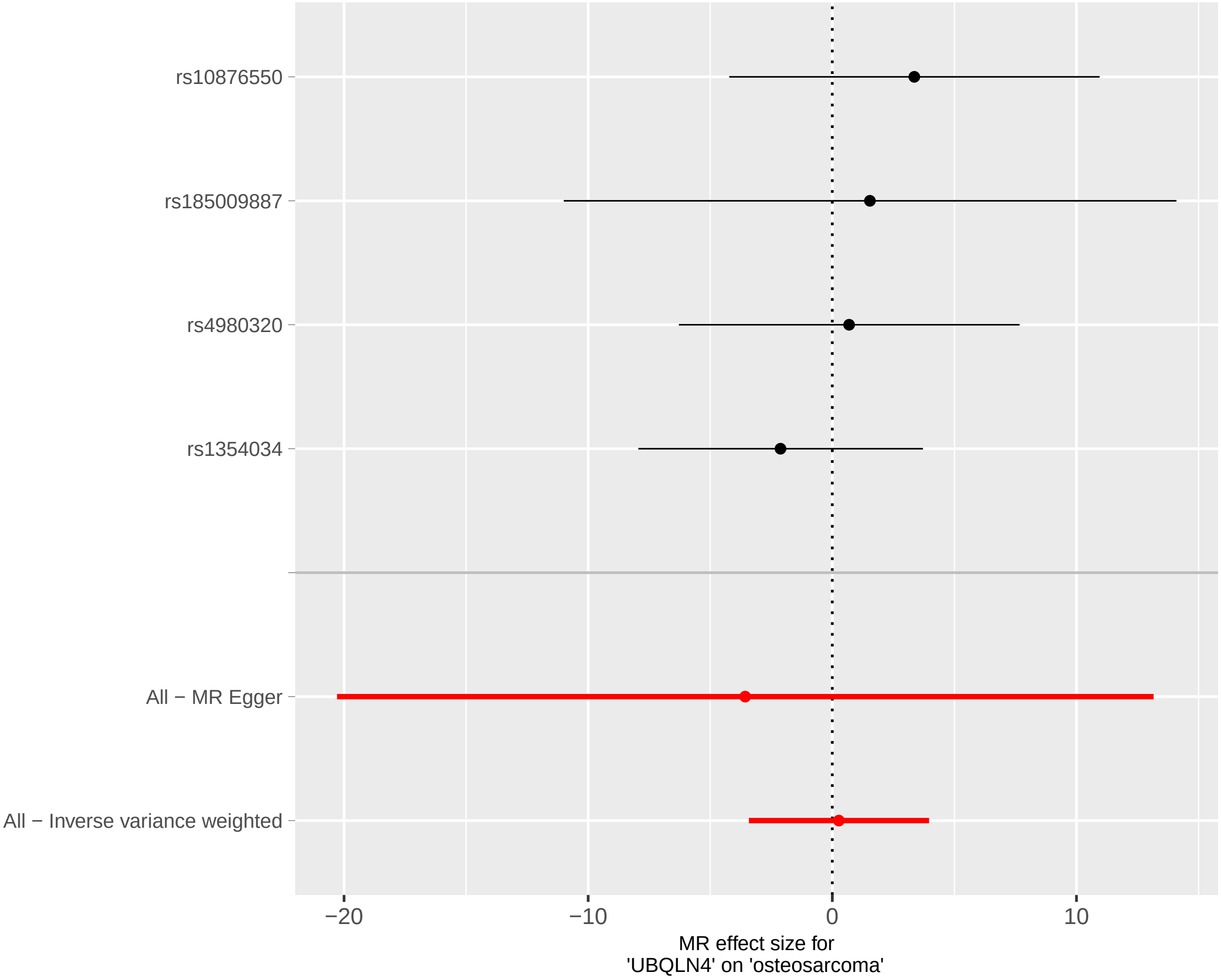
rs10876550
rs185009887
rs4980320
rs1354034
All − MR Egger
All − Inverse variance weighted
−20
−10
0
10
MR effect size for
'UBQLN4' on 'osteosarcoma'

Insufficient number of SNPs

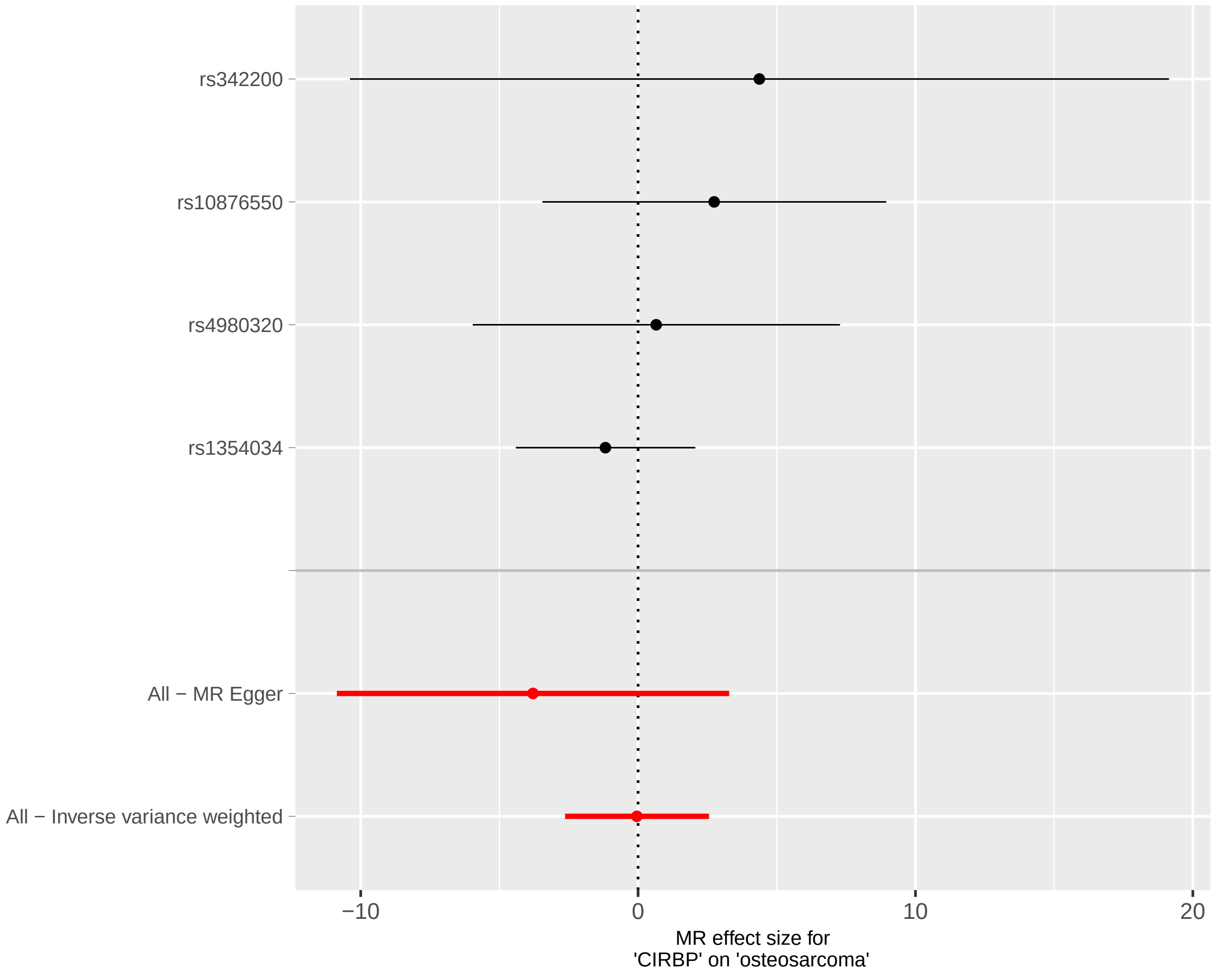

rs342200
rs10876550
rs4980320
rs1354034
All − MR Egger
All − Inverse variance weighted
−10
0
10
20
MR effect size for
'CIRBP' on 'osteosarcoma'

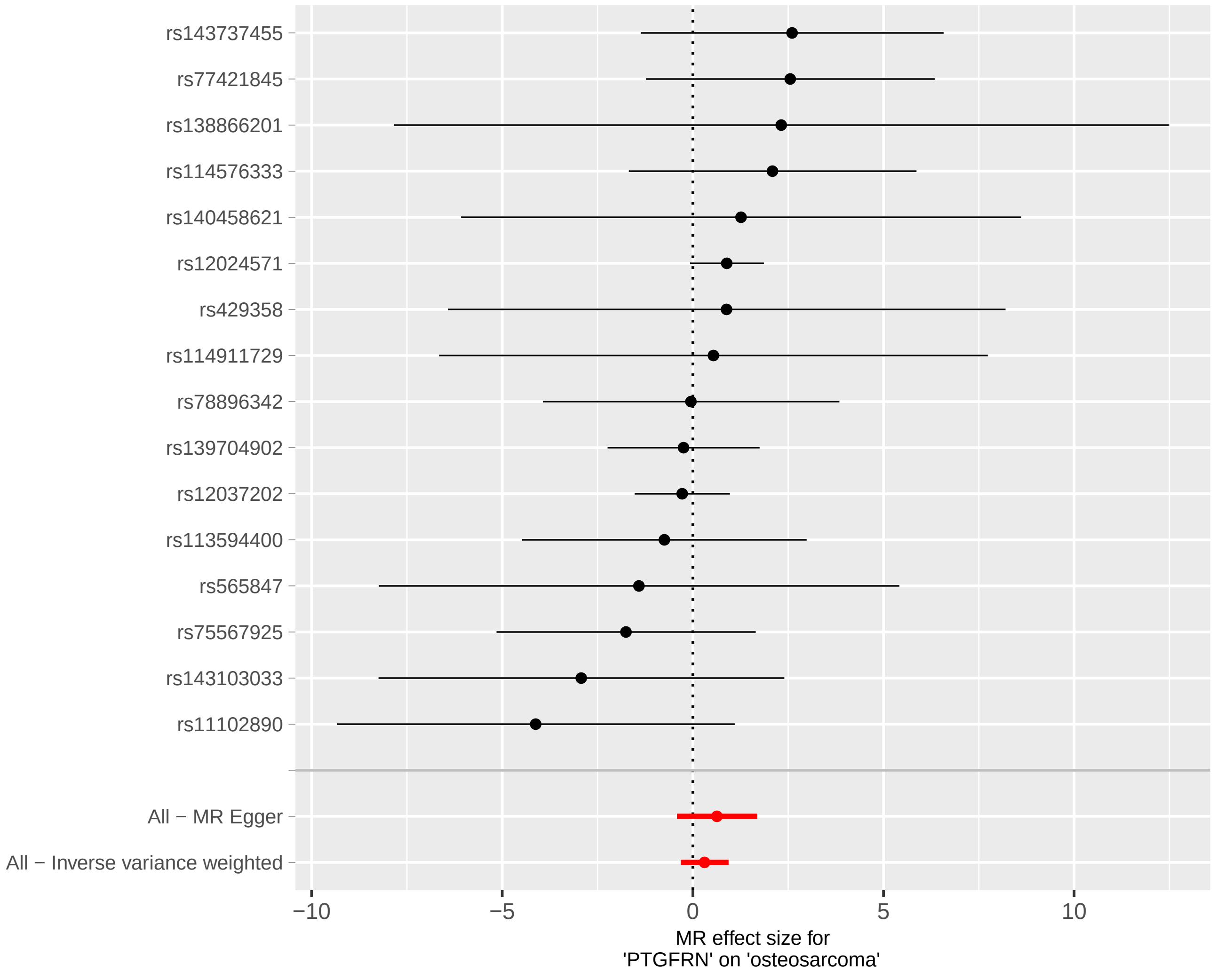
rs143737455
rs77421845
rs138866201
rs114576333
rs140458621
rs12024571
rs429358
rs114911729
rs78896342
rs139704902
rs12037202
rs113594400
rs565847
rs75567925
rs143103033
rs11102890
All − MR Egger
All − Inverse variance weighted
−10
−5
0
5
10
MR effect size for
'PTGFRN' on 'osteosarcoma'

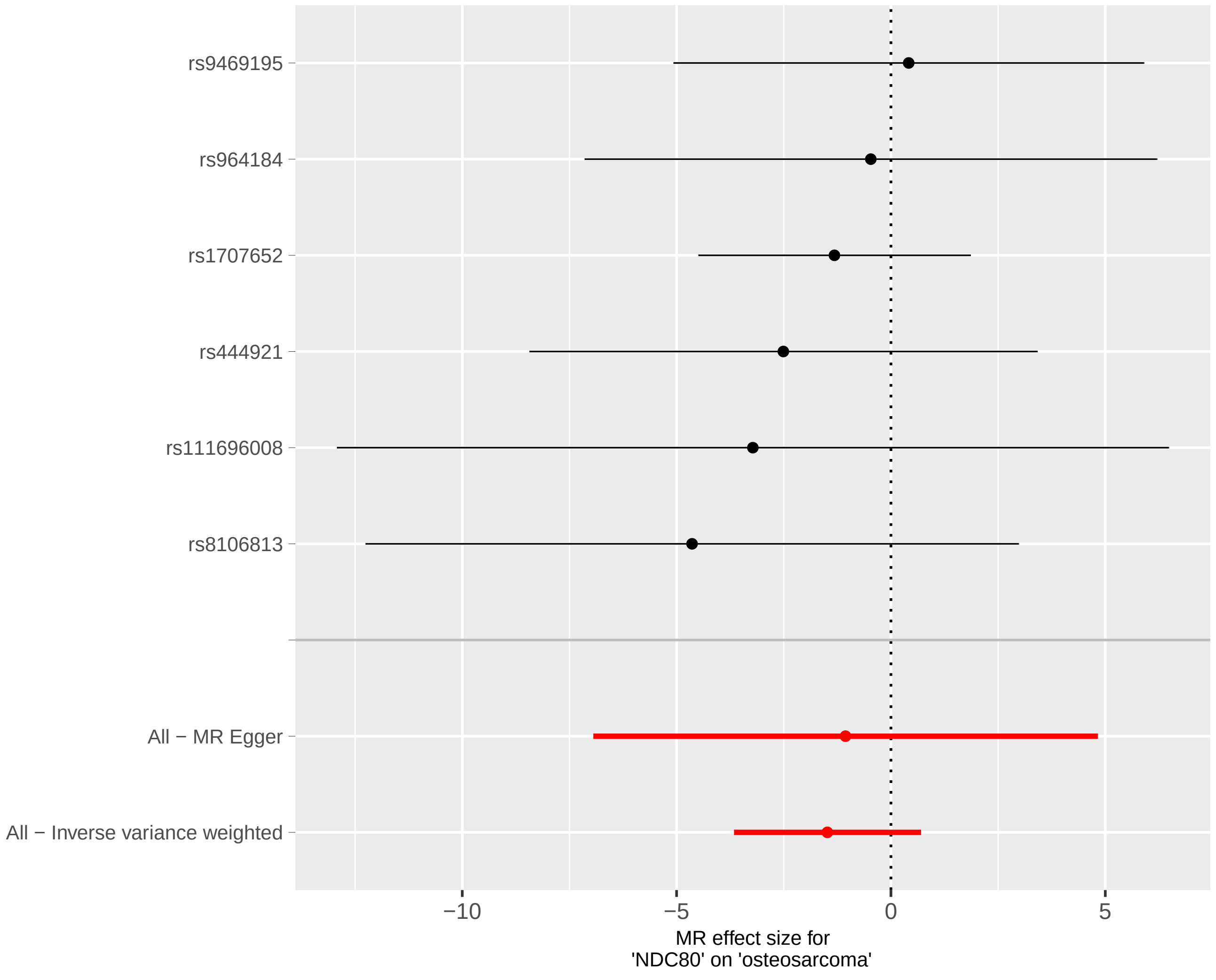
rs9469195
rs964184
rs1707652
rs444921
rs111696008
rs8106813
All − MR Egger
All − Inverse variance weighted
−10
−5
0
5
MR effect size for
'NDC80' on 'osteosarcoma'

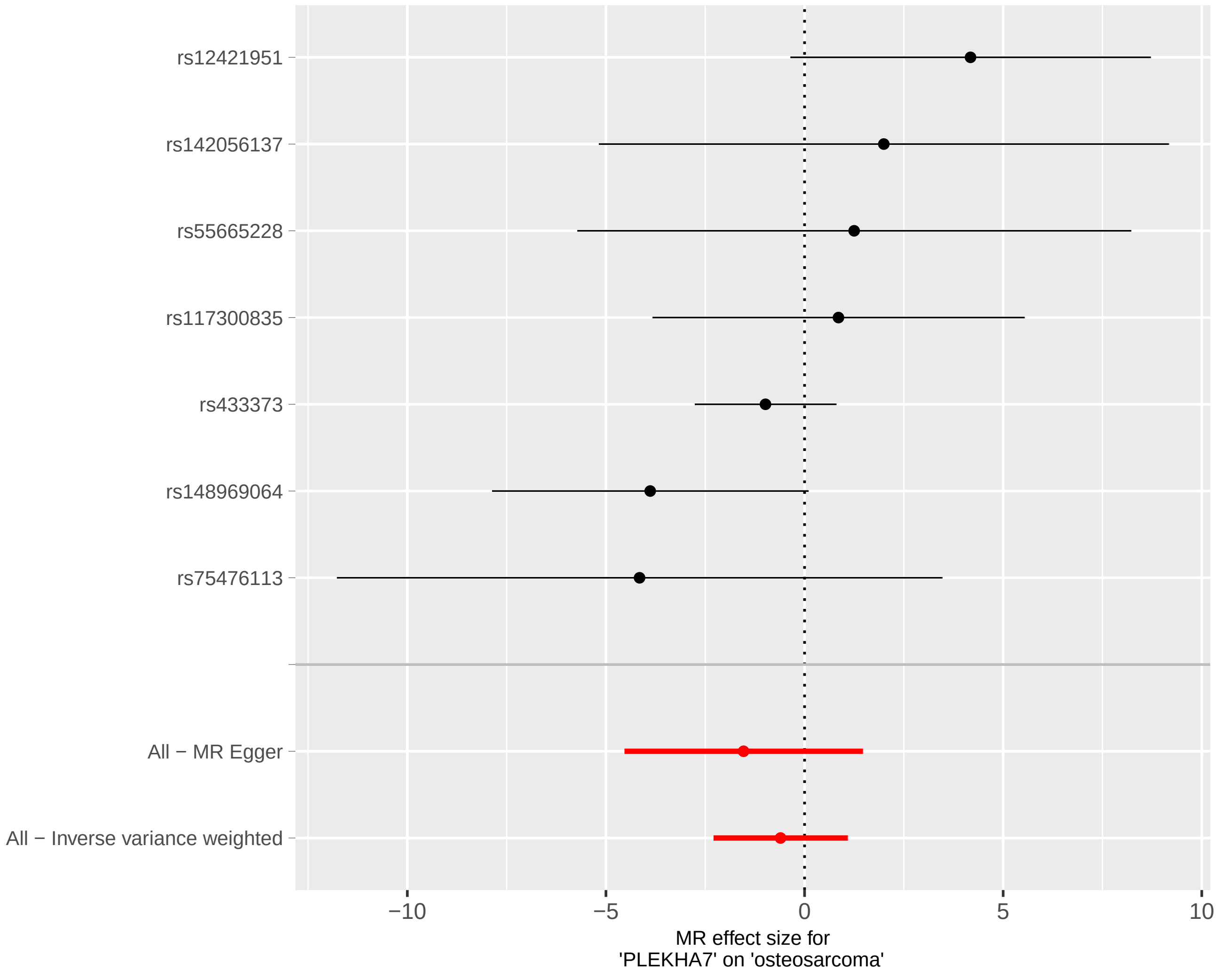

rs12421951
rs142056137
rs55665228
rs117300835
rs433373
rs148969064
rs75476113
All − MR Egger
All − Inverse variance weighted
−10
−5
0
5
10
MR effect size for
'PLEKHA7' on 'osteosarcoma'

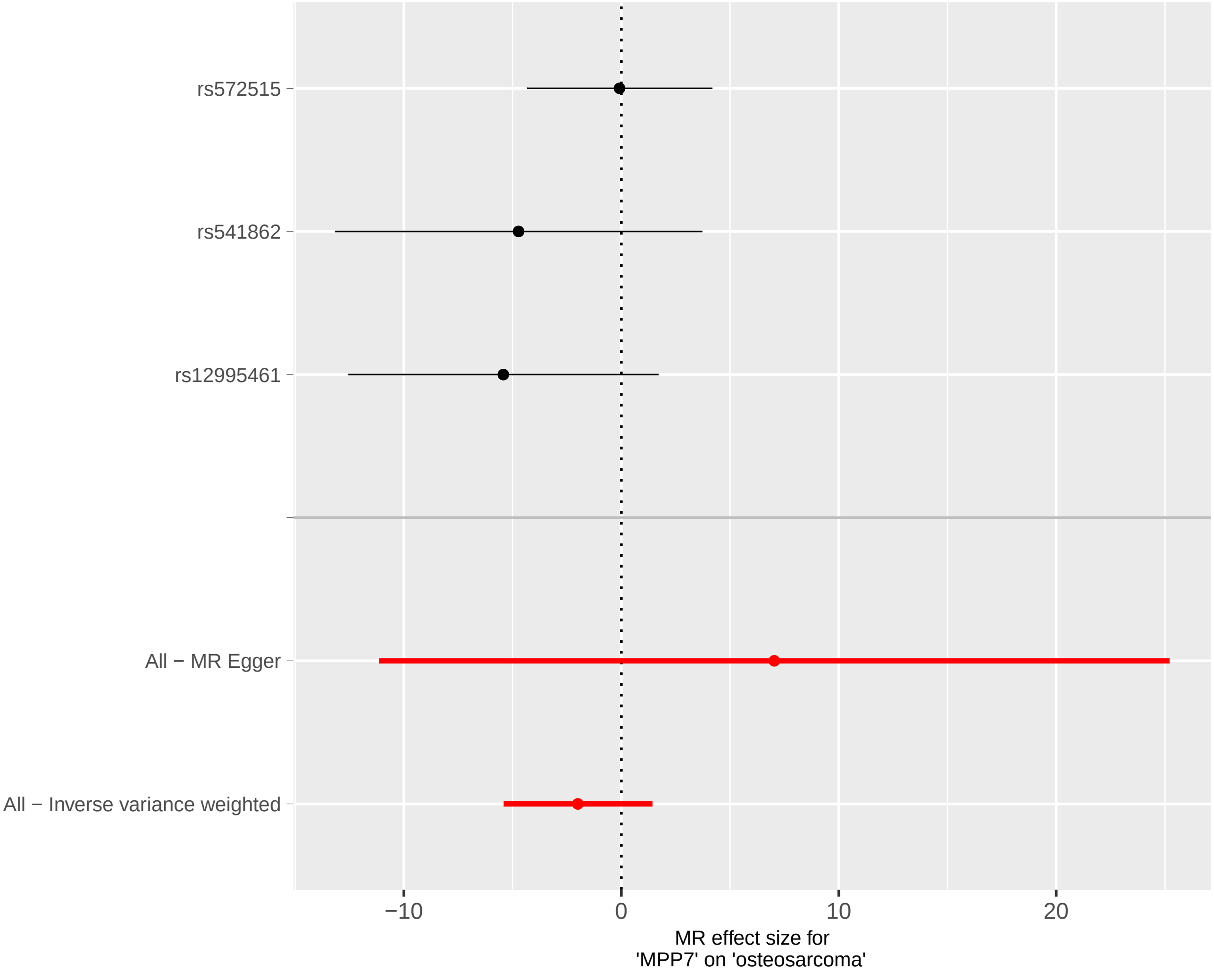
rs572515
rs541862
rs12995461
All − MR Egger
All − Inverse variance weighted
−10
0
10
20
MR effect size for
'MPP7' on 'osteosarcoma'

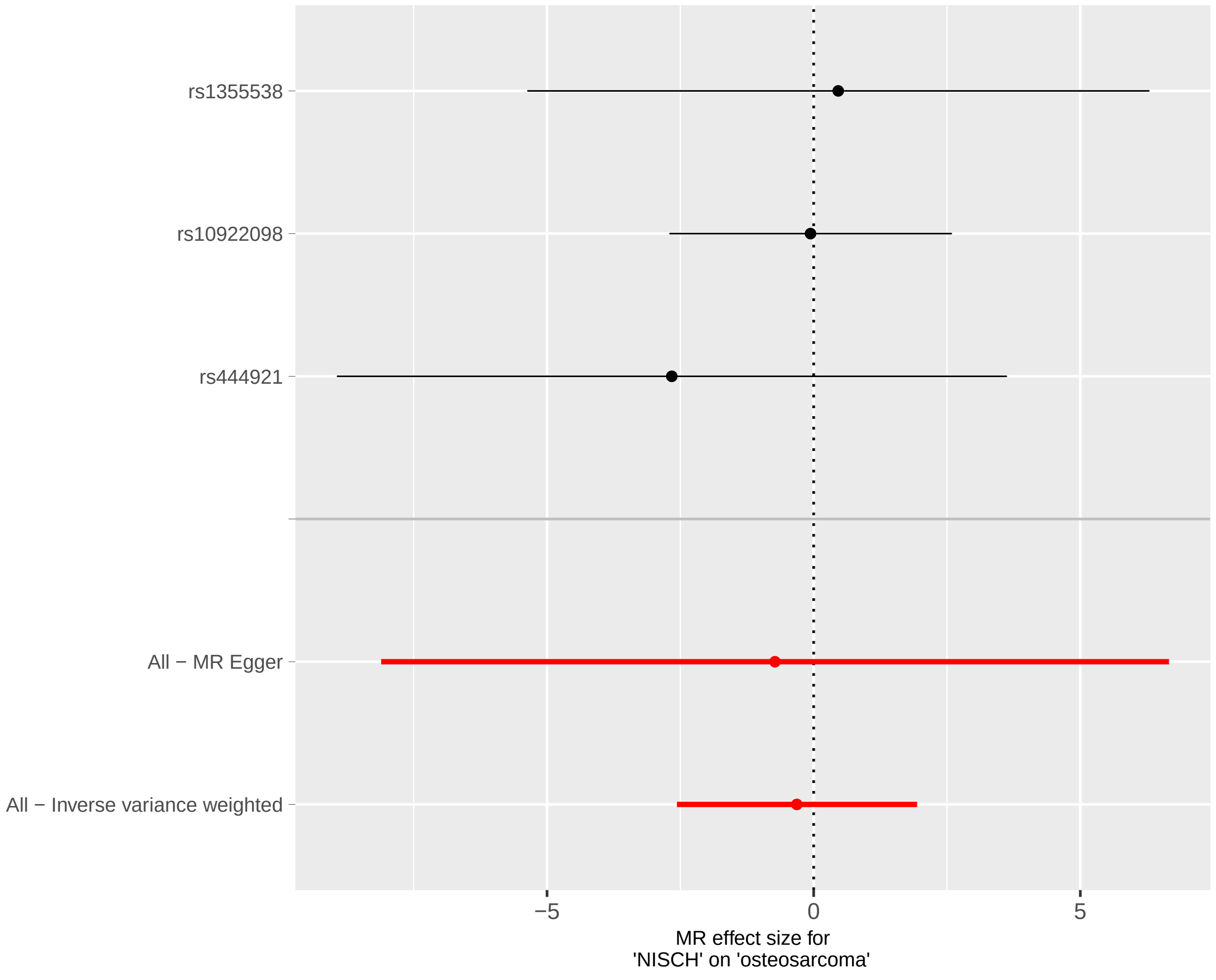

rs1355538
rs10922098
rs444921
All − MR Egger
All − Inverse variance weighted
−5
0
5
MR effect size for
'NISCH' on 'osteosarcoma'

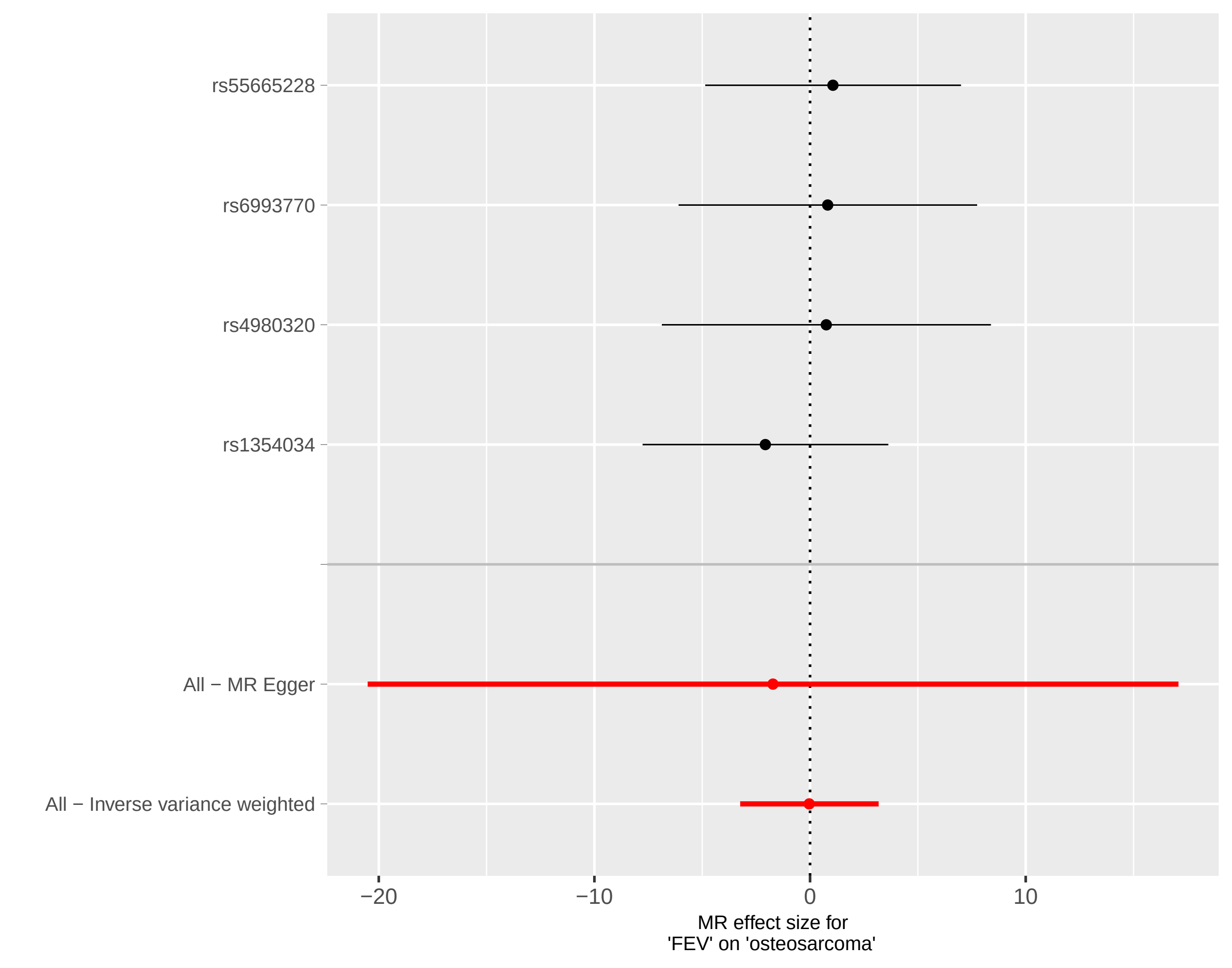
rs55665228
rs6993770
rs4980320
rs1354034
All − MR Egger
All − Inverse variance weighted
−20
−10
0
10
MR effect size for
'FEV' on 'osteosarcoma'

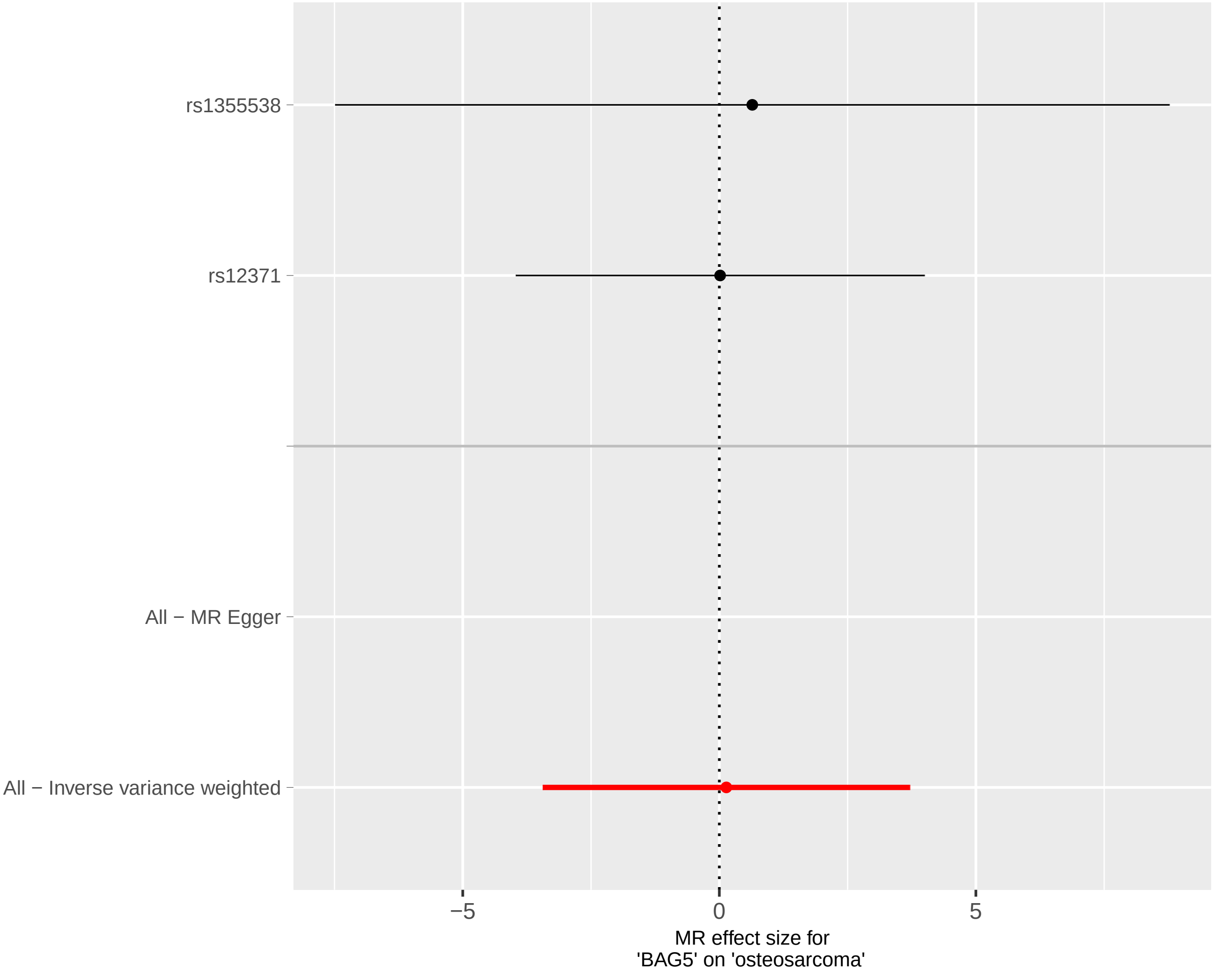
rs1355538
rs12371
All − MR Egger
All − Inverse variance weighted
−5
0
5
MR effect size for
'BAG5' on 'osteosarcoma'

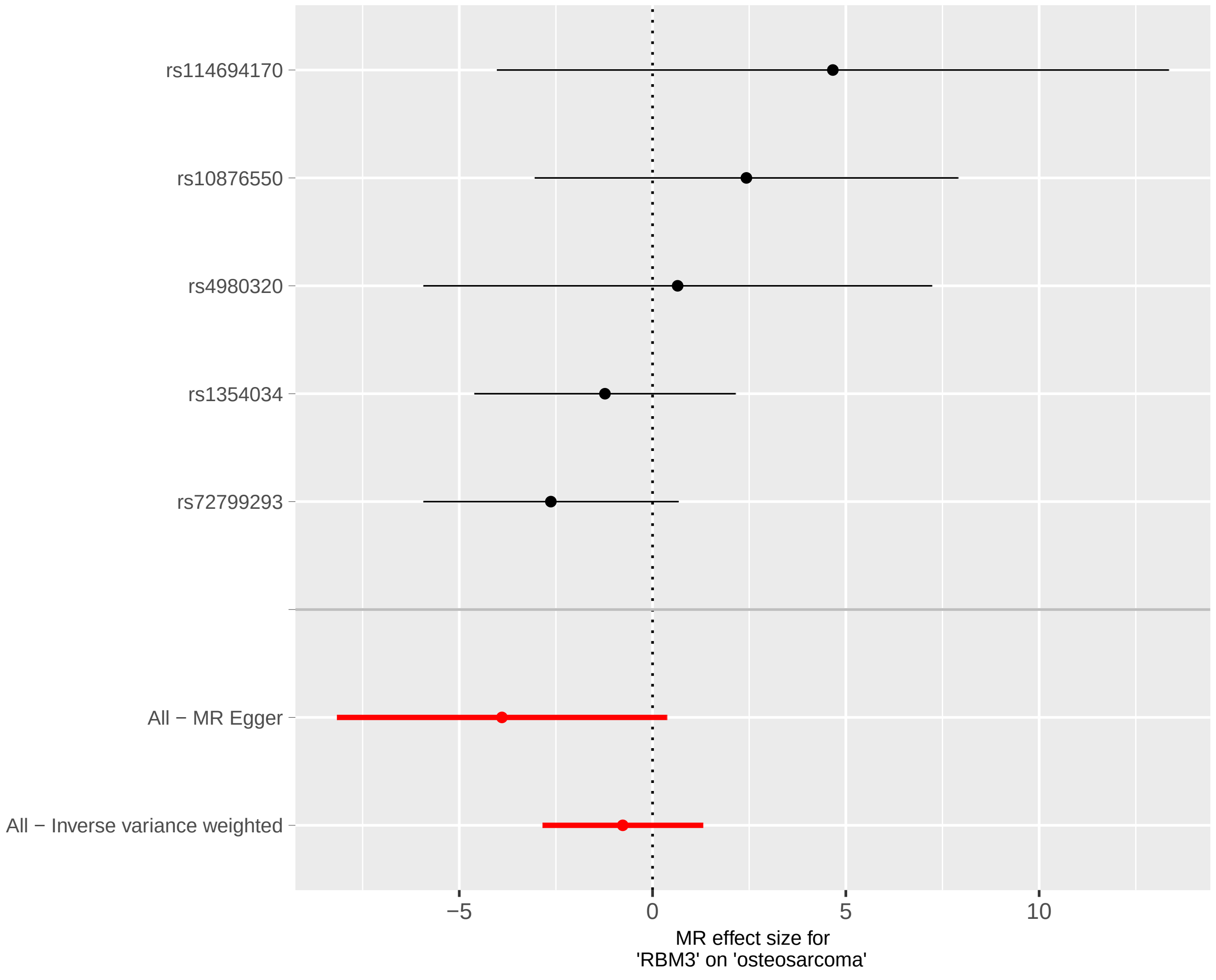
rs114694170
rs10876550
rs4980320
rs1354034
rs72799293
All − MR Egger
All − Inverse variance weighted
−5
0
5
10
MR effect size for
'RBM3' on 'osteosarcoma'

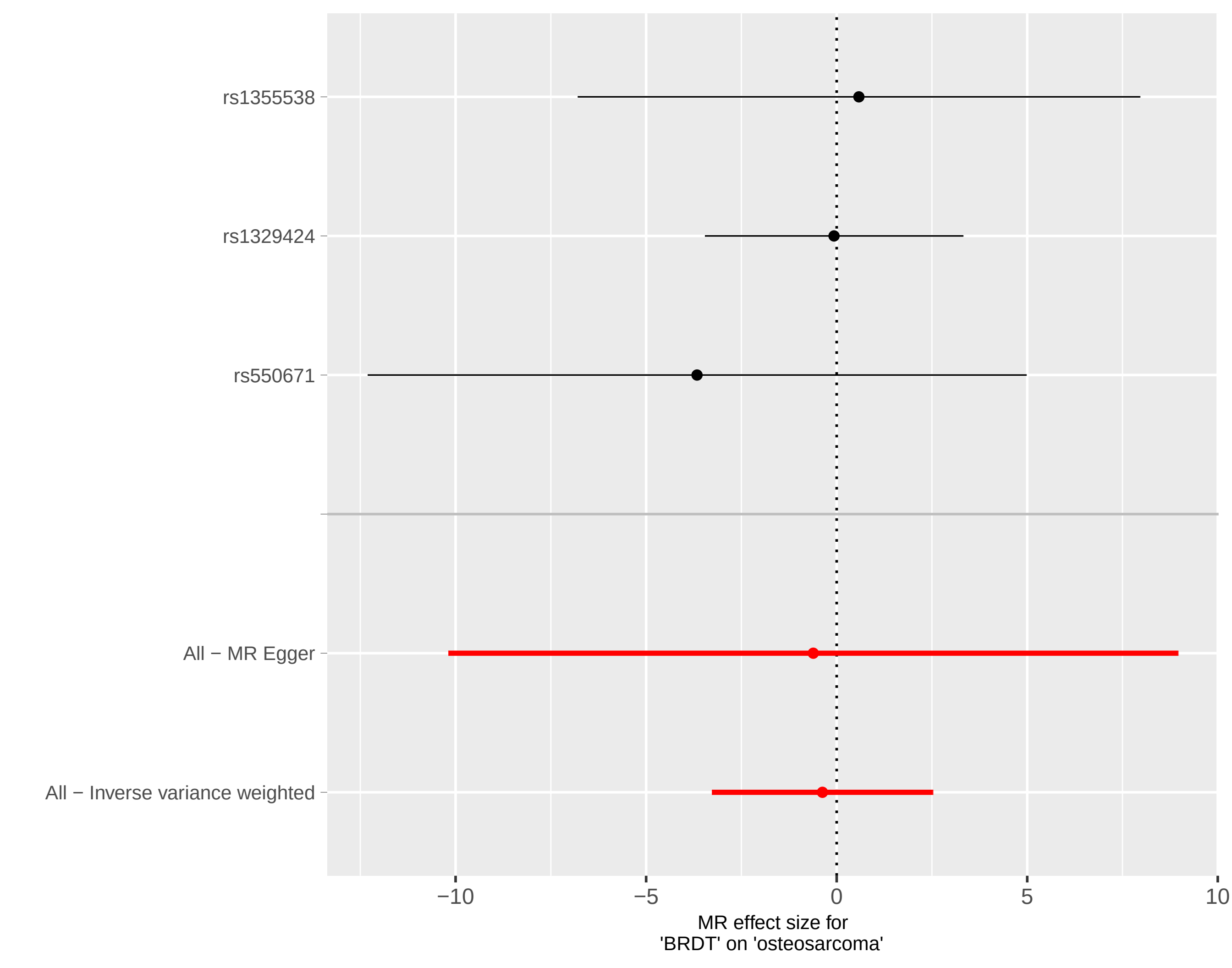
rs1355538
rs1329424
rs550671
All − MR Egger
All − Inverse variance weighted
−10
−5
0
5
10
MR effect size for
'BRDT' on 'osteosarcoma'

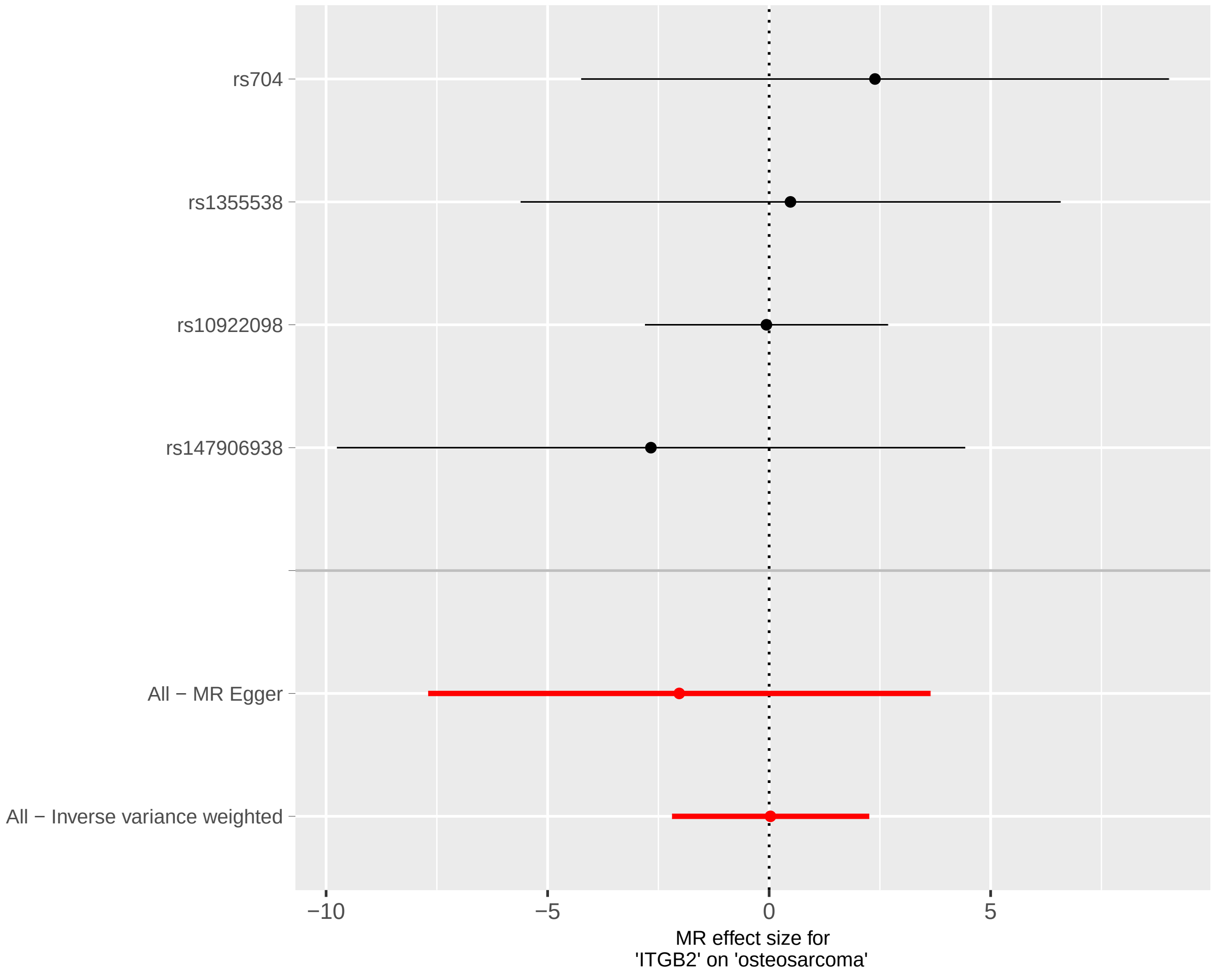
rs704
rs1355538
rs10922098
rs147906938
All − MR Egger
All − Inverse variance weighted
−10
−5
0
5
MR effect size for
'ITGB2' on 'osteosarcoma'

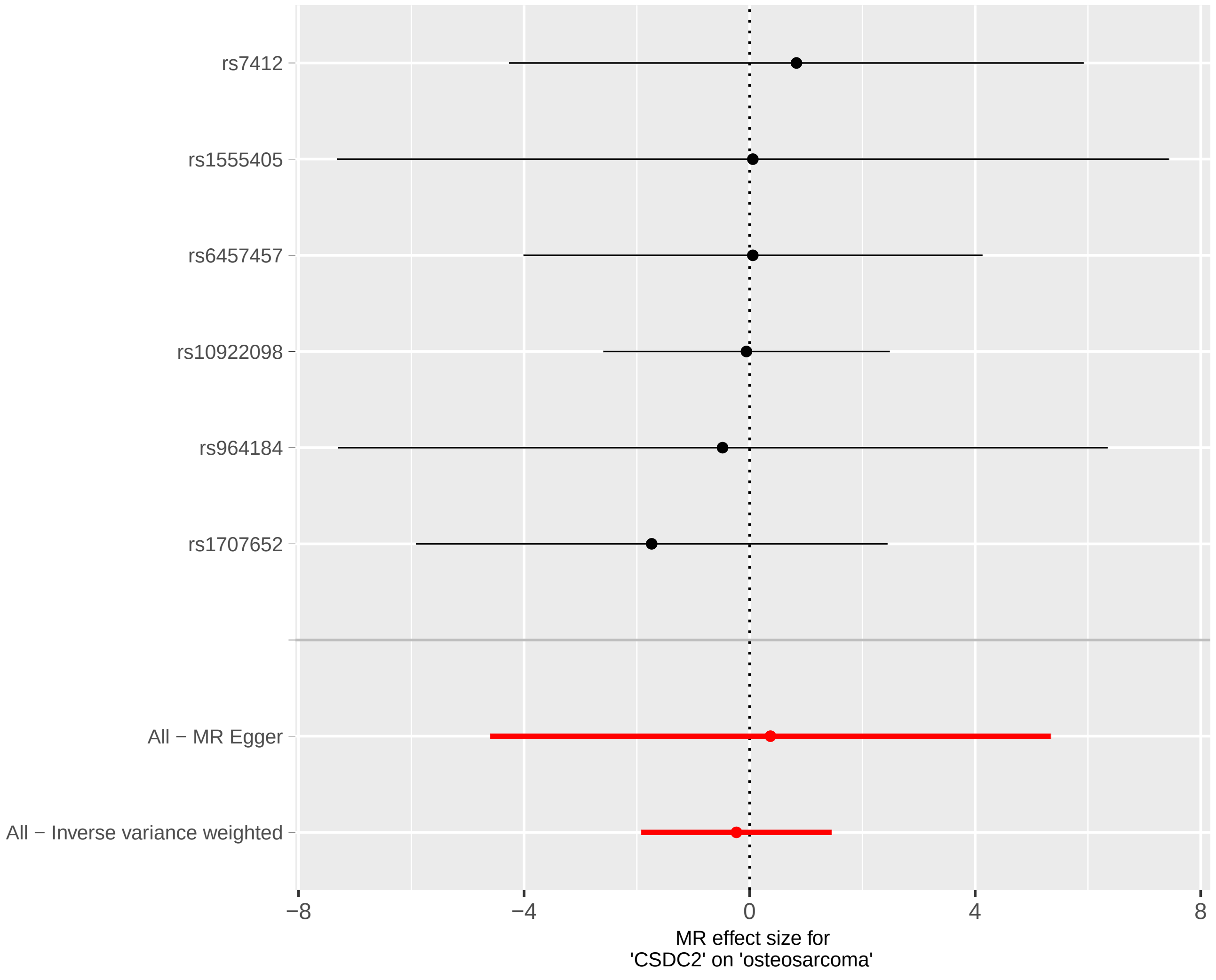
rs7412
rs1555405
rs6457457
rs10922098
rs964184
rs1707652
All − MR Egger
All − Inverse variance weighted
−8
−4
0
4
8
MR effect size for
'CSDC2' on 'osteosarcoma'

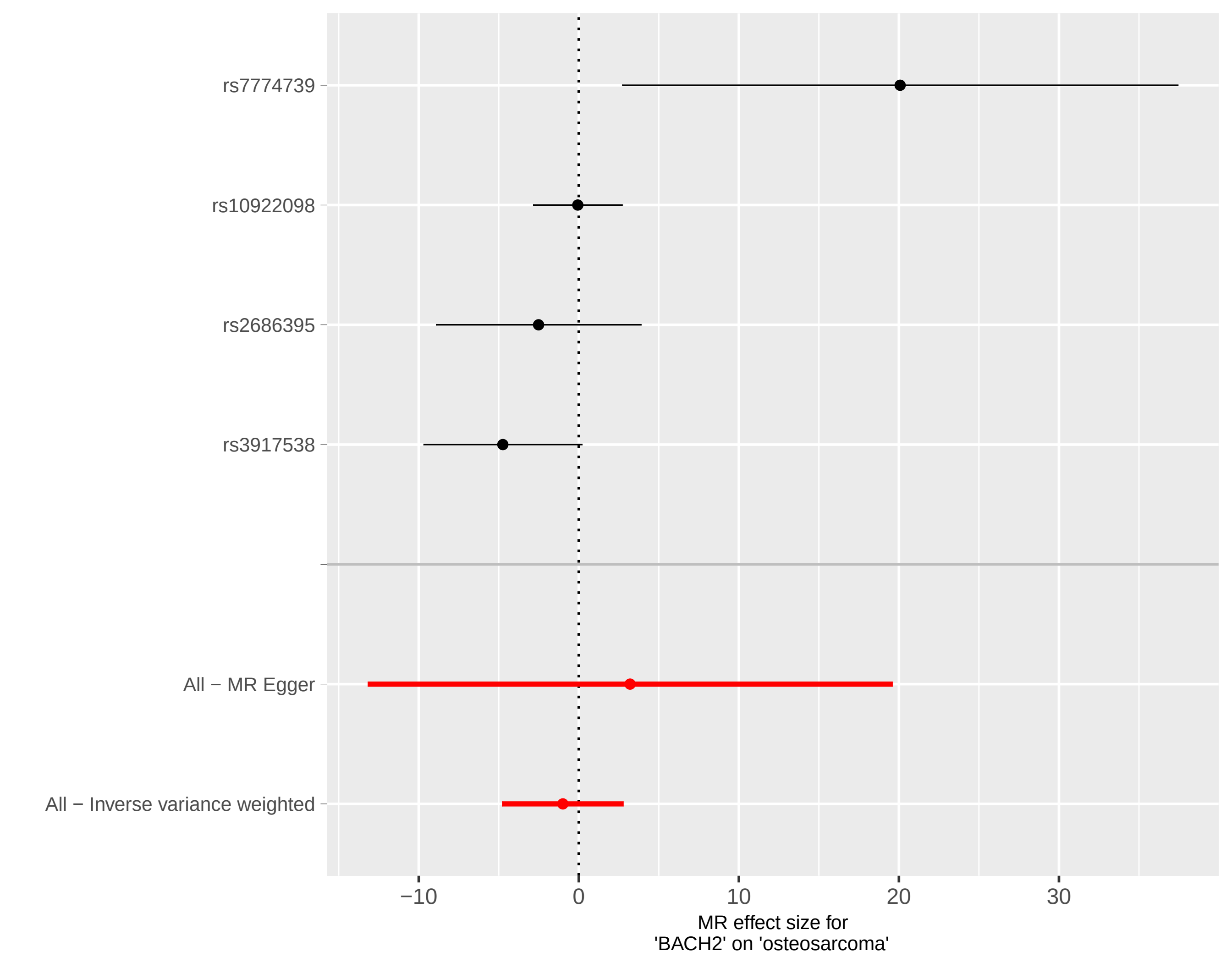
rs7774739
rs10922098
rs2686395
rs3917538
All − MR Egger
All − Inverse variance weighted
−10
0
10
20
30
MR effect size for
'BACH2' on 'osteosarcoma'

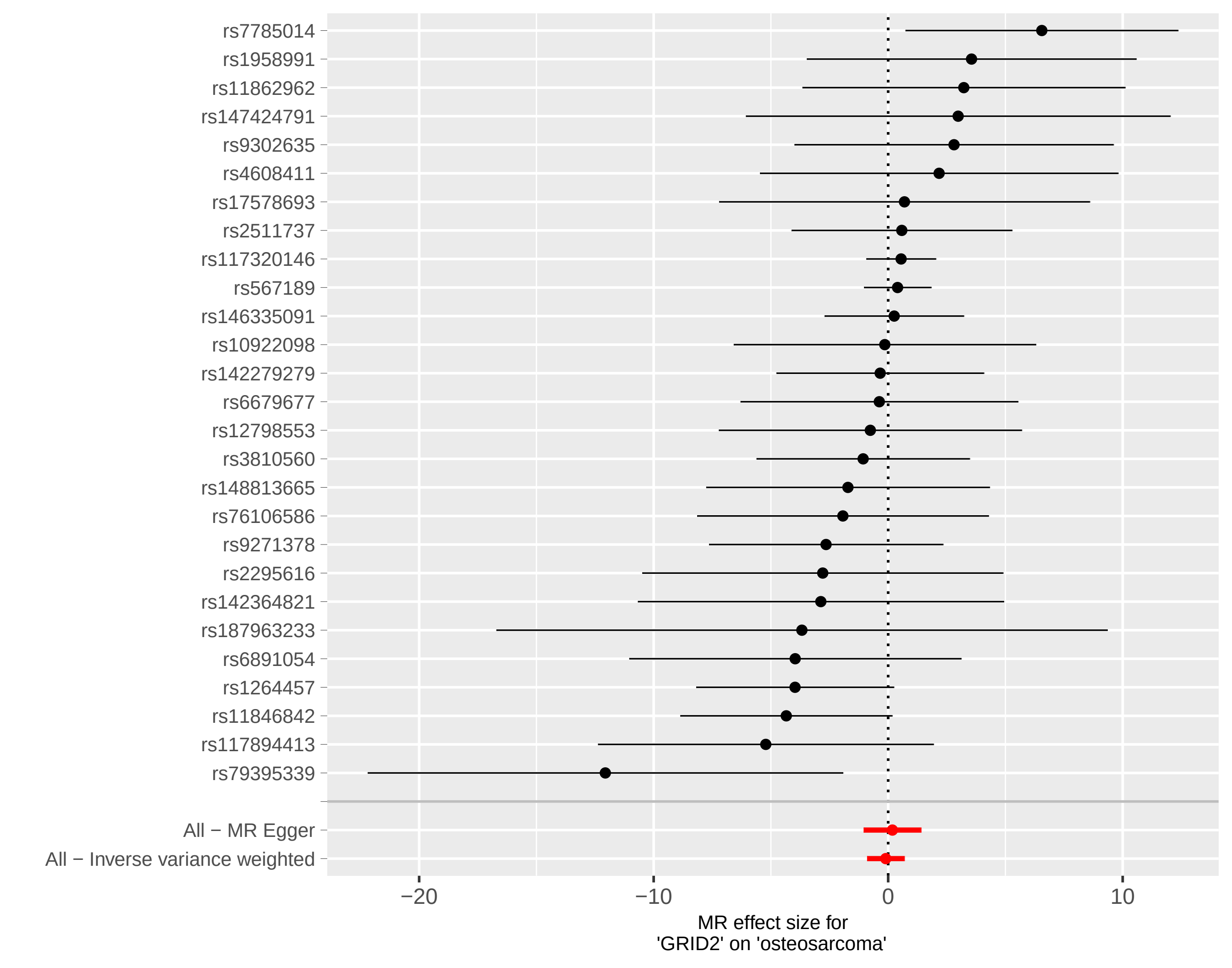

rs7785014
rs1958991
rs11862962
rs147424791
rs9302635
rs4608411
rs17578693
rs2511737
rs117320146
rs567189
rs146335091
rs10922098
rs142279279
rs6679677
rs12798553
rs3810560
rs148813665
rs76106586
rs9271378
rs2295616
rs142364821
rs187963233
rs6891054
rs1264457
rs11846842
rs117894413
rs79395339
All − MR Egger
All − Inverse variance weighted
−20
−10
0
10
MR effect size for
'GRID2' on 'osteosarcoma'

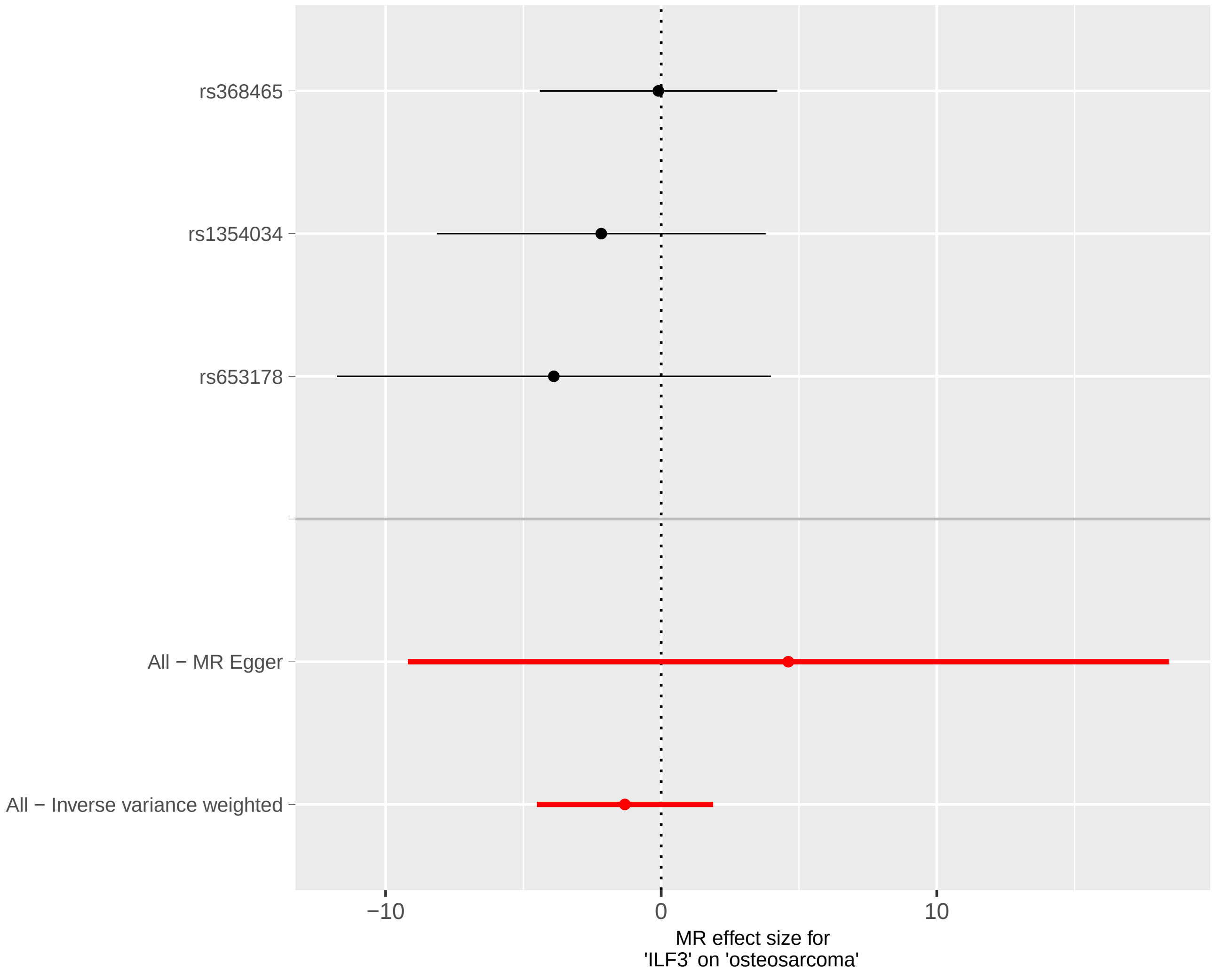

rs368465
rs1354034
rs653178
All − MR Egger
All − Inverse variance weighted
−10
0
10
MR effect size for
'ILF3' on 'osteosarcoma'

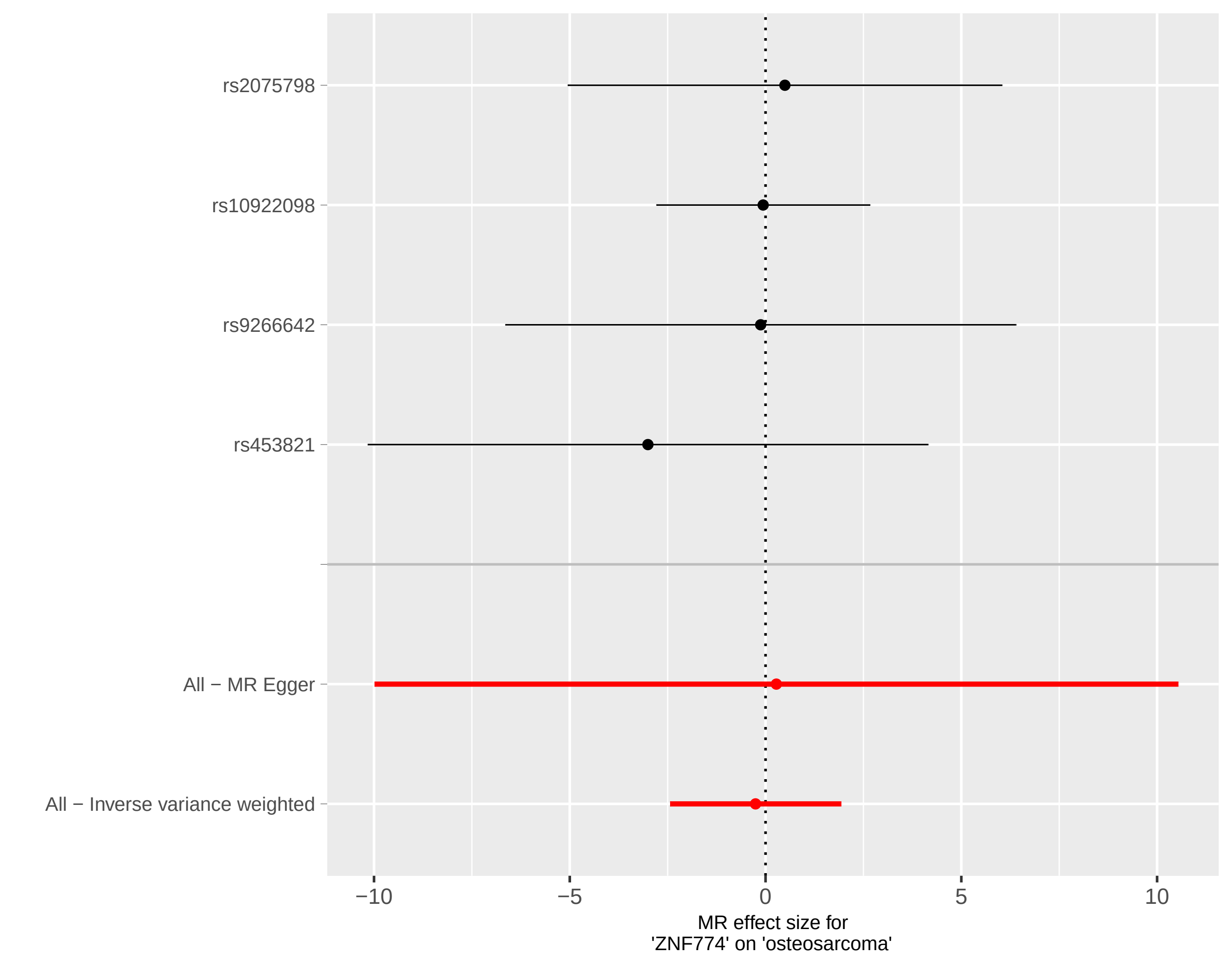
rs2075798
rs10922098
rs9266642
rs453821
All − MR Egger
All − Inverse variance weighted
−10
−5
0
5
10
MR effect size for
'ZNF774' on 'osteosarcoma'

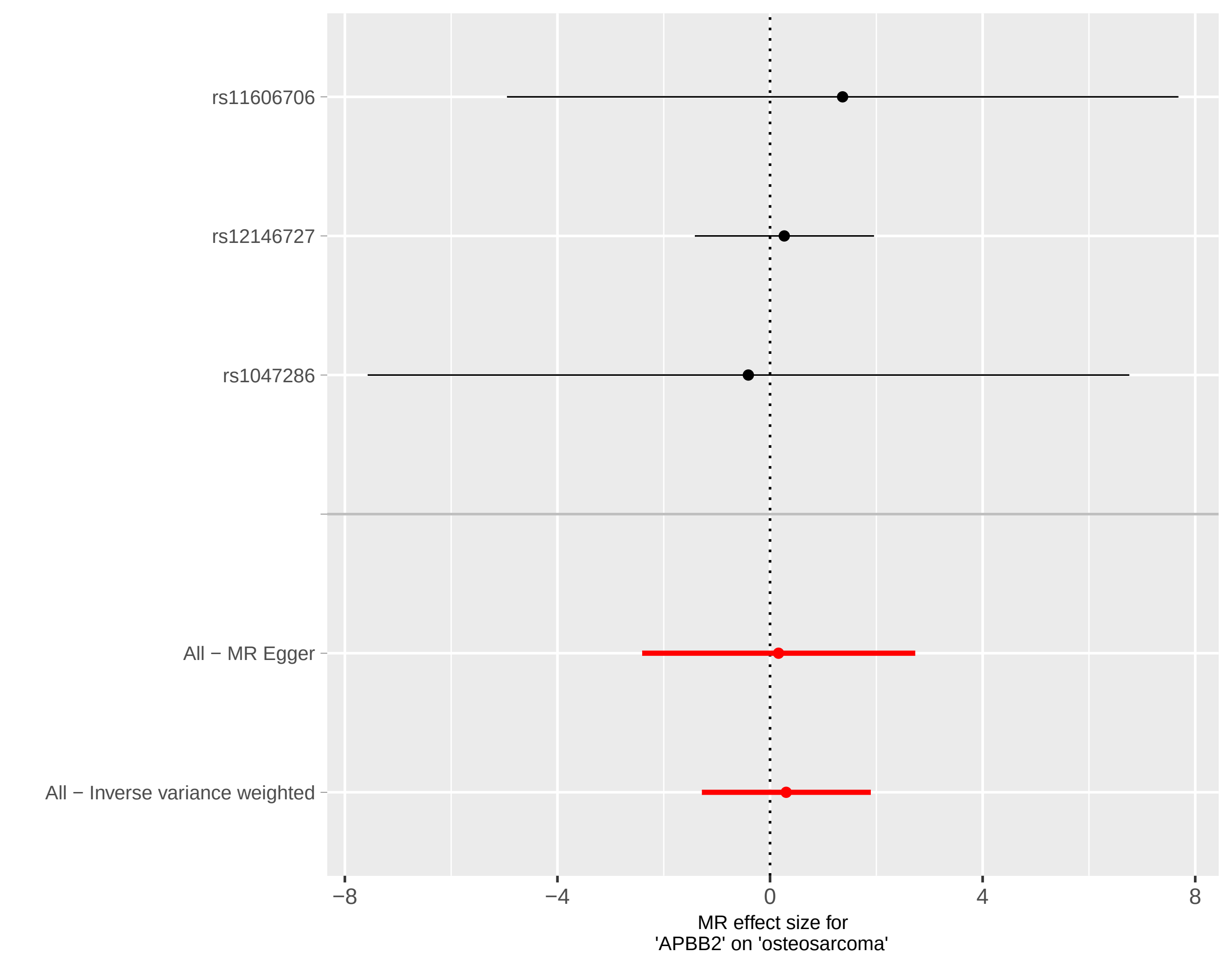

rs11606706
rs12146727
rs1047286
All − MR Egger
All − Inverse variance weighted
−8
−4
0
4
8
MR effect size for
'APBB2' on 'osteosarcoma'

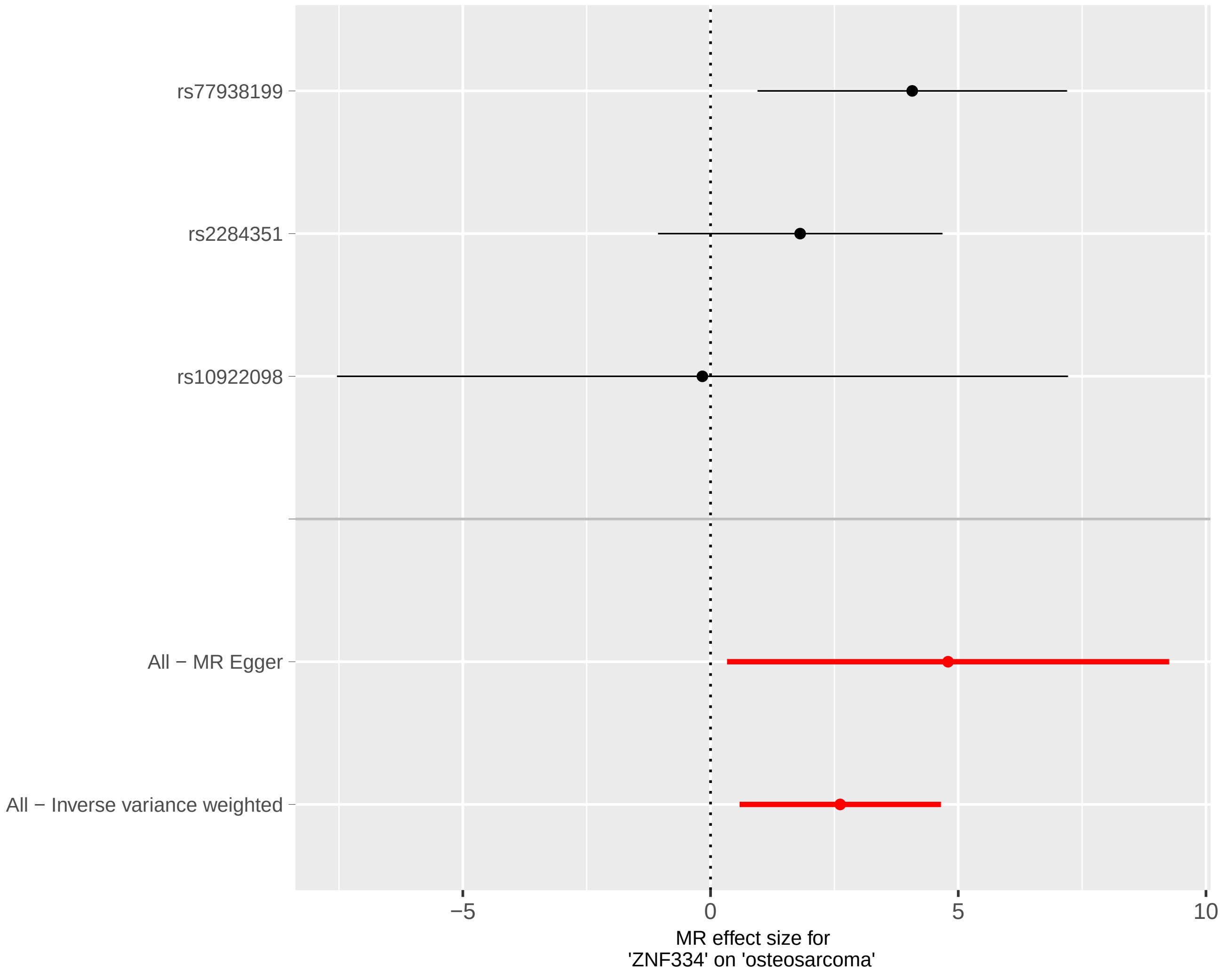

rs77938199
rs2284351
rs10922098
All − MR Egger
All − Inverse variance weighted
−5
0
5
10
MR effect size for
'ZNF334' on 'osteosarcoma'

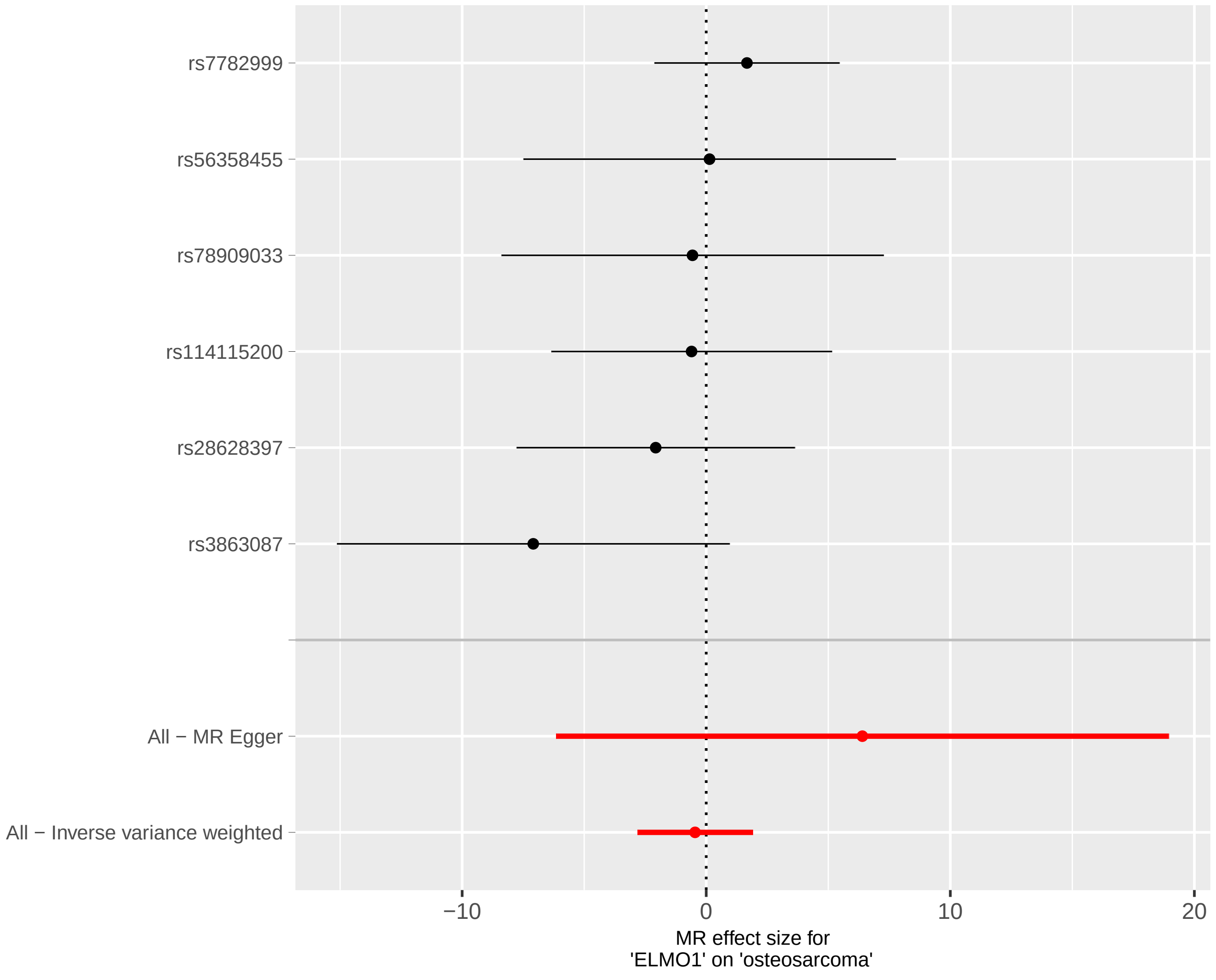

rs7782999
rs56358455
rs78909033
rs114115200
rs28628397
rs3863087
All − MR Egger
All − Inverse variance weighted
−10
0
10
20
MR effect size for
'ELMO1' on 'osteosarcoma'

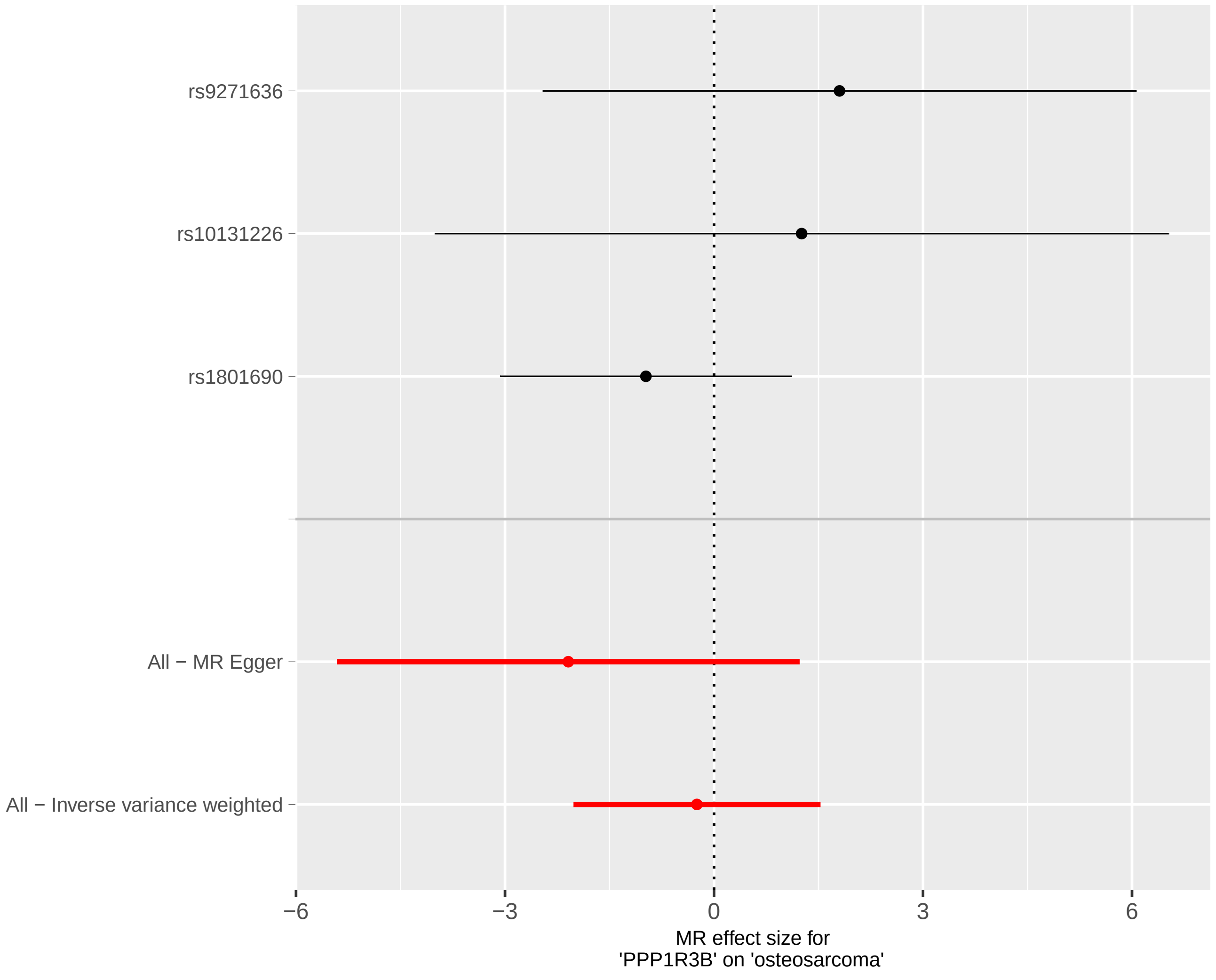

rs9271636
rs10131226
rs1801690
All − MR Egger
All − Inverse variance weighted
−6
−3
0
3
6
MR effect size for
'PPP1R3B' on 'osteosarcoma'

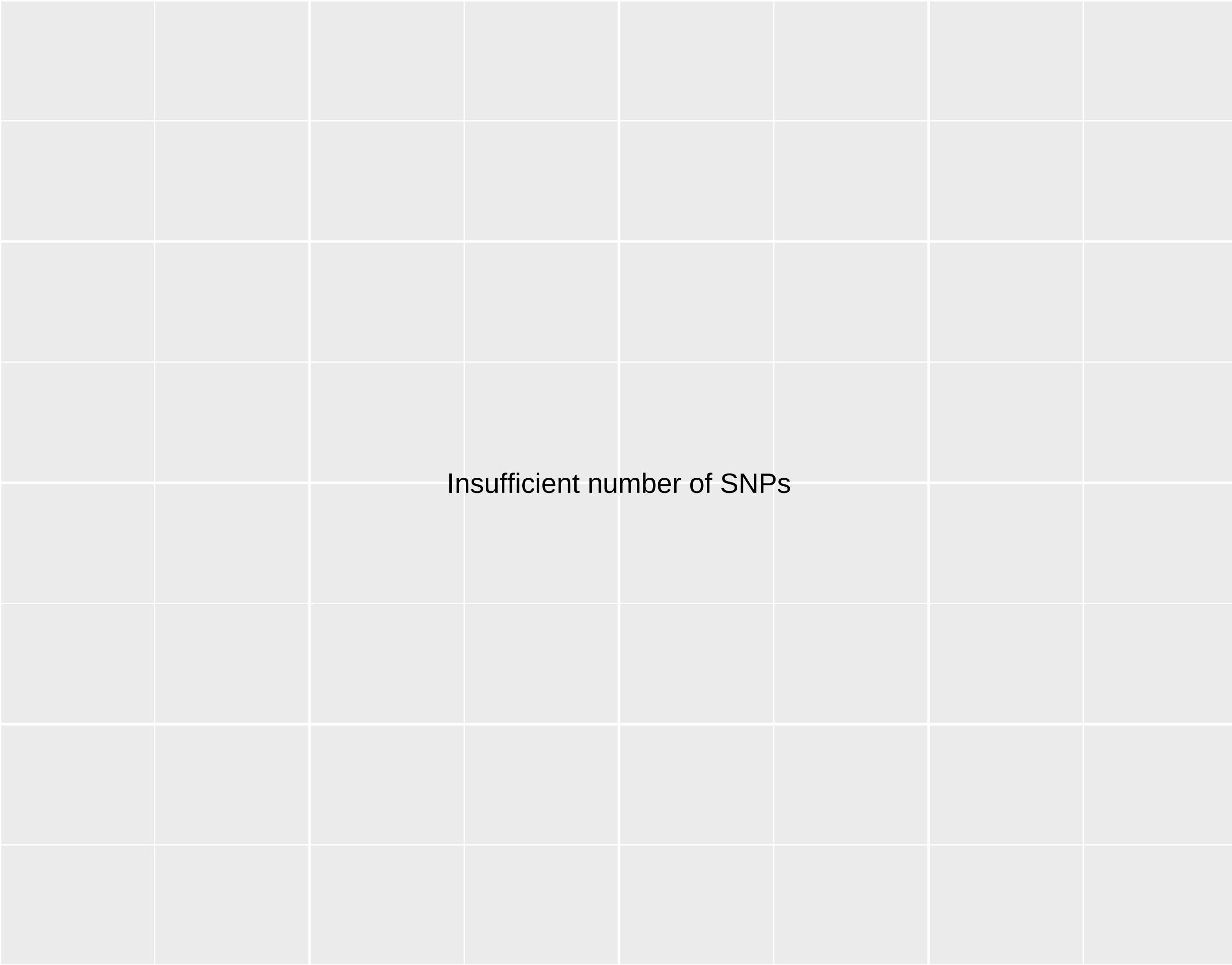
Insufficient number of SNPs

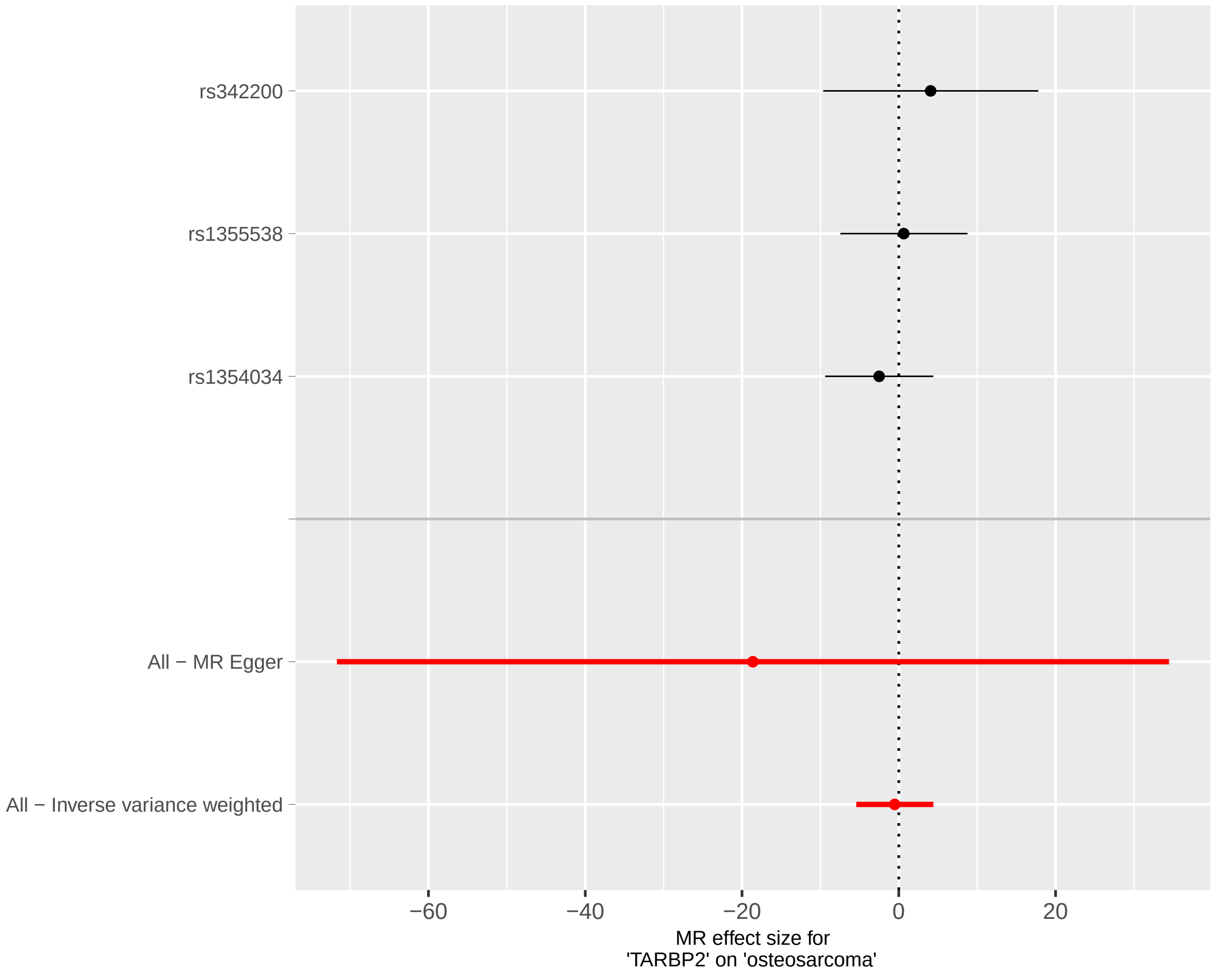

rs342200
rs1355538
rs1354034
All − MR Egger
All − Inverse variance weighted
−60
−40
−20
0
20
MR effect size for
'TARBP2' on 'osteosarcoma'

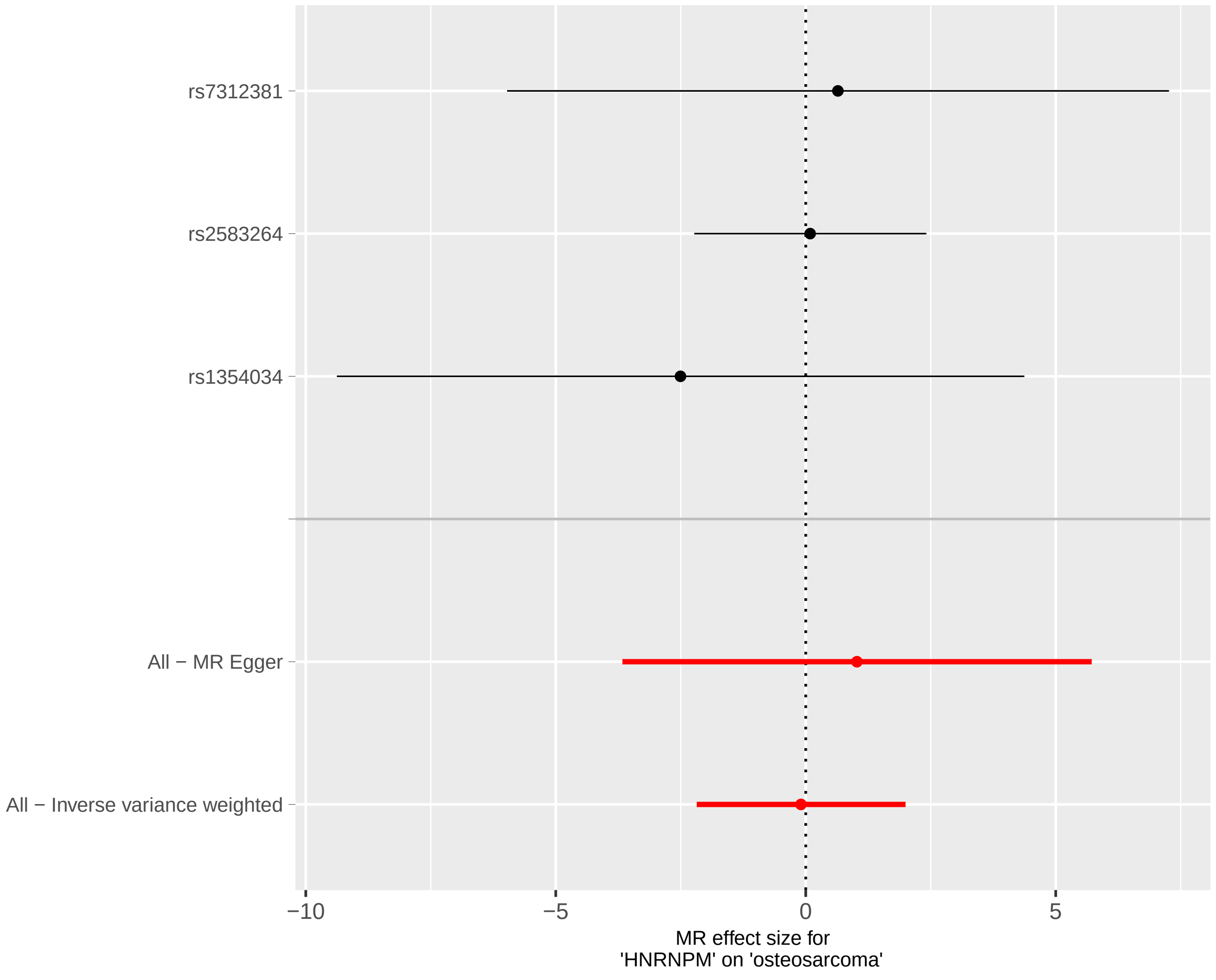

rs7312381
rs2583264
rs1354034
All − MR Egger
All − Inverse variance weighted
−10
−5
0
5
MR effect size for
'HNRNPM' on 'osteosarcoma'

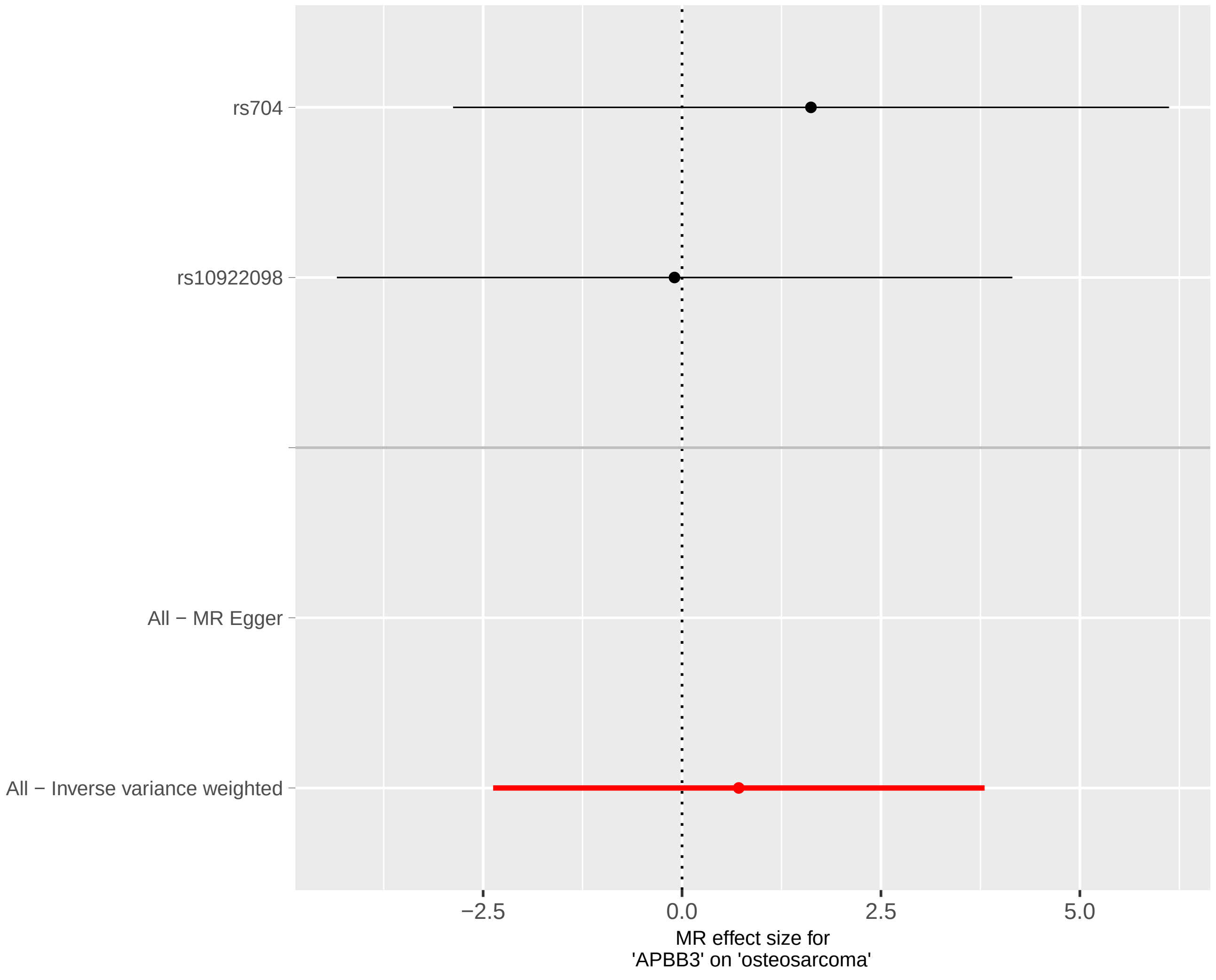

rs704
rs10922098
All − MR Egger
All − Inverse variance weighted
−2.5
0.0
2.5
5.0
MR effect size for
'APBB3' on 'osteosarcoma'

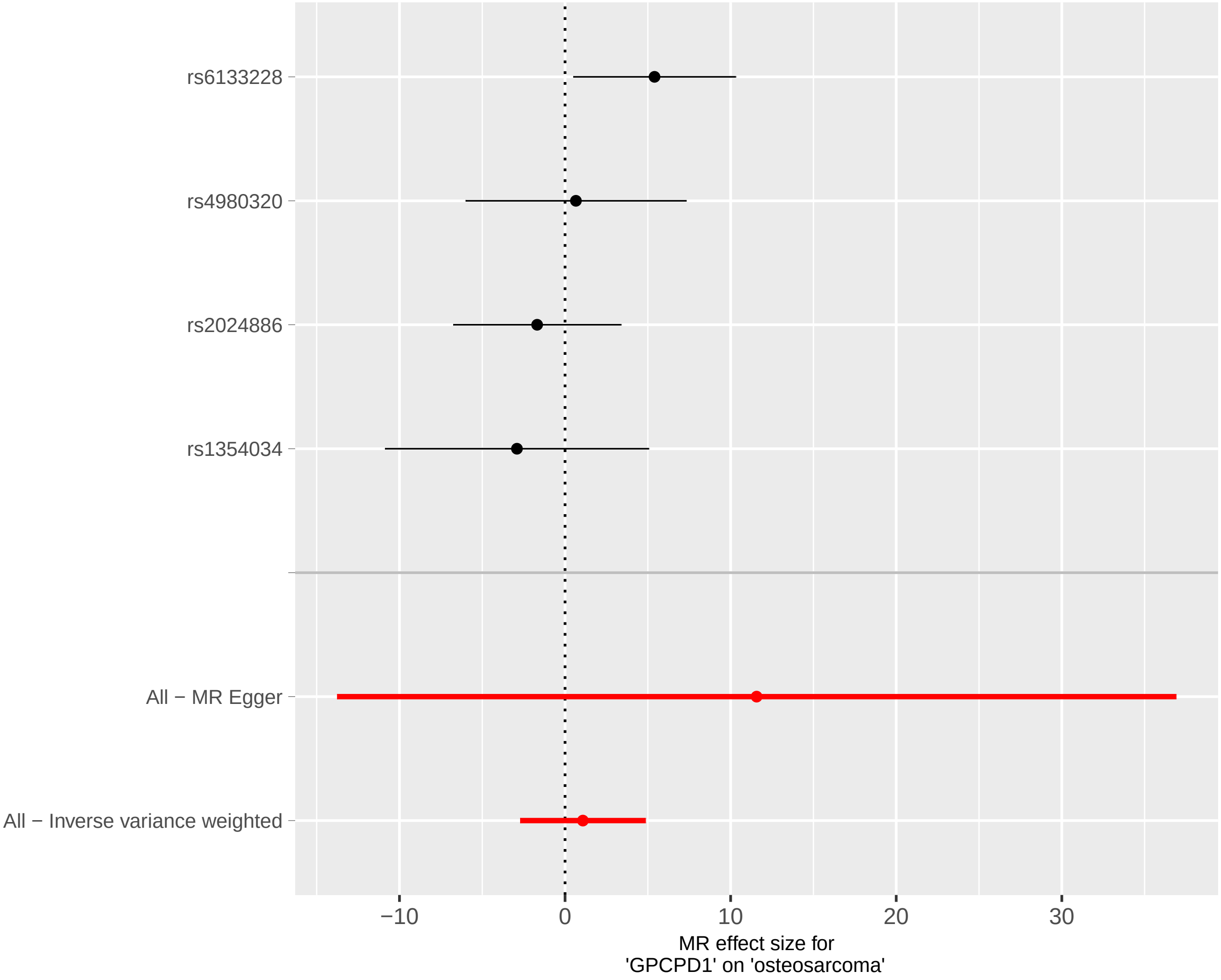
rs6133228
rs4980320
rs2024886
rs1354034
All − MR Egger
All − Inverse variance weighted
−10
0
10
20
30
MR effect size for
'GPCPD1' on 'osteosarcoma'

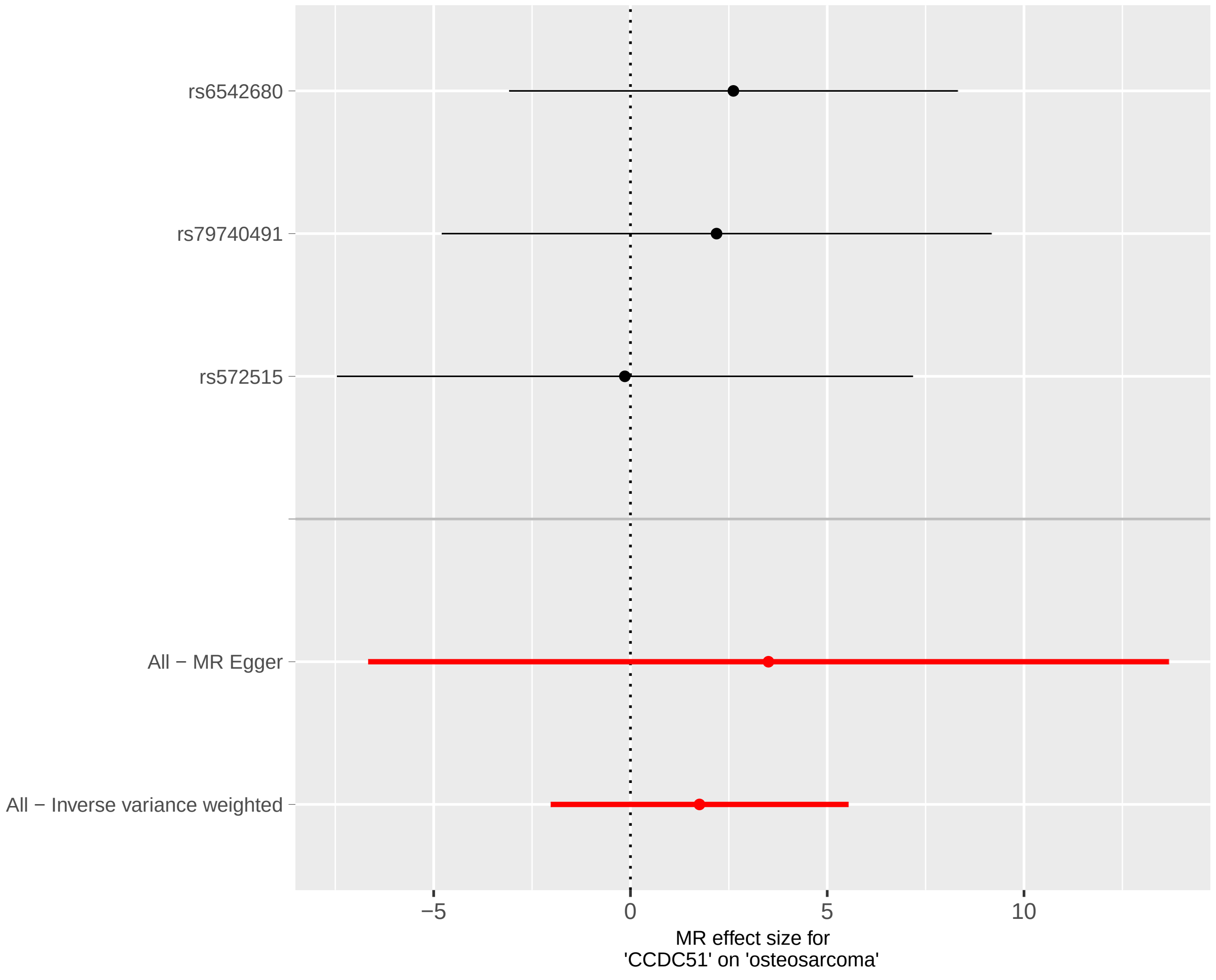
rs6542680
rs79740491
rs572515
All − MR Egger
All − Inverse variance weighted
−5
0
5
10
MR effect size for
'CCDC51' on 'osteosarcoma'

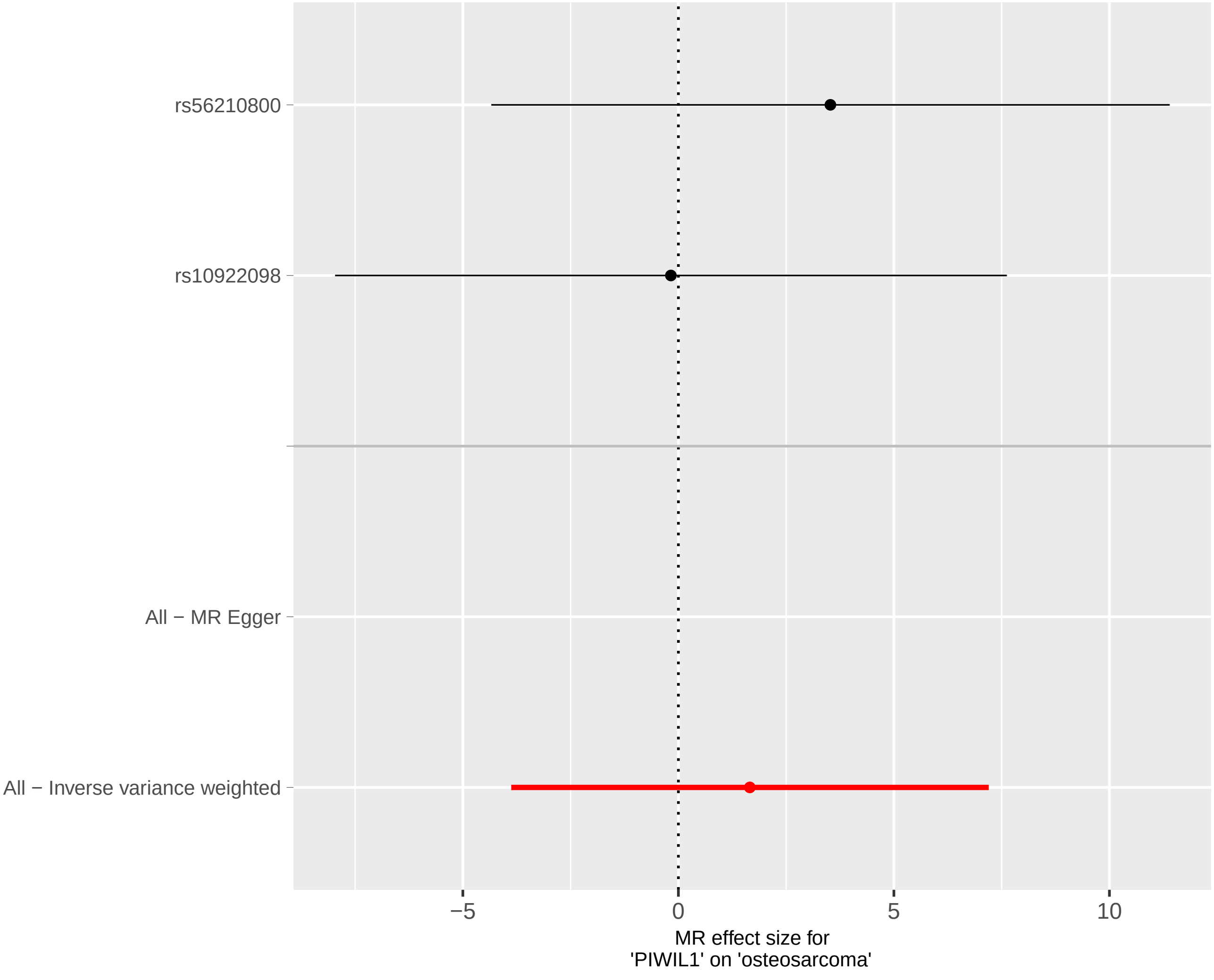

rs56210800
rs10922098
All − MR Egger
All − Inverse variance weighted
−5
0
5
10
MR effect size for
'PIWIL1' on 'osteosarcoma'

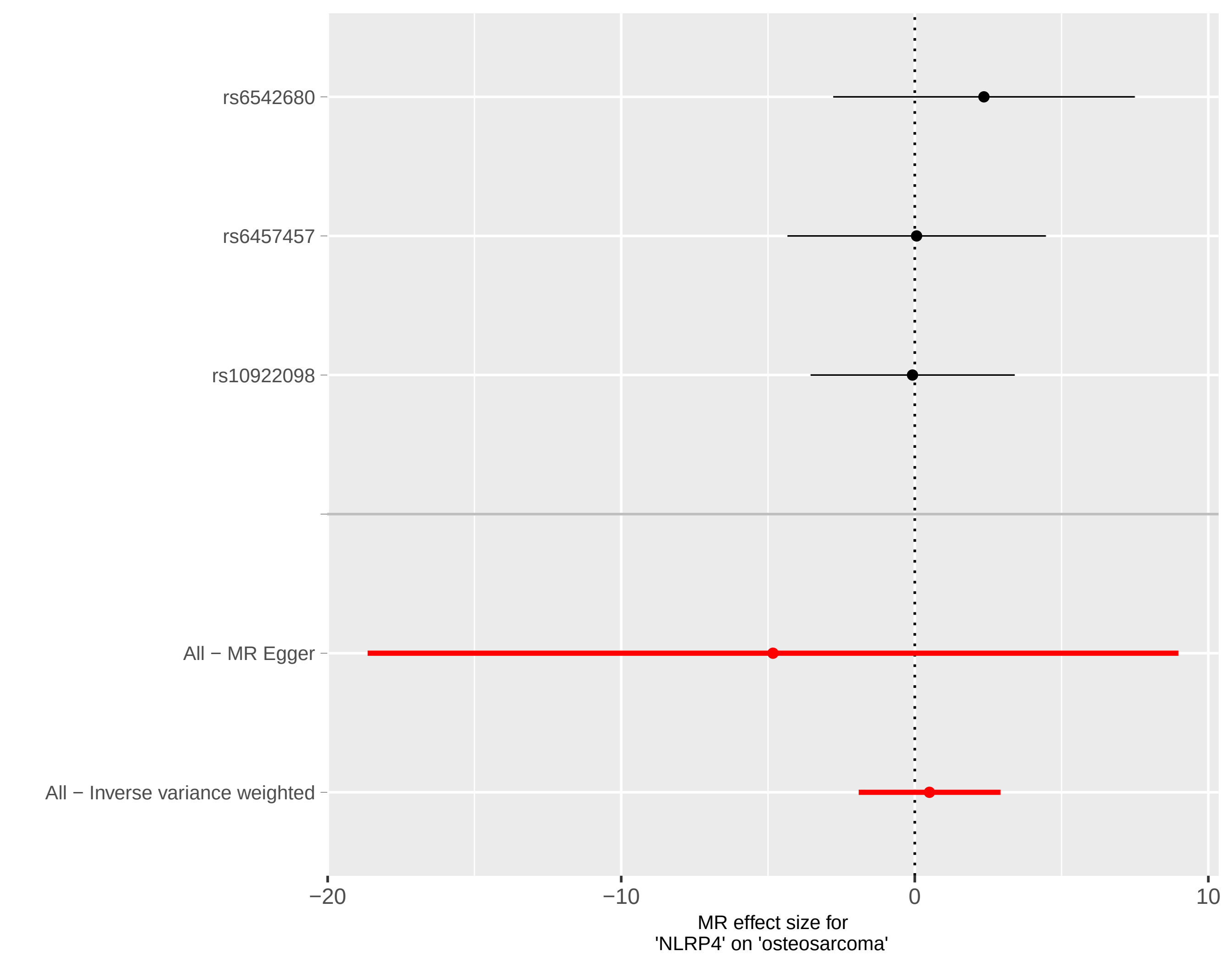

rs6542680
rs6457457
rs10922098
All − MR Egger
All − Inverse variance weighted
−20
−10
0
10
MR effect size for
'NLRP4' on 'osteosarcoma'

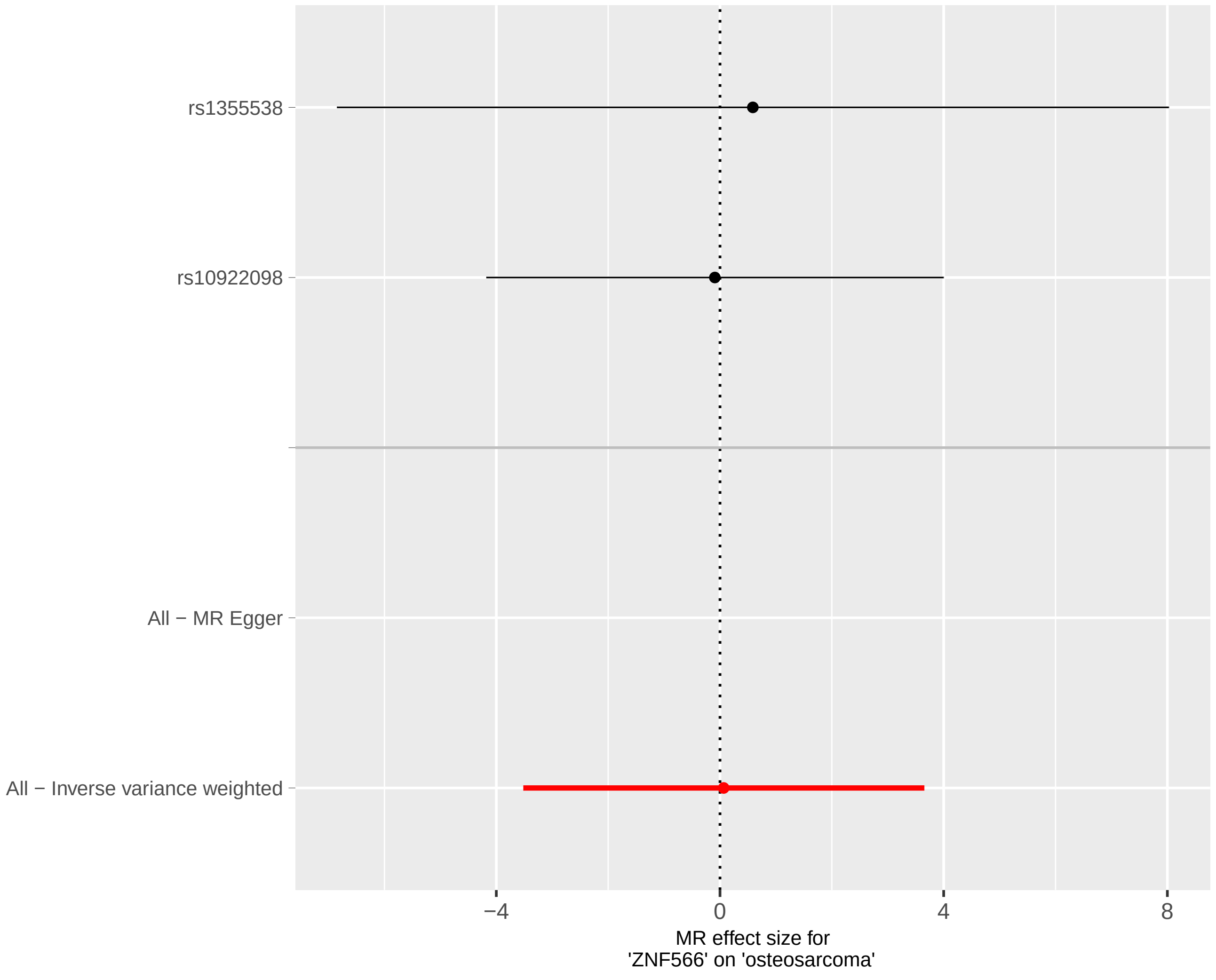
rs1355538
rs10922098
All − MR Egger
All − Inverse variance weighted
−4
0
4
8
MR effect size for
'ZNF566' on 'osteosarcoma'

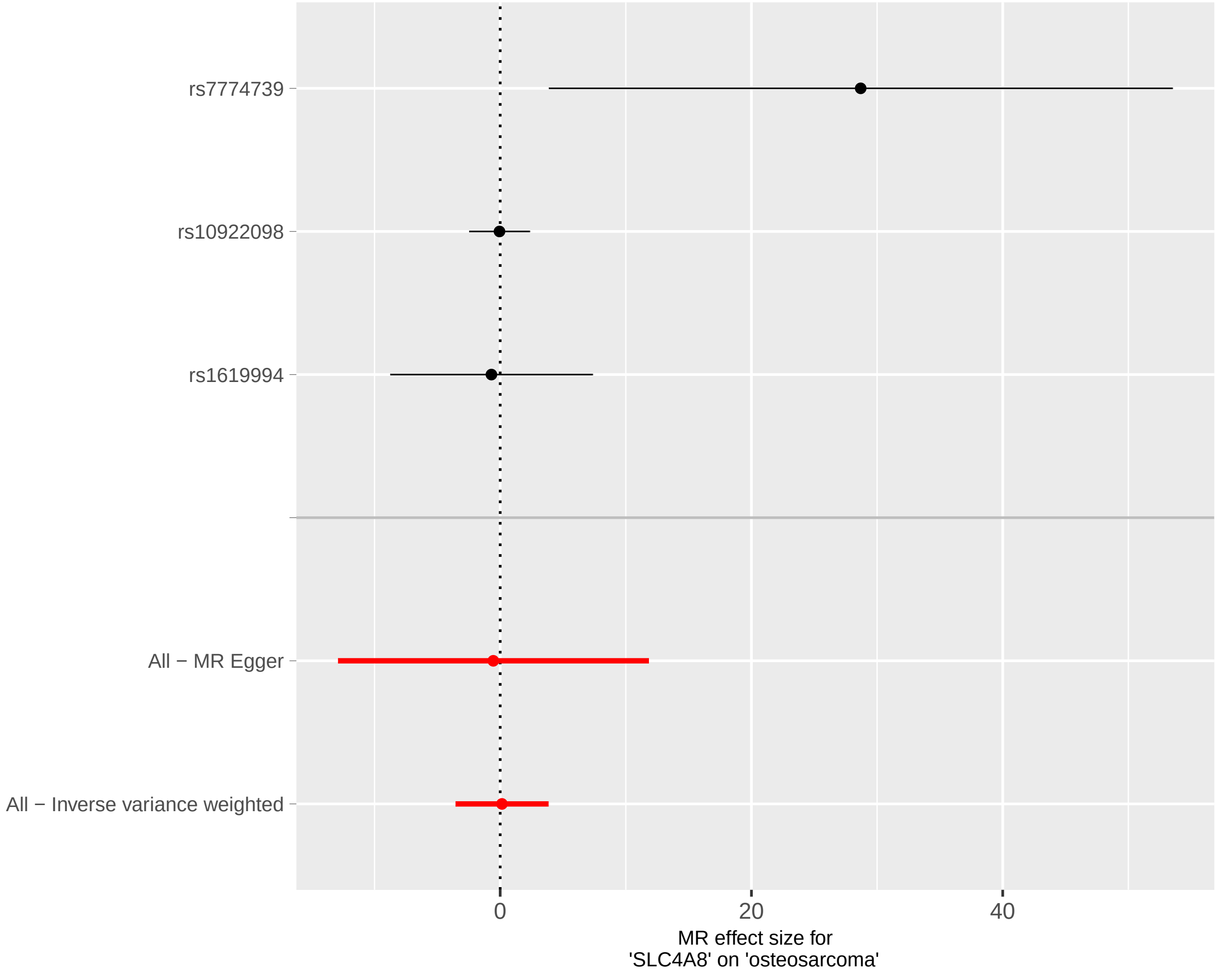

rs7774739
rs10922098
rs1619994
All − MR Egger
All − Inverse variance weighted
0
20
40
MR effect size for
'SLC4A8' on 'osteosarcoma'

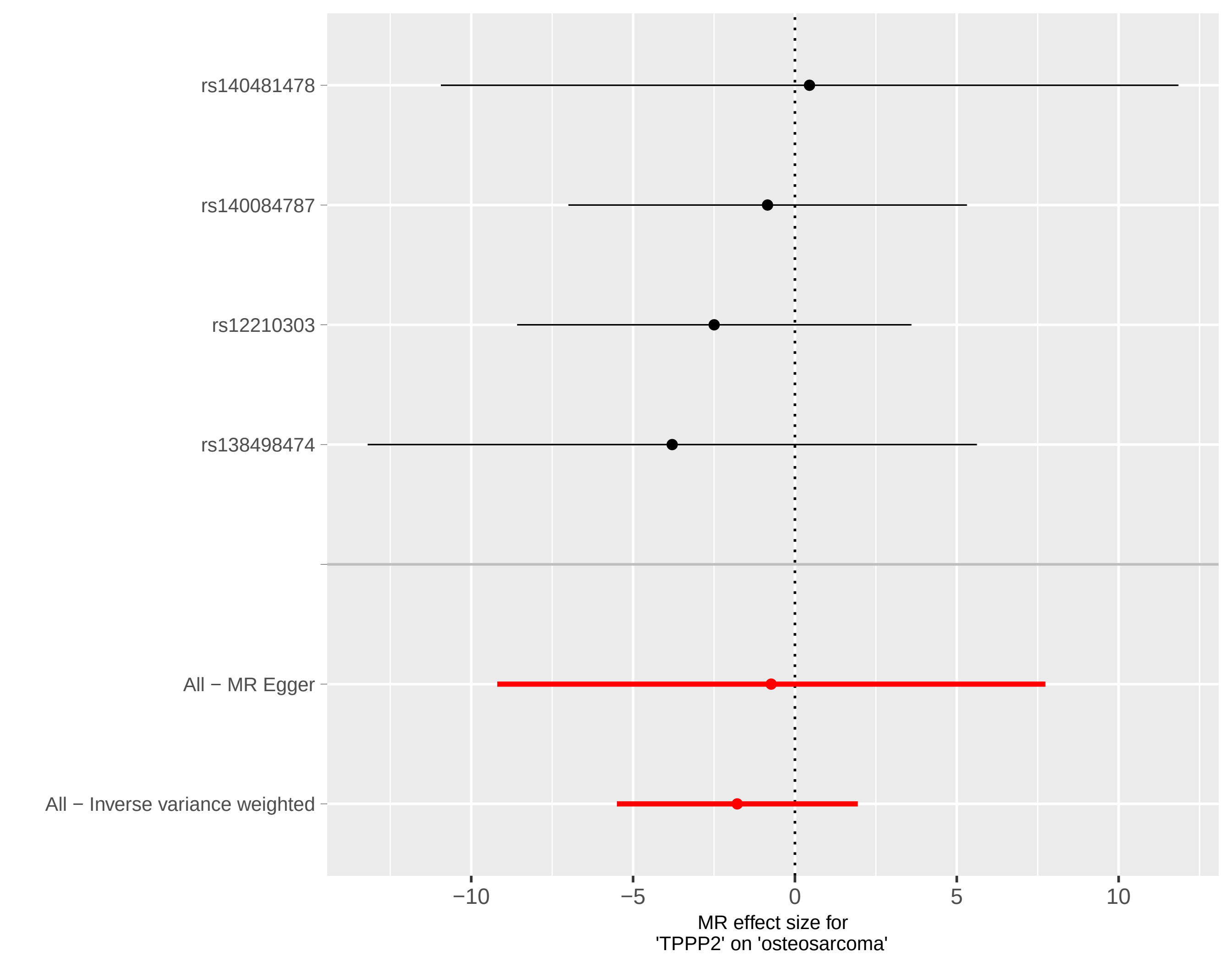
rs140481478
rs140084787
rs12210303
rs138498474
All − MR Egger
All − Inverse variance weighted
−10
−5
0
5
10
MR effect size for
'TPPP2' on 'osteosarcoma'

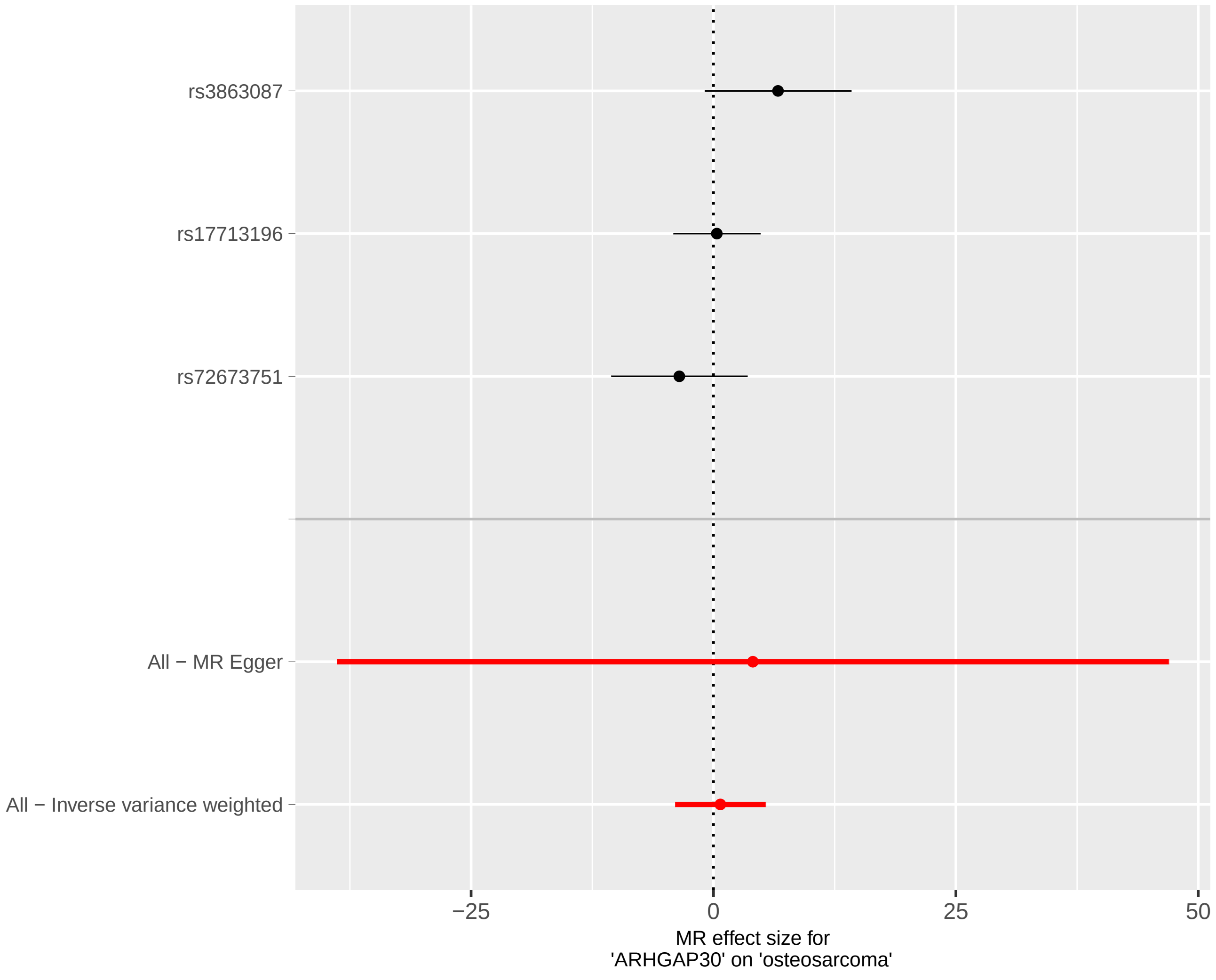

rs3863087
rs17713196
rs72673751
All − MR Egger
All − Inverse variance weighted
−25
0
25
50
MR effect size for
'ARHGAP30' on 'osteosarcoma'

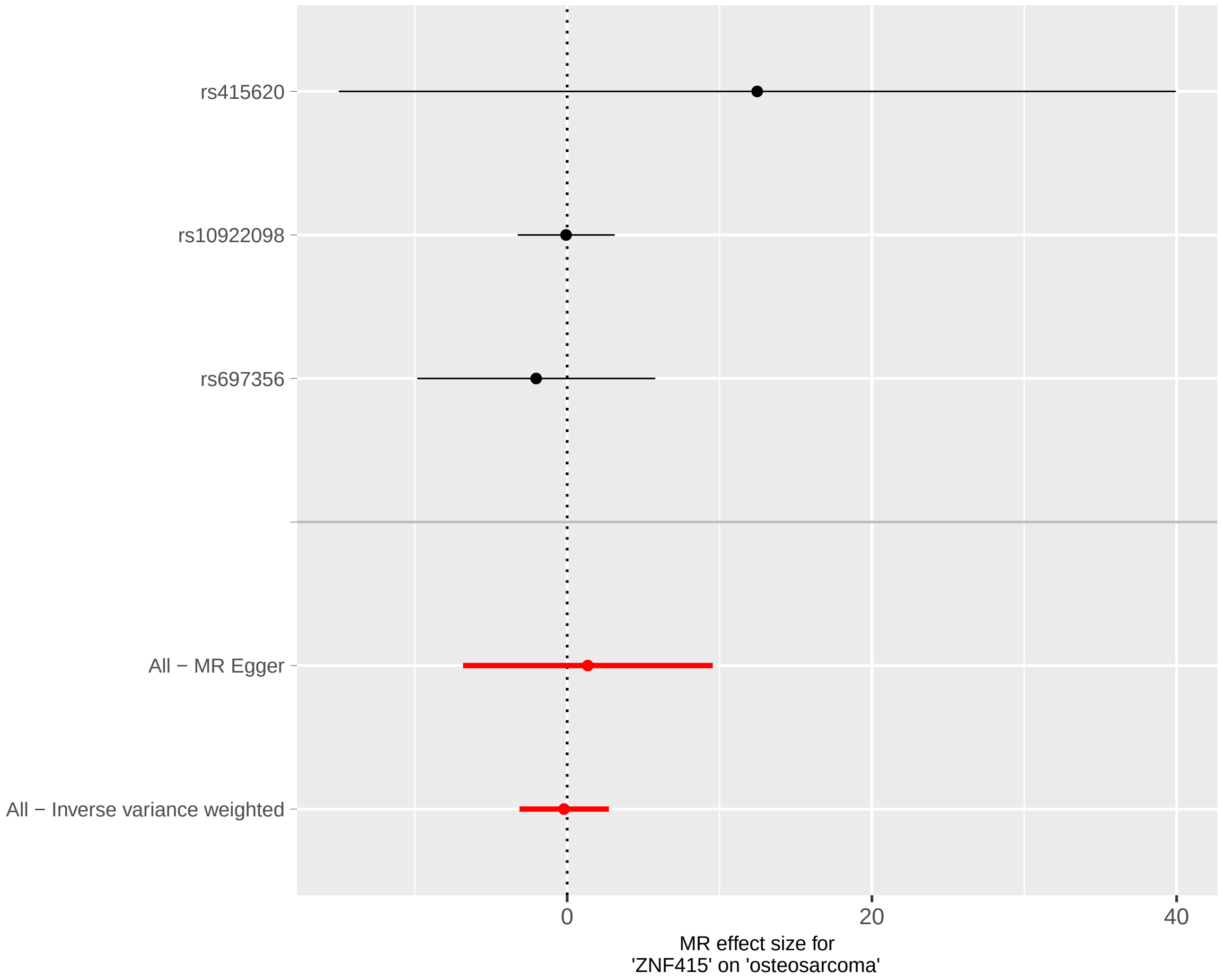
rs415620
rs10922098
rs697356
All − MR Egger
All − Inverse variance weighted
0
20
40
MR effect size for
'ZNF415' on 'osteosarcoma'

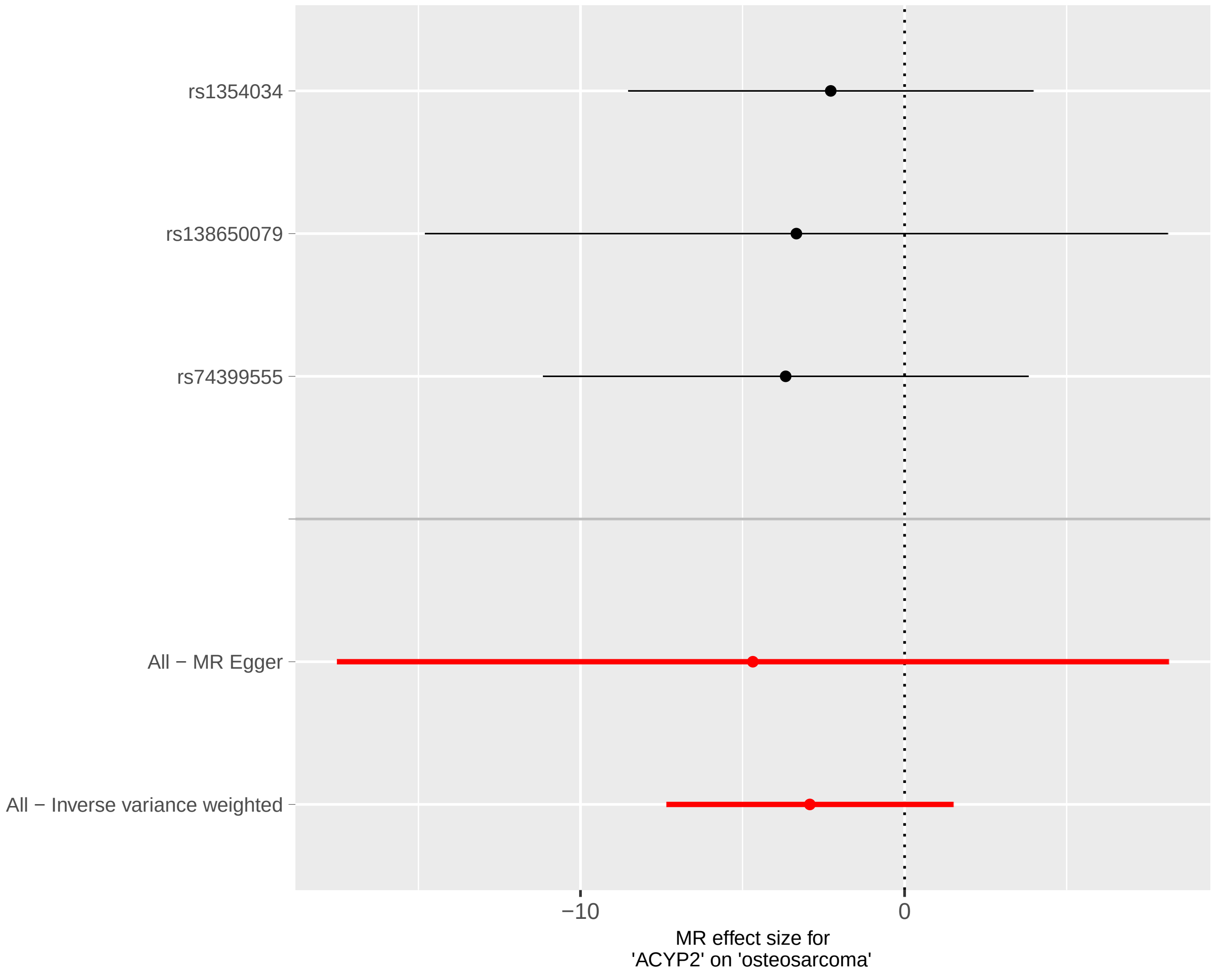

rs1354034
rs138650079
rs74399555
All − MR Egger
All − Inverse variance weighted
−10
0
MR effect size for
'ACYP2' on 'osteosarcoma'

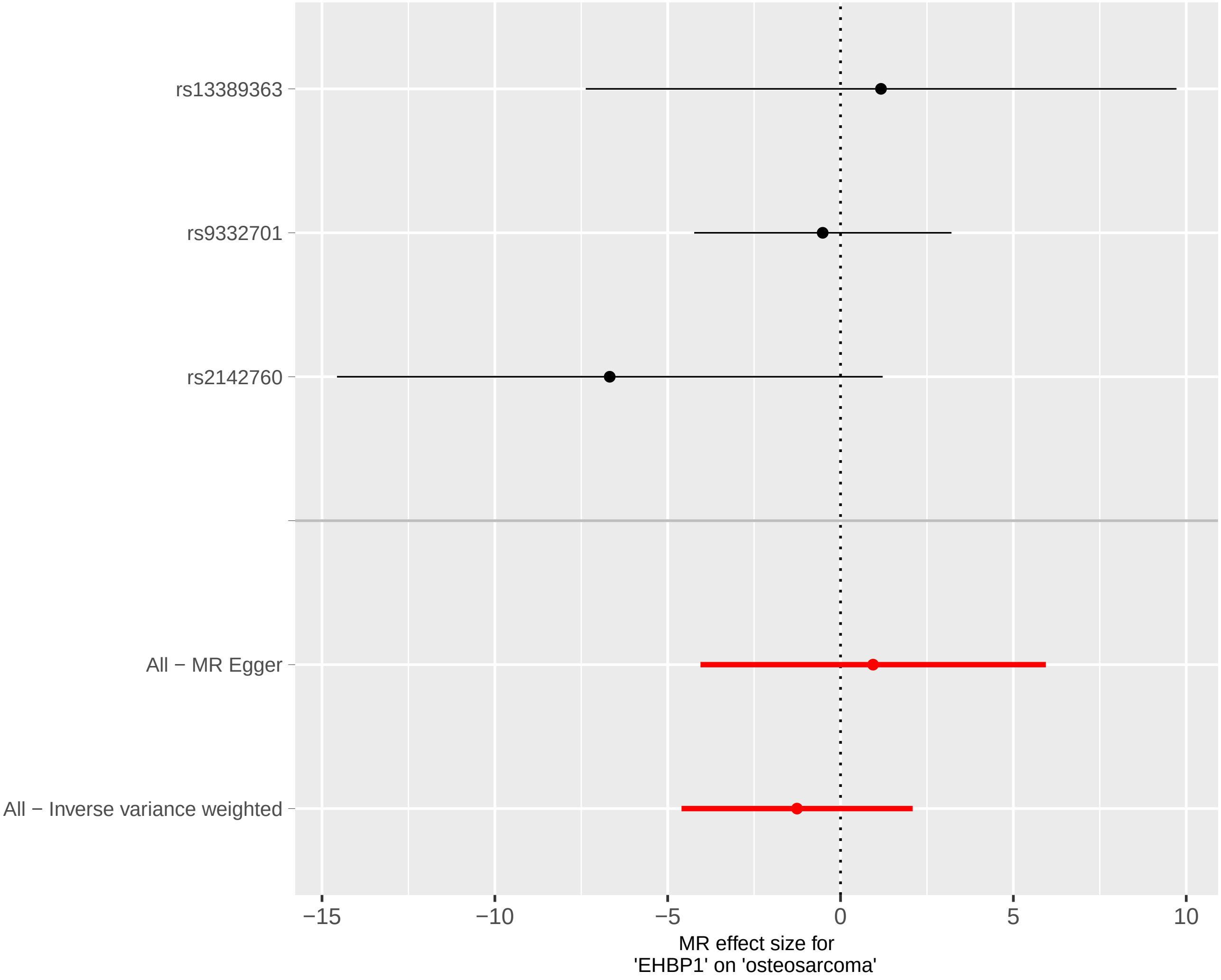

rs13389363
rs9332701
rs2142760
All − MR Egger
All − Inverse variance weighted
−15
−10
−5
0
5
10
MR effect size for
'EHBP1' on 'osteosarcoma'

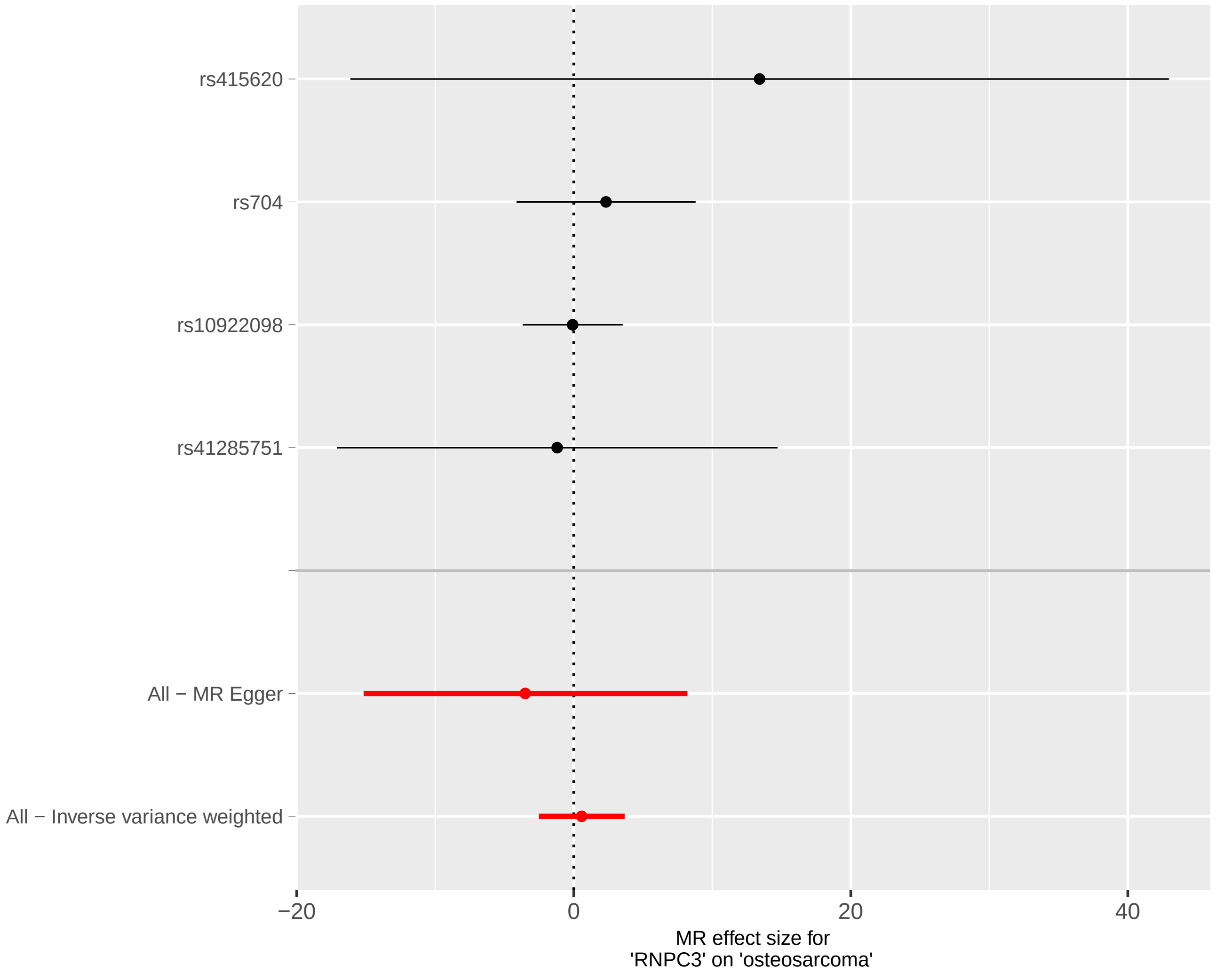

rs415620
rs704
rs10922098
rs41285751
All − MR Egger
All − Inverse variance weighted
−20
0
20
40
MR effect size for
'RNPC3' on 'osteosarcoma'

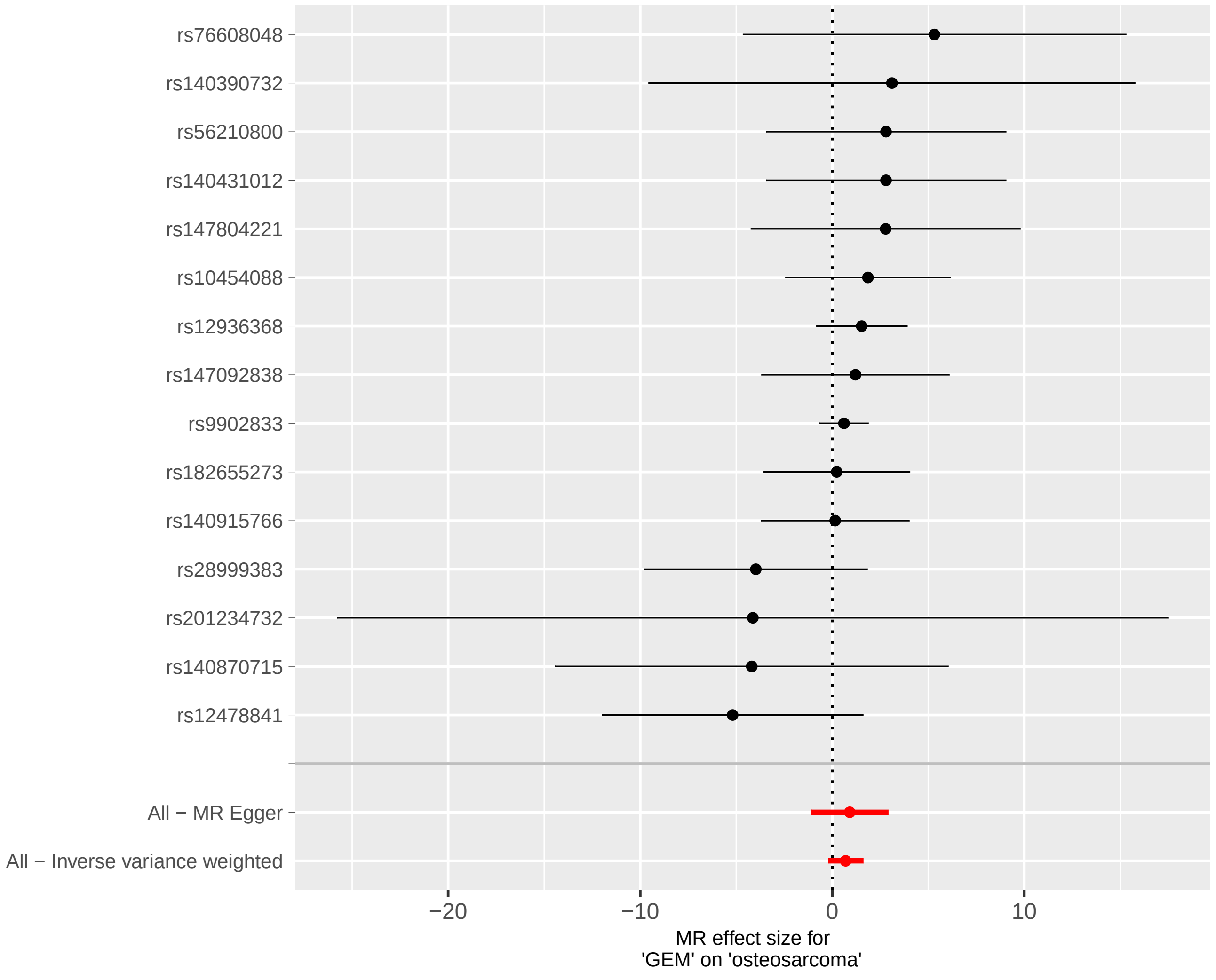
rs76608048
rs140390732
rs56210800
rs140431012
rs147804221
rs10454088
rs12936368
rs147092838
rs9902833
rs182655273
rs140915766
rs28999383
rs201234732
rs140870715
rs12478841
All − MR Egger
All − Inverse variance weighted
−20
−10
0
10
MR effect size for
'GEM' on 'osteosarcoma'

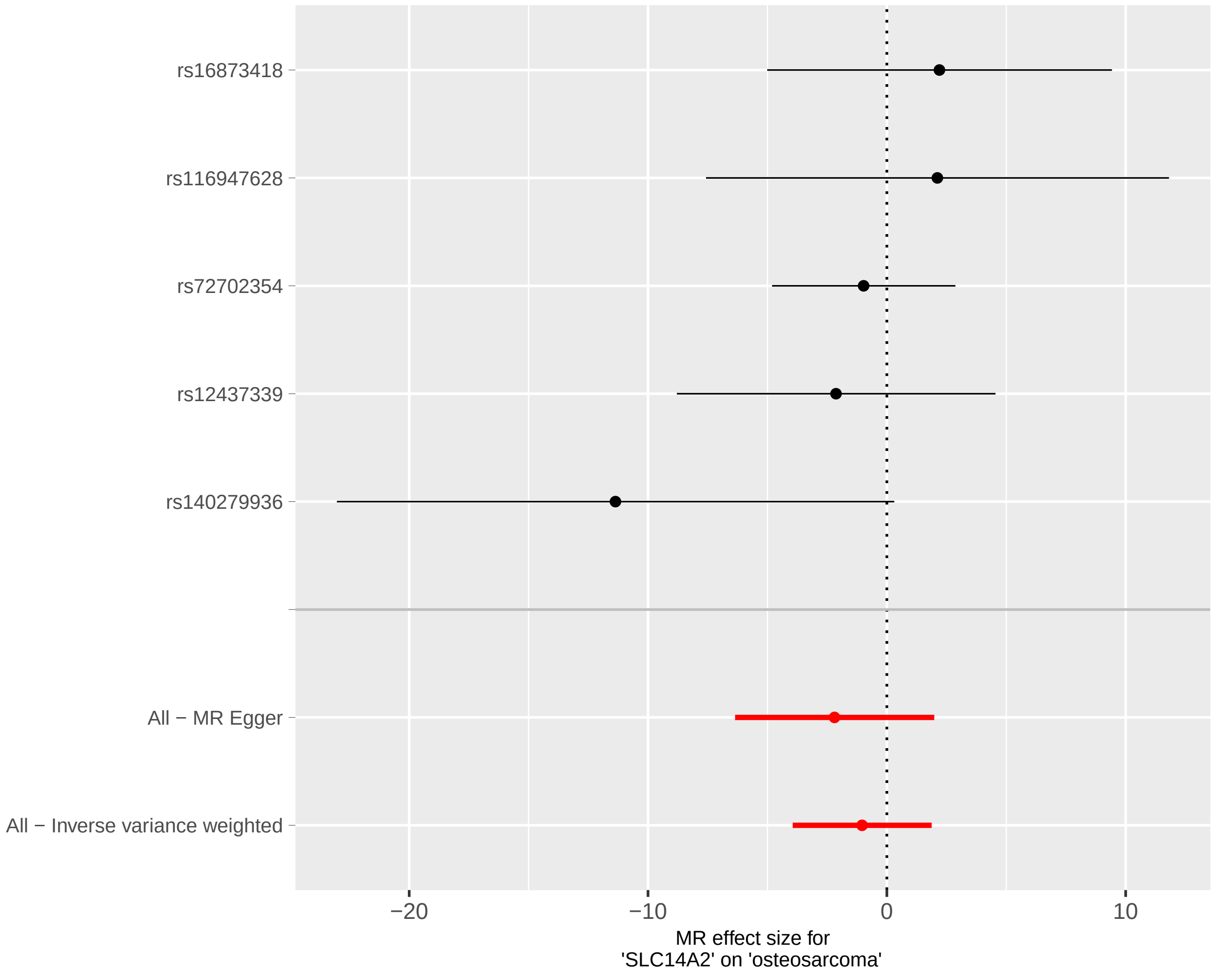

rs16873418
rs116947628
rs72702354
rs12437339
rs140279936
All − MR Egger
All − Inverse variance weighted
−20
−10
0
10
MR effect size for
'SLC14A2' on 'osteosarcoma'

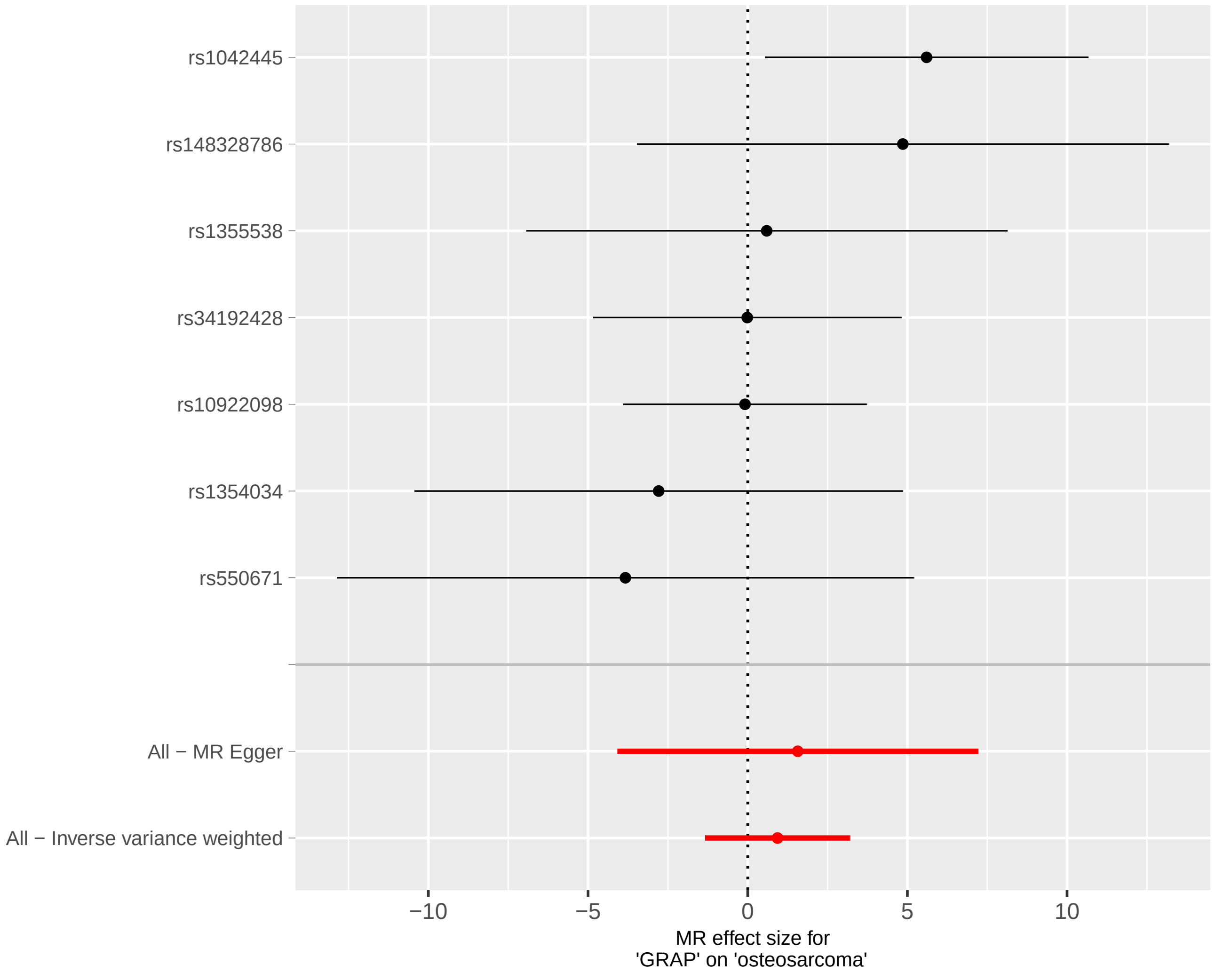

rs1042445
rs148328786
rs1355538
rs34192428
rs10922098
rs1354034
rs550671
All − MR Egger
All − Inverse variance weighted
−10
−5
0
5
10
MR effect size for
'GRAP' on 'osteosarcoma'

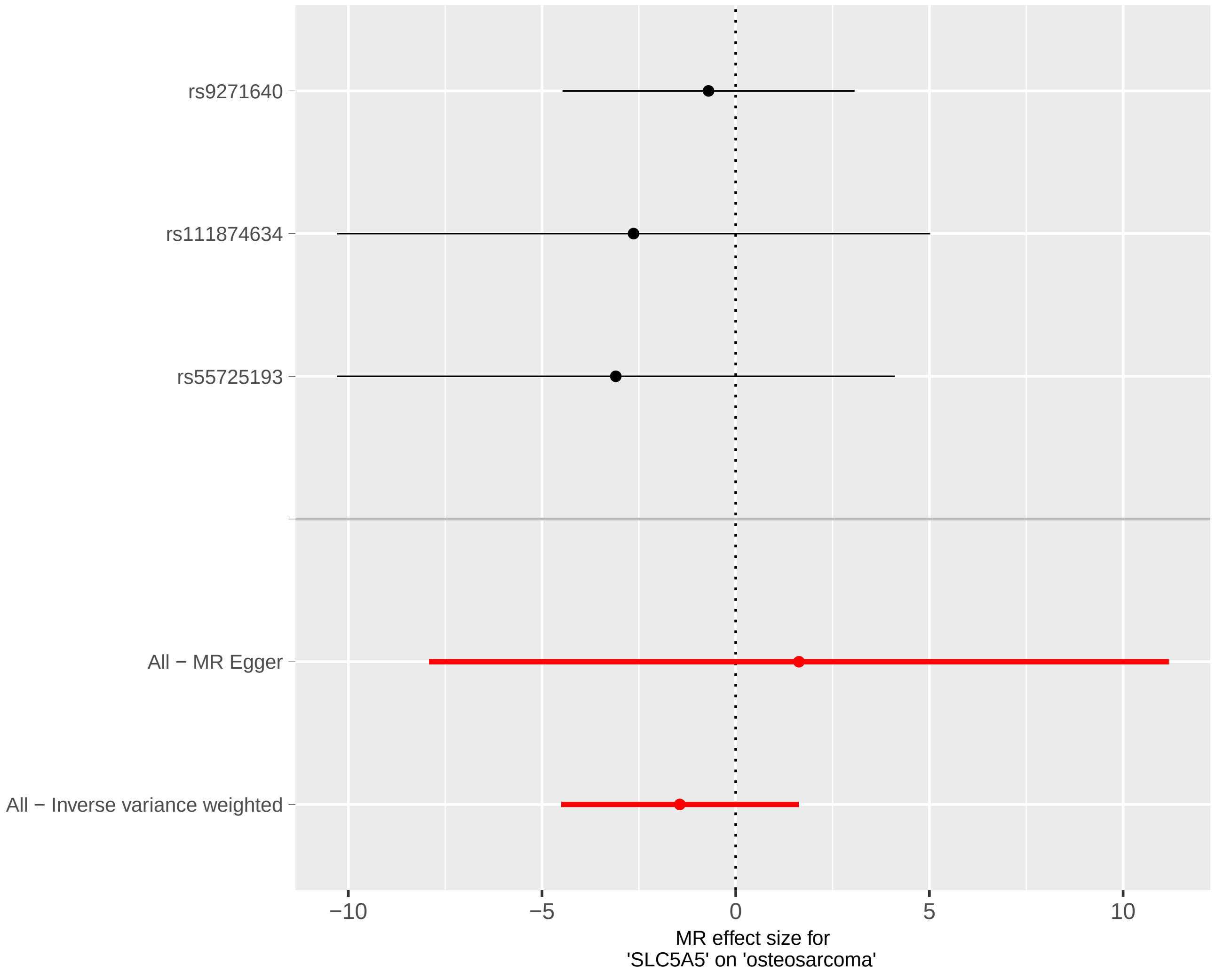

rs9271640
rs111874634
rs55725193
All − MR Egger
All − Inverse variance weighted
−10
−5
0
5
10
MR effect size for
'SLC5A5' on 'osteosarcoma'

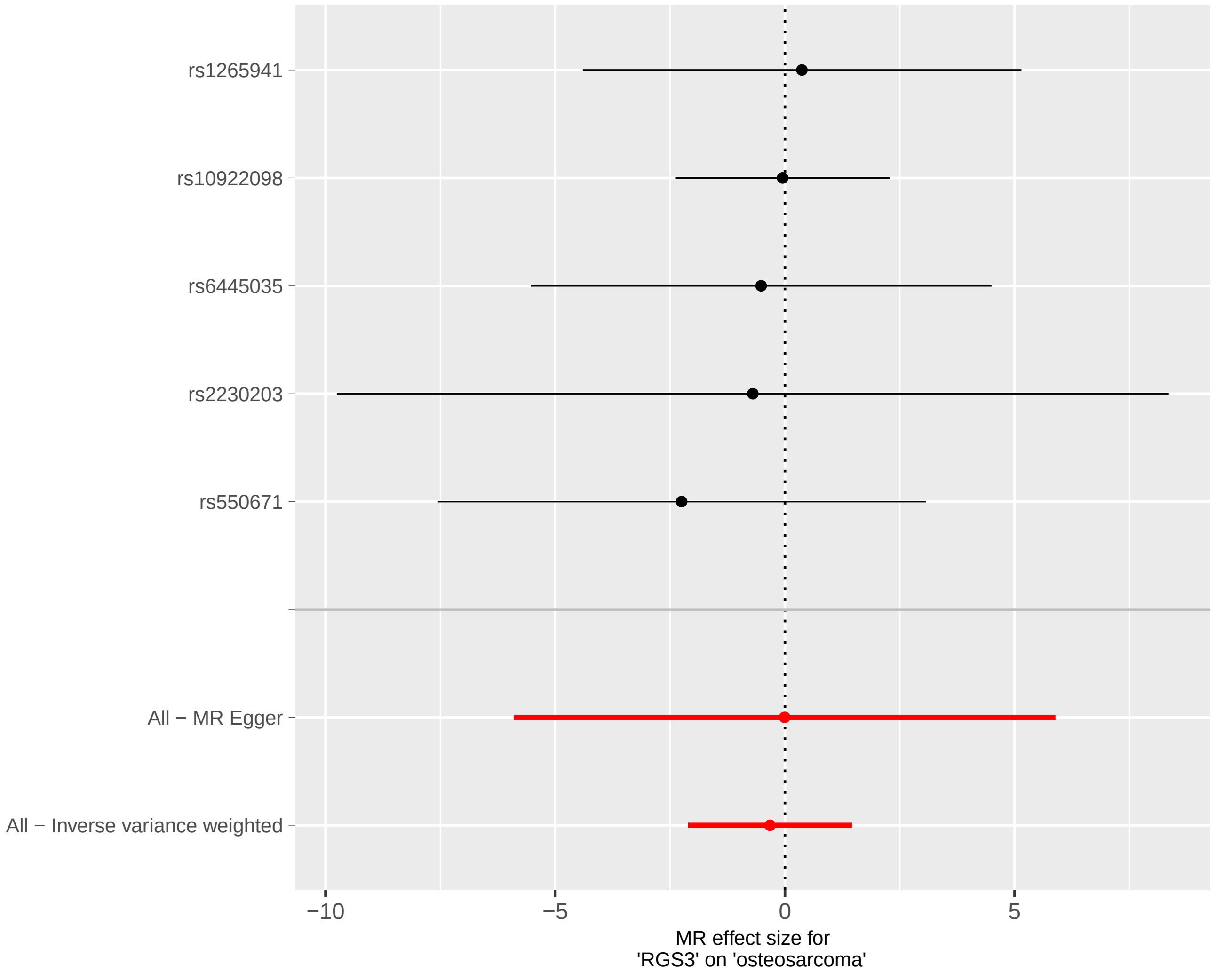

rs1265941
rs10922098
rs6445035
rs2230203
rs550671
All − MR Egger
All − Inverse variance weighted
−10
−5
0
5
MR effect size for
'RGS3' on 'osteosarcoma'

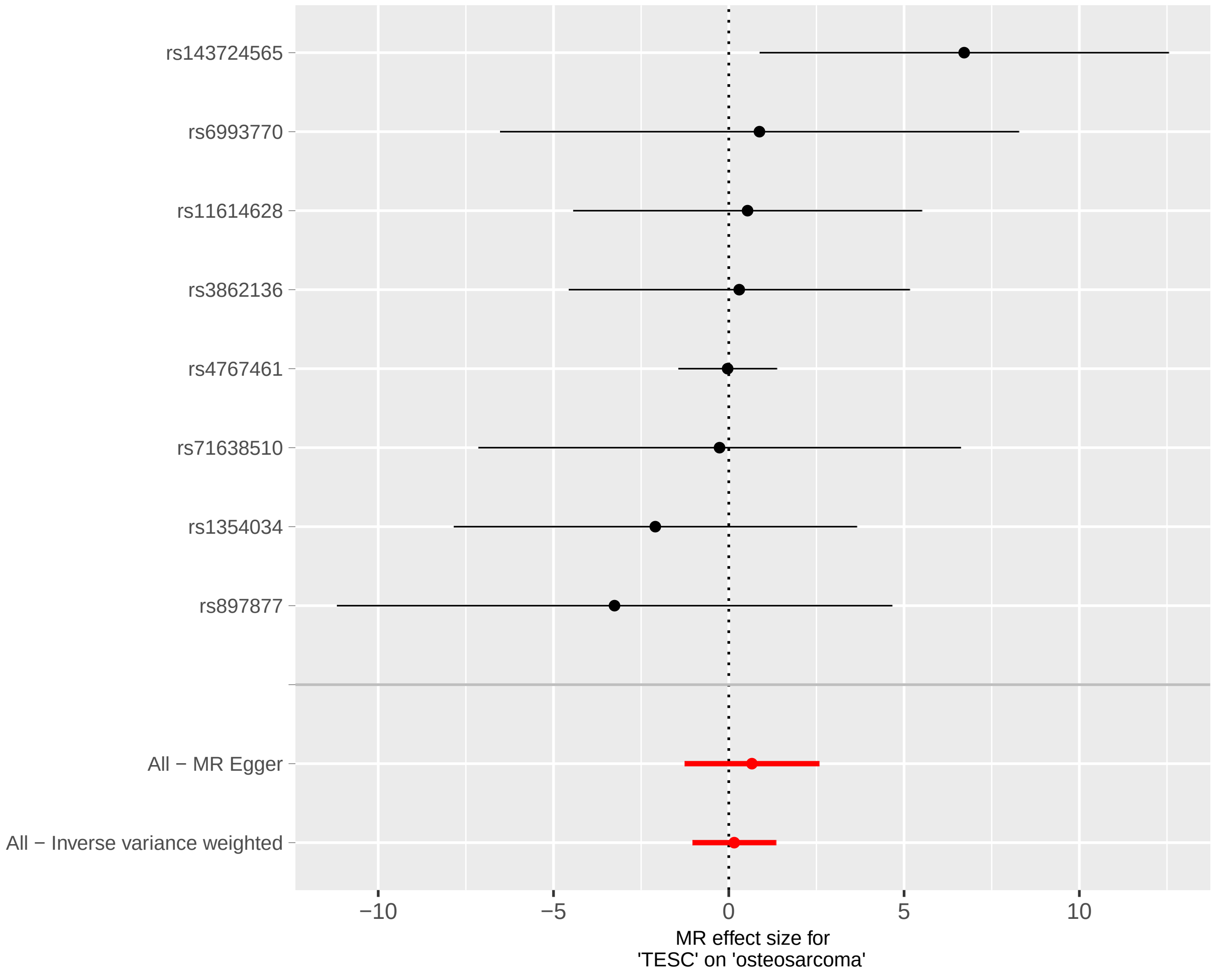

rs143724565
rs6993770
rs11614628
rs3862136
rs4767461
rs71638510
rs1354034
rs897877
All − MR Egger
All − Inverse variance weighted
−10
−5
0
5
10
MR effect size for
'TESC' on 'osteosarcoma'

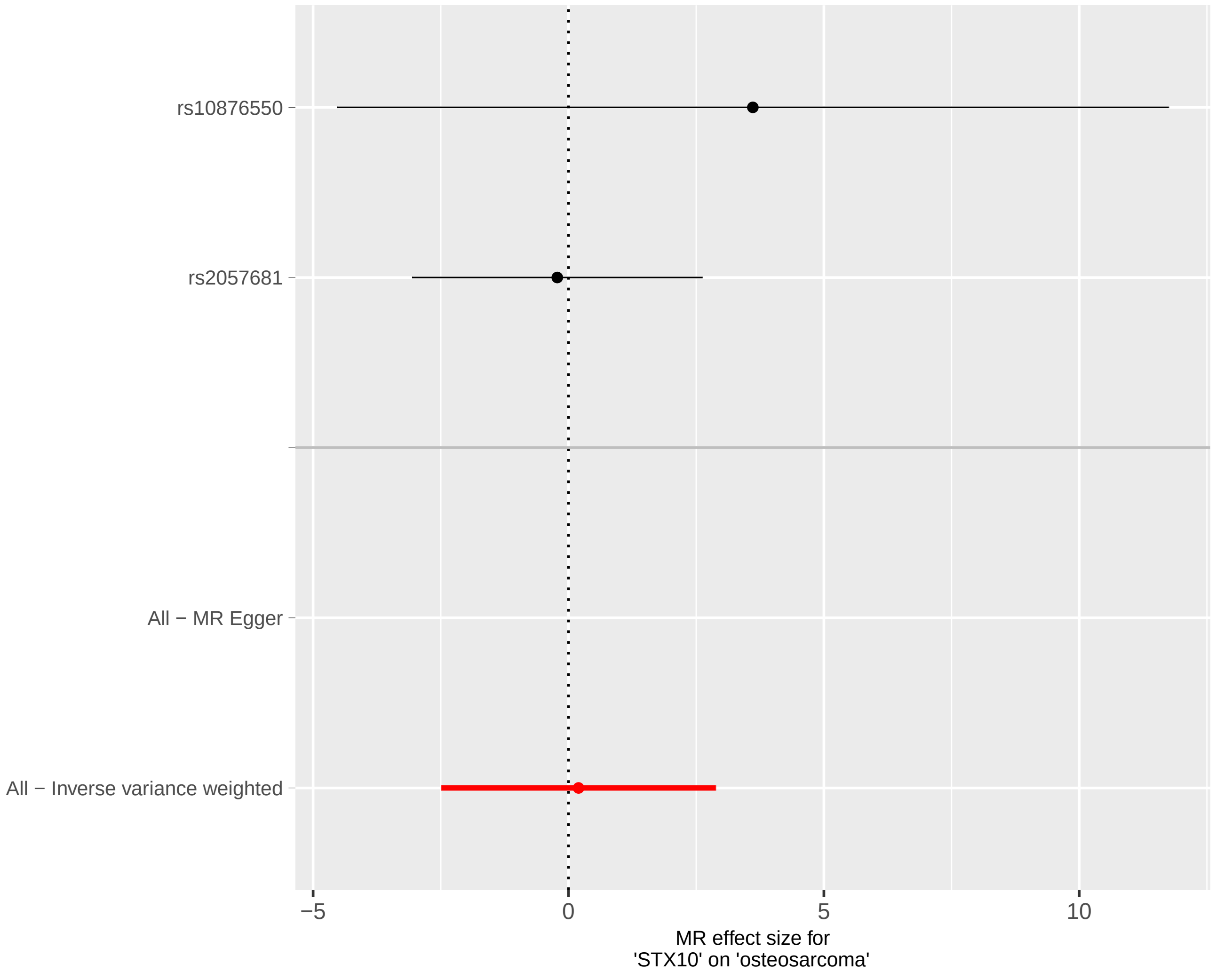
rs10876550
rs2057681
All − MR Egger
All − Inverse variance weighted
−5
0
5
10
MR effect size for
'STX10' on 'osteosarcoma'

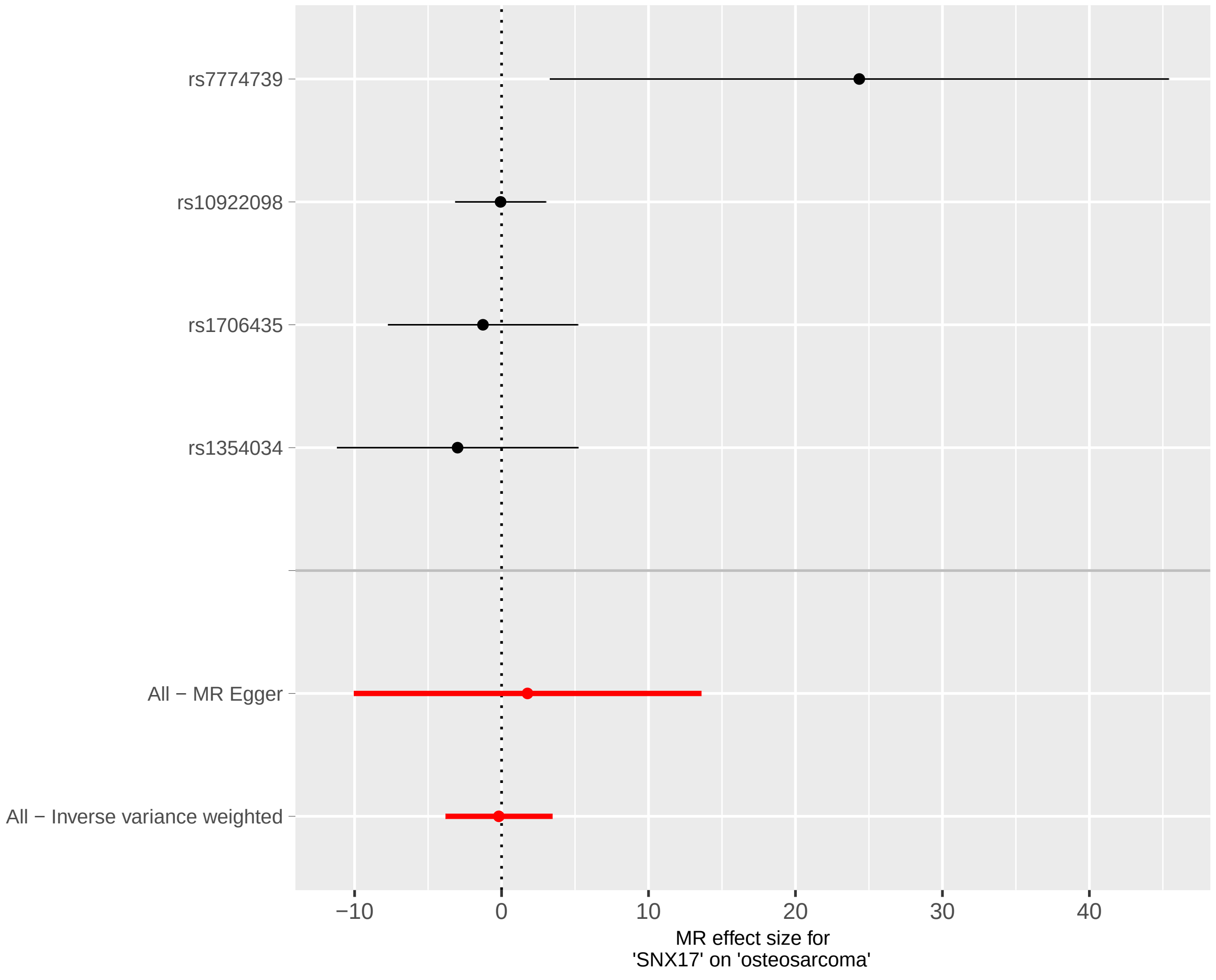
rs7774739
rs10922098
rs1706435
rs1354034
All − MR Egger
All − Inverse variance weighted
−10
0
10
20
30
40
MR effect size for
'SNX17' on 'osteosarcoma'

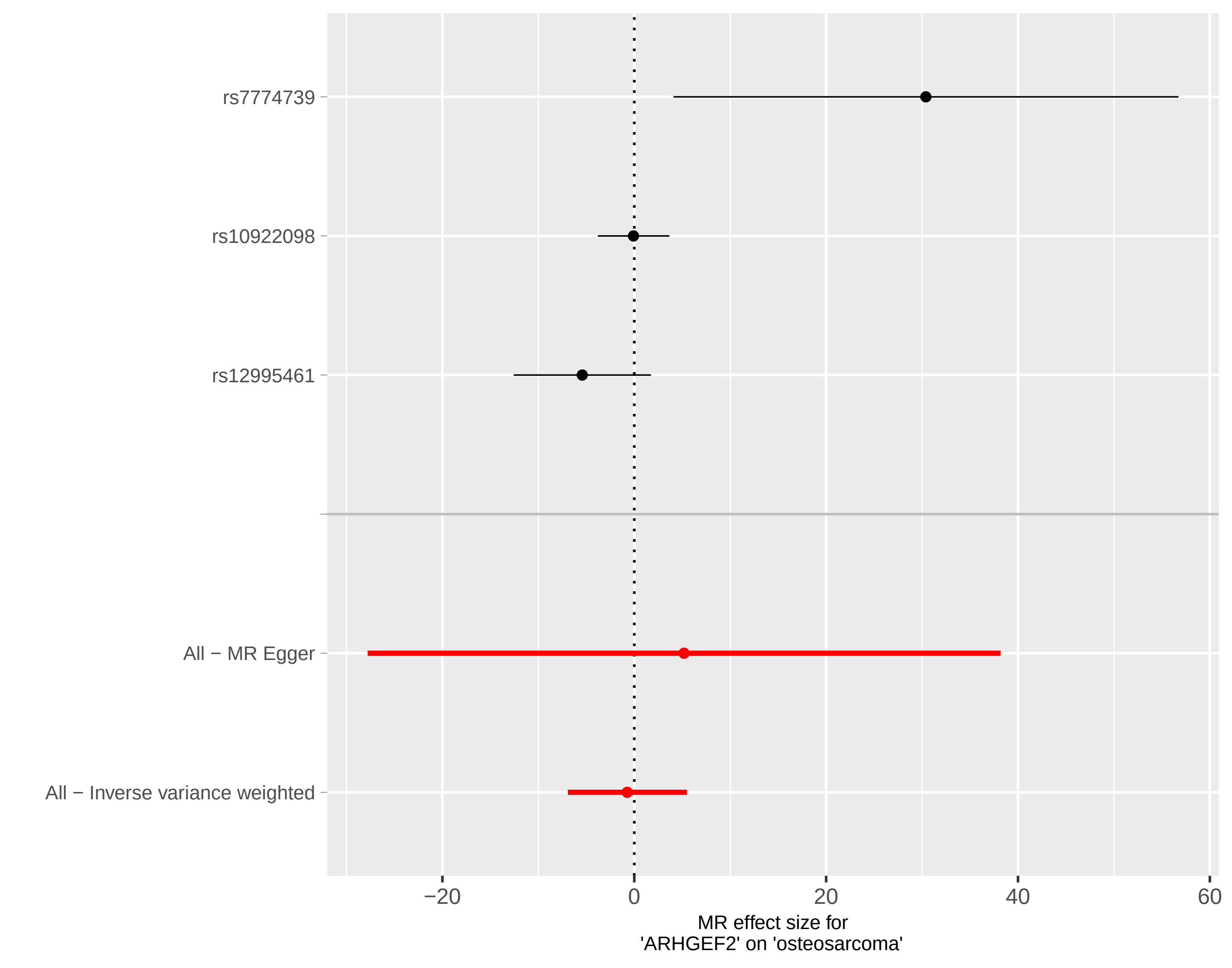

rs7774739
rs10922098
rs12995461
All − MR Egger
All − Inverse variance weighted
−20
0
20
40
60
MR effect size for
'ARHGEF2' on 'osteosarcoma'

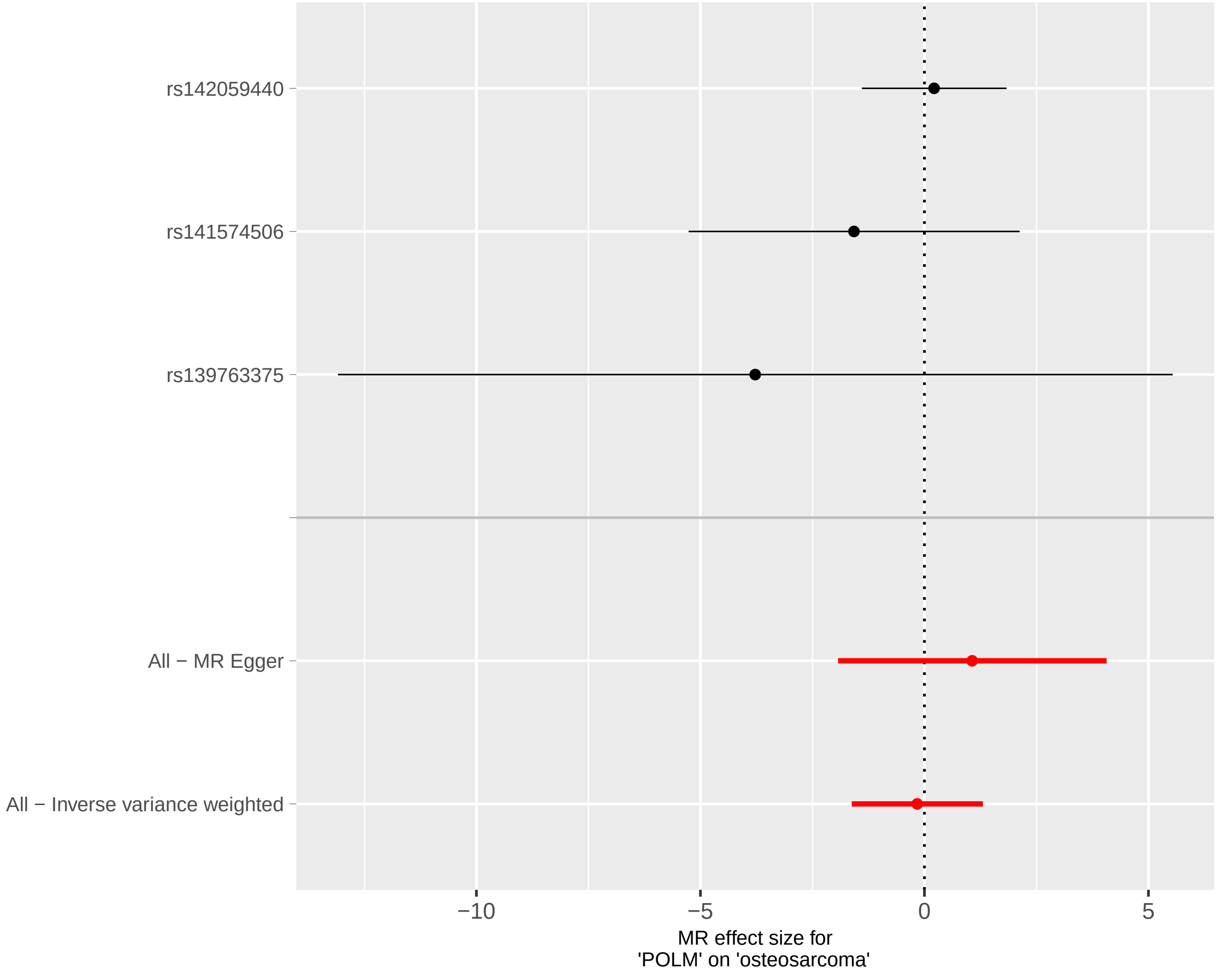

rs142059440
rs141574506
rs139763375
All − MR Egger
All − Inverse variance weighted
−10
−5
0
5
MR effect size for
'POLM' on 'osteosarcoma'

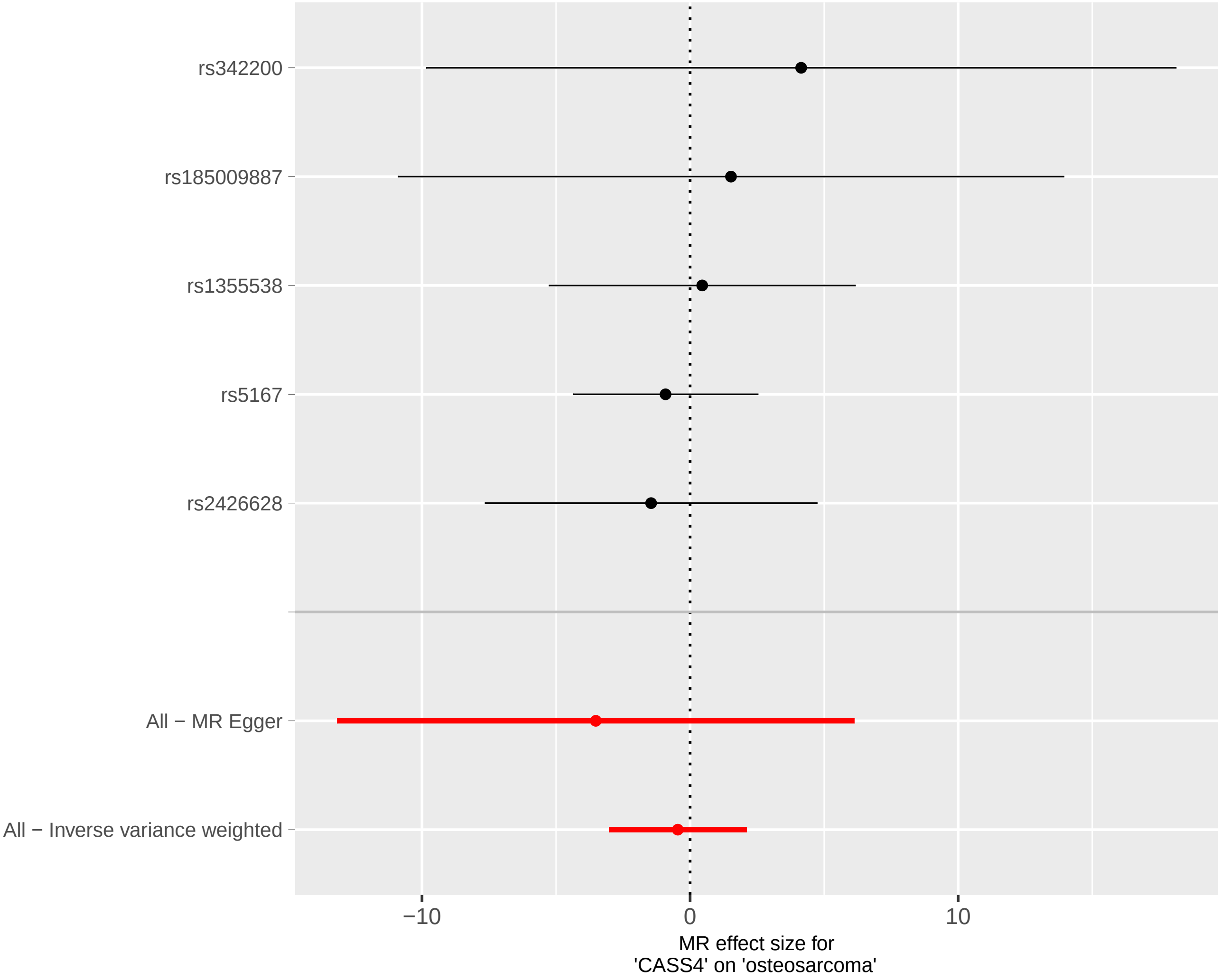
rs342200
rs185009887
rs1355538
rs5167
rs2426628
All − MR Egger
All − Inverse variance weighted
−10
0
10
MR effect size for
'CASS4' on 'osteosarcoma'

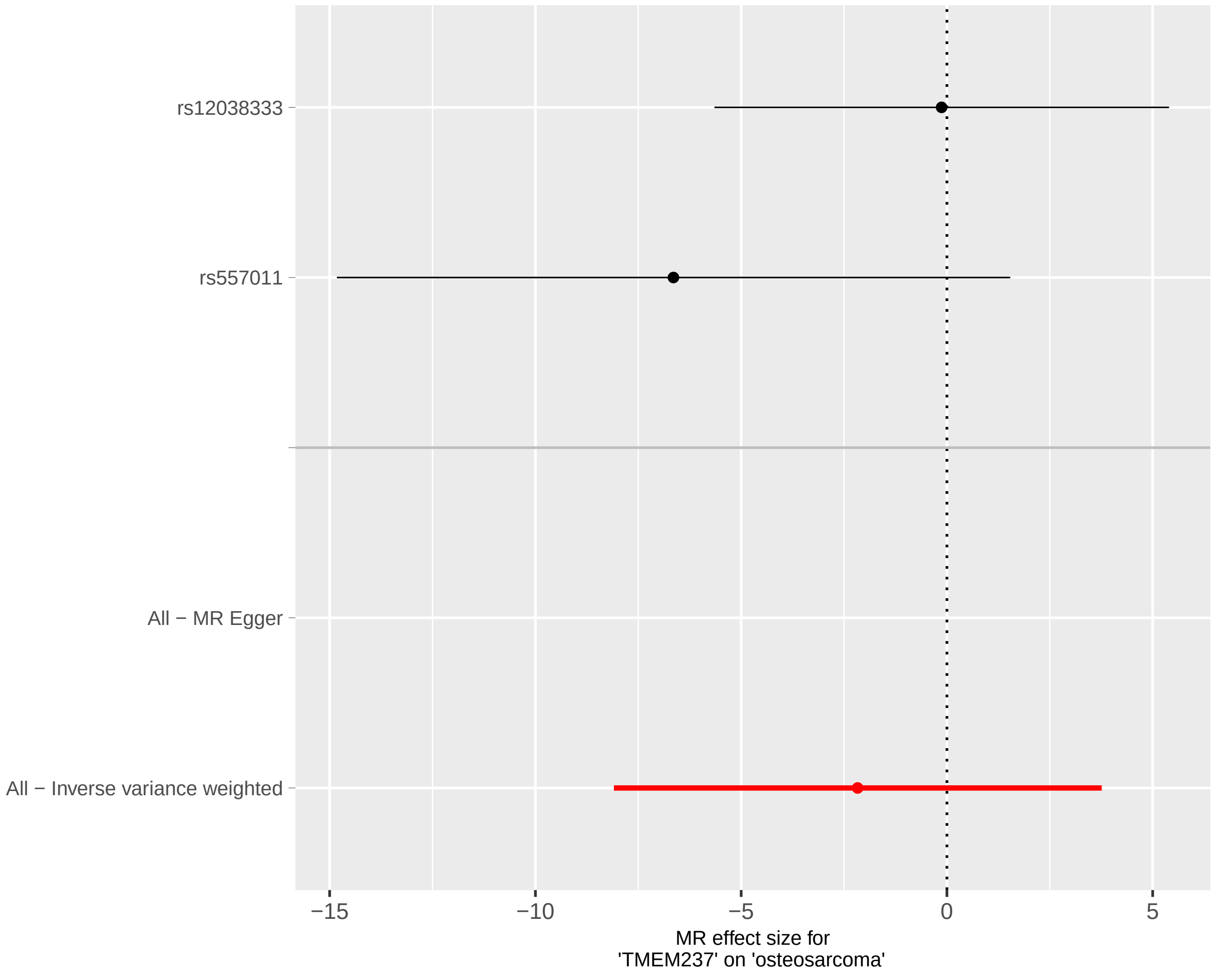
rs12038333
rs557011
All − MR Egger
All − Inverse variance weighted
−15
−10
−5
0
5
MR effect size for
'TMEM237' on 'osteosarcoma'

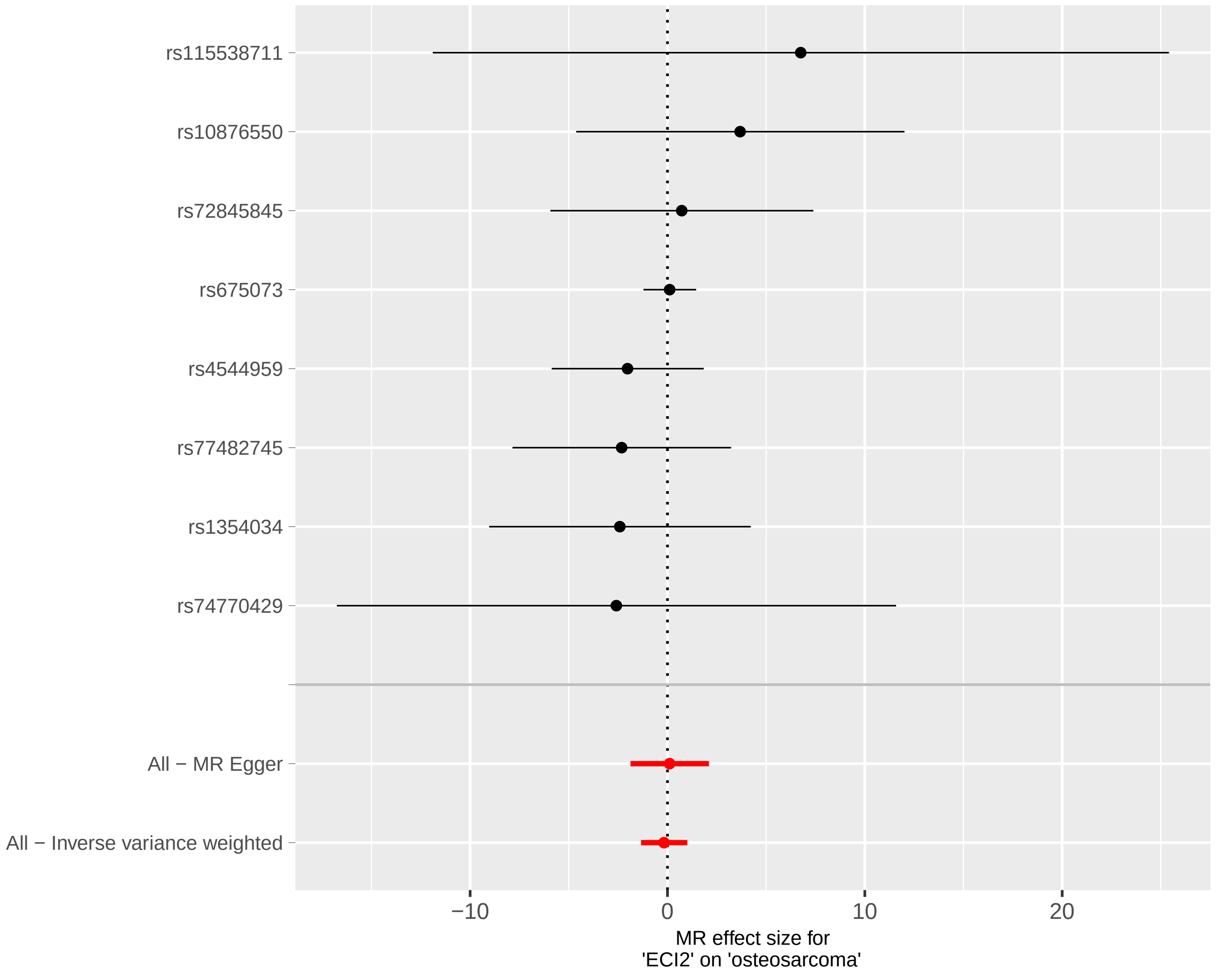
rs115538711
rs10876550
rs72845845
rs675073
rs4544959
rs77482745
rs1354034
rs74770429
All − MR Egger
All − Inverse variance weighted
−10
0
10
20
MR effect size for
'ECI2' on 'osteosarcoma'

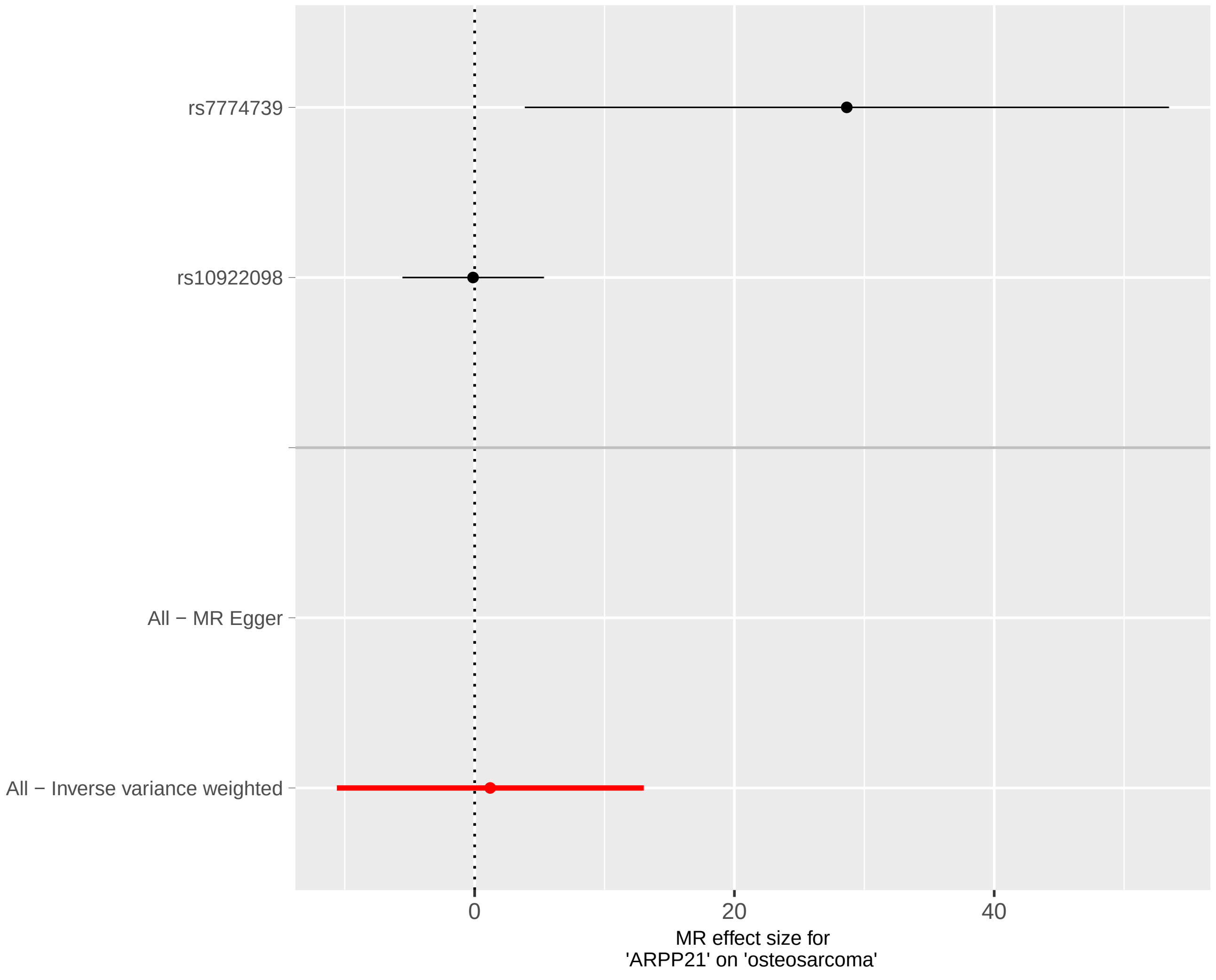
rs7774739
rs10922098
All − MR Egger
All − Inverse variance weighted
0
20
40
MR effect size for
'ARPP21' on 'osteosarcoma'

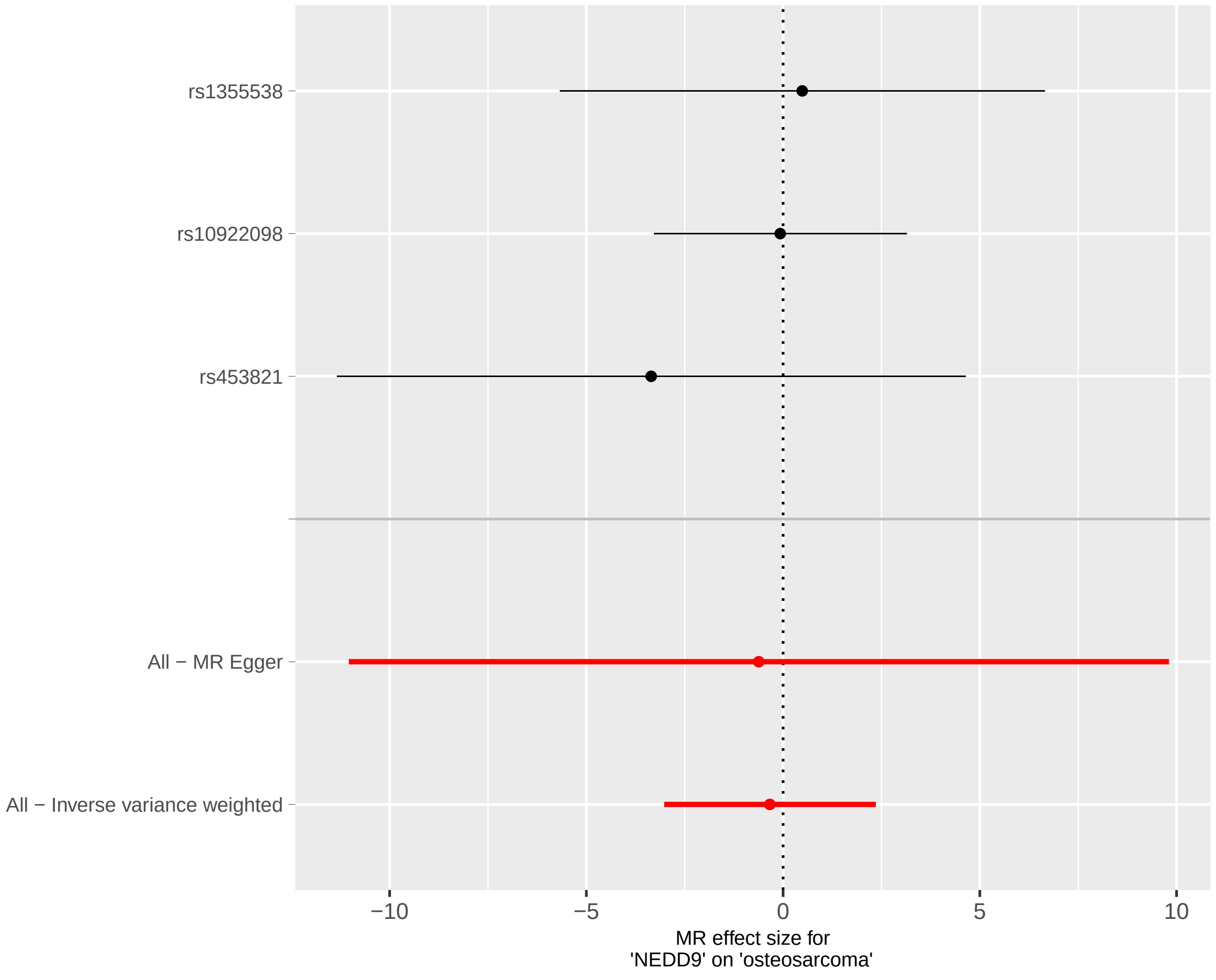

rs1355538
rs10922098
rs453821
All − MR Egger
All − Inverse variance weighted
−10
−5
0
5
10
MR effect size for
'NEDD9' on 'osteosarcoma'

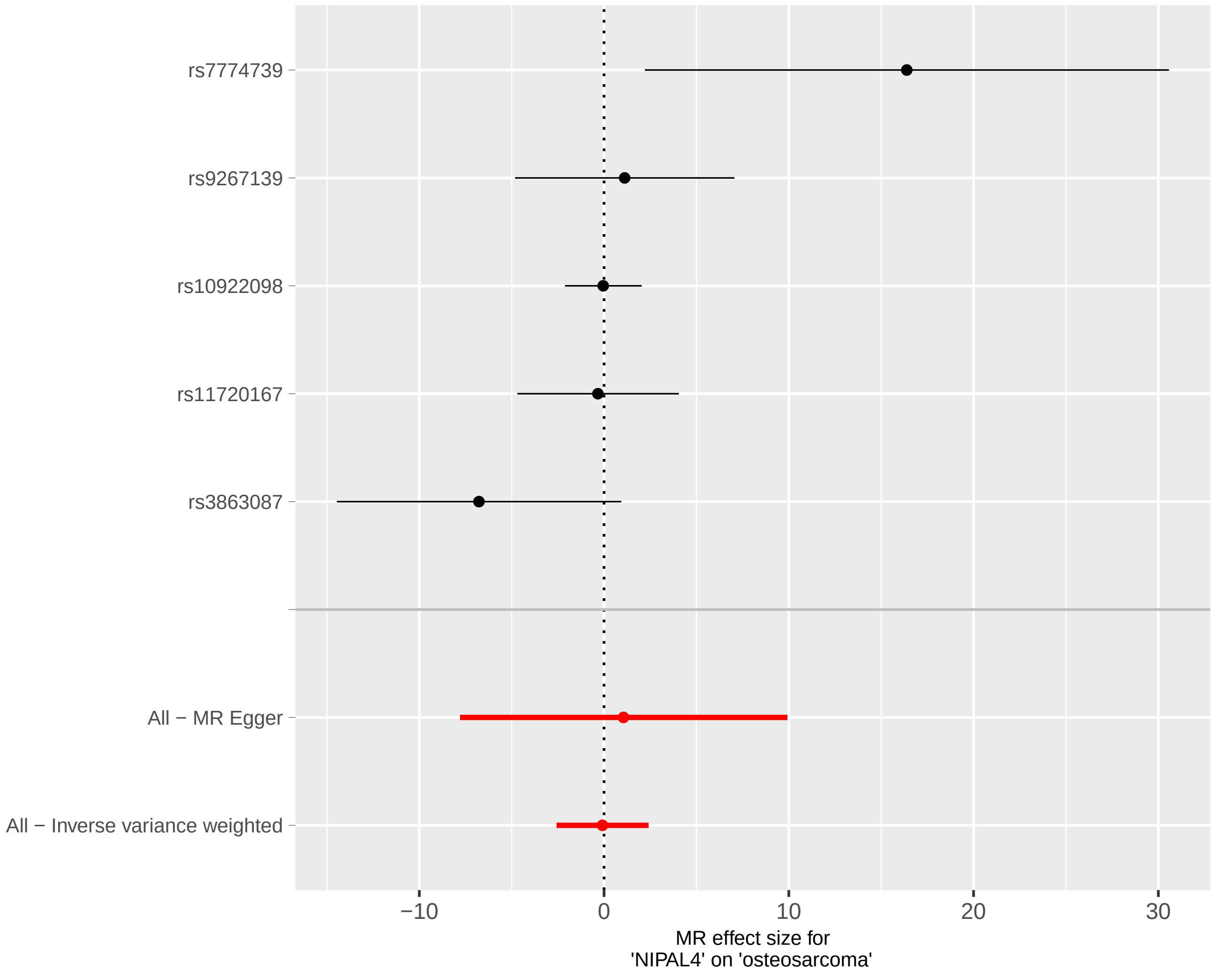

rs7774739
rs9267139
rs10922098
rs11720167
rs3863087
All − MR Egger
All − Inverse variance weighted
−10
0
10
20
30
MR effect size for
'NIPAL4' on 'osteosarcoma'

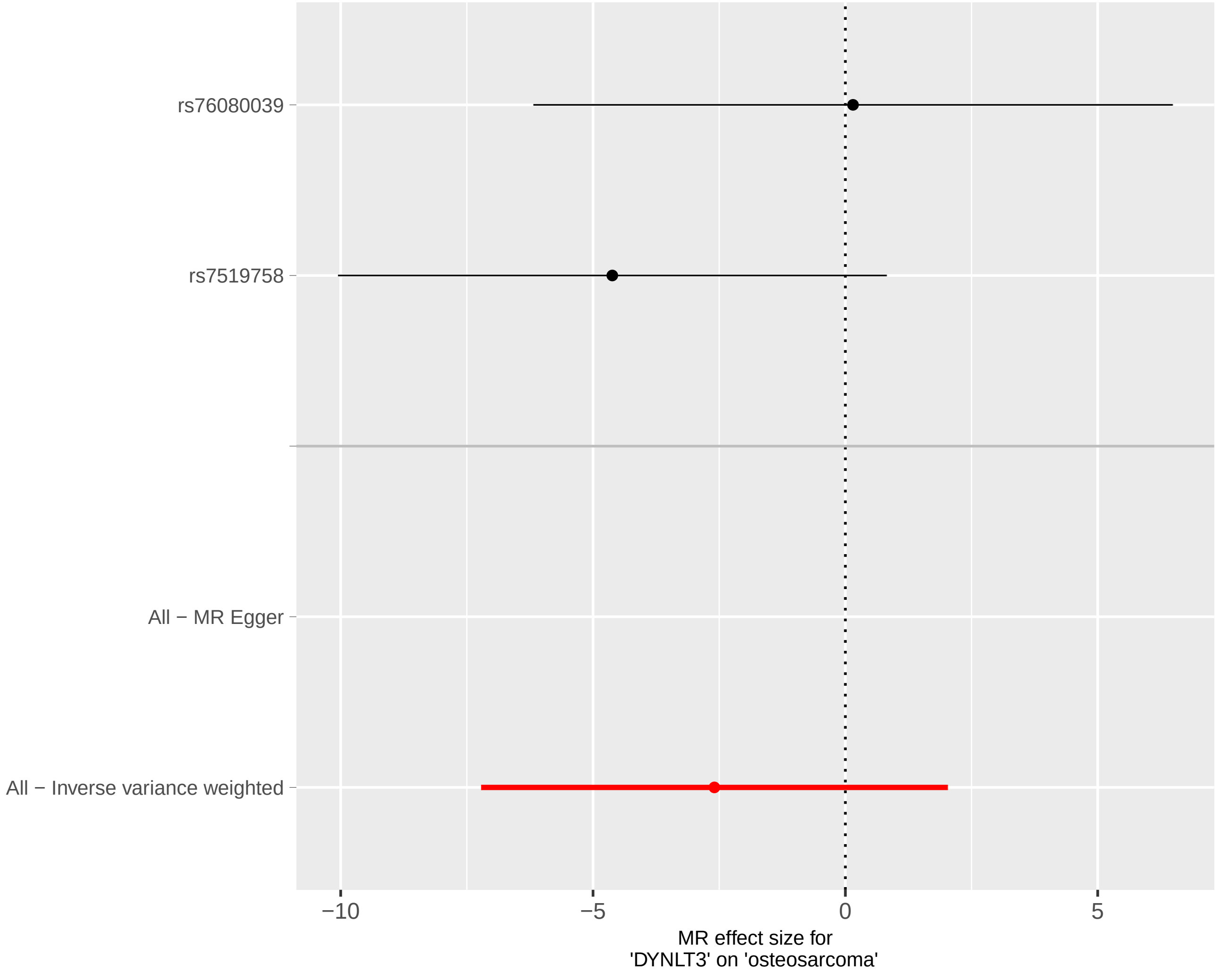
rs76080039
rs7519758
All − MR Egger
All − Inverse variance weighted
−10
−5
0
5
MR effect size for
'DYNLT3' on 'osteosarcoma'

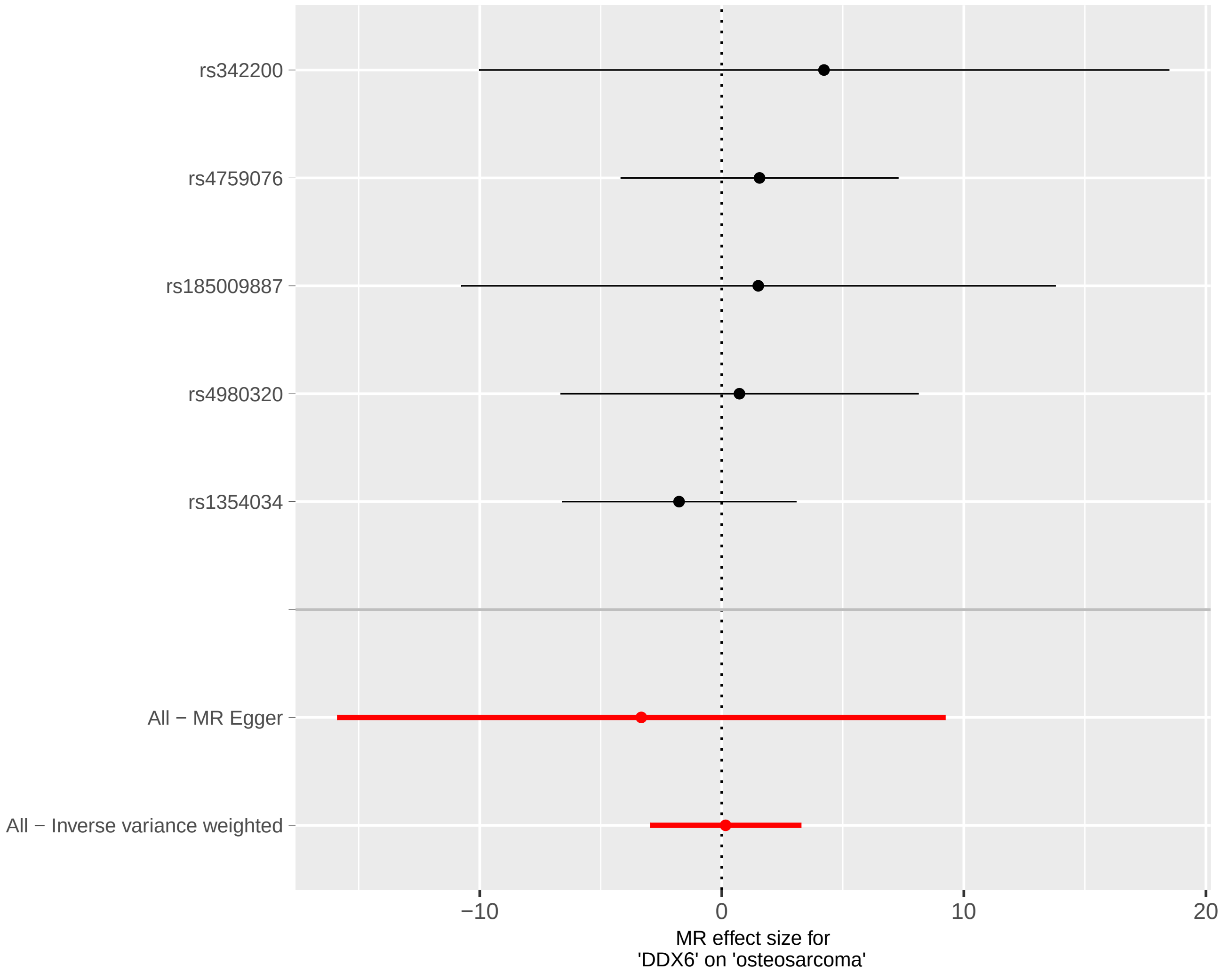
rs342200
rs4759076
rs185009887
rs4980320
rs1354034
All − MR Egger
All − Inverse variance weighted
−10
0
10
20
MR effect size for
'DDX6' on 'osteosarcoma'

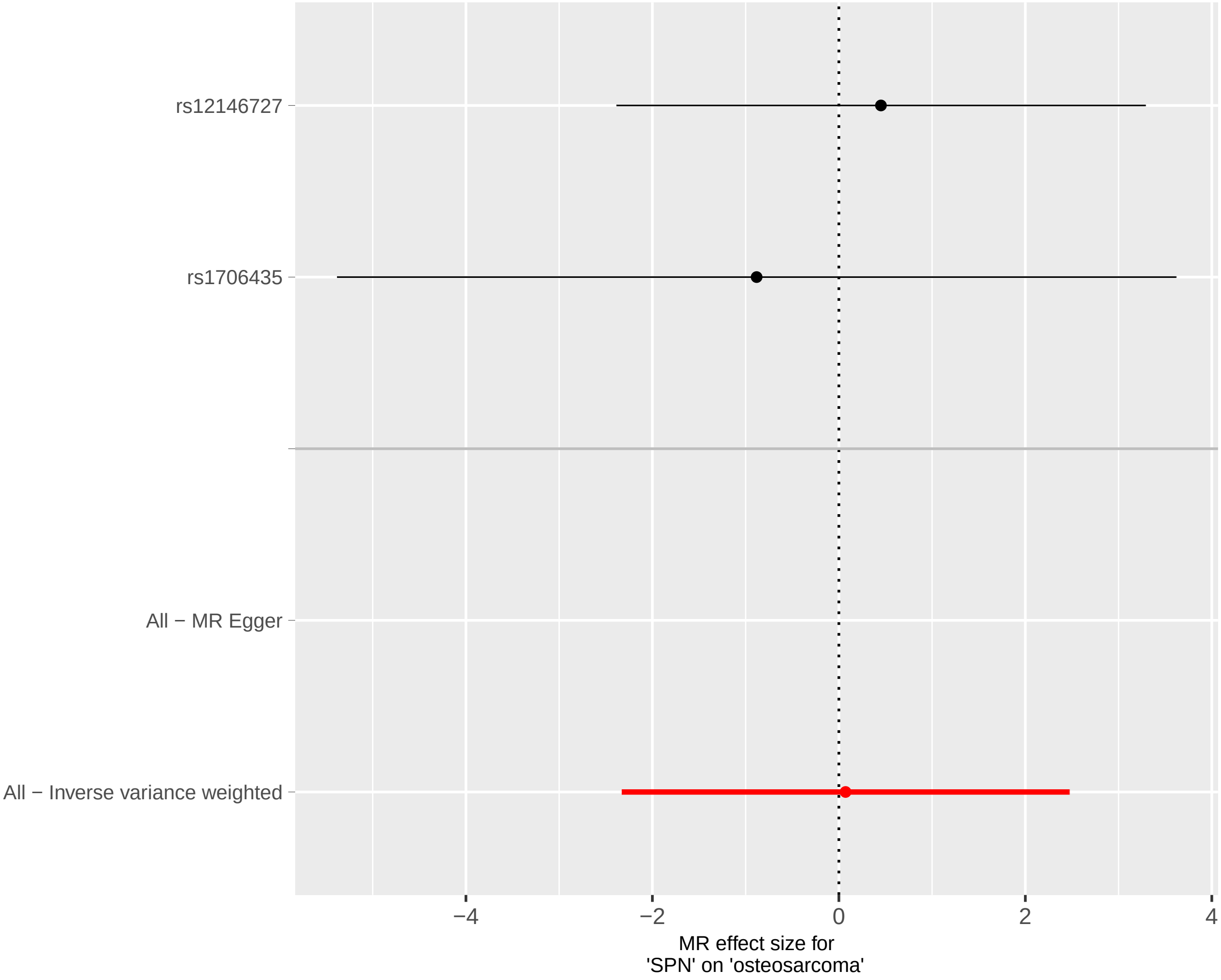
rs12146727
rs1706435
All − MR Egger
All − Inverse variance weighted
−4
−2
0
2
4
MR effect size for
'SPN' on 'osteosarcoma'

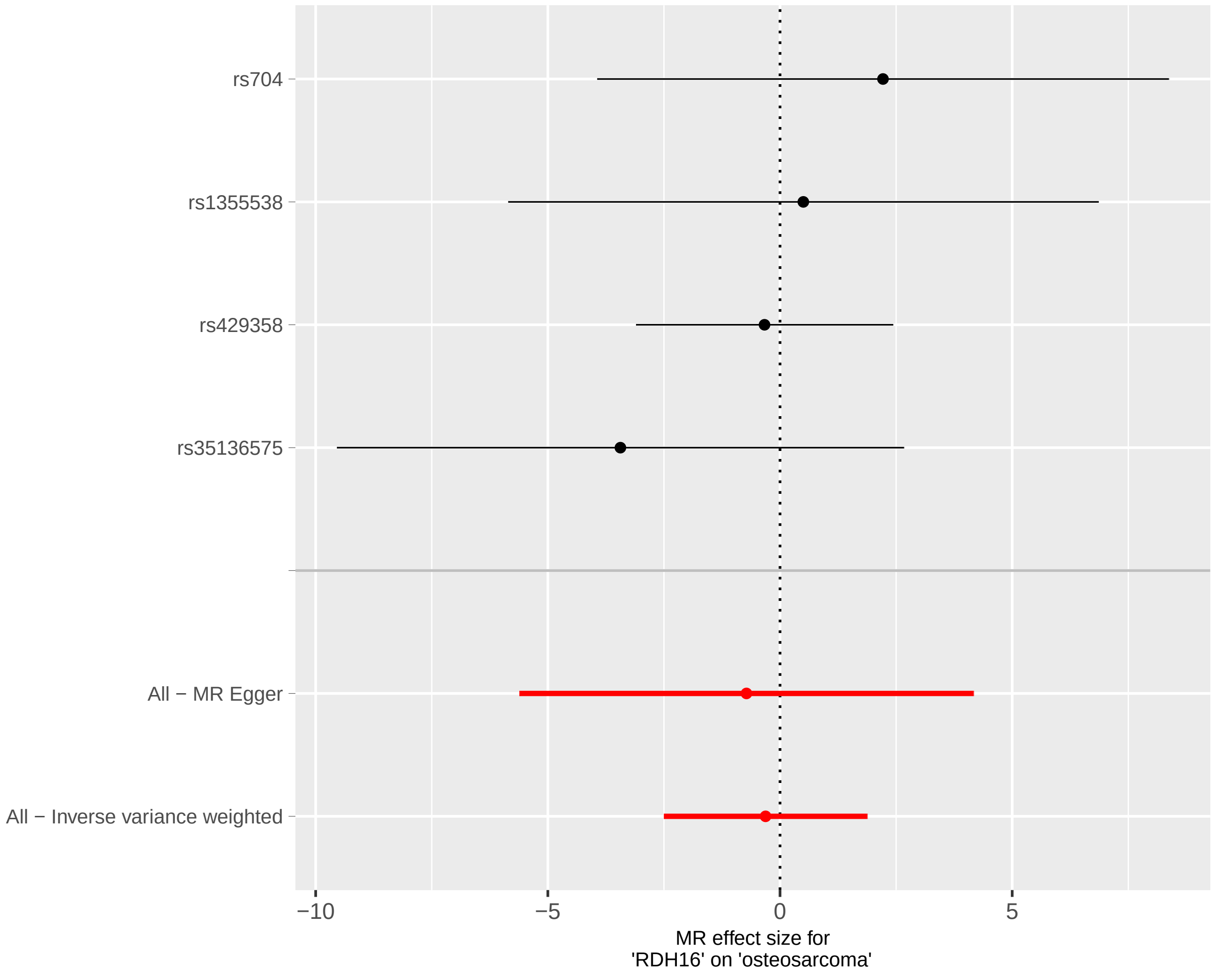
rs704
rs1355538
rs429358
rs35136575
All − MR Egger
All − Inverse variance weighted
−10
−5
0
5
MR effect size for
'RDH16' on 'osteosarcoma'

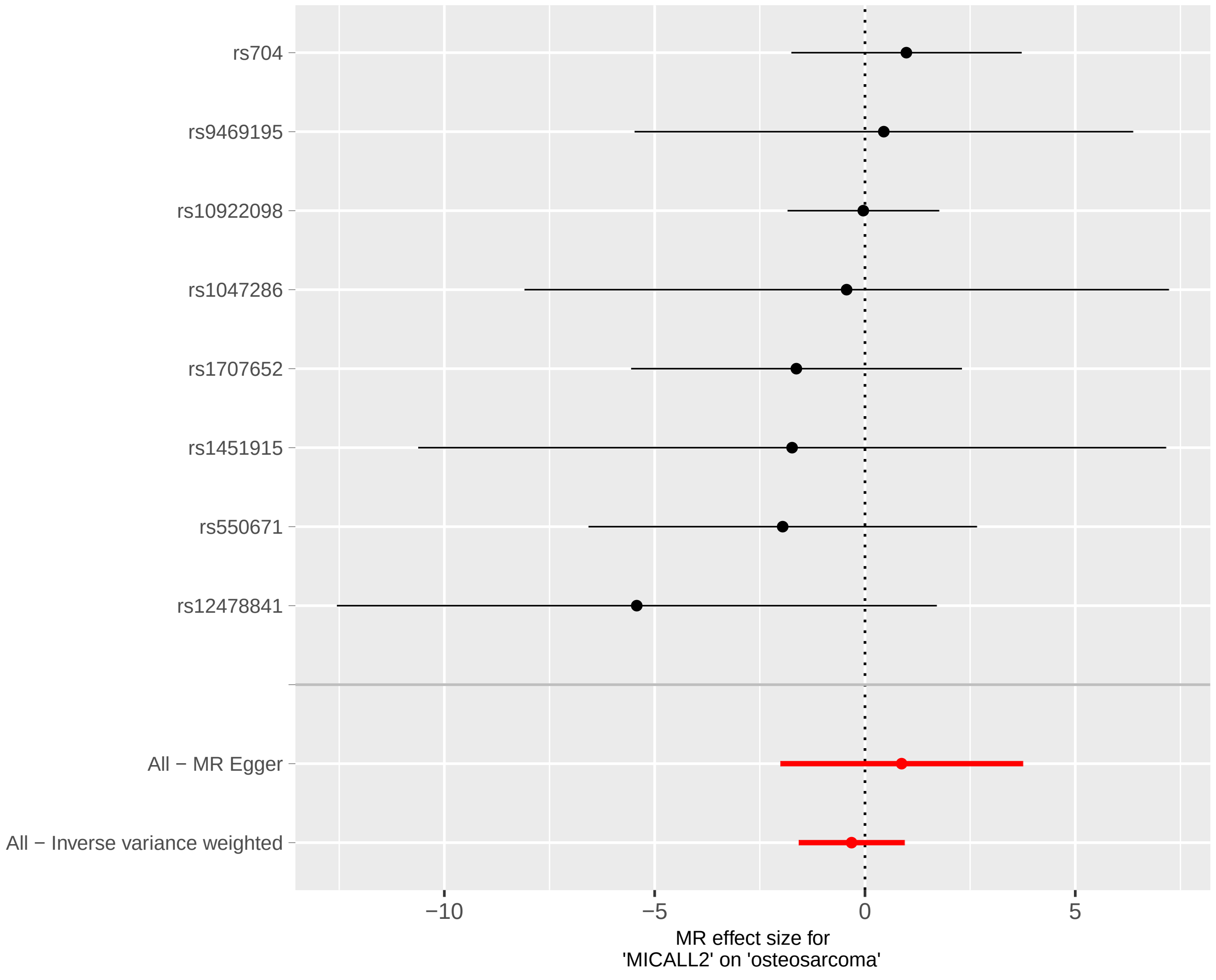
rs704
rs9469195
rs10922098
rs1047286
rs1707652
rs1451915
rs550671
rs12478841
All − MR Egger
All − Inverse variance weighted
−10
−5
0
5
MR effect size for
'MICALL2' on 'osteosarcoma'

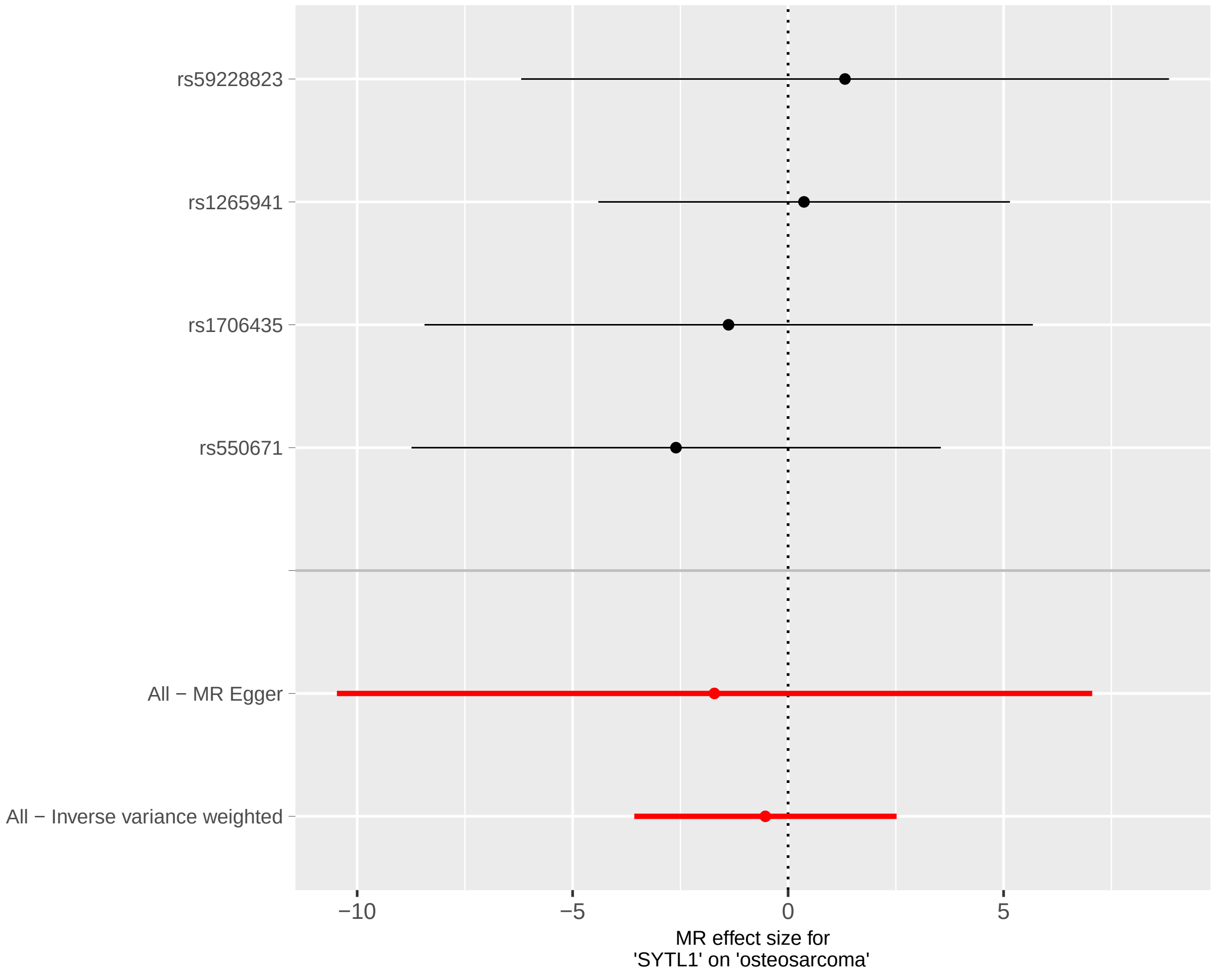
rs59228823
rs1265941
rs1706435
rs550671
All − MR Egger
All − Inverse variance weighted
−10
−5
0
5
MR effect size for
'SYTL1' on 'osteosarcoma'

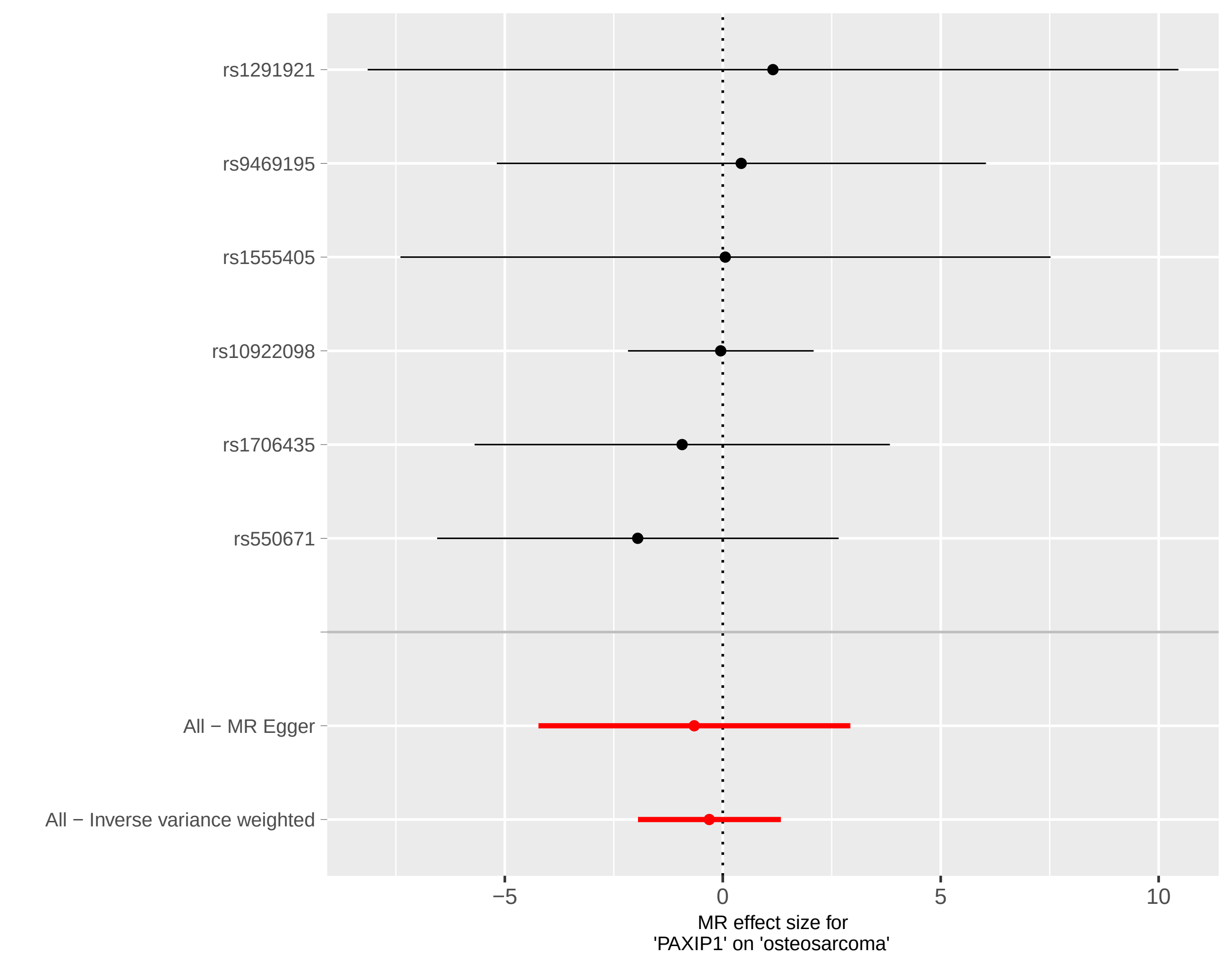

rs1291921
rs9469195
rs1555405
rs10922098
rs1706435
rs550671
All − MR Egger
All − Inverse variance weighted
−5
0
5
10
MR effect size for
'PAXIP1' on 'osteosarcoma'

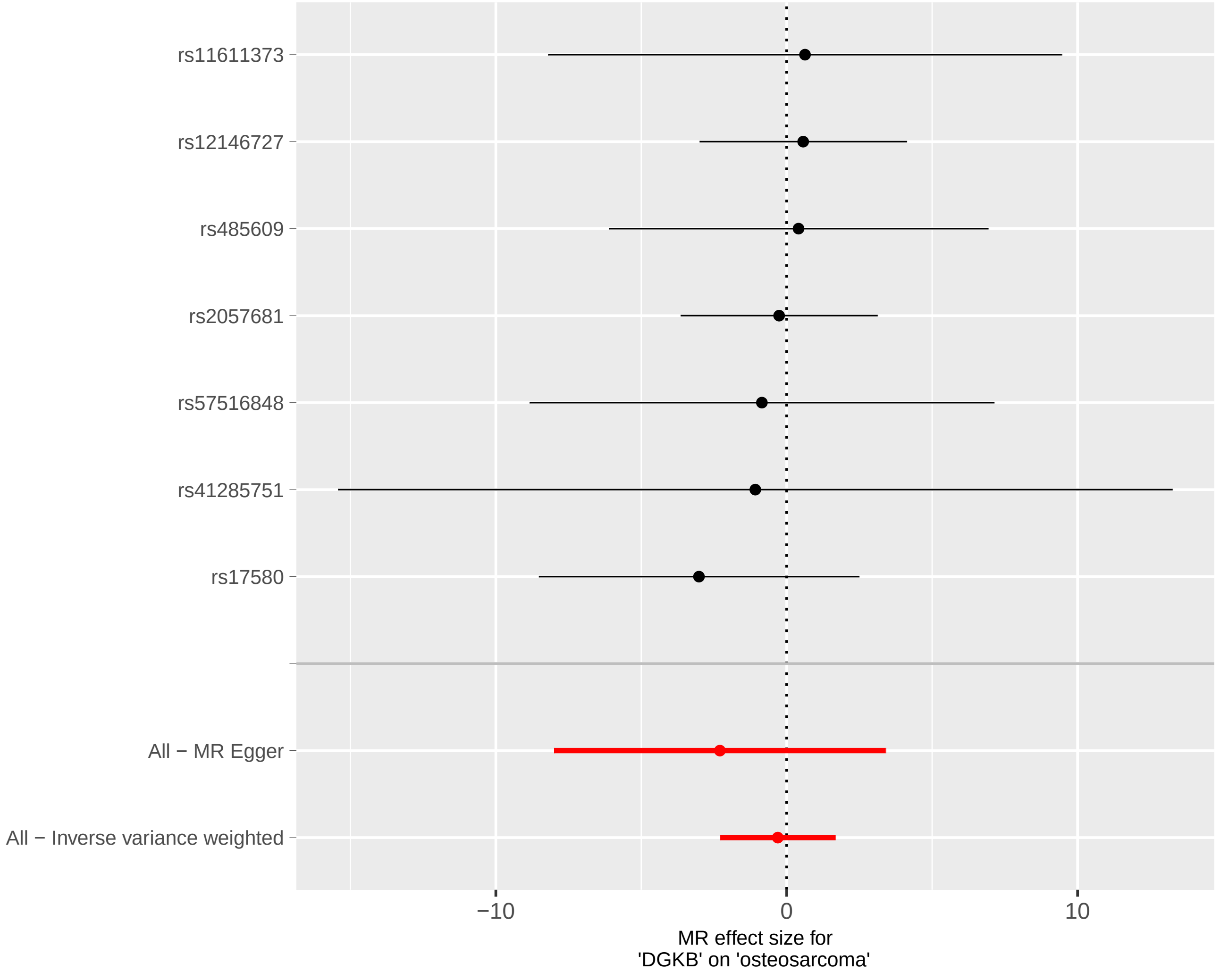

rs11611373
rs12146727
rs485609
rs2057681
rs57516848
rs41285751
rs17580
All − MR Egger
All − Inverse variance weighted
−10
0
10
MR effect size for
'DGKB' on 'osteosarcoma'

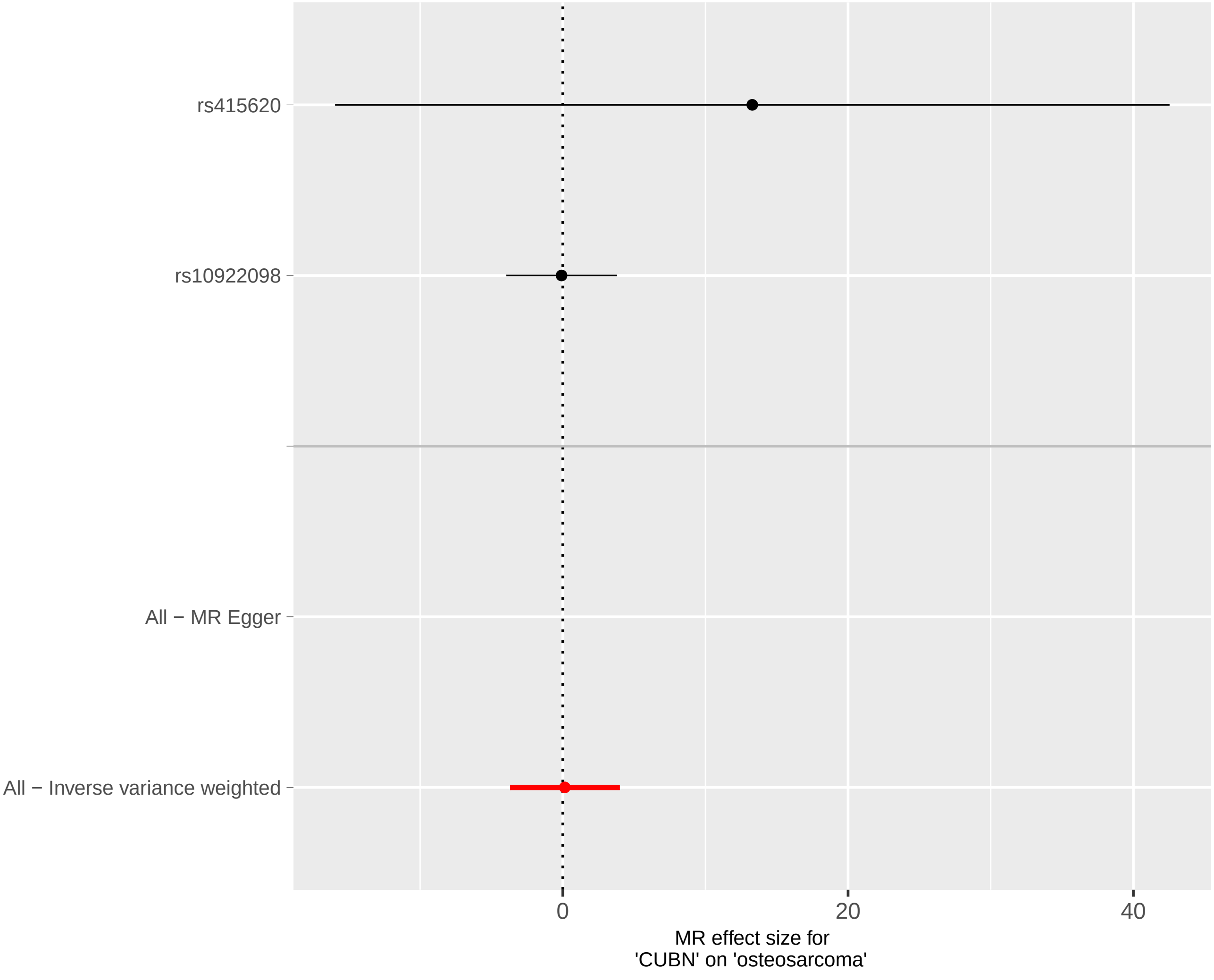
rs415620
rs10922098
All − MR Egger
All − Inverse variance weighted
0
20
40
MR effect size for
'CUBN' on 'osteosarcoma'

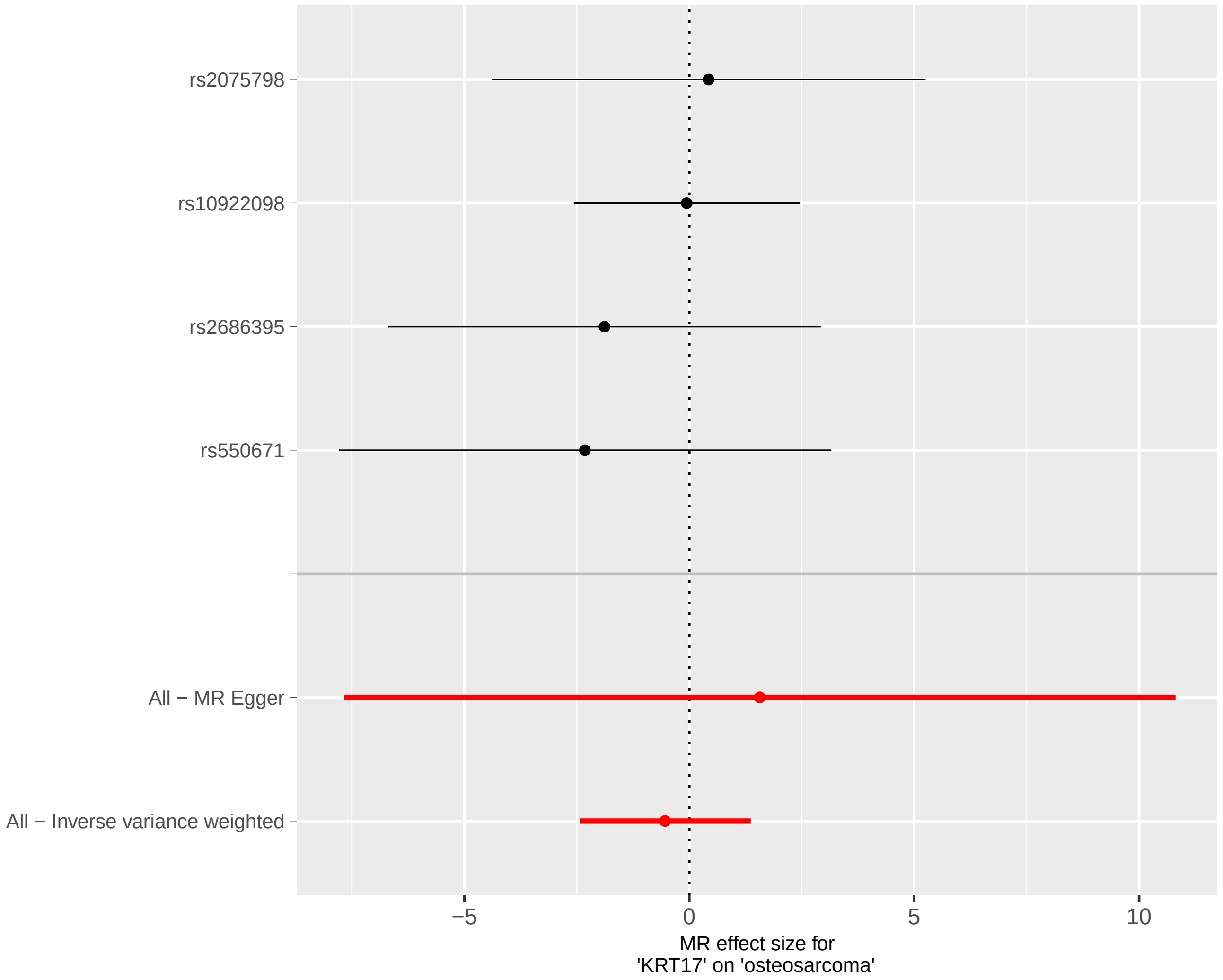
rs2075798
rs10922098
rs2686395
rs550671
All − MR Egger
All − Inverse variance weighted
−5
0
5
10
MR effect size for
'KRT17' on 'osteosarcoma'

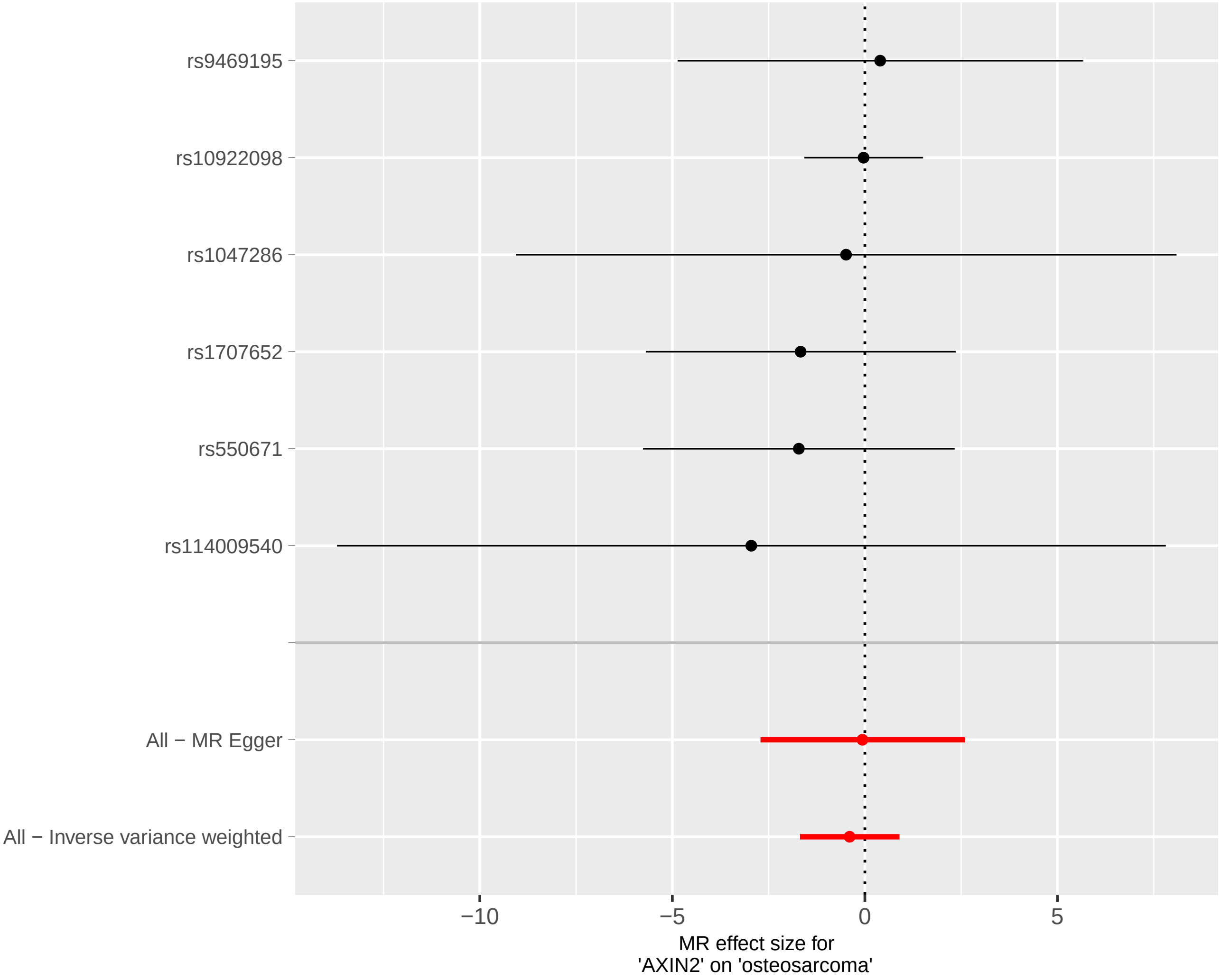
rs9469195
rs10922098
rs1047286
rs1707652
rs550671
rs114009540
All − MR Egger
All − Inverse variance weighted
−10
−5
0
5
MR effect size for
'AXIN2' on 'osteosarcoma'

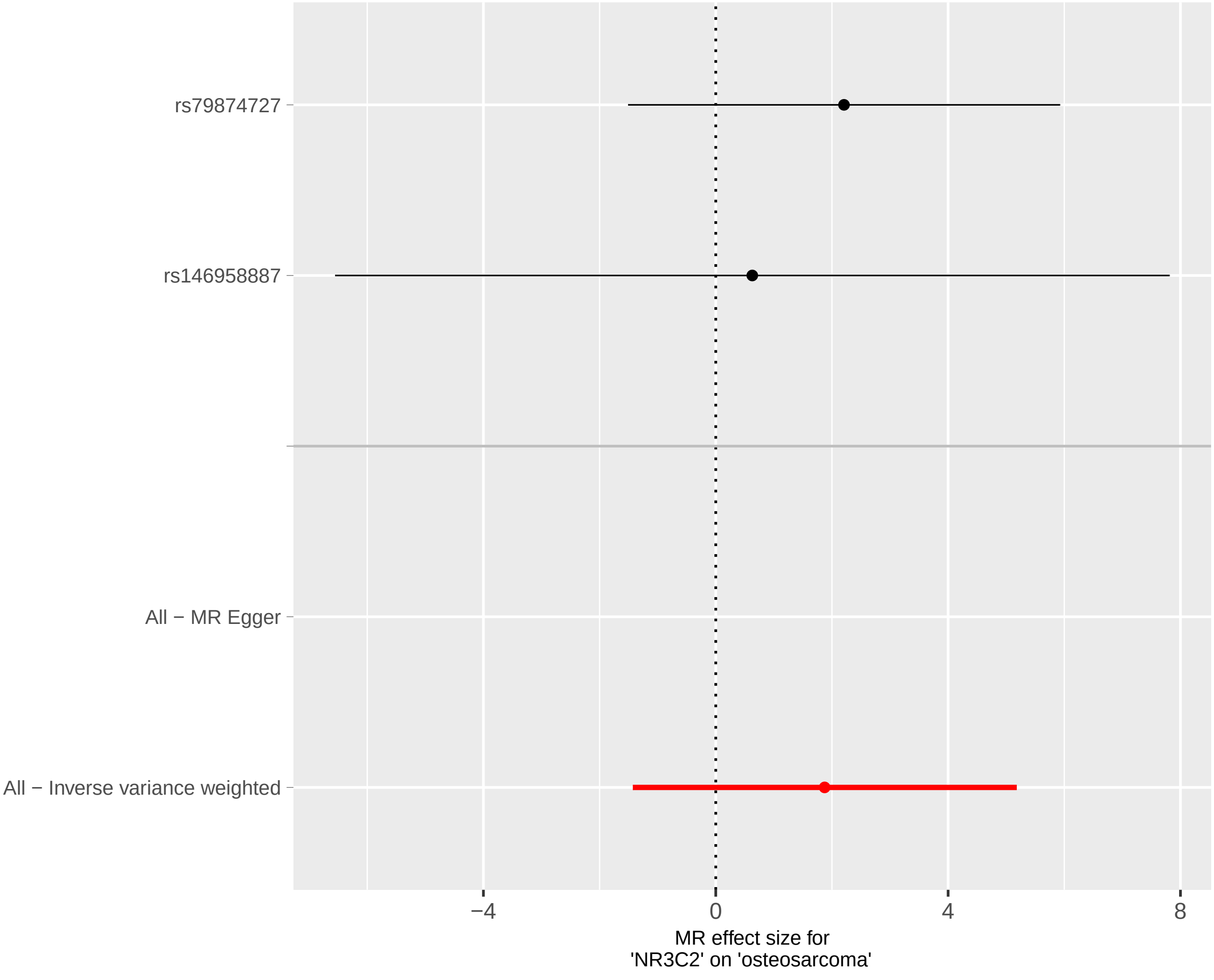
rs79874727
rs146958887
All − MR Egger
All − Inverse variance weighted
−4
0
4
8
MR effect size for
'NR3C2' on 'osteosarcoma'

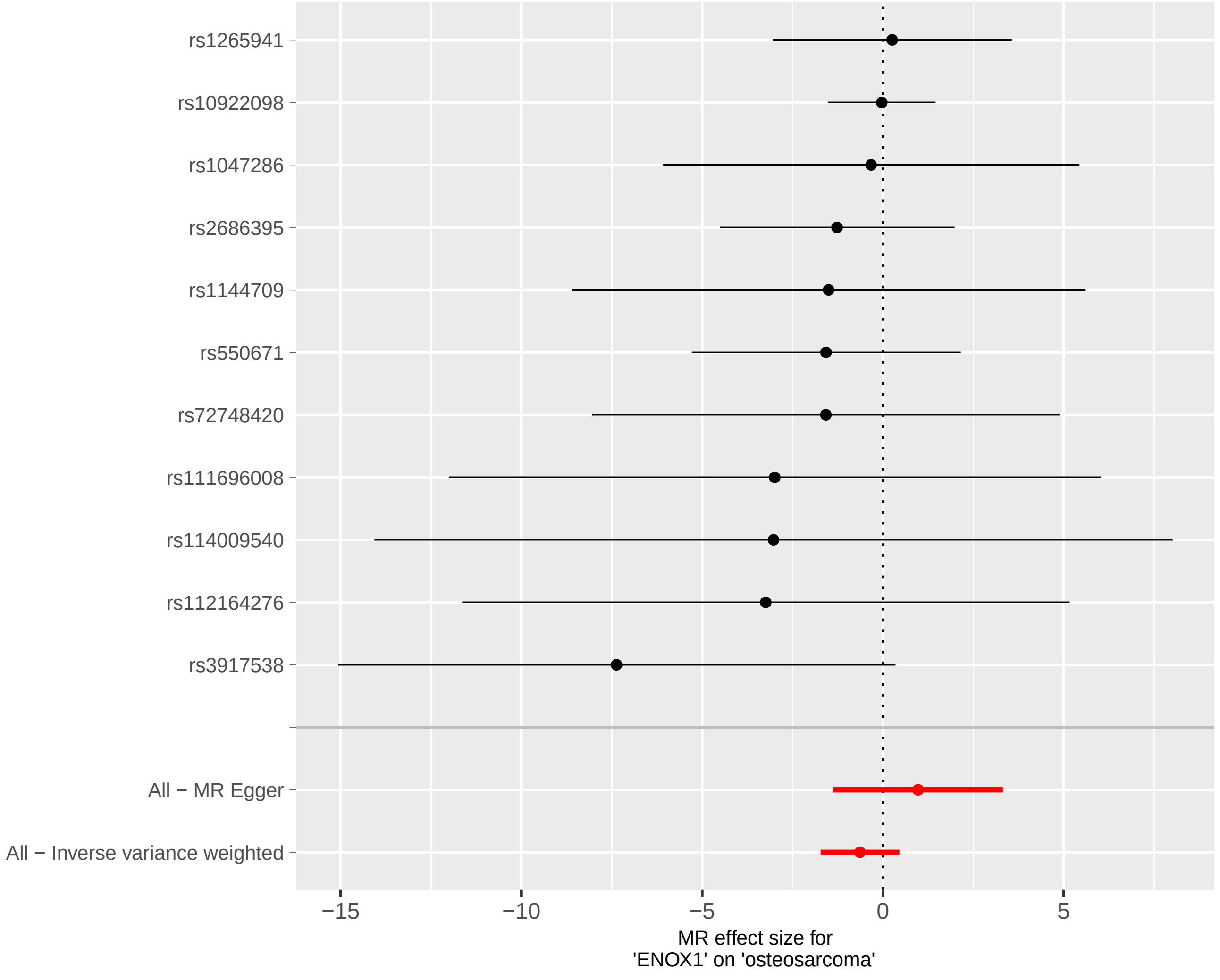
rs1265941
rs10922098
rs1047286
rs2686395
rs1144709
rs550671
rs72748420
rs111696008
rs114009540
rs112164276
rs3917538
All − MR Egger
All − Inverse variance weighted
−15
−10
−5
0
5
MR effect size for
'ENOX1' on 'osteosarcoma'

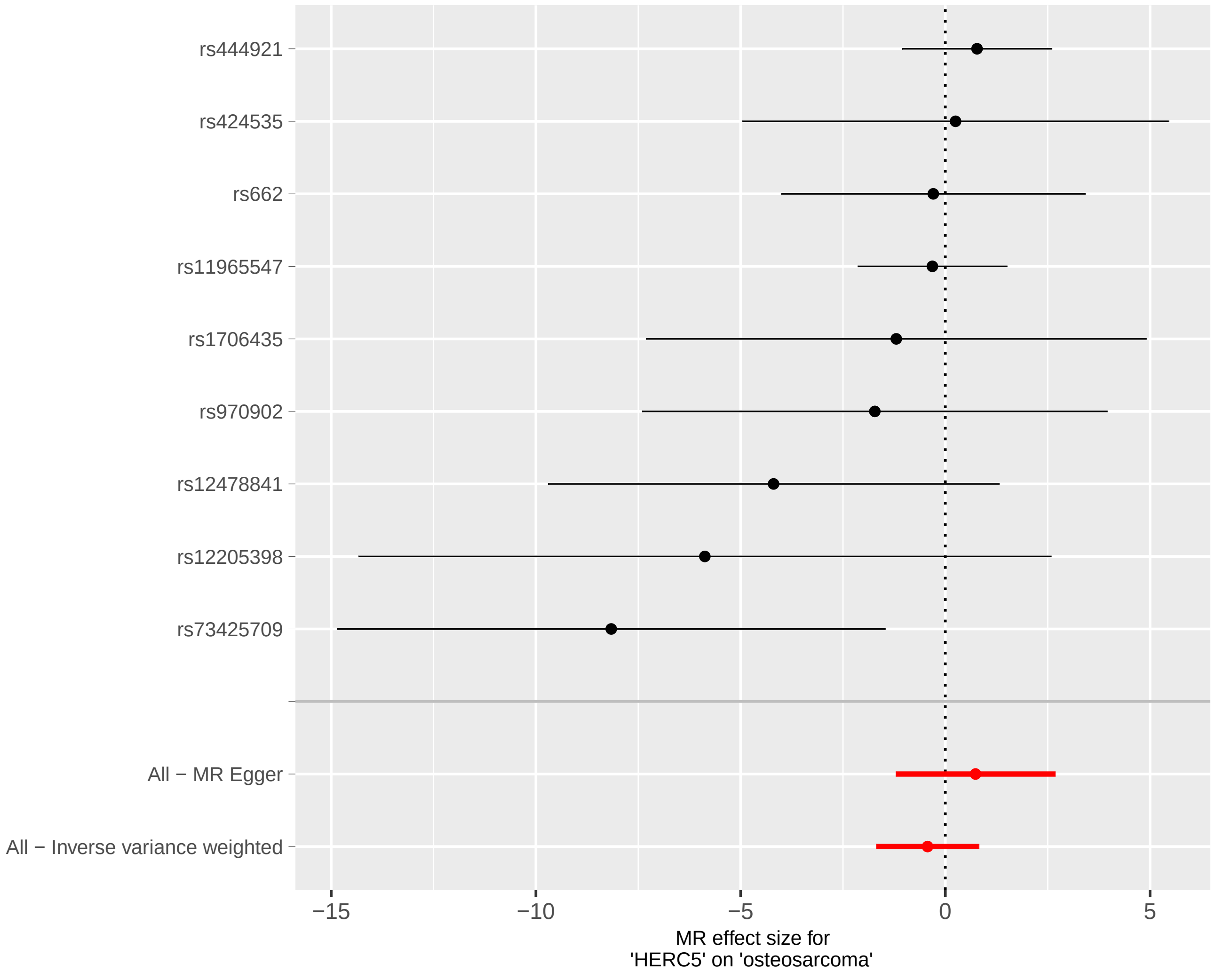

rs444921
rs424535
rs662
rs11965547
rs1706435
rs970902
rs12478841
rs12205398
rs73425709
All − MR Egger
All − Inverse variance weighted
−15
−10
−5
0
5
MR effect size for
'HERC5' on 'osteosarcoma'

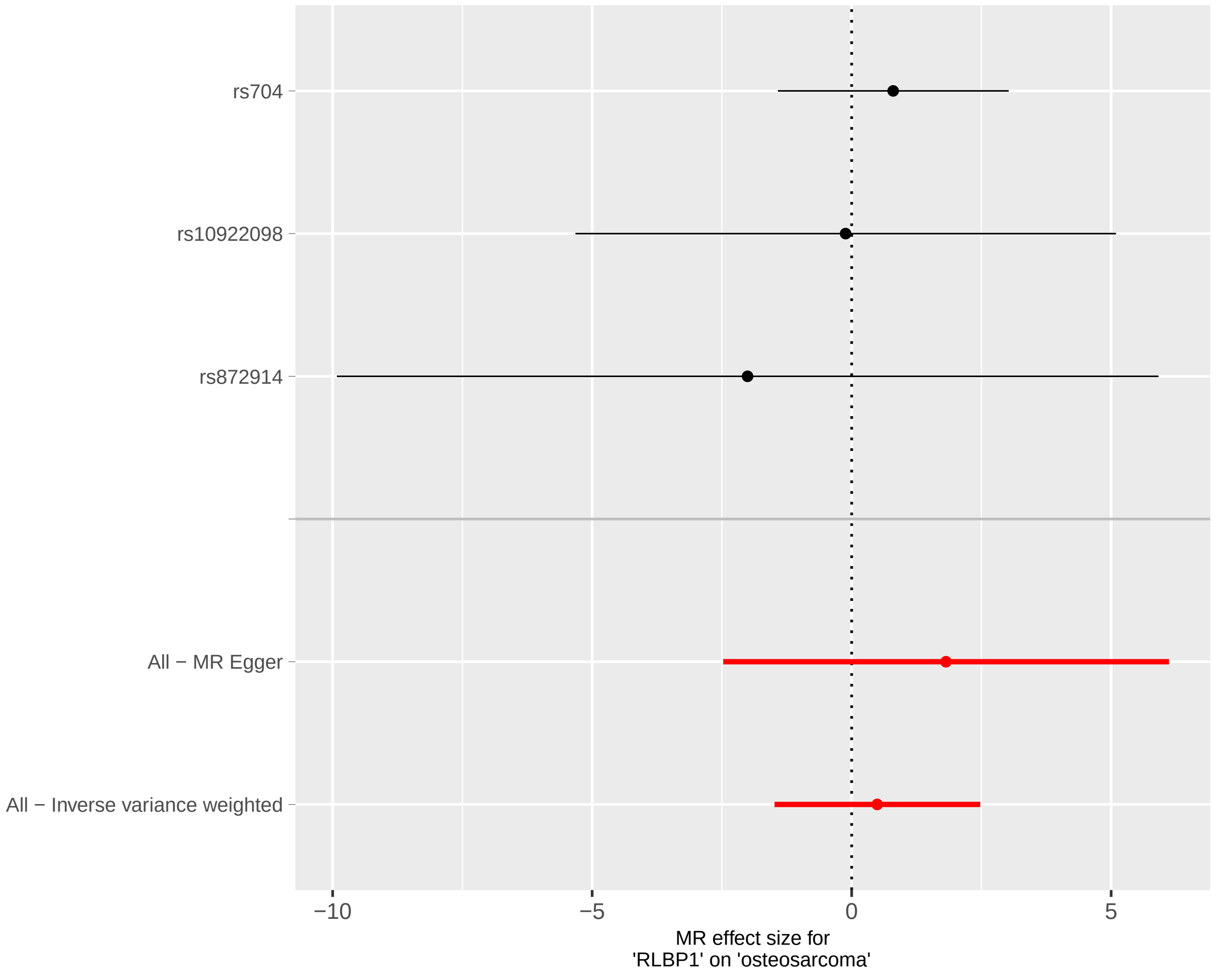

rs704
rs10922098
rs872914
All − MR Egger
All − Inverse variance weighted
−10
−5
0
5
MR effect size for
'RLBP1' on 'osteosarcoma'

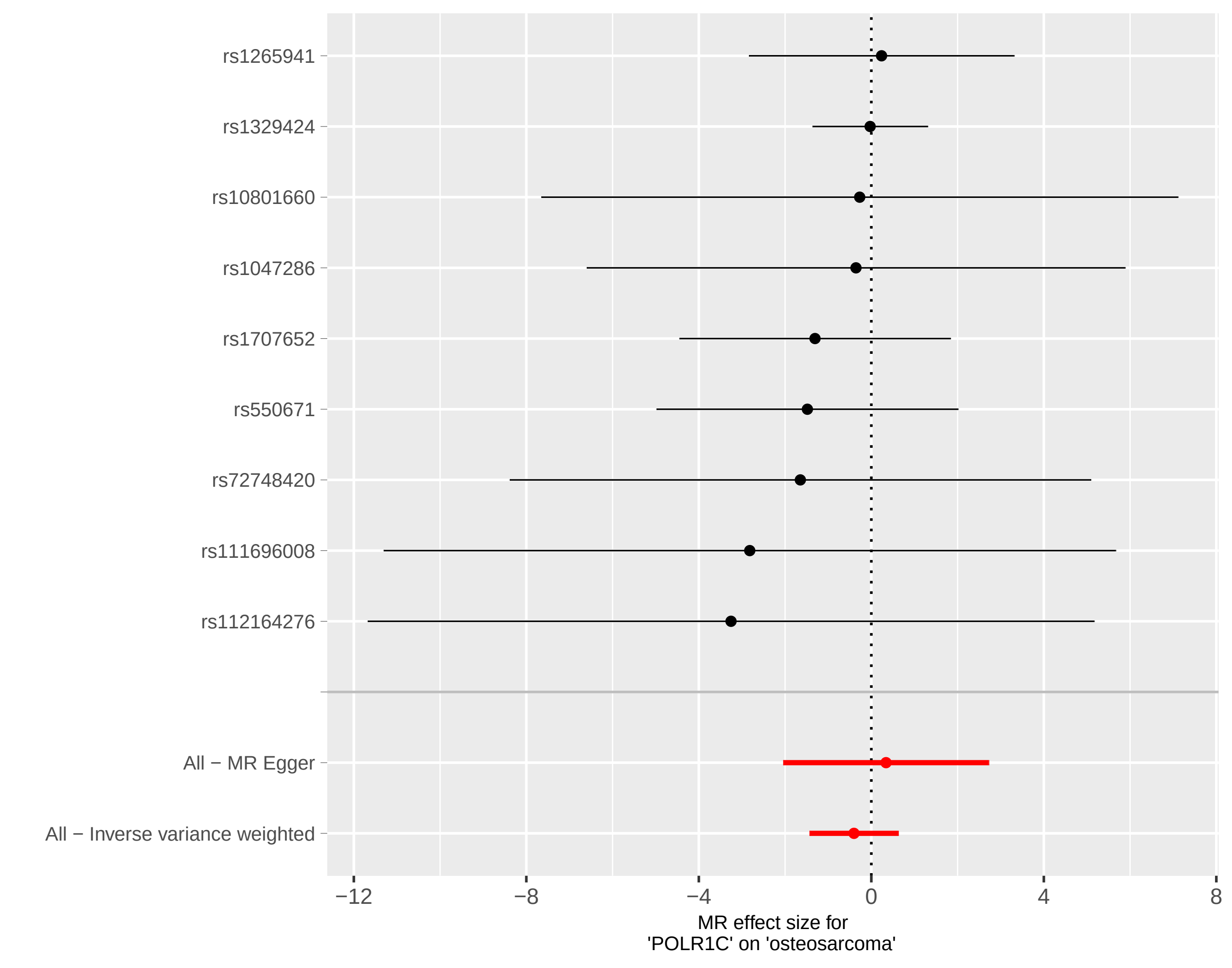

rs1265941
rs1329424
rs10801660
rs1047286
rs1707652
rs550671
rs72748420
rs111696008
rs112164276
All − MR Egger
All − Inverse variance weighted
−12
−8
−4
0
4
8
MR effect size for
'POLR1C' on 'osteosarcoma'

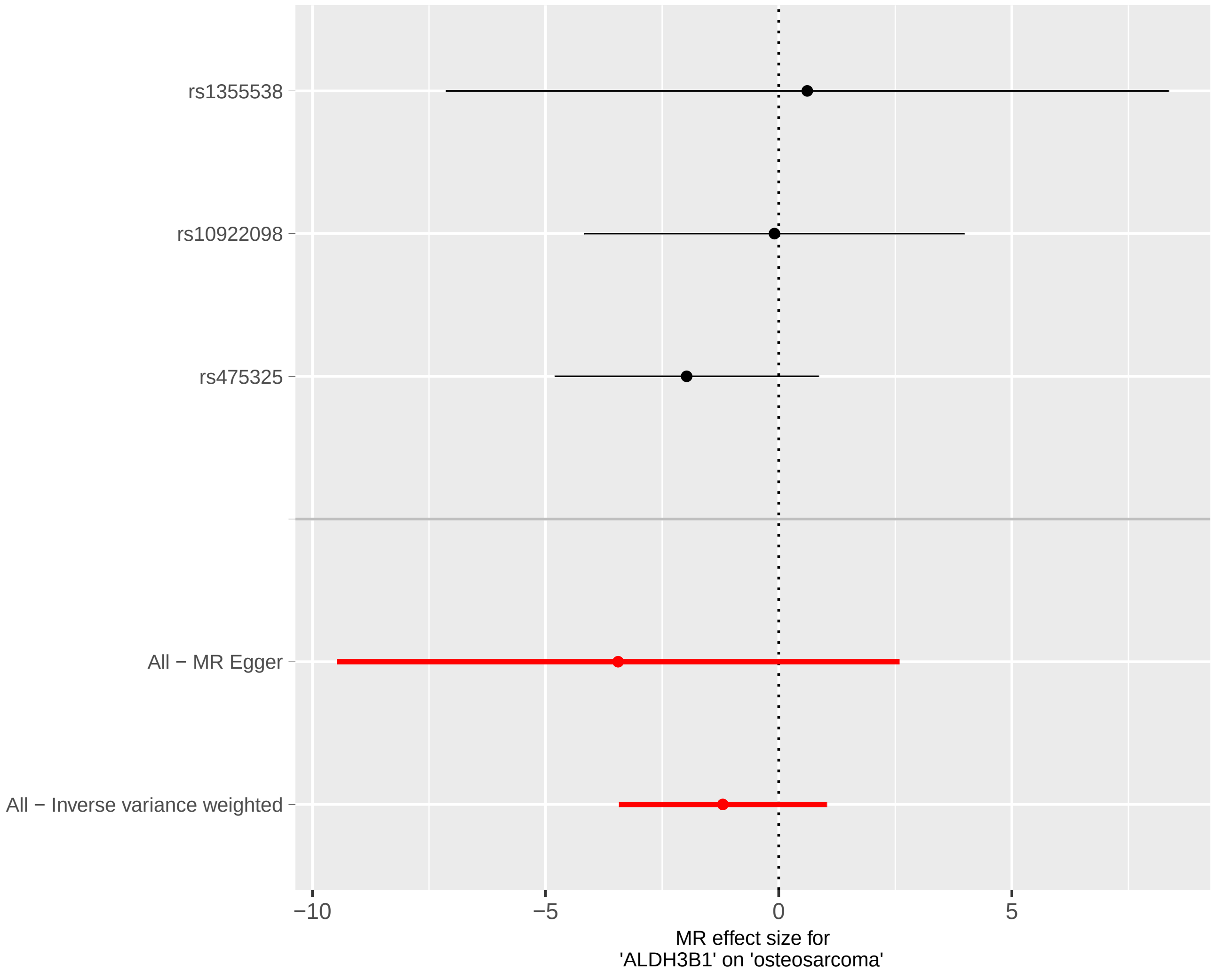
rs1355538
rs10922098
rs475325
All − MR Egger
All − Inverse variance weighted
−10
−5
0
5
MR effect size for
'ALDH3B1' on 'osteosarcoma'

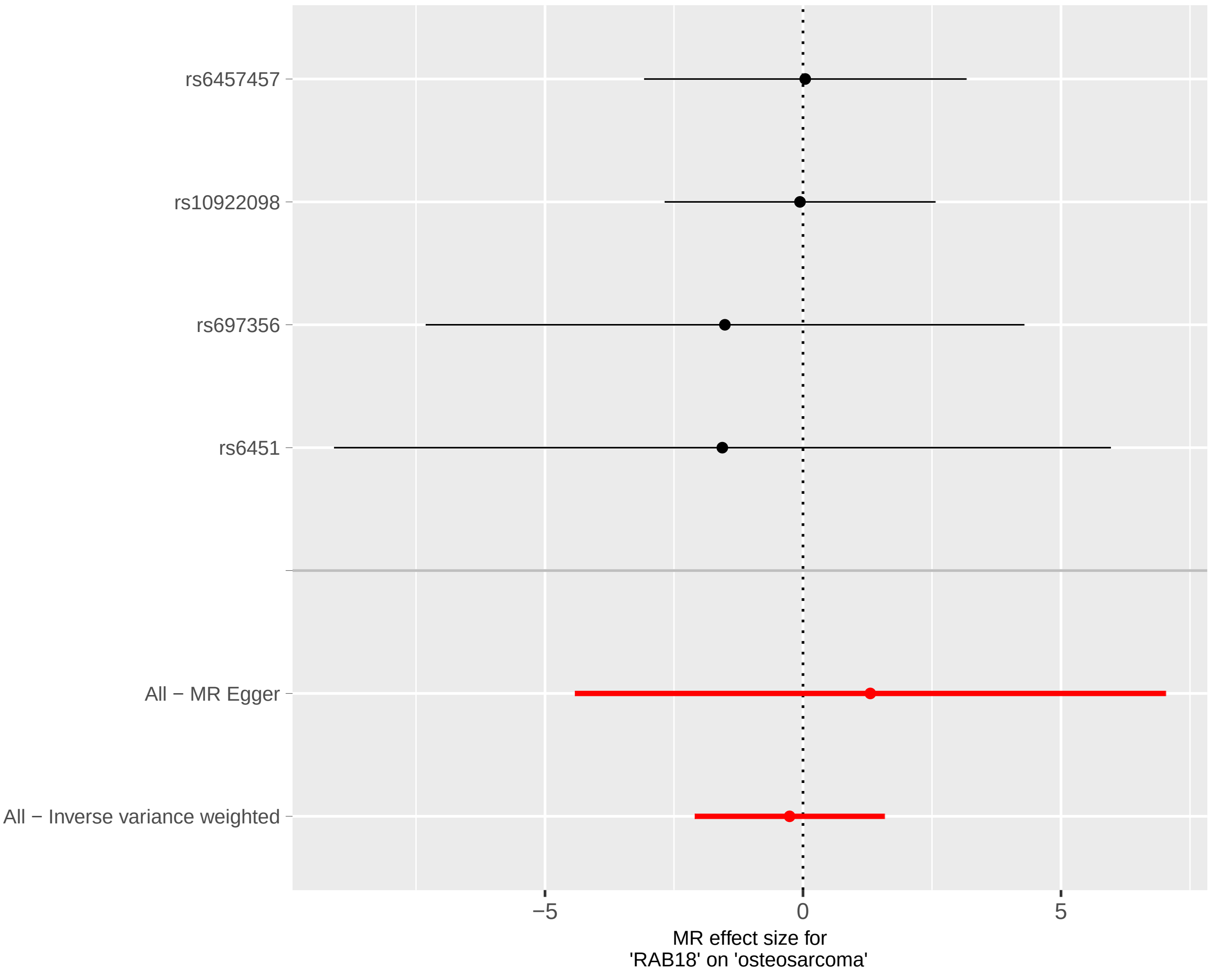
rs6457457
rs10922098
rs697356
rs6451
All − MR Egger
All − Inverse variance weighted
−5
0
5
MR effect size for
'RAB18' on 'osteosarcoma'

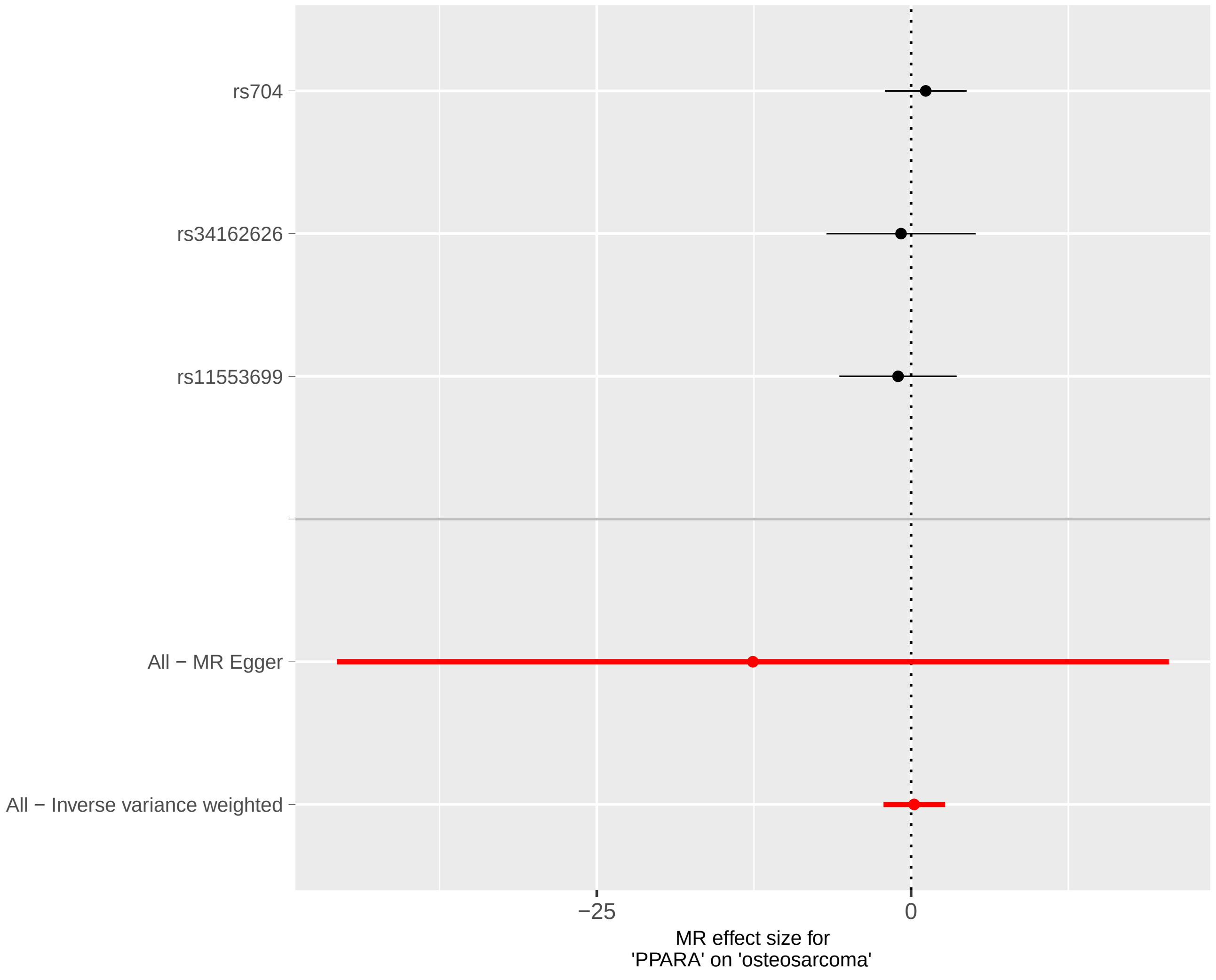
rs704
rs34162626
rs11553699
All − MR Egger
All − Inverse variance weighted
−25
0
MR effect size for
'PPARA' on 'osteosarcoma'

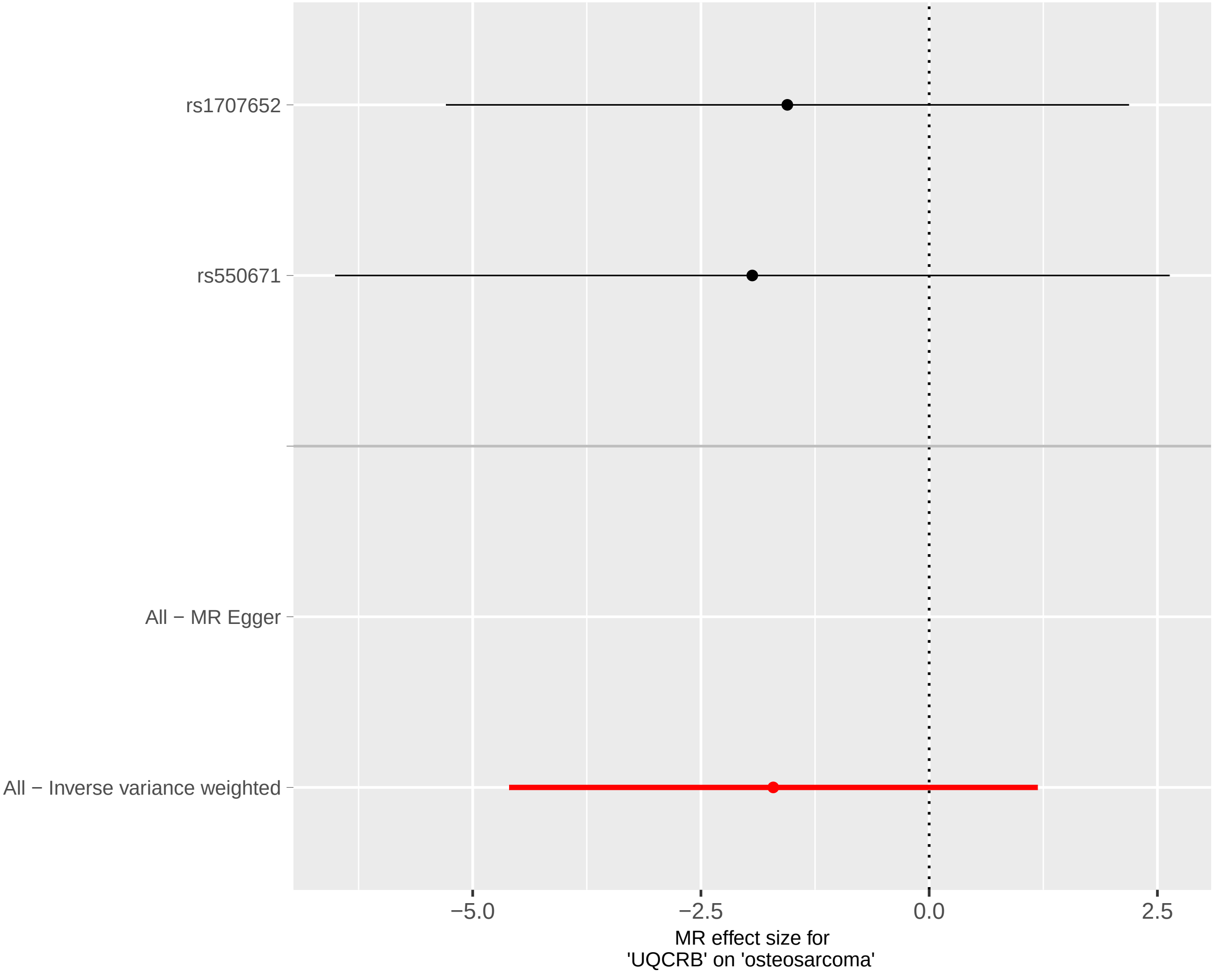
rs1707652
rs550671
All − MR Egger
All − Inverse variance weighted
−5.0
−2.5
0.0
2.5
MR effect size for
'UQCRB' on 'osteosarcoma'

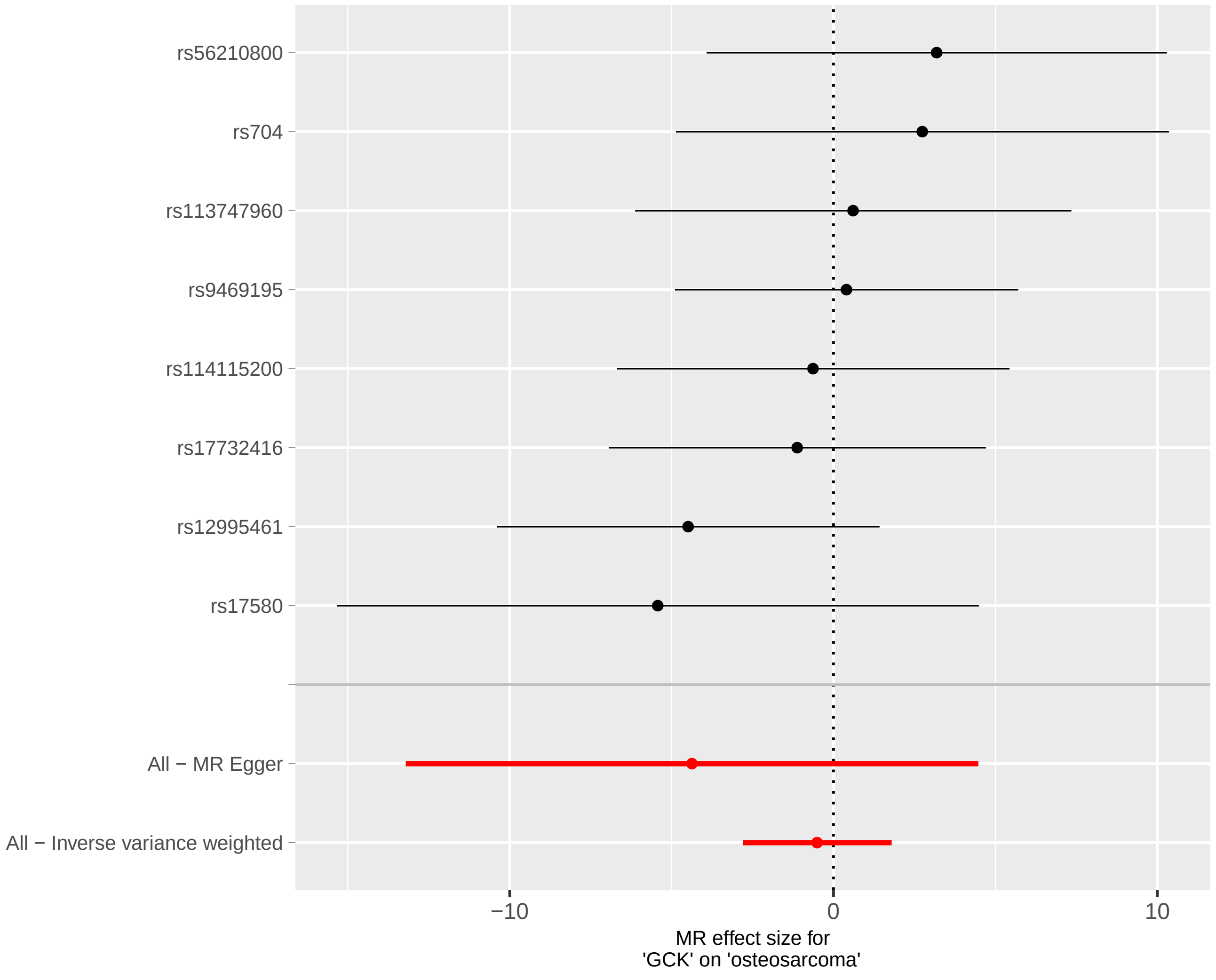

rs56210800
rs704
rs113747960
rs9469195
rs114115200
rs17732416
rs12995461
rs17580
All − MR Egger
All − Inverse variance weighted
−10
0
10
MR effect size for
'GCK' on 'osteosarcoma'

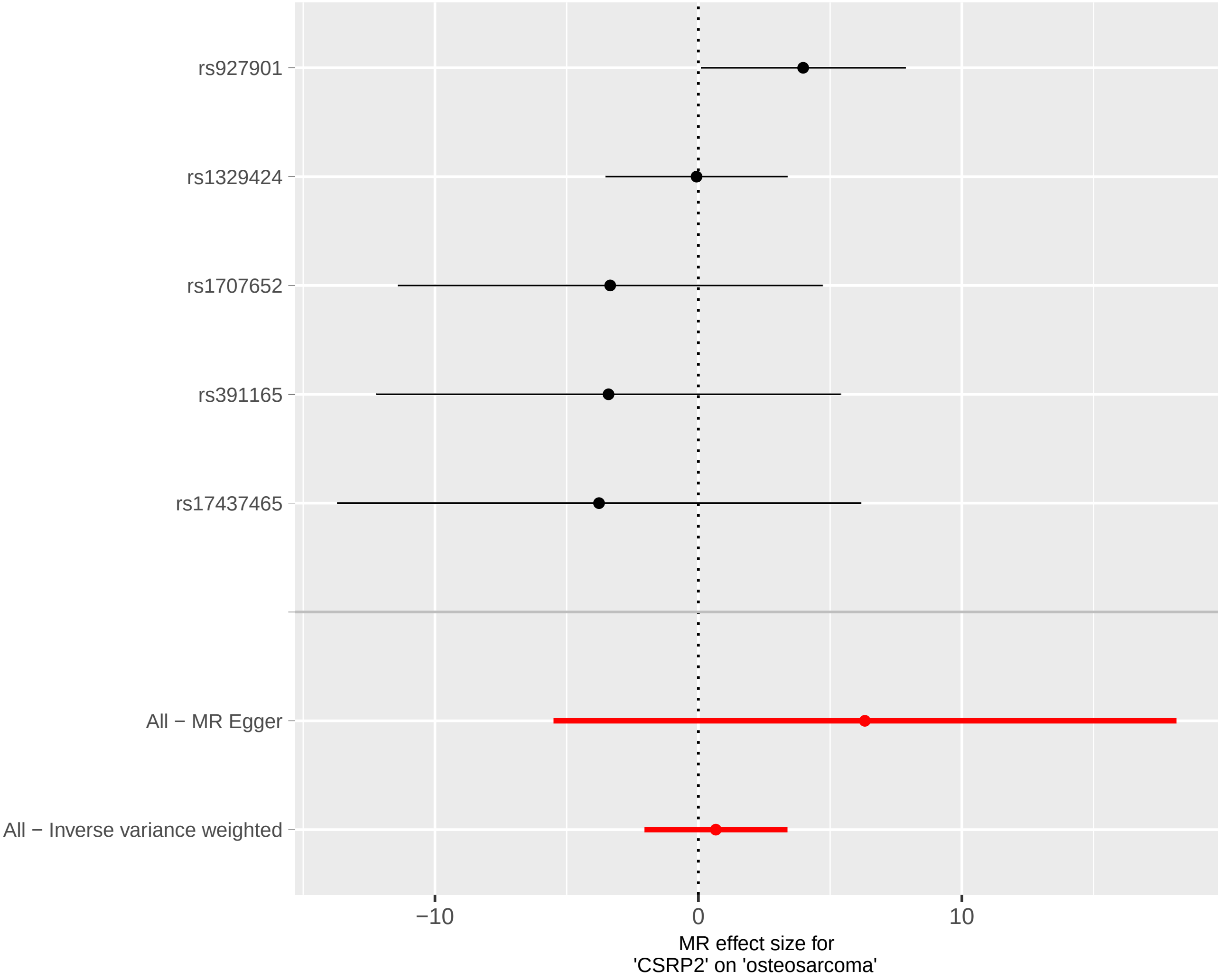

rs927901
rs1329424
rs1707652
rs391165
rs17437465
All − MR Egger
All − Inverse variance weighted
−10
0
10
MR effect size for
'CSRP2' on 'osteosarcoma'

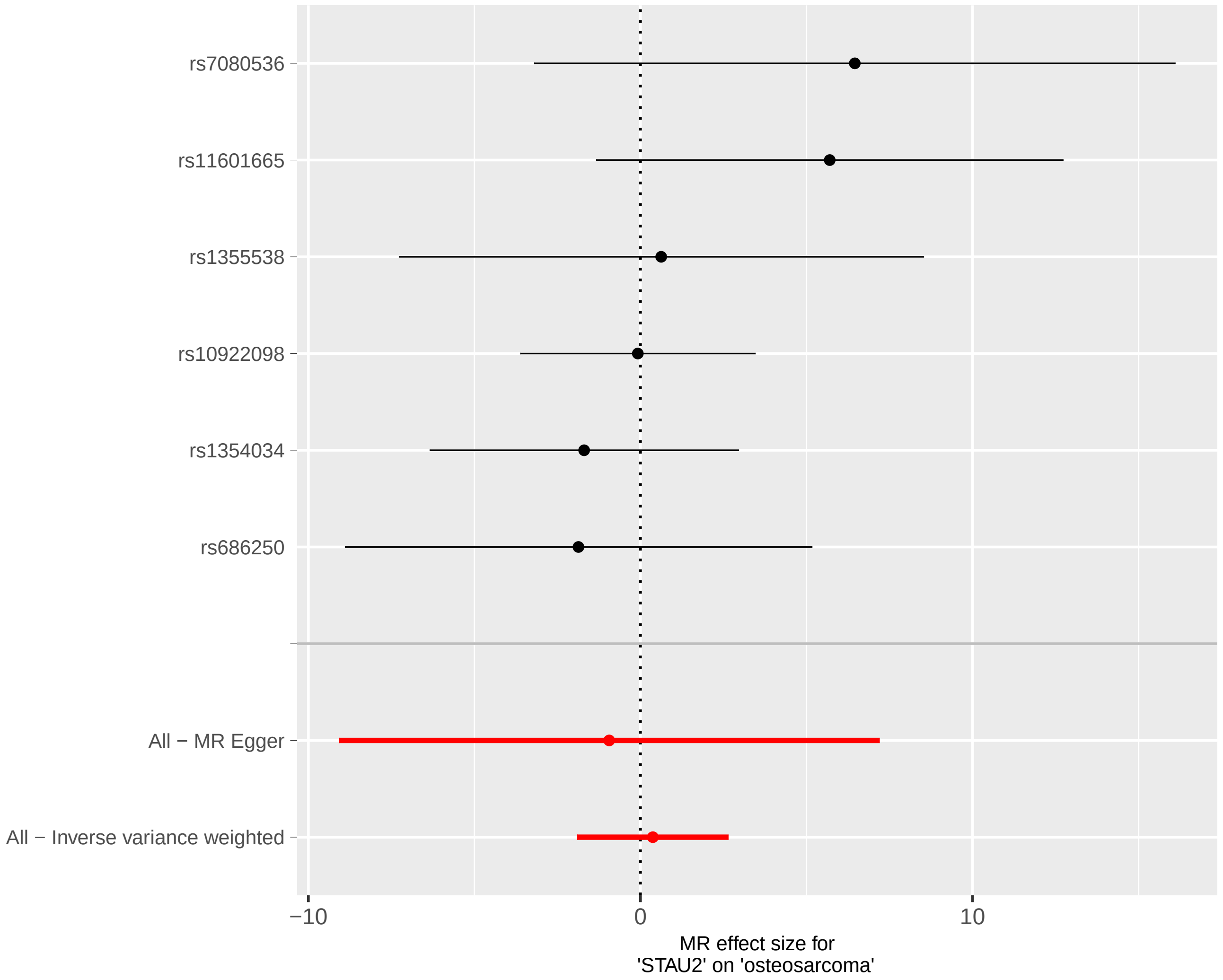
rs7080536
rs11601665
rs1355538
rs10922098
rs1354034
rs686250
All − MR Egger
All − Inverse variance weighted
−10
0
10
MR effect size for
'STAU2' on 'osteosarcoma'

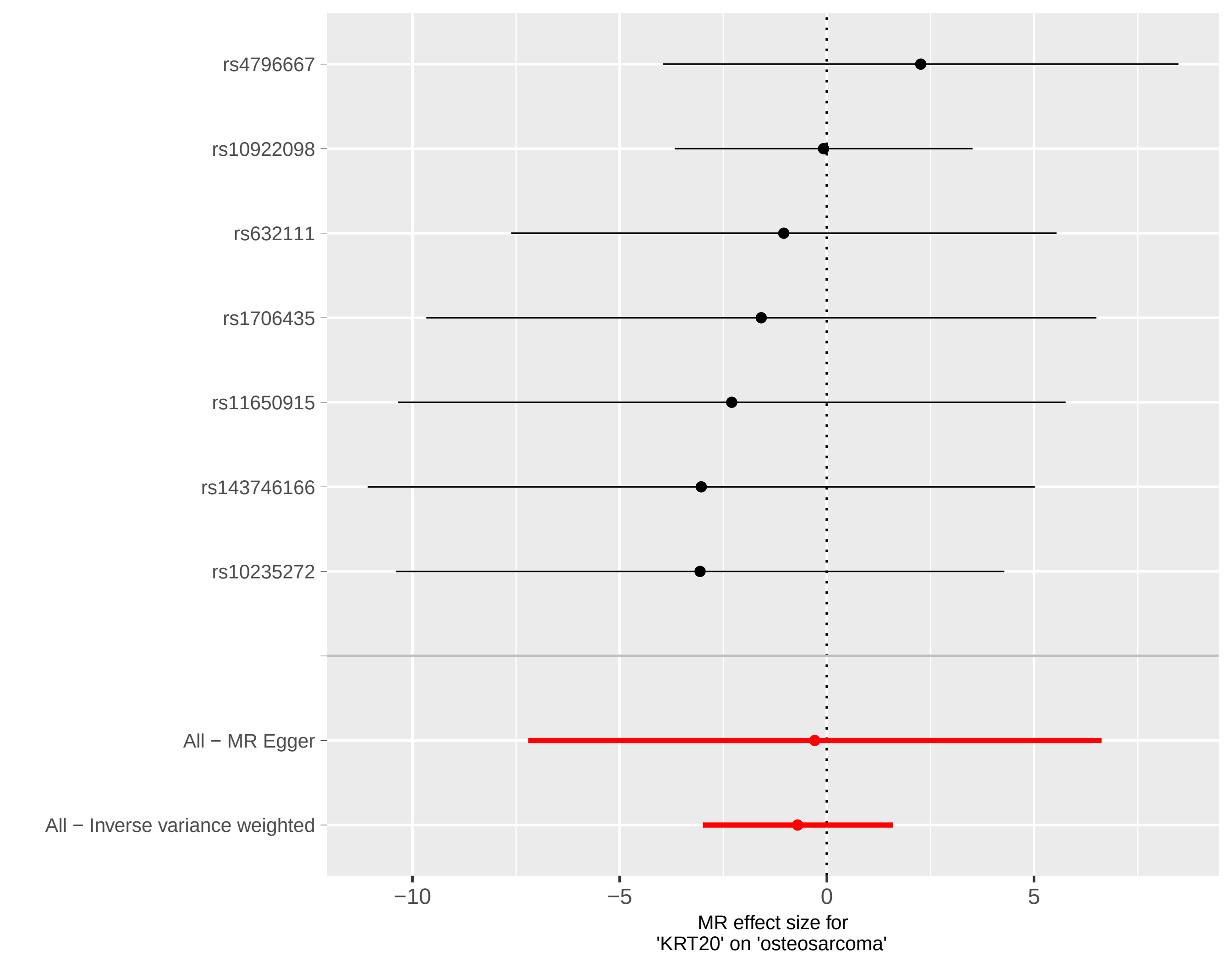

rs4796667
rs10922098
rs632111
rs1706435
rs11650915
rs143746166
rs10235272
All − MR Egger
All − Inverse variance weighted
−10
−5
0
5
MR effect size for
'KRT20' on 'osteosarcoma'

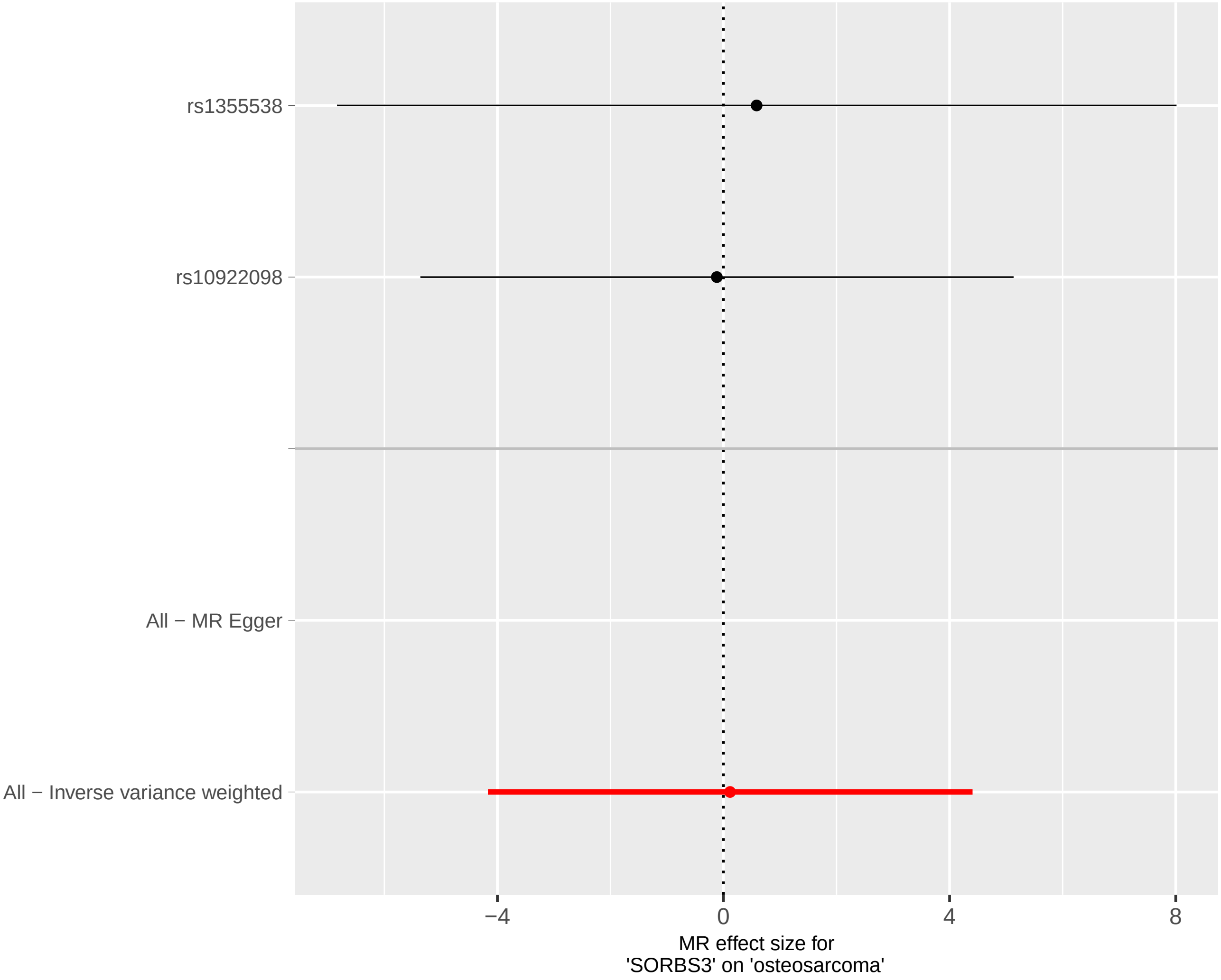

rs1355538
rs10922098
All − MR Egger
All − Inverse variance weighted
−4
0
4
8
MR effect size for
'SORBS3' on 'osteosarcoma'

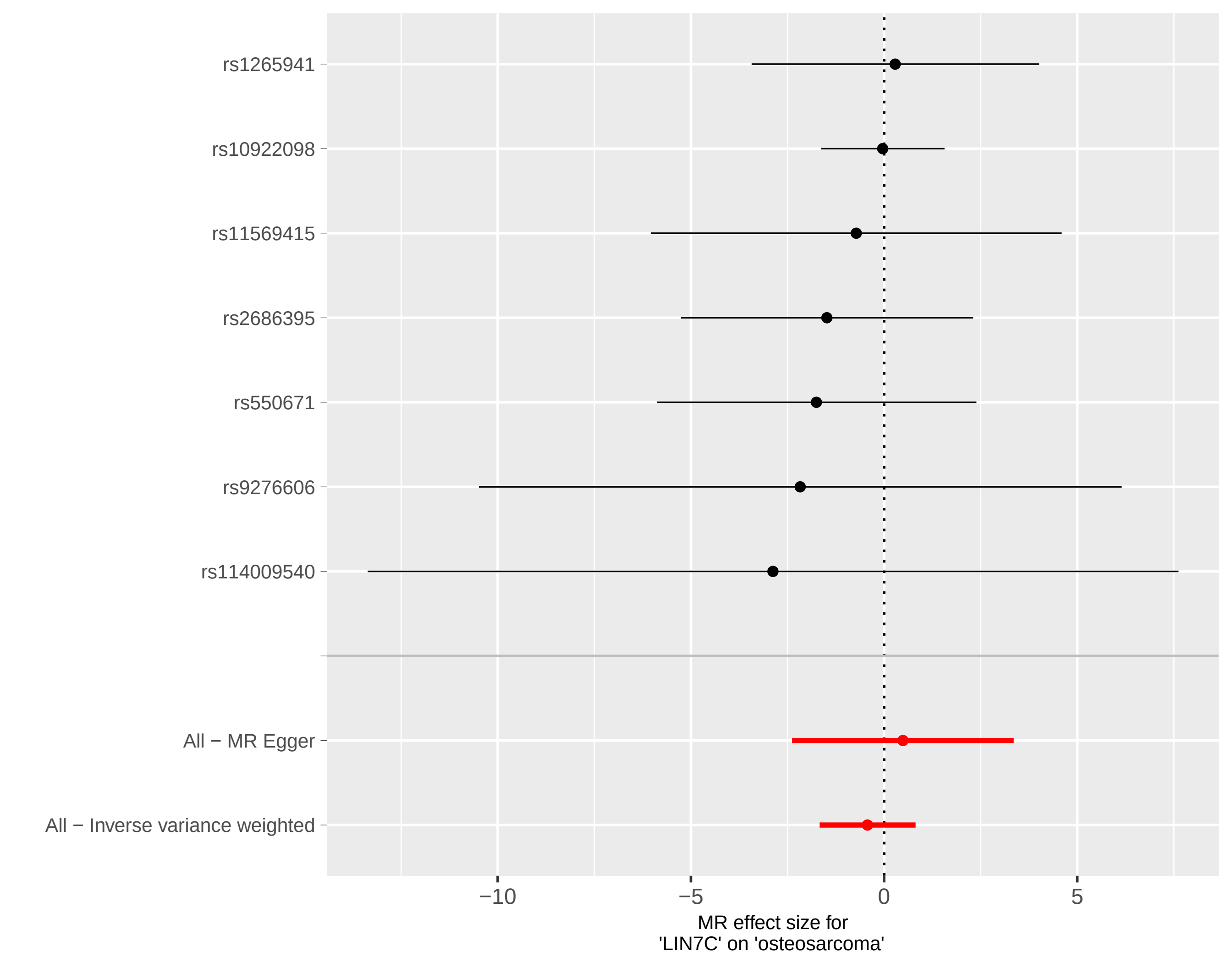
rs1265941
rs10922098
rs11569415
rs2686395
rs550671
rs9276606
rs114009540
All − MR Egger
All − Inverse variance weighted
−10
−5
0
5
MR effect size for
'LIN7C' on 'osteosarcoma'

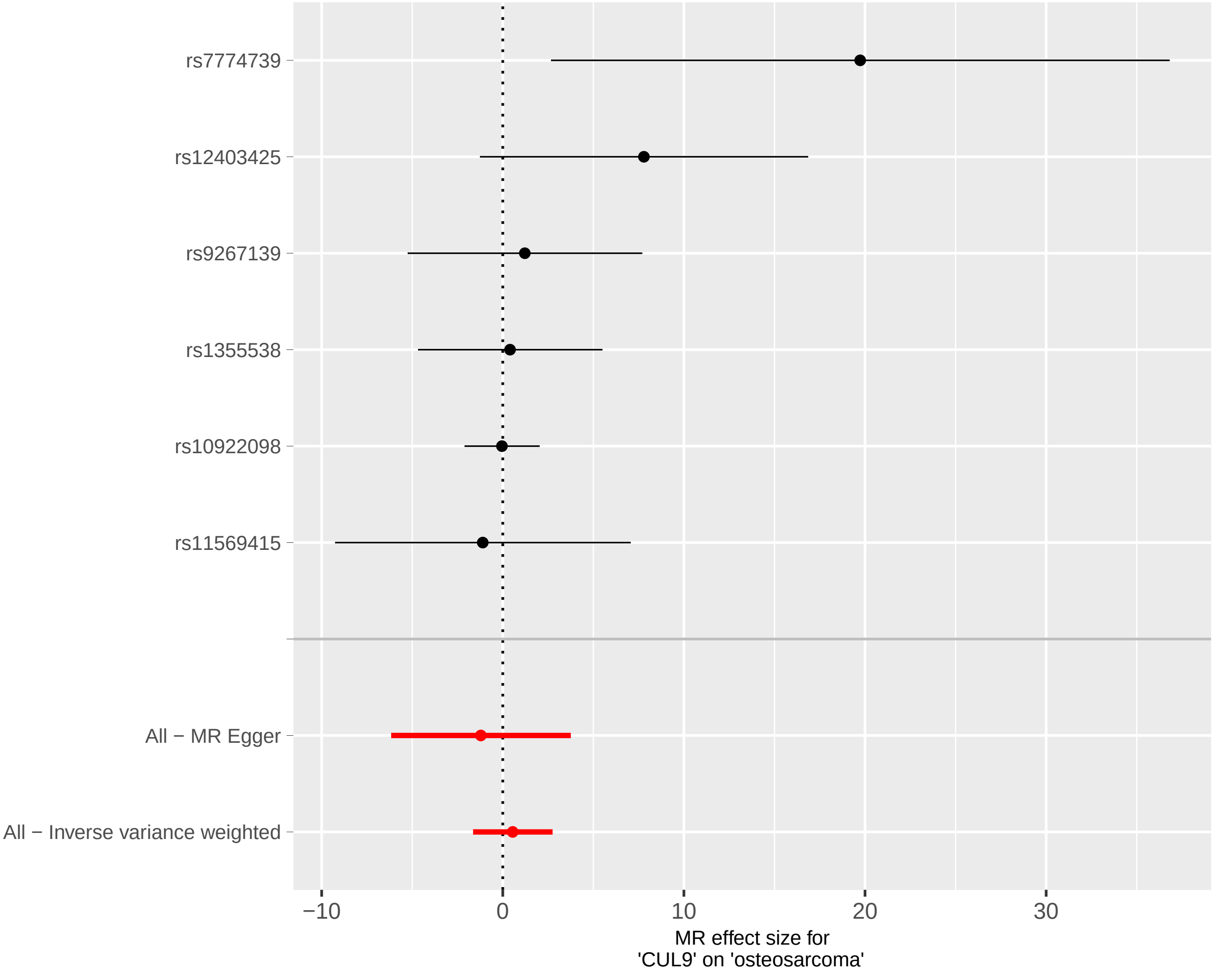
rs7774739
rs12403425
rs9267139
rs1355538
rs10922098
rs11569415
All − MR Egger
All − Inverse variance weighted
−10
0
10
20
30
MR effect size for
'CUL9' on 'osteosarcoma'

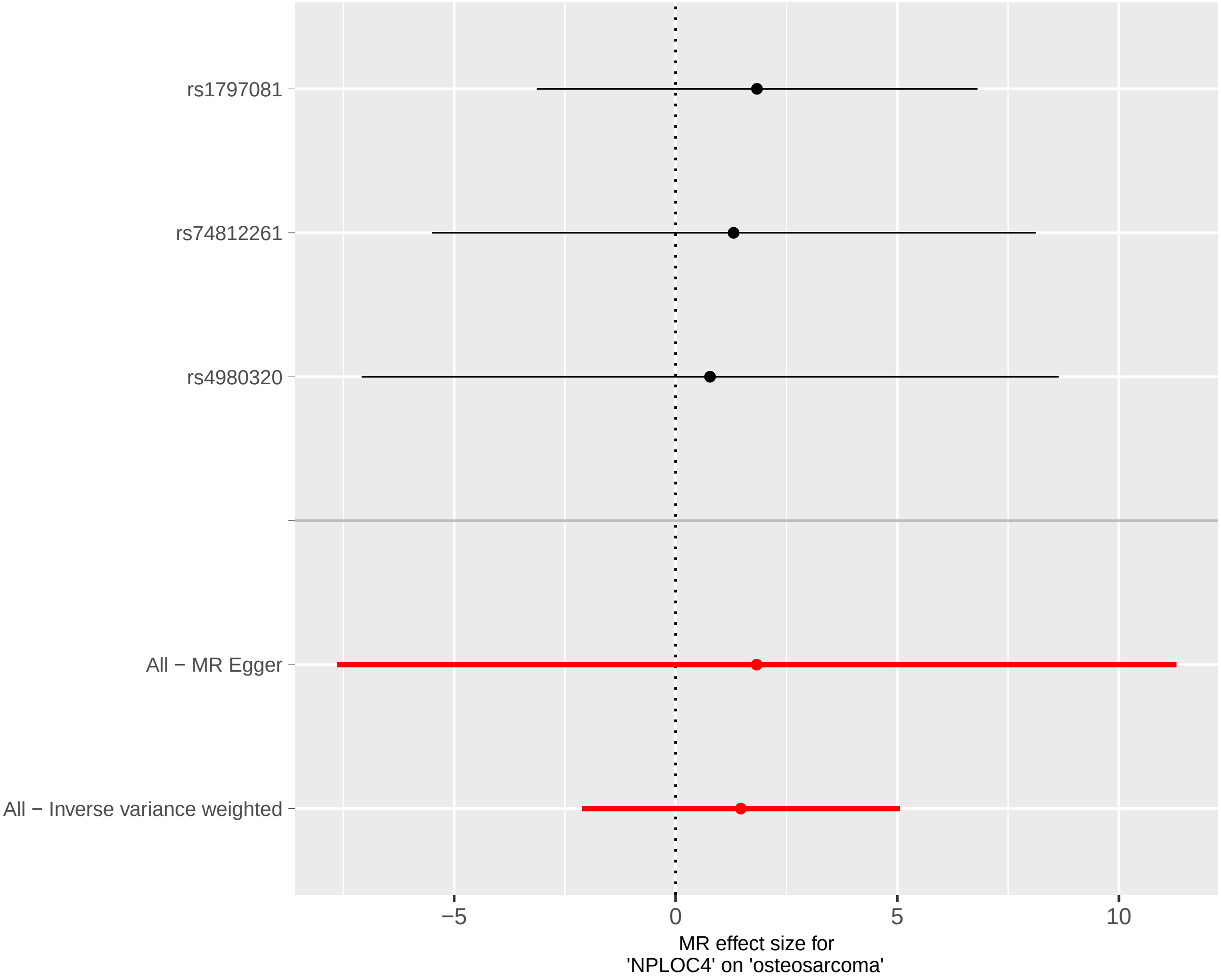

rs1797081
rs74812261
rs4980320
All − MR Egger
All − Inverse variance weighted
−5
0
5
10
MR effect size for
'NPLOC4' on 'osteosarcoma'

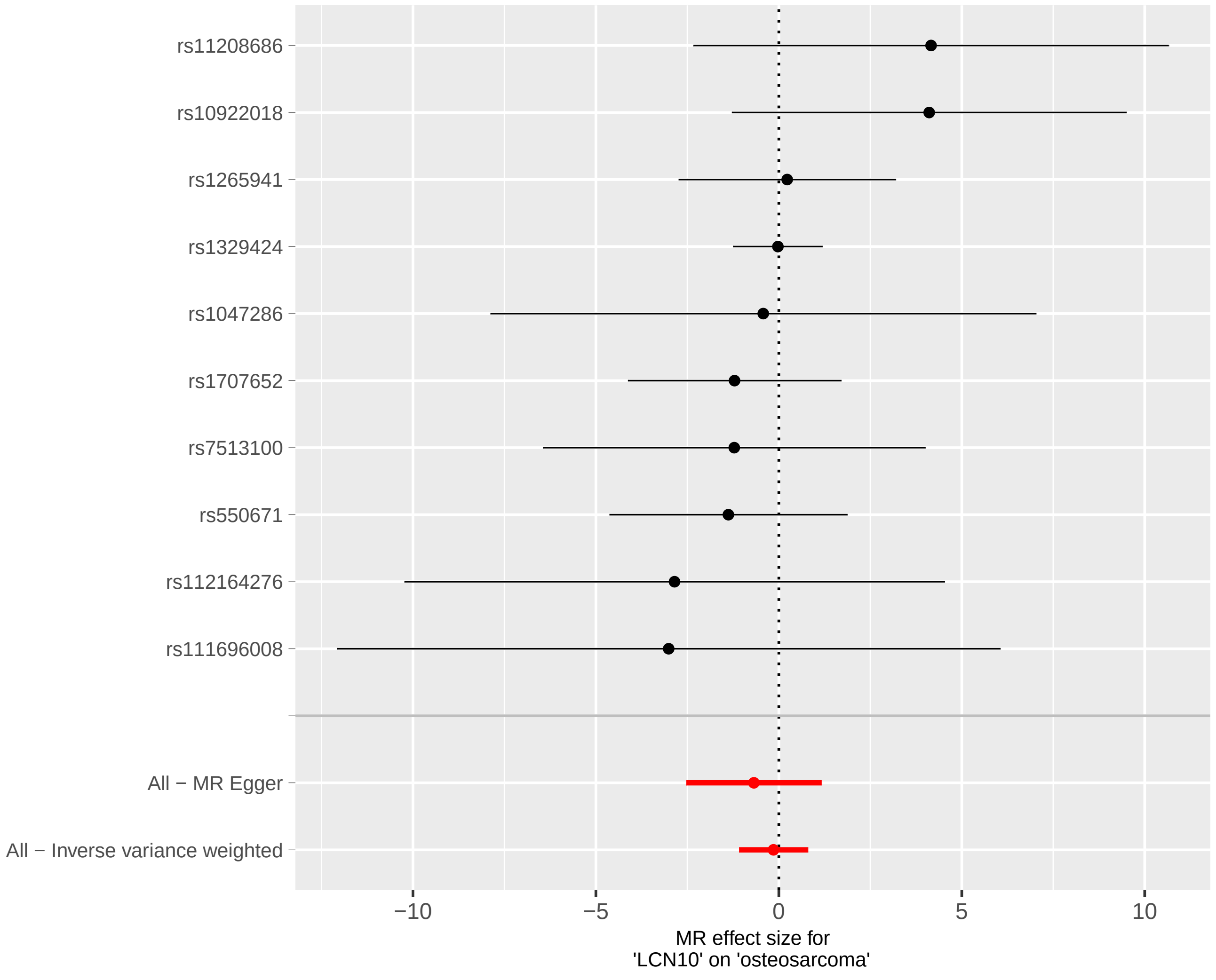
rs11208686
rs10922018
rs1265941
rs1329424
rs1047286
rs1707652
rs7513100
rs550671
rs112164276
rs111696008
All − MR Egger
All − Inverse variance weighted
−10
−5
0
5
10
MR effect size for
'LCN10' on 'osteosarcoma'

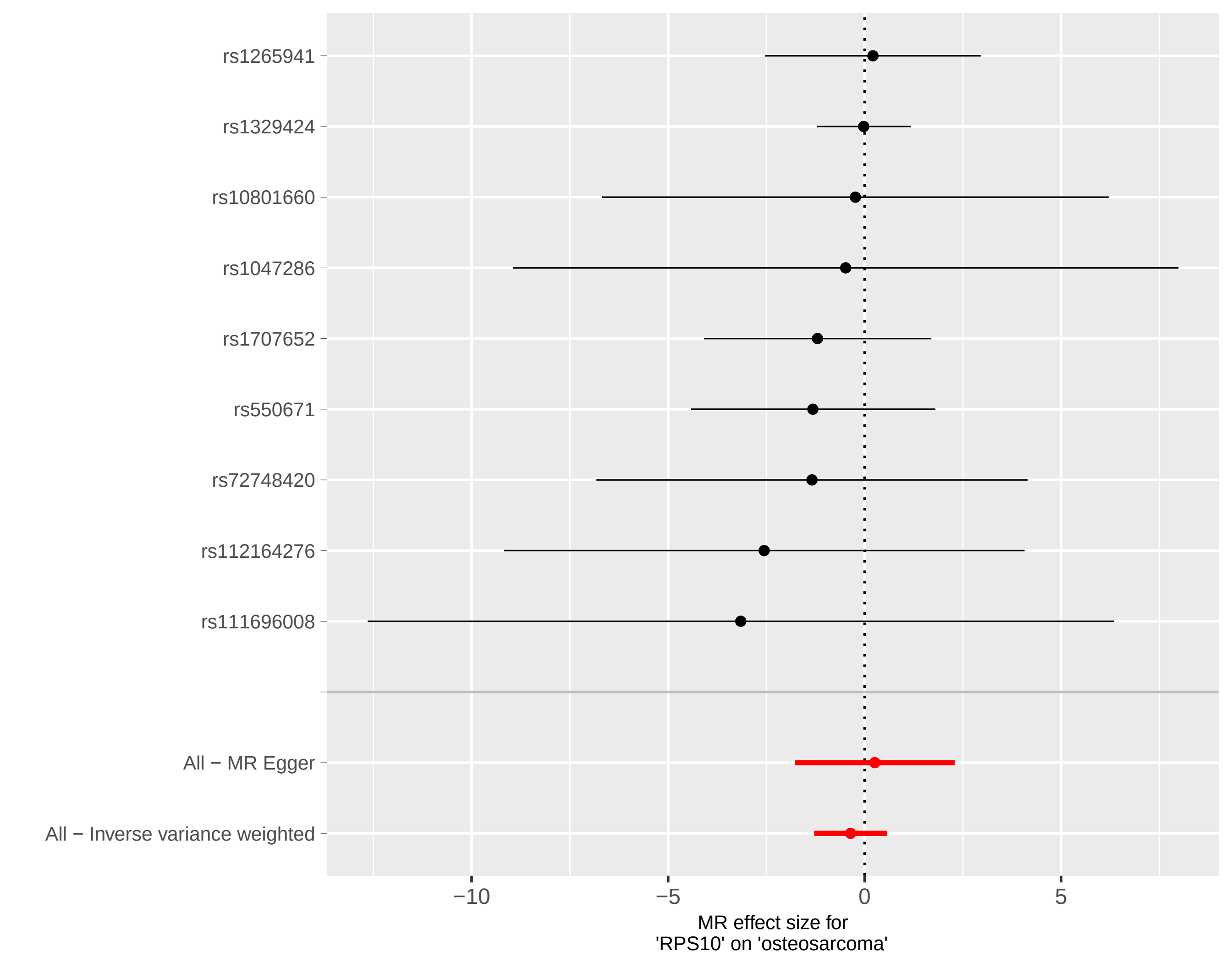

rs1265941
rs1329424
rs10801660
rs1047286
rs1707652
rs550671
rs72748420
rs112164276
rs111696008
All − MR Egger
All − Inverse variance weighted
−10
−5
0
5
MR effect size for
'RPS10' on 'osteosarcoma'

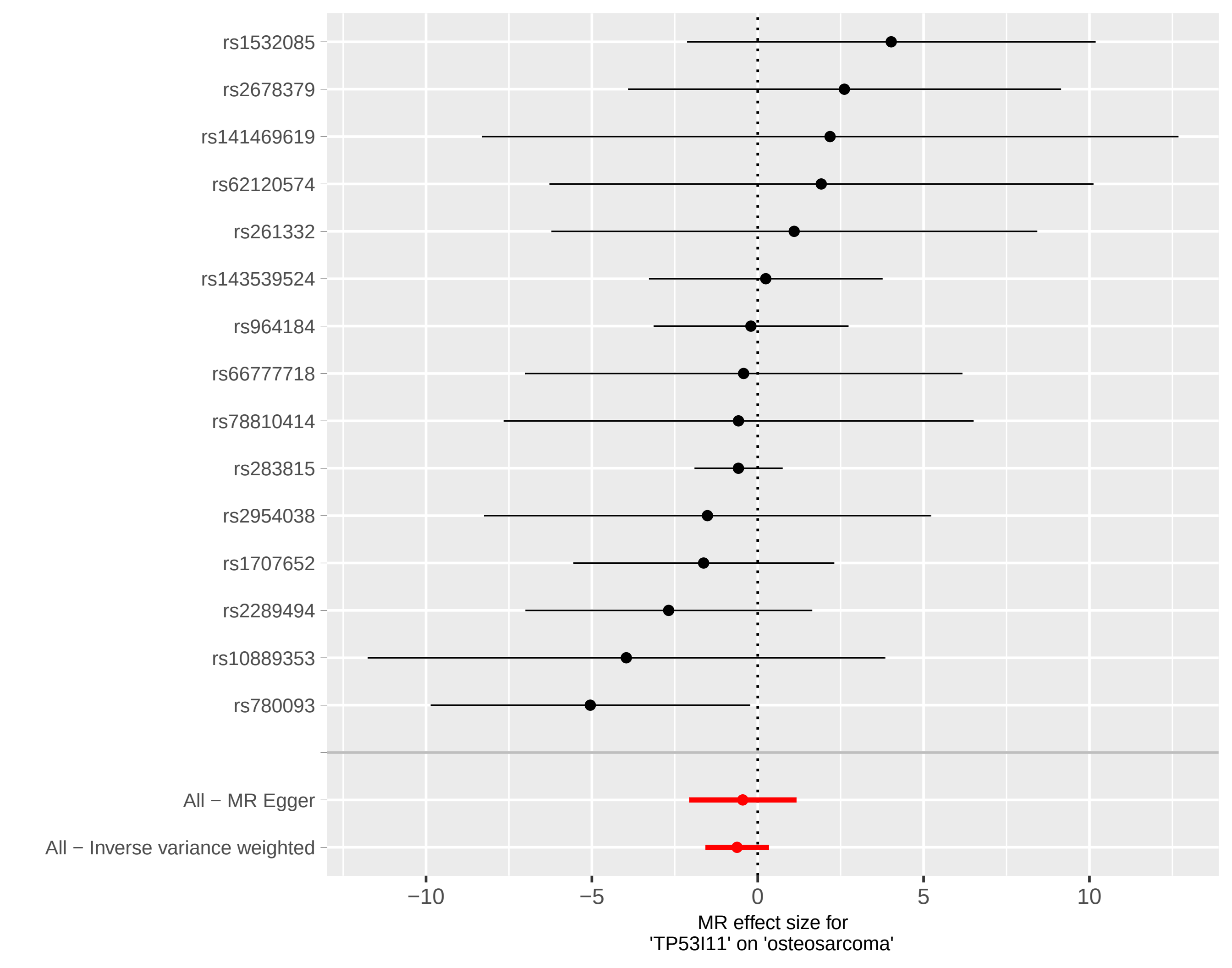
rs1532085
rs2678379
rs141469619
rs62120574
rs261332
rs143539524
rs964184
rs66777718
rs78810414
rs283815
rs2954038
rs1707652
rs2289494
rs10889353
rs780093
All − MR Egger
All − Inverse variance weighted
−10
−5
0
5
10
MR effect size for
'TP53I11' on 'osteosarcoma'

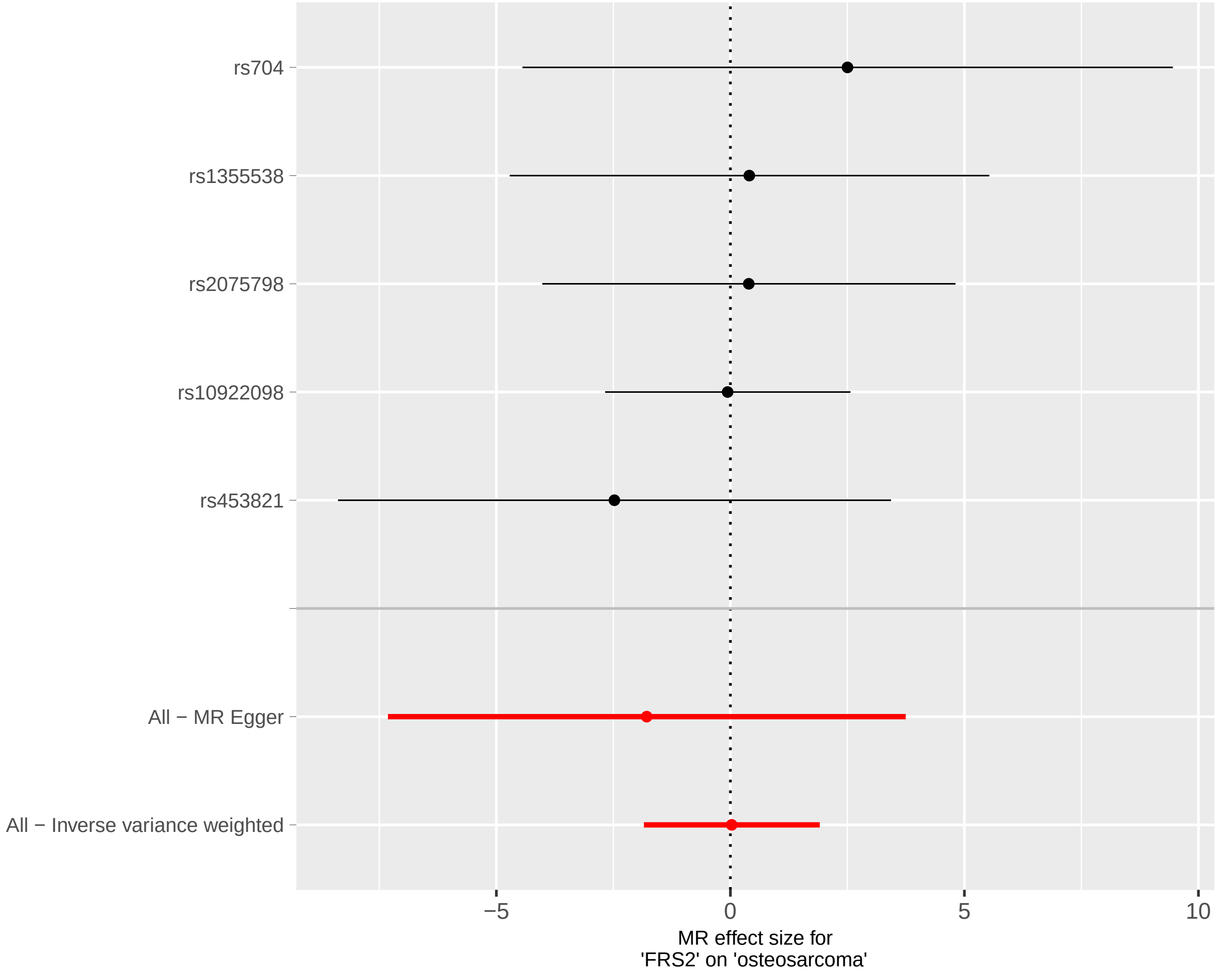

rs704
rs1355538
rs2075798
rs10922098
rs453821
All − MR Egger
All − Inverse variance weighted
−5
0
5
10
MR effect size for
'FRS2' on 'osteosarcoma'

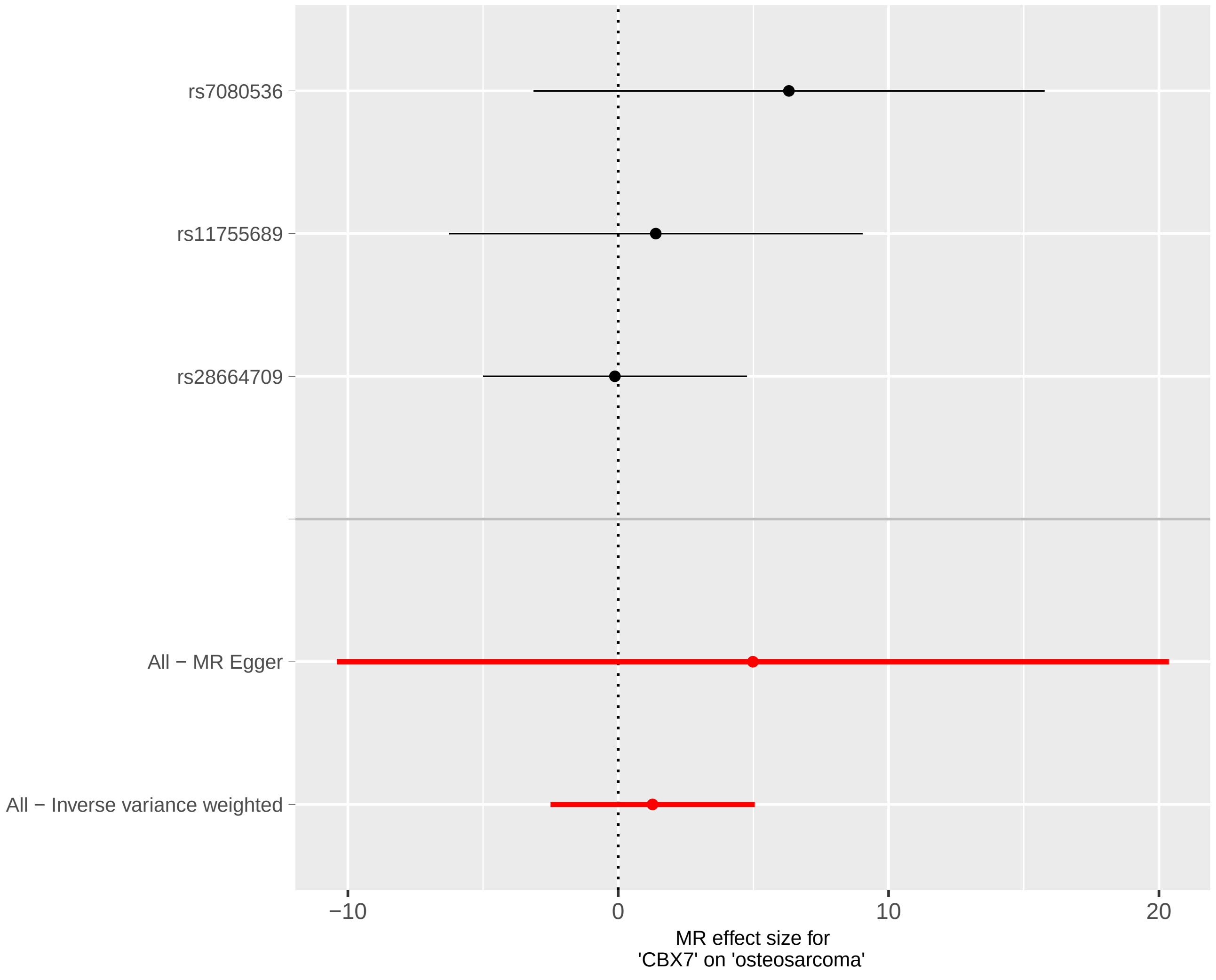
rs7080536
rs11755689
rs28664709
All − MR Egger
All − Inverse variance weighted
−10
0
10
20
MR effect size for
'CBX7' on 'osteosarcoma'

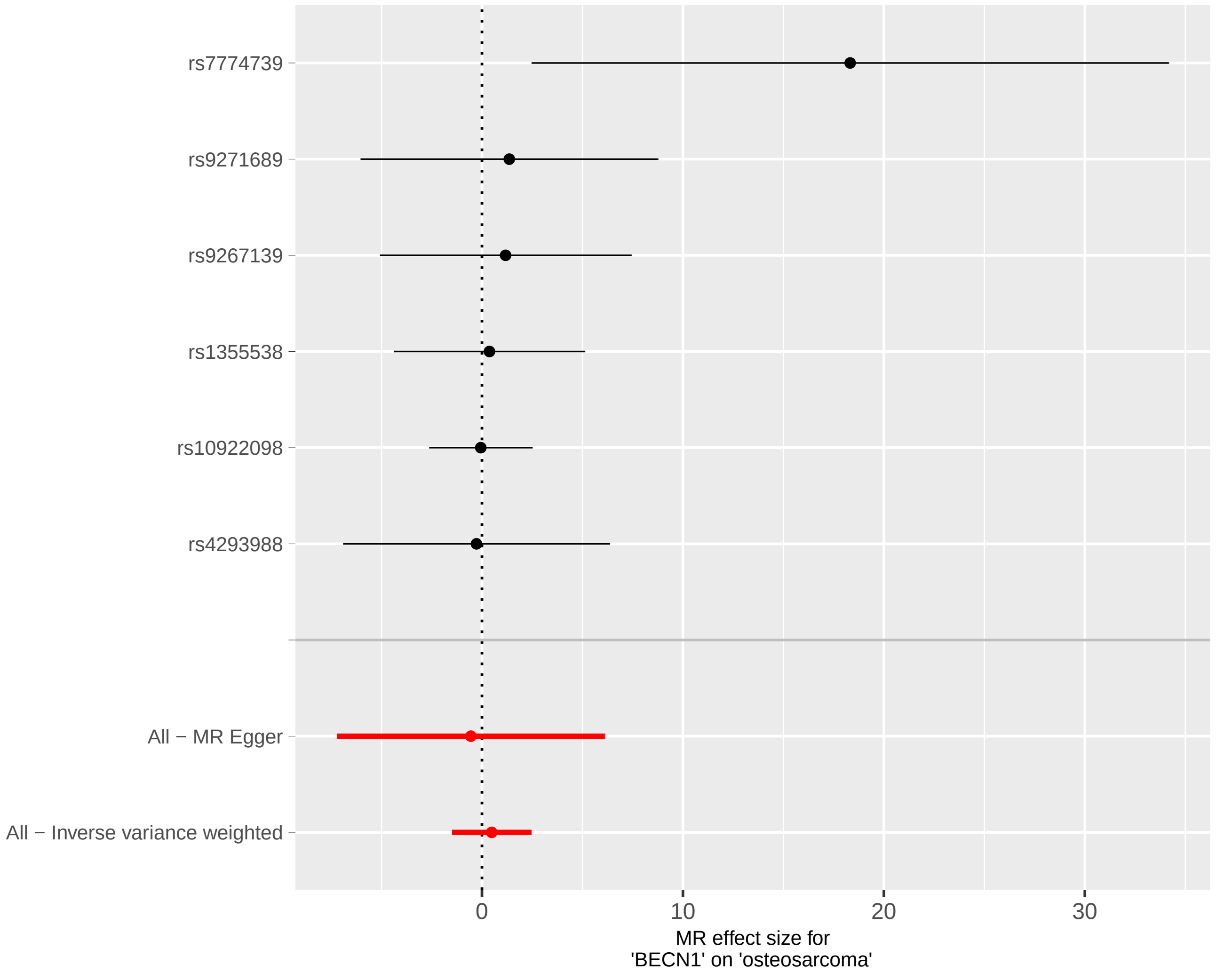
rs7774739
rs9271689
rs9267139
rs1355538
rs10922098
rs4293988
All − MR Egger
All − Inverse variance weighted
0
10
20
30
MR effect size for
'BECN1' on 'osteosarcoma'

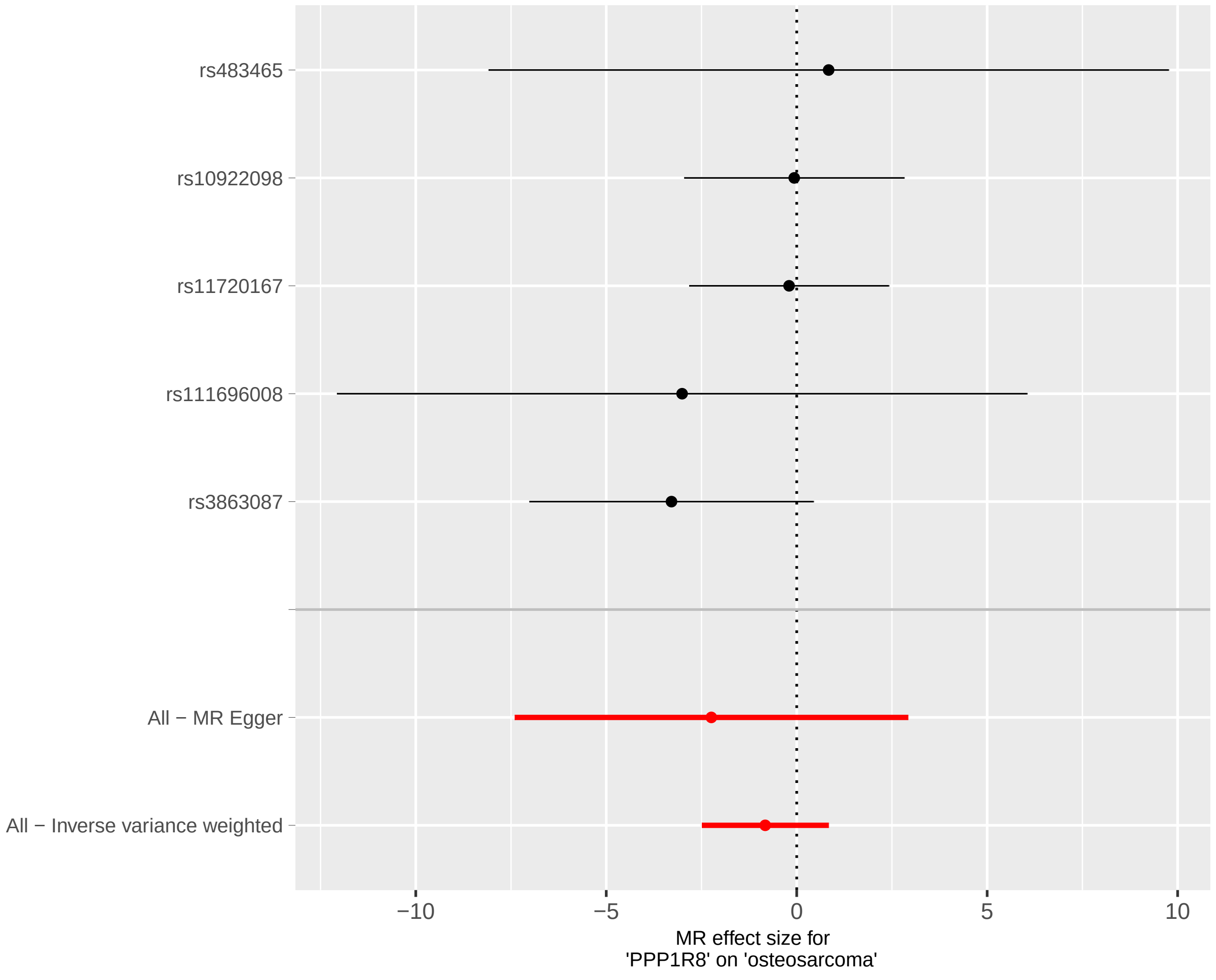
rs483465
rs10922098
rs11720167
rs111696008
rs3863087
All − MR Egger
All − Inverse variance weighted
−10
−5
0
5
10
MR effect size for
'PPP1R8' on 'osteosarcoma'

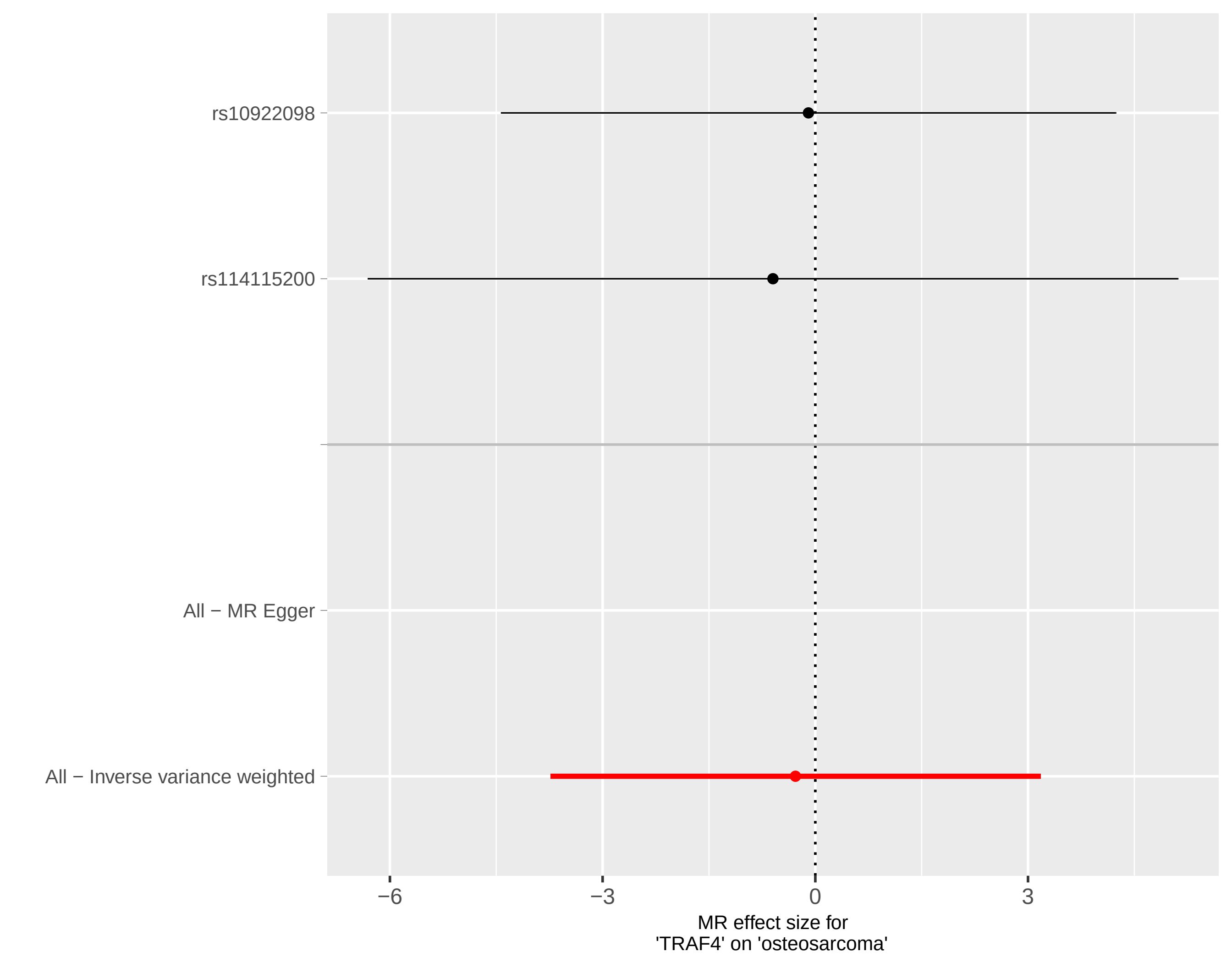

rs10922098
rs114115200
All − MR Egger
All − Inverse variance weighted
−6
−3
0
3
MR effect size for
'TRAF4' on 'osteosarcoma'

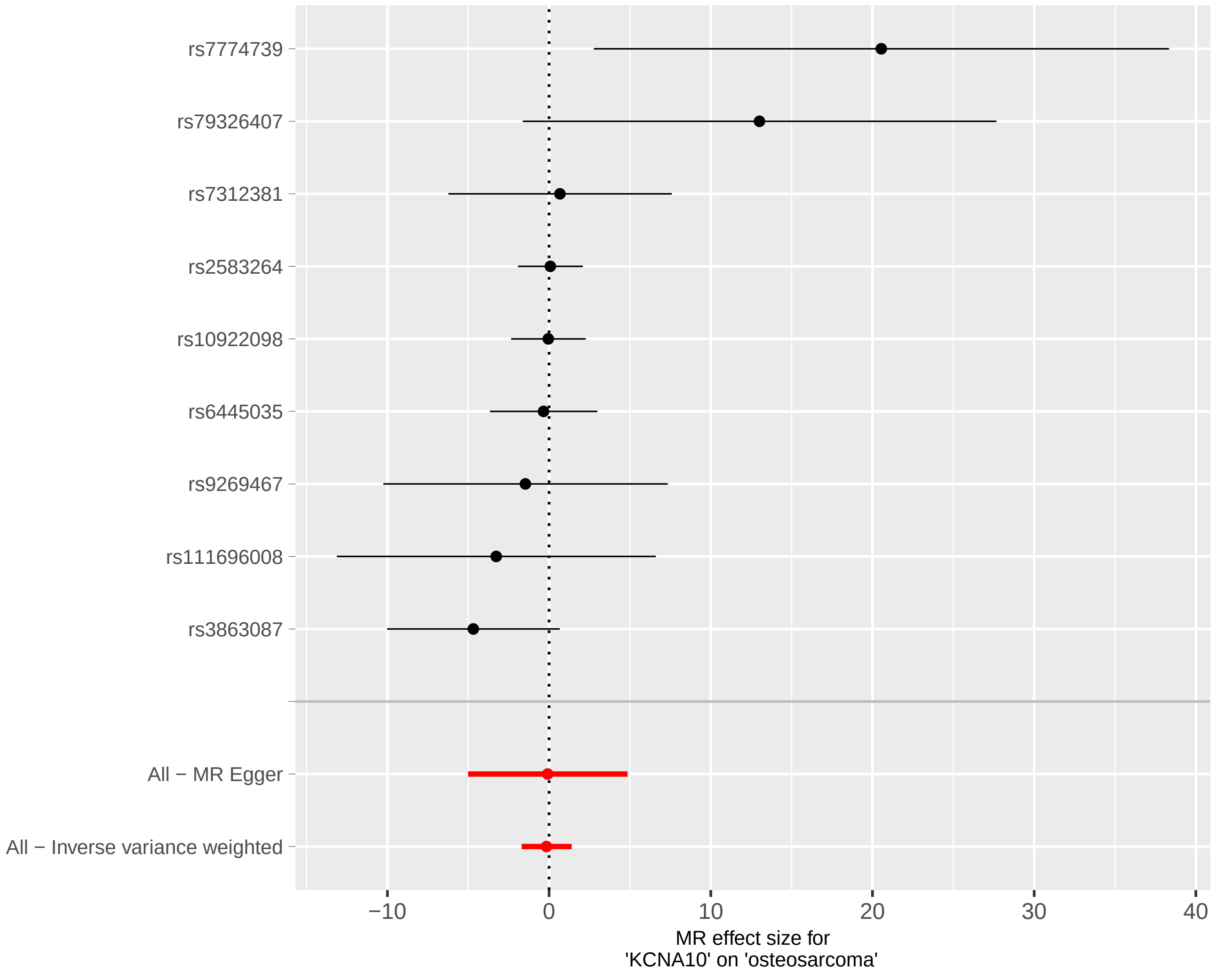
rs7774739
rs79326407
rs7312381
rs2583264
rs10922098
rs6445035
rs9269467
rs111696008
rs3863087
All − MR Egger
All − Inverse variance weighted
−10
0
10
20
30
40
MR effect size for
'KCNA10' on 'osteosarcoma'

Insufficient number of SNPs

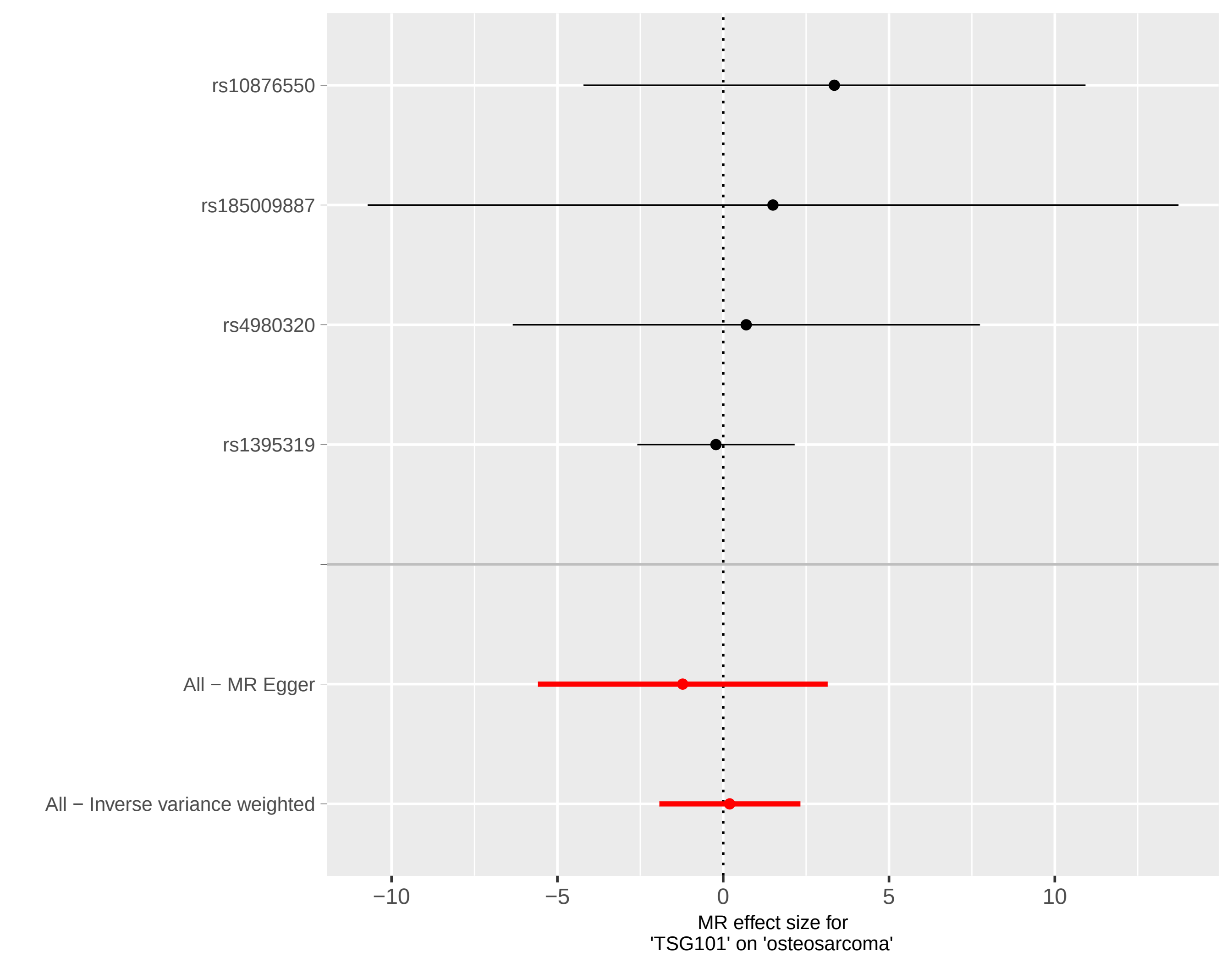
rs10876550
rs185009887
rs4980320
rs1395319
All − MR Egger
All − Inverse variance weighted
−10
−5
0
5
10
MR effect size for
'TSG101' on 'osteosarcoma'

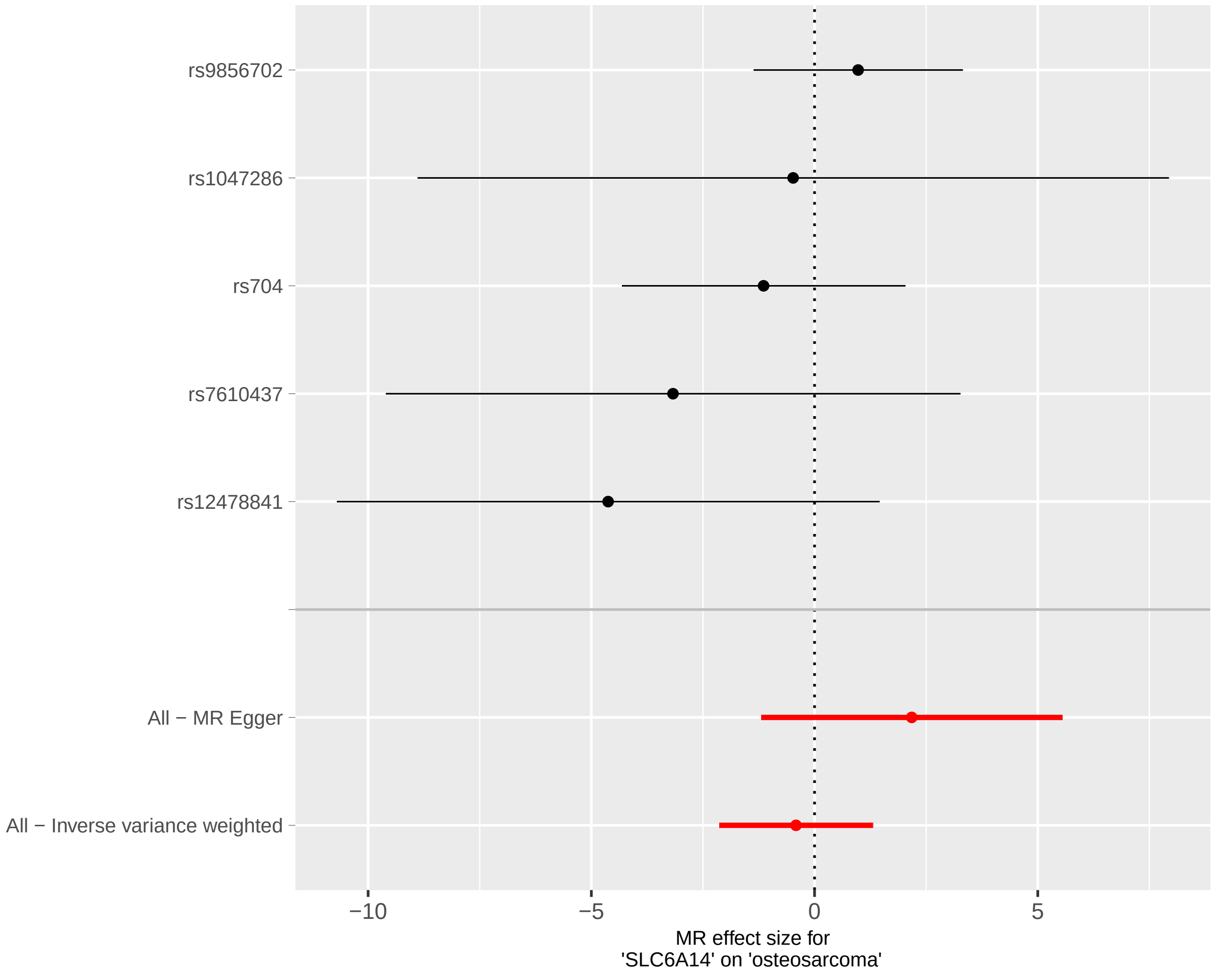

rs9856702
rs1047286
rs704
rs7610437
rs12478841
All − MR Egger
All − Inverse variance weighted
−10
−5
0
5
MR effect size for
'SLC6A14' on 'osteosarcoma'

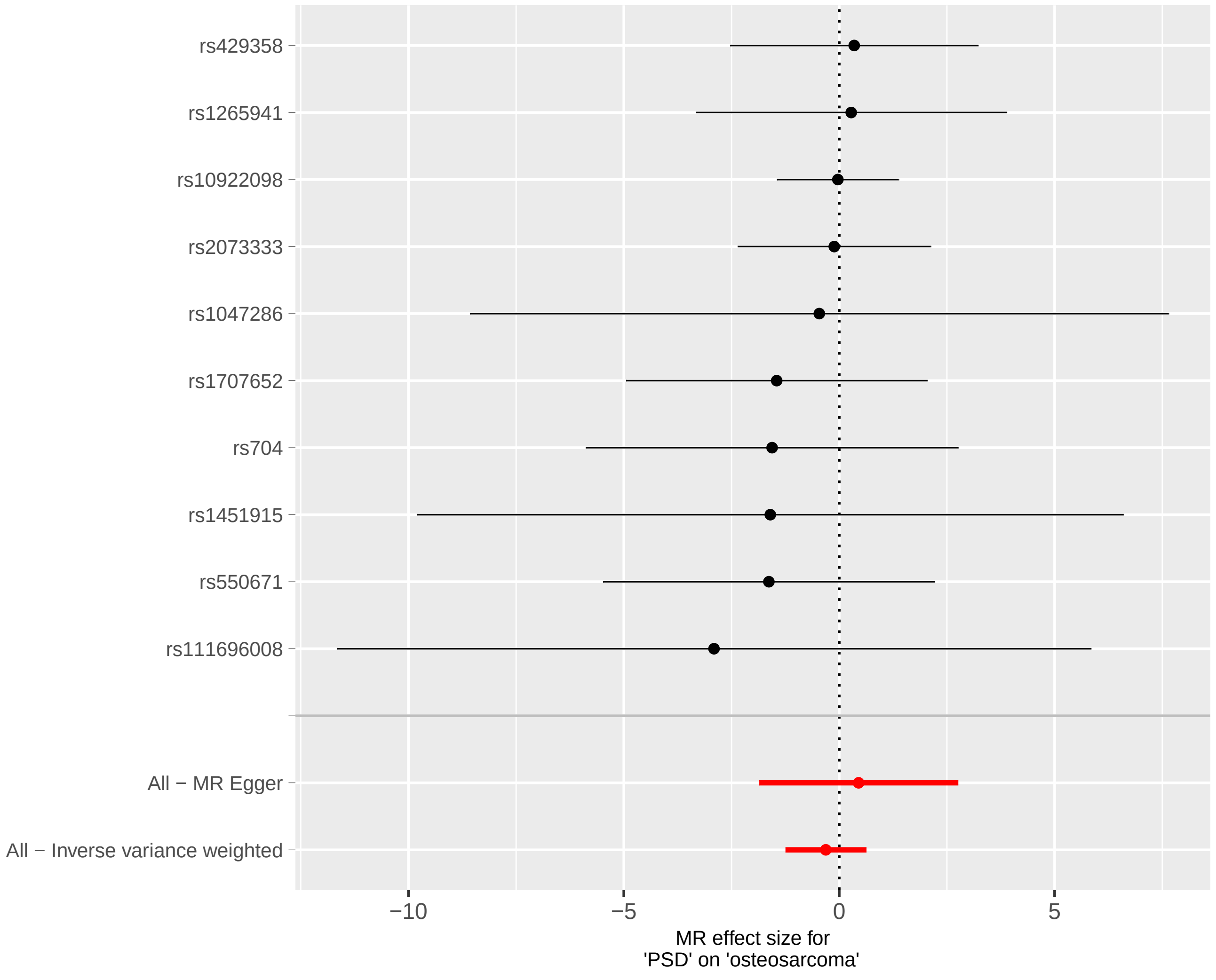

rs429358
rs1265941
rs10922098
rs2073333
rs1047286
rs1707652
rs704
rs1451915
rs550671
rs111696008
All − MR Egger
All − Inverse variance weighted
−10
−5
0
5
MR effect size for
'PSD' on 'osteosarcoma'

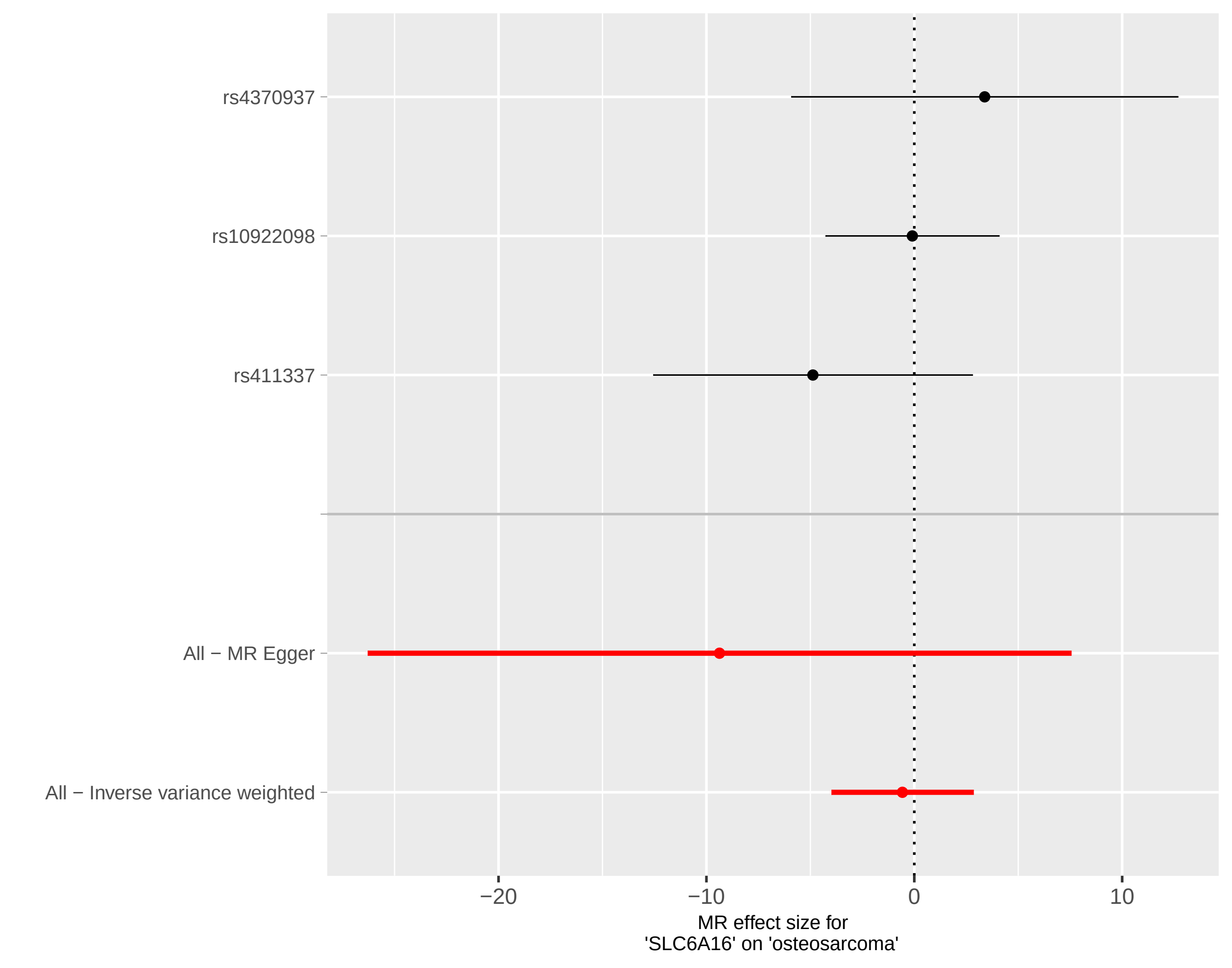
rs4370937
rs10922098
rs411337
All − MR Egger
All − Inverse variance weighted
−20
−10
0
10
MR effect size for
'SLC6A16' on 'osteosarcoma'

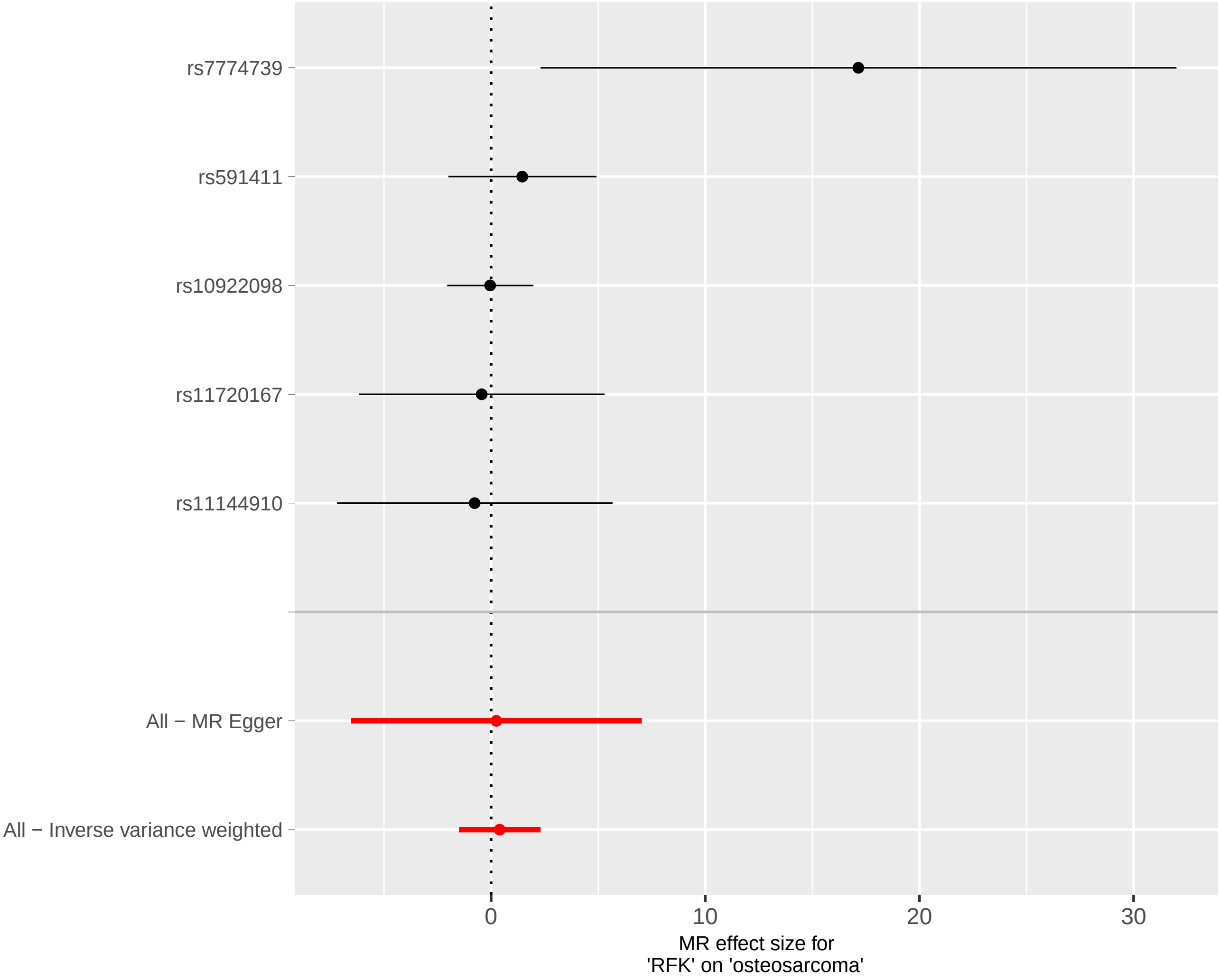

rs7774739
rs591411
rs10922098
rs11720167
rs11144910
All − MR Egger
All − Inverse variance weighted
0
10
20
30
MR effect size for
'RFK' on 'osteosarcoma'

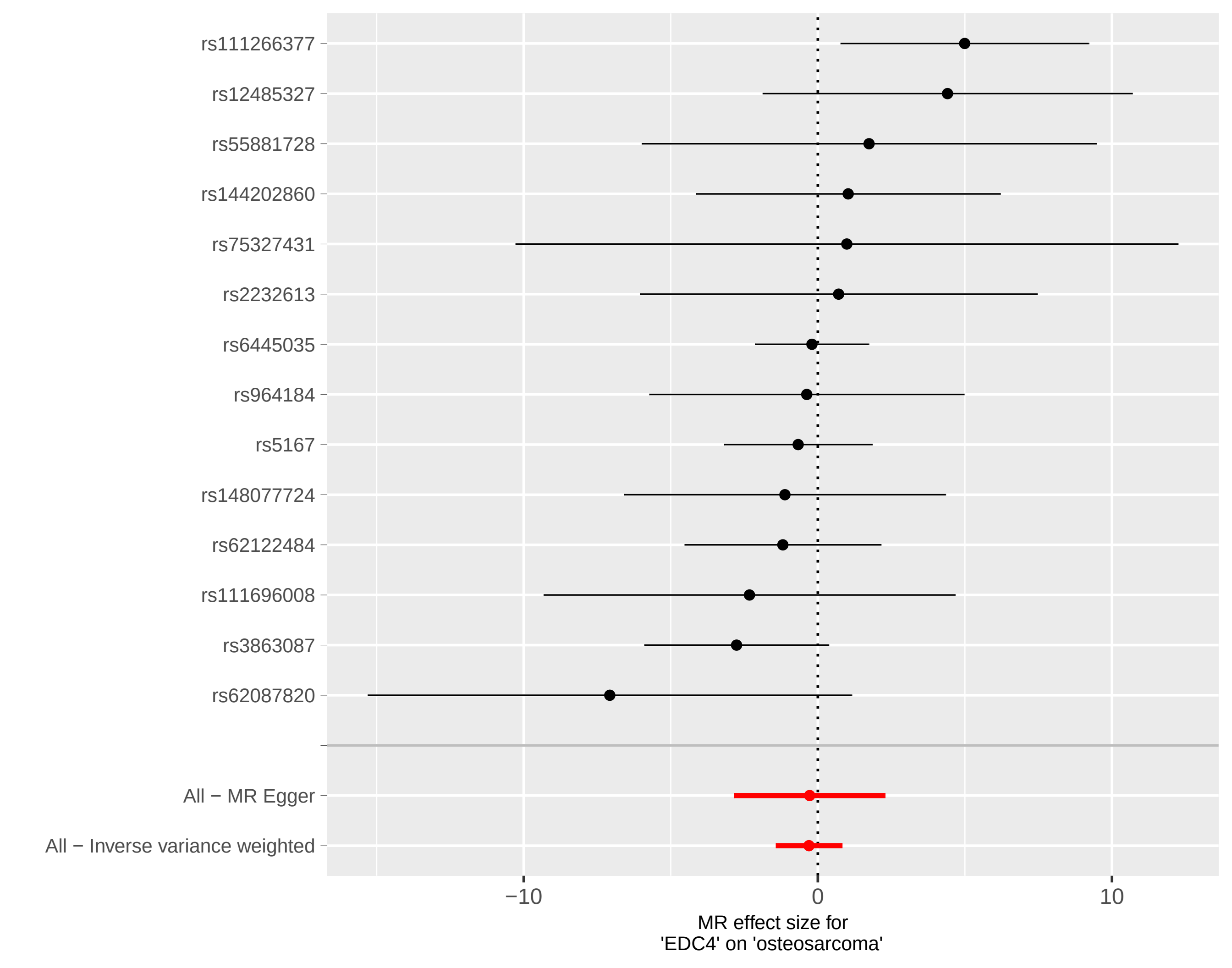
rs111266377
rs12485327
rs55881728
rs144202860
rs75327431
rs2232613
rs6445035
rs964184
rs5167
rs148077724
rs62122484
rs111696008
rs3863087
rs62087820
All − MR Egger
All − Inverse variance weighted
−10
0
10
MR effect size for
'EDC4' on 'osteosarcoma'

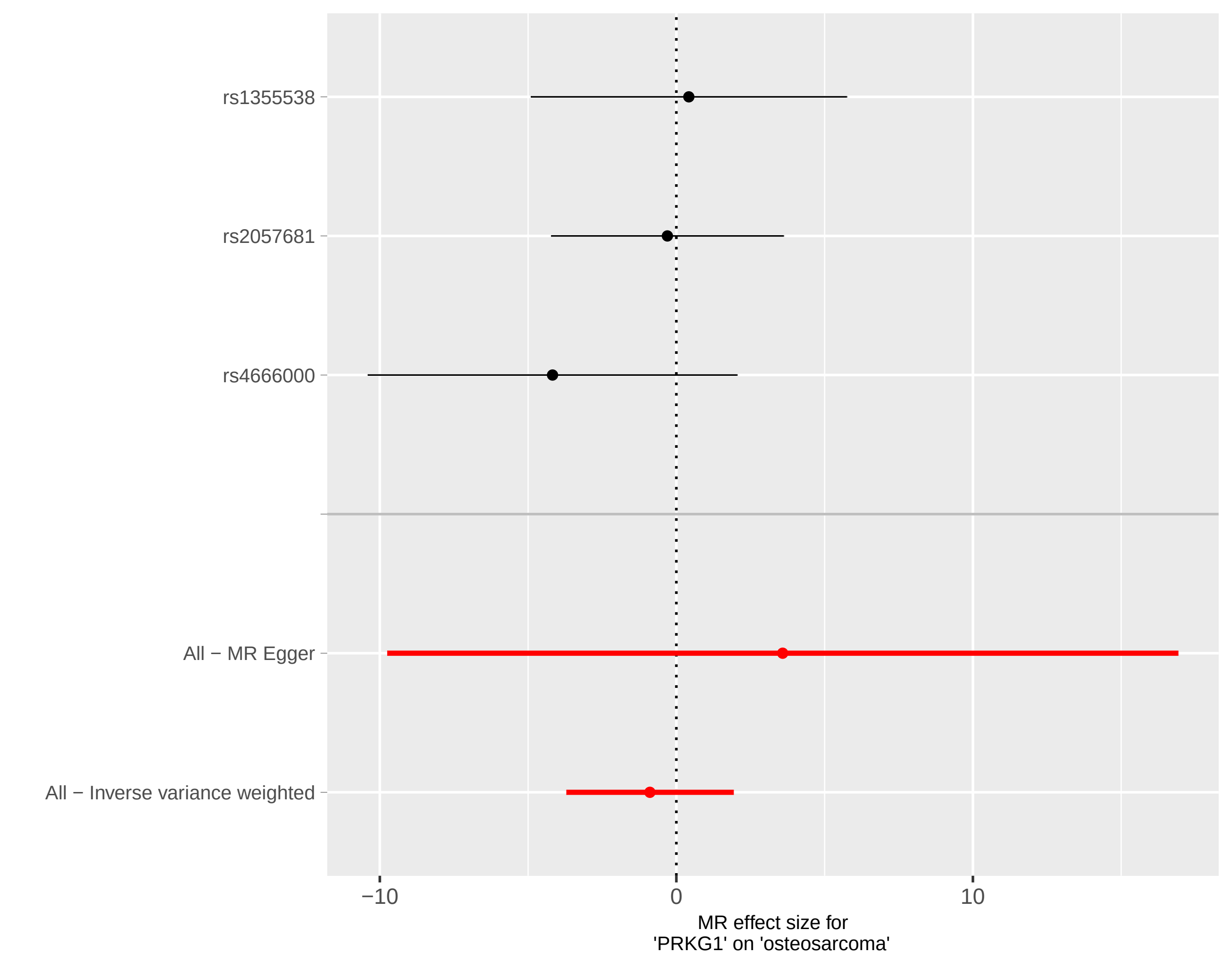
rs1355538
rs2057681
rs4666000
All − MR Egger
All − Inverse variance weighted
−10
0
10
MR effect size for
'PRKG1' on 'osteosarcoma'

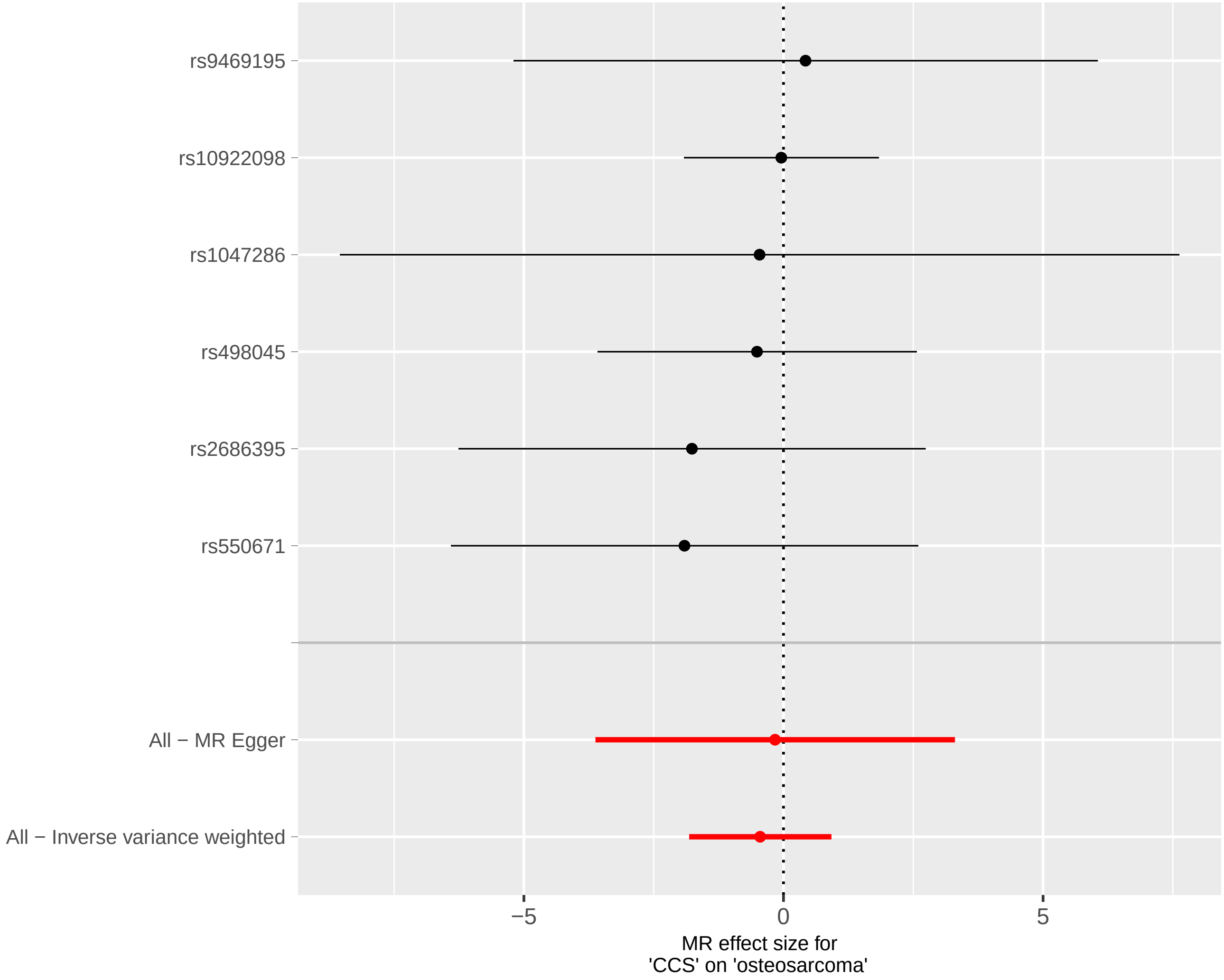
rs9469195
rs10922098
rs1047286
rs498045
rs2686395
rs550671
All − MR Egger
All − Inverse variance weighted
−5
0
5
MR effect size for
'CCS' on 'osteosarcoma'

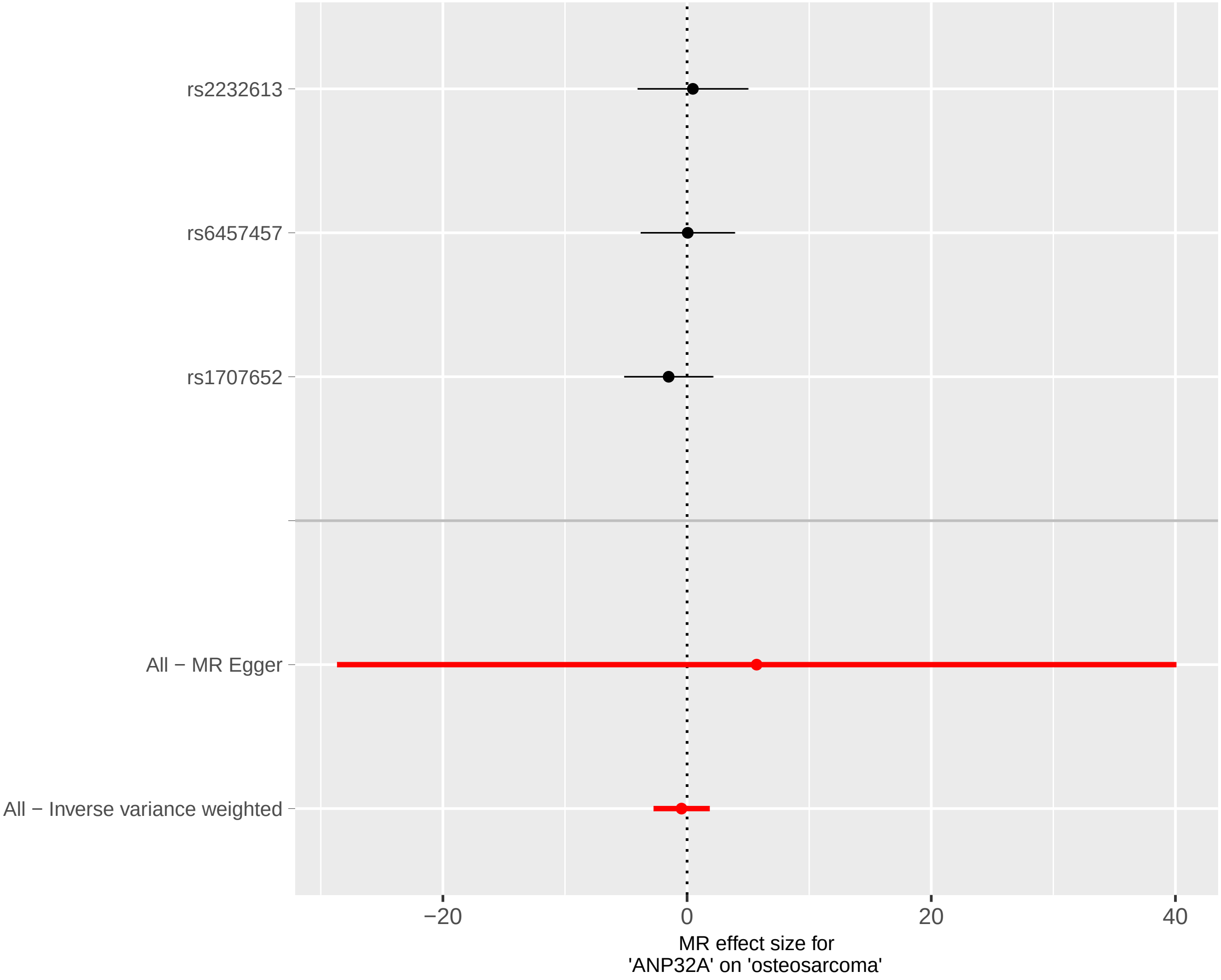
rs2232613
rs6457457
rs1707652
All − MR Egger
All − Inverse variance weighted
−20
0
20
40
MR effect size for
'ANP32A' on 'osteosarcoma'

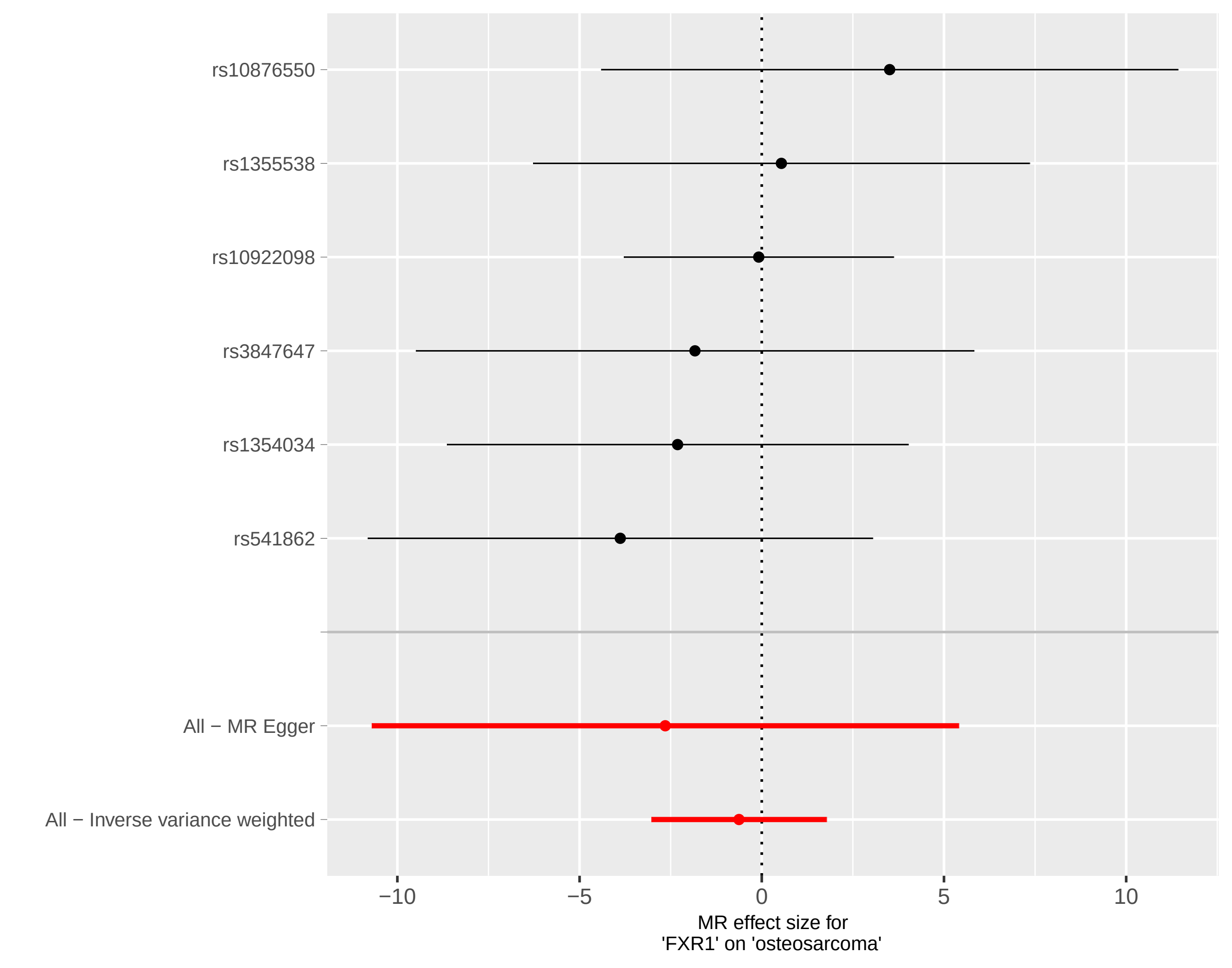
rs10876550
rs1355538
rs10922098
rs3847647
rs1354034
rs541862
All − MR Egger
All − Inverse variance weighted
−10
−5
0
5
10
MR effect size for
'FXR1' on 'osteosarcoma'

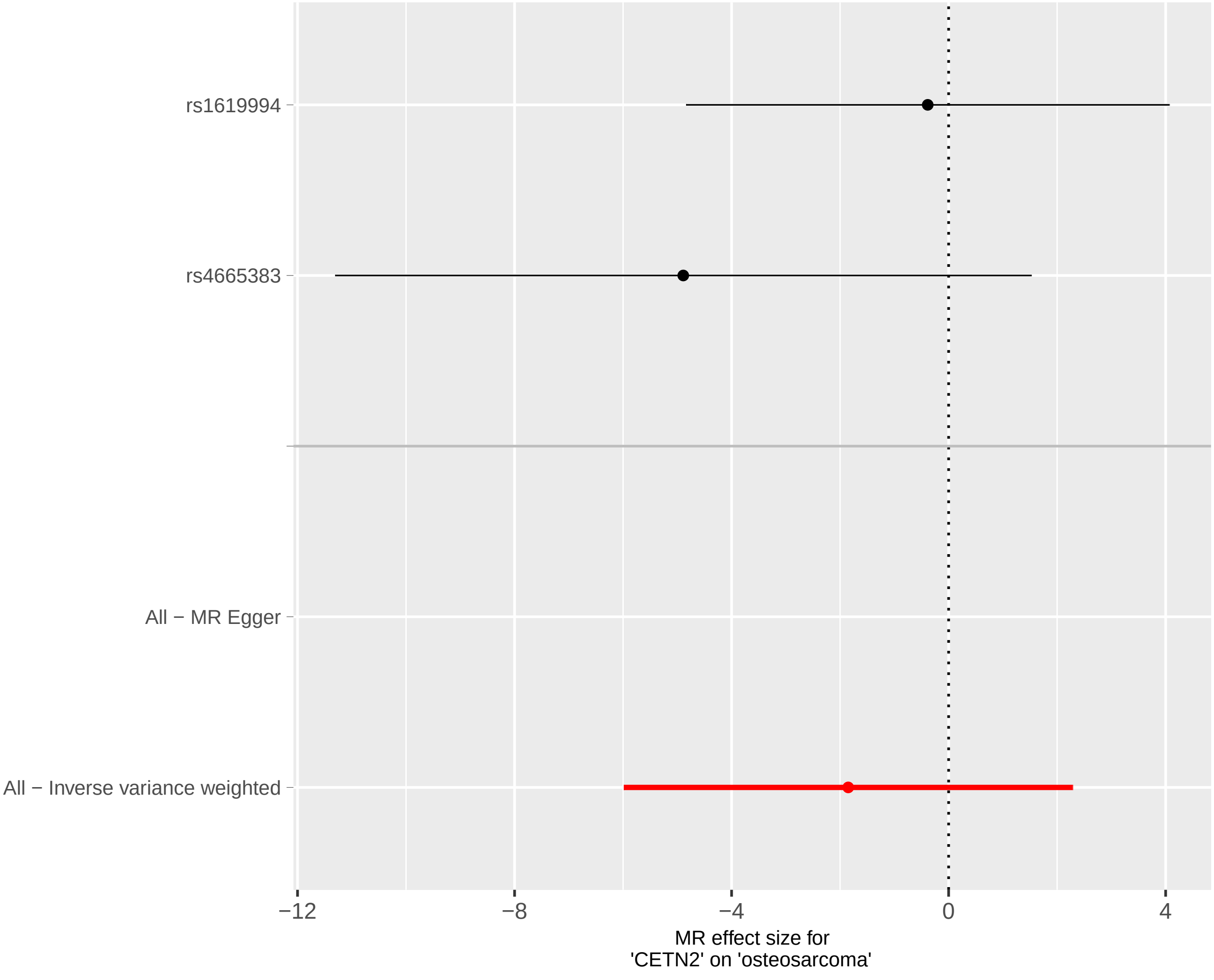
rs1619994
rs4665383
All − MR Egger
All − Inverse variance weighted
−12
−8
−4
0
4
MR effect size for
'CETN2' on 'osteosarcoma'

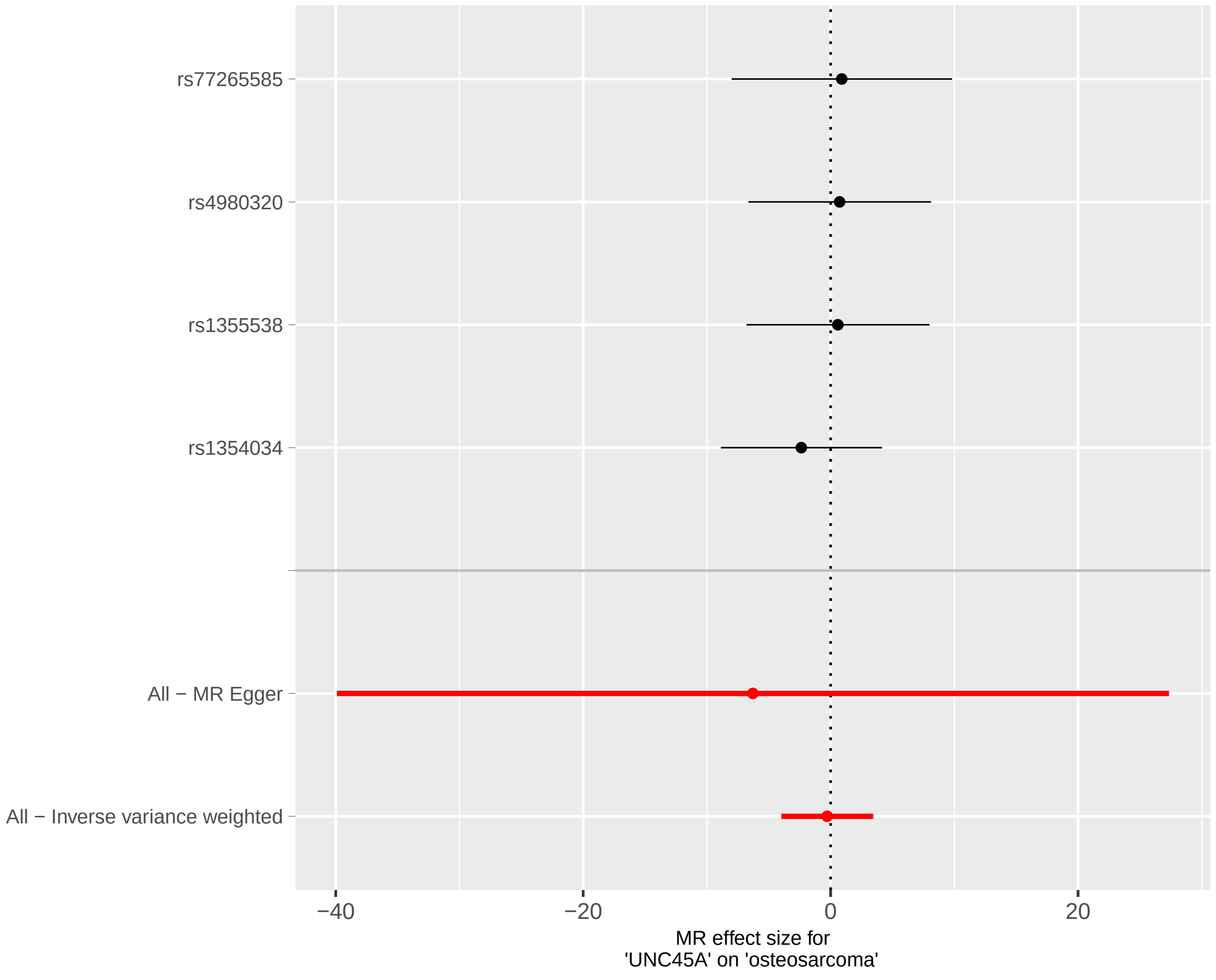
rs77265585
rs4980320
rs1355538
rs1354034
All − MR Egger
All − Inverse variance weighted
−40
−20
0
20
MR effect size for
'UNC45A' on 'osteosarcoma'

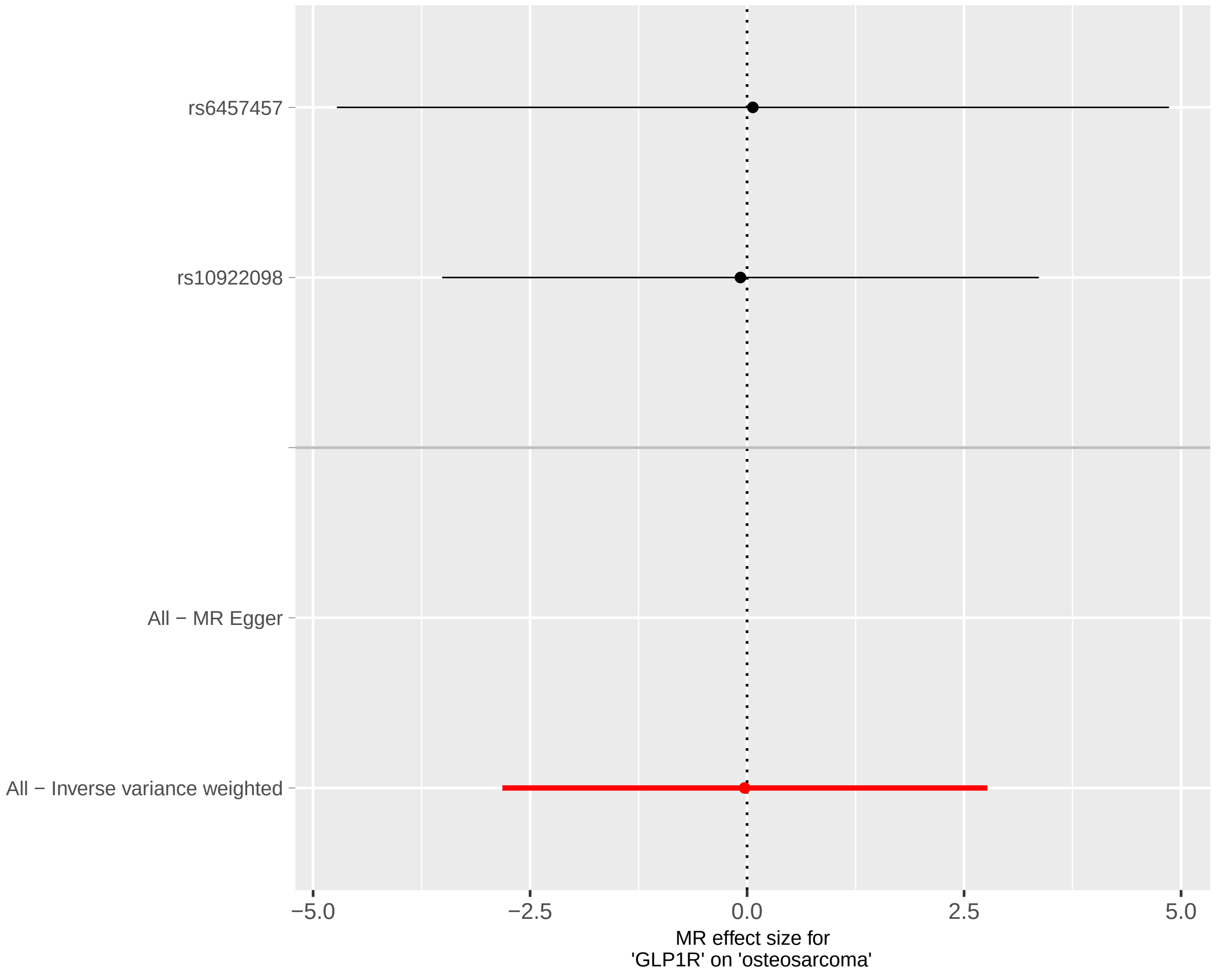

rs6457457
rs10922098
All − MR Egger
All − Inverse variance weighted
−5.0
−2.5
0.0
2.5
5.0
MR effect size for
'GLP1R' on 'osteosarcoma'

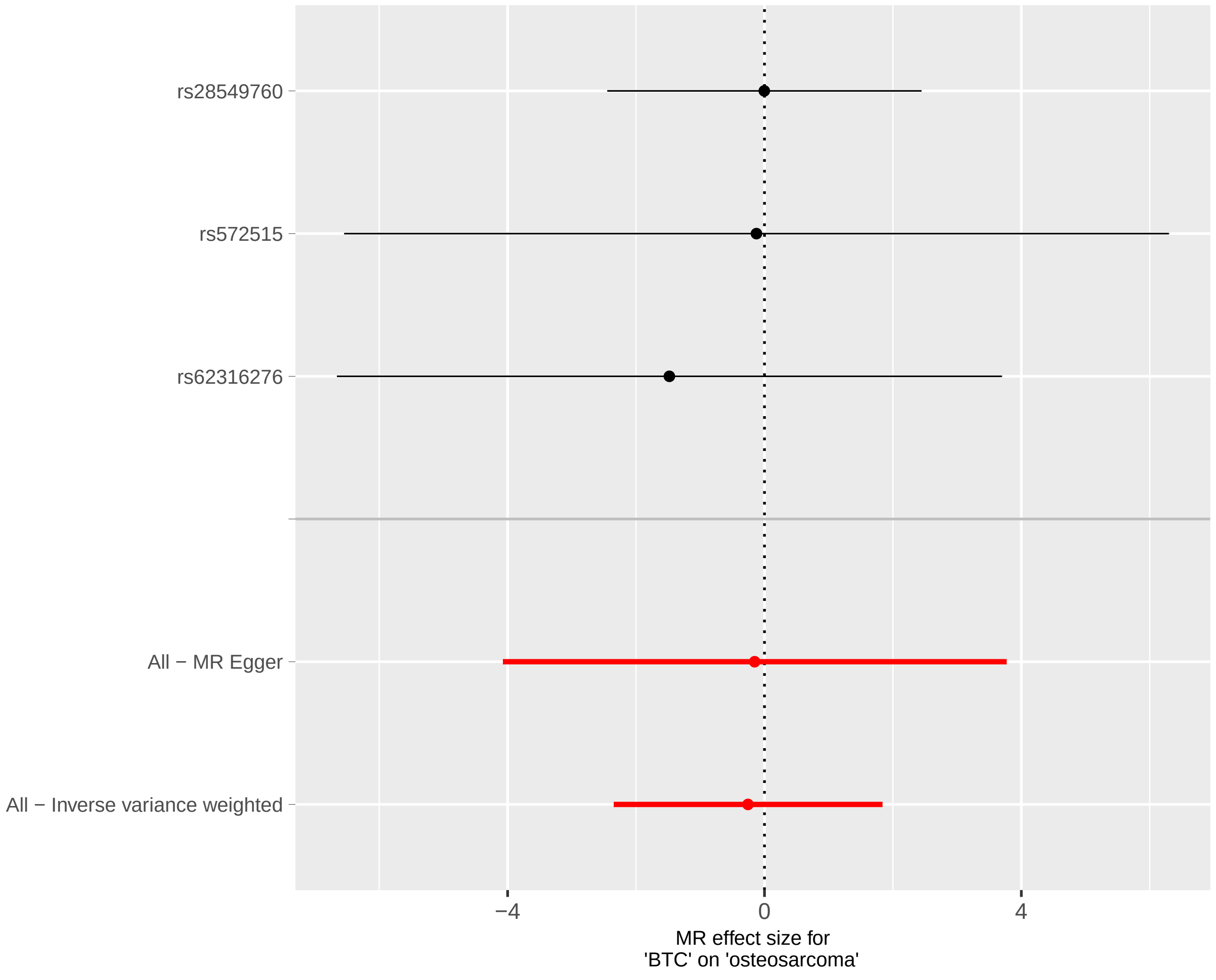
rs28549760
rs572515
rs62316276
All − MR Egger
All − Inverse variance weighted
−4
0
4
MR effect size for
'BTC' on 'osteosarcoma'

Insufficient number of SNPs

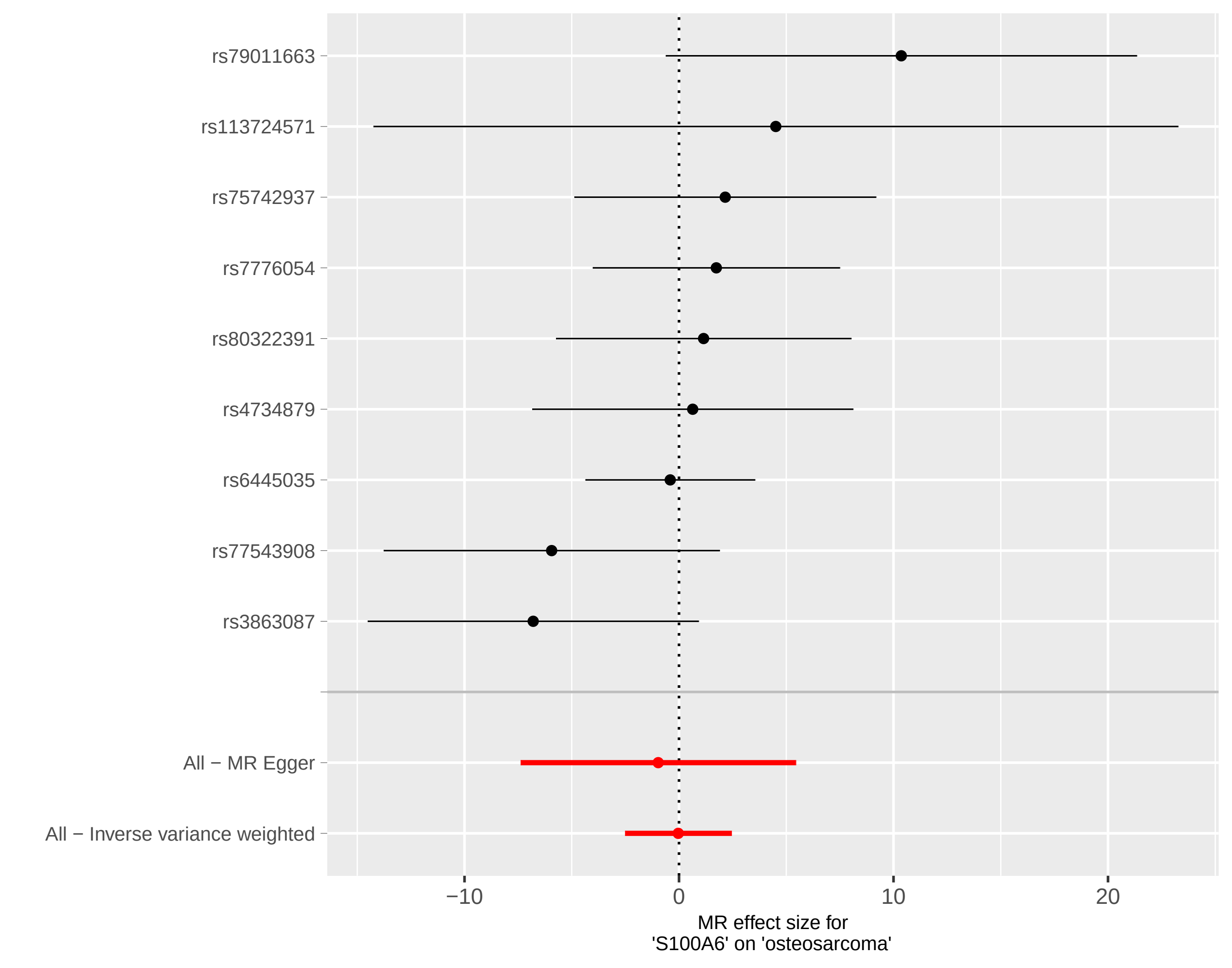

rs79011663
rs113724571
rs75742937
rs7776054
rs80322391
rs4734879
rs6445035
rs77543908
rs3863087
All − MR Egger
All − Inverse variance weighted
−10
0
10
20
MR effect size for
'S100A6' on 'osteosarcoma'

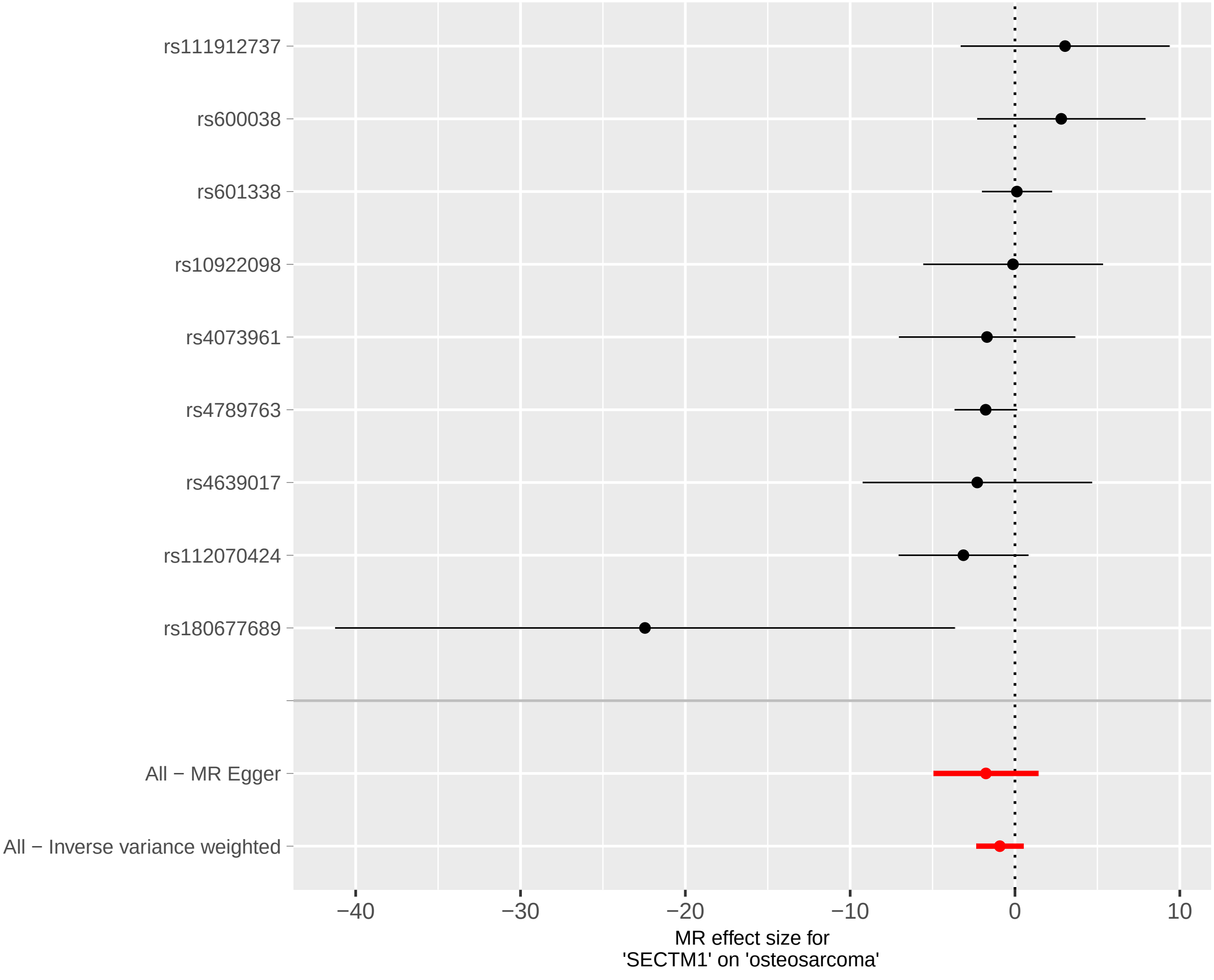

rs111912737
rs600038
rs601338
rs10922098
rs4073961
rs4789763
rs4639017
rs112070424
rs180677689
All − MR Egger
All − Inverse variance weighted
−40
−30
−20
−10
0
10
MR effect size for
'SECTM1' on 'osteosarcoma'

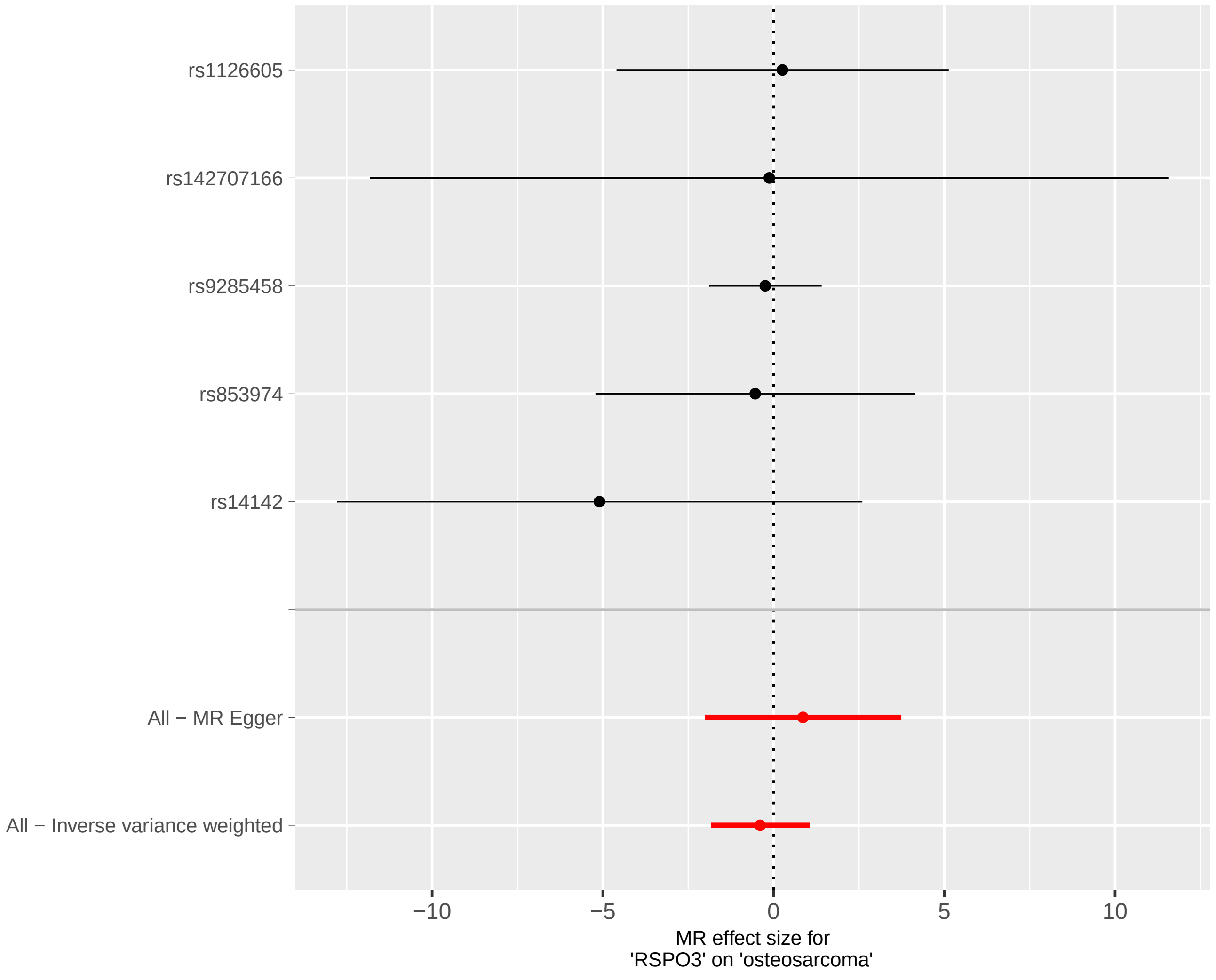

rs1126605
rs142707166
rs9285458
rs853974
rs14142
All − MR Egger
All − Inverse variance weighted
−10
−5
0
5
10
MR effect size for
'RSPO3' on 'osteosarcoma'

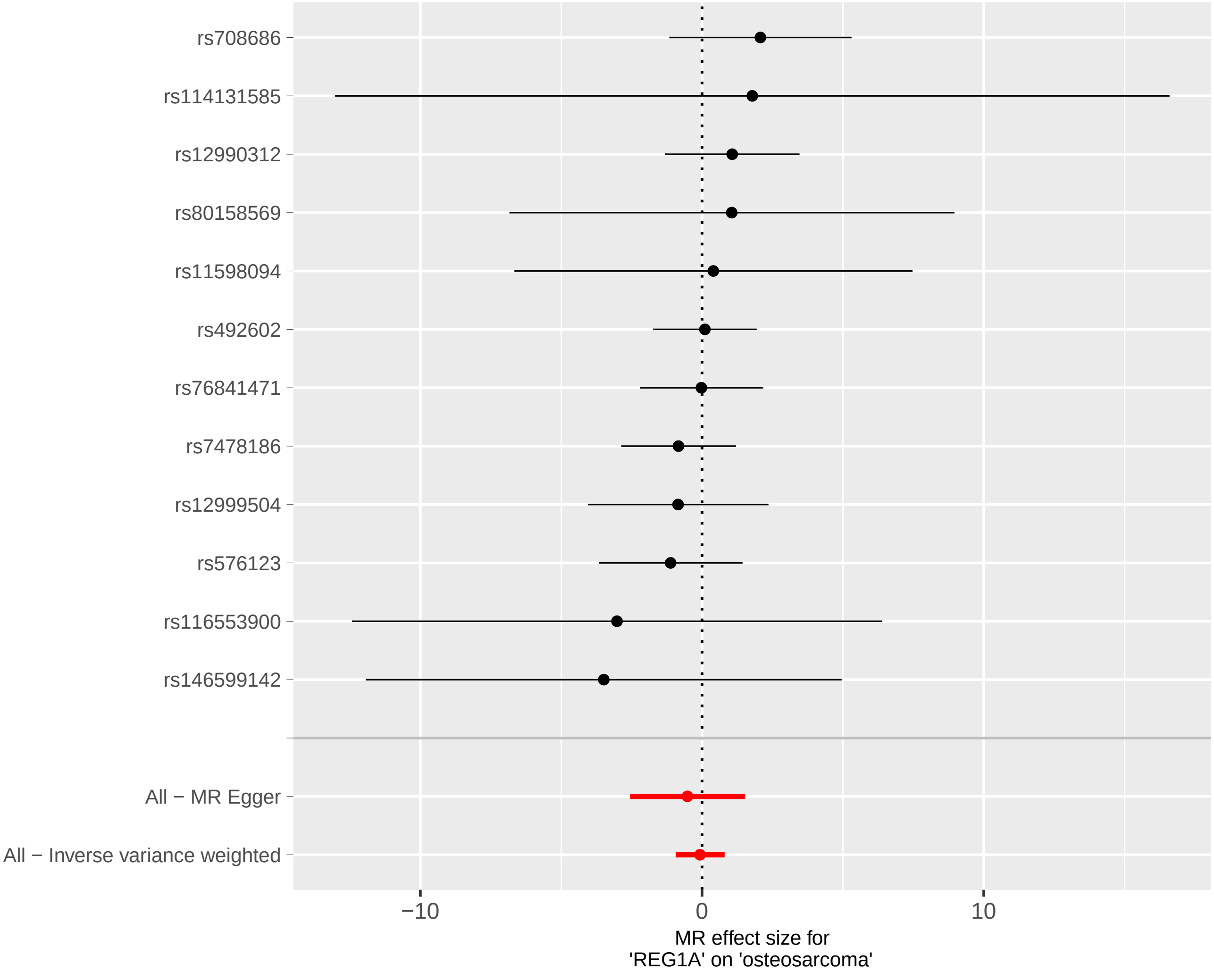

rs708686
rs114131585
rs12990312
rs80158569
rs11598094
rs492602
rs76841471
rs7478186
rs12999504
rs576123
rs116553900
rs146599142
All − MR Egger
All − Inverse variance weighted
−10
0
10
MR effect size for
'REG1A' on 'osteosarcoma'

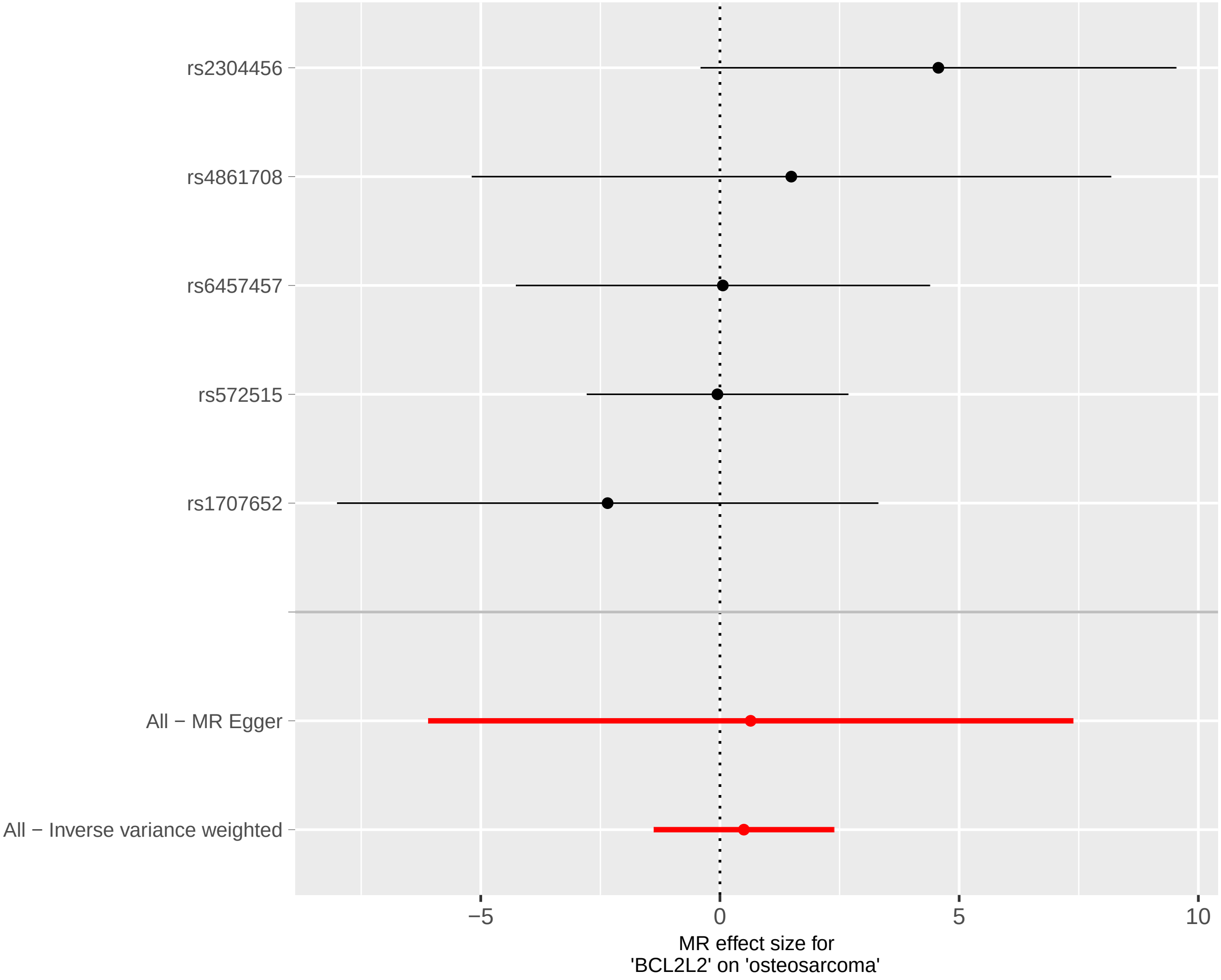
rs2304456
rs4861708
rs6457457
rs572515
rs1707652
All − MR Egger
All − Inverse variance weighted
−5
0
5
10
MR effect size for
'BCL2L2' on 'osteosarcoma'

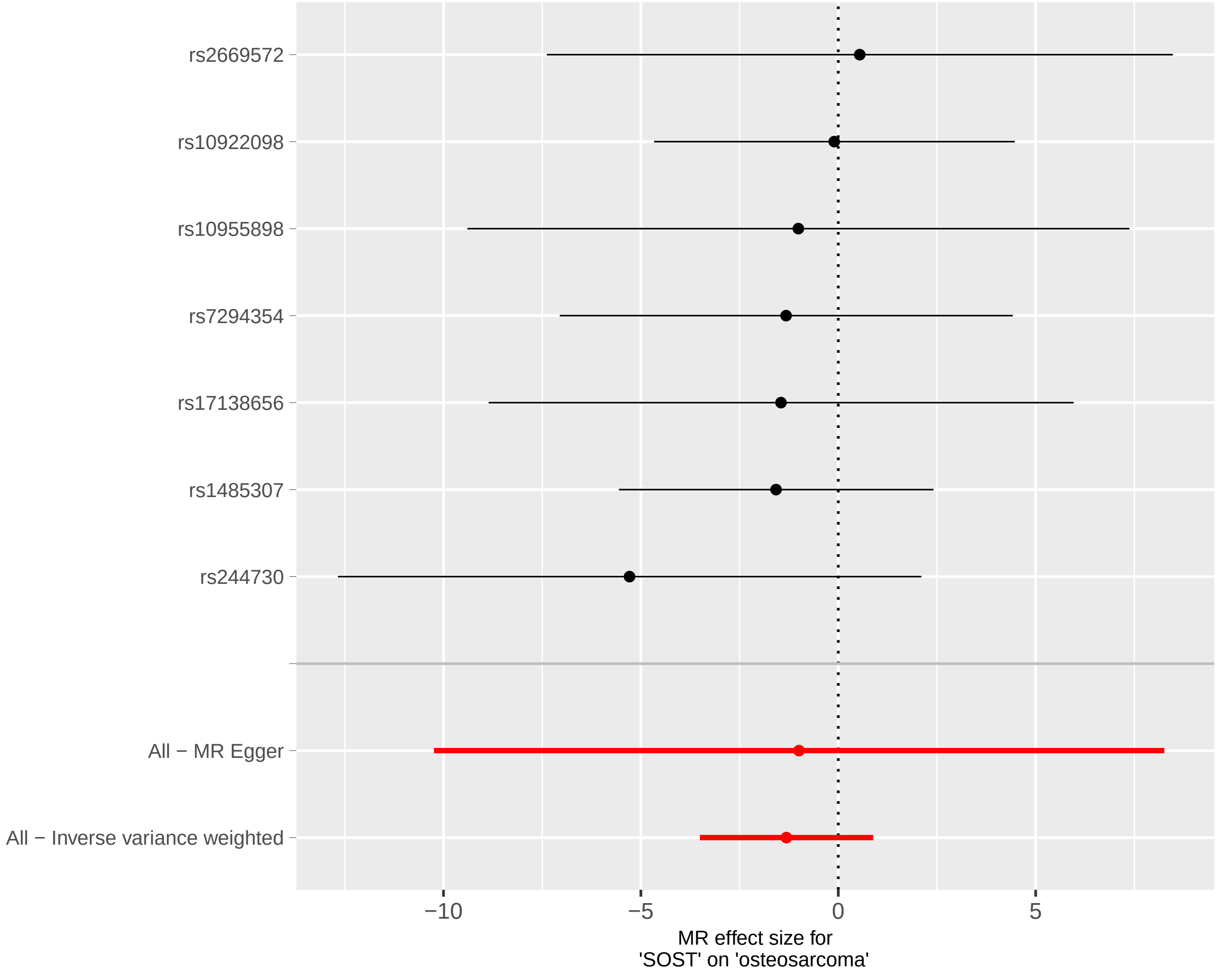
rs2669572
rs10922098
rs10955898
rs7294354
rs17138656
rs1485307
rs244730
All − MR Egger
All − Inverse variance weighted
−10
−5
0
5
MR effect size for
'SOST' on 'osteosarcoma'

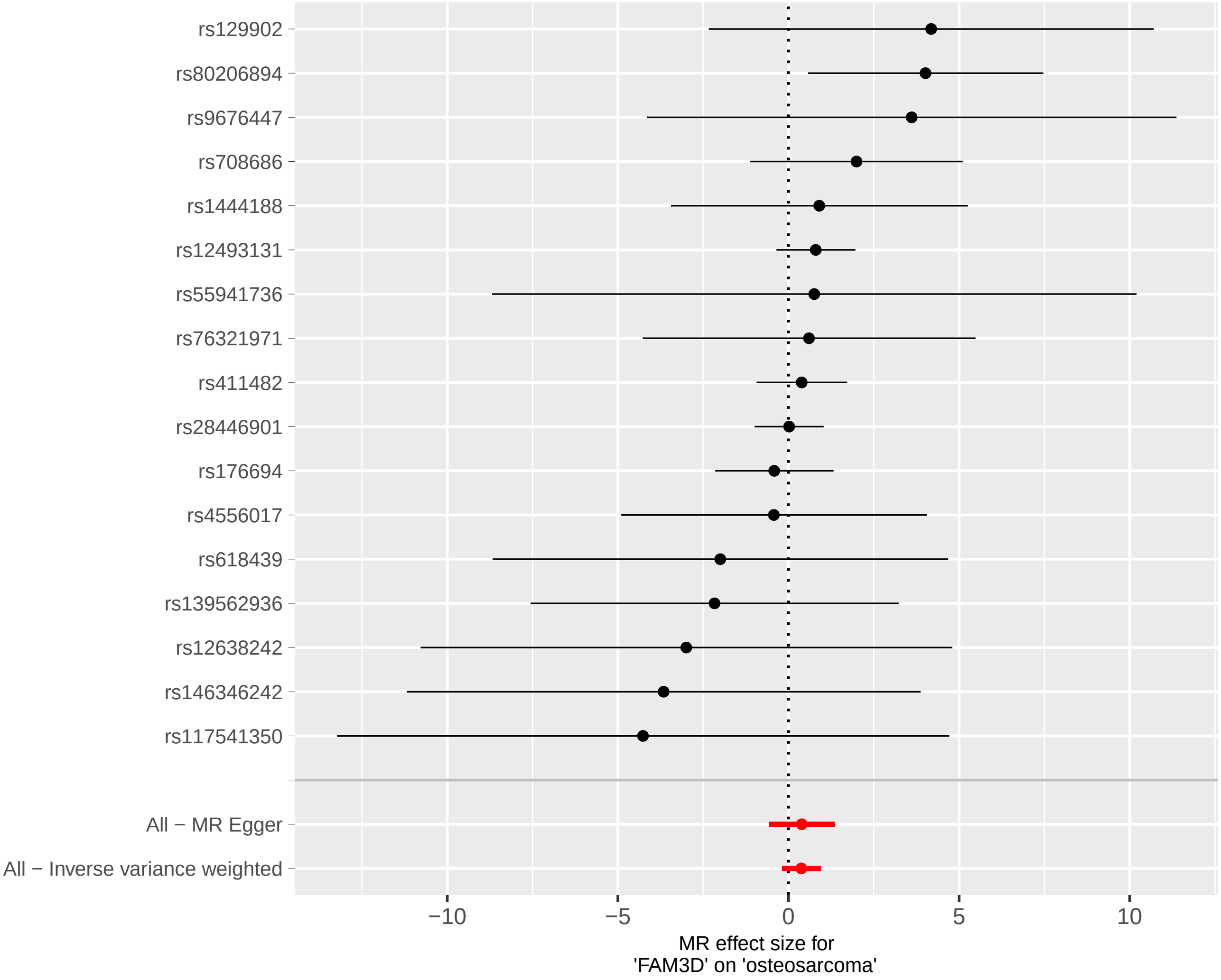

rs129902
rs80206894
rs9676447
rs708686
rs1444188
rs12493131
rs55941736
rs76321971
rs411482
rs28446901
rs176694
rs4556017
rs618439
rs139562936
rs12638242
rs146346242
rs117541350
All − MR Egger
All − Inverse variance weighted
−10
−5
0
5
10
MR effect size for
'FAM3D' on 'osteosarcoma'

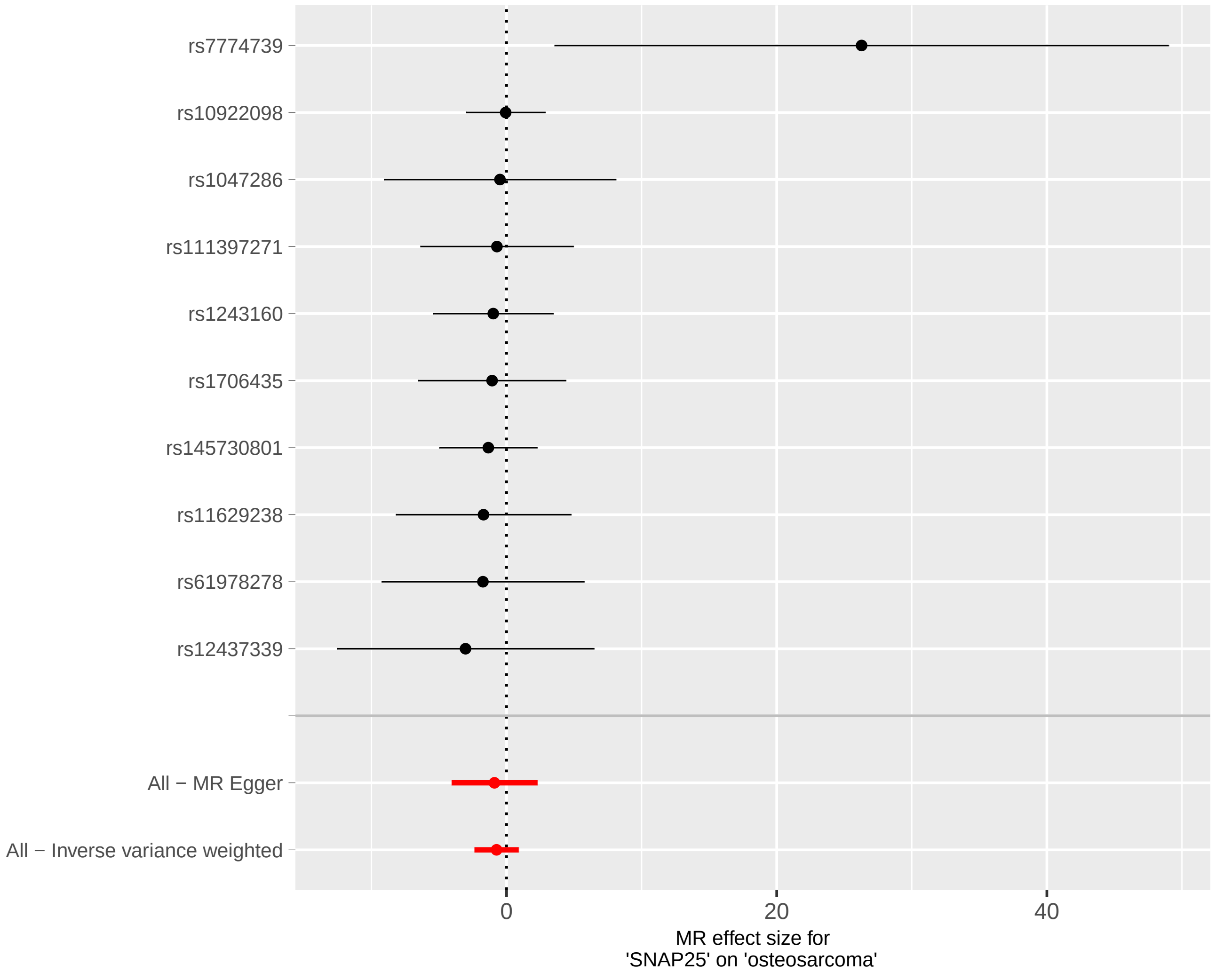
rs7774739
rs10922098
rs1047286
rs111397271
rs1243160
rs1706435
rs145730801
rs11629238
rs61978278
rs12437339
All − MR Egger
All − Inverse variance weighted
0
20
40
MR effect size for
'SNAP25' on 'osteosarcoma'

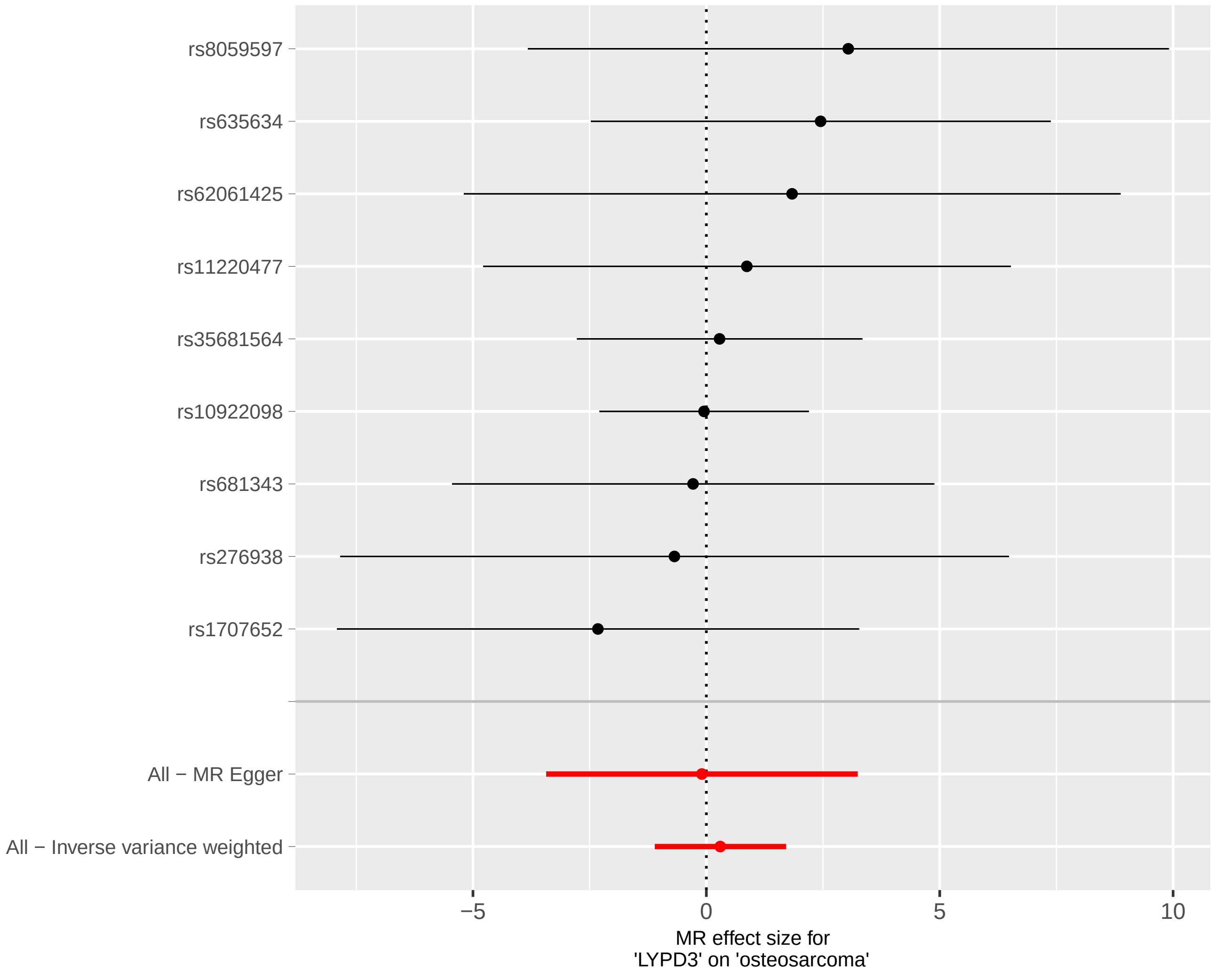

rs8059597
rs635634
rs62061425
rs11220477
rs35681564
rs10922098
rs681343
rs276938
rs1707652
All − MR Egger
All − Inverse variance weighted
−5
0
5
10
MR effect size for
'LYPD3' on 'osteosarcoma'

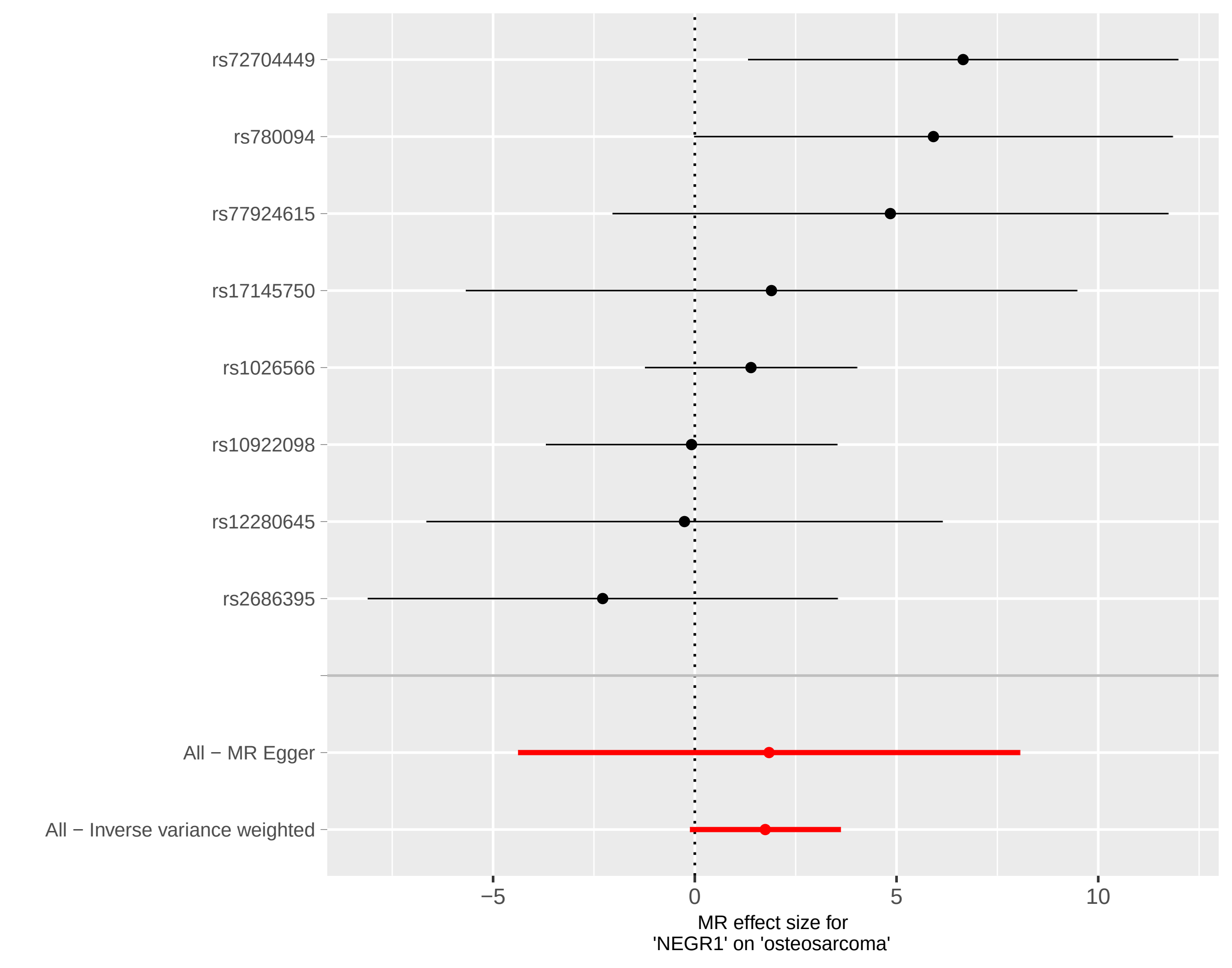
rs72704449
rs780094
rs77924615
rs17145750
rs1026566
rs10922098
rs12280645
rs2686395
All − MR Egger
All − Inverse variance weighted
−5
0
5
10
MR effect size for
'NEGR1' on 'osteosarcoma'

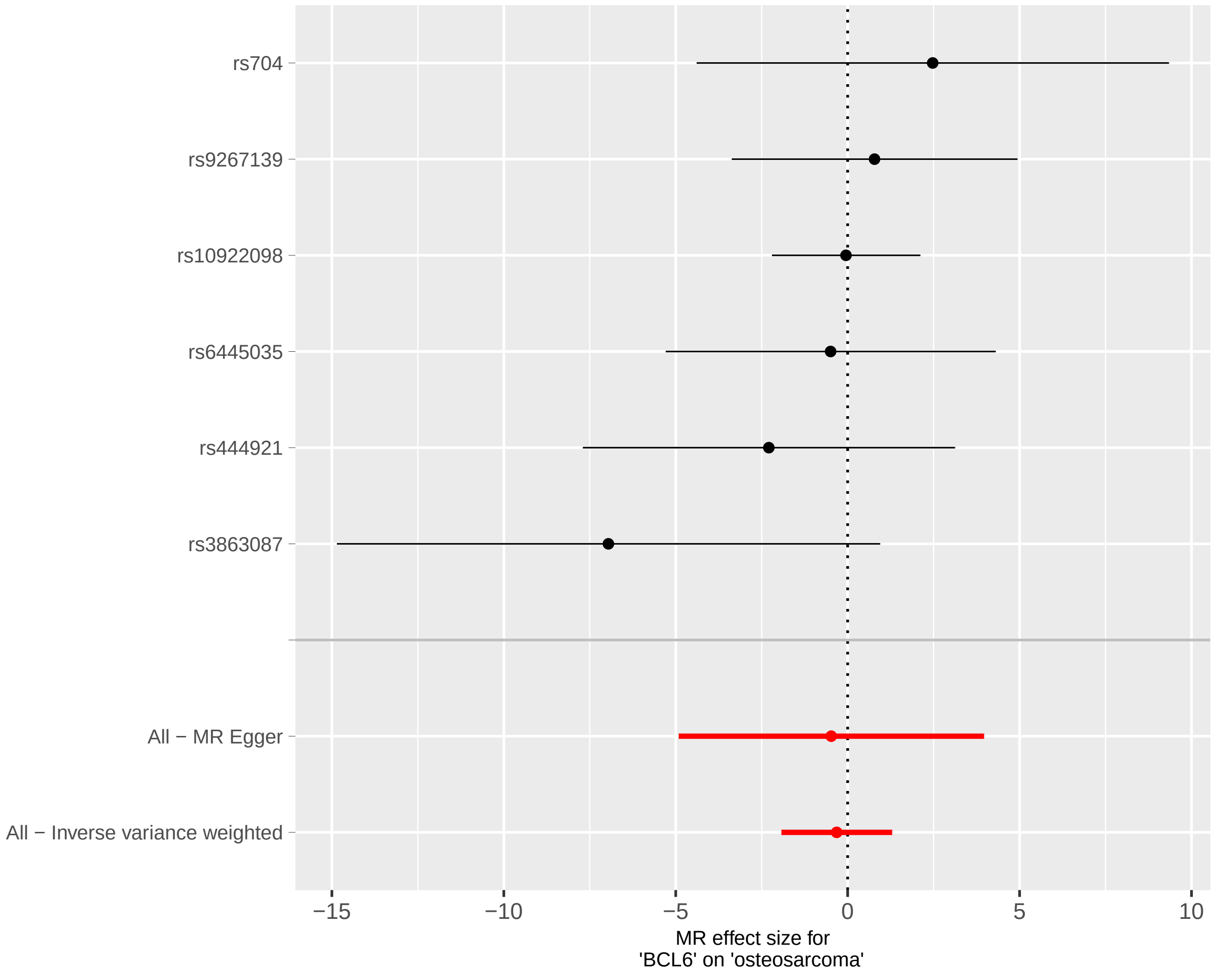

rs704
rs9267139
rs10922098
rs6445035
rs444921
rs3863087
All − MR Egger
All − Inverse variance weighted
−15
−10
−5
0
5
10
MR effect size for
'BCL6' on 'osteosarcoma'

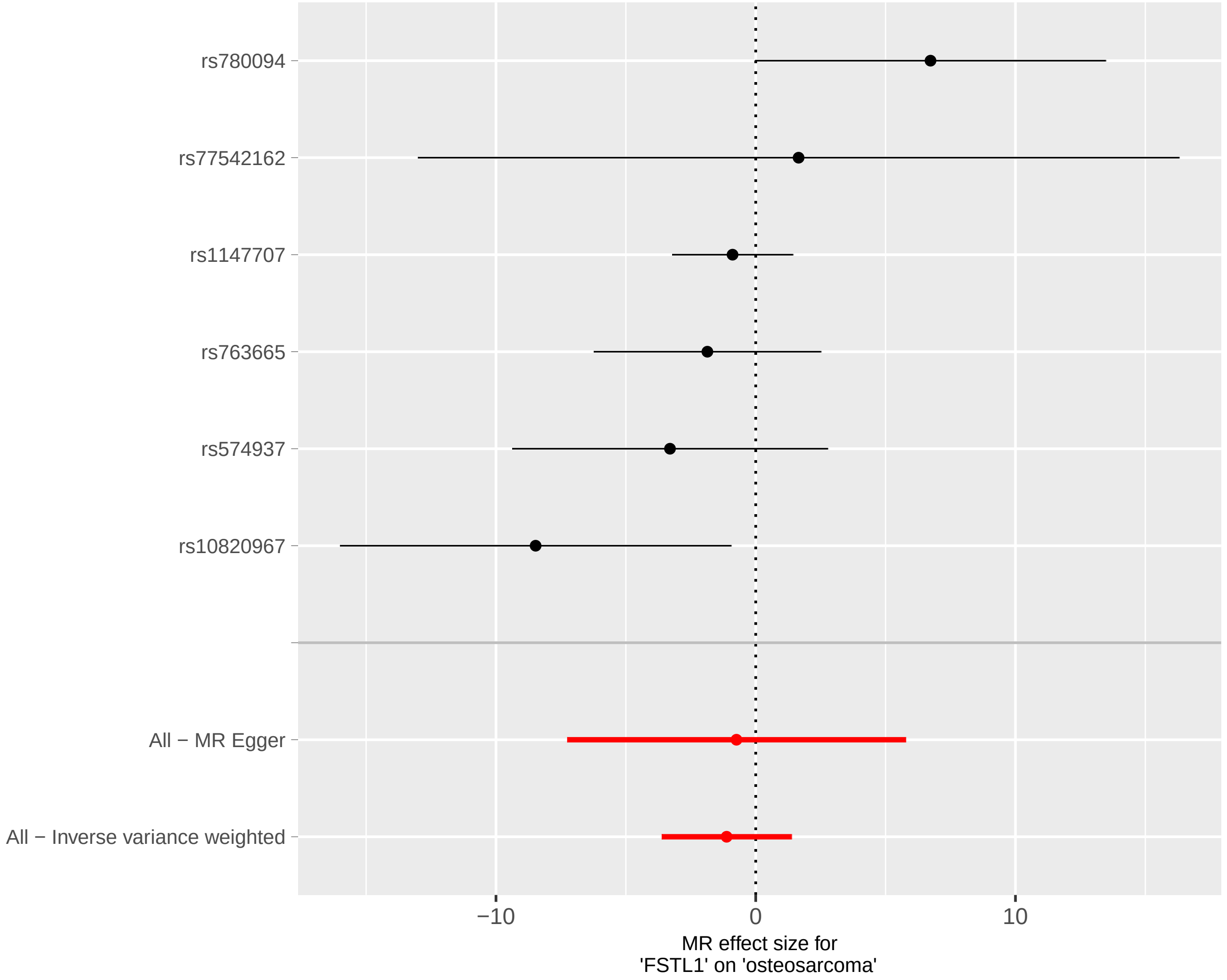

rs780094
rs77542162
rs1147707
rs763665
rs574937
rs10820967
All − MR Egger
All − Inverse variance weighted
−10
0
10
MR effect size for
'FSTL1' on 'osteosarcoma'

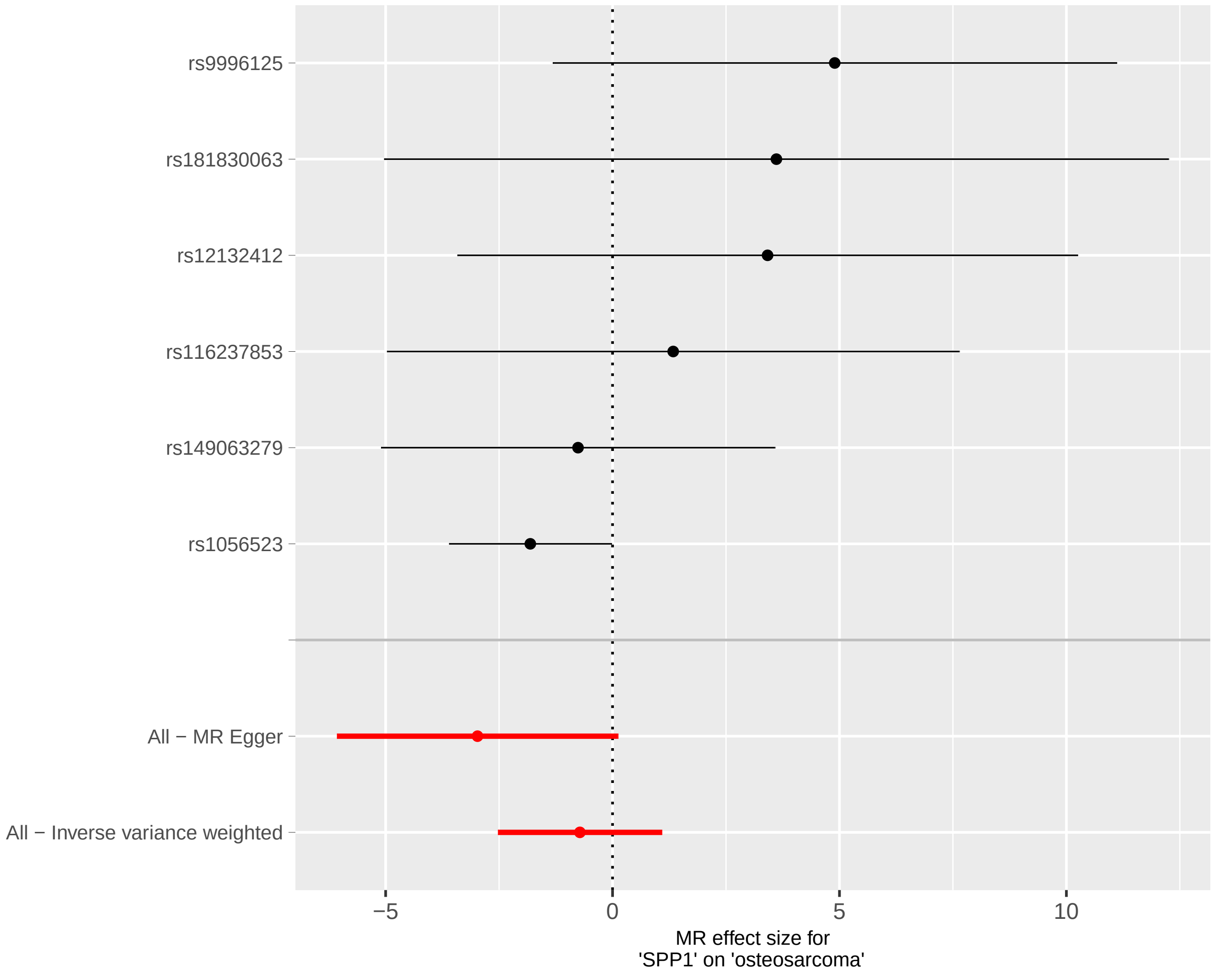

rs9996125
rs181830063
rs12132412
rs116237853
rs149063279
rs1056523
All − MR Egger
All − Inverse variance weighted
−5
0
5
10
MR effect size for
'SPP1' on 'osteosarcoma'

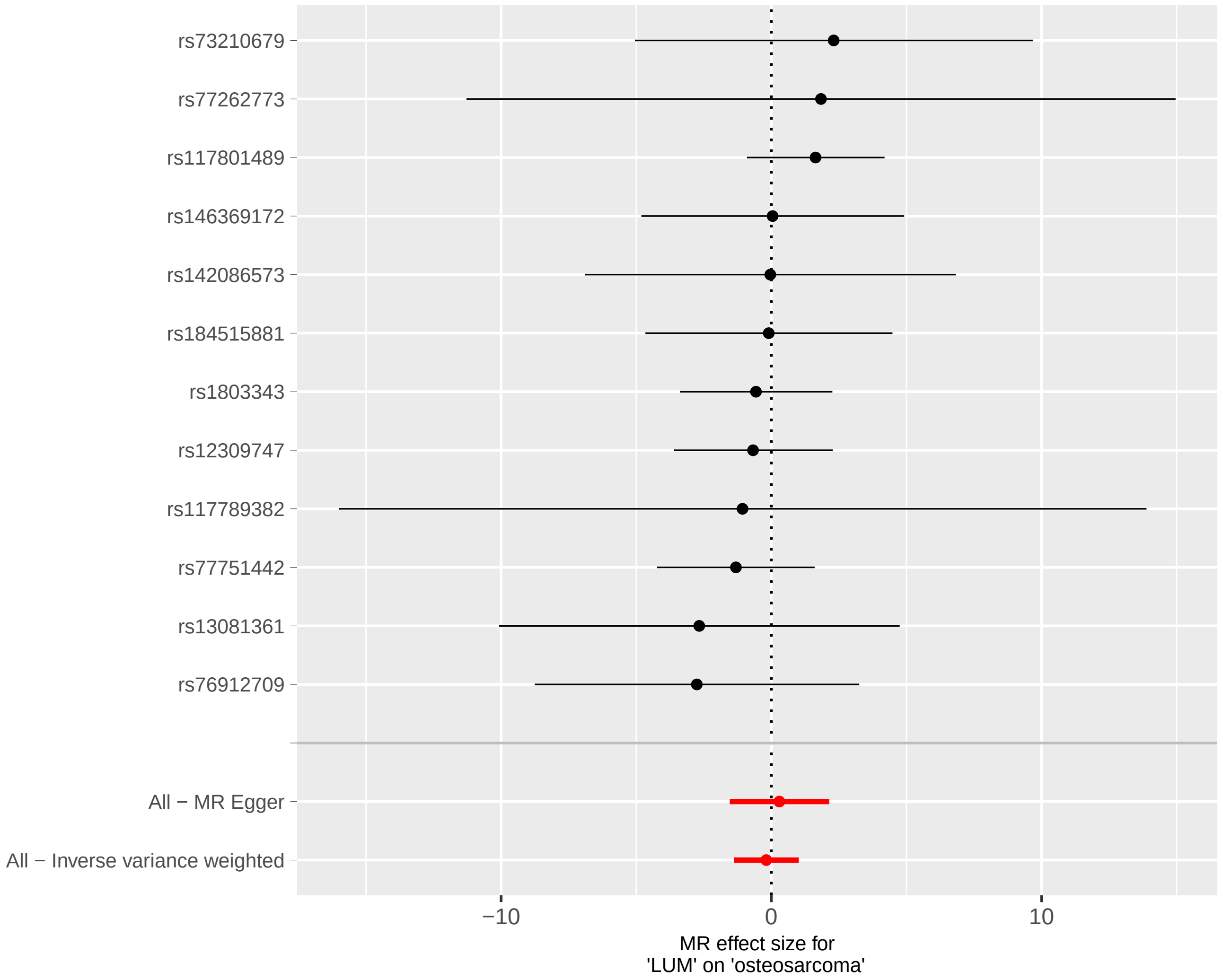

rs73210679
rs77262773
rs117801489
rs146369172
rs142086573
rs184515881
rs1803343
rs12309747
rs117789382
rs77751442
rs13081361
rs76912709
All − MR Egger
All − Inverse variance weighted
−10
0
10
MR effect size for
'LUM' on 'osteosarcoma'

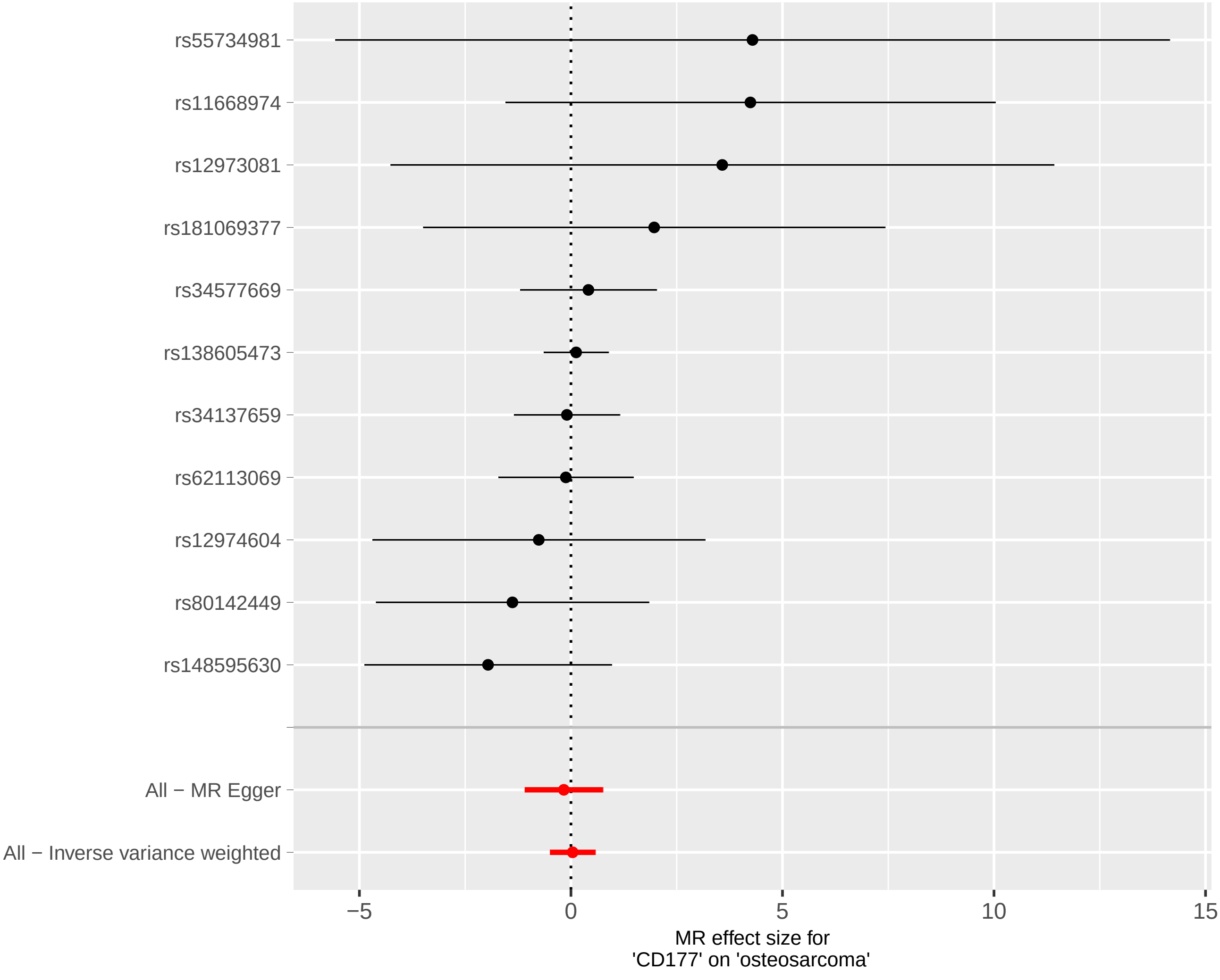
rs55734981
rs11668974
rs12973081
rs181069377
rs34577669
rs138605473
rs34137659
rs62113069
rs12974604
rs80142449
rs148595630
All − MR Egger
All − Inverse variance weighted
−5
0
5
10
15
MR effect size for
'CD177' on 'osteosarcoma'

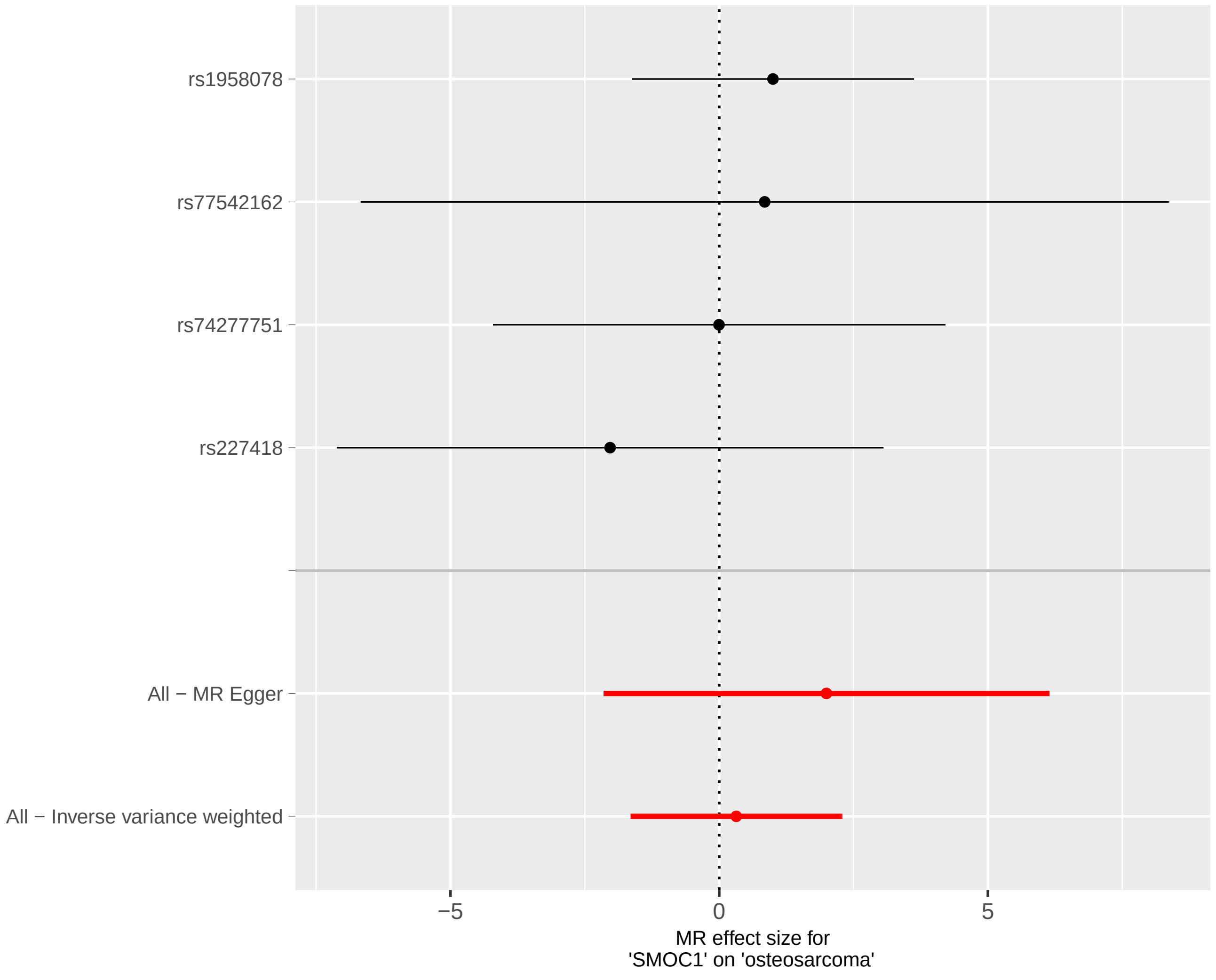

rs1958078
rs77542162
rs74277751
rs227418
All − MR Egger
All − Inverse variance weighted
−5
0
5
MR effect size for
'SMOC1' on 'osteosarcoma'

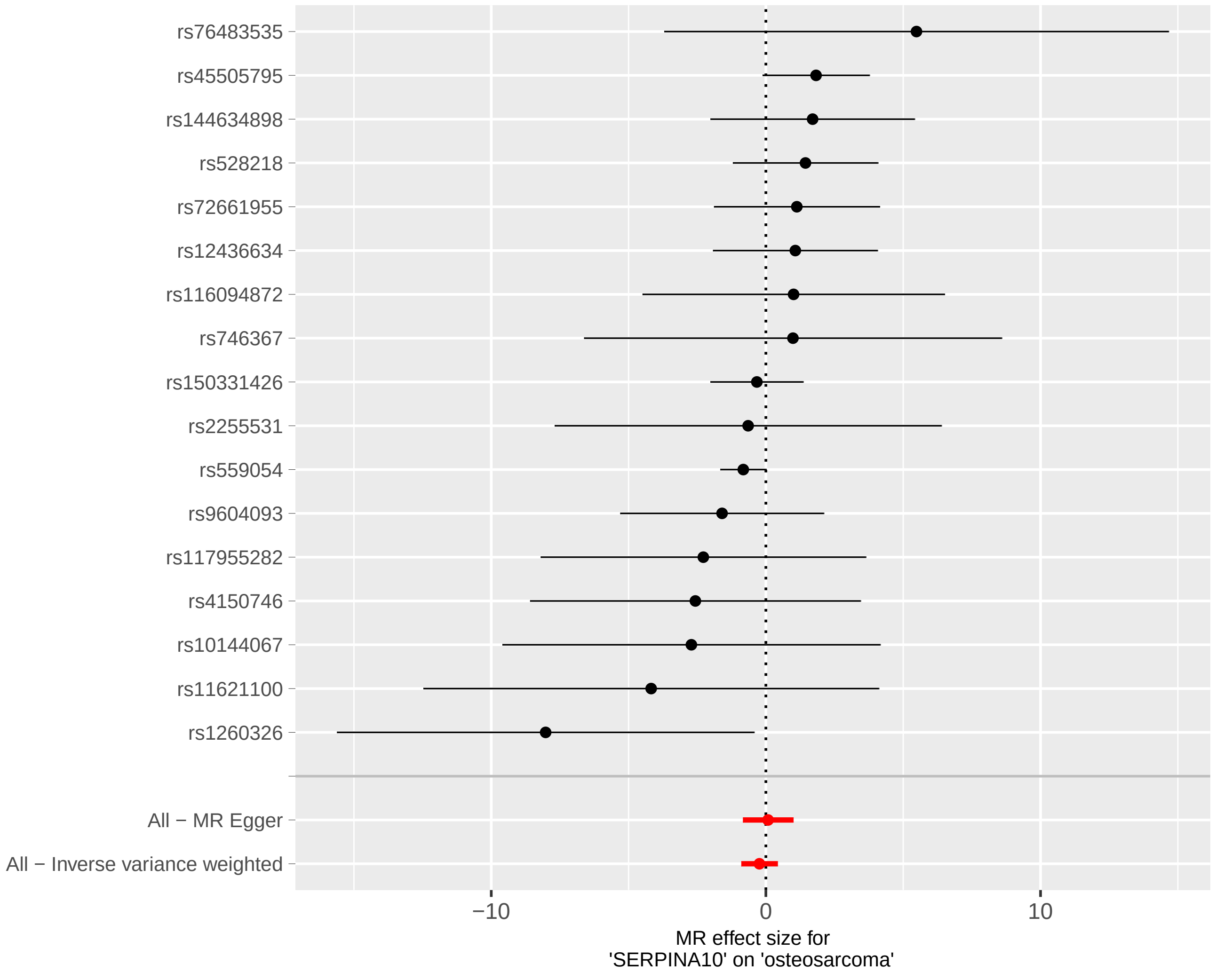

rs76483535
rs45505795
rs144634898
rs528218
rs72661955
rs12436634
rs116094872
rs746367
rs150331426
rs2255531
rs559054
rs9604093
rs117955282
rs4150746
rs10144067
rs11621100
rs1260326
All − MR Egger
All − Inverse variance weighted
−10
0
10
MR effect size for
'SERPINA10' on 'osteosarcoma'

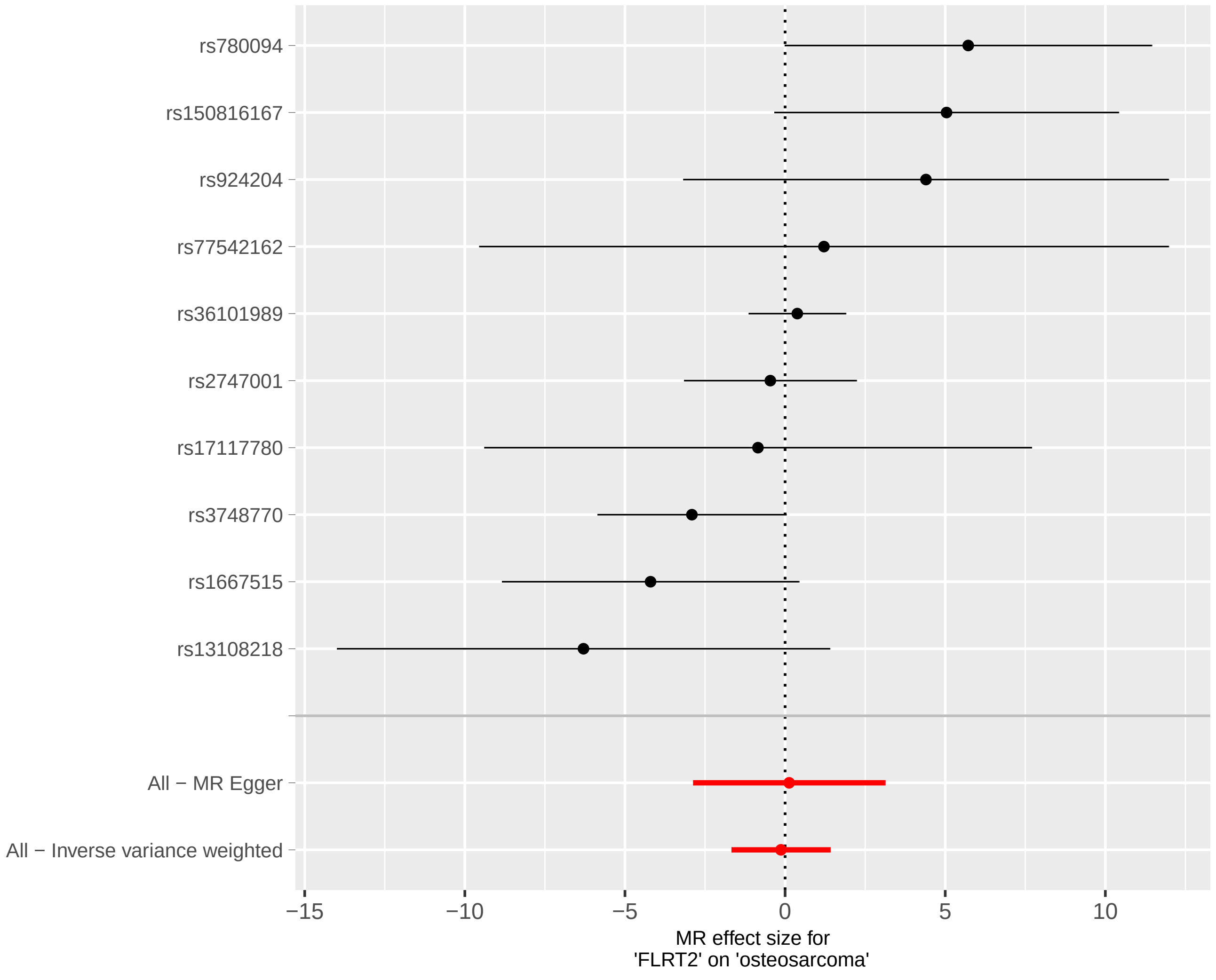
rs780094
rs150816167
rs924204
rs77542162
rs36101989
rs2747001
rs17117780
rs3748770
rs1667515
rs13108218
All − MR Egger
All − Inverse variance weighted
−15
−10
−5
0
5
10
MR effect size for
'FLRT2' on 'osteosarcoma'

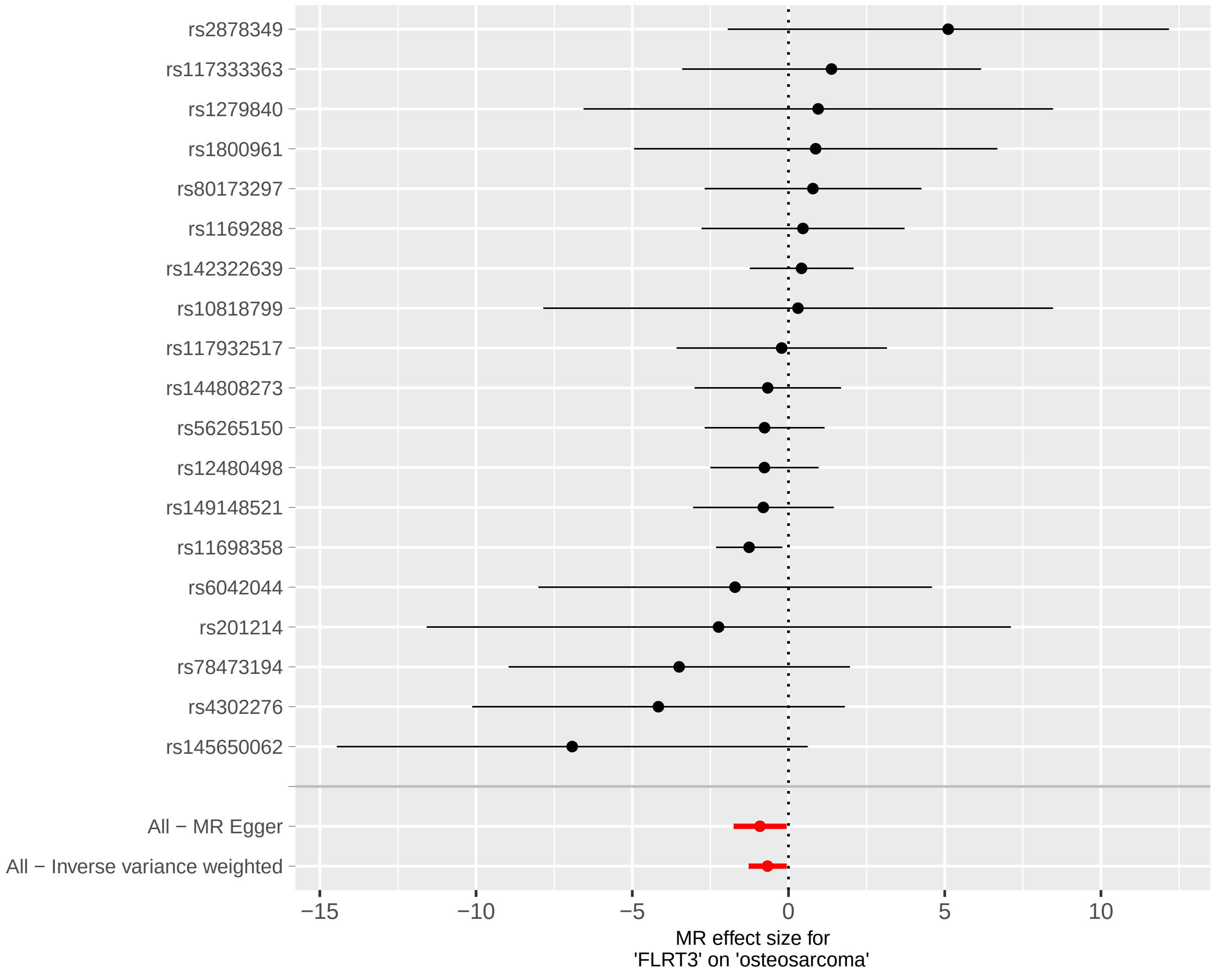
rs2878349
rs117333363
rs1279840
rs1800961
rs80173297
rs1169288
rs142322639
rs10818799
rs117932517
rs144808273
rs56265150
rs12480498
rs149148521
rs11698358
rs6042044
rs201214
rs78473194
rs4302276
rs145650062
All − MR Egger
All − Inverse variance weighted
−15
−10
−5
0
5
10
MR effect size for
'FLRT3' on 'osteosarcoma'

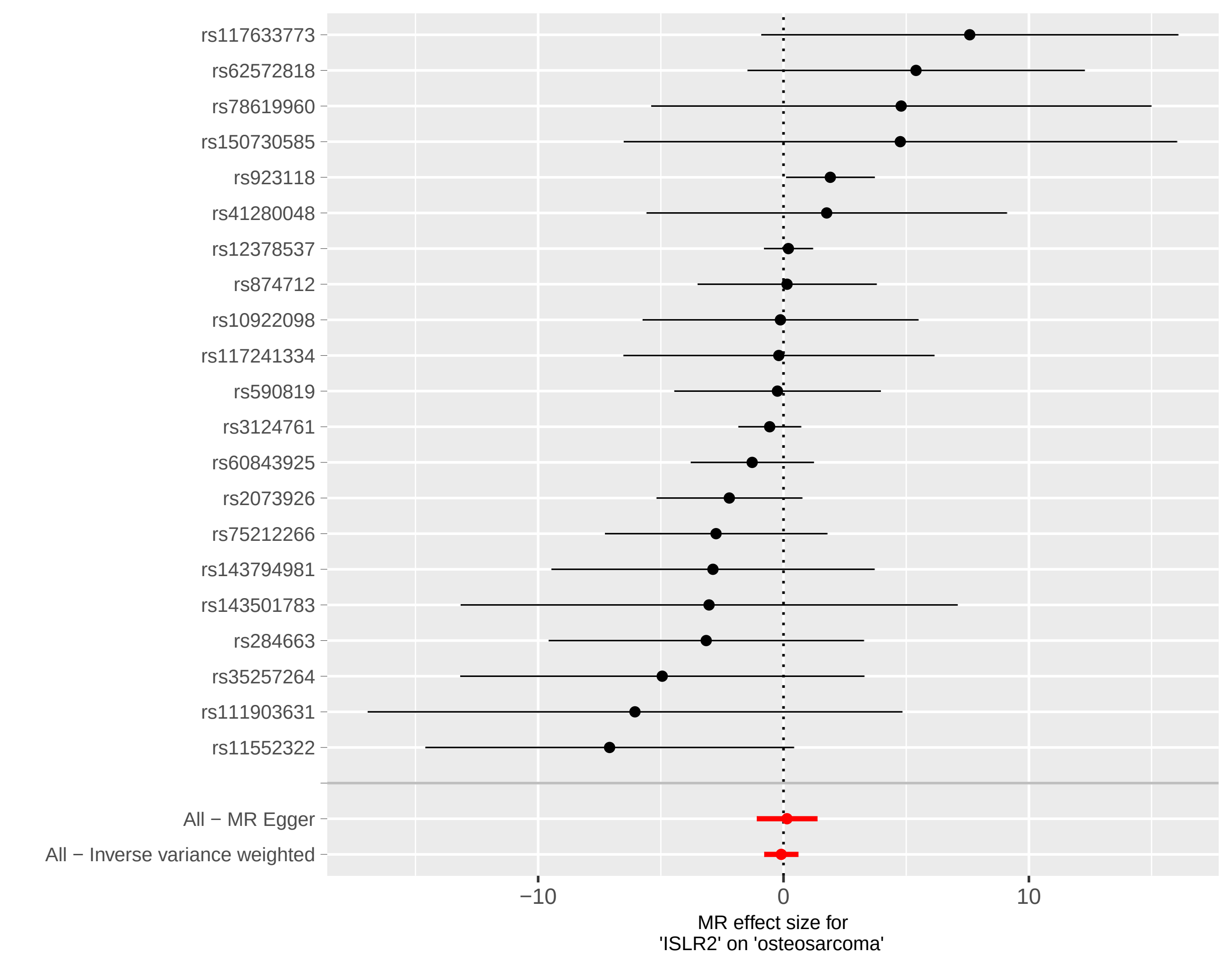
rs117633773
rs62572818
rs78619960
rs150730585
rs923118
rs41280048
rs12378537
rs874712
rs10922098
rs117241334
rs590819
rs3124761
rs60843925
rs2073926
rs75212266
rs143794981
rs143501783
rs284663
rs35257264
rs111903631
rs11552322
All − MR Egger
All − Inverse variance weighted
−10
0
10
MR effect size for
'ISLR2' on 'osteosarcoma'

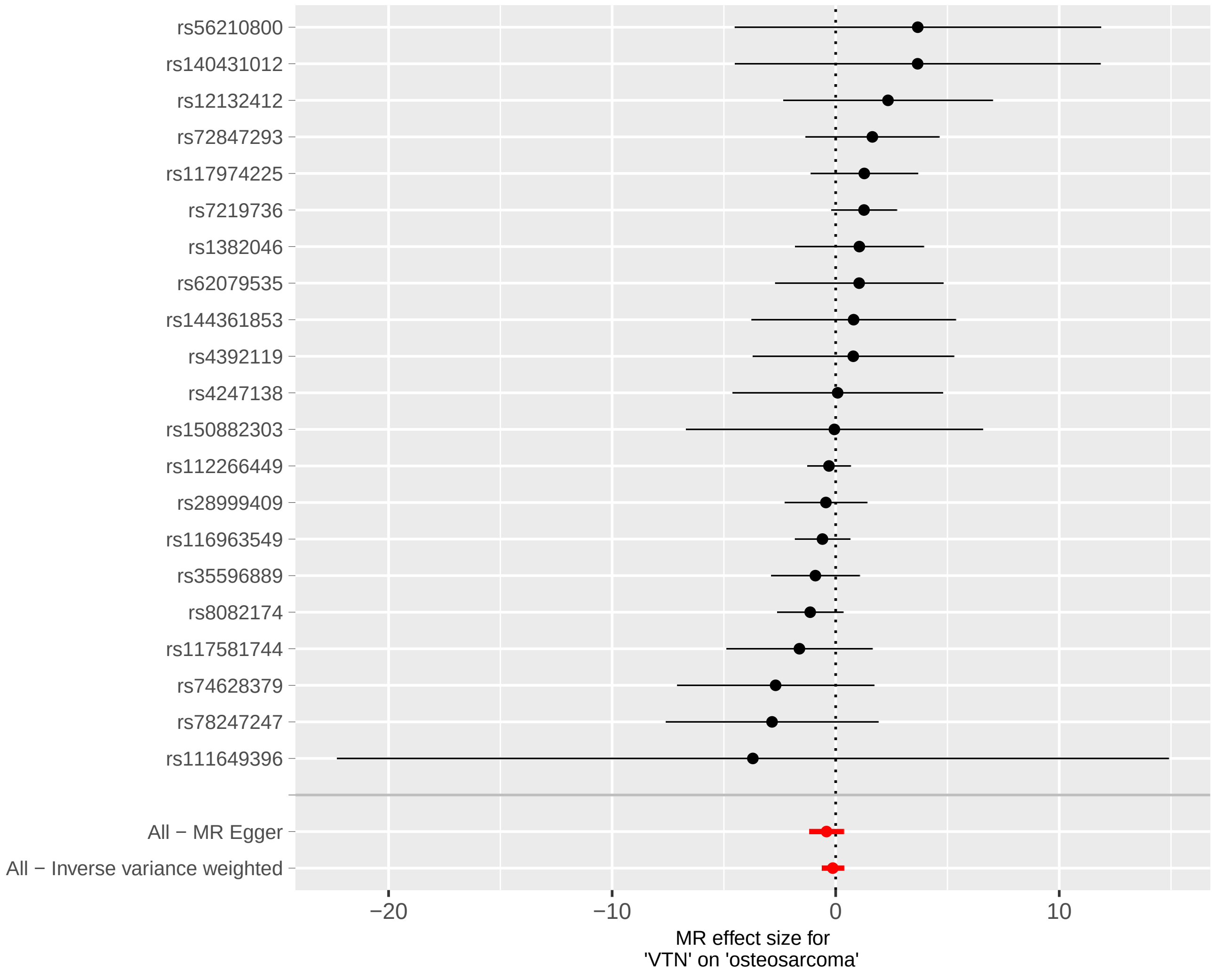

rs56210800
rs140431012
rs12132412
rs72847293
rs117974225
rs7219736
rs1382046
rs62079535
rs144361853
rs4392119
rs4247138
rs150882303
rs112266449
rs28999409
rs116963549
rs35596889
rs8082174
rs117581744
rs74628379
rs78247247
rs111649396
All − MR Egger
All − Inverse variance weighted
−20
−10
0
10
MR effect size for
'VTN' on 'osteosarcoma'

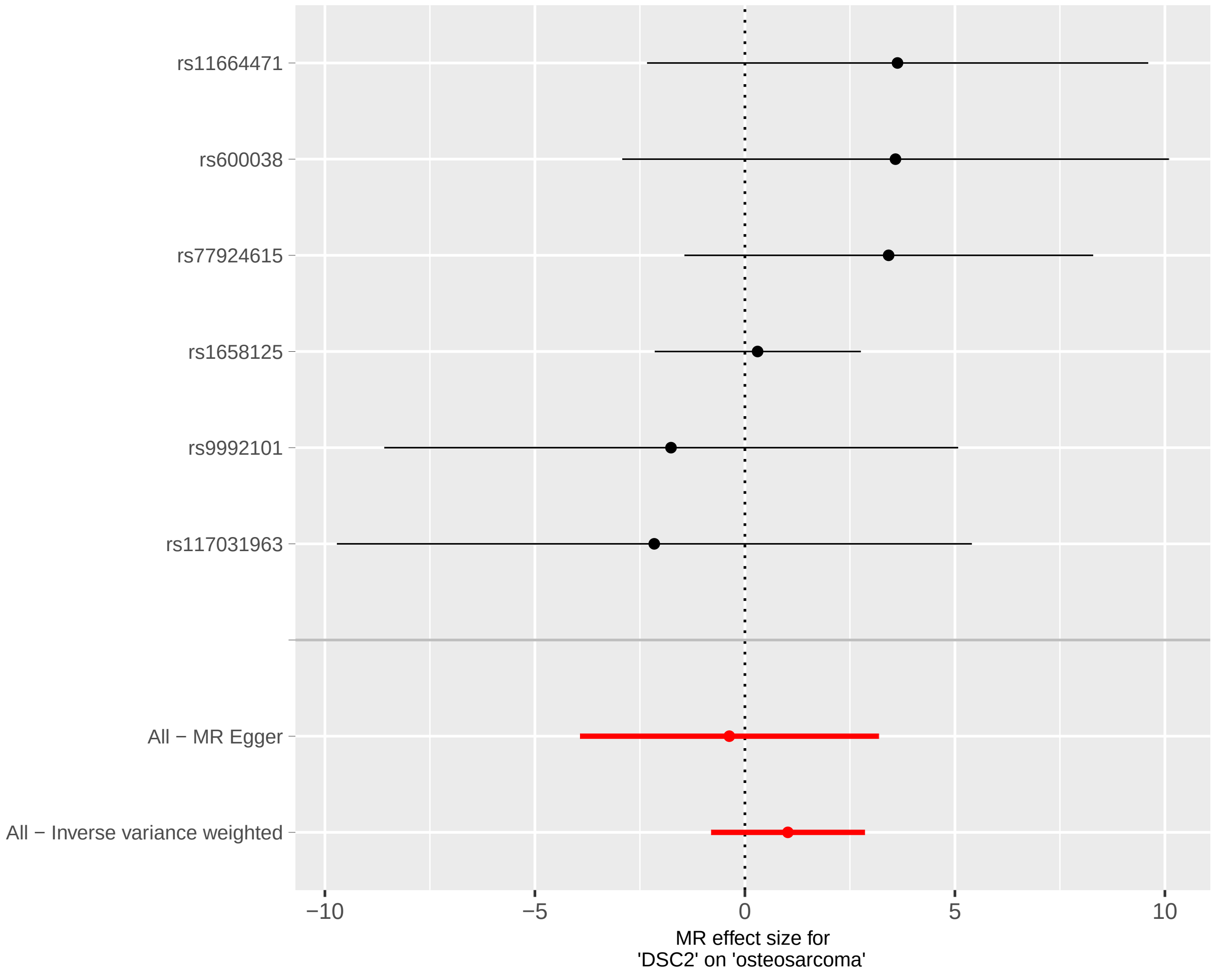

rs11664471
rs600038
rs77924615
rs1658125
rs9992101
rs117031963
All − MR Egger
All − Inverse variance weighted
−10
−5
0
5
10
MR effect size for
'DSC2' on 'osteosarcoma'

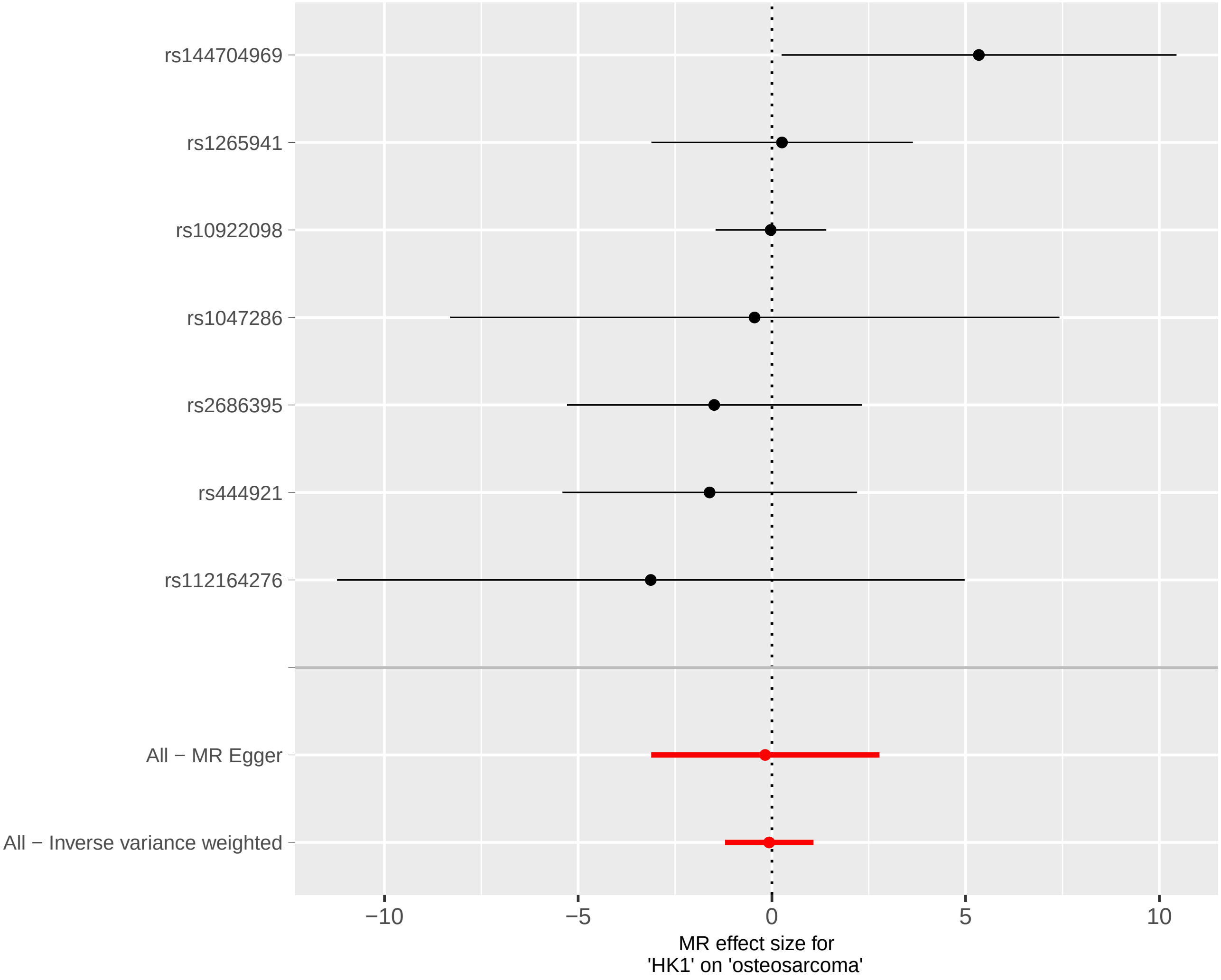

rs144704969
rs1265941
rs10922098
rs1047286
rs2686395
rs444921
rs112164276
All − MR Egger
All − Inverse variance weighted
−10
−5
0
5
10
MR effect size for
'HK1' on 'osteosarcoma'

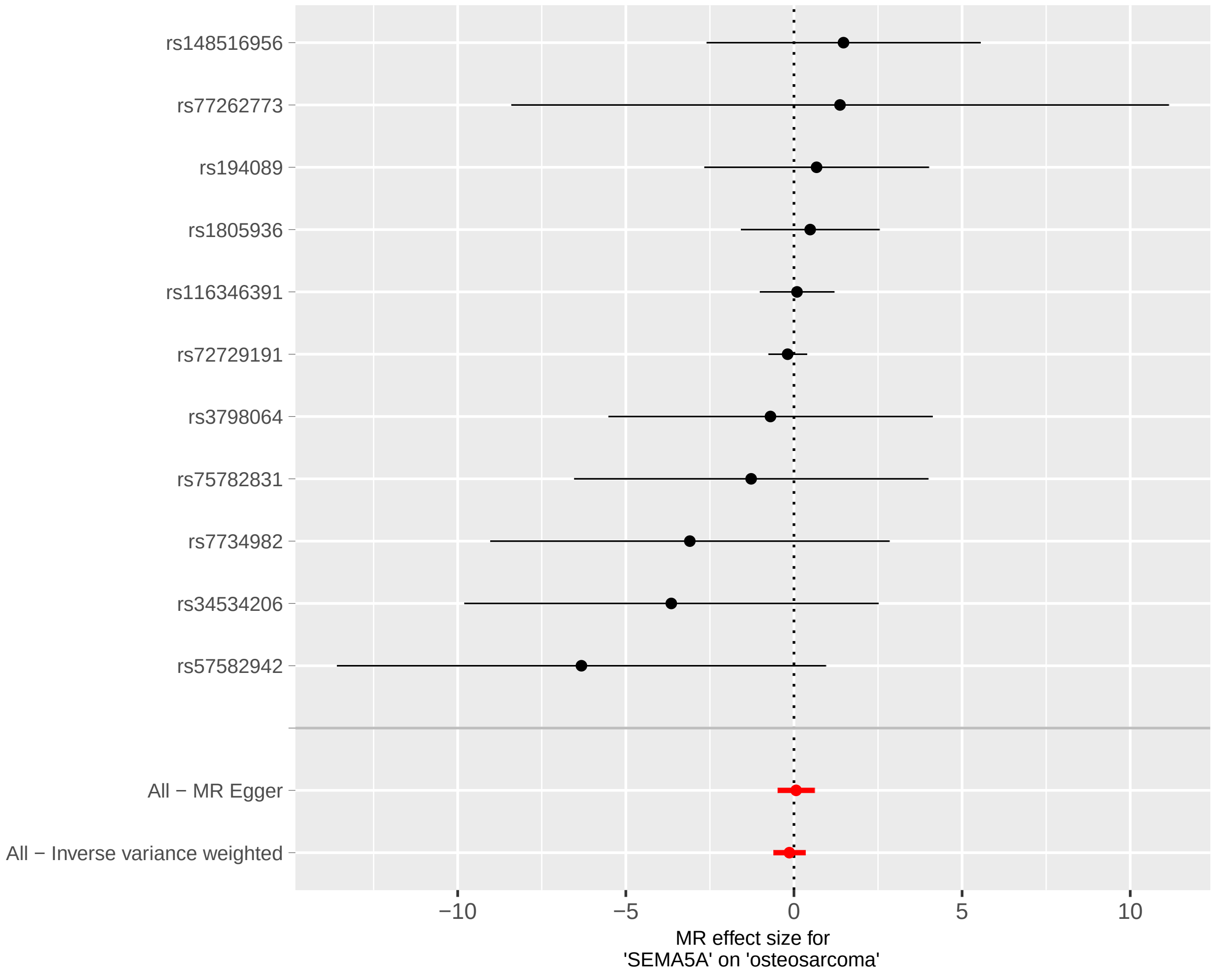
rs148516956
rs77262773
rs194089
rs1805936
rs116346391
rs72729191
rs3798064
rs75782831
rs7734982
rs34534206
rs57582942
All − MR Egger
All − Inverse variance weighted
−10
−5
0
5
10
MR effect size for
'SEMA5A' on 'osteosarcoma'

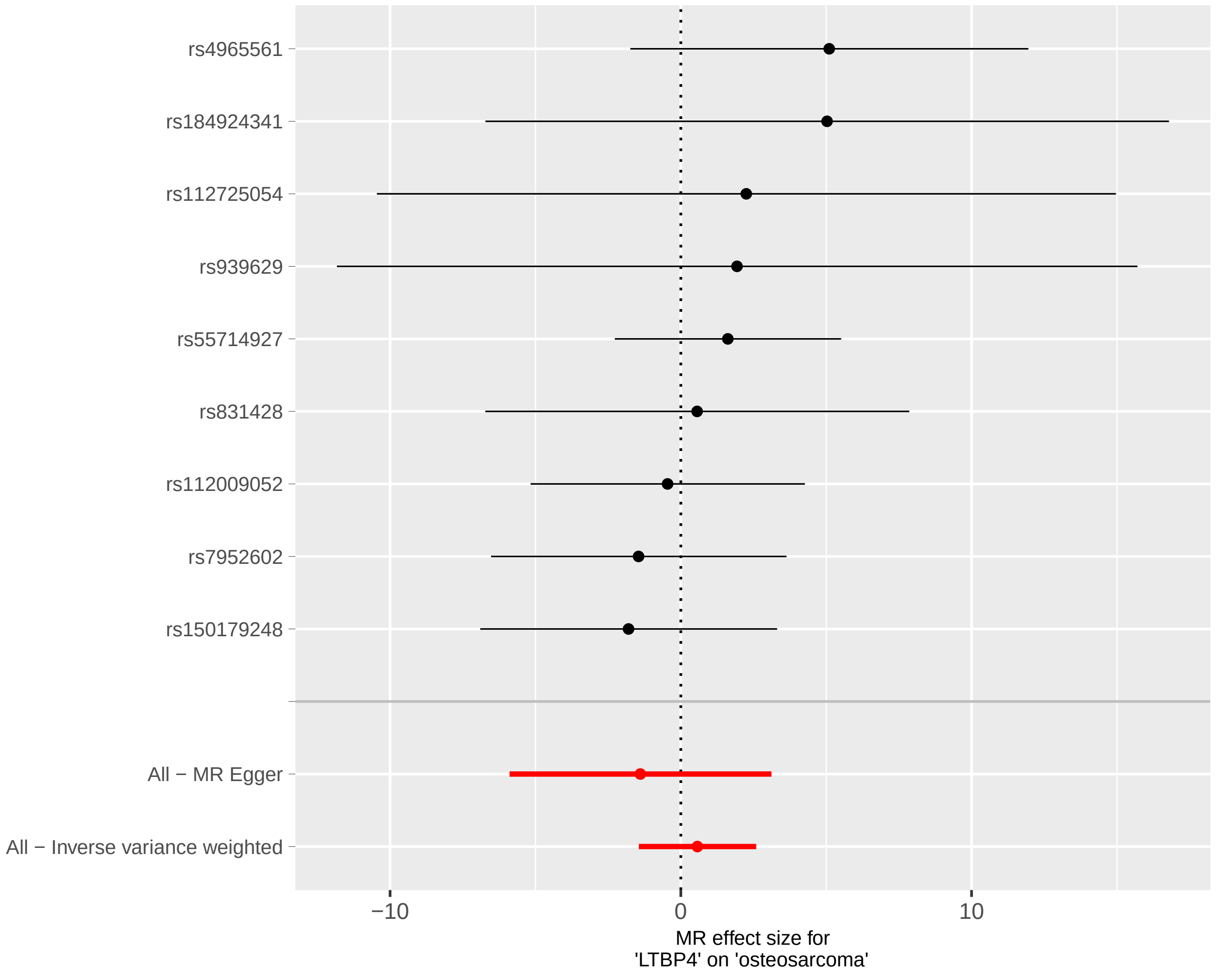
rs4965561
rs184924341
rs112725054
rs939629
rs55714927
rs831428
rs112009052
rs7952602
rs150179248
All − MR Egger
All − Inverse variance weighted
−10
0
10
MR effect size for
'LTBP4' on 'osteosarcoma'

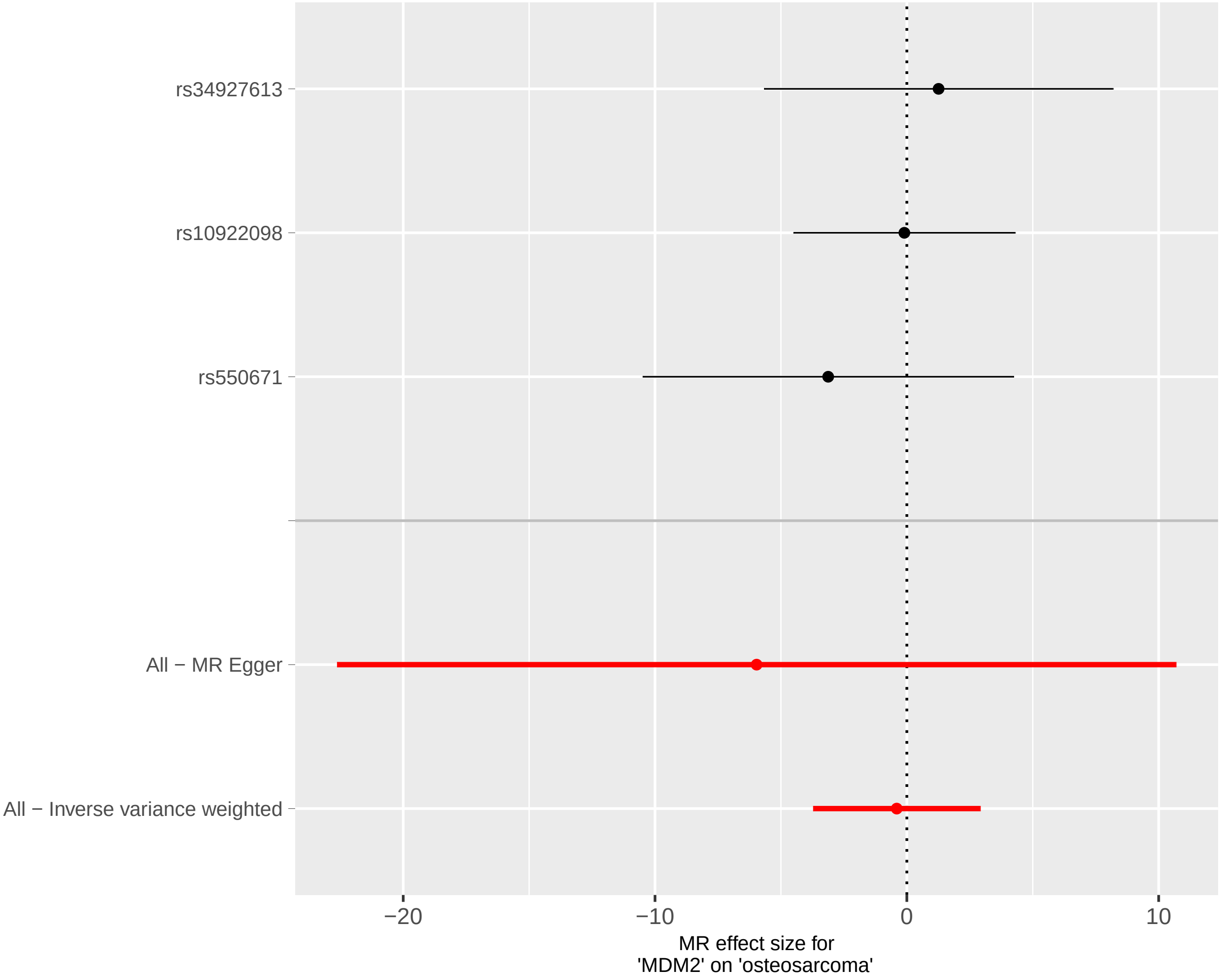
rs34927613
rs10922098
rs550671
All − MR Egger
All − Inverse variance weighted
−20
−10
0
10
MR effect size for
'MDM2' on 'osteosarcoma'

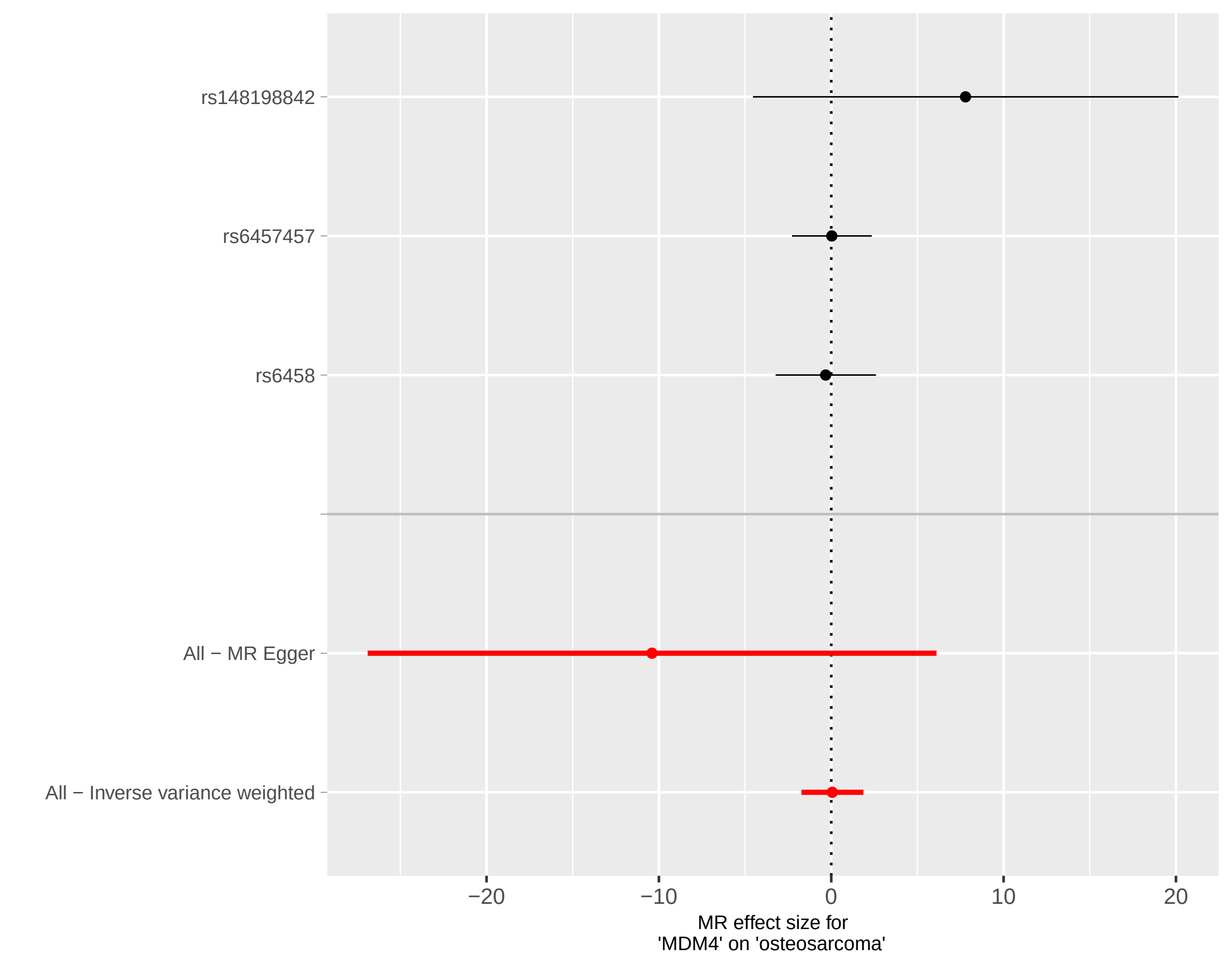

rs148198842
rs6457457
rs6458
All − MR Egger
All − Inverse variance weighted
−20
−10
0
10
20
MR effect size for
'MDM4' on 'osteosarcoma'

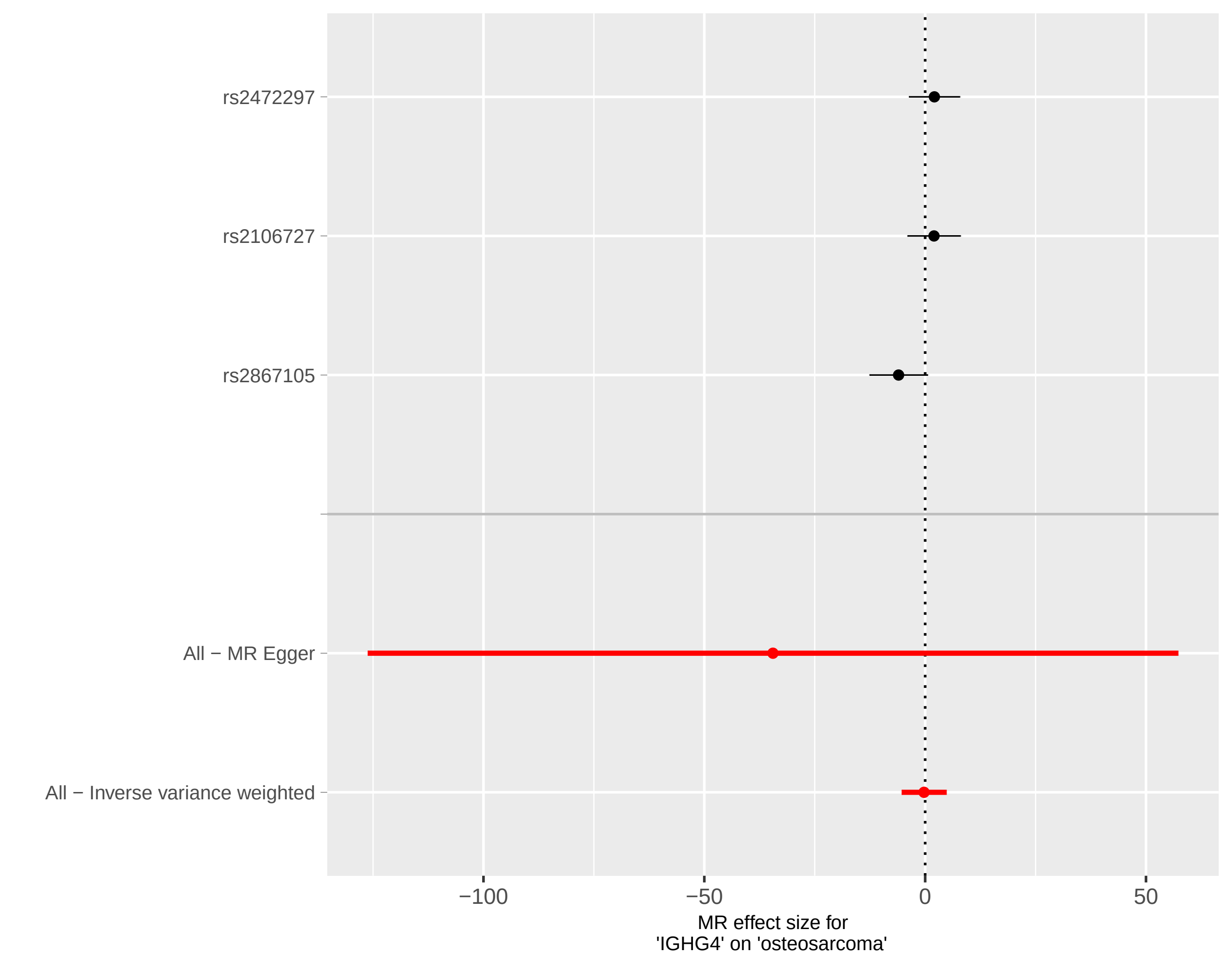
rs2472297
rs2106727
rs2867105
All − MR Egger
All − Inverse variance weighted
−100
−50
0
50
MR effect size for
'IGHG4' on 'osteosarcoma'

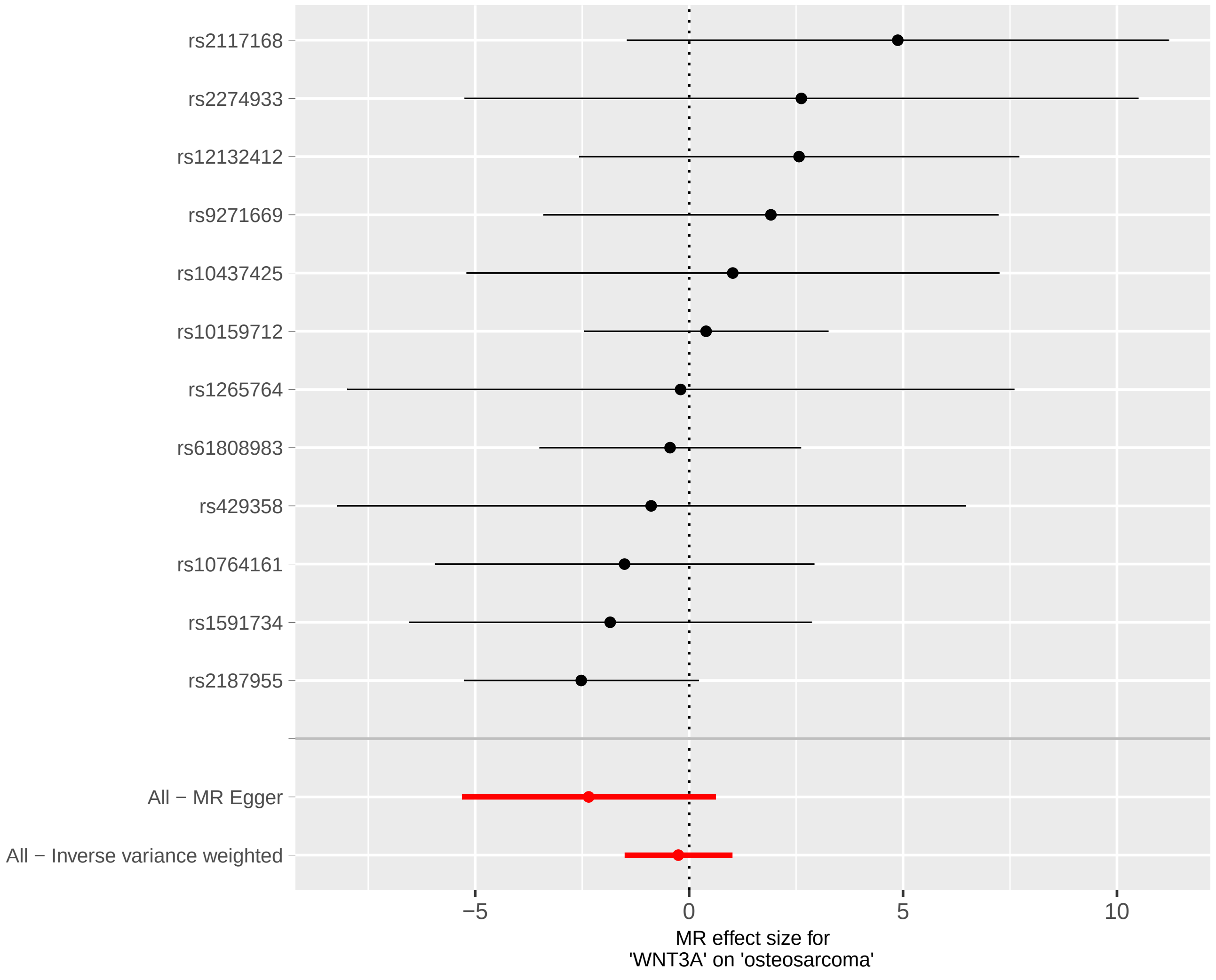
rs2117168
rs2274933
rs12132412
rs9271669
rs10437425
rs10159712
rs1265764
rs61808983
rs429358
rs10764161
rs1591734
rs2187955
All − MR Egger
All − Inverse variance weighted
−5
0
5
10
MR effect size for
'WNT3A' on 'osteosarcoma'

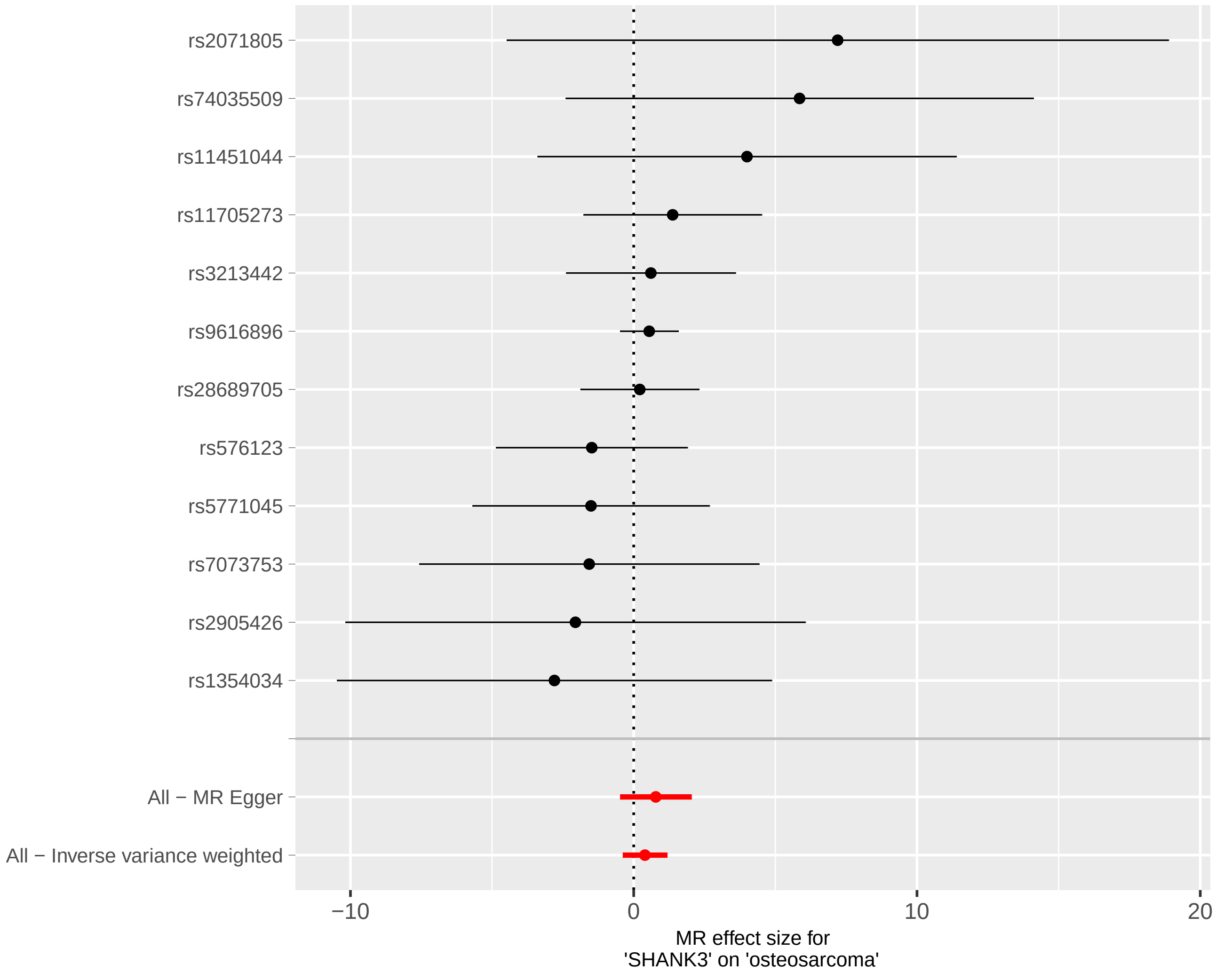
rs2071805
rs74035509
rs11451044
rs11705273
rs3213442
rs9616896
rs28689705
rs576123
rs5771045
rs7073753
rs2905426
rs1354034
All − MR Egger
All − Inverse variance weighted
−10
0
10
20
MR effect size for
'SHANK3' on 'osteosarcoma'

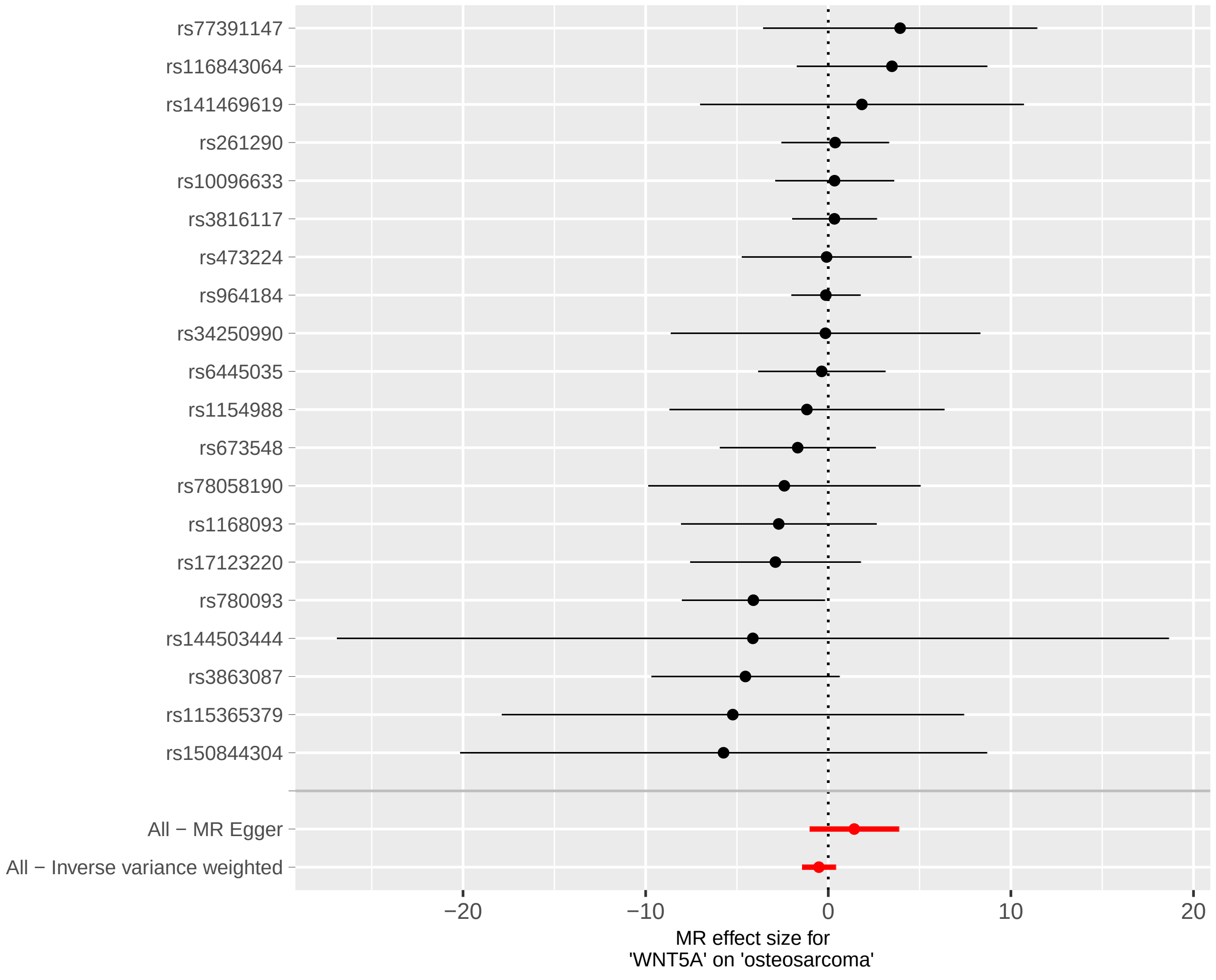

rs77391147
rs116843064
rs141469619
rs261290
rs10096633
rs3816117
rs473224
rs964184
rs34250990
rs6445035
rs1154988
rs673548
rs78058190
rs1168093
rs17123220
rs780093
rs144503444
rs3863087
rs115365379
rs150844304
All − MR Egger
All − Inverse variance weighted
−20
−10
0
10
20
MR effect size for
'WNT5A' on 'osteosarcoma'

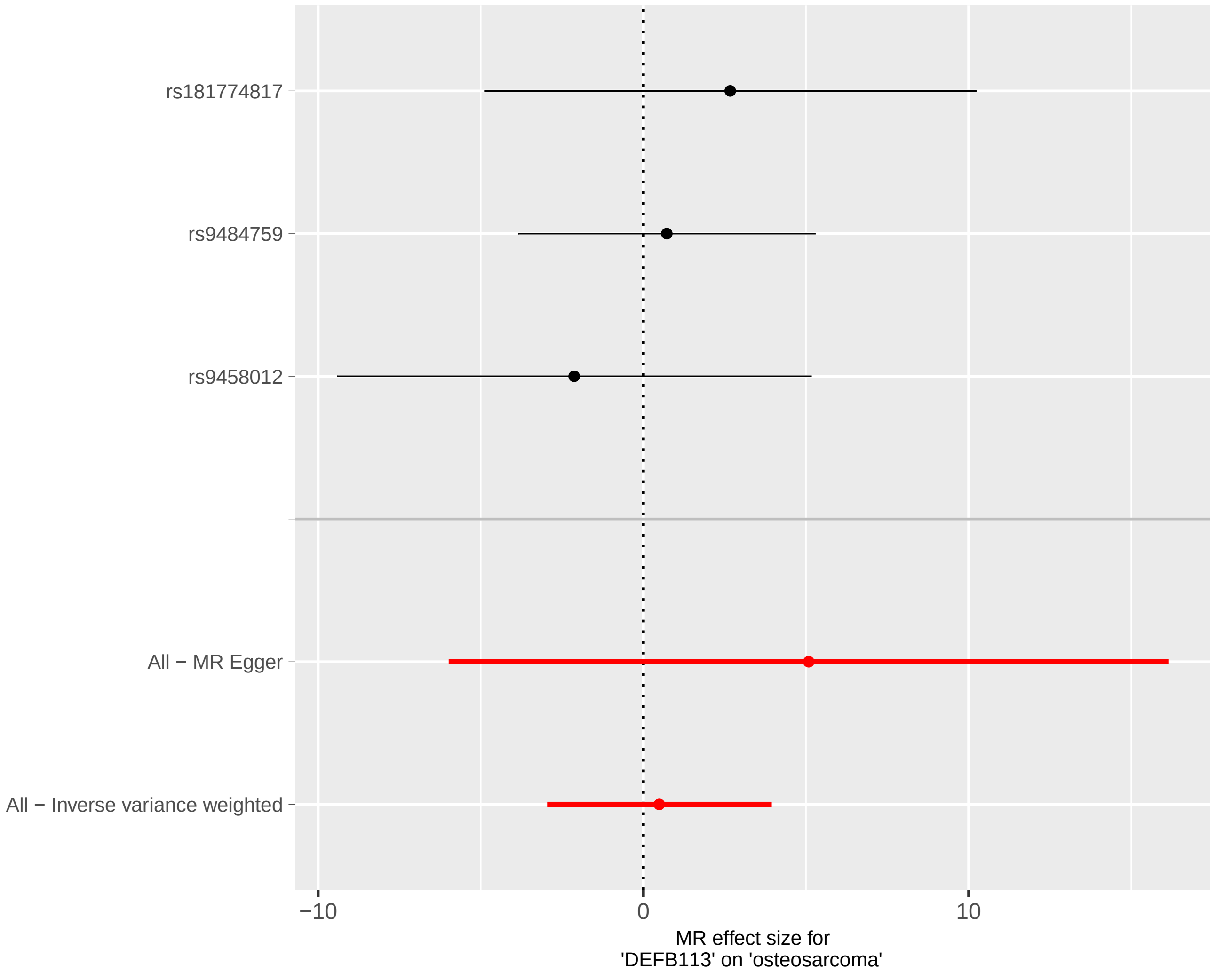

rs181774817
rs9484759
rs9458012
All − MR Egger
All − Inverse variance weighted
−10
0
10
MR effect size for
'DEFB113' on 'osteosarcoma'

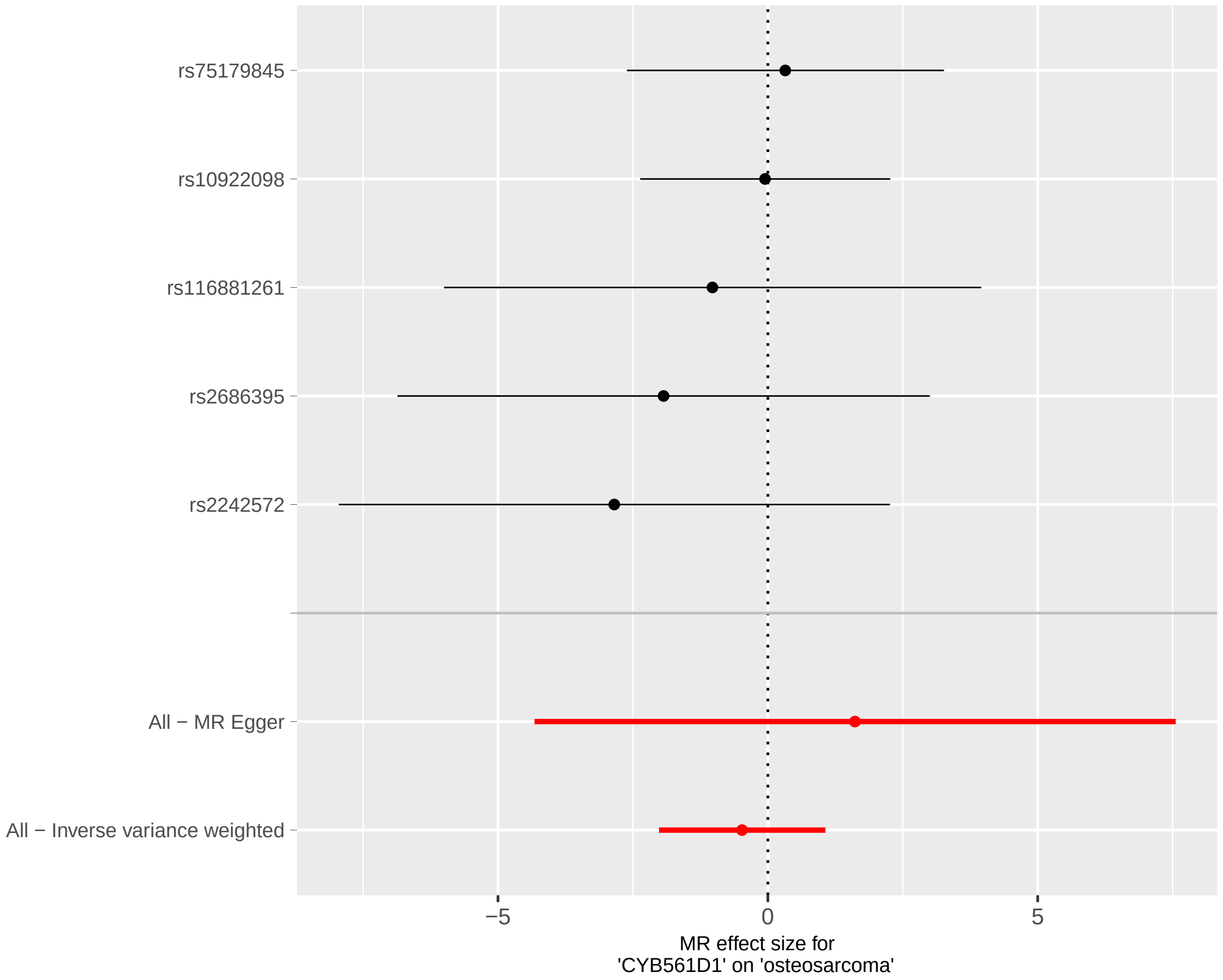
rs75179845
rs10922098
rs116881261
rs2686395
rs2242572
All − MR Egger
All − Inverse variance weighted
−5
0
5
MR effect size for
'CYB561D1' on 'osteosarcoma'

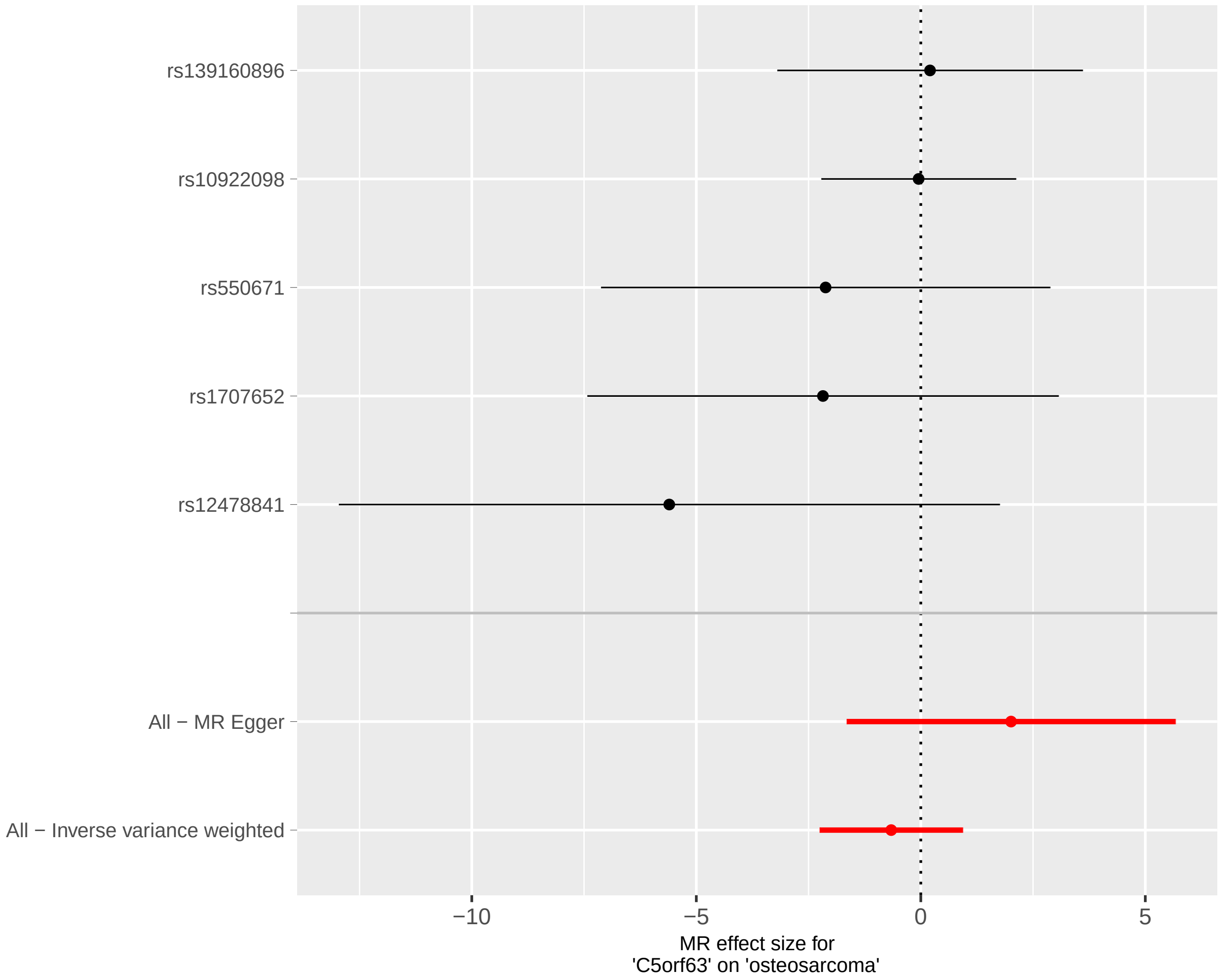
rs139160896
rs10922098
rs550671
rs1707652
rs12478841
All − MR Egger
All − Inverse variance weighted
−10
−5
0
5
MR effect size for
'C5orf63' on 'osteosarcoma'

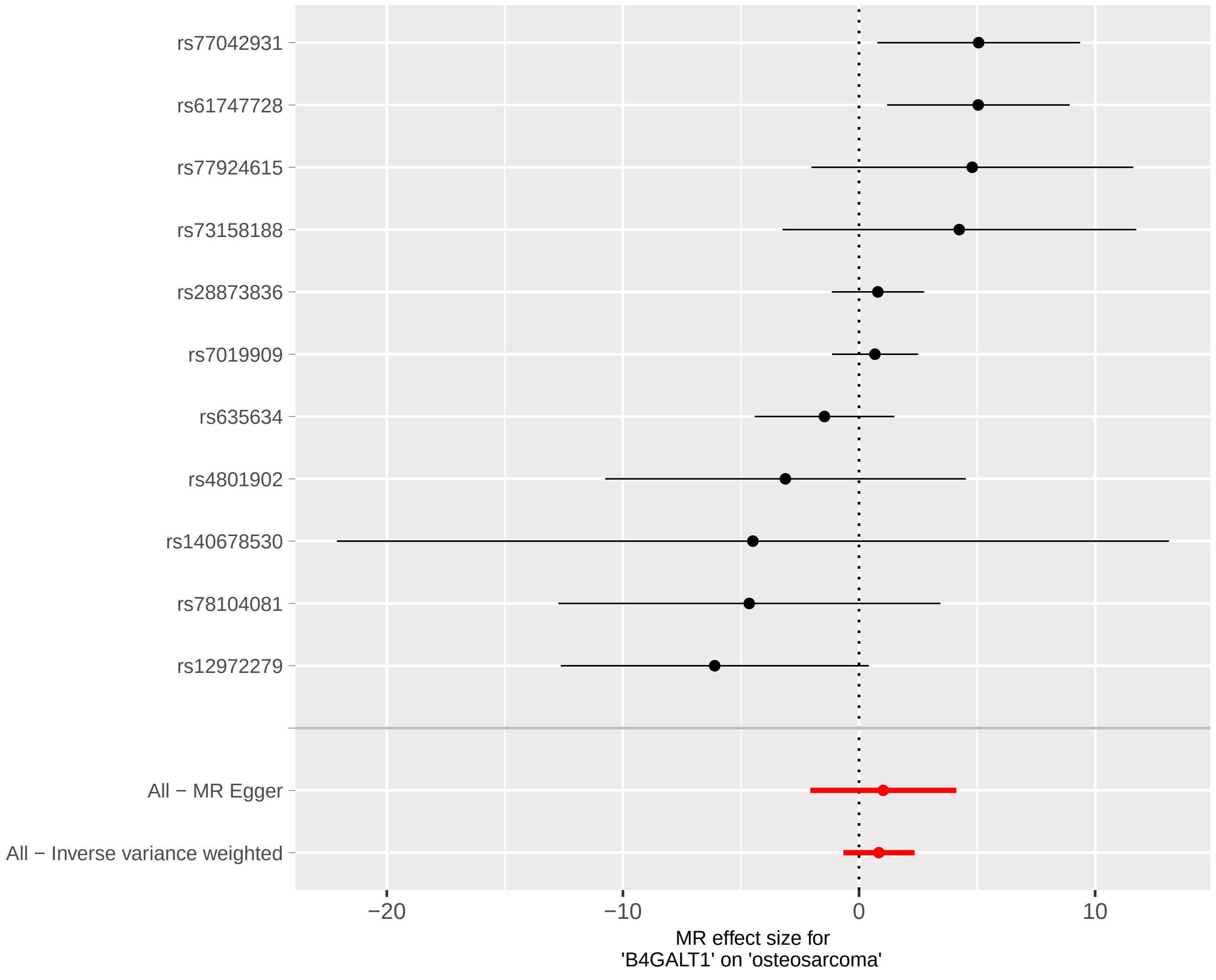

rs77042931
rs61747728
rs77924615
rs73158188
rs28873836
rs7019909
rs635634
rs4801902
rs140678530
rs78104081
rs12972279
All − MR Egger
All − Inverse variance weighted
−20
−10
0
10
MR effect size for
'B4GALT1' on 'osteosarcoma'

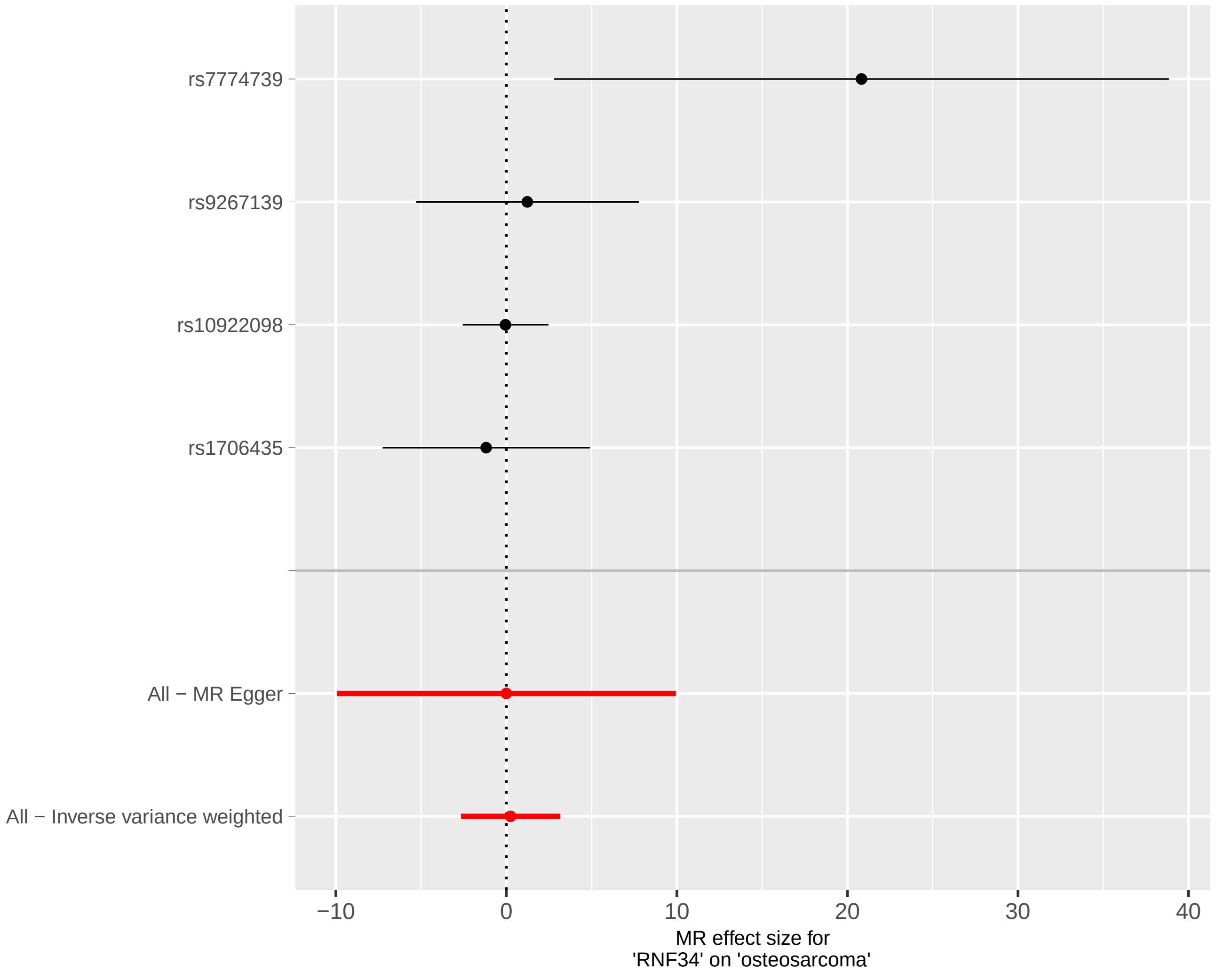

rs7774739
rs9267139
rs10922098
rs1706435
All − MR Egger
All − Inverse variance weighted
−10
0
10
20
30
40
MR effect size for
'RNF34' on 'osteosarcoma'

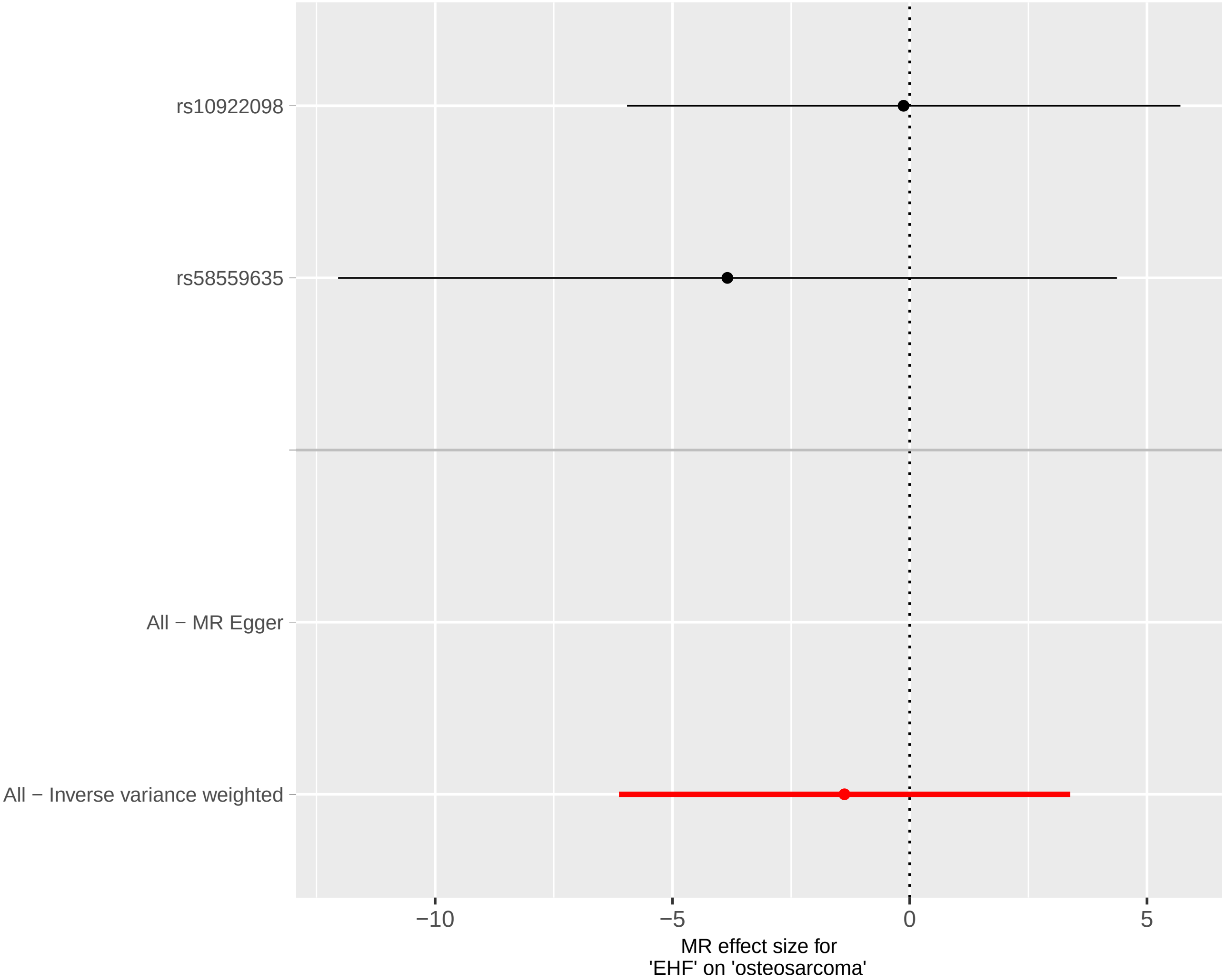

rs10922098
rs58559635
All − MR Egger
All − Inverse variance weighted
−10
−5
0
5
MR effect size for
'EHF' on 'osteosarcoma'

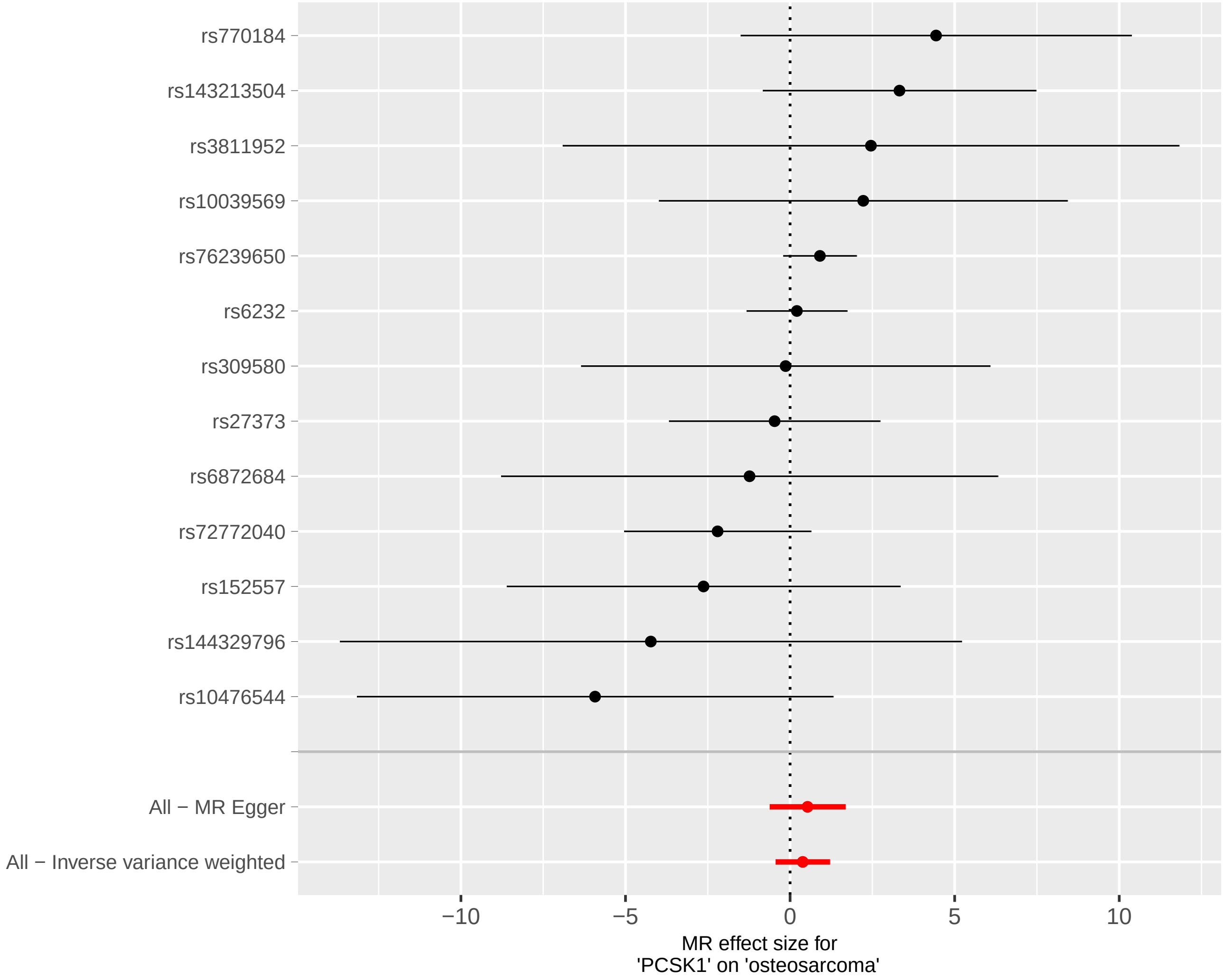
rs770184
rs143213504
rs3811952
rs10039569
rs76239650
rs6232
rs309580
rs27373
rs6872684
rs72772040
rs152557
rs144329796
rs10476544
All − MR Egger
All − Inverse variance weighted
−10
−5
0
5
10
MR effect size for
'PCSK1' on 'osteosarcoma'

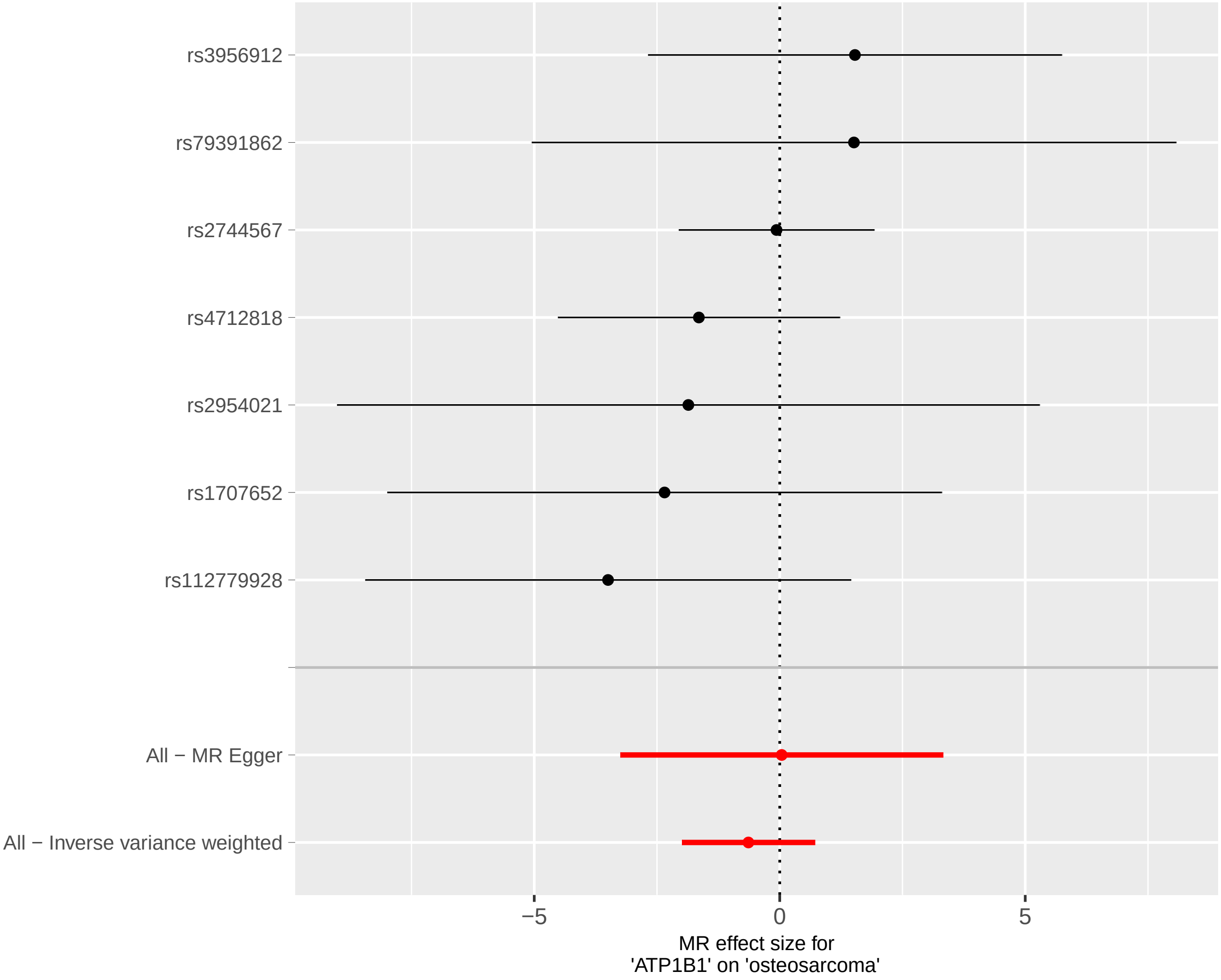
rs3956912
rs79391862
rs2744567
rs4712818
rs2954021
rs1707652
rs112779928
All − MR Egger
All − Inverse variance weighted
−5
0
5
MR effect size for
'ATP1B1' on 'osteosarcoma'

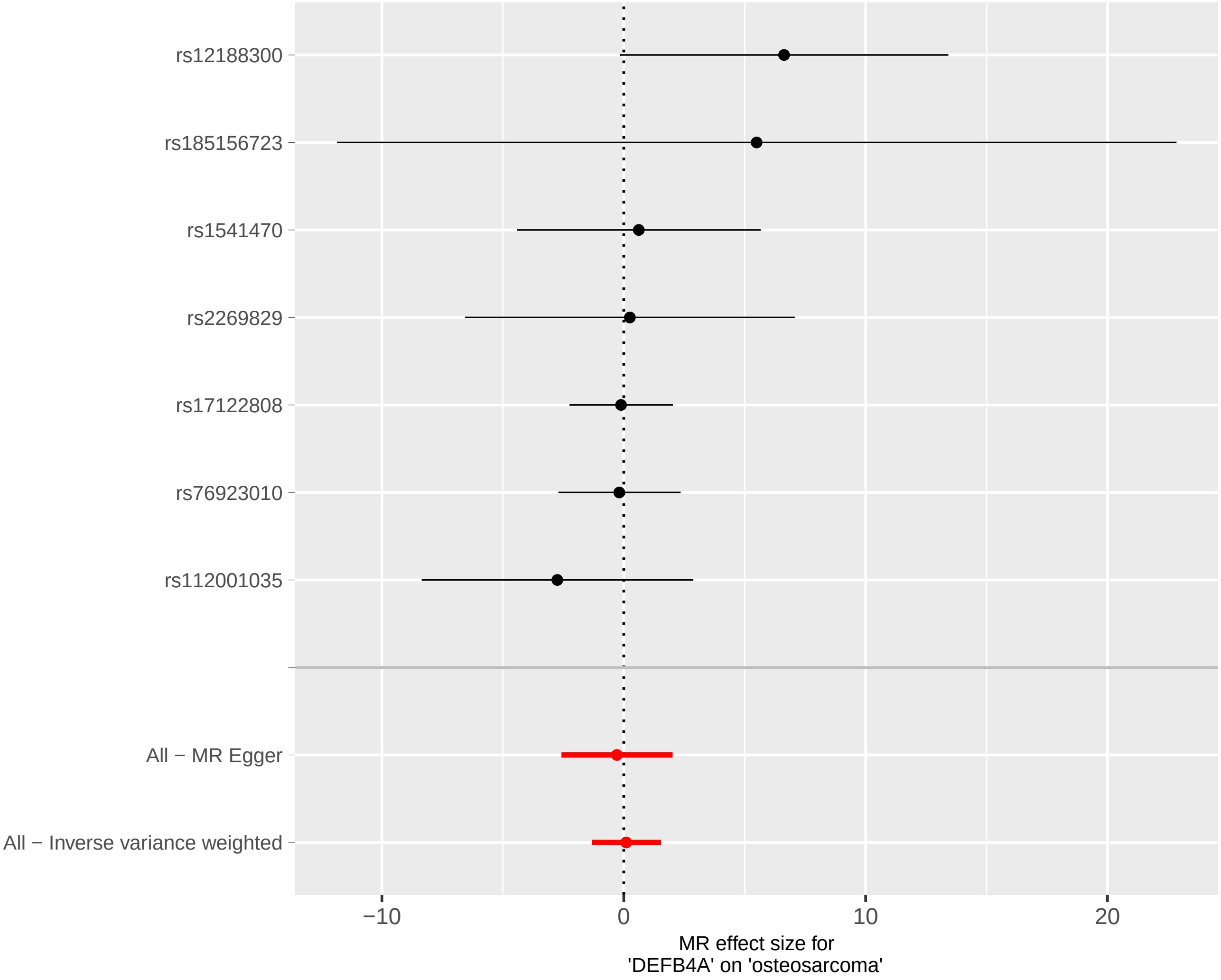
rs12188300
rs185156723
rs1541470
rs2269829
rs17122808
rs76923010
rs112001035
All − MR Egger
All − Inverse variance weighted
−10
0
10
20
MR effect size for
'DEFB4A' on 'osteosarcoma'

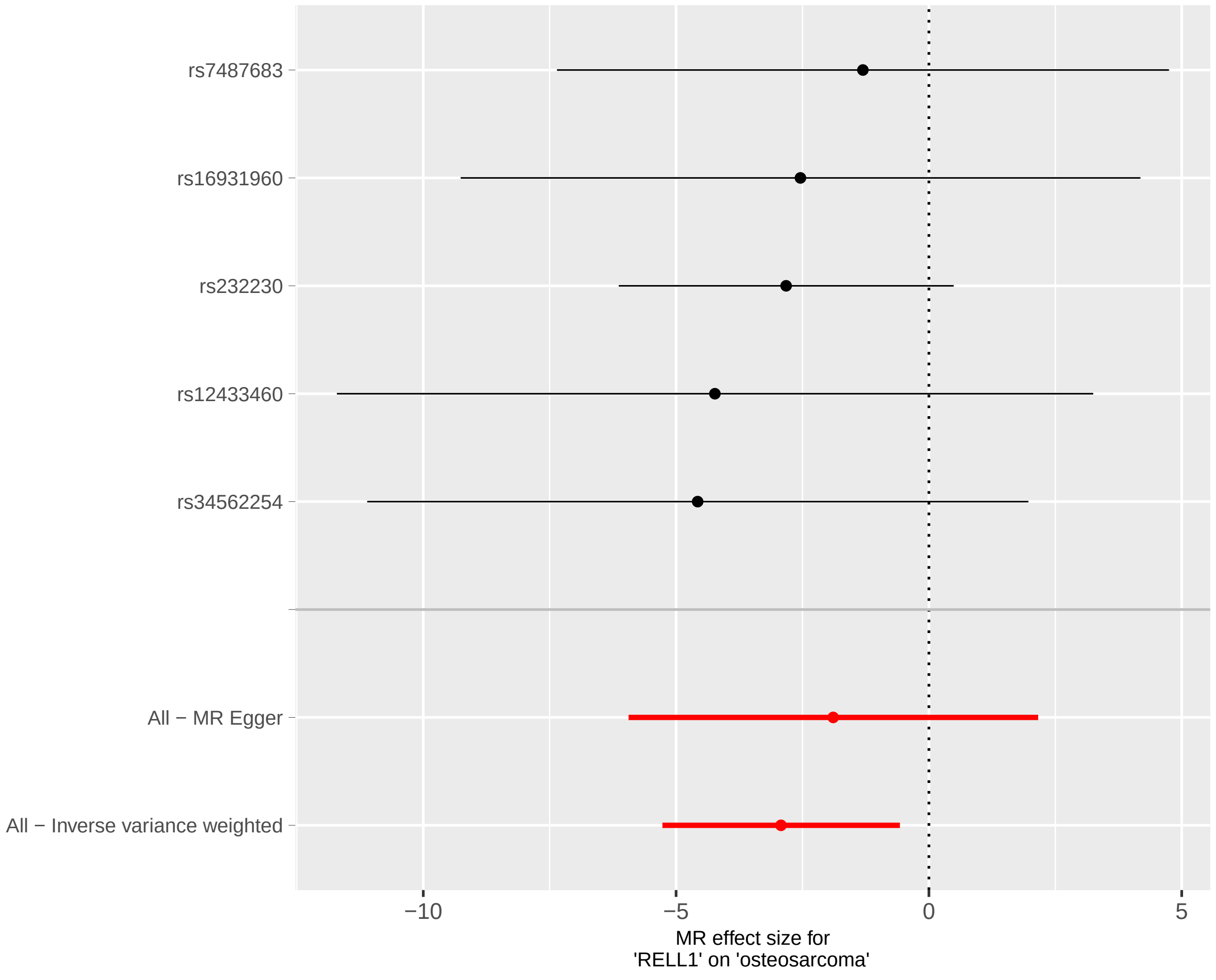
rs7487683
rs16931960
rs232230
rs12433460
rs34562254
All − MR Egger
All − Inverse variance weighted
−10
−5
0
5
MR effect size for
'RELL1' on 'osteosarcoma'

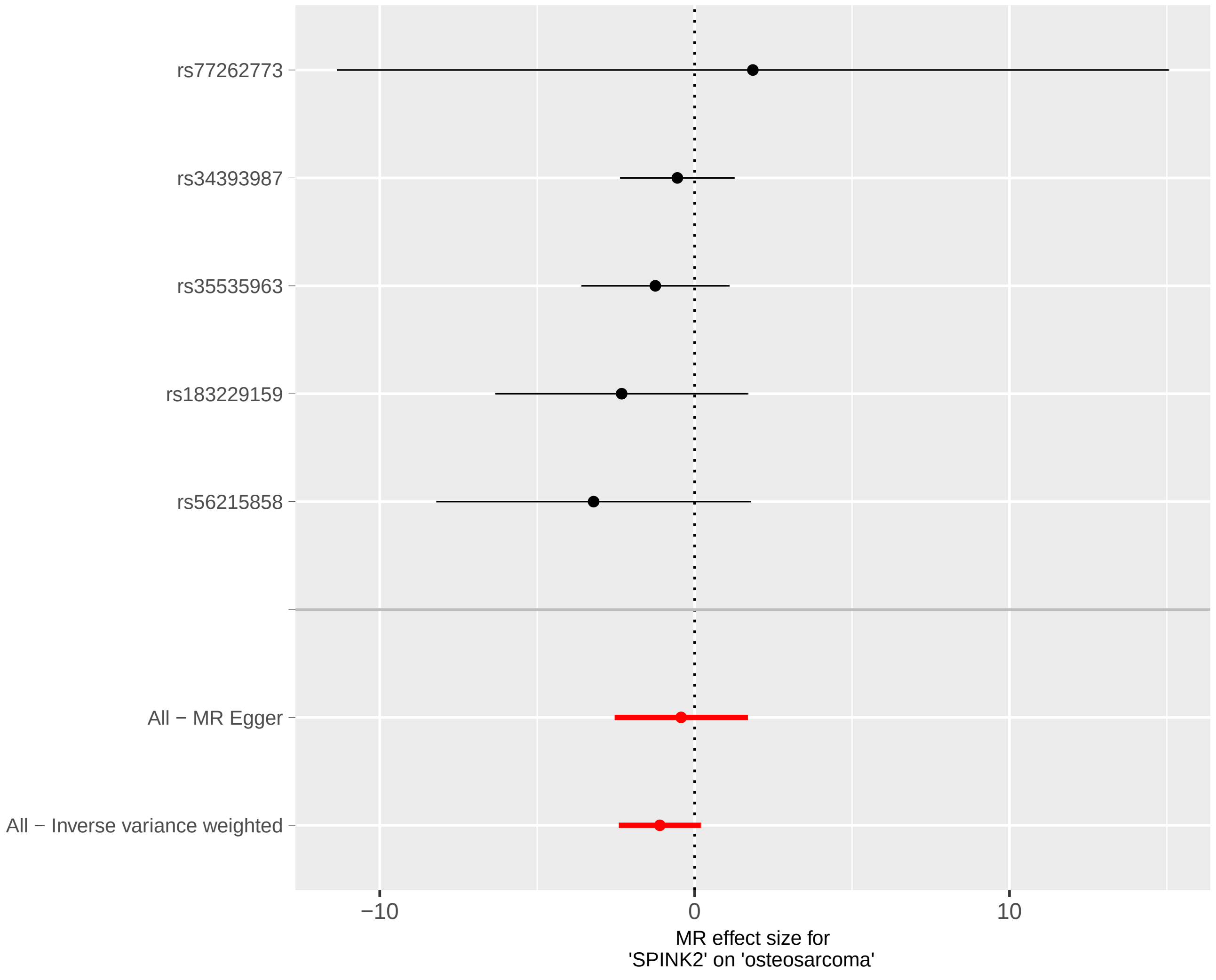

rs77262773
rs34393987
rs35535963
rs183229159
rs56215858
All − MR Egger
All − Inverse variance weighted
−10
0
10
MR effect size for
'SPINK2' on 'osteosarcoma'

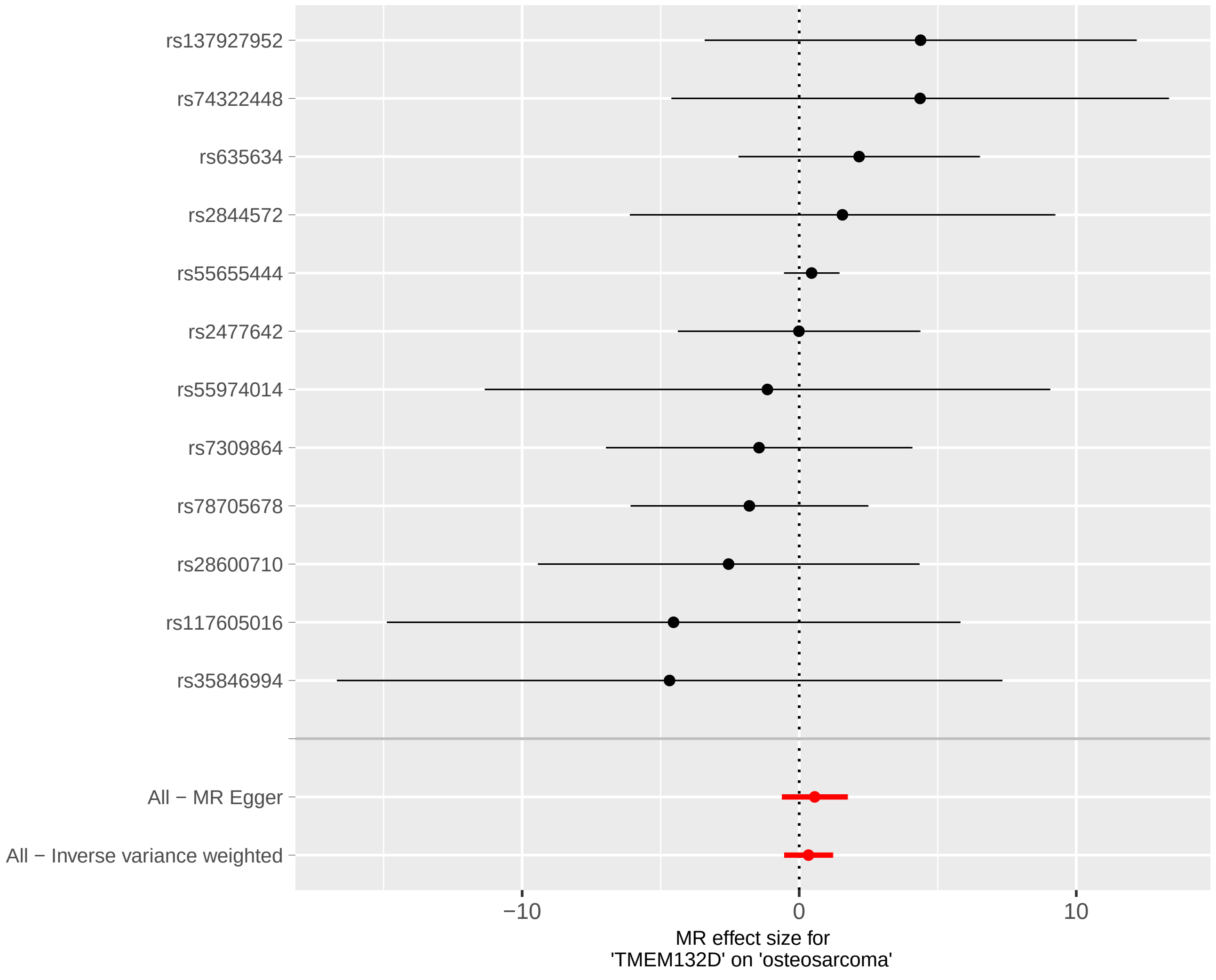
rs137927952
rs74322448
rs635634
rs2844572
rs55655444
rs2477642
rs55974014
rs7309864
rs78705678
rs28600710
rs117605016
rs35846994
All − MR Egger
All − Inverse variance weighted
−10
0
10
MR effect size for
'TMEM132D' on 'osteosarcoma'

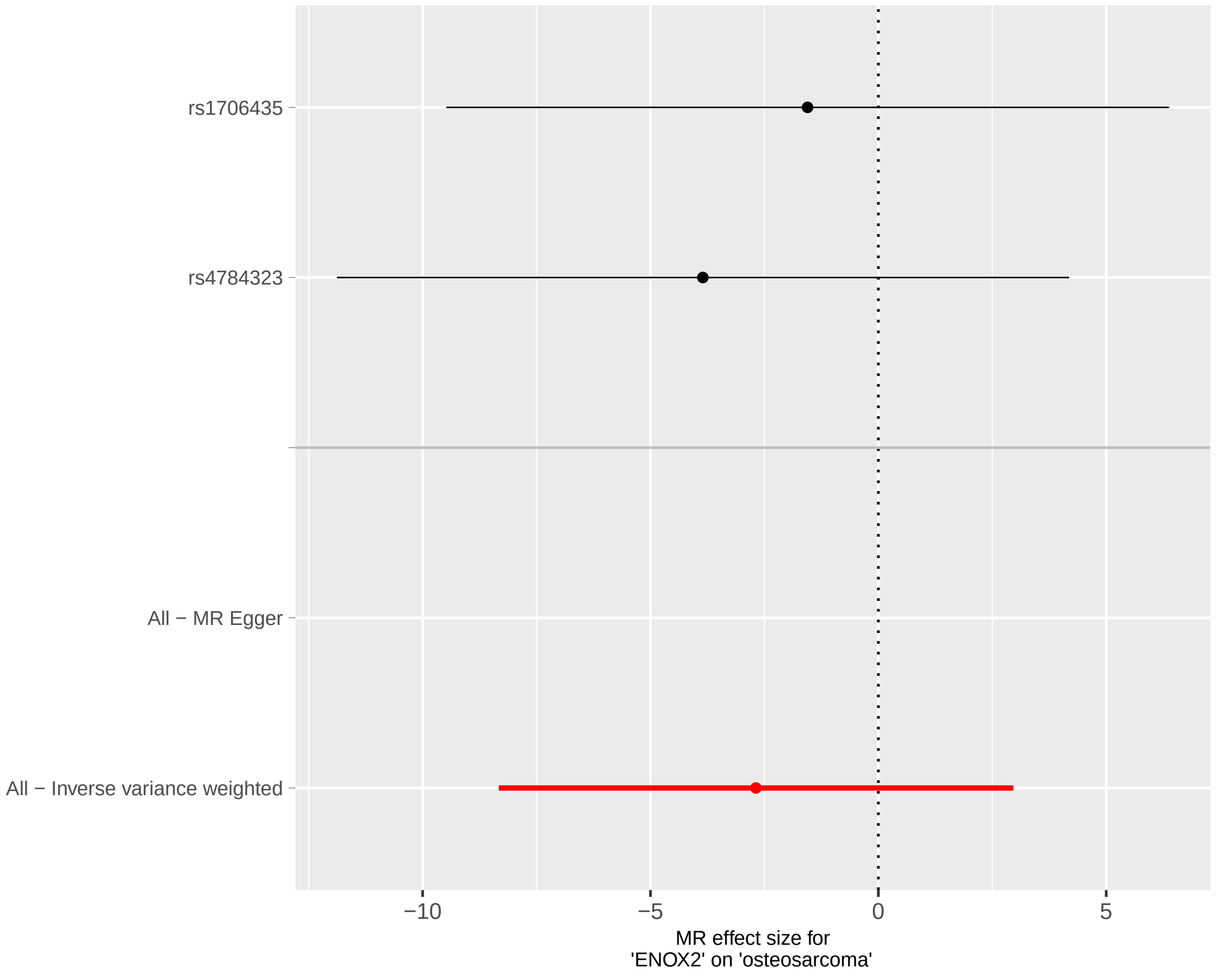

rs1706435
rs4784323
All − MR Egger
All − Inverse variance weighted
−10
−5
0
5
MR effect size for
'ENOX2' on 'osteosarcoma'

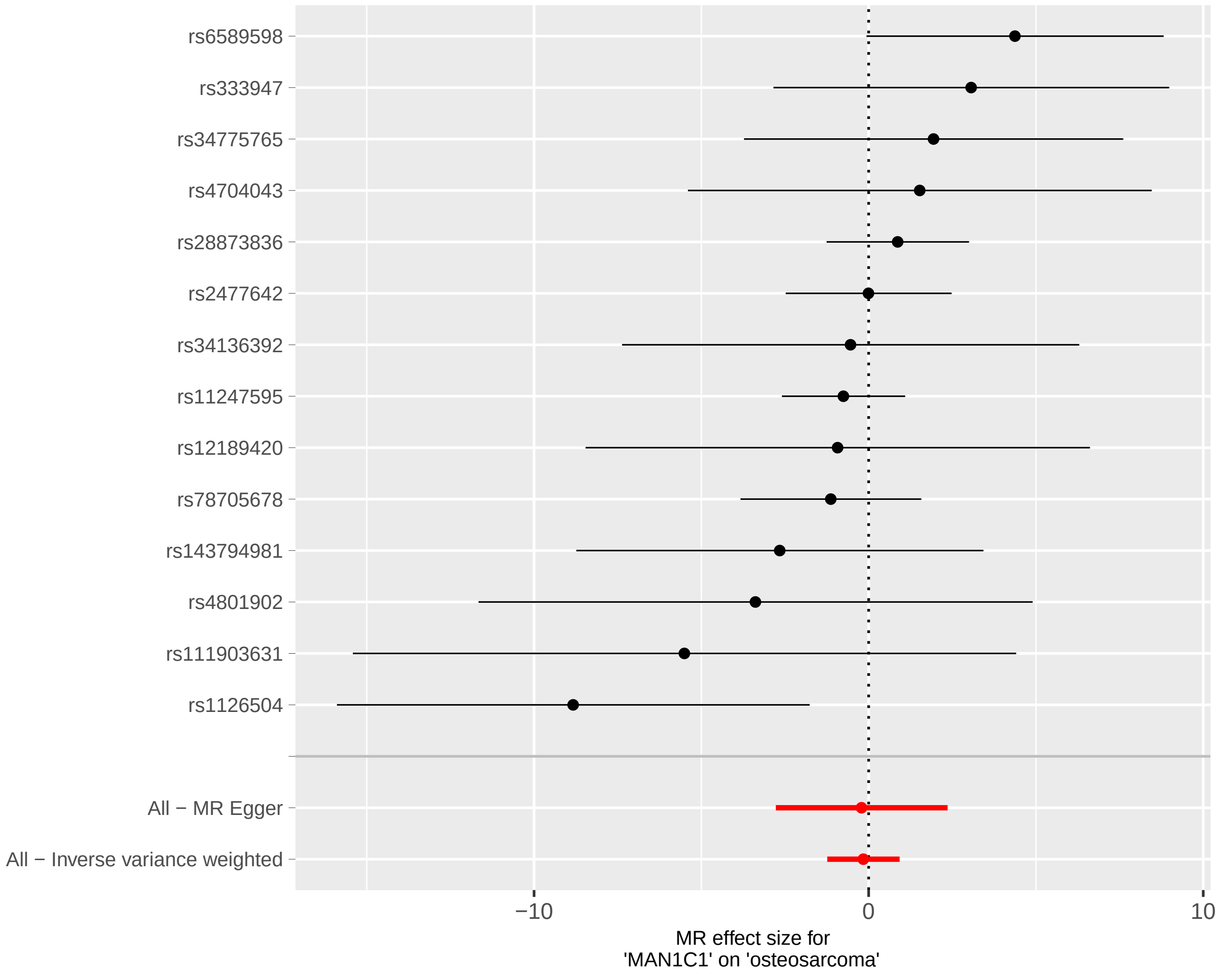

rs6589598
rs333947
rs34775765
rs4704043
rs28873836
rs2477642
rs34136392
rs11247595
rs12189420
rs78705678
rs143794981
rs4801902
rs111903631
rs1126504
All − MR Egger
All − Inverse variance weighted
−10
0
10
MR effect size for
'MAN1C1' on 'osteosarcoma'

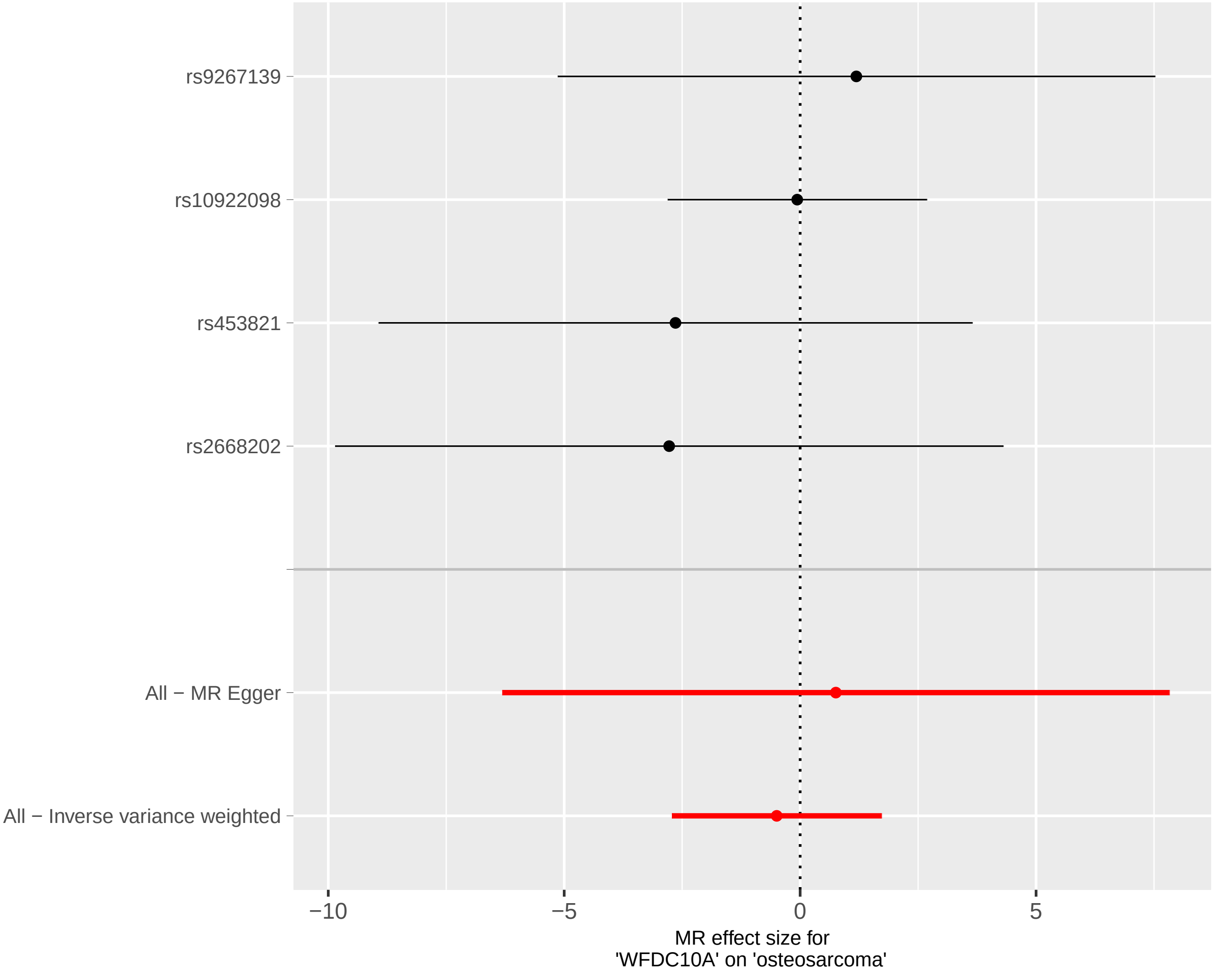

rs9267139
rs10922098
rs453821
rs2668202
All − MR Egger
All − Inverse variance weighted
−10
−5
0
5
MR effect size for
'WFDC10A' on 'osteosarcoma'

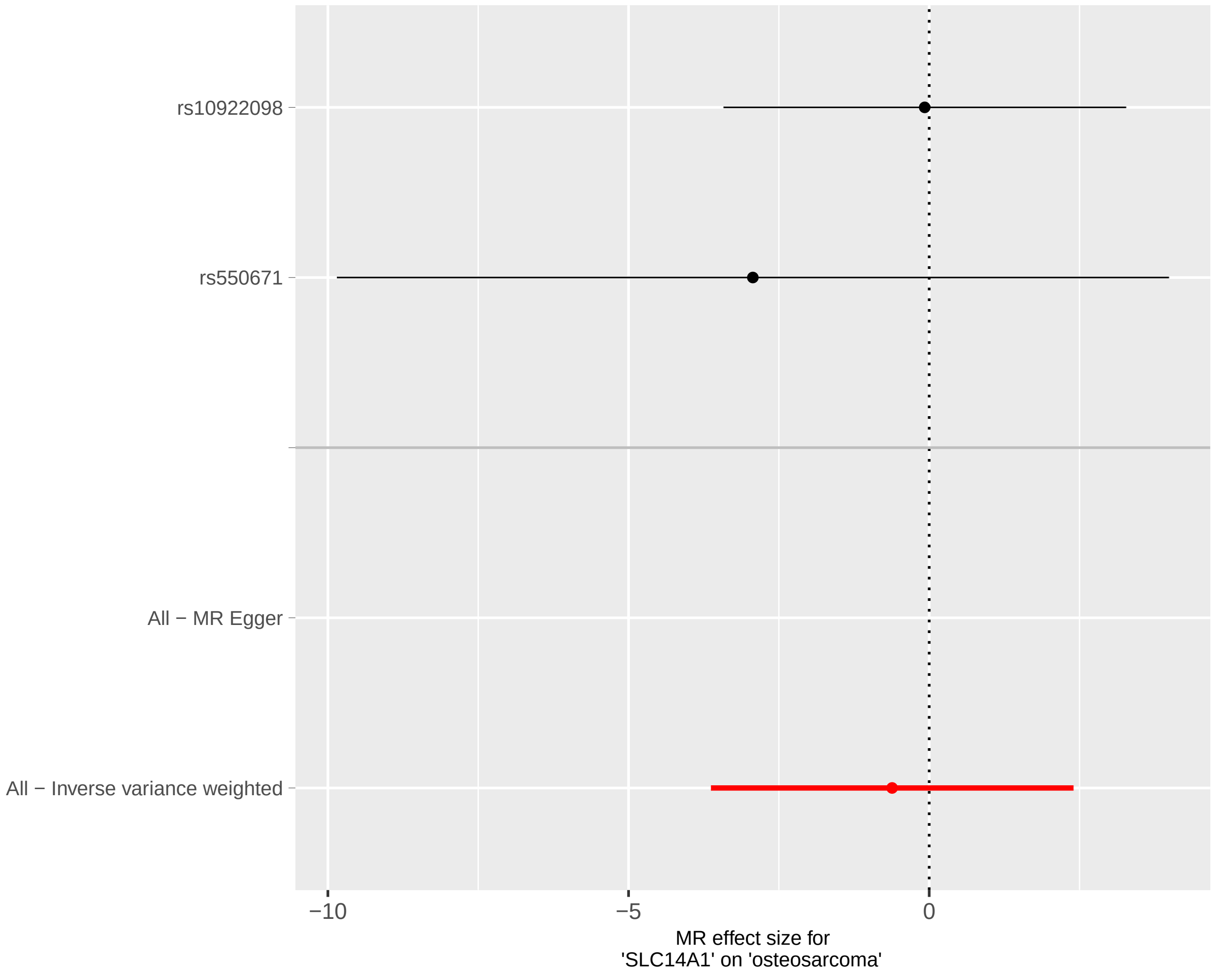
rs10922098
rs550671
All − MR Egger
All − Inverse variance weighted
−10
−5
0
MR effect size for
'SLC14A1' on 'osteosarcoma'

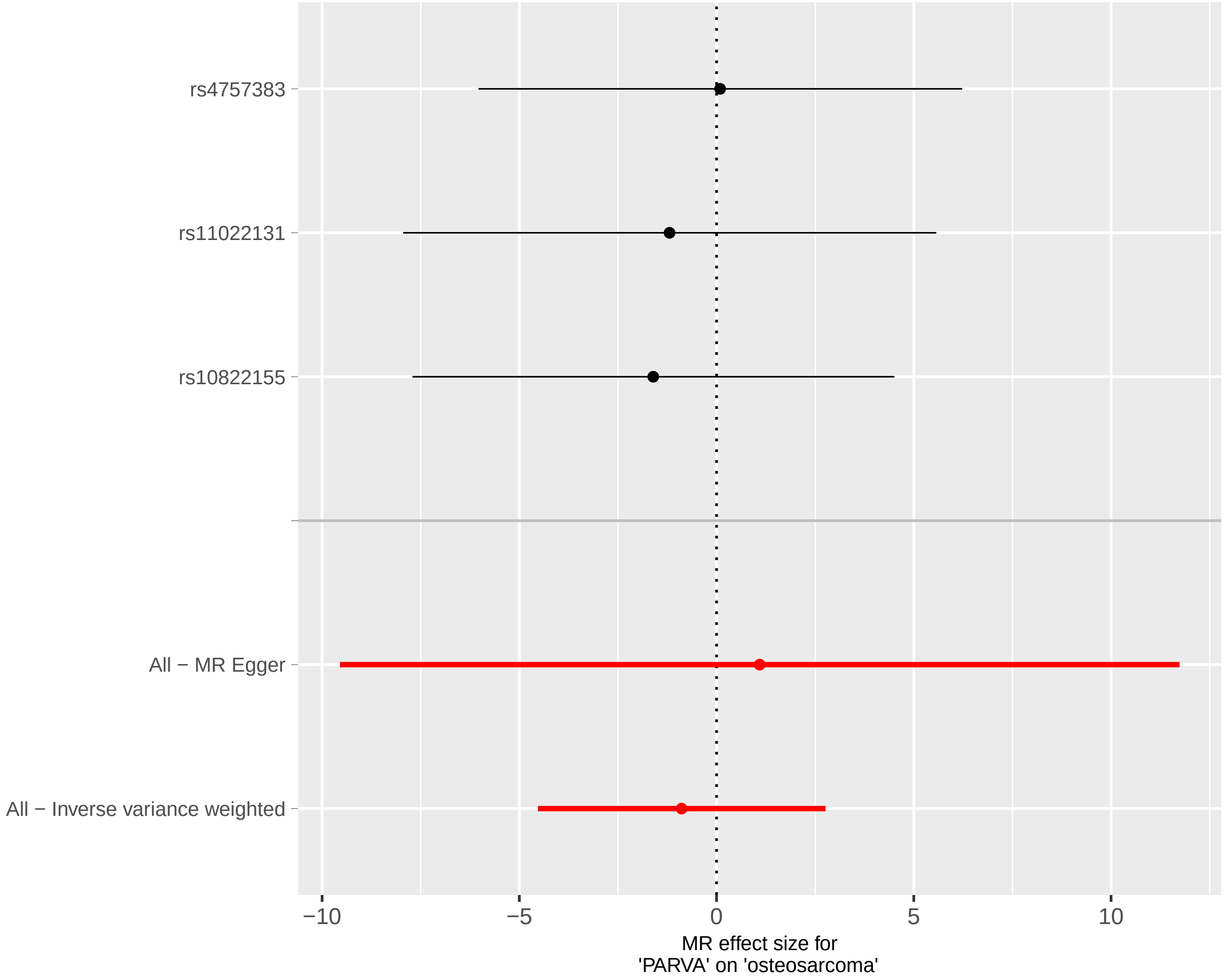

rs4757383
rs11022131
rs10822155
All − MR Egger
All − Inverse variance weighted
−10
−5
0
5
10
MR effect size for
'PARVA' on 'osteosarcoma'

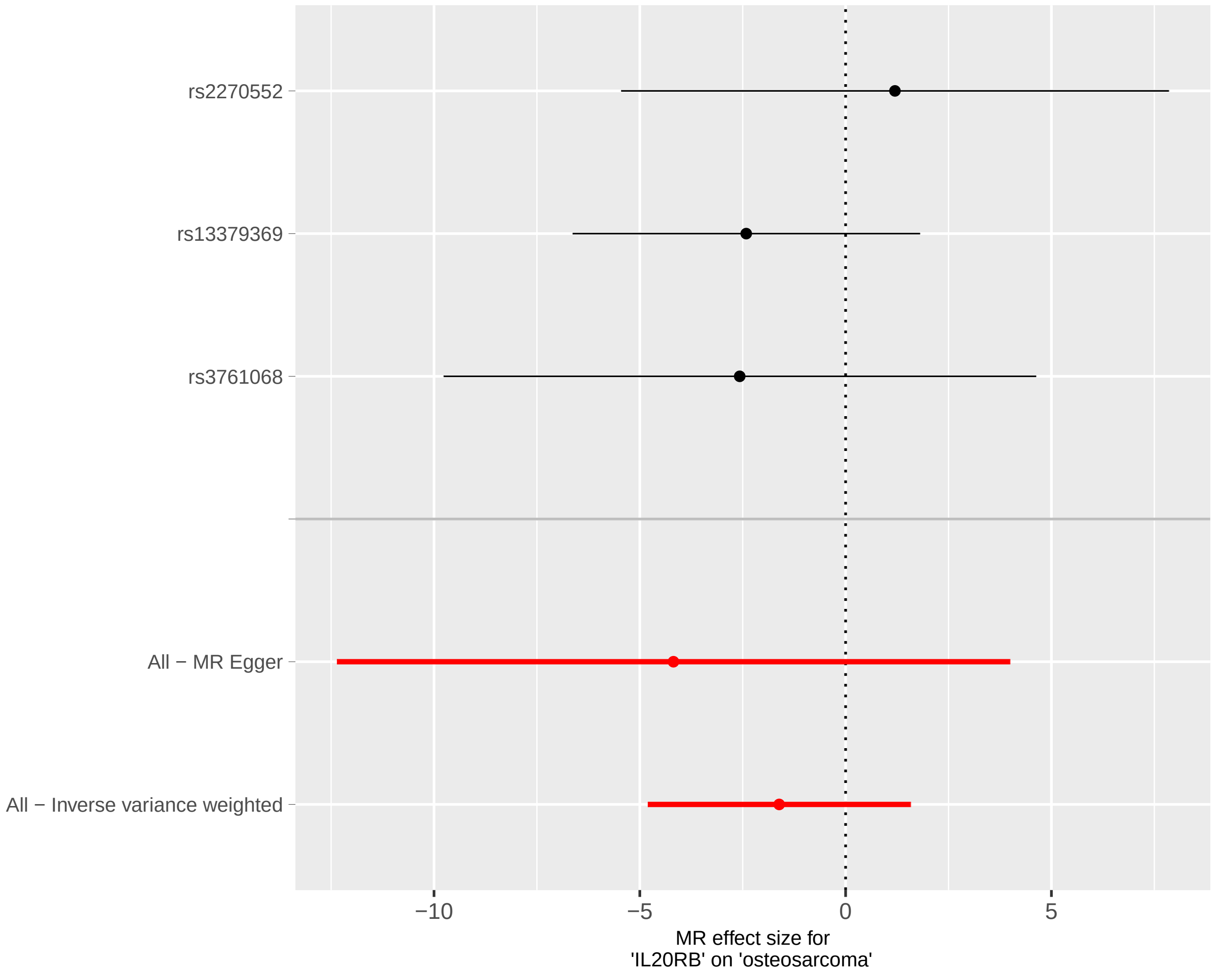
rs2270552
rs13379369
rs3761068
All − MR Egger
All − Inverse variance weighted
−10
−5
0
5
MR effect size for
'IL20RB' on 'osteosarcoma'

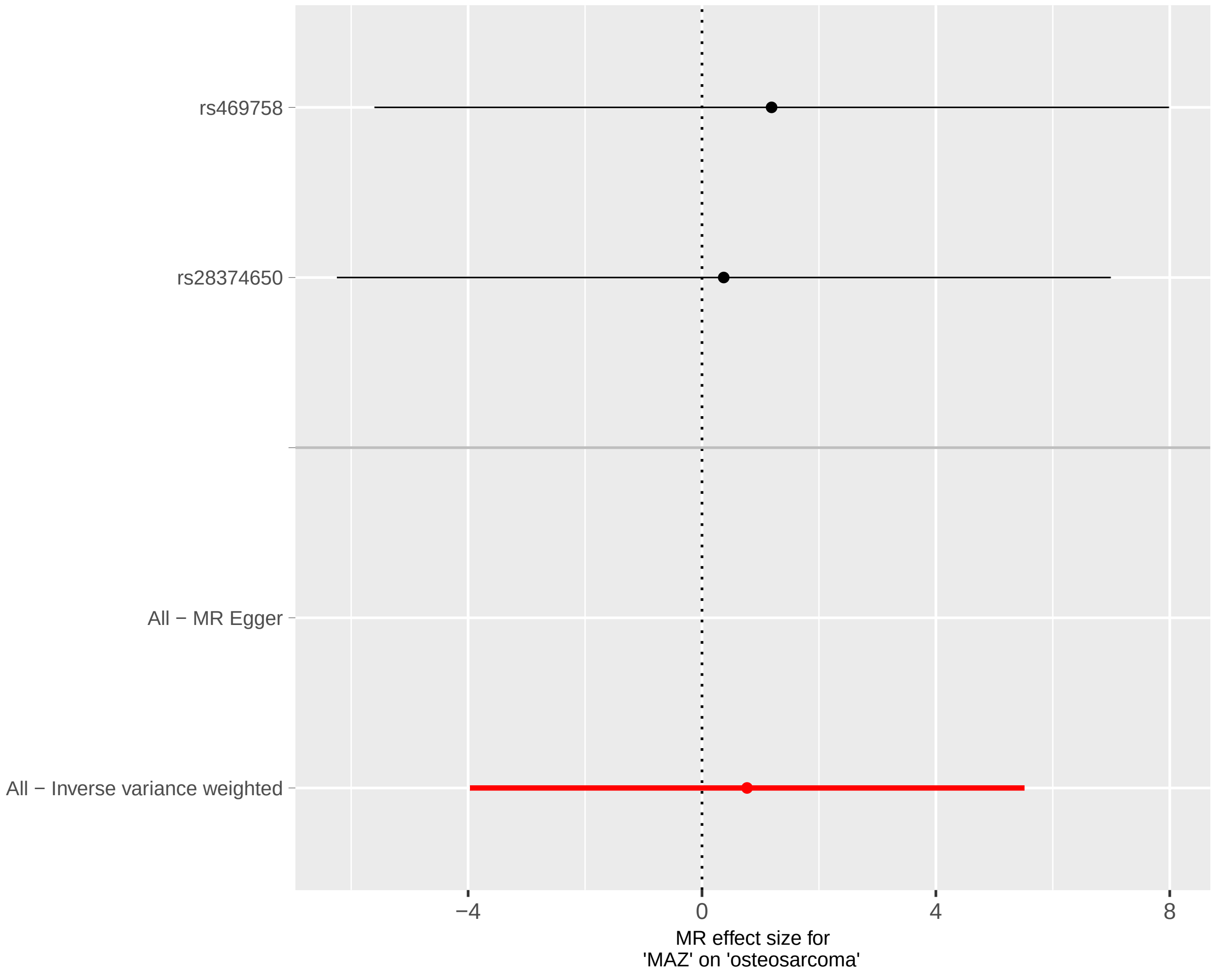

rs469758
rs28374650
All − MR Egger
All − Inverse variance weighted
−4
0
4
8
MR effect size for
'MAZ' on 'osteosarcoma'

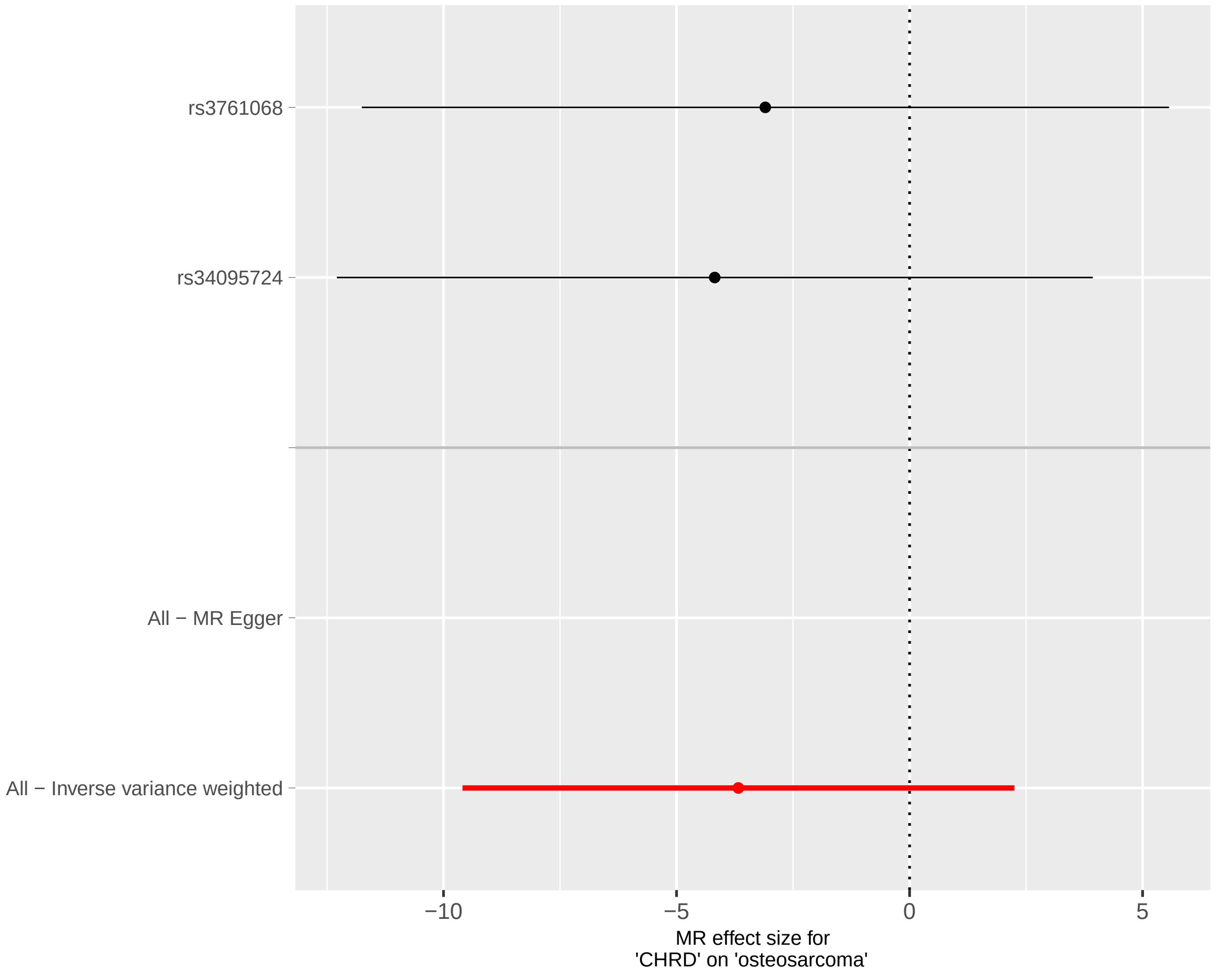

rs3761068
rs34095724
All − MR Egger
All − Inverse variance weighted
−10
−5
0
5
MR effect size for
'CHRD' on 'osteosarcoma'

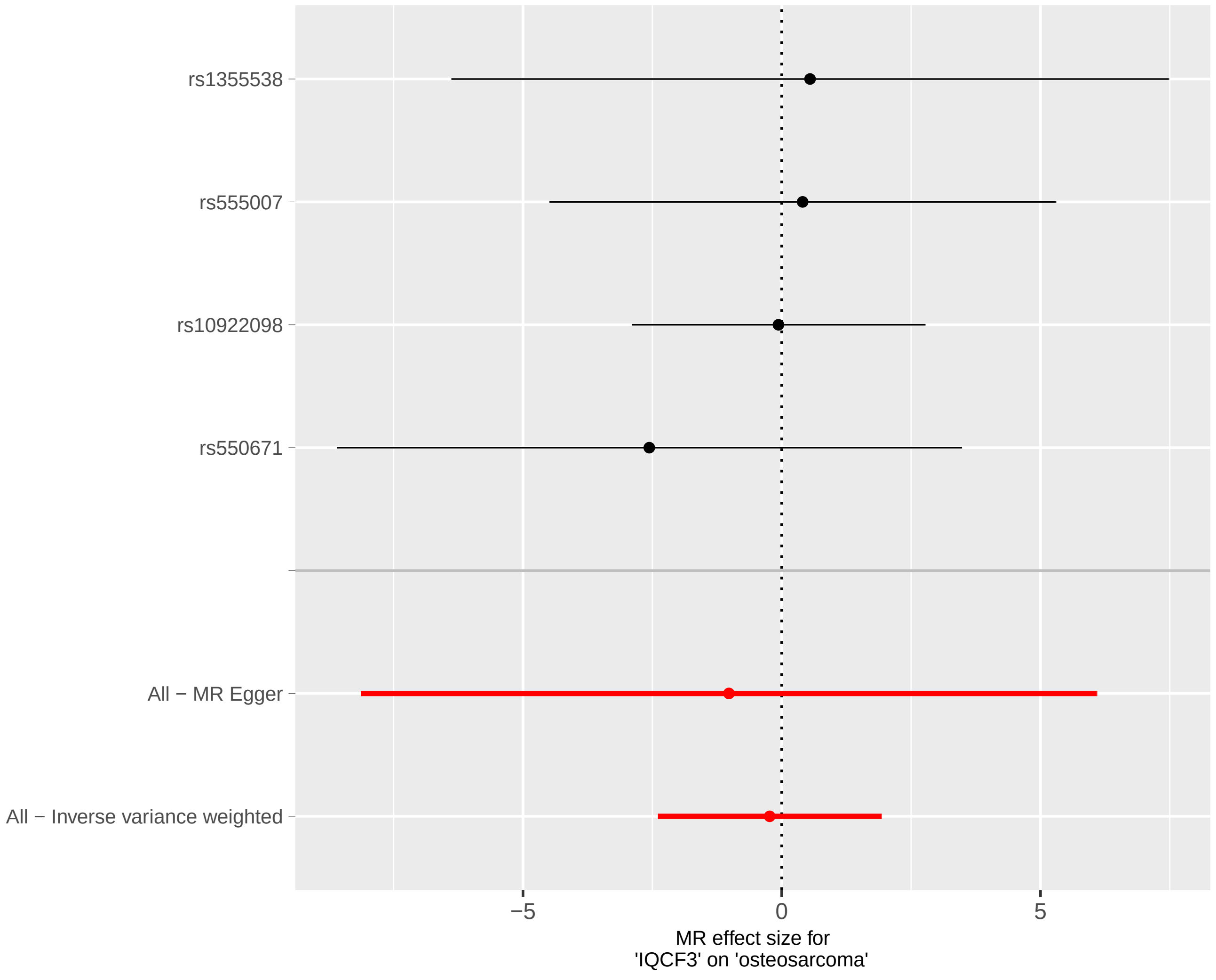
rs1355538
rs555007
rs10922098
rs550671
All − MR Egger
All − Inverse variance weighted
−5
0
5
MR effect size for
'IQCF3' on 'osteosarcoma'

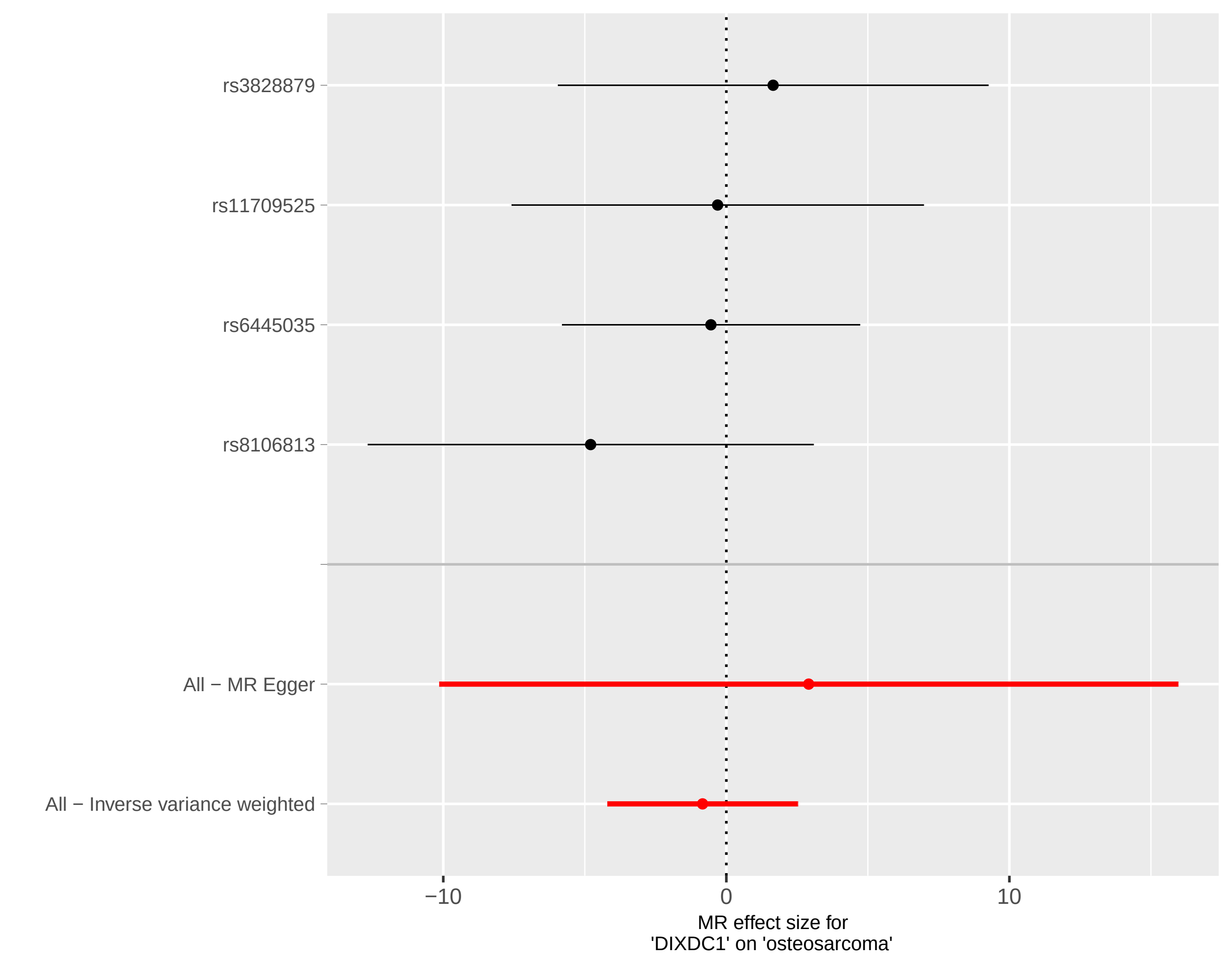

rs3828879
rs11709525
rs6445035
rs8106813
All − MR Egger
All − Inverse variance weighted
−10
0
10
MR effect size for
'DIXDC1' on 'osteosarcoma'

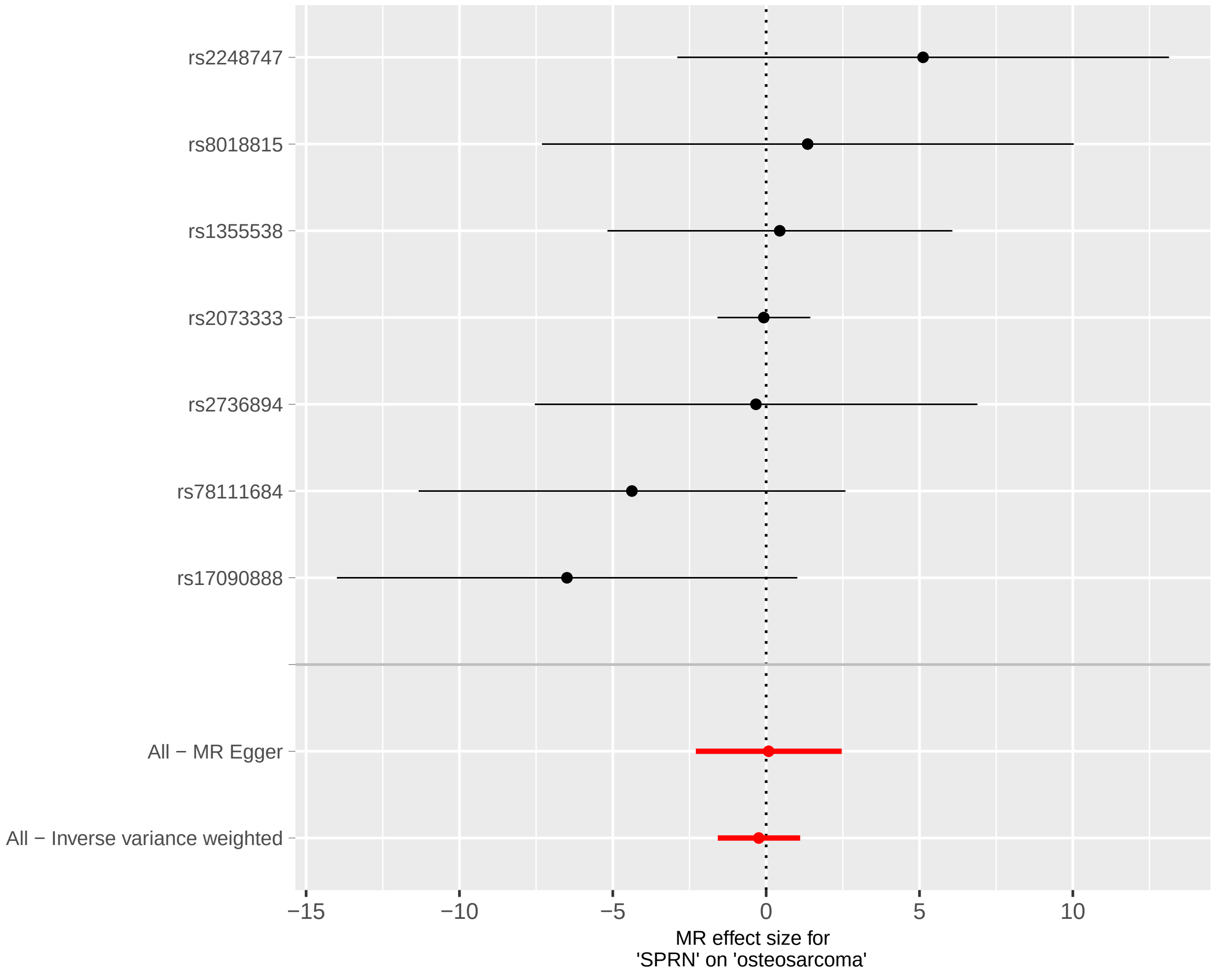
rs2248747
rs8018815
rs1355538
rs2073333
rs2736894
rs78111684
rs17090888
All − MR Egger
All − Inverse variance weighted
−15
−10
−5
0
5
10
MR effect size for
'SPRN' on 'osteosarcoma'

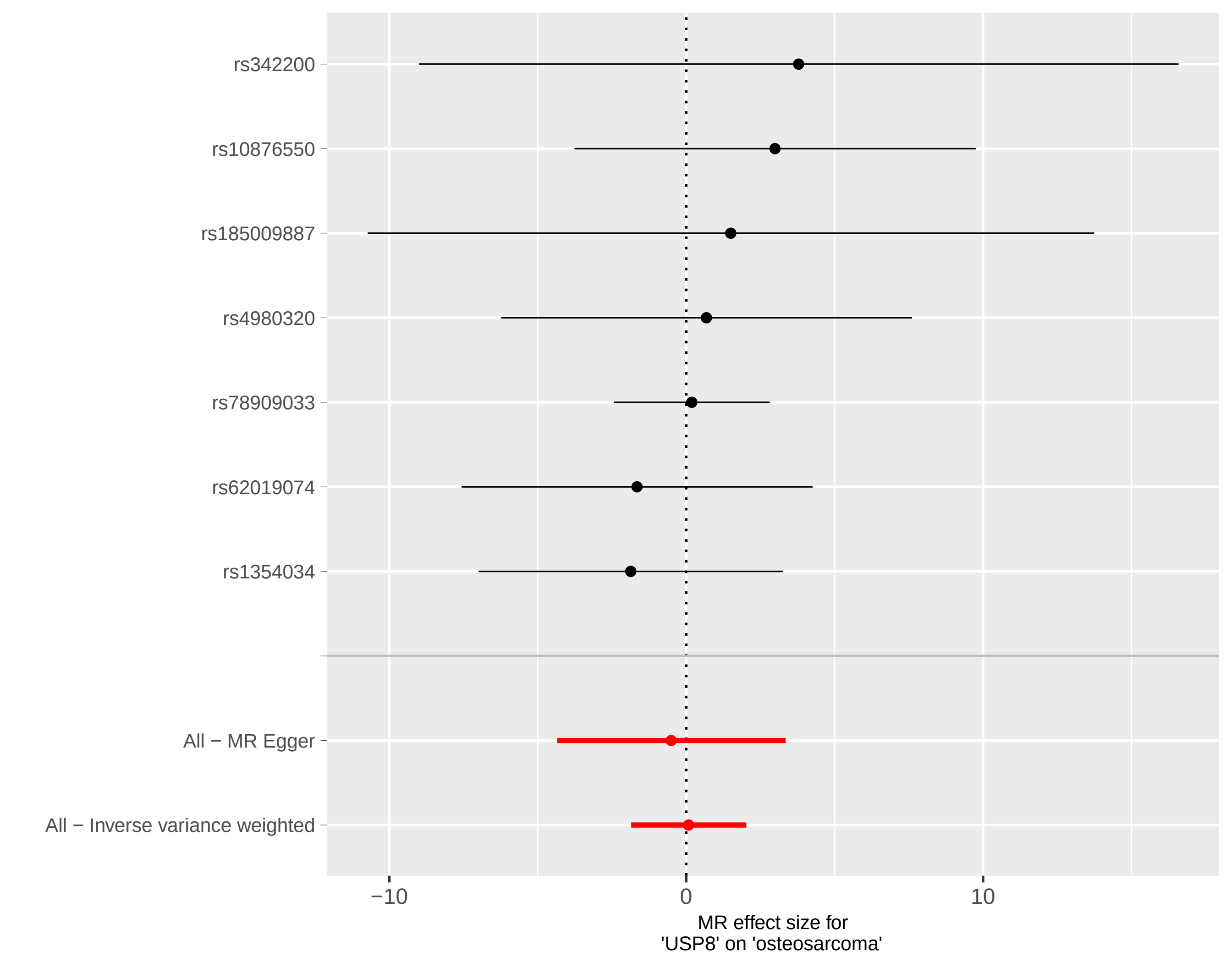

rs342200
rs10876550
rs185009887
rs4980320
rs78909033
rs62019074
rs1354034
All − MR Egger
All − Inverse variance weighted
−10
0
10
MR effect size for
'USP8' on 'osteosarcoma'

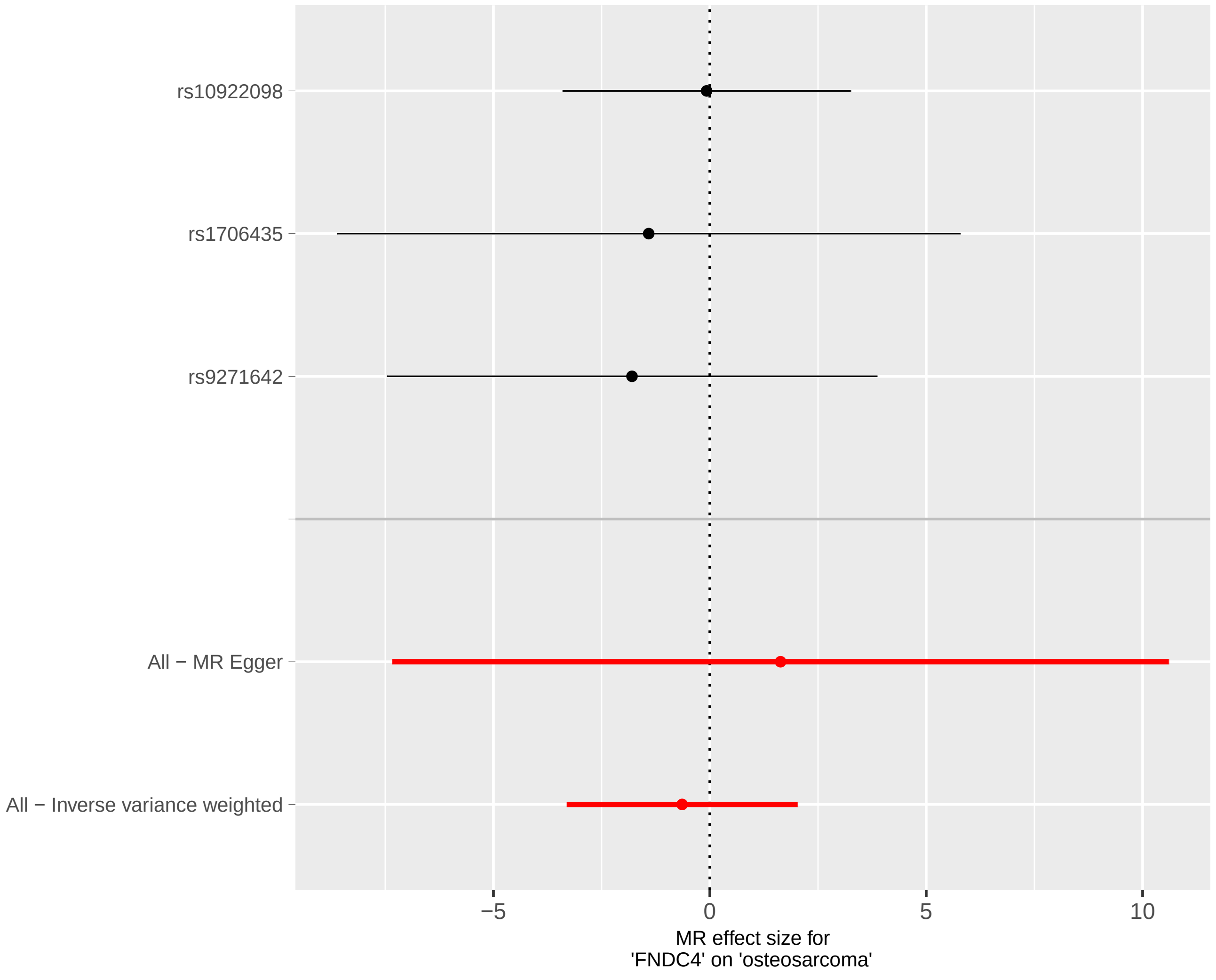

rs10922098
rs1706435
rs9271642
All − MR Egger
All − Inverse variance weighted
−5
0
5
10
MR effect size for
'FNDC4' on 'osteosarcoma'

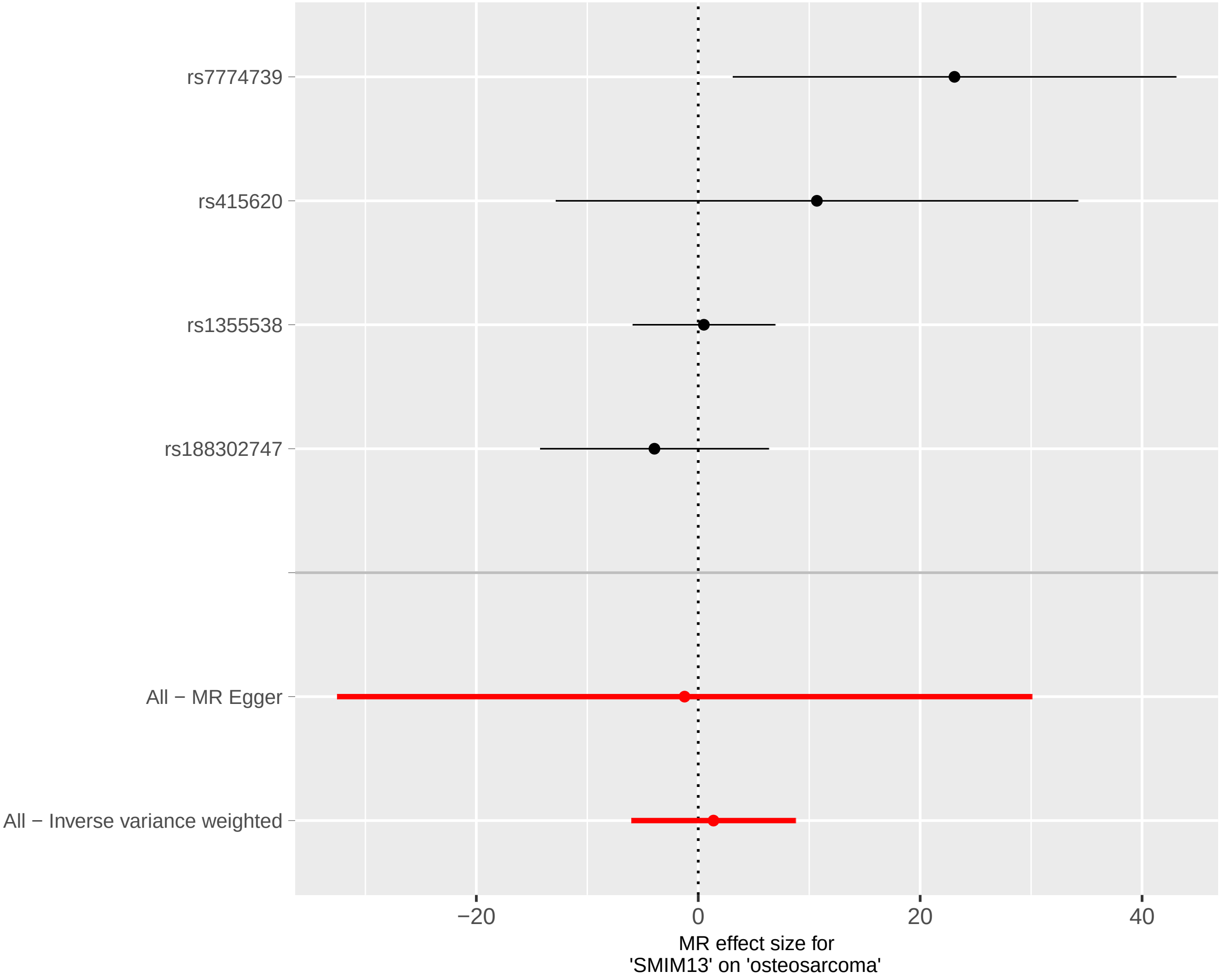
rs7774739
rs415620
rs1355538
rs188302747
All − MR Egger
All − Inverse variance weighted
−20
0
20
40
MR effect size for
'SMIM13' on 'osteosarcoma'

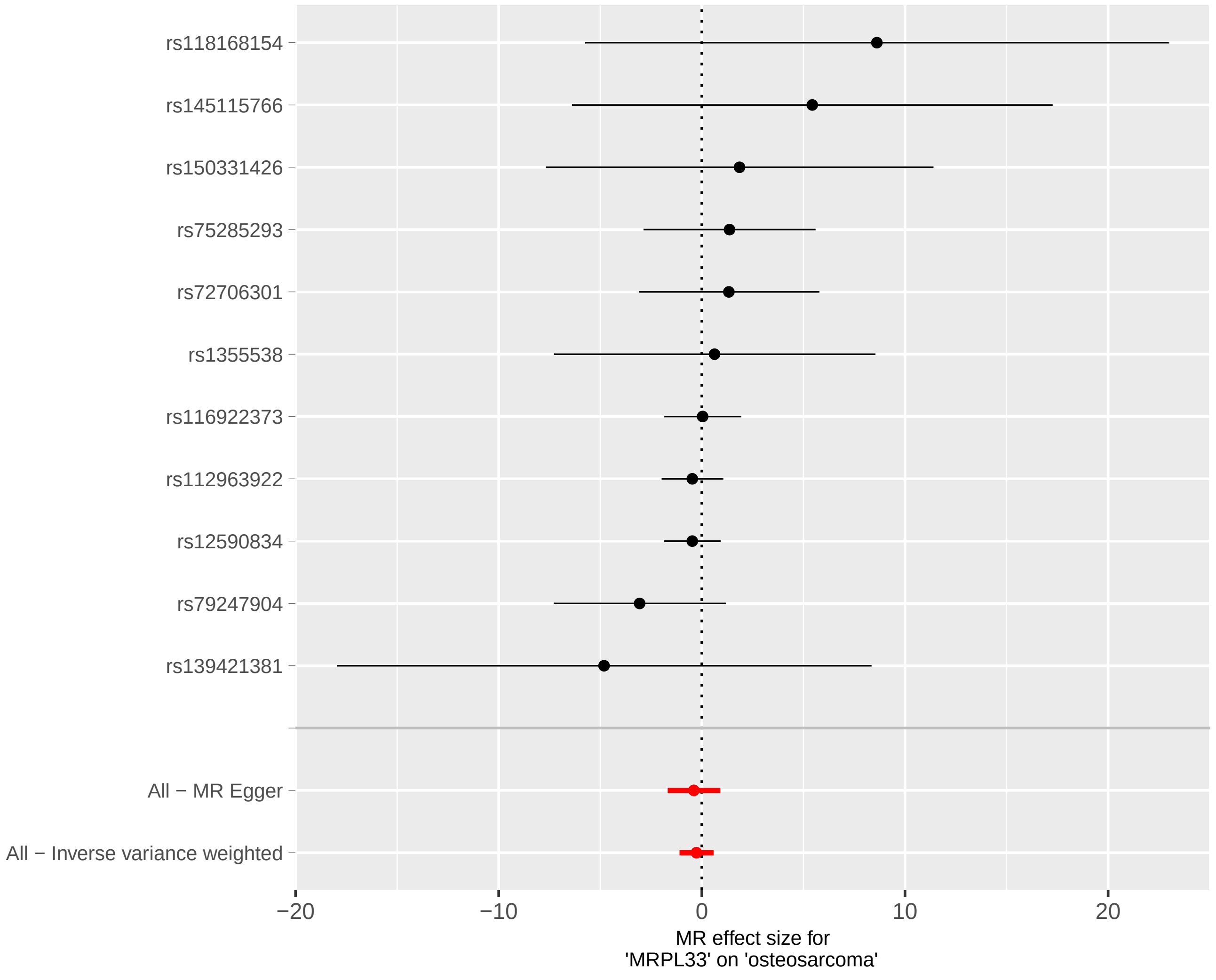

rs118168154
rs145115766
rs150331426
rs75285293
rs72706301
rs1355538
rs116922373
rs112963922
rs12590834
rs79247904
rs139421381
All − MR Egger
All − Inverse variance weighted
−20
−10
0
10
20
MR effect size for
'MRPL33' on 'osteosarcoma'

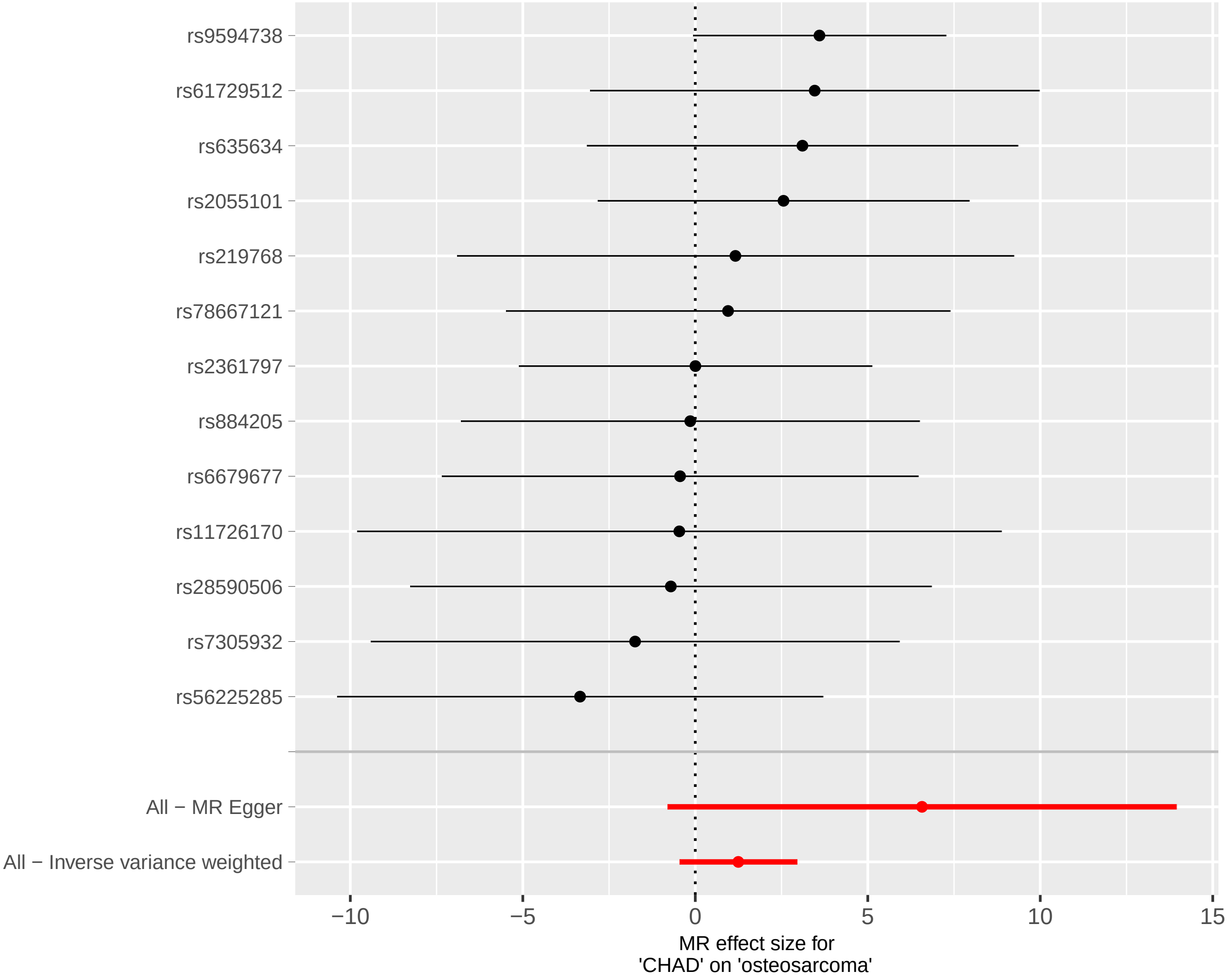
rs9594738
rs61729512
rs635634
rs2055101
rs219768
rs78667121
rs2361797
rs884205
rs6679677
rs11726170
rs28590506
rs7305932
rs56225285
All − MR Egger
All − Inverse variance weighted
−10
−5
0
5
10
15
MR effect size for
'CHAD' on 'osteosarcoma'

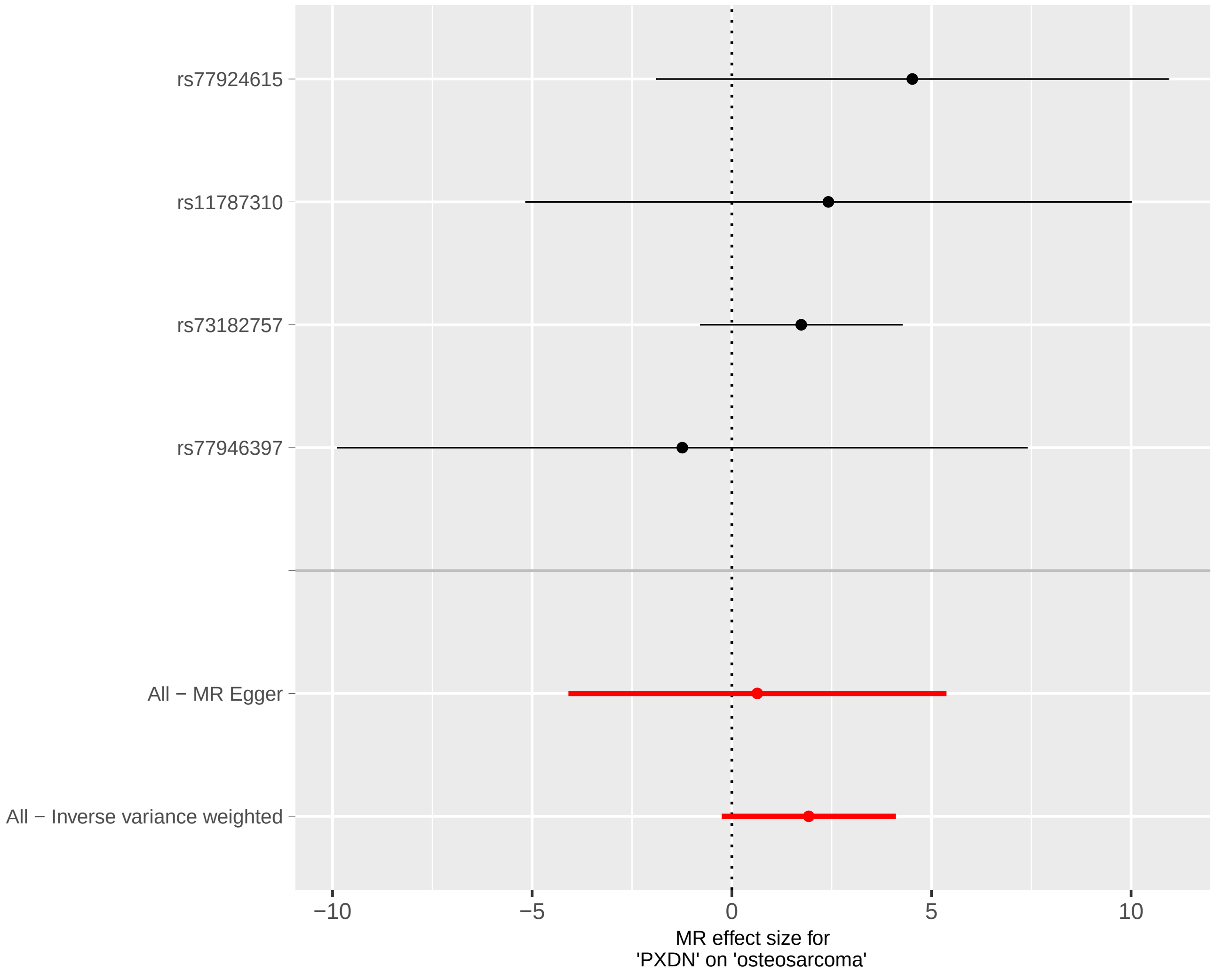
rs77924615
rs11787310
rs73182757
rs77946397
All − MR Egger
All − Inverse variance weighted
−10
−5
0
5
10
MR effect size for
'PXDN' on 'osteosarcoma'

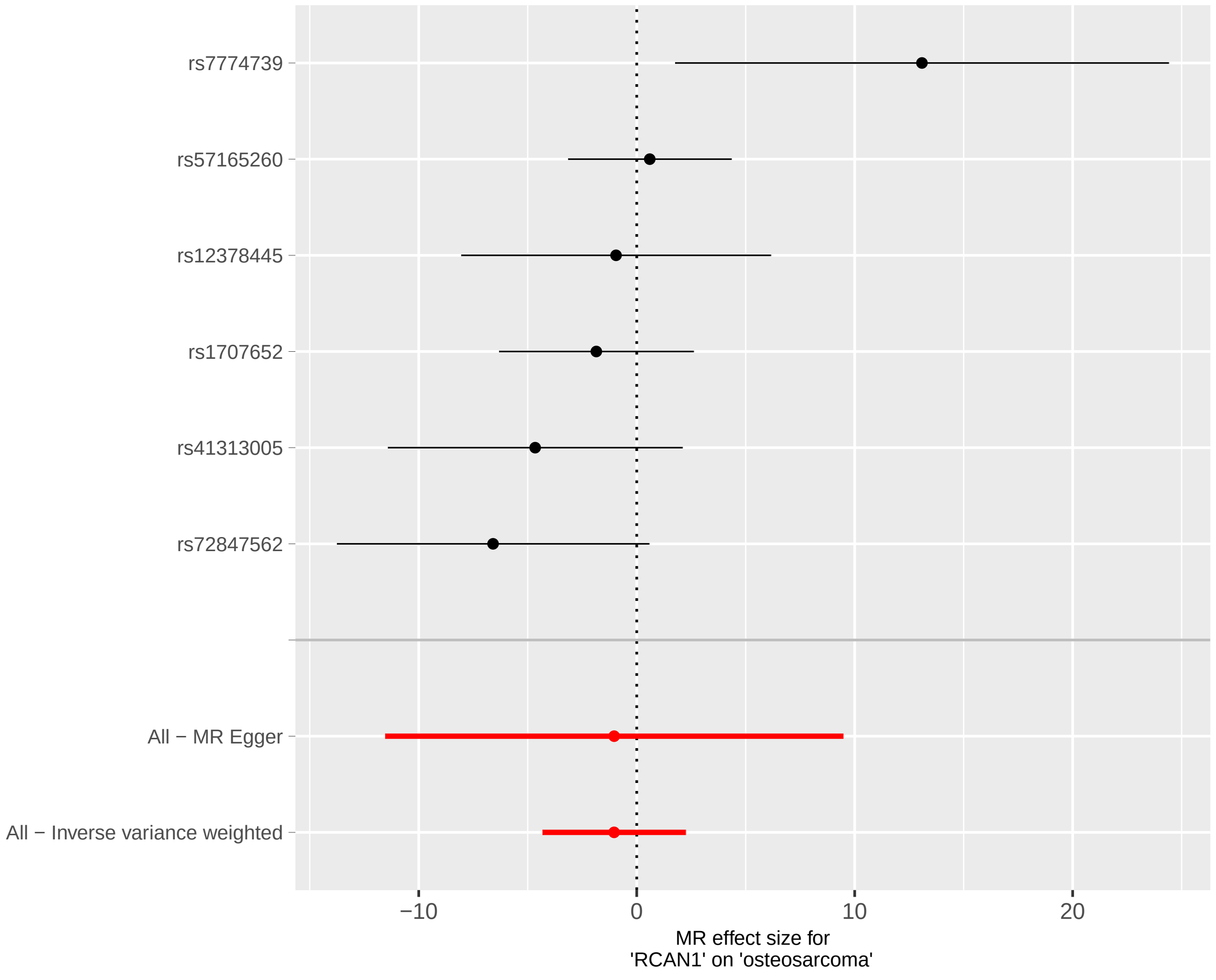

rs7774739
rs57165260
rs12378445
rs1707652
rs41313005
rs72847562
All − MR Egger
All − Inverse variance weighted
−10
0
10
20
MR effect size for
'RCAN1' on 'osteosarcoma'

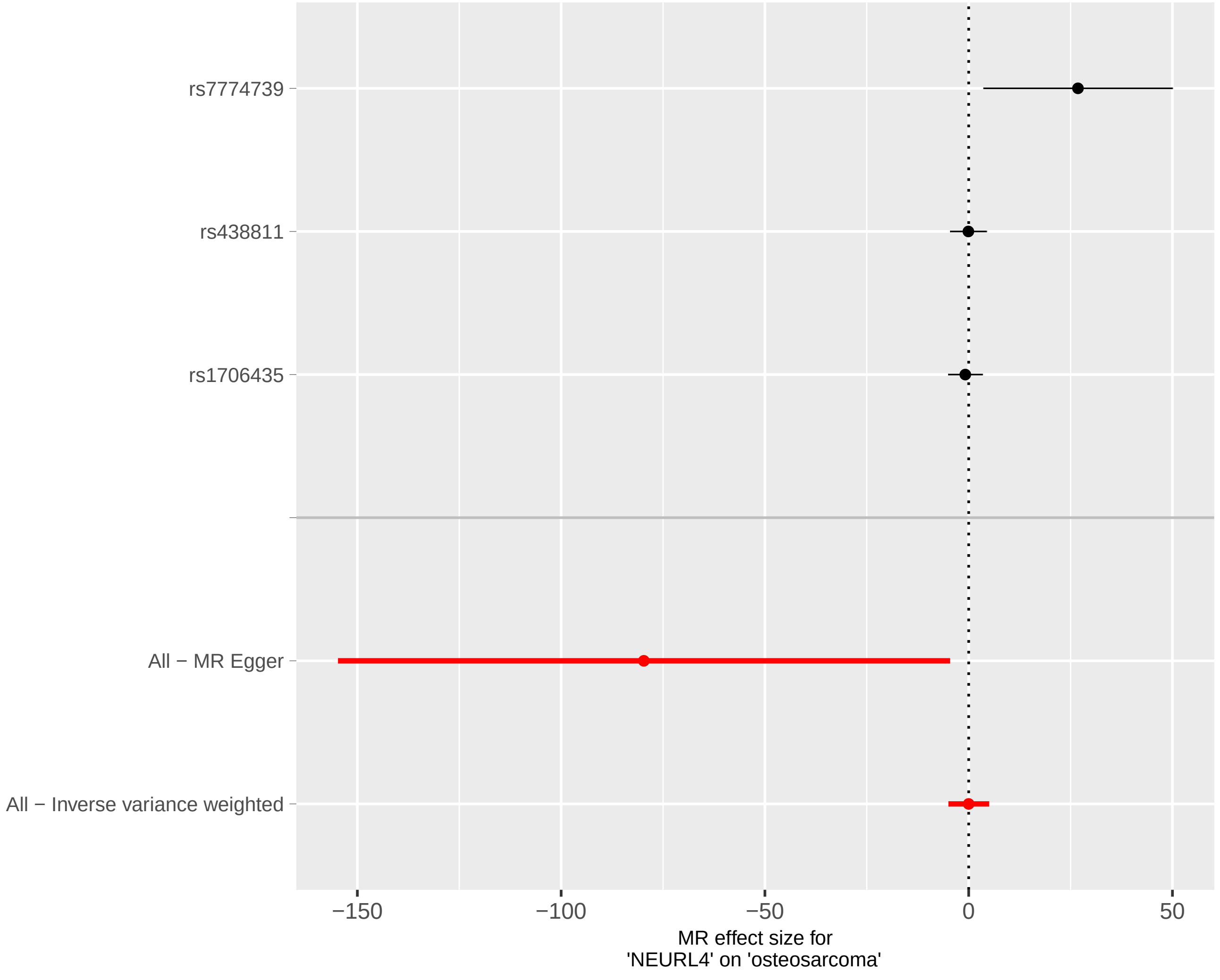
rs7774739
rs438811
rs1706435
All − MR Egger
All − Inverse variance weighted
−150
−100
−50
0
50
MR effect size for
'NEURL4' on 'osteosarcoma'

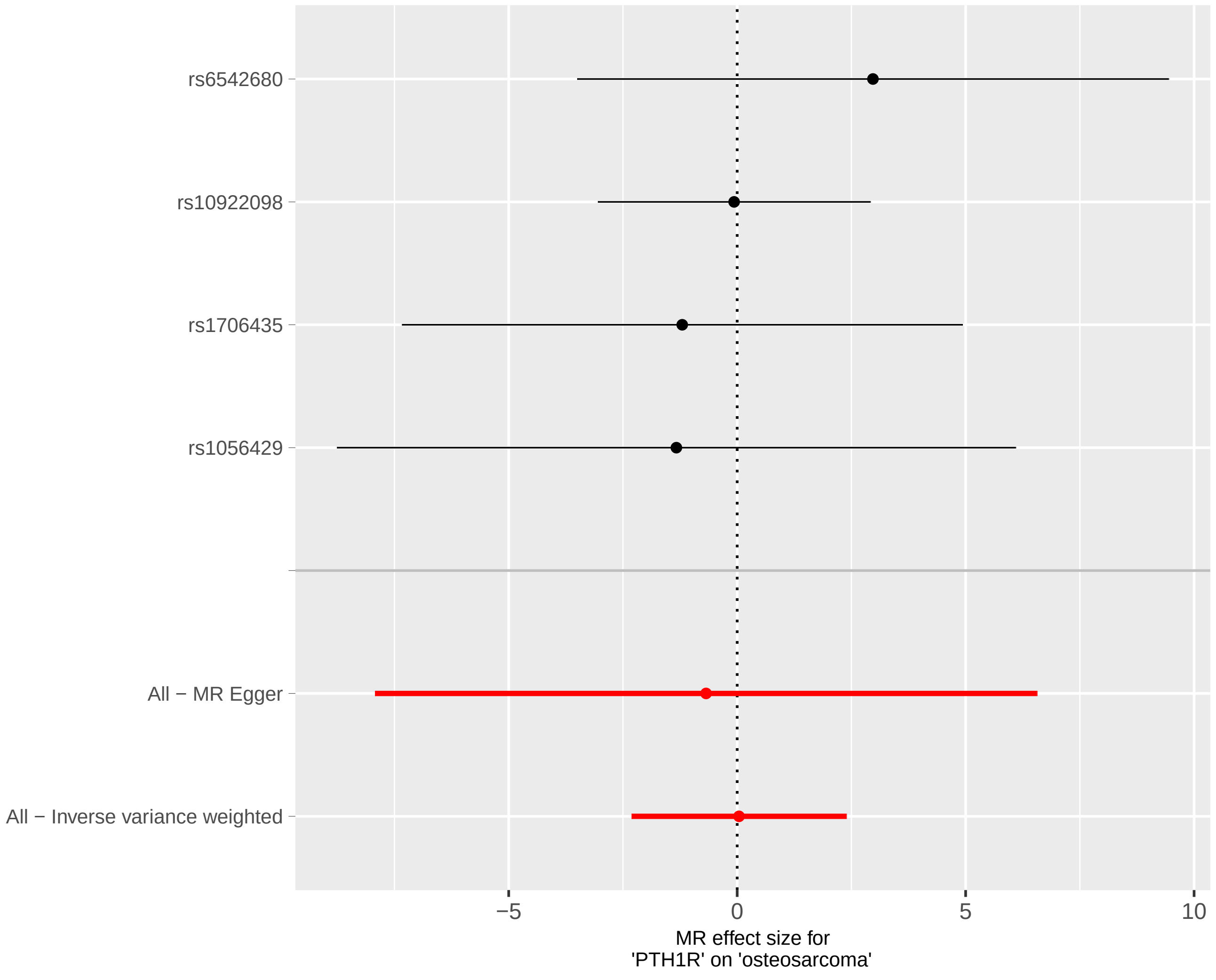

rs6542680
rs10922098
rs1706435
rs1056429
All − MR Egger
All − Inverse variance weighted
−5
0
5
10
MR effect size for
'PTH1R' on 'osteosarcoma'

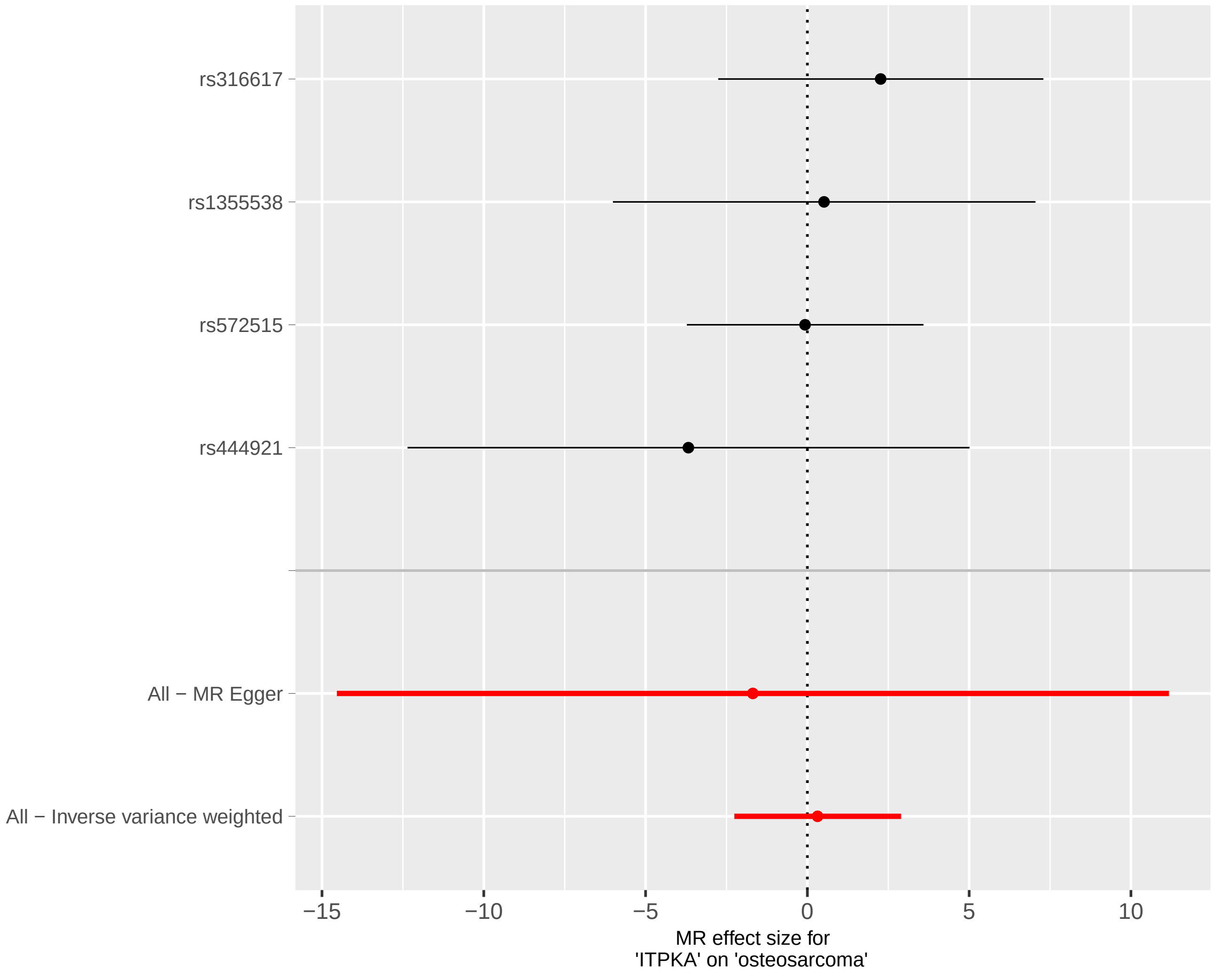

rs316617
rs1355538
rs572515
rs444921
All − MR Egger
All − Inverse variance weighted
−15
−10
−5
0
5
10
MR effect size for
'ITPKA' on 'osteosarcoma'

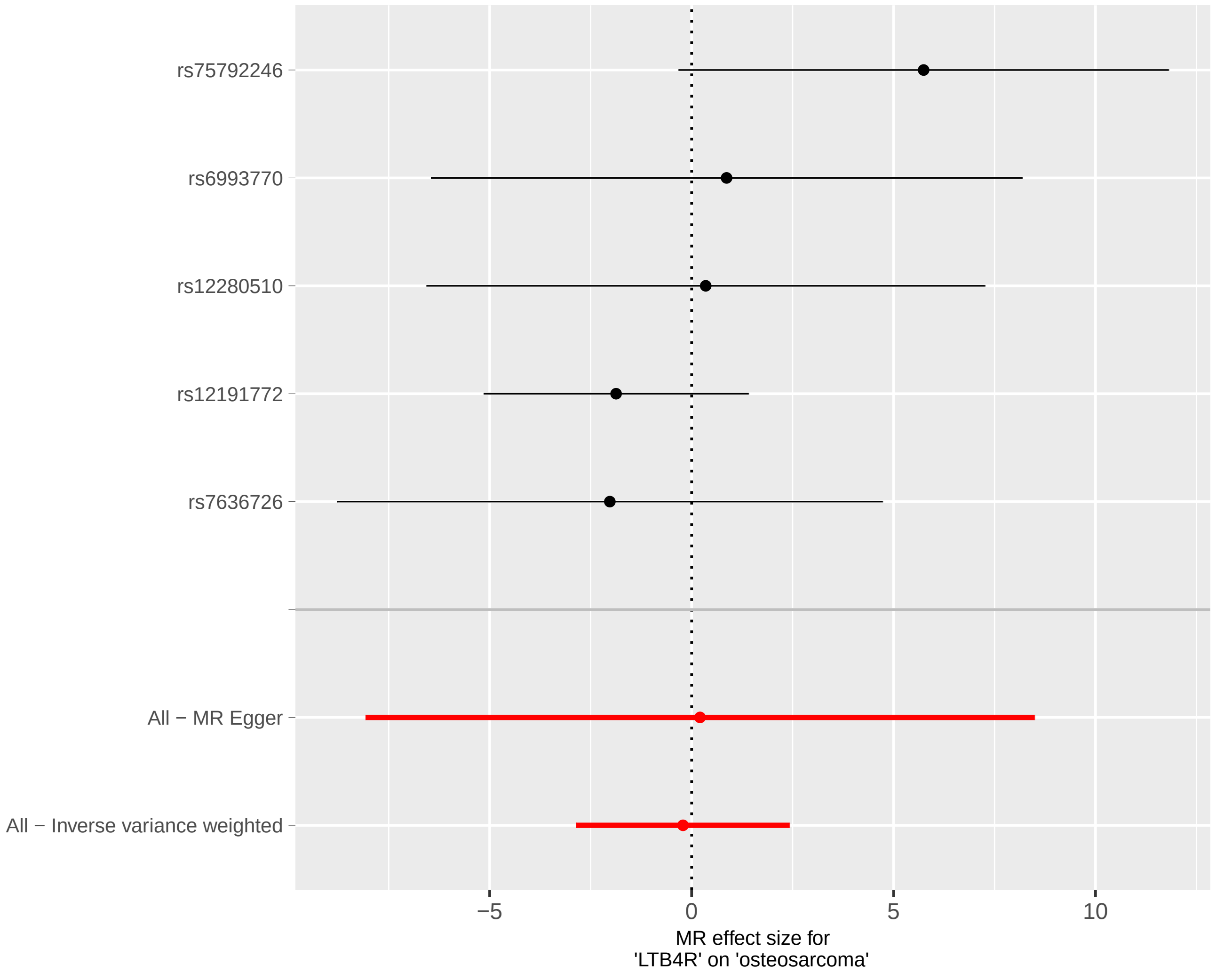
rs75792246
rs6993770
rs12280510
rs12191772
rs7636726
All − MR Egger
All − Inverse variance weighted
−5
0
5
10
MR effect size for
'LTB4R' on 'osteosarcoma'

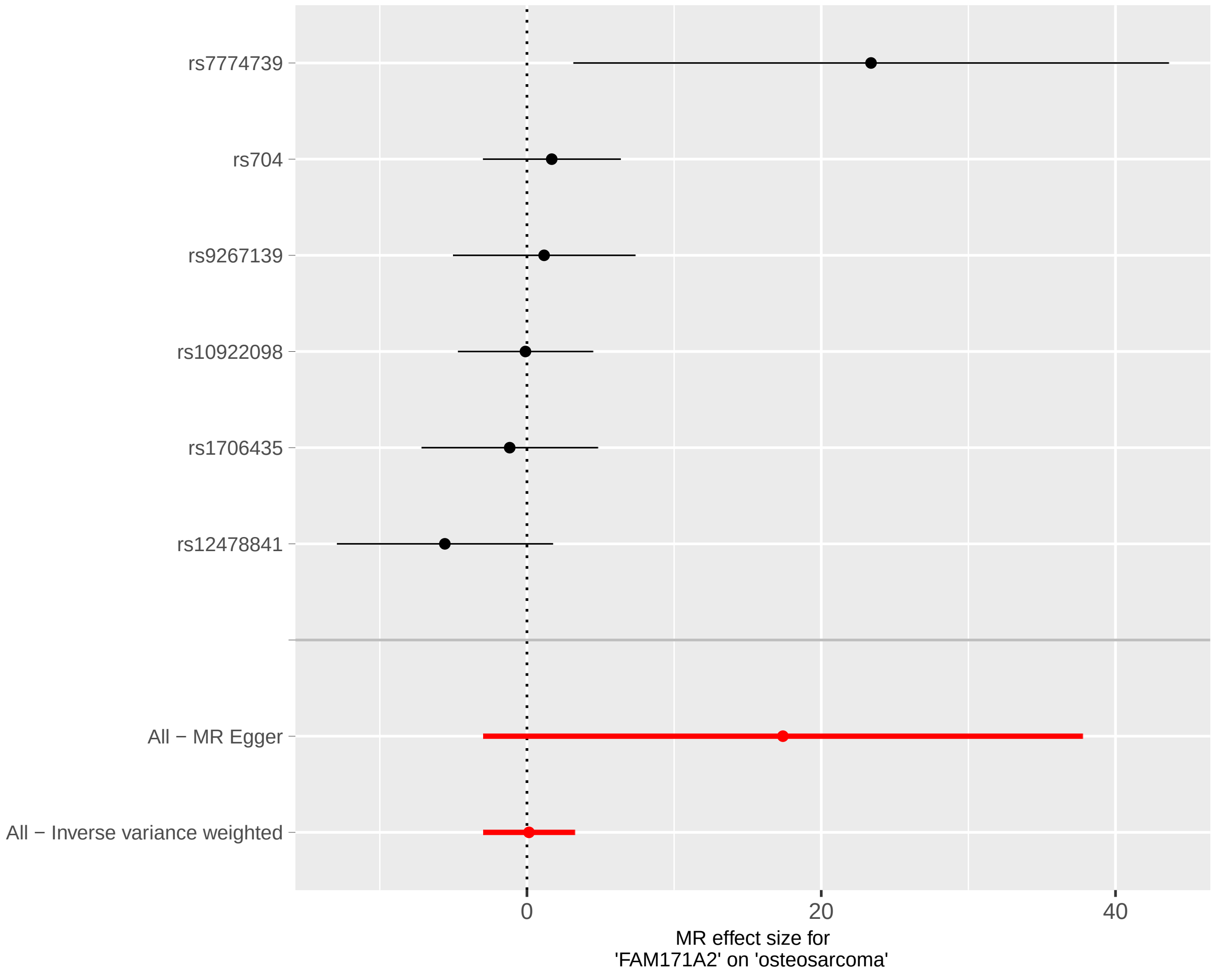
rs7774739
rs704
rs9267139
rs10922098
rs1706435
rs12478841
All − MR Egger
All − Inverse variance weighted
0
20
40
MR effect size for
'FAM171A2' on 'osteosarcoma'

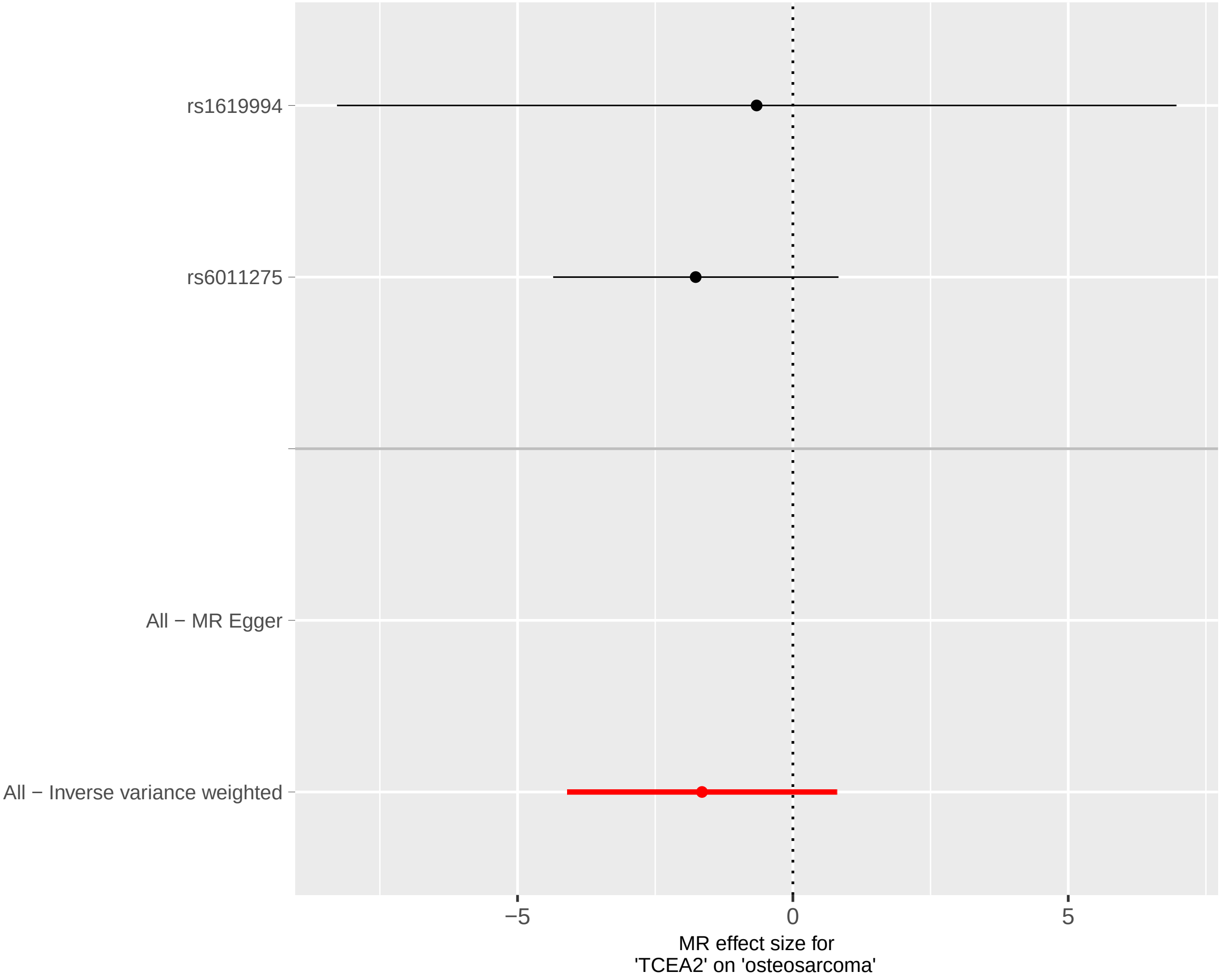
rs1619994
rs6011275
All − MR Egger
All − Inverse variance weighted
−5
0
5
MR effect size for
'TCEA2' on 'osteosarcoma'

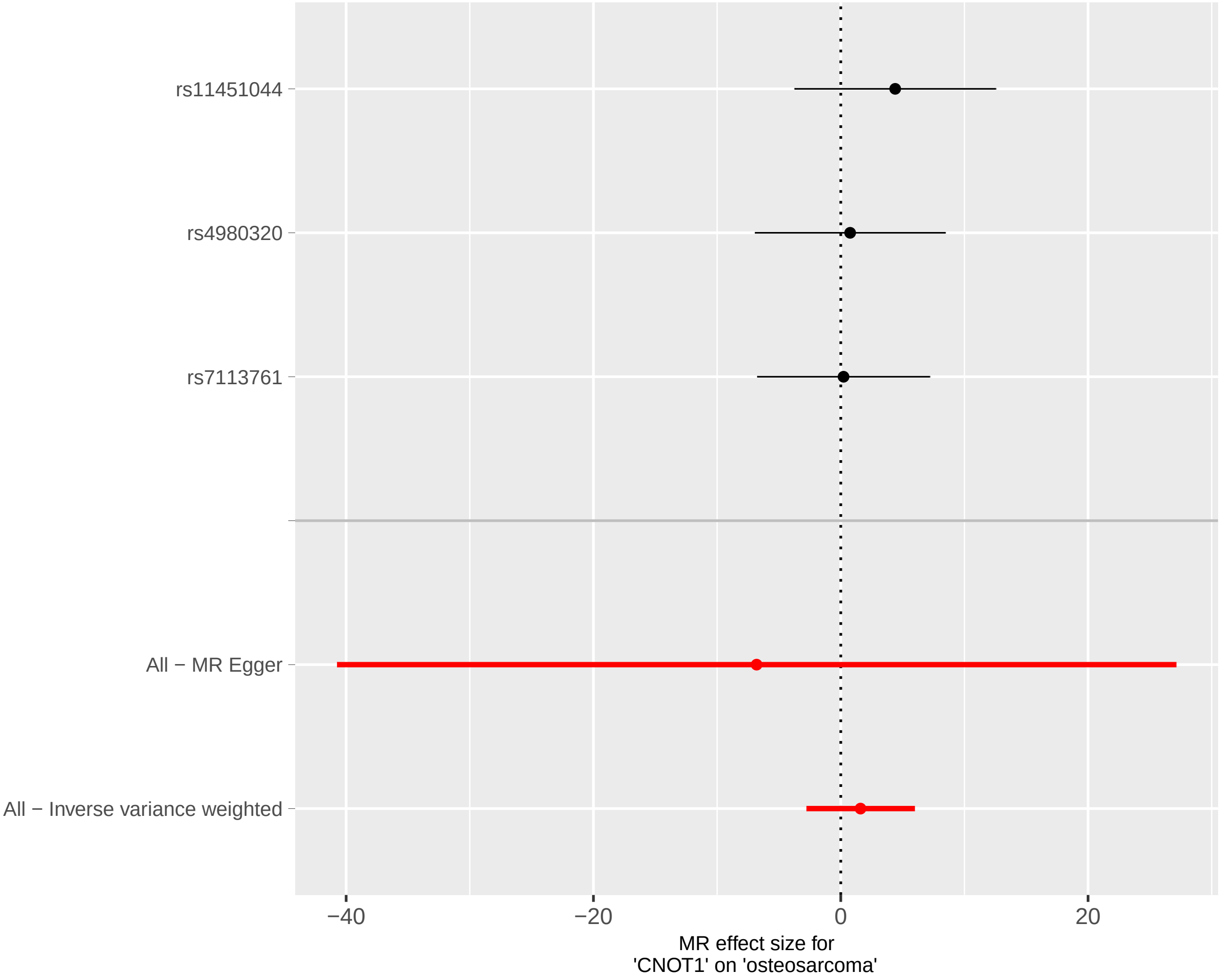

rs11451044
rs4980320
rs7113761
All − MR Egger
All − Inverse variance weighted
−40
−20
0
20
MR effect size for
'CNOT1' on 'osteosarcoma'

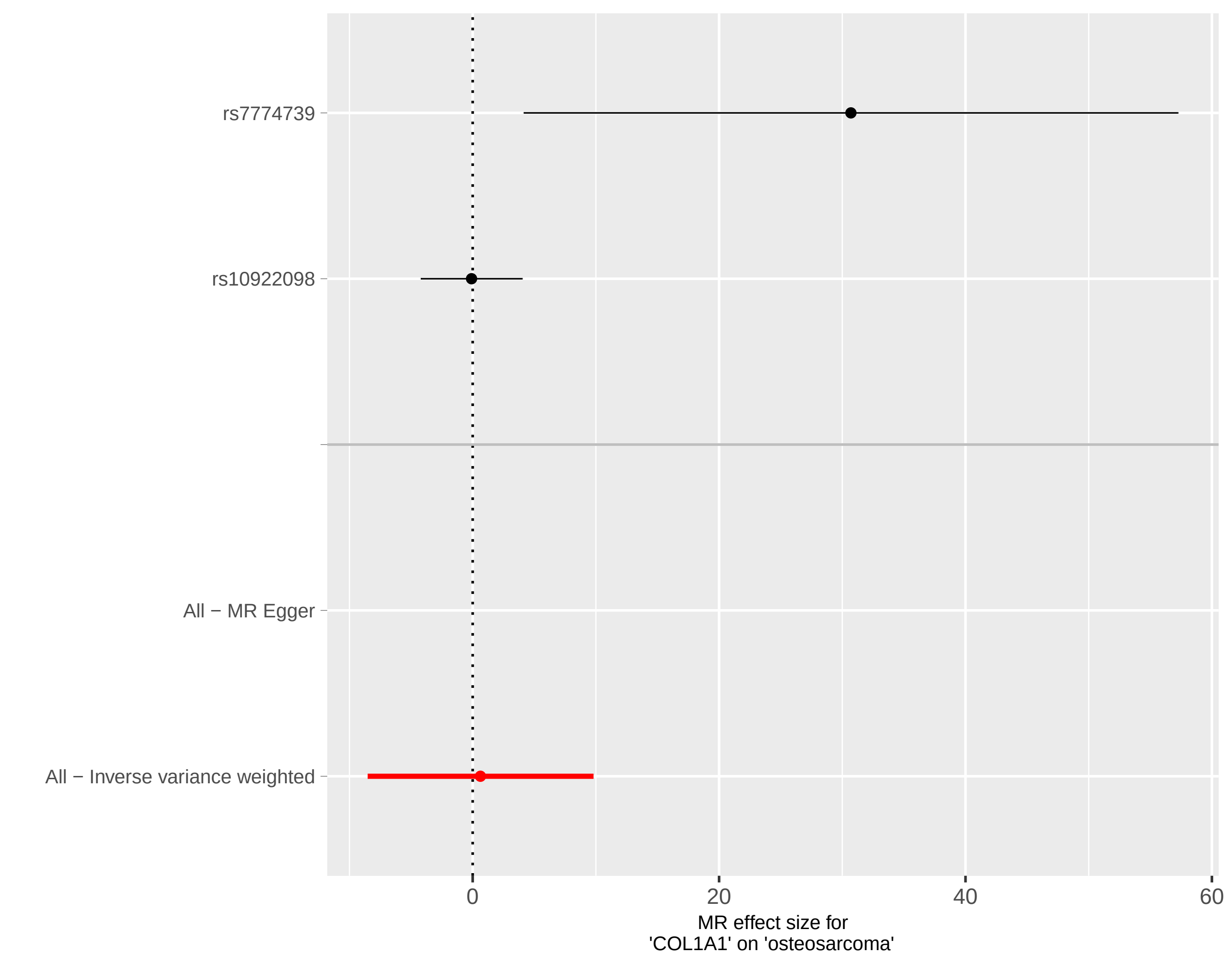
rs7774739
rs10922098
All − MR Egger
All − Inverse variance weighted
0
20
40
60
MR effect size for
'COL1A1' on 'osteosarcoma'

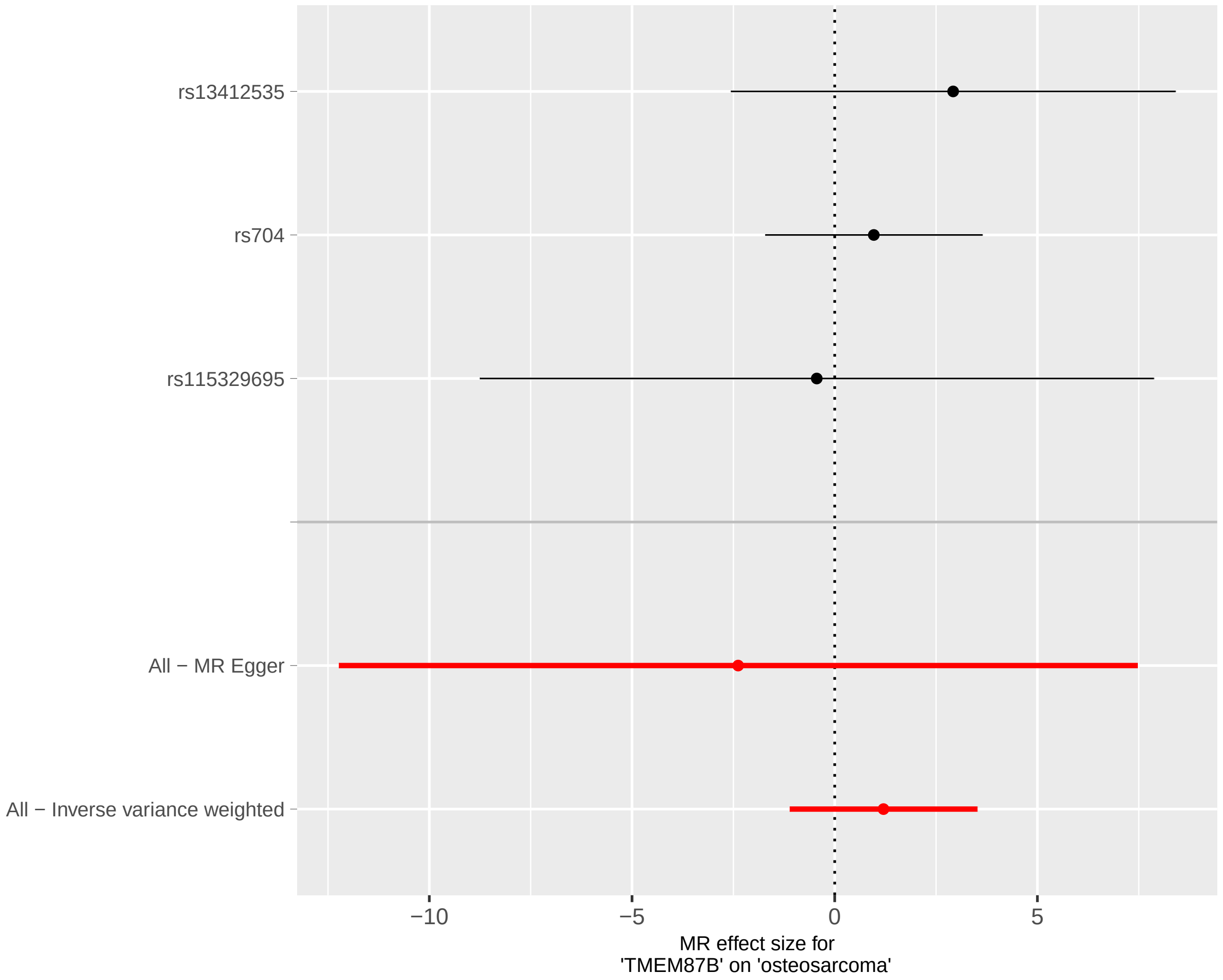

rs13412535
rs704
rs115329695
All − MR Egger
All − Inverse variance weighted
−10
−5
0
5
MR effect size for
'TMEM87B' on 'osteosarcoma'

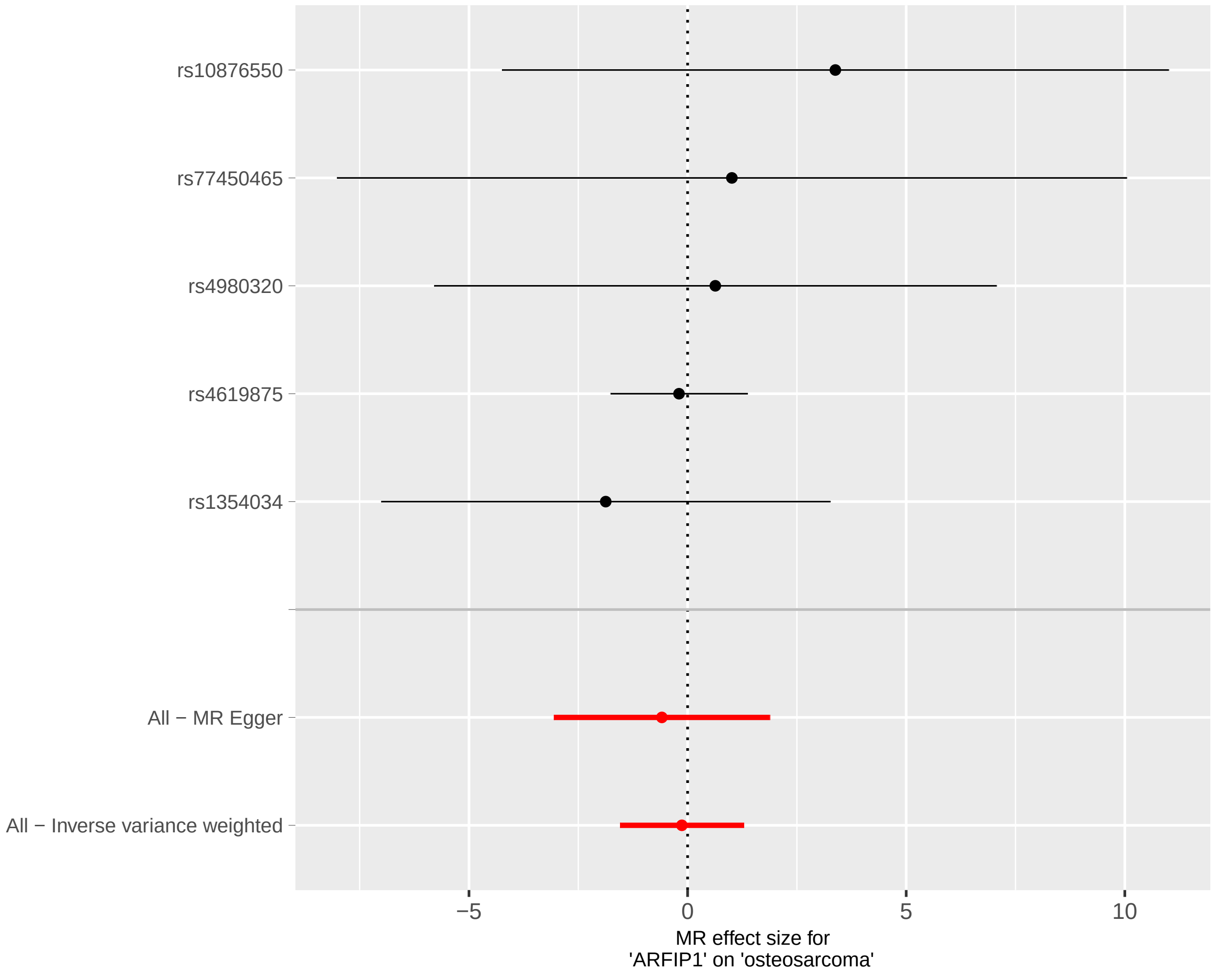
rs10876550
rs77450465
rs4980320
rs4619875
rs1354034
All − MR Egger
All − Inverse variance weighted
−5
0
5
10
MR effect size for
'ARFIP1' on 'osteosarcoma'

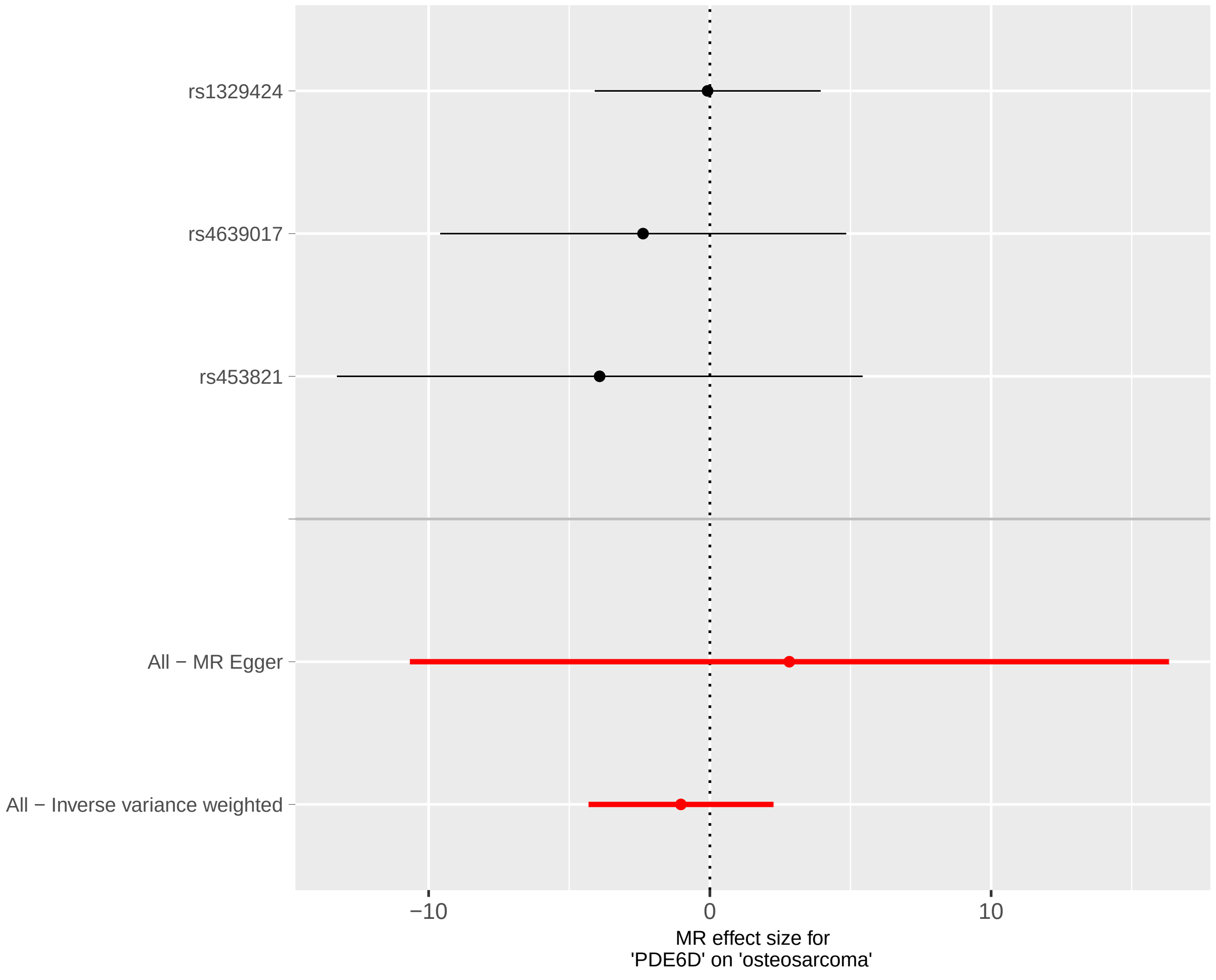
rs1329424
rs4639017
rs453821
All − MR Egger
All − Inverse variance weighted
−10
0
10
MR effect size for
'PDE6D' on 'osteosarcoma'

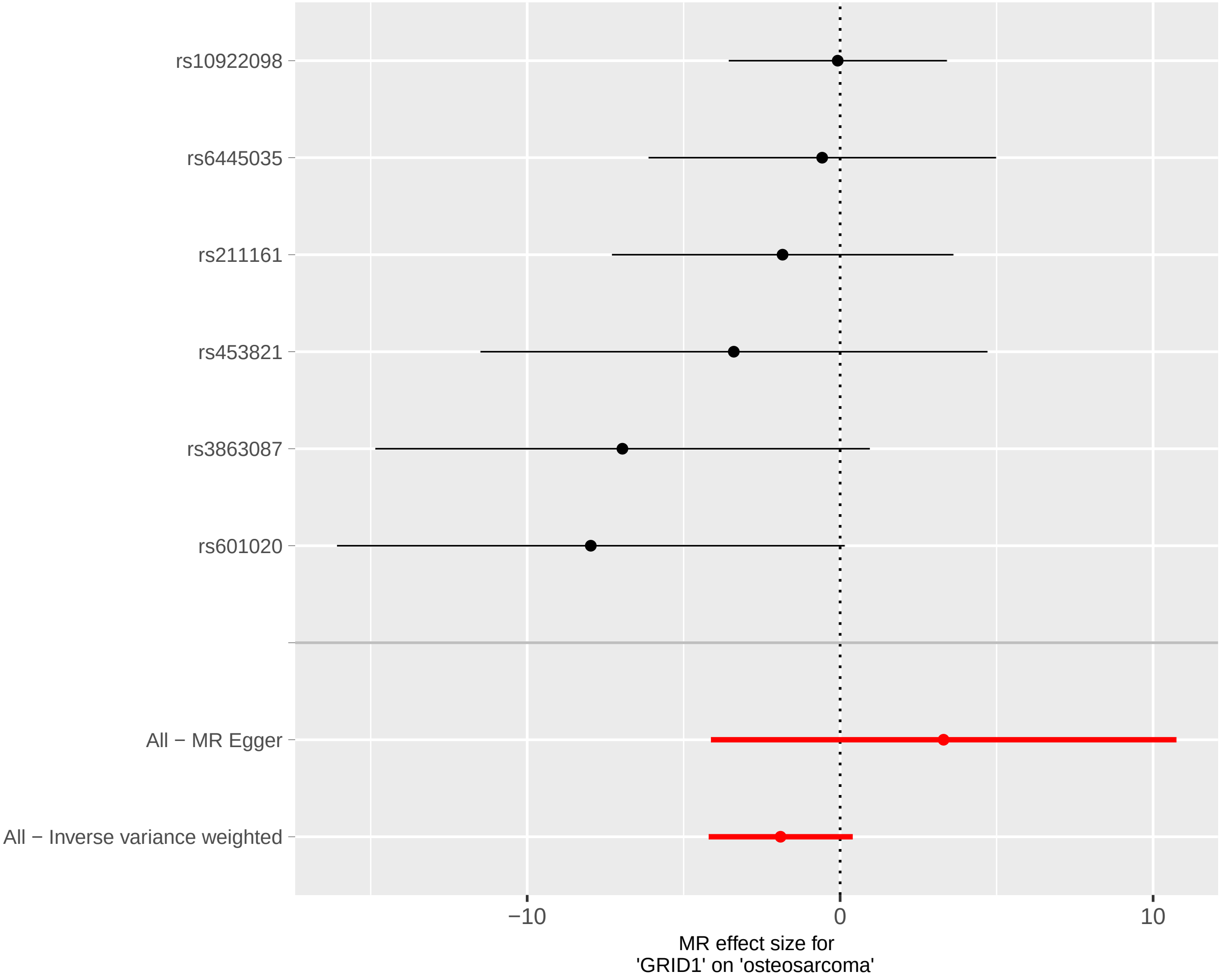
rs10922098
rs6445035
rs211161
rs453821
rs3863087
rs601020
All − MR Egger
All − Inverse variance weighted
−10
0
10
MR effect size for
'GRID1' on 'osteosarcoma'

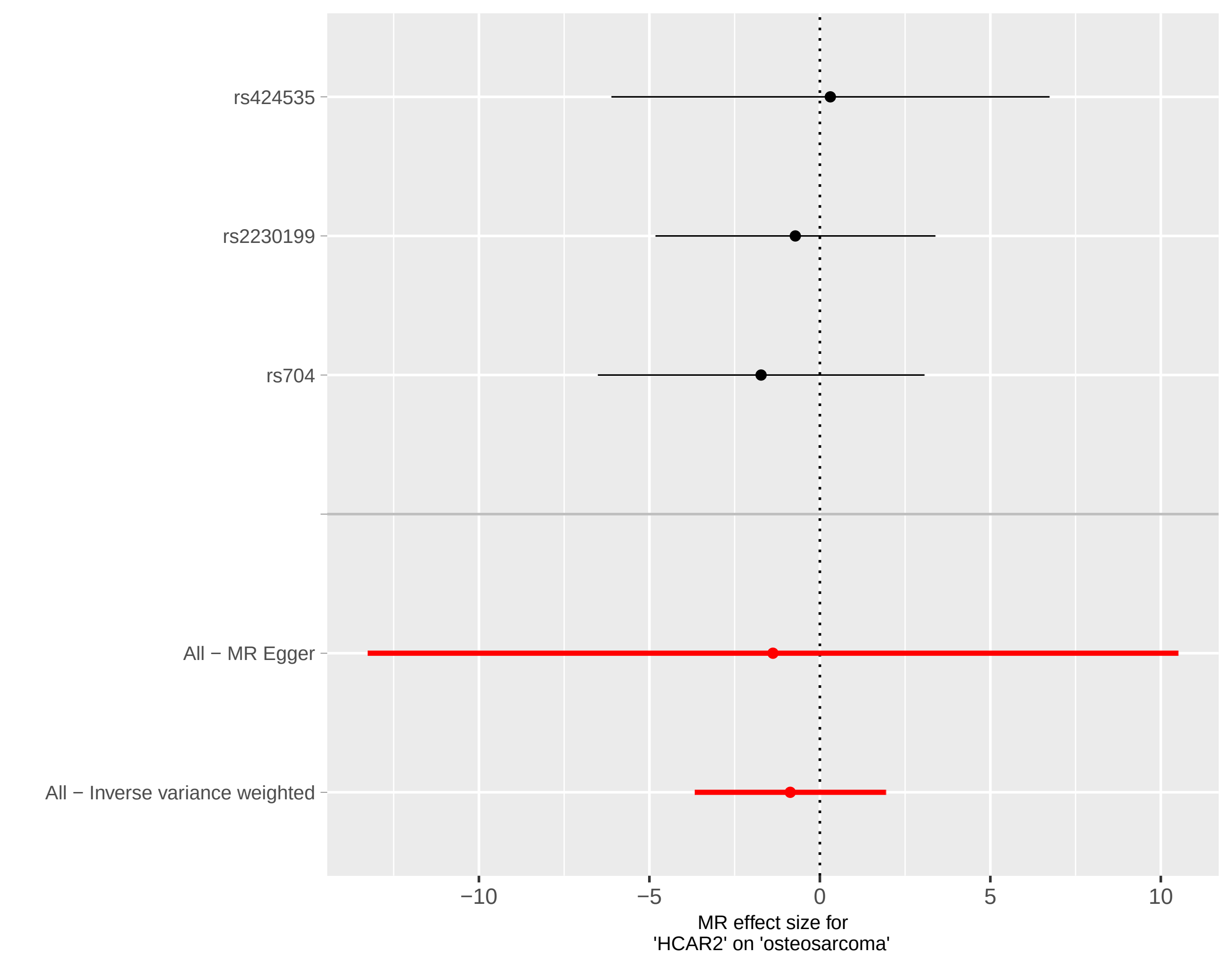
rs424535
rs2230199
rs704
All − MR Egger
All − Inverse variance weighted
−10
−5
0
5
10
MR effect size for
'HCAR2' on 'osteosarcoma'

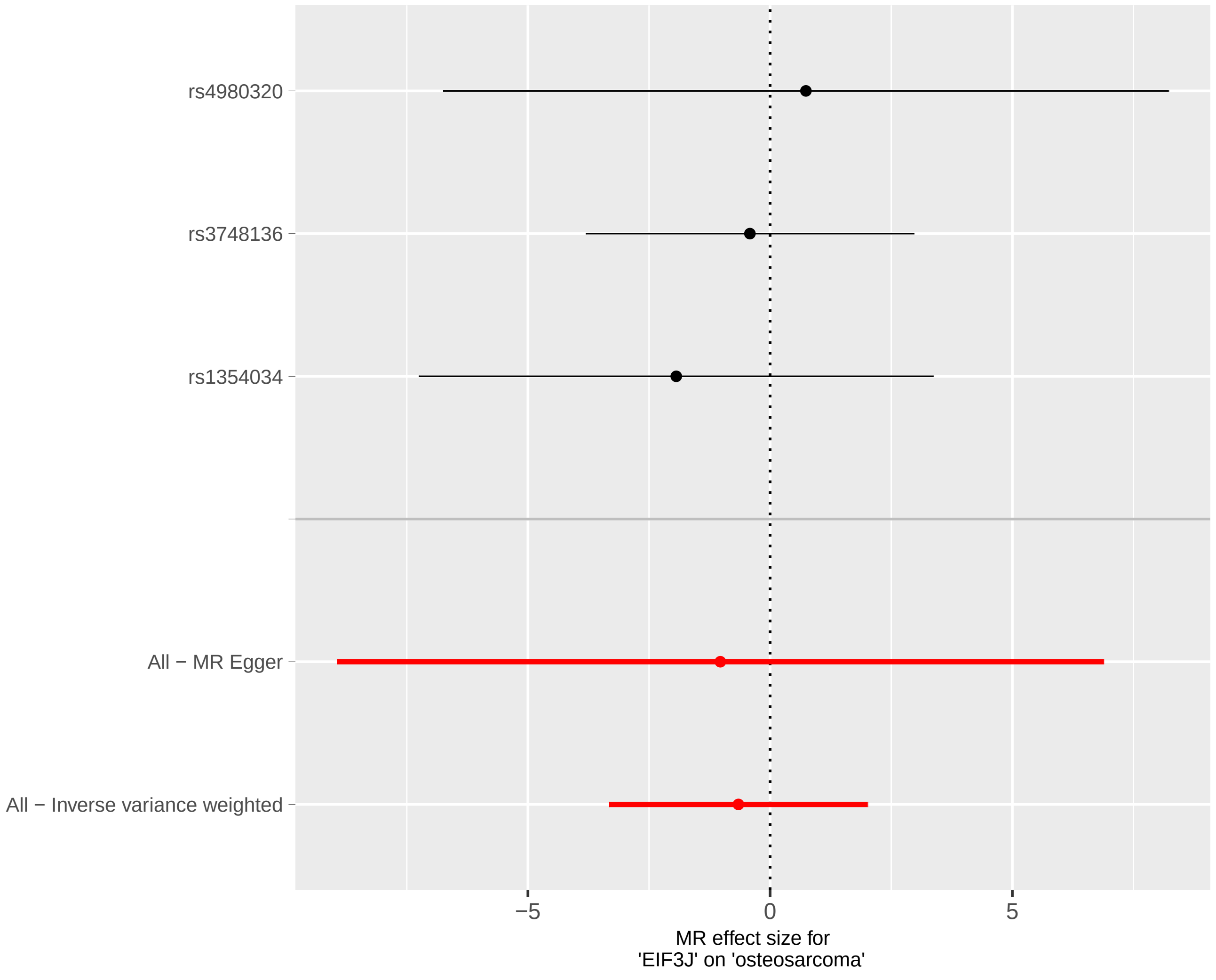
rs4980320
rs3748136
rs1354034
All − MR Egger
All − Inverse variance weighted
−5
0
5
MR effect size for
'EIF3J' on 'osteosarcoma'

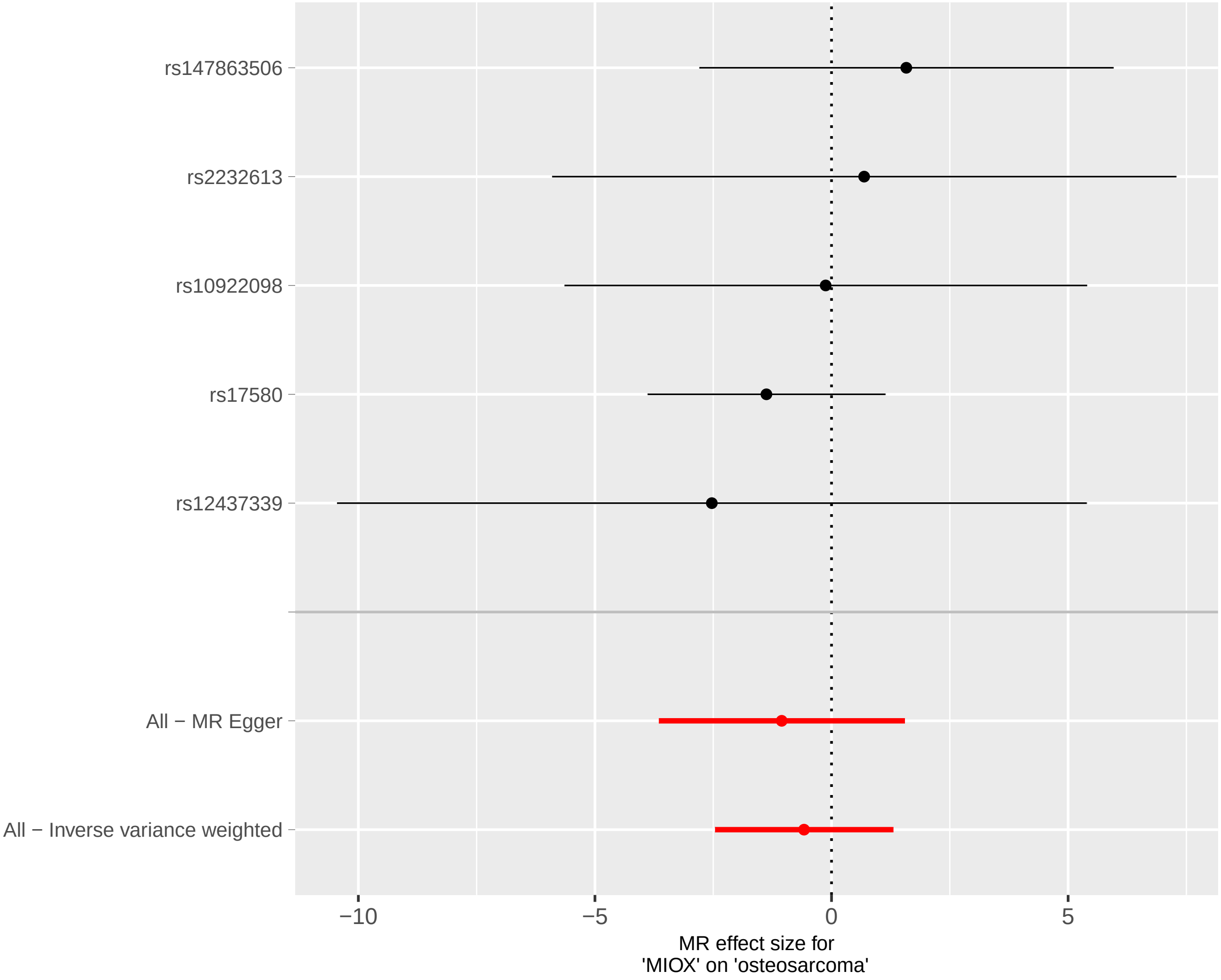
rs147863506
rs2232613
rs10922098
rs17580
rs12437339
All − MR Egger
All − Inverse variance weighted
−10
−5
0
5
MR effect size for
'MIOX' on 'osteosarcoma'

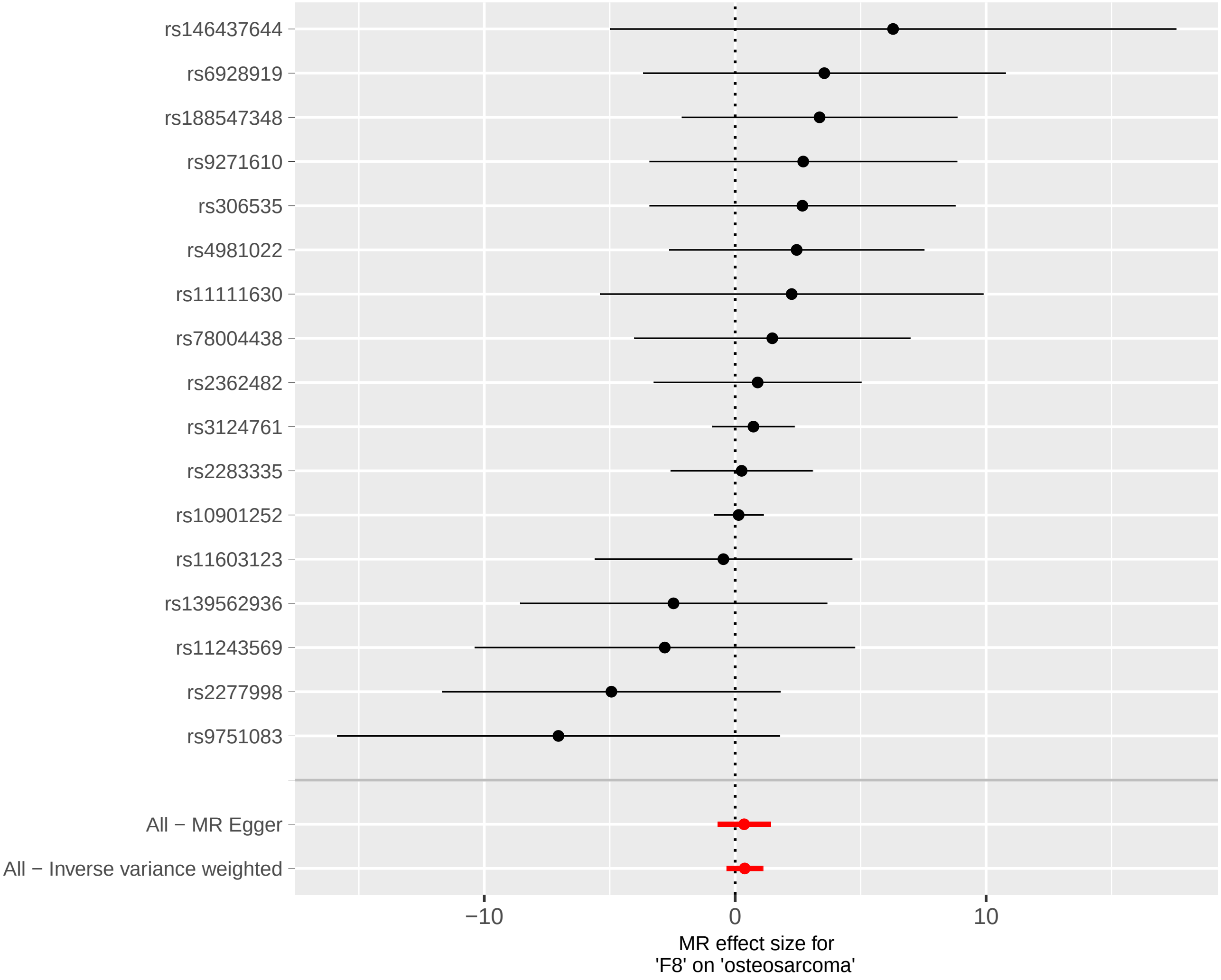

rs146437644
rs6928919
rs188547348
rs9271610
rs306535
rs4981022
rs11111630
rs78004438
rs2362482
rs3124761
rs2283335
rs10901252
rs11603123
rs139562936
rs11243569
rs2277998
rs9751083
All − MR Egger
All − Inverse variance weighted
−10
0
10
MR effect size for
'F8' on 'osteosarcoma'

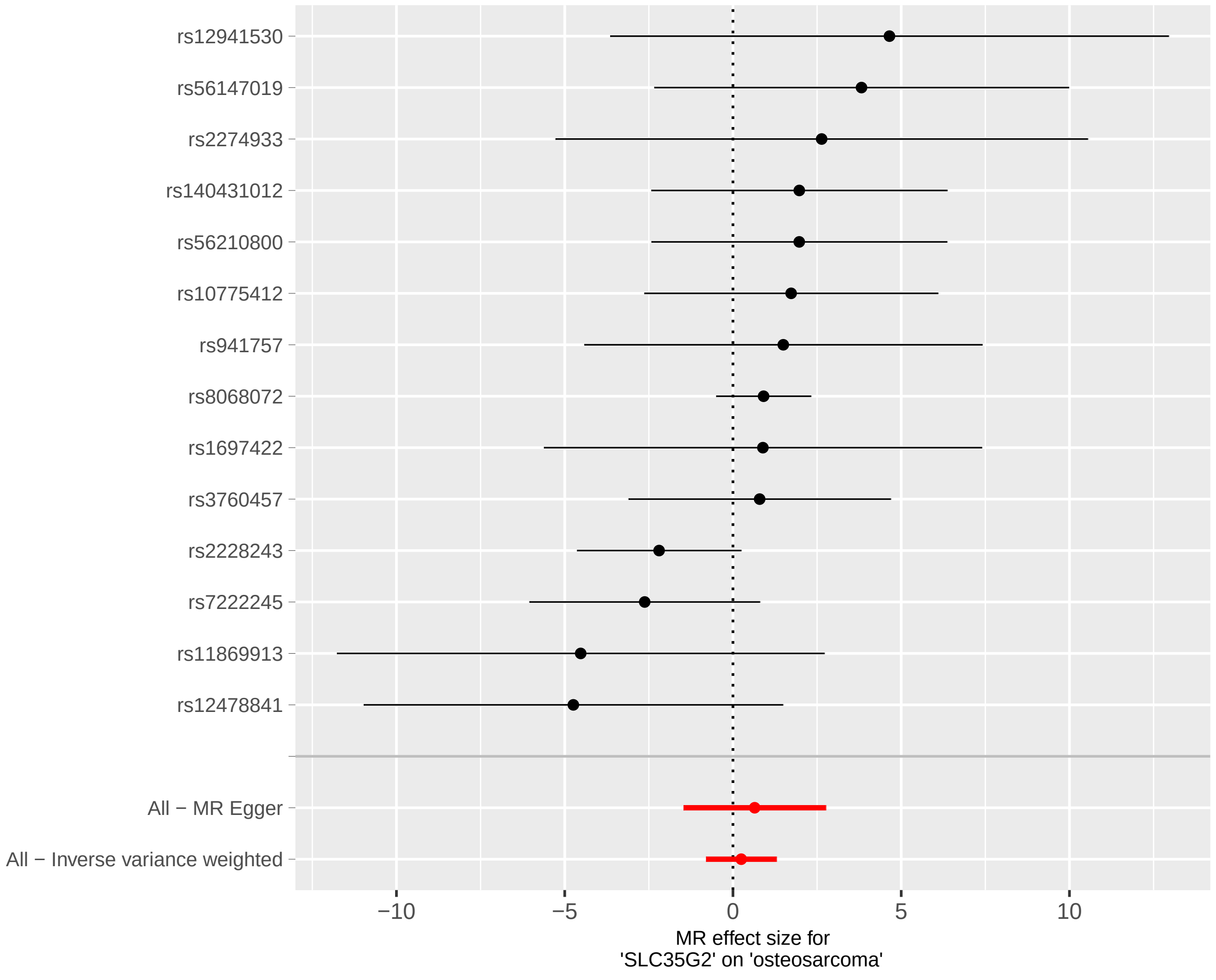
rs12941530
rs56147019
rs2274933
rs140431012
rs56210800
rs10775412
rs941757
rs8068072
rs1697422
rs3760457
rs2228243
rs7222245
rs11869913
rs12478841
All − MR Egger
All − Inverse variance weighted
−10
−5
0
5
10
MR effect size for
'SLC35G2' on 'osteosarcoma'

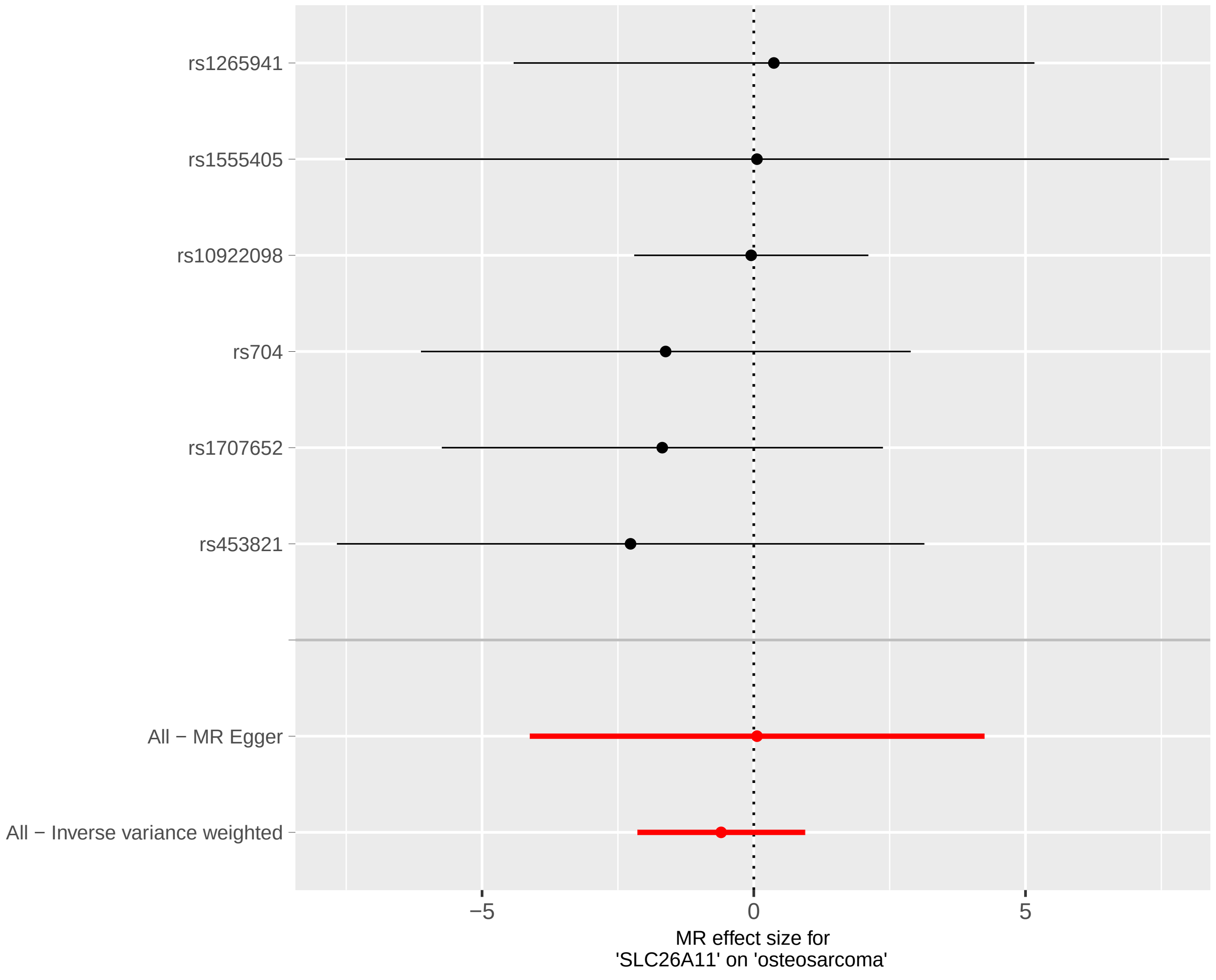
rs1265941
rs1555405
rs10922098
rs704
rs1707652
rs453821
All − MR Egger
All − Inverse variance weighted
−5
0
5
MR effect size for
'SLC26A11' on 'osteosarcoma'

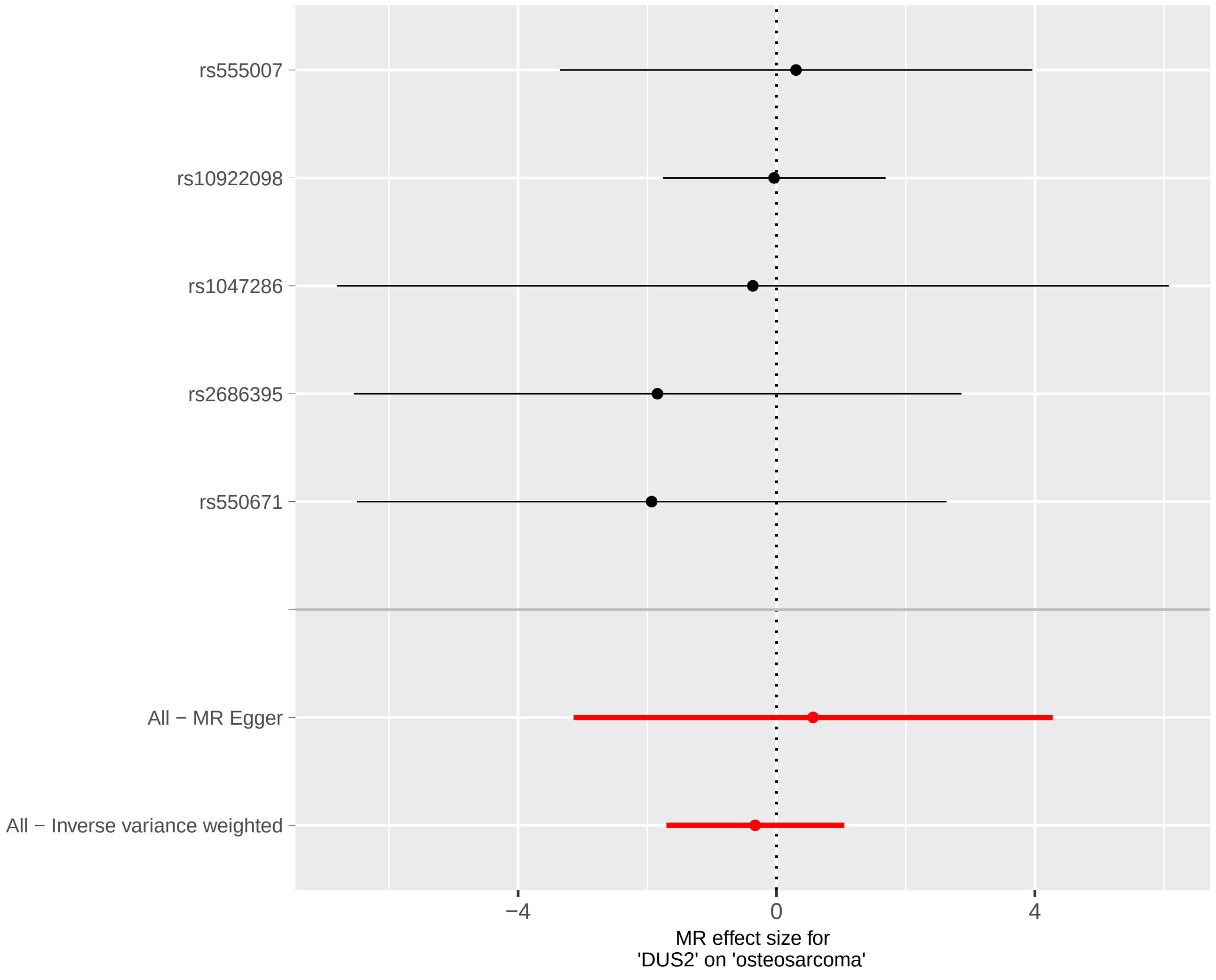
rs555007
rs10922098
rs1047286
rs2686395
rs550671
All − MR Egger
All − Inverse variance weighted
−4
0
4
MR effect size for
'DUS2' on 'osteosarcoma'

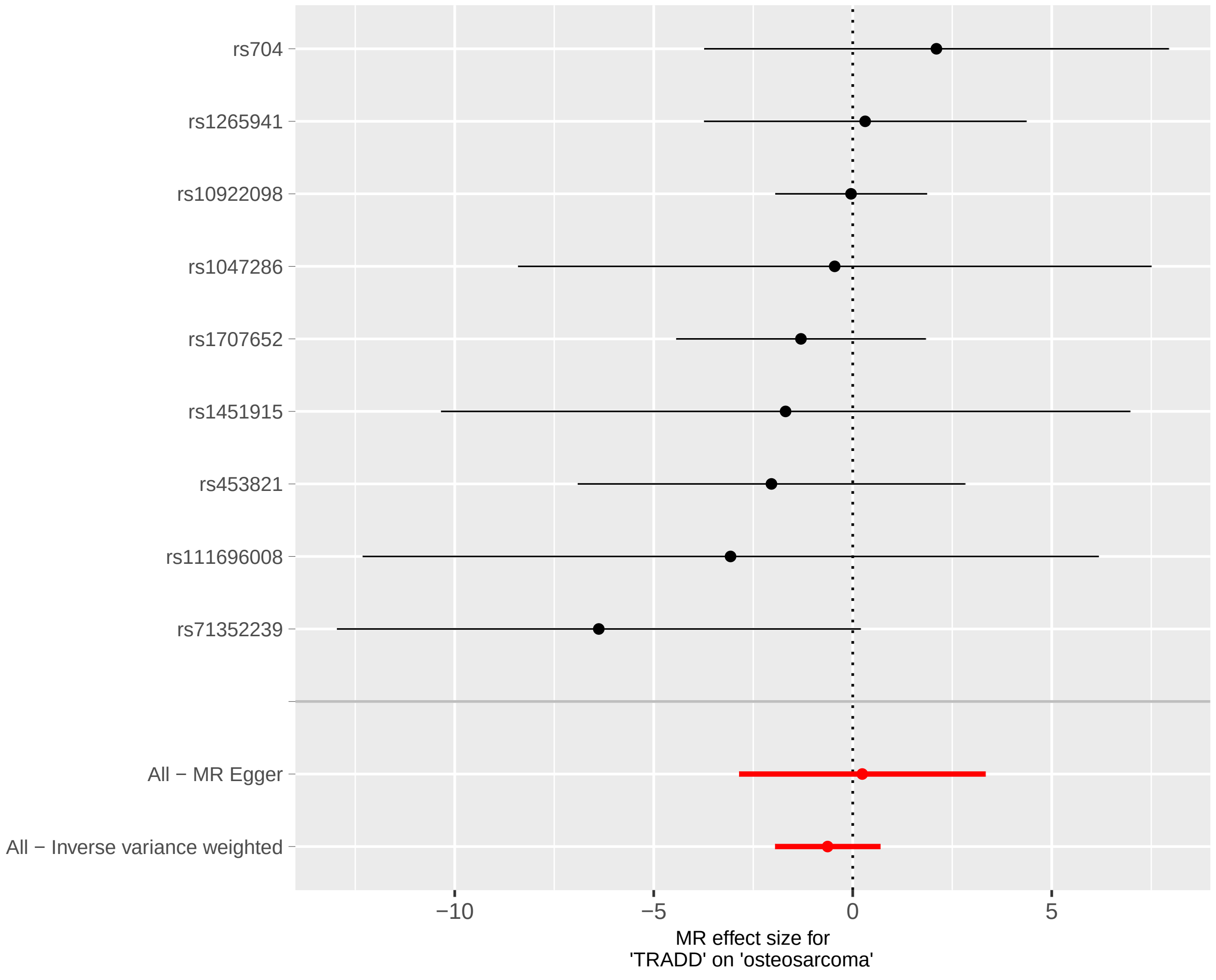

rs704
rs1265941
rs10922098
rs1047286
rs1707652
rs1451915
rs453821
rs111696008
rs71352239
All − MR Egger
All − Inverse variance weighted
−10
−5
0
5
MR effect size for
'TRADD' on 'osteosarcoma'

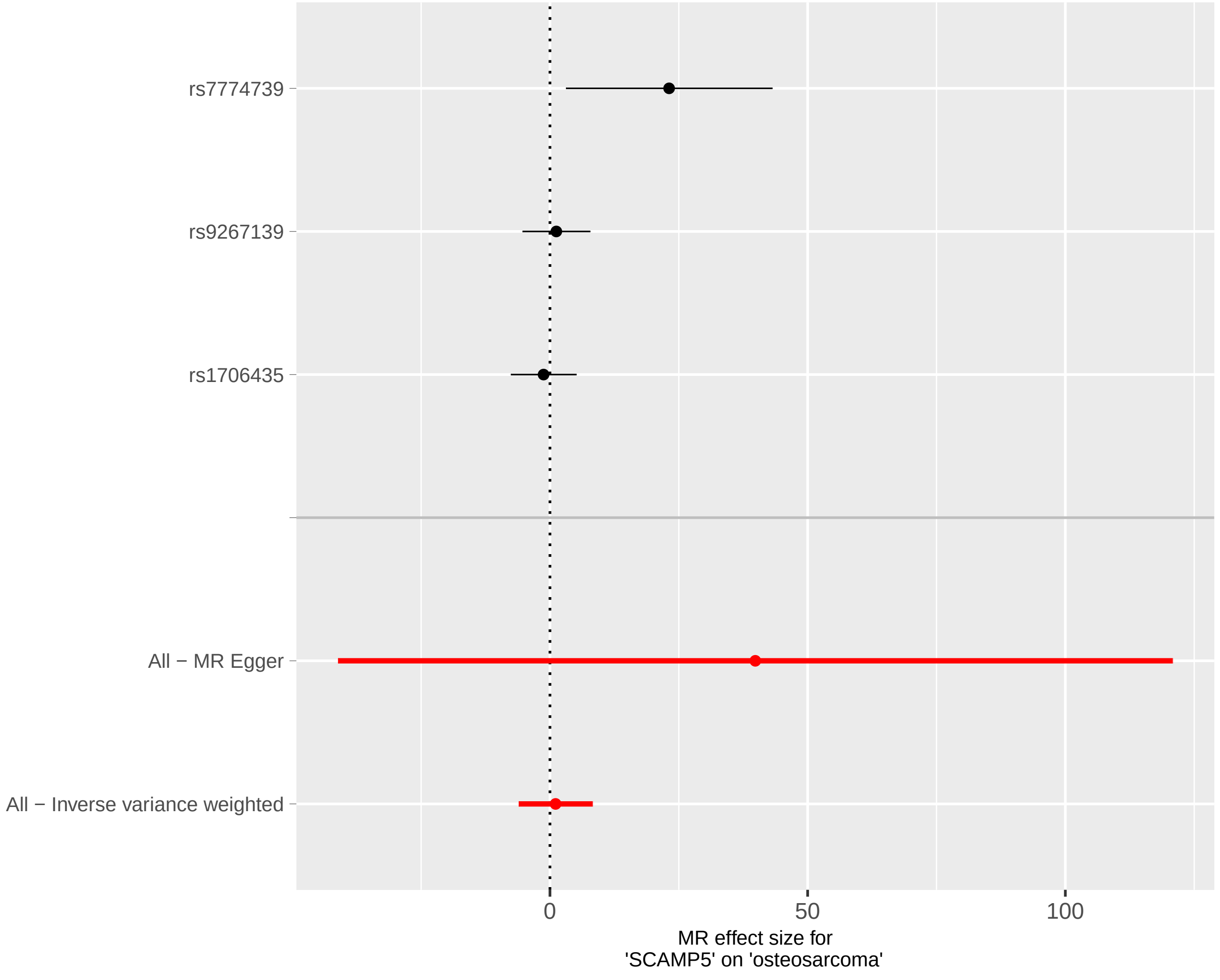

rs7774739
rs9267139
rs1706435
All − MR Egger
All − Inverse variance weighted
0
50
100
MR effect size for
'SCAMP5' on 'osteosarcoma'

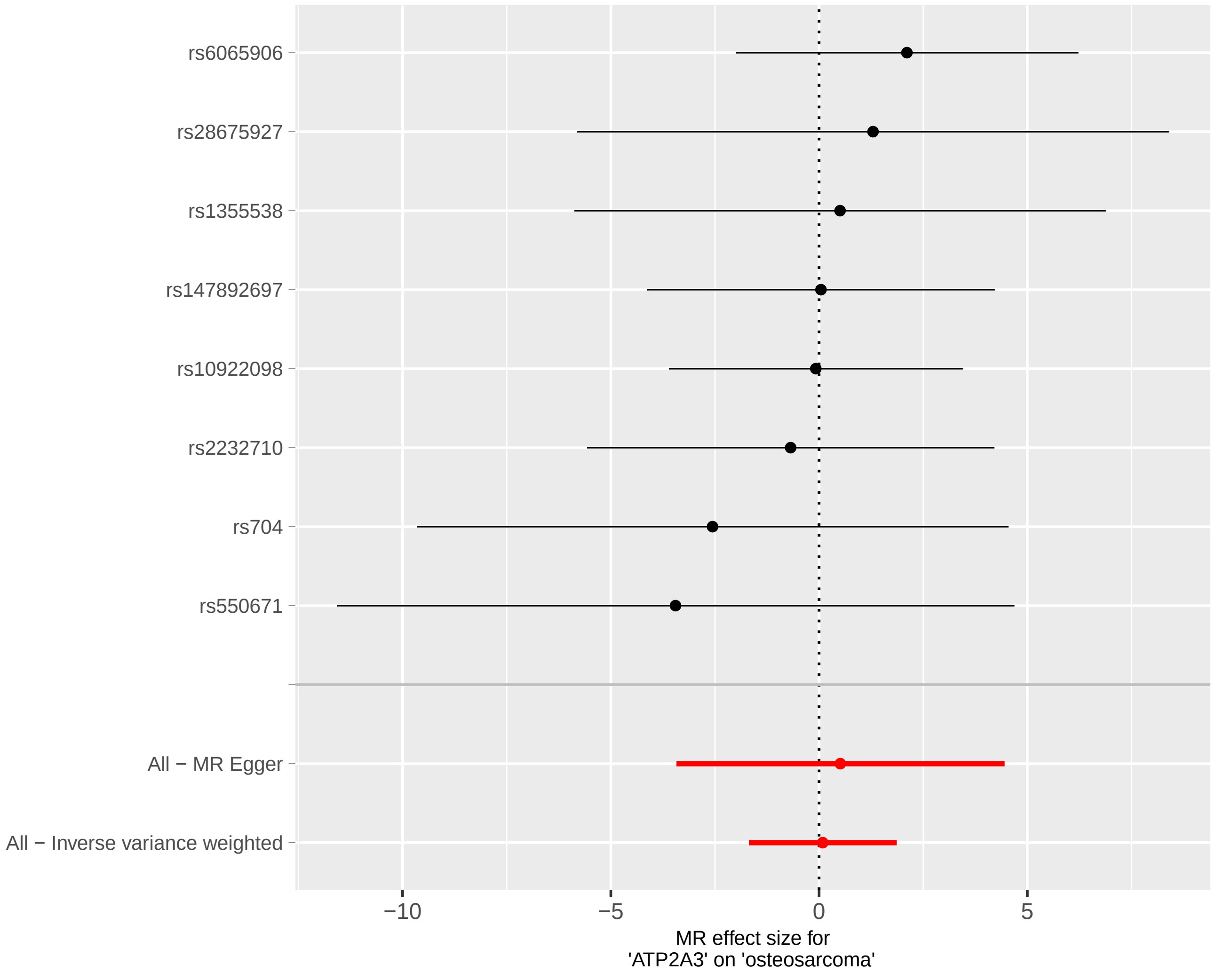

rs6065906
rs28675927
rs1355538
rs147892697
rs10922098
rs2232710
rs704
rs550671
All − MR Egger
All − Inverse variance weighted
−10
−5
0
5
MR effect size for
'ATP2A3' on 'osteosarcoma'

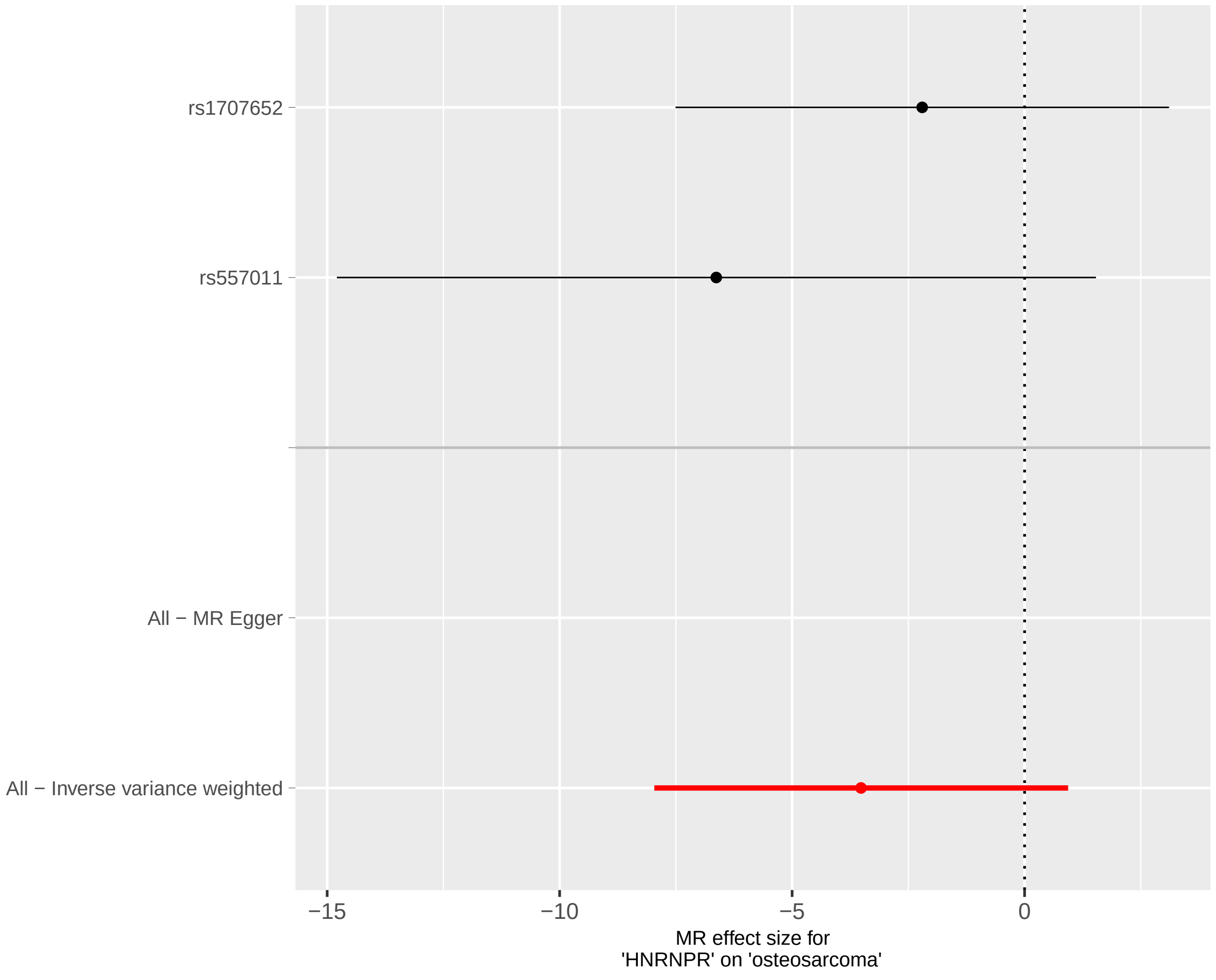
rs1707652
rs557011
All − MR Egger
All − Inverse variance weighted
−15
−10
−5
0
MR effect size for
'HNRNPR' on 'osteosarcoma'

Insufficient number of SNPs

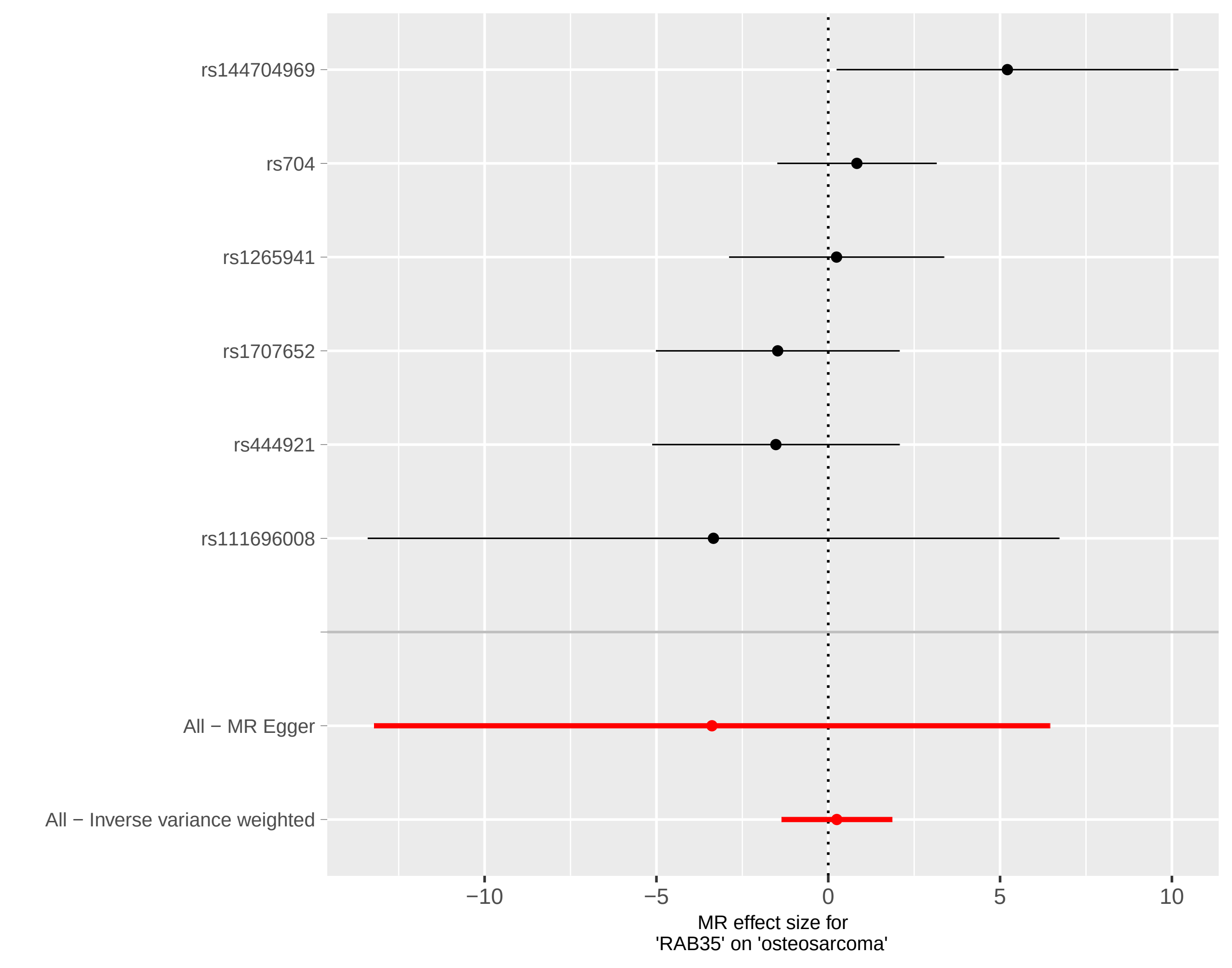

rs144704969
rs704
rs1265941
rs1707652
rs444921
rs111696008
All − MR Egger
All − Inverse variance weighted
−10
−5
0
5
10
MR effect size for
'RAB35' on 'osteosarcoma'

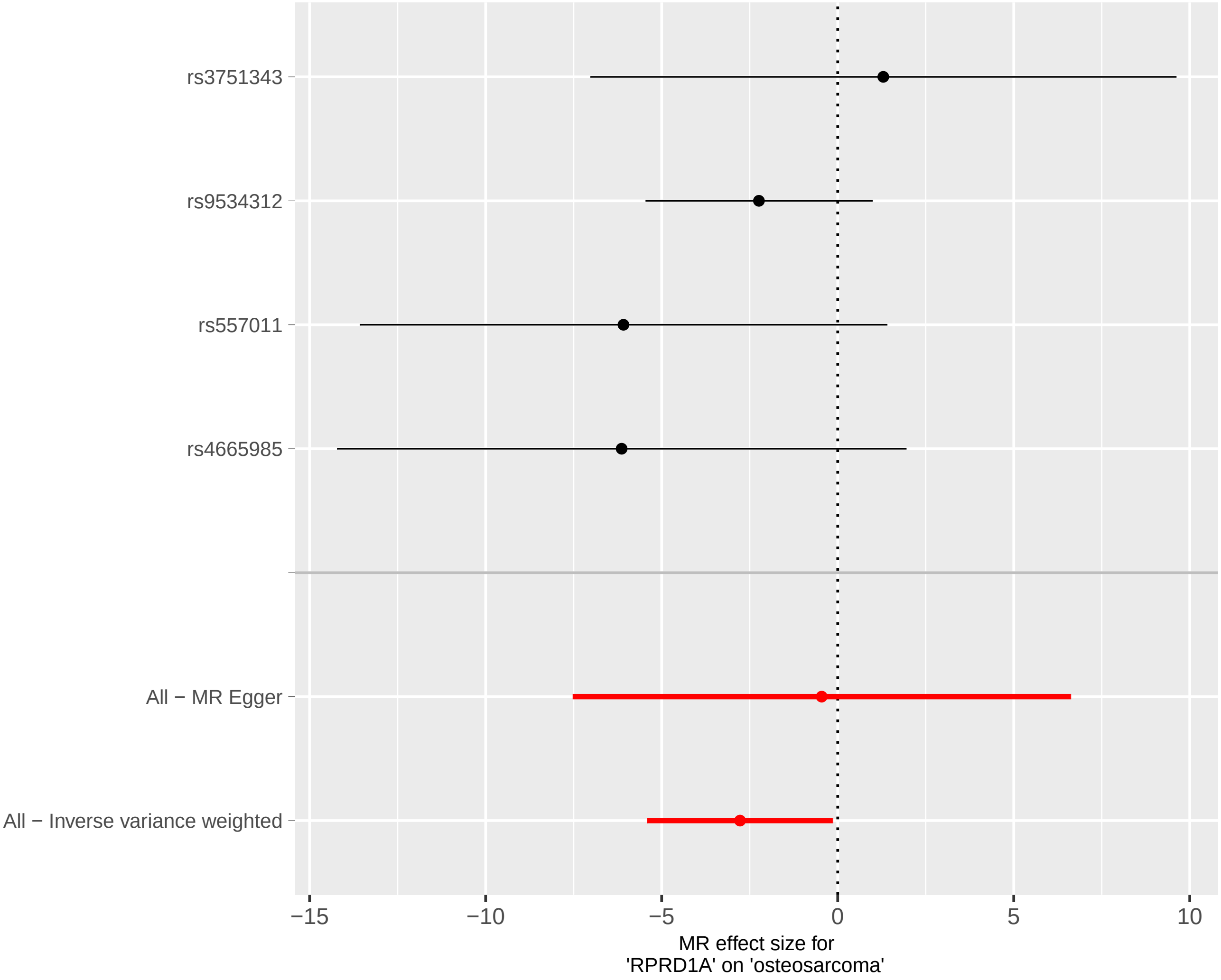

rs3751343
rs9534312
rs557011
rs4665985
All − MR Egger
All − Inverse variance weighted
−15
−10
−5
0
5
10
MR effect size for
'RPRD1A' on 'osteosarcoma'

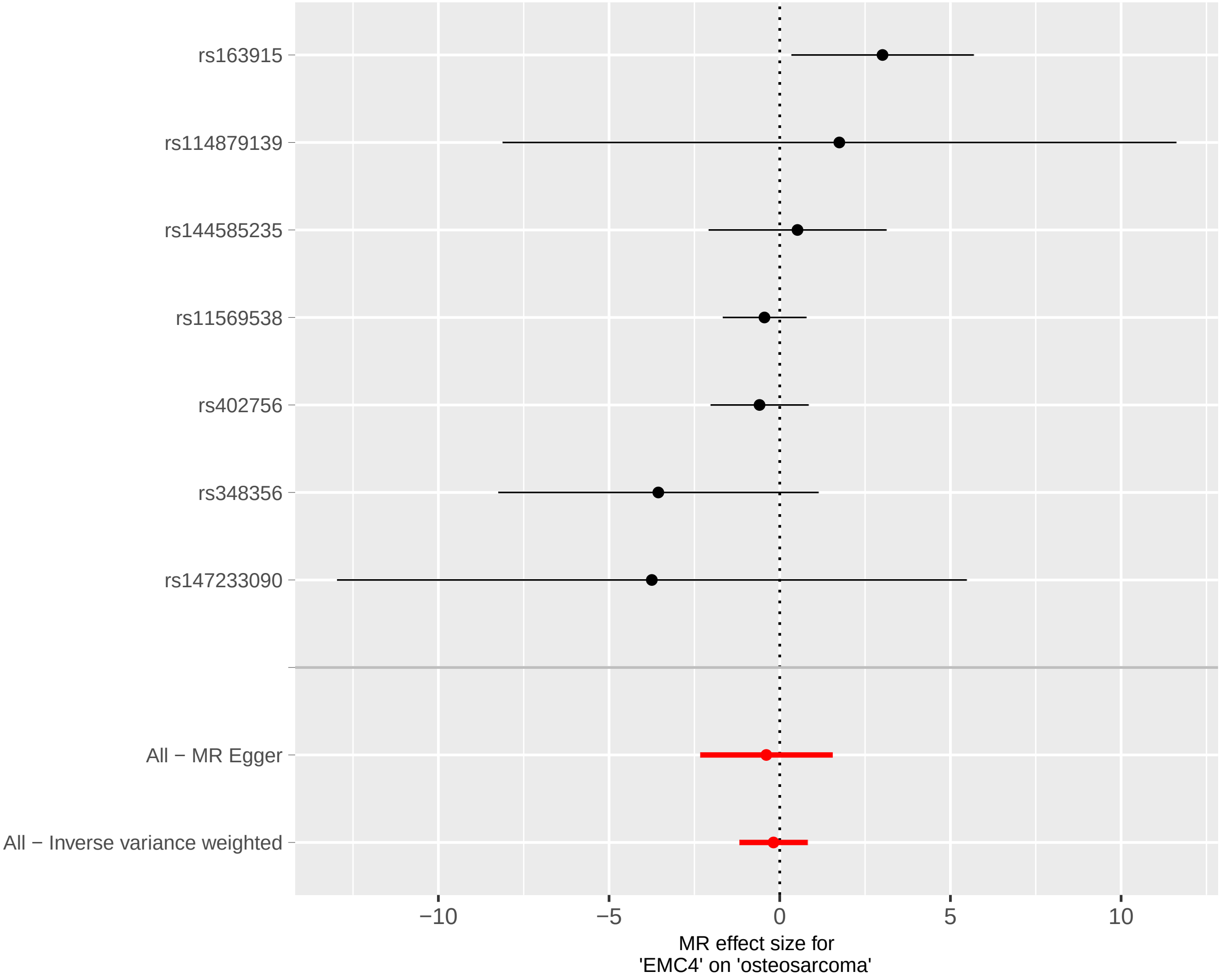
rs163915
rs114879139
rs144585235
rs11569538
rs402756
rs348356
rs147233090
All − MR Egger
All − Inverse variance weighted
−10
−5
0
5
10
MR effect size for
'EMC4' on 'osteosarcoma'

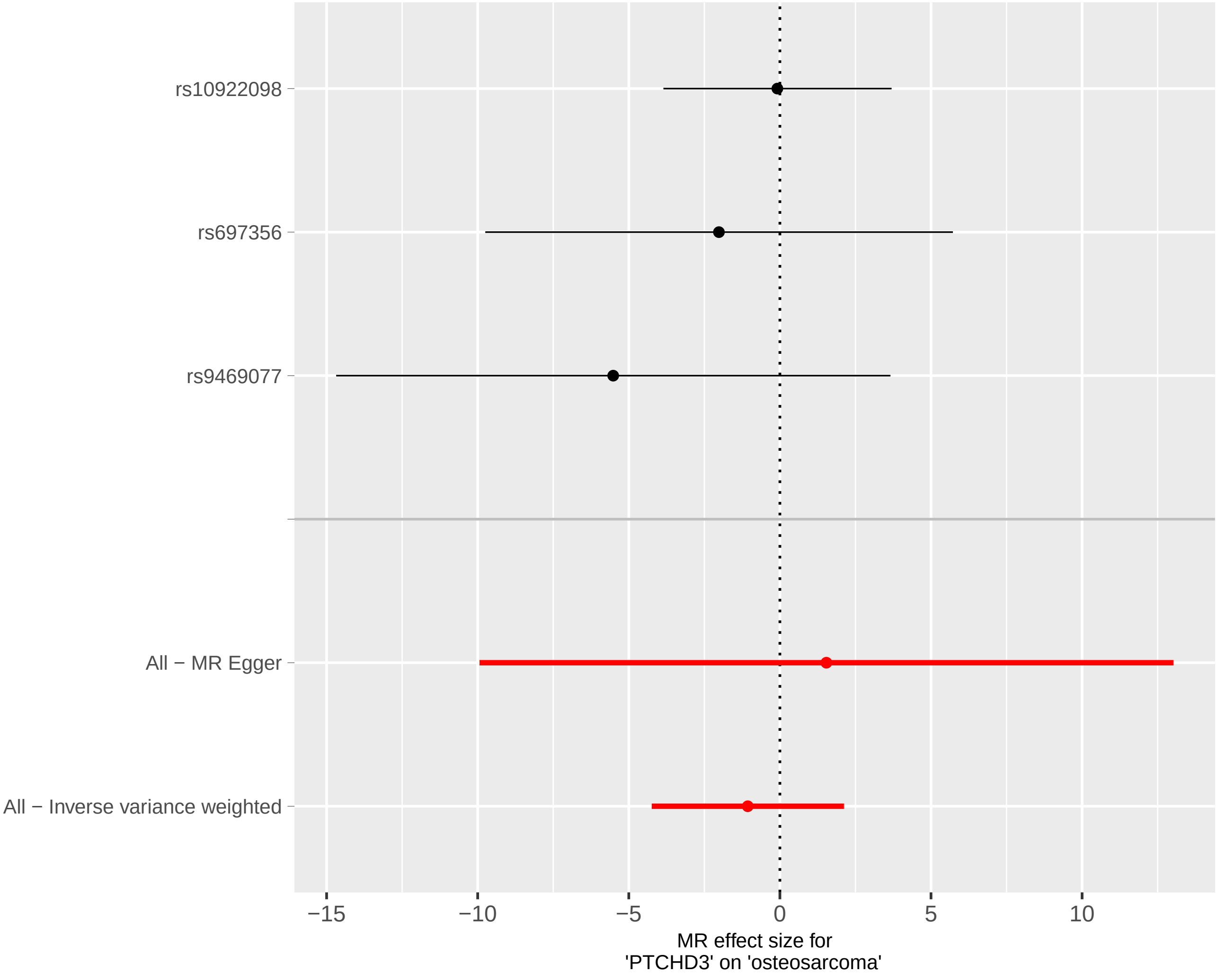
rs10922098
rs697356
rs9469077
All − MR Egger
All − Inverse variance weighted
−15
−10
−5
0
5
10
MR effect size for
'PTCHD3' on 'osteosarcoma'

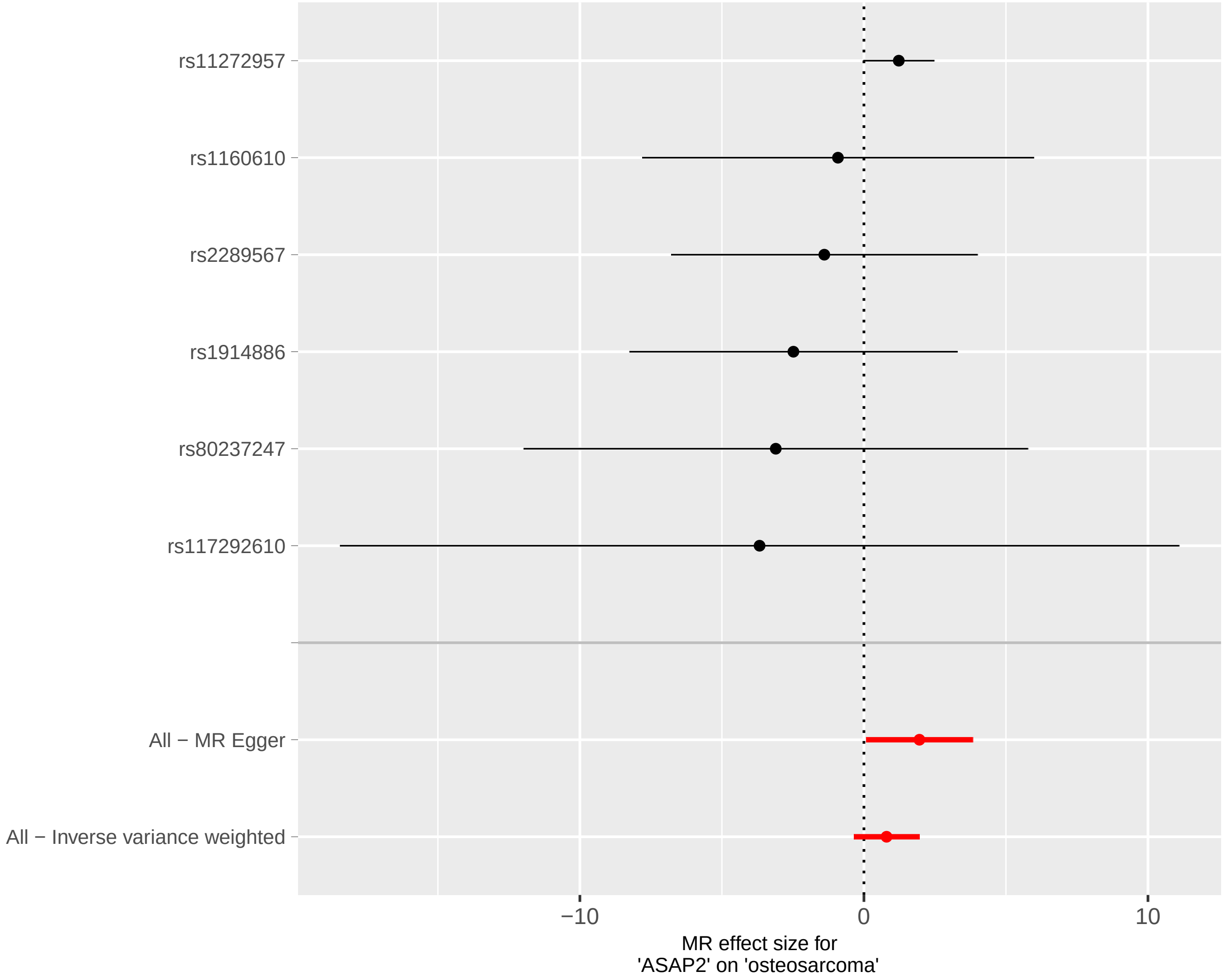

rs11272957
rs1160610
rs2289567
rs1914886
rs80237247
rs117292610
All − MR Egger
All − Inverse variance weighted
−10
0
10
MR effect size for
'ASAP2' on 'osteosarcoma'

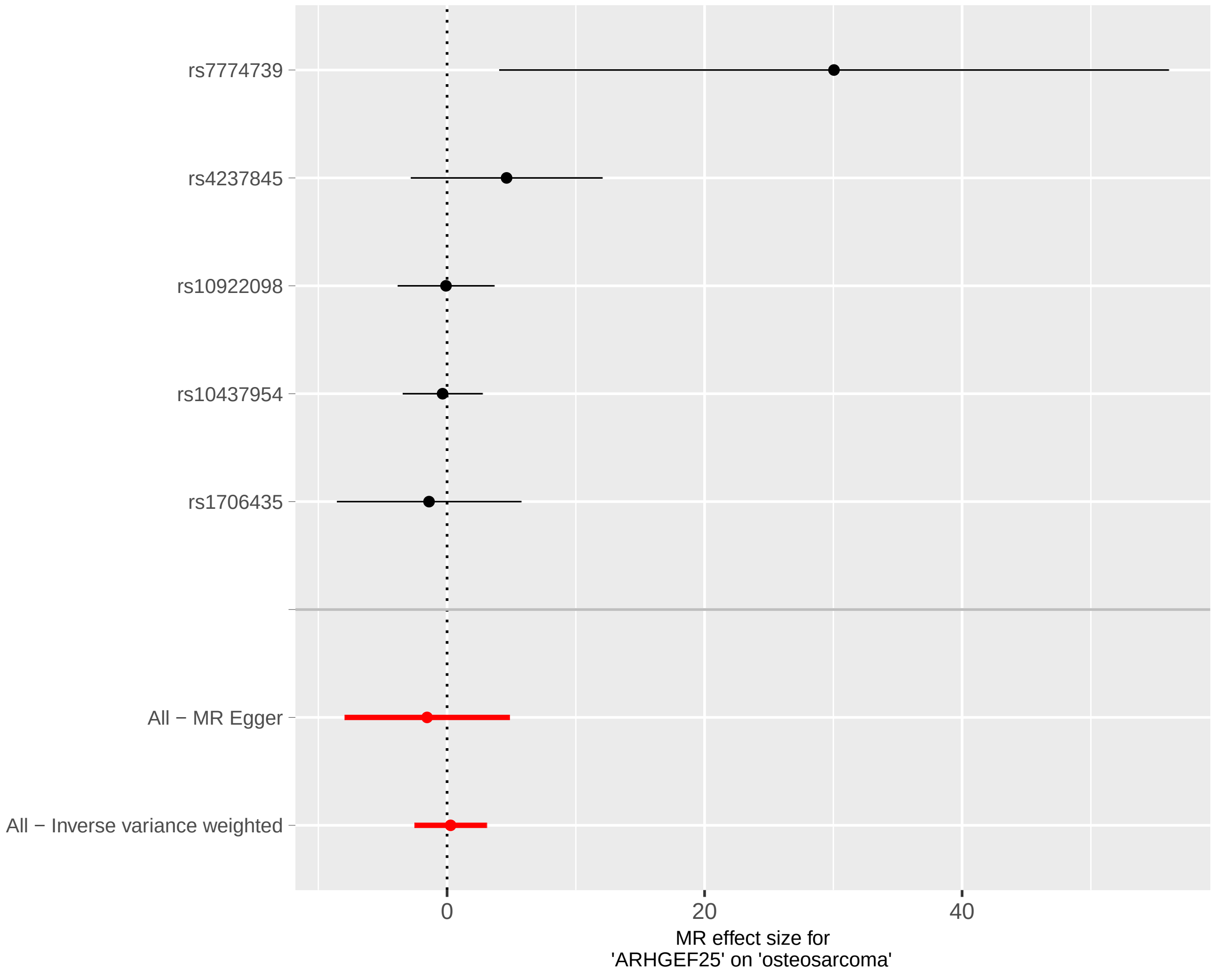

rs7774739
rs4237845
rs10922098
rs10437954
rs1706435
All − MR Egger
All − Inverse variance weighted
0
20
40
MR effect size for
'ARHGEF25' on 'osteosarcoma'

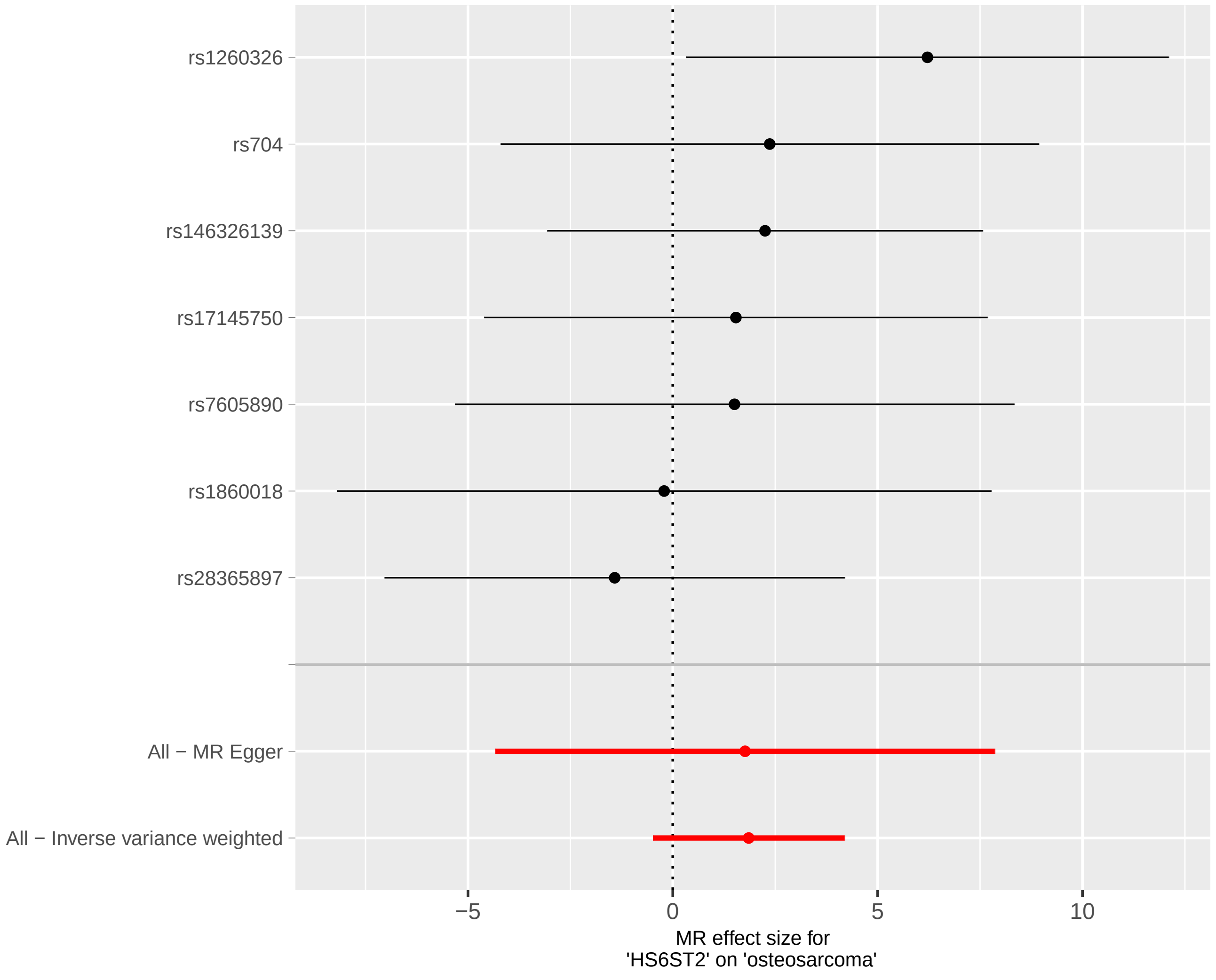

rs1260326
rs704
rs146326139
rs17145750
rs7605890
rs1860018
rs28365897
All − MR Egger
All − Inverse variance weighted
−5
0
5
10
MR effect size for
'HS6ST2' on 'osteosarcoma'

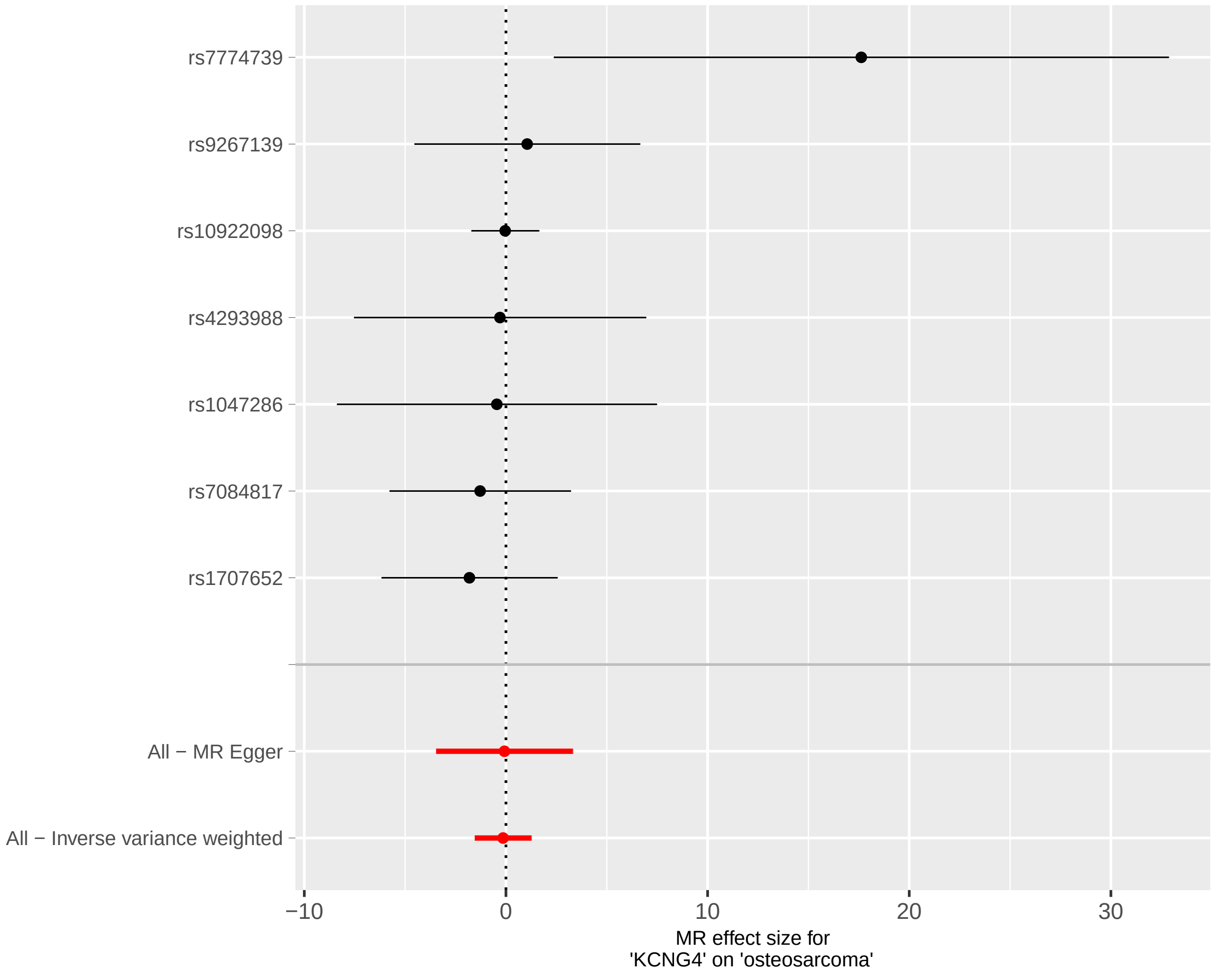

rs7774739
rs9267139
rs10922098
rs4293988
rs1047286
rs7084817
rs1707652
All − MR Egger
All − Inverse variance weighted
−10
0
10
20
30
MR effect size for
'KCNG4' on 'osteosarcoma'

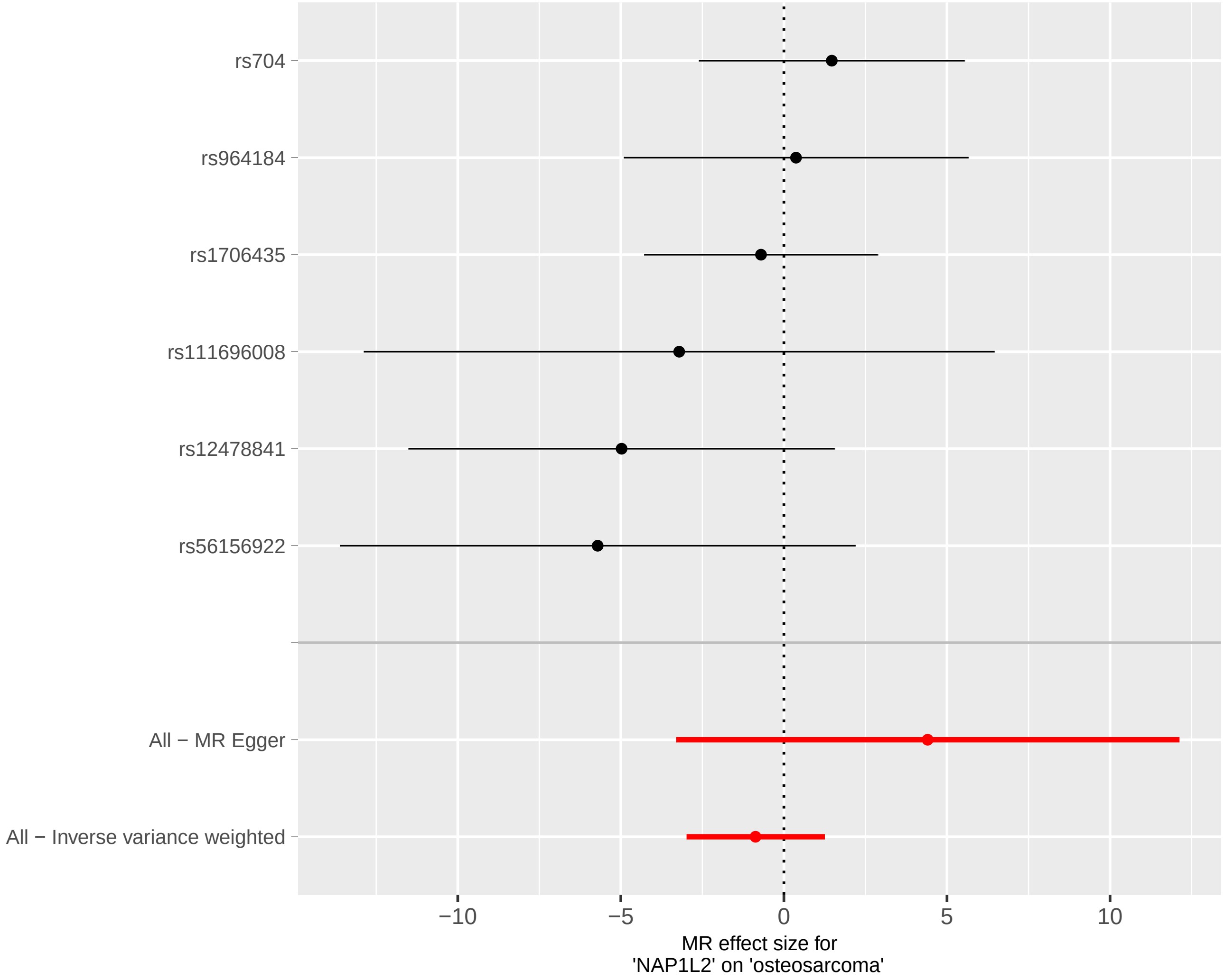

rs704
rs964184
rs1706435
rs111696008
rs12478841
rs56156922
All − MR Egger
All − Inverse variance weighted
−10
−5
0
5
10
MR effect size for
'NAP1L2' on 'osteosarcoma'

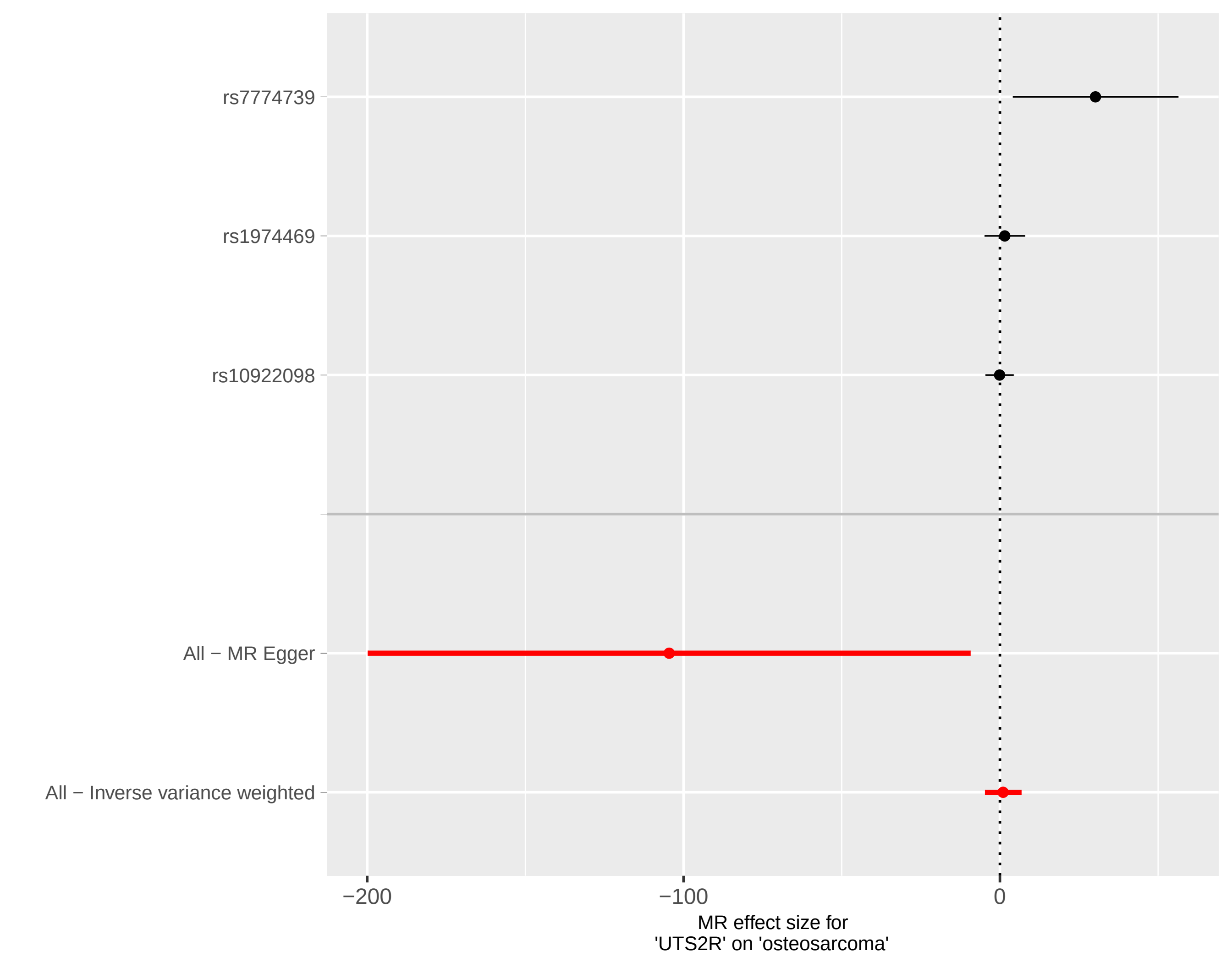

rs7774739
rs1974469
rs10922098
All − MR Egger
All − Inverse variance weighted
−200
−100
0
MR effect size for
'UTS2R' on 'osteosarcoma'

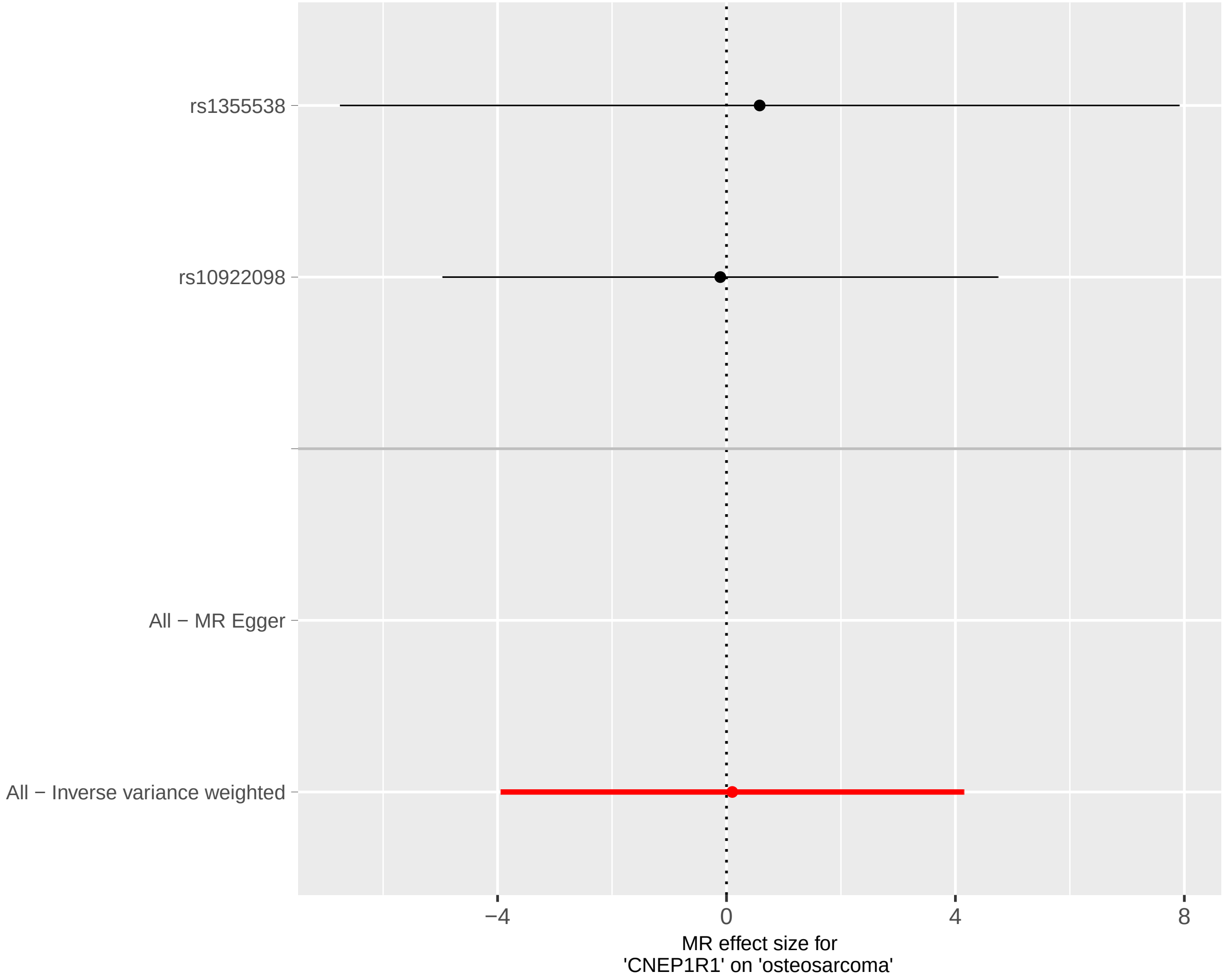
rs1355538
rs10922098
All − MR Egger
All − Inverse variance weighted
−4
0
4
8
MR effect size for
'CNEP1R1' on 'osteosarcoma'

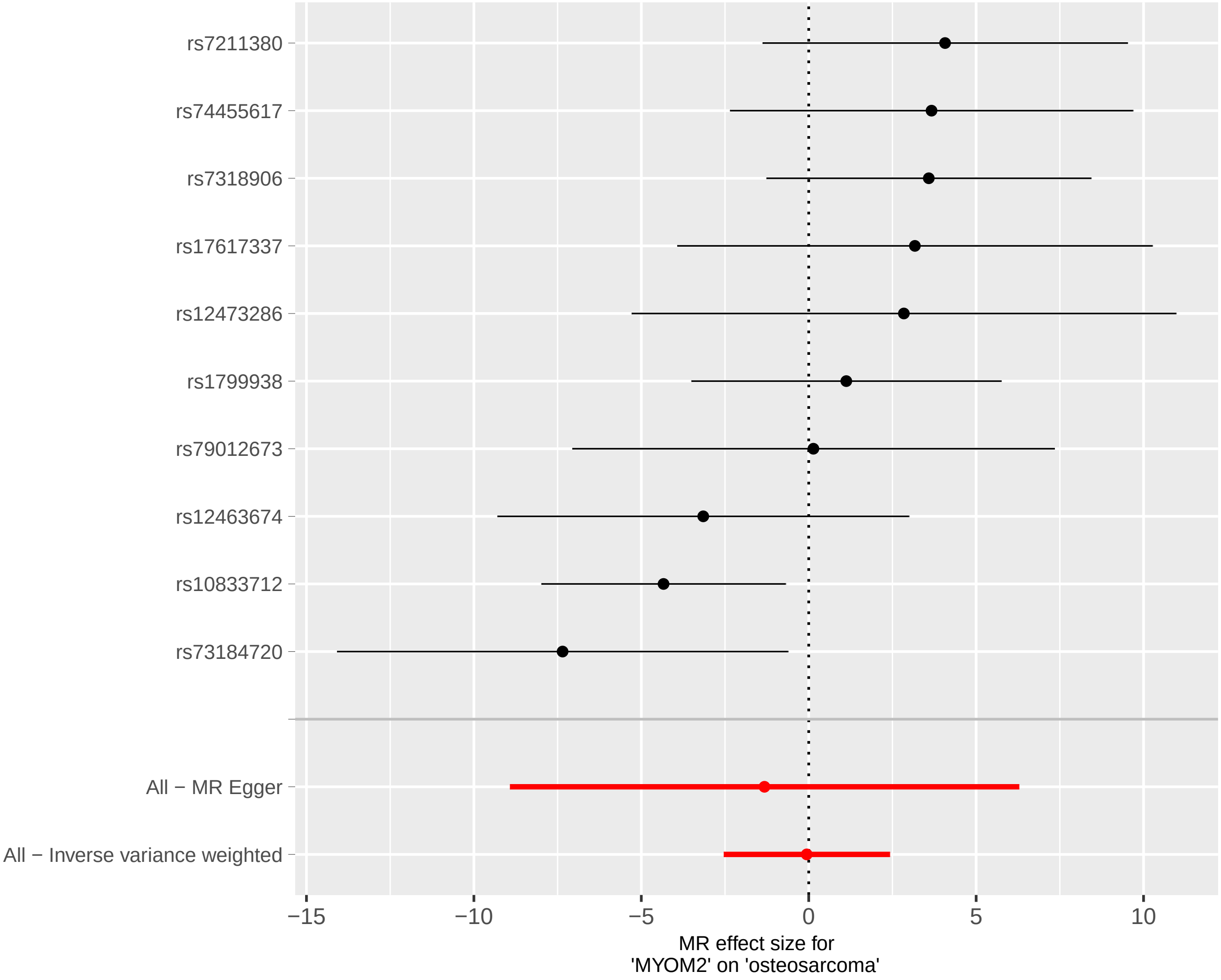

rs7211380
rs74455617
rs7318906
rs17617337
rs12473286
rs1799938
rs79012673
rs12463674
rs10833712
rs73184720
All − MR Egger
All − Inverse variance weighted
−15
−10
−5
0
5
10
MR effect size for
'MYOM2' on 'osteosarcoma'

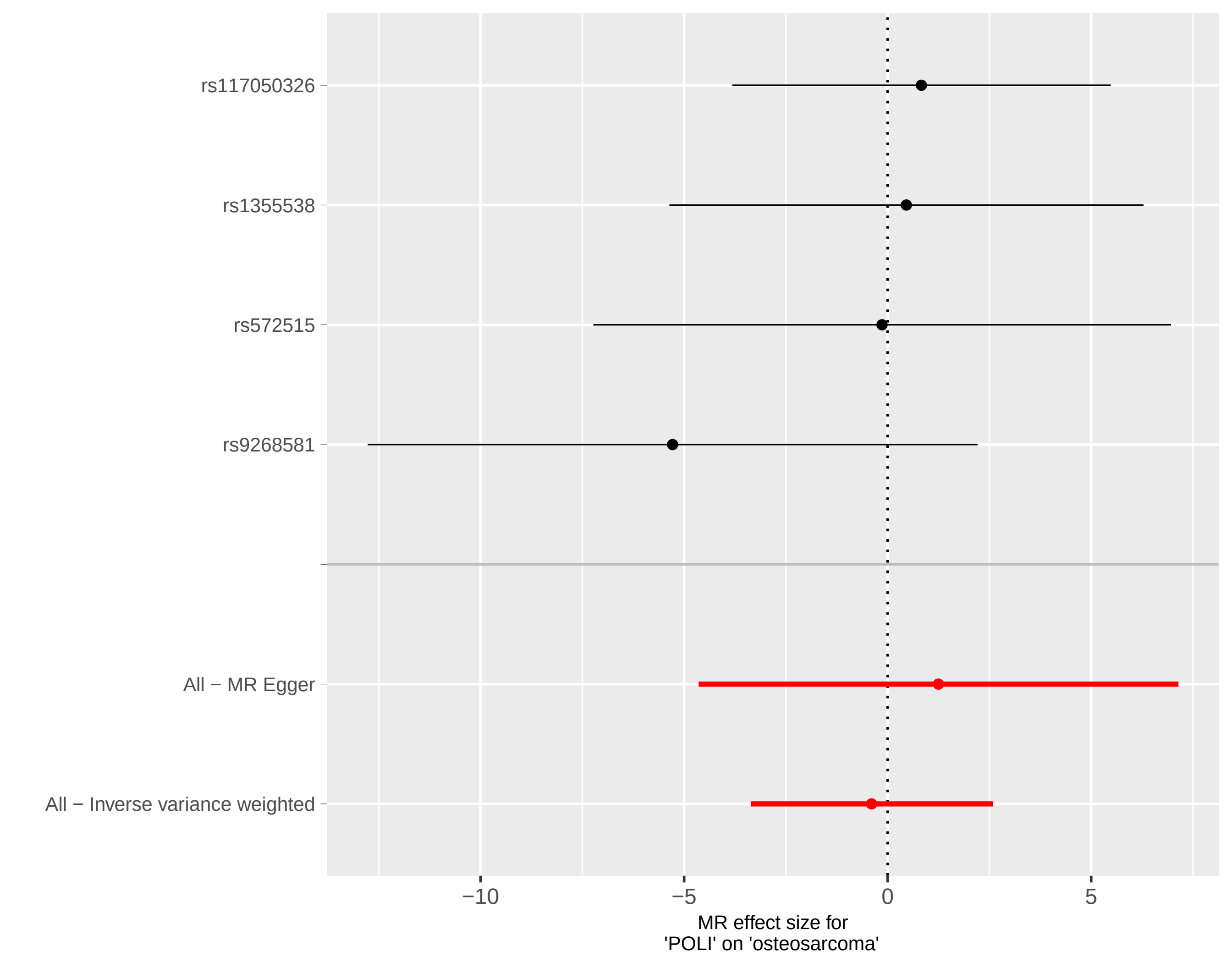

rs117050326
rs1355538
rs572515
rs9268581
All − MR Egger
All − Inverse variance weighted
−10
−5
0
5
MR effect size for
'POLI' on 'osteosarcoma'

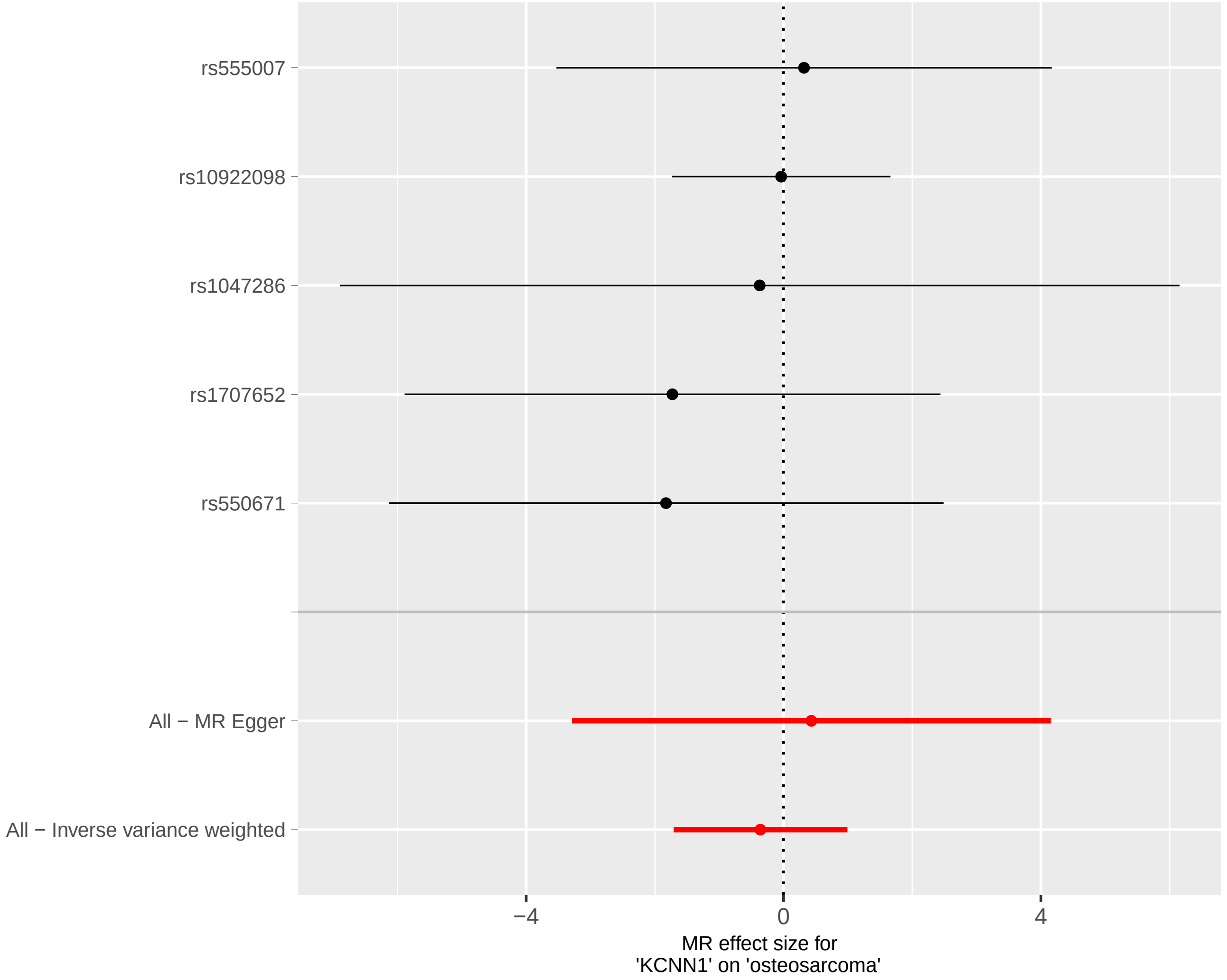
rs555007
rs10922098
rs1047286
rs1707652
rs550671
All − MR Egger
All − Inverse variance weighted
−4
0
4
MR effect size for
'KCNN1' on 'osteosarcoma'

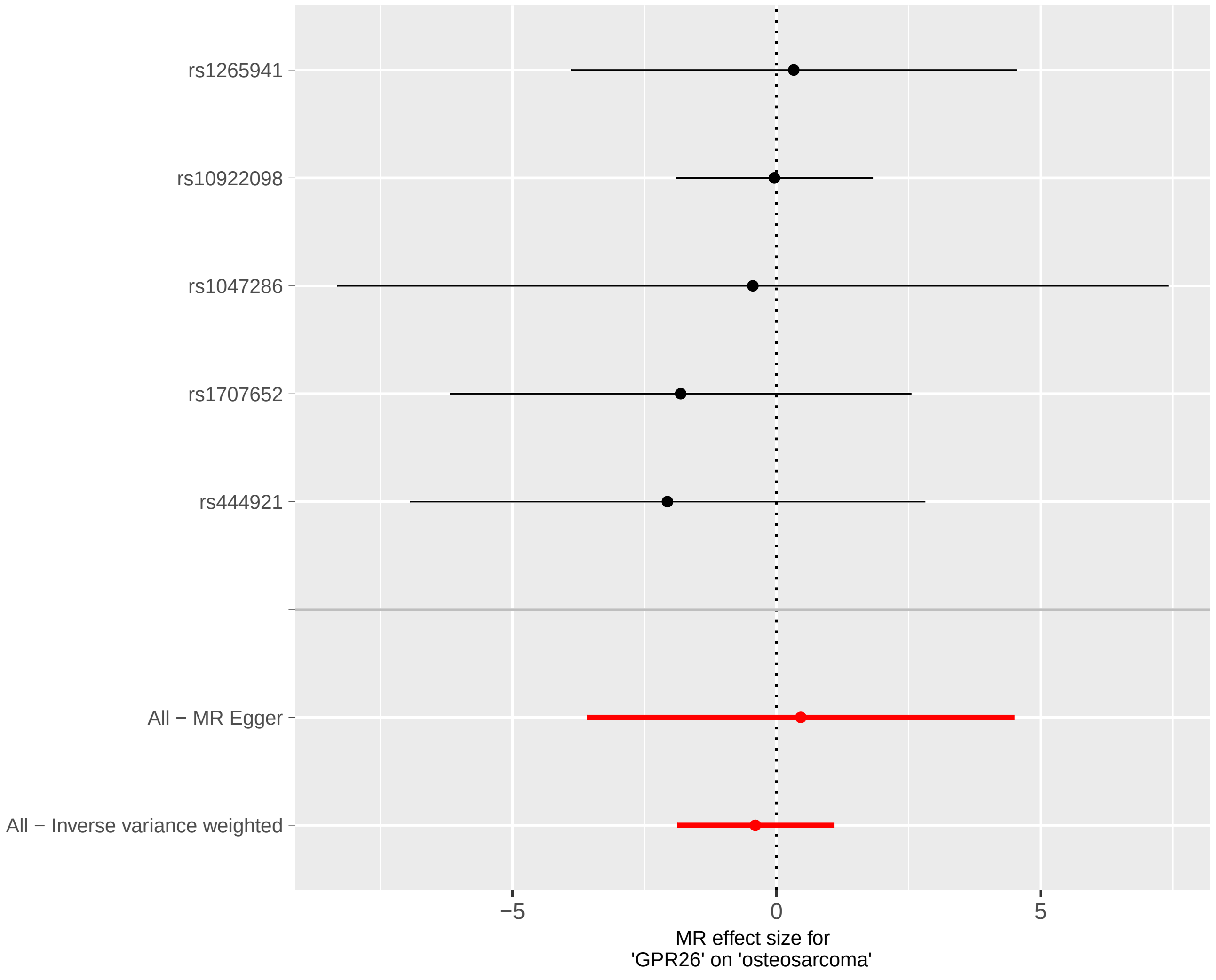
rs1265941
rs10922098
rs1047286
rs1707652
rs444921
All − MR Egger
All − Inverse variance weighted
−5
0
5
MR effect size for
'GPR26' on 'osteosarcoma'

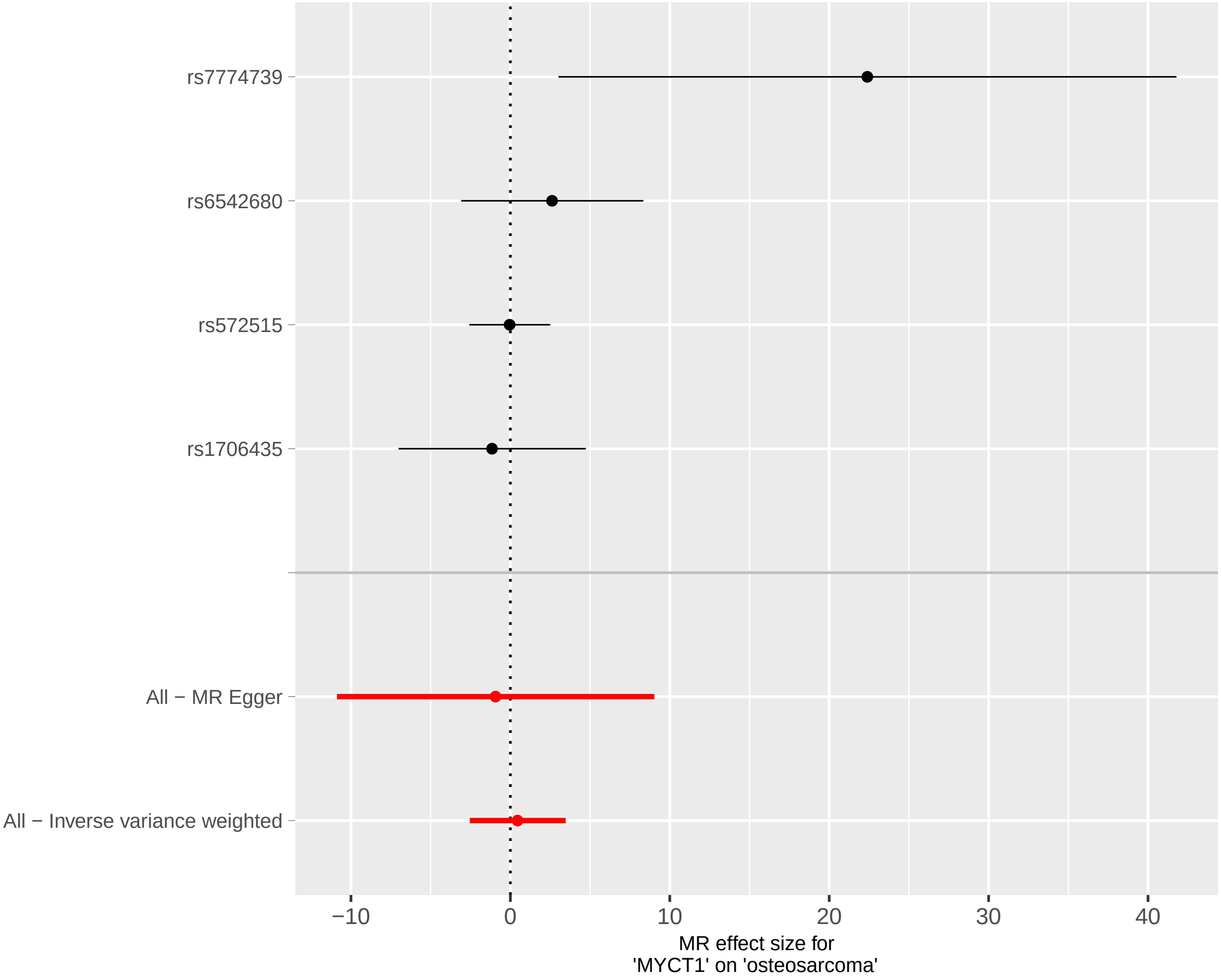

rs7774739
rs6542680
rs572515
rs1706435
All − MR Egger
All − Inverse variance weighted
−10
0
10
20
30
40
MR effect size for
'MYCT1' on 'osteosarcoma'

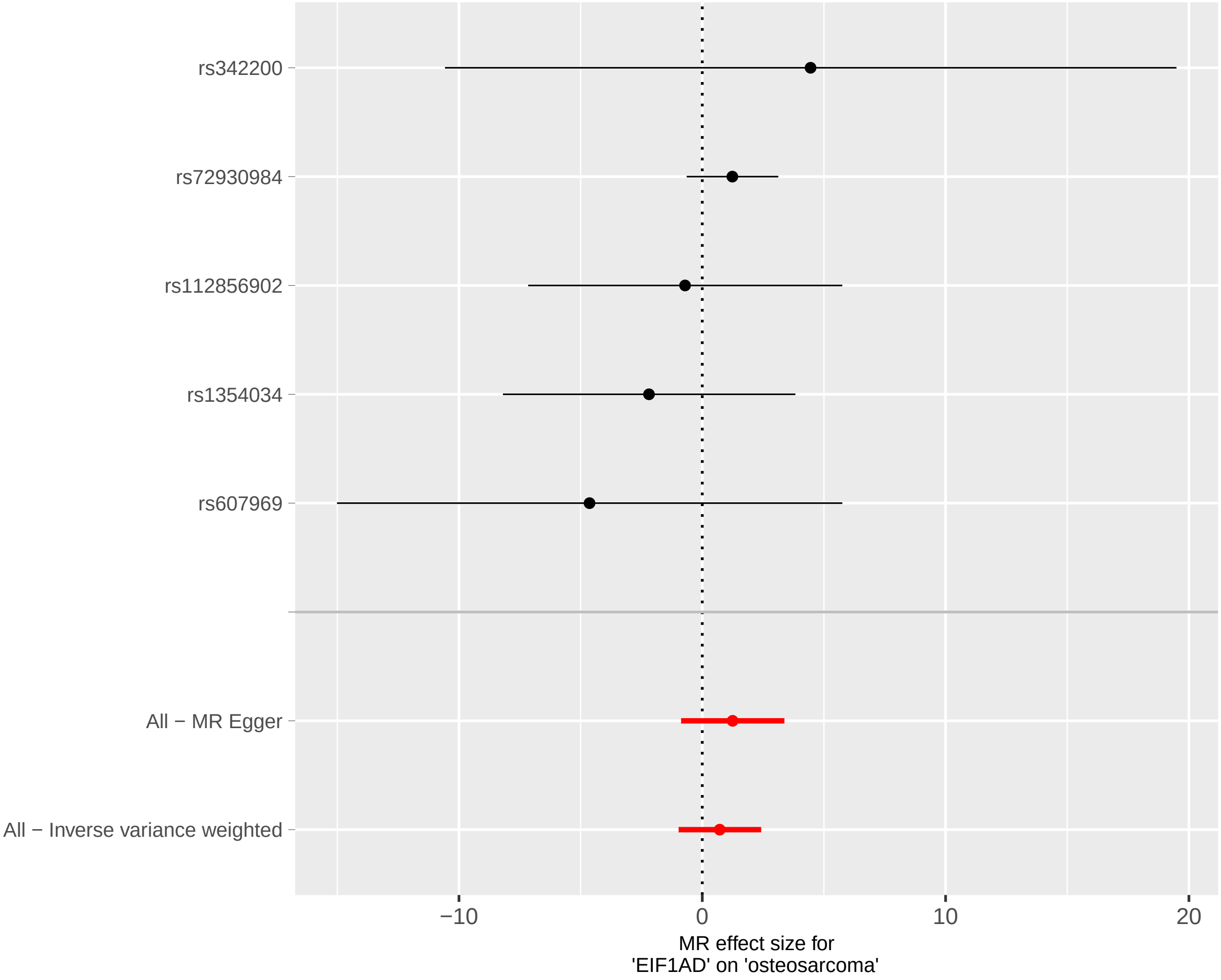
rs342200
rs72930984
rs112856902
rs1354034
rs607969
All − MR Egger
All − Inverse variance weighted
−10
0
10
20
MR effect size for
'EIF1AD' on 'osteosarcoma'

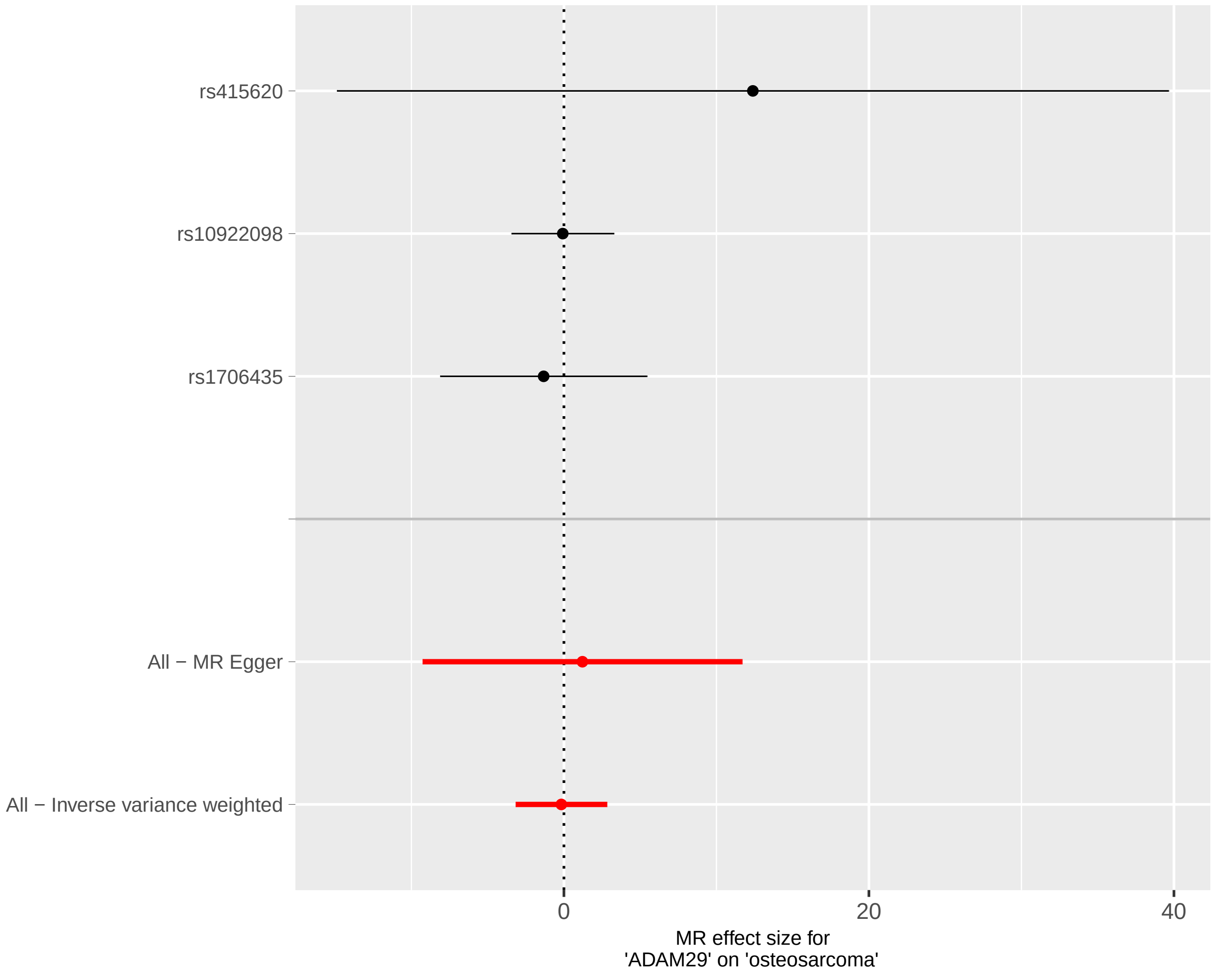

rs415620
rs10922098
rs1706435
All − MR Egger
All − Inverse variance weighted
0
20
40
MR effect size for
'ADAM29' on 'osteosarcoma'

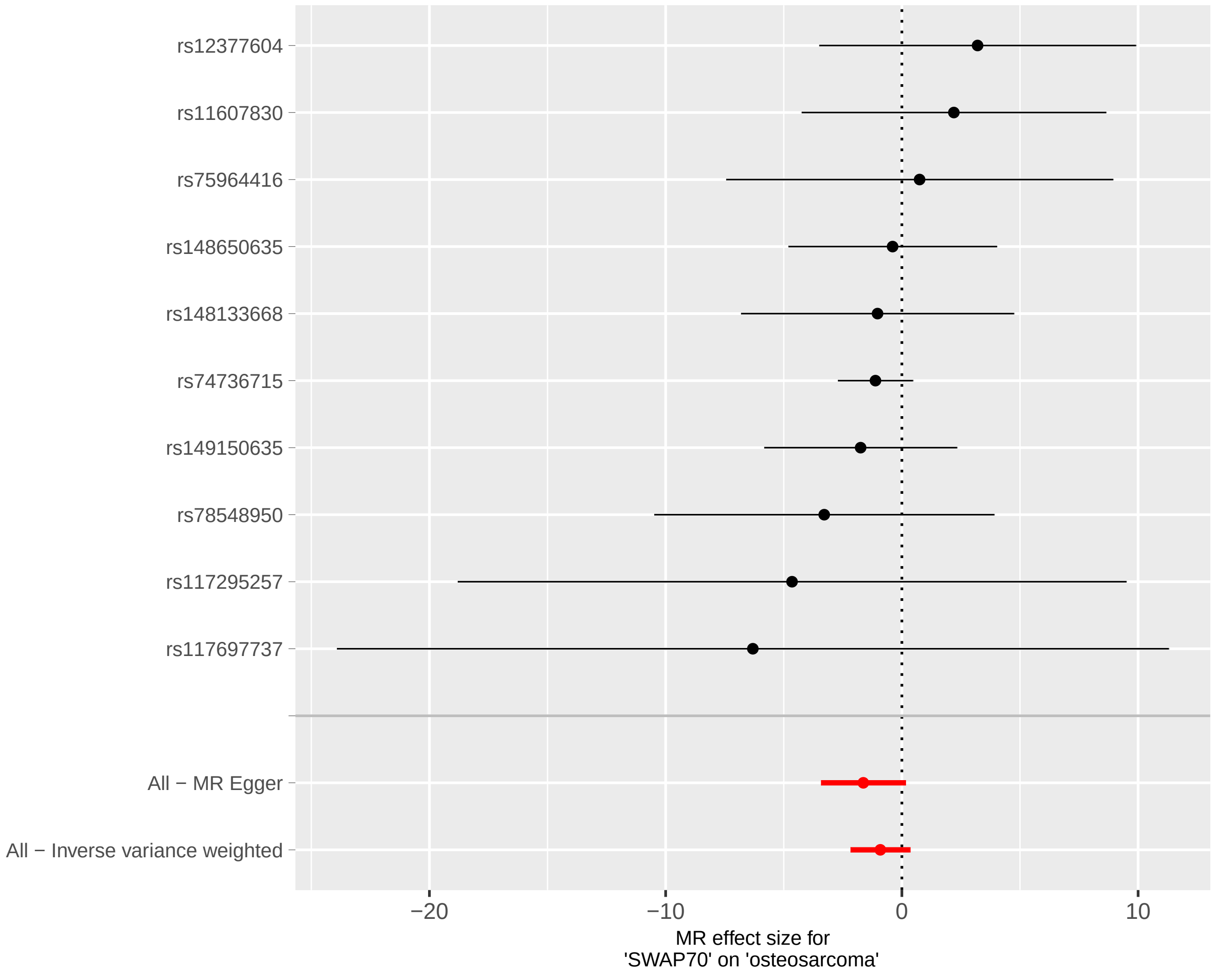
rs12377604
rs11607830
rs75964416
rs148650635
rs148133668
rs74736715
rs149150635
rs78548950
rs117295257
rs117697737
All − MR Egger
All − Inverse variance weighted
−20
−10
0
10
MR effect size for
'SWAP70' on 'osteosarcoma'

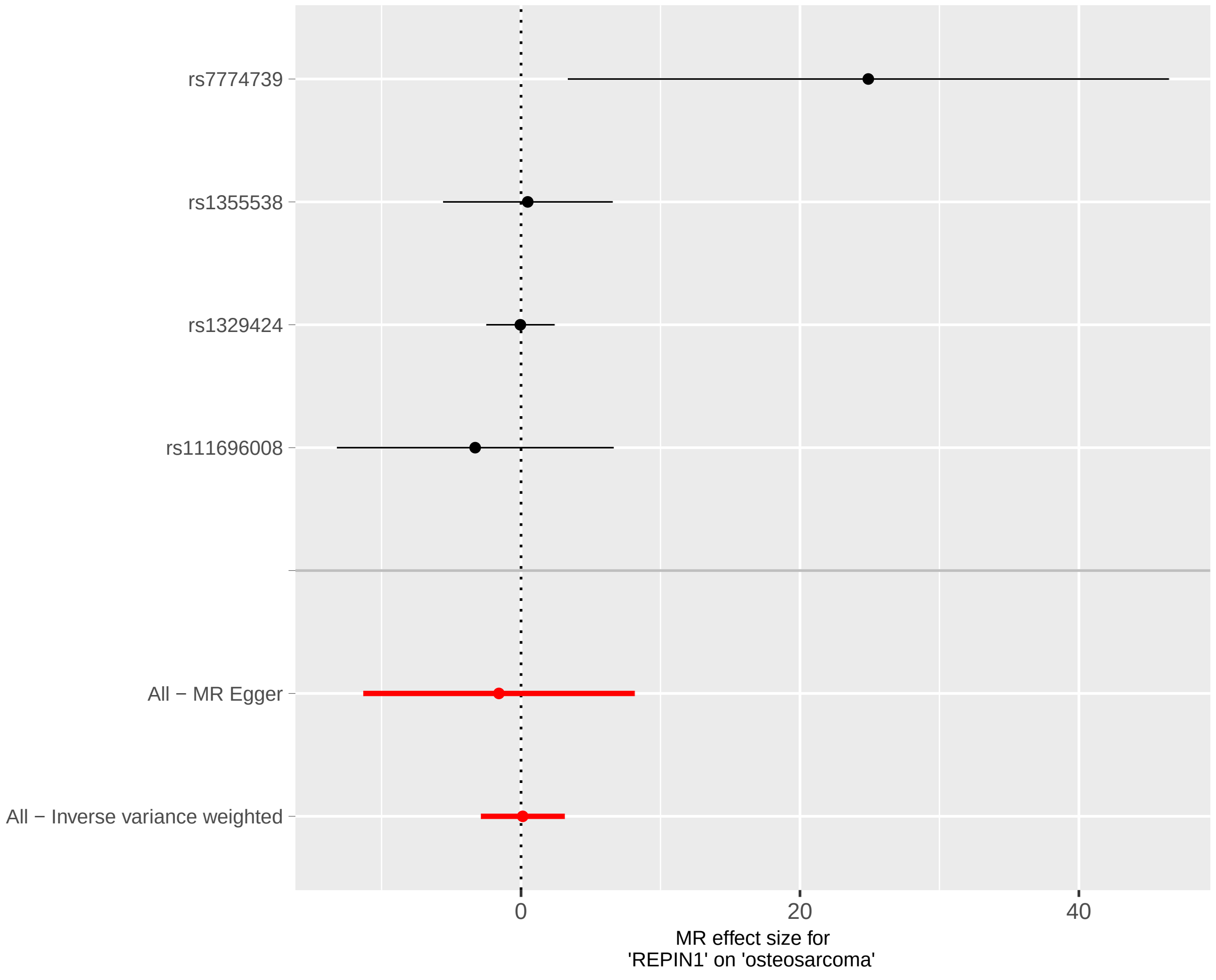
rs7774739
rs1355538
rs1329424
rs111696008
All − MR Egger
All − Inverse variance weighted
0
20
40
MR effect size for
'REPIN1' on 'osteosarcoma'

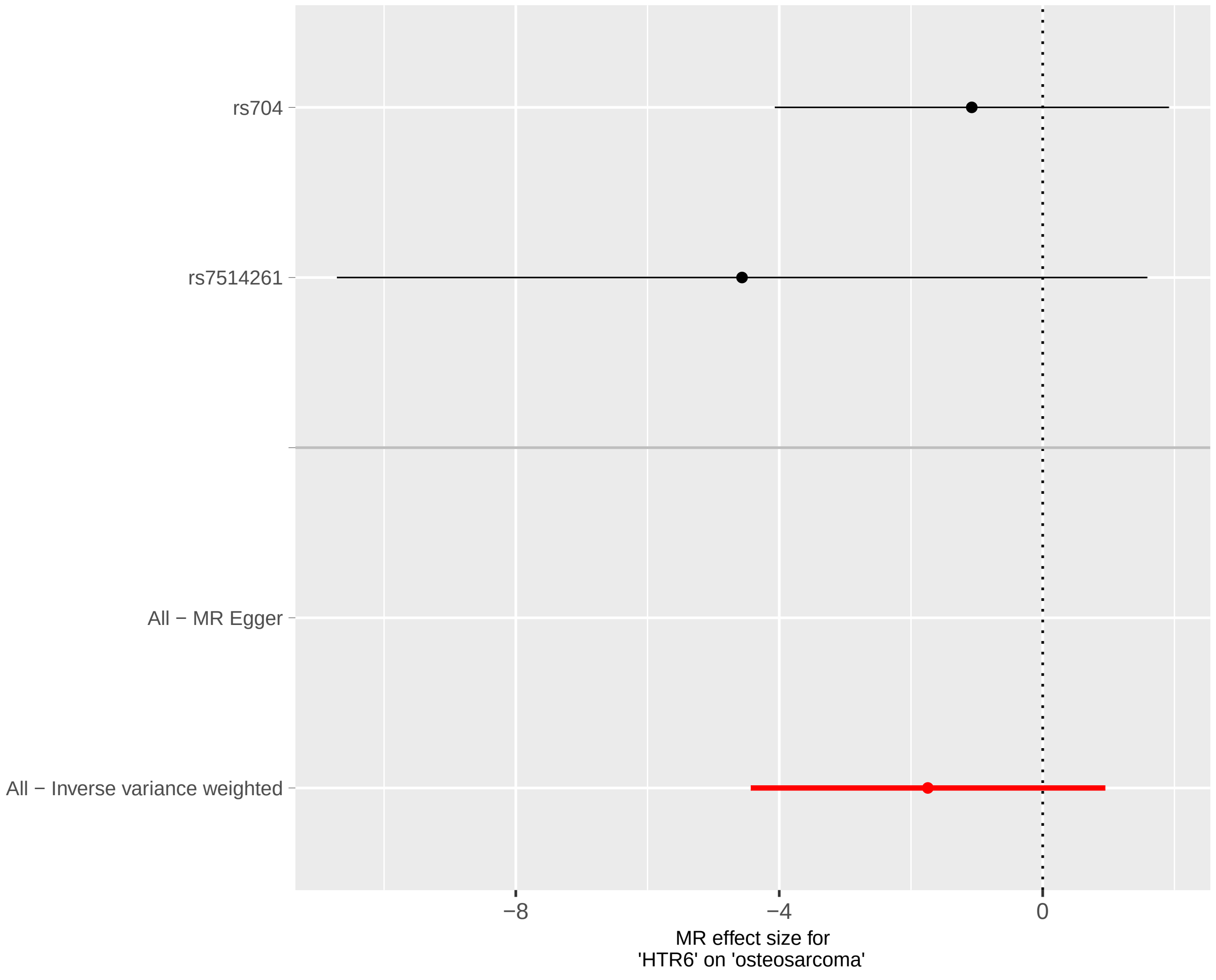

rs704
rs7514261
All − MR Egger
All − Inverse variance weighted
−8
−4
0
MR effect size for
'HTR6' on 'osteosarcoma'

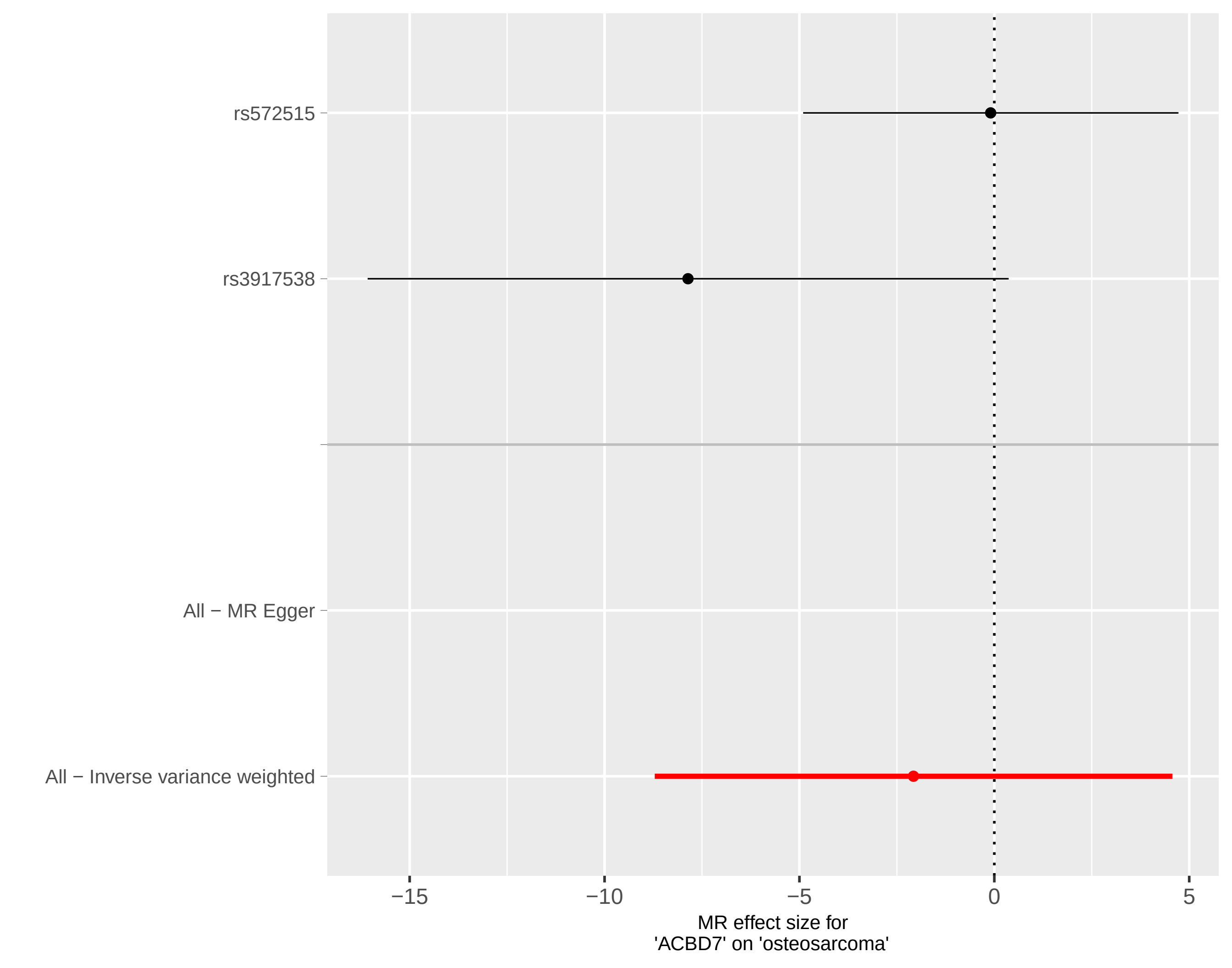
rs572515
rs3917538
All − MR Egger
All − Inverse variance weighted
−15
−10
−5
0
5
MR effect size for
'ACBD7' on 'osteosarcoma'

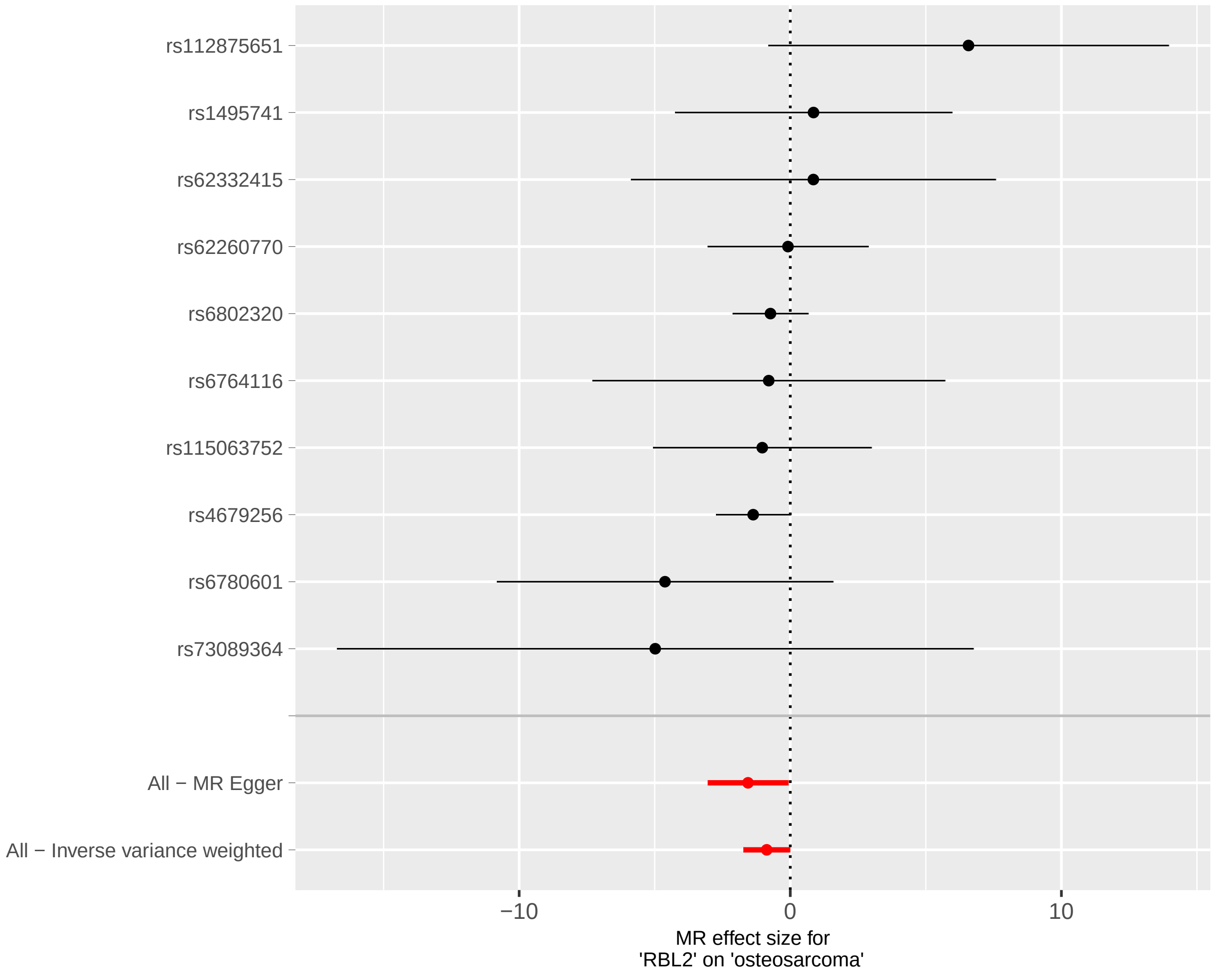
rs112875651
rs1495741
rs62332415
rs62260770
rs6802320
rs6764116
rs115063752
rs4679256
rs6780601
rs73089364
All − MR Egger
All − Inverse variance weighted
−10
0
10
MR effect size for
'RBL2' on 'osteosarcoma'

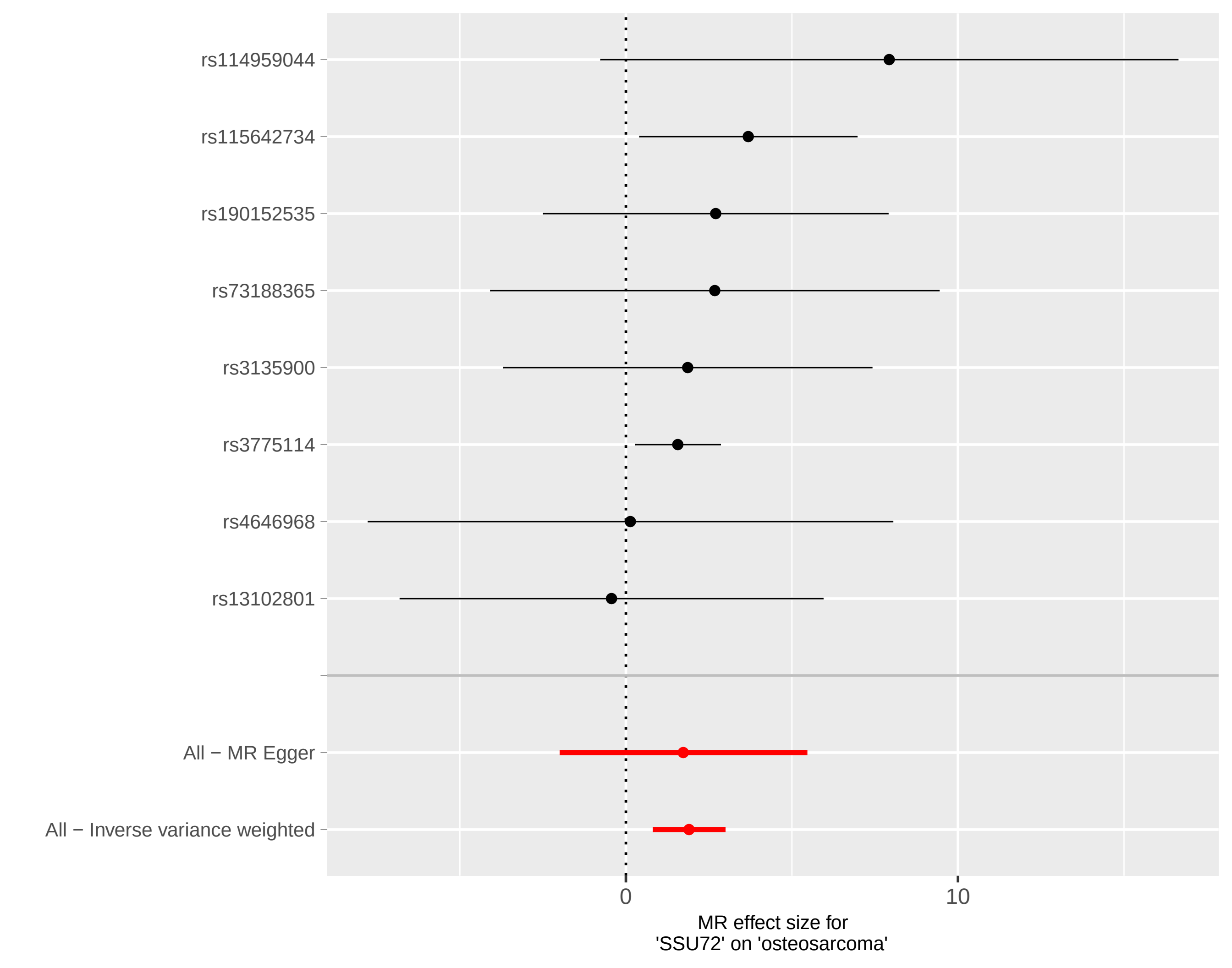

rs114959044
rs115642734
rs190152535
rs73188365
rs3135900
rs3775114
rs4646968
rs13102801
All − MR Egger
All − Inverse variance weighted
0
10
MR effect size for
'SSU72' on 'osteosarcoma'

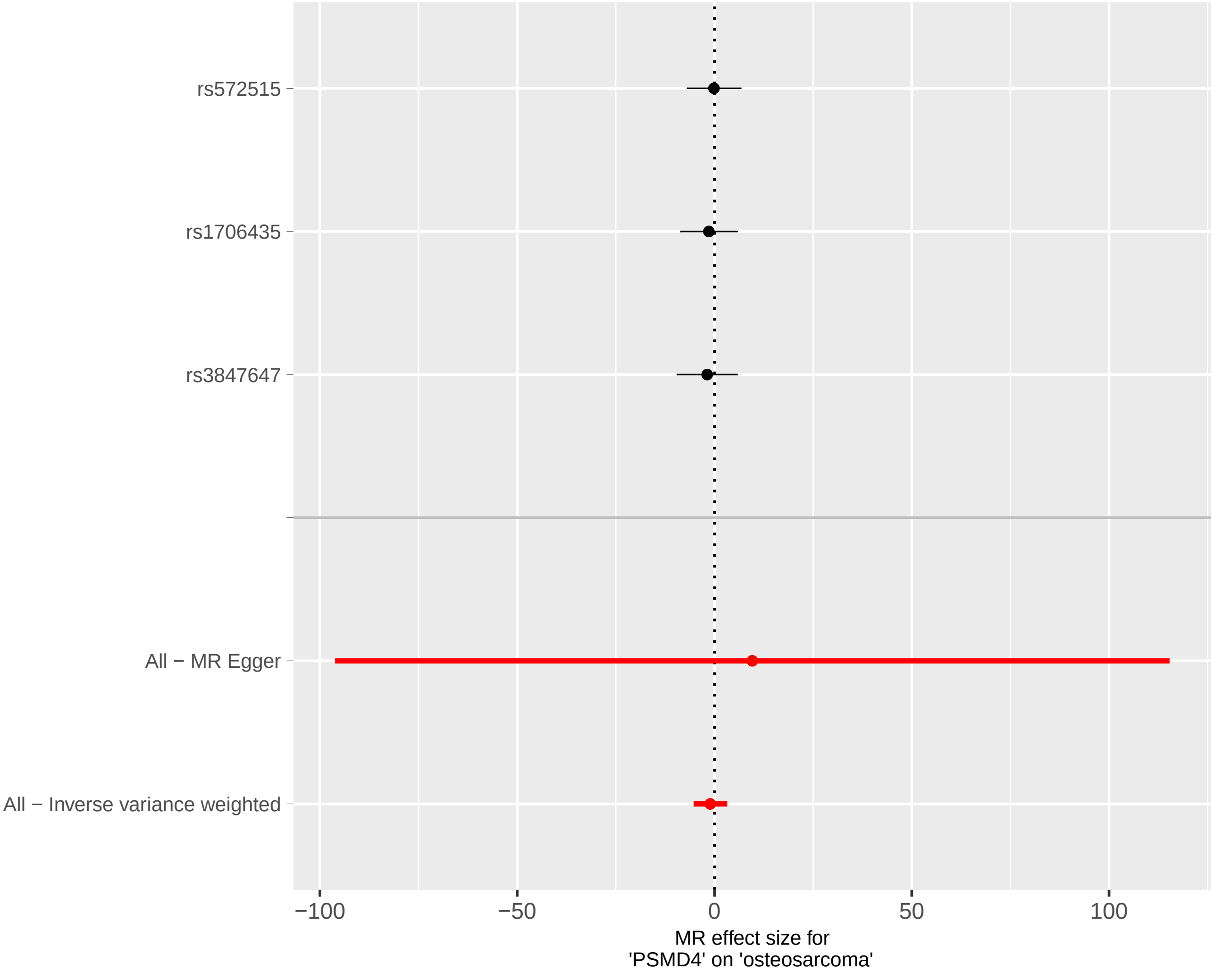
rs572515
rs1706435
rs3847647
All − MR Egger
All − Inverse variance weighted
−100
−50
0
50
100
MR effect size for
'PSMD4' on 'osteosarcoma'

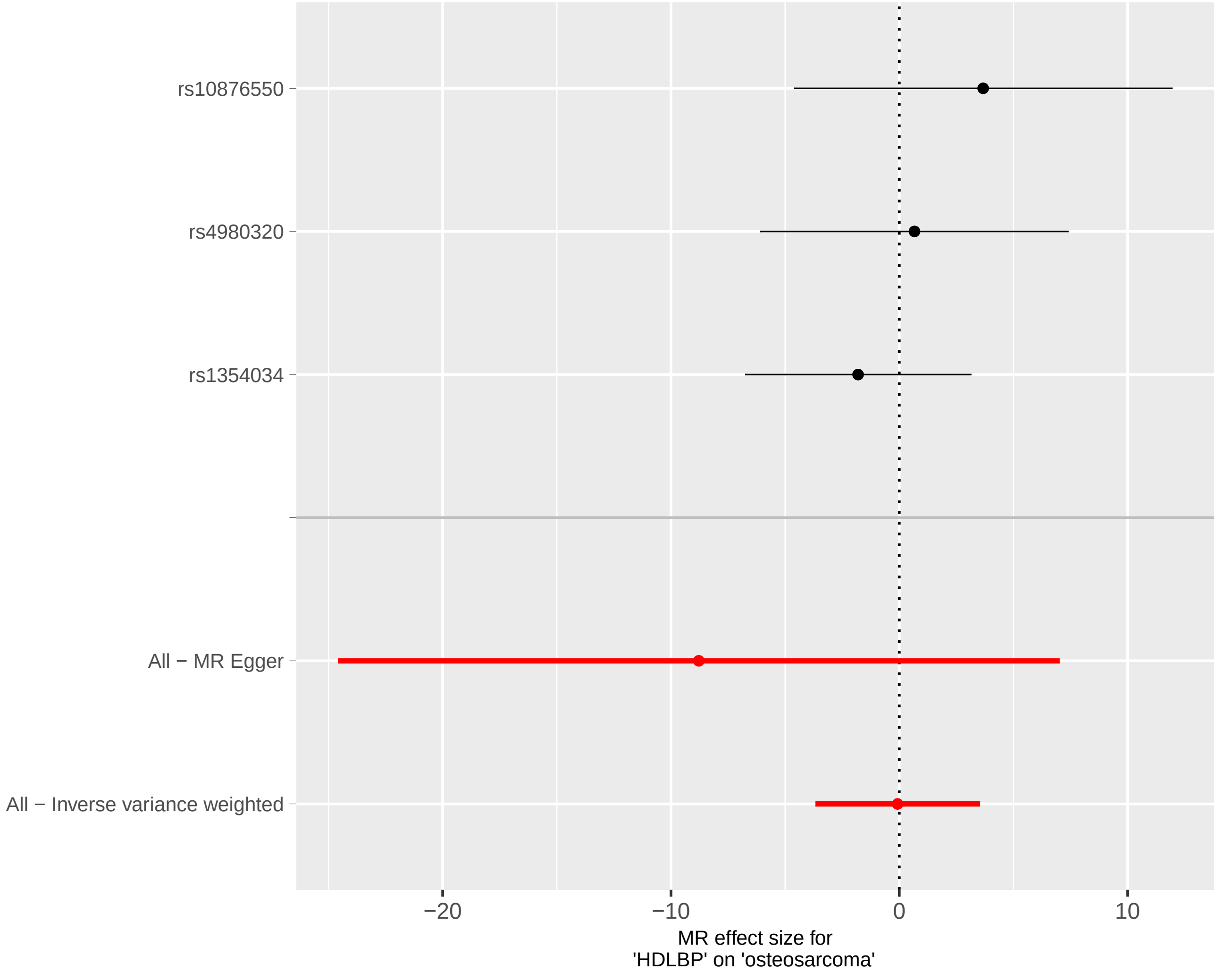
rs10876550
rs4980320
rs1354034
All − MR Egger
All − Inverse variance weighted
−20
−10
0
10
MR effect size for
'HDLBP' on 'osteosarcoma'

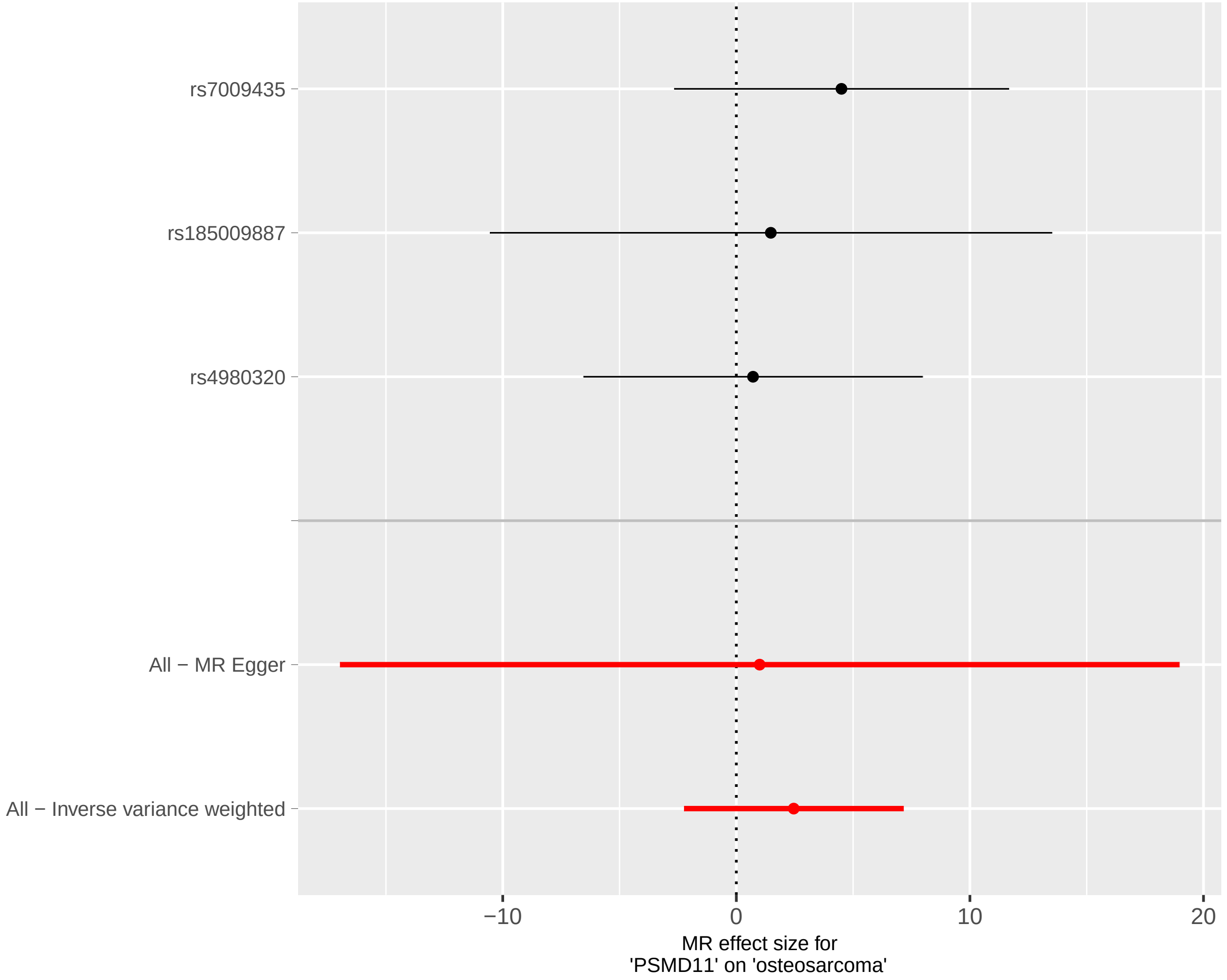
rs7009435
rs185009887
rs4980320
All − MR Egger
All − Inverse variance weighted
−10
0
10
20
MR effect size for
'PSMD11' on 'osteosarcoma'

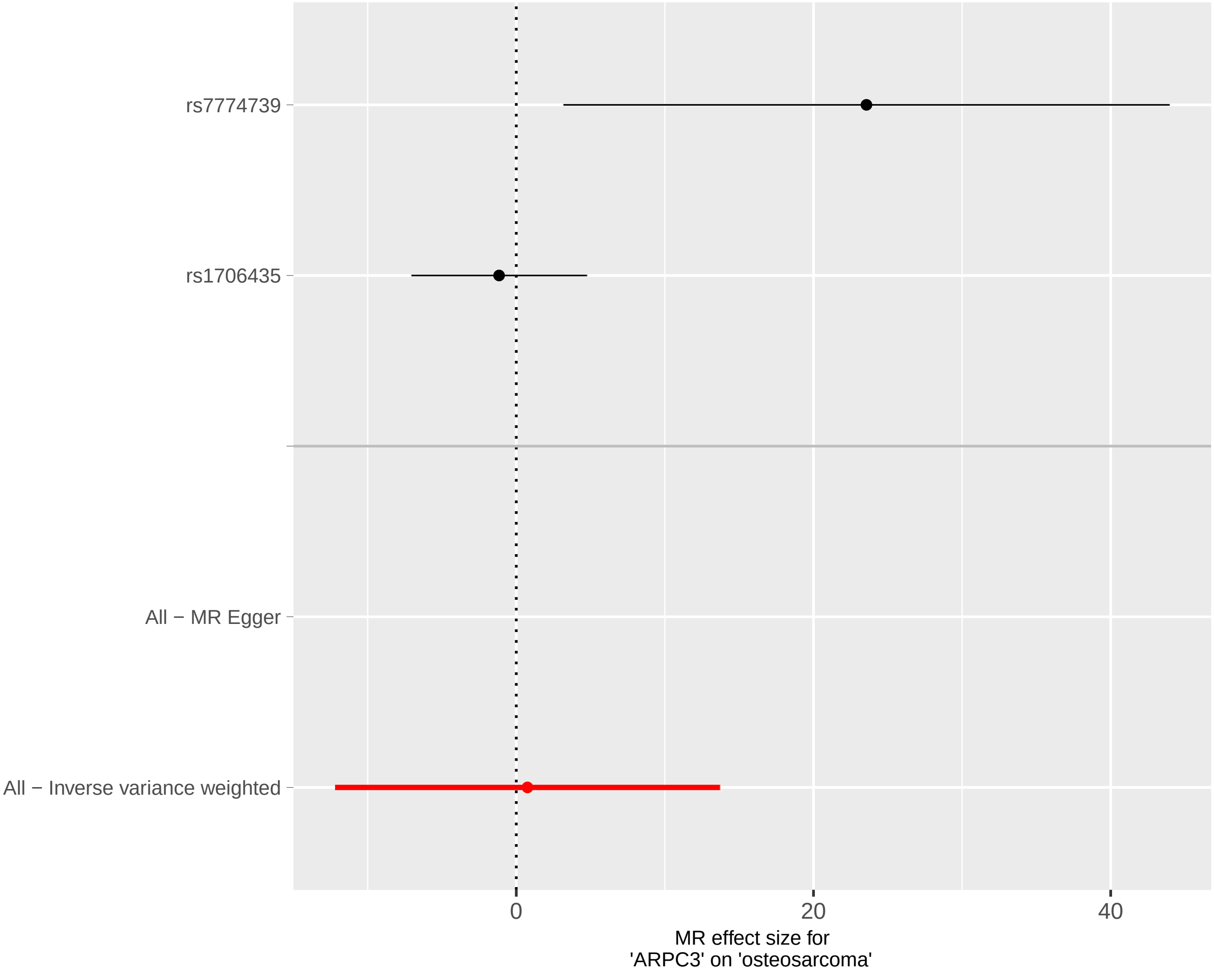
rs7774739
rs1706435
All − MR Egger
All − Inverse variance weighted
0
20
40
MR effect size for
'ARPC3' on 'osteosarcoma'

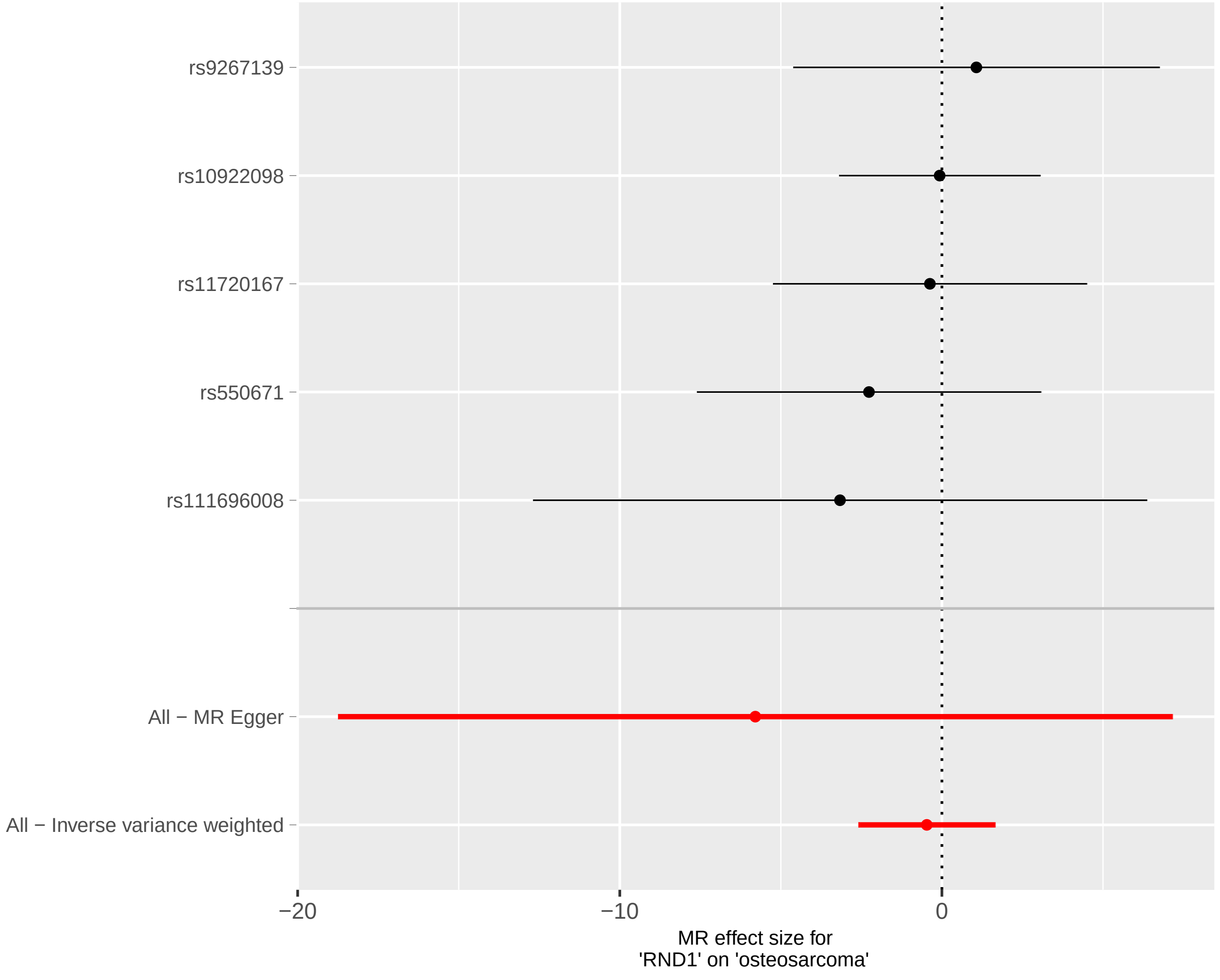
rs9267139
rs10922098
rs11720167
rs550671
rs111696008
All − MR Egger
All − Inverse variance weighted
−20
−10
0
MR effect size for
'RND1' on 'osteosarcoma'

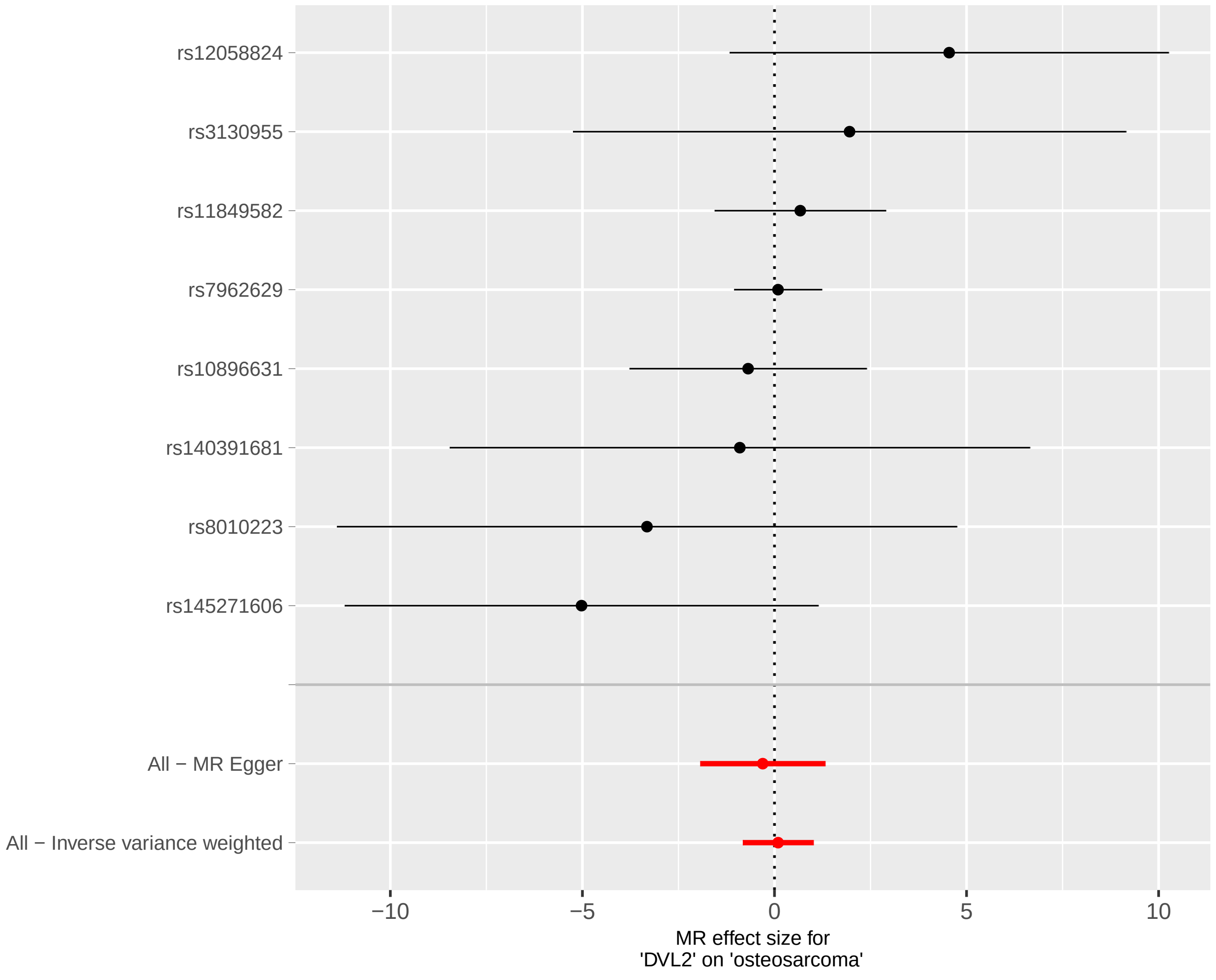
rs12058824
rs3130955
rs11849582
rs7962629
rs10896631
rs140391681
rs8010223
rs145271606
All − MR Egger
All − Inverse variance weighted
−10
−5
0
5
10
MR effect size for
'DVL2' on 'osteosarcoma'

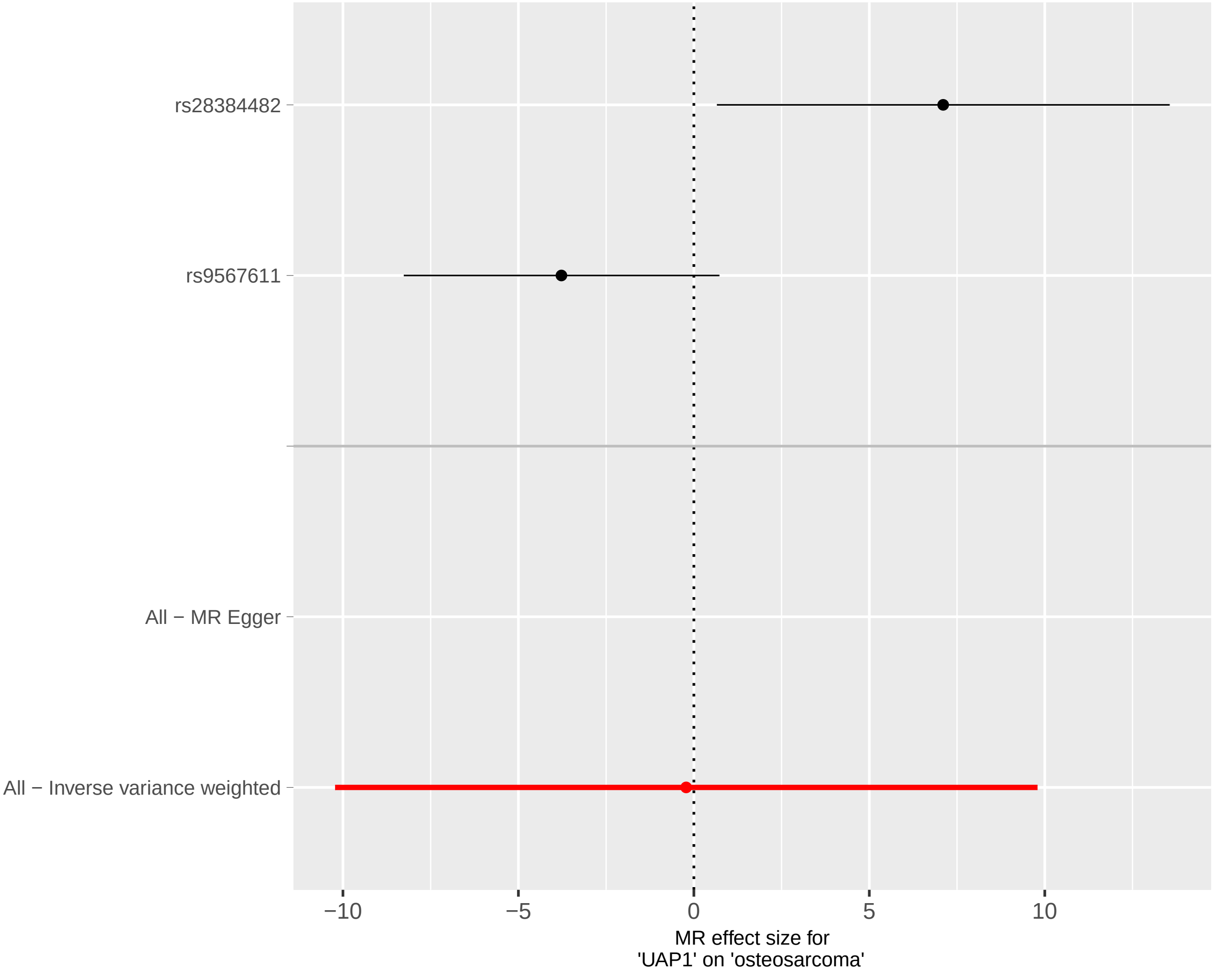

rs28384482
rs9567611
All − MR Egger
All − Inverse variance weighted
−10
−5
0
5
10
MR effect size for
'UAP1' on 'osteosarcoma'

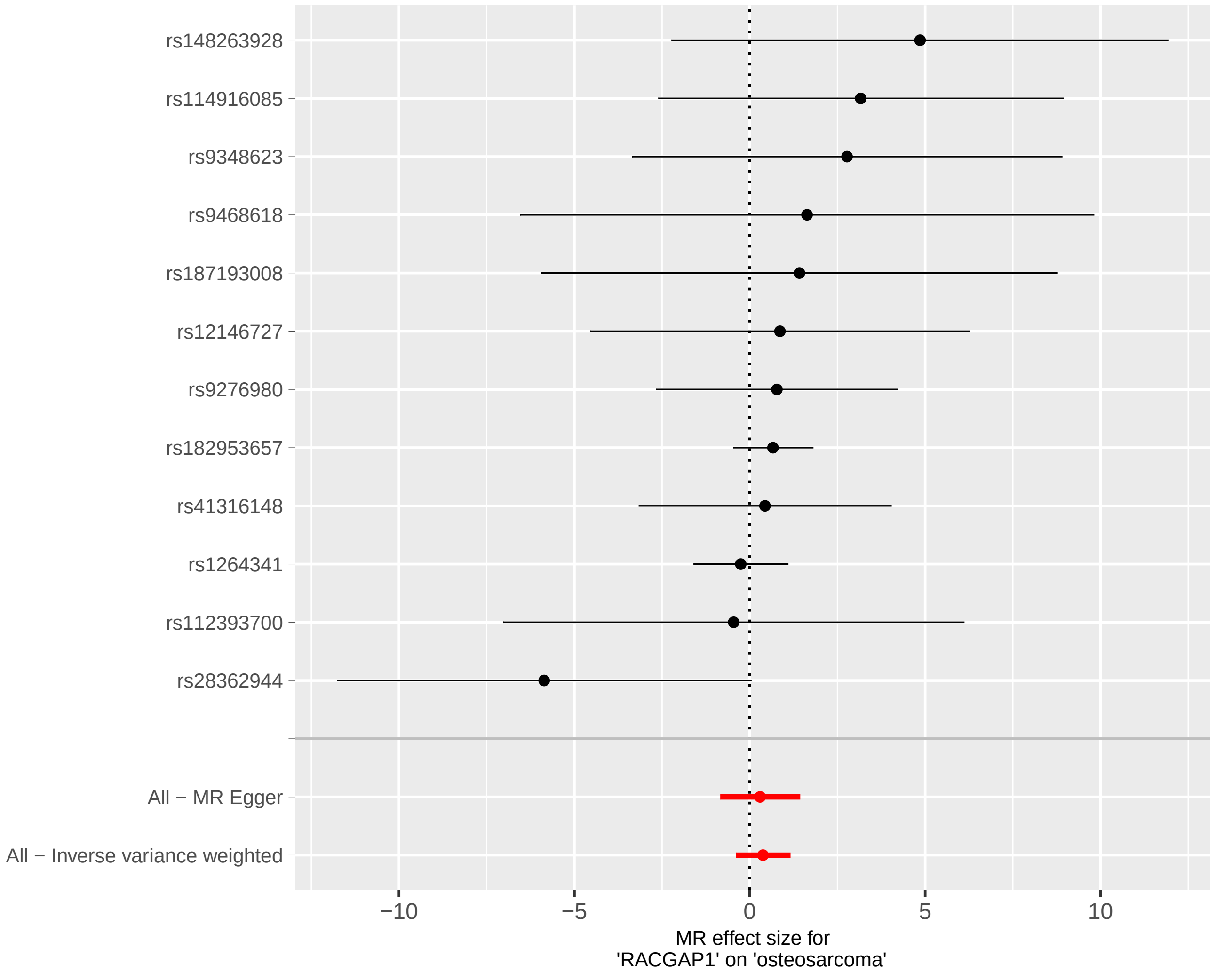

rs148263928
rs114916085
rs9348623
rs9468618
rs187193008
rs12146727
rs9276980
rs182953657
rs41316148
rs1264341
rs112393700
rs28362944
All − MR Egger
All − Inverse variance weighted
−10
−5
0
5
10
MR effect size for
'RACGAP1' on 'osteosarcoma'

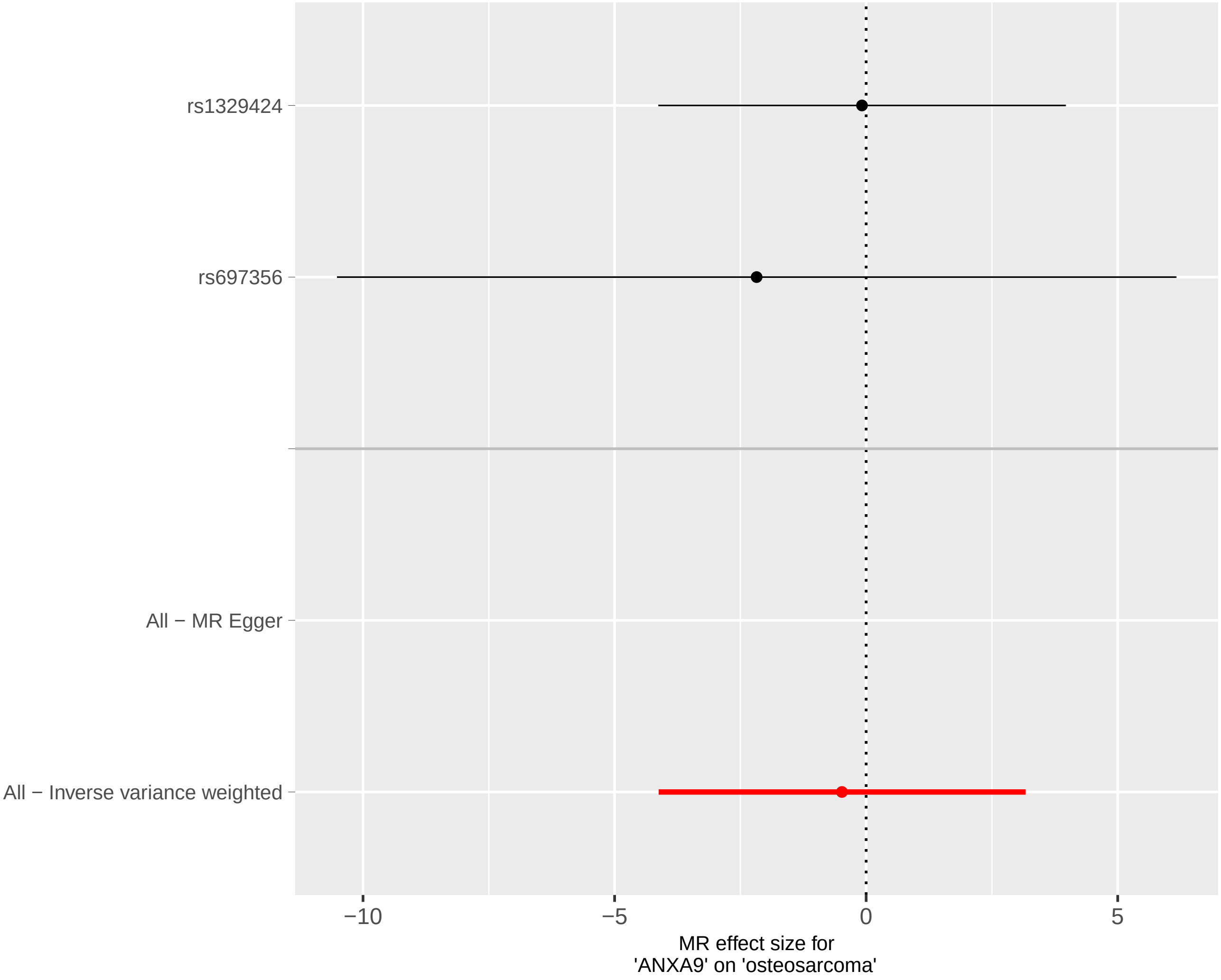

rs1329424
rs697356
All − MR Egger
All − Inverse variance weighted
−10
−5
0
5
MR effect size for
'ANXA9' on 'osteosarcoma'

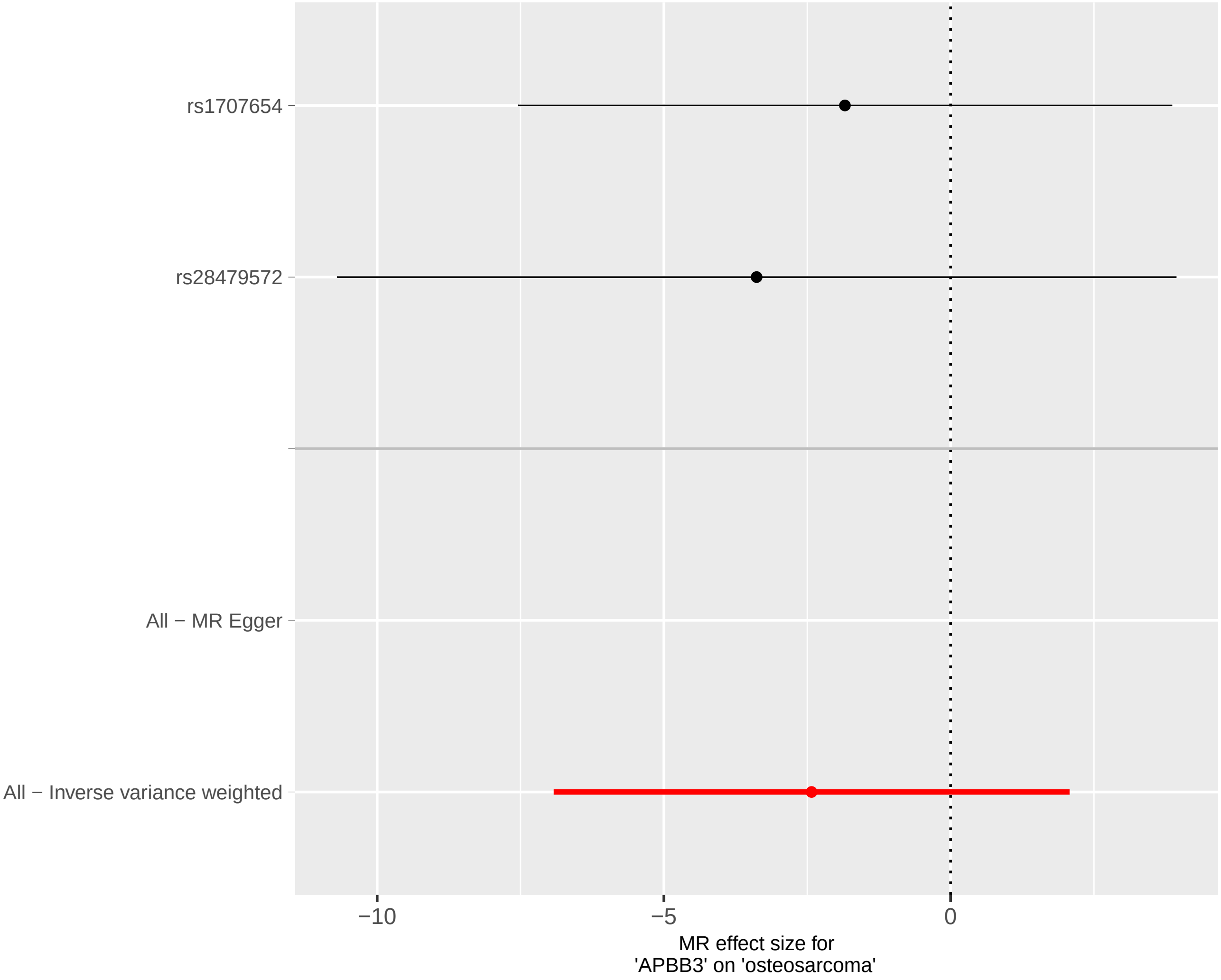

rs1707654
rs28479572
All − MR Egger
All − Inverse variance weighted
−10
−5
0
MR effect size for
'APBB3' on 'osteosarcoma'

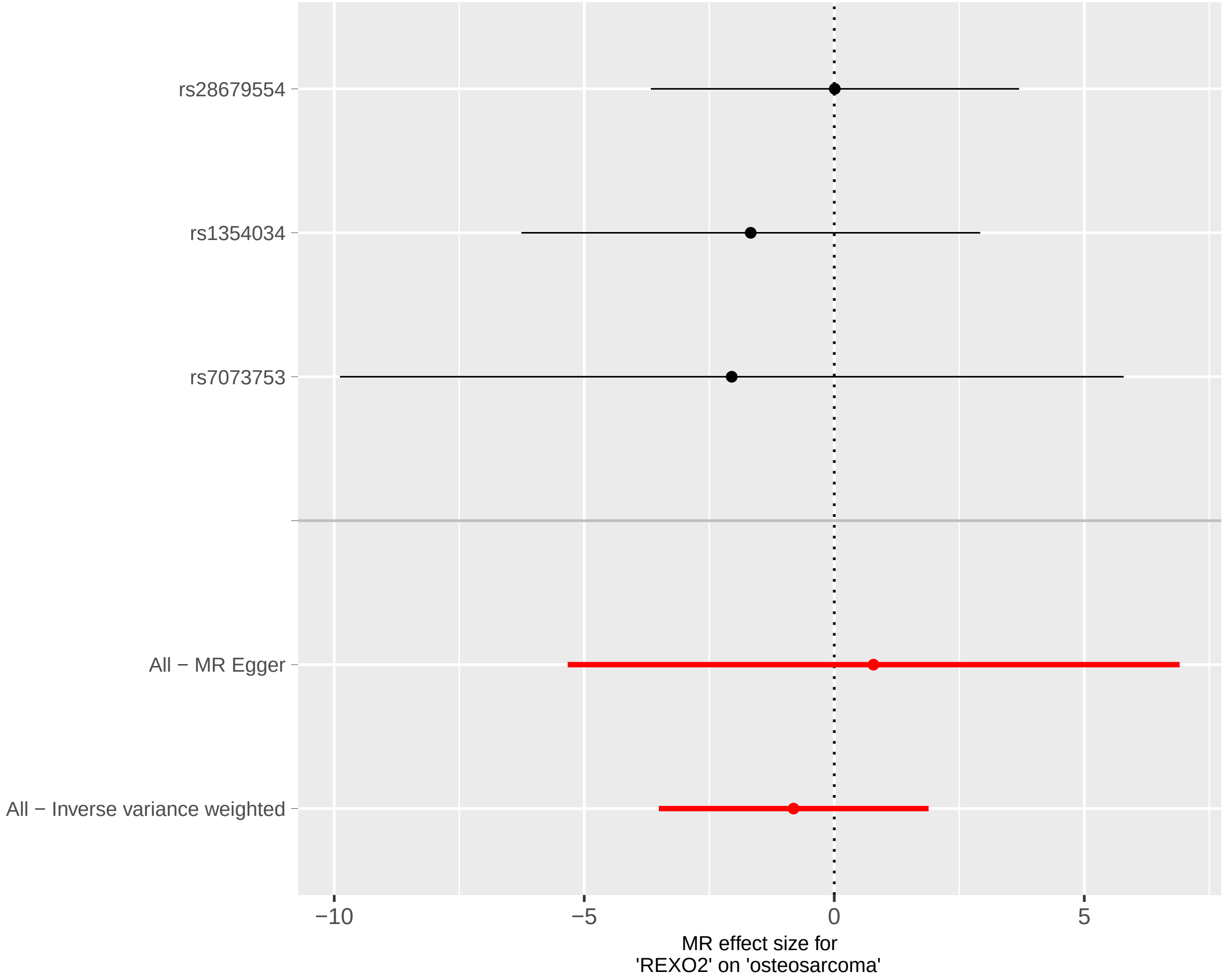

rs28679554
rs1354034
rs7073753
All − MR Egger
All − Inverse variance weighted
−10
−5
0
5
MR effect size for
'REXO2' on 'osteosarcoma'

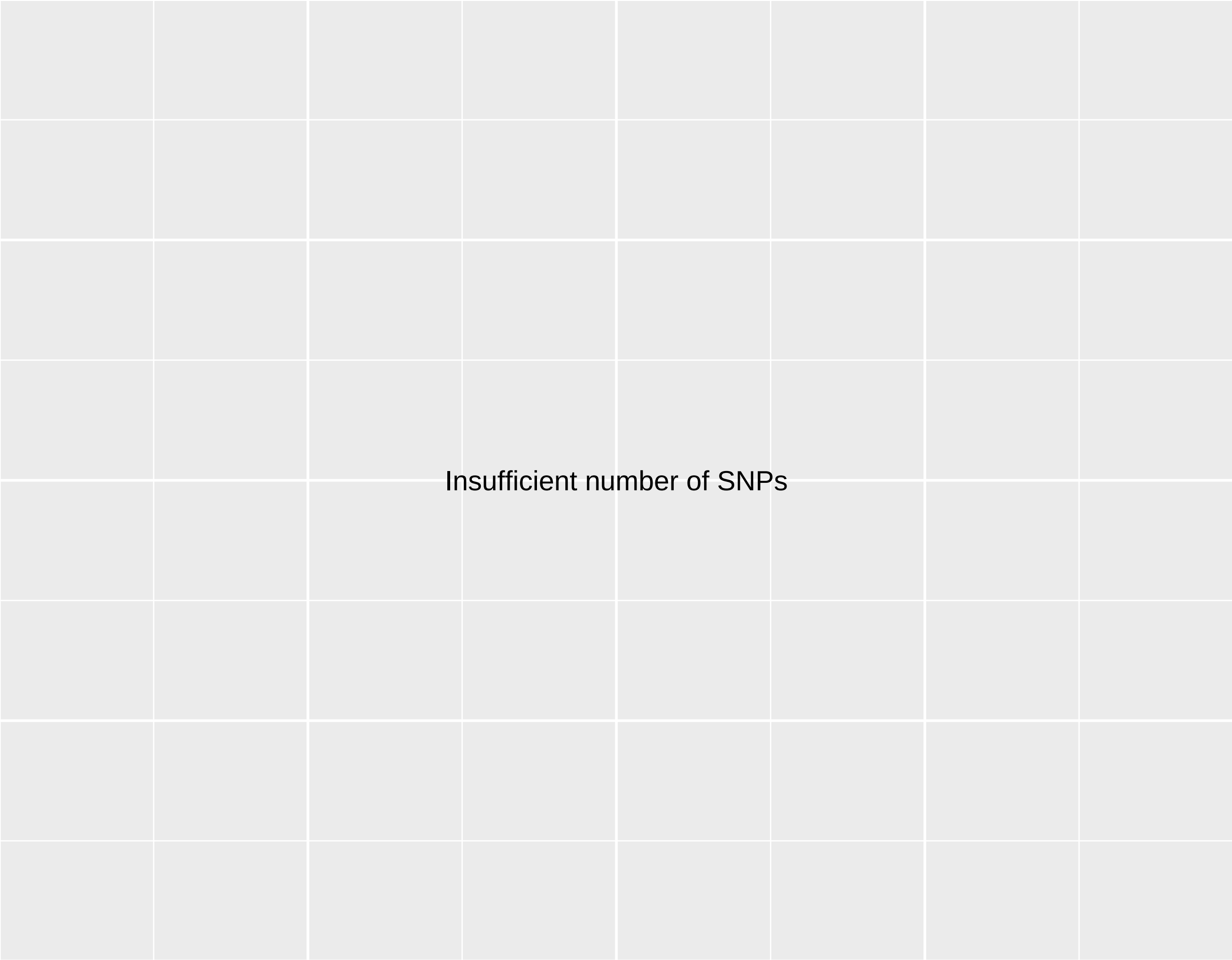
Insufficient number of SNPs

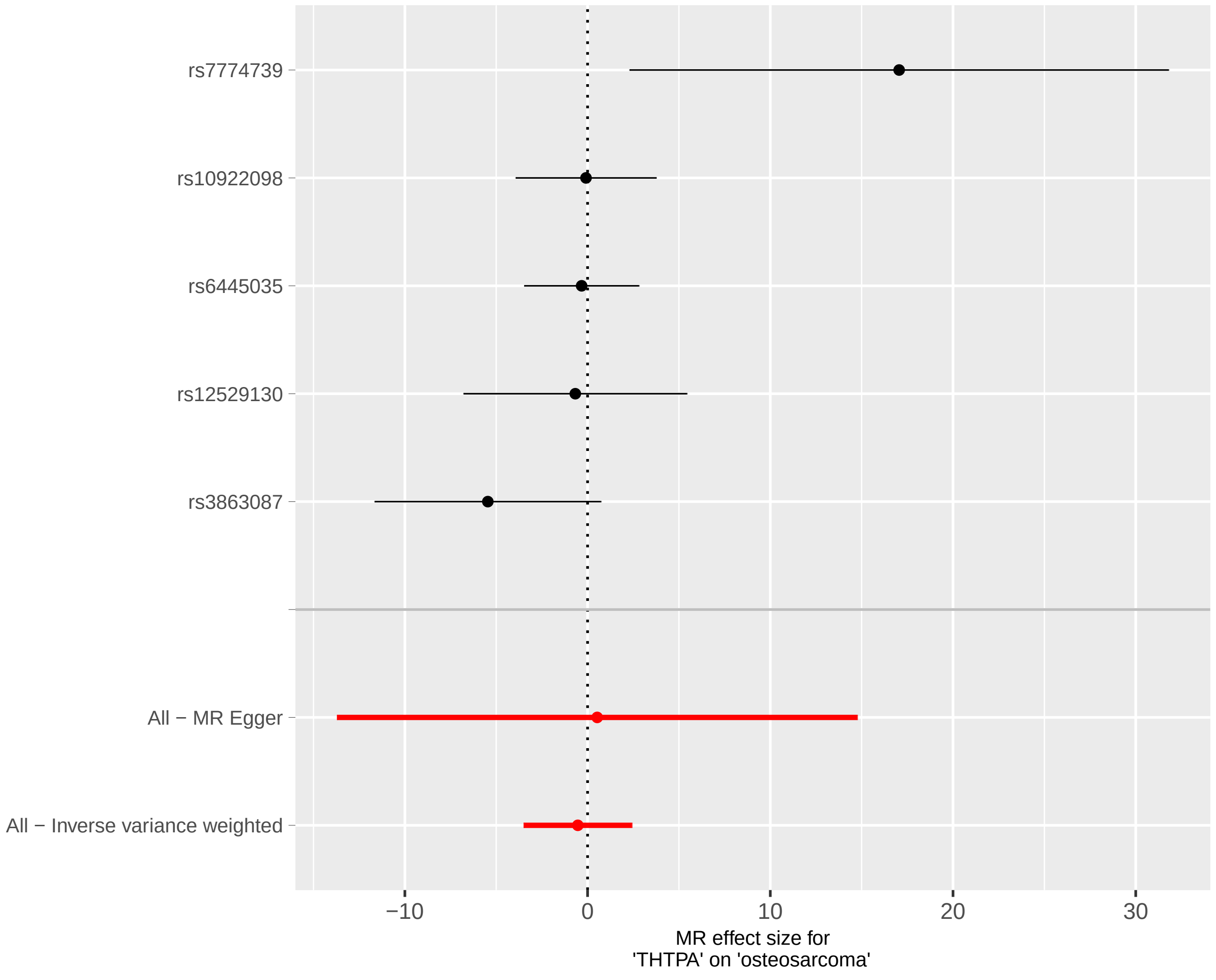

rs7774739
rs10922098
rs6445035
rs12529130
rs3863087
All − MR Egger
All − Inverse variance weighted
−10
0
10
20
30
MR effect size for
'THTPA' on 'osteosarcoma'

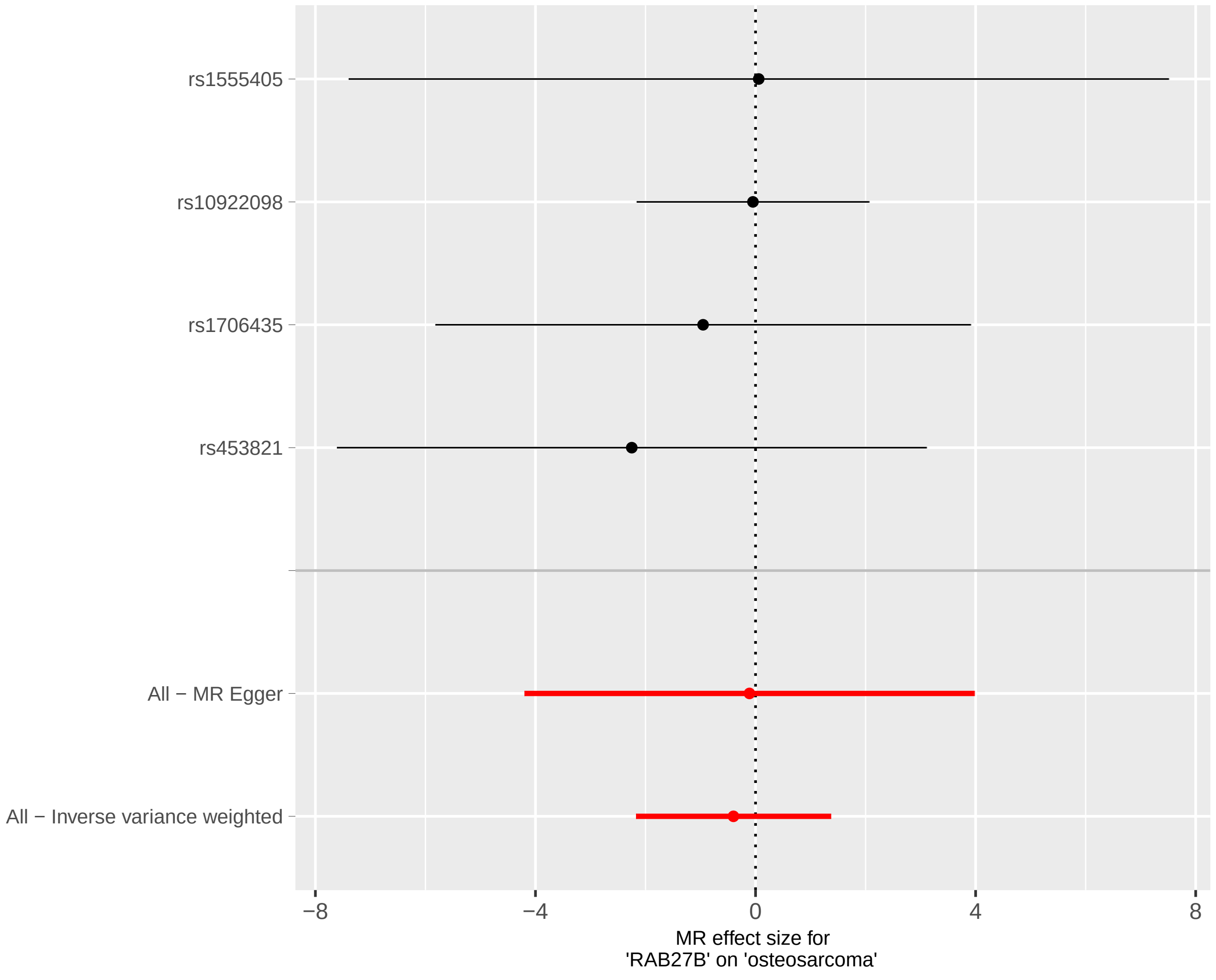
rs1555405
rs10922098
rs1706435
rs453821
All − MR Egger
All − Inverse variance weighted
−8
−4
0
4
8
MR effect size for
'RAB27B' on 'osteosarcoma'

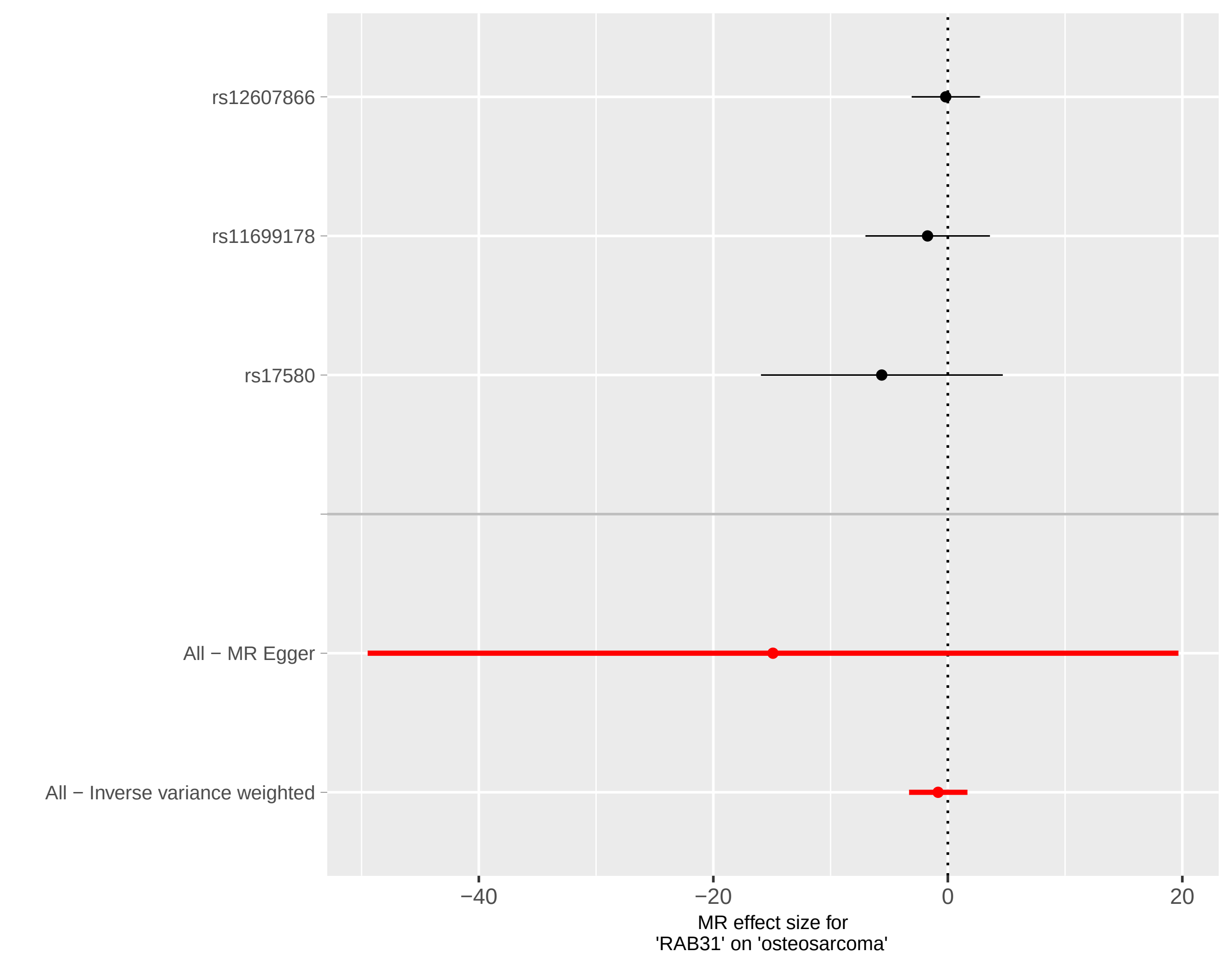

rs12607866
rs11699178
rs17580
All − MR Egger
All − Inverse variance weighted
−40
−20
0
20
MR effect size for
'RAB31' on 'osteosarcoma'

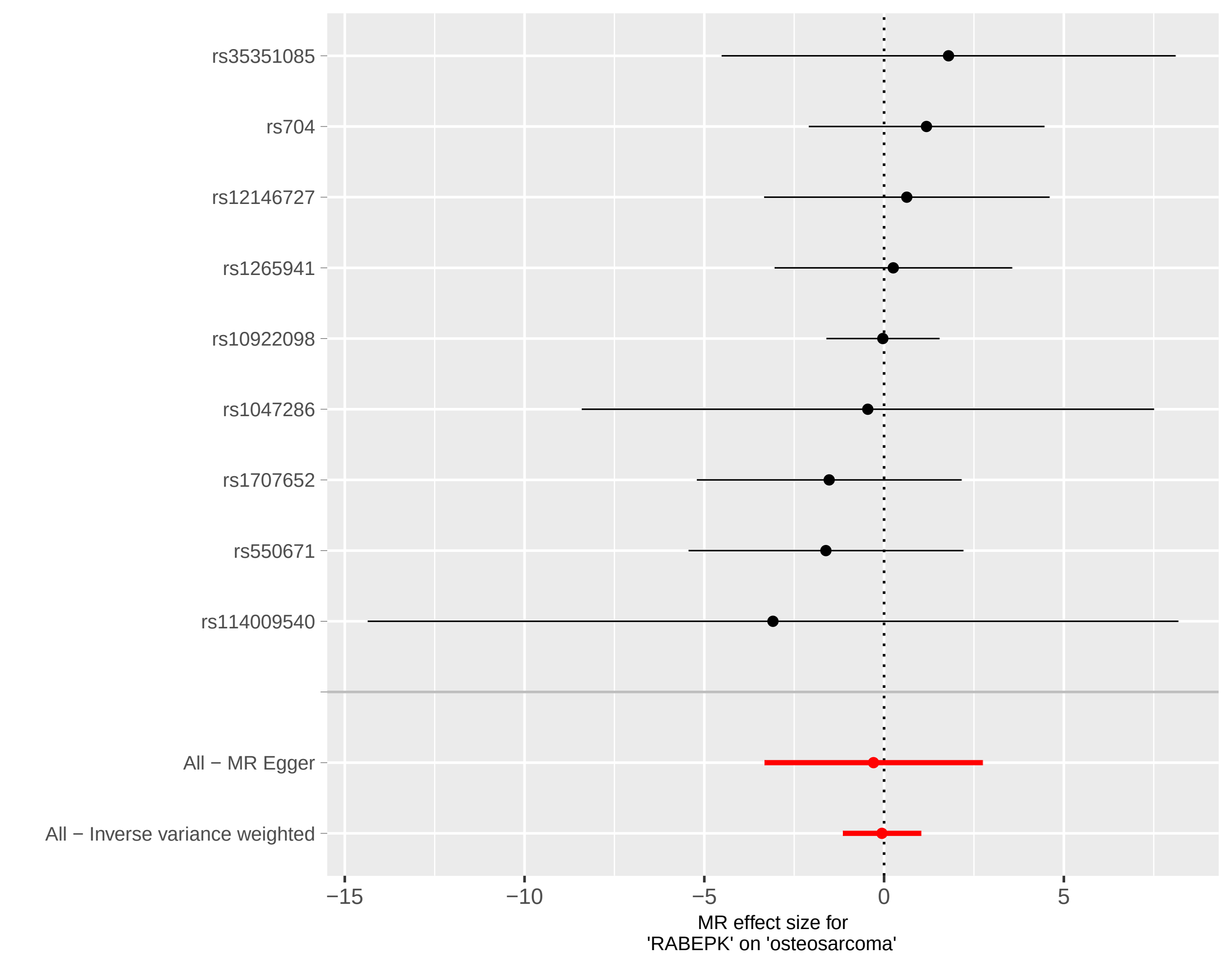
rs35351085
rs704
rs12146727
rs1265941
rs10922098
rs1047286
rs1707652
rs550671
rs114009540
All − MR Egger
All − Inverse variance weighted
−15
−10
−5
0
5
MR effect size for
'RABEPK' on 'osteosarcoma'

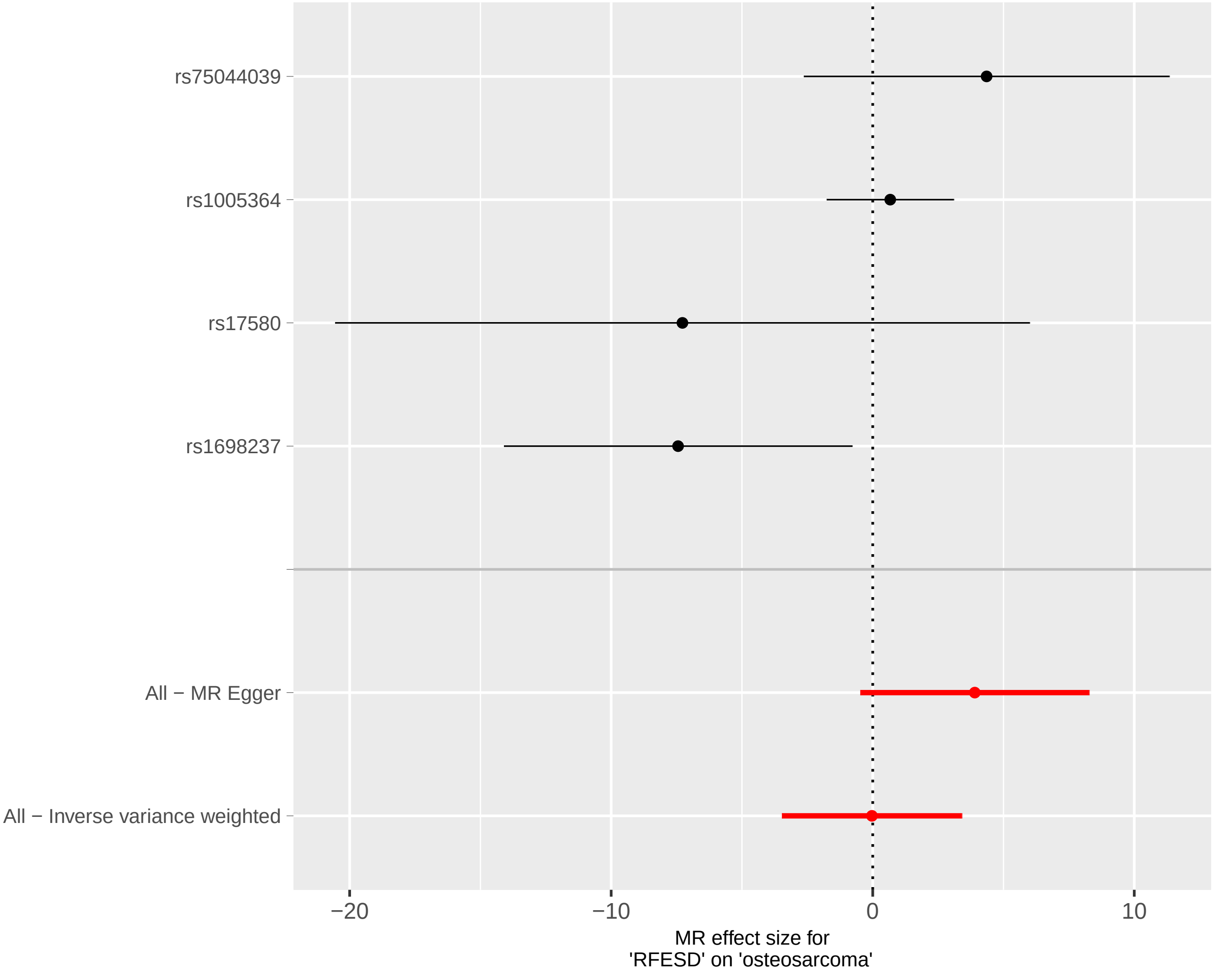

rs75044039
rs1005364
rs17580
rs1698237
All − MR Egger
All − Inverse variance weighted
−20
−10
0
10
MR effect size for
'RFESD' on 'osteosarcoma'

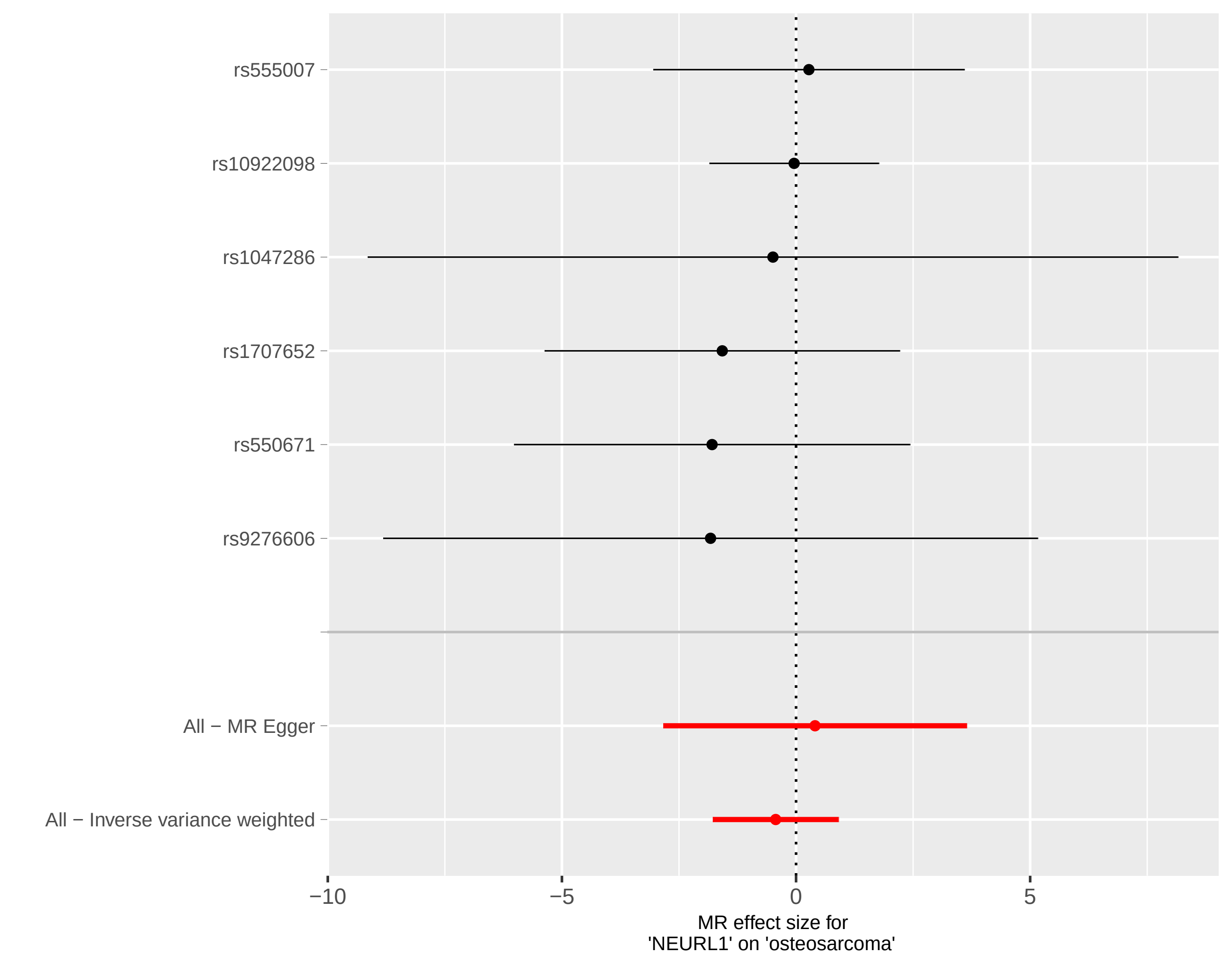
rs555007
rs10922098
rs1047286
rs1707652
rs550671
rs9276606
All − MR Egger
All − Inverse variance weighted
−10
−5
0
5
MR effect size for
'NEURL1' on 'osteosarcoma'

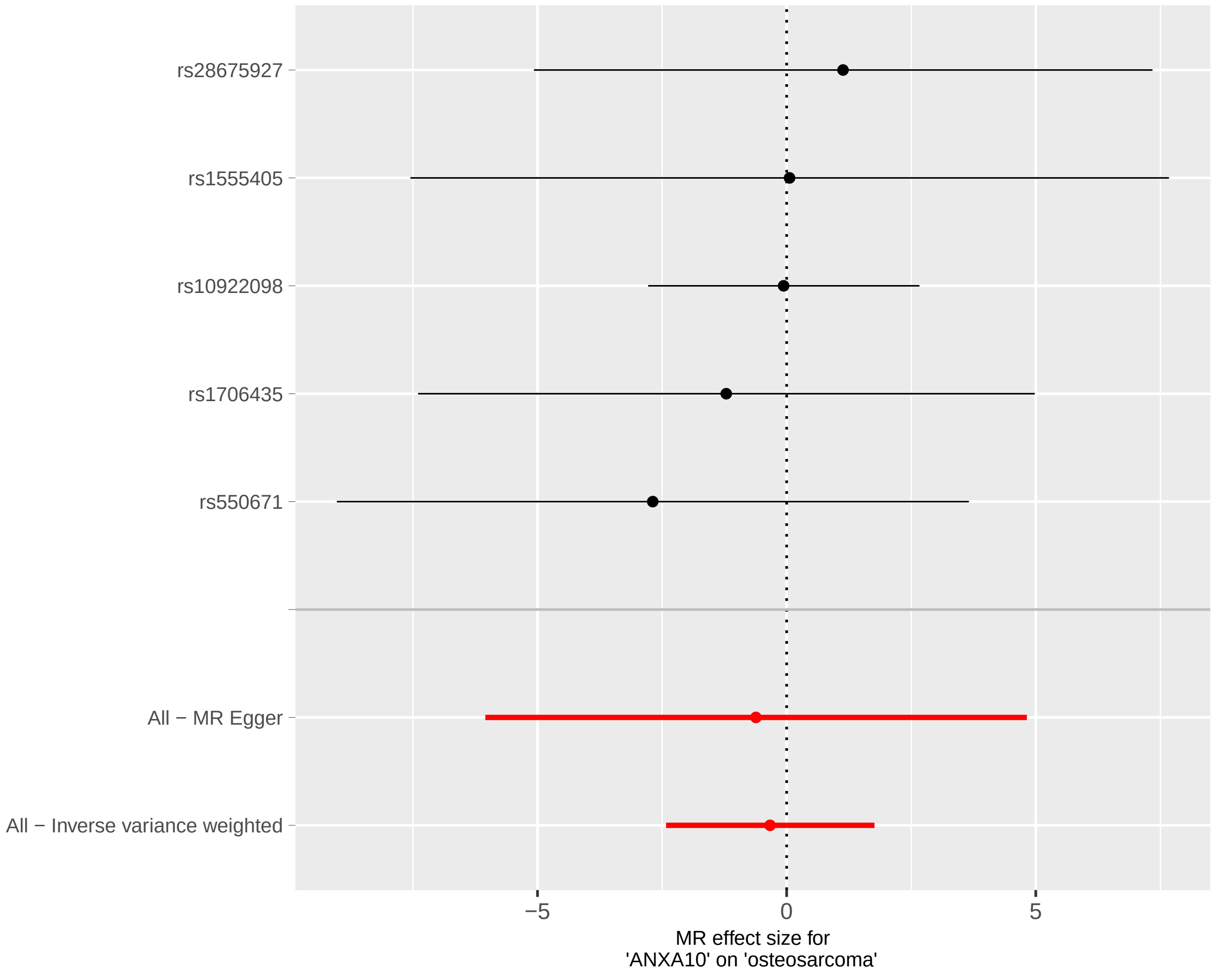
rs28675927
rs1555405
rs10922098
rs1706435
rs550671
All − MR Egger
All − Inverse variance weighted
−5
0
5
MR effect size for
'ANXA10' on 'osteosarcoma'

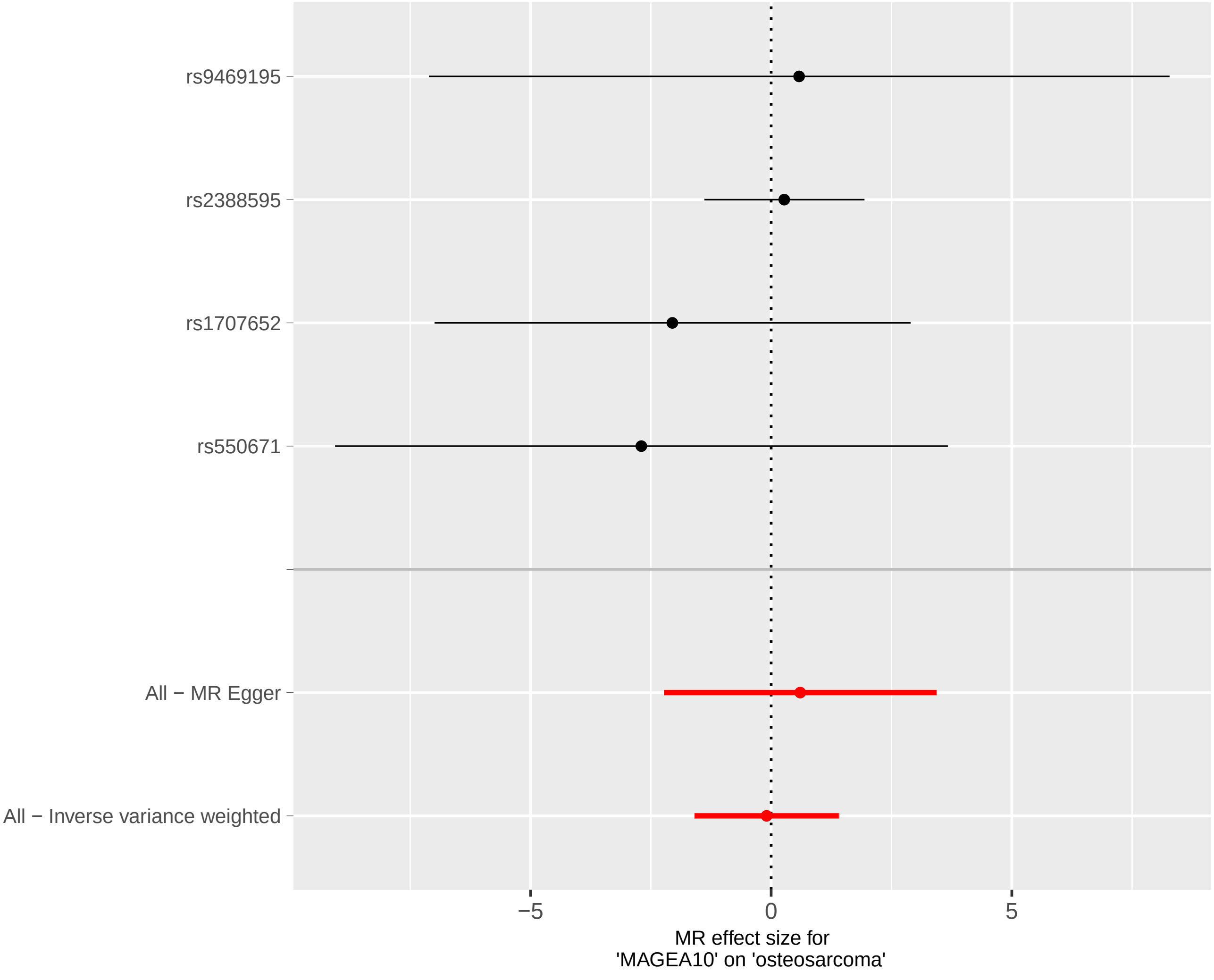

rs9469195
rs2388595
rs1707652
rs550671
All − MR Egger
All − Inverse variance weighted
−5
0
5
MR effect size for
'MAGEA10' on 'osteosarcoma'

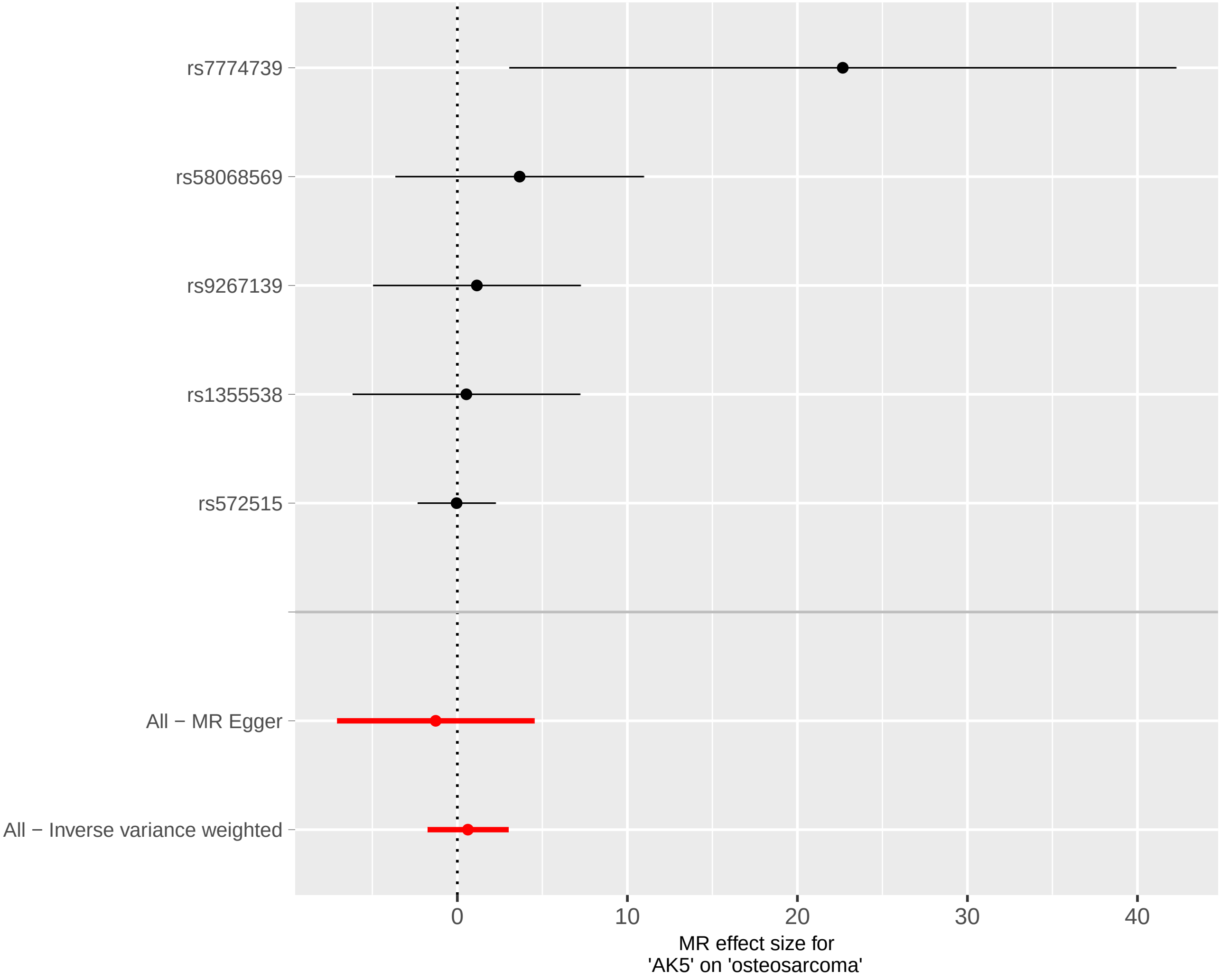
rs7774739
rs58068569
rs9267139
rs1355538
rs572515
All − MR Egger
All − Inverse variance weighted
0
10
20
30
40
MR effect size for
'AK5' on 'osteosarcoma'

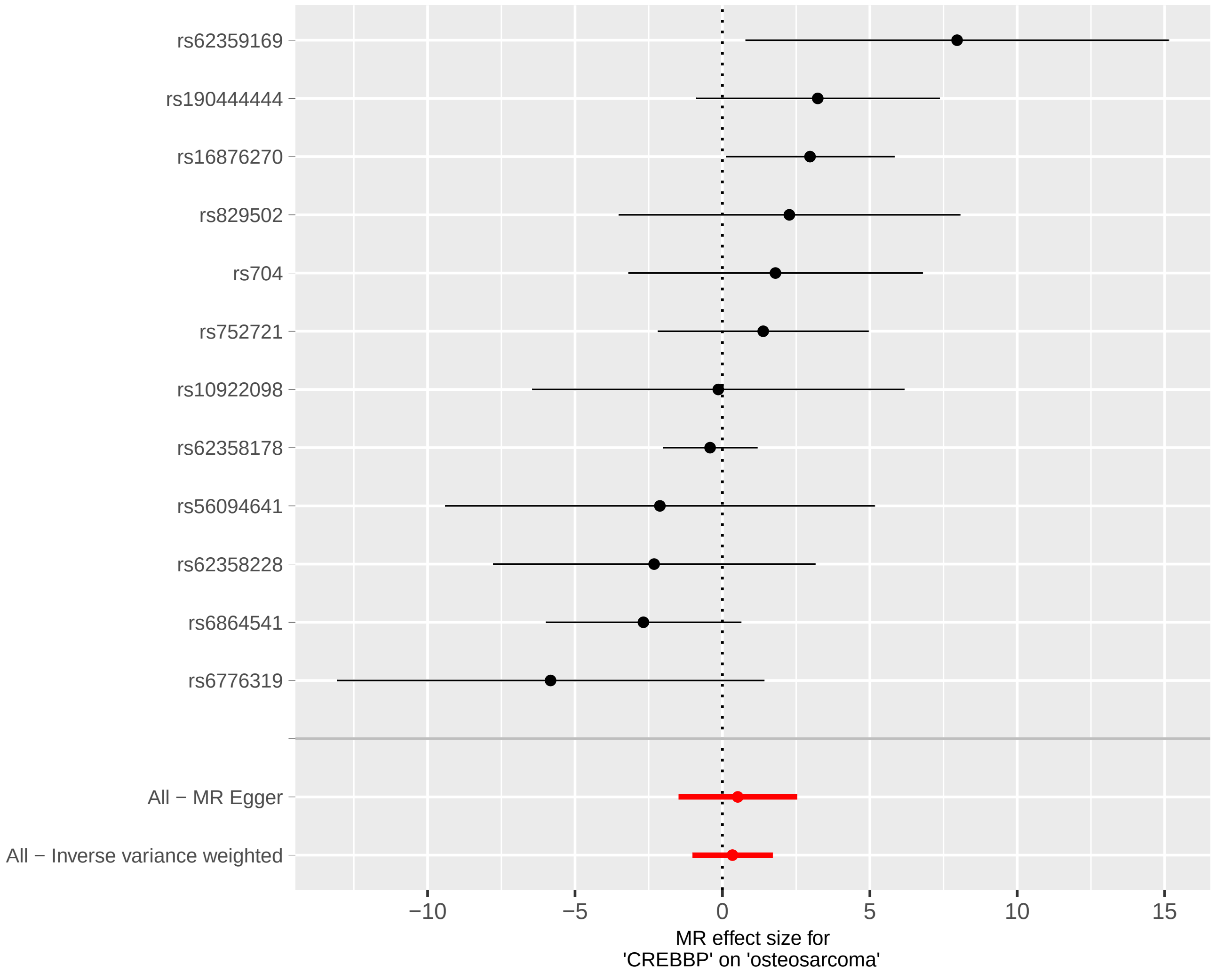

rs62359169
rs190444444
rs16876270
rs829502
rs704
rs752721
rs10922098
rs62358178
rs56094641
rs62358228
rs6864541
rs6776319
All − MR Egger
All − Inverse variance weighted
−10
−5
0
5
10
15
MR effect size for
'CREBBP' on 'osteosarcoma'

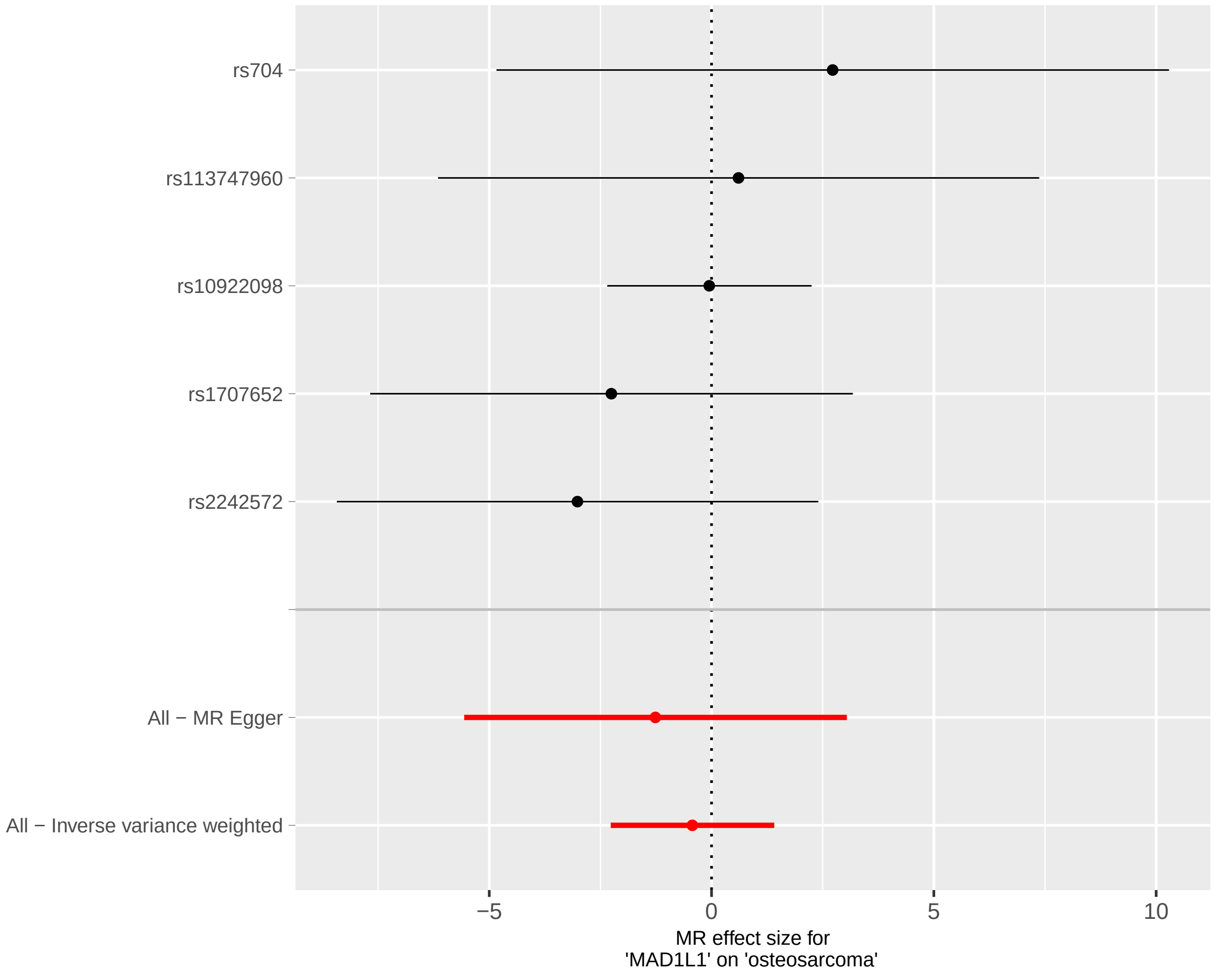

rs704
rs113747960
rs10922098
rs1707652
rs2242572
All − MR Egger
All − Inverse variance weighted
−5
0
5
10
MR effect size for
'MAD1L1' on 'osteosarcoma'

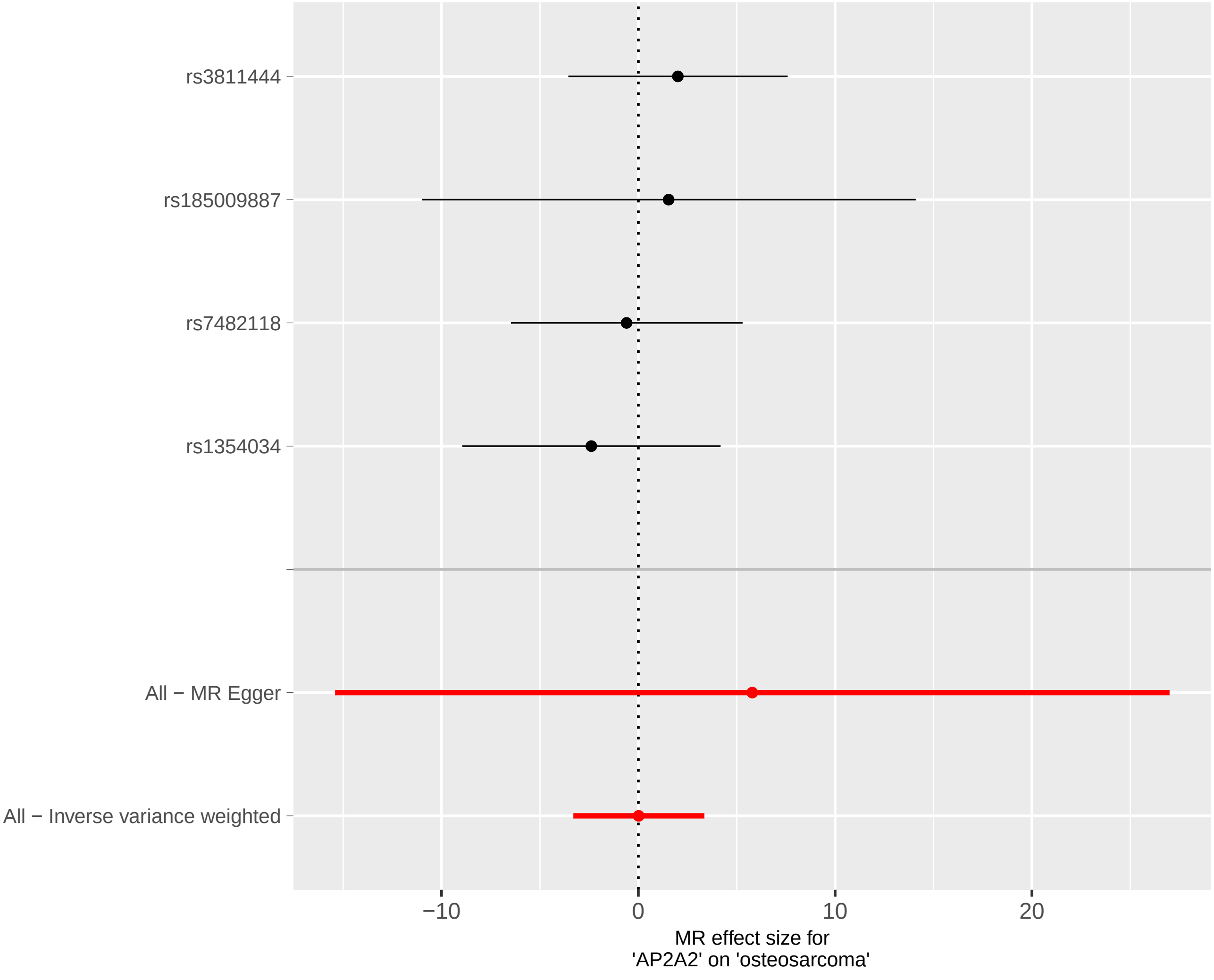

rs3811444
rs185009887
rs7482118
rs1354034
All − MR Egger
All − Inverse variance weighted
−10
0
10
20
MR effect size for
'AP2A2' on 'osteosarcoma'

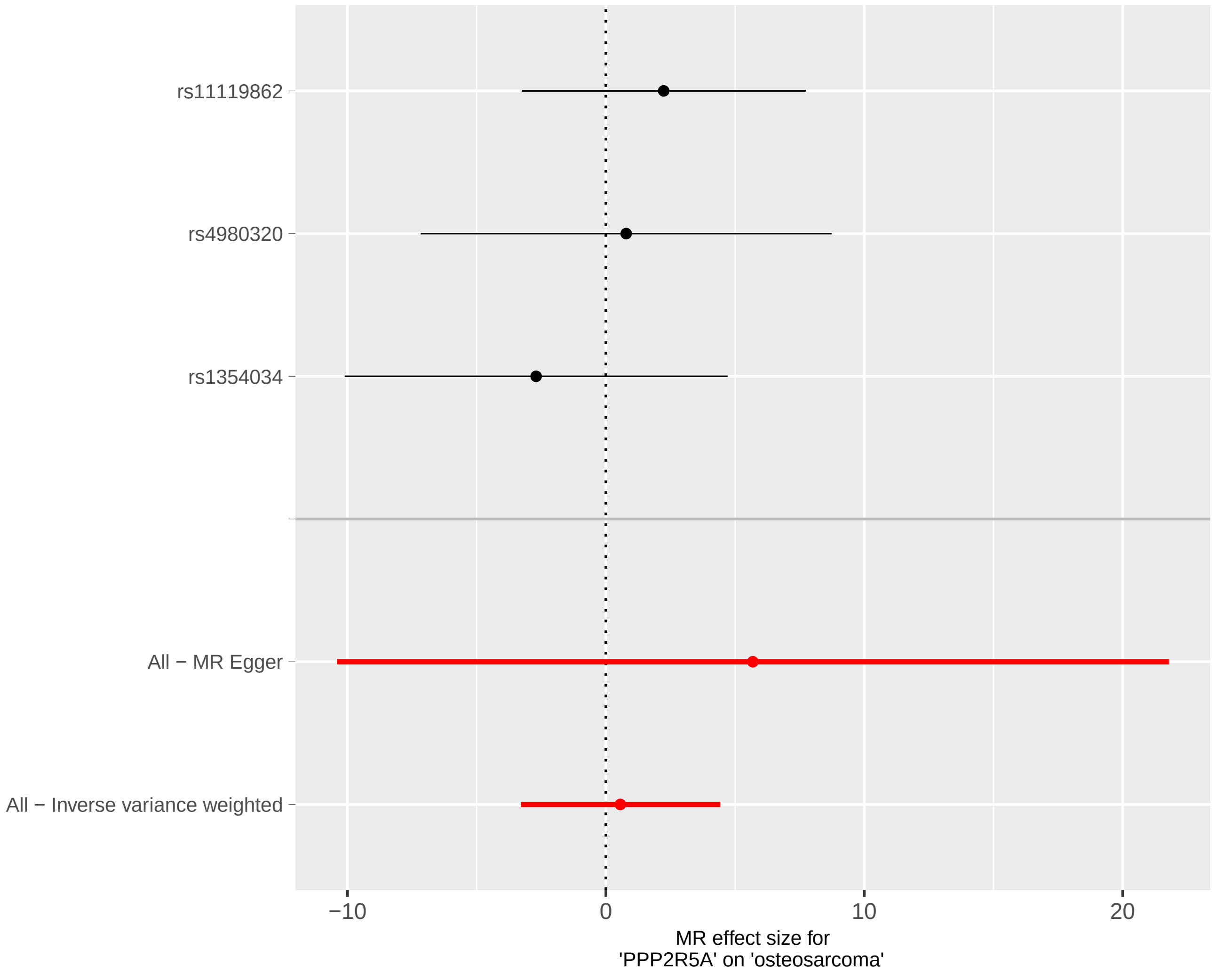
rs11119862
rs4980320
rs1354034
All − MR Egger
All − Inverse variance weighted
−10
0
10
20
MR effect size for
'PPP2R5A' on 'osteosarcoma'

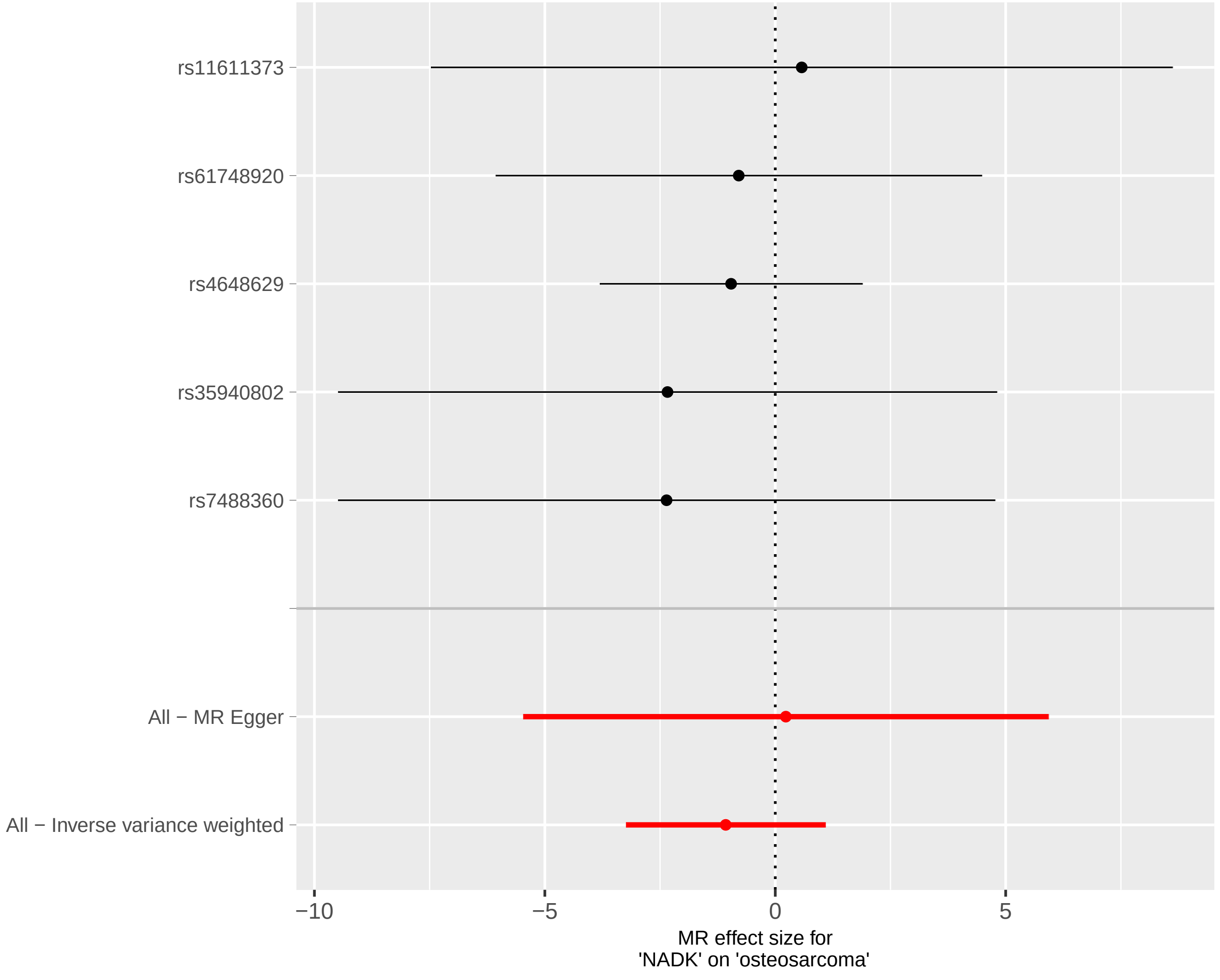
rs11611373
rs61748920
rs4648629
rs35940802
rs7488360
All − MR Egger
All − Inverse variance weighted
−10
−5
0
5
MR effect size for
'NADK' on 'osteosarcoma'

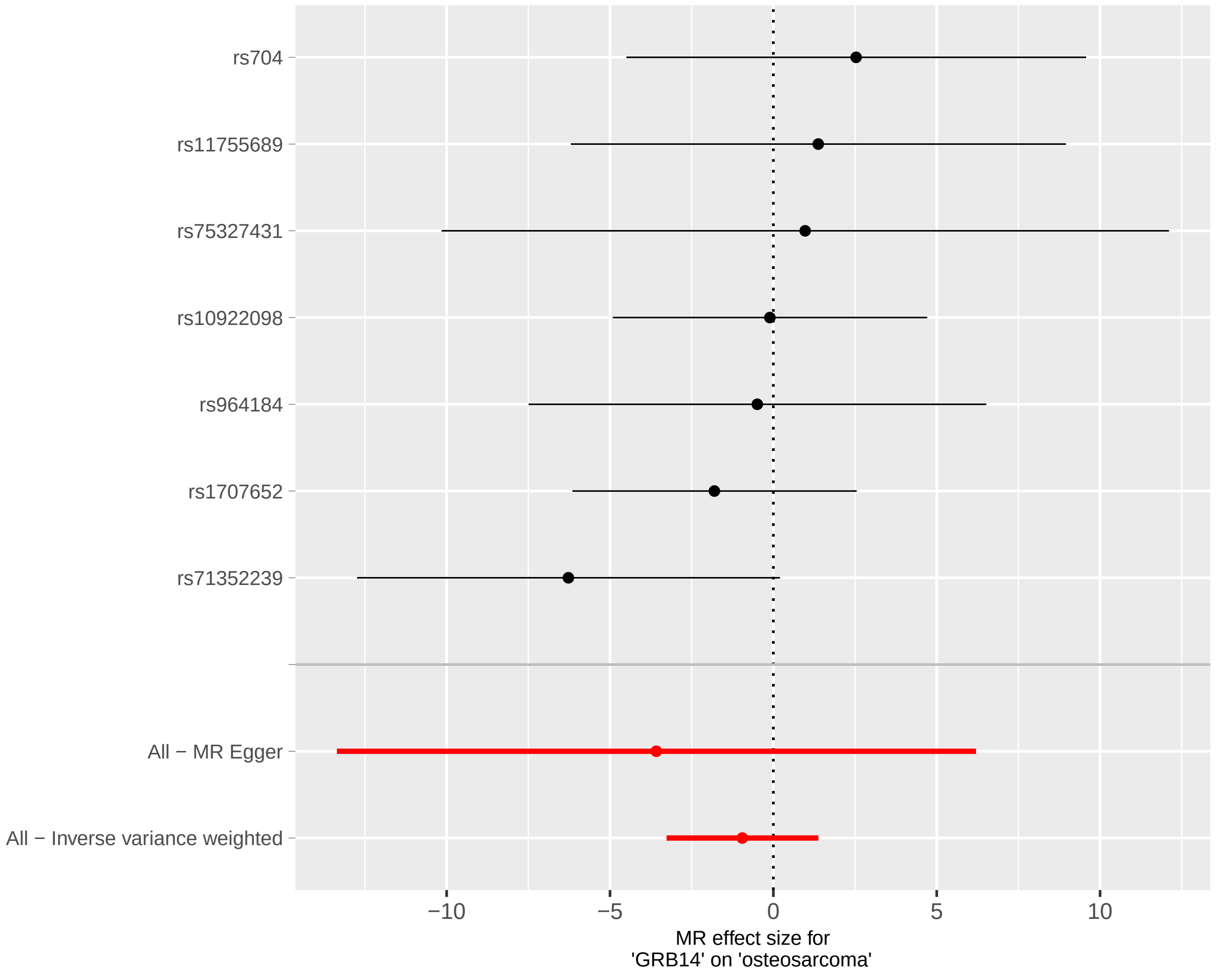

rs704
rs11755689
rs75327431
rs10922098
rs964184
rs1707652
rs71352239
All − MR Egger
All − Inverse variance weighted
−10
−5
0
5
10
MR effect size for
'GRB14' on 'osteosarcoma'

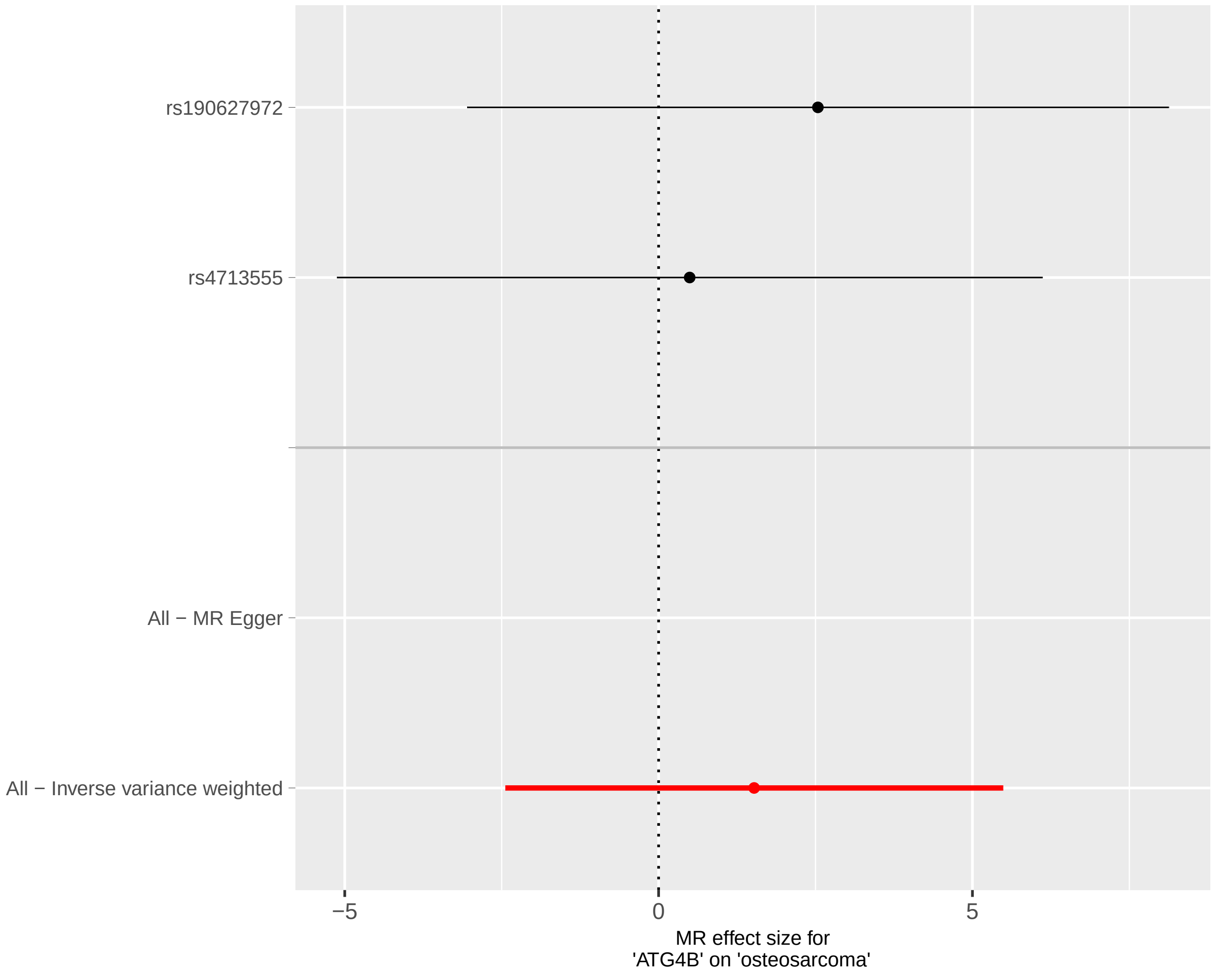

rs190627972
rs4713555
All − MR Egger
All − Inverse variance weighted
−5
0
5
MR effect size for
'ATG4B' on 'osteosarcoma'

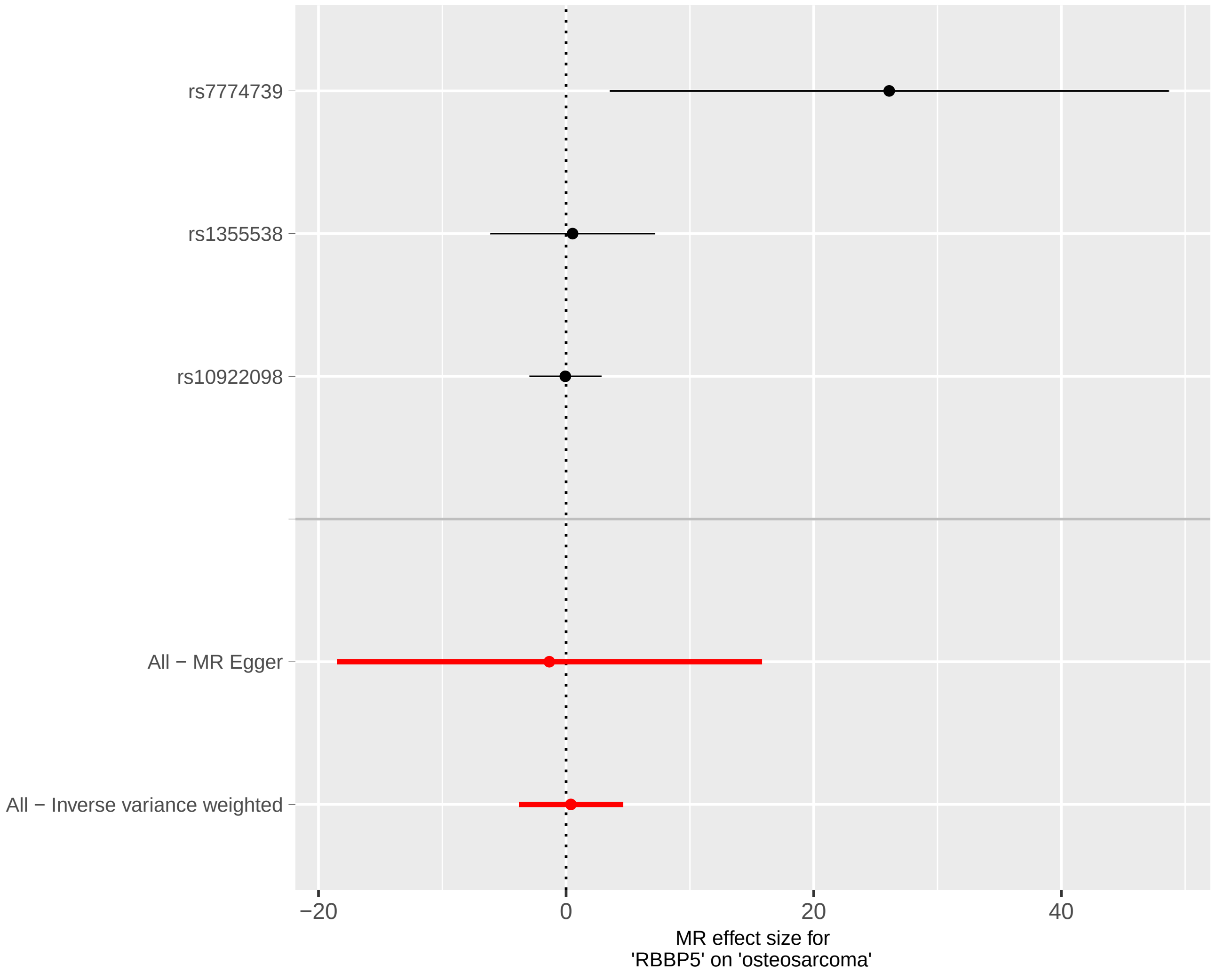

rs7774739
rs1355538
rs10922098
All − MR Egger
All − Inverse variance weighted
−20
0
20
40
MR effect size for
'RBBP5' on 'osteosarcoma'

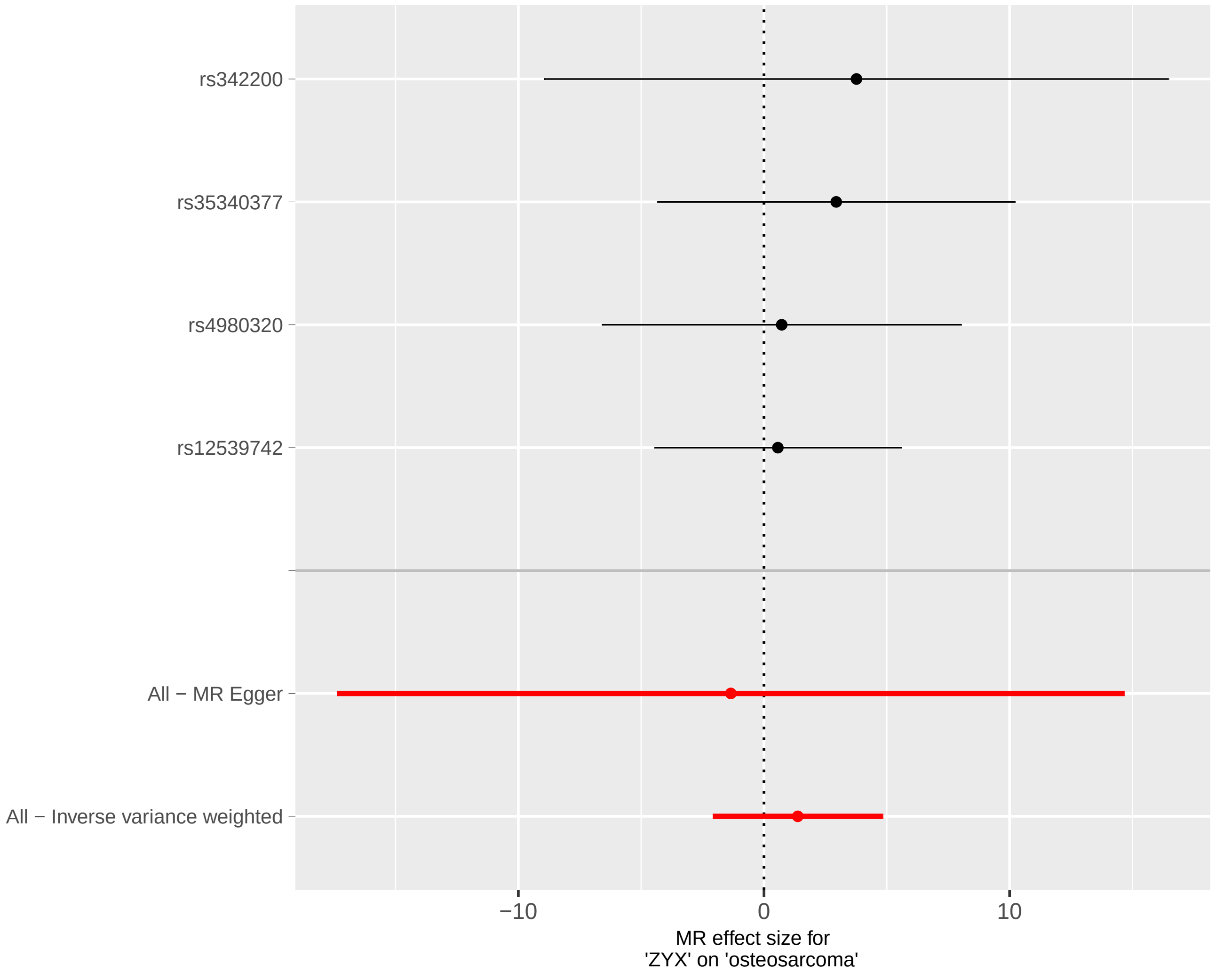

rs342200
rs35340377
rs4980320
rs12539742
All − MR Egger
All − Inverse variance weighted
−10
0
10
MR effect size for
'ZYX' on 'osteosarcoma'

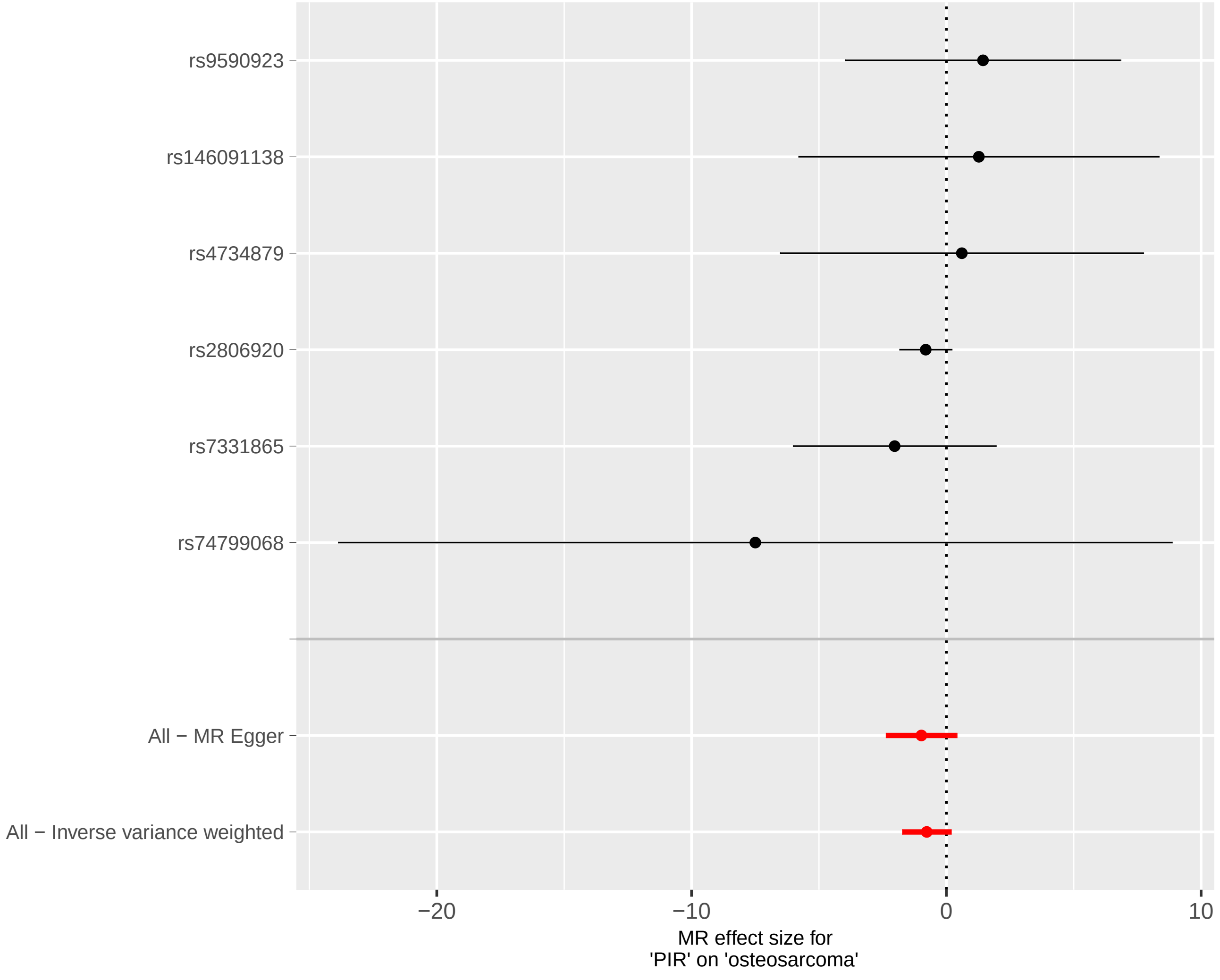

rs9590923
rs146091138
rs4734879
rs2806920
rs7331865
rs74799068
All − MR Egger
All − Inverse variance weighted
−20
−10
0
10
MR effect size for
'PIR' on 'osteosarcoma'

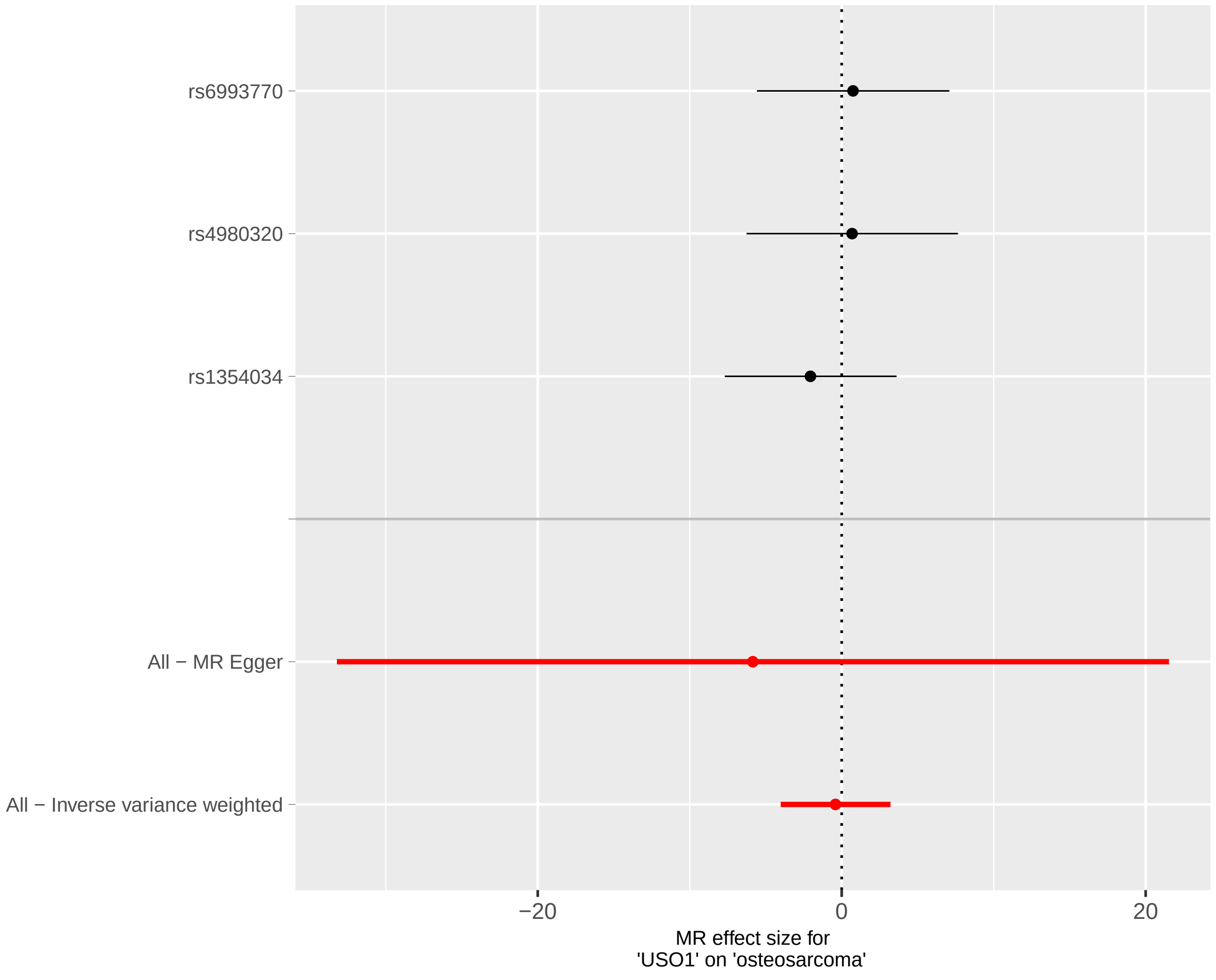
rs6993770
rs4980320
rs1354034
All − MR Egger
All − Inverse variance weighted
−20
0
20
MR effect size for
'USO1' on 'osteosarcoma'

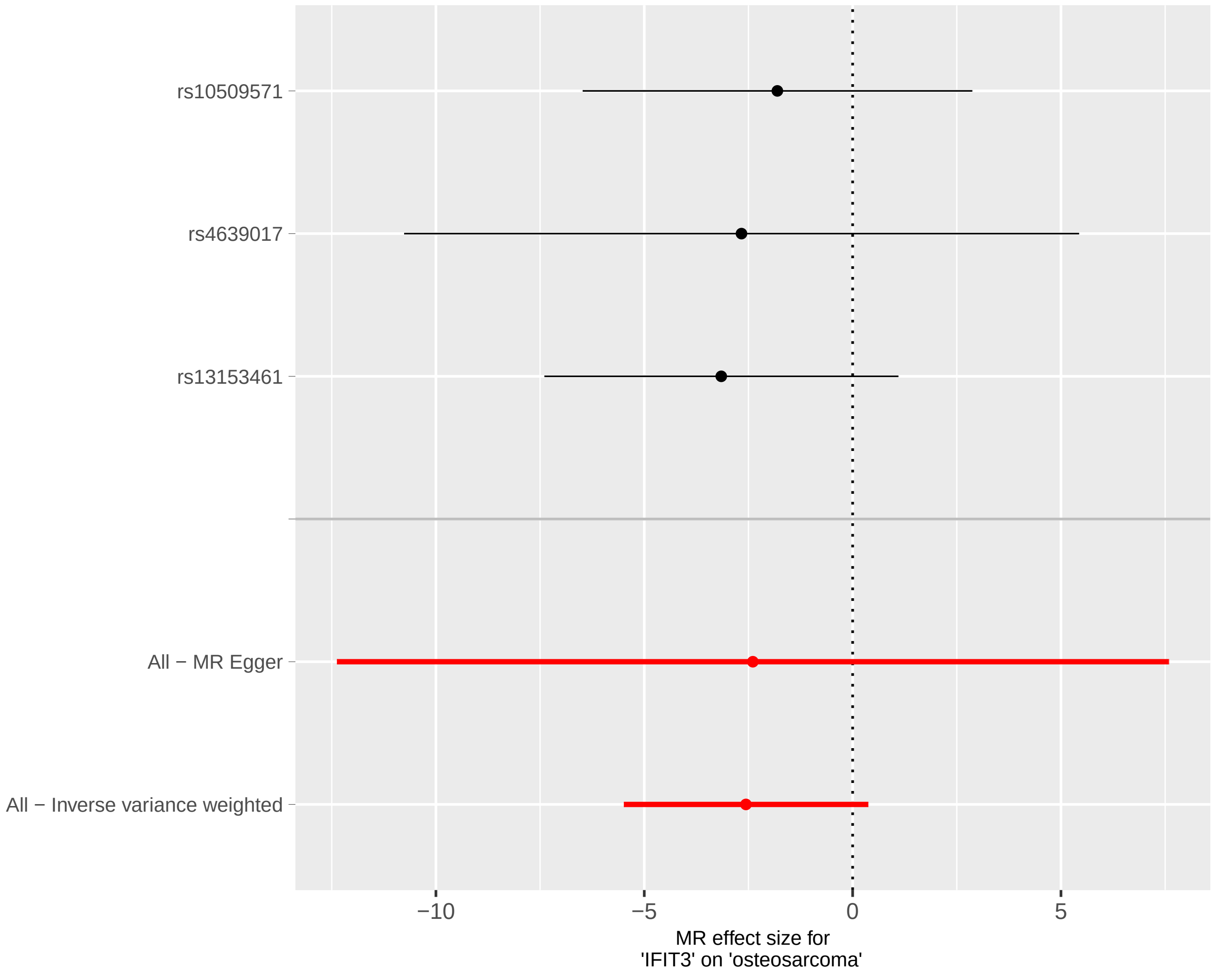
rs10509571
rs4639017
rs13153461
All − MR Egger
All − Inverse variance weighted
−10
−5
0
5
MR effect size for
'IFIT3' on 'osteosarcoma'

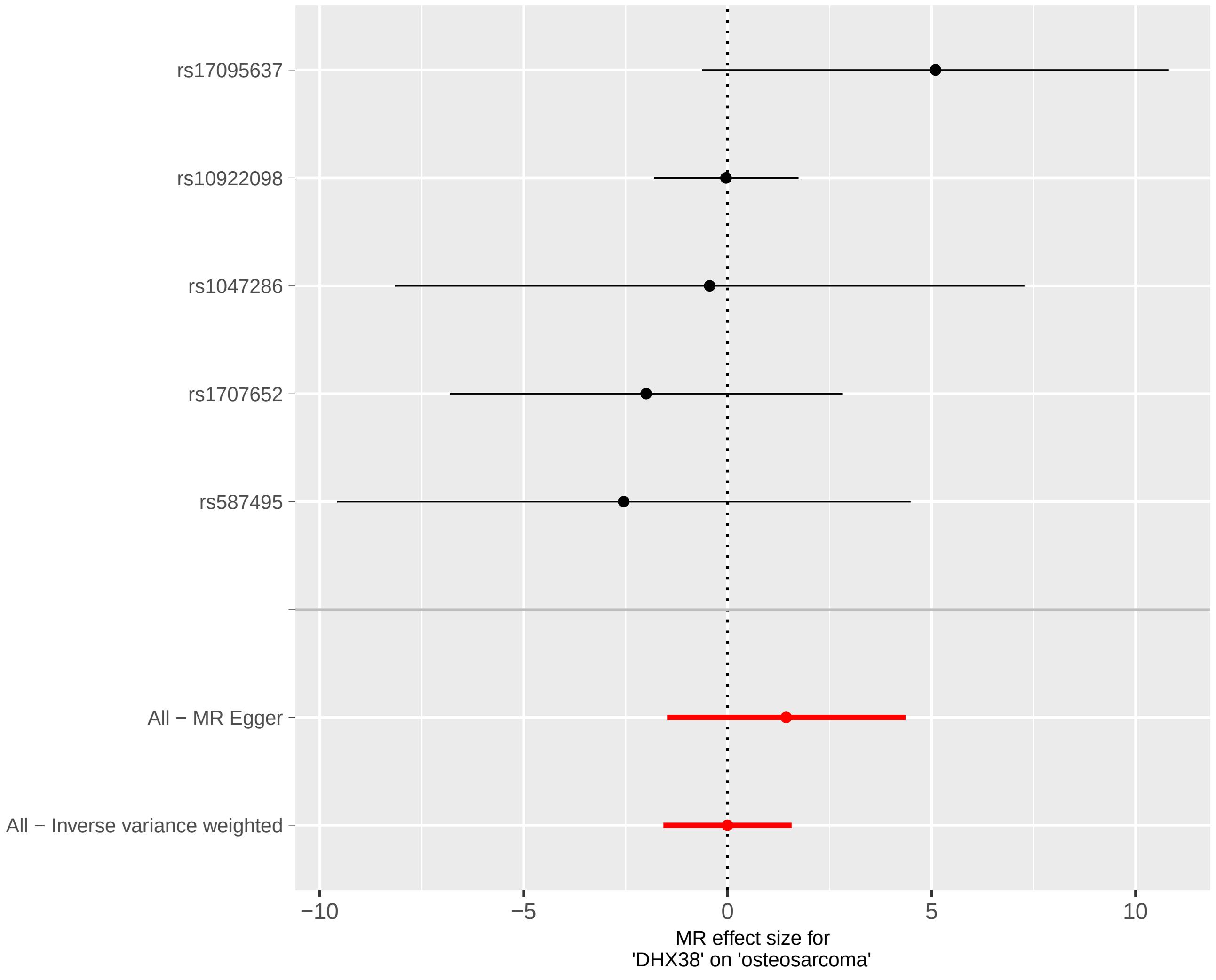

rs17095637
rs10922098
rs1047286
rs1707652
rs587495
All − MR Egger
All − Inverse variance weighted
−10
−5
0
5
10
MR effect size for
'DHX38' on 'osteosarcoma'

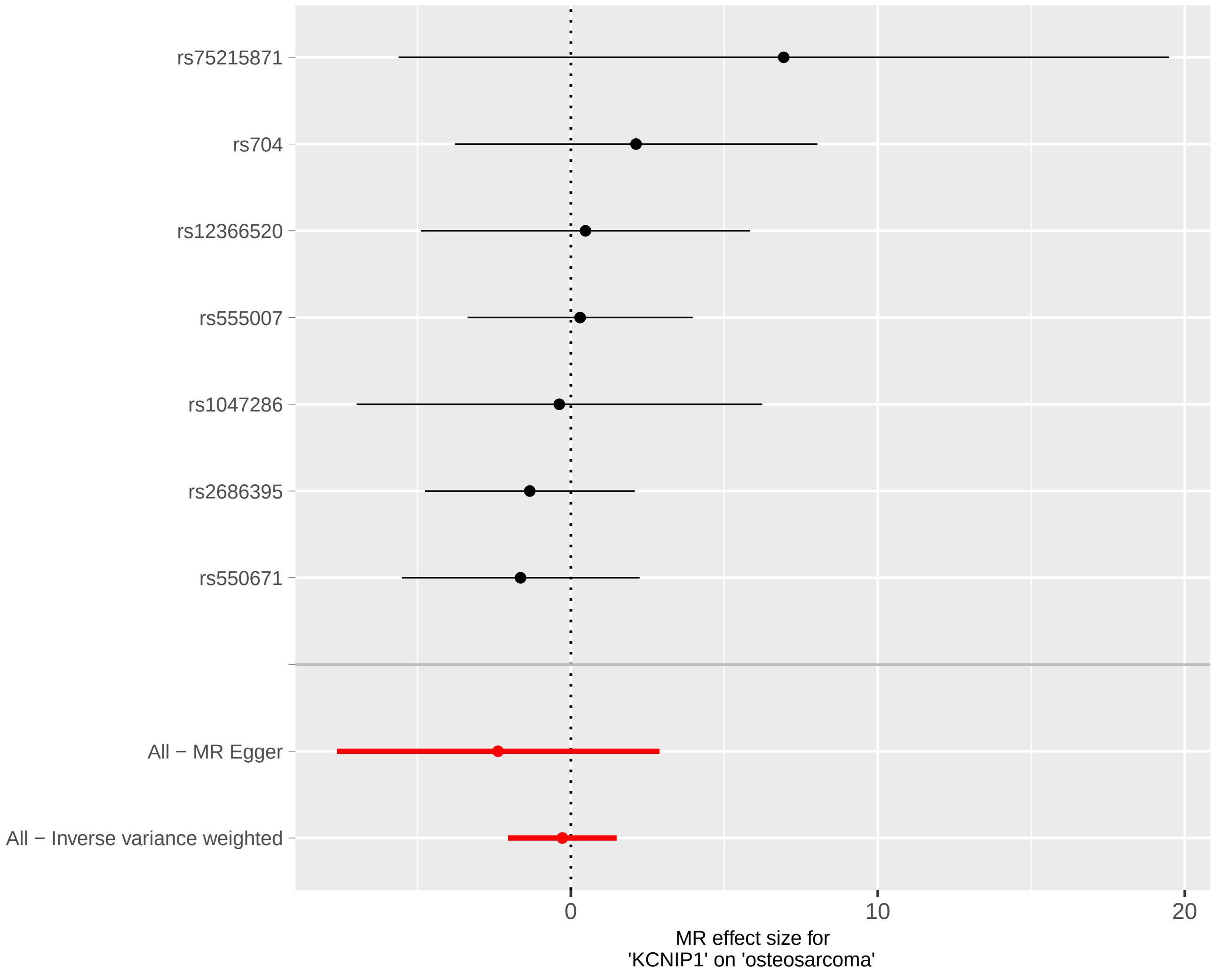

rs75215871
rs704
rs12366520
rs555007
rs1047286
rs2686395
rs550671
All − MR Egger
All − Inverse variance weighted
0
10
20
MR effect size for
'KCNIP1' on 'osteosarcoma'

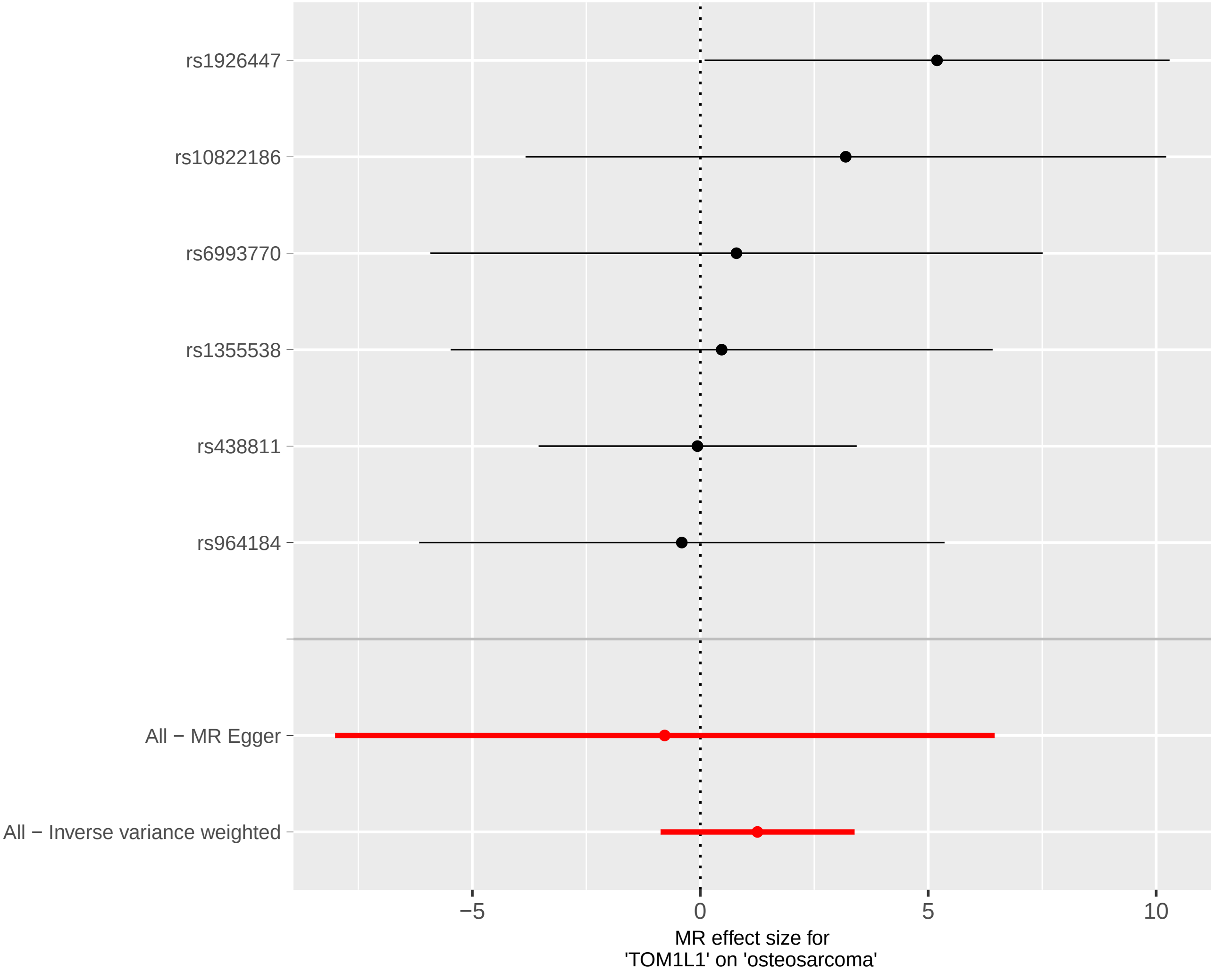

rs1926447
rs10822186
rs6993770
rs1355538
rs438811
rs964184
All − MR Egger
All − Inverse variance weighted
−5
0
5
10
MR effect size for
'TOM1L1' on 'osteosarcoma'

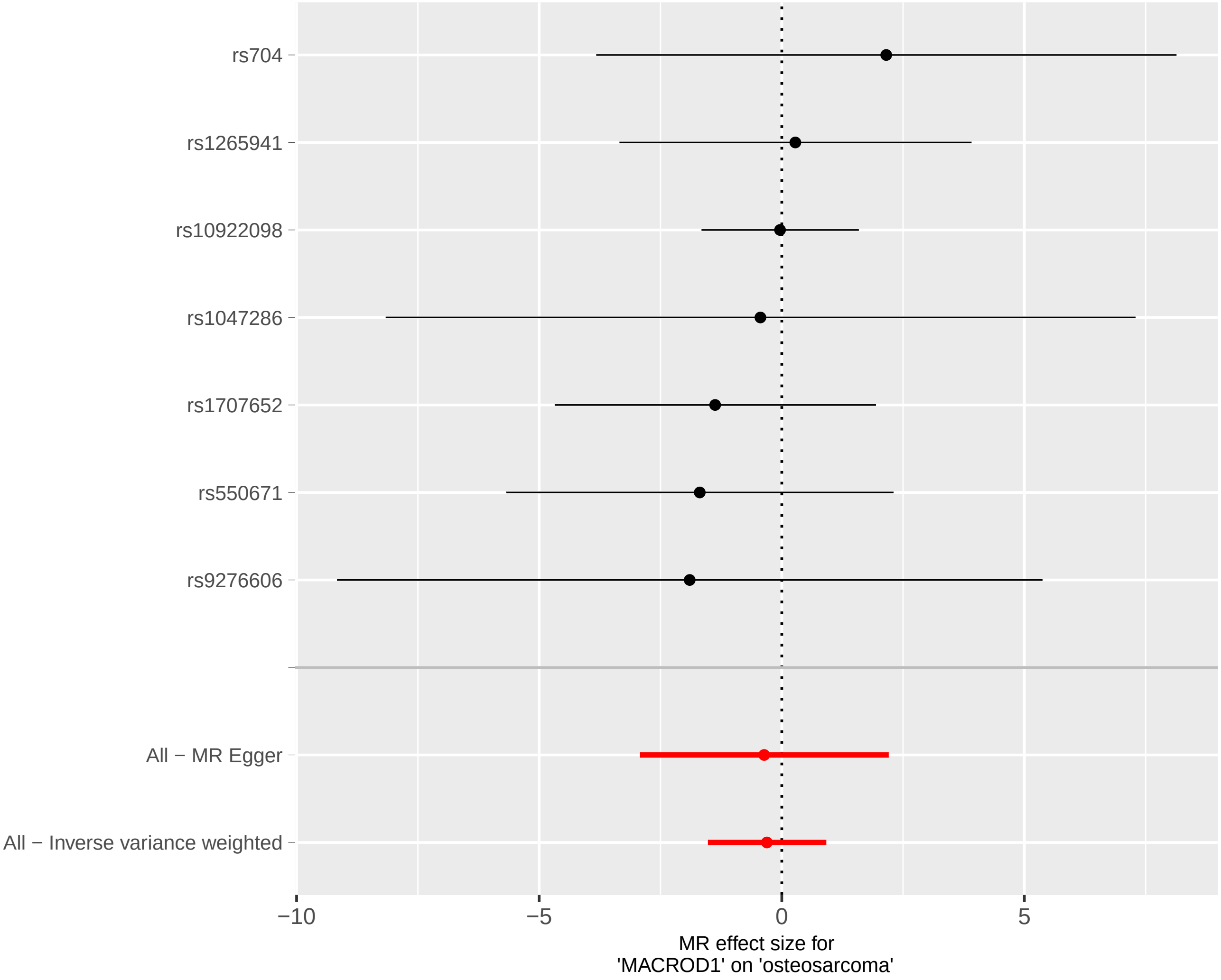

rs704
rs1265941
rs10922098
rs1047286
rs1707652
rs550671
rs9276606
All − MR Egger
All − Inverse variance weighted
−10
−5
0
5
MR effect size for
'MACROD1' on 'osteosarcoma'

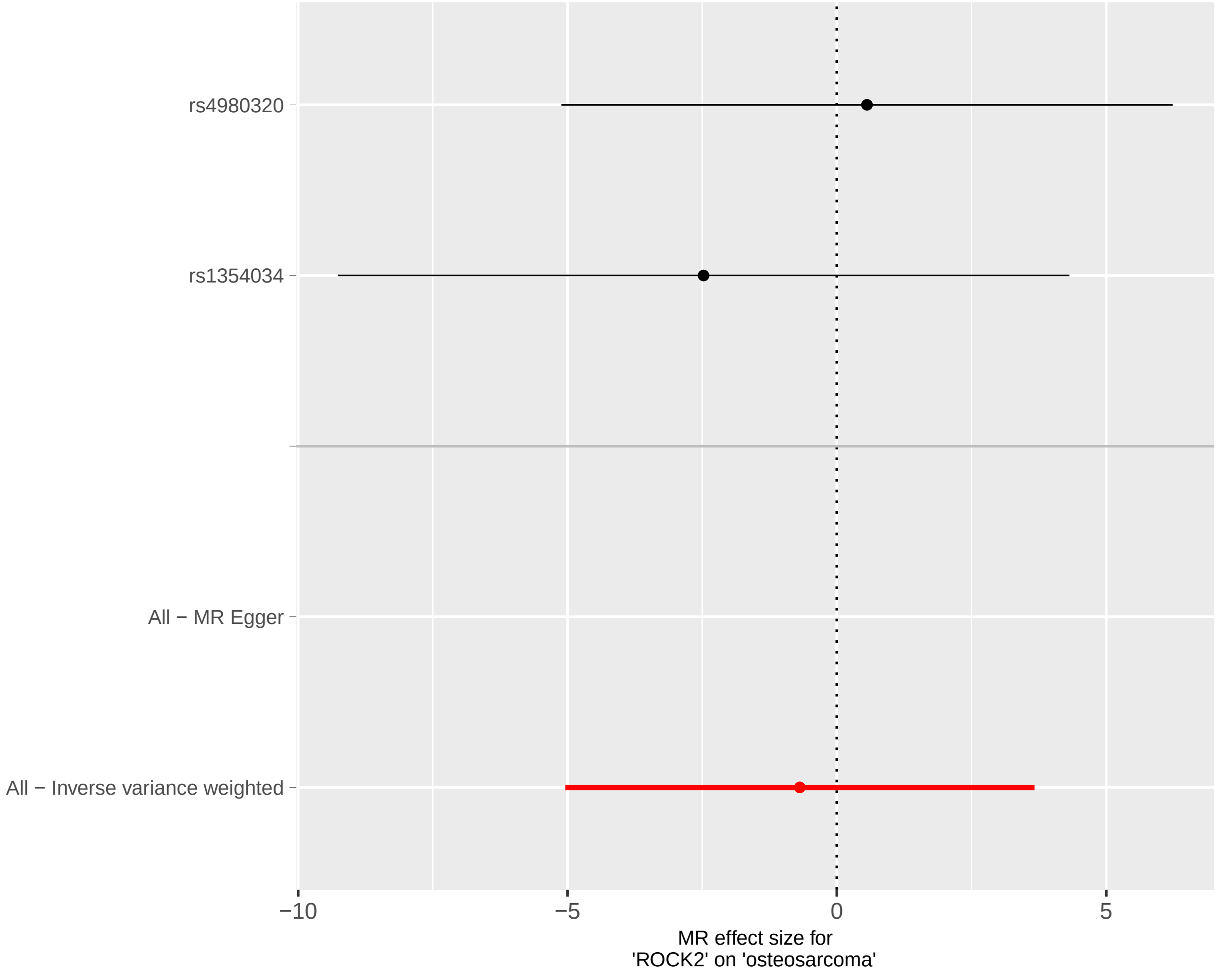
rs4980320
rs1354034
All − MR Egger
All − Inverse variance weighted
−10
−5
0
5
MR effect size for
'ROCK2' on 'osteosarcoma'

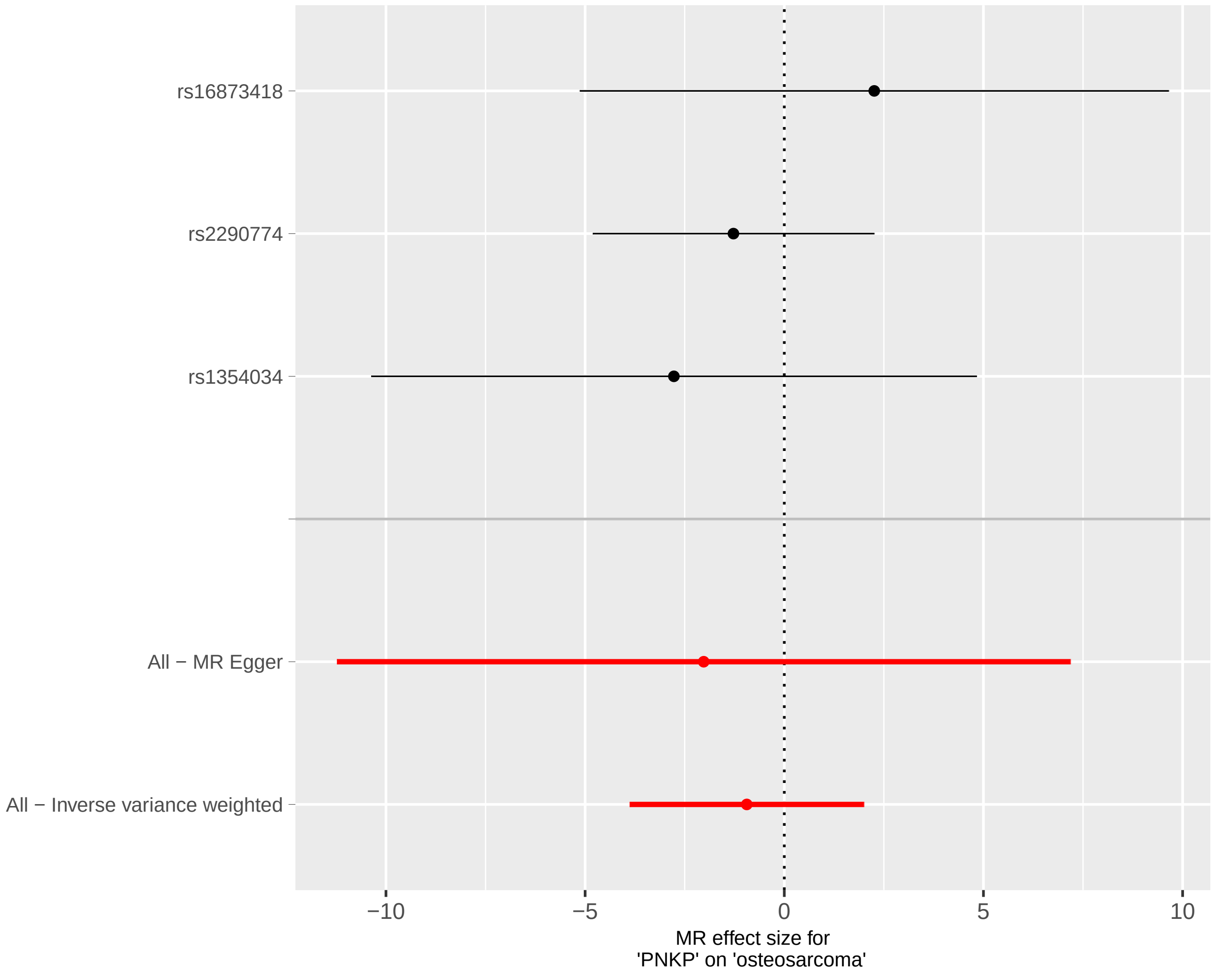

rs16873418
rs2290774
rs1354034
All − MR Egger
All − Inverse variance weighted
−10
−5
0
5
10
MR effect size for
'PNKP' on 'osteosarcoma'

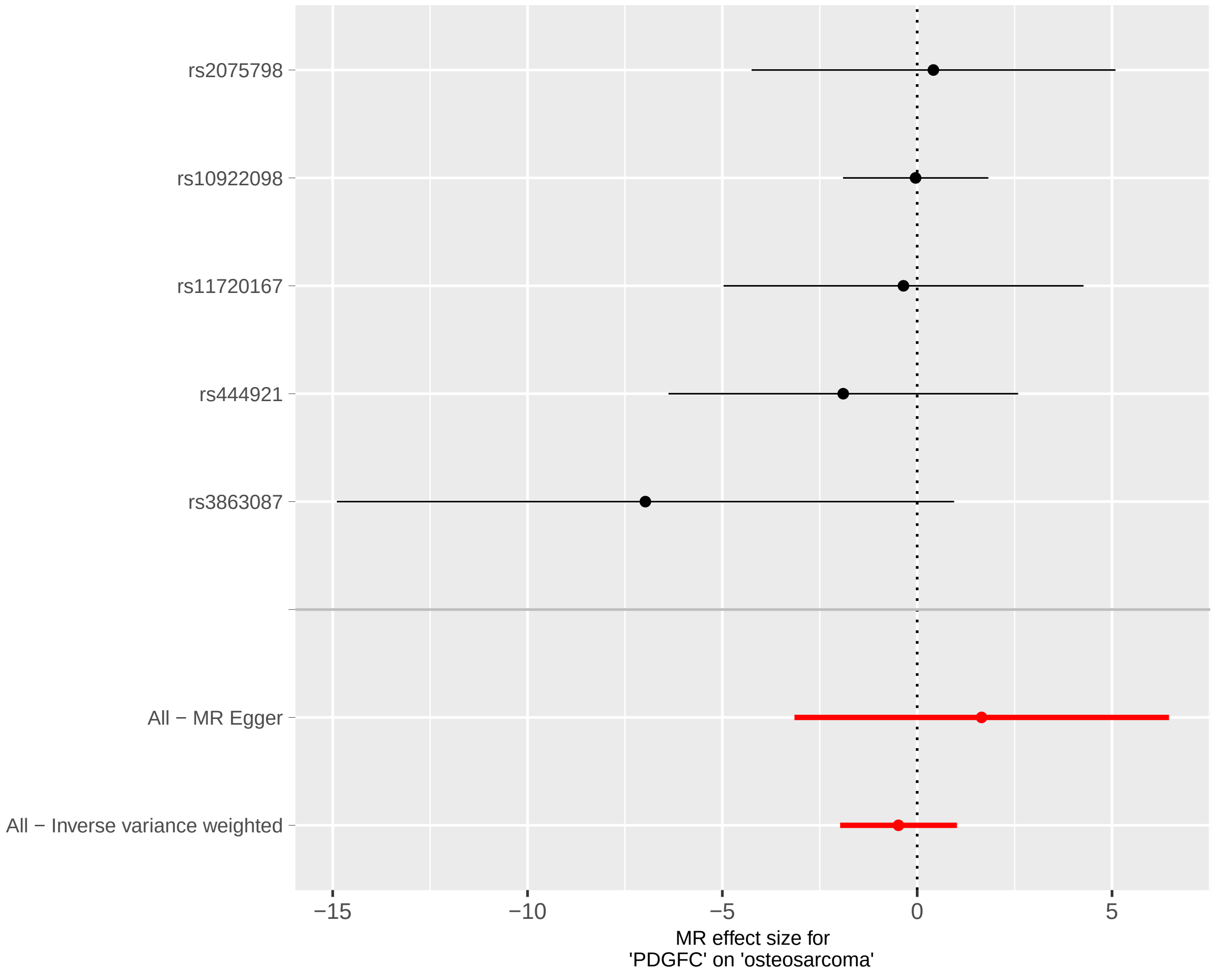

rs2075798
rs10922098
rs11720167
rs444921
rs3863087
All − MR Egger
All − Inverse variance weighted
−15
−10
−5
0
5
MR effect size for
'PDGFC' on 'osteosarcoma'

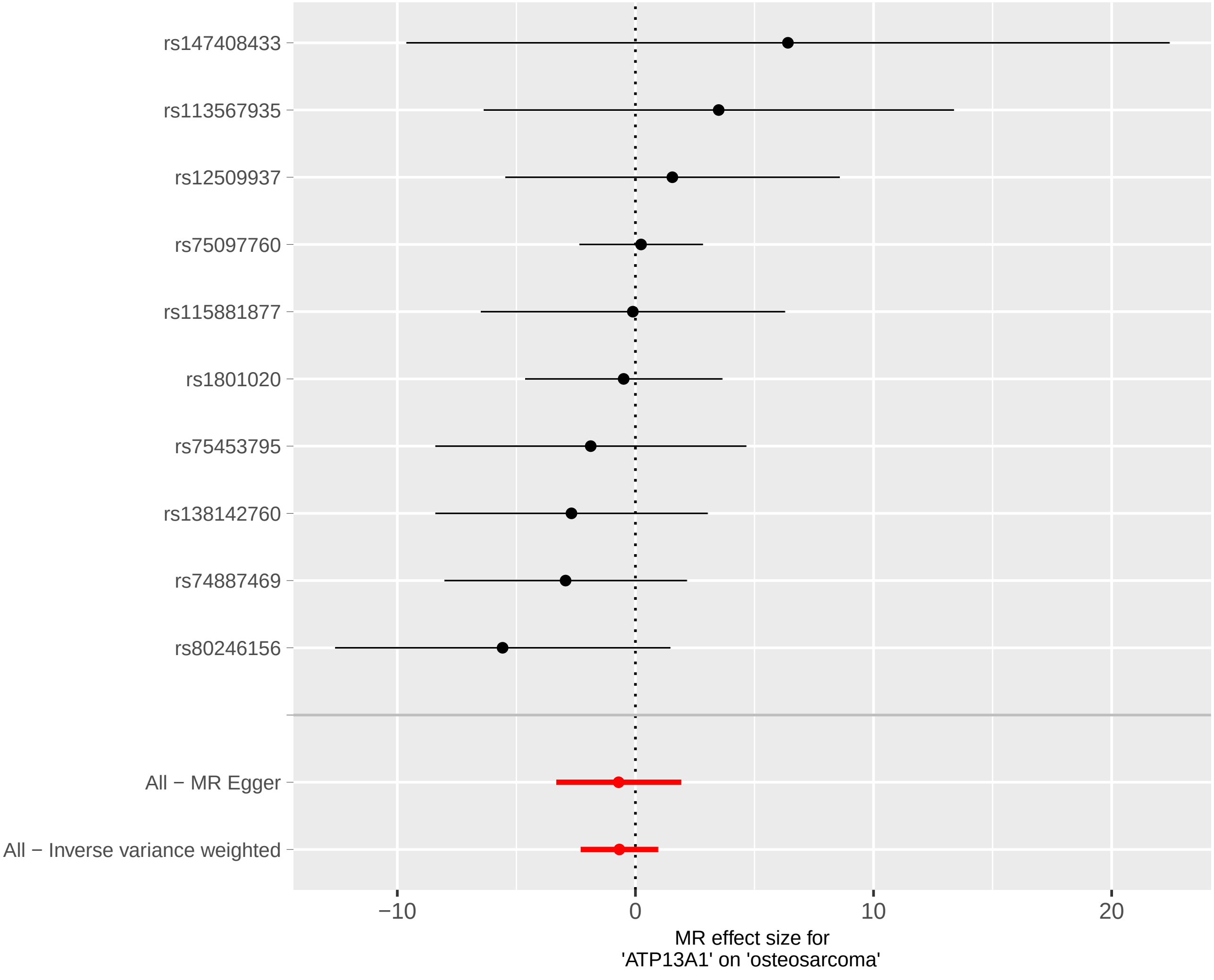

rs147408433
rs113567935
rs12509937
rs75097760
rs115881877
rs1801020
rs75453795
rs138142760
rs74887469
rs80246156
All − MR Egger
All − Inverse variance weighted
−10
0
10
20
MR effect size for
'ATP13A1' on 'osteosarcoma'

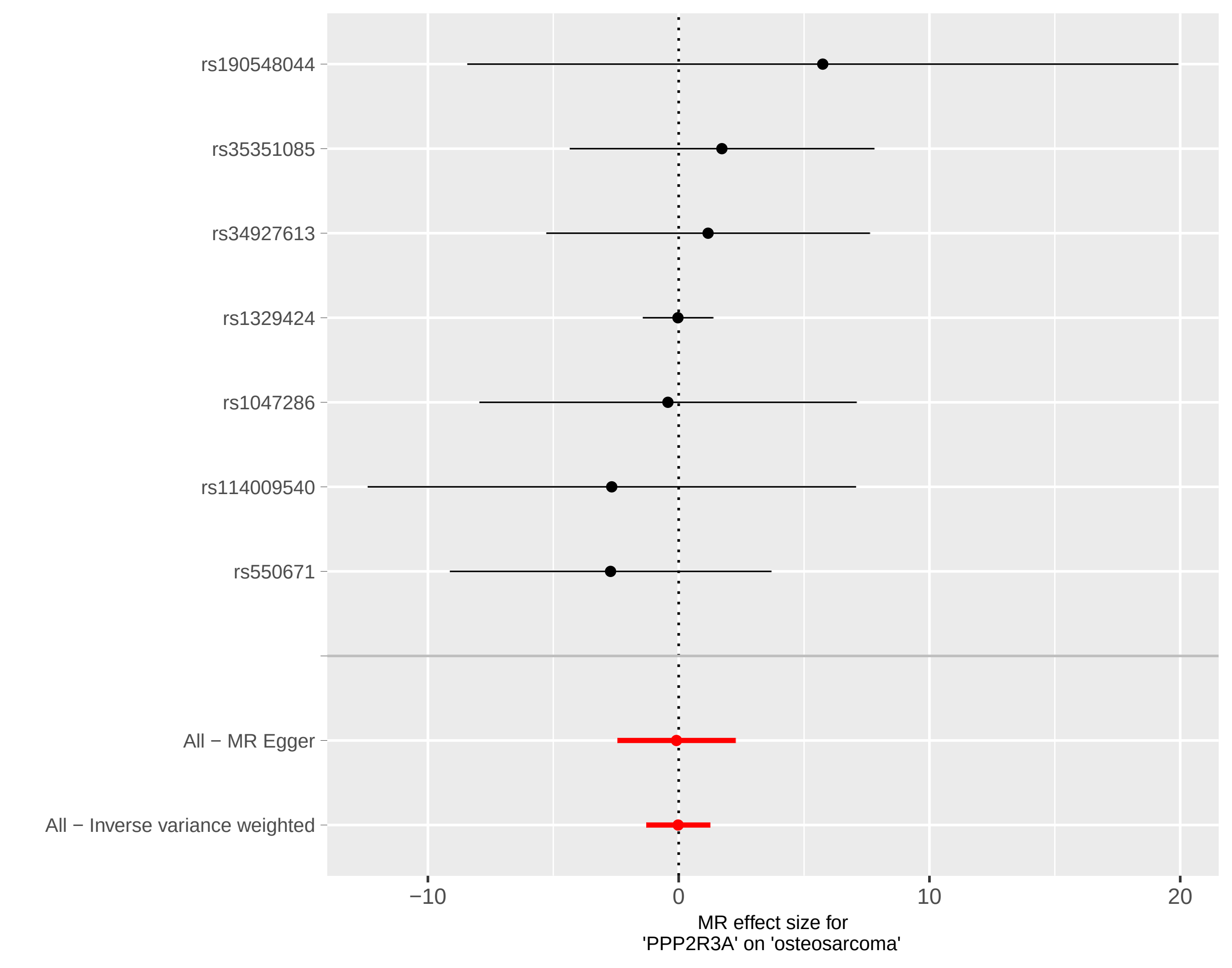

rs190548044
rs35351085
rs34927613
rs1329424
rs1047286
rs114009540
rs550671
All − MR Egger
All − Inverse variance weighted
−10
0
10
20
MR effect size for
'PPP2R3A' on 'osteosarcoma'

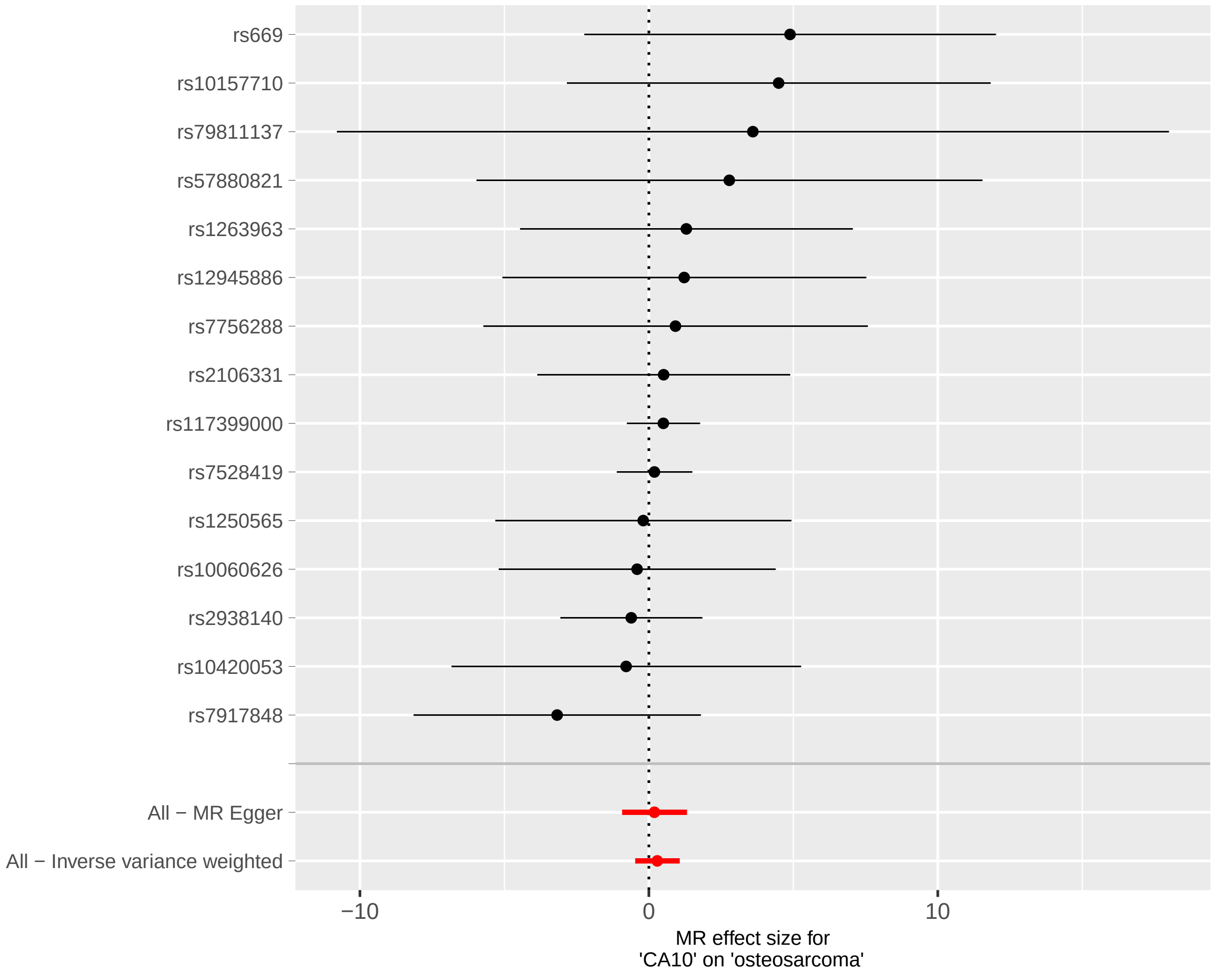
rs669
rs10157710
rs79811137
rs57880821
rs1263963
rs12945886
rs7756288
rs2106331
rs117399000
rs7528419
rs1250565
rs10060626
rs2938140
rs10420053
rs7917848
All − MR Egger
All − Inverse variance weighted
−10
0
10
MR effect size for
'CA10' on 'osteosarcoma'

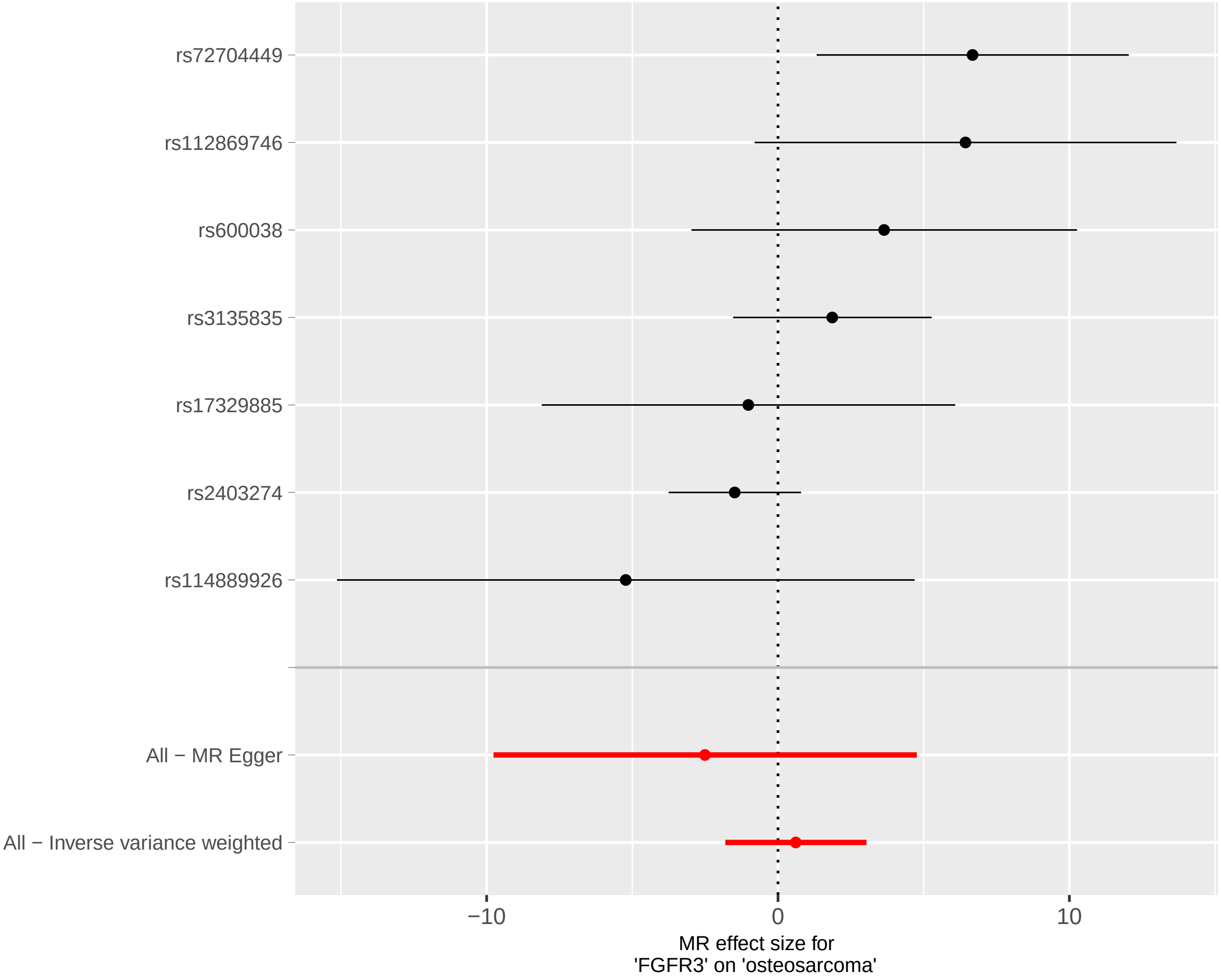
rs72704449
rs112869746
rs600038
rs3135835
rs17329885
rs2403274
rs114889926
All − MR Egger
All − Inverse variance weighted
−10
0
10
MR effect size for
'FGFR3' on 'osteosarcoma'

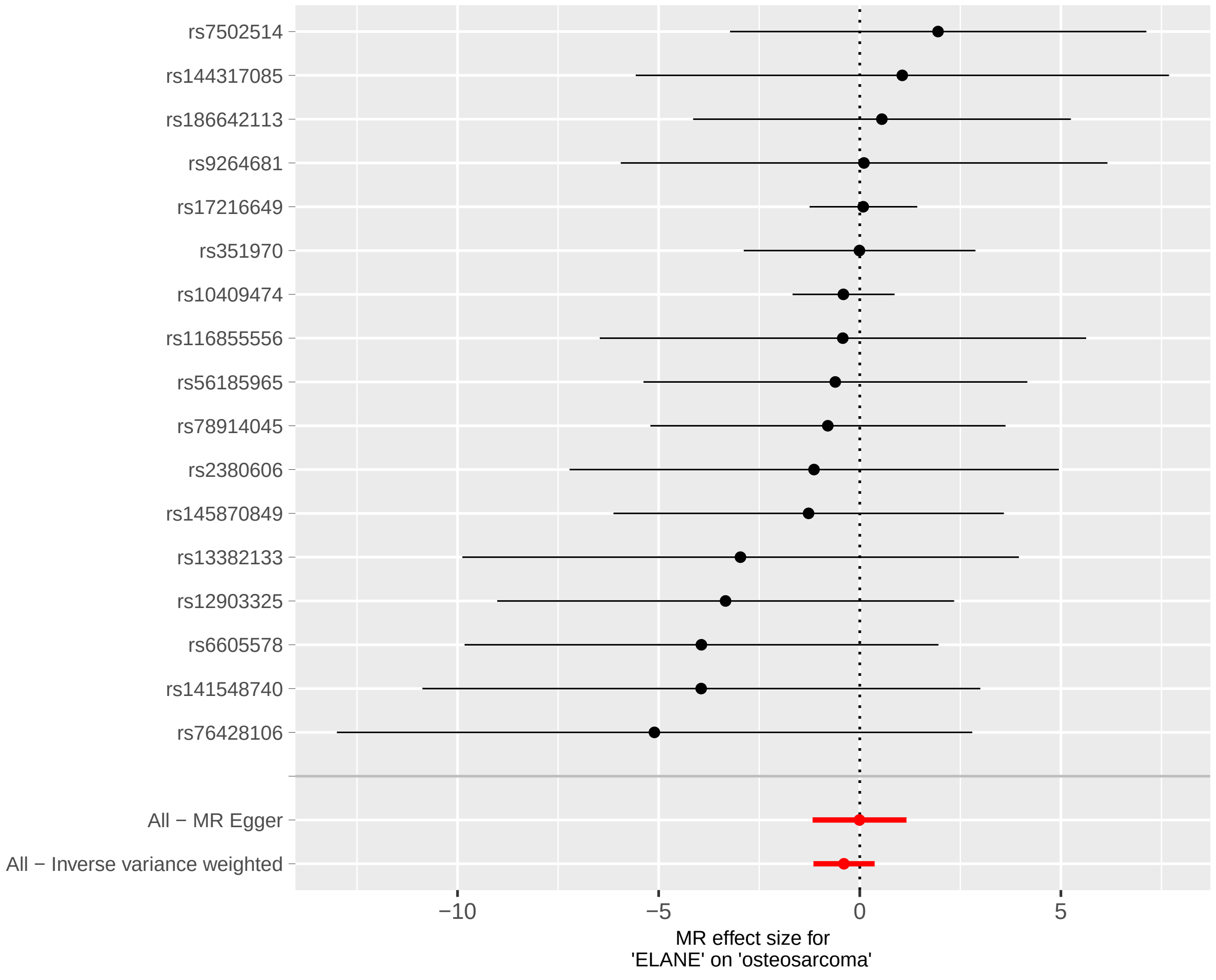
rs7502514
rs144317085
rs186642113
rs9264681
rs17216649
rs351970
rs10409474
rs116855556
rs56185965
rs78914045
rs2380606
rs145870849
rs13382133
rs12903325
rs6605578
rs141548740
rs76428106
All − MR Egger
All − Inverse variance weighted
−10
−5
0
5
MR effect size for
'ELANE' on 'osteosarcoma'

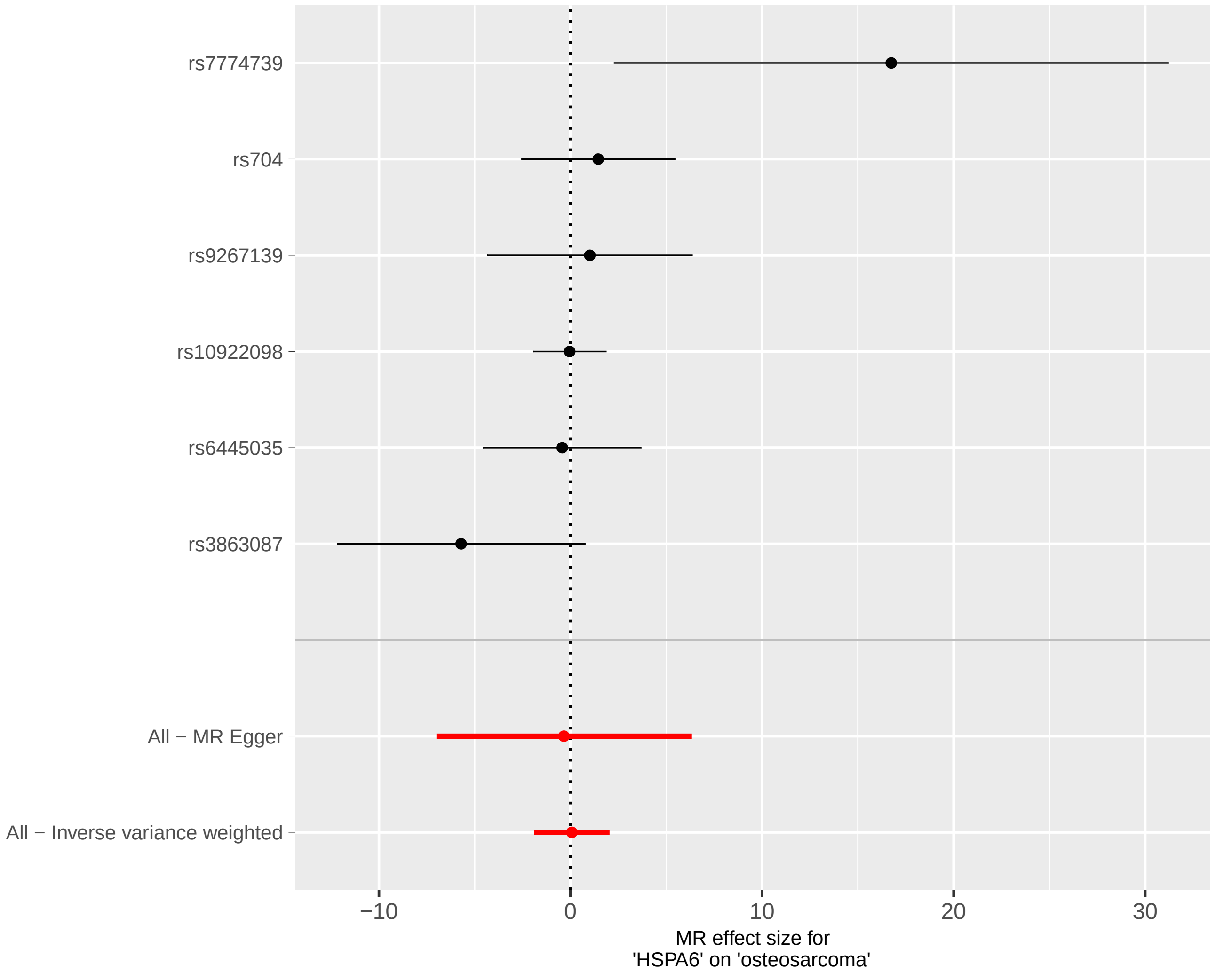
rs7774739
rs704
rs9267139
rs10922098
rs6445035
rs3863087
All − MR Egger
All − Inverse variance weighted
−10
0
10
20
30
MR effect size for
'HSPA6' on 'osteosarcoma'

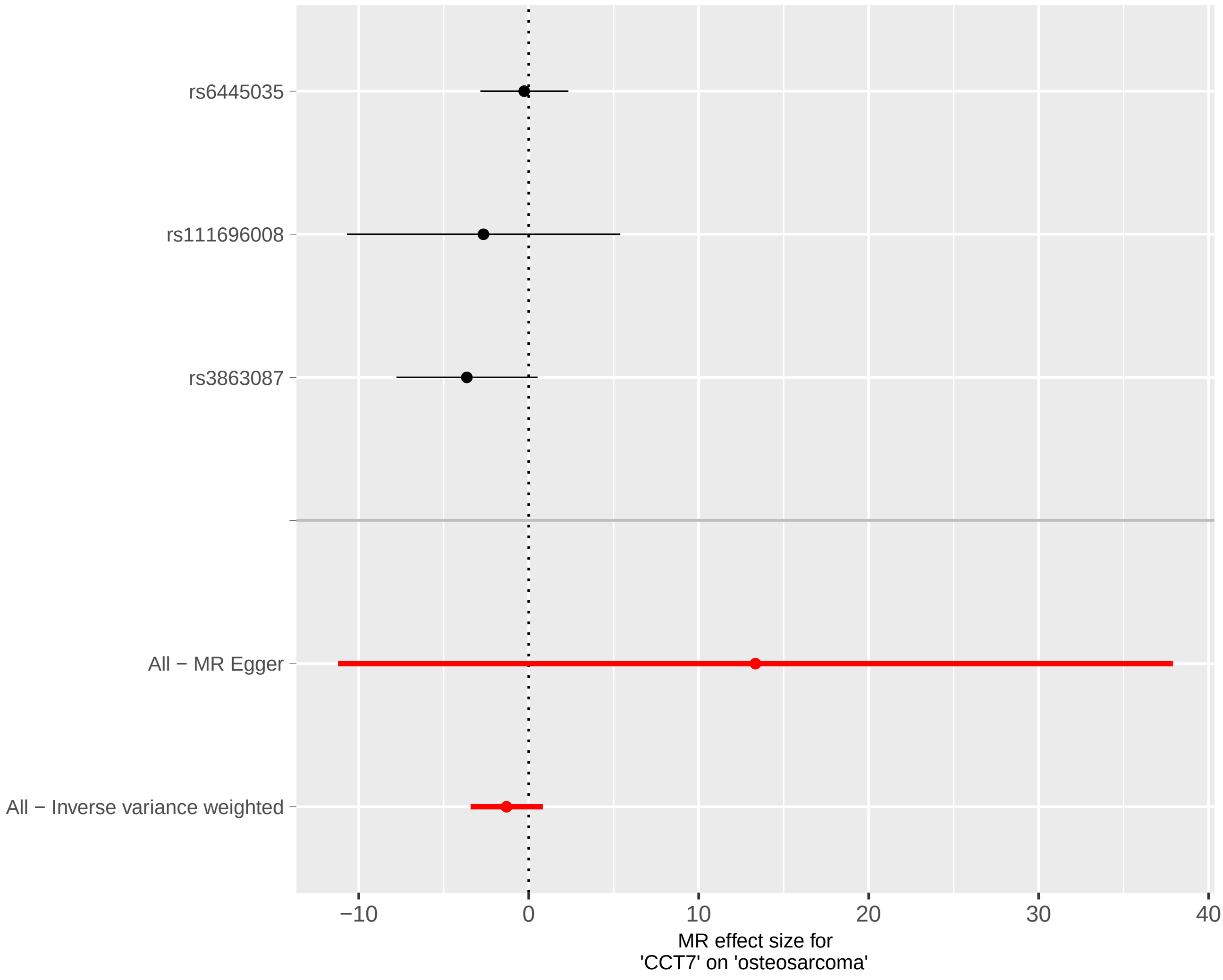
rs6445035
rs111696008
rs3863087
All − MR Egger
All − Inverse variance weighted
−10
0
10
20
30
40
MR effect size for
'CCT7' on 'osteosarcoma'

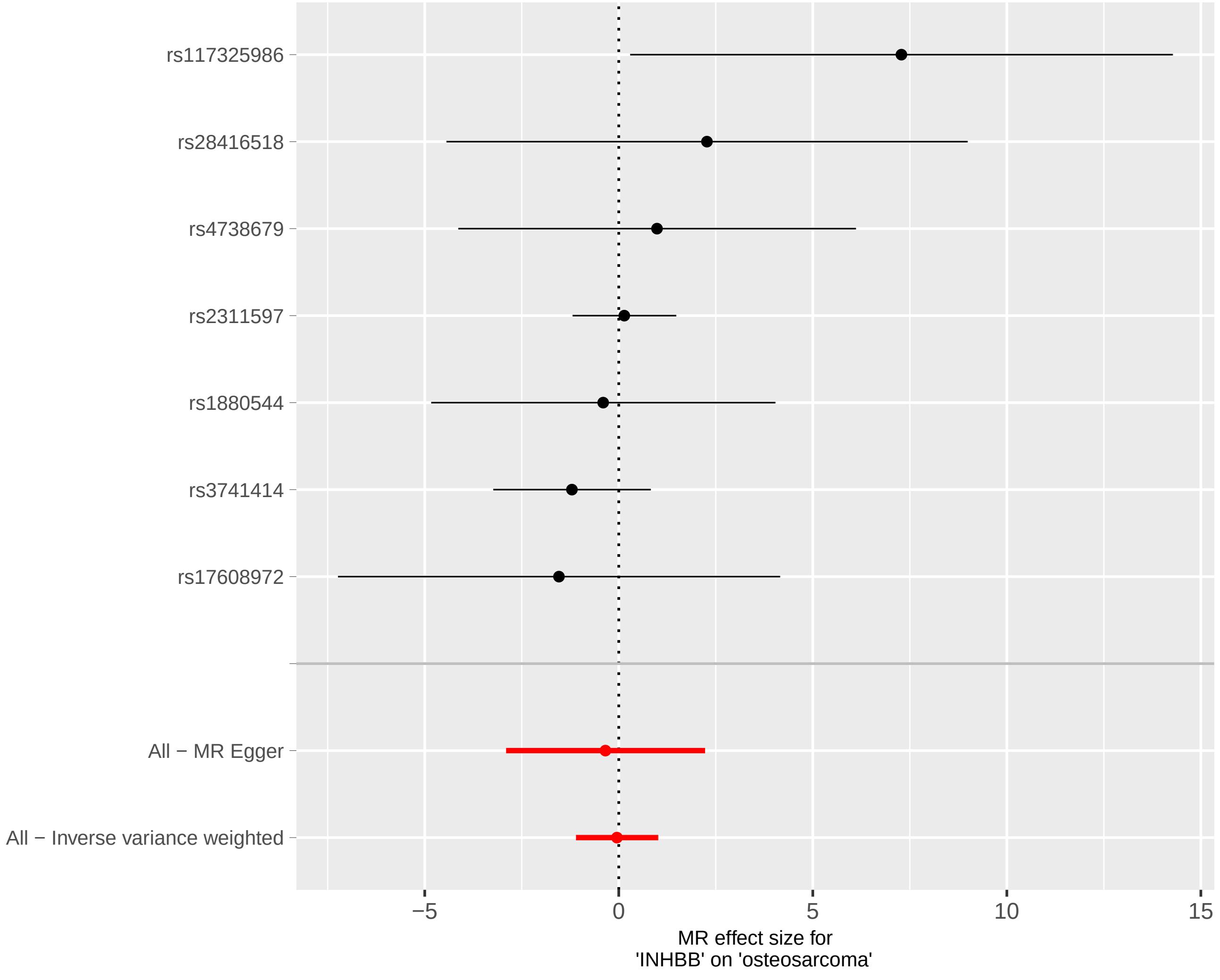
rs117325986
rs28416518
rs4738679
rs2311597
rs1880544
rs3741414
rs17608972
All − MR Egger
All − Inverse variance weighted
−5
0
5
10
15
MR effect size for
'INHBB' on 'osteosarcoma'

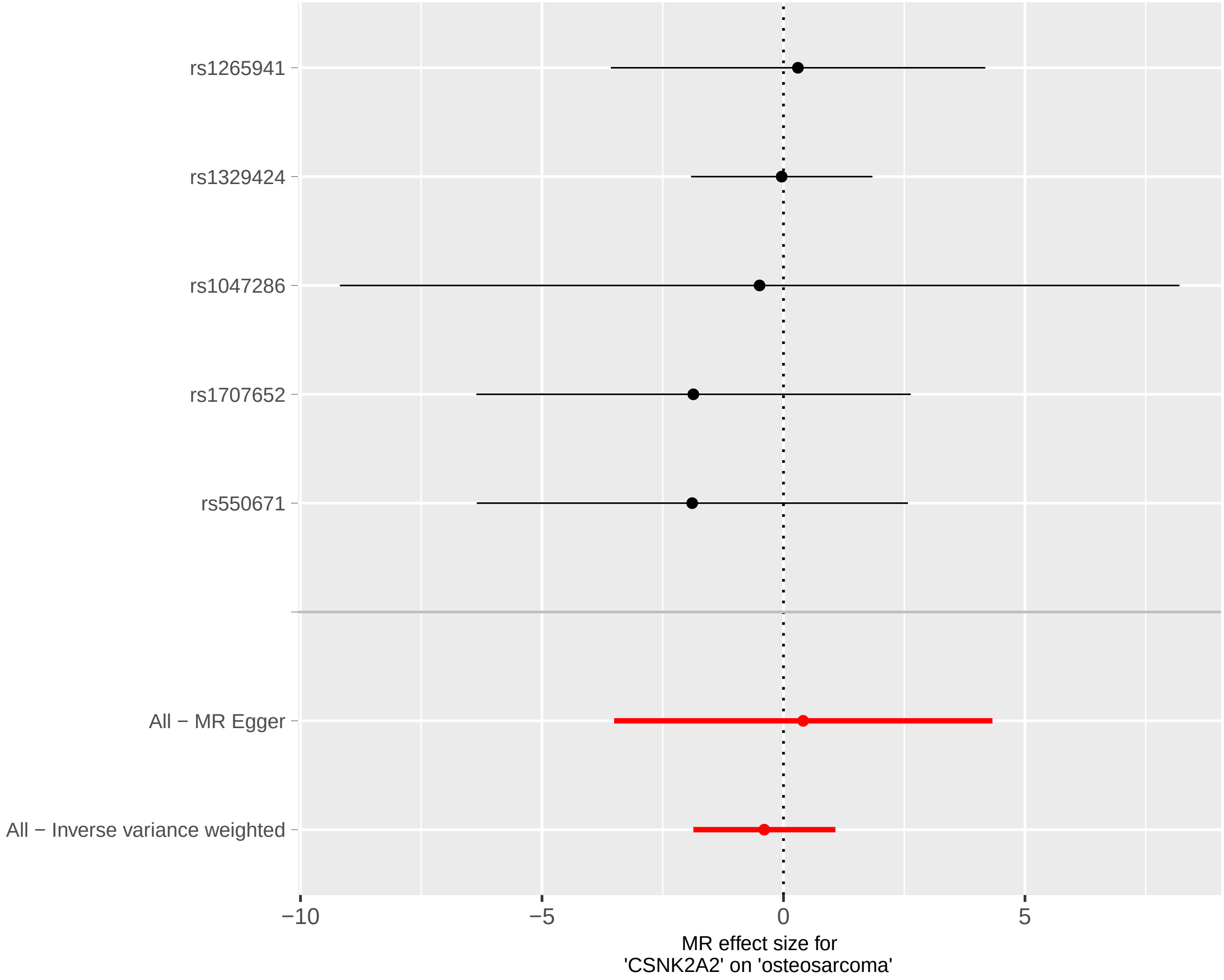

rs1265941
rs1329424
rs1047286
rs1707652
rs550671
All − MR Egger
All − Inverse variance weighted
−10
−5
0
5
MR effect size for
'CSNK2A2' on 'osteosarcoma'

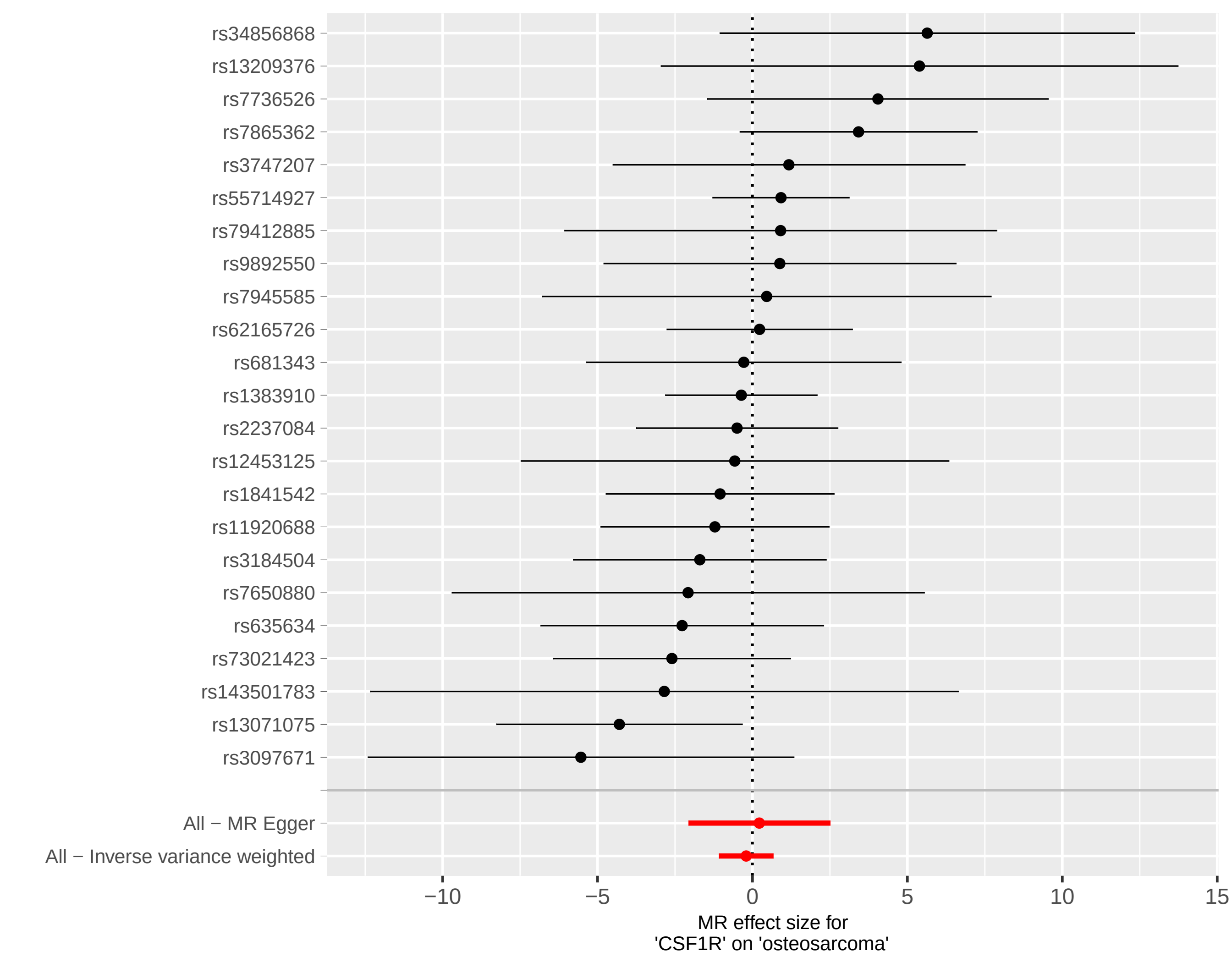
rs34856868
rs13209376
rs7736526
rs7865362
rs3747207
rs55714927
rs79412885
rs9892550
rs7945585
rs62165726
rs681343
rs1383910
rs2237084
rs12453125
rs1841542
rs11920688
rs3184504
rs7650880
rs635634
rs73021423
rs143501783
rs13071075
rs3097671
All − MR Egger
All − Inverse variance weighted
−10
−5
0
5
10
15
MR effect size for
'CSF1R' on 'osteosarcoma'

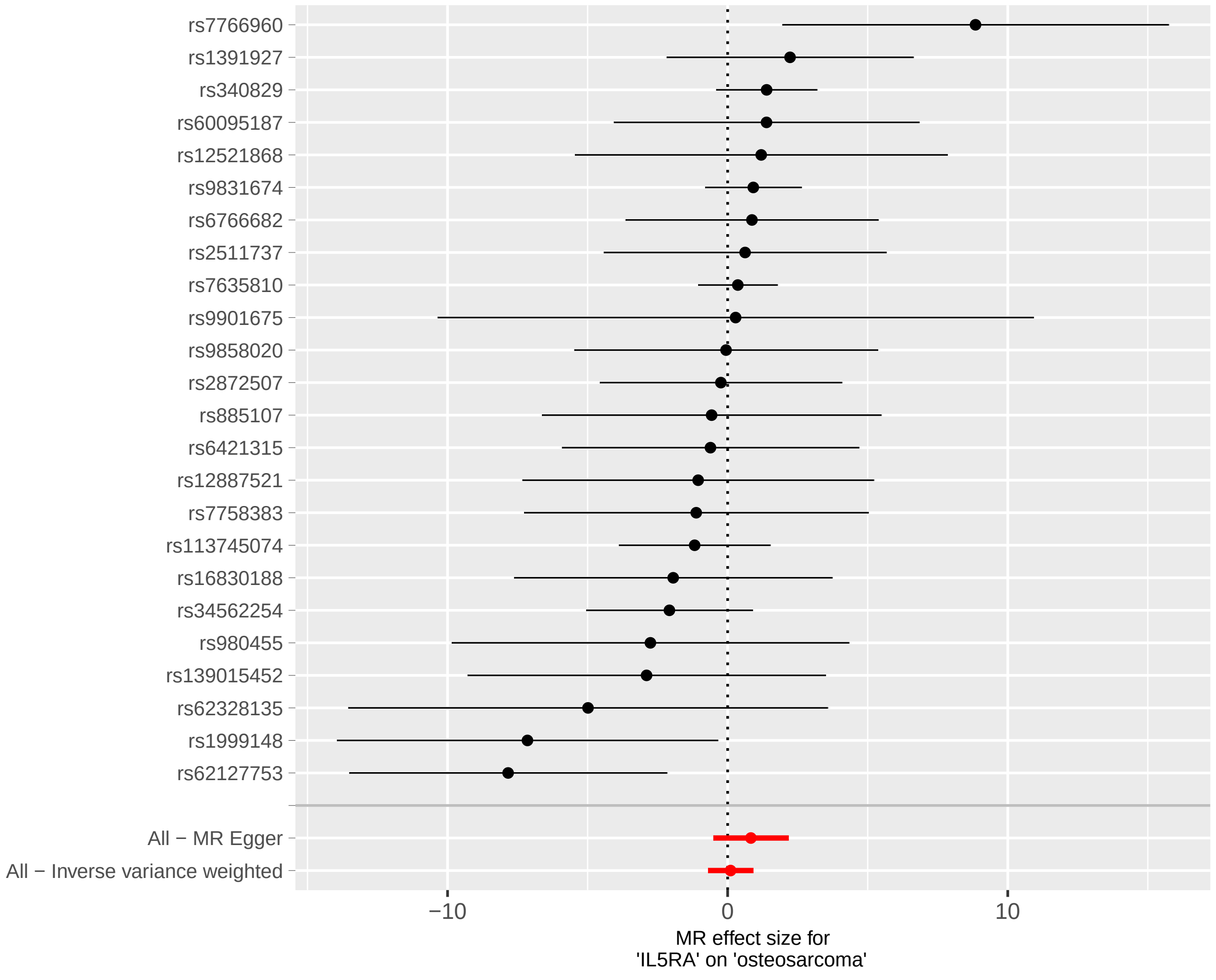

rs7766960
rs1391927
rs340829
rs60095187
rs12521868
rs9831674
rs6766682
rs2511737
rs7635810
rs9901675
rs9858020
rs2872507
rs885107
rs6421315
rs12887521
rs7758383
rs113745074
rs16830188
rs34562254
rs980455
rs139015452
rs62328135
rs1999148
rs62127753
All − MR Egger
All − Inverse variance weighted
−10
0
10
MR effect size for
'IL5RA' on 'osteosarcoma'

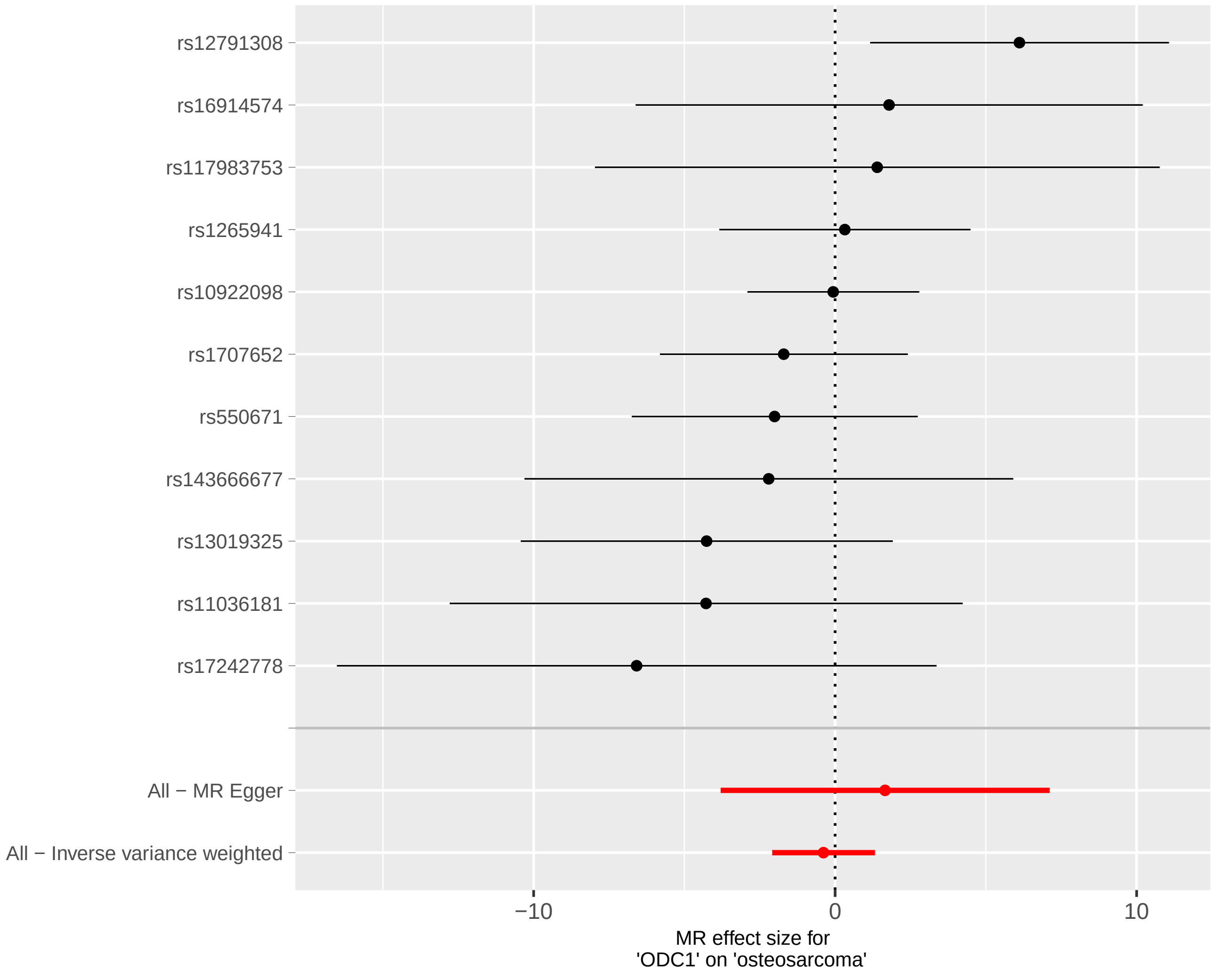
rs12791308
rs16914574
rs117983753
rs1265941
rs10922098
rs1707652
rs550671
rs143666677
rs13019325
rs11036181
rs17242778
All − MR Egger
All − Inverse variance weighted
−10
0
10
MR effect size for
'ODC1' on 'osteosarcoma'

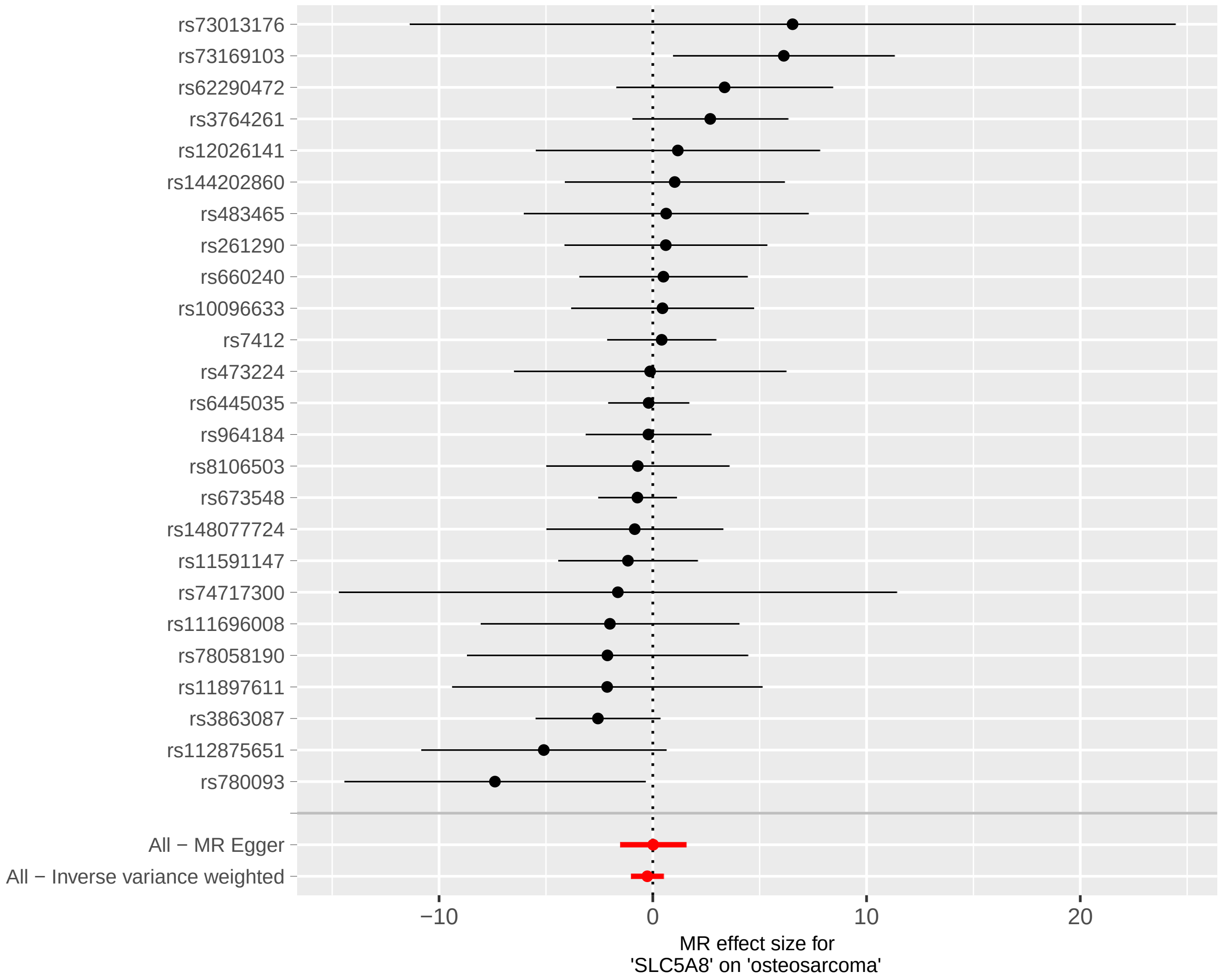
rs73013176
rs73169103
rs62290472
rs3764261
rs12026141
rs144202860
rs483465
rs261290
rs660240
rs10096633
rs7412
rs473224
rs6445035
rs964184
rs8106503
rs673548
rs148077724
rs11591147
rs74717300
rs111696008
rs78058190
rs11897611
rs3863087
rs112875651
rs780093
All − MR Egger
All − Inverse variance weighted
−10
0
10
20
MR effect size for
'SLC5A8' on 'osteosarcoma'

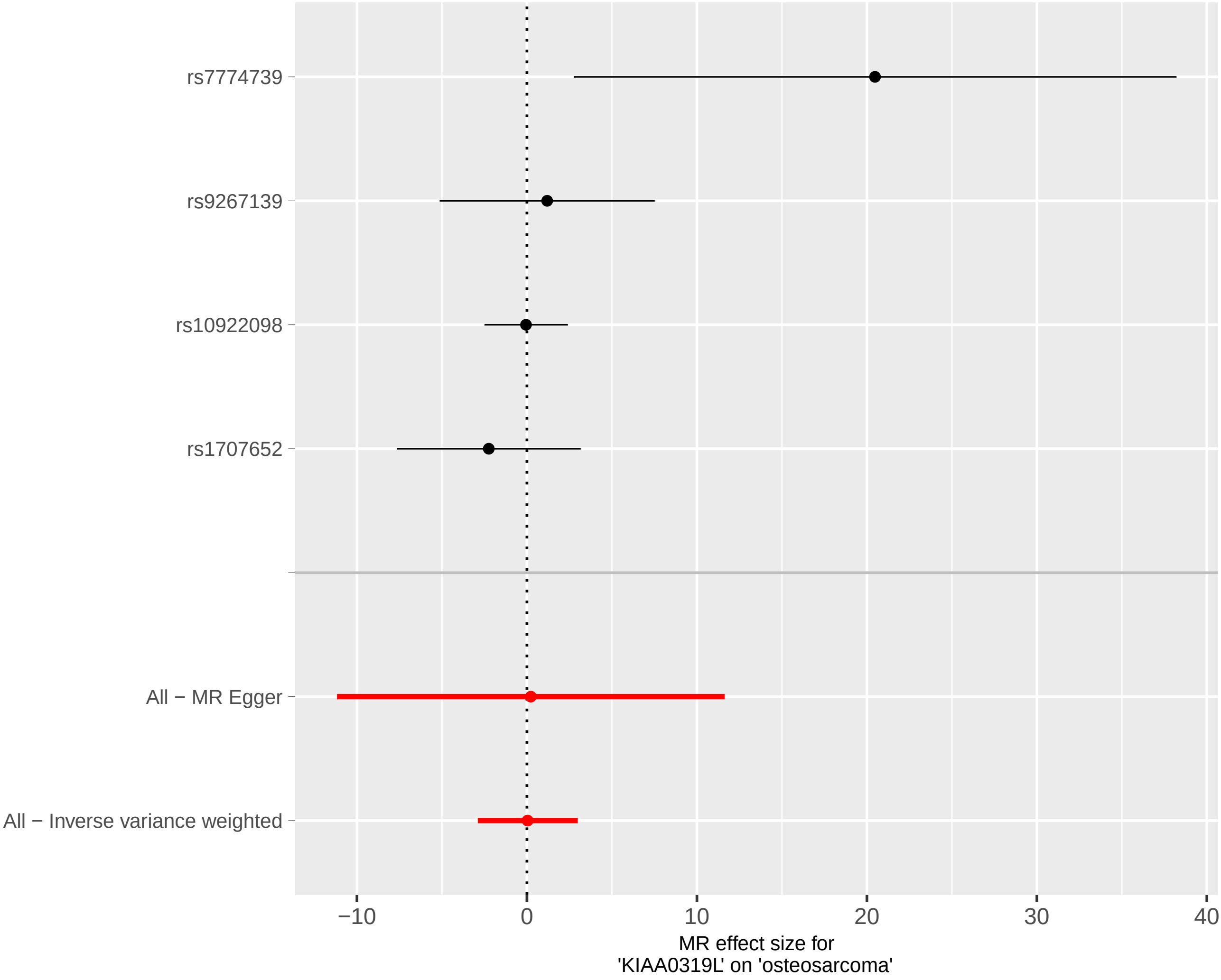

rs7774739
rs9267139
rs10922098
rs1707652
All − MR Egger
All − Inverse variance weighted
−10
0
10
20
30
40
MR effect size for
'KIAA0319L' on 'osteosarcoma'

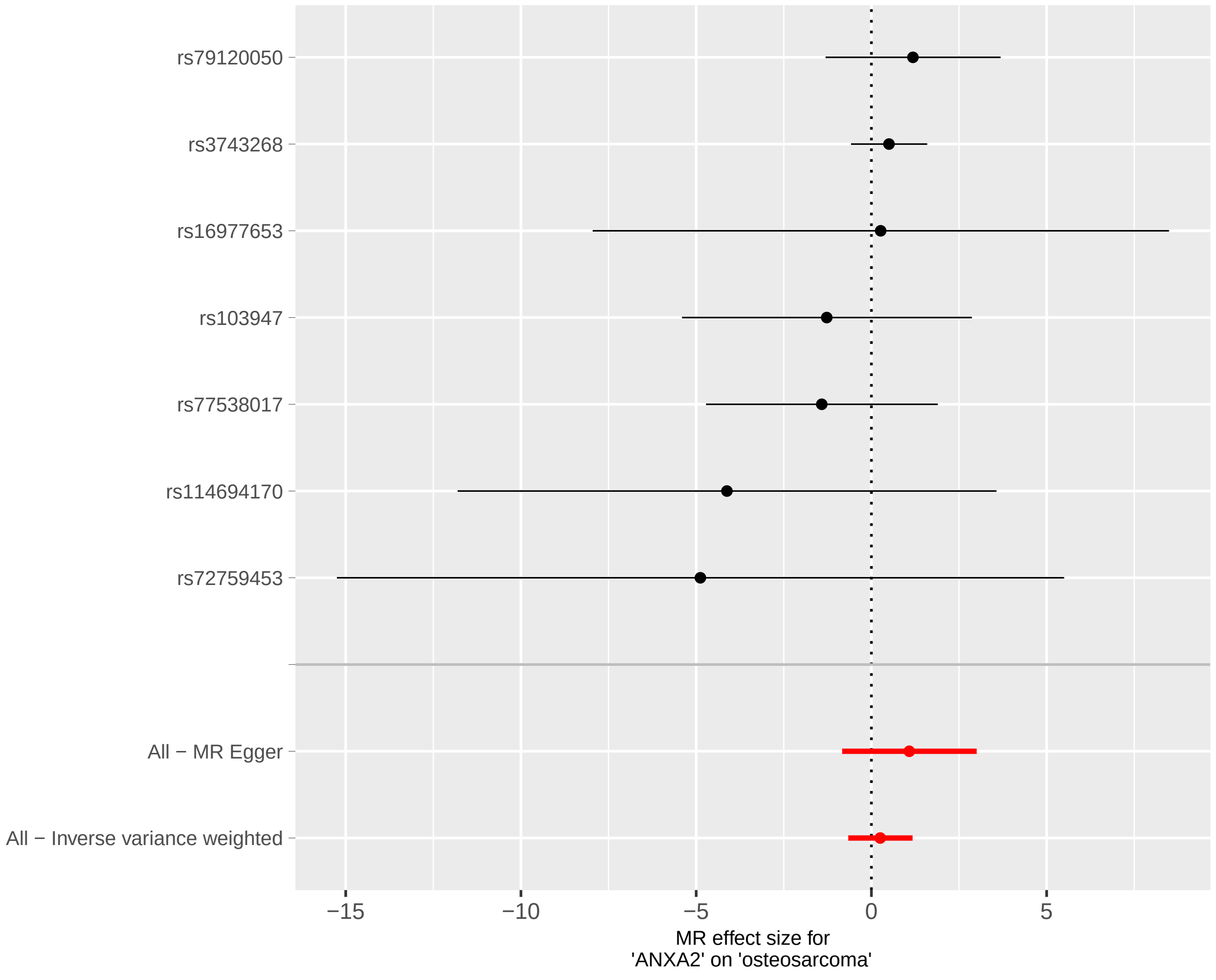

rs79120050
rs3743268
rs16977653
rs103947
rs77538017
rs114694170
rs72759453
All − MR Egger
All − Inverse variance weighted
−15
−10
−5
0
5
MR effect size for
'ANXA2' on 'osteosarcoma'

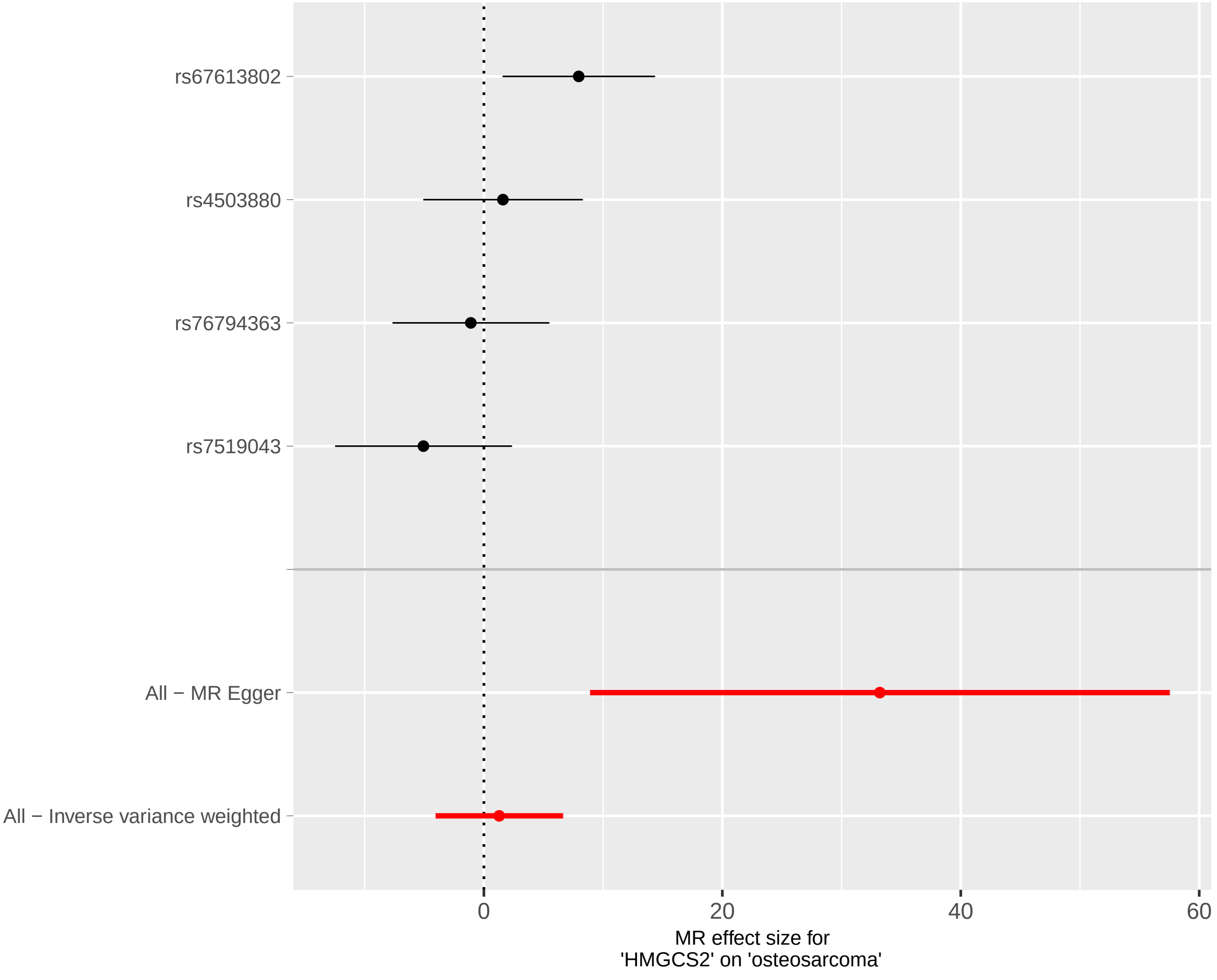

rs67613802
rs4503880
rs76794363
rs7519043
All − MR Egger
All − Inverse variance weighted
0
20
40
60
MR effect size for
'HMGCS2' on 'osteosarcoma'

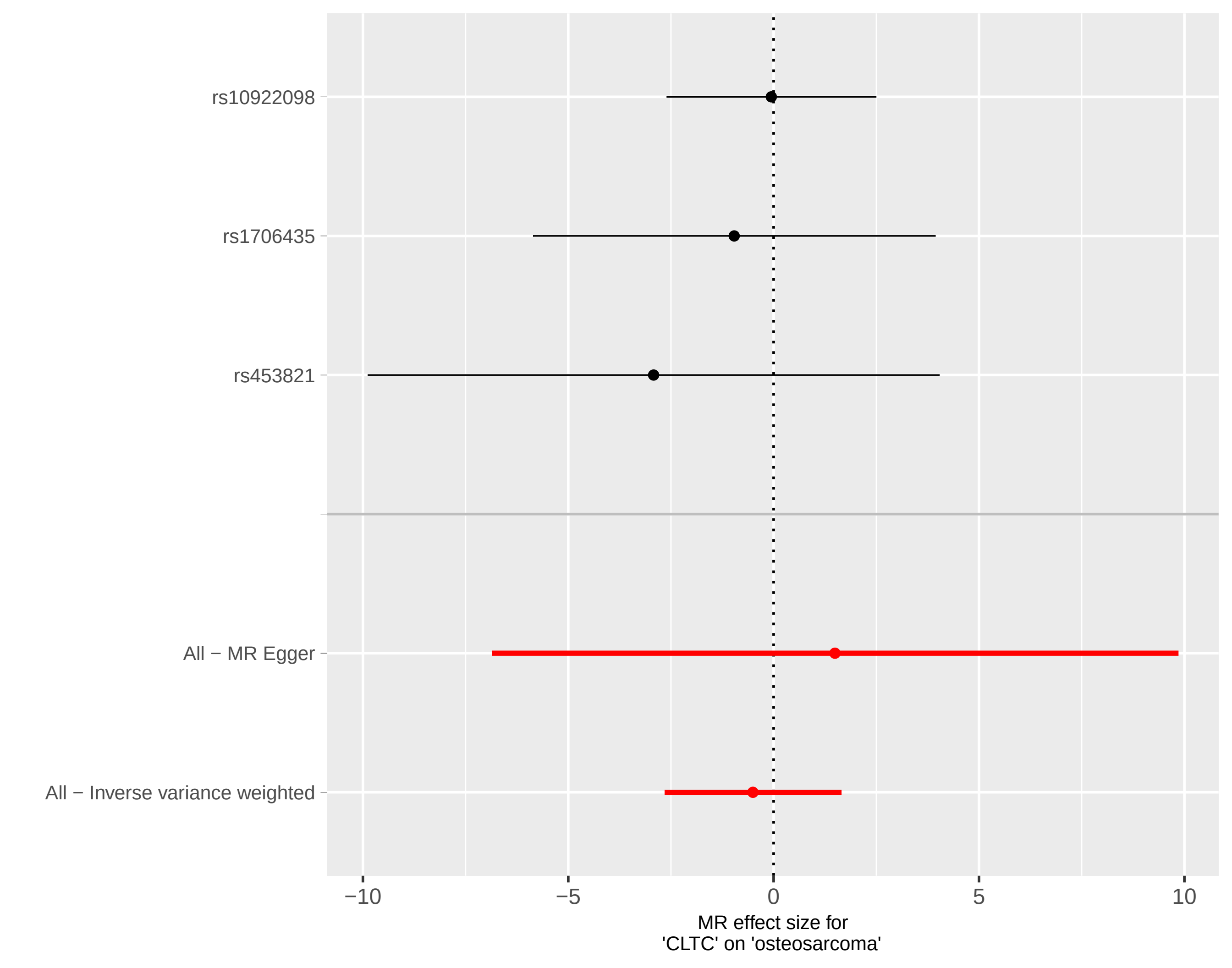
rs10922098
rs1706435
rs453821
All − MR Egger
All − Inverse variance weighted
−10
−5
0
5
10
MR effect size for
'CLTC' on 'osteosarcoma'

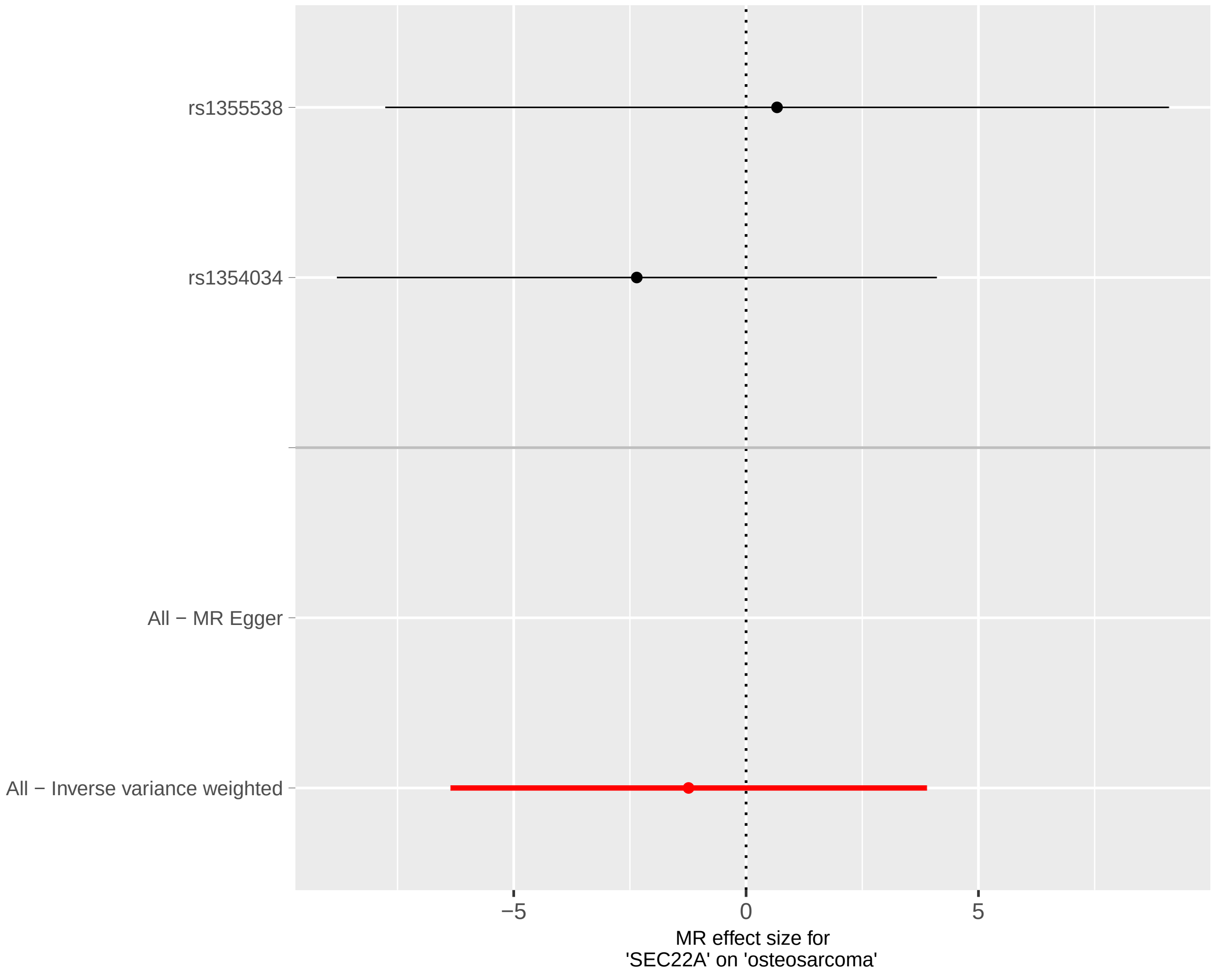

rs1355538
rs1354034
All − MR Egger
All − Inverse variance weighted
−5
0
5
MR effect size for
'SEC22A' on 'osteosarcoma'

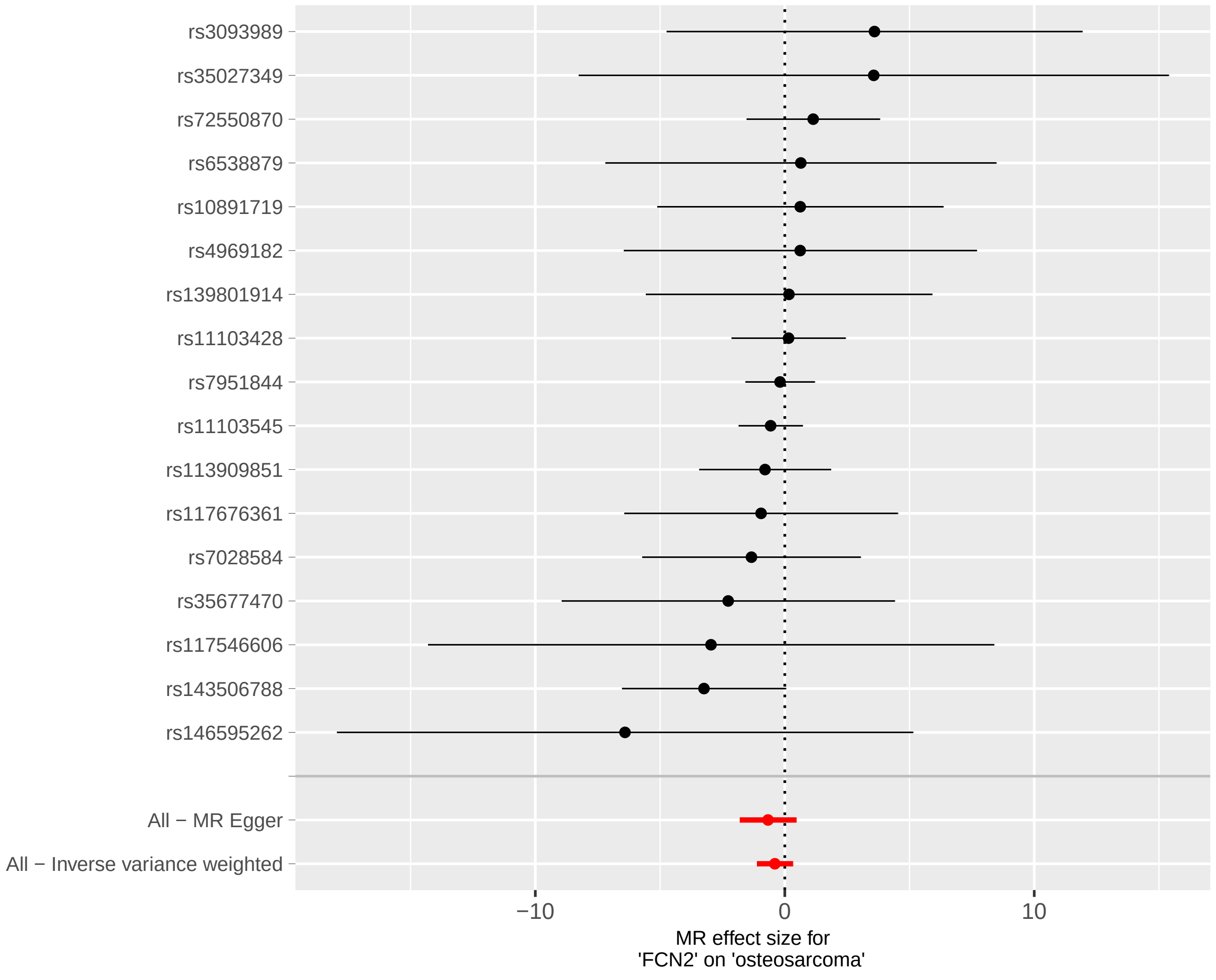

rs3093989
rs35027349
rs72550870
rs6538879
rs10891719
rs4969182
rs139801914
rs11103428
rs7951844
rs11103545
rs113909851
rs117676361
rs7028584
rs35677470
rs117546606
rs143506788
rs146595262
All − MR Egger
All − Inverse variance weighted
−10
0
10
MR effect size for
'FCN2' on 'osteosarcoma'

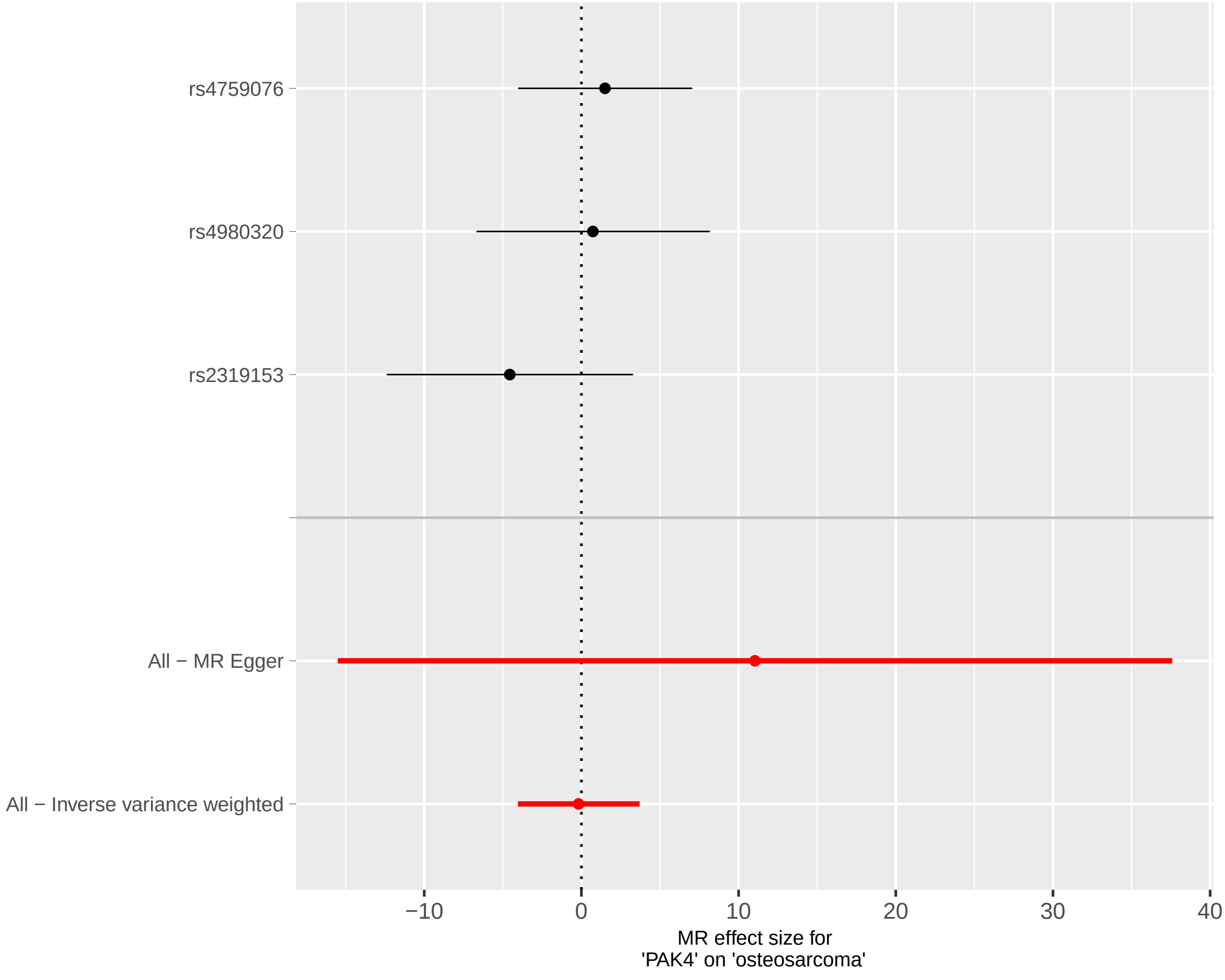

rs4759076
rs4980320
rs2319153
All − MR Egger
All − Inverse variance weighted
−10
0
10
20
30
40
MR effect size for
'PAK4' on 'osteosarcoma'

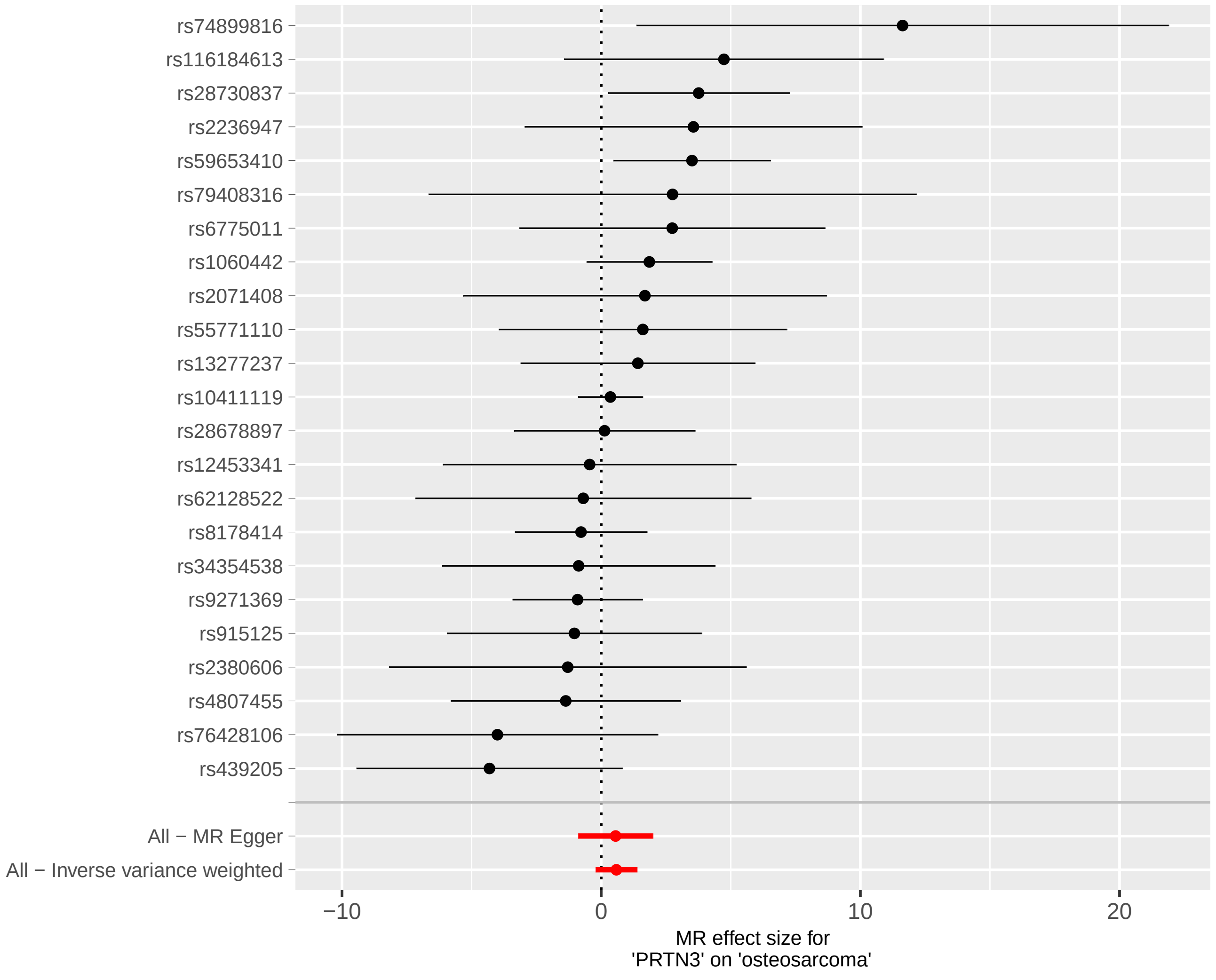

rs74899816
rs116184613
rs28730837
rs2236947
rs59653410
rs79408316
rs6775011
rs1060442
rs2071408
rs55771110
rs13277237
rs10411119
rs28678897
rs12453341
rs62128522
rs8178414
rs34354538
rs9271369
rs915125
rs2380606
rs4807455
rs76428106
rs439205
All − MR Egger
All − Inverse variance weighted
−10
0
10
20
MR effect size for
'PRTN3' on 'osteosarcoma'

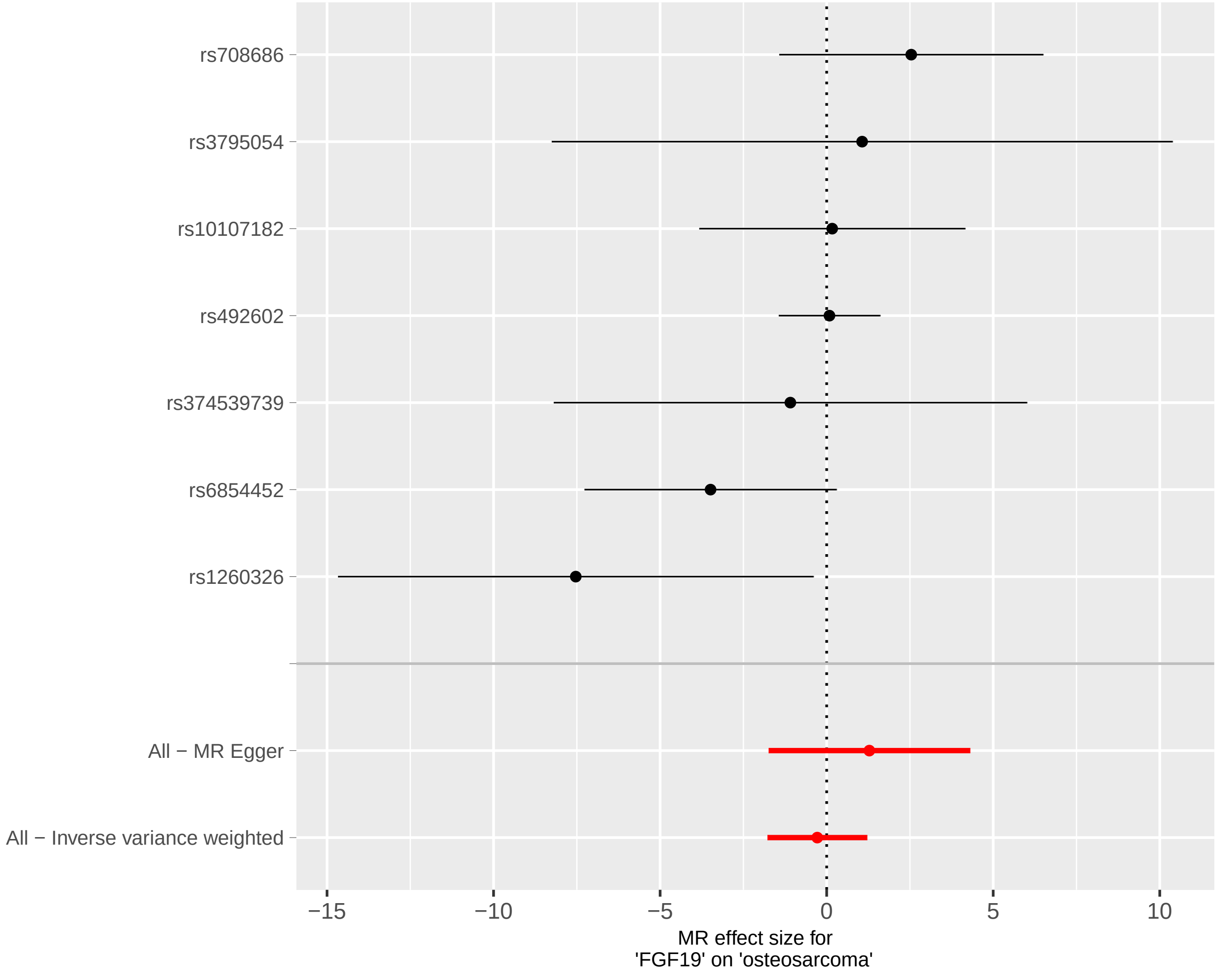

rs708686
rs3795054
rs10107182
rs492602
rs374539739
rs6854452
rs1260326
All − MR Egger
All − Inverse variance weighted
−15
−10
−5
0
5
10
MR effect size for
'FGF19' on 'osteosarcoma'

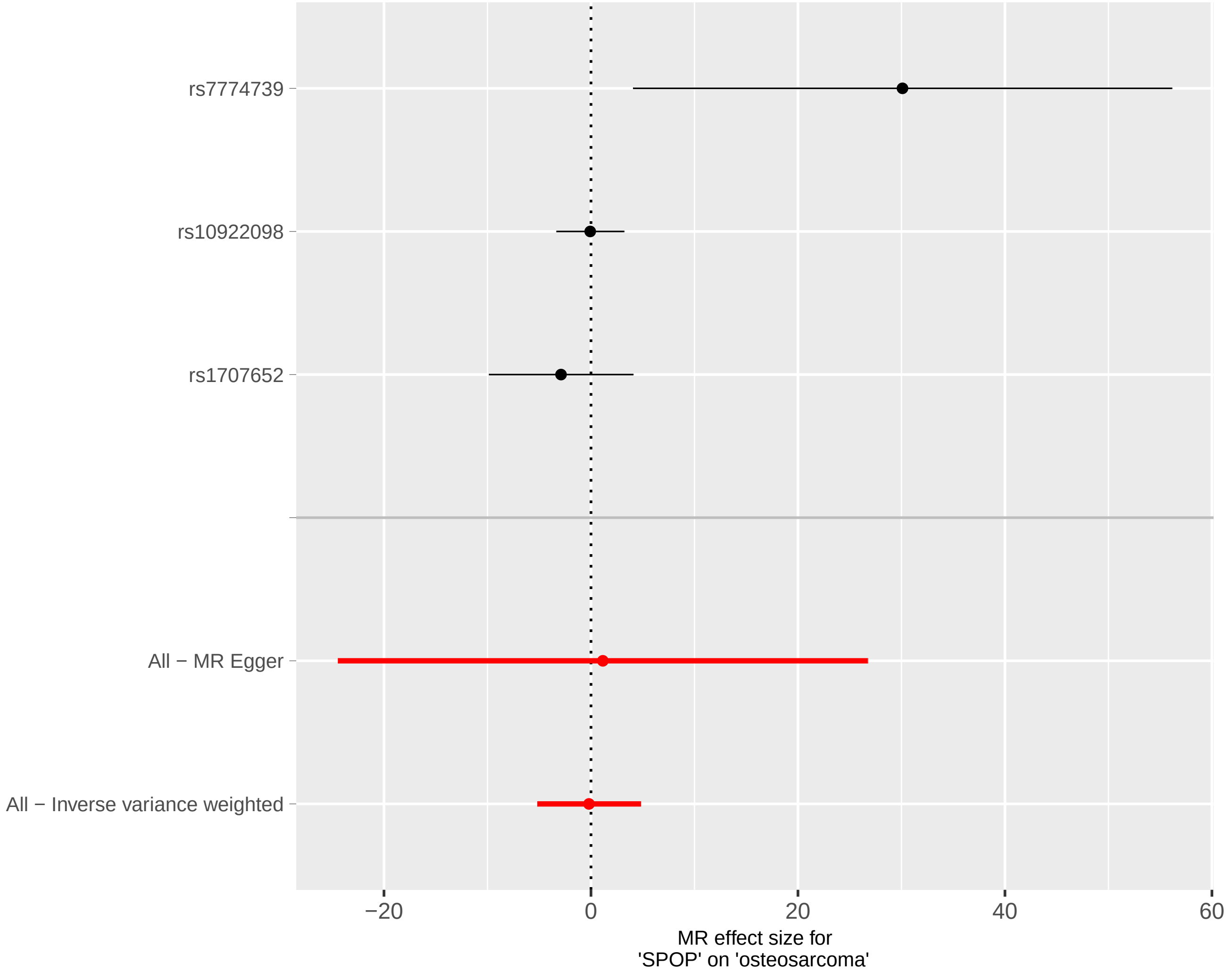

rs7774739
rs10922098
rs1707652
All − MR Egger
All − Inverse variance weighted
−20
0
20
40
60
MR effect size for
'SPOP' on 'osteosarcoma'

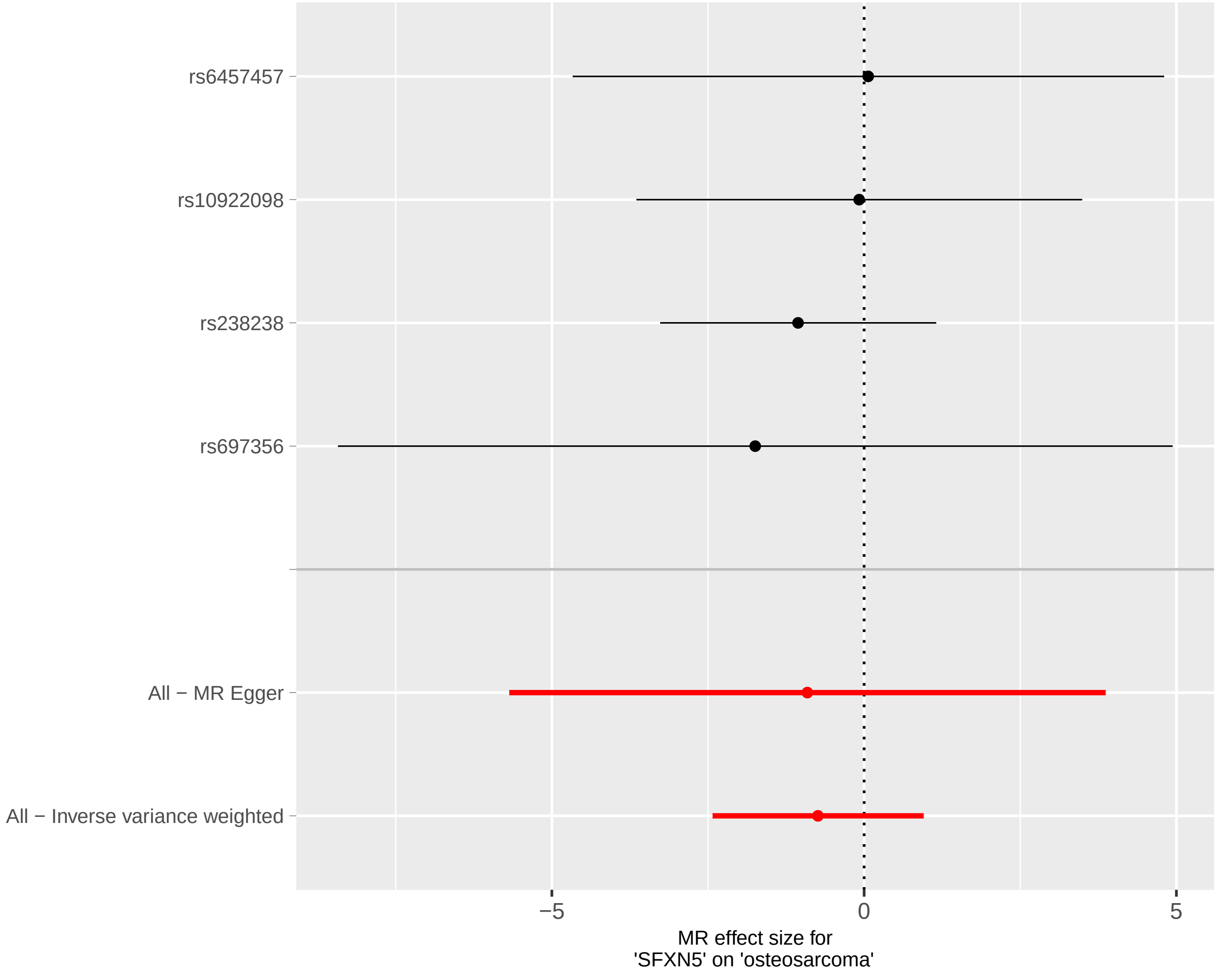

rs6457457
rs10922098
rs238238
rs697356
All − MR Egger
All − Inverse variance weighted
−5
0
5
MR effect size for
'SFXN5' on 'osteosarcoma'

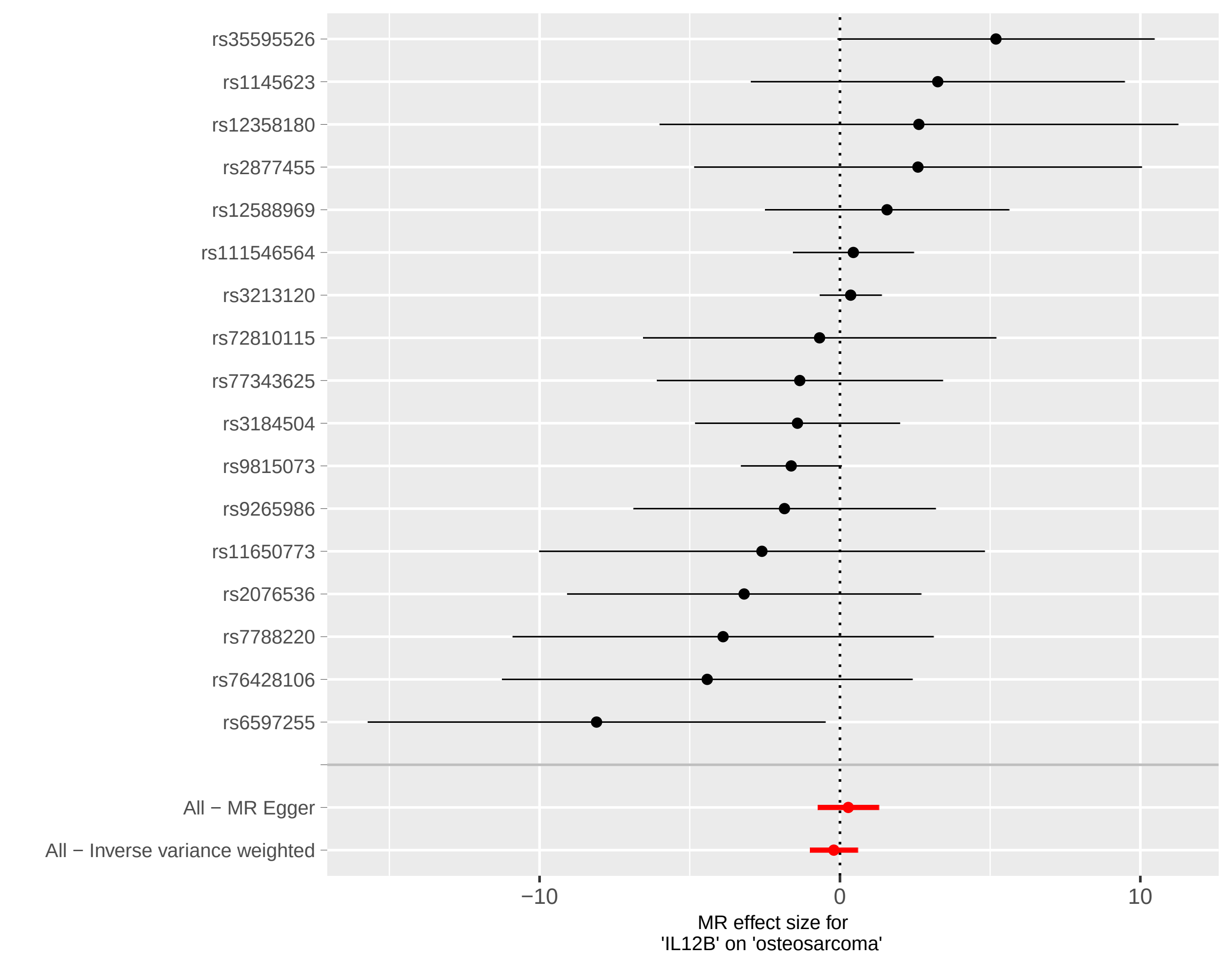
rs35595526
rs1145623
rs12358180
rs2877455
rs12588969
rs111546564
rs3213120
rs72810115
rs77343625
rs3184504
rs9815073
rs9265986
rs11650773
rs2076536
rs7788220
rs76428106
rs6597255
All − MR Egger
All − Inverse variance weighted
−10
0
10
MR effect size for
'IL12B' on 'osteosarcoma'

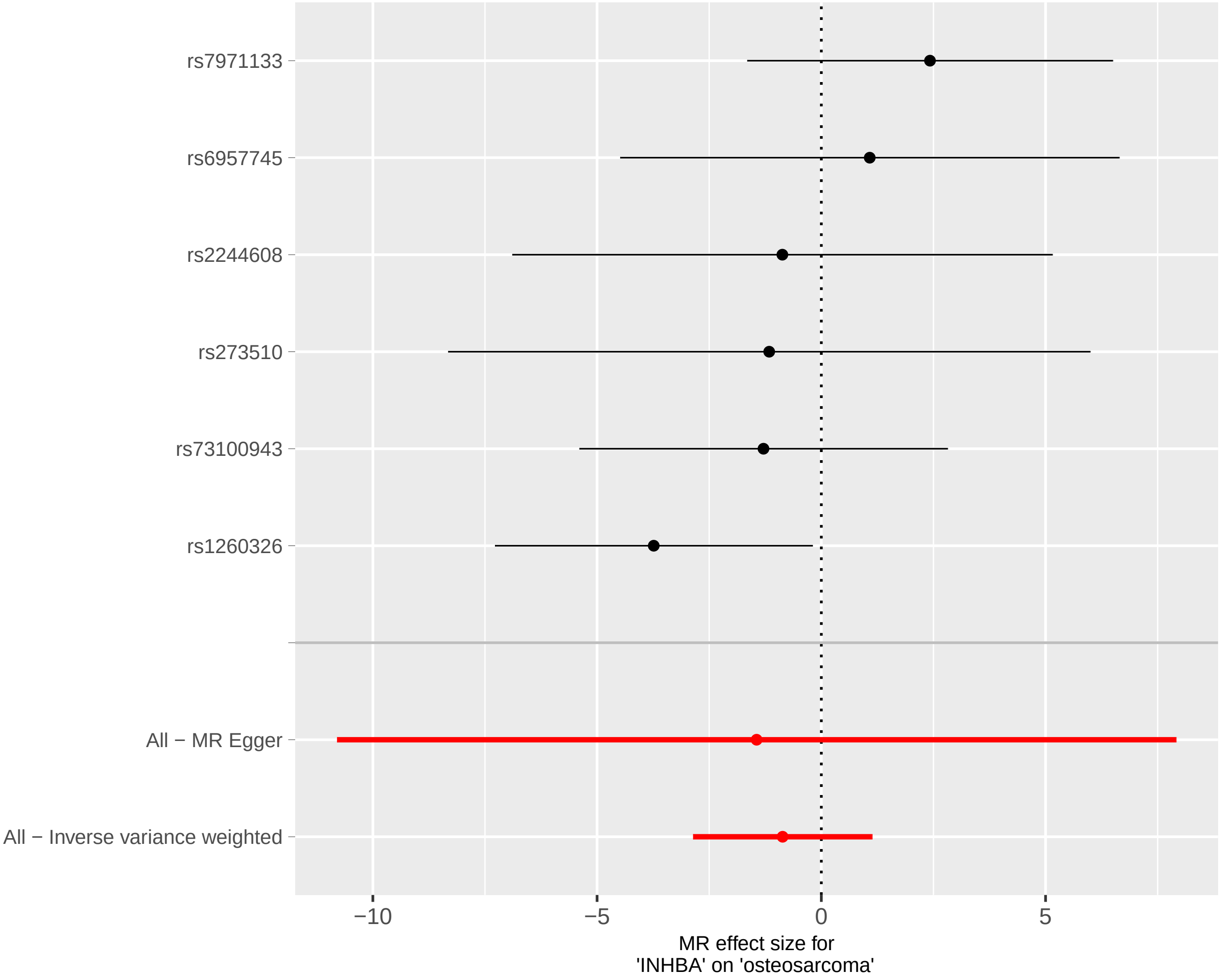

rs7971133
rs6957745
rs2244608
rs273510
rs73100943
rs1260326
All − MR Egger
All − Inverse variance weighted
−10
−5
0
5
MR effect size for
'INHBA' on 'osteosarcoma'

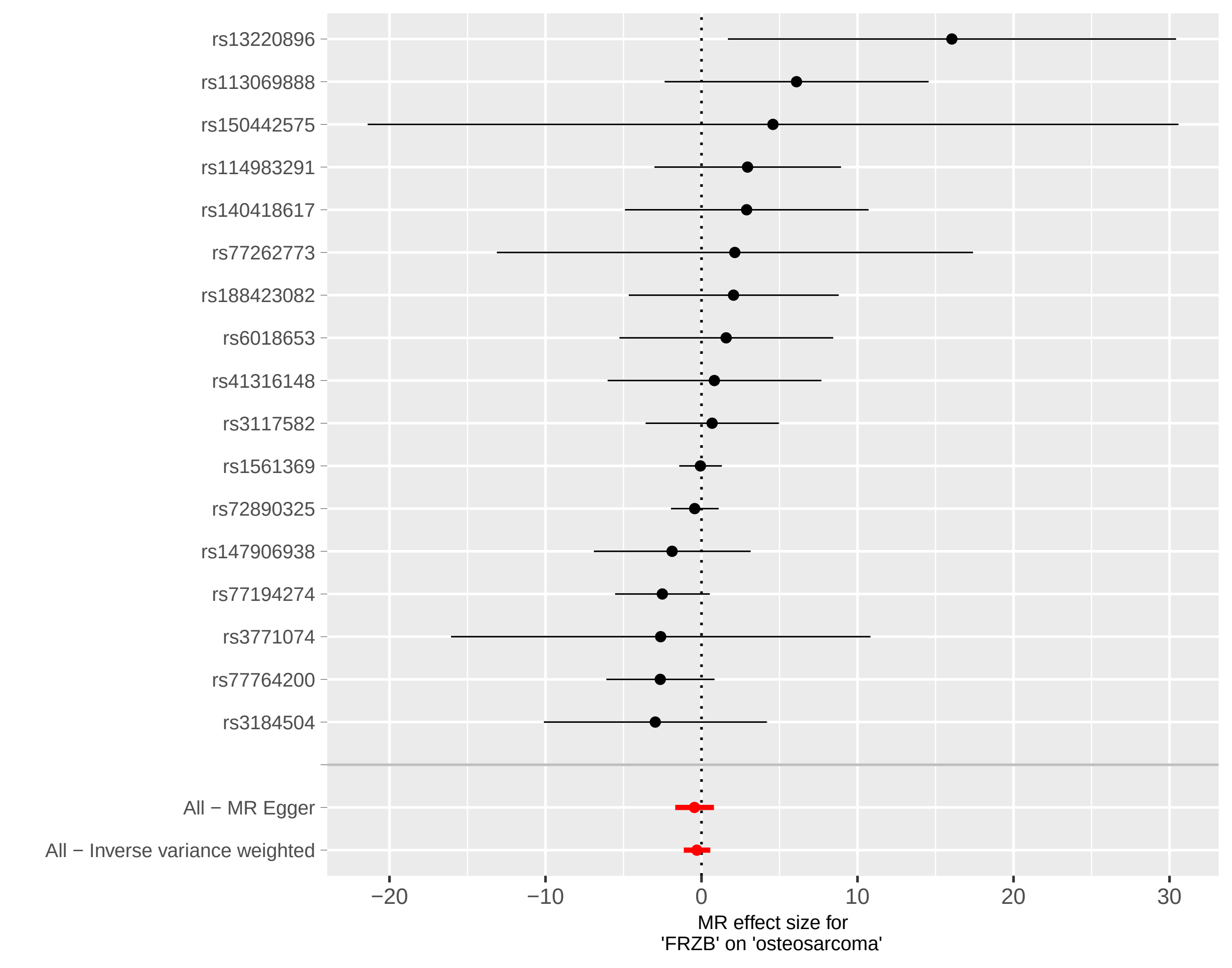

rs13220896
rs113069888
rs150442575
rs114983291
rs140418617
rs77262773
rs188423082
rs6018653
rs41316148
rs3117582
rs1561369
rs72890325
rs147906938
rs77194274
rs3771074
rs77764200
rs3184504
All − MR Egger
All − Inverse variance weighted
−20
−10
0
10
20
30
MR effect size for
'FRZB' on 'osteosarcoma'

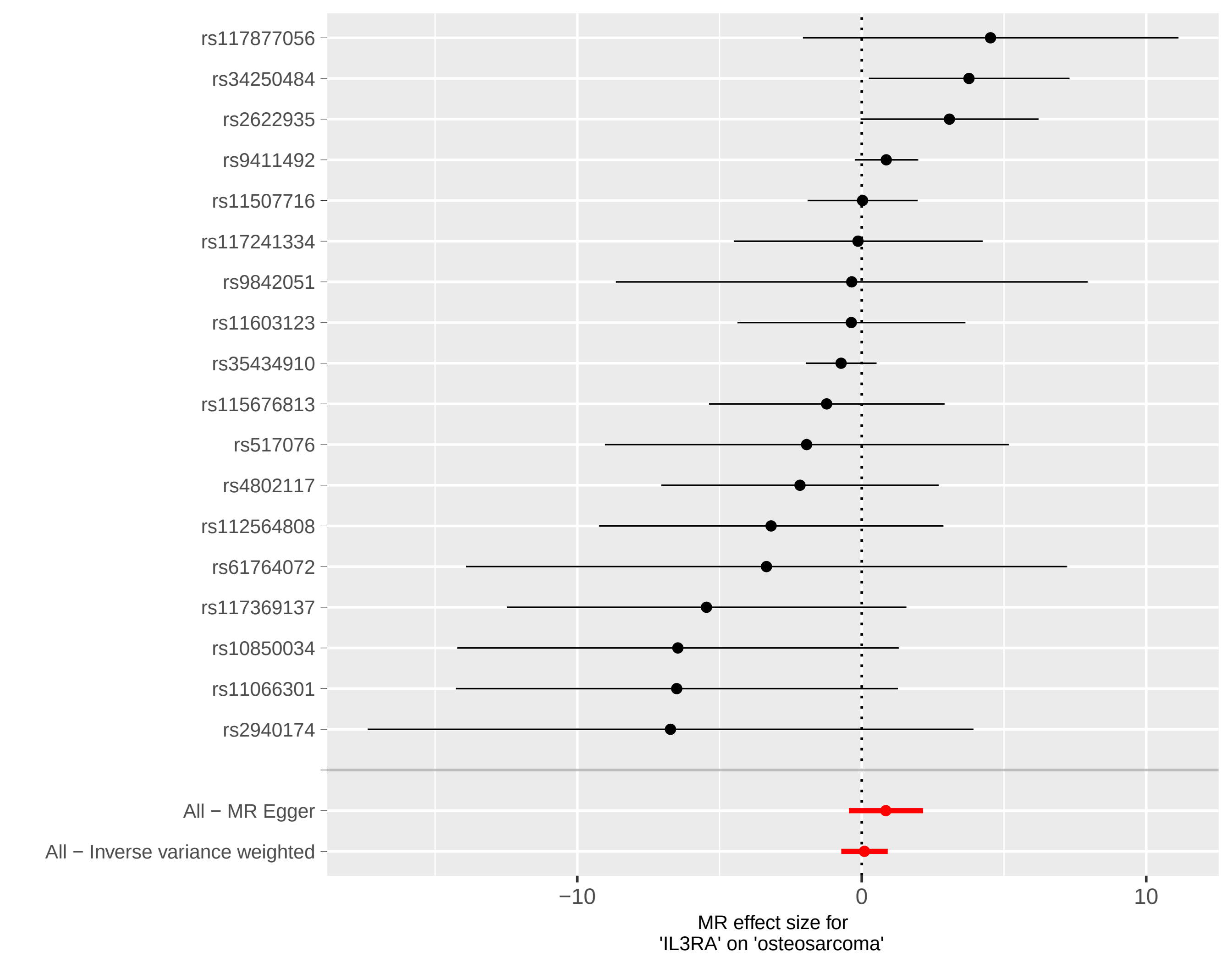
rs117877056
rs34250484
rs2622935
rs9411492
rs11507716
rs117241334
rs9842051
rs11603123
rs35434910
rs115676813
rs517076
rs4802117
rs112564808
rs61764072
rs117369137
rs10850034
rs11066301
rs2940174
All − MR Egger
All − Inverse variance weighted
−10
0
10
MR effect size for
'IL3RA' on 'osteosarcoma'

Insufficient number of SNPs

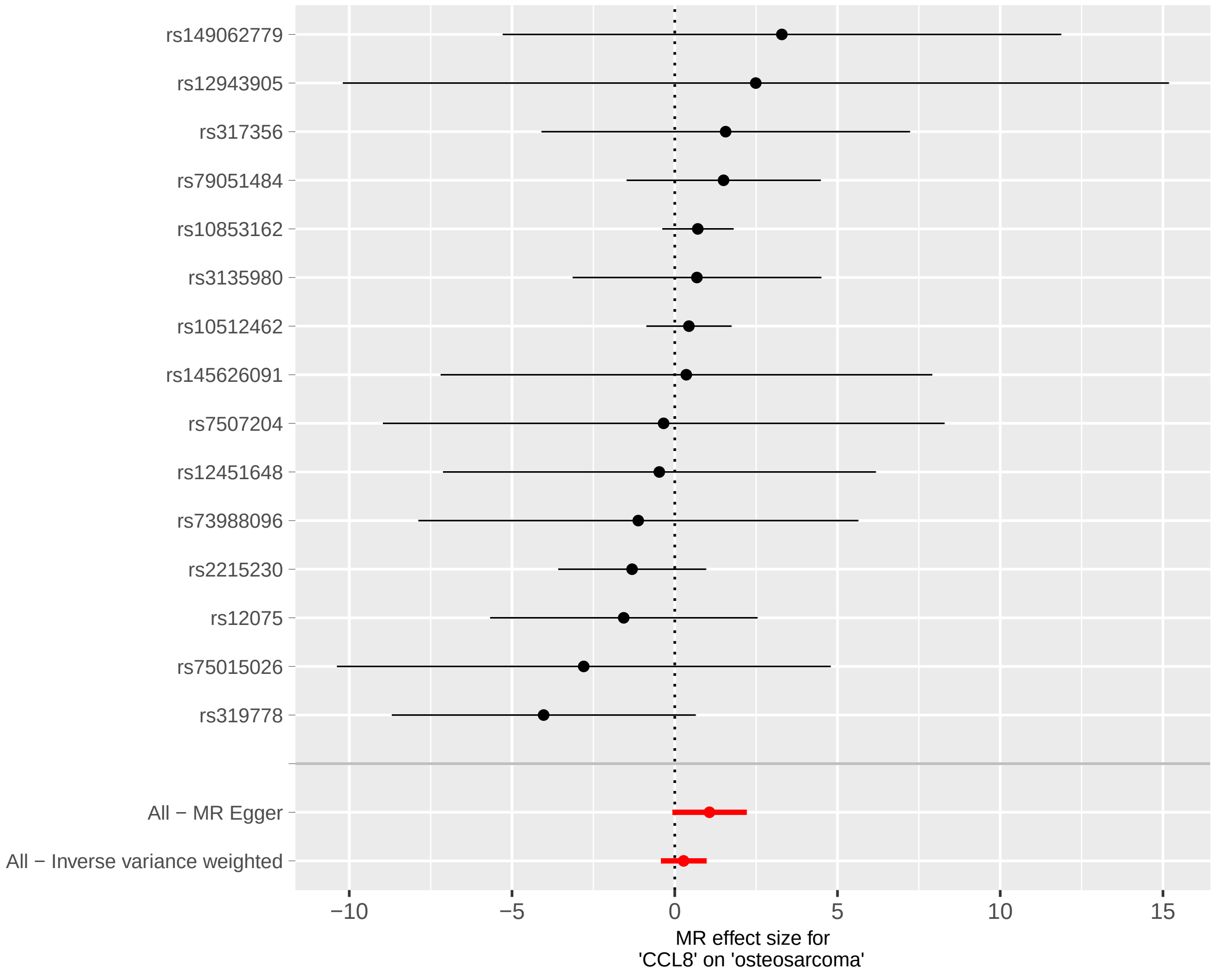

rs149062779
rs12943905
rs317356
rs79051484
rs10853162
rs3135980
rs10512462
rs145626091
rs7507204
rs12451648
rs73988096
rs2215230
rs12075
rs75015026
rs319778
All − MR Egger
All − Inverse variance weighted
−10
−5
0
5
10
15
MR effect size for
'CCL8' on 'osteosarcoma'

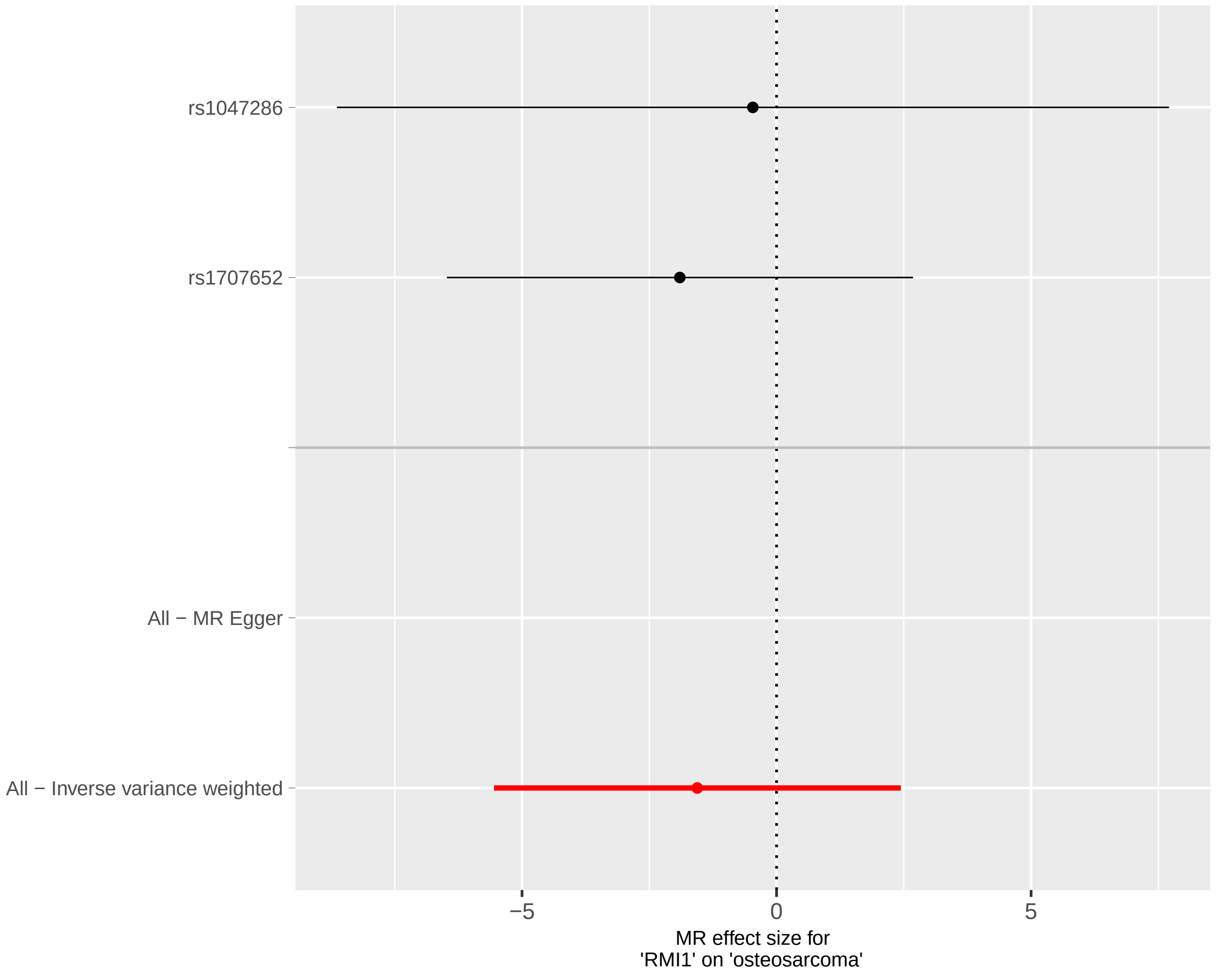
rs1047286
rs1707652
All − MR Egger
All − Inverse variance weighted
−5
0
5
MR effect size for
'RMI1' on 'osteosarcoma'

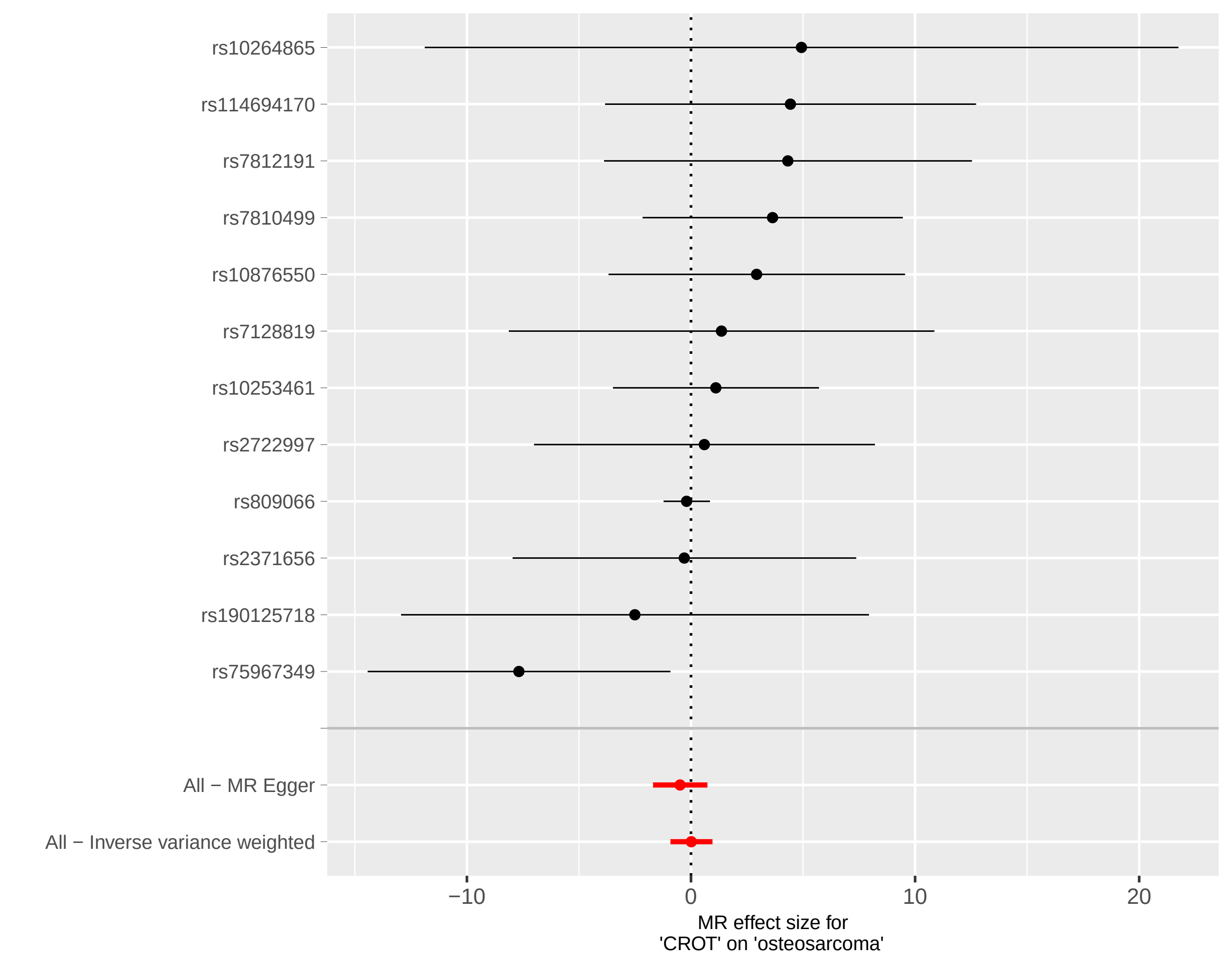
rs10264865
rs114694170
rs7812191
rs7810499
rs10876550
rs7128819
rs10253461
rs2722997
rs809066
rs2371656
rs190125718
rs75967349
All − MR Egger
All − Inverse variance weighted
−10
0
10
20
MR effect size for
'CROT' on 'osteosarcoma'

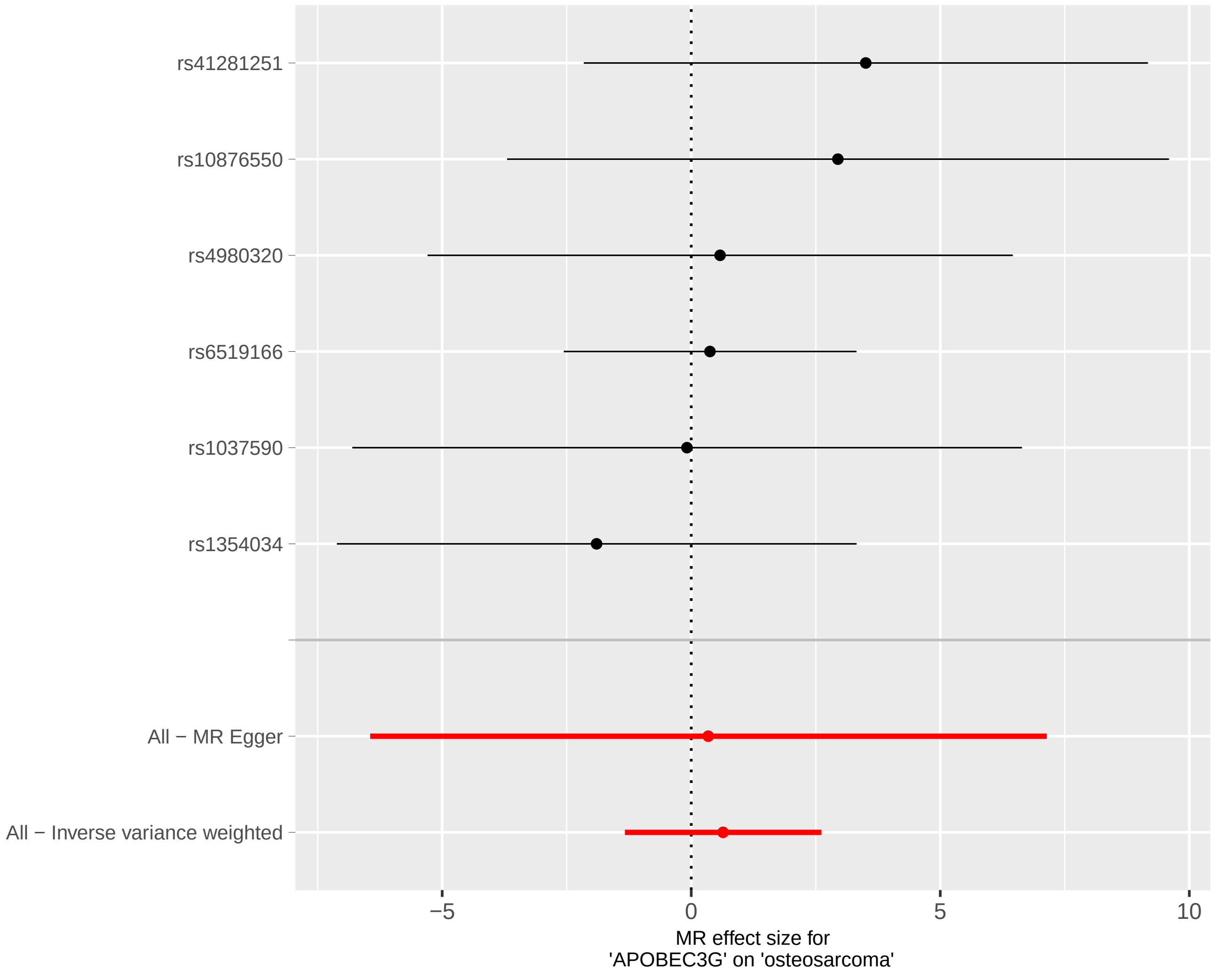

rs41281251
rs10876550
rs4980320
rs6519166
rs1037590
rs1354034
All − MR Egger
All − Inverse variance weighted
−5
0
5
10
MR effect size for
'APOBEC3G' on 'osteosarcoma'

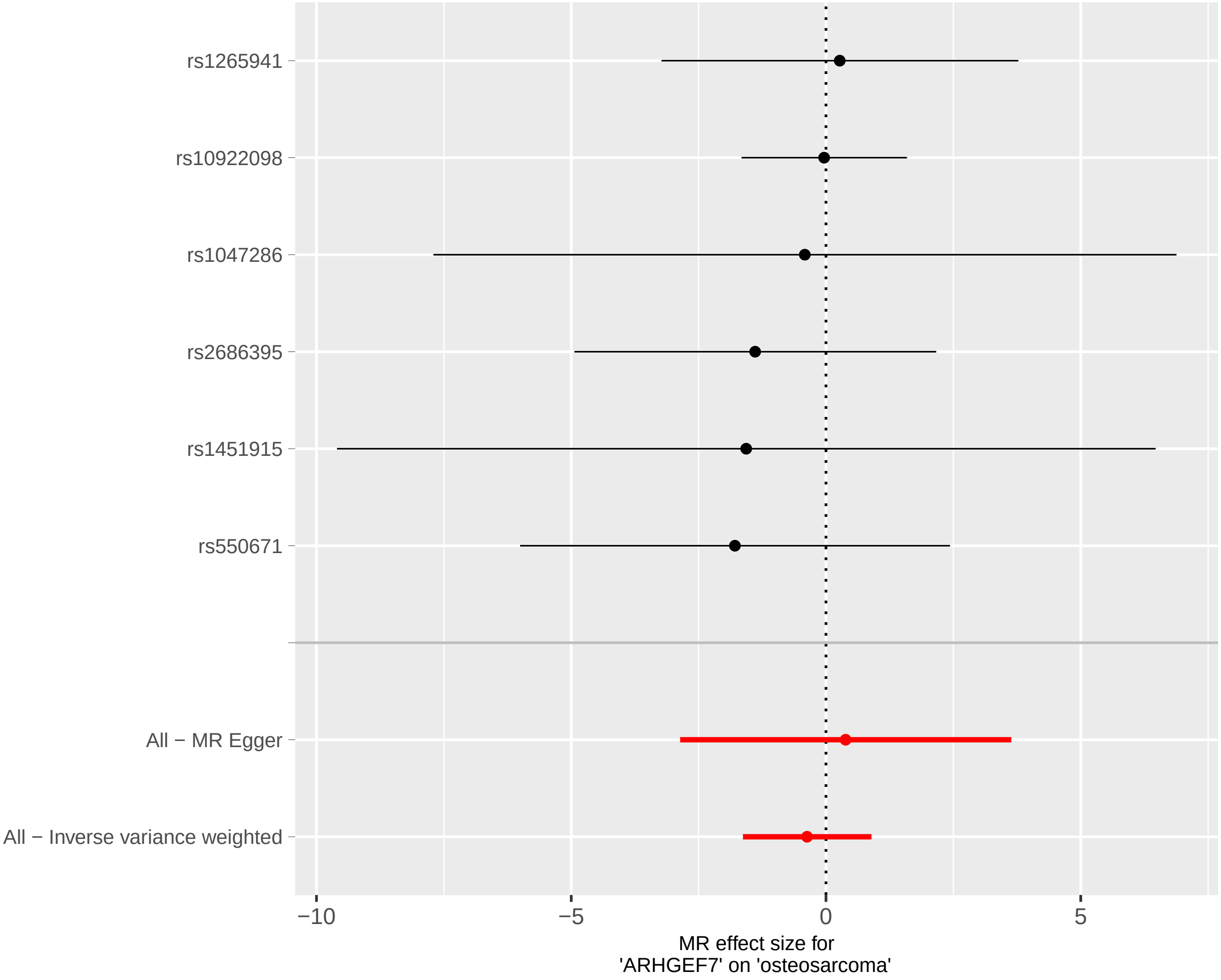

rs1265941
rs10922098
rs1047286
rs2686395
rs1451915
rs550671
All − MR Egger
All − Inverse variance weighted
−10
−5
0
5
MR effect size for
'ARHGEF7' on 'osteosarcoma'

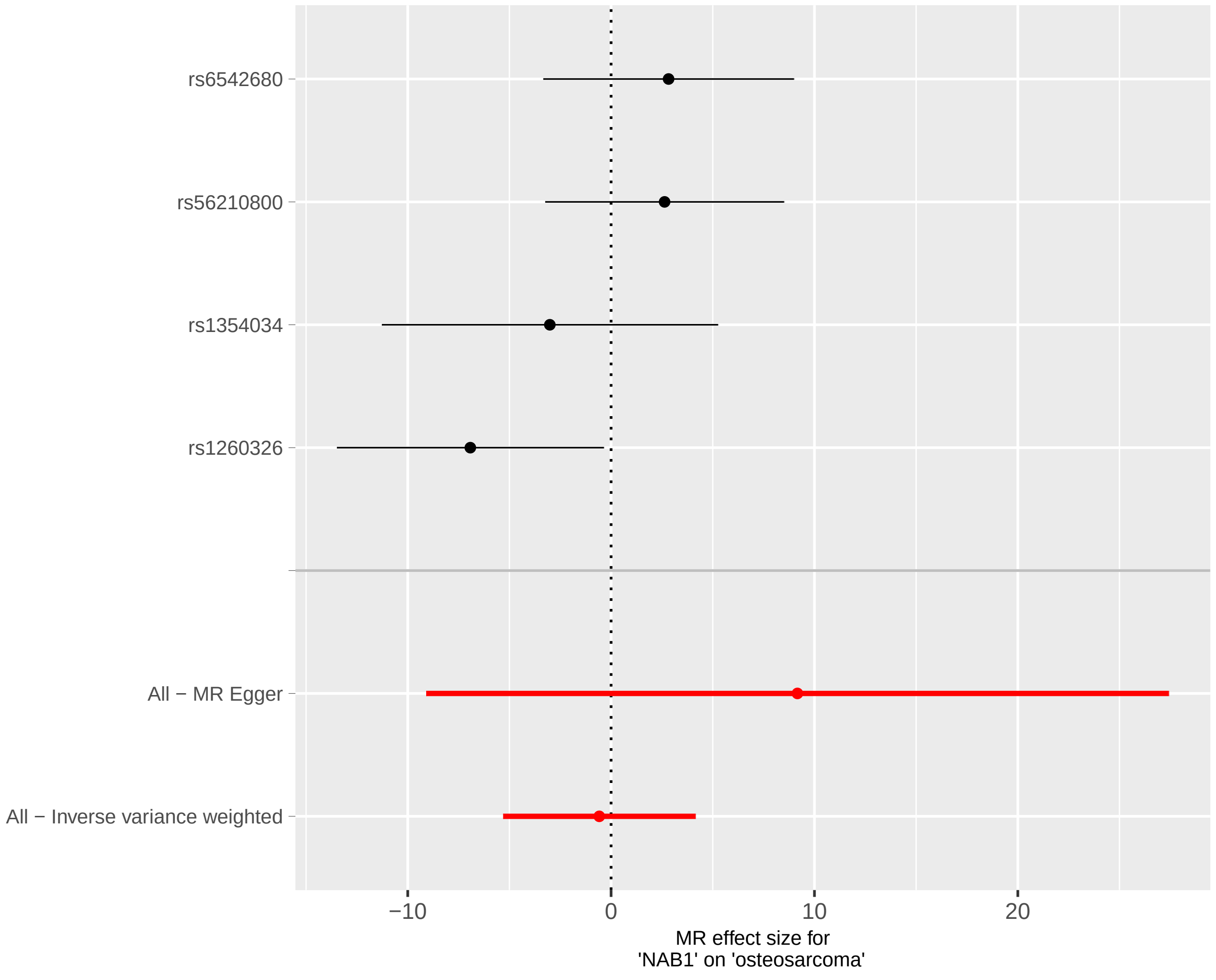

rs6542680
rs56210800
rs1354034
rs1260326
All − MR Egger
All − Inverse variance weighted
−10
0
10
20
MR effect size for
'NAB1' on 'osteosarcoma'

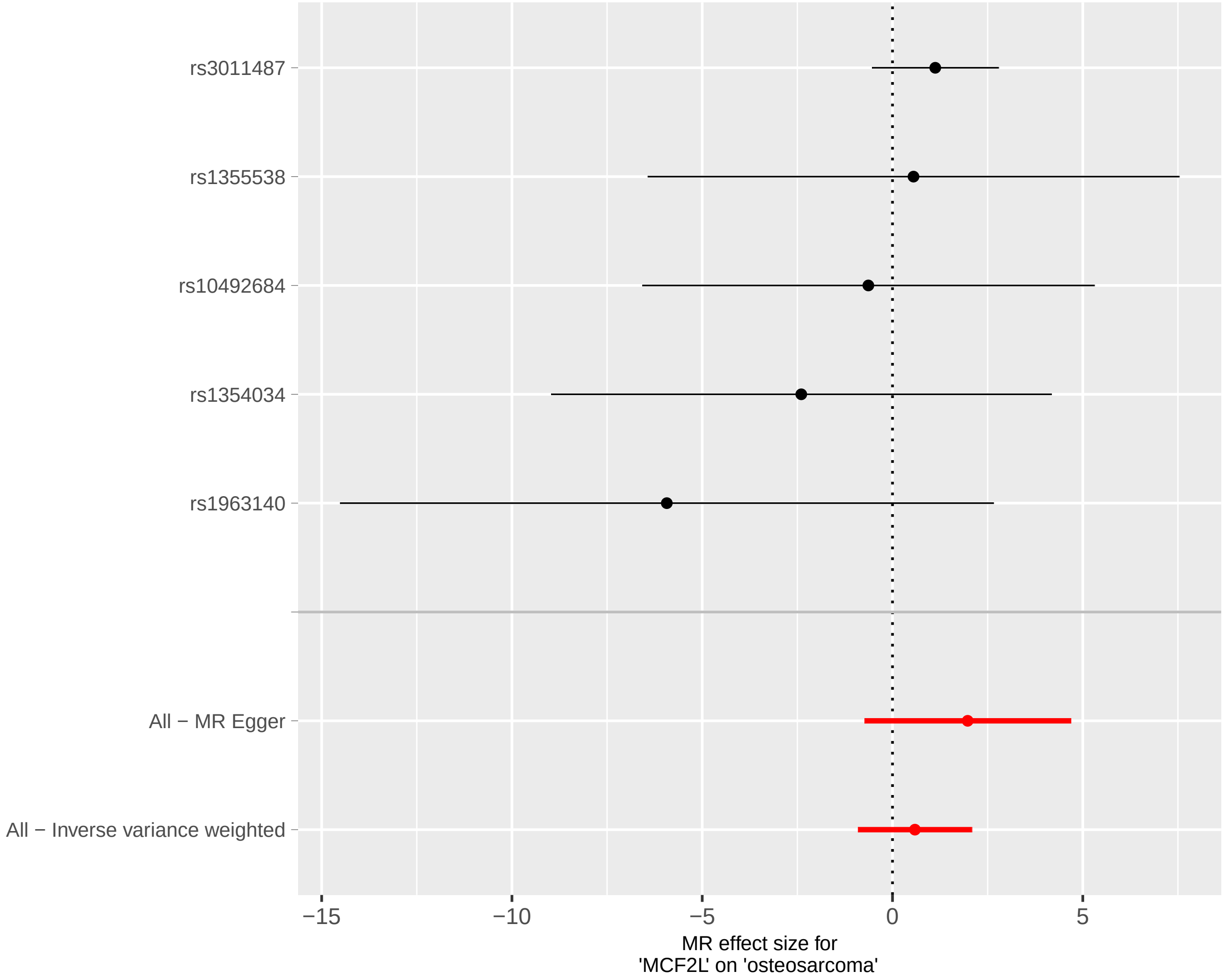

rs3011487
rs1355538
rs10492684
rs1354034
rs1963140
All − MR Egger
All − Inverse variance weighted
−15
−10
−5
0
5
MR effect size for
'MCF2L' on 'osteosarcoma'

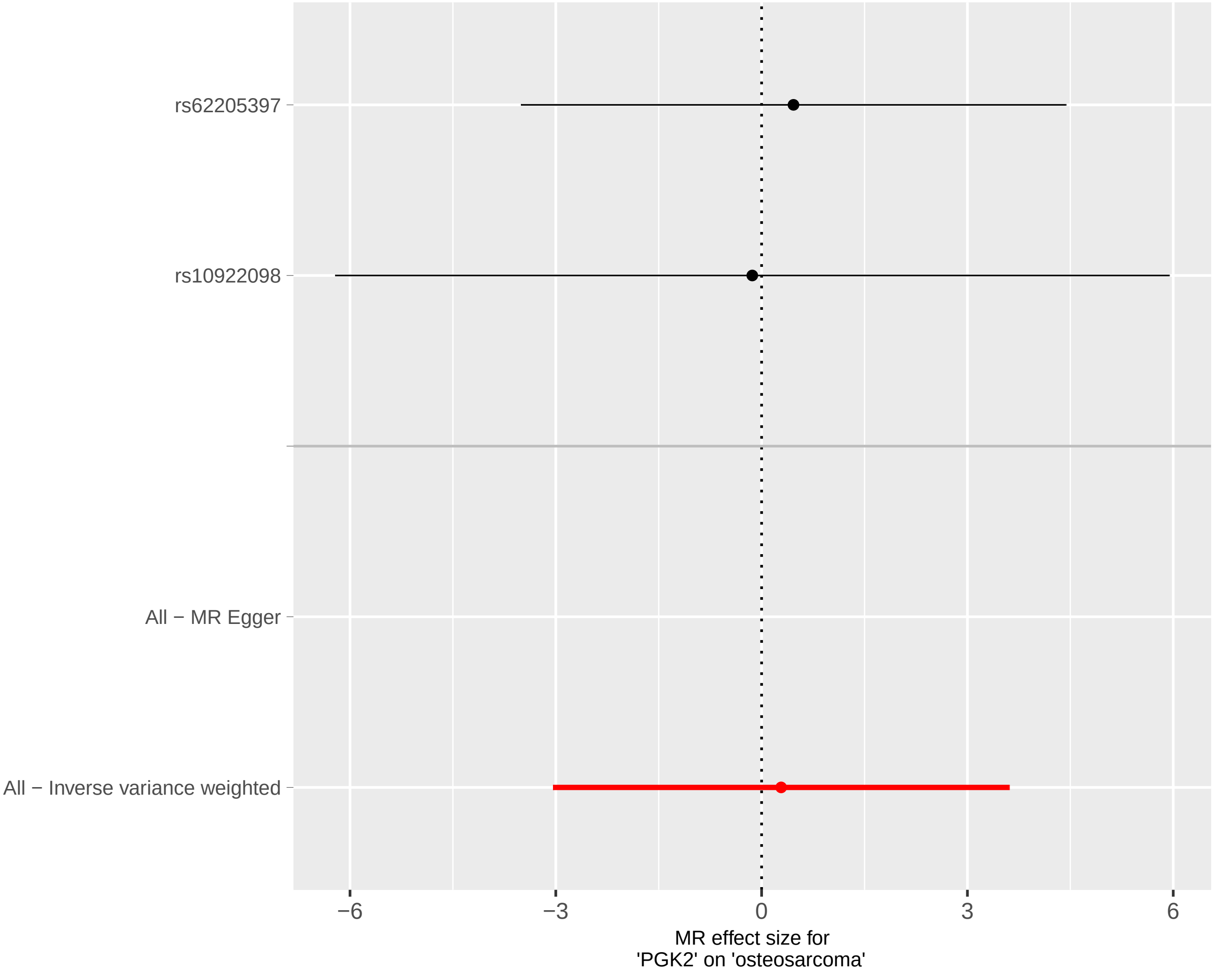
rs62205397
rs10922098
All − MR Egger
All − Inverse variance weighted
−6
−3
0
3
6
MR effect size for
'PGK2' on 'osteosarcoma'

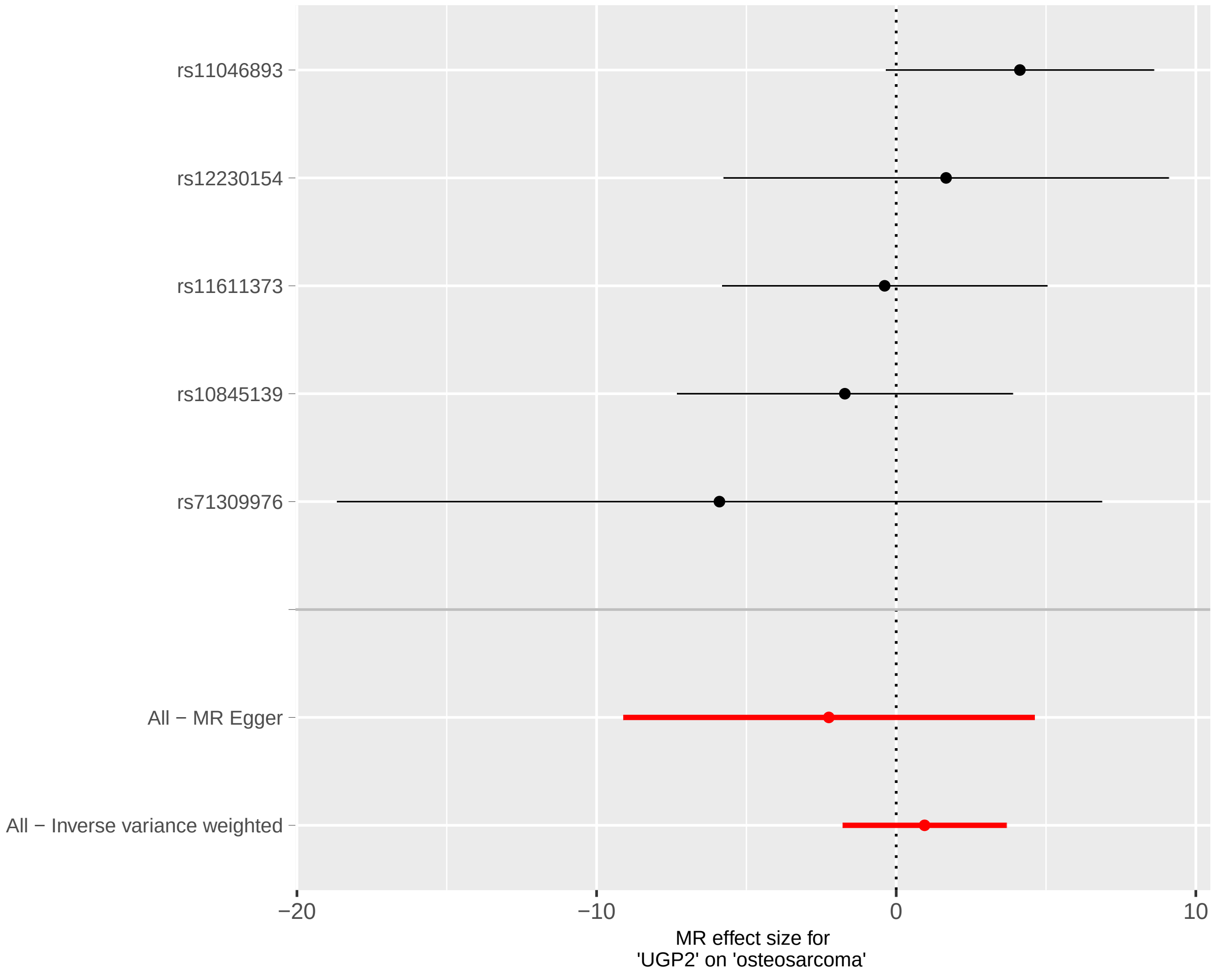
rs11046893
rs12230154
rs11611373
rs10845139
rs71309976
All − MR Egger
All − Inverse variance weighted
−20
−10
0
10
MR effect size for
'UGP2' on 'osteosarcoma'

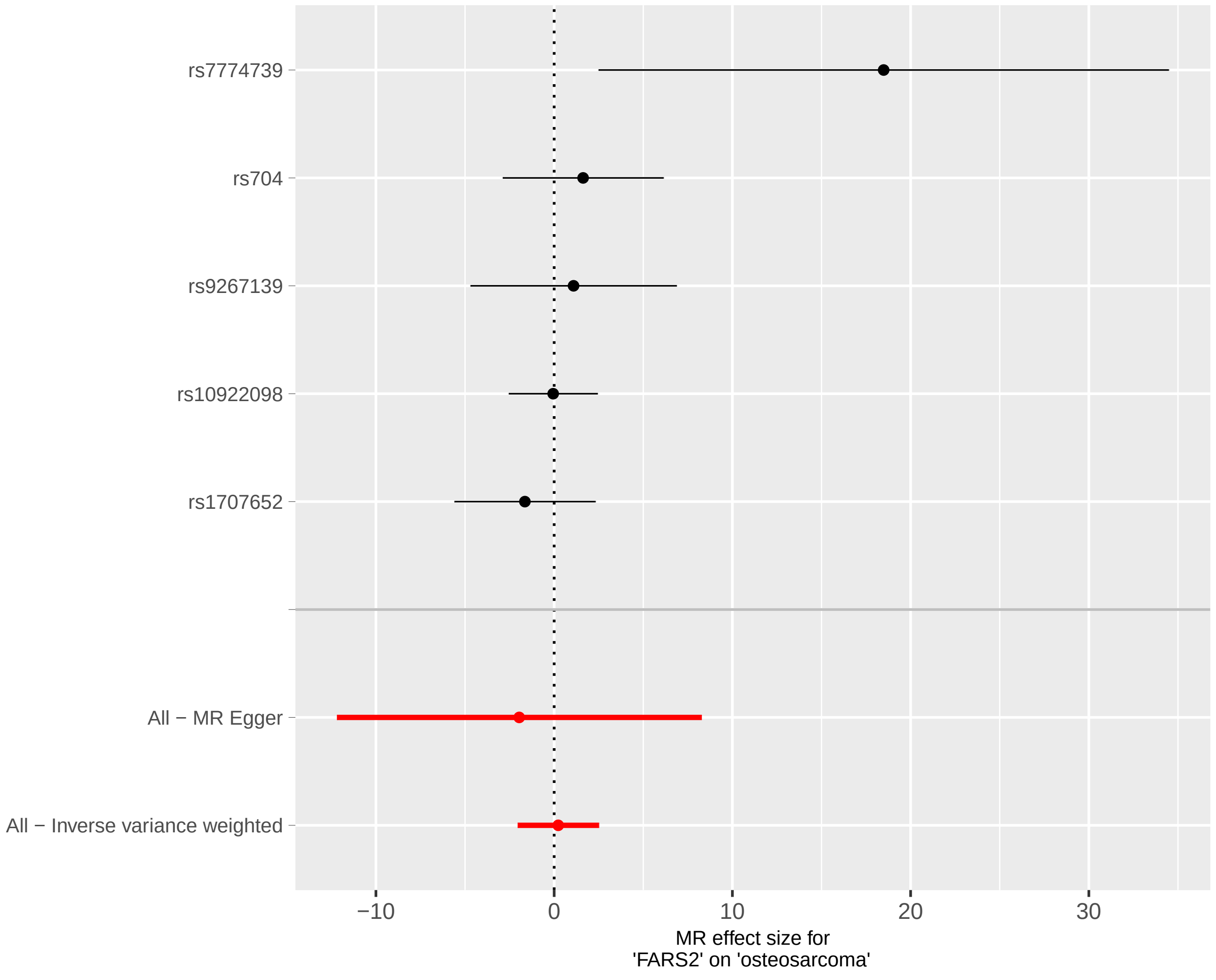

rs7774739
rs704
rs9267139
rs10922098
rs1707652
All − MR Egger
All − Inverse variance weighted
−10
0
10
20
30
MR effect size for
'FARS2' on 'osteosarcoma'

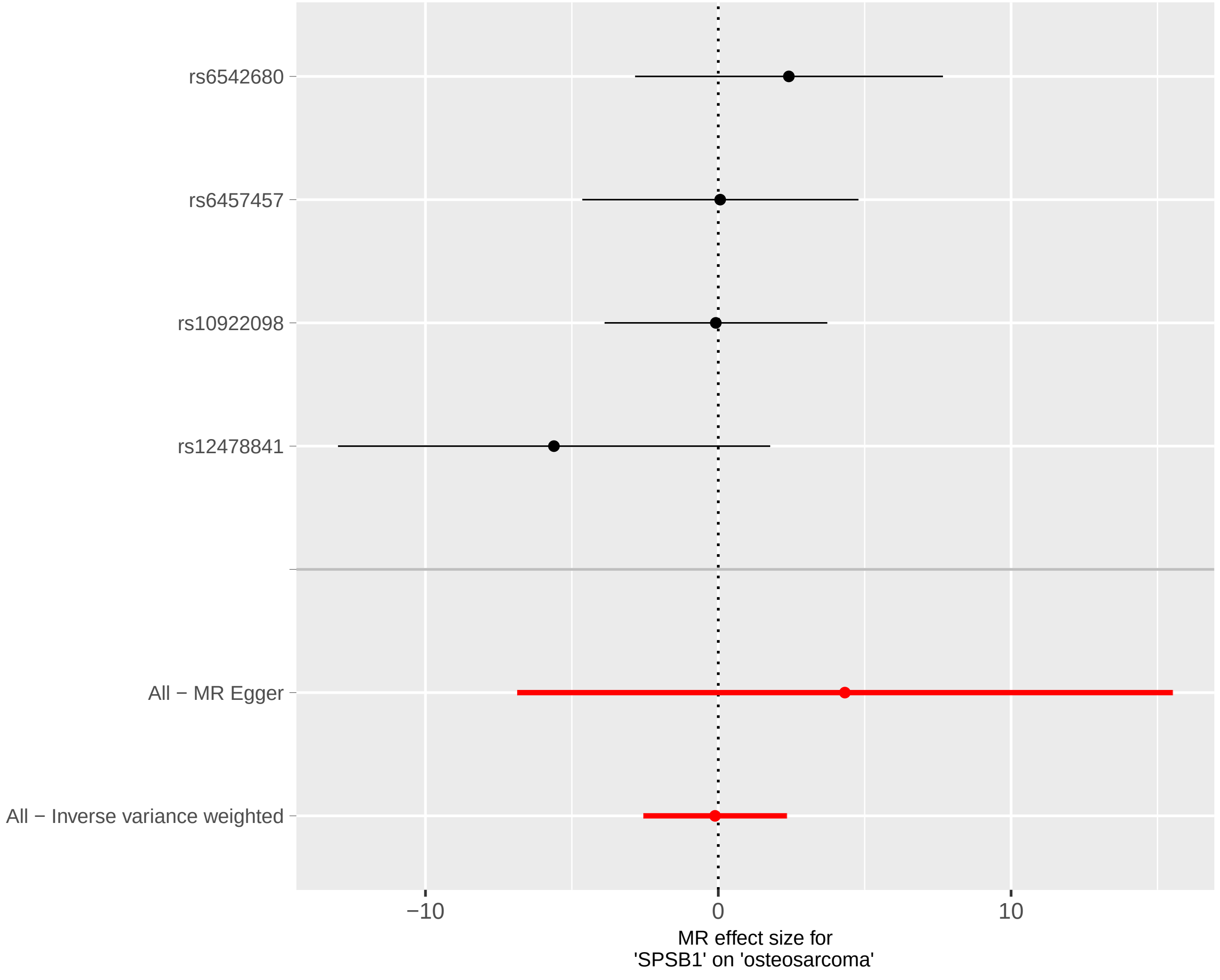
rs6542680
rs6457457
rs10922098
rs12478841
All − MR Egger
All − Inverse variance weighted
−10
0
10
MR effect size for
'SPSB1' on 'osteosarcoma'

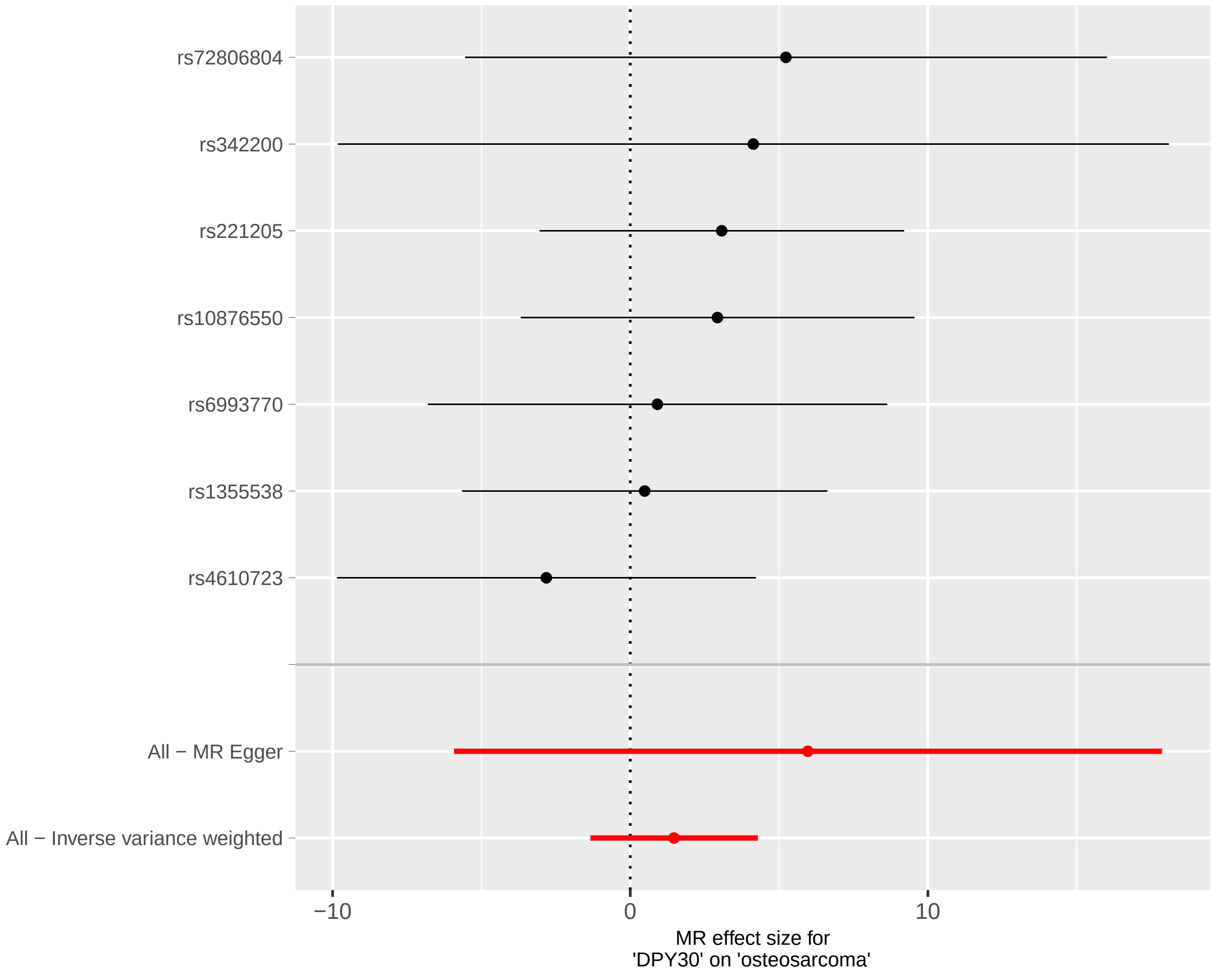
rs72806804
rs342200
rs221205
rs10876550
rs6993770
rs1355538
rs4610723
All − MR Egger
All − Inverse variance weighted
−10
0
10
MR effect size for
'DPY30' on 'osteosarcoma'

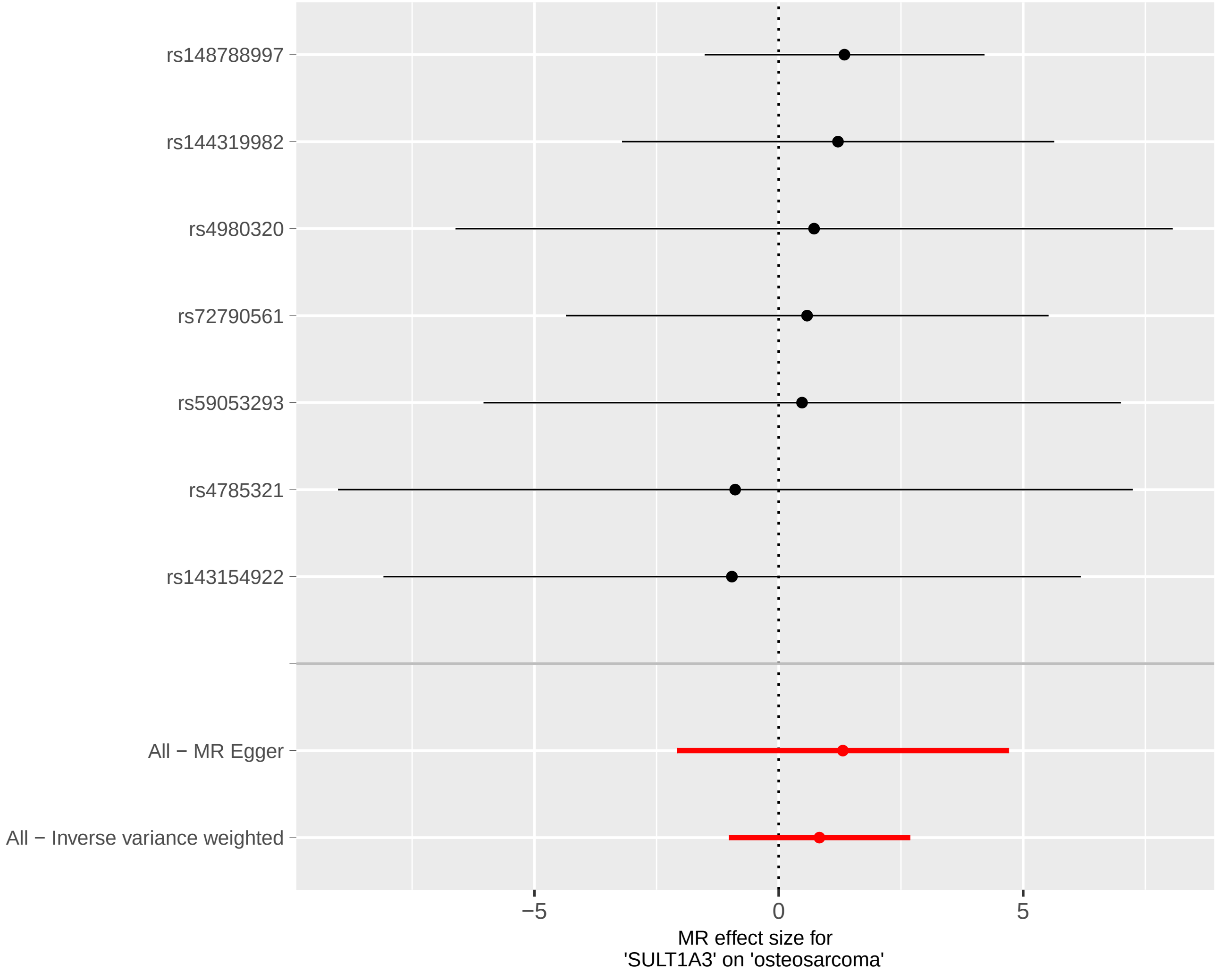

rs148788997
rs144319982
rs4980320
rs72790561
rs59053293
rs4785321
rs143154922
All − MR Egger
All − Inverse variance weighted
−5
0
5
MR effect size for
'SULT1A3' on 'osteosarcoma'

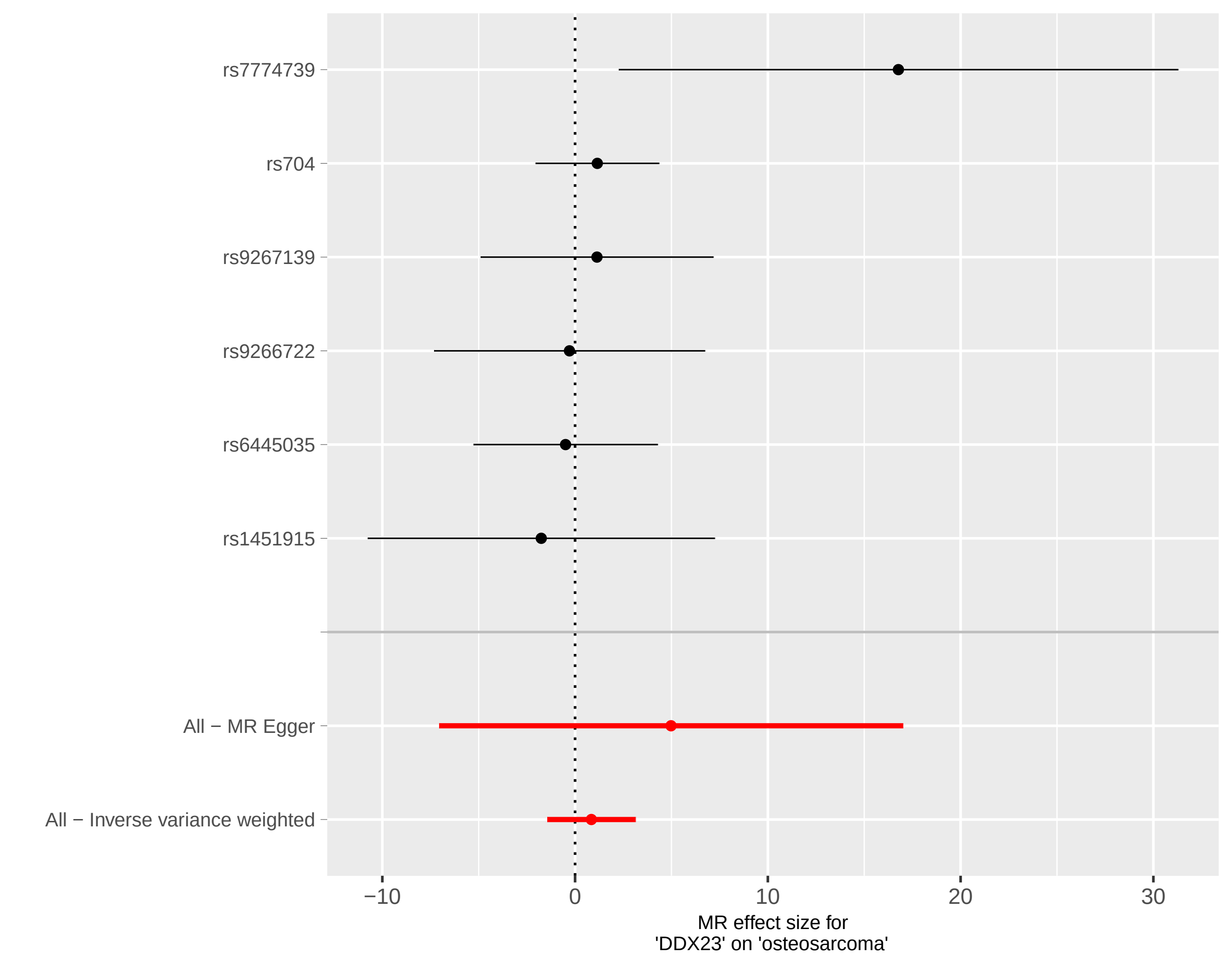

rs7774739
rs704
rs9267139
rs9266722
rs6445035
rs1451915
All − MR Egger
All − Inverse variance weighted
−10
0
10
20
30
MR effect size for
'DDX23' on 'osteosarcoma'

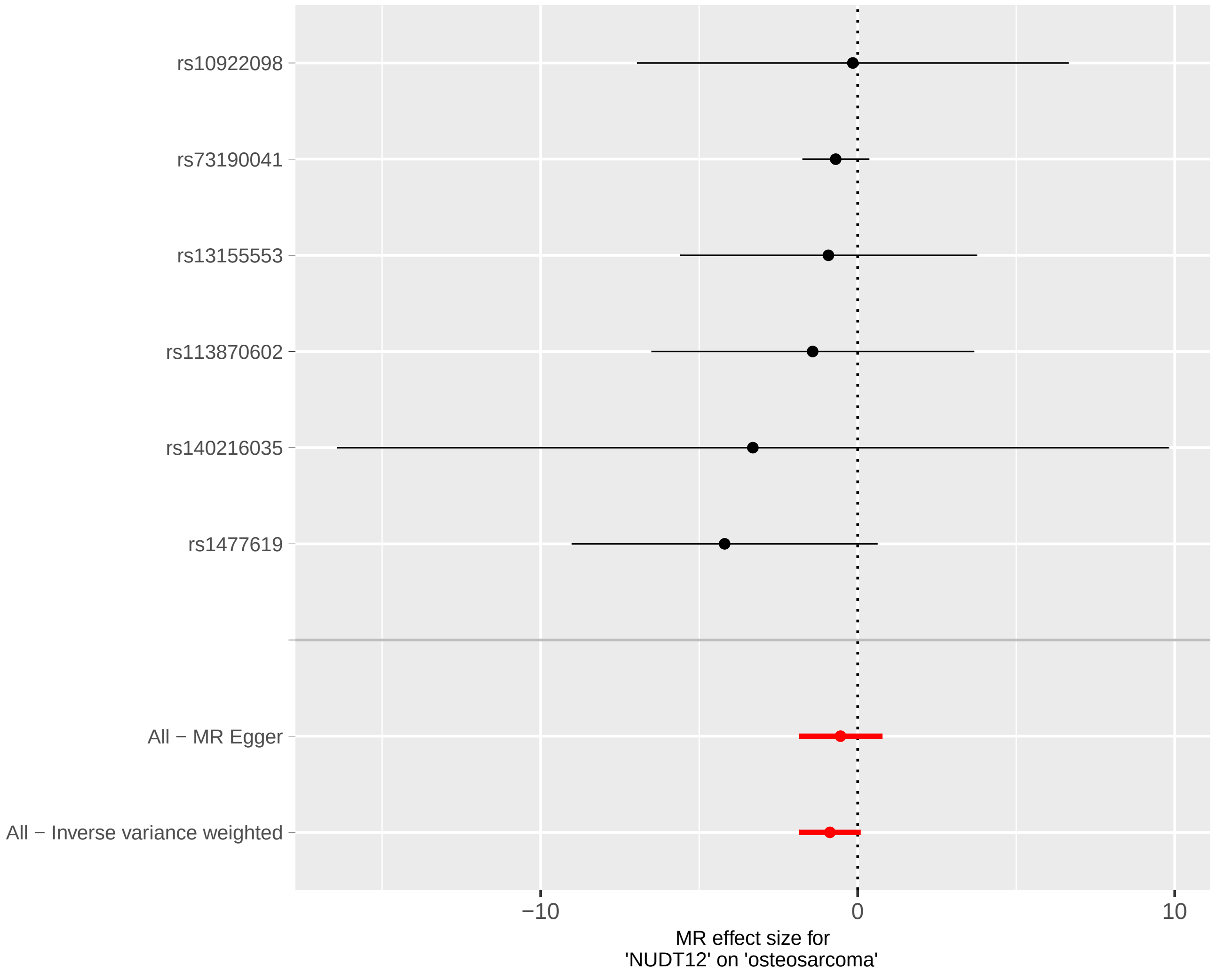

rs10922098
rs73190041
rs13155553
rs113870602
rs140216035
rs1477619
All − MR Egger
All − Inverse variance weighted
−10
0
10
MR effect size for
'NUDT12' on 'osteosarcoma'

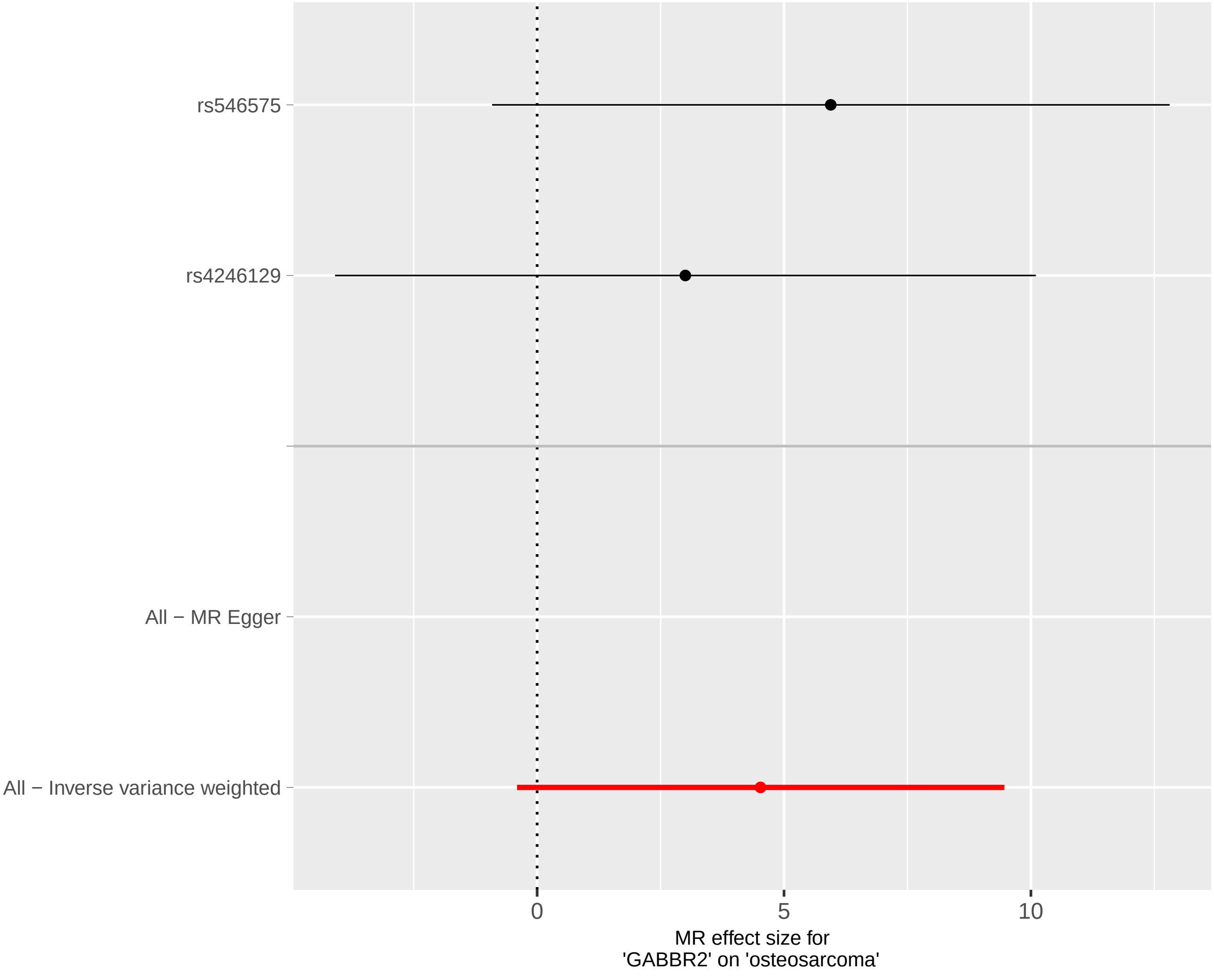

rs546575
rs4246129
All − MR Egger
All − Inverse variance weighted
0
5
10
MR effect size for
'GABBR2' on 'osteosarcoma'

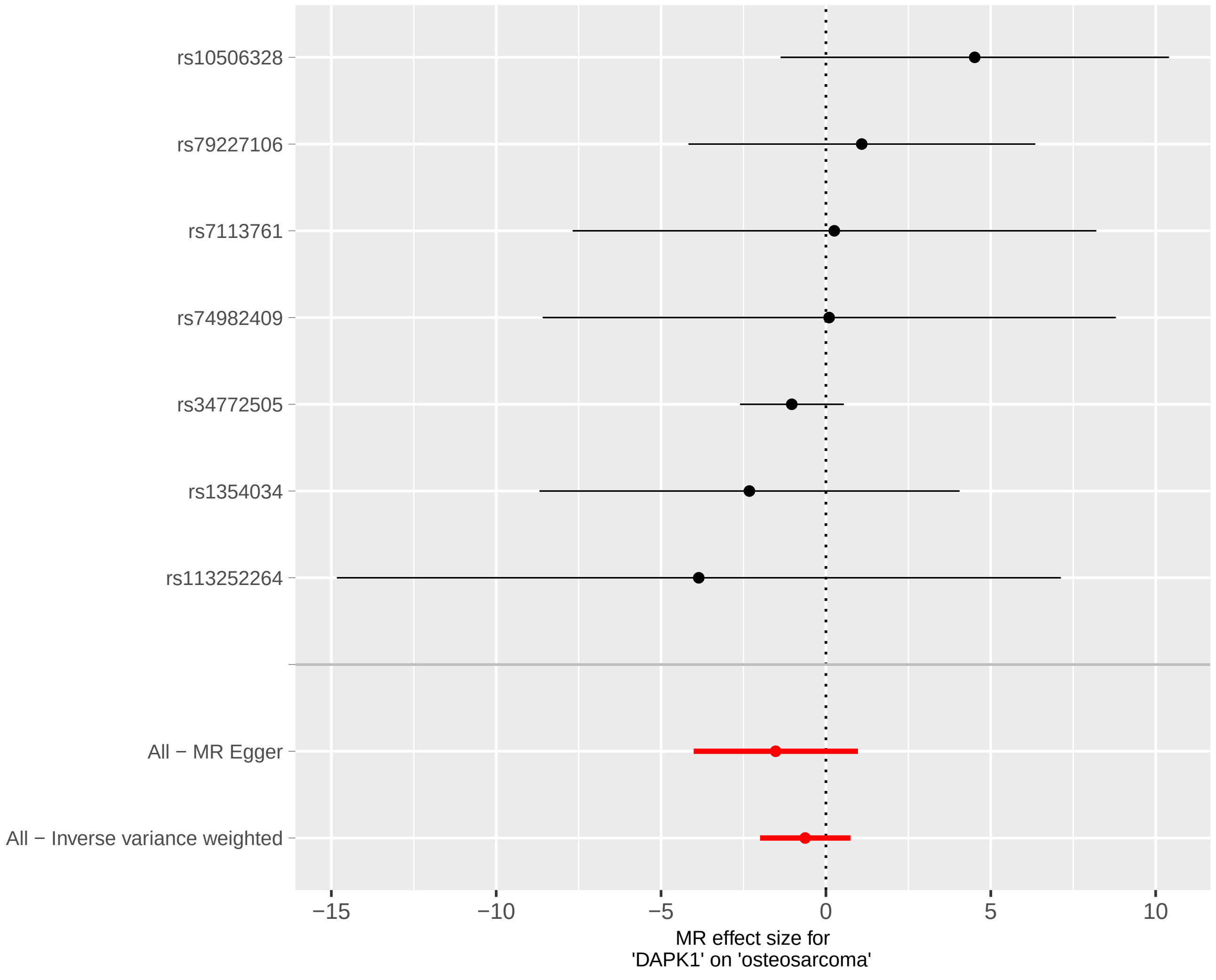
rs10506328
rs79227106
rs7113761
rs74982409
rs34772505
rs1354034
rs113252264
All − MR Egger
All − Inverse variance weighted
−15
−10
−5
0
5
10
MR effect size for
'DAPK1' on 'osteosarcoma'

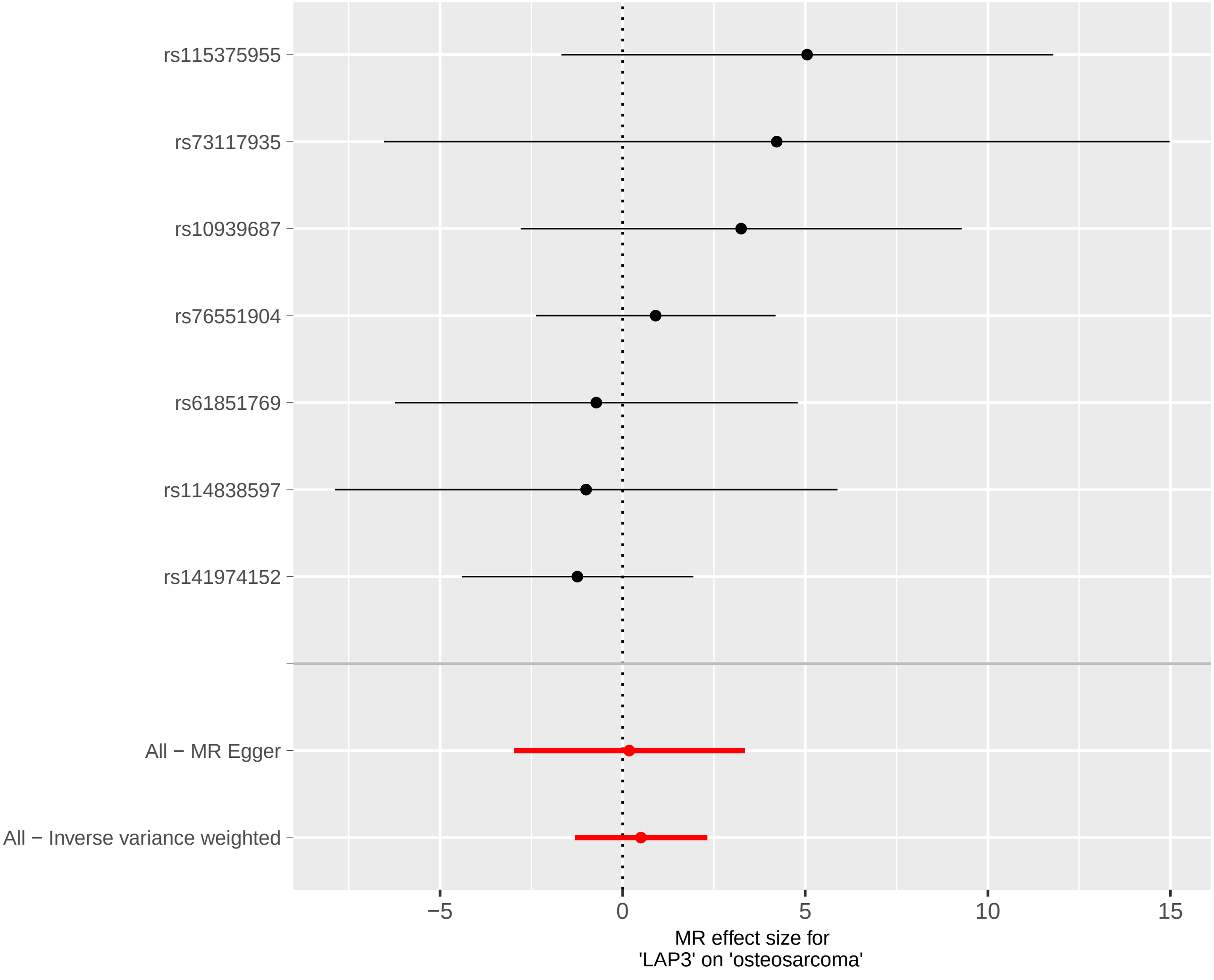
rs115375955
rs73117935
rs10939687
rs76551904
rs61851769
rs114838597
rs141974152
All − MR Egger
All − Inverse variance weighted
−5
0
5
10
15
MR effect size for
'LAP3' on 'osteosarcoma'

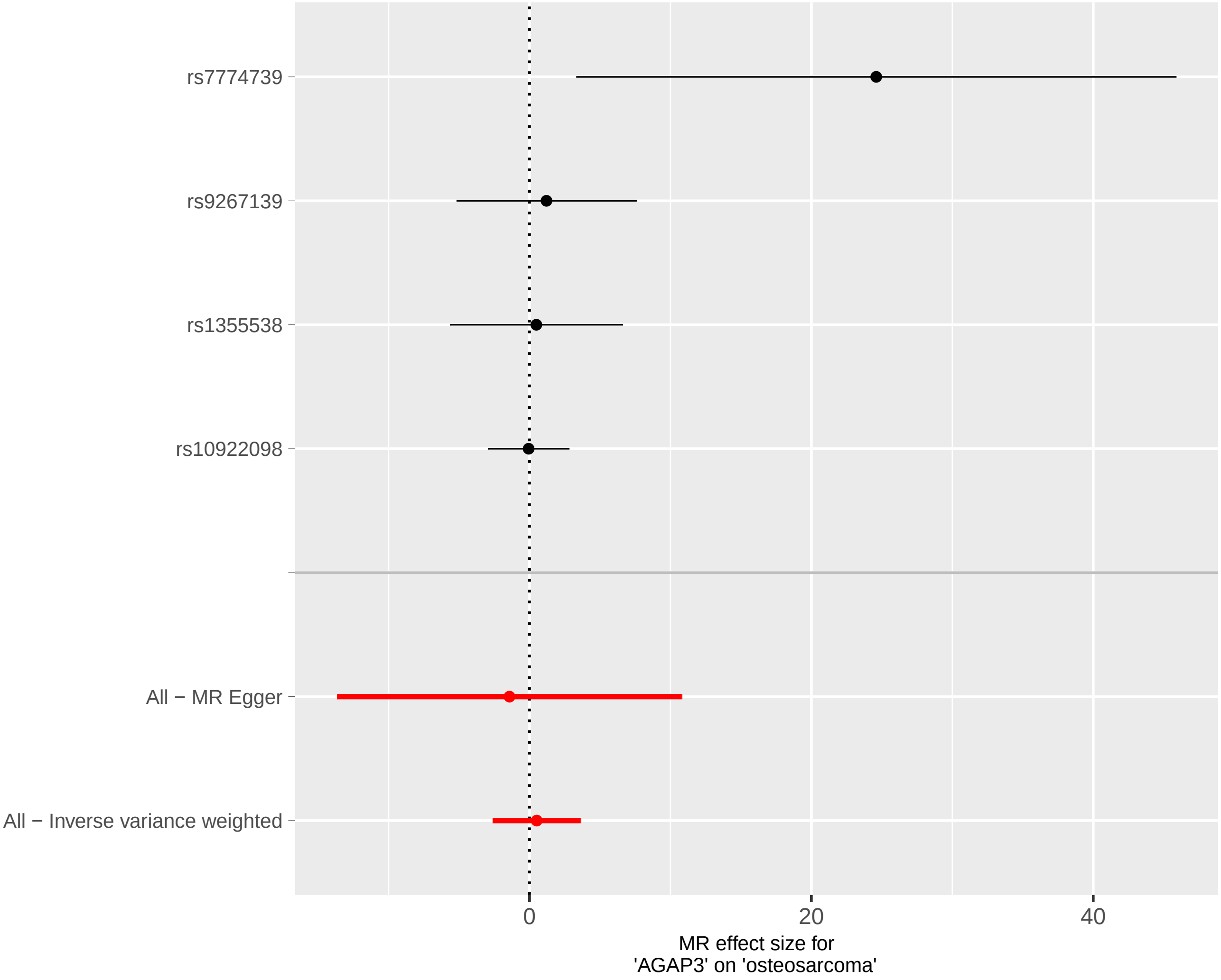

rs7774739
rs9267139
rs1355538
rs10922098
All − MR Egger
All − Inverse variance weighted
0
20
40
MR effect size for
'AGAP3' on 'osteosarcoma'

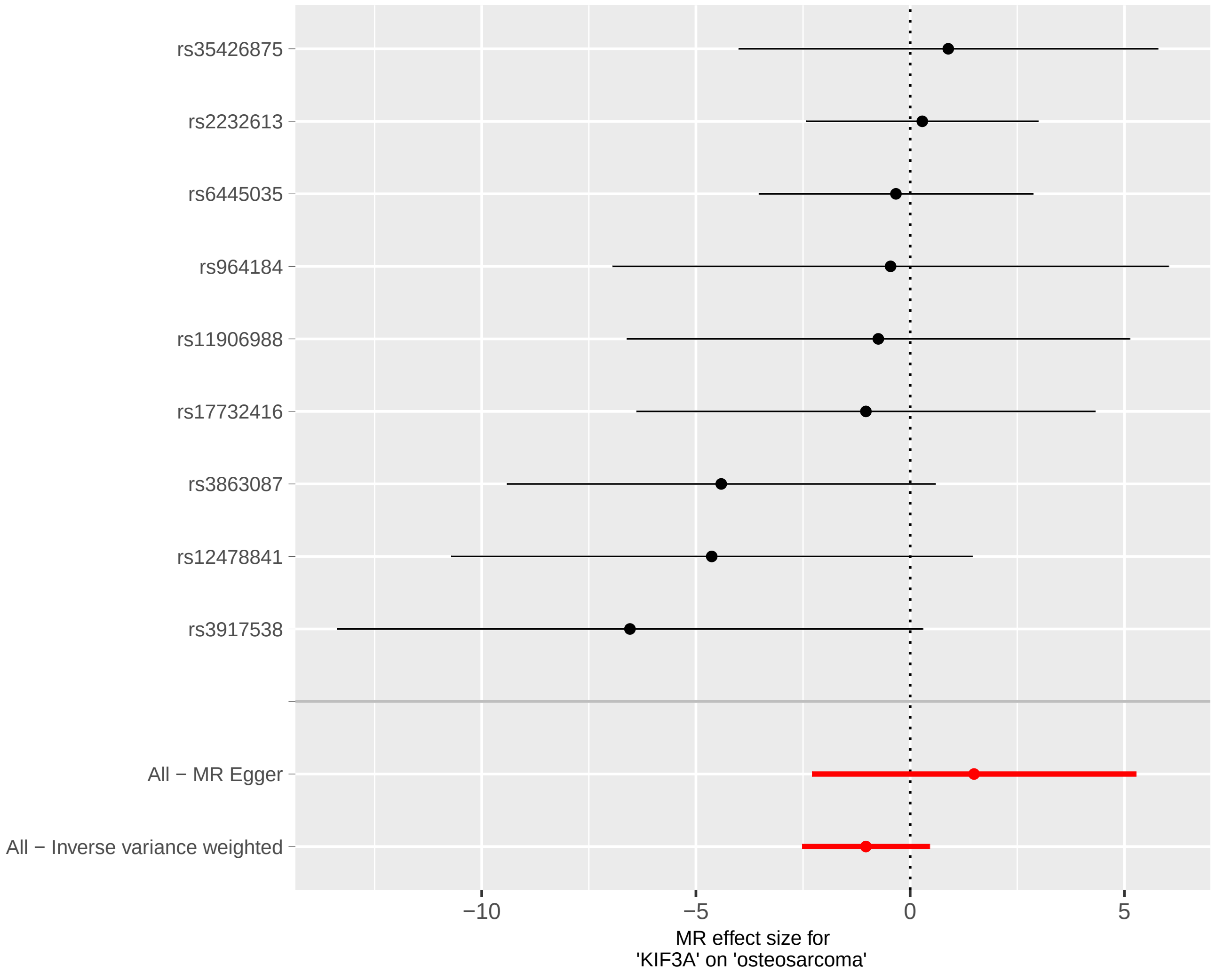

rs35426875
rs2232613
rs6445035
rs964184
rs11906988
rs17732416
rs3863087
rs12478841
rs3917538
All − MR Egger
All − Inverse variance weighted
−10
−5
0
5
MR effect size for
'KIF3A' on 'osteosarcoma'

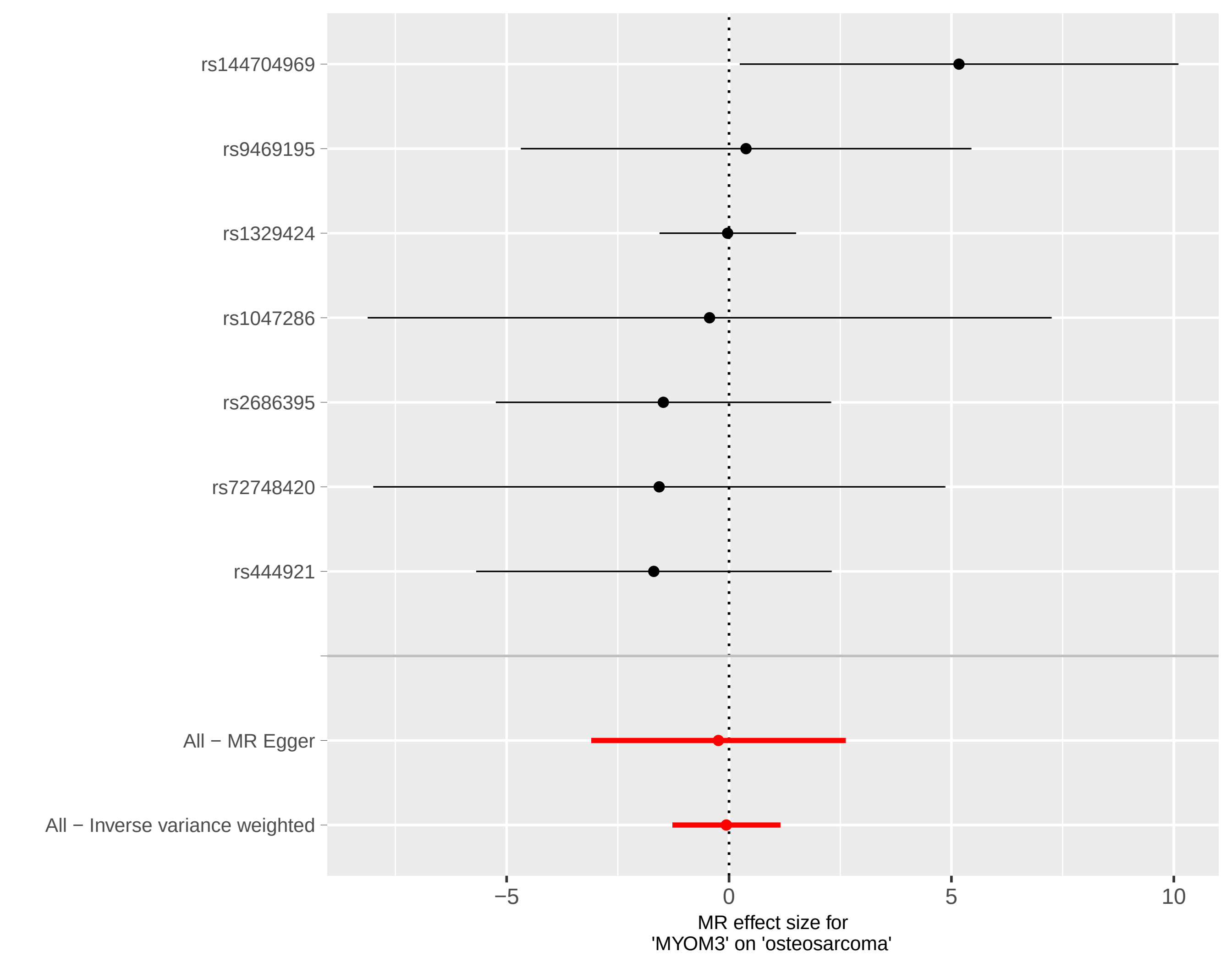
rs144704969
rs9469195
rs1329424
rs1047286
rs2686395
rs72748420
rs444921
All − MR Egger
All − Inverse variance weighted
−5
0
5
10
MR effect size for
'MYOM3' on 'osteosarcoma'

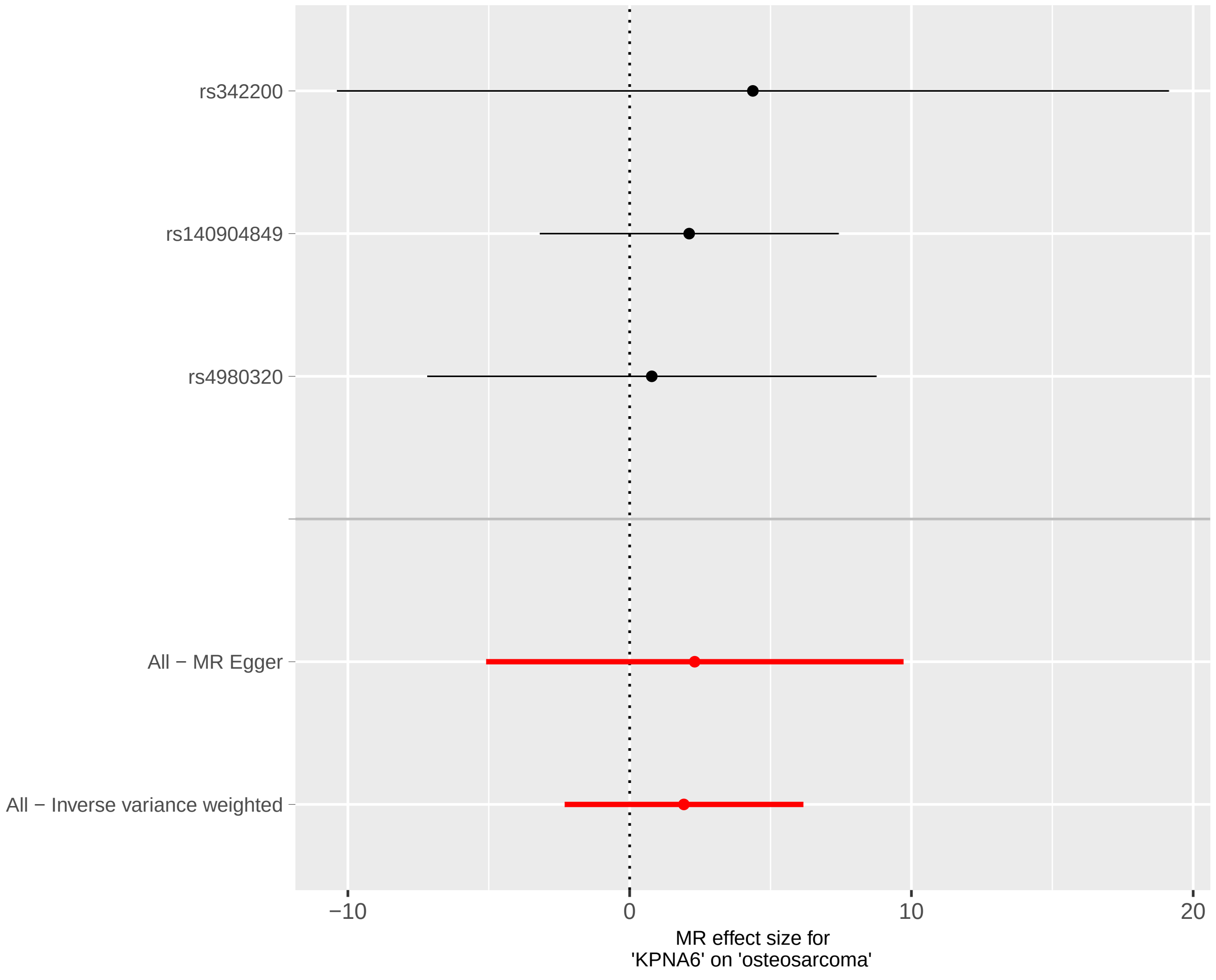
rs342200
rs140904849
rs4980320
All − MR Egger
All − Inverse variance weighted
−10
0
10
20
MR effect size for
'KPNA6' on 'osteosarcoma'

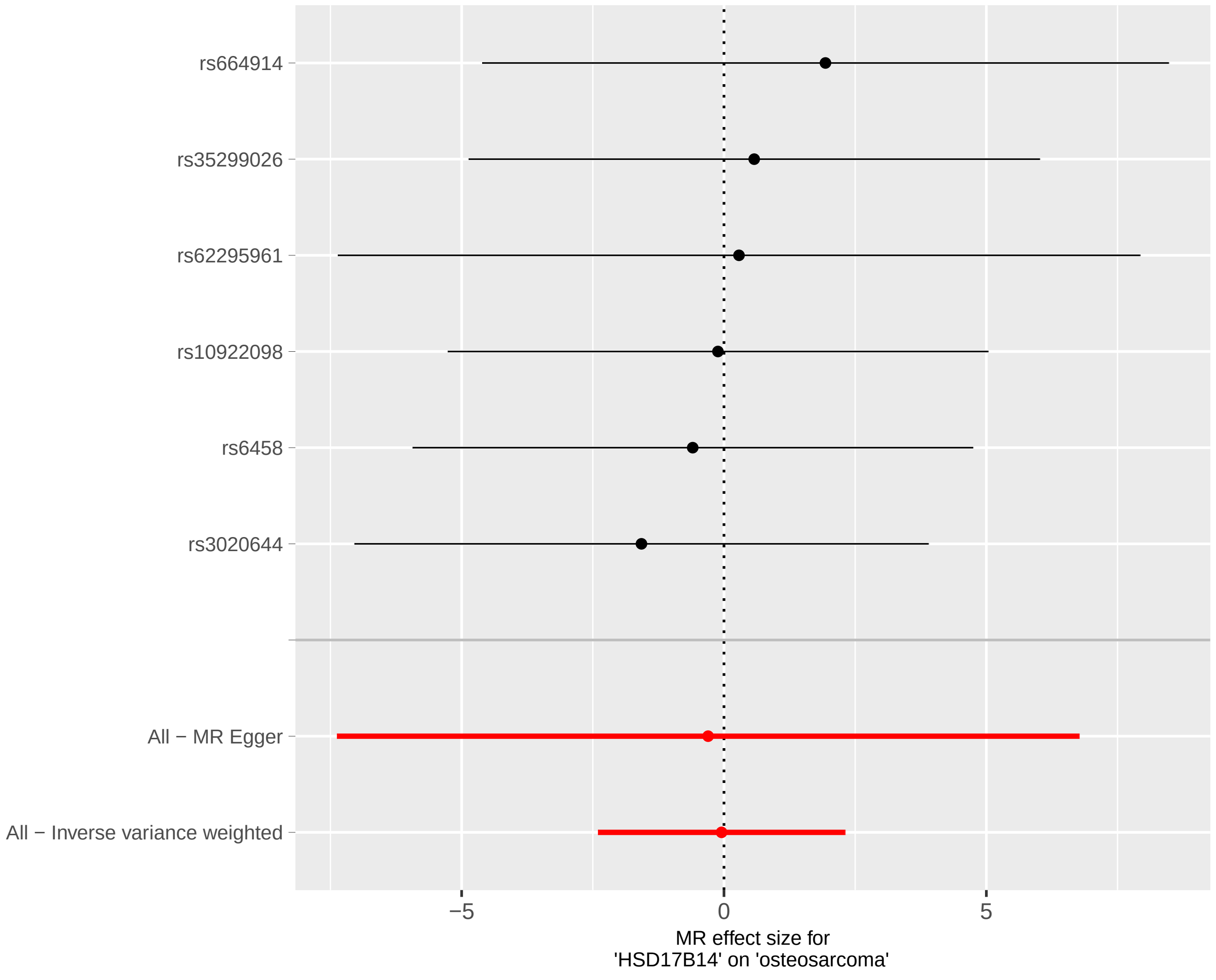

rs664914
rs35299026
rs62295961
rs10922098
rs6458
rs3020644
All − MR Egger
All − Inverse variance weighted
−5
0
5
MR effect size for
'HSD17B14' on 'osteosarcoma'

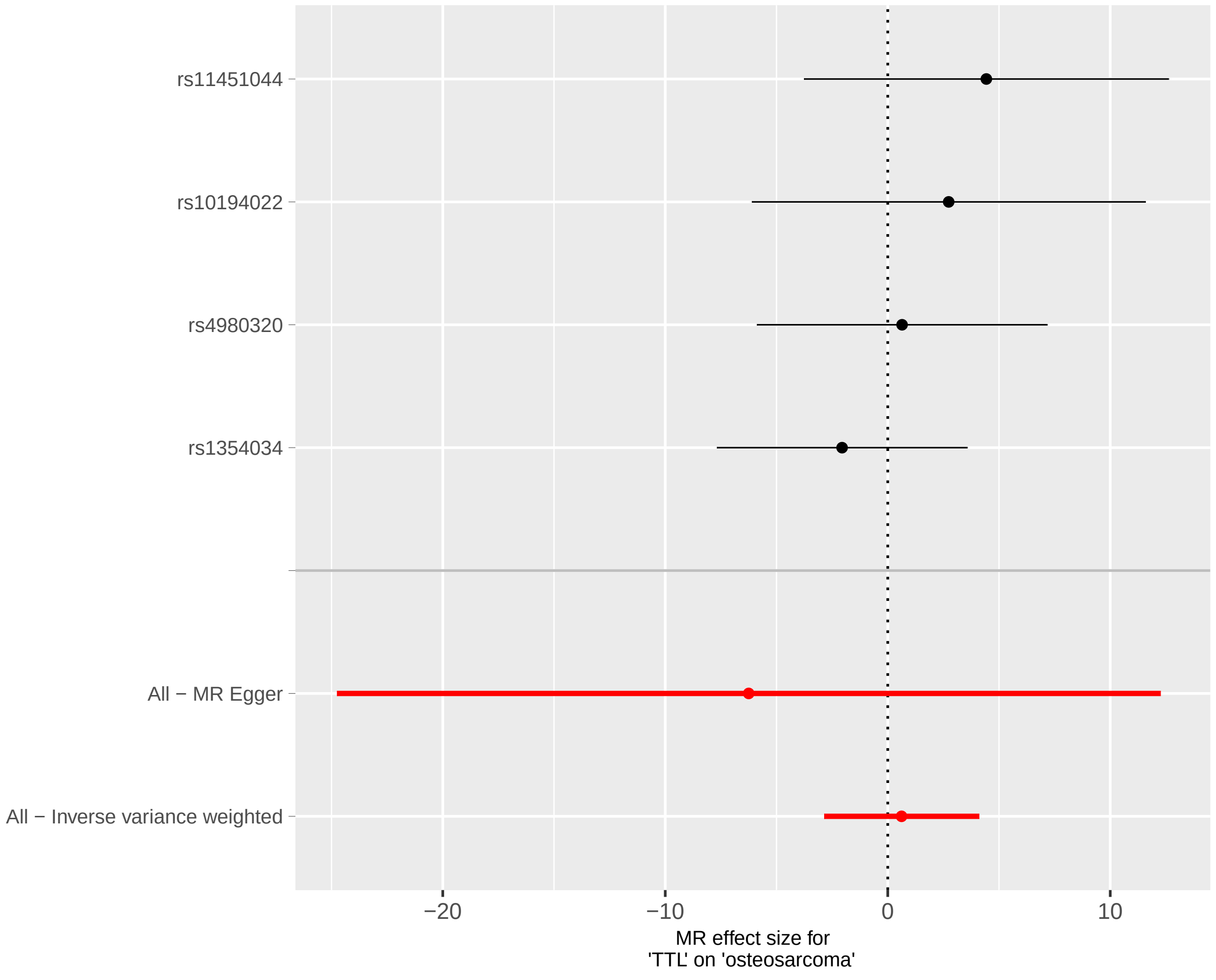
rs11451044
rs10194022
rs4980320
rs1354034
All − MR Egger
All − Inverse variance weighted
−20
−10
0
10
MR effect size for
'TTL' on 'osteosarcoma'

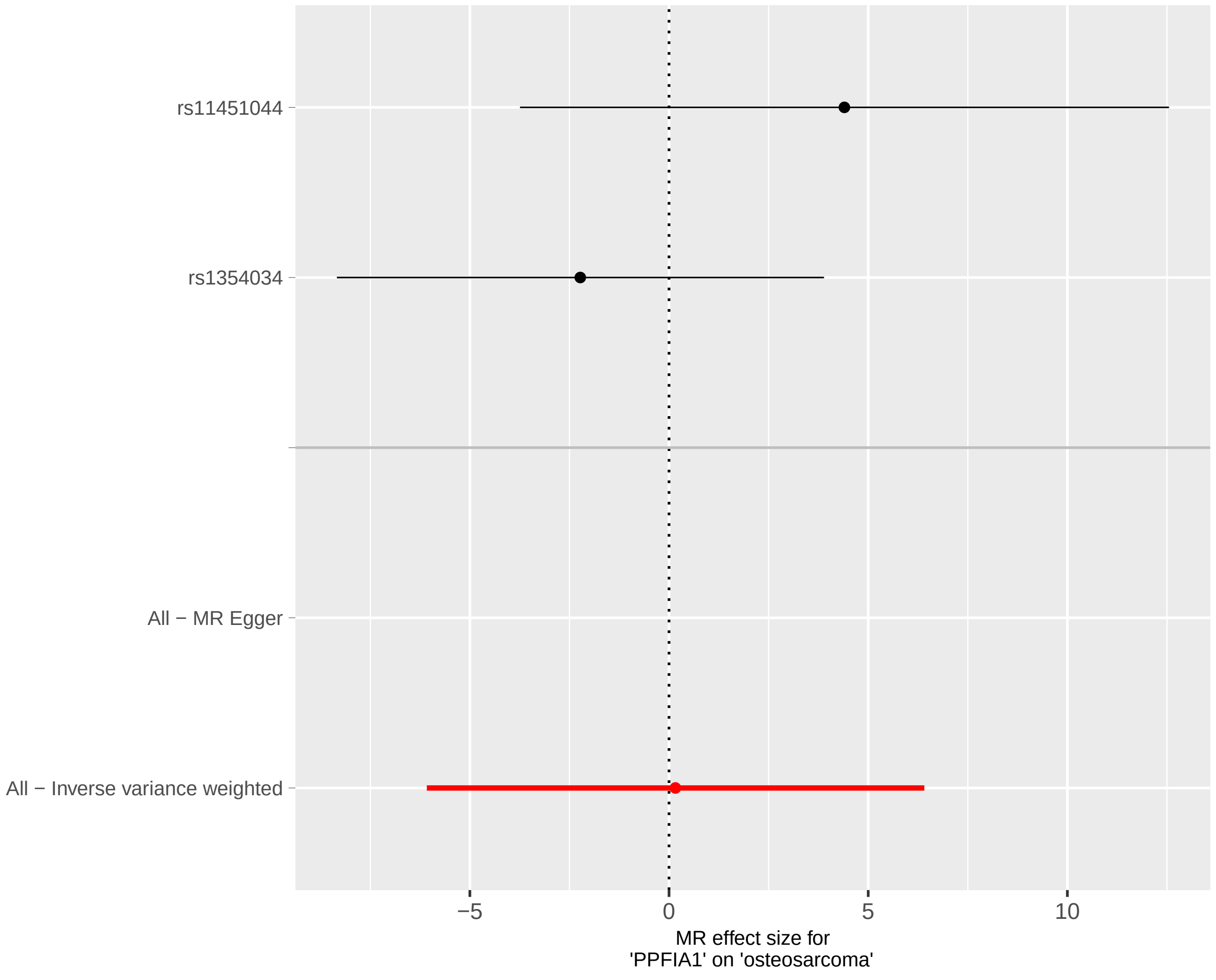

rs11451044
rs1354034
All − MR Egger
All − Inverse variance weighted
−5
0
5
10
MR effect size for
'PPFIA1' on 'osteosarcoma'

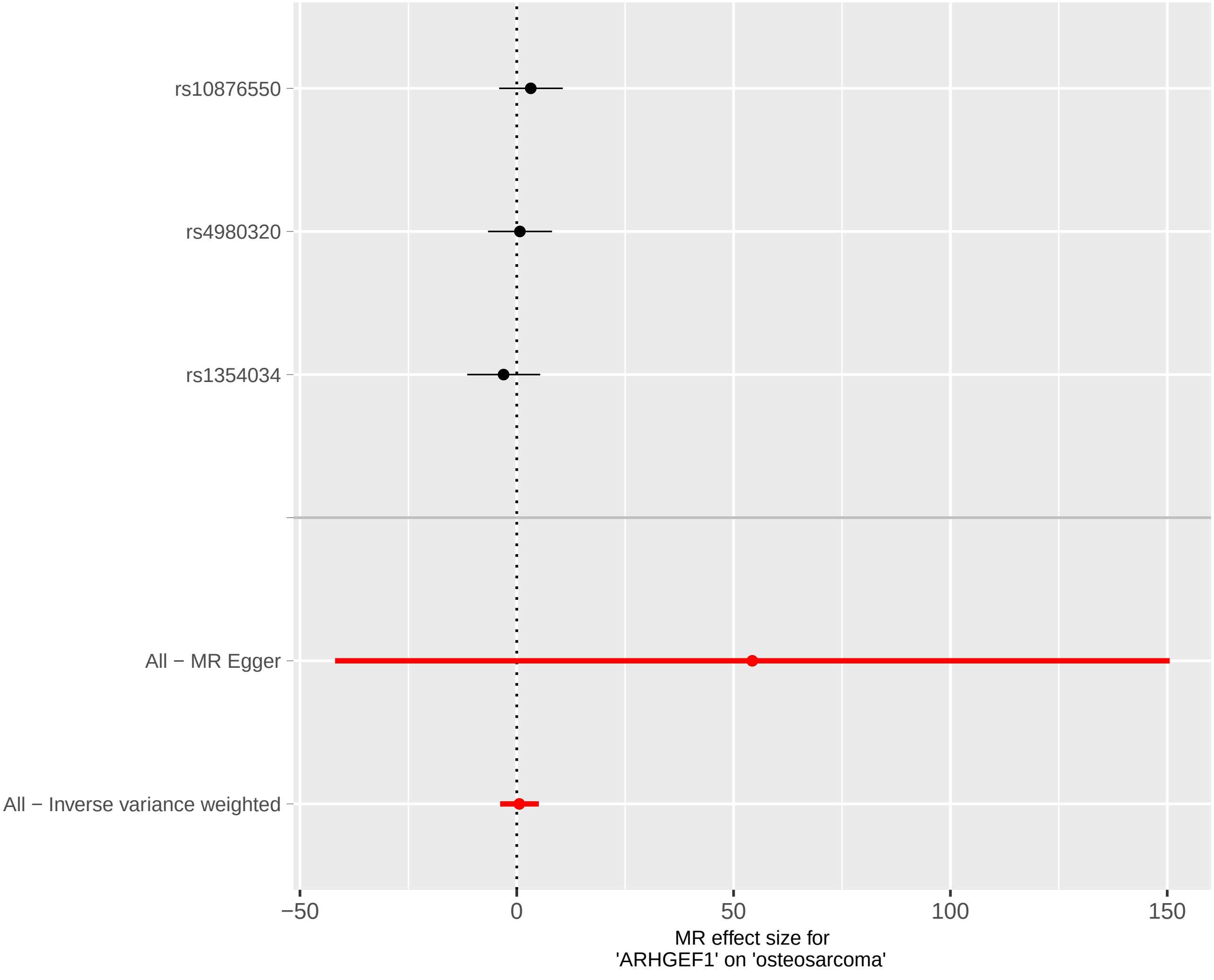

rs10876550
rs4980320
rs1354034
All − MR Egger
All − Inverse variance weighted
−50
0
50
100
150
MR effect size for
'ARHGEF1' on 'osteosarcoma'

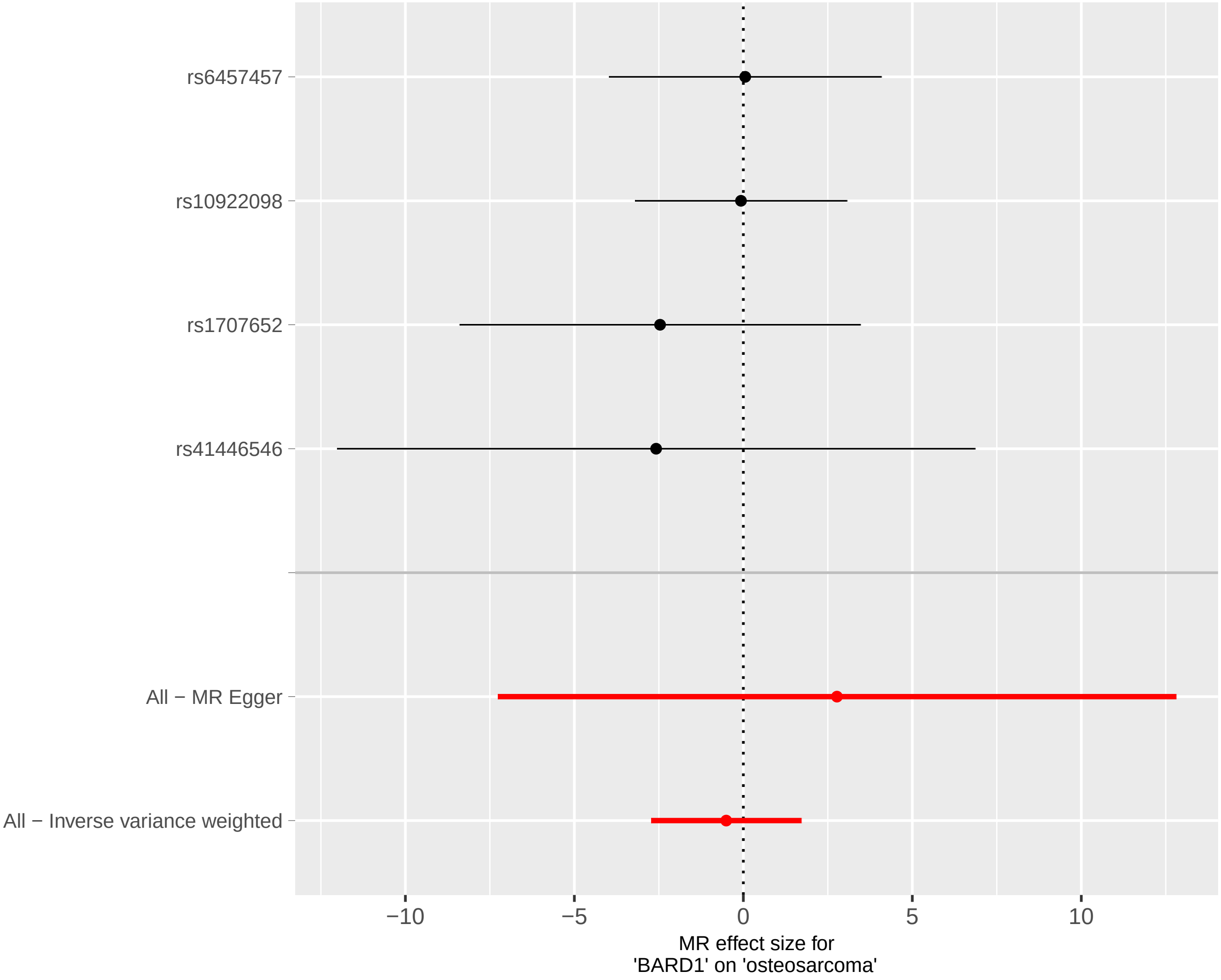
rs6457457
rs10922098
rs1707652
rs41446546
All − MR Egger
All − Inverse variance weighted
−10
−5
0
5
10
MR effect size for
'BARD1' on 'osteosarcoma'

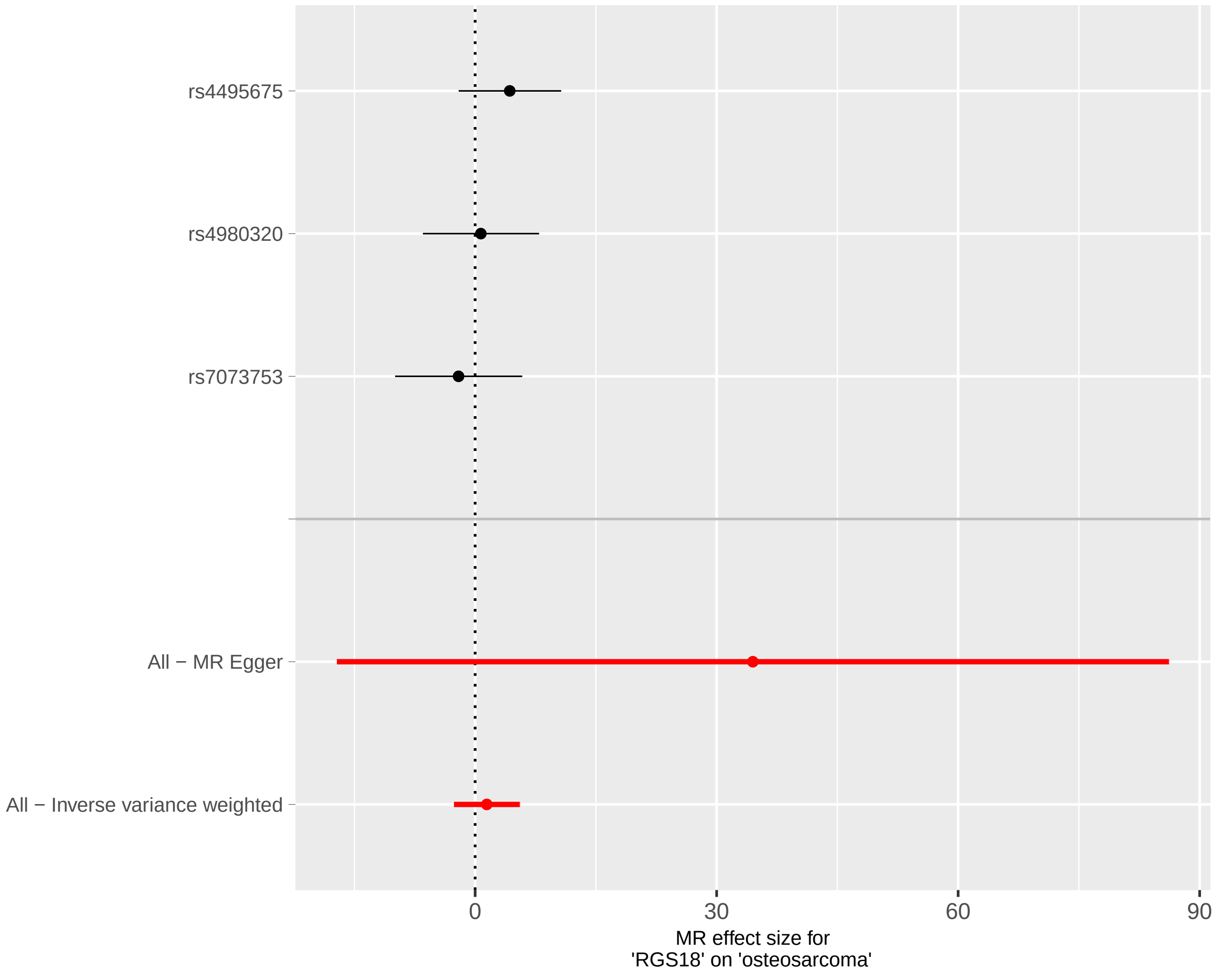
rs4495675
rs4980320
rs7073753
All − MR Egger
All − Inverse variance weighted
0
30
60
90
MR effect size for
'RGS18' on 'osteosarcoma'

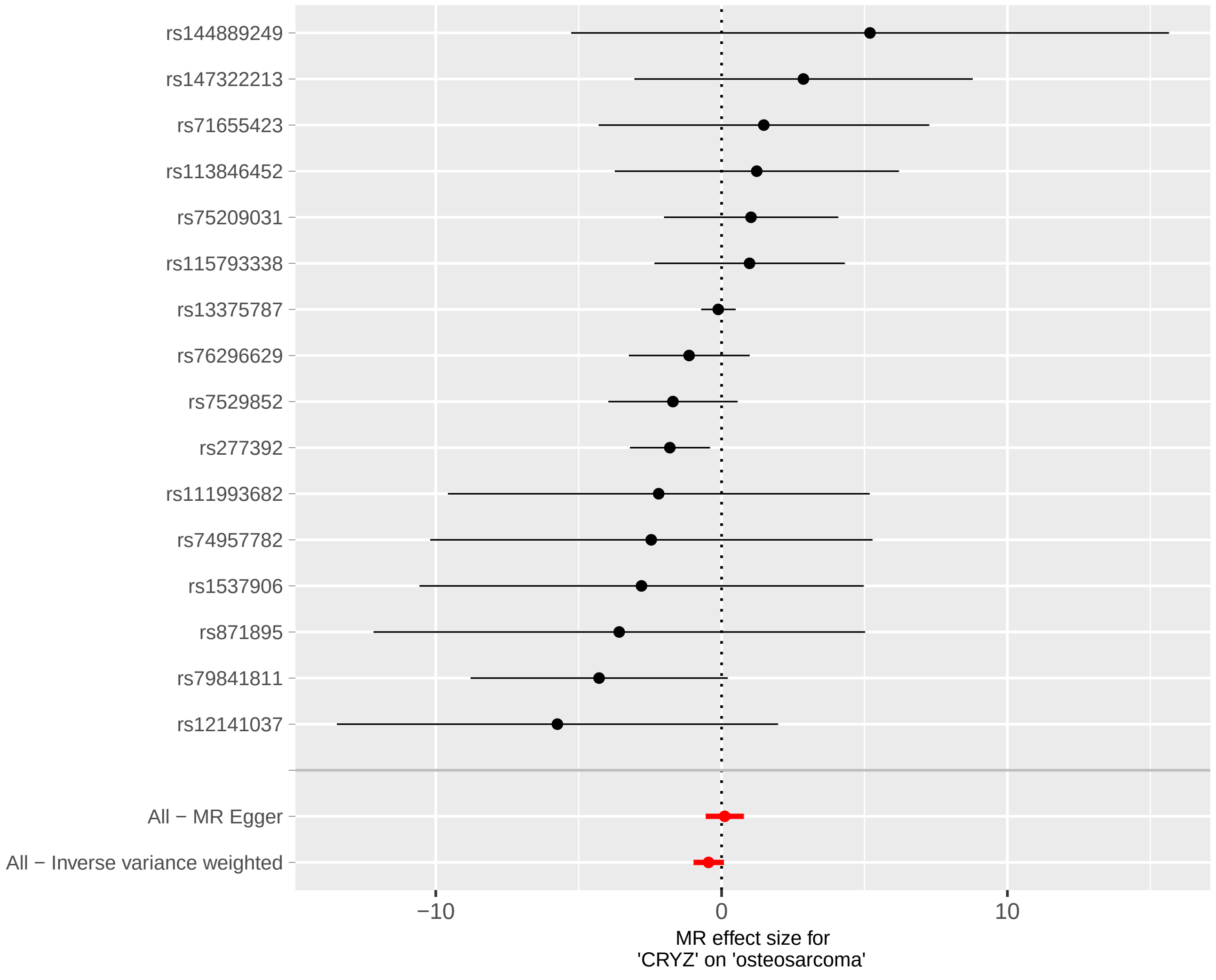
rs144889249
rs147322213
rs71655423
rs113846452
rs75209031
rs115793338
rs13375787
rs76296629
rs7529852
rs277392
rs111993682
rs74957782
rs1537906
rs871895
rs79841811
rs12141037
All − MR Egger
All − Inverse variance weighted
−10
0
10
MR effect size for
'CRYZ' on 'osteosarcoma'

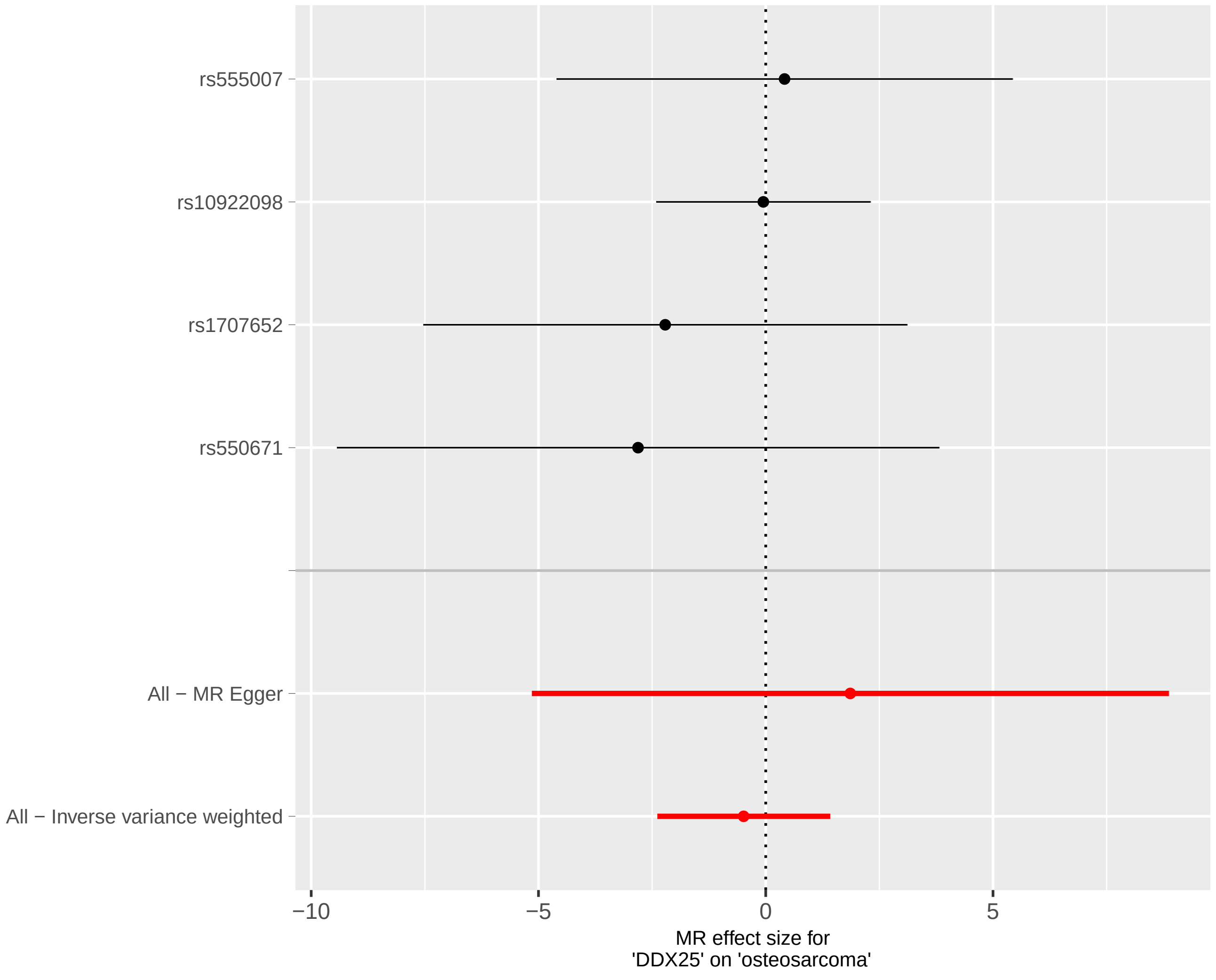

rs555007
rs10922098
rs1707652
rs550671
All − MR Egger
All − Inverse variance weighted
−10
−5
0
5
MR effect size for
'DDX25' on 'osteosarcoma'

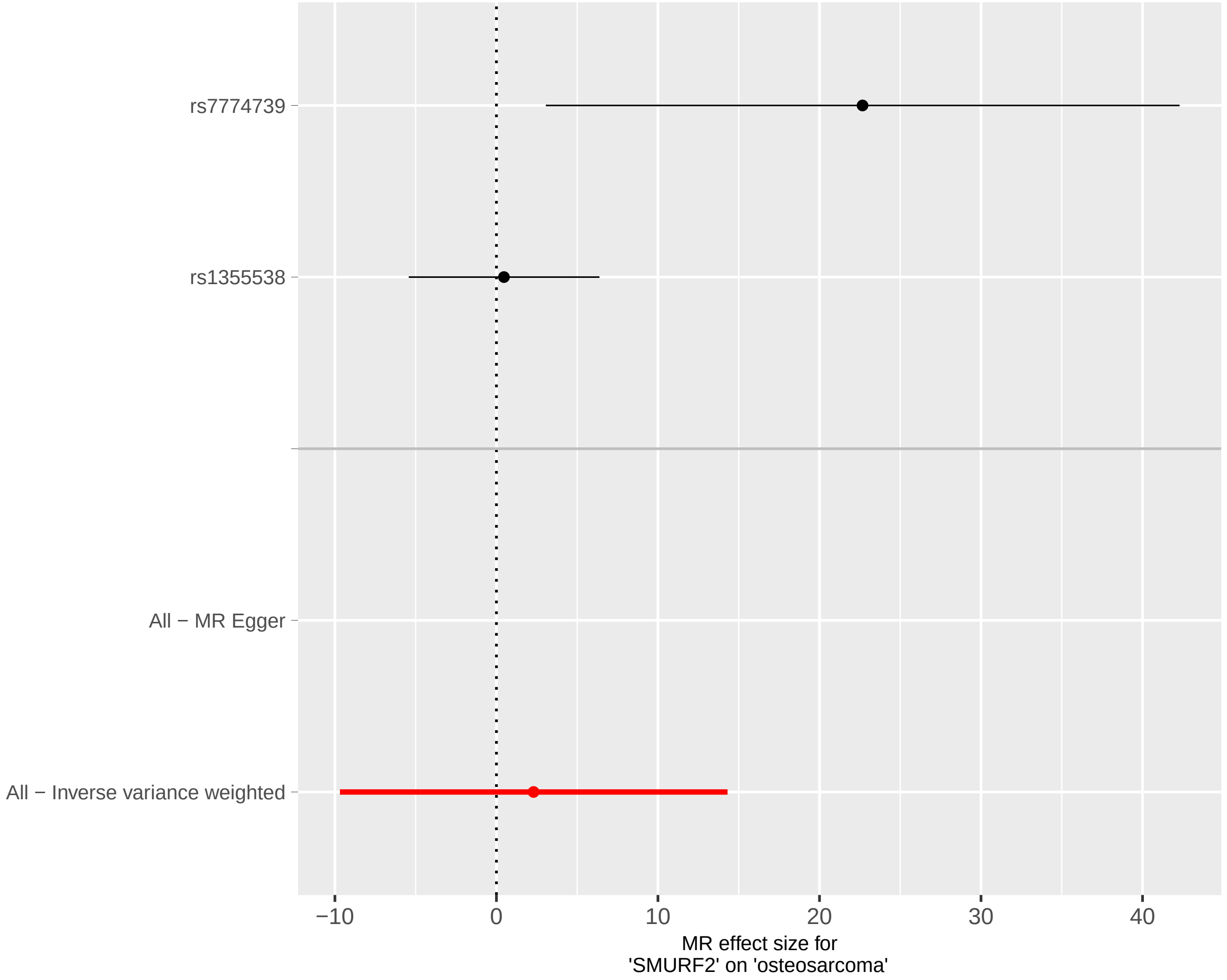

rs7774739
rs1355538
All − MR Egger
All − Inverse variance weighted
−10
0
10
20
30
40
MR effect size for
'SMURF2' on 'osteosarcoma'

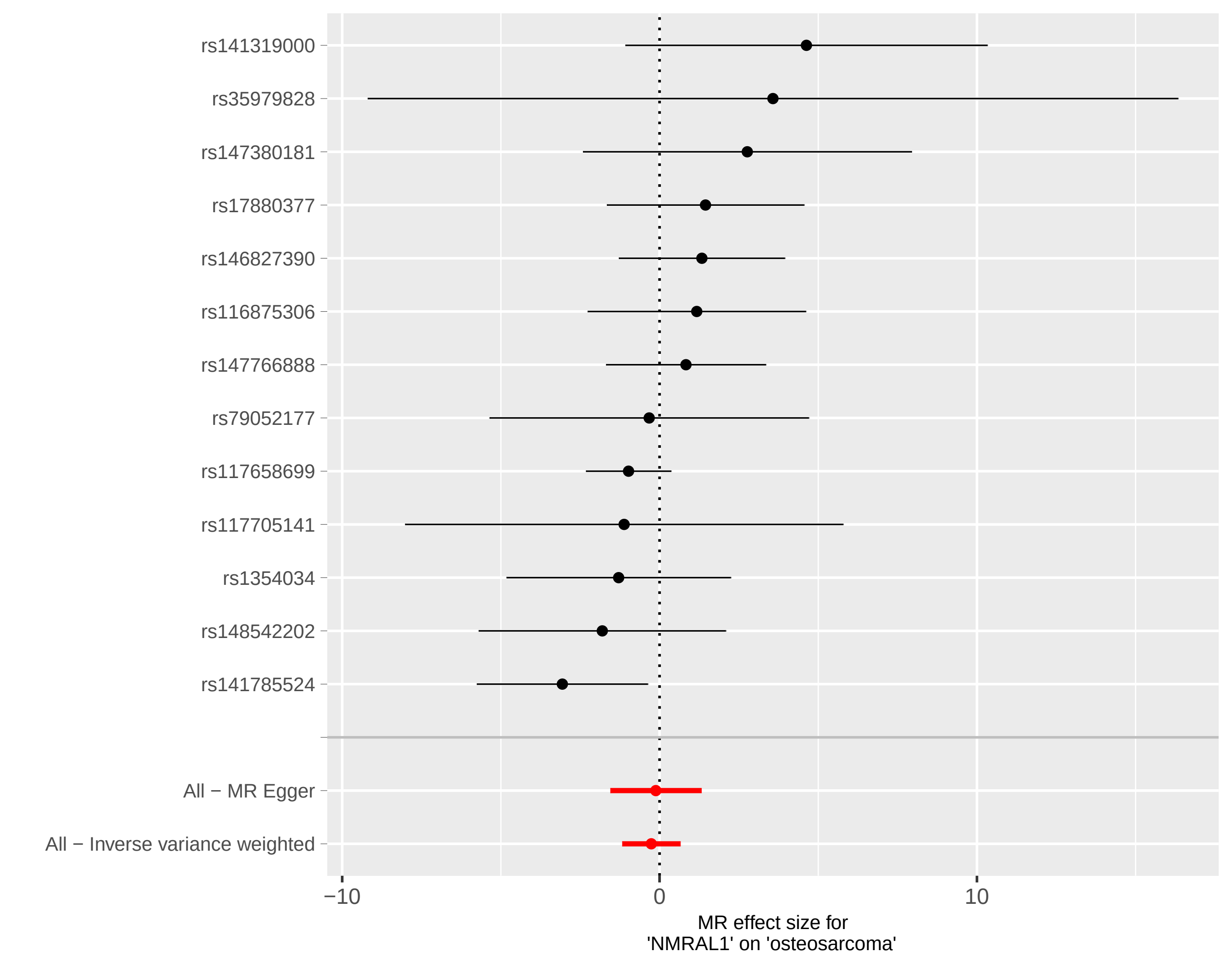

rs141319000
rs35979828
rs147380181
rs17880377
rs146827390
rs116875306
rs147766888
rs79052177
rs117658699
rs117705141
rs1354034
rs148542202
rs141785524
All − MR Egger
All − Inverse variance weighted
−10
0
10
MR effect size for
'NMRAL1' on 'osteosarcoma'

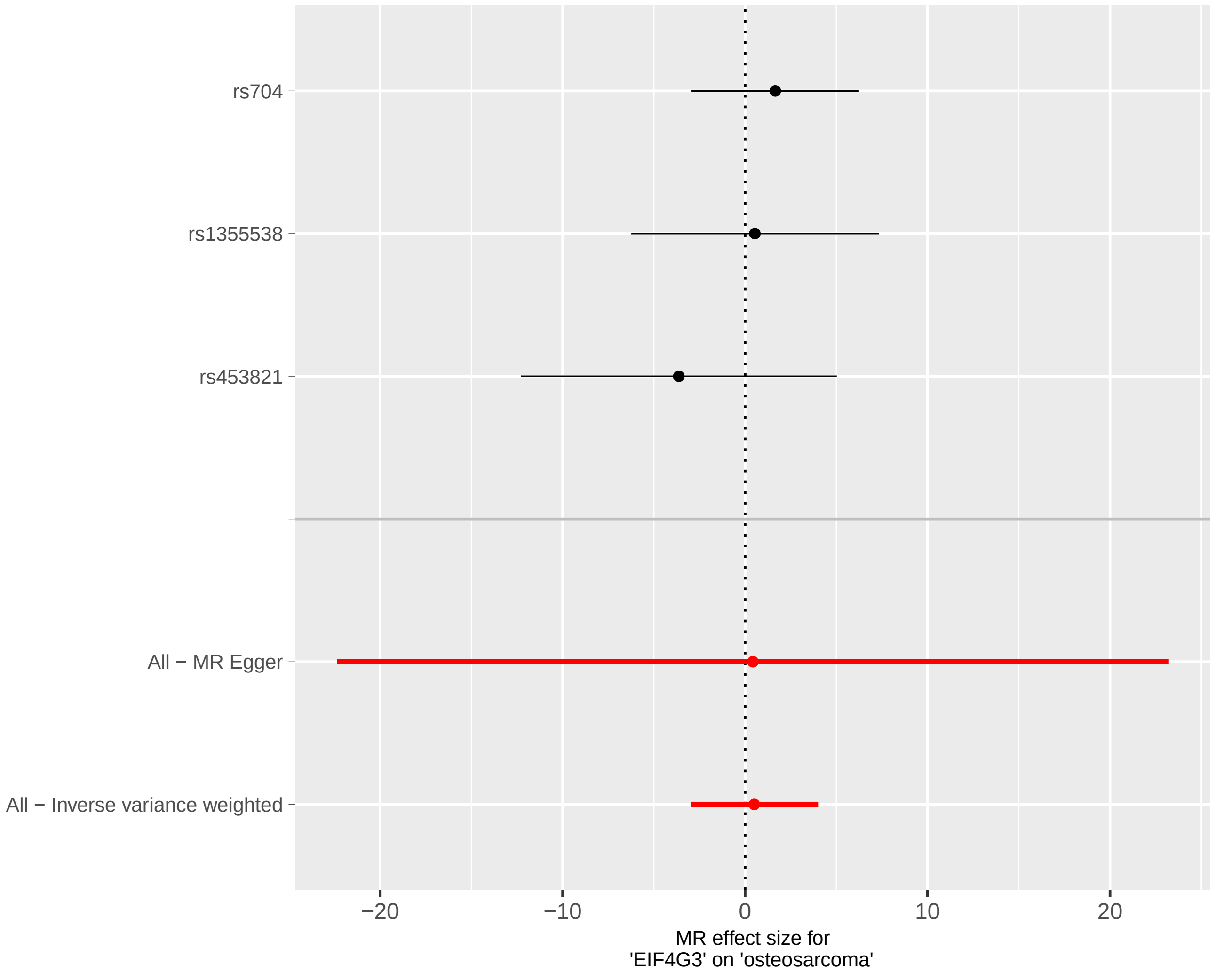

rs704
rs1355538
rs453821
All − MR Egger
All − Inverse variance weighted
−20
−10
0
10
20
MR effect size for
'EIF4G3' on 'osteosarcoma'

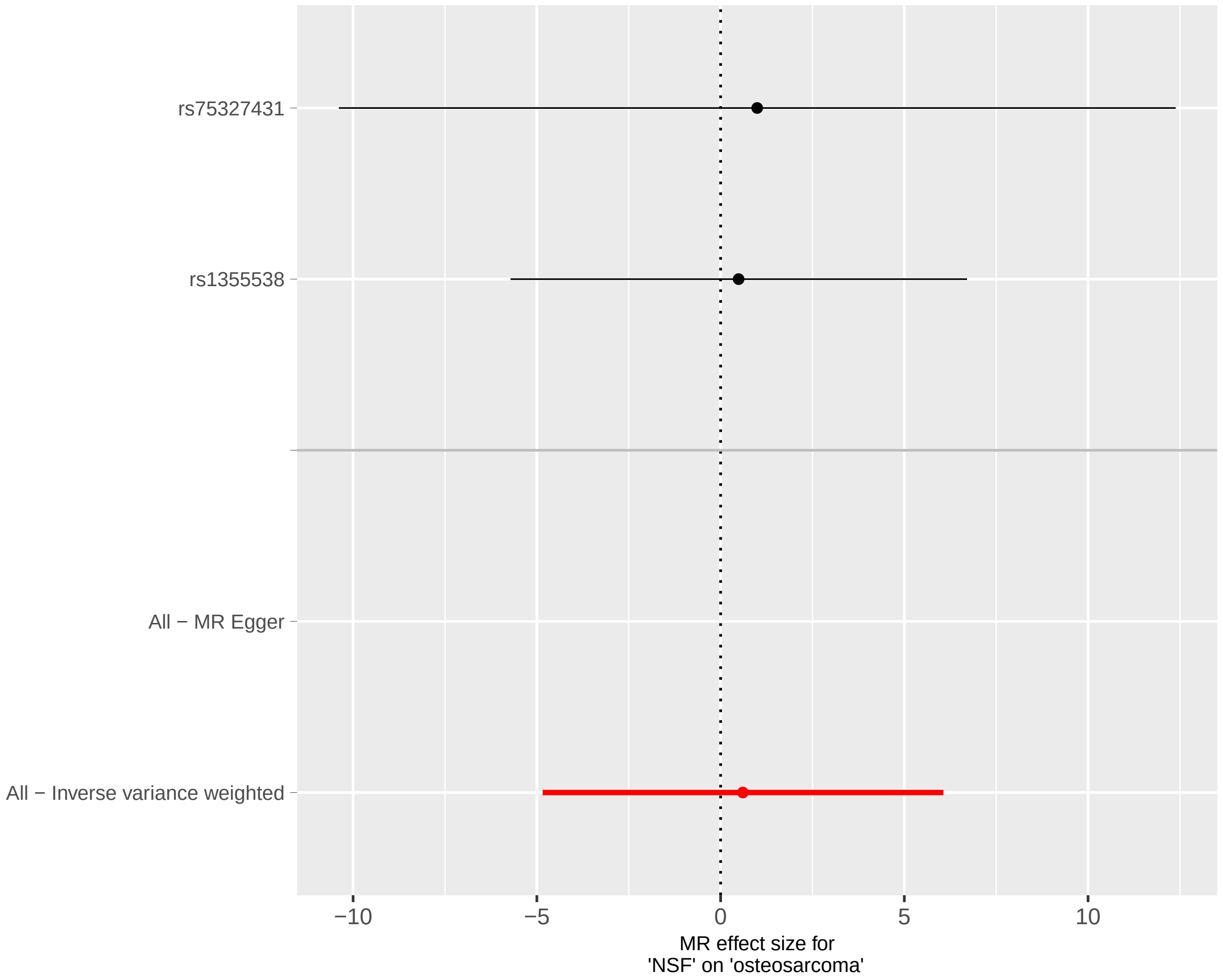
rs75327431
rs1355538
All − MR Egger
All − Inverse variance weighted
−10
−5
0
5
10
MR effect size for
'NSF' on 'osteosarcoma'

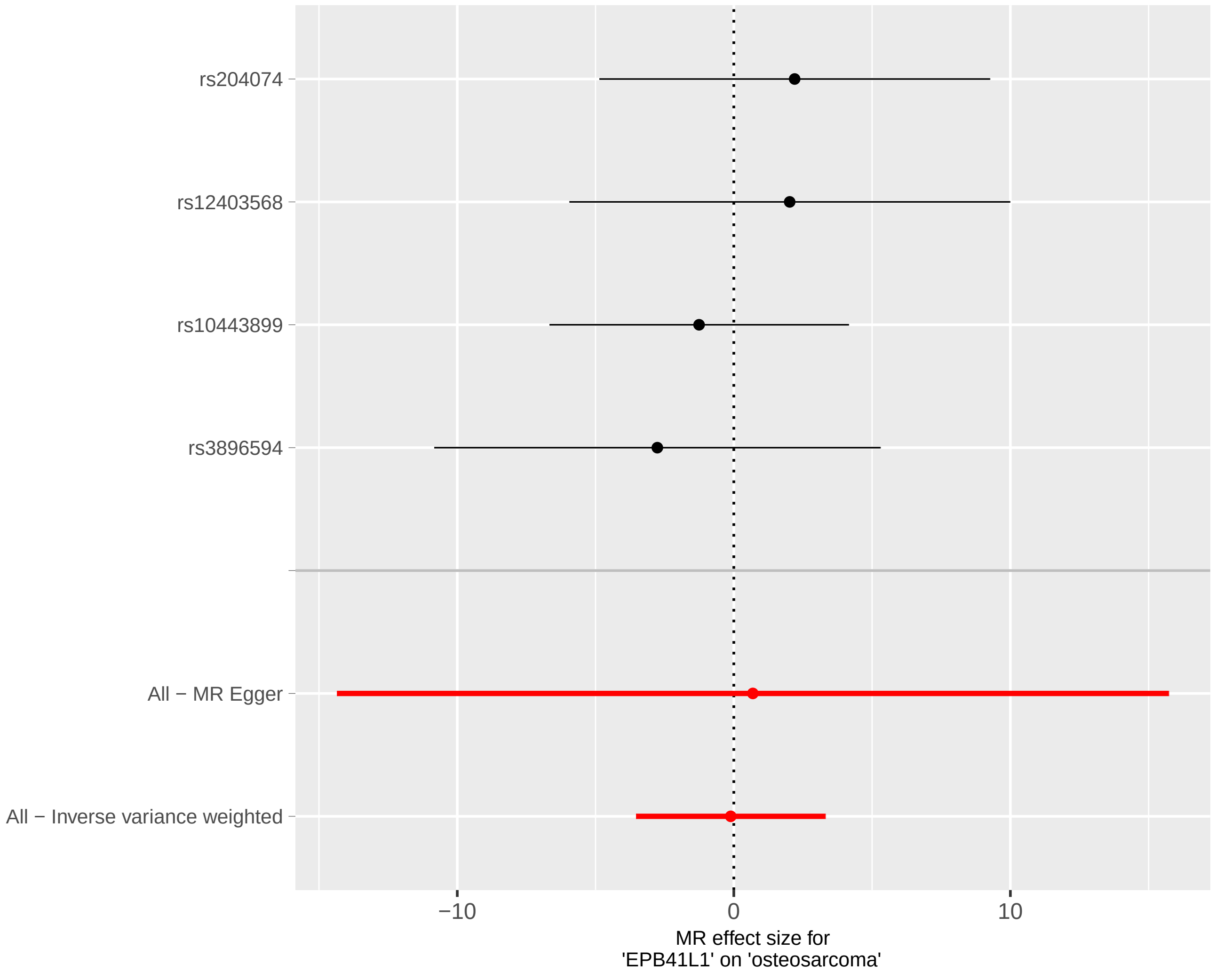
rs204074
rs12403568
rs10443899
rs3896594
All − MR Egger
All − Inverse variance weighted
−10
0
10
MR effect size for
'EPB41L1' on 'osteosarcoma'

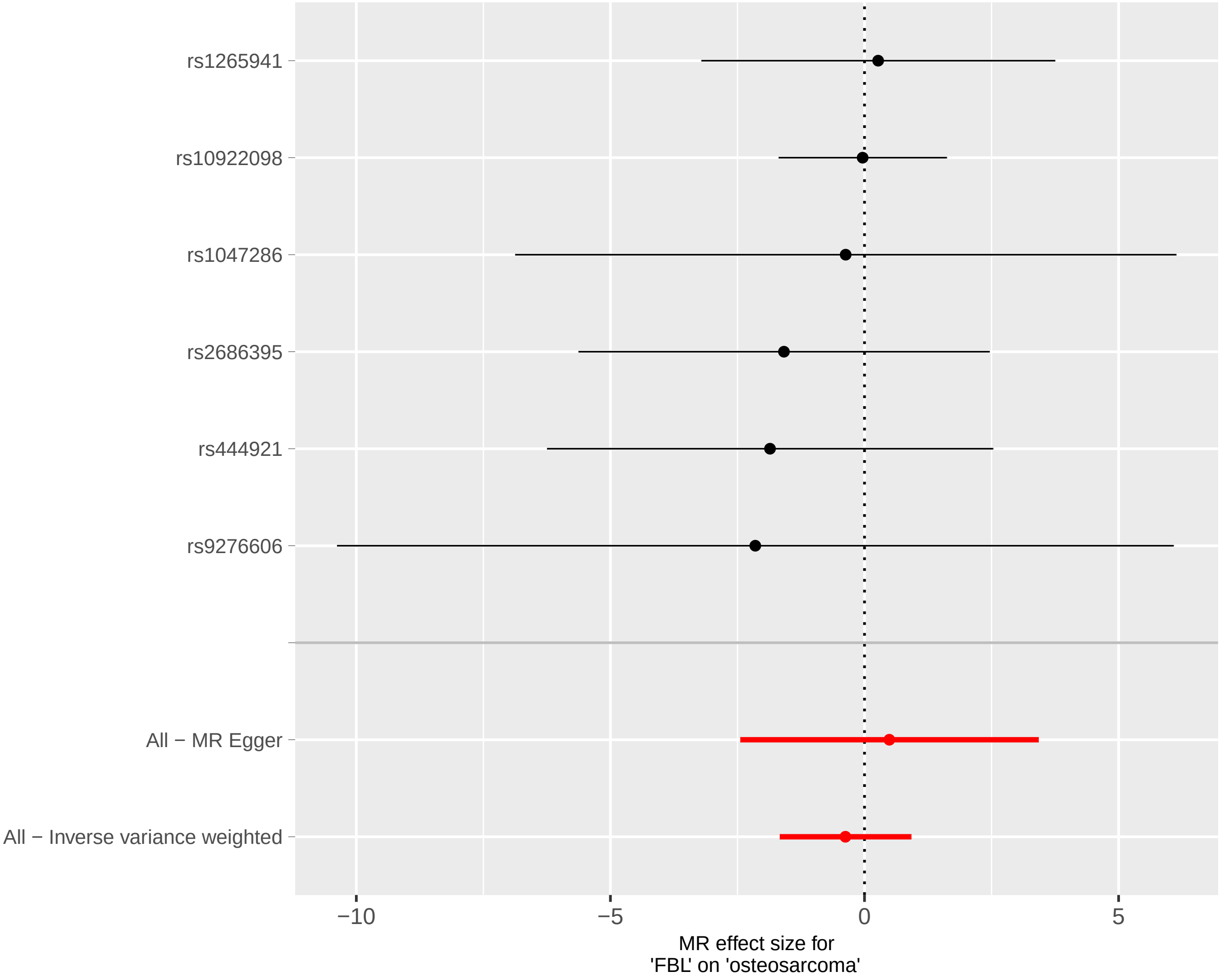

rs1265941
rs10922098
rs1047286
rs2686395
rs444921
rs9276606
All − MR Egger
All − Inverse variance weighted
−10
−5
0
5
MR effect size for
'FBL' on 'osteosarcoma'

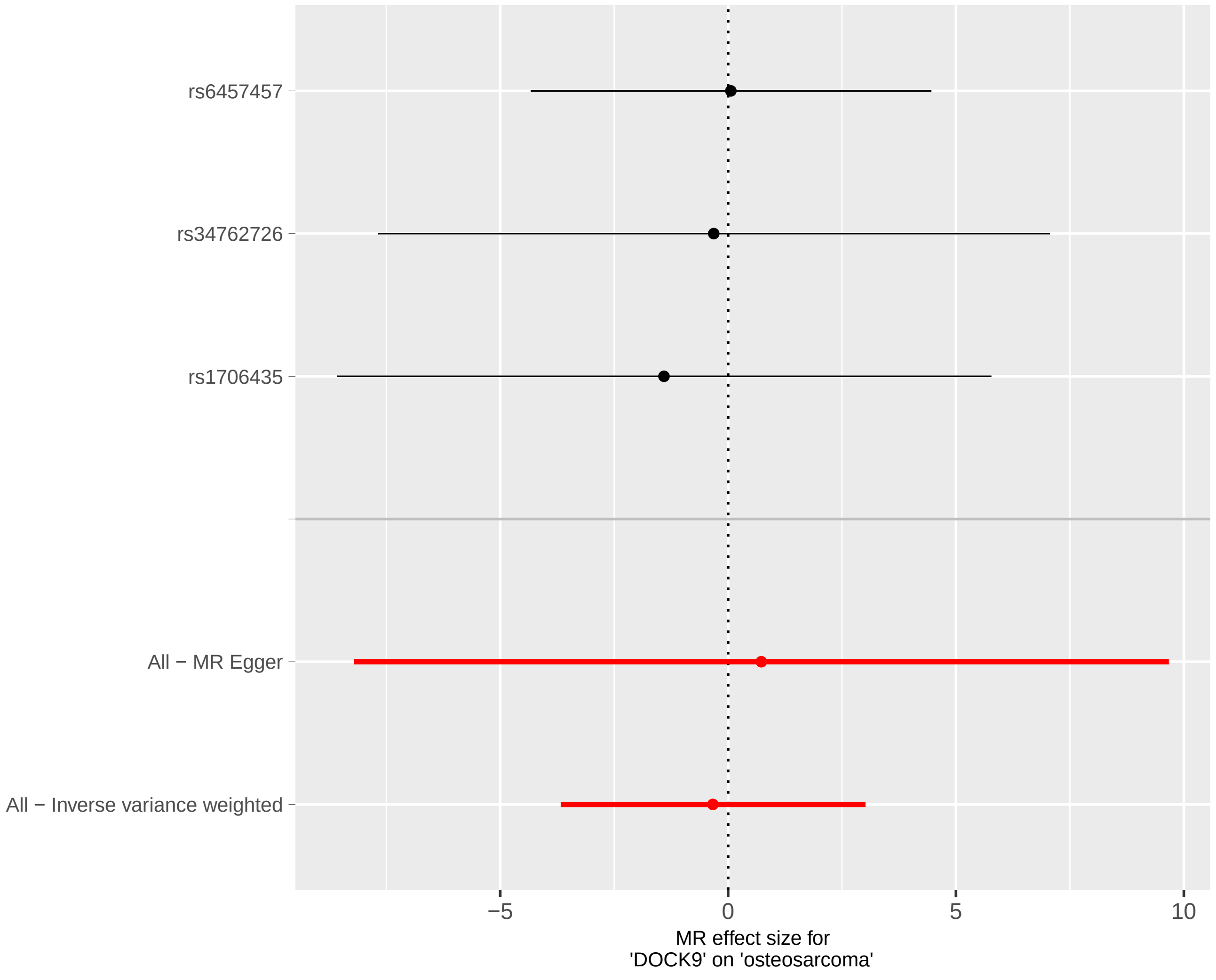
rs6457457
rs34762726
rs1706435
All − MR Egger
All − Inverse variance weighted
−5
0
5
10
MR effect size for
'DOCK9' on 'osteosarcoma'

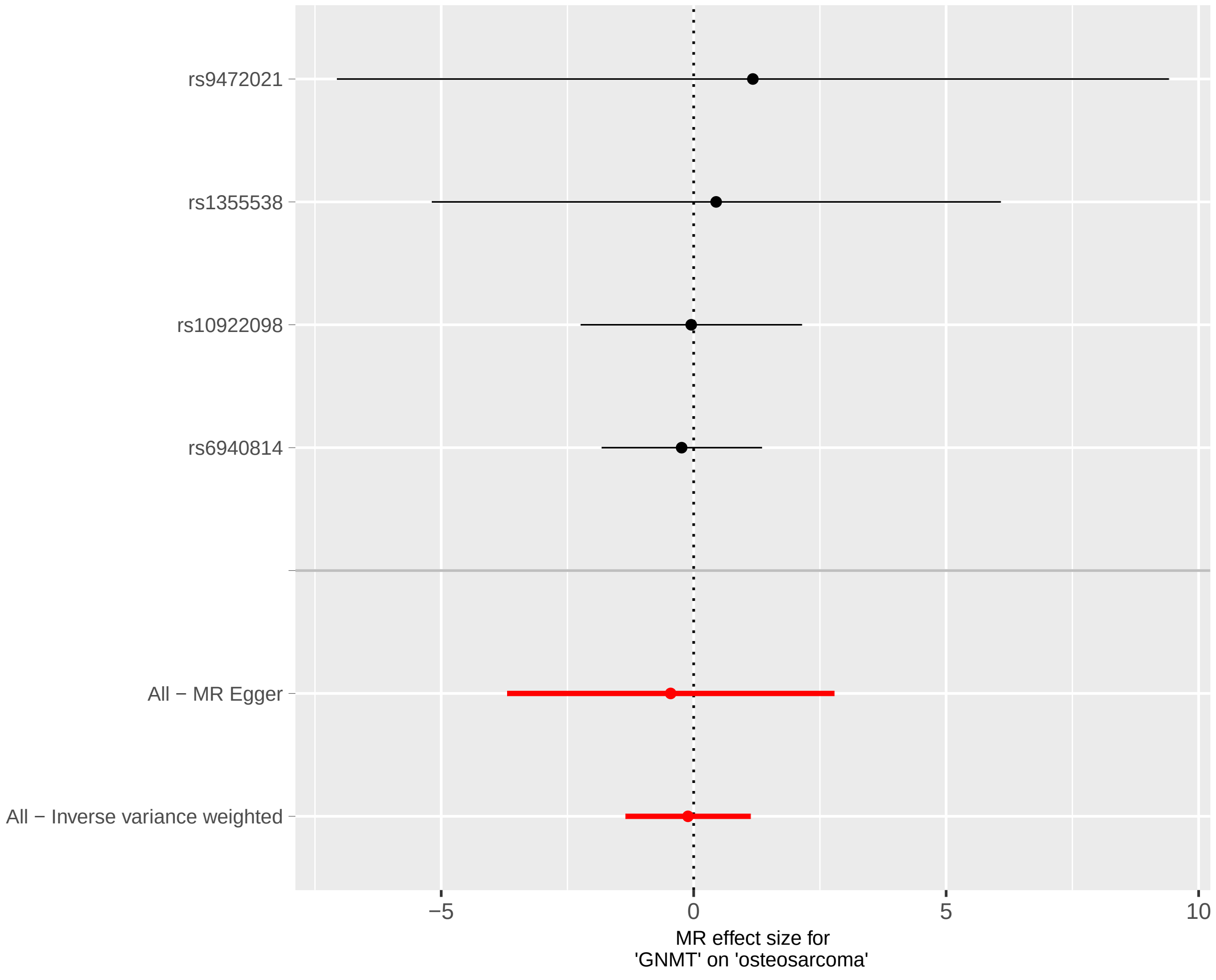
rs9472021
rs1355538
rs10922098
rs6940814
All − MR Egger
All − Inverse variance weighted
−5
0
5
10
MR effect size for
'GNMT' on 'osteosarcoma'

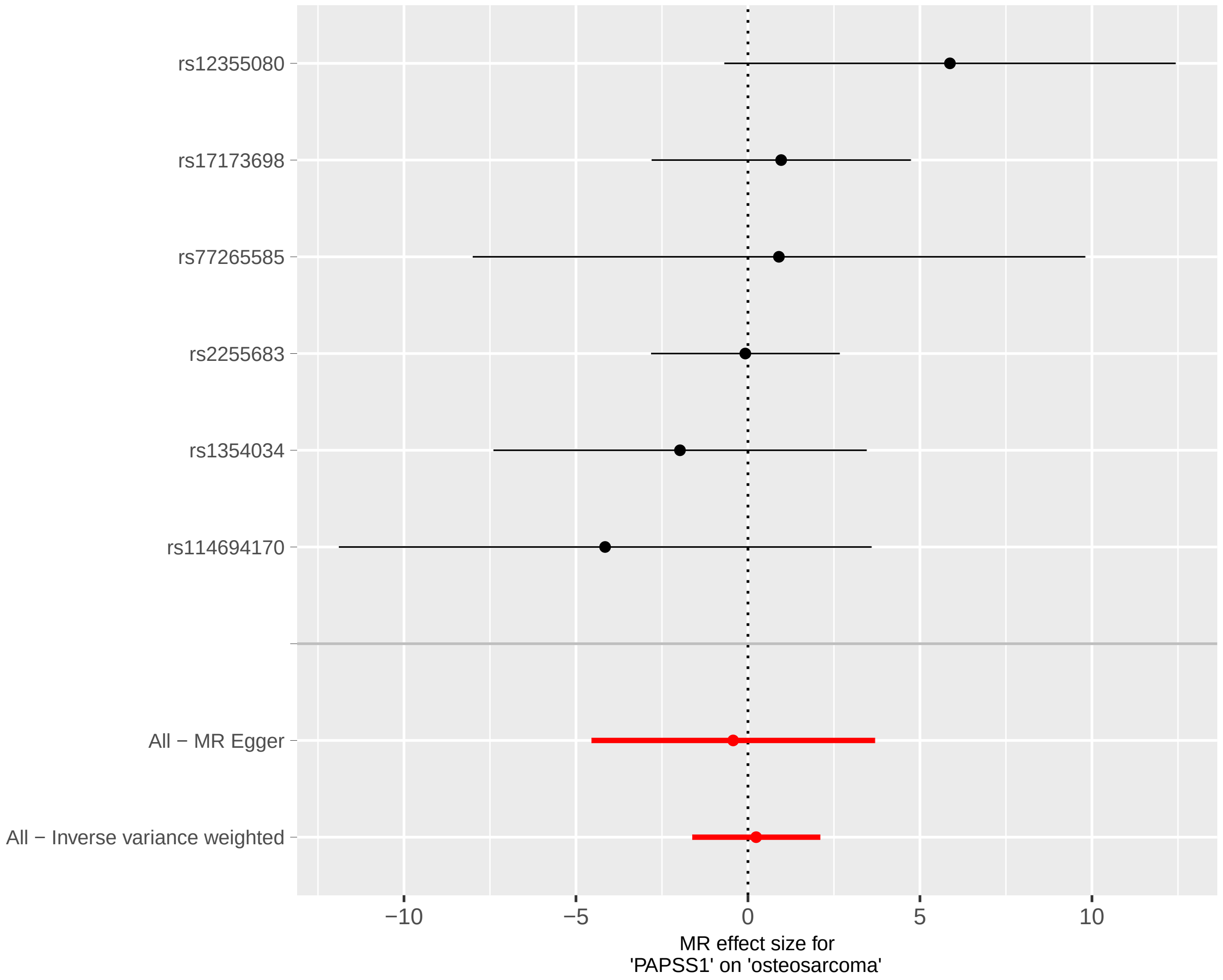

rs12355080
rs17173698
rs77265585
rs2255683
rs1354034
rs114694170
All − MR Egger
All − Inverse variance weighted
−10
−5
0
5
10
MR effect size for
'PAPSS1' on 'osteosarcoma'

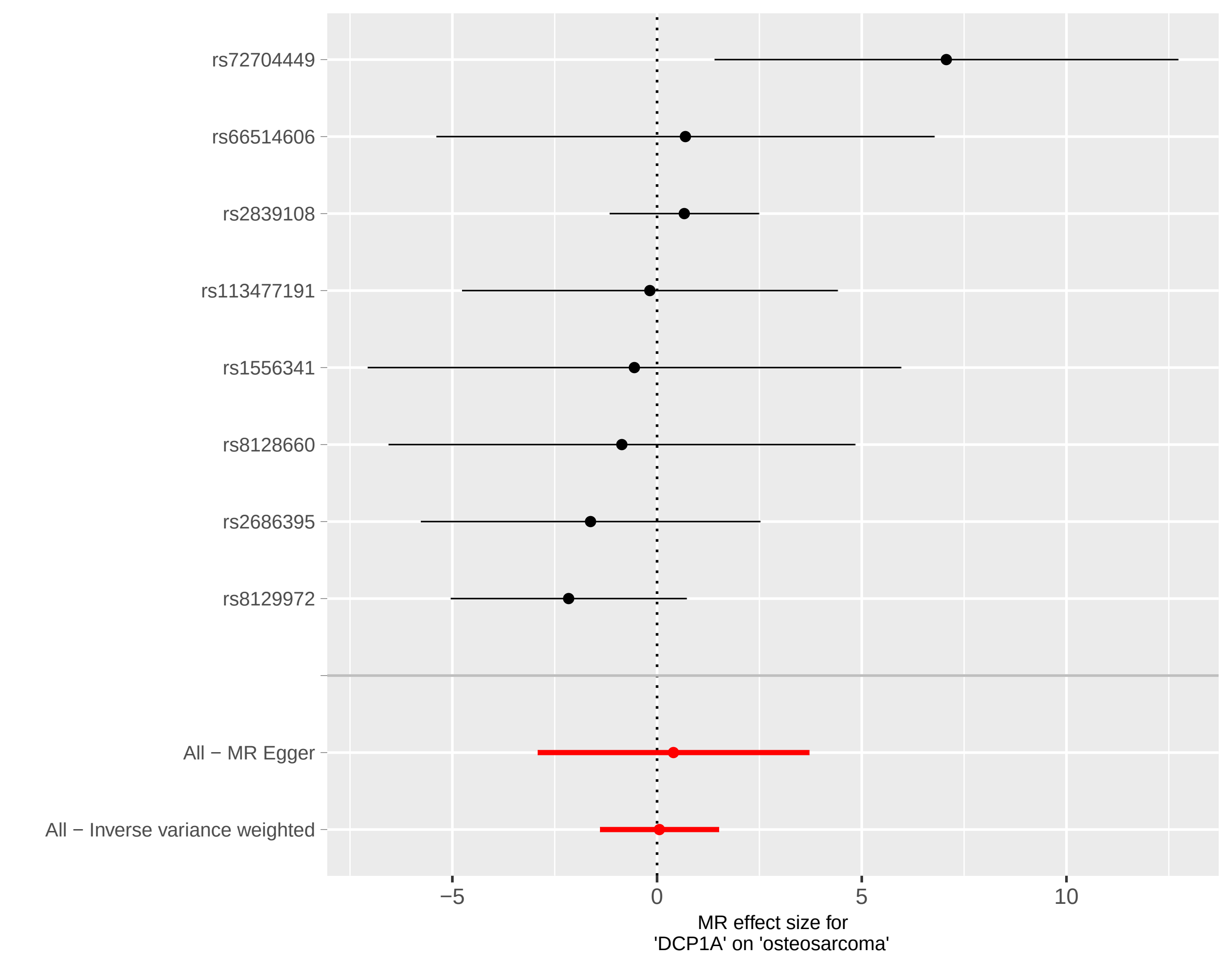

rs72704449
rs66514606
rs2839108
rs113477191
rs1556341
rs8128660
rs2686395
rs8129972
All − MR Egger
All − Inverse variance weighted
−5
0
5
10
MR effect size for
'DCP1A' on 'osteosarcoma'

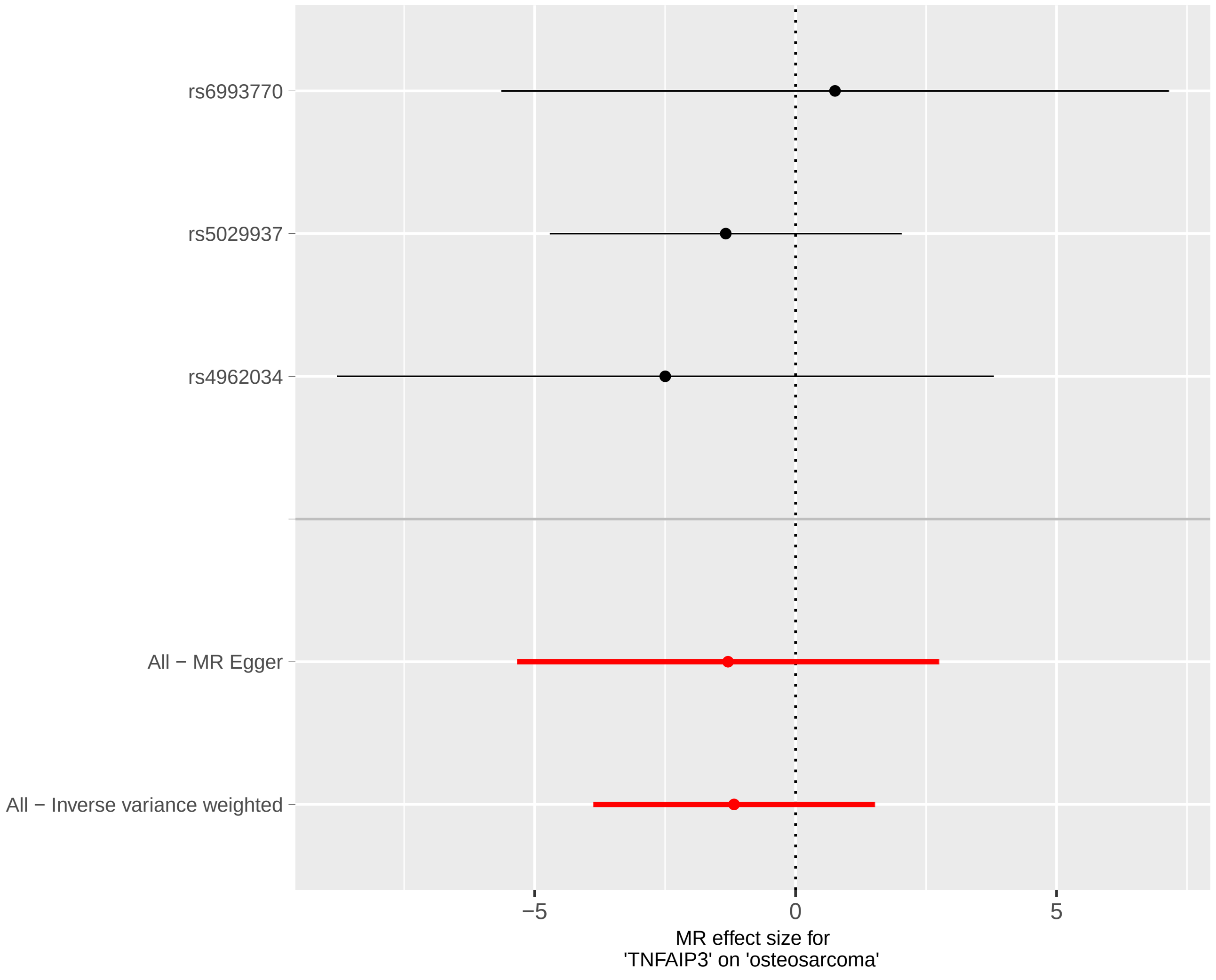

rs6993770
rs5029937
rs4962034
All − MR Egger
All − Inverse variance weighted
−5
0
5
MR effect size for
'TNFAIP3' on 'osteosarcoma'

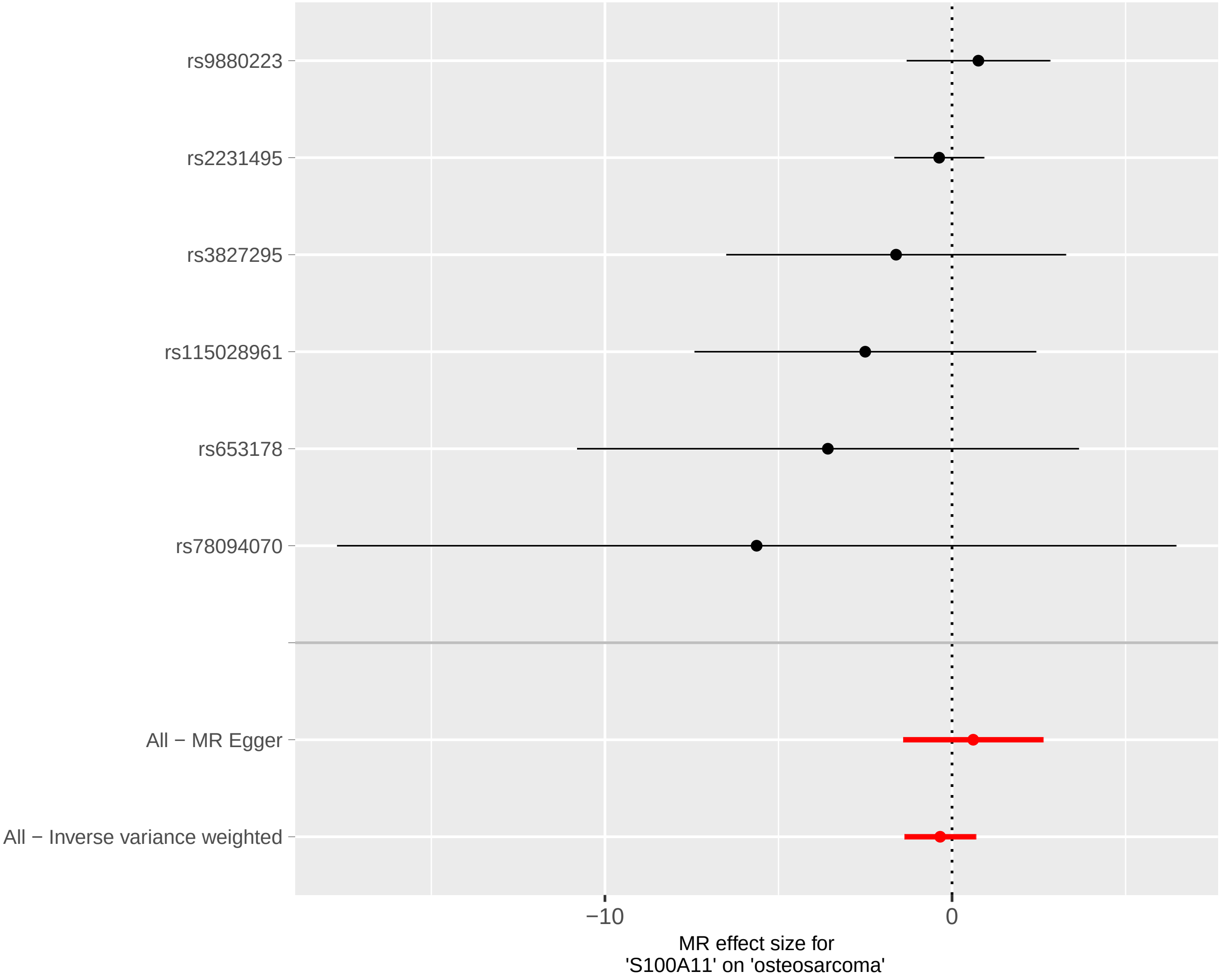

rs9880223
rs2231495
rs3827295
rs115028961
rs653178
rs78094070
All − MR Egger
All − Inverse variance weighted
−10
0
MR effect size for
'S100A11' on 'osteosarcoma'

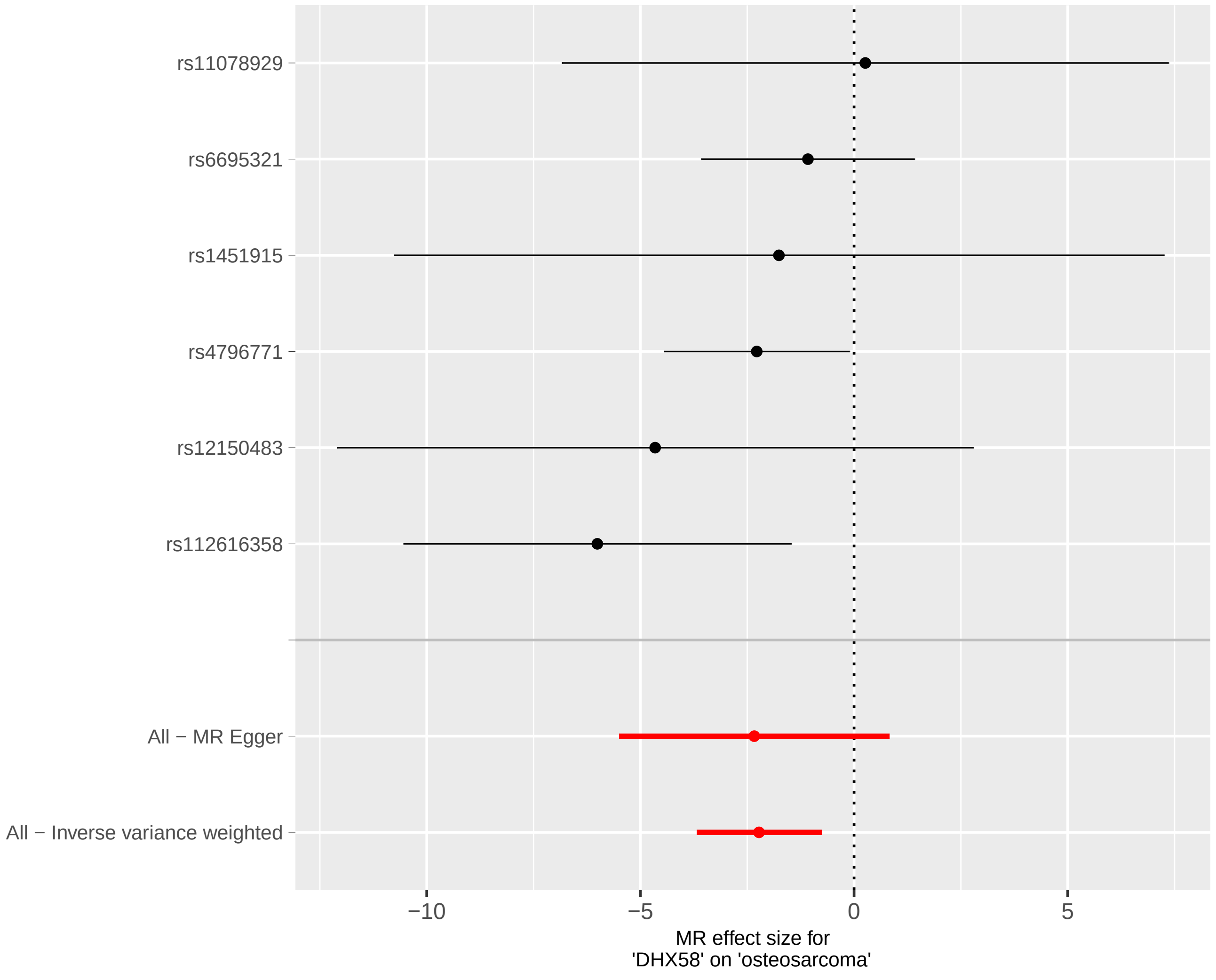

rs11078929
rs6695321
rs1451915
rs4796771
rs12150483
rs112616358
All − MR Egger
All − Inverse variance weighted
−10
−5
0
5
MR effect size for
'DHX58' on 'osteosarcoma'

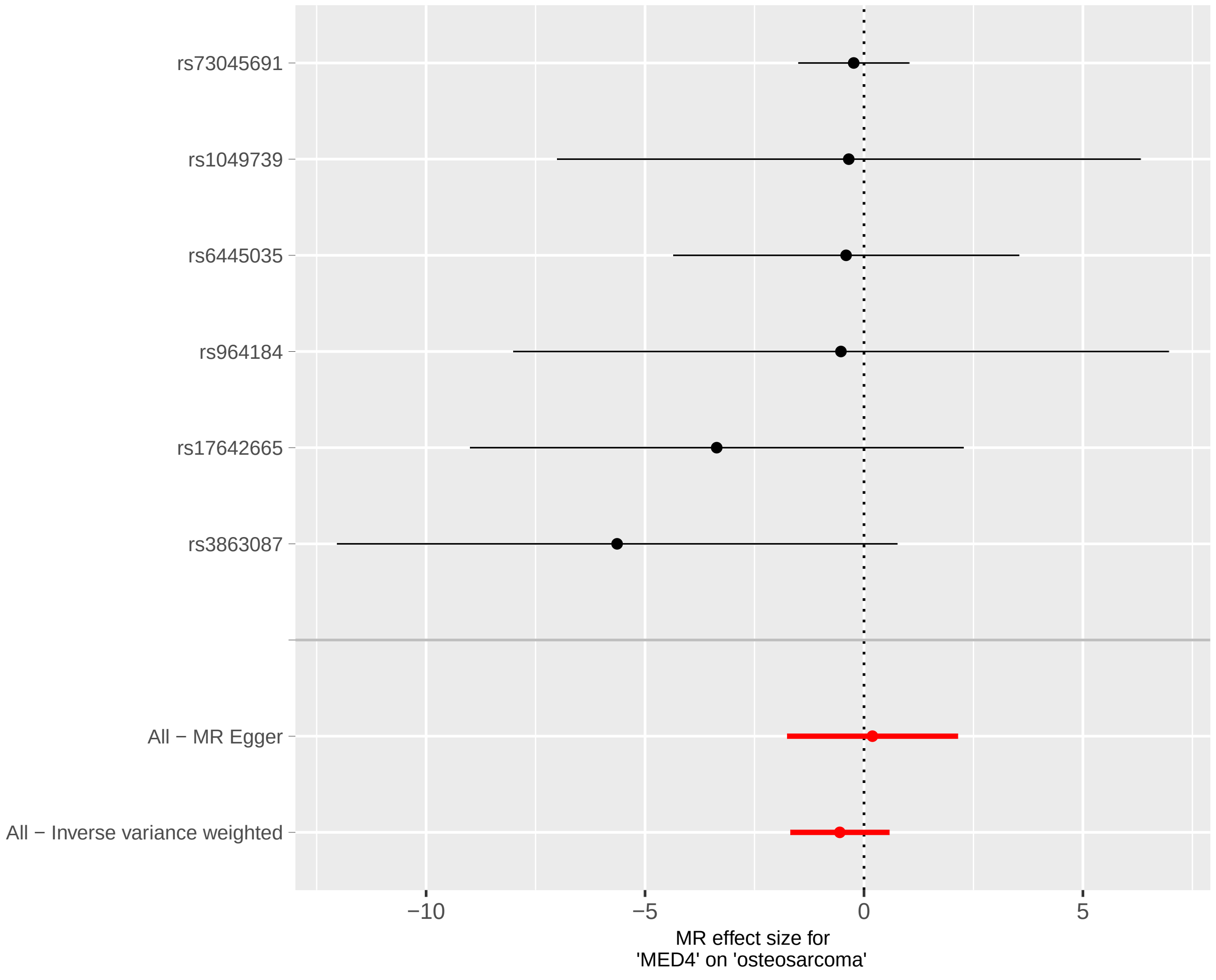
rs73045691
rs1049739
rs6445035
rs964184
rs17642665
rs3863087
All − MR Egger
All − Inverse variance weighted
−10
−5
0
5
MR effect size for
'MED4' on 'osteosarcoma'

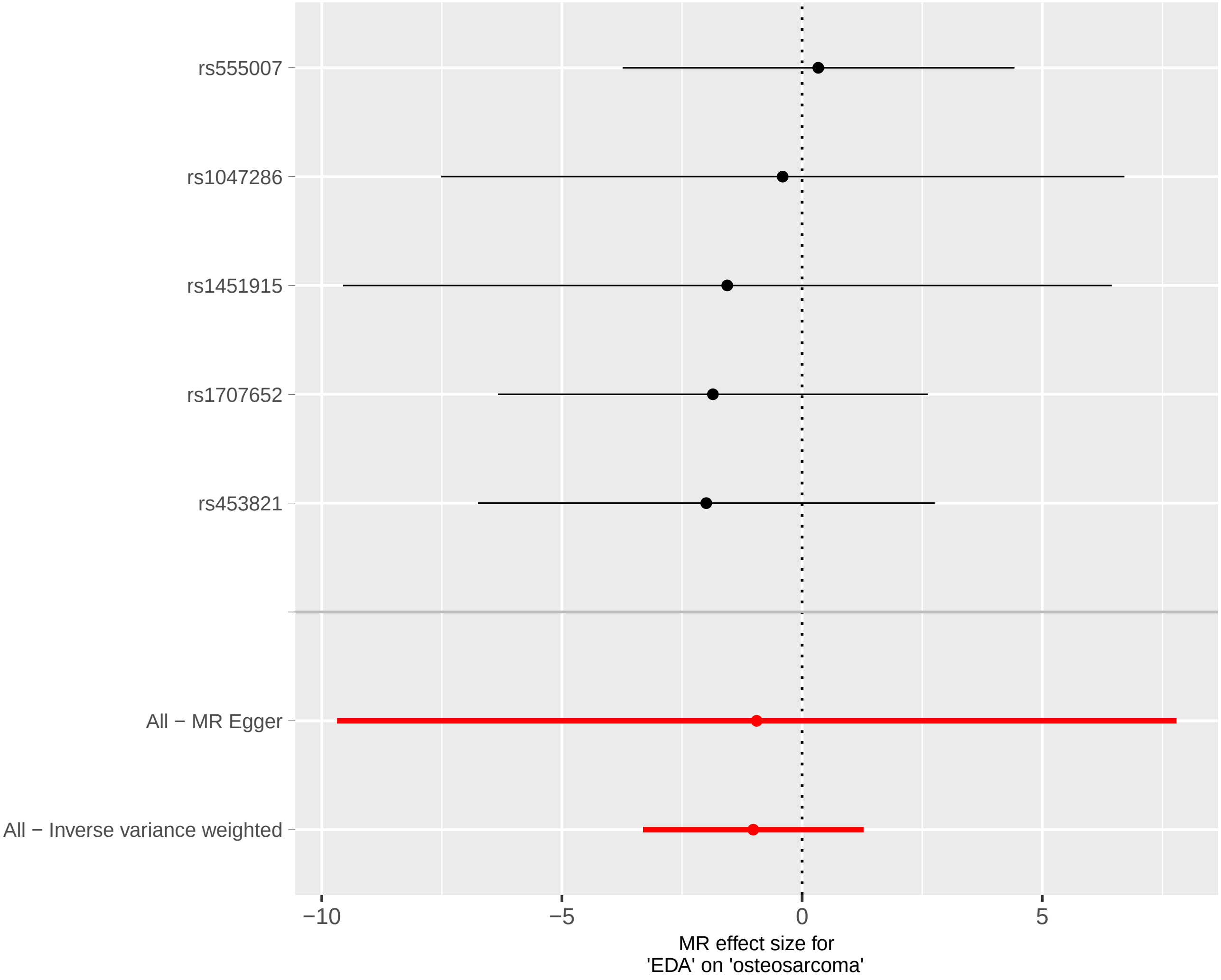

rs555007
rs1047286
rs1451915
rs1707652
rs453821
All − MR Egger
All − Inverse variance weighted
−10
−5
0
5
MR effect size for
'EDA' on 'osteosarcoma'

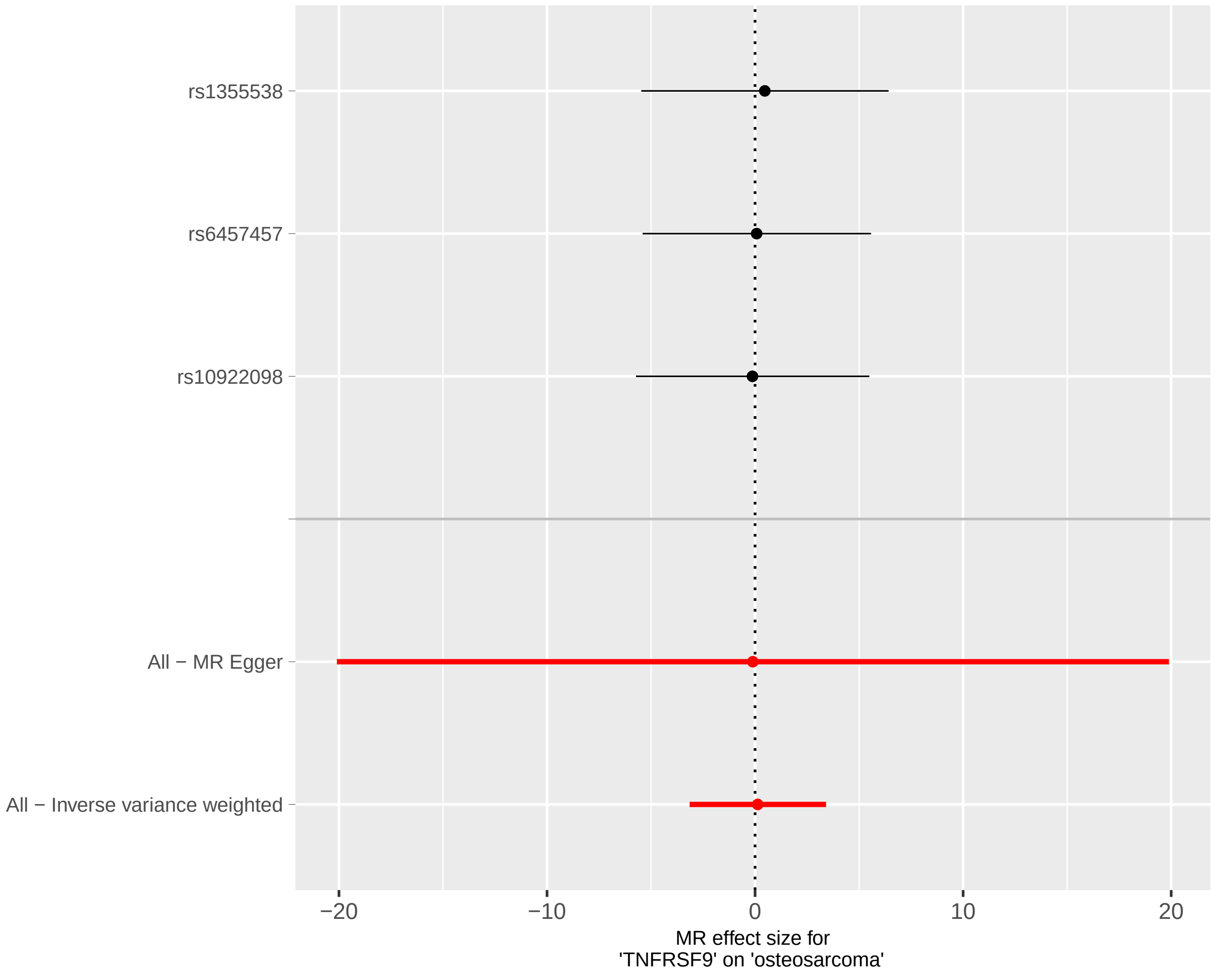

rs1355538
rs6457457
rs10922098
All − MR Egger
All − Inverse variance weighted
−20
−10
0
10
20
MR effect size for
'TNFRSF9' on 'osteosarcoma'

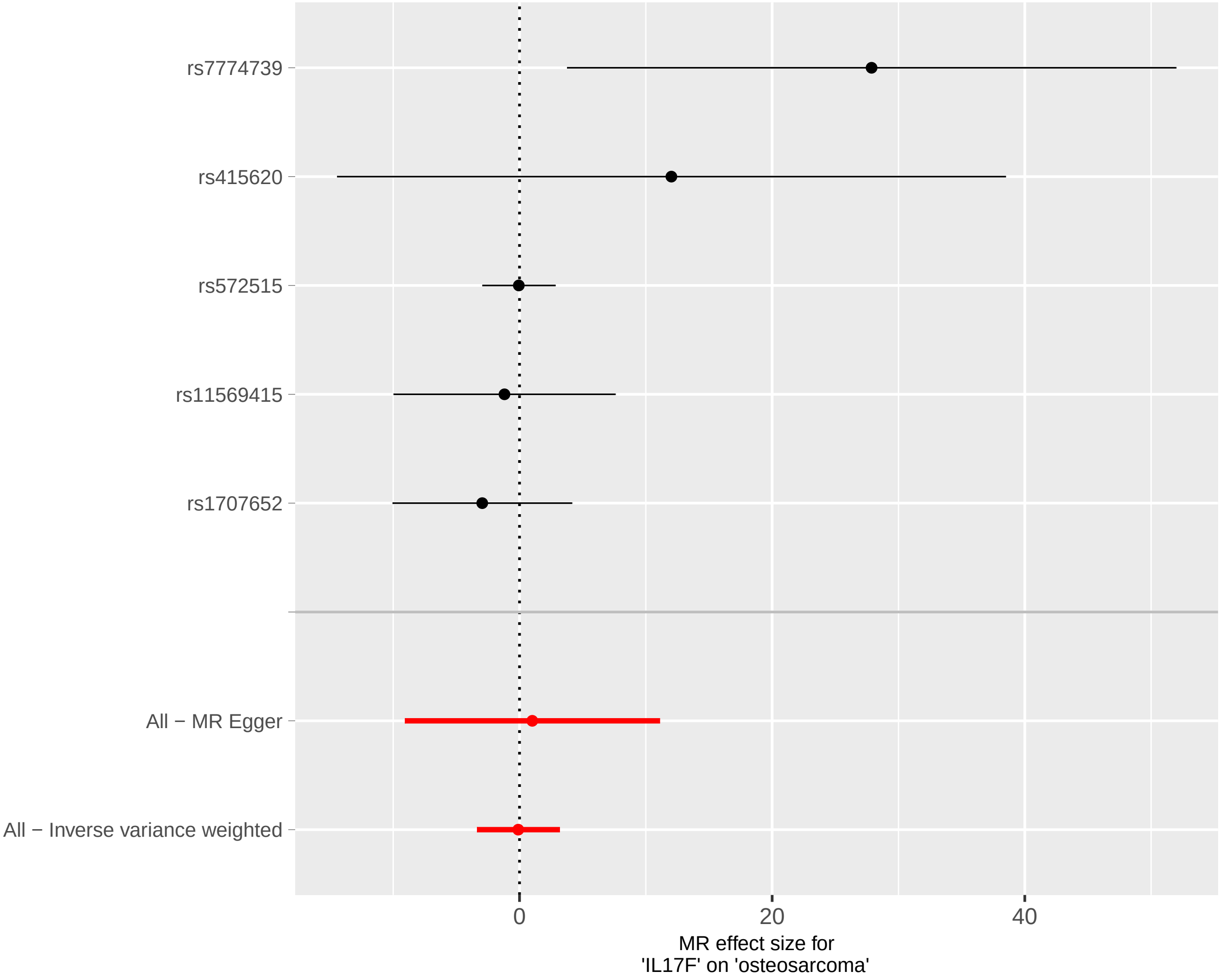

rs7774739
rs415620
rs572515
rs11569415
rs1707652
All − MR Egger
All − Inverse variance weighted
0
20
40
MR effect size for
'IL17F' on 'osteosarcoma'

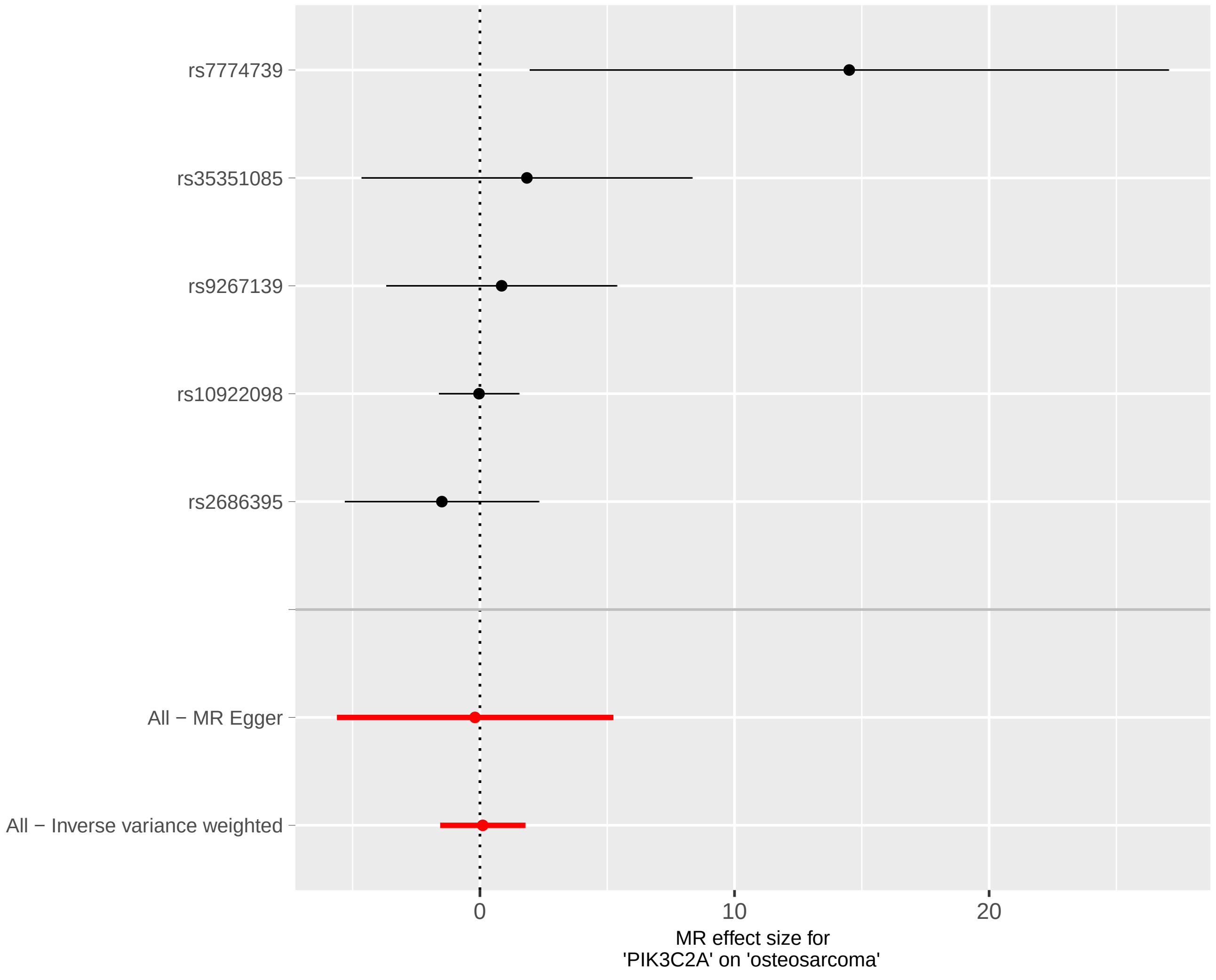
rs7774739
rs35351085
rs9267139
rs10922098
rs2686395
All − MR Egger
All − Inverse variance weighted
0
10
20
MR effect size for
'PIK3C2A' on 'osteosarcoma'

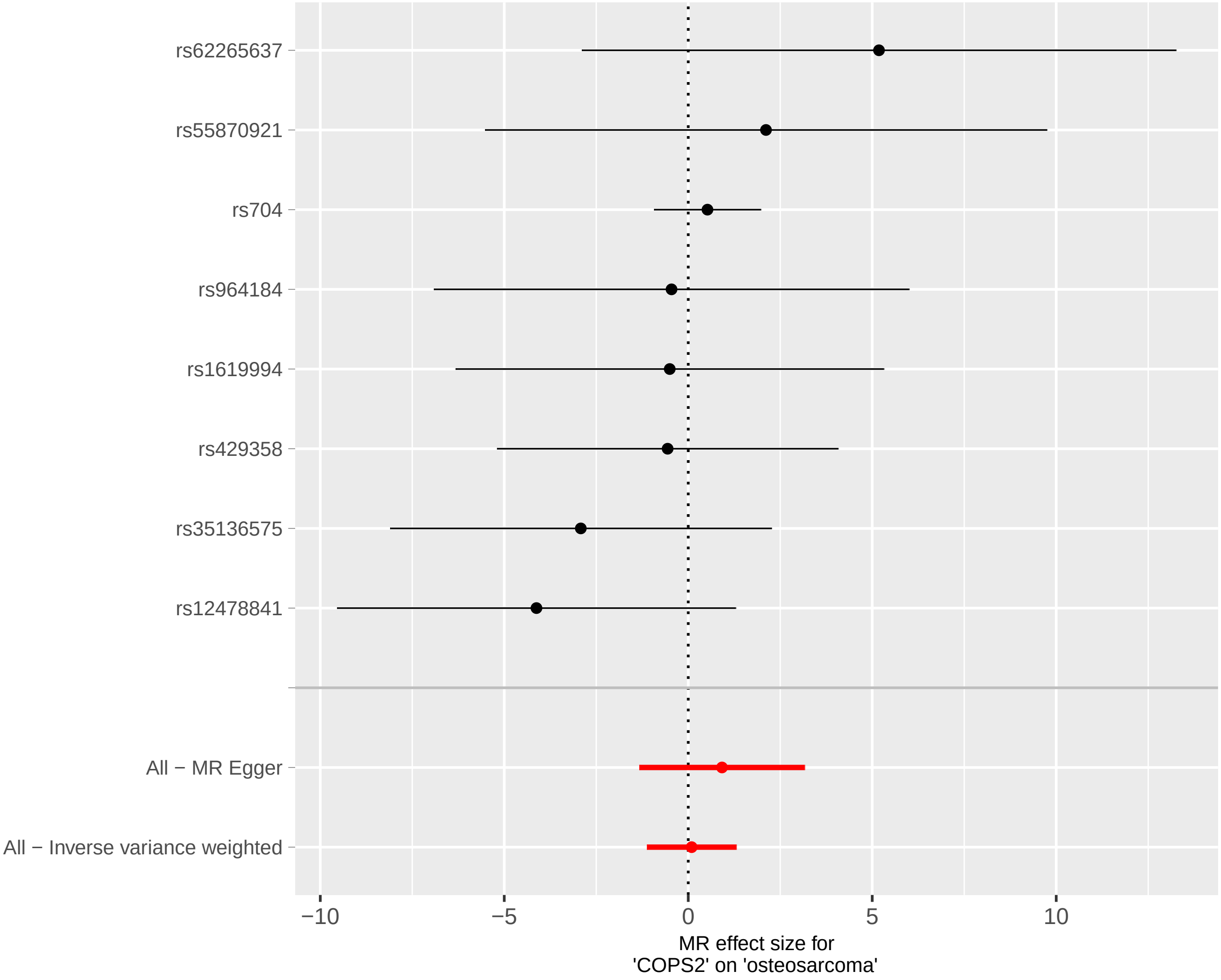

rs62265637
rs55870921
rs704
rs964184
rs1619994
rs429358
rs35136575
rs12478841
All − MR Egger
All − Inverse variance weighted
−10
−5
0
5
10
MR effect size for
'COPS2' on 'osteosarcoma'

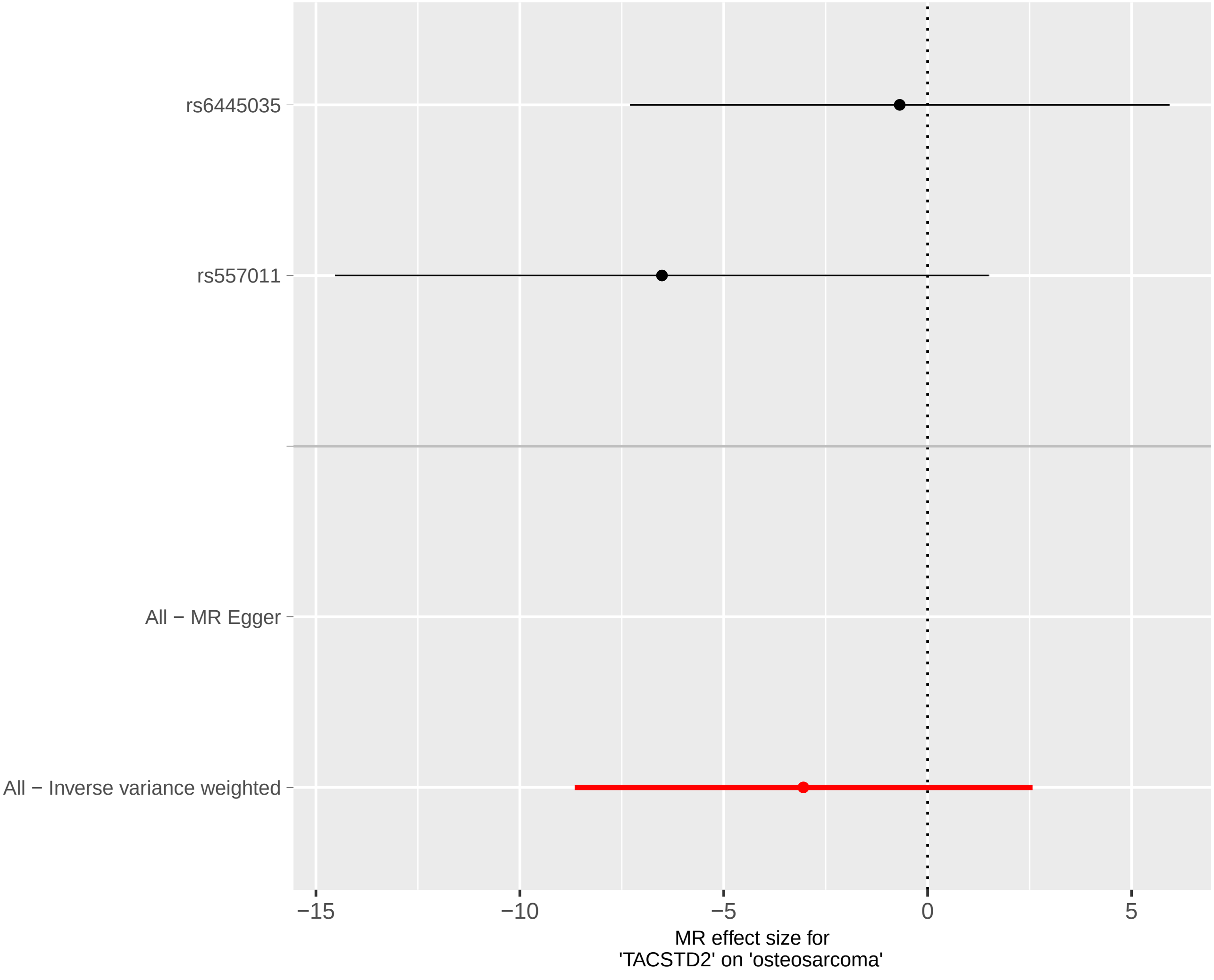

rs6445035
rs557011
All − MR Egger
All − Inverse variance weighted
−15
−10
−5
0
5
MR effect size for
'TACSTD2' on 'osteosarcoma'

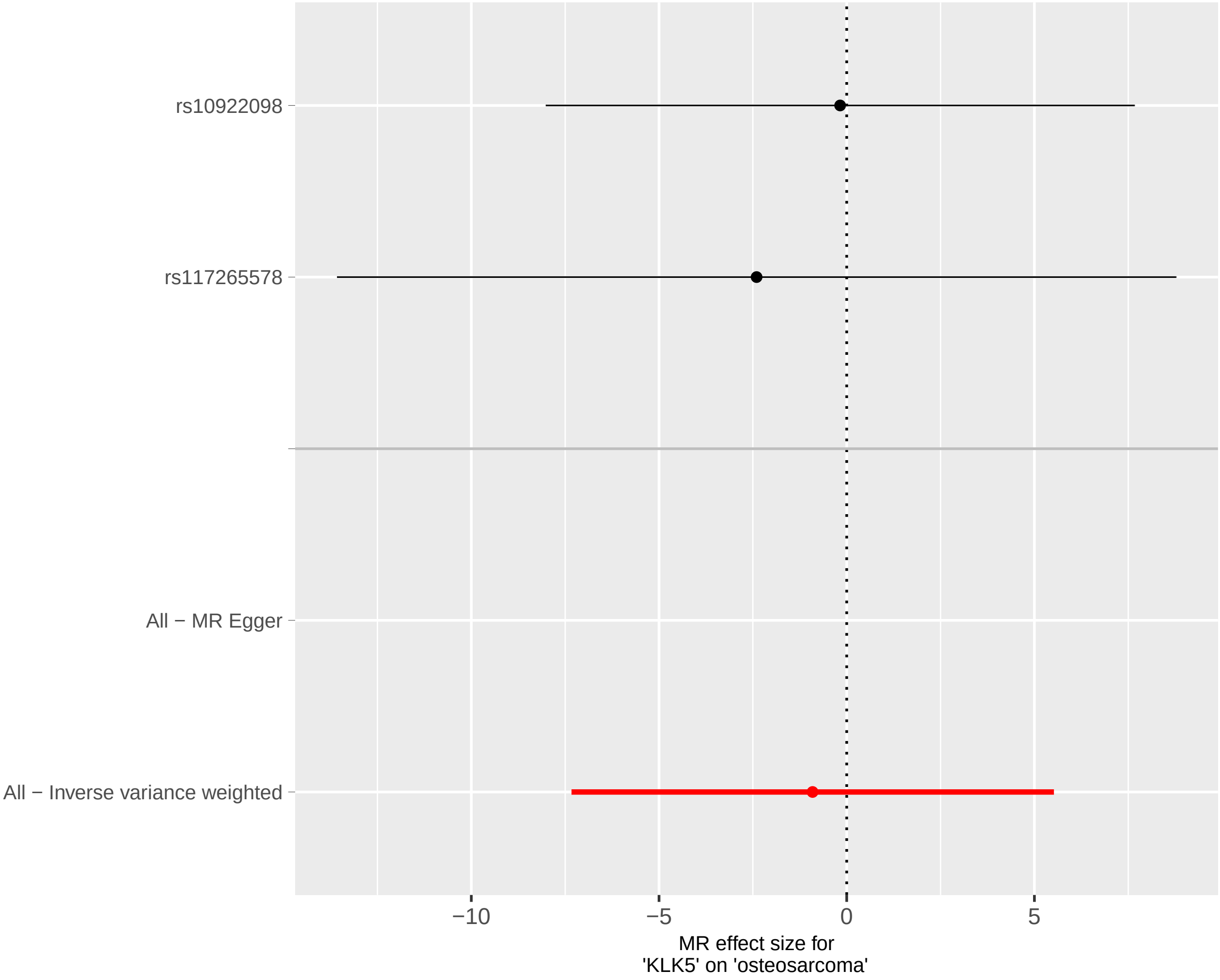

rs10922098
rs117265578
All − MR Egger
All − Inverse variance weighted
−10
−5
0
5
MR effect size for
'KLK5' on 'osteosarcoma'

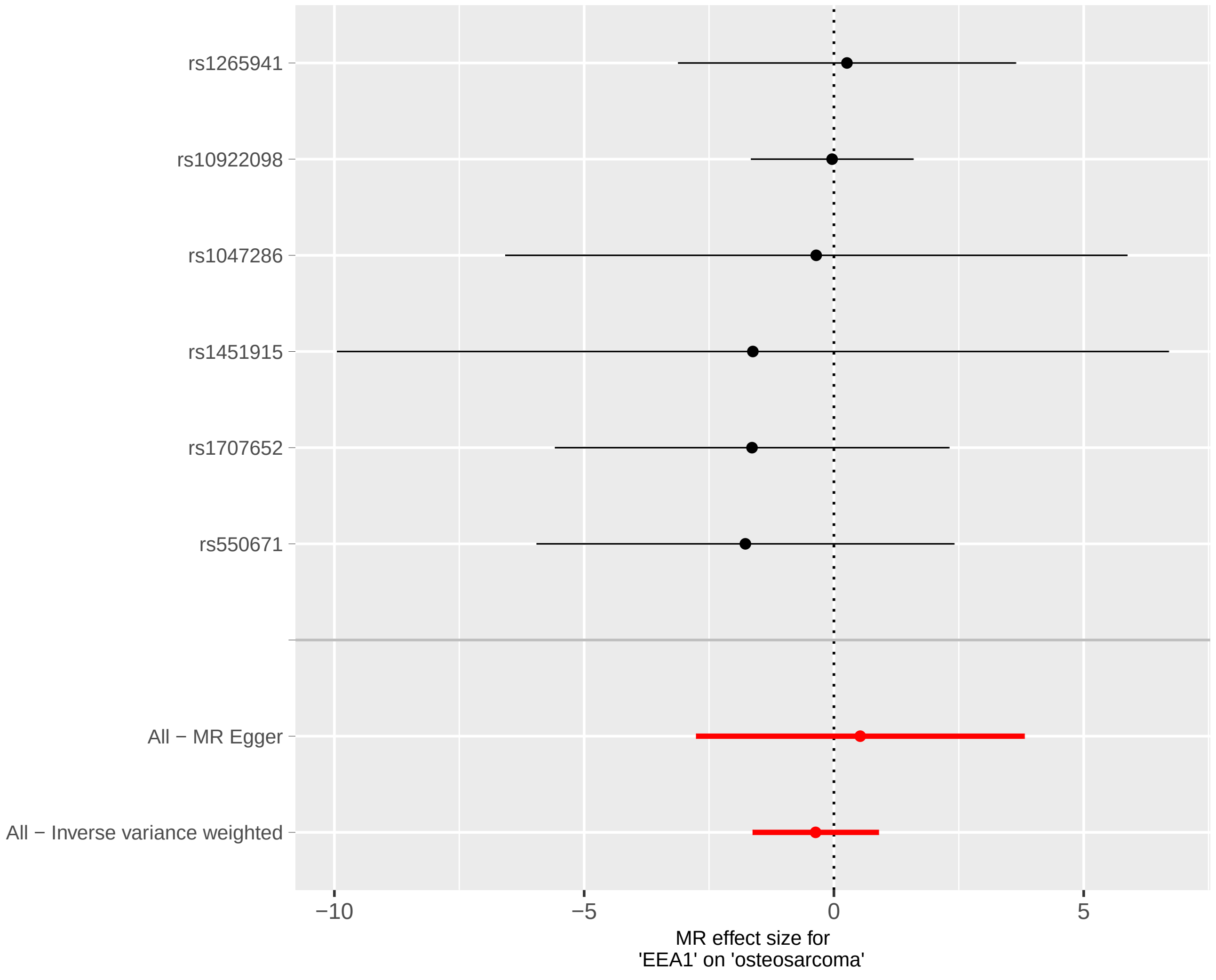

rs1265941
rs10922098
rs1047286
rs1451915
rs1707652
rs550671
All − MR Egger
All − Inverse variance weighted
−10
−5
0
5
MR effect size for
'EEA1' on 'osteosarcoma'

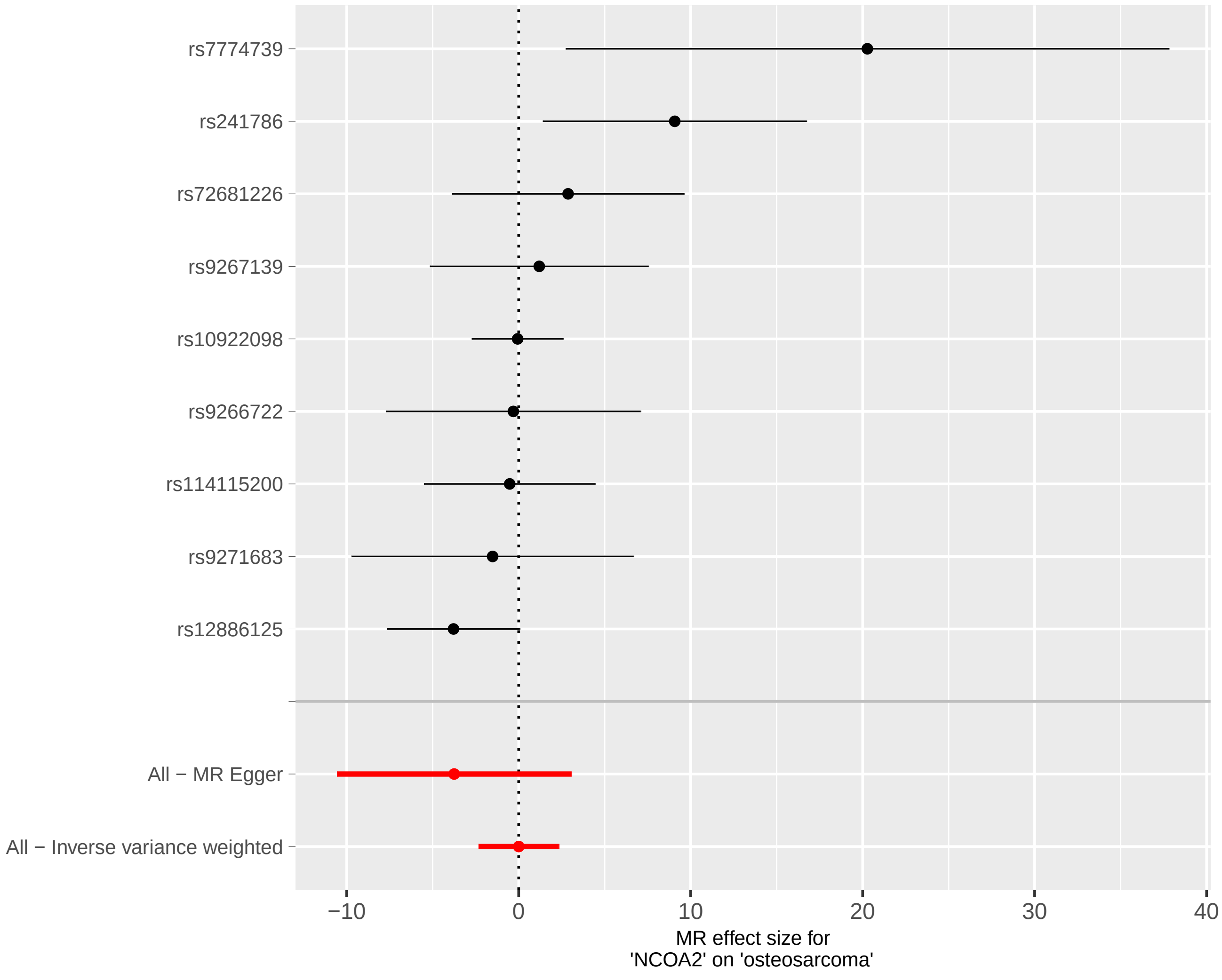

rs7774739
rs241786
rs72681226
rs9267139
rs10922098
rs9266722
rs114115200
rs9271683
rs12886125
All − MR Egger
All − Inverse variance weighted
−10
0
10
20
30
40
MR effect size for
'NCOA2' on 'osteosarcoma'

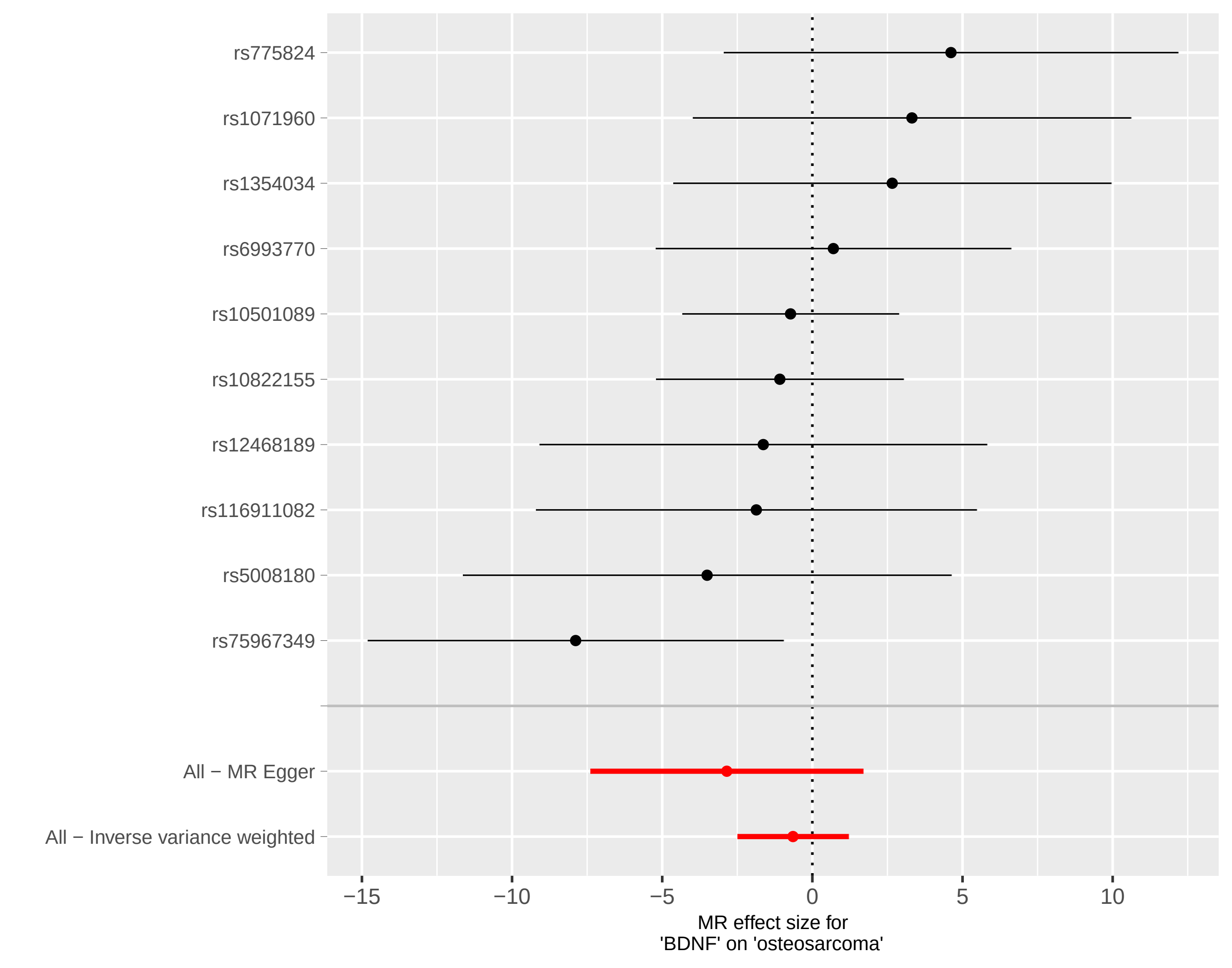

rs775824
rs1071960
rs1354034
rs6993770
rs10501089
rs10822155
rs12468189
rs116911082
rs5008180
rs75967349
All − MR Egger
All − Inverse variance weighted
−15
−10
−5
0
5
10
MR effect size for
'BDNF' on 'osteosarcoma'

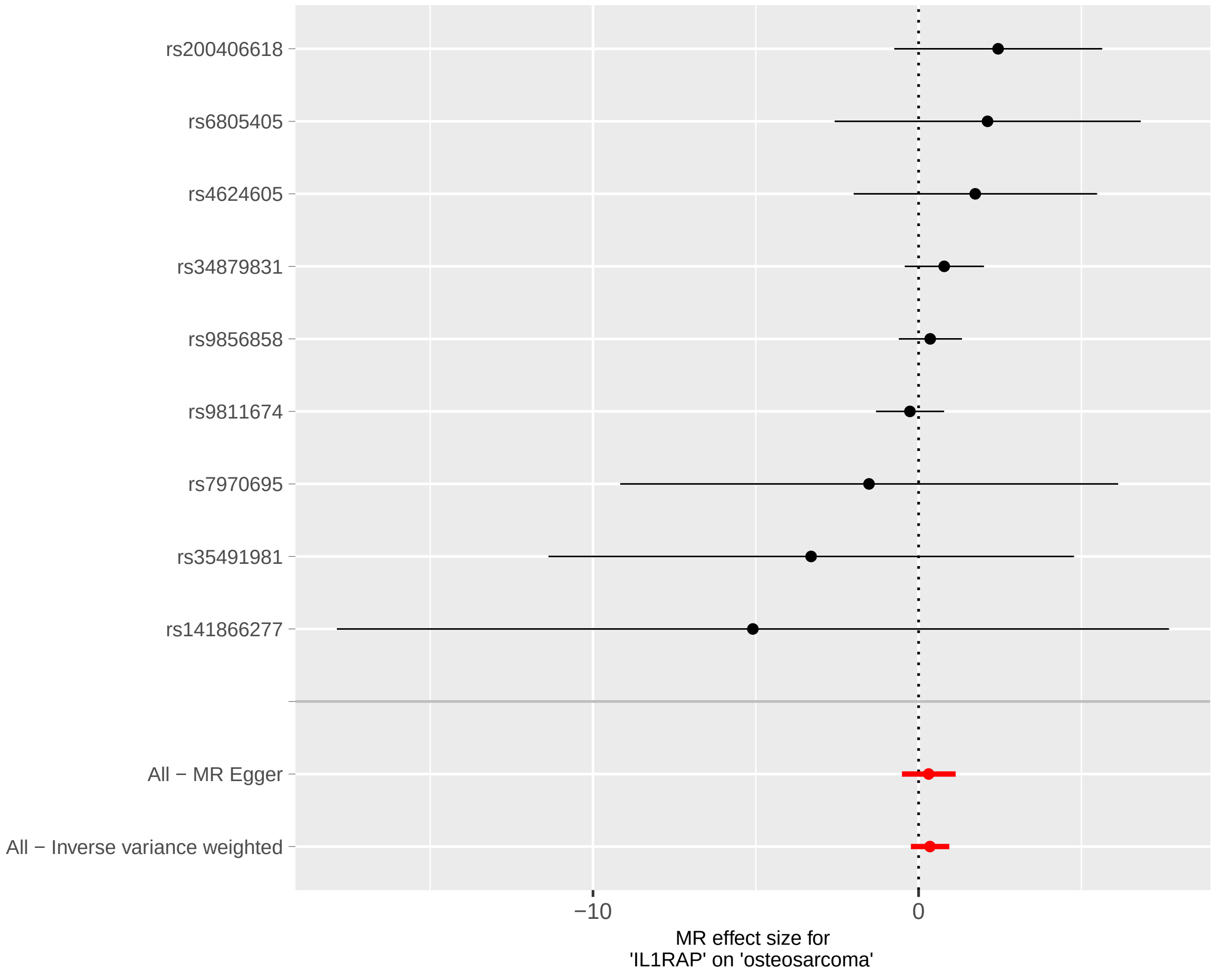
rs200406618
rs6805405
rs4624605
rs34879831
rs9856858
rs9811674
rs7970695
rs35491981
rs141866277
All − MR Egger
All − Inverse variance weighted
−10
0
MR effect size for
'IL1RAP' on 'osteosarcoma'

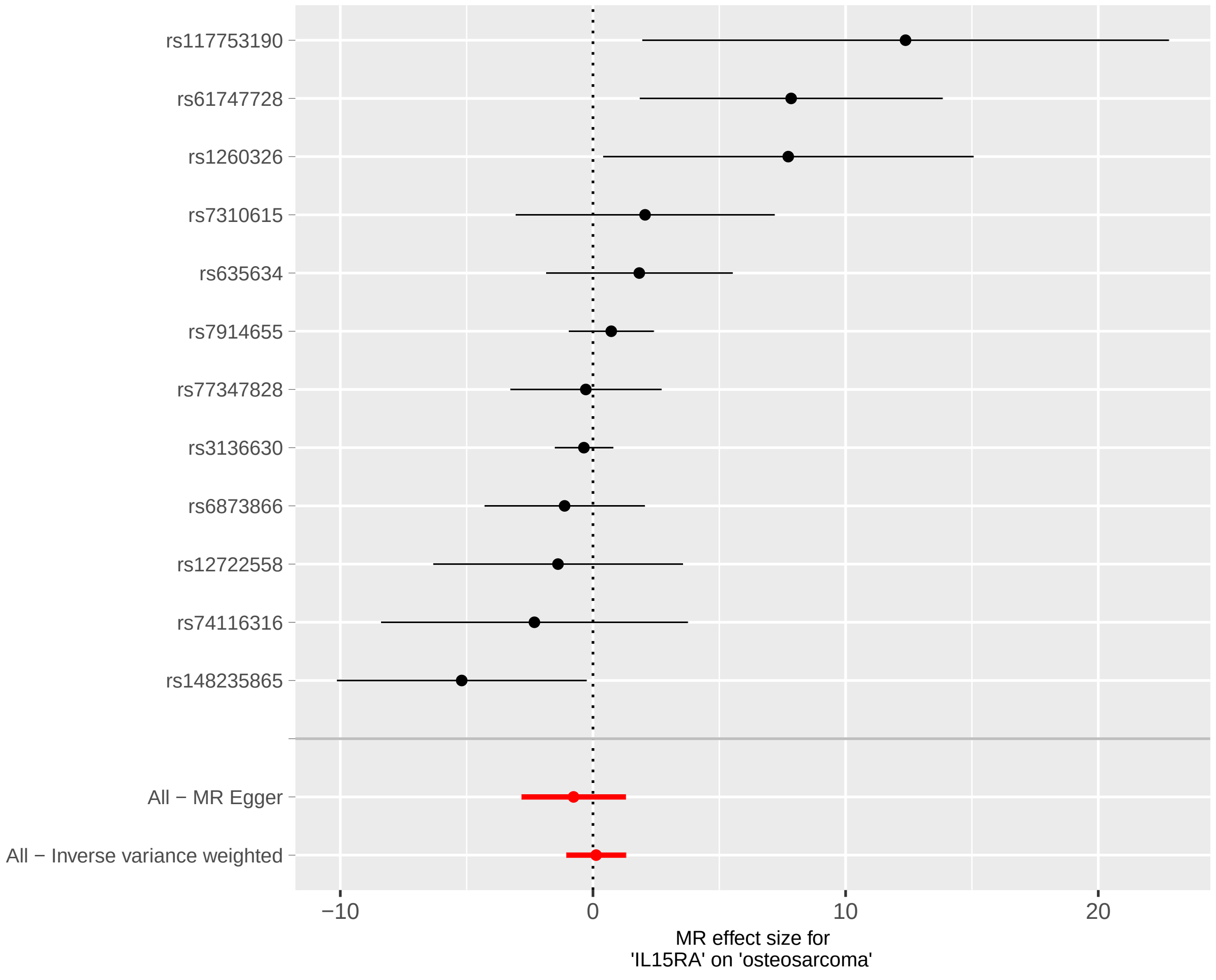

rs117753190
rs61747728
rs1260326
rs7310615
rs635634
rs7914655
rs77347828
rs3136630
rs6873866
rs12722558
rs74116316
rs148235865
All − MR Egger
All − Inverse variance weighted
−10
0
10
20
MR effect size for
'IL15RA' on 'osteosarcoma'

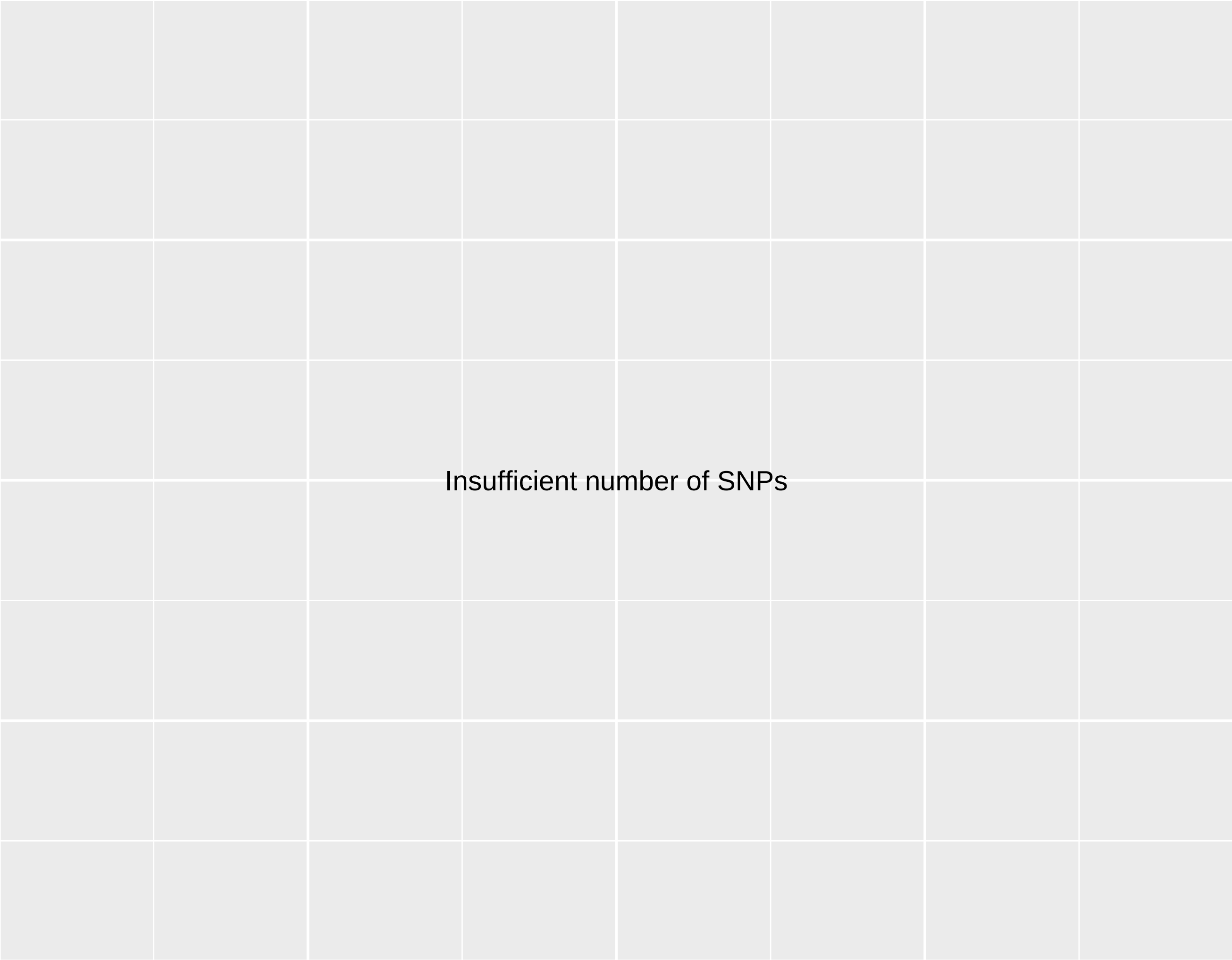
Insufficient number of SNPs

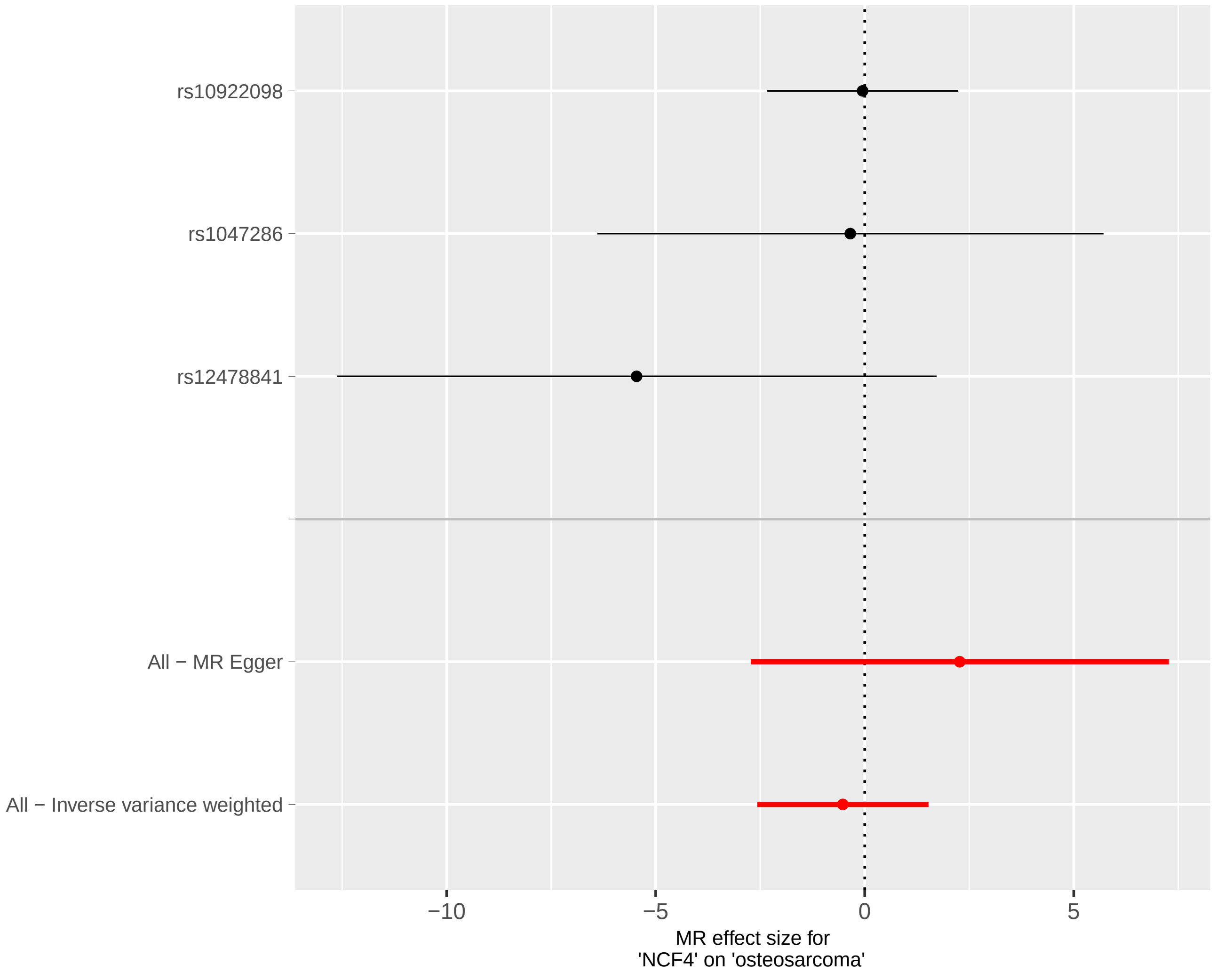

rs10922098
rs1047286
rs12478841
All − MR Egger
All − Inverse variance weighted
−10
−5
0
5
MR effect size for
'NCF4' on 'osteosarcoma'

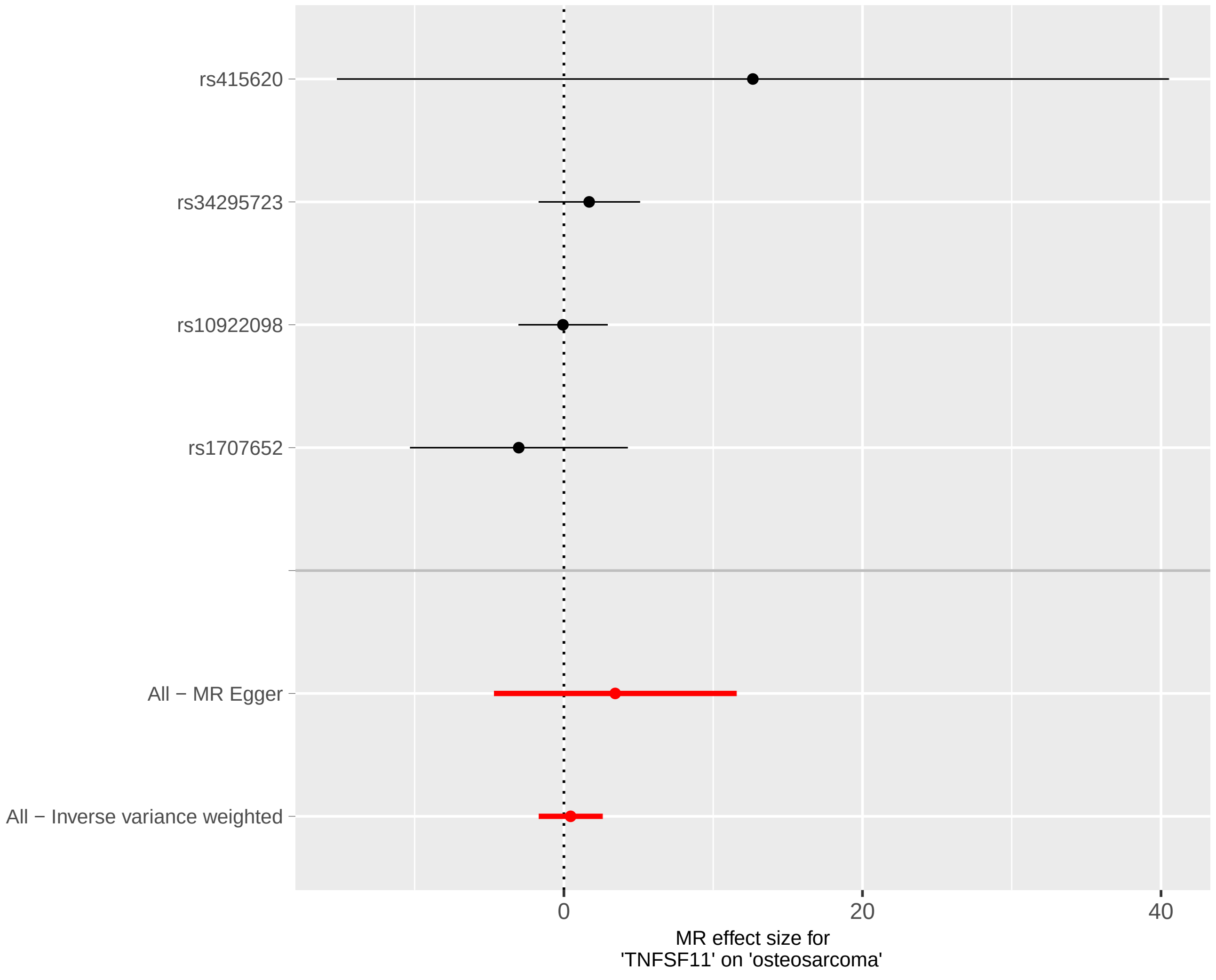

rs415620
rs34295723
rs10922098
rs1707652
All − MR Egger
All − Inverse variance weighted
0
20
40
MR effect size for
'TNFSF11' on 'osteosarcoma'

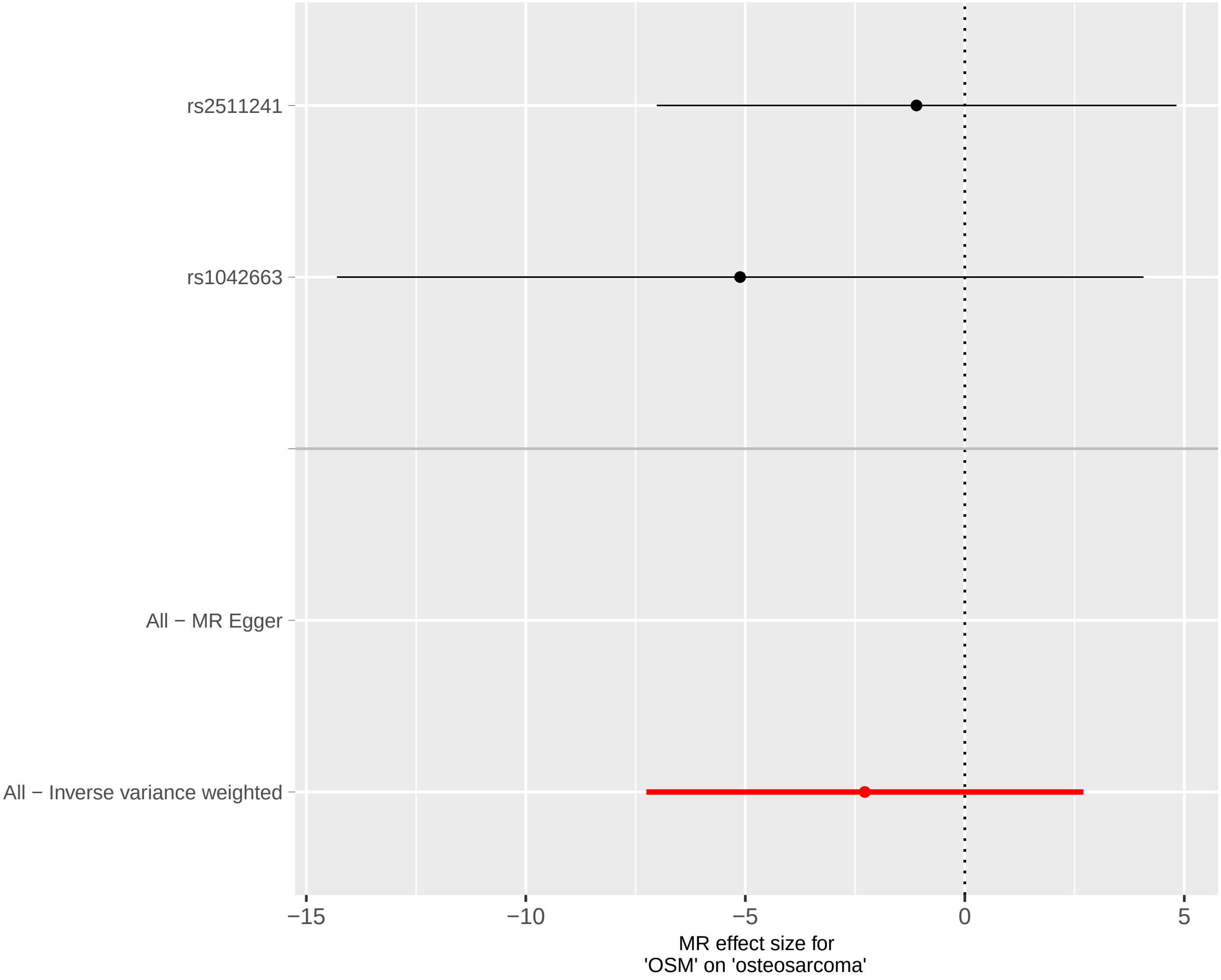

rs2511241
rs1042663
All − MR Egger
All − Inverse variance weighted
−15
−10
−5
0
5
MR effect size for
'OSM' on 'osteosarcoma'

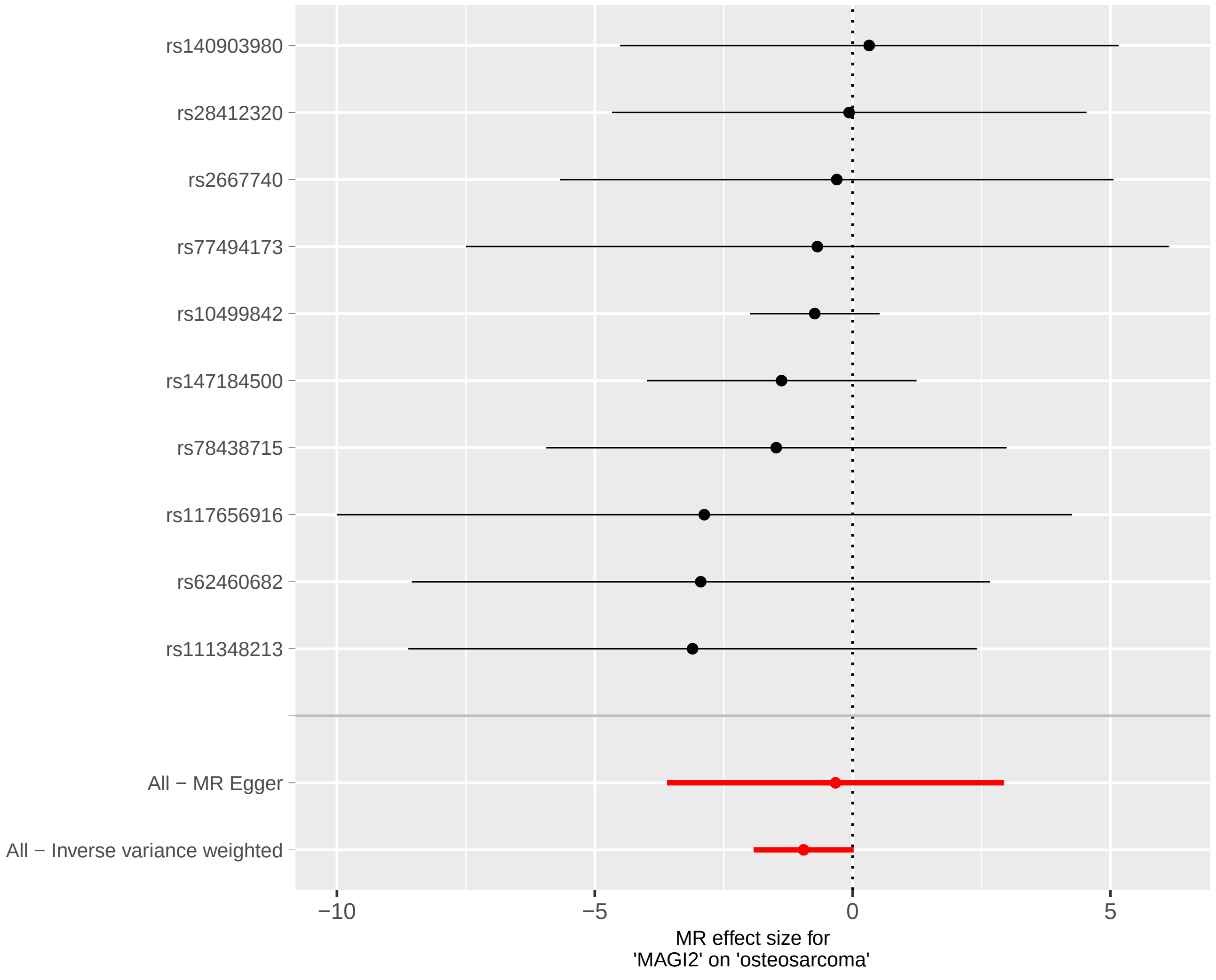
rs140903980
rs28412320
rs2667740
rs77494173
rs10499842
rs147184500
rs78438715
rs117656916
rs62460682
rs111348213
All − MR Egger
All − Inverse variance weighted
−10
−5
0
5
MR effect size for
'MAGI2' on 'osteosarcoma'

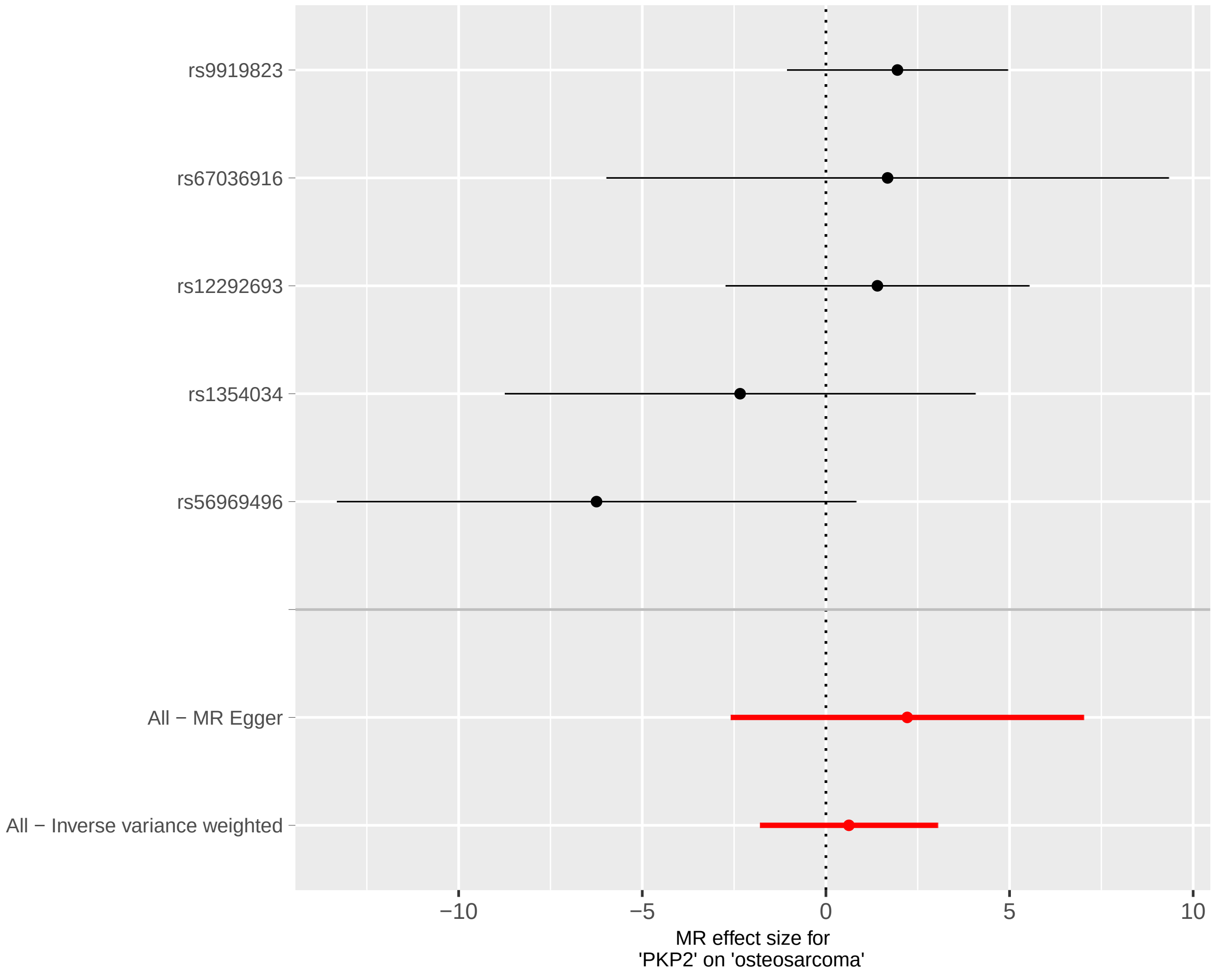
rs9919823
rs67036916
rs12292693
rs1354034
rs56969496
All − MR Egger
All − Inverse variance weighted
−10
−5
0
5
10
MR effect size for
'PKP2' on 'osteosarcoma'

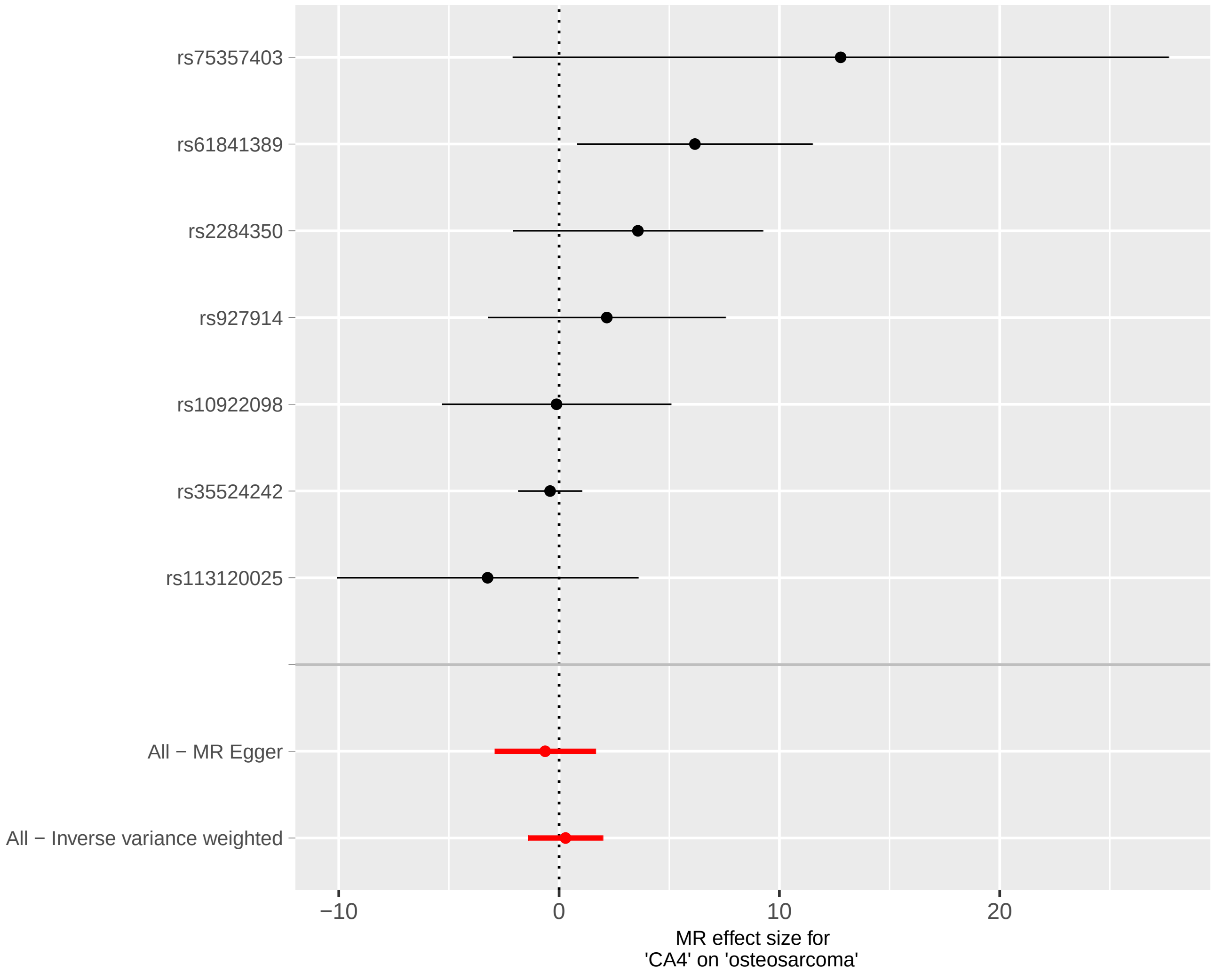
rs75357403
rs61841389
rs2284350
rs927914
rs10922098
rs35524242
rs113120025
All − MR Egger
All − Inverse variance weighted
−10
0
10
20
MR effect size for
'CA4' on 'osteosarcoma'

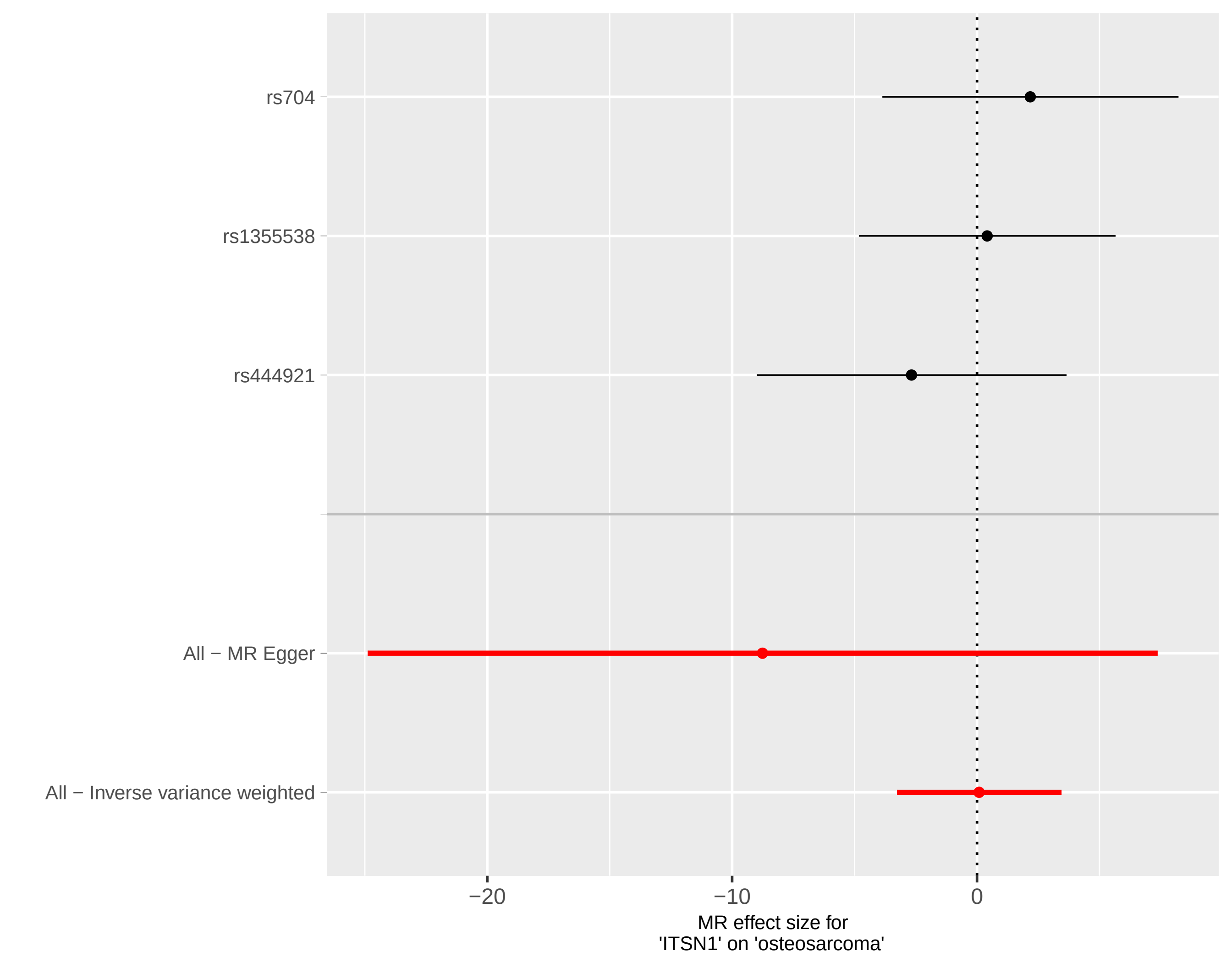
rs704
rs1355538
rs444921
All − MR Egger
All − Inverse variance weighted
−20
−10
0
MR effect size for
'ITSN1' on 'osteosarcoma'

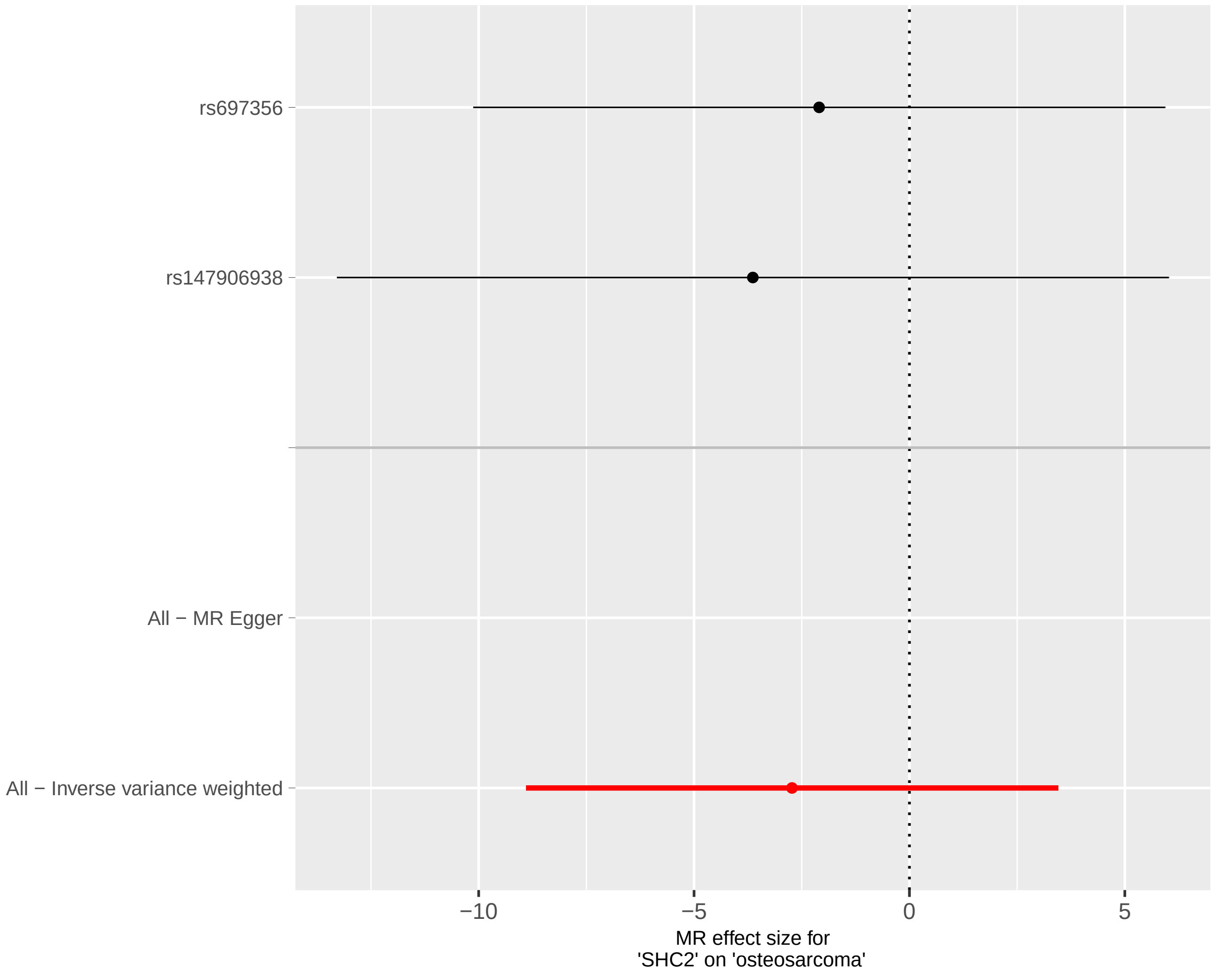

rs697356
rs147906938
All − MR Egger
All − Inverse variance weighted
−10
−5
0
5
MR effect size for
'SHC2' on 'osteosarcoma'

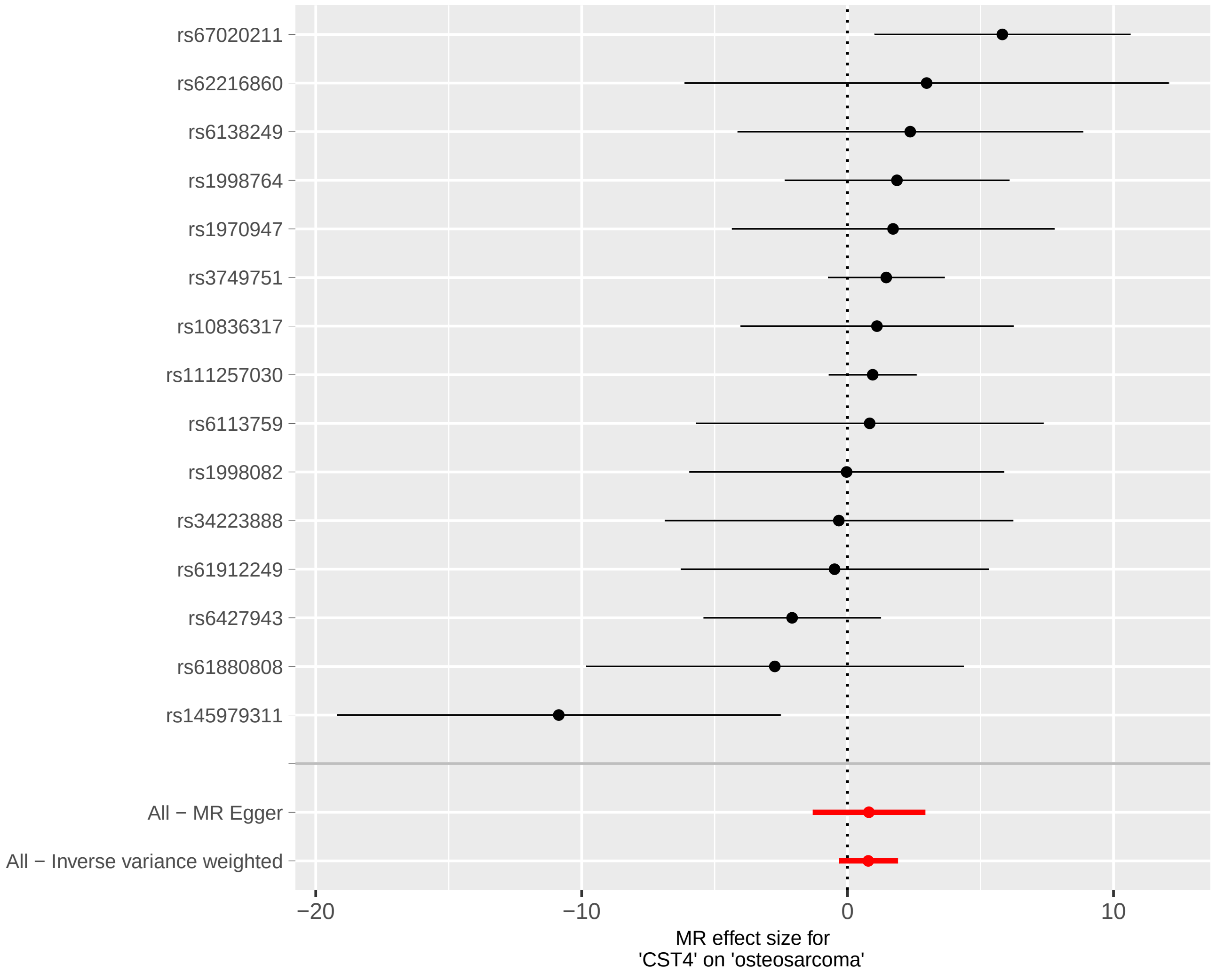
rs67020211
rs62216860
rs6138249
rs1998764
rs1970947
rs3749751
rs10836317
rs111257030
rs6113759
rs1998082
rs34223888
rs61912249
rs6427943
rs61880808
rs145979311
All − MR Egger
All − Inverse variance weighted
−20
−10
0
10
MR effect size for
'CST4' on 'osteosarcoma'

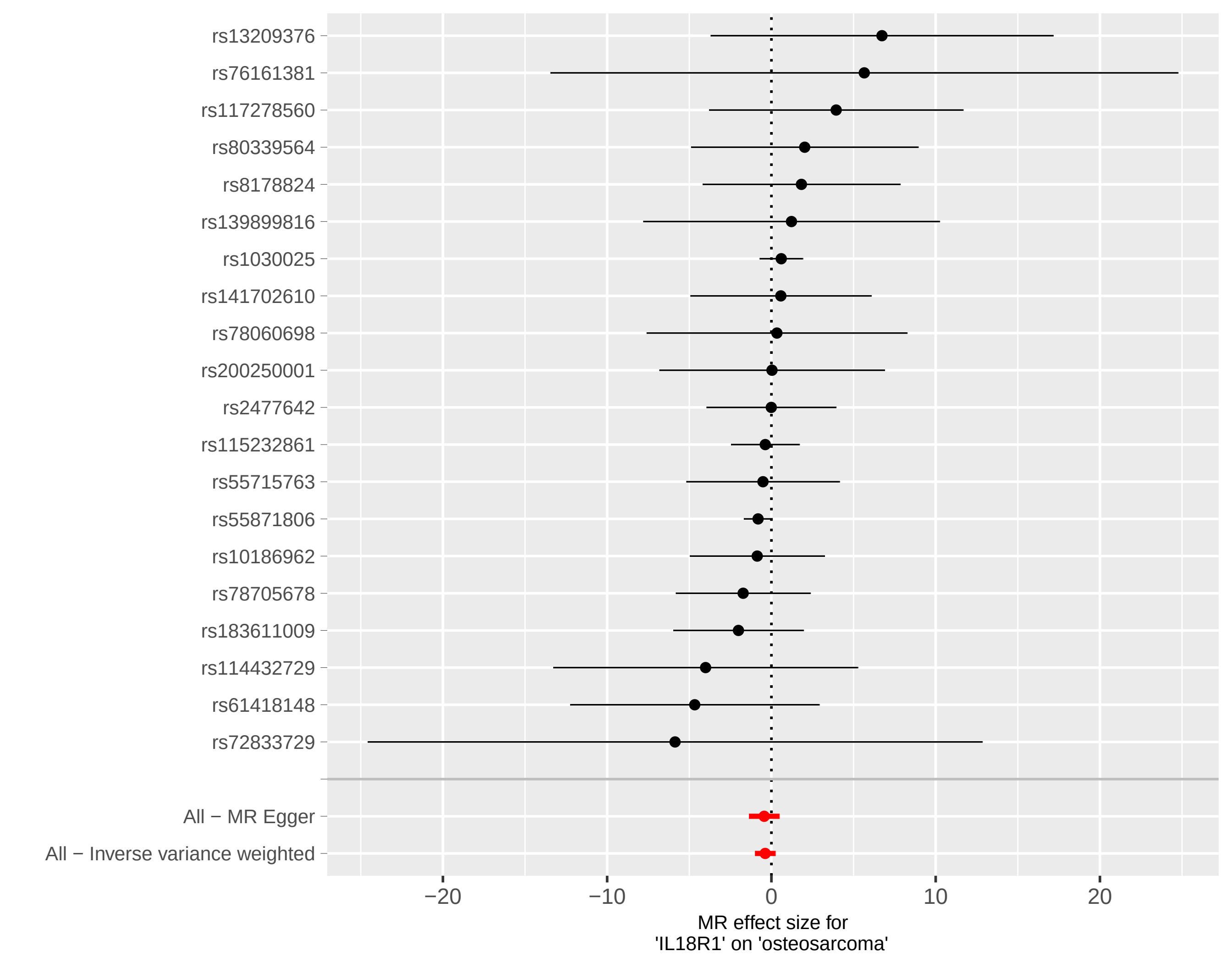
rs13209376
rs76161381
rs117278560
rs80339564
rs8178824
rs139899816
rs1030025
rs141702610
rs78060698
rs200250001
rs2477642
rs115232861
rs55715763
rs55871806
rs10186962
rs78705678
rs183611009
rs114432729
rs61418148
rs72833729
All − MR Egger
All − Inverse variance weighted
−20
−10
0
10
20
MR effect size for
'IL18R1' on 'osteosarcoma'

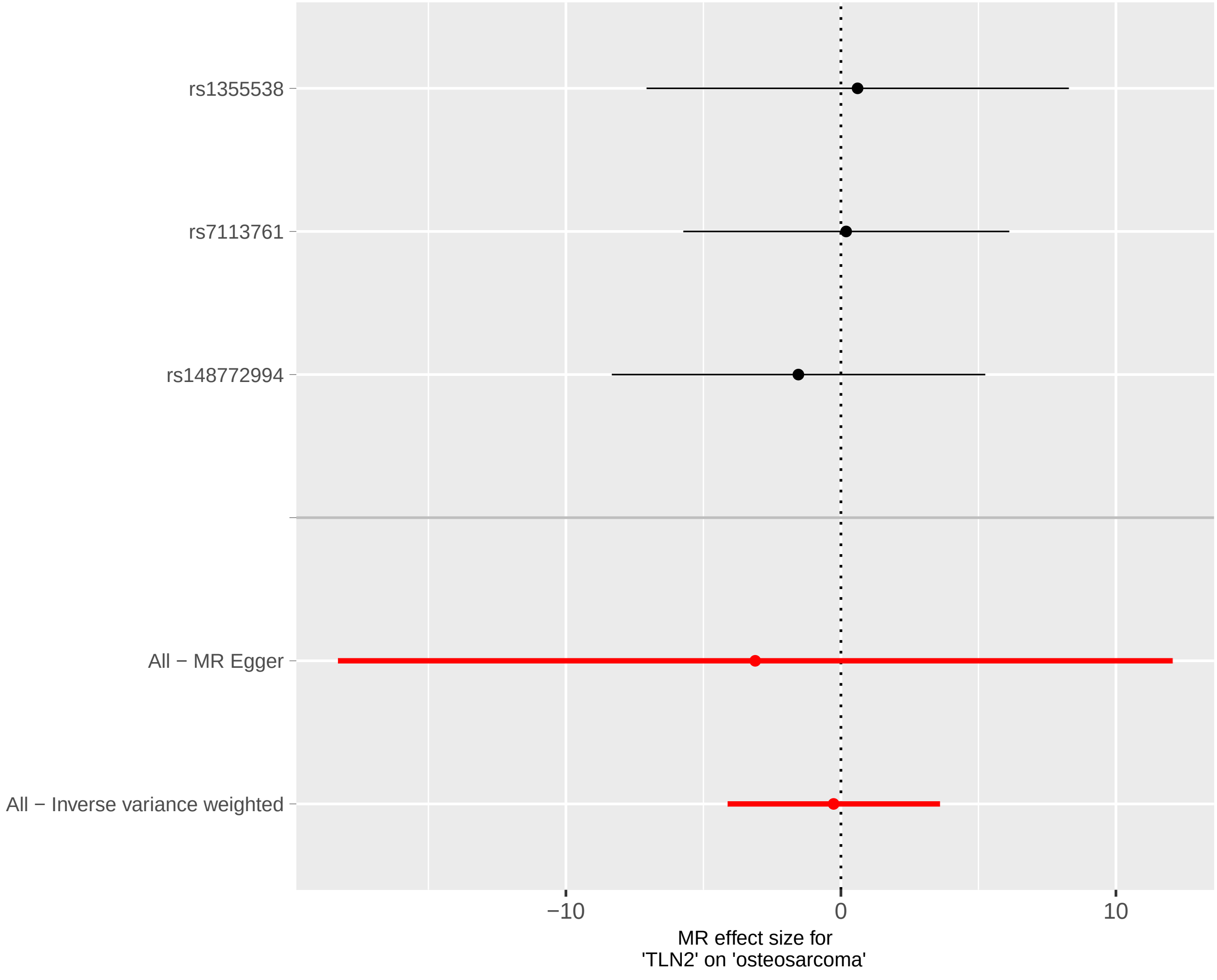

rs1355538
rs7113761
rs148772994
All − MR Egger
All − Inverse variance weighted
−10
0
10
MR effect size for
'TLN2' on 'osteosarcoma'

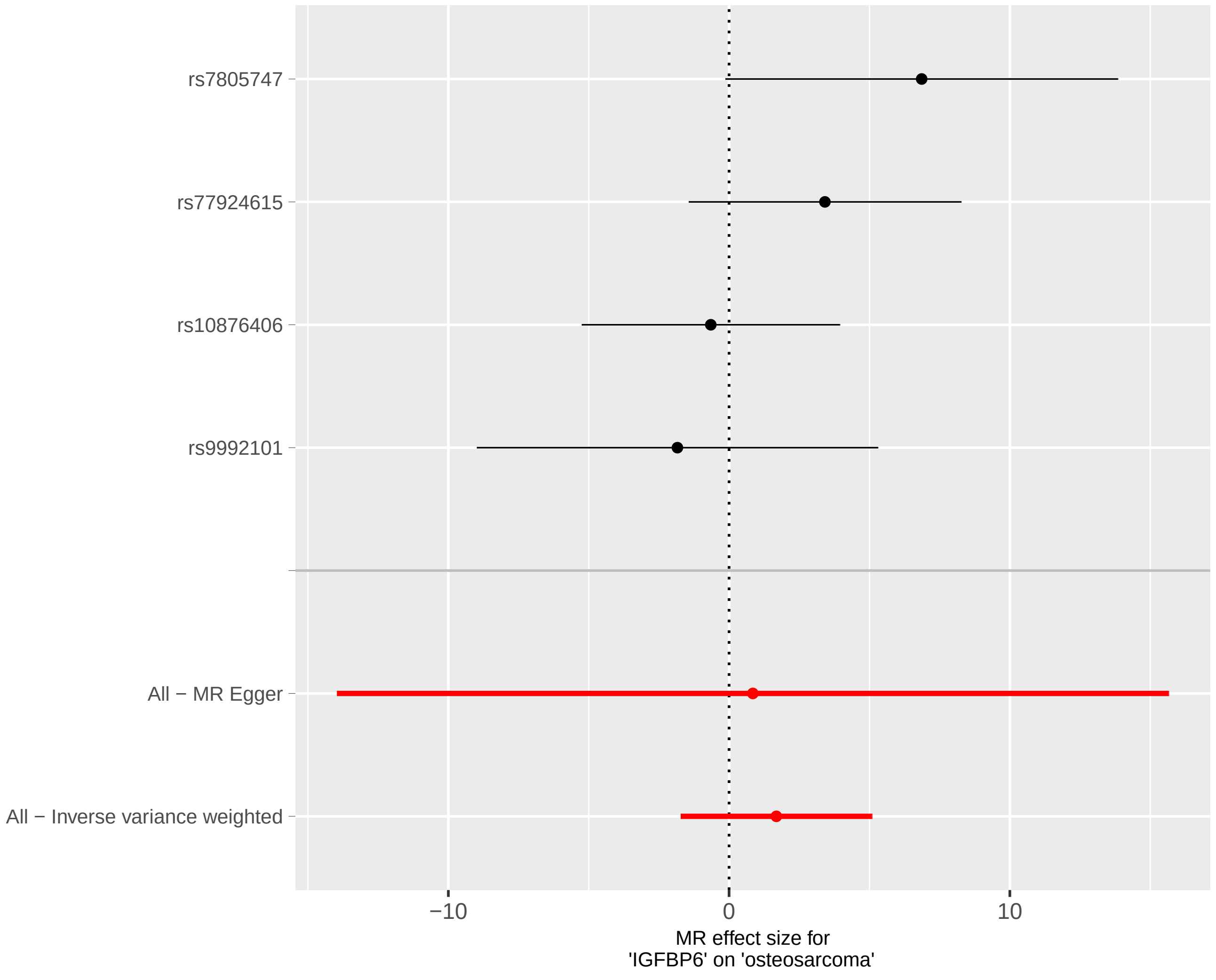
rs7805747
rs77924615
rs10876406
rs9992101
All − MR Egger
All − Inverse variance weighted
−10
0
10
MR effect size for
'IGFBP6' on 'osteosarcoma'

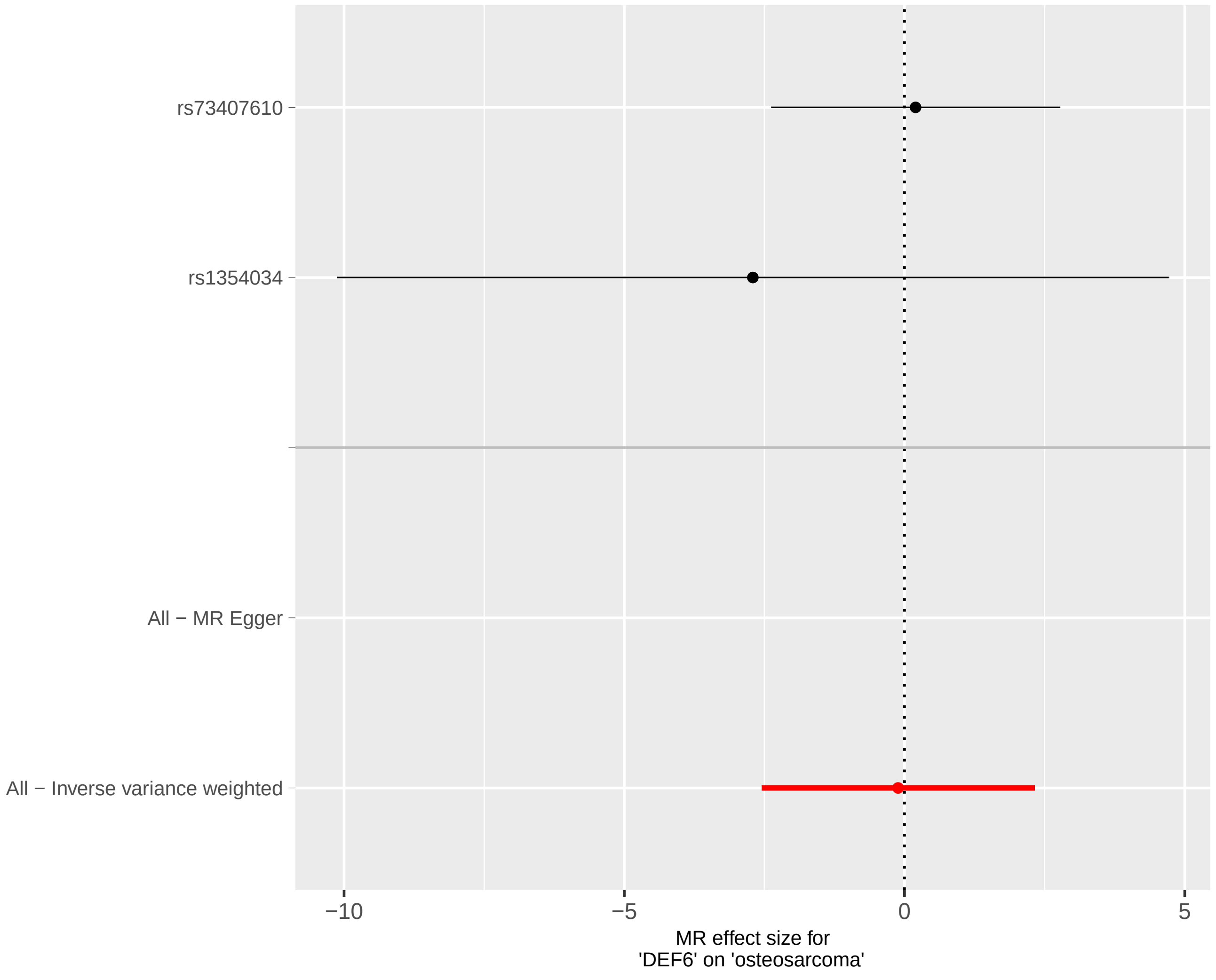
rs73407610
rs1354034
All − MR Egger
All − Inverse variance weighted
−10
−5
0
5
MR effect size for
'DEF6' on 'osteosarcoma'

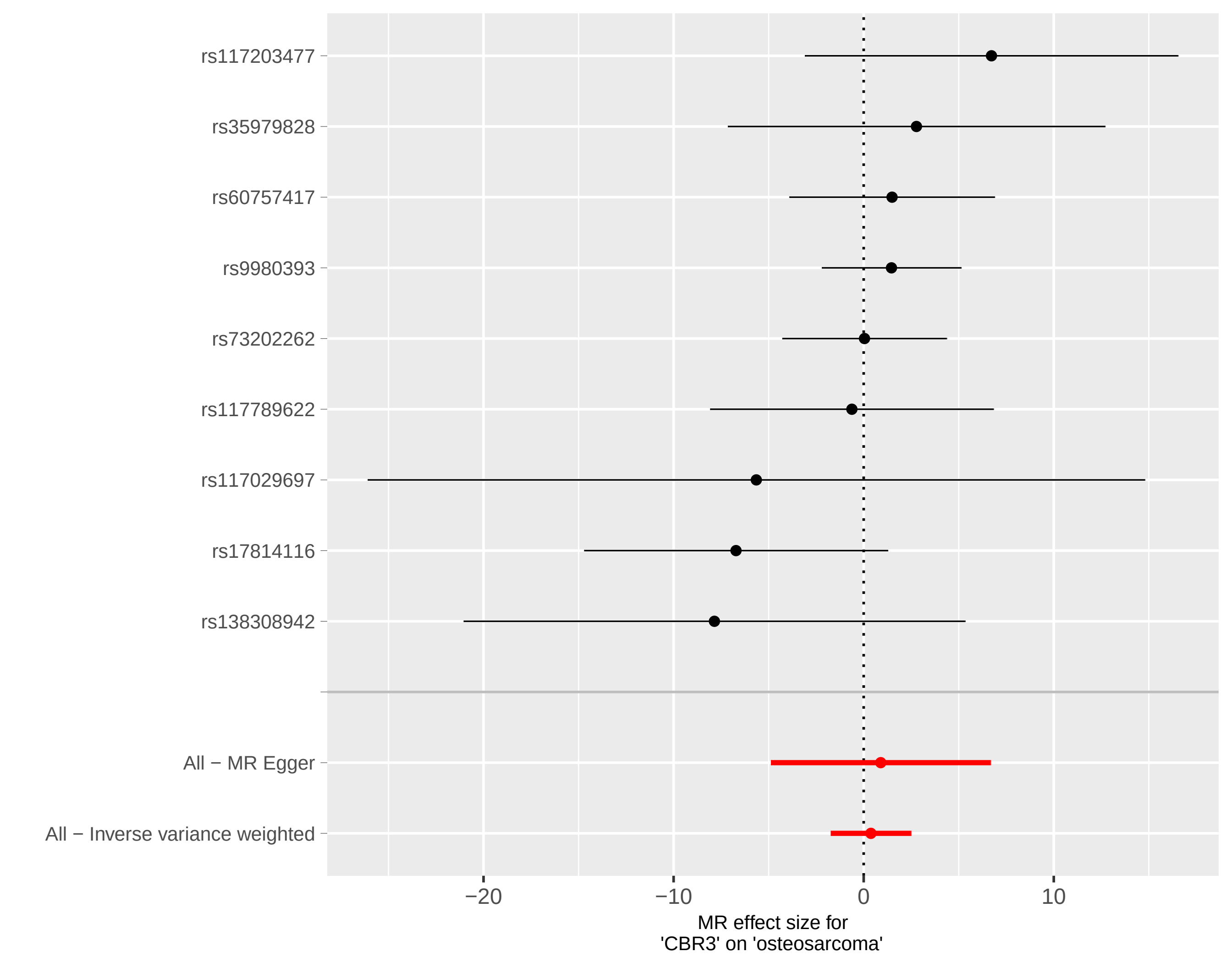
rs117203477
rs35979828
rs60757417
rs9980393
rs73202262
rs117789622
rs117029697
rs17814116
rs138308942
All − MR Egger
All − Inverse variance weighted
−20
−10
0
10
MR effect size for
'CBR3' on 'osteosarcoma'

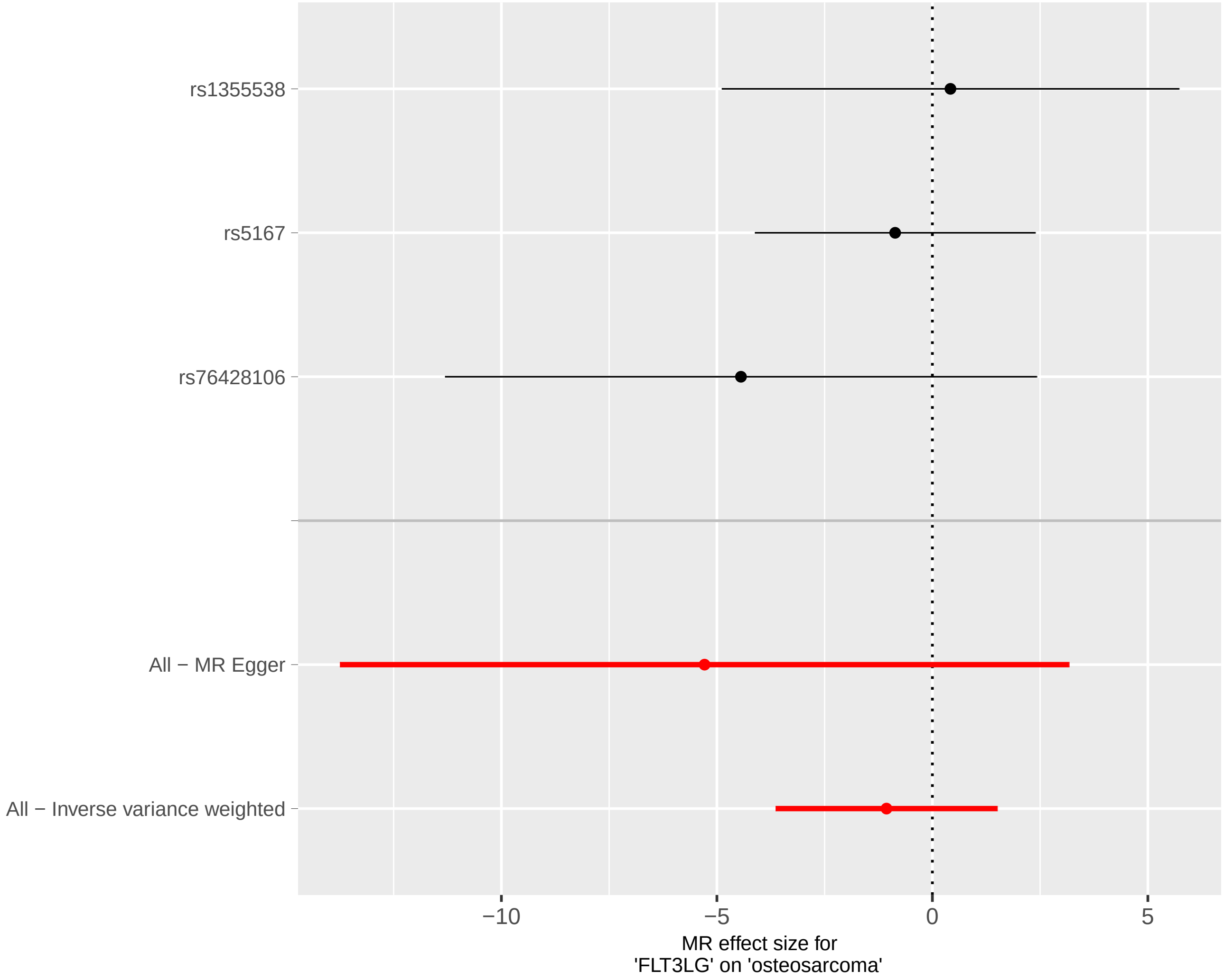

rs1355538
rs5167
rs76428106
All − MR Egger
All − Inverse variance weighted
−10
−5
0
5
MR effect size for
'FLT3LG' on 'osteosarcoma'

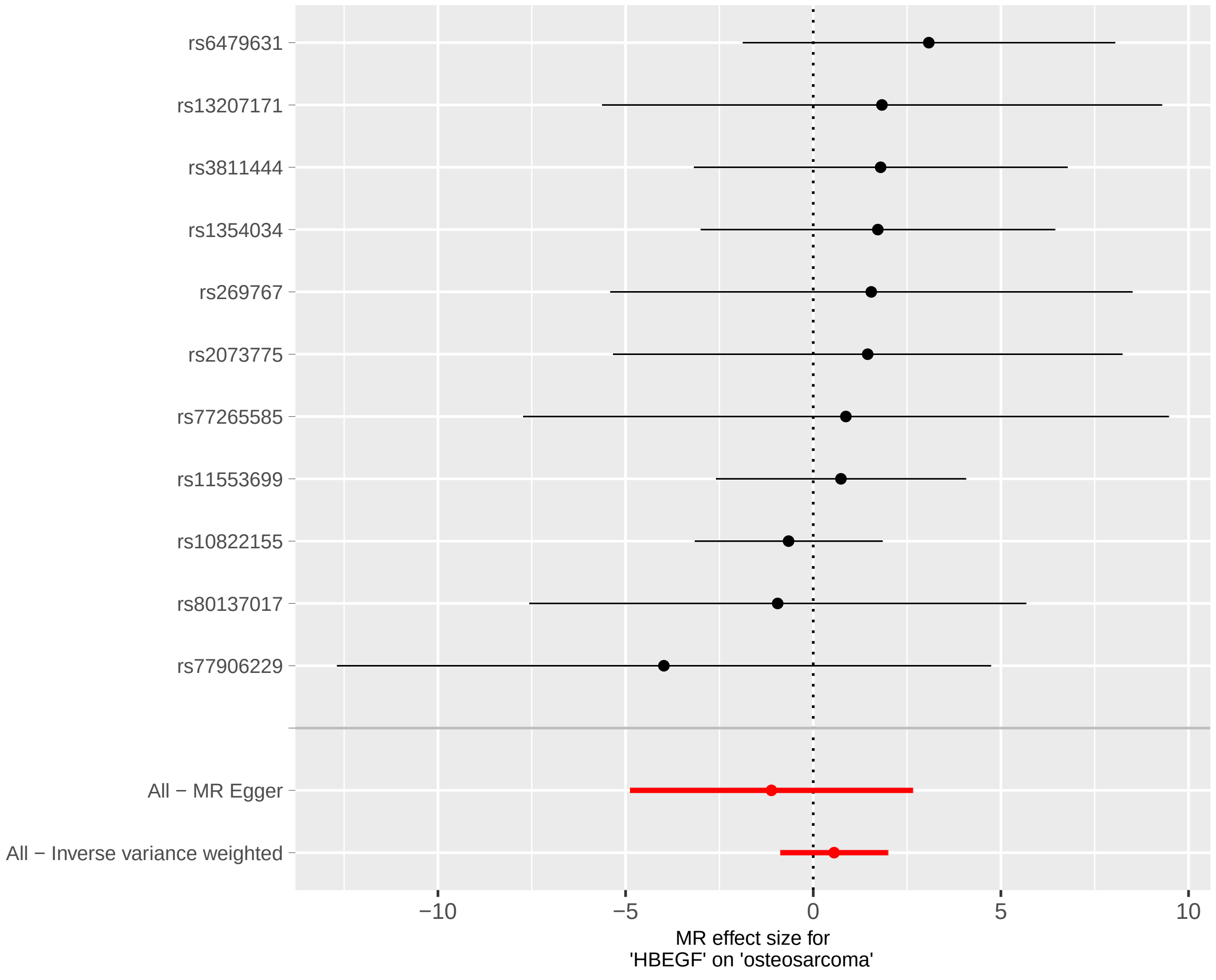
rs6479631
rs13207171
rs3811444
rs1354034
rs269767
rs2073775
rs77265585
rs11553699
rs10822155
rs80137017
rs77906229
All − MR Egger
All − Inverse variance weighted
−10
−5
0
5
10
MR effect size for
'HBEGF' on 'osteosarcoma'

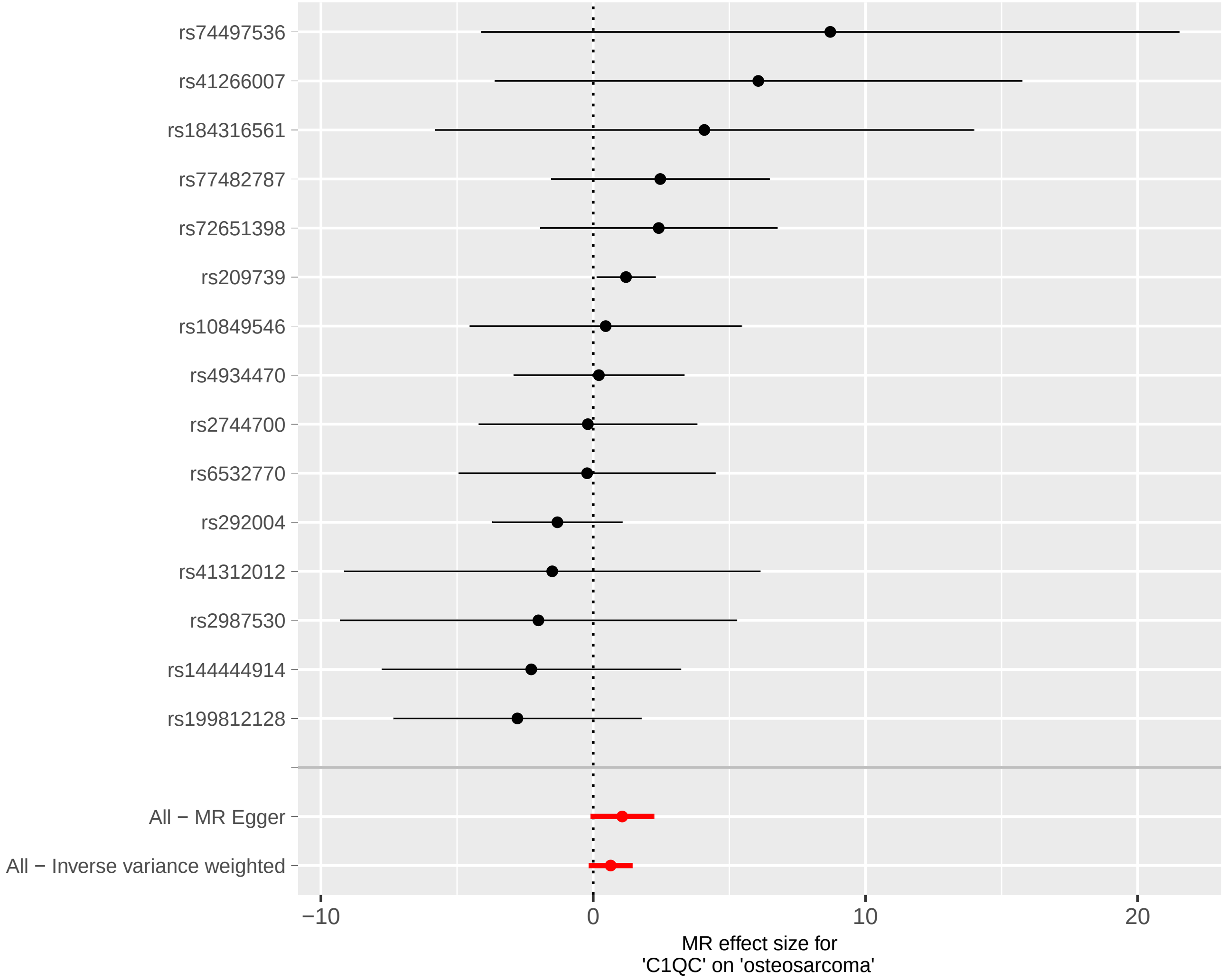

rs74497536
rs41266007
rs184316561
rs77482787
rs72651398
rs209739
rs10849546
rs4934470
rs2744700
rs6532770
rs292004
rs41312012
rs2987530
rs144444914
rs199812128
All − MR Egger
All − Inverse variance weighted
−10
0
10
20
MR effect size for
'C1QC' on 'osteosarcoma'

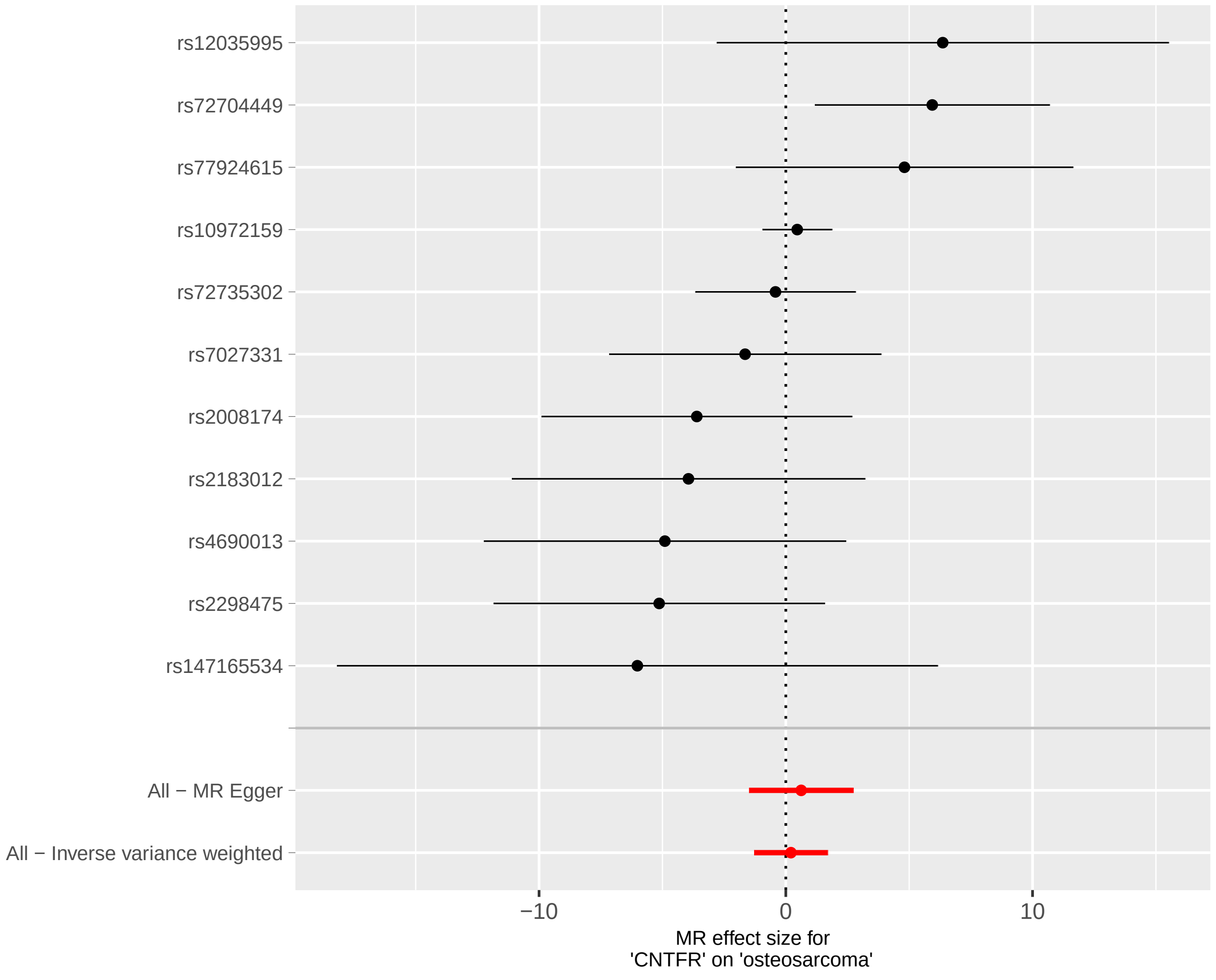

rs12035995
rs72704449
rs77924615
rs10972159
rs72735302
rs7027331
rs2008174
rs2183012
rs4690013
rs2298475
rs147165534
All − MR Egger
All − Inverse variance weighted
−10
0
10
MR effect size for
'CNTFR' on 'osteosarcoma'

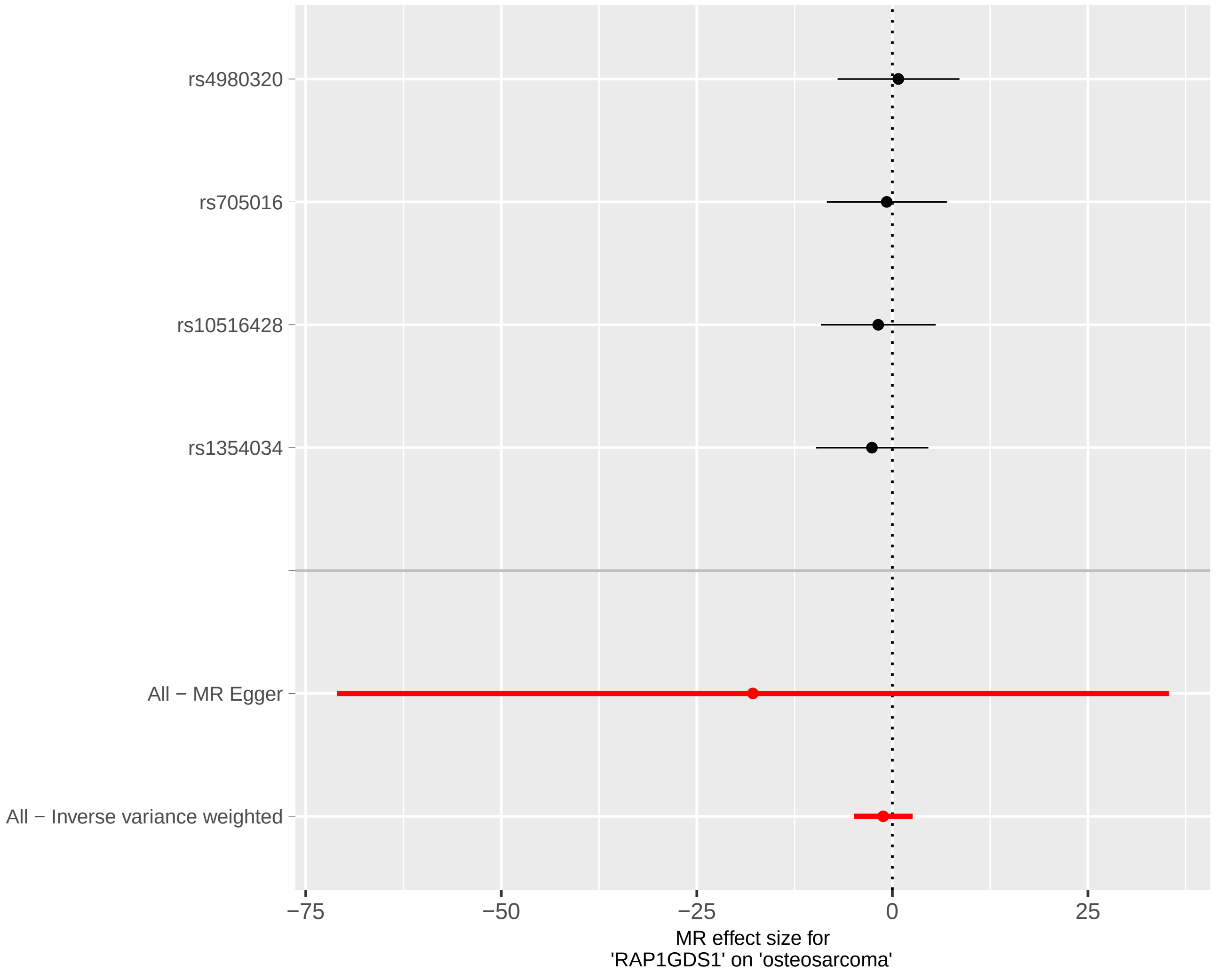
rs4980320
rs705016
rs10516428
rs1354034
All − MR Egger
All − Inverse variance weighted
−75
−50
−25
0
25
MR effect size for
'RAP1GDS1' on 'osteosarcoma'

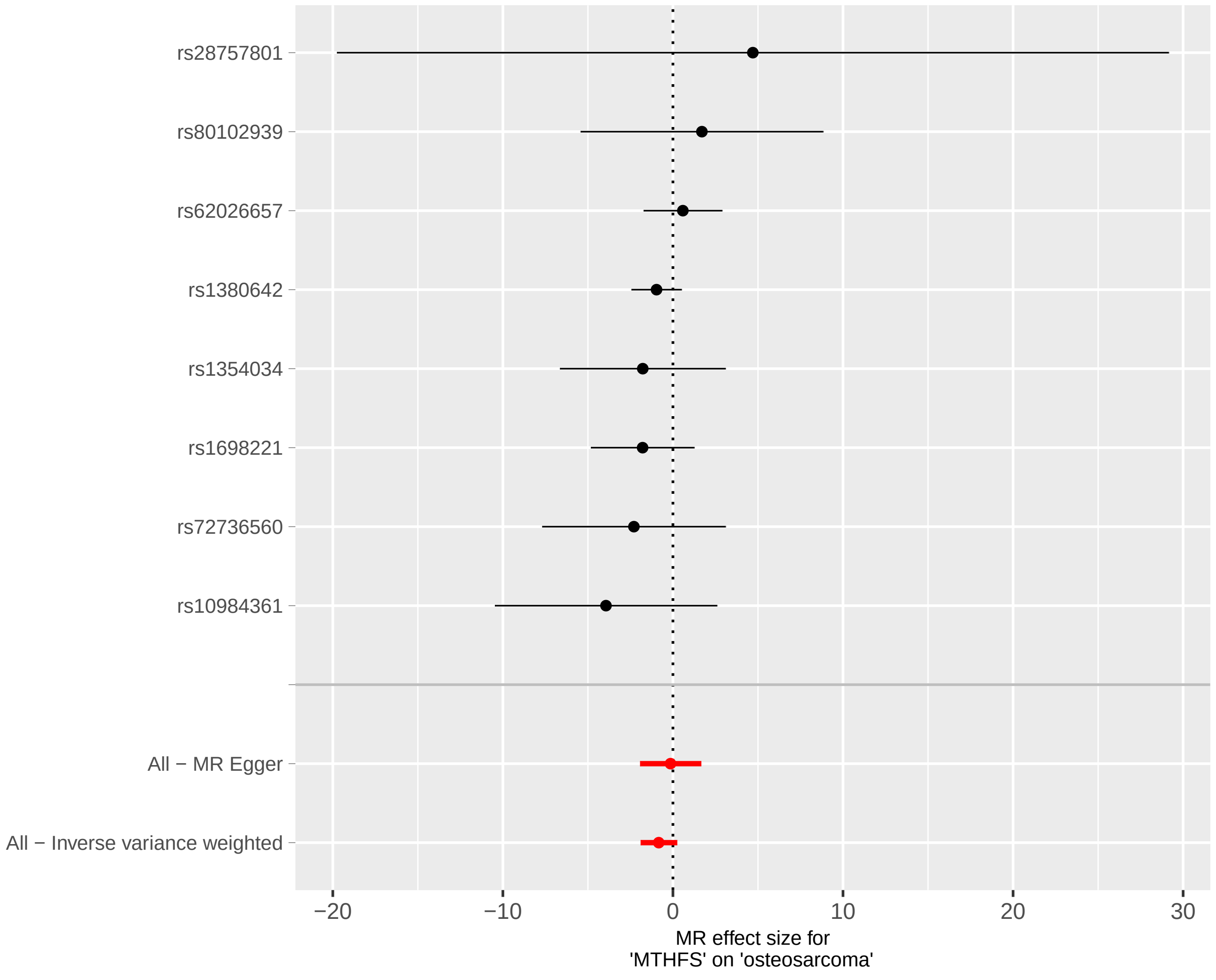

rs28757801
rs80102939
rs62026657
rs1380642
rs1354034
rs1698221
rs72736560
rs10984361
All − MR Egger
All − Inverse variance weighted
−20
−10
0
10
20
30
MR effect size for
'MTHFS' on 'osteosarcoma'

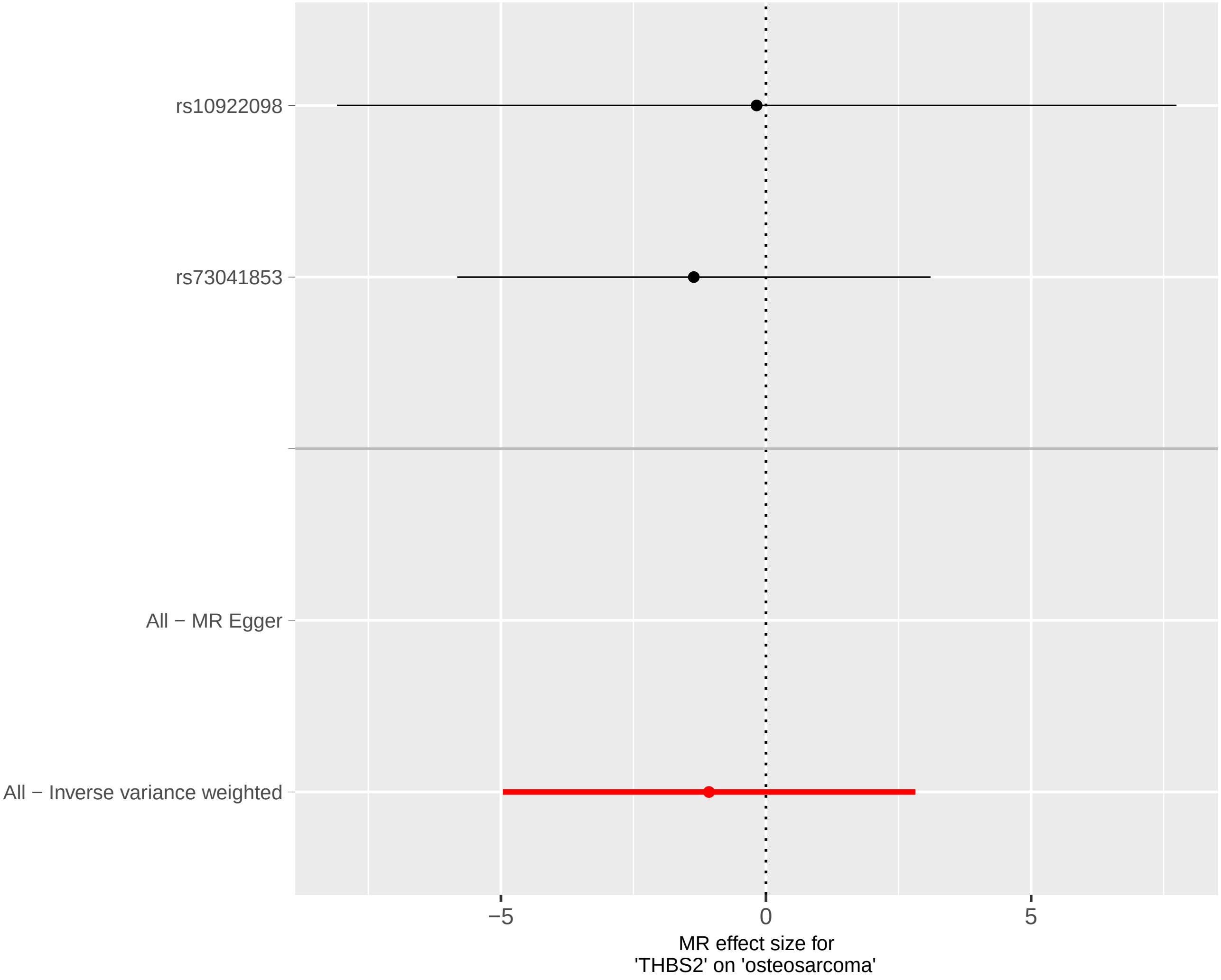
rs10922098
rs73041853
All − MR Egger
All − Inverse variance weighted
−5
0
5
MR effect size for
'THBS2' on 'osteosarcoma'

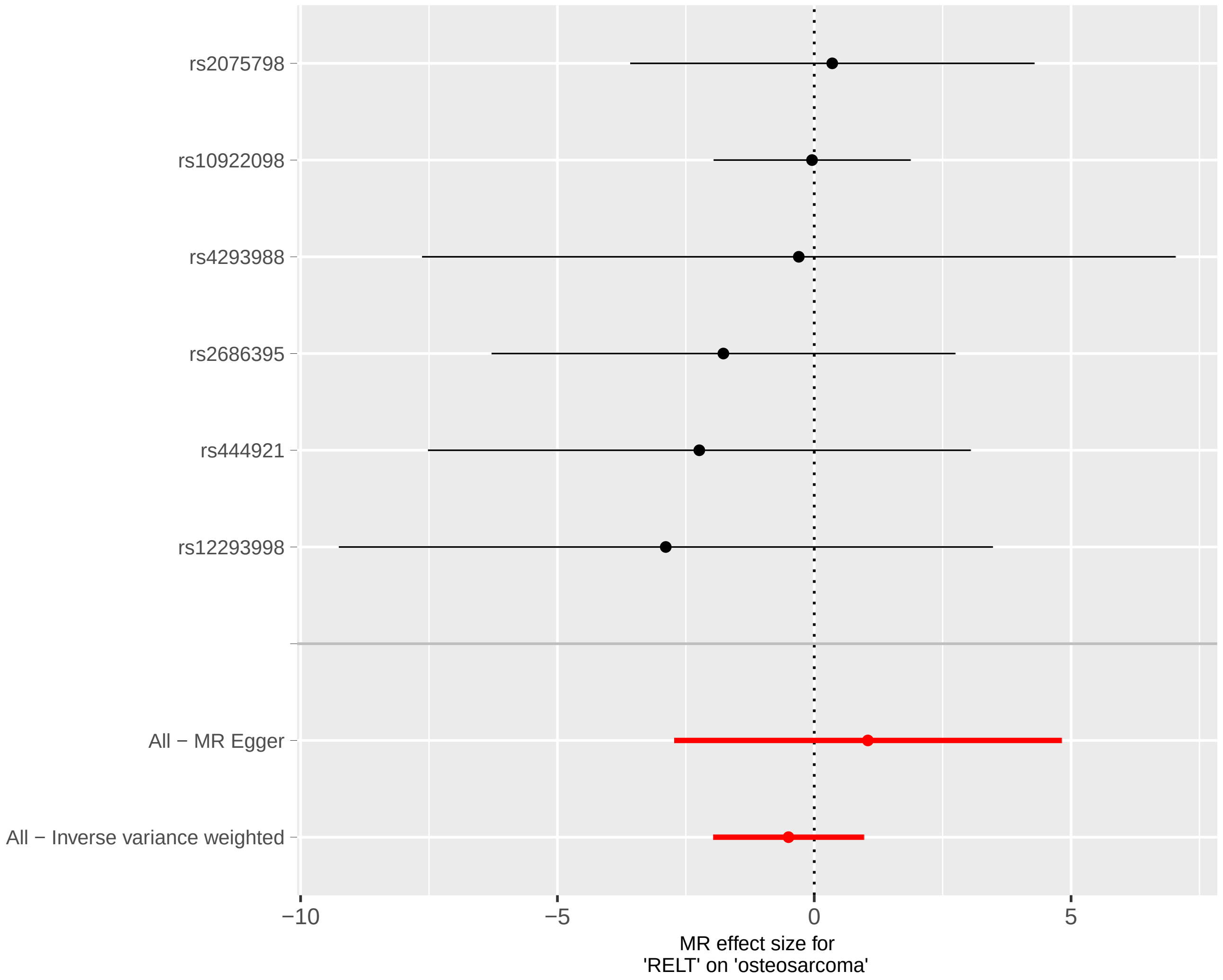

rs2075798
rs10922098
rs4293988
rs2686395
rs444921
rs12293998
All − MR Egger
All − Inverse variance weighted
−10
−5
0
5
MR effect size for
'RELT' on 'osteosarcoma'

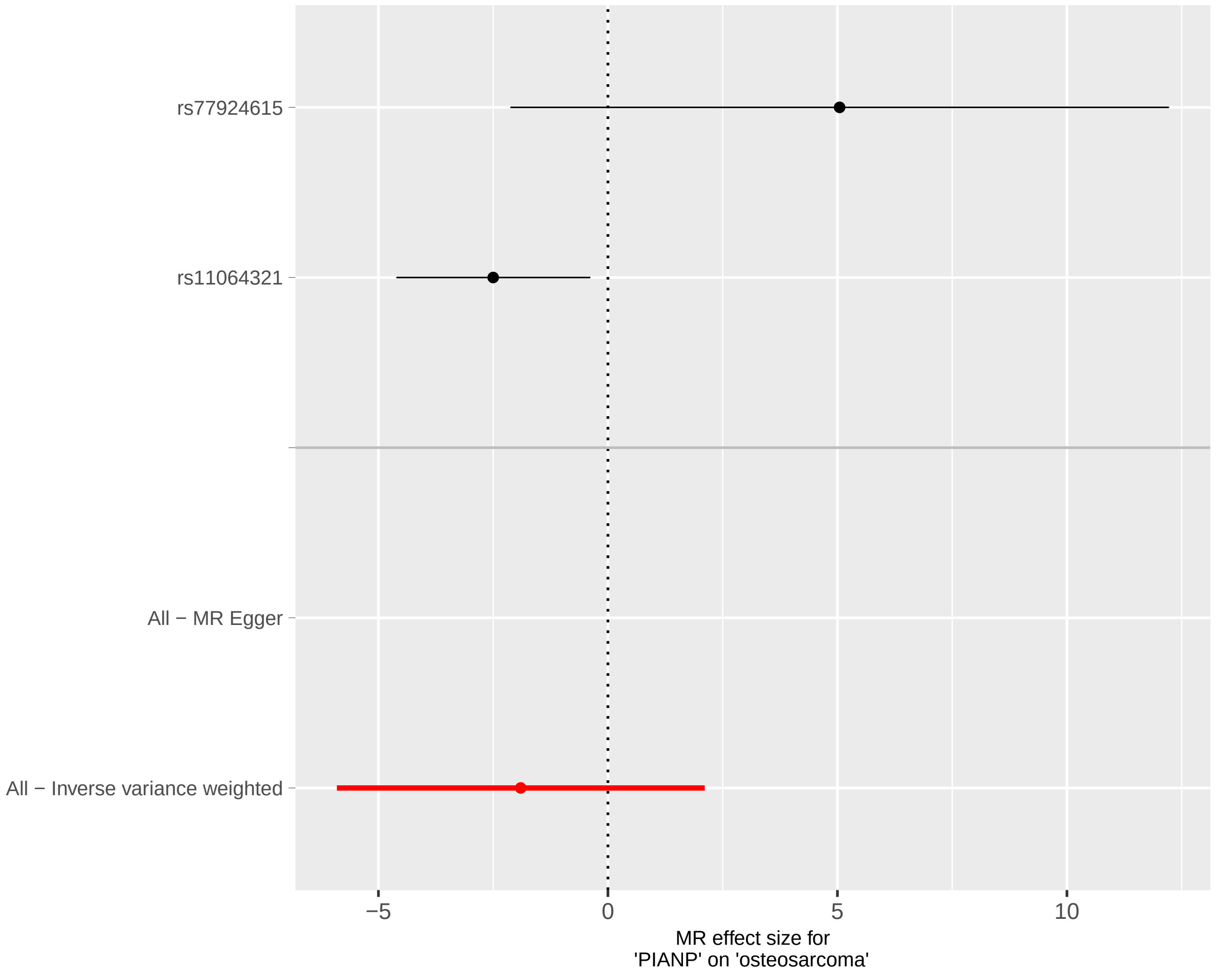
rs77924615
rs11064321
All − MR Egger
All − Inverse variance weighted
−5
0
5
10
MR effect size for
'PIANP' on 'osteosarcoma'

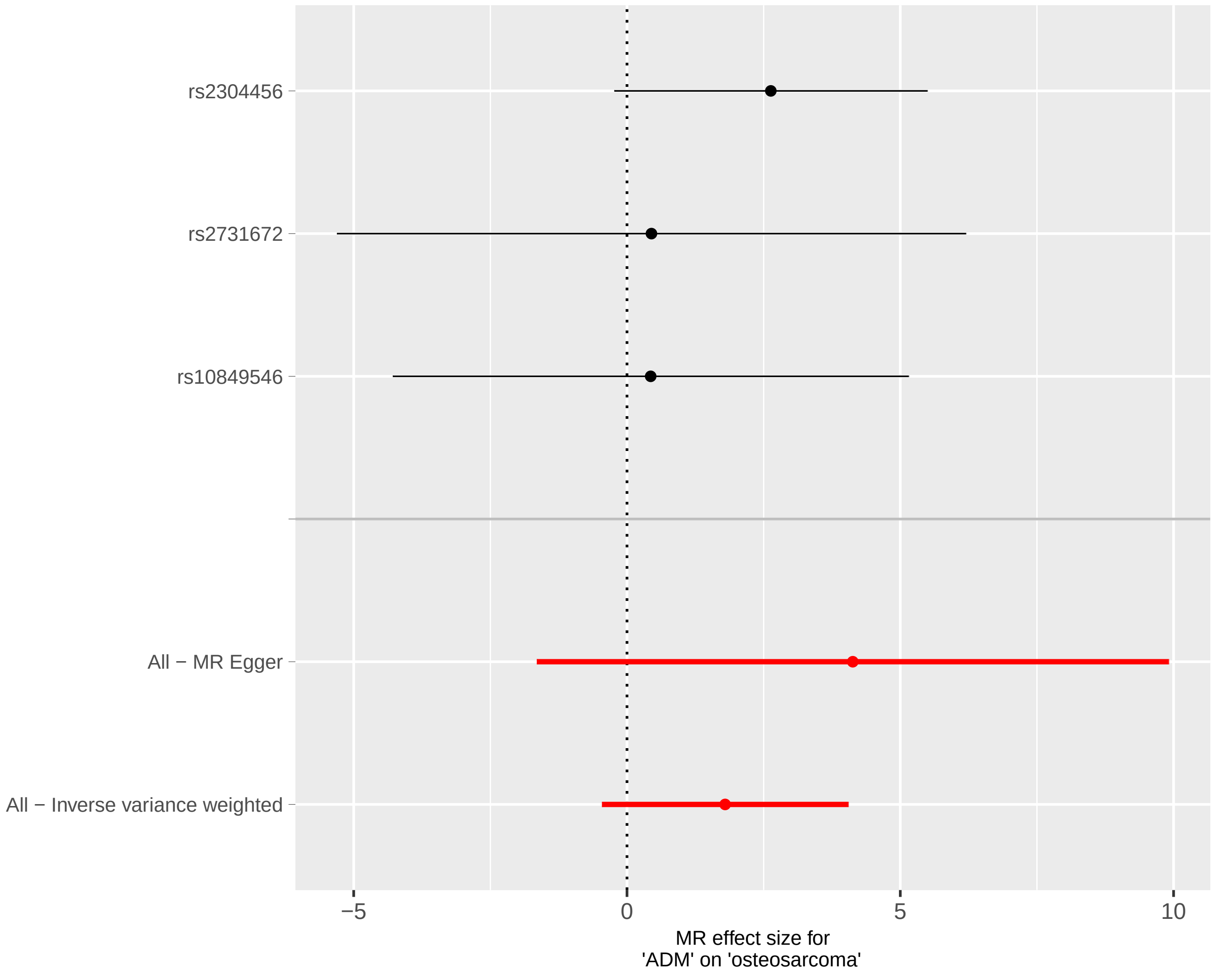
rs2304456
rs2731672
rs10849546
All − MR Egger
All − Inverse variance weighted
−5
0
5
10
MR effect size for
'ADM' on 'osteosarcoma'

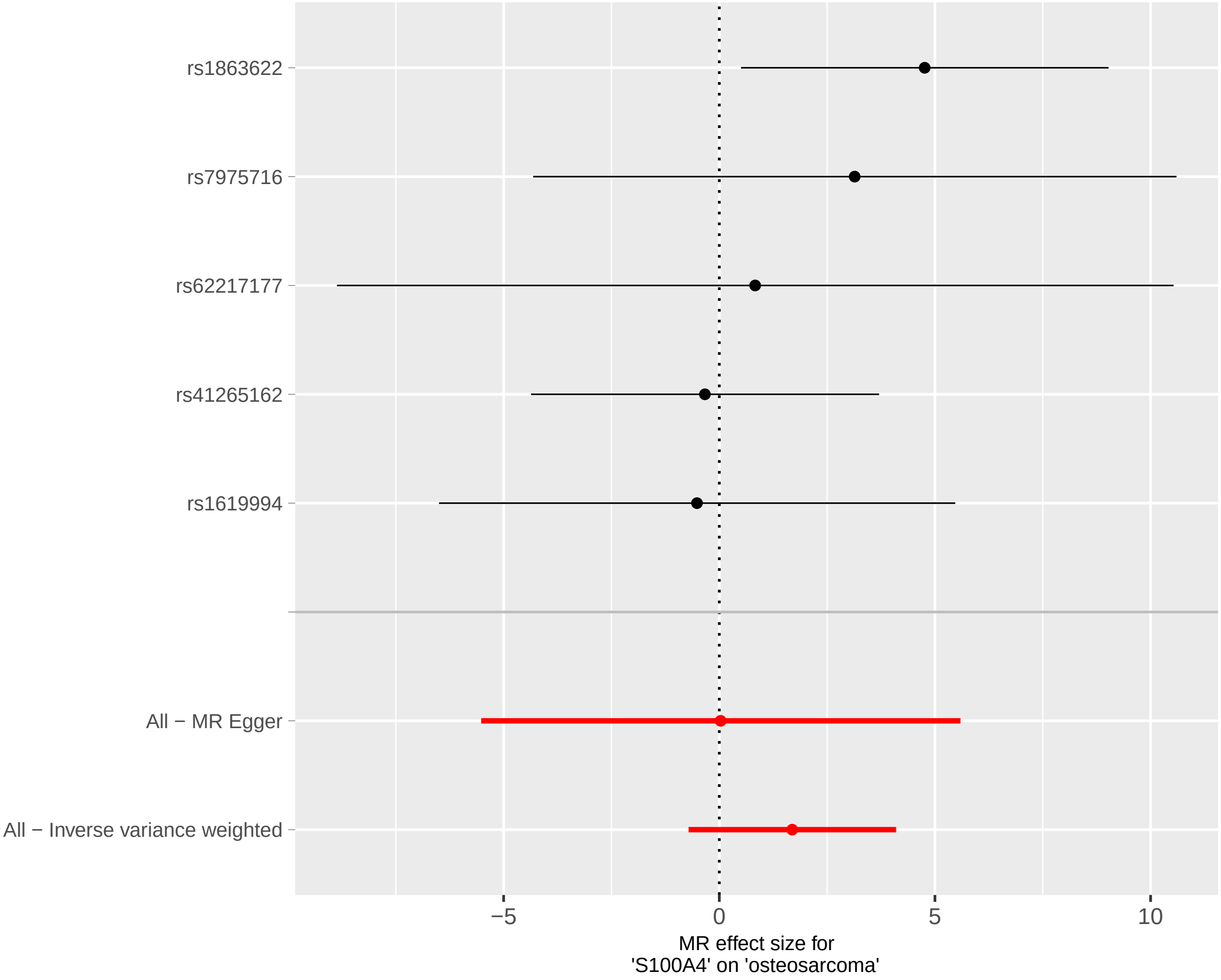
rs1863622
rs7975716
rs62217177
rs41265162
rs1619994
All − MR Egger
All − Inverse variance weighted
−5
0
5
10
MR effect size for
'S100A4' on 'osteosarcoma'

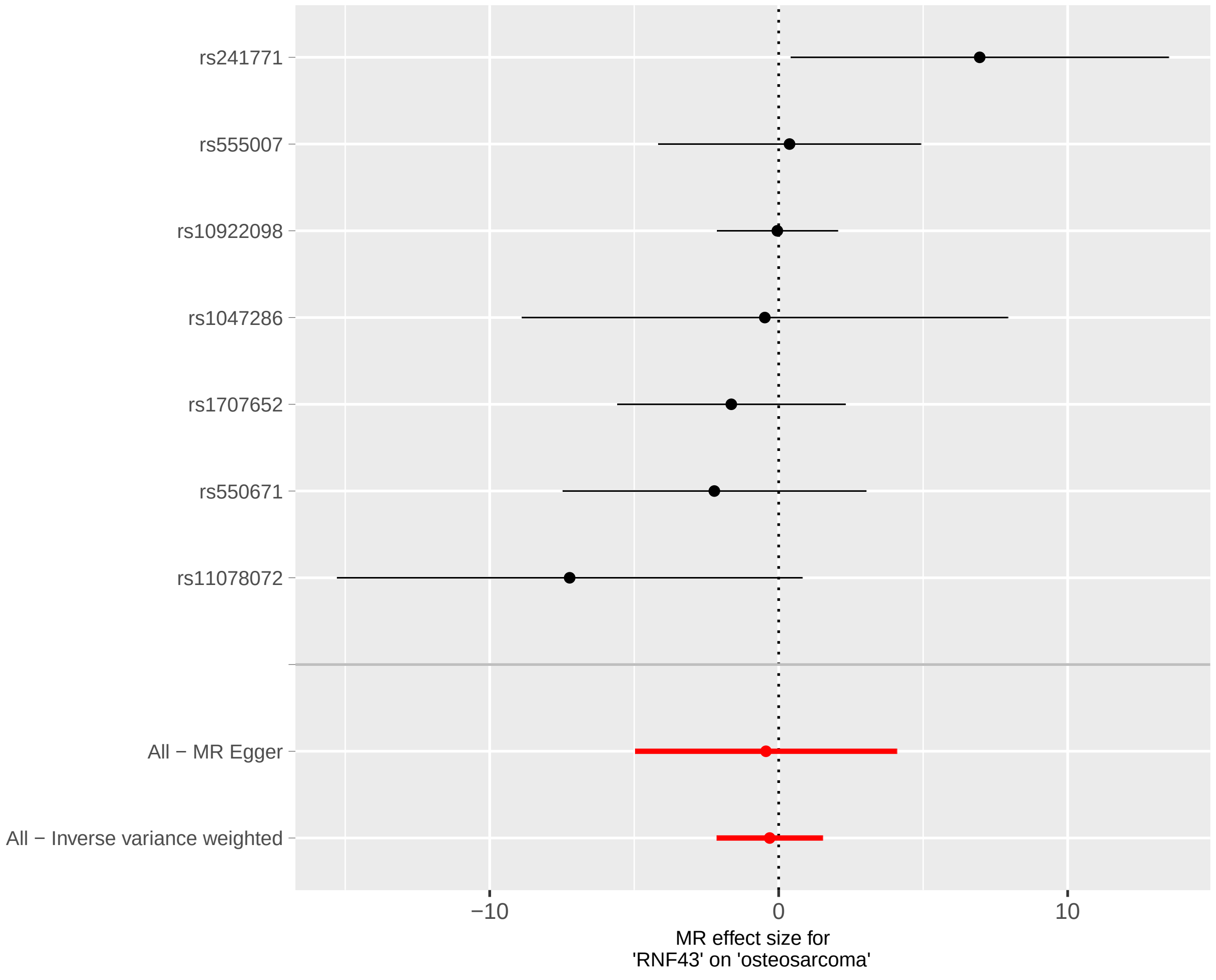
rs241771
rs555007
rs10922098
rs1047286
rs1707652
rs550671
rs11078072
All − MR Egger
All − Inverse variance weighted
−10
0
10
MR effect size for
'RNF43' on 'osteosarcoma'

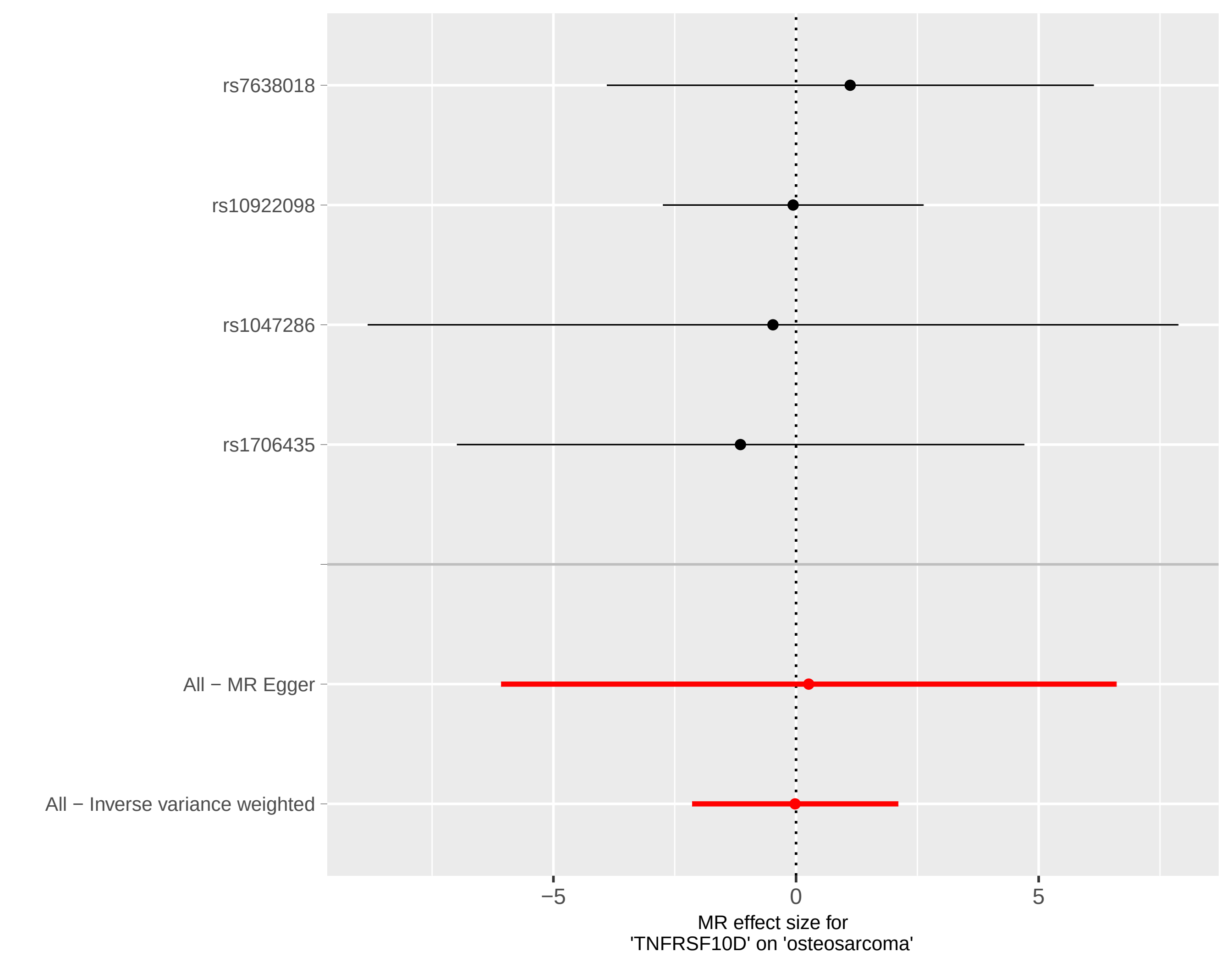
rs7638018
rs10922098
rs1047286
rs1706435
All − MR Egger
All − Inverse variance weighted
−5
0
5
MR effect size for
'TNFRSF10D' on 'osteosarcoma'

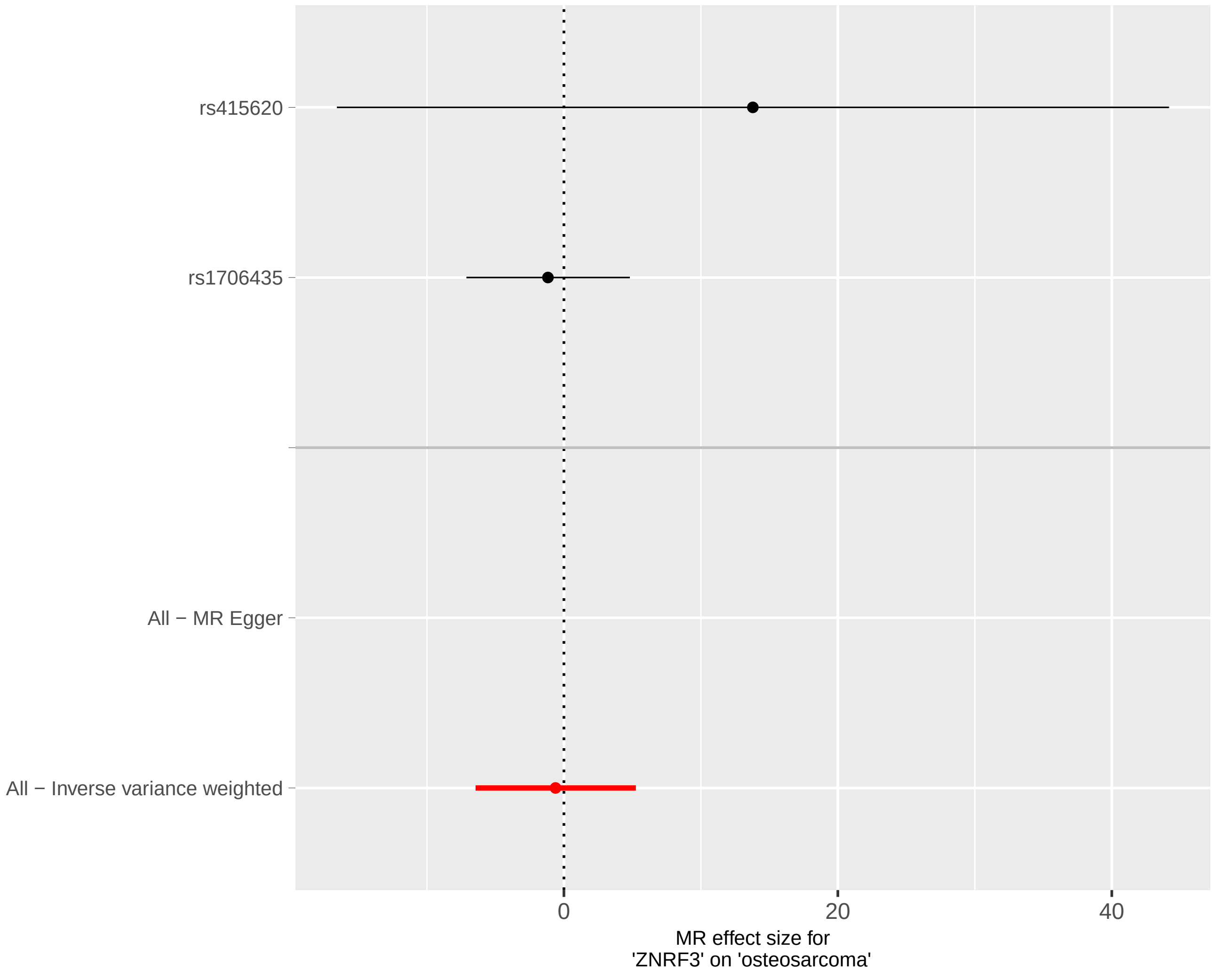
rs415620
rs1706435
All − MR Egger
All − Inverse variance weighted
0
20
40
MR effect size for
'ZNRF3' on 'osteosarcoma'

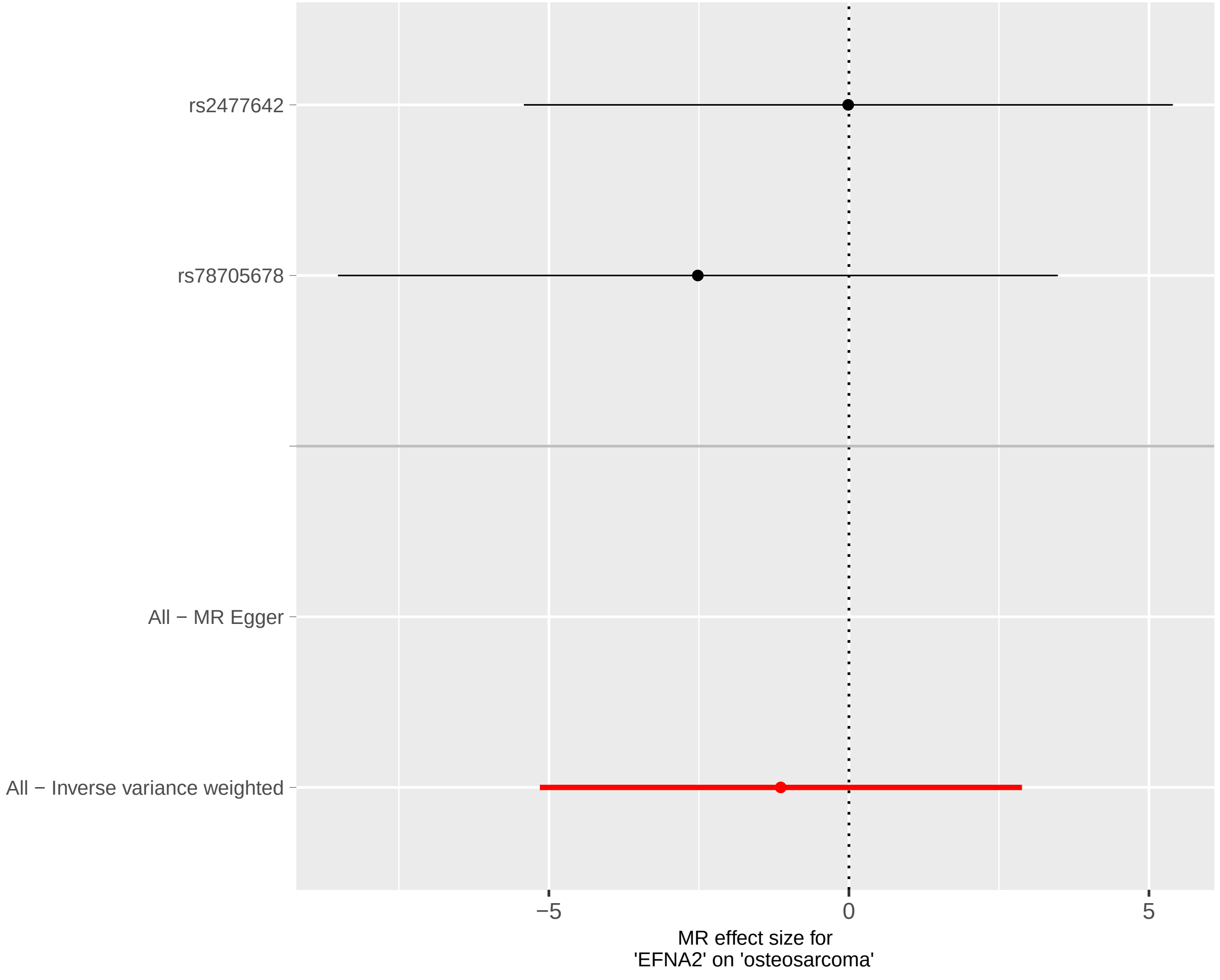
rs2477642
rs78705678
All − MR Egger
All − Inverse variance weighted
−5
0
5
MR effect size for
'EFNA2' on 'osteosarcoma'

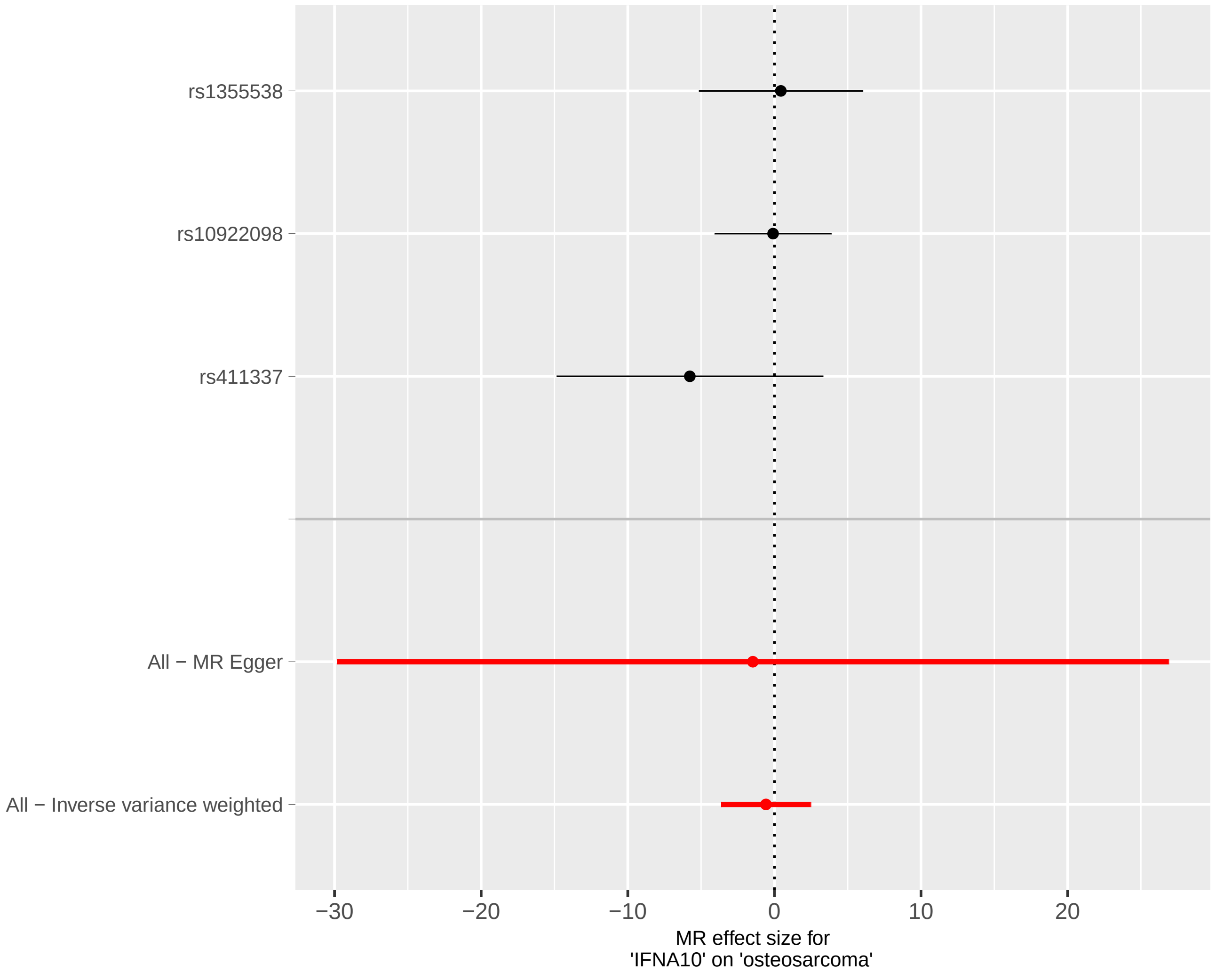

rs1355538
rs10922098
rs411337
All − MR Egger
All − Inverse variance weighted
−30
−20
−10
0
10
20
MR effect size for
'IFNA10' on 'osteosarcoma'

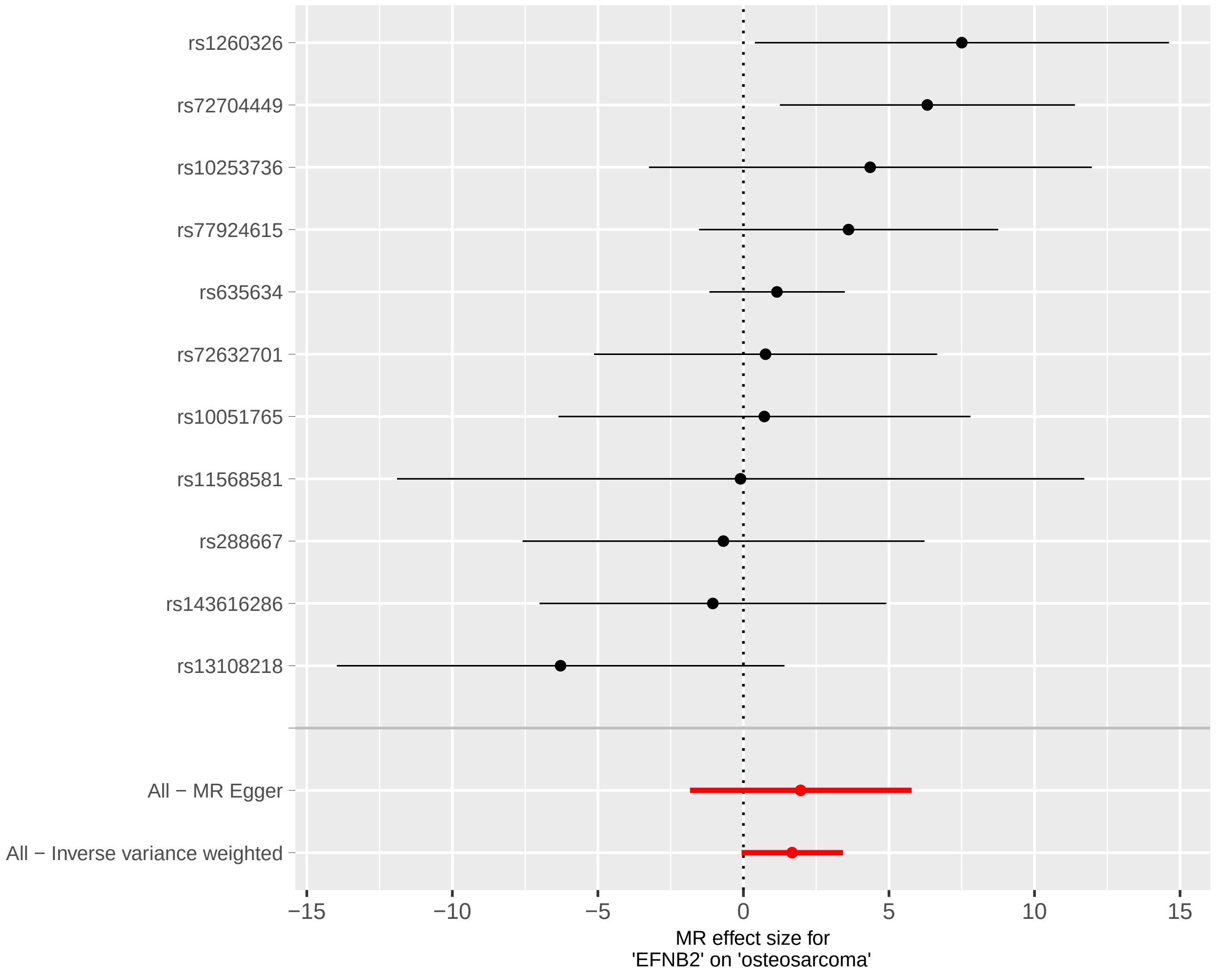

rs1260326
rs72704449
rs10253736
rs77924615
rs635634
rs72632701
rs10051765
rs11568581
rs288667
rs143616286
rs13108218
All − MR Egger
All − Inverse variance weighted
−15
−10
−5
0
5
10
15
MR effect size for
'EFNB2' on 'osteosarcoma'

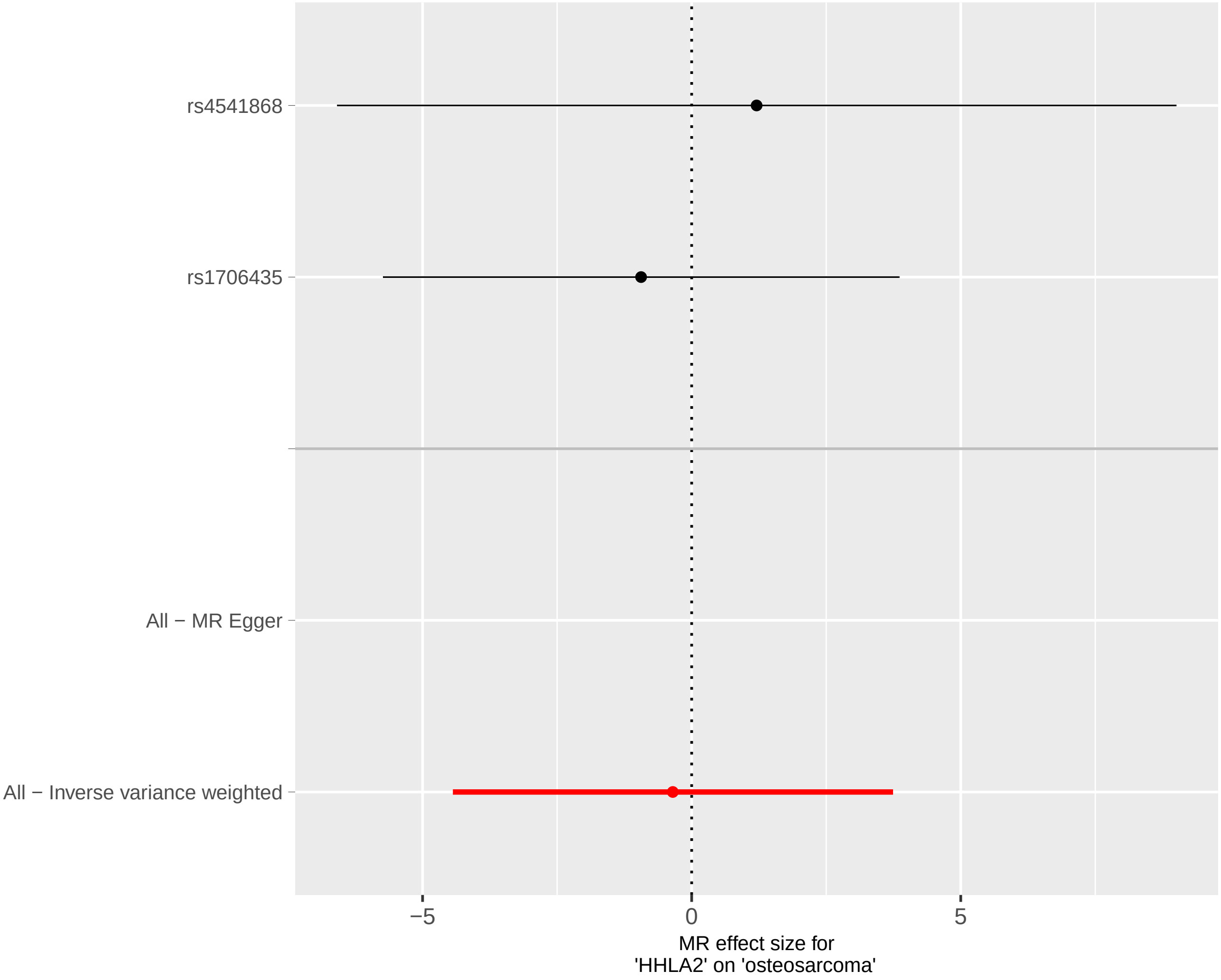

rs4541868
rs1706435
All − MR Egger
All − Inverse variance weighted
−5
0
5
MR effect size for
'HHLA2' on 'osteosarcoma'

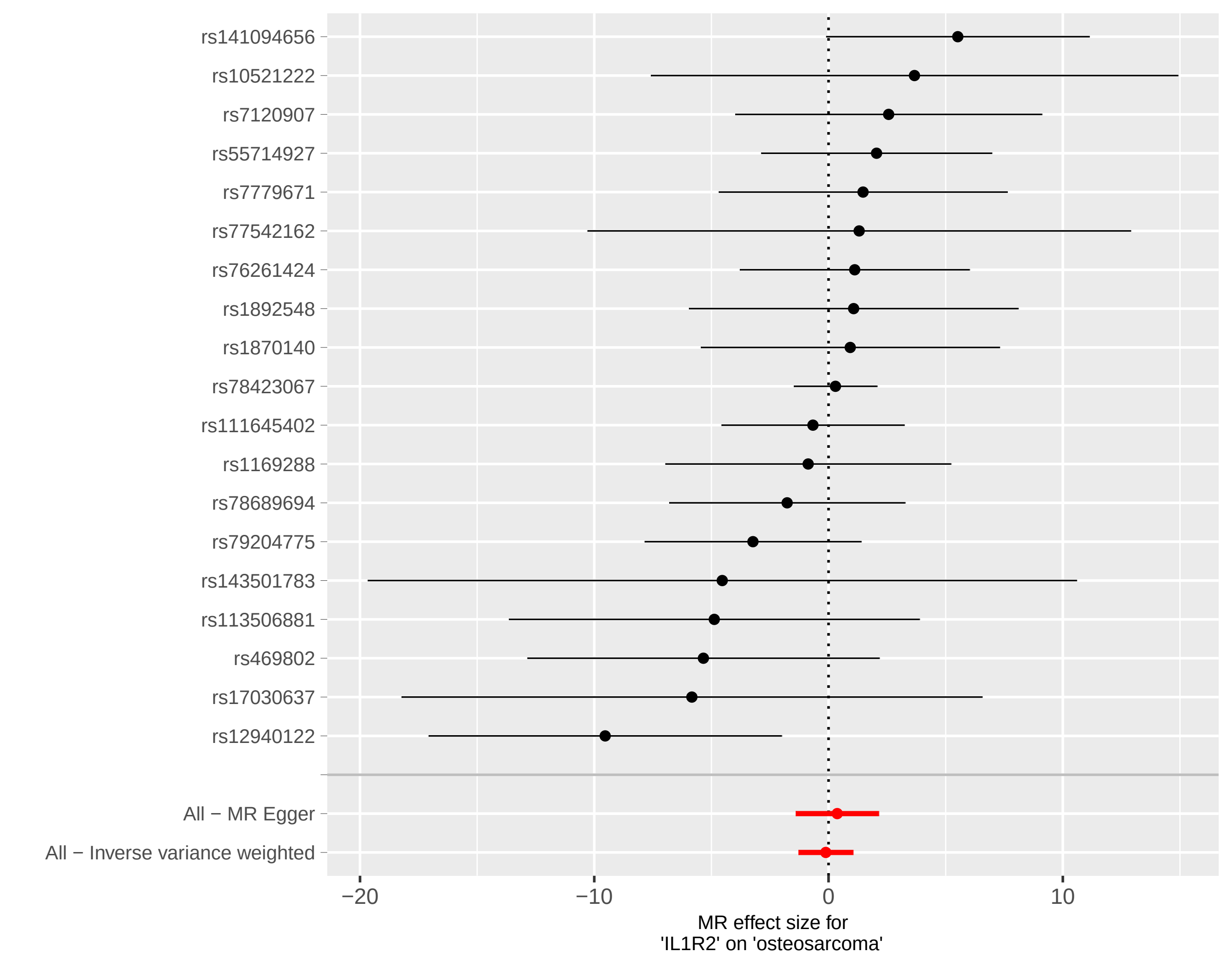

rs141094656
rs10521222
rs7120907
rs55714927
rs7779671
rs77542162
rs76261424
rs1892548
rs1870140
rs78423067
rs111645402
rs1169288
rs78689694
rs79204775
rs143501783
rs113506881
rs469802
rs17030637
rs12940122
All − MR Egger
All − Inverse variance weighted
−20
−10
0
10
MR effect size for
'IL1R2' on 'osteosarcoma'

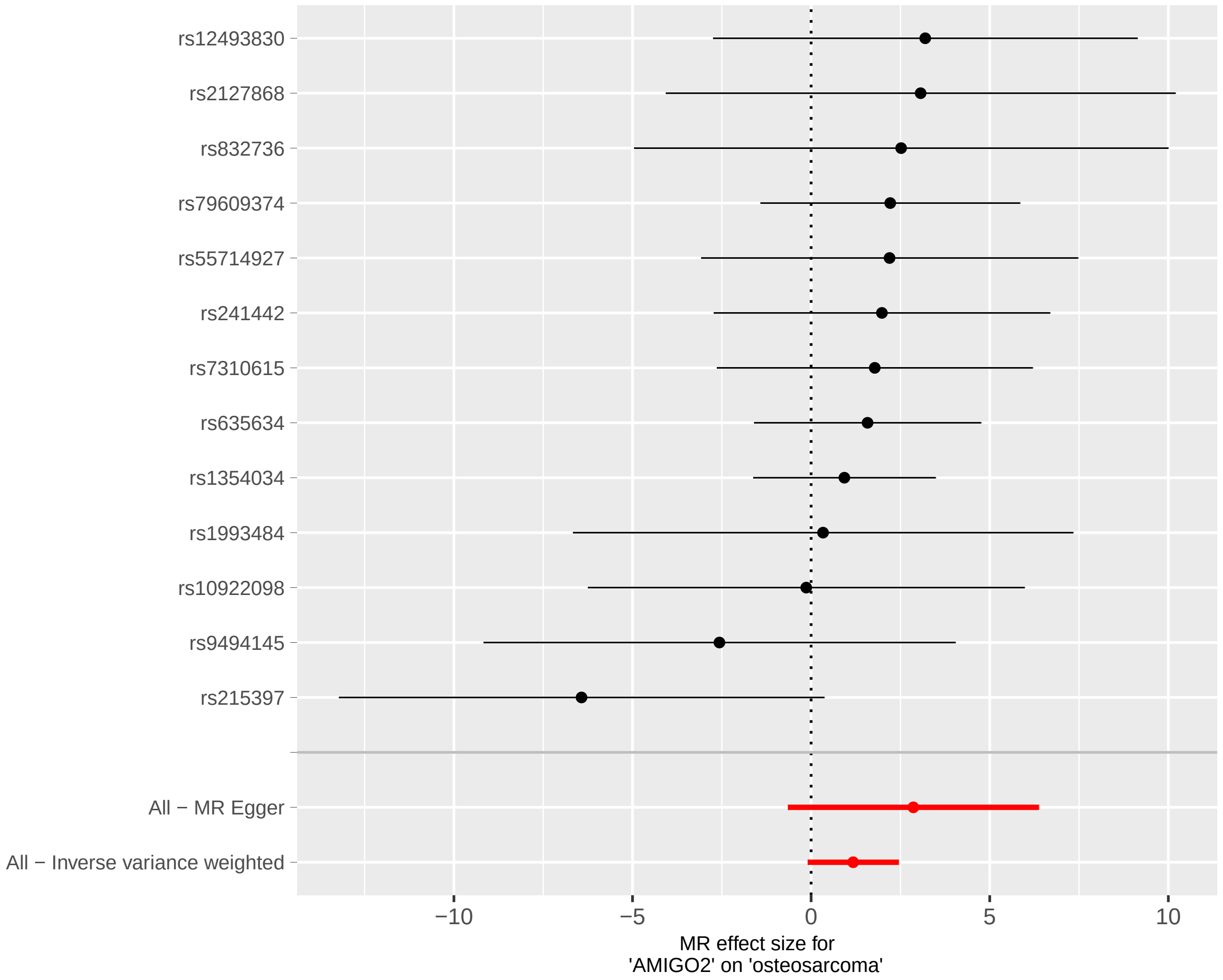

rs12493830
rs2127868
rs832736
rs79609374
rs55714927
rs241442
rs7310615
rs635634
rs1354034
rs1993484
rs10922098
rs9494145
rs215397
All − MR Egger
All − Inverse variance weighted
−10
−5
0
5
10
MR effect size for
'AMIGO2' on 'osteosarcoma'

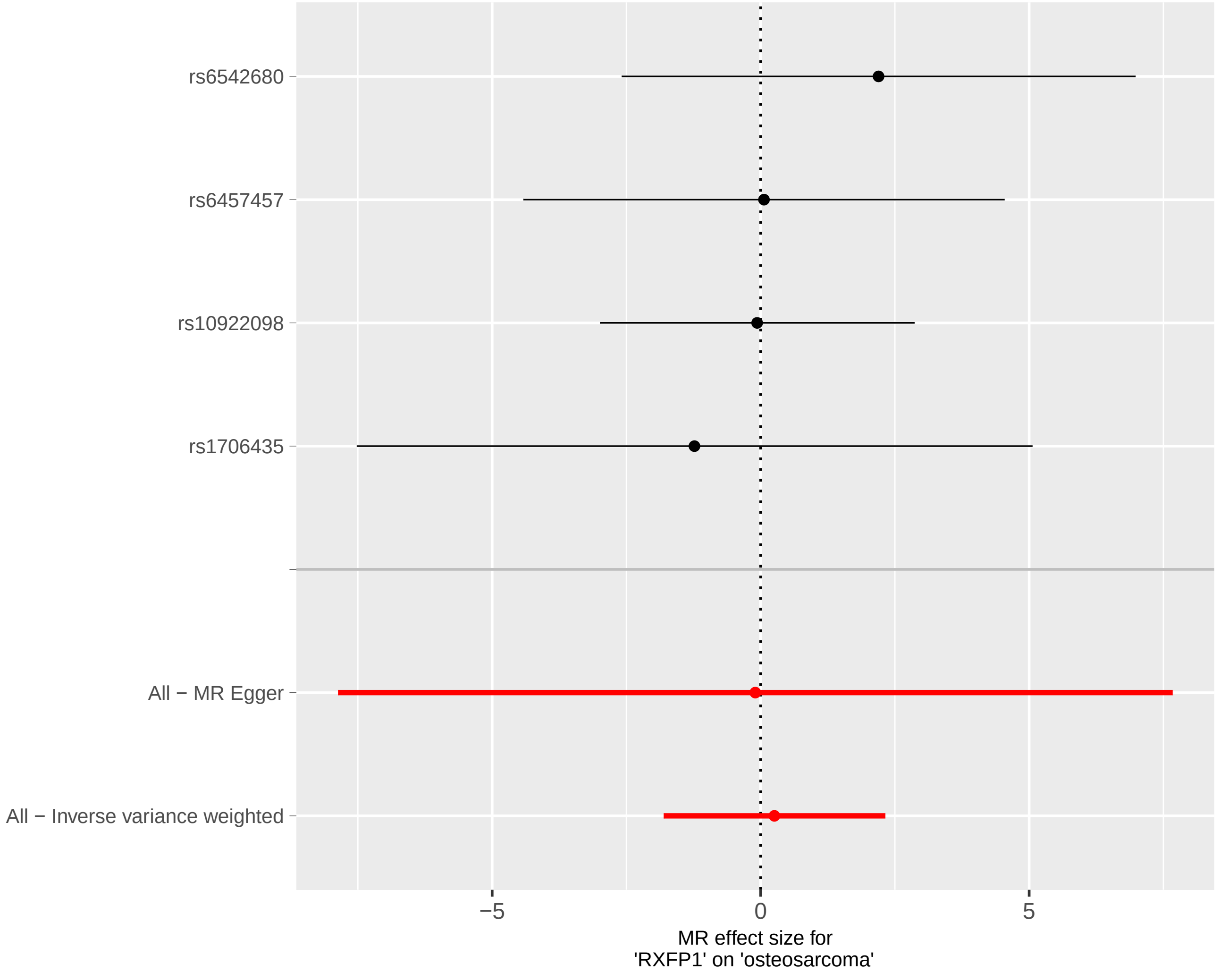
rs6542680
rs6457457
rs10922098
rs1706435
All − MR Egger
All − Inverse variance weighted
−5
0
5
MR effect size for
'RXFP1' on 'osteosarcoma'

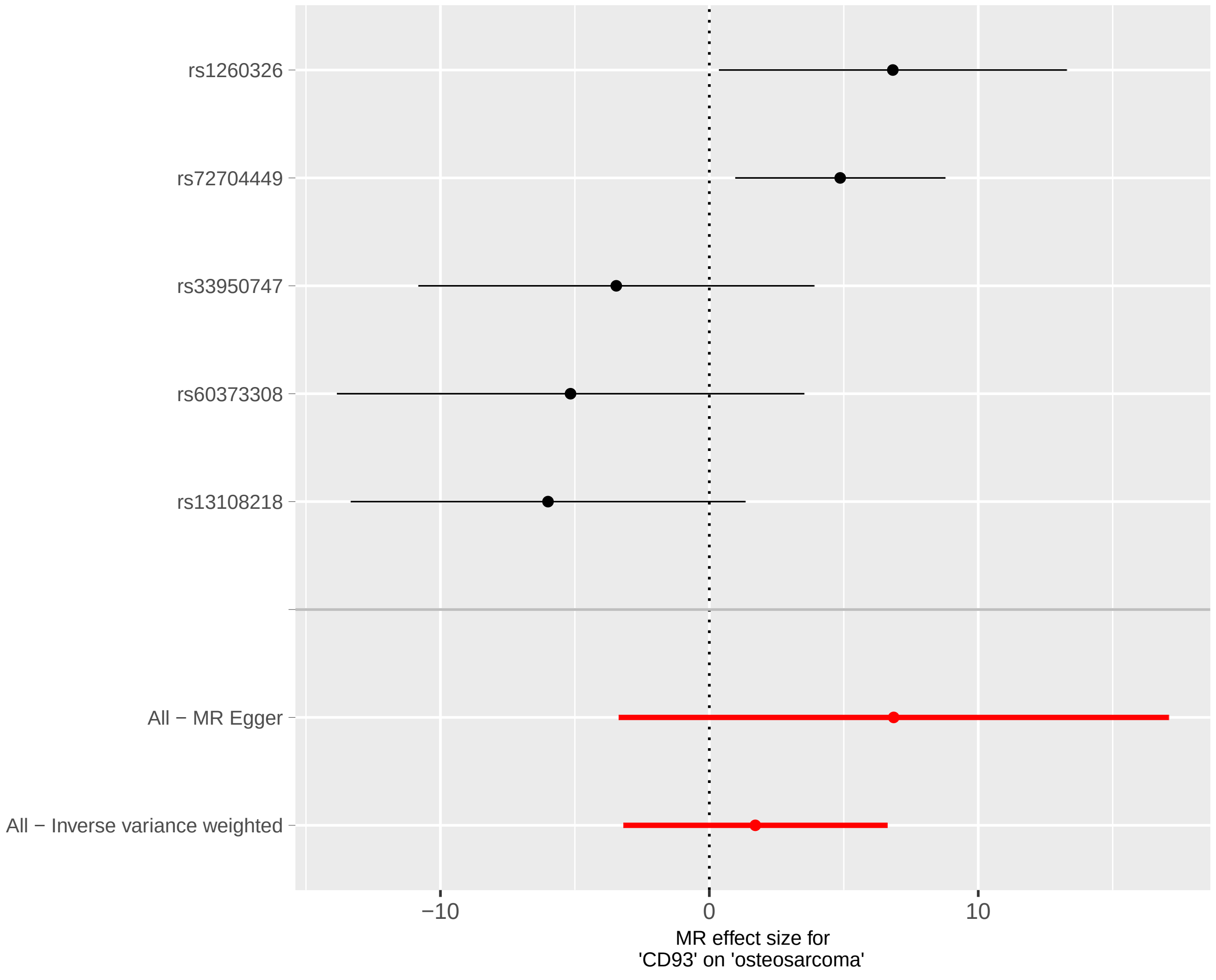

rs1260326
rs72704449
rs33950747
rs60373308
rs13108218
All − MR Egger
All − Inverse variance weighted
−10
0
10
MR effect size for
'CD93' on 'osteosarcoma'

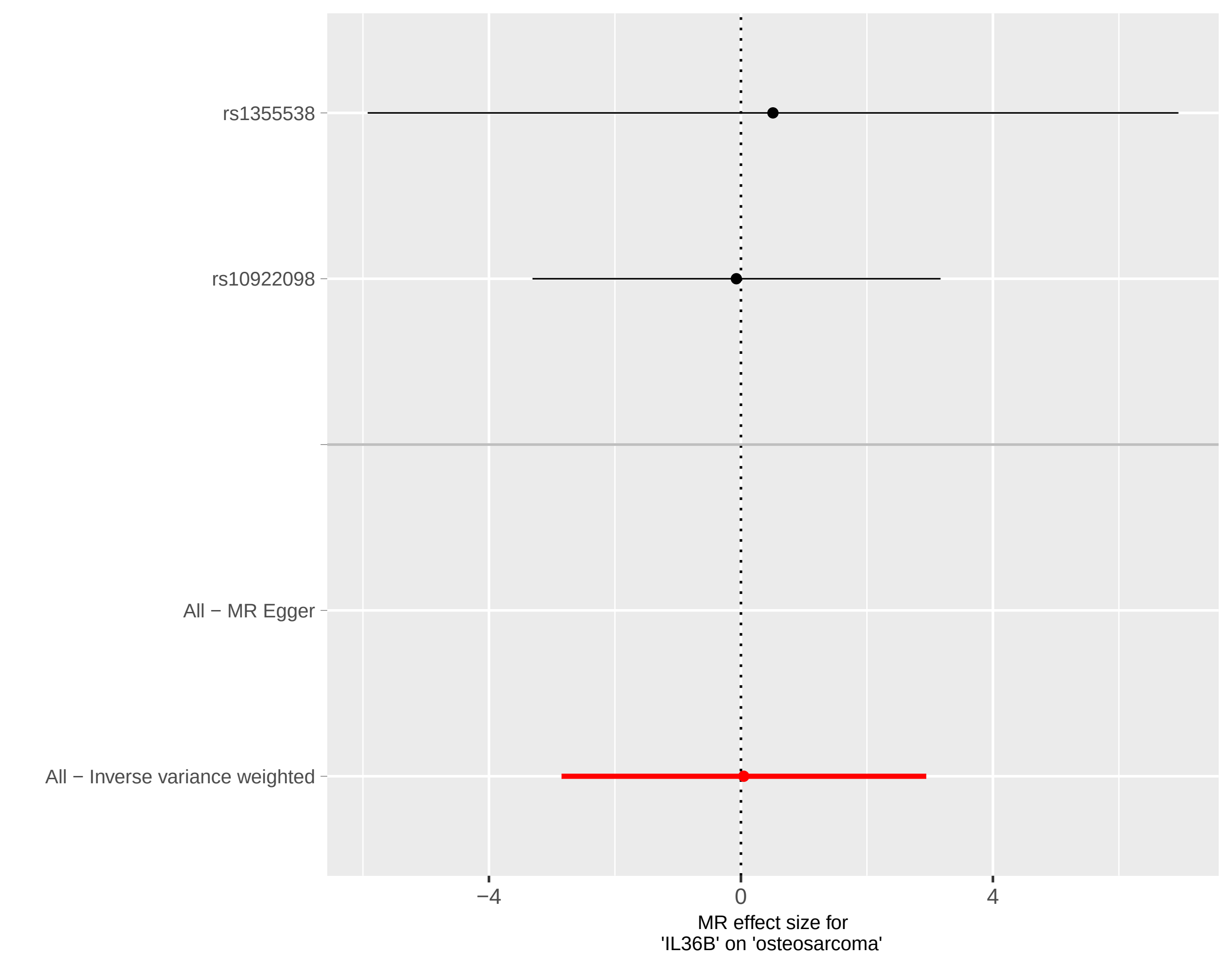
rs1355538
rs10922098
All − MR Egger
All − Inverse variance weighted
−4
0
4
MR effect size for
'IL36B' on 'osteosarcoma'

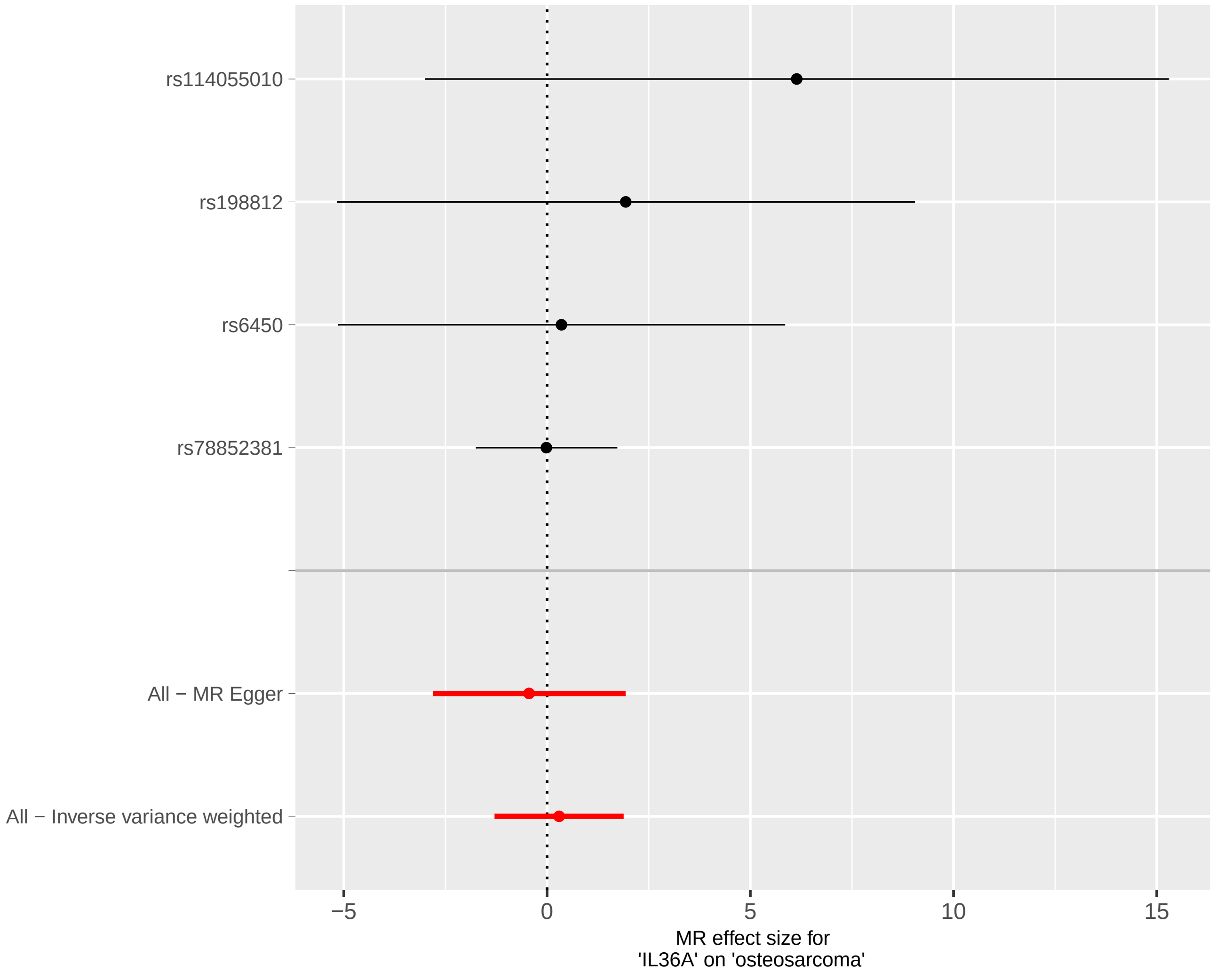
rs114055010
rs198812
rs6450
rs78852381
All − MR Egger
All − Inverse variance weighted
−5
0
5
10
15
MR effect size for
'IL36A' on 'osteosarcoma'

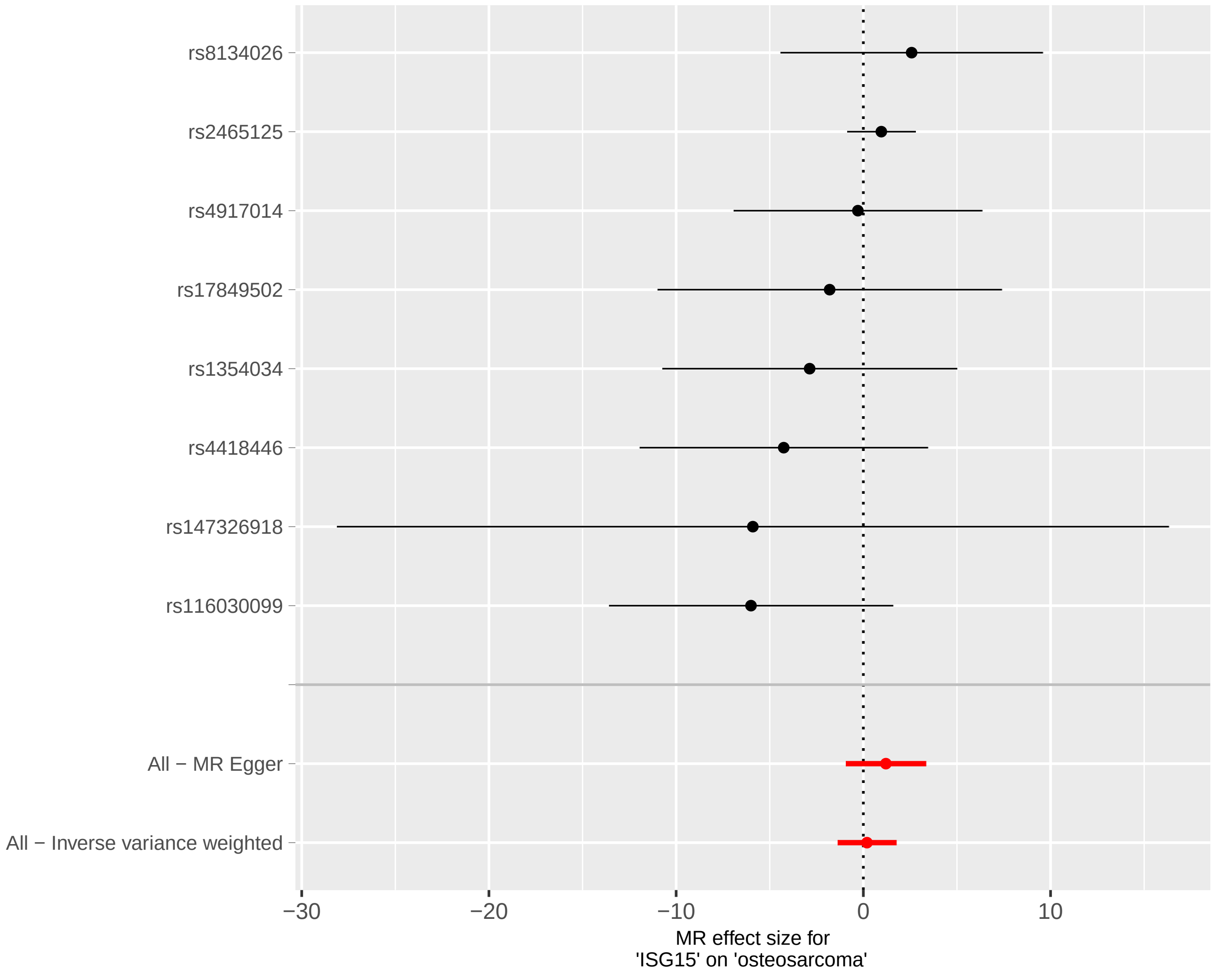

rs8134026
rs2465125
rs4917014
rs17849502
rs1354034
rs4418446
rs147326918
rs116030099
All − MR Egger
All − Inverse variance weighted
−30
−20
−10
0
10
MR effect size for
'ISG15' on 'osteosarcoma'

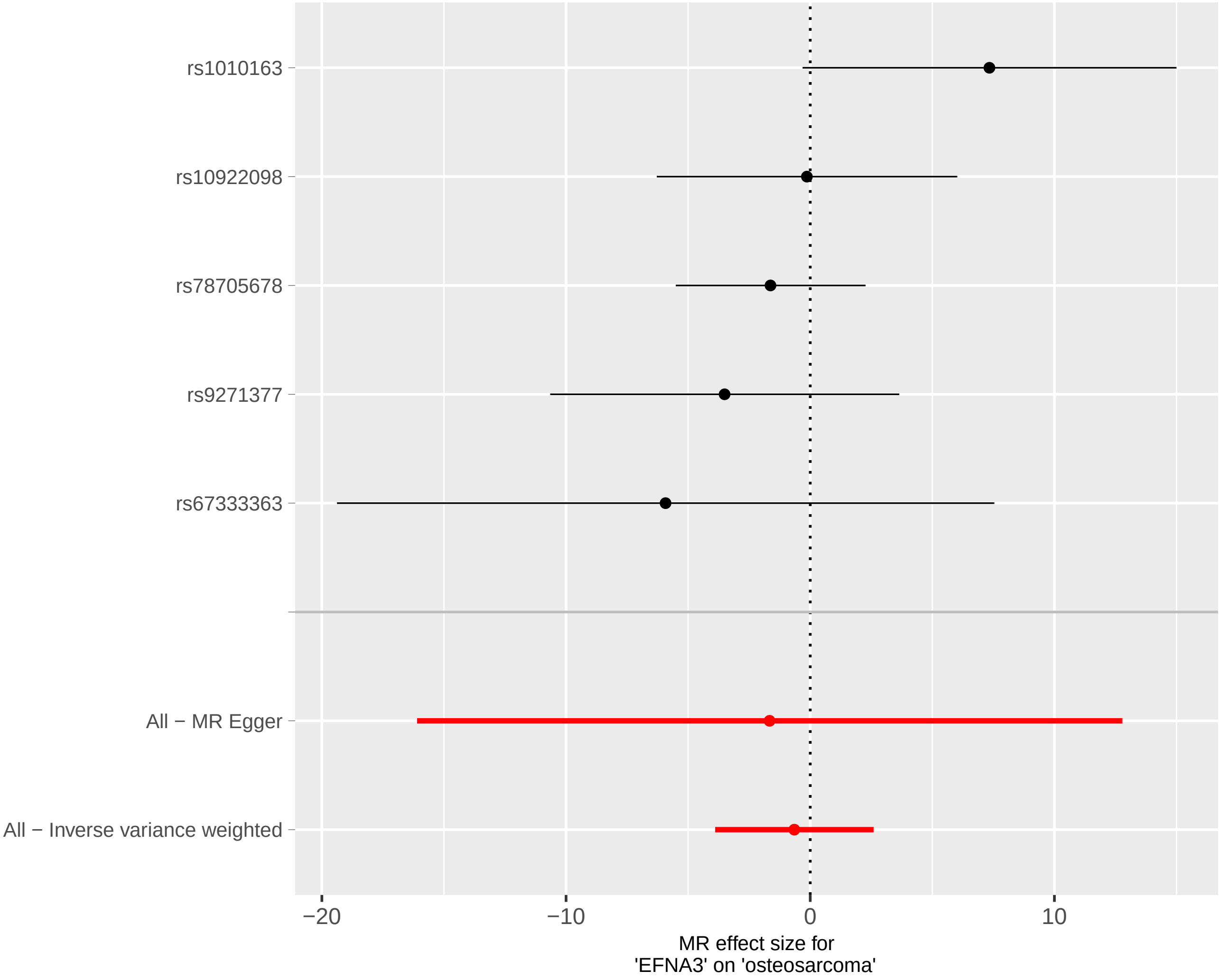
rs1010163
rs10922098
rs78705678
rs9271377
rs67333363
All − MR Egger
All − Inverse variance weighted
−20
−10
0
10
MR effect size for
'EFNA3' on 'osteosarcoma'

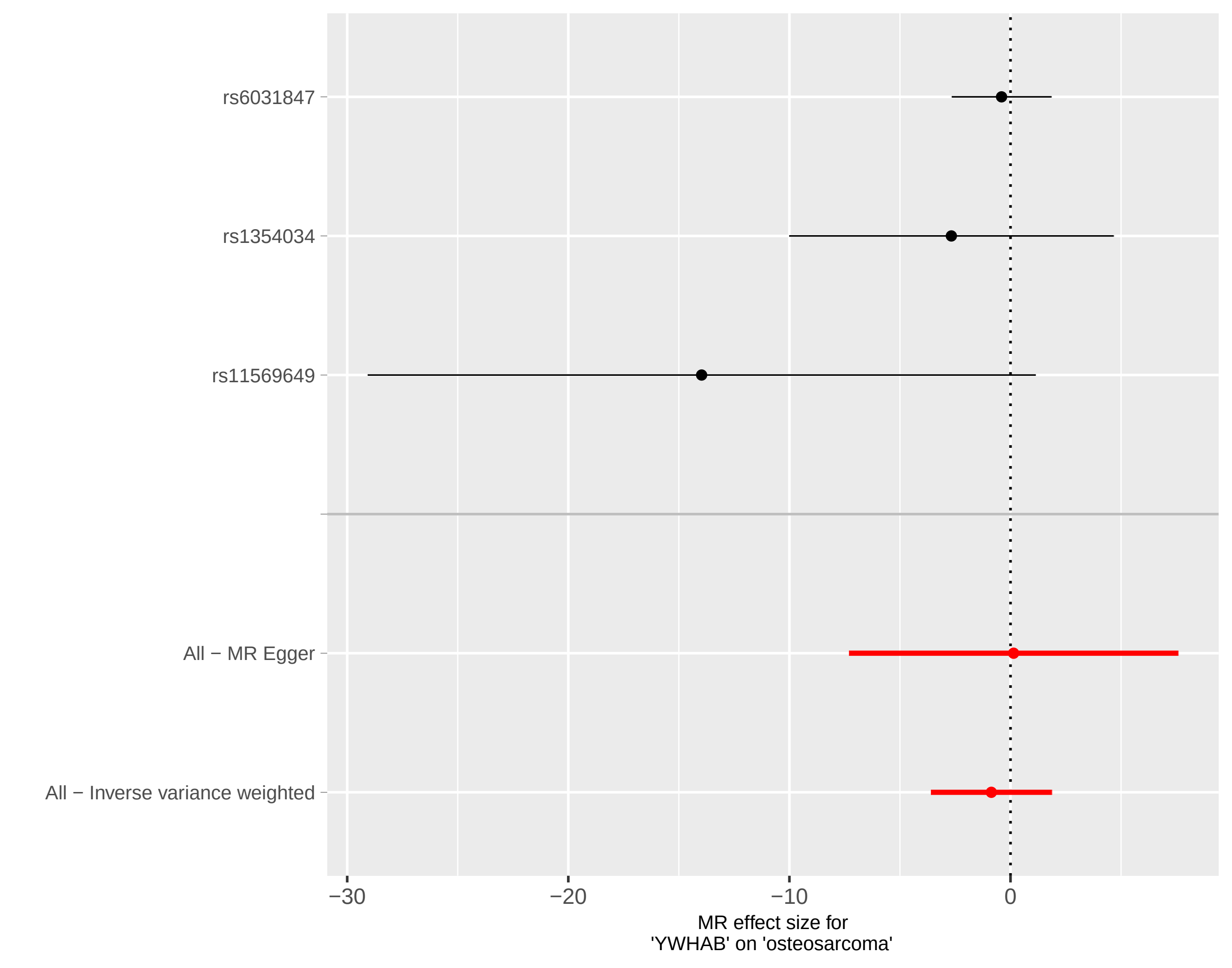

rs6031847
rs1354034
rs11569649
All − MR Egger
All − Inverse variance weighted
−30
−20
−10
0
MR effect size for
'YWHAB' on 'osteosarcoma'

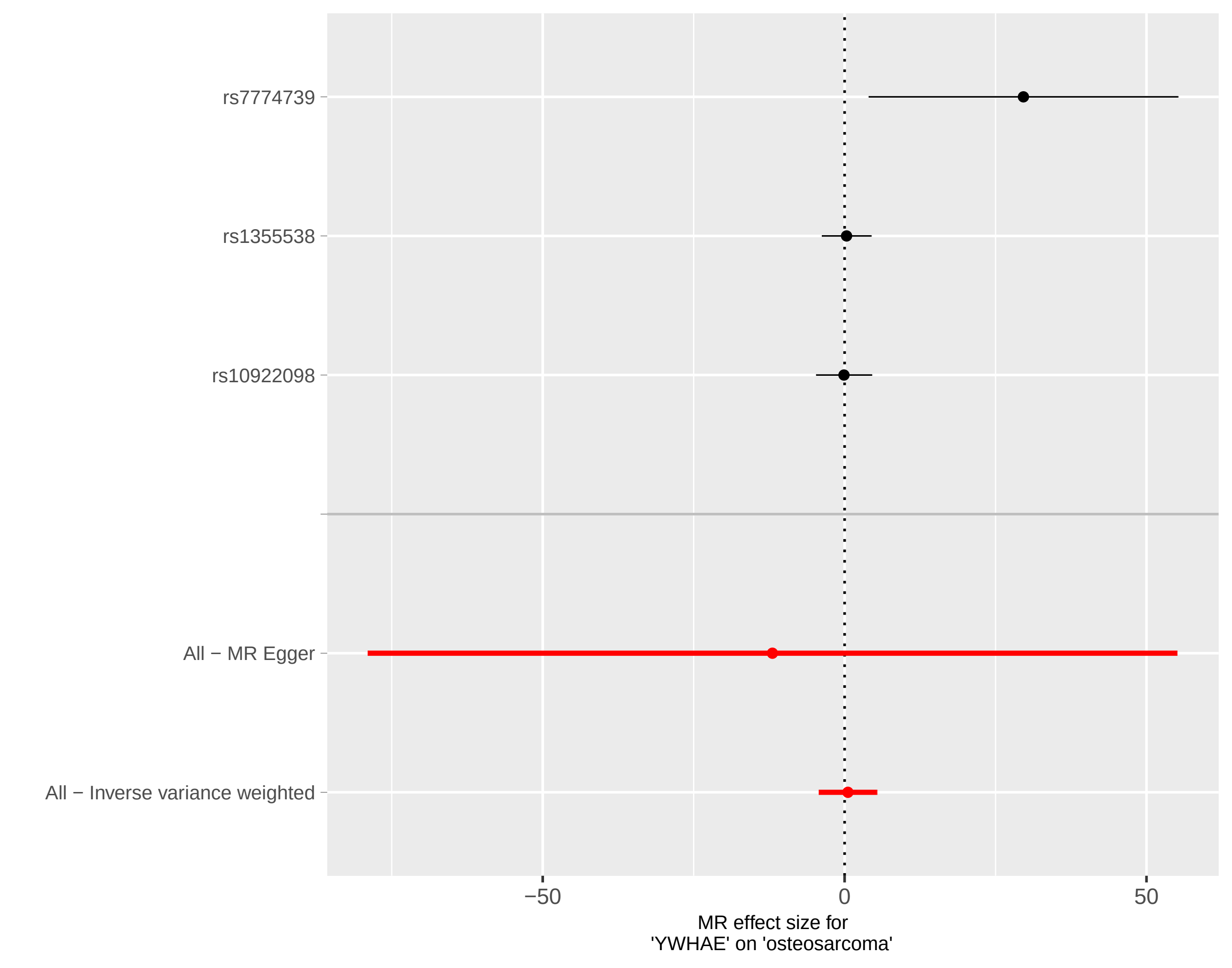
rs7774739
rs1355538
rs10922098
All − MR Egger
All − Inverse variance weighted
−50
0
50
MR effect size for
'YWHAE' on 'osteosarcoma'

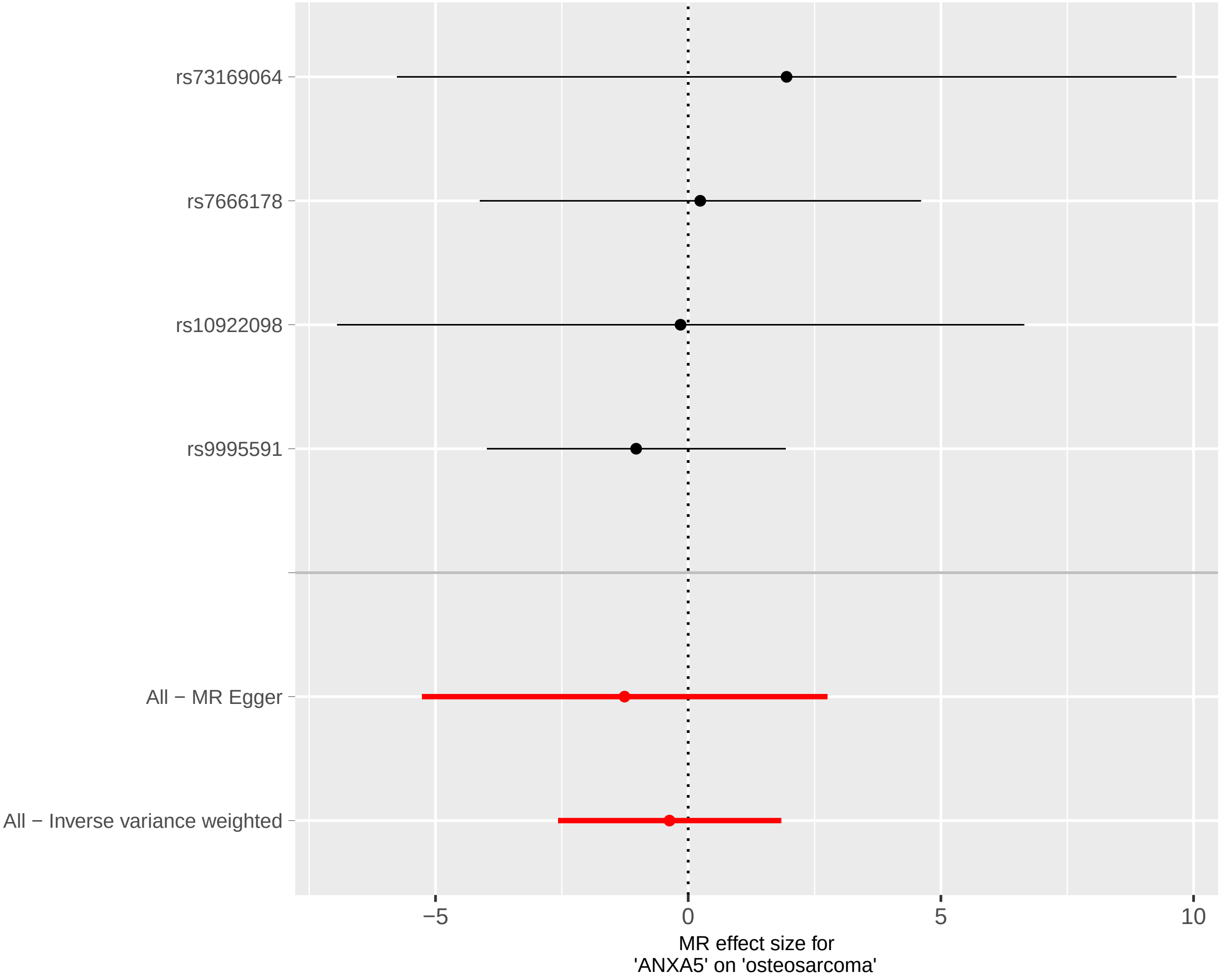
rs73169064
rs7666178
rs10922098
rs9995591
All − MR Egger
All − Inverse variance weighted
−5
0
5
10
MR effect size for
'ANXA5' on 'osteosarcoma'

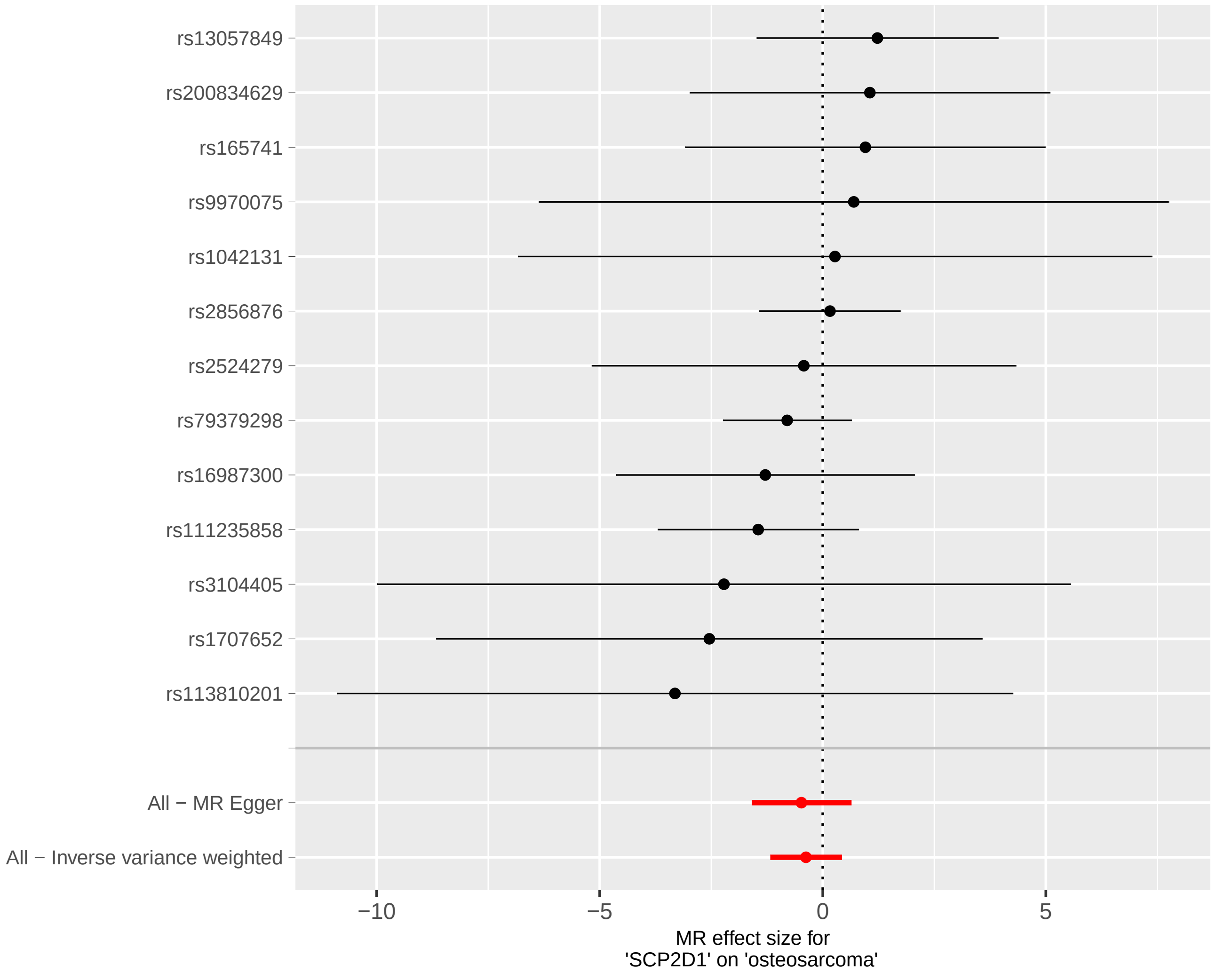
rs13057849
rs200834629
rs165741
rs9970075
rs1042131
rs2856876
rs2524279
rs79379298
rs16987300
rs111235858
rs3104405
rs1707652
rs113810201
All − MR Egger
All − Inverse variance weighted
−10
−5
0
5
MR effect size for
'SCP2D1' on 'osteosarcoma'

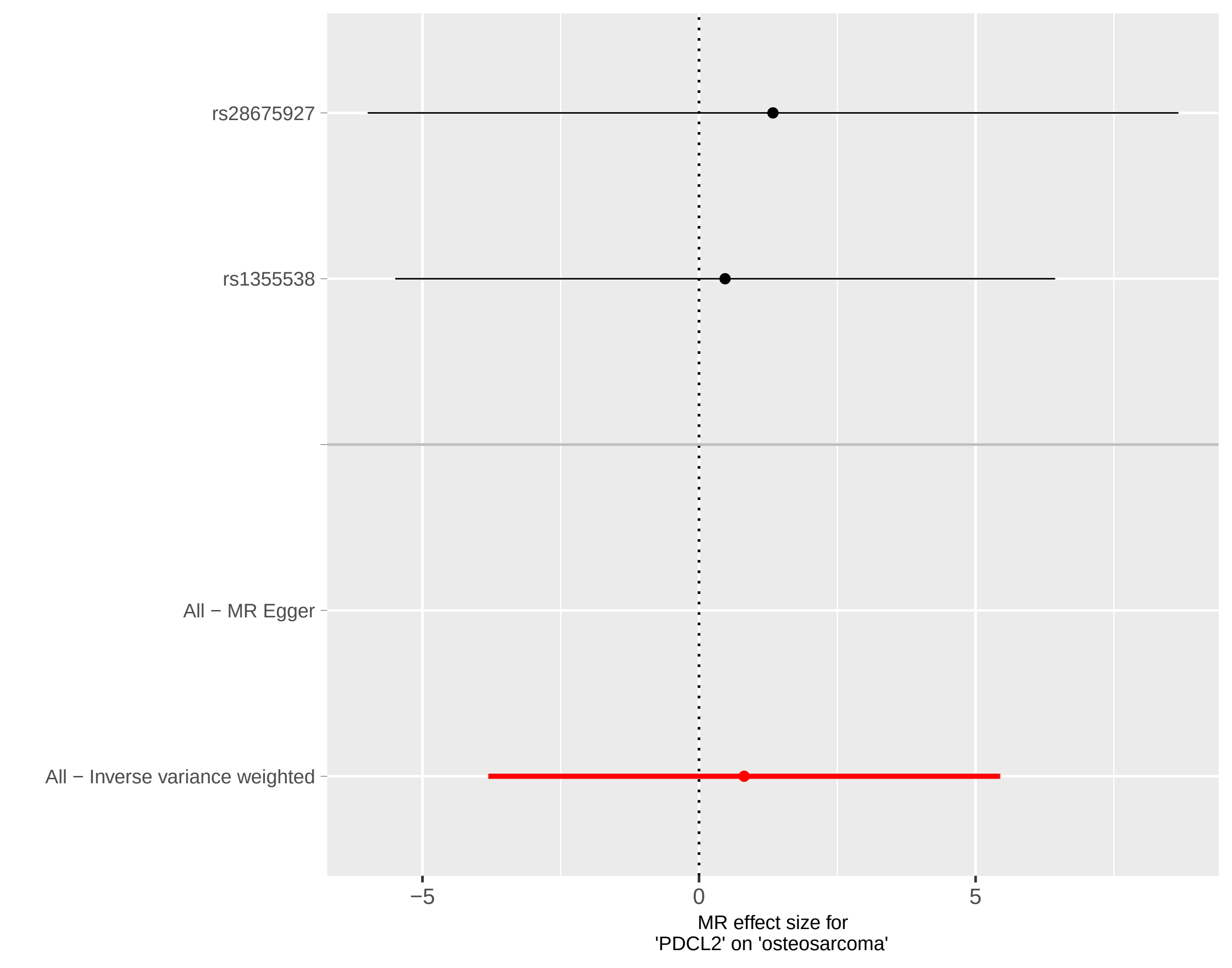
rs28675927
rs1355538
All − MR Egger
All − Inverse variance weighted
−5
0
5
MR effect size for
'PDCL2' on 'osteosarcoma'

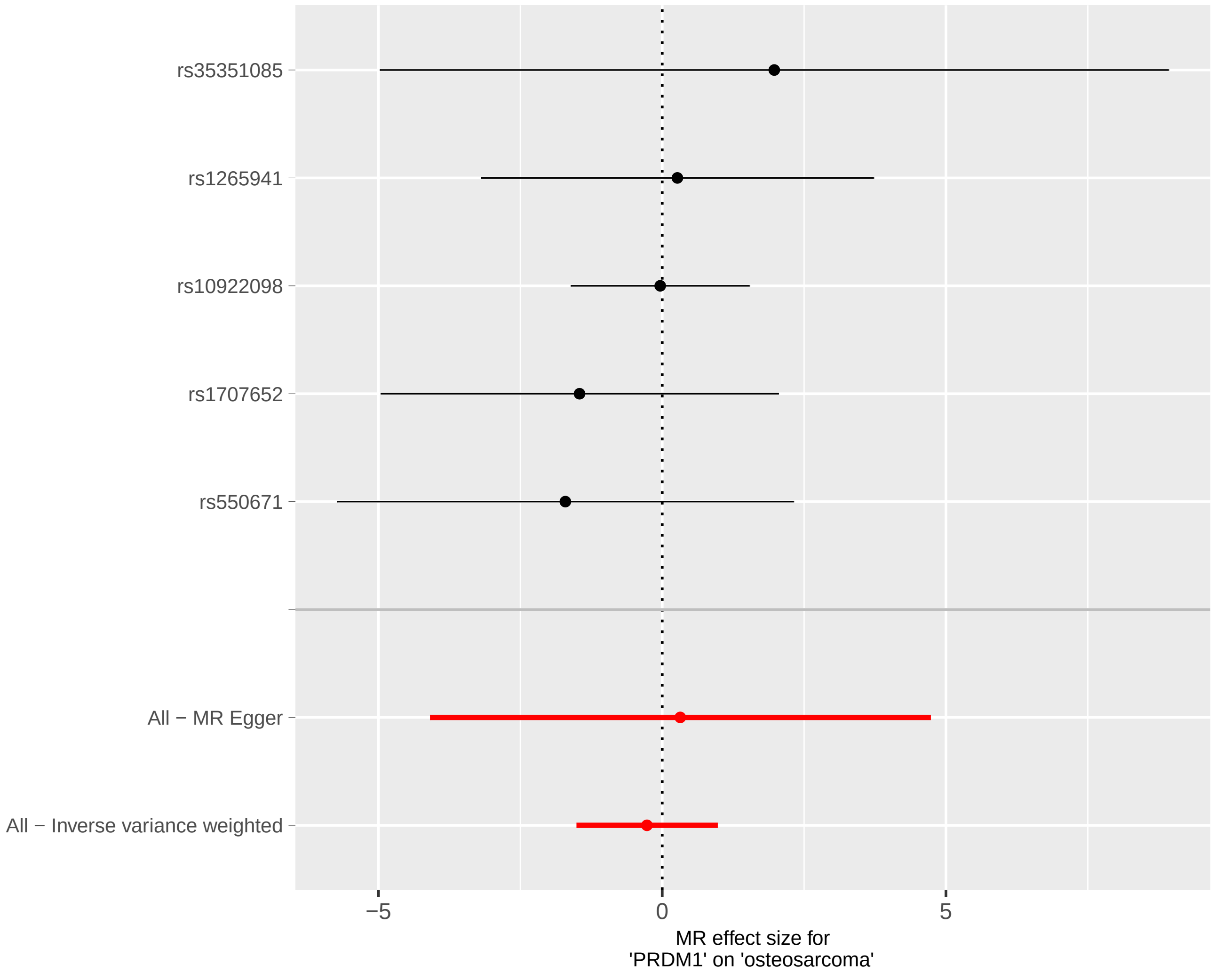
rs35351085
rs1265941
rs10922098
rs1707652
rs550671
All − MR Egger
All − Inverse variance weighted
−5
0
5
MR effect size for
'PRDM1' on 'osteosarcoma'

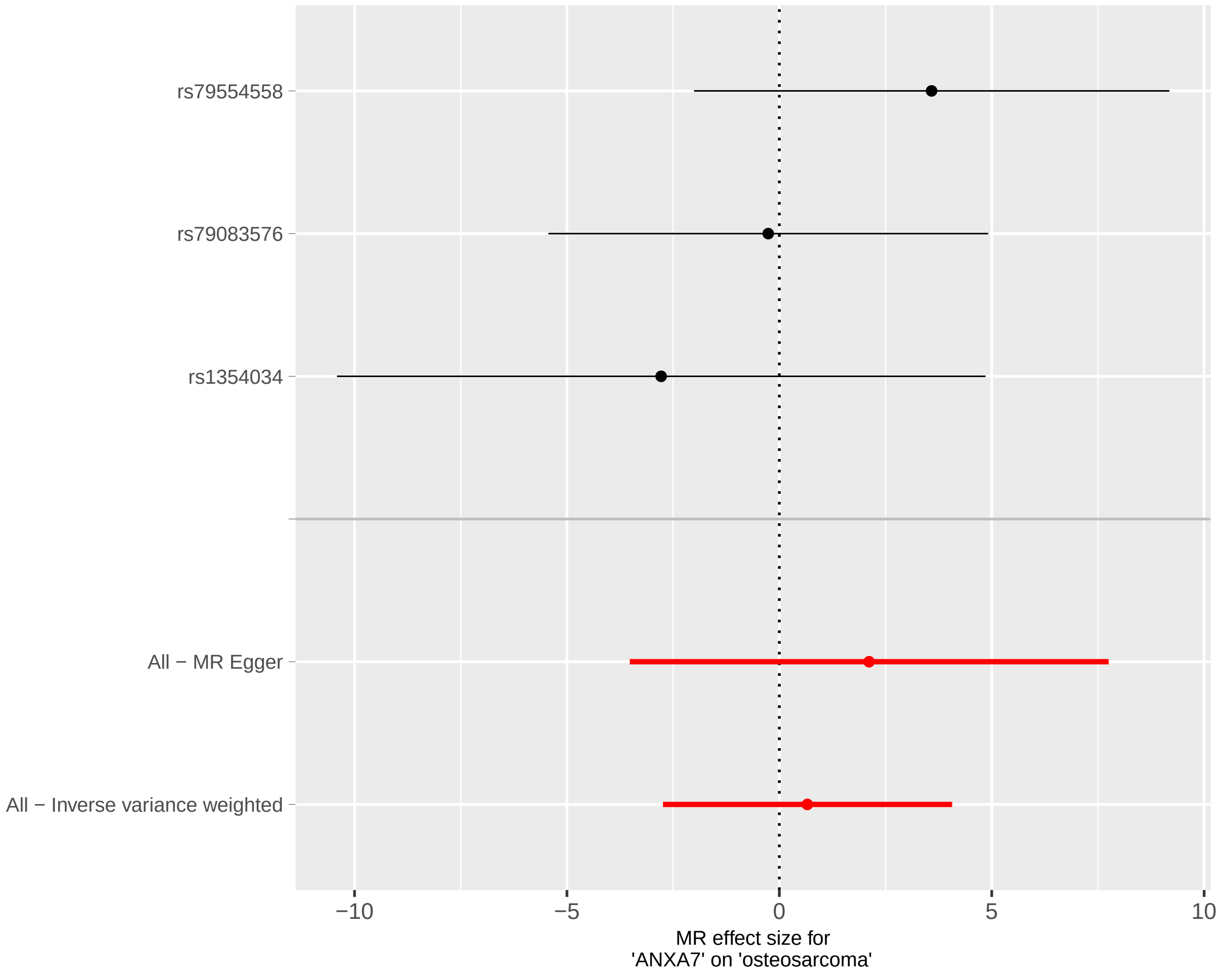
rs79554558
rs79083576
rs1354034
All − MR Egger
All − Inverse variance weighted
−10
−5
0
5
10
MR effect size for
'ANXA7' on 'osteosarcoma'

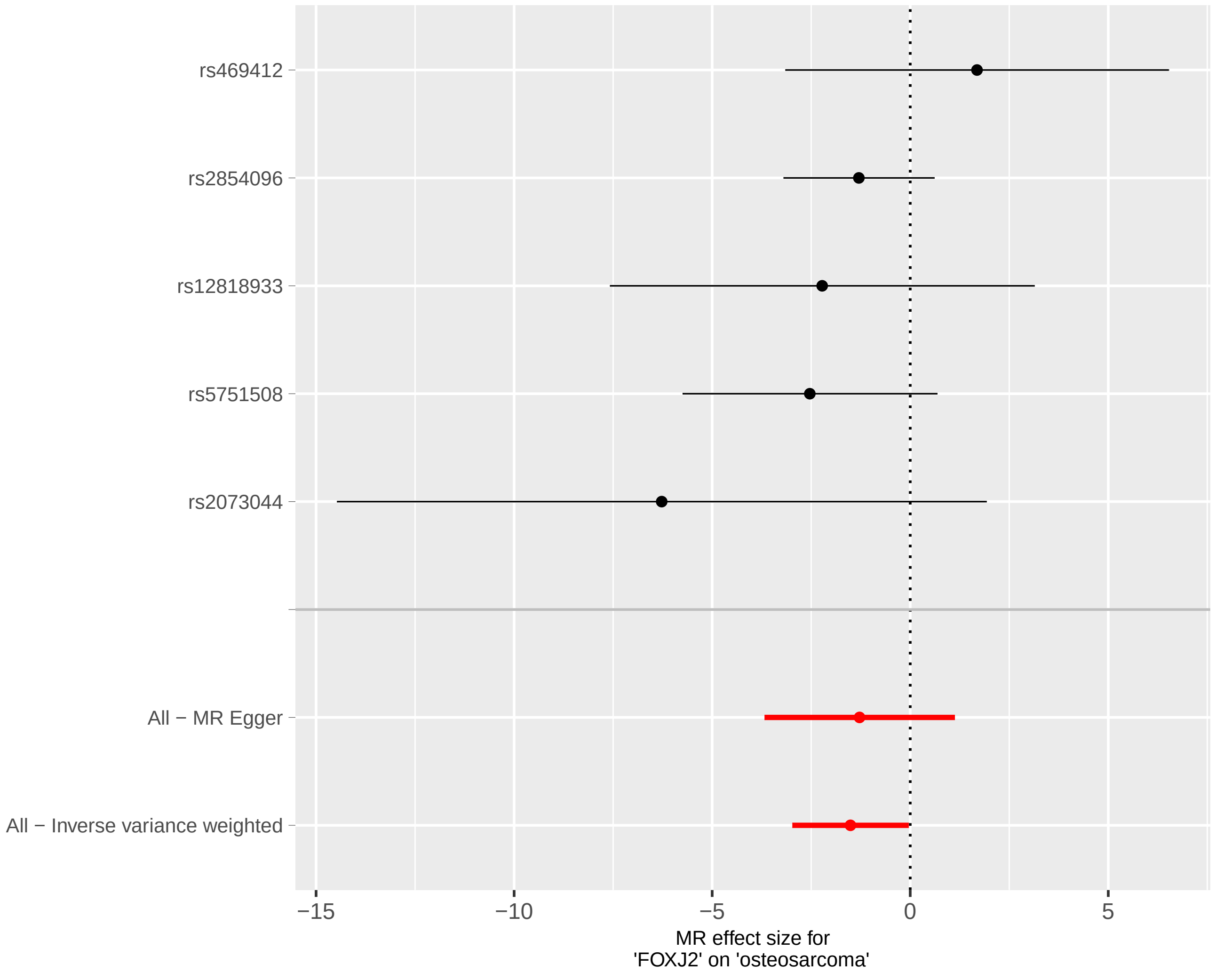
rs469412
rs2854096
rs12818933
rs5751508
rs2073044
All − MR Egger
All − Inverse variance weighted
−15
−10
−5
0
5
MR effect size for
'FOXJ2' on 'osteosarcoma'

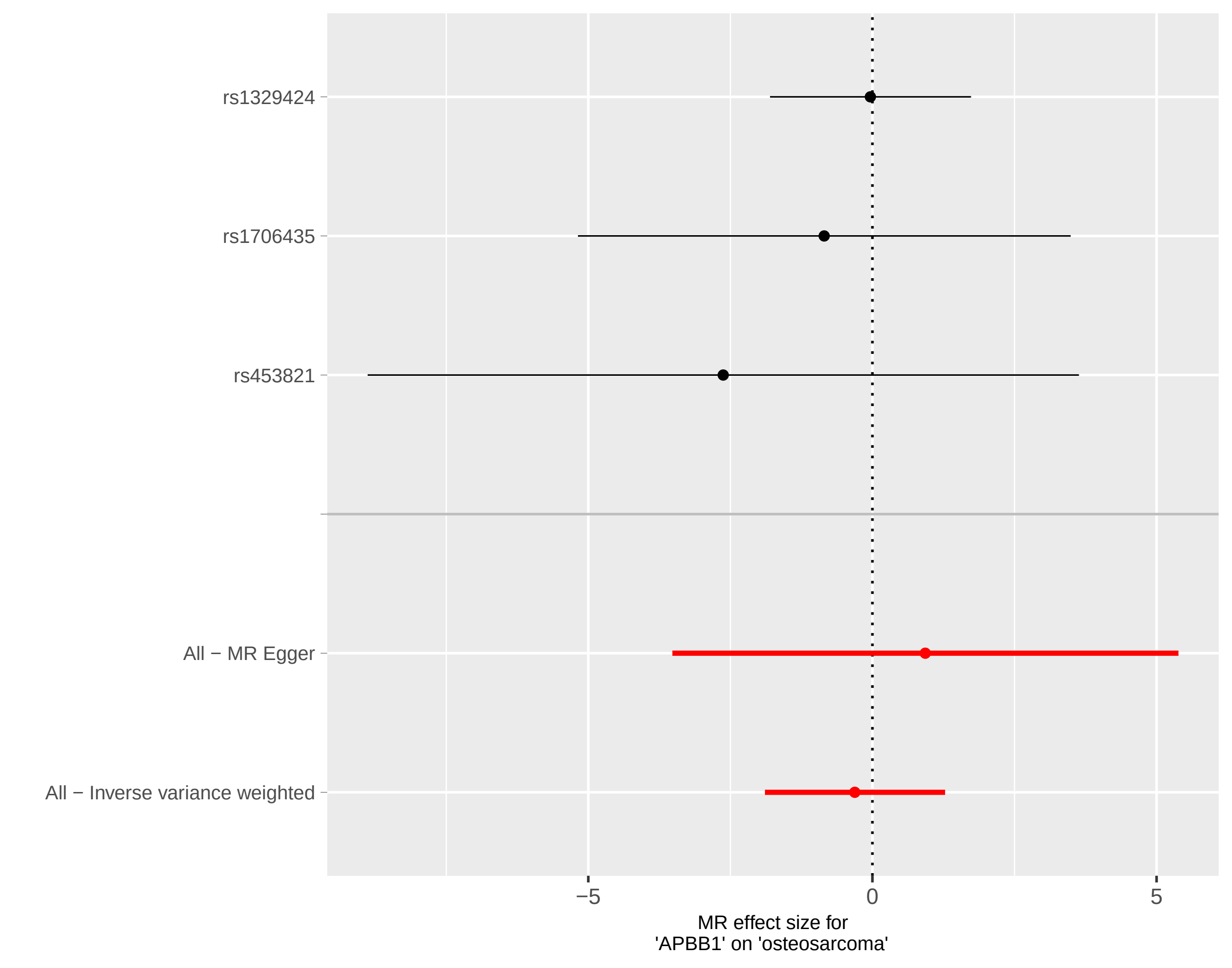
rs1329424
rs1706435
rs453821
All − MR Egger
All − Inverse variance weighted
−5
0
5
MR effect size for
'APBB1' on 'osteosarcoma'

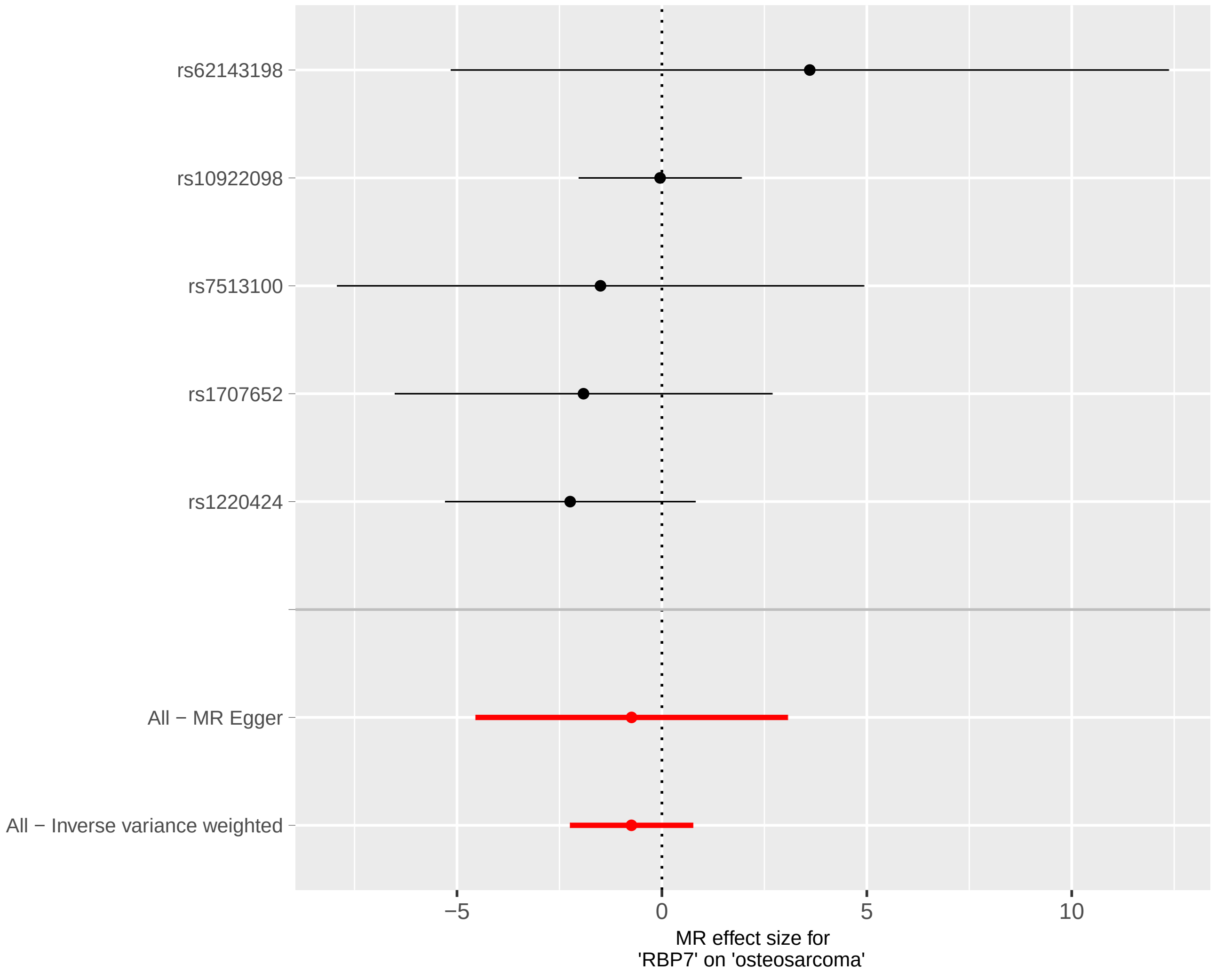

rs62143198
rs10922098
rs7513100
rs1707652
rs1220424
All − MR Egger
All − Inverse variance weighted
−5
0
5
10
MR effect size for
'RBP7' on 'osteosarcoma'

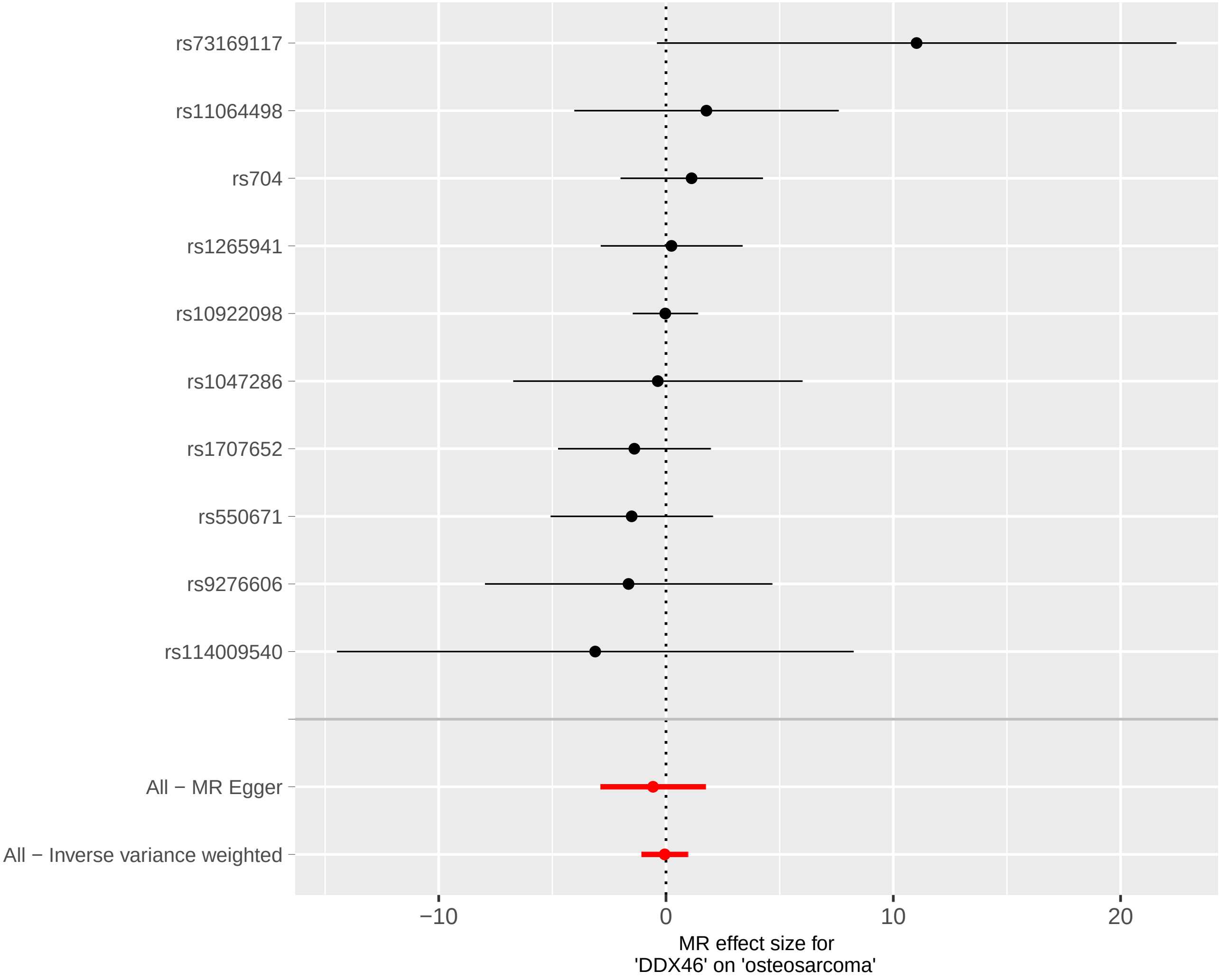
rs73169117
rs11064498
rs704
rs1265941
rs10922098
rs1047286
rs1707652
rs550671
rs9276606
rs114009540
All − MR Egger
All − Inverse variance weighted
−10
0
10
20
MR effect size for
'DDX46' on 'osteosarcoma'

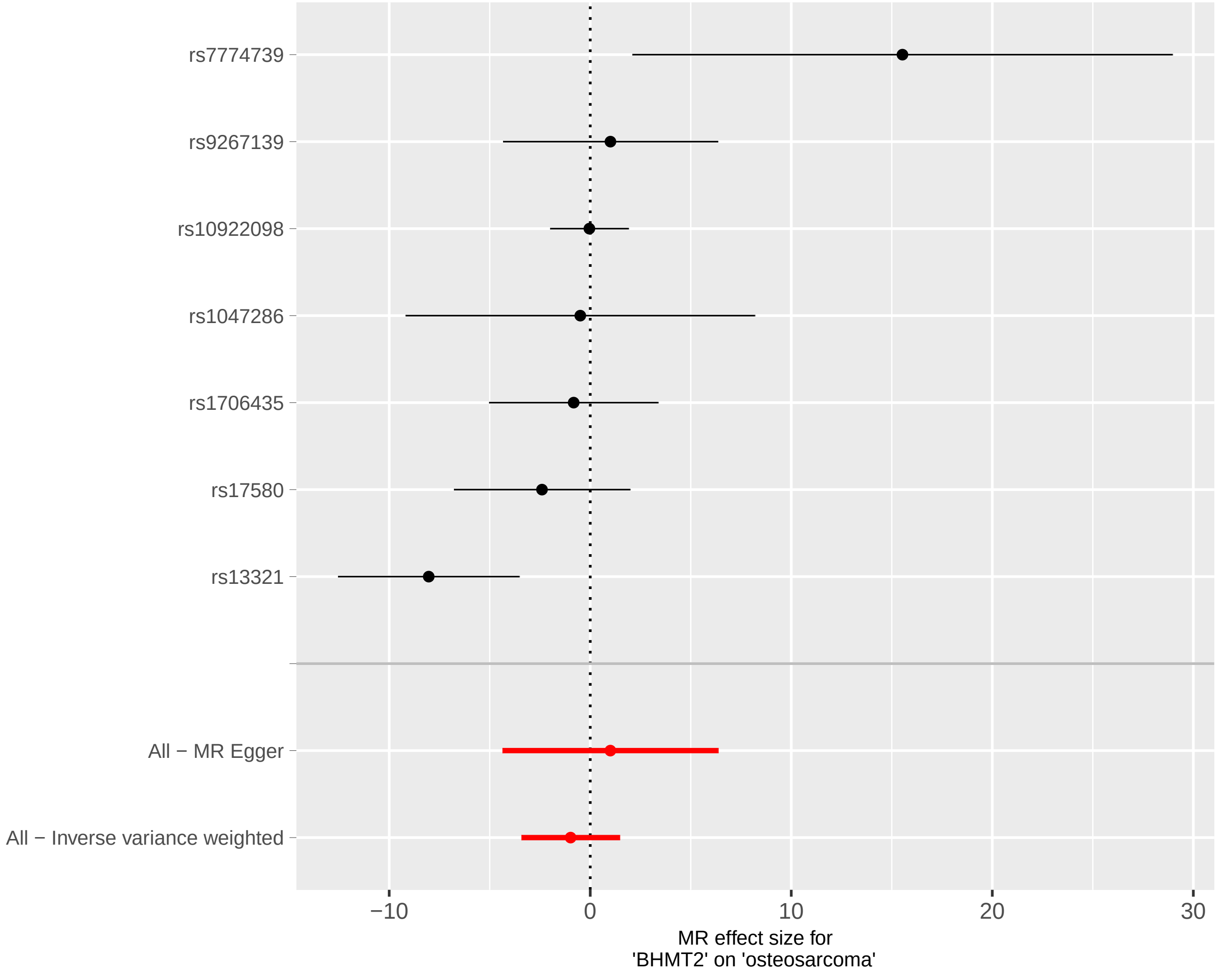
rs7774739
rs9267139
rs10922098
rs1047286
rs1706435
rs17580
rs13321
All − MR Egger
All − Inverse variance weighted
−10
0
10
20
30
MR effect size for
'BHMT2' on 'osteosarcoma'

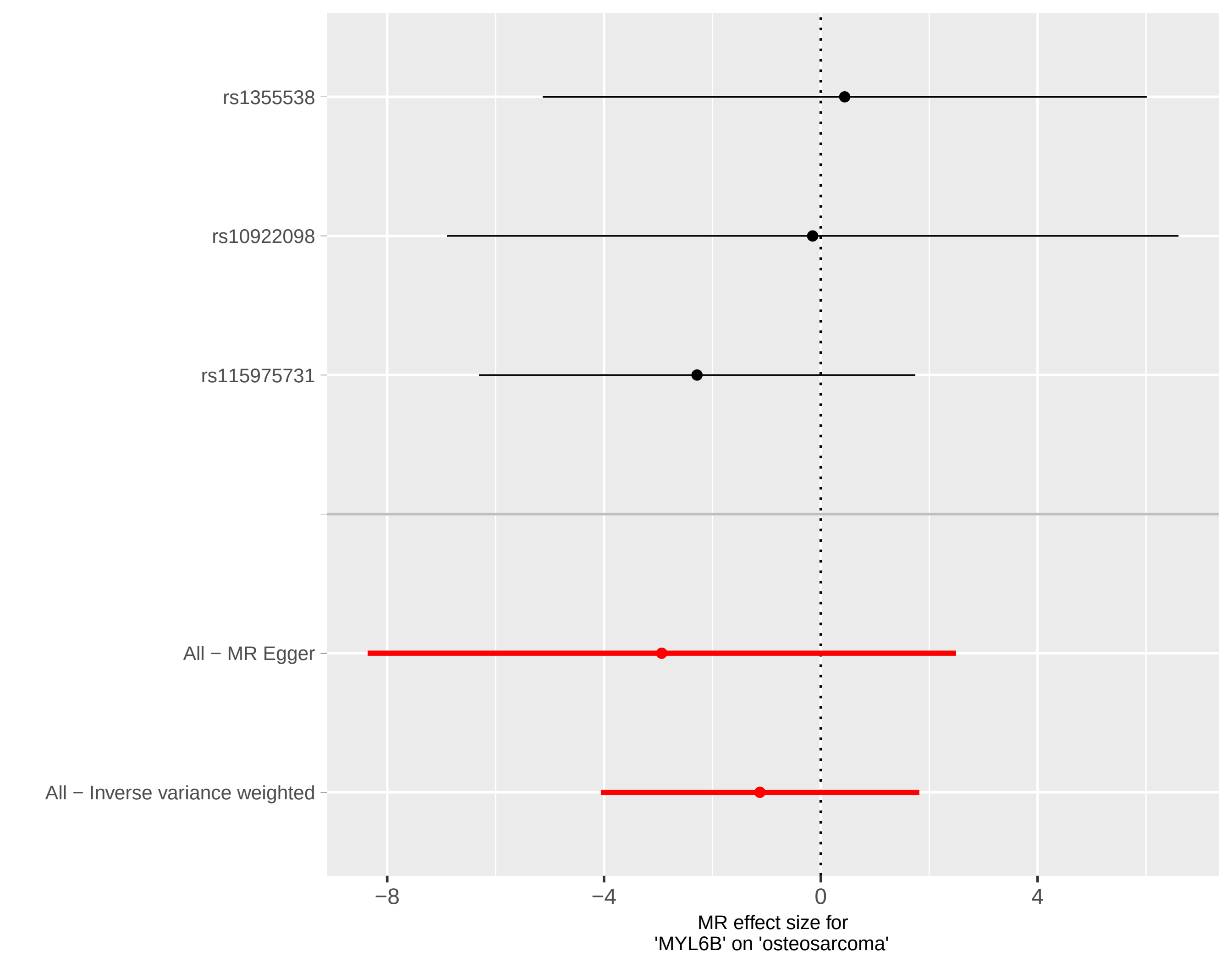
rs1355538
rs10922098
rs115975731
All − MR Egger
All − Inverse variance weighted
−8
−4
0
4
MR effect size for
'MYL6B' on 'osteosarcoma'

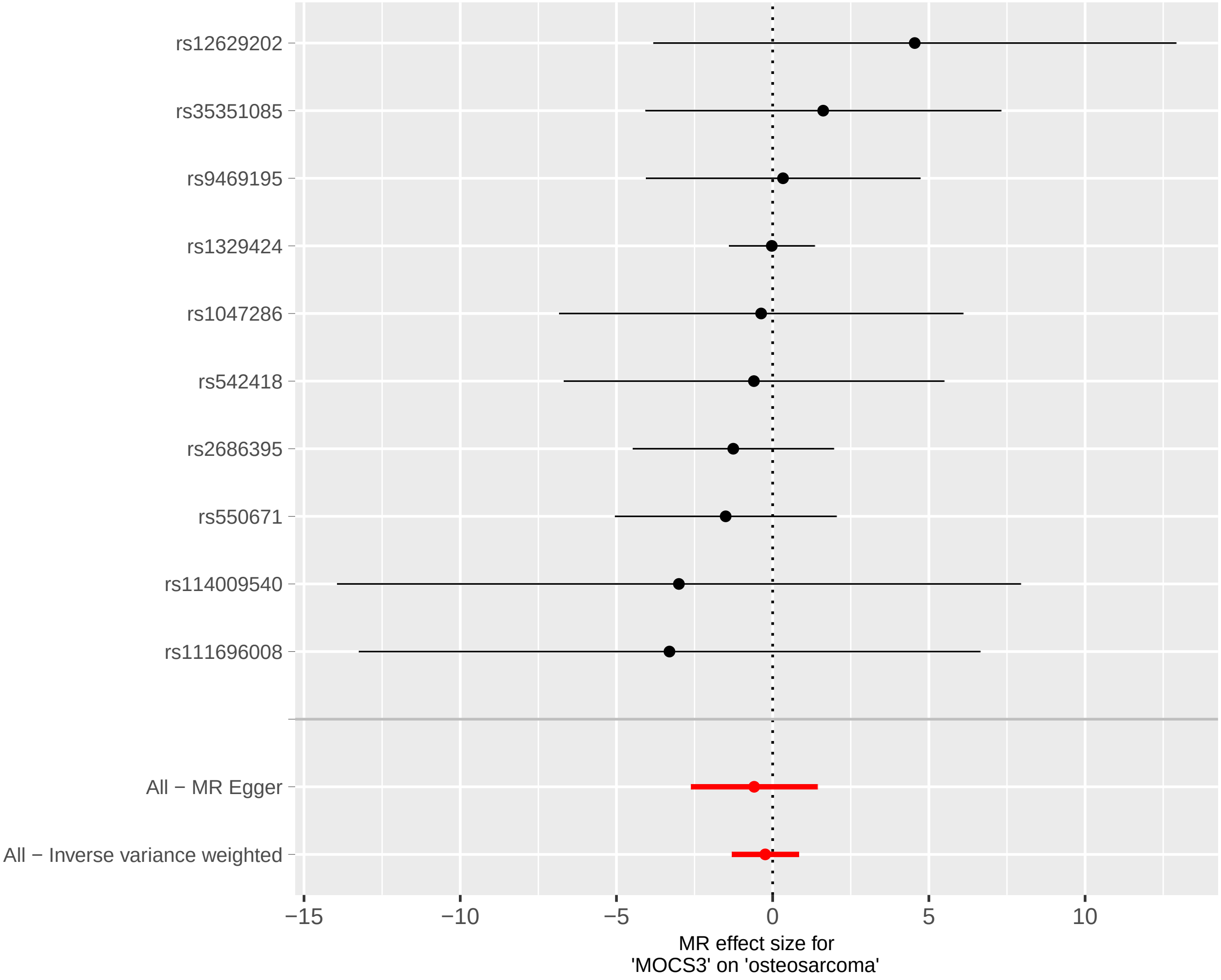
rs12629202
rs35351085
rs9469195
rs1329424
rs1047286
rs542418
rs2686395
rs550671
rs114009540
rs111696008
All − MR Egger
All − Inverse variance weighted
−15
−10
−5
0
5
10
MR effect size for
'MOCS3' on 'osteosarcoma'

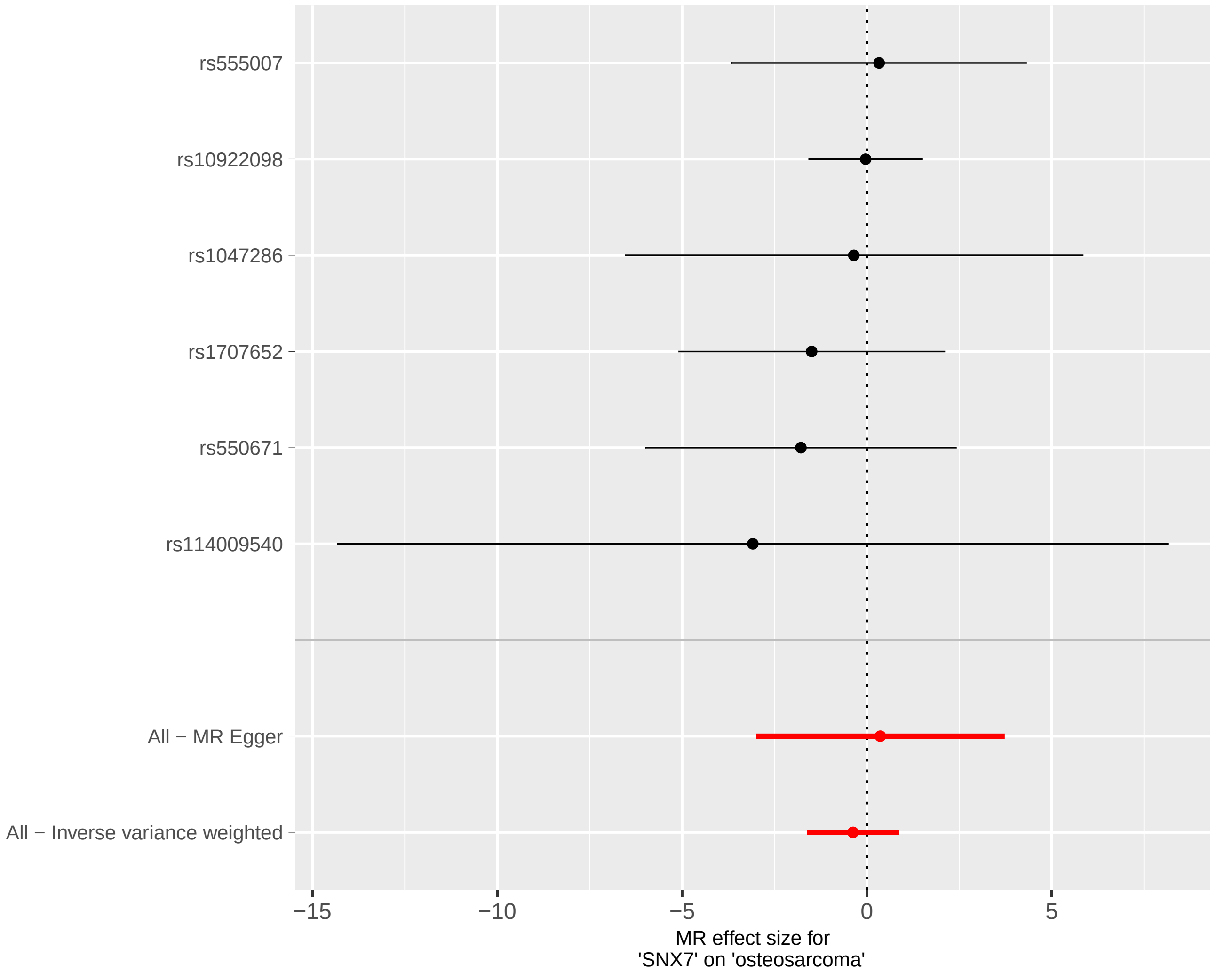

rs555007
rs10922098
rs1047286
rs1707652
rs550671
rs114009540
All − MR Egger
All − Inverse variance weighted
−15
−10
−5
0
5
MR effect size for
'SNX7' on 'osteosarcoma'

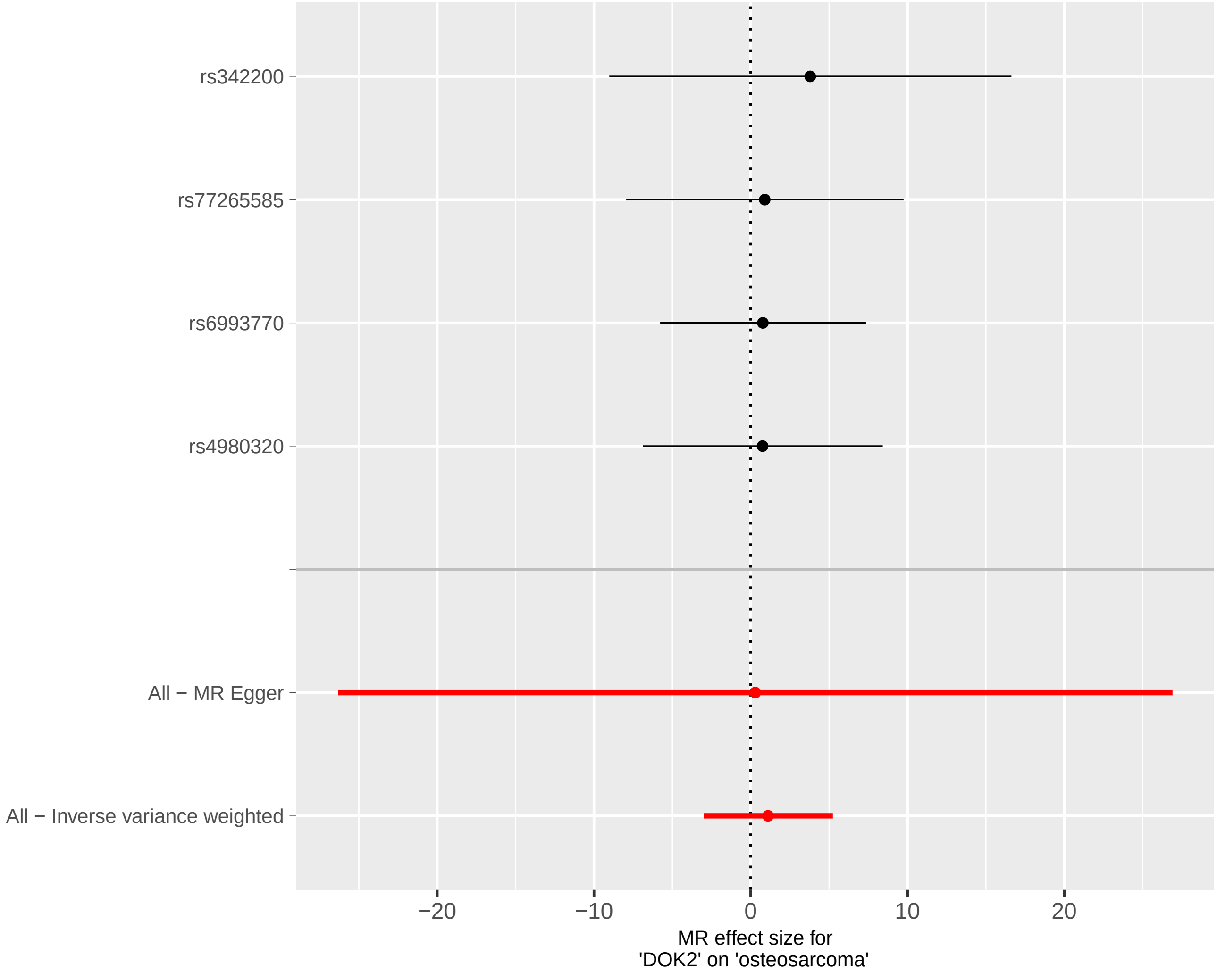

rs342200
rs77265585
rs6993770
rs4980320
All − MR Egger
All − Inverse variance weighted
−20
−10
0
10
20
MR effect size for
'DOK2' on 'osteosarcoma'

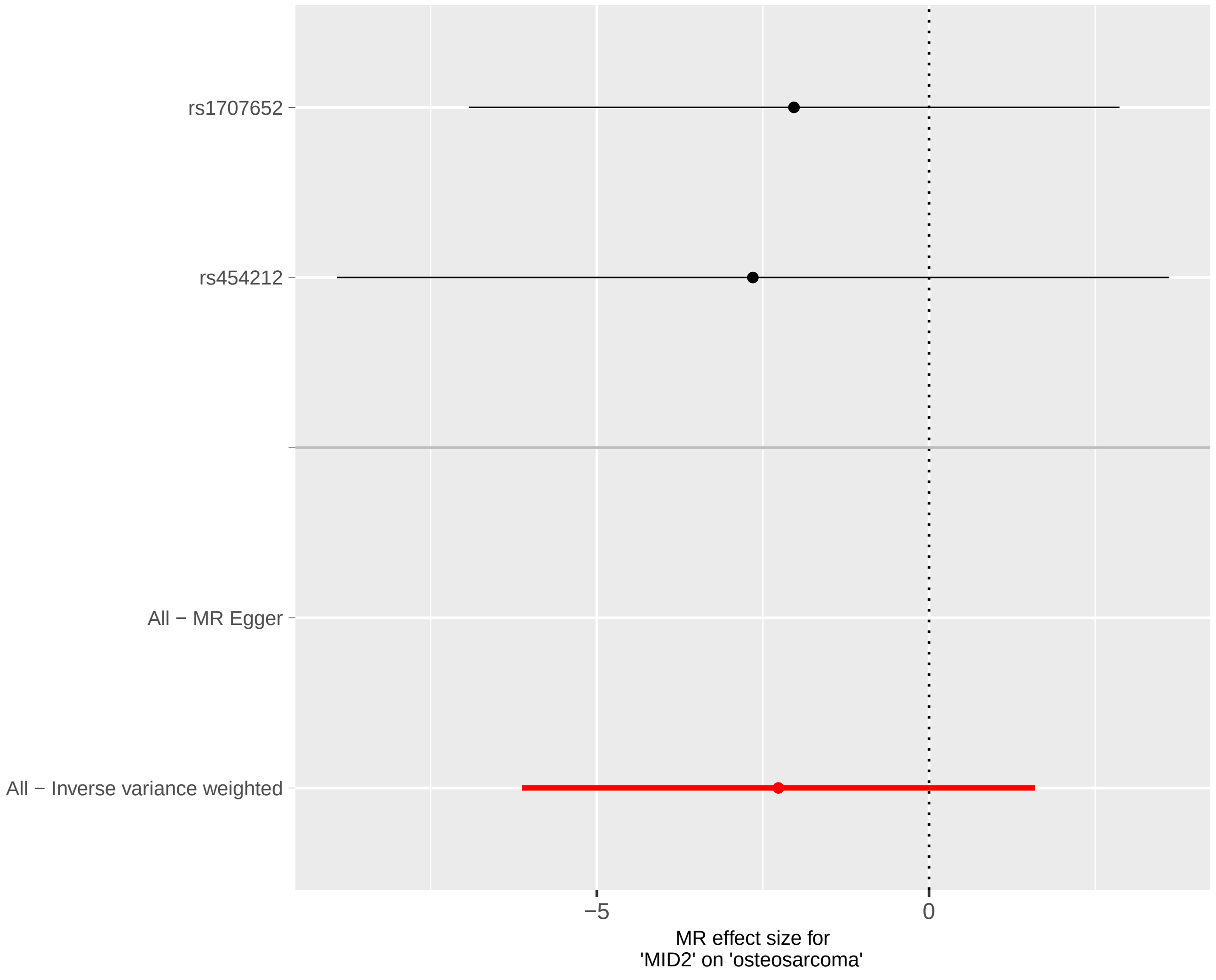

rs1707652
rs454212
All − MR Egger
All − Inverse variance weighted
−5
0
MR effect size for
'MID2' on 'osteosarcoma'

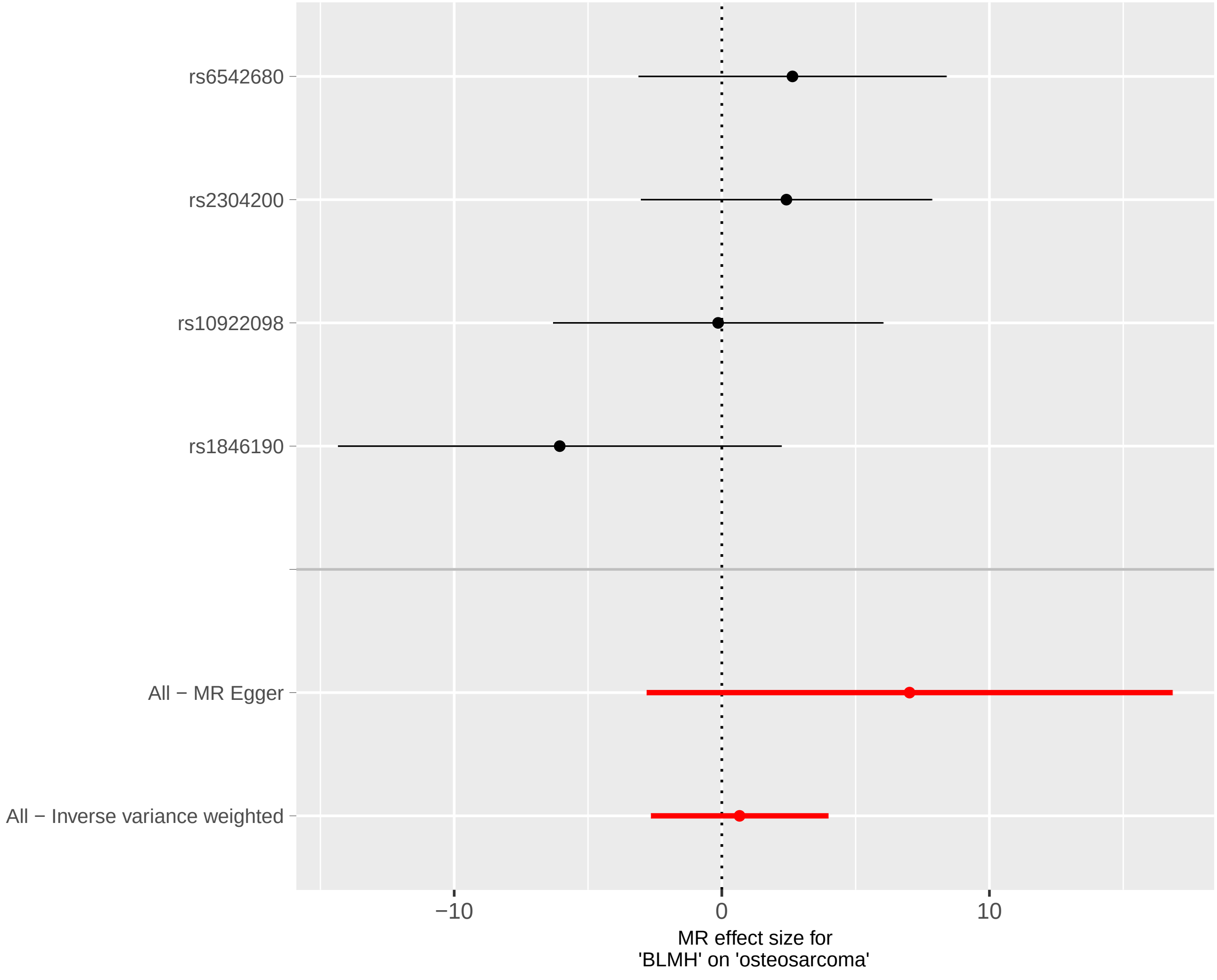

rs6542680
rs2304200
rs10922098
rs1846190
All − MR Egger
All − Inverse variance weighted
−10
0
10
MR effect size for
'BLMH' on 'osteosarcoma'

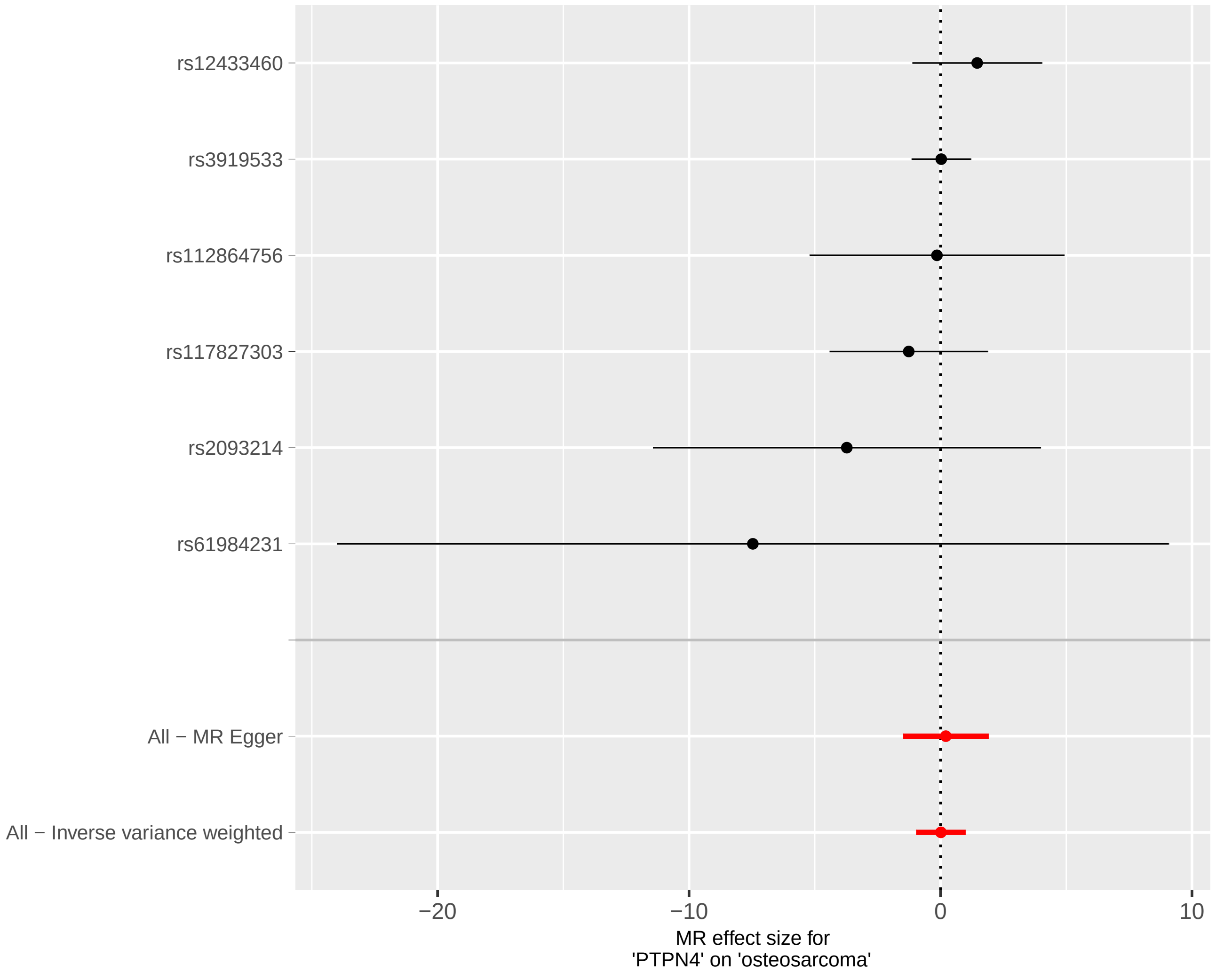
rs12433460
rs3919533
rs112864756
rs117827303
rs2093214
rs61984231
All − MR Egger
All − Inverse variance weighted
−20
−10
0
10
MR effect size for
'PTPN4' on 'osteosarcoma'

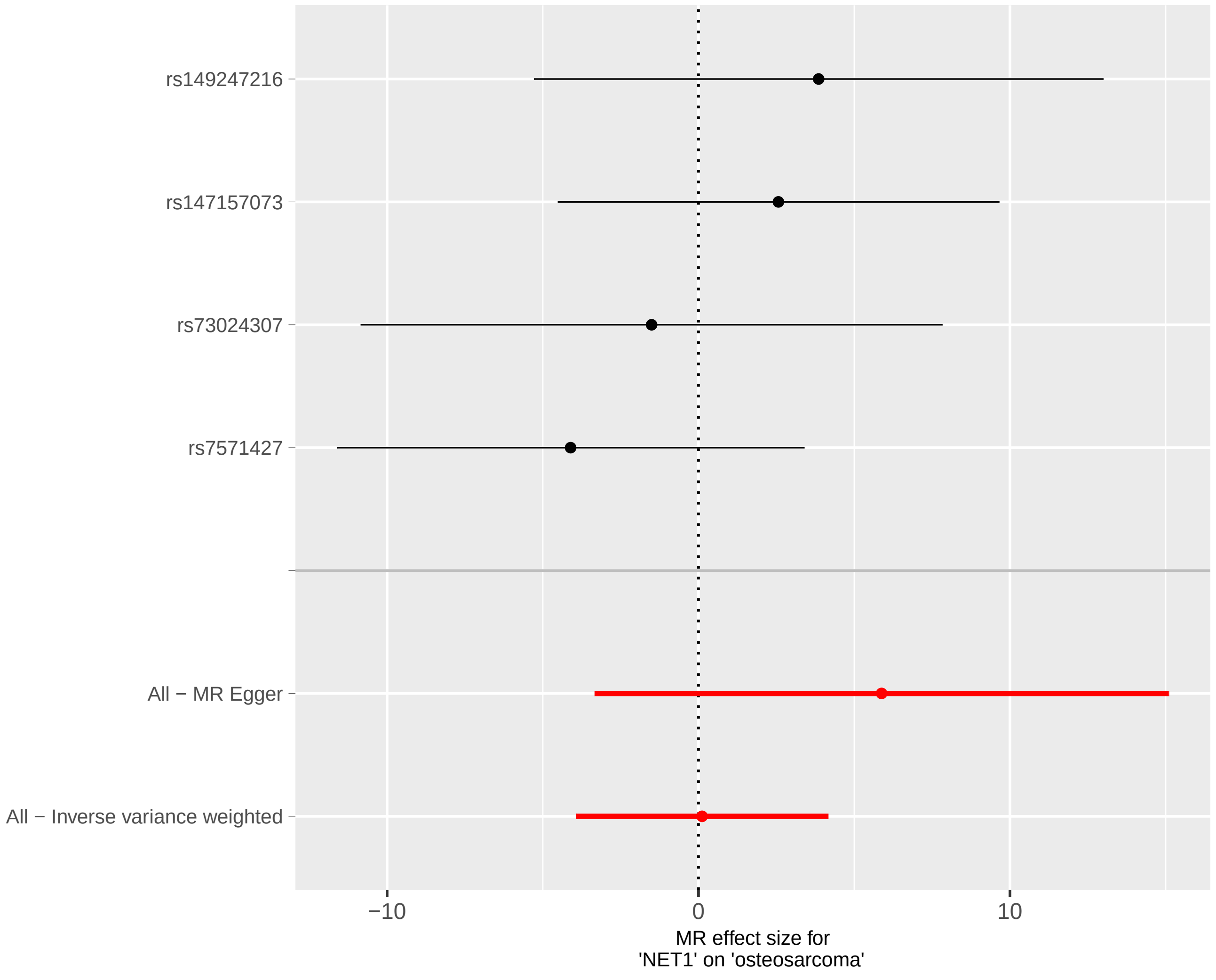
rs149247216
rs147157073
rs73024307
rs7571427
All − MR Egger
All − Inverse variance weighted
−10
0
10
MR effect size for
'NET1' on 'osteosarcoma'

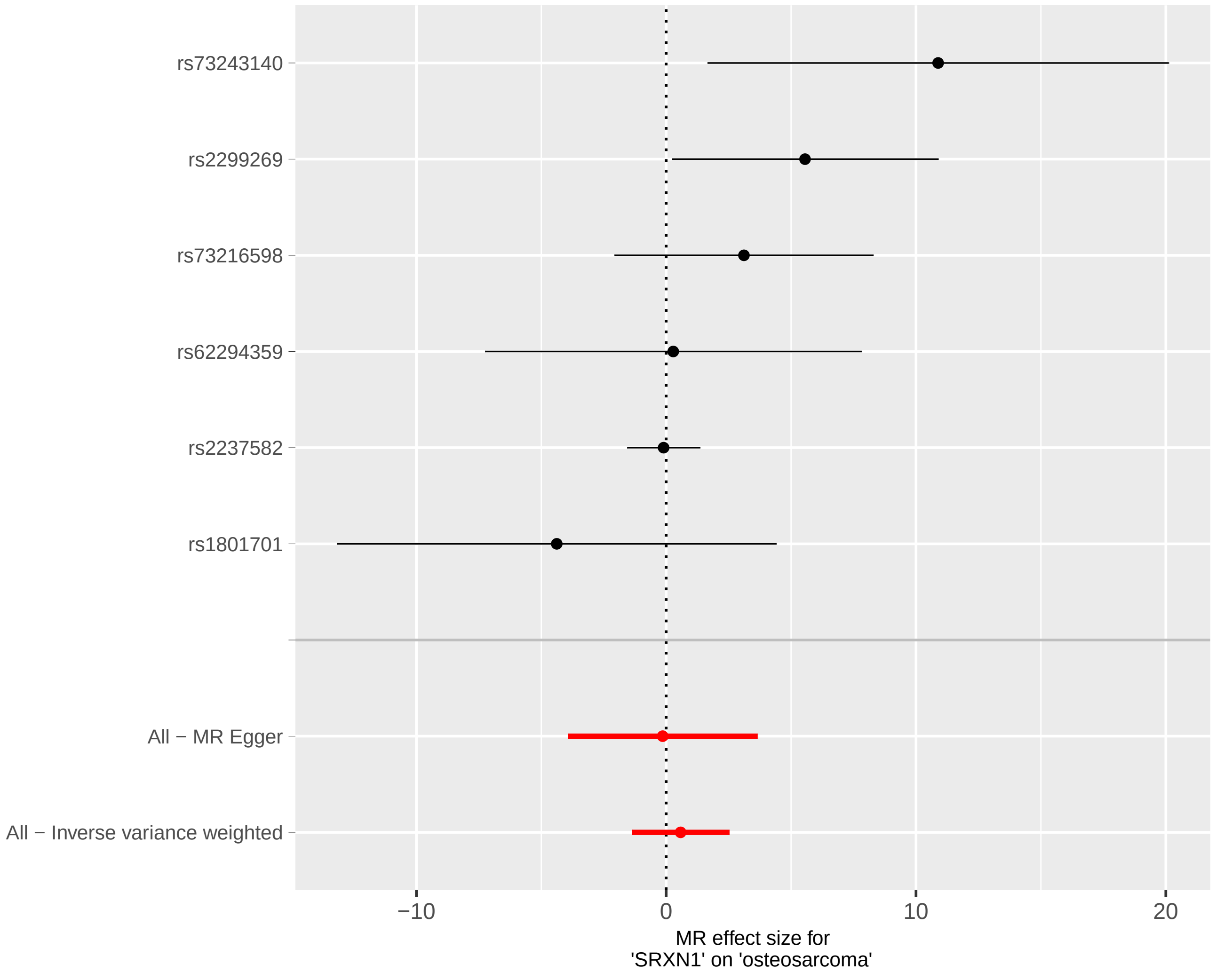
rs73243140
rs2299269
rs73216598
rs62294359
rs2237582
rs1801701
All − MR Egger
All − Inverse variance weighted
−10
0
10
20
MR effect size for
'SRXN1' on 'osteosarcoma'

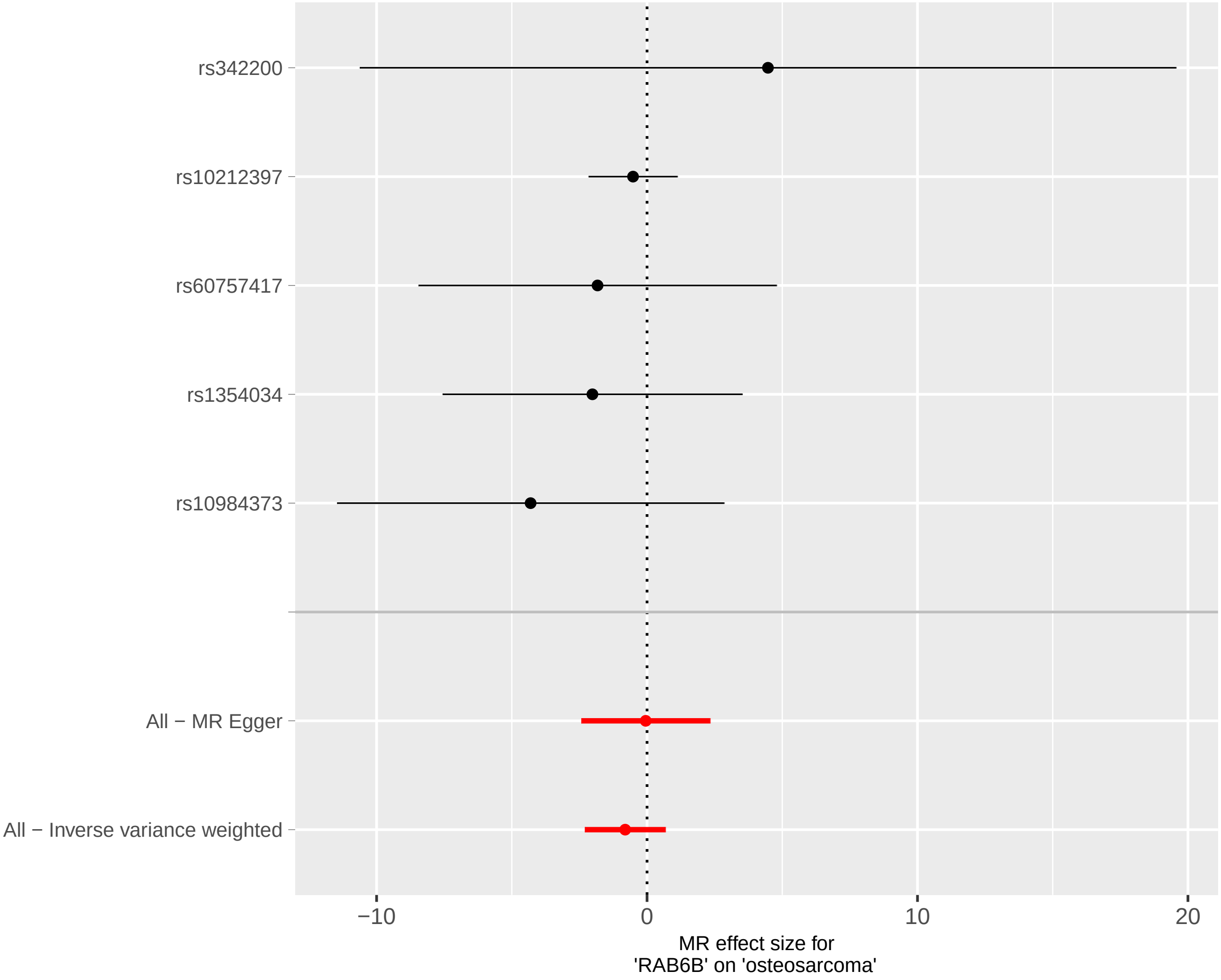

rs342200
rs10212397
rs60757417
rs1354034
rs10984373
All − MR Egger
All − Inverse variance weighted
−10
0
10
20
MR effect size for
'RAB6B' on 'osteosarcoma'

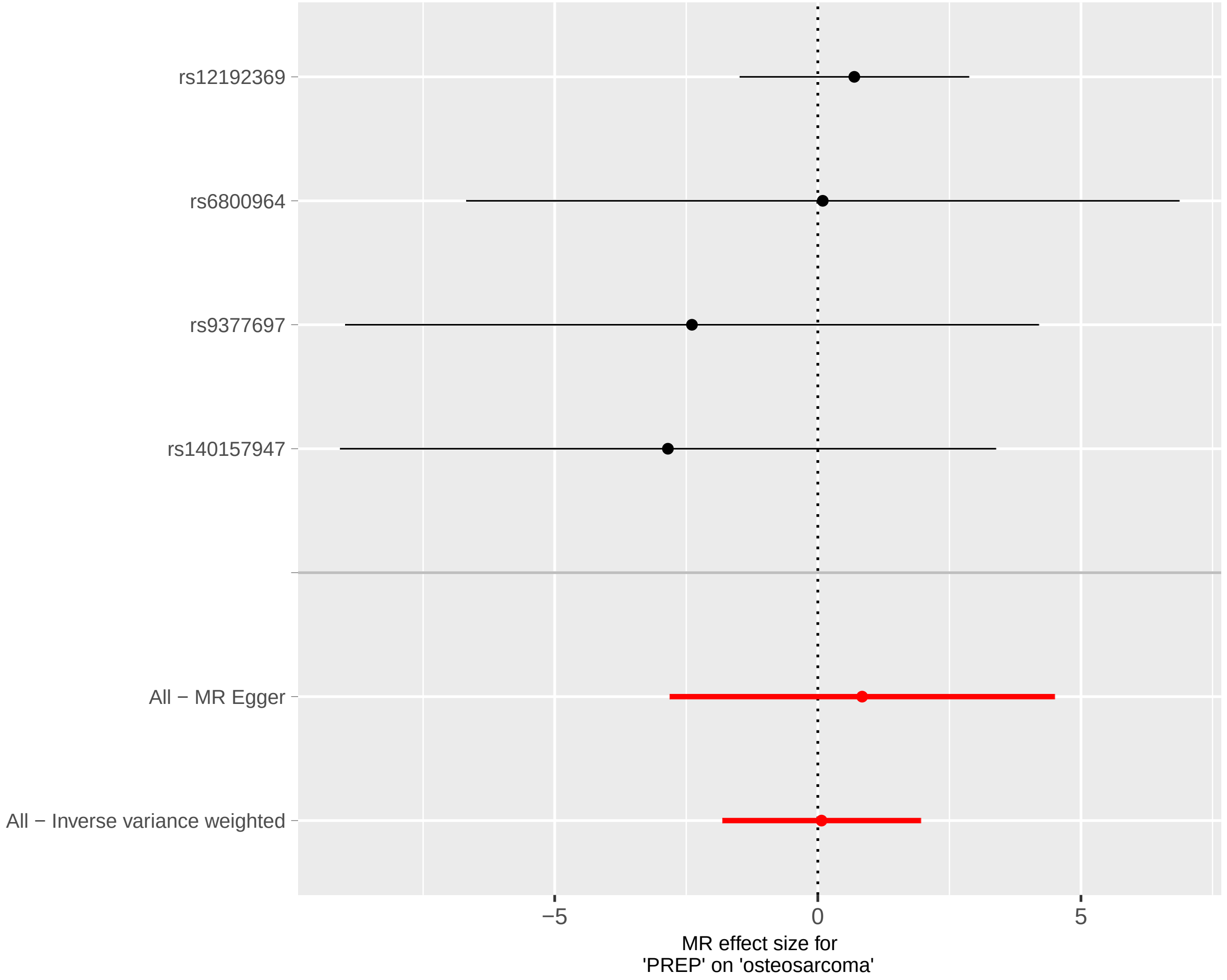
rs12192369
rs6800964
rs9377697
rs140157947
All − MR Egger
All − Inverse variance weighted
−5
0
5
MR effect size for
'PREP' on 'osteosarcoma'

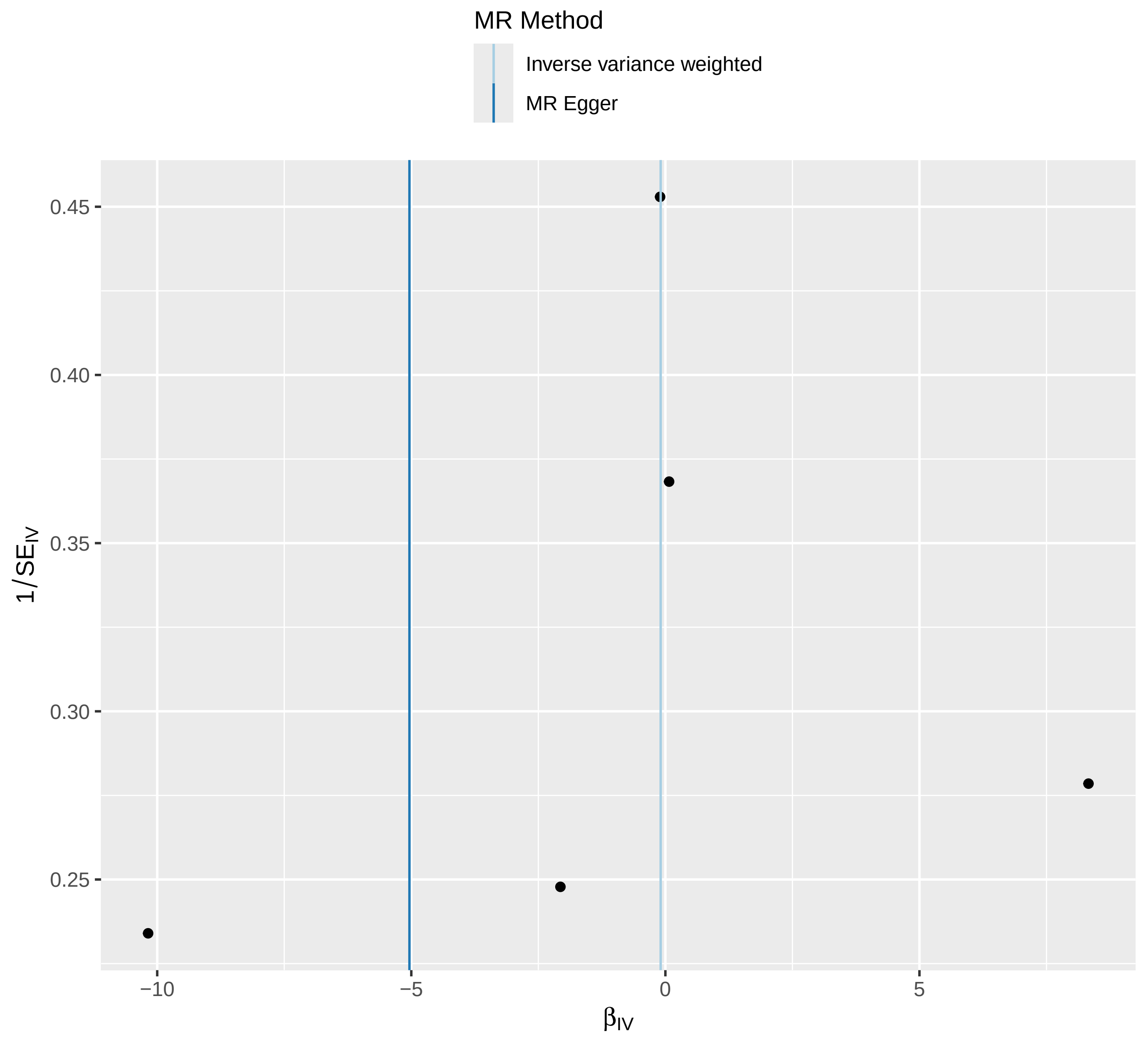
MR Method
Inverse variance weighted
MR Egger
0.45
0.40
0.35
0.30
0.25
1/SE_IV
−10
−5
0
5
β_IV

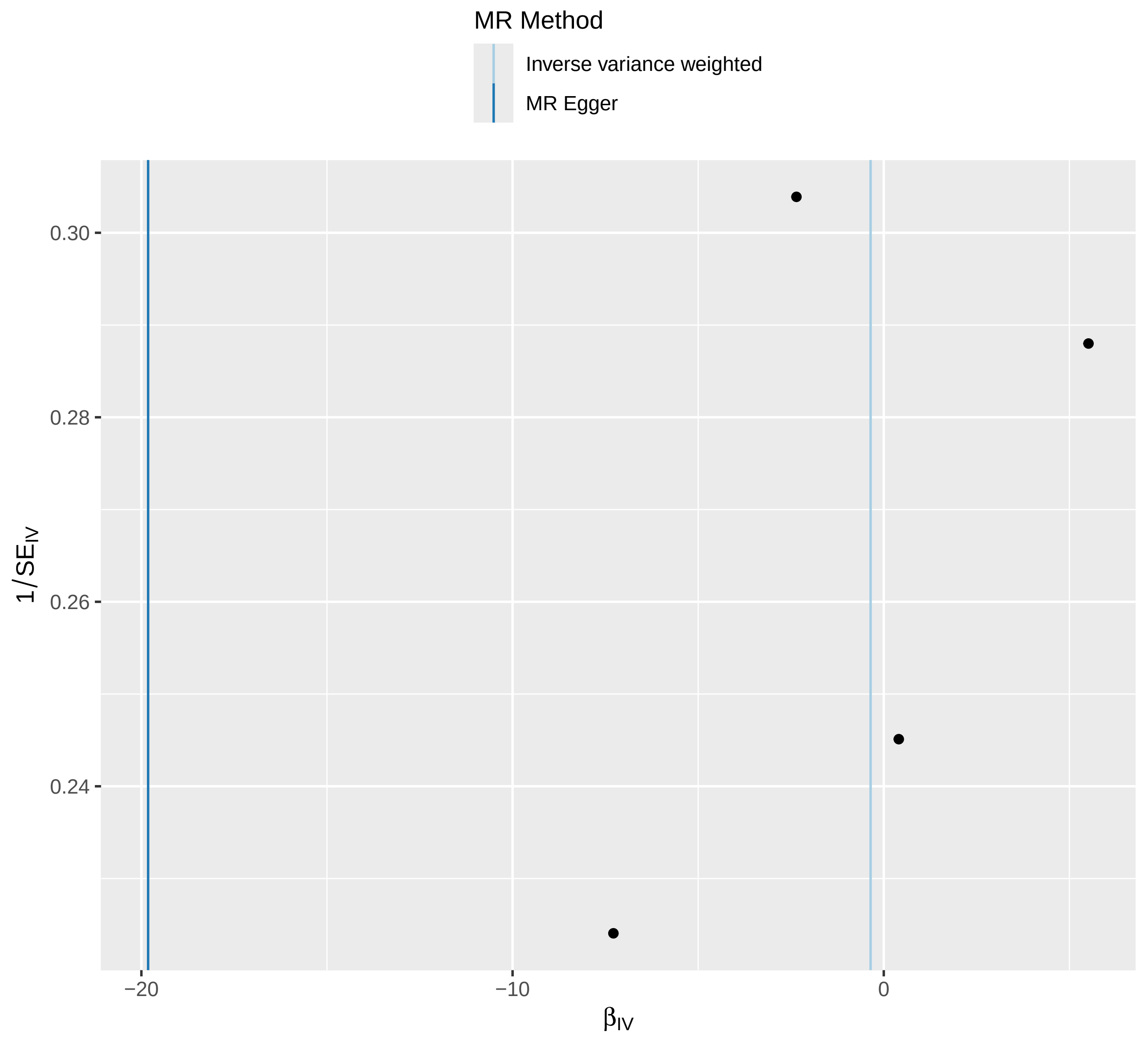

MR Method
Inverse variance weighted
MR Egger
0.30
0.28
0.26
0.24
1/SE_IV
−20
−10
0
β_IV

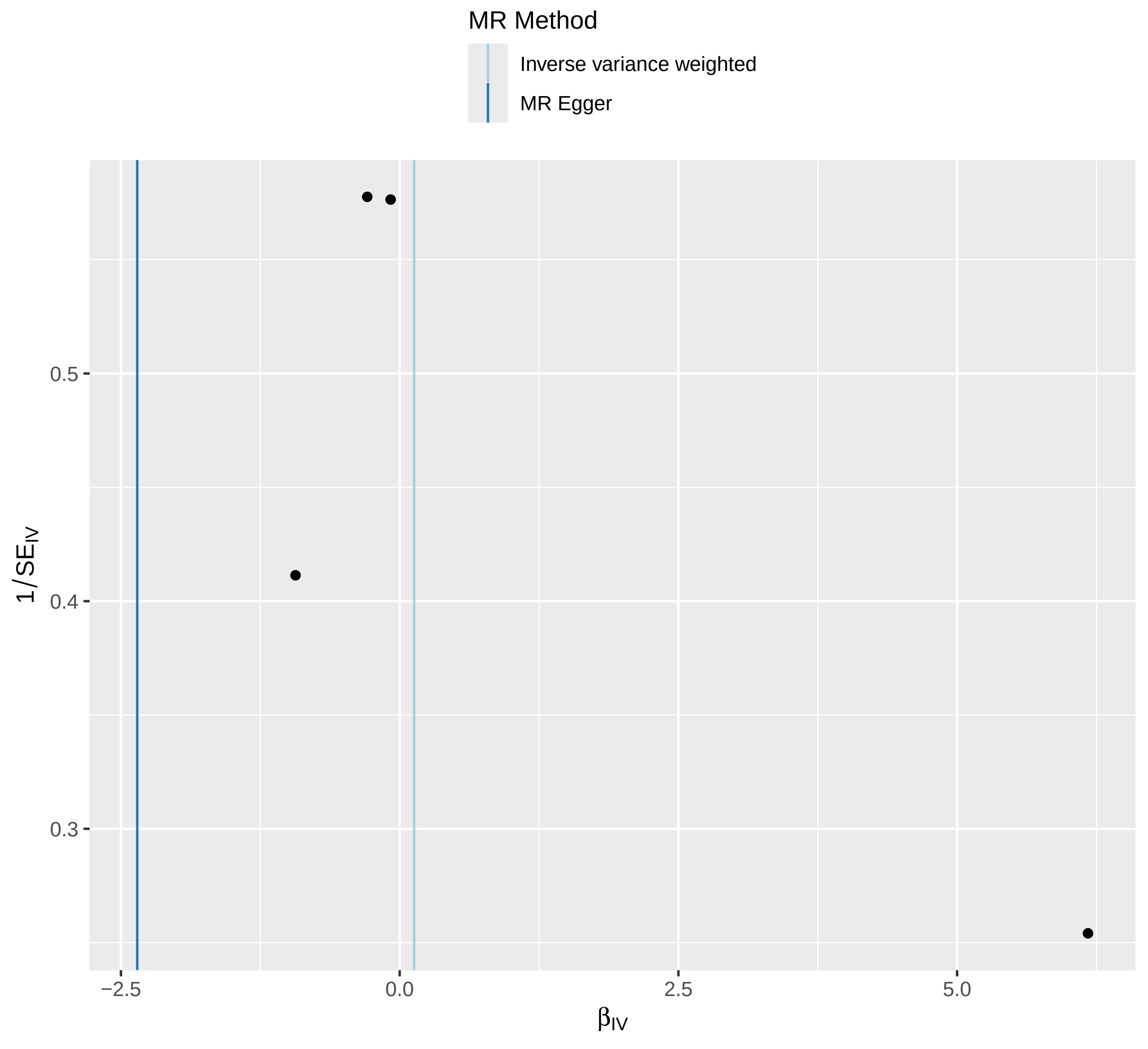
MR Method
Inverse variance weighted
MR Egger
0.5
0.4
0.3
1/SE_IV
−2.5
0.0
2.5
5.0
β_IV

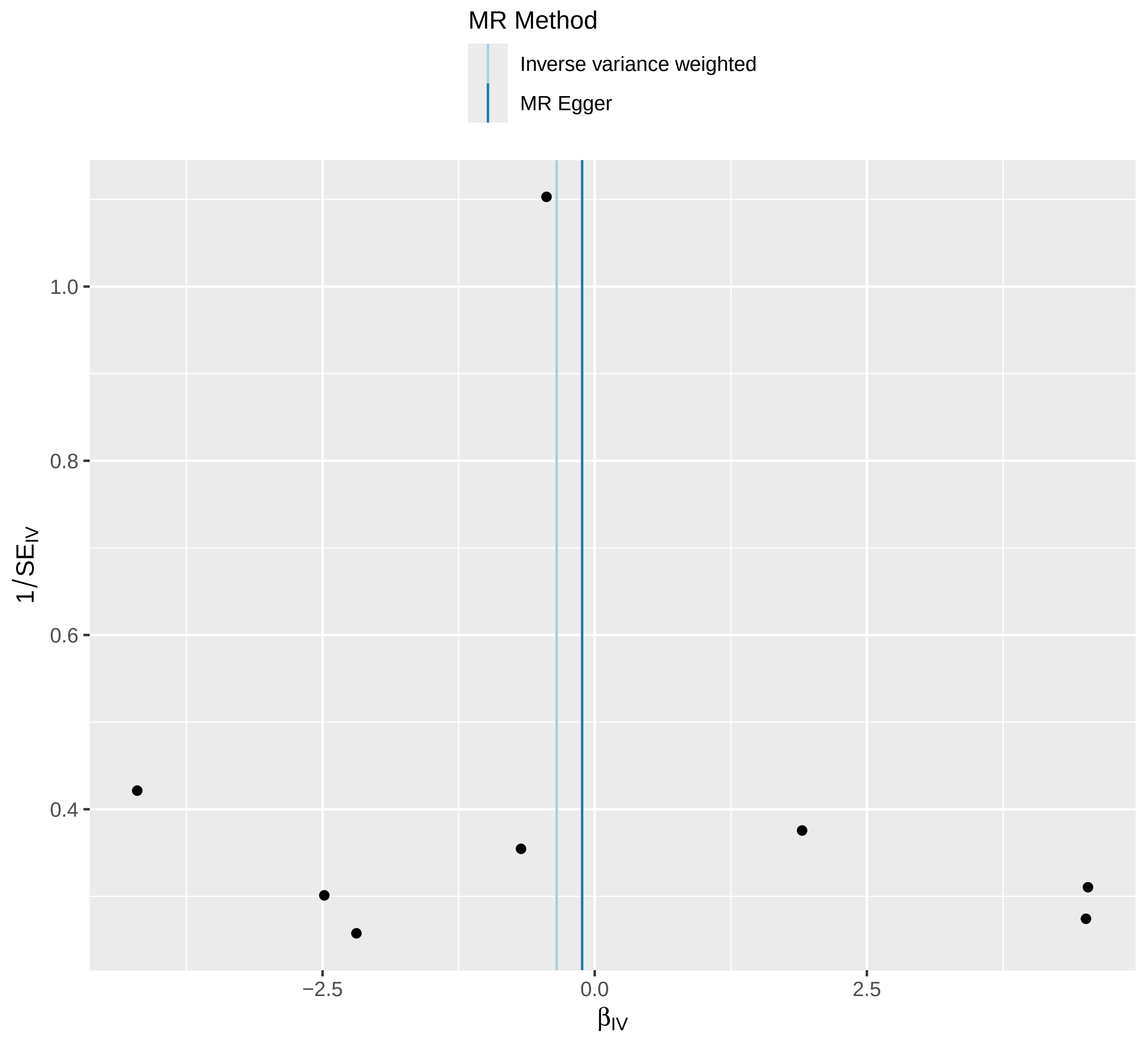
MR Method
Inverse variance weighted
MR Egger
1.0
0.8
0.6
0.4
1/SE_IV
−2.5
0.0
2.5
β_IV

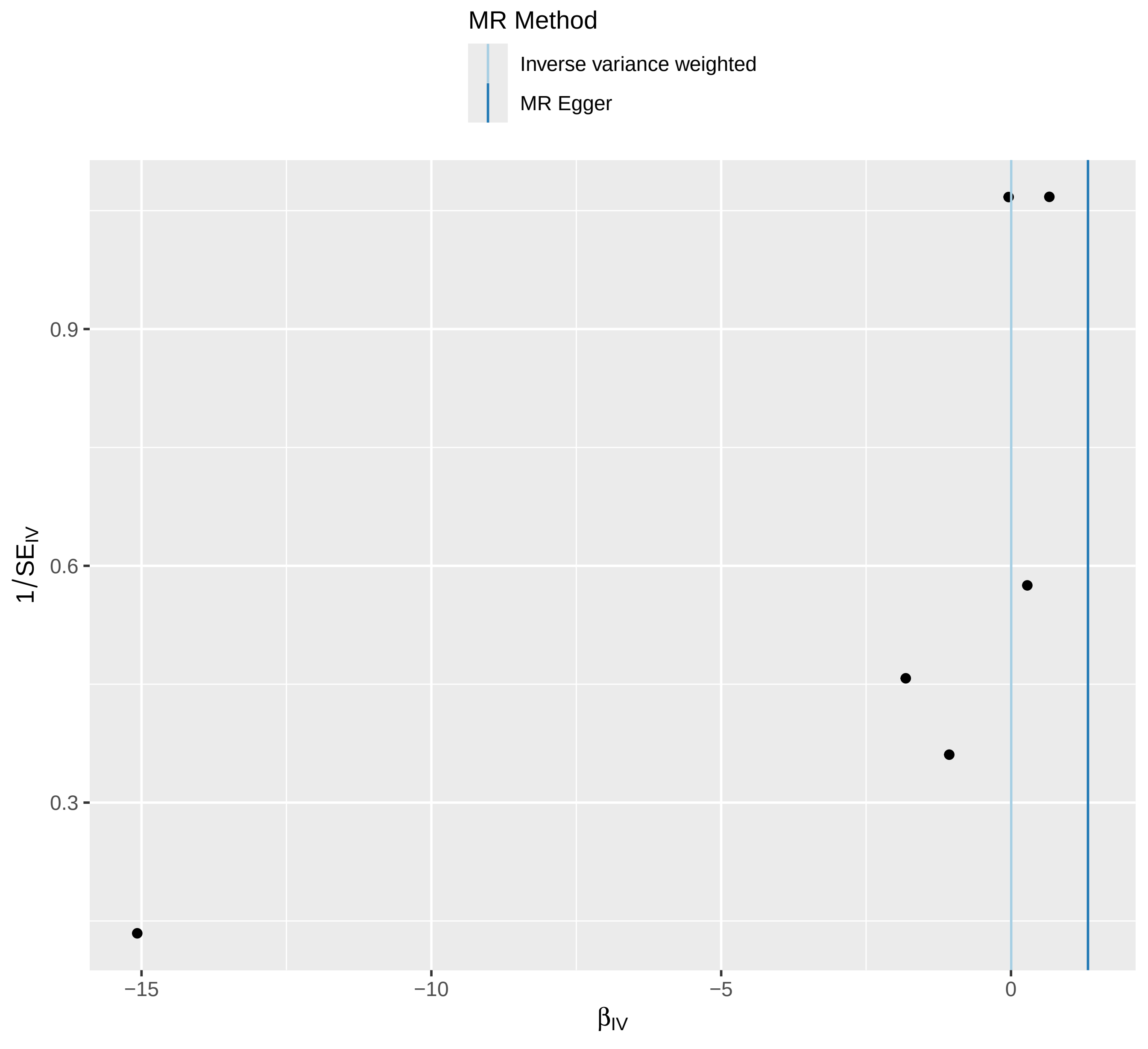

MR Method
Inverse variance weighted
MR Egger
0.9
0.6
0.3
1/SE_IV
−15
−10
−5
0
β_IV

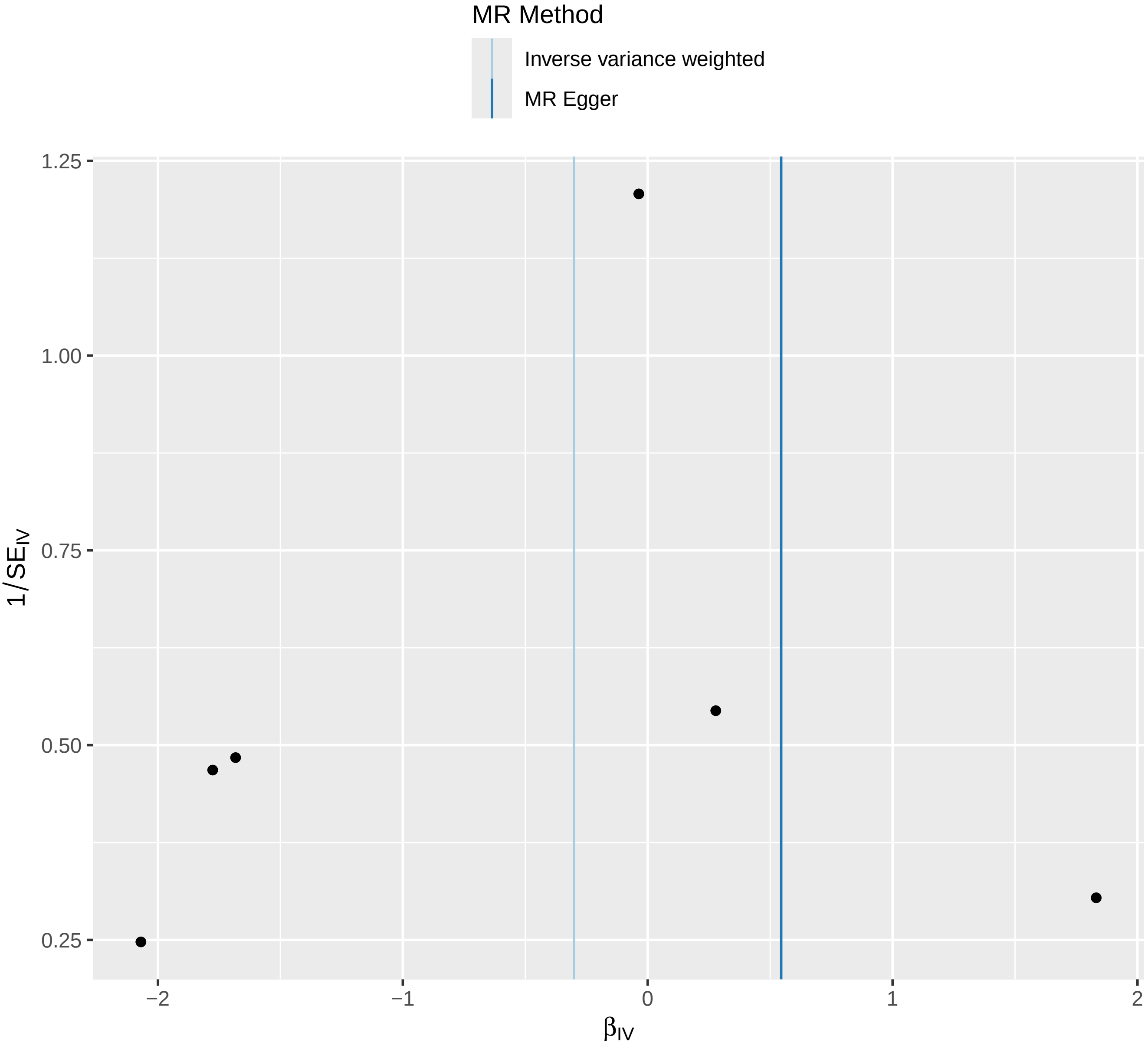
MR Method
Inverse variance weighted
MR Egger
1.25
1.00
0.75
0.50
0.25
1/SE_IV
−2
−1
0
1
2
β_IV

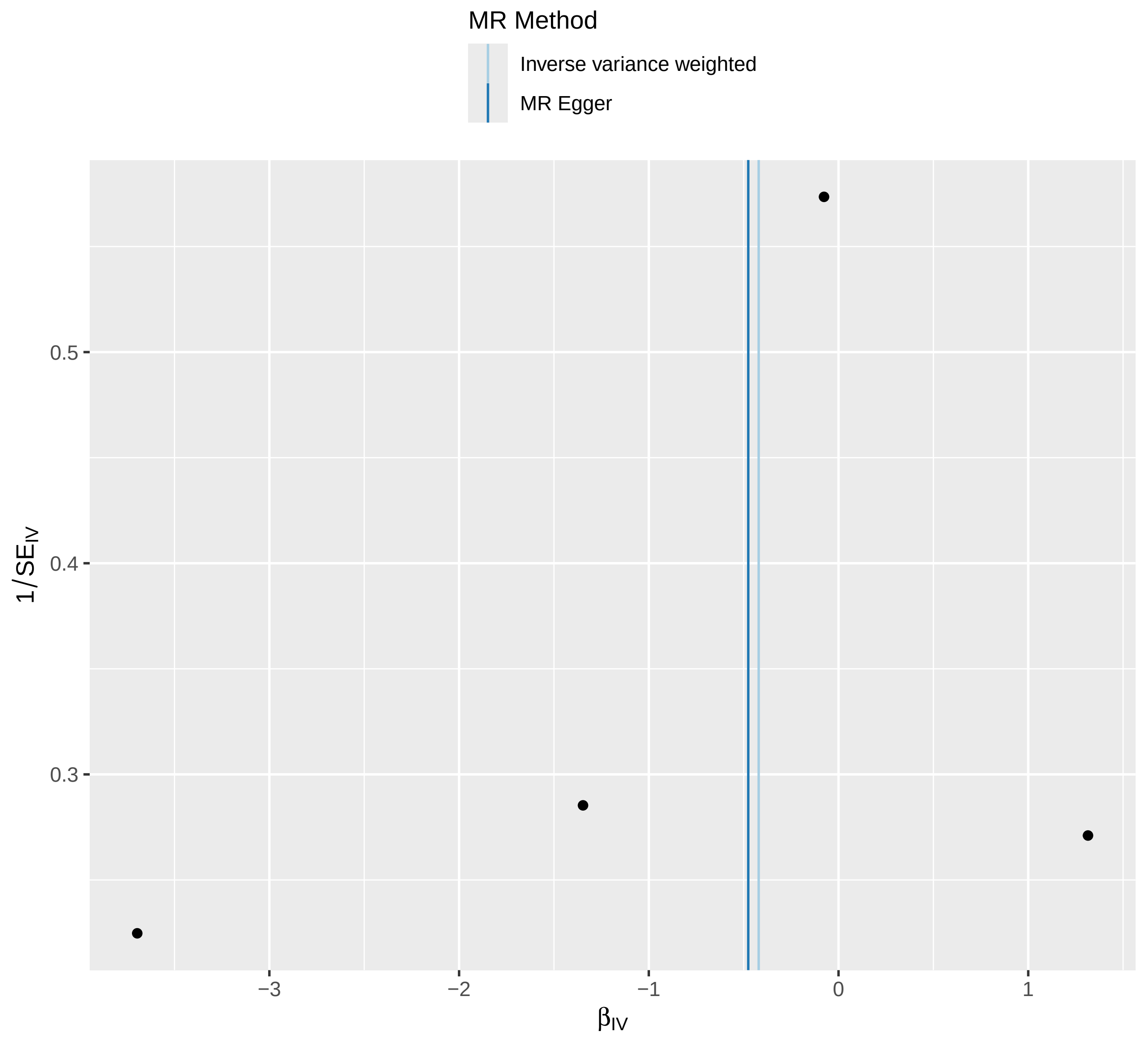
MR Method
Inverse variance weighted
MR Egger
0.5
0.4
0.3
1/SE$_{IV}$
−3
−2
−1
0
1
β$_{IV}$

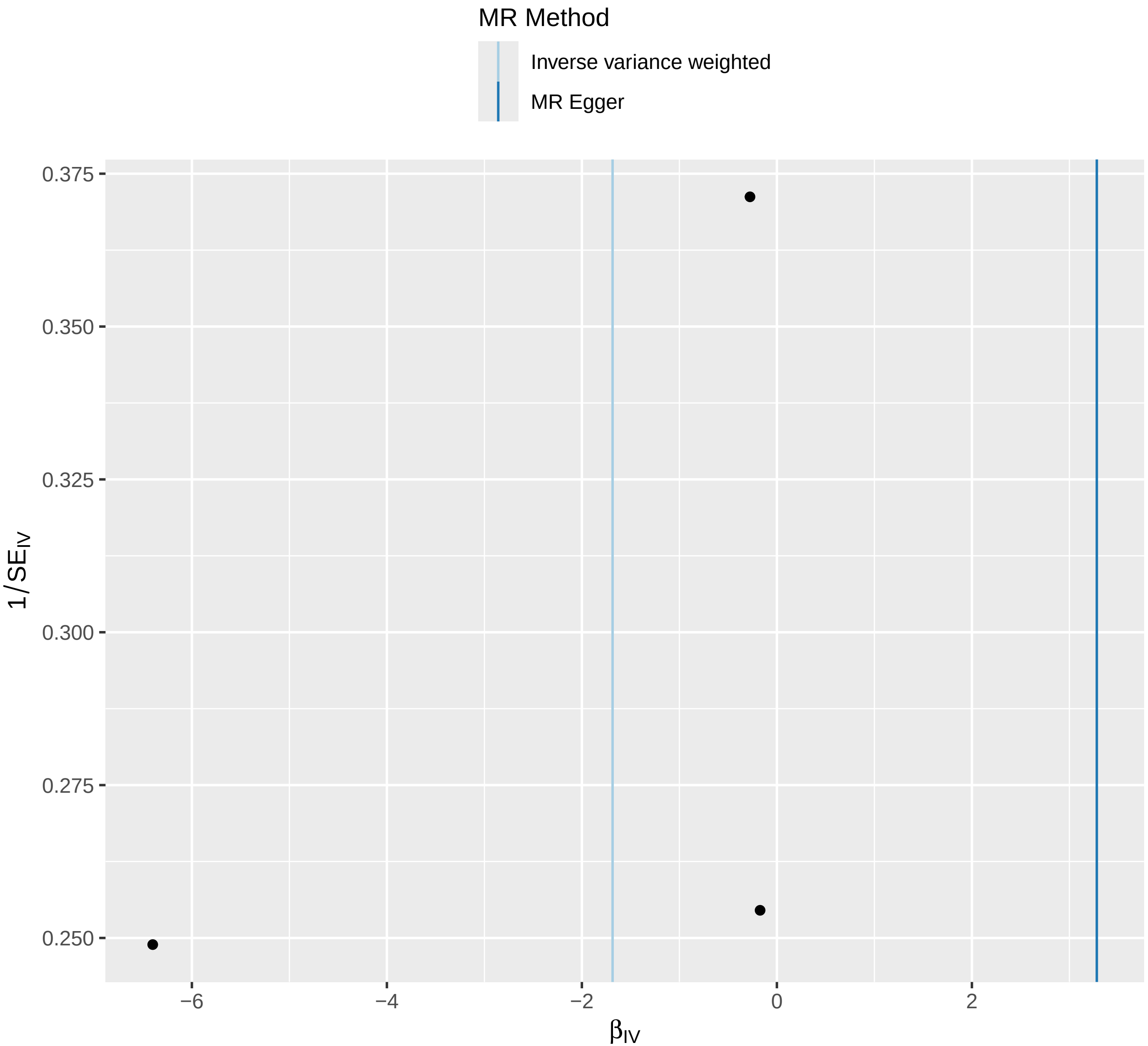
MR Method
Inverse variance weighted
MR Egger
0.375
0.350
0.325
0.300
0.275
0.250
1/SE_IV
−6
−4
−2
0
2
β_IV

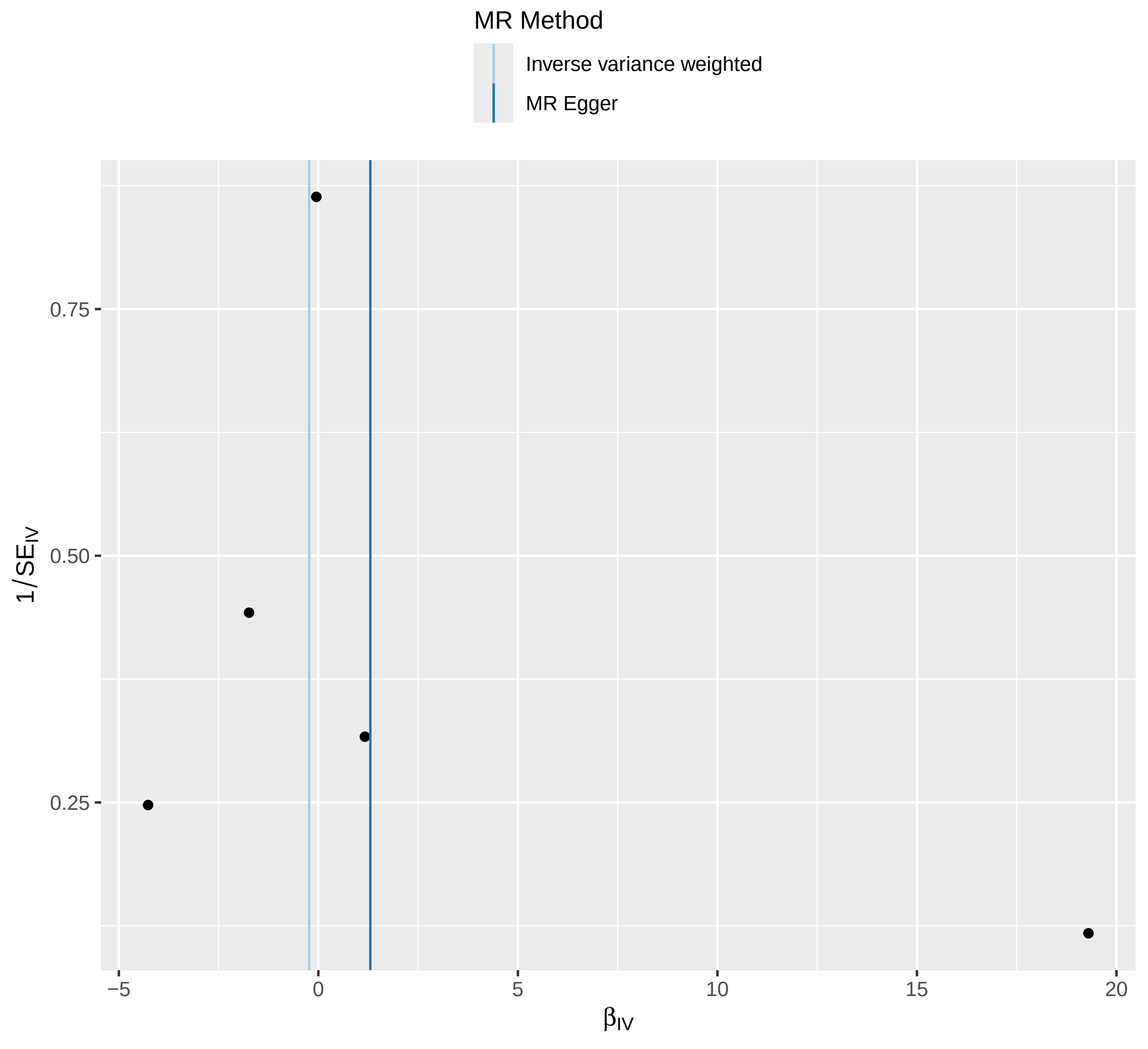
MR Method
Inverse variance weighted
MR Egger
0.75
0.50
0.25
1/SE$_{IV}$
−5
0
5
10
15
20
β_{IV}

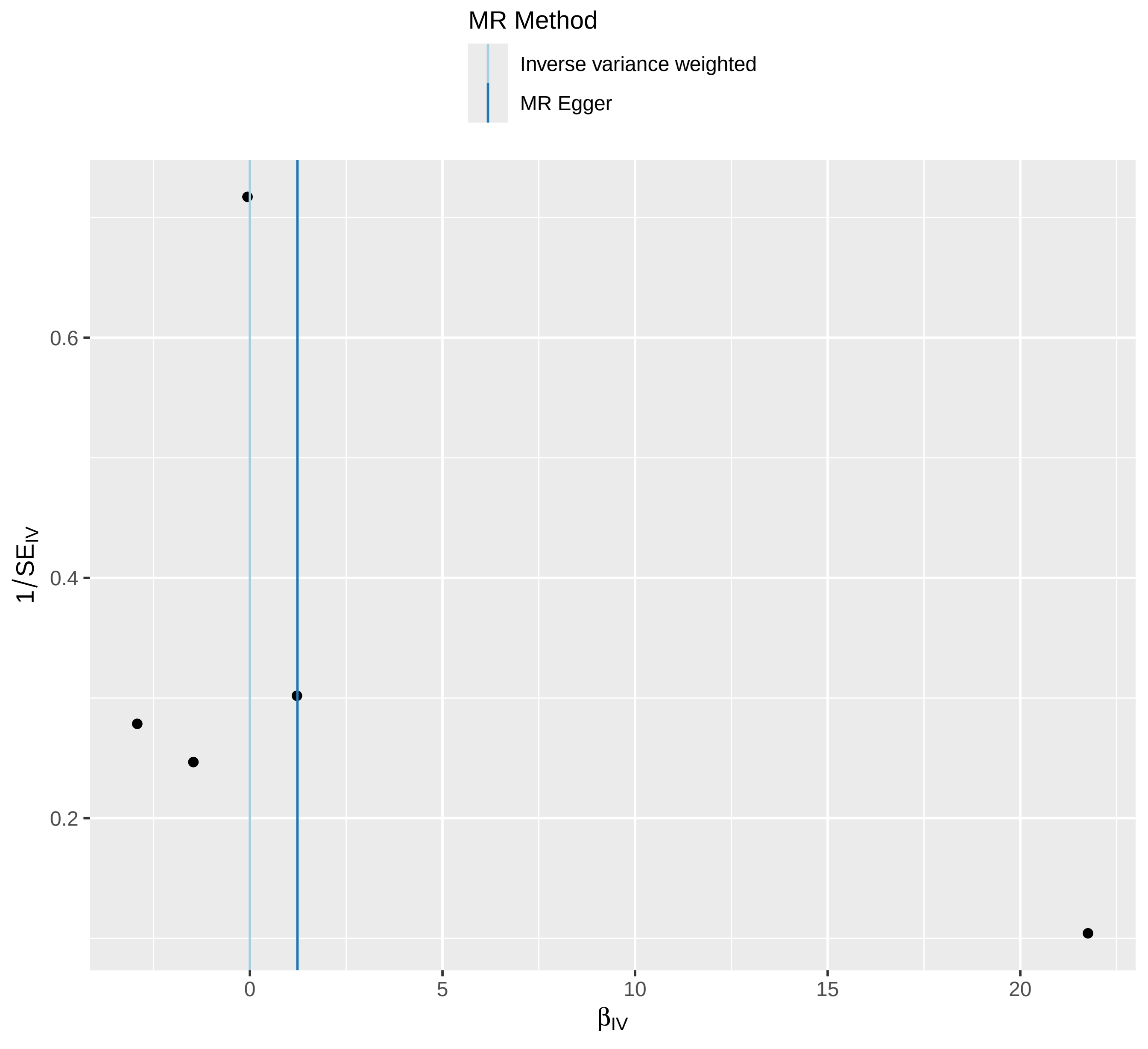
MR Method
Inverse variance weighted
MR Egger
1/SE_IV
0.6
0.4
0.2
0
5
10
15
20
β_IV

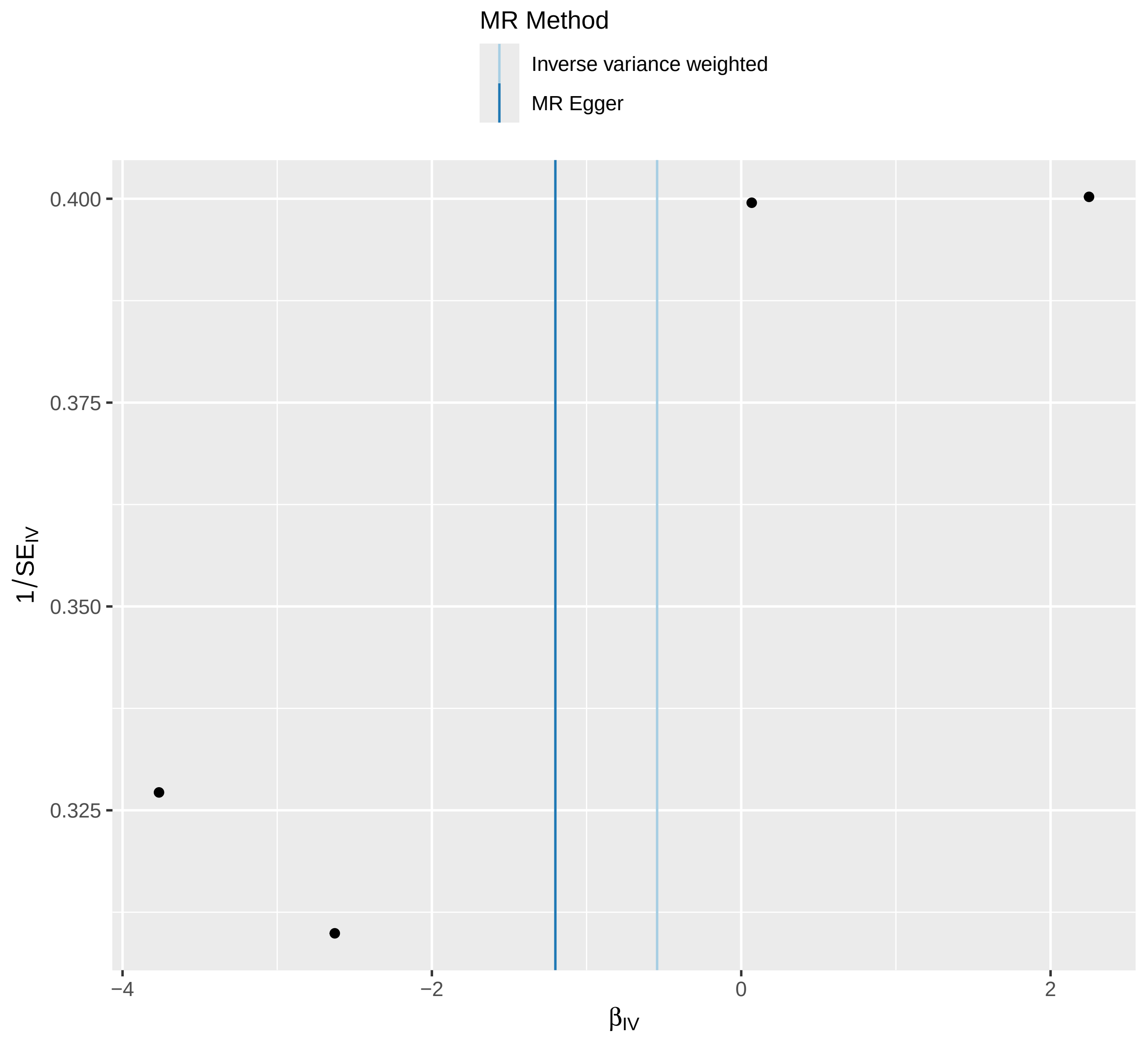

MR Method
Inverse variance weighted
MR Egger
0.400
0.375
0.350
0.325
1/SE_IV
−4
−2
0
2
β_IV

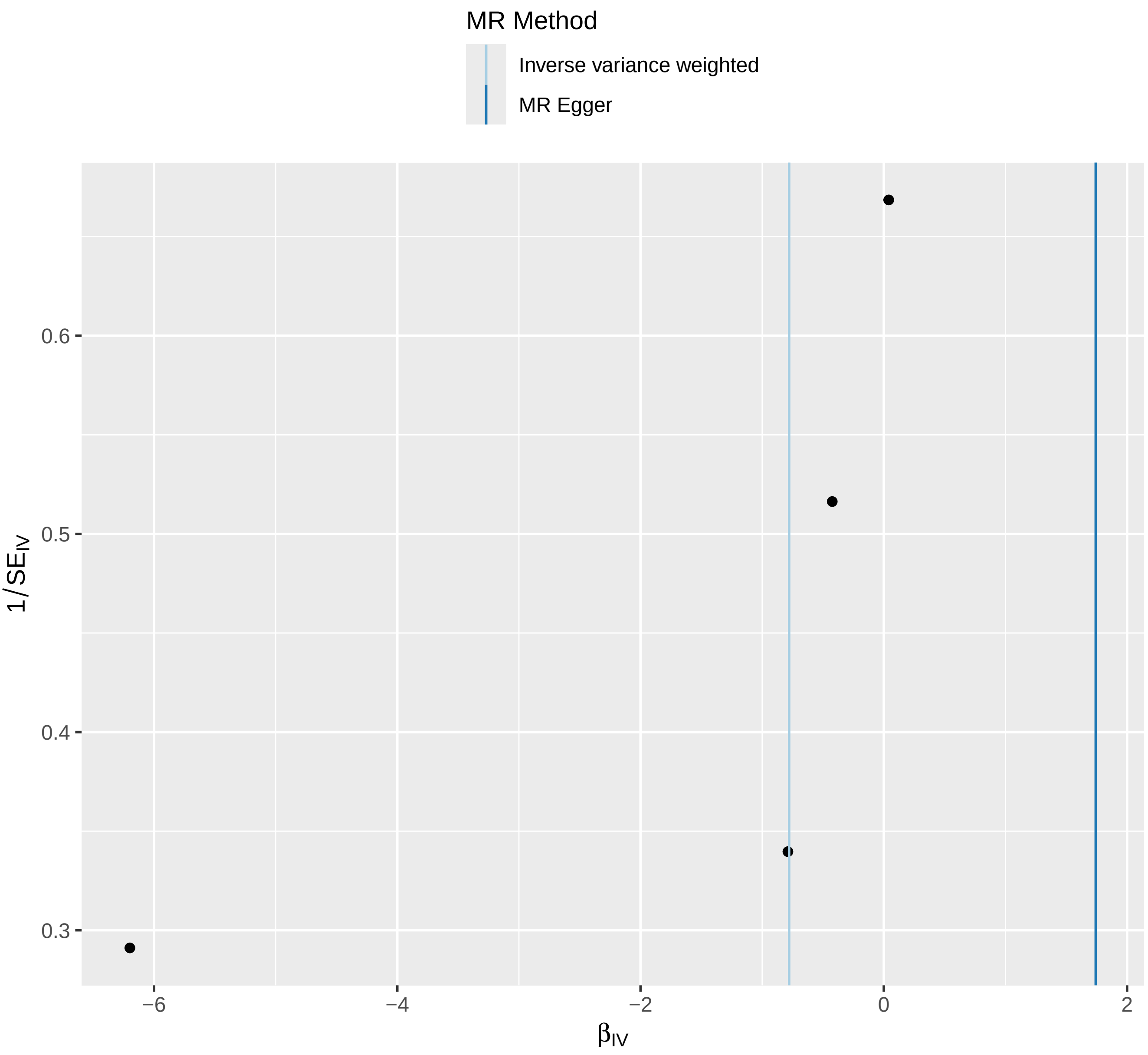

MR Method
Inverse variance weighted
MR Egger
0.6
0.5
0.4
0.3
1/SE_IV
−6
−4
−2
0
2
β_IV

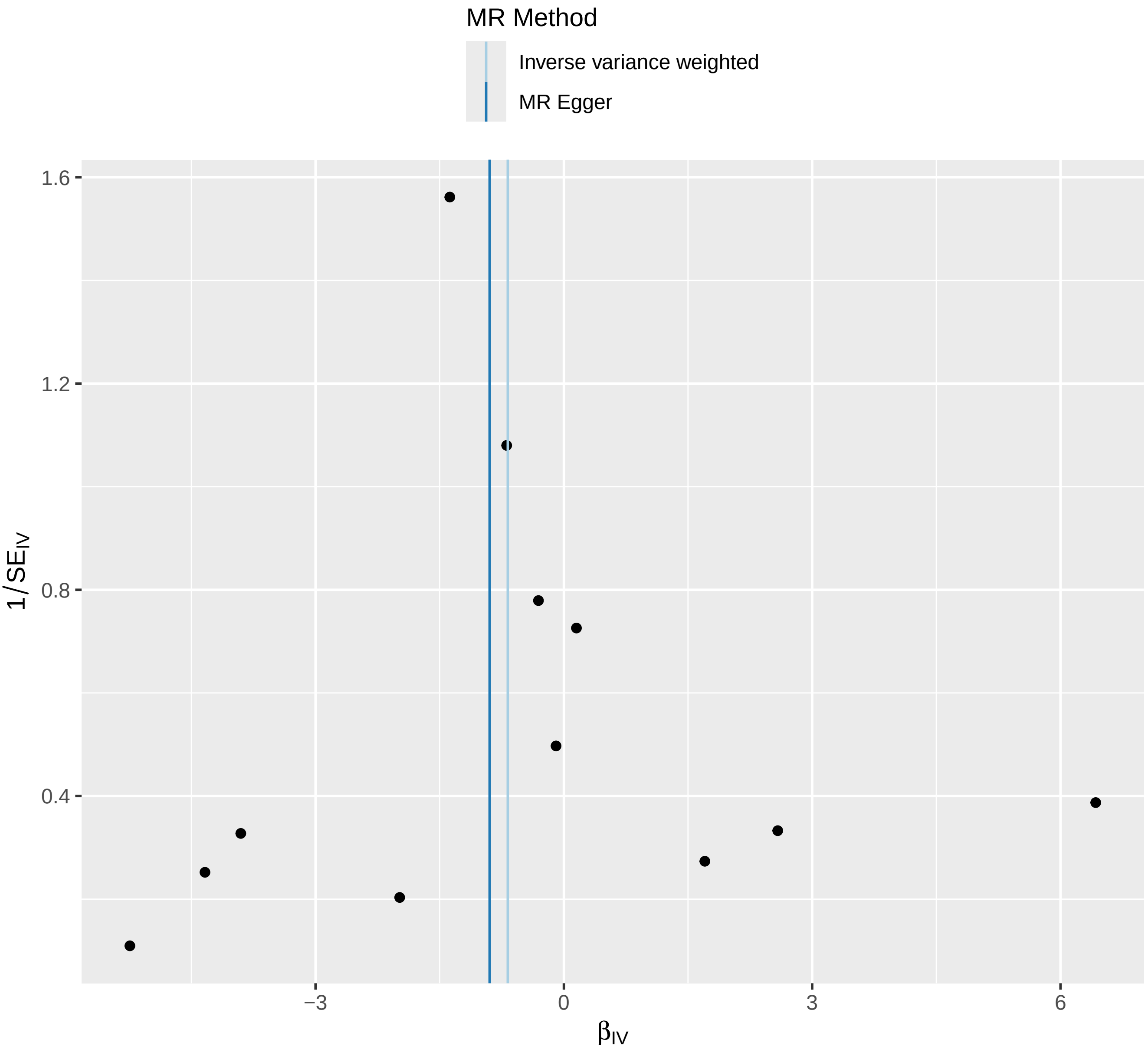

MR Method
Inverse variance weighted
MR Egger
1.6
1.2
0.8
0.4
1/SE_IV
−3
0
3
6
β_IV

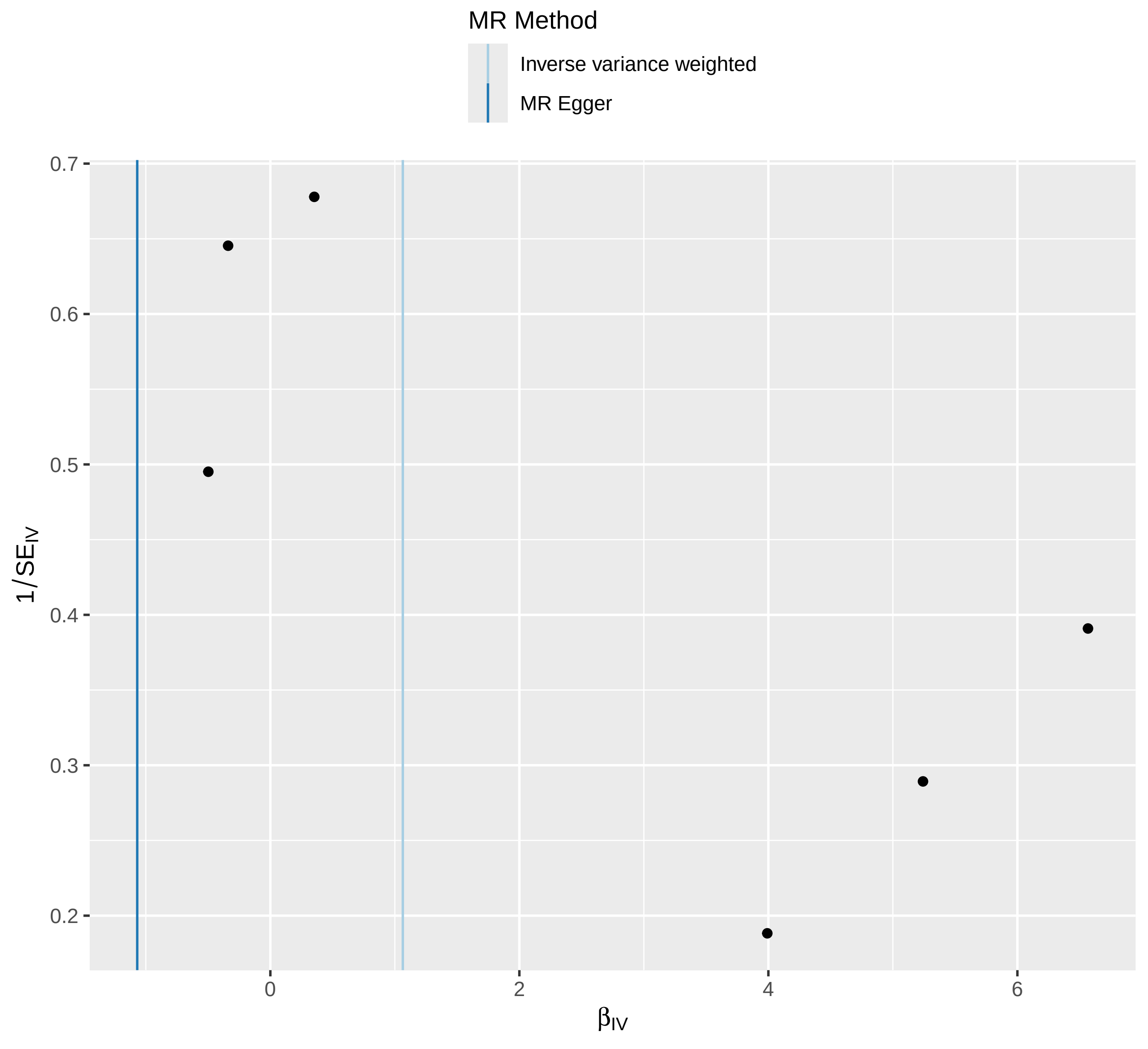
MR Method
Inverse variance weighted
MR Egger
0.7
0.6
0.5
0.4
0.3
0.2
1/SE_IV
0
2
4
6
β_IV

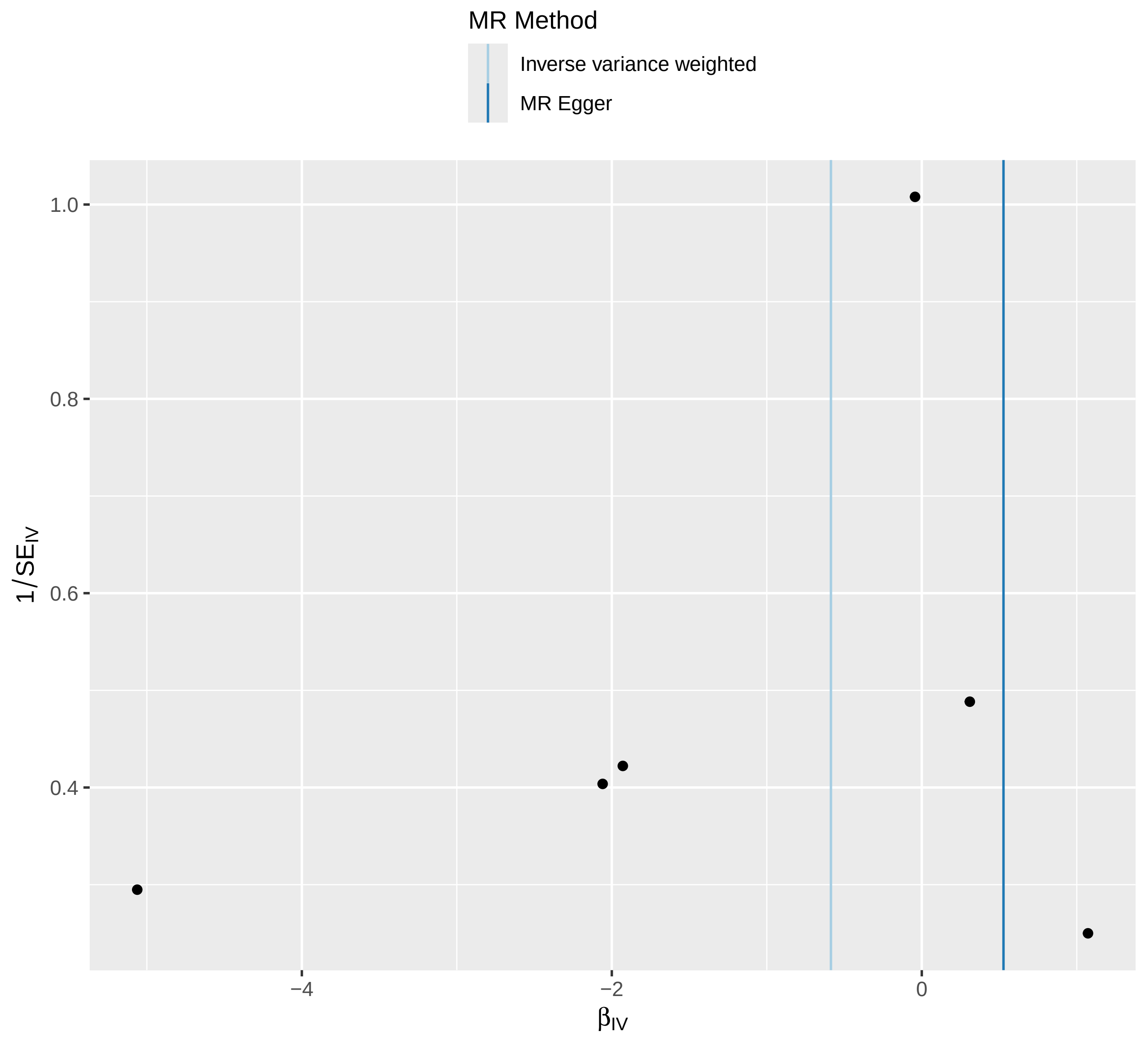

MR Method
Inverse variance weighted
MR Egger
1.0
0.8
0.6
0.4
1/SE_IV
−4
−2
0
β_IV

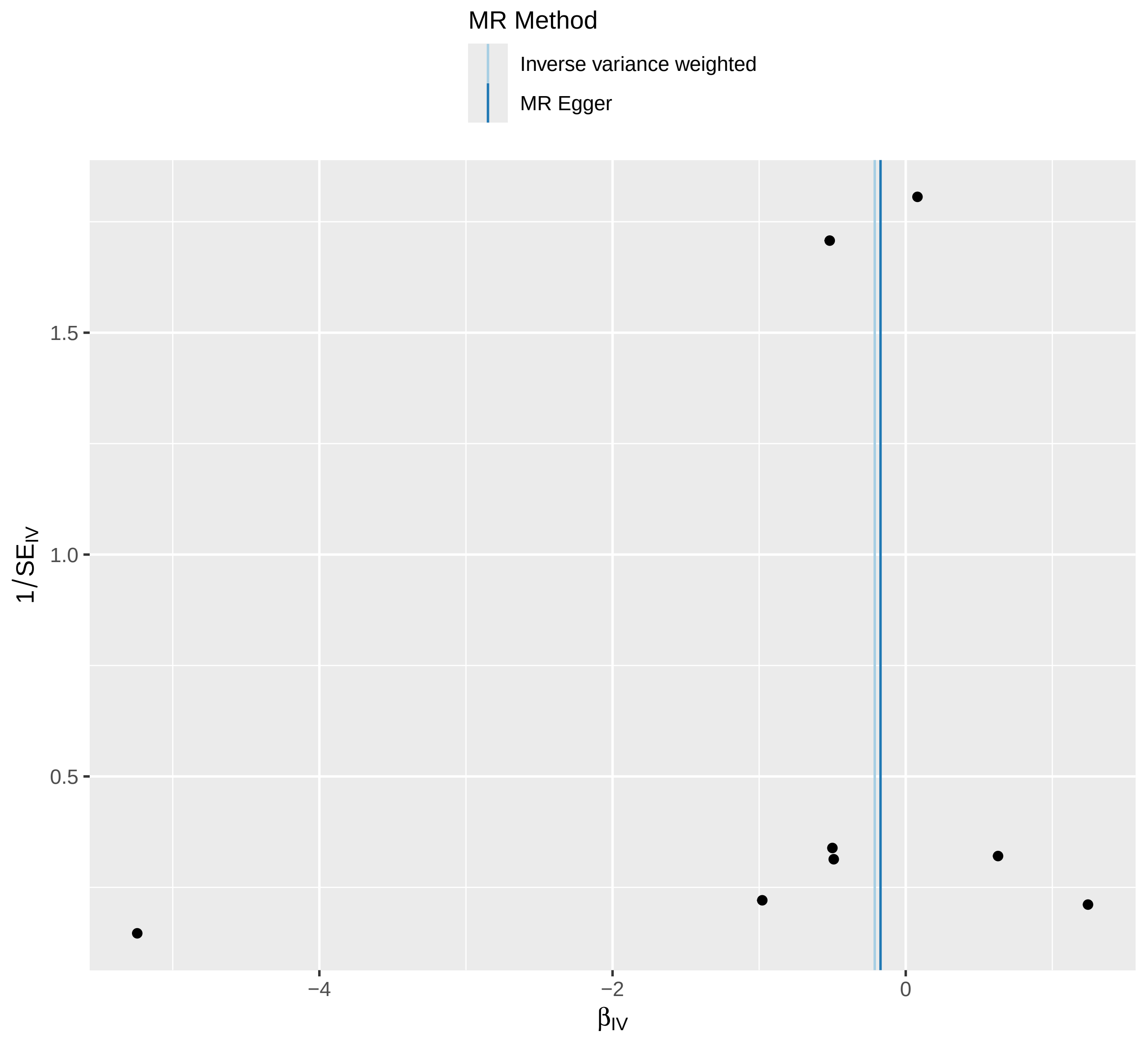
MR Method
Inverse variance weighted
MR Egger
1.5
1.0
0.5
−4
−2
0
1/SE_IV
β_IV

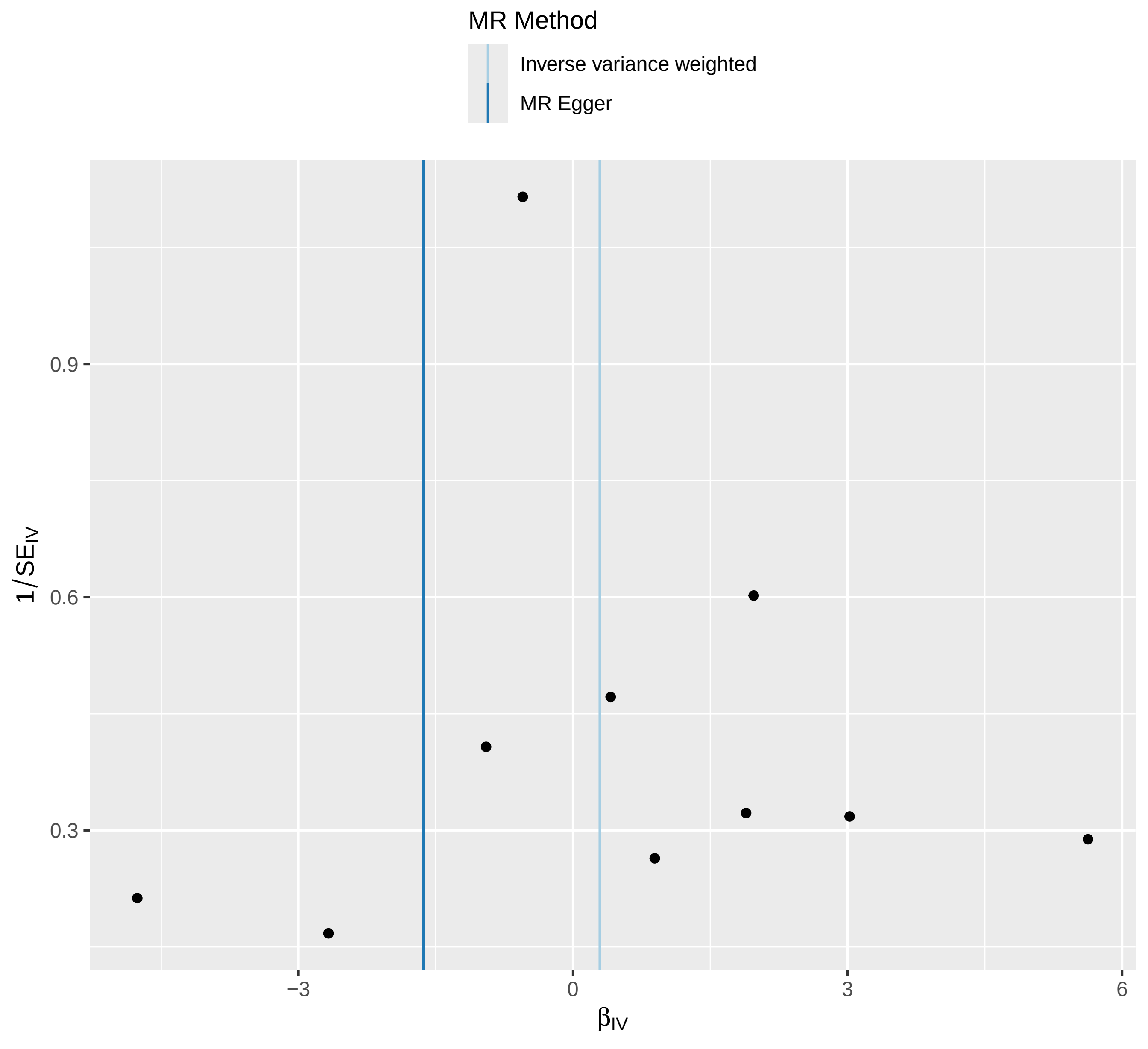
MR Method
Inverse variance weighted
MR Egger
0.9
0.6
0.3
1/SE_IV
−3
0
3
6
β_IV

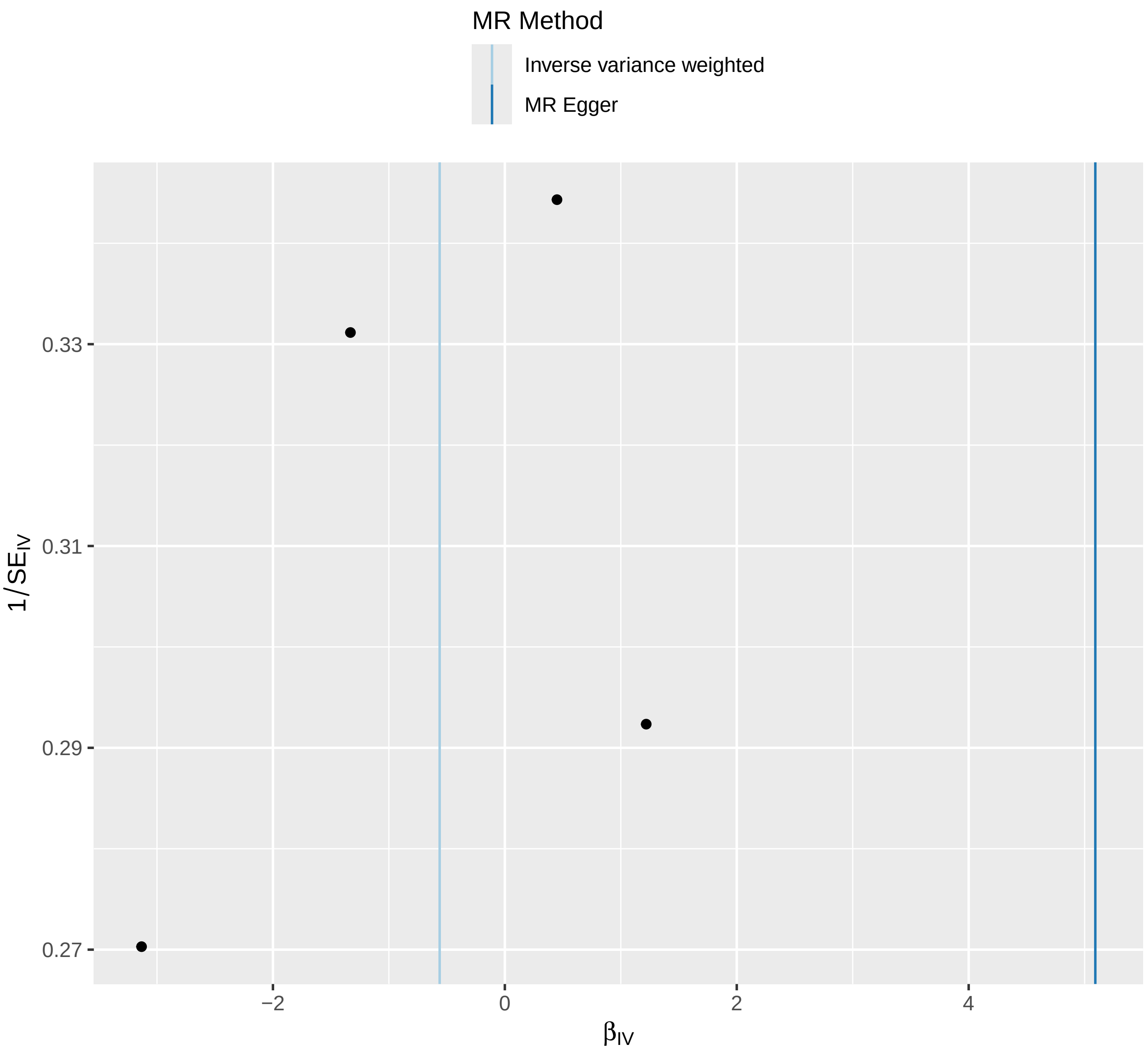

MR Method
Inverse variance weighted
MR Egger
0.33
0.31
0.29
0.27
1/SE_IV
−2
0
2
4
β_IV

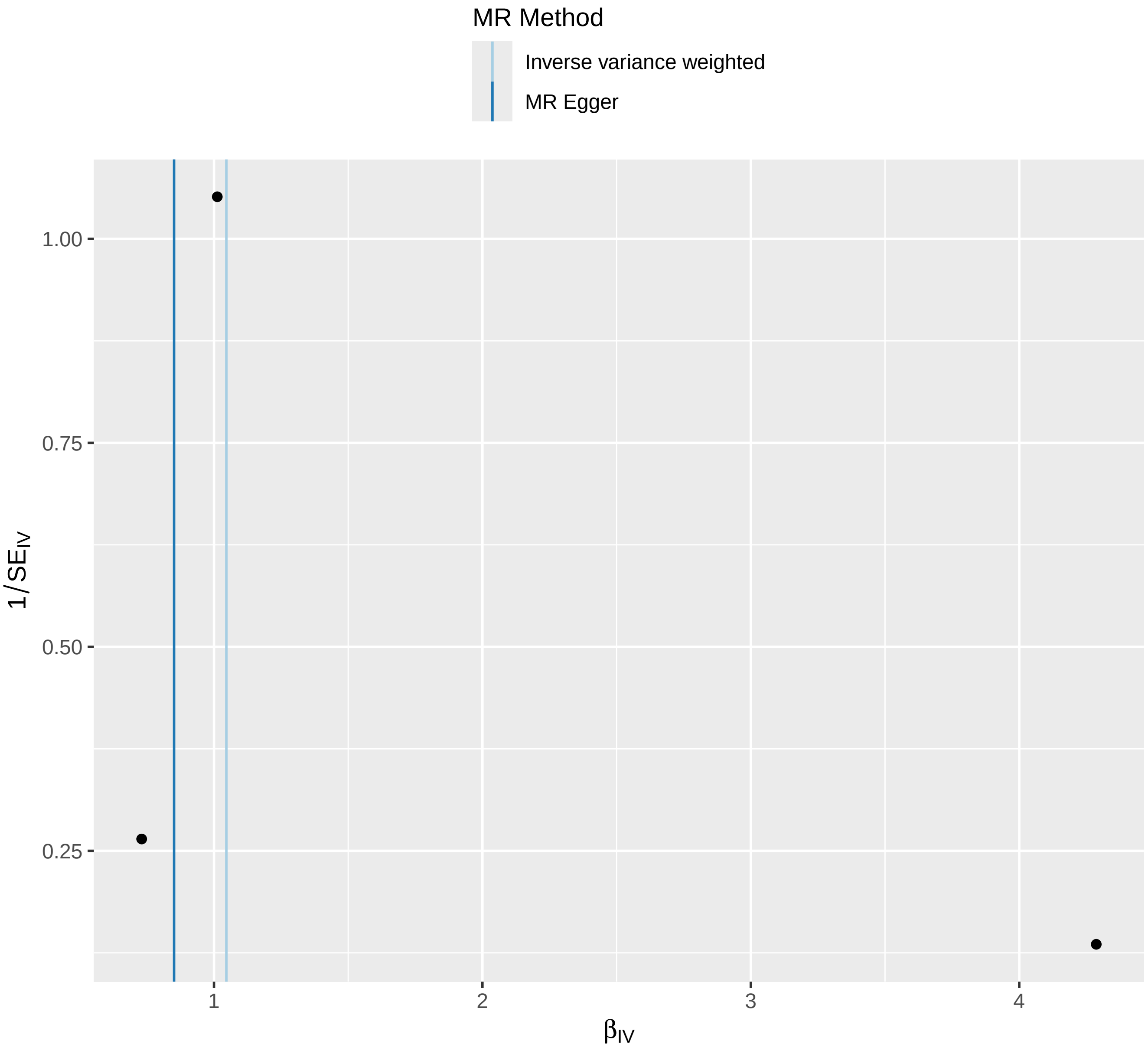
MR Method
Inverse variance weighted
MR Egger
1.00
0.75
0.50
0.25
1/SE$_{IV}$
1
2
3
4
β$_{IV}$

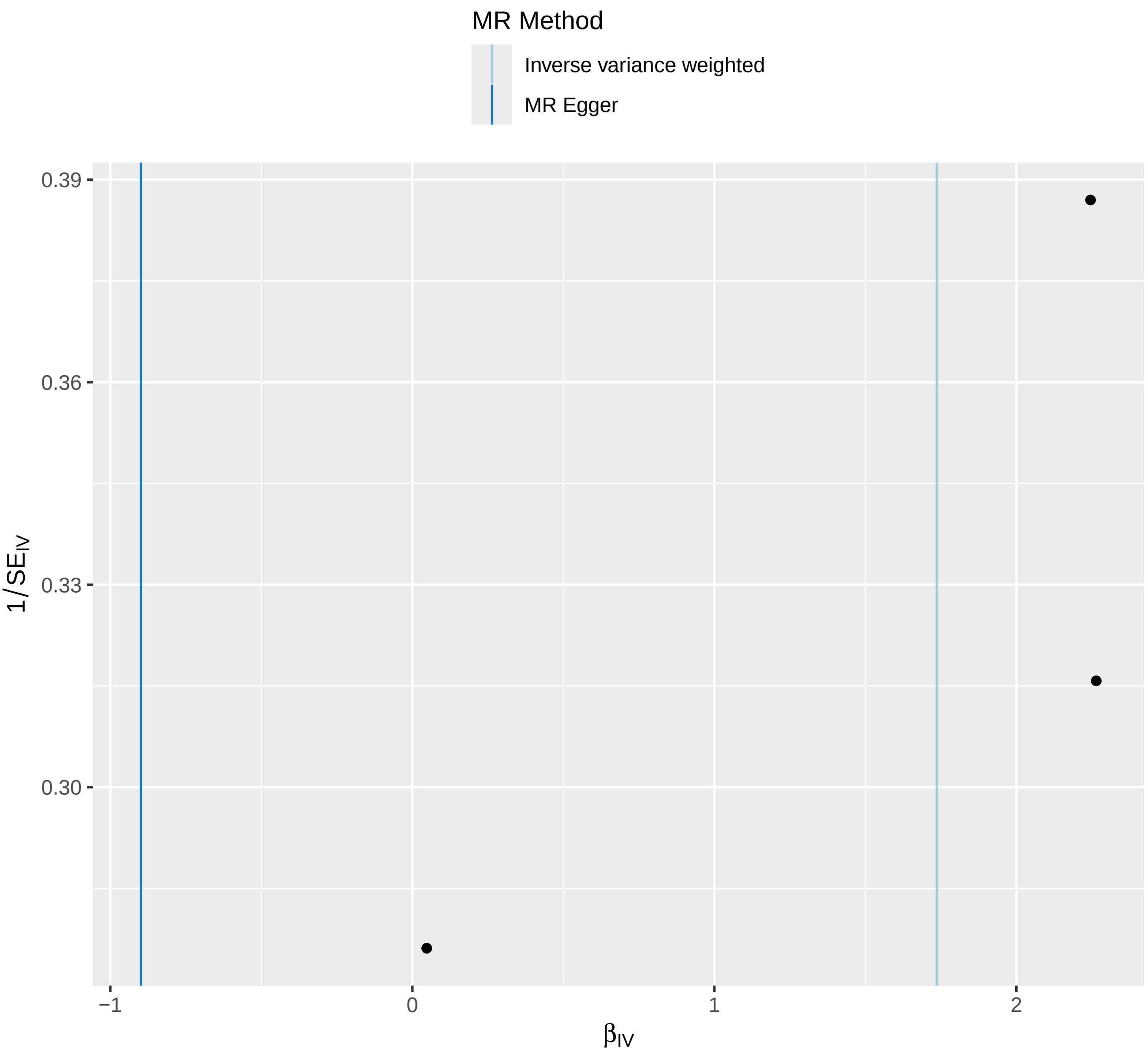

MR Method
Inverse variance weighted
MR Egger
0.39
0.36
0.33
0.30
$1/SE_{IV}$
−1
0
1
2
β_{IV}

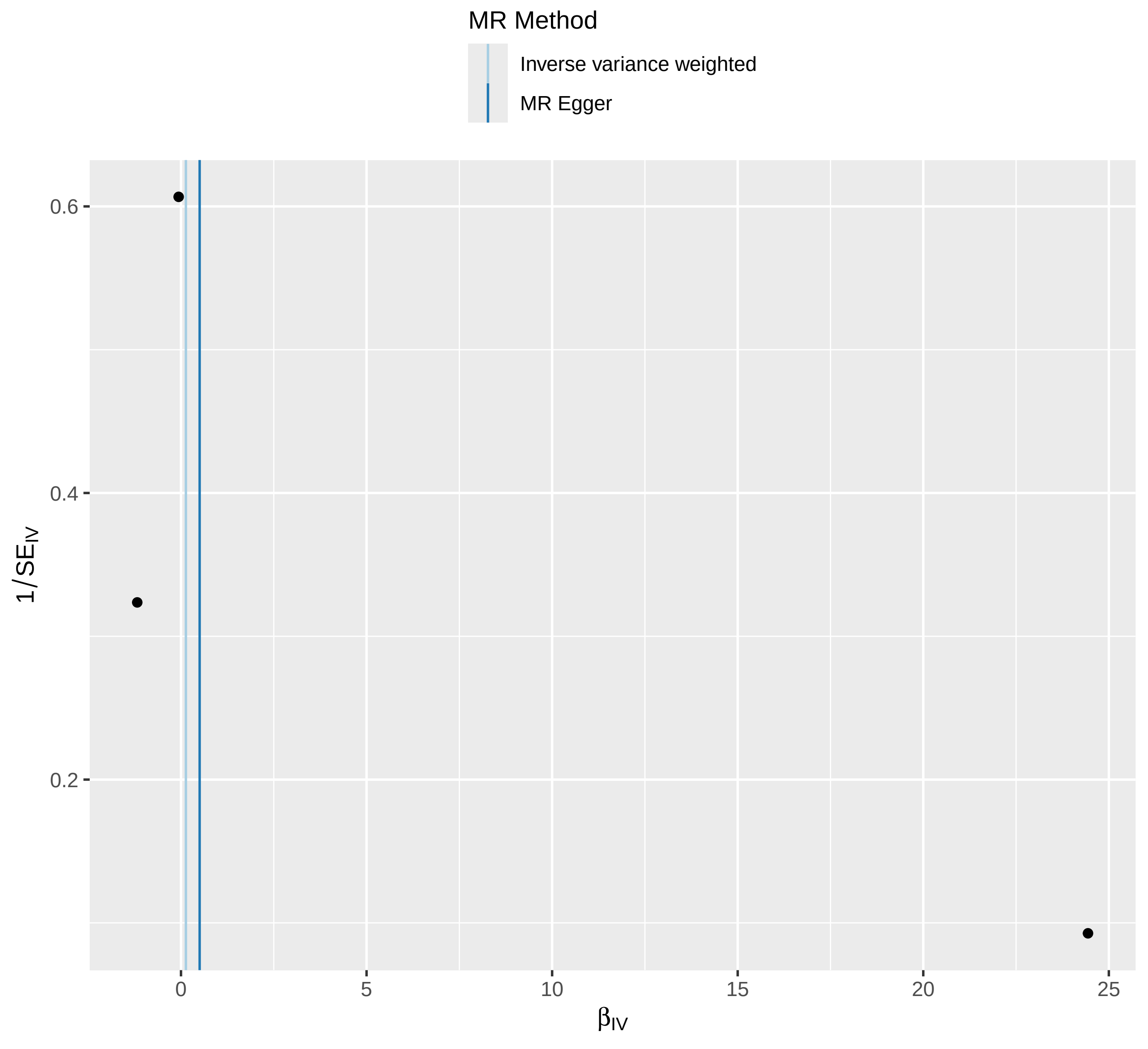

MR Method
Inverse variance weighted
MR Egger
0.6
0.4
0.2
1/SE_IV
0
5
10
15
20
25
β_IV

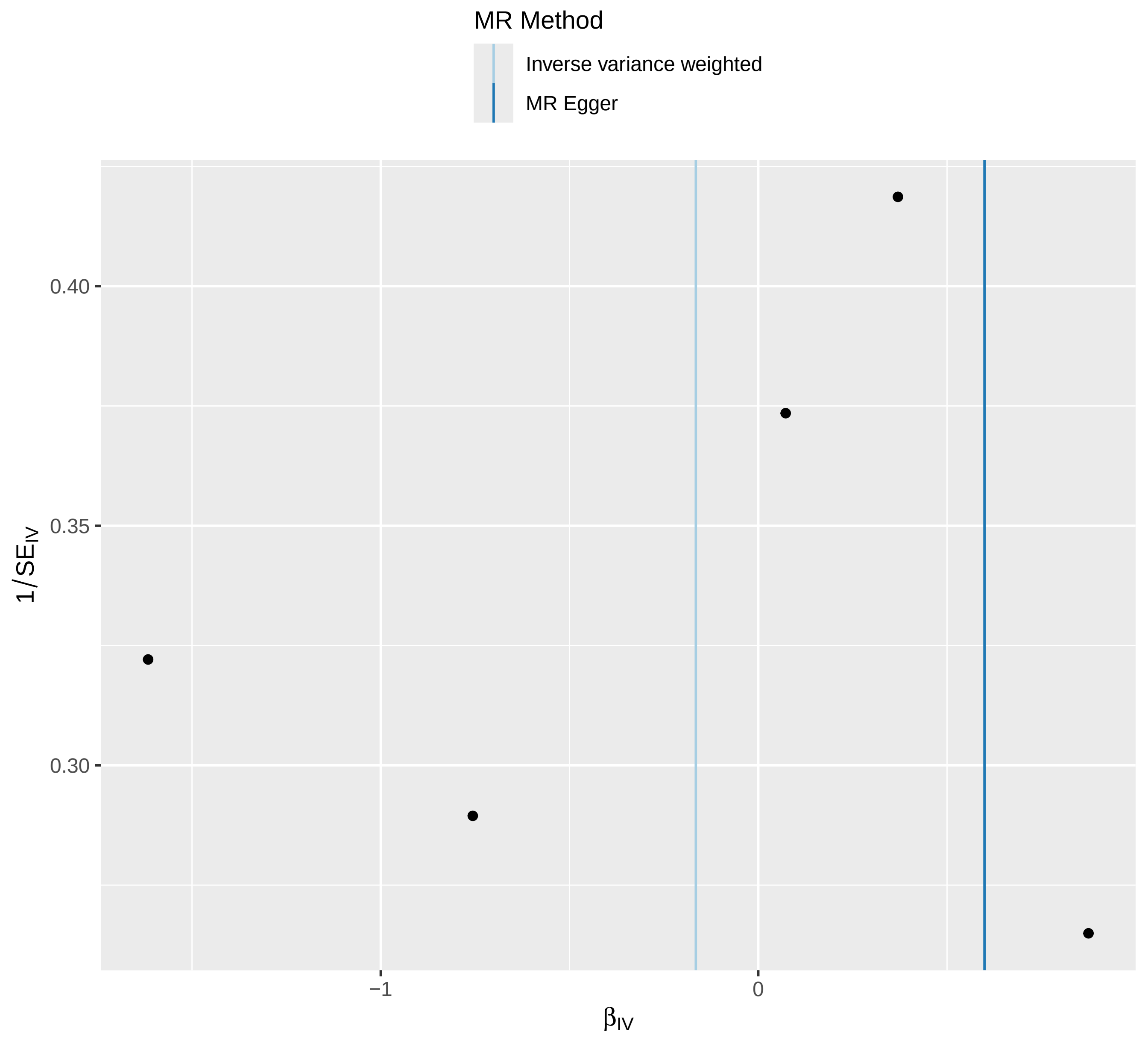
MR Method
Inverse variance weighted
MR Egger
0.40
0.35
0.30
1/SE_IV
−1
0
β_IV

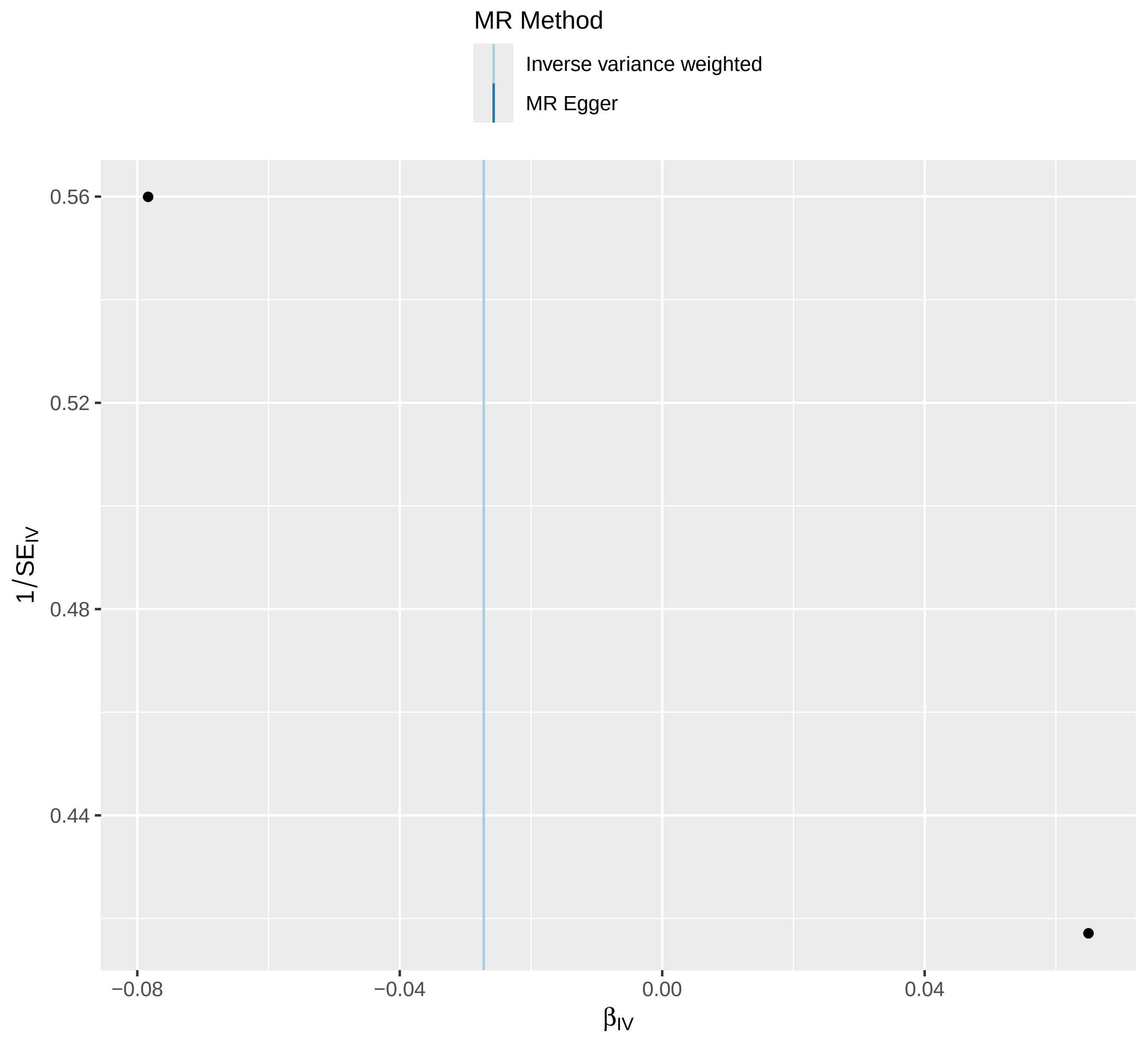

MR Method
Inverse variance weighted
MR Egger
0.56
0.52
0.48
0.44
1/SE_IV
−0.08
−0.04
0.00
0.04
β_IV

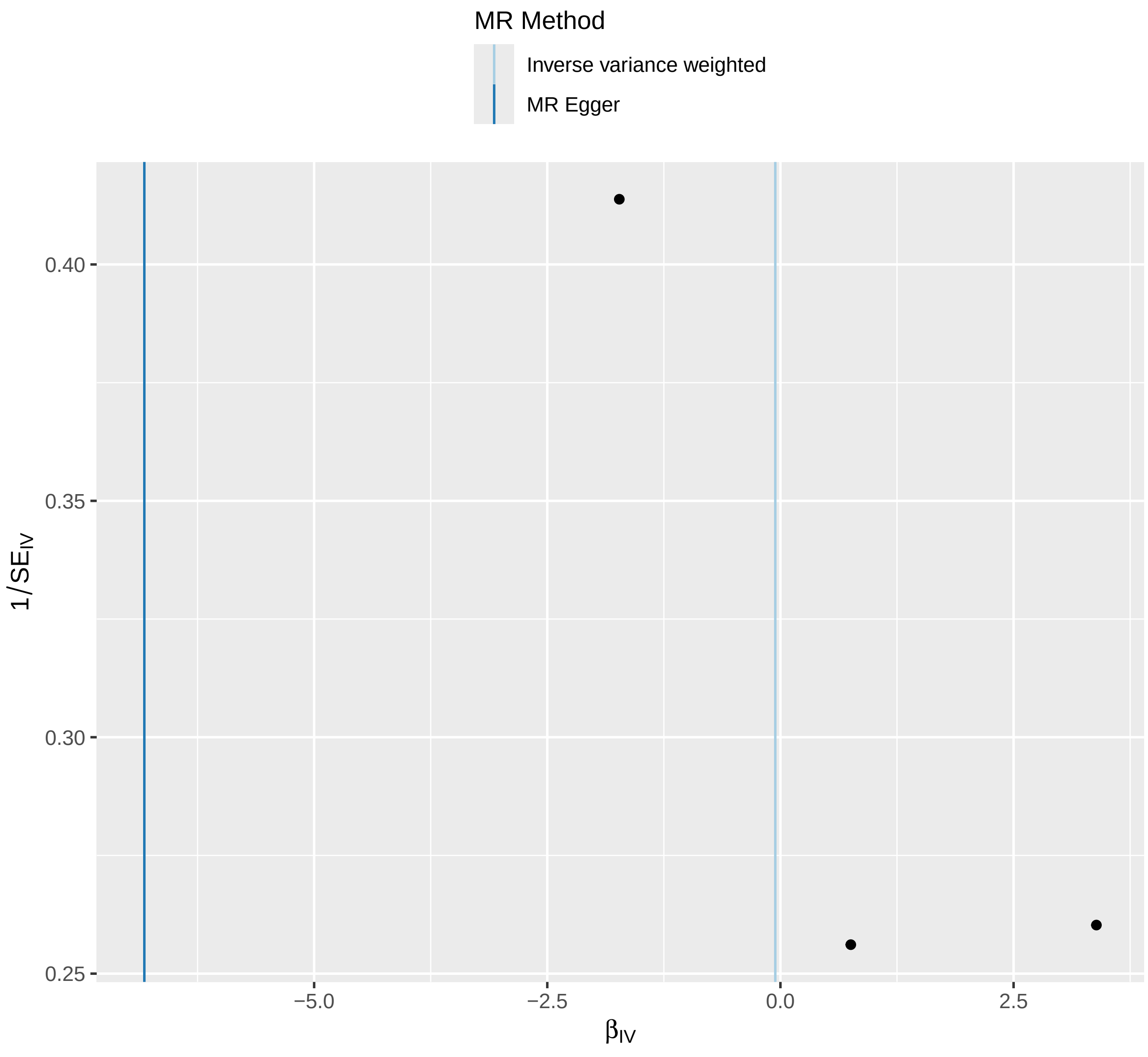
MR Method
Inverse variance weighted
MR Egger
0.40
0.35
0.30
0.25
1/SE_IV
−5.0
−2.5
0.0
2.5
β_IV

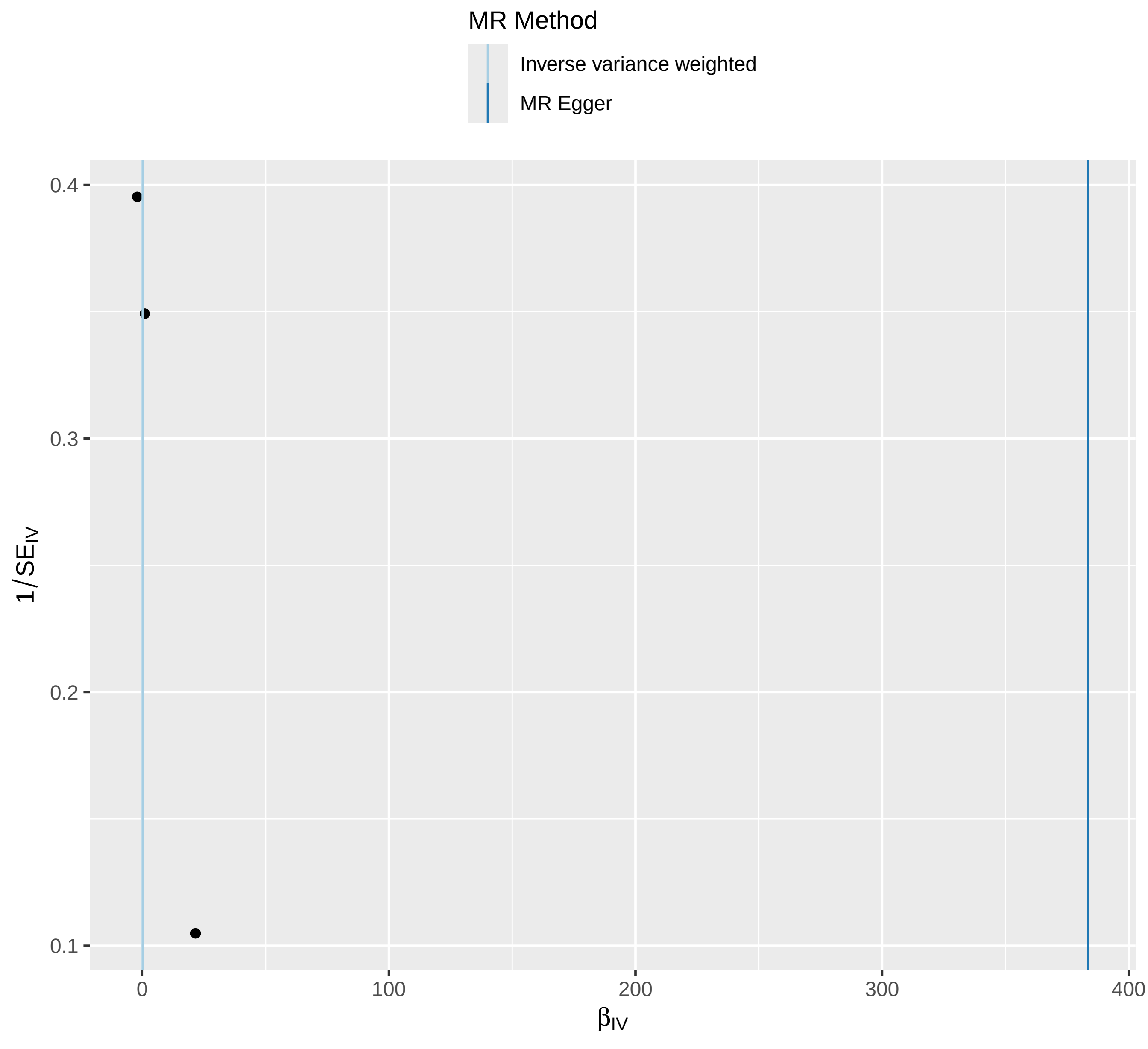
MR Method
Inverse variance weighted
MR Egger
1/SE_IV
0.4
0.3
0.2
0.1
0
100
200
300
400
β_IV

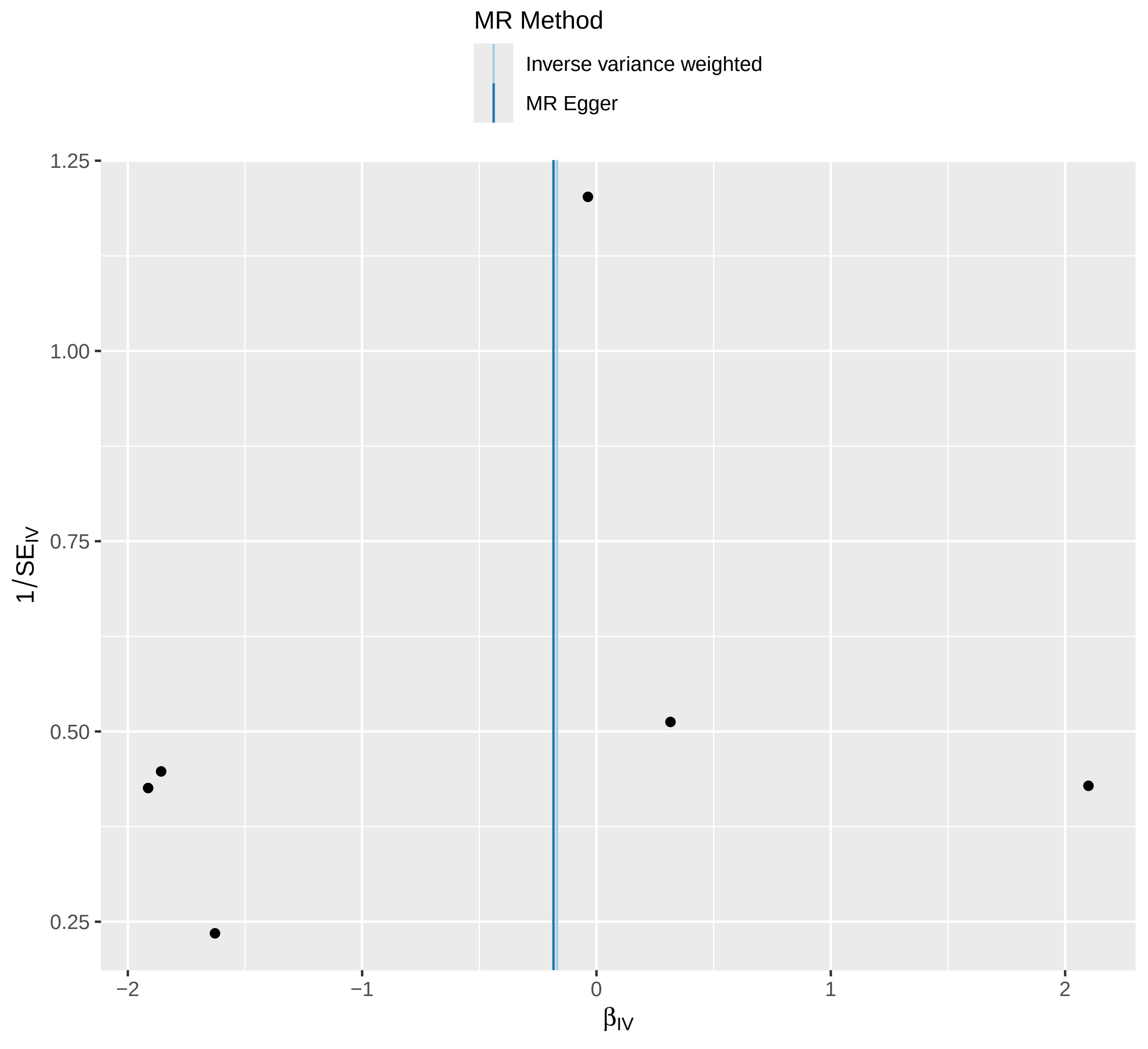
MR Method
Inverse variance weighted
MR Egger
1.25
1.00
0.75
0.50
0.25
1/SE_IV
−2
−1
0
1
2
β_IV

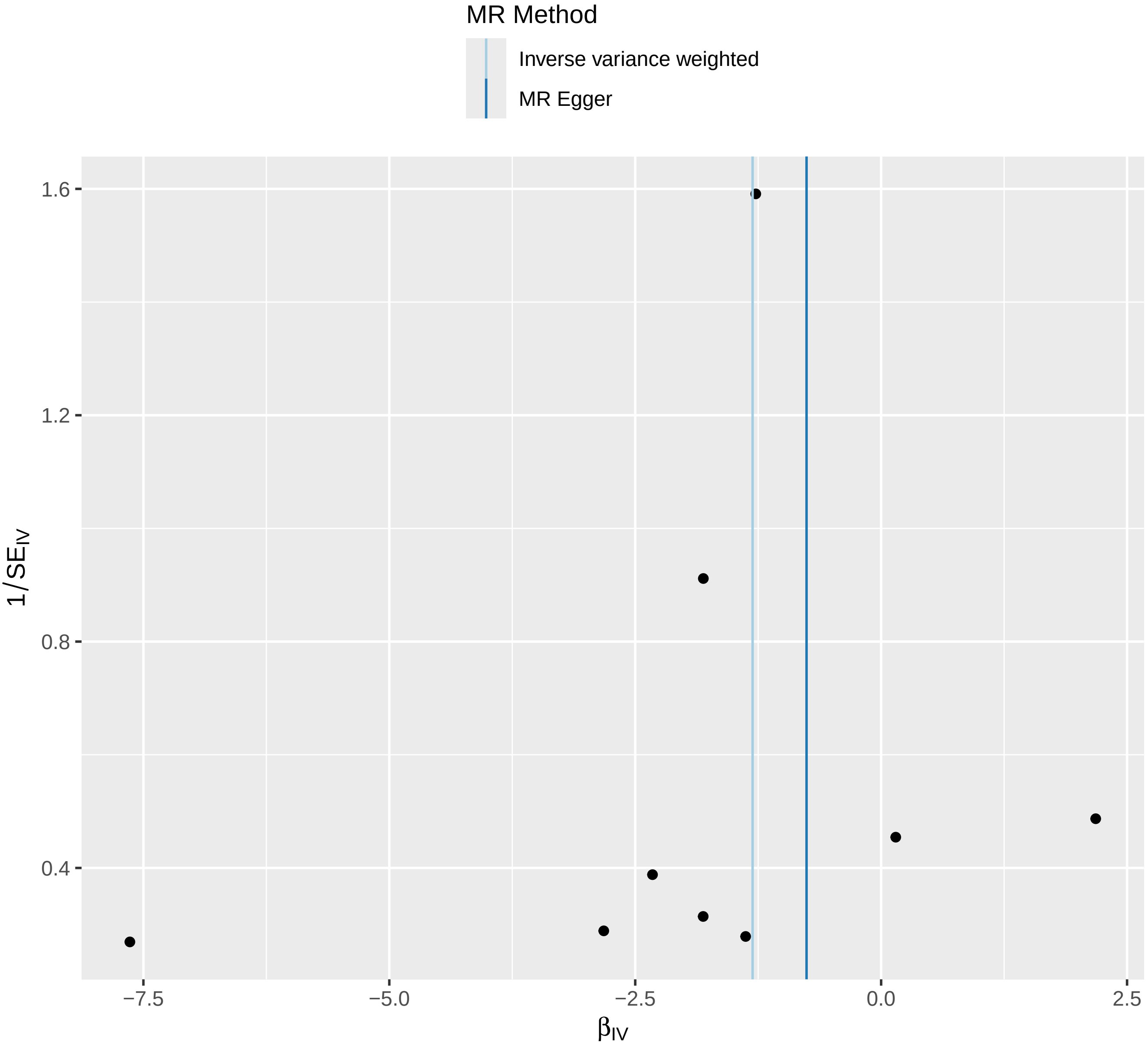
MR Method
Inverse variance weighted
MR Egger
1.6
1.2
0.8
0.4
1/SE_IV
−7.5
−5.0
−2.5
0.0
2.5
β_IV

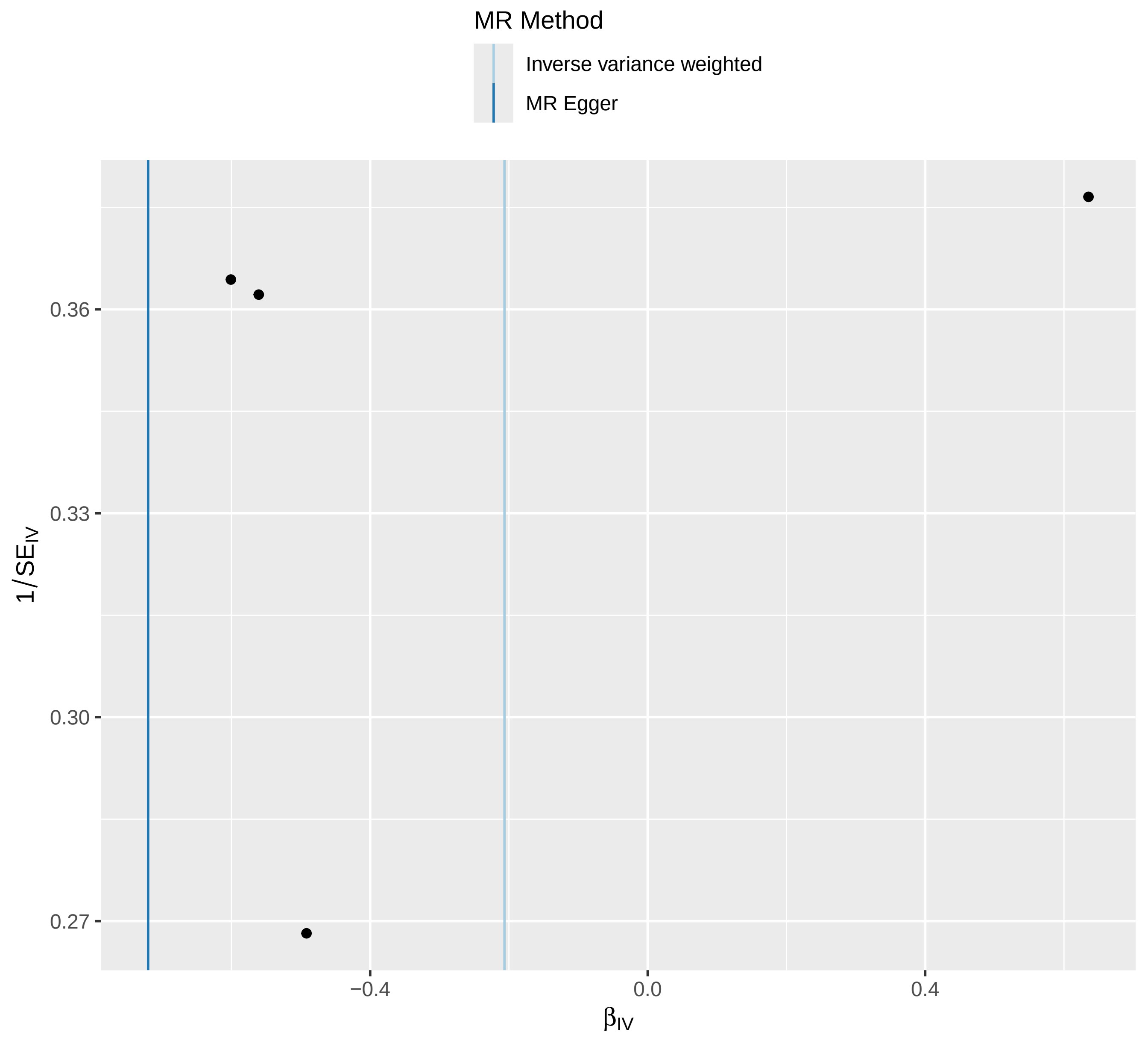
MR Method
Inverse variance weighted
MR Egger
0.36
0.33
0.30
0.27
$1/SE_{IV}$
−0.4
0.0
0.4
β_{IV}

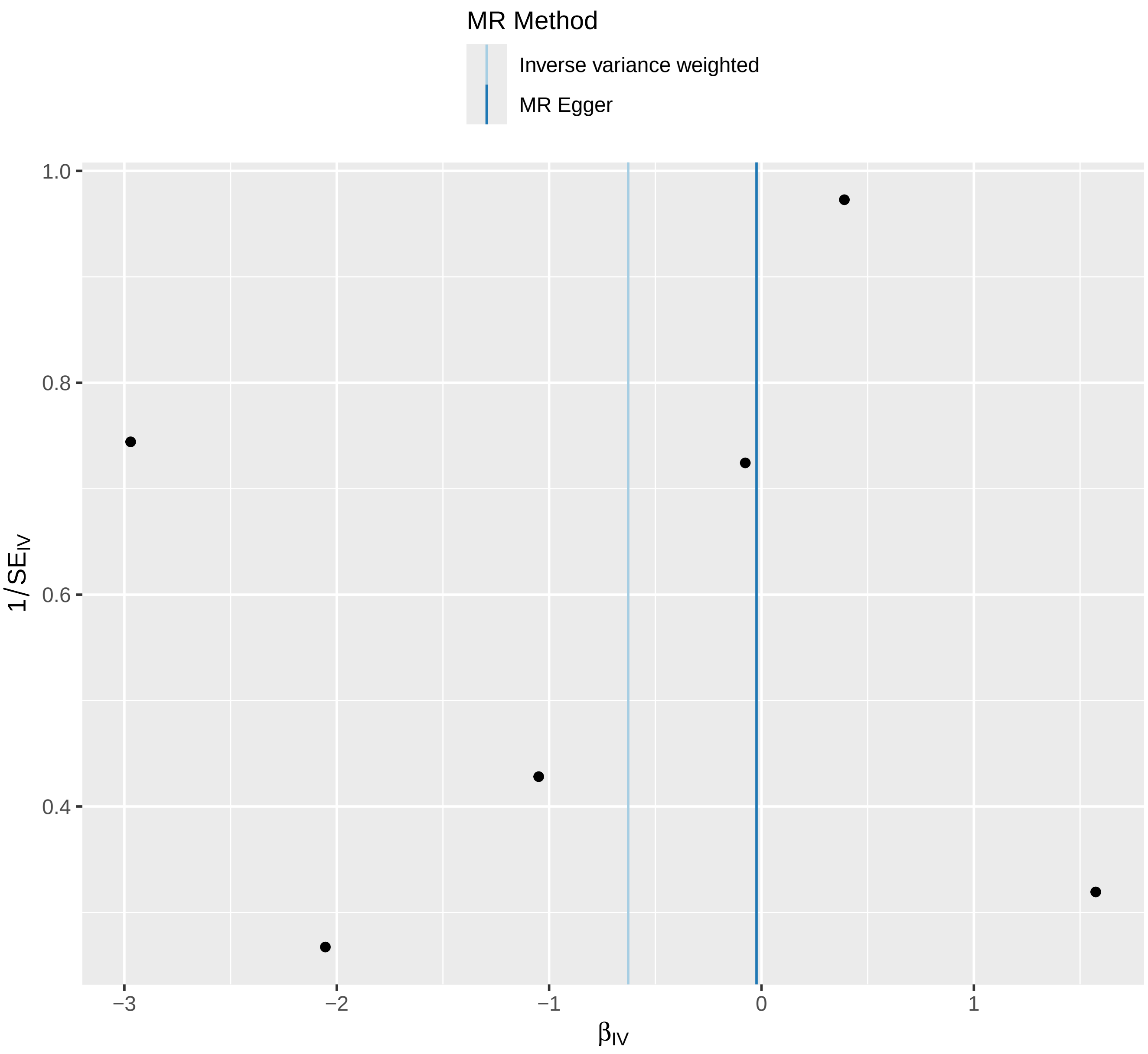
MR Method
Inverse variance weighted
MR Egger
1.0
0.8
0.6
0.4
1/SE_IV
−3
−2
−1
0
1
β_IV

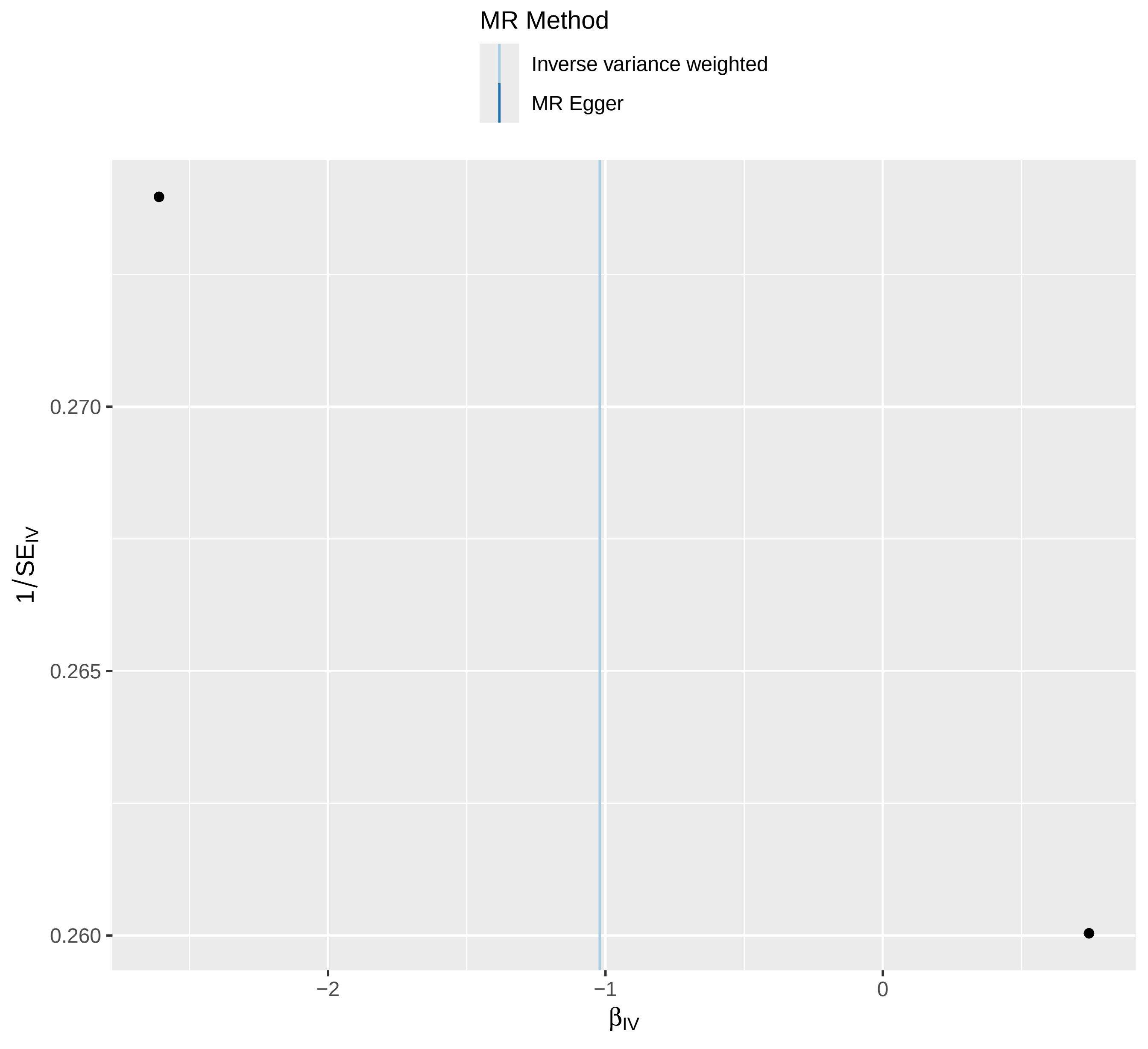
MR Method
Inverse variance weighted
MR Egger
0.270
0.265
0.260
1/SE_IV
−2
−1
0
β_IV

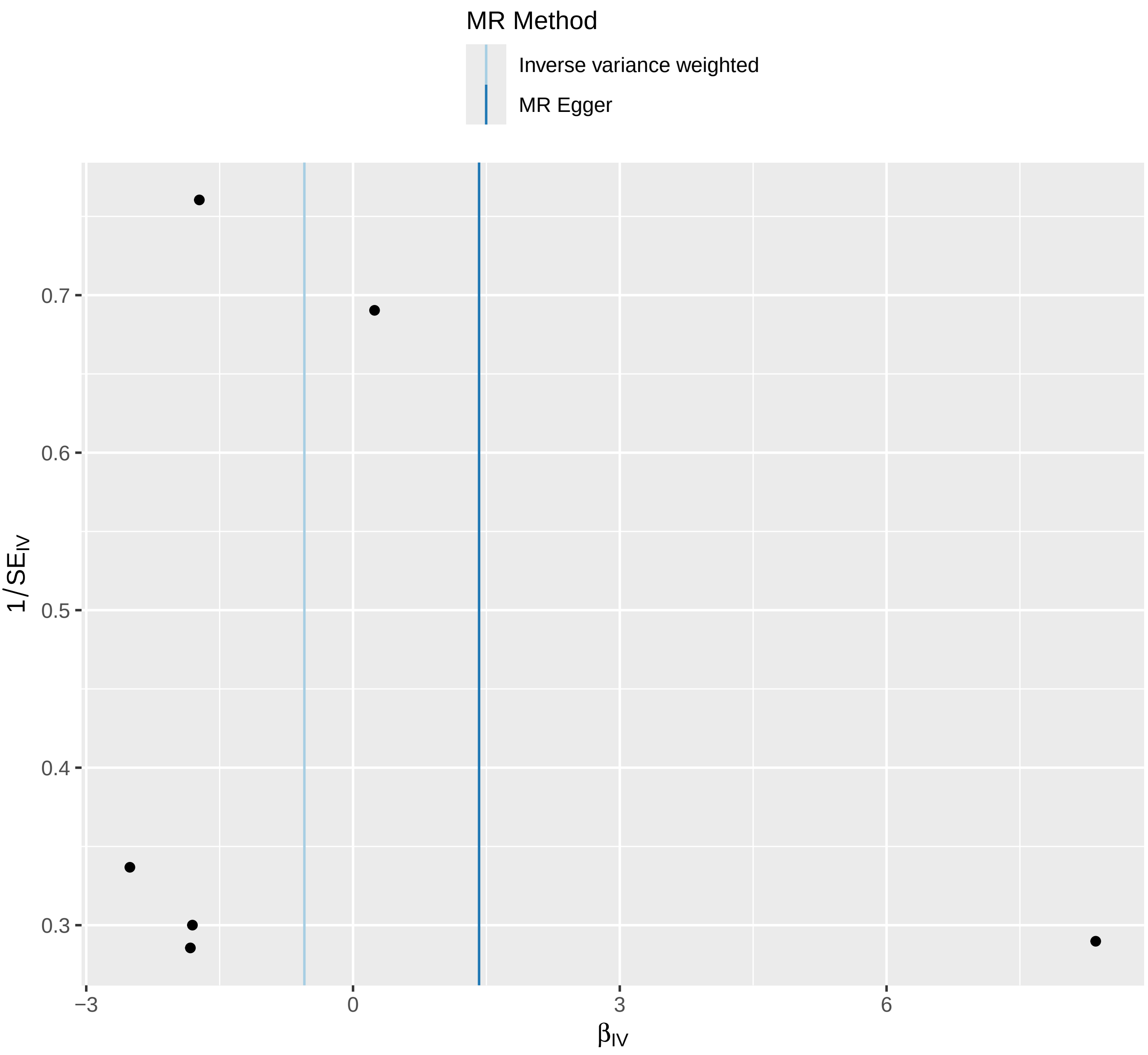
MR Method
Inverse variance weighted
MR Egger
0.7
0.6
0.5
0.4
0.3
1/SE_IV
−3
0
3
6
β_IV

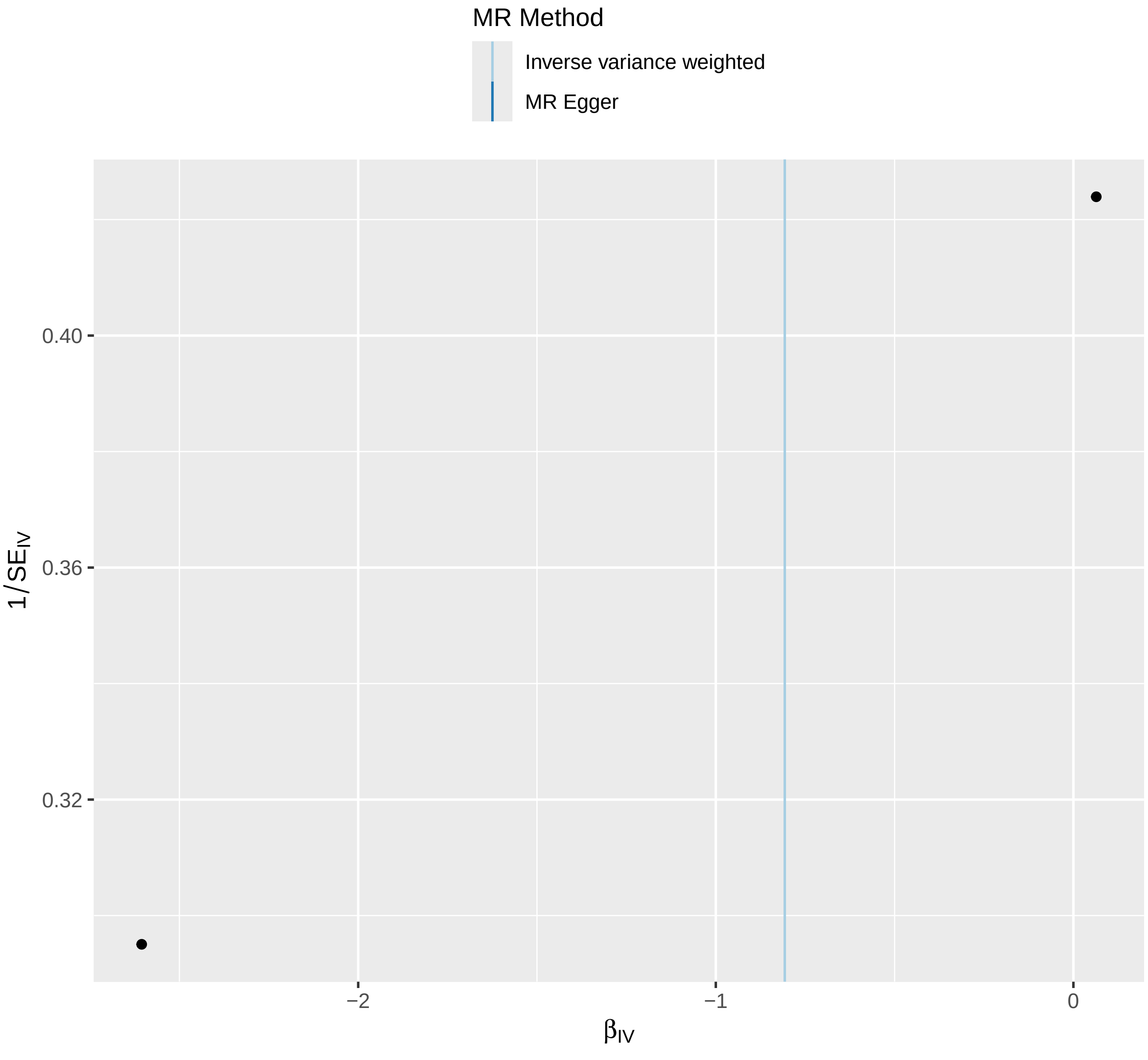
MR Method
Inverse variance weighted
MR Egger
0.40
0.36
0.32
1/SE_IV
−2
−1
0
β_IV

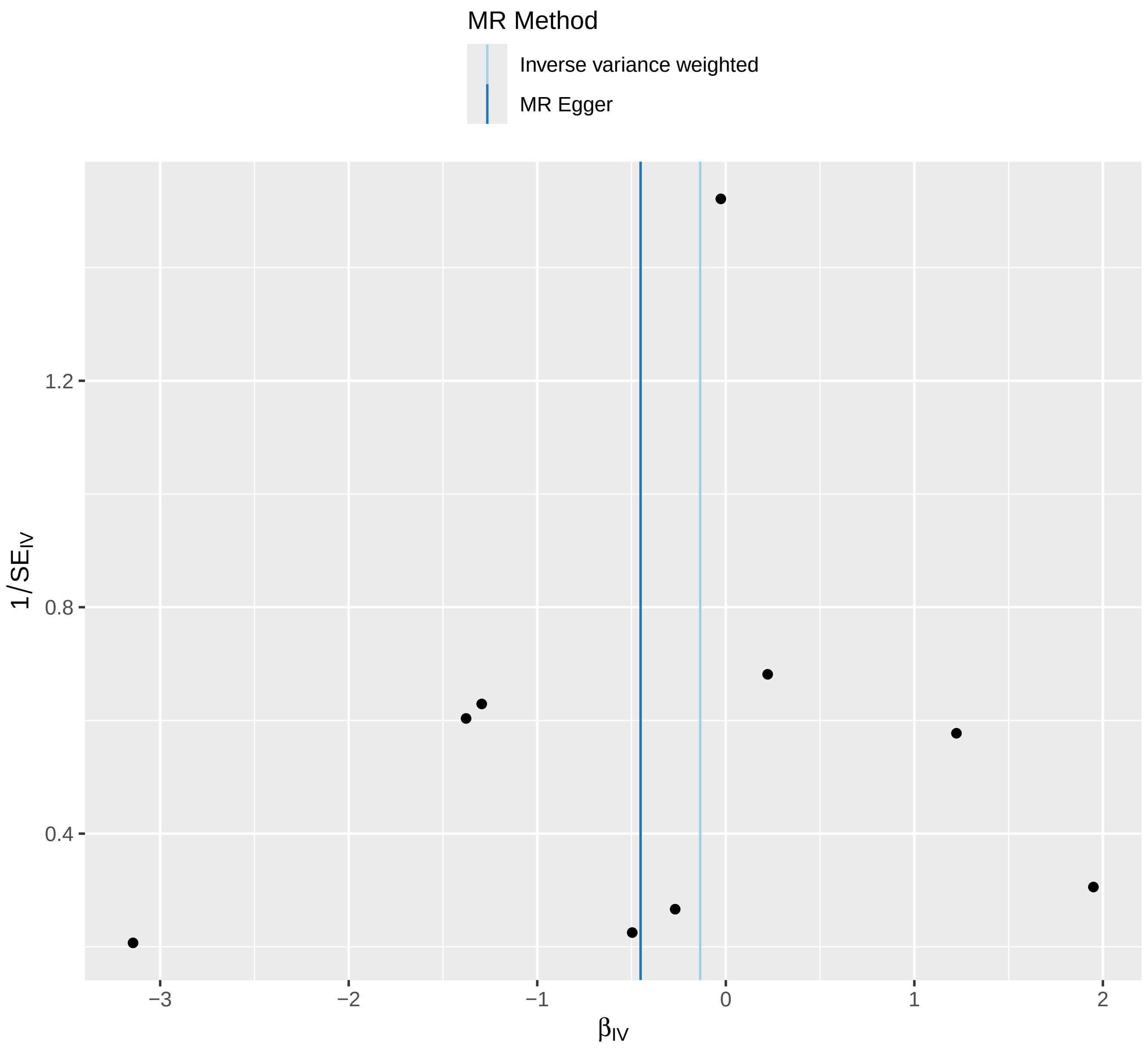
MR Method
Inverse variance weighted
MR Egger
1/SE$_{IV}$
1.2
0.8
0.4
−3
−2
−1
0
1
2
β$_{IV}$

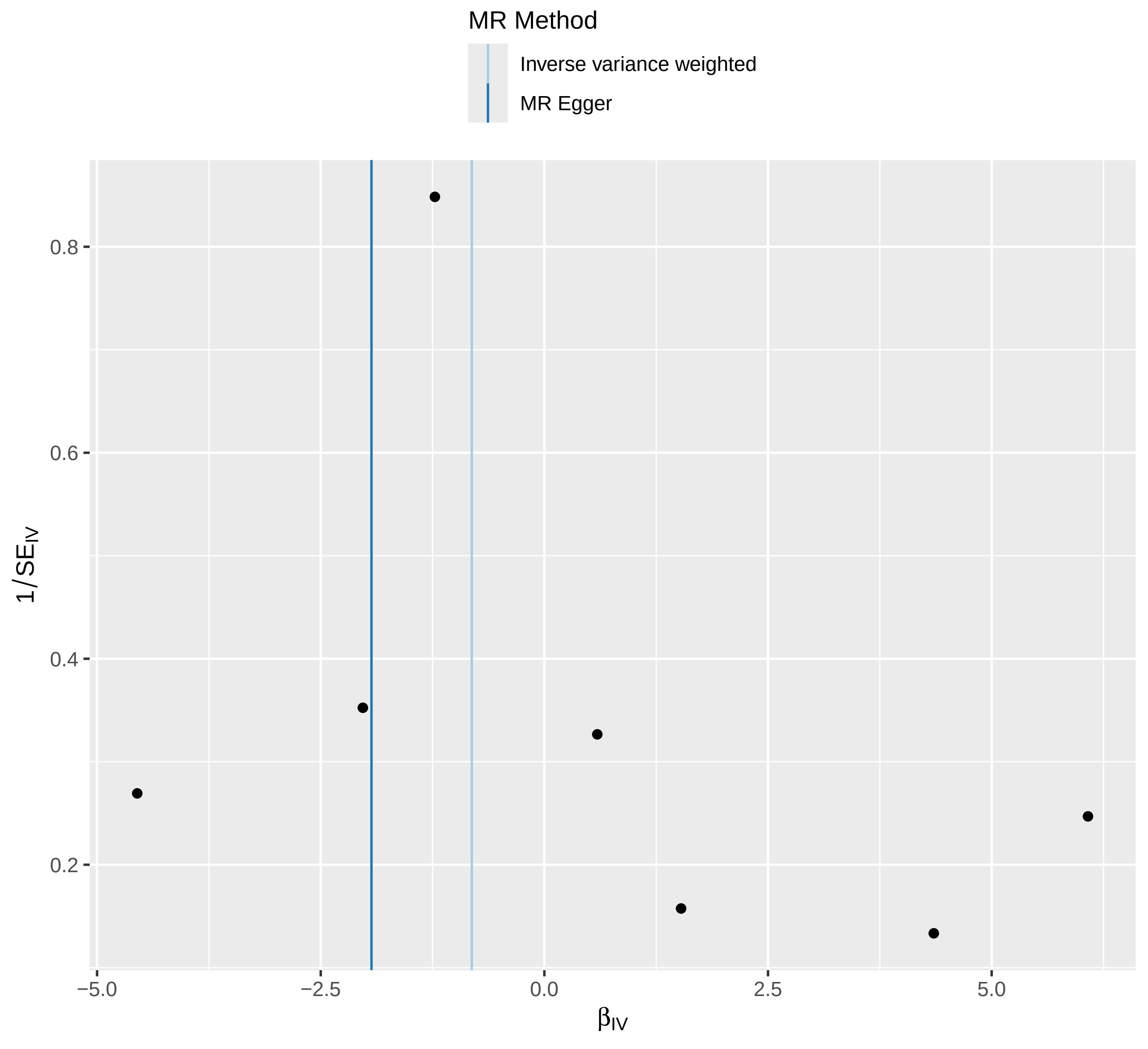

MR Method
Inverse variance weighted
MR Egger
0.8
0.6
0.4
0.2
1/SE_IV
−5.0
−2.5
0.0
2.5
5.0
β_IV

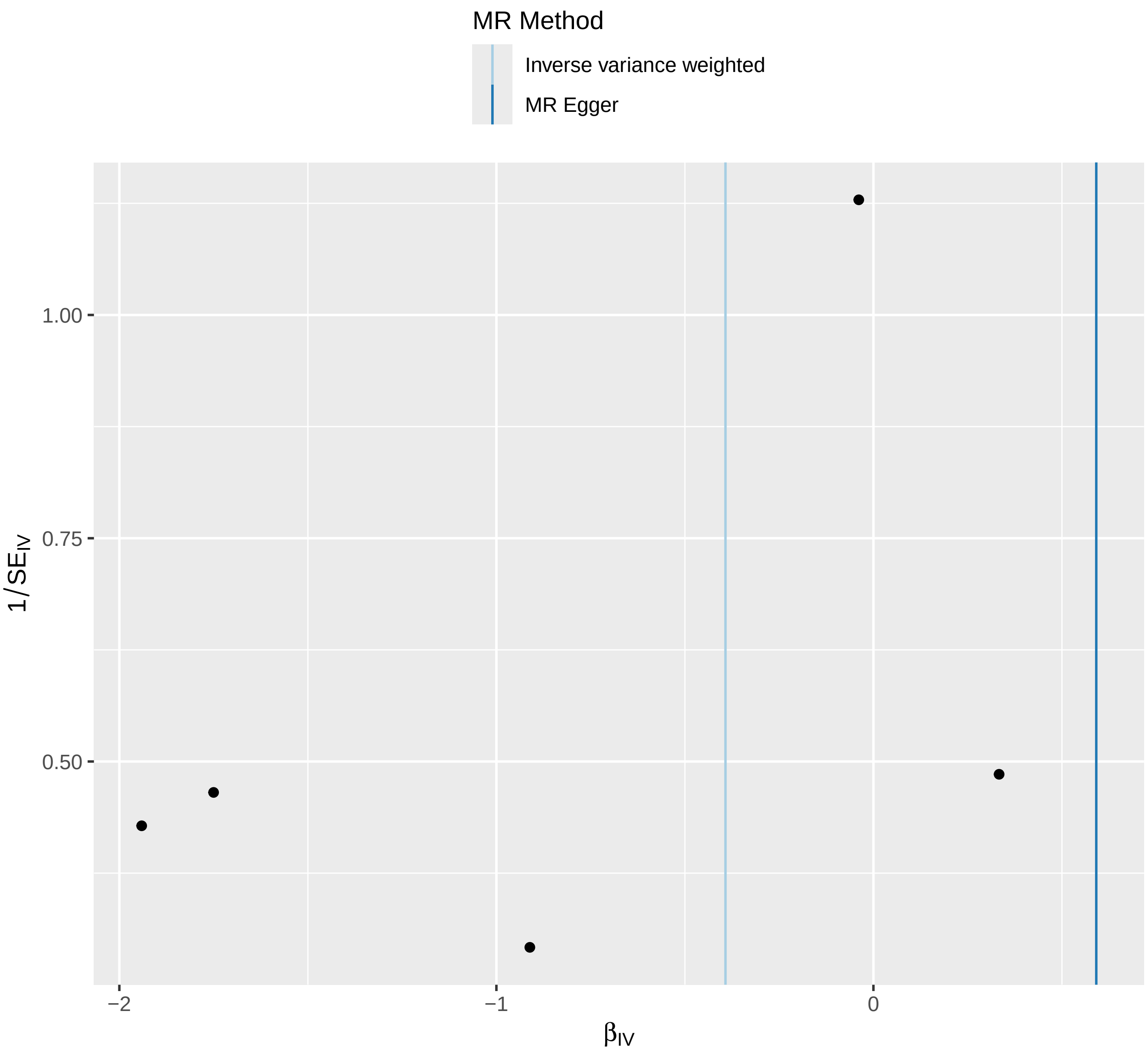

MR Method
Inverse variance weighted
MR Egger
1.00
0.75
0.50
1/SE_IV
−2
−1
0
β_IV

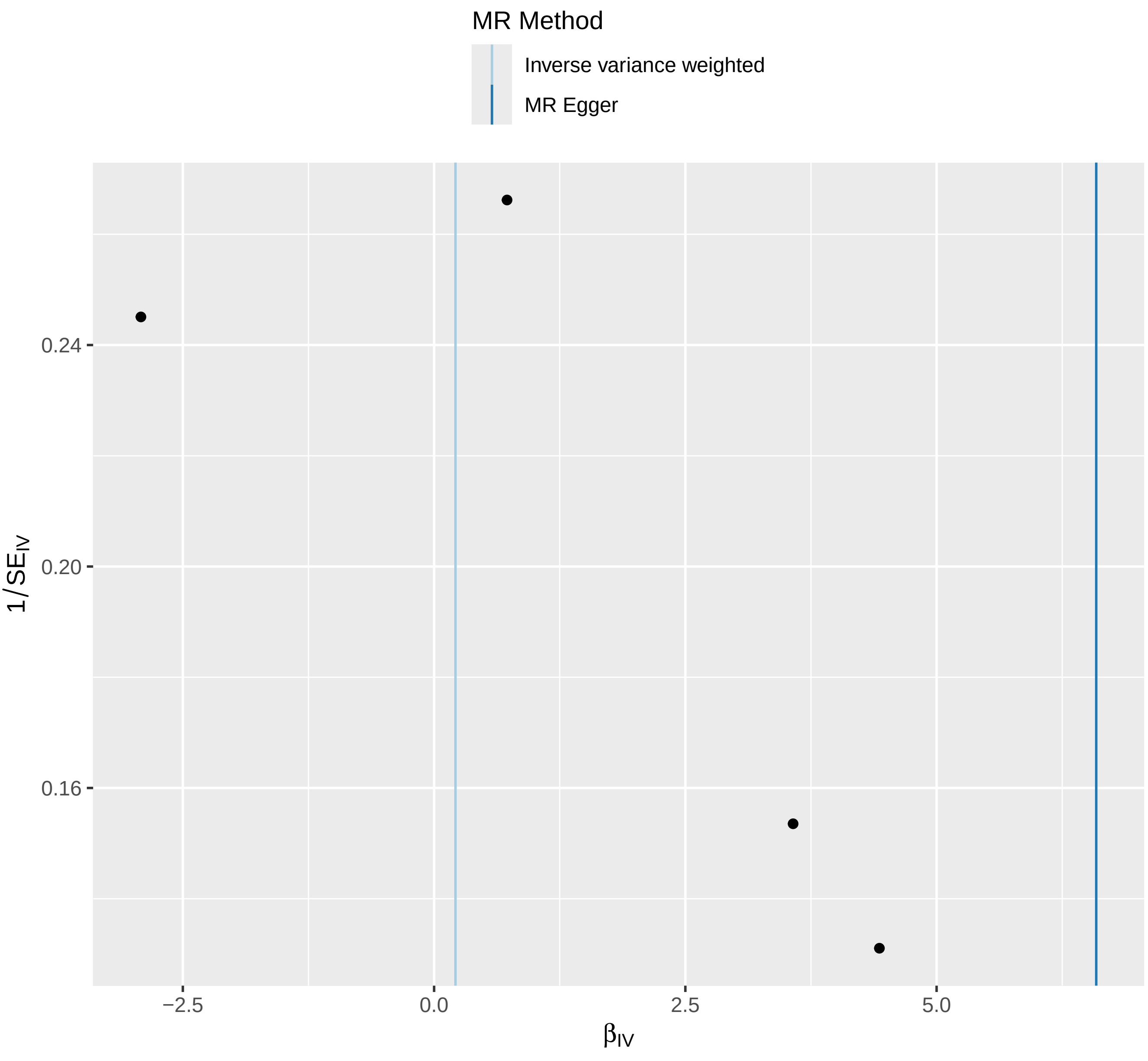
MR Method
Inverse variance weighted
MR Egger
0.24
0.20
0.16
1/SE$_{IV}$
−2.5
0.0
2.5
5.0
β$_{IV}$

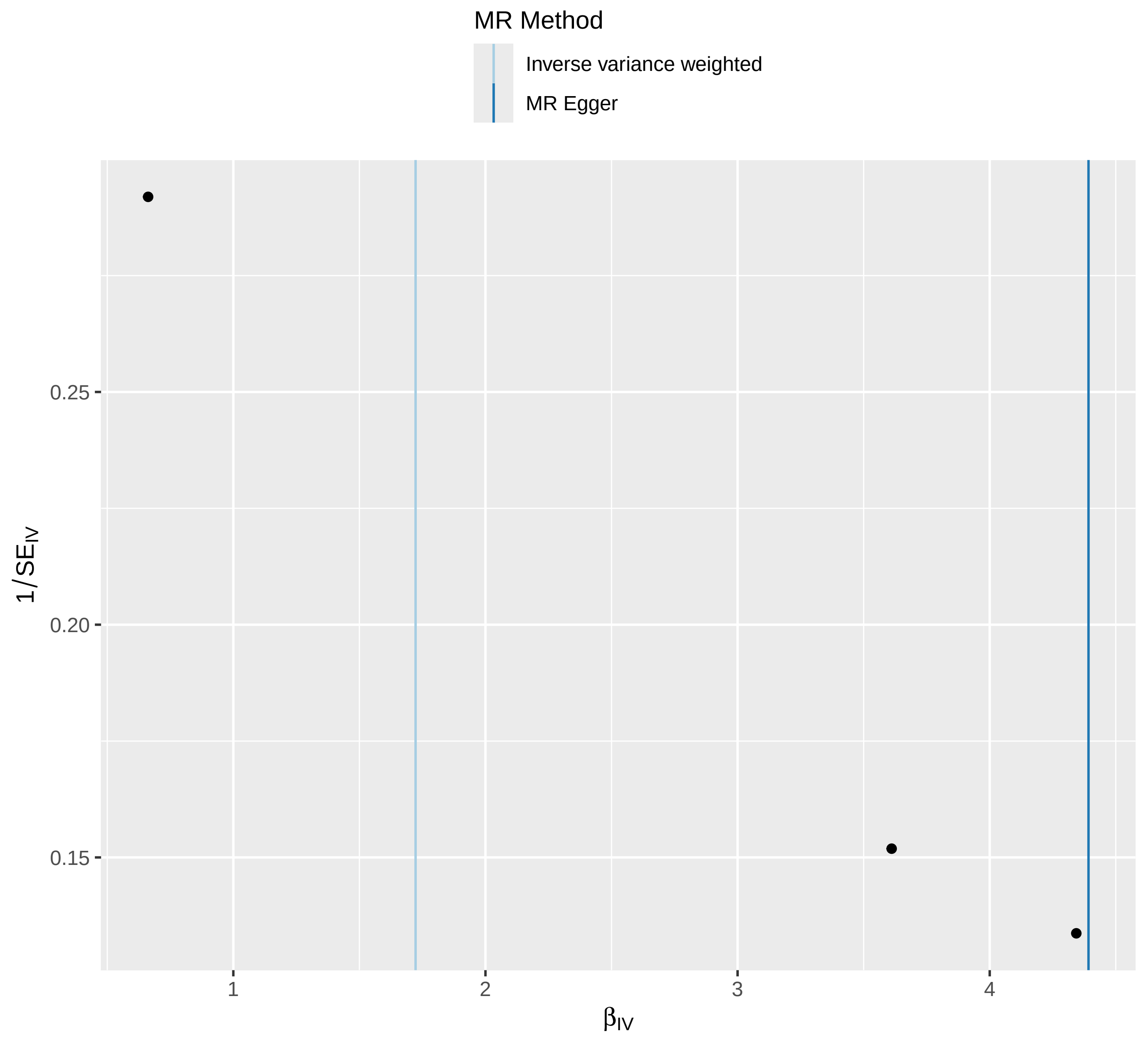
MR Method
Inverse variance weighted
MR Egger
0.25
0.20
0.15
1/SE_IV
1
2
3
4
β_IV

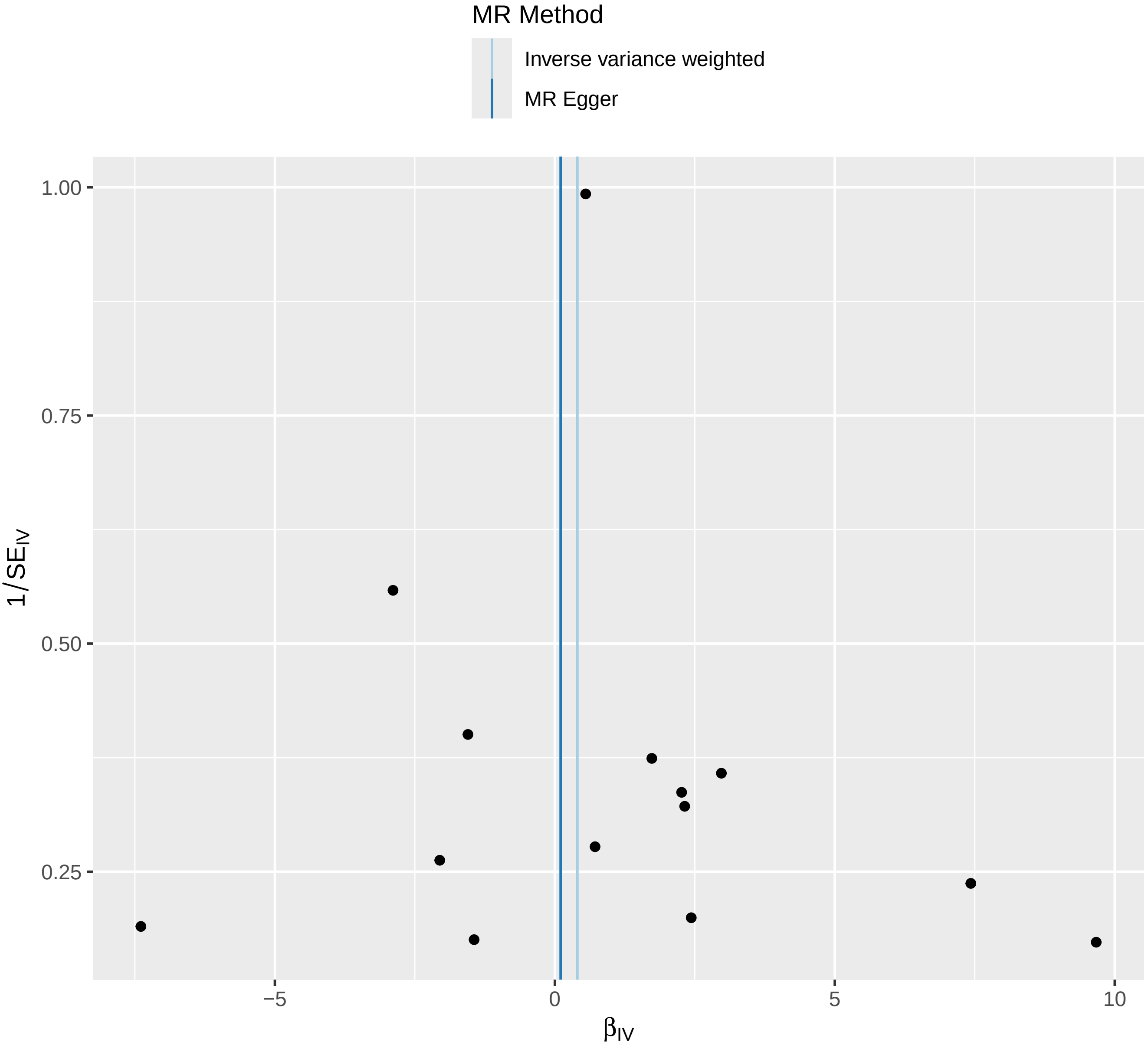
MR Method
Inverse variance weighted
MR Egger
1.00
0.75
0.50
0.25
−5
0
5
10
$1/SE_{IV}$
β_{IV}

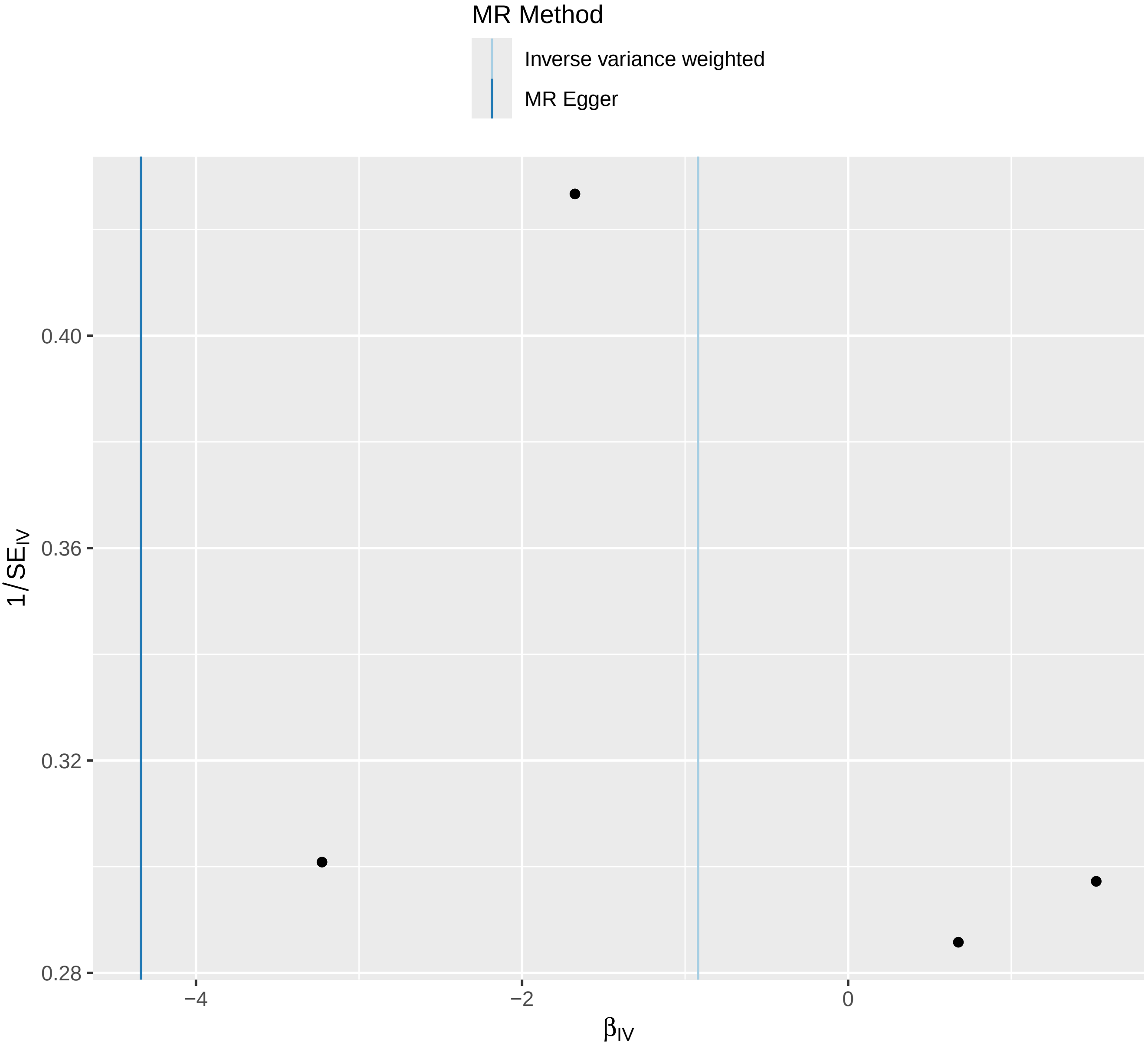

MR Method
Inverse variance weighted
MR Egger
0.40
0.36
0.32
0.28
1/SE_IV
−4
−2
0
β_IV

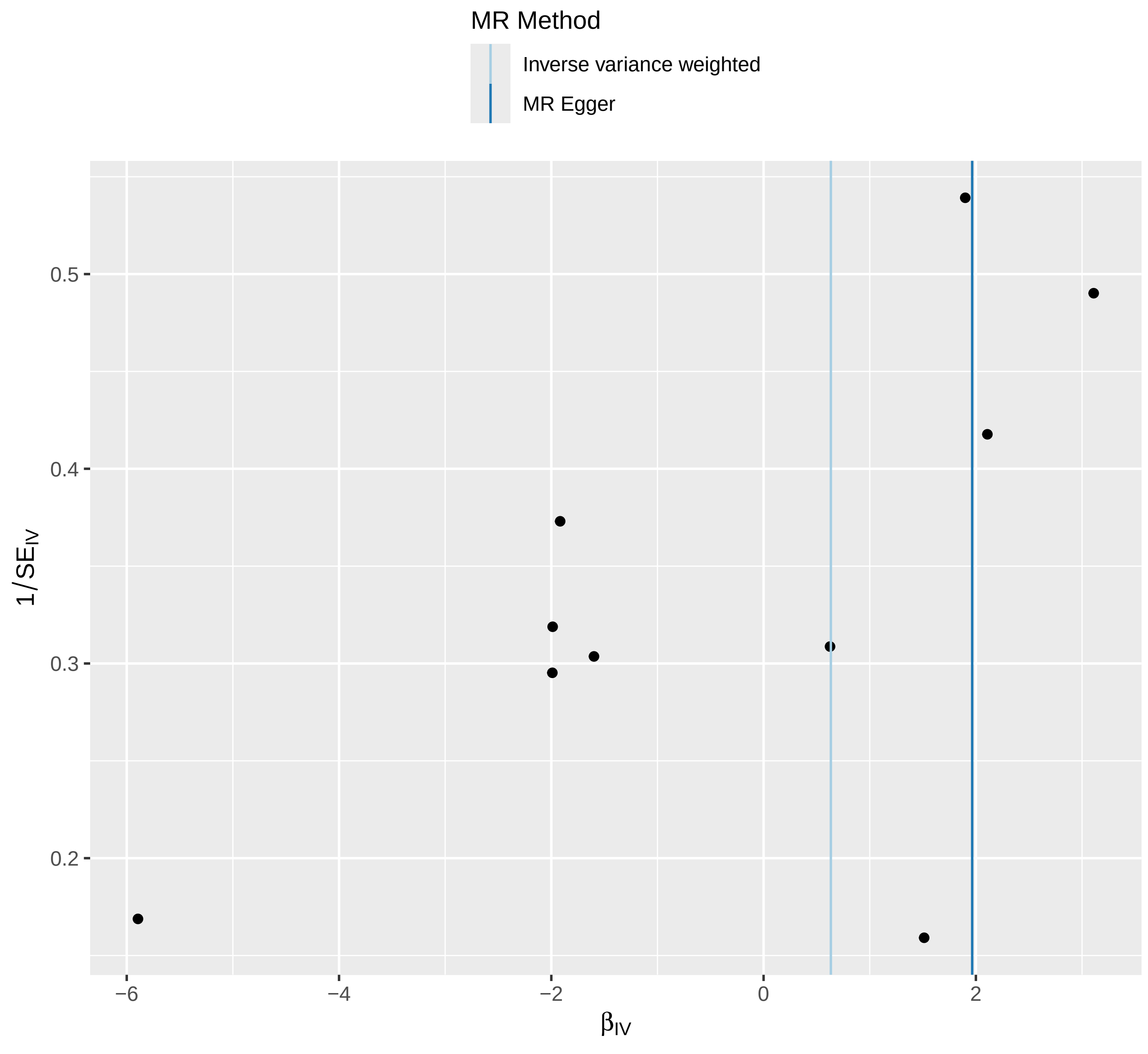
MR Method
Inverse variance weighted
MR Egger
0.5
0.4
0.3
0.2
1/SE_IV
−6
−4
−2
0
2
β_IV

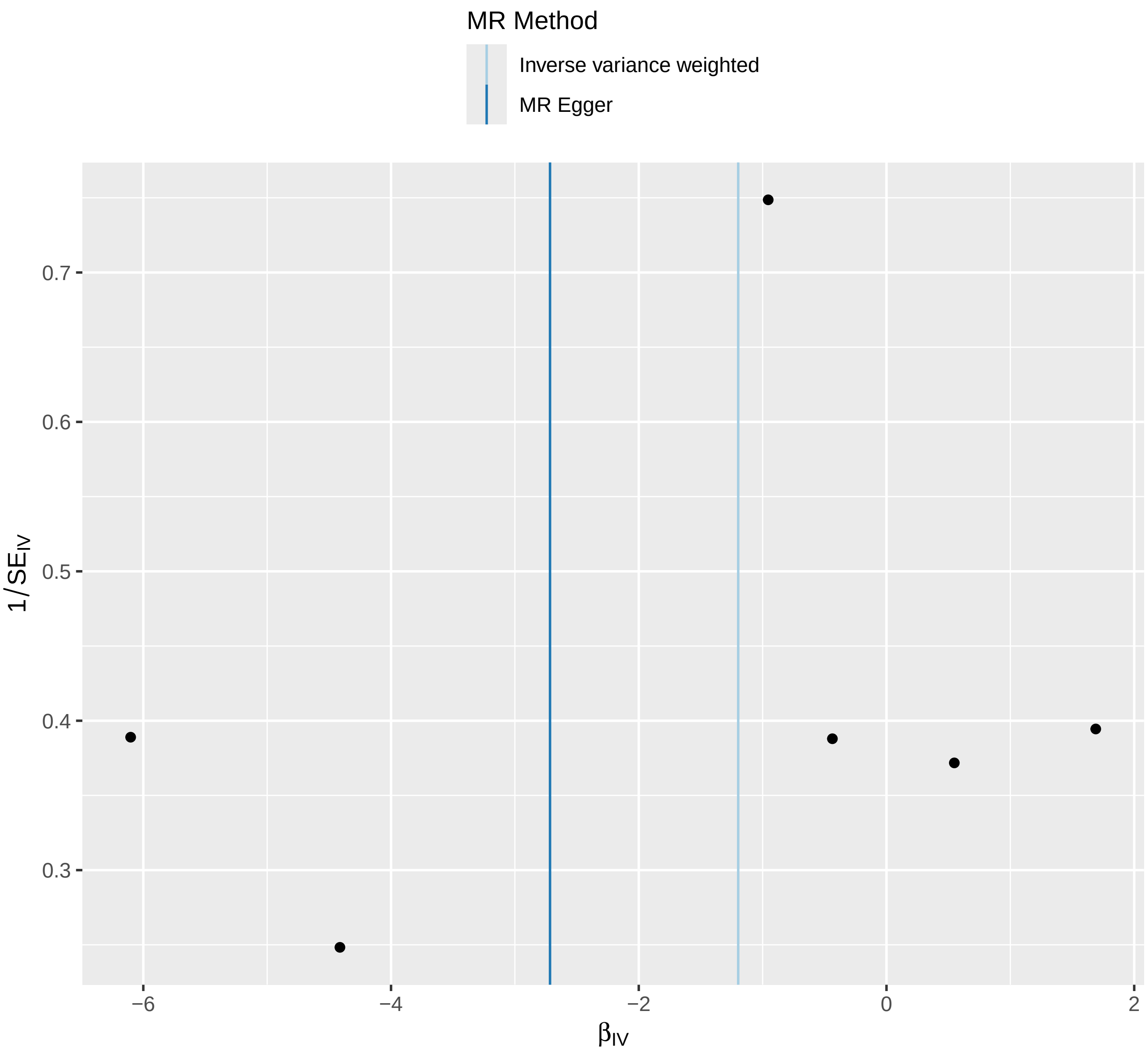

MR Method
Inverse variance weighted
MR Egger
0.7
0.6
0.5
0.4
0.3
1/SE_IV
−6
−4
−2
0
2
β_IV

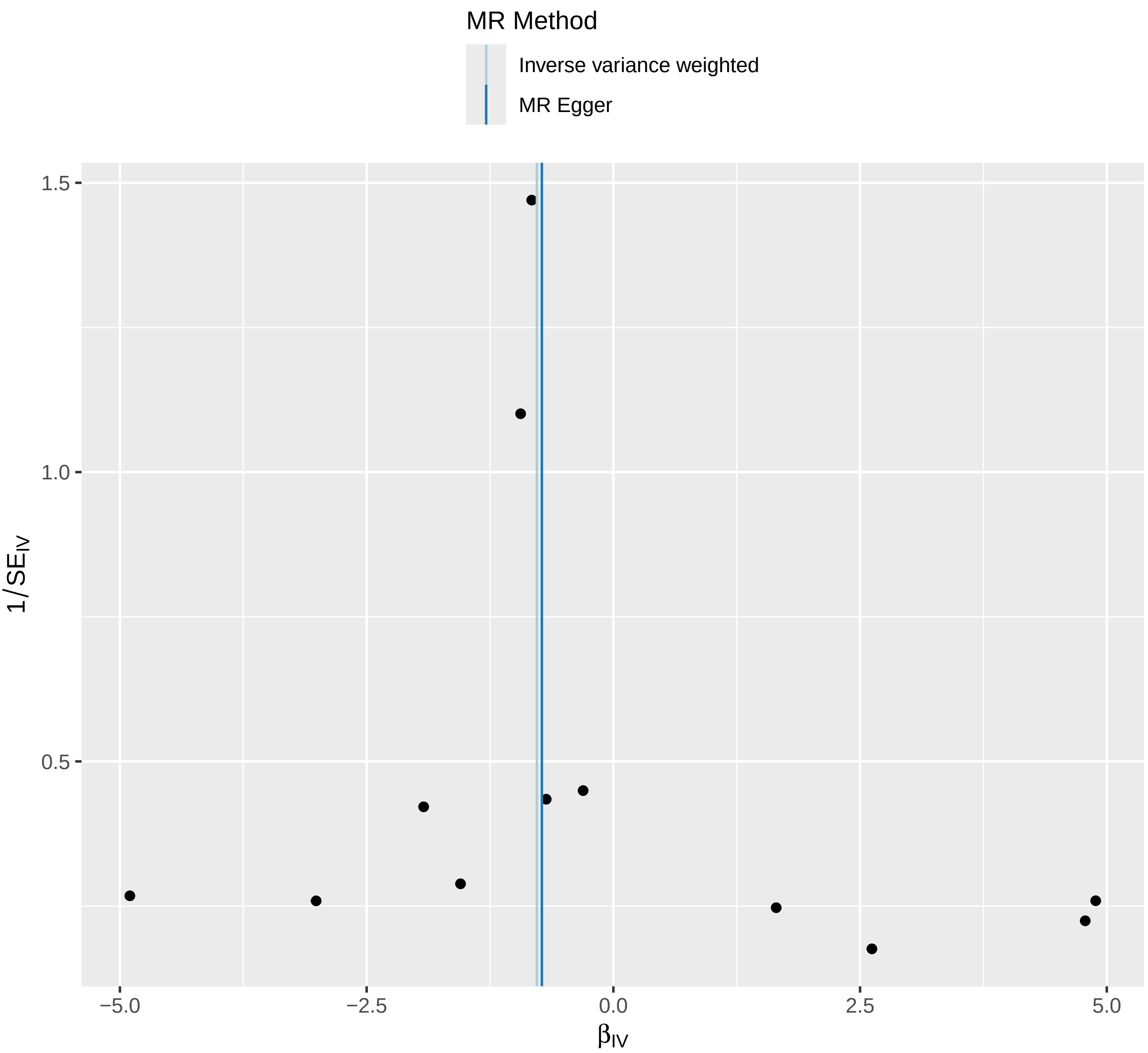

MR Method
Inverse variance weighted
MR Egger
1.5
1.0
0.5
1/SE_IV
−5.0
−2.5
0.0
2.5
5.0
β_IV

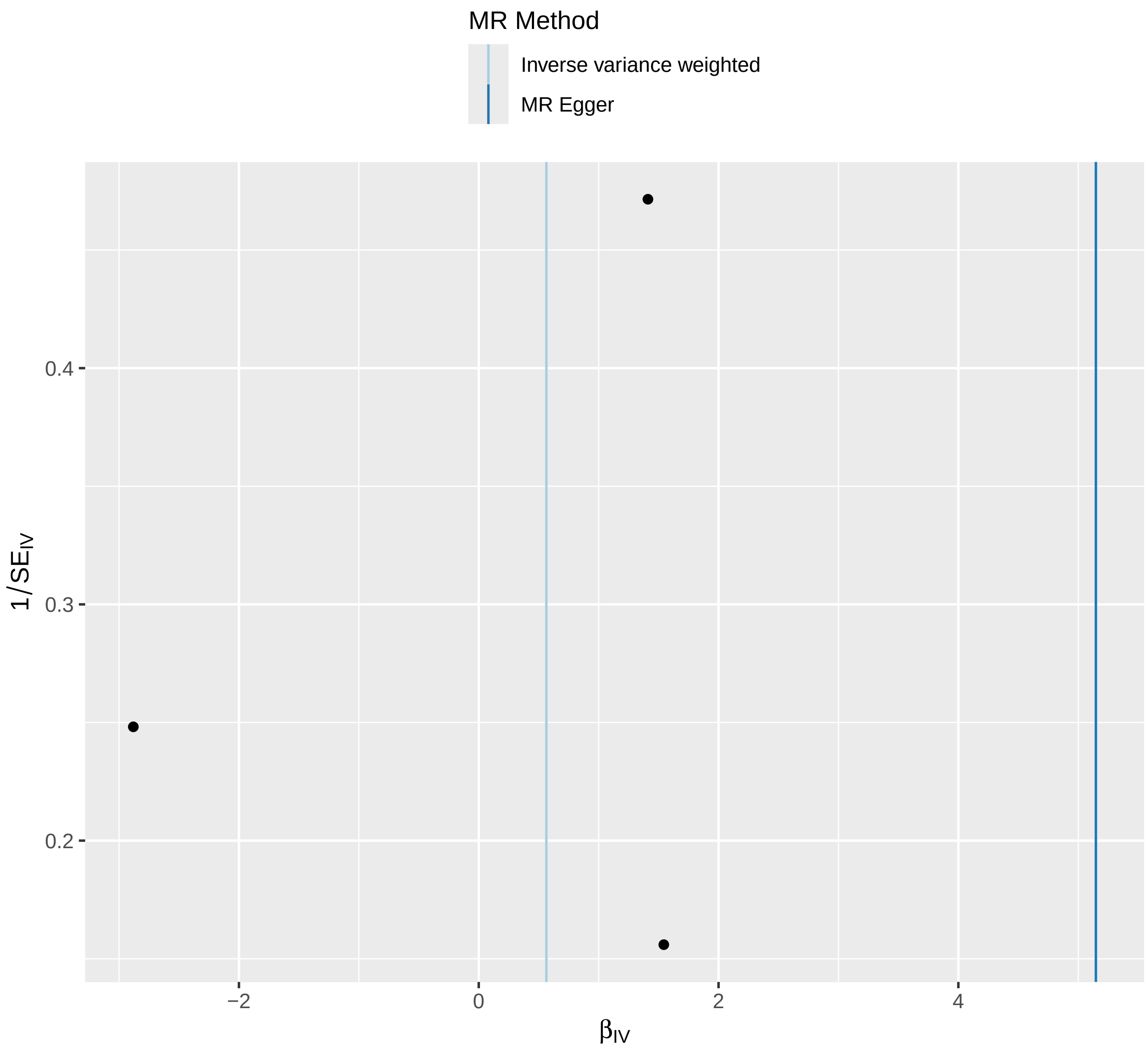
MR Method
Inverse variance weighted
MR Egger
0.4
0.3
0.2
1/SE$_{IV}$
−2
0
2
4
β$_{IV}$

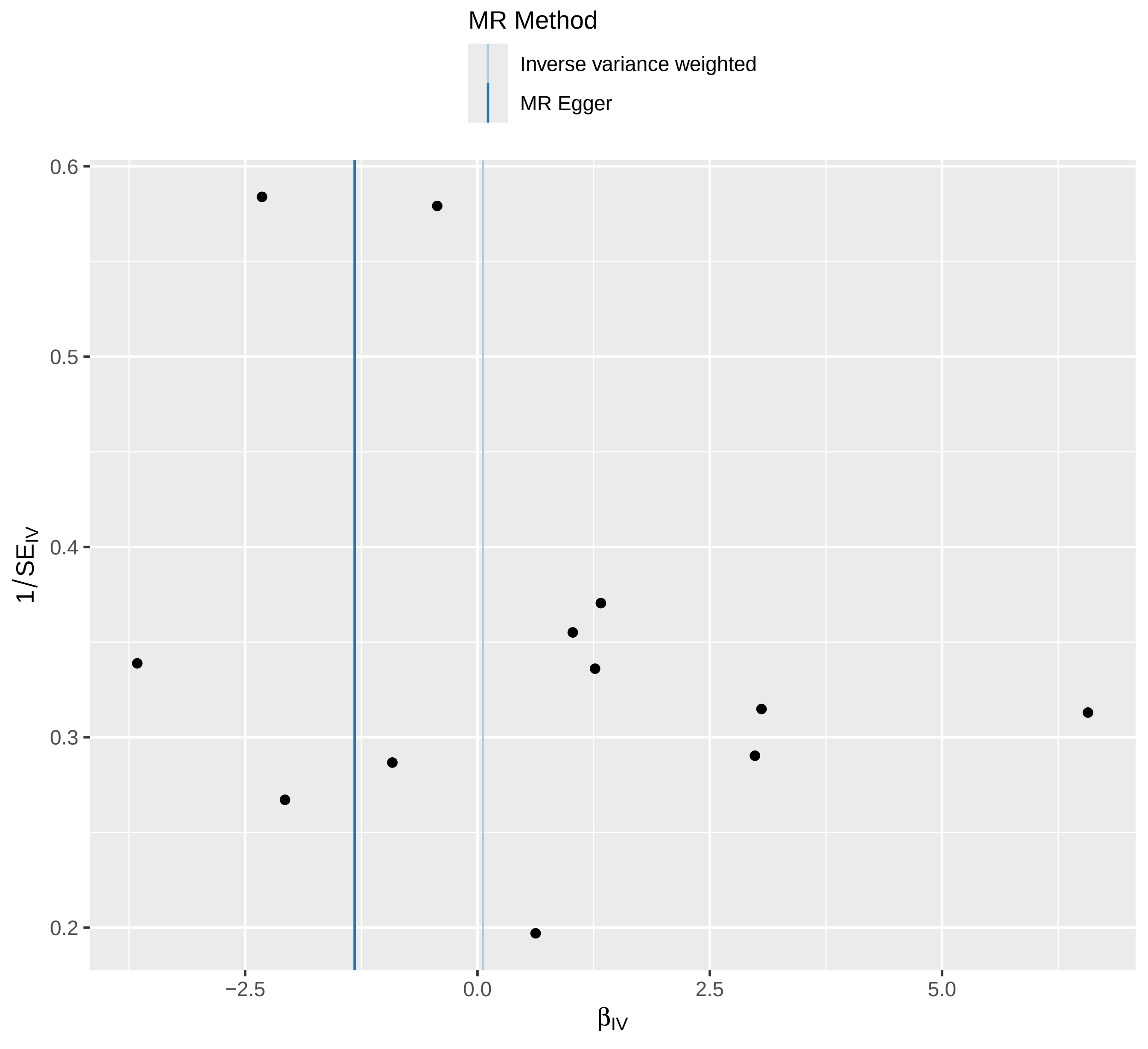

MR Method
Inverse variance weighted
MR Egger
0.6
0.5
0.4
0.3
0.2
$1/SE_{IV}$
−2.5
0.0
2.5
5.0
β_{IV}

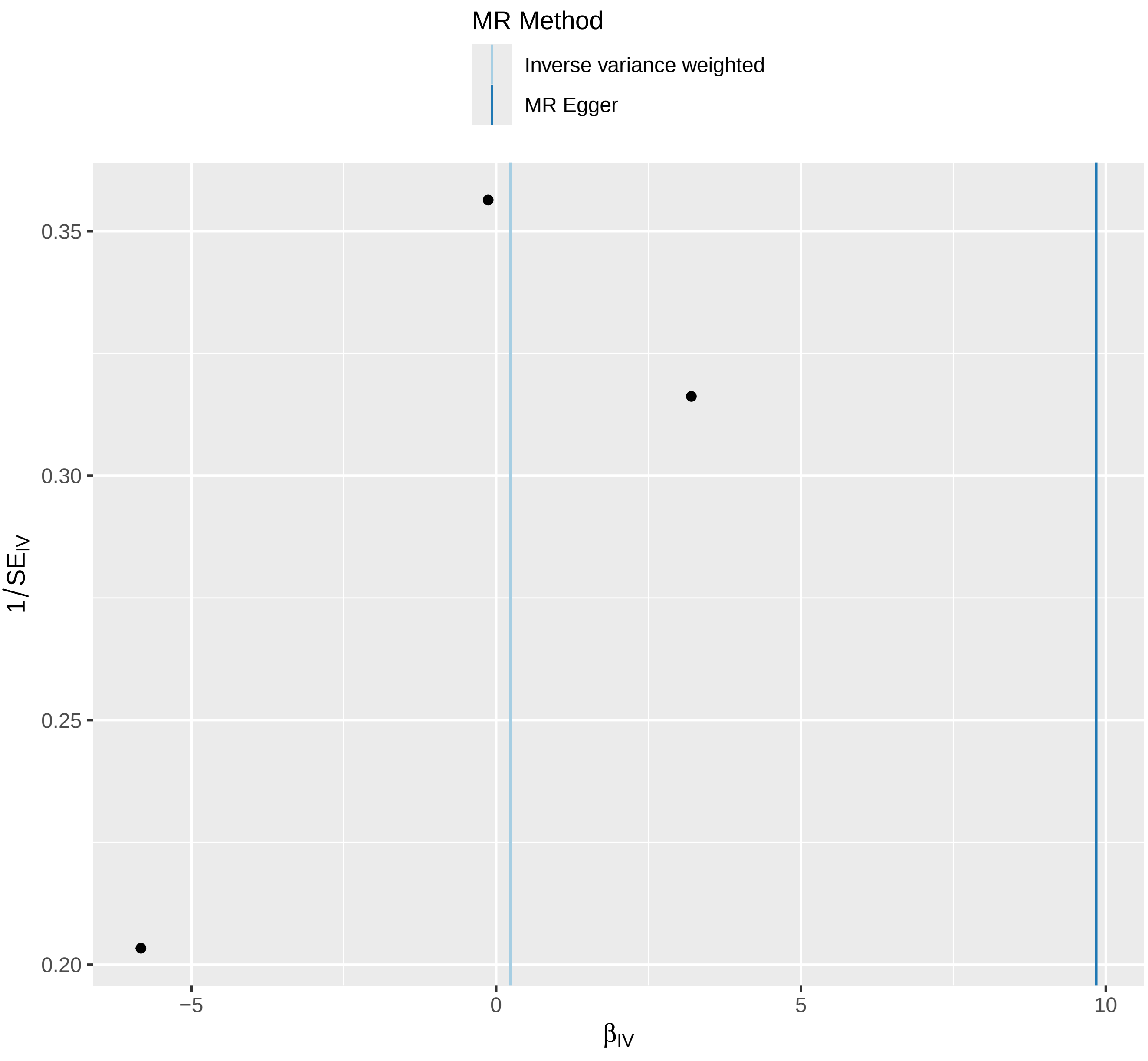

MR Method
Inverse variance weighted
MR Egger
0.35
0.30
0.25
0.20
1/SE_IV
−5
0
5
10
β_IV

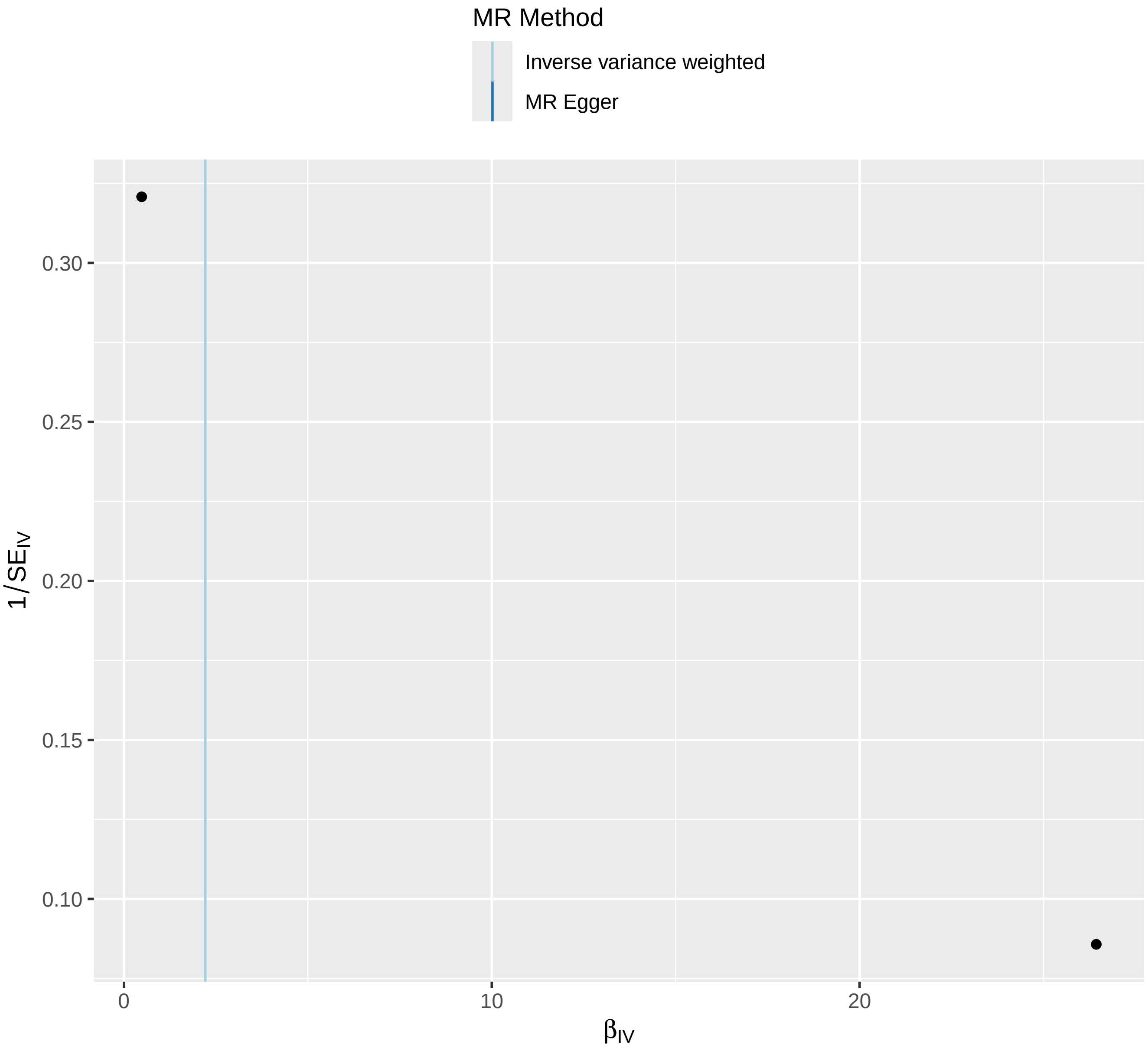
MR Method
Inverse variance weighted
MR Egger
0.30
0.25
0.20
0.15
0.10
$1/SE_{IV}$
0
10
20
β_{IV}

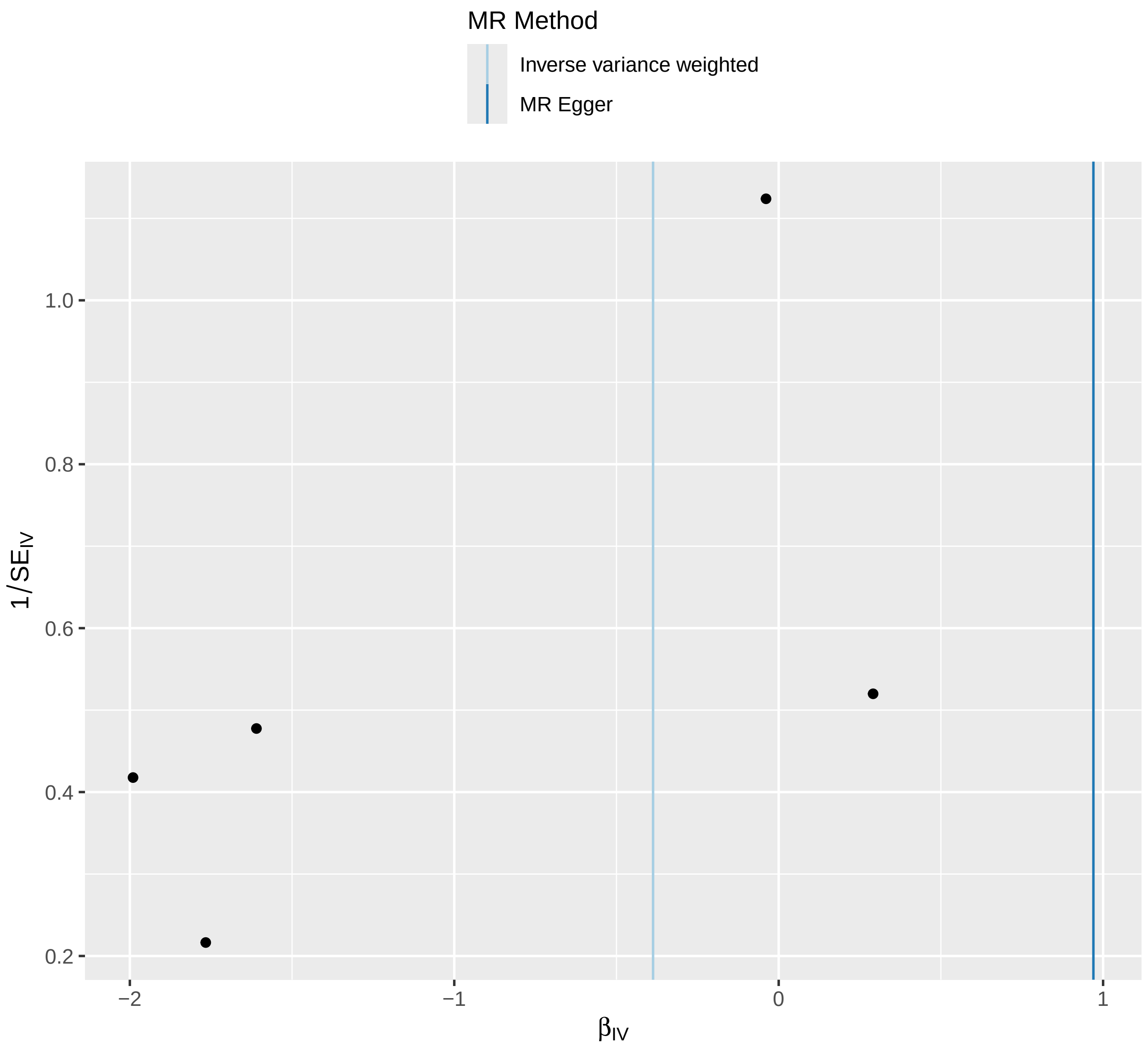
MR Method
Inverse variance weighted
MR Egger
1.0
0.8
0.6
0.4
0.2
1/SE_IV
−2
−1
0
1
β_IV

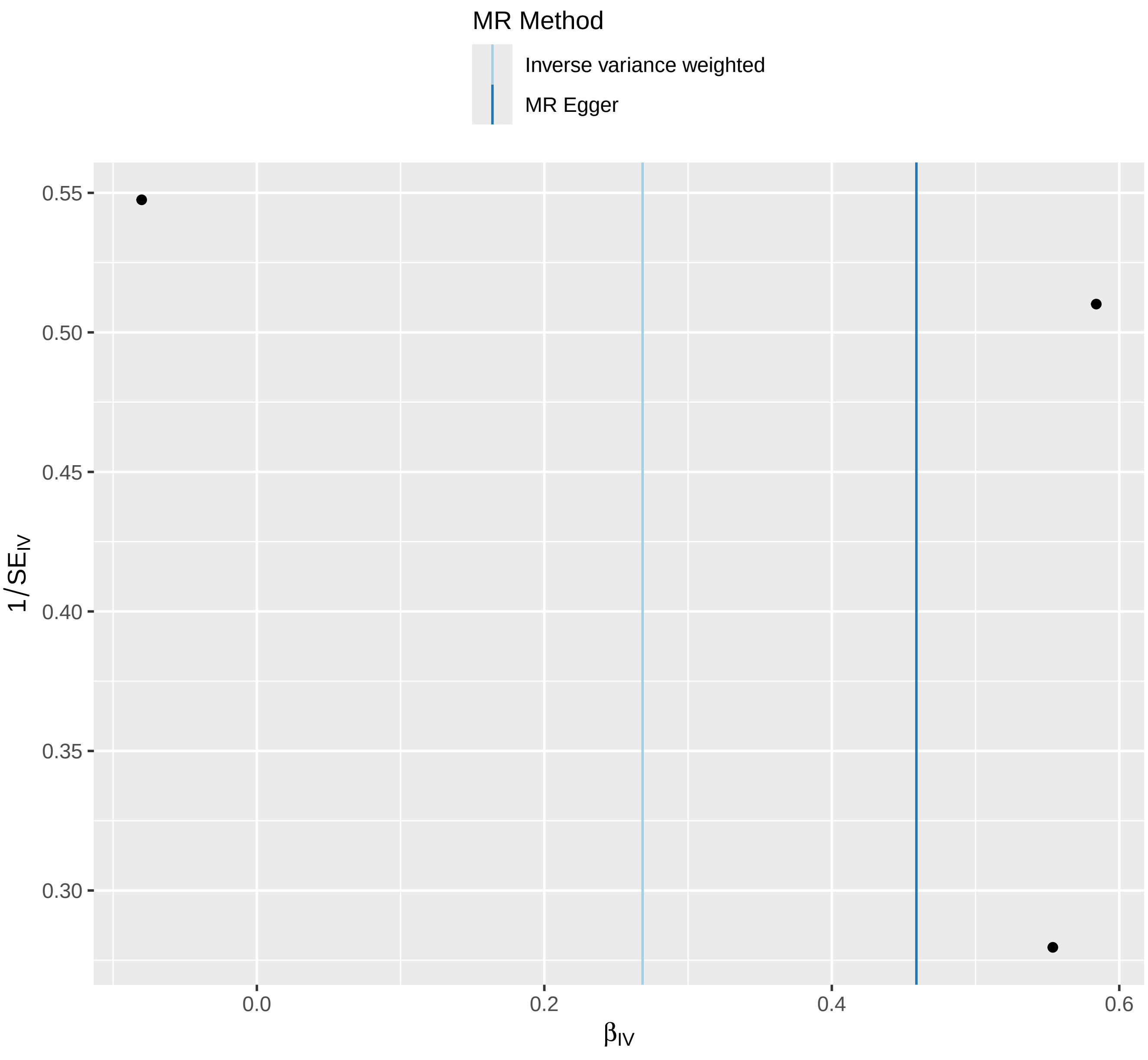
MR Method
Inverse variance weighted
MR Egger
0.55
0.50
0.45
0.40
0.35
0.30
1/SE_IV
0.0
0.2
0.4
0.6
β_IV

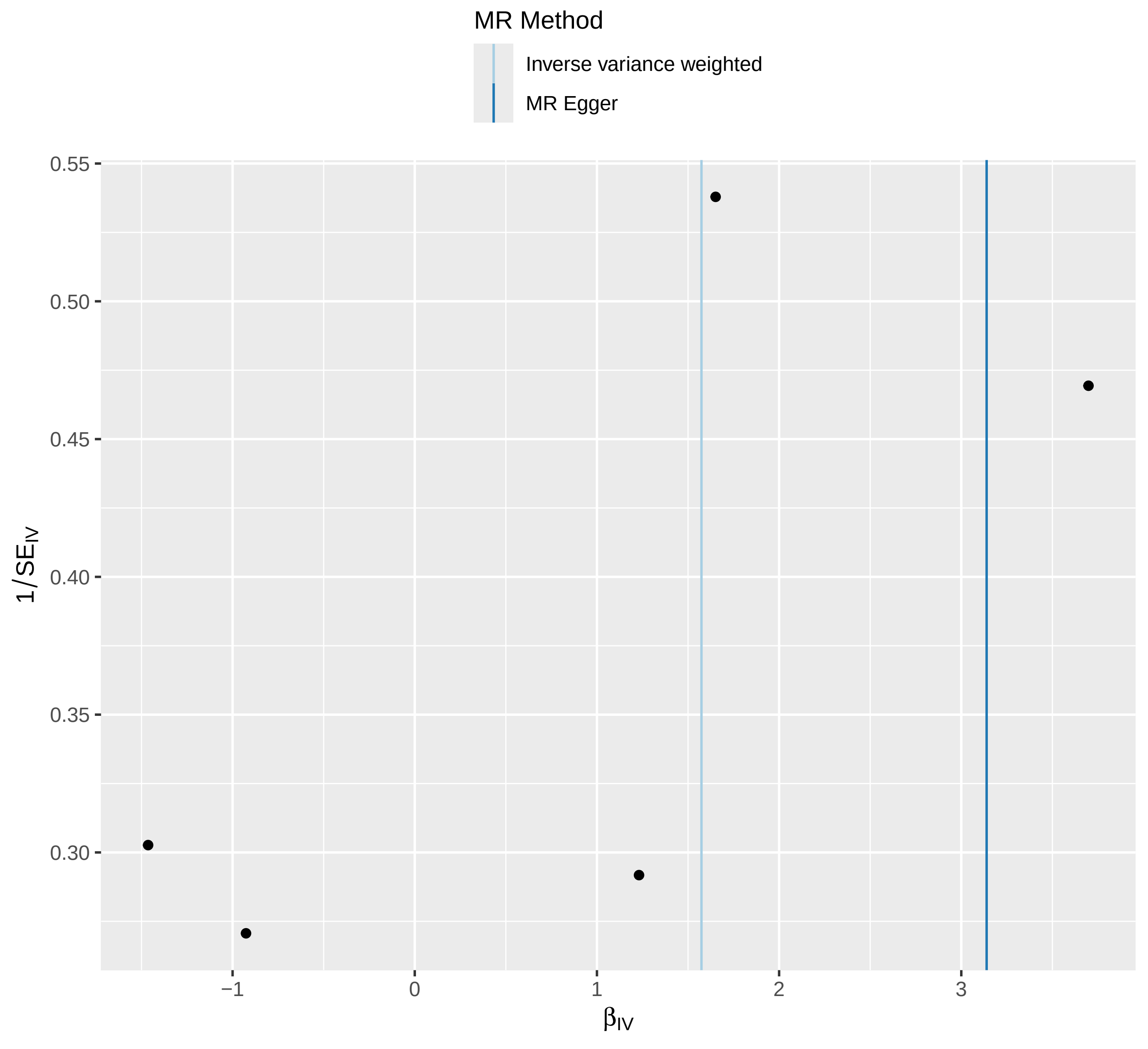

MR Method
Inverse variance weighted
MR Egger
0.55
0.50
0.45
0.40
0.35
0.30
$1/SE_{IV}$
−1
0
1
2
3
β_{IV}

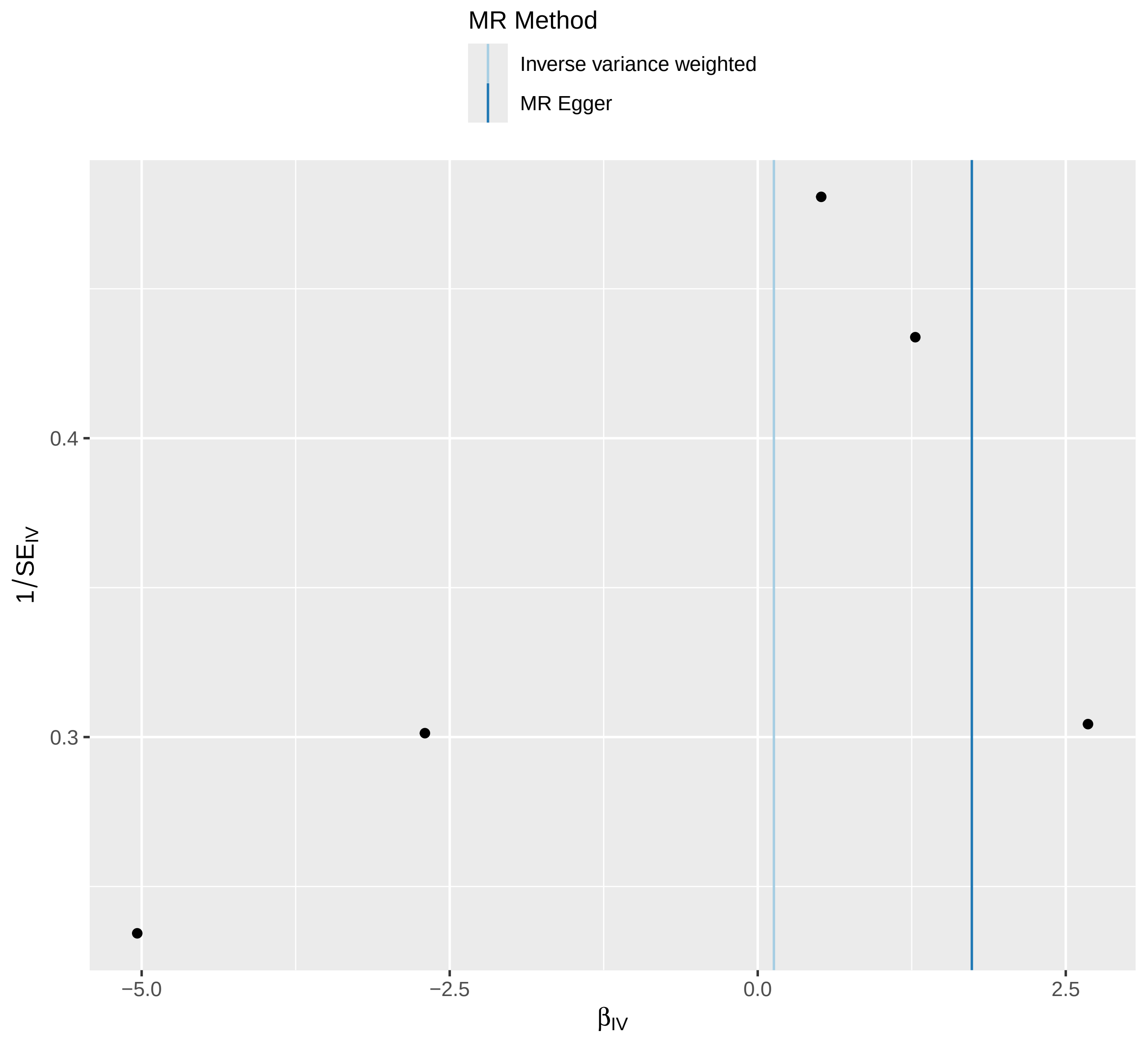
MR Method
Inverse variance weighted
MR Egger
0.4
0.3
1/SE$_{IV}$
−5.0
−2.5
0.0
2.5
β$_{IV}$

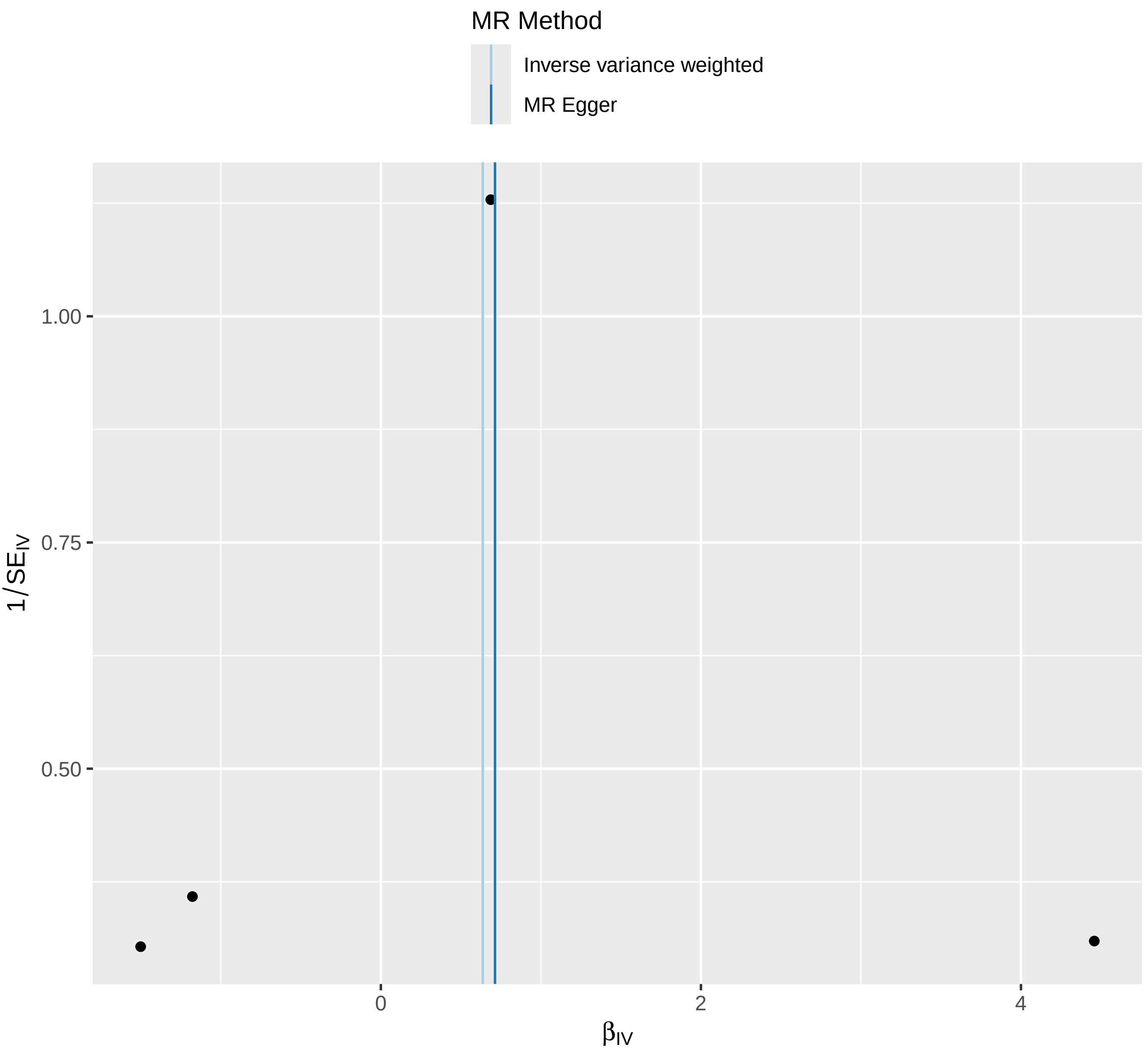
MR Method
Inverse variance weighted
MR Egger
1.00
0.75
0.50
1/SE_IV
0
2
4
β_IV

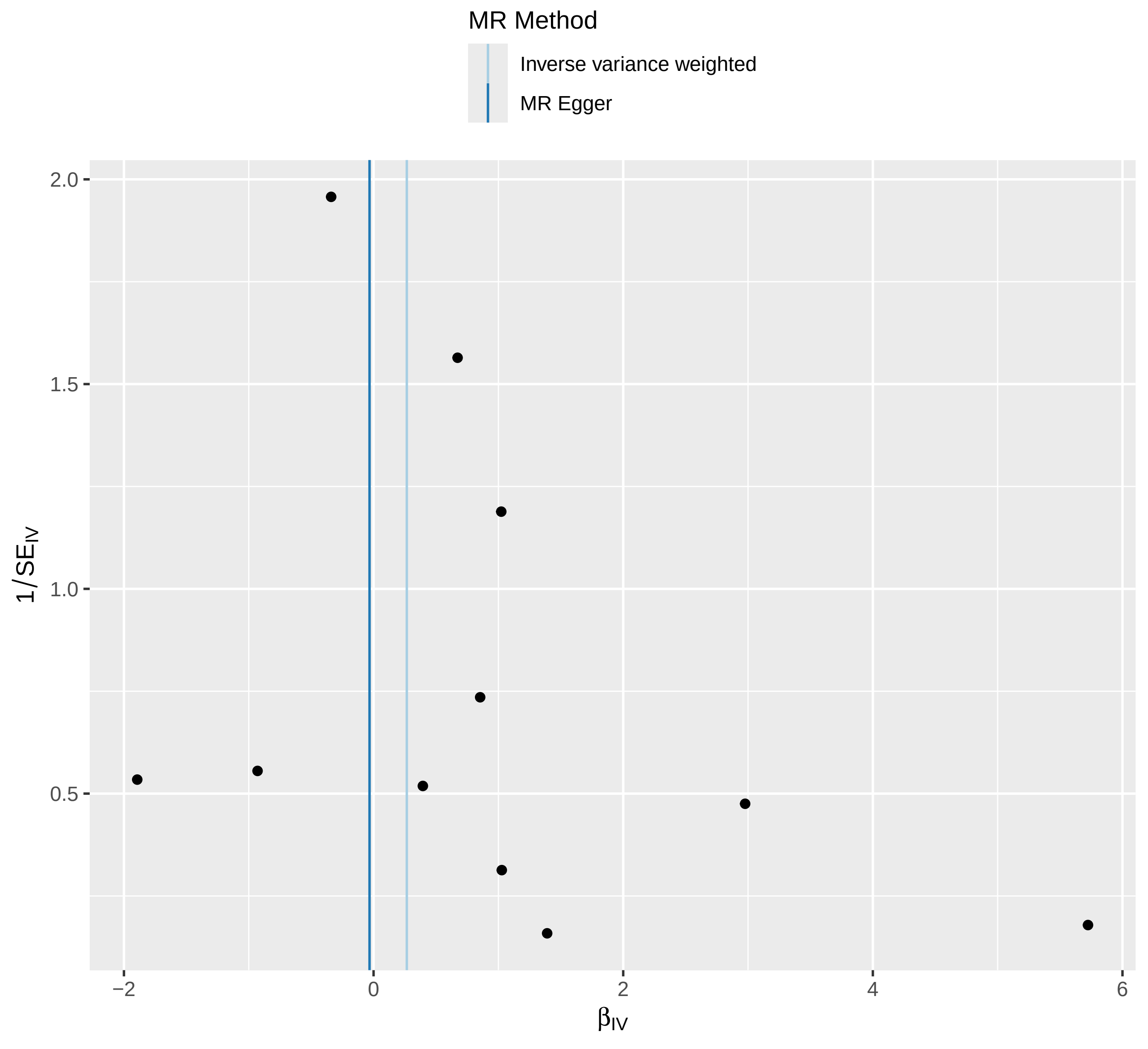
MR Method
Inverse variance weighted
MR Egger
2.0
1.5
1.0
0.5
$1/SE_{IV}$
−2
0
2
4
6
β_{IV}

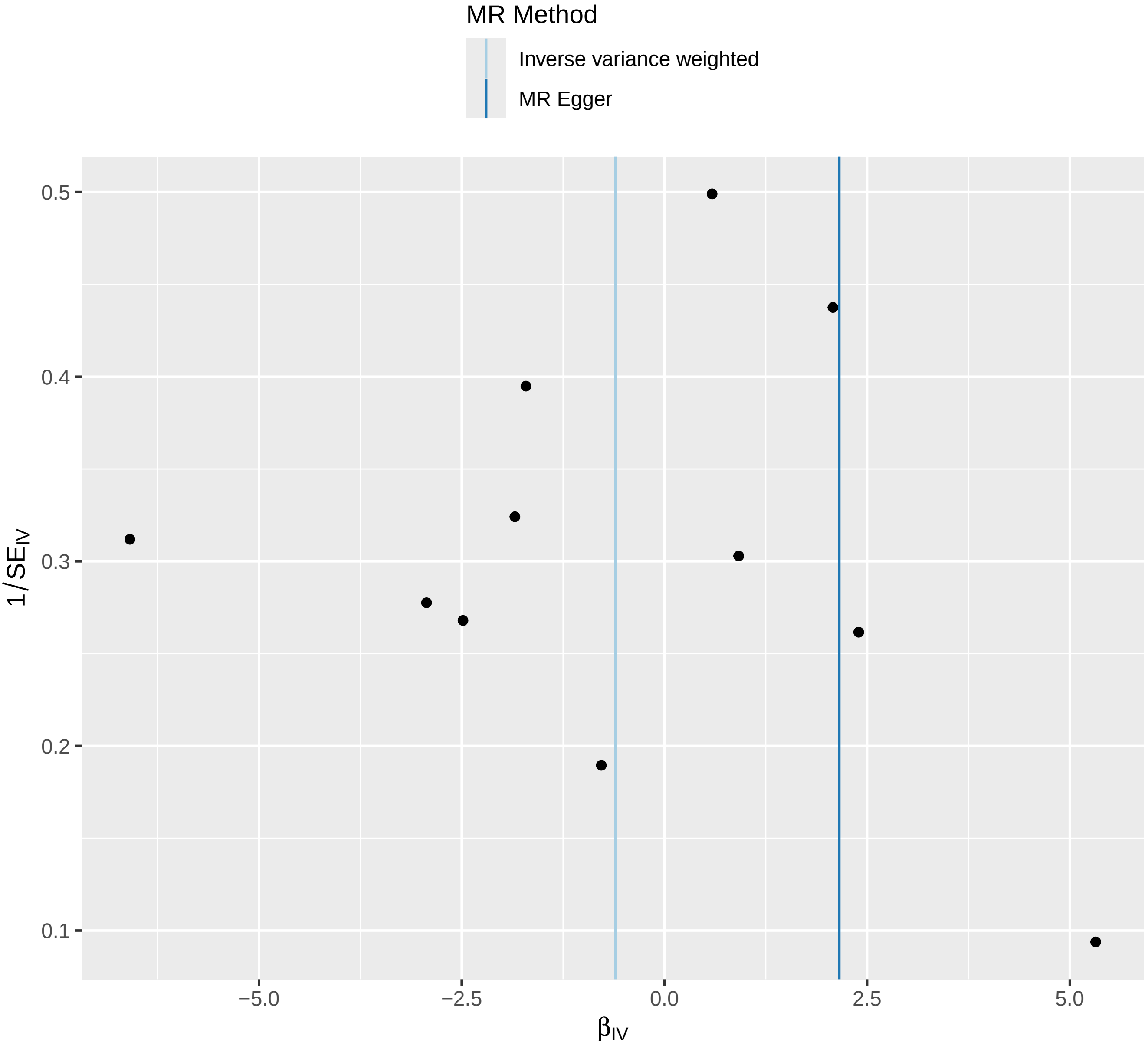

MR Method
Inverse variance weighted
MR Egger
0.5
0.4
0.3
0.2
0.1
1/SE$_{IV}$
−5.0
−2.5
0.0
2.5
5.0
β$_{IV}$

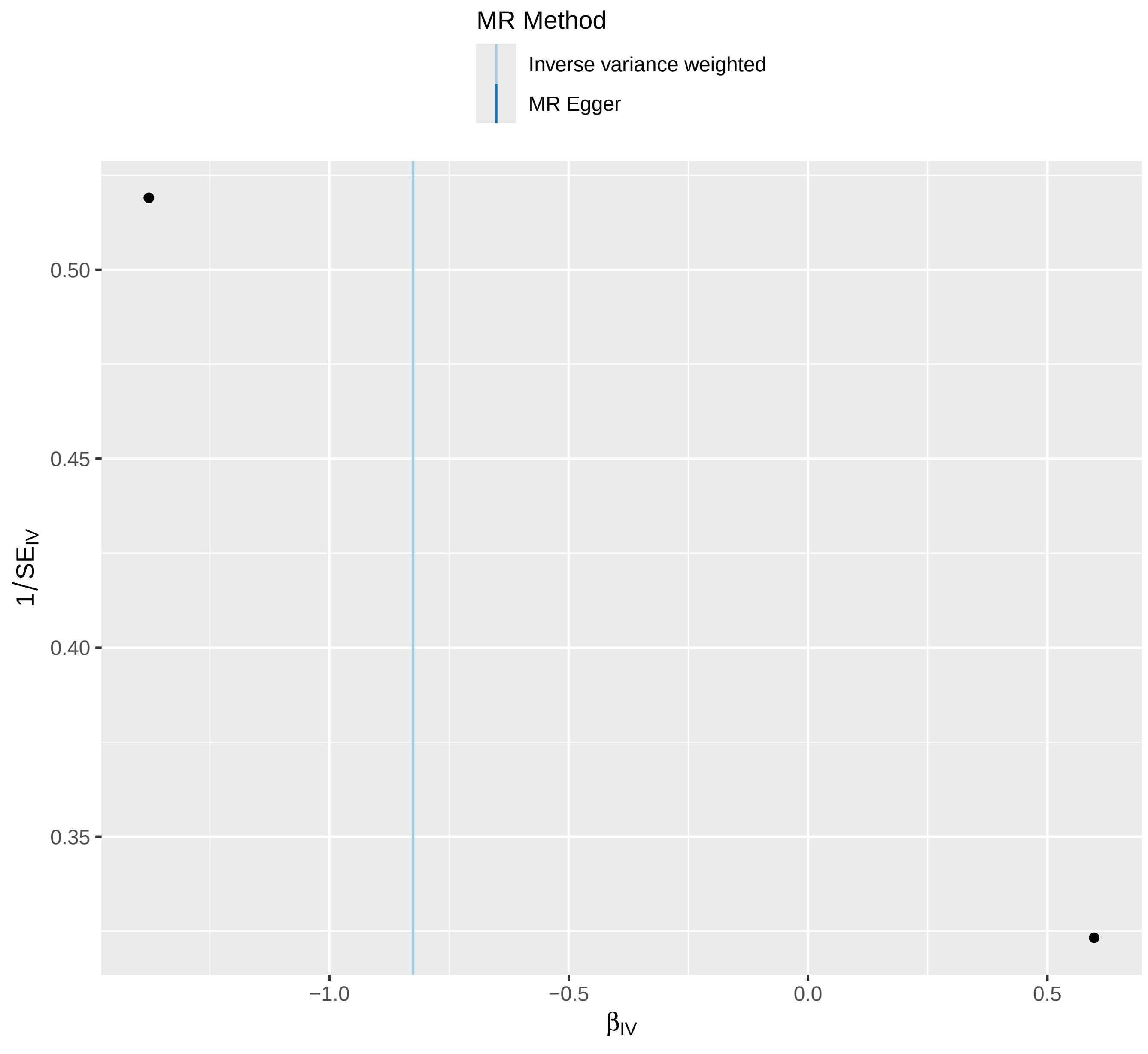
MR Method
Inverse variance weighted
MR Egger
0.50
0.45
0.40
0.35
$1/SE_{IV}$
−1.0
−0.5
0.0
0.5
β_{IV}

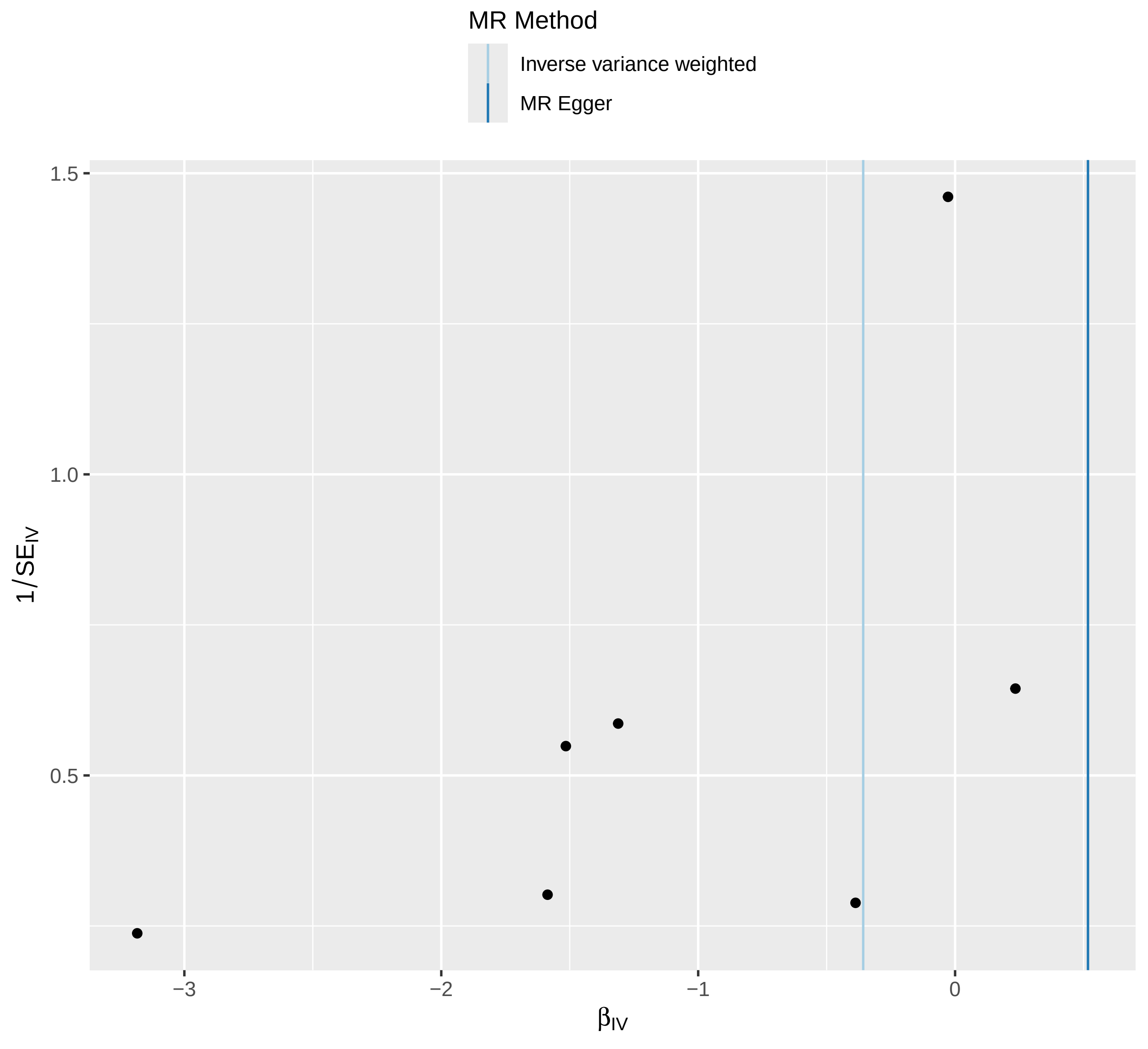

MR Method
Inverse variance weighted
MR Egger
1.5
1.0
0.5
1/SE_IV
−3
−2
−1
0
β_IV

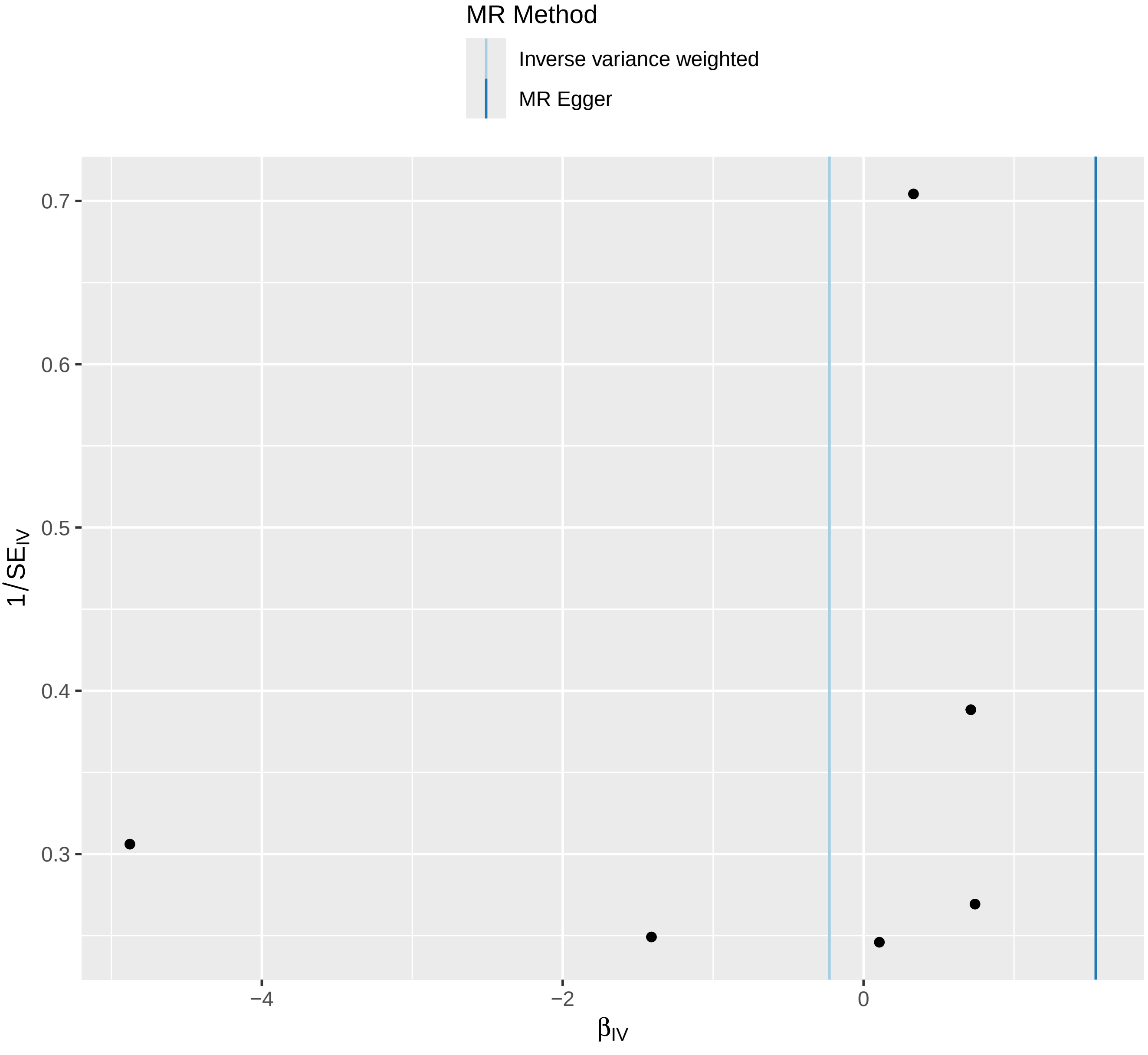
MR Method
Inverse variance weighted
MR Egger
0.7
0.6
0.5
0.4
0.3
1/SE_IV
−4
−2
0
β_IV

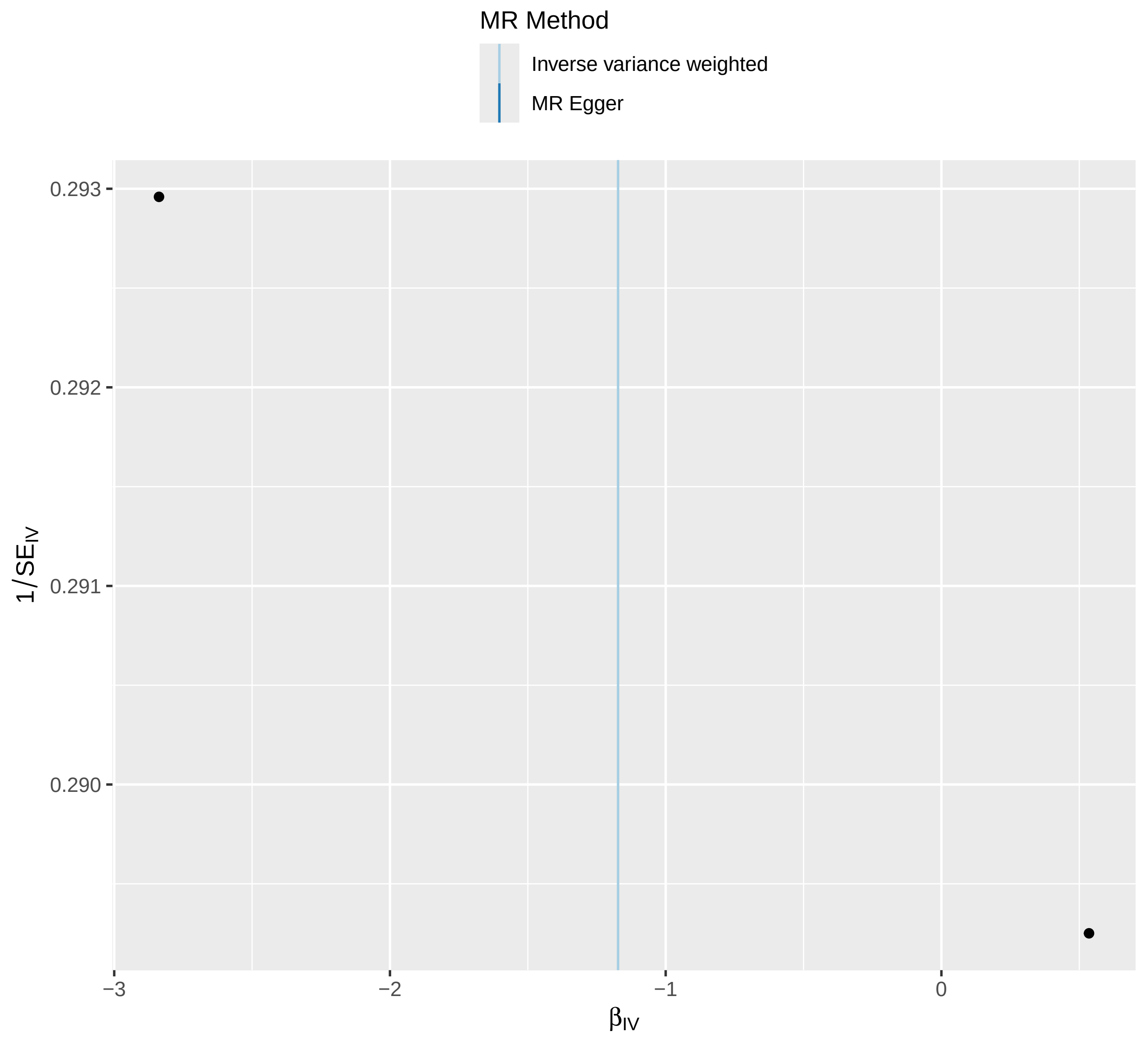
MR Method
Inverse variance weighted
MR Egger
0.293
0.292
0.291
0.290
1/SE_IV
−3
−2
−1
0
β_IV

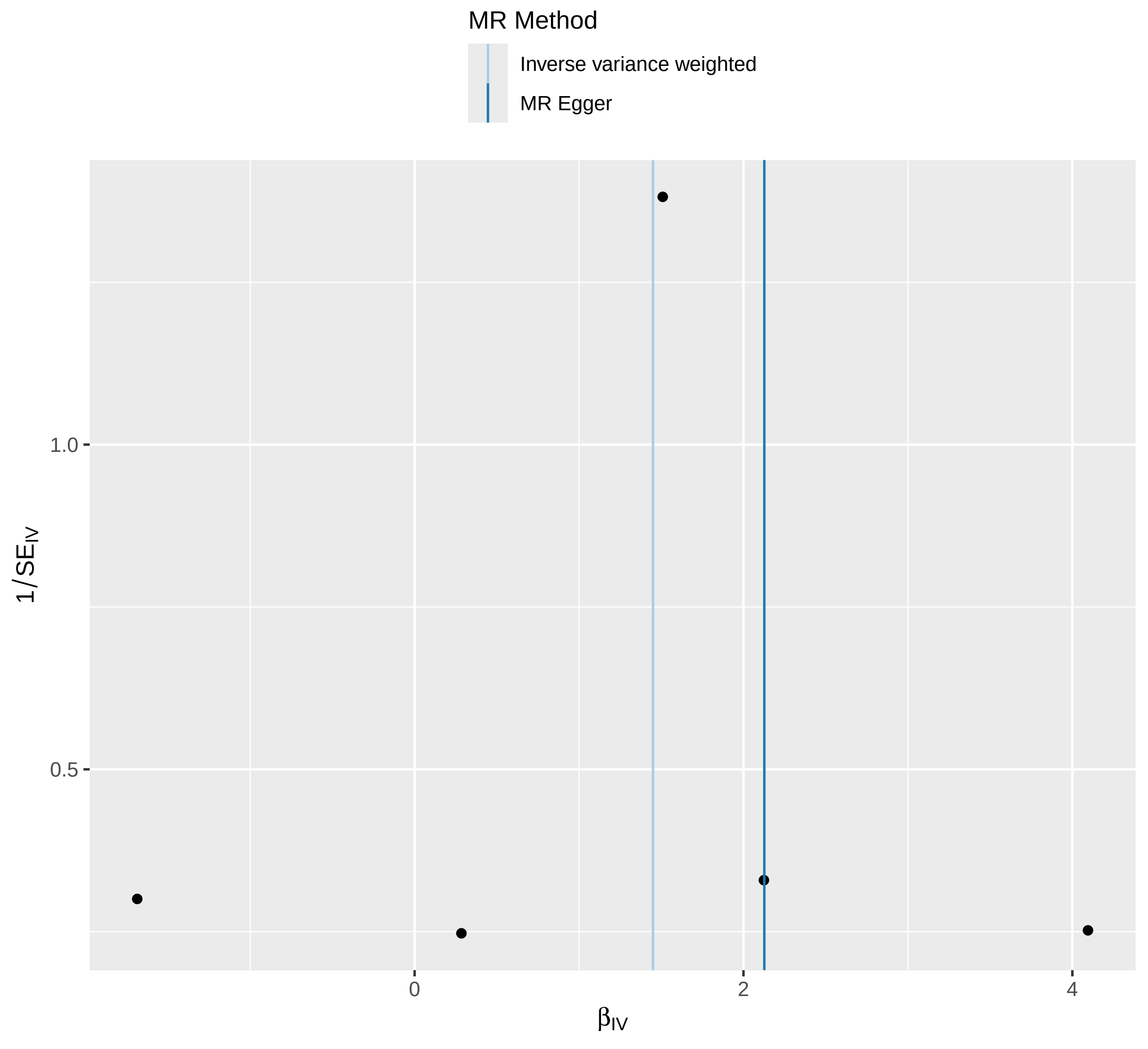

MR Method
Inverse variance weighted
MR Egger
1.0
0.5
0
2
4
$1/SE_{IV}$
β_{IV}

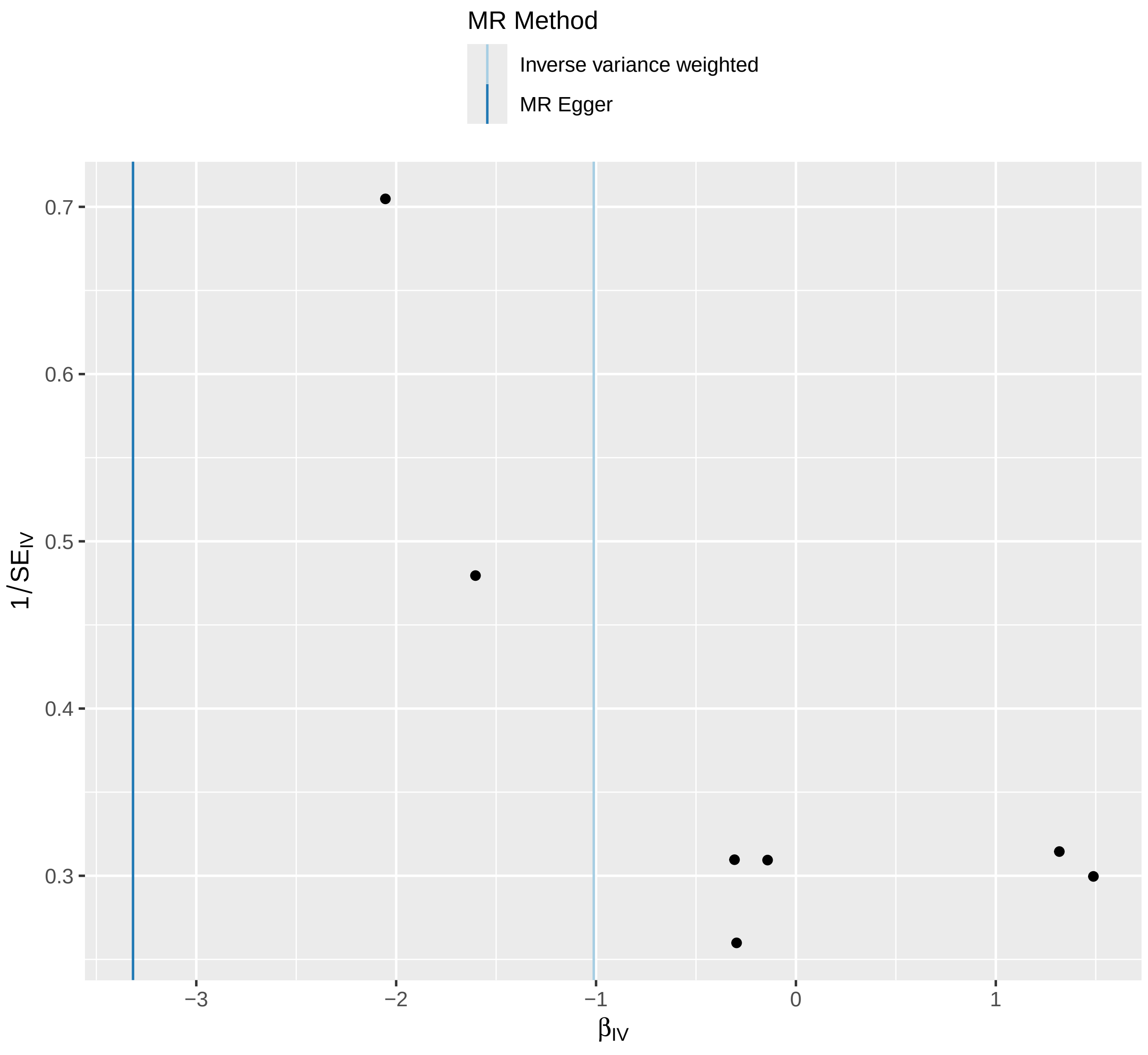
MR Method
Inverse variance weighted
MR Egger
0.7
0.6
0.5
0.4
0.3
1/SE_IV
−3
−2
−1
0
1
β_IV

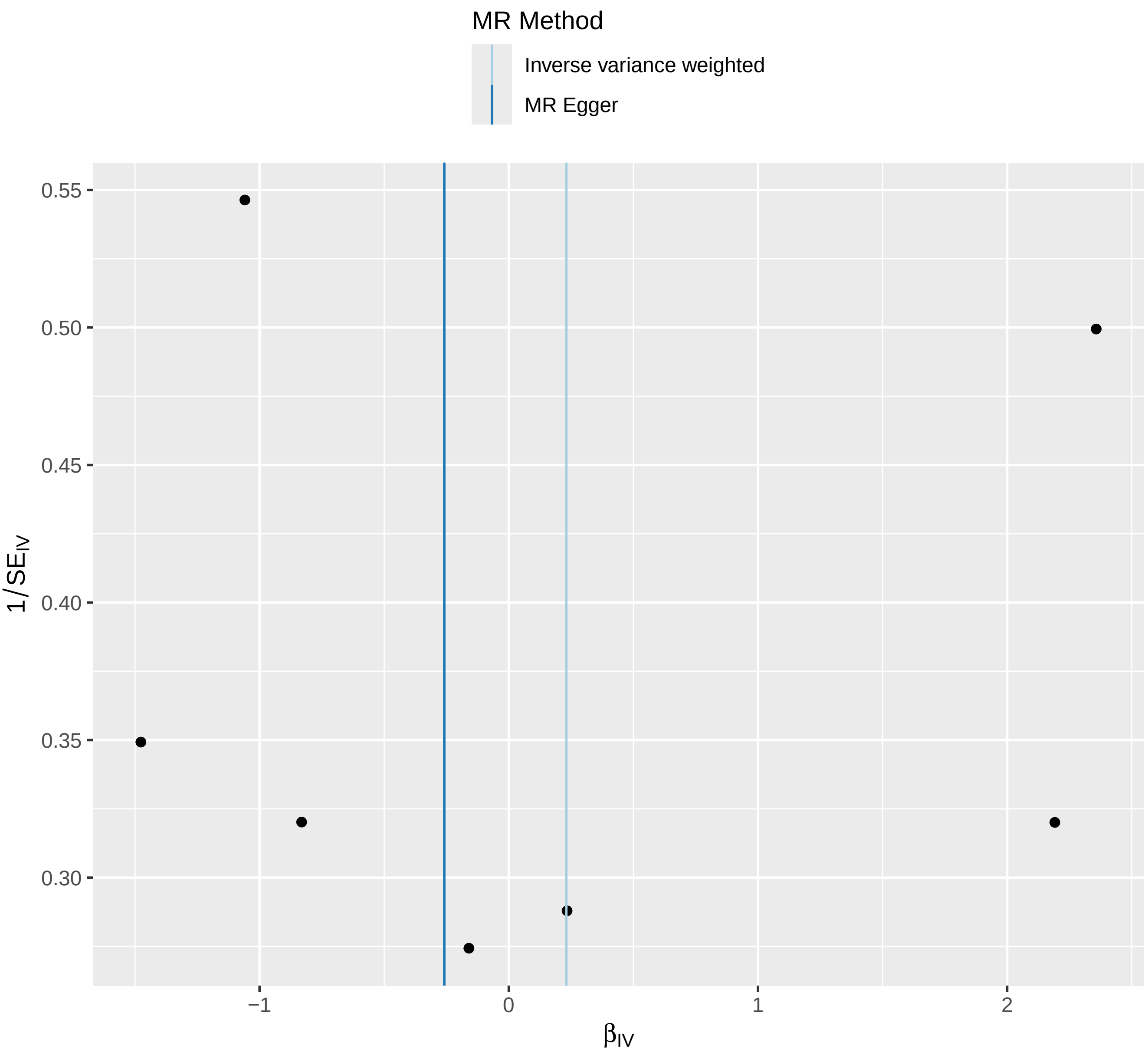

MR Method
Inverse variance weighted
MR Egger
0.55
0.50
0.45
0.40
0.35
0.30
1/SE_IV
−1
0
1
2
β_IV

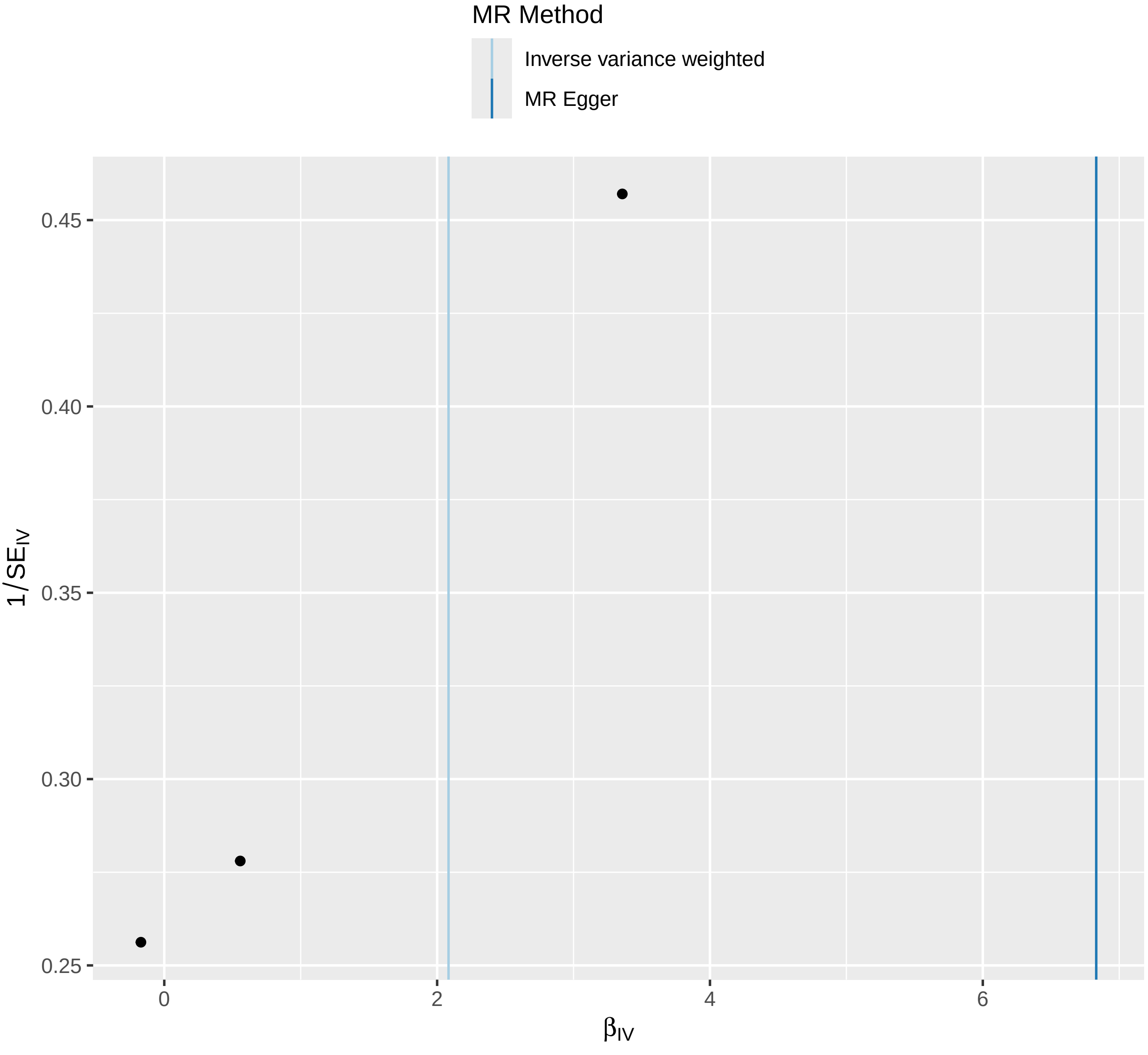
MR Method
Inverse variance weighted
MR Egger
0.45
0.40
0.35
0.30
0.25
$1/SE_{IV}$
0
2
4
6
β_{IV}

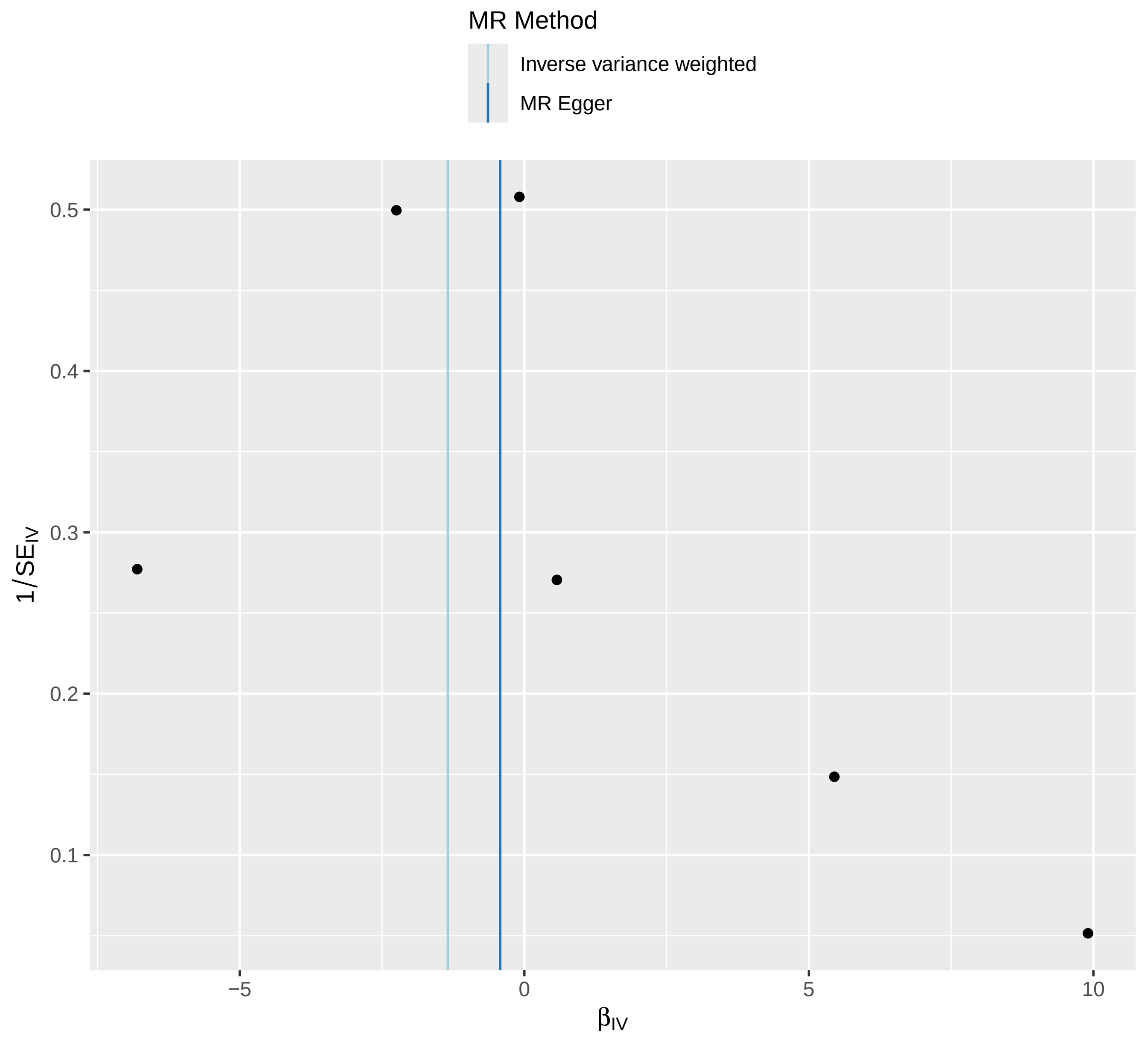

MR Method
Inverse variance weighted
MR Egger
0.5
0.4
0.3
0.2
0.1
1/SE_IV
−5
0
5
10
β_IV

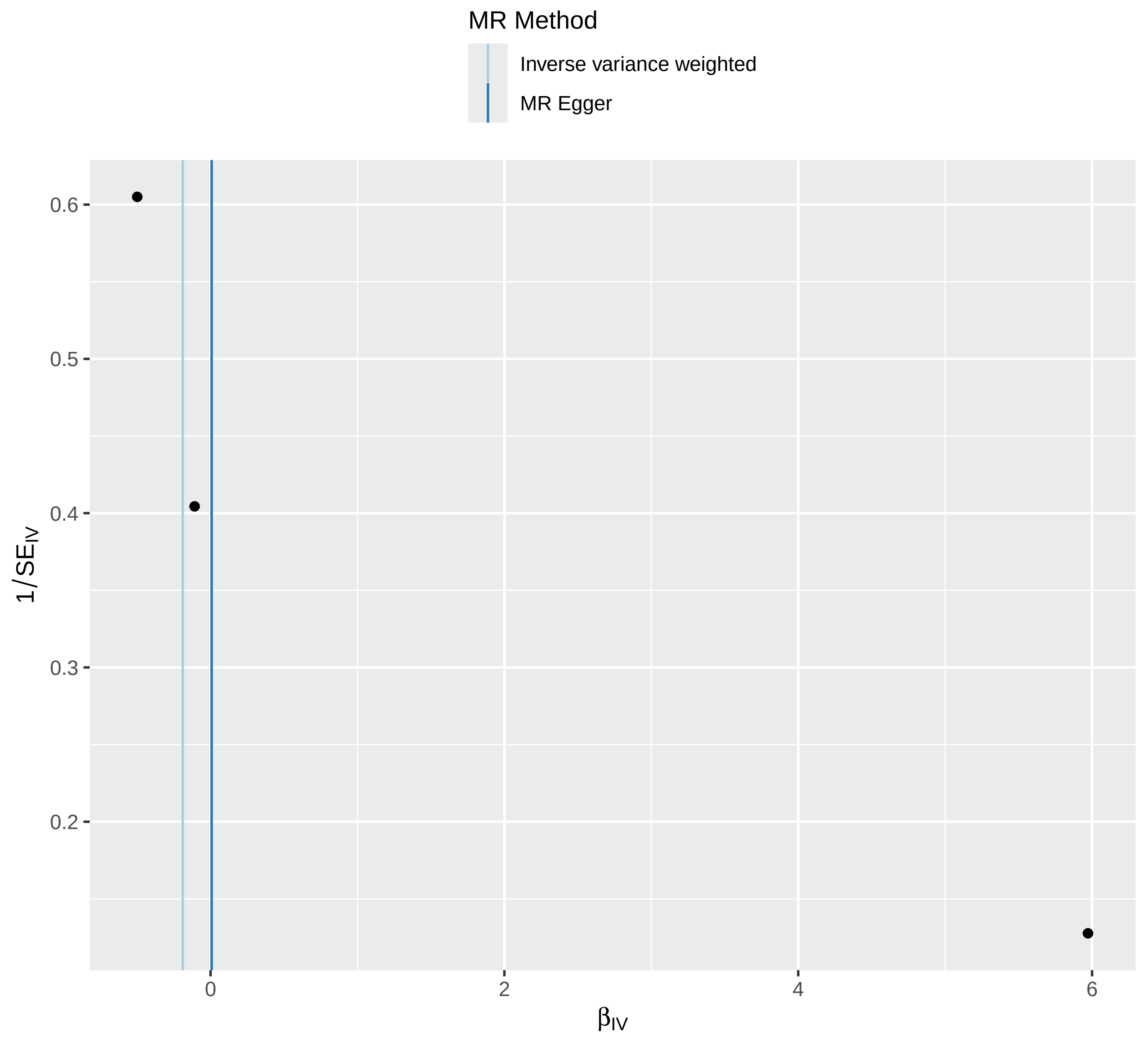
MR Method
Inverse variance weighted
MR Egger
0.6
0.5
0.4
0.3
0.2
$1/SE_{IV}$
0
2
4
6
β_{IV}

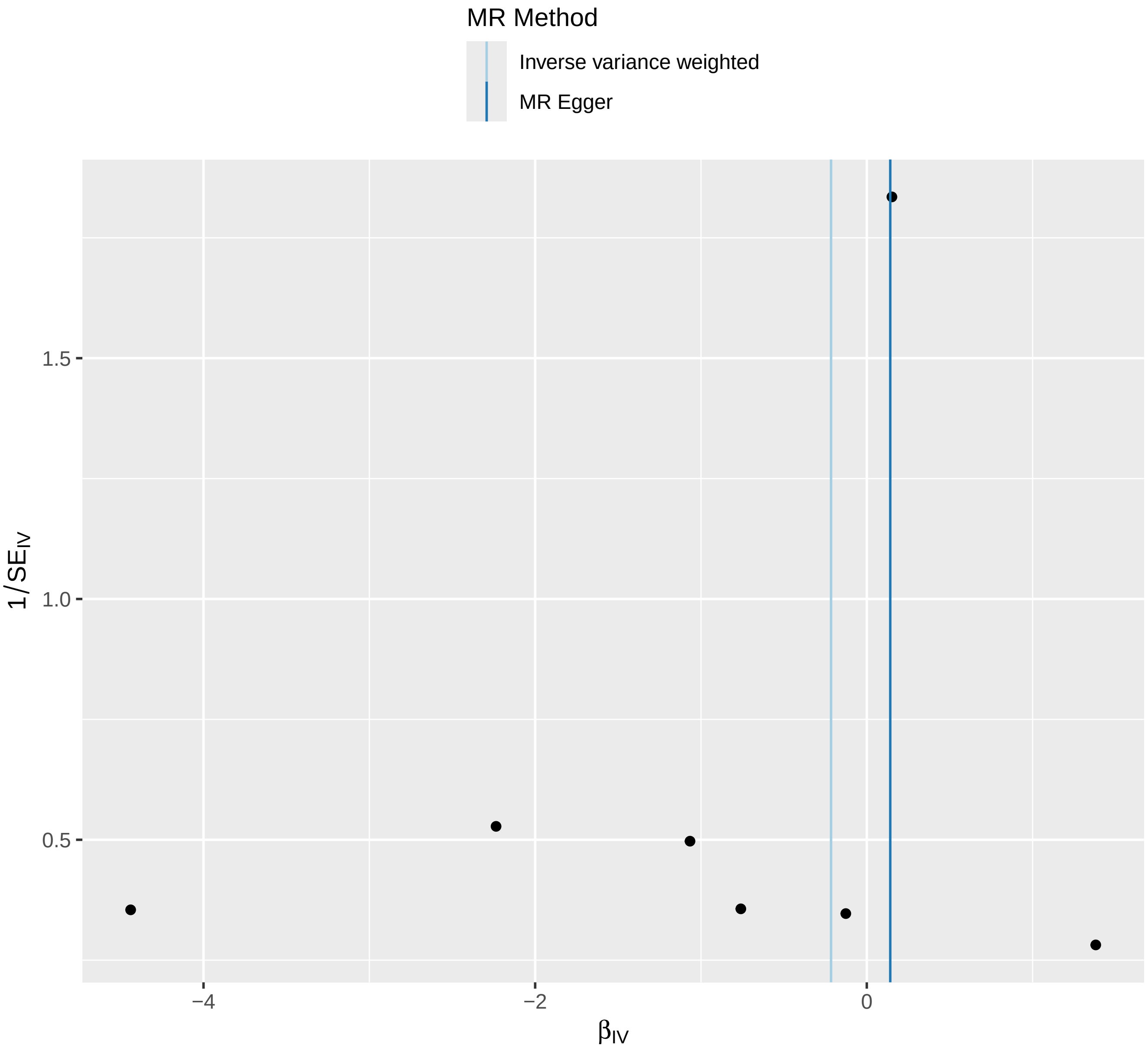
MR Method
Inverse variance weighted
MR Egger
1.5
1.0
0.5
−4
−2
0
β_{IV}
$1/SE_{IV}$

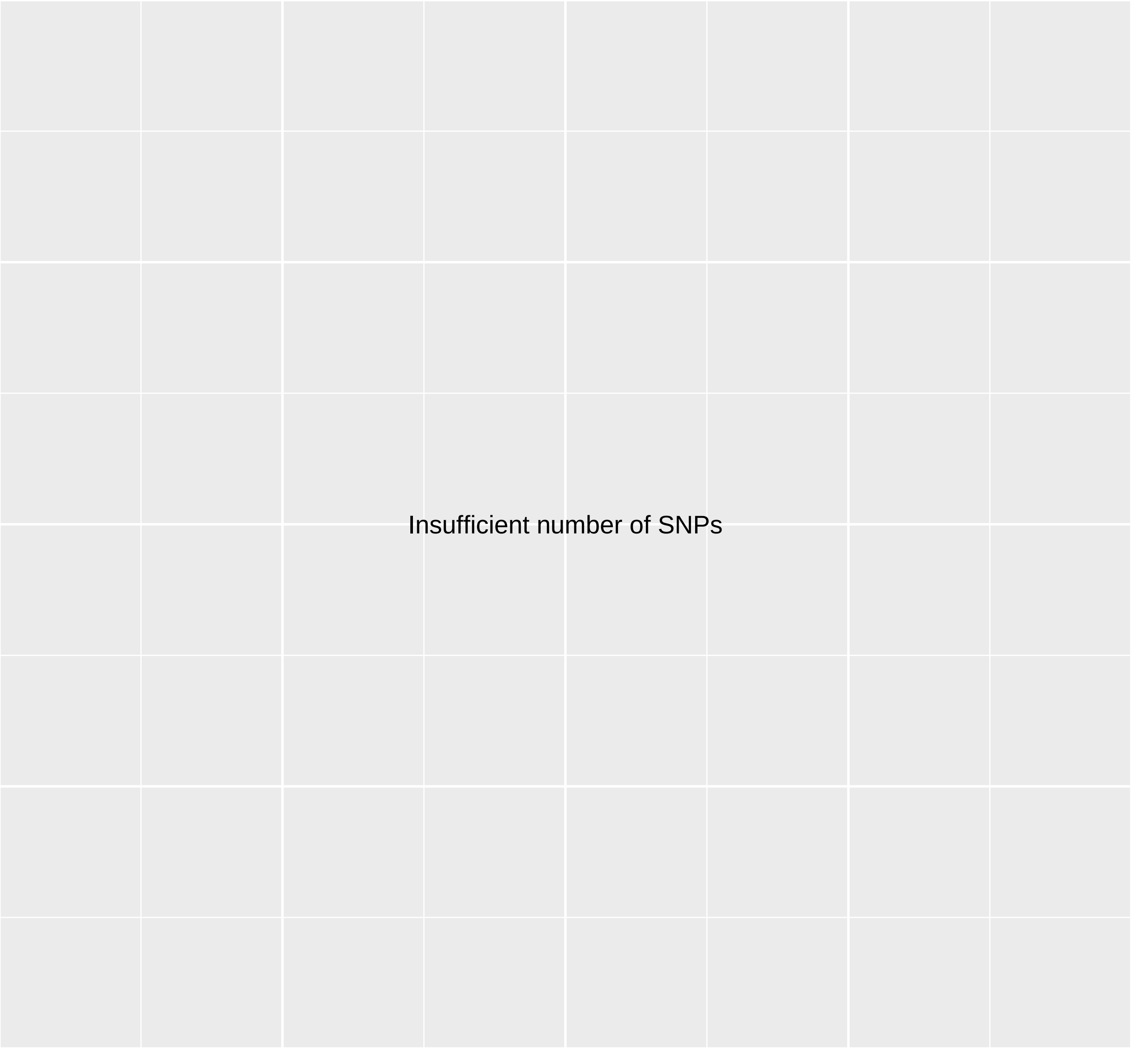
Insufficient number of SNPs

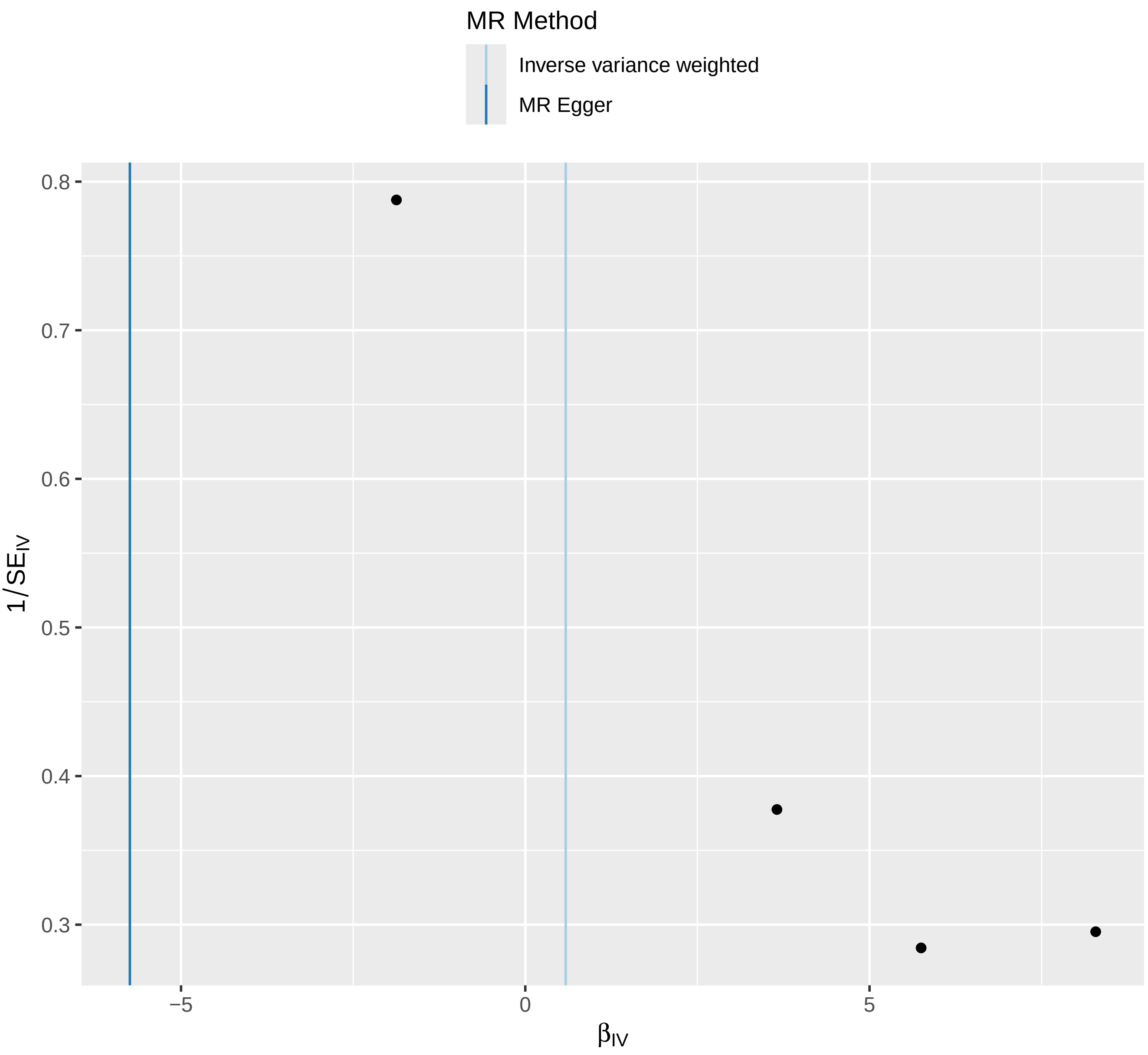
MR Method
Inverse variance weighted
MR Egger
0.8
0.7
0.6
0.5
0.4
0.3
1/SE_IV
−5
0
5
β_IV

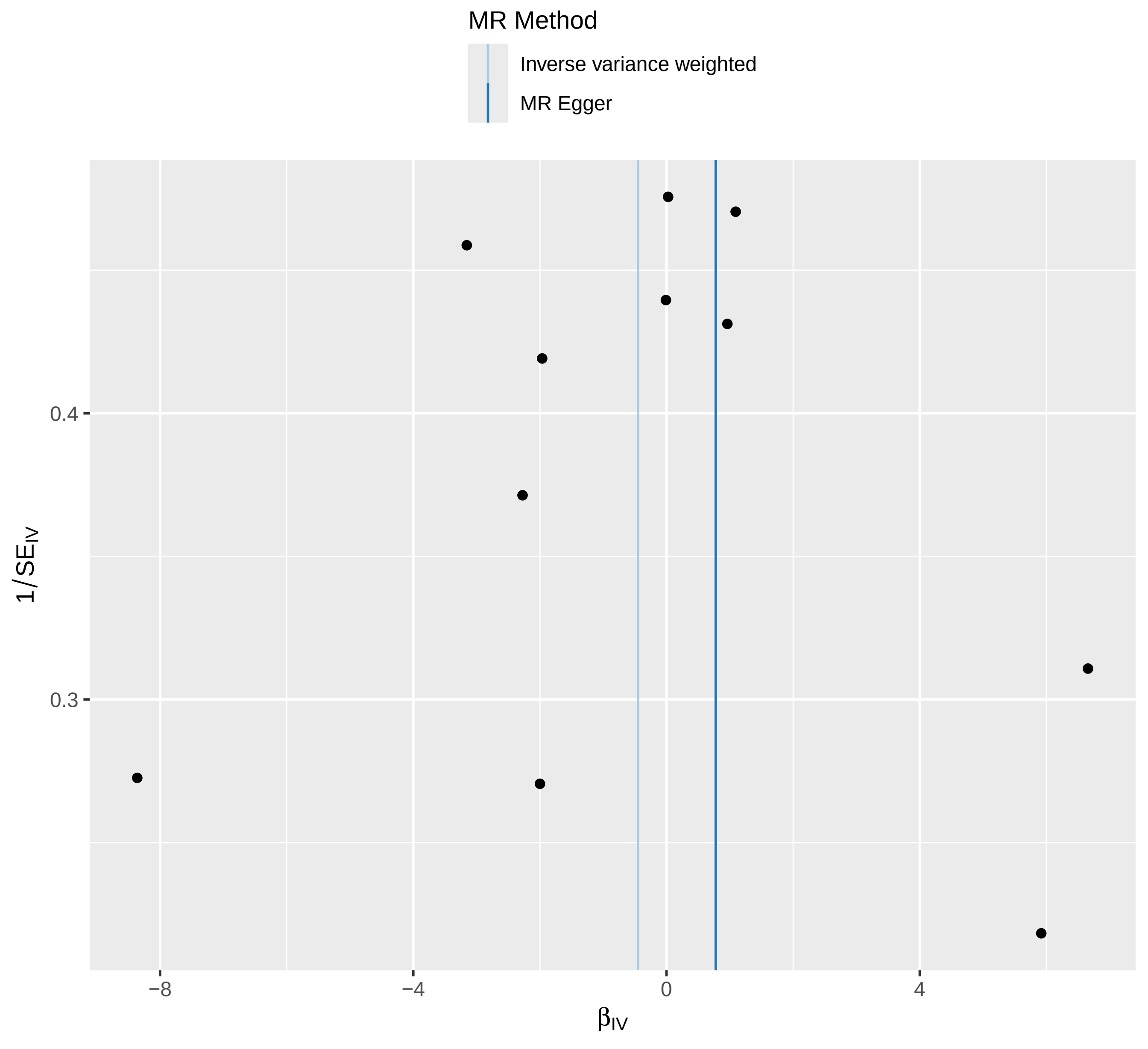
MR Method
Inverse variance weighted
MR Egger
0.4
0.3
1/SE_IV
−8
−4
0
4
β_IV

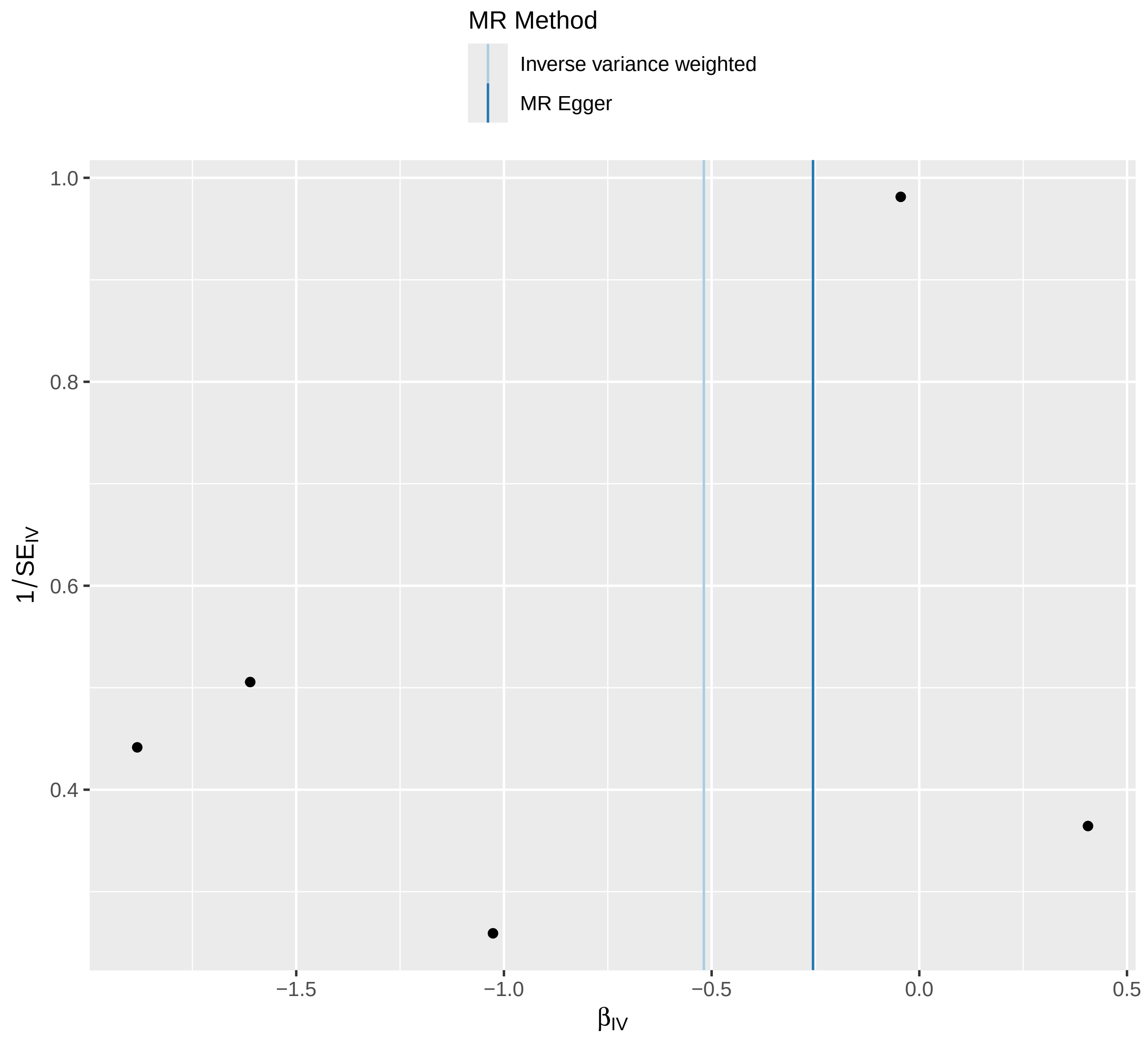
MR Method
Inverse variance weighted
MR Egger
1.0
0.8
0.6
0.4
1/SE_IV
−1.5
−1.0
−0.5
0.0
0.5
β_IV

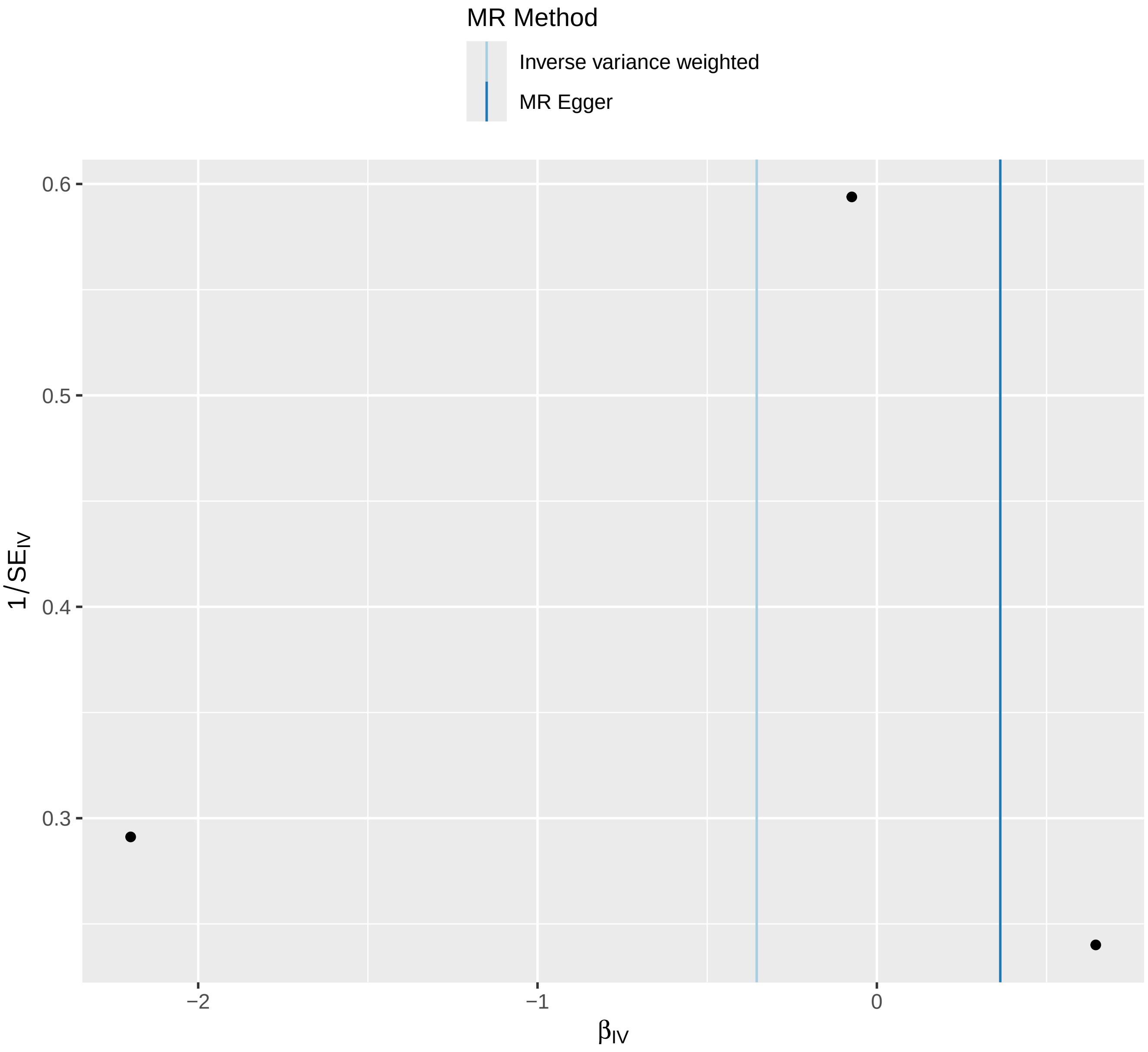
MR Method
Inverse variance weighted
MR Egger
0.6
0.5
0.4
0.3
1/SE_IV
−2
−1
0
β_IV

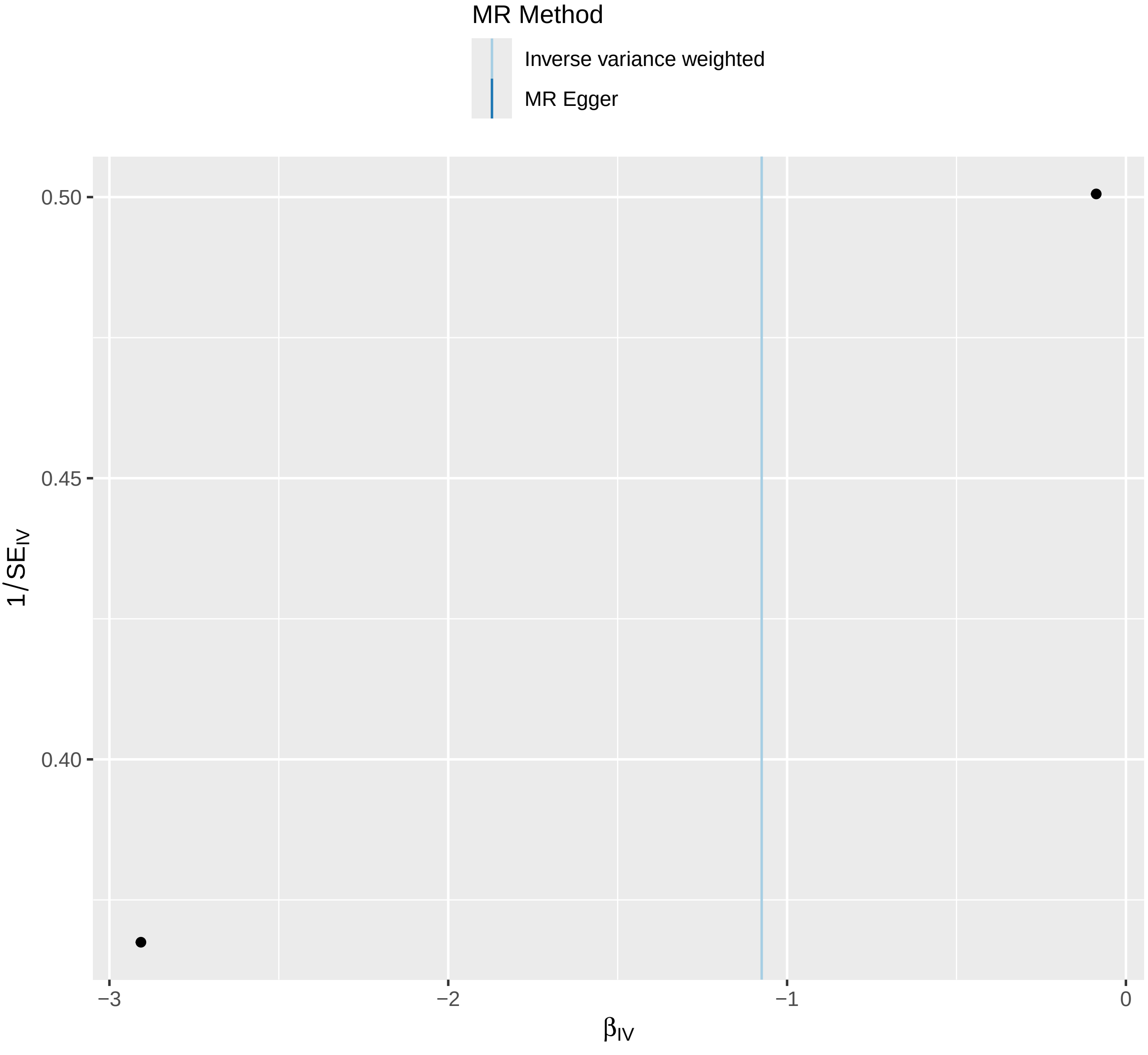
MR Method
Inverse variance weighted
MR Egger
0.50
0.45
0.40
1/SE$_{IV}$
−3
−2
−1
0
β$_{IV}$

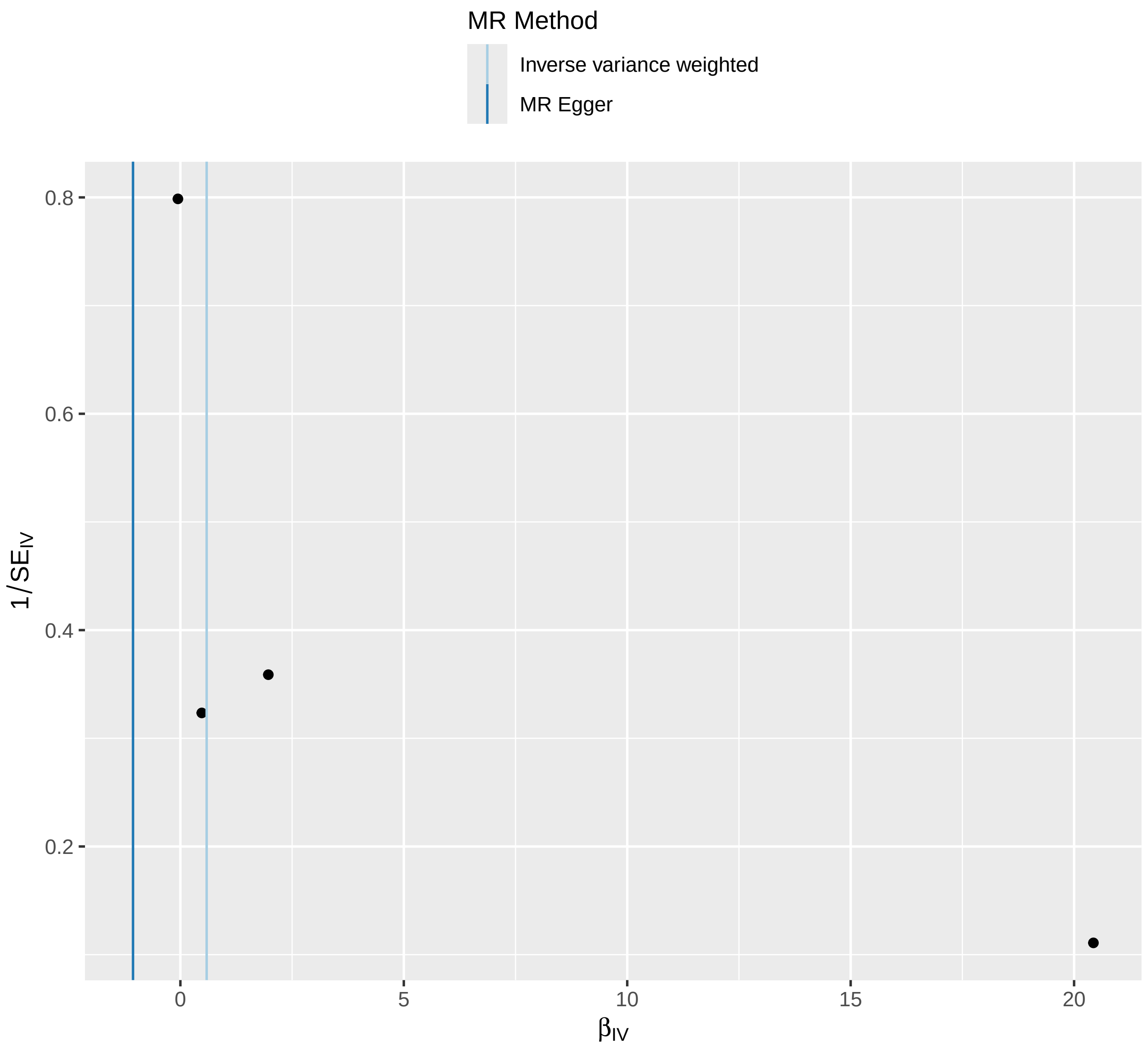
MR Method
Inverse variance weighted
MR Egger
0.8
0.6
0.4
0.2
1/SE_IV
0
5
10
15
20
β_IV

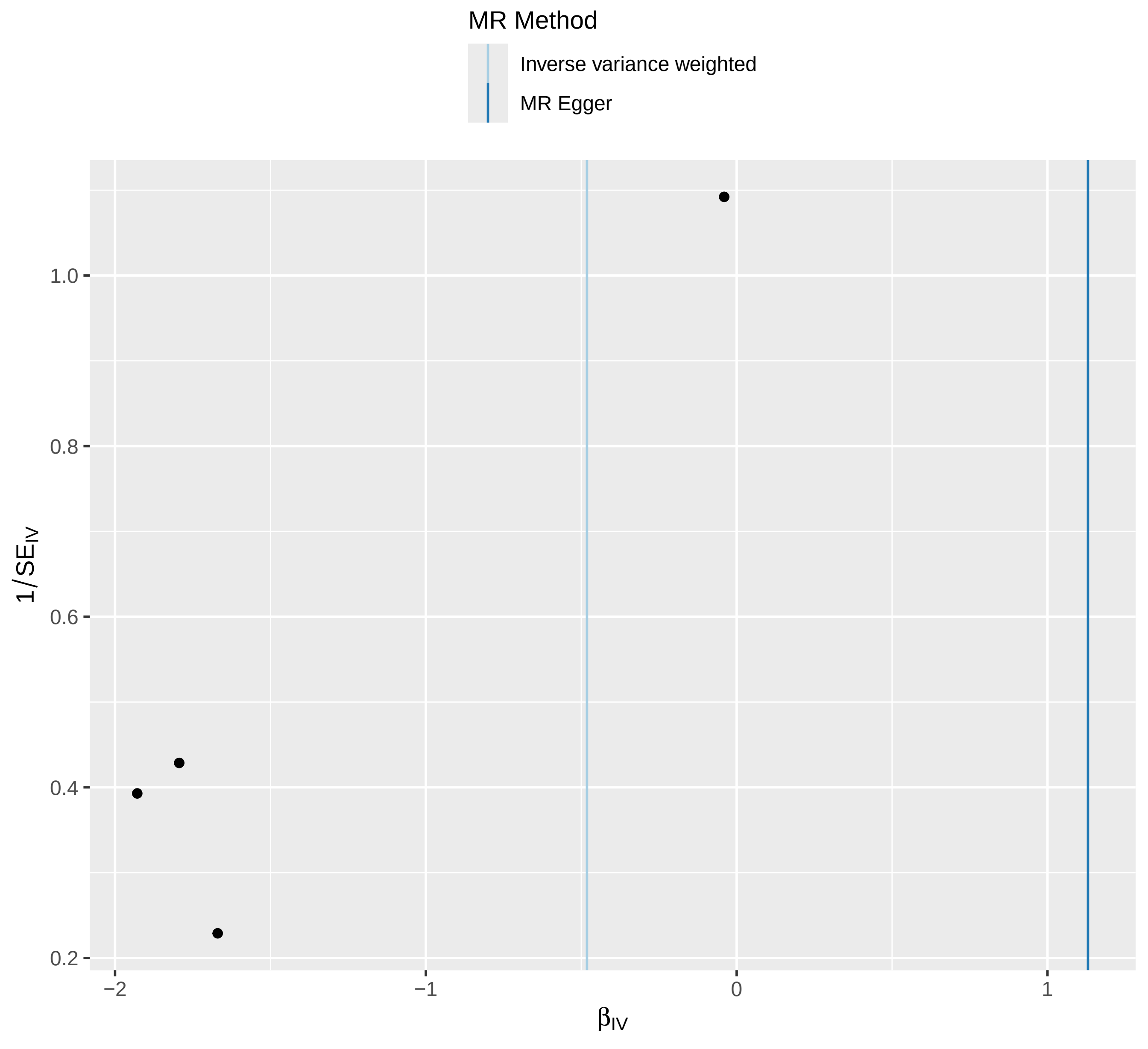
MR Method
Inverse variance weighted
MR Egger
1.0
0.8
0.6
0.4
0.2
$1/SE_{IV}$
−2
−1
0
1
β_{IV}

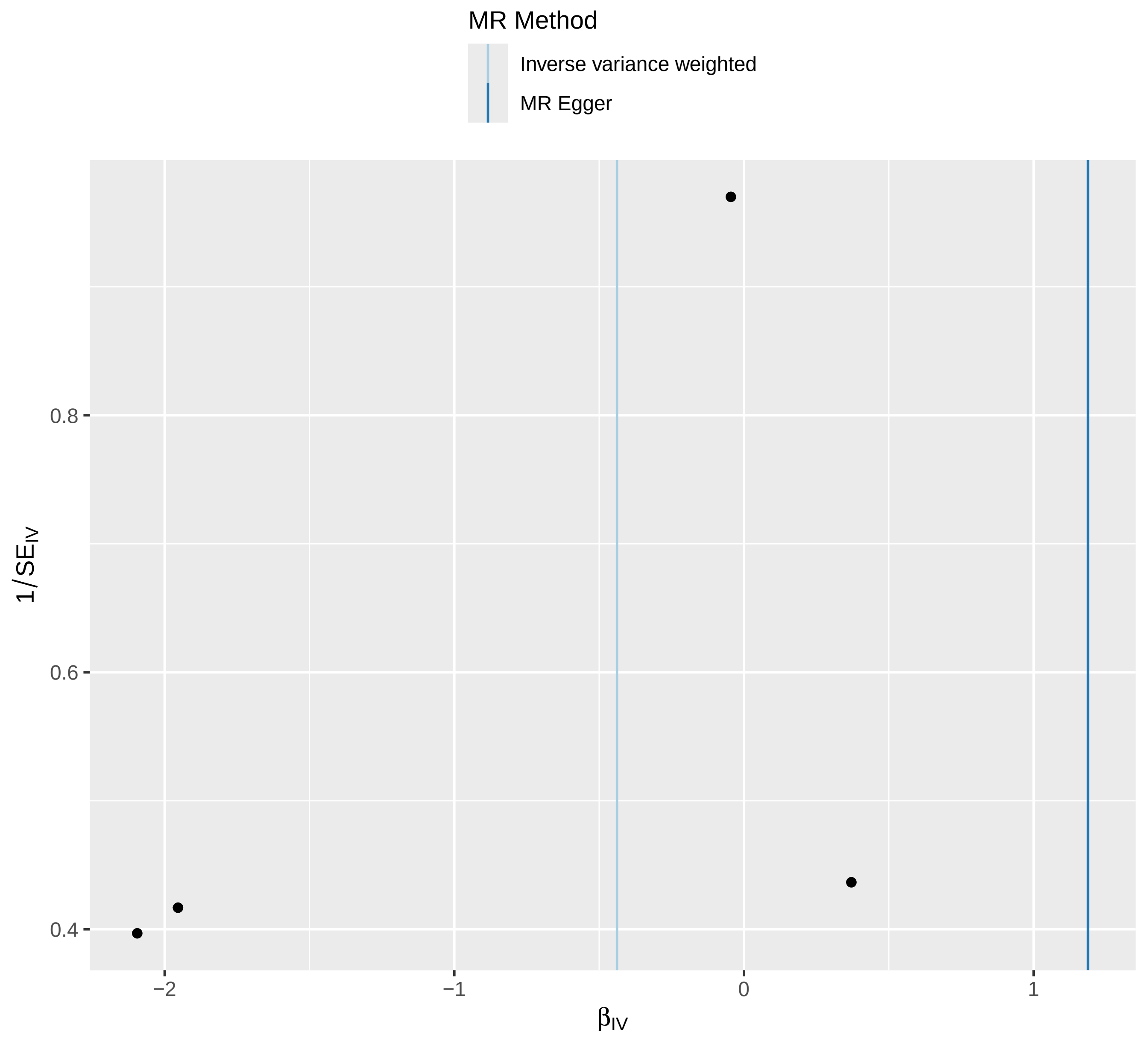
MR Method
Inverse variance weighted
MR Egger
0.8
0.6
0.4
1/SE_IV
−2
−1
0
1
β_IV

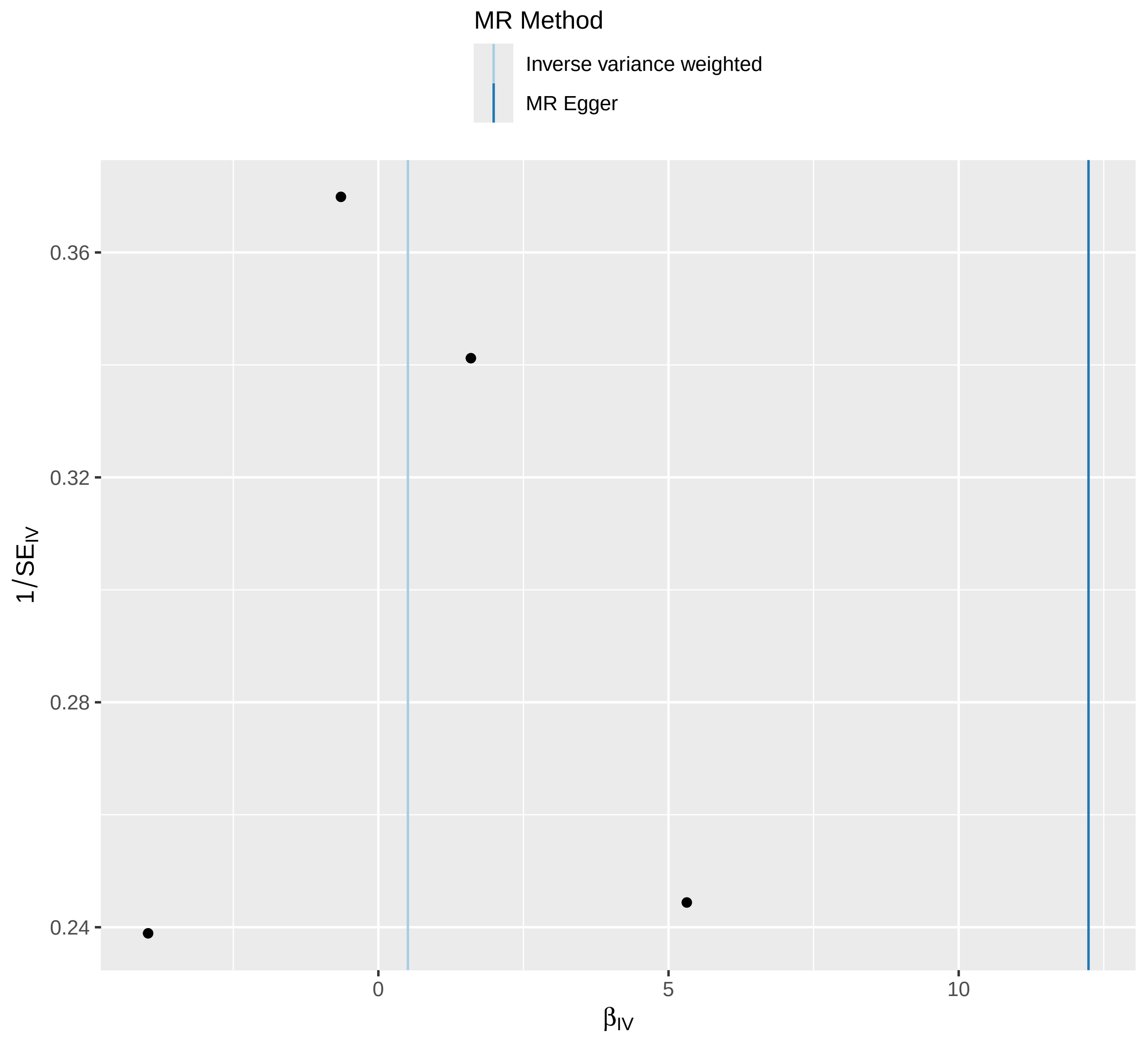

MR Method
Inverse variance weighted
MR Egger
0.36
0.32
0.28
0.24
1/SE_IV
0
5
10
β_IV

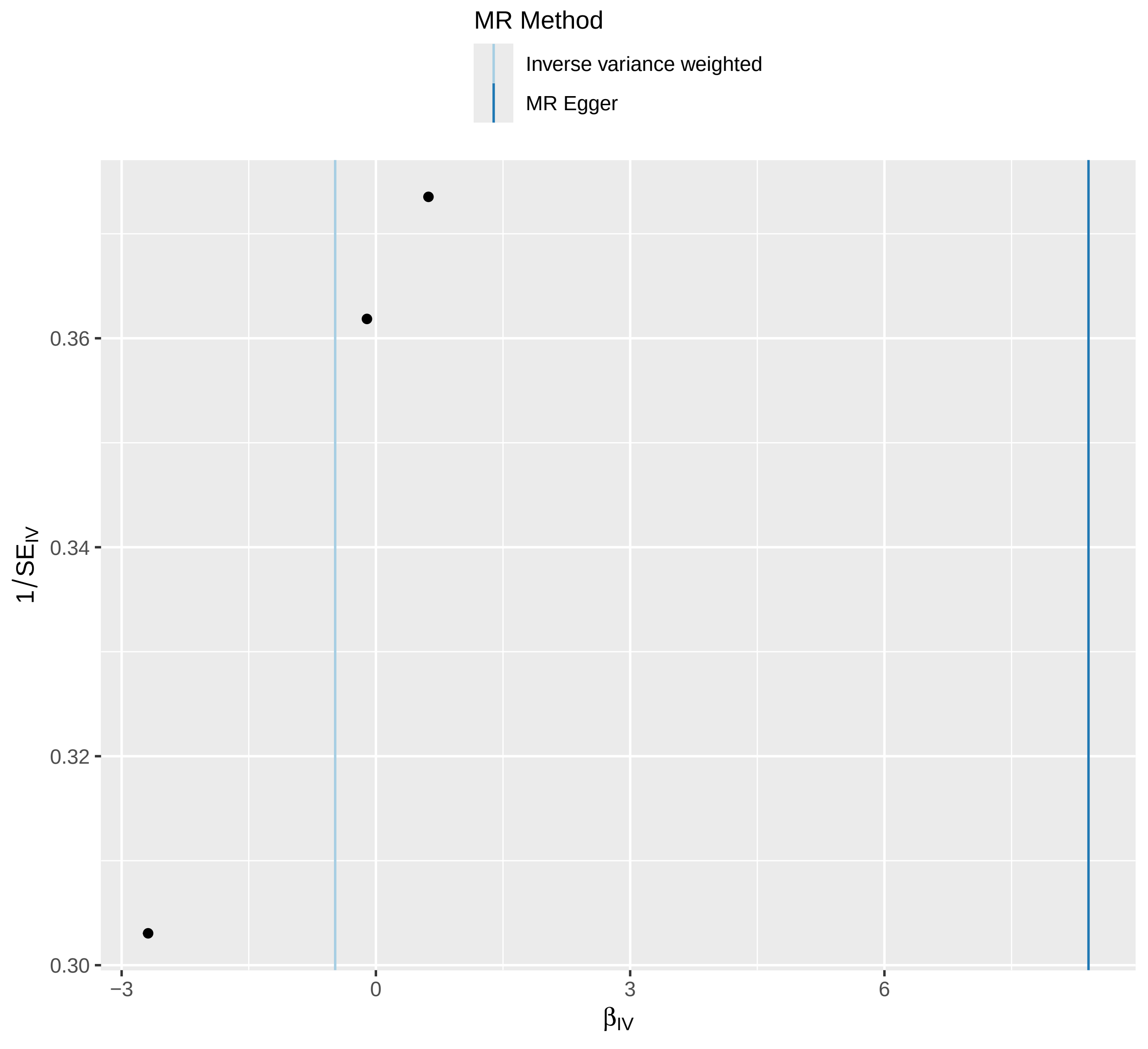
MR Method
Inverse variance weighted
MR Egger
0.36
0.34
0.32
0.30
1/SE_IV
−3
0
3
6
β_IV

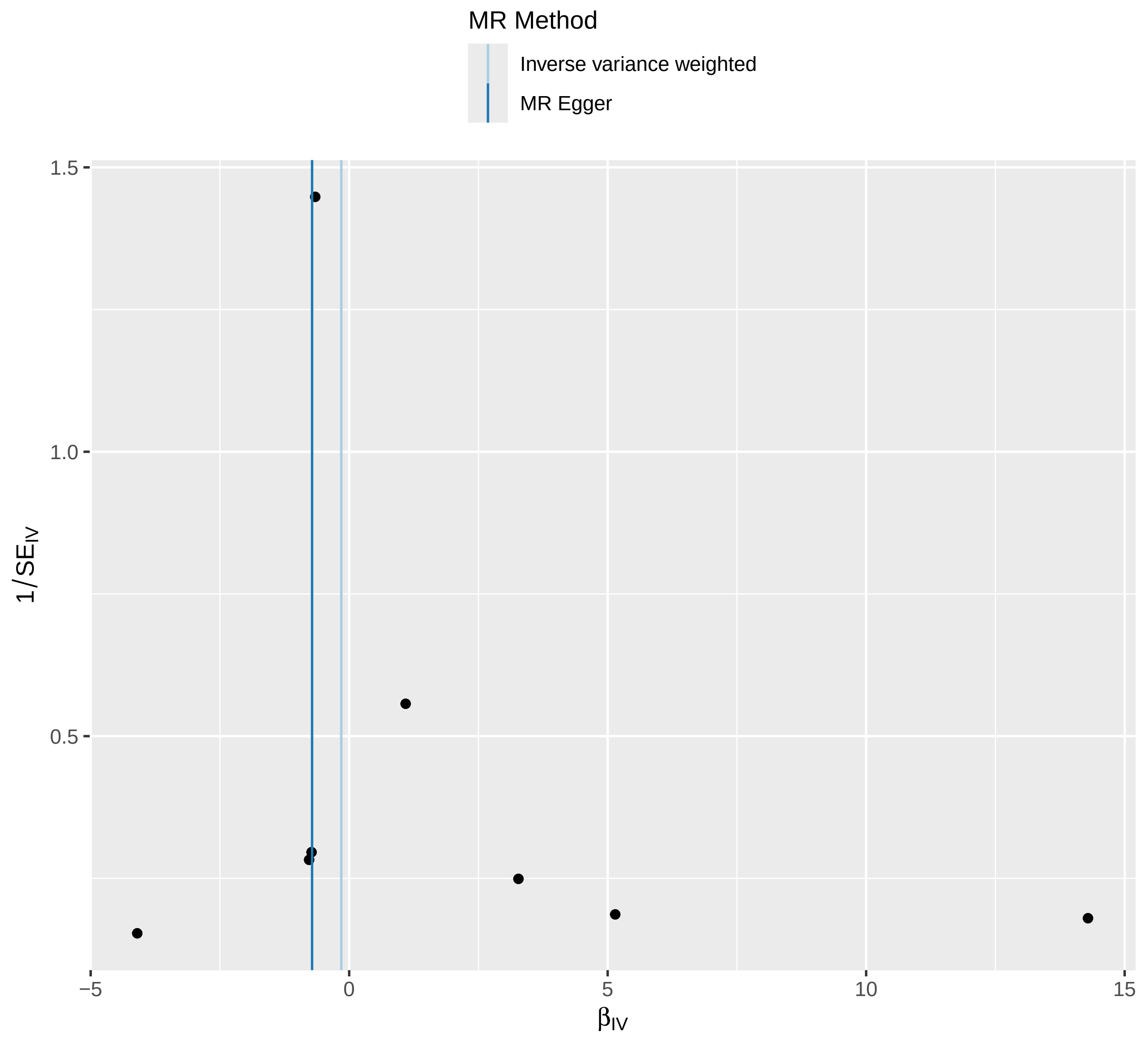
MR Method
Inverse variance weighted
MR Egger
1.5
1.0
0.5
1/SE_IV
−5
0
5
10
15
β_IV

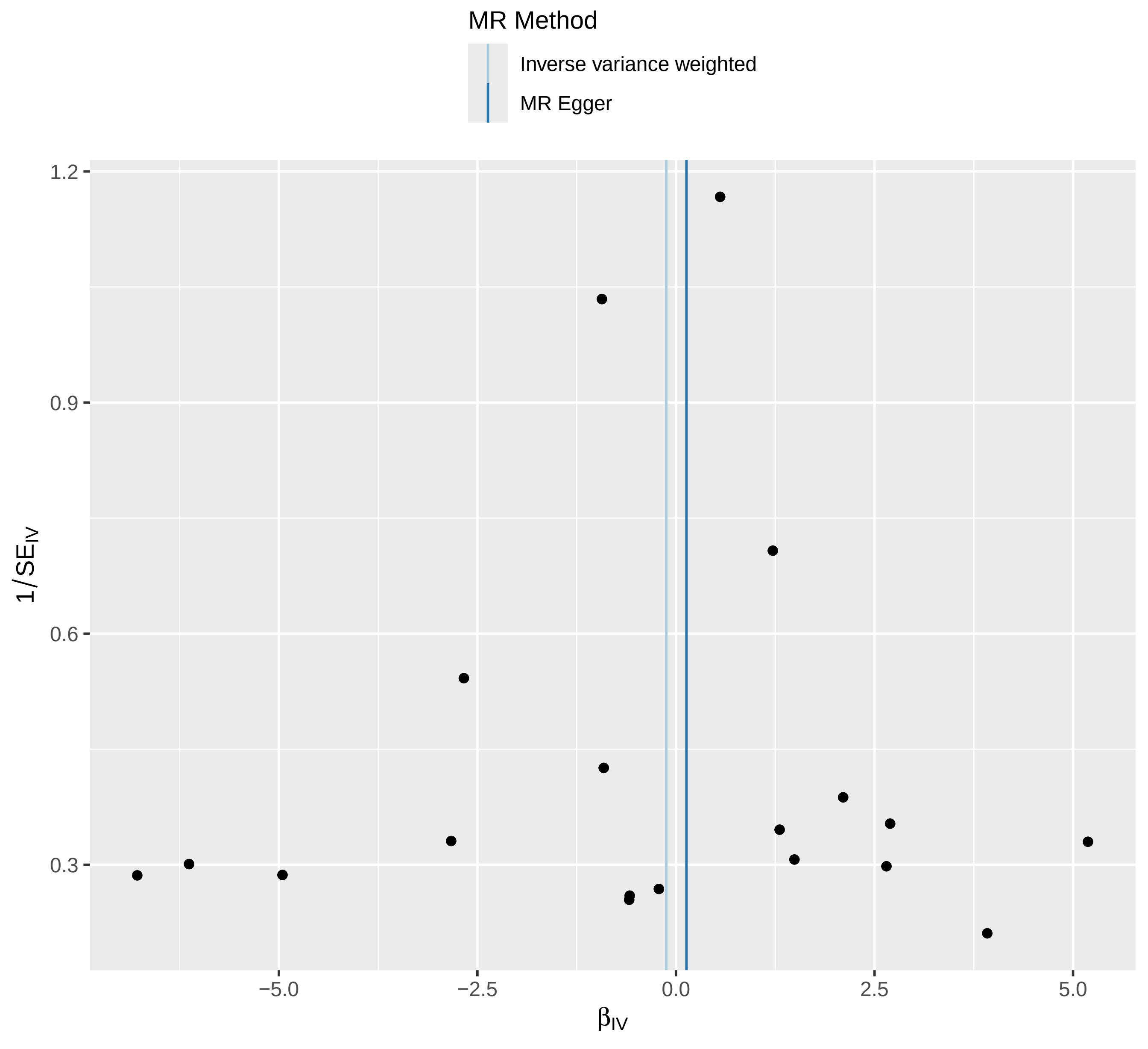
MR Method
Inverse variance weighted
MR Egger
1.2
0.9
0.6
0.3
1/SE_IV
−5.0
−2.5
0.0
2.5
5.0
β_IV

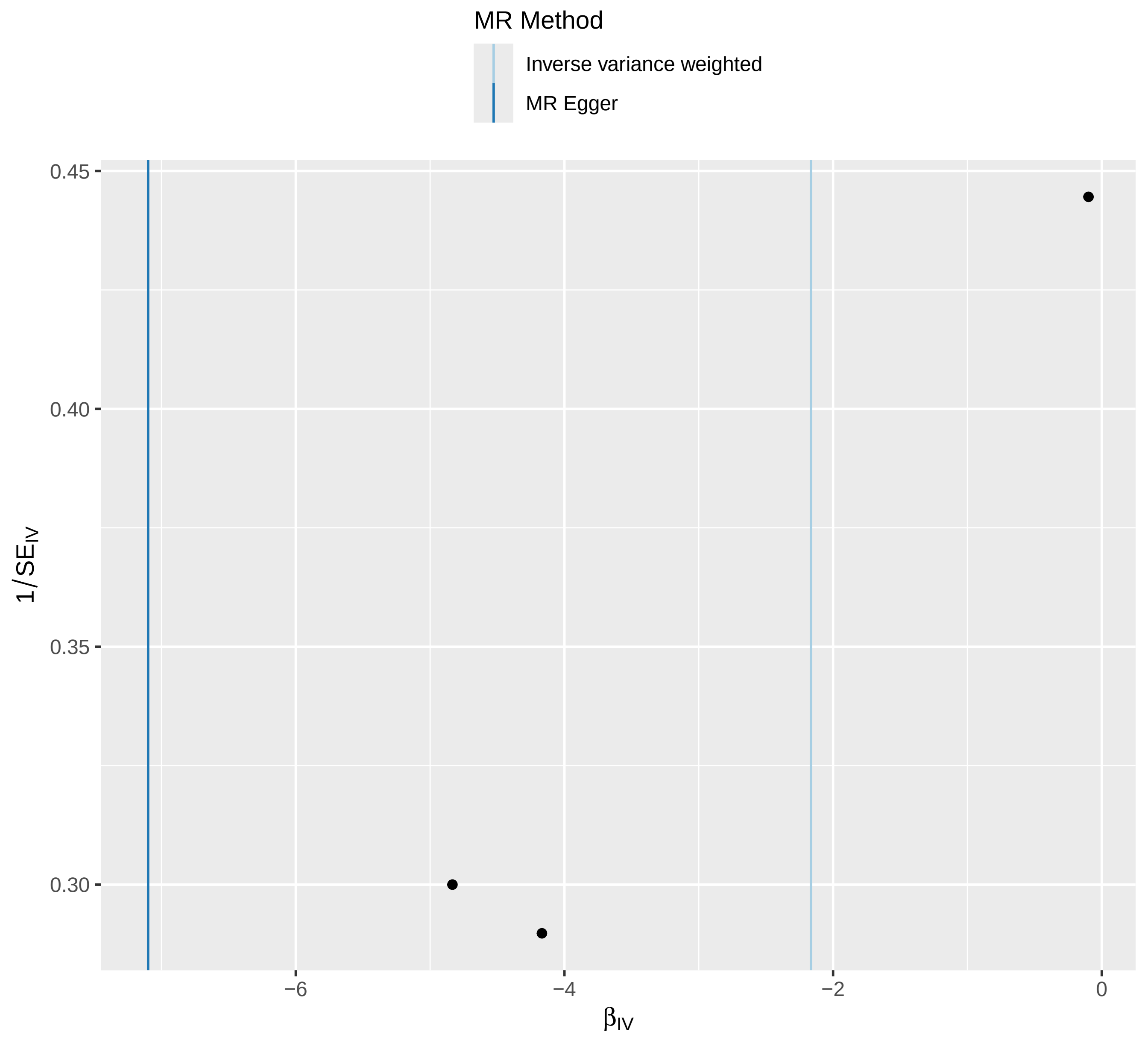
MR Method
Inverse variance weighted
MR Egger
0.45
0.40
0.35
0.30
1/SE$_{IV}$
−6
−4
−2
0
β$_{IV}$

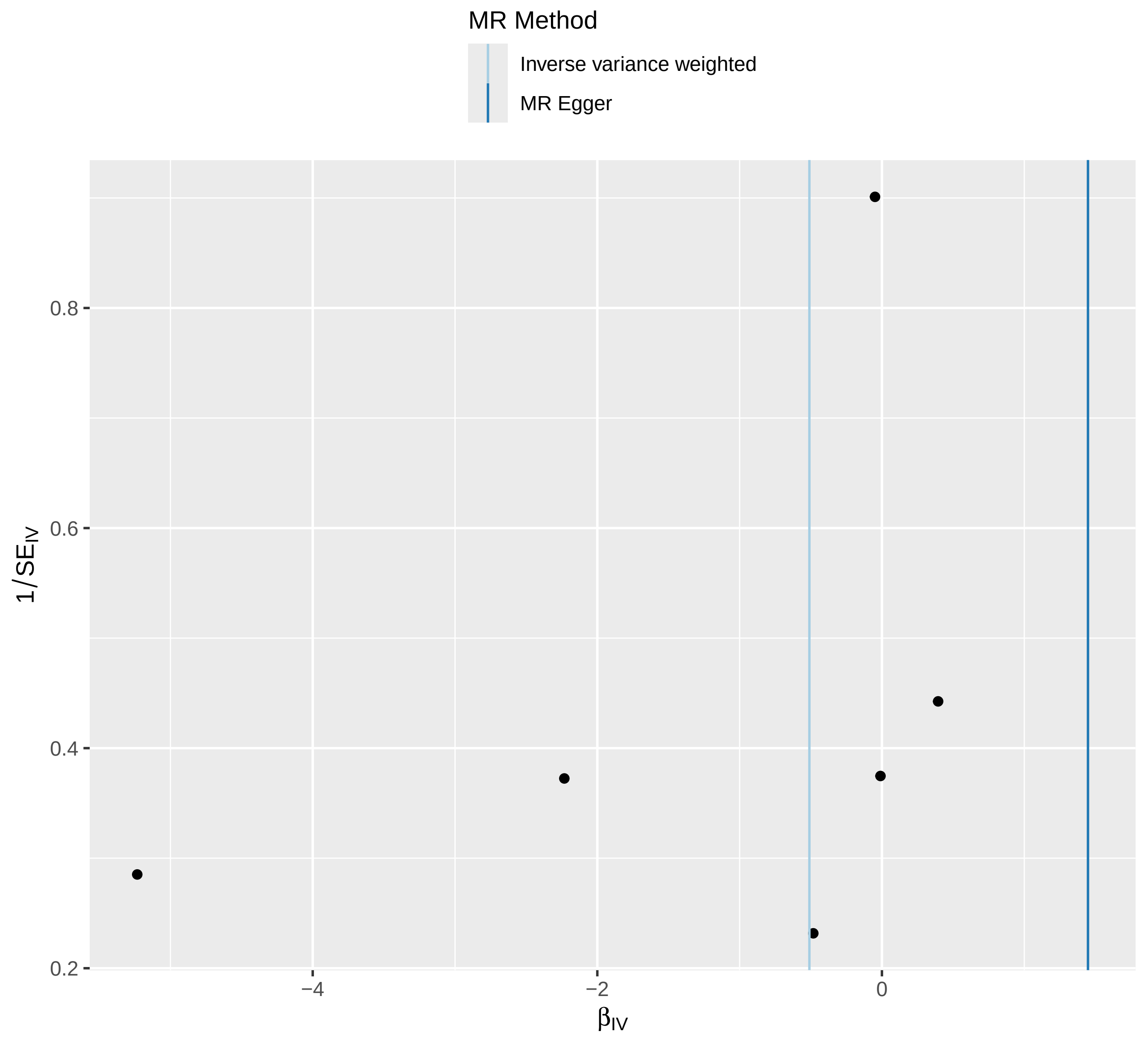
MR Method
Inverse variance weighted
MR Egger
0.8
0.6
0.4
0.2
1/SE$_{IV}$
−4
−2
0
β$_{IV}$

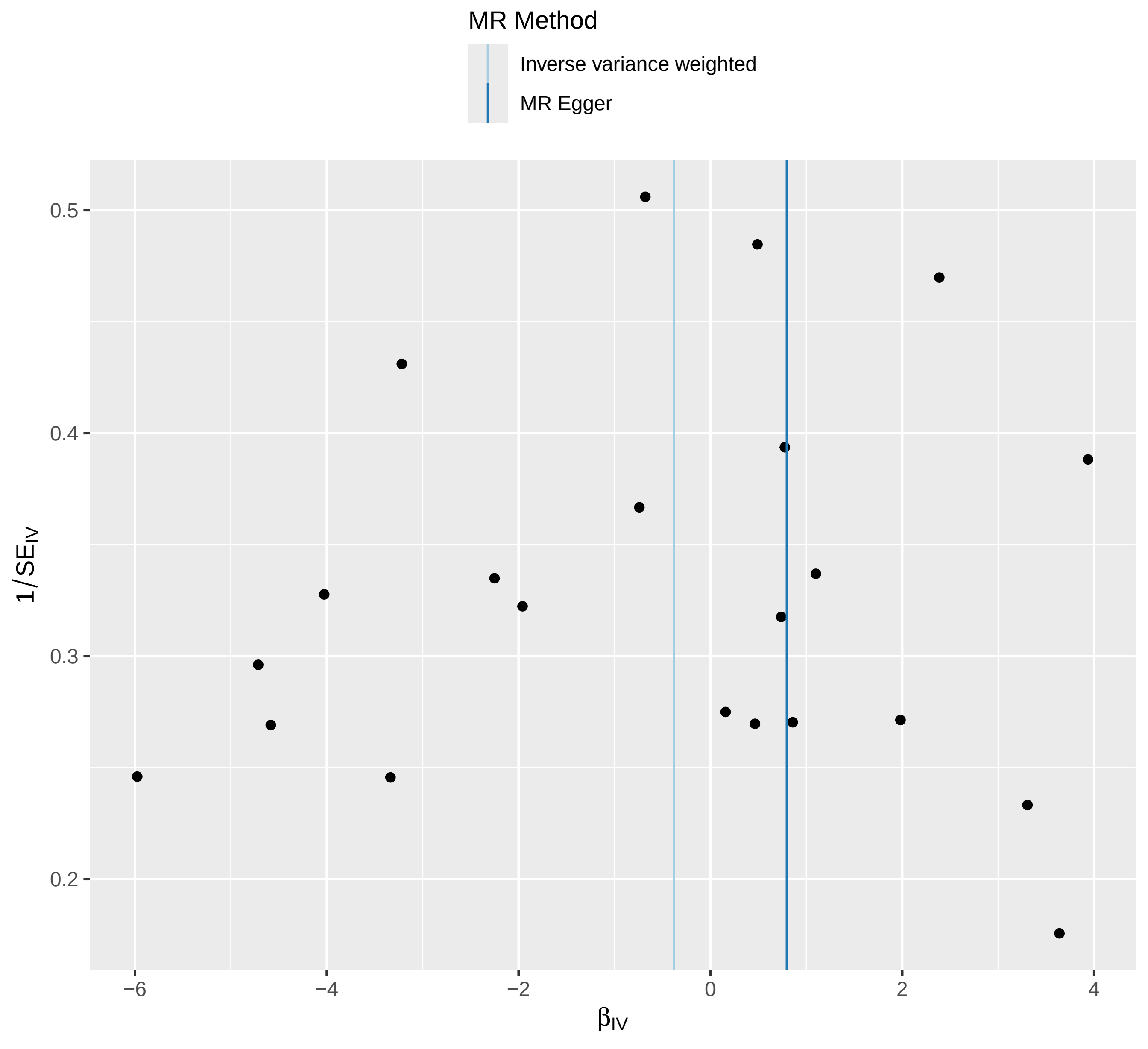

MR Method
Inverse variance weighted
MR Egger
0.5
0.4
0.3
0.2
1/SE_IV
−6
−4
−2
0
2
4
β_IV

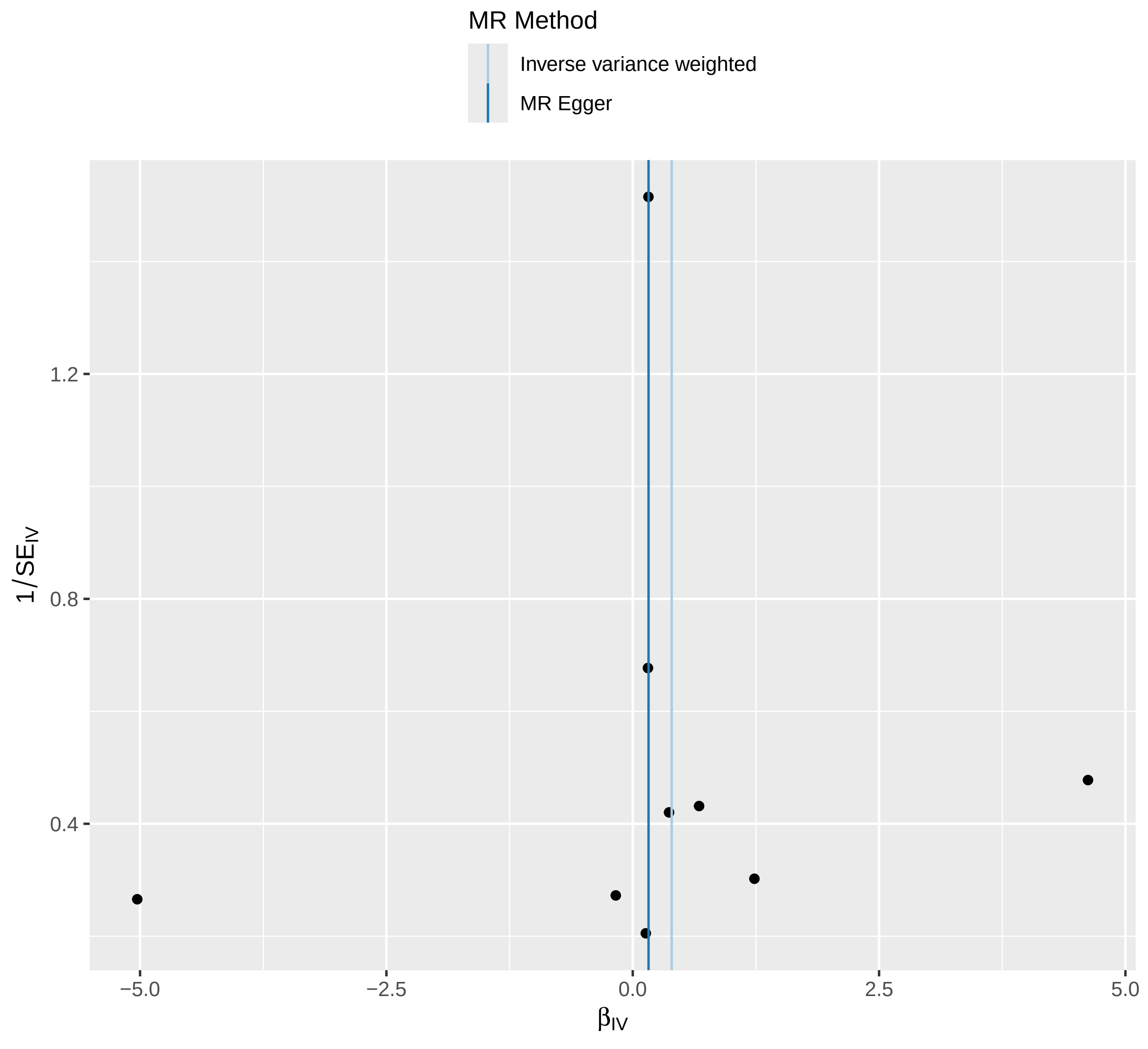
MR Method
Inverse variance weighted
MR Egger
1.2
0.8
0.4
$1/SE_{IV}$
−5.0
−2.5
0.0
2.5
5.0
β_{IV}

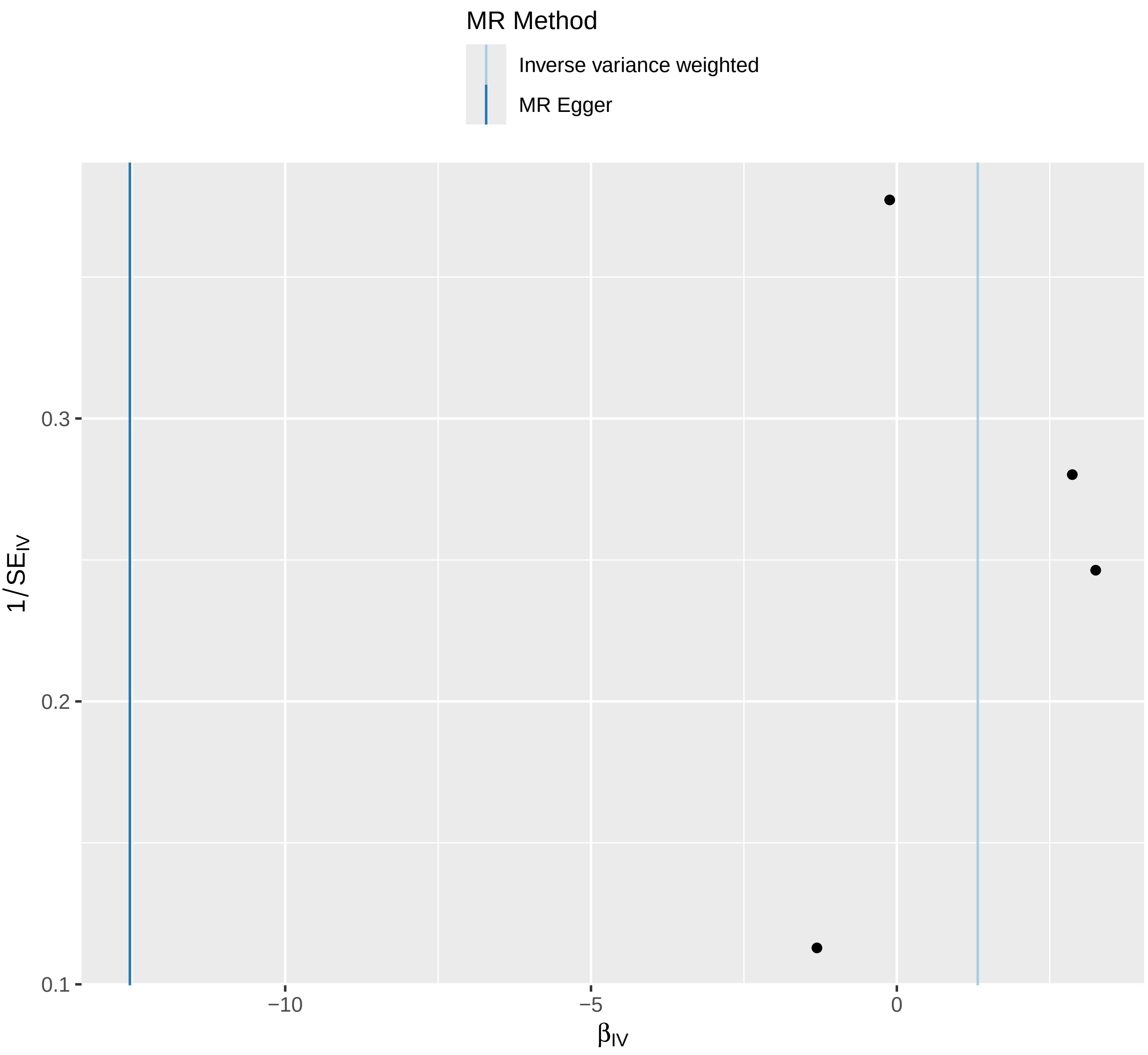
MR Method
Inverse variance weighted
MR Egger
0.3
0.2
0.1
−10
−5
0
1/SE_IV
β_IV

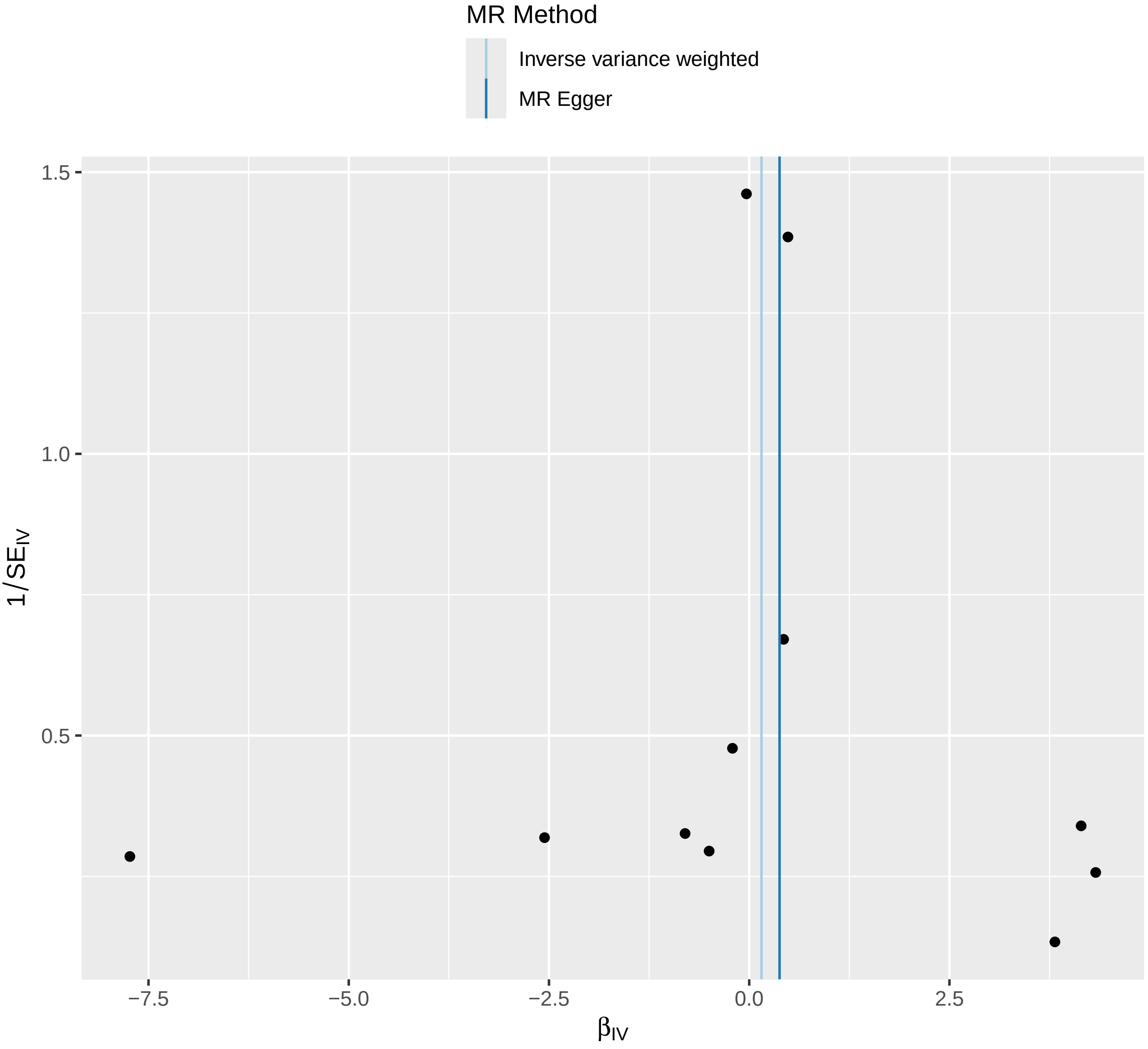

MR Method
Inverse variance weighted
MR Egger
1.5
1.0
0.5
1/SE$_{IV}$
−7.5
−5.0
−2.5
0.0
2.5
β_{IV}

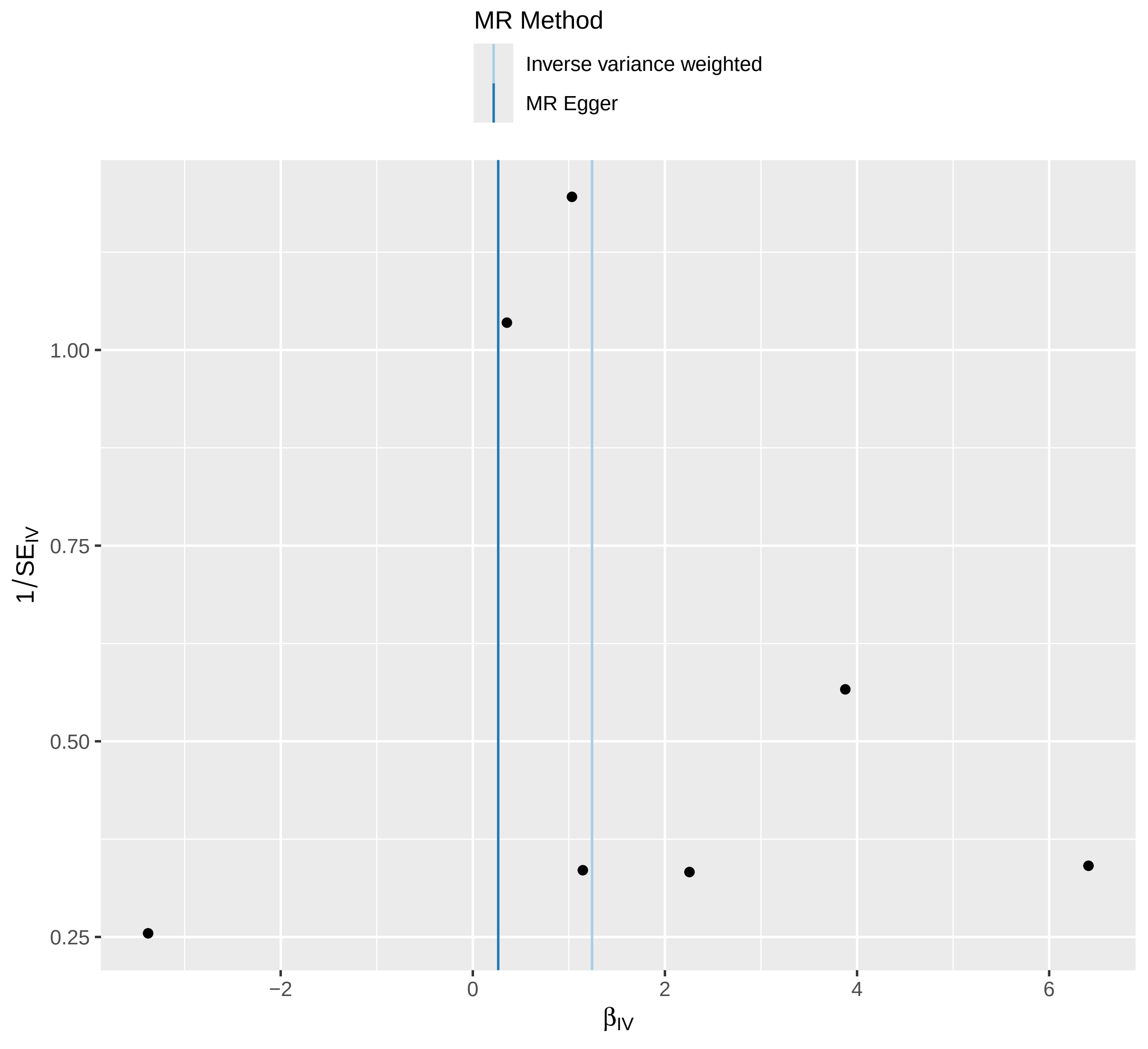

MR Method
Inverse variance weighted
MR Egger
1.00
0.75
0.50
0.25
$1/SE_{IV}$
−2
0
2
4
6
β_{IV}

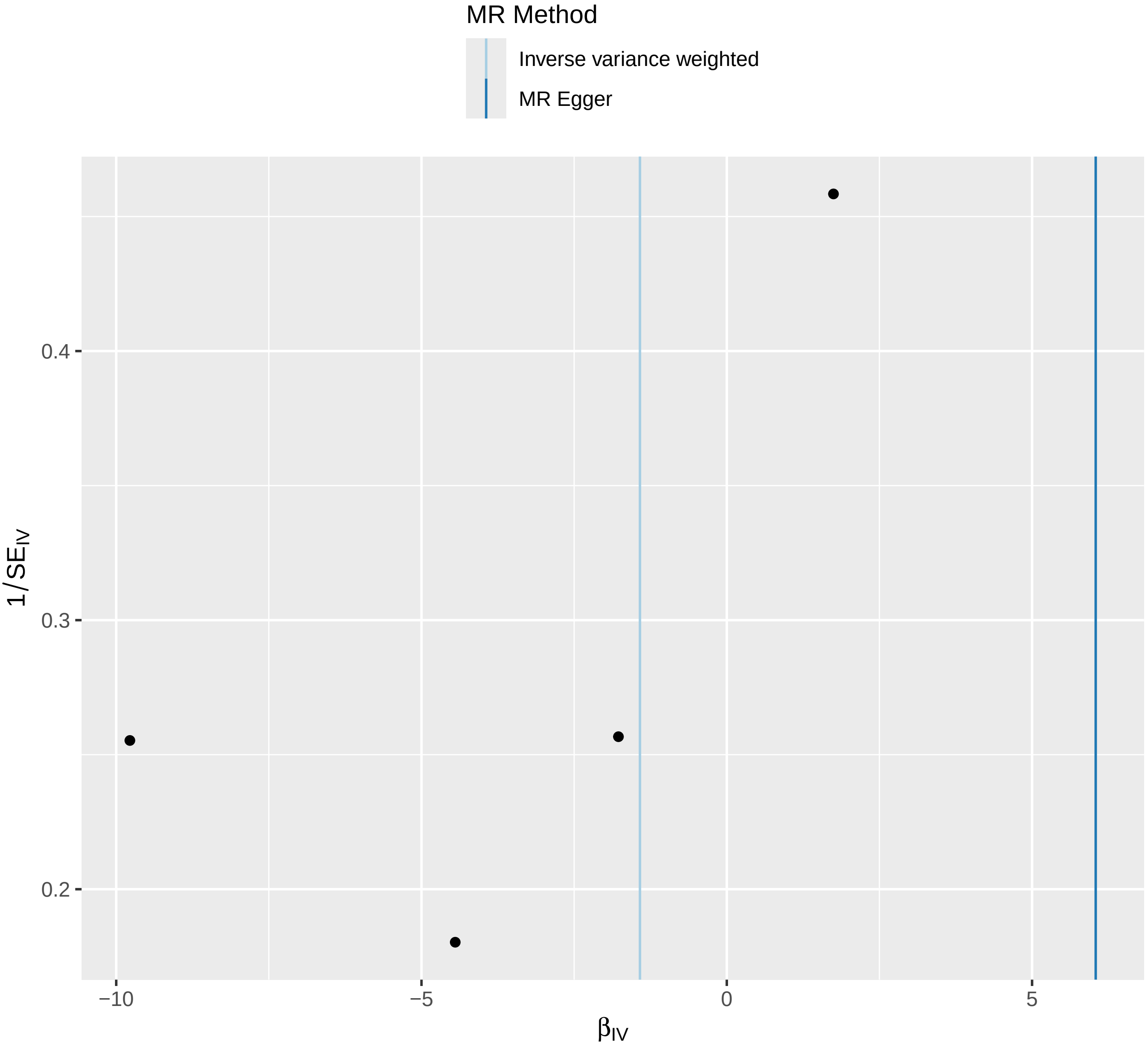
MR Method
Inverse variance weighted
MR Egger
0.4
0.3
0.2
1/SE_IV
−10
−5
0
5
β_IV

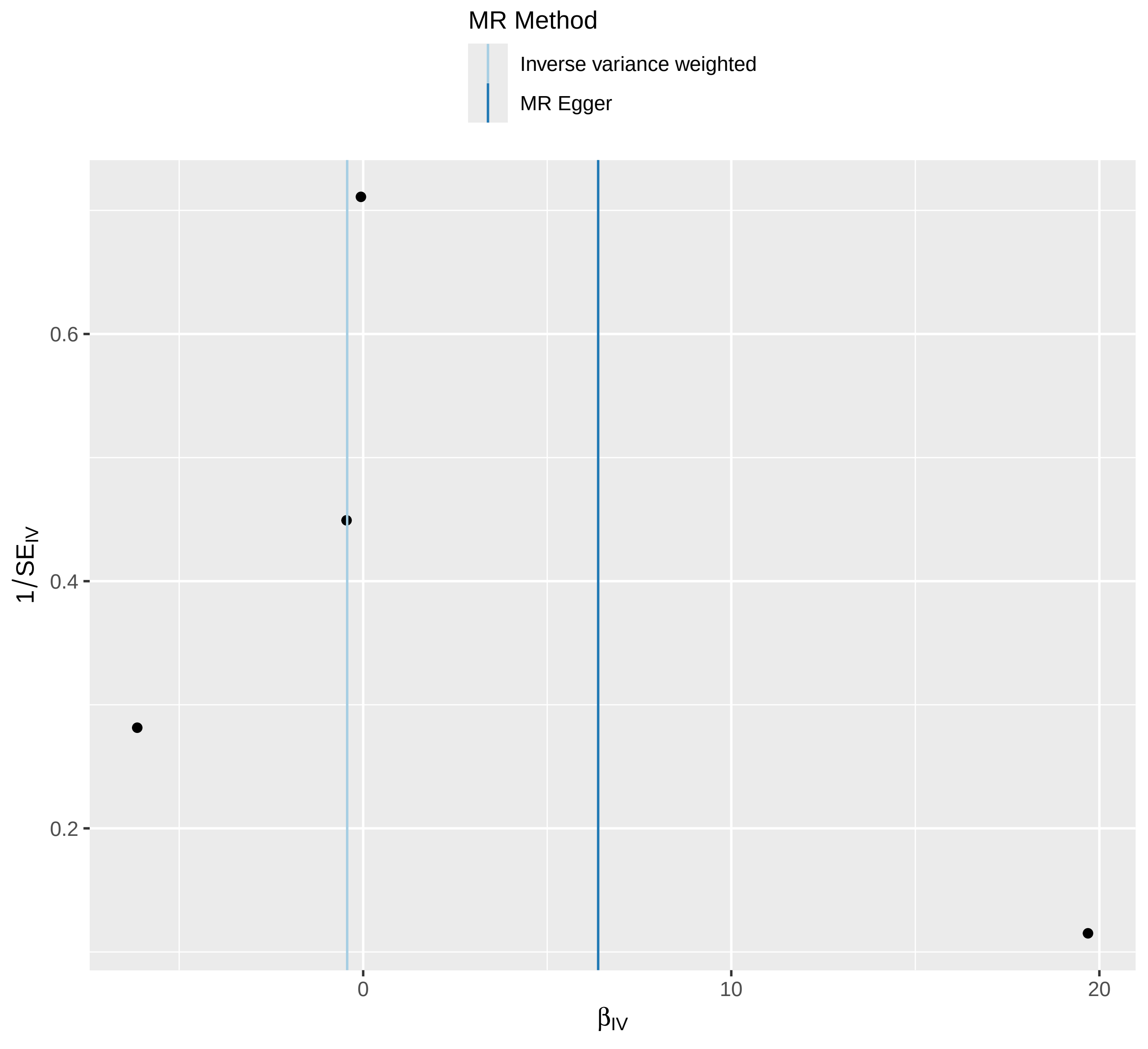
MR Method
Inverse variance weighted
MR Egger
0.6
0.4
0.2
1/SE_IV
0
10
20
β_IV

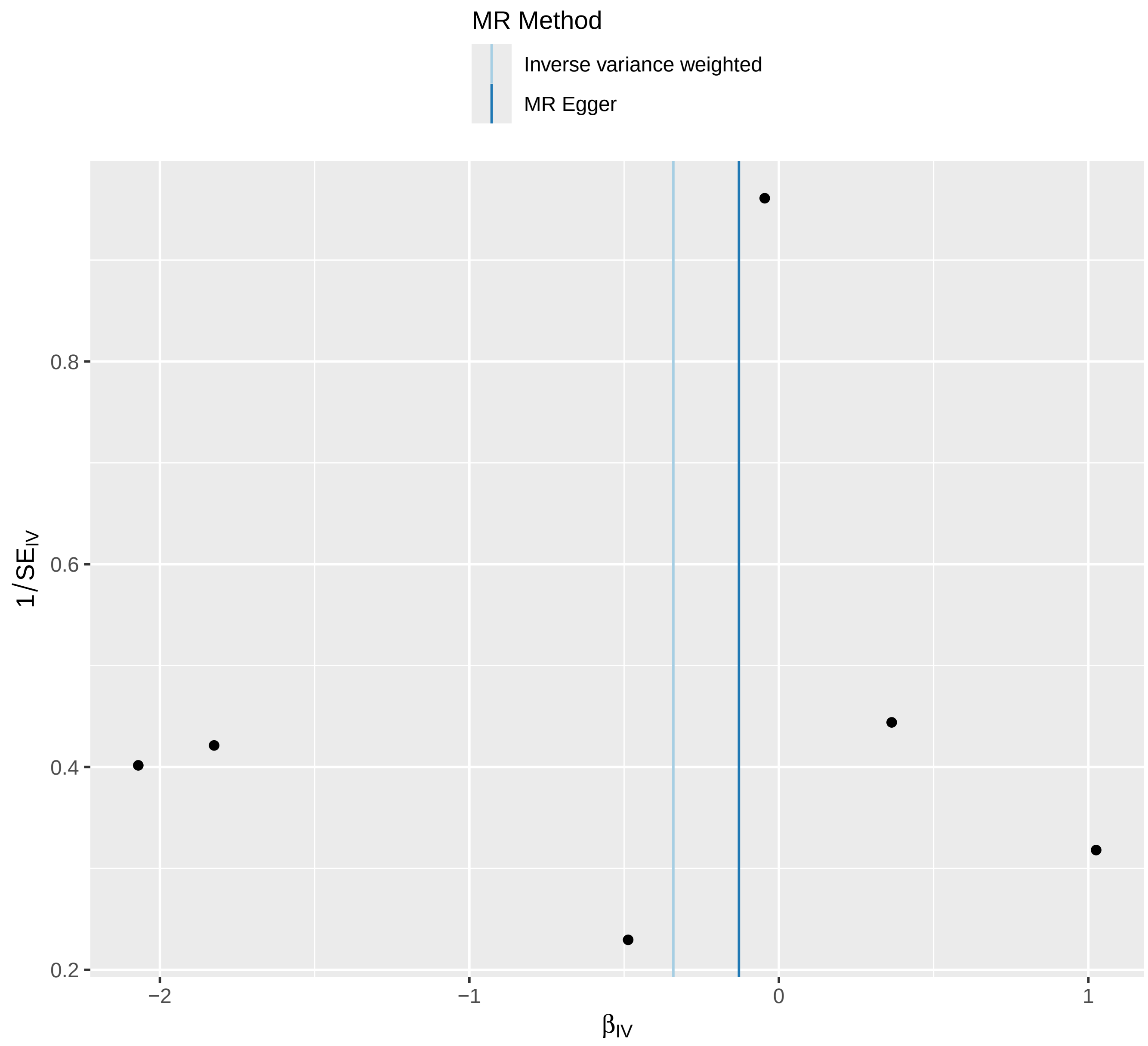
MR Method
Inverse variance weighted
MR Egger
0.8
0.6
0.4
0.2
1/SE_IV
−2
−1
0
1
β_IV

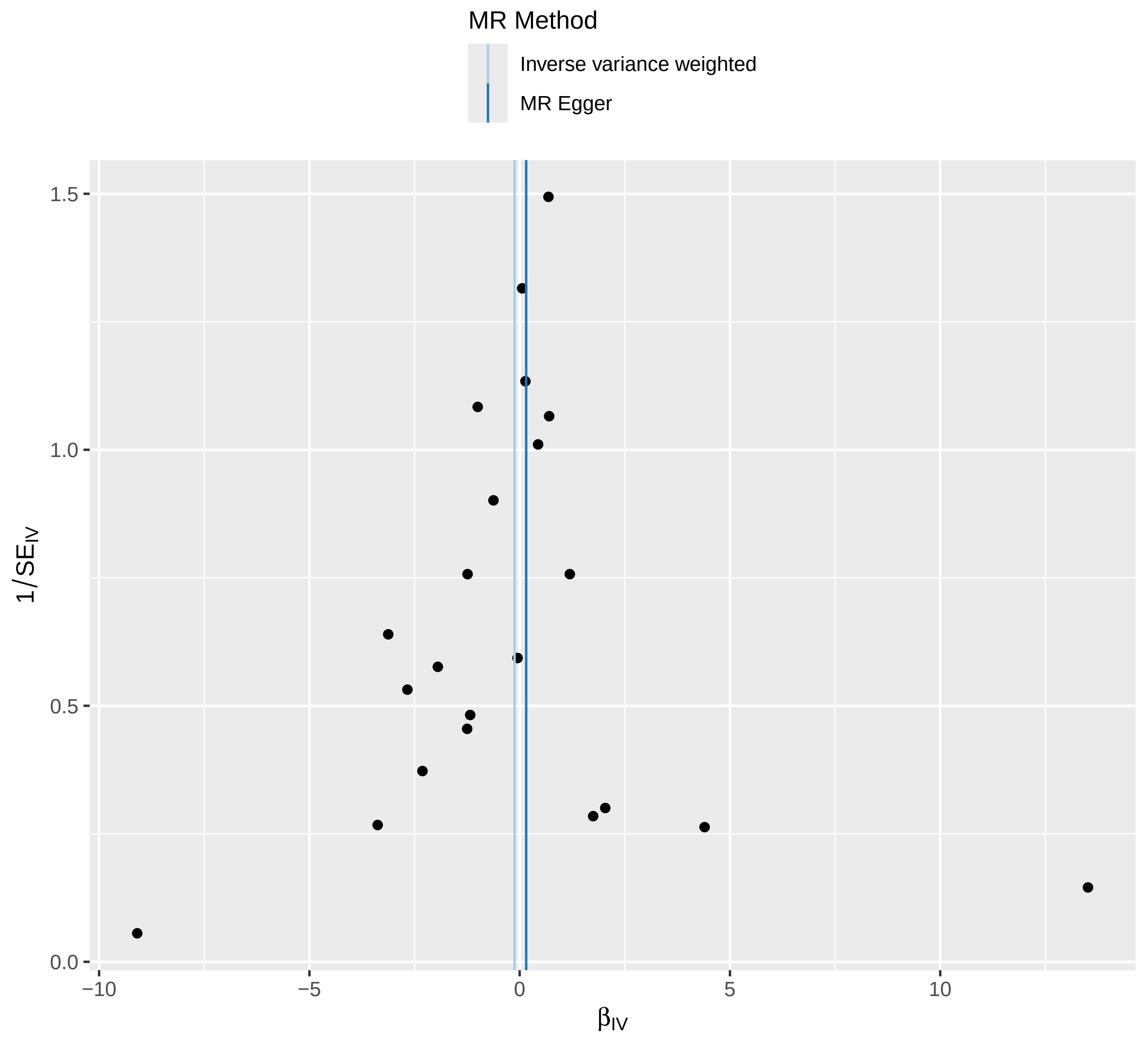
MR Method
Inverse variance weighted
MR Egger
1.5
1.0
0.5
0.0
$1/SE_{IV}$
−10
−5
0
5
10
β_{IV}

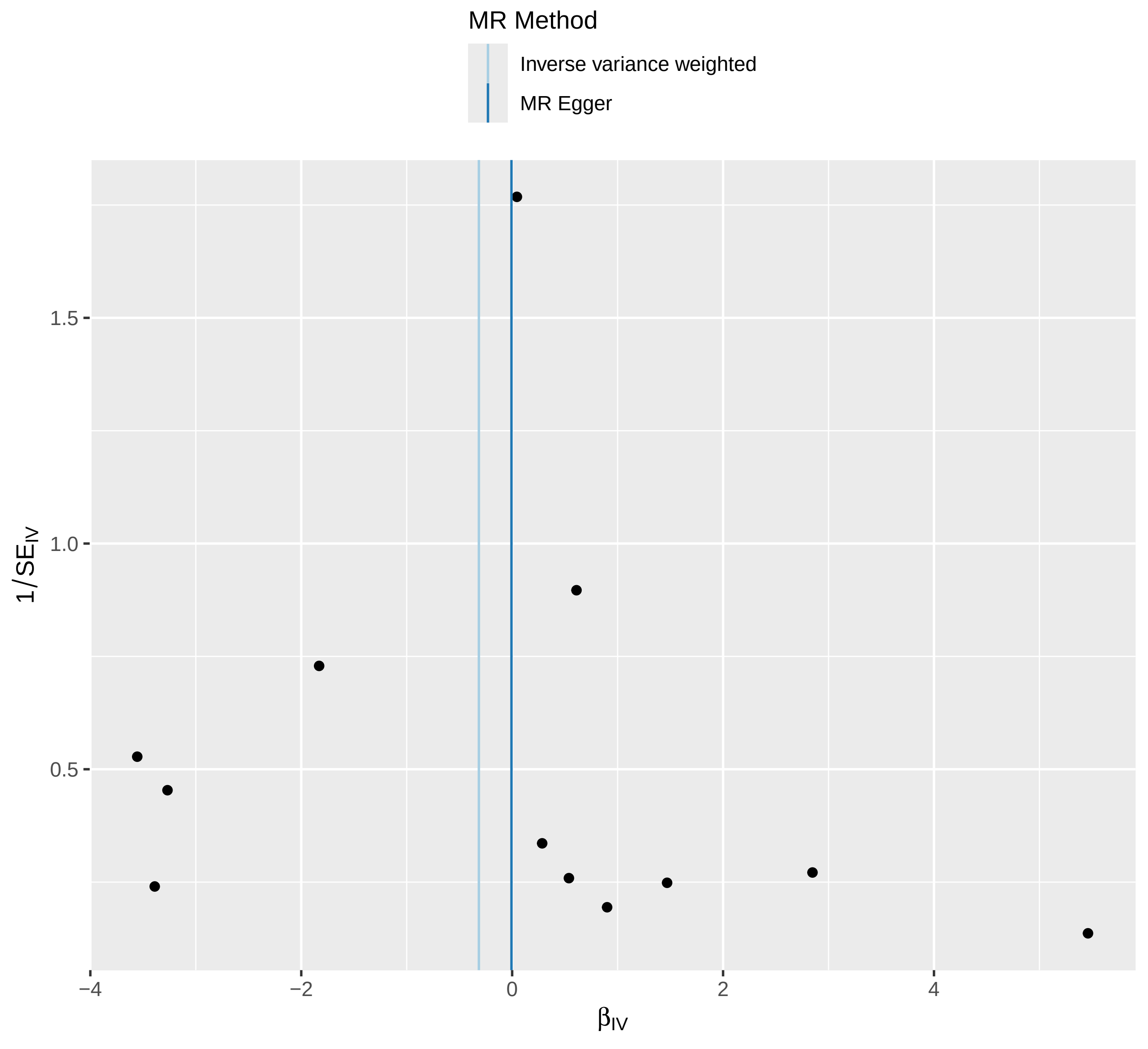
MR Method
Inverse variance weighted
MR Egger
1.5
1.0
0.5
1/SE_IV
−4
−2
0
2
4
β_IV

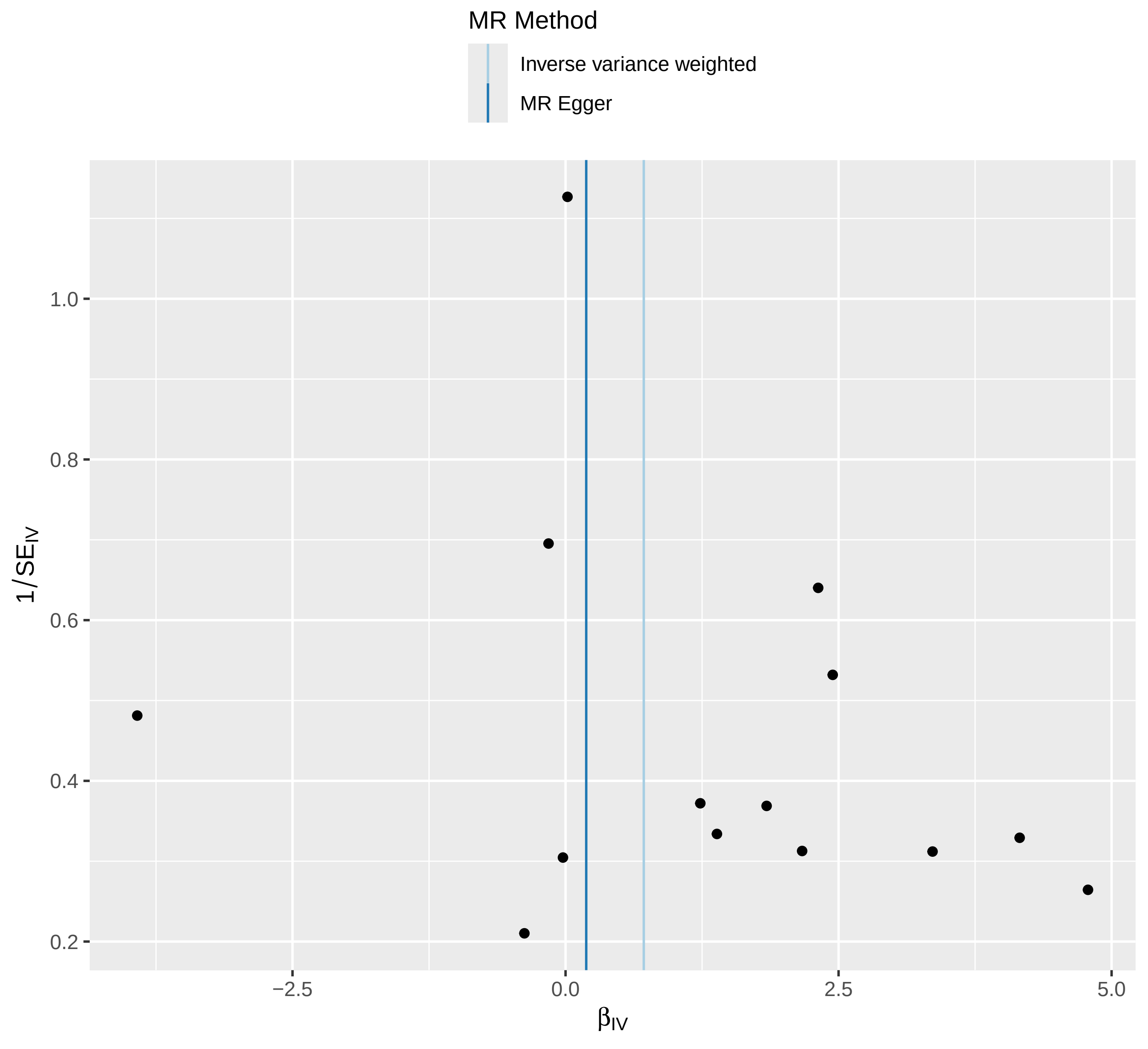
MR Method
Inverse variance weighted
MR Egger
1.0
0.8
0.6
0.4
0.2
1/SE_IV
−2.5
0.0
2.5
5.0
β_IV

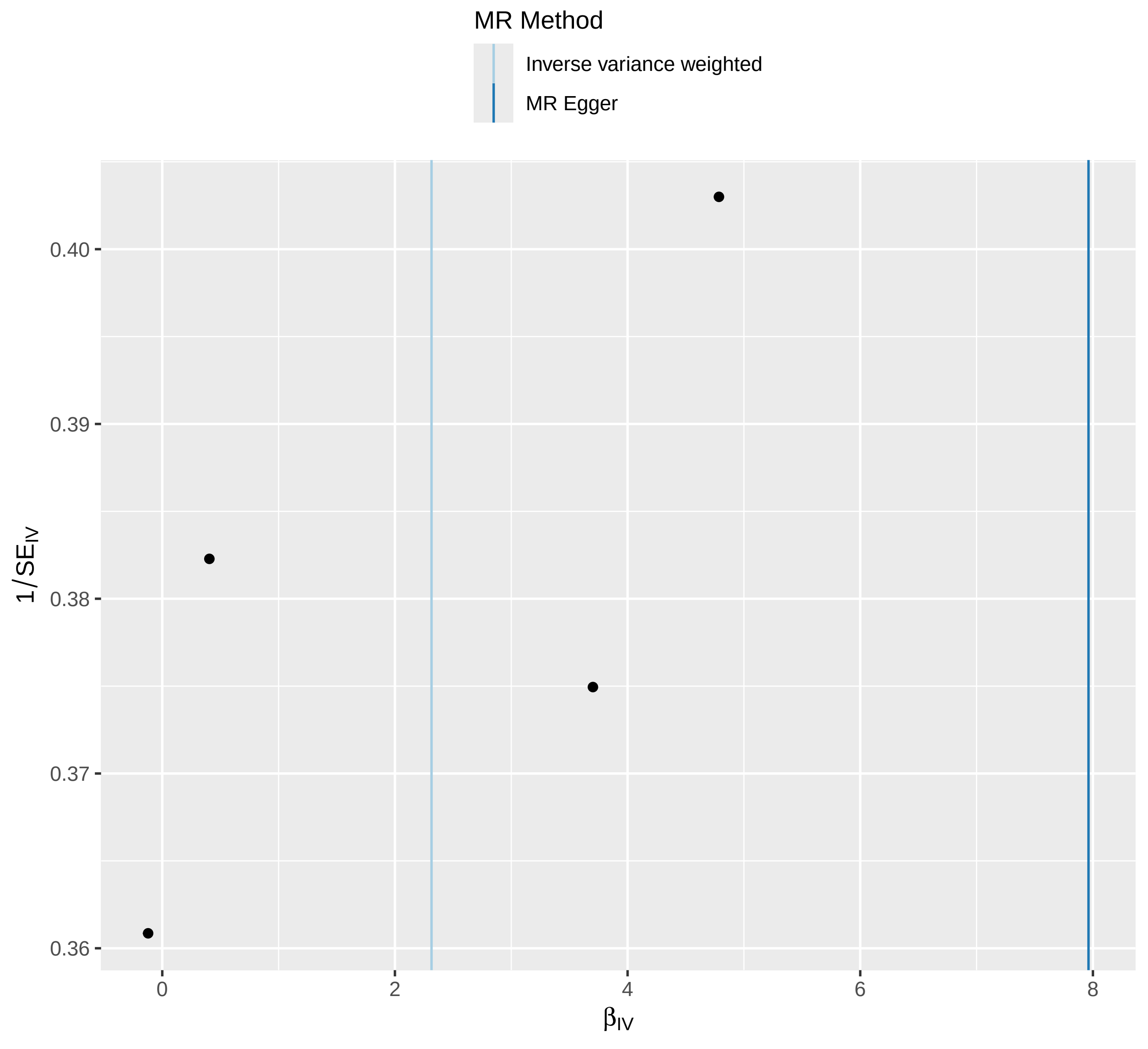
MR Method
Inverse variance weighted
MR Egger
0.40
0.39
0.38
0.37
0.36
1/SE_IV
0
2
4
6
8
β_IV

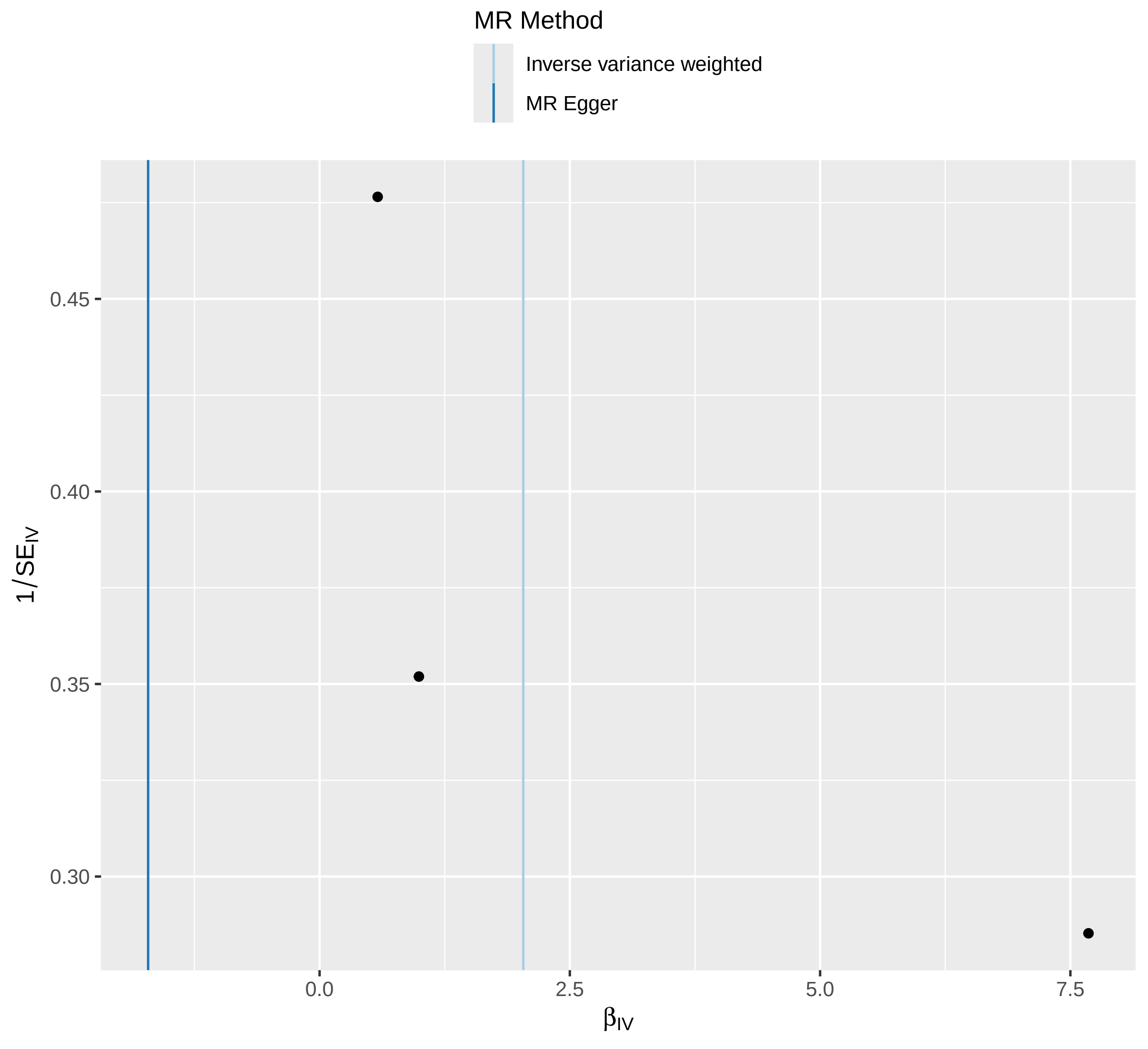
MR Method
Inverse variance weighted
MR Egger
0.45
0.40
0.35
0.30
1/SE_IV
0.0
2.5
5.0
7.5
β_IV

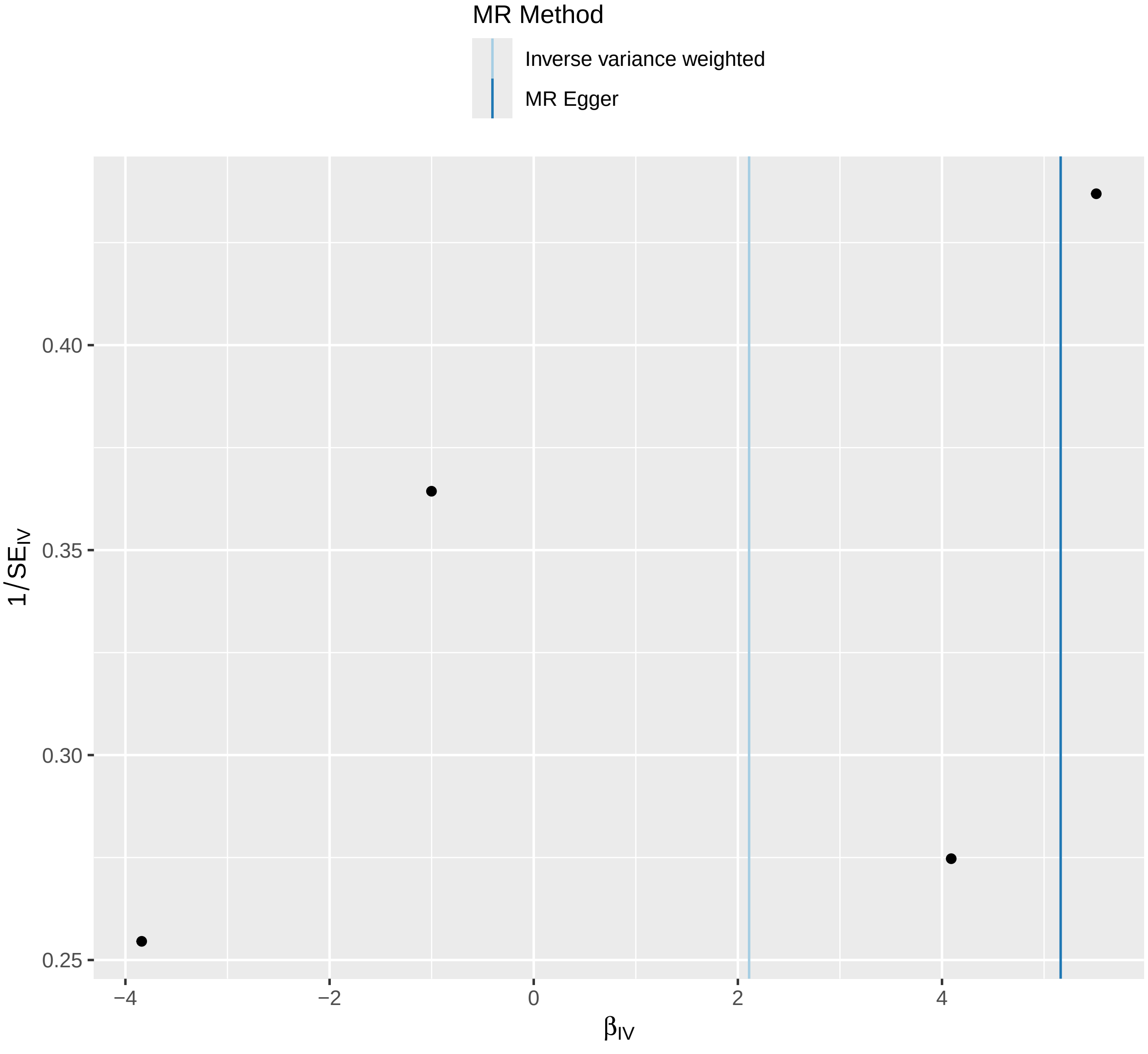
MR Method
Inverse variance weighted
MR Egger
1/SE_IV
0.40
0.35
0.30
0.25
−4
−2
0
2
4
β_IV

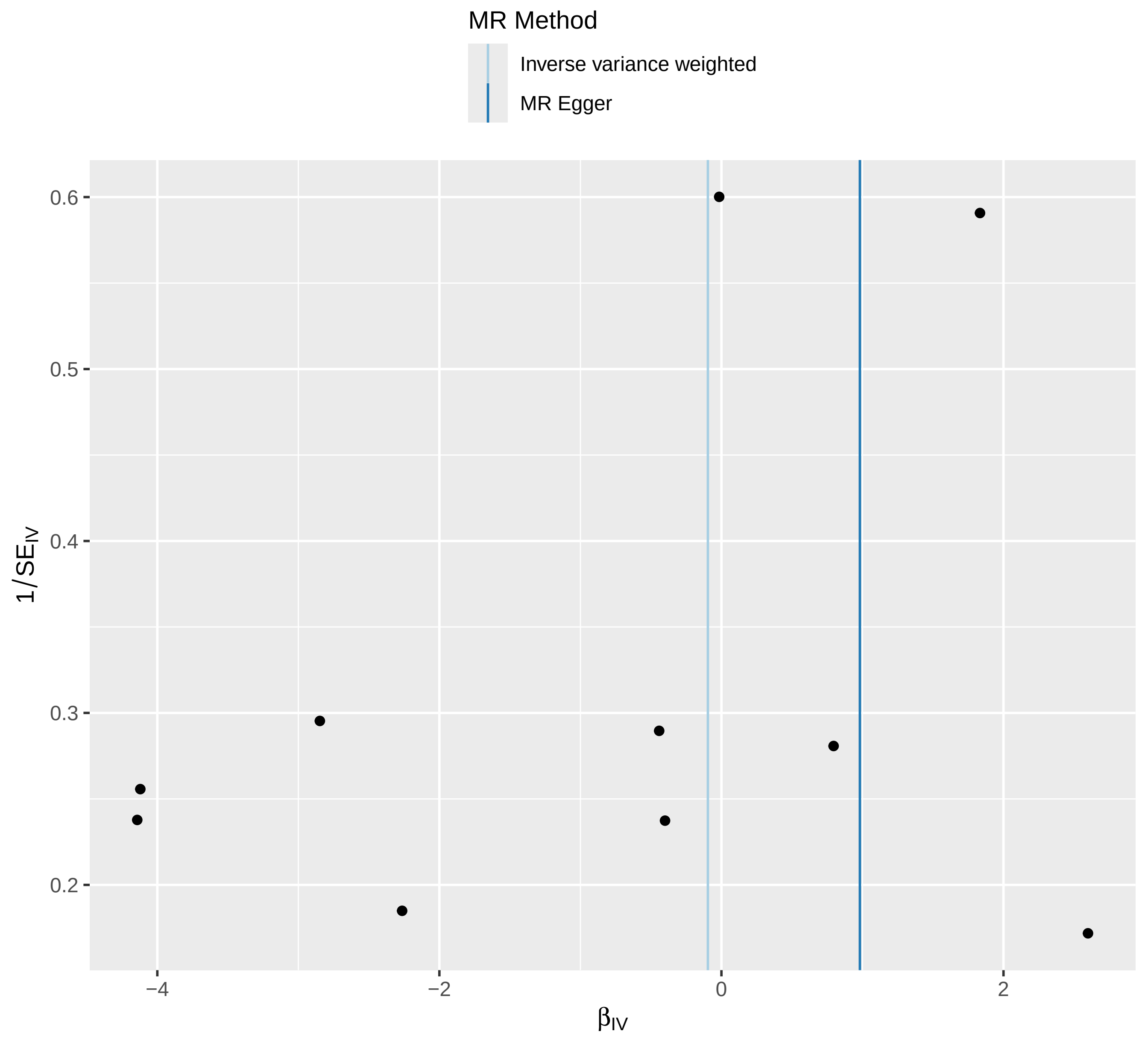

MR Method
Inverse variance weighted
MR Egger
0.6
0.5
0.4
0.3
0.2
1/SE_IV
−4
−2
0
2
β_IV

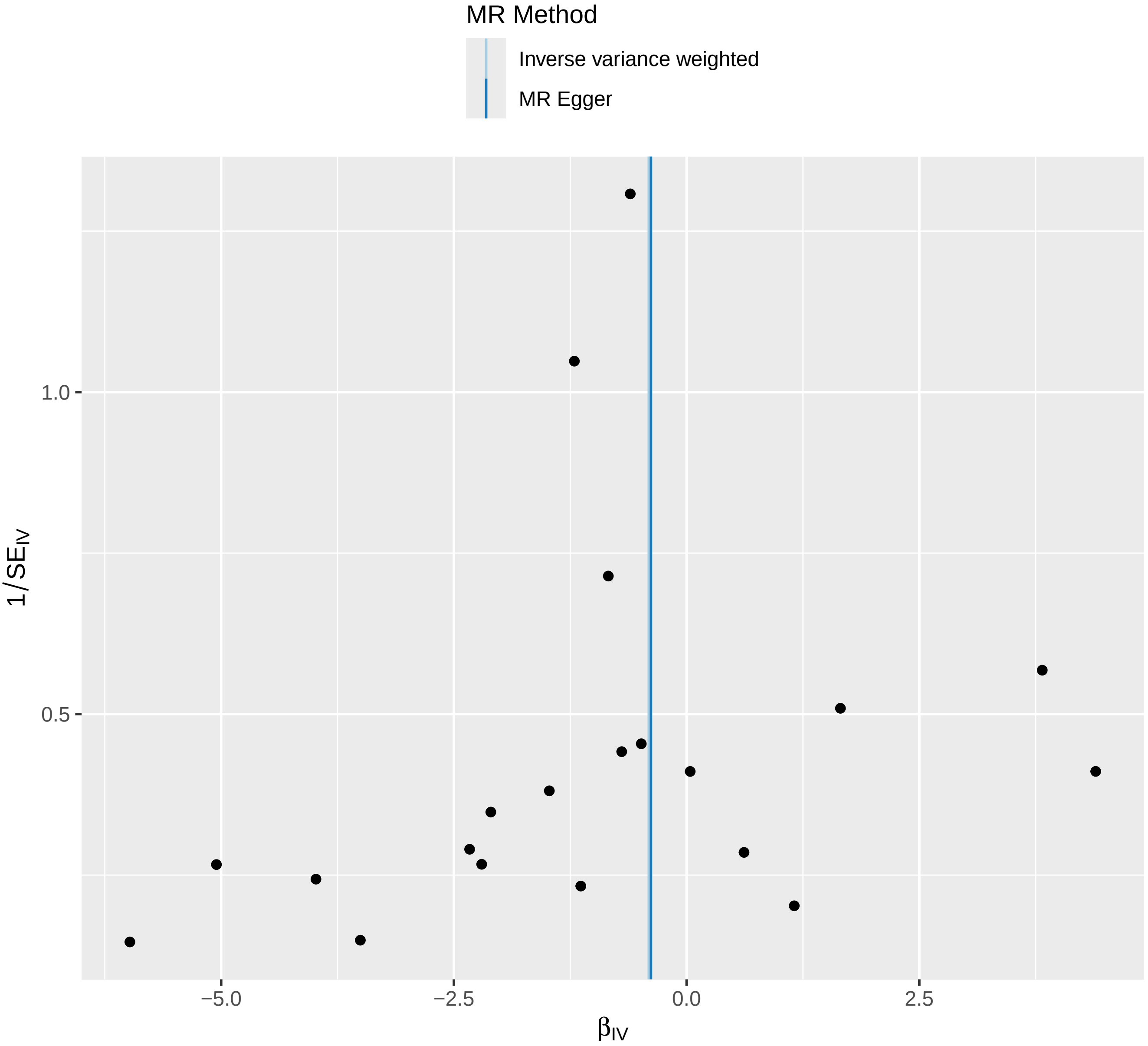

MR Method
Inverse variance weighted
MR Egger
1.0
0.5
1/SE_IV
−5.0
−2.5
0.0
2.5
β_IV

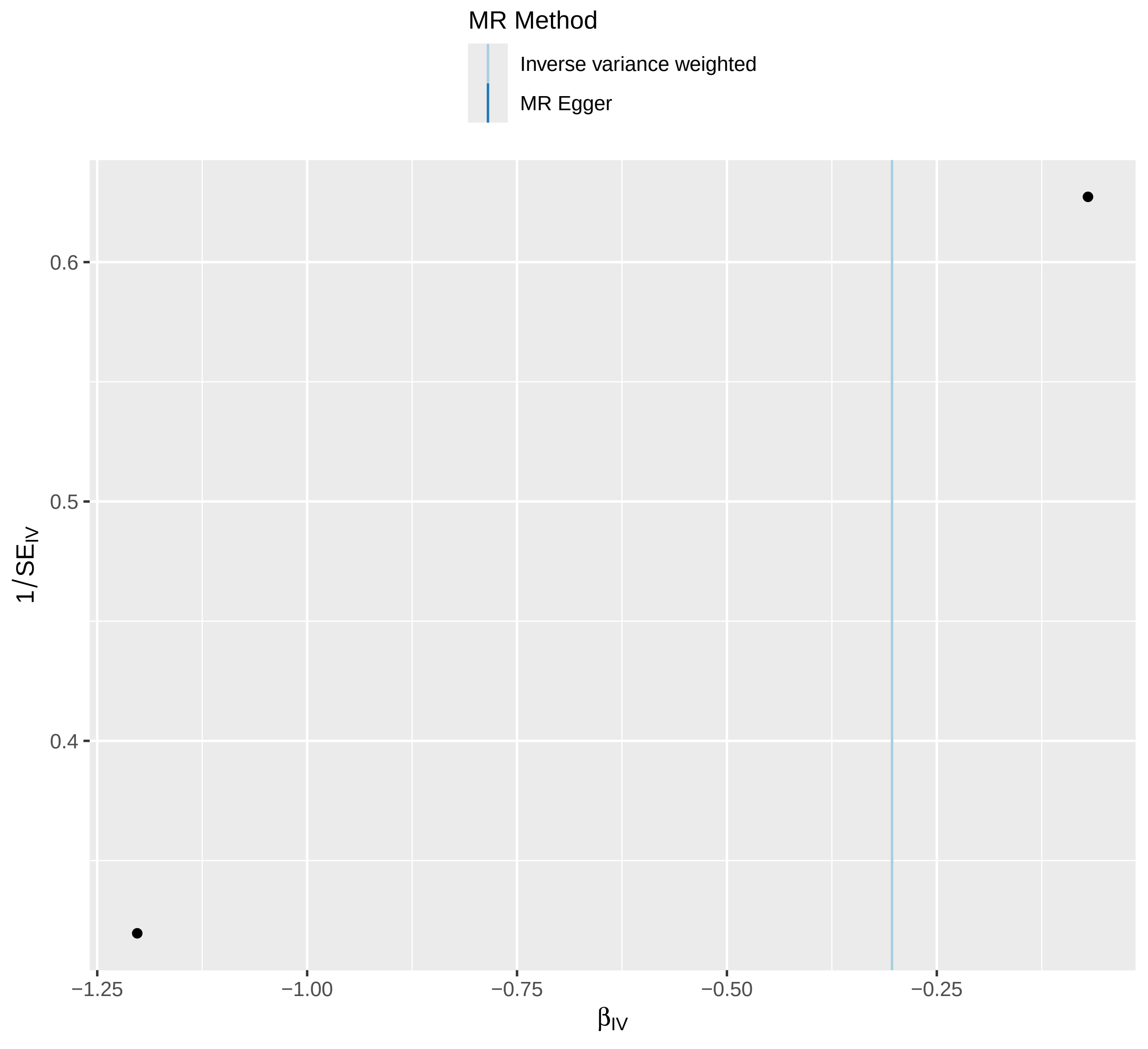
MR Method
Inverse variance weighted
MR Egger
0.6
0.5
0.4
1/SE_IV
−1.25
−1.00
−0.75
−0.50
−0.25
β_IV

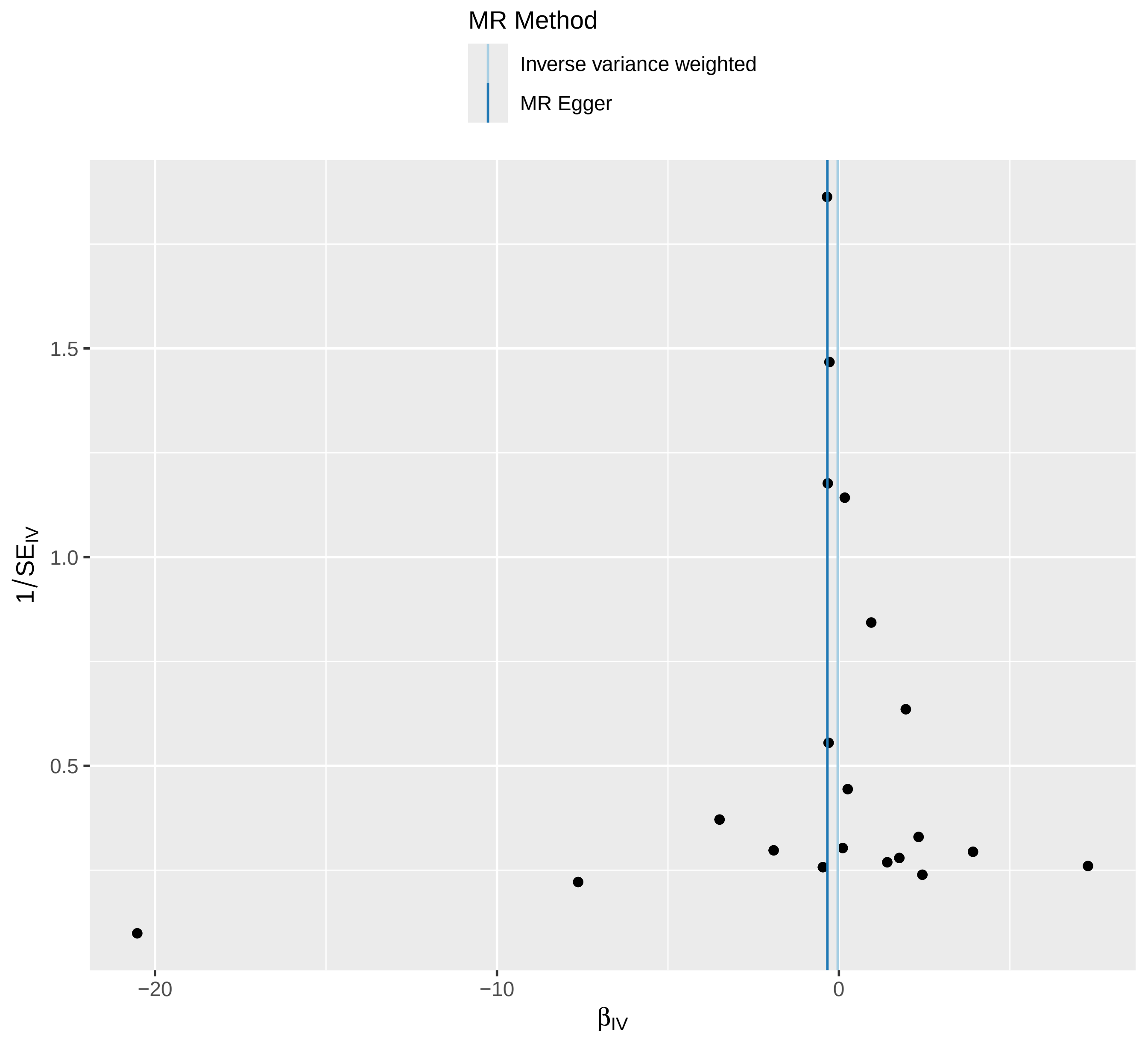

MR Method
Inverse variance weighted
MR Egger
1.5
1.0
0.5
1/SE$_{IV}$
−20
−10
0
β$_{IV}$

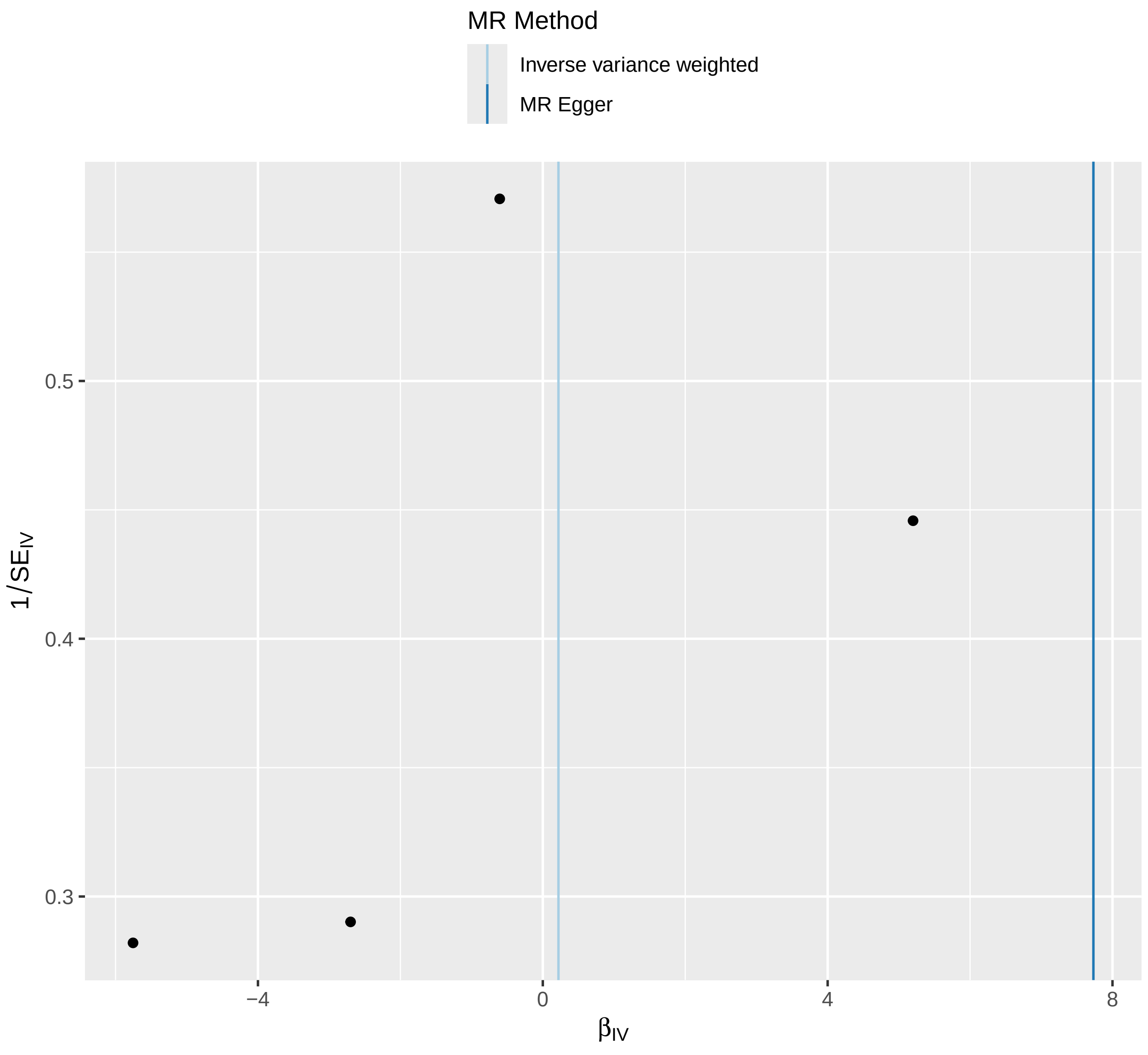
MR Method
Inverse variance weighted
MR Egger
0.5
0.4
0.3
$1/SE_{IV}$
−4
0
4
8
β_{IV}

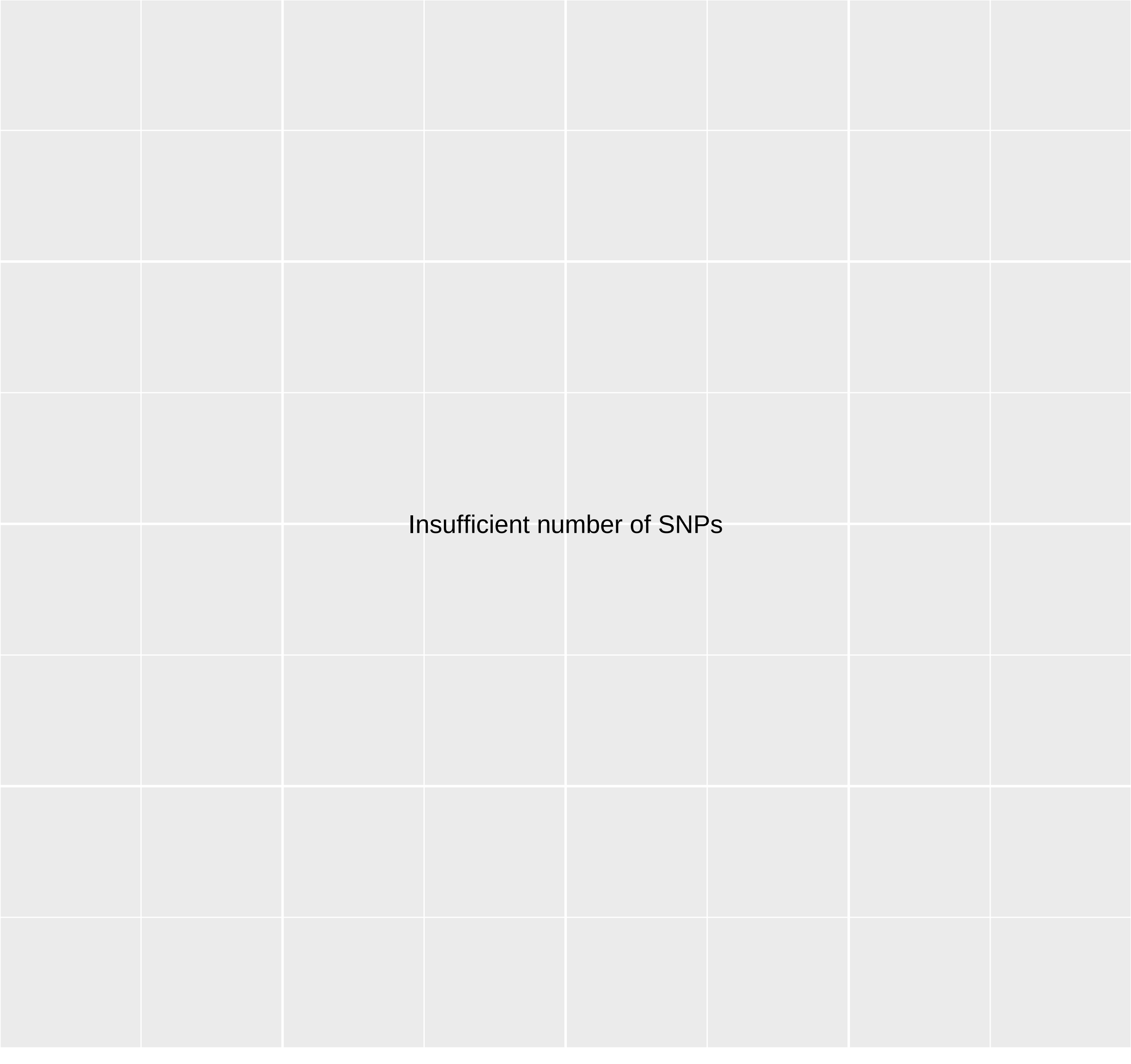
Insufficient number of SNPs

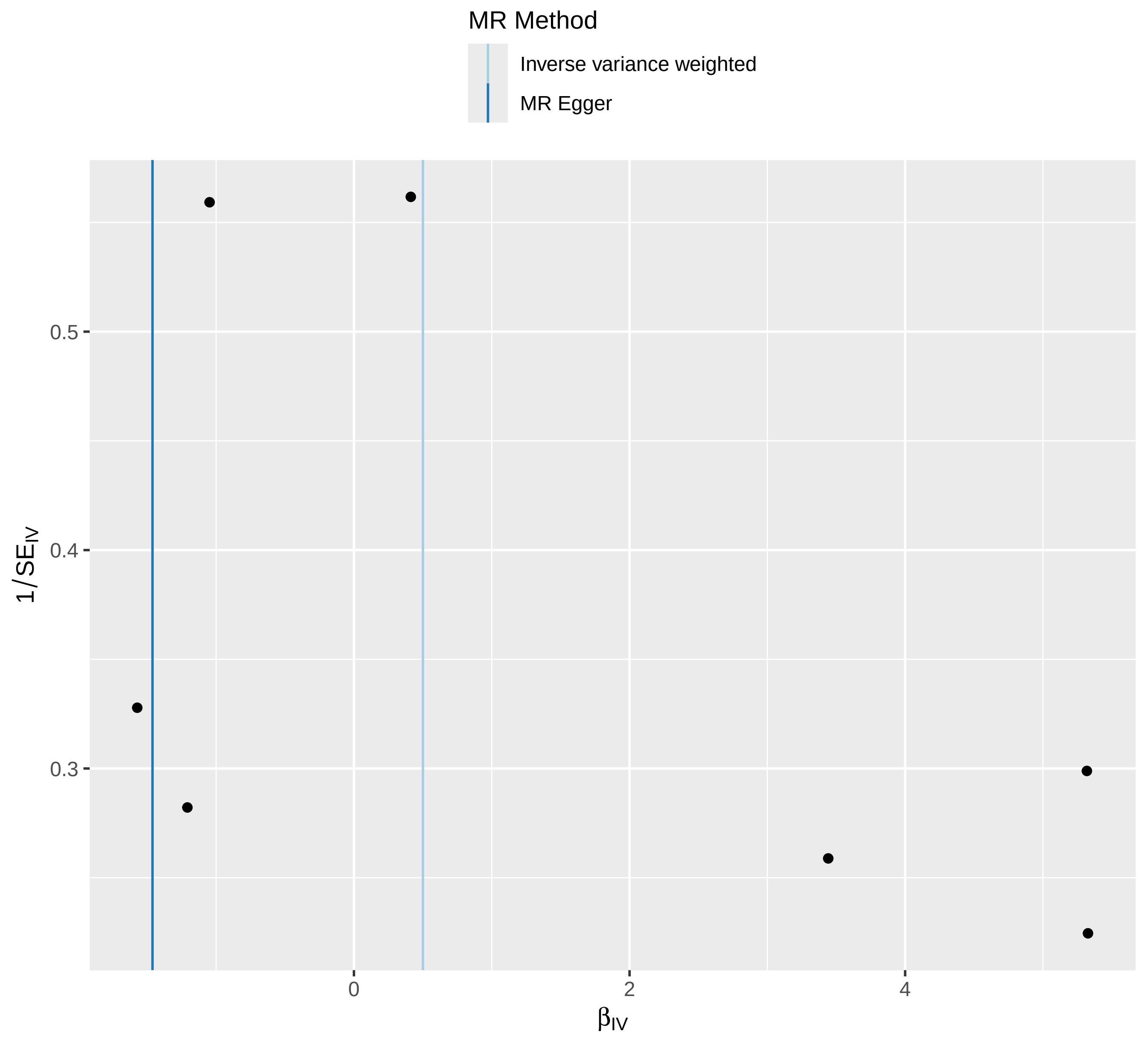
MR Method
Inverse variance weighted
MR Egger
0.5
0.4
0.3
1/SE_IV
0
2
4
β_IV

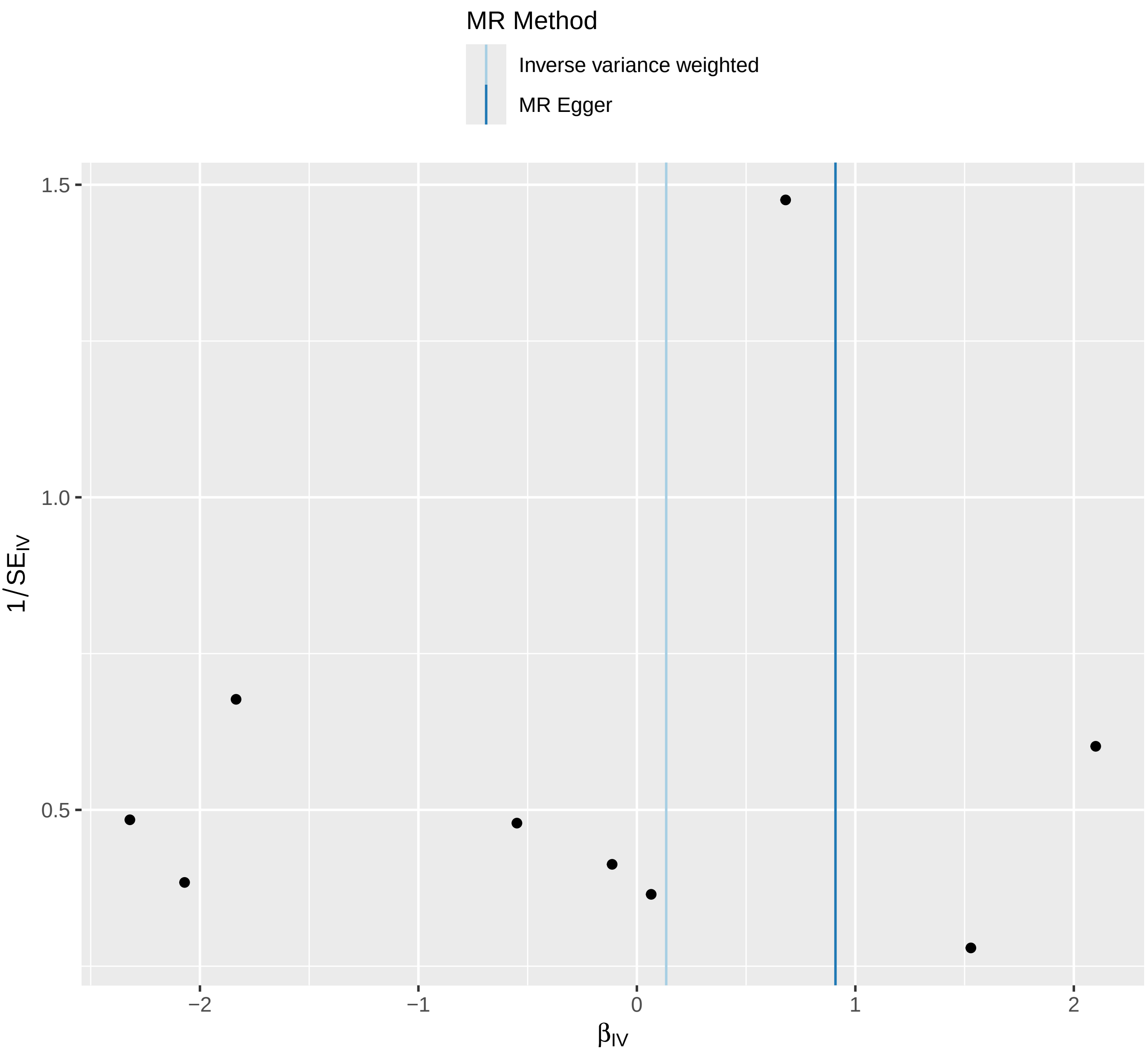
MR Method
Inverse variance weighted
MR Egger
1.5
1.0
0.5
1/SE_IV
−2
−1
0
1
2
β_IV

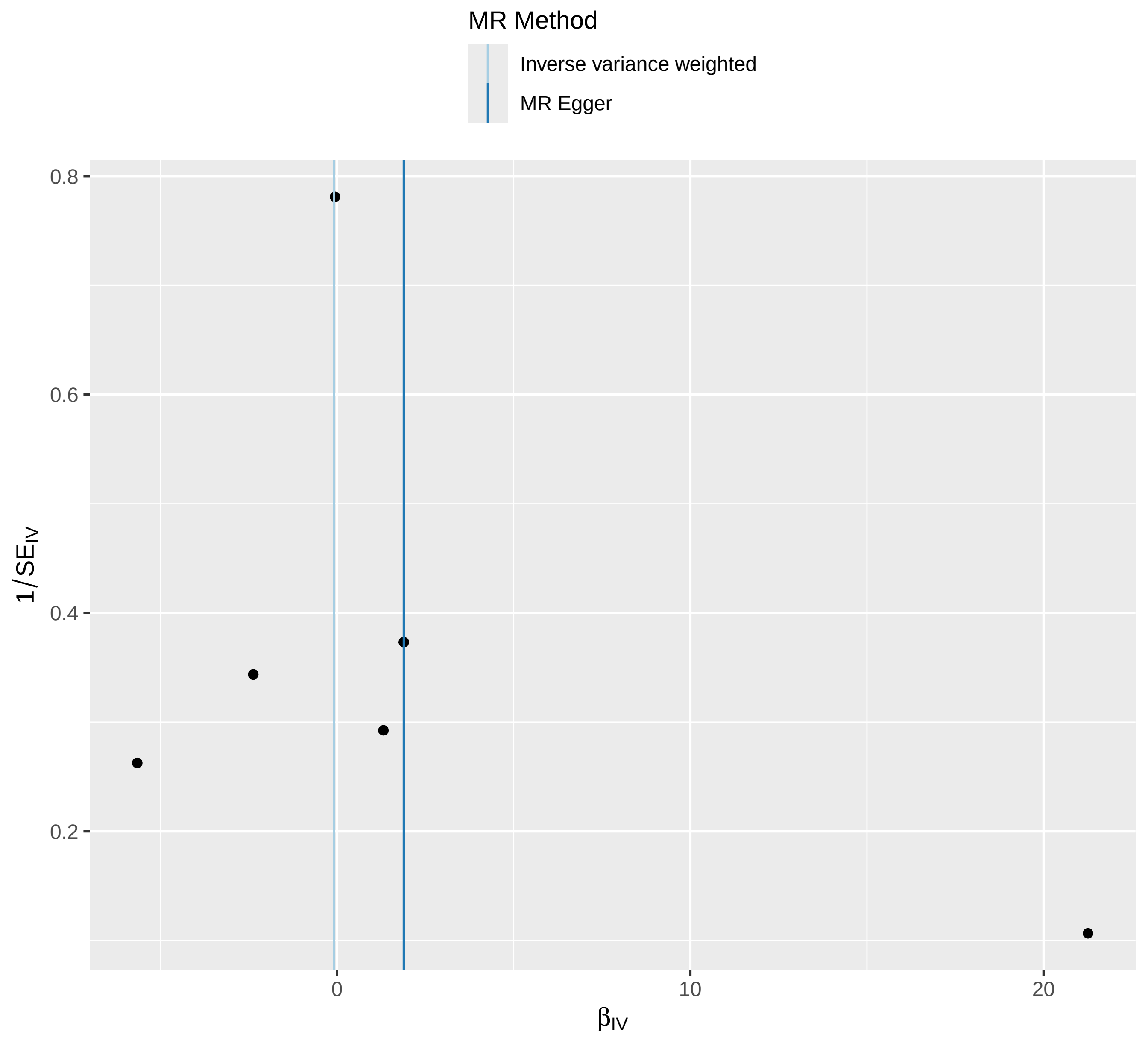
MR Method
Inverse variance weighted
MR Egger
0.8
0.6
0.4
0.2
1/SE_IV
0
10
20
β_IV

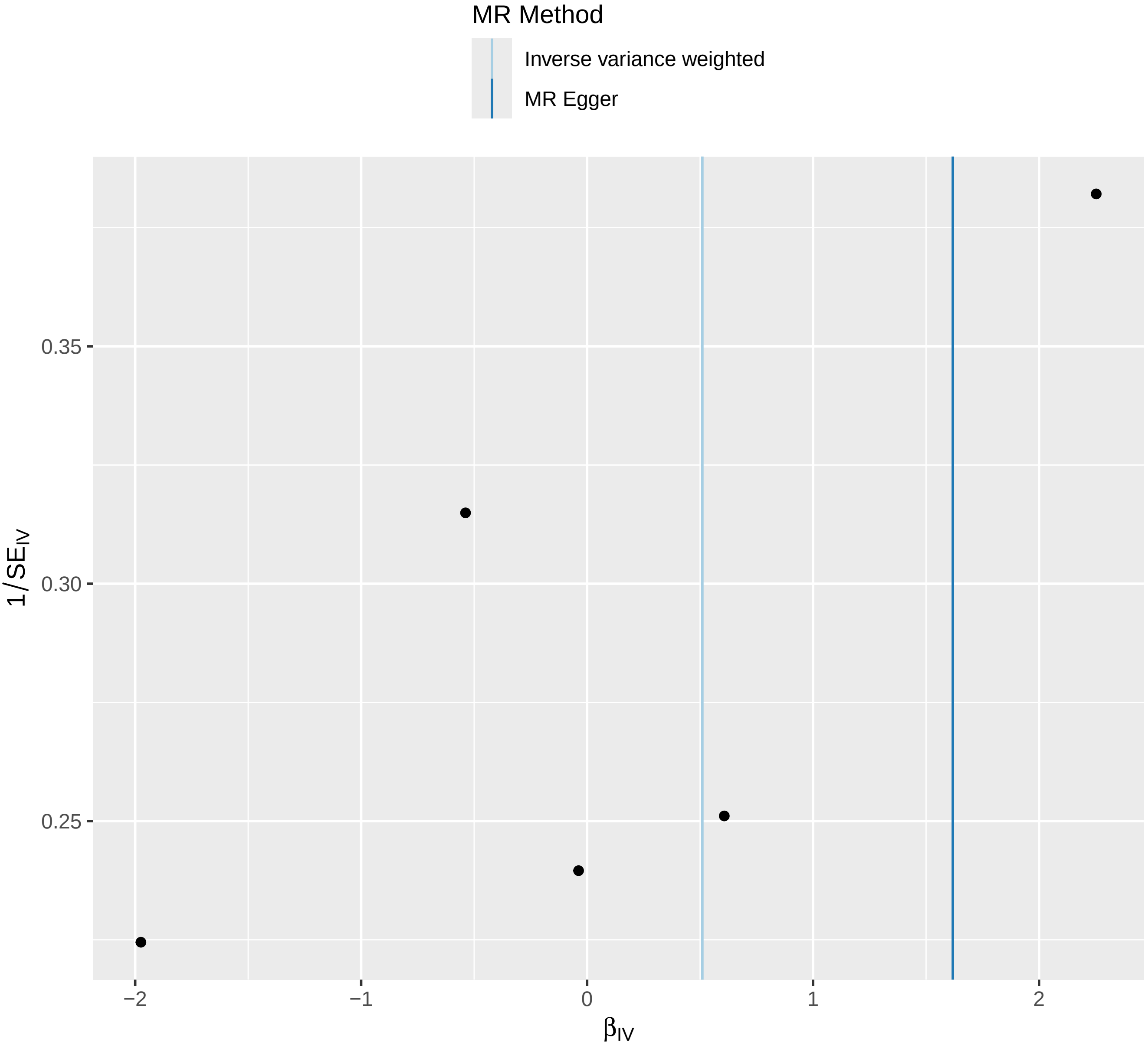

MR Method
Inverse variance weighted
MR Egger
0.35
0.30
0.25
1/SE_IV
−2
−1
0
1
2
β_IV

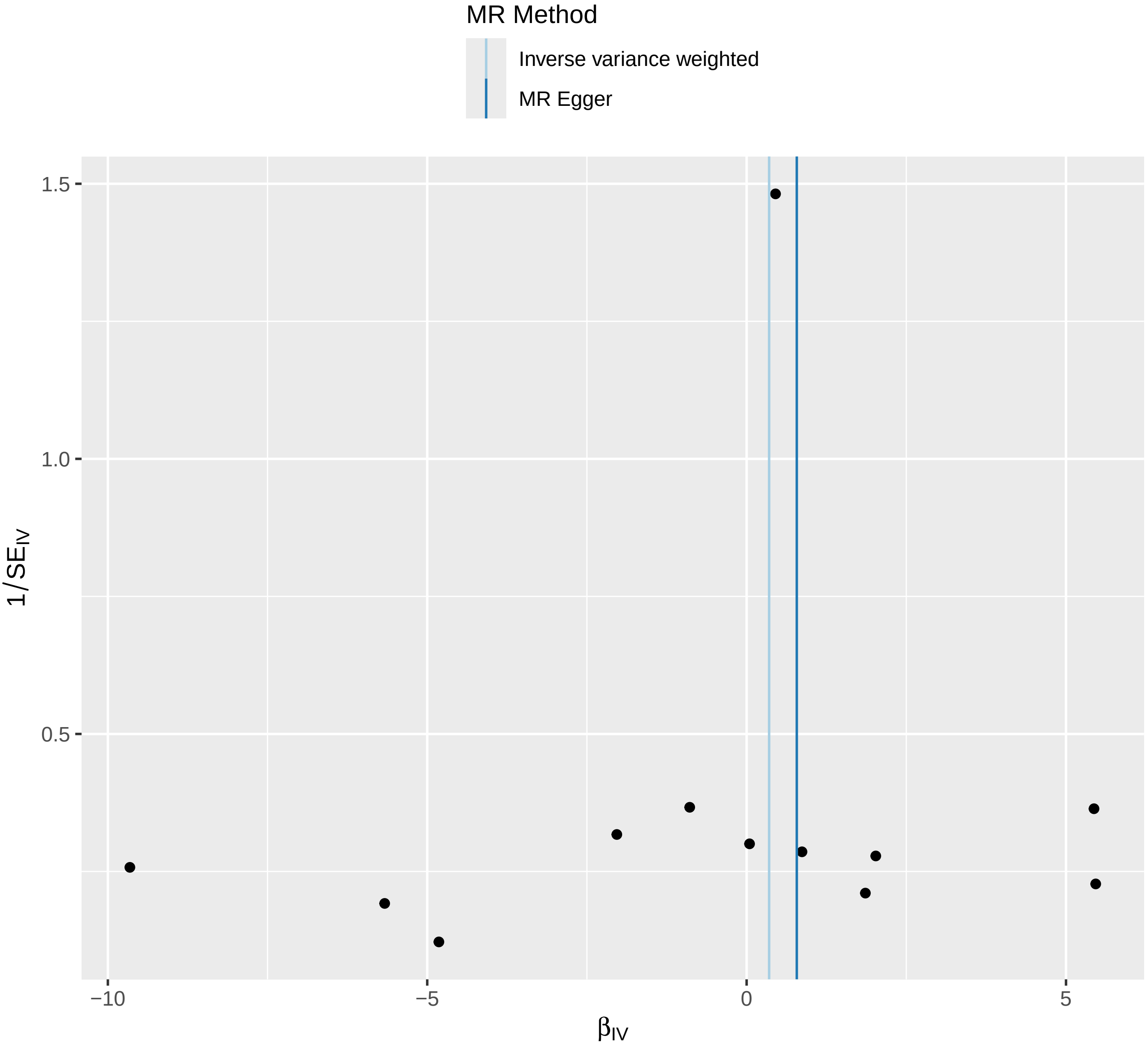
MR Method
Inverse variance weighted
MR Egger
1.5
1.0
0.5
1/SE_IV
−10
−5
0
5
β_IV

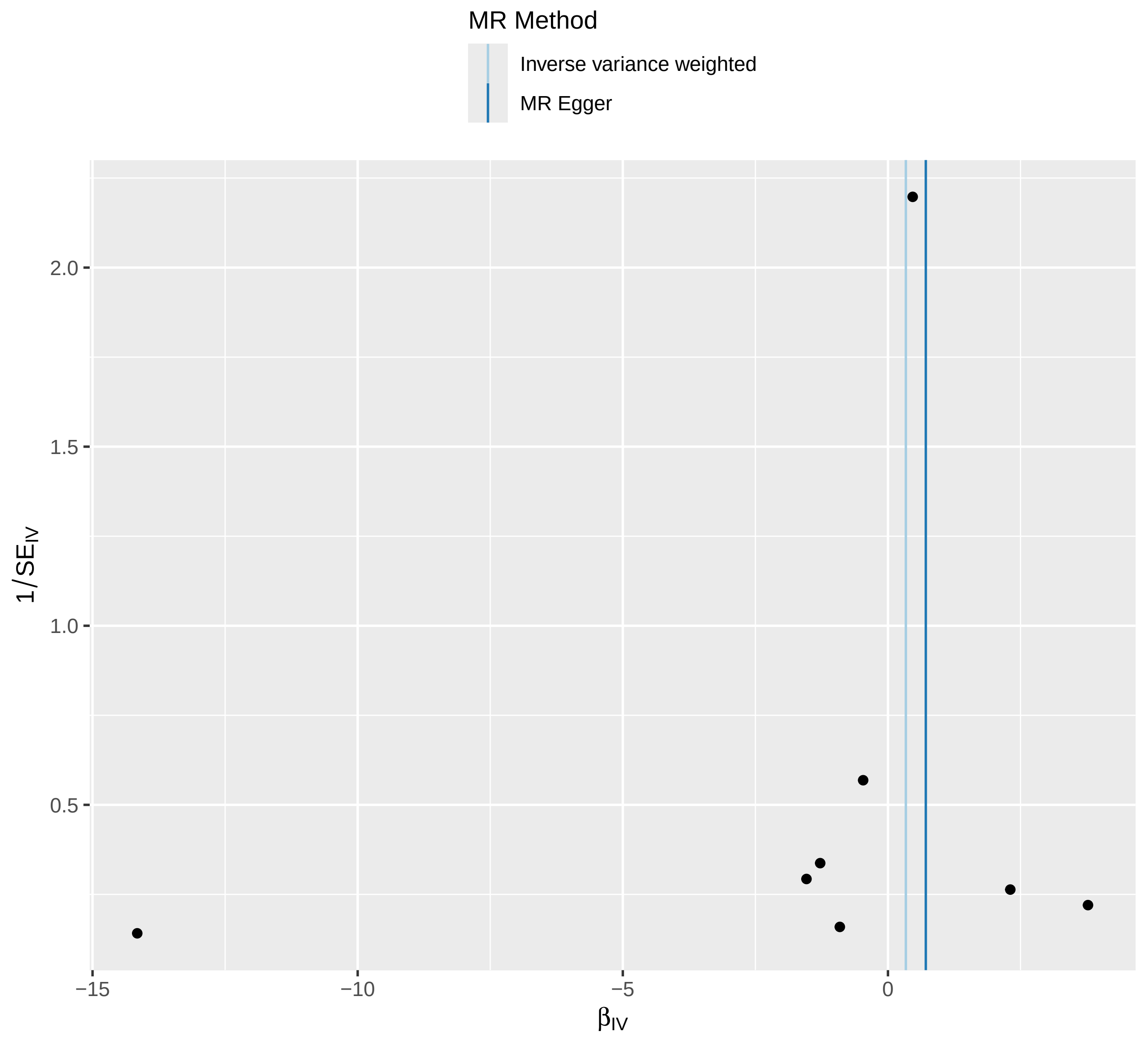

MR Method
Inverse variance weighted
MR Egger
2.0
1.5
1.0
0.5
1/SE$_{IV}$
−15
−10
−5
0
β$_{IV}$

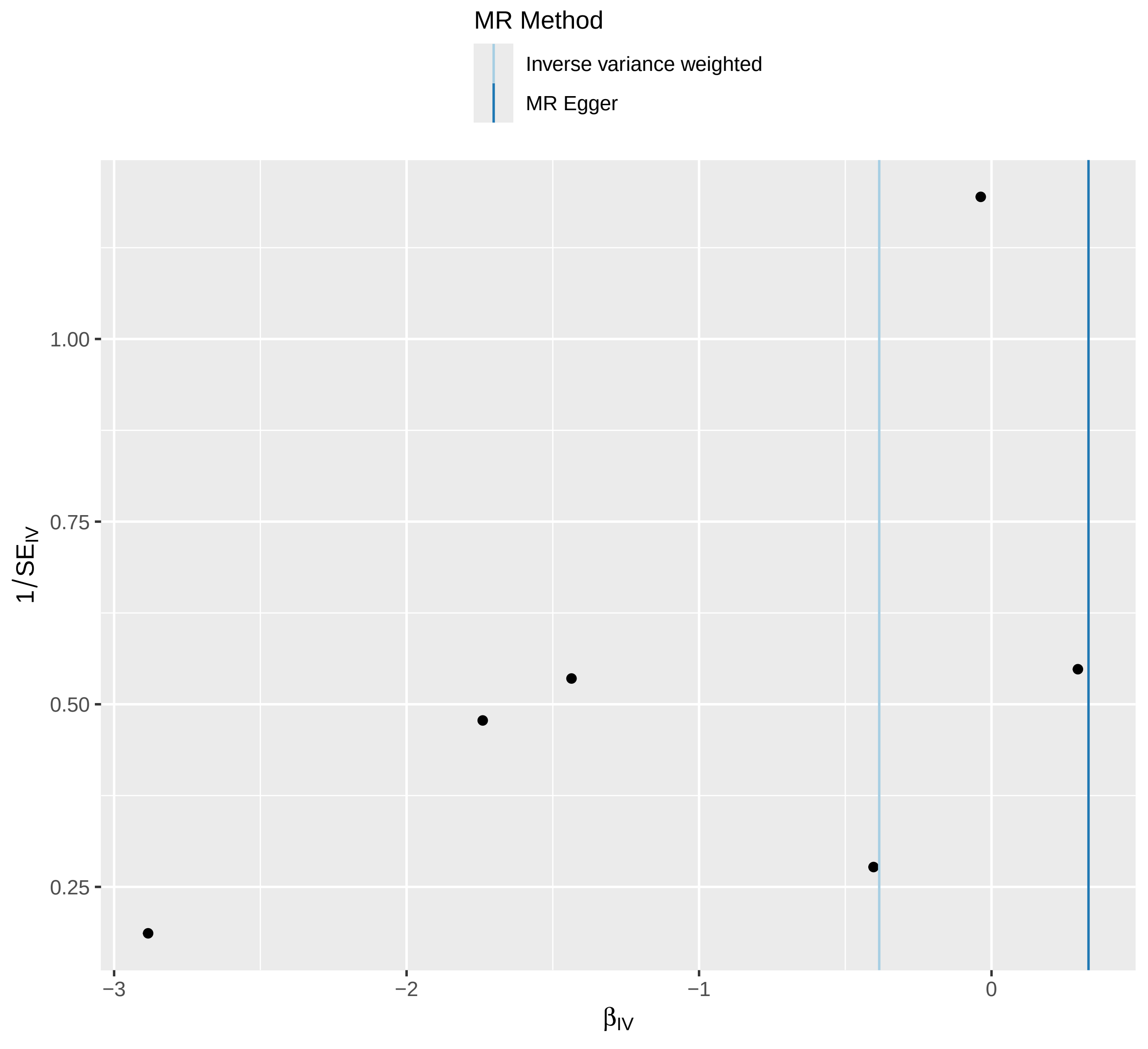

MR Method
Inverse variance weighted
MR Egger
1.00
0.75
0.50
0.25
1/SE$_{IV}$
−3
−2
−1
0
β$_{IV}$

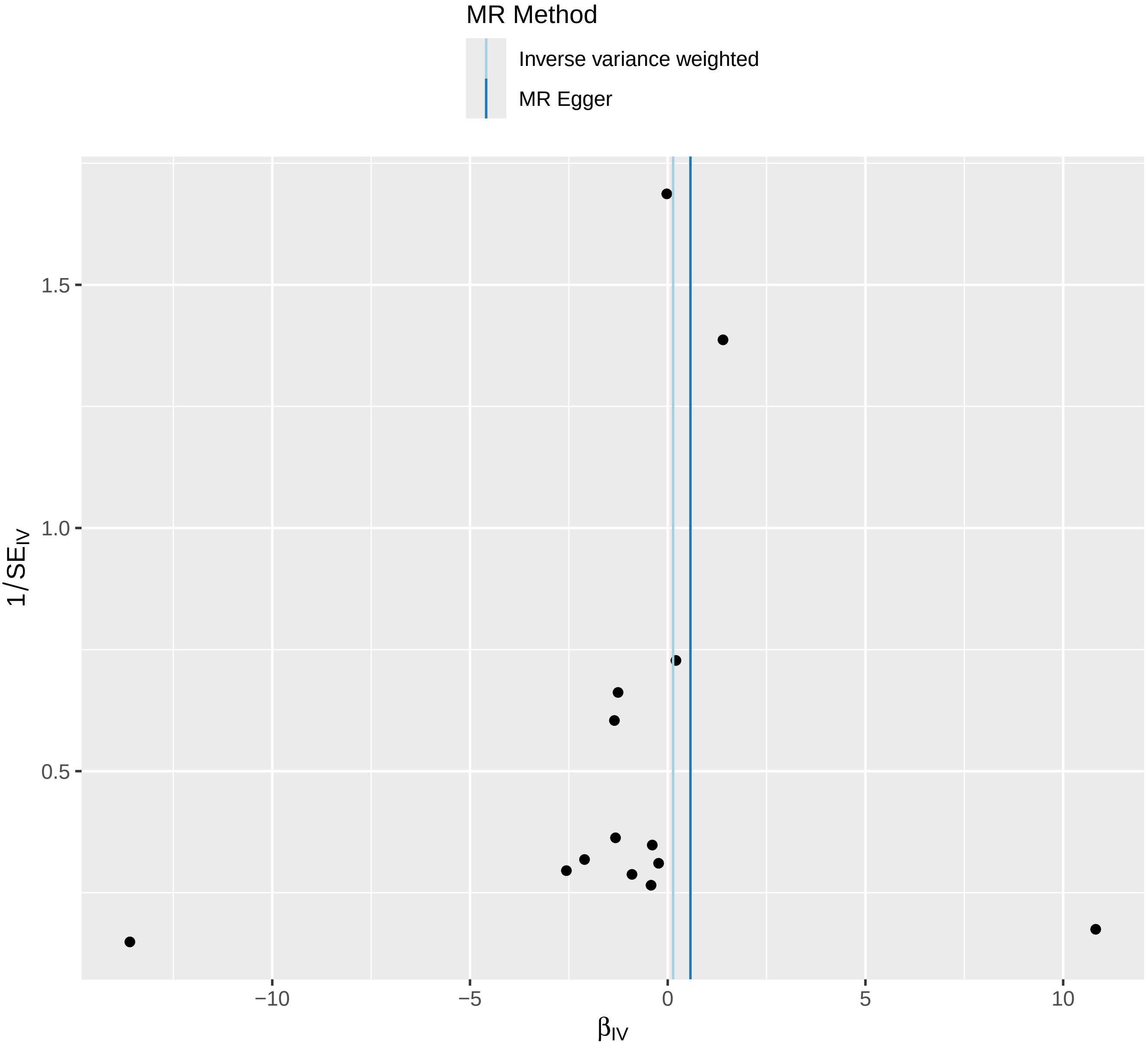
MR Method
Inverse variance weighted
MR Egger
1.5
1.0
0.5
$1/SE_{IV}$
−10
−5
0
5
10
β_{IV}

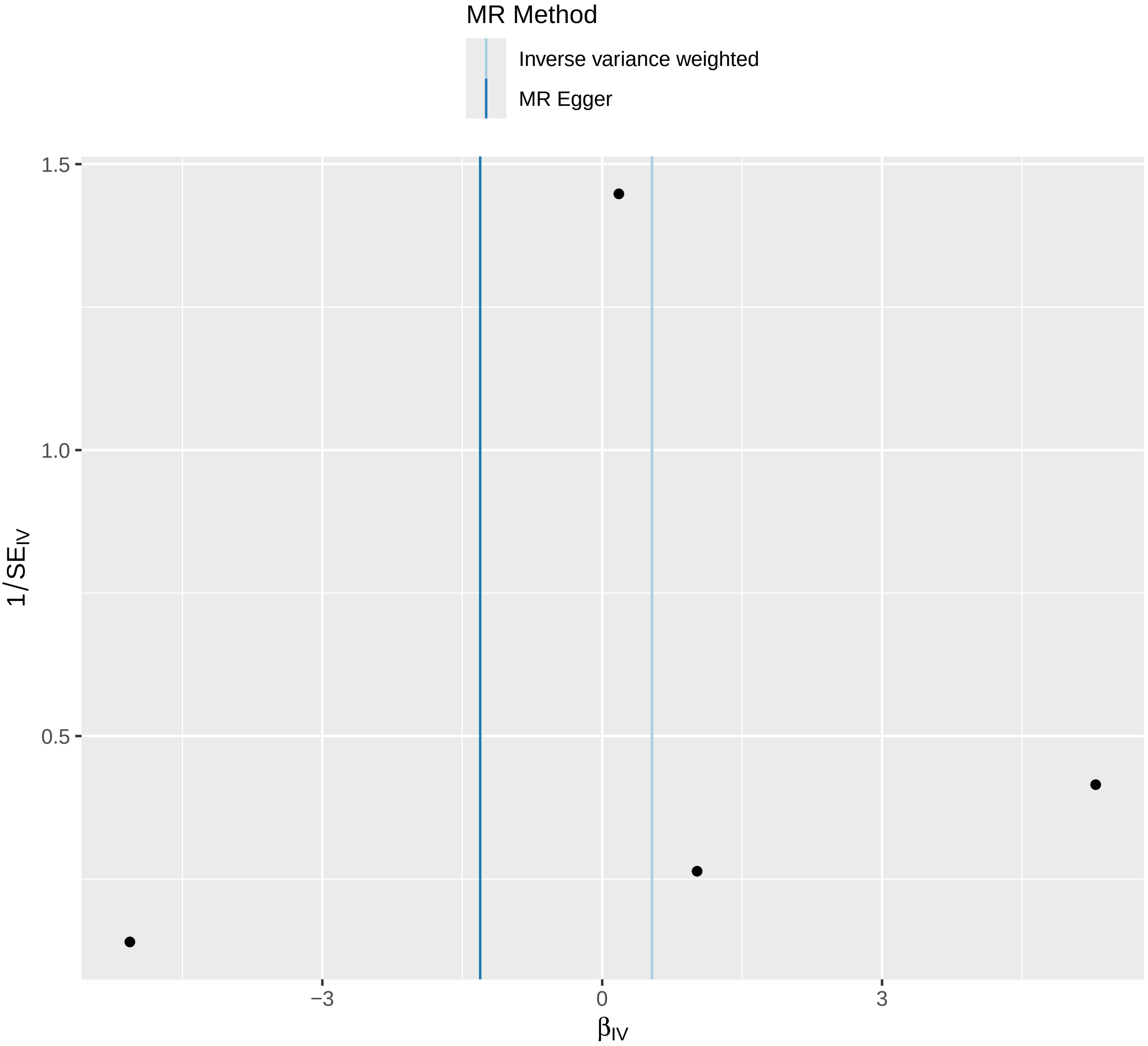
MR Method
Inverse variance weighted
MR Egger
1.5
1.0
0.5
1/SE_IV
−3
0
3
β_IV

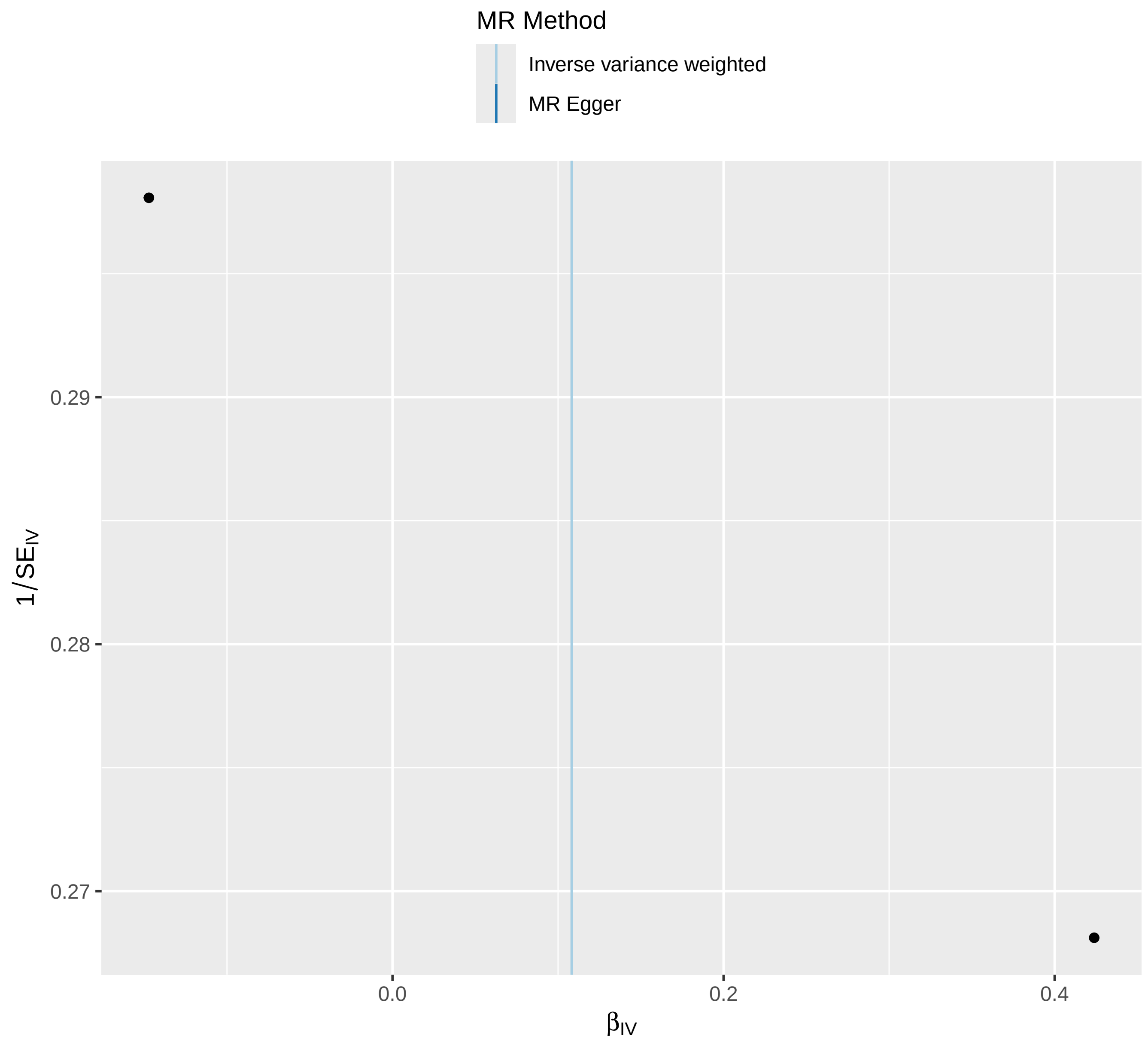

MR Method
Inverse variance weighted
MR Egger
0.29
0.28
0.27
1/SE_IV
0.0
0.2
0.4
β_IV

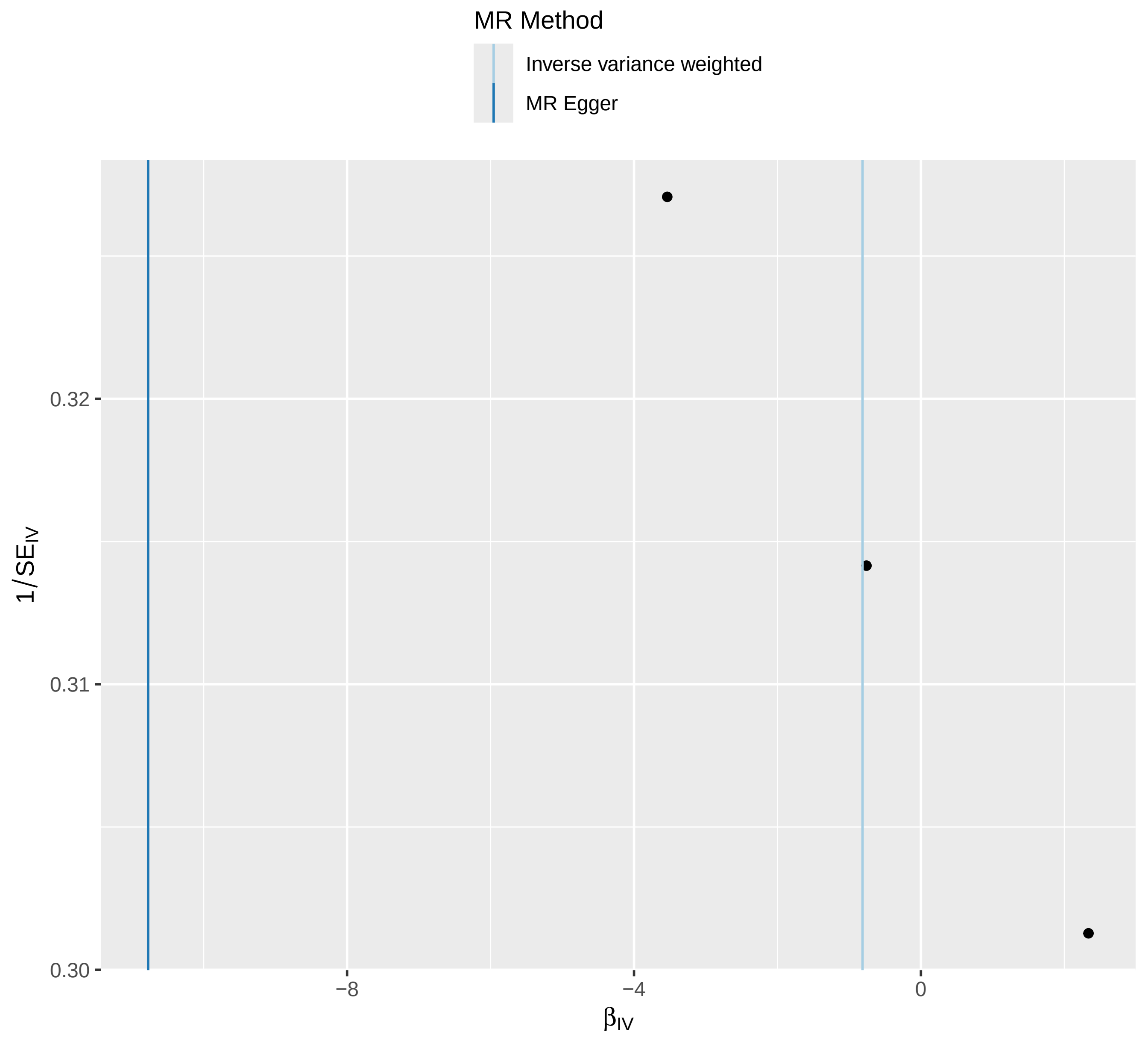

MR Method
Inverse variance weighted
MR Egger
0.32
0.31
0.30
1/SE_IV
−8
−4
0
β_IV

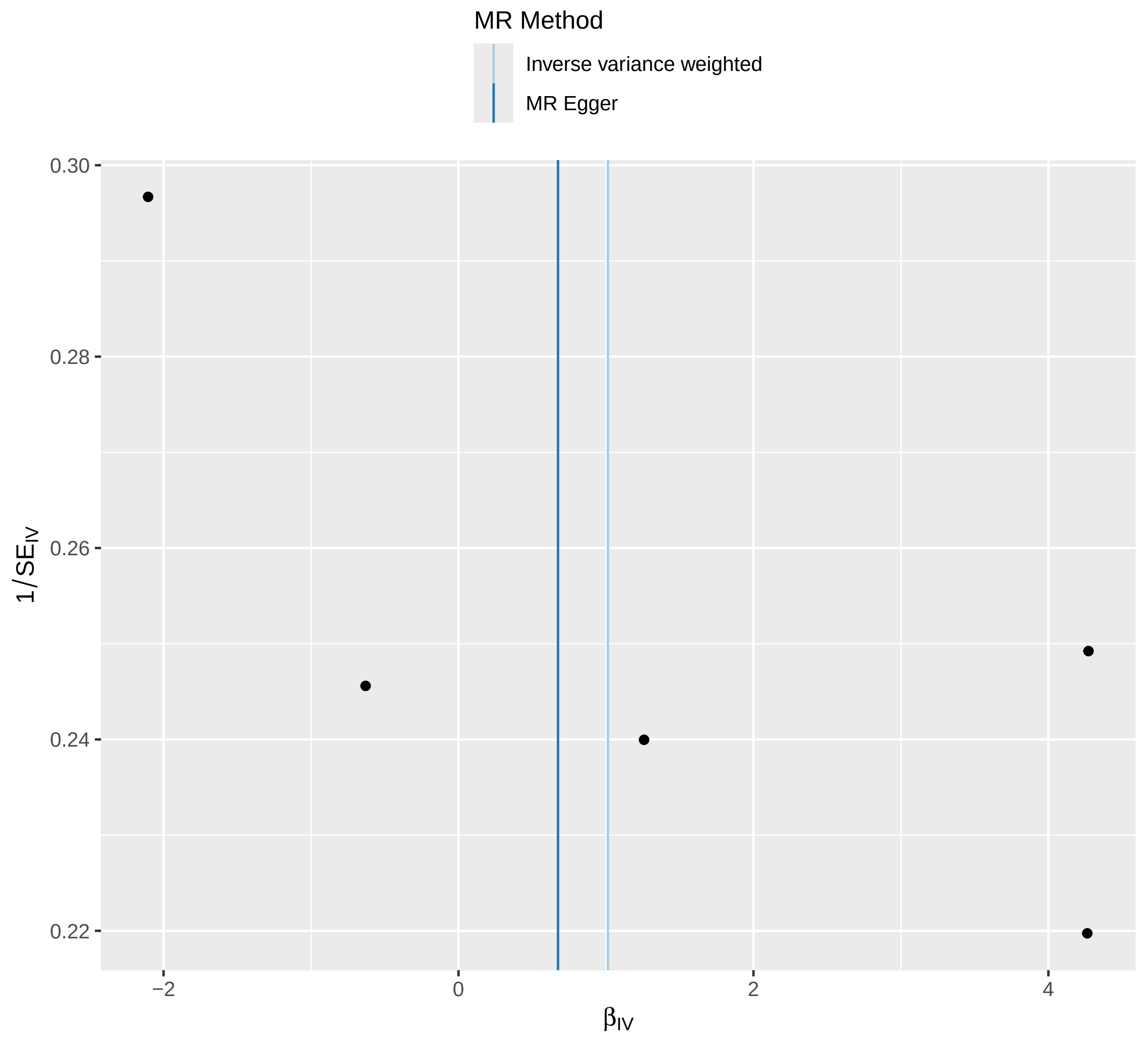

MR Method
Inverse variance weighted
MR Egger
0.30
0.28
0.26
0.24
0.22
1/SE_IV
−2
0
2
4
β_IV

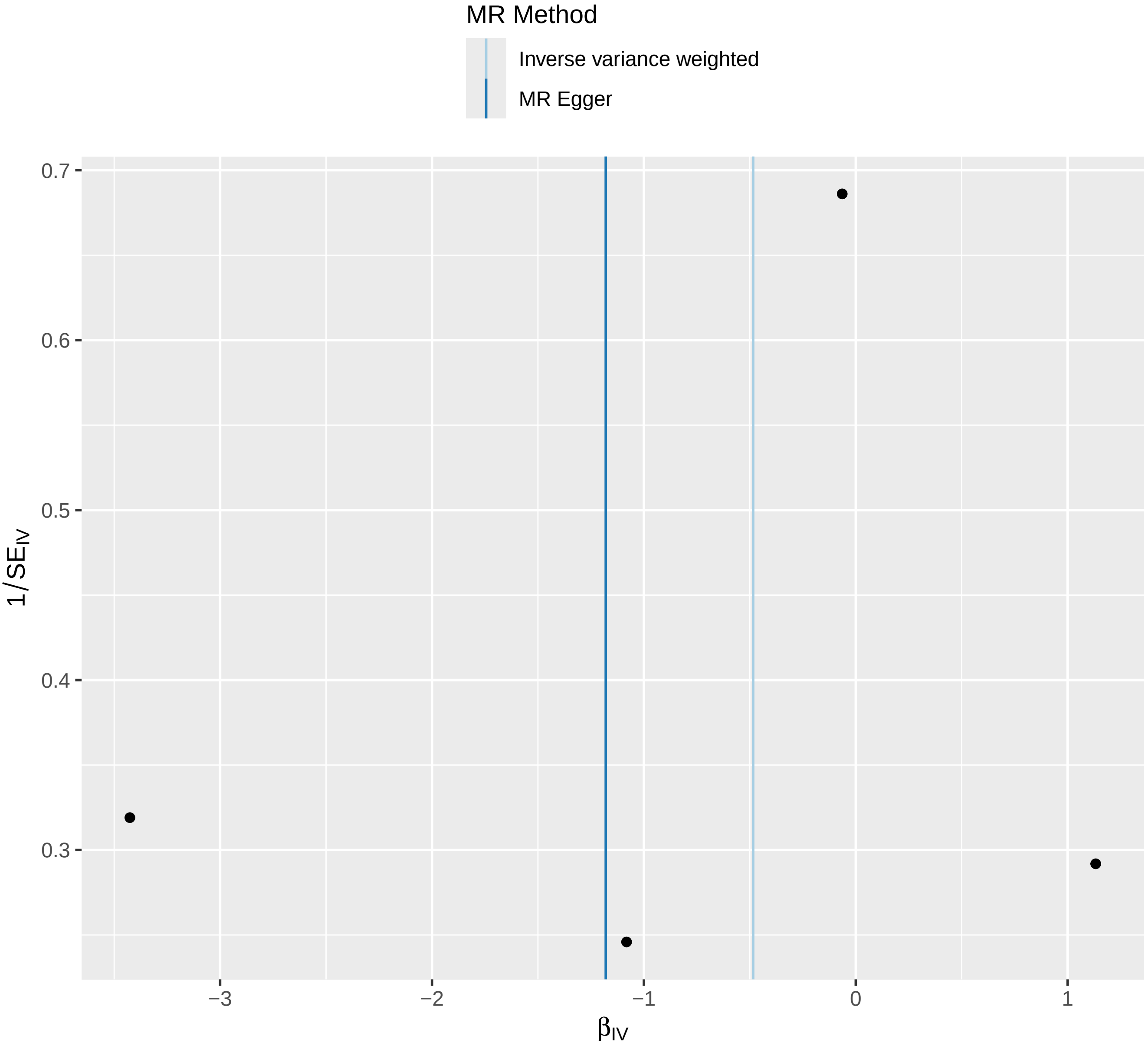

MR Method
Inverse variance weighted
MR Egger
0.7
0.6
0.5
0.4
0.3
1/SE$_{IV}$
−3
−2
−1
0
1
β$_{IV}$

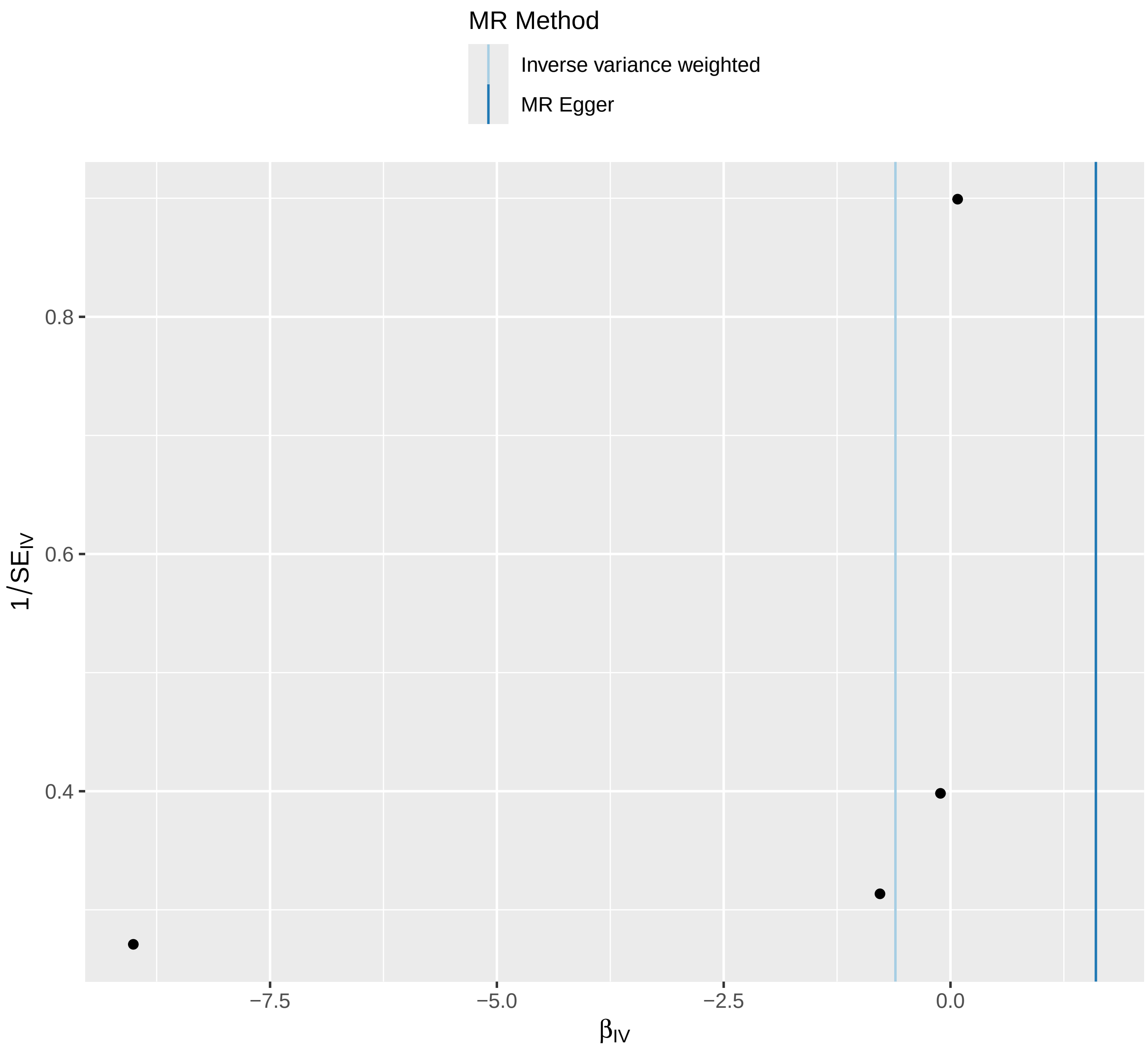
MR Method
Inverse variance weighted
MR Egger
0.8
0.6
0.4
1/SE$_{IV}$
−7.5
−5.0
−2.5
0.0
β$_{IV}$

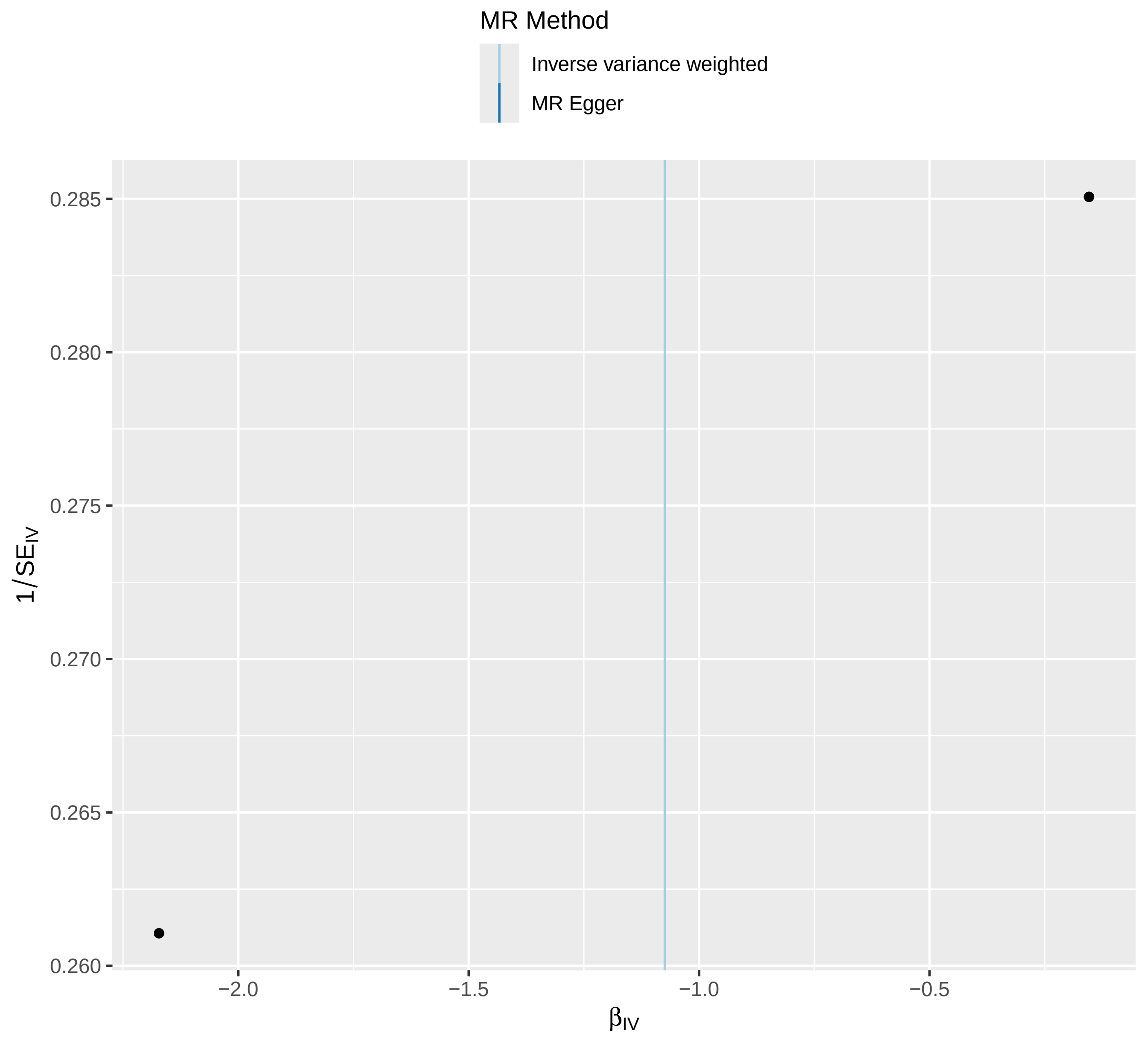
MR Method
Inverse variance weighted
MR Egger
0.285
0.280
0.275
0.270
0.265
0.260
1/SE_IV
−2.0
−1.5
−1.0
−0.5
β_IV

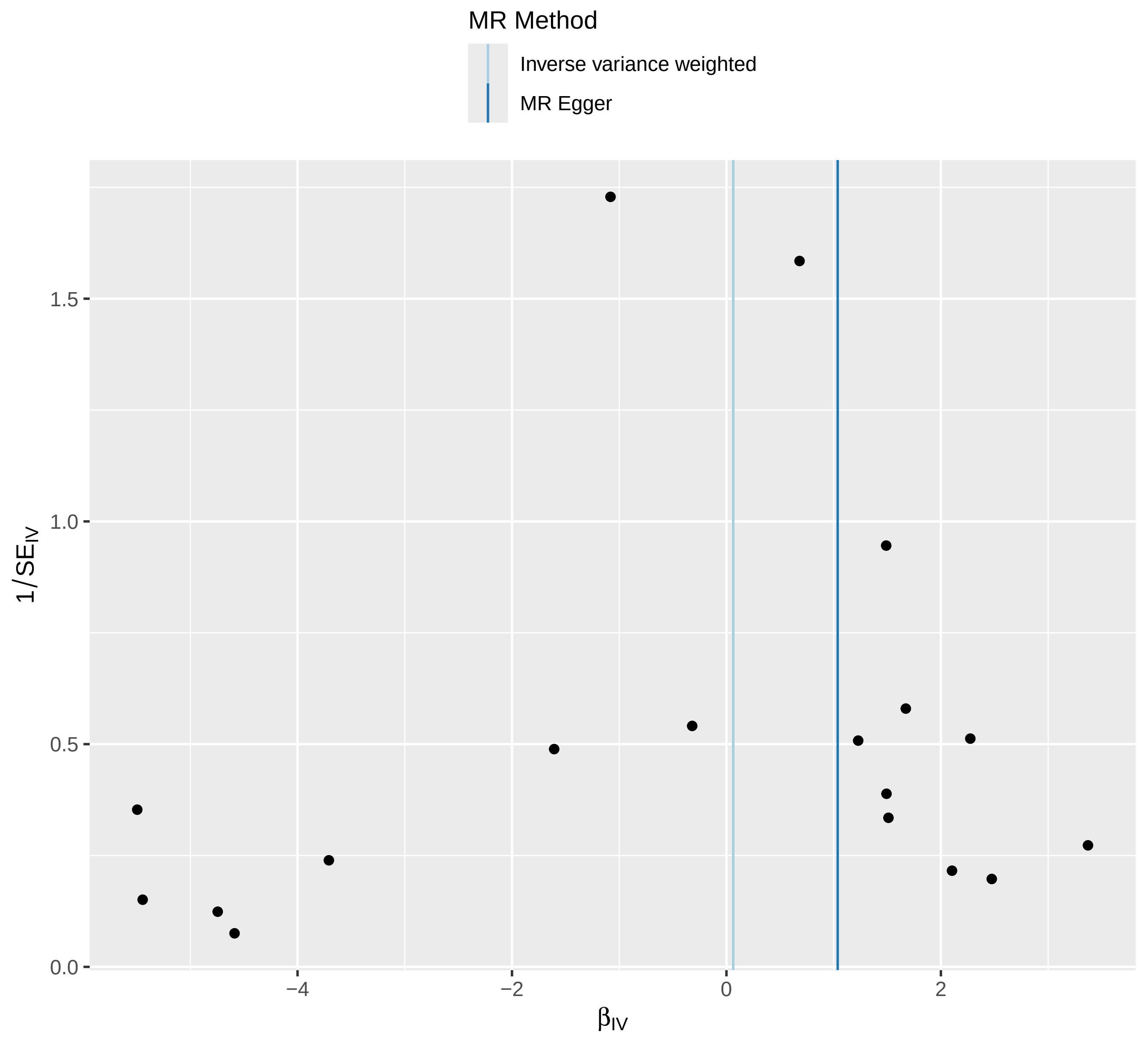

MR Method
Inverse variance weighted
MR Egger
1.5
1.0
0.5
0.0
−4
−2
0
2
1/SE_IV
β_IV

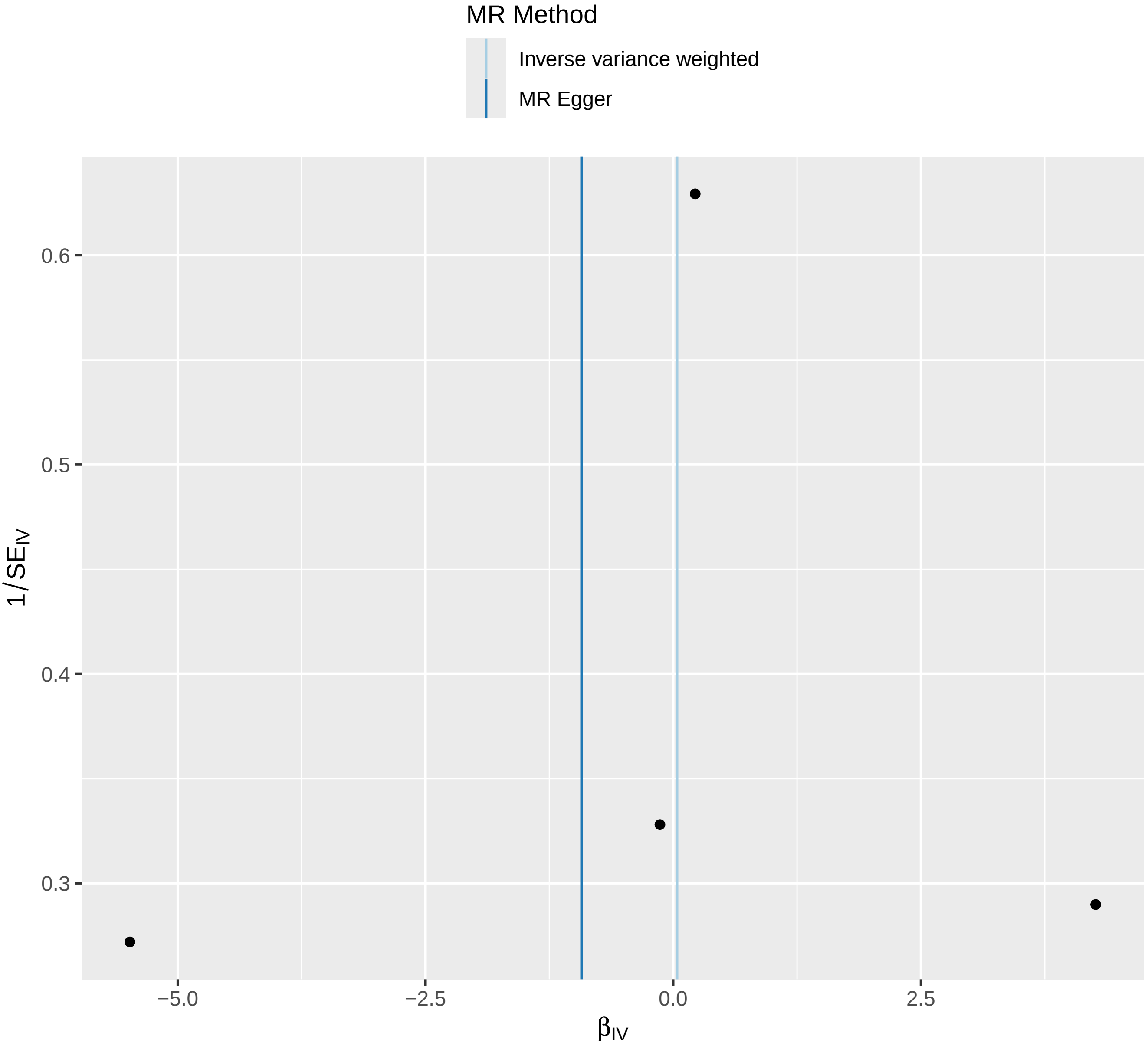

MR Method
Inverse variance weighted
MR Egger
0.6
0.5
0.4
0.3
1/SE_IV
−5.0
−2.5
0.0
2.5
β_IV

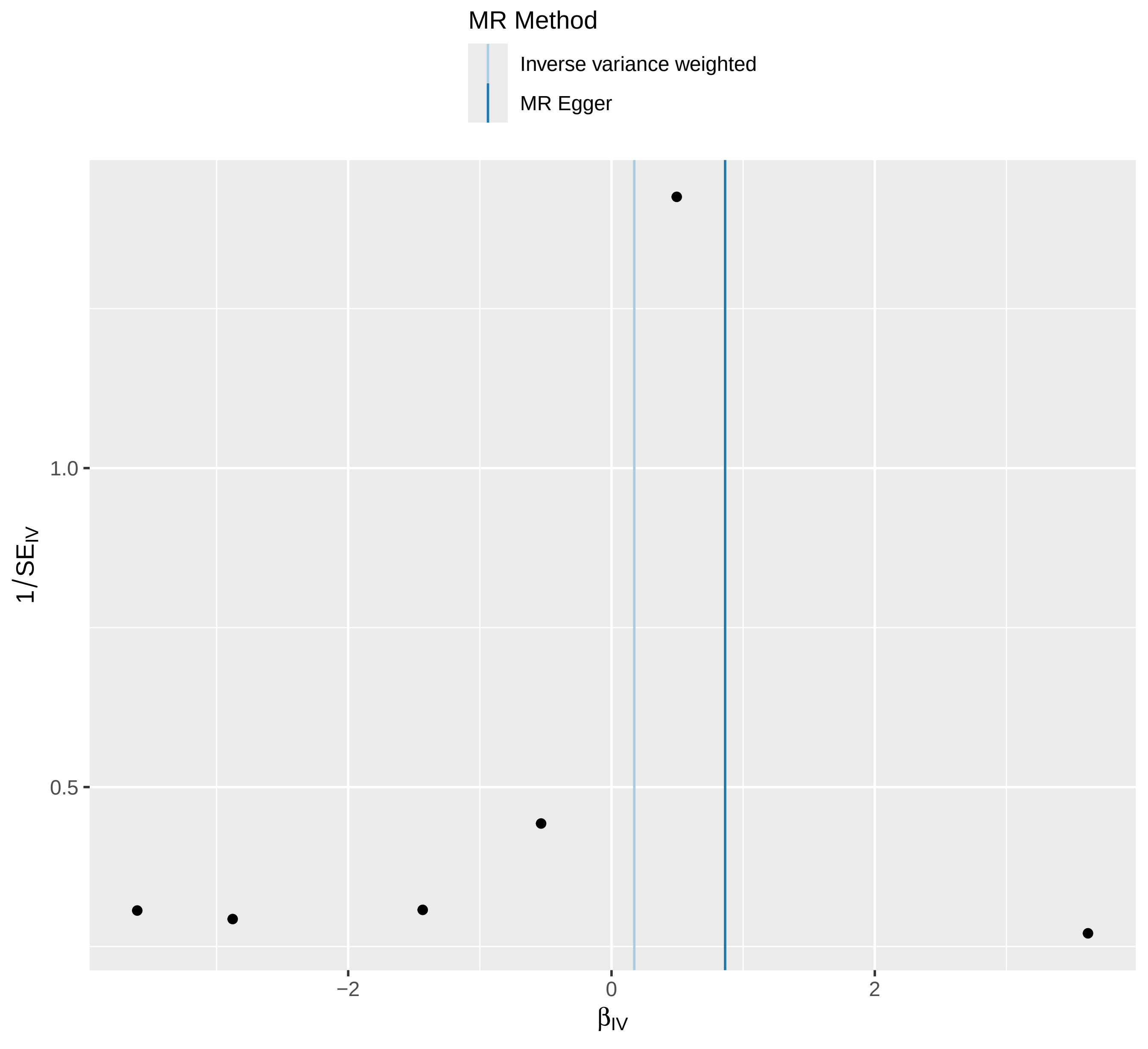

MR Method
Inverse variance weighted
MR Egger
1.0
0.5
1/SE_IV
−2
0
2
β_IV

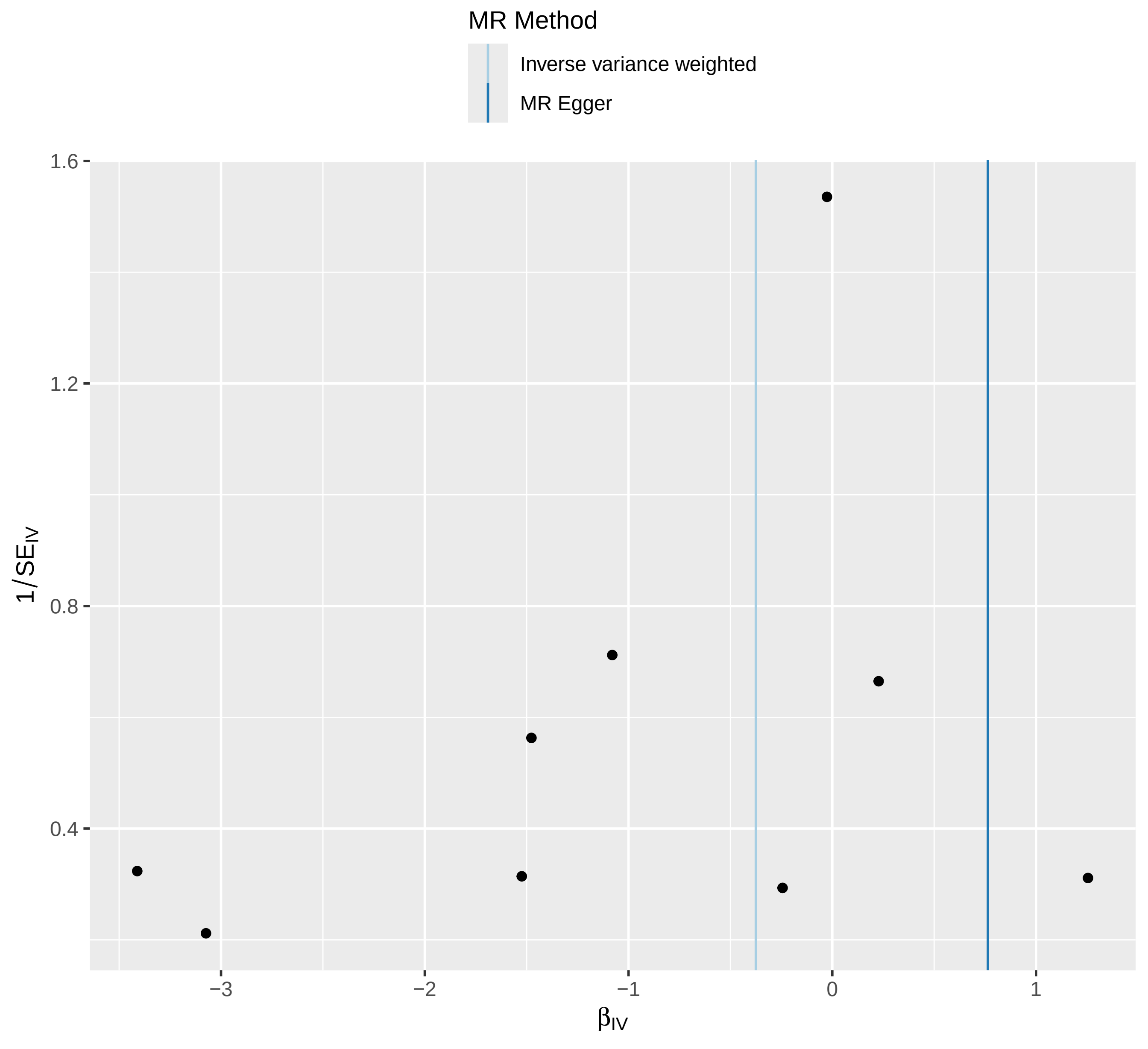
MR Method
Inverse variance weighted
MR Egger
1.6
1.2
0.8
0.4
$1/SE_{IV}$
−3
−2
−1
0
1
β_{IV}

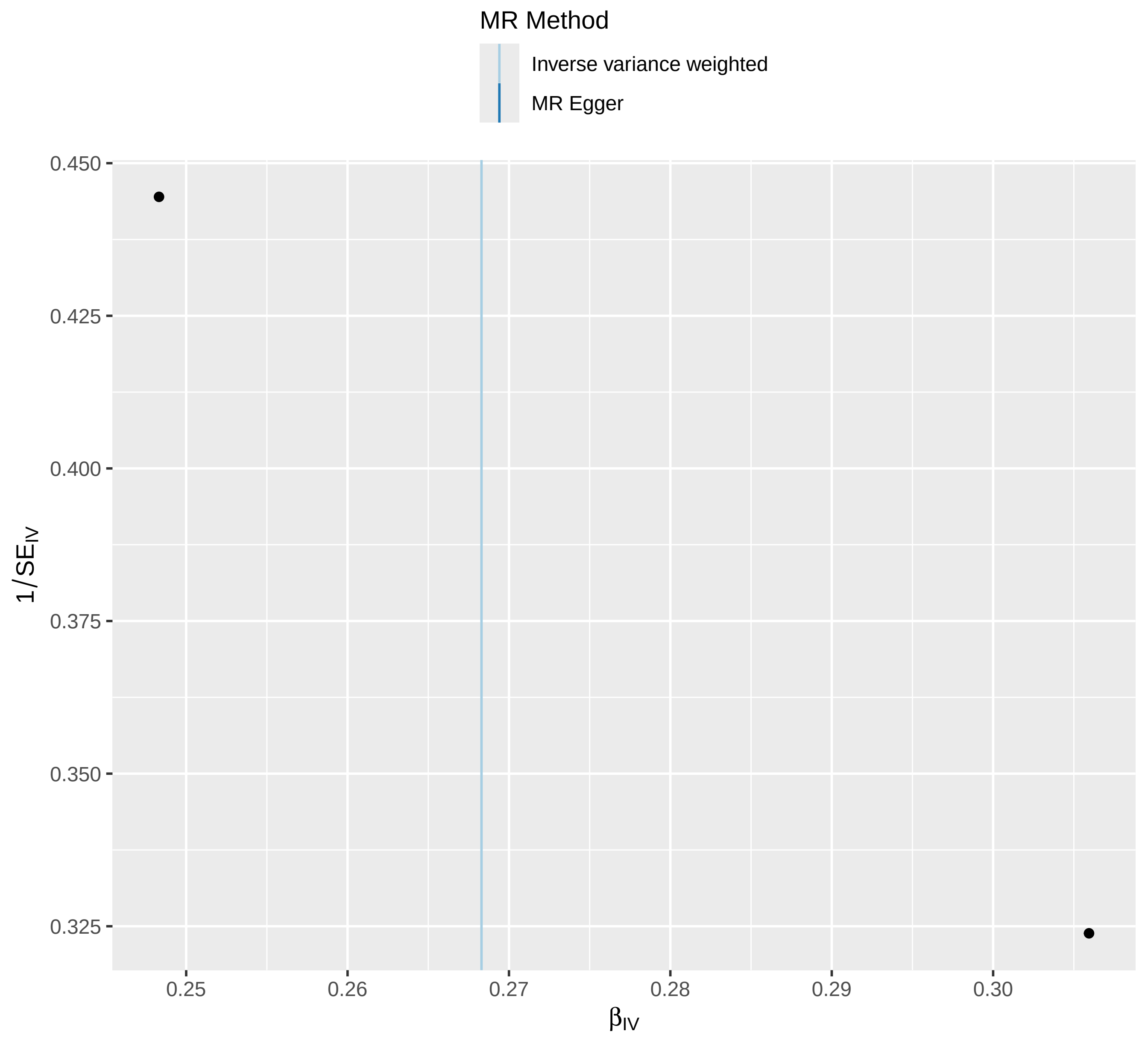

MR Method
Inverse variance weighted
MR Egger
0.450
0.425
0.400
0.375
0.350
0.325
1/SE_IV
0.25
0.26
0.27
0.28
0.29
0.30
β_IV

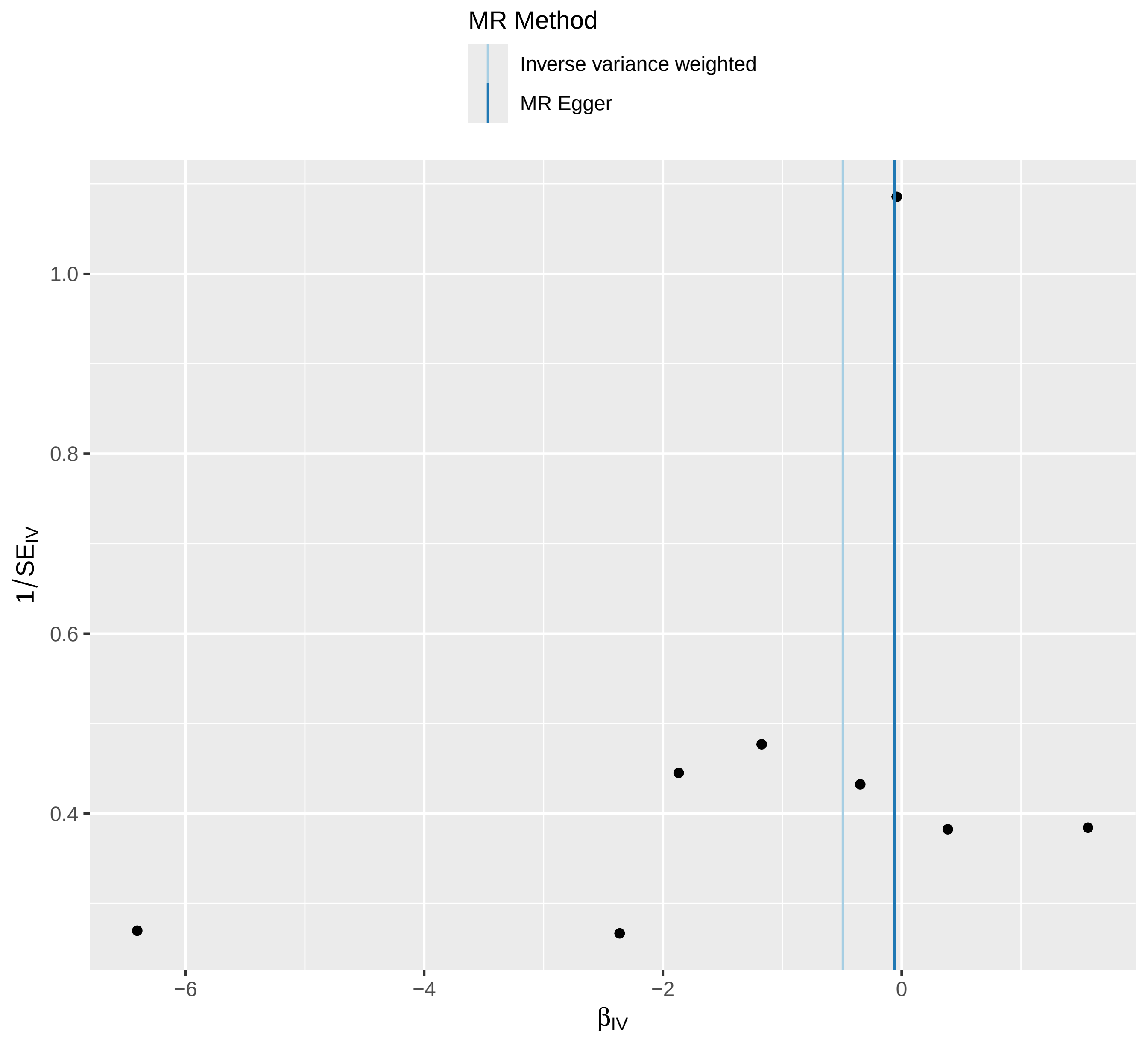
MR Method
Inverse variance weighted
MR Egger
1.0
0.8
0.6
0.4
1/SE_IV
−6
−4
−2
0
β_IV

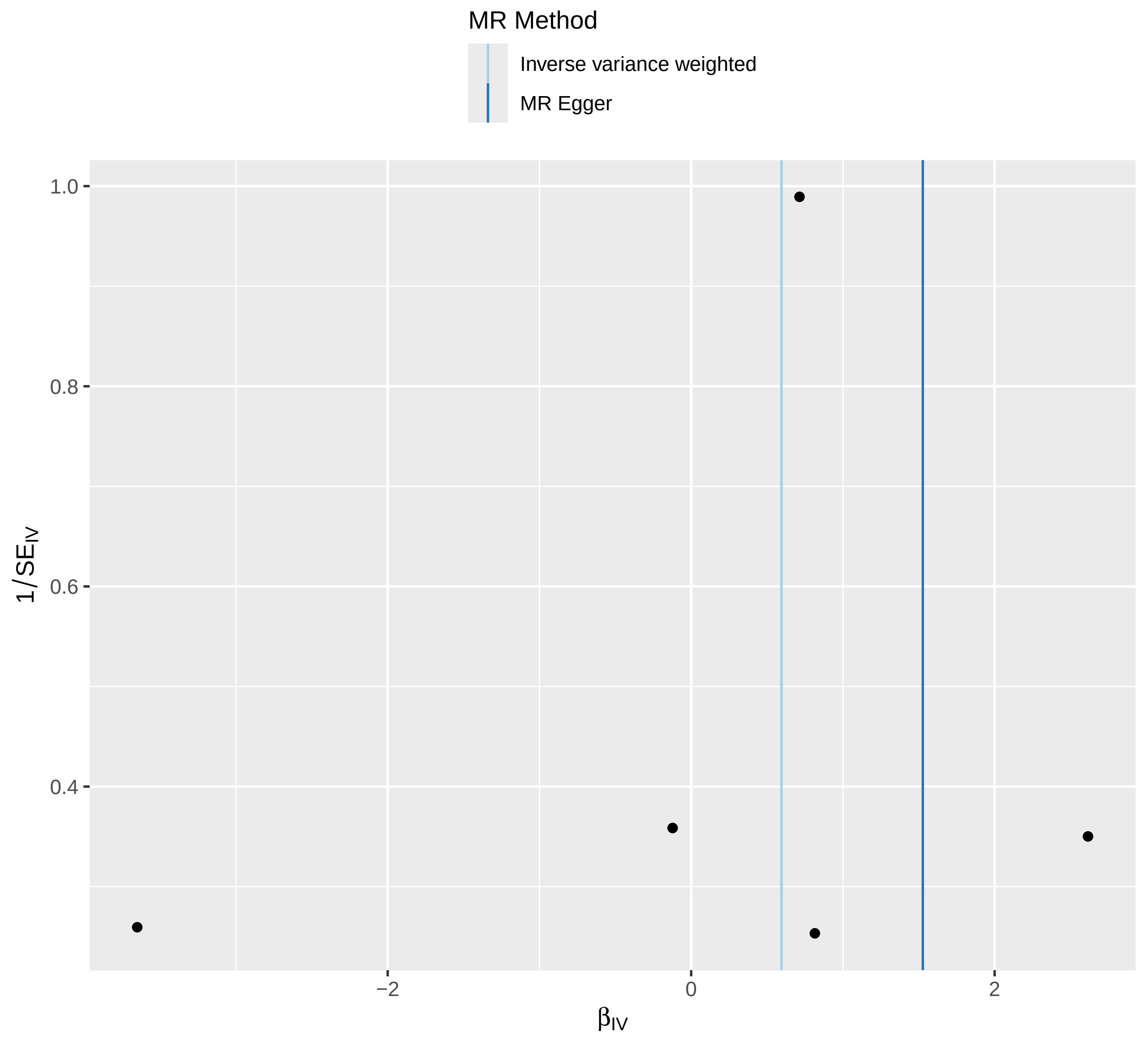
MR Method
Inverse variance weighted
MR Egger
1.0
0.8
0.6
0.4
1/SE_IV
−2
0
2
β_IV

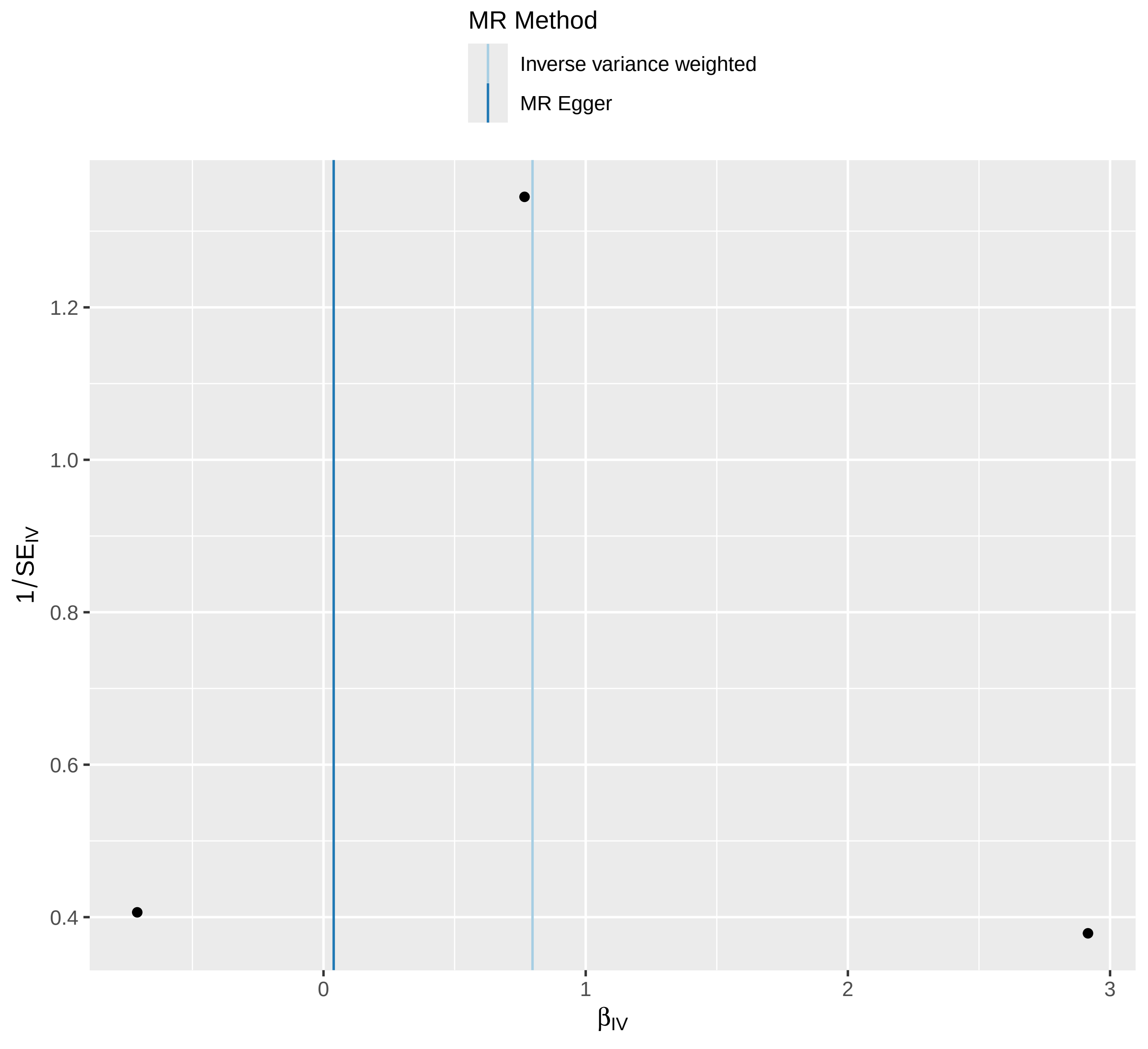
MR Method
Inverse variance weighted
MR Egger
1.2
1.0
0.8
0.6
0.4
0
1
2
3
1/SE_IV
β_IV

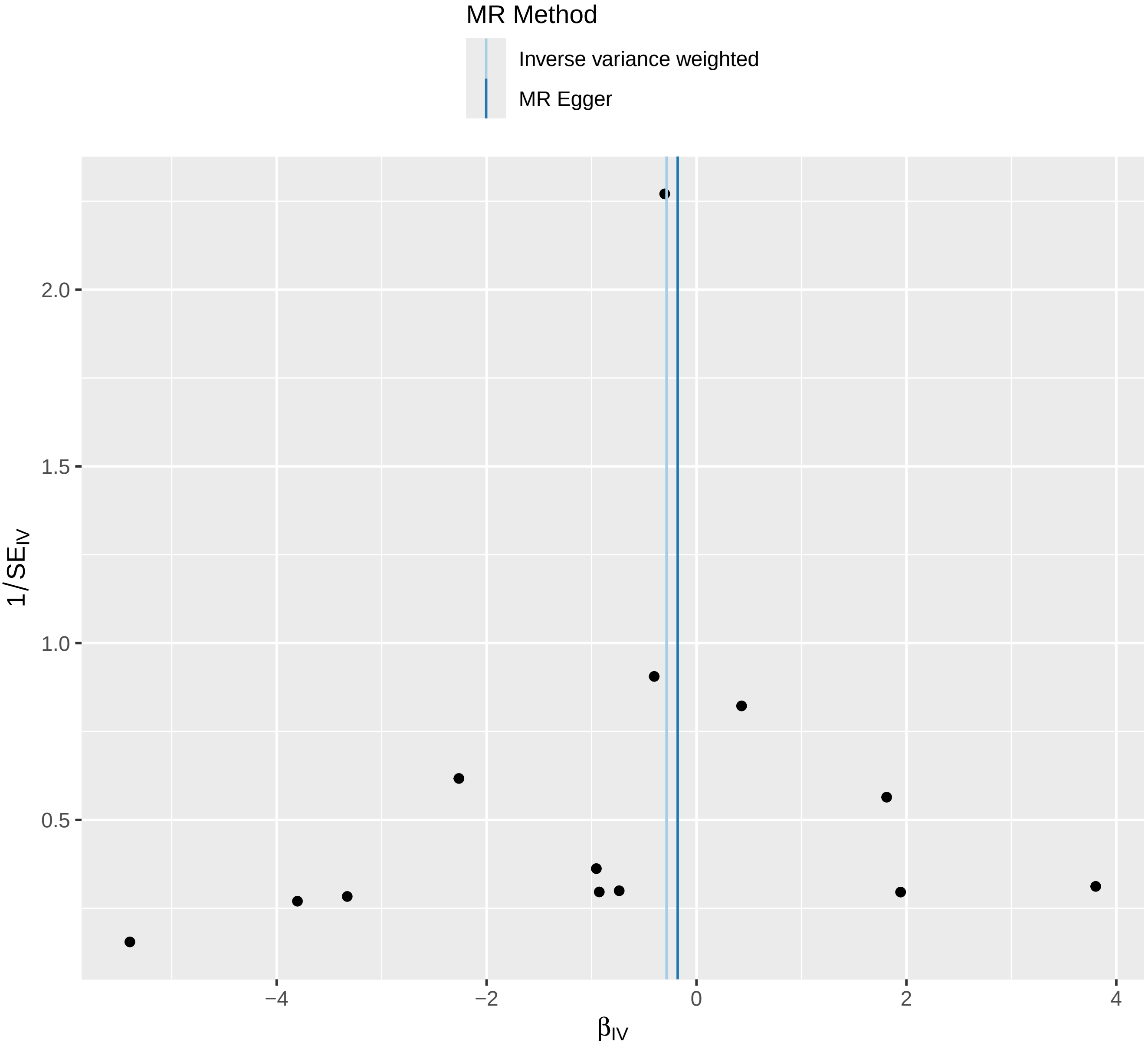

MR Method
Inverse variance weighted
MR Egger
2.0
1.5
1.0
0.5
1/SE_IV
−4
−2
0
2
4
β_IV

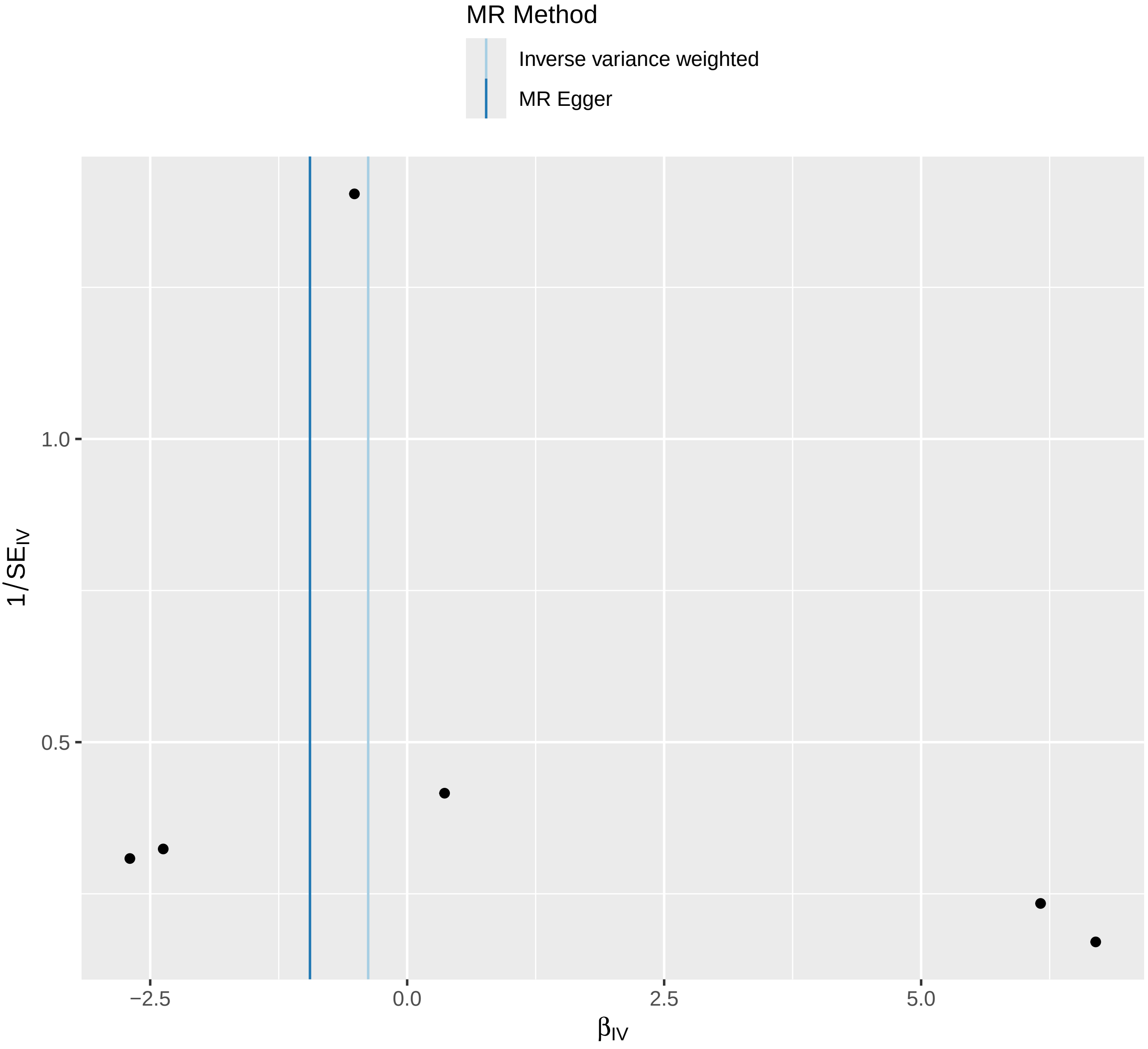

MR Method
Inverse variance weighted
MR Egger
1.0
0.5
1/SE_IV
−2.5
0.0
2.5
5.0
β_IV

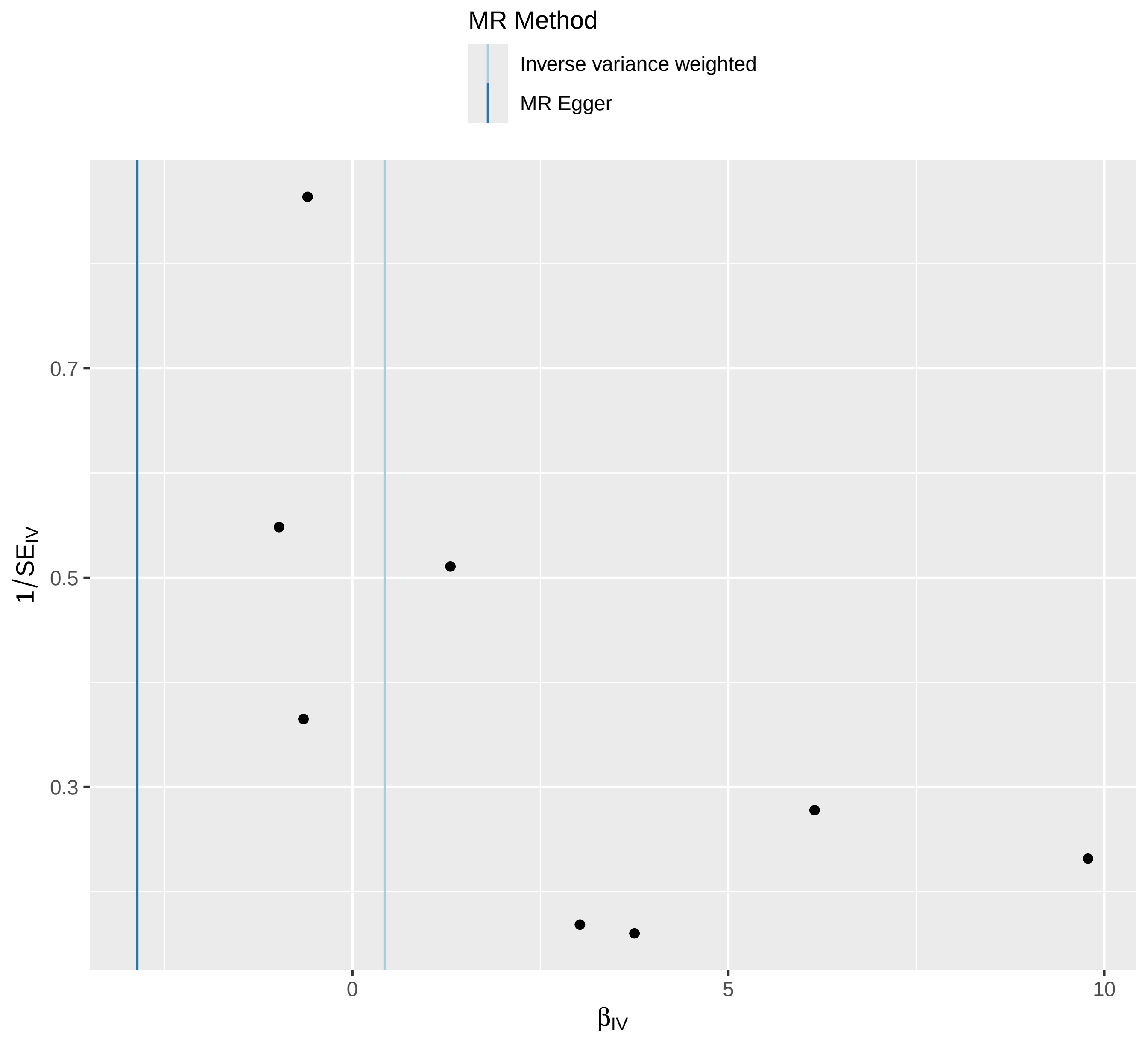

MR Method
Inverse variance weighted
MR Egger
0.7
0.5
0.3
1/SE_IV
0
5
10
β_IV

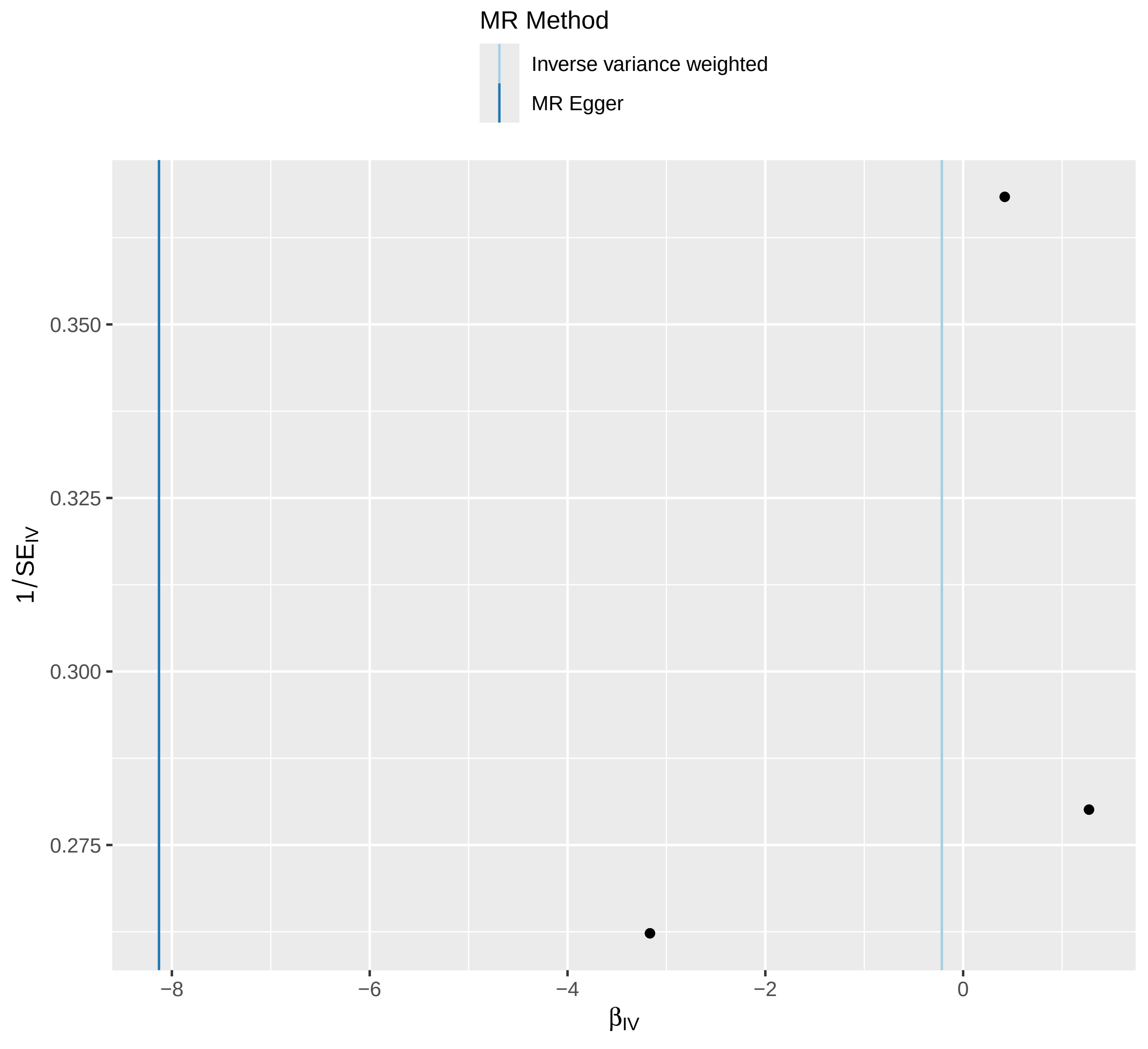
MR Method
Inverse variance weighted
MR Egger
0.350
0.325
0.300
0.275
1/SE$_{IV}$
−8
−6
−4
−2
0
β$_{IV}$

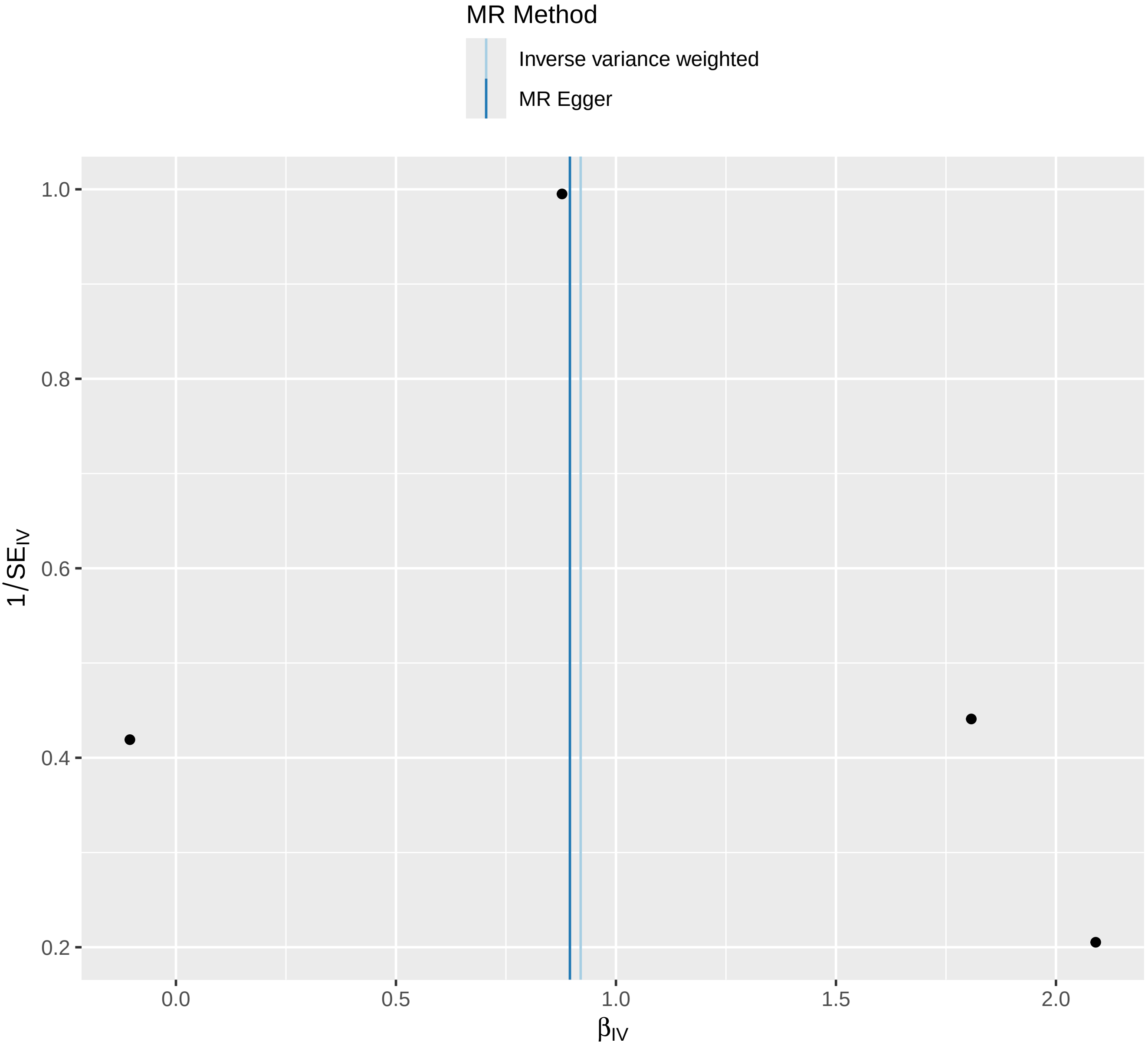

MR Method
Inverse variance weighted
MR Egger
1.0
0.8
0.6
0.4
0.2
1/SE_IV
0.0
0.5
1.0
1.5
2.0
β_IV

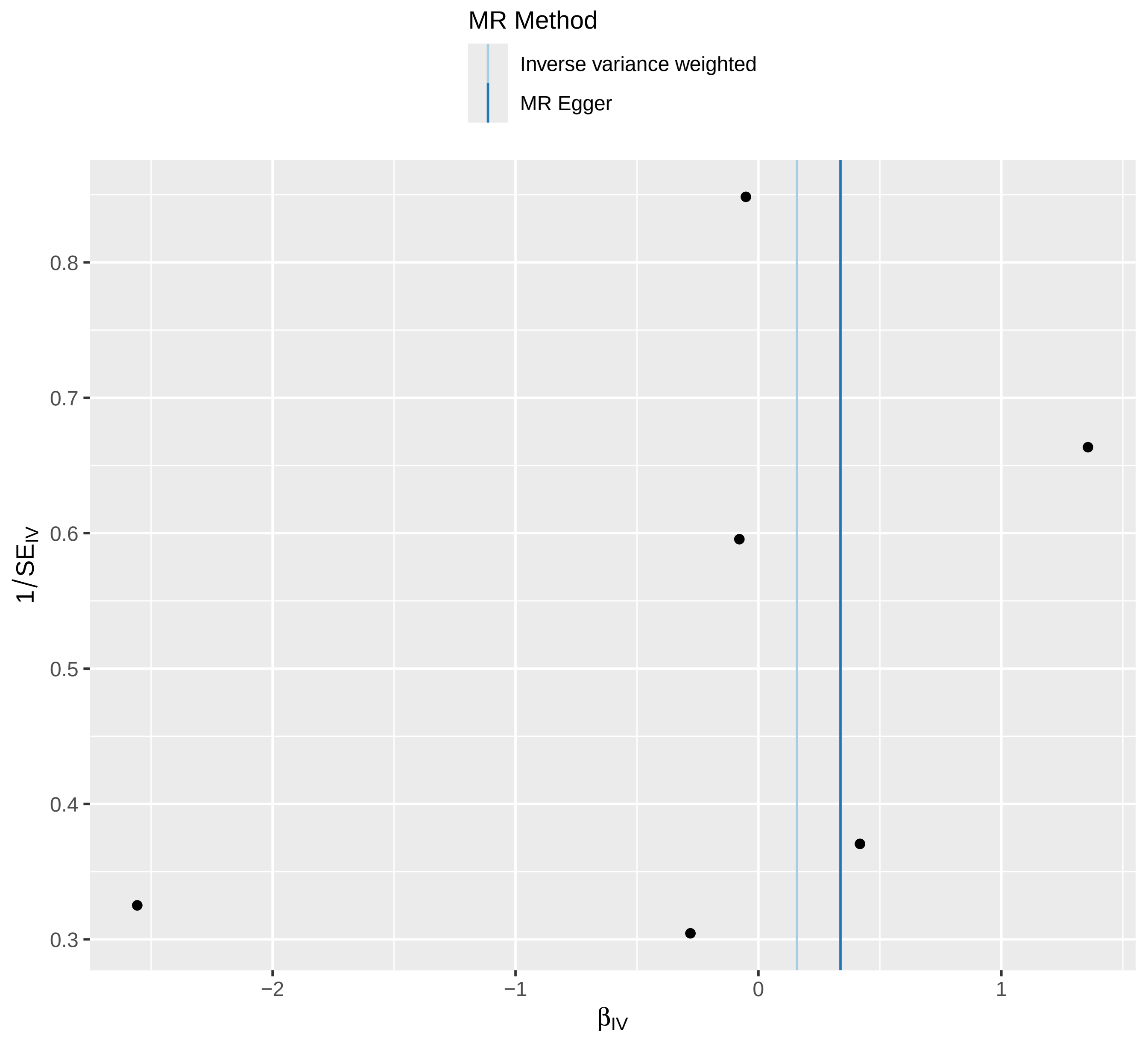

MR Method
Inverse variance weighted
MR Egger
1/SE$_{IV}$
0.8
0.7
0.6
0.5
0.4
0.3
−2
−1
0
1
β$_{IV}$

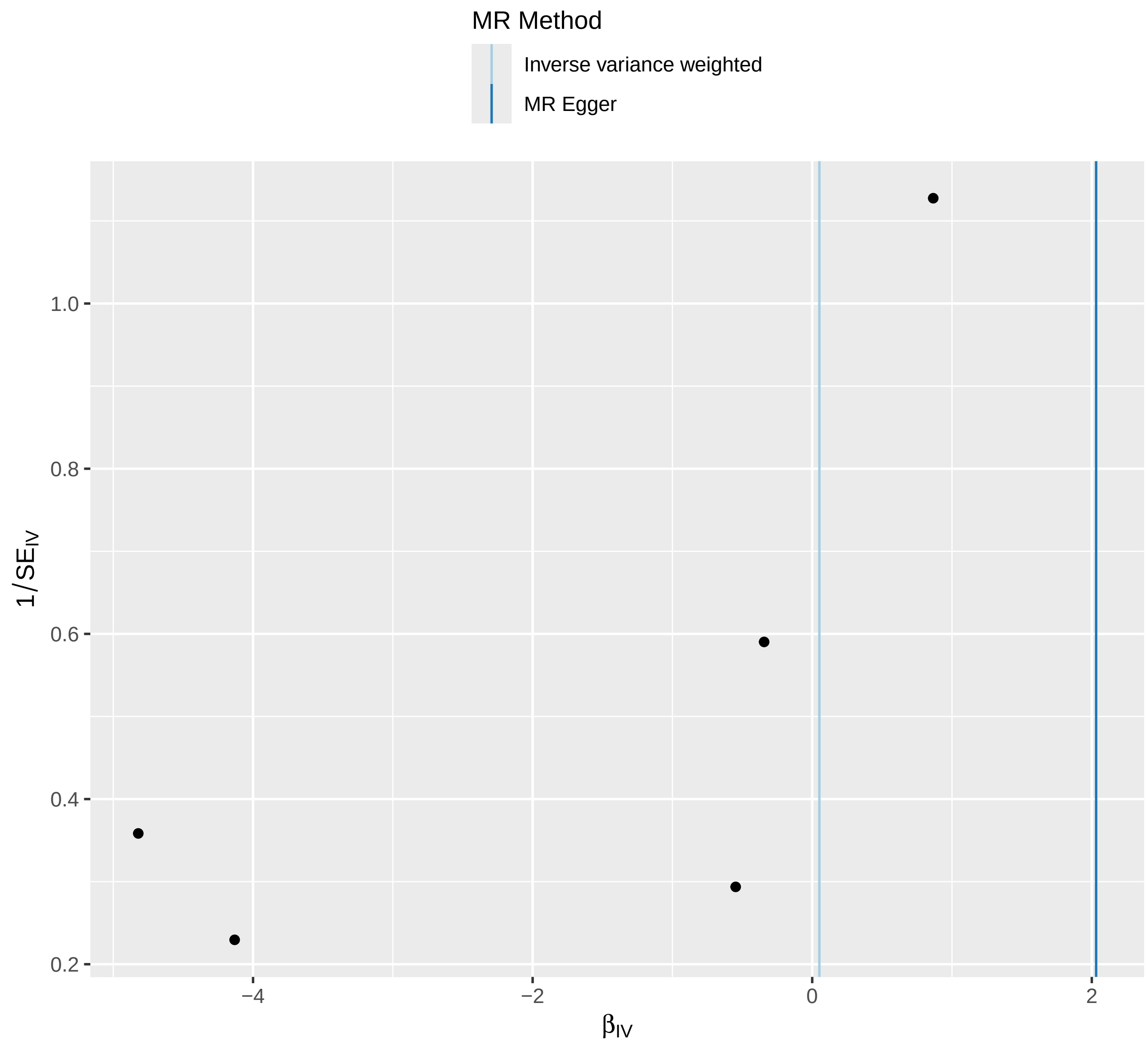
MR Method
Inverse variance weighted
MR Egger
1.0
0.8
0.6
0.4
0.2
$1/SE_{IV}$
−4
−2
0
2
β_{IV}

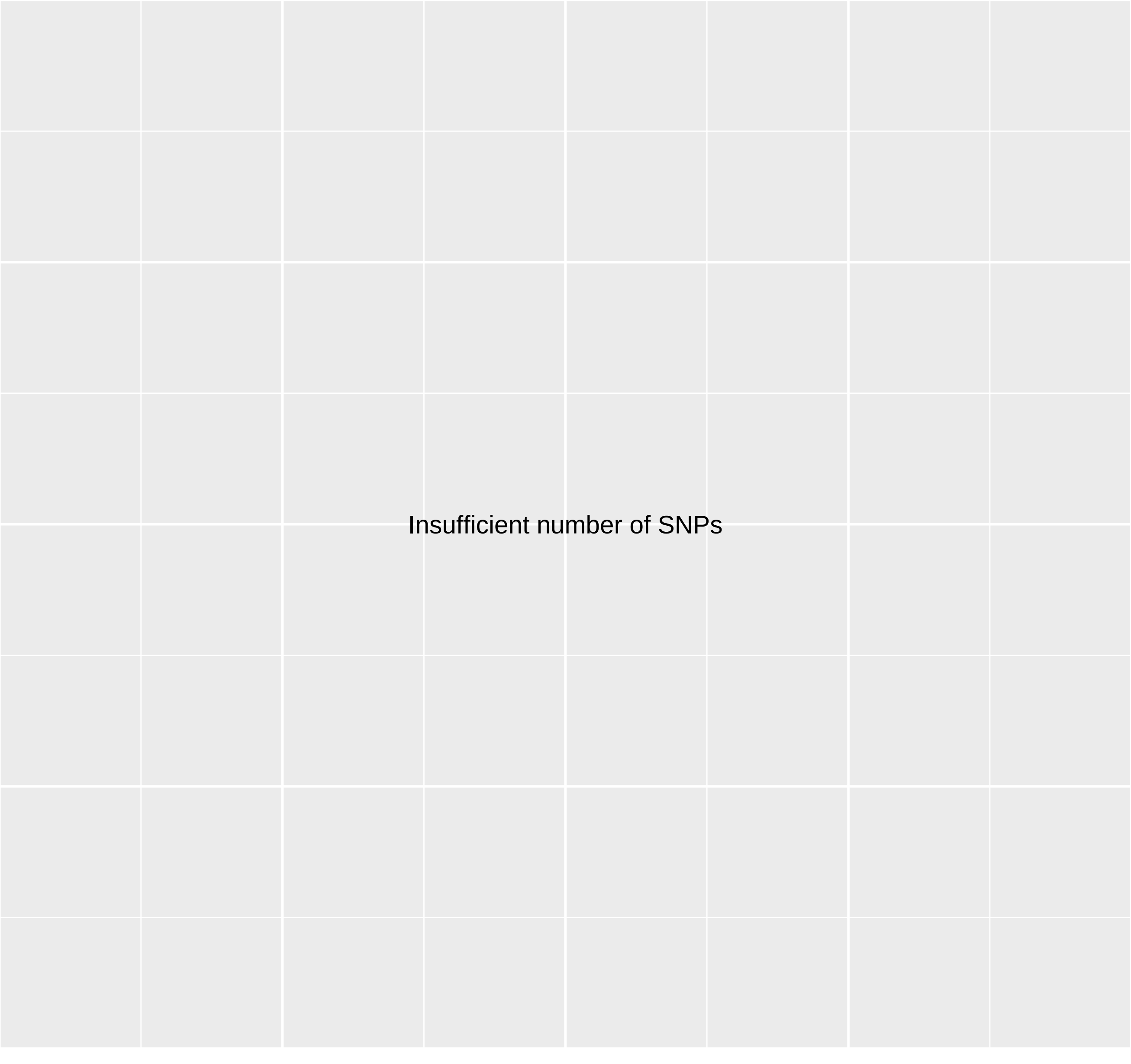
Insufficient number of SNPs

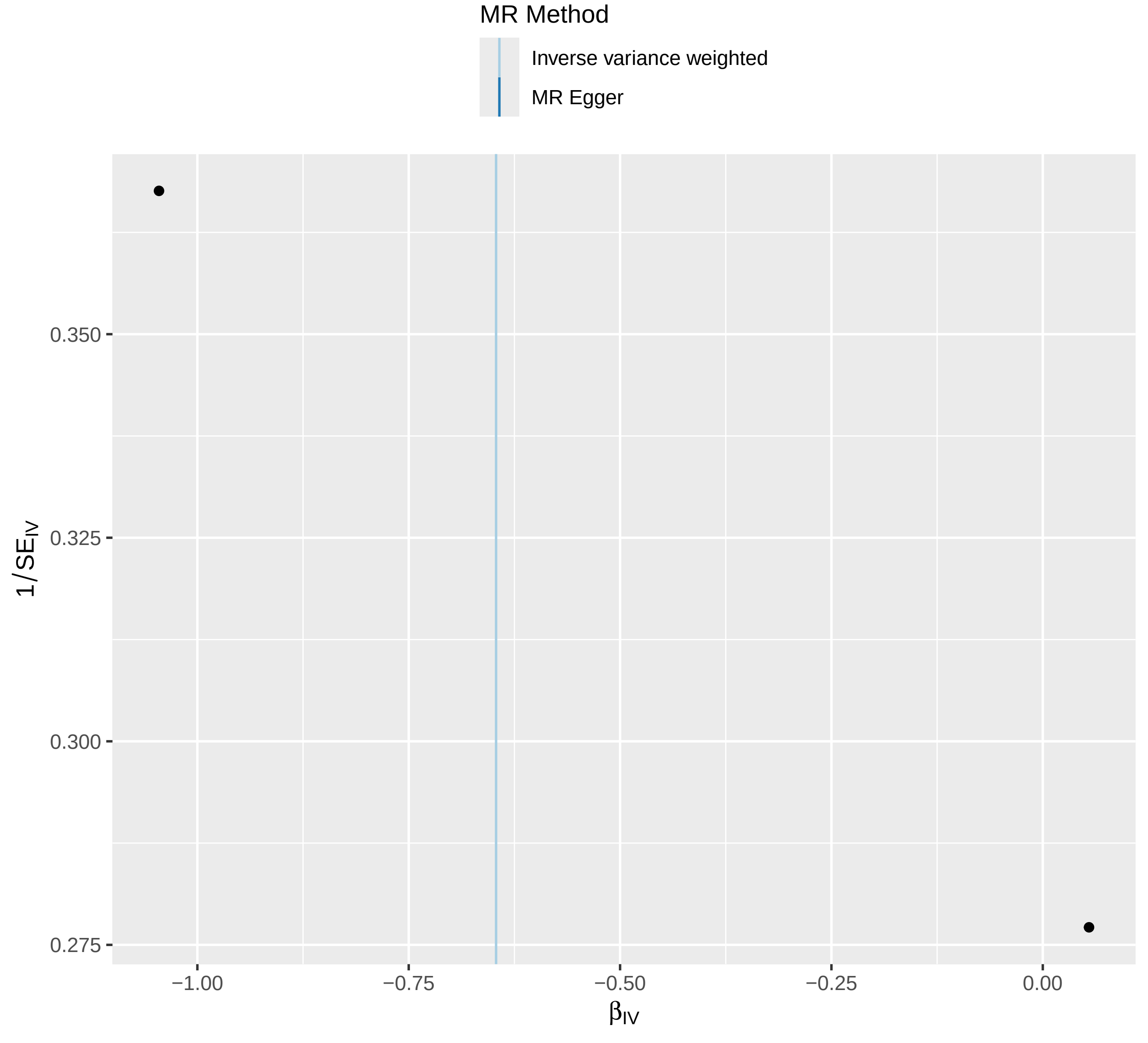
MR Method
Inverse variance weighted
MR Egger
0.350
0.325
0.300
0.275
1/SE_IV
−1.00
−0.75
−0.50
−0.25
0.00
β_IV

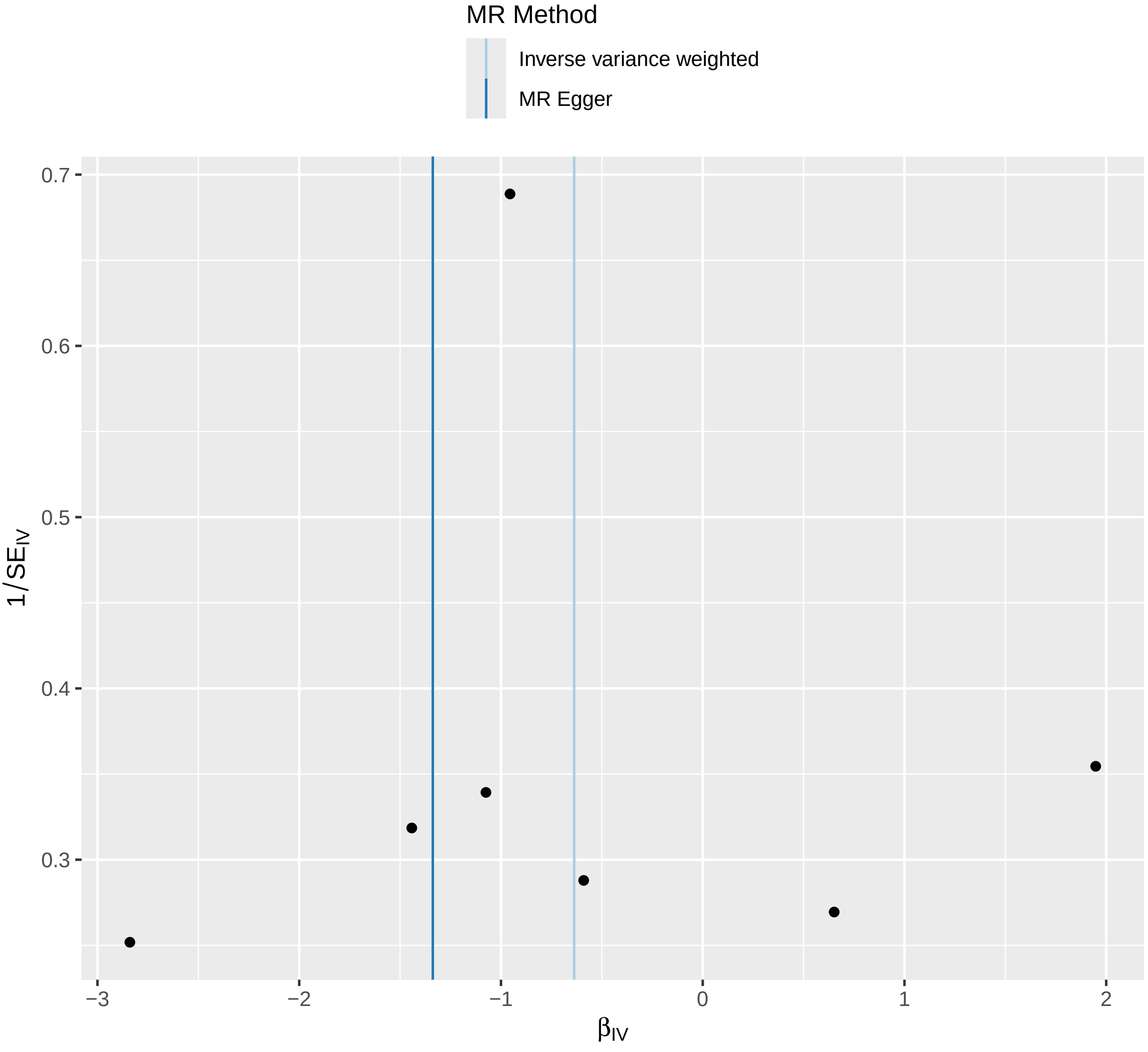

MR Method
Inverse variance weighted
MR Egger
0.7
0.6
0.5
0.4
0.3
1/SE$_{IV}$
−3
−2
−1
0
1
2
β$_{IV}$

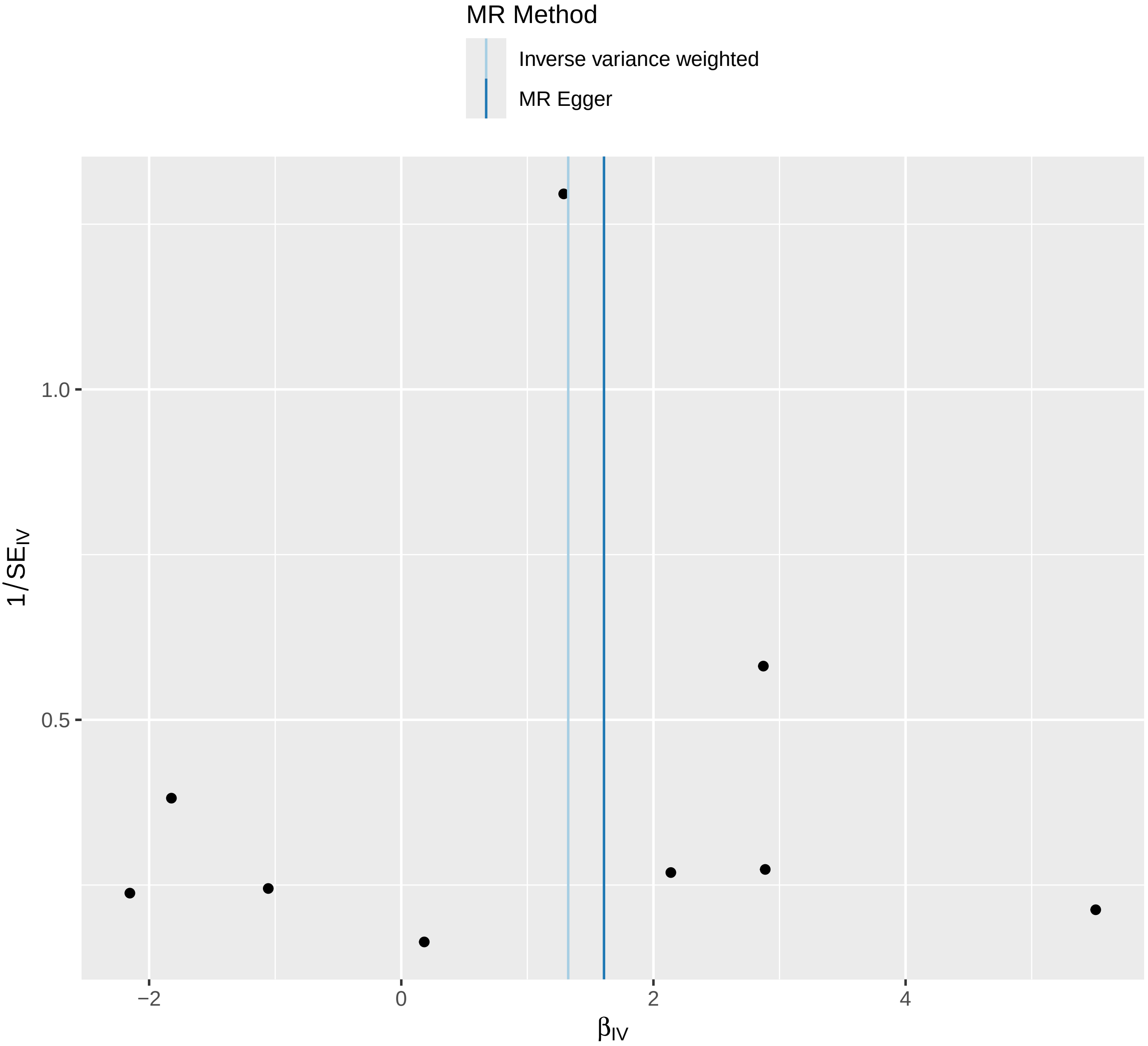

MR Method
Inverse variance weighted
MR Egger
1.0
0.5
1/SE_IV
−2
0
2
4
β_IV

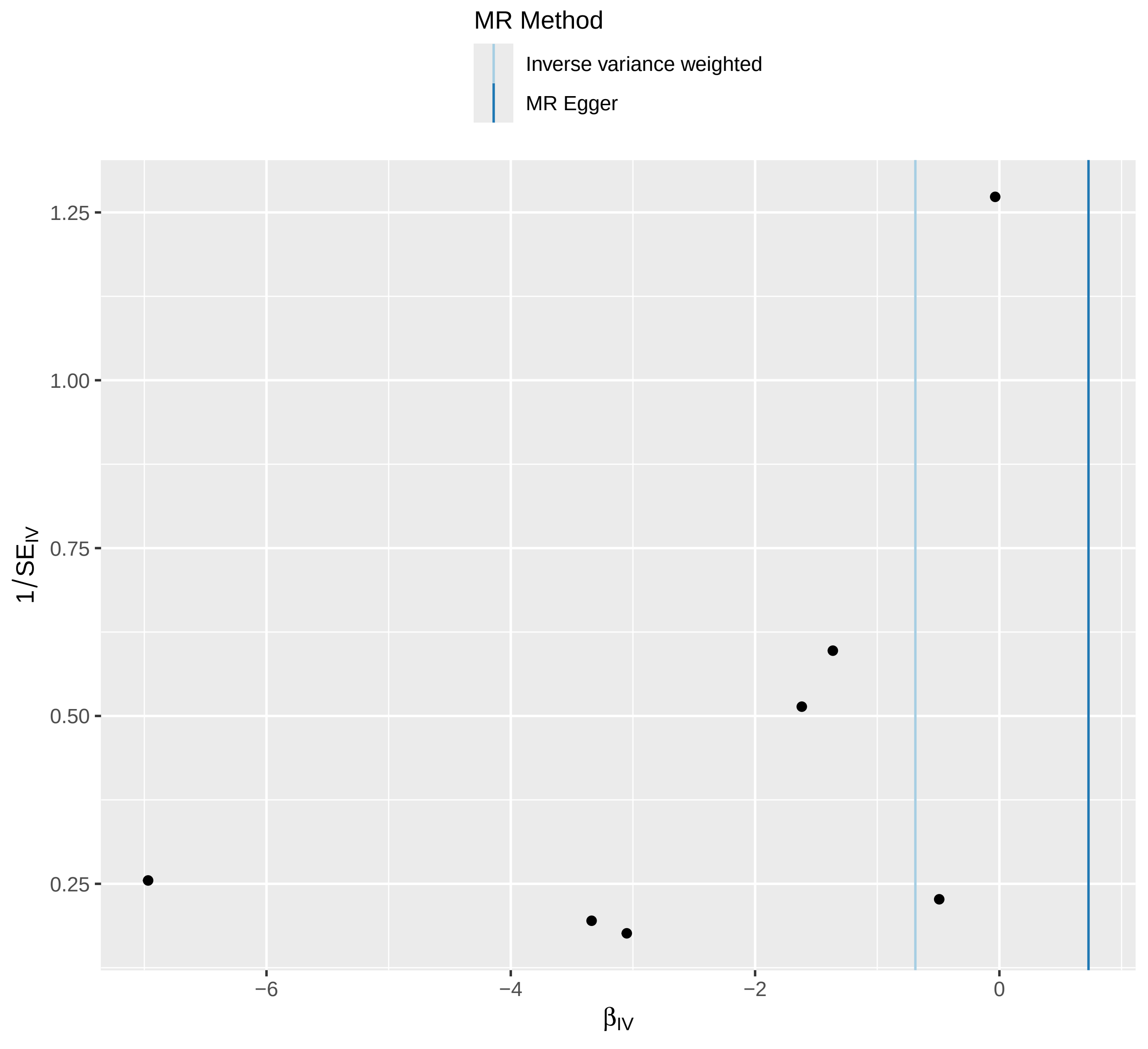
MR Method
Inverse variance weighted
MR Egger
1.25
1.00
0.75
0.50
0.25
$1/SE_{IV}$
−6
−4
−2
0
β_{IV}

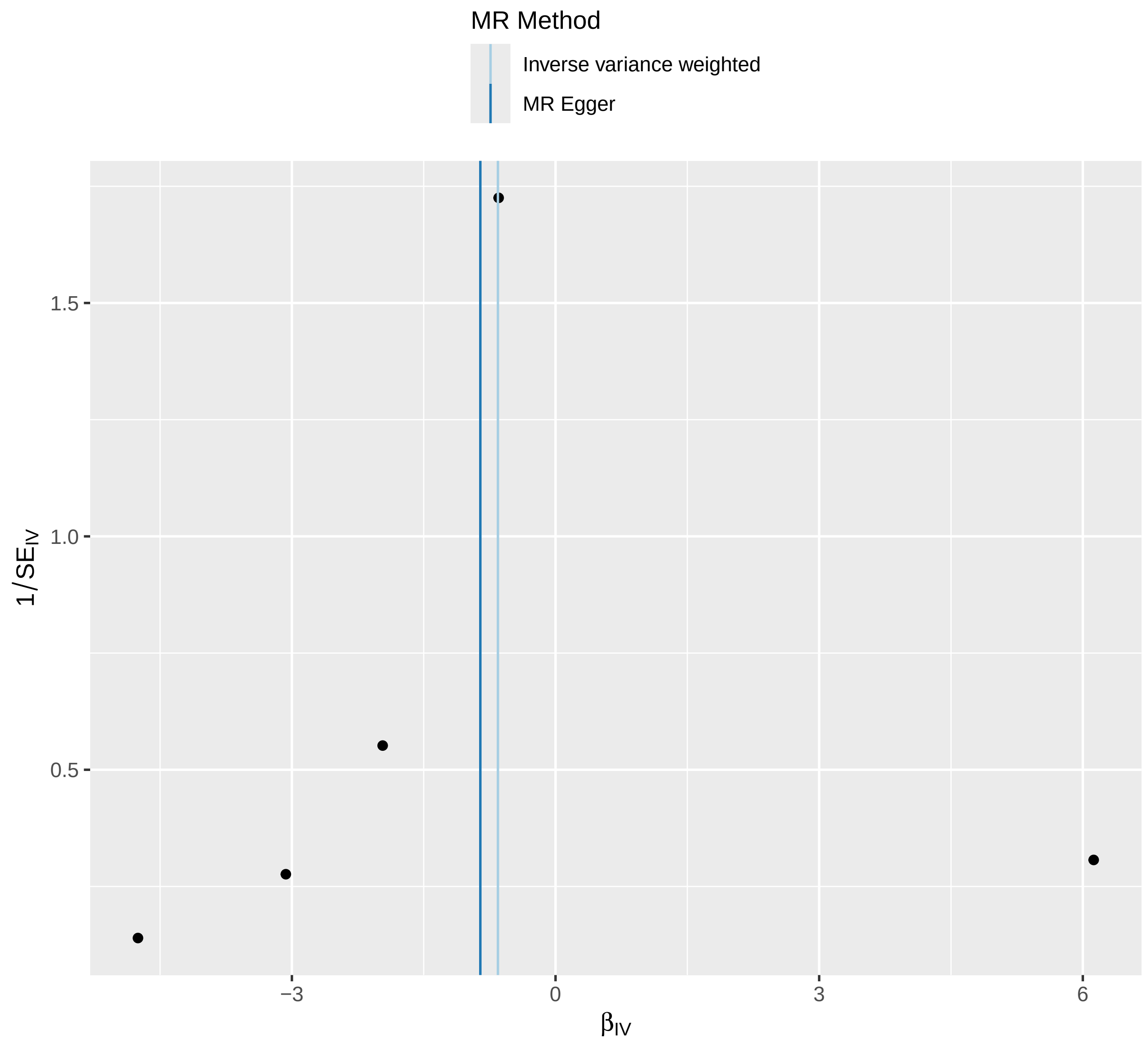

MR Method
Inverse variance weighted
MR Egger
1.5
1.0
0.5
1/SE_IV
−3
0
3
6
β_IV

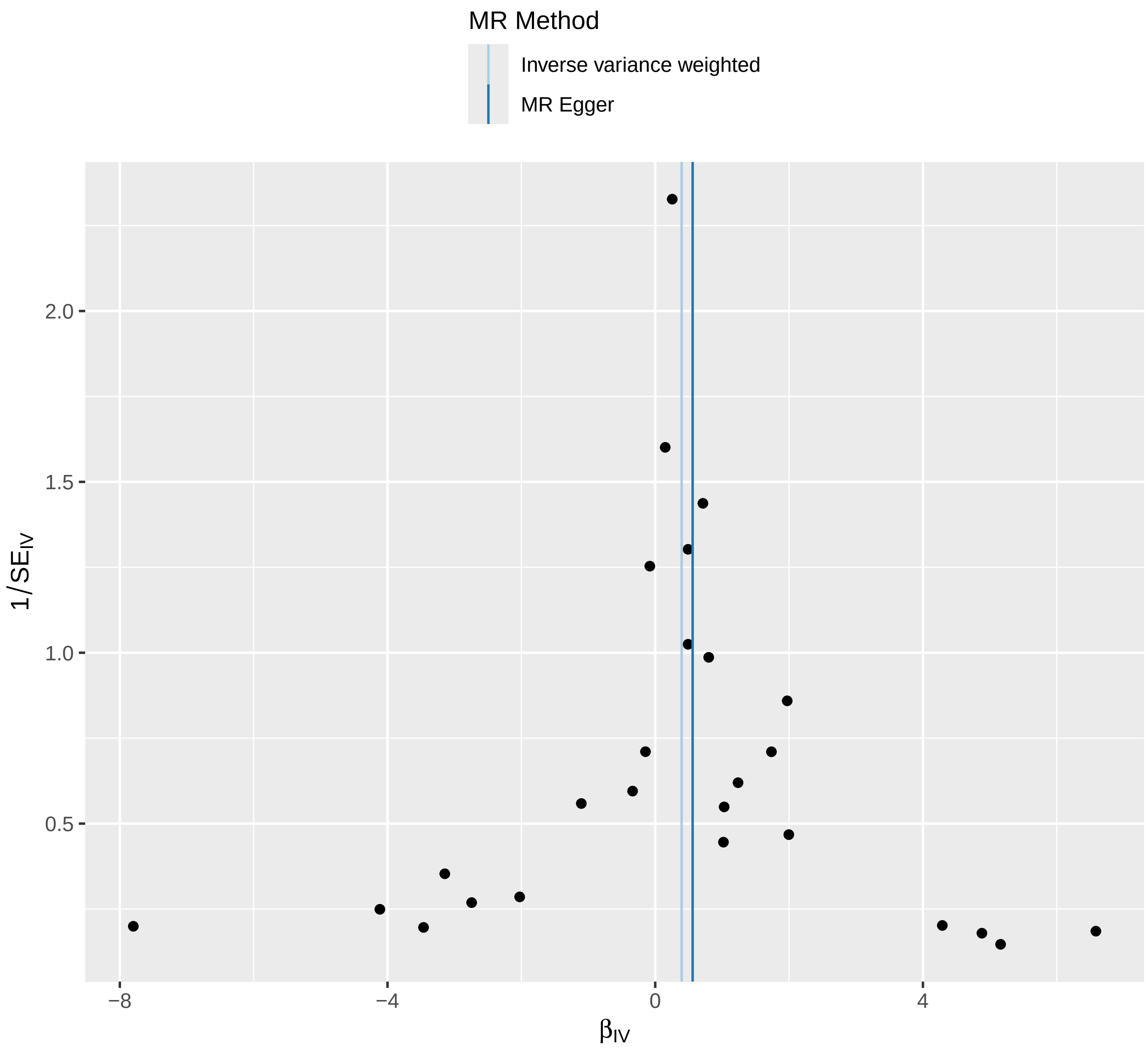

MR Method
Inverse variance weighted
MR Egger
2.0
1.5
1.0
0.5
$1/SE_{IV}$
−8
−4
0
4
β_{IV}

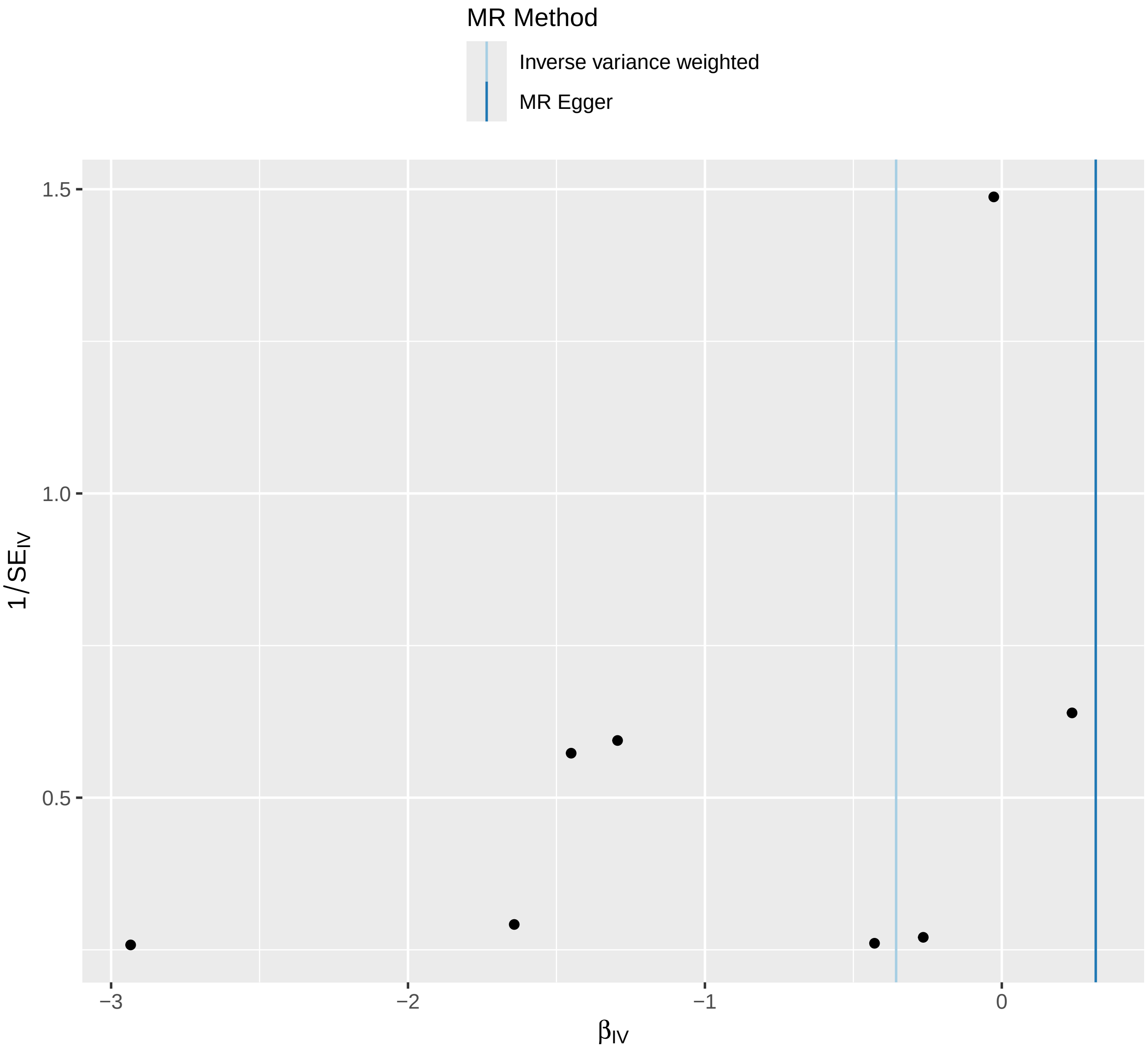
MR Method
Inverse variance weighted
MR Egger
1.5
1.0
0.5
1/SE$_{IV}$
−3
−2
−1
0
β$_{IV}$

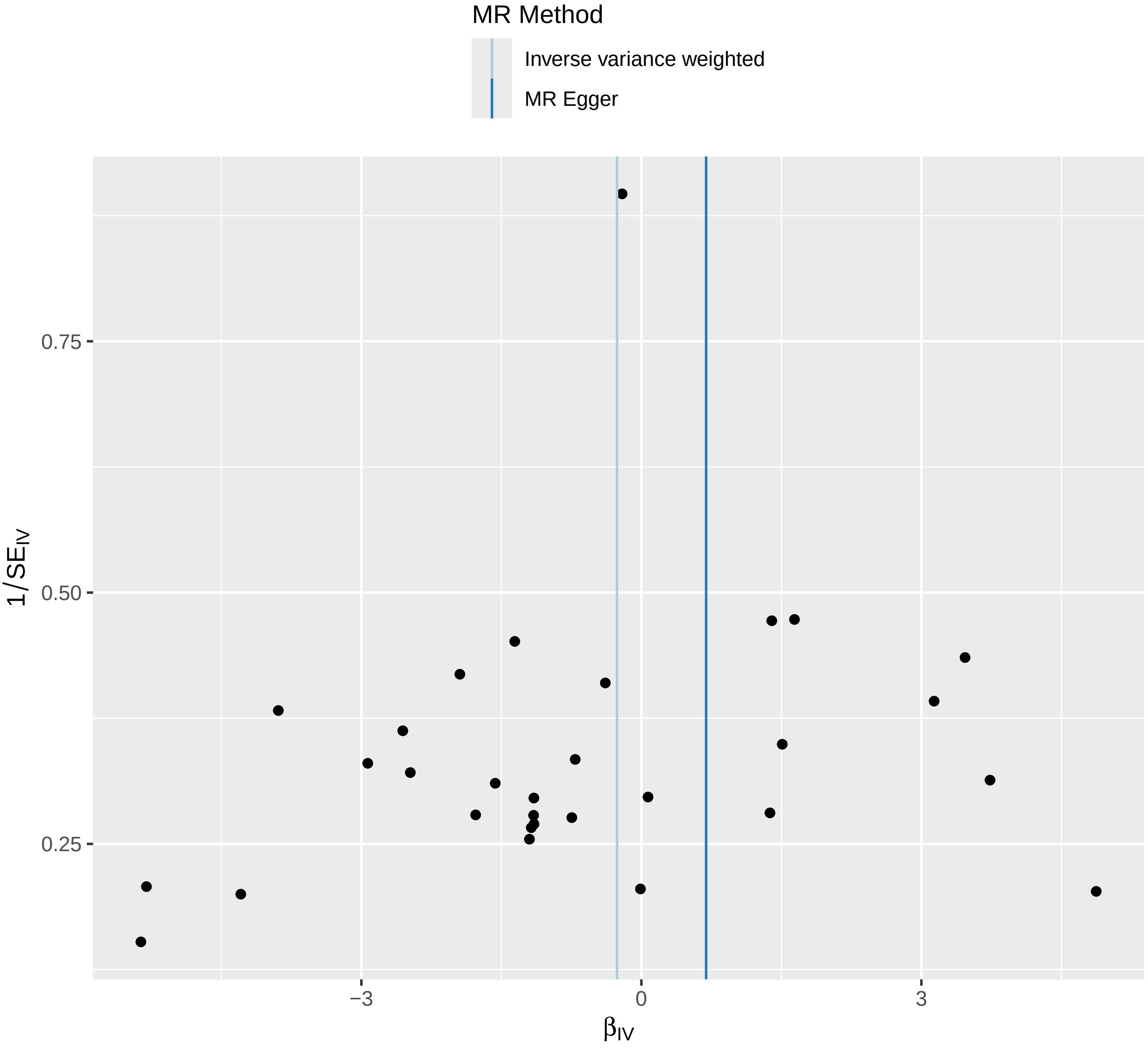
MR Method
Inverse variance weighted
MR Egger
0.75
0.50
0.25
−3
0
3
$1/SE_{IV}$
β_{IV}

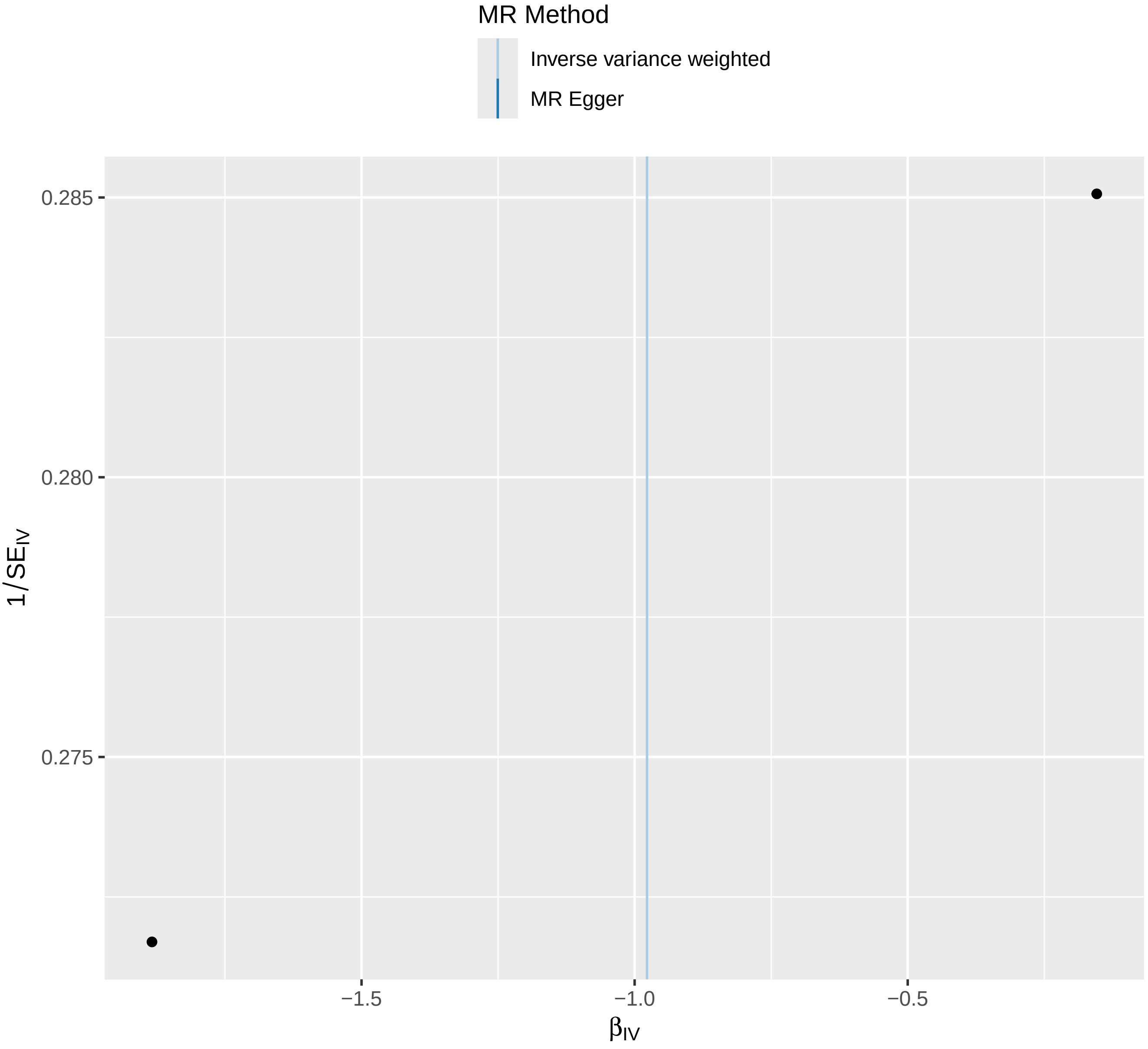

MR Method
Inverse variance weighted
MR Egger
0.285
0.280
0.275
$1/SE_{IV}$
−1.5
−1.0
−0.5
β_{IV}

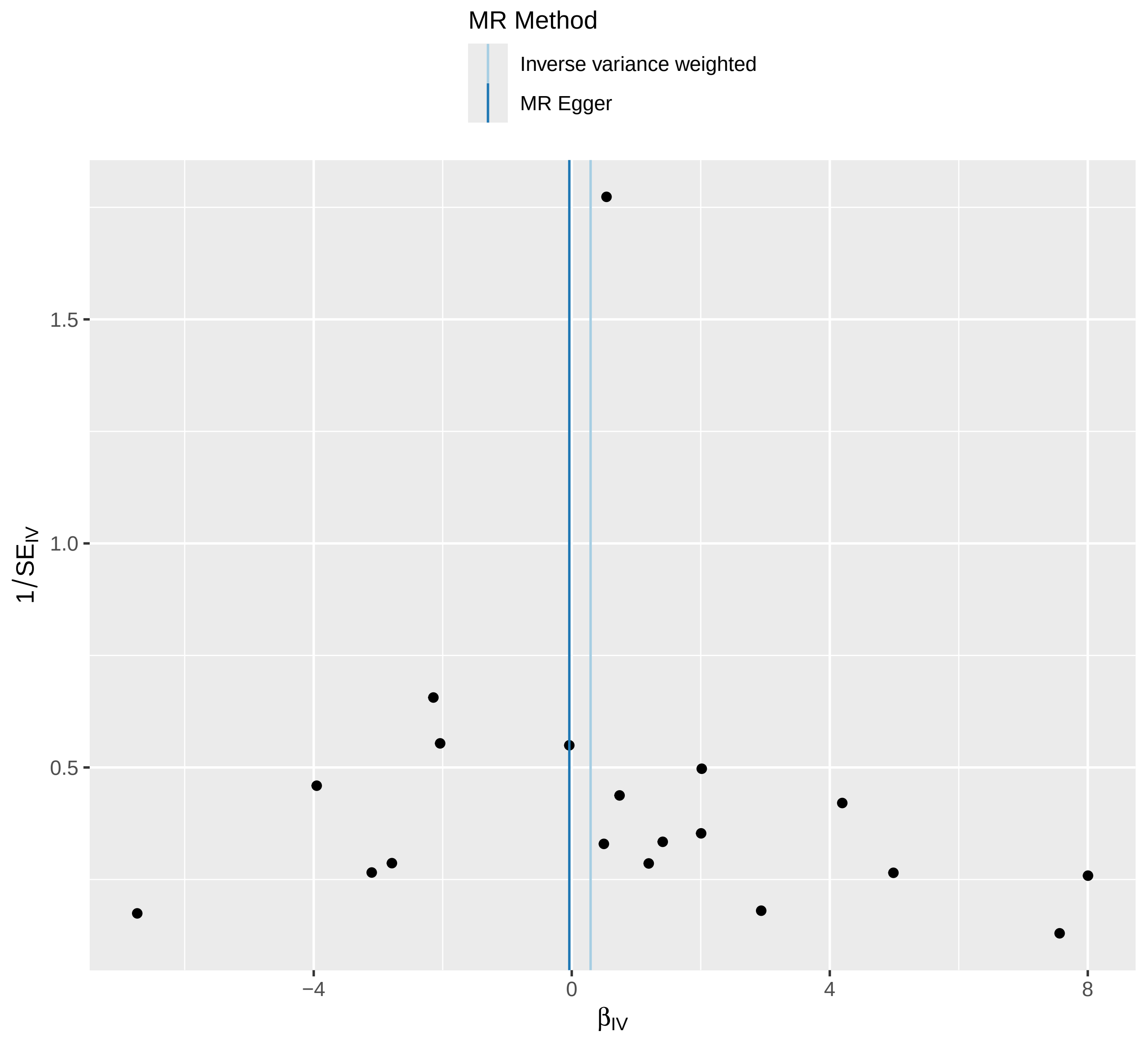
MR Method
Inverse variance weighted
MR Egger
1.5
1.0
0.5
1/SE_IV
−4
0
4
8
β_IV

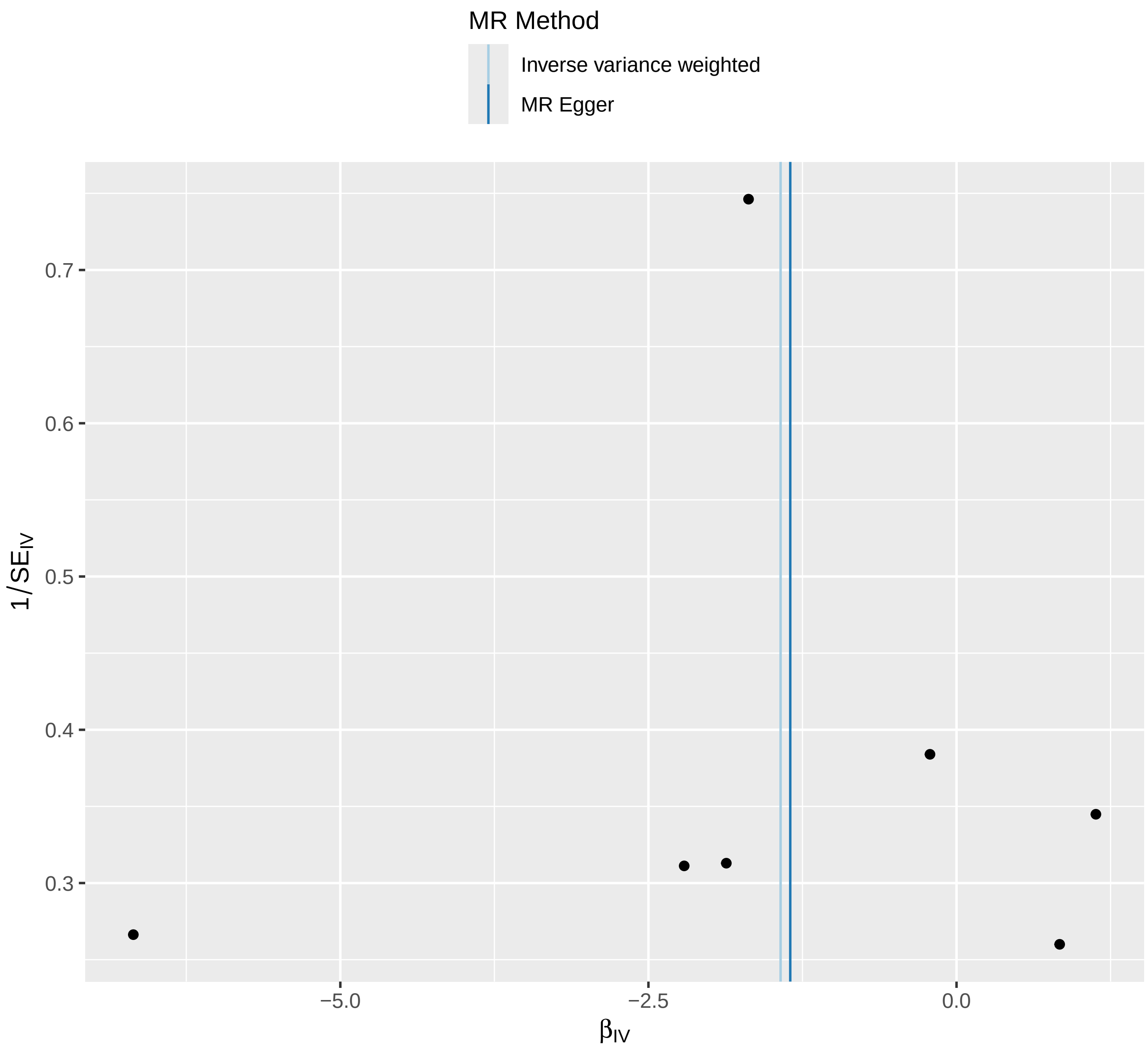
MR Method
Inverse variance weighted
MR Egger
0.7
0.6
0.5
0.4
0.3
1/SE$_{IV}$
−5.0
−2.5
0.0
β$_{IV}$

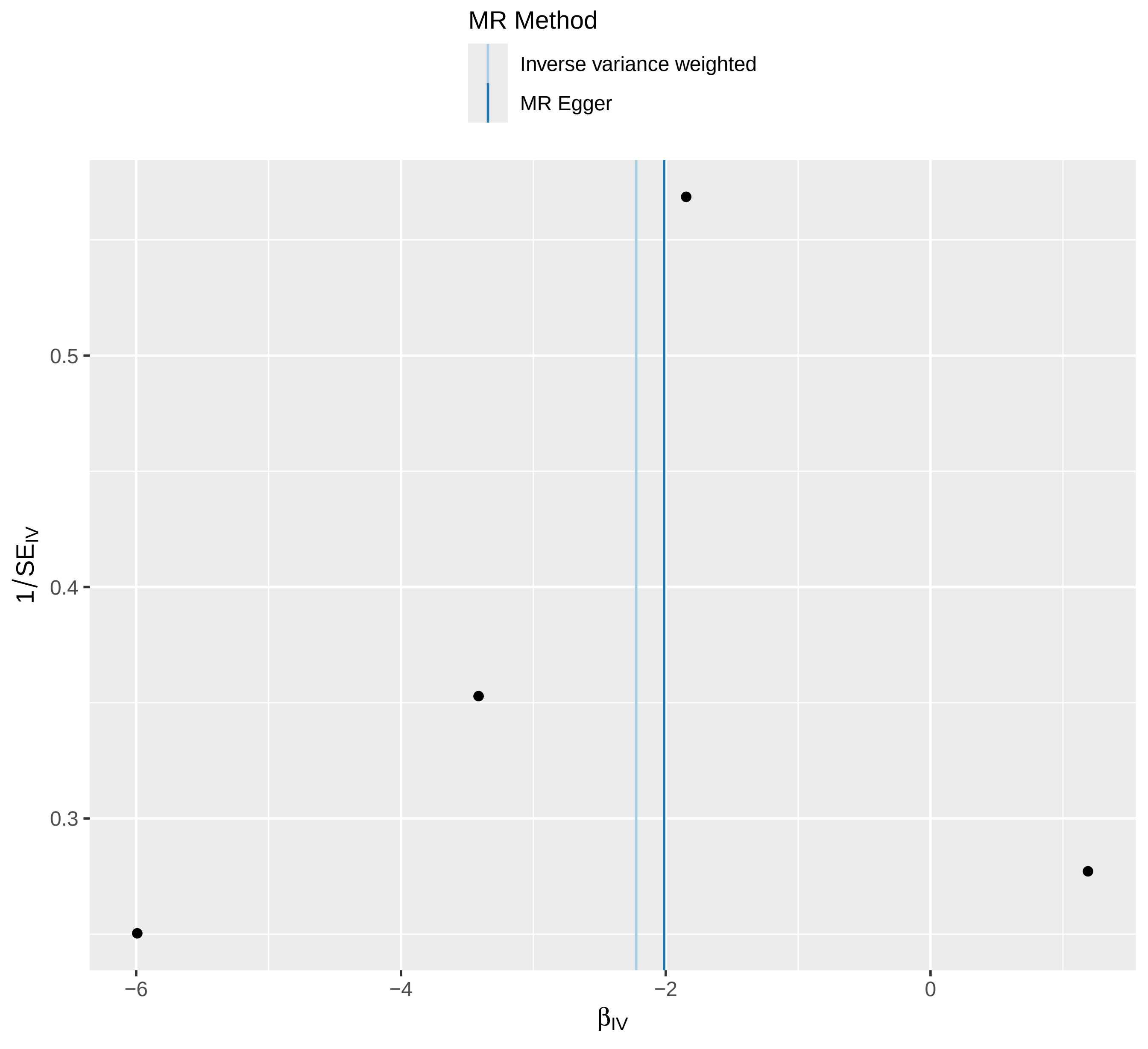

MR Method
Inverse variance weighted
MR Egger
0.5
0.4
0.3
1/SE_IV
−6
−4
−2
0
β_IV

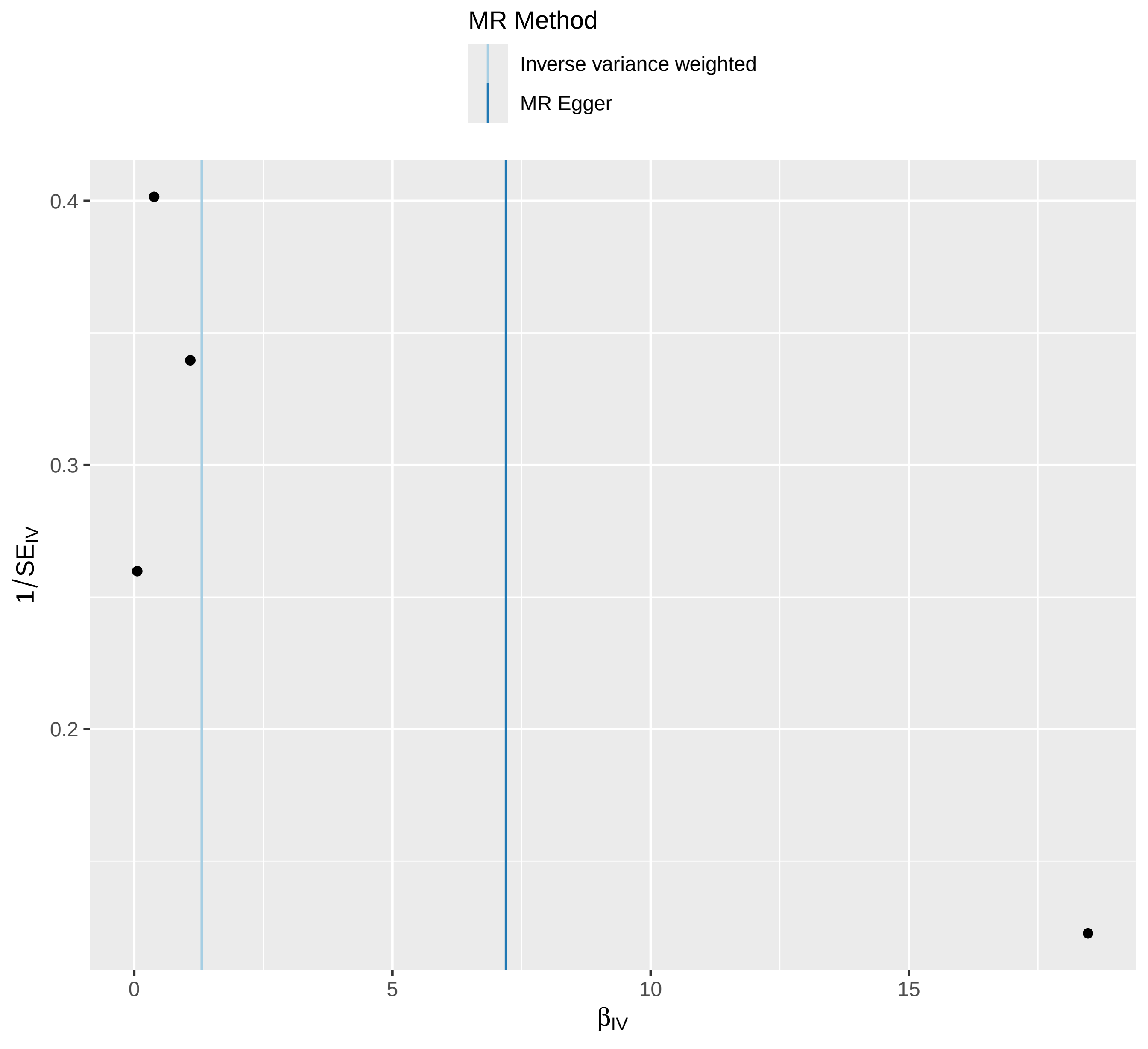

MR Method
Inverse variance weighted
MR Egger
0.4
0.3
0.2
0
5
10
15
1/SE_IV
β_IV

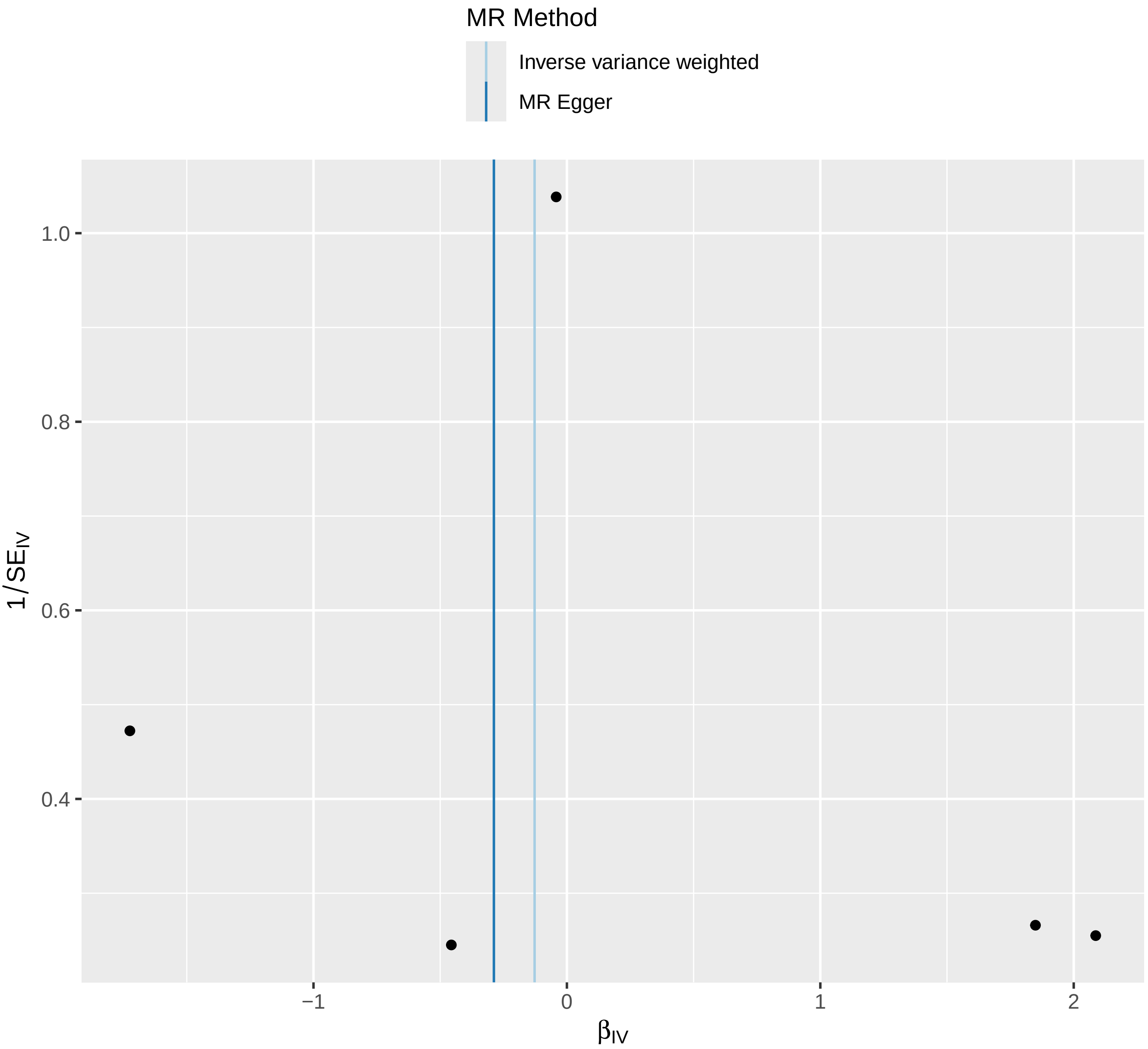
MR Method
Inverse variance weighted
MR Egger
1.0
0.8
0.6
0.4
−1
0
1
2
β_{IV}
$1/SE_{IV}$

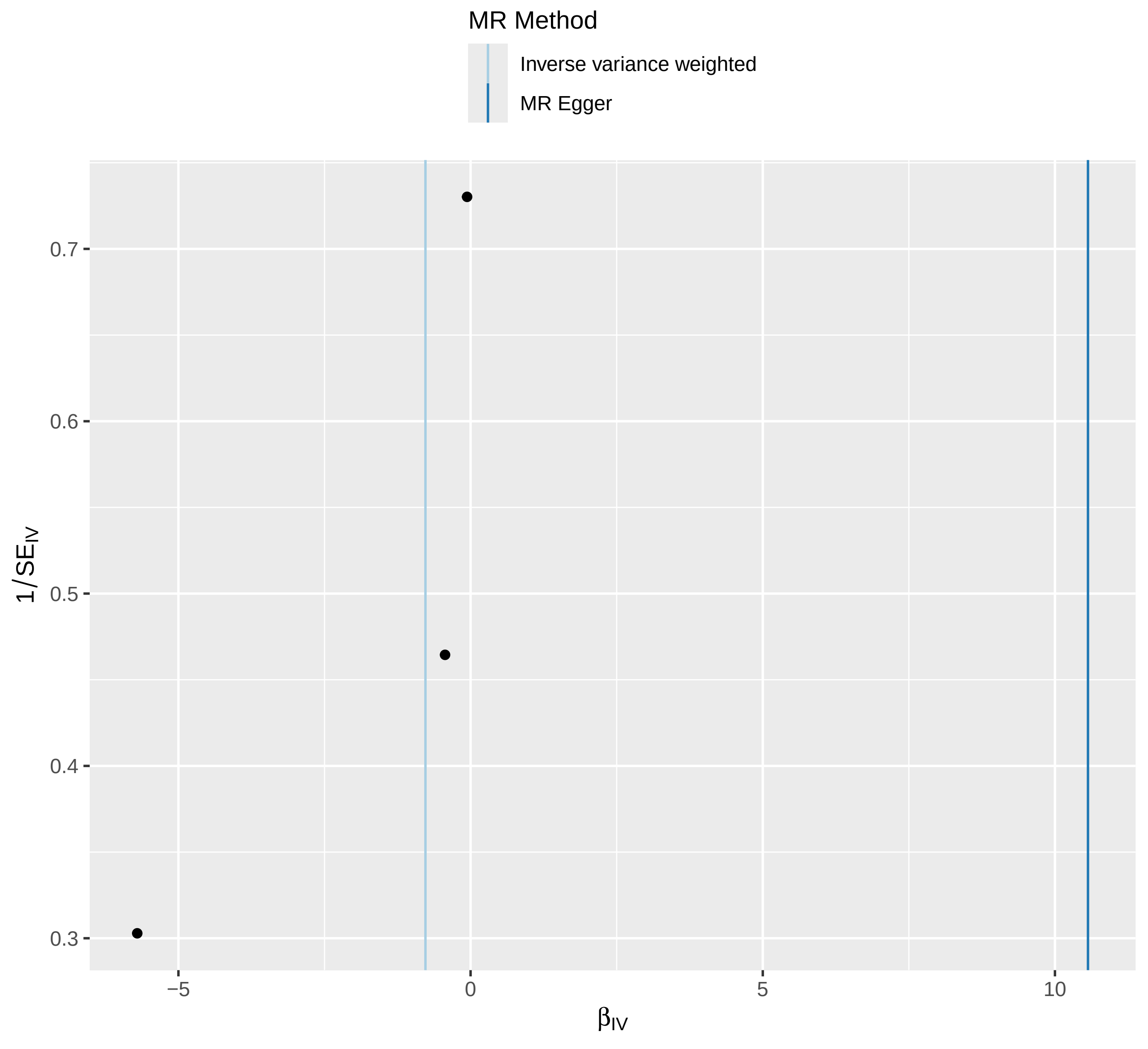
MR Method
Inverse variance weighted
MR Egger
0.7
0.6
0.5
0.4
0.3
1/SE_IV
−5
0
5
10
β_IV

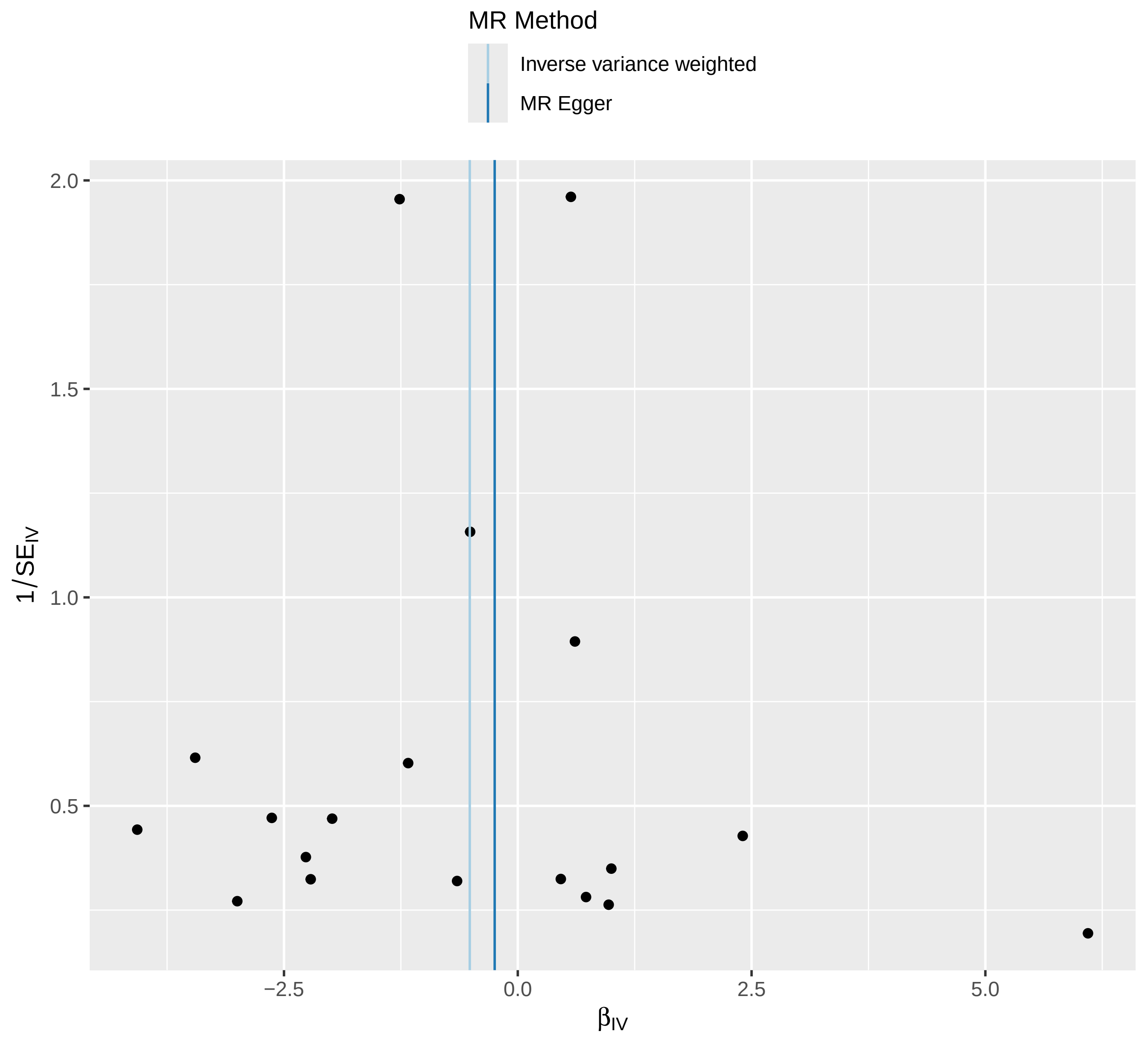
MR Method
Inverse variance weighted
MR Egger
2.0
1.5
1.0
0.5
1/SE$_{IV}$
−2.5
0.0
2.5
5.0
β$_{IV}$

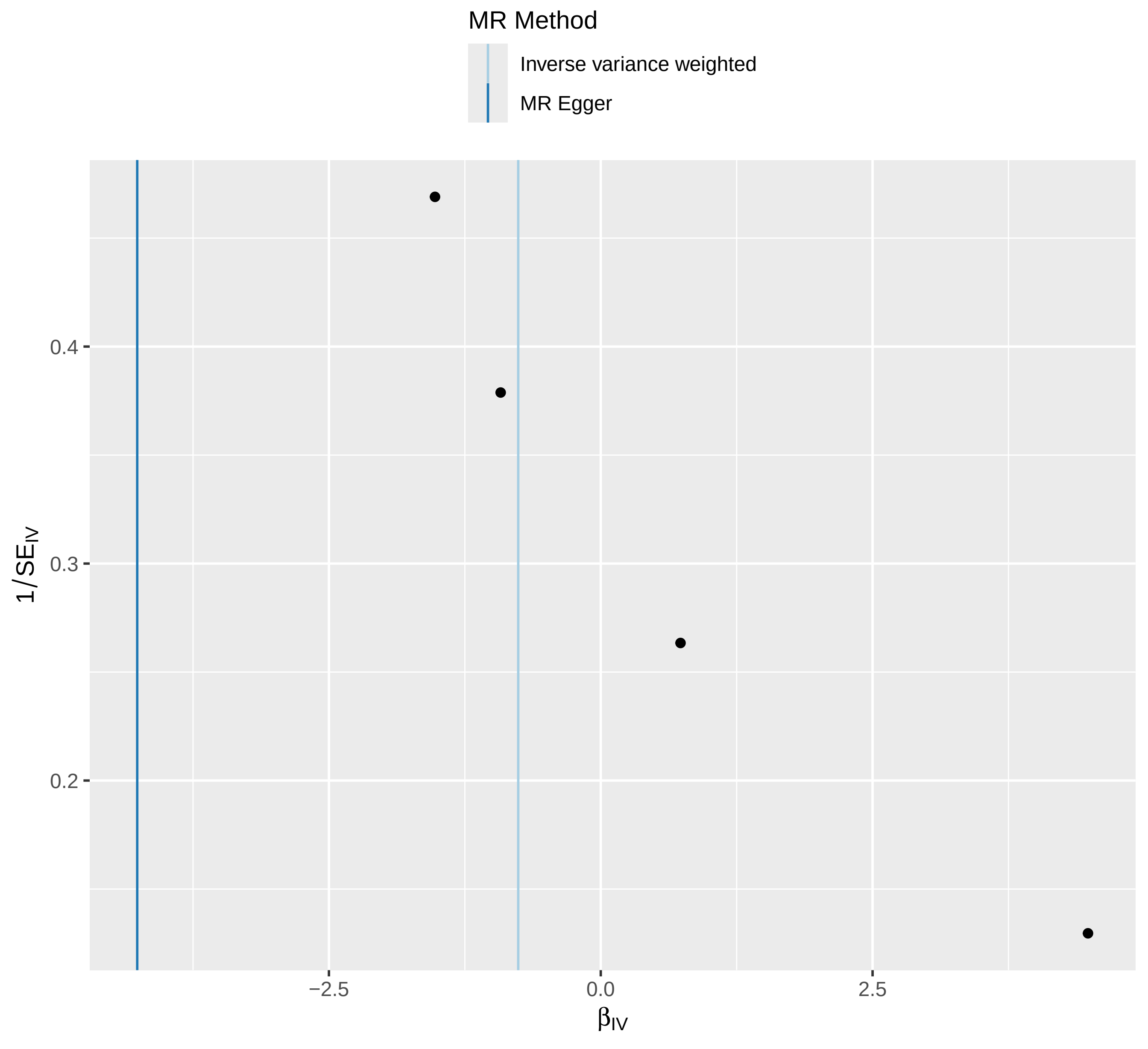
MR Method
Inverse variance weighted
MR Egger
0.4
0.3
0.2
$1/SE_{IV}$
−2.5
0.0
2.5
β_{IV}

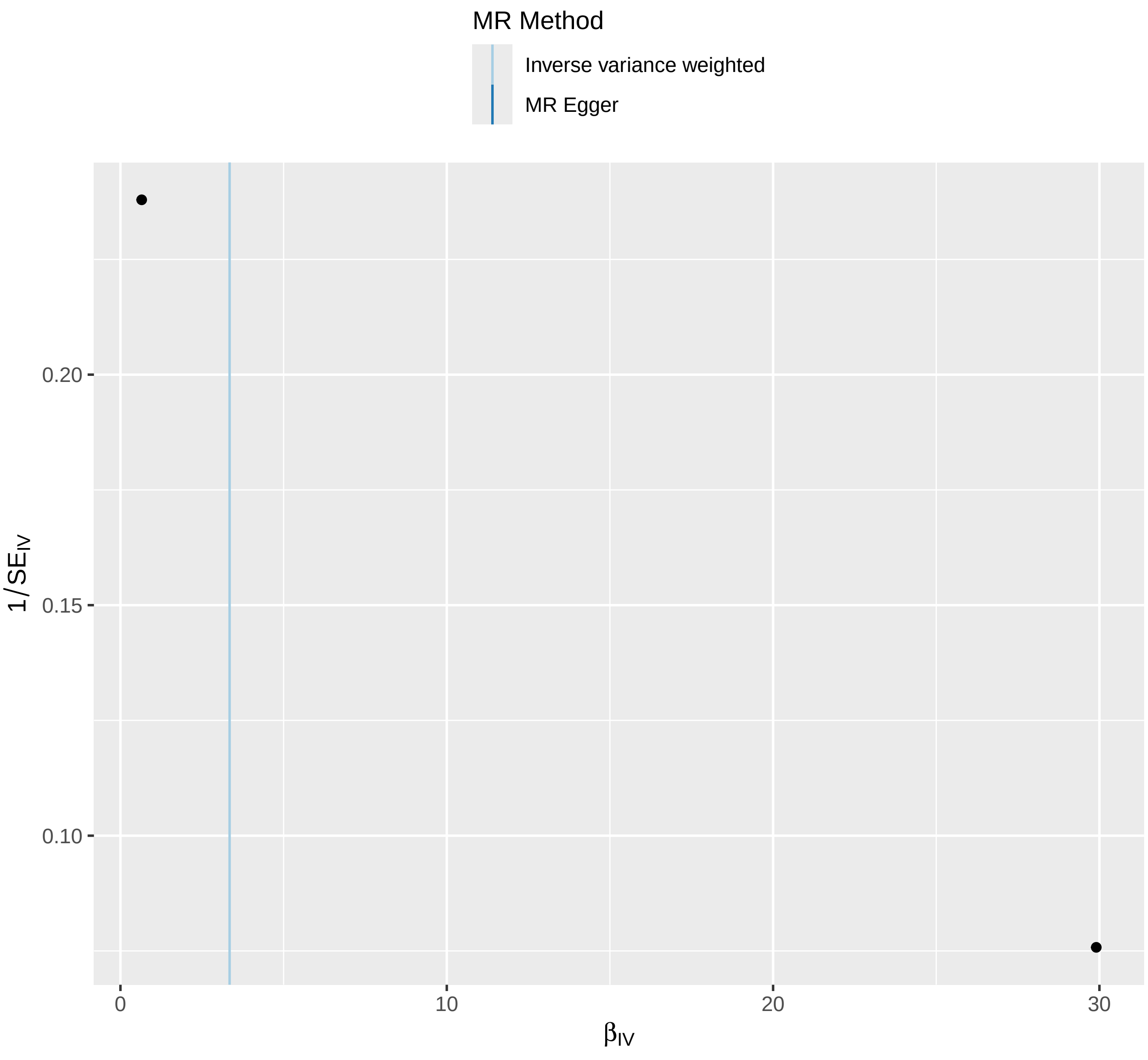
MR Method
Inverse variance weighted
MR Egger
0.20
0.15
0.10
0
10
20
30
$1/SE_{IV}$
β_{IV}

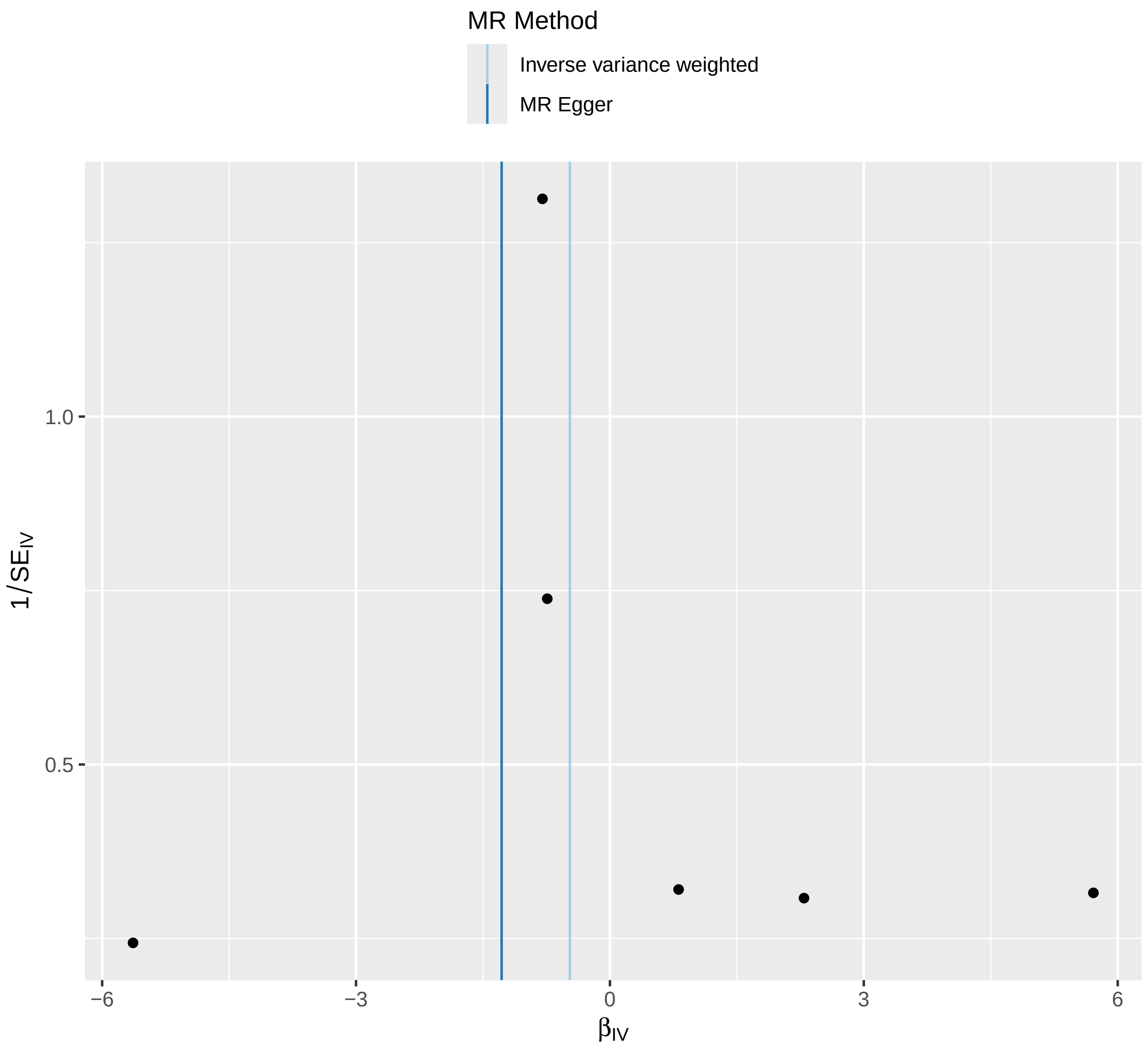
MR Method
Inverse variance weighted
MR Egger
1/SE_IV
1.0
0.5
−6
−3
0
3
6
β_IV

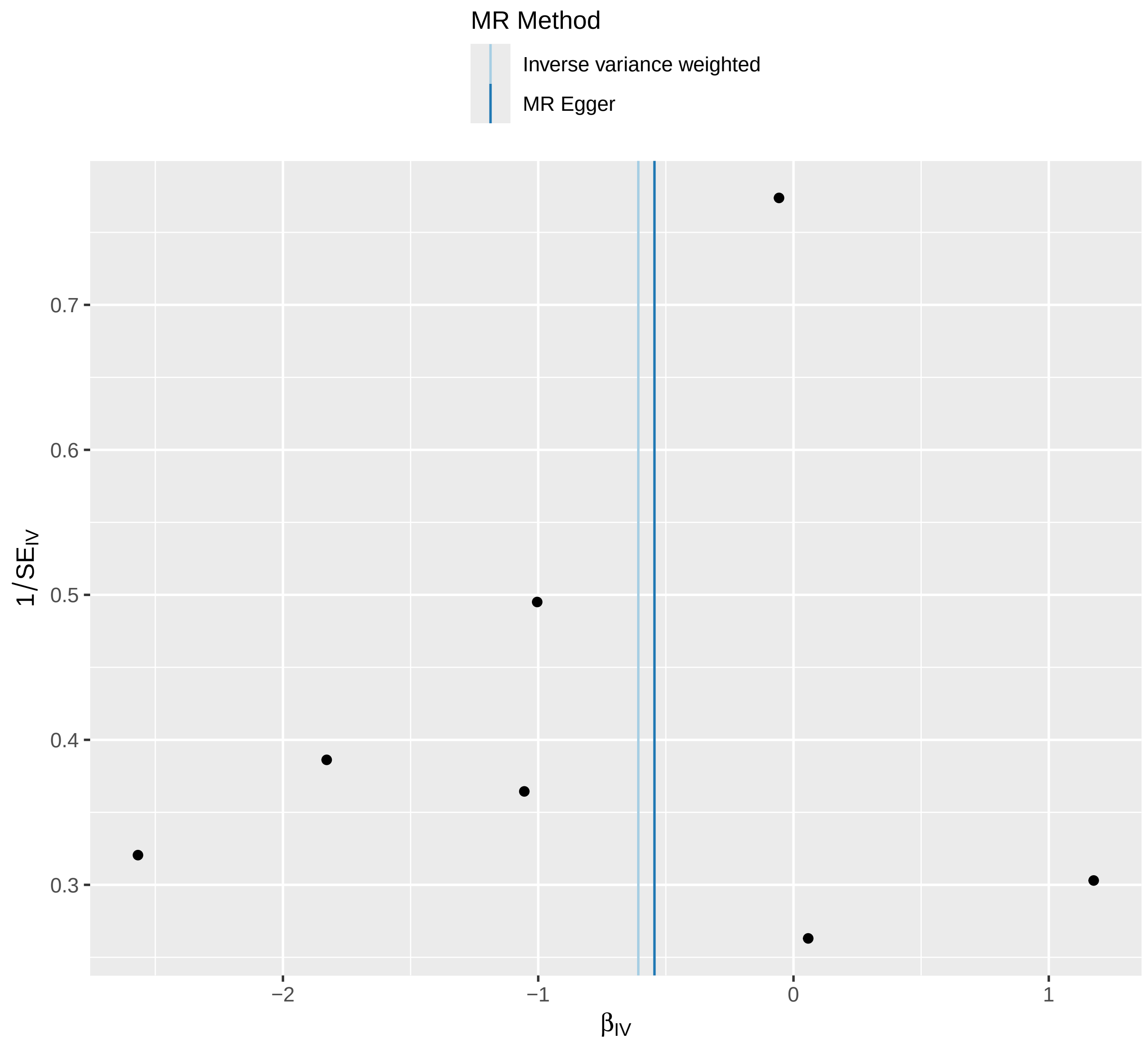
MR Method
Inverse variance weighted
MR Egger
0.7
0.6
0.5
0.4
0.3
1/SE$_{IV}$
−2
−1
0
1
β$_{IV}$

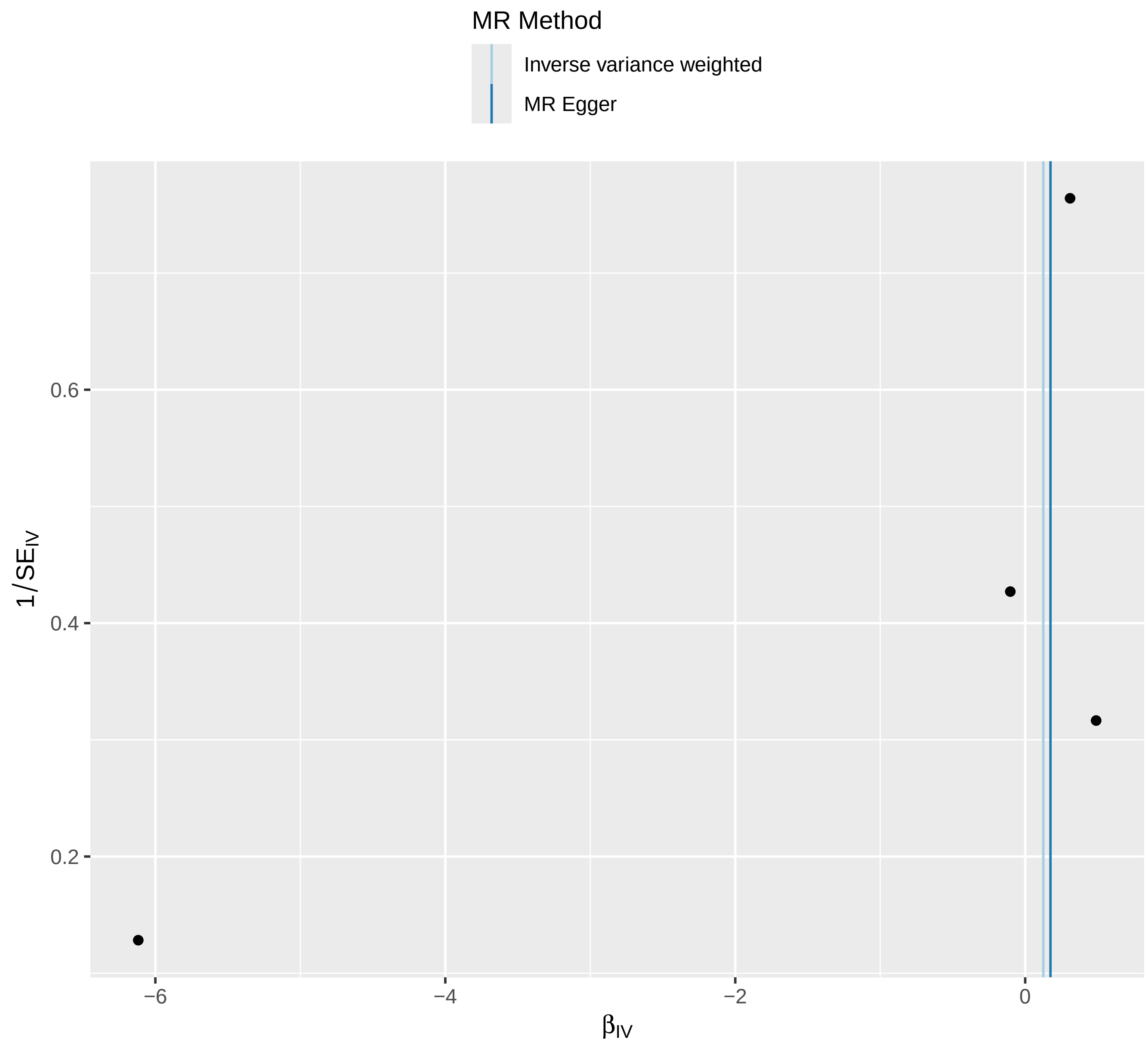
MR Method
Inverse variance weighted
MR Egger
0.6
0.4
0.2
1/SE_IV
−6
−4
−2
0
β_IV

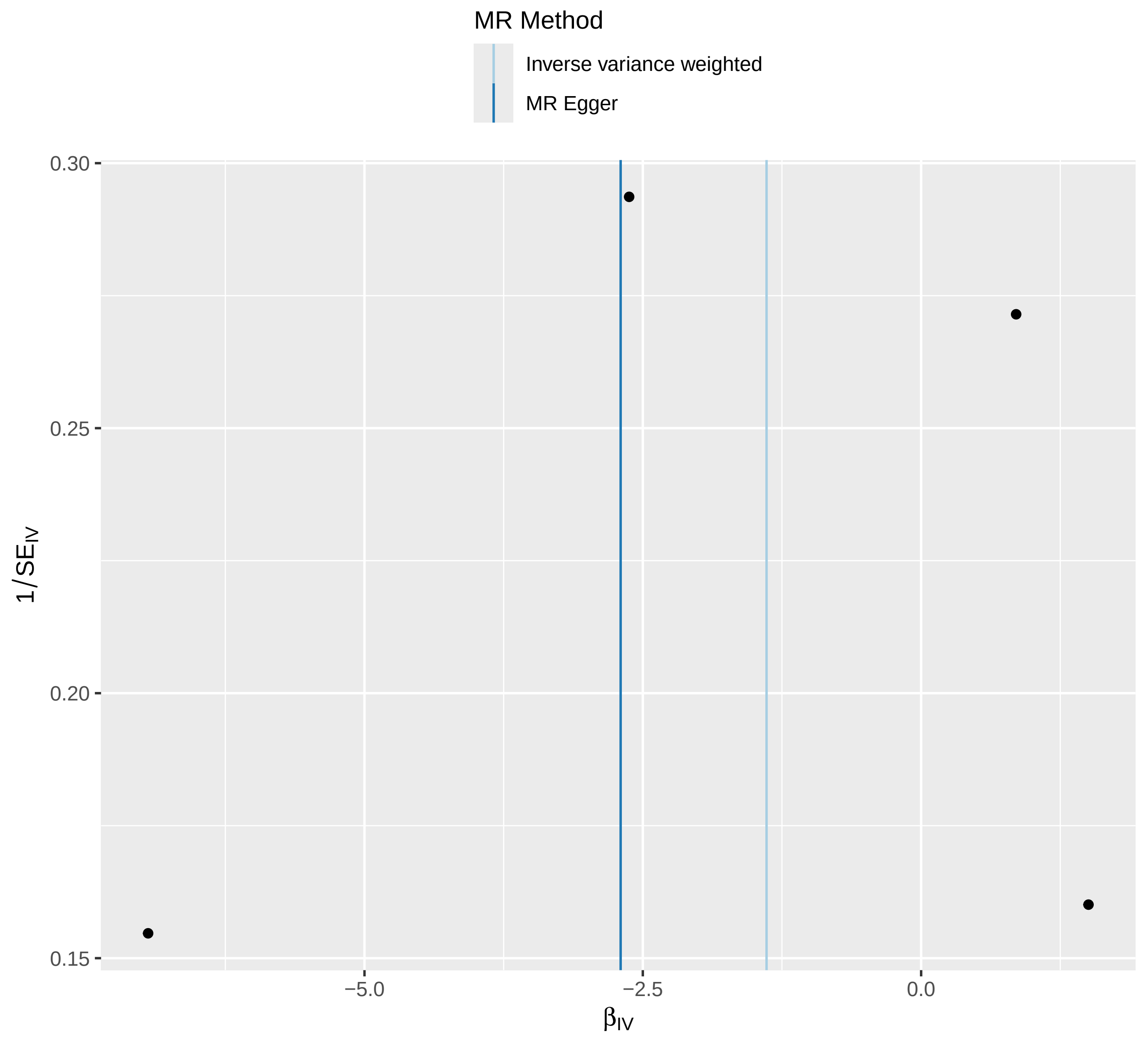
MR Method
Inverse variance weighted
MR Egger
0.30
0.25
0.20
0.15
−5.0
−2.5
0.0
$1/SE_{IV}$
β_{IV}

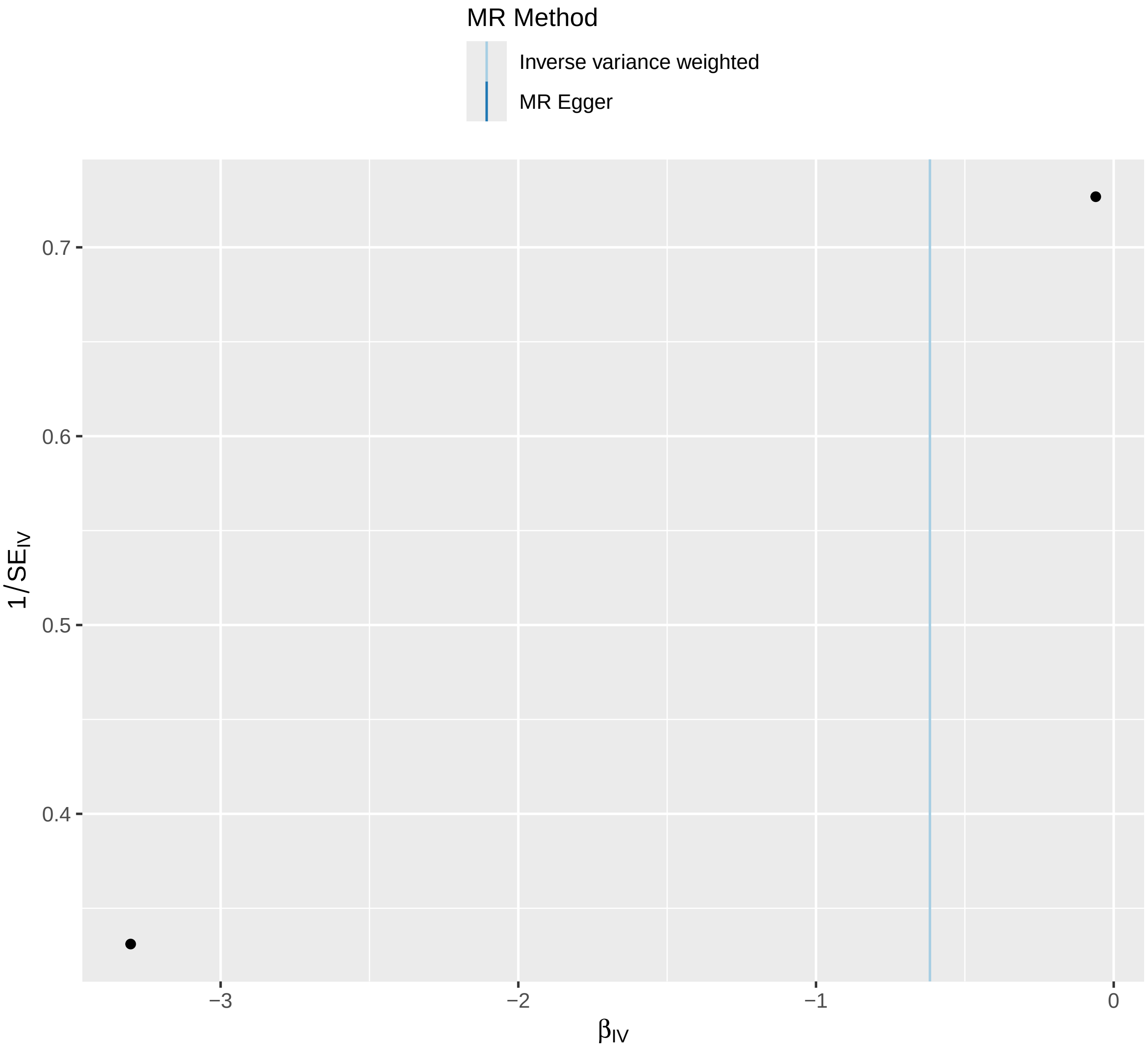
MR Method
Inverse variance weighted
MR Egger
0.7
0.6
0.5
0.4
1/SE_IV
−3
−2
−1
0
β_IV

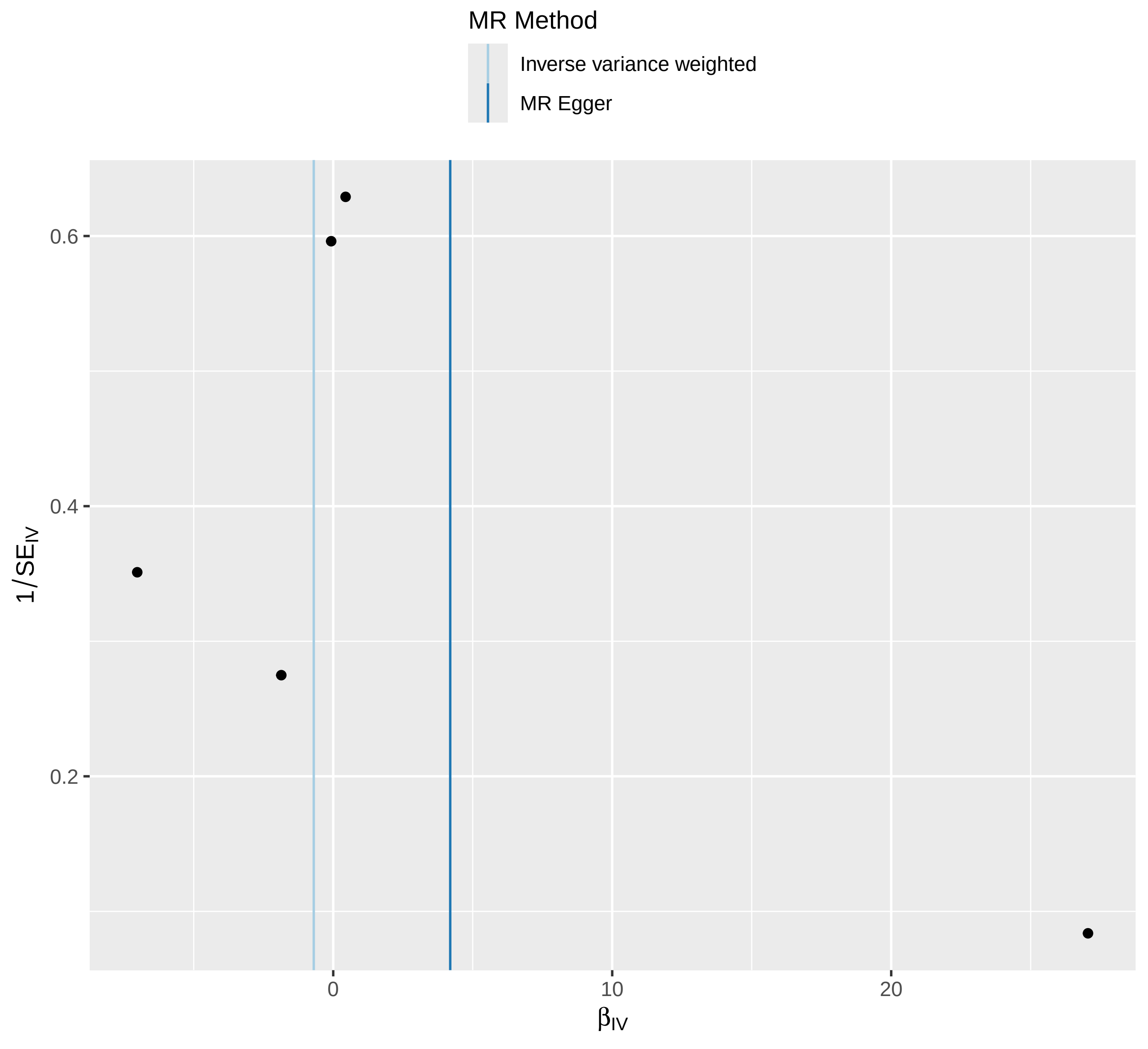
MR Method
Inverse variance weighted
MR Egger
0.6
0.4
0.2
$1/SE_{IV}$
0
10
20
β_{IV}

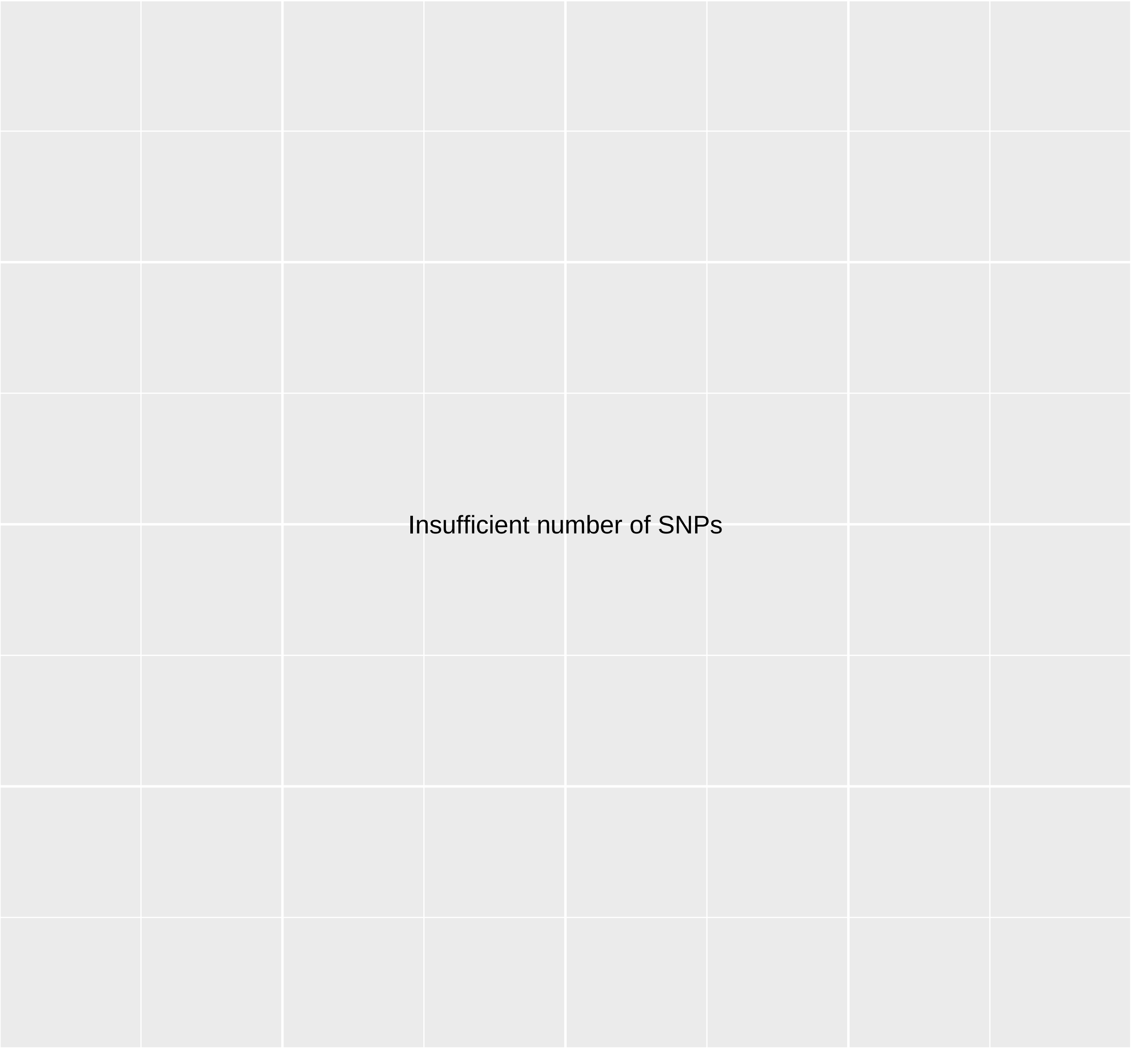
Insufficient number of SNPs

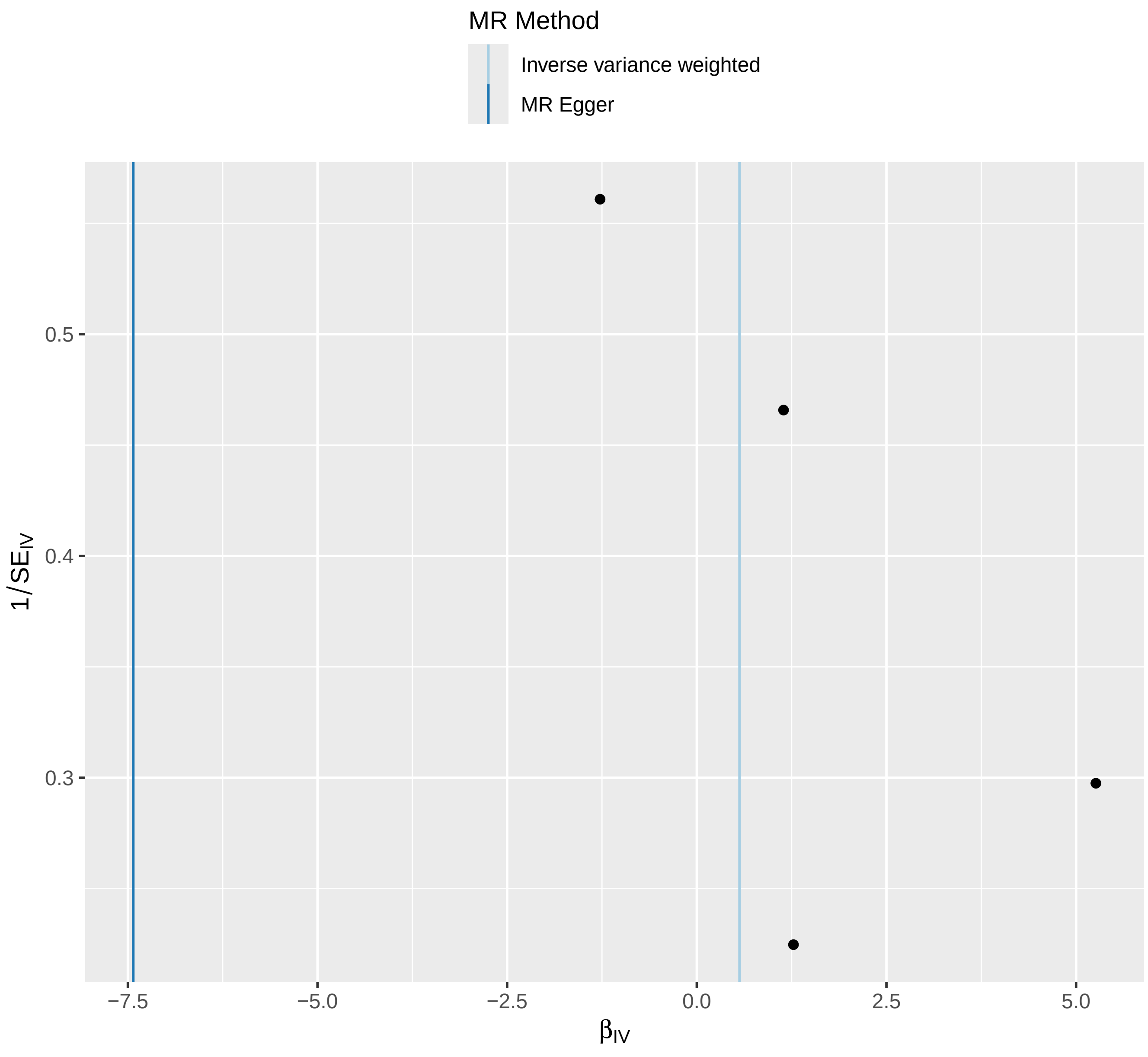
MR Method
Inverse variance weighted
MR Egger
0.5
0.4
0.3
1/SE_IV
−7.5
−5.0
−2.5
0.0
2.5
5.0
β_IV

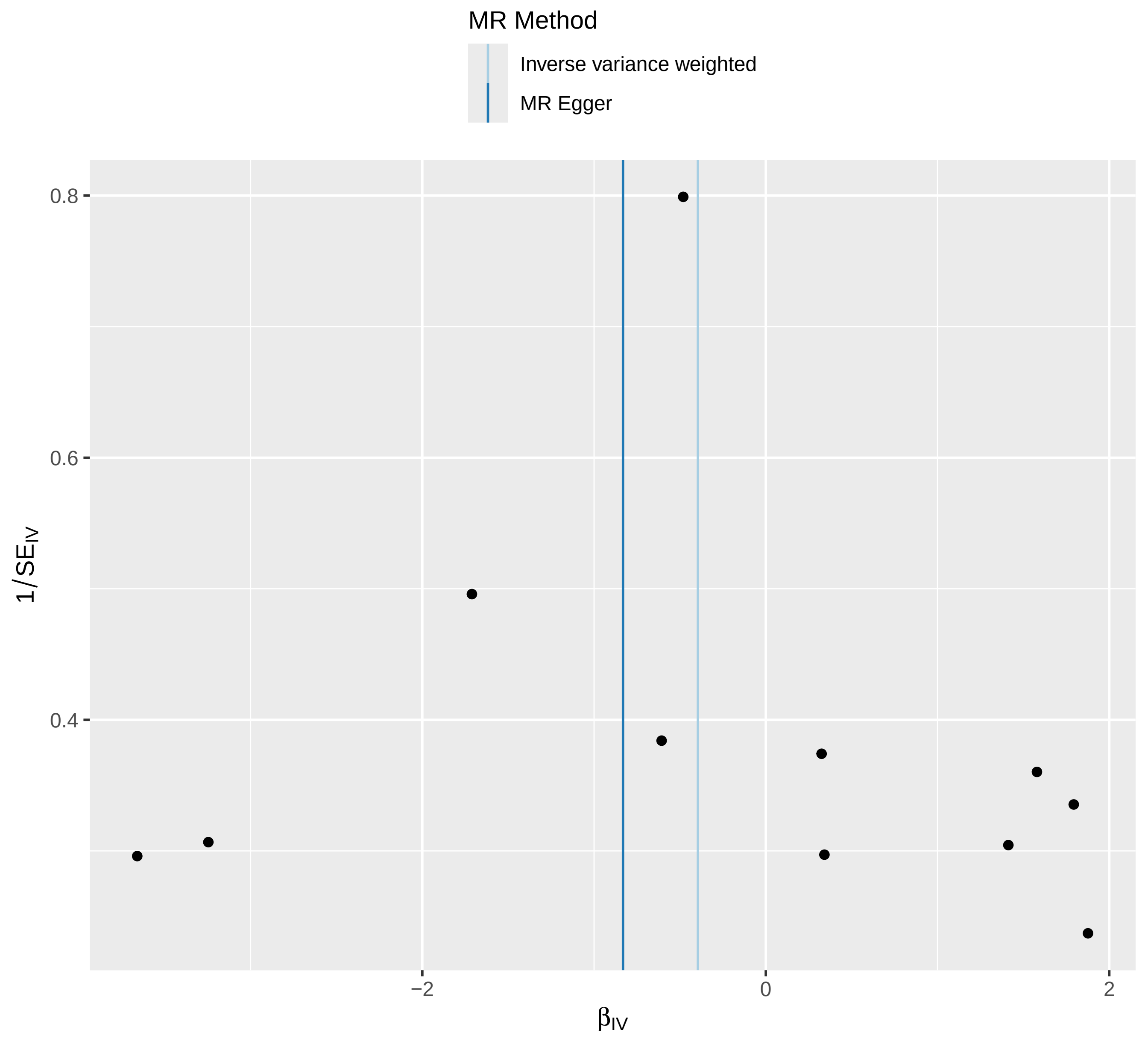

MR Method
Inverse variance weighted
MR Egger
0.8
0.6
0.4
1/SE_IV
−2
0
2
β_IV

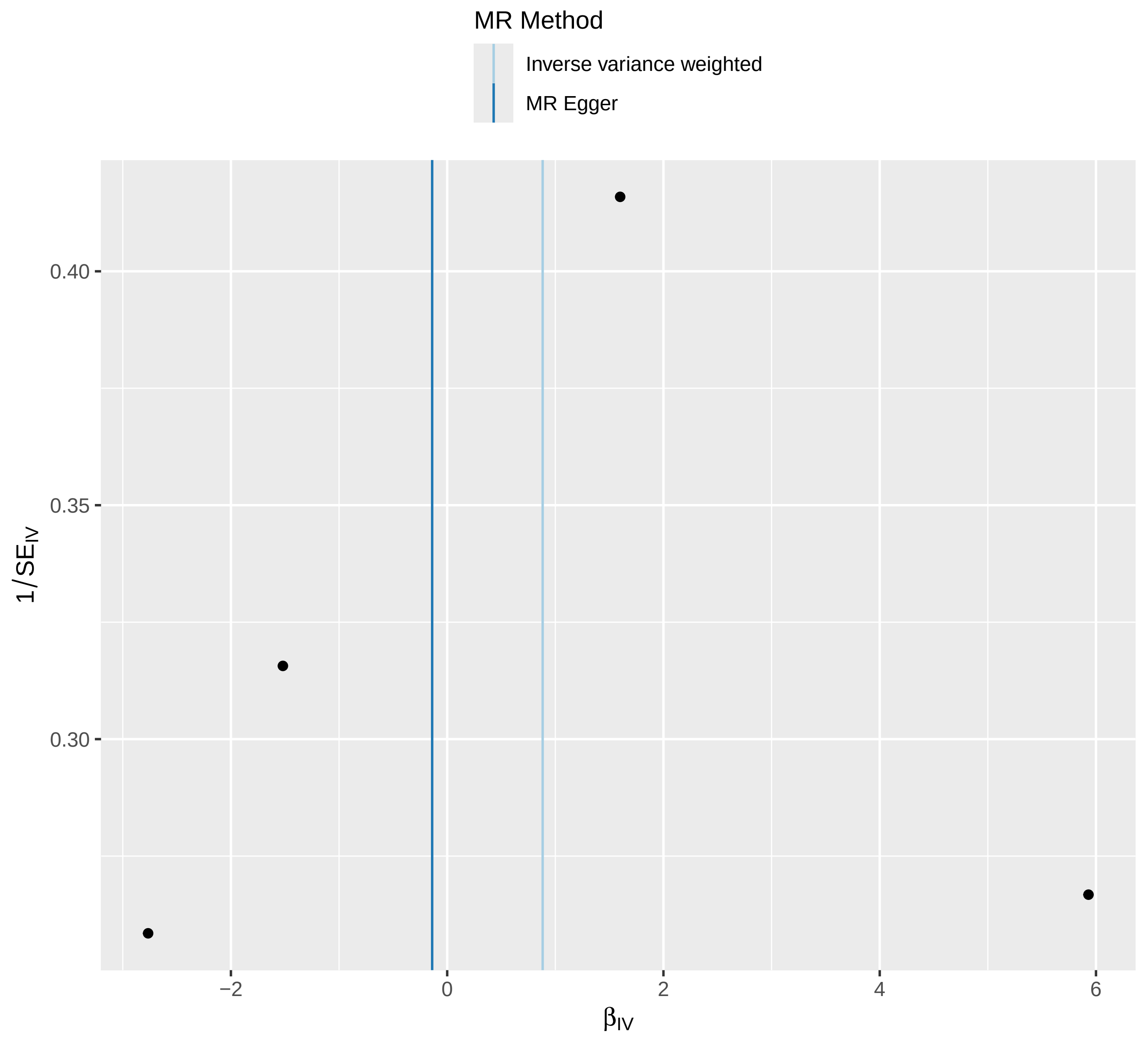

MR Method
Inverse variance weighted
MR Egger
0.40
0.35
0.30
1/SE_IV
−2
0
2
4
6
β_IV

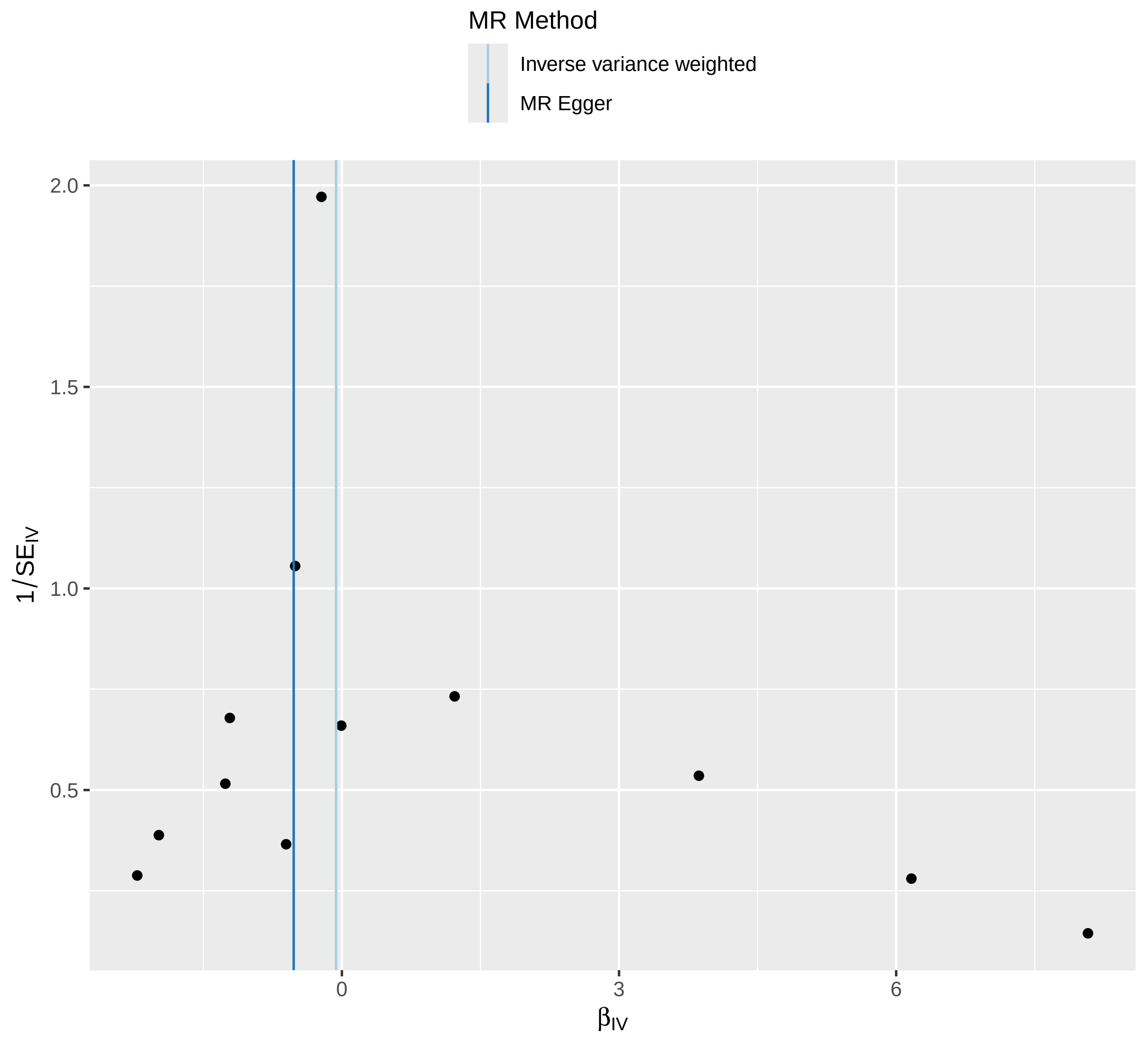
MR Method
Inverse variance weighted
MR Egger
2.0
1.5
1.0
0.5
0
3
6
$1/SE_{IV}$
β_{IV}

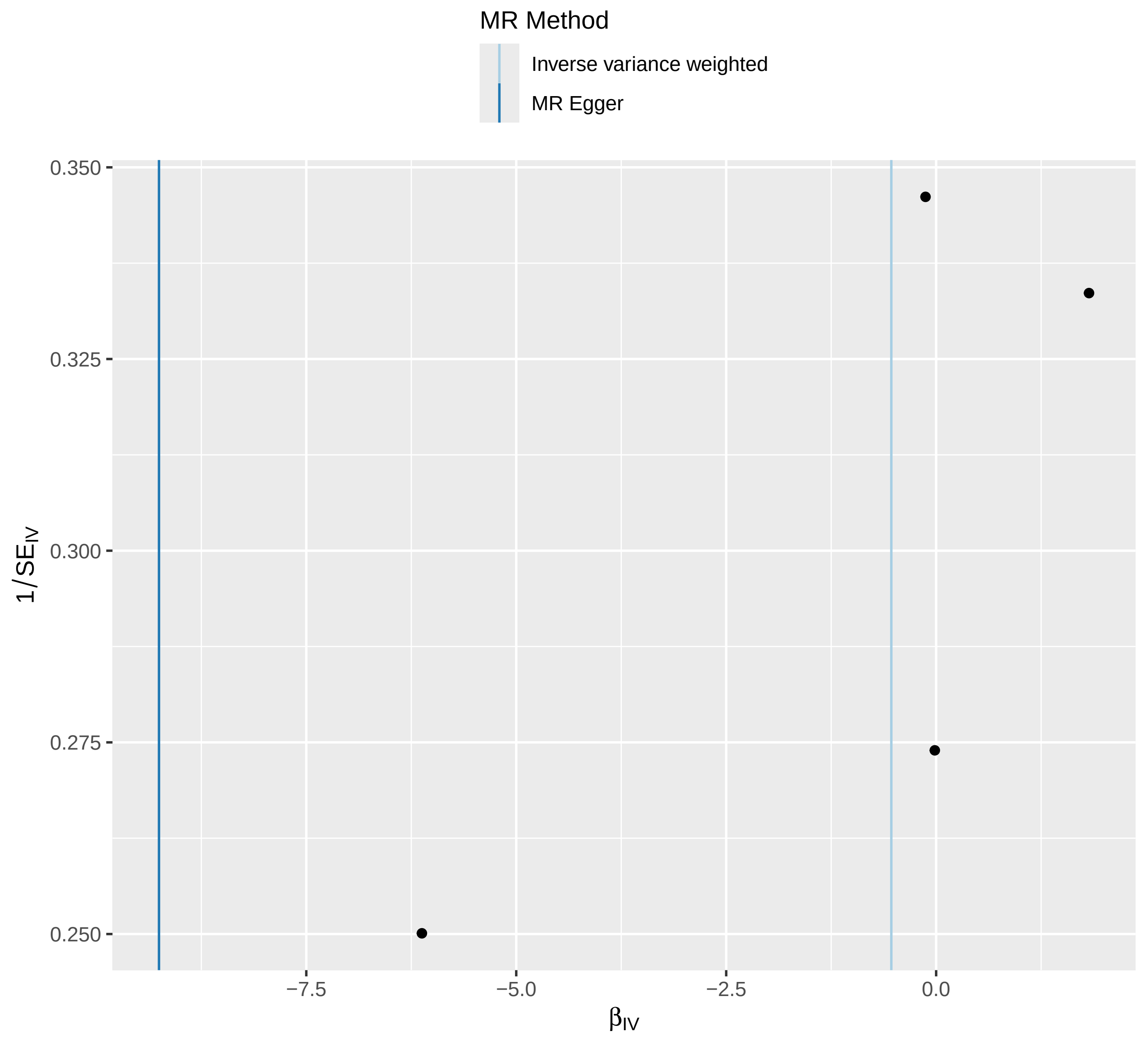
MR Method
Inverse variance weighted
MR Egger
0.350
0.325
0.300
0.275
0.250
1/SE_IV
−7.5
−5.0
−2.5
0.0
β_IV

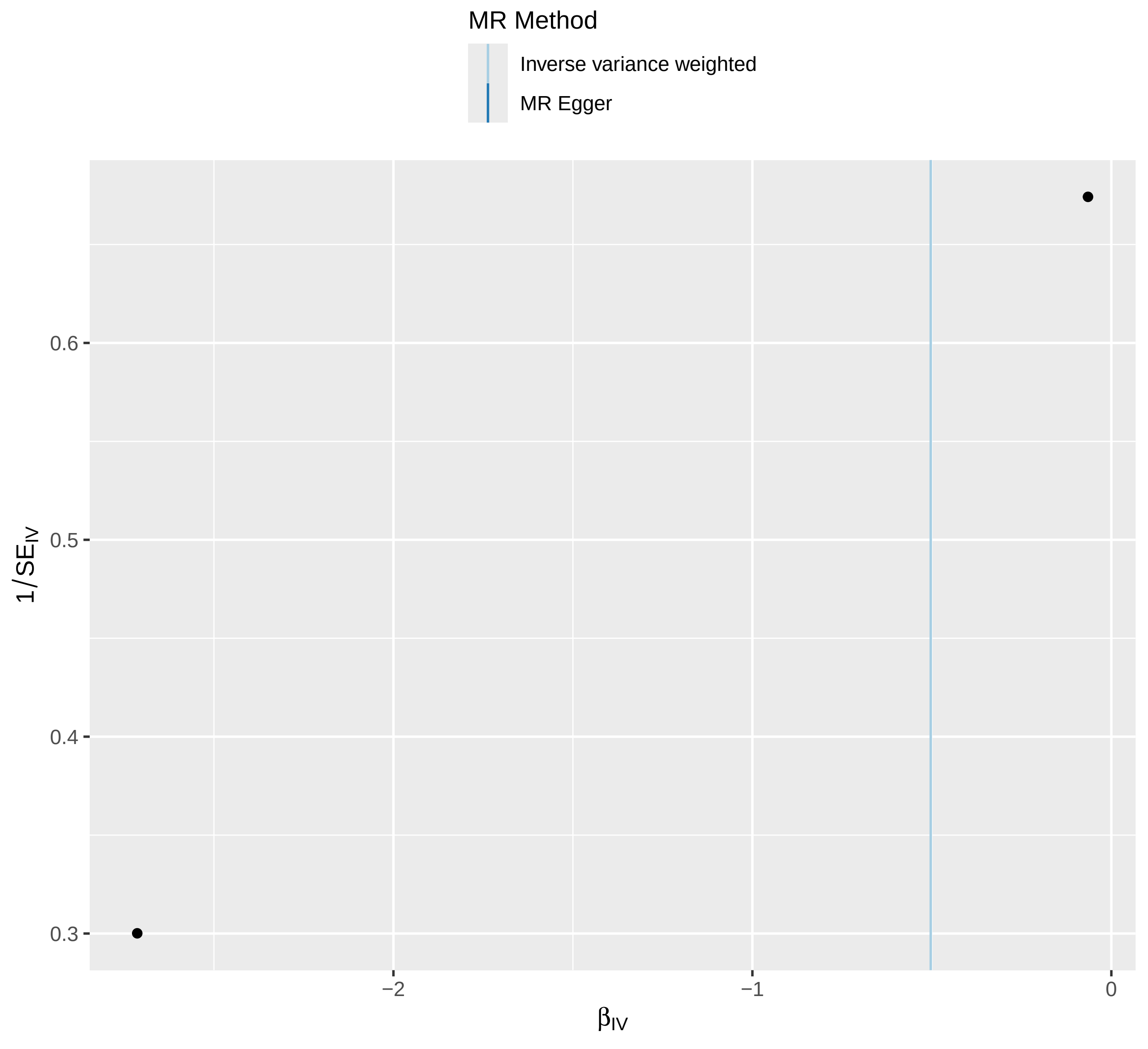
MR Method
Inverse variance weighted
MR Egger
0.6
0.5
0.4
0.3
1/SE$_{IV}$
−2
−1
0
β$_{IV}$

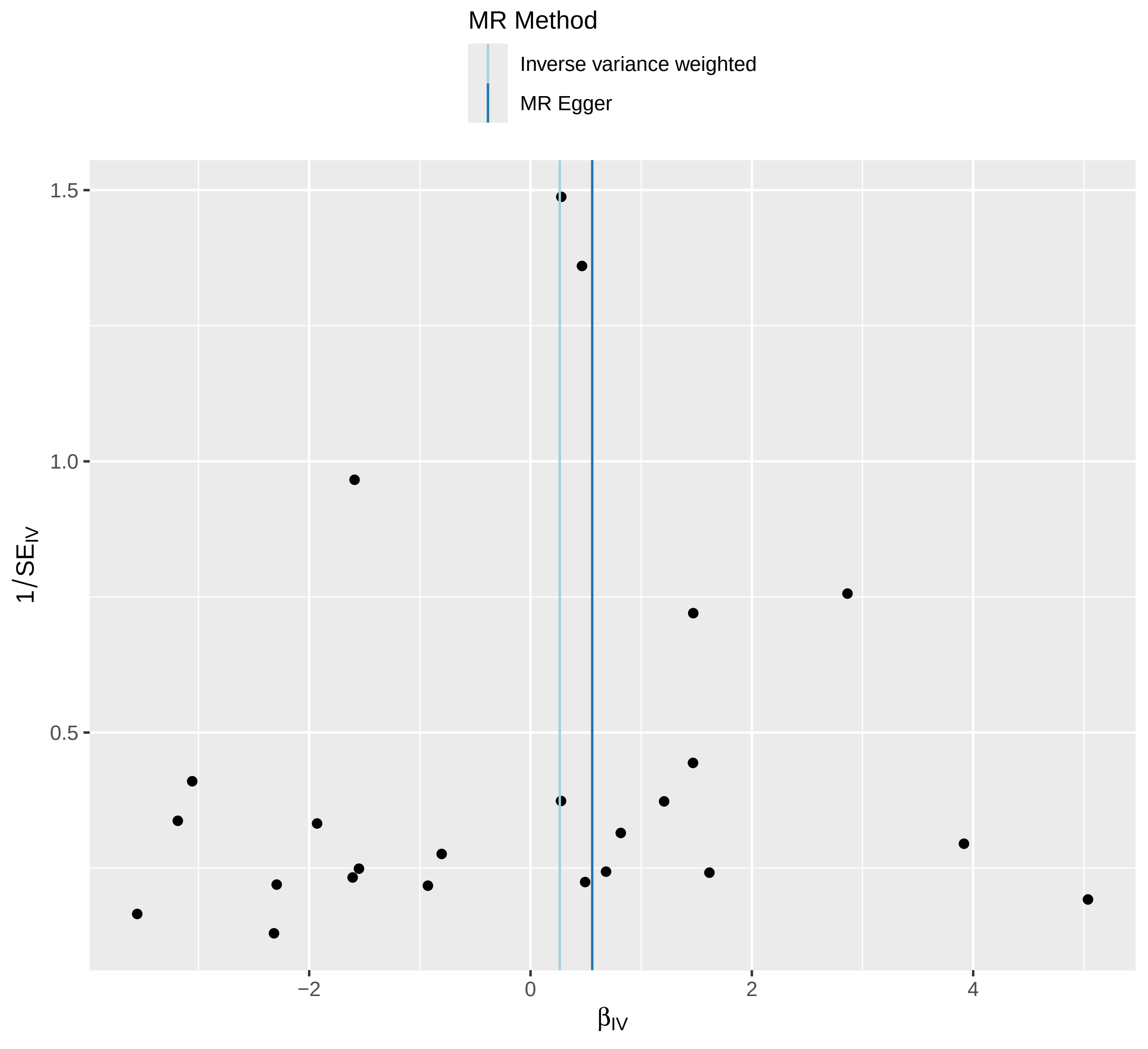

MR Method
Inverse variance weighted
MR Egger
1.5
1.0
0.5
1/SE_IV
−2
0
2
4
β_IV

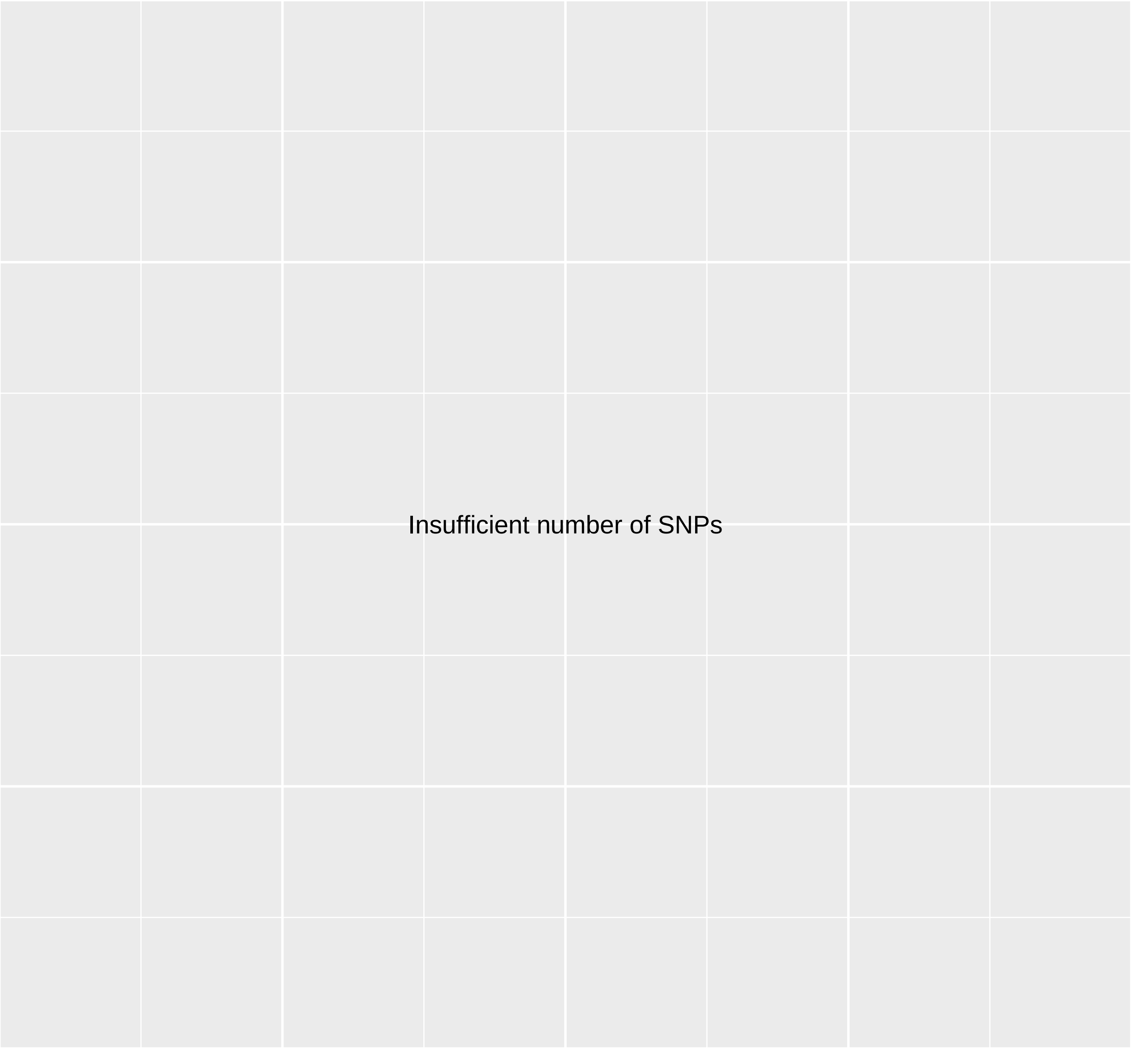
Insufficient number of SNPs

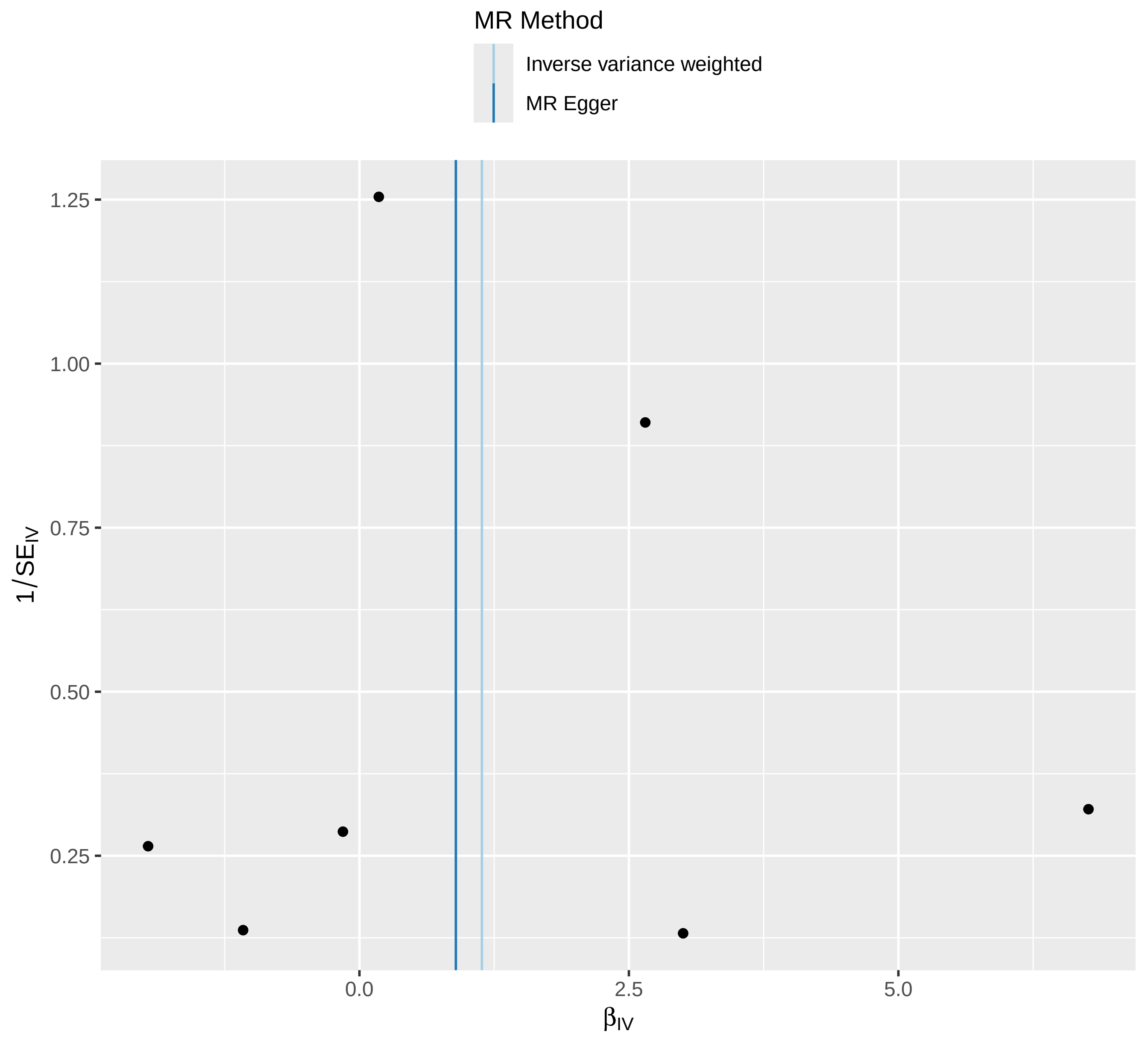
MR Method
Inverse variance weighted
MR Egger
1.25
1.00
0.75
0.50
0.25
1/SE_IV
0.0
2.5
5.0
β_IV

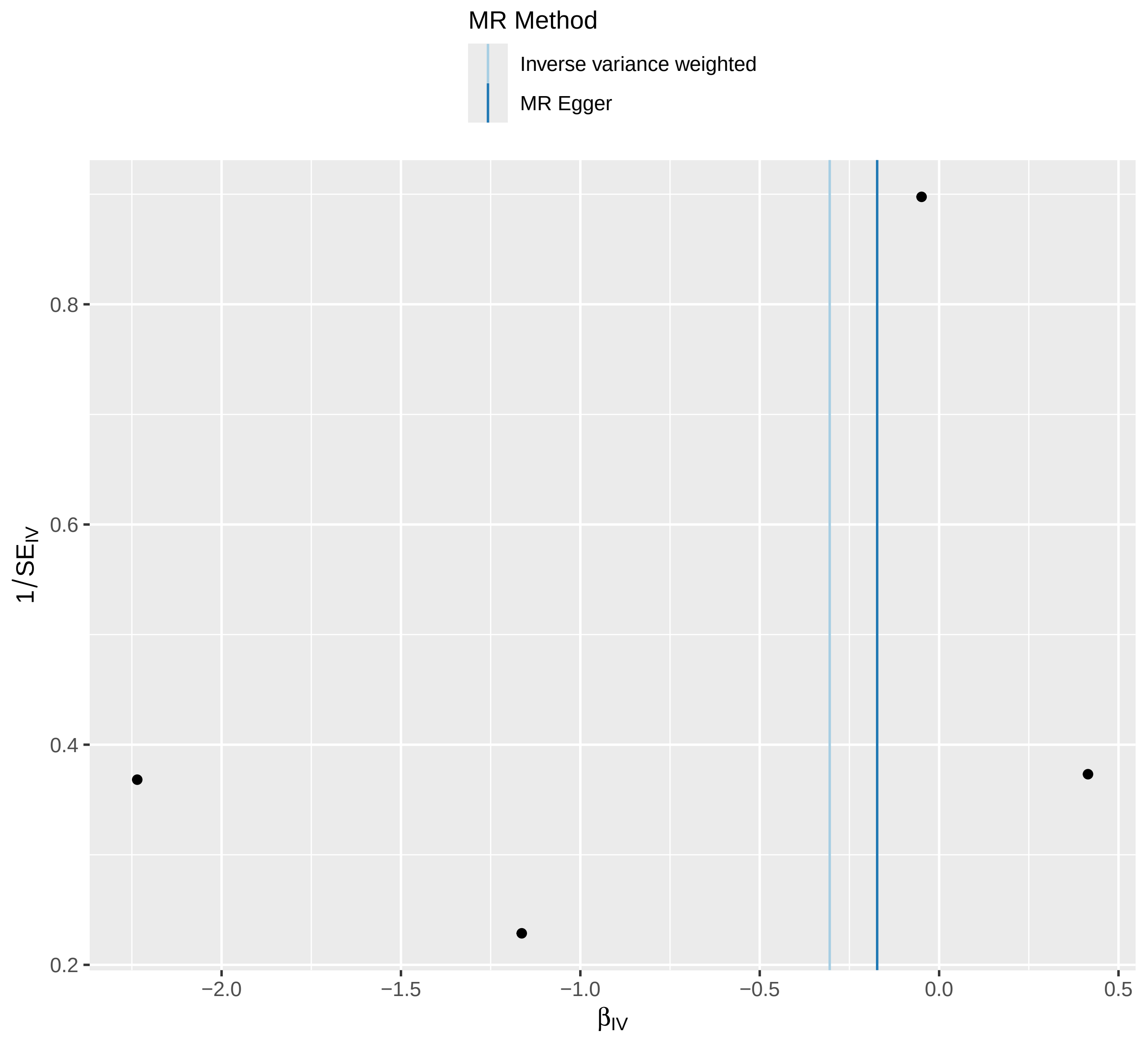

MR Method
Inverse variance weighted
MR Egger
0.8
0.6
0.4
0.2
1/SE$_{IV}$
−2.0
−1.5
−1.0
−0.5
0.0
0.5
β$_{IV}$

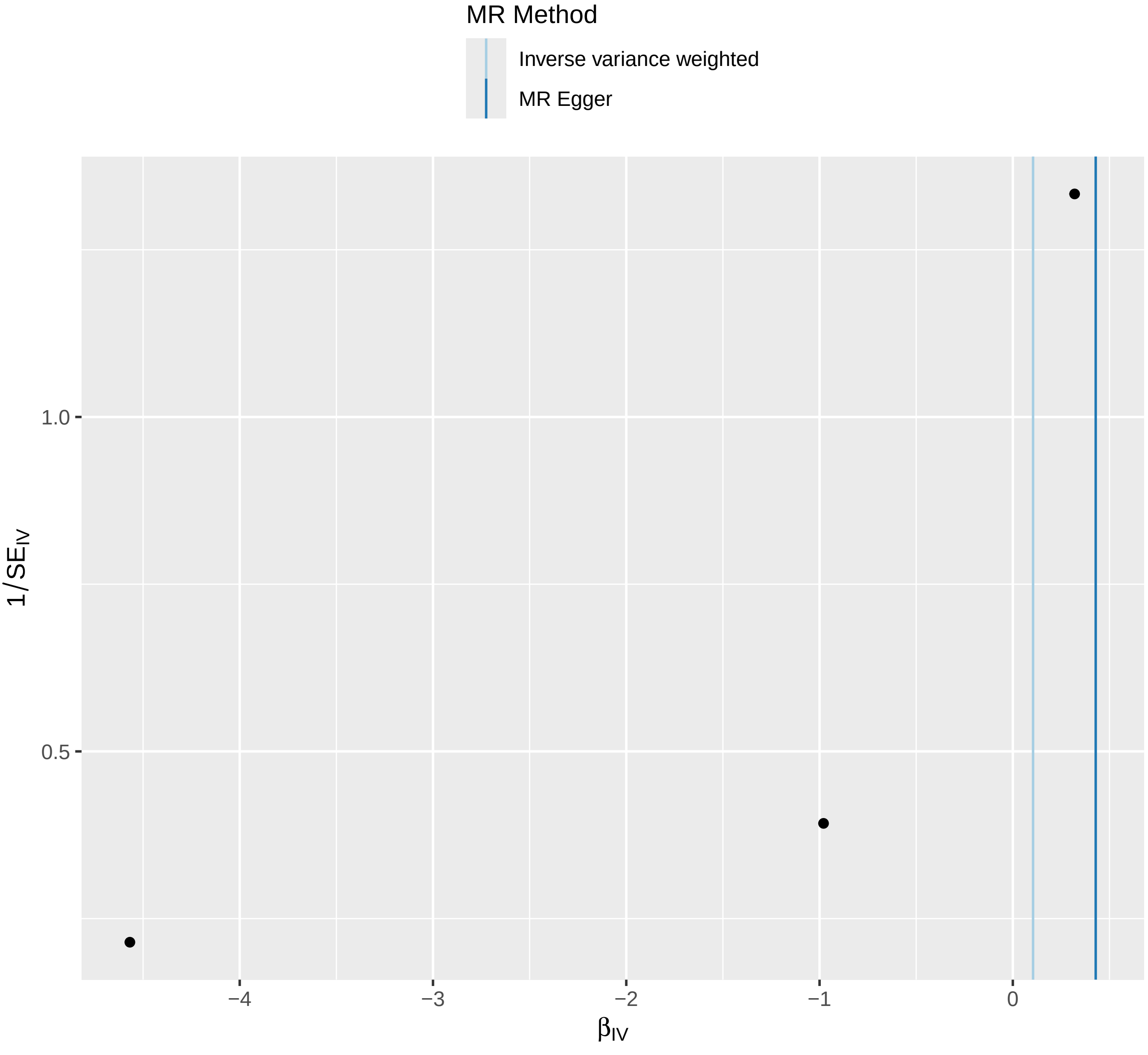

MR Method
Inverse variance weighted
MR Egger
1.0
0.5
1/SE$_{IV}$
−4
−3
−2
−1
0
β$_{IV}$

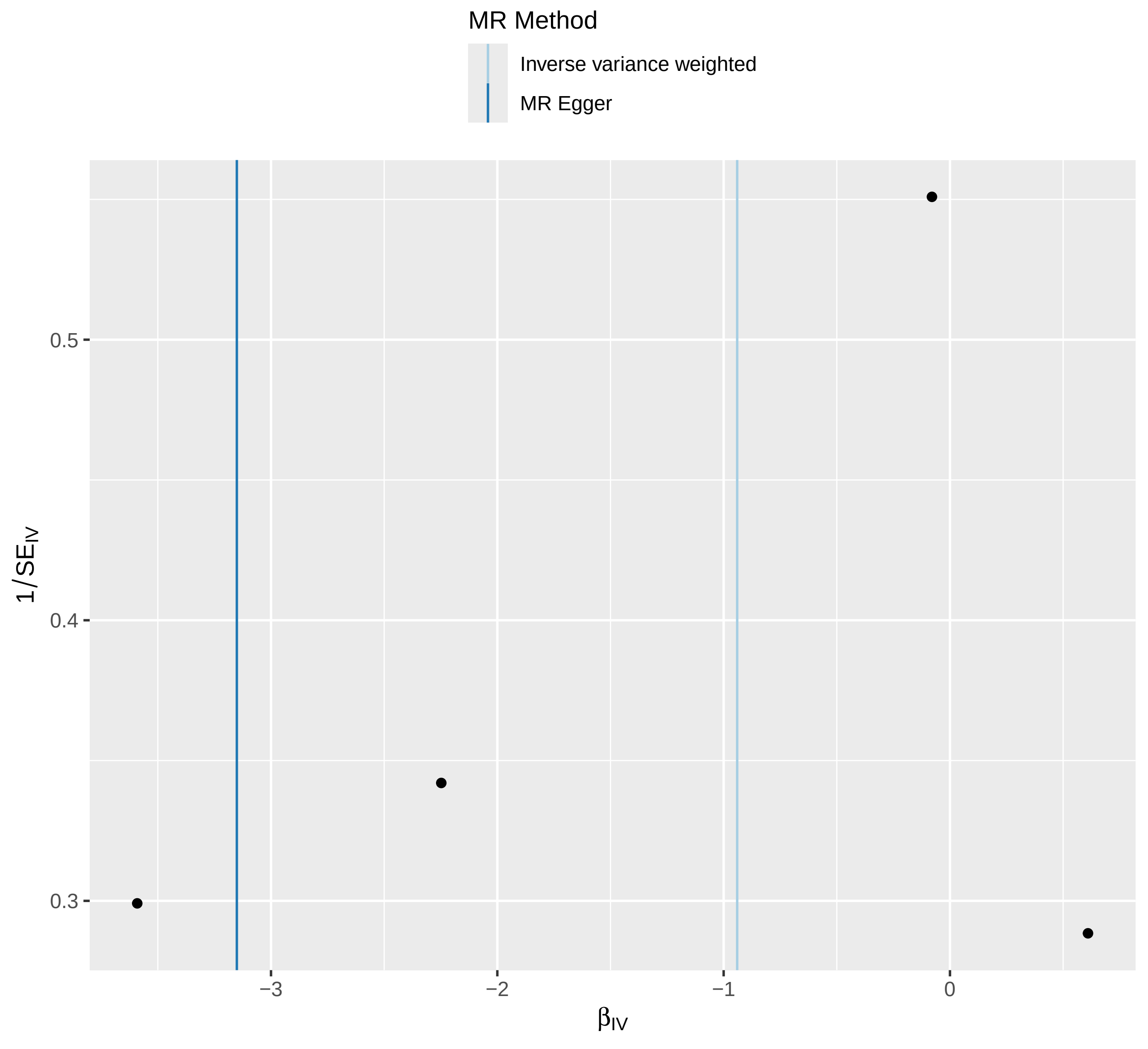

MR Method
Inverse variance weighted
MR Egger
0.5
0.4
0.3
1/SE_IV
−3
−2
−1
0
β_IV

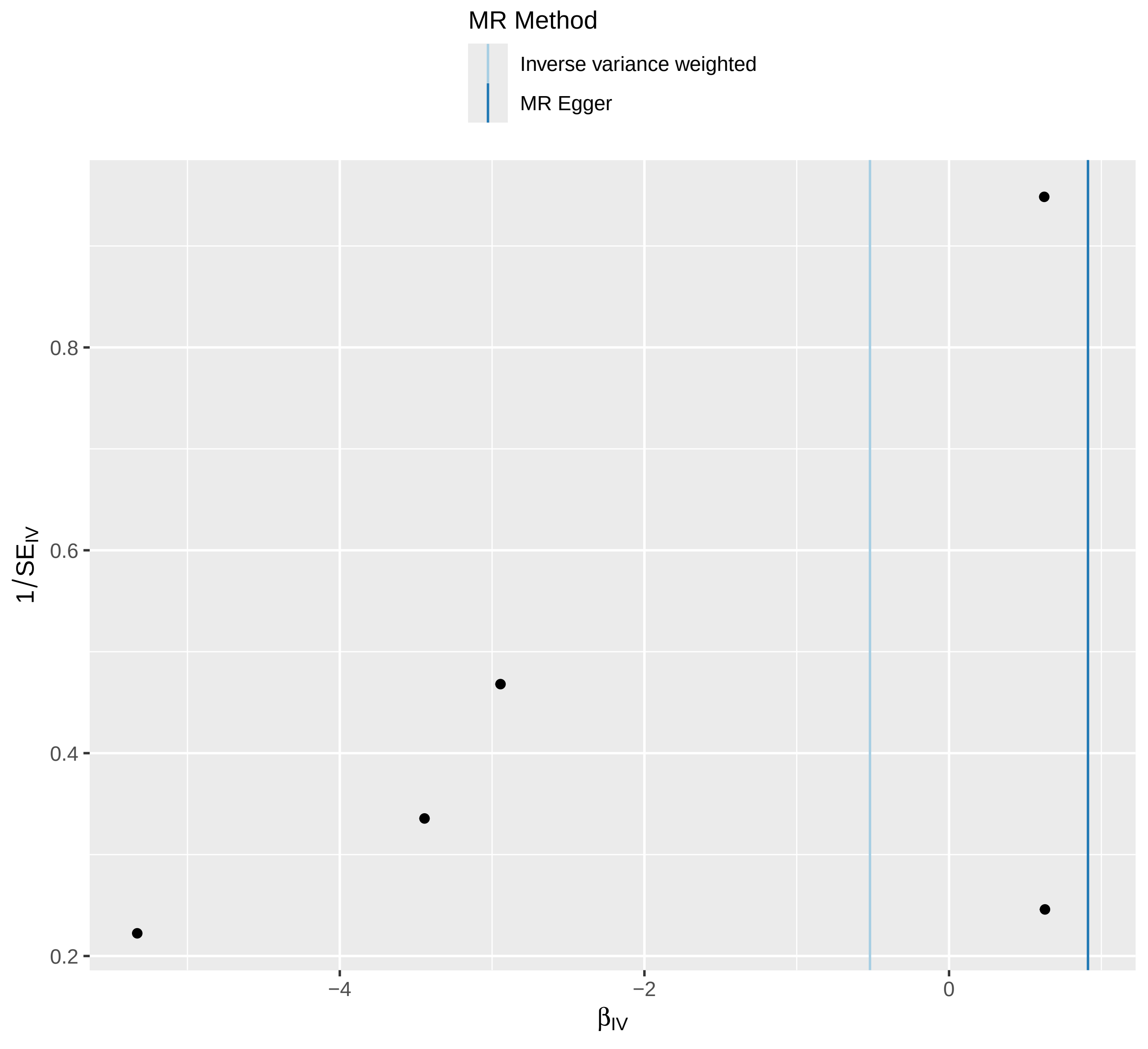

MR Method
Inverse variance weighted
MR Egger
0.8
0.6
0.4
0.2
1/SE_IV
−4
−2
0
β_IV

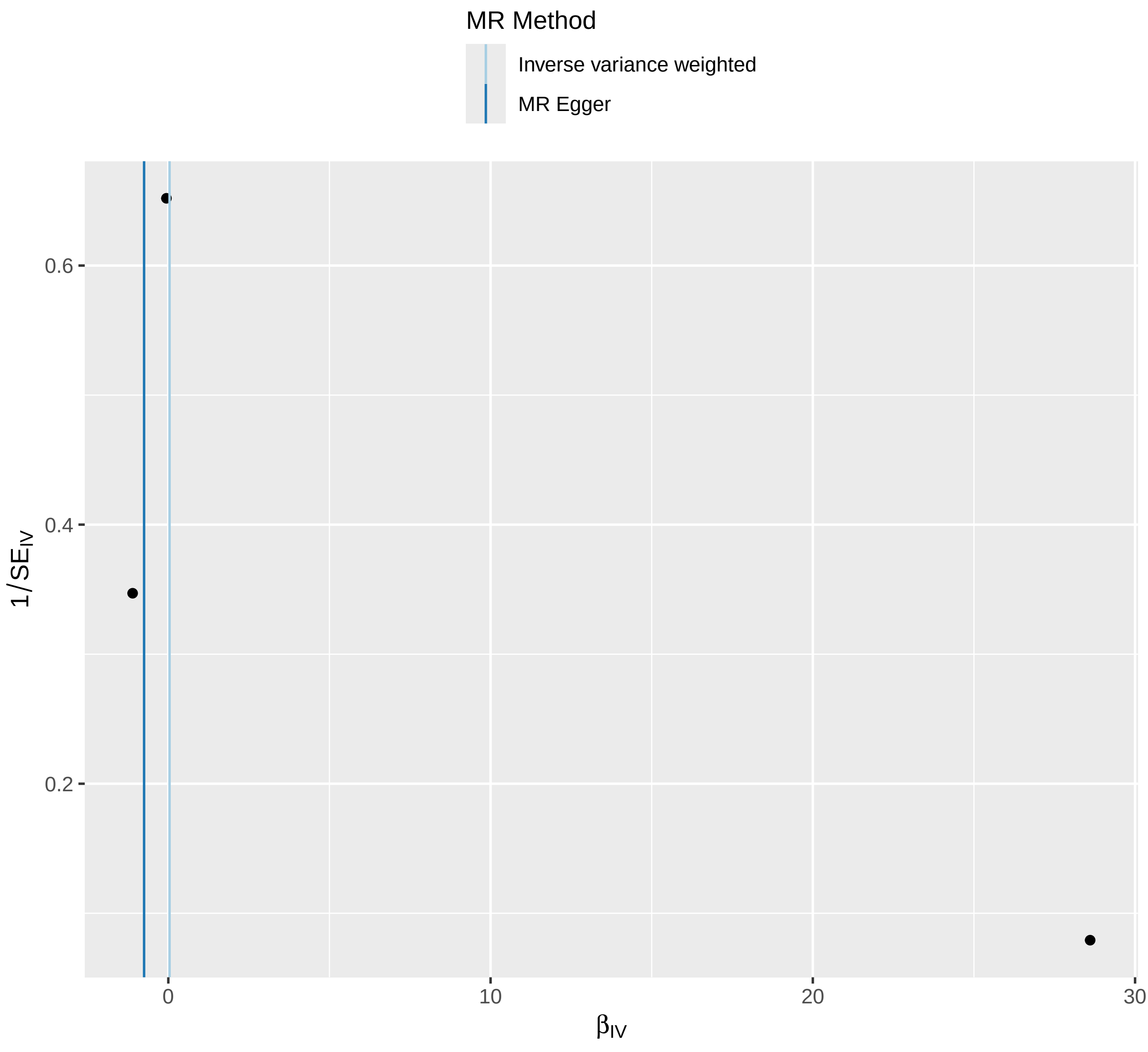
MR Method
Inverse variance weighted
MR Egger
0.6
0.4
0.2
1/SE_IV
0
10
20
30
β_IV

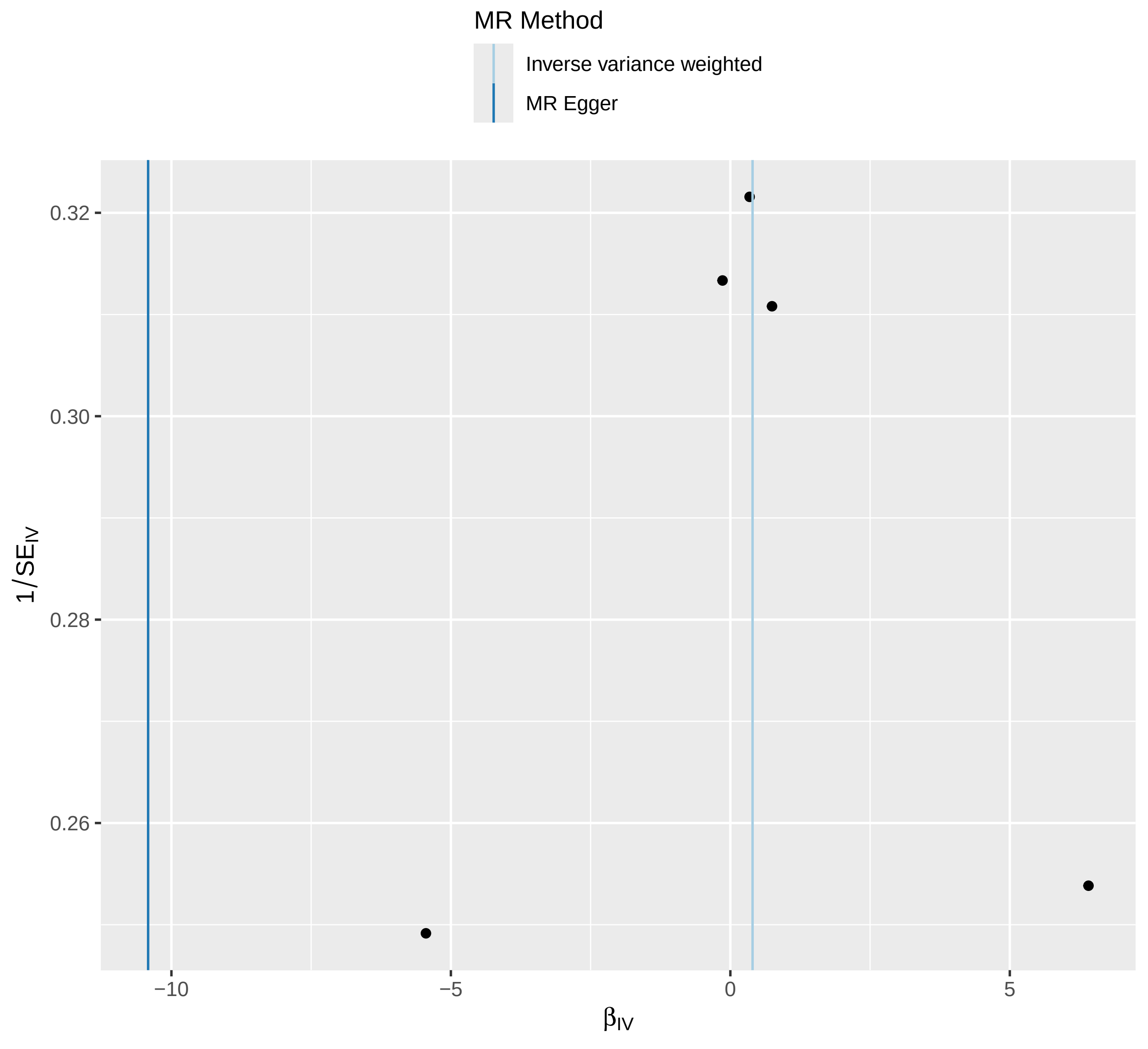
MR Method
Inverse variance weighted
MR Egger
0.32
0.30
0.28
0.26
1/SE_IV
−10
−5
0
5
β_IV

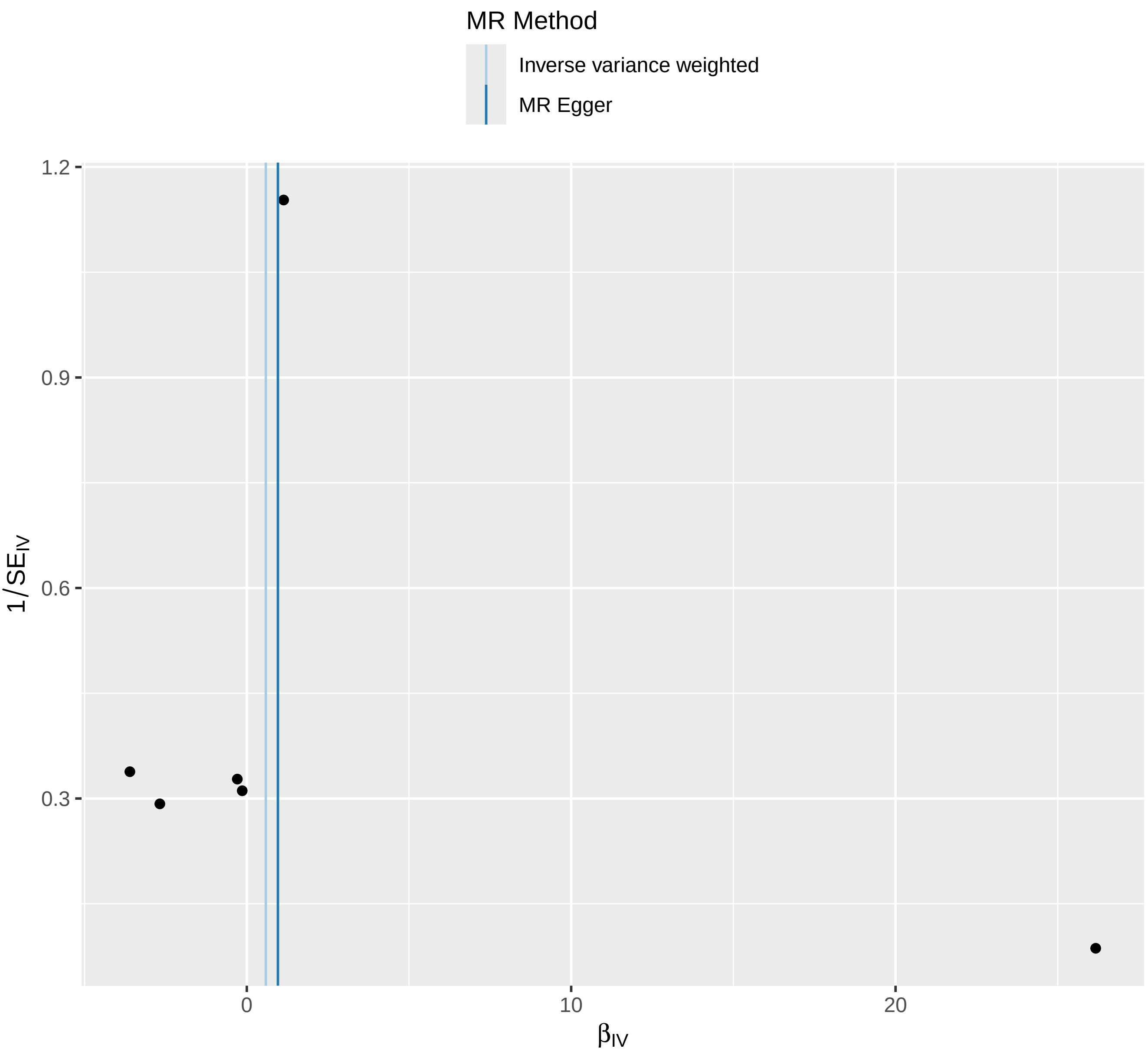
MR Method
Inverse variance weighted
MR Egger
1.2
0.9
0.6
0.3
1/SE_IV
0
10
20
β_IV

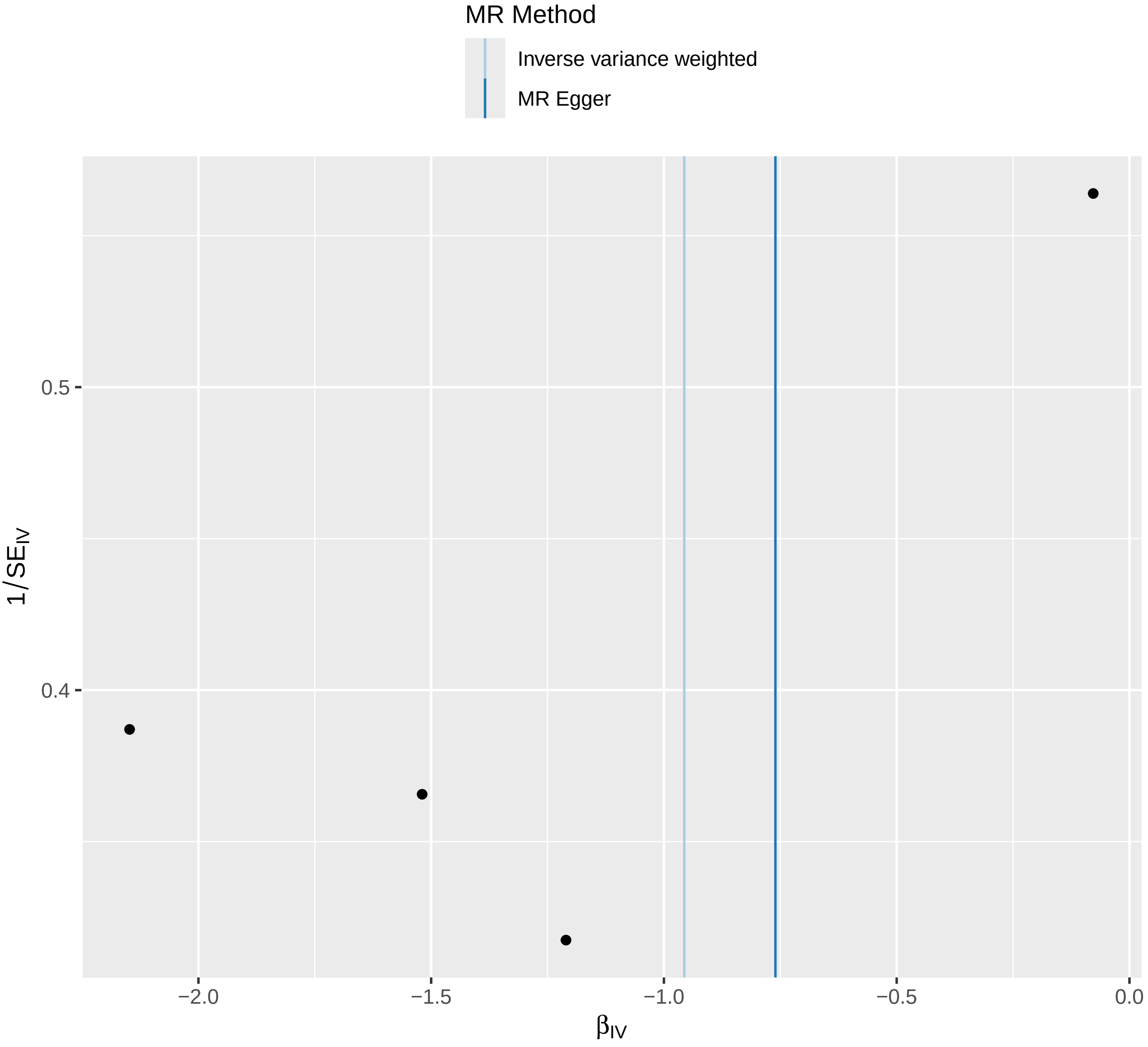

MR Method
Inverse variance weighted
MR Egger
0.5
0.4
1/SE_IV
−2.0
−1.5
−1.0
−0.5
0.0
β_IV

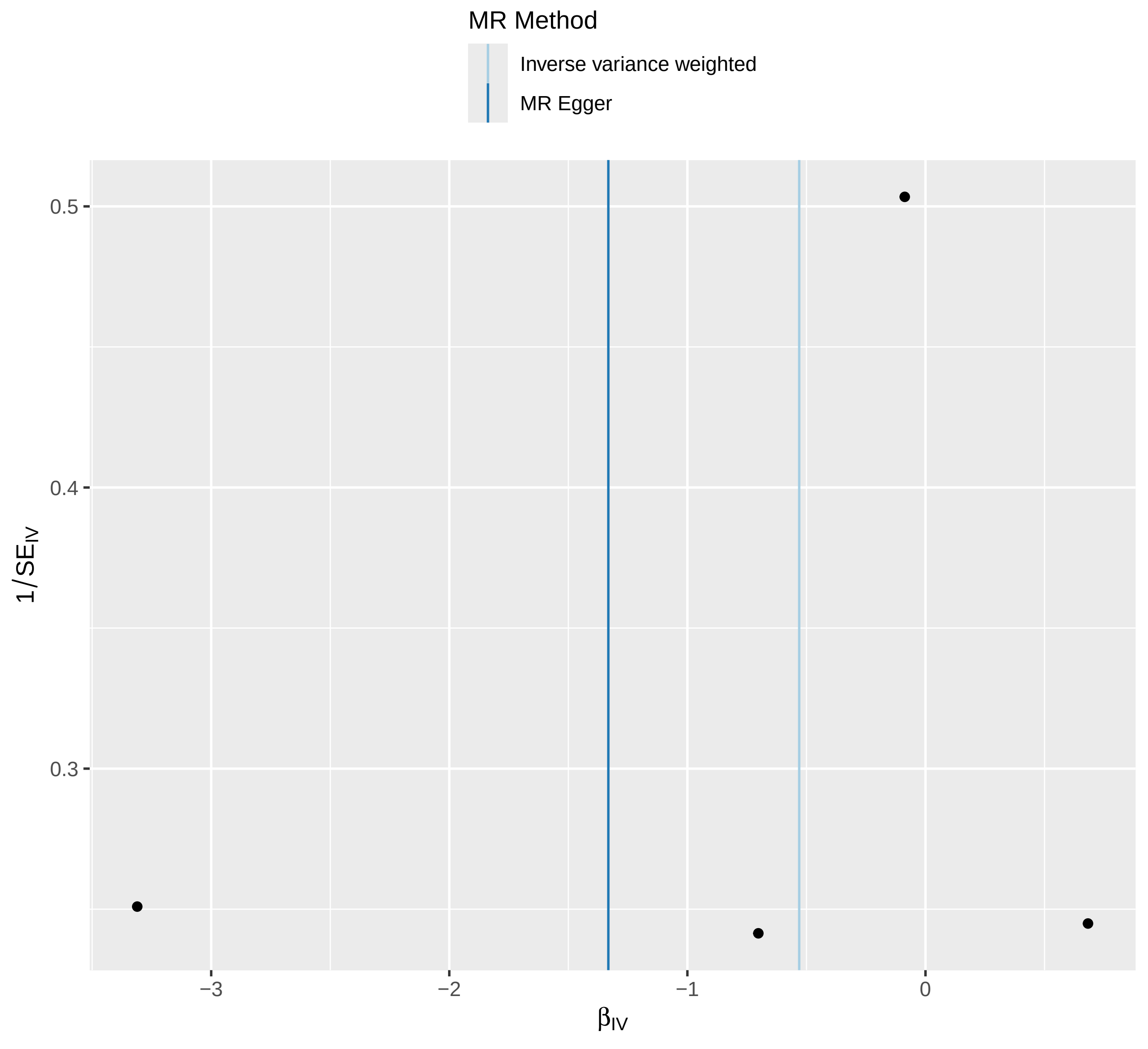

MR Method
Inverse variance weighted
MR Egger
0.5
0.4
0.3
1/SE$_{IV}$
−3
−2
−1
0
β$_{IV}$

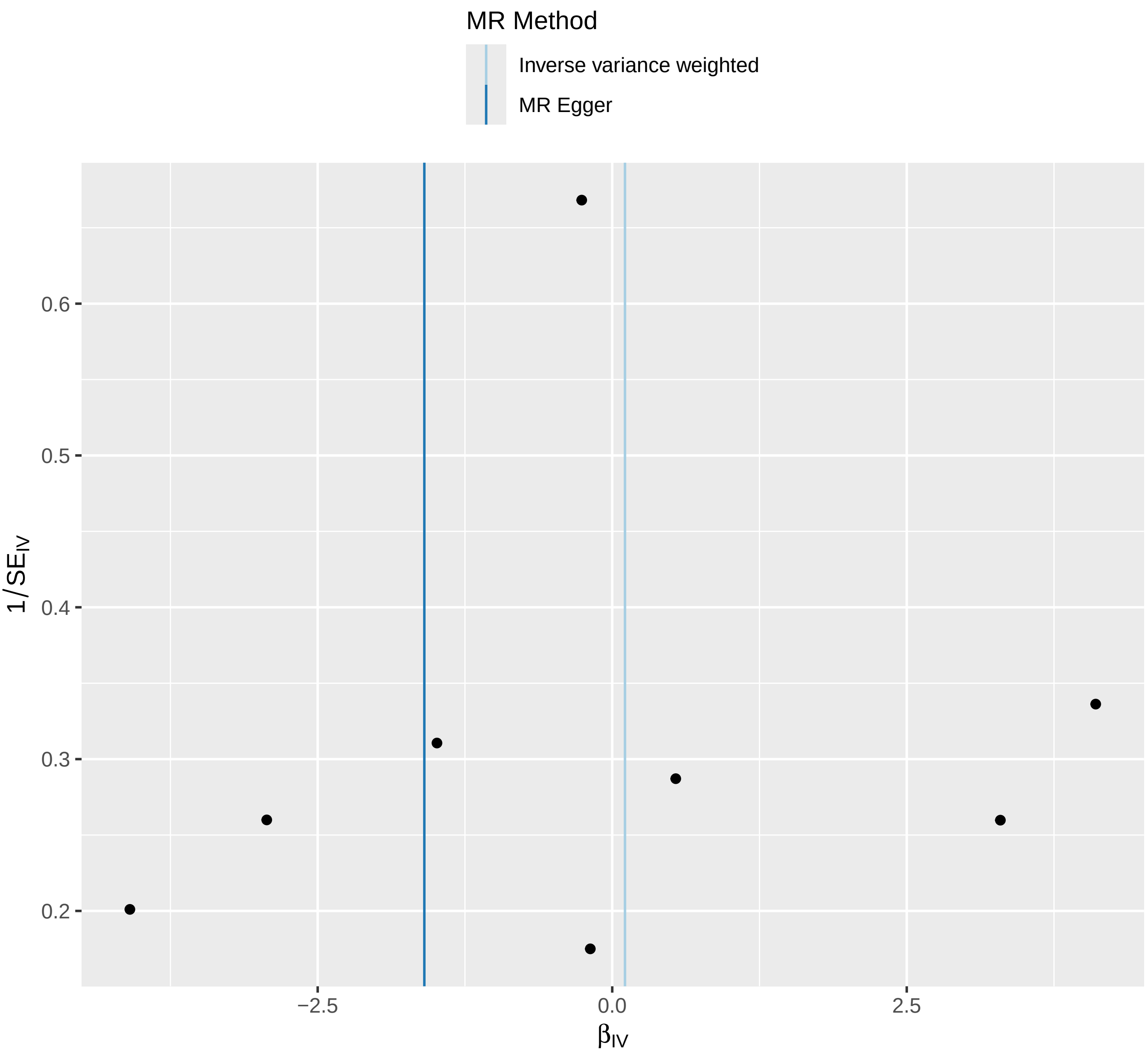

MR Method
Inverse variance weighted
MR Egger
0.6
0.5
0.4
0.3
0.2
1/SE$_{IV}$
−2.5
0.0
2.5
β$_{IV}$

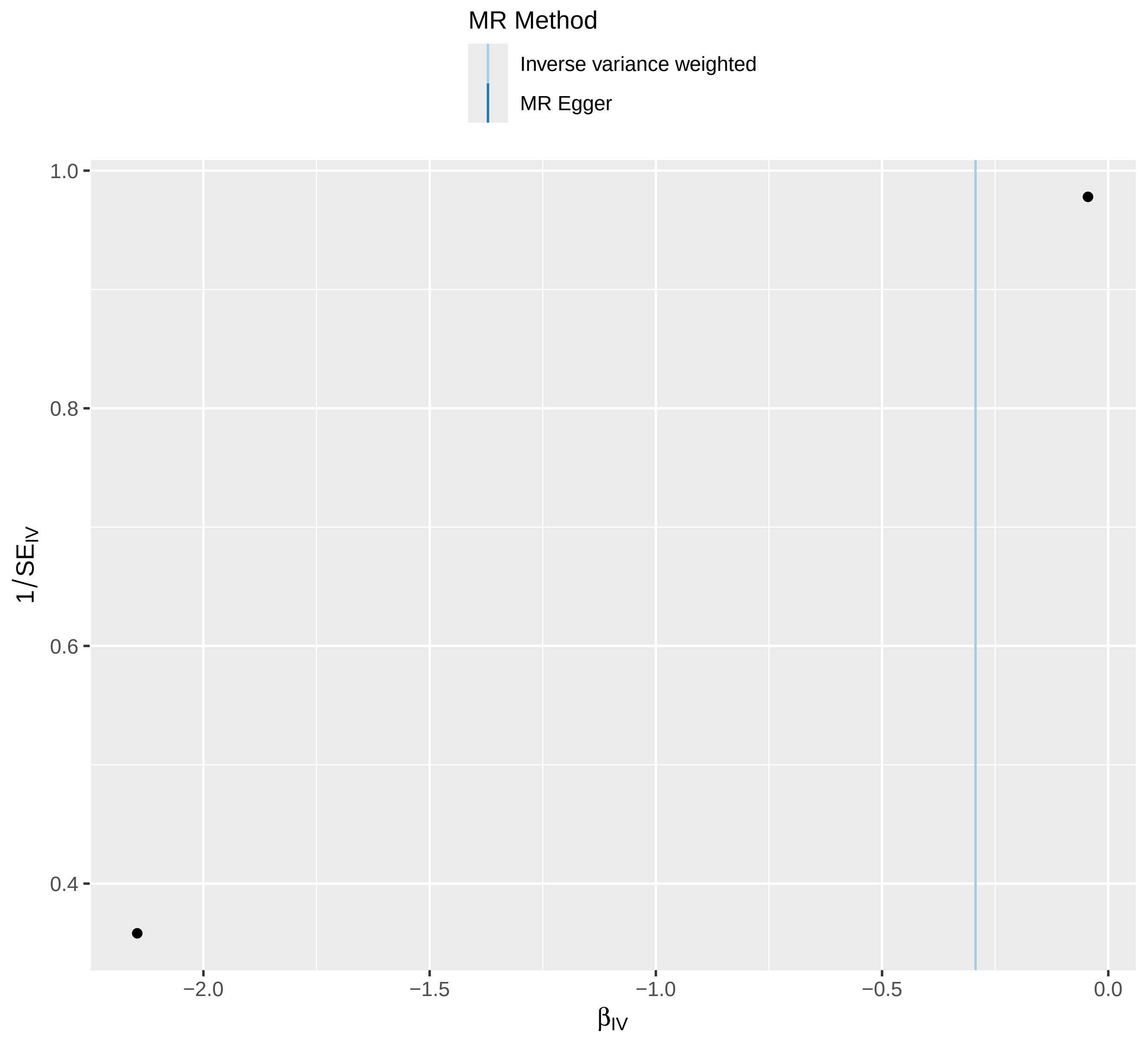
MR Method
Inverse variance weighted
MR Egger
1.0
0.8
0.6
0.4
1/SE$_{IV}$
−2.0
−1.5
−1.0
−0.5
0.0
β$_{IV}$

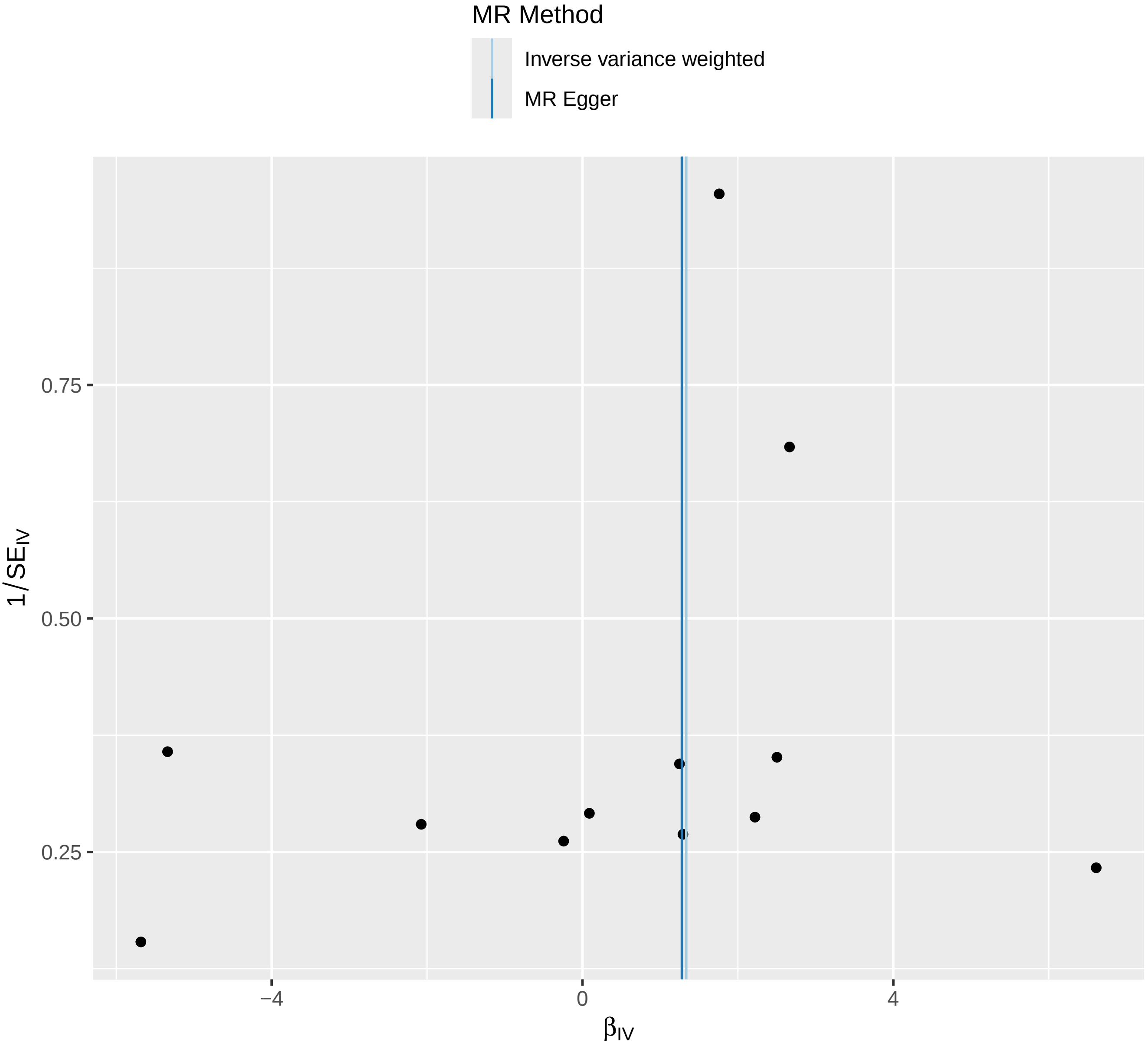
MR Method
Inverse variance weighted
MR Egger
0.75
0.50
0.25
1/SE_IV
−4
0
4
β_IV

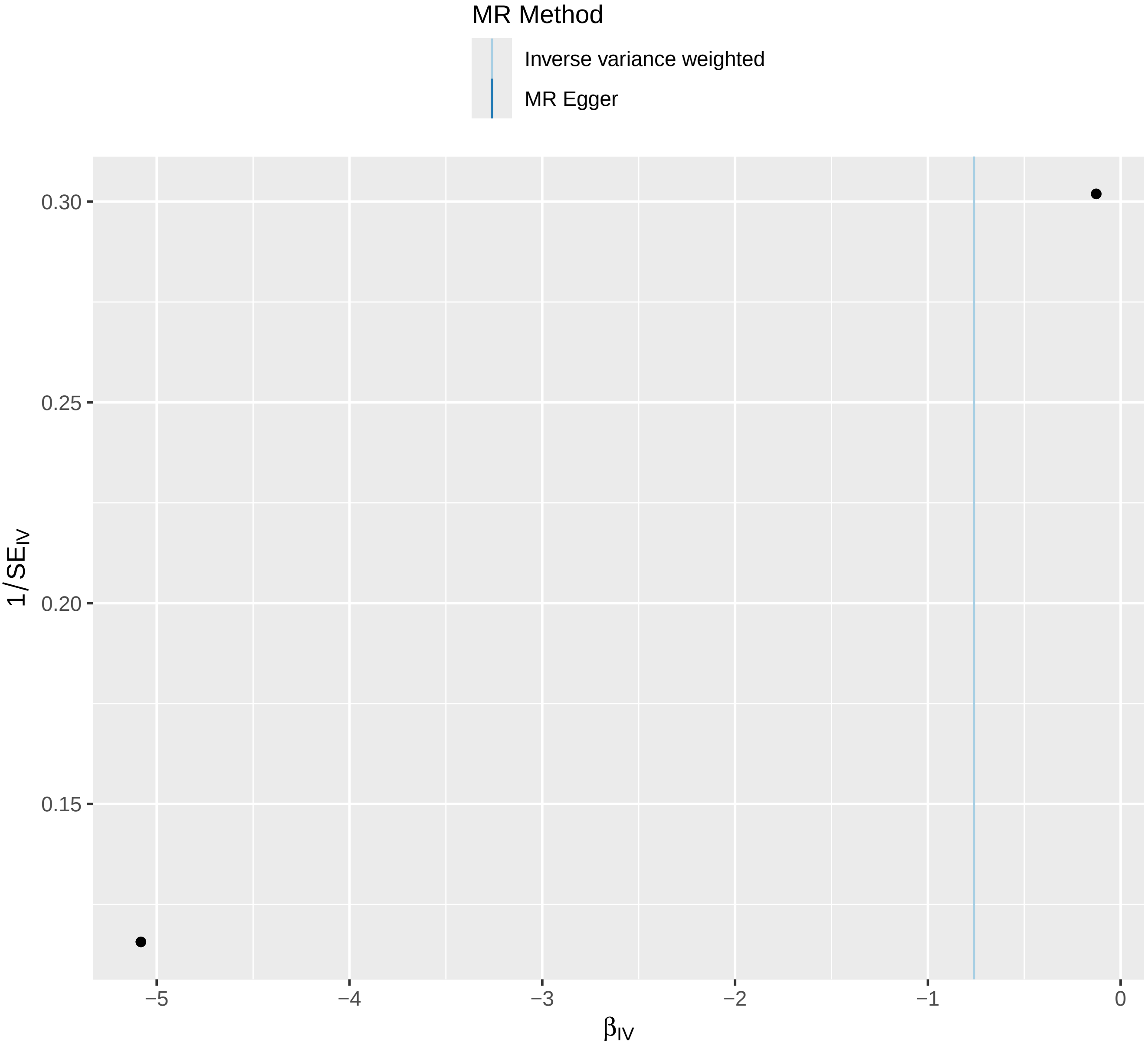
MR Method
Inverse variance weighted
MR Egger
0.30
0.25
0.20
0.15
1/SE_IV
−5
−4
−3
−2
−1
0
β_IV

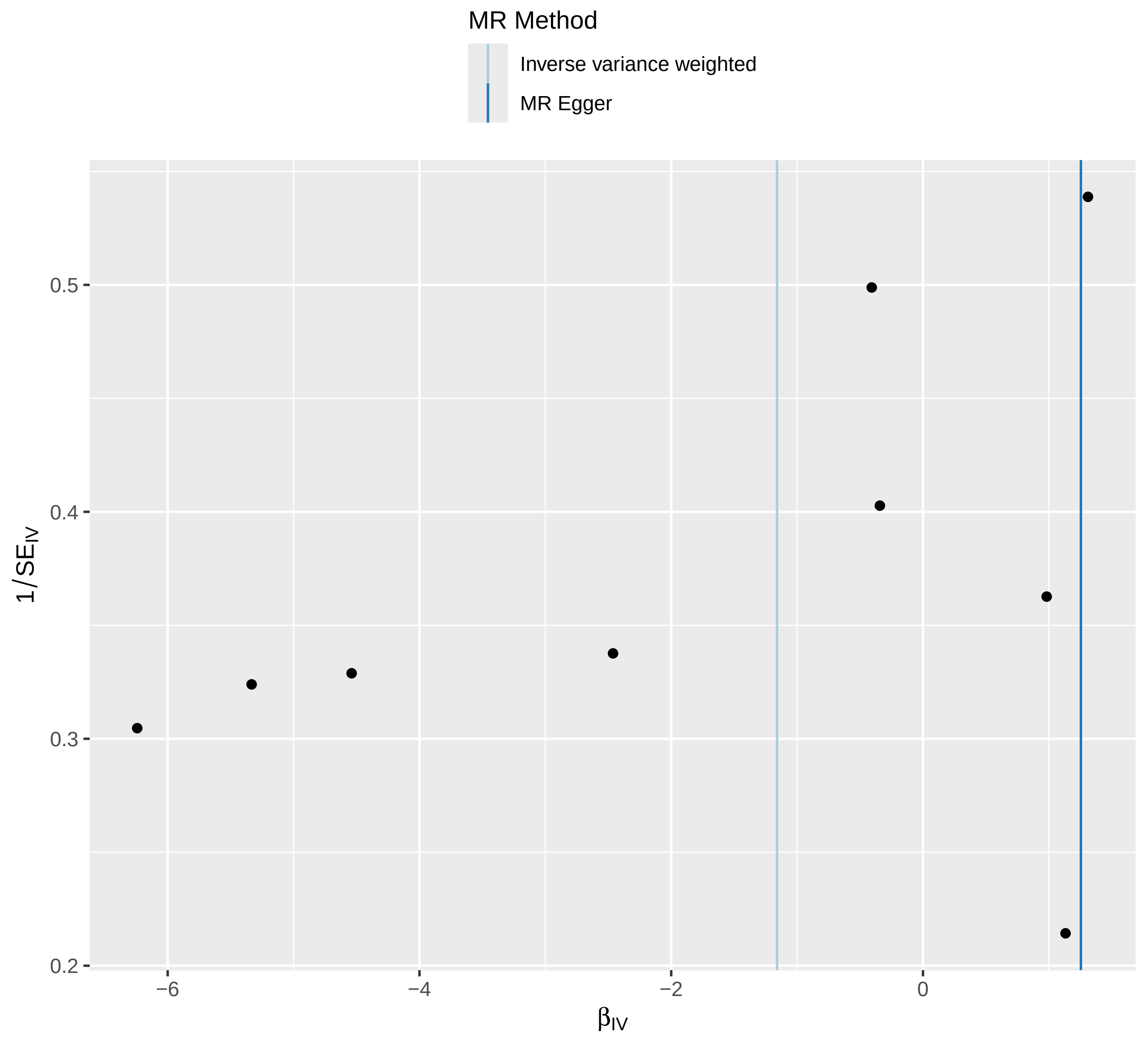
MR Method
Inverse variance weighted
MR Egger
0.5
0.4
0.3
0.2
$1/SE_{IV}$
−6
−4
−2
0
β_{IV}

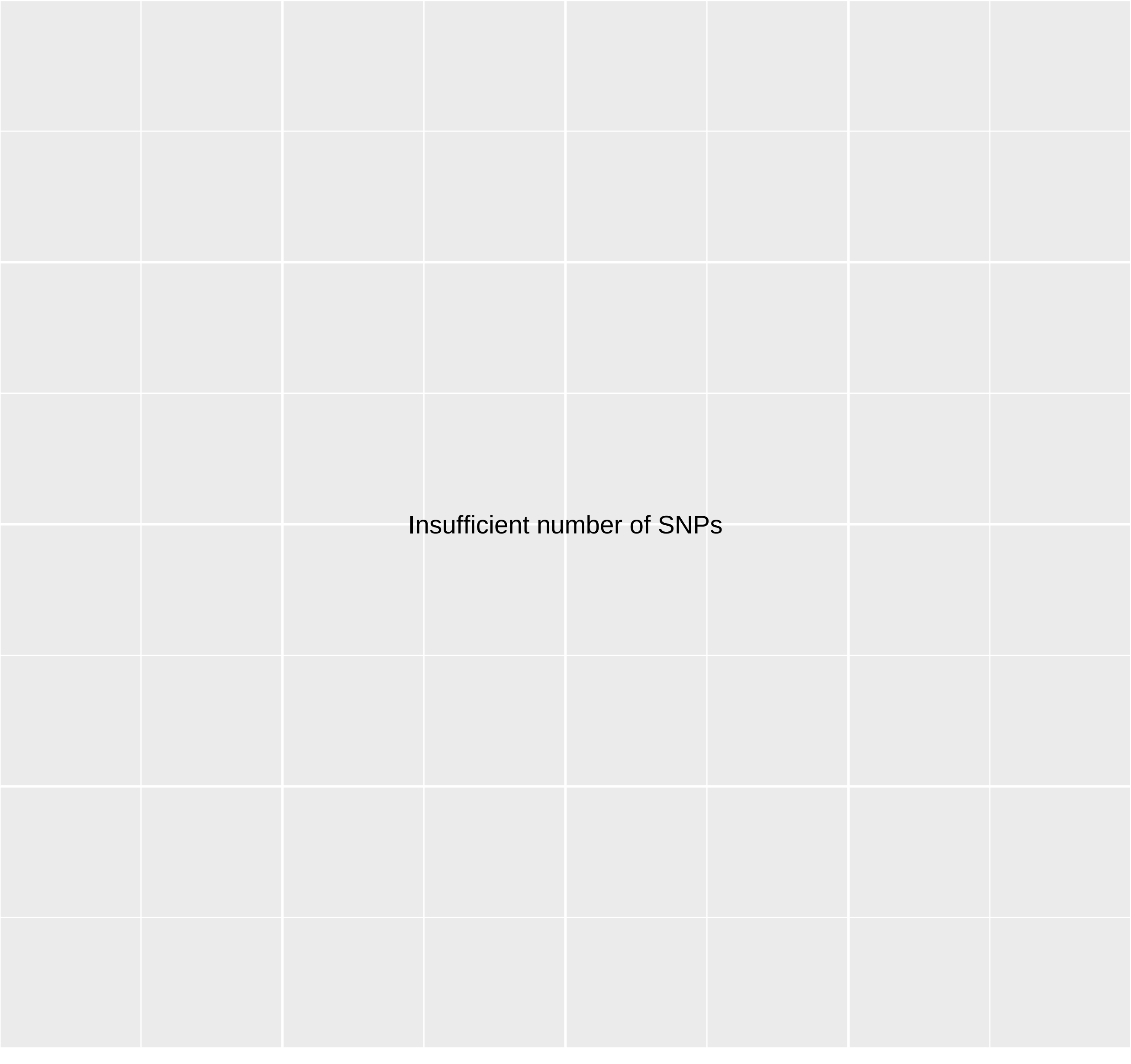
Insufficient number of SNPs

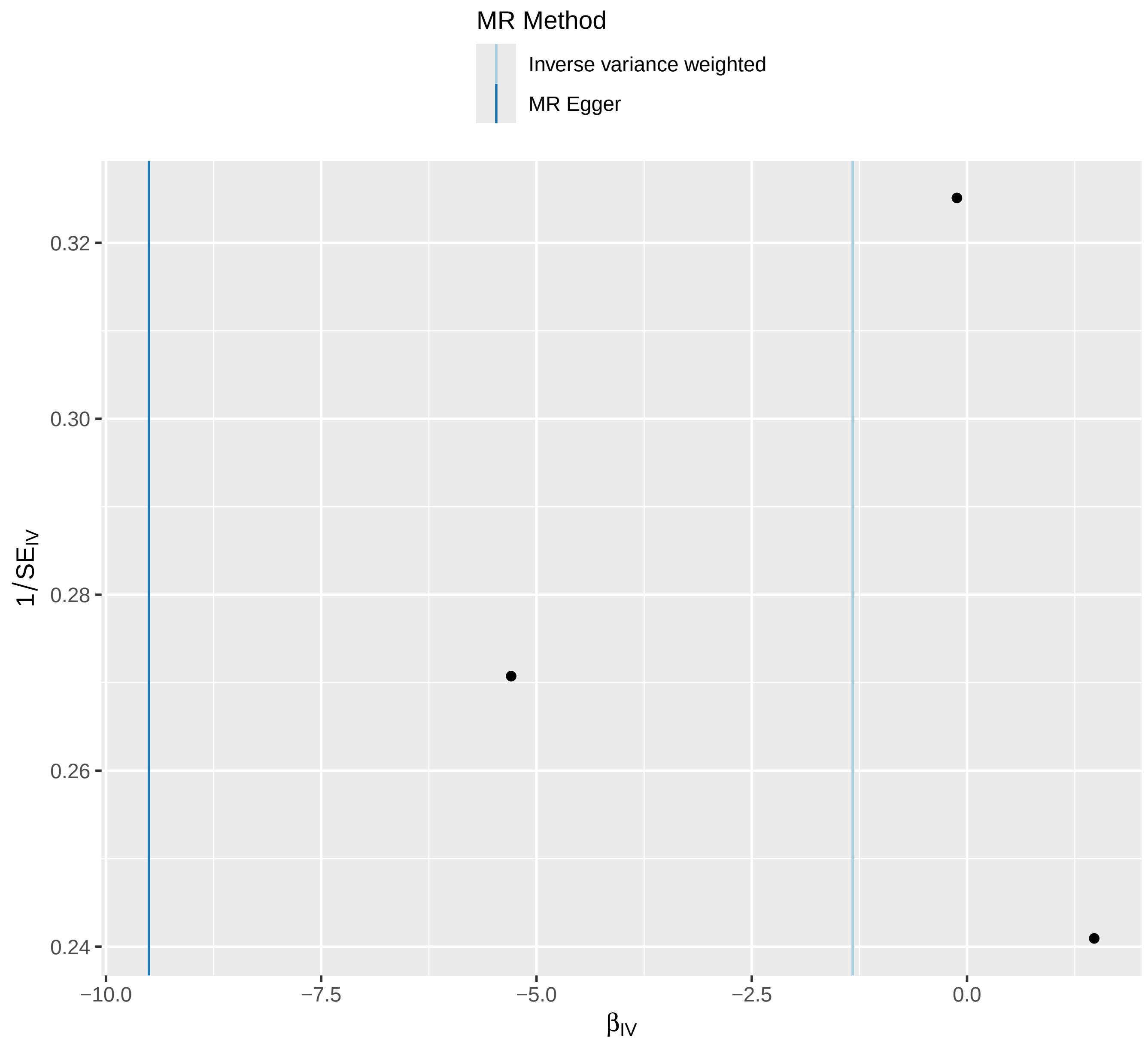
MR Method
Inverse variance weighted
MR Egger
0.32
0.30
0.28
0.26
0.24
1/SE_IV
−10.0
−7.5
−5.0
−2.5
0.0
β_IV

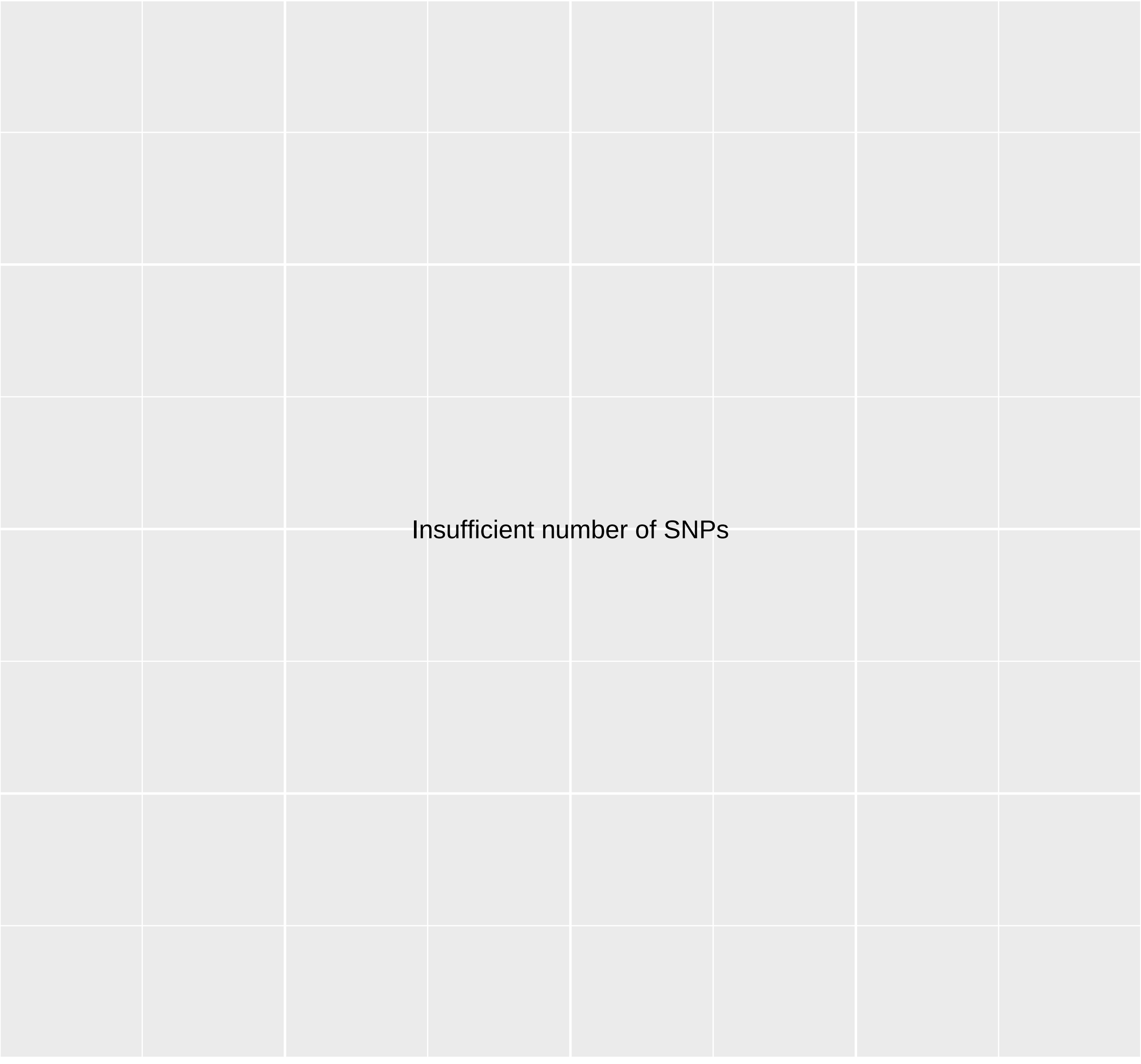
Insufficient number of SNPs

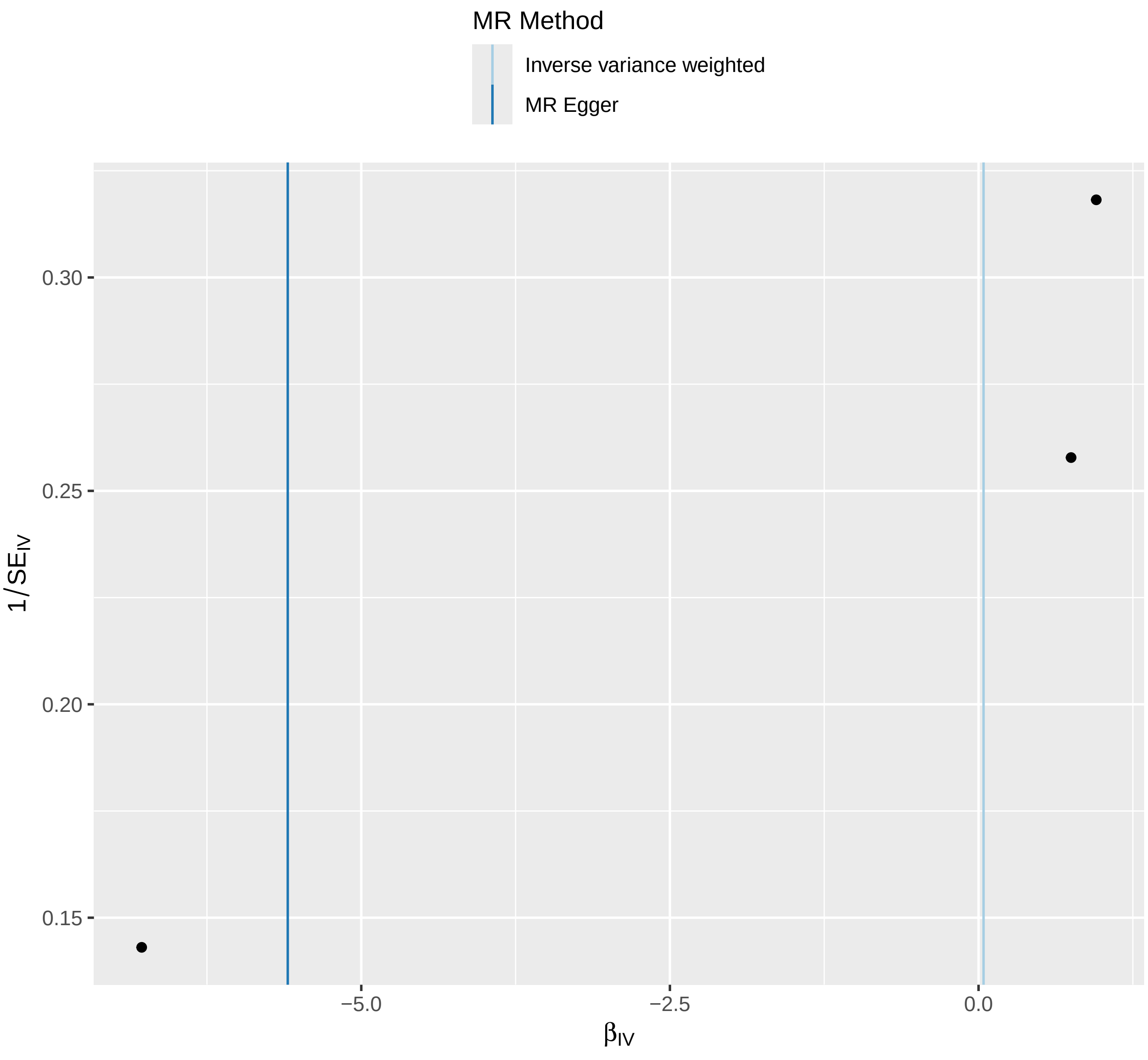
MR Method
Inverse variance weighted
MR Egger
0.30
0.25
0.20
0.15
1/SE$_{IV}$
−5.0
−2.5
0.0
β$_{IV}$

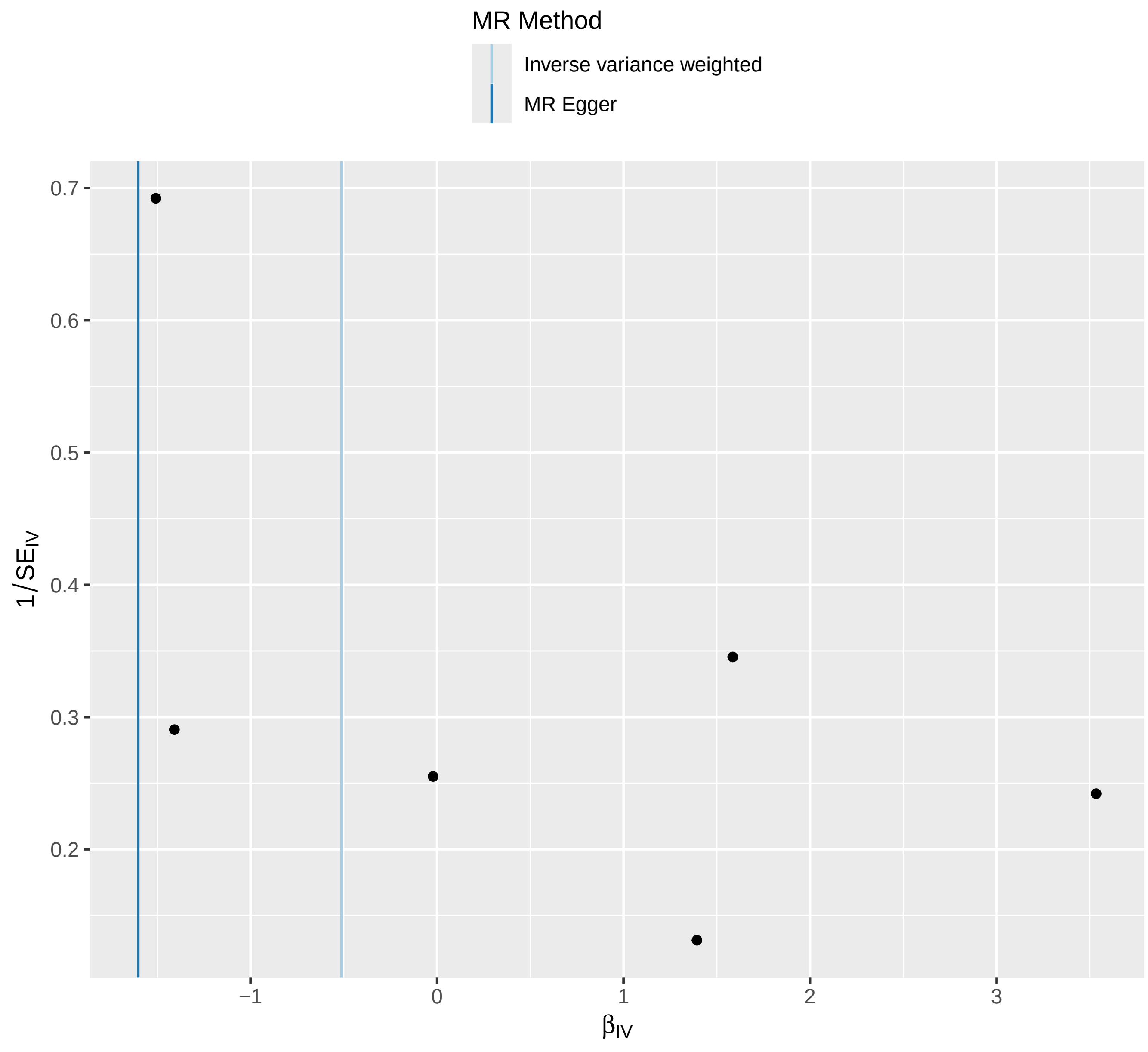

MR Method
Inverse variance weighted
MR Egger
0.7
0.6
0.5
0.4
0.3
0.2
$1/SE_{IV}$
−1
0
1
2
3
β_{IV}

Insufficient number of SNPs

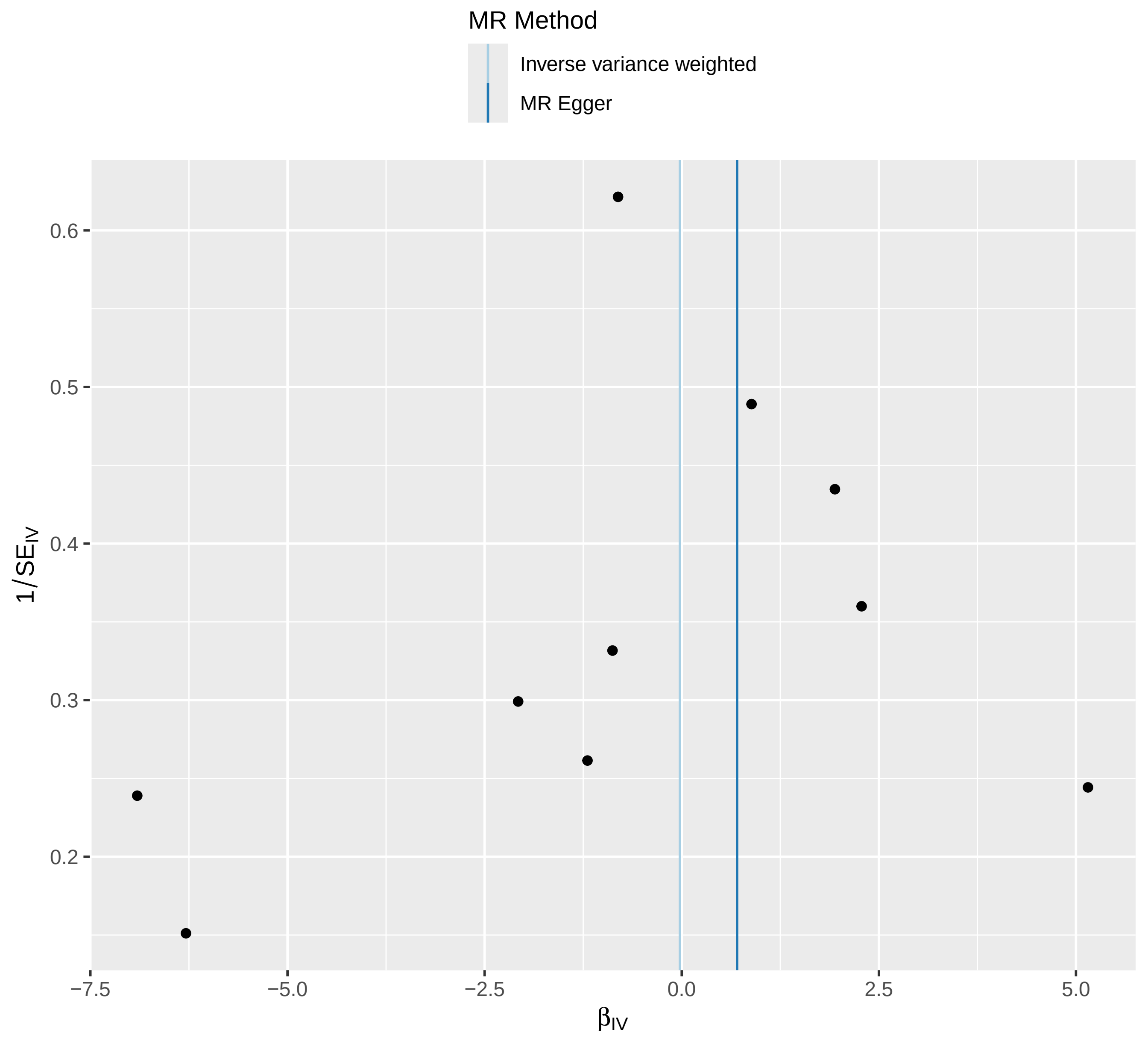
MR Method
Inverse variance weighted
MR Egger
1/SE_IV
β_IV
0.6
0.5
0.4
0.3
0.2
−7.5
−5.0
−2.5
0.0
2.5
5.0

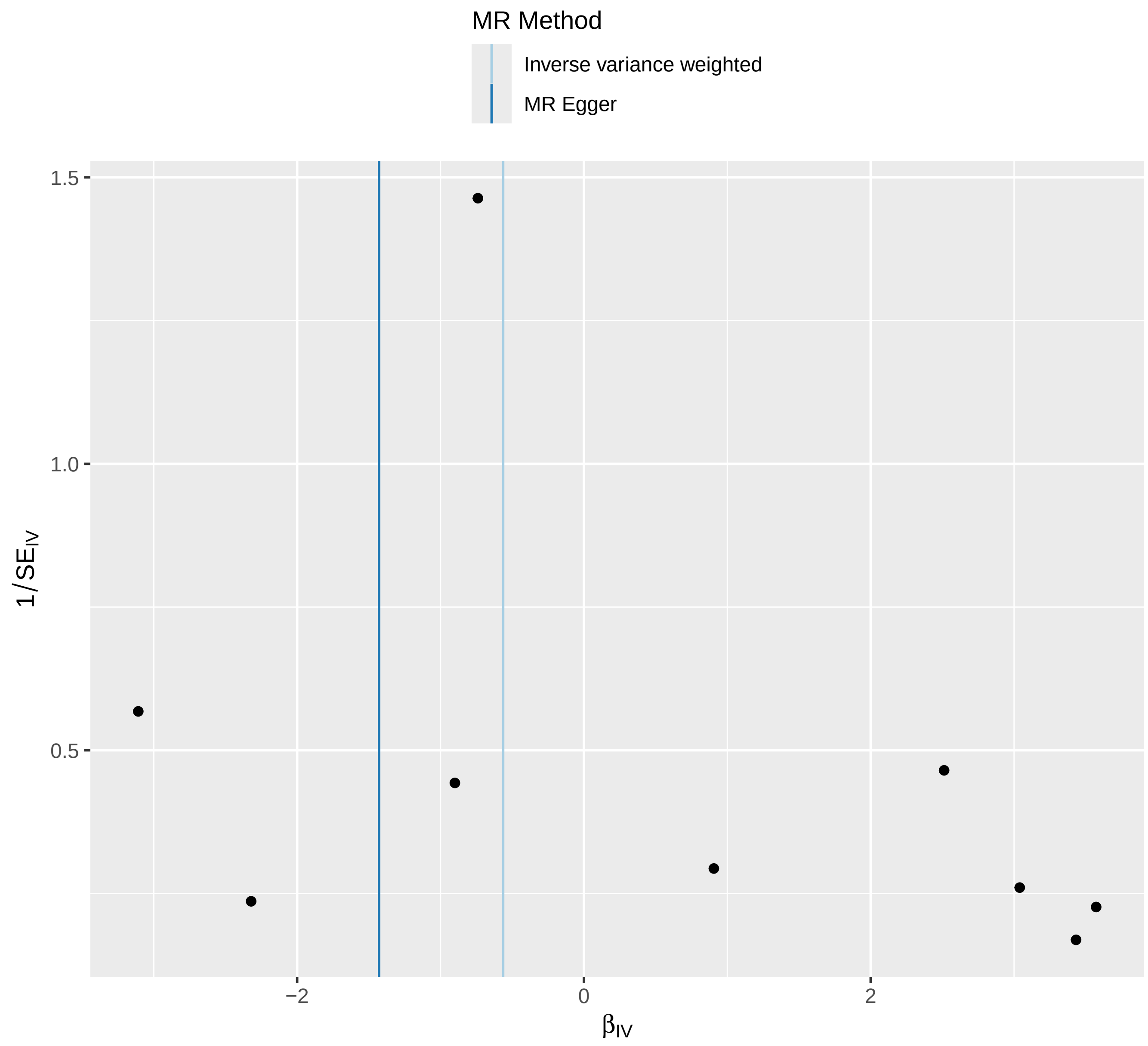

MR Method
Inverse variance weighted
MR Egger
1.5
1.0
0.5
−2
0
2
$1/SE_{IV}$
β_{IV}

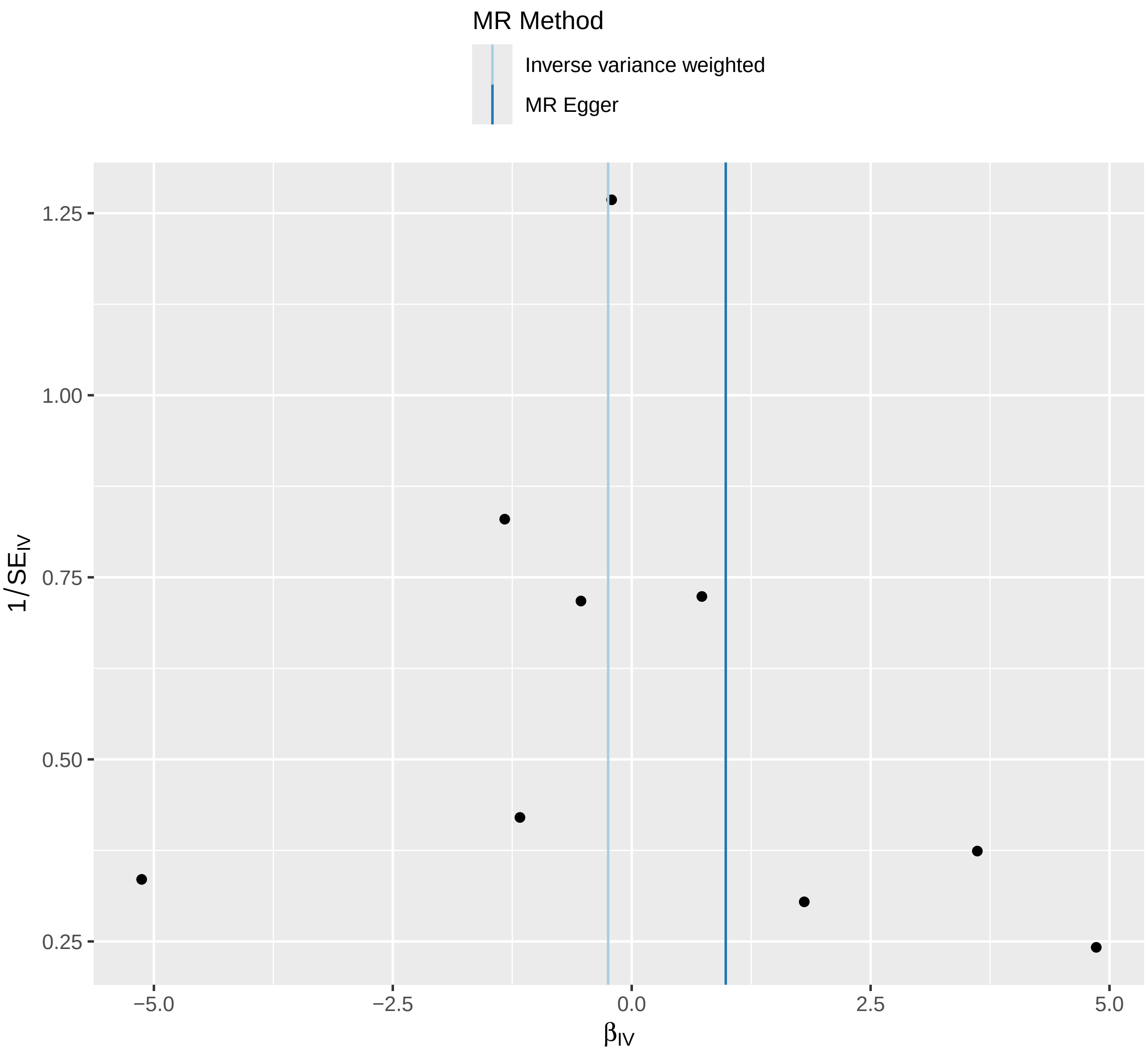
MR Method
Inverse variance weighted
MR Egger
1.25
1.00
0.75
0.50
0.25
1/SE_IV
−5.0
−2.5
0.0
2.5
5.0
β_IV

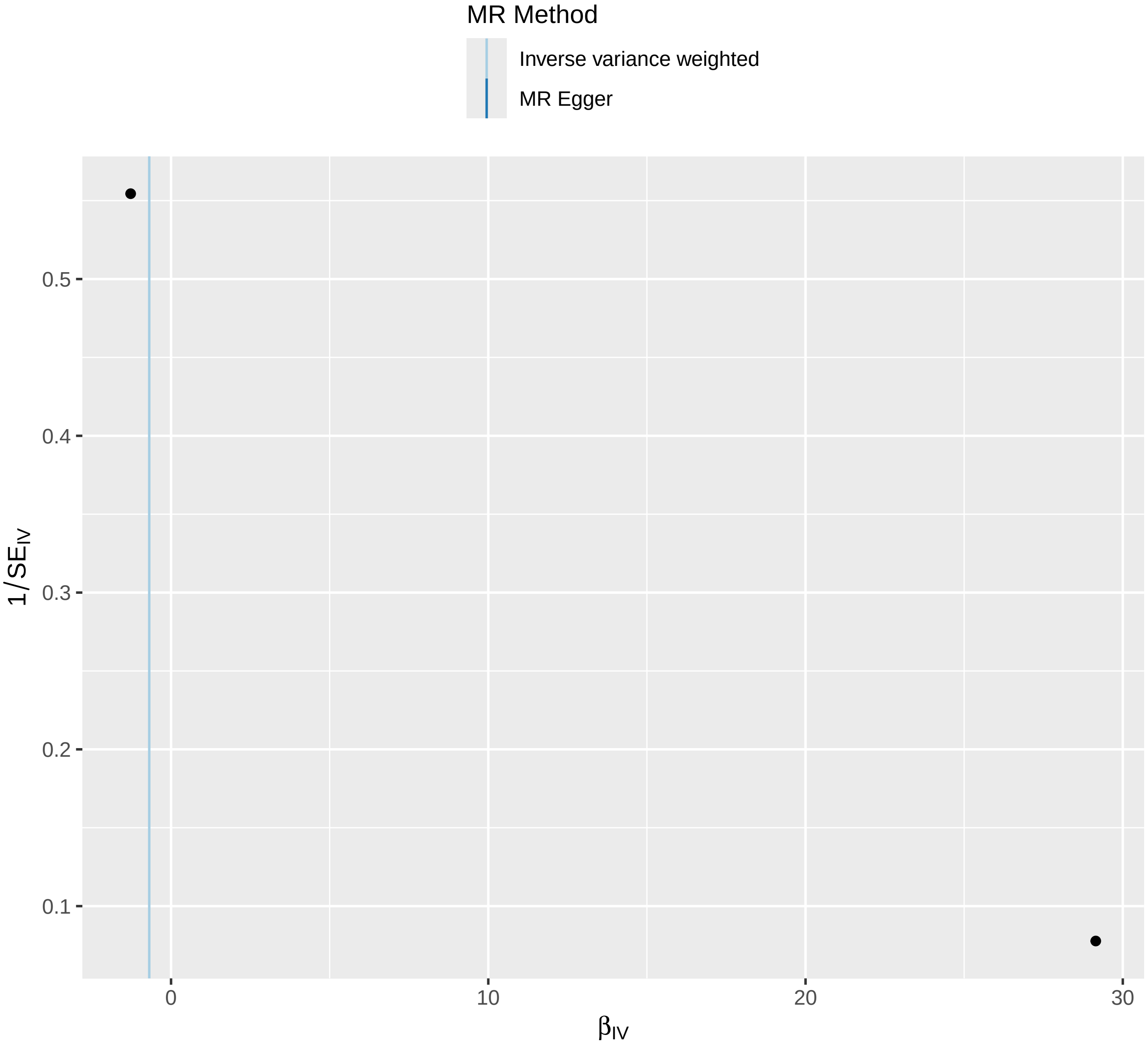
MR Method
Inverse variance weighted
MR Egger
0.5
0.4
0.3
0.2
0.1
$1/SE_{IV}$
0
10
20
30
β_{IV}

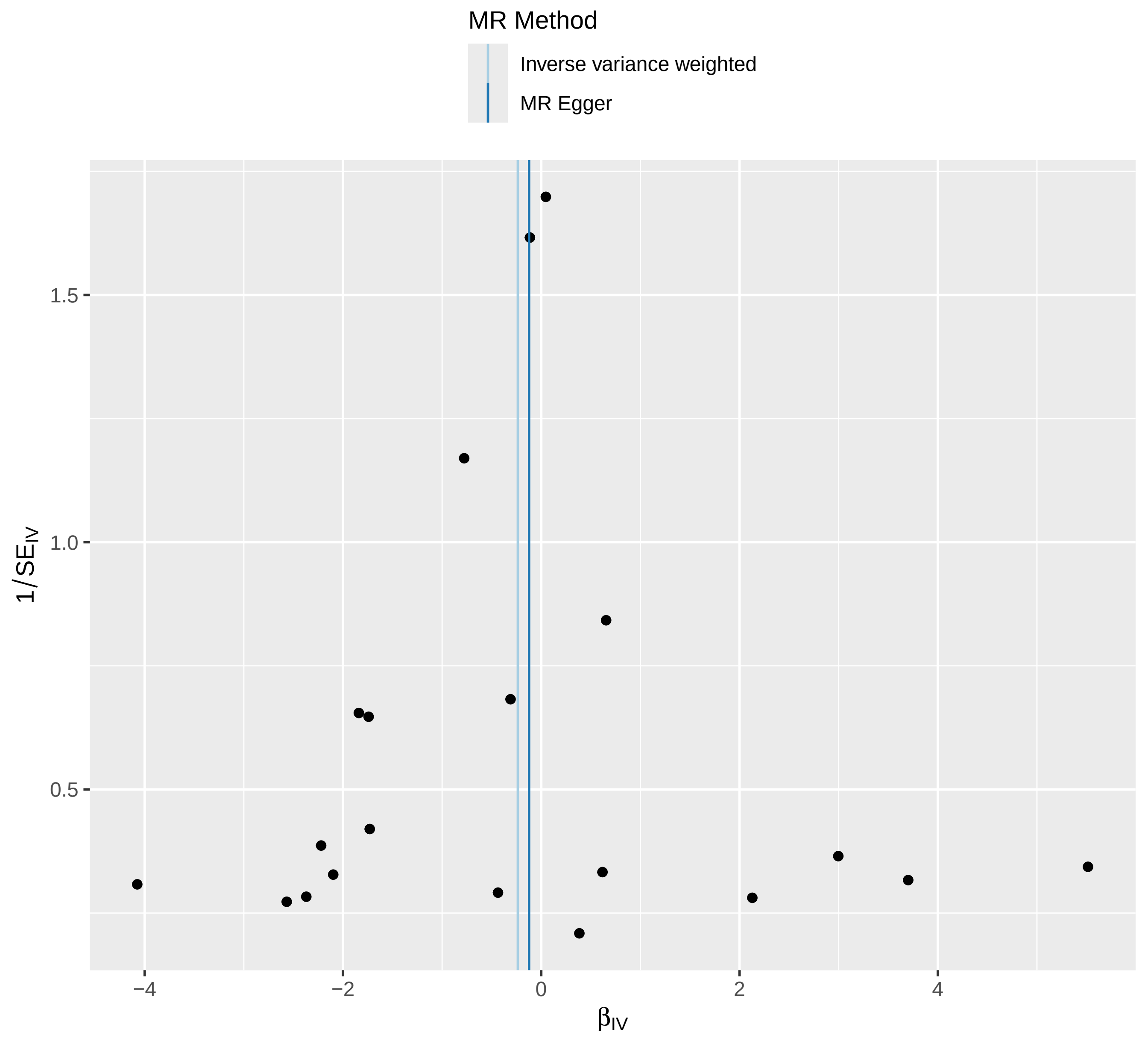

MR Method
Inverse variance weighted
MR Egger
1.5
1.0
0.5
1/SE_IV
−4
−2
0
2
4
β_IV

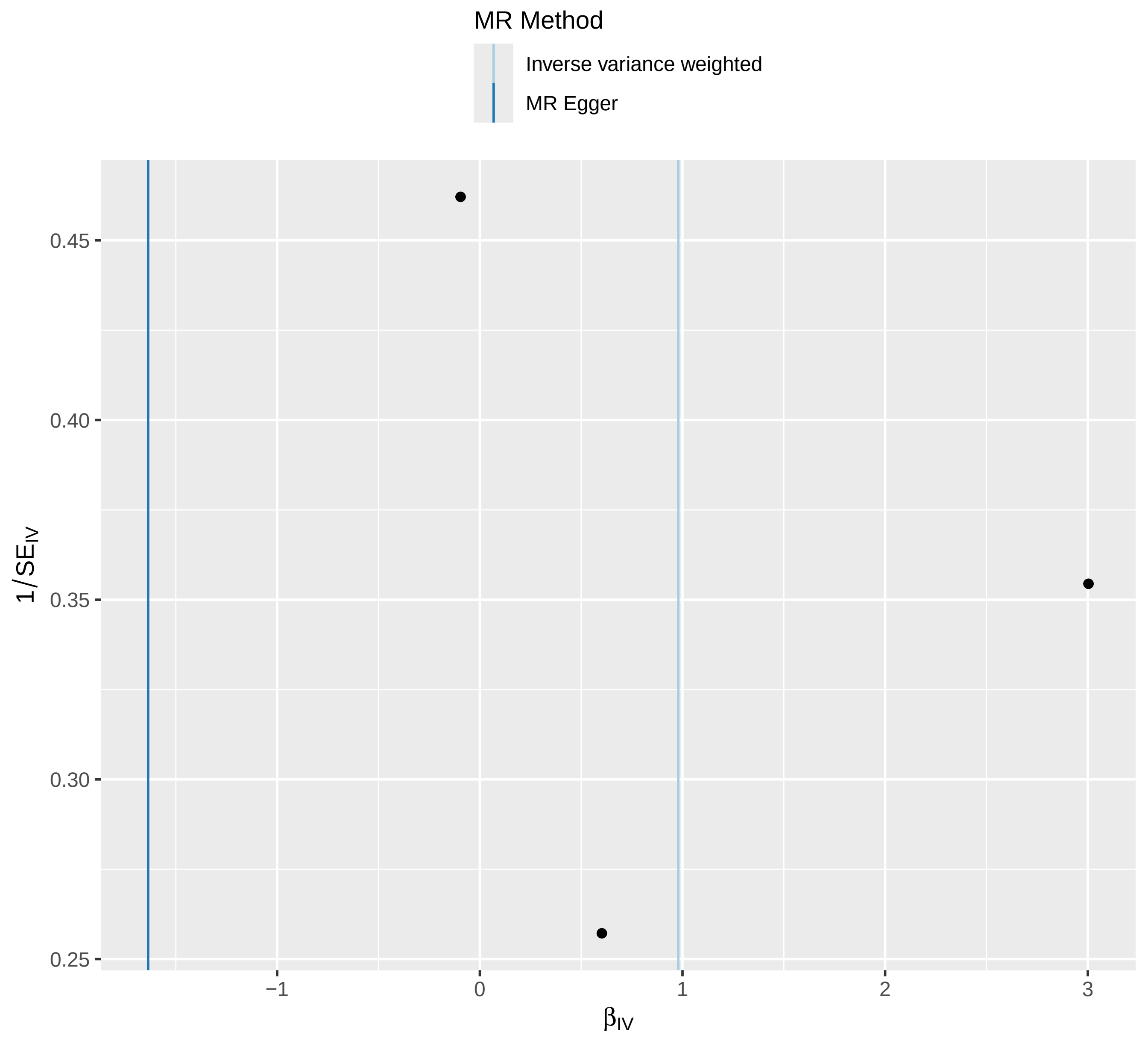
MR Method
Inverse variance weighted
MR Egger
1/SE$_{IV}$
0.45
0.40
0.35
0.30
0.25
−1
0
1
2
3
β$_{IV}$

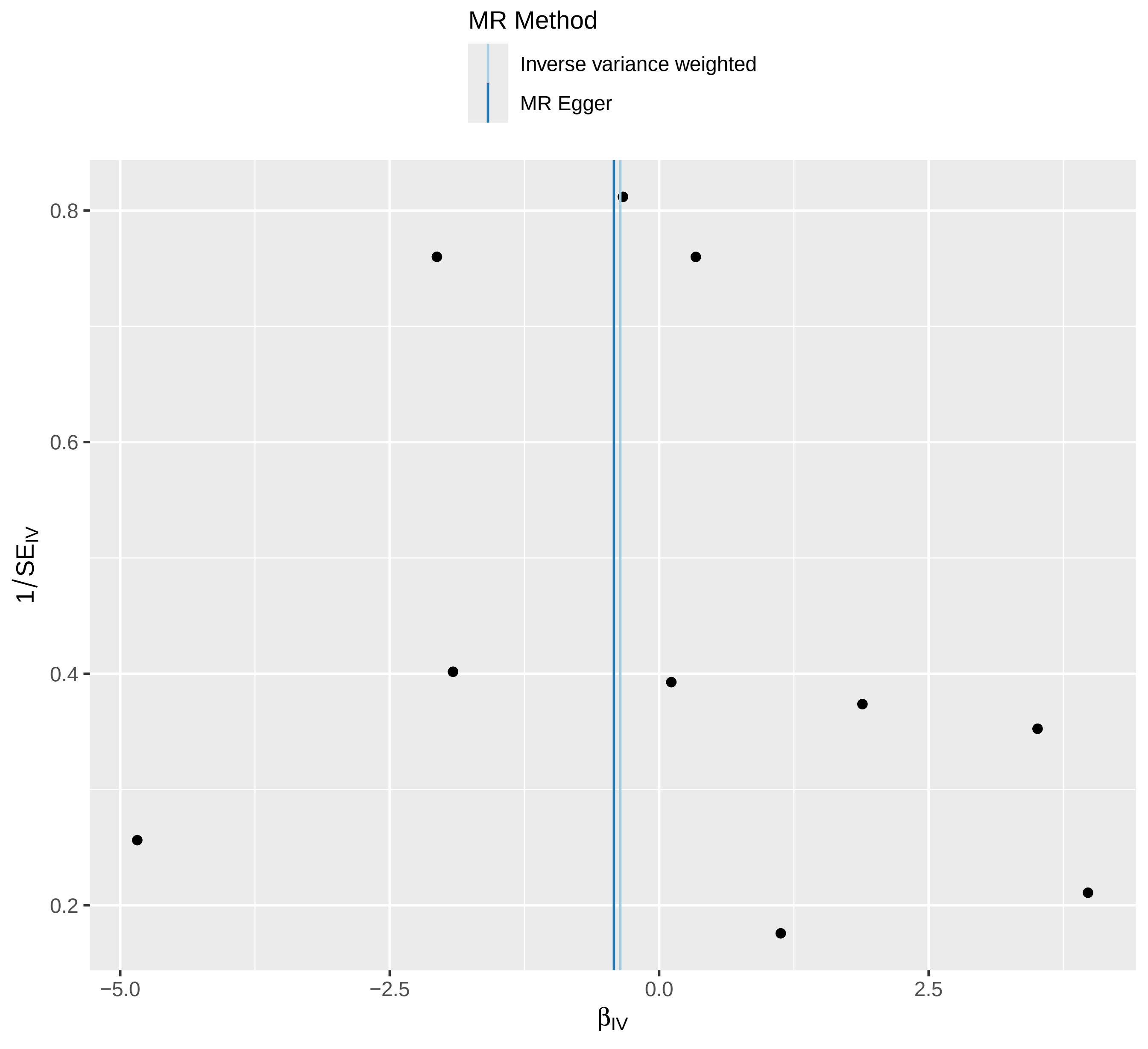
MR Method
Inverse variance weighted
MR Egger
1/SE_IV
0.8
0.6
0.4
0.2
−5.0
−2.5
0.0
2.5
β_IV

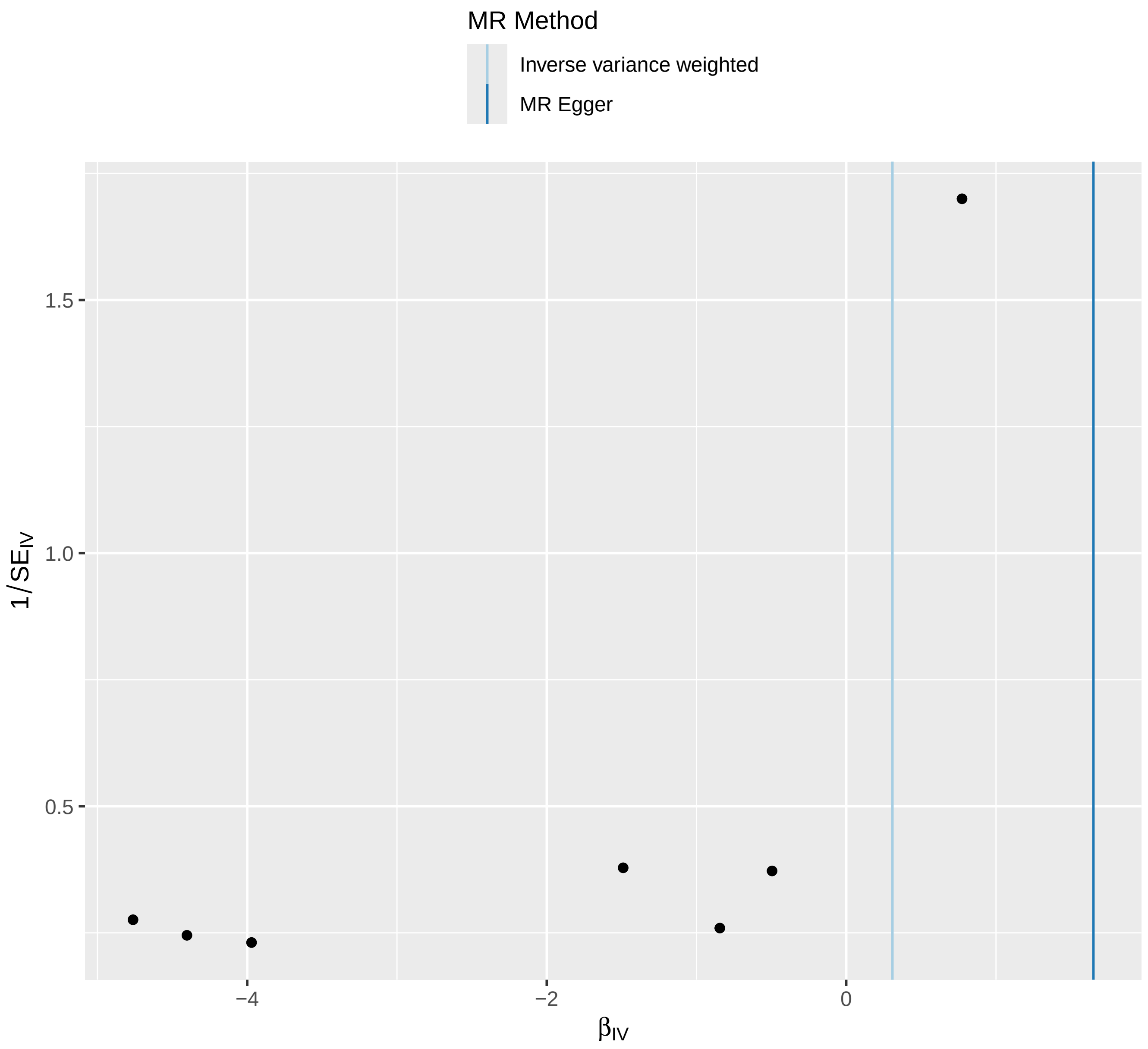
MR Method
Inverse variance weighted
MR Egger
1.5
1.0
0.5
1/SE_IV
−4
−2
0
β_IV

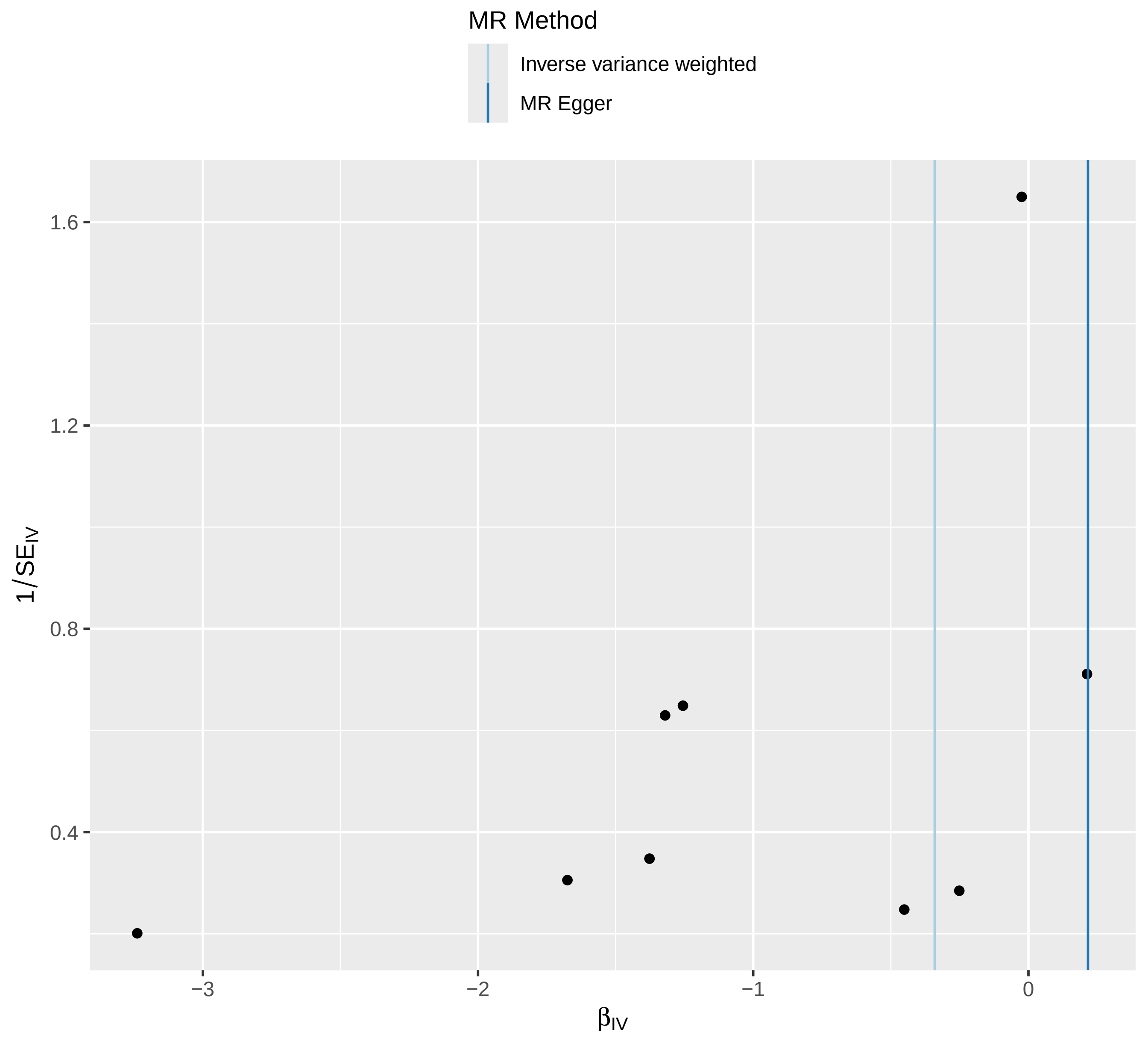
MR Method
Inverse variance weighted
MR Egger
1.6
1.2
0.8
0.4
1/SE_IV
−3
−2
−1
0
β_IV

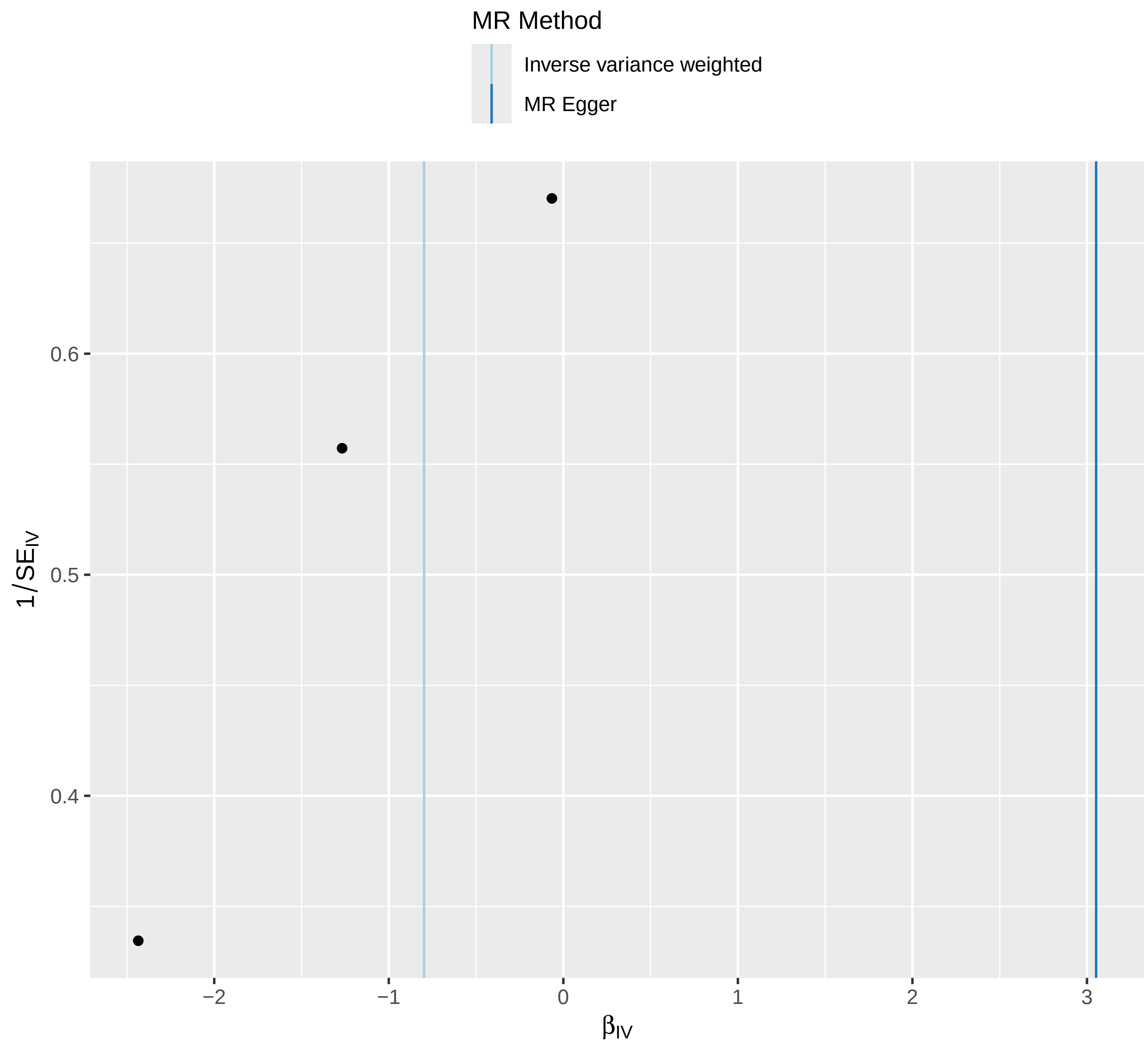

MR Method
Inverse variance weighted
MR Egger
0.6
0.5
0.4
1/SE_IV
−2
−1
0
1
2
3
β_IV

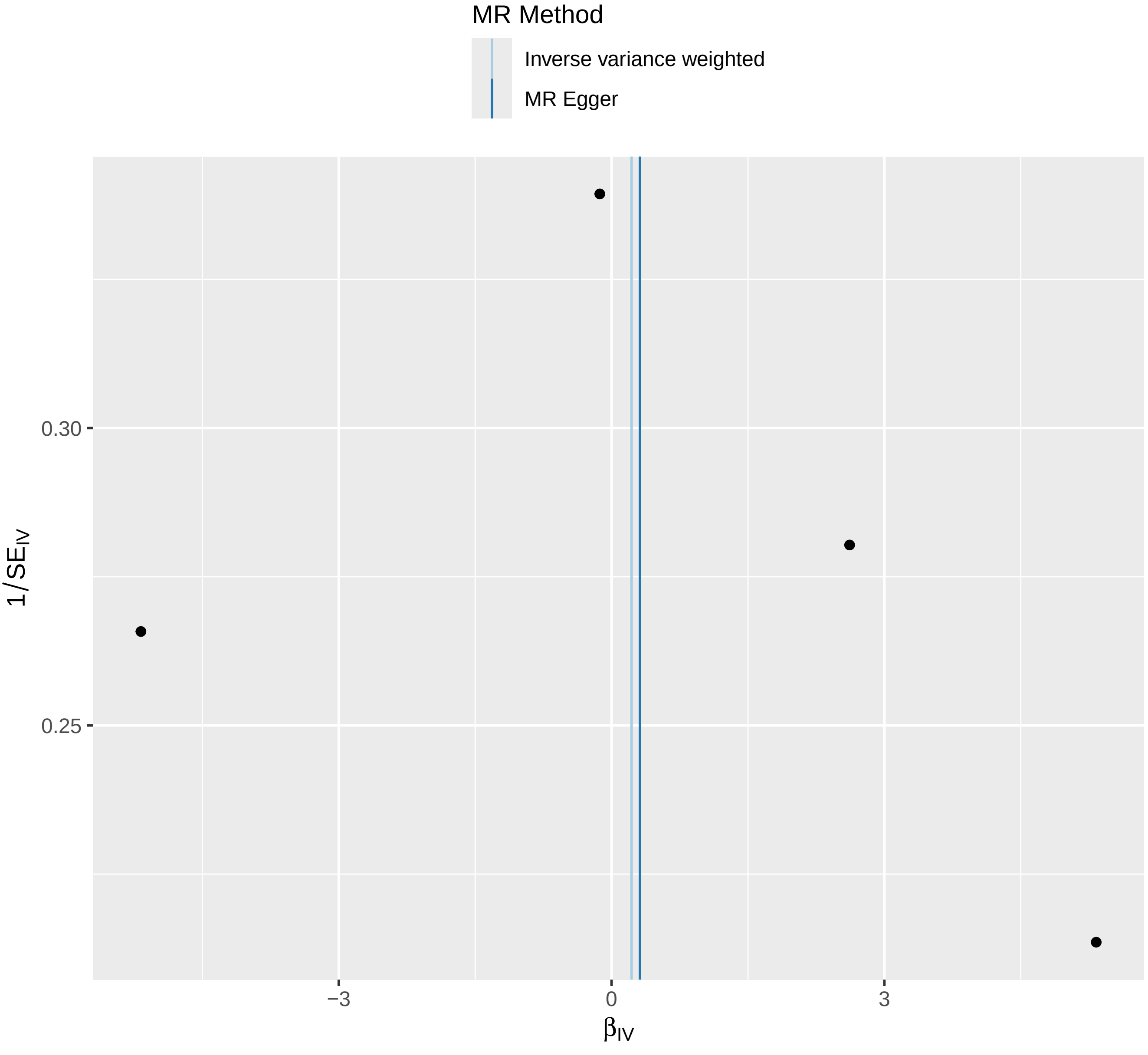
MR Method
Inverse variance weighted
MR Egger
0.30
0.25
1/SE_IV
−3
0
3
β_IV

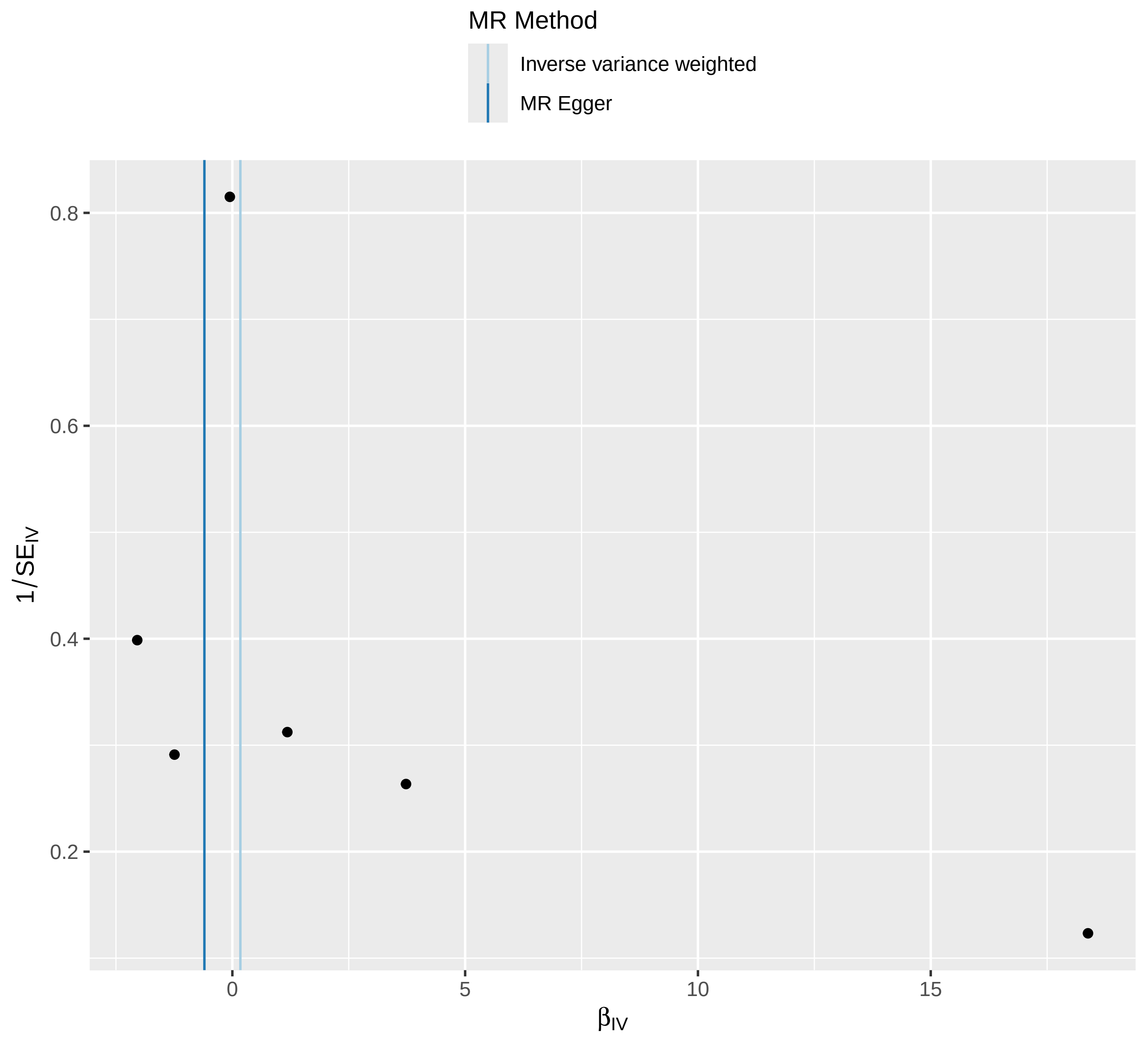
MR Method
Inverse variance weighted
MR Egger
0.8
0.6
0.4
0.2
$1/SE_{IV}$
0
5
10
15
β_{IV}

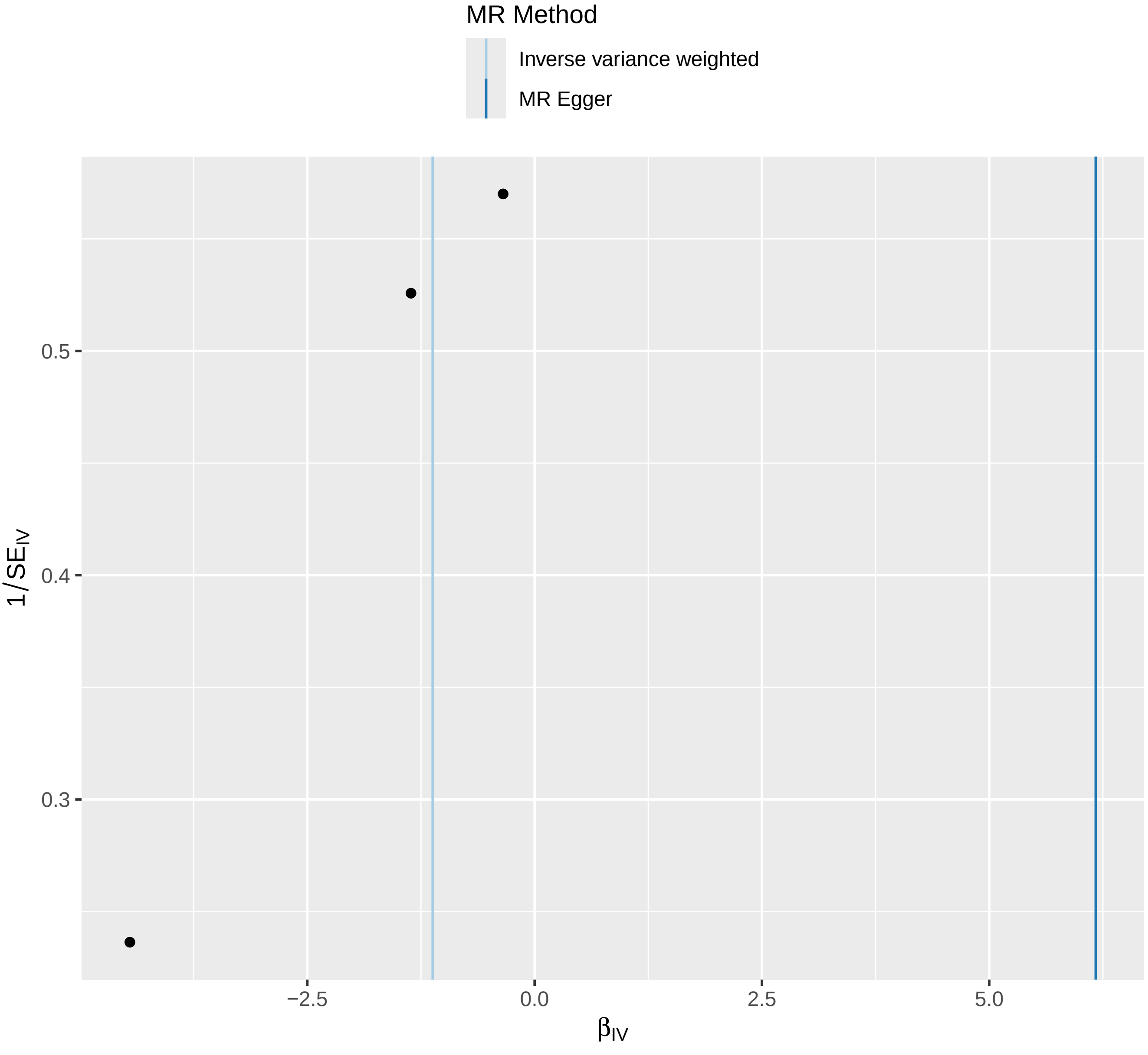
MR Method
Inverse variance weighted
MR Egger
0.5
0.4
0.3
1/SE$_{IV}$
−2.5
0.0
2.5
5.0
β$_{IV}$

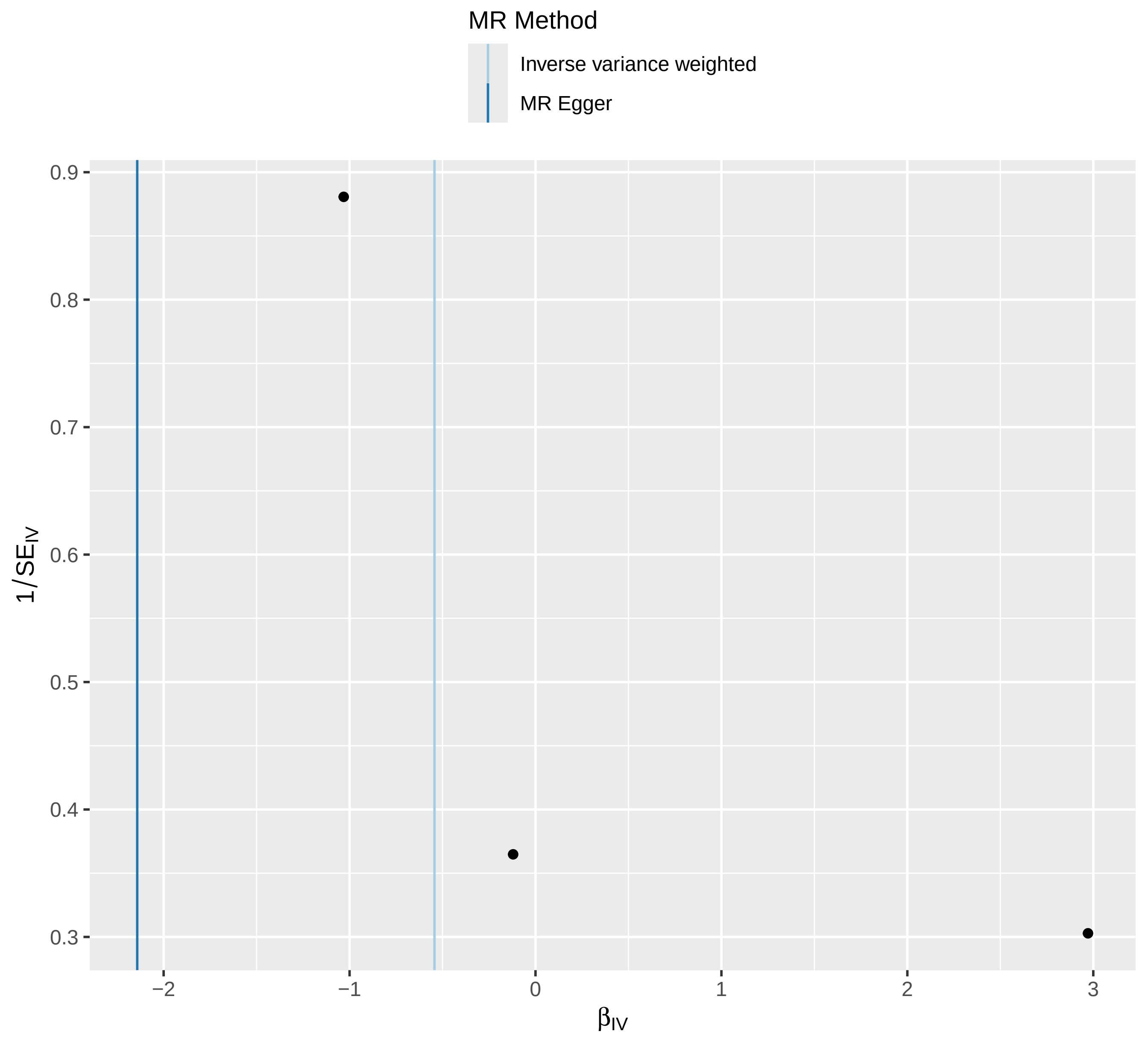
MR Method
Inverse variance weighted
MR Egger
0.9
0.8
0.7
0.6
0.5
0.4
0.3
1/SE_IV
−2
−1
0
1
2
3
β_IV

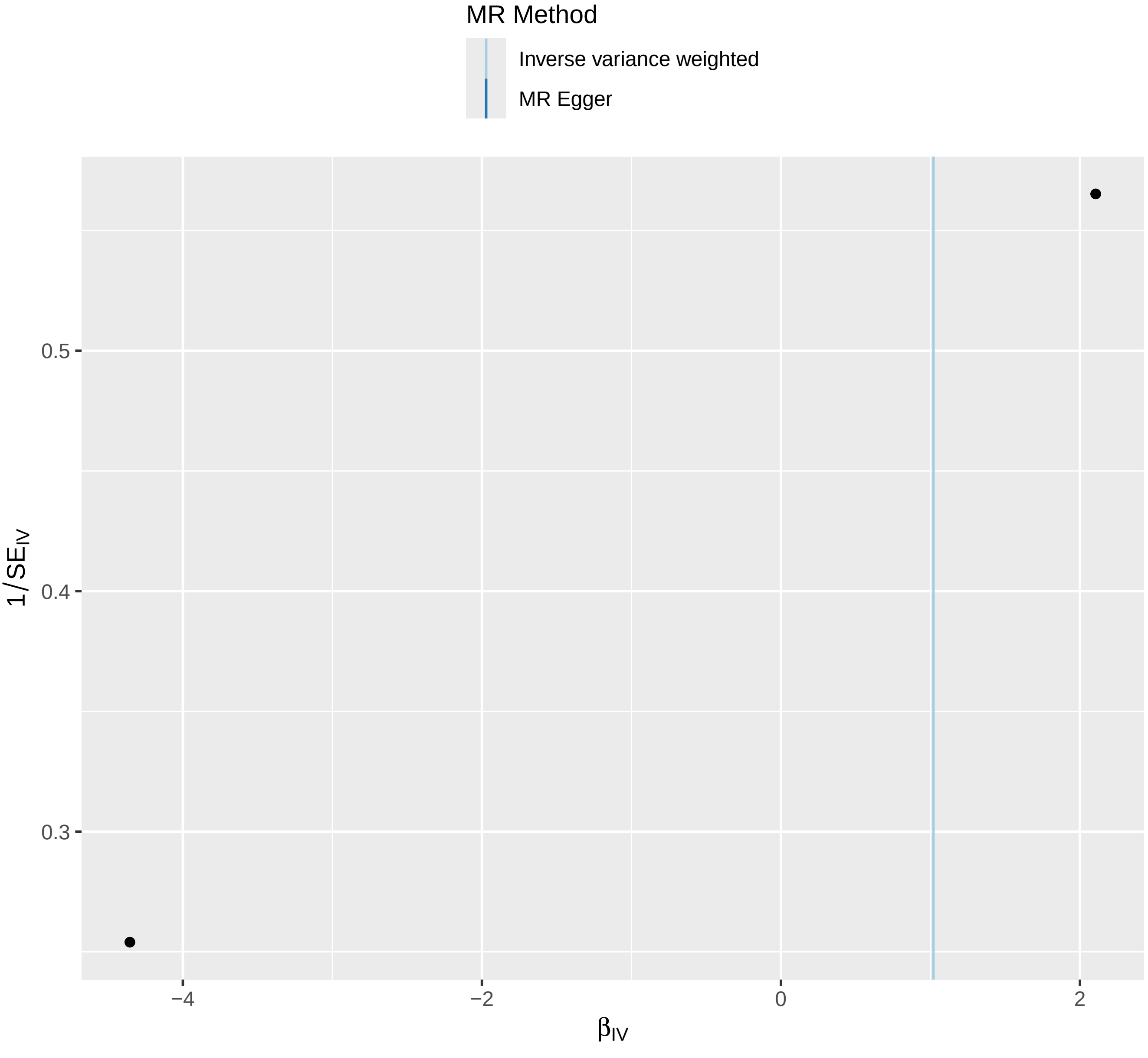

MR Method
Inverse variance weighted
MR Egger
1/SE_IV
0.5
0.4
0.3
−4
−2
0
2
β_IV

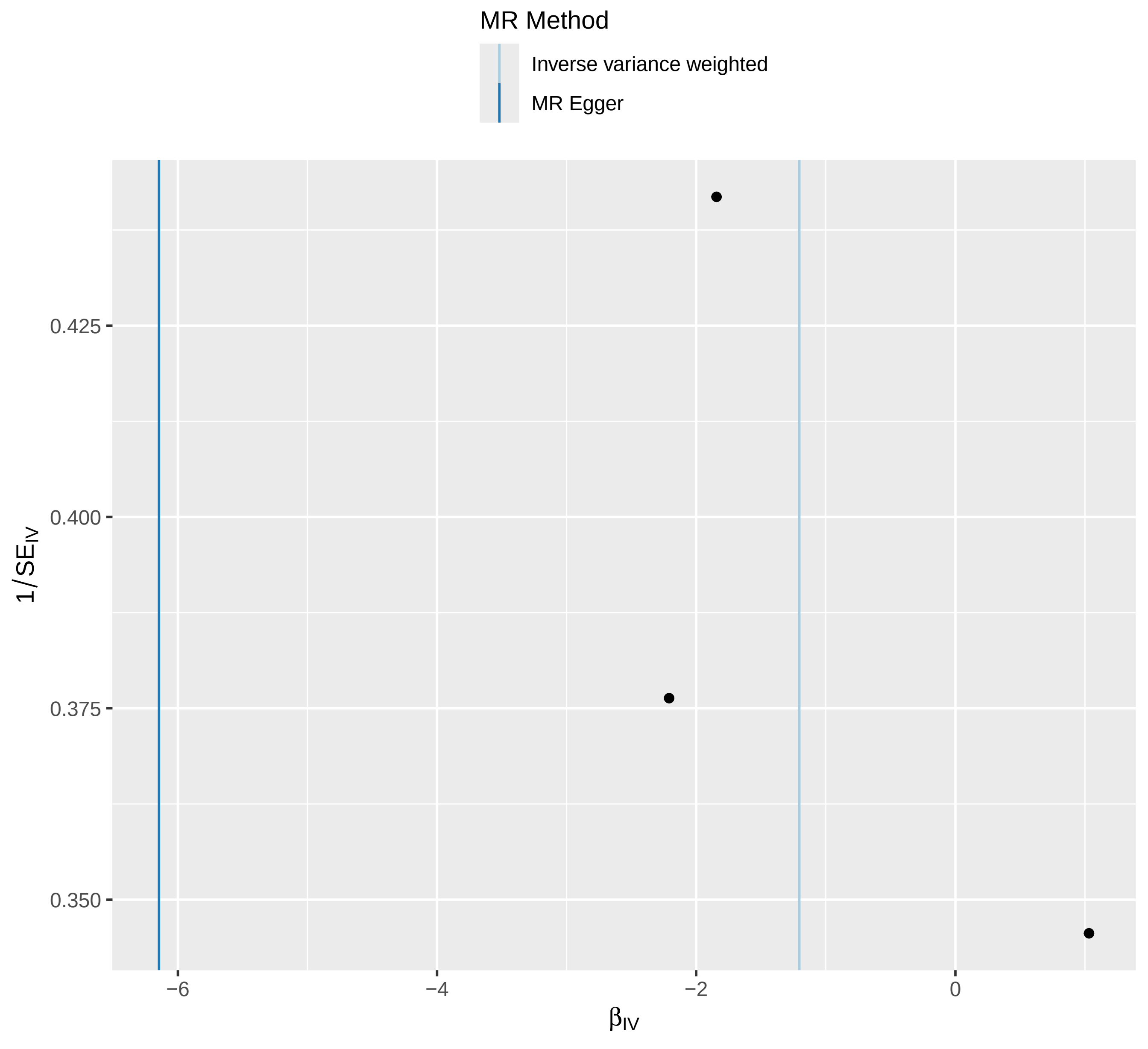
MR Method
Inverse variance weighted
MR Egger
0.425
0.400
0.375
0.350
1/SE$_{IV}$
−6
−4
−2
0
β$_{IV}$

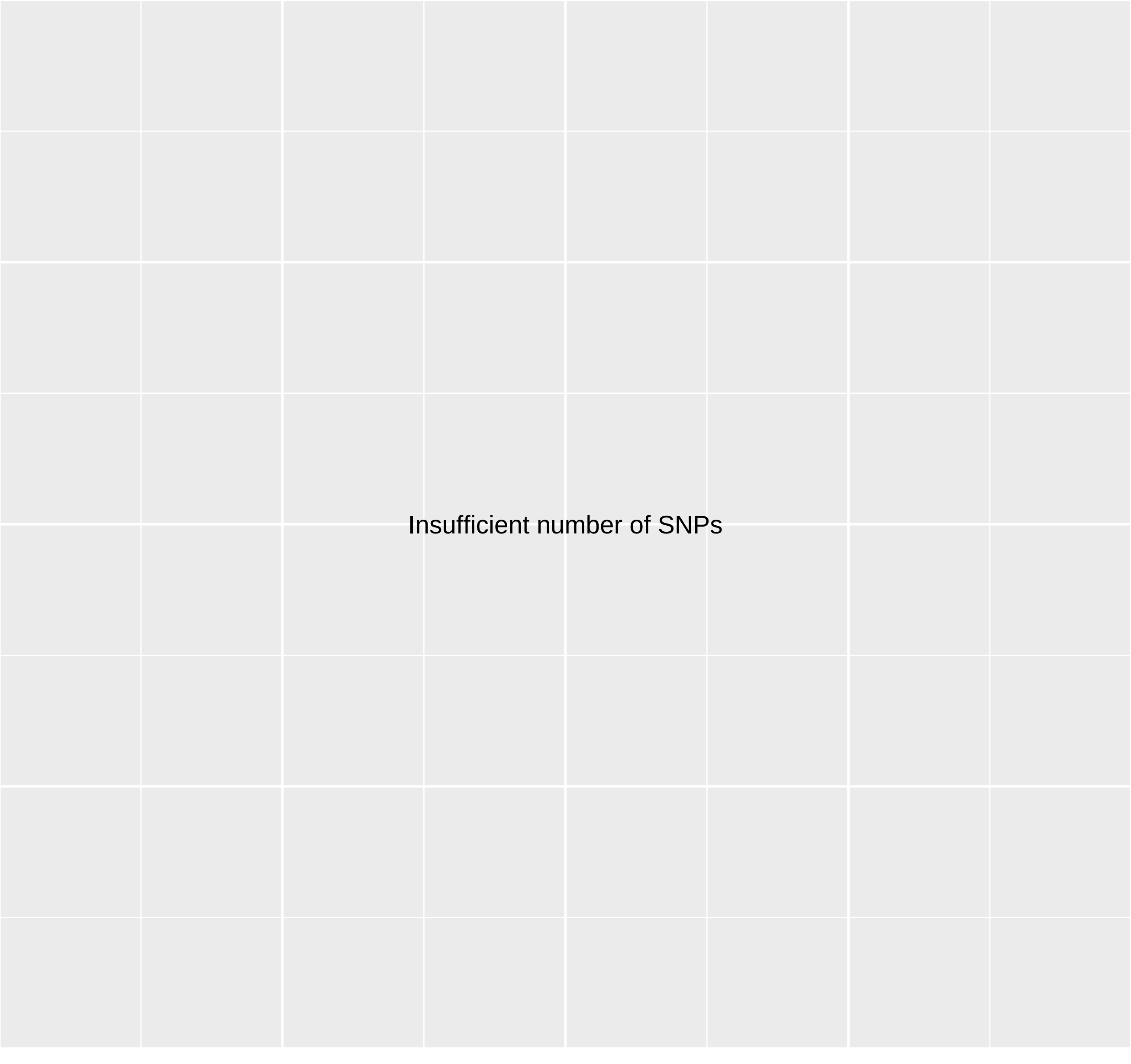
Insufficient number of SNPs

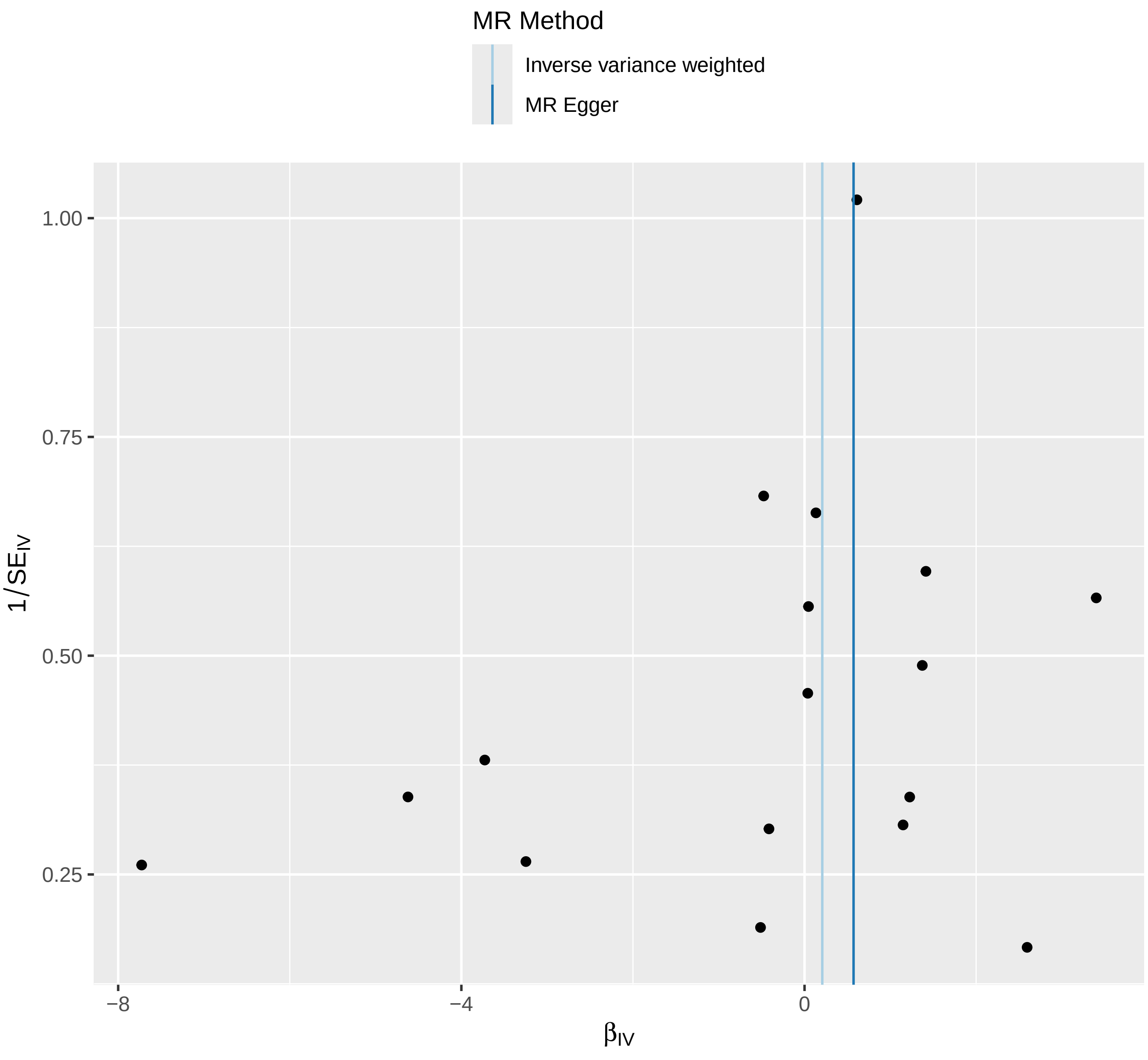
MR Method
Inverse variance weighted
MR Egger
1.00
0.75
0.50
0.25
1/SE$_{IV}$
−8
−4
0
β_{IV}

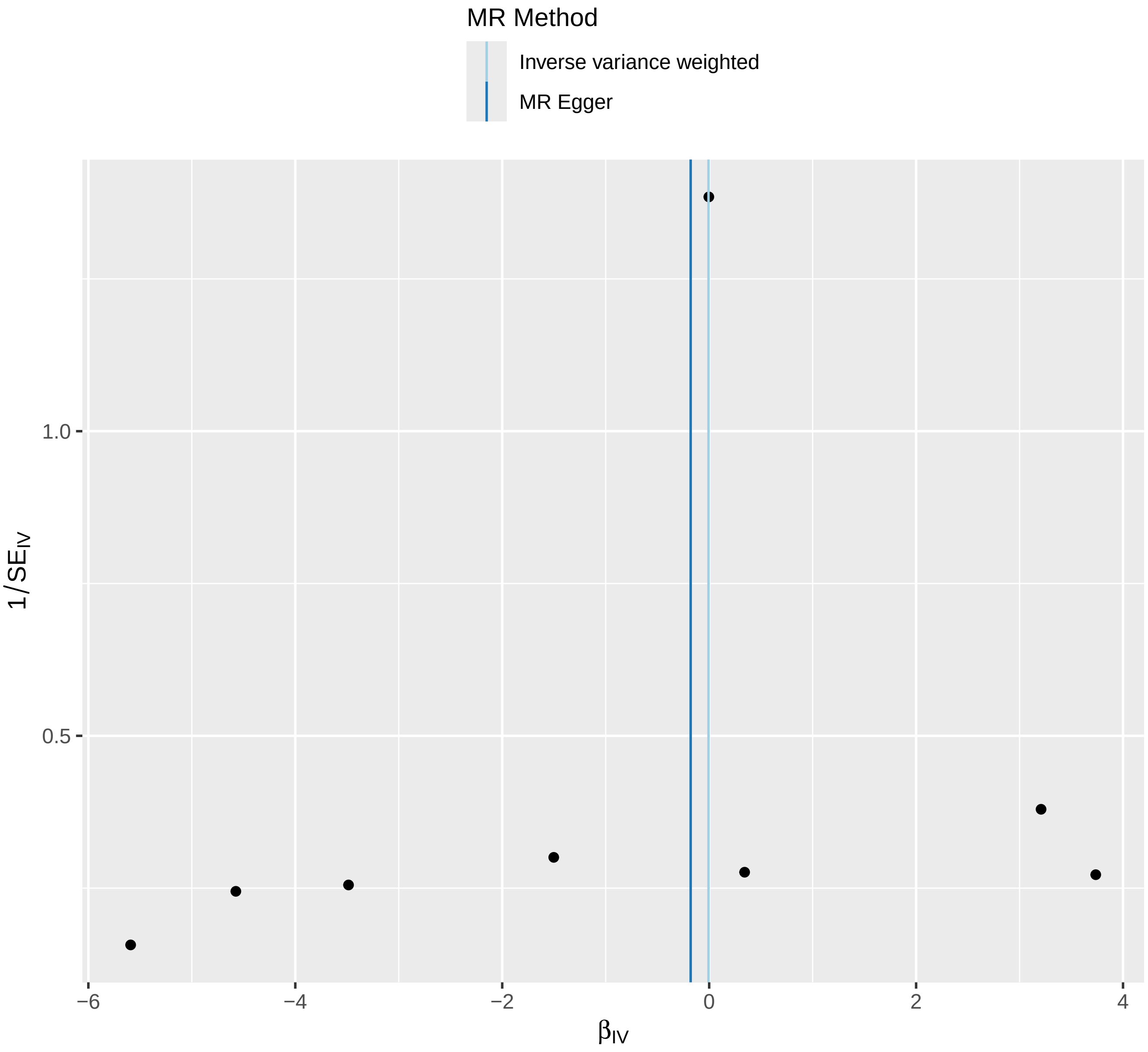
MR Method
Inverse variance weighted
MR Egger
1/SE$_{IV}$
1.0
0.5
−6
−4
−2
0
2
4
β$_{IV}$

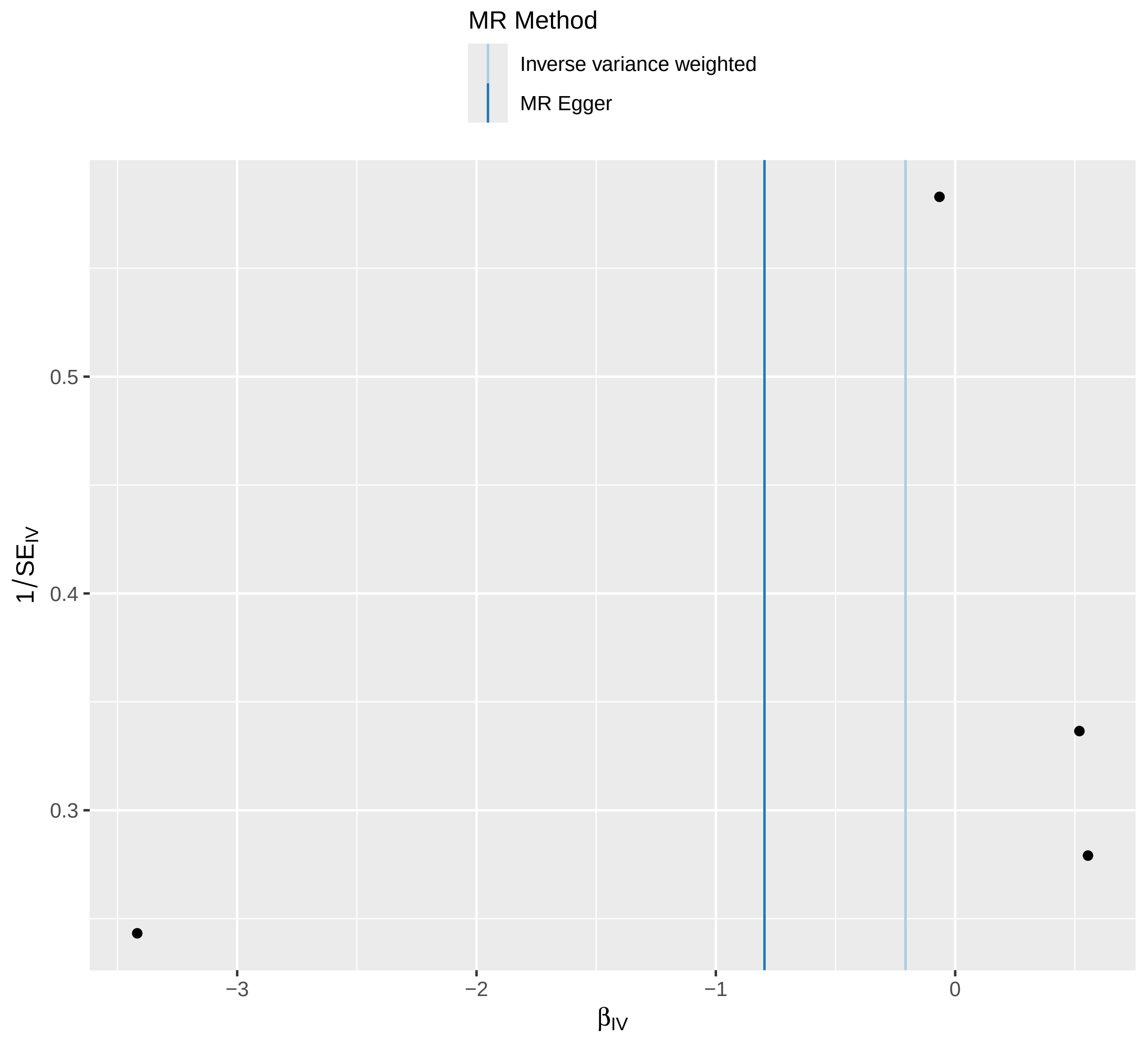
MR Method
Inverse variance weighted
MR Egger
0.5
0.4
0.3
1/SE_IV
−3
−2
−1
0
β_IV

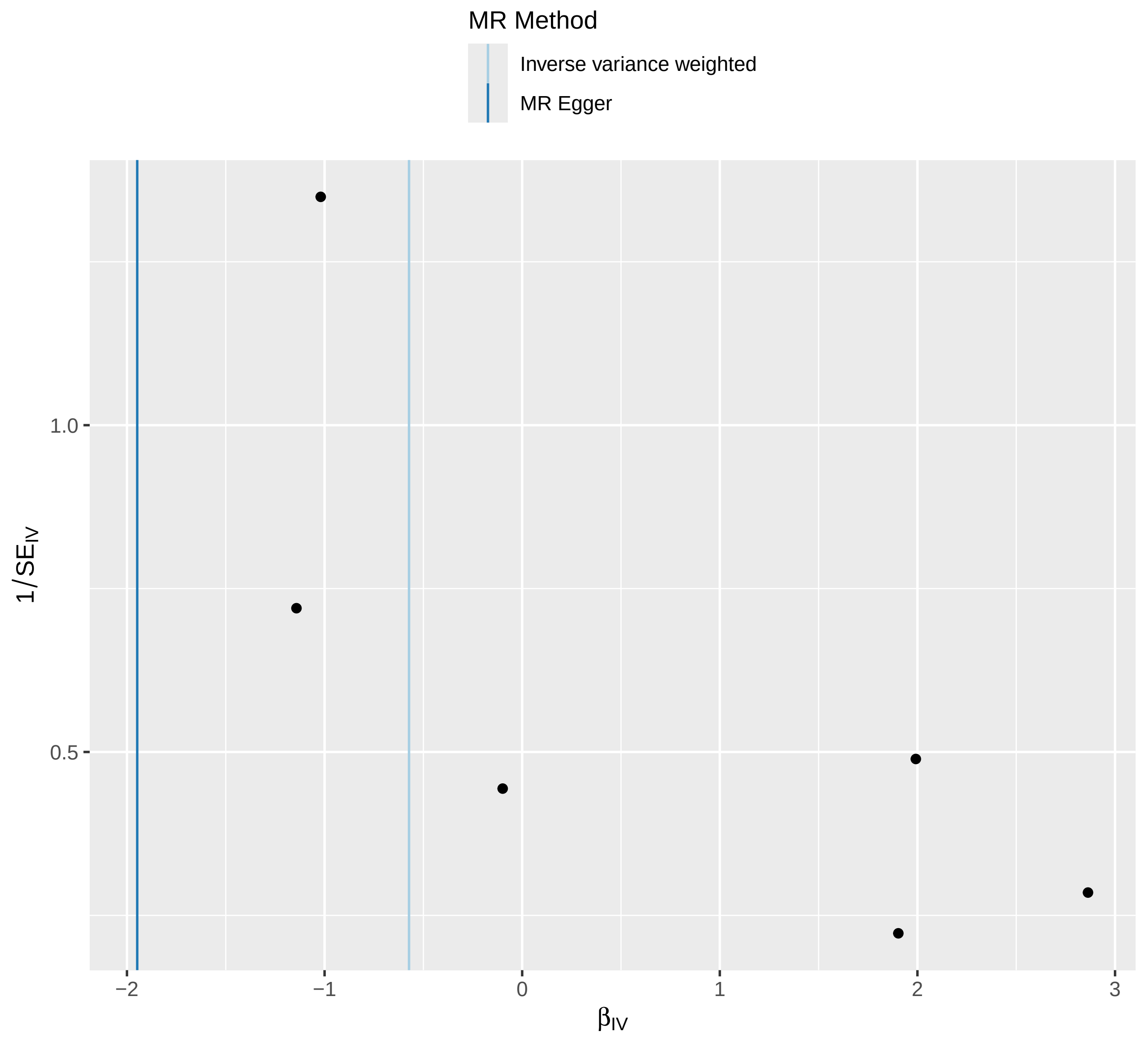

MR Method
Inverse variance weighted
MR Egger
1.0
0.5
1/SE_IV
−2
−1
0
1
2
3
β_IV

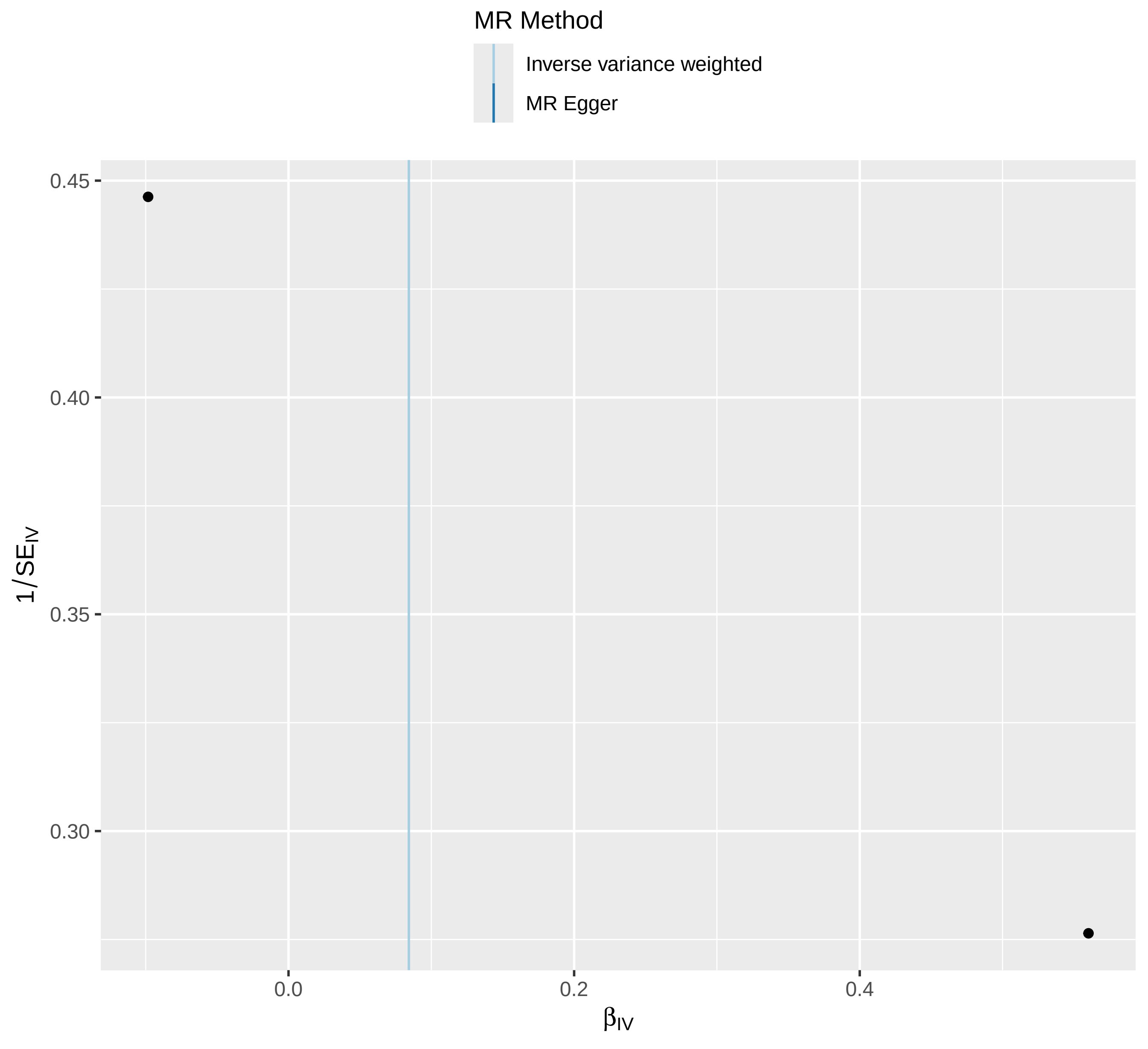
MR Method
Inverse variance weighted
MR Egger
0.45
0.40
0.35
0.30
$1/SE_{IV}$
0.0
0.2
0.4
β_{IV}

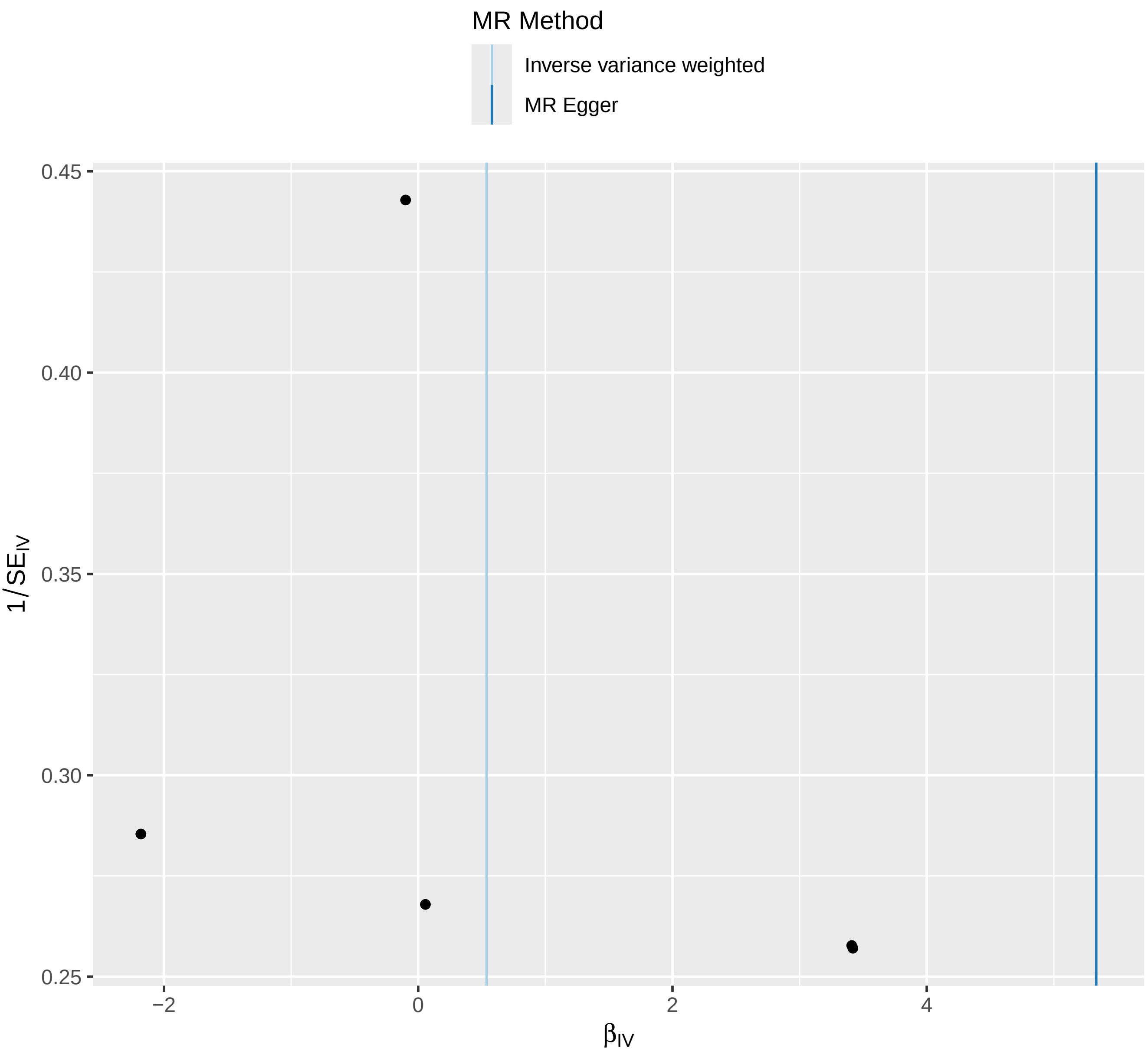

MR Method
Inverse variance weighted
MR Egger
0.45
0.40
0.35
0.30
0.25
1/SE_IV
−2
0
2
4
β_IV

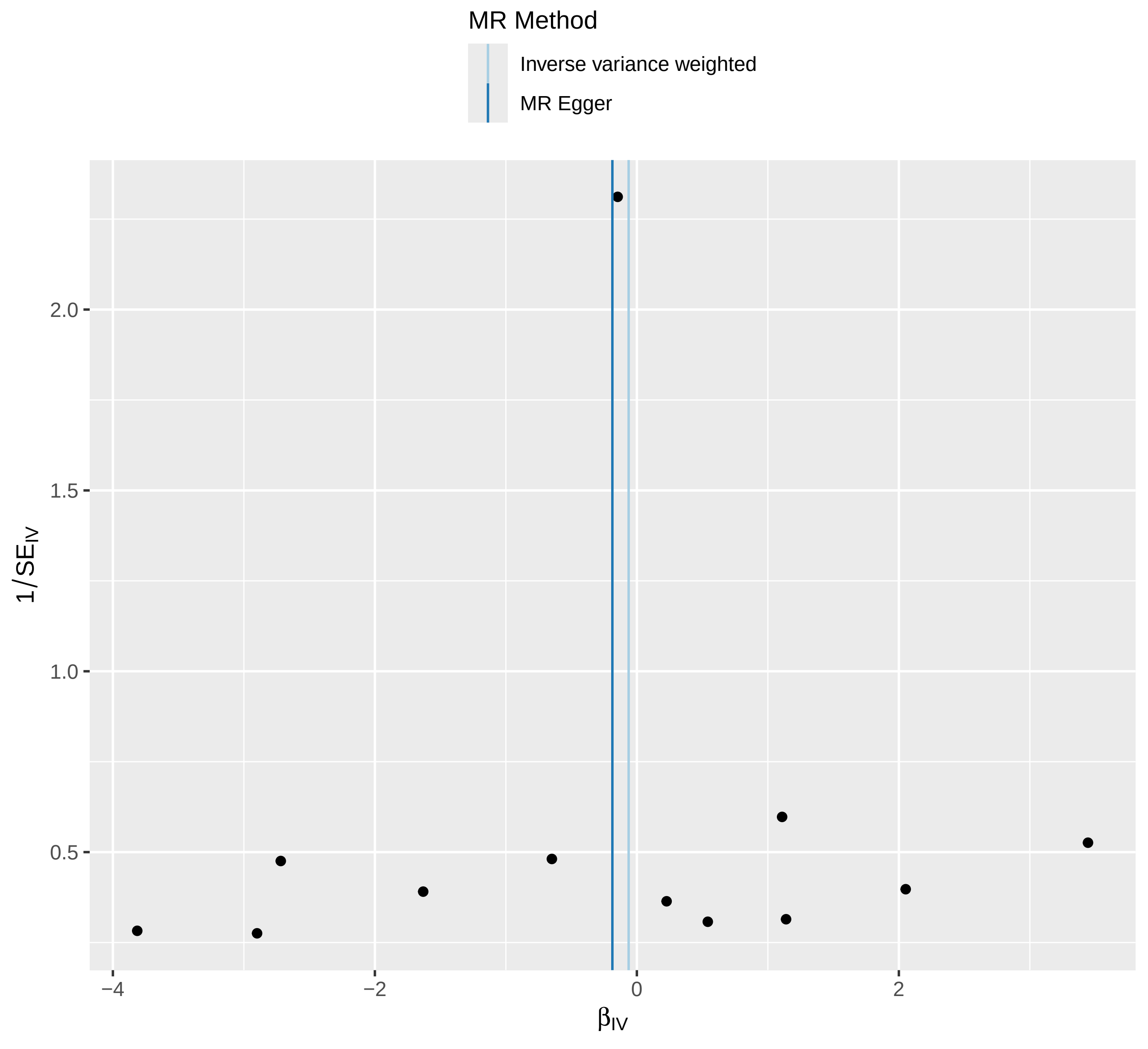
MR Method
Inverse variance weighted
MR Egger
2.0
1.5
1.0
0.5
1/SE_IV
−4
−2
0
2
β_IV

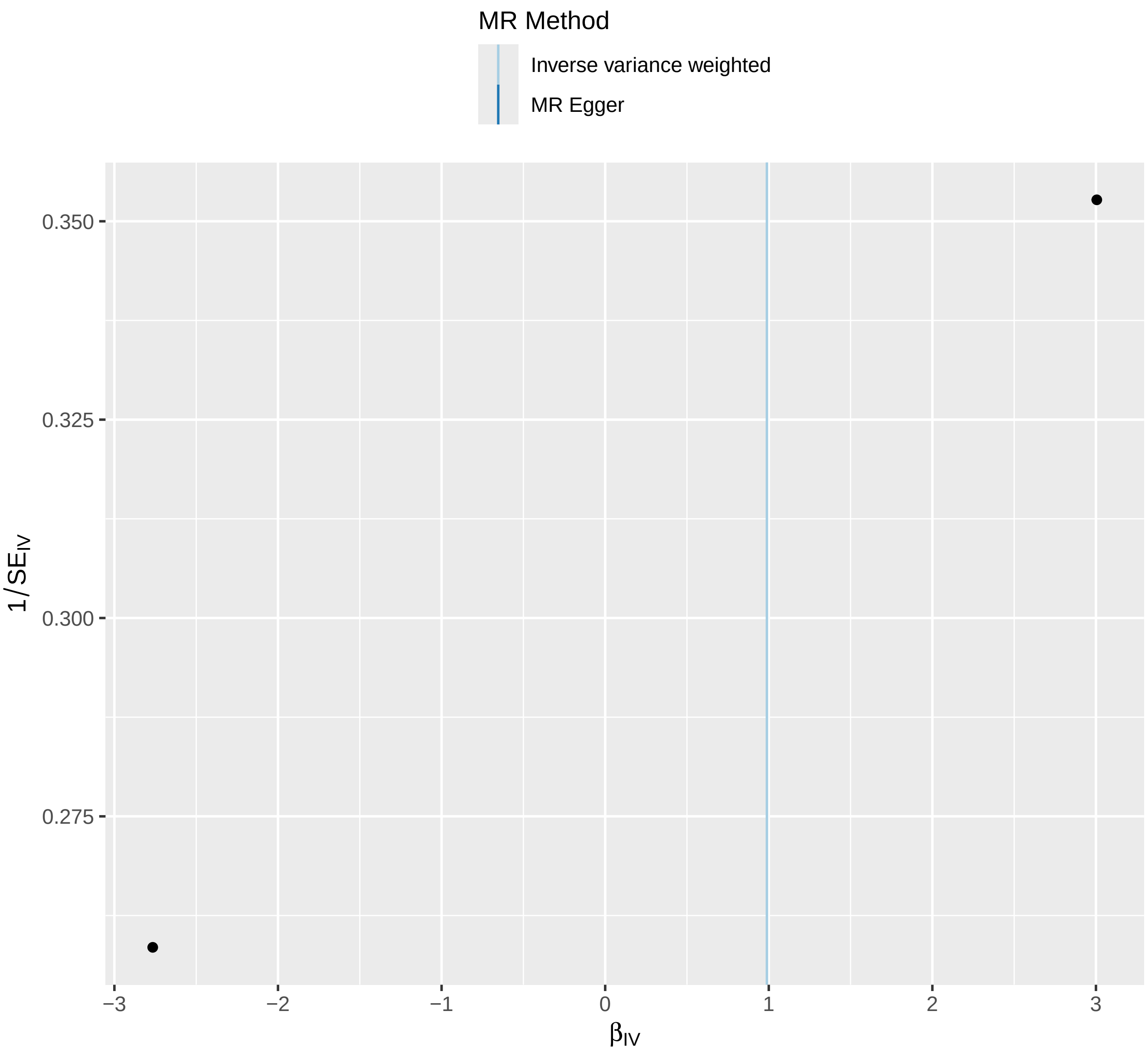
MR Method
Inverse variance weighted
MR Egger
0.350
0.325
0.300
0.275
1/SE$_{IV}$
−3
−2
−1
0
1
2
3
β$_{IV}$

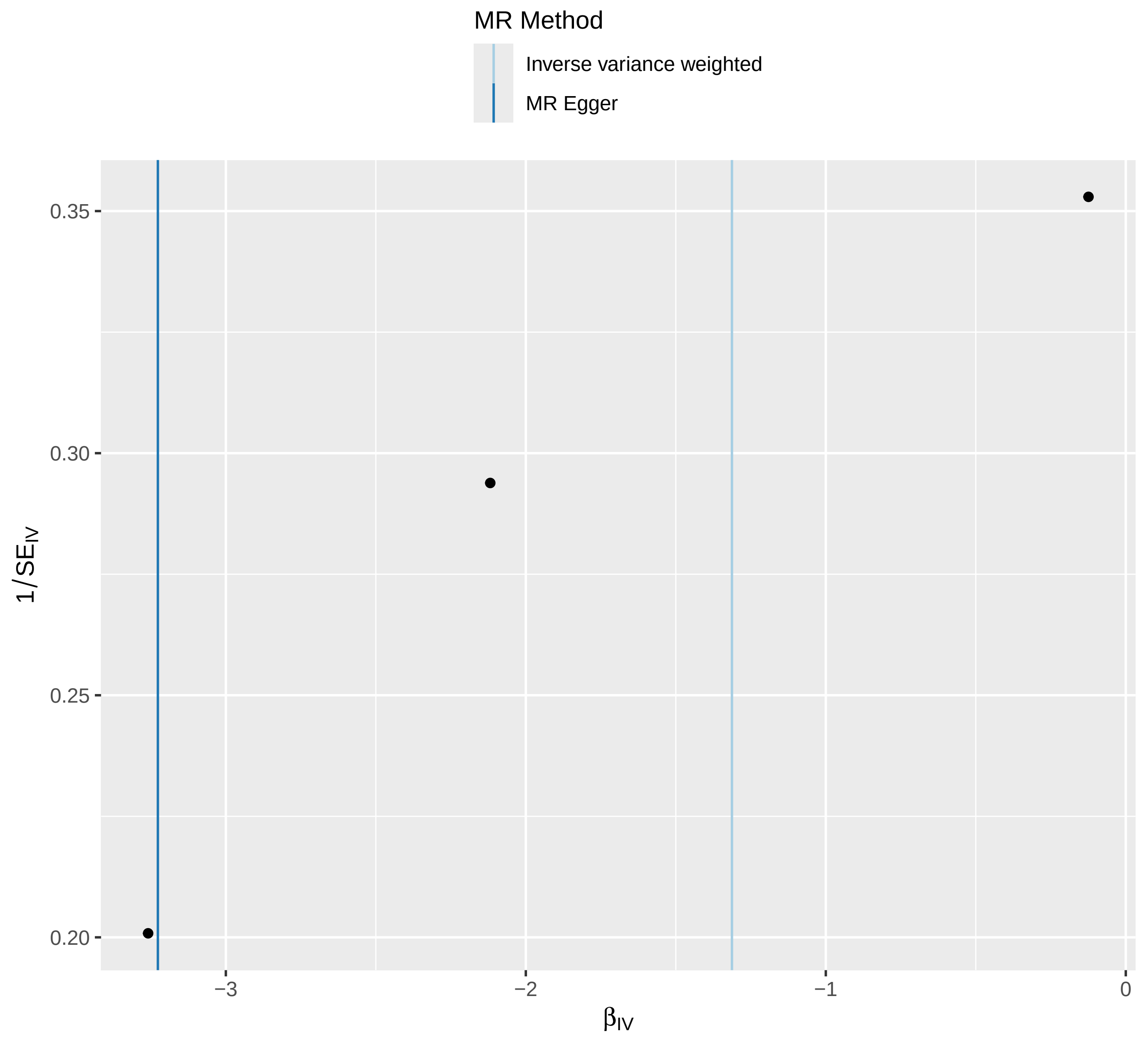
MR Method
Inverse variance weighted
MR Egger
0.35
0.30
0.25
0.20
1/SE_IV
−3
−2
−1
0
β_IV

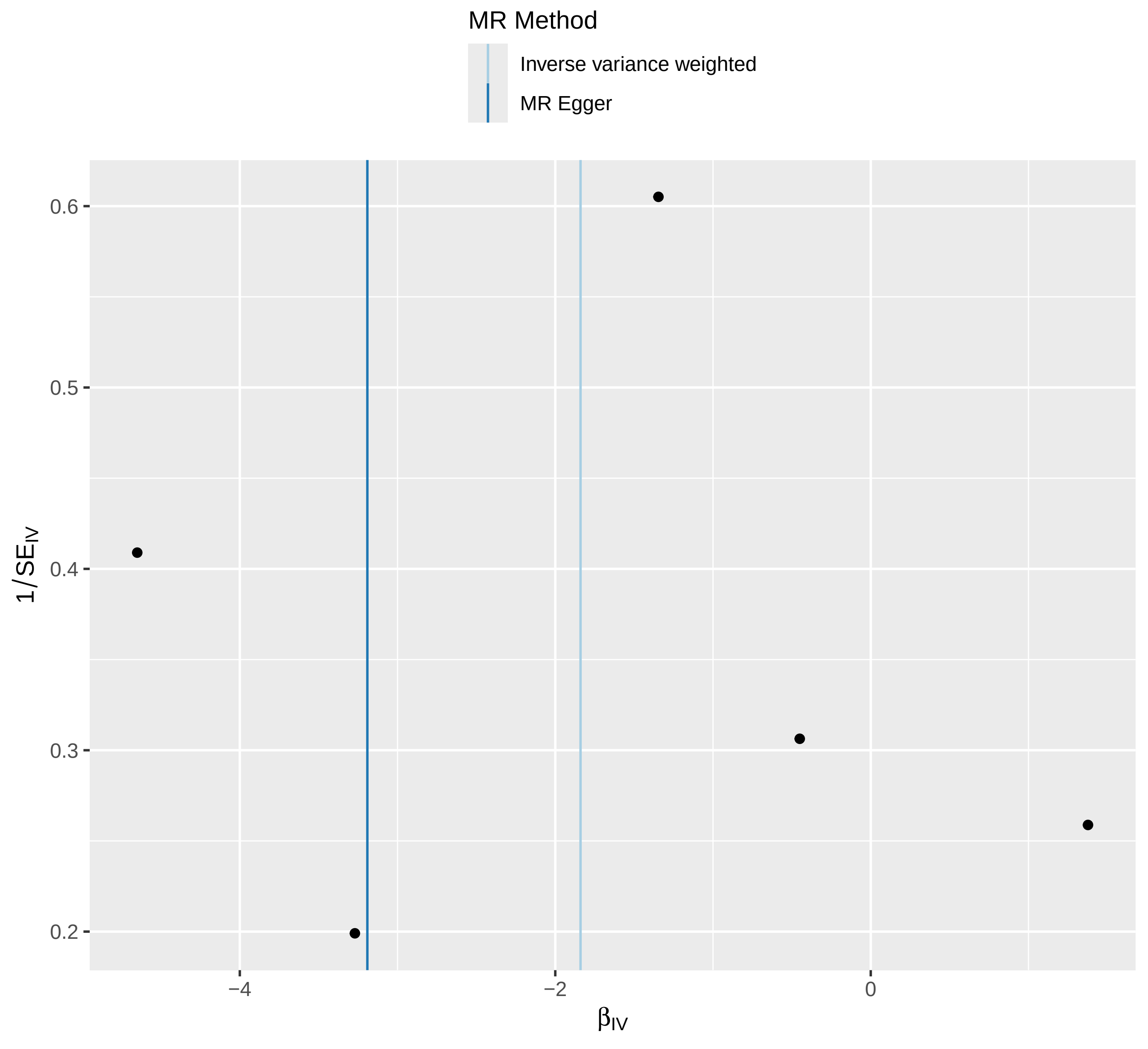
MR Method
Inverse variance weighted
MR Egger
0.6
0.5
0.4
0.3
0.2
1/SE_IV
−4
−2
0
β_IV

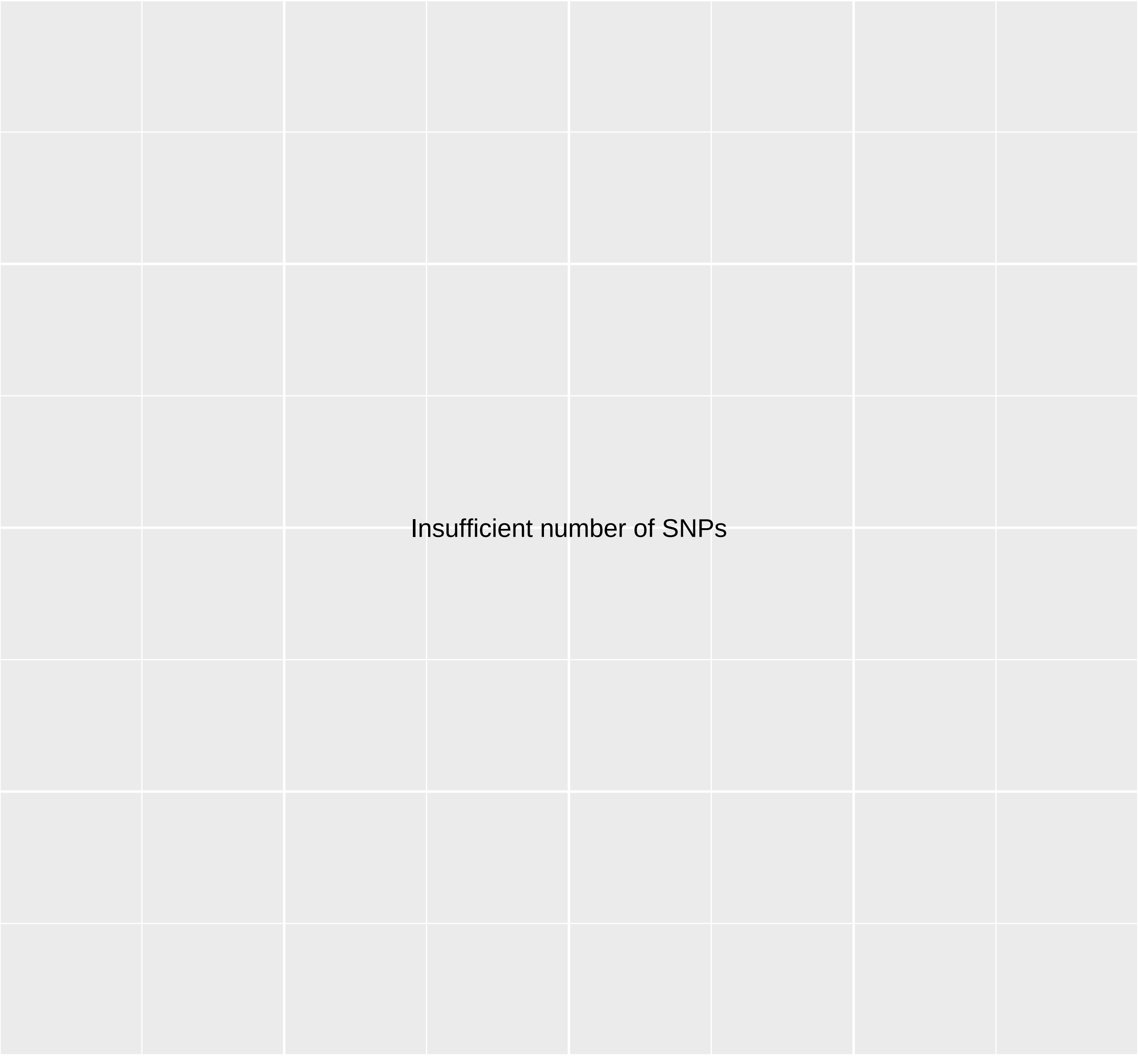
Insufficient number of SNPs

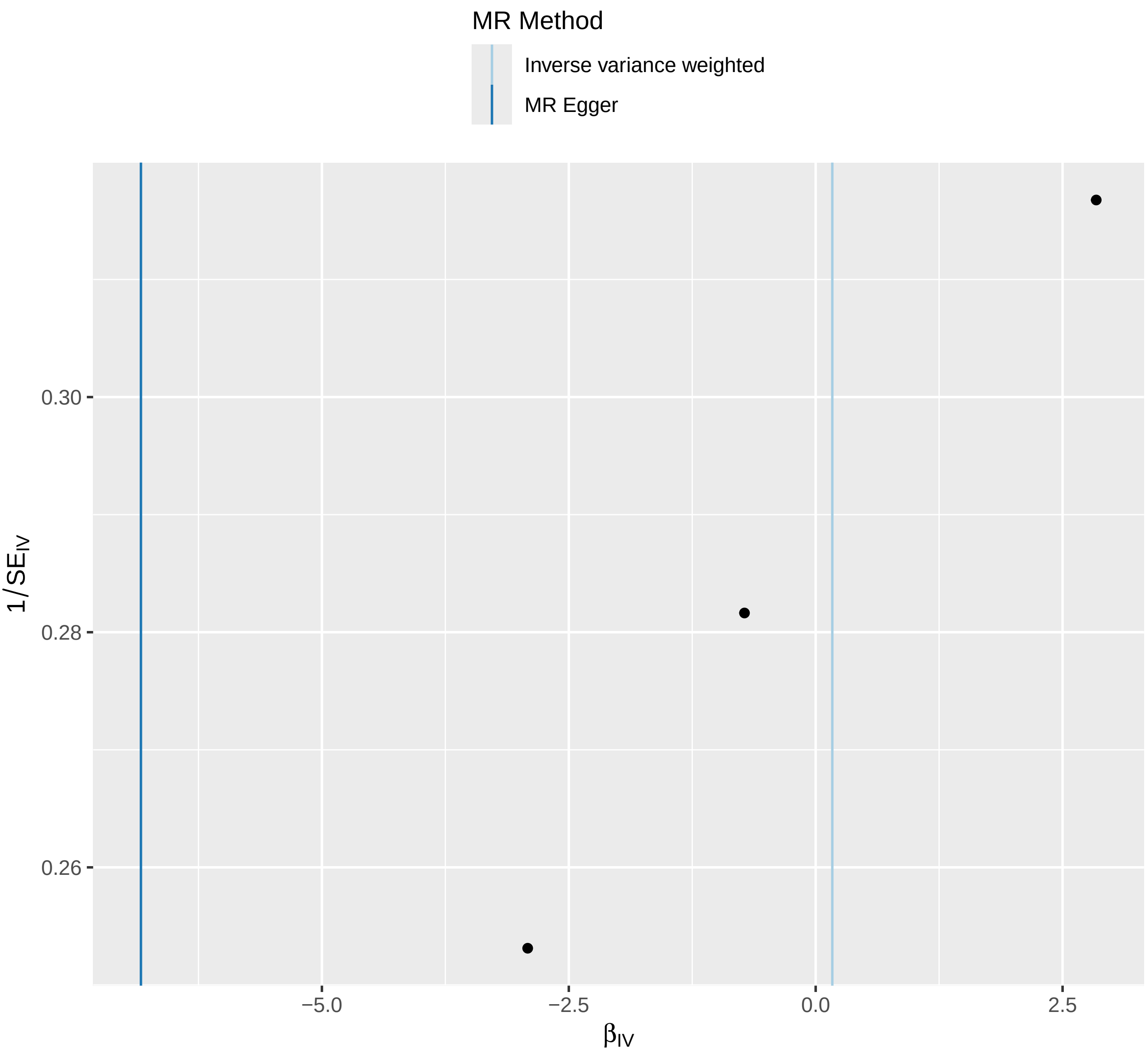
MR Method
Inverse variance weighted
MR Egger
0.30
0.28
0.26
$1/SE_{IV}$
−5.0
−2.5
0.0
2.5
β_{IV}

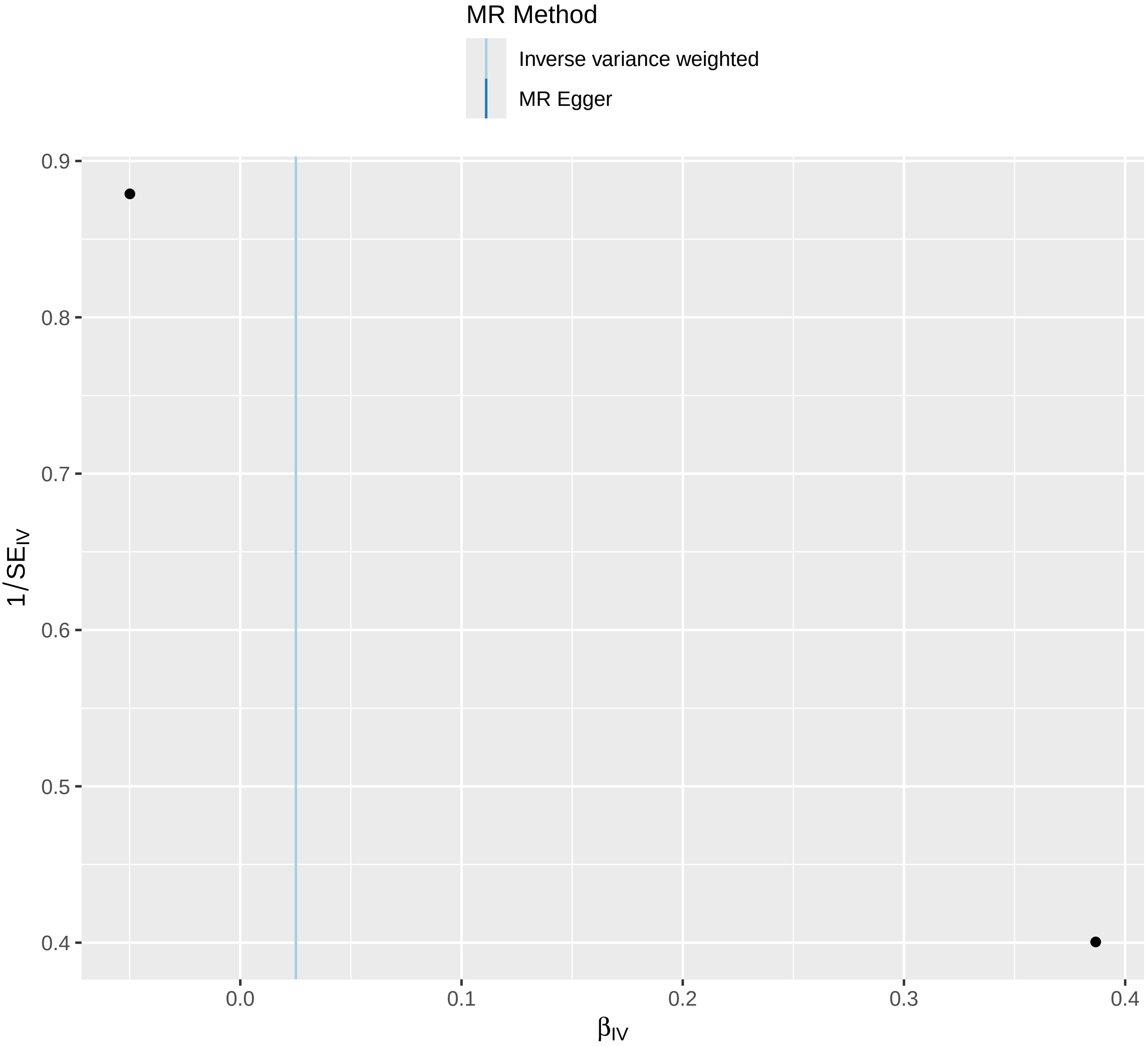
MR Method
Inverse variance weighted
MR Egger
0.9
0.8
0.7
0.6
0.5
0.4
$1/SE_{IV}$
0.0
0.1
0.2
0.3
0.4
β_{IV}

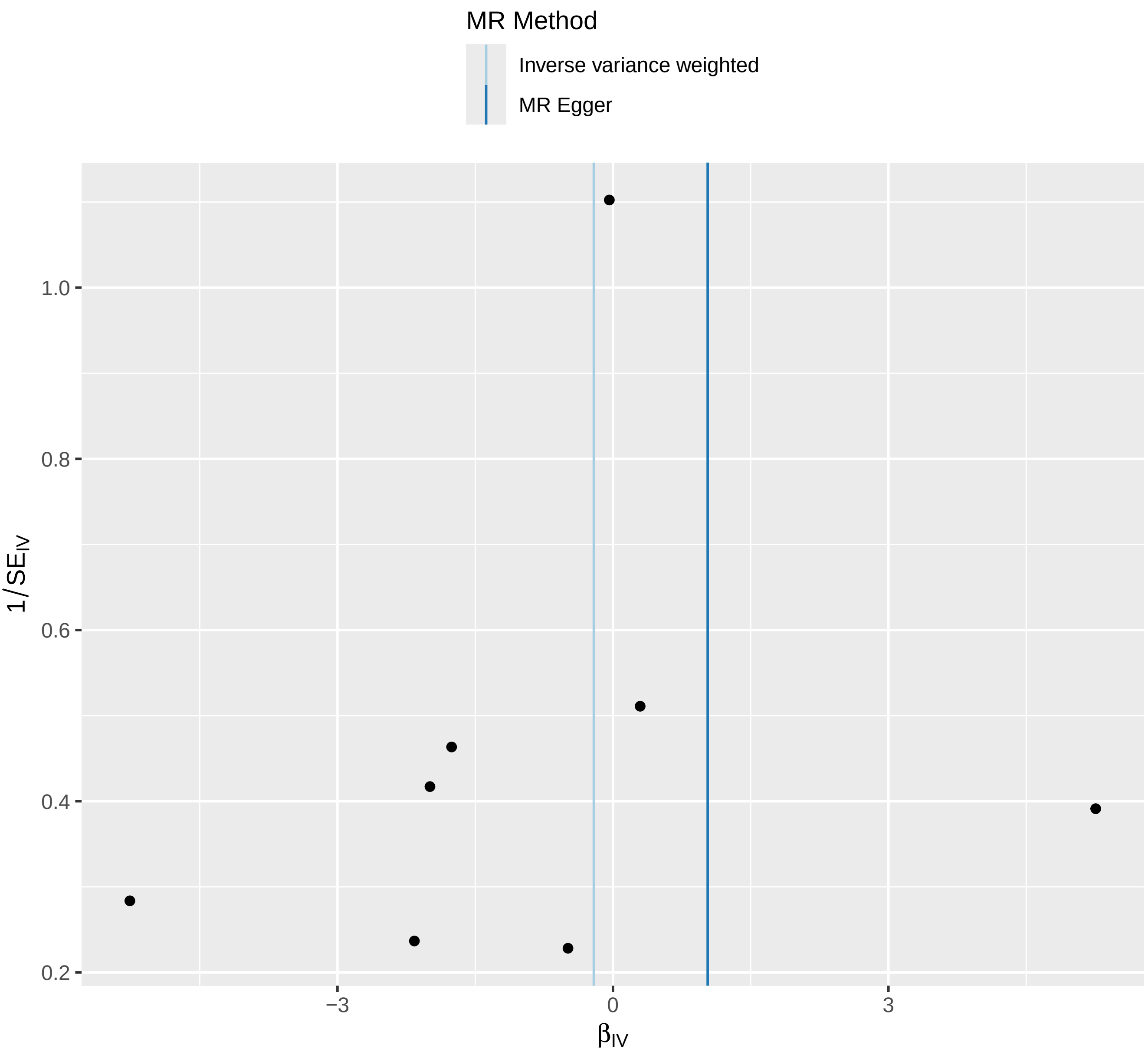
MR Method
Inverse variance weighted
MR Egger
1.0
0.8
0.6
0.4
0.2
−3
0
3
$1/SE_{IV}$
β_{IV}

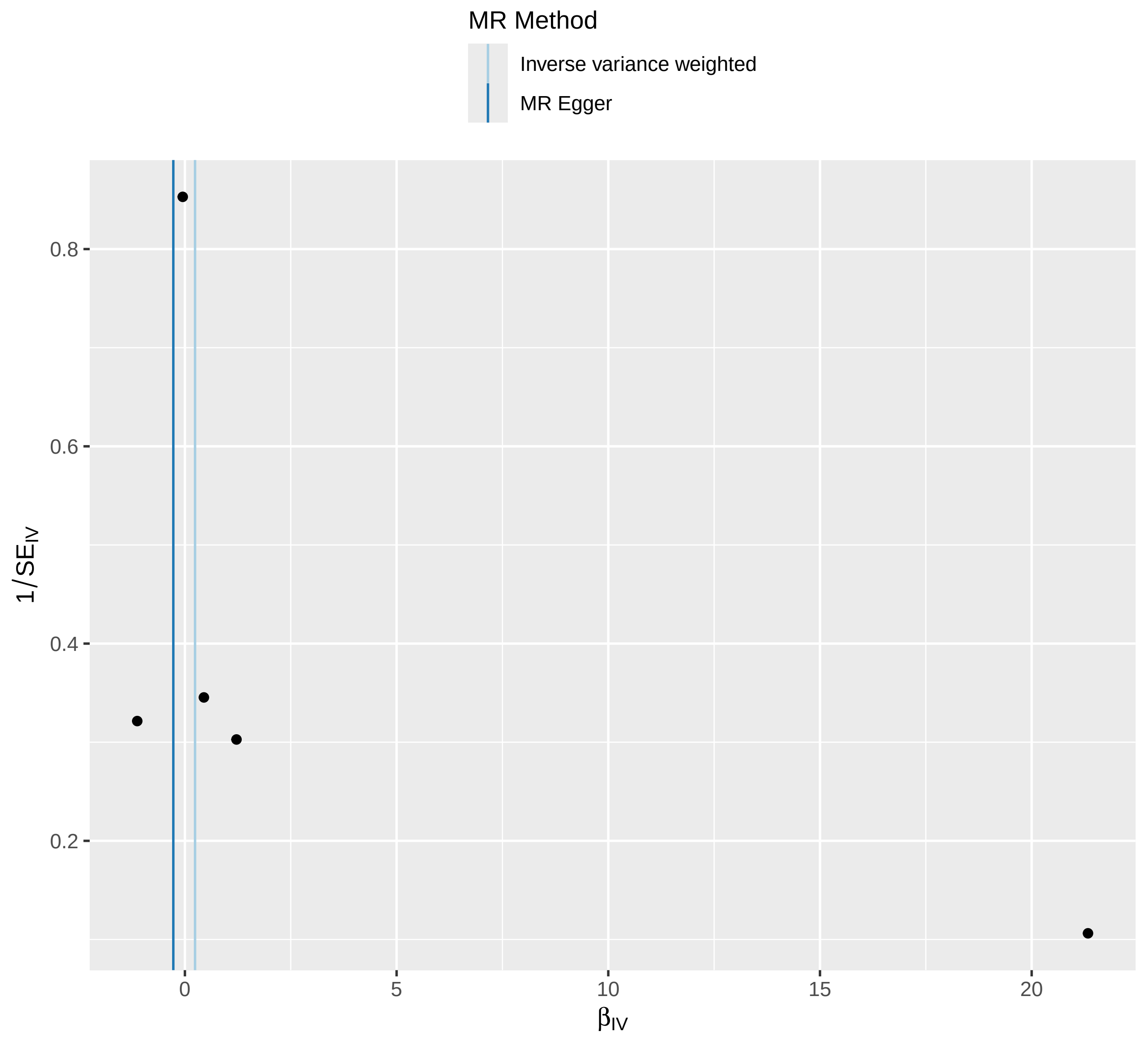

MR Method
Inverse variance weighted
MR Egger
0.8
0.6
0.4
0.2
1/SE_IV
0
5
10
15
20
β_IV

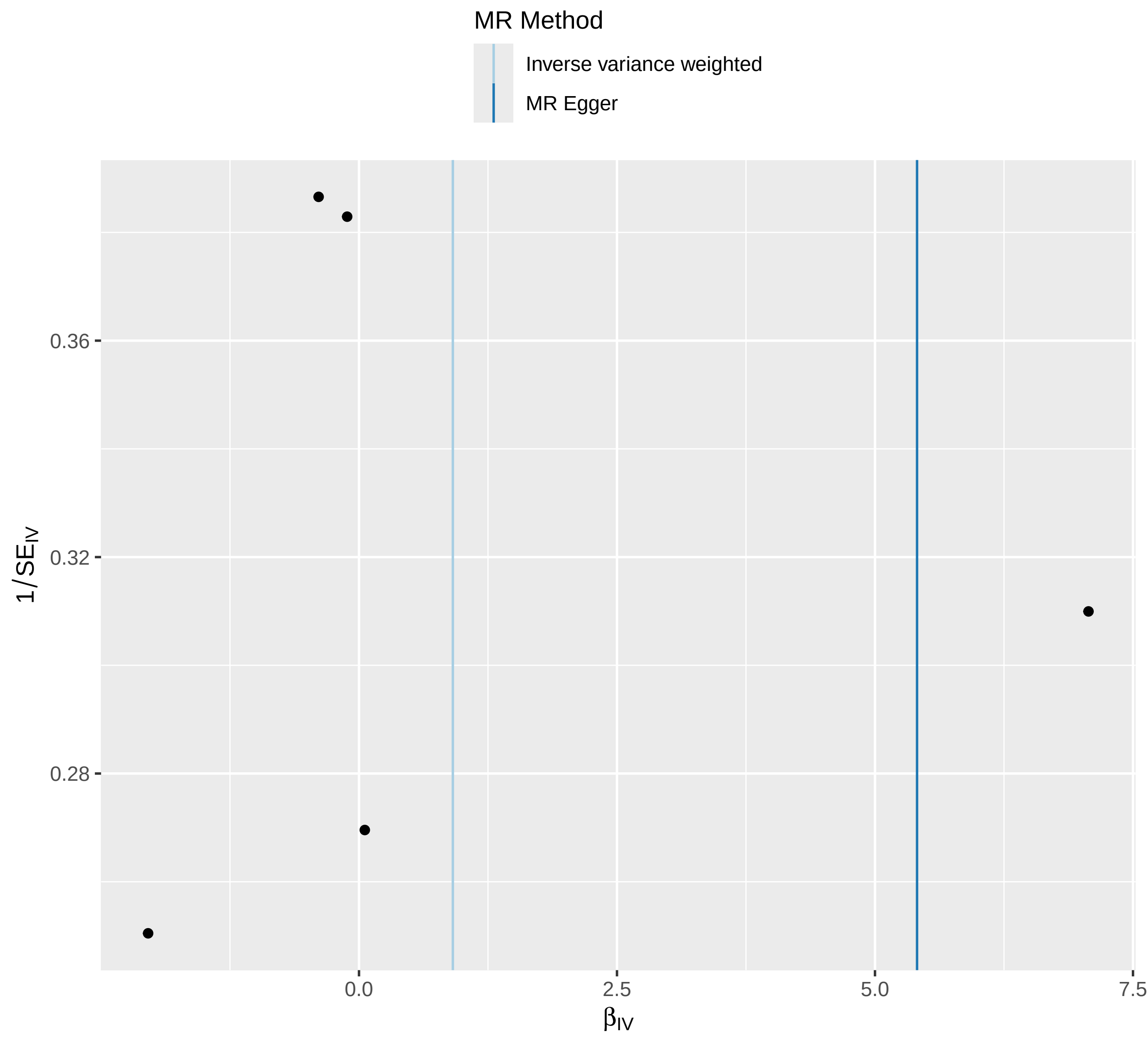

MR Method
Inverse variance weighted
MR Egger
0.36
0.32
0.28
$1/SE_{IV}$
0.0
2.5
5.0
7.5
β_{IV}

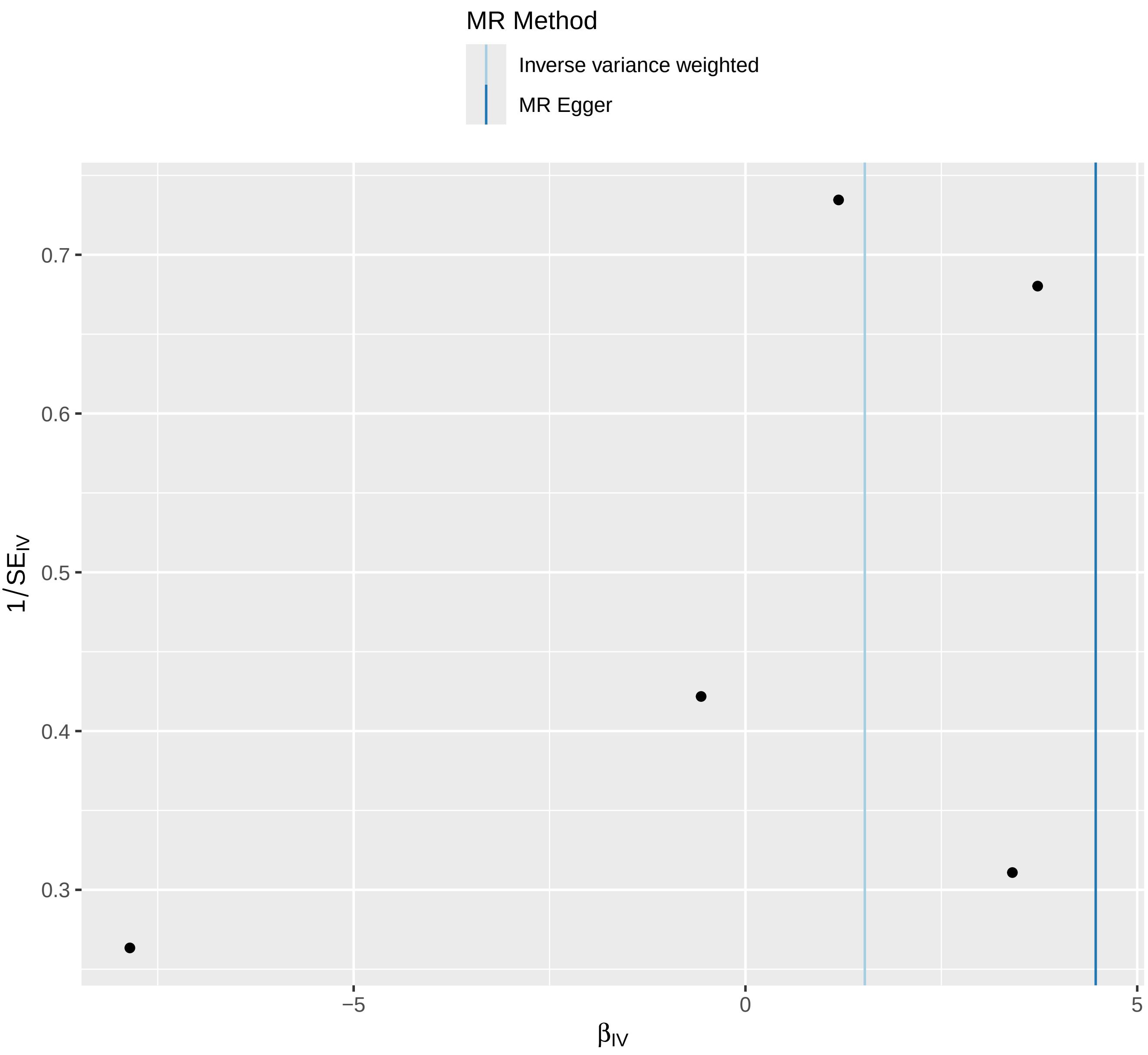

MR Method
Inverse variance weighted
MR Egger
0.7
0.6
0.5
0.4
0.3
1/SE$_{IV}$
−5
0
5
β$_{IV}$

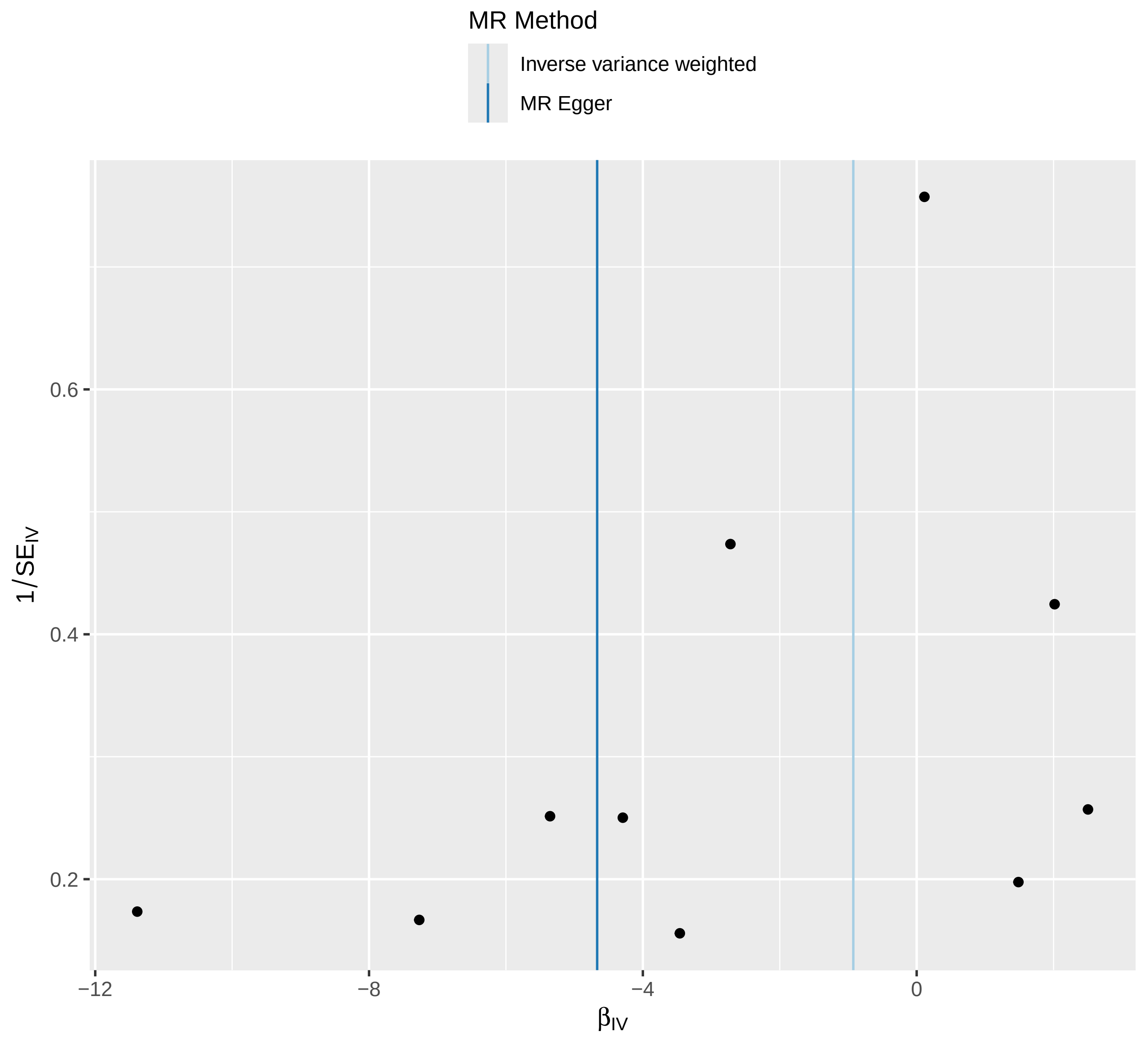

MR Method
Inverse variance weighted
MR Egger
0.6
0.4
0.2
1/SE$_{IV}$
−12
−8
−4
0
β$_{IV}$

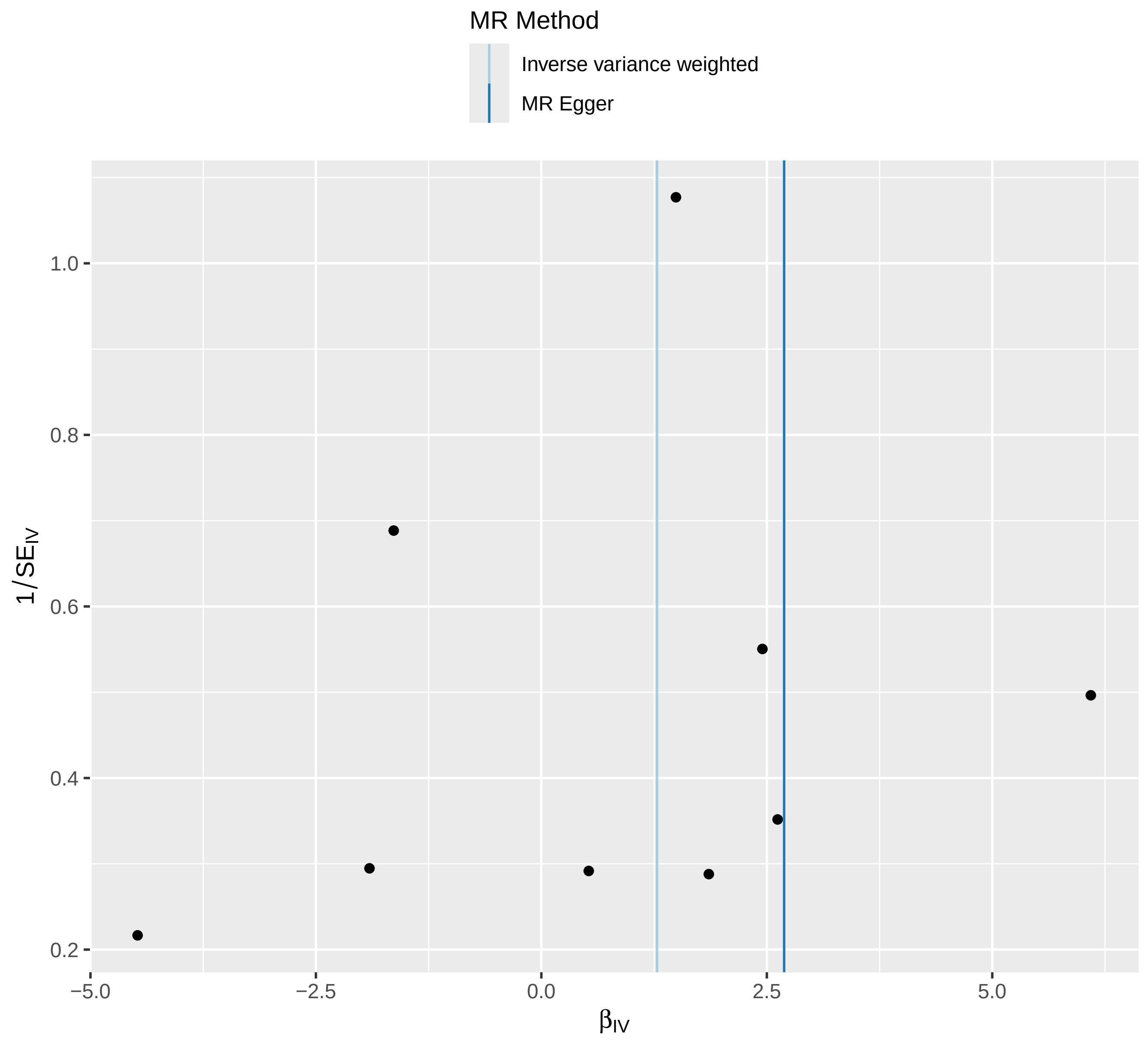
MR Method
Inverse variance weighted
MR Egger
1.0
0.8
0.6
0.4
0.2
1/SE_IV
−5.0
−2.5
0.0
2.5
5.0
β_IV

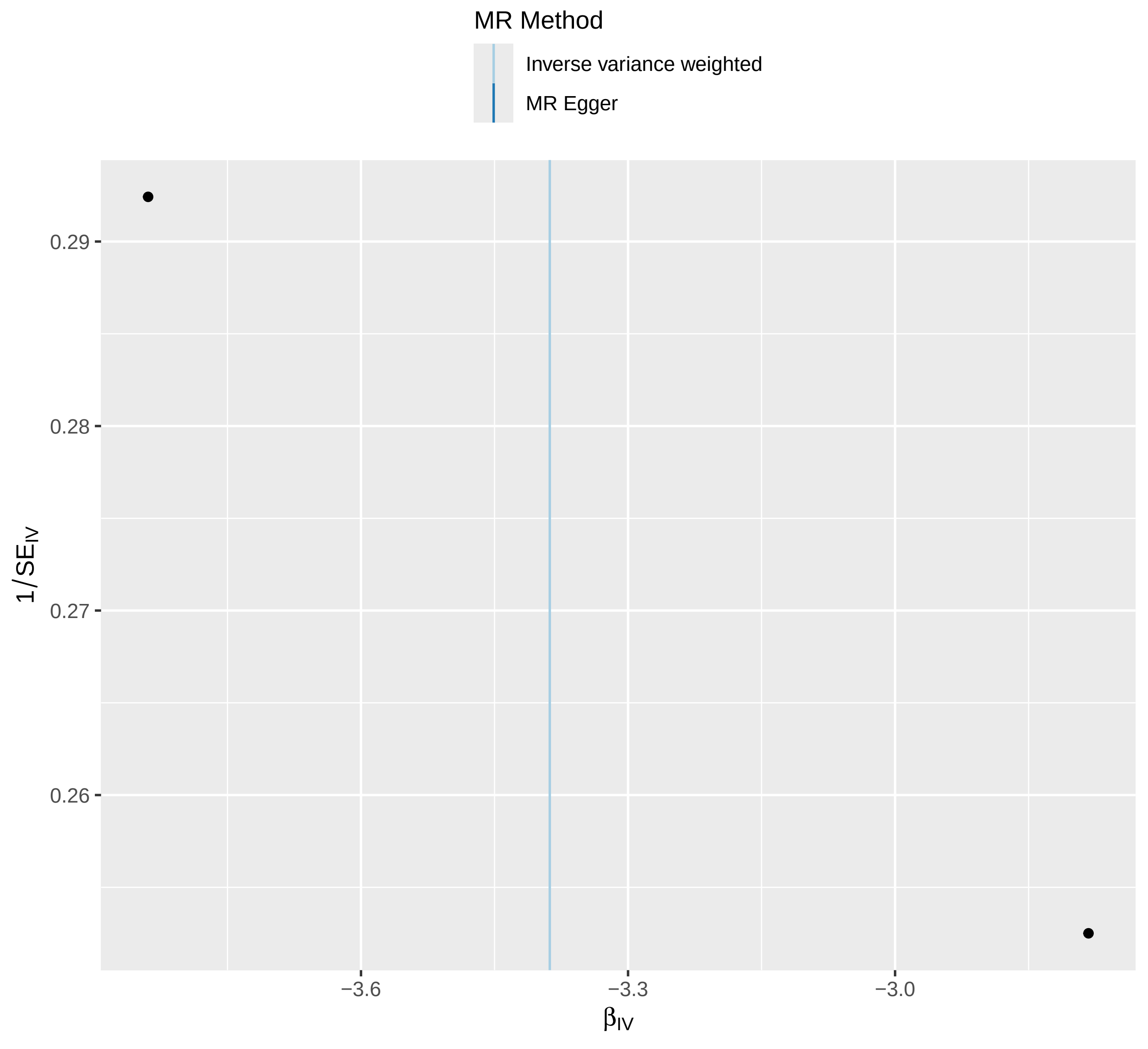

MR Method
Inverse variance weighted
MR Egger
0.29
0.28
0.27
0.26
1/SE_IV
−3.6
−3.3
−3.0
β_IV

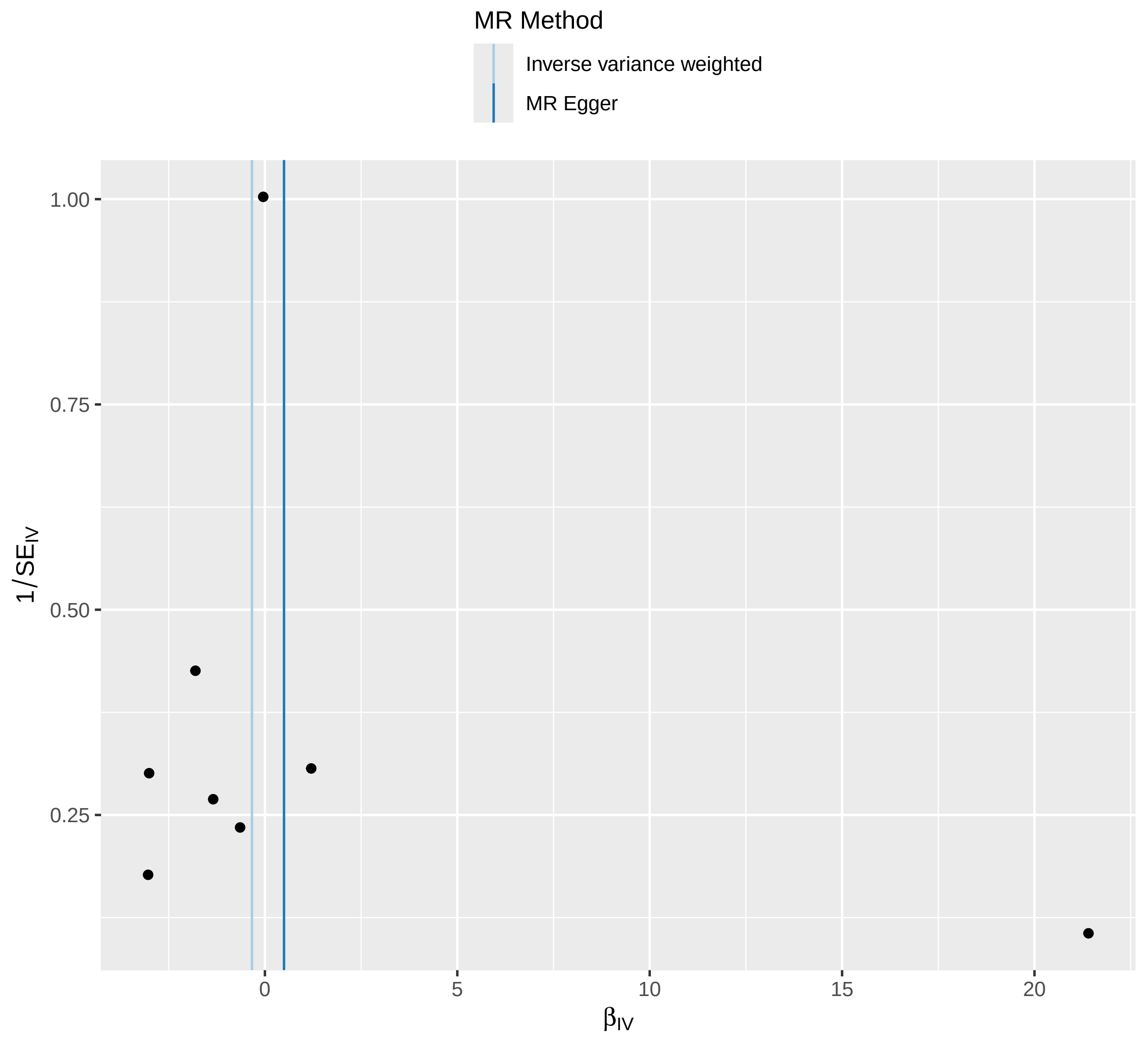
MR Method
Inverse variance weighted
MR Egger
1.00
0.75
0.50
0.25
1/SE_IV
0
5
10
15
20
β_IV

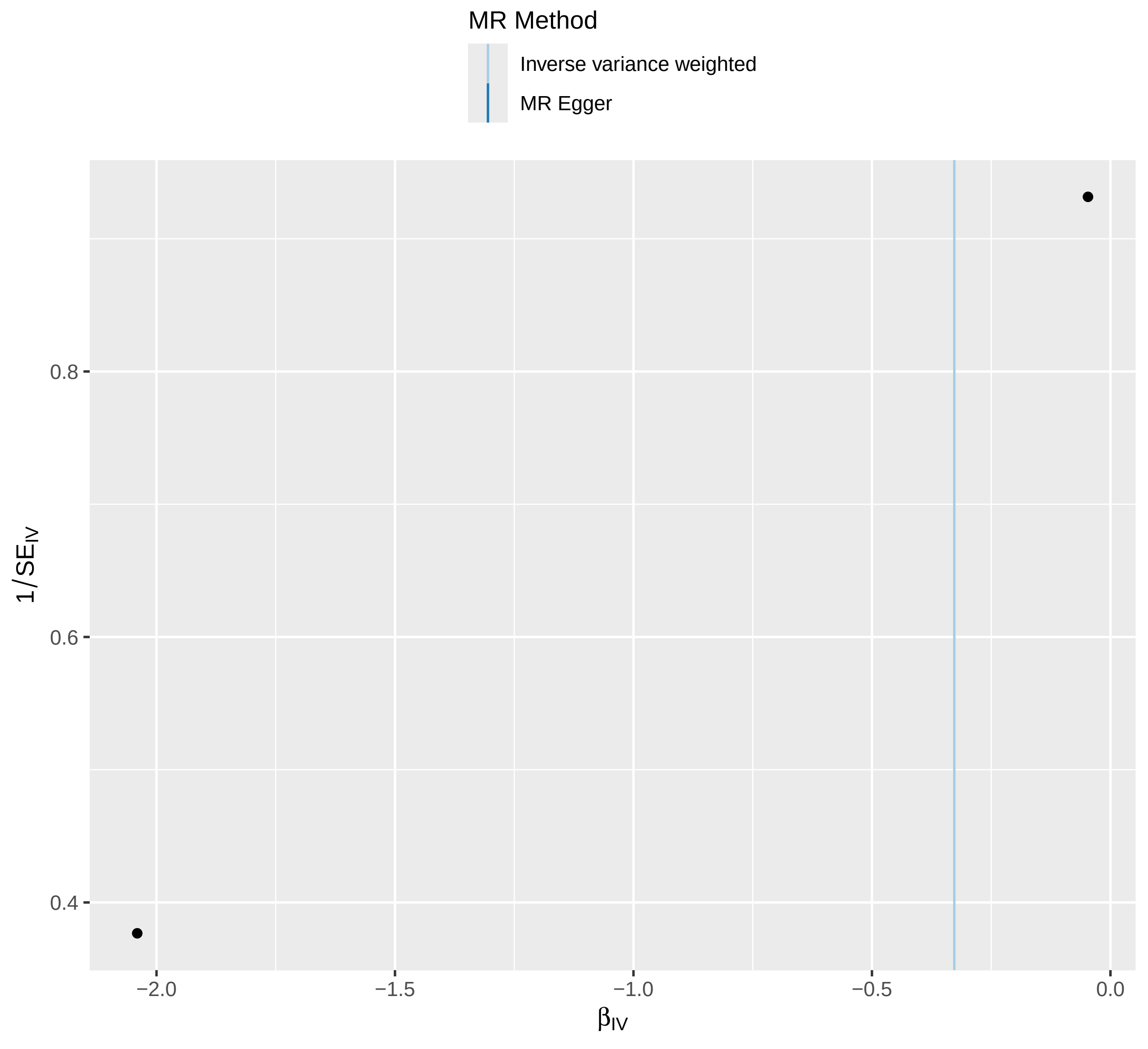
MR Method
Inverse variance weighted
MR Egger
0.8
0.6
0.4
1/SE_IV
−2.0
−1.5
−1.0
−0.5
0.0
β_IV

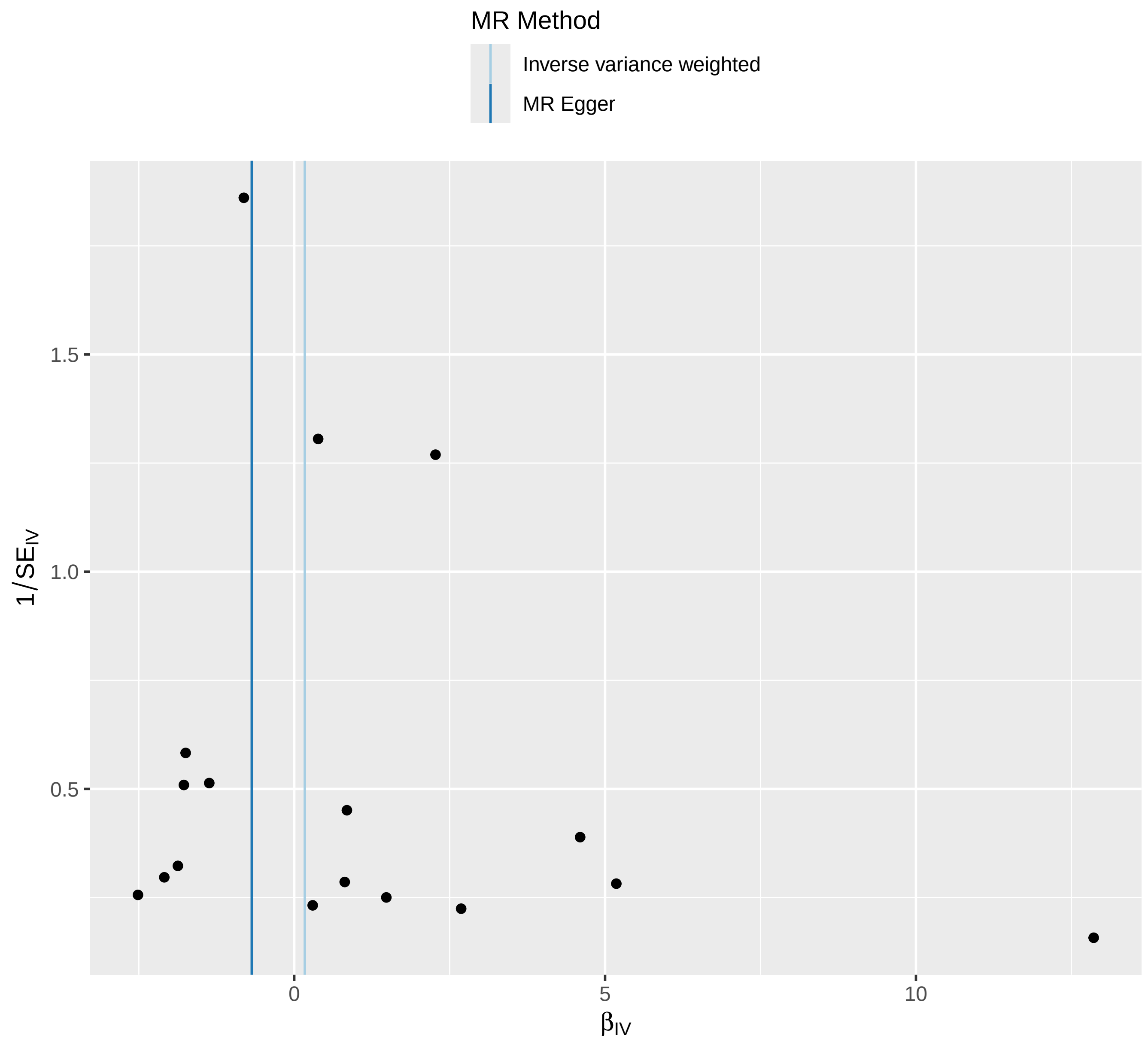
MR Method
Inverse variance weighted
MR Egger
1.5
1.0
0.5
1/SE$_{IV}$
0
5
10
β$_{IV}$

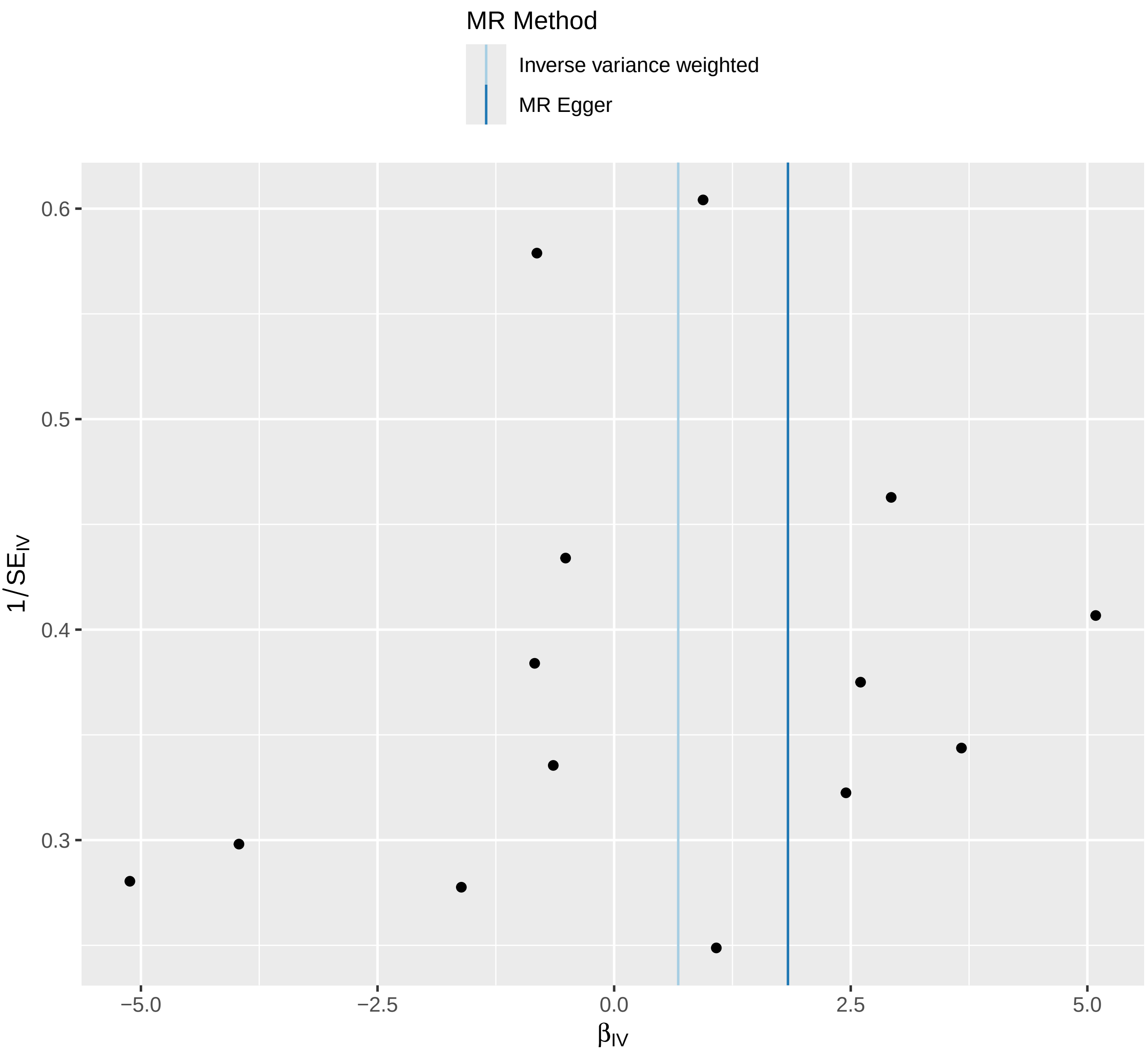
MR Method
Inverse variance weighted
MR Egger
0.6
0.5
0.4
0.3
1/SE$_{IV}$
−5.0
−2.5
0.0
2.5
5.0
β$_{IV}$

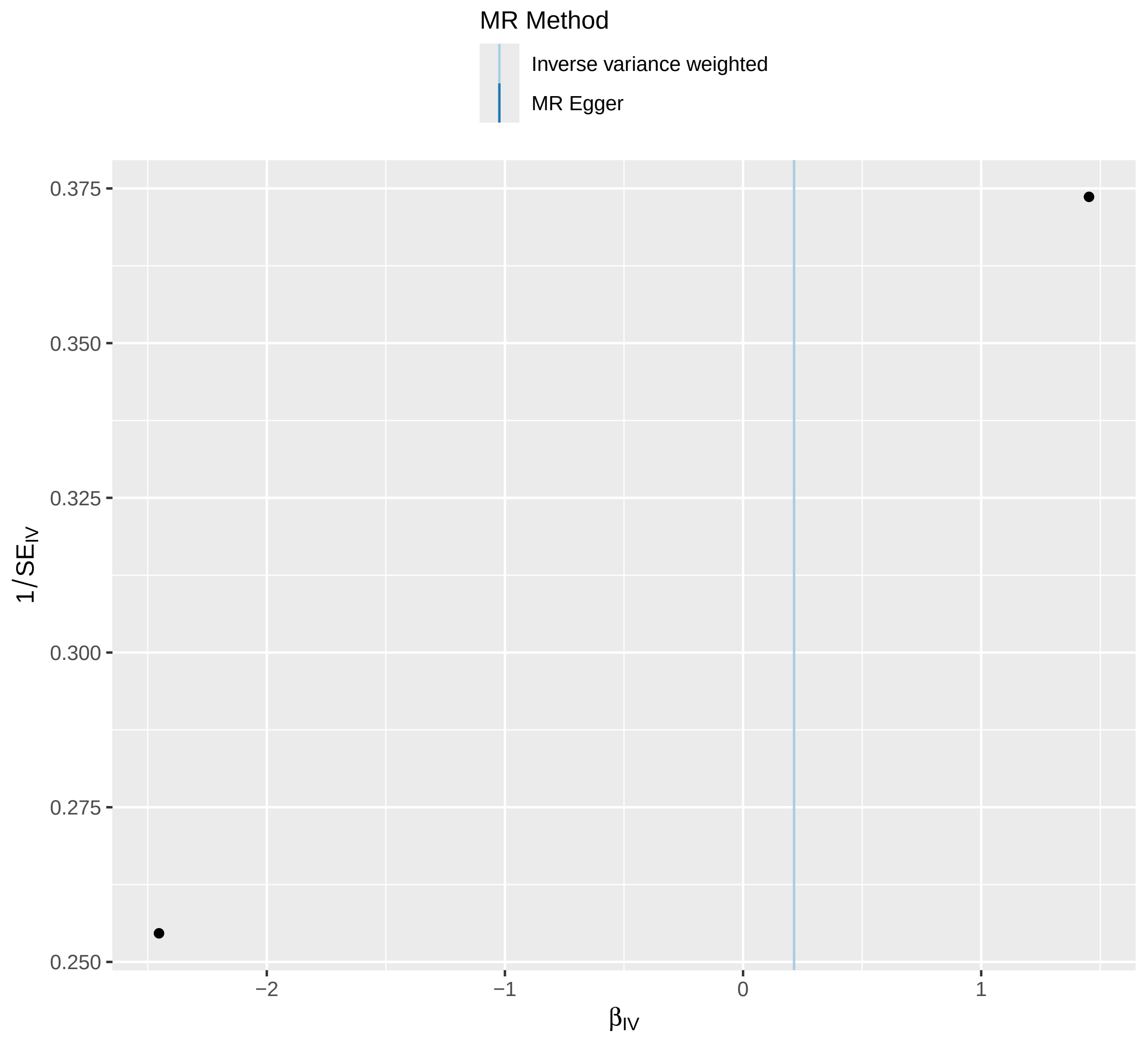
MR Method
Inverse variance weighted
MR Egger
0.375
0.350
0.325
0.300
0.275
0.250
1/SE_IV
−2
−1
0
1
β_IV

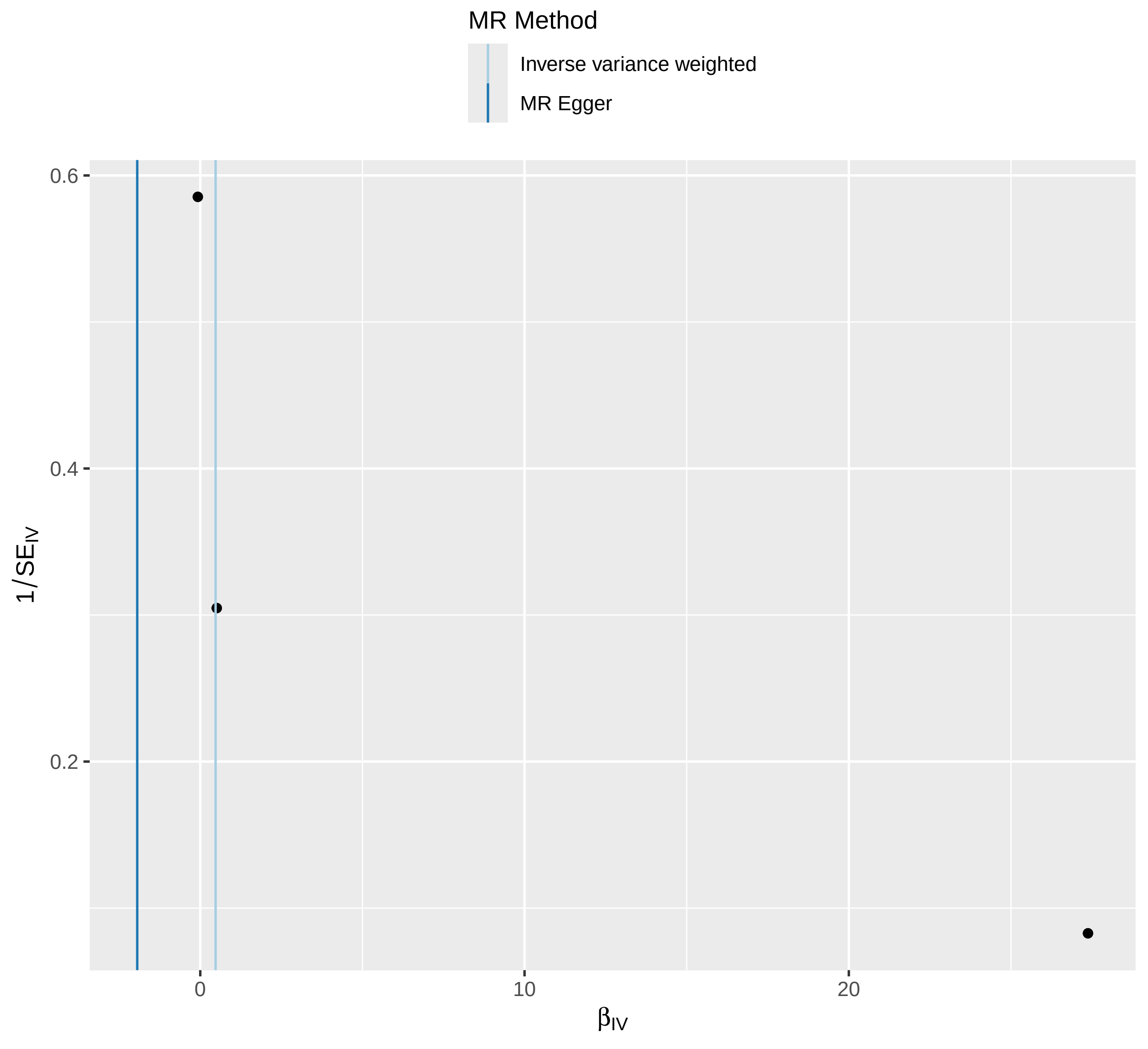
MR Method
Inverse variance weighted
MR Egger
0.6
0.4
0.2
1/SE$_{IV}$
0
10
20
β_{IV}

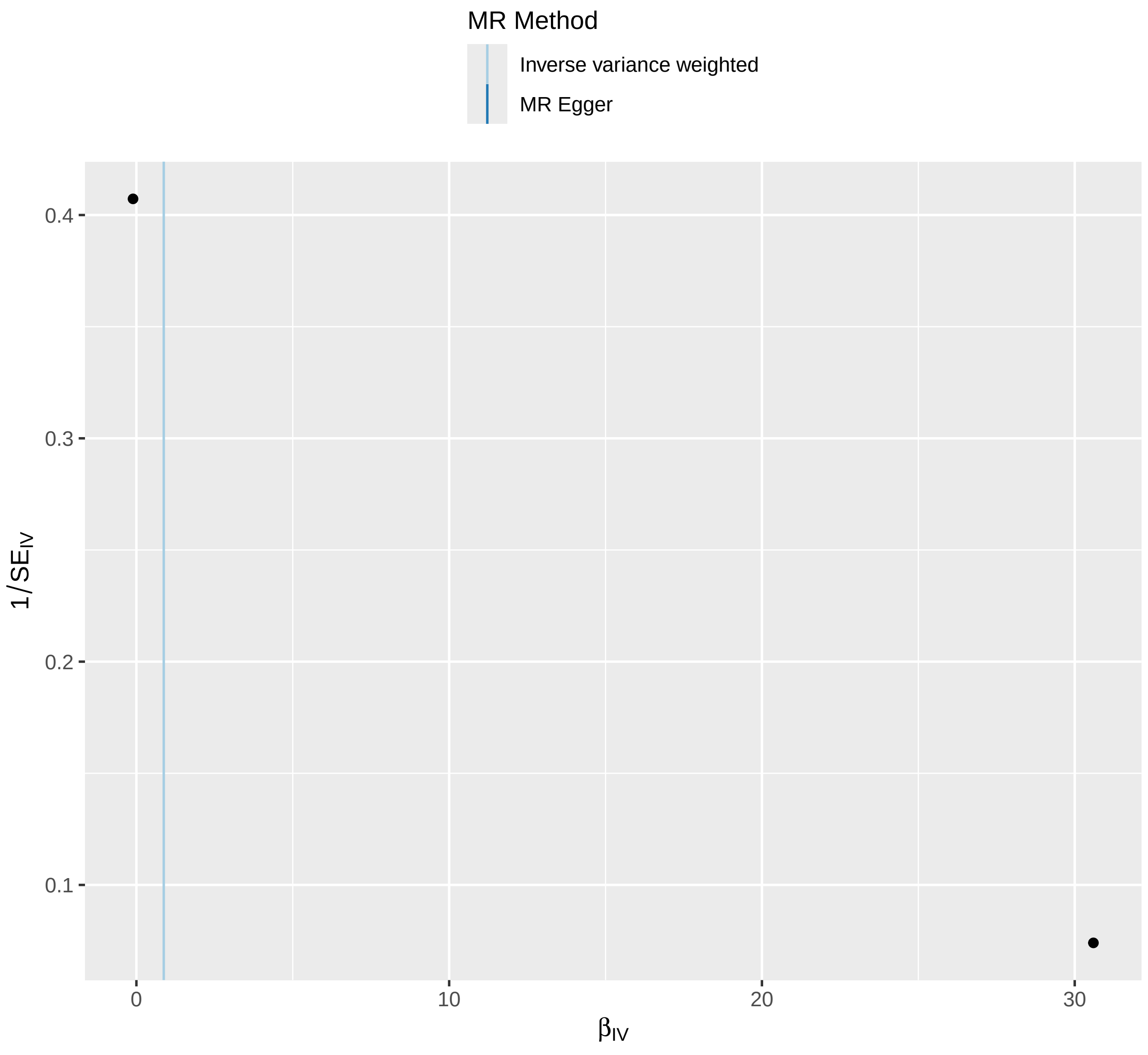
MR Method
Inverse variance weighted
MR Egger
0.4
0.3
0.2
0.1
1/SE_IV
0
10
20
30
β_IV

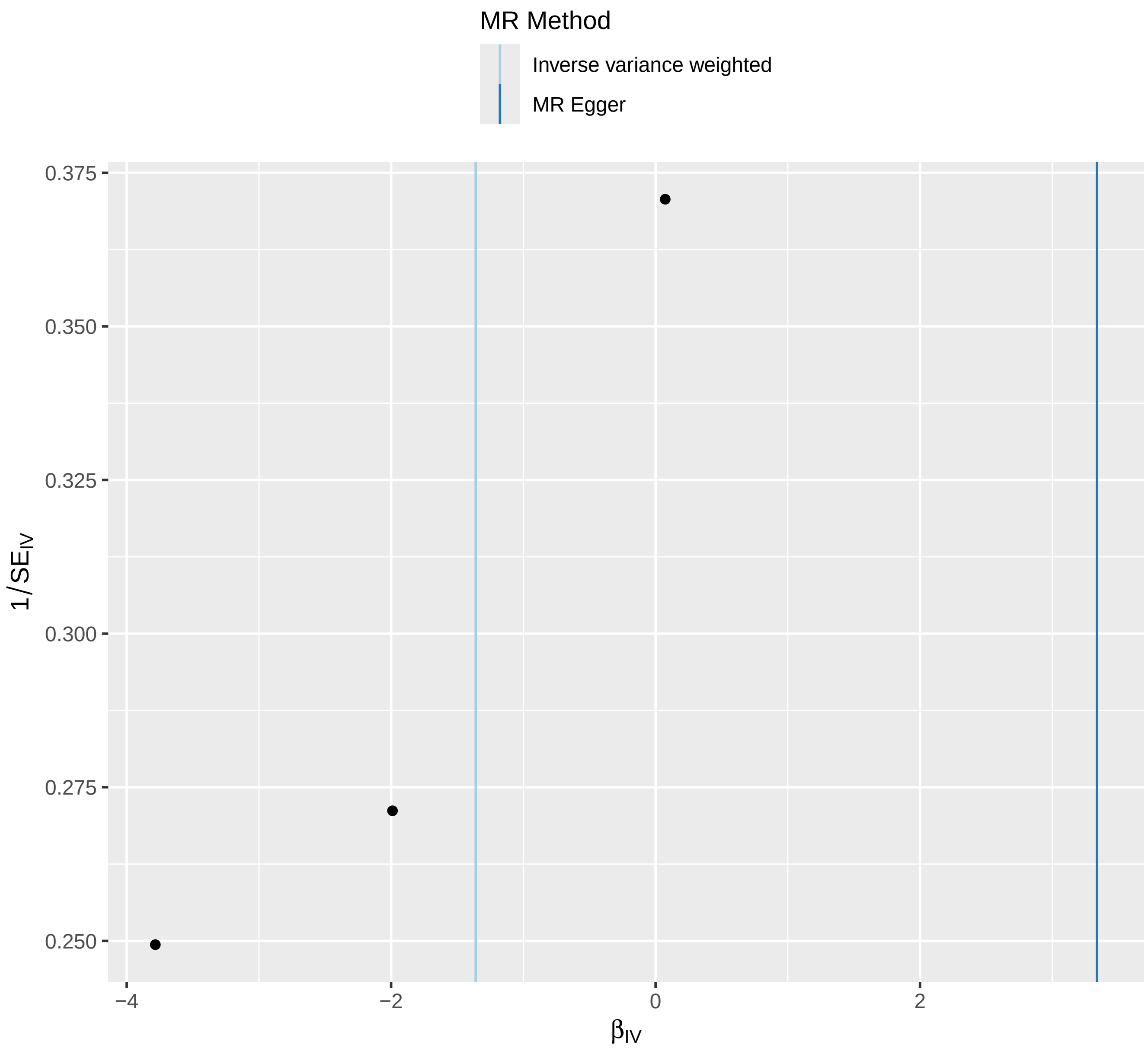

MR Method
Inverse variance weighted
MR Egger
0.375
0.350
0.325
0.300
0.275
0.250
1/SE_IV
−4
−2
0
2
β_IV

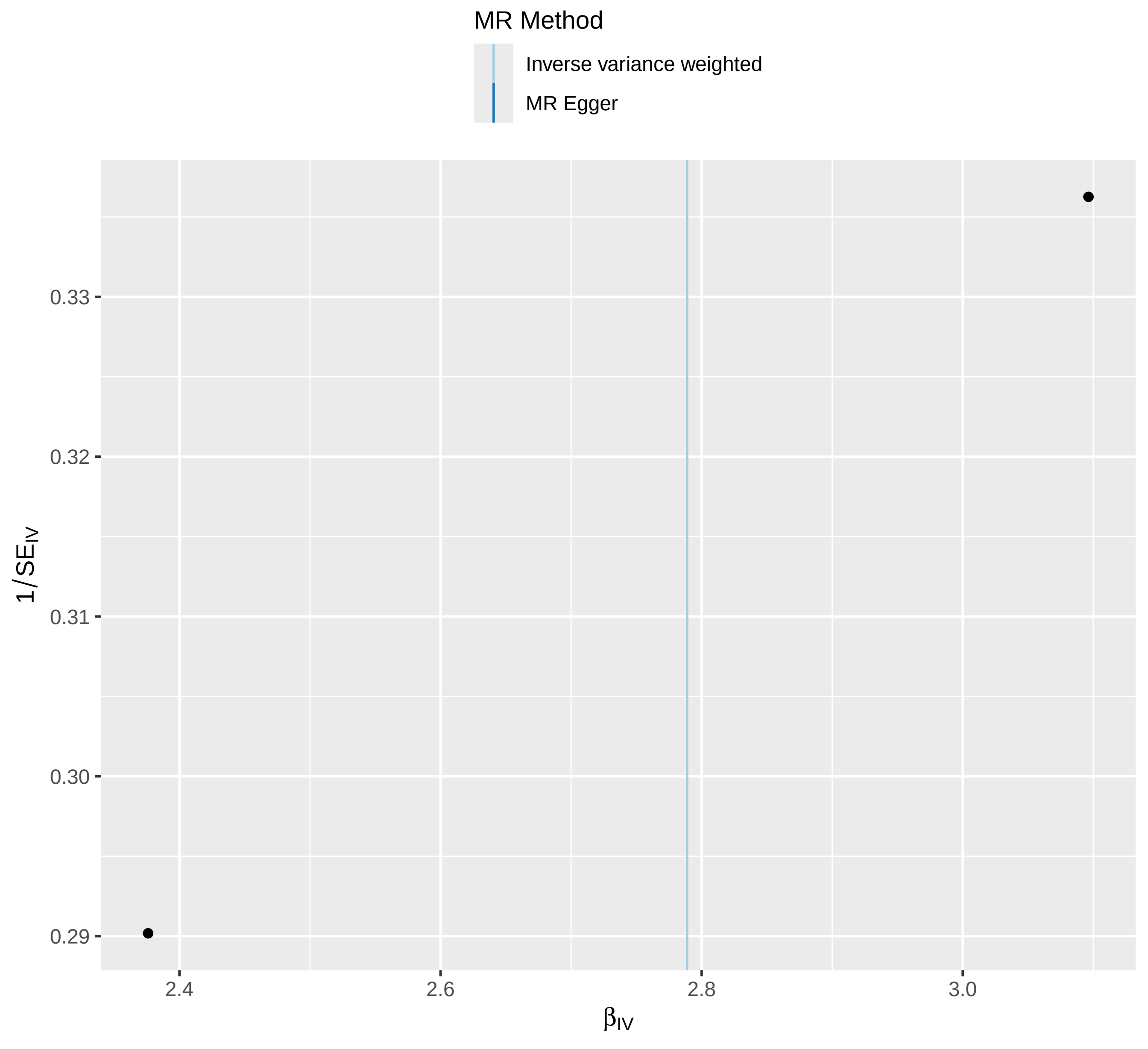
MR Method
Inverse variance weighted
MR Egger
0.33
0.32
0.31
0.30
0.29
1/SE_IV
2.4
2.6
2.8
3.0
β_IV

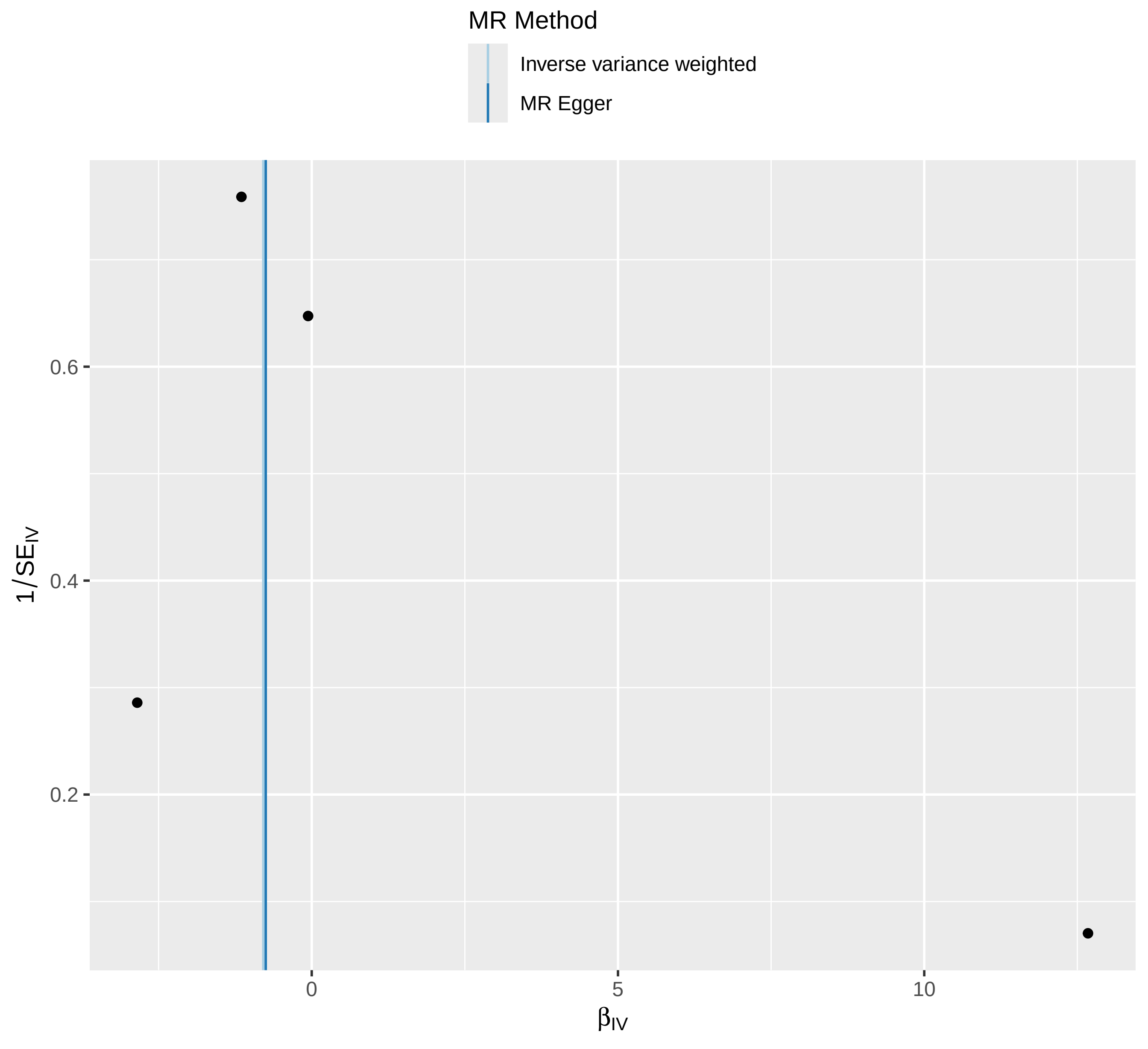
MR Method
Inverse variance weighted
MR Egger
0.6
0.4
0.2
1/SE$_{IV}$
0
5
10
β$_{IV}$

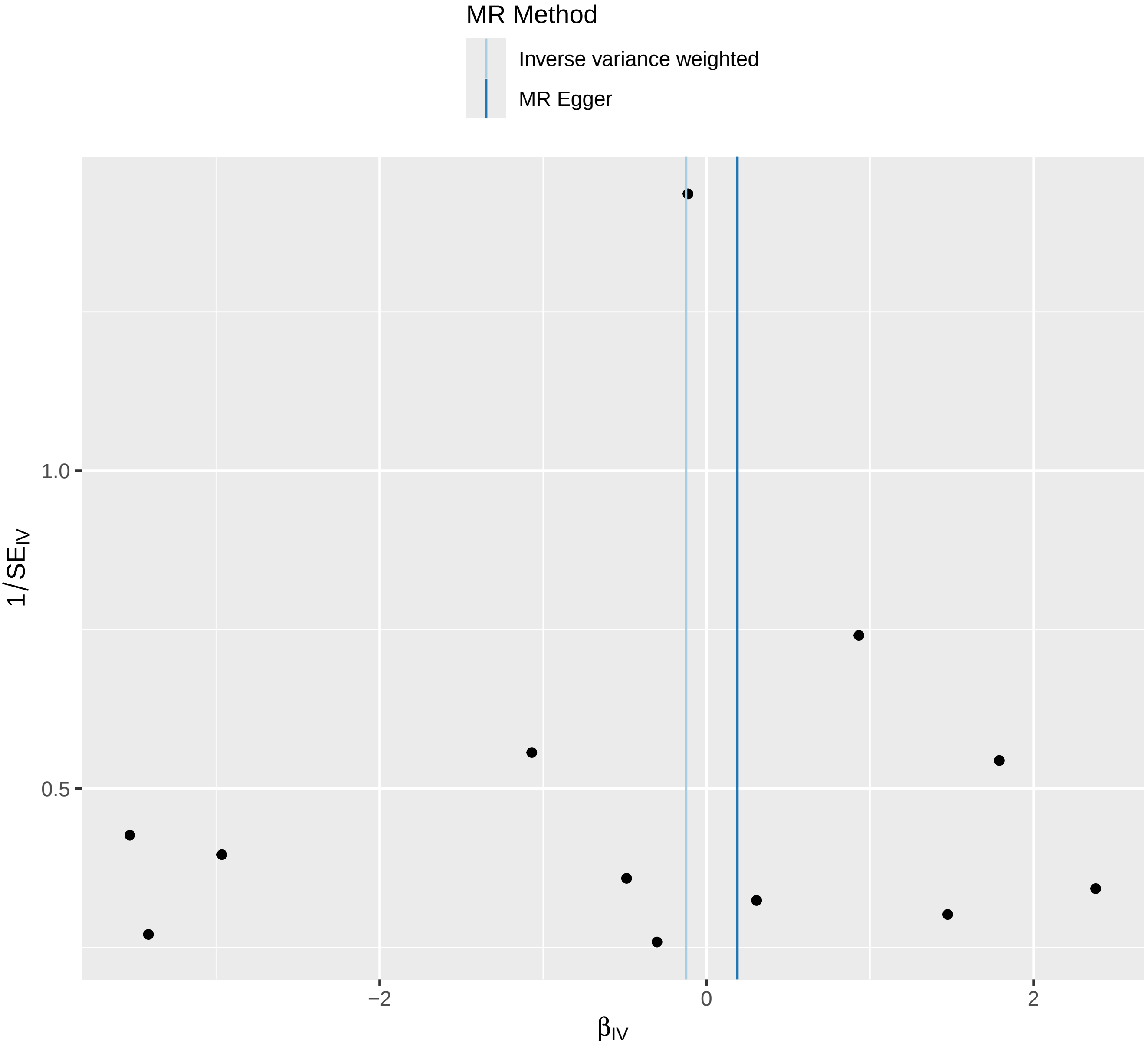

MR Method
Inverse variance weighted
MR Egger
1.0
0.5
1/SE_IV
−2
0
2
β_IV

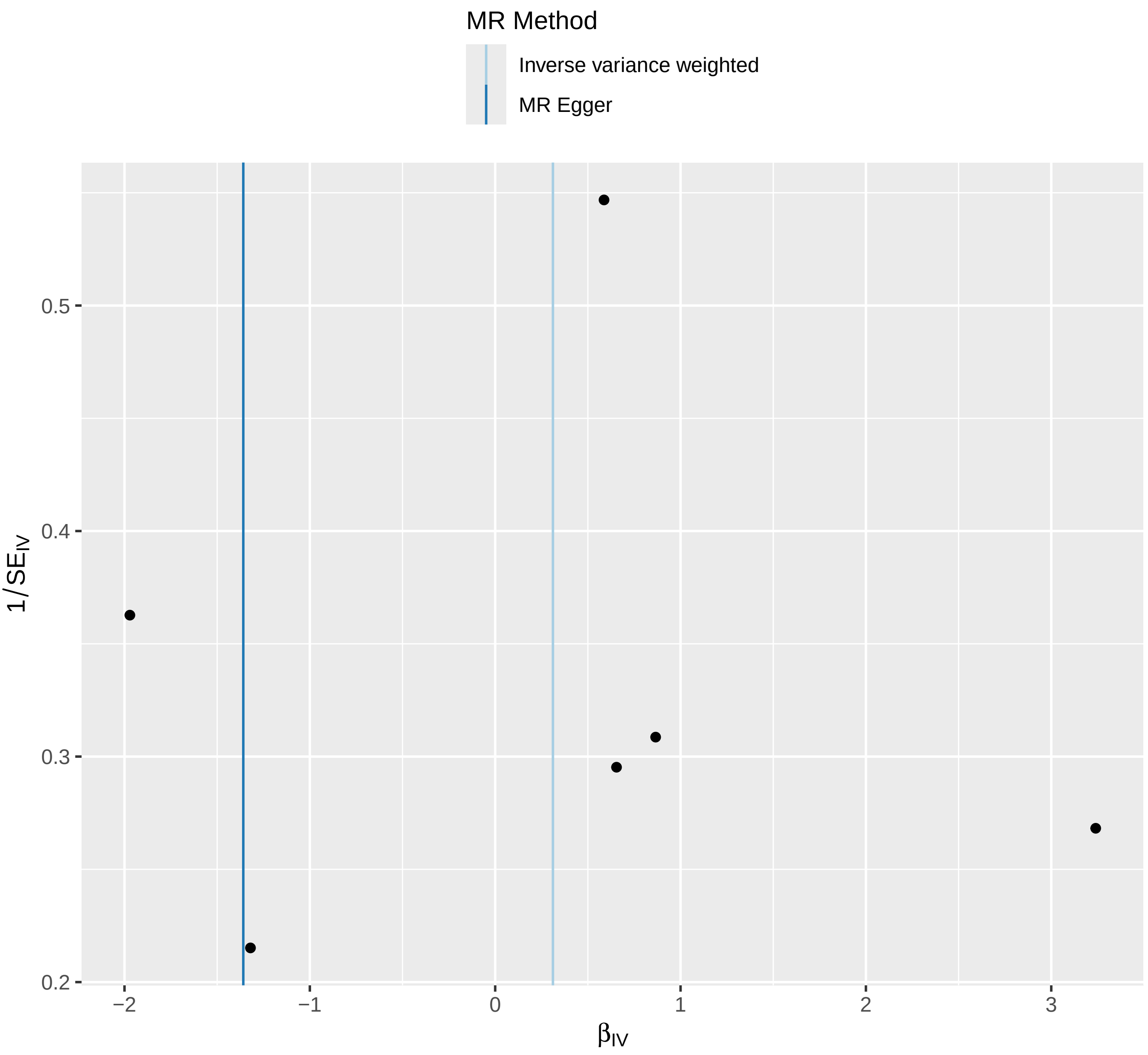
MR Method
Inverse variance weighted
MR Egger
0.5
0.4
0.3
0.2
1/SE_IV
−2
−1
0
1
2
3
β_IV

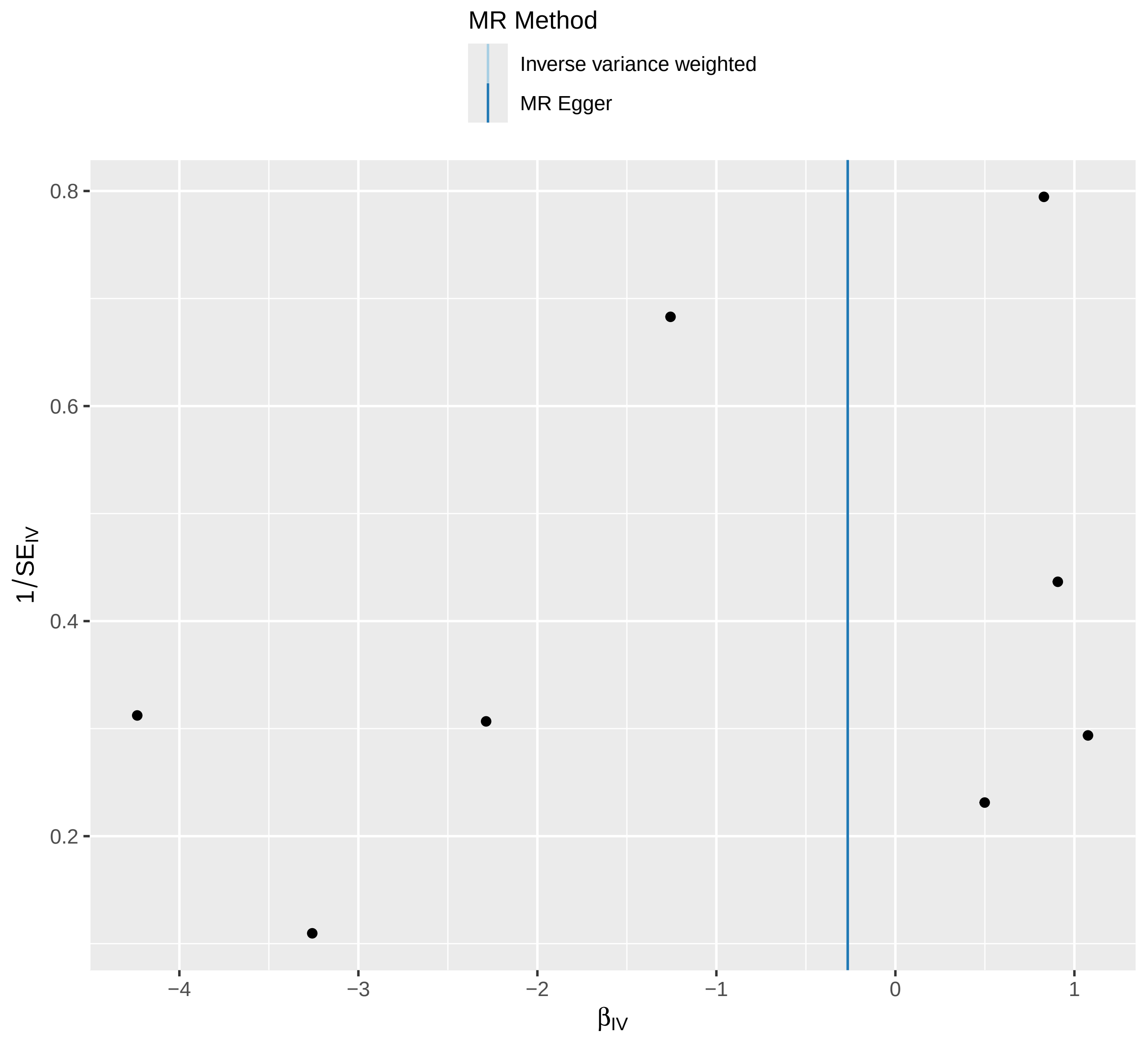
MR Method
Inverse variance weighted
MR Egger
0.8
0.6
0.4
0.2
1/SE_IV
−4
−3
−2
−1
0
1
β_IV

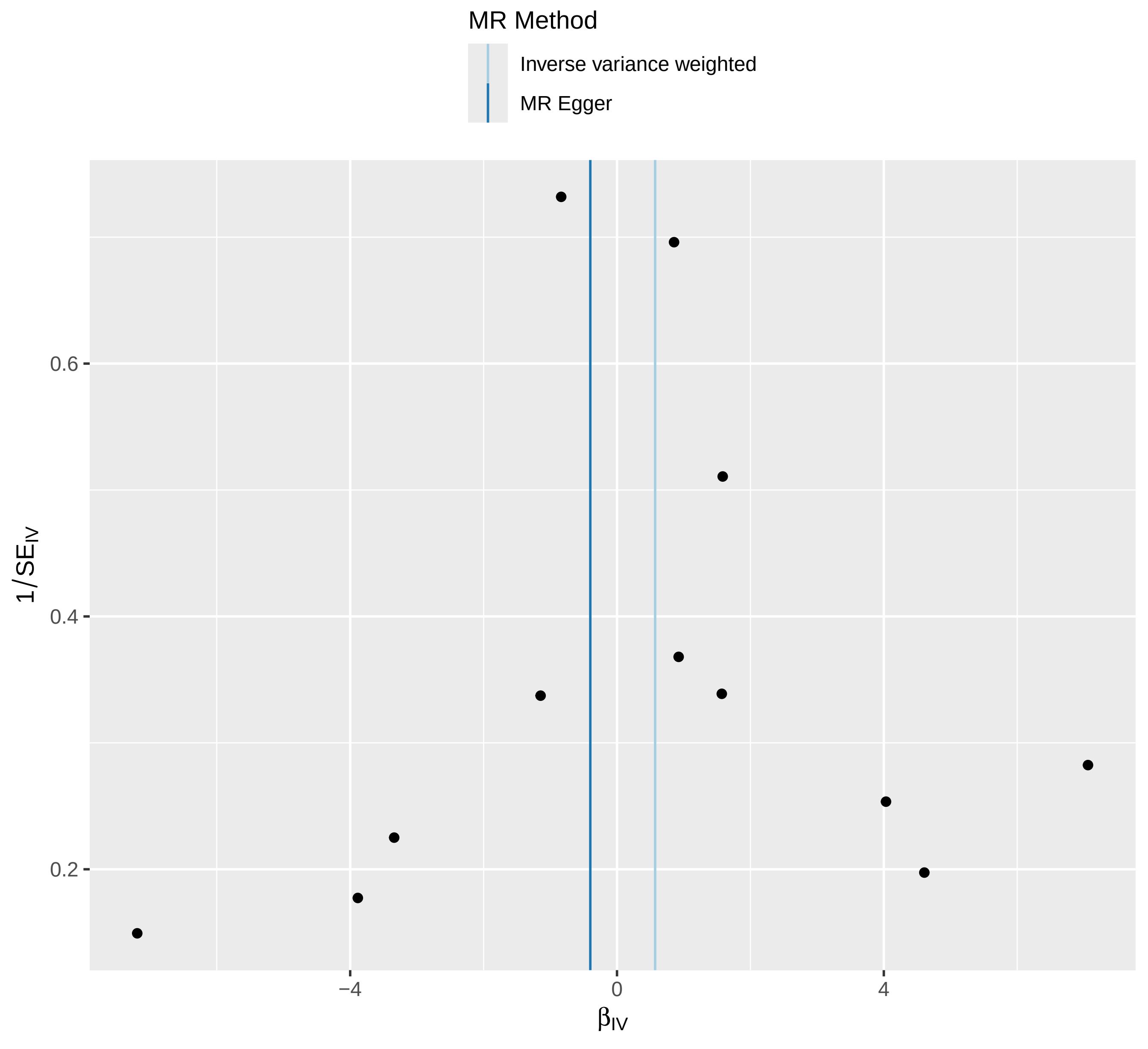
MR Method
Inverse variance weighted
MR Egger
1/SE_IV
0.6
0.4
0.2
−4
0
4
β_IV

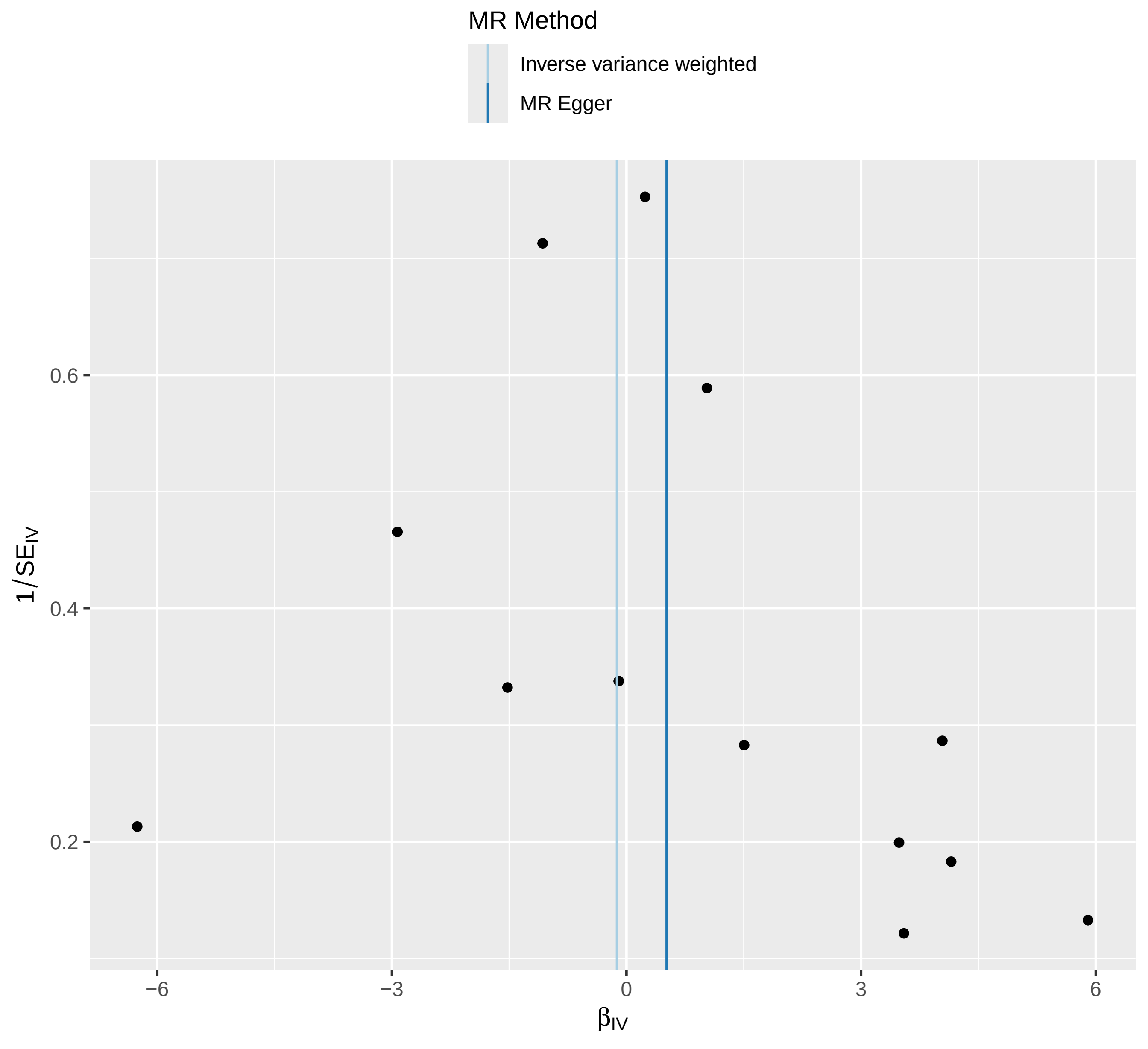
MR Method
Inverse variance weighted
MR Egger
0.6
0.4
0.2
1/SE$_{IV}$
−6
−3
0
3
6
β_{IV}

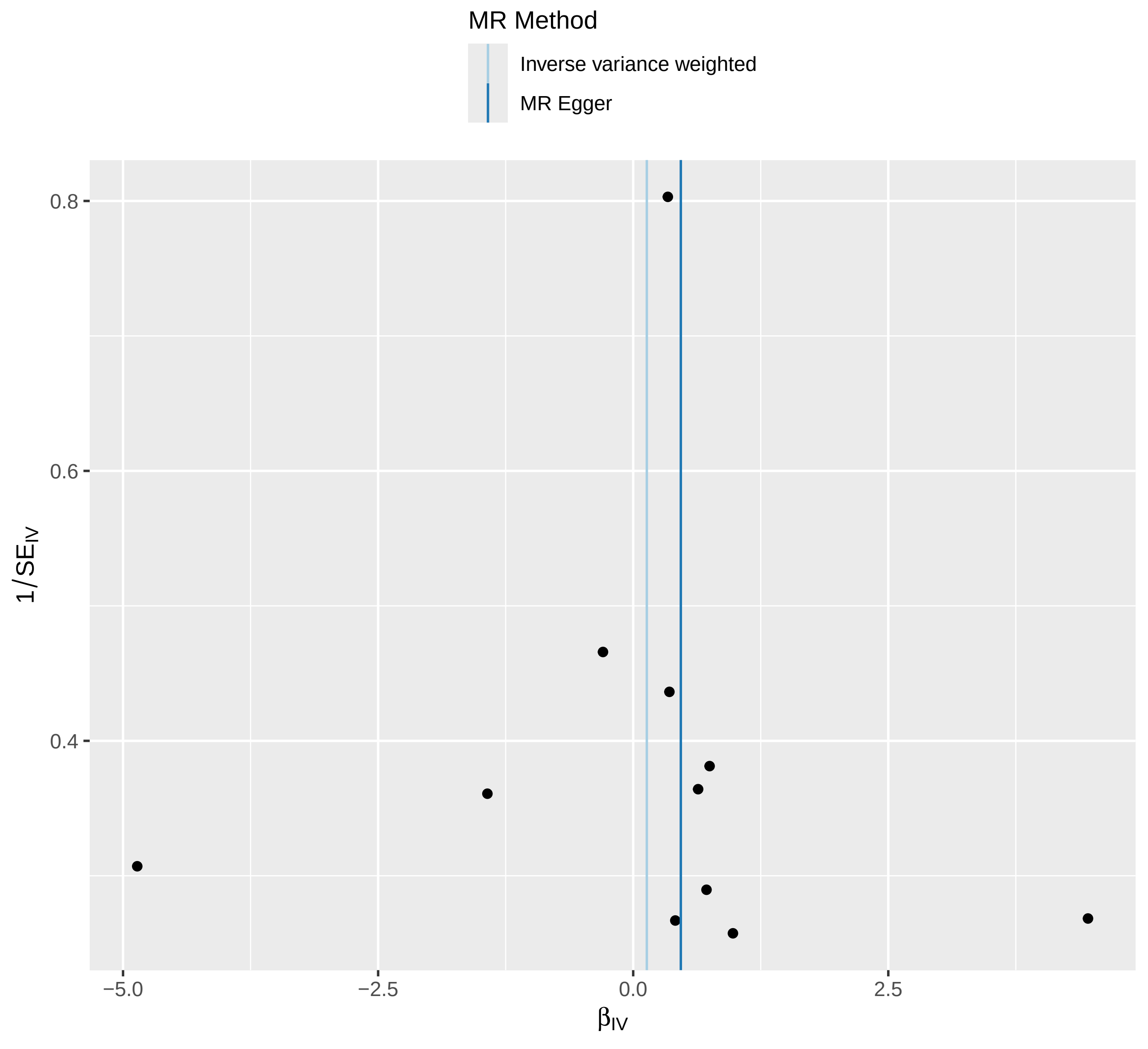

MR Method
Inverse variance weighted
MR Egger
0.8
0.6
0.4
1/SE_IV
−5.0
−2.5
0.0
2.5
β_IV

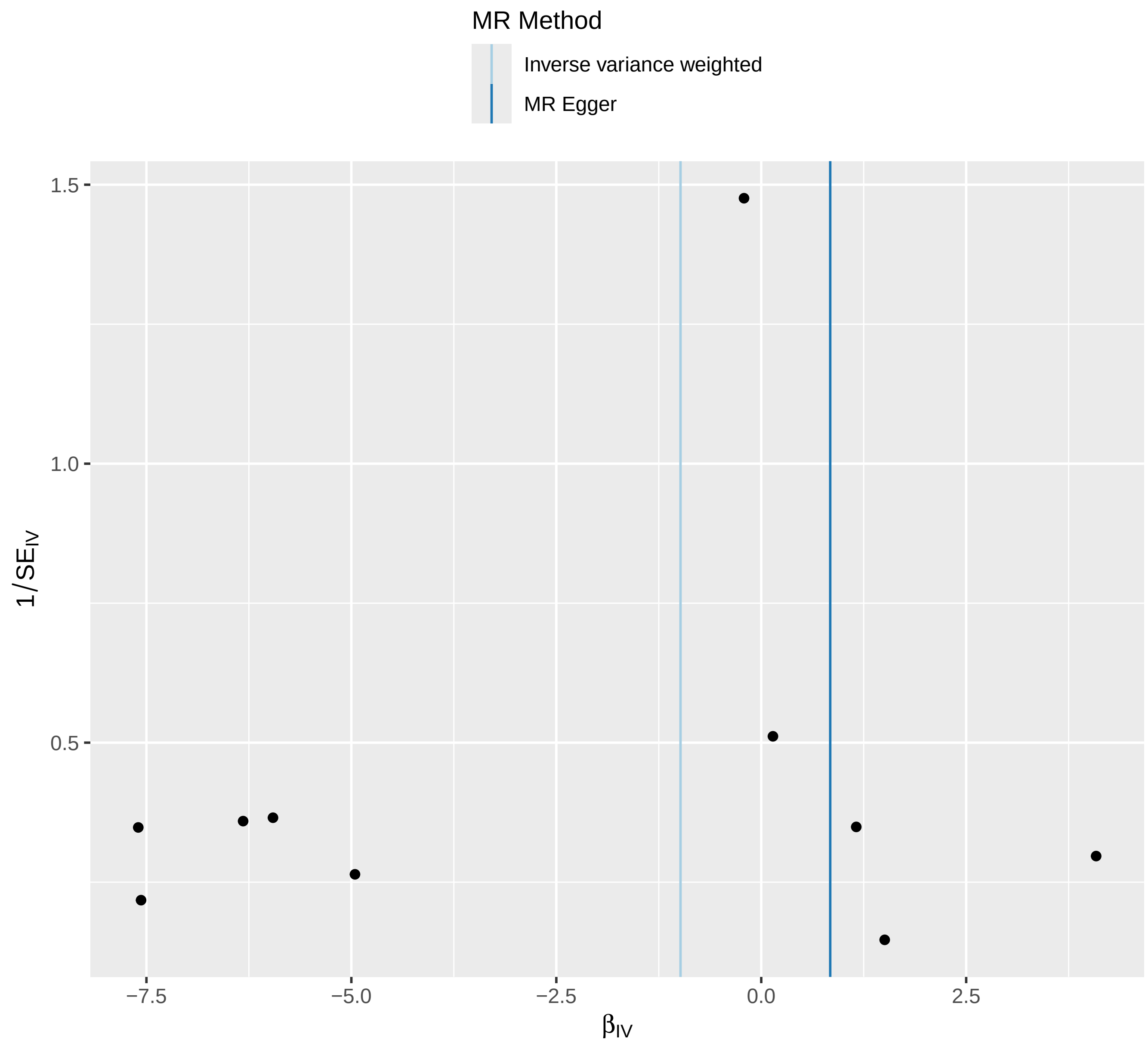
MR Method
Inverse variance weighted
MR Egger
1.5
1.0
0.5
1/SE$_{IV}$
−7.5
−5.0
−2.5
0.0
2.5
β$_{IV}$

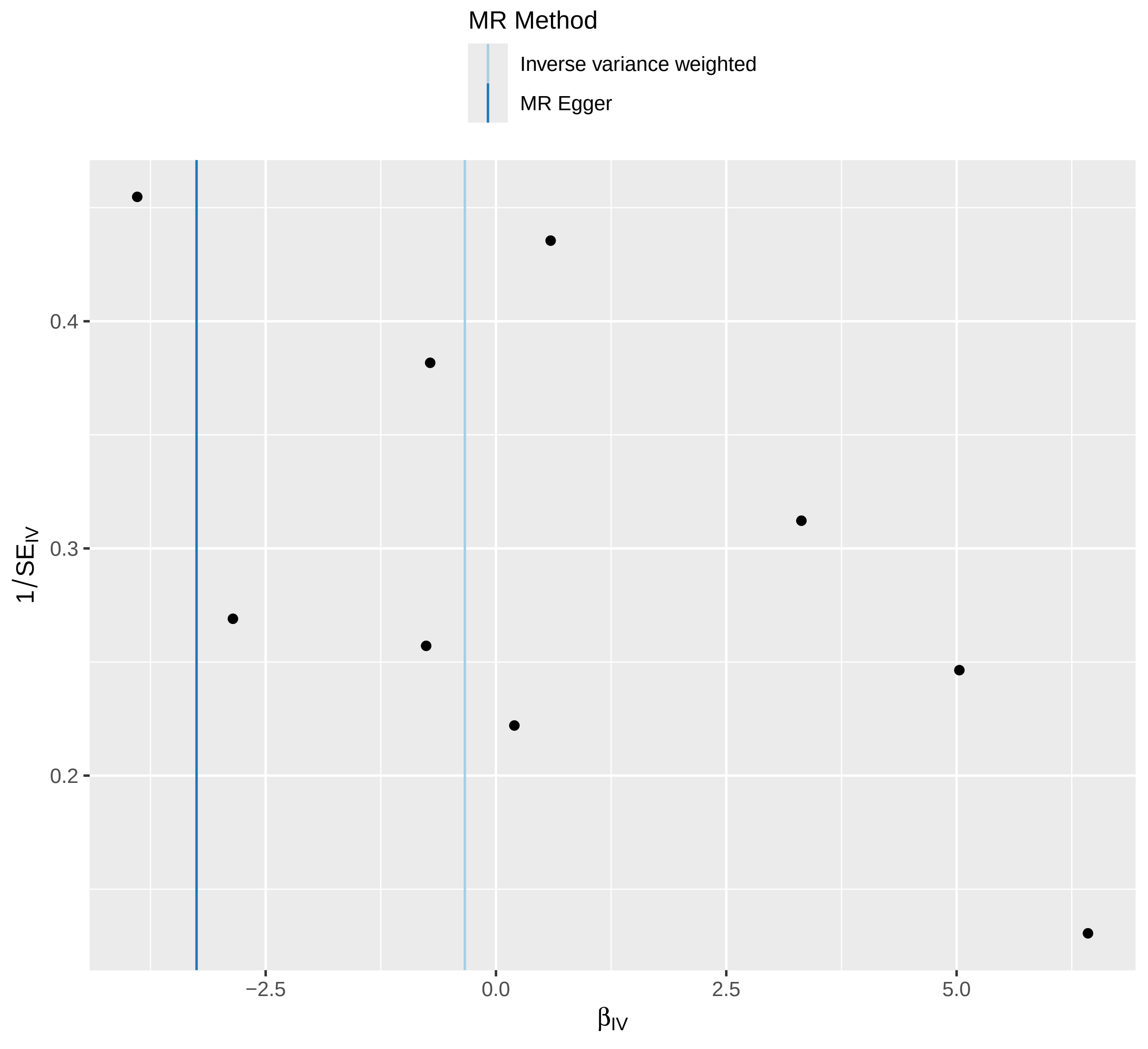
MR Method
Inverse variance weighted
MR Egger
0.4
0.3
0.2
1/SE$_{IV}$
−2.5
0.0
2.5
5.0
β$_{IV}$

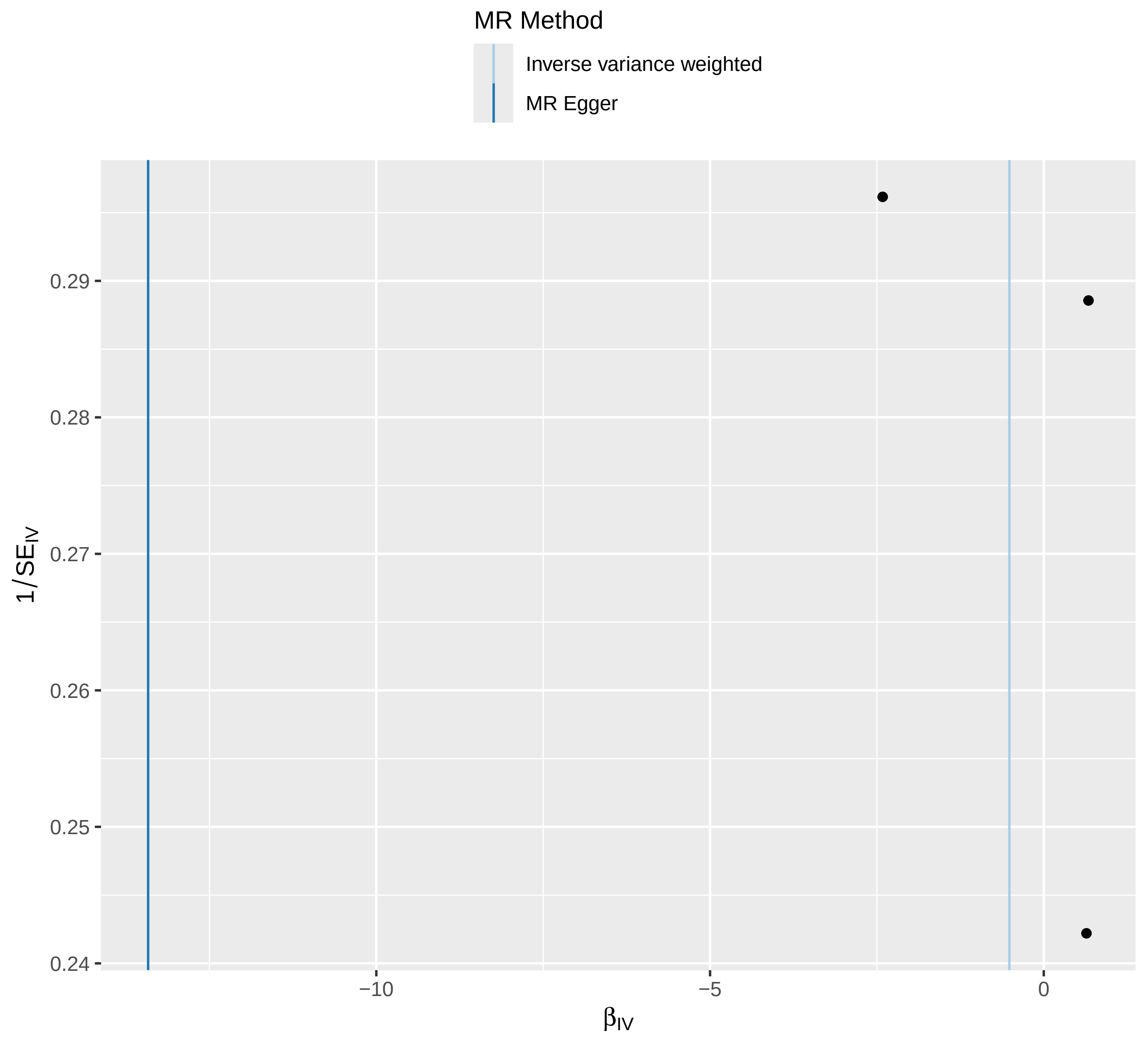

MR Method
Inverse variance weighted
MR Egger
0.29
0.28
0.27
0.26
0.25
0.24
1/SE_IV
−10
−5
0
β_IV

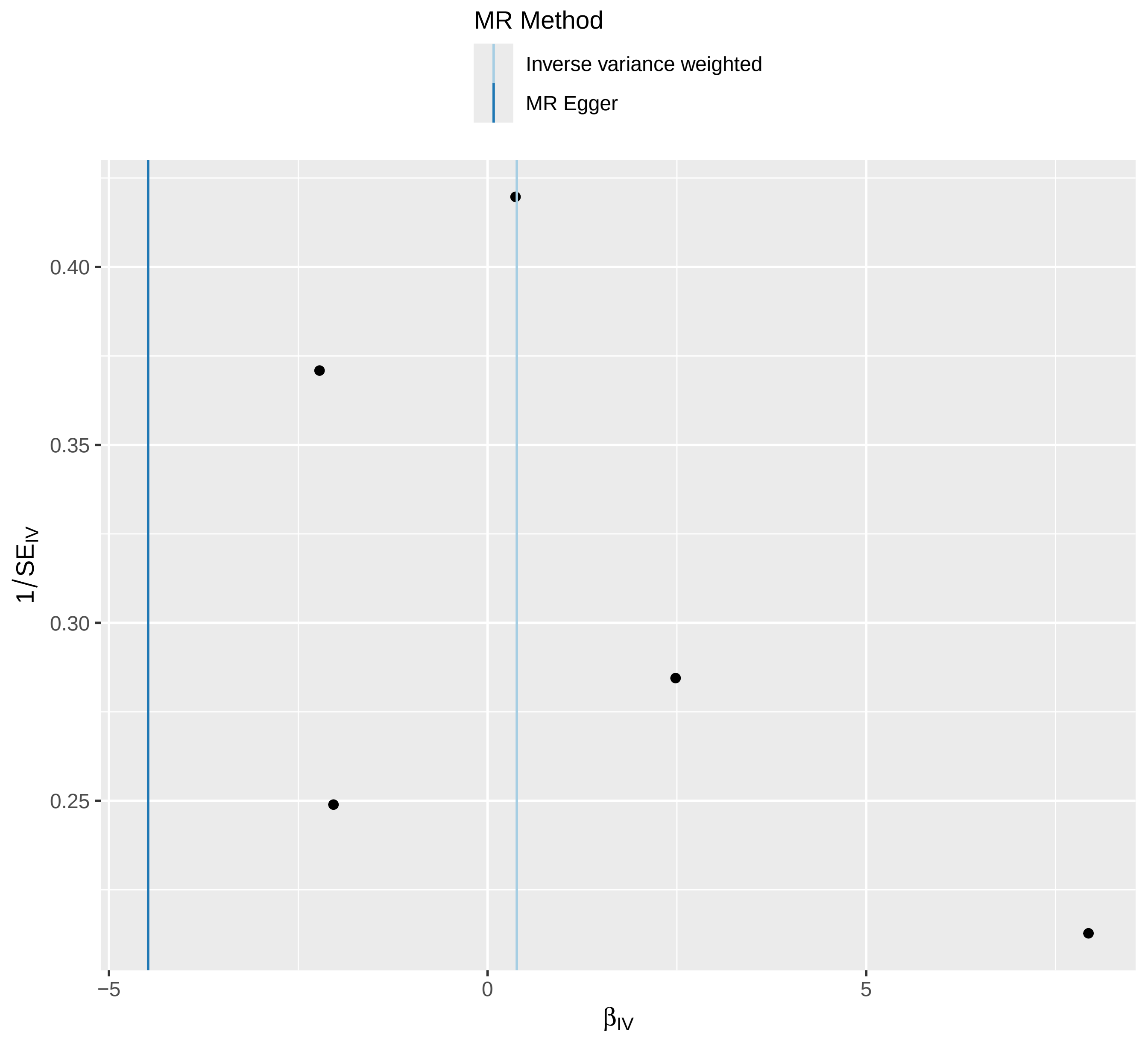

MR Method
Inverse variance weighted
MR Egger
0.40
0.35
0.30
0.25
1/SE_IV
−5
0
5
β_IV

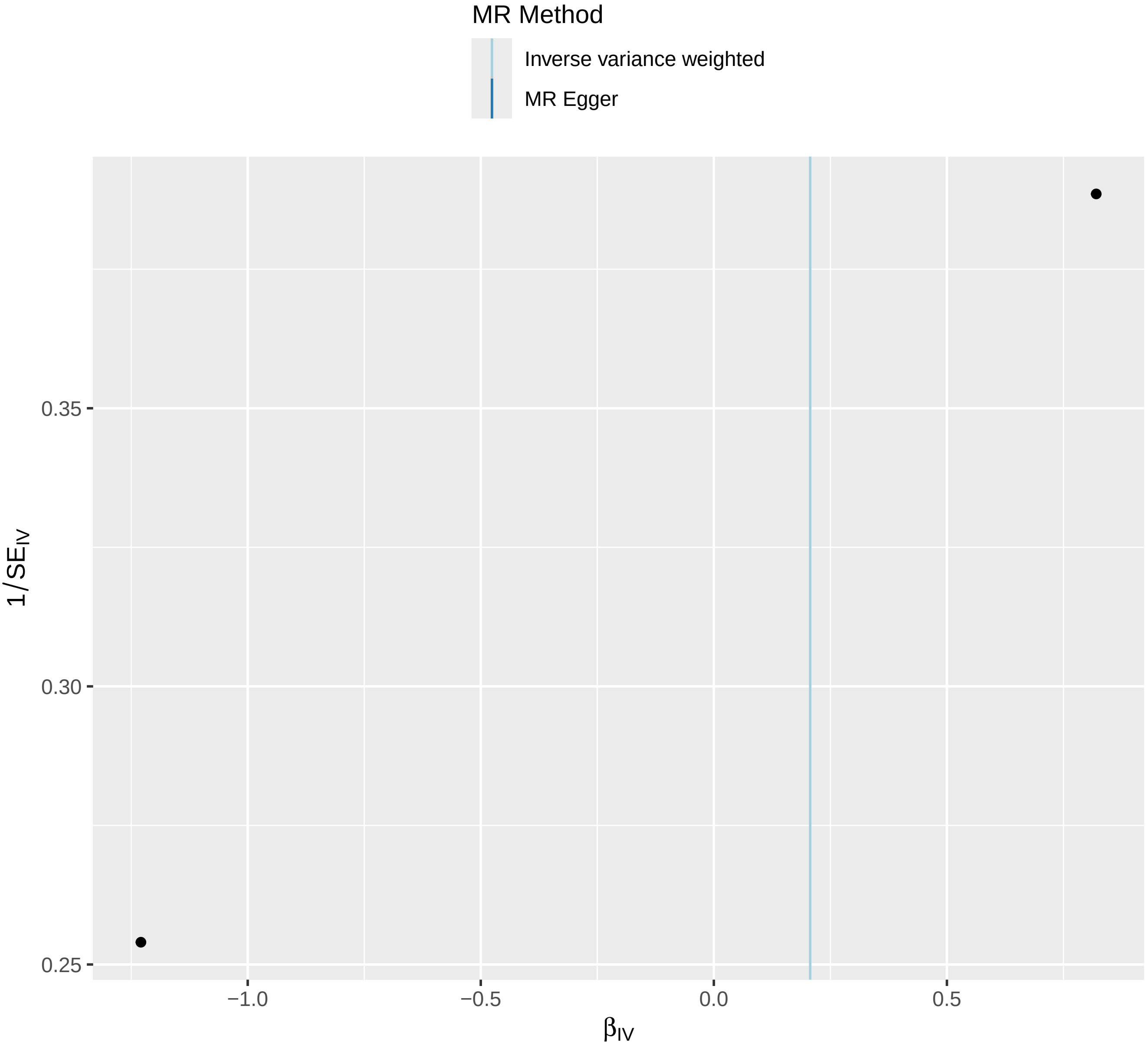
MR Method
Inverse variance weighted
MR Egger
0.35
0.30
0.25
1/SE_IV
−1.0
−0.5
0.0
0.5
β_IV

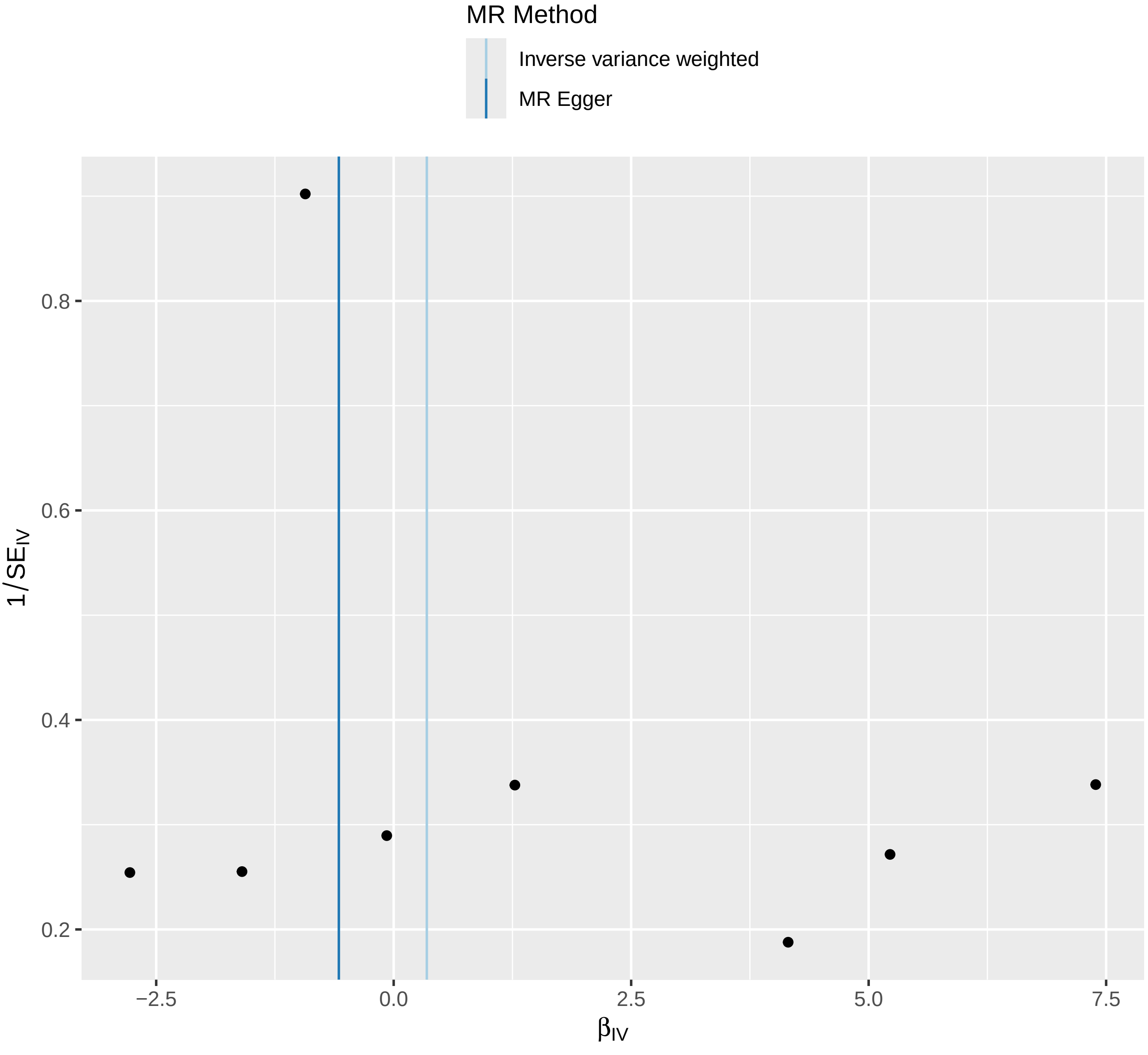
MR Method
Inverse variance weighted
MR Egger
0.8
0.6
0.4
0.2
1/SE_IV
−2.5
0.0
2.5
5.0
7.5
β_IV

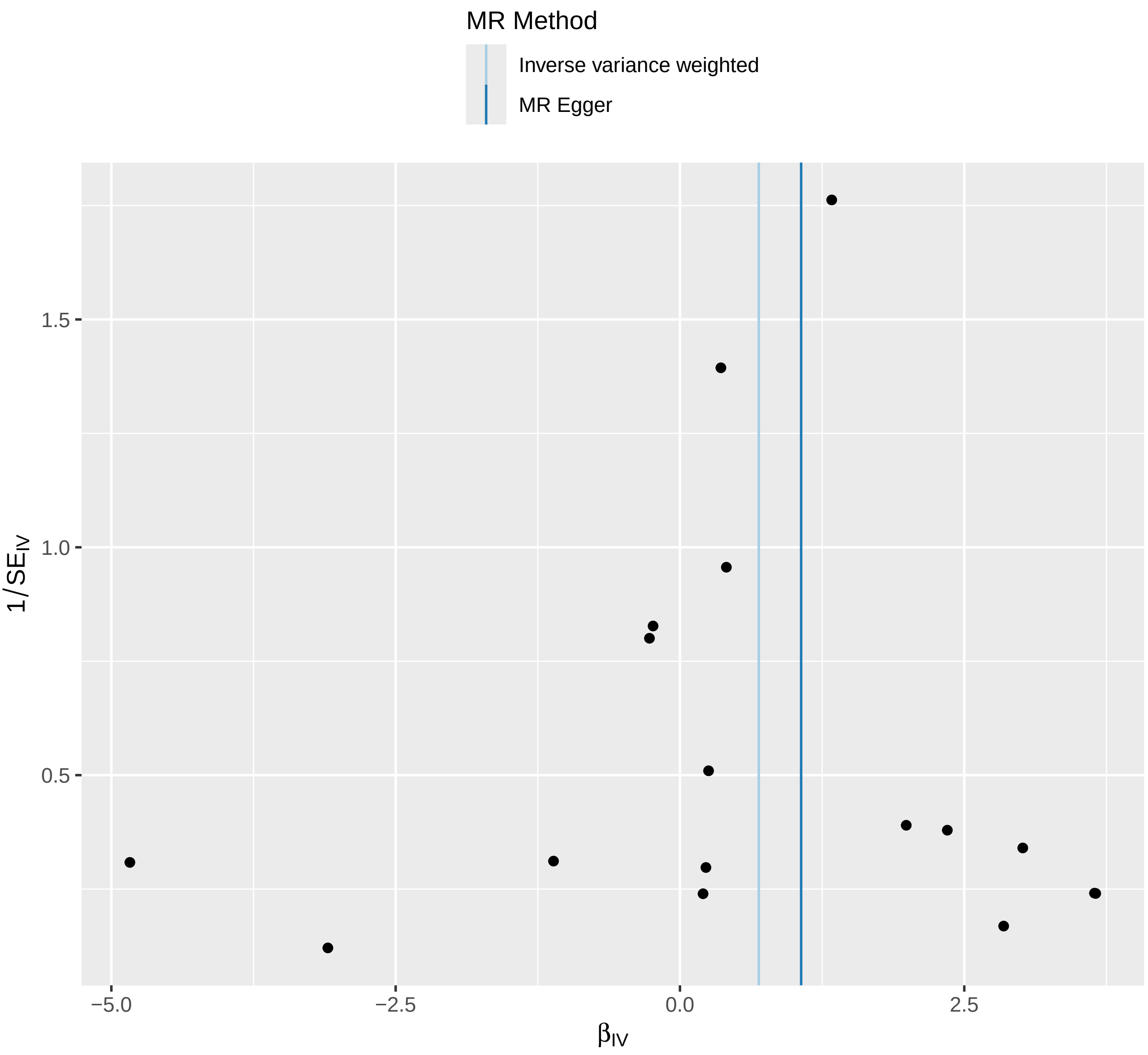
MR Method
Inverse variance weighted
MR Egger
1.5
1.0
0.5
$1/SE_{IV}$
−5.0
−2.5
0.0
2.5
β_{IV}

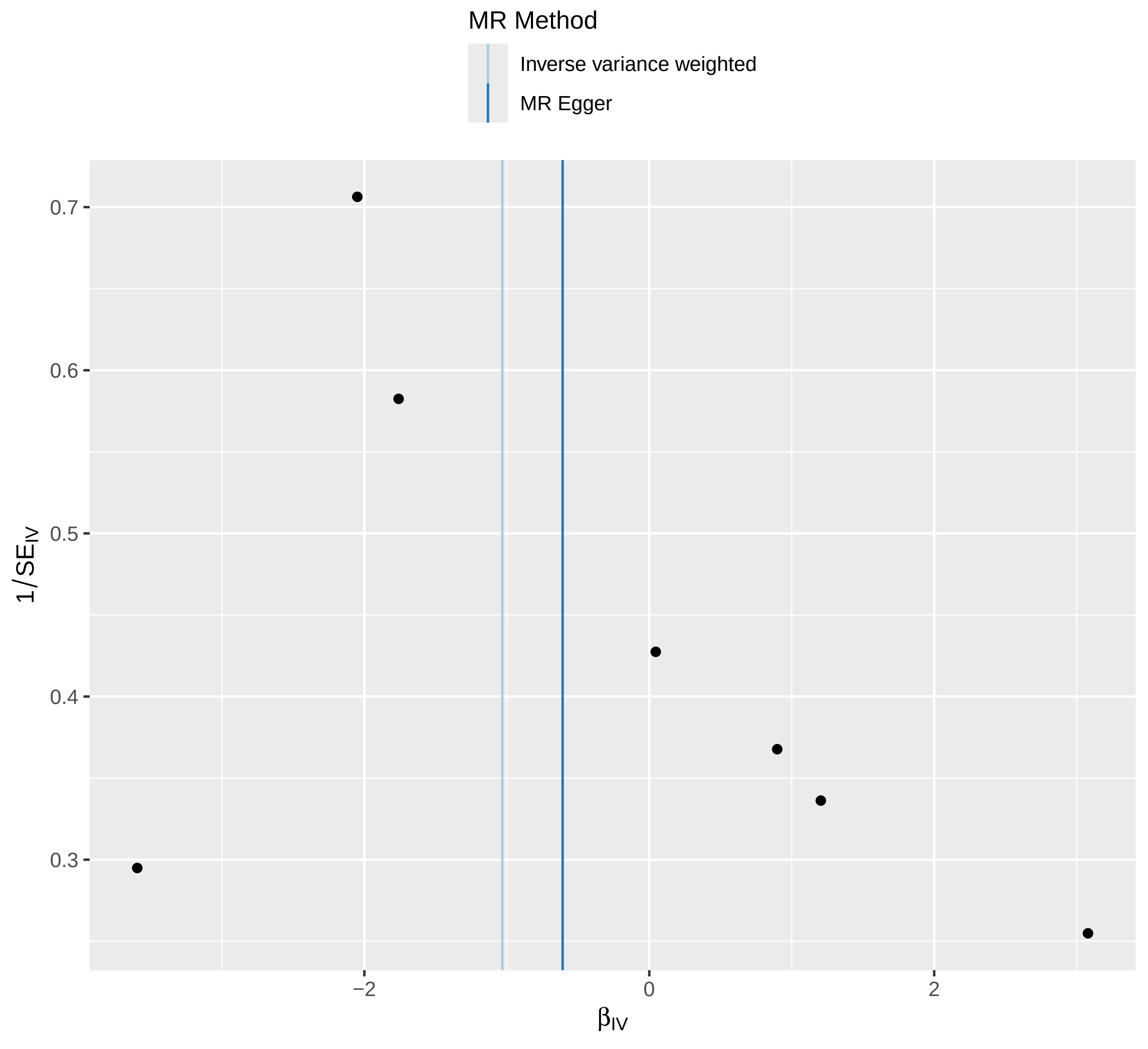
MR Method
Inverse variance weighted
MR Egger
0.7
0.6
0.5
0.4
0.3
1/SE$_{IV}$
−2
0
2
β$_{IV}$

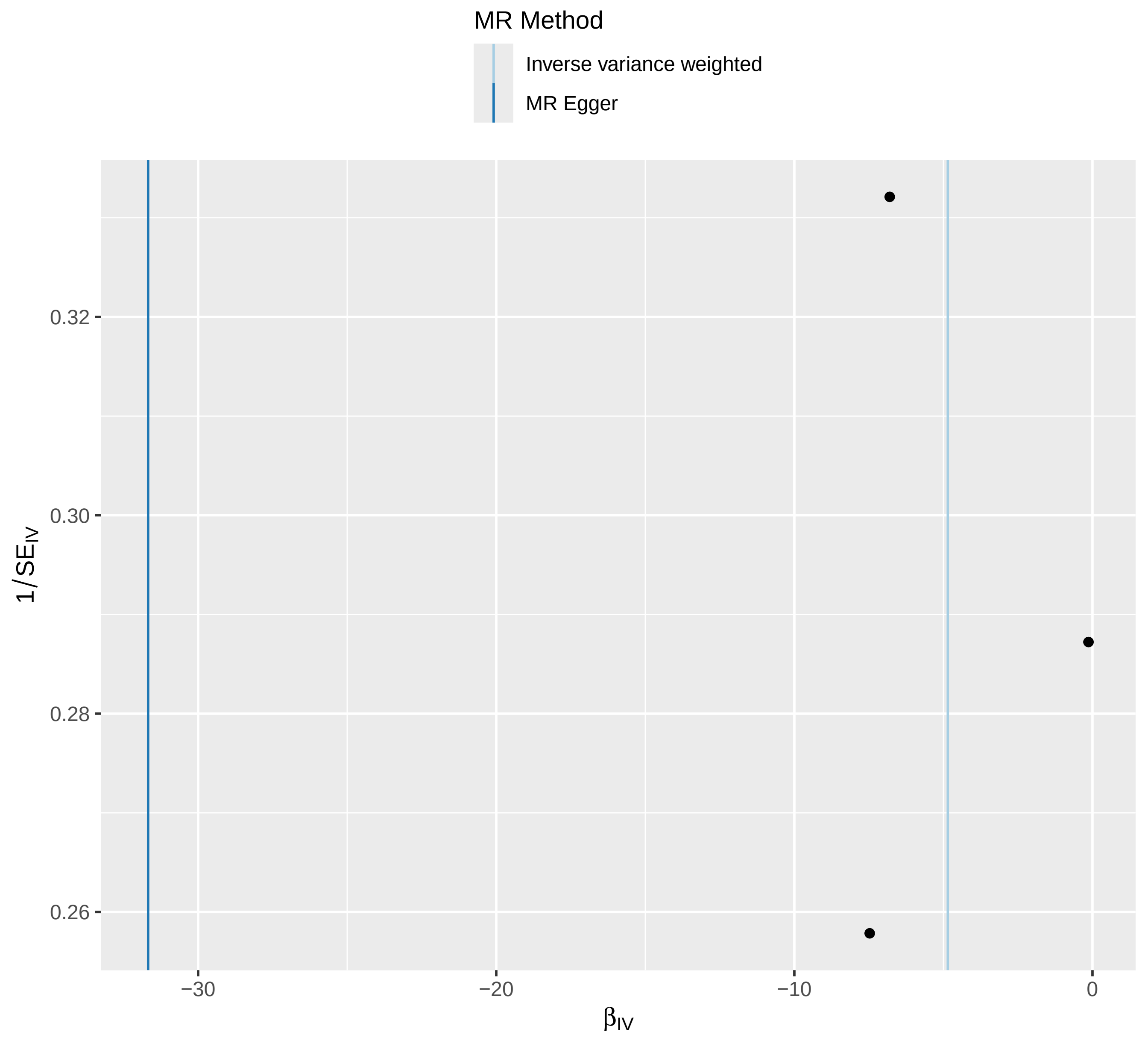

MR Method
Inverse variance weighted
MR Egger
0.32
0.30
0.28
0.26
1/SE_IV
−30
−20
−10
0
β_IV

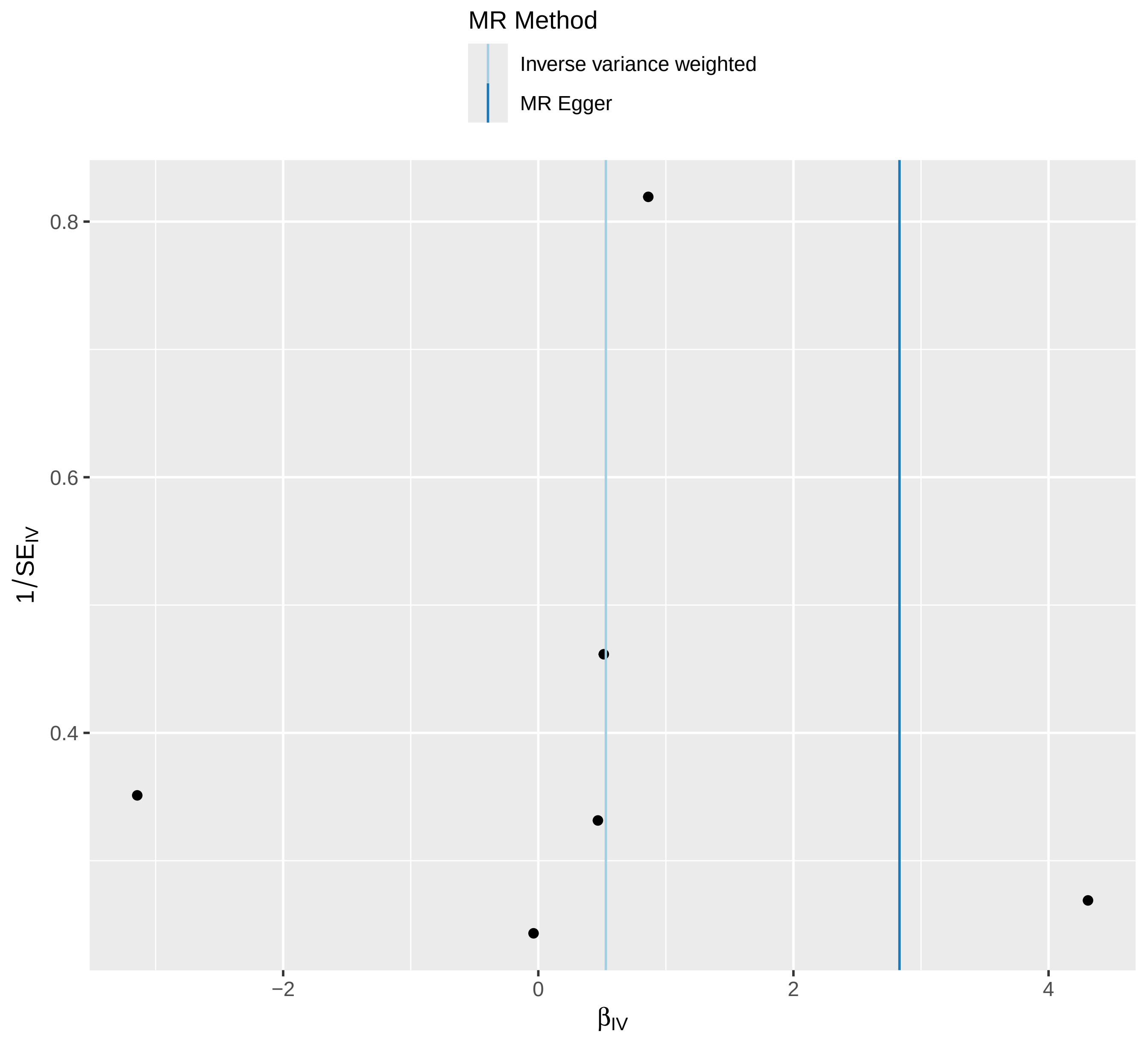

MR Method
Inverse variance weighted
MR Egger
0.8
0.6
0.4
$1/SE_{IV}$
−2
0
2
4
β_{IV}

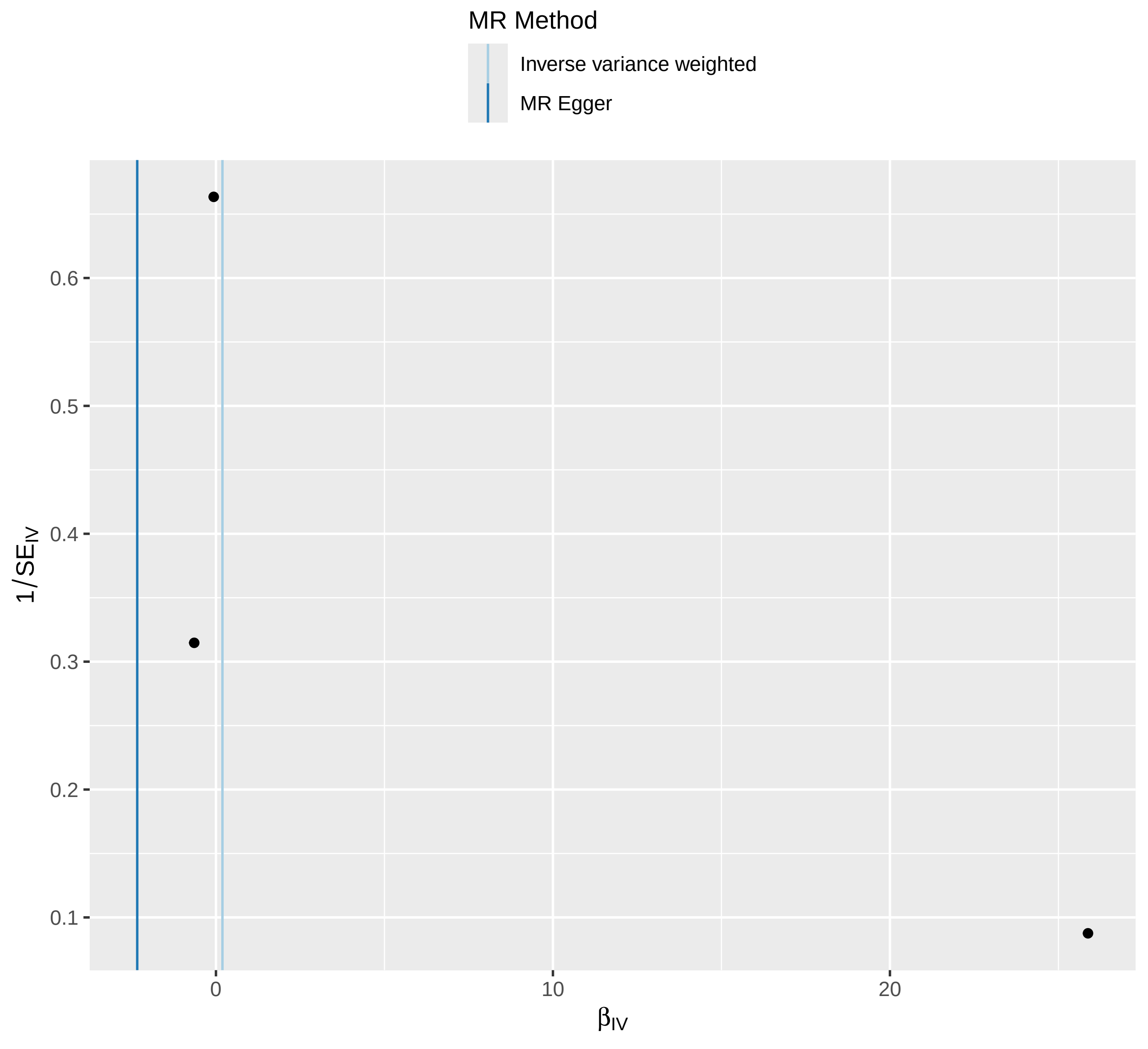
MR Method
Inverse variance weighted
MR Egger
0.6
0.5
0.4
0.3
0.2
0.1
1/SE_IV
0
10
20
β_IV

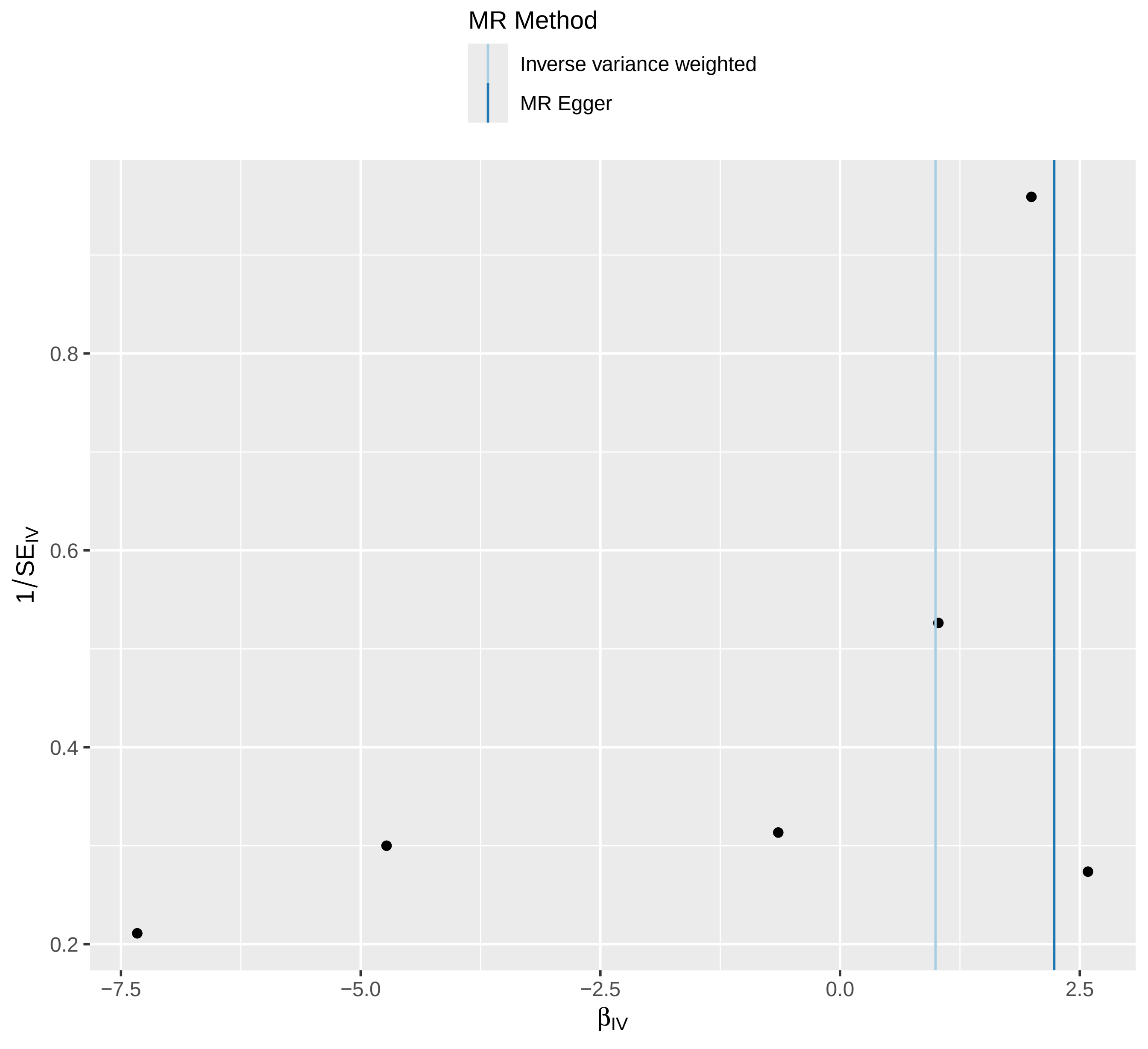
MR Method
Inverse variance weighted
MR Egger
0.8
0.6
0.4
0.2
$1/SE_{IV}$
−7.5
−5.0
−2.5
0.0
2.5
β_{IV}

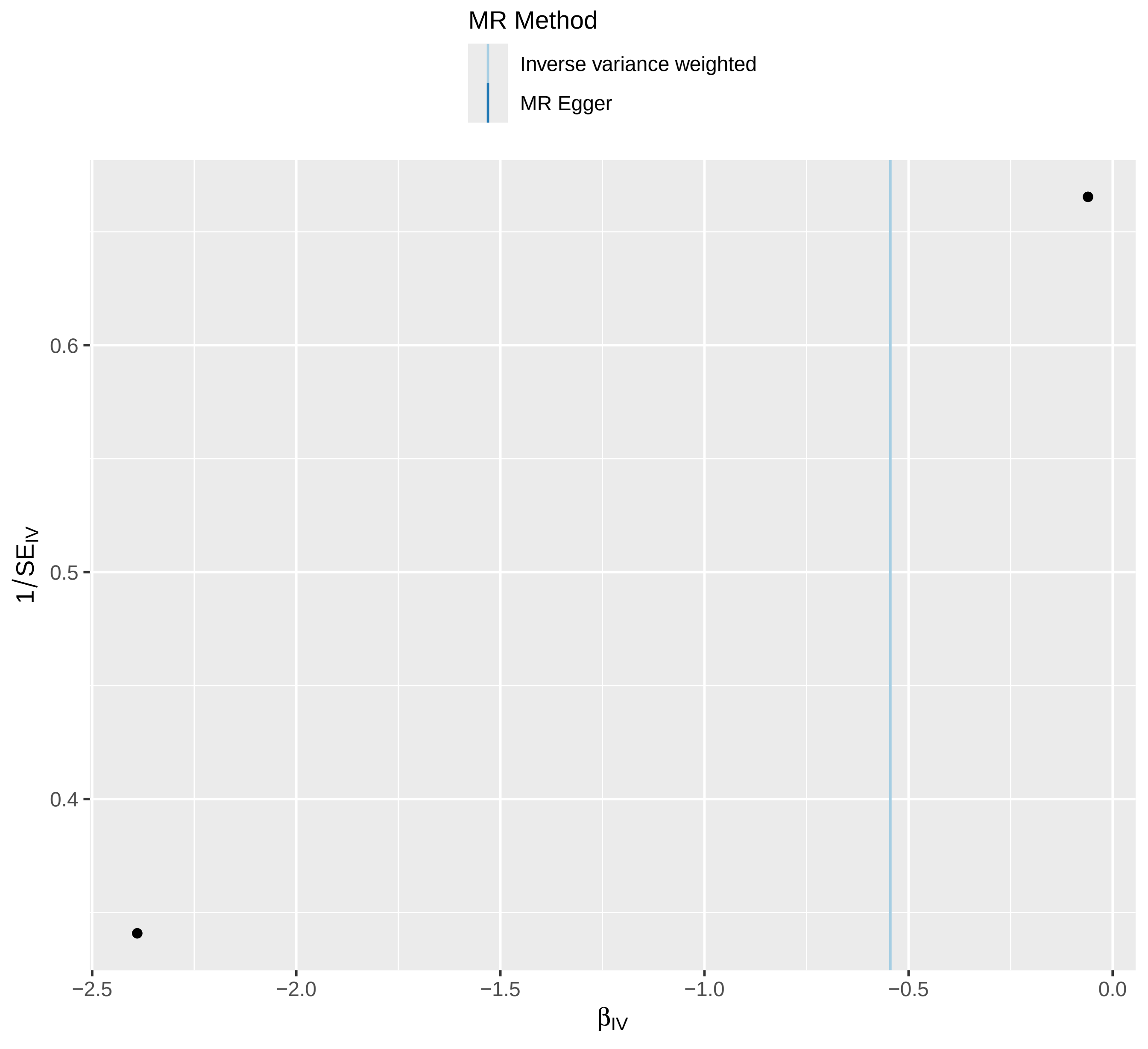
MR Method
Inverse variance weighted
MR Egger
0.6
0.5
0.4
1/SE$_{IV}$
−2.5
−2.0
−1.5
−1.0
−0.5
0.0
β$_{IV}$

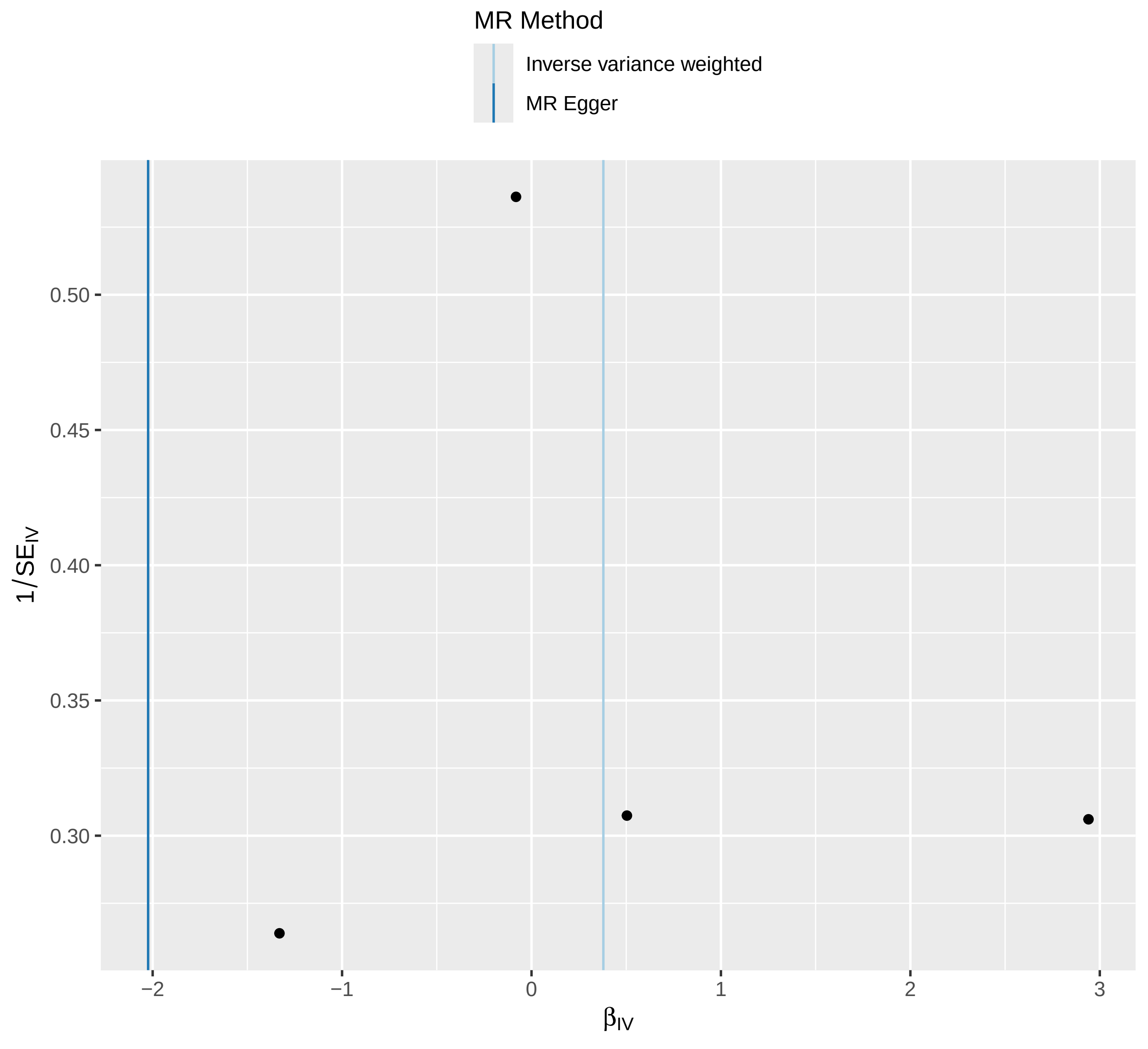

MR Method
Inverse variance weighted
MR Egger
0.50
0.45
0.40
0.35
0.30
$1/SE_{IV}$
−2
−1
0
1
2
3
β_{IV}

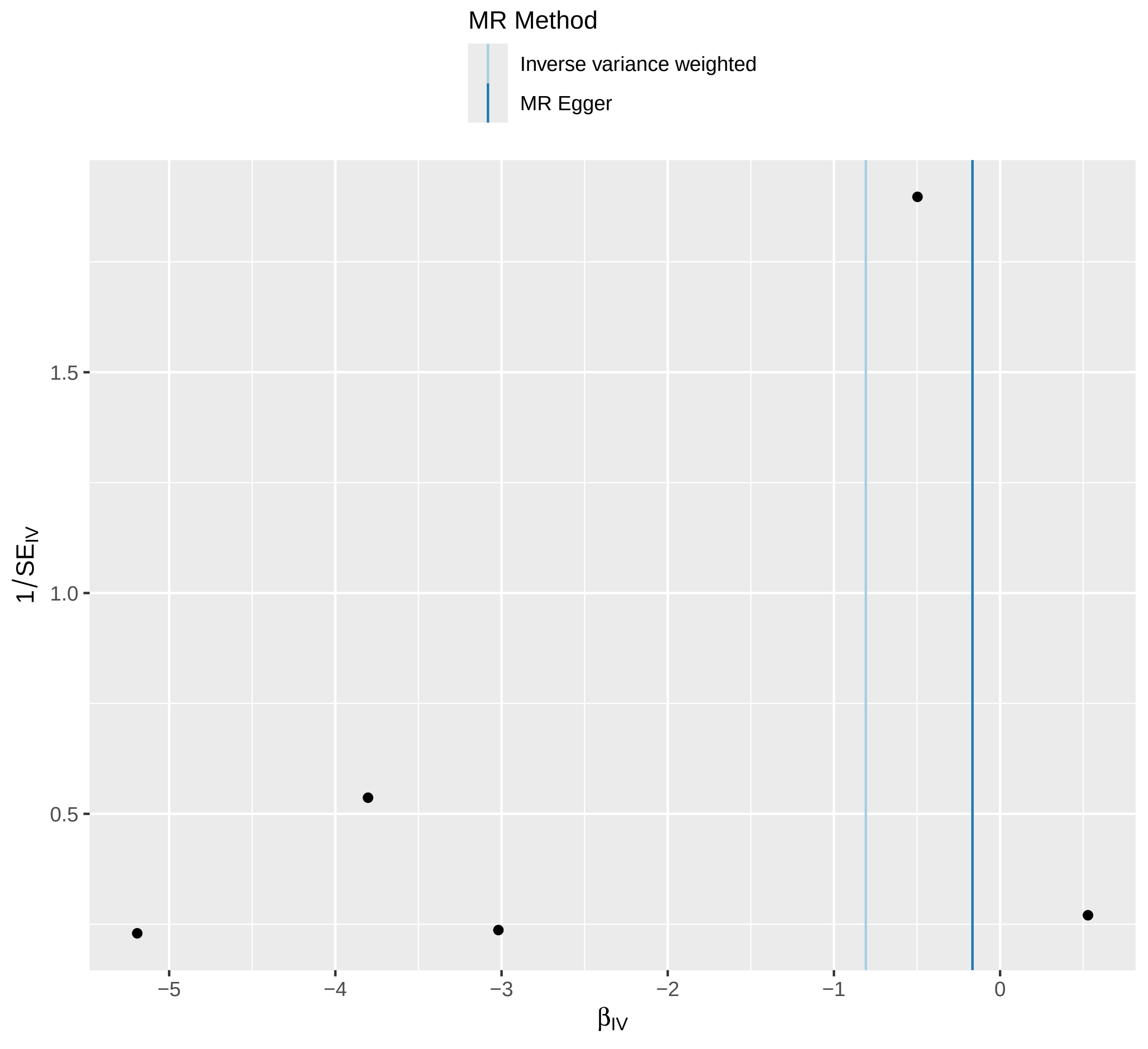
MR Method
Inverse variance weighted
MR Egger
1.5
1.0
0.5
1/SE$_{IV}$
−5
−4
−3
−2
−1
0
β$_{IV}$

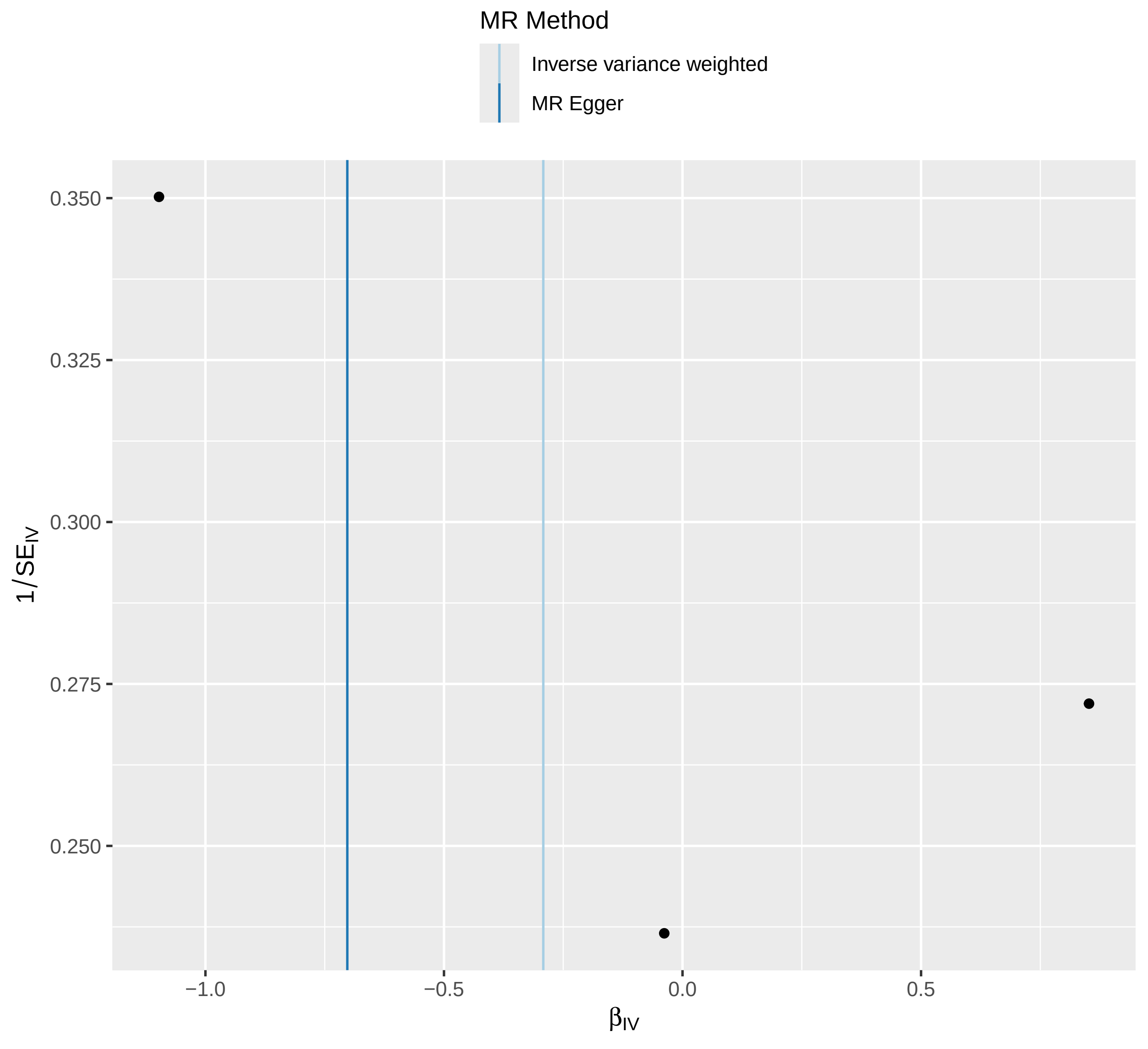
MR Method
Inverse variance weighted
MR Egger
0.350
0.325
0.300
0.275
0.250
$1/SE_{IV}$
−1.0
−0.5
0.0
0.5
β_{IV}

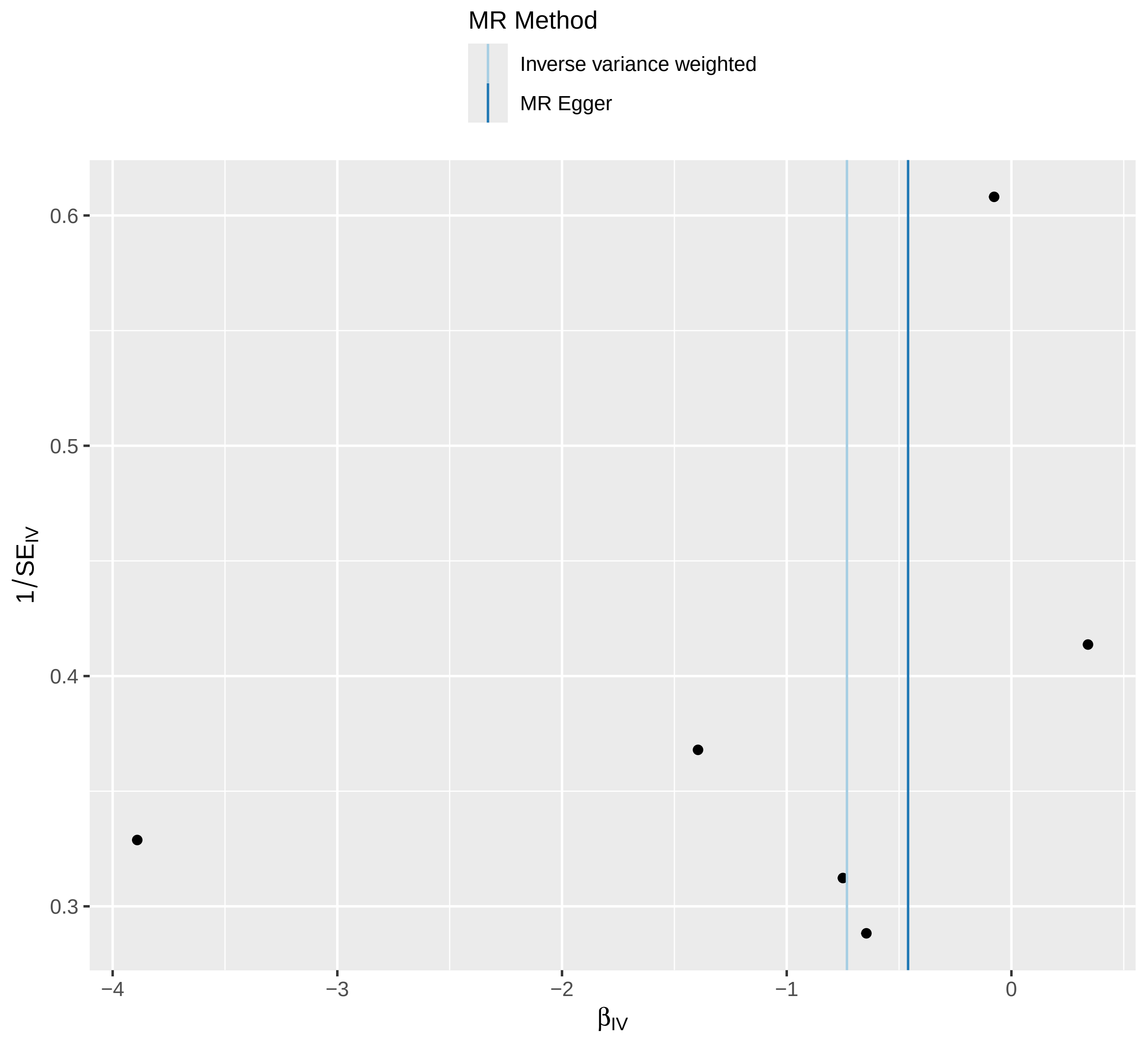
MR Method
Inverse variance weighted
MR Egger
0.6
0.5
0.4
0.3
1/SE_IV
−4
−3
−2
−1
0
β_IV

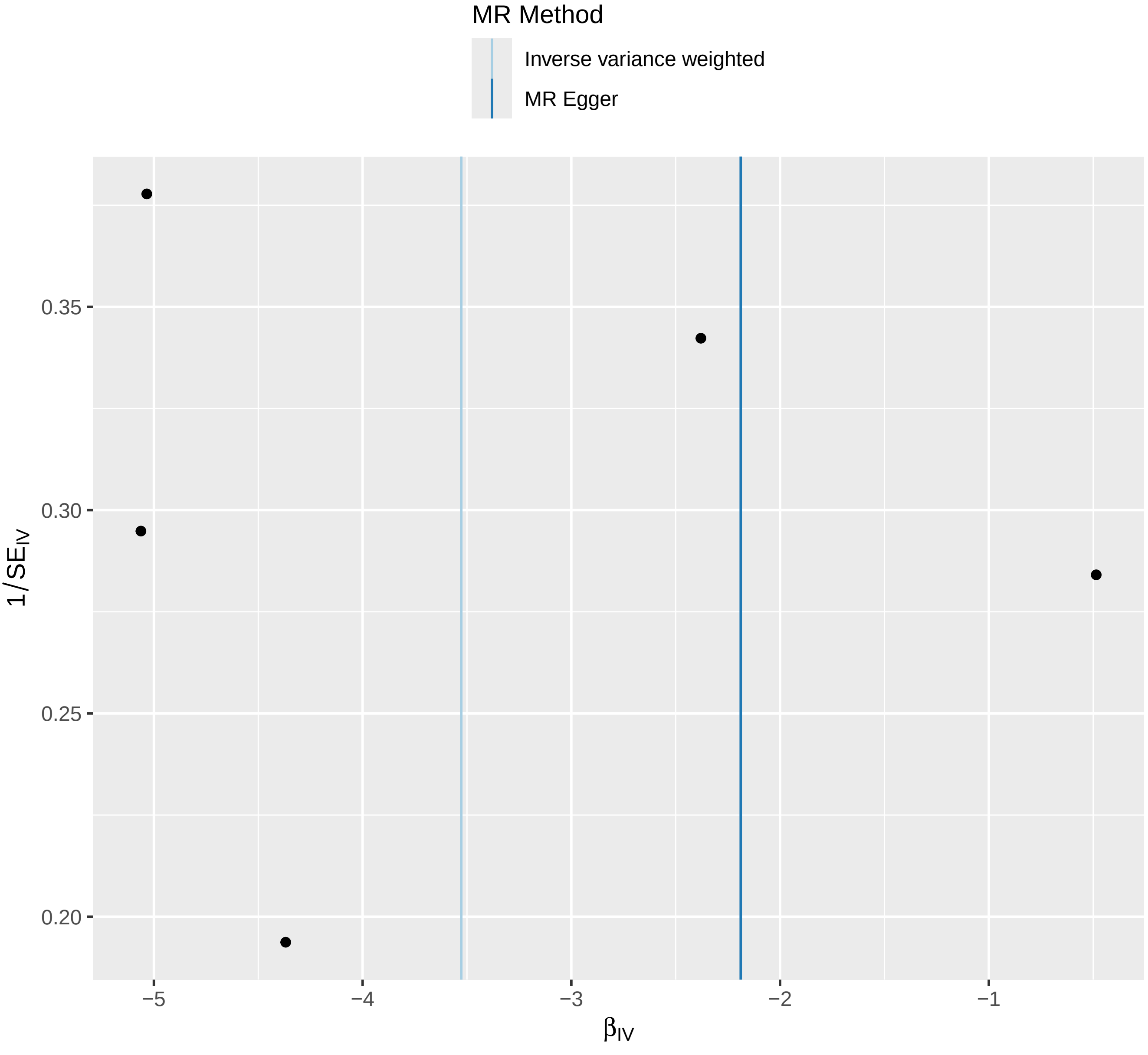
MR Method
Inverse variance weighted
MR Egger
0.35
0.30
0.25
0.20
1/SE$_{IV}$
−5
−4
−3
−2
−1
β$_{IV}$

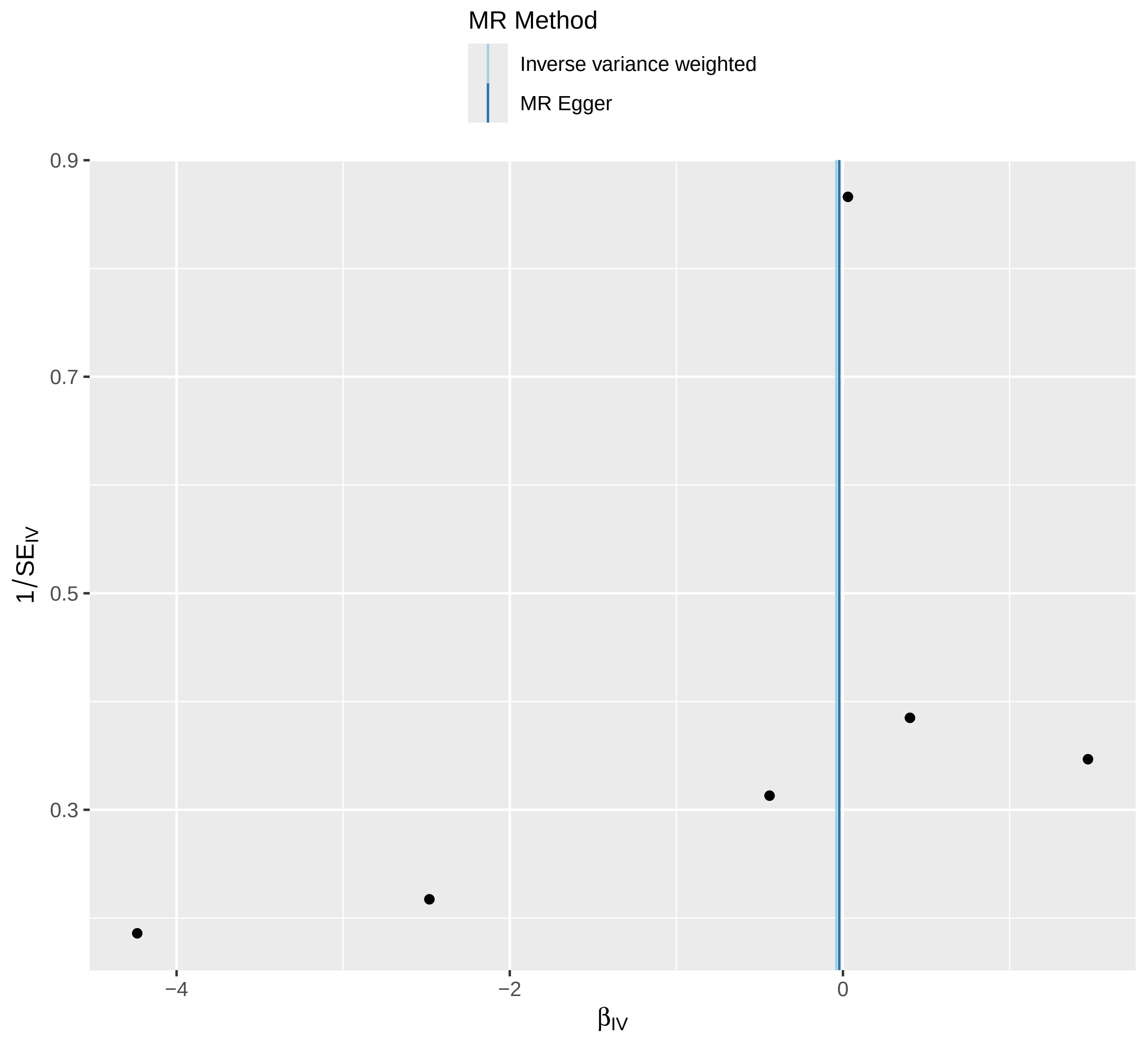
MR Method
Inverse variance weighted
MR Egger
0.9
0.7
0.5
0.3
1/SE_IV
−4
−2
0
β_IV

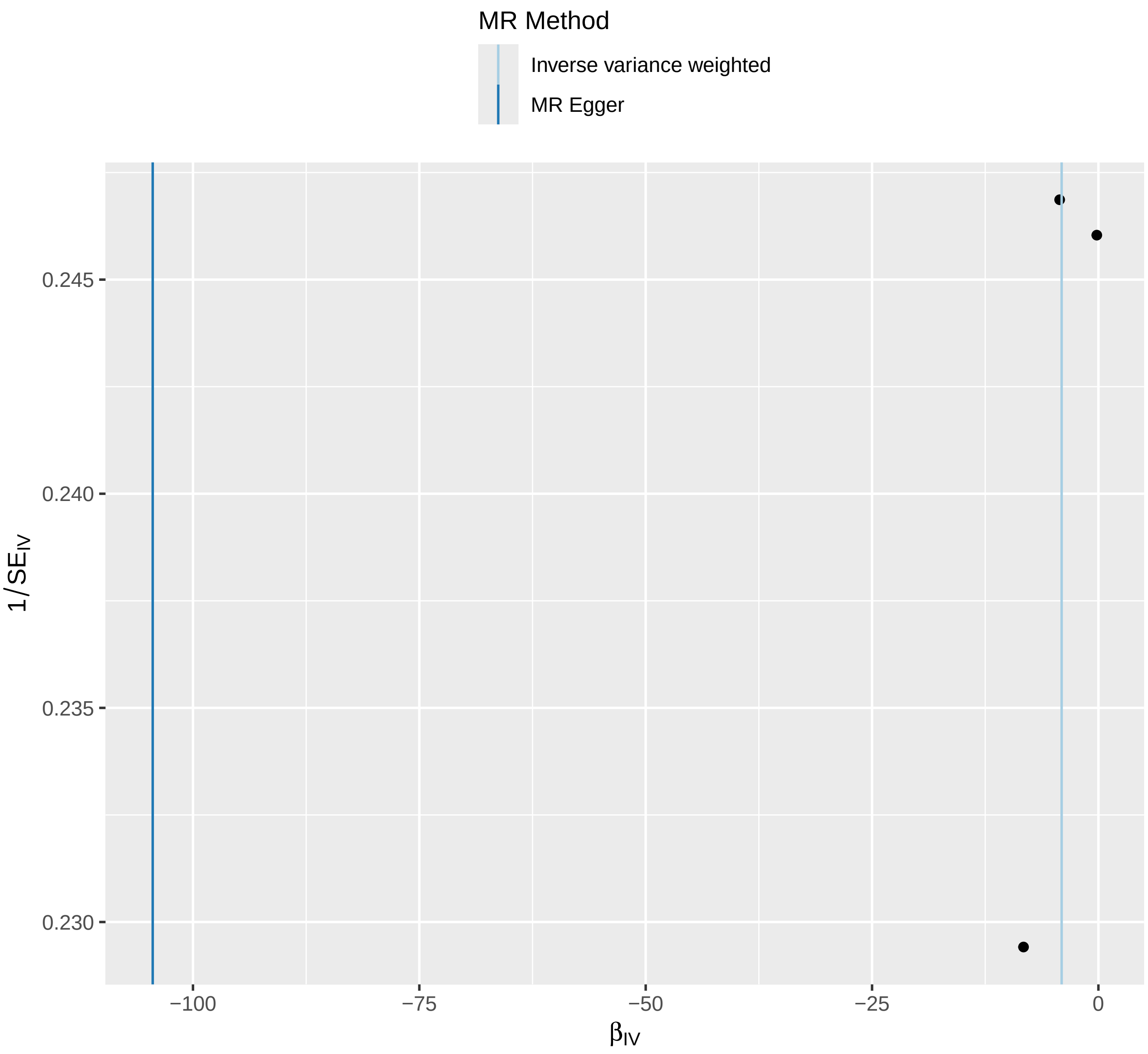

MR Method
Inverse variance weighted
MR Egger
0.245
0.240
0.235
0.230
1/SE_IV
−100
−75
−50
−25
0
β_IV

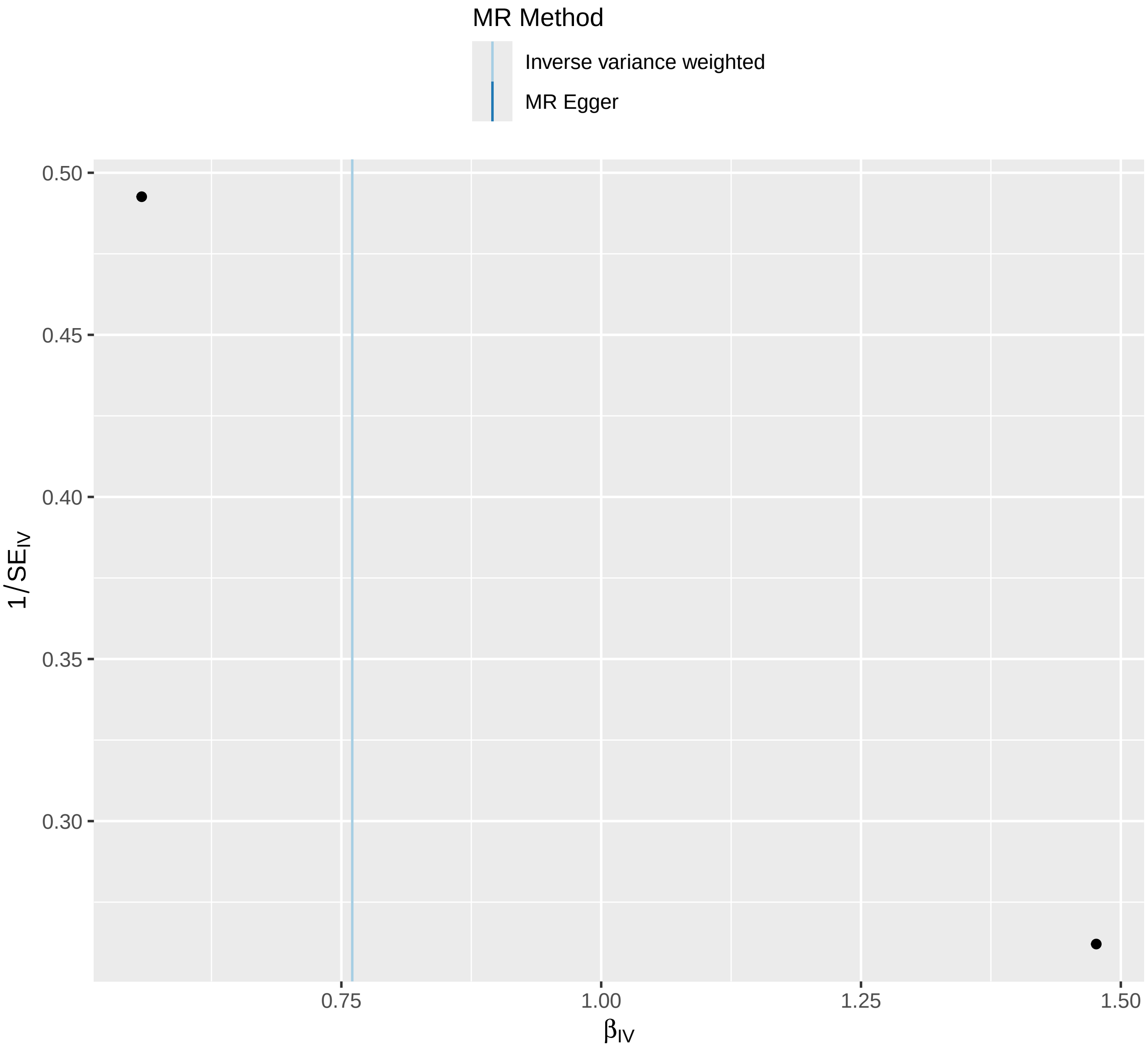
MR Method
Inverse variance weighted
MR Egger
0.50
0.45
0.40
0.35
0.30
$1/SE_{IV}$
0.75
1.00
1.25
1.50
β_{IV}

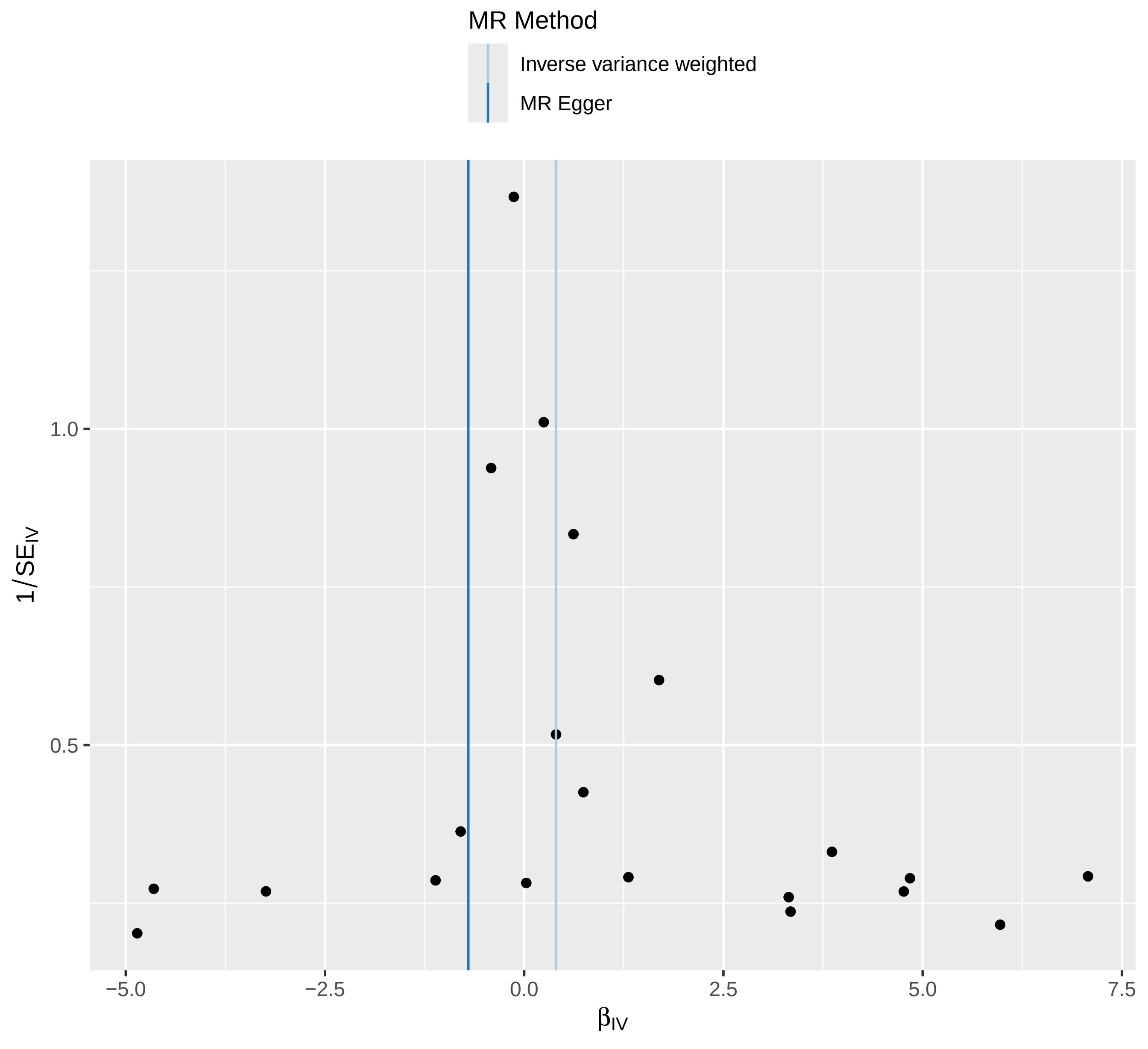
MR Method
Inverse variance weighted
MR Egger
1.0
0.5
1/SE$_{IV}$
−5.0
−2.5
0.0
2.5
5.0
7.5
β$_{IV}$

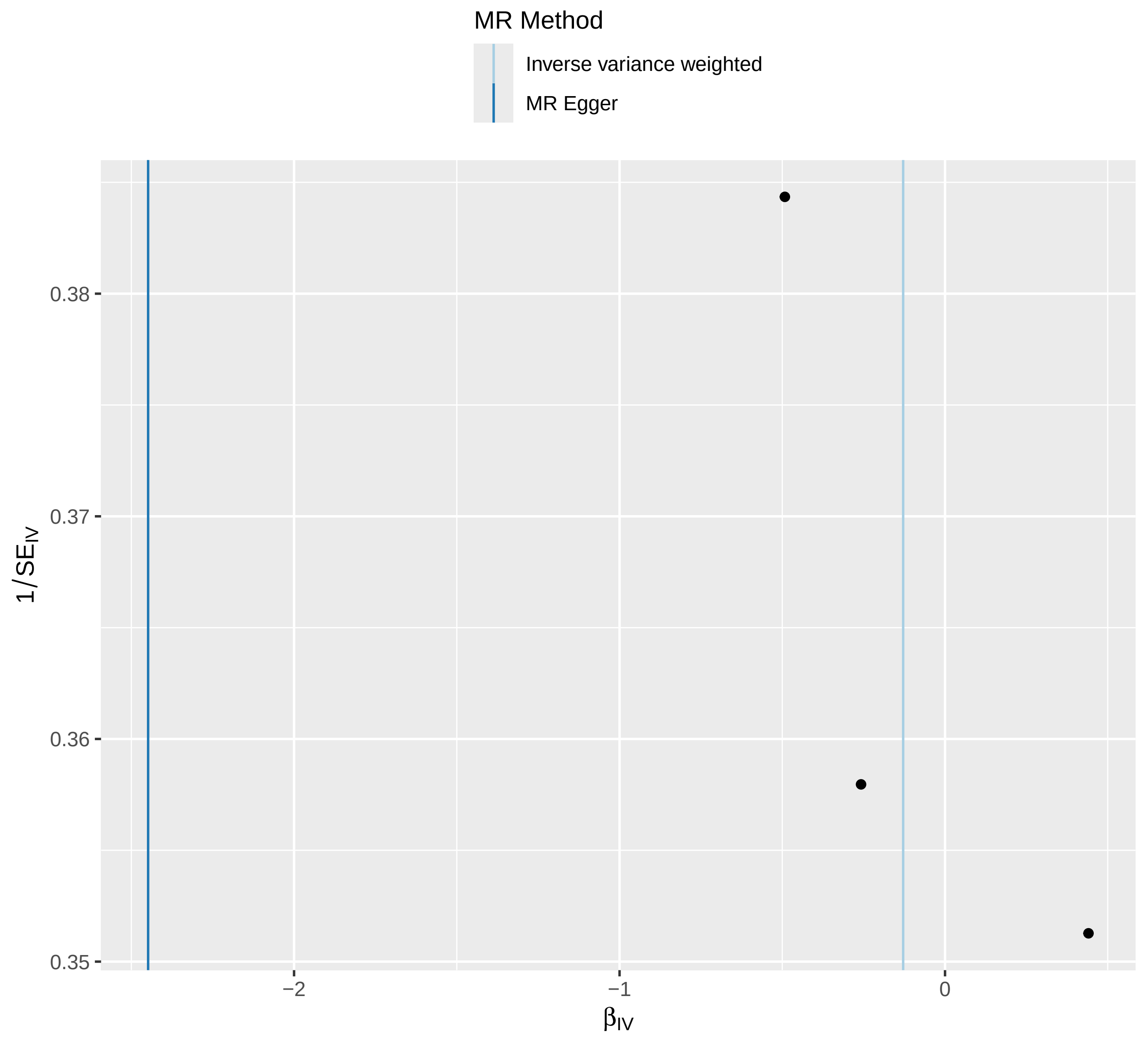
MR Method
Inverse variance weighted
MR Egger
0.38
0.37
0.36
0.35
1/SE_IV
−2
−1
0
β_IV

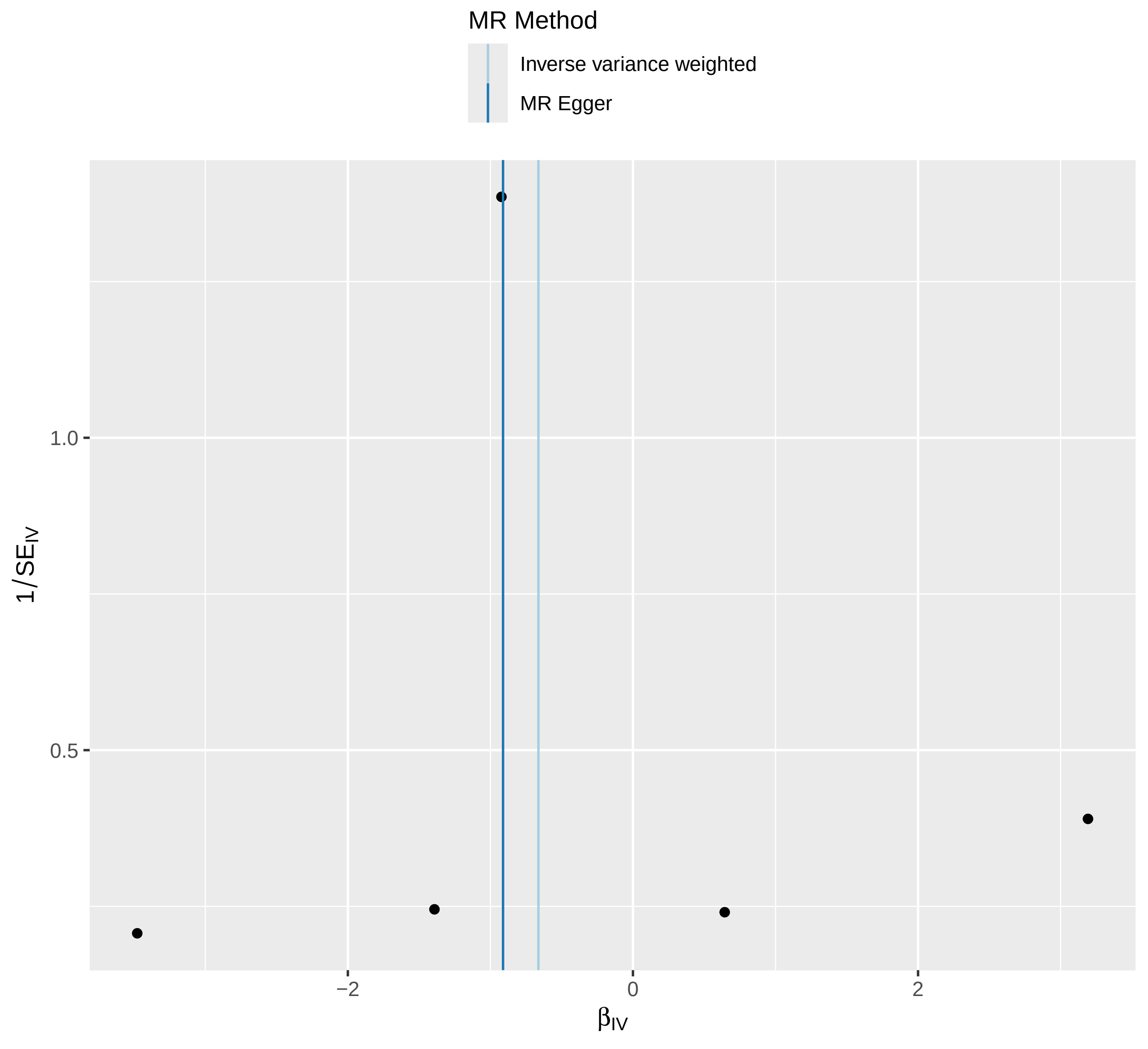
MR Method
Inverse variance weighted
MR Egger
1.0
0.5
1/SE_IV
−2
0
2
β_IV

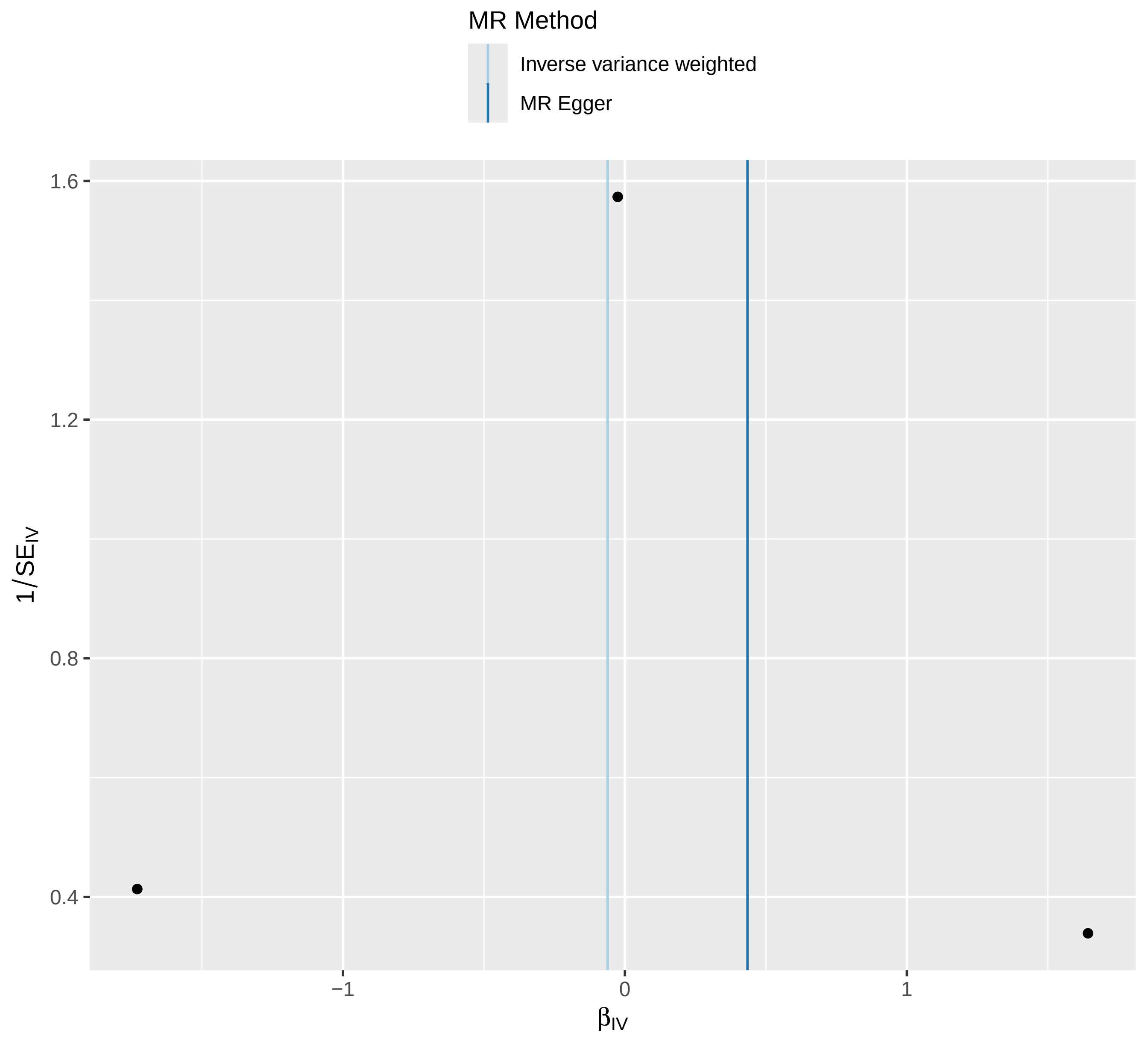
MR Method
Inverse variance weighted
MR Egger
1.6
1.2
0.8
0.4
1/SE_IV
−1
0
1
β_IV

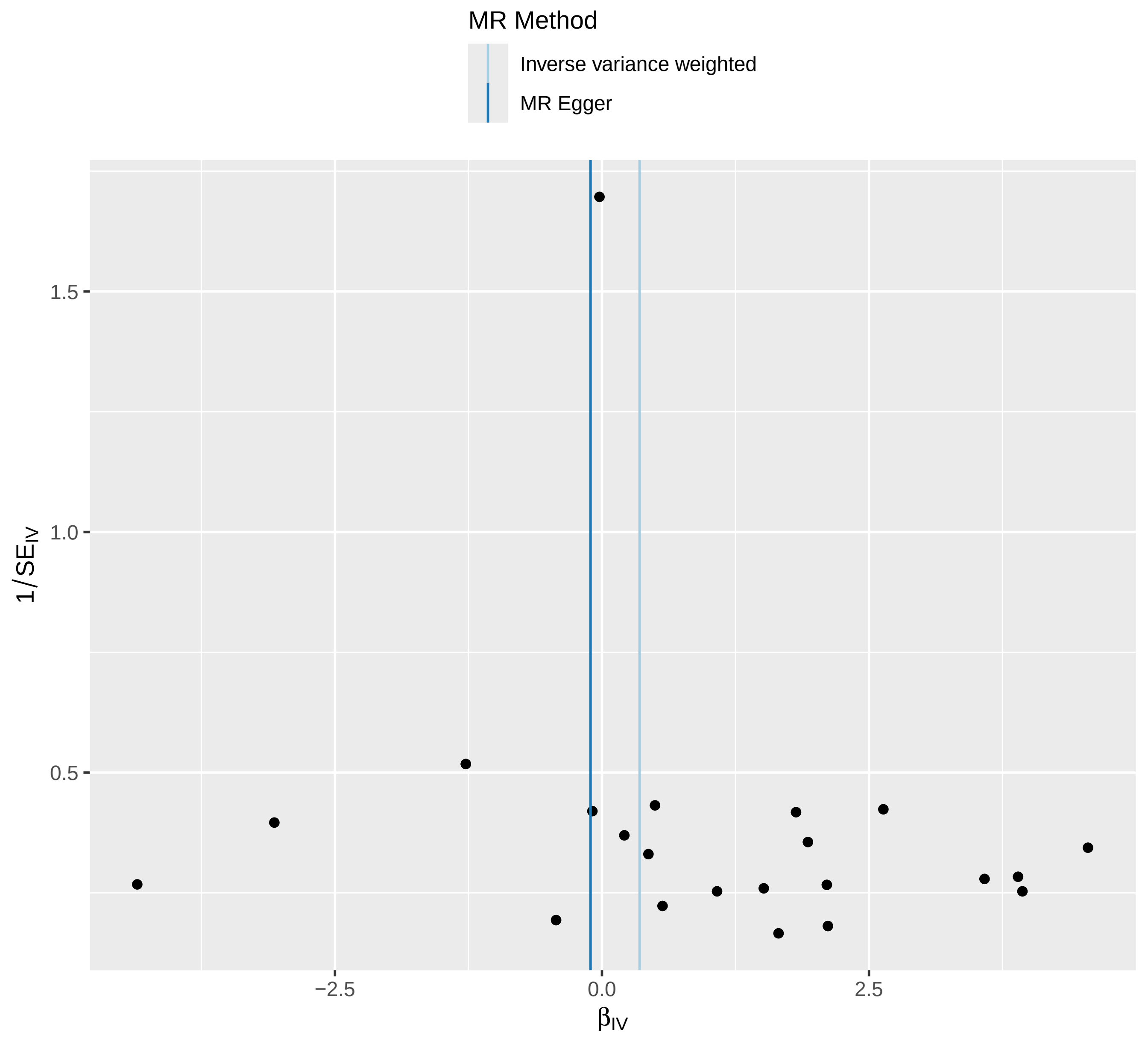
MR Method
Inverse variance weighted
MR Egger
1.5
1.0
0.5
1/SE_IV
−2.5
0.0
2.5
β_IV

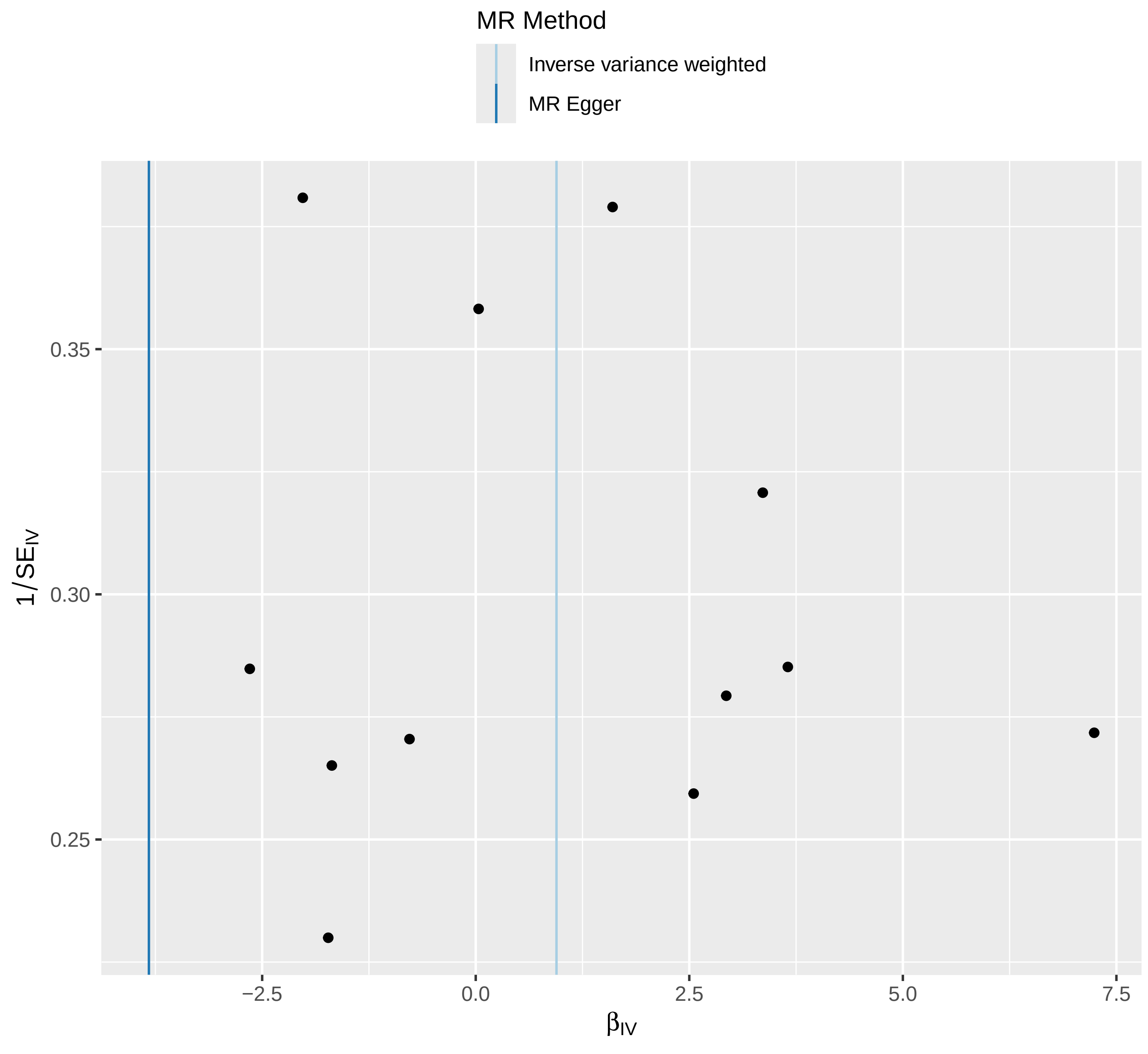
MR Method
Inverse variance weighted
MR Egger
0.35
0.30
0.25
1/SE$_{IV}$
−2.5
0.0
2.5
5.0
7.5
β_{IV}

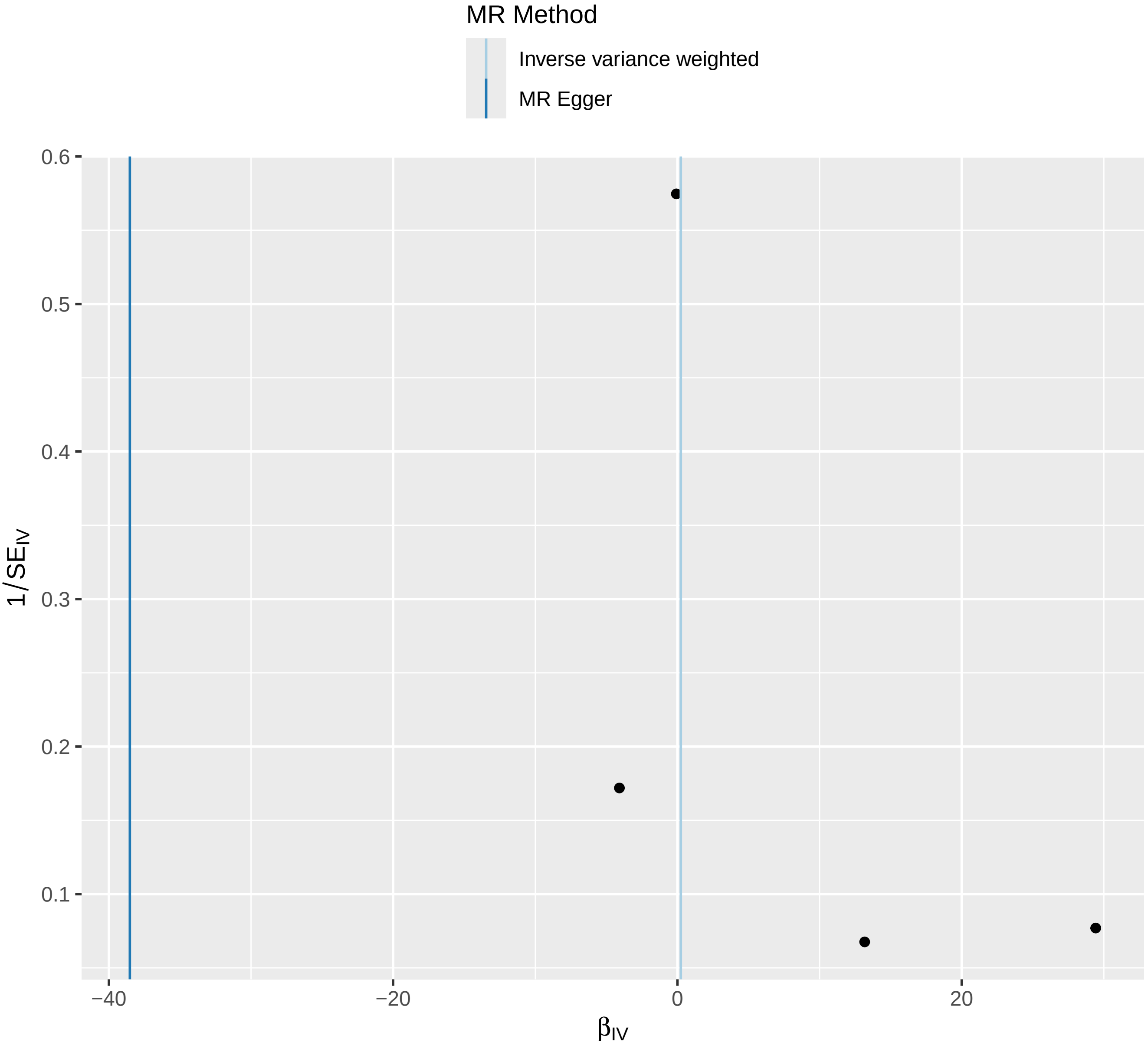
MR Method
Inverse variance weighted
MR Egger
0.6
0.5
0.4
0.3
0.2
0.1
1/SE_IV
−40
−20
0
20
β_IV

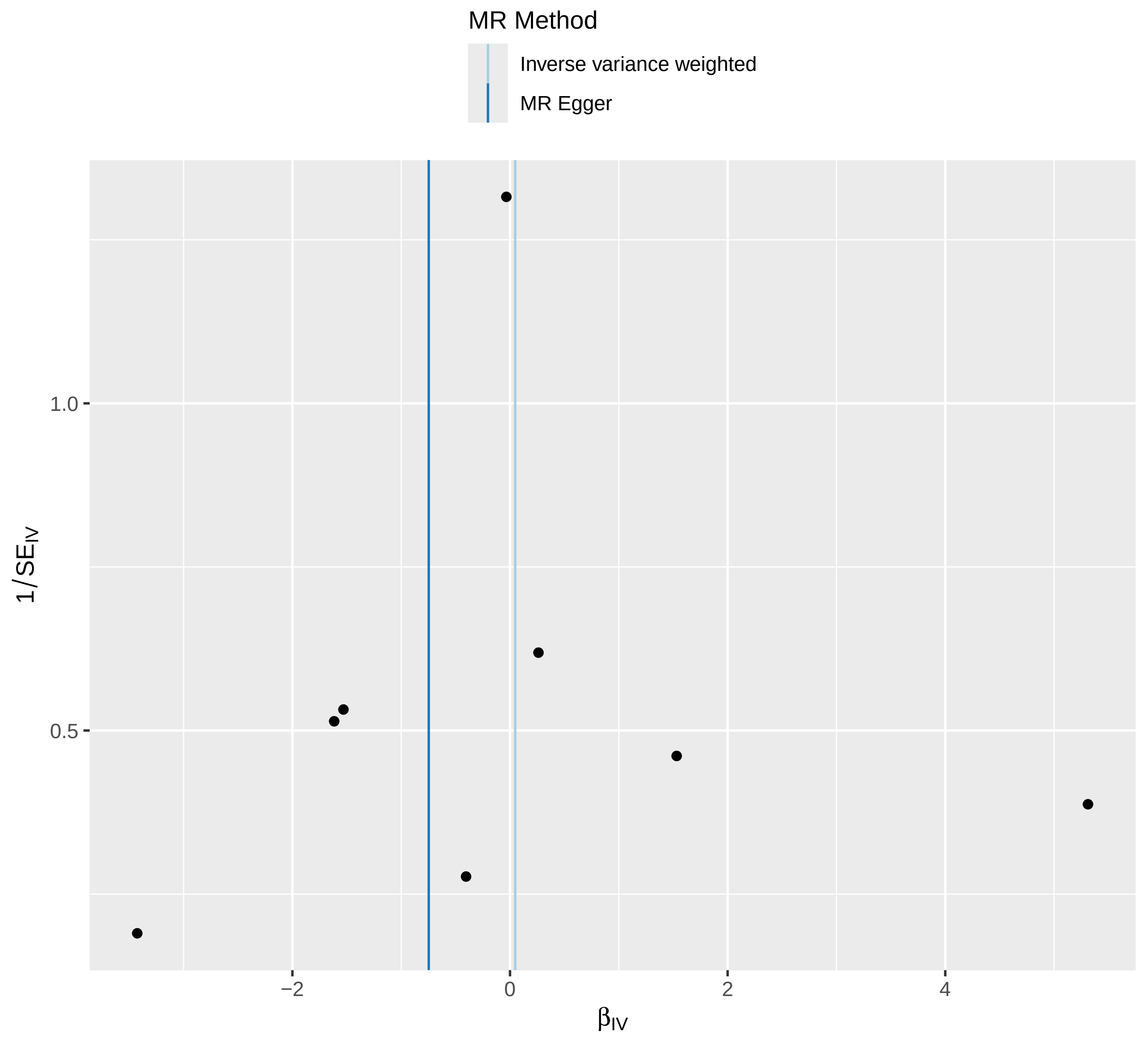
MR Method
Inverse variance weighted
MR Egger
1.0
0.5
1/SE_IV
−2
0
2
4
β_IV

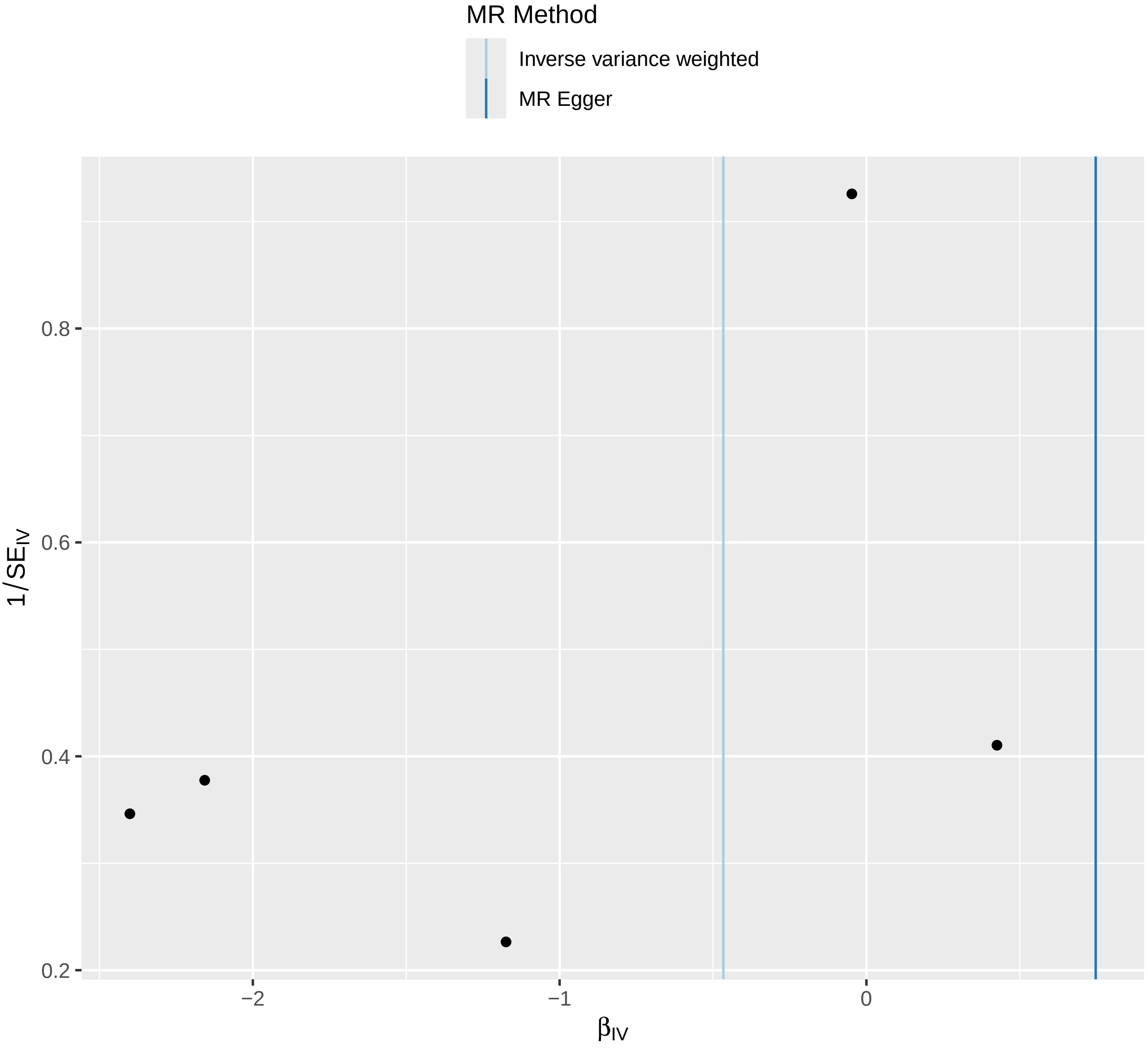
MR Method
Inverse variance weighted
MR Egger
0.8
0.6
0.4
0.2
1/SE_IV
−2
−1
0
β_IV

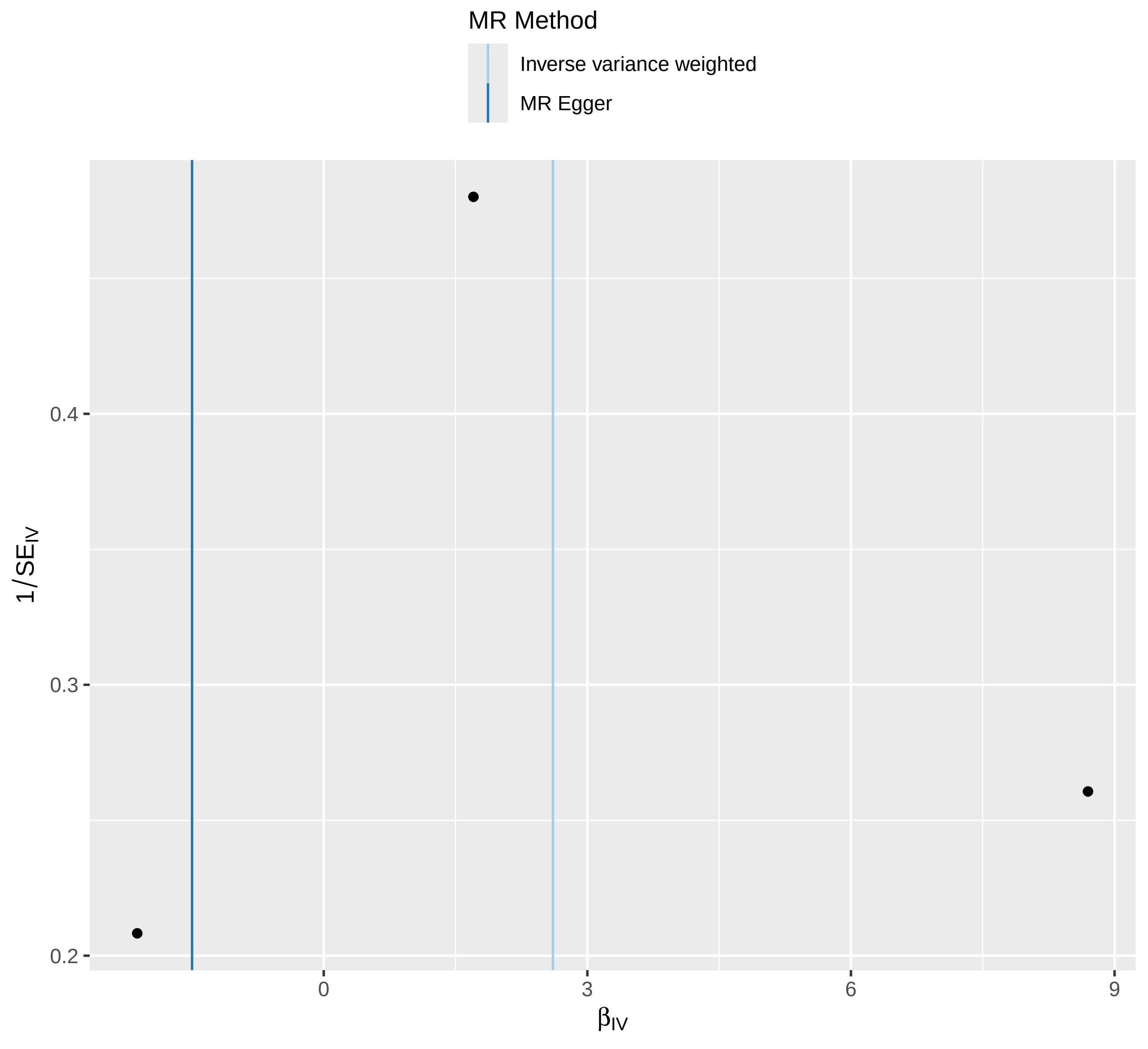
MR Method
Inverse variance weighted
MR Egger
0.4
0.3
0.2
0
3
6
9
1/SE_IV
β_IV

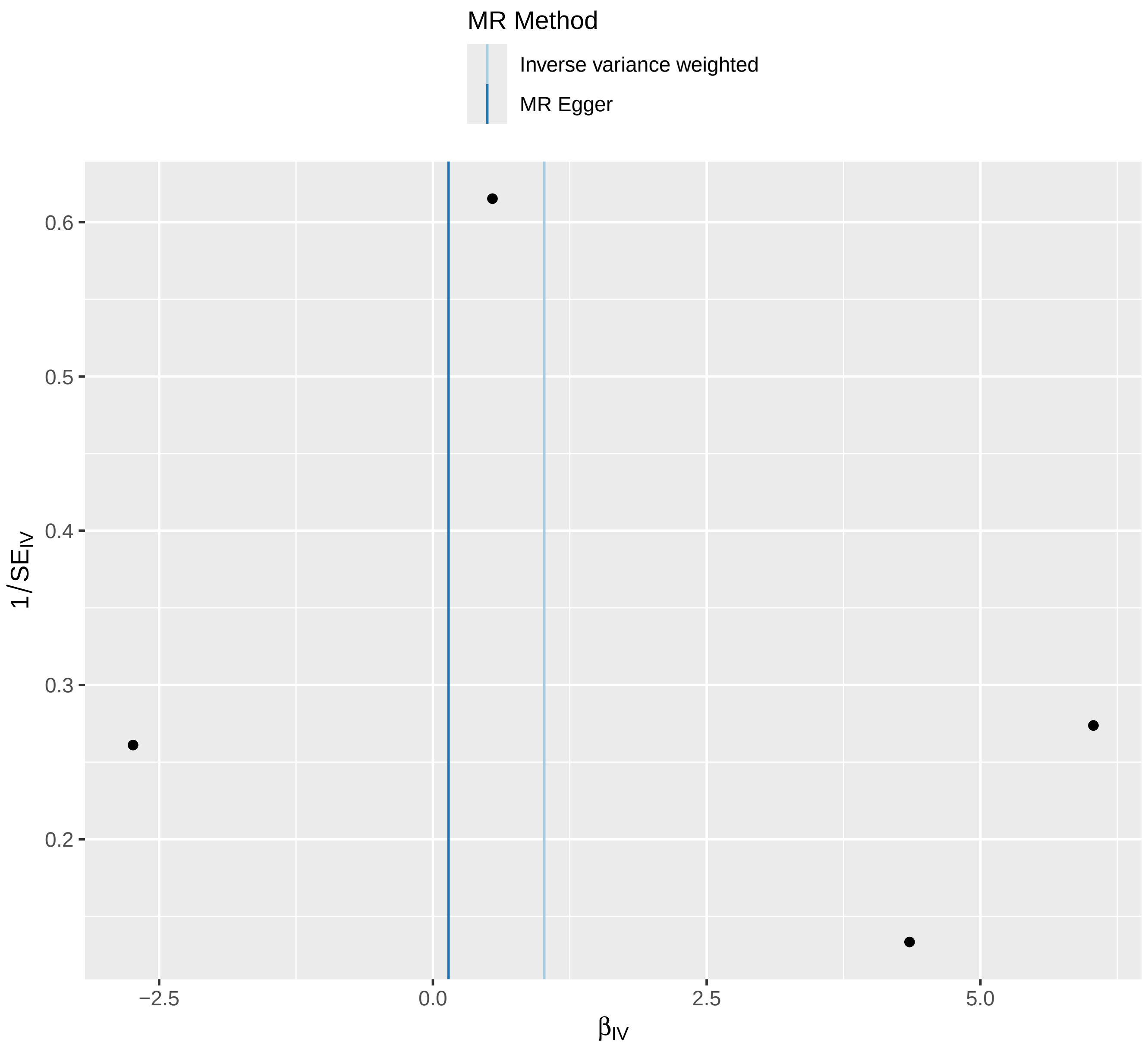
MR Method
Inverse variance weighted
MR Egger
0.6
0.5
0.4
0.3
0.2
$1/SE_{IV}$
−2.5
0.0
2.5
5.0
β_{IV}

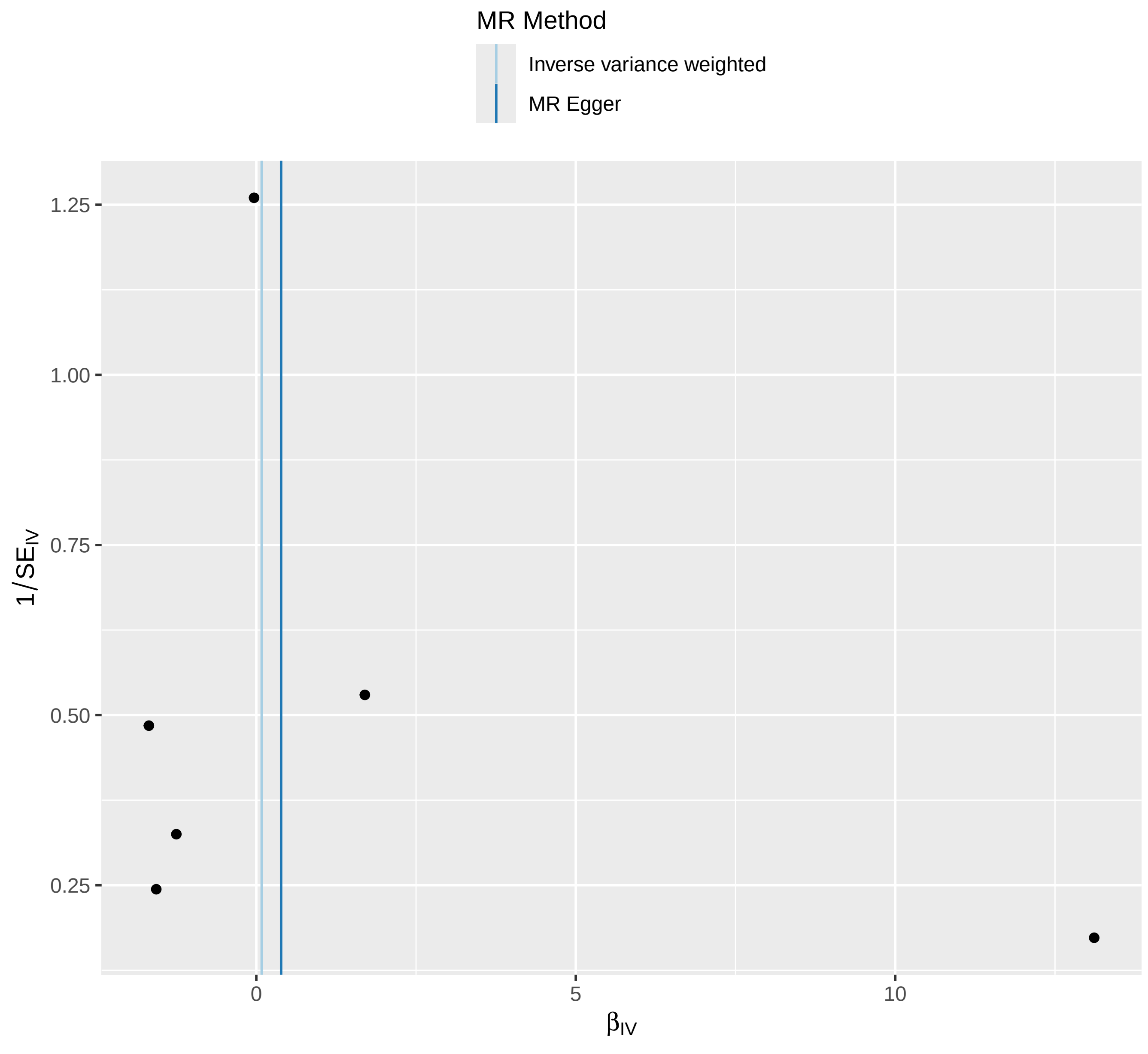
MR Method
Inverse variance weighted
MR Egger
1.25
1.00
0.75
0.50
0.25
$1/SE_{IV}$
0
5
10
β_{IV}

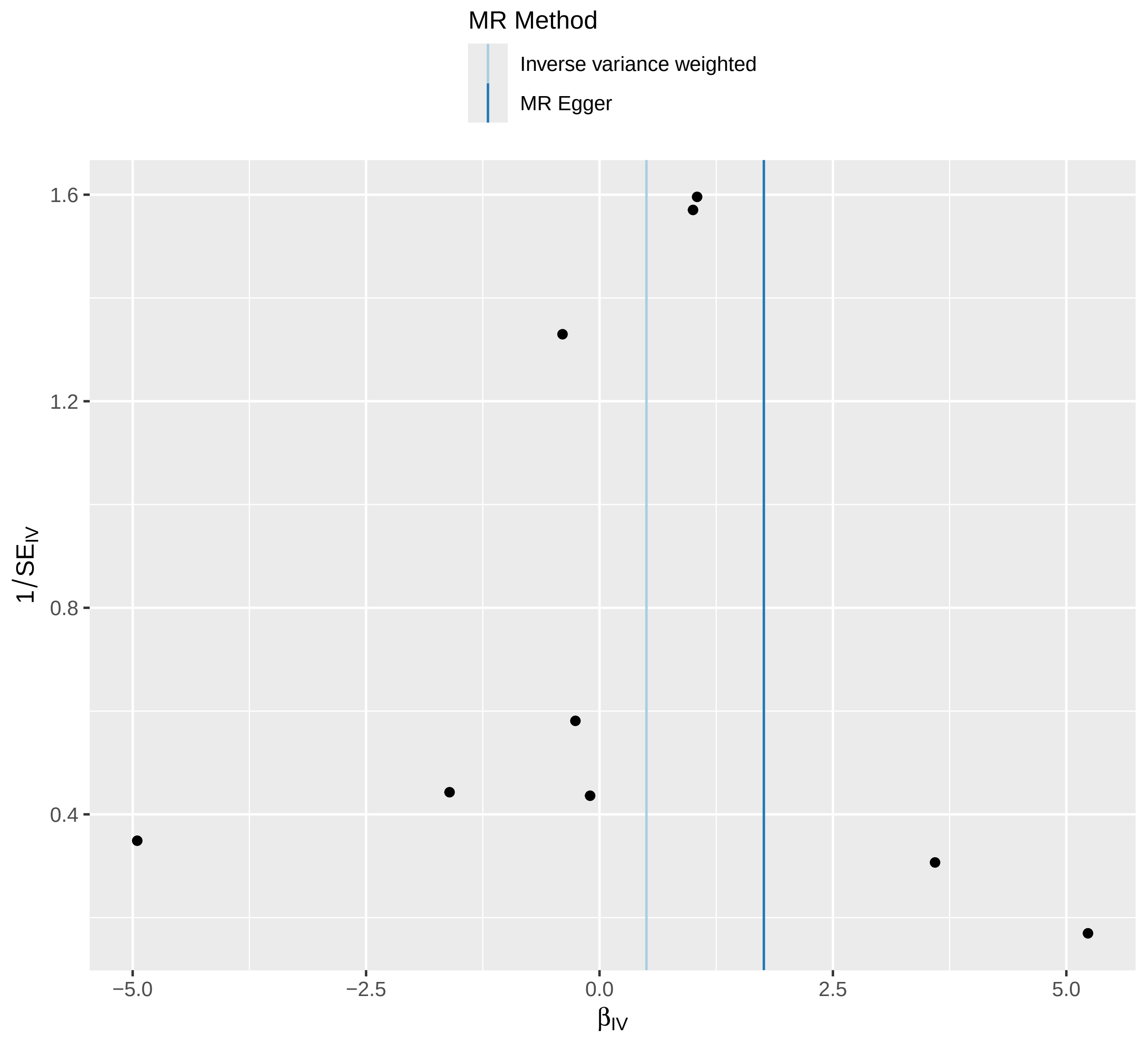

MR Method
Inverse variance weighted
MR Egger
1.6
1.2
0.8
0.4
1/SE_IV
−5.0
−2.5
0.0
2.5
5.0
β_IV

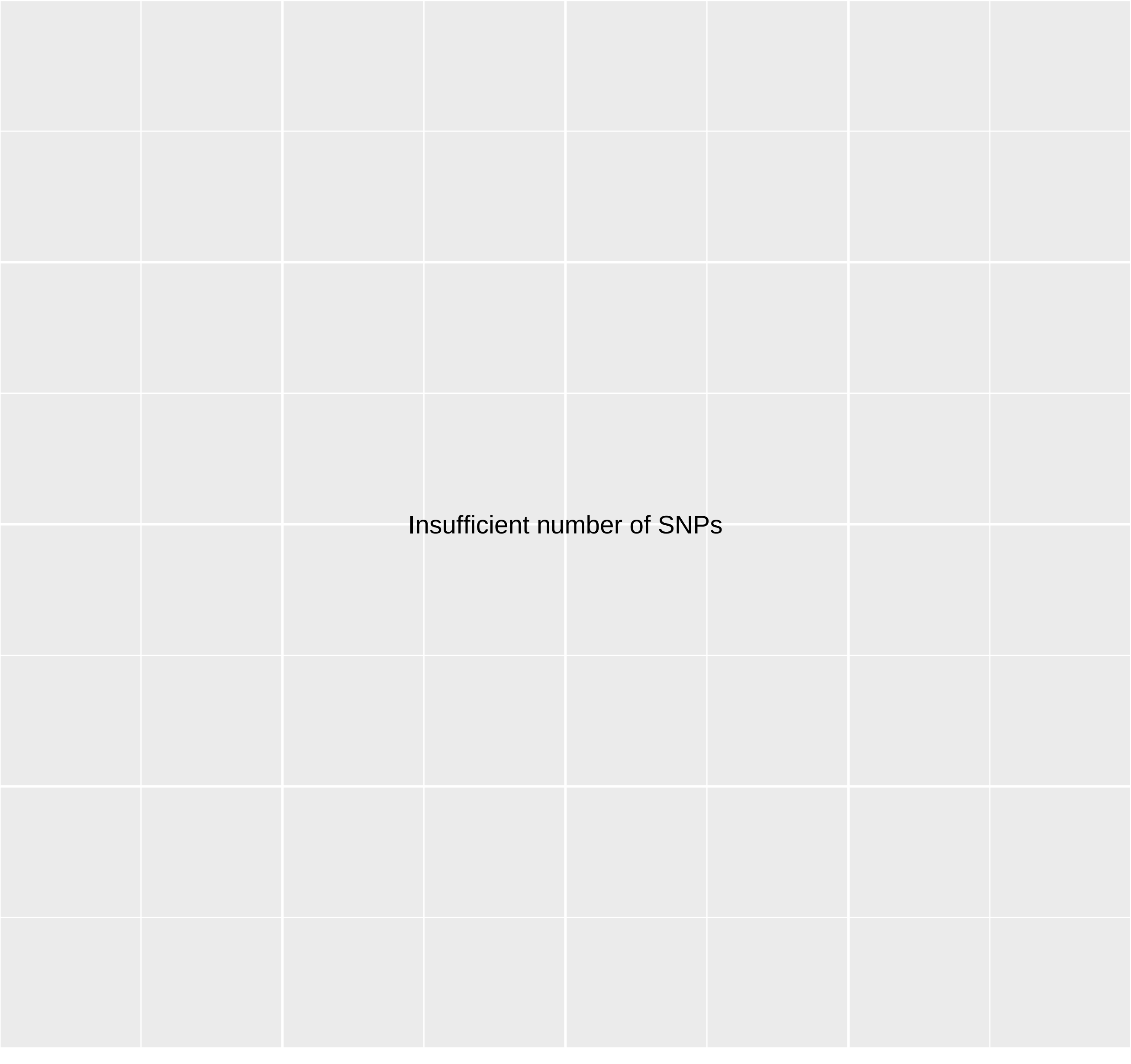
Insufficient number of SNPs

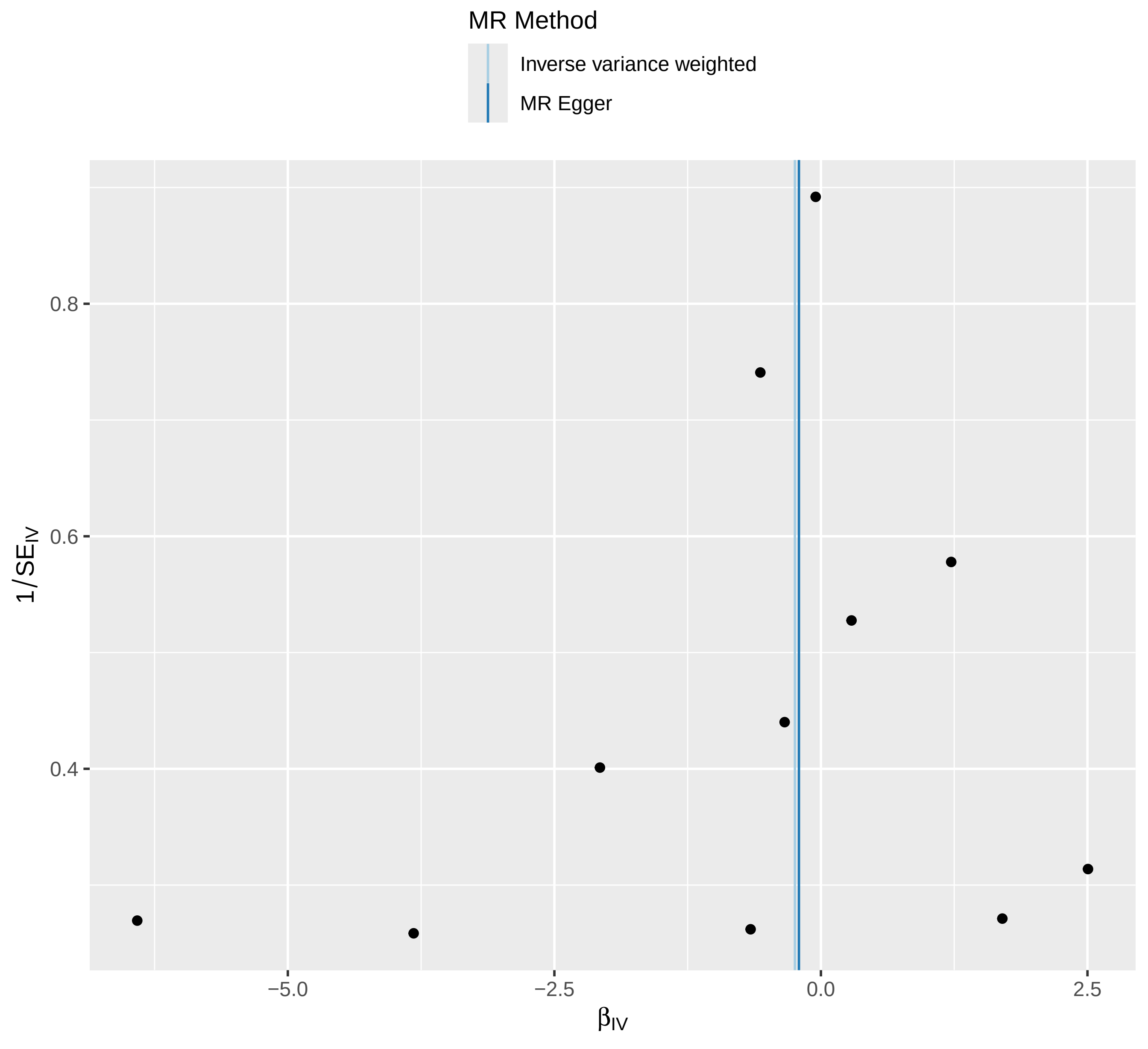
MR Method
Inverse variance weighted
MR Egger
0.8
0.6
0.4
$1/SE_{IV}$
−5.0
−2.5
0.0
2.5
β_{IV}

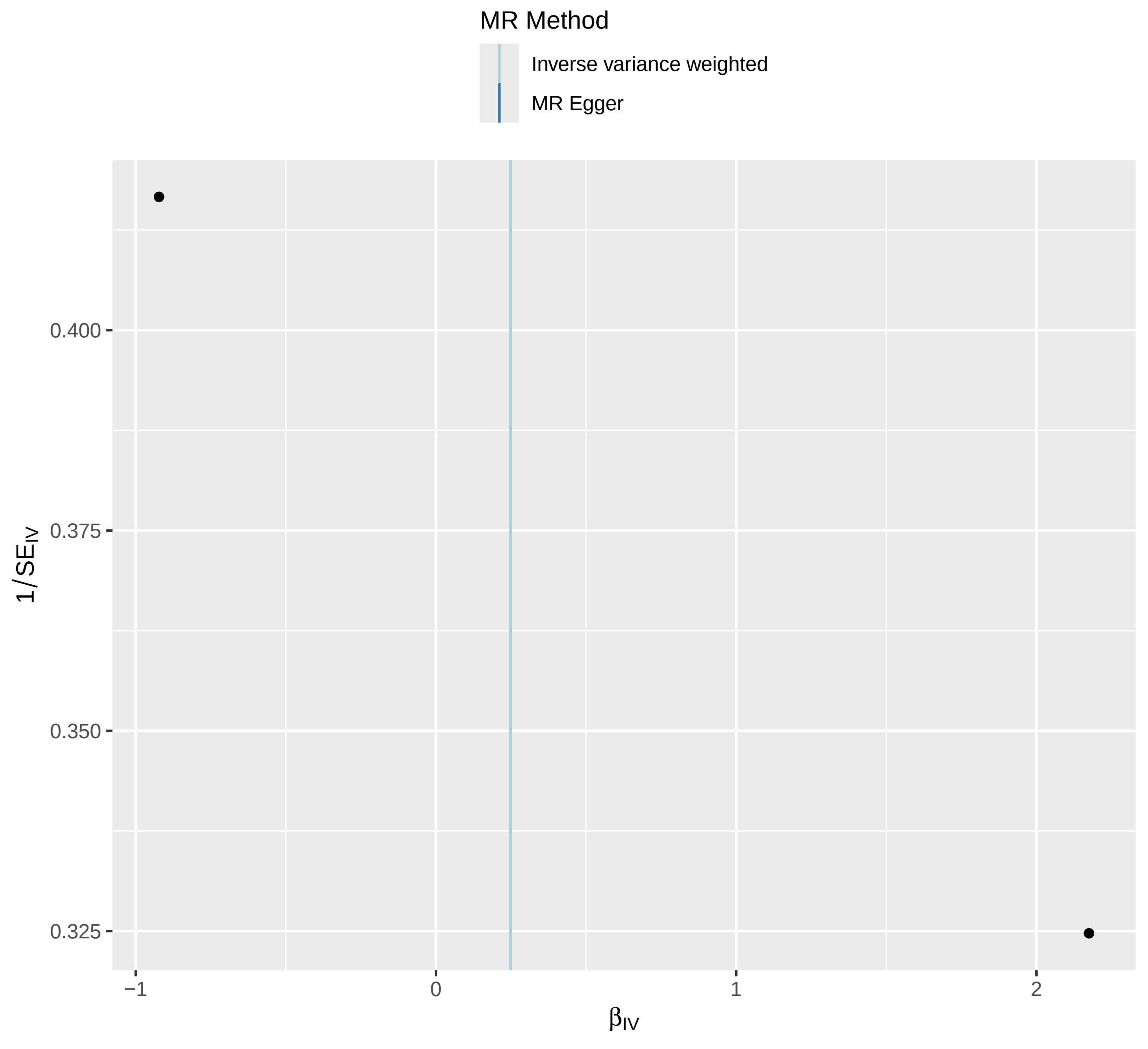
MR Method
Inverse variance weighted
MR Egger
0.400
0.375
0.350
0.325
1/SE_IV
−1
0
1
2
β_IV

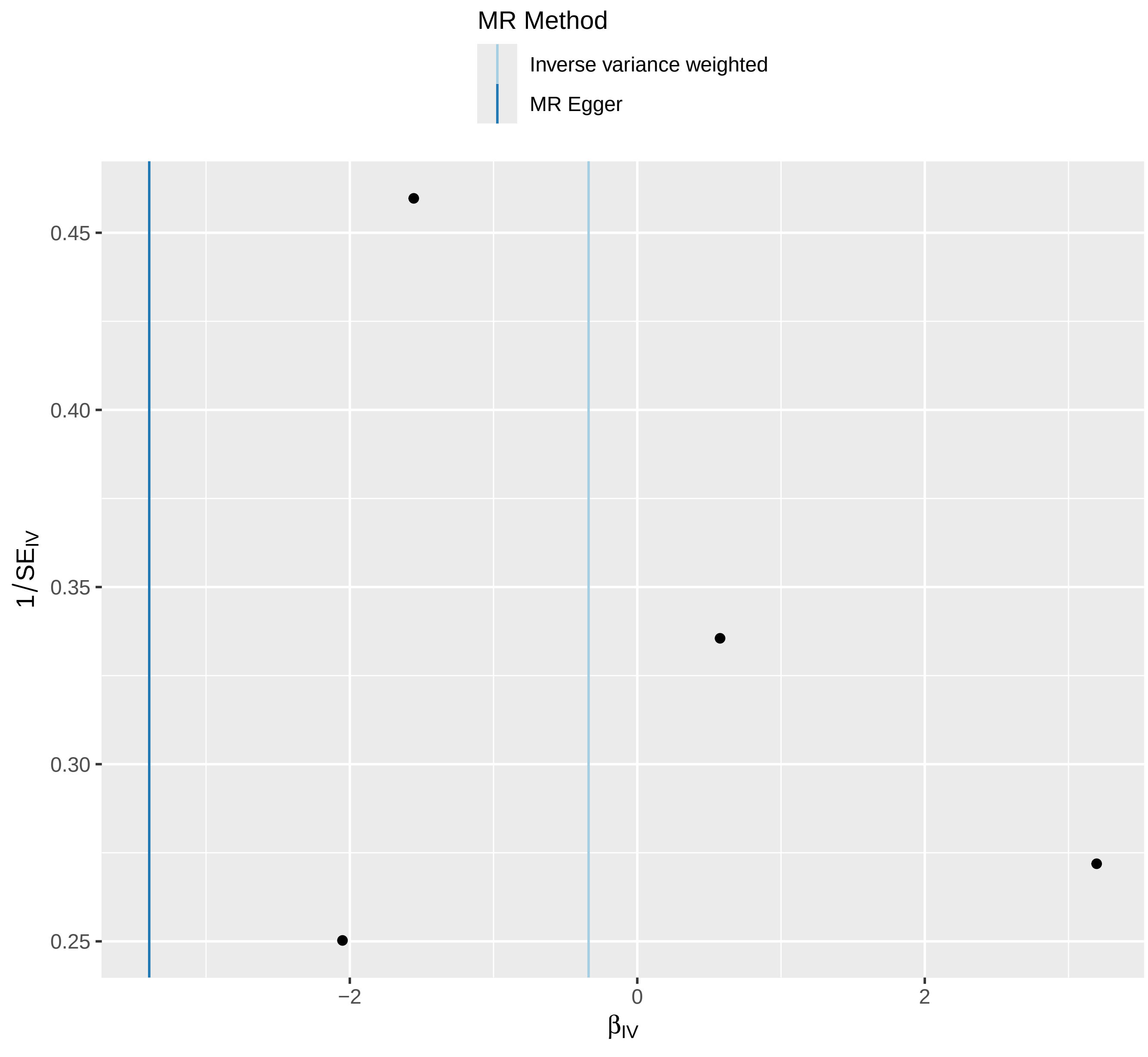
MR Method
Inverse variance weighted
MR Egger
0.45
0.40
0.35
0.30
0.25
$1/SE_{IV}$
−2
0
2
β_{IV}

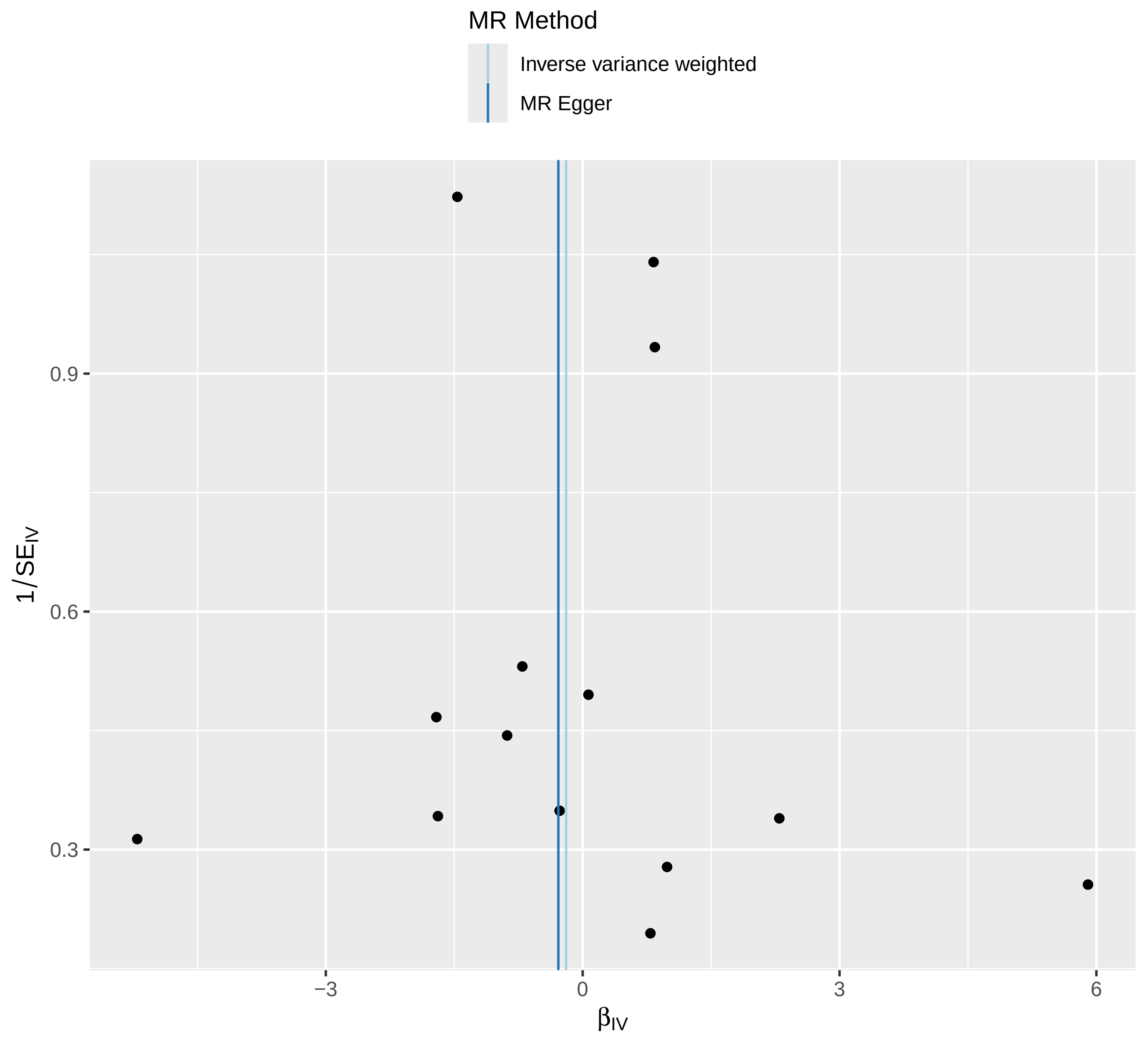
MR Method
Inverse variance weighted
MR Egger
1/SE$_{IV}$
0.9
0.6
0.3
−3
0
3
6
β$_{IV}$

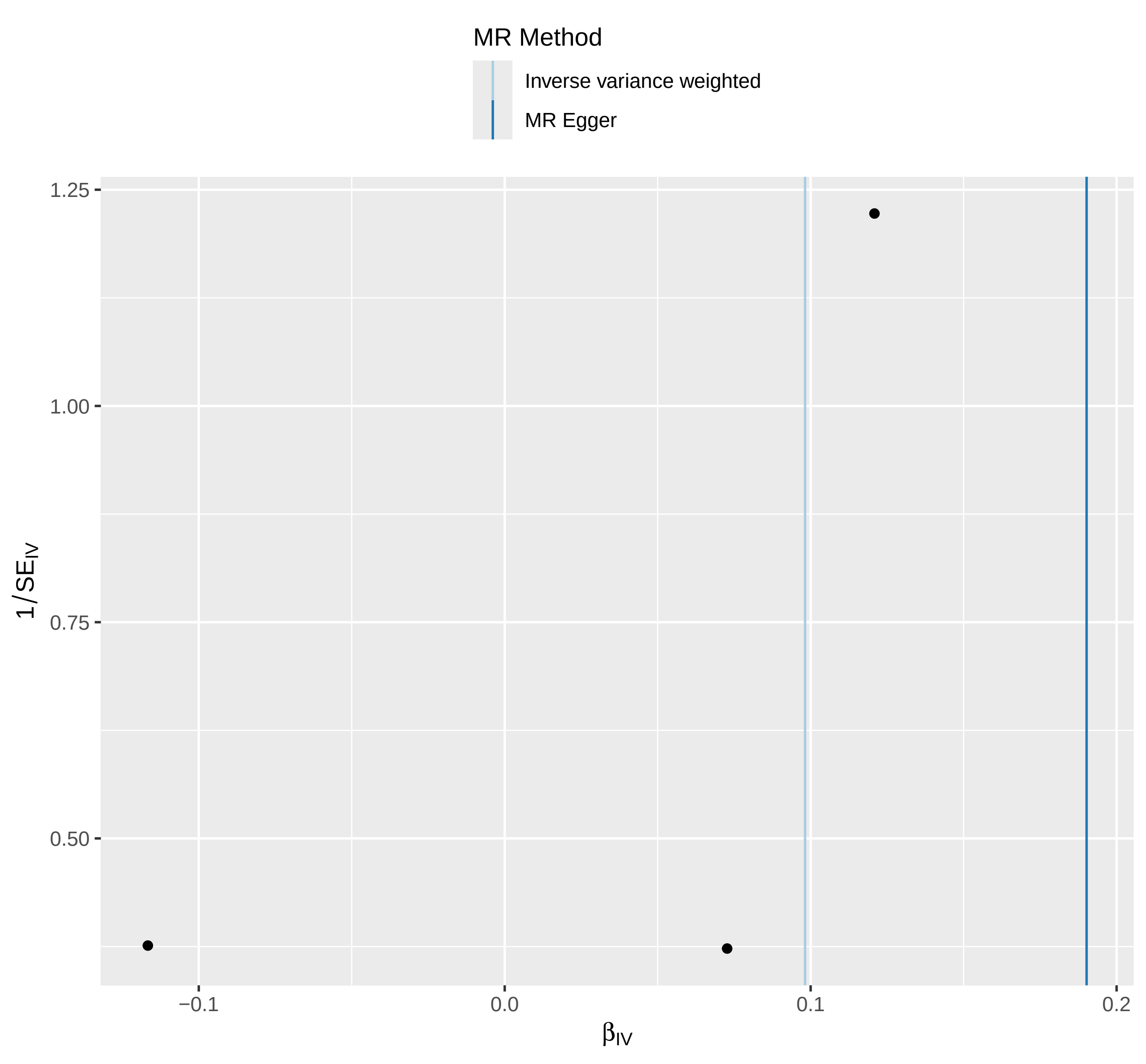
MR Method
Inverse variance weighted
MR Egger
1.25
1.00
0.75
0.50
1/SE_IV
−0.1
0.0
0.1
0.2
β_IV

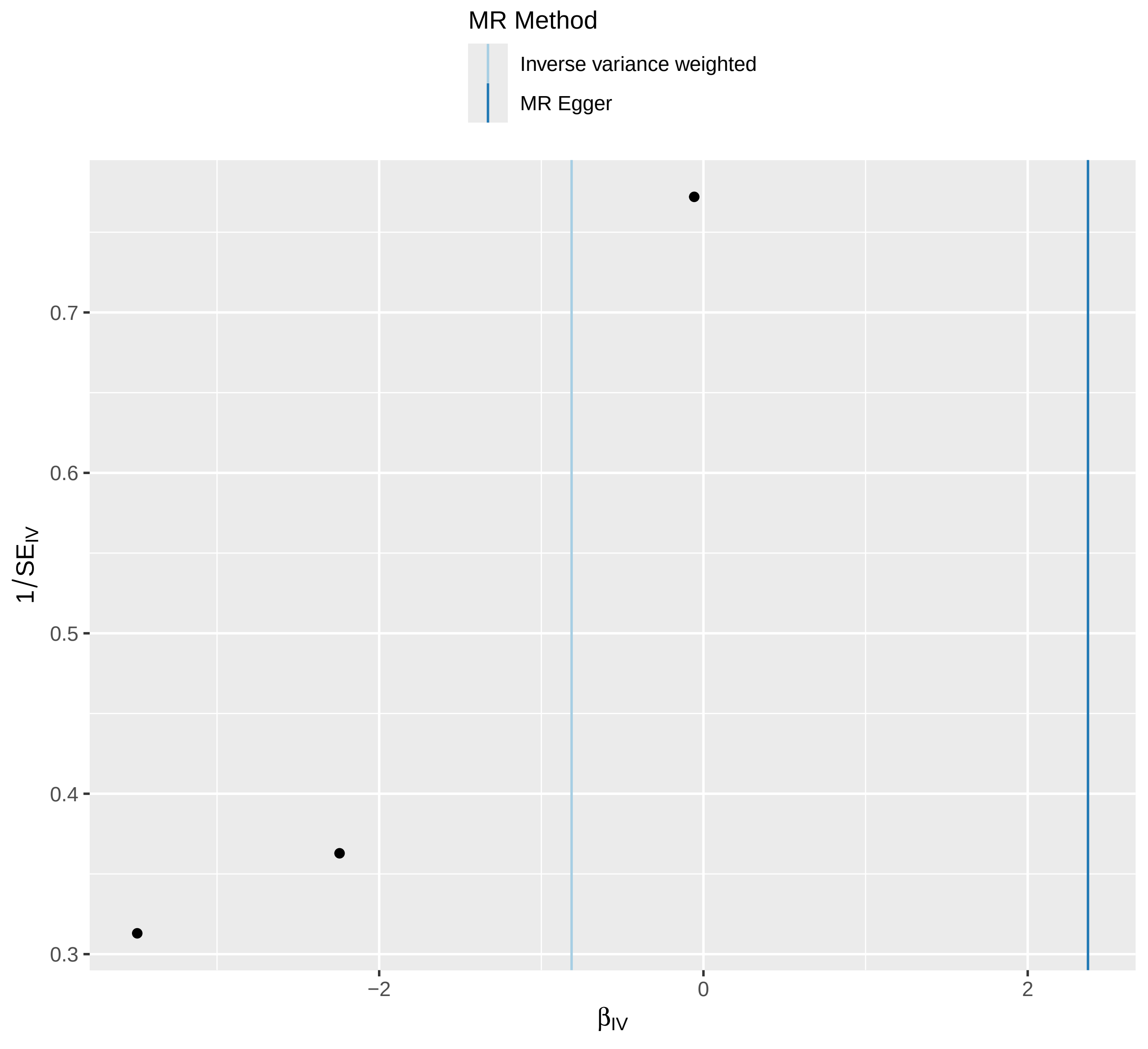
MR Method
Inverse variance weighted
MR Egger
0.7
0.6
0.5
0.4
0.3
1/SE_IV
−2
0
2
β_IV

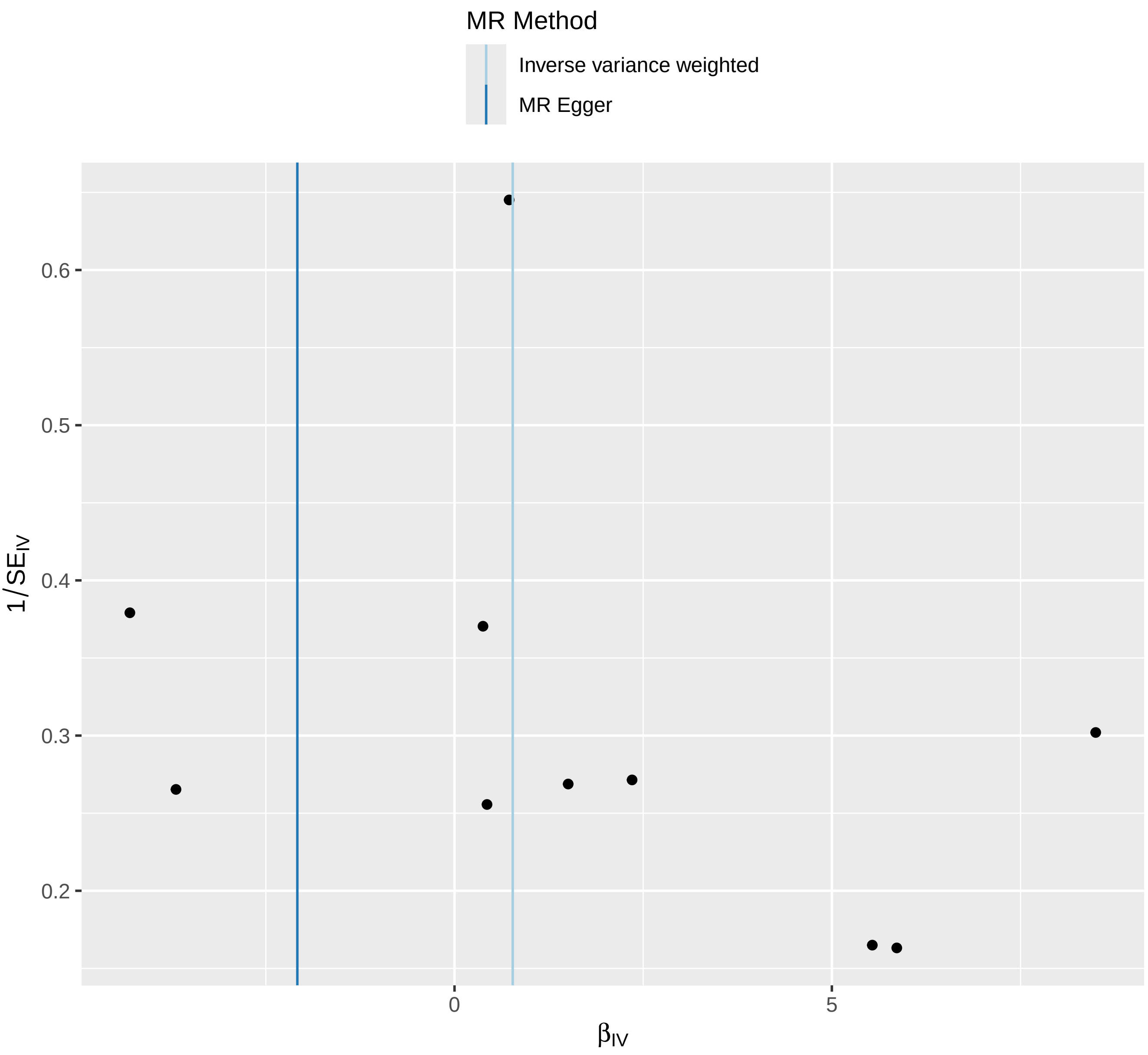

MR Method
Inverse variance weighted
MR Egger
0.6
0.5
0.4
0.3
0.2
1/SE$_{IV}$
0
5
β$_{IV}$

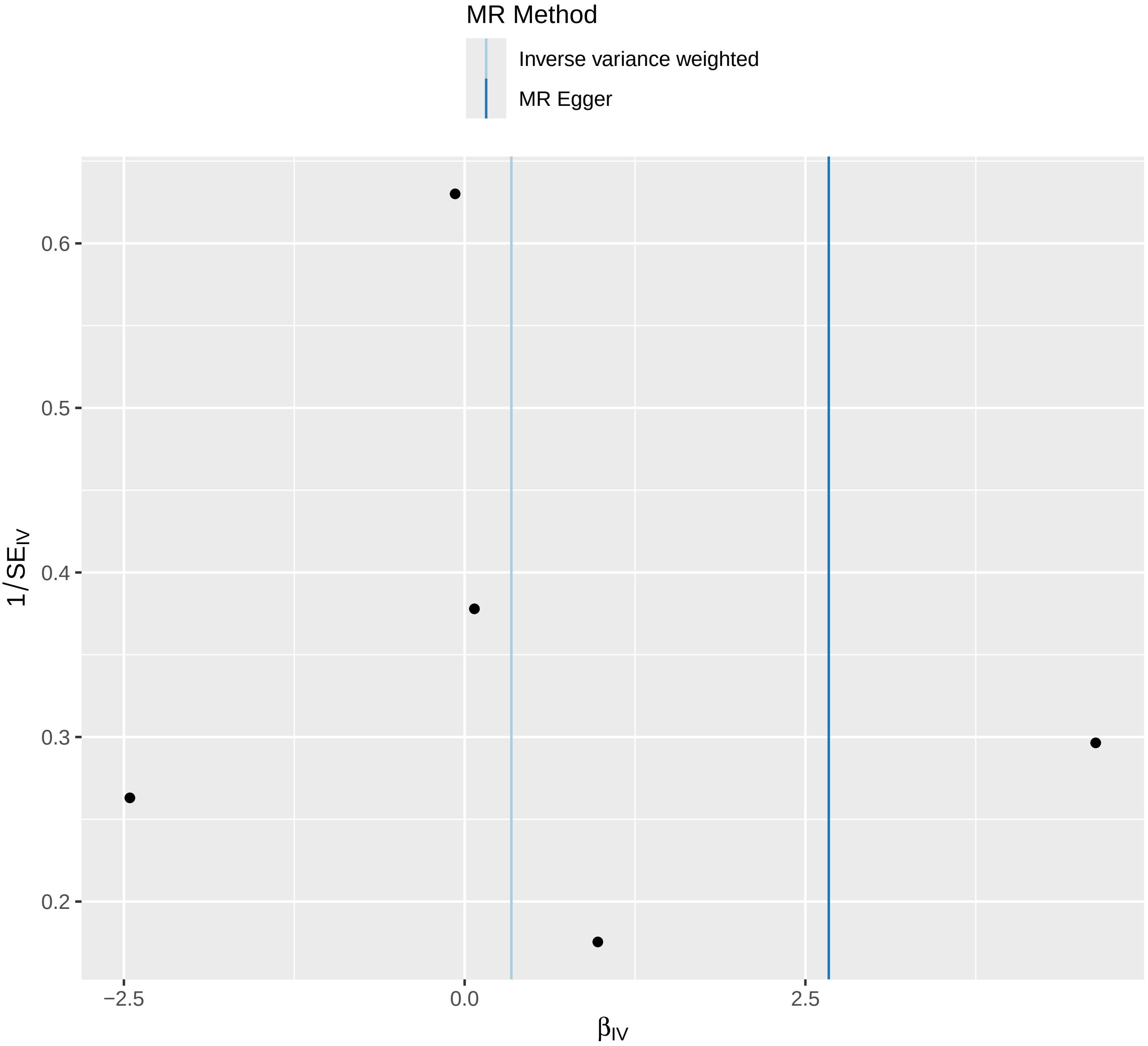
MR Method
Inverse variance weighted
MR Egger
0.6
0.5
0.4
0.3
0.2
1/SE$_{IV}$
−2.5
0.0
2.5
β$_{IV}$

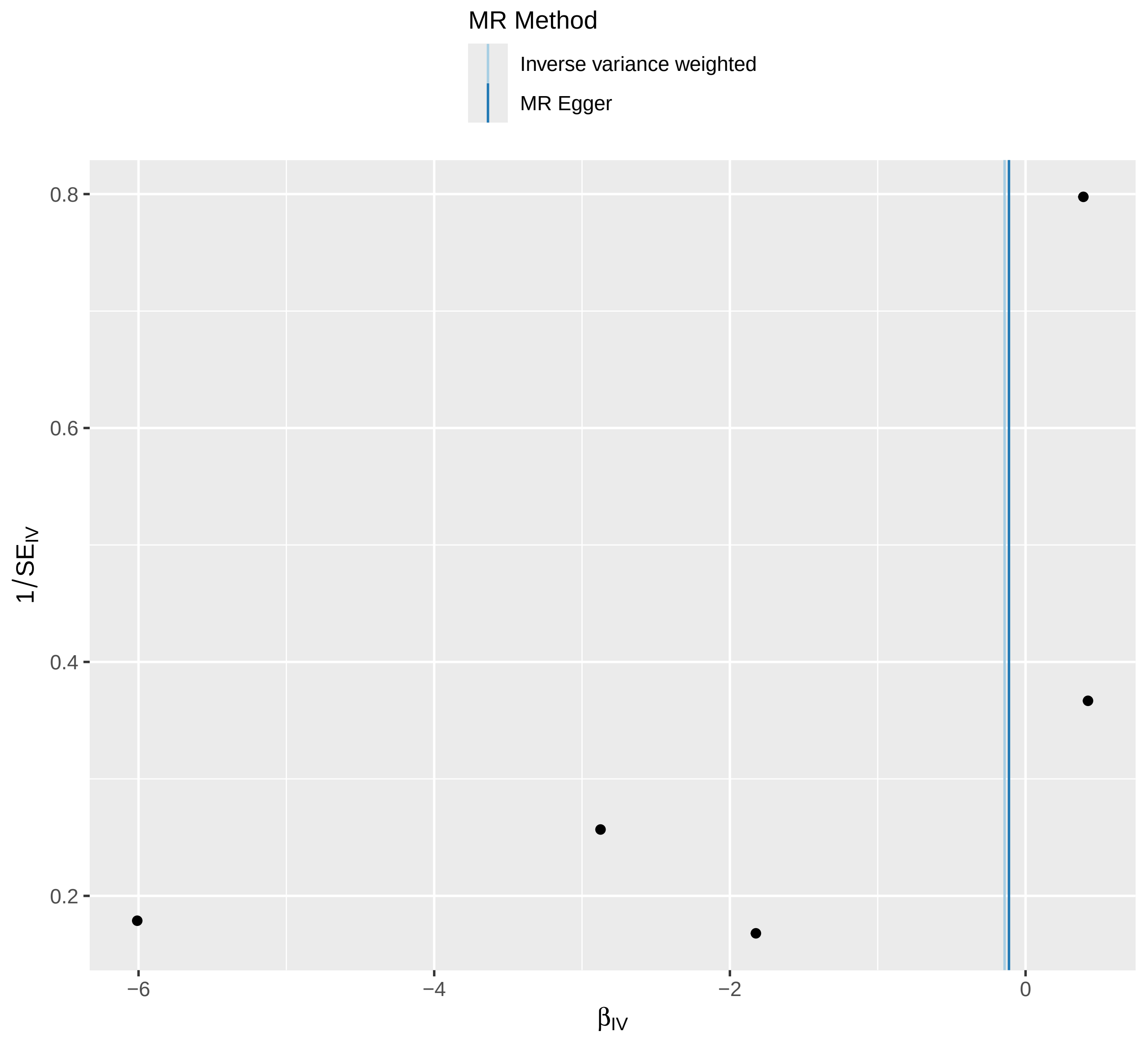
MR Method
Inverse variance weighted
MR Egger
0.8
0.6
0.4
0.2
1/SE_IV
−6
−4
−2
0
β_IV

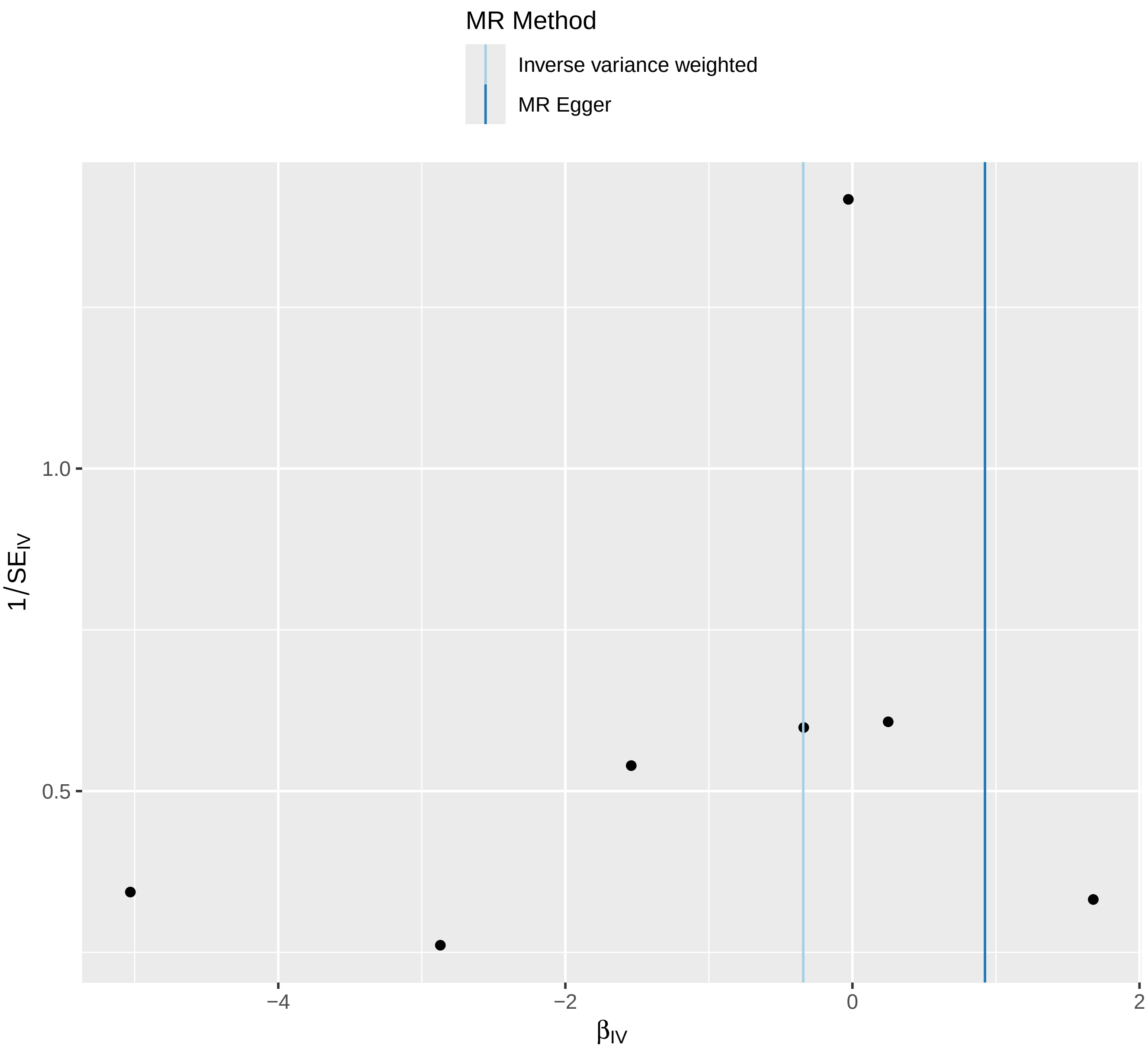
MR Method
Inverse variance weighted
MR Egger
1.0
0.5
1/SE_IV
−4
−2
0
2
β_IV

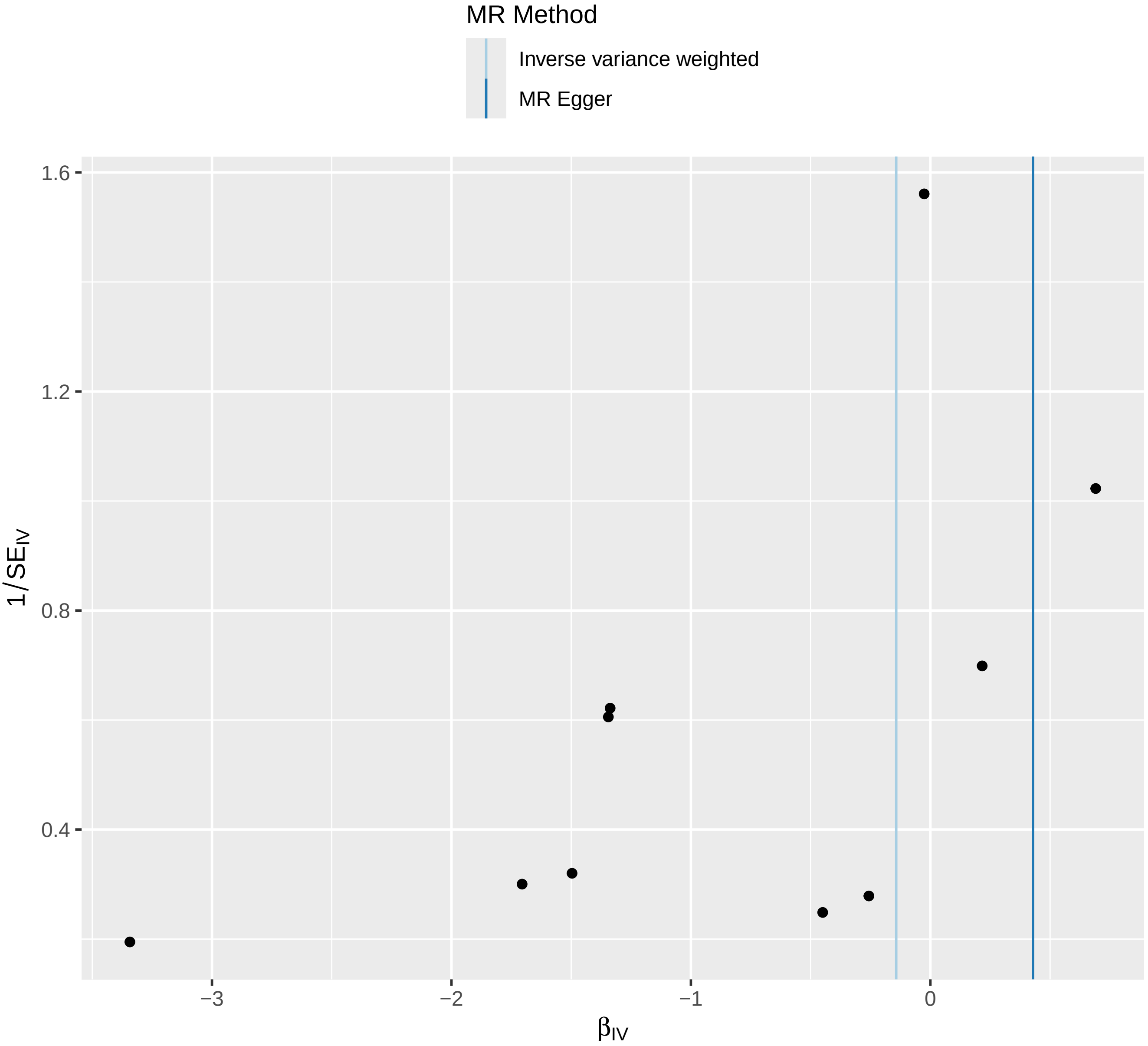
MR Method
Inverse variance weighted
MR Egger
1.6
1.2
0.8
0.4
1/SE_IV
−3
−2
−1
0
β_IV

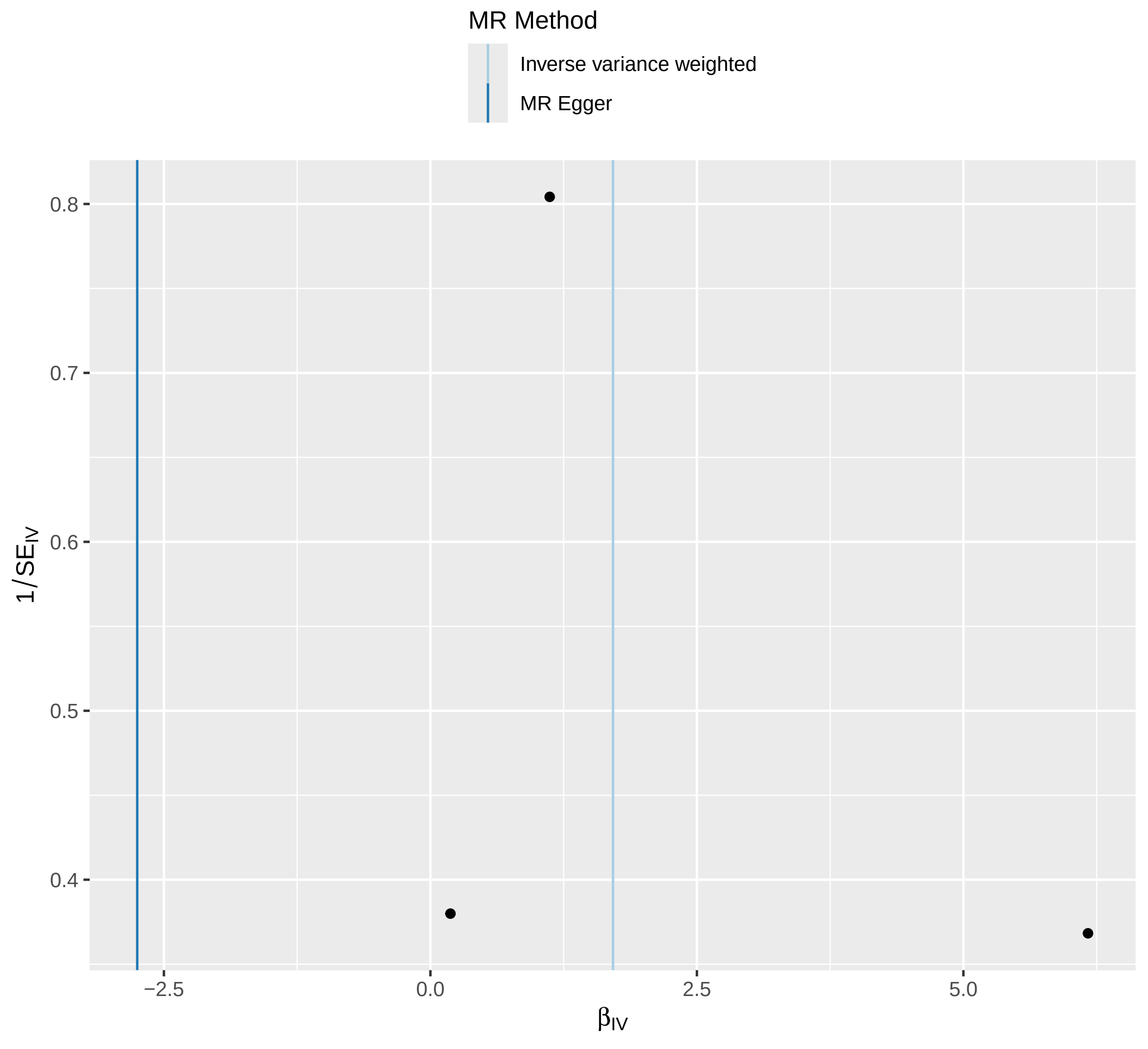
MR Method
Inverse variance weighted
MR Egger
0.8
0.7
0.6
0.5
0.4
1/SE_IV
−2.5
0.0
2.5
5.0
β_IV

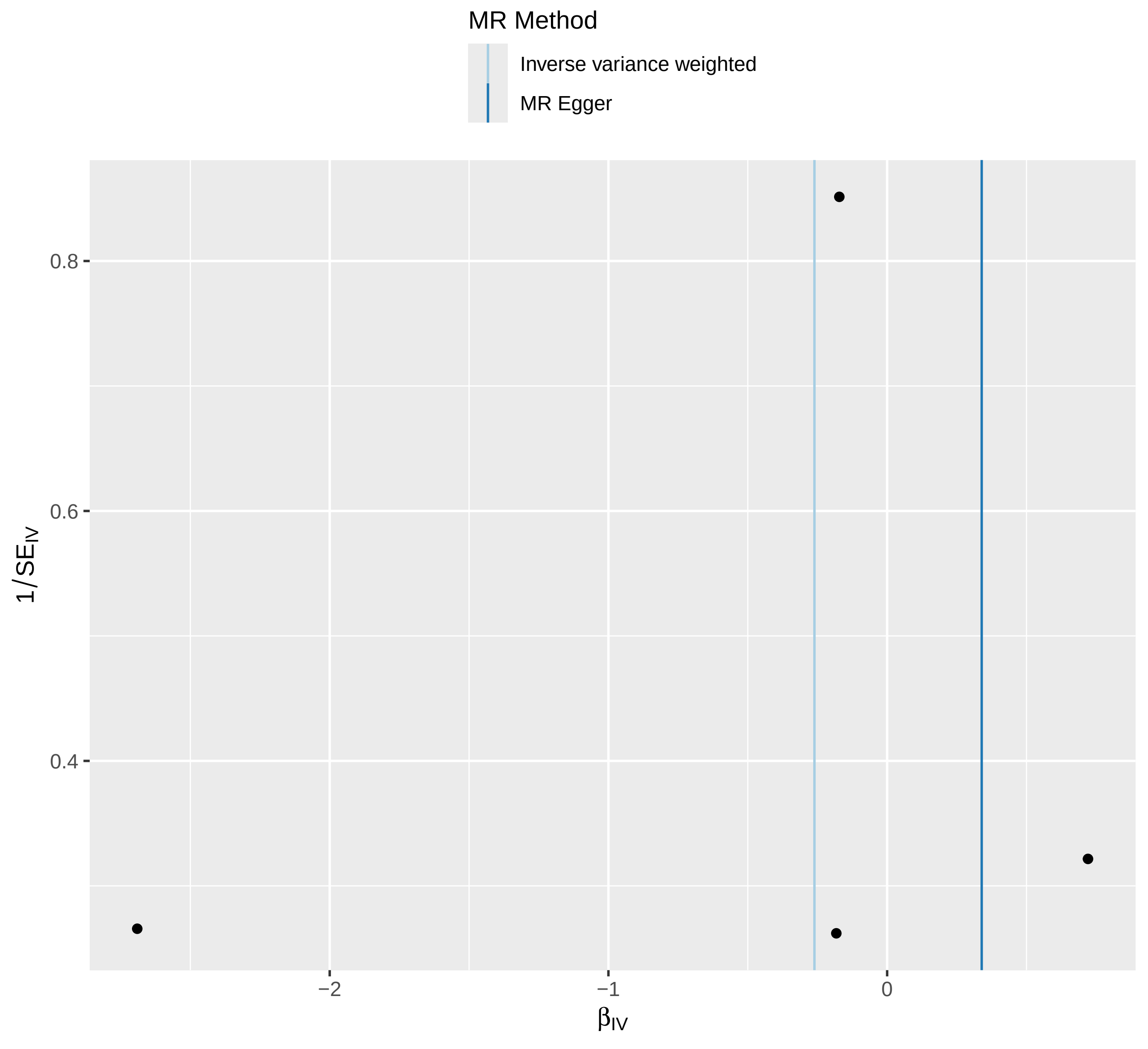
MR Method
Inverse variance weighted
MR Egger
0.8
0.6
0.4
1/SE$_{IV}$
−2
−1
0
β$_{IV}$

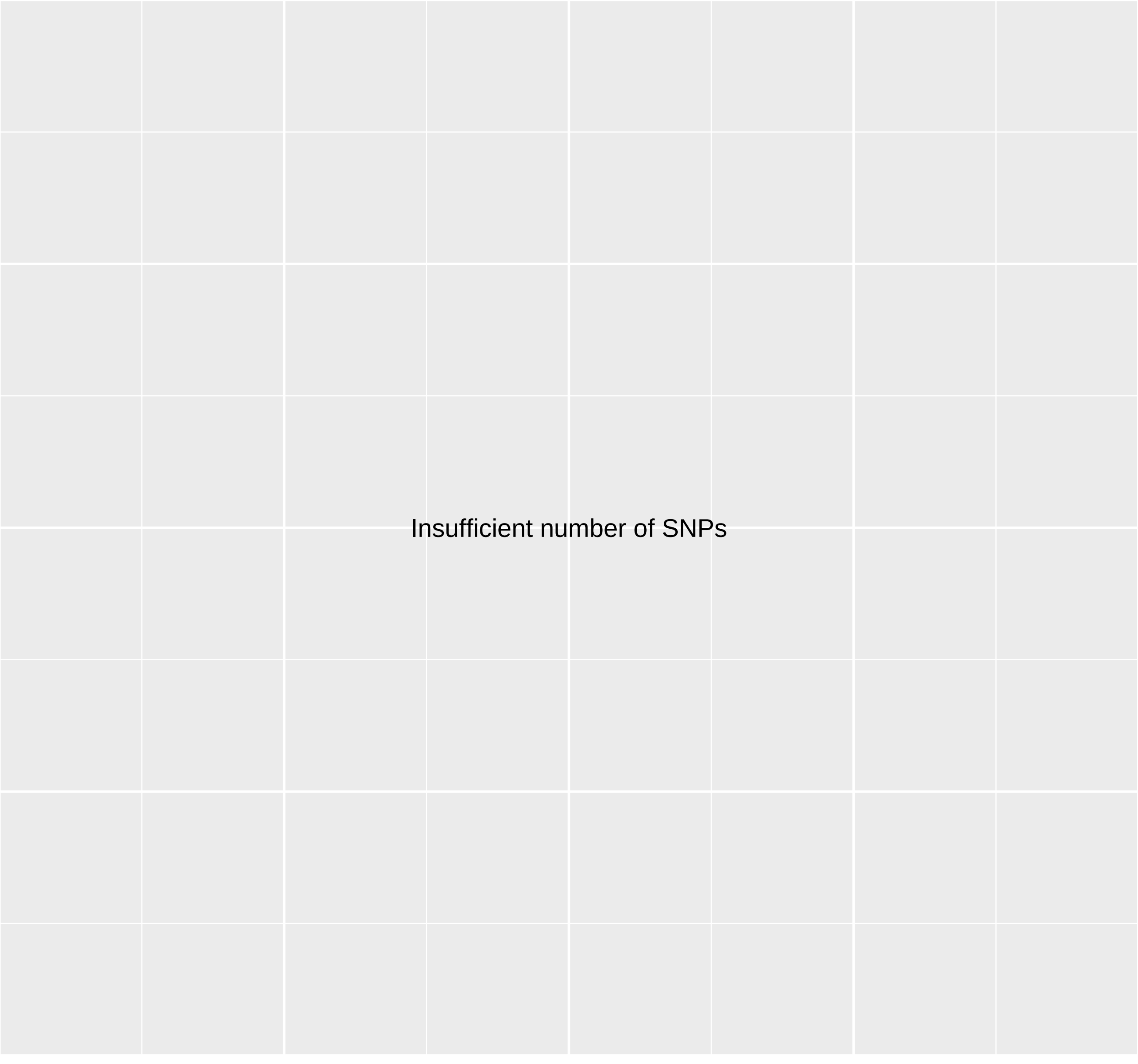
Insufficient number of SNPs

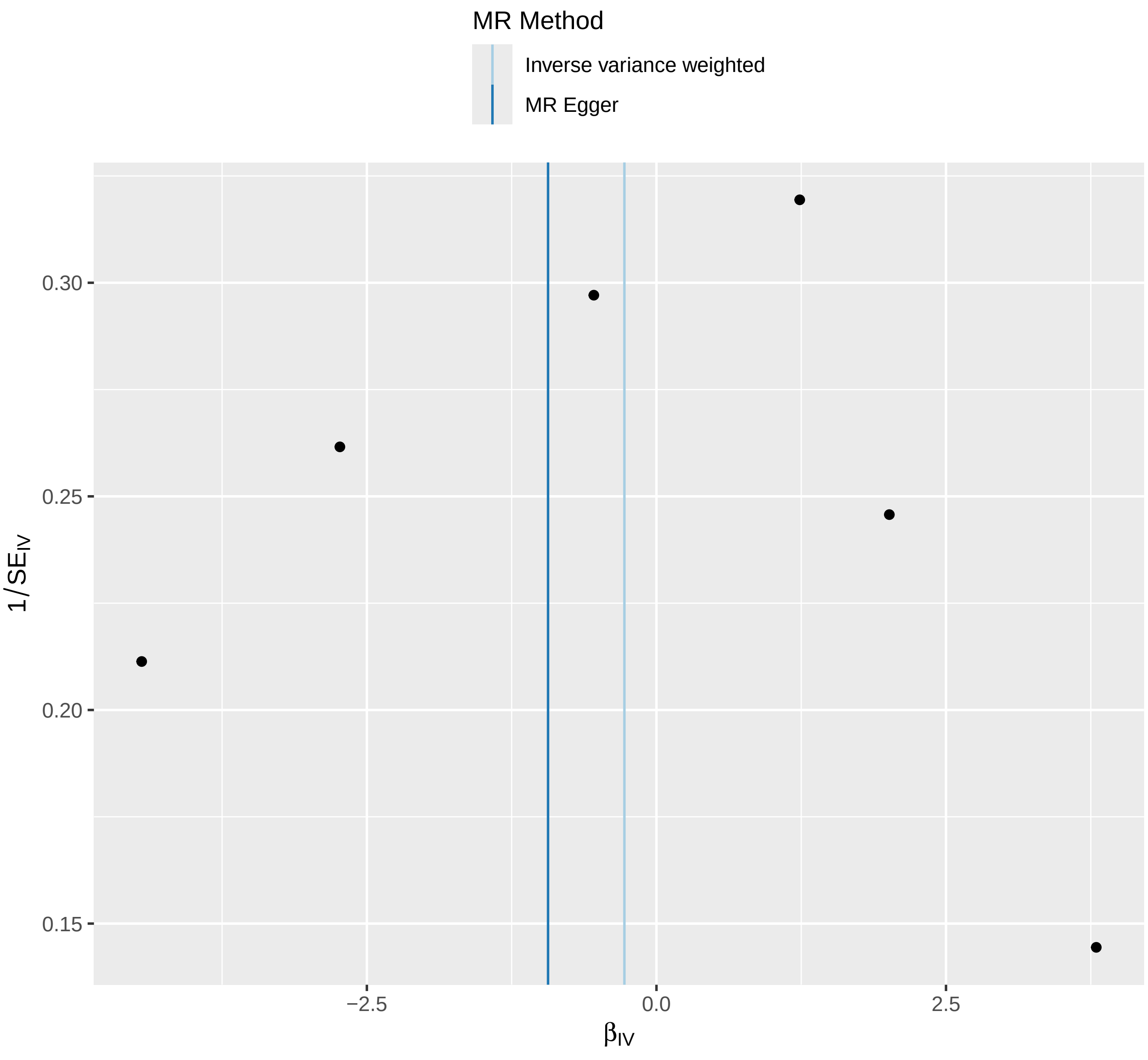

MR Method
Inverse variance weighted
MR Egger
0.30
0.25
0.20
0.15
1/SE_IV
−2.5
0.0
2.5
β_IV

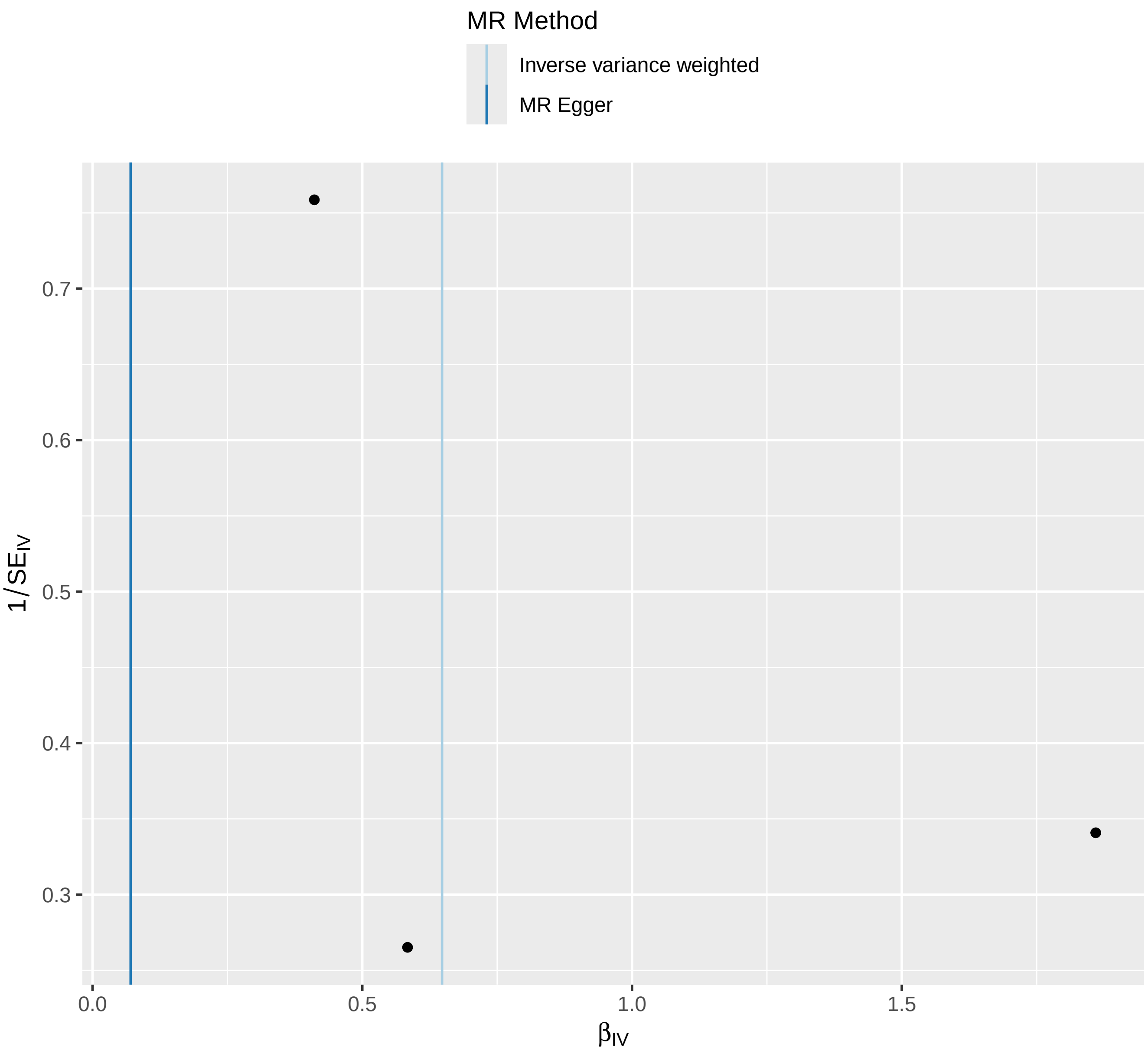
MR Method
Inverse variance weighted
MR Egger
0.7
0.6
0.5
0.4
0.3
$1/SE_{IV}$
0.0
0.5
1.0
1.5
β_{IV}

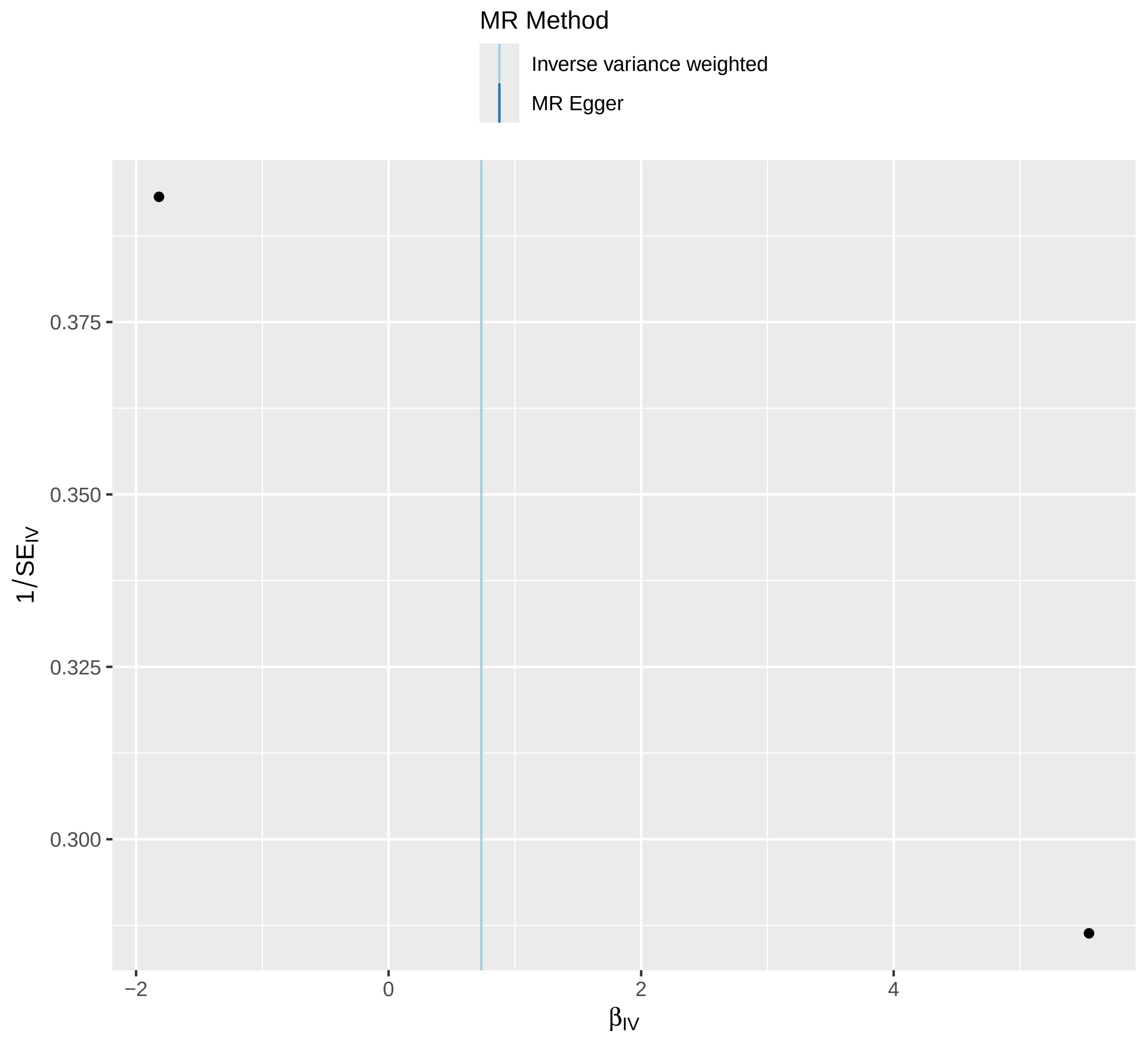
MR Method
Inverse variance weighted
MR Egger
0.375
0.350
0.325
0.300
1/SE$_{IV}$
−2
0
2
4
β$_{IV}$

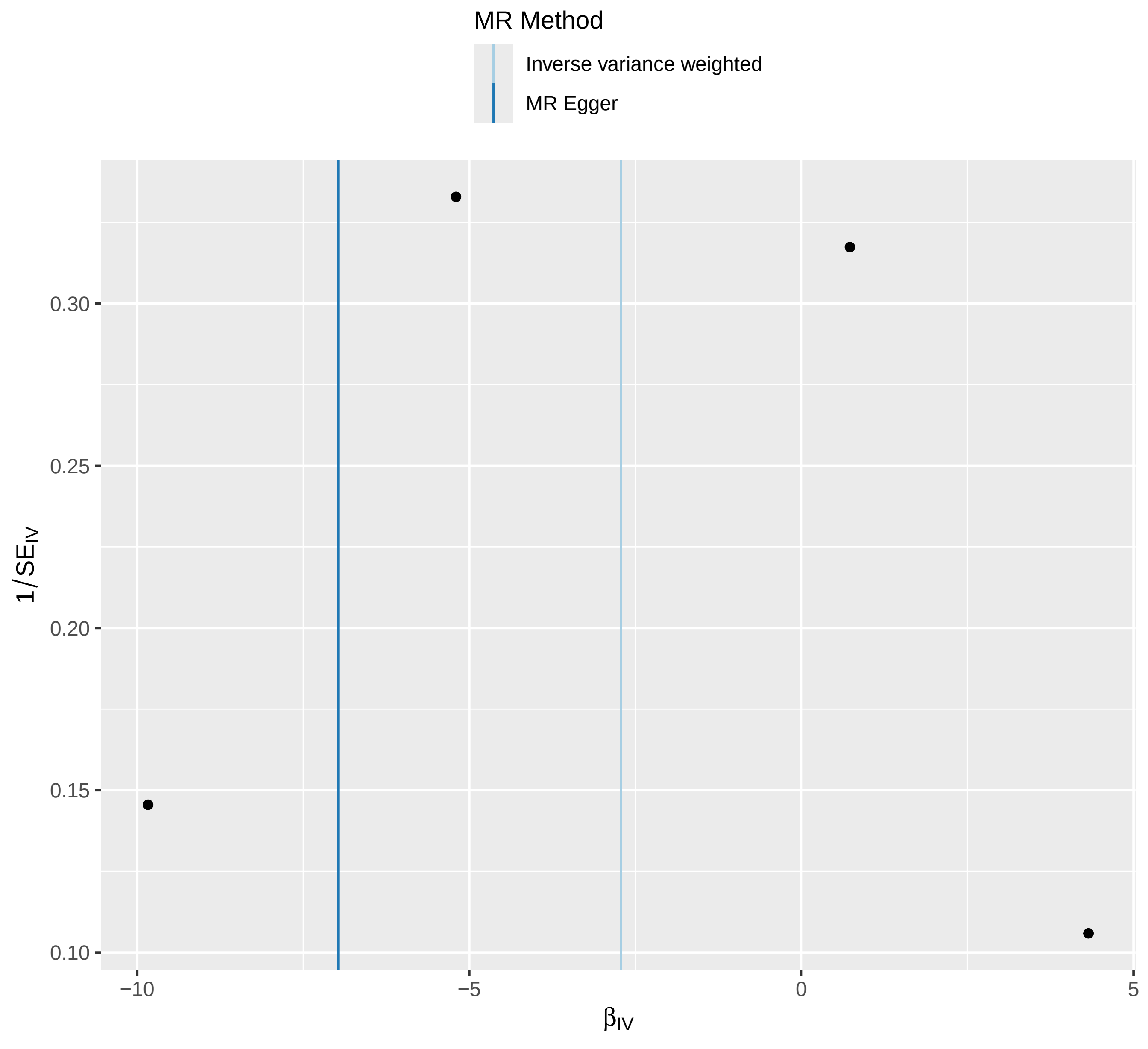
MR Method
Inverse variance weighted
MR Egger
0.30
0.25
0.20
0.15
0.10
1/SE_IV
−10
−5
0
5
β_IV

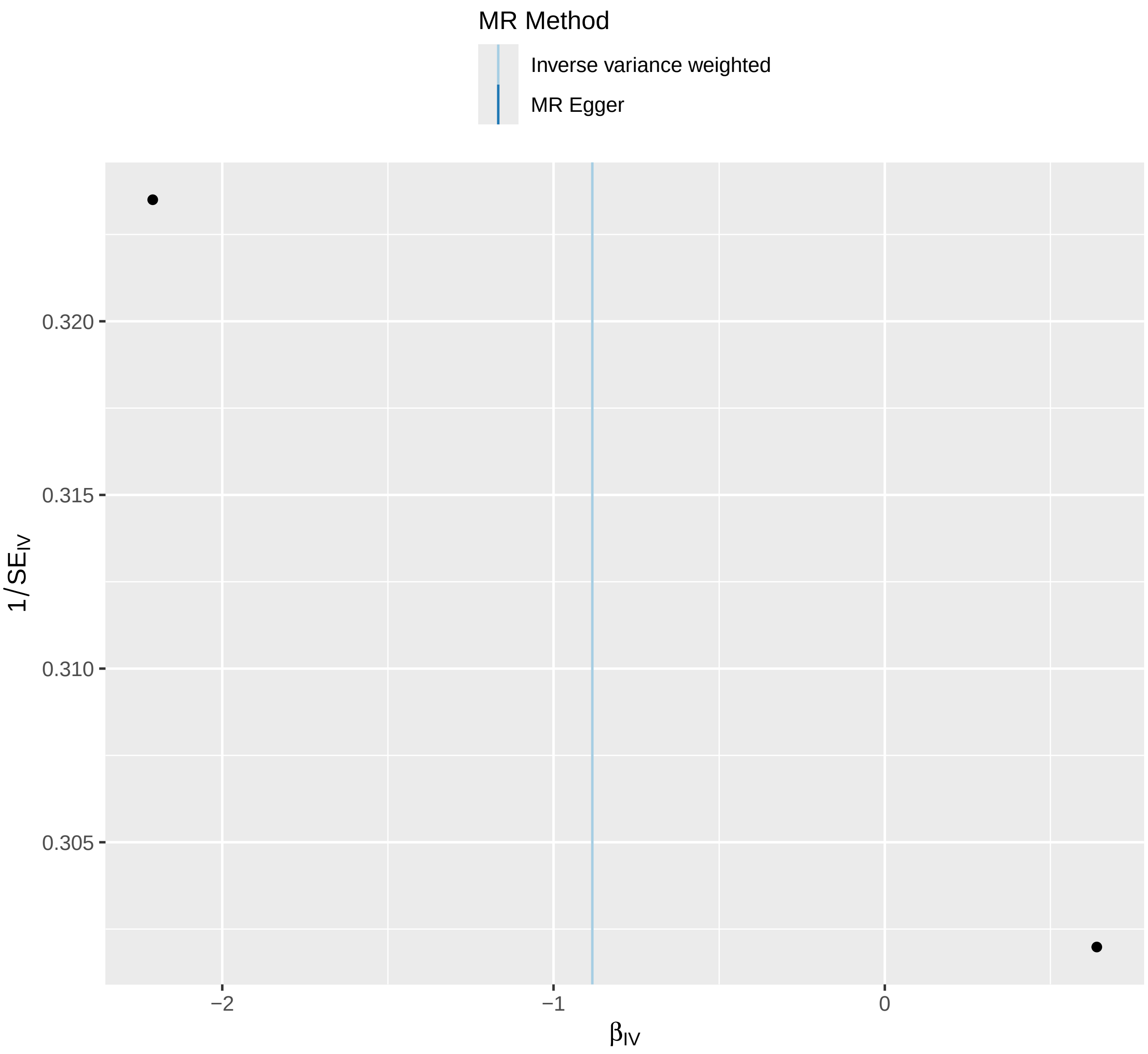
MR Method
Inverse variance weighted
MR Egger
0.320
0.315
0.310
0.305
1/SE_IV
−2
−1
0
β_IV

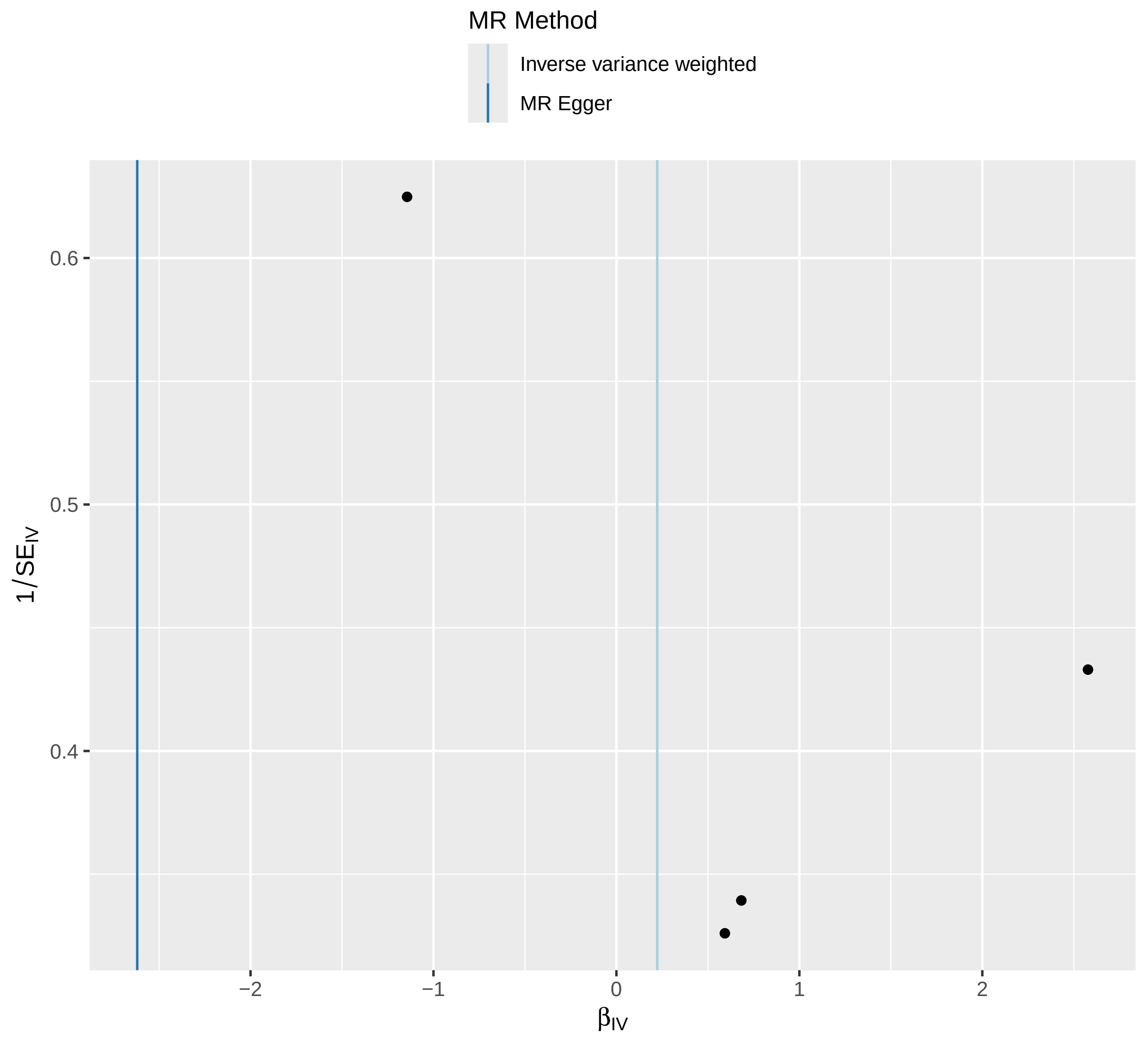
MR Method
Inverse variance weighted
MR Egger
0.6
0.5
0.4
1/SE_IV
−2
−1
0
1
2
β_IV

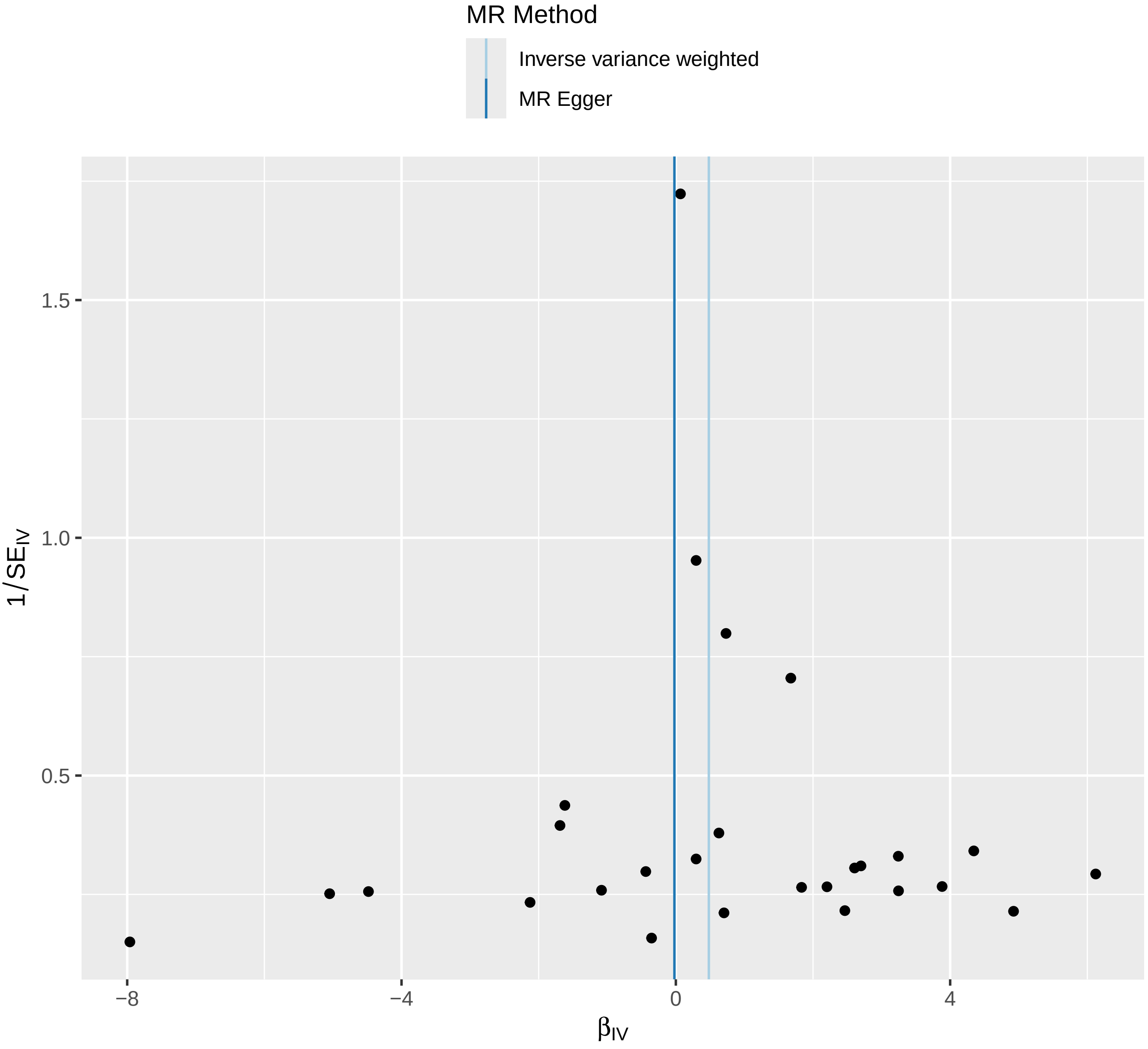
MR Method
Inverse variance weighted
MR Egger
1.5
1.0
0.5
1/SE$_{IV}$
−8
−4
0
4
β$_{IV}$

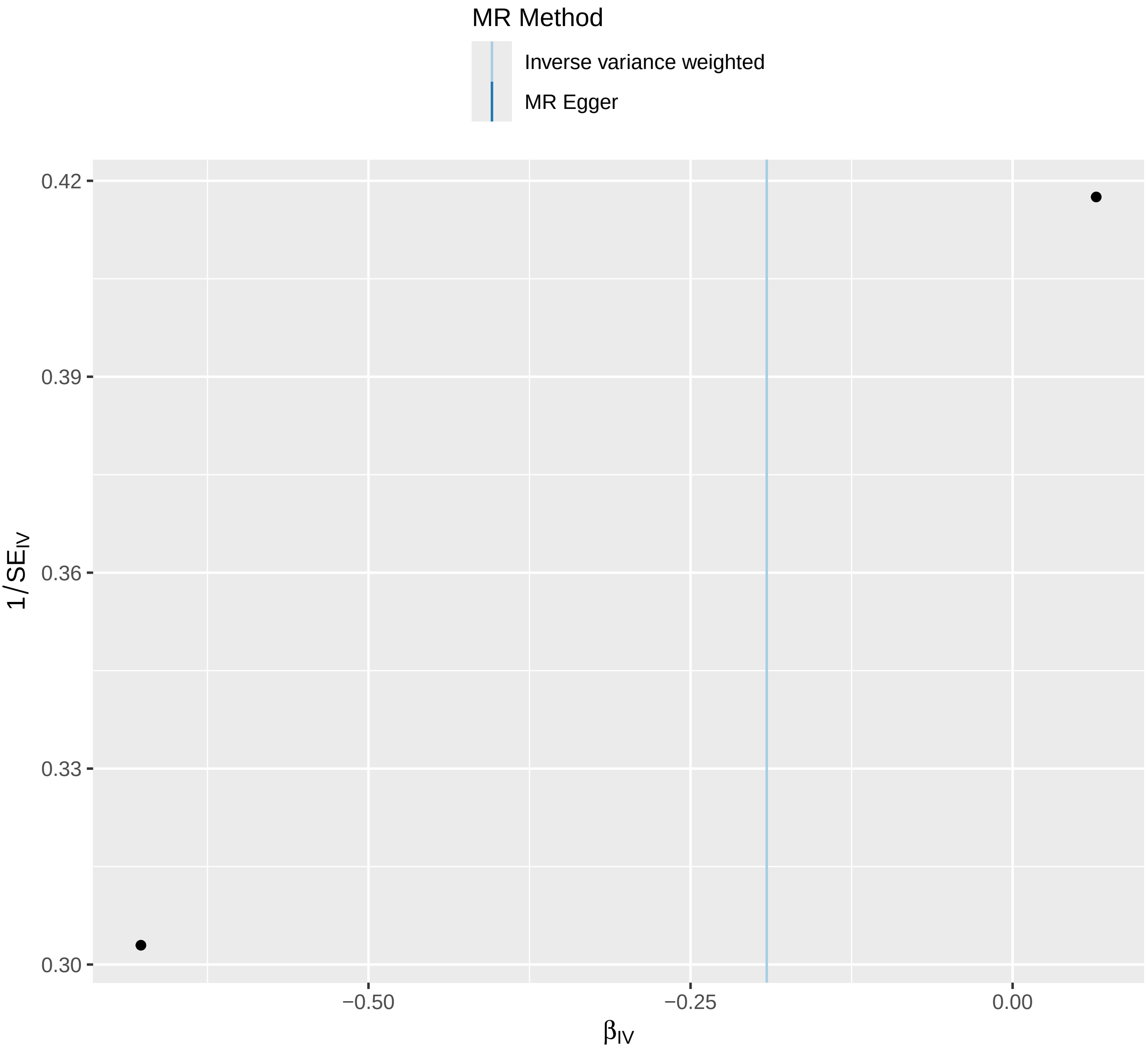
MR Method
Inverse variance weighted
MR Egger
0.42
0.39
0.36
0.33
0.30
−0.50
−0.25
0.00
$1/SE_{IV}$
β_{IV}

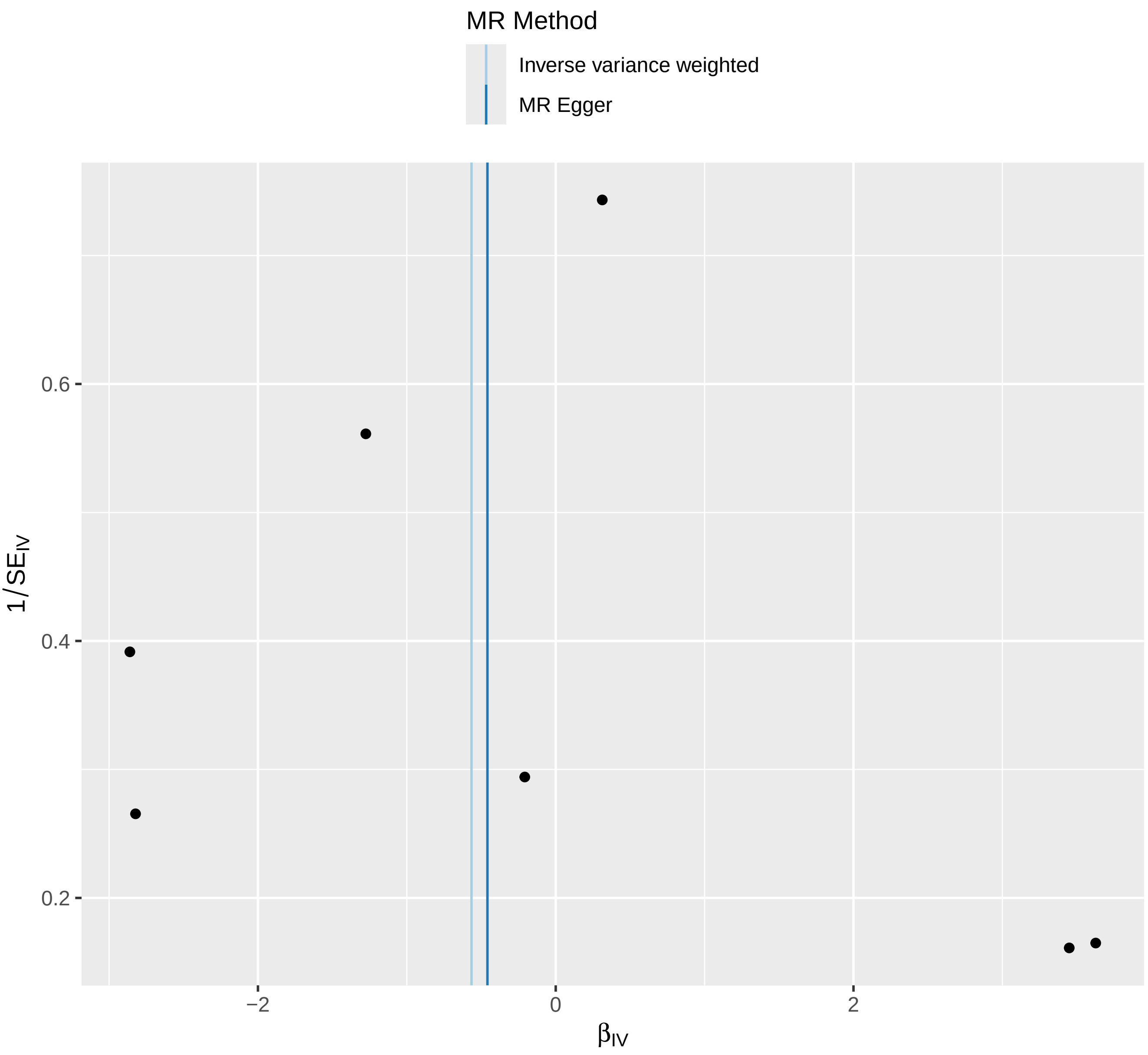
MR Method
Inverse variance weighted
MR Egger
0.6
0.4
0.2
−2
0
2
β_{IV}
$1/SE_{IV}$

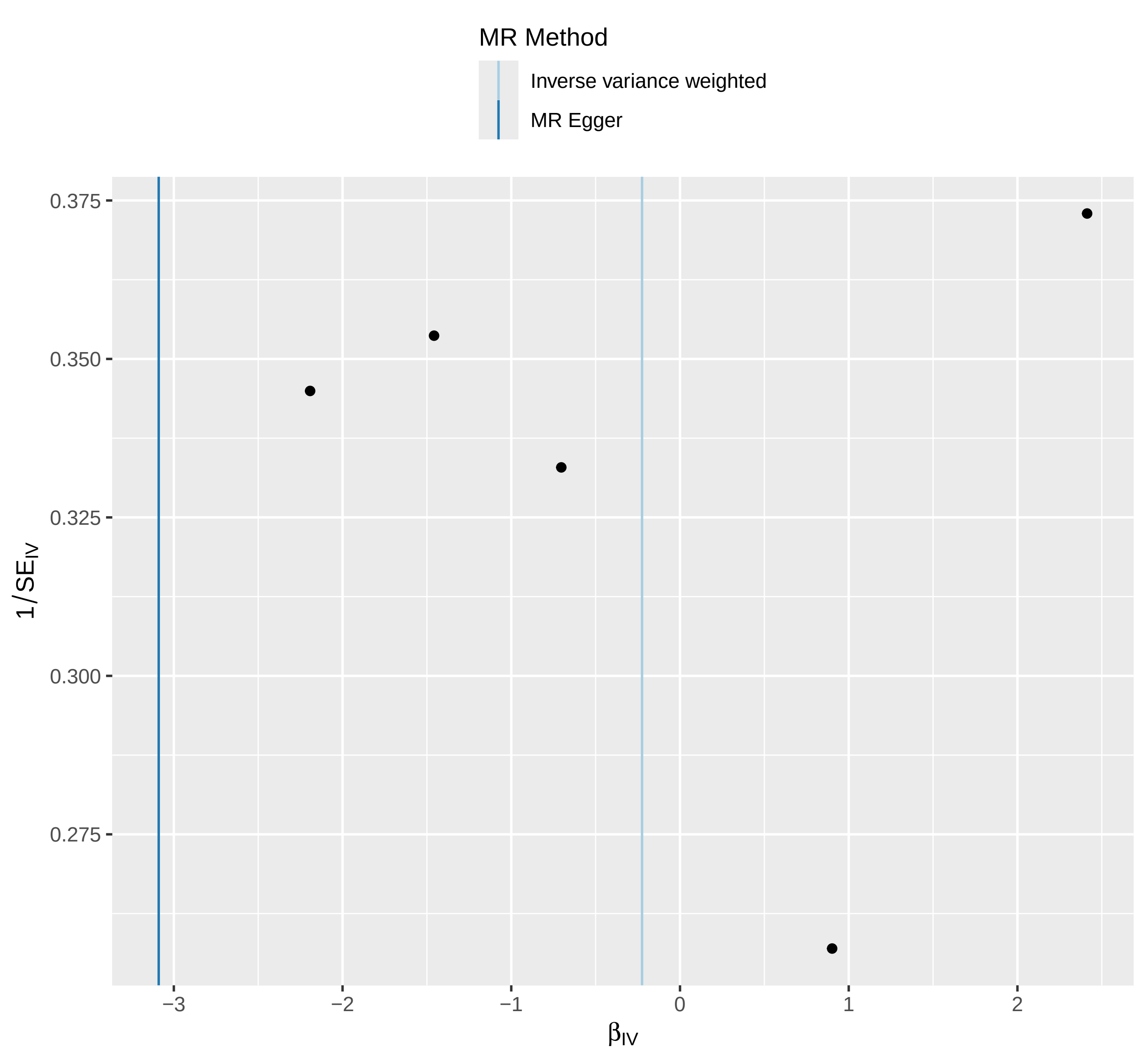

MR Method
Inverse variance weighted
MR Egger
0.375
0.350
0.325
0.300
0.275
1/SE_IV
−3
−2
−1
0
1
2
β_IV

Insufficient number of SNPs

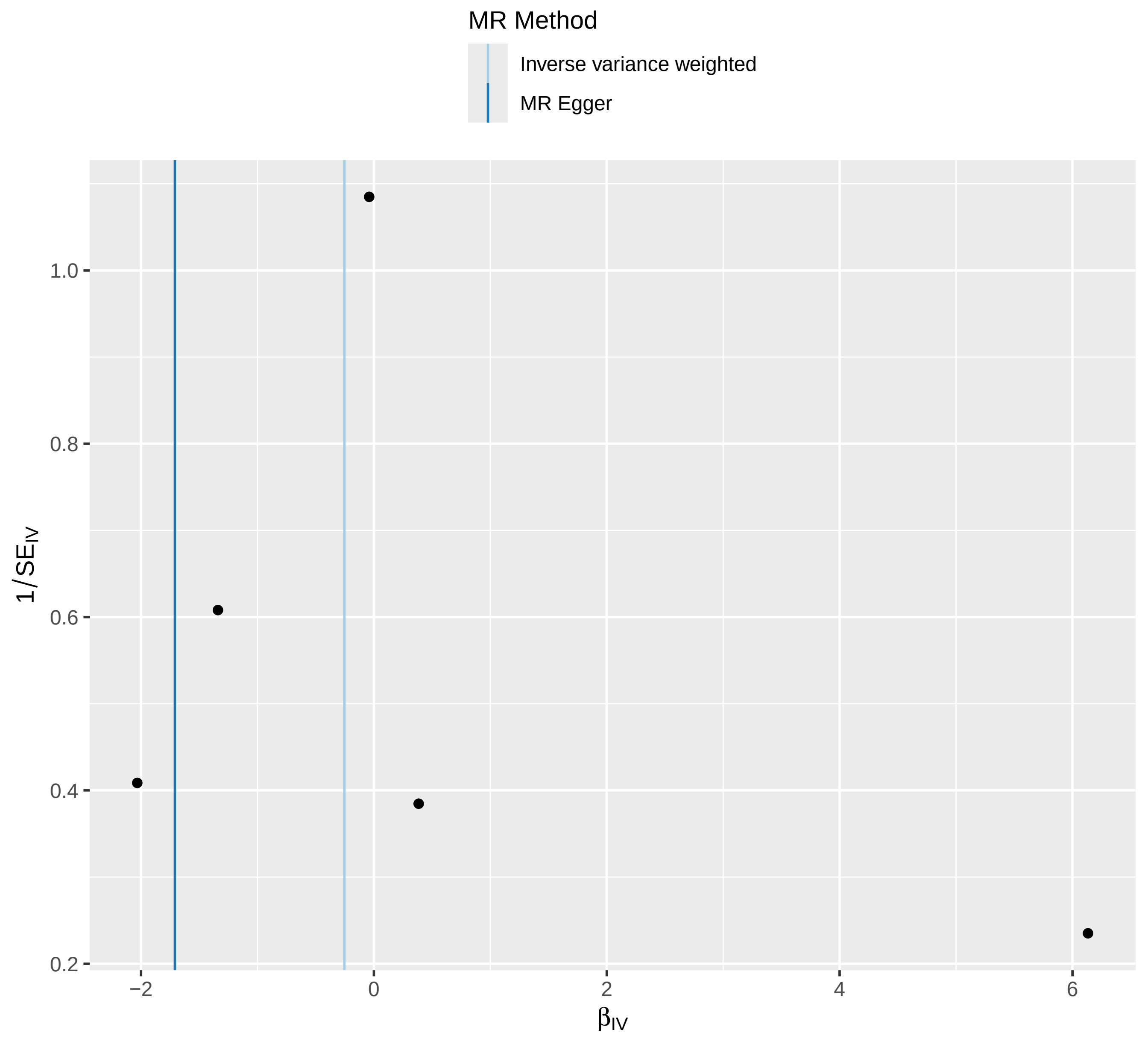
MR Method
Inverse variance weighted
MR Egger
1.0
0.8
0.6
0.4
0.2
1/SE_IV
−2
0
2
4
6
β_IV

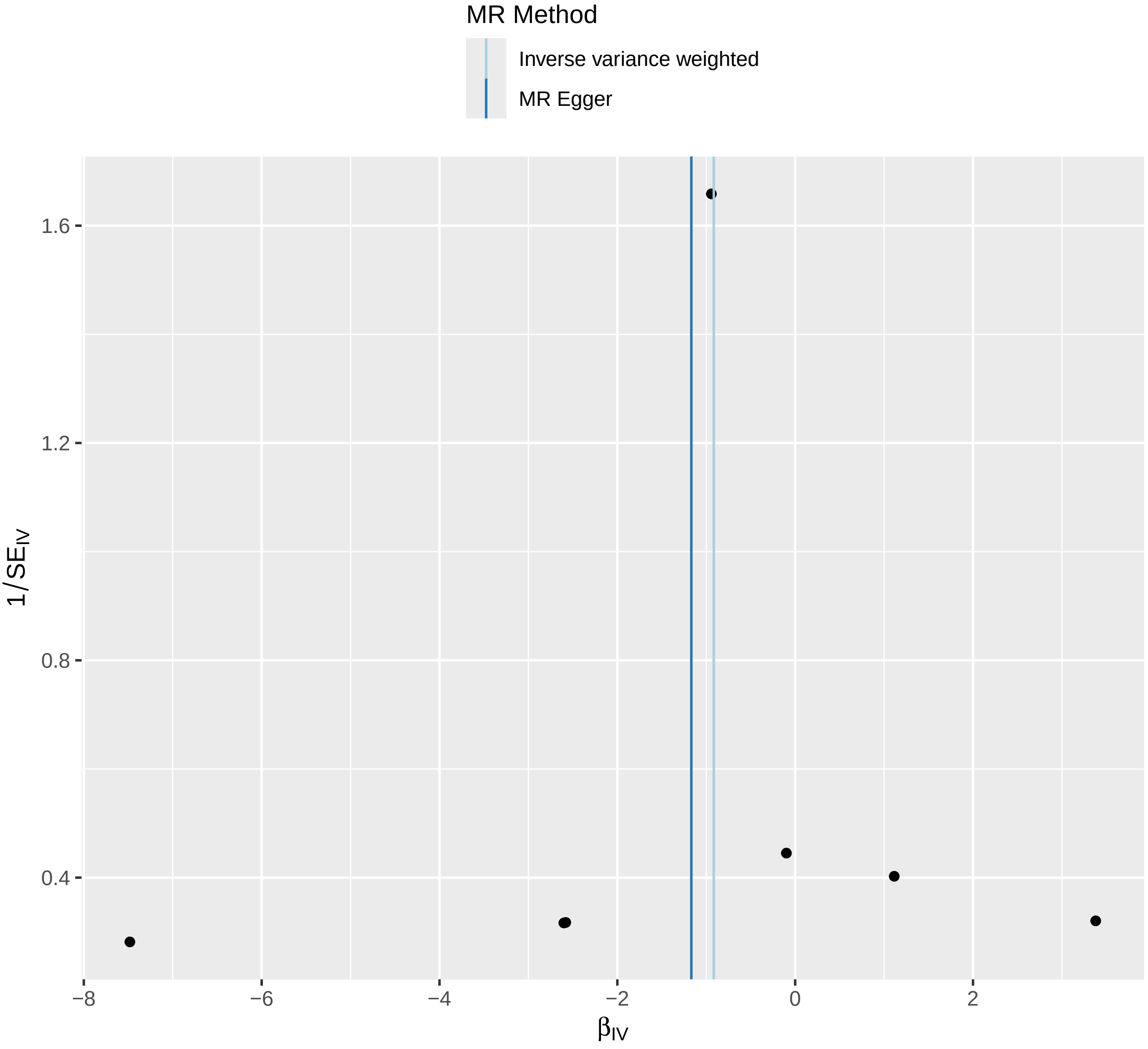
MR Method
Inverse variance weighted
MR Egger
1.6
1.2
0.8
0.4
1/SE_IV
−8
−6
−4
−2
0
2
β_IV

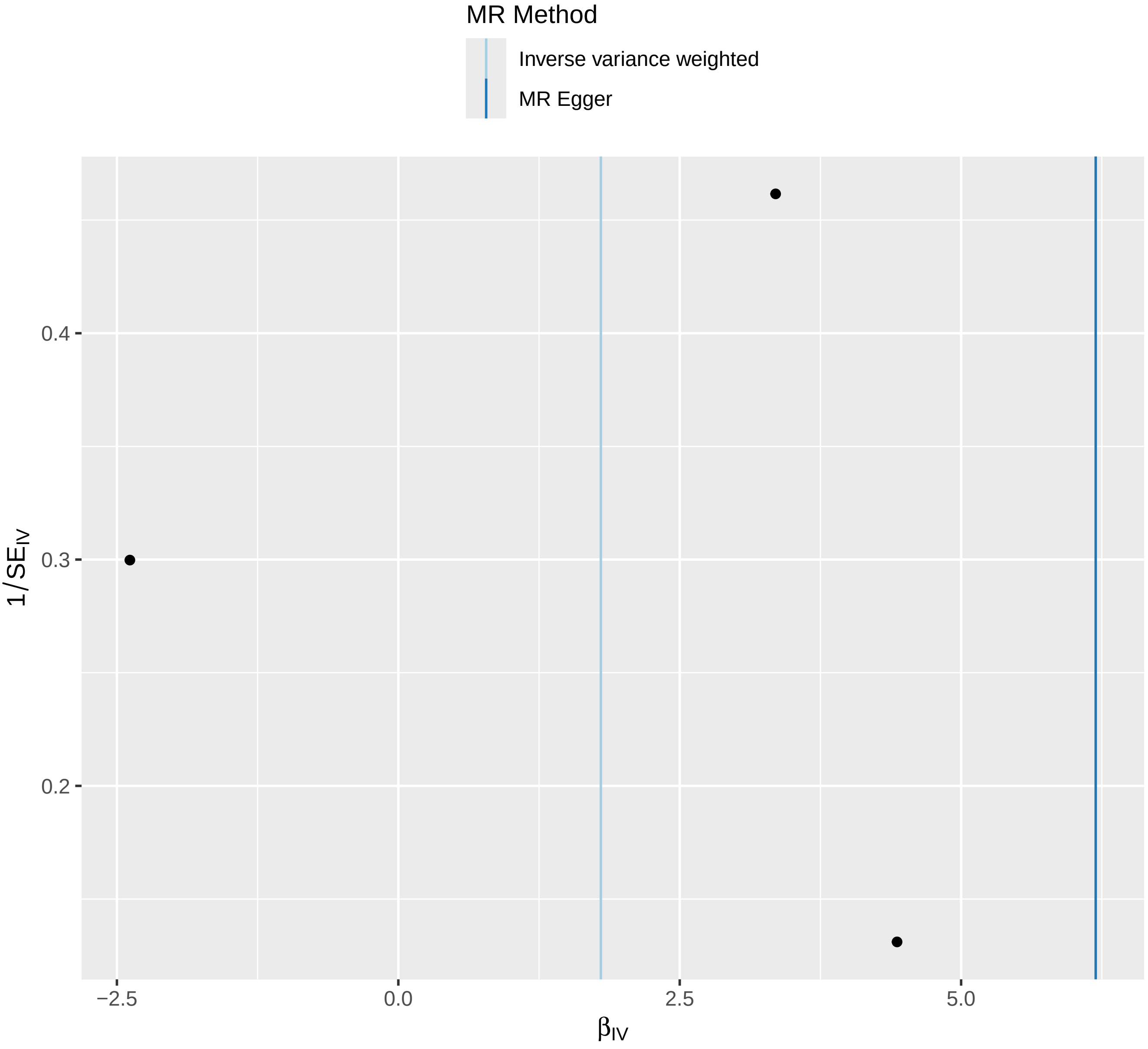
MR Method
Inverse variance weighted
MR Egger
0.4
0.3
0.2
1/SE_IV
−2.5
0.0
2.5
5.0
β_IV

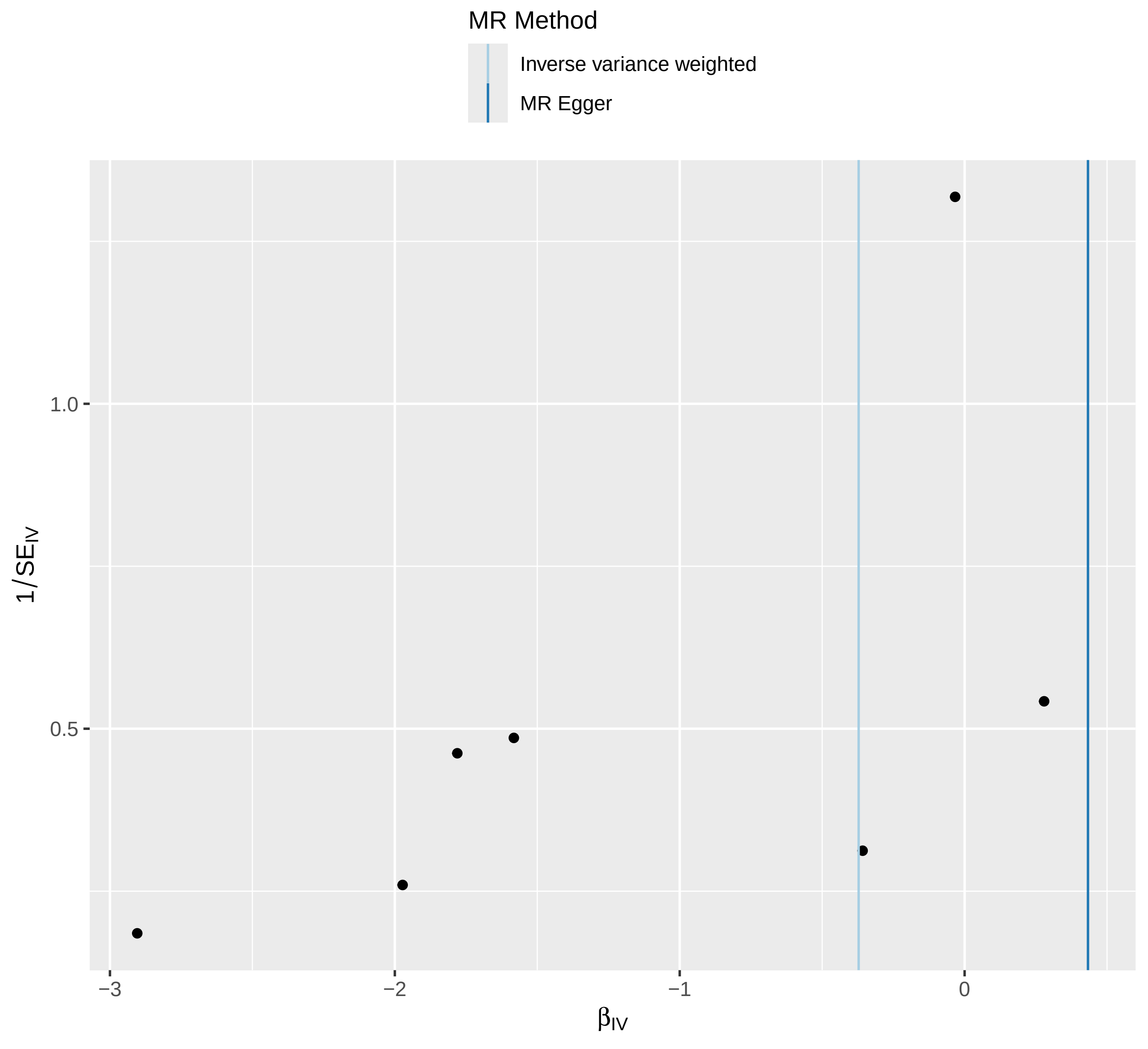
MR Method
Inverse variance weighted
MR Egger
1.0
0.5
1/SE_IV
−3
−2
−1
0
β_IV

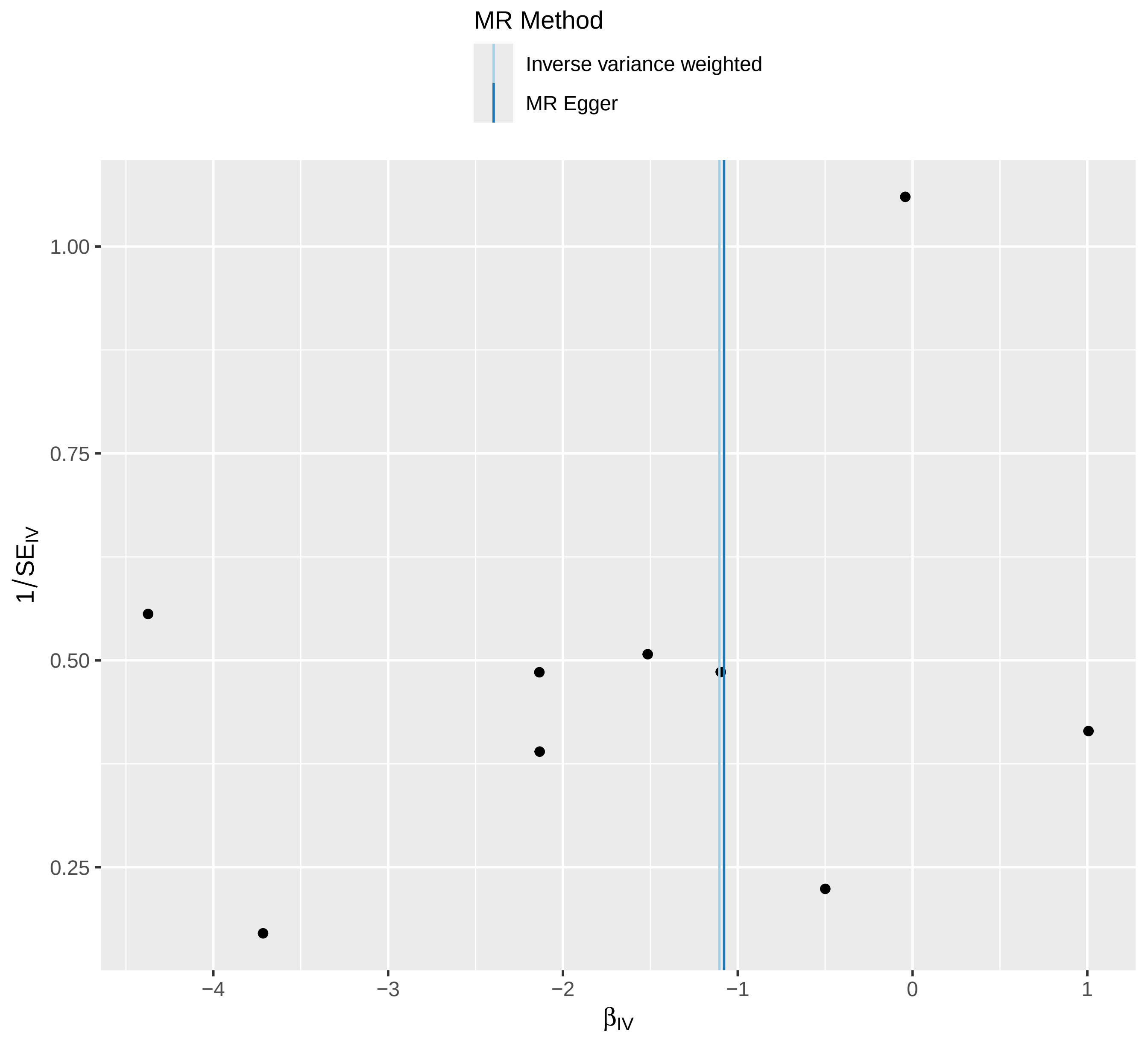

MR Method
Inverse variance weighted
MR Egger
1.00
0.75
0.50
0.25
1/SE_IV
−4
−3
−2
−1
0
1
β_IV

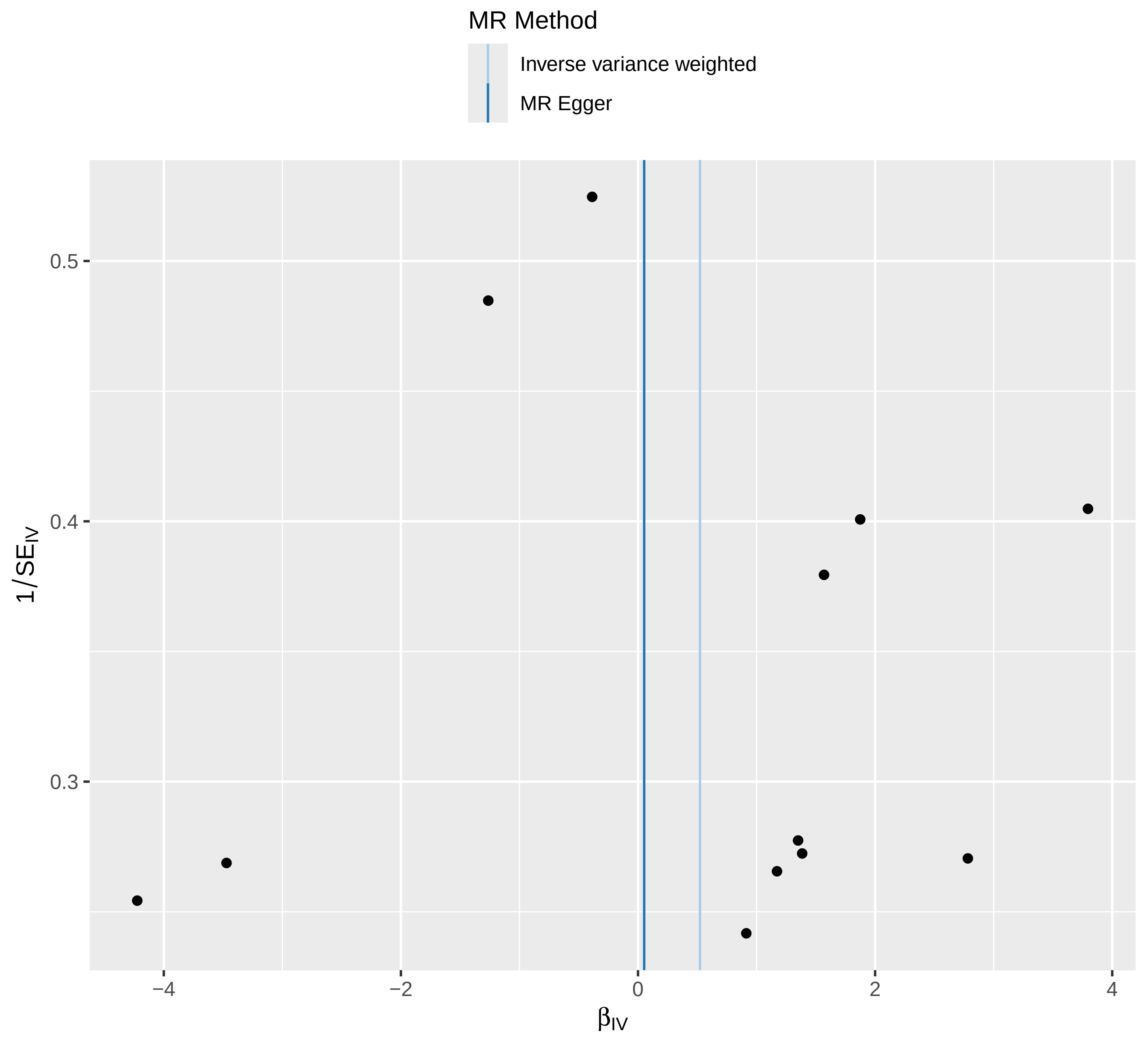

MR Method
Inverse variance weighted
MR Egger
0.5
0.4
0.3
$1/SE_{IV}$
−4
−2
0
2
4
β_{IV}

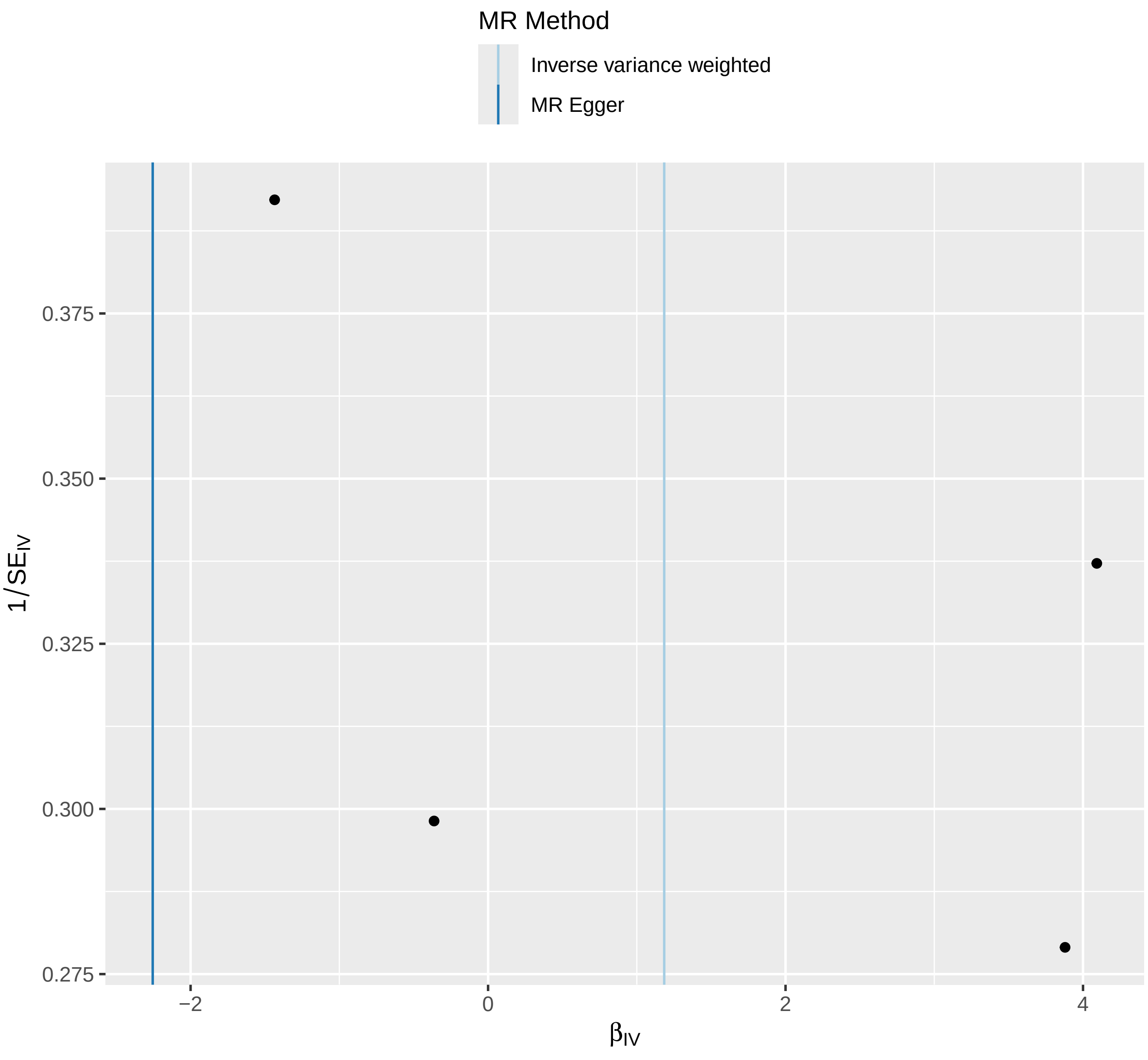
MR Method
Inverse variance weighted
MR Egger
0.375
0.350
0.325
0.300
0.275
$1/SE_{IV}$
−2
0
2
4
β_{IV}

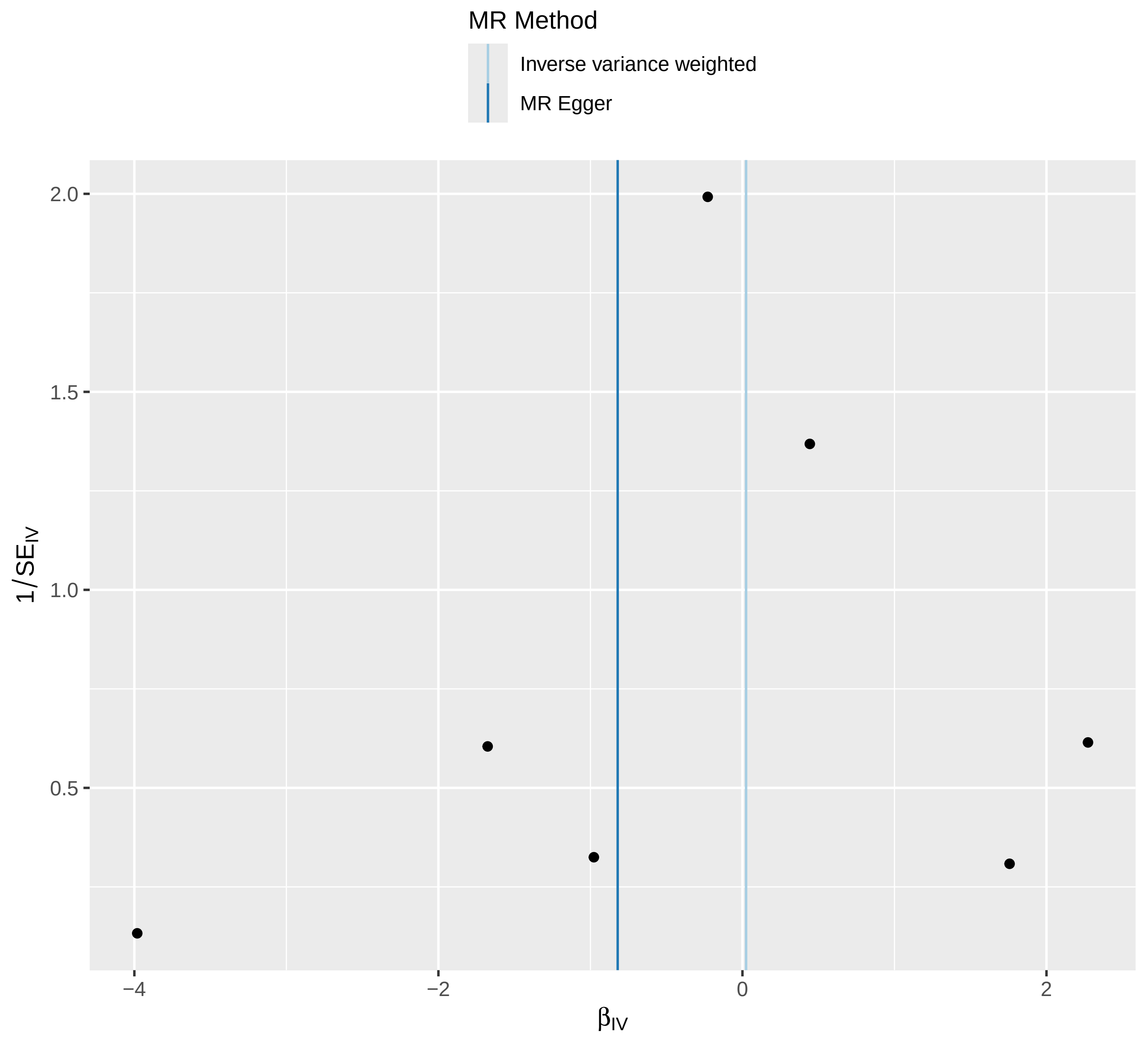
MR Method
Inverse variance weighted
MR Egger
2.0
1.5
1.0
0.5
1/SE_IV
−4
−2
0
2
β_IV

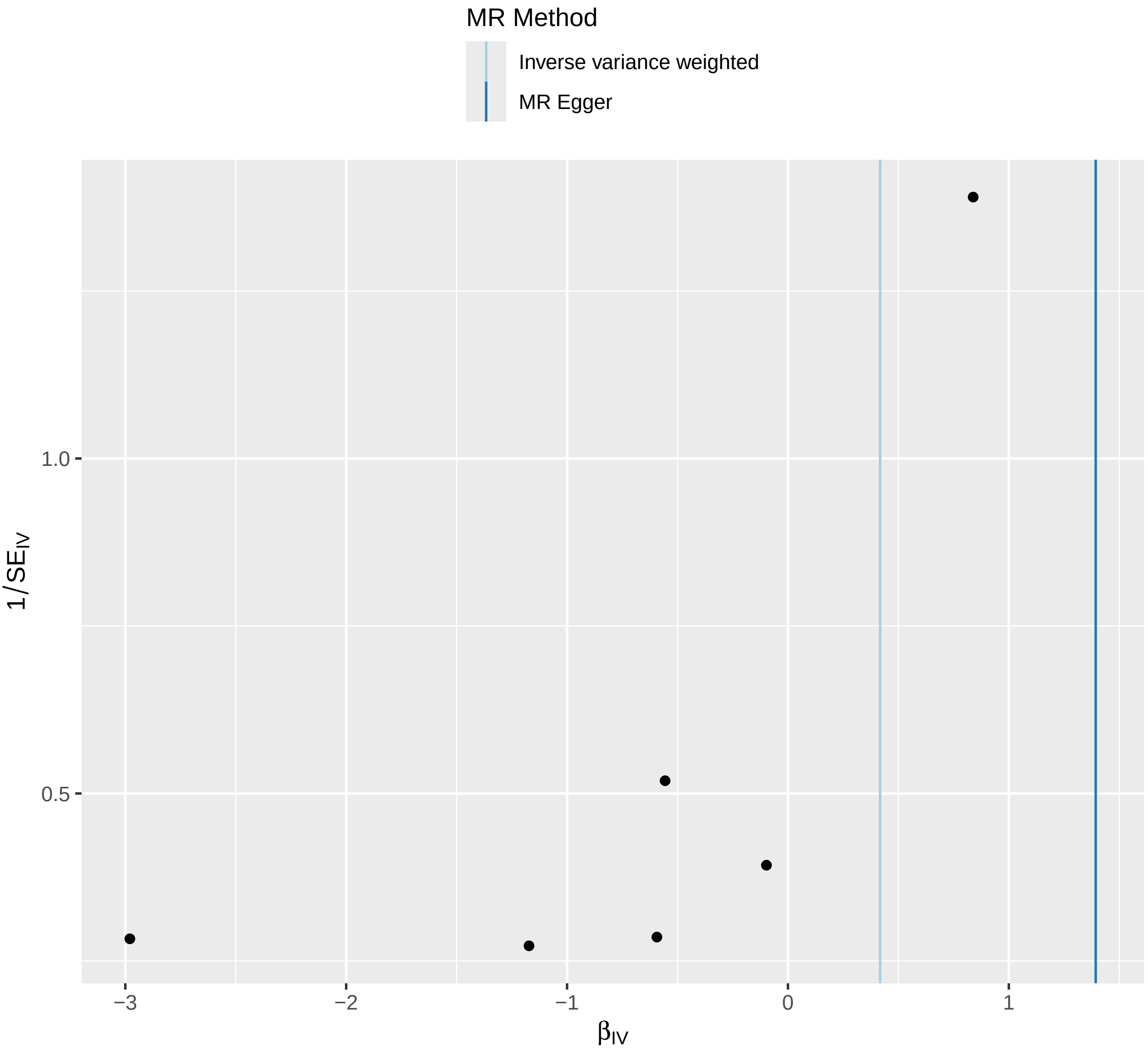

MR Method
Inverse variance weighted
MR Egger
1.0
0.5
1/SE_IV
−3
−2
−1
0
1
β_IV

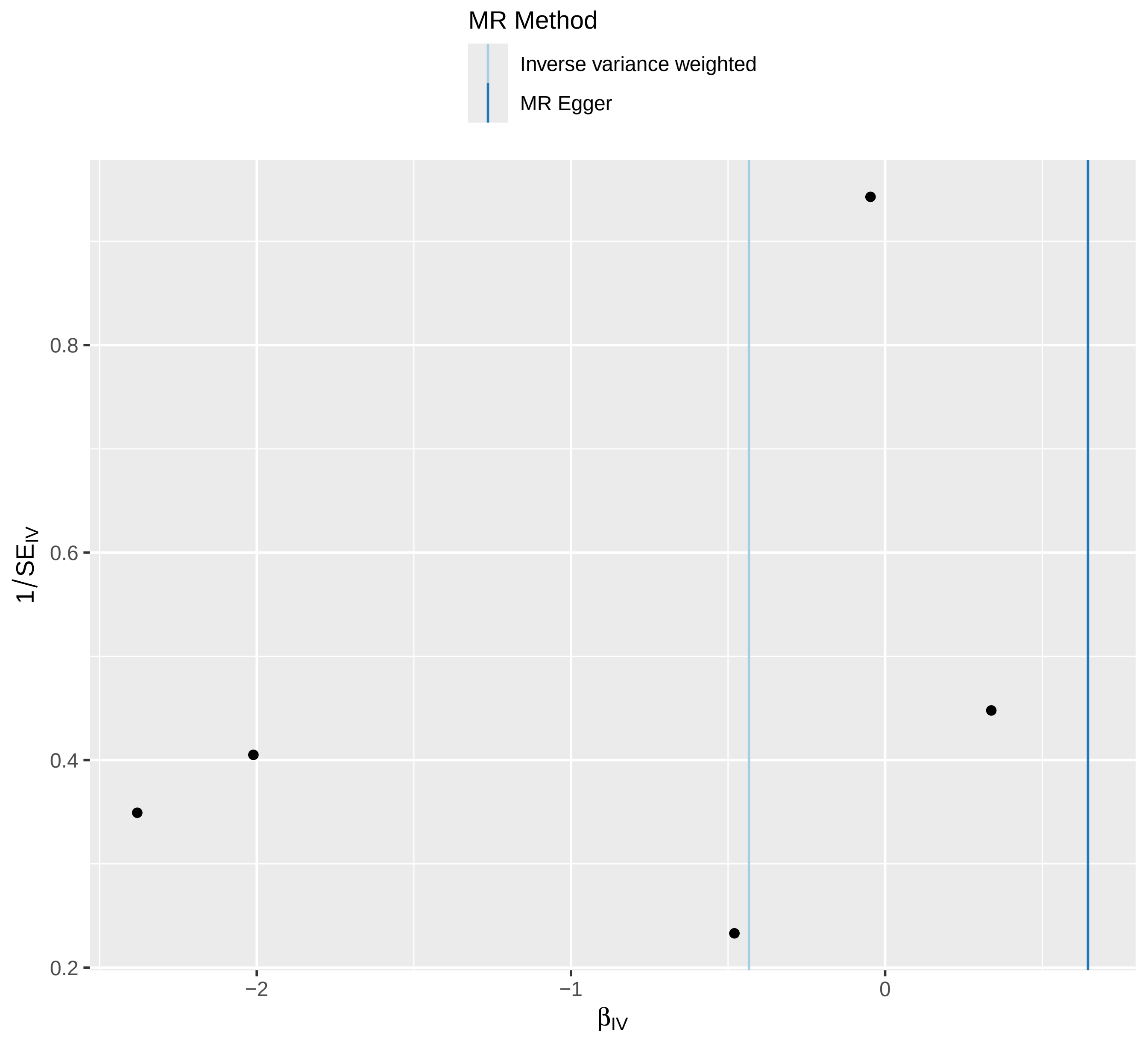
MR Method
Inverse variance weighted
MR Egger
0.8
0.6
0.4
0.2
−2
−1
0
1/SE_IV
β_IV

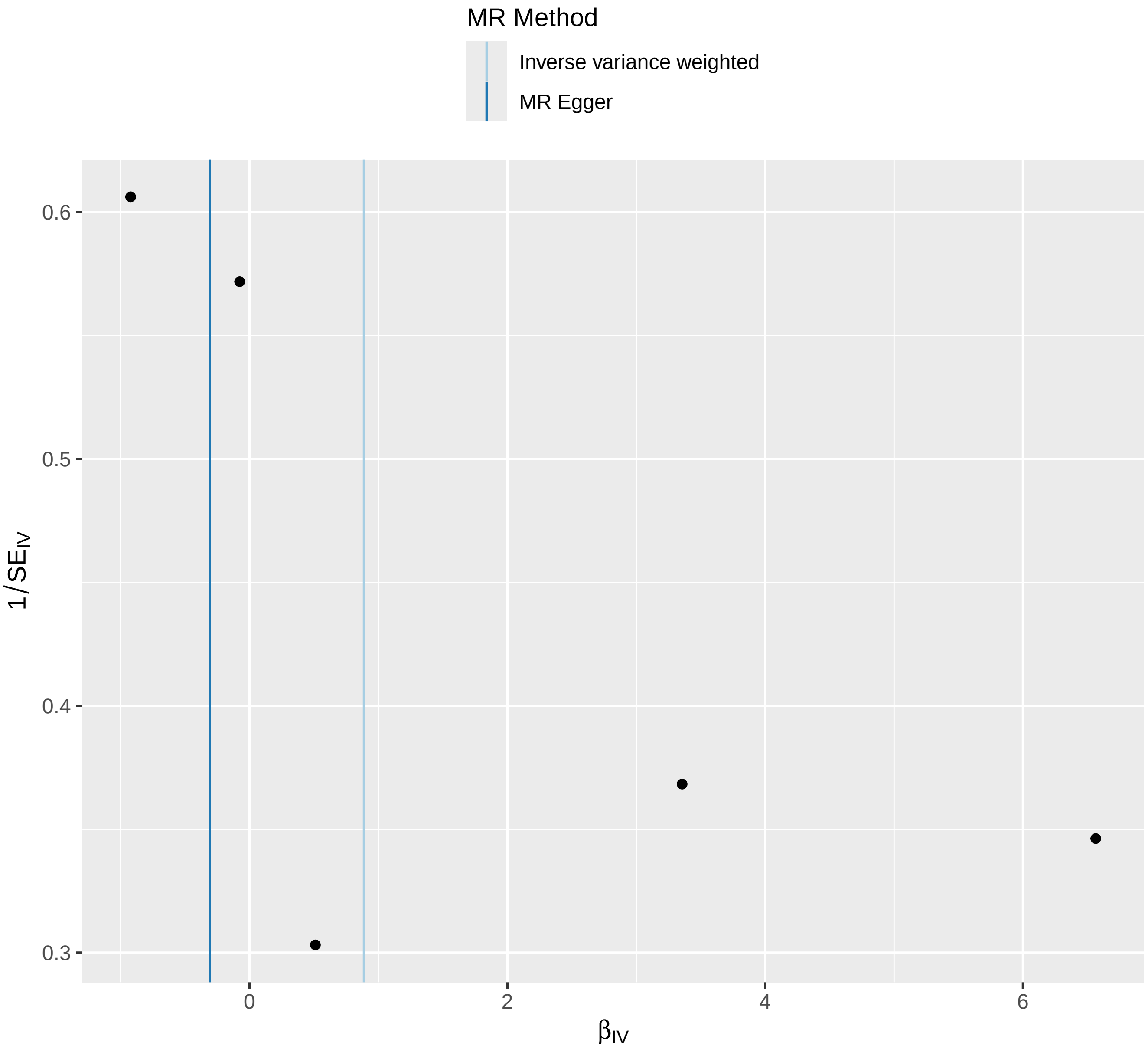

MR Method
Inverse variance weighted
MR Egger
0.6
0.5
0.4
0.3
0
2
4
6
$1/SE_{IV}$
β_{IV}

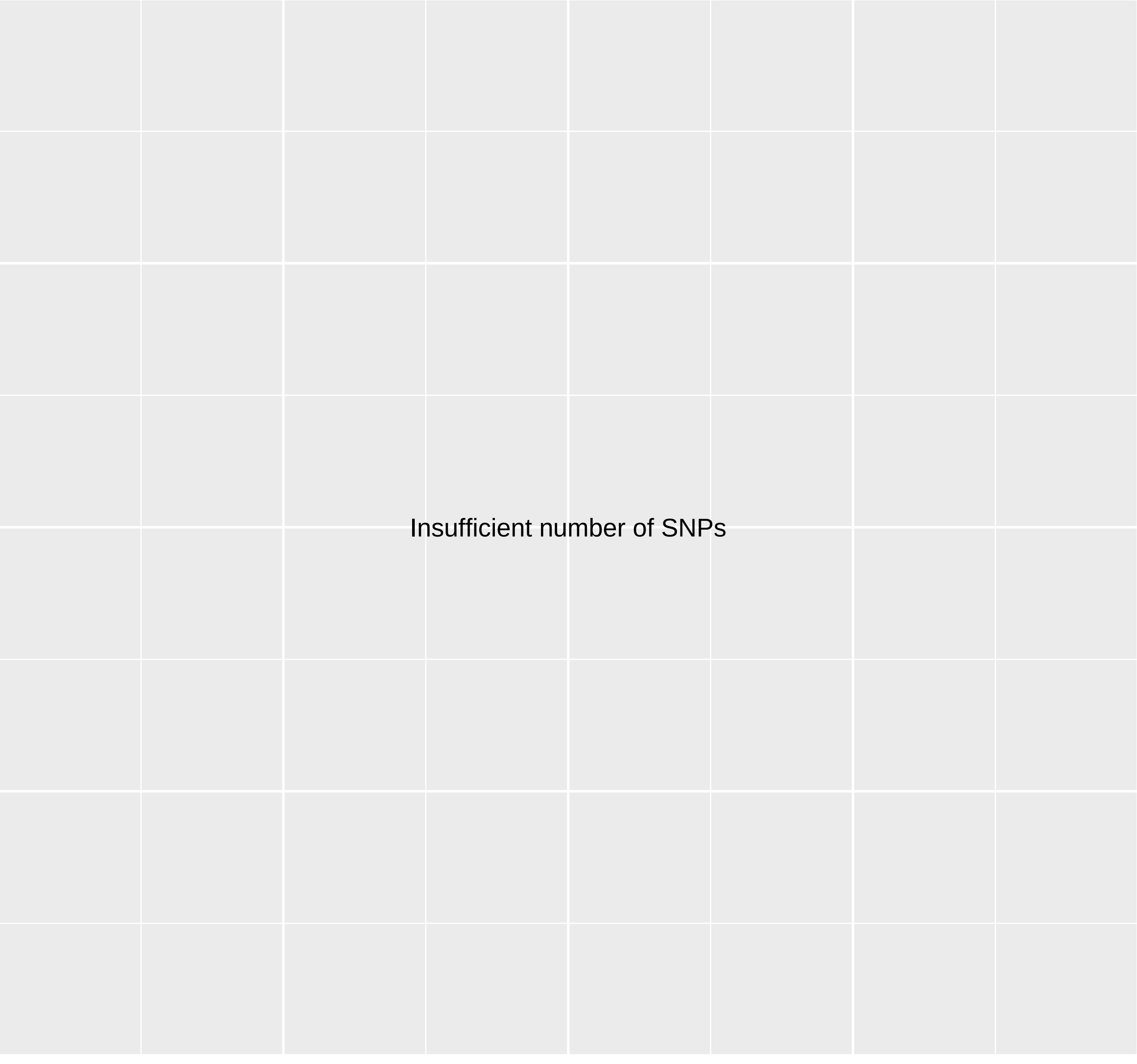
Insufficient number of SNPs

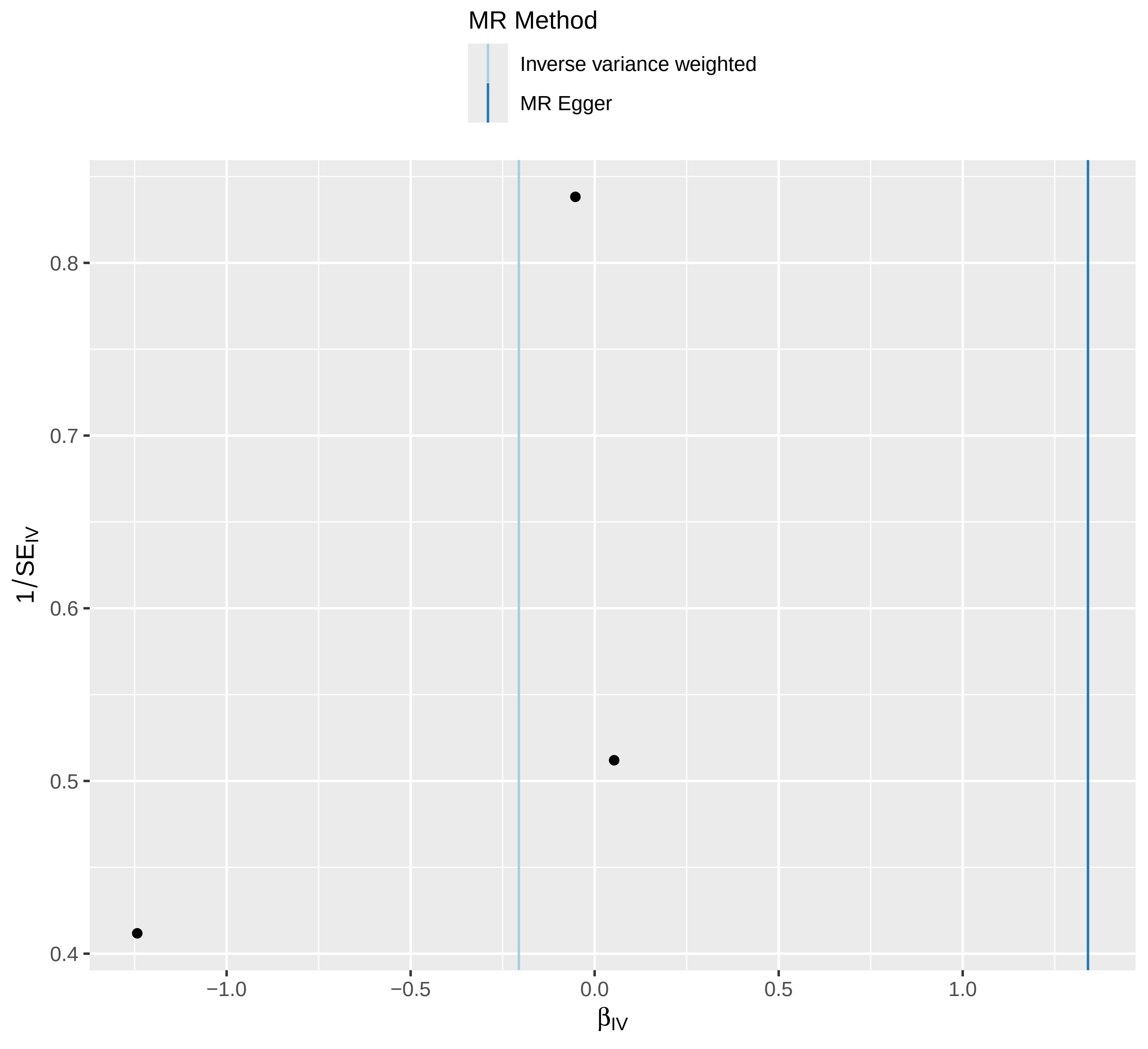

MR Method
Inverse variance weighted
MR Egger
0.8
0.7
0.6
0.5
0.4
1/SE$_{IV}$
−1.0
−0.5
0.0
0.5
1.0
β$_{IV}$

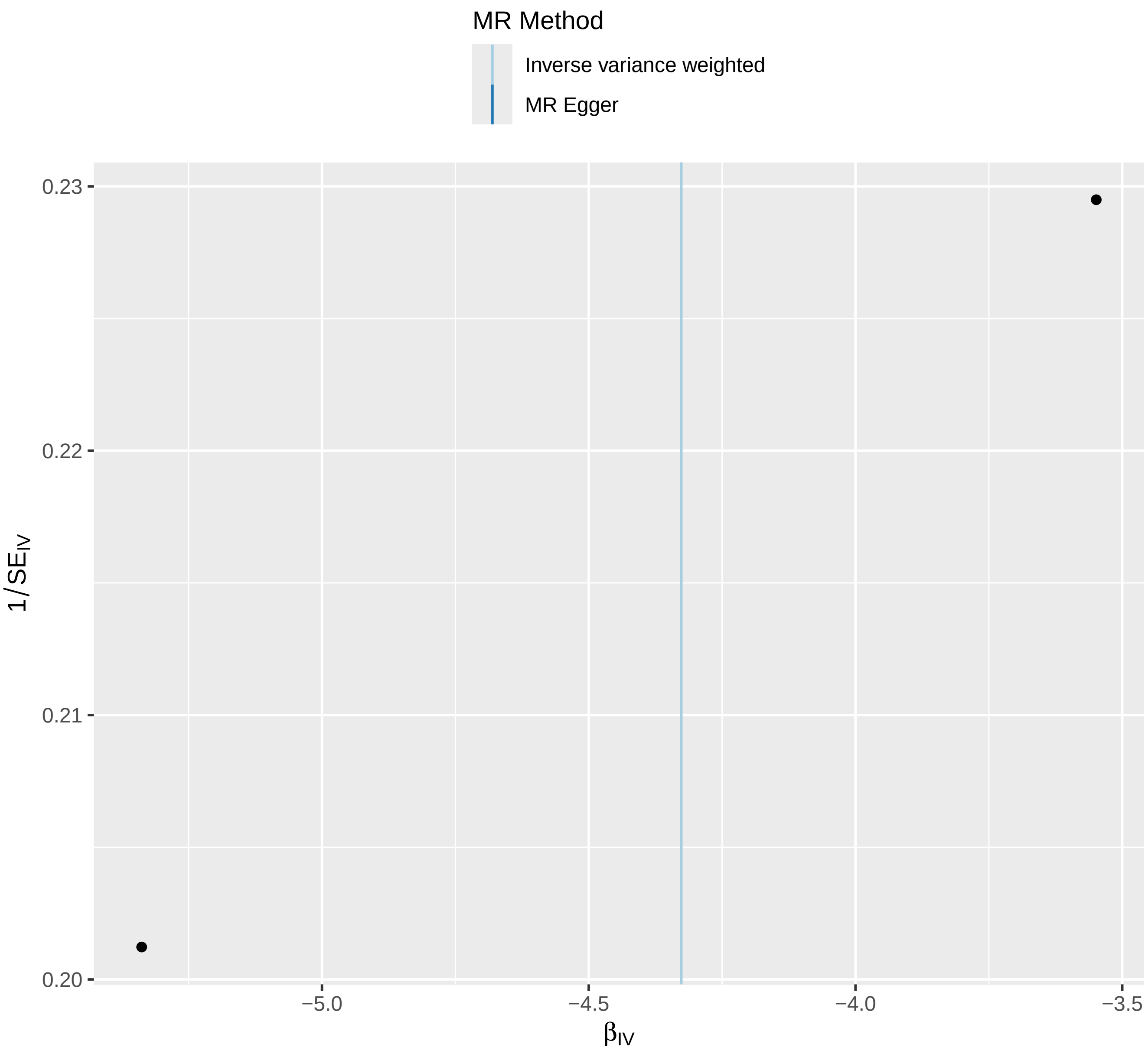

MR Method
Inverse variance weighted
MR Egger
0.23
0.22
0.21
0.20
1/SE_IV
−5.0
−4.5
−4.0
−3.5
β_IV

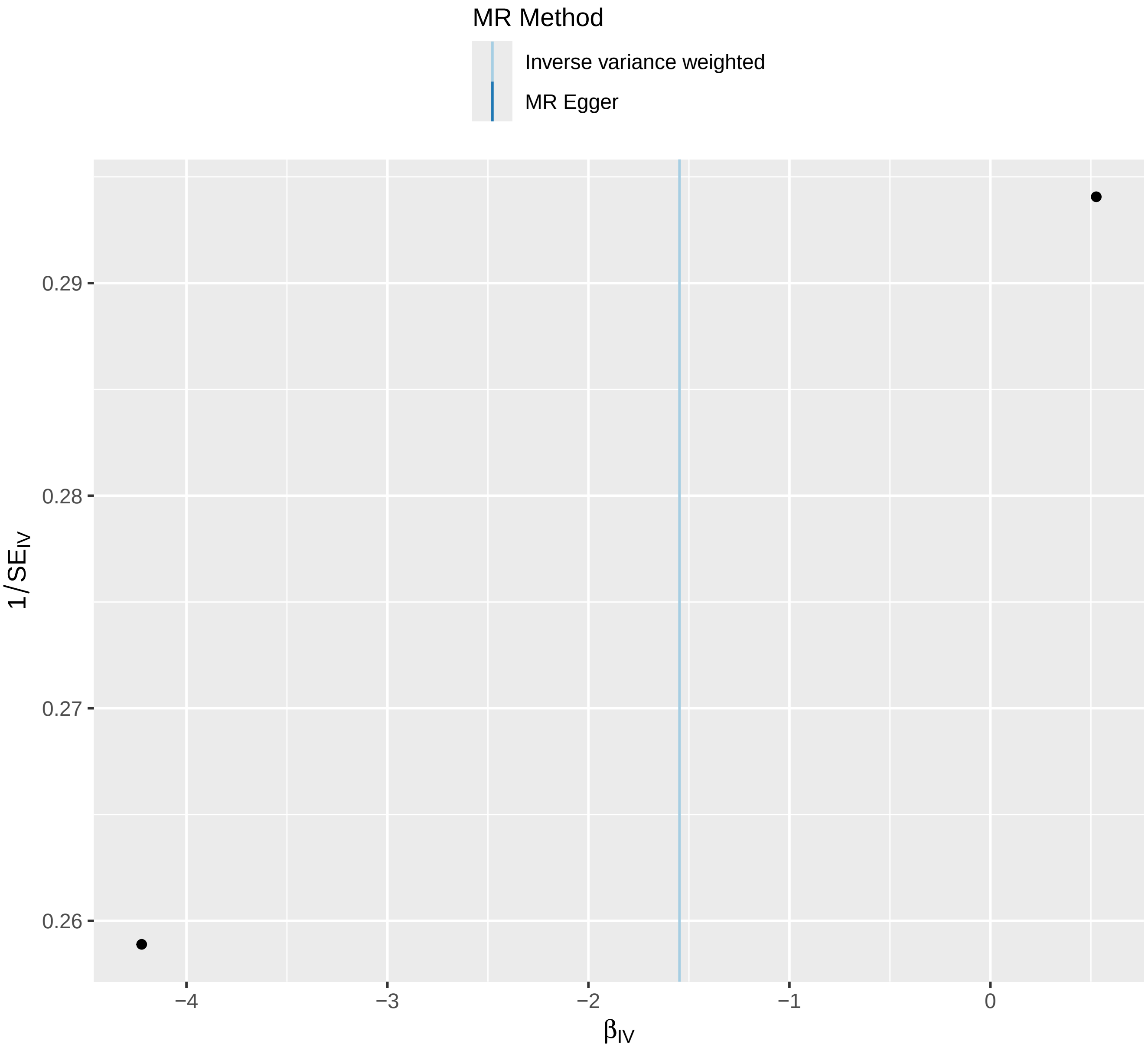
MR Method
Inverse variance weighted
MR Egger
0.29
0.28
0.27
0.26
1/SE_IV
−4
−3
−2
−1
0
β_IV

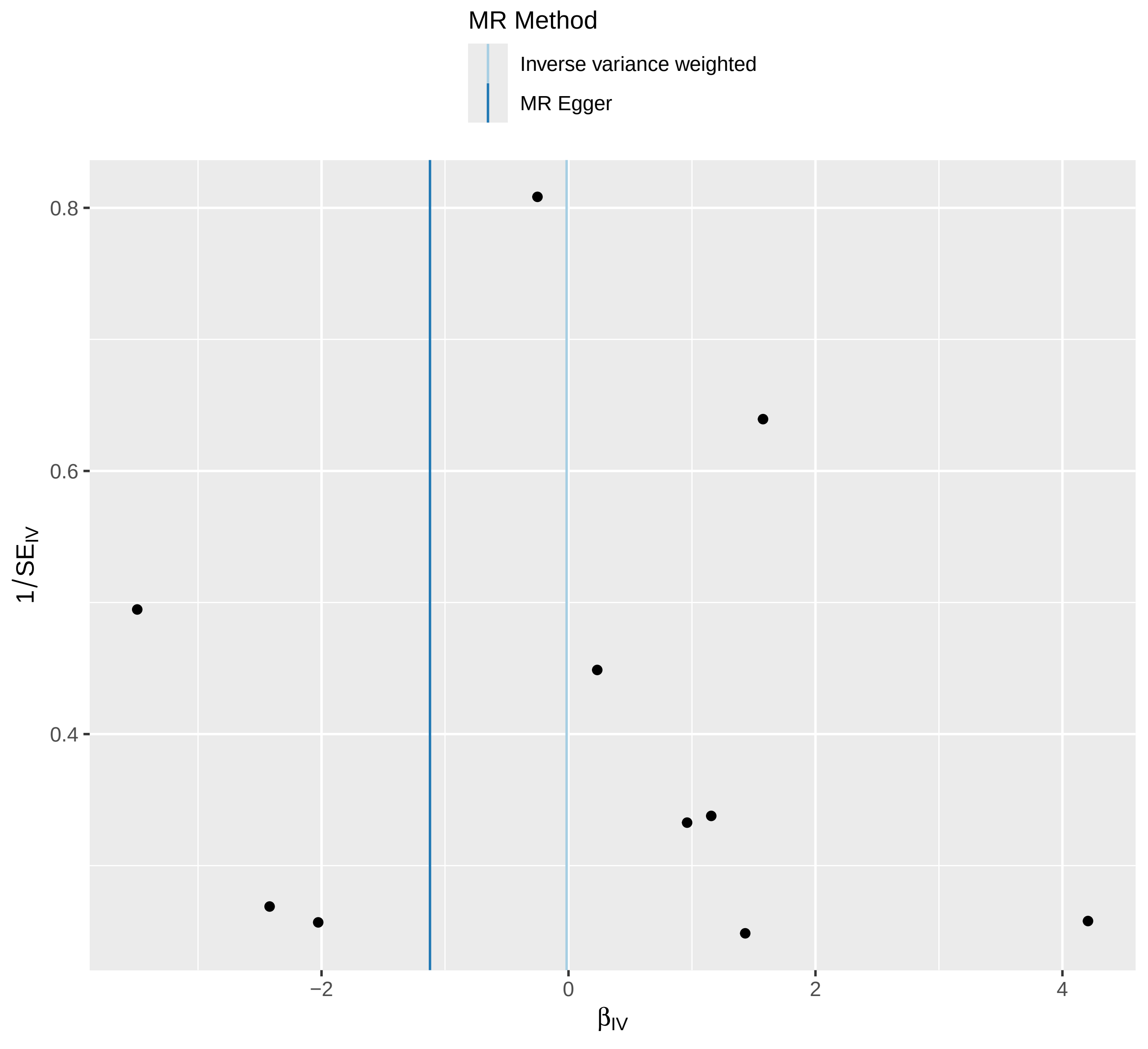
MR Method
Inverse variance weighted
MR Egger
0.8
0.6
0.4
1/SE$_{IV}$
−2
0
2
4
β$_{IV}$

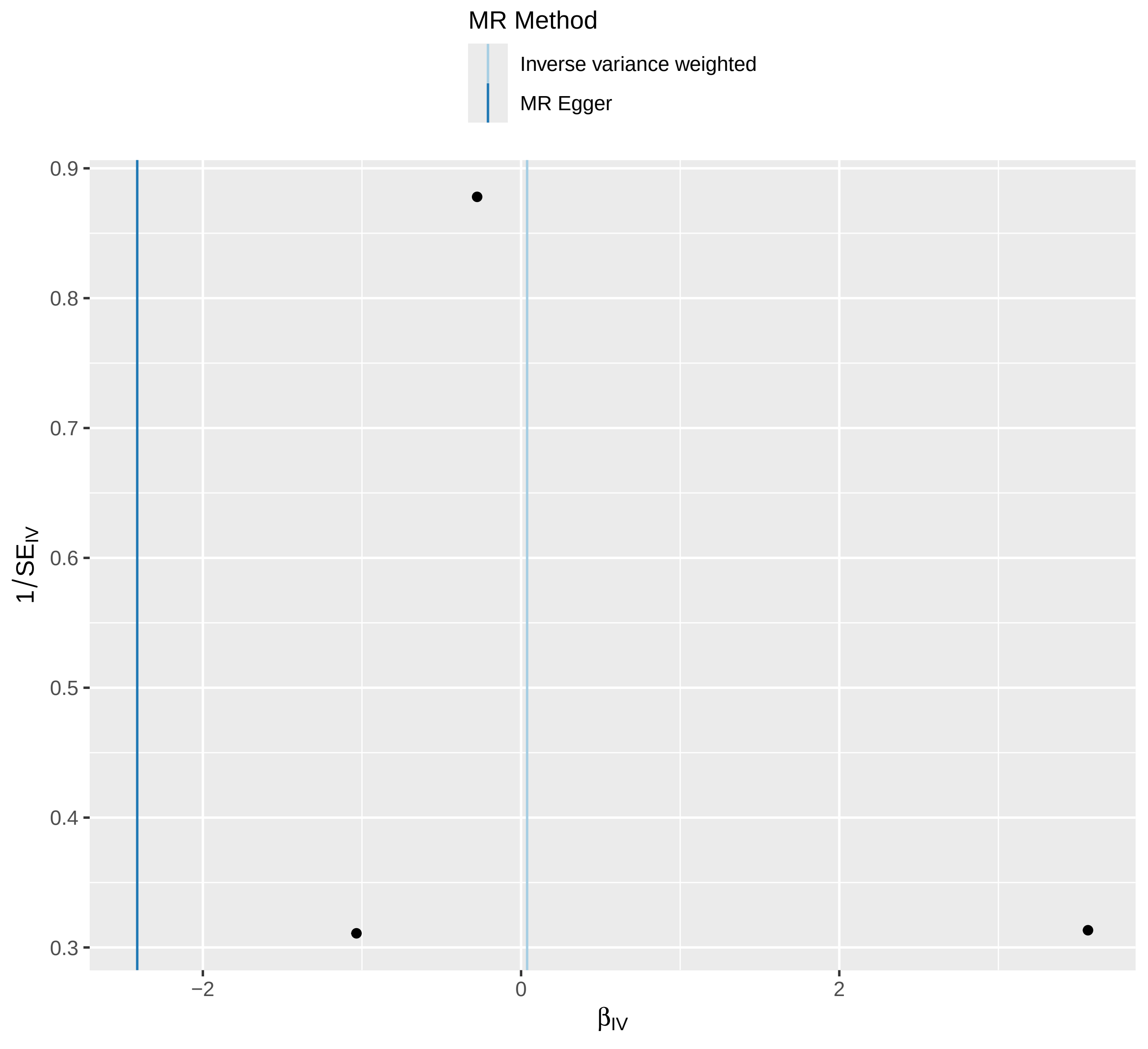
MR Method
Inverse variance weighted
MR Egger
0.9
0.8
0.7
0.6
0.5
0.4
0.3
1/SE_IV
−2
0
2
β_IV

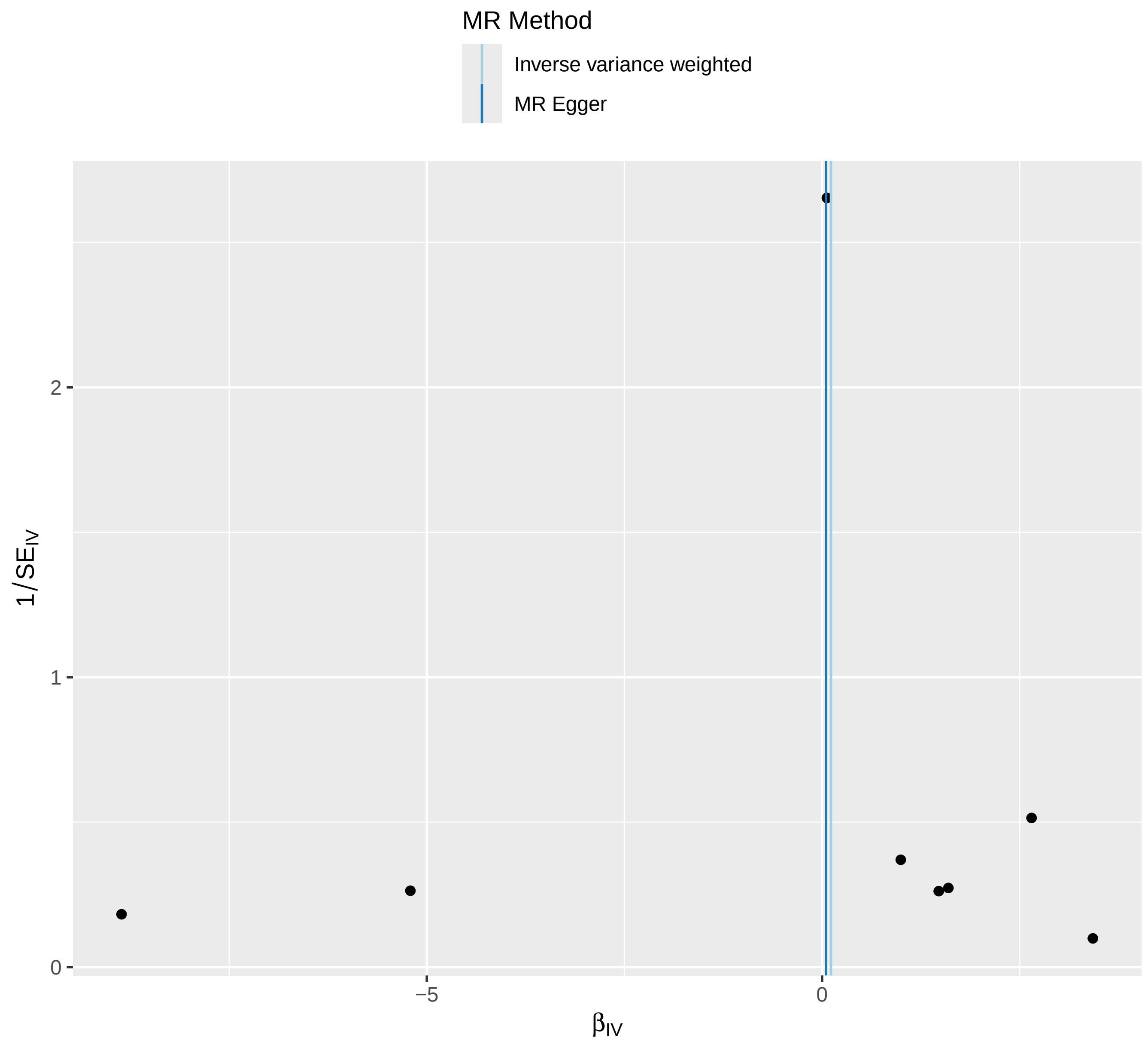
MR Method
Inverse variance weighted
MR Egger
2
1
0
1/SE$_{IV}$
−5
0
β$_{IV}$

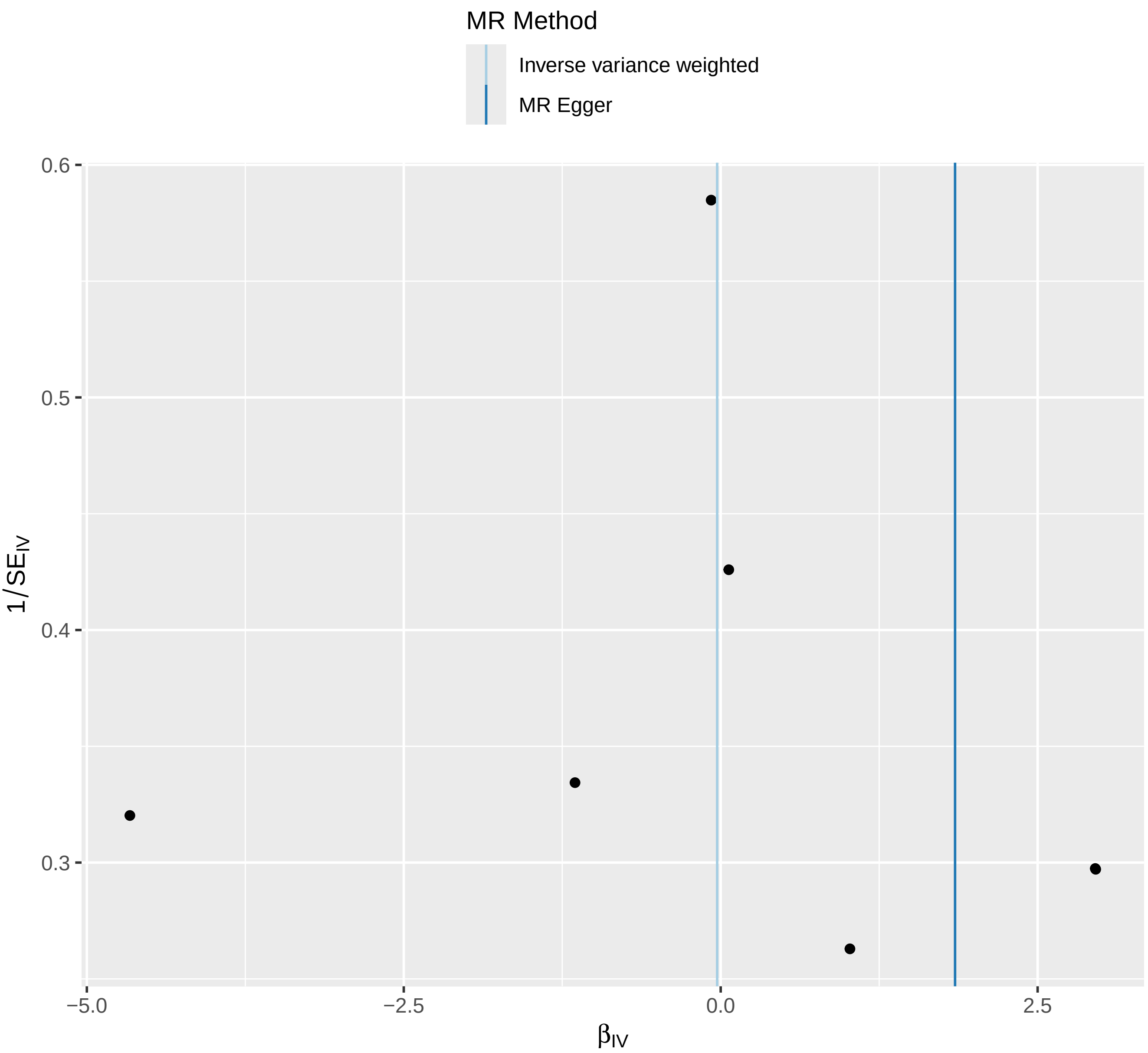
MR Method
Inverse variance weighted
MR Egger
0.6
0.5
0.4
0.3
1/SE_IV
−5.0
−2.5
0.0
2.5
β_IV

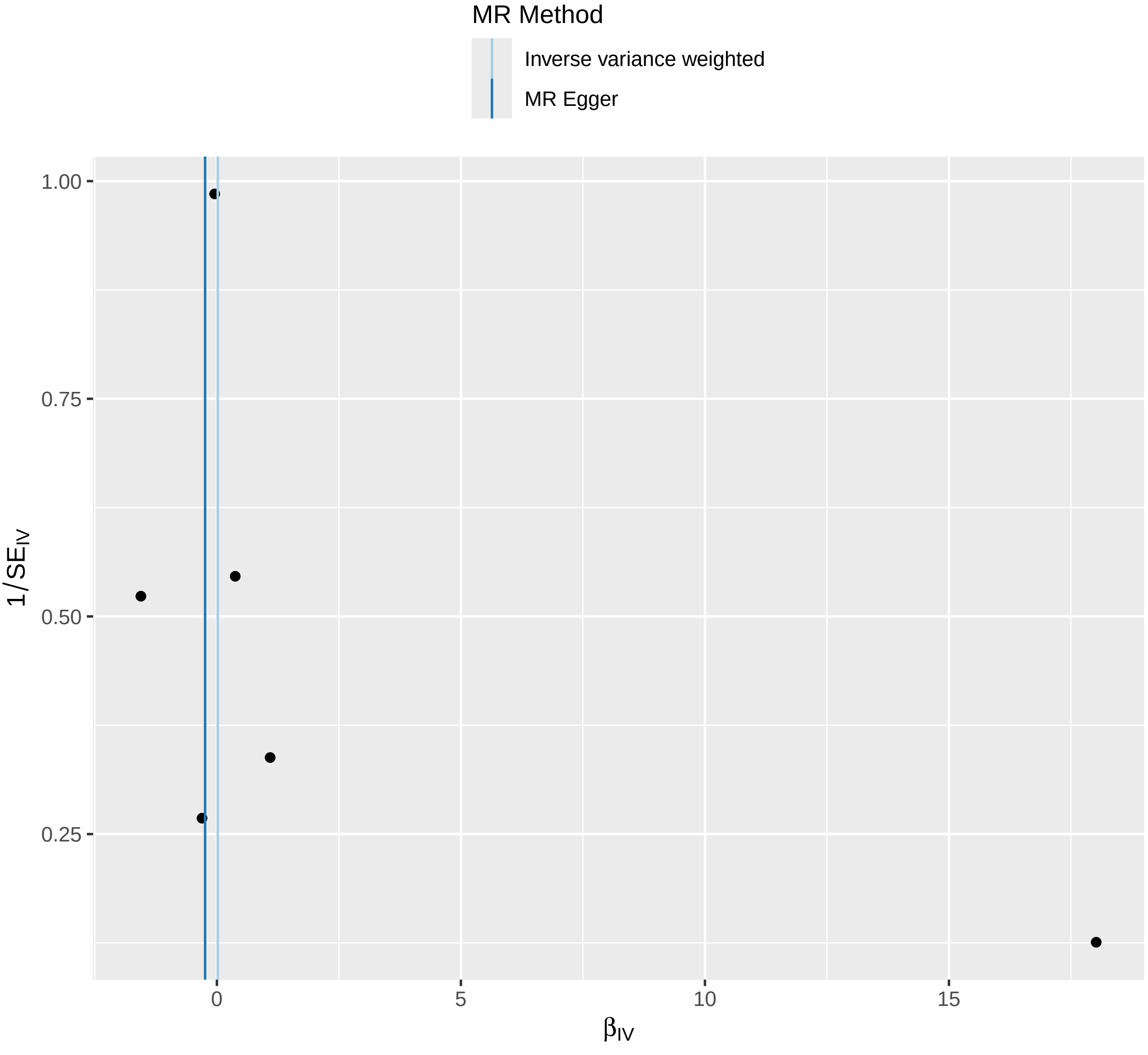
MR Method
Inverse variance weighted
MR Egger
1/SE_IV
1.00
0.75
0.50
0.25
0
5
10
15
β_IV

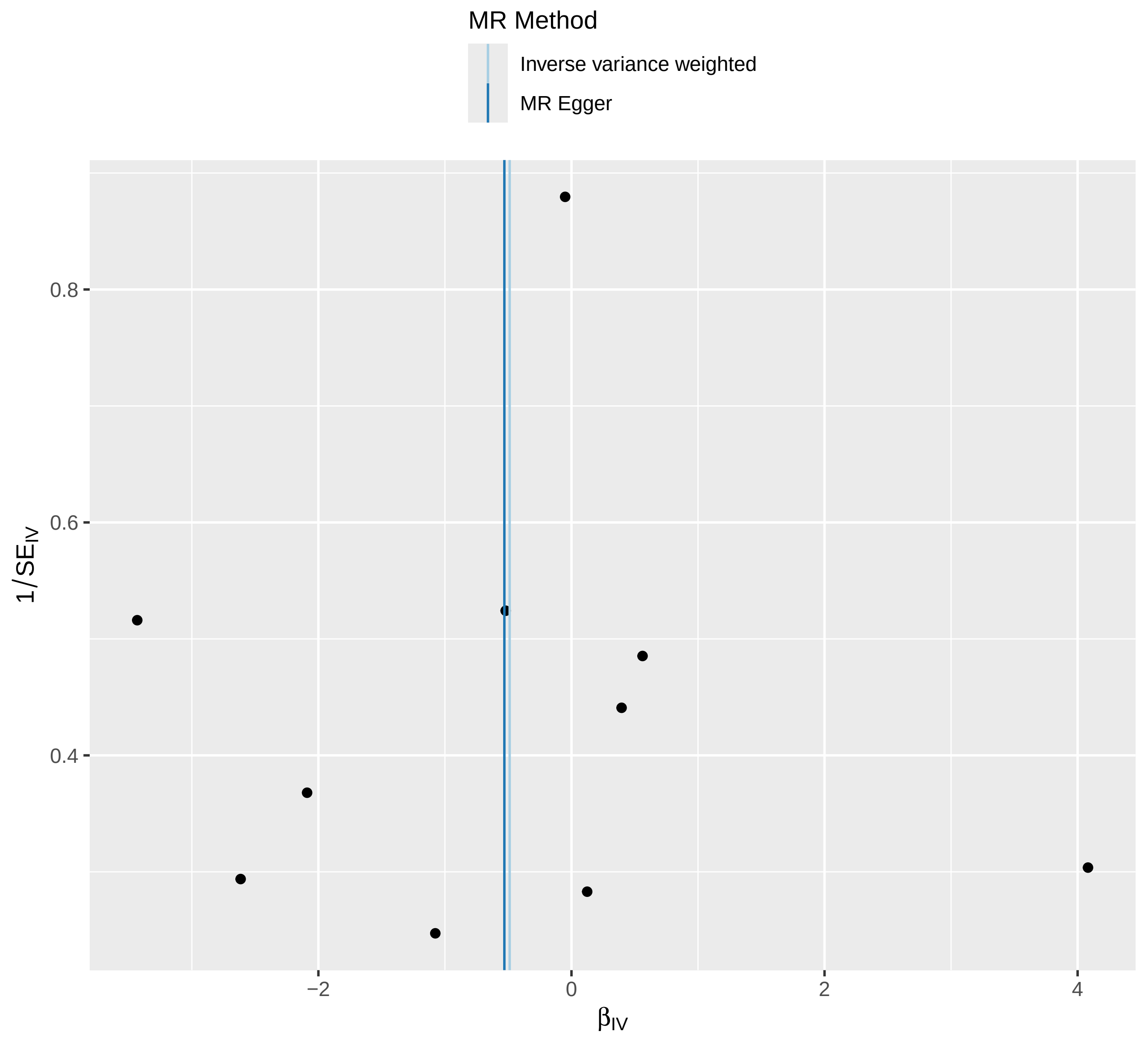

MR Method
Inverse variance weighted
MR Egger
0.8
0.6
0.4
1/SE_IV
−2
0
2
4
β_IV

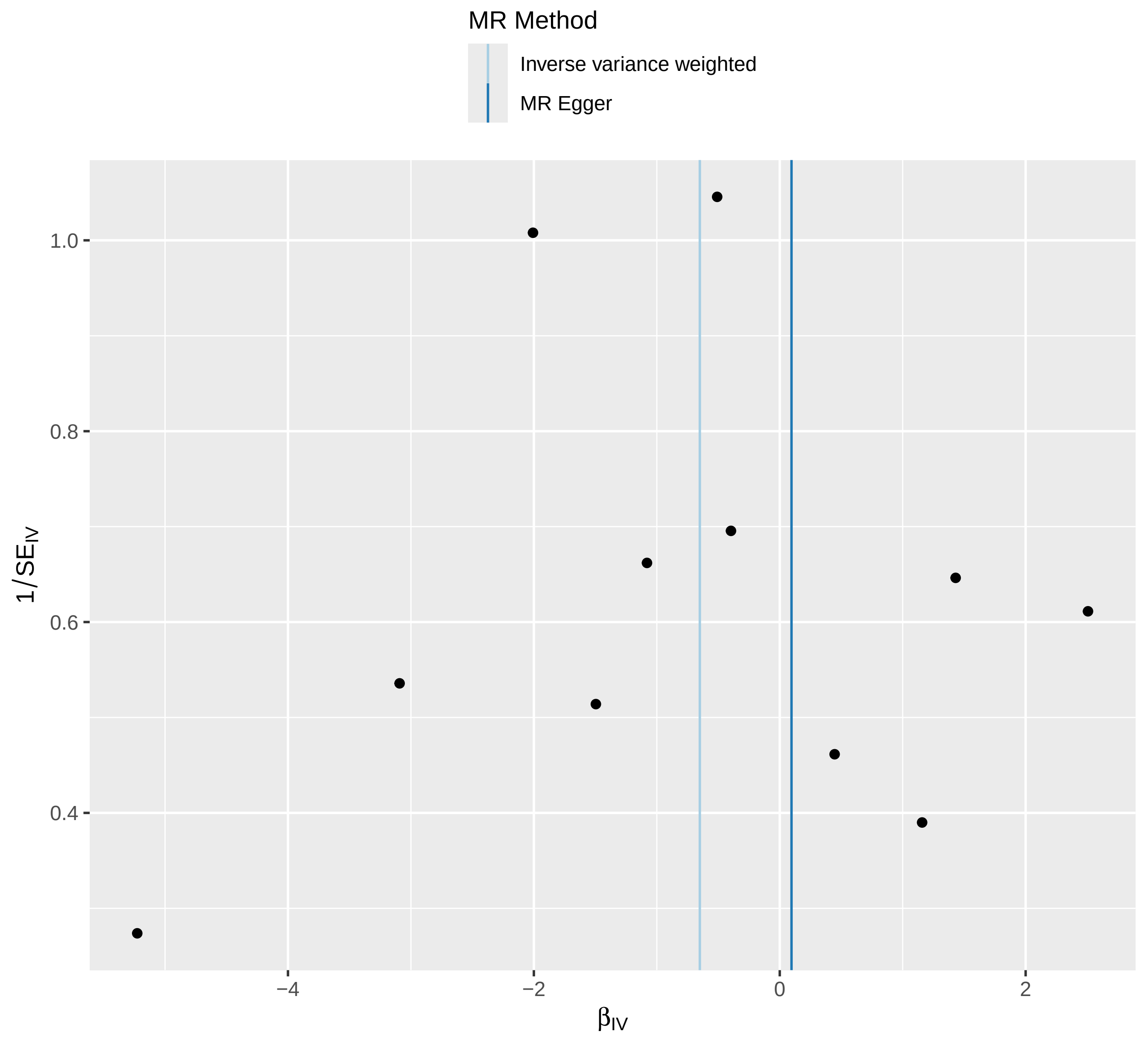
MR Method
Inverse variance weighted
MR Egger
1.0
0.8
0.6
0.4
1/SE_IV
−4
−2
0
2
β_IV

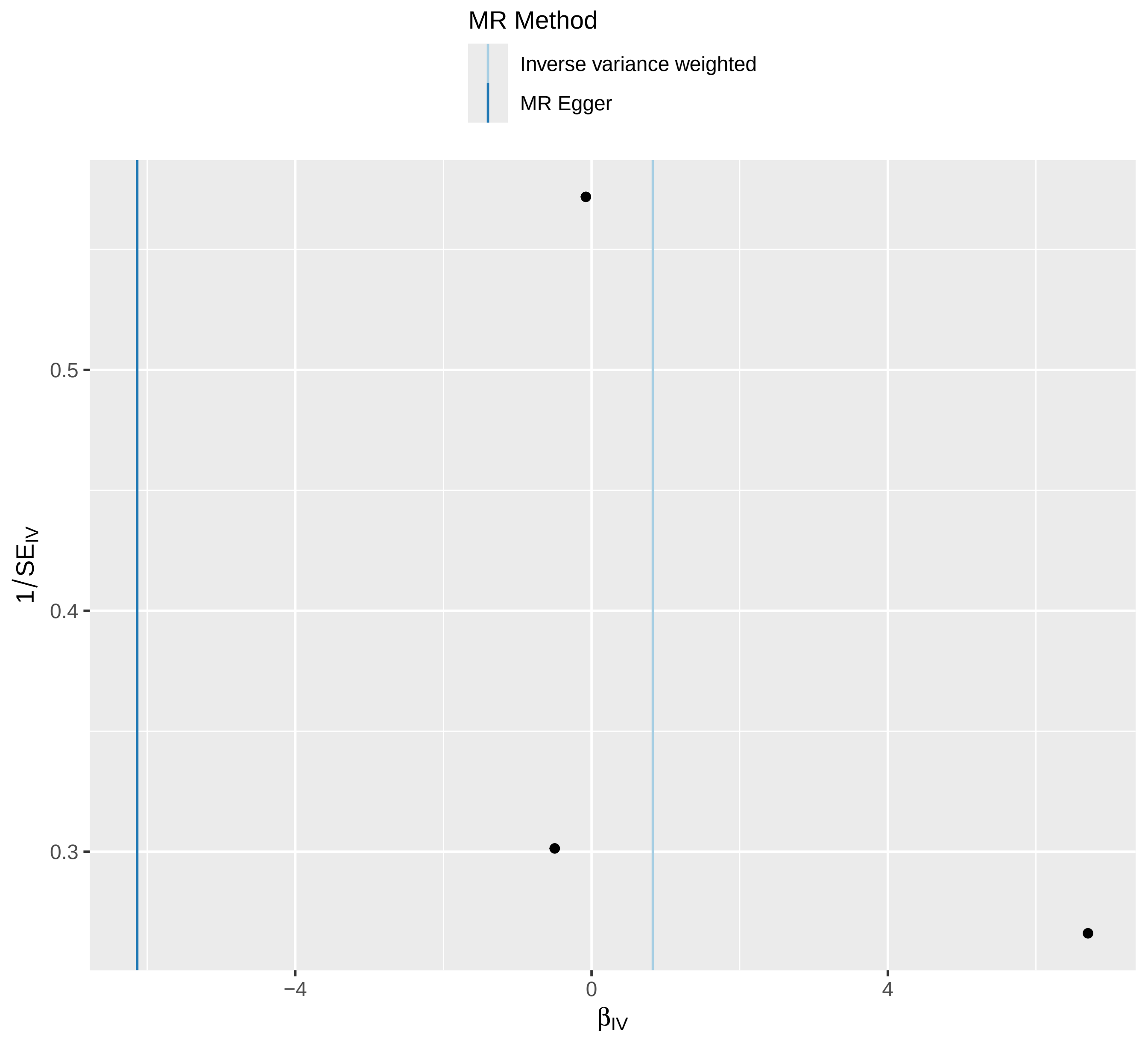
MR Method
Inverse variance weighted
MR Egger
0.5
0.4
0.3
1/SE_IV
−4
0
4
β_IV

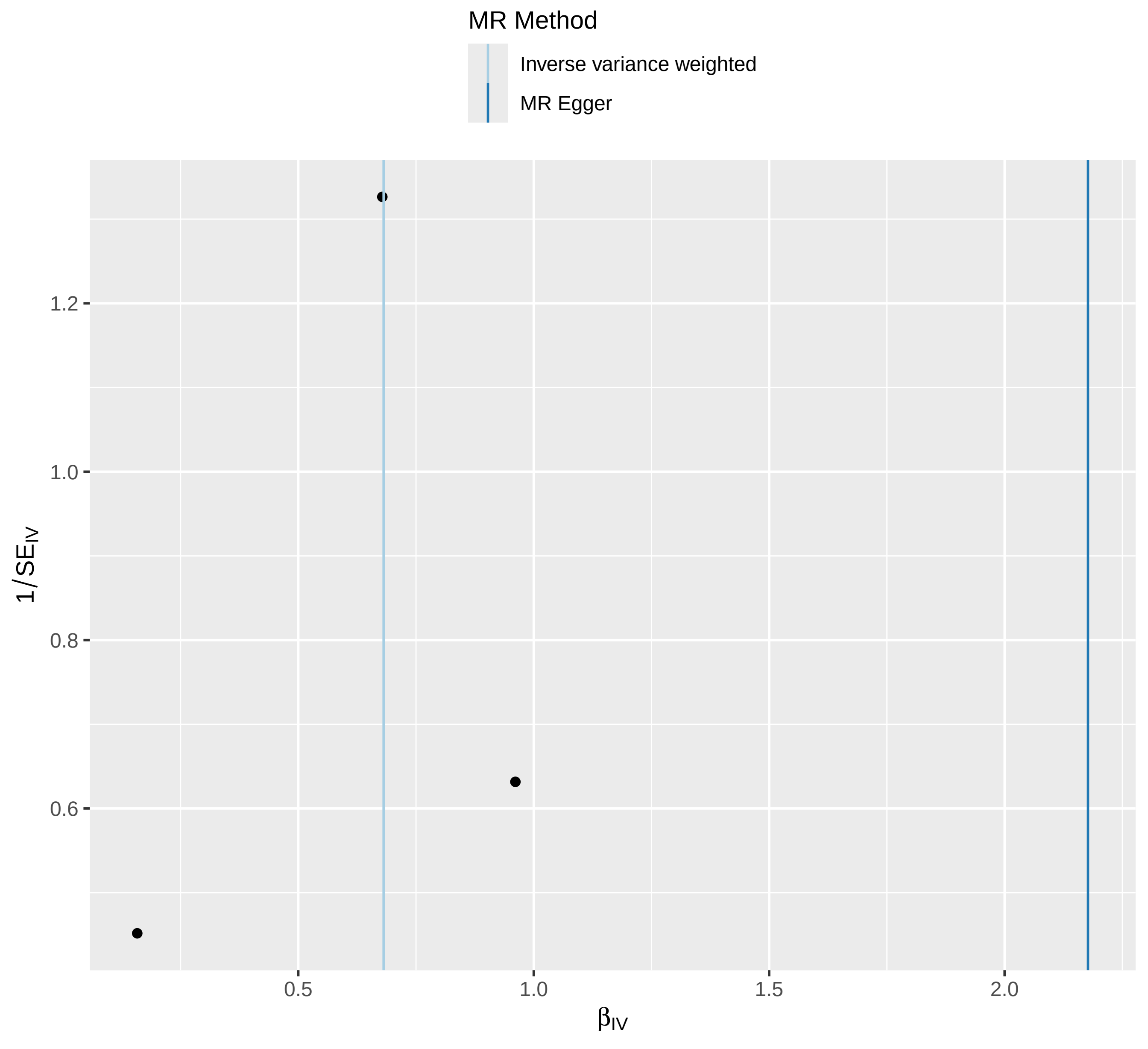
MR Method
Inverse variance weighted
MR Egger
1.2
1.0
0.8
0.6
1/SE_IV
0.5
1.0
1.5
2.0
β_IV

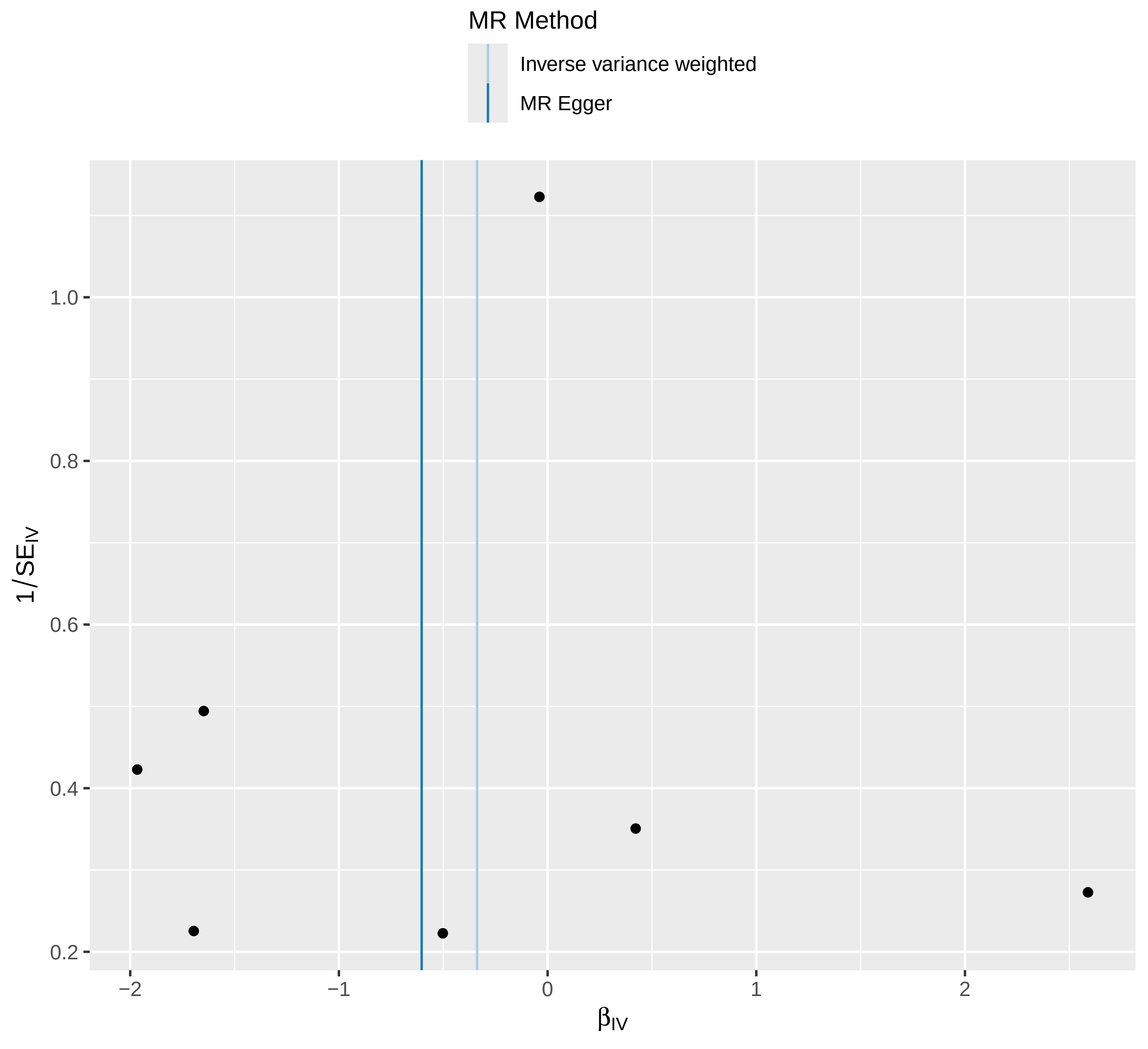
MR Method
Inverse variance weighted
MR Egger
1/SE$_{IV}$
1.0
0.8
0.6
0.4
0.2
−2
−1
0
1
2
β$_{IV}$

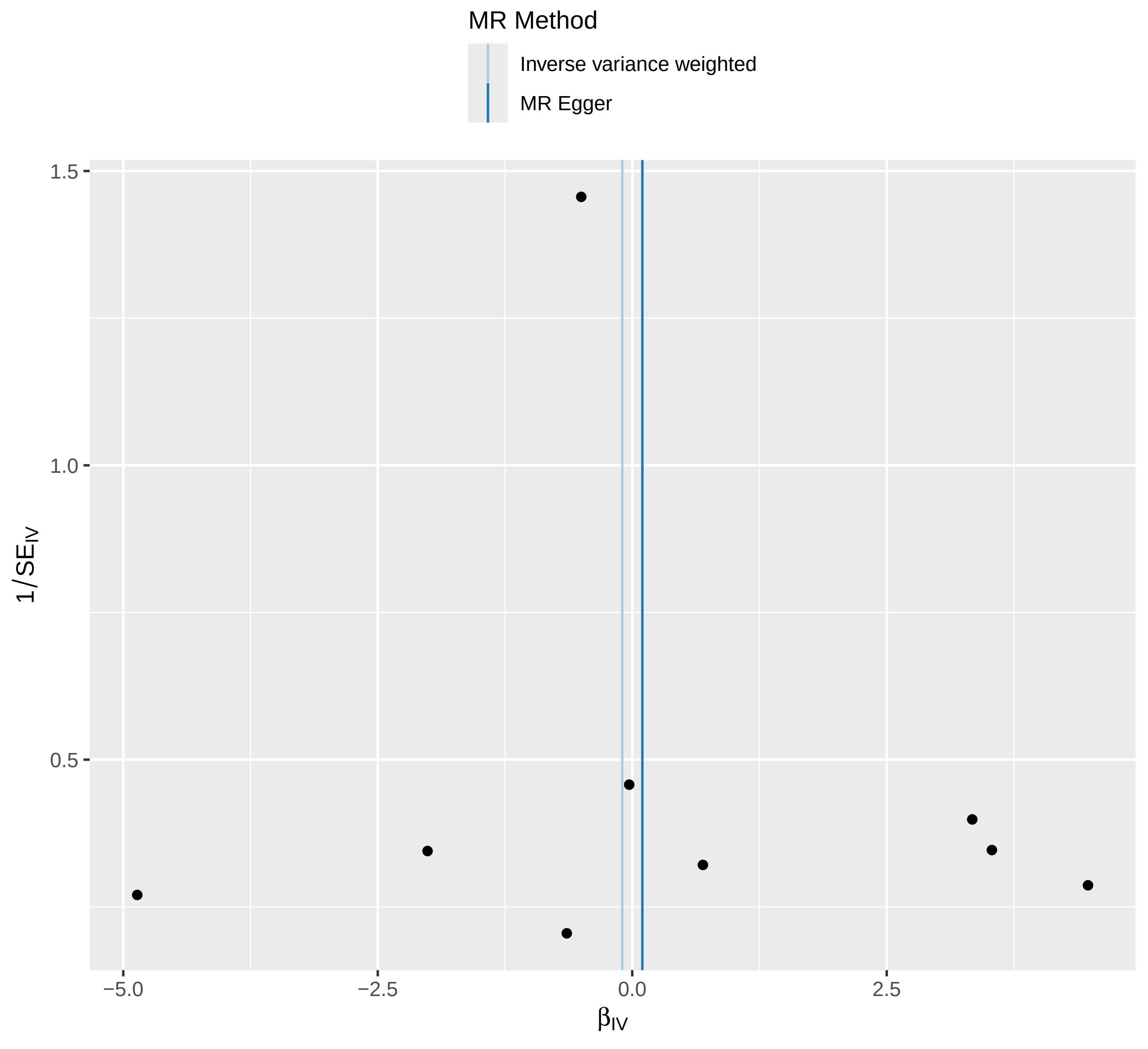

MR Method
Inverse variance weighted
MR Egger
1.5
1.0
0.5
1/SE$_{IV}$
−5.0
−2.5
0.0
2.5
β$_{IV}$

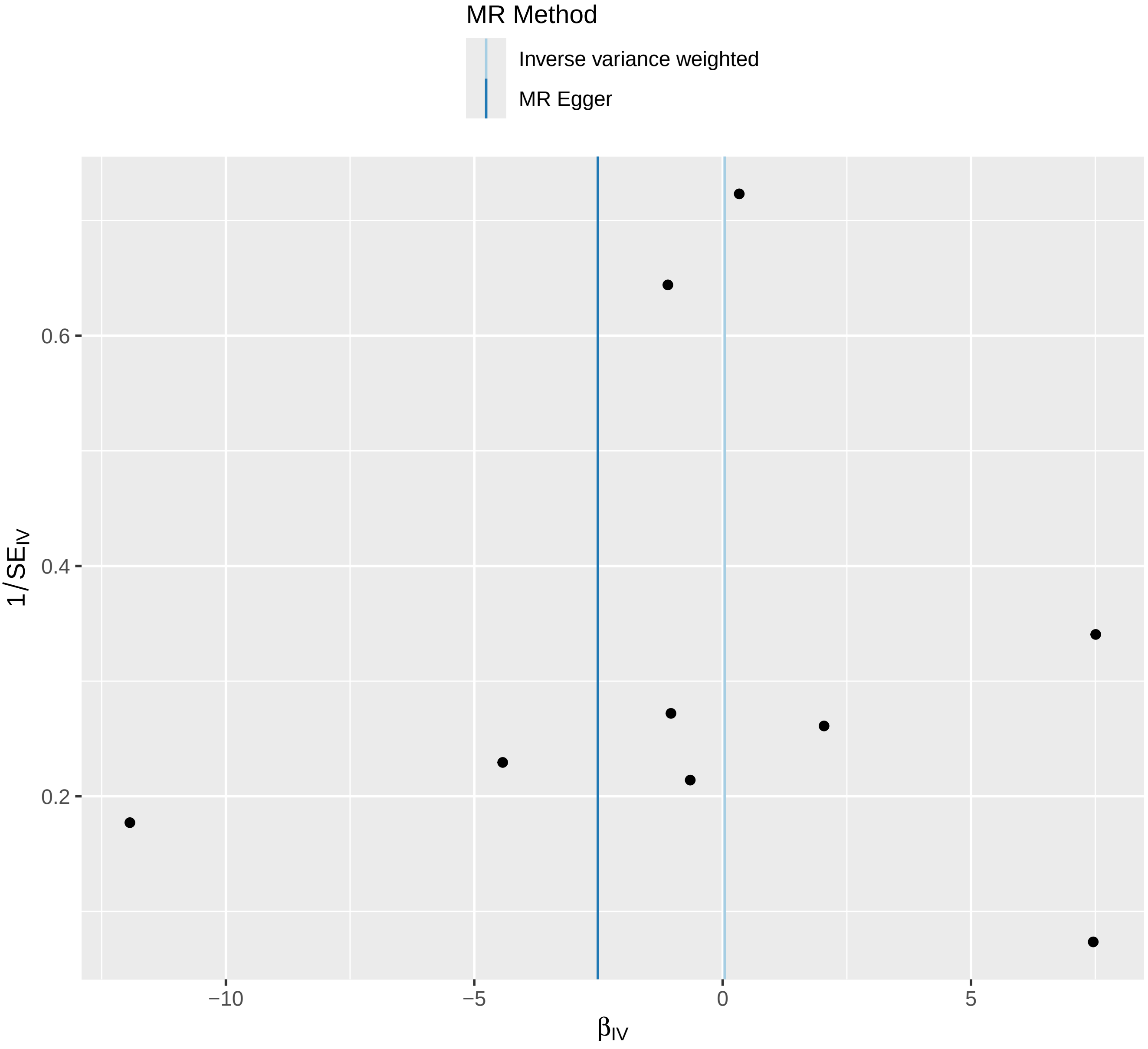
MR Method
Inverse variance weighted
MR Egger
0.6
0.4
0.2
1/SE_IV
−10
−5
0
5
β_IV

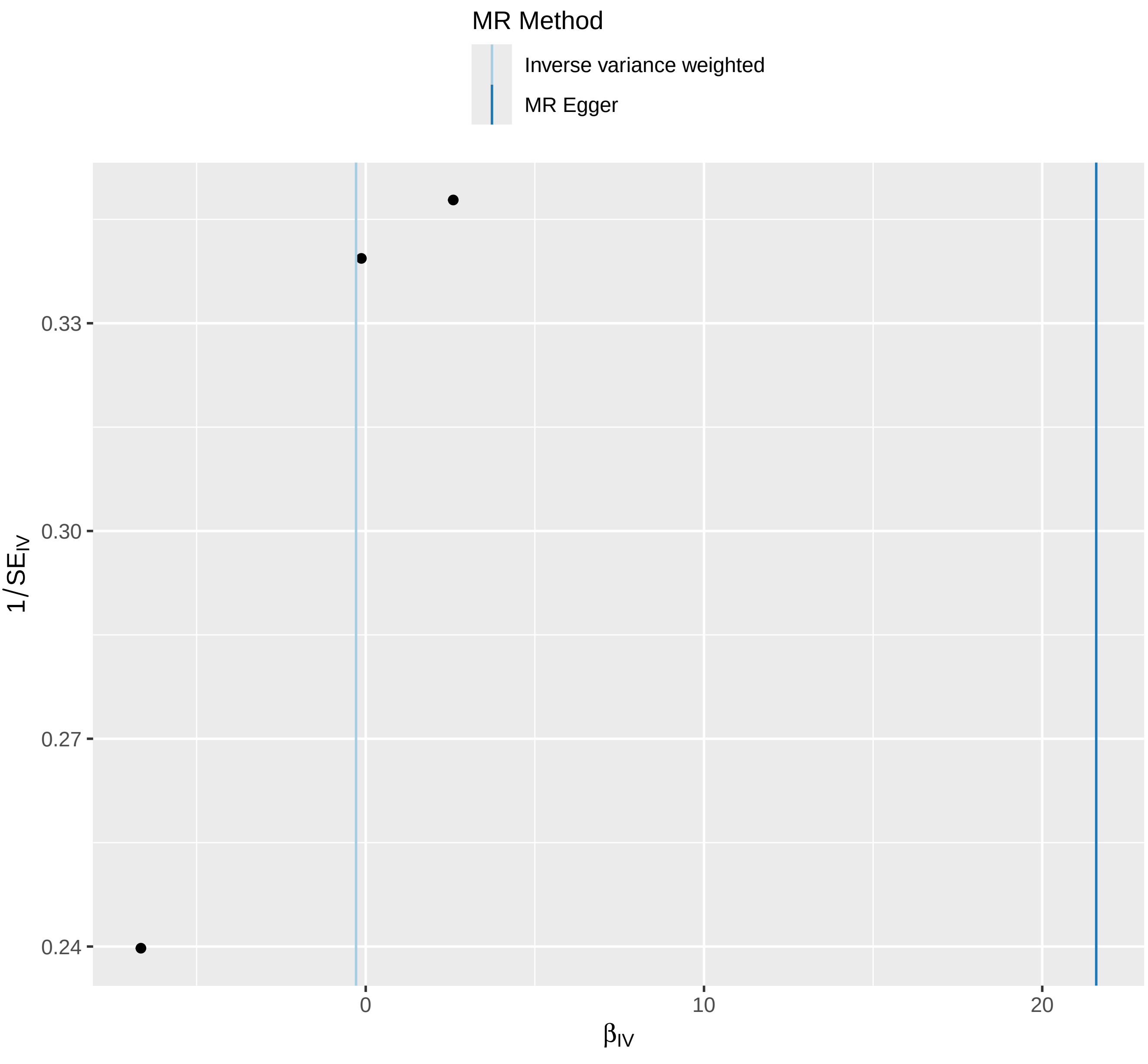

MR Method
Inverse variance weighted
MR Egger
0.33
0.30
0.27
0.24
1/SE_IV
0
10
20
β_IV

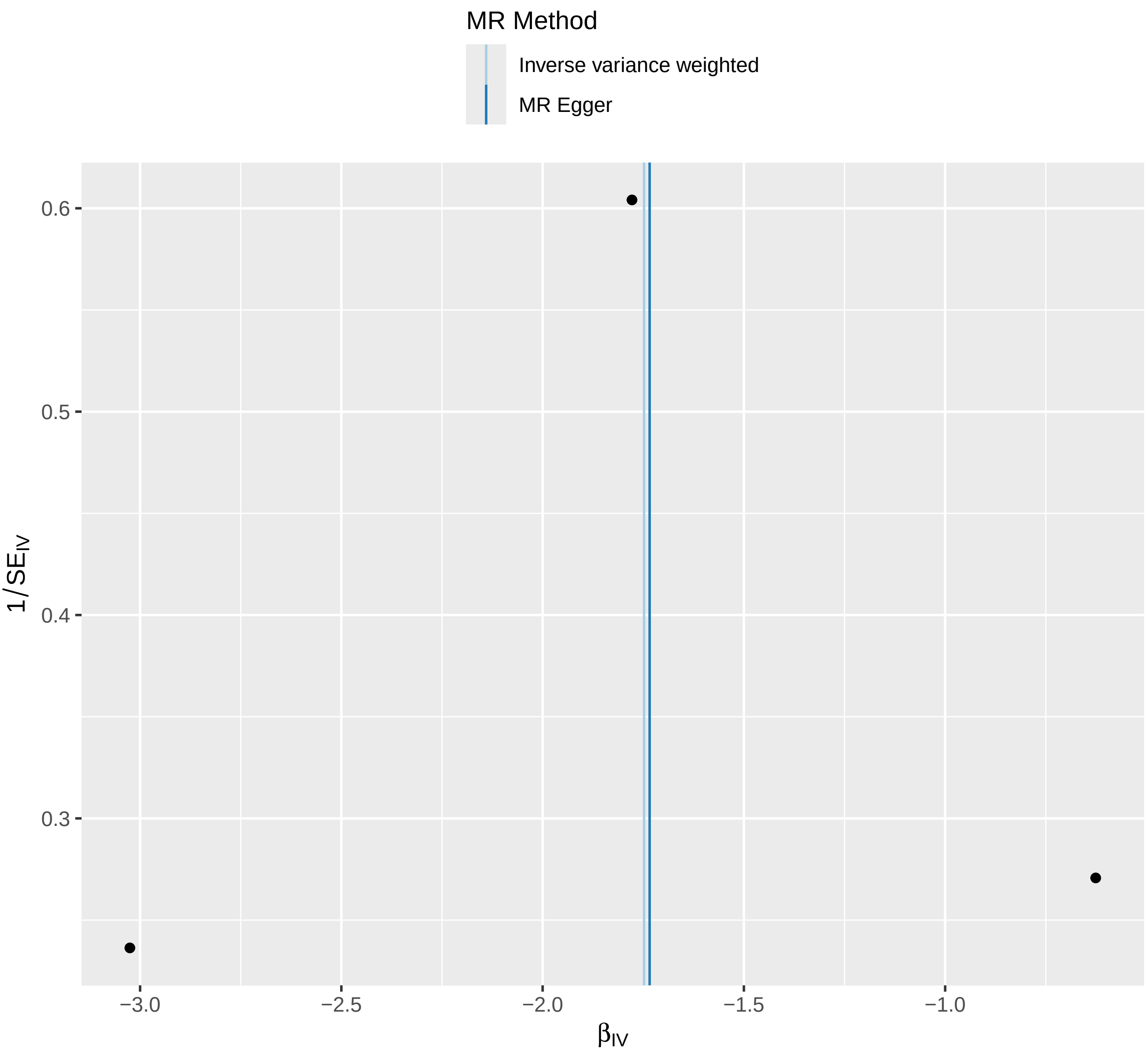
MR Method
Inverse variance weighted
MR Egger
0.6
0.5
0.4
0.3
$1/SE_{IV}$
−3.0
−2.5
−2.0
−1.5
−1.0
β_{IV}

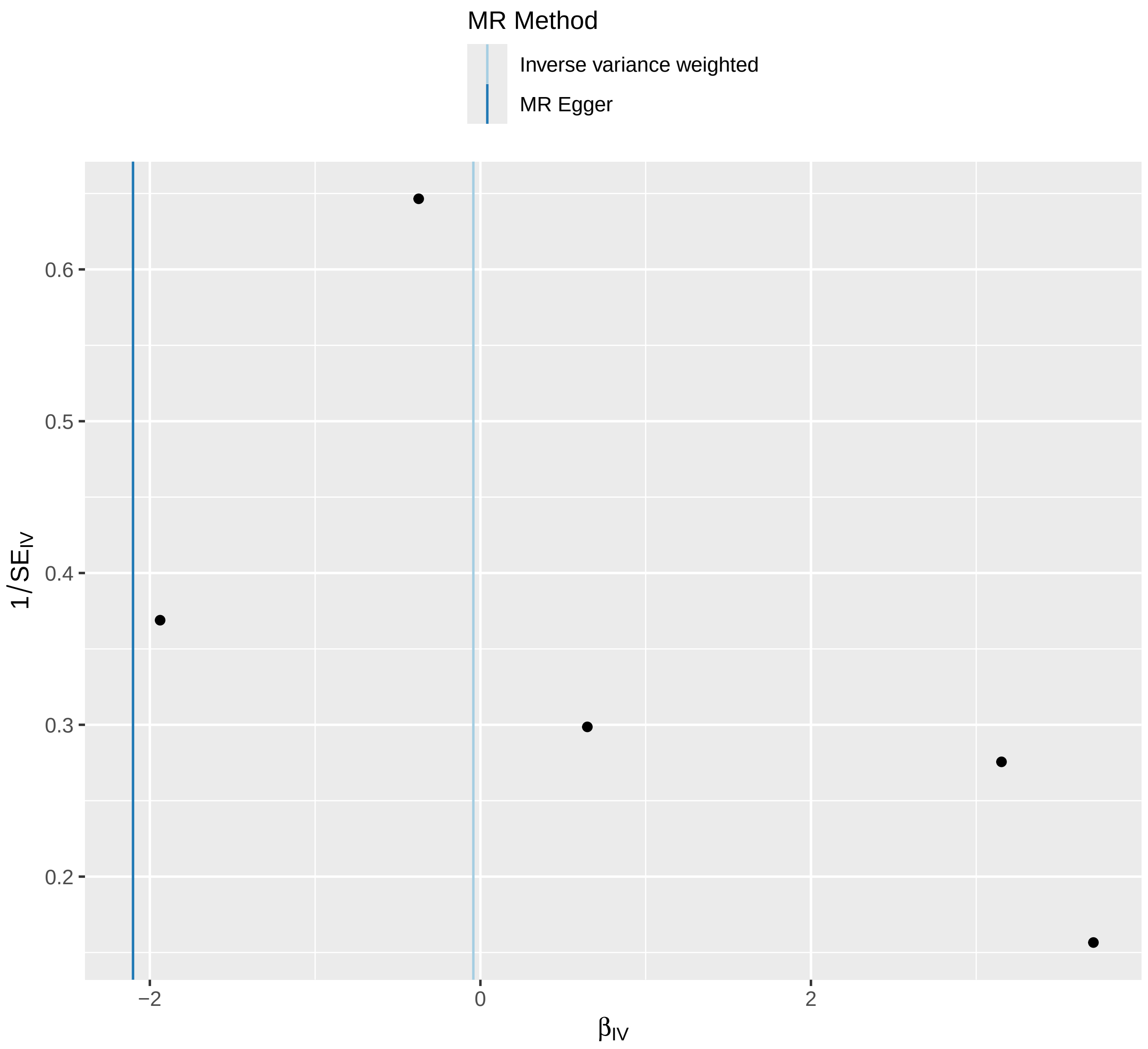
MR Method
Inverse variance weighted
MR Egger
0.6
0.5
0.4
0.3
0.2
1/SE_IV
−2
0
2
β_IV

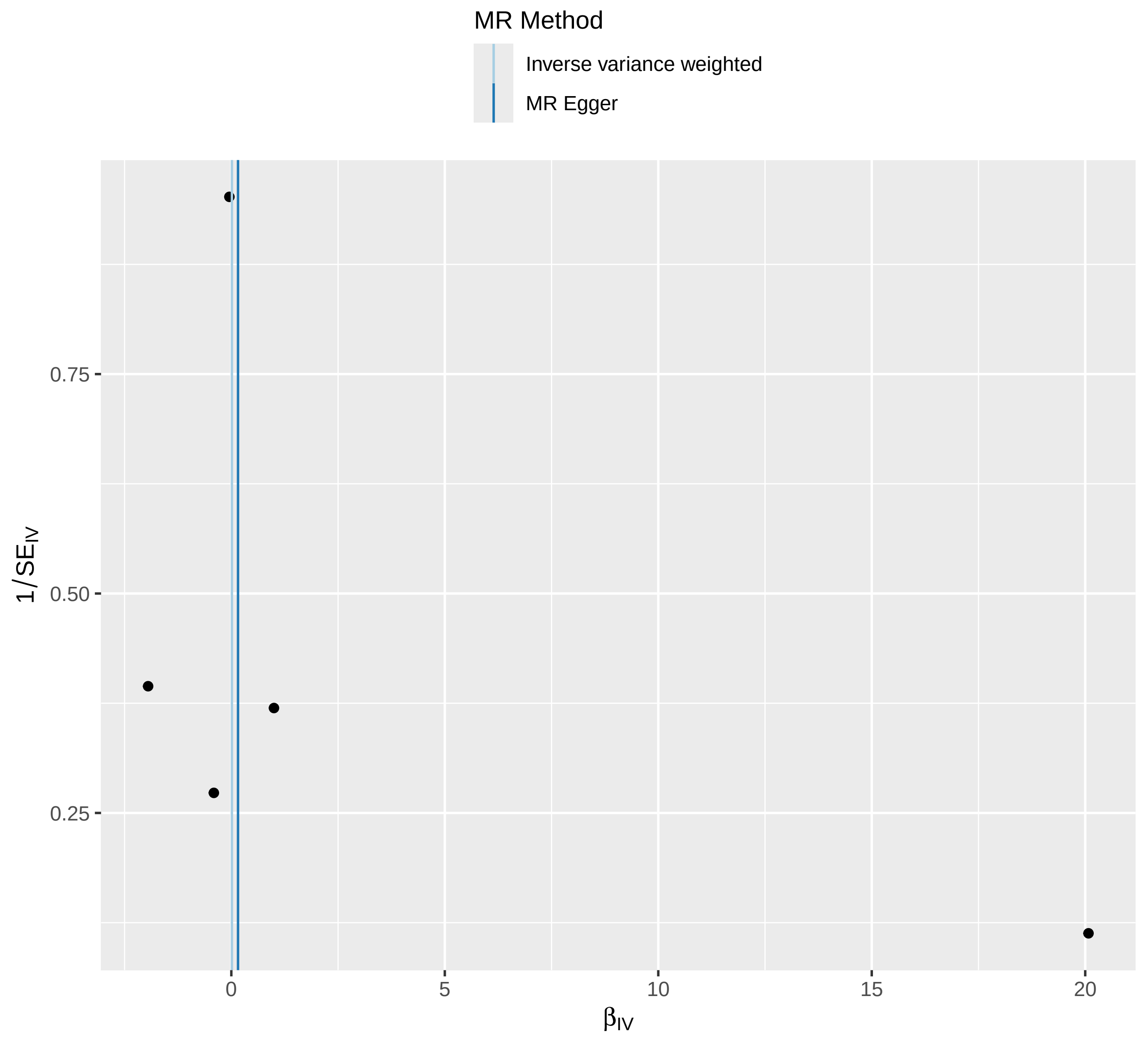

MR Method
Inverse variance weighted
MR Egger
1/SE_IV
0.75
0.50
0.25
0
5
10
15
20
β_IV

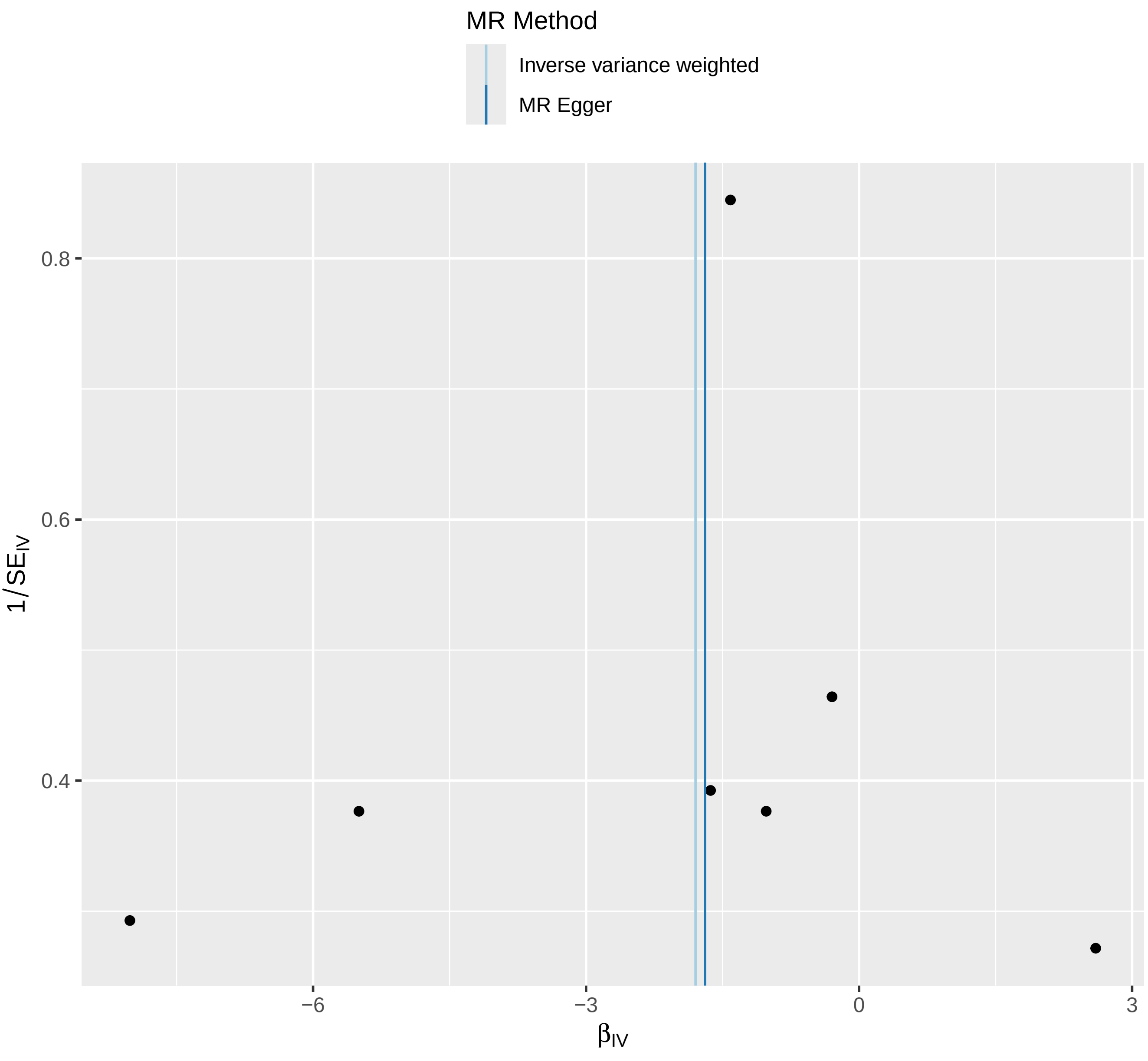

MR Method
Inverse variance weighted
MR Egger
0.8
0.6
0.4
1/SE$_{IV}$
−6
−3
0
3
β$_{IV}$

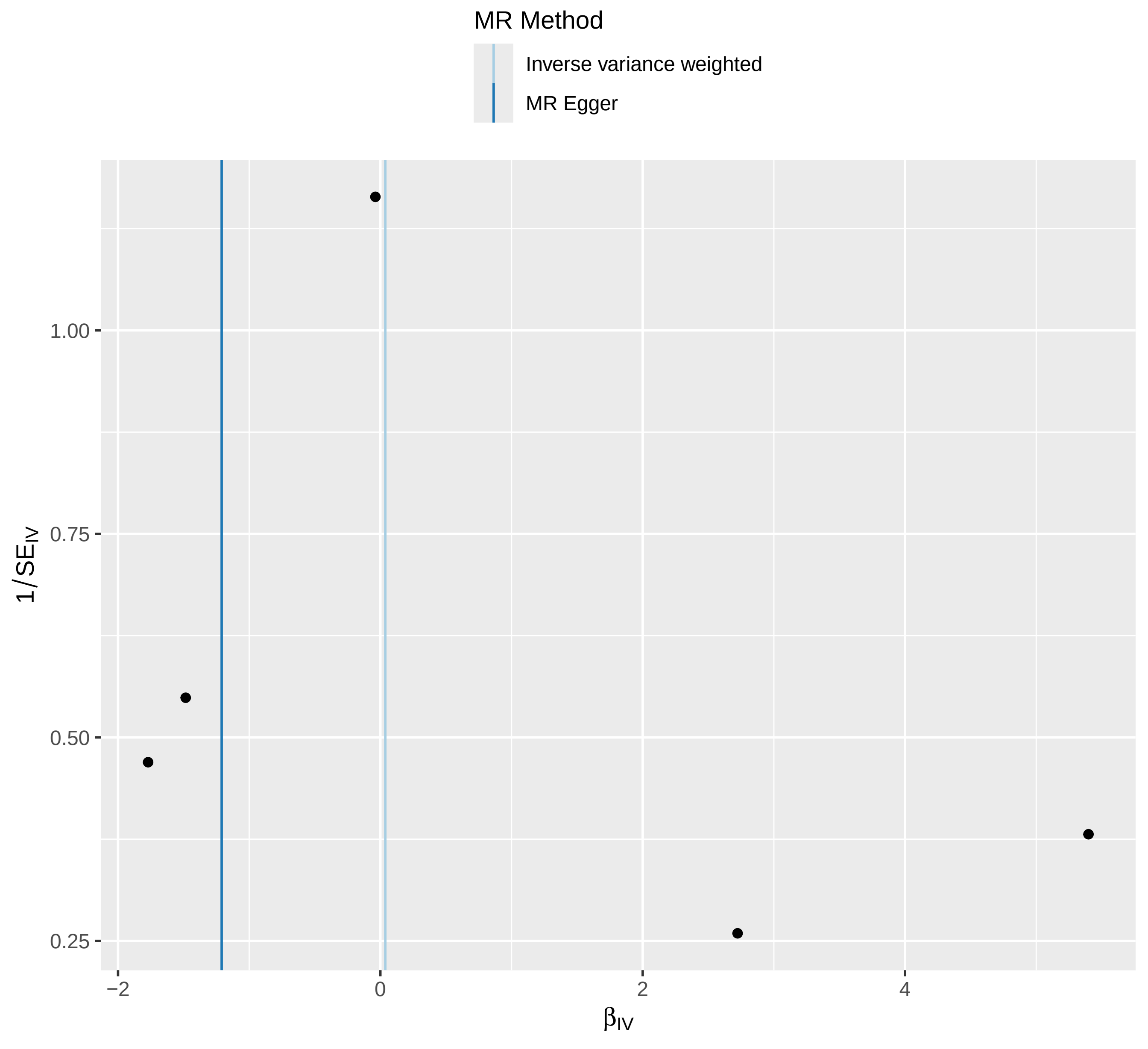
MR Method
Inverse variance weighted
MR Egger
1.00
0.75
0.50
0.25
1/SE_IV
−2
0
2
4
β_IV

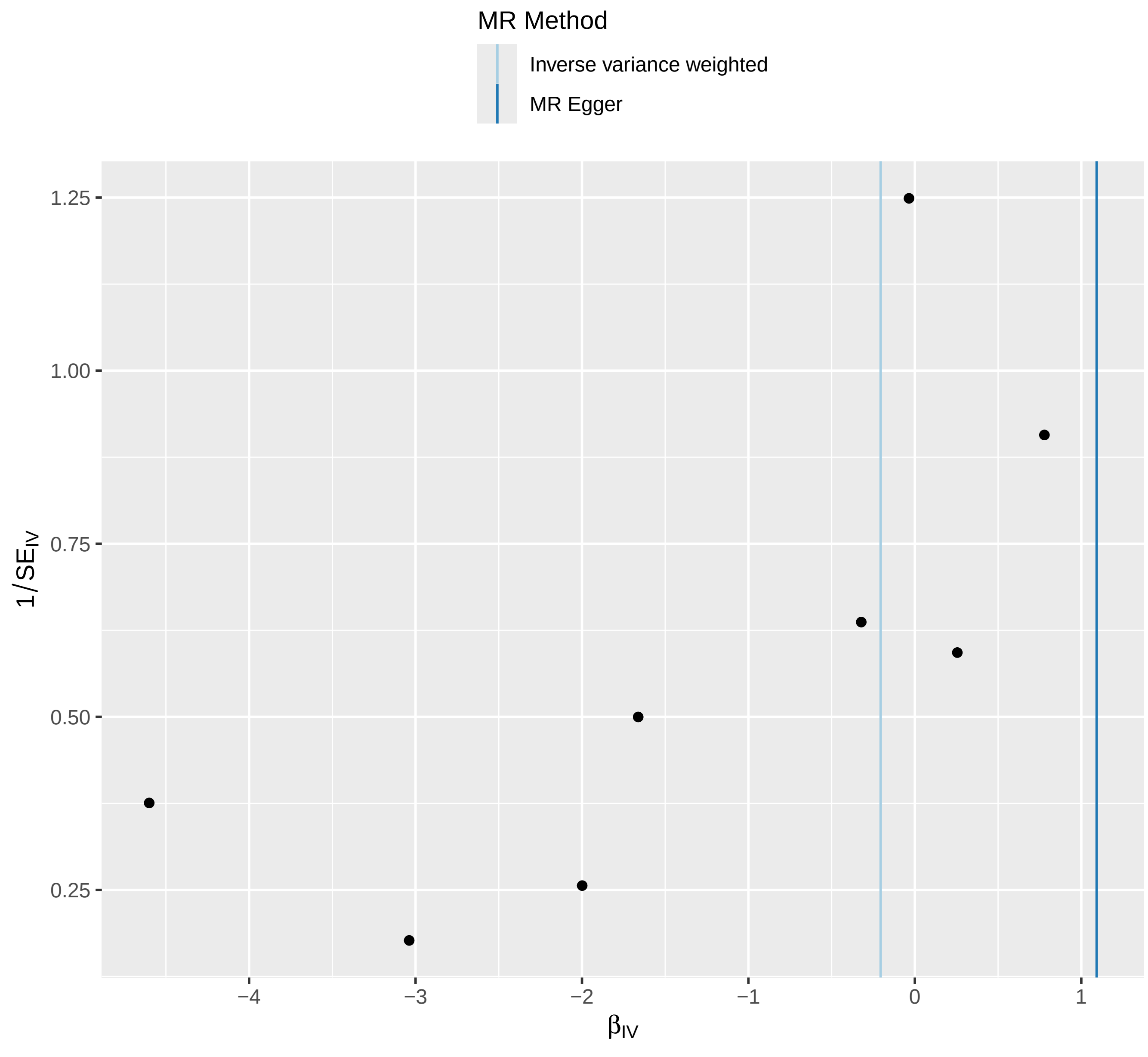

MR Method
Inverse variance weighted
MR Egger
1.25
1.00
0.75
0.50
0.25
$1/SE_{IV}$
−4
−3
−2
−1
0
1
β_{IV}

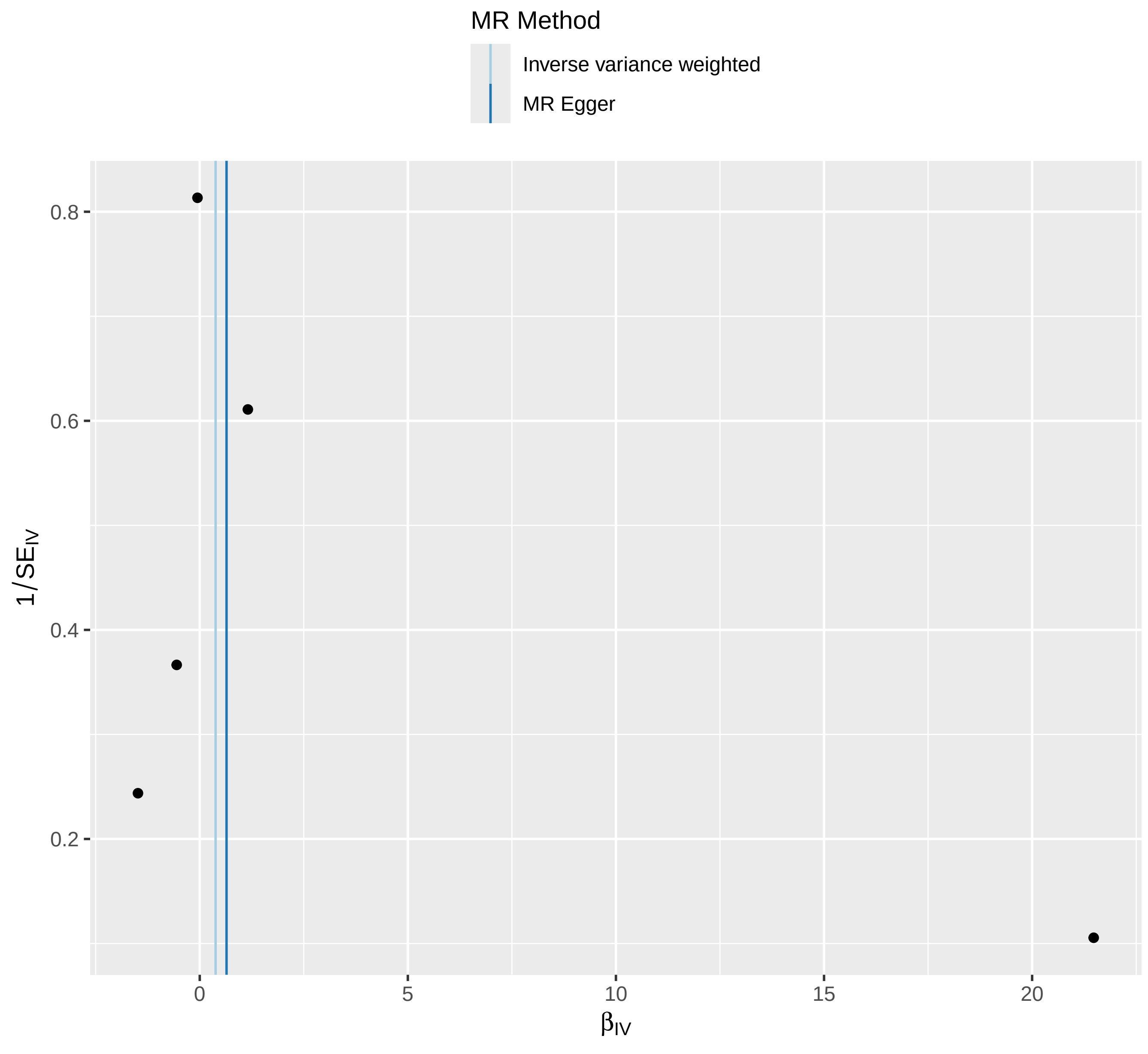
MR Method
Inverse variance weighted
MR Egger
0.8
0.6
0.4
0.2
1/SE$_{IV}$
0
5
10
15
20
β$_{IV}$

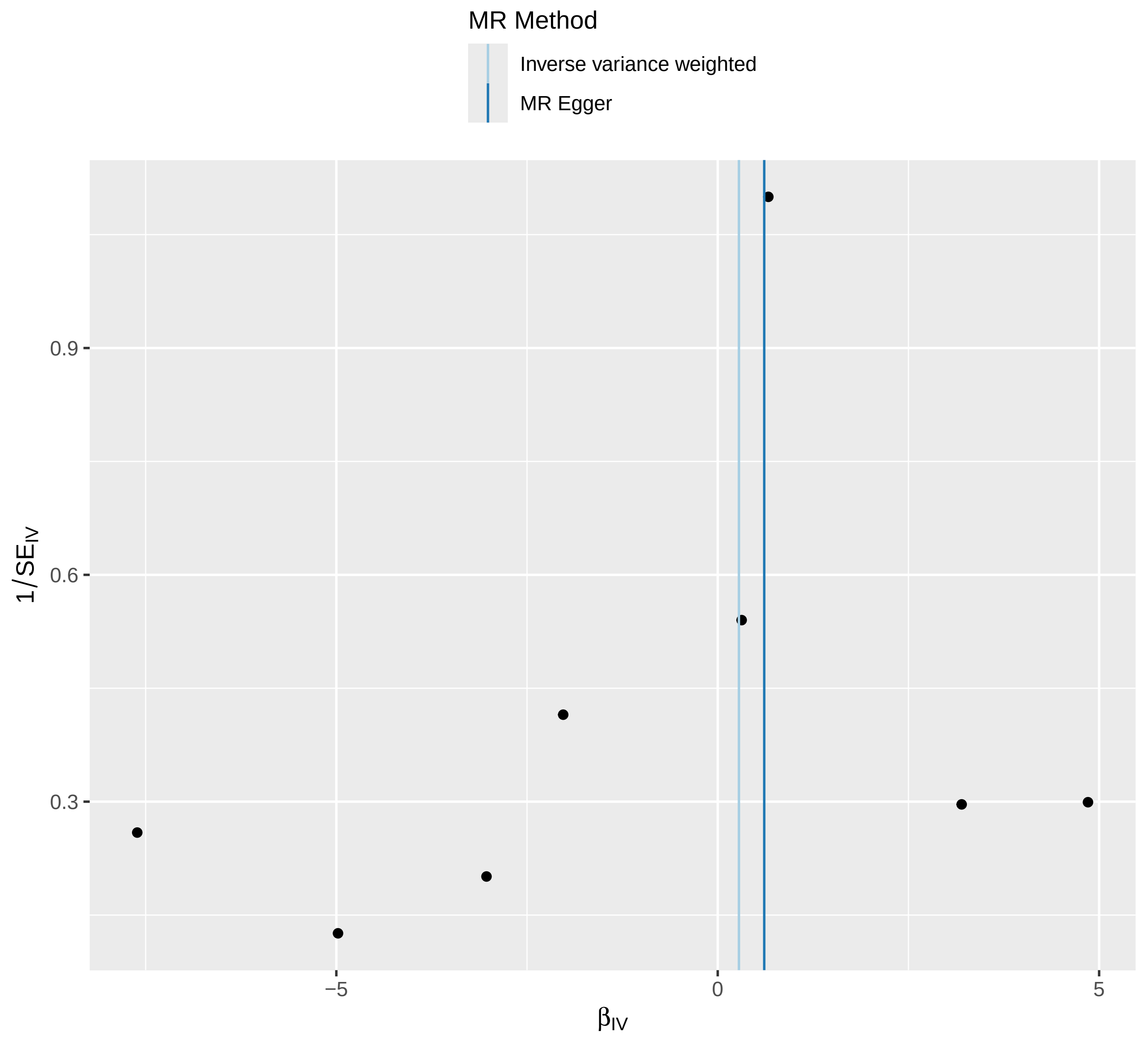
MR Method
Inverse variance weighted
MR Egger
1/SE$_{IV}$
0.9
0.6
0.3
−5
0
5
β$_{IV}$

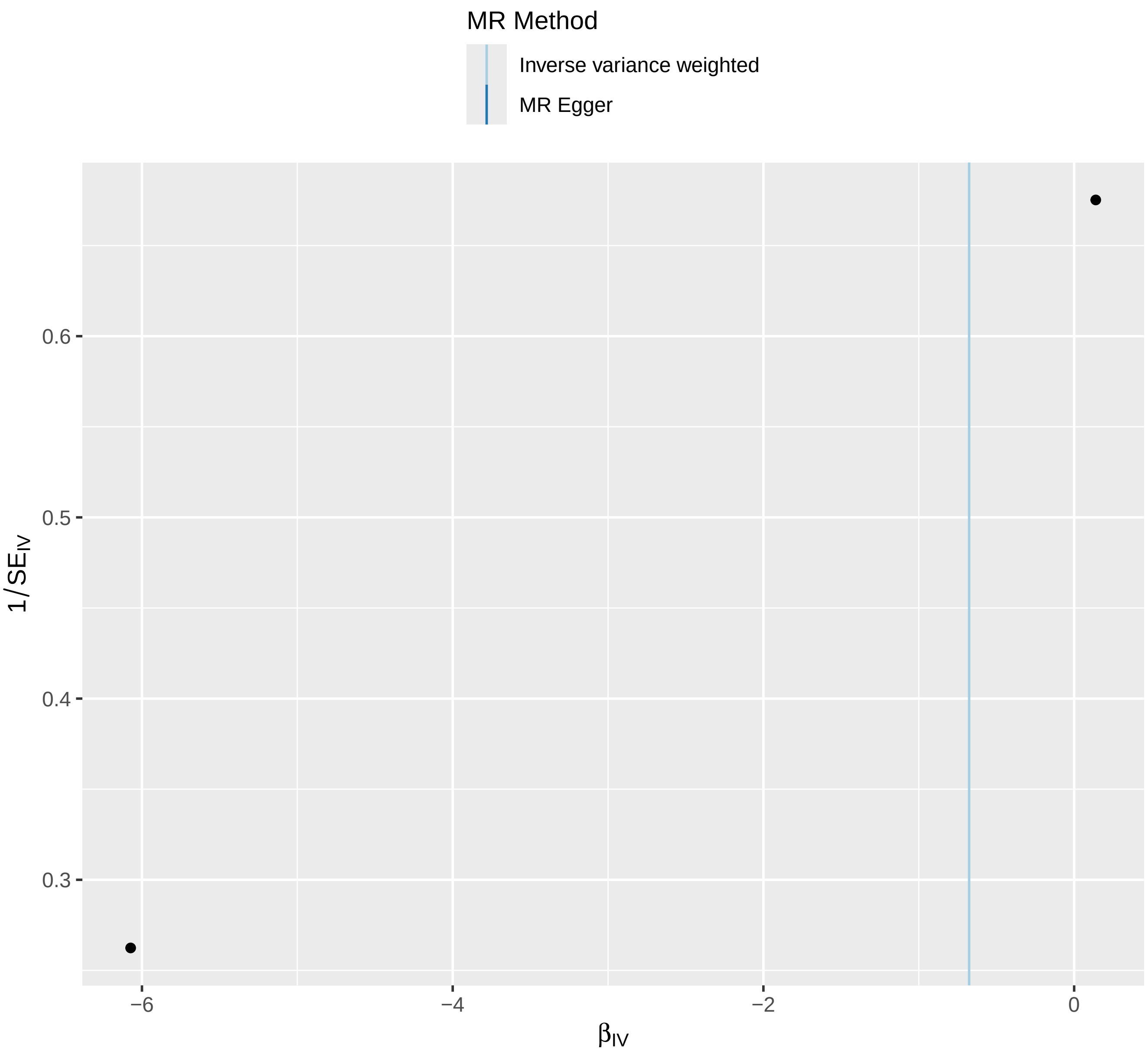
MR Method
Inverse variance weighted
MR Egger
0.6
0.5
0.4
0.3
1/SE_IV
−6
−4
−2
0
β_IV

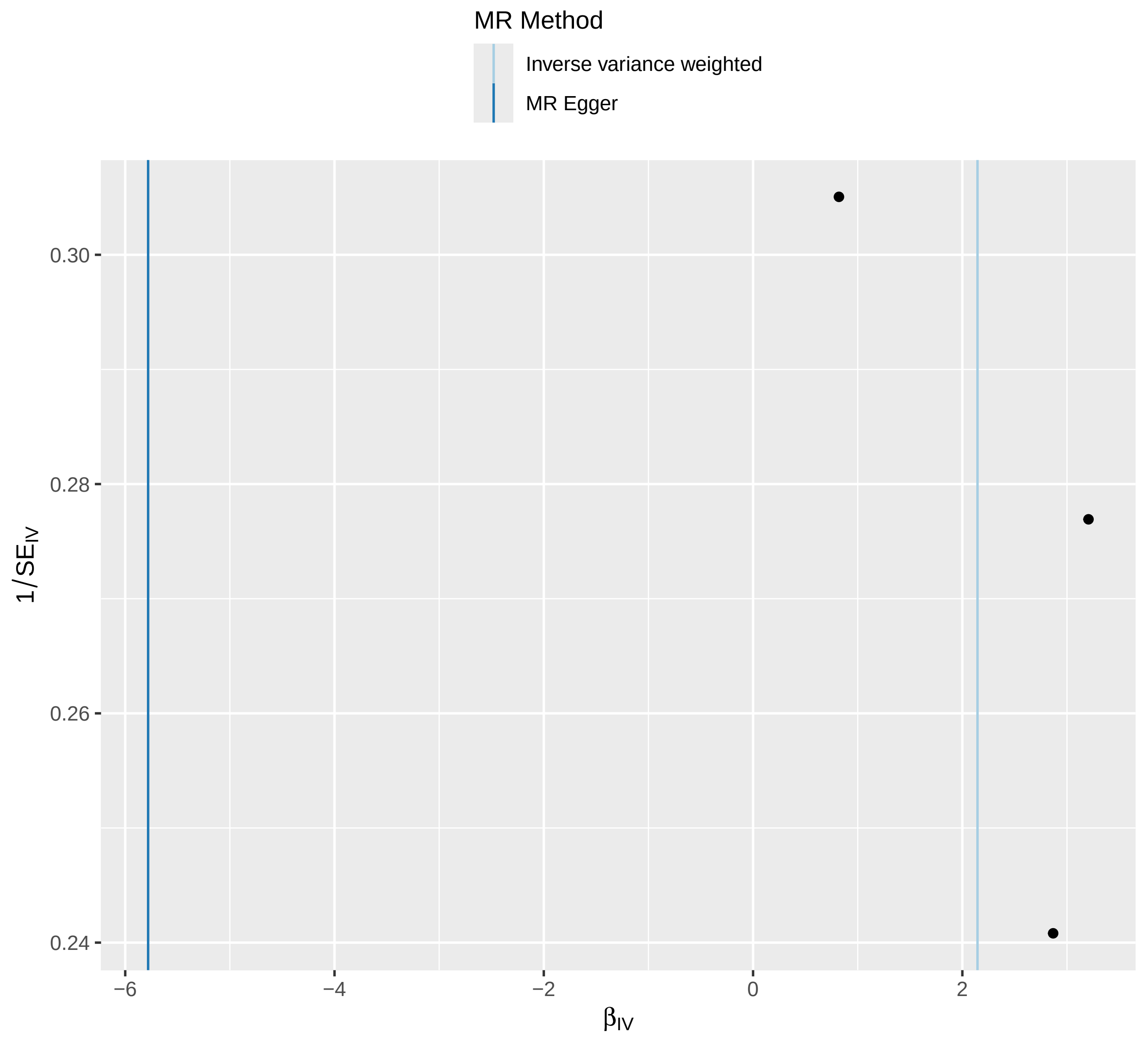
MR Method
Inverse variance weighted
MR Egger
0.30
0.28
0.26
0.24
−6
−4
−2
0
2
$1/SE_{IV}$
β_{IV}

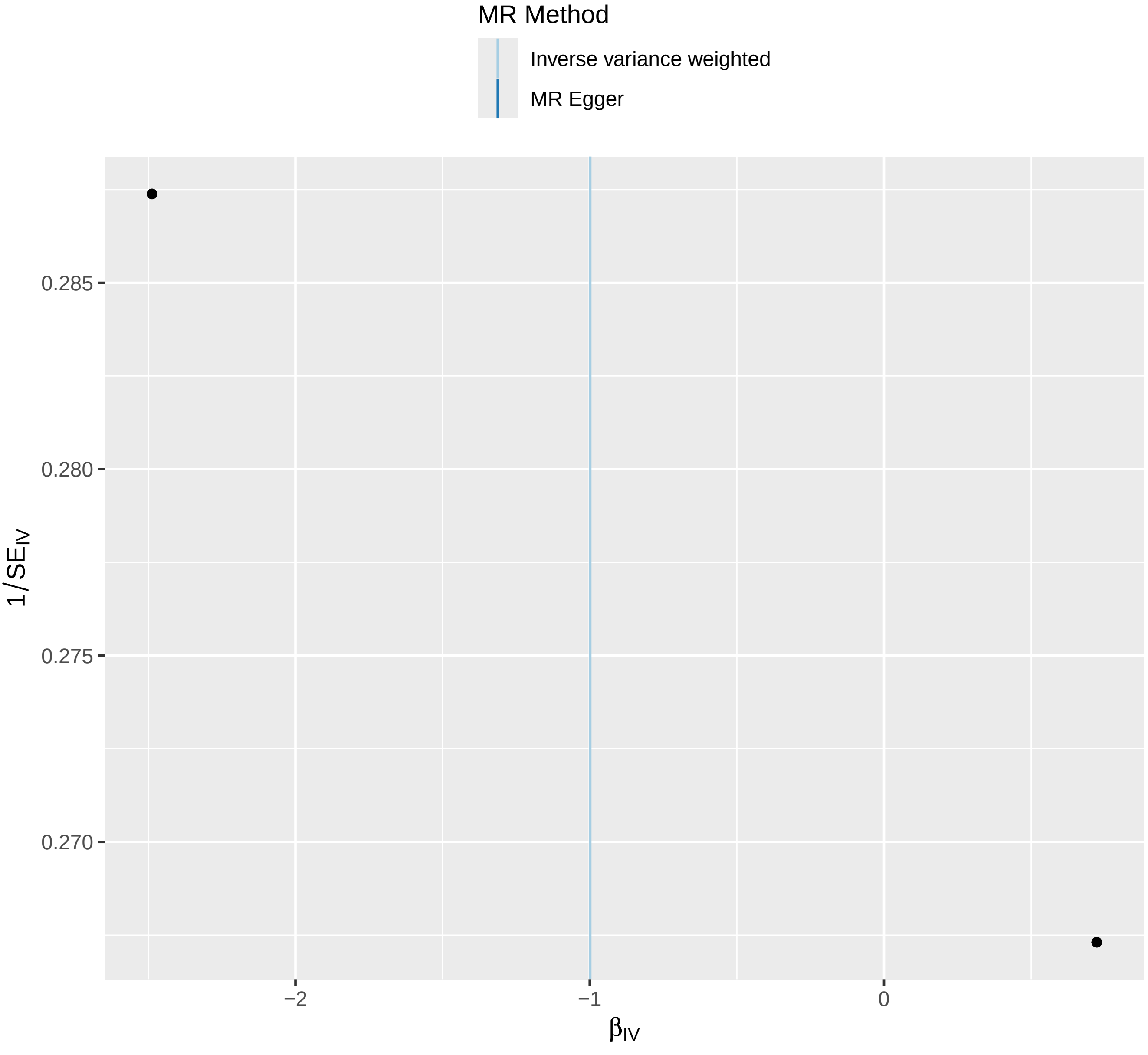
MR Method
Inverse variance weighted
MR Egger
0.285
0.280
0.275
0.270
1/SE_IV
−2
−1
0
β_IV

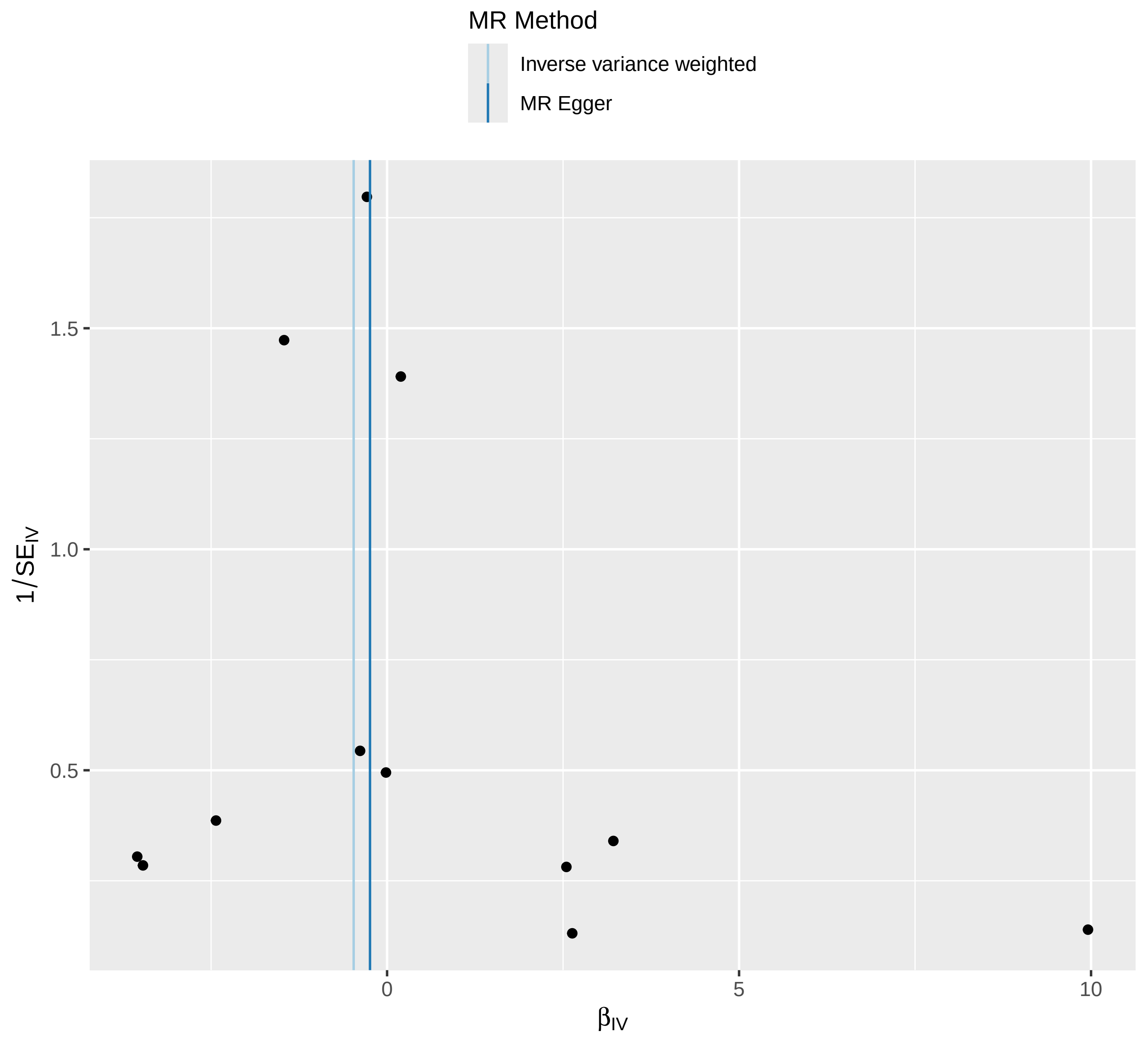
MR Method
Inverse variance weighted
MR Egger
1.5
1.0
0.5
$1/SE_{IV}$
0
5
10
β_{IV}

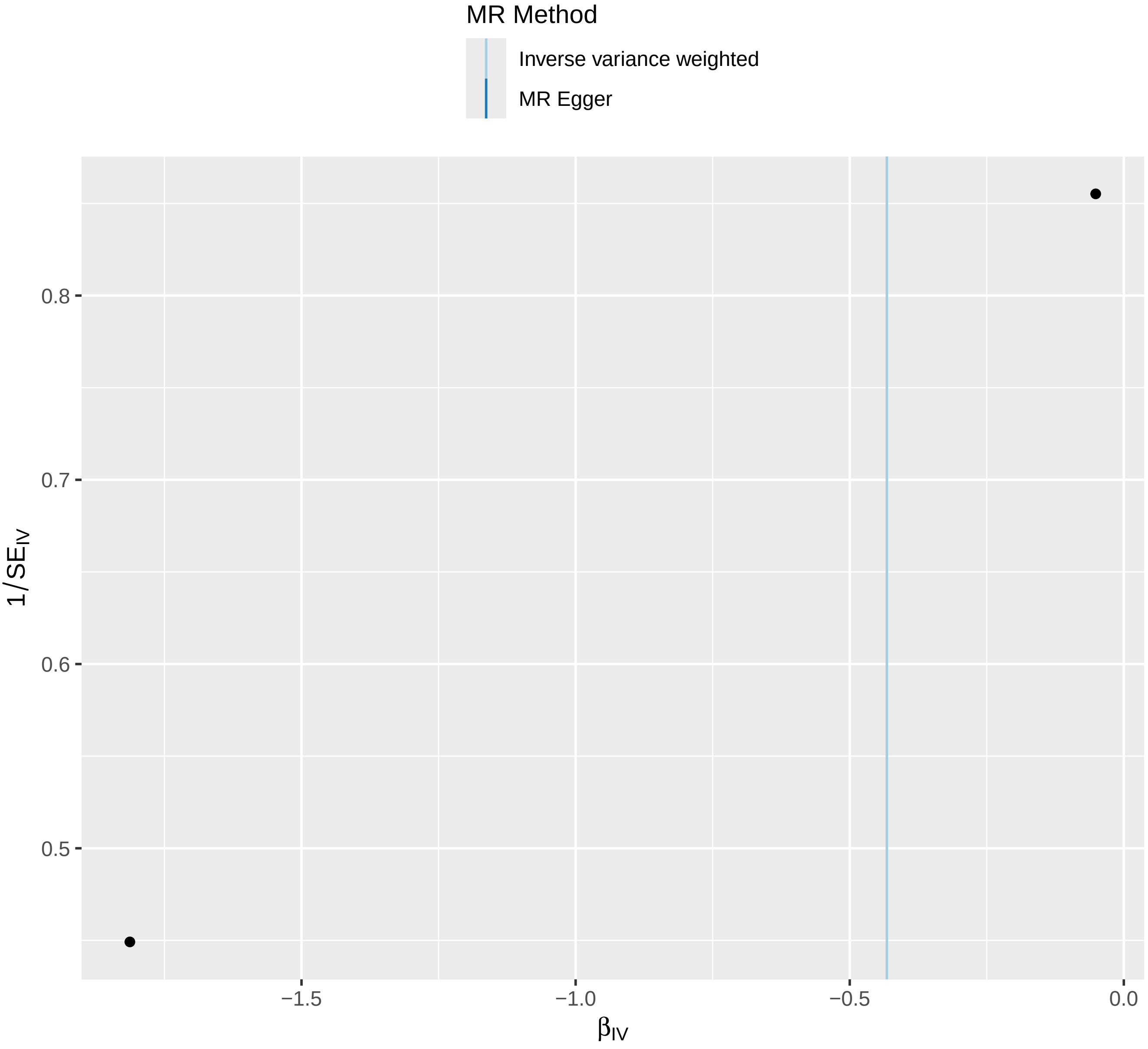
MR Method
Inverse variance weighted
MR Egger
0.8
0.7
0.6
0.5
1/SE_IV
−1.5
−1.0
−0.5
0.0
β_IV

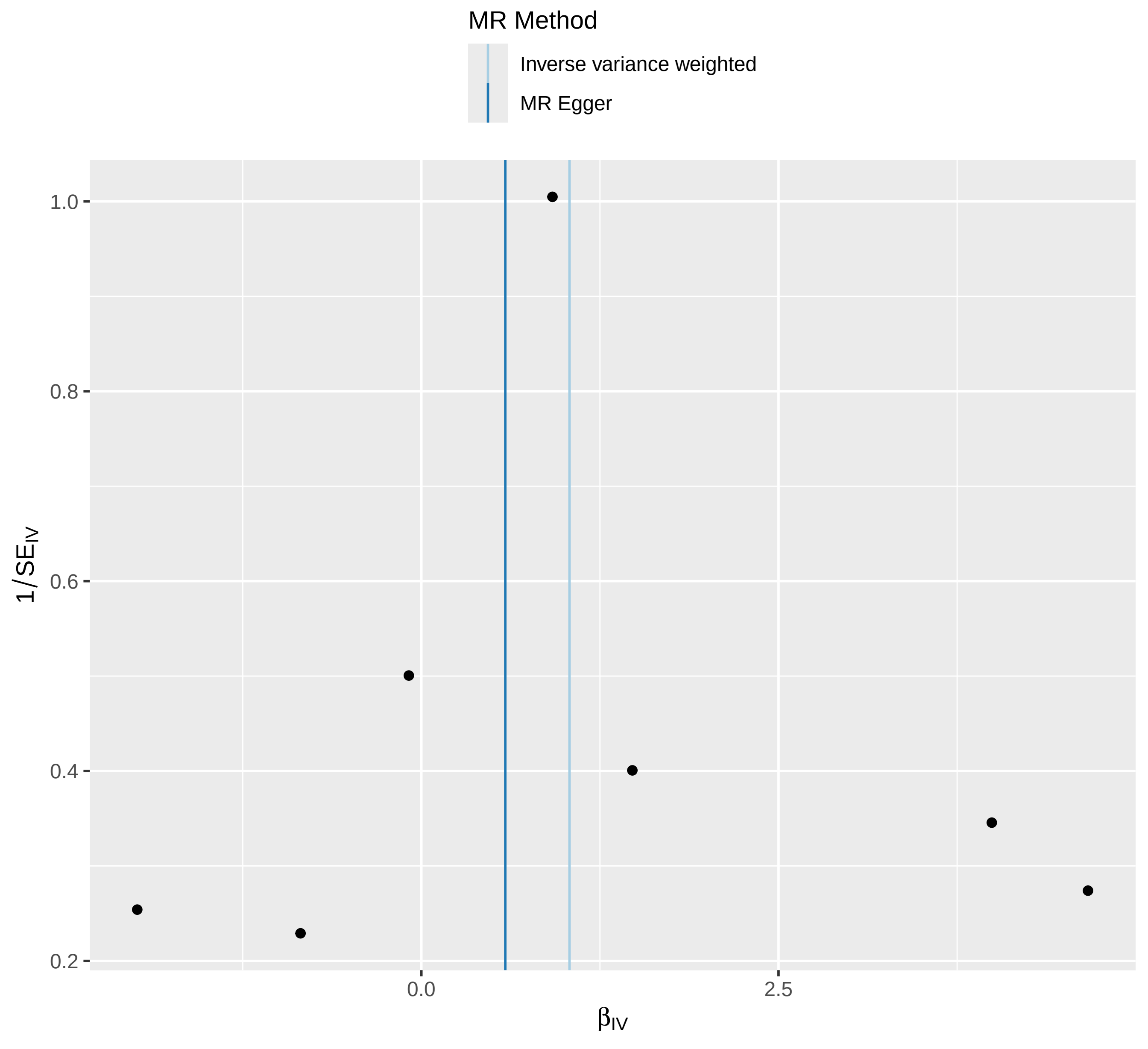
MR Method
Inverse variance weighted
MR Egger
1.0
0.8
0.6
0.4
0.2
1/SE_IV
0.0
2.5
β_IV

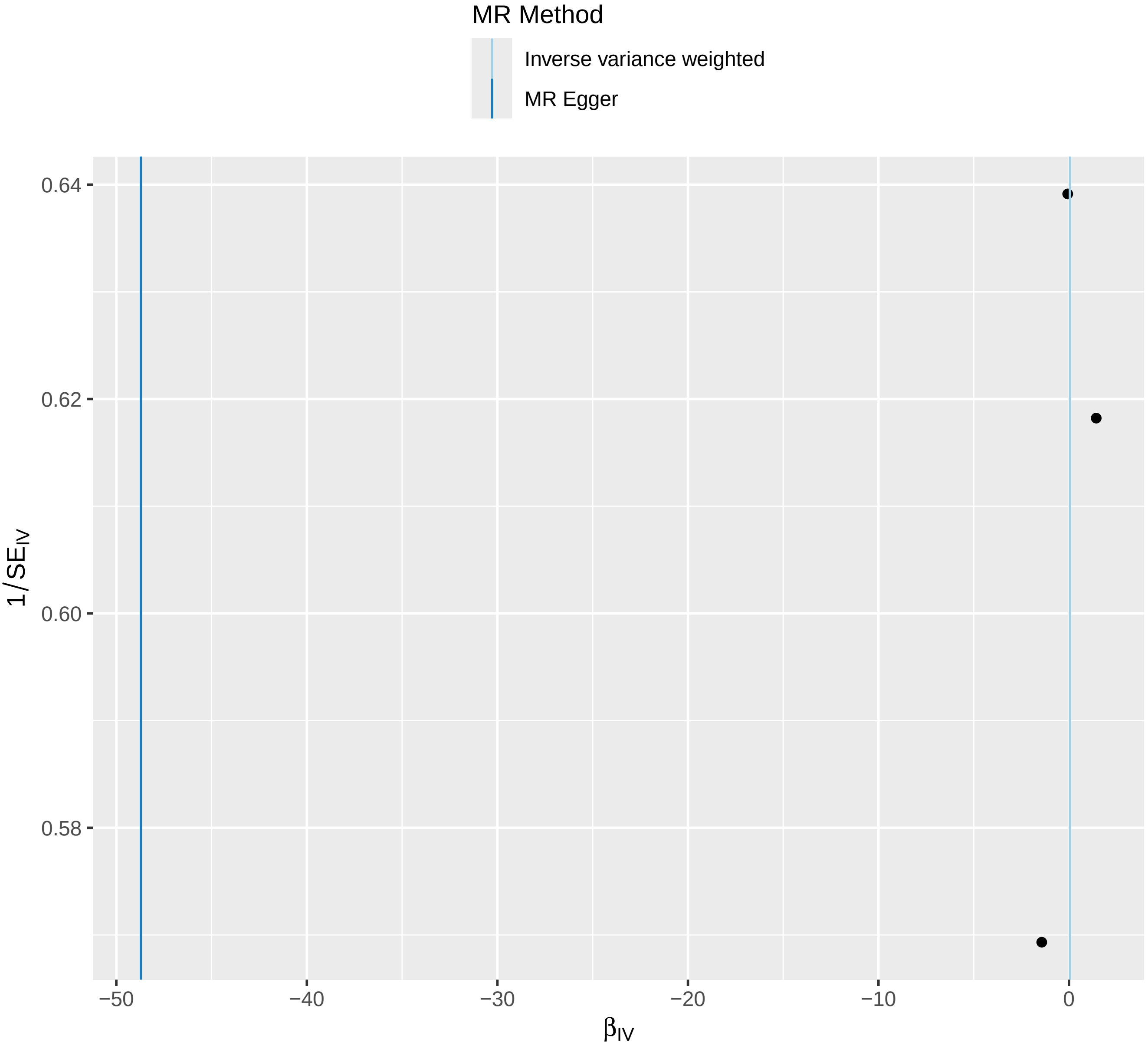
MR Method
Inverse variance weighted
MR Egger
0.64
0.62
0.60
0.58
$1/SE_{IV}$
−50
−40
−30
−20
−10
0
β_{IV}

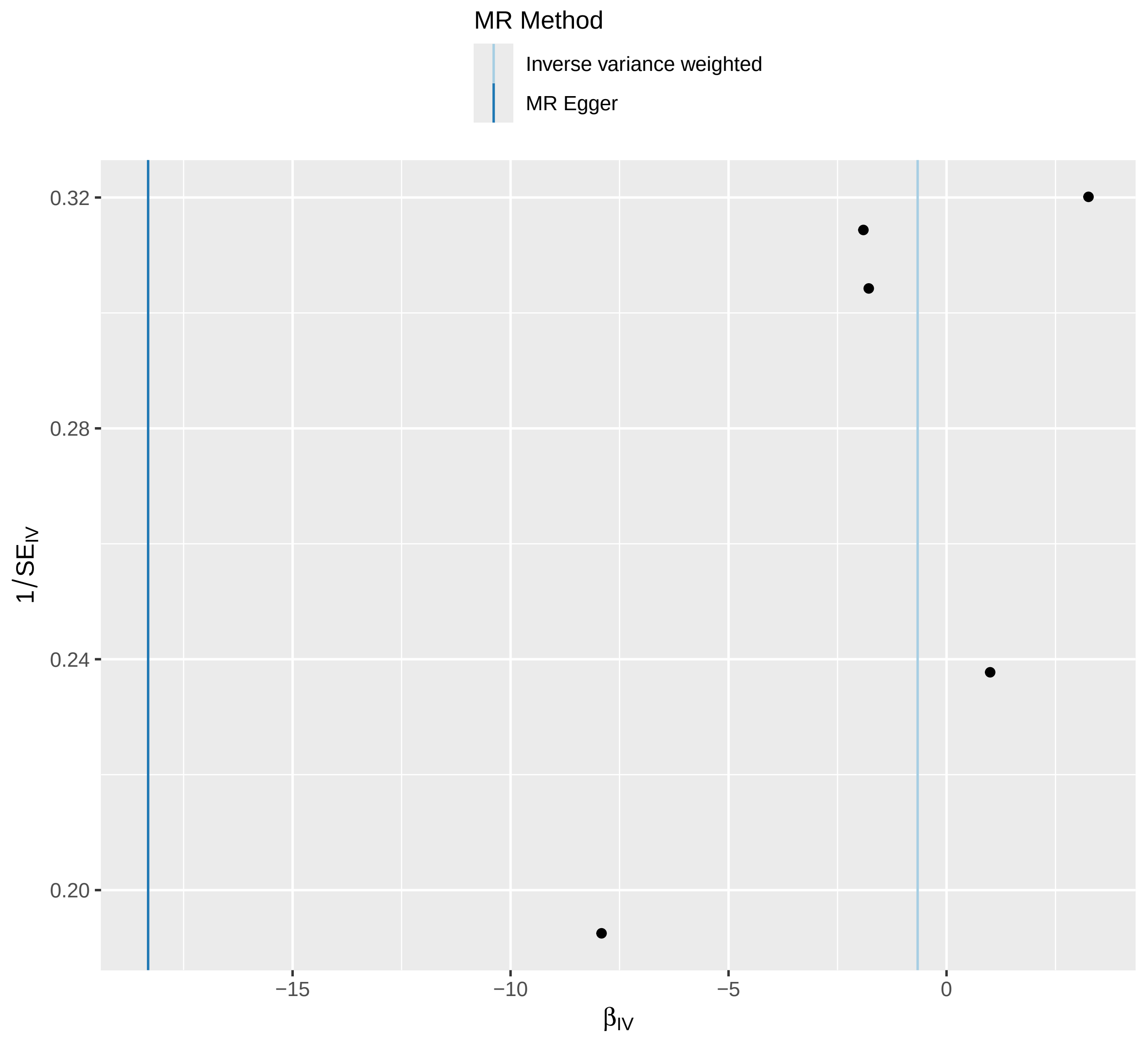
MR Method
Inverse variance weighted
MR Egger
0.32
0.28
0.24
0.20
1/SE$_{IV}$
−15
−10
−5
0
β$_{IV}$

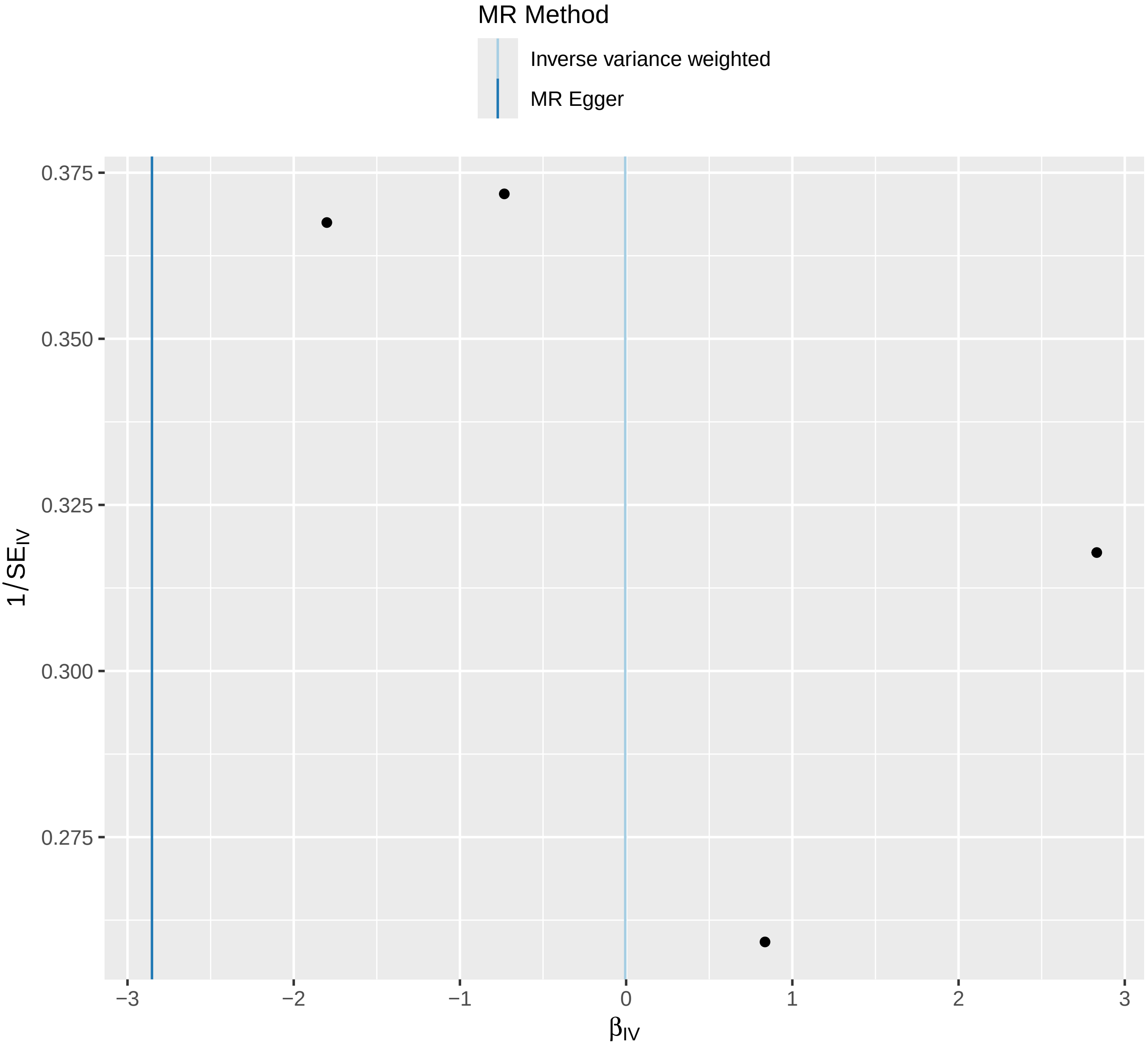
MR Method
Inverse variance weighted
MR Egger
0.375
0.350
0.325
0.300
0.275
1/SE_IV
−3
−2
−1
0
1
2
3
β_IV

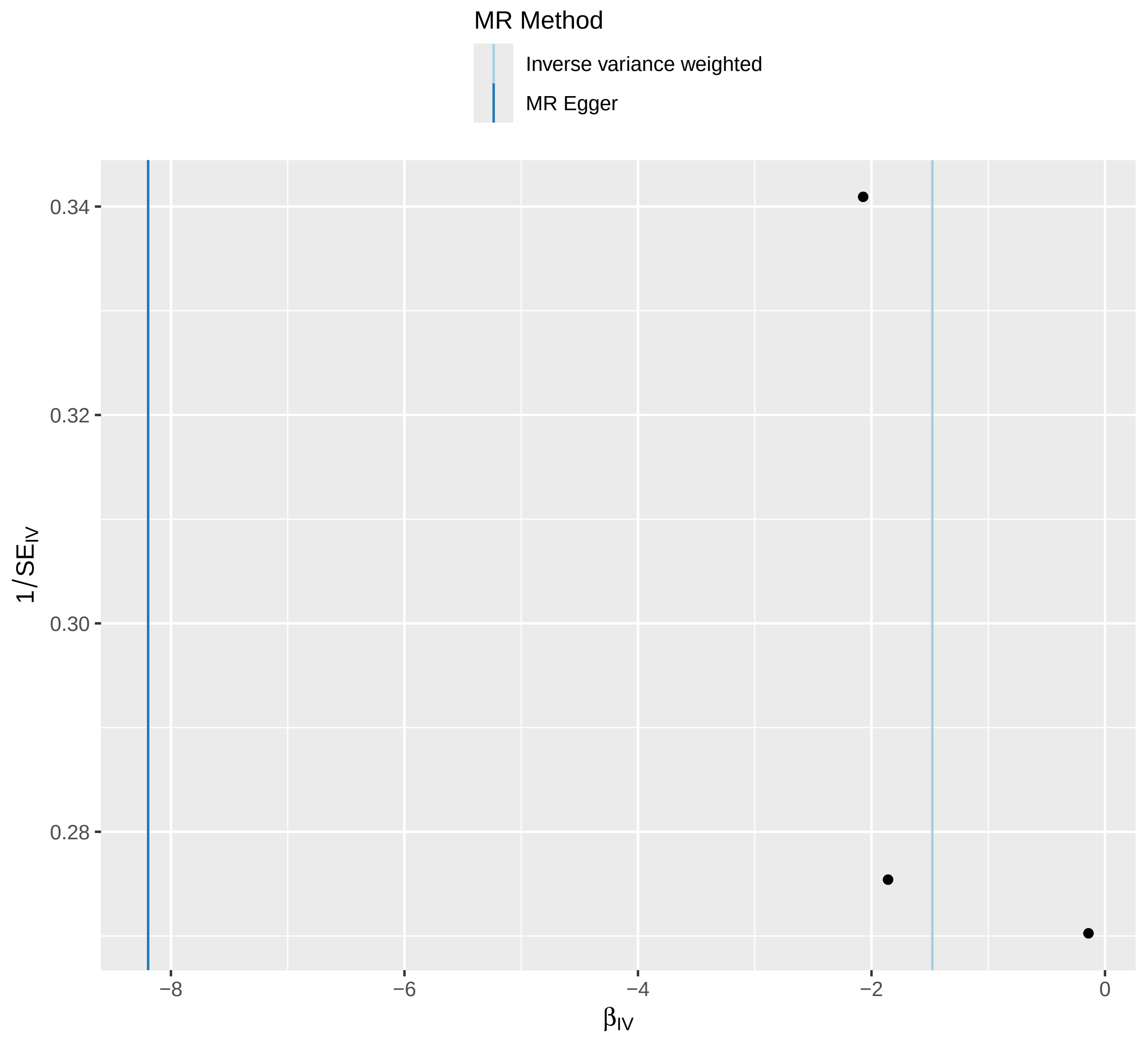
MR Method
Inverse variance weighted
MR Egger
0.34
0.32
0.30
0.28
$1/SE_{IV}$
−8
−6
−4
−2
0
β_{IV}

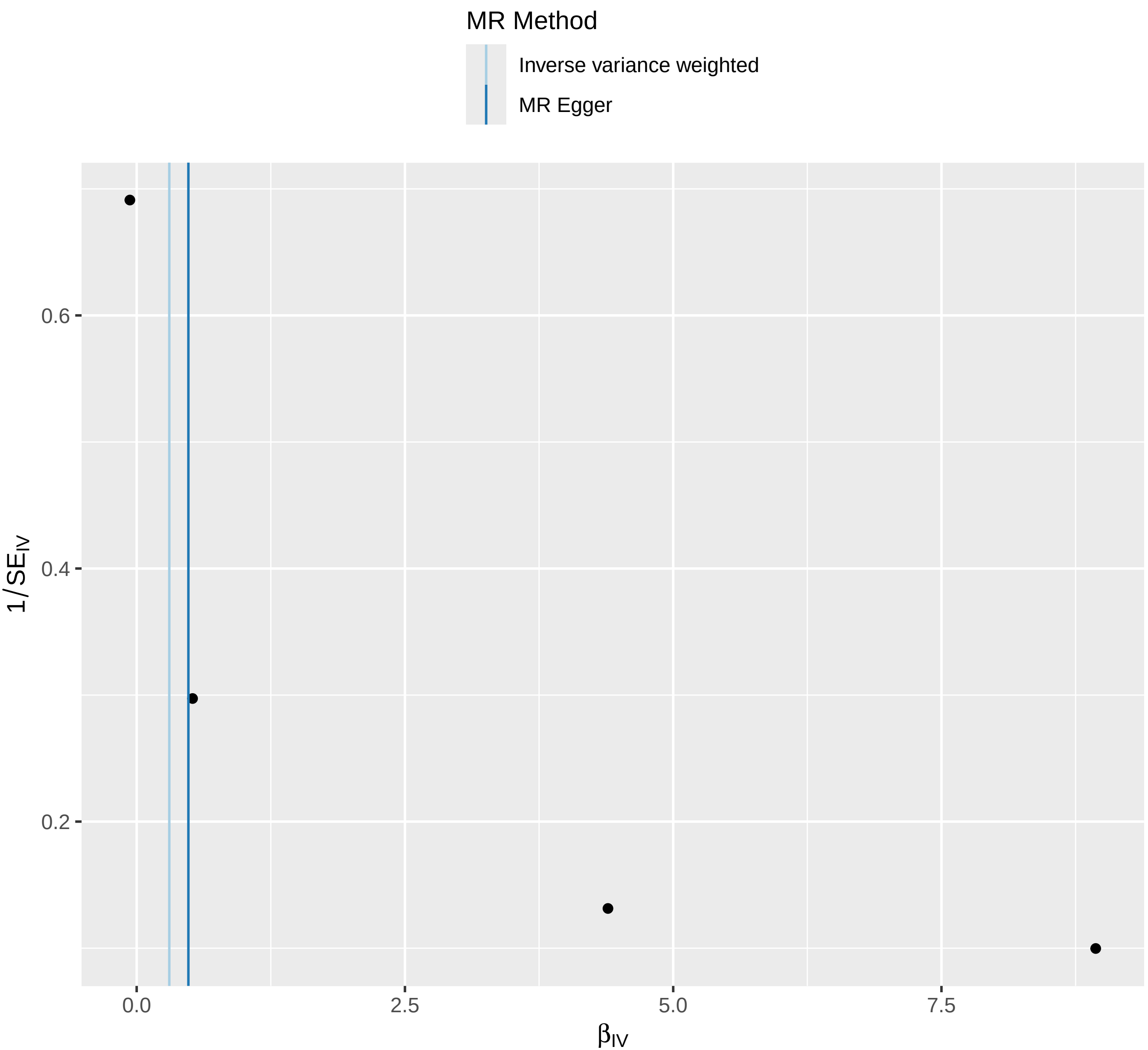

MR Method
Inverse variance weighted
MR Egger
0.6
0.4
0.2
1/SE_{IV}
0.0
2.5
5.0
7.5
β_{IV}

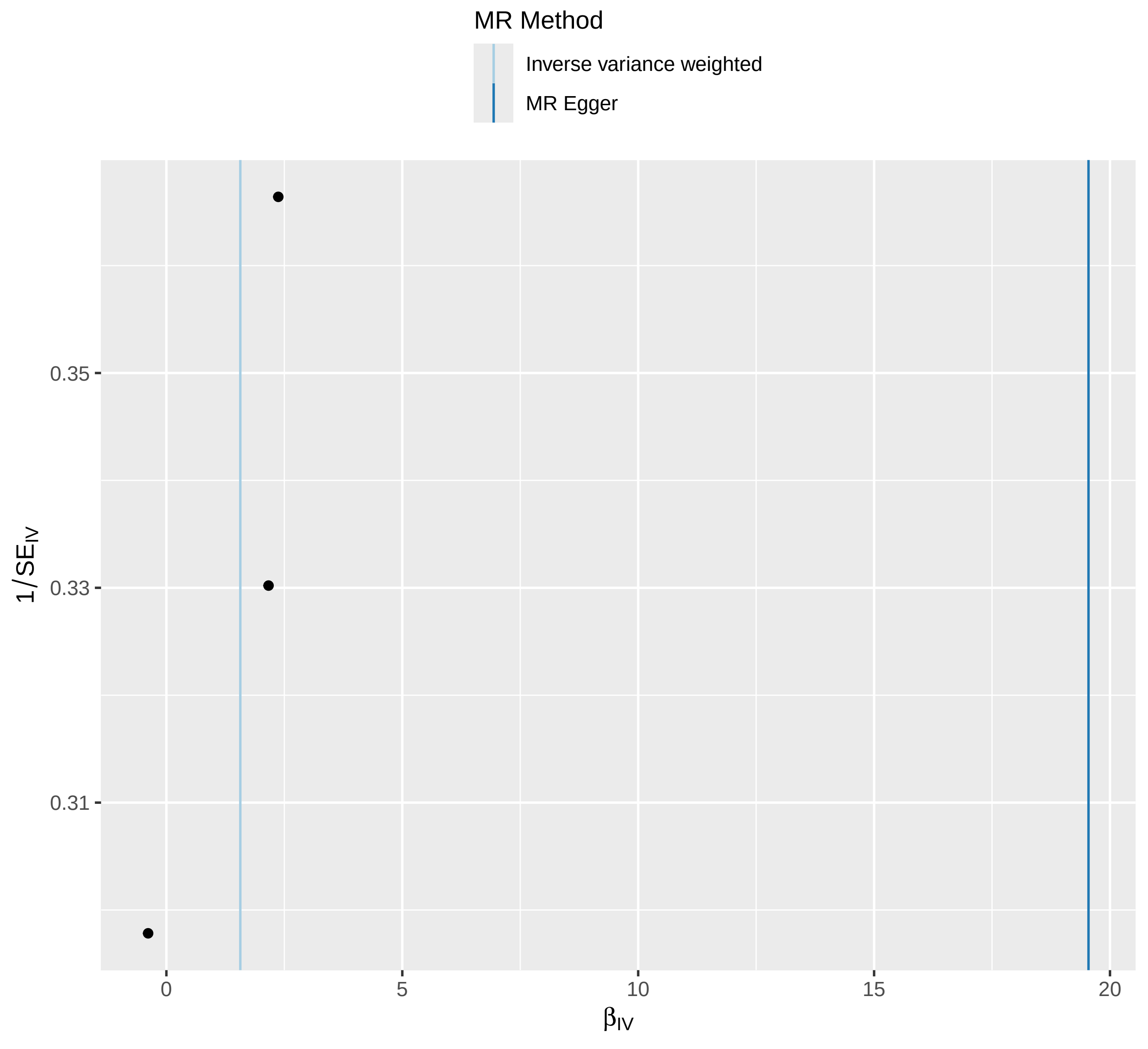
MR Method
Inverse variance weighted
MR Egger
0.35
0.33
0.31
1/SE_IV
0
5
10
15
20
β_IV

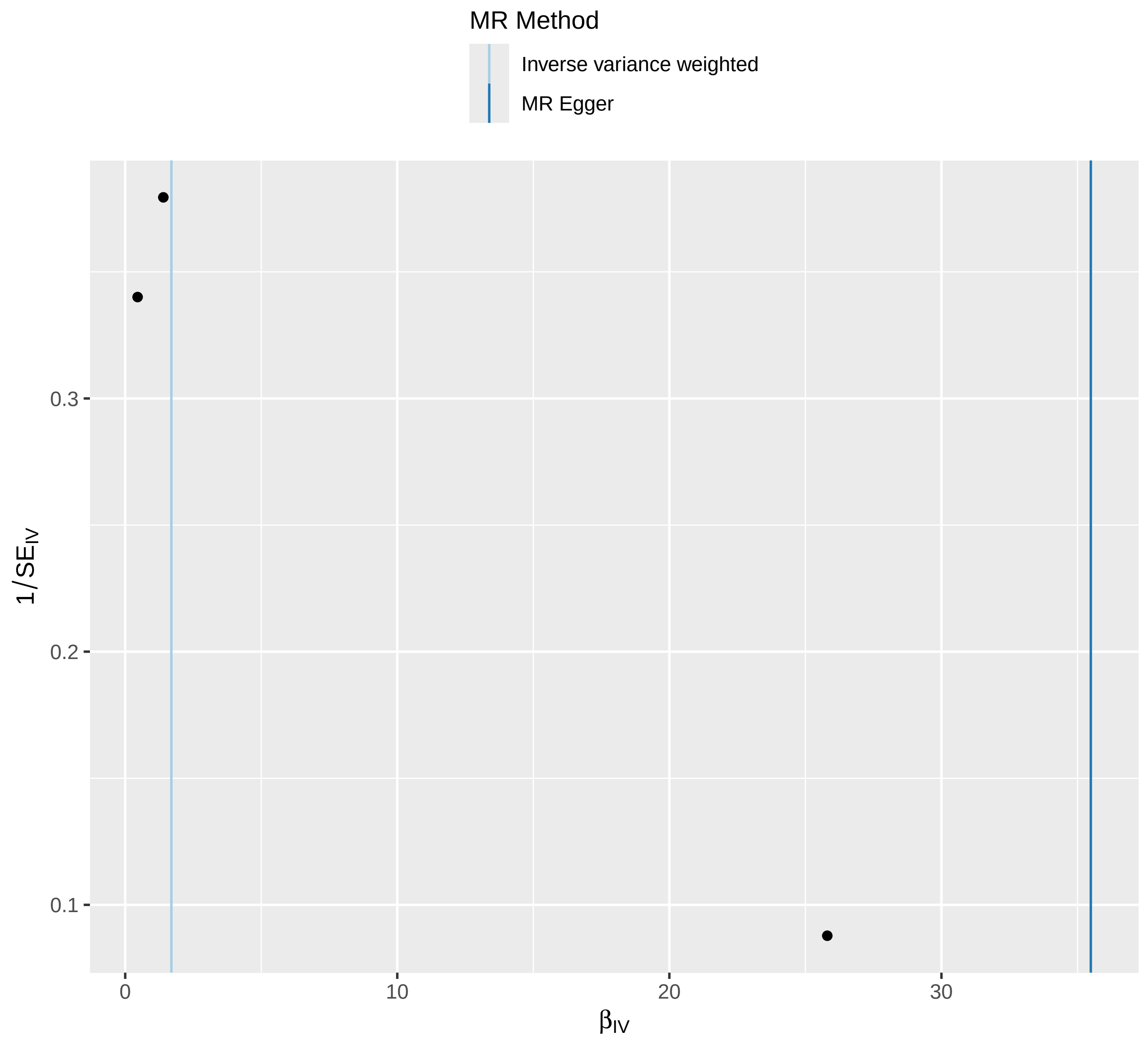

MR Method
Inverse variance weighted
MR Egger
0.3
0.2
0.1
$1/SE_{IV}$
0
10
20
30
β_{IV}

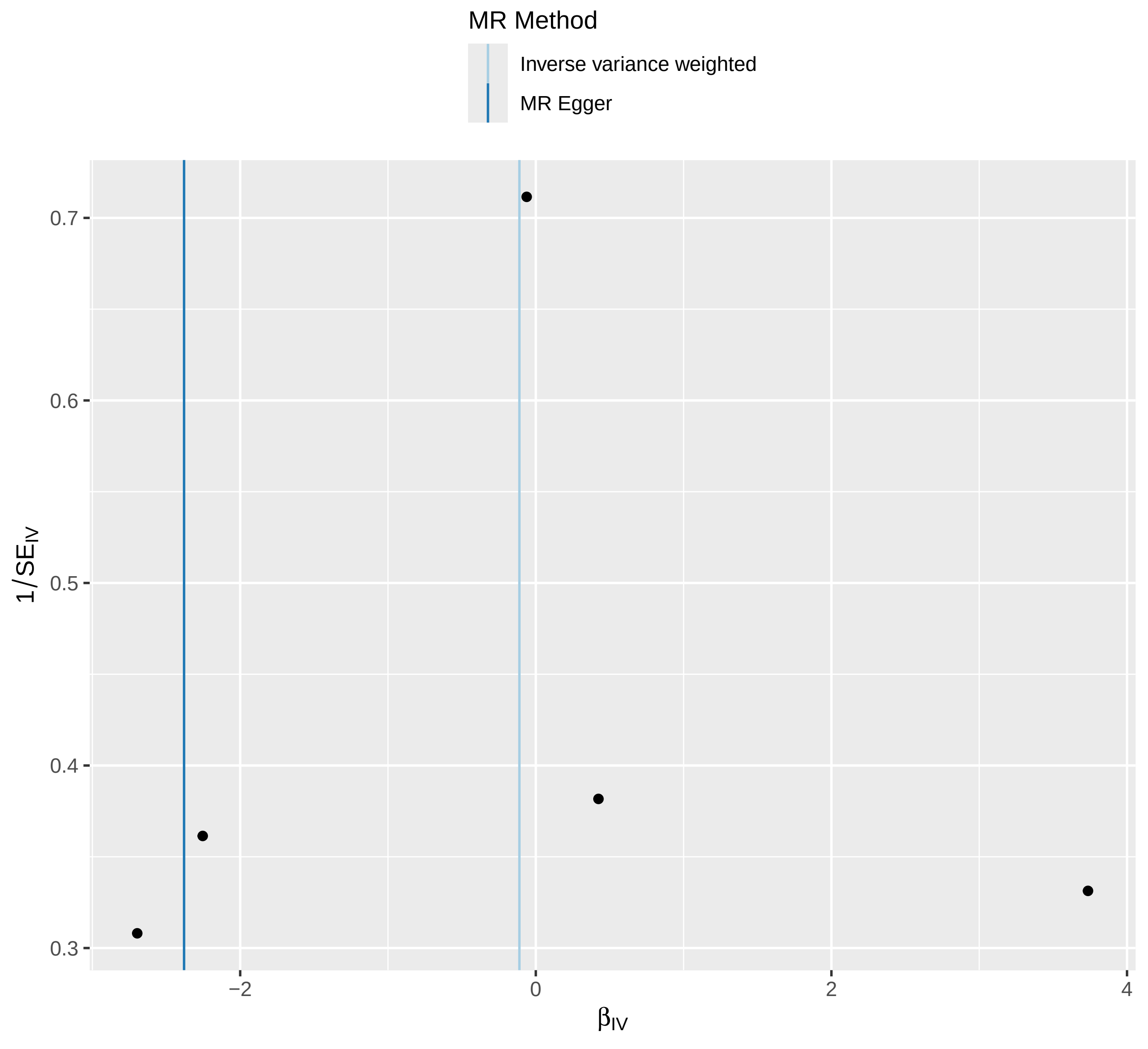
MR Method
Inverse variance weighted
MR Egger
0.7
0.6
0.5
0.4
0.3
1/SE$_{IV}$
−2
0
2
4
β$_{IV}$

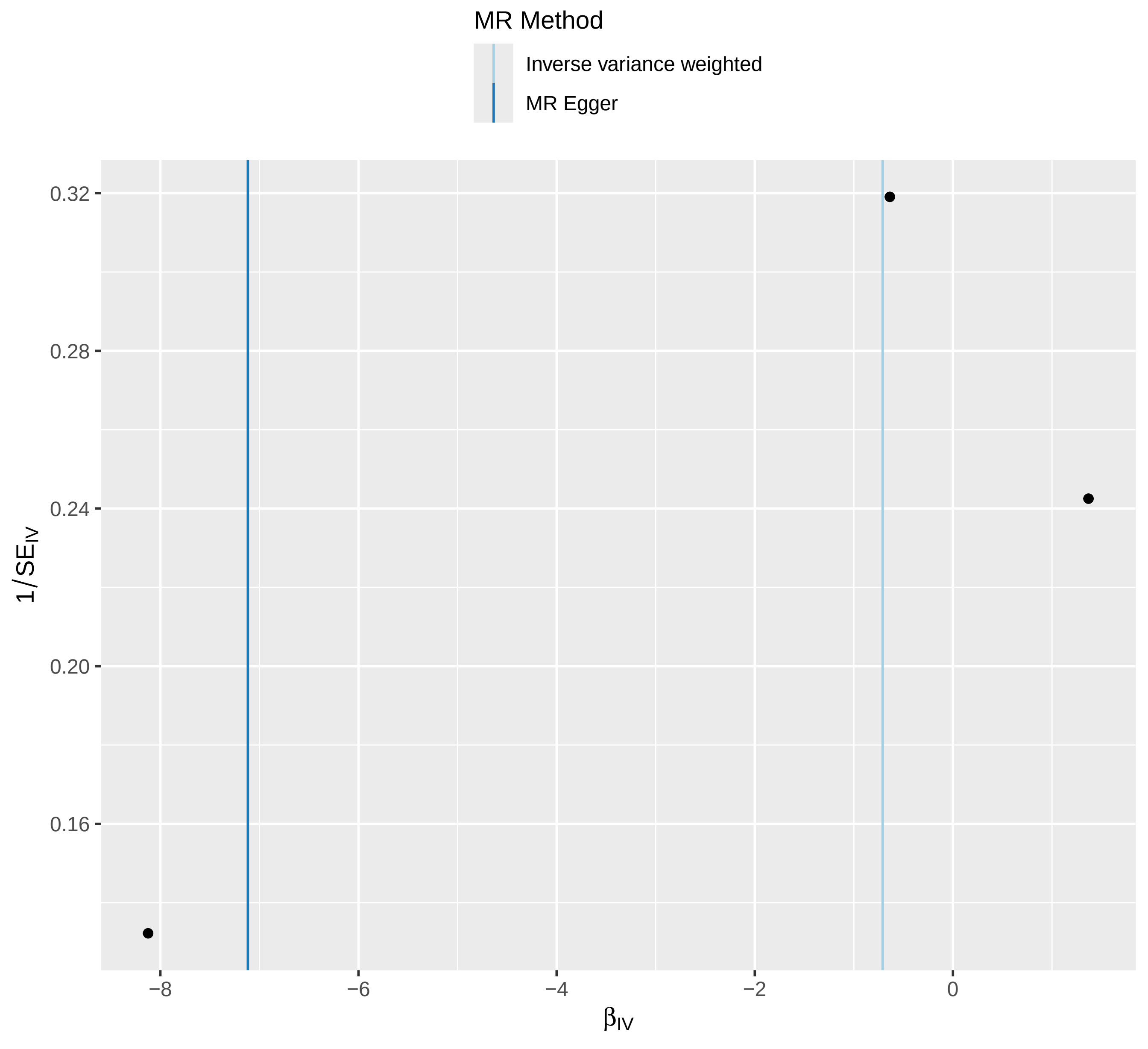

MR Method
Inverse variance weighted
MR Egger
0.32
0.28
0.24
0.20
0.16
1/SE$_{IV}$
−8
−6
−4
−2
0
β$_{IV}$

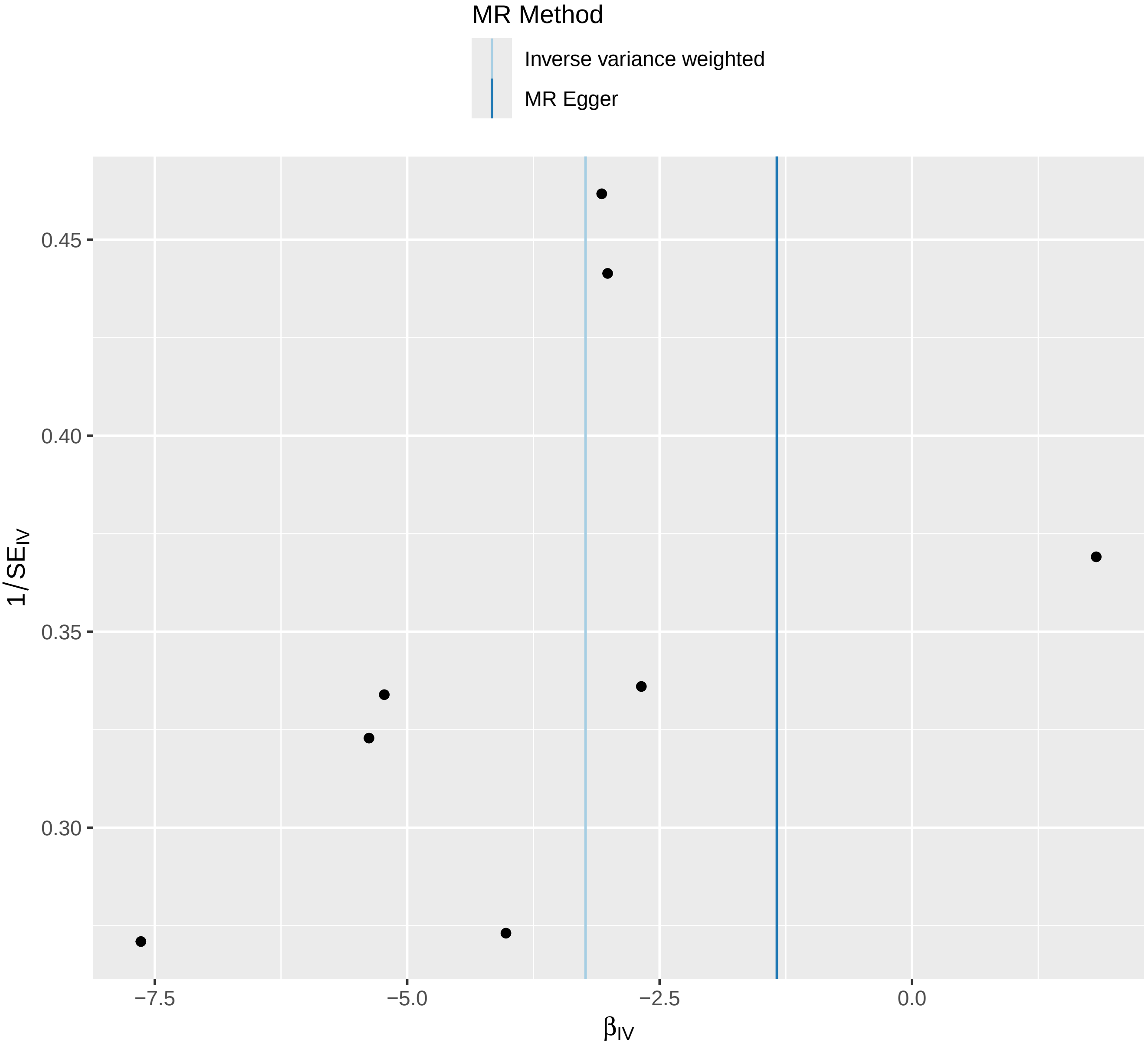
MR Method
Inverse variance weighted
MR Egger
0.45
0.40
0.35
0.30
1/SE_IV
−7.5
−5.0
−2.5
0.0
β_IV

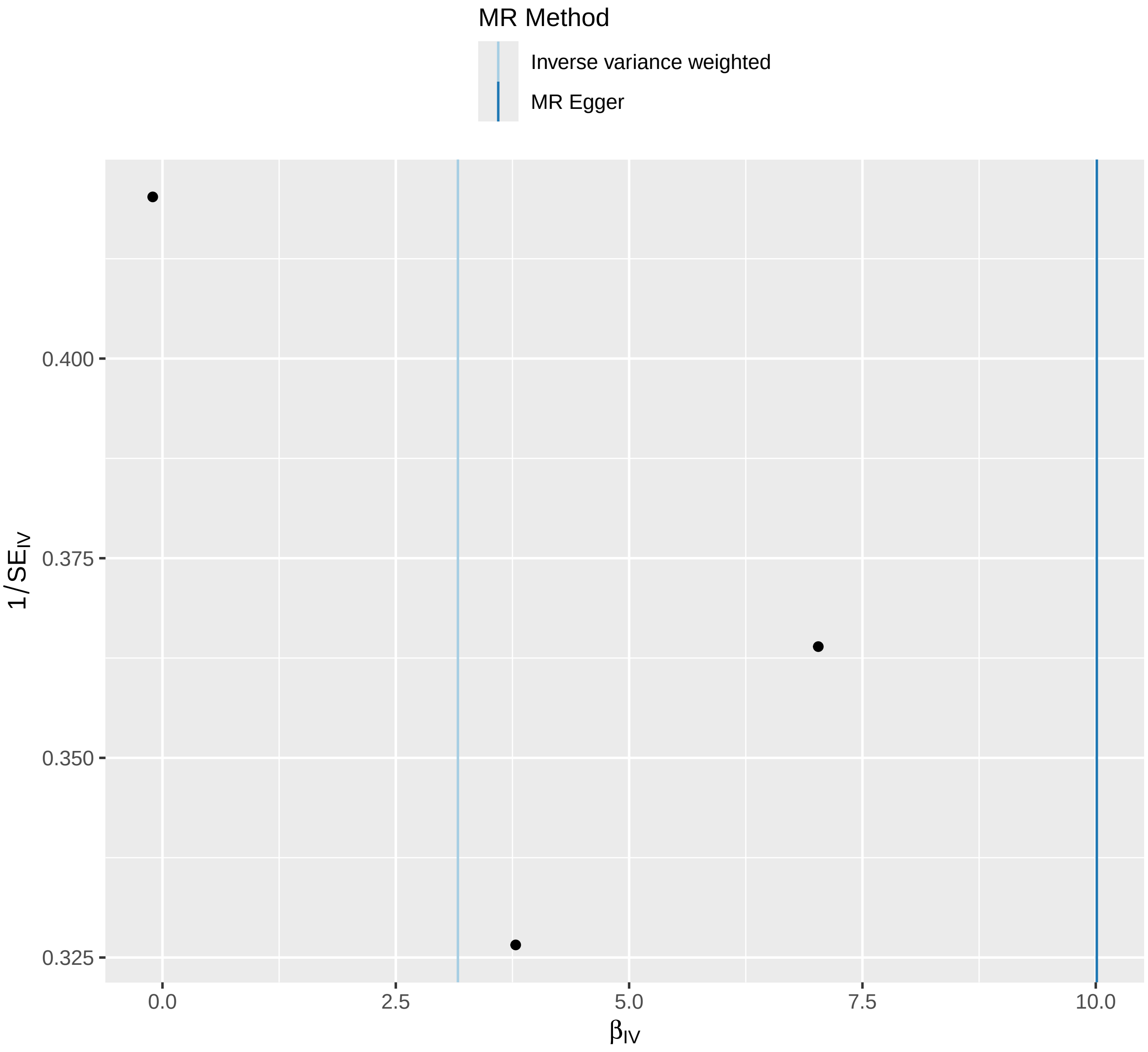
MR Method
Inverse variance weighted
MR Egger
0.400
0.375
0.350
0.325
$1/SE_{IV}$
0.0
2.5
5.0
7.5
10.0
β_{IV}

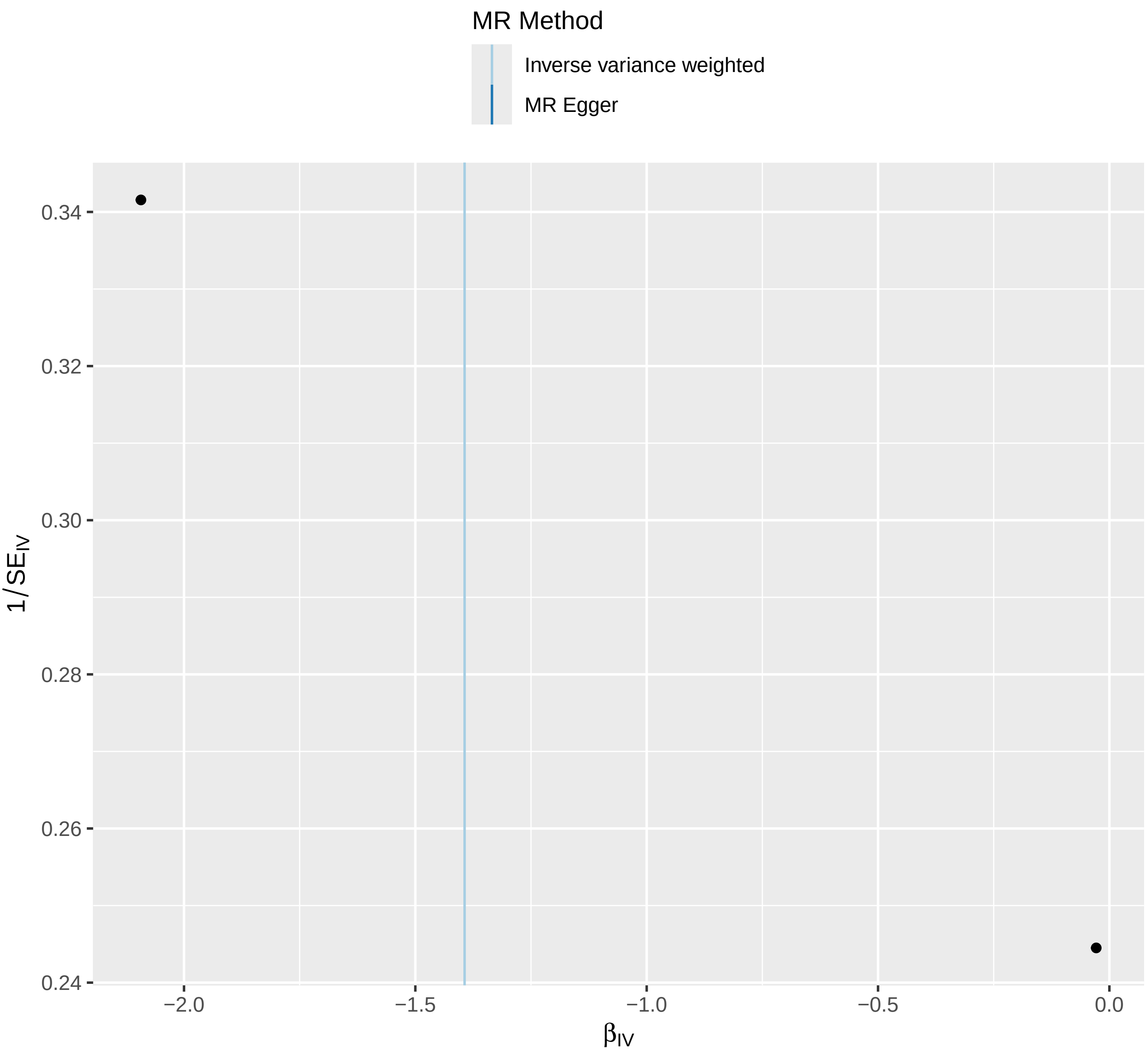
MR Method
Inverse variance weighted
MR Egger
0.34
0.32
0.30
0.28
0.26
0.24
1/SE_IV
−2.0
−1.5
−1.0
−0.5
0.0
β_IV

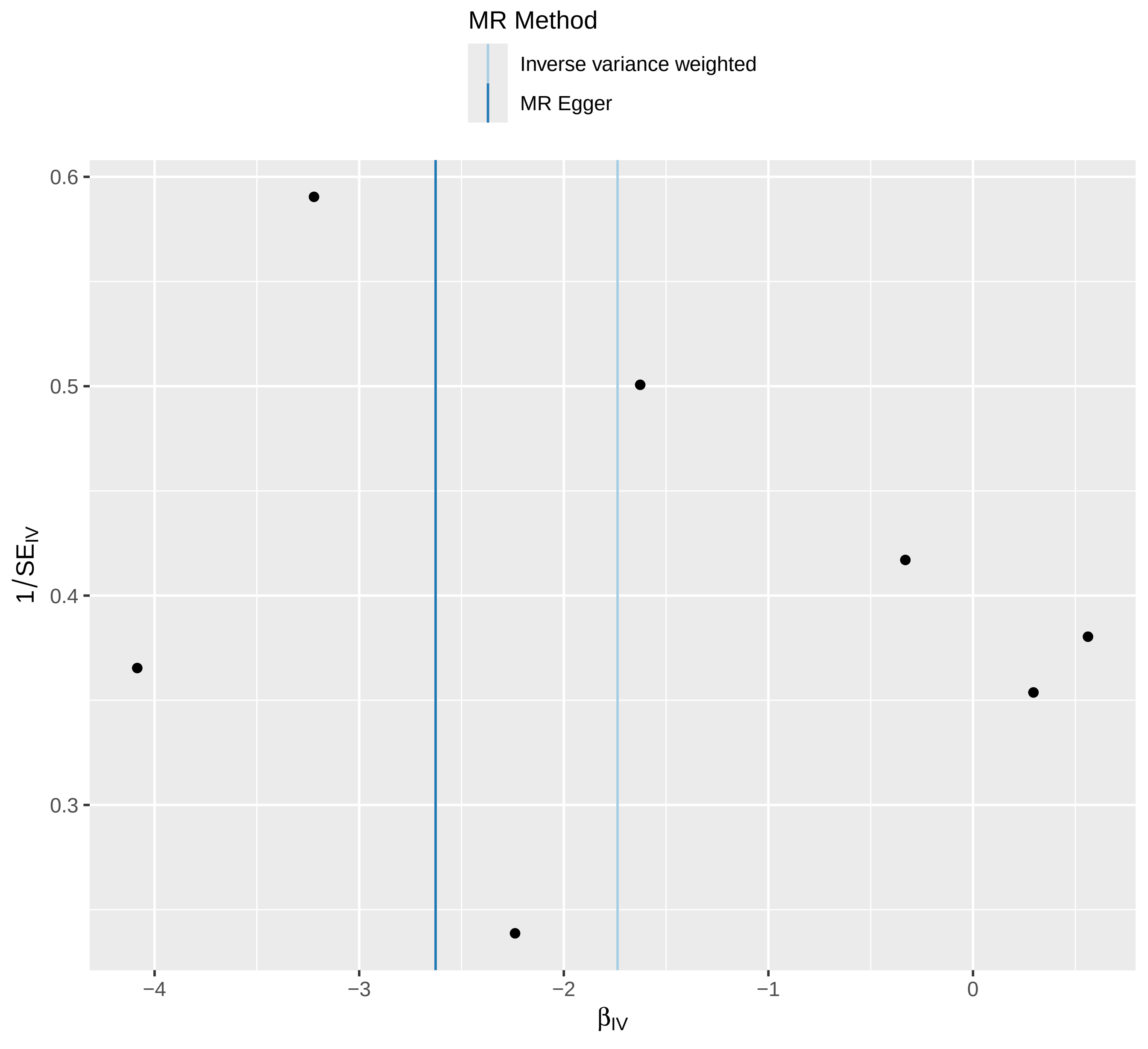
MR Method
Inverse variance weighted
MR Egger
0.6
0.5
0.4
0.3
$1/SE_{IV}$
−4
−3
−2
−1
0
β_{IV}

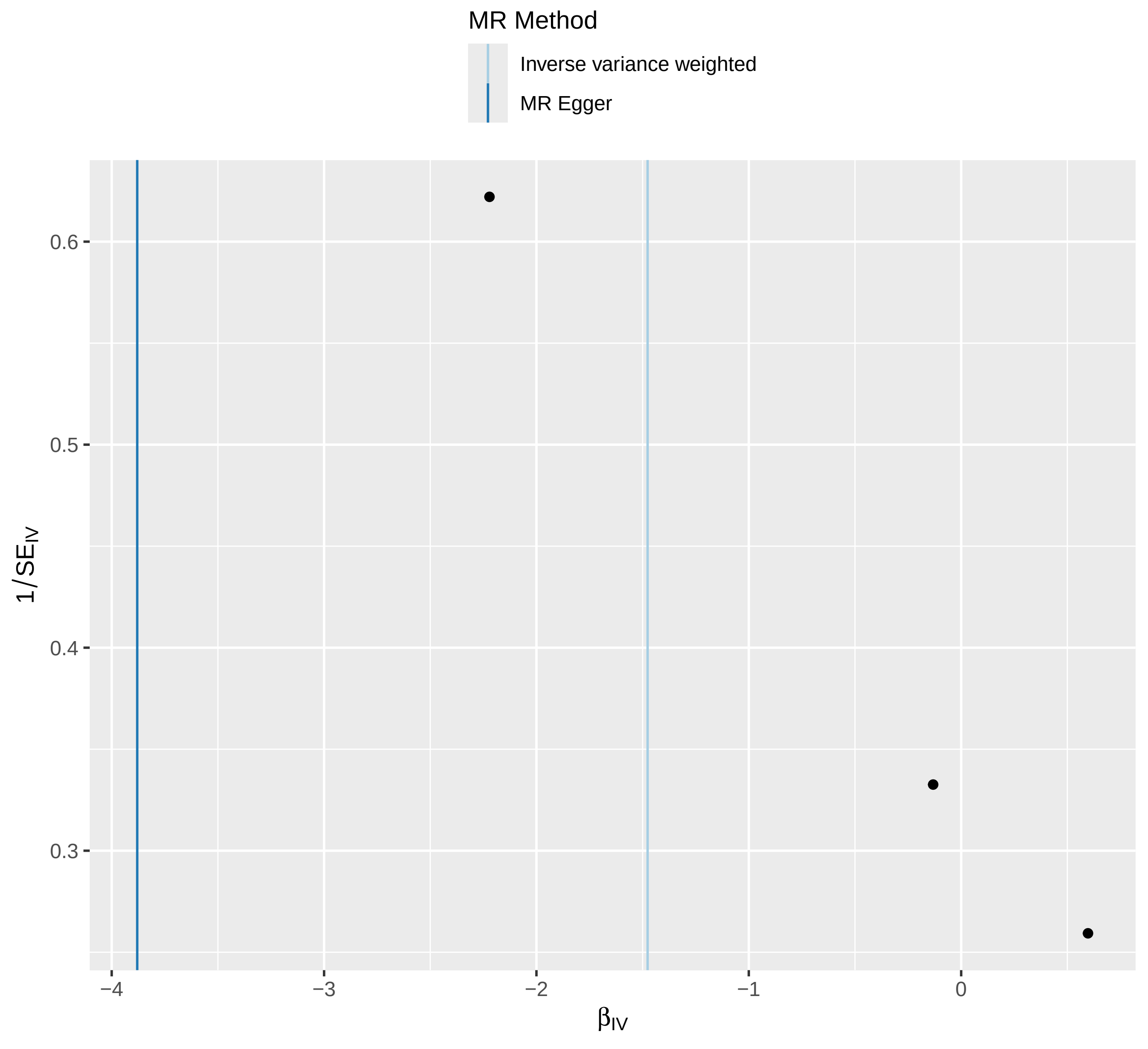
MR Method
Inverse variance weighted
MR Egger
0.6
0.5
0.4
0.3
1/SE$_{IV}$
−4
−3
−2
−1
0
β$_{IV}$

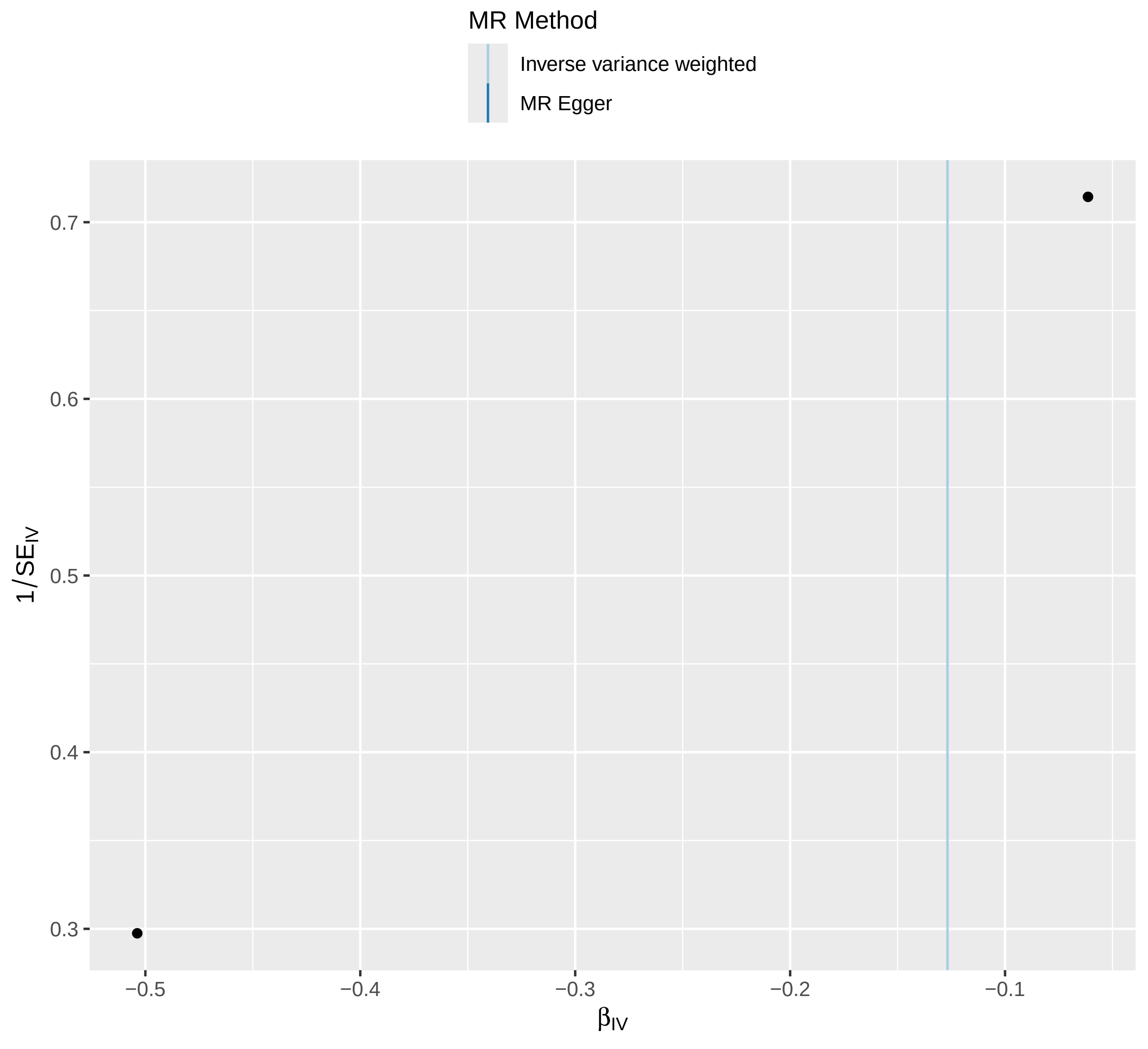

MR Method
Inverse variance weighted
MR Egger
1/SE_IV
0.7
0.6
0.5
0.4
0.3
−0.5
−0.4
−0.3
−0.2
−0.1
β_IV

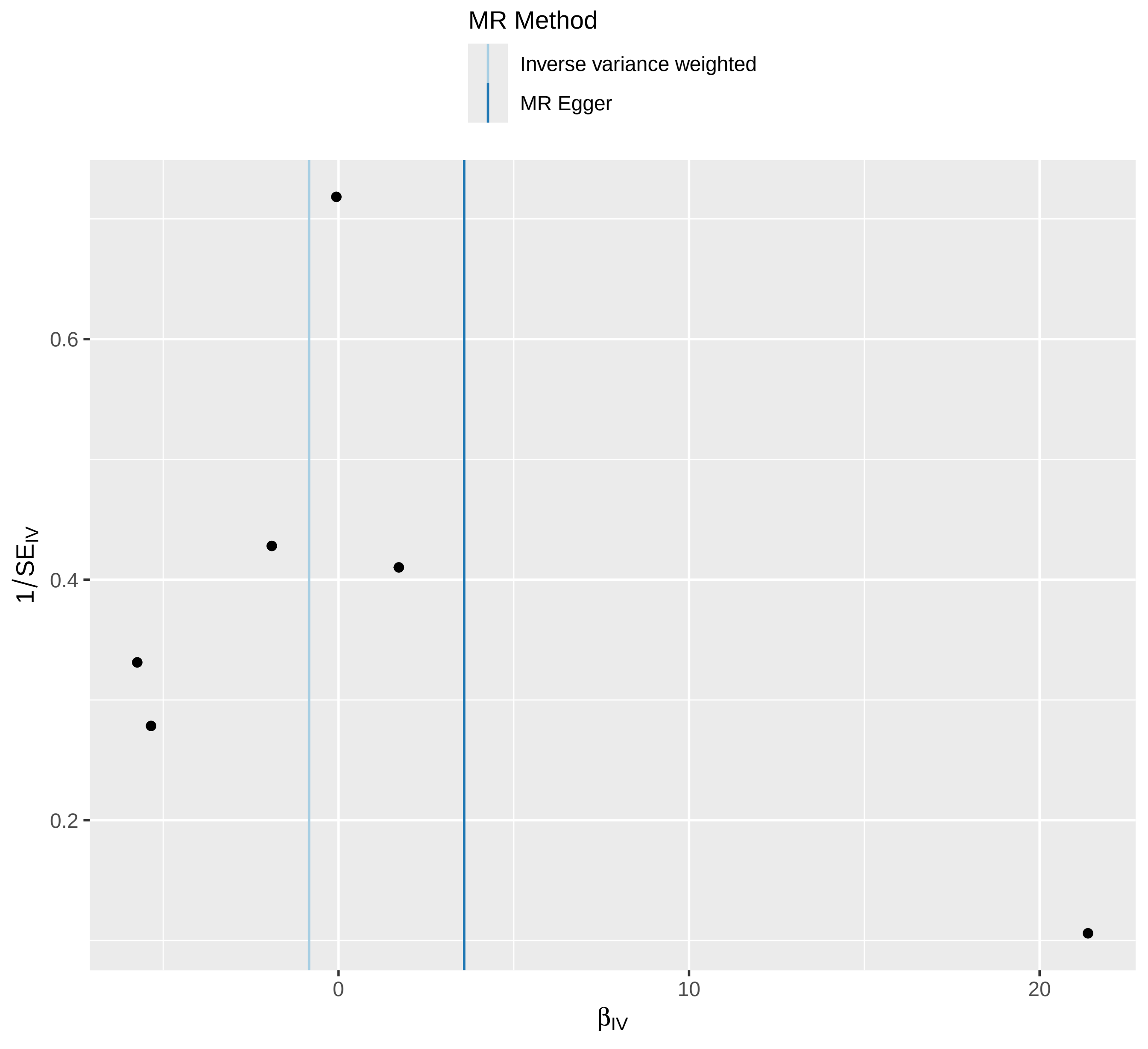
MR Method
Inverse variance weighted
MR Egger
0.6
0.4
0.2
1/SE_IV
0
10
20
β_IV

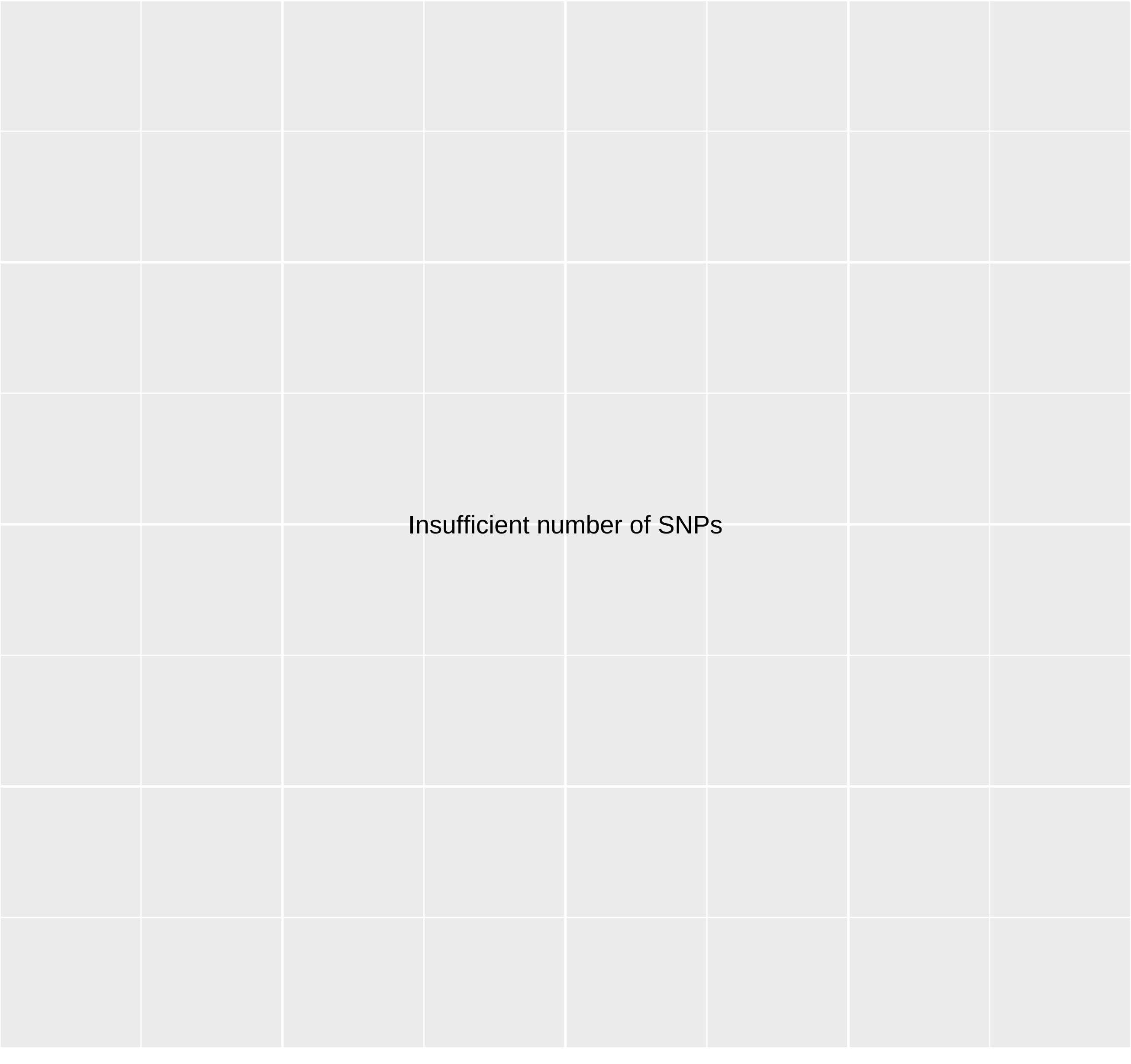
Insufficient number of SNPs

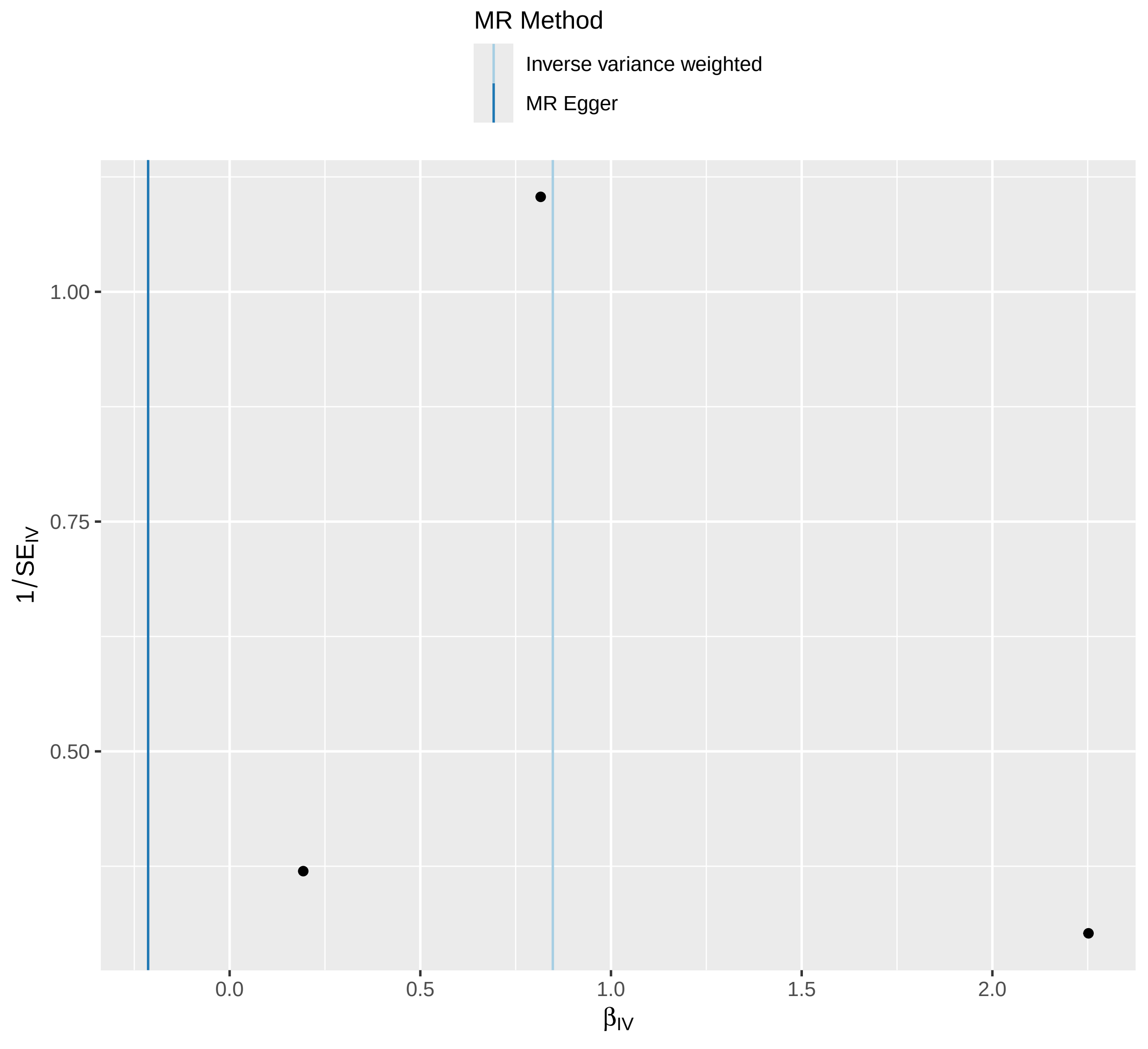

MR Method
Inverse variance weighted
MR Egger
1.00
0.75
0.50
0.0
0.5
1.0
1.5
2.0
$1/SE_{IV}$
β_{IV}

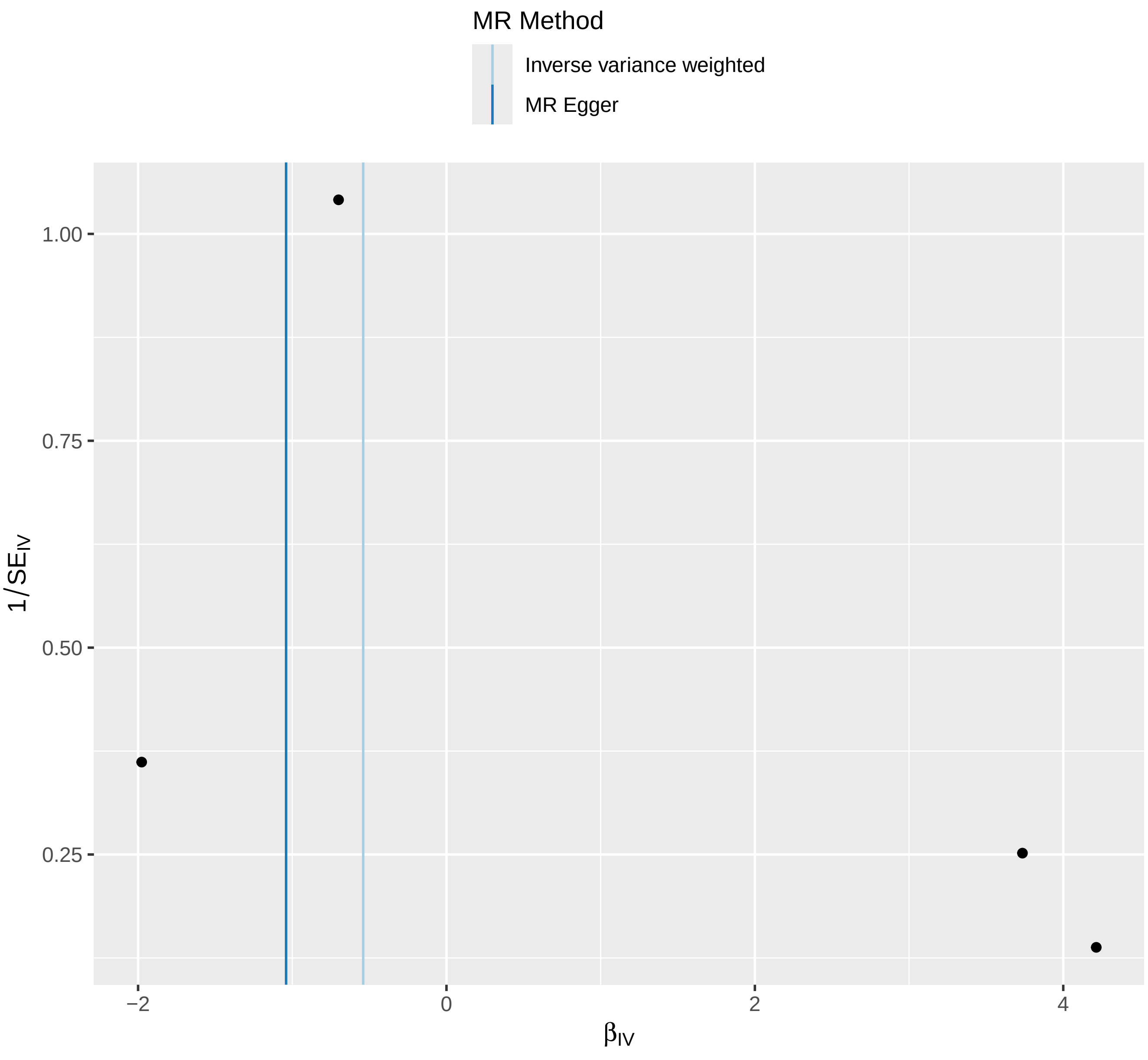
MR Method
Inverse variance weighted
MR Egger
1/SE_IV
1.00
0.75
0.50
0.25
−2
0
2
4
β_IV

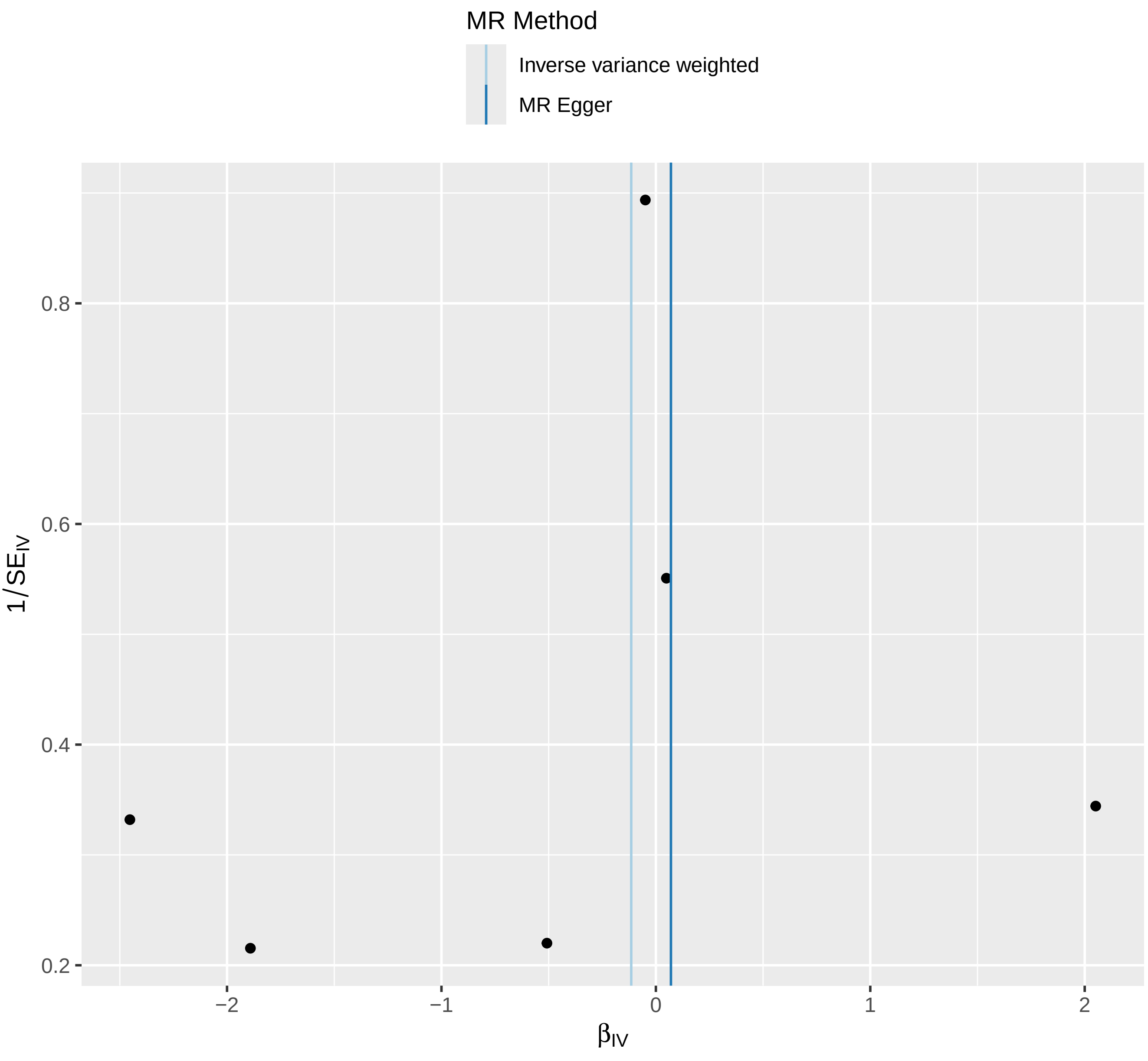
MR Method
Inverse variance weighted
MR Egger
0.8
0.6
0.4
0.2
1/SE$_{IV}$
−2
−1
0
1
2
β$_{IV}$

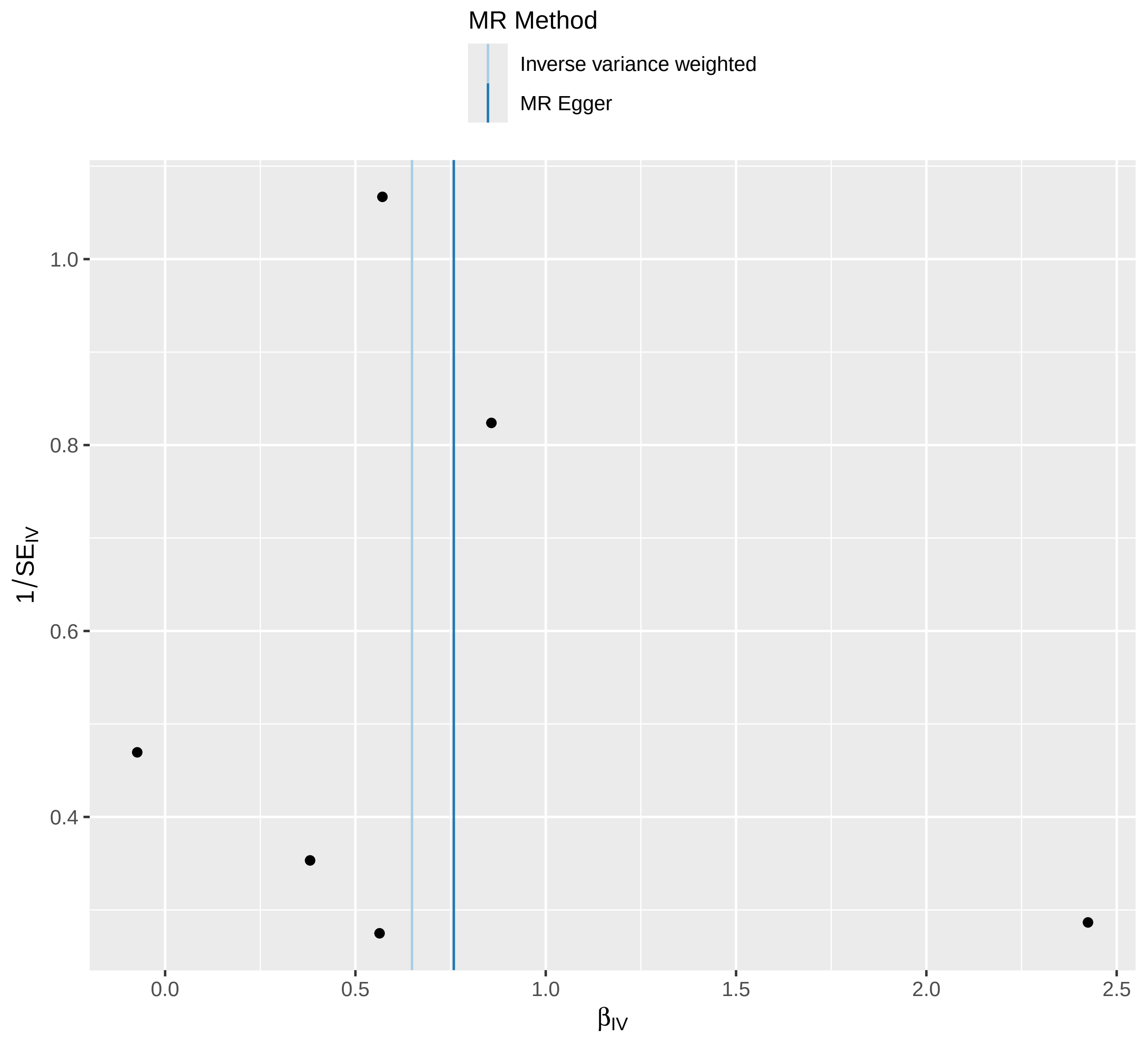
MR Method
Inverse variance weighted
MR Egger
1.0
0.8
0.6
0.4
1/SE_{IV}
0.0
0.5
1.0
1.5
2.0
2.5
$β_{IV}$

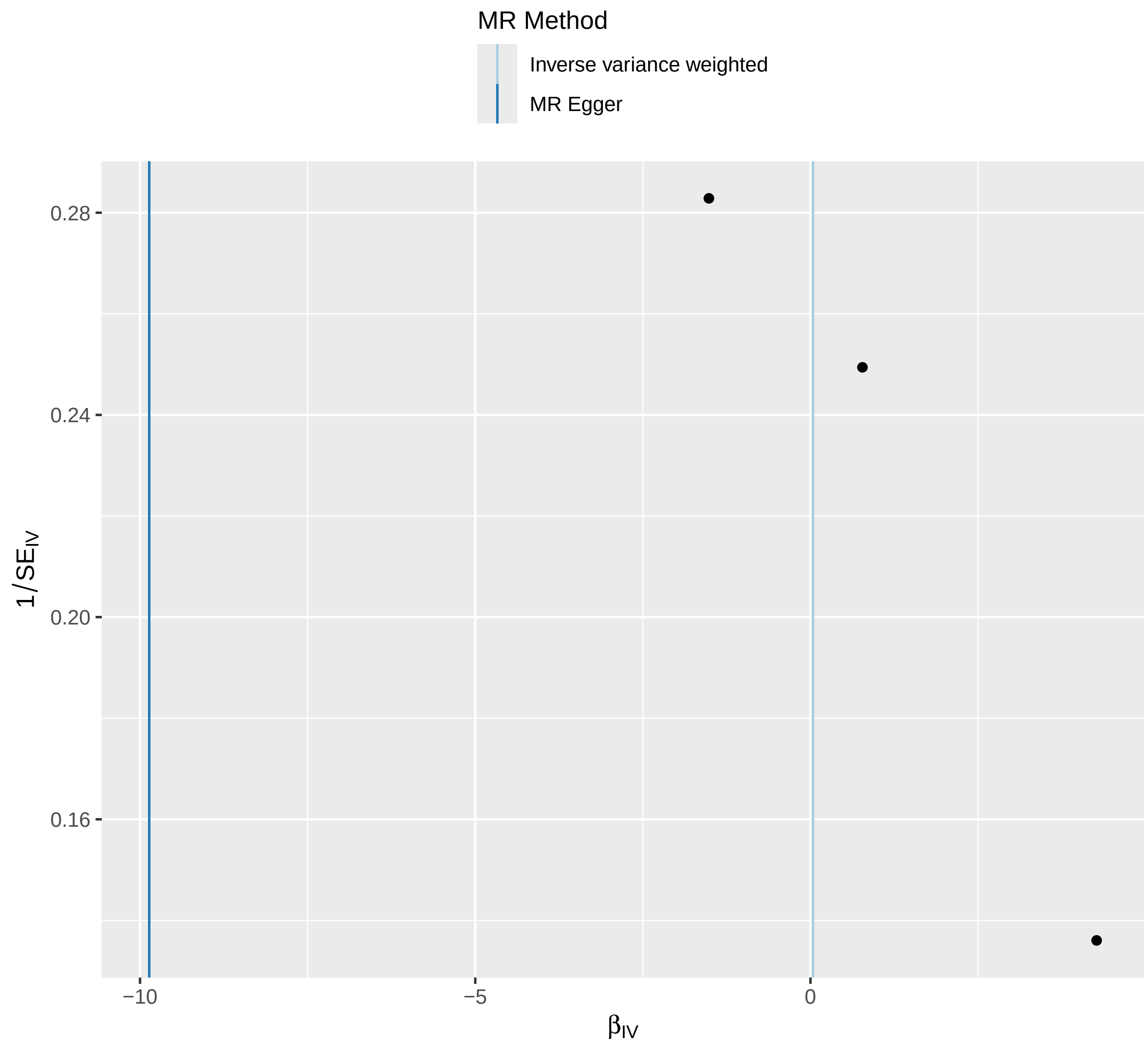

MR Method
Inverse variance weighted
MR Egger
0.28
0.24
0.20
0.16
$1/SE_{IV}$
−10
−5
0
β_{IV}

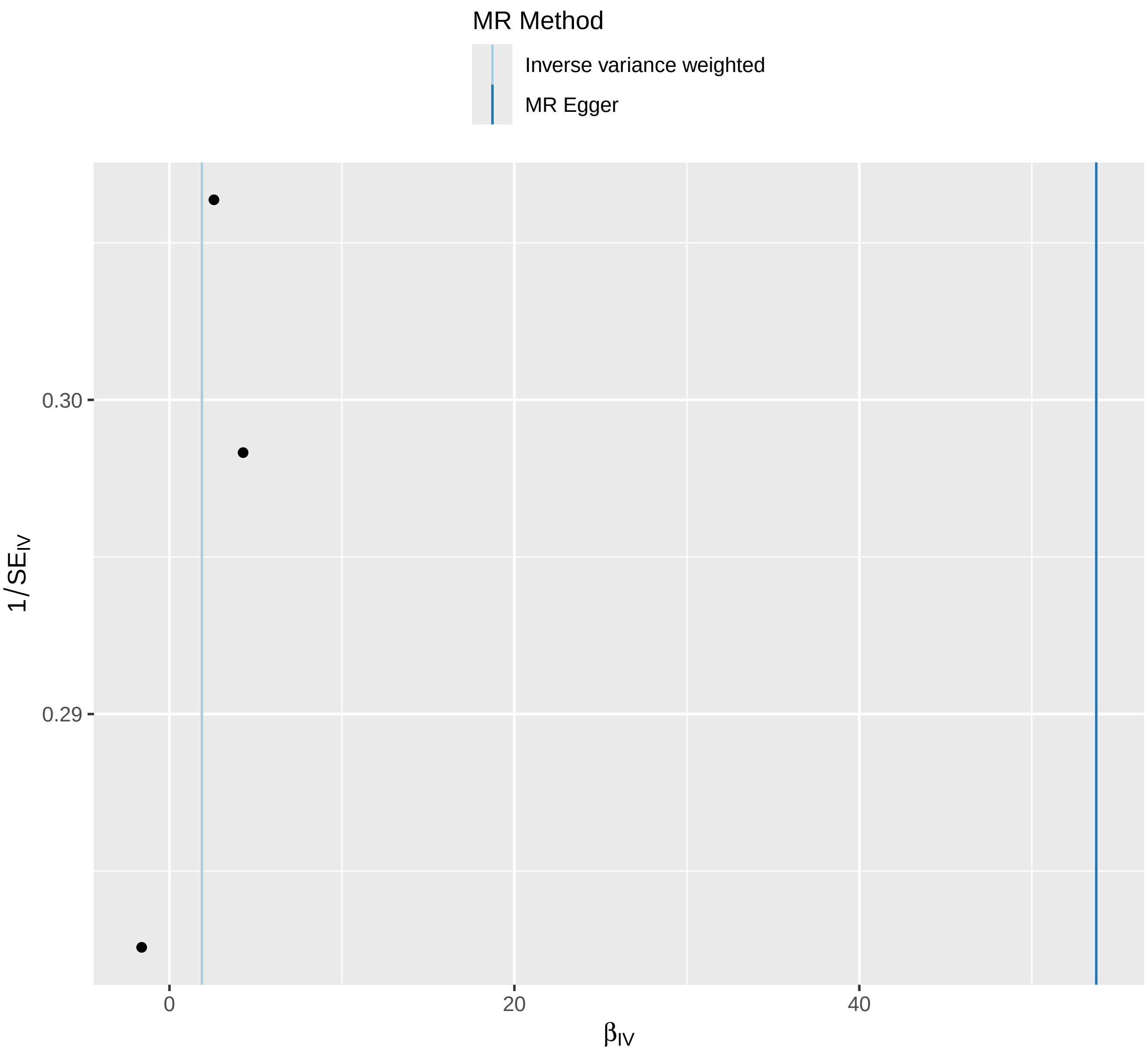
MR Method
Inverse variance weighted
MR Egger
0.30
0.29
1/SE_IV
0
20
40
β_IV

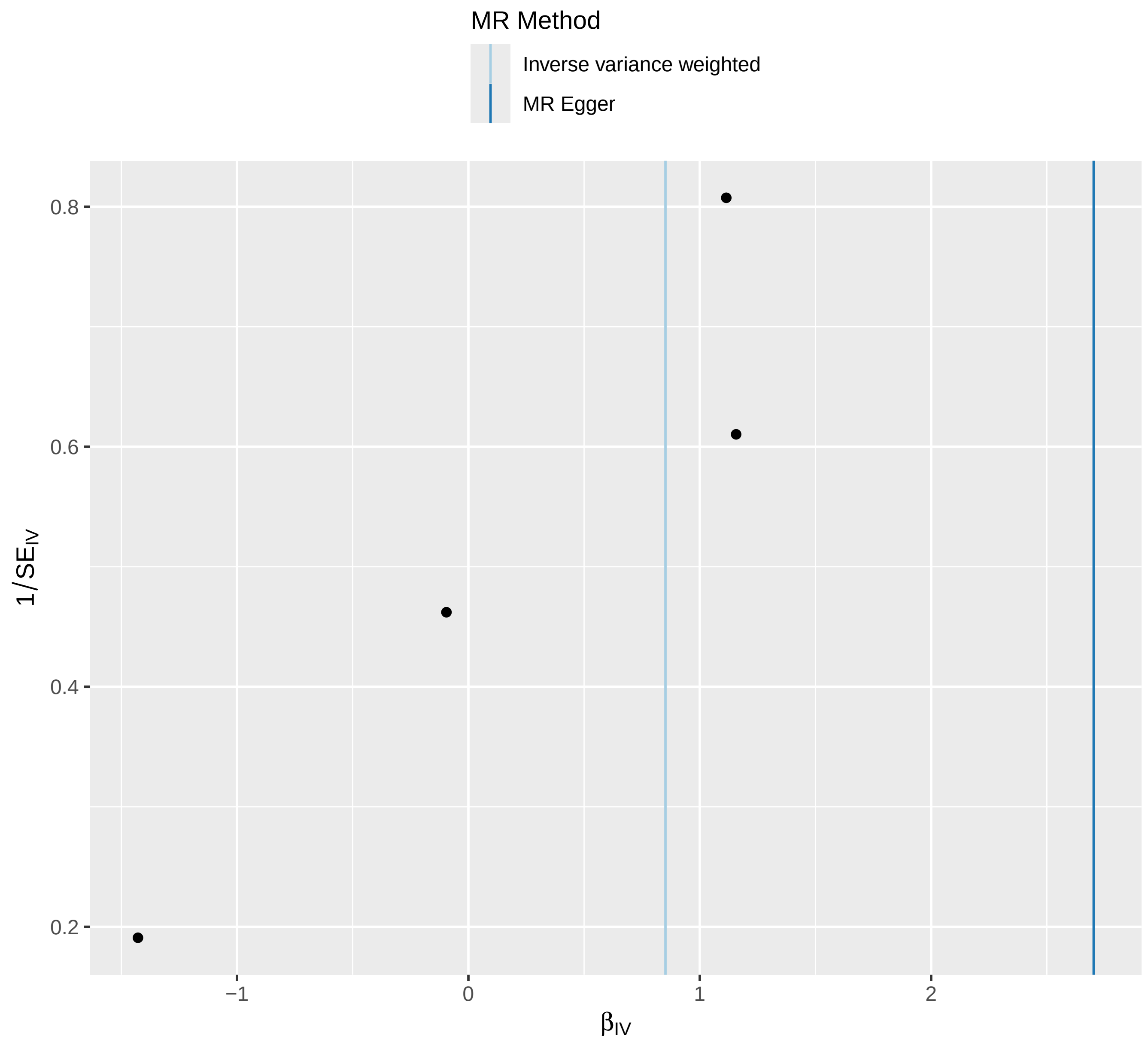
MR Method
Inverse variance weighted
MR Egger
0.8
0.6
0.4
0.2
1/SE_IV
−1
0
1
2
β_IV

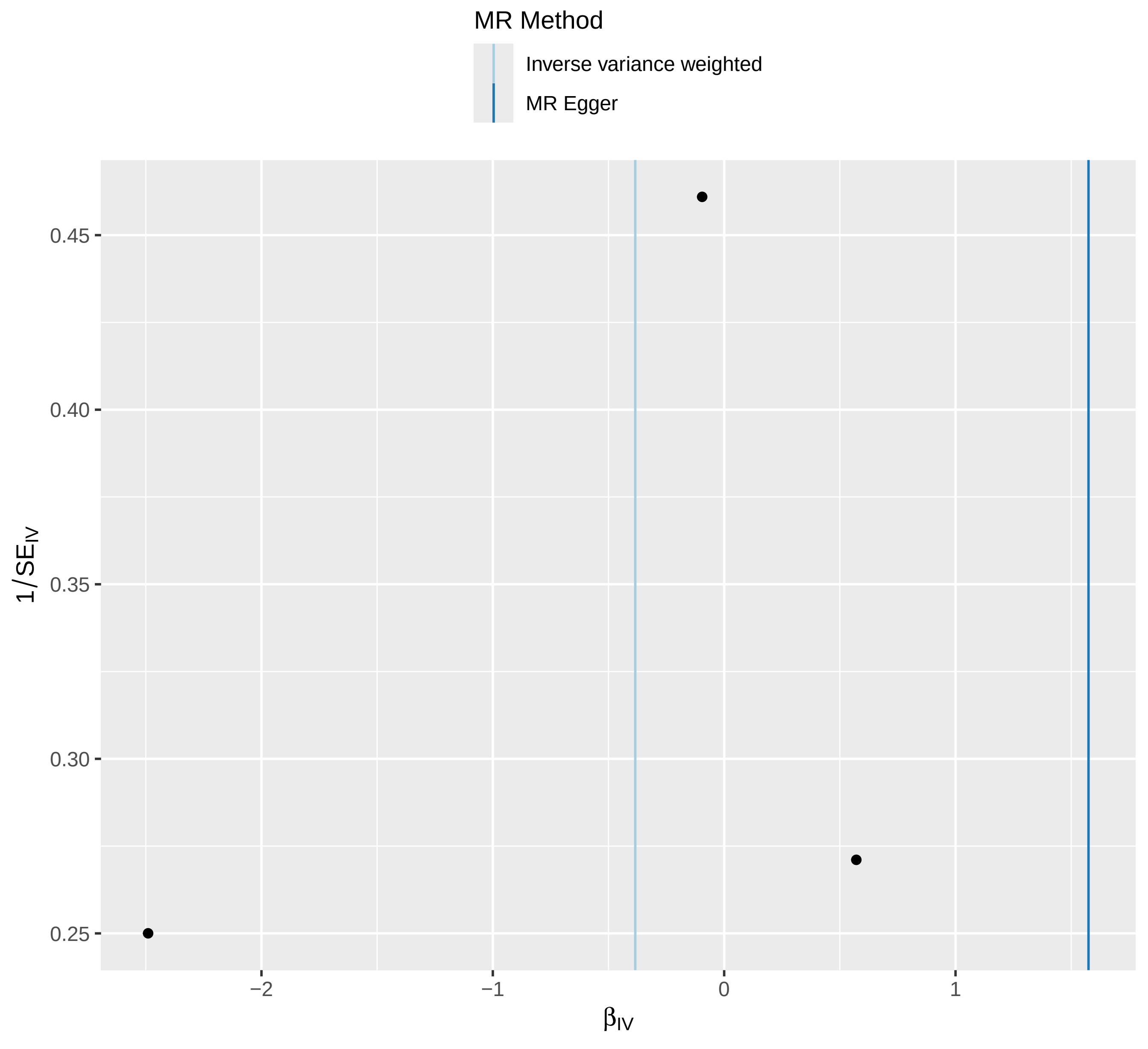
MR Method
Inverse variance weighted
MR Egger
0.45
0.40
0.35
0.30
0.25
1/SE_IV
−2
−1
0
1
β_IV

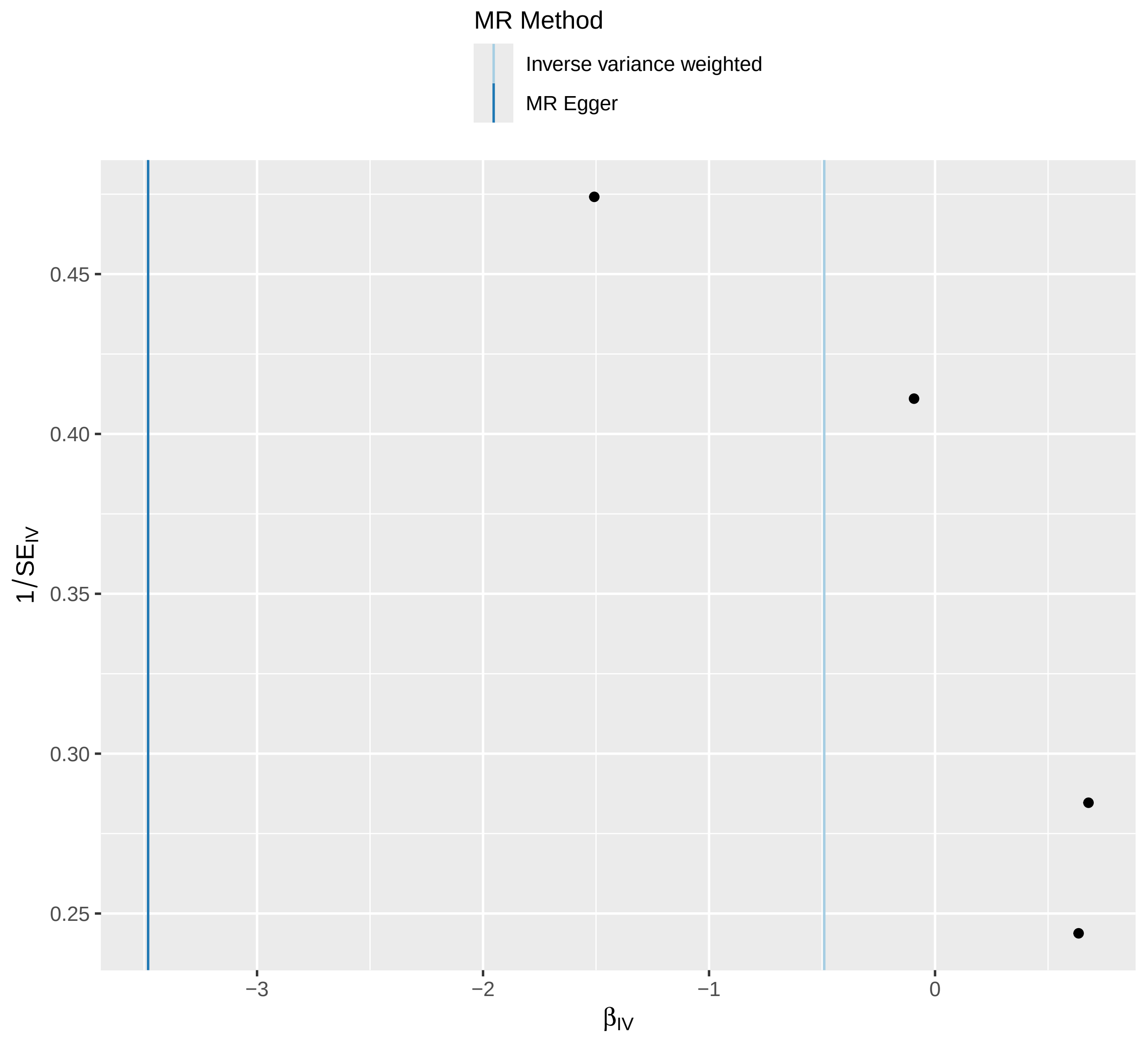
MR Method
Inverse variance weighted
MR Egger
0.45
0.40
0.35
0.30
0.25
1/SE_IV
−3
−2
−1
0
β_IV

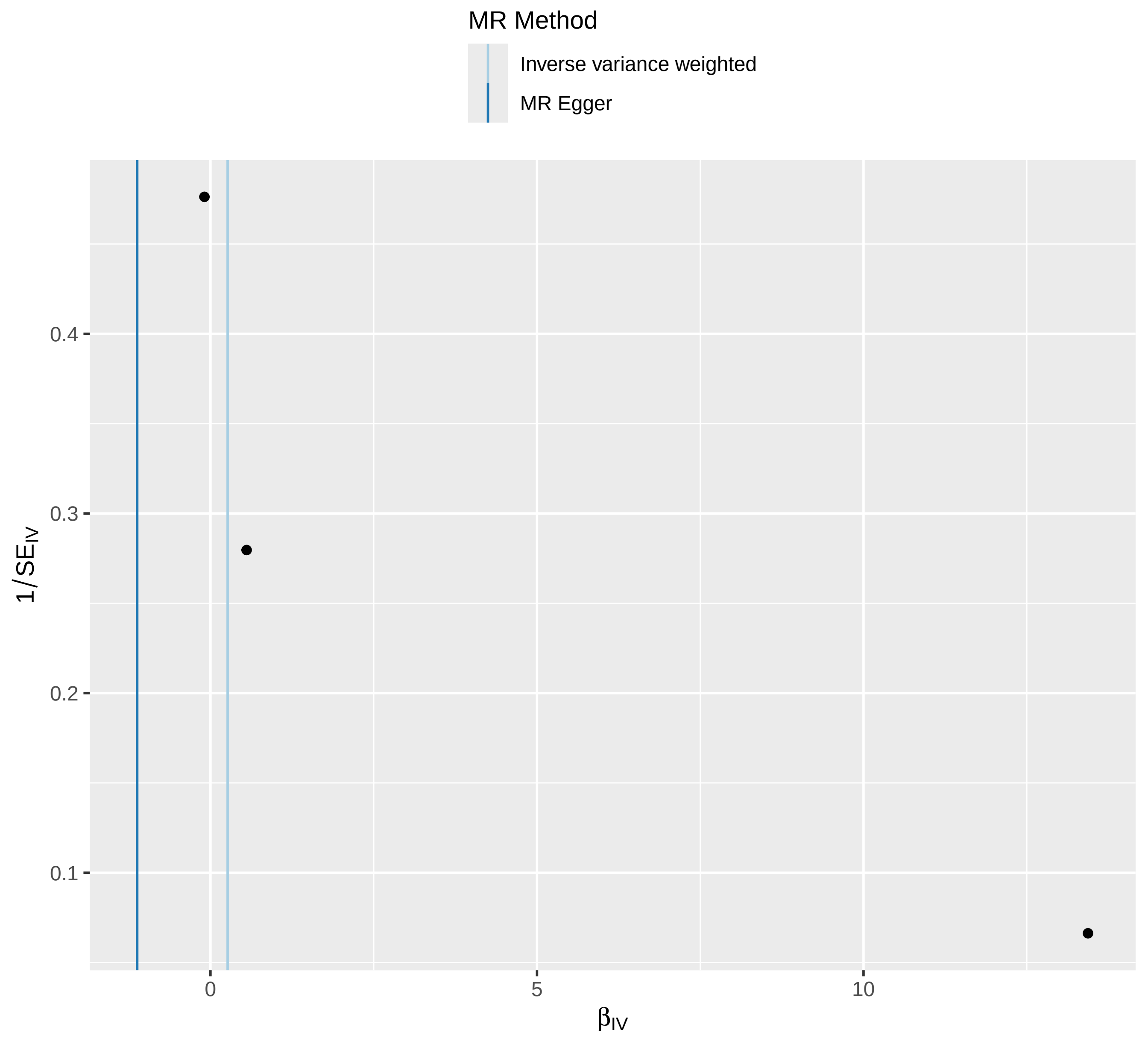
MR Method
Inverse variance weighted
MR Egger
0.1
0.2
0.3
0.4
0
5
10
$1/SE_{IV}$
β_{IV}

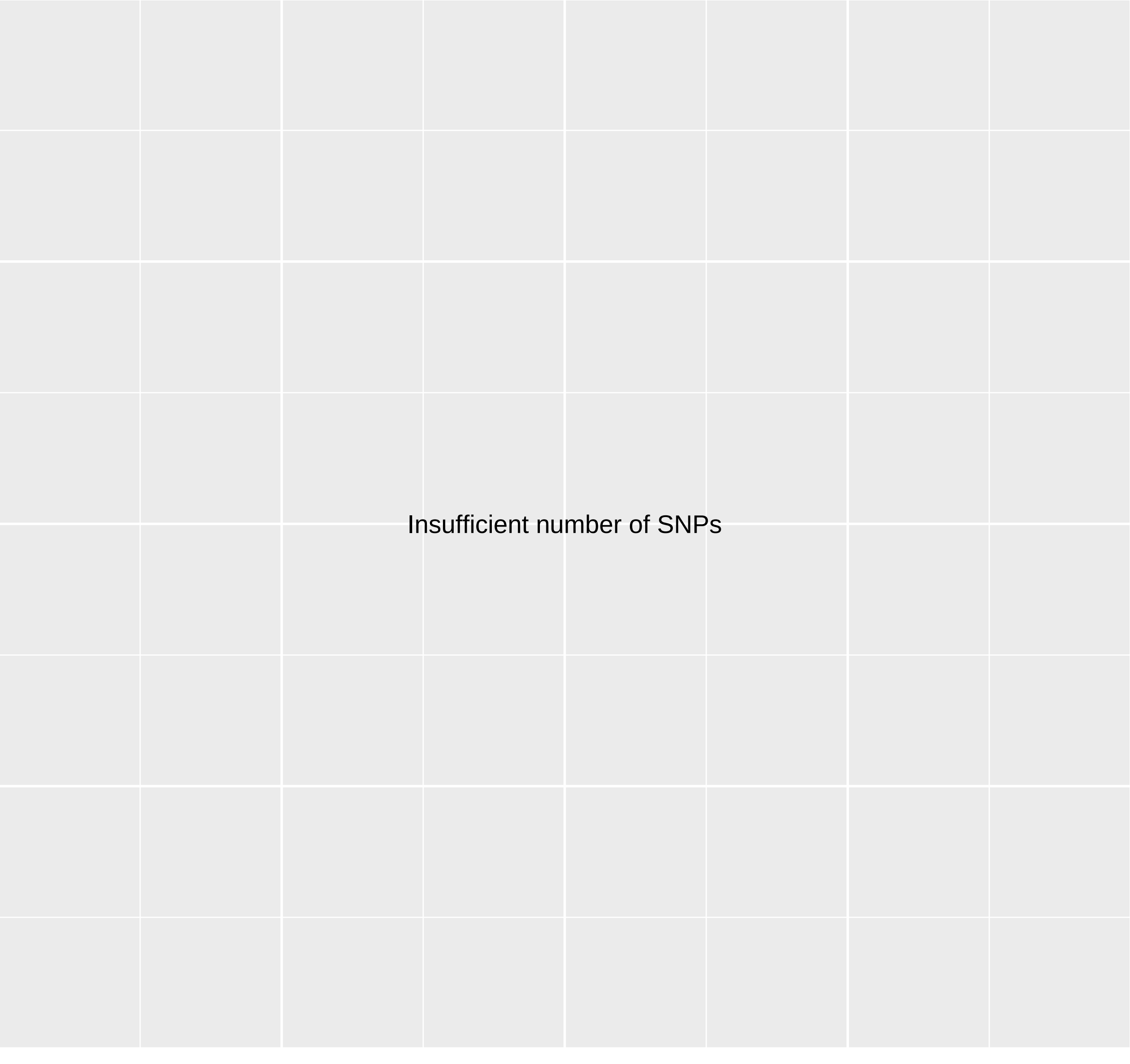
Insufficient number of SNPs

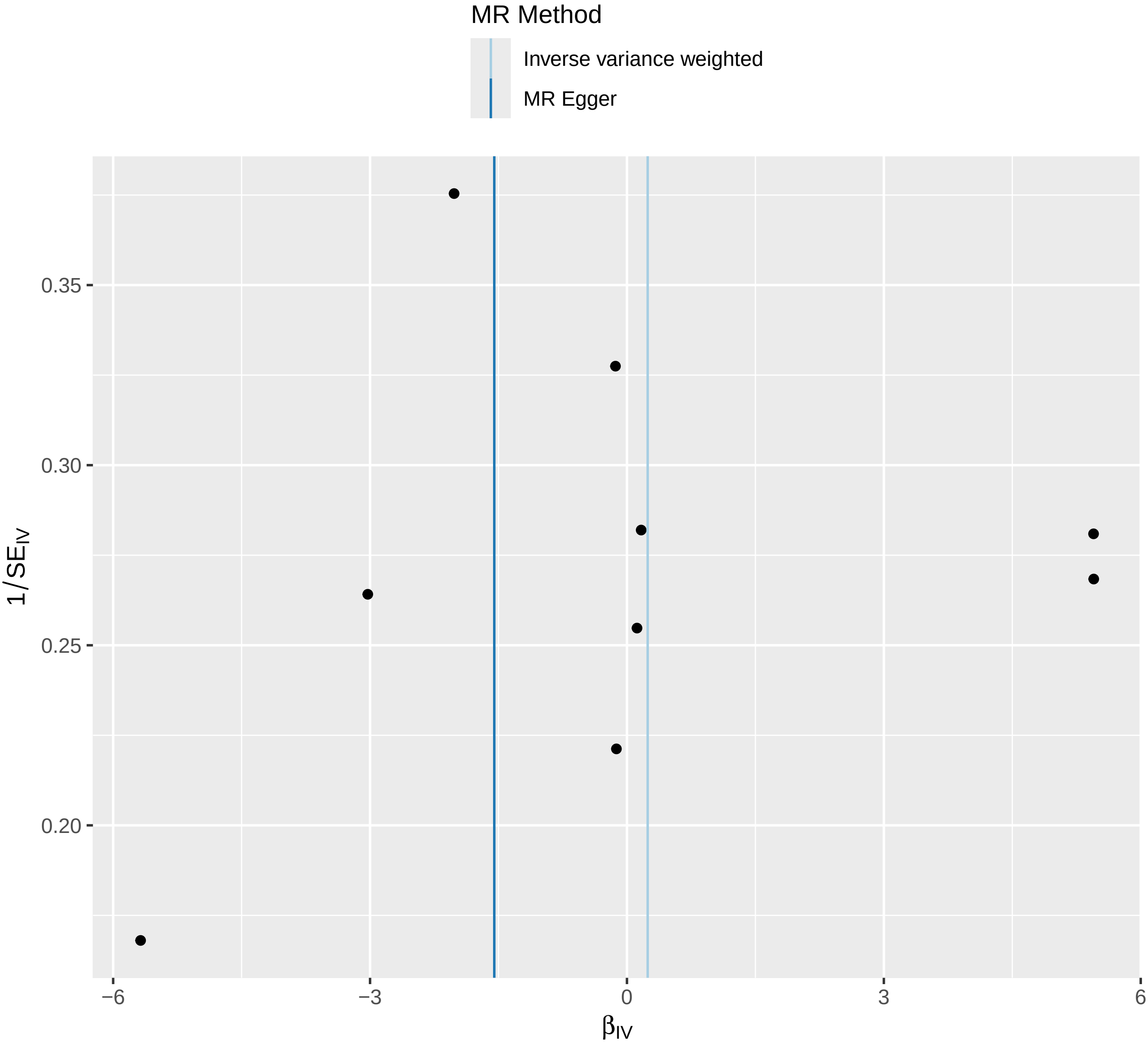

MR Method
Inverse variance weighted
MR Egger
0.35
0.30
0.25
0.20
1/SE_IV
−6
−3
0
3
6
β_IV

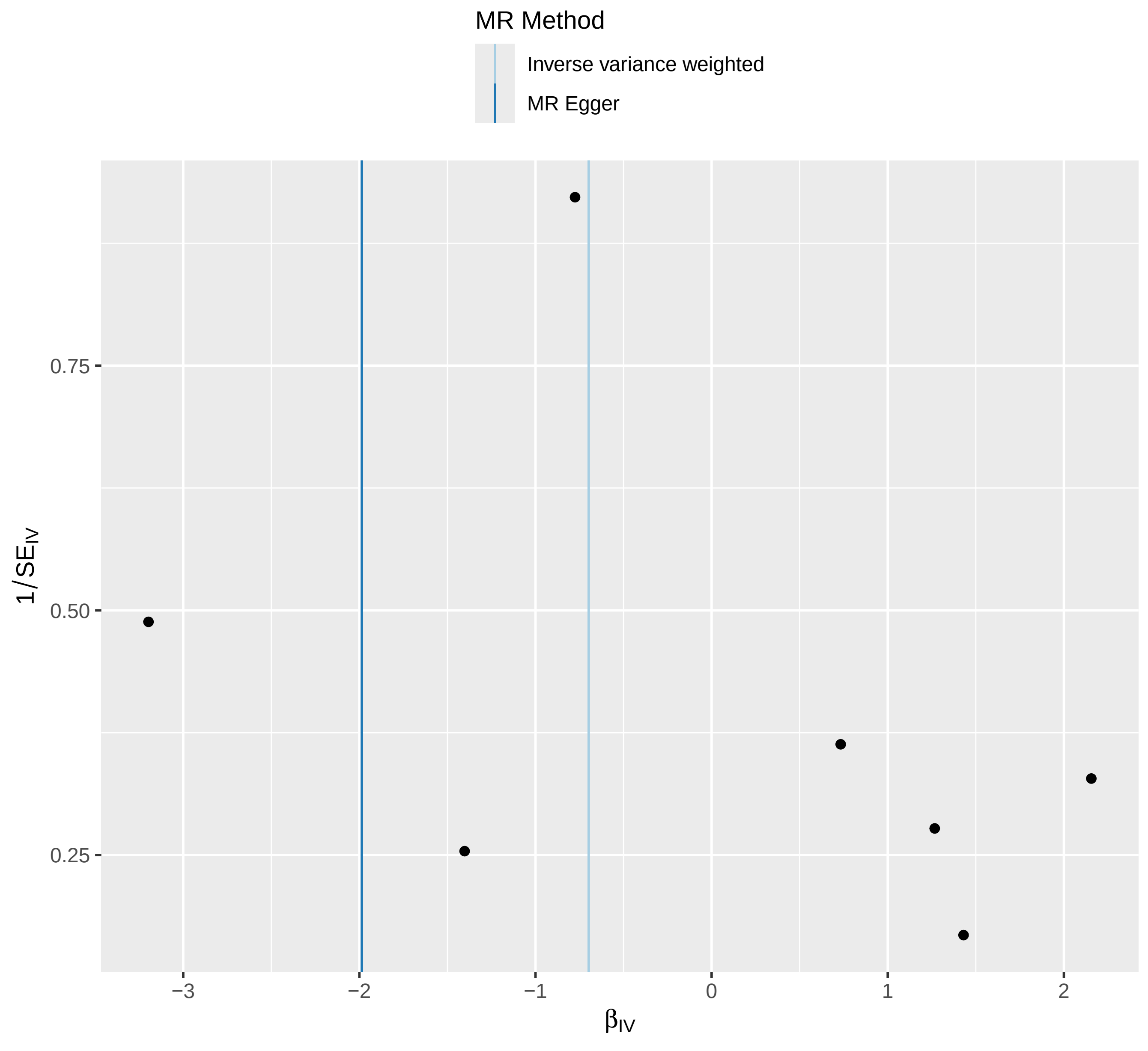
MR Method
Inverse variance weighted
MR Egger
0.75
0.50
0.25
1/SE_IV
−3
−2
−1
0
1
2
β_IV

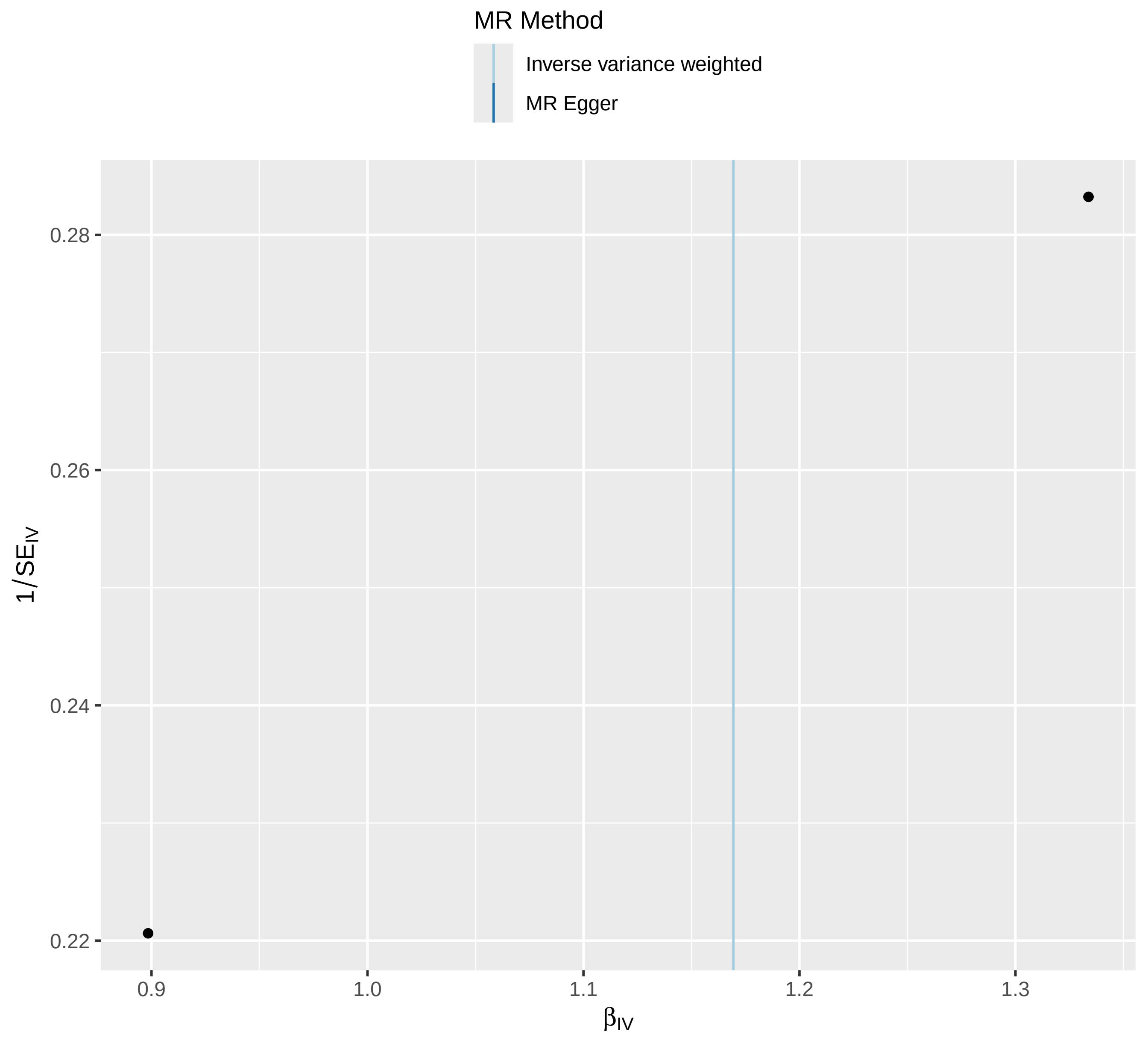

MR Method
Inverse variance weighted
MR Egger
0.28
0.26
0.24
0.22
0.9
1.0
1.1
1.2
1.3
$1/SE_{IV}$
β_{IV}

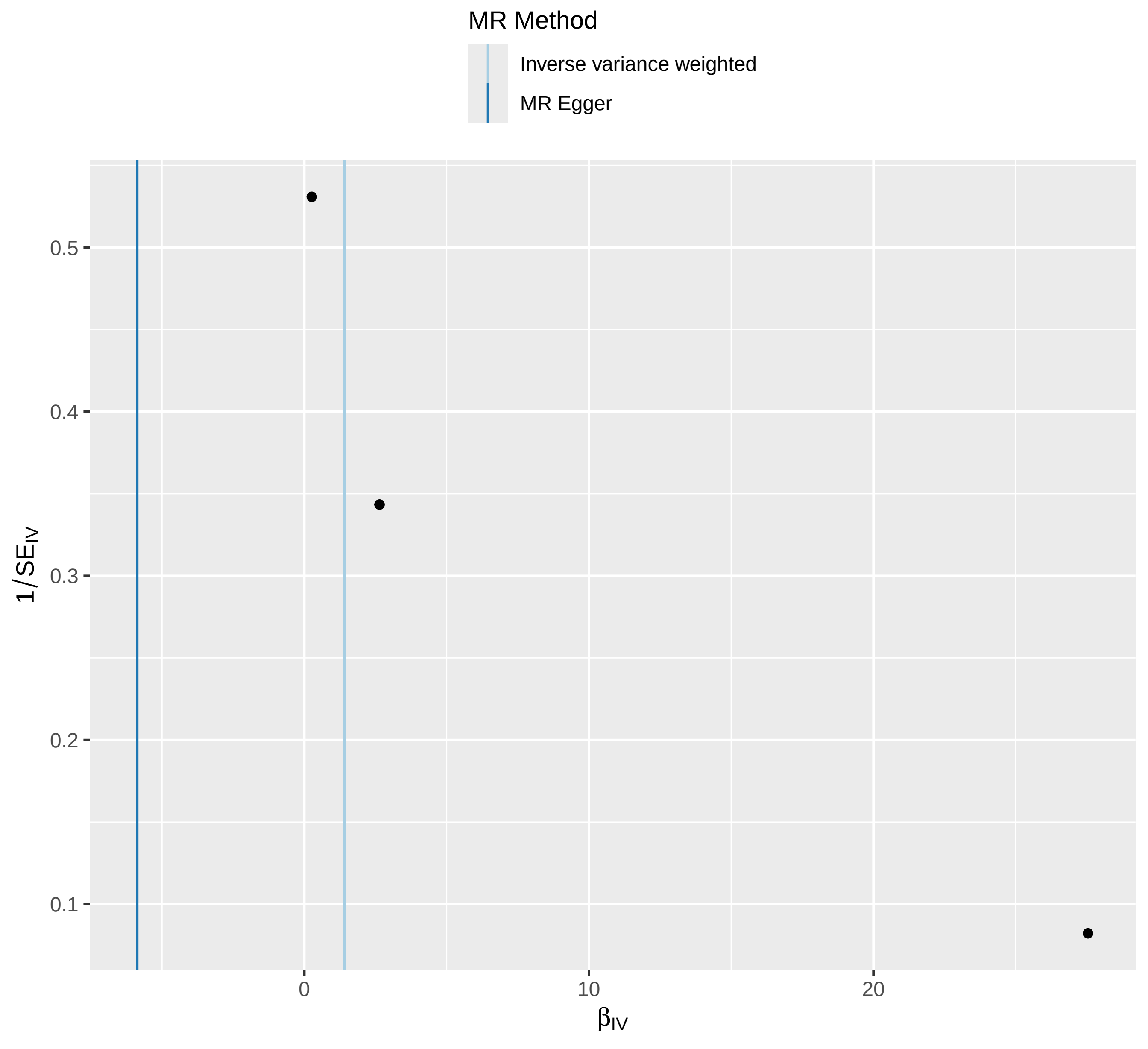
MR Method
Inverse variance weighted
MR Egger
0.5
0.4
0.3
0.2
0.1
0
10
20
1/SE_IV
β_IV

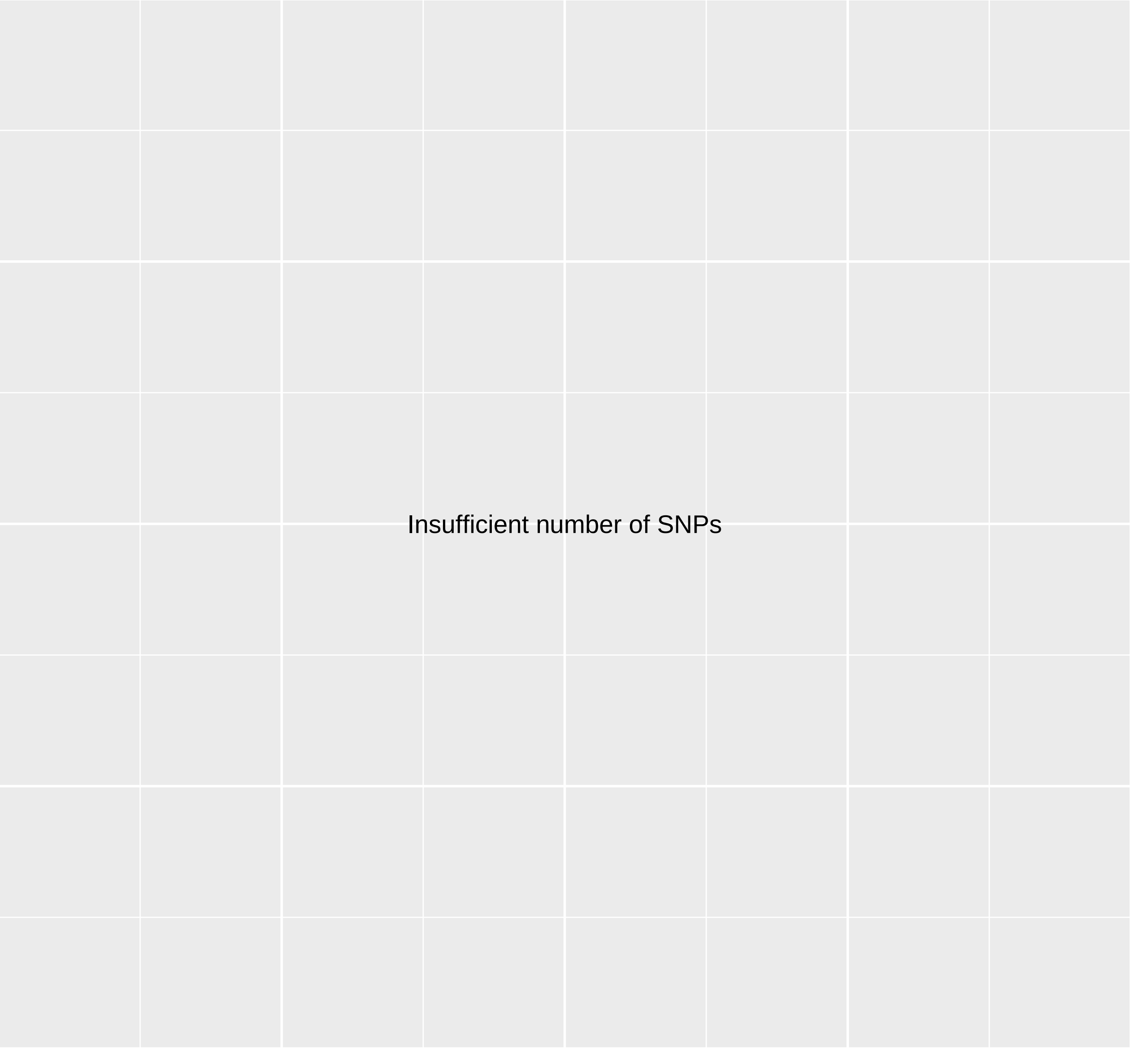
Insufficient number of SNPs

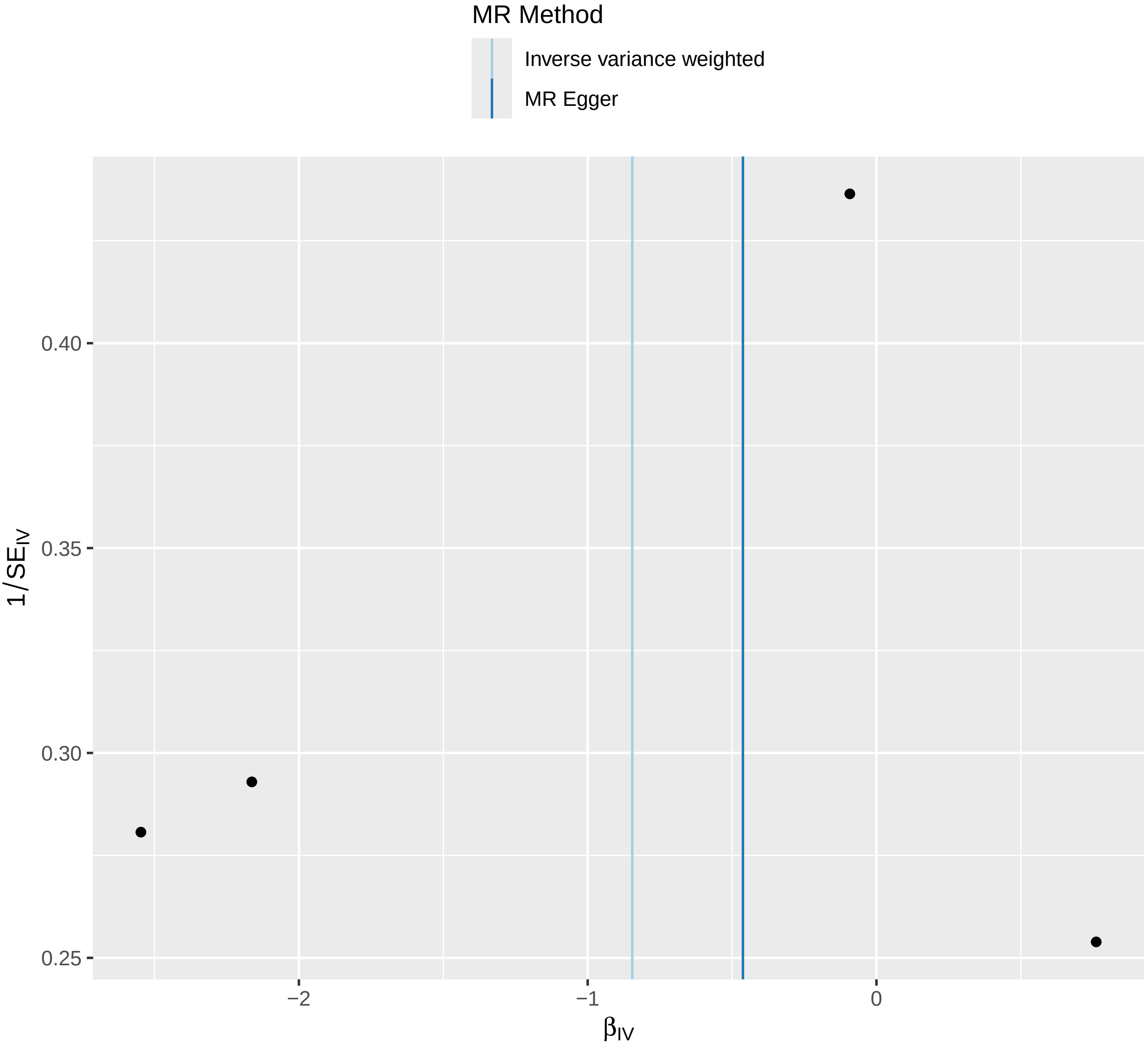

MR Method
Inverse variance weighted
MR Egger
0.40
0.35
0.30
0.25
1/SE_IV
−2
−1
0
β_IV

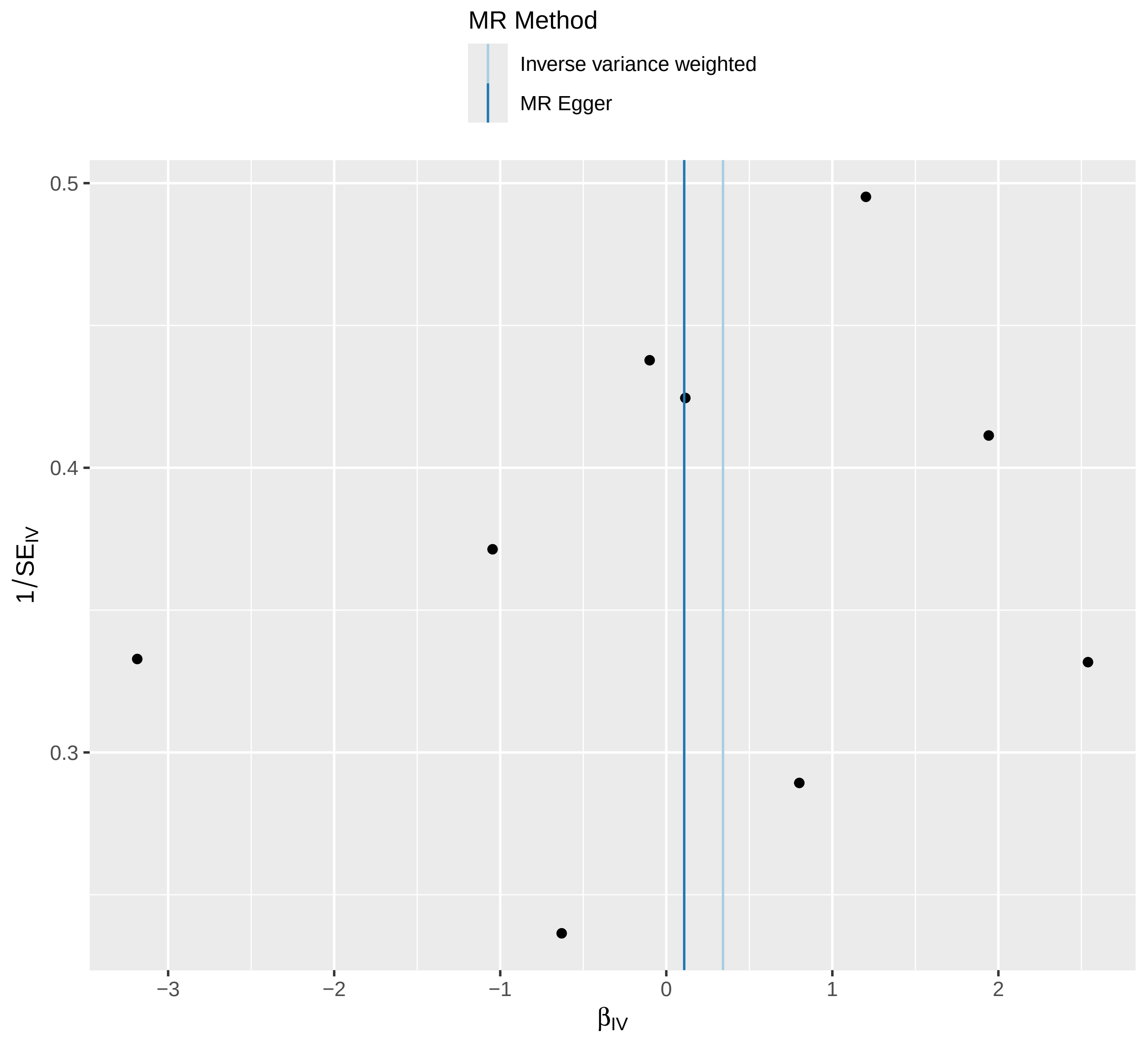
MR Method
Inverse variance weighted
MR Egger
1/SE_{IV}
0.5
0.4
0.3
−3
−2
−1
0
1
2
β_{IV}

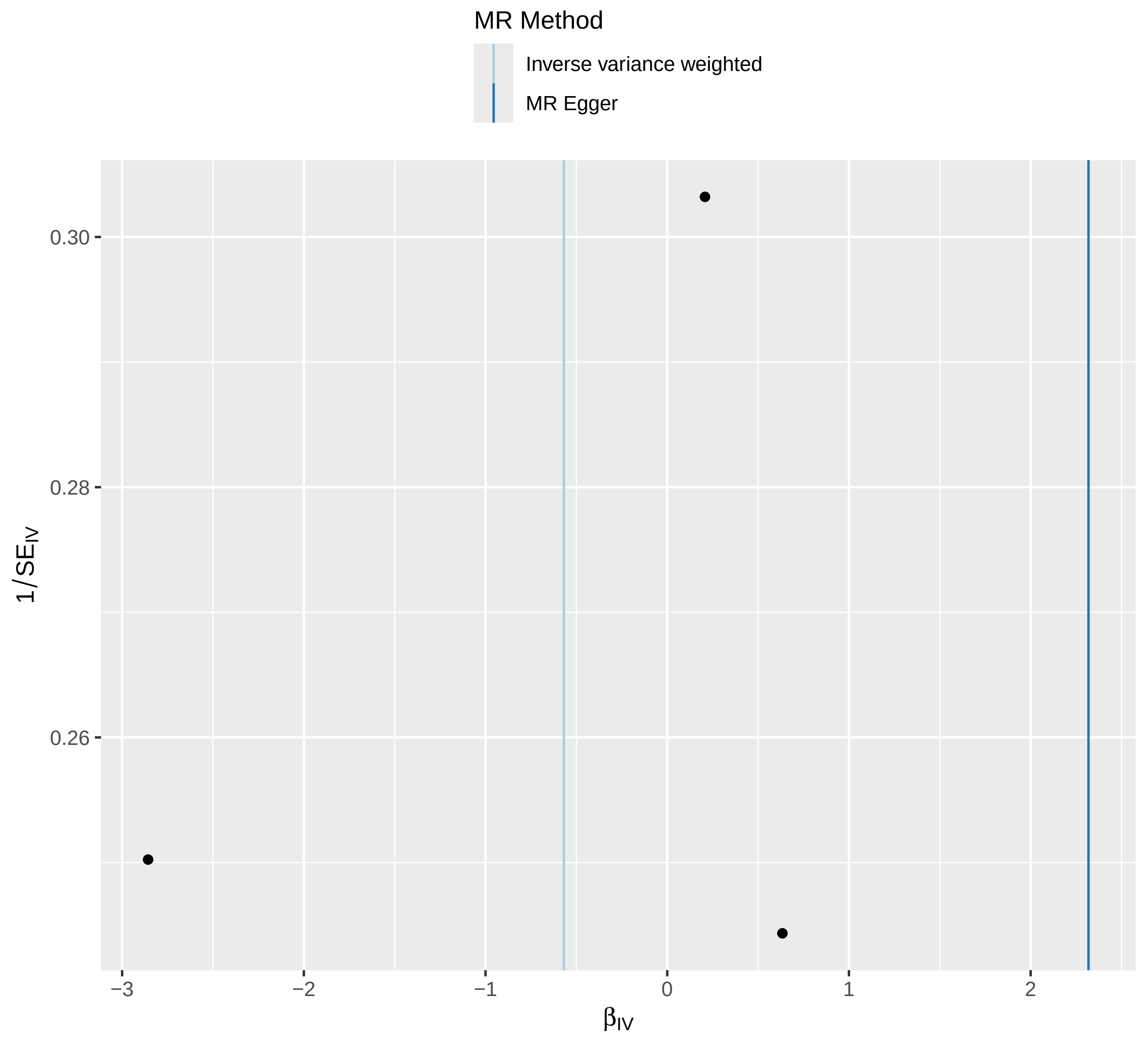

MR Method
Inverse variance weighted
MR Egger
0.30
0.28
0.26
$1/SE_{IV}$
−3
−2
−1
0
1
2
β_{IV}

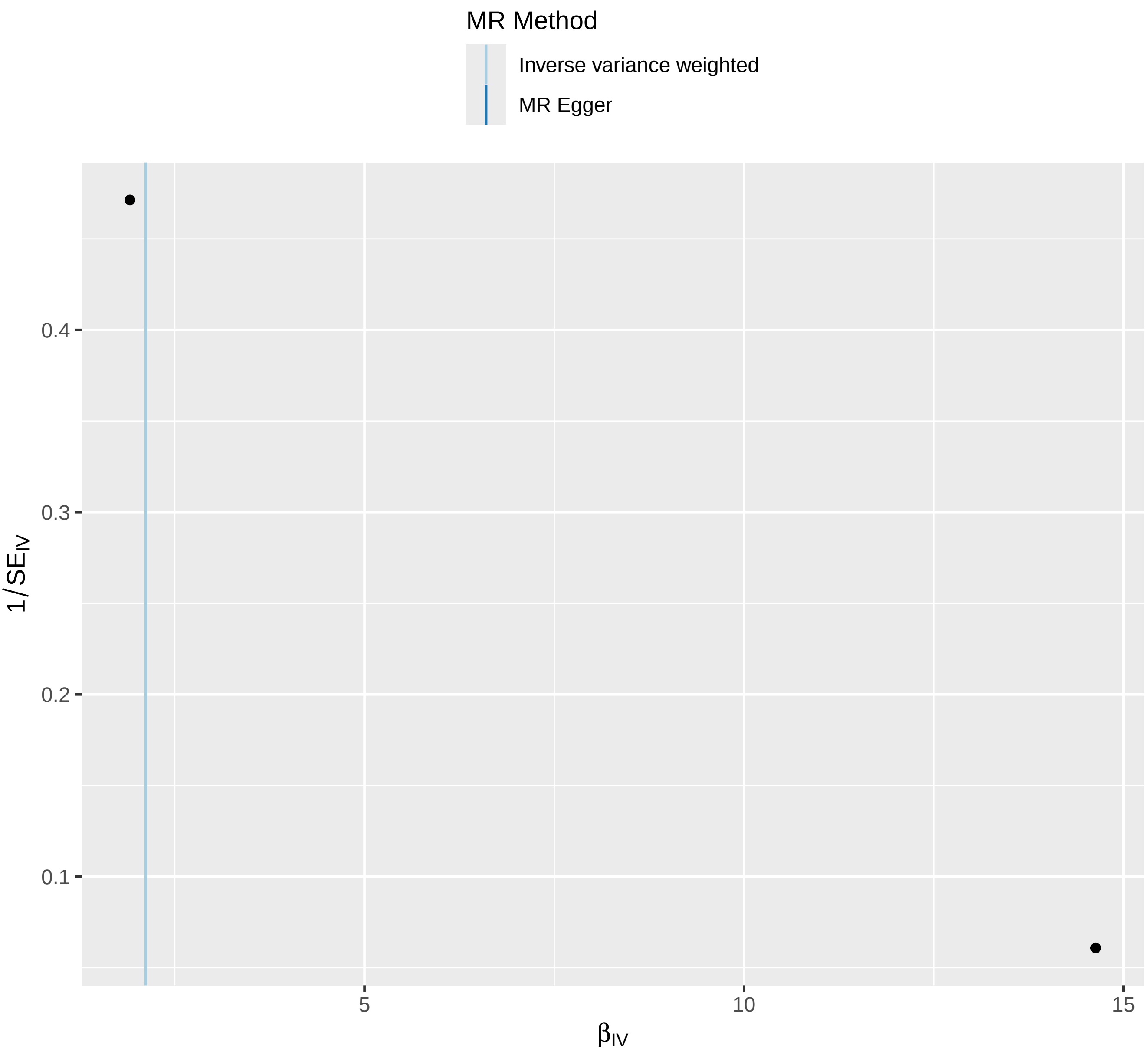
MR Method
Inverse variance weighted
MR Egger
0.4
0.3
0.2
0.1
1/SE_IV
5
10
15
β_IV

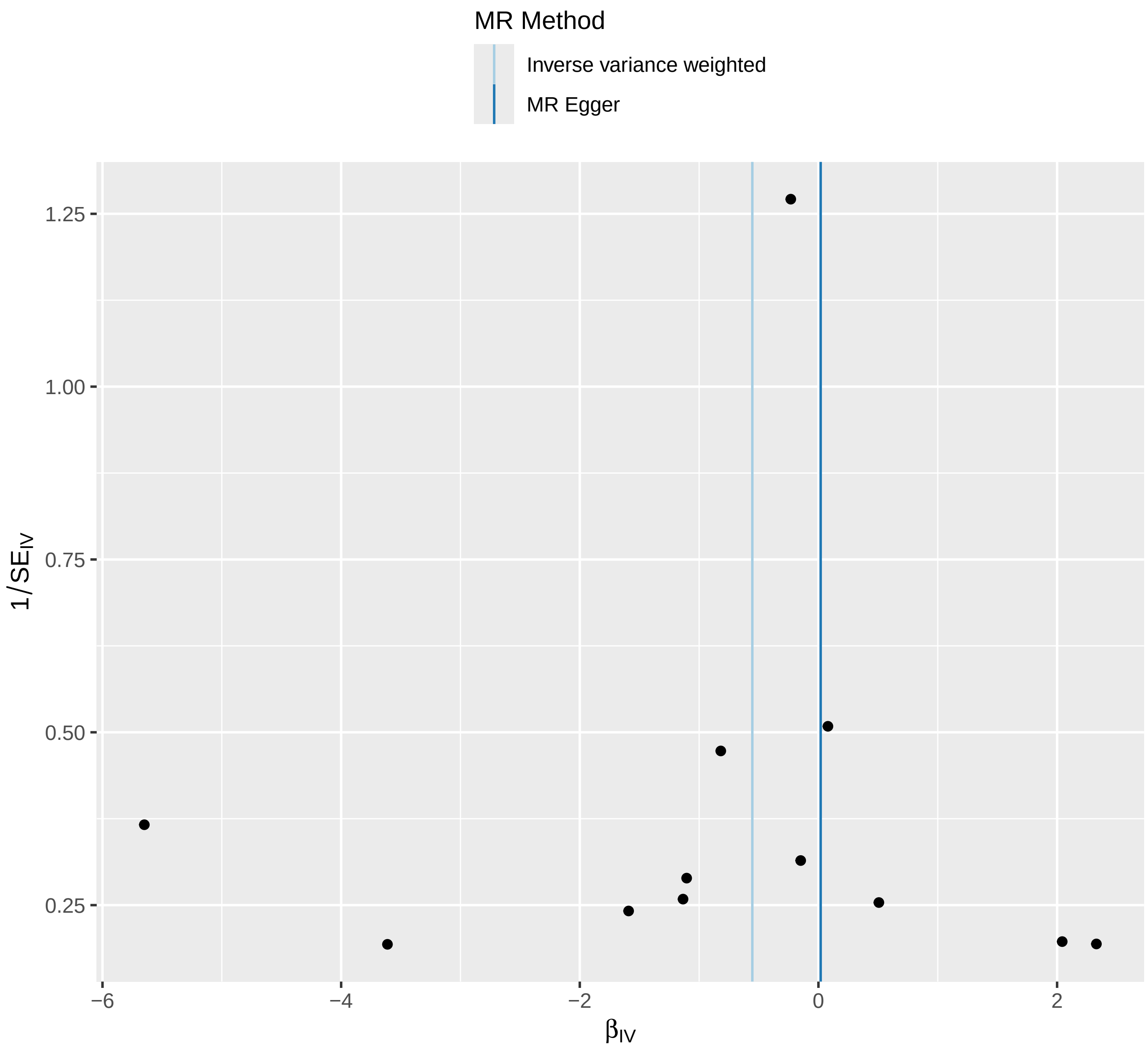

MR Method
Inverse variance weighted
MR Egger
1.25
1.00
0.75
0.50
0.25
$1/SE_{IV}$
−6
−4
−2
0
2
β_{IV}

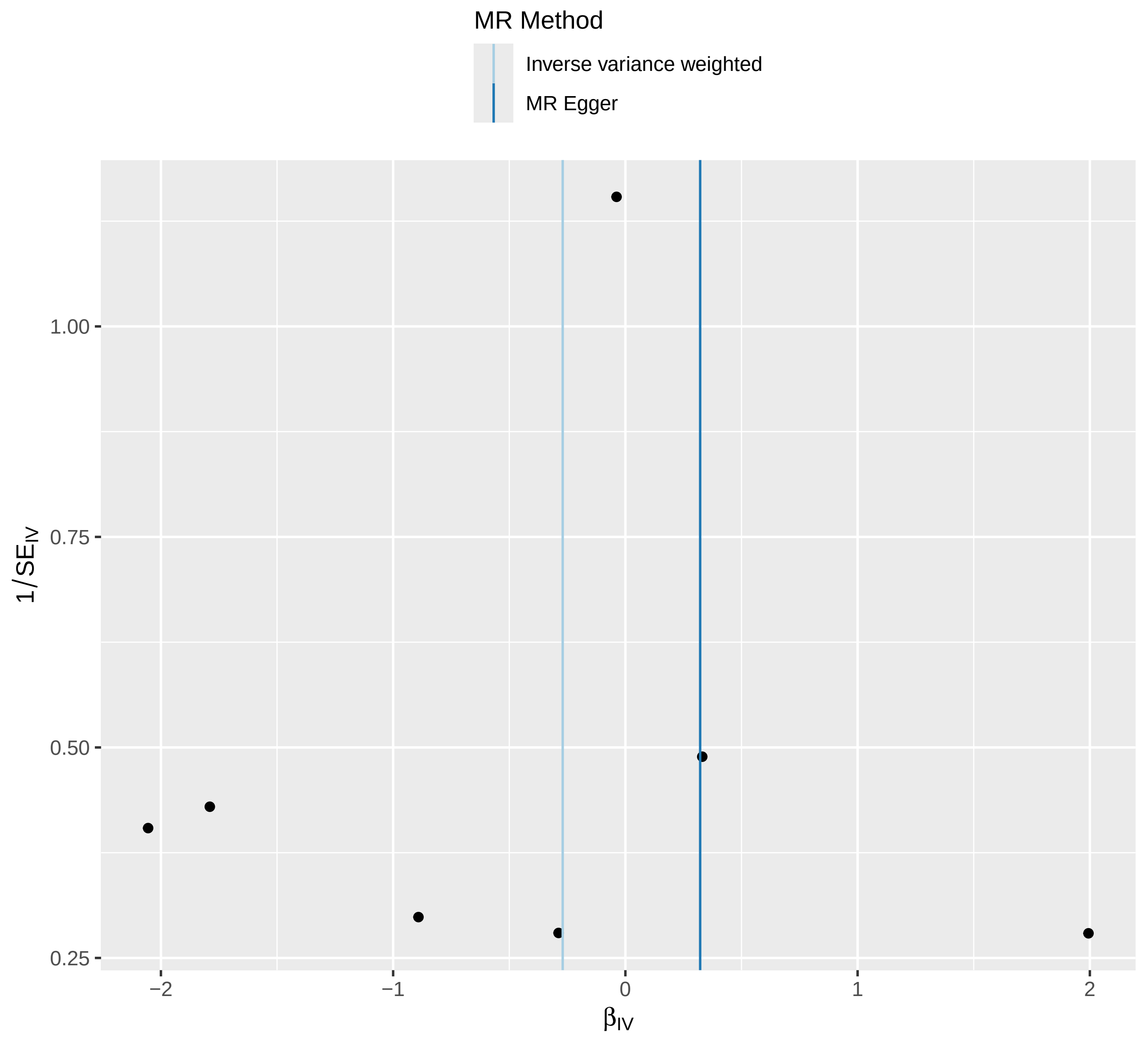
MR Method
Inverse variance weighted
MR Egger
1/SE_IV
1.00
0.75
0.50
0.25
−2
−1
0
1
2
β_IV

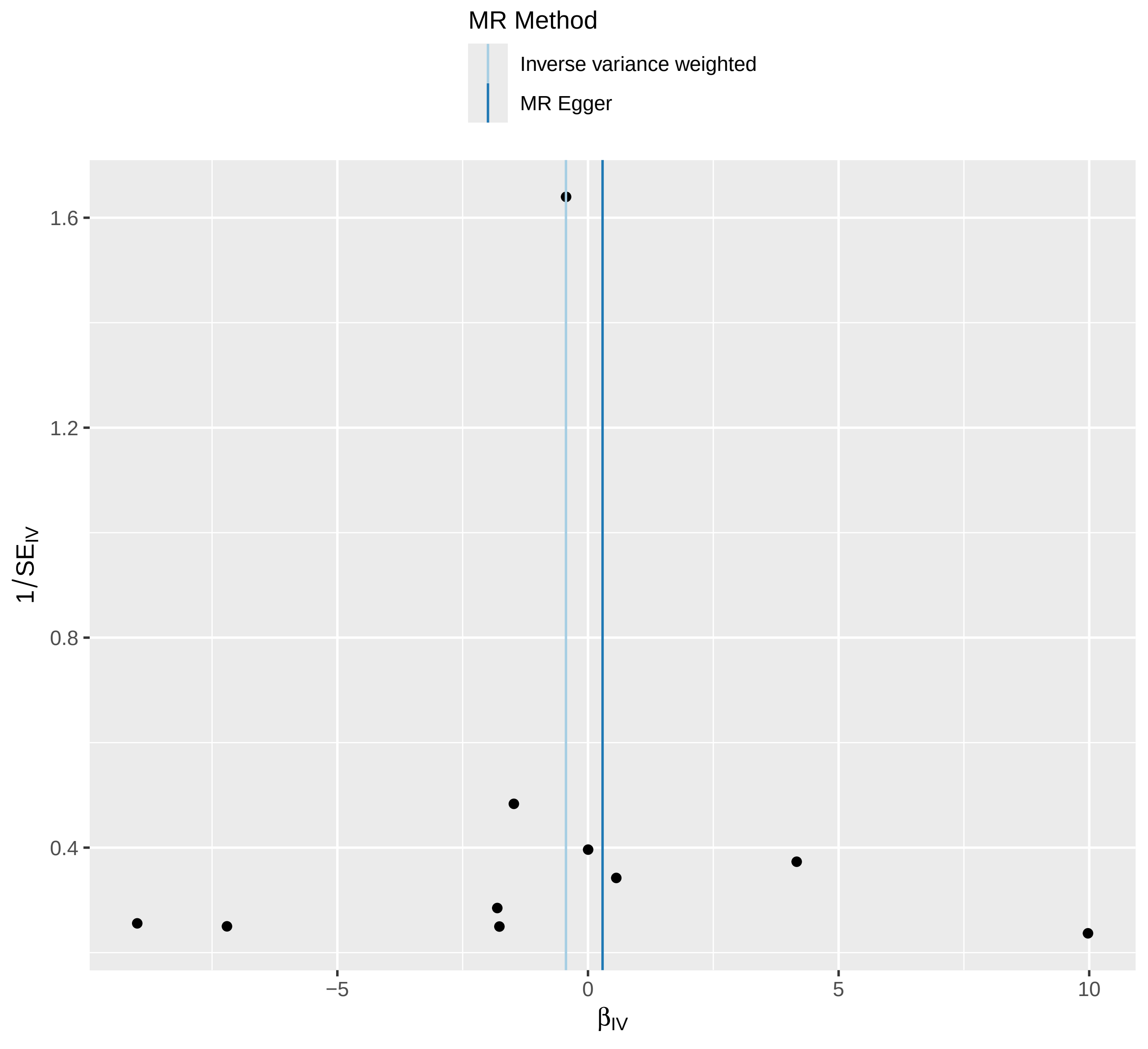
MR Method
Inverse variance weighted
MR Egger
1.6
1.2
0.8
0.4
1/SE$_{IV}$
−5
0
5
10
β$_{IV}$

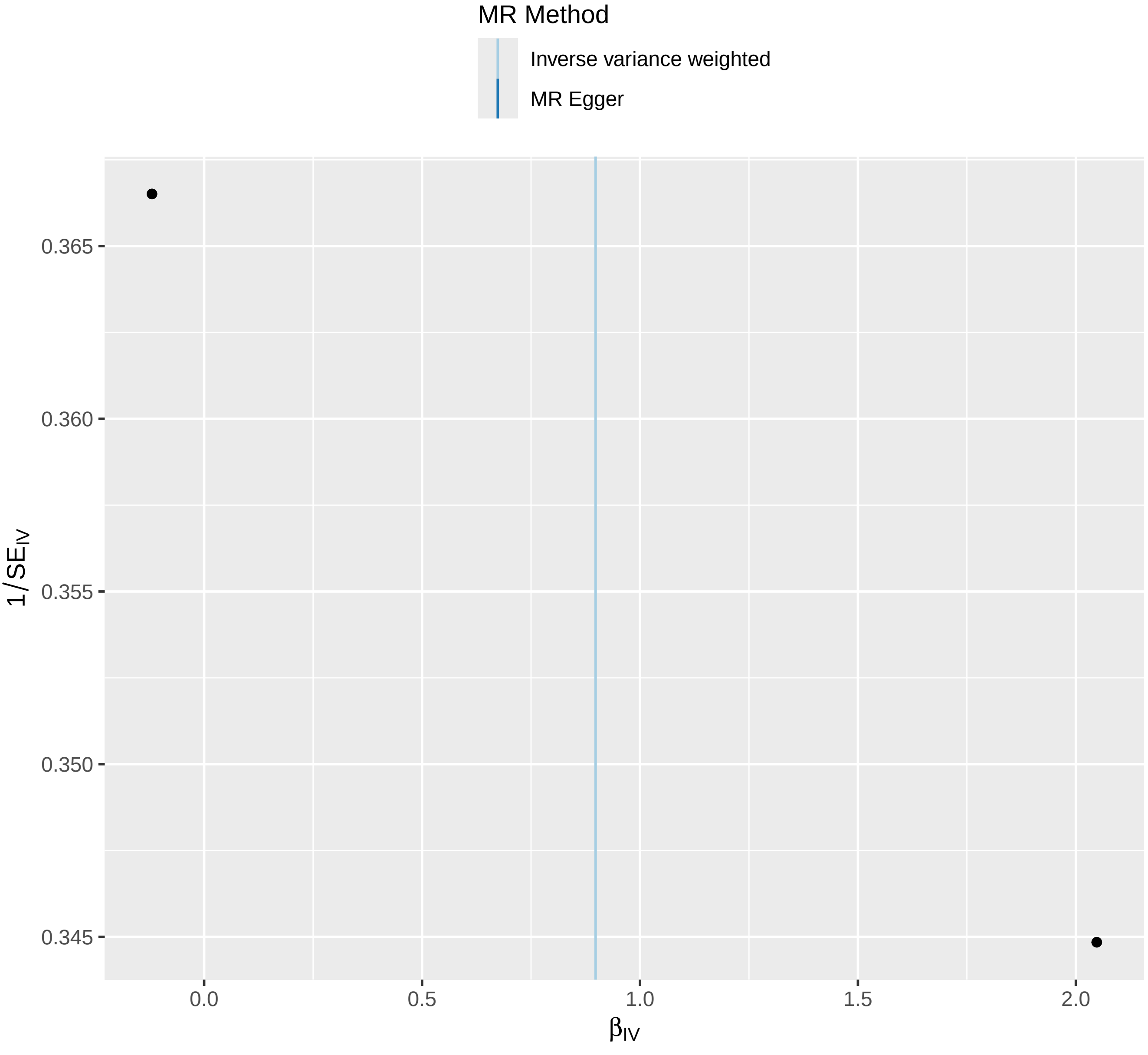
MR Method
Inverse variance weighted
MR Egger
0.365
0.360
0.355
0.350
0.345
$1/SE_{IV}$
0.0
0.5
1.0
1.5
2.0
β_{IV}

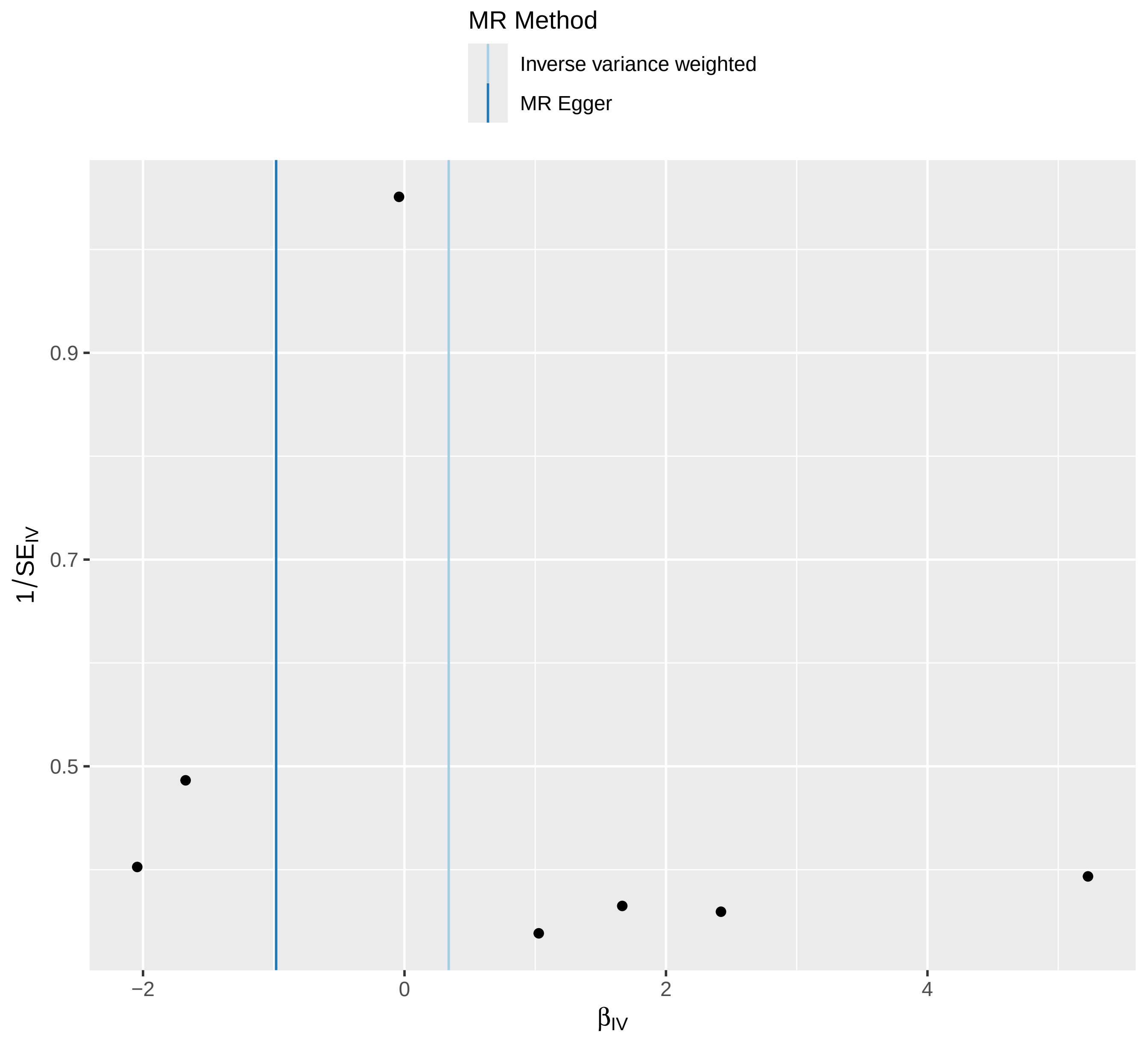

MR Method
Inverse variance weighted
MR Egger
0.9
0.7
0.5
1/SE$_{IV}$
−2
0
2
4
β$_{IV}$

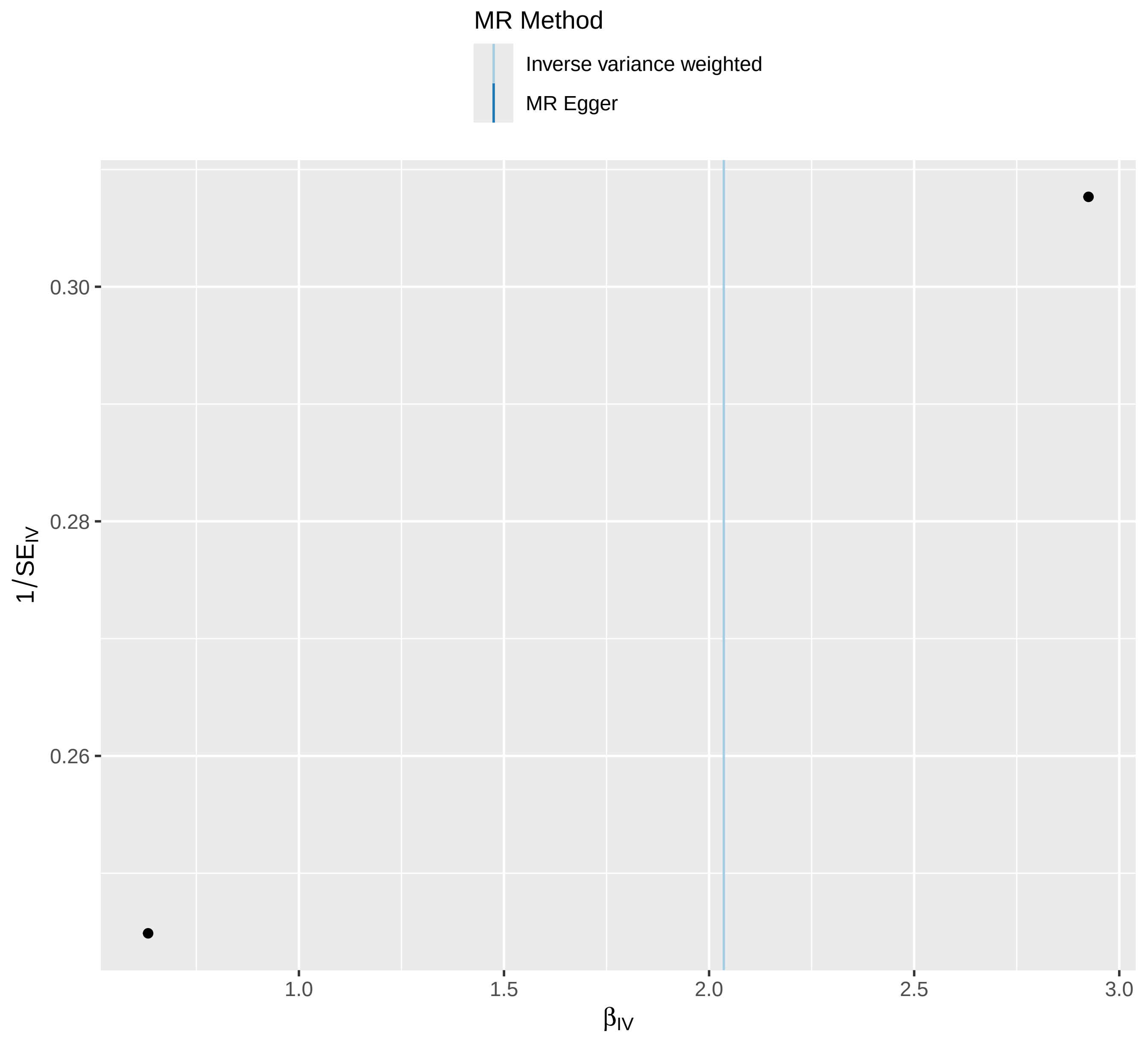

MR Method
Inverse variance weighted
MR Egger
0.30
0.28
0.26
$1/SE_{IV}$
1.0
1.5
2.0
2.5
3.0
β_{IV}

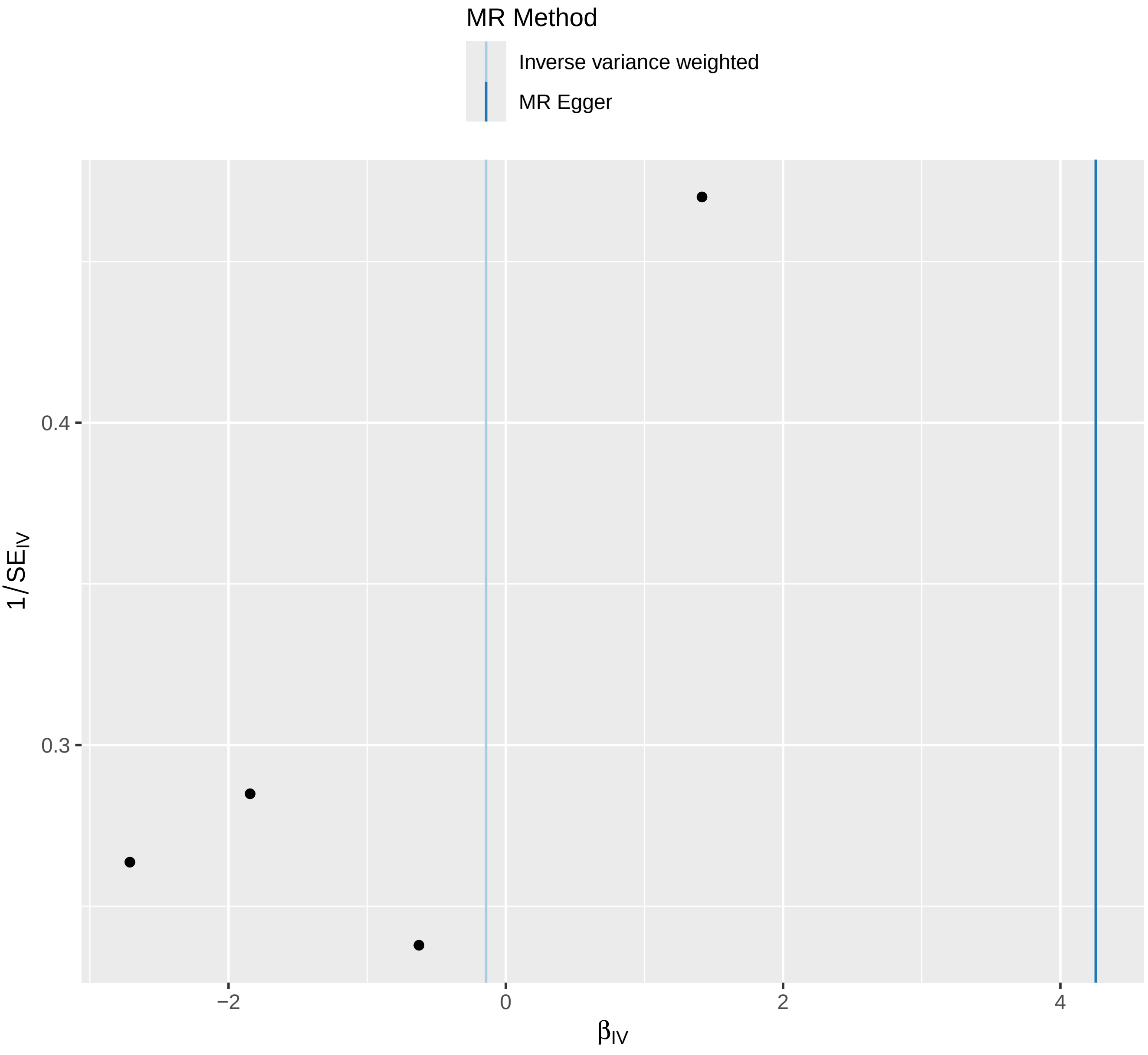

MR Method
Inverse variance weighted
MR Egger
0.4
0.3
−2
0
2
4
1/SE_IV
β_IV

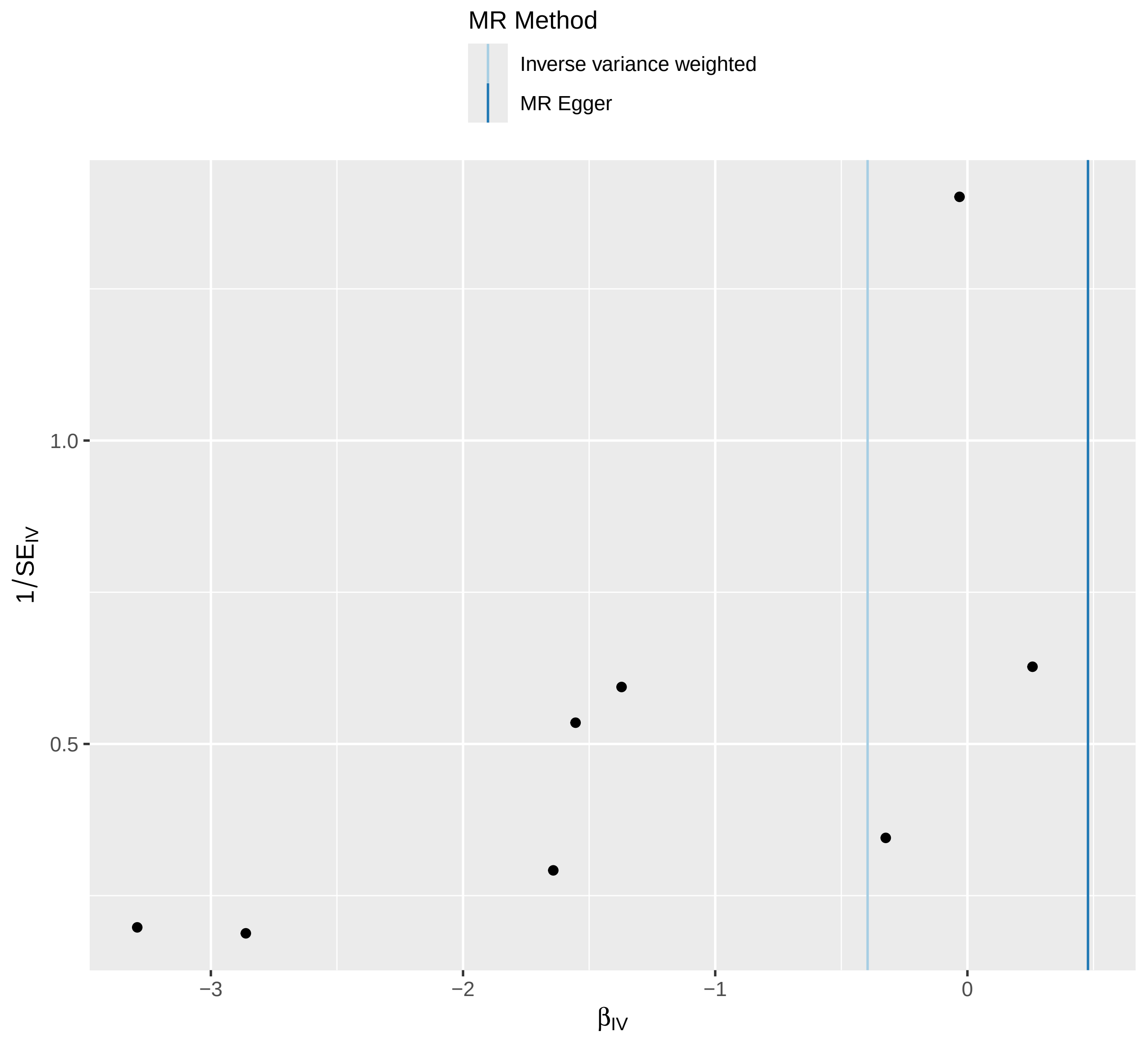
MR Method
Inverse variance weighted
MR Egger
1.0
0.5
1/SE_IV
−3
−2
−1
0
β_IV

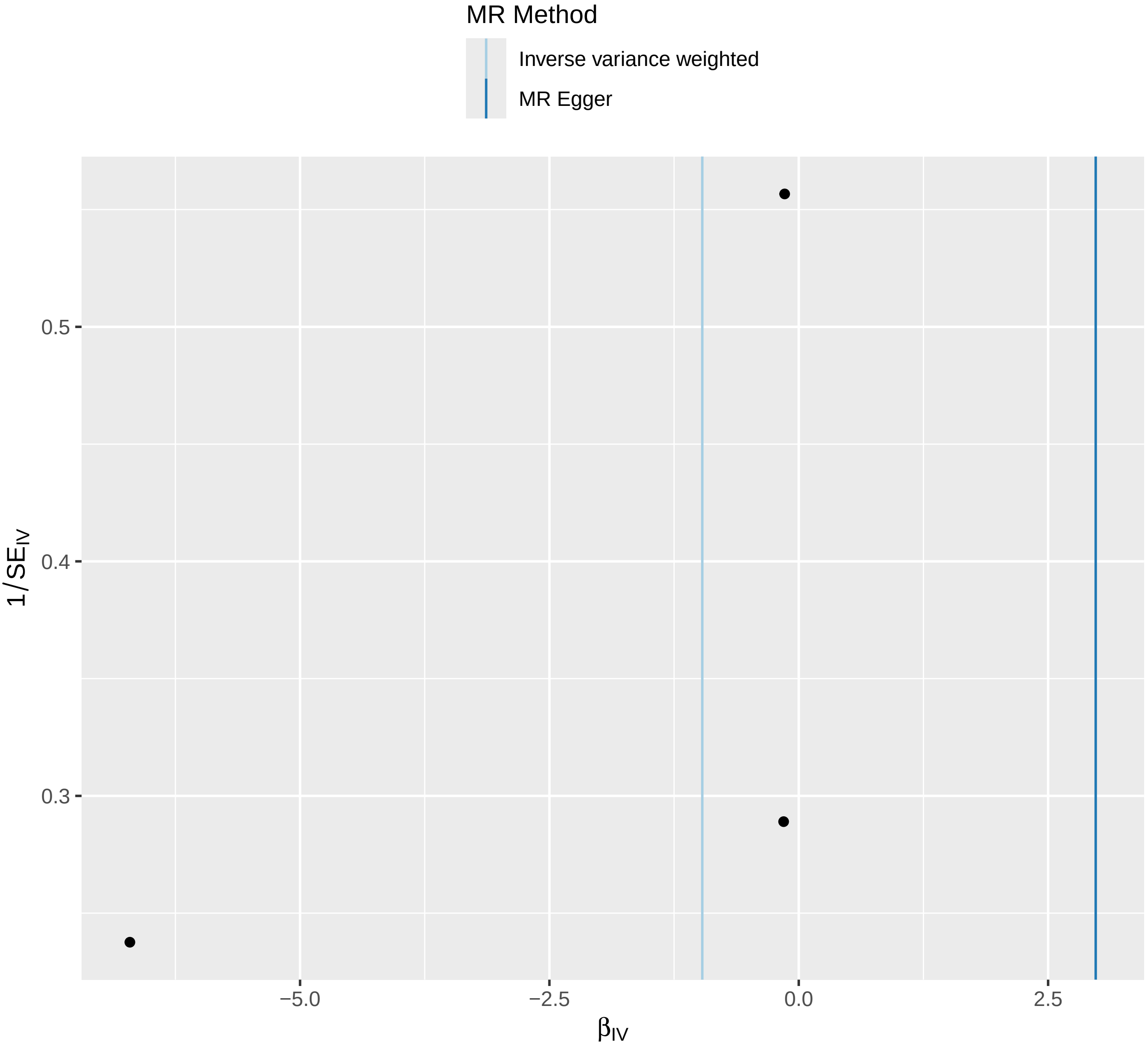
MR Method
Inverse variance weighted
MR Egger
0.5
0.4
0.3
1/SE_IV
−5.0
−2.5
0.0
2.5
β_IV

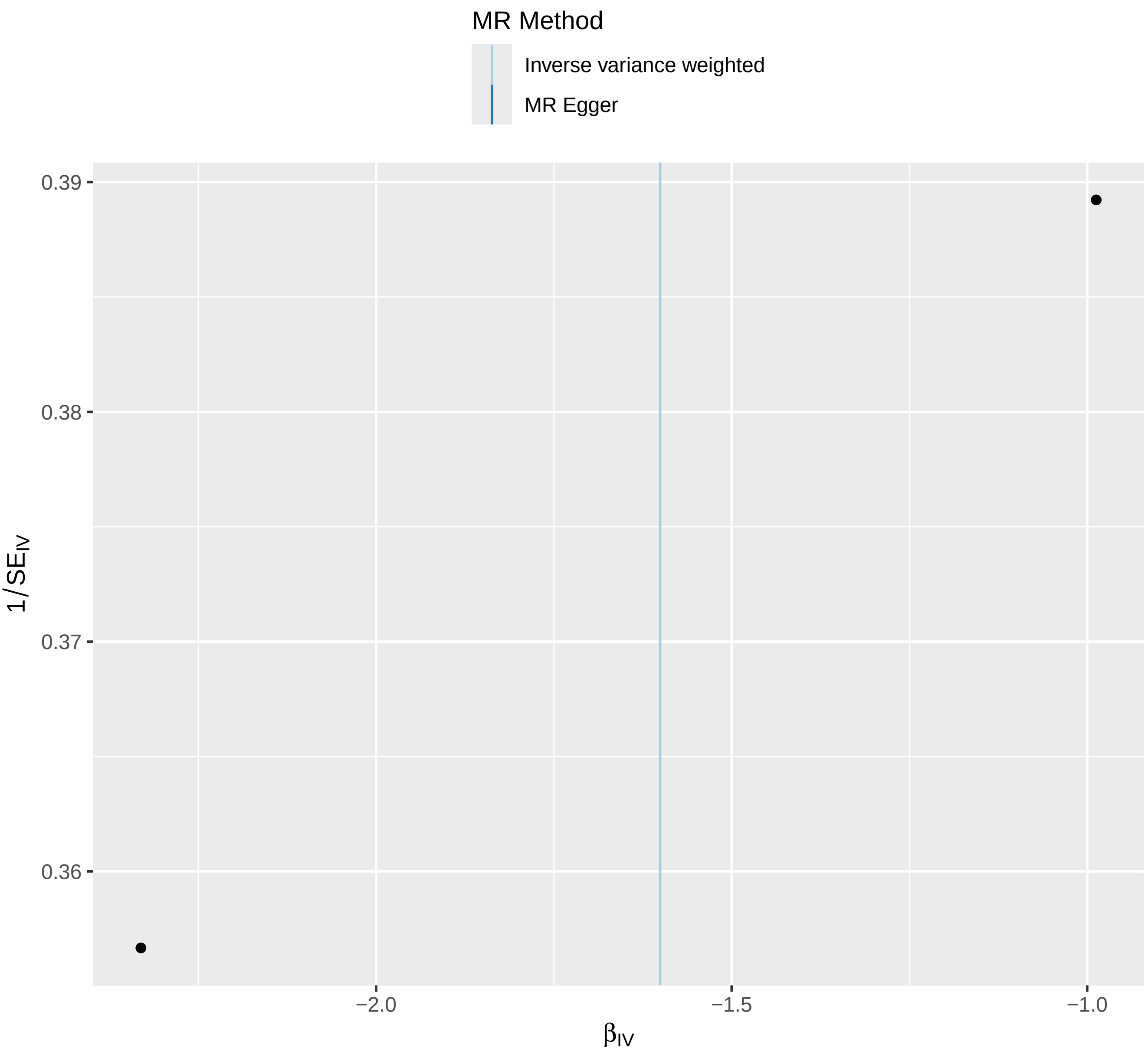
MR Method
Inverse variance weighted
MR Egger
0.39
0.38
0.37
0.36
1/SE_IV
−2.0
−1.5
−1.0
β_IV

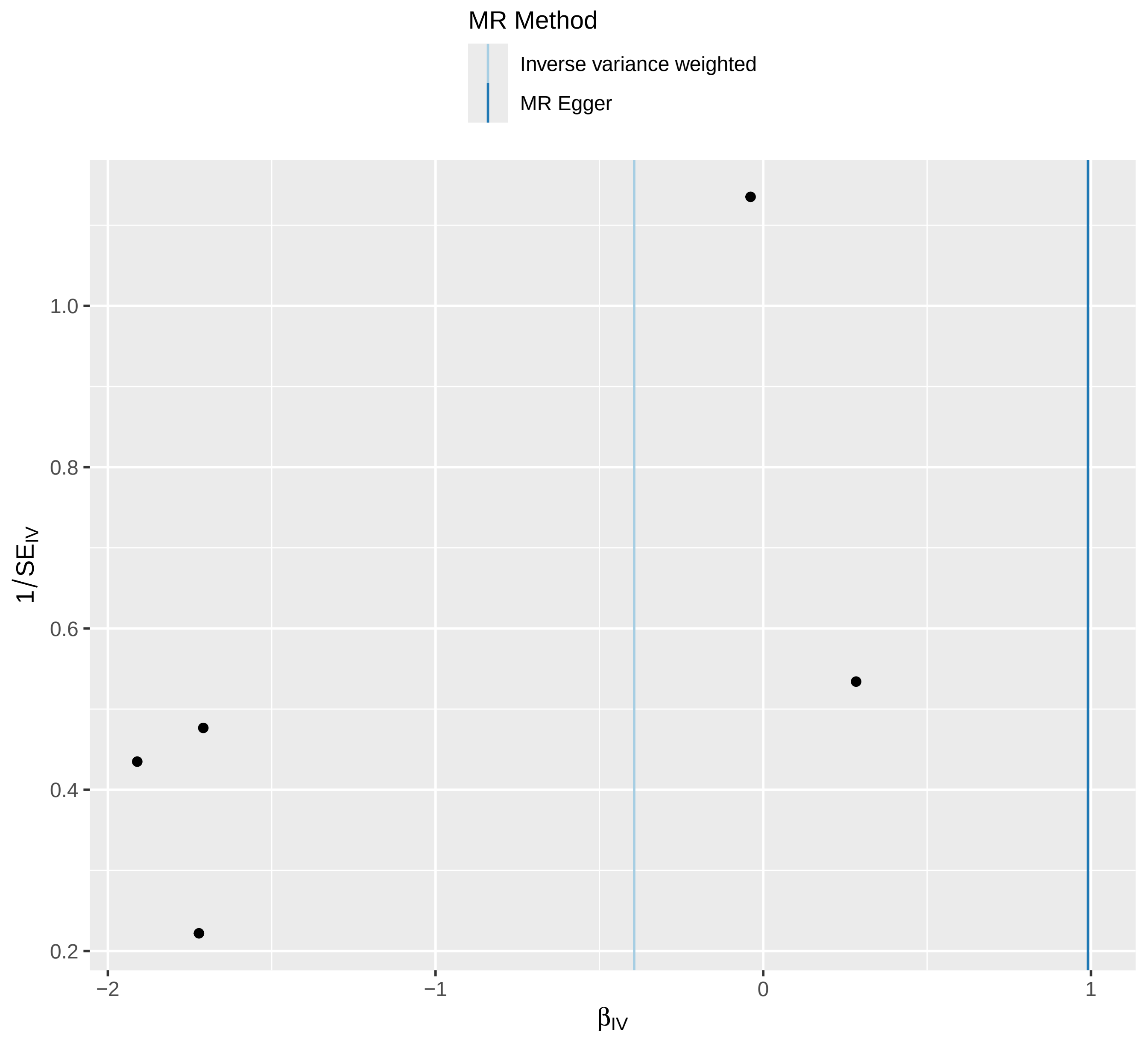
MR Method
Inverse variance weighted
MR Egger
1.0
0.8
0.6
0.4
0.2
$1/SE_{IV}$
−2
−1
0
1
β_{IV}

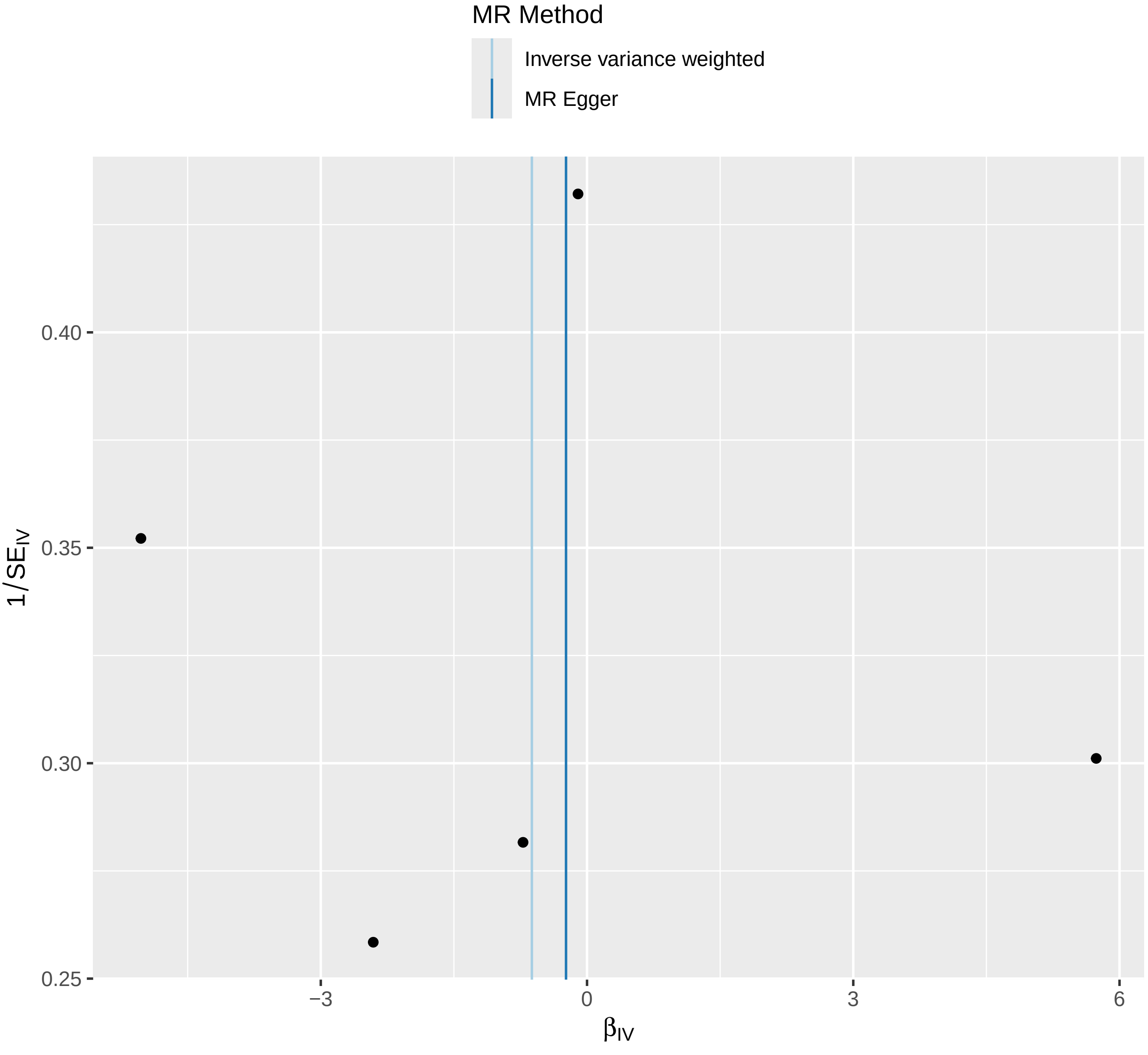
MR Method
Inverse variance weighted
MR Egger
0.40
0.35
0.30
0.25
1/SE_IV
−3
0
3
6
β_IV

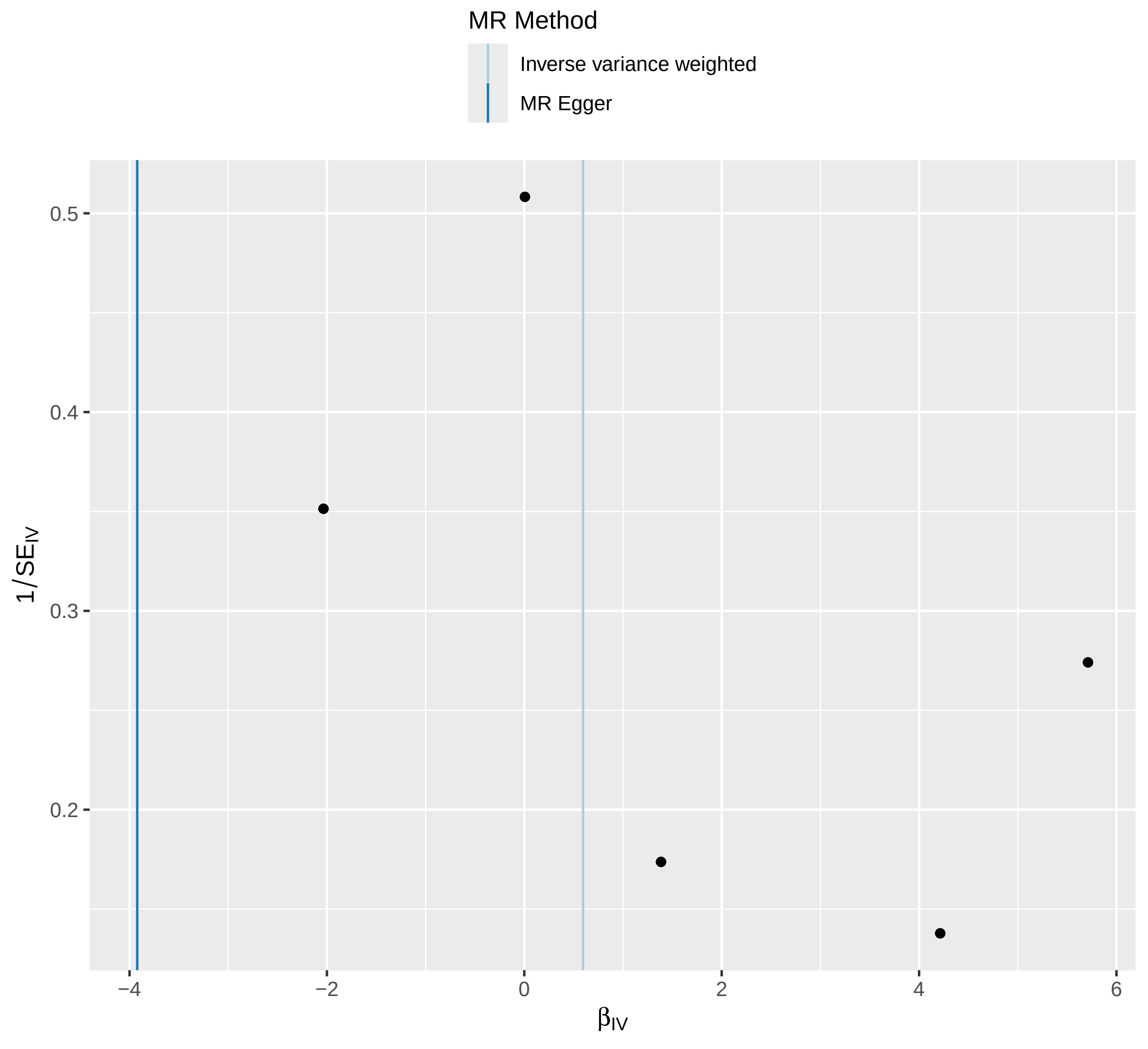
MR Method
Inverse variance weighted
MR Egger
0.5
0.4
0.3
0.2
1/SE$_{IV}$
−4
−2
0
2
4
6
β$_{IV}$

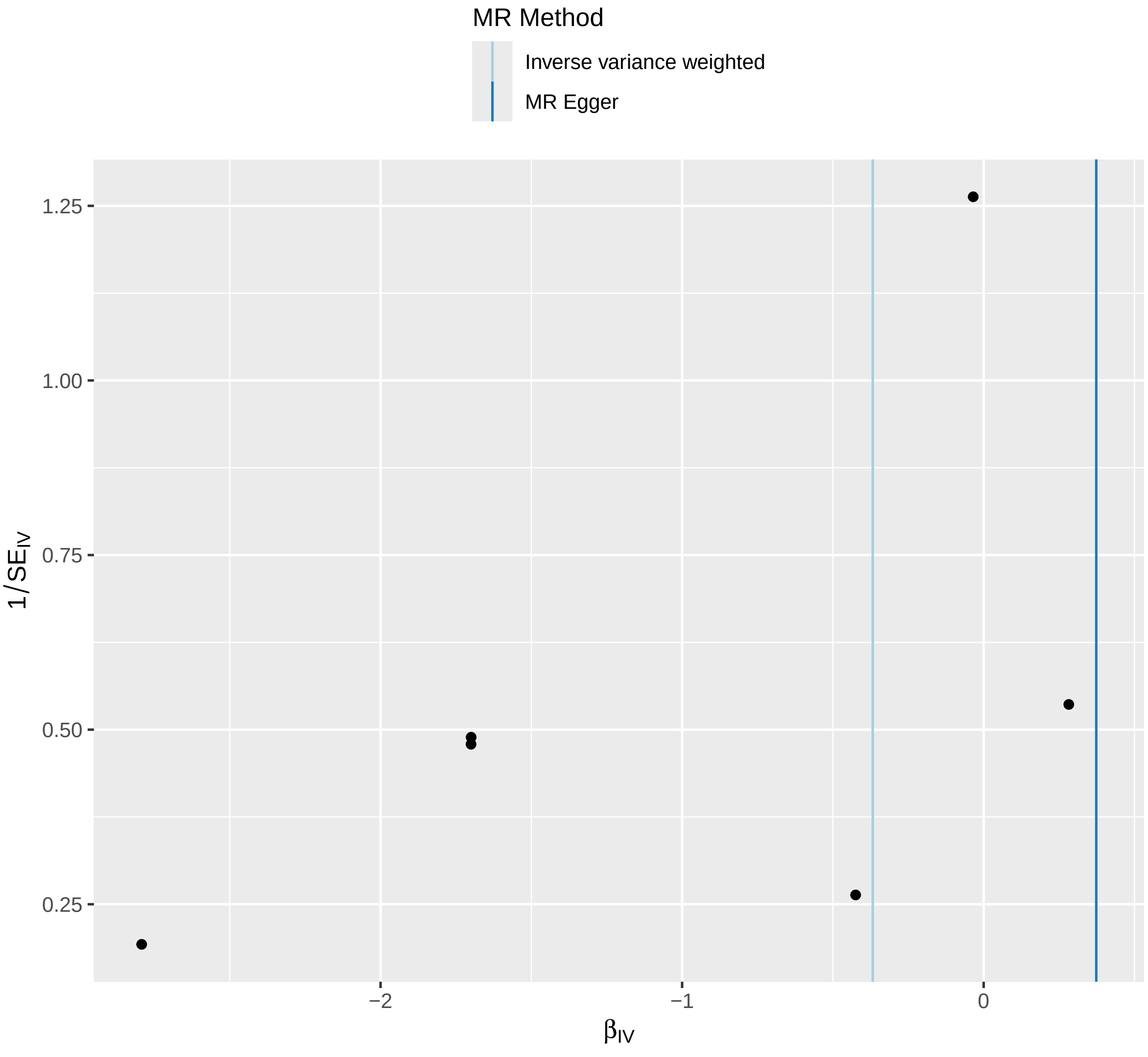
MR Method
Inverse variance weighted
MR Egger
1.25
1.00
0.75
0.50
0.25
1/SE_IV
−2
−1
0
β_IV

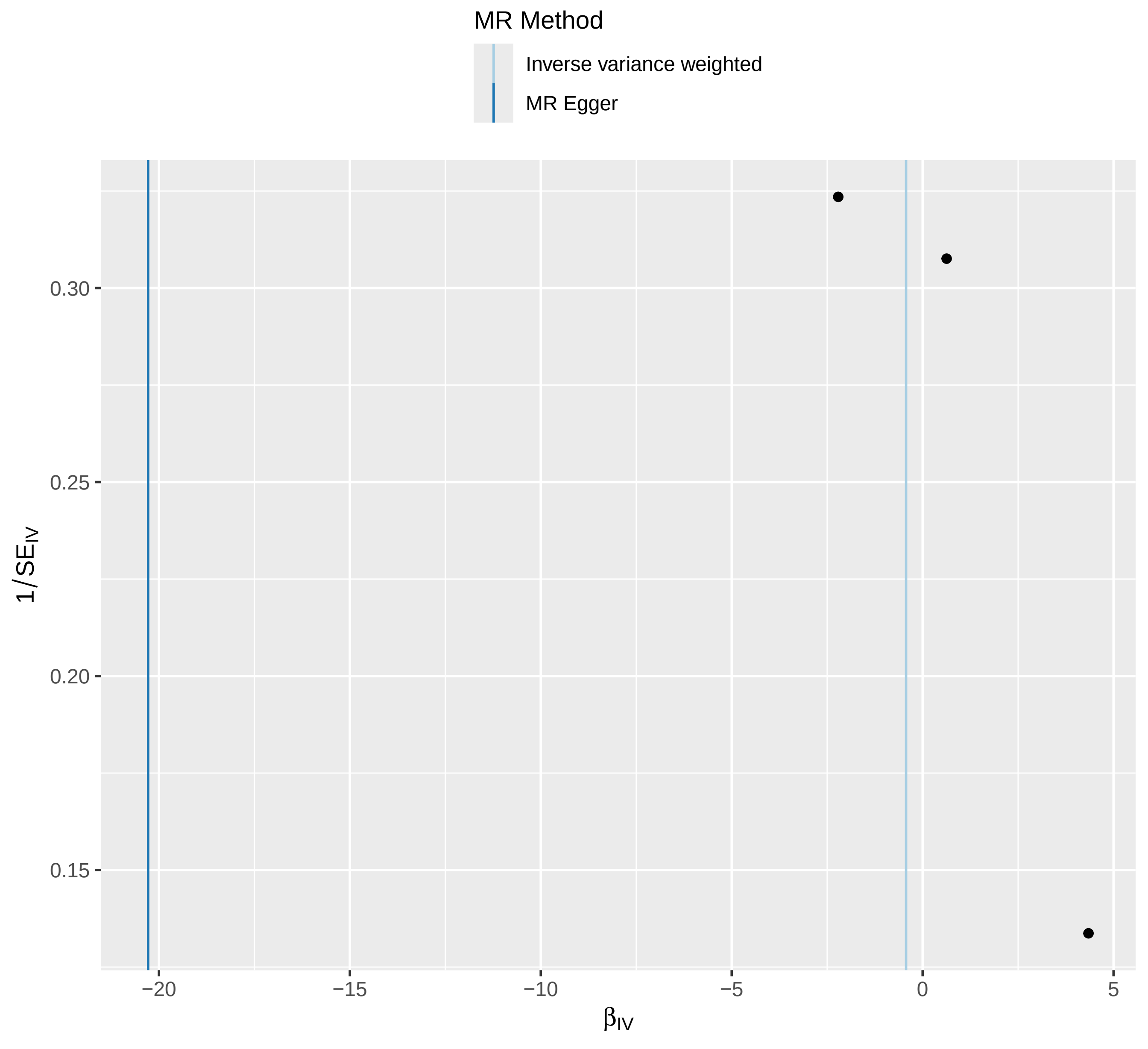
MR Method
Inverse variance weighted
MR Egger
0.30
0.25
0.20
0.15
1/SE$_{IV}$
−20
−15
−10
−5
0
5
β_{IV}

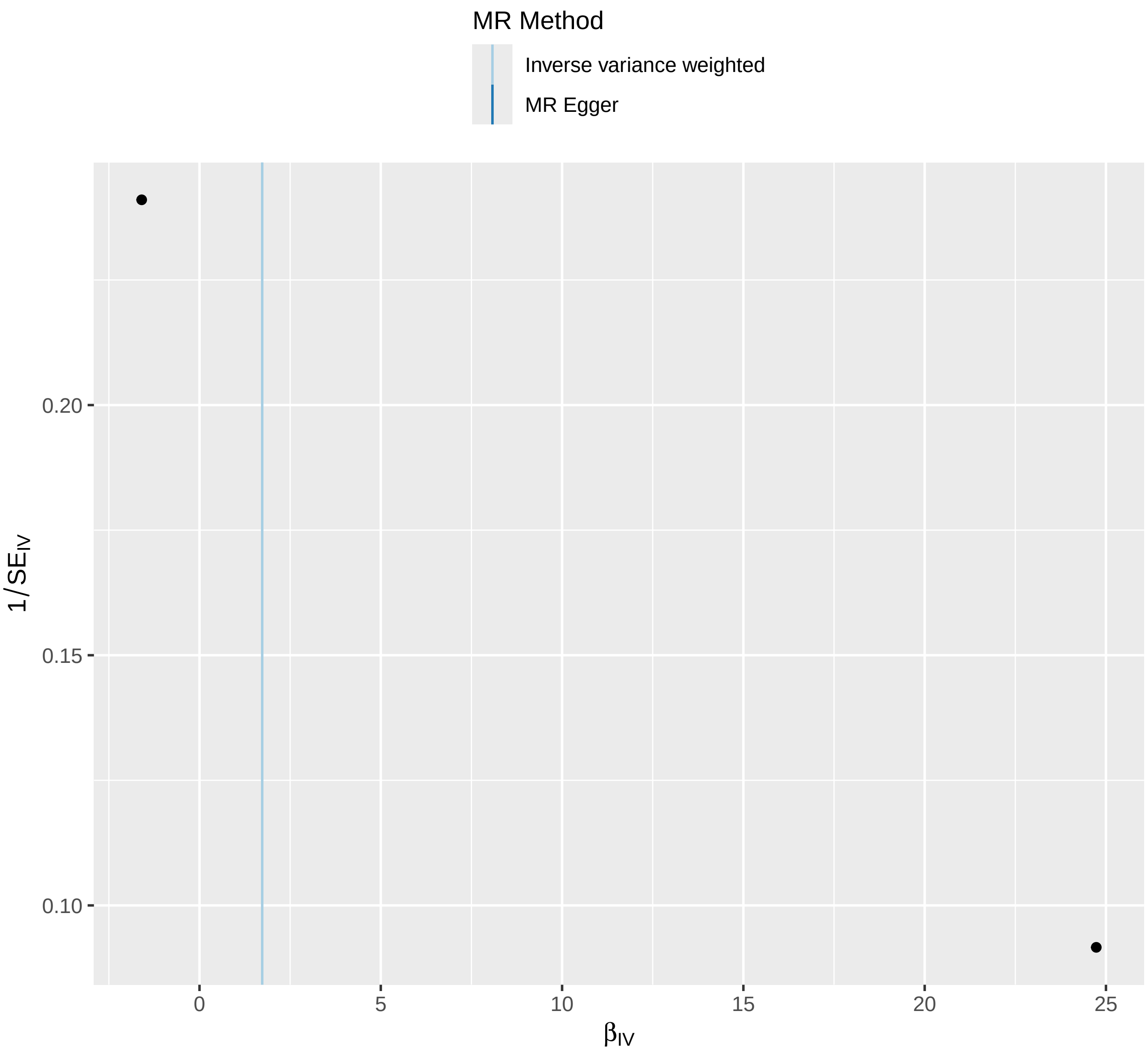

MR Method
Inverse variance weighted
MR Egger
0.20
0.15
0.10
1/SE$_{IV}$
0
5
10
15
20
25
β_{IV}

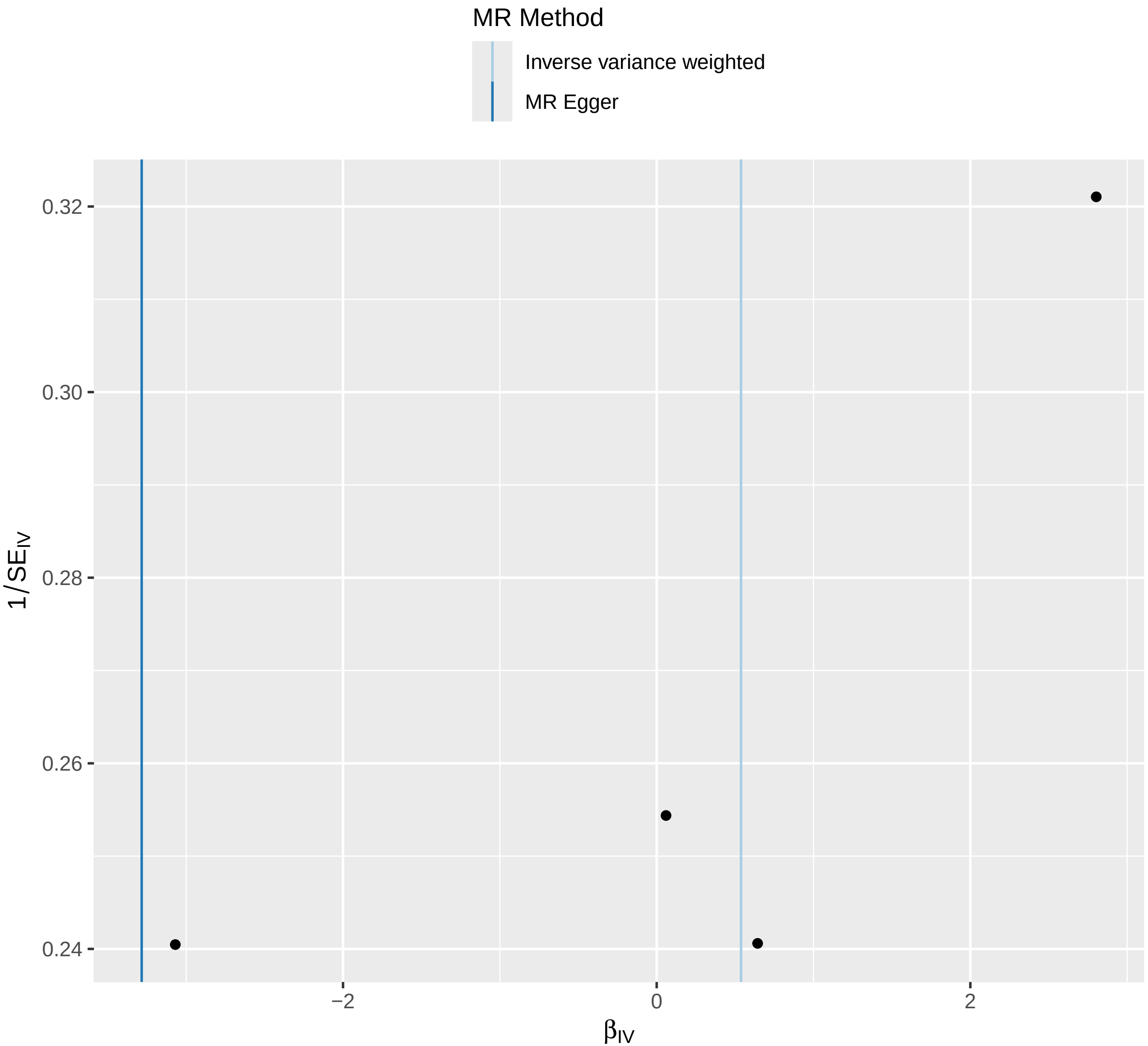
MR Method
Inverse variance weighted
MR Egger
0.32
0.30
0.28
0.26
0.24
1/SE_IV
−2
0
2
β_IV

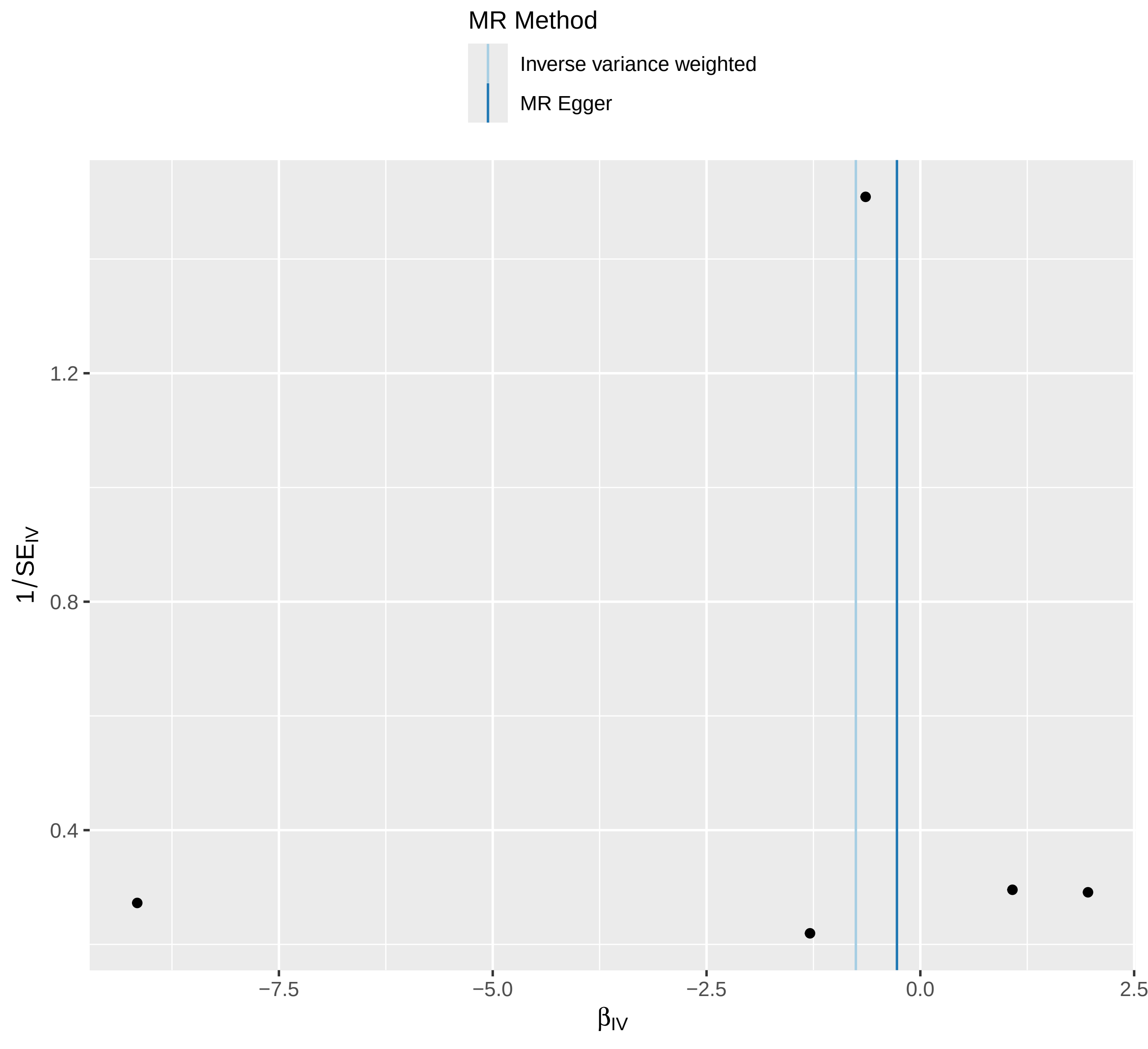
MR Method
Inverse variance weighted
MR Egger
1.2
0.8
0.4
1/SE_IV
−7.5
−5.0
−2.5
0.0
2.5
β_IV

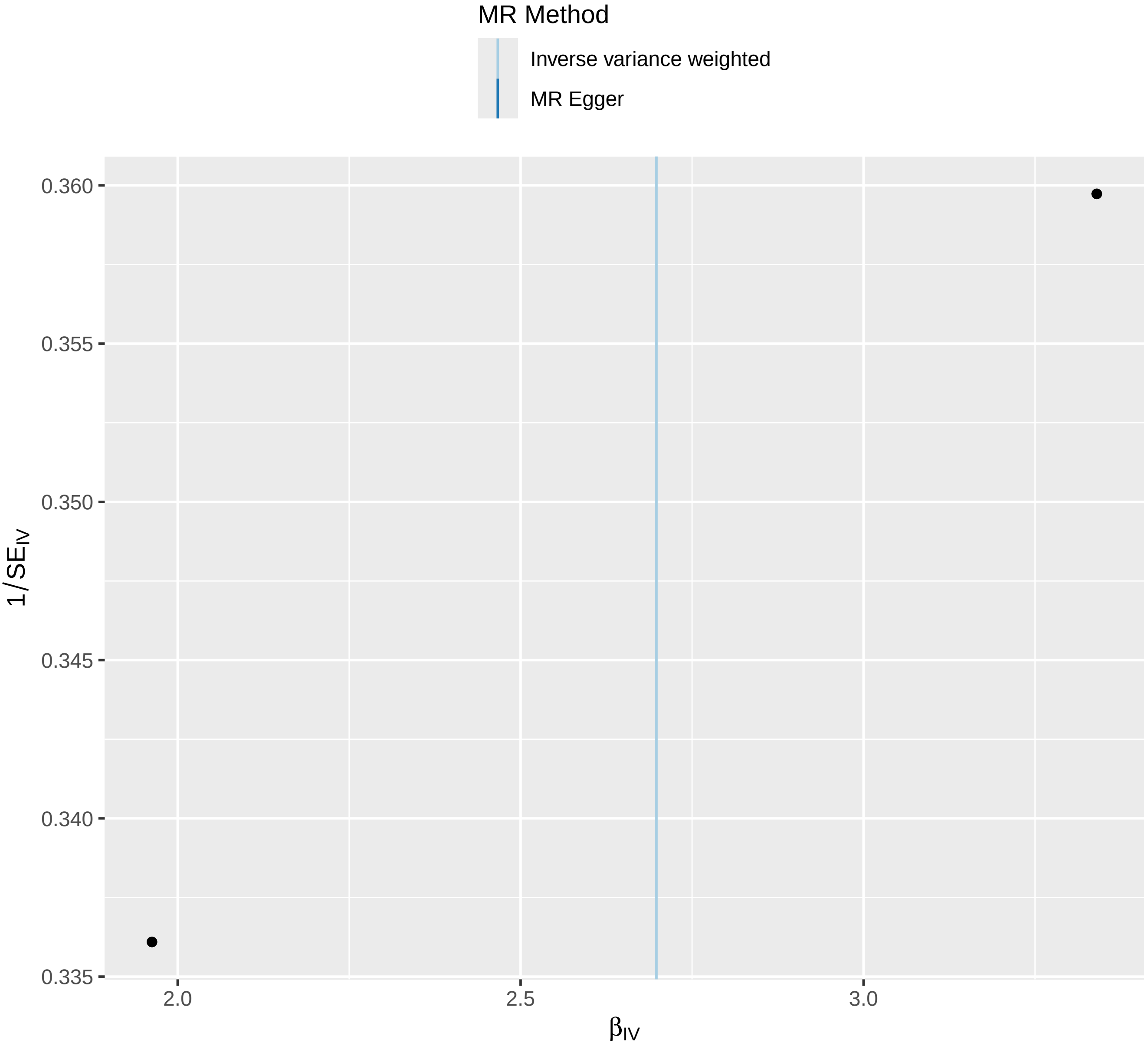
MR Method
Inverse variance weighted
MR Egger
0.360
0.355
0.350
0.345
0.340
0.335
2.0
2.5
3.0
β_{IV}
$1/SE_{IV}$

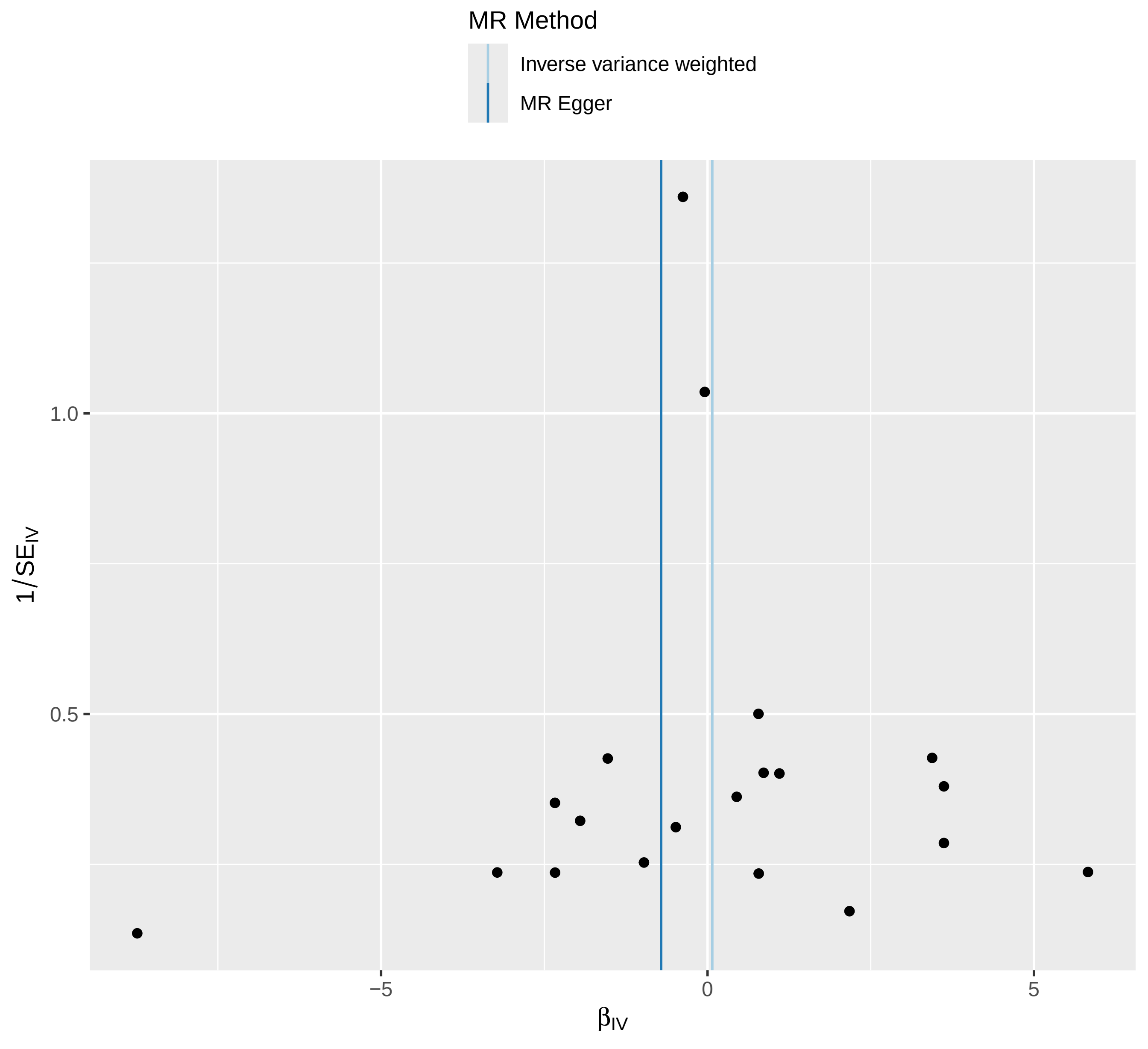

MR Method
Inverse variance weighted
MR Egger
1.0
0.5
1/SE$_{IV}$
−5
0
5
β$_{IV}$

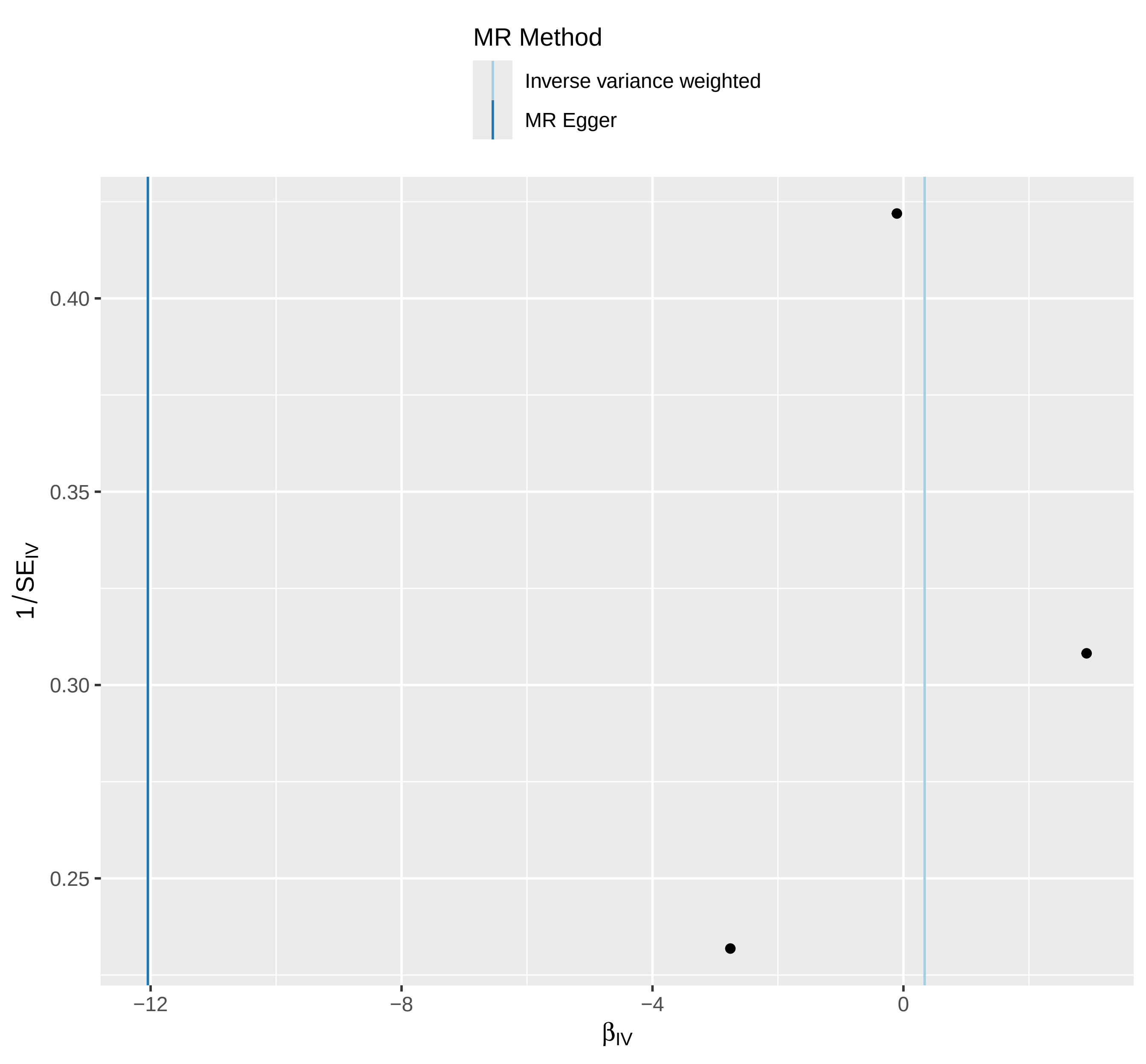
MR Method
Inverse variance weighted
MR Egger
0.40
0.35
0.30
0.25
1/SE_IV
−12
−8
−4
0
β_IV

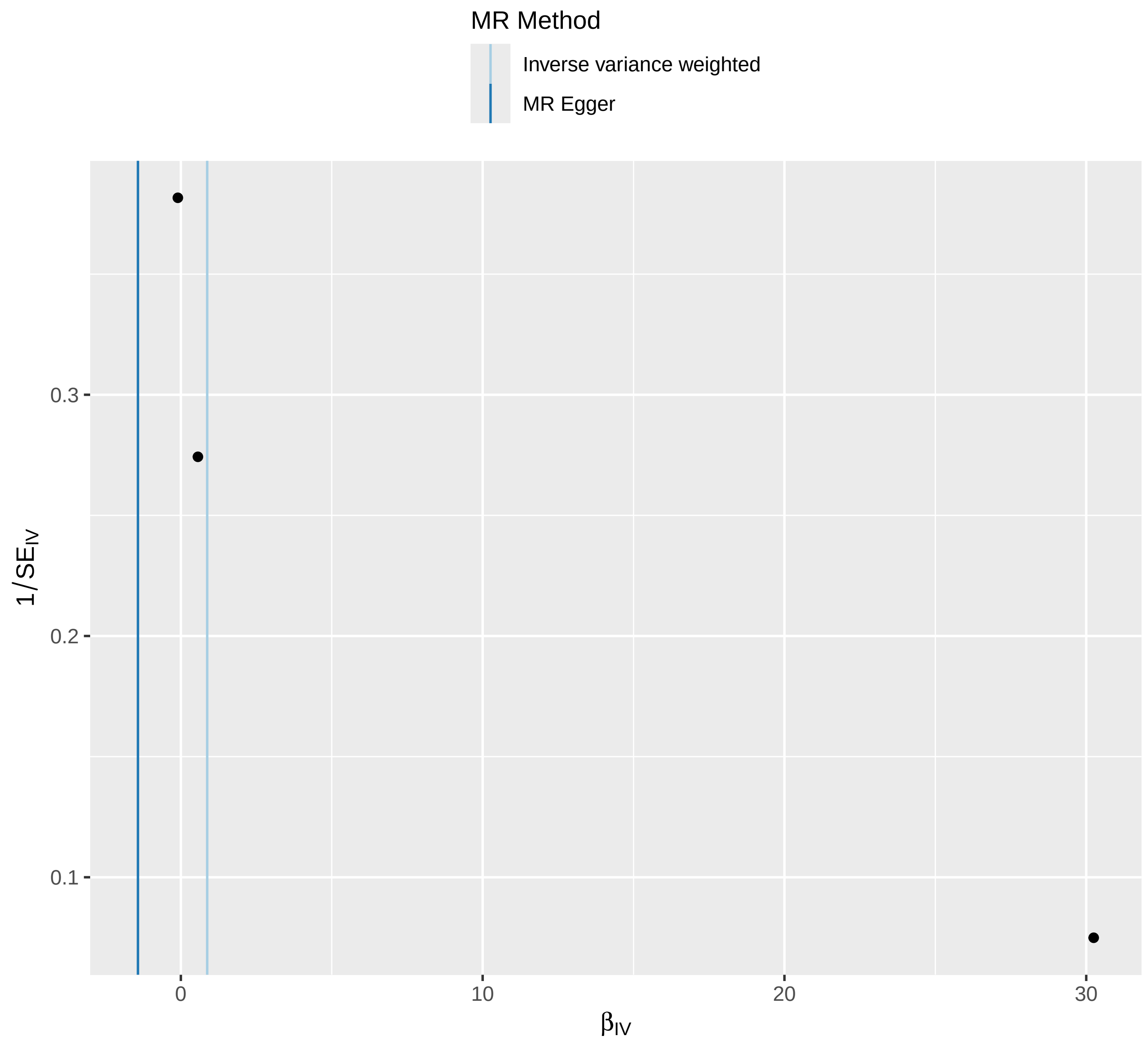
MR Method
Inverse variance weighted
MR Egger
0.3
0.2
0.1
$1/SE_{IV}$
0
10
20
30
β_{IV}

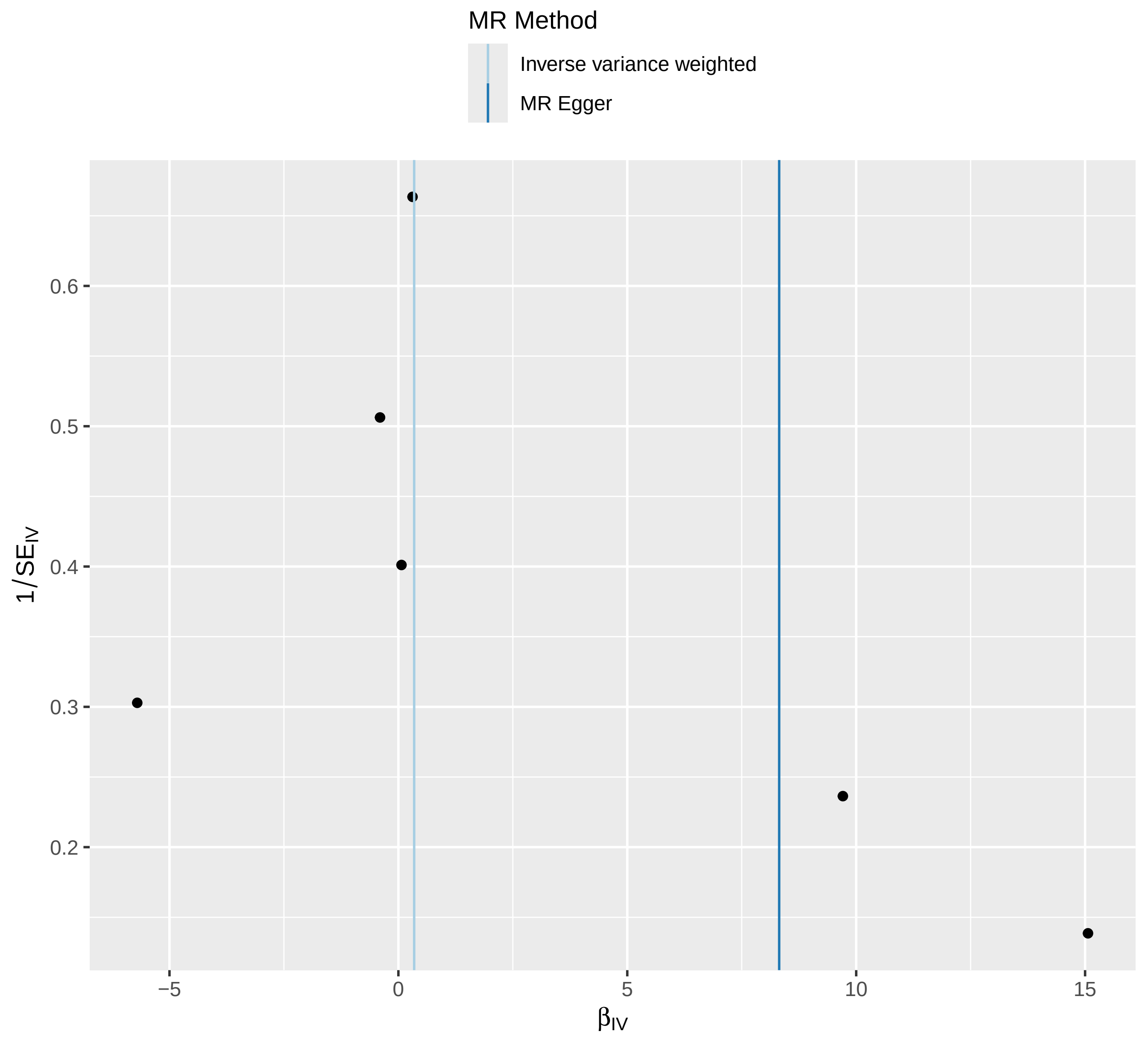

MR Method
Inverse variance weighted
MR Egger
0.6
0.5
0.4
0.3
0.2
1/SE_IV
−5
0
5
10
15
β_IV

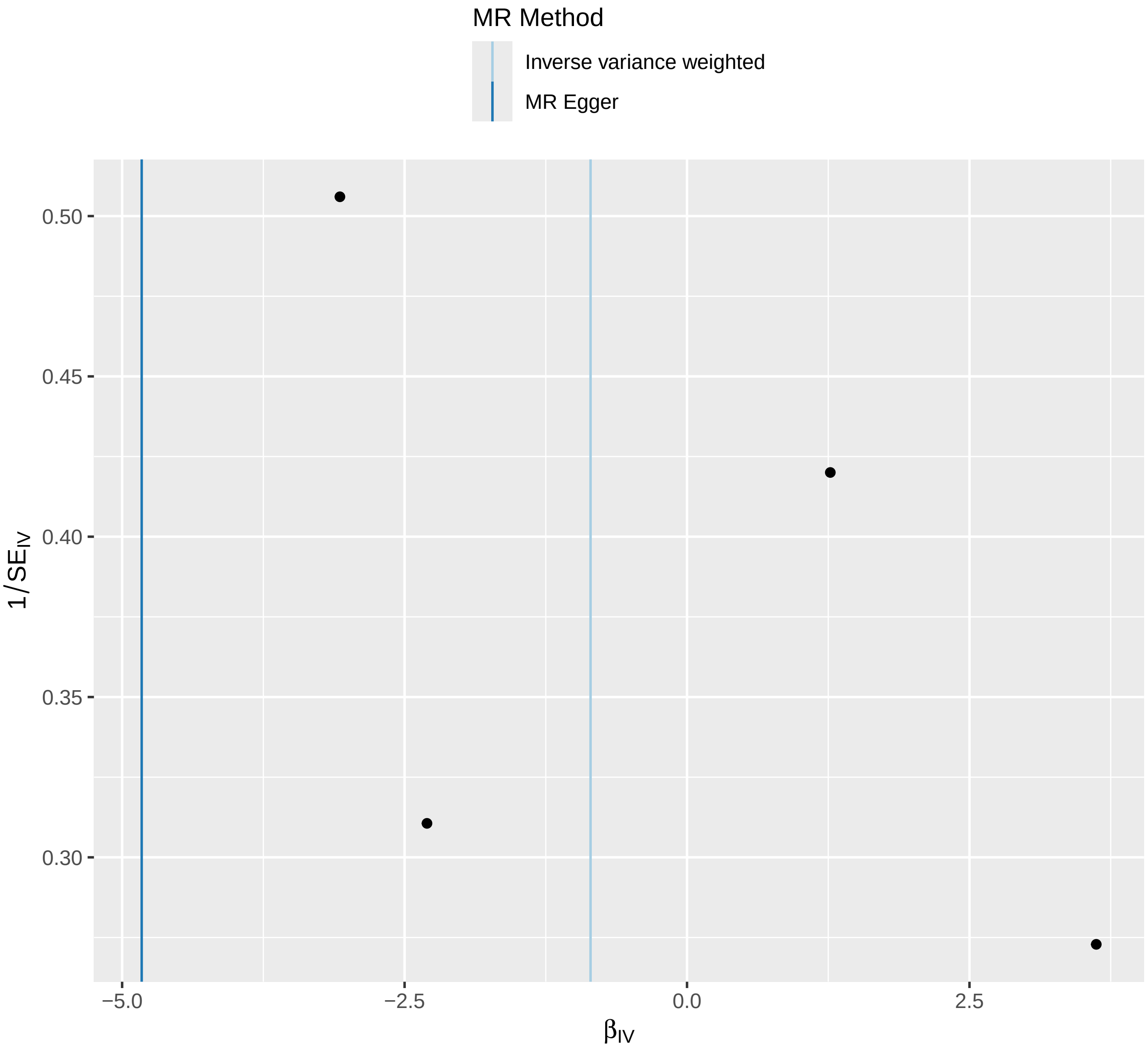

MR Method
Inverse variance weighted
MR Egger
0.50
0.45
0.40
0.35
0.30
$1/SE_{IV}$
−5.0
−2.5
0.0
2.5
β_{IV}

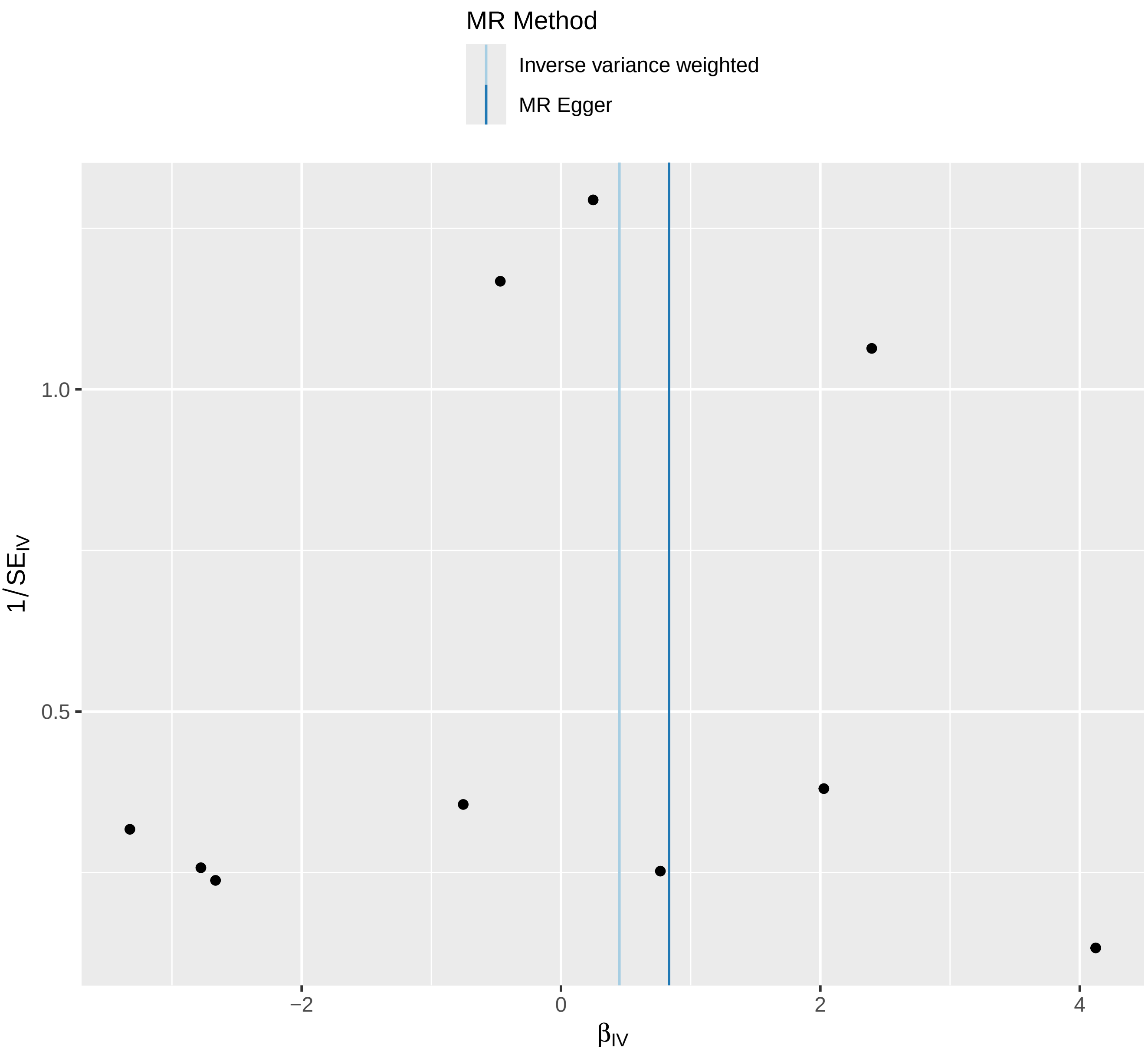
MR Method
Inverse variance weighted
MR Egger
1.0
0.5
1/SE_{IV}
−2
0
2
4
β_{IV}

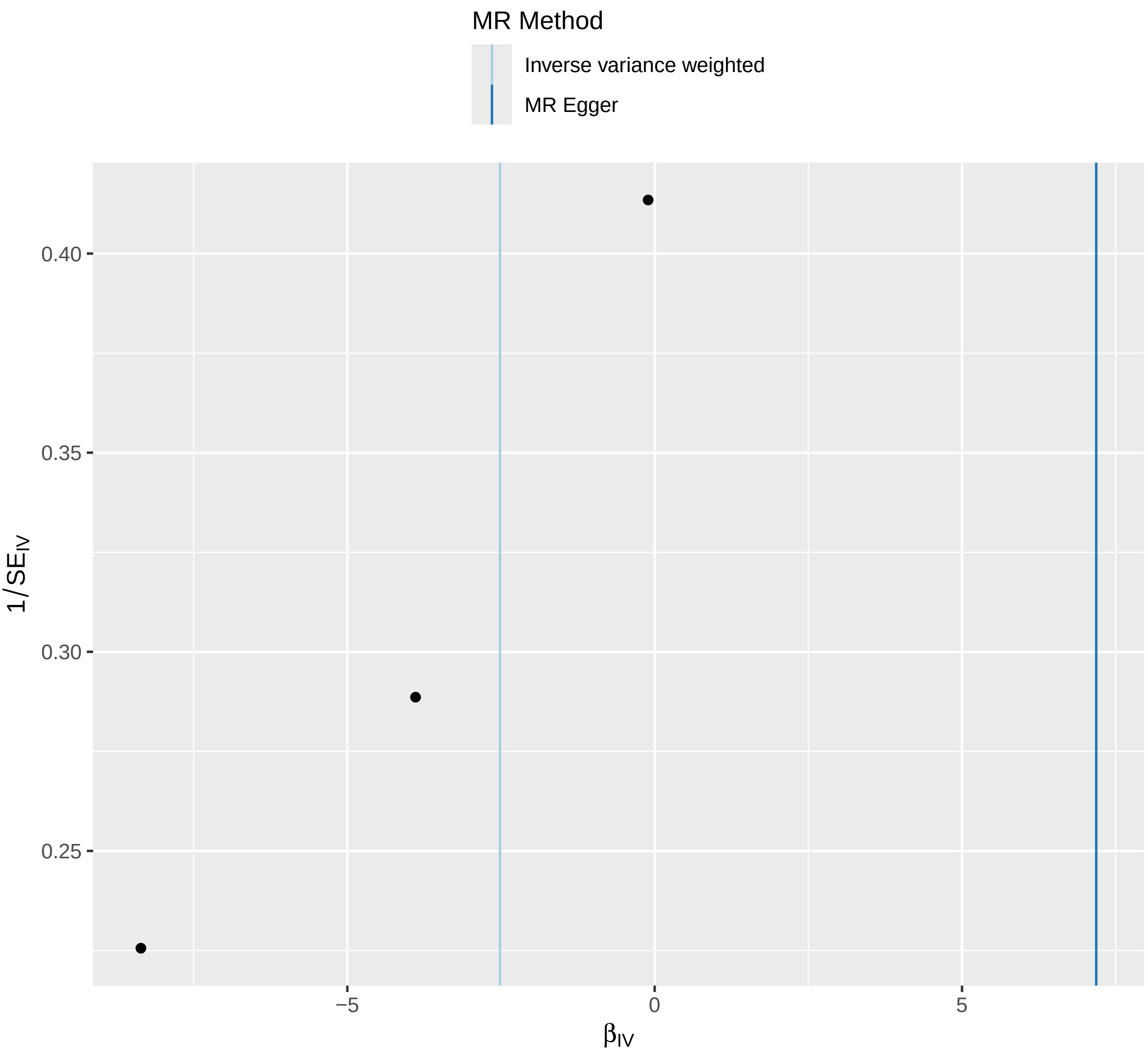
MR Method
Inverse variance weighted
MR Egger
0.40
0.35
0.30
0.25
1/SE$_{IV}$
−5
0
5
β$_{IV}$

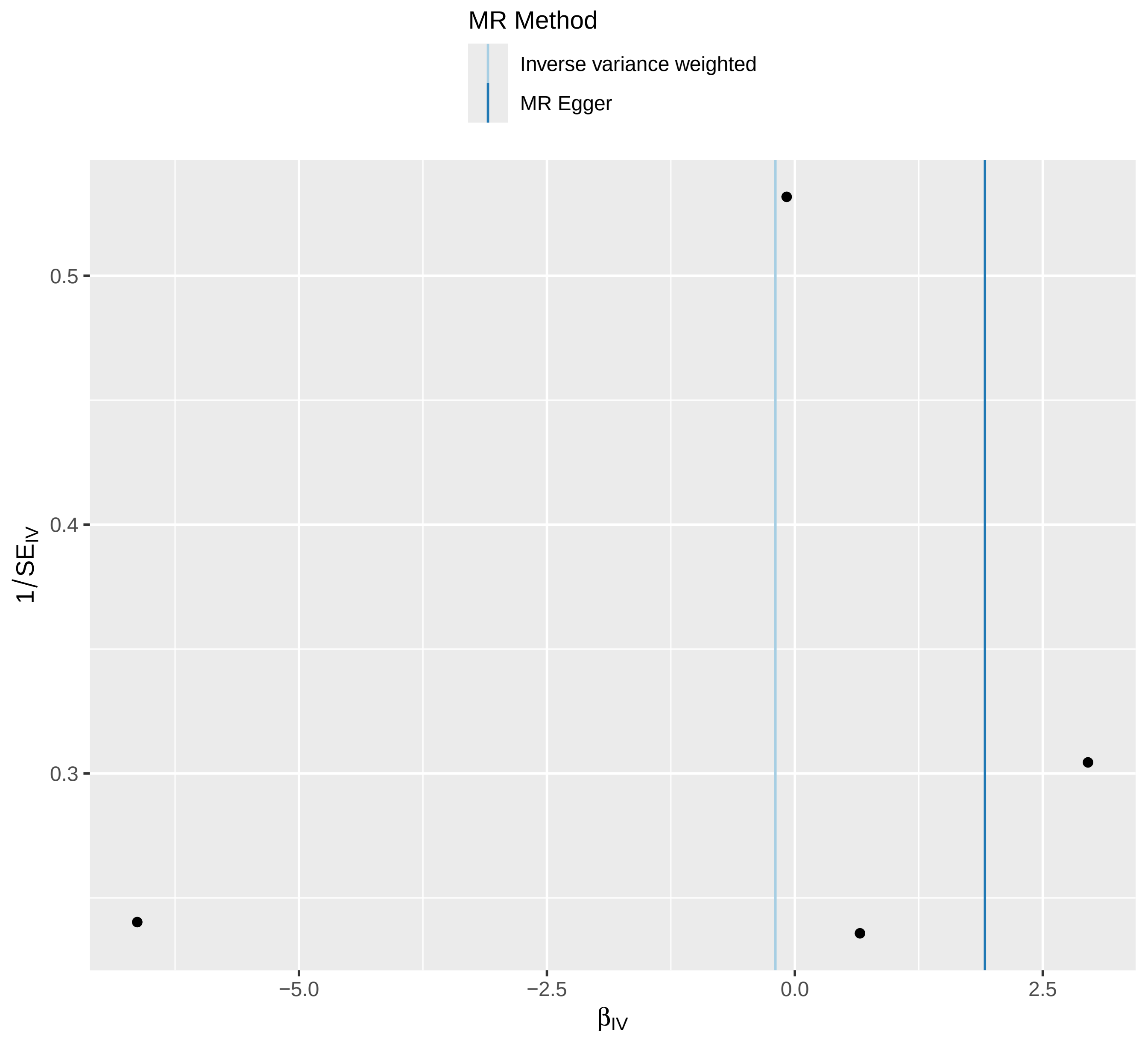

MR Method
Inverse variance weighted
MR Egger
0.5
0.4
0.3
1/SE_IV
−5.0
−2.5
0.0
2.5
β_IV

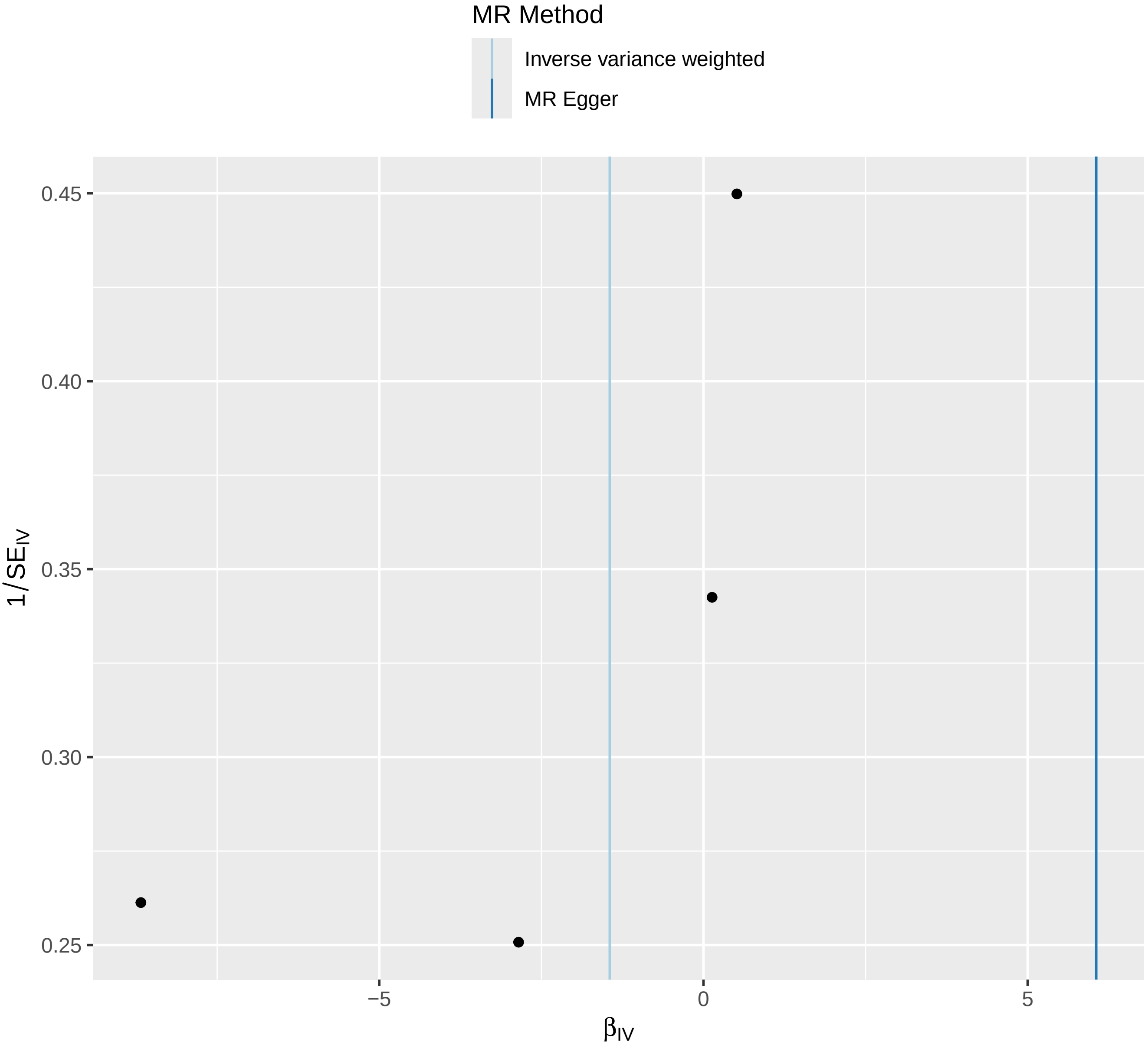

MR Method
Inverse variance weighted
MR Egger
0.45
0.40
0.35
0.30
0.25
1/SE_IV
−5
0
5
β_IV

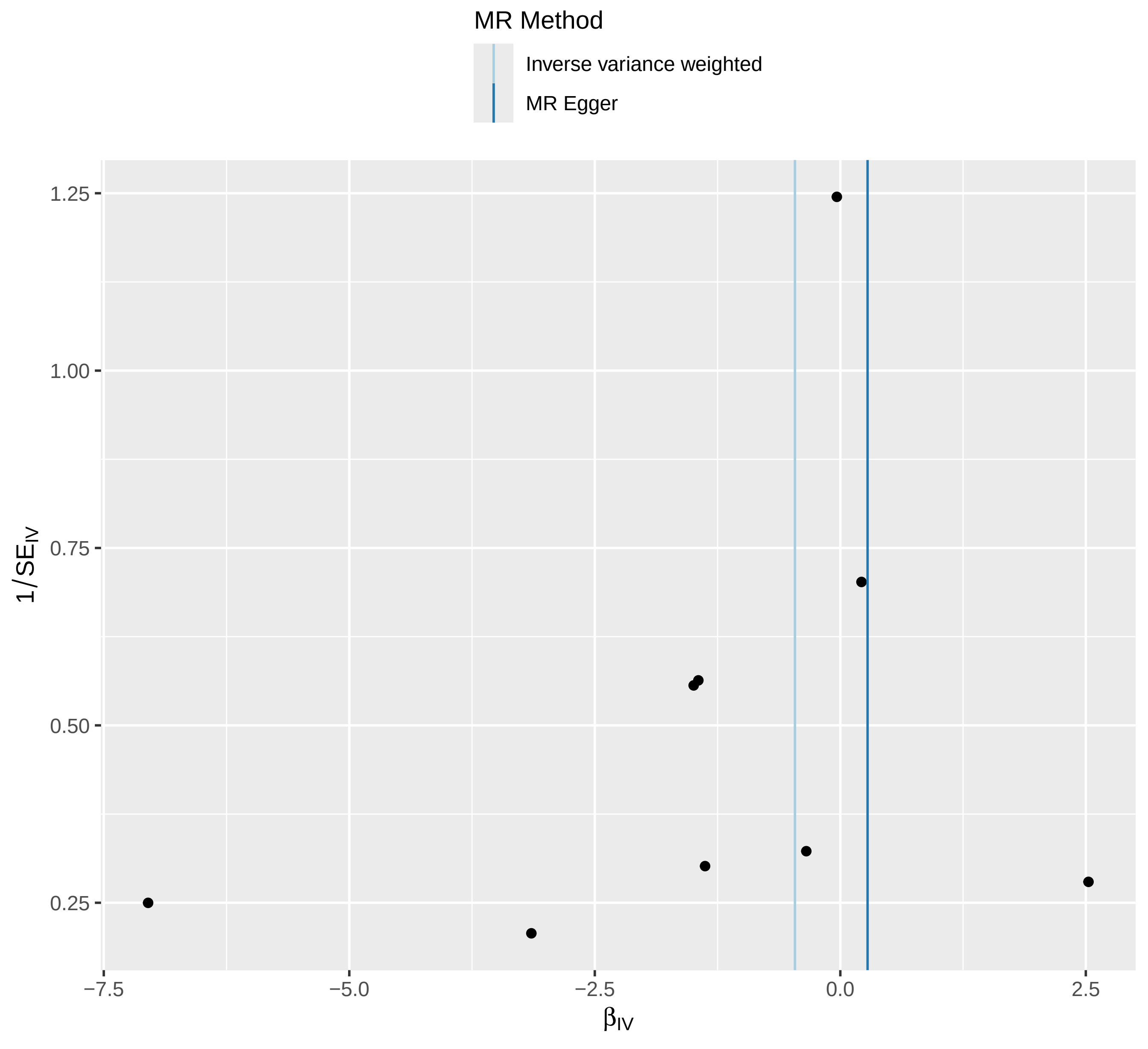
MR Method
Inverse variance weighted
MR Egger
1.25
1.00
0.75
0.50
0.25
1/SE_IV
−7.5
−5.0
−2.5
0.0
2.5
β_IV

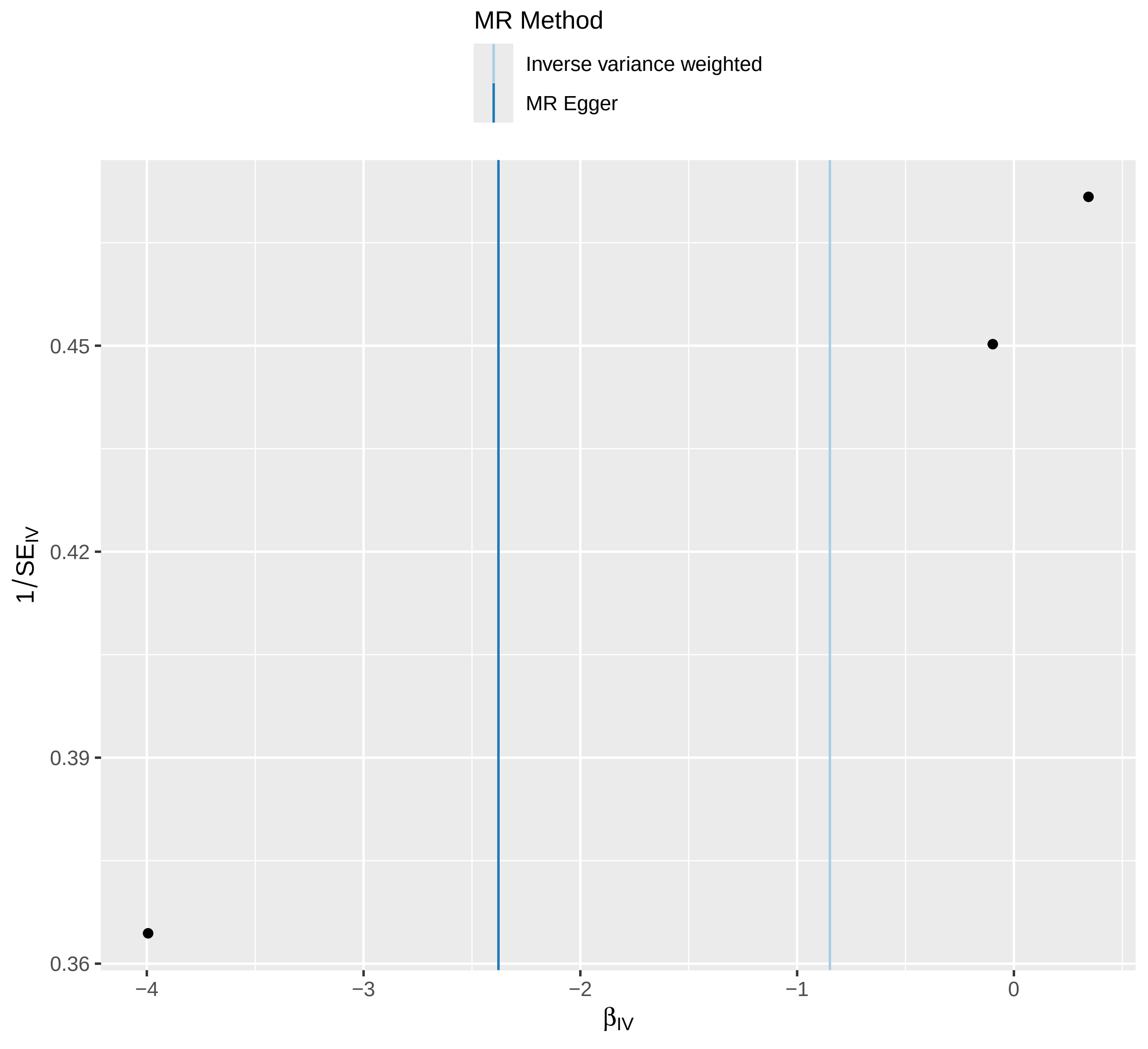
MR Method
Inverse variance weighted
MR Egger
1/SE_{IV}
0.45
0.42
0.39
0.36
−4
−3
−2
−1
0
β_{IV}

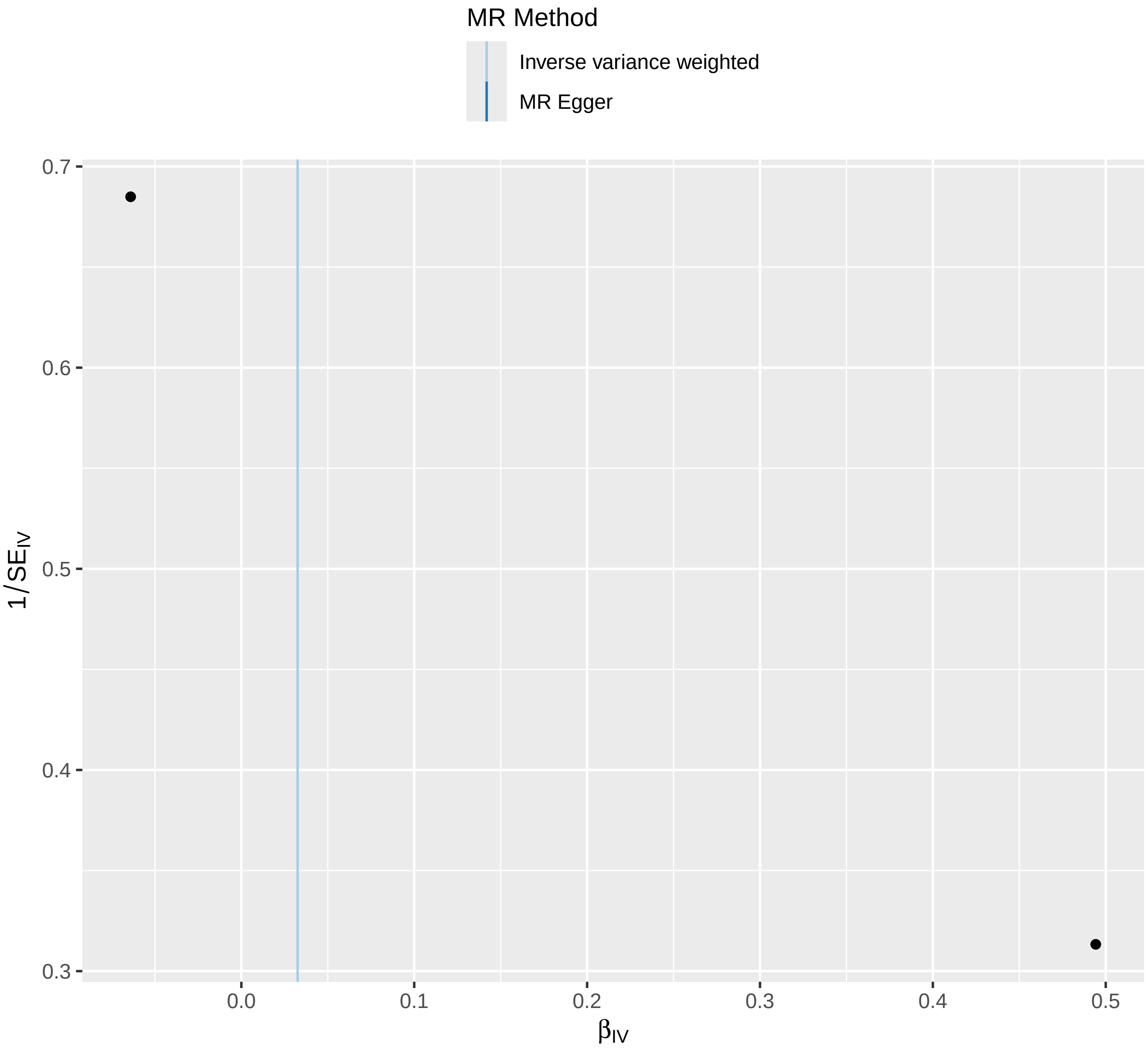
MR Method
Inverse variance weighted
MR Egger
0.7
0.6
0.5
0.4
0.3
$1/SE_{IV}$
0.0
0.1
0.2
0.3
0.4
0.5
β_{IV}

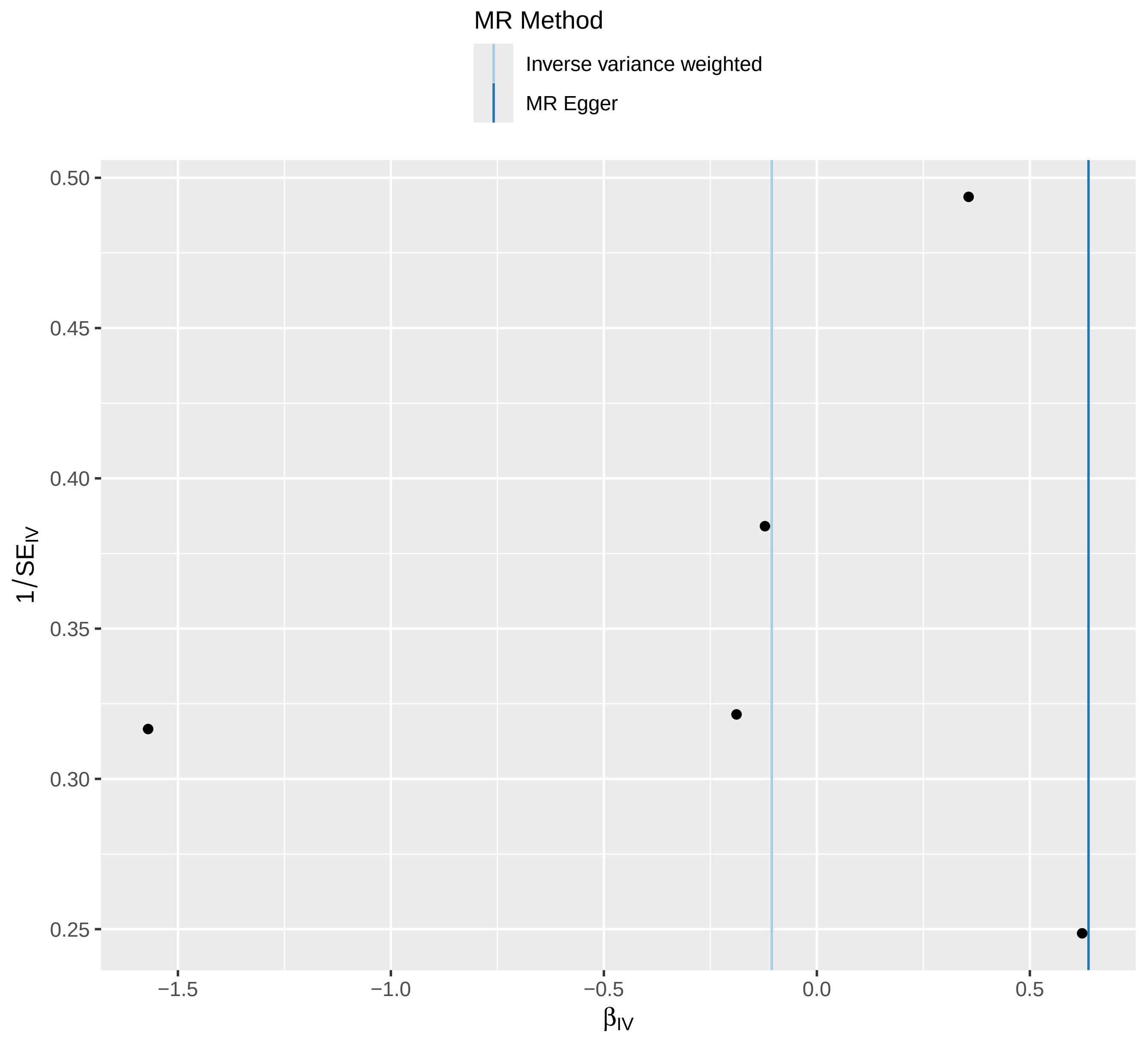
MR Method
Inverse variance weighted
MR Egger
0.50
0.45
0.40
0.35
0.30
0.25
$1/SE_{IV}$
−1.5
−1.0
−0.5
0.0
0.5
β_{IV}

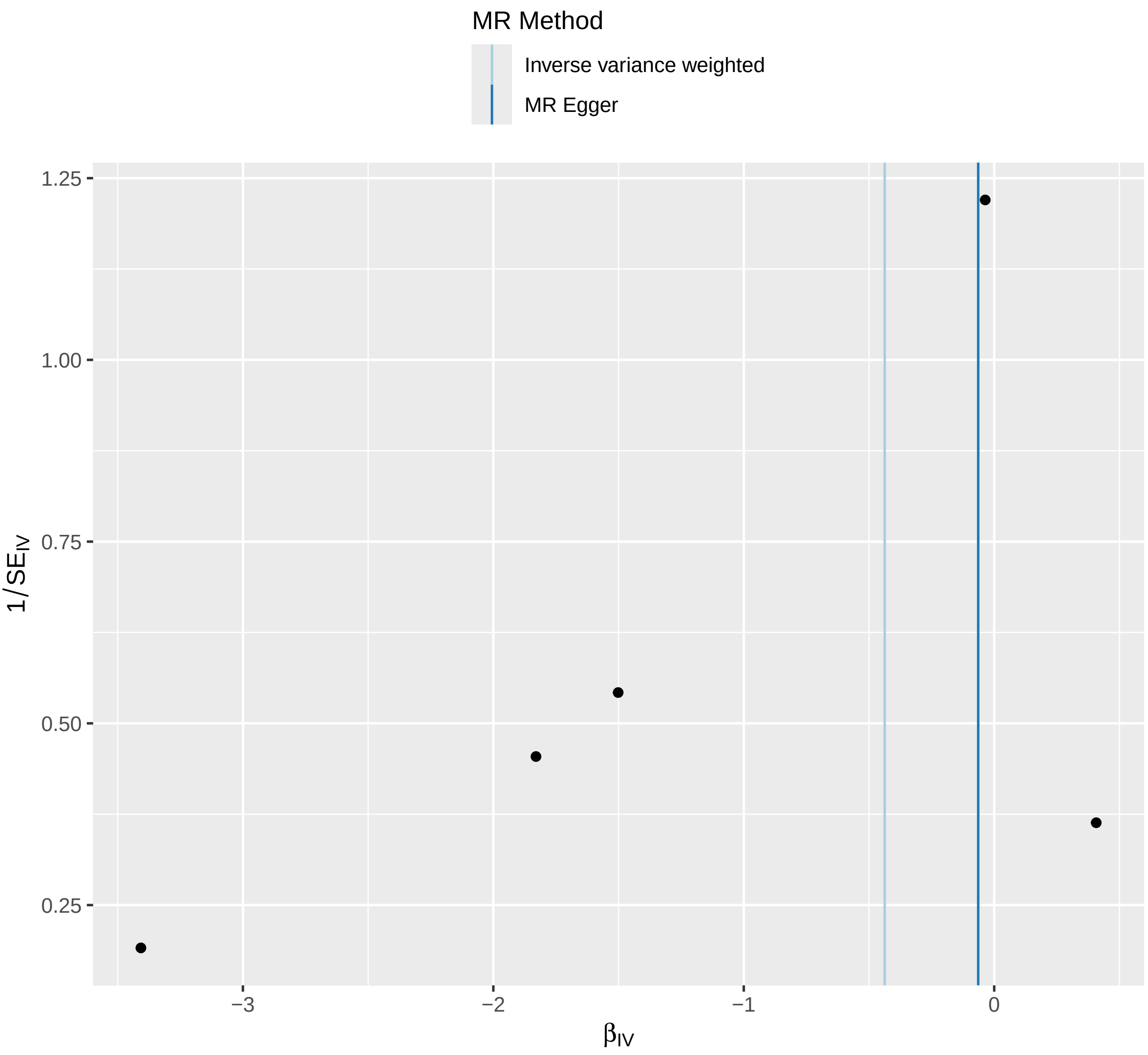
MR Method
Inverse variance weighted
MR Egger
1.25
1.00
0.75
0.50
0.25
1/SE_IV
−3
−2
−1
0
β_IV

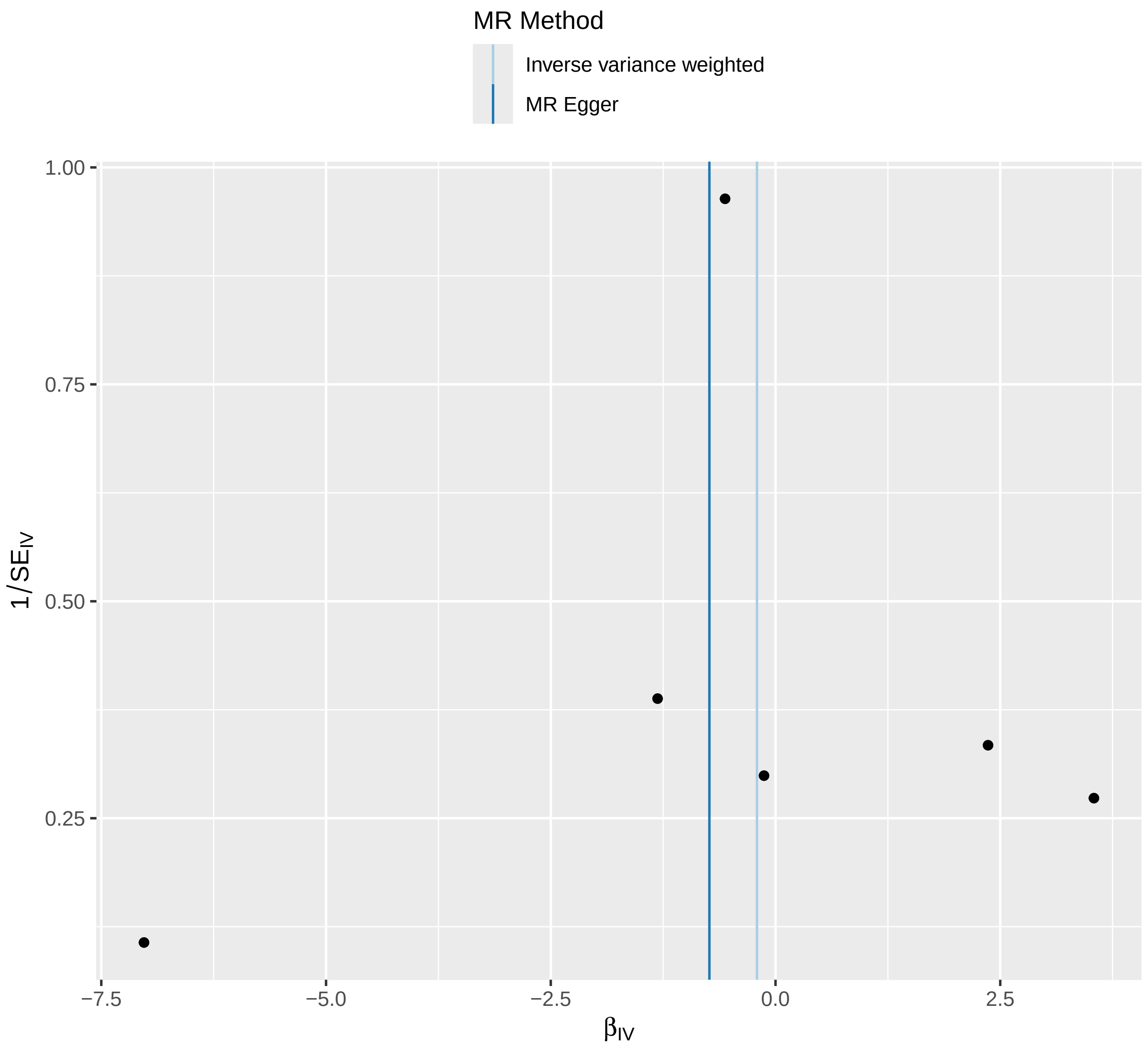
MR Method
Inverse variance weighted
MR Egger
1.00
0.75
0.50
0.25
1/SE_IV
−7.5
−5.0
−2.5
0.0
2.5
β_IV

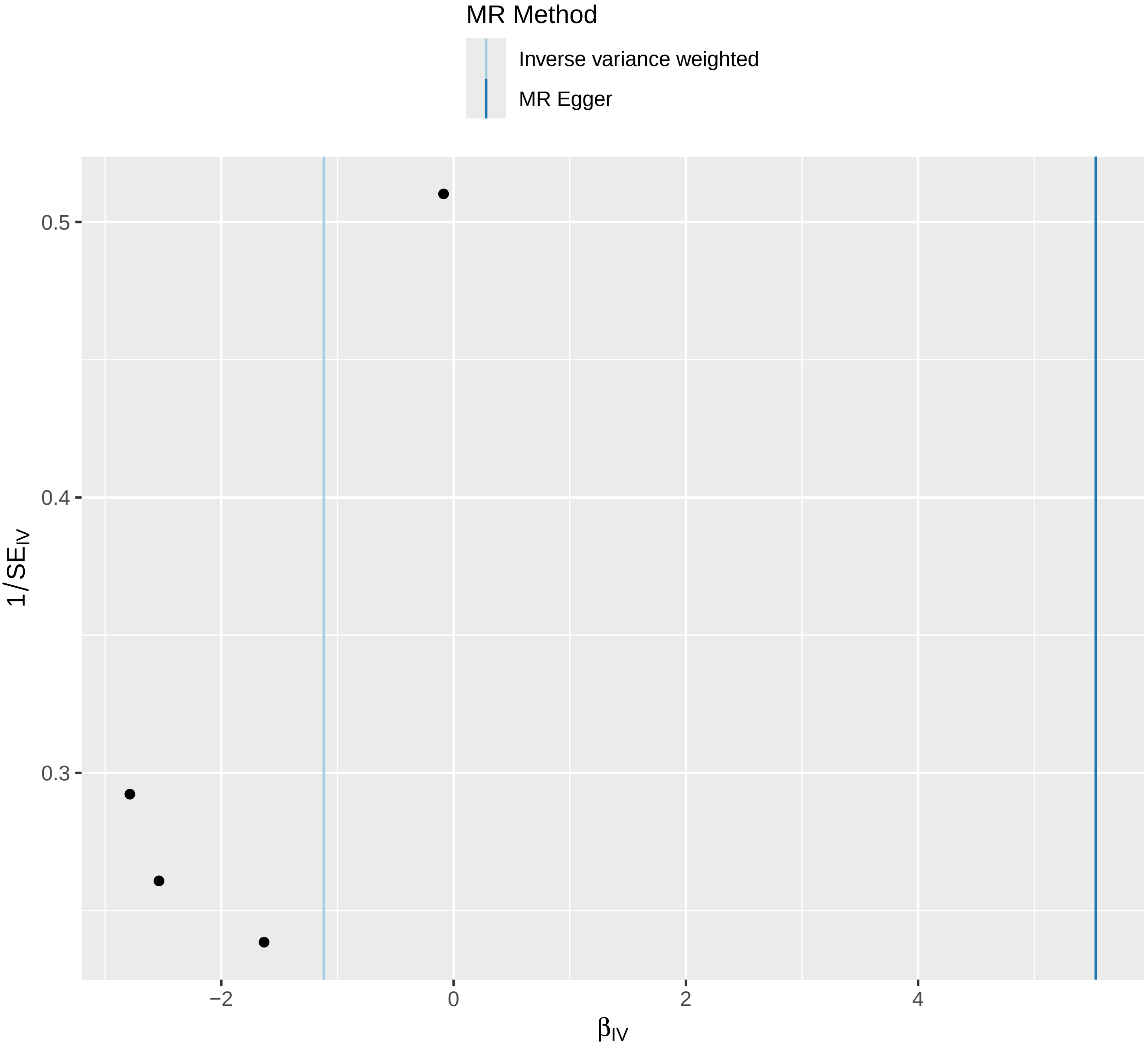
MR Method
Inverse variance weighted
MR Egger
0.5
0.4
0.3
1/SE_IV
−2
0
2
4
β_IV

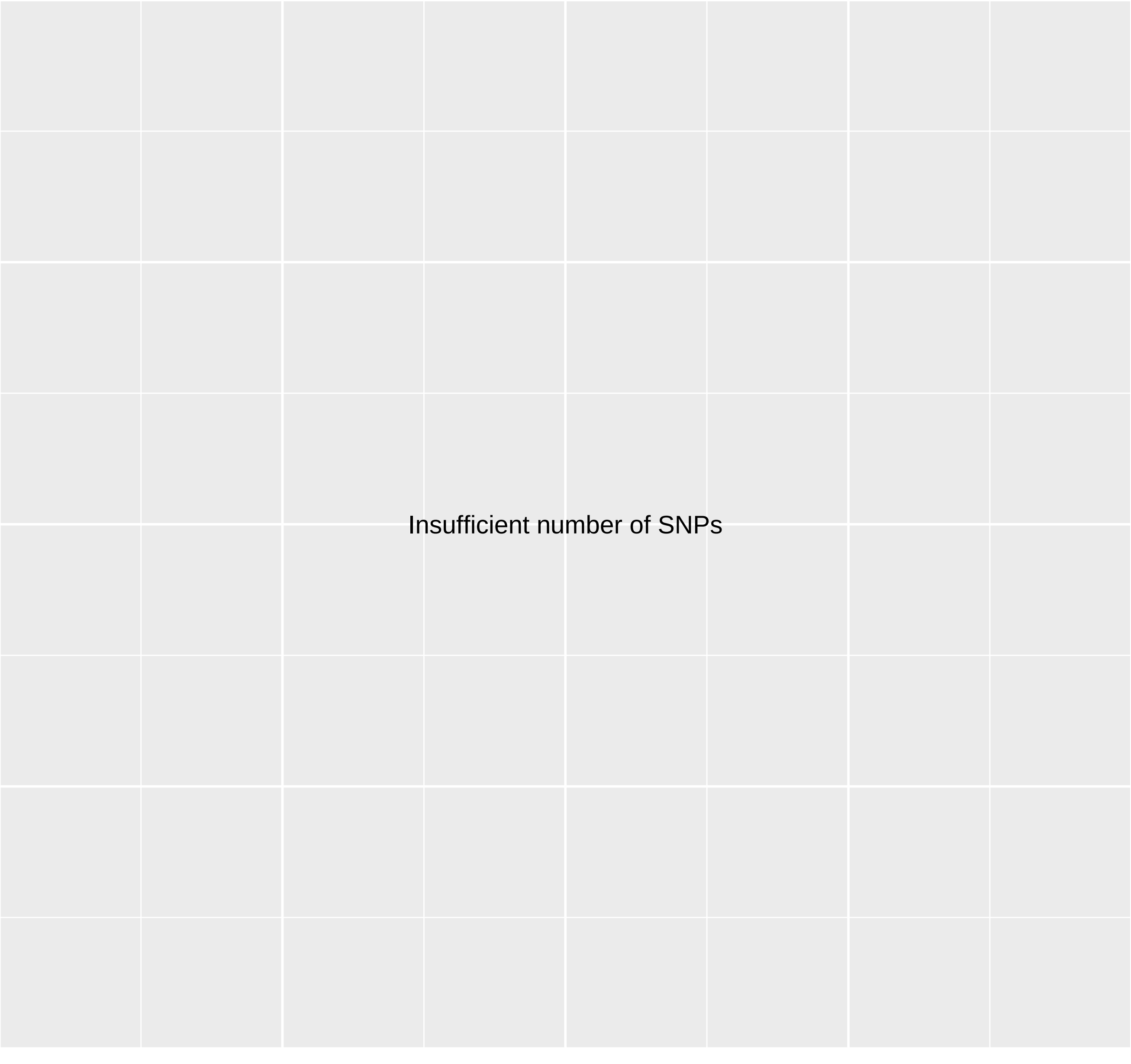
Insufficient number of SNPs

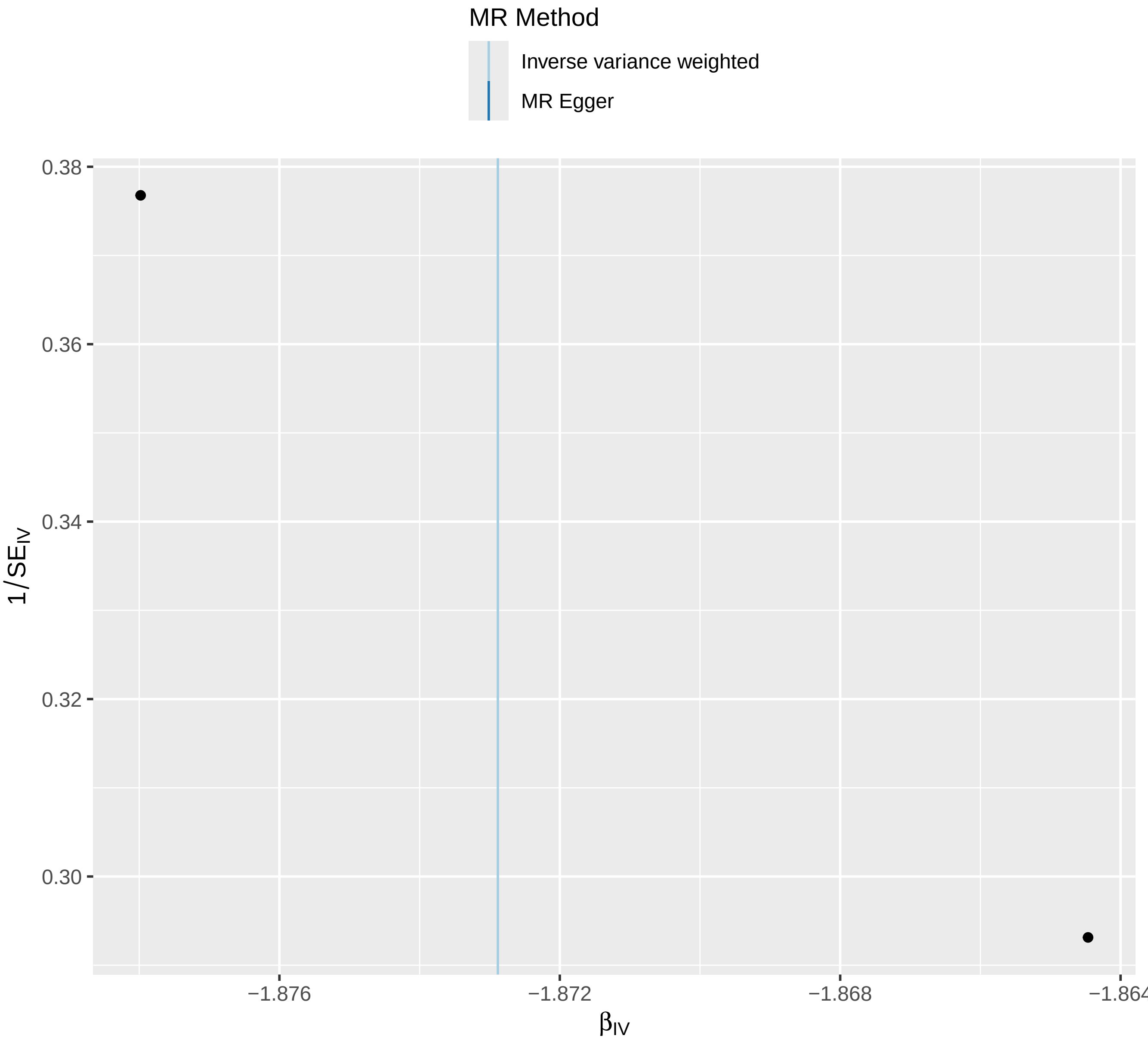
MR Method
Inverse variance weighted
MR Egger
0.38
0.36
0.34
0.32
0.30
1/SE_IV
−1.876
−1.872
−1.868
−1.864
β_IV

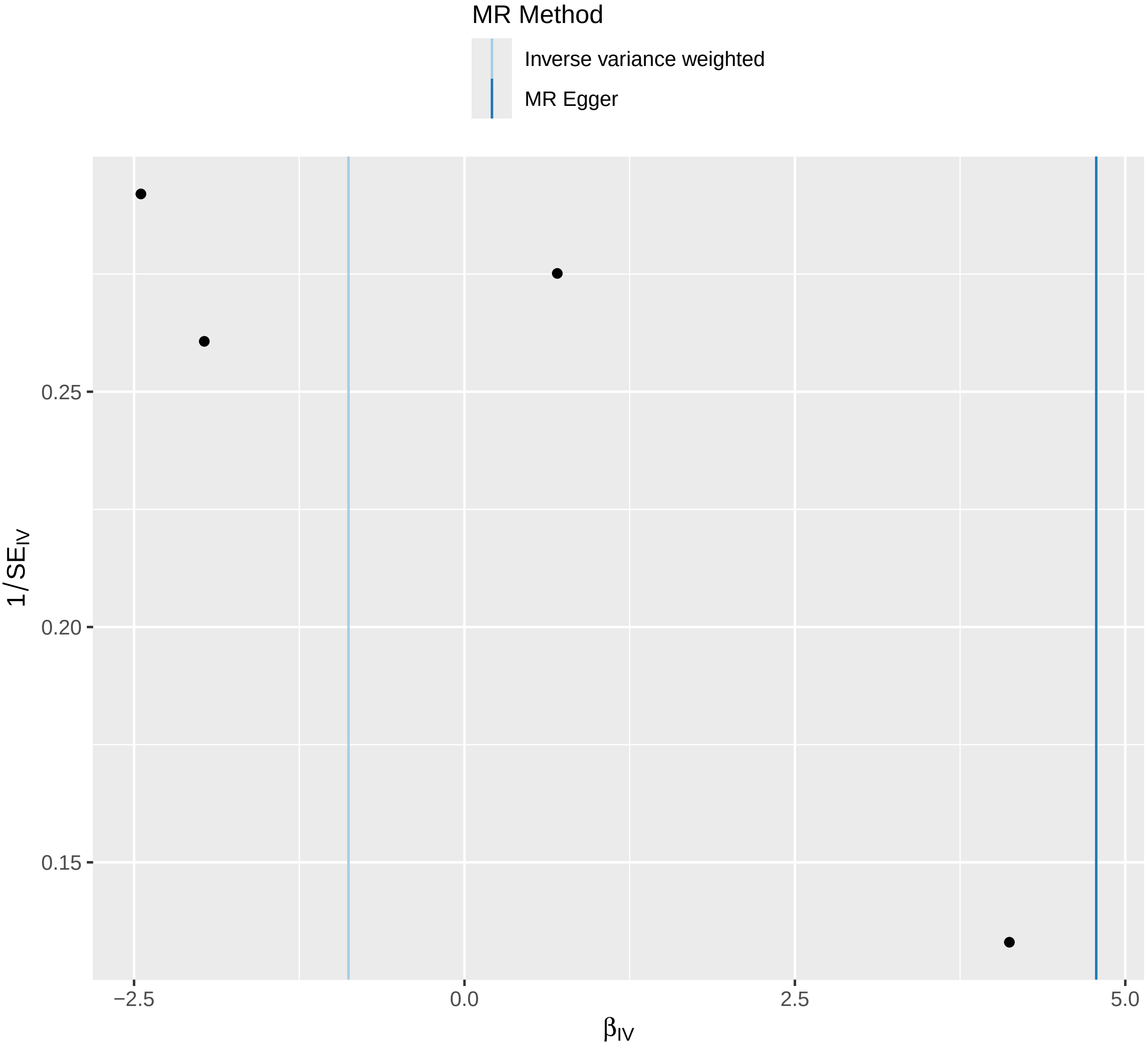
MR Method
Inverse variance weighted
MR Egger
0.25
0.20
0.15
1/SE_IV
−2.5
0.0
2.5
5.0
β_IV

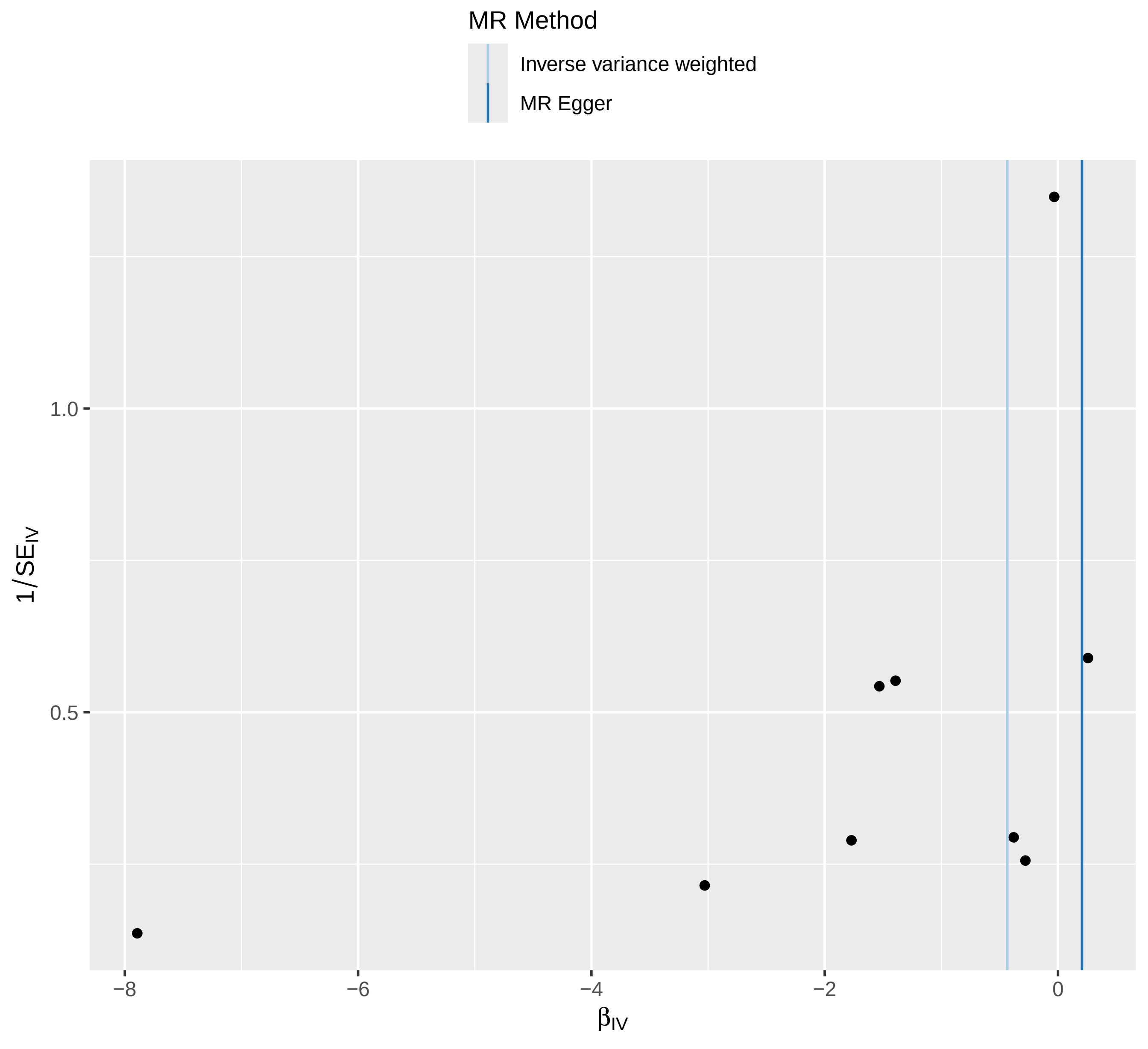
MR Method
Inverse variance weighted
MR Egger
1.0
0.5
1/SE_IV
−8
−6
−4
−2
0
β_IV

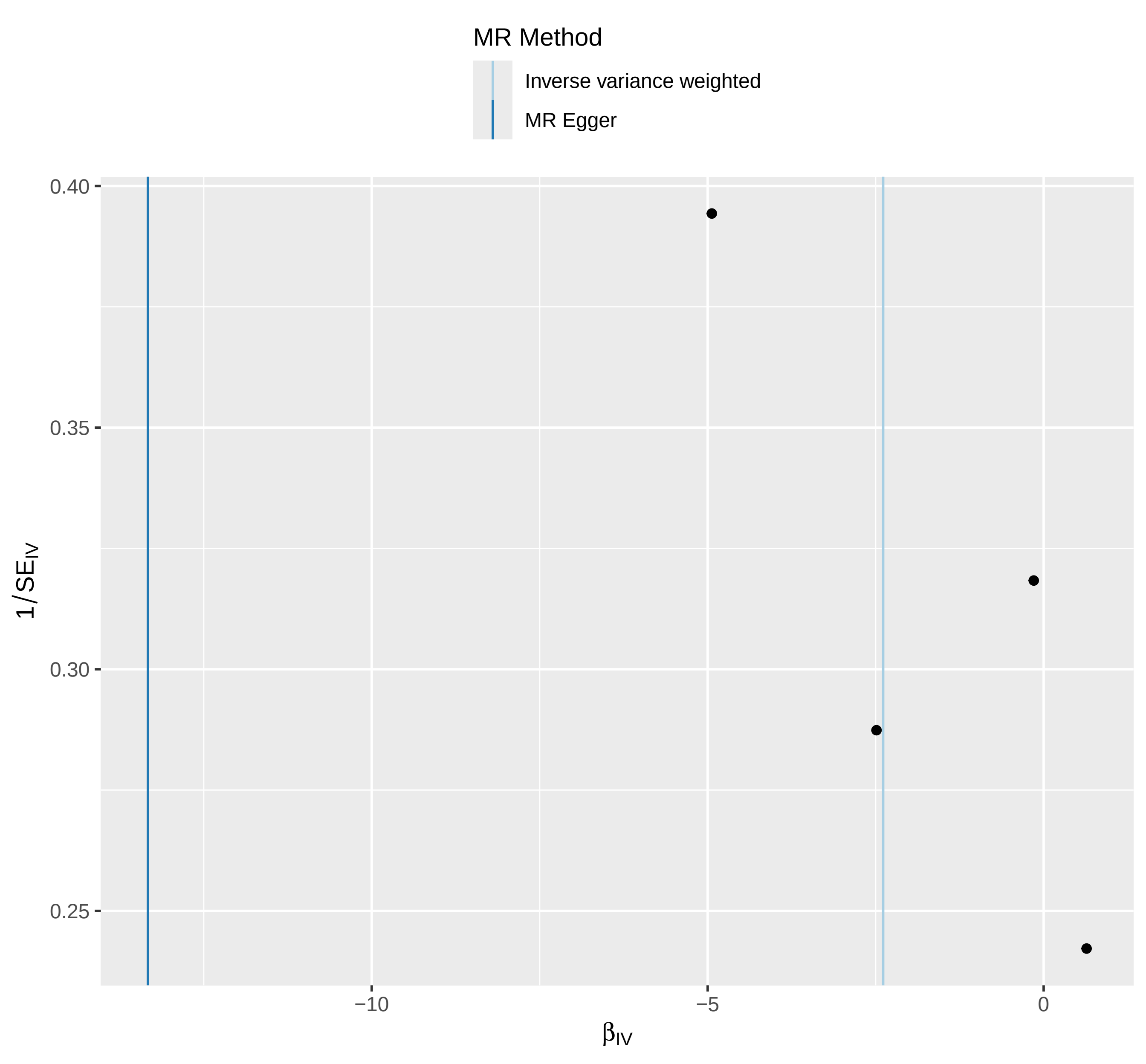
MR Method
Inverse variance weighted
MR Egger
0.40
0.35
0.30
0.25
1/SE_IV
−10
−5
0
β_IV

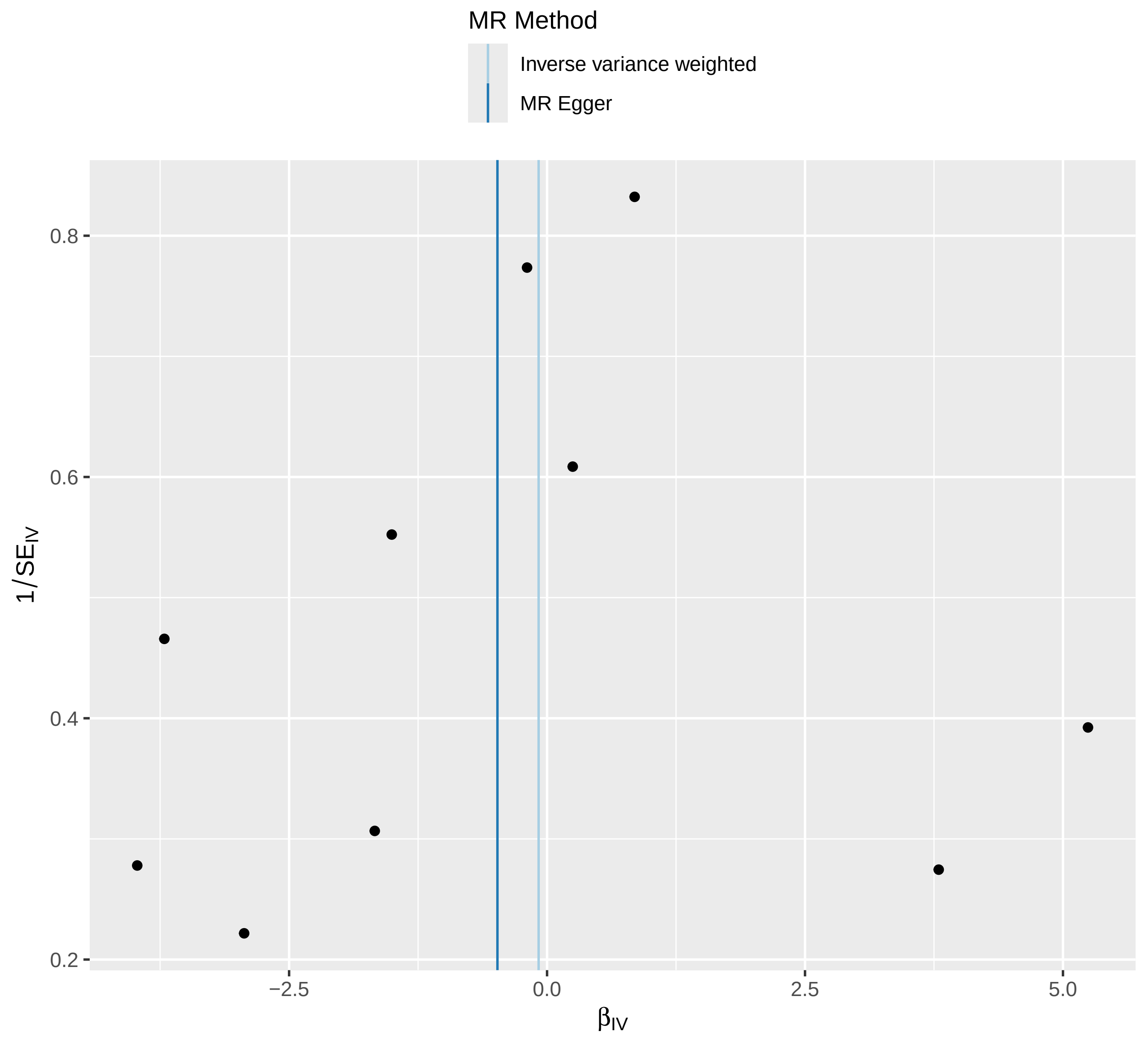
MR Method
Inverse variance weighted
MR Egger
0.8
0.6
0.4
0.2
1/SE$_{IV}$
−2.5
0.0
2.5
5.0
β$_{IV}$

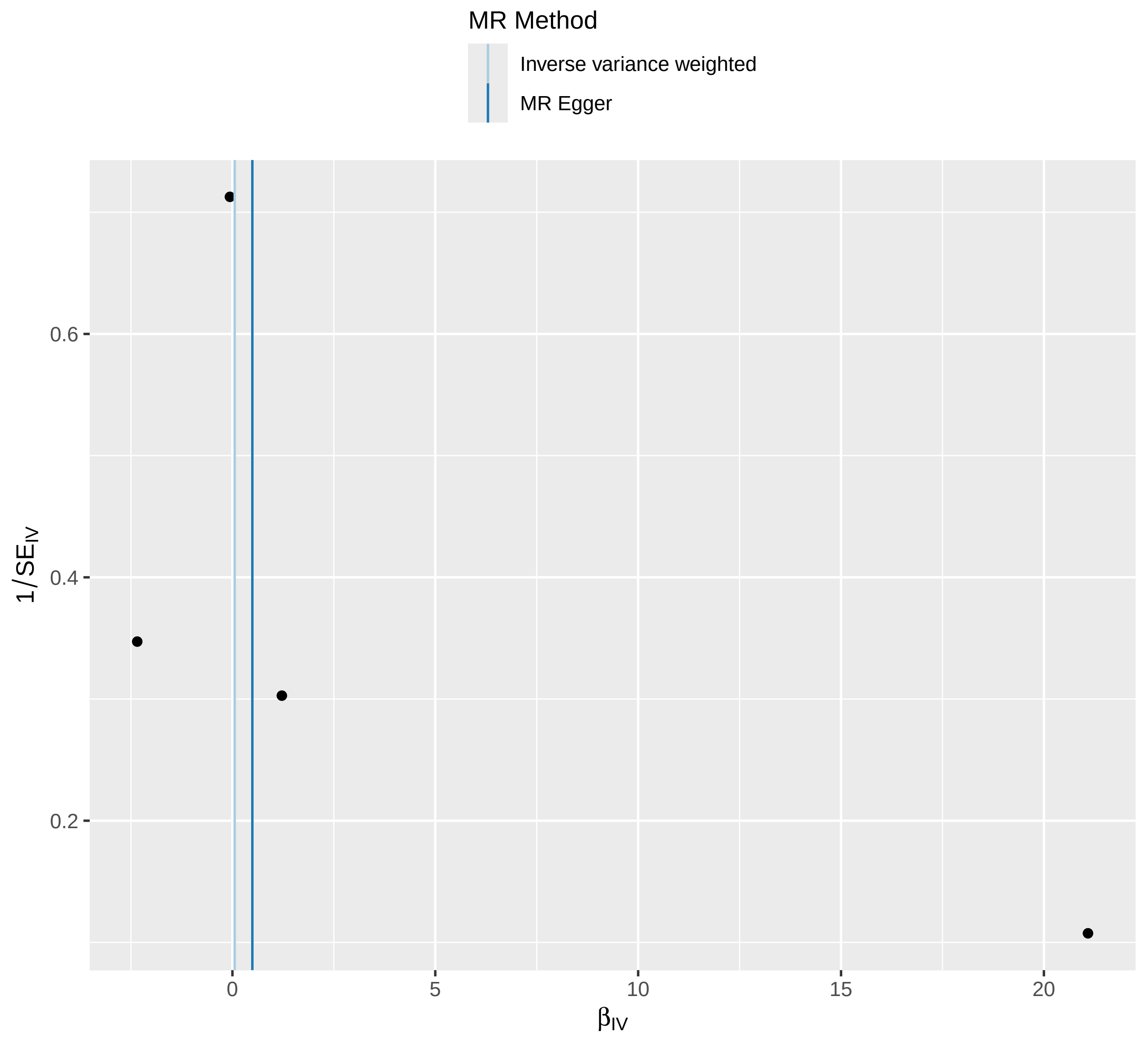
MR Method
Inverse variance weighted
MR Egger
0.6
0.4
0.2
1/SE_IV
0
5
10
15
20
β_IV

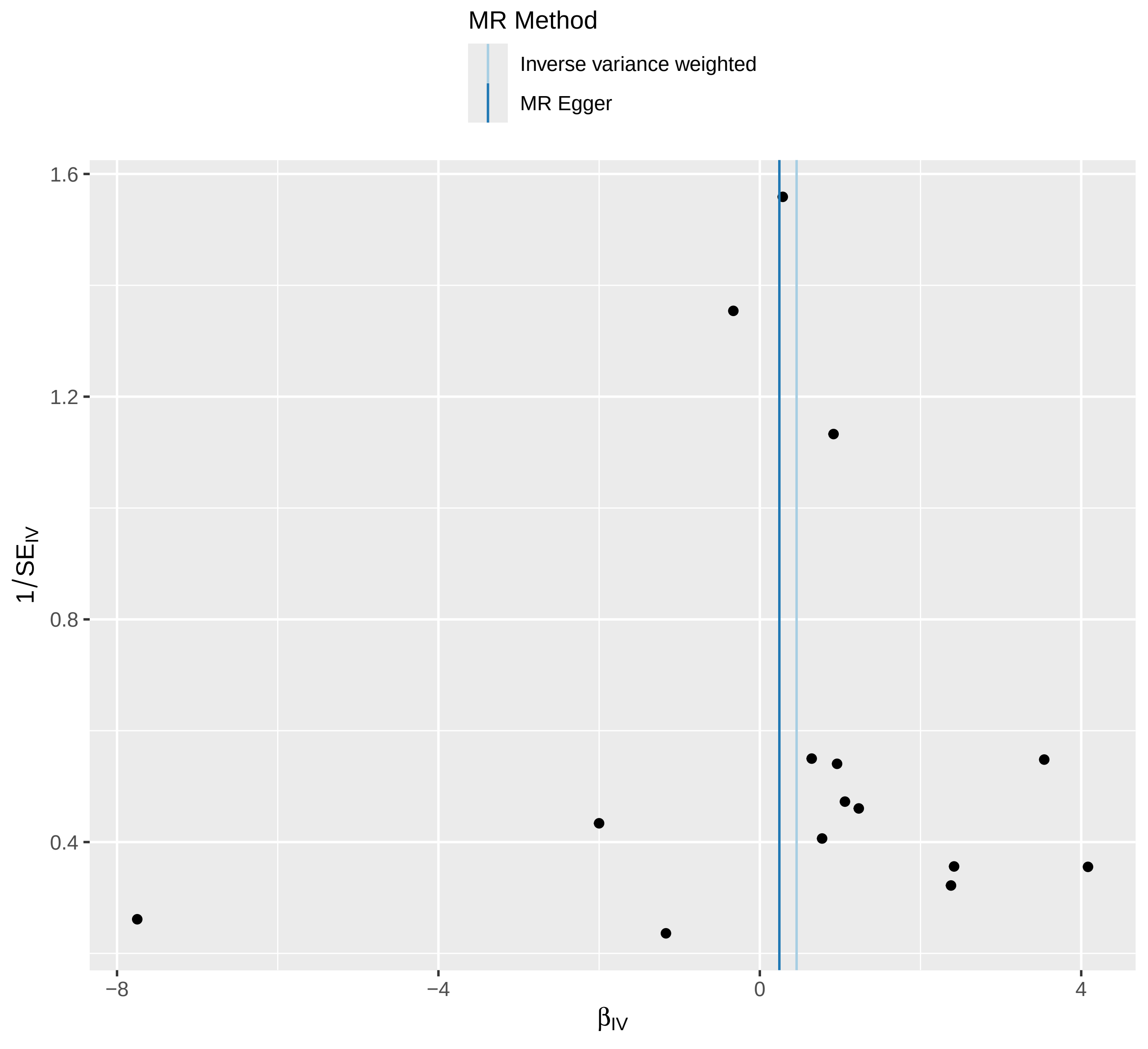

MR Method
Inverse variance weighted
MR Egger
1.6
1.2
0.8
0.4
$1/SE_{IV}$
−8
−4
0
4
β_{IV}

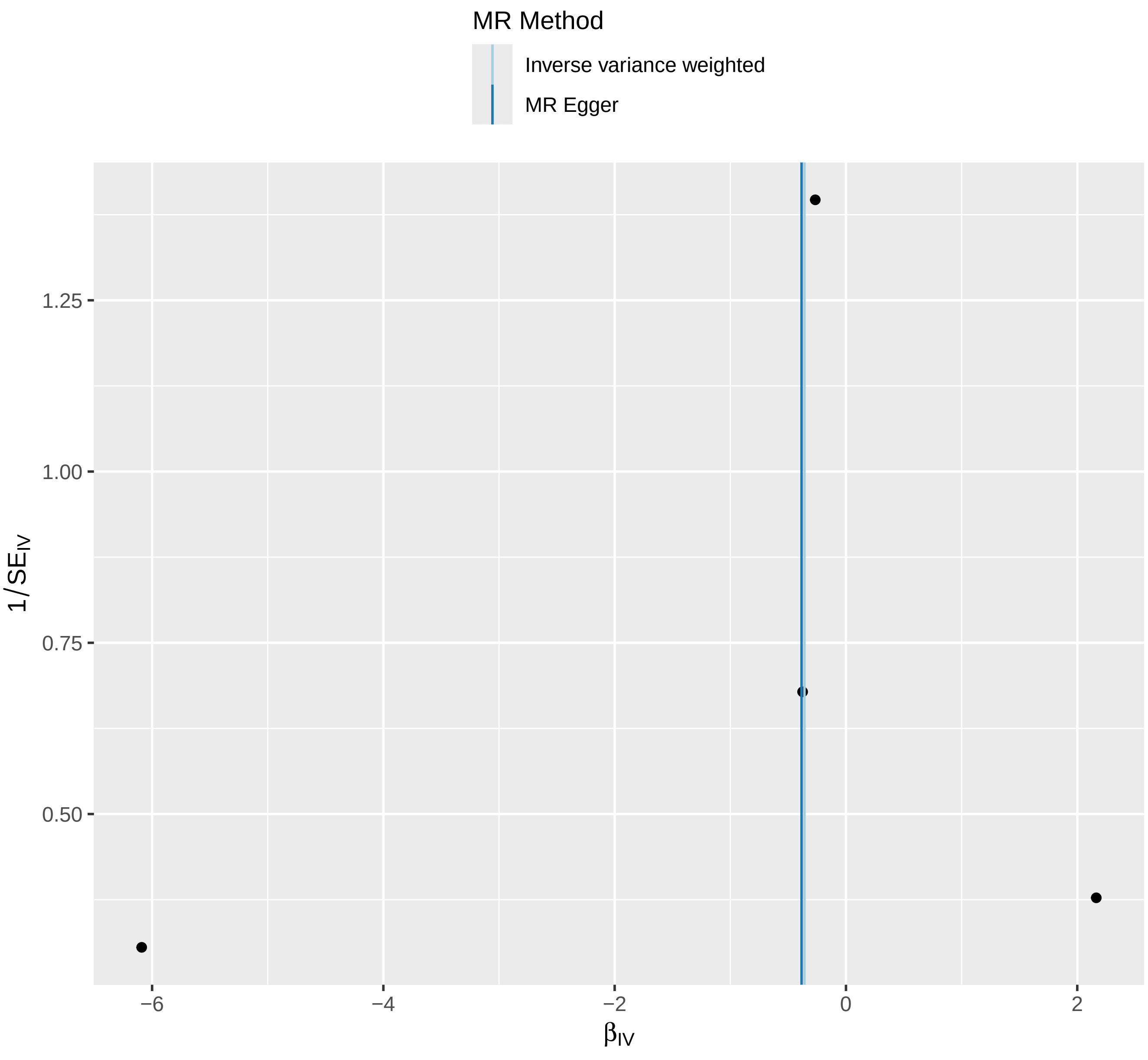

MR Method
Inverse variance weighted
MR Egger
1.25
1.00
0.75
0.50
1/SE_IV
−6
−4
−2
0
2
β_IV

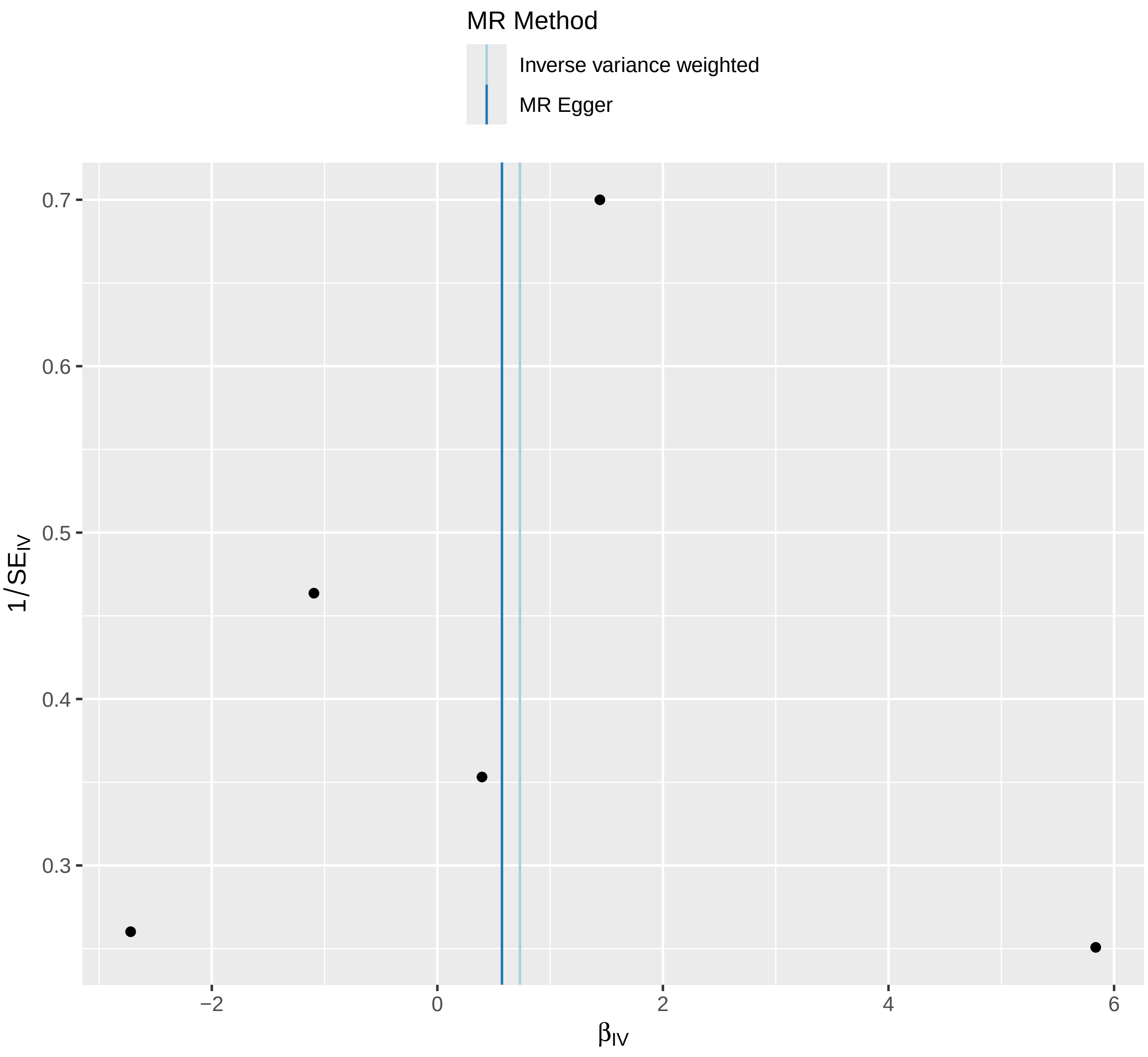
MR Method
Inverse variance weighted
MR Egger
0.7
0.6
0.5
0.4
0.3
$1/SE_{IV}$
−2
0
2
4
6
β_{IV}

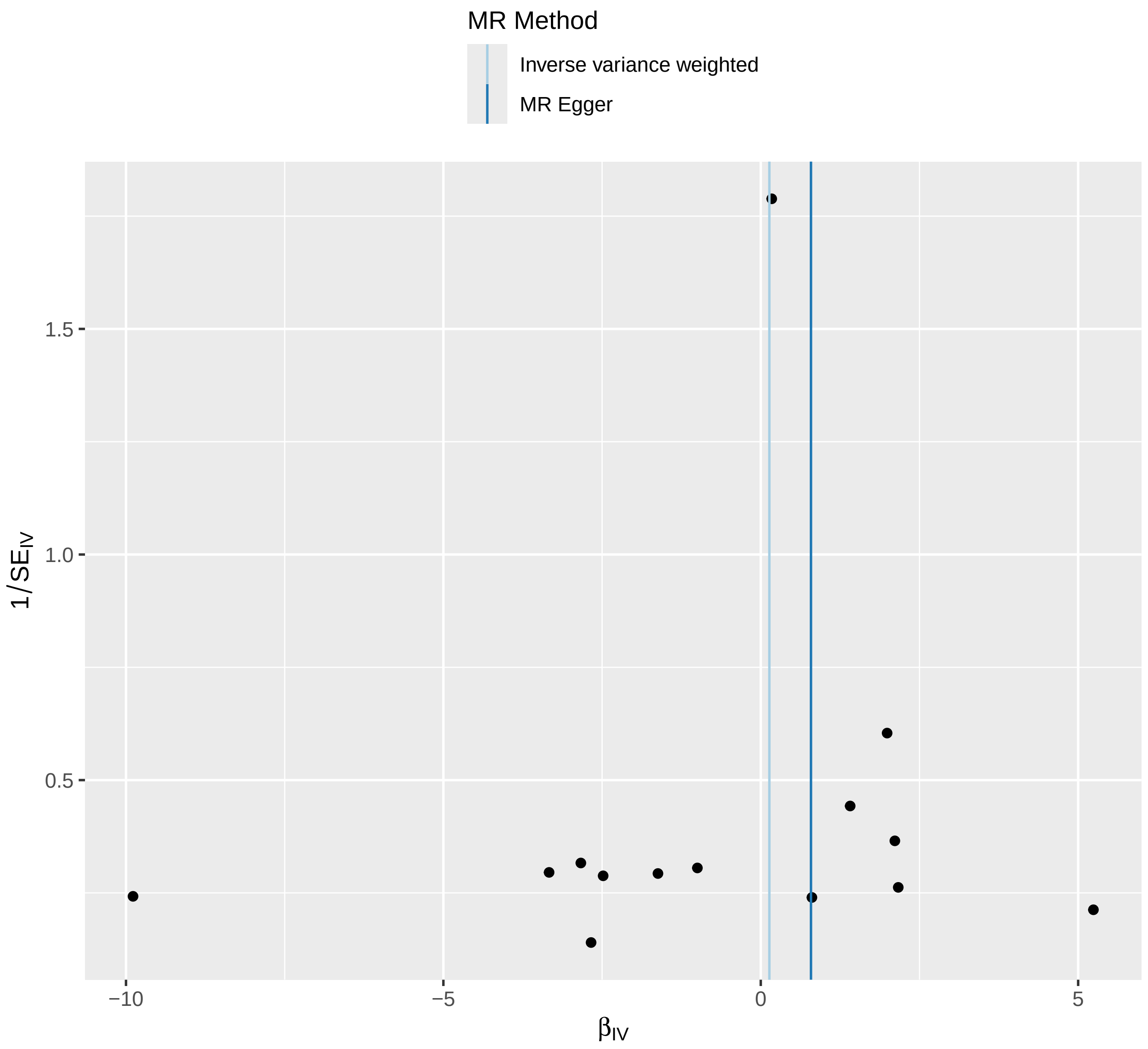
MR Method
Inverse variance weighted
MR Egger
1.5
1.0
0.5
1/SE_IV
−10
−5
0
5
β_IV

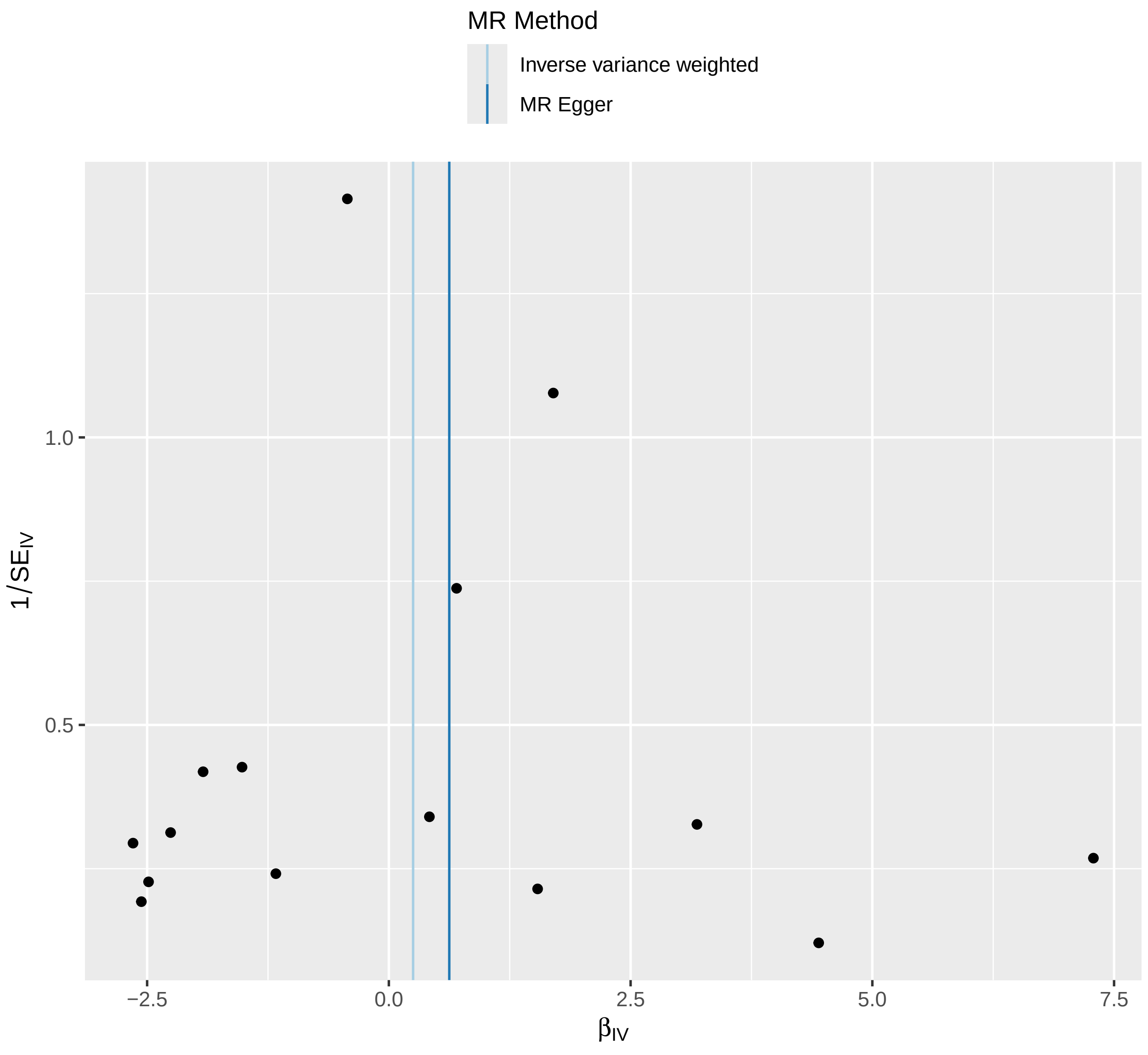
MR Method
Inverse variance weighted
MR Egger
1/SE_{IV}
1.0
0.5
−2.5
0.0
2.5
5.0
7.5
β_{IV}

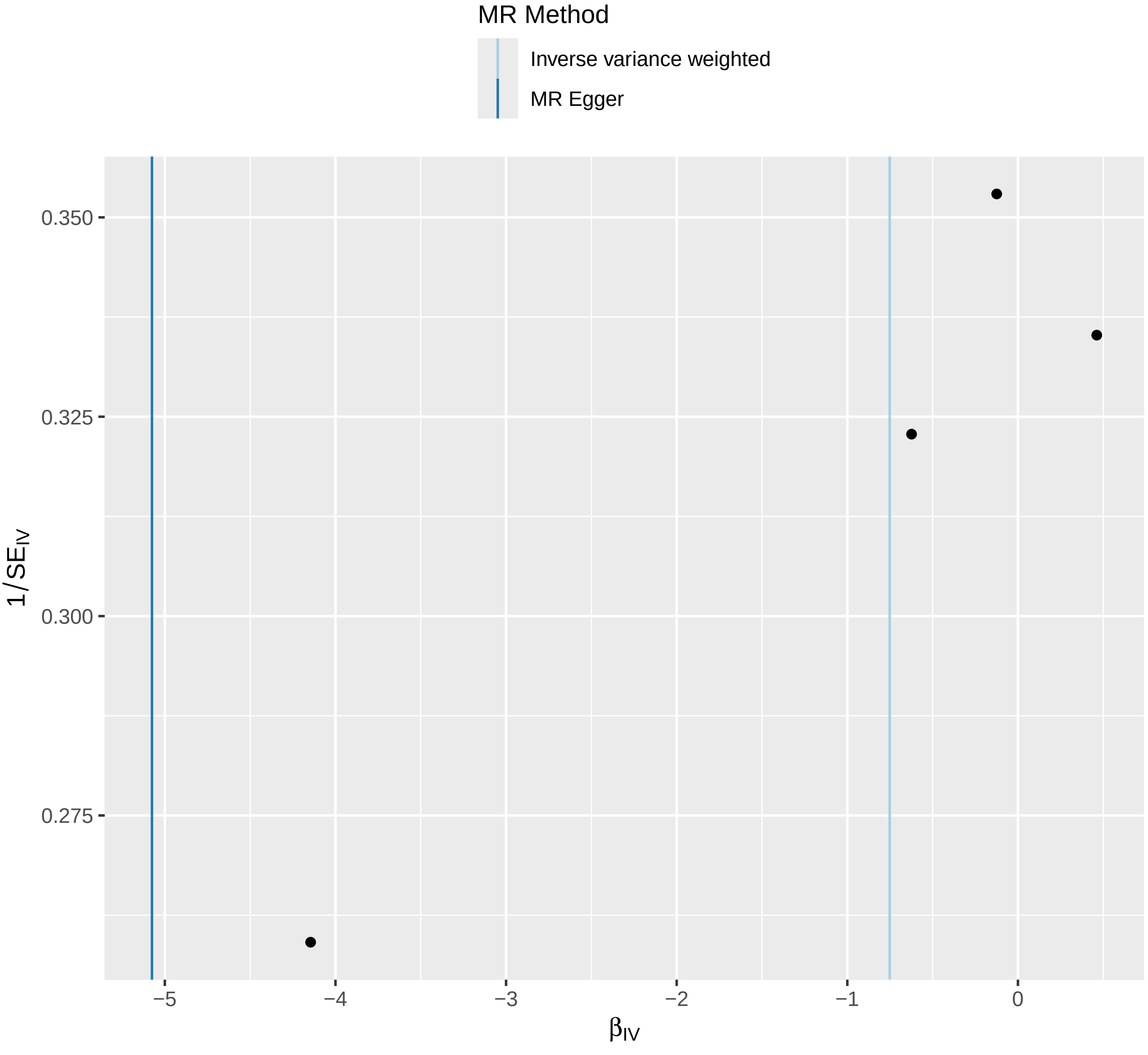
MR Method
Inverse variance weighted
MR Egger
0.350
0.325
0.300
0.275
$1/SE_{IV}$
−5
−4
−3
−2
−1
0
β_{IV}

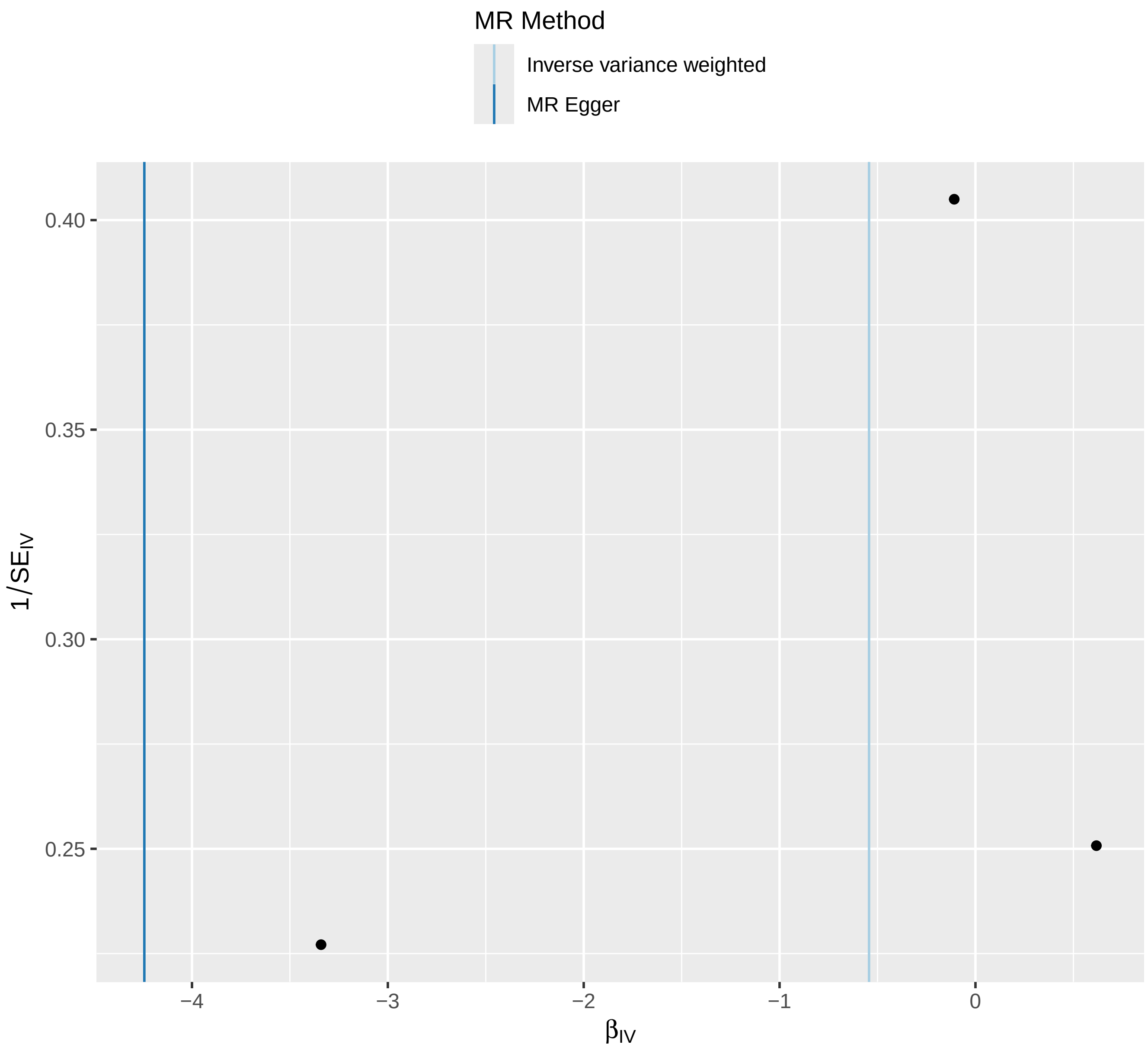
MR Method
Inverse variance weighted
MR Egger
0.40
0.35
0.30
0.25
1/SE_IV
−4
−3
−2
−1
0
β_IV

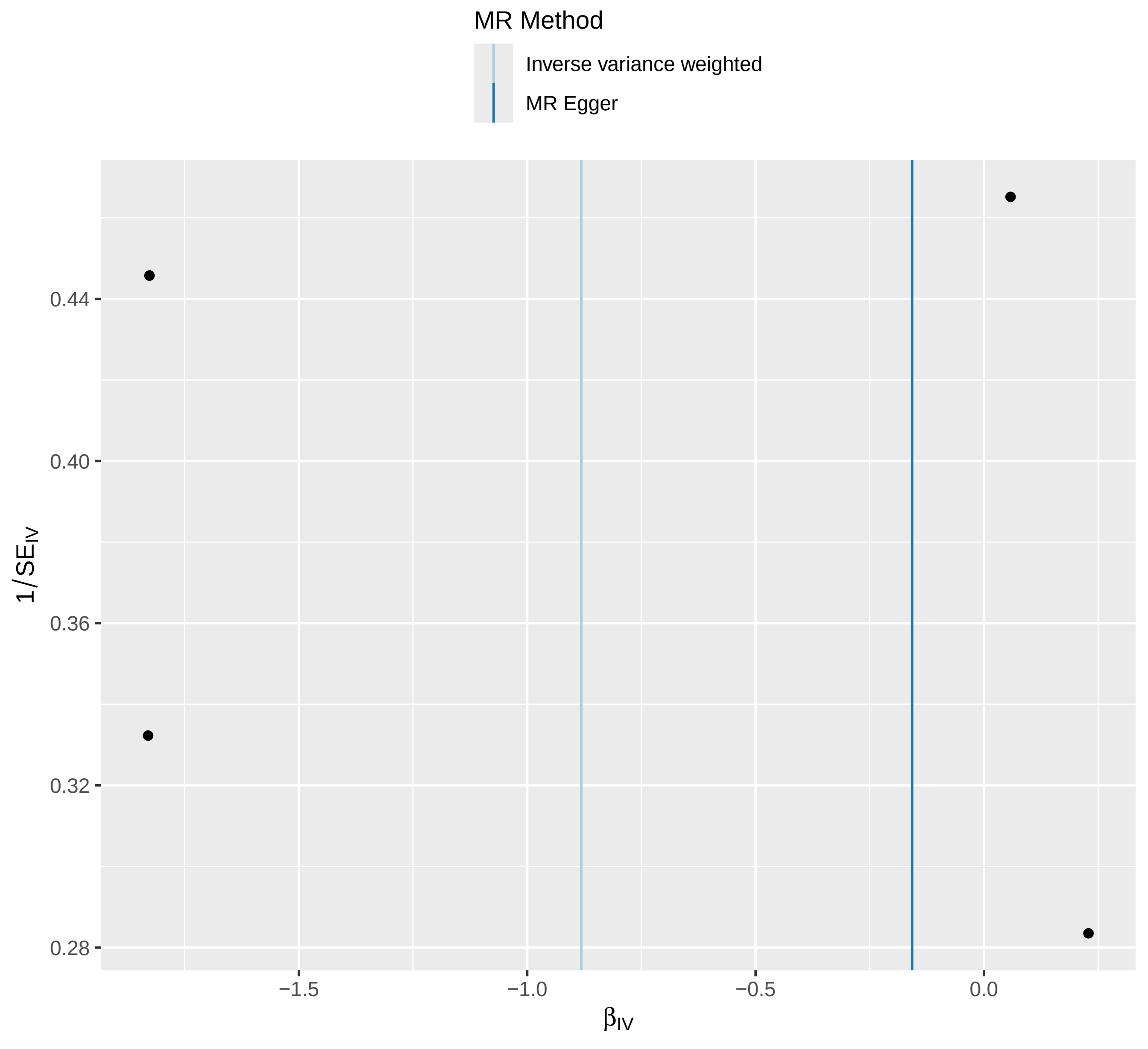
MR Method
Inverse variance weighted
MR Egger
0.44
0.40
0.36
0.32
0.28
1/SE_IV
−1.5
−1.0
−0.5
0.0
β_IV

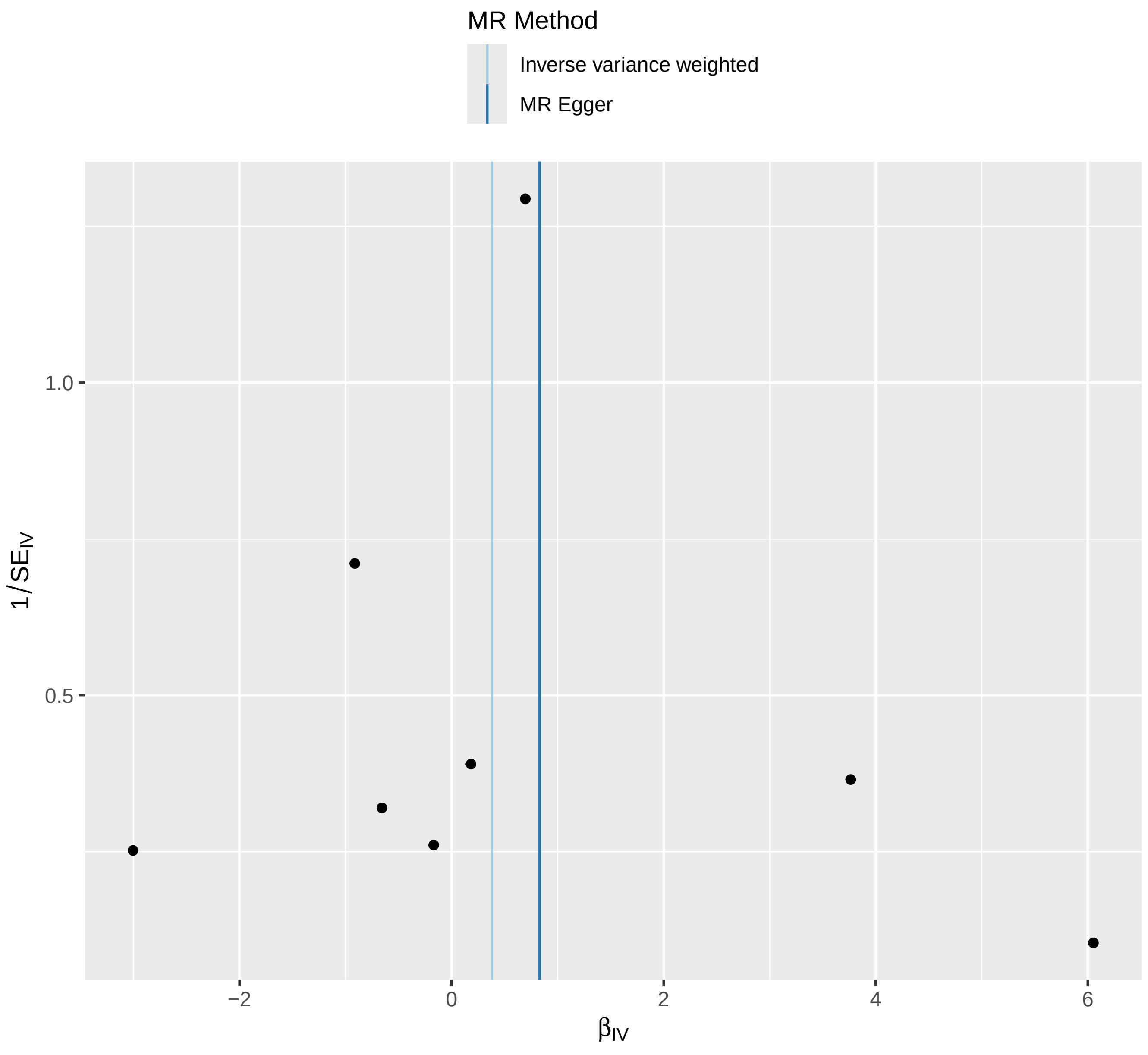
MR Method
Inverse variance weighted
MR Egger
1.0
0.5
1/SE_IV
−2
0
2
4
6
β_IV

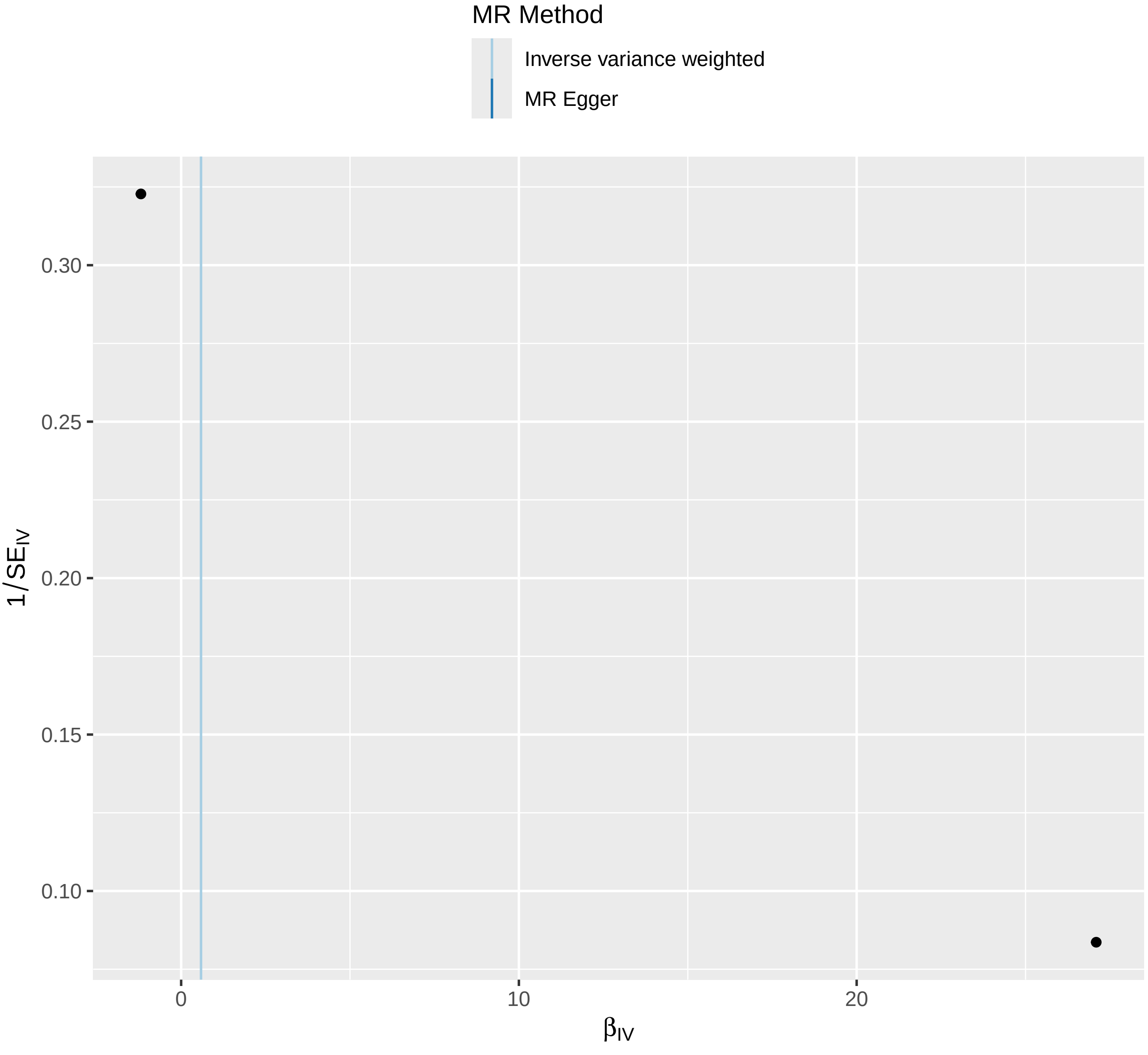

MR Method
Inverse variance weighted
MR Egger
0.30
0.25
0.20
0.15
0.10
$1/SE_{IV}$
0
10
20
β_{IV}

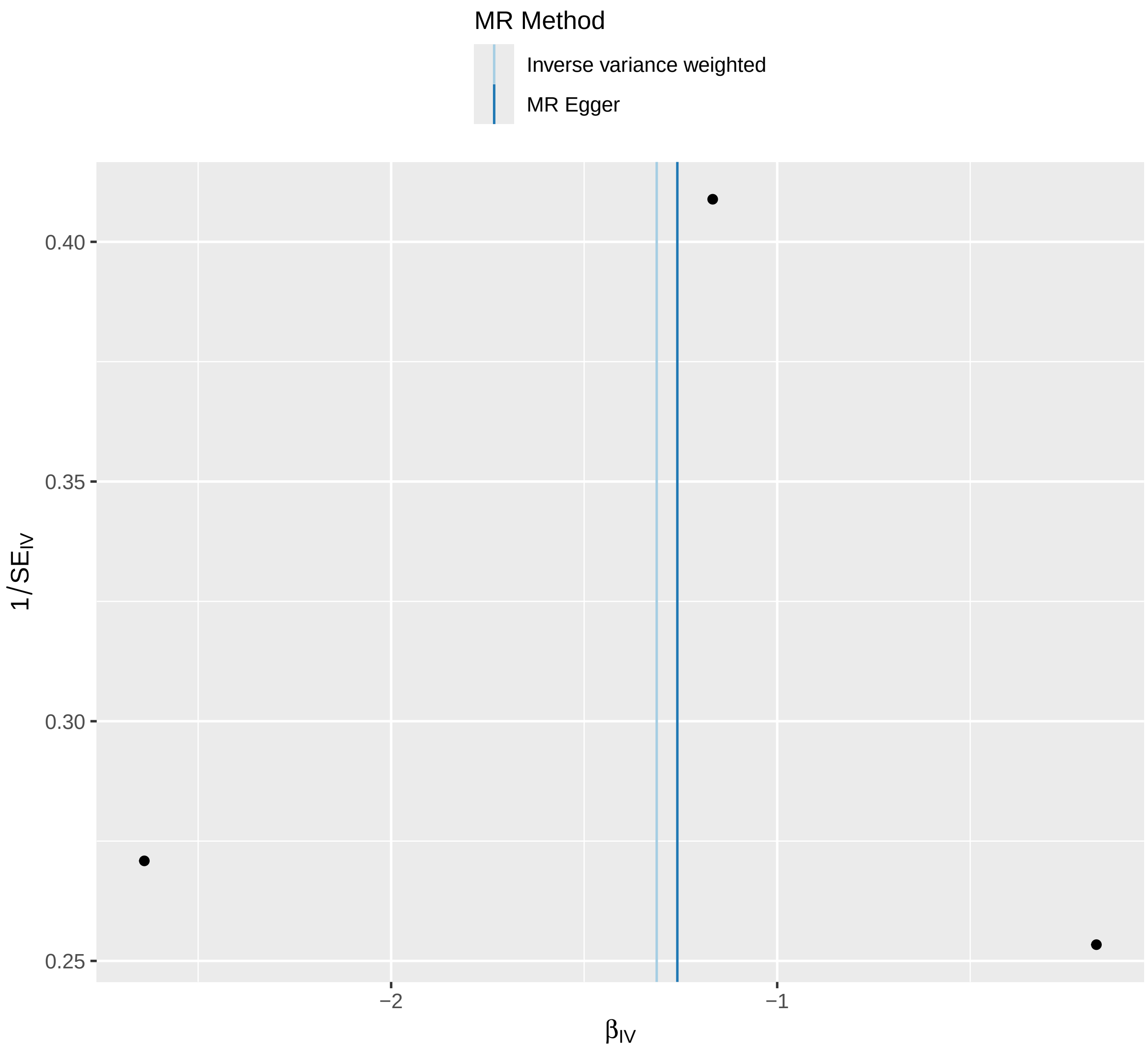
MR Method
Inverse variance weighted
MR Egger
0.40
0.35
0.30
0.25
1/SE$_{IV}$
−2
−1
β$_{IV}$

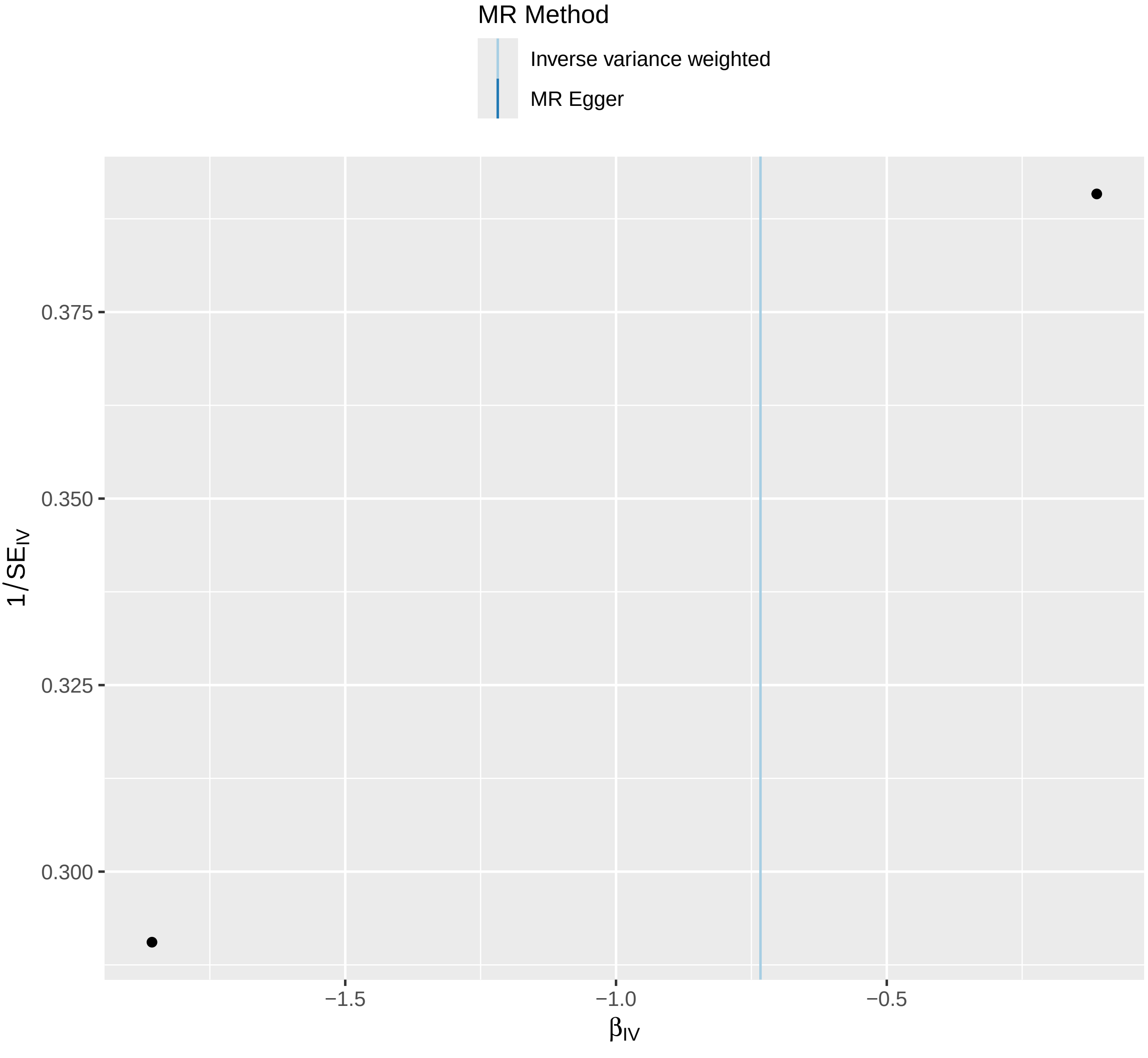
MR Method
Inverse variance weighted
MR Egger
0.375
0.350
0.325
0.300
1/SE_IV
−1.5
−1.0
−0.5
β_IV

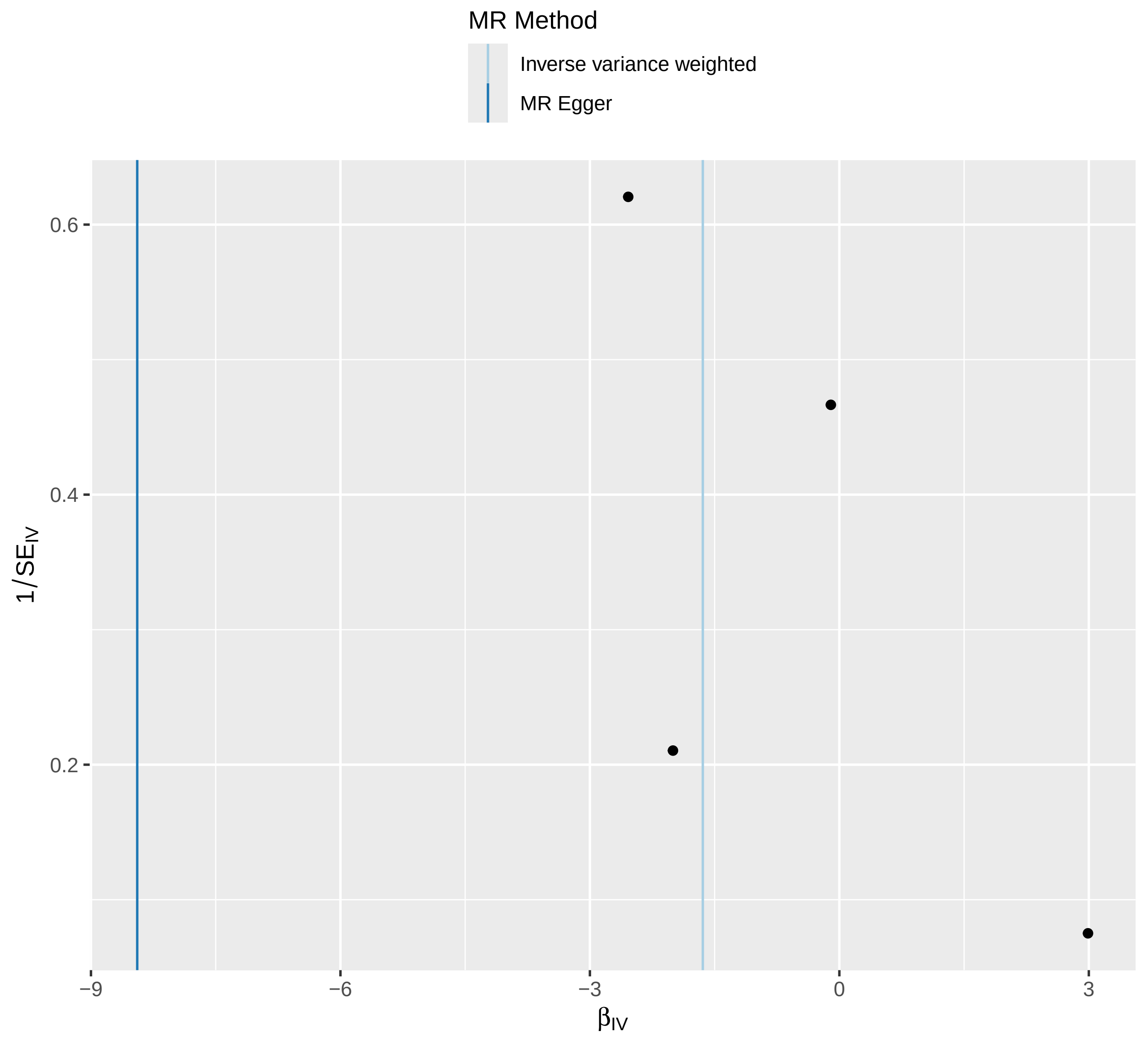
MR Method
Inverse variance weighted
MR Egger
0.6
0.4
0.2
1/SE$_{IV}$
−9
−6
−3
0
3
β$_{IV}$

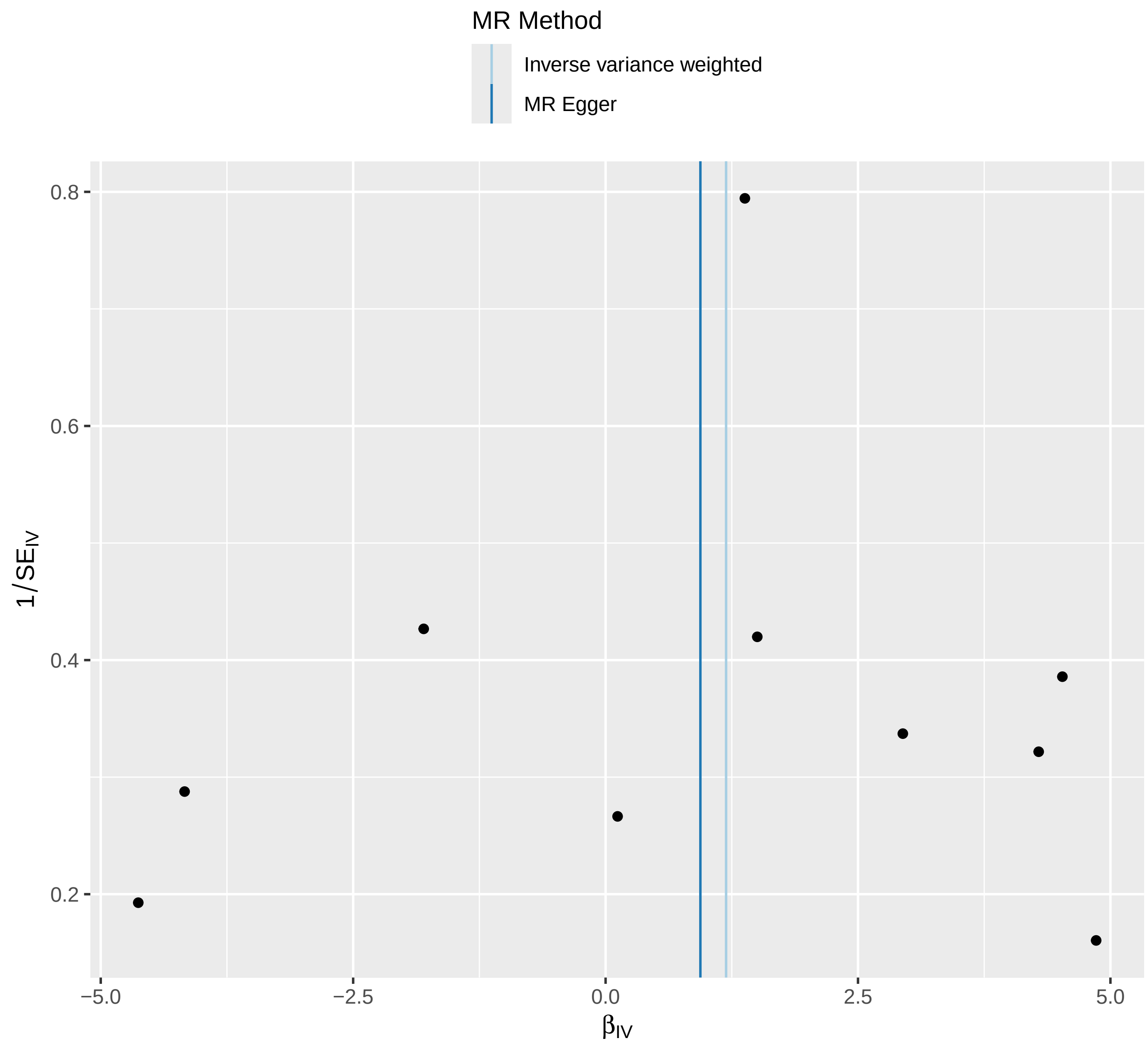
MR Method
Inverse variance weighted
MR Egger
0.8
0.6
0.4
0.2
1/SE_IV
−5.0
−2.5
0.0
2.5
5.0
β_IV

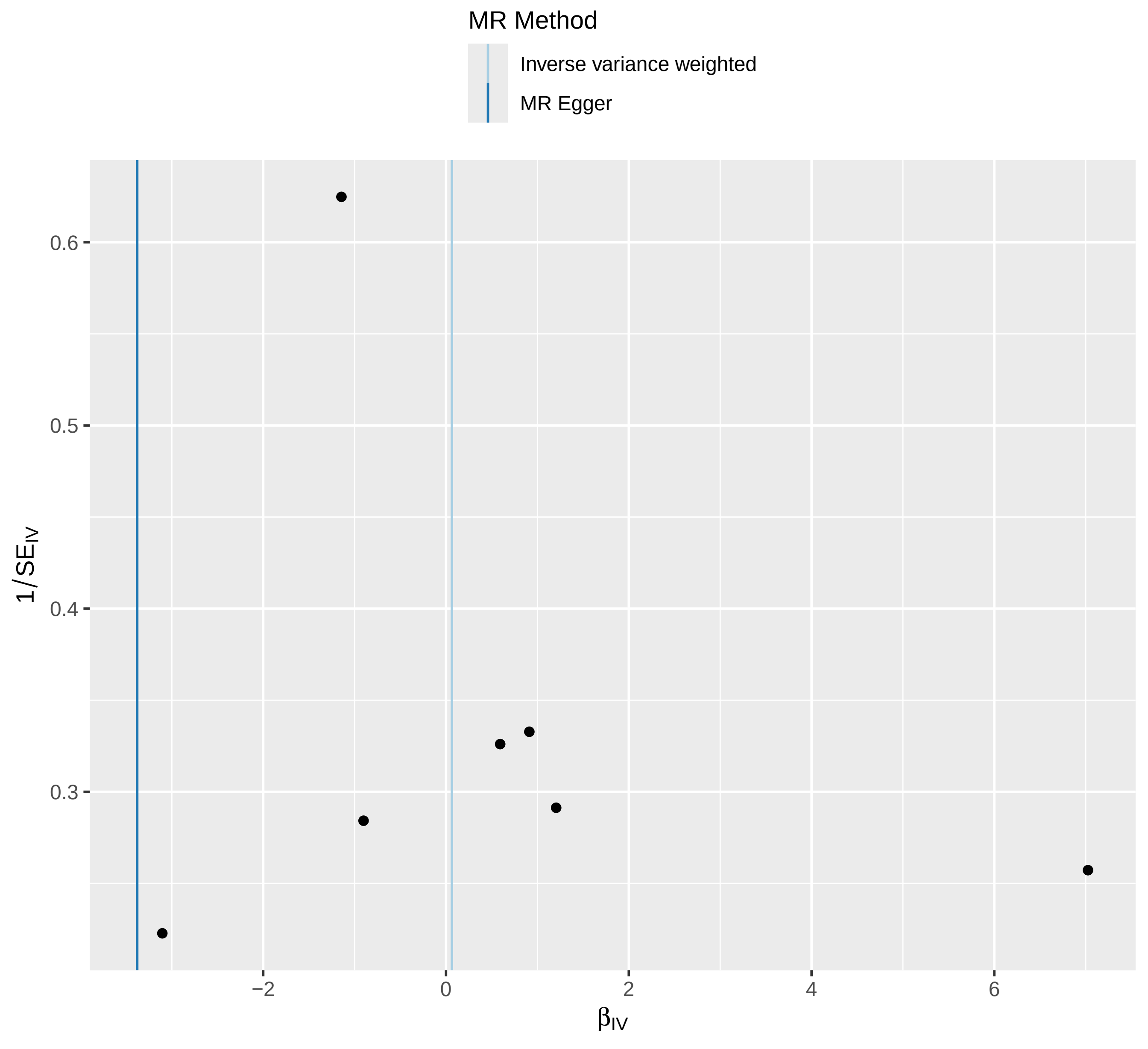
MR Method
Inverse variance weighted
MR Egger
0.6
0.5
0.4
0.3
1/SE$_{IV}$
−2
0
2
4
6
β$_{IV}$

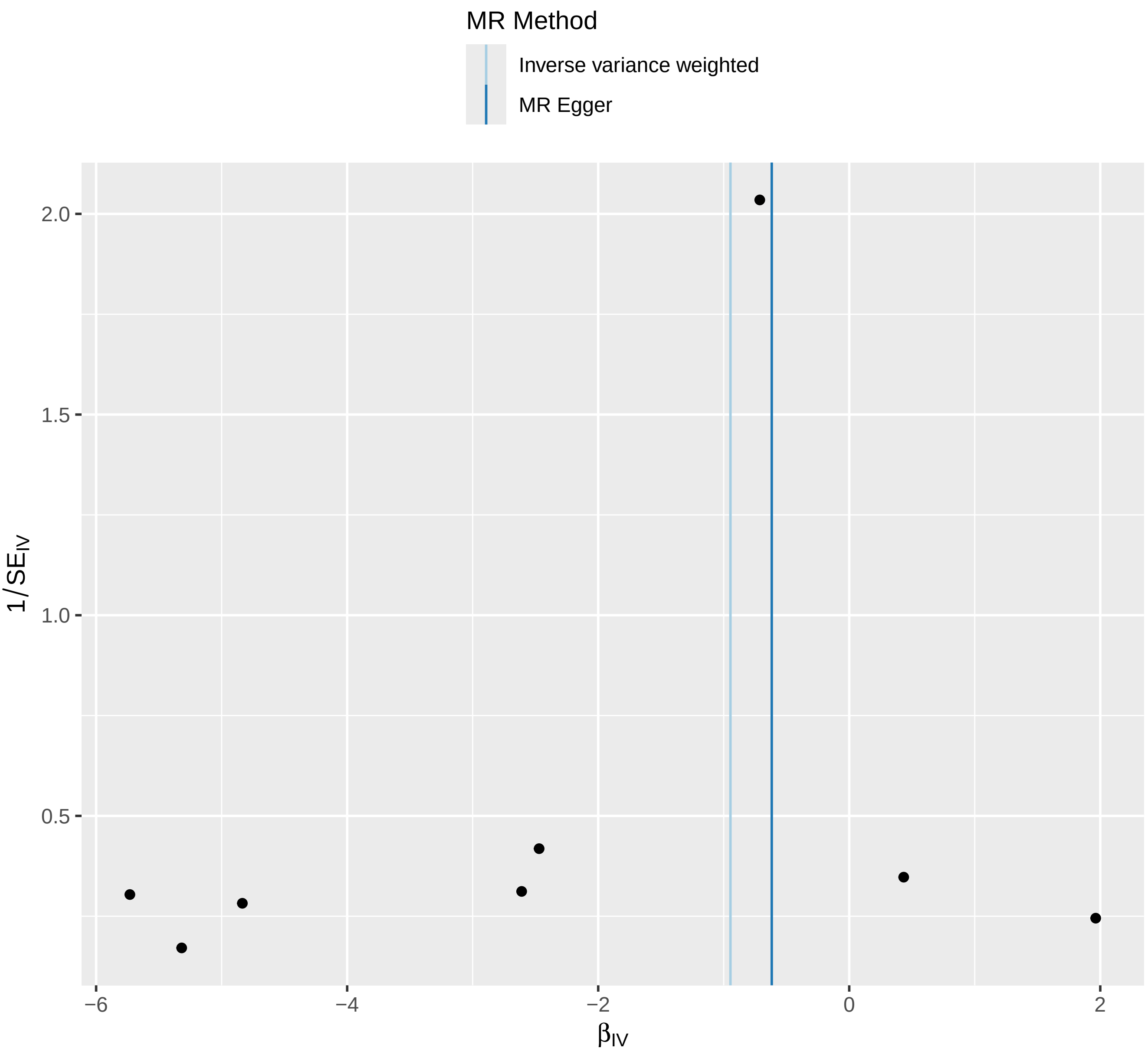

MR Method
Inverse variance weighted
MR Egger
2.0
1.5
1.0
0.5
1/SE_IV
−6
−4
−2
0
2
β_IV

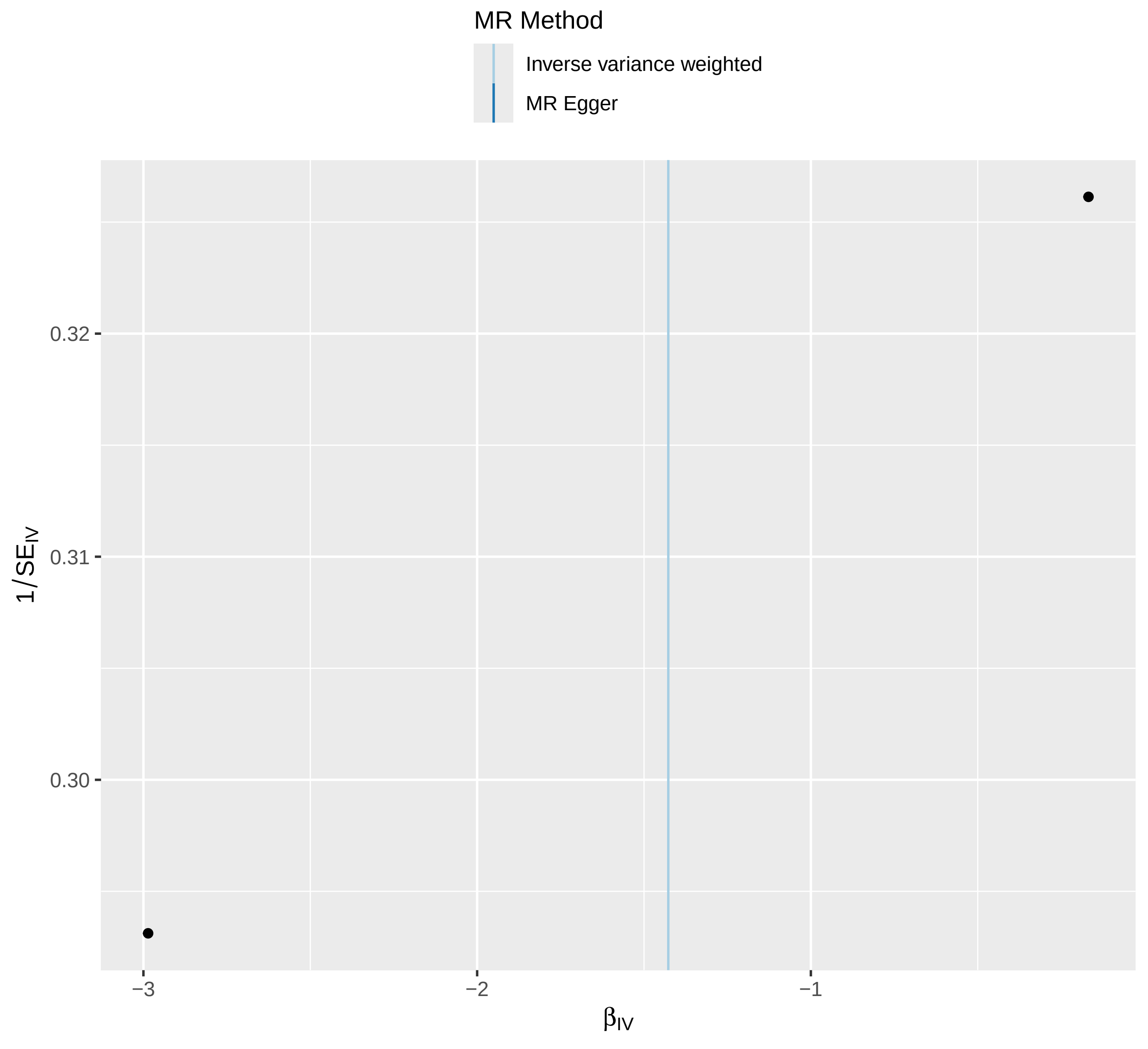

MR Method
Inverse variance weighted
MR Egger
0.32
0.31
0.30
1/SE$_{IV}$
−3
−2
−1
β$_{IV}$

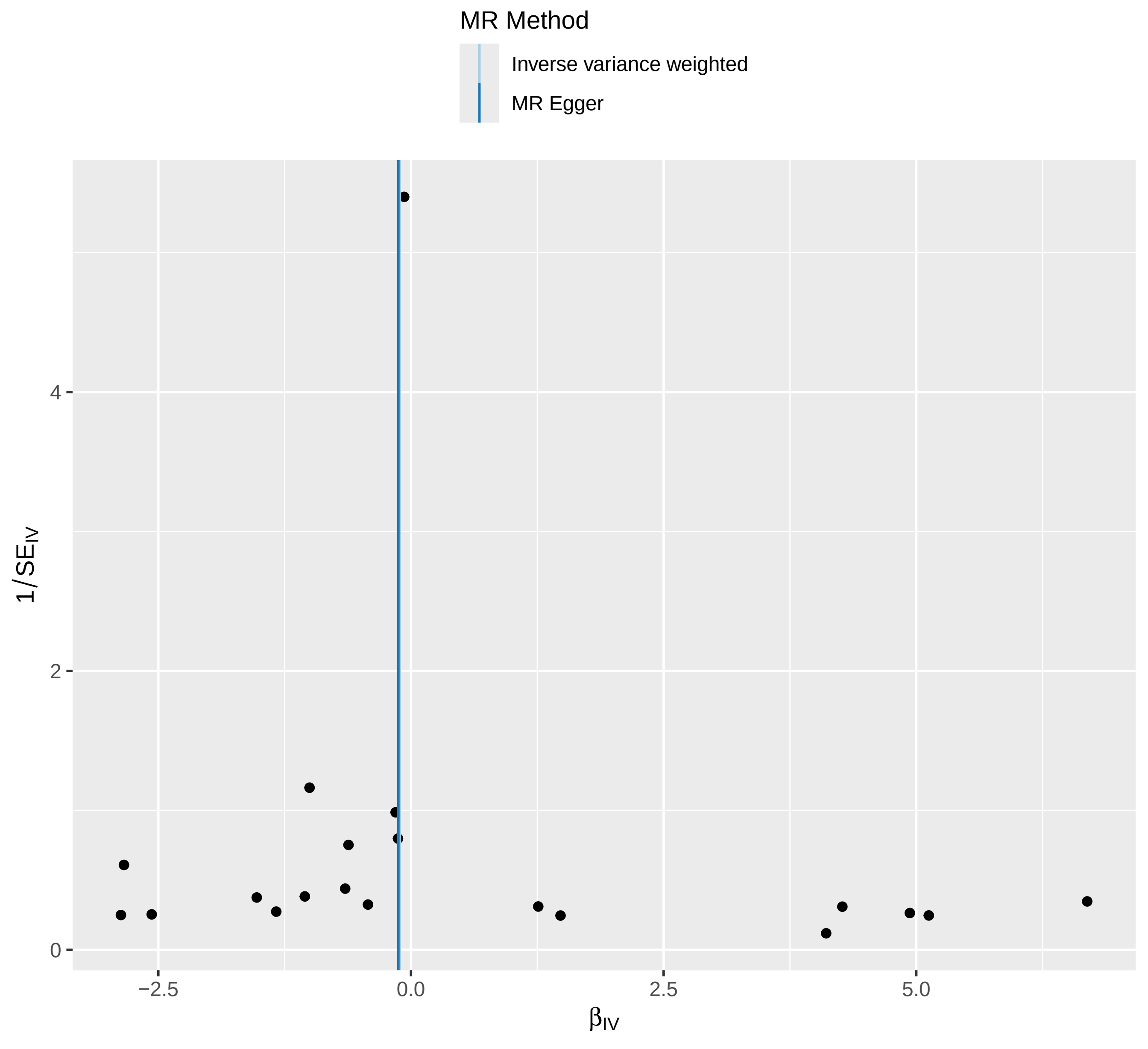

MR Method
Inverse variance weighted
MR Egger
1/SE_IV
4
2
0
−2.5
0.0
2.5
5.0
β_IV

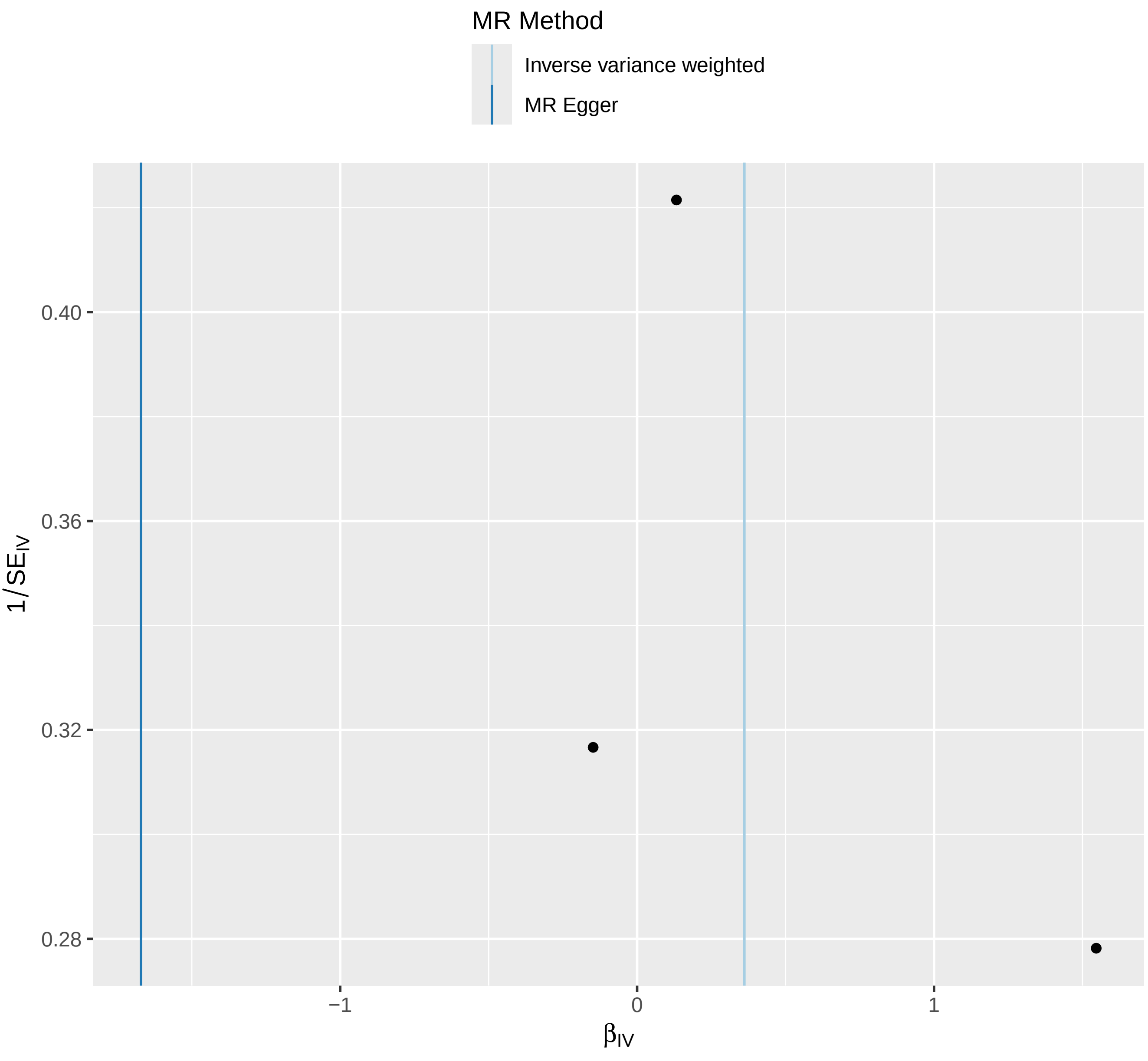
MR Method
Inverse variance weighted
MR Egger
0.40
0.36
0.32
0.28
1/SE_IV
−1
0
1
β_IV

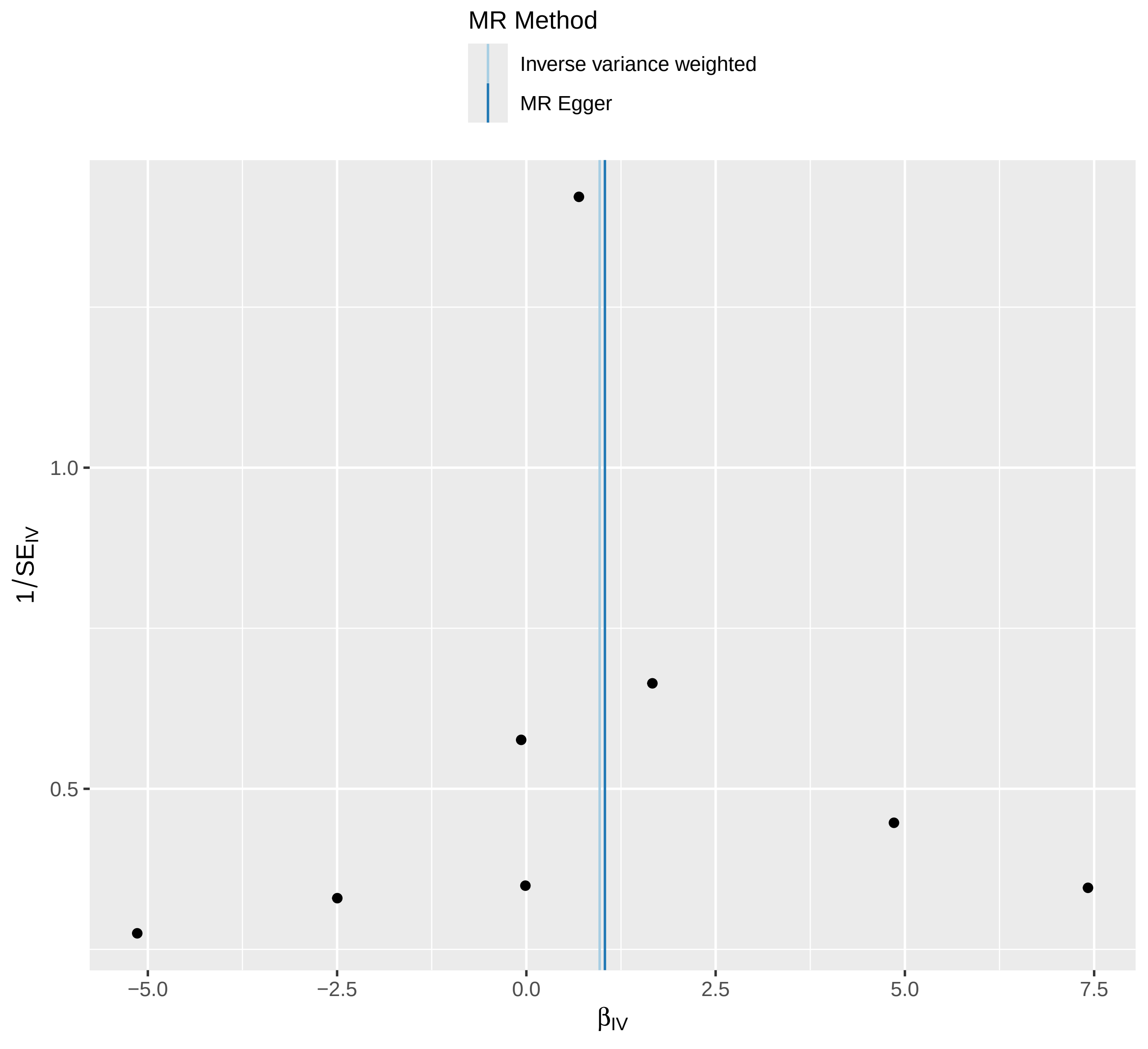
MR Method
Inverse variance weighted
MR Egger
1.0
0.5
1/SE$_{IV}$
−5.0
−2.5
0.0
2.5
5.0
7.5
β$_{IV}$

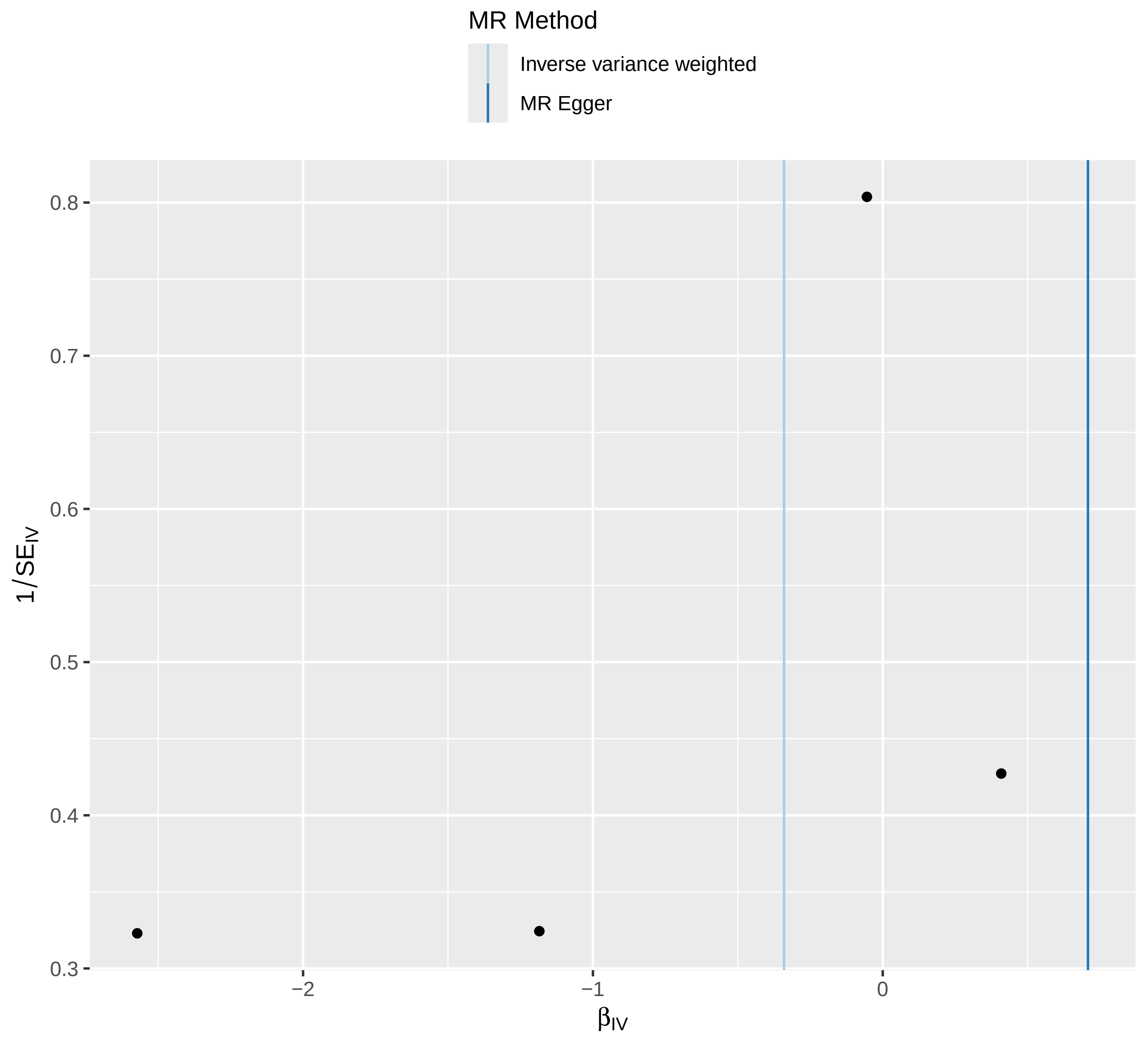
MR Method
Inverse variance weighted
MR Egger
0.8
0.7
0.6
0.5
0.4
0.3
1/SE_IV
−2
−1
0
β_IV

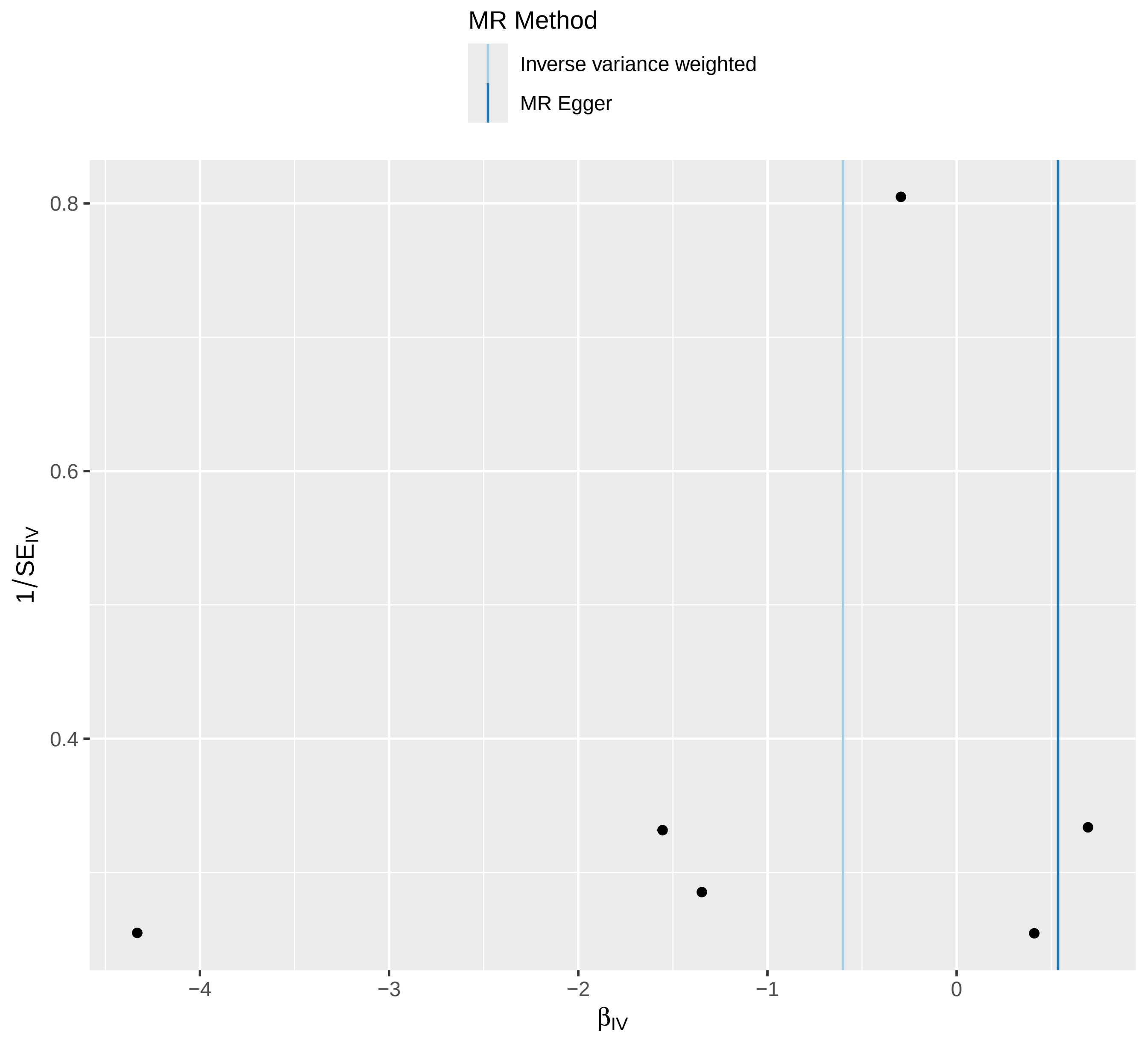
MR Method
Inverse variance weighted
MR Egger
0.8
0.6
0.4
1/SE_IV
−4
−3
−2
−1
0
β_IV

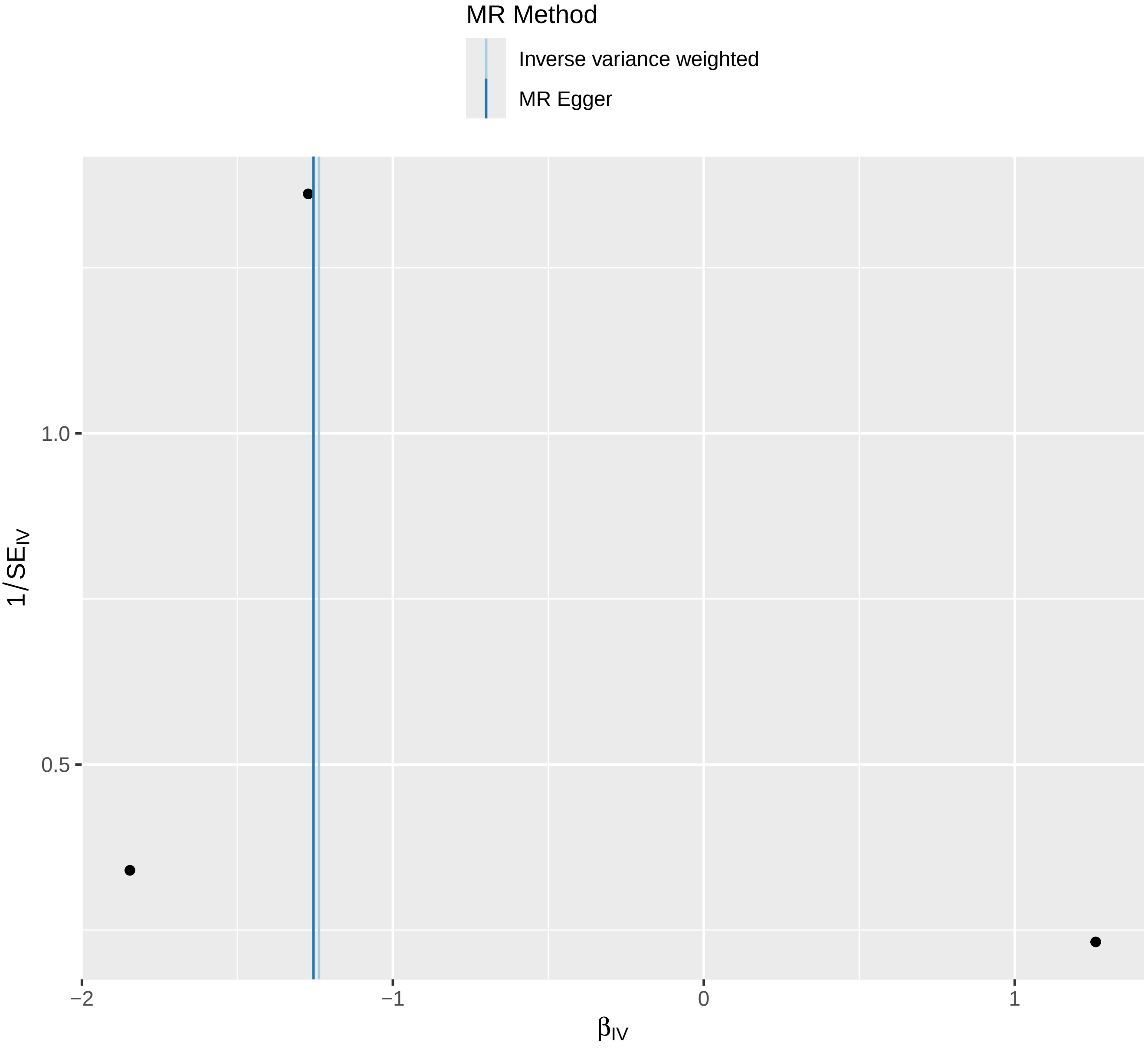

MR Method
Inverse variance weighted
MR Egger
1.0
0.5
$1/SE_{IV}$
−2
−1
0
1
β_{IV}

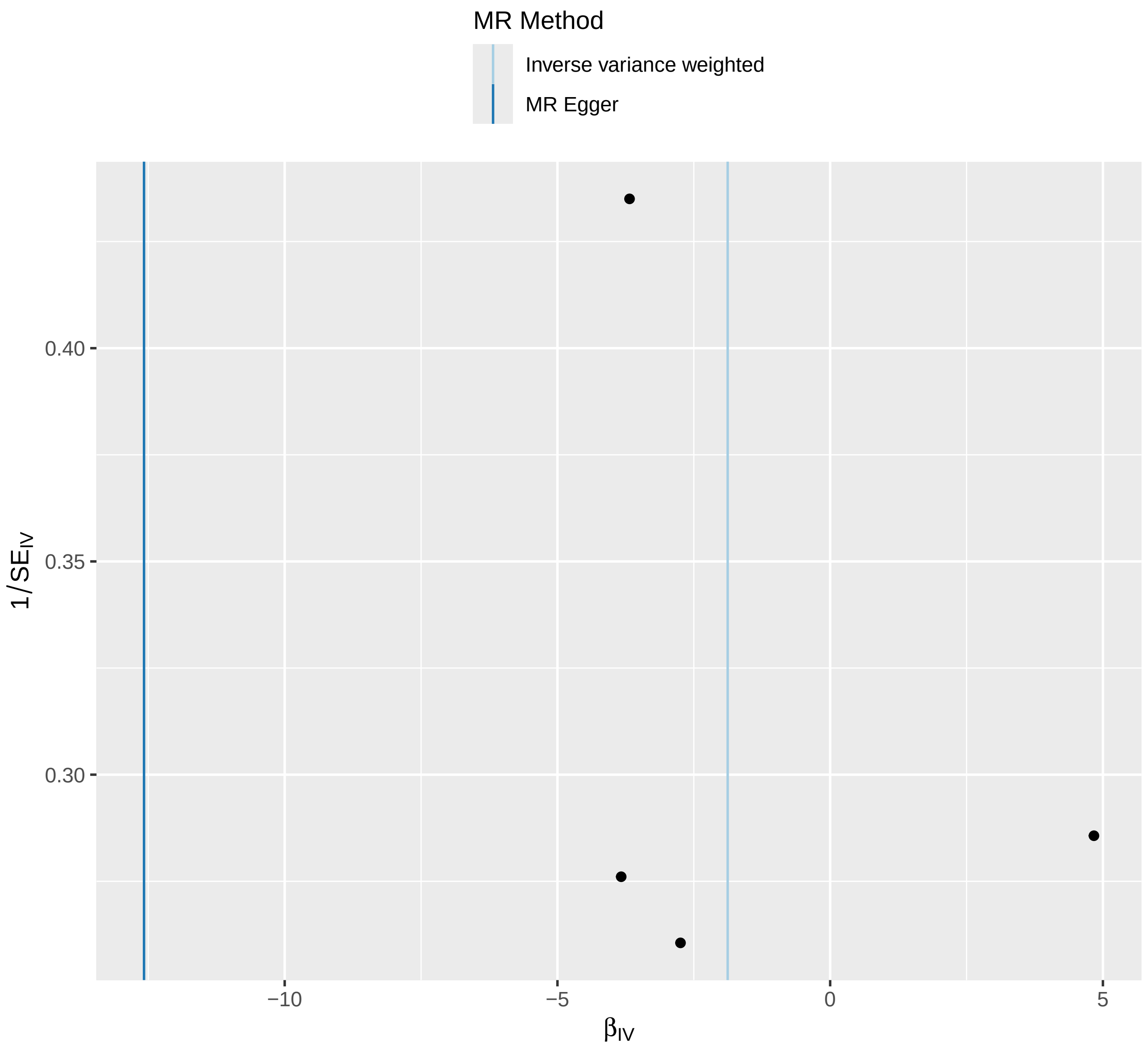

MR Method
Inverse variance weighted
MR Egger
0.40
0.35
0.30
1/SE_IV
−10
−5
0
5
β_IV

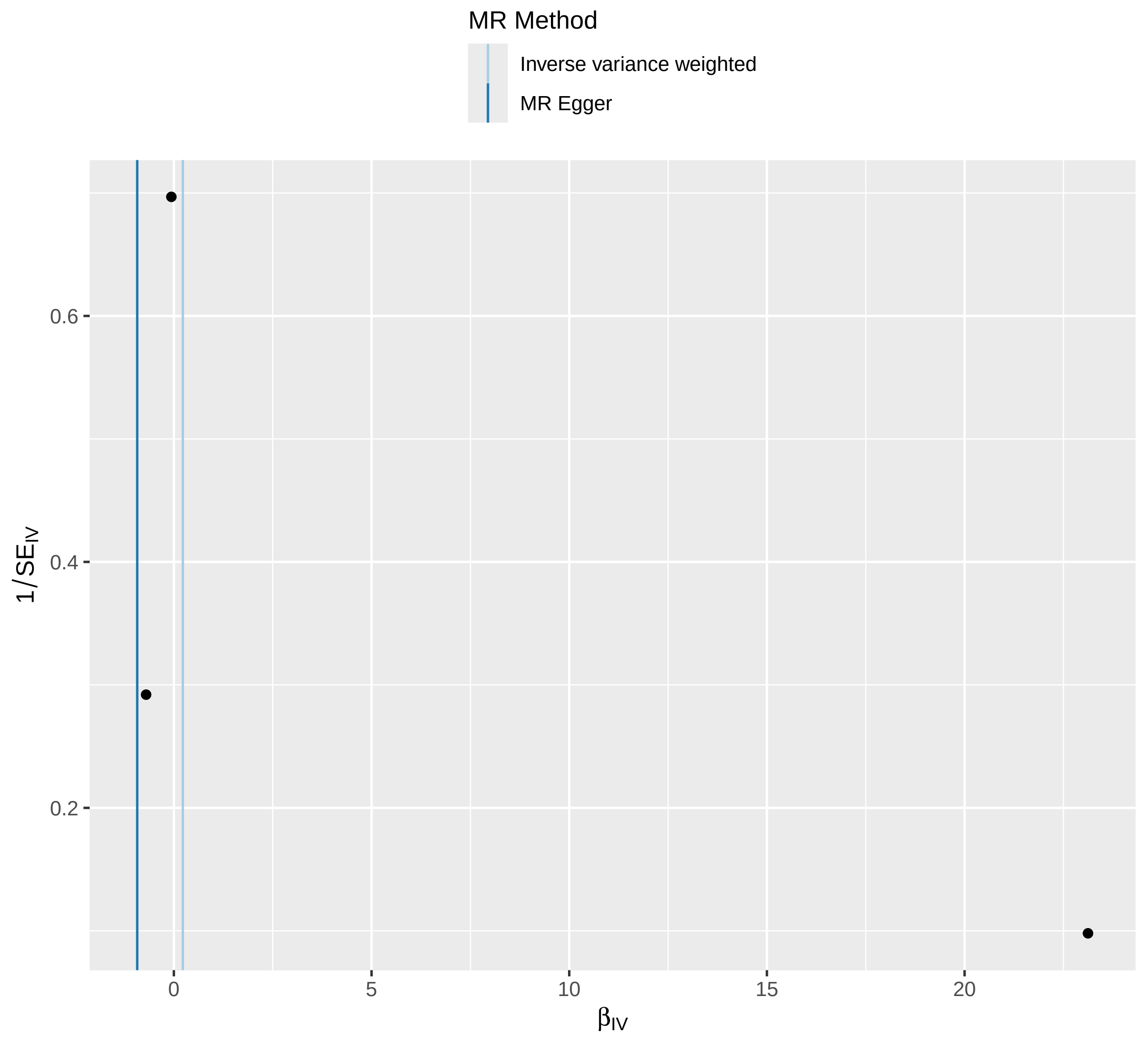
MR Method
Inverse variance weighted
MR Egger
0.6
0.4
0.2
1/SE_IV
0
5
10
15
20
β_IV

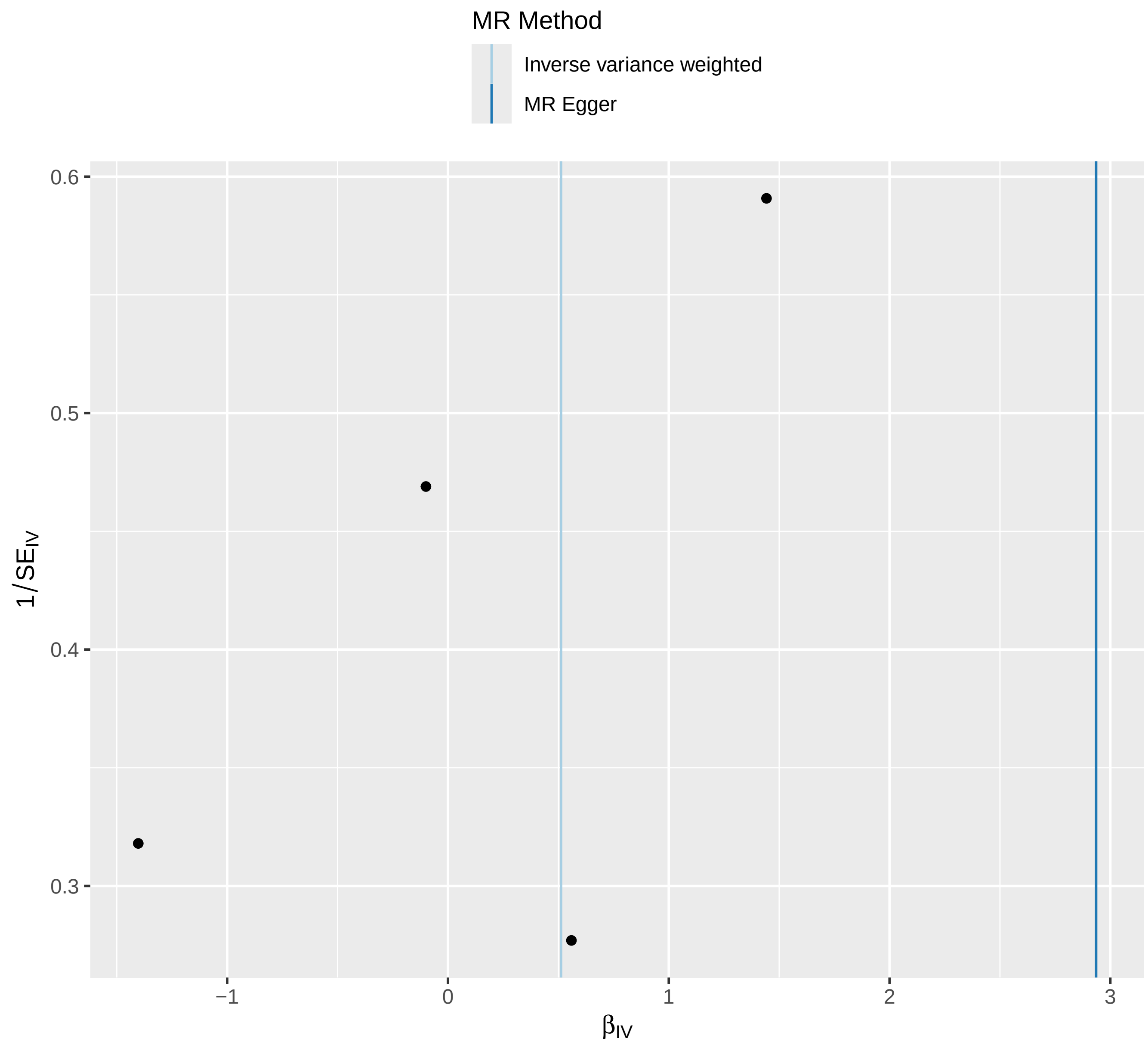
MR Method
Inverse variance weighted
MR Egger
0.6
0.5
0.4
0.3
1/SE_IV
−1
0
1
2
3
β_IV

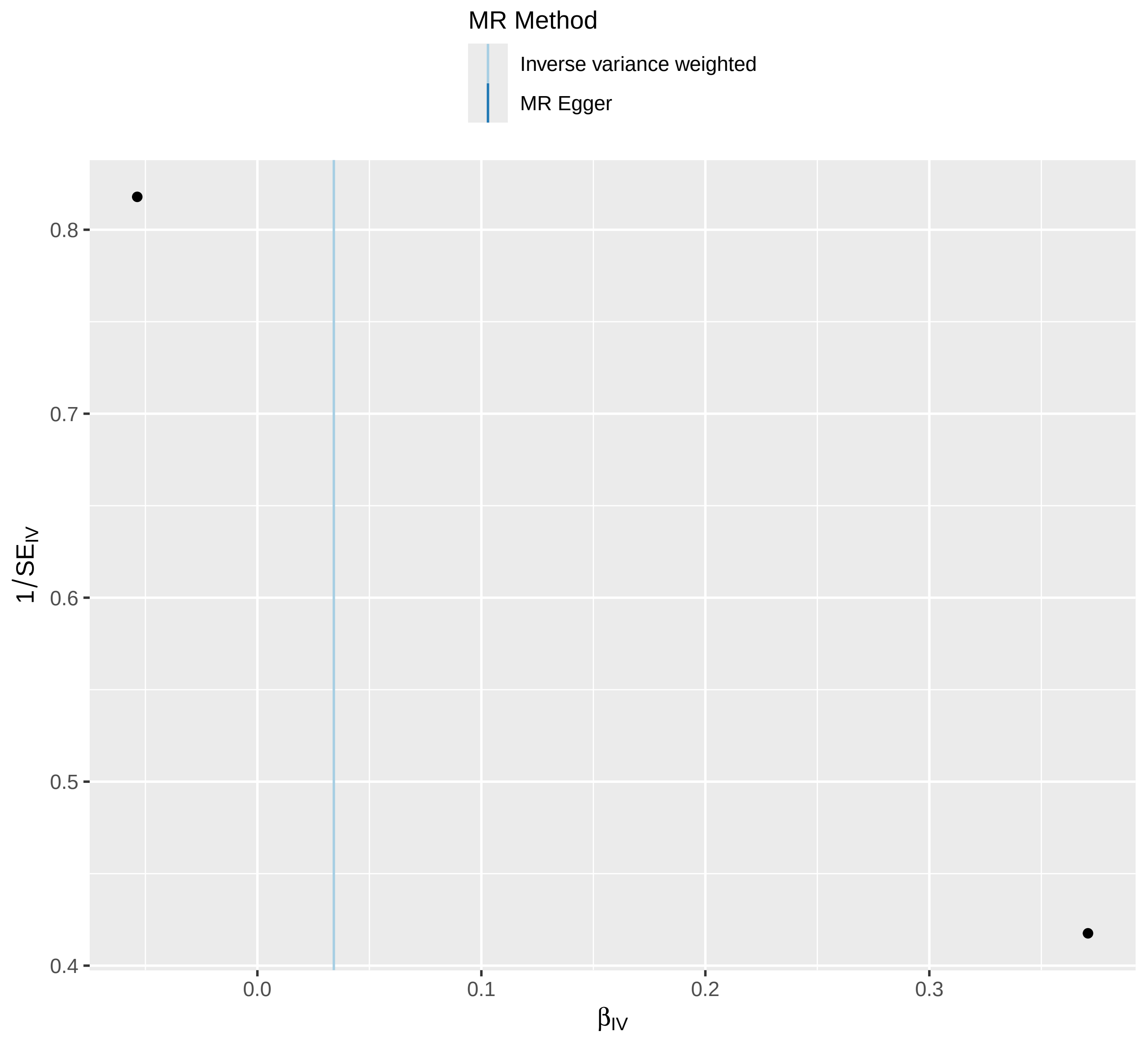
MR Method
Inverse variance weighted
MR Egger
0.8
0.7
0.6
0.5
0.4
$1/SE_{IV}$
0.0
0.1
0.2
0.3
β_{IV}

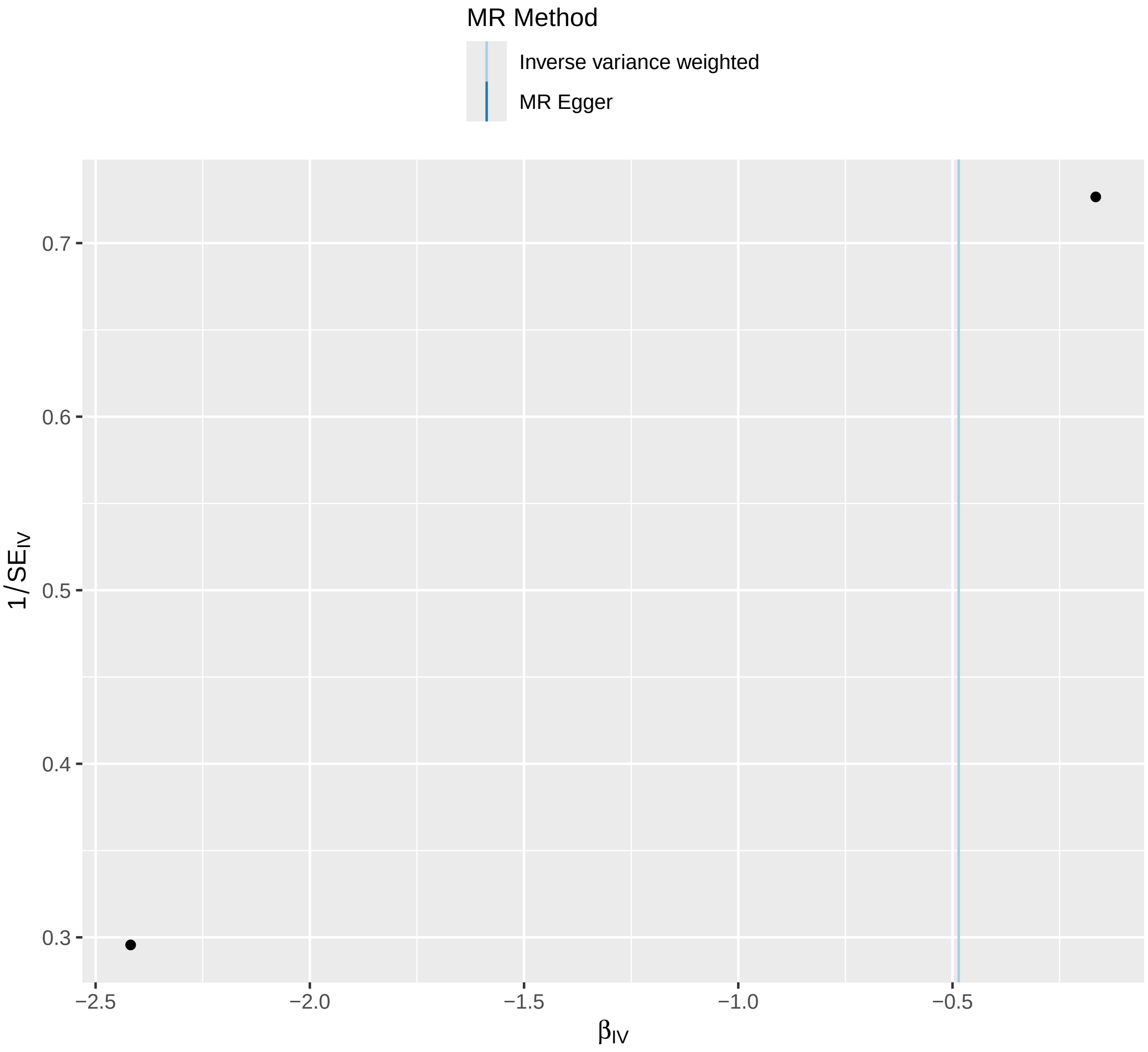

MR Method
Inverse variance weighted
MR Egger
0.7
0.6
0.5
0.4
0.3
1/SE_IV
−2.5
−2.0
−1.5
−1.0
−0.5
β_IV

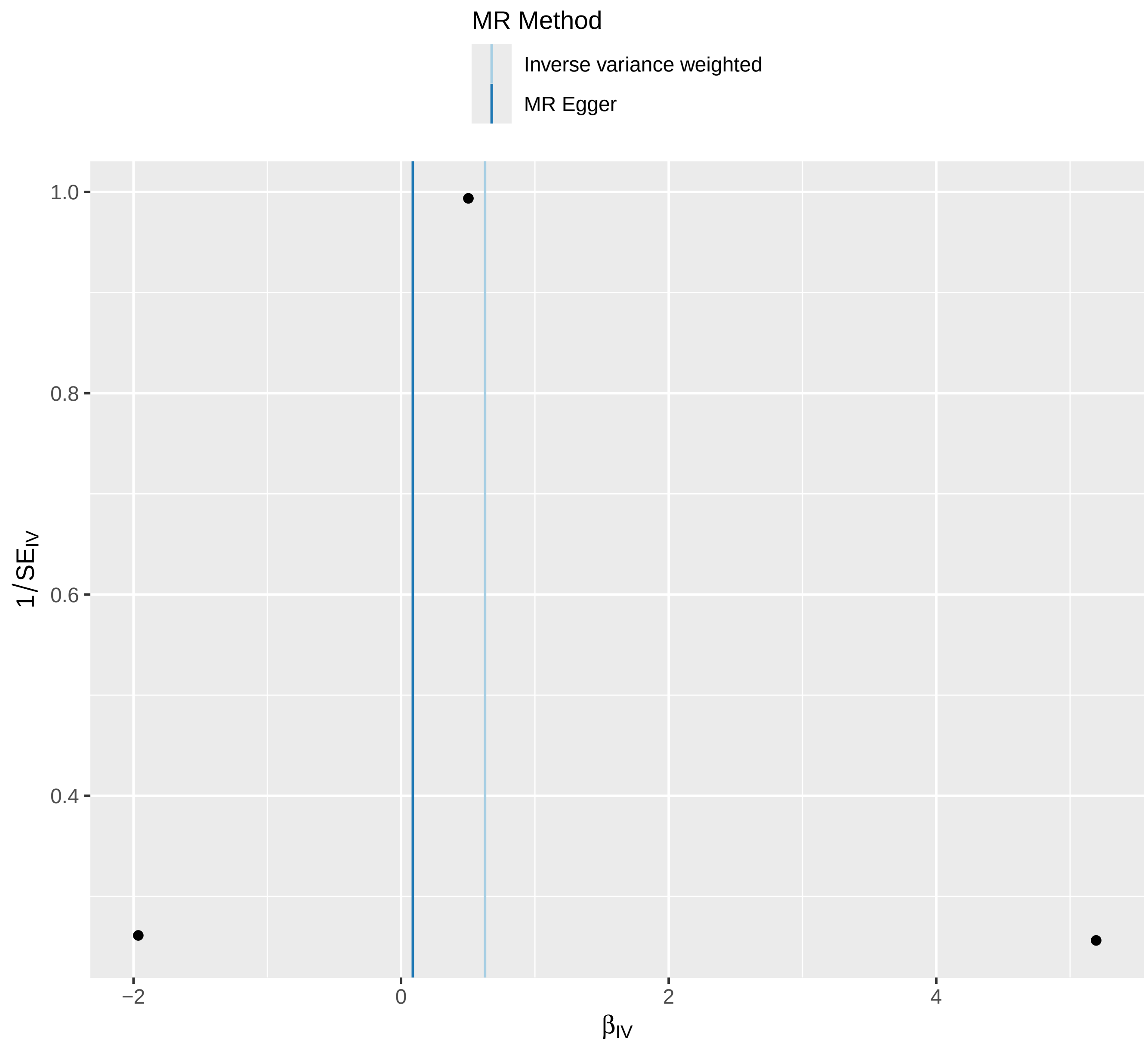
MR Method
Inverse variance weighted
MR Egger
1.0
0.8
0.6
0.4
1/SE_IV
−2
0
2
4
β_IV

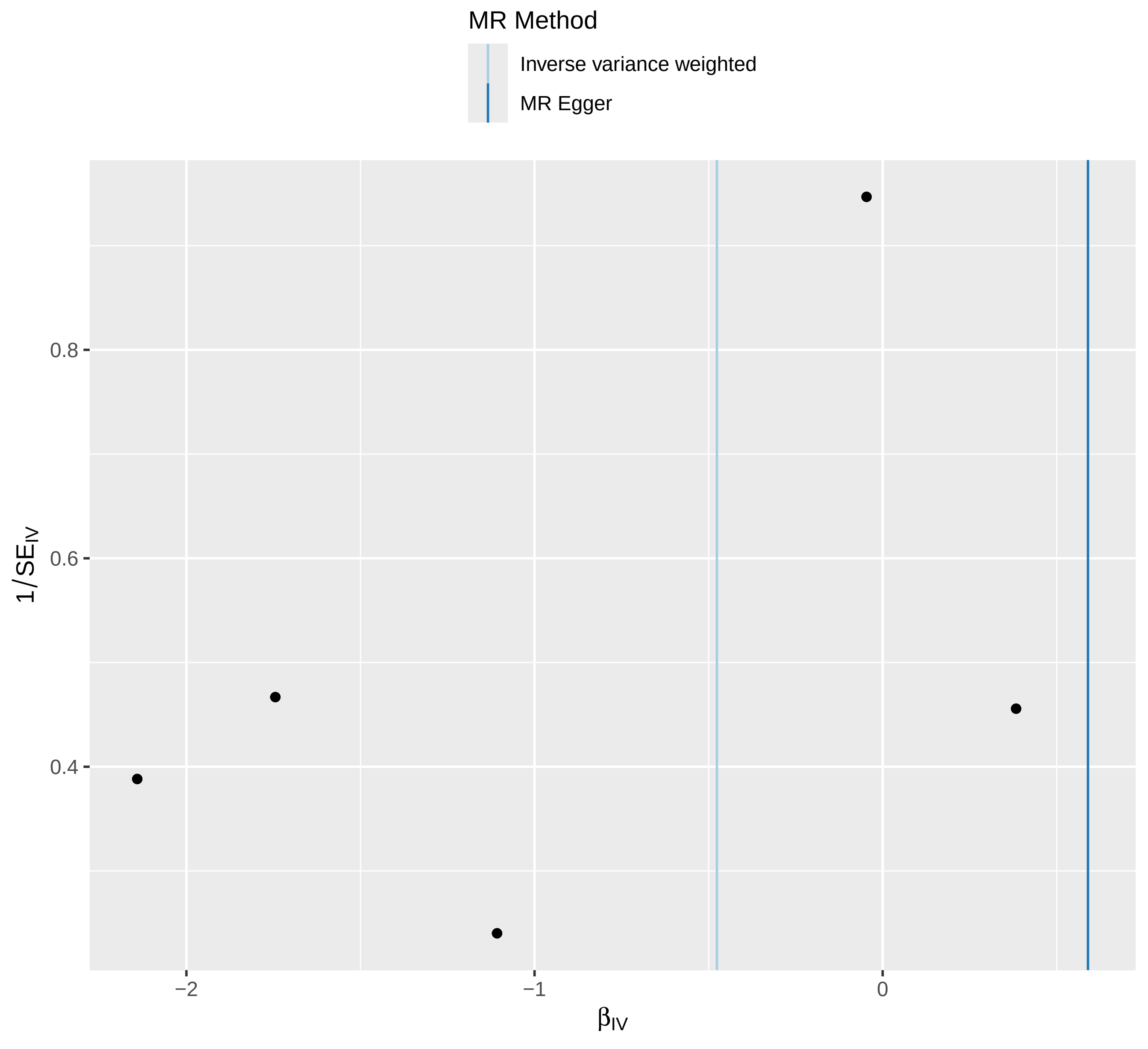
MR Method
Inverse variance weighted
MR Egger
0.8
0.6
0.4
1/SE_IV
−2
−1
0
β_IV

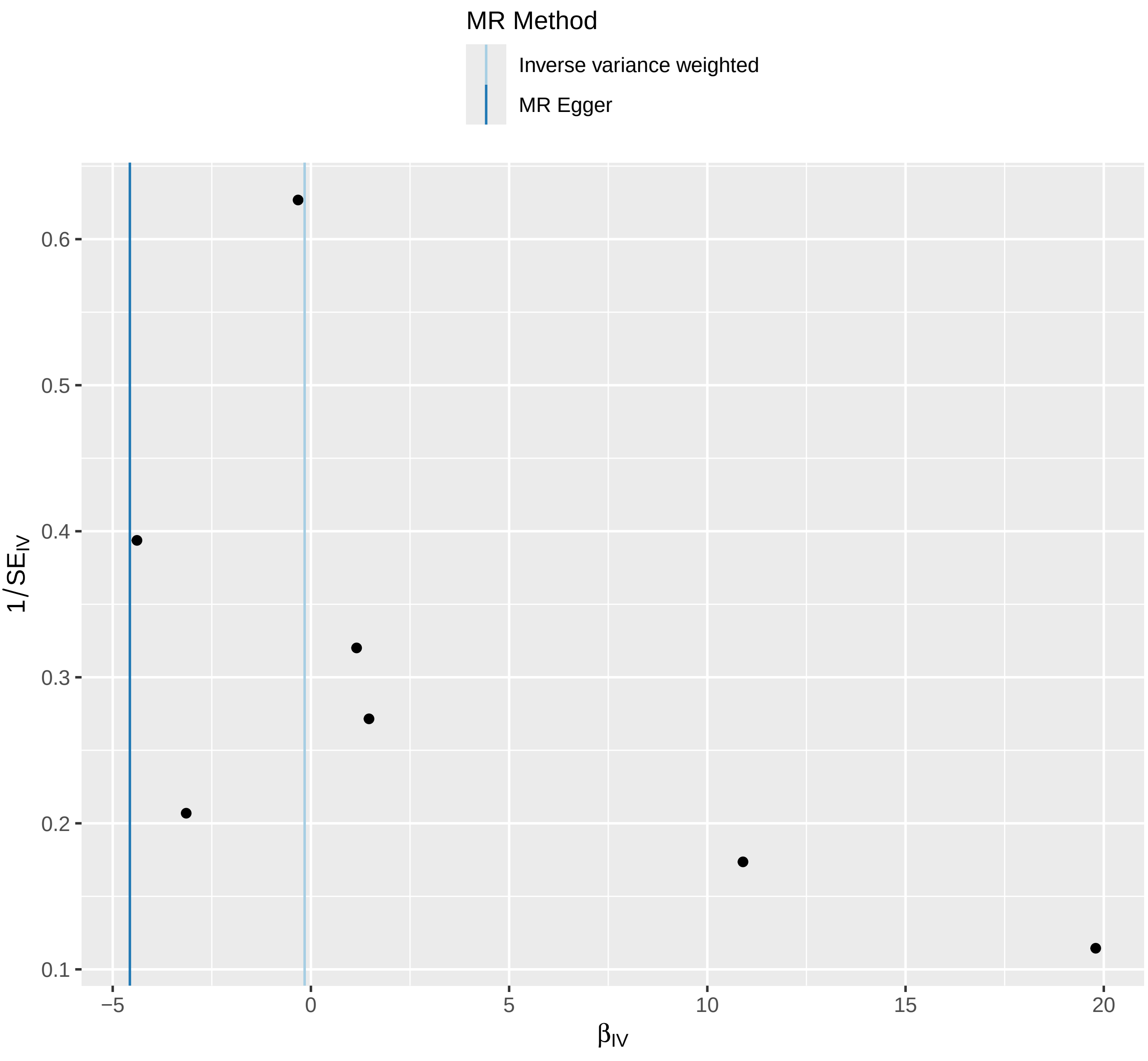
MR Method
Inverse variance weighted
MR Egger
0.6
0.5
0.4
0.3
0.2
0.1
1/SE_IV
−5
0
5
10
15
20
β_IV

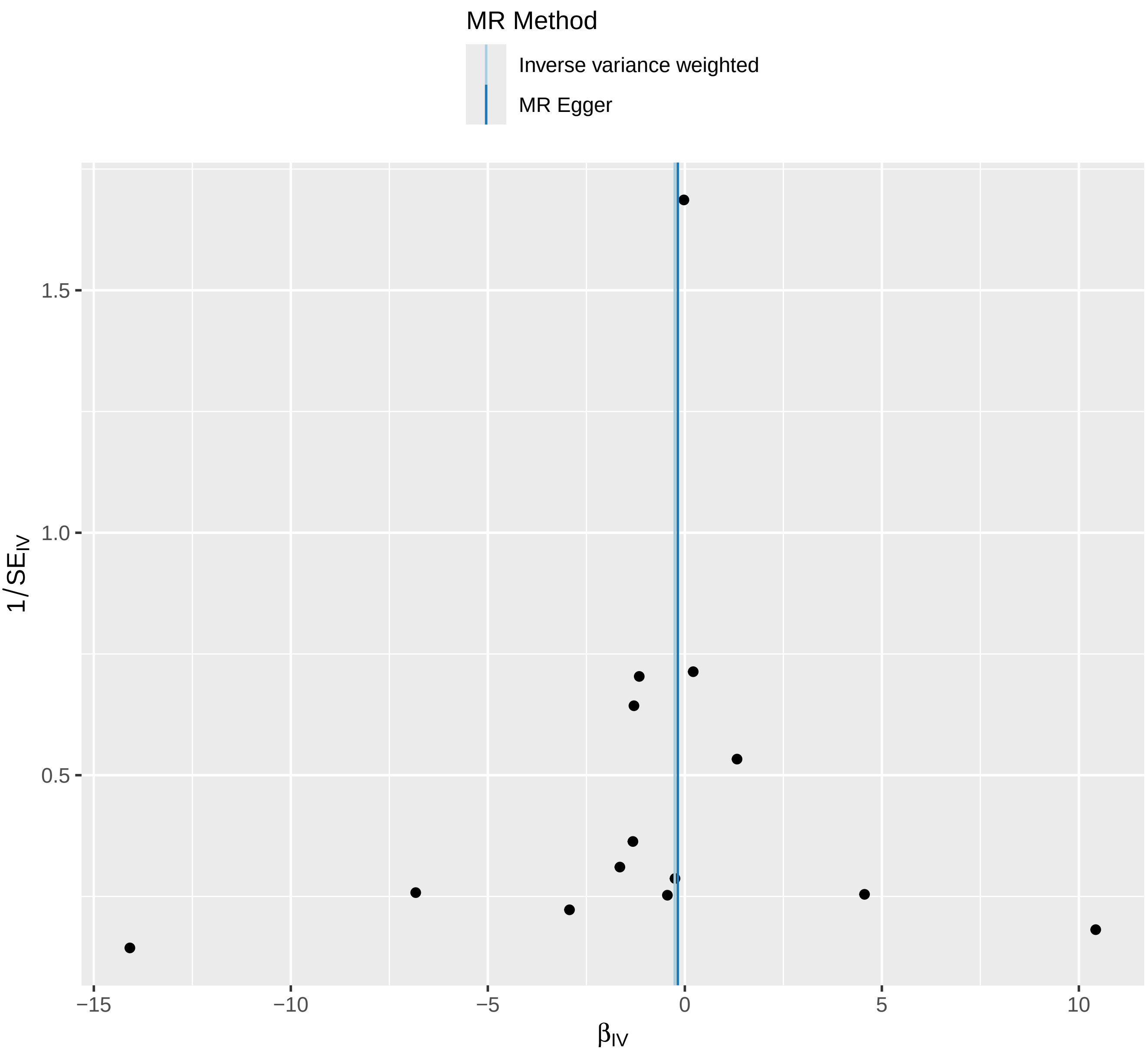
MR Method
Inverse variance weighted
MR Egger
1.5
1.0
0.5
$1/SE_{IV}$
−15
−10
−5
0
5
10
β_{IV}

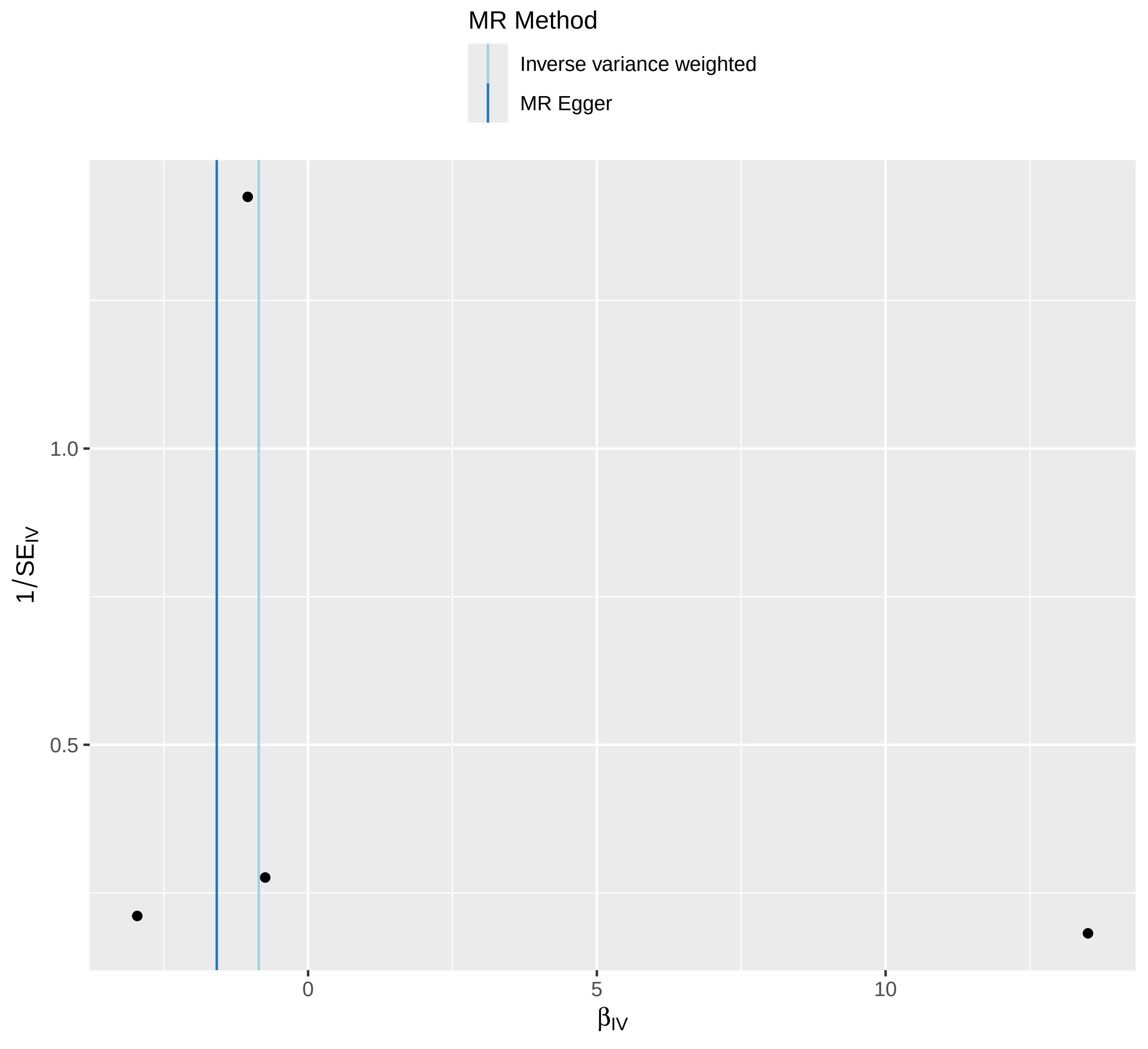
MR Method
Inverse variance weighted
MR Egger
1.0
0.5
1/SE_IV
0
5
10
β_IV

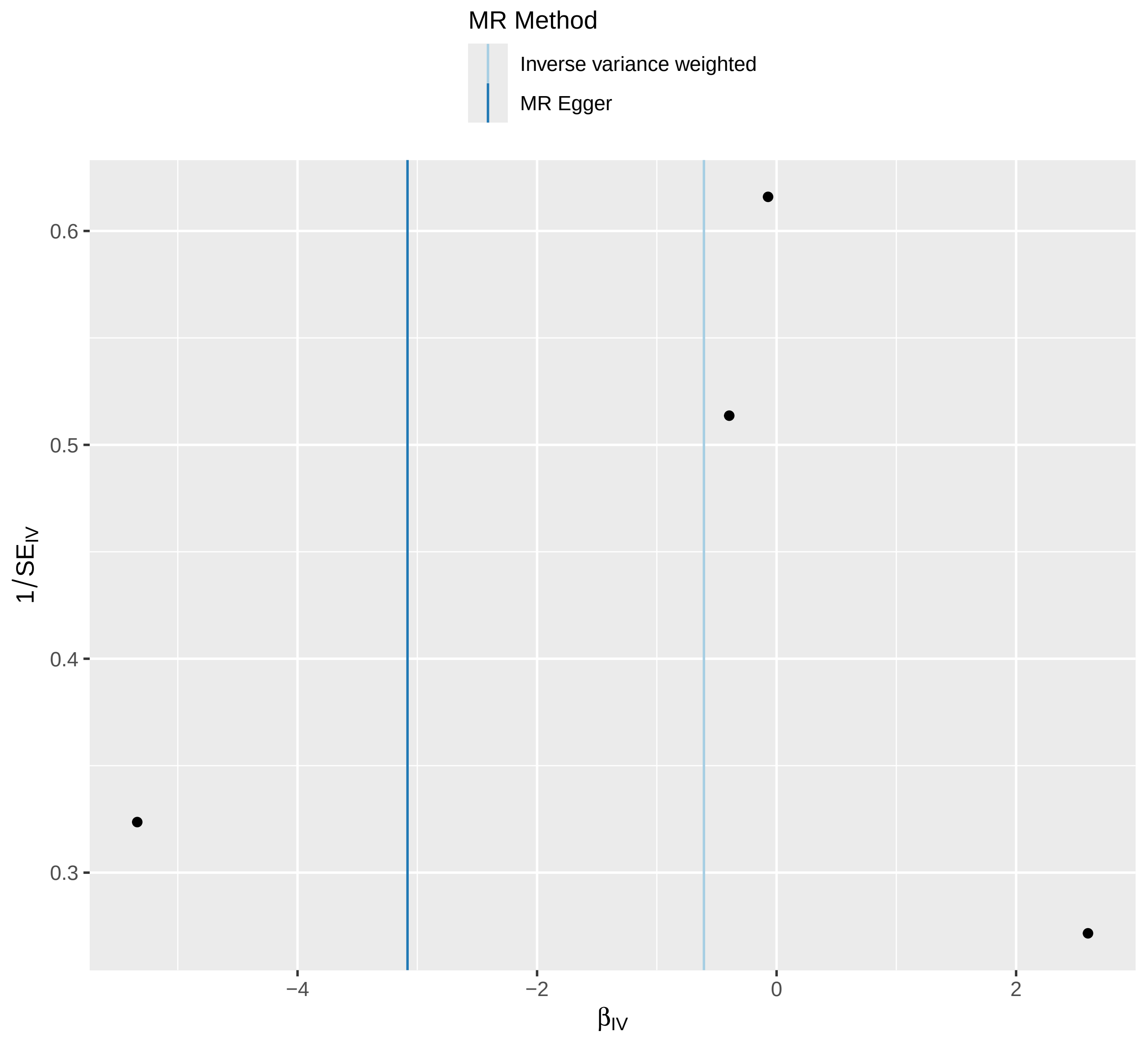

MR Method
Inverse variance weighted
MR Egger
0.6
0.5
0.4
0.3
1/SE_IV
−4
−2
0
2
β_IV

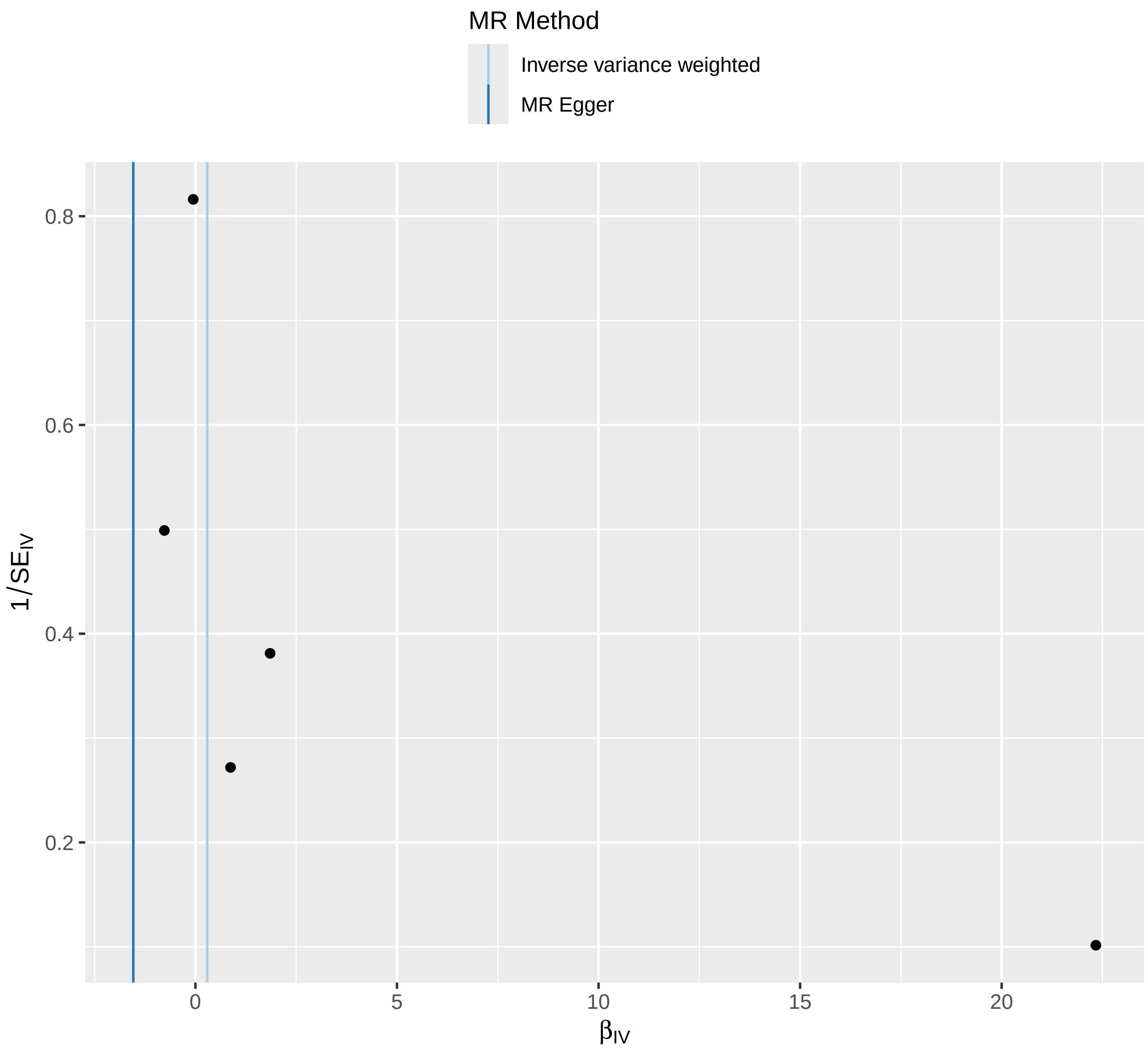
MR Method
Inverse variance weighted
MR Egger
0.8
0.6
0.4
0.2
1/SE_IV
0
5
10
15
20
β_IV

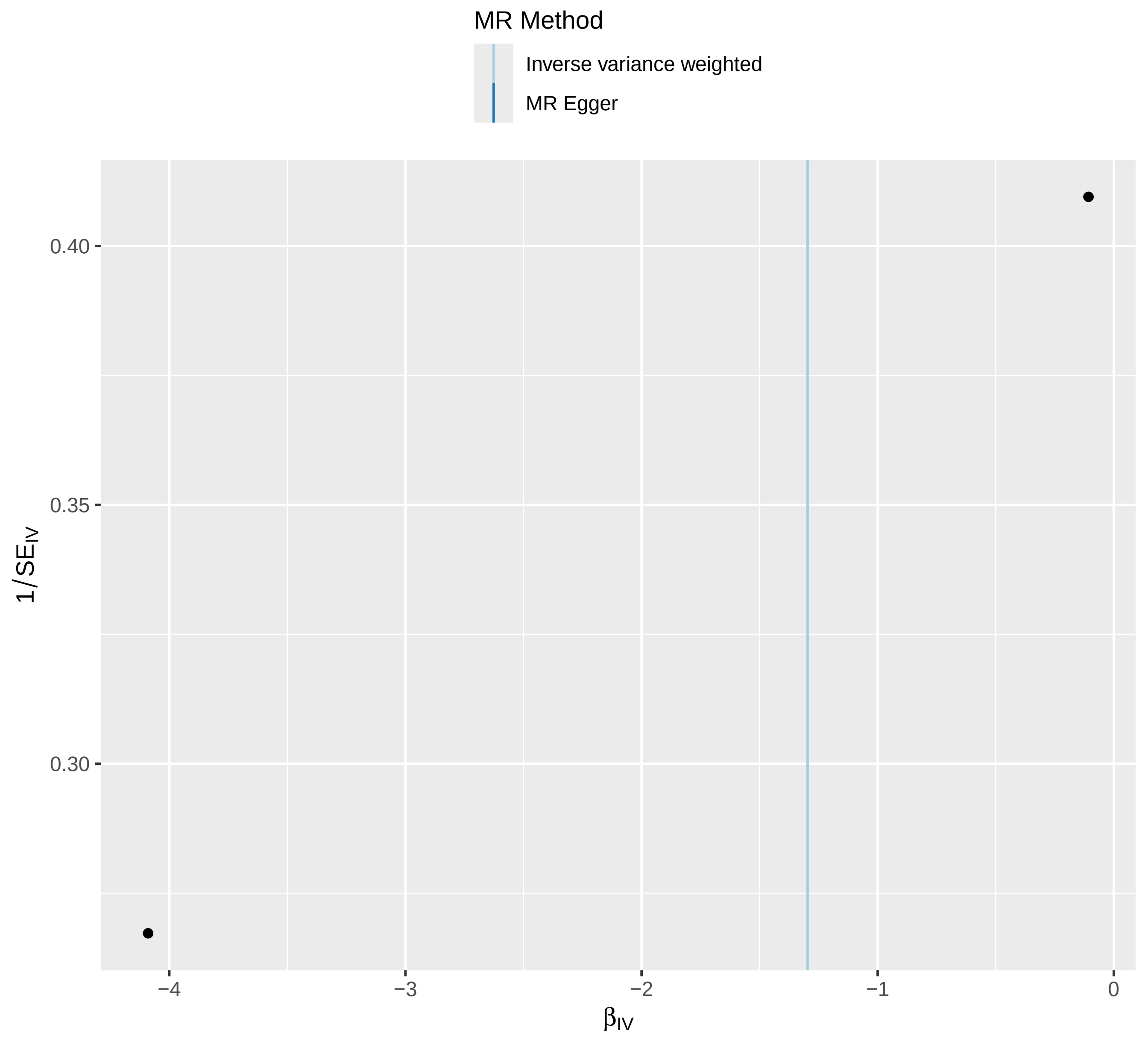
MR Method
Inverse variance weighted
MR Egger
0.40
0.35
0.30
1/SE_IV
−4
−3
−2
−1
0
β_IV

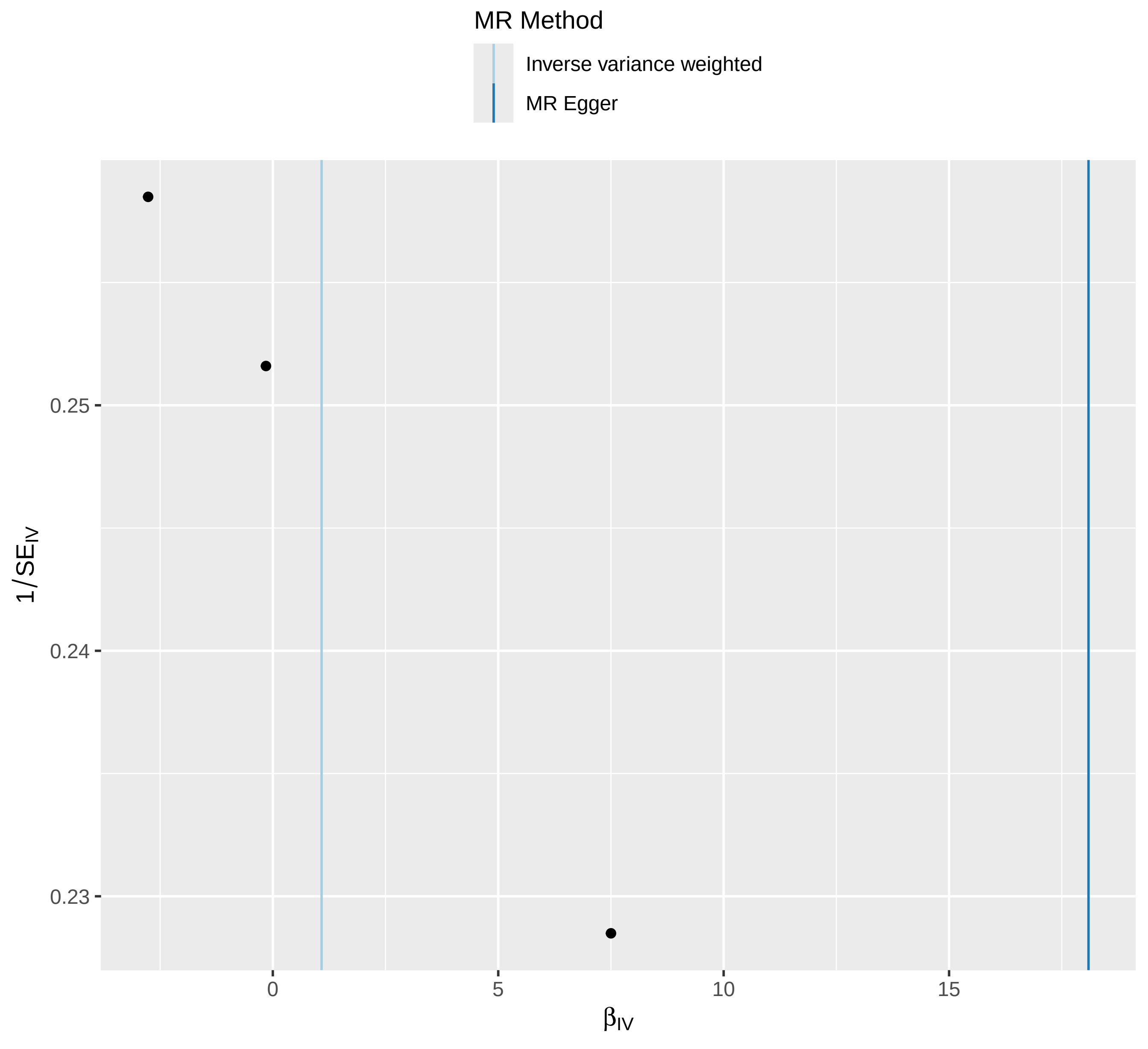
MR Method
Inverse variance weighted
MR Egger
0.25
0.24
0.23
1/SE_IV
0
5
10
15
β_IV

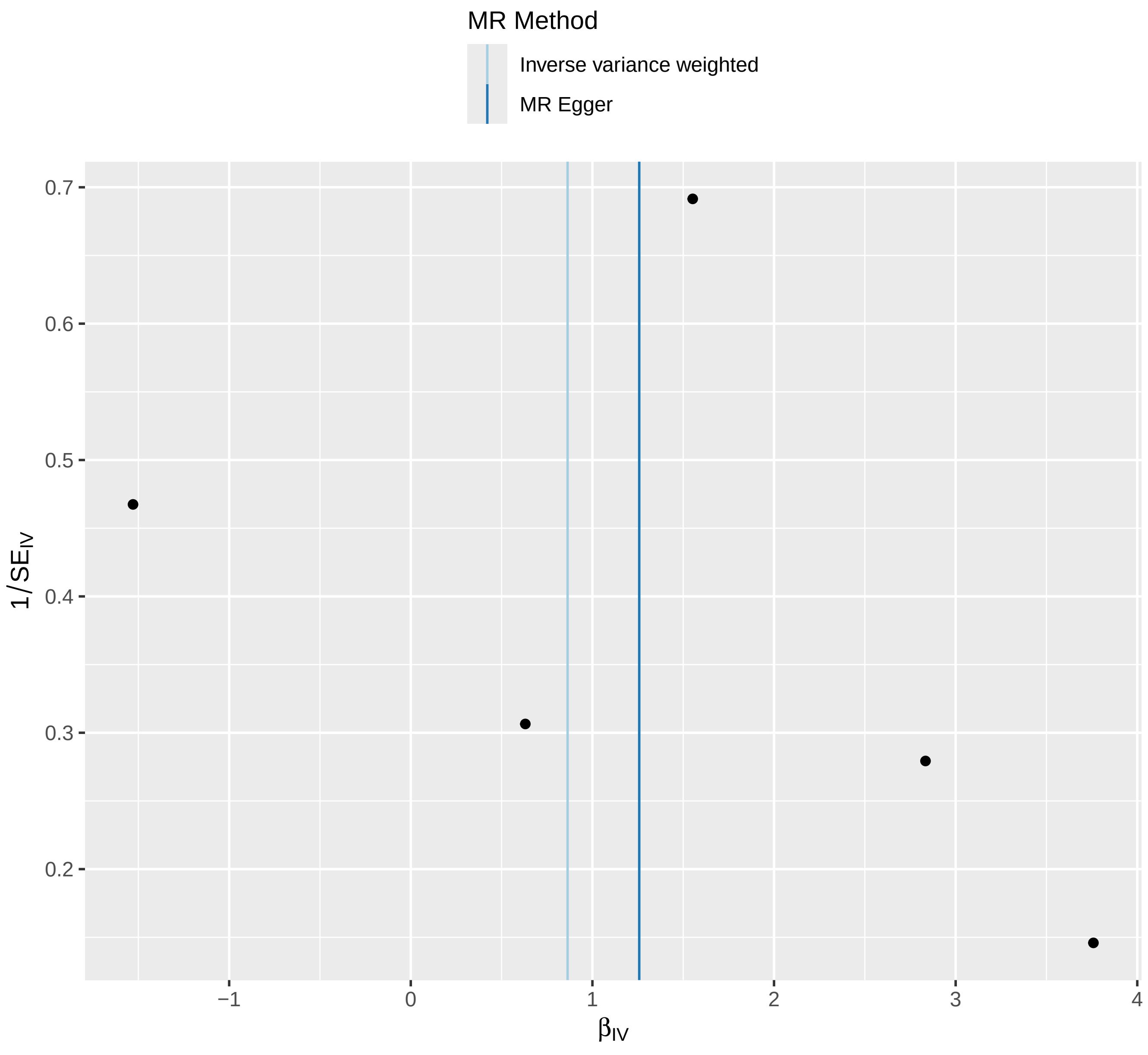
MR Method
Inverse variance weighted
MR Egger
0.7
0.6
0.5
0.4
0.3
0.2
$1/SE_{IV}$
−1
0
1
2
3
4
β_{IV}

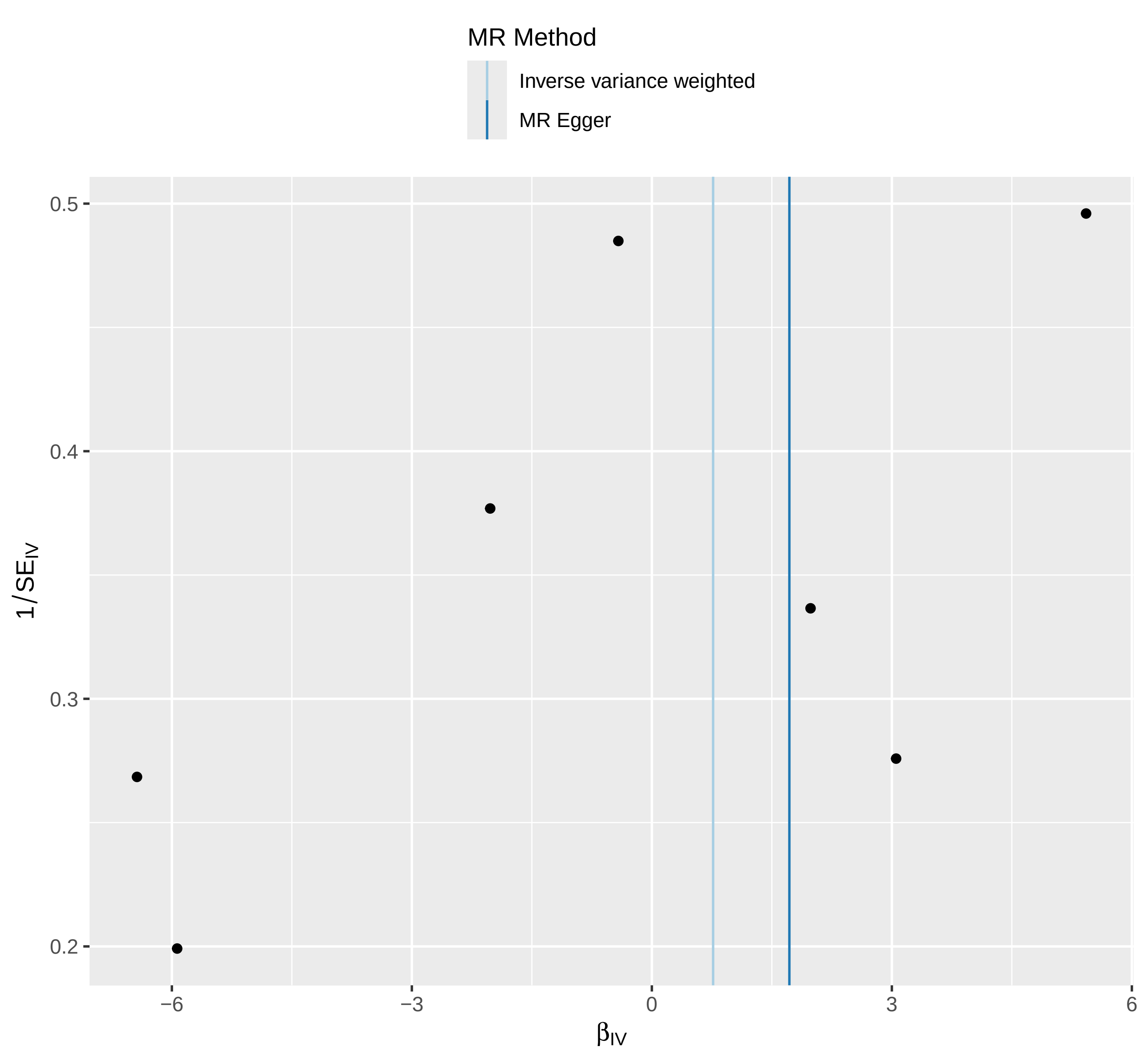
MR Method
Inverse variance weighted
MR Egger
0.5
0.4
0.3
0.2
1/SE_IV
−6
−3
0
3
6
β_IV

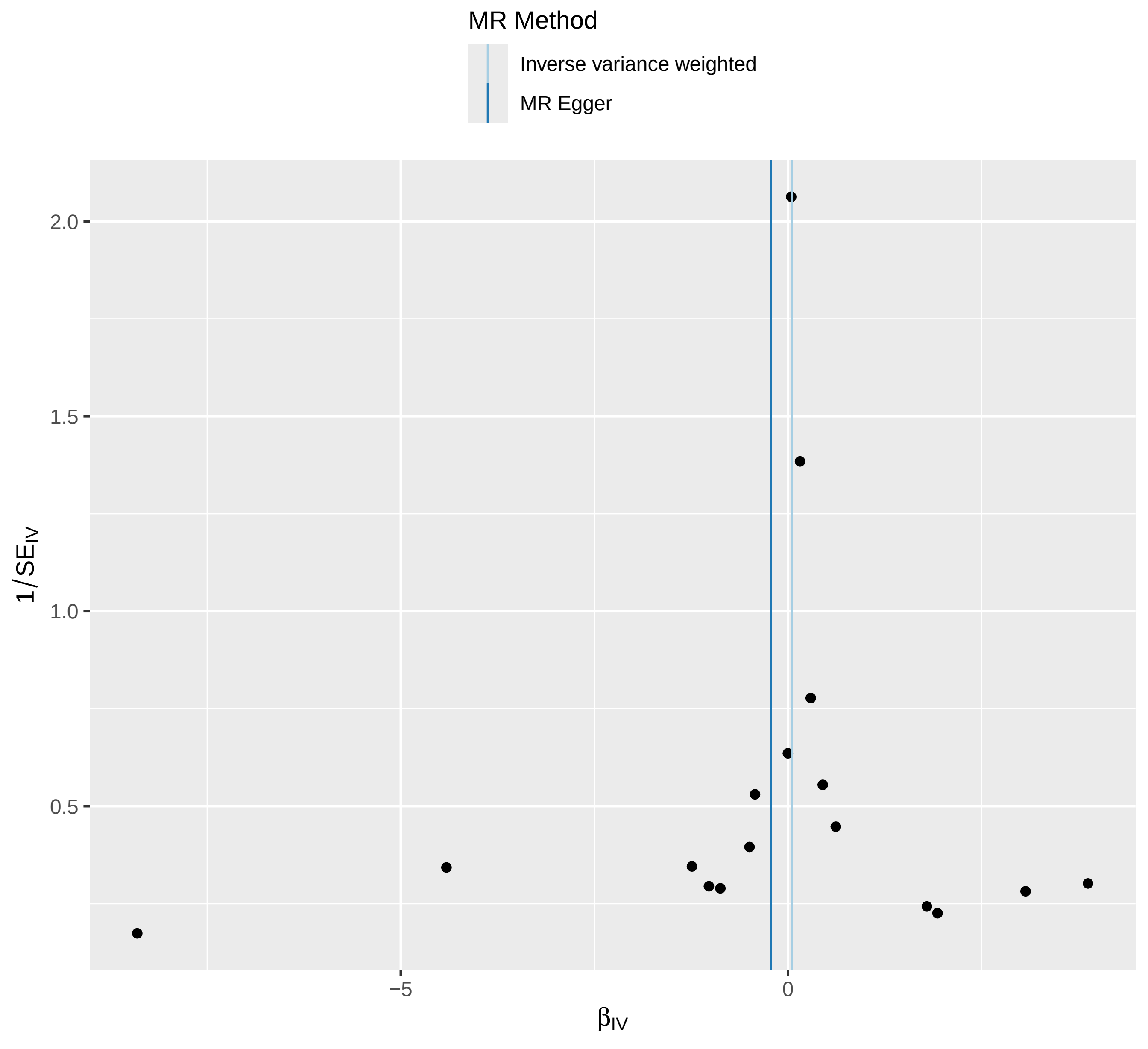
MR Method
Inverse variance weighted
MR Egger
2.0
1.5
1.0
0.5
1/SE_IV
−5
0
β_IV

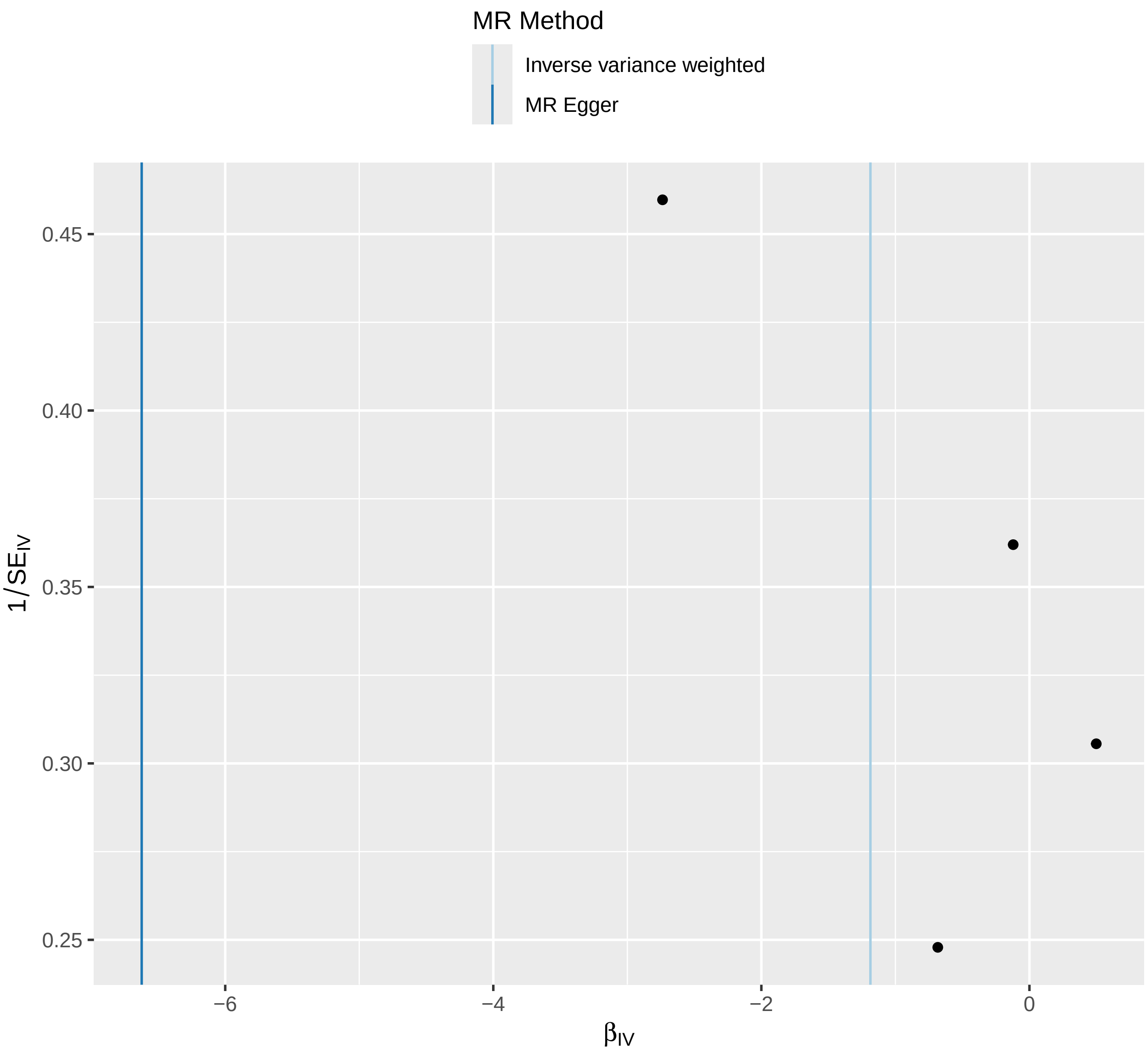
MR Method
Inverse variance weighted
MR Egger
0.45
0.40
0.35
0.30
0.25
1/SE_IV
−6
−4
−2
0
β_IV

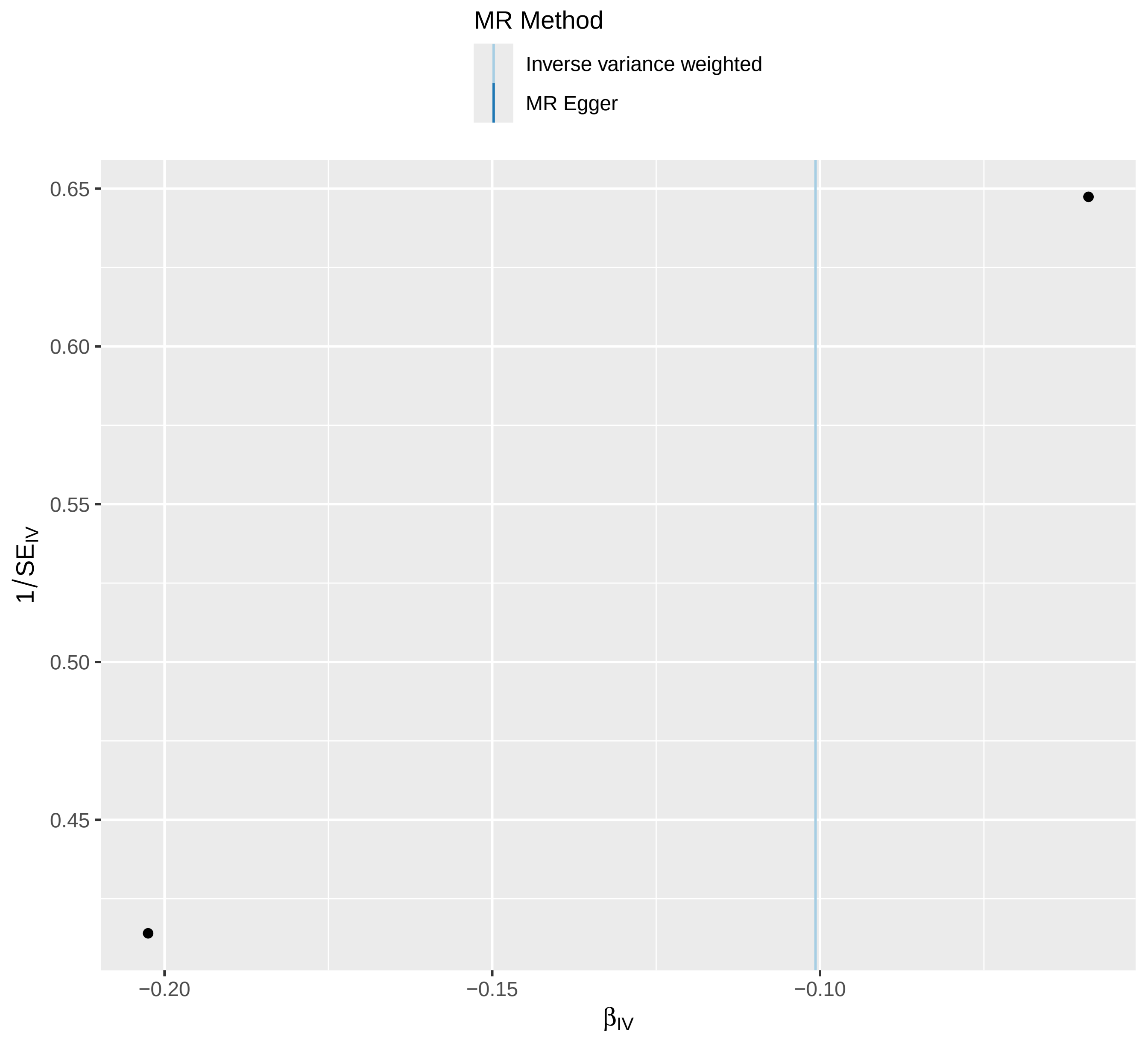
MR Method
Inverse variance weighted
MR Egger
0.65
0.60
0.55
0.50
0.45
1/SE_IV
−0.20
−0.15
−0.10
β_IV

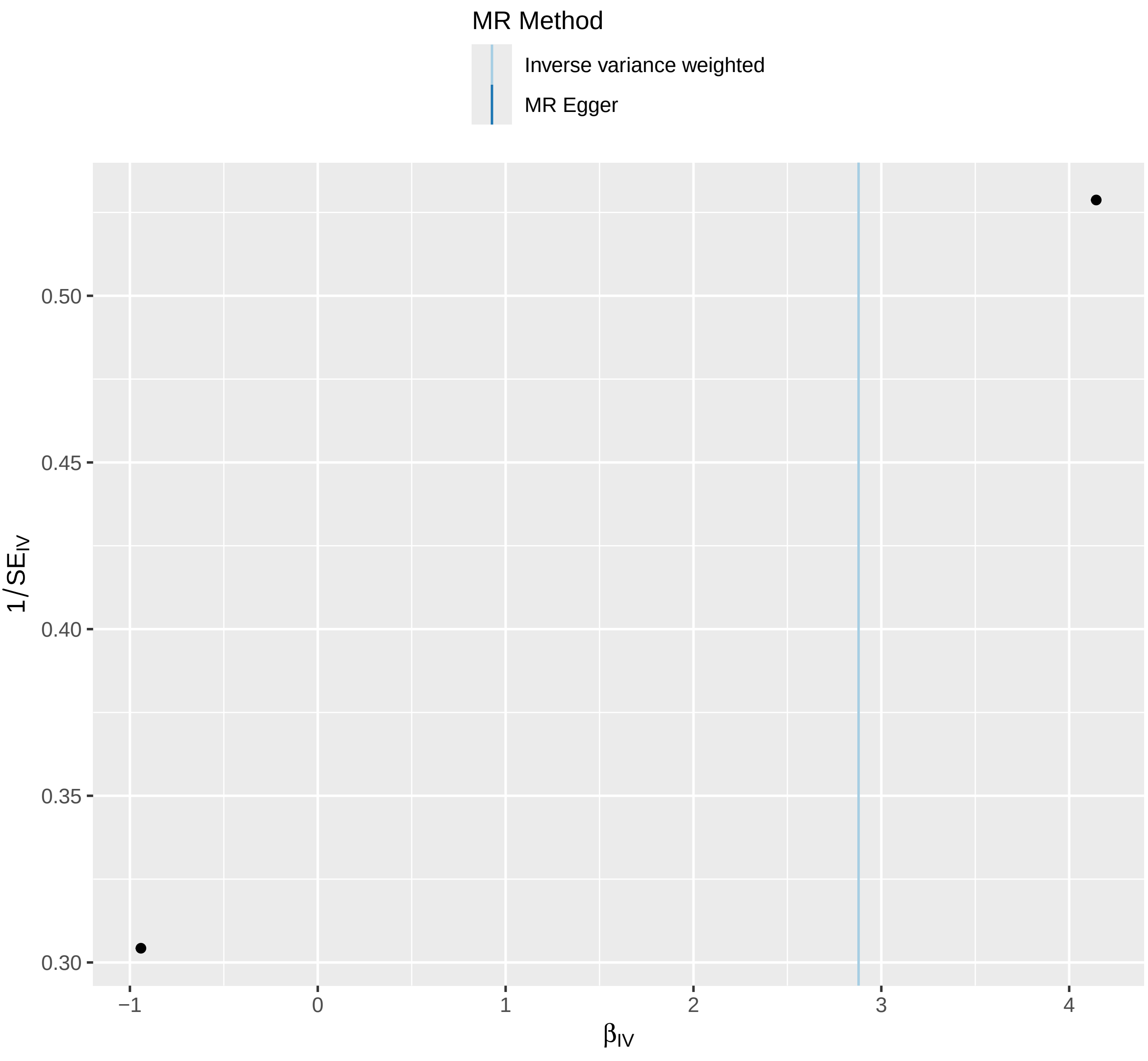
MR Method
Inverse variance weighted
MR Egger
0.50
0.45
0.40
0.35
0.30
1/SE_IV
−1
0
1
2
3
4
β_IV

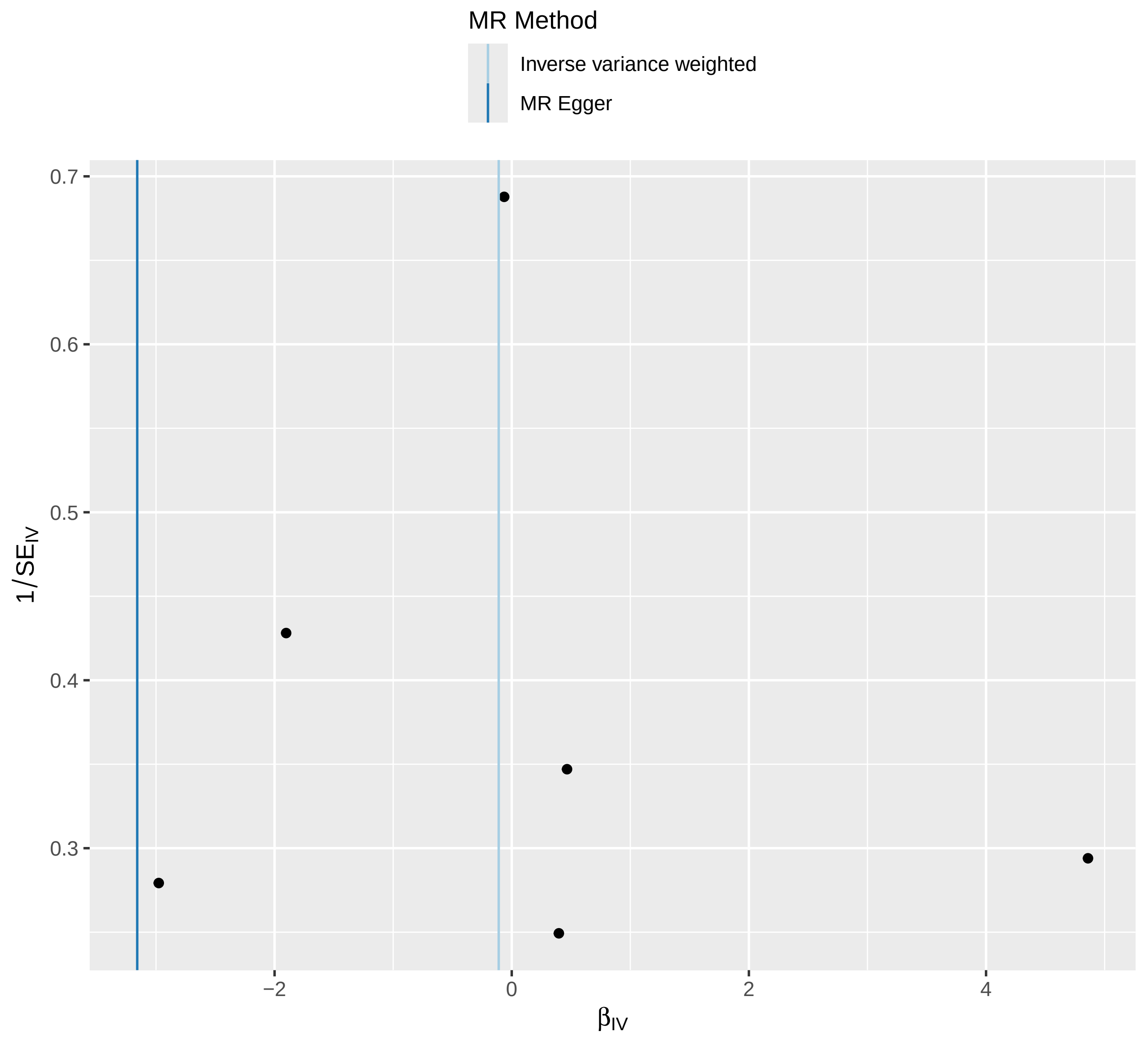

MR Method
Inverse variance weighted
MR Egger
0.7
0.6
0.5
0.4
0.3
1/SE_IV
−2
0
2
4
β_IV

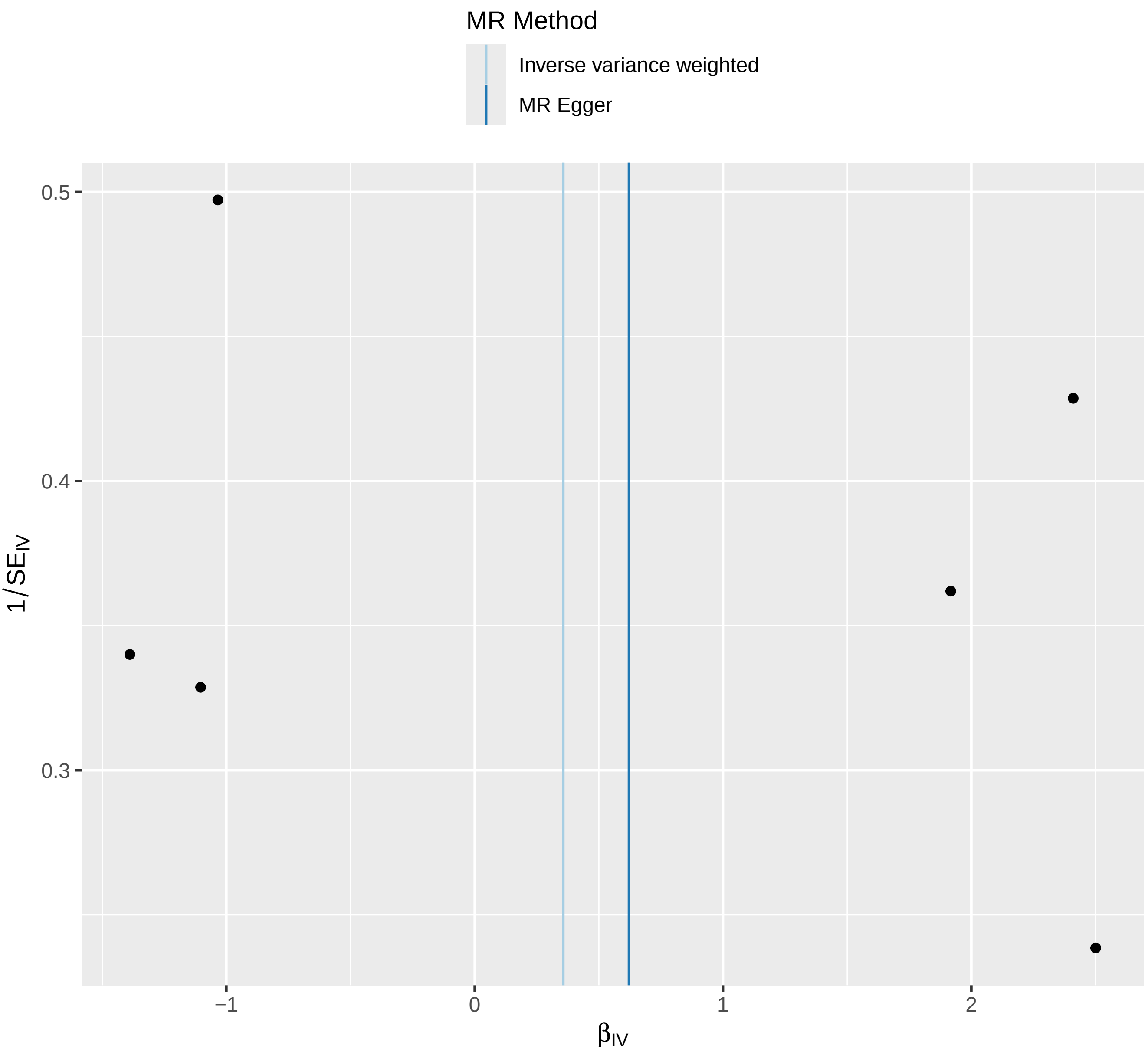

MR Method
Inverse variance weighted
MR Egger
0.5
0.4
0.3
1/SE_IV
−1
0
1
2
β_IV

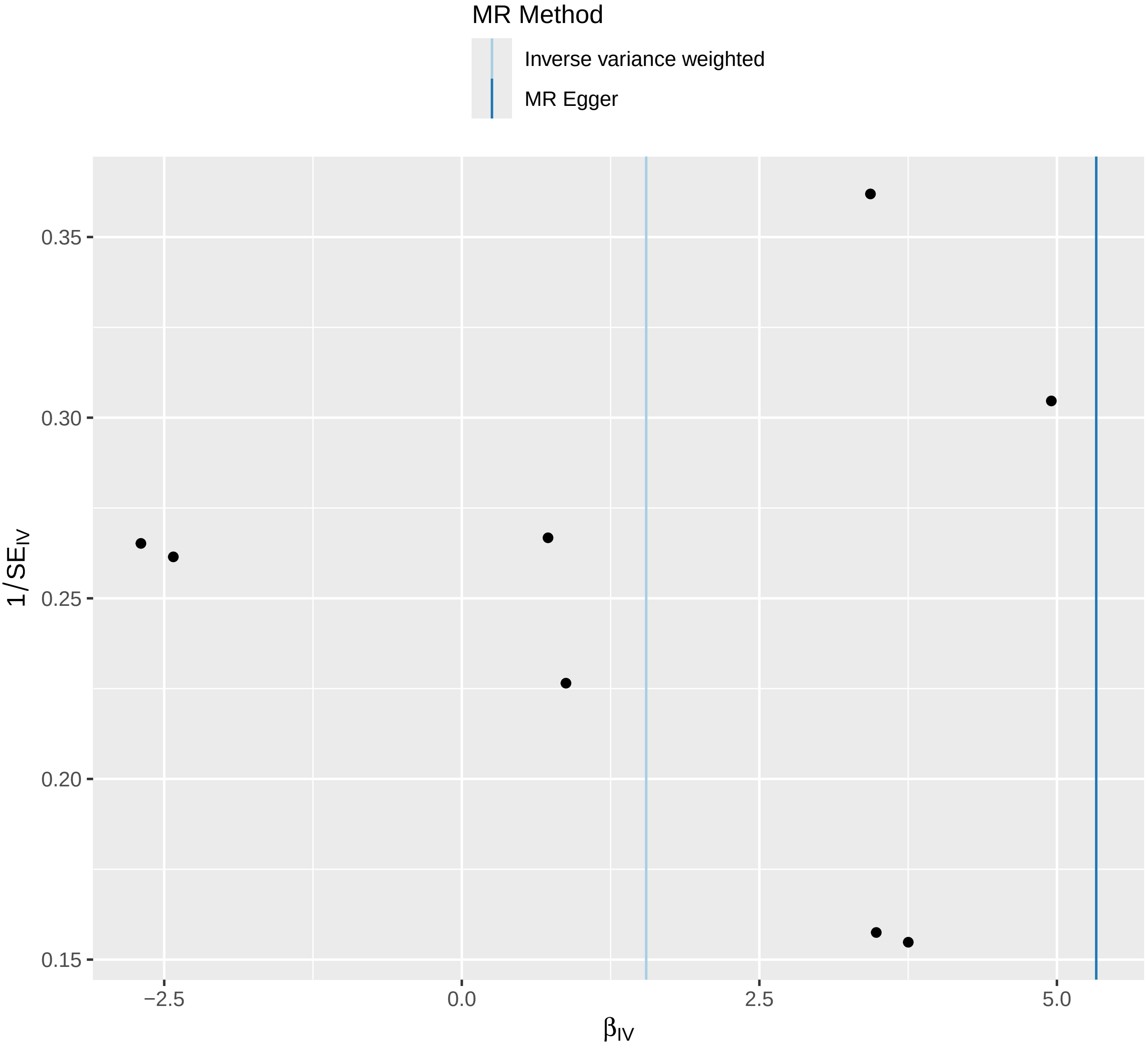
MR Method
Inverse variance weighted
MR Egger
0.35
0.30
0.25
0.20
0.15
1/SE_IV
−2.5
0.0
2.5
5.0
β_IV

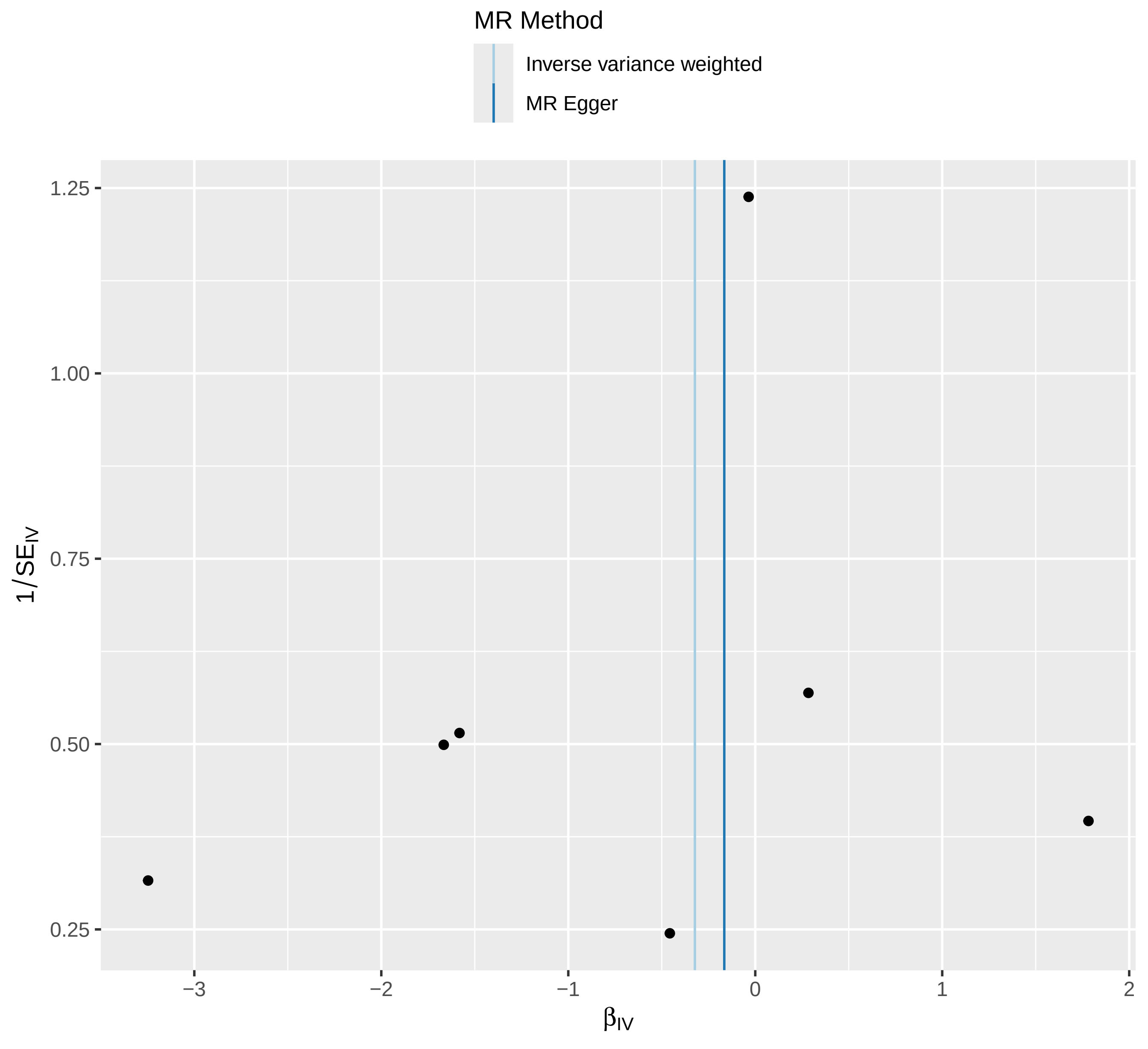

MR Method
Inverse variance weighted
MR Egger
1.25
1.00
0.75
0.50
0.25
1/SE_IV
−3
−2
−1
0
1
2
β_IV

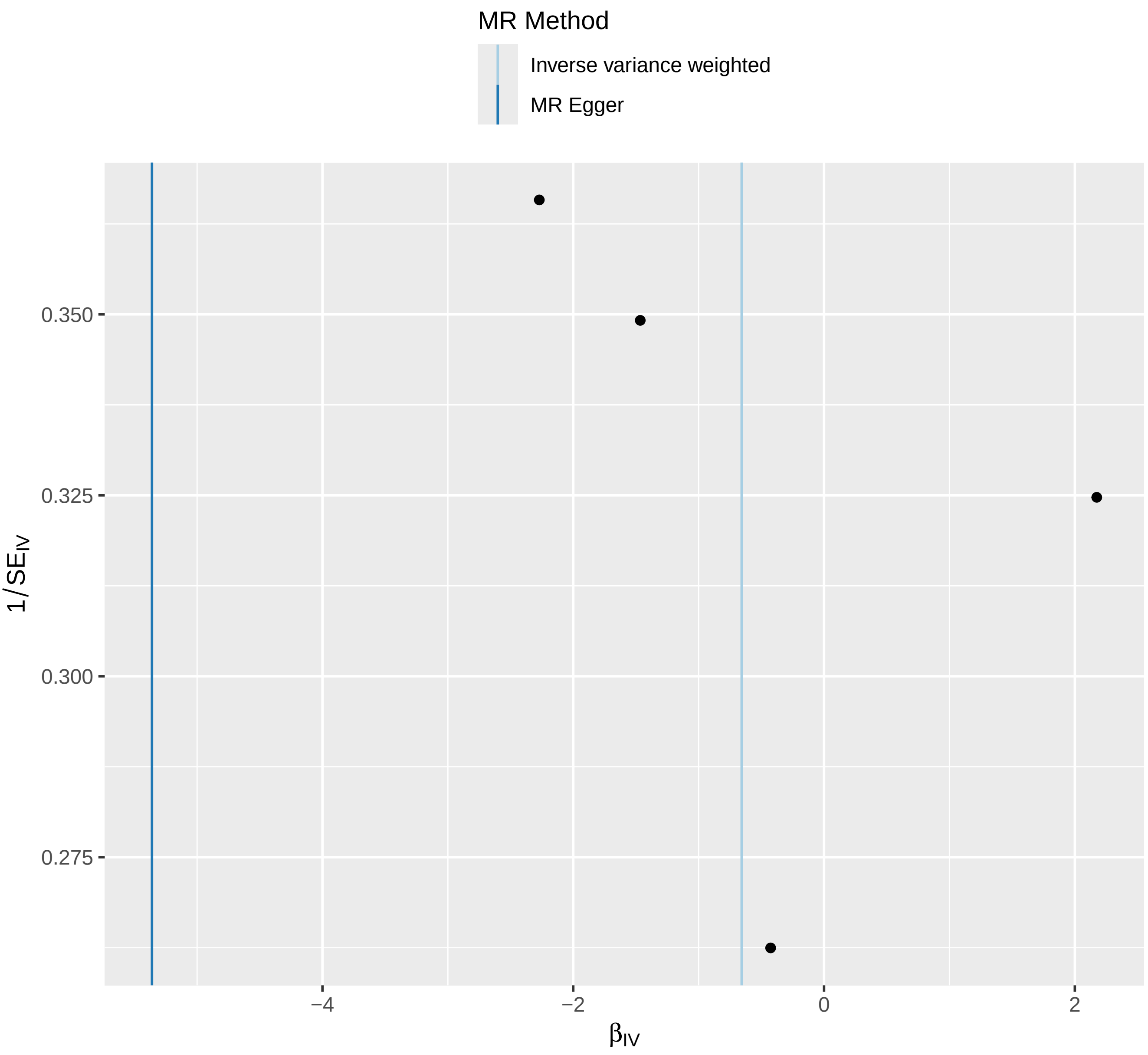
MR Method
Inverse variance weighted
MR Egger
0.350
0.325
0.300
0.275
1/SE_IV
−4
−2
0
2
β_IV

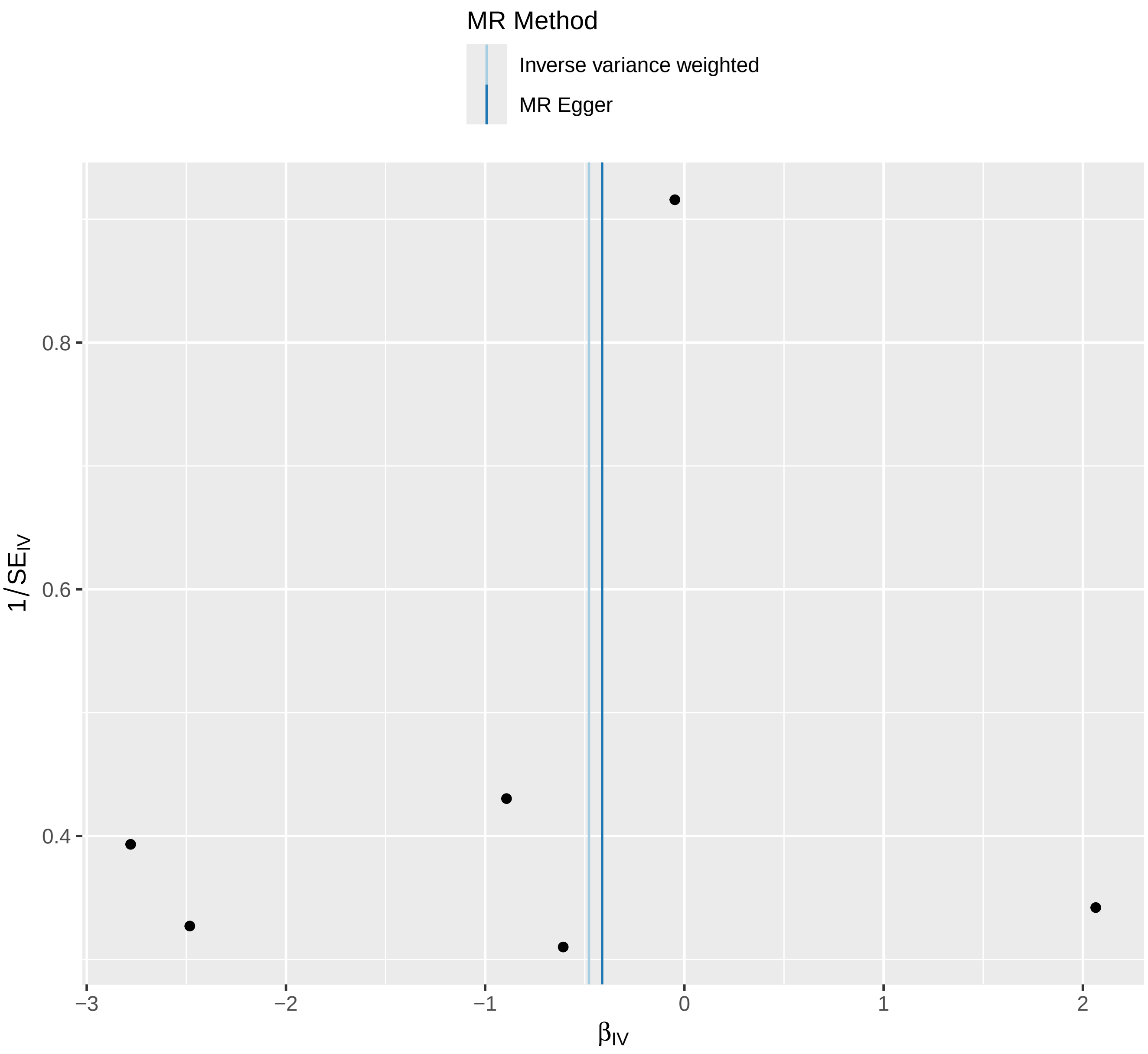
MR Method
Inverse variance weighted
MR Egger
0.8
0.6
0.4
1/SE$_{IV}$
−3
−2
−1
0
1
2
β$_{IV}$

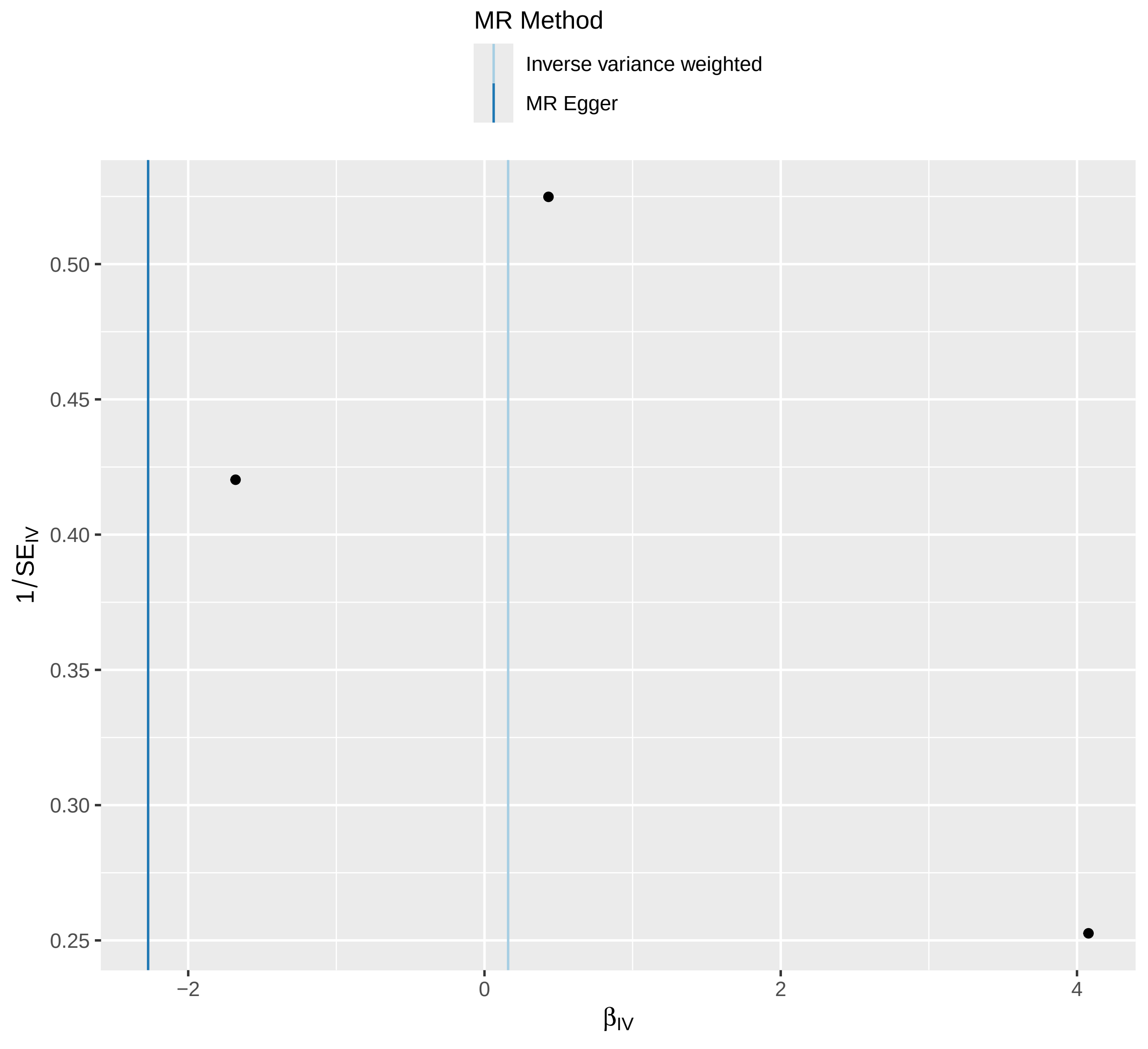
MR Method
Inverse variance weighted
MR Egger
0.50
0.45
0.40
0.35
0.30
0.25
1/SE_IV
−2
0
2
4
β_IV

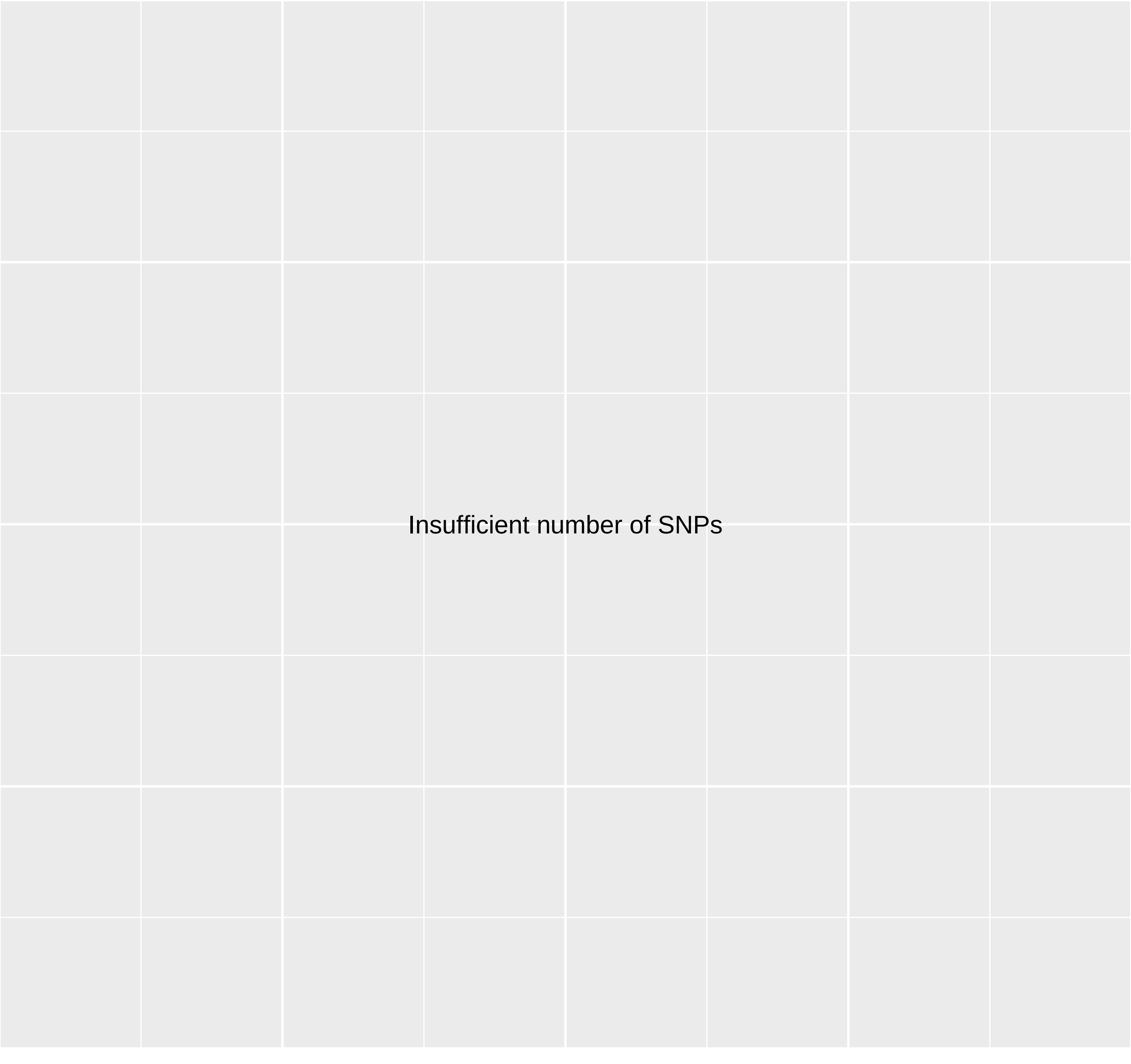
Insufficient number of SNPs

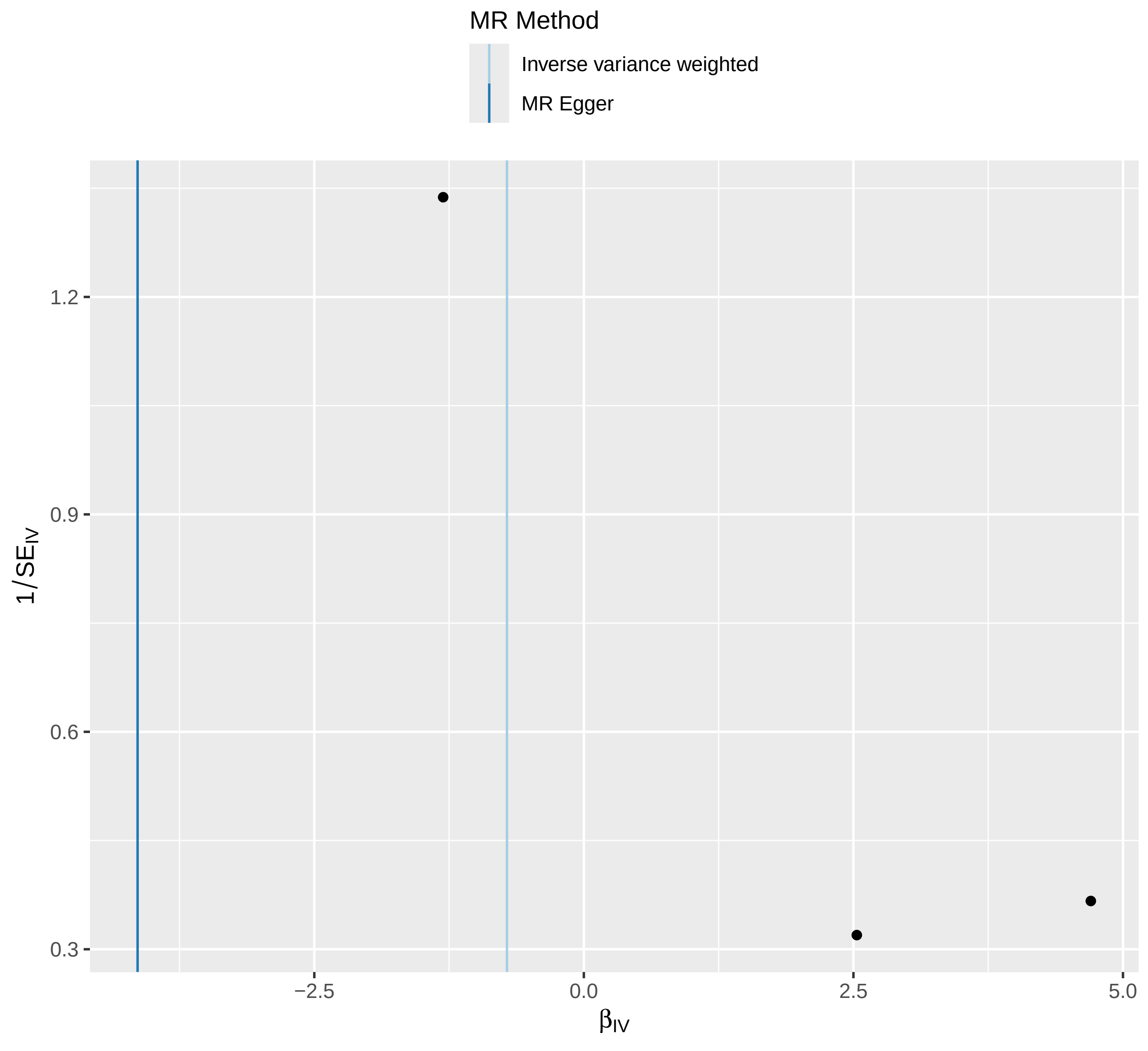

MR Method
Inverse variance weighted
MR Egger
1.2
0.9
0.6
0.3
1/SE$_{IV}$
−2.5
0.0
2.5
5.0
β$_{IV}$

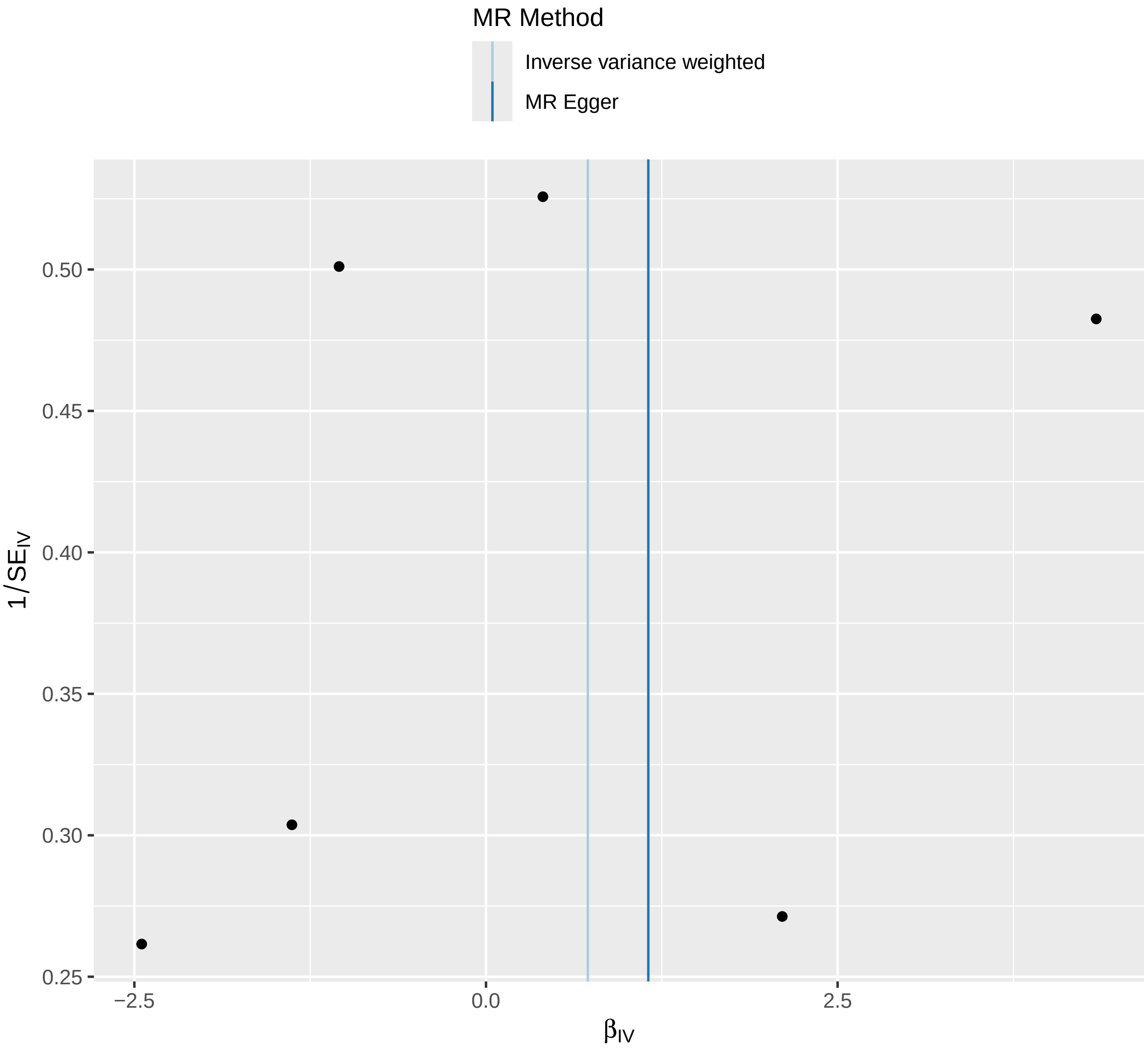
MR Method
Inverse variance weighted
MR Egger
0.50
0.45
0.40
0.35
0.30
0.25
1/SE$_{IV}$
−2.5
0.0
2.5
β$_{IV}$

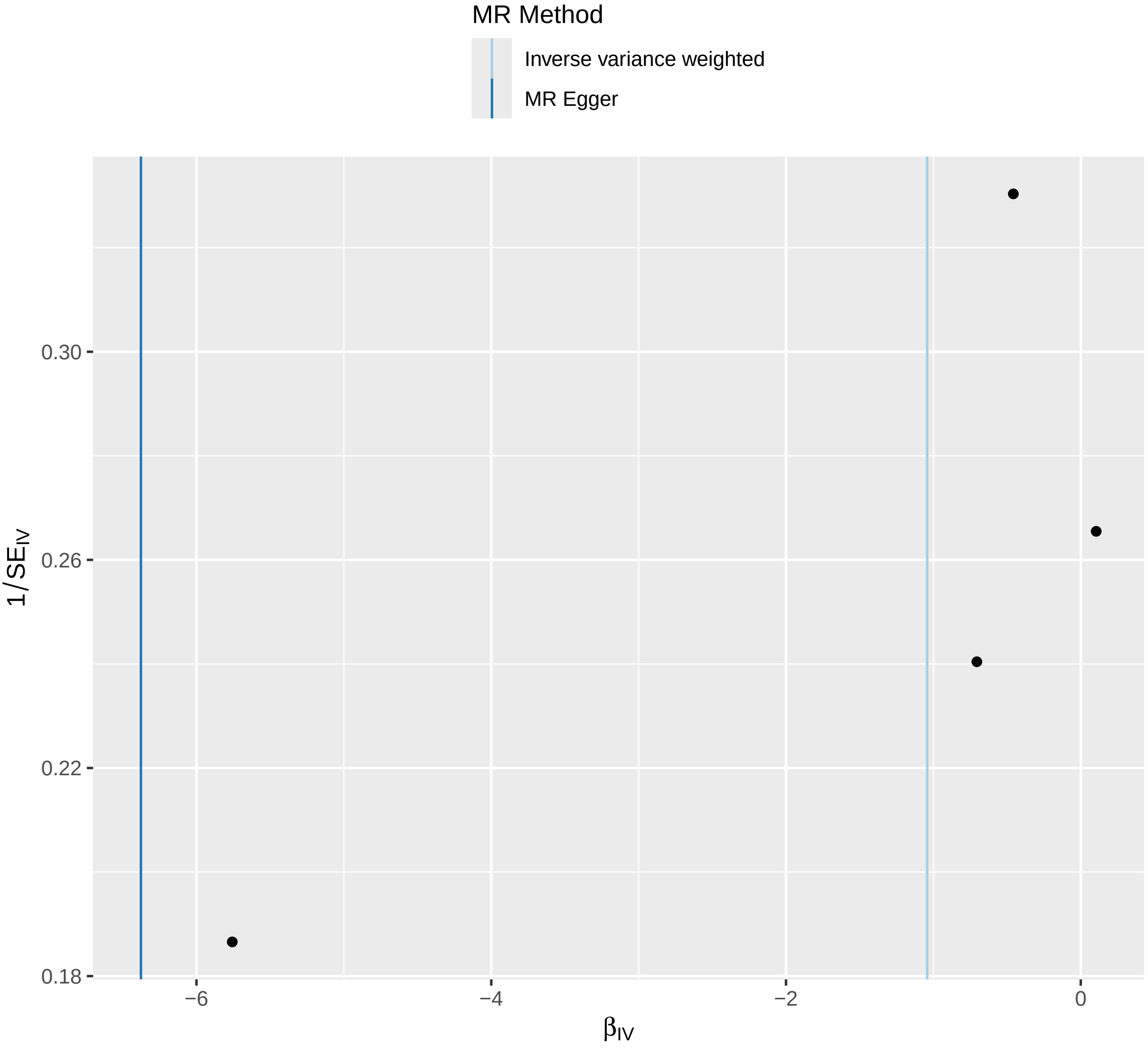
MR Method
Inverse variance weighted
MR Egger
0.30
0.26
0.22
0.18
$1/SE_{IV}$
−6
−4
−2
0
β_{IV}

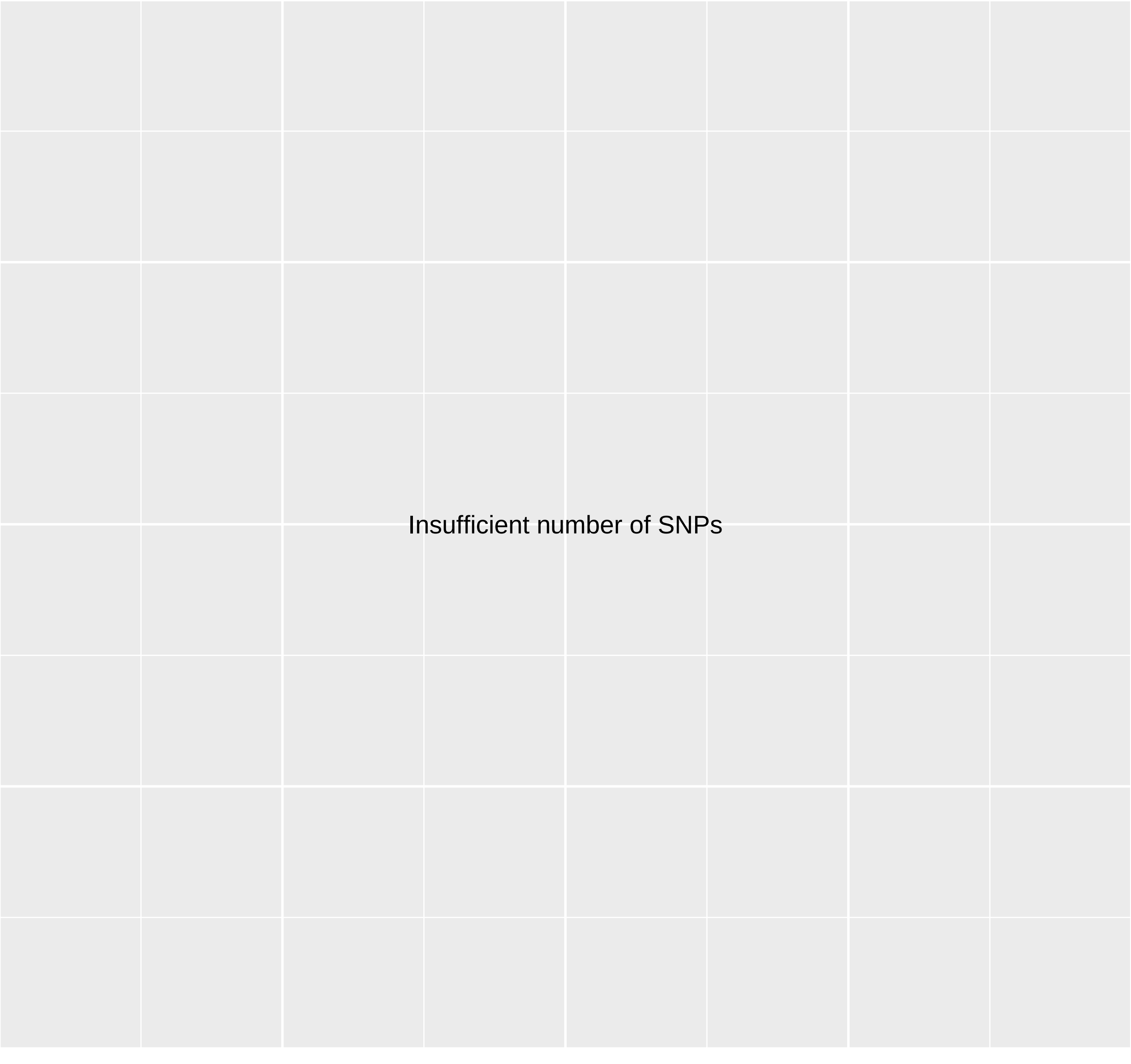
Insufficient number of SNPs

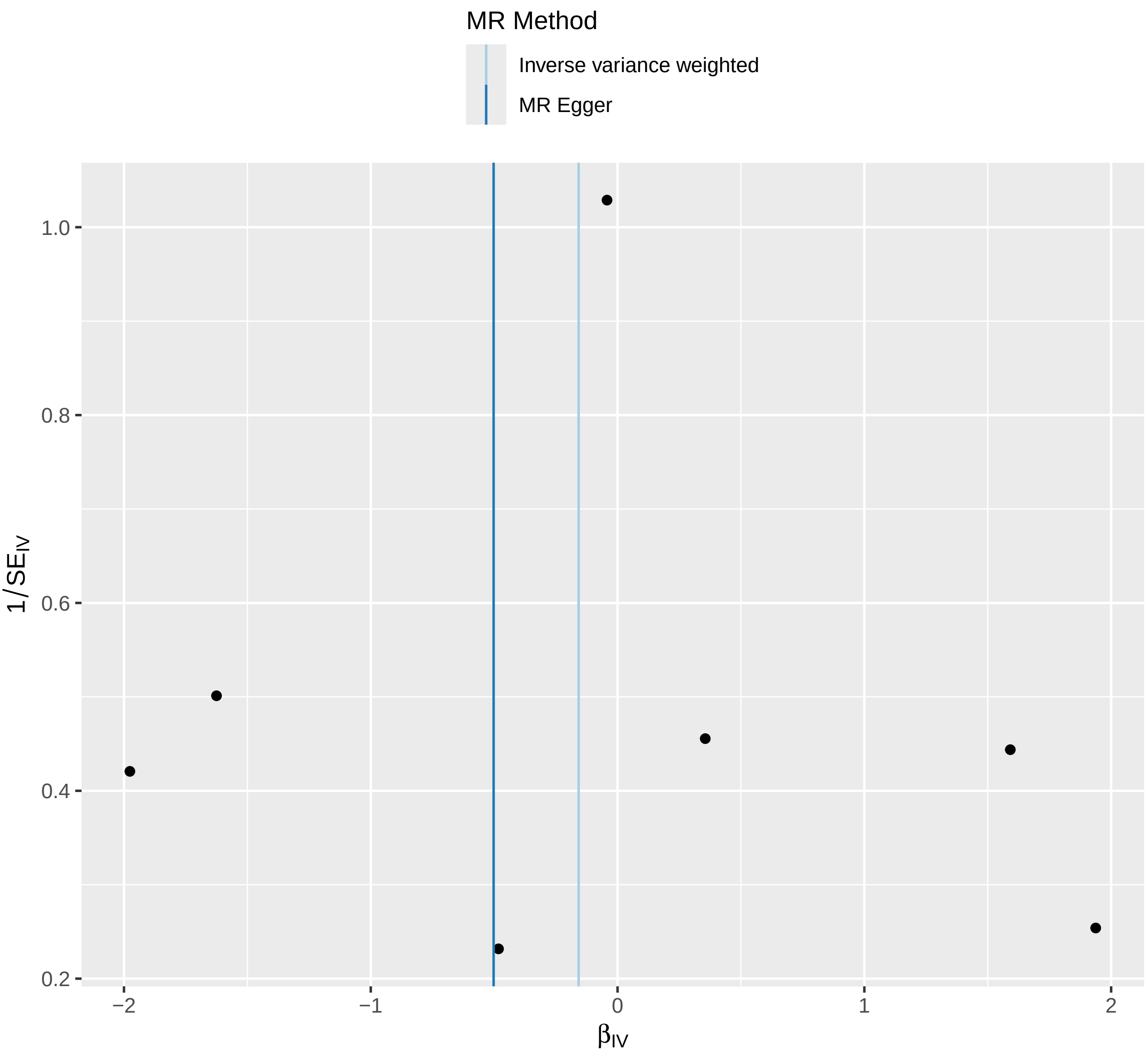
MR Method
Inverse variance weighted
MR Egger
1/SE_IV
1.0
0.8
0.6
0.4
0.2
−2
−1
0
1
2
β_IV

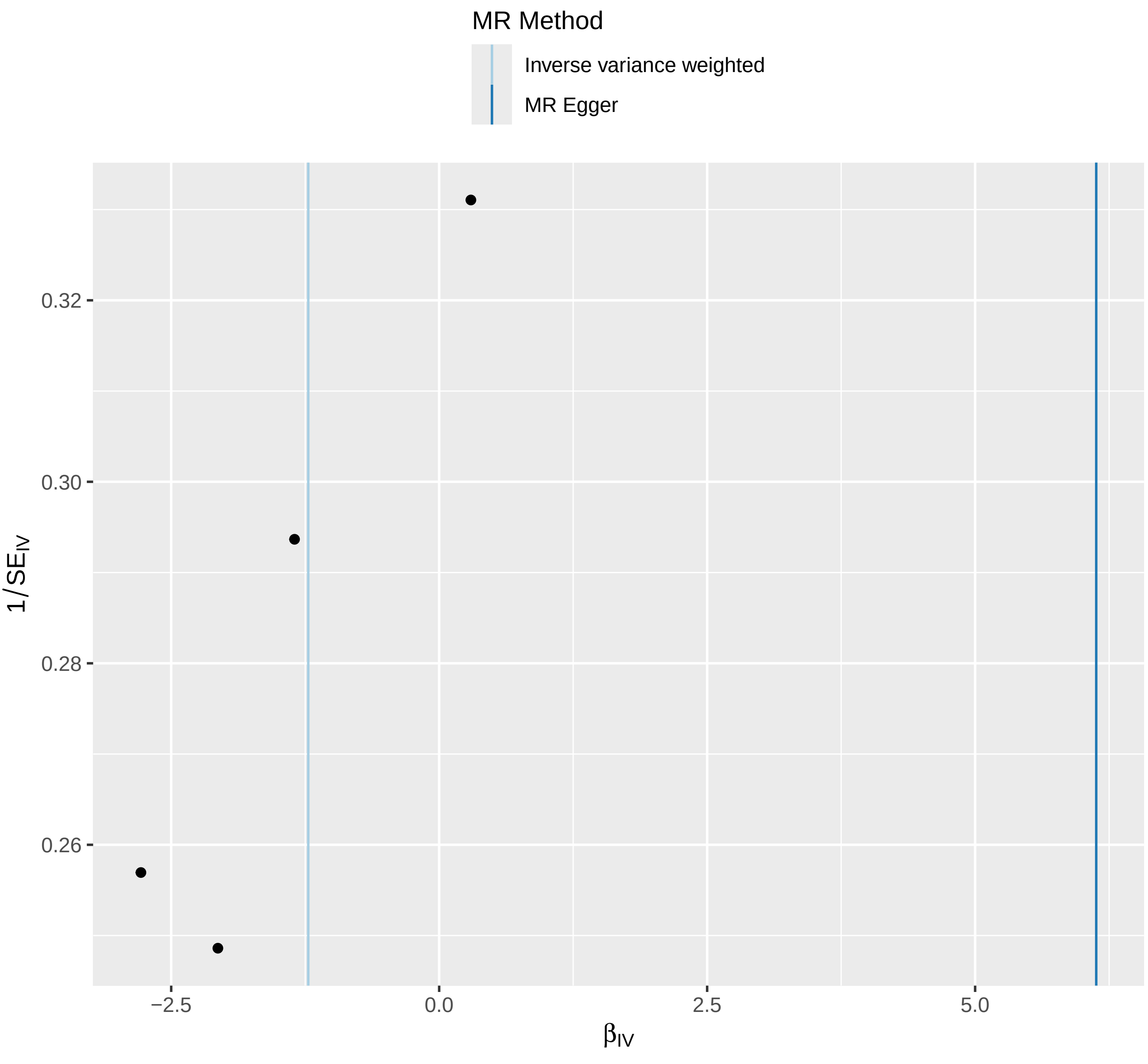

MR Method
Inverse variance weighted
MR Egger
0.32
0.30
0.28
0.26
1/SE_IV
−2.5
0.0
2.5
5.0
β_IV

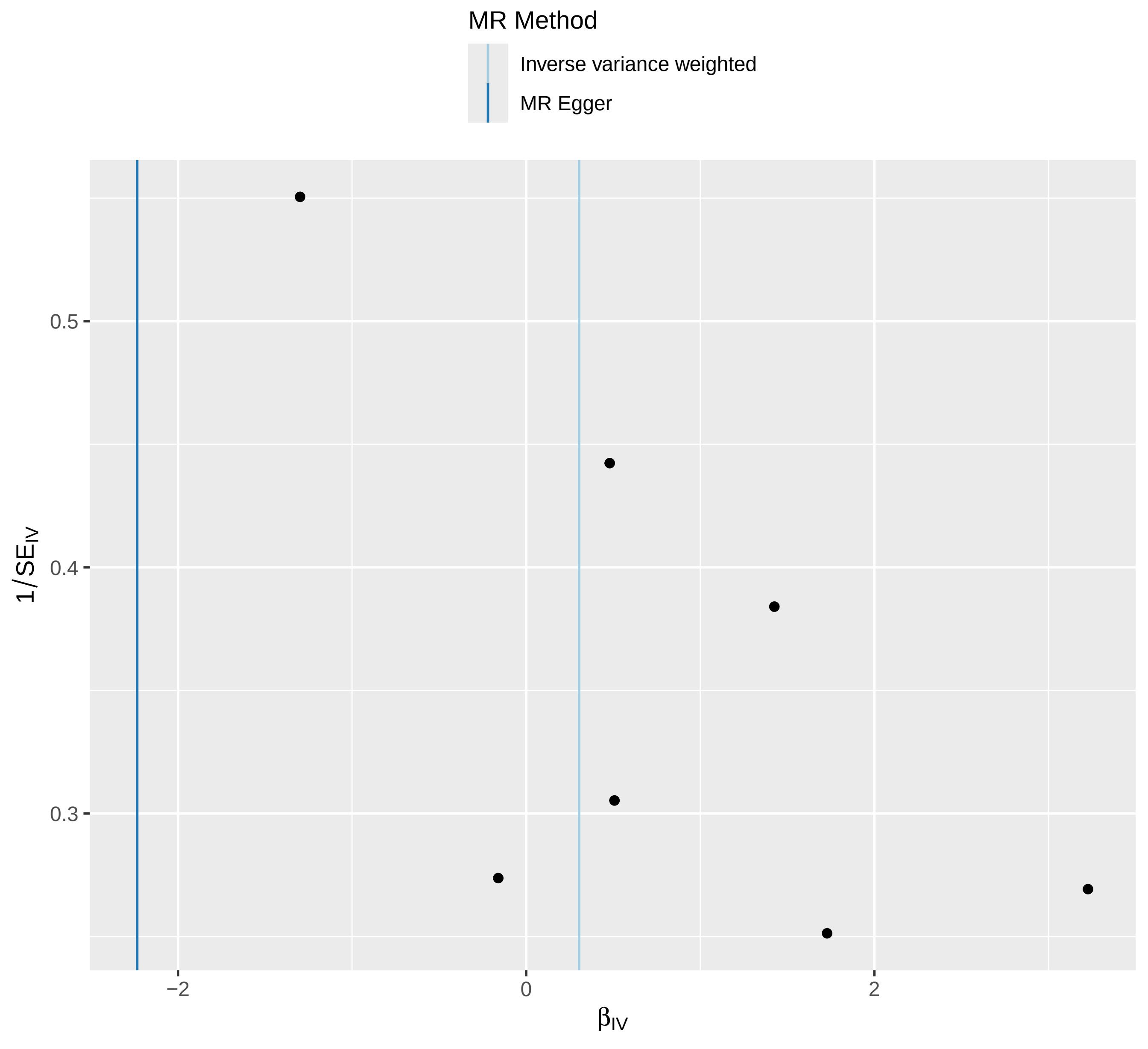
MR Method
Inverse variance weighted
MR Egger
1/SE_IV
0.5
0.4
0.3
−2
0
2
β_IV

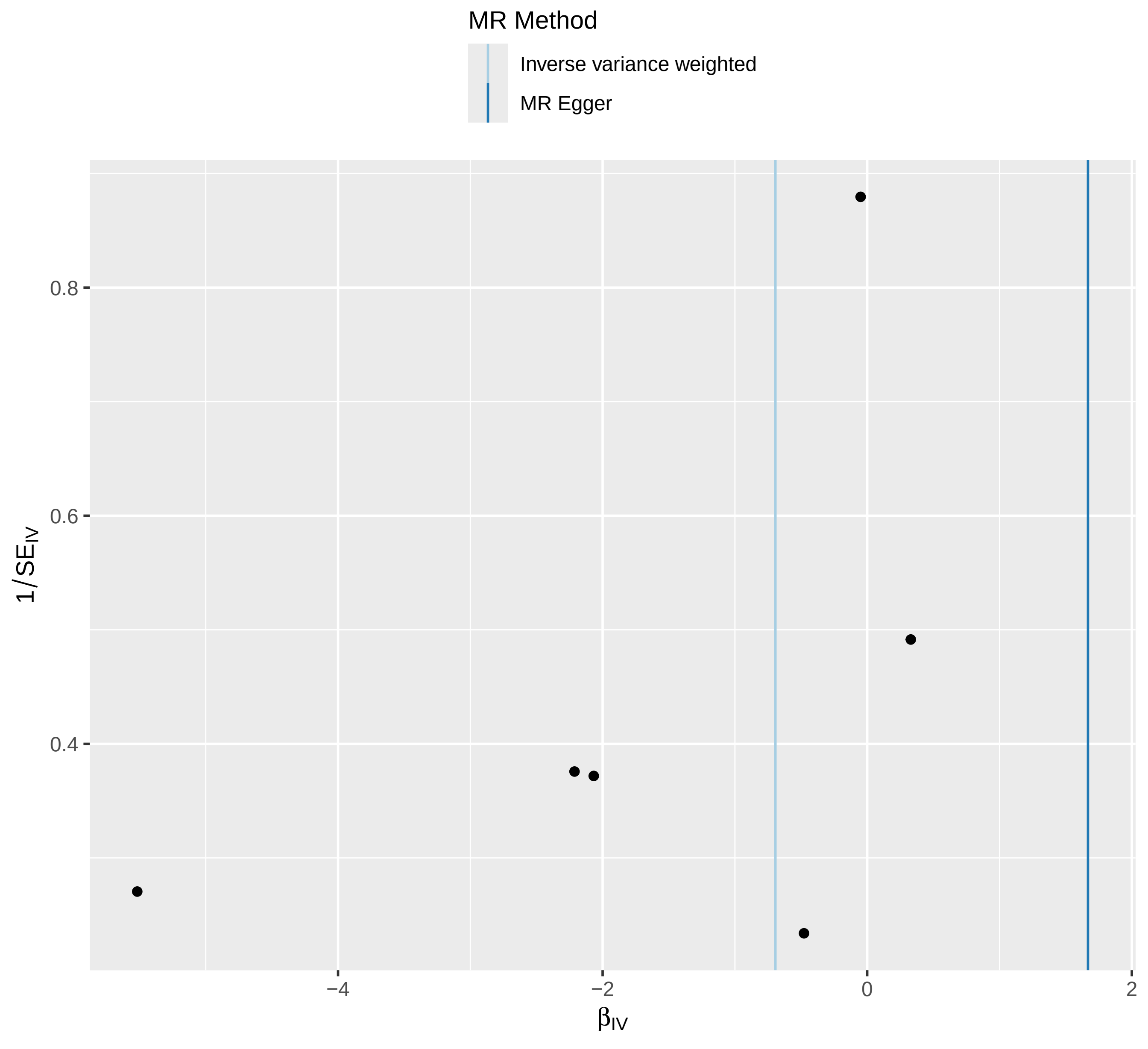
MR Method
Inverse variance weighted
MR Egger
0.8
0.6
0.4
1/SE$_{IV}$
−4
−2
0
2
β$_{IV}$

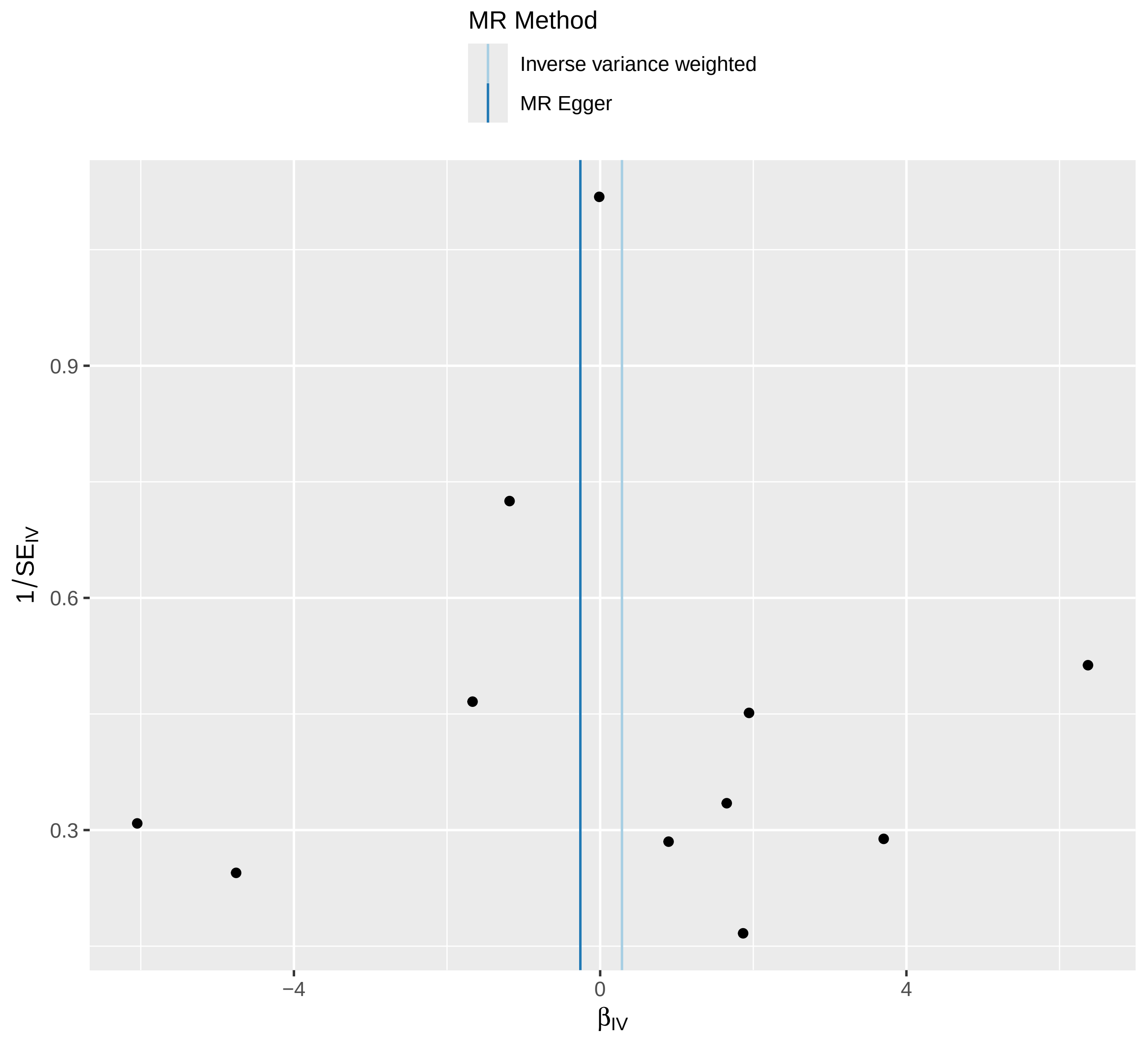
MR Method
Inverse variance weighted
MR Egger
0.9
0.6
0.3
1/SE$_{IV}$
−4
0
4
β$_{IV}$

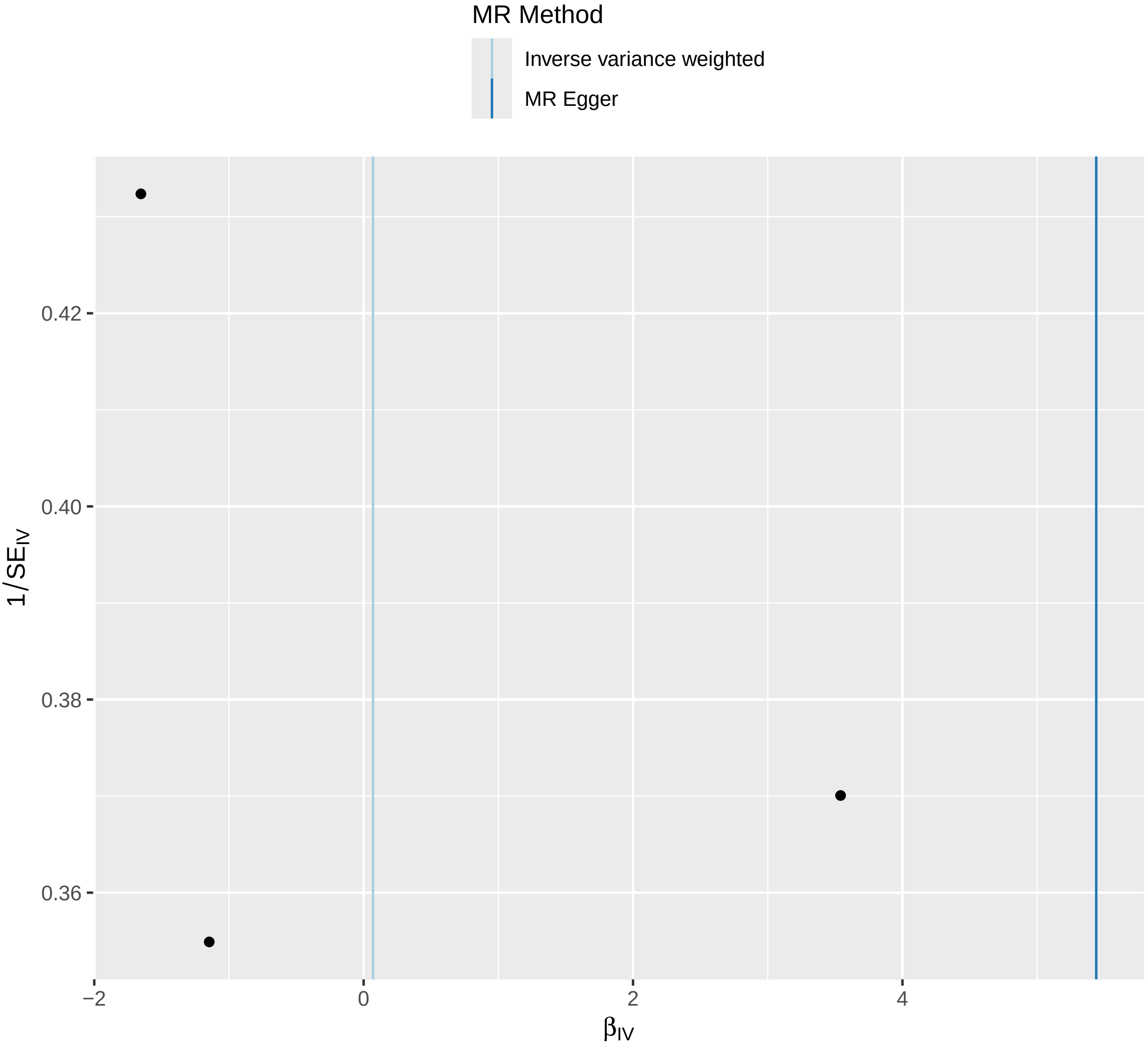
MR Method
Inverse variance weighted
MR Egger
0.42
0.40
0.38
0.36
1/SE_IV
−2
0
2
4
β_IV

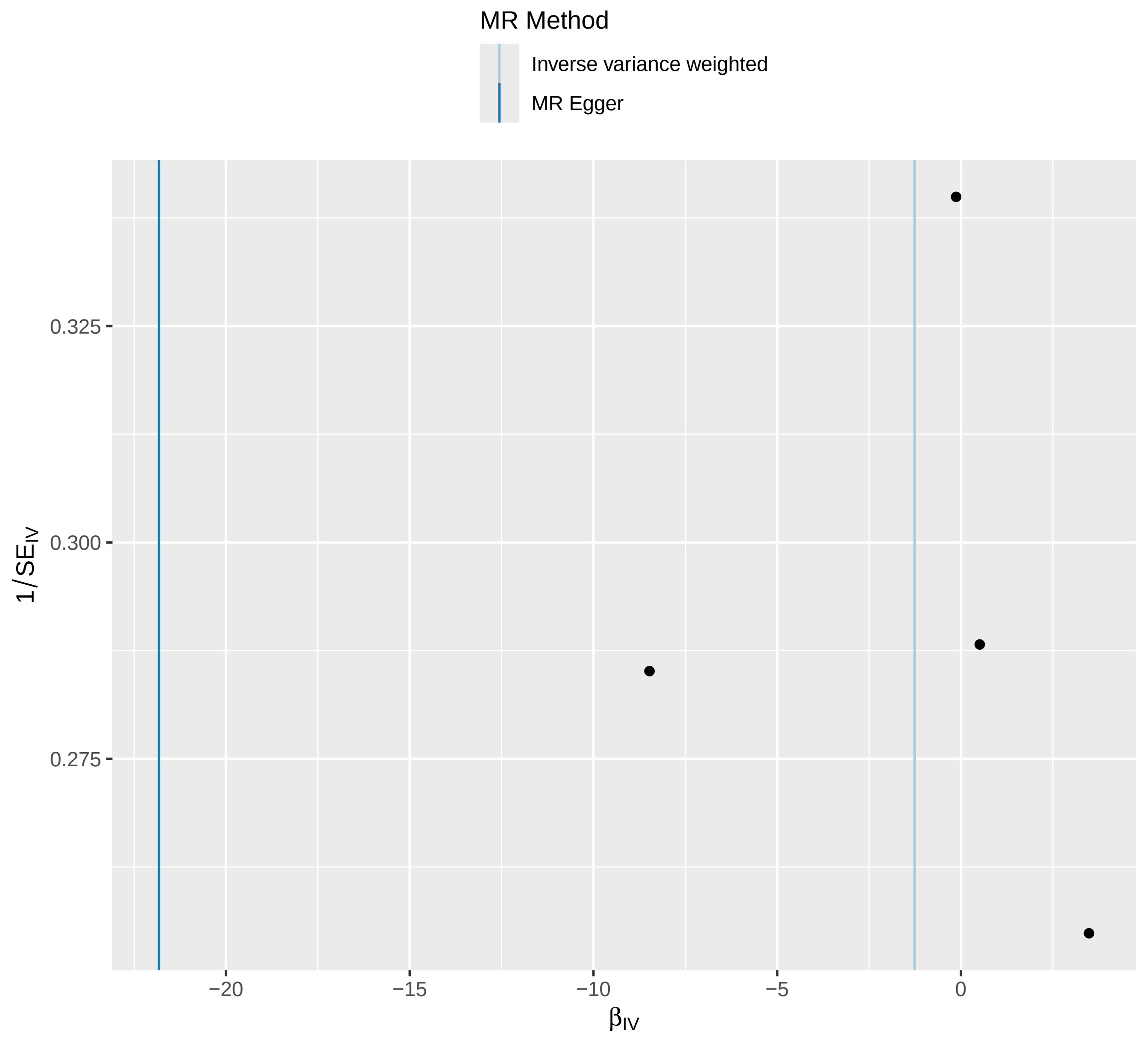
MR Method
Inverse variance weighted
MR Egger
0.325
0.300
0.275
1/SE_IV
−20
−15
−10
−5
0
β_IV

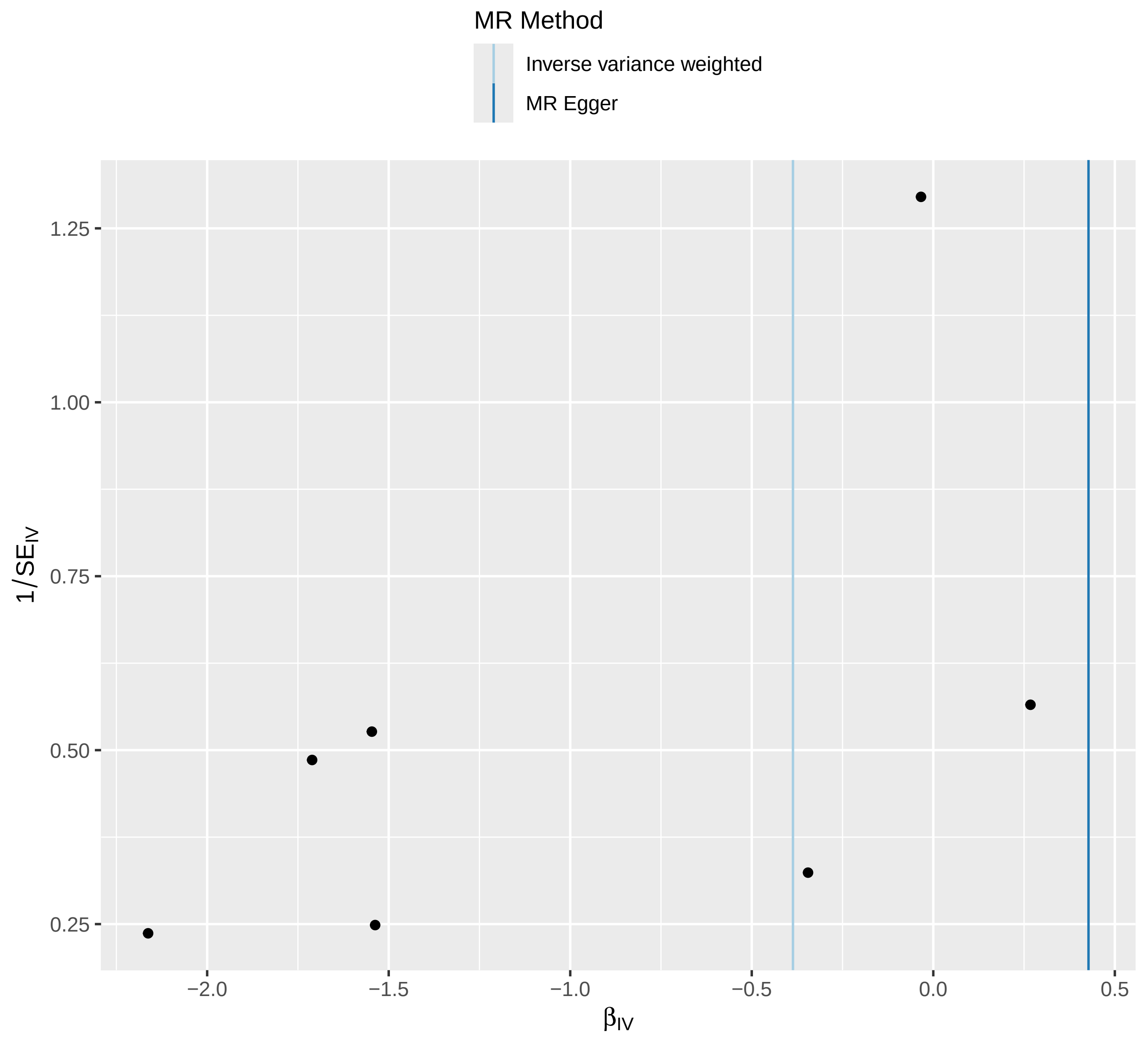

MR Method
Inverse variance weighted
MR Egger
1.25
1.00
0.75
0.50
0.25
1/SE_{IV}
−2.0
−1.5
−1.0
−0.5
0.0
0.5
β_{IV}

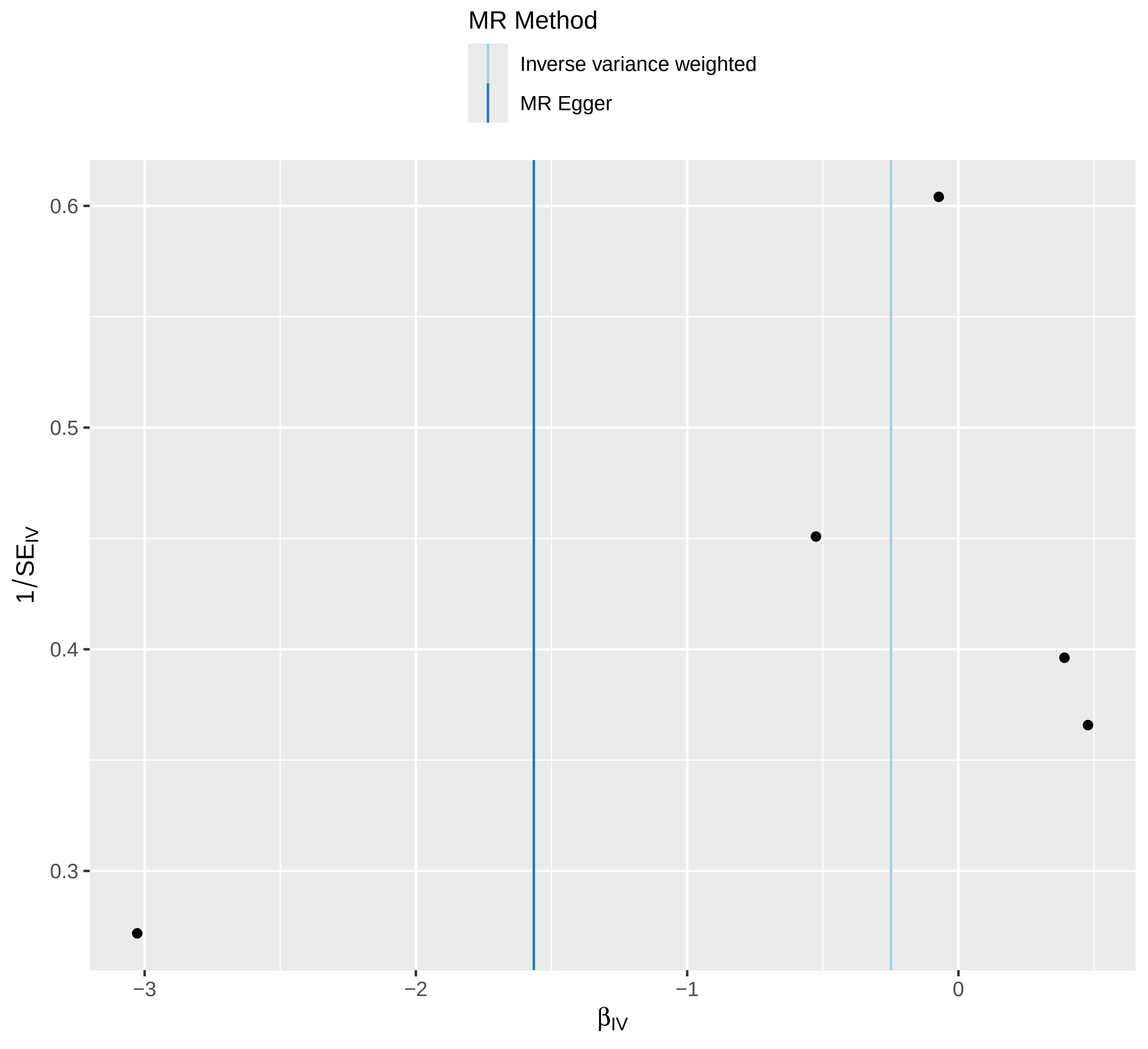
MR Method
Inverse variance weighted
MR Egger
0.6
0.5
0.4
0.3
1/SE$_{IV}$
−3
−2
−1
0
β$_{IV}$

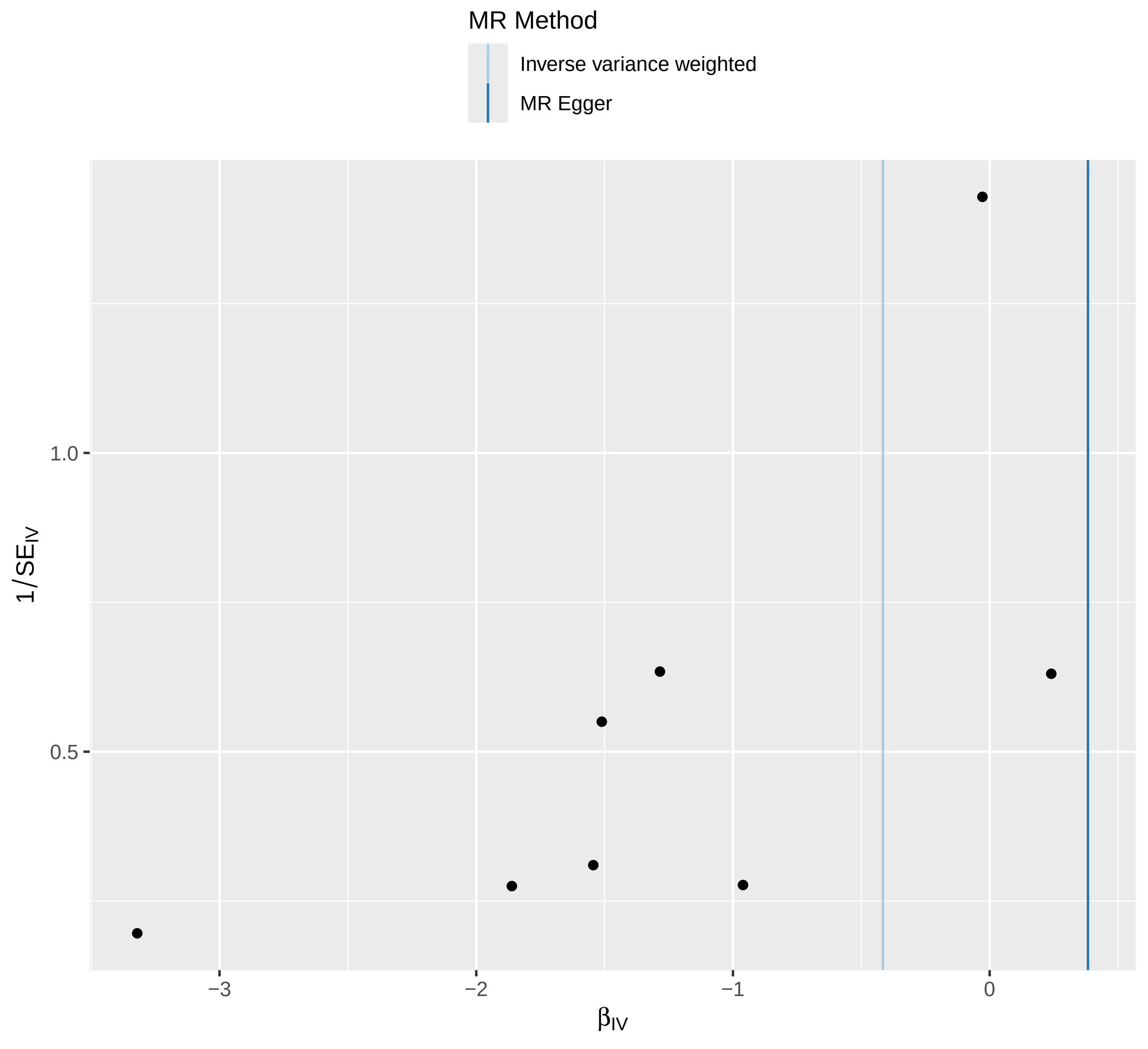
MR Method
Inverse variance weighted
MR Egger
1.0
0.5
1/SE$_{IV}$
−3
−2
−1
0
β$_{IV}$

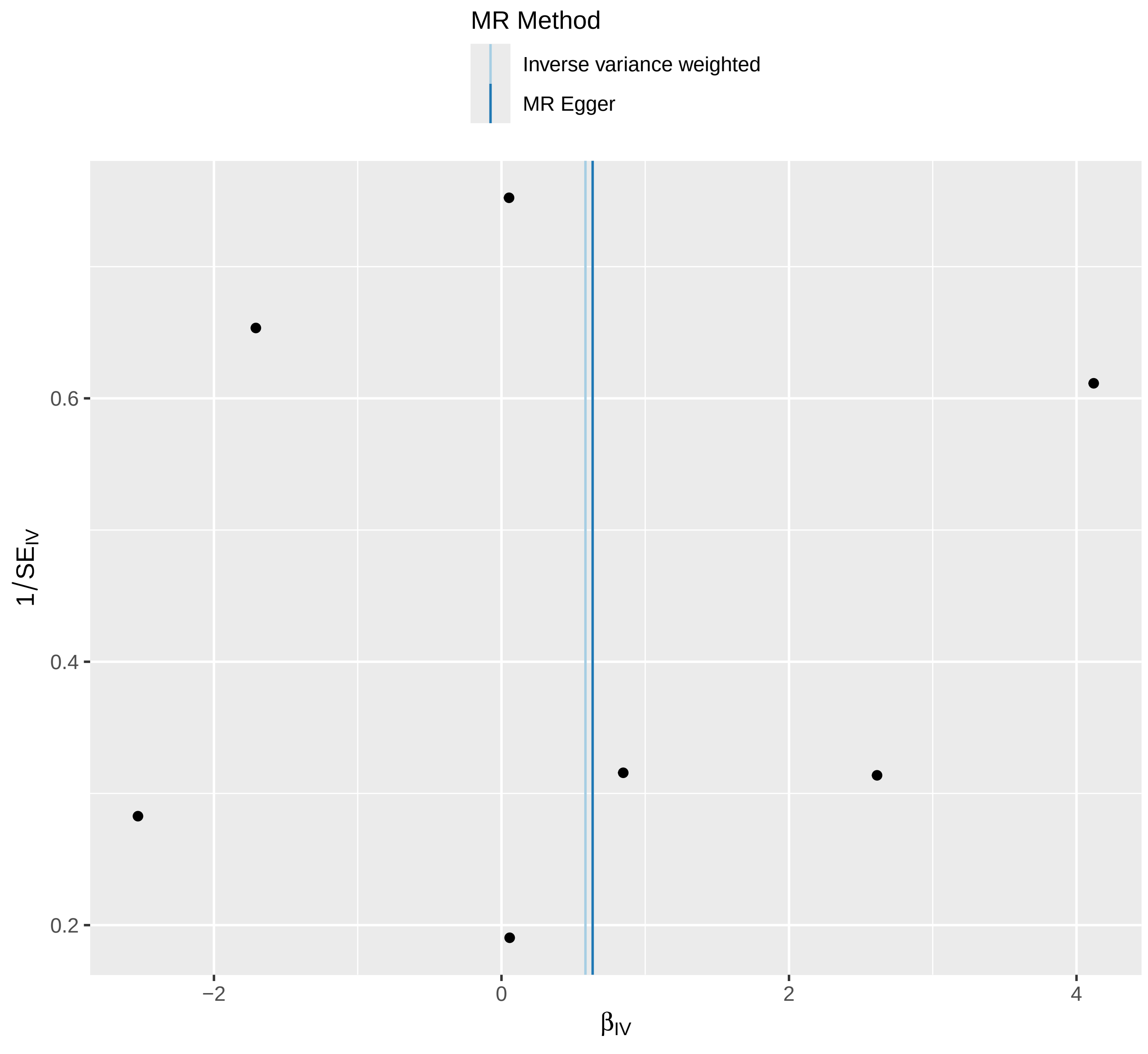
MR Method
Inverse variance weighted
MR Egger
1/SE_IV
0.6
0.4
0.2
−2
0
2
4
β_IV

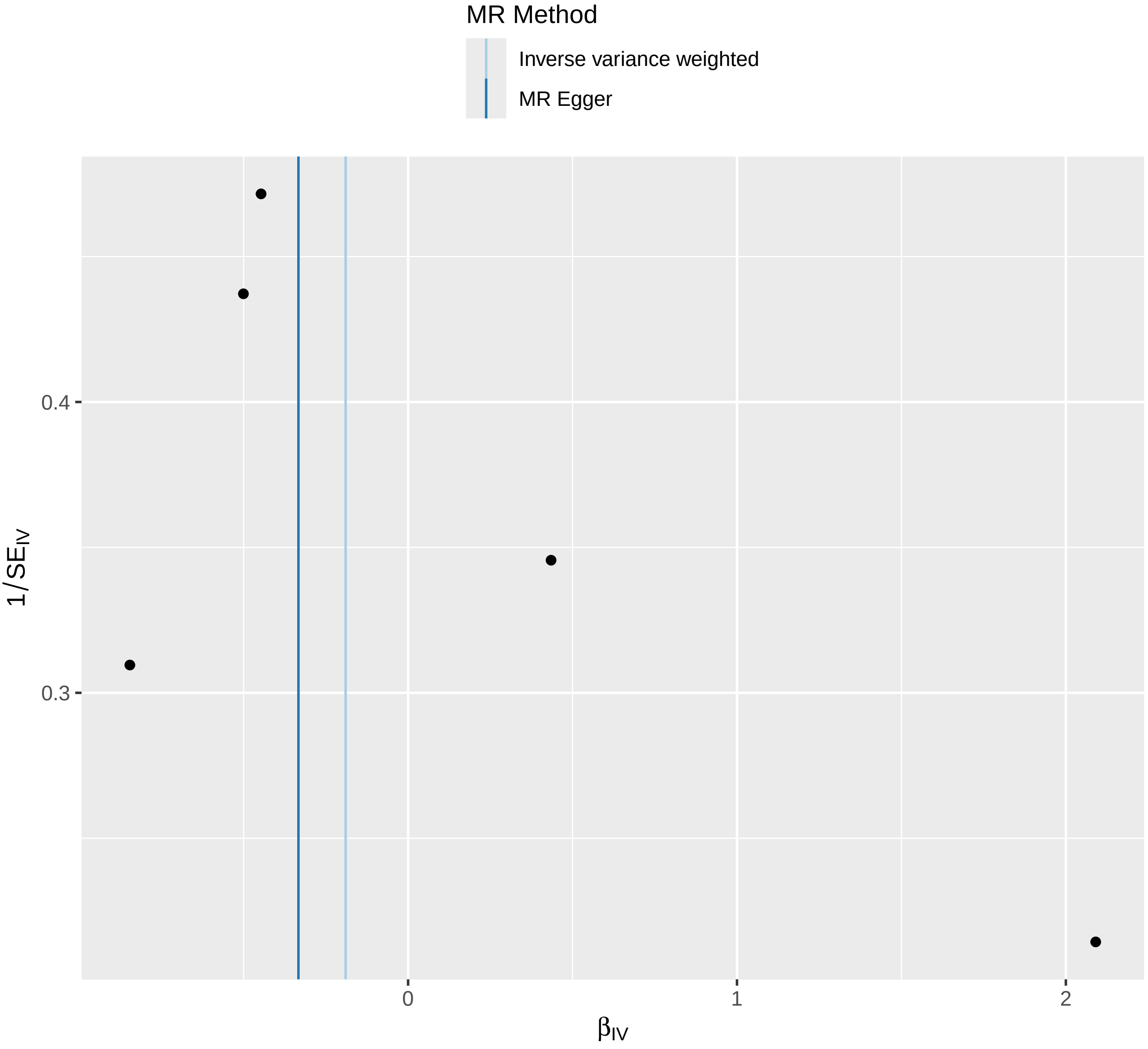
MR Method
Inverse variance weighted
MR Egger
0.4
0.3
1/SE_IV
0
1
2
β_IV

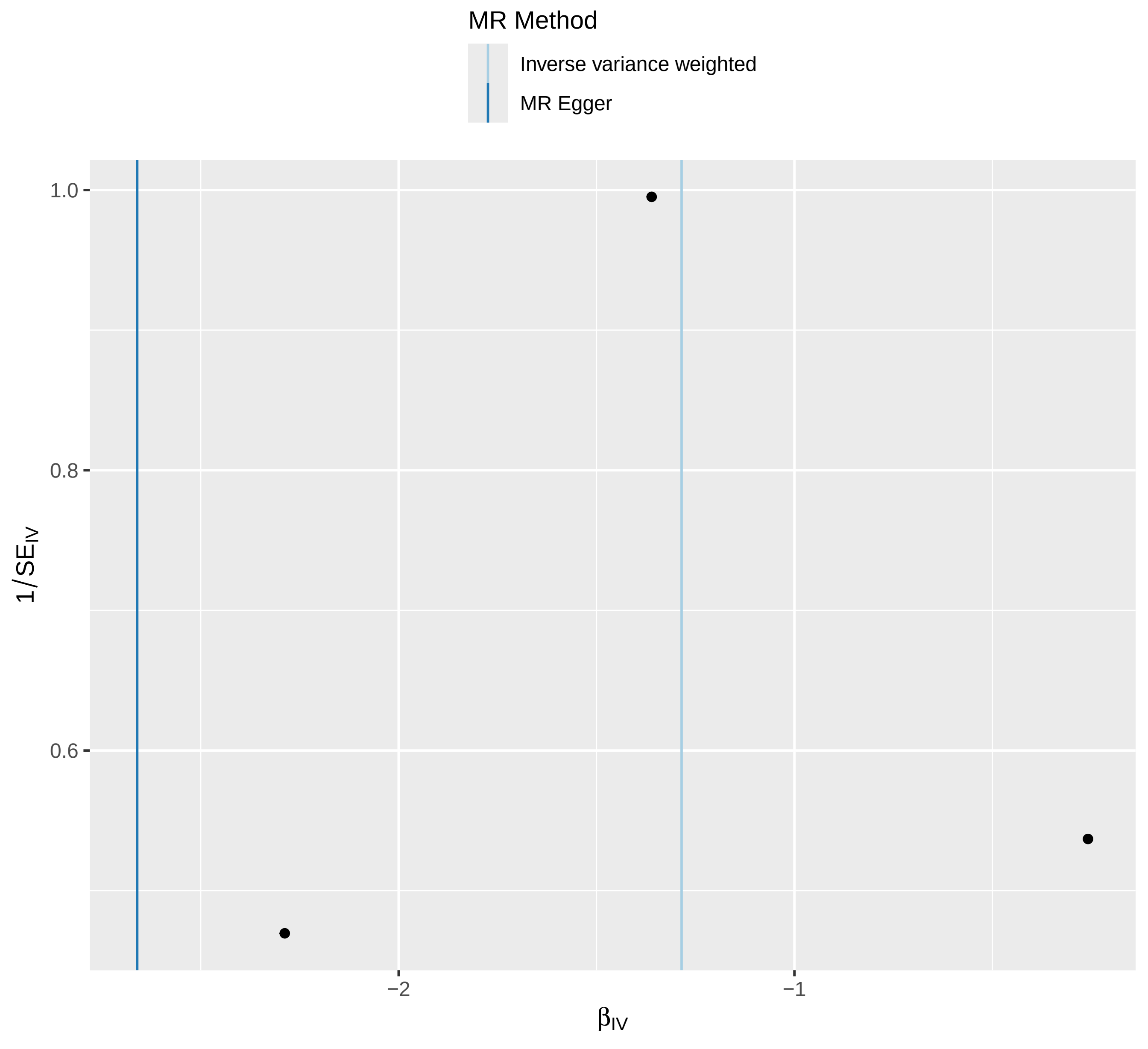
MR Method
Inverse variance weighted
MR Egger
1.0
0.8
0.6
1/SE$_{IV}$
−2
−1
β$_{IV}$

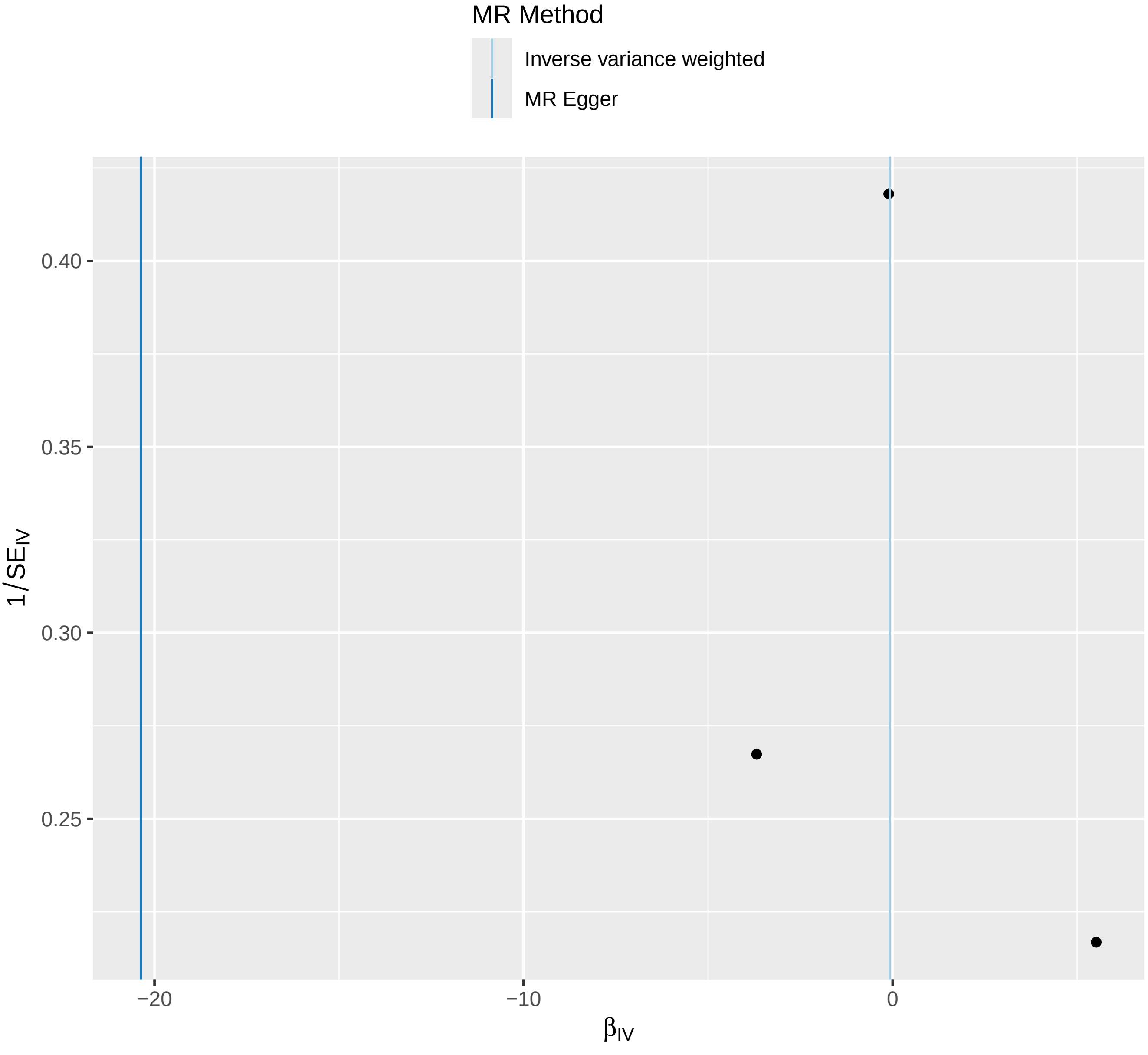
MR Method
Inverse variance weighted
MR Egger
0.40
0.35
0.30
0.25
1/SE_IV
−20
−10
0
β_IV

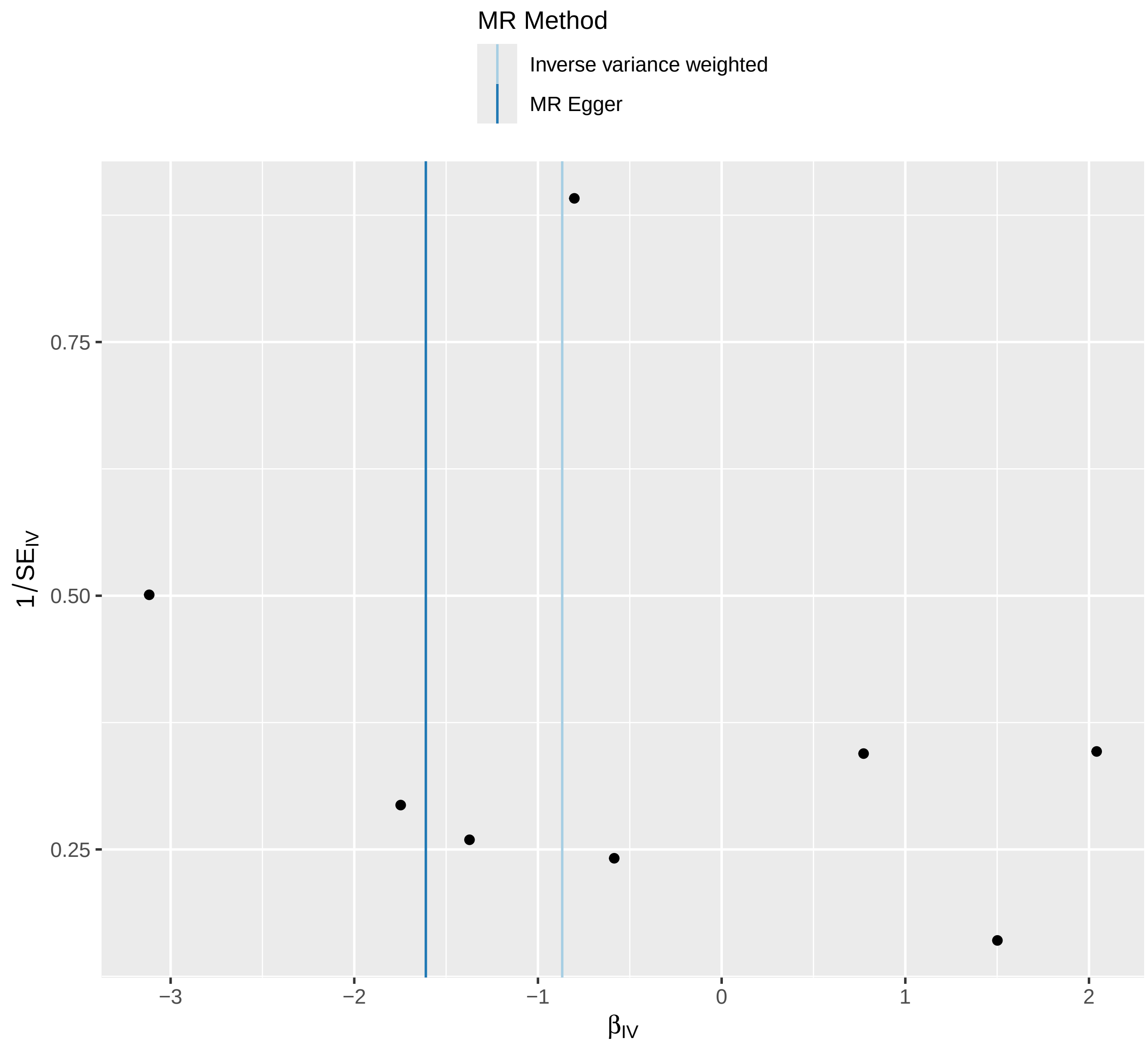

MR Method
Inverse variance weighted
MR Egger
0.75
0.50
0.25
1/SE$_{IV}$
−3
−2
−1
0
1
2
β$_{IV}$

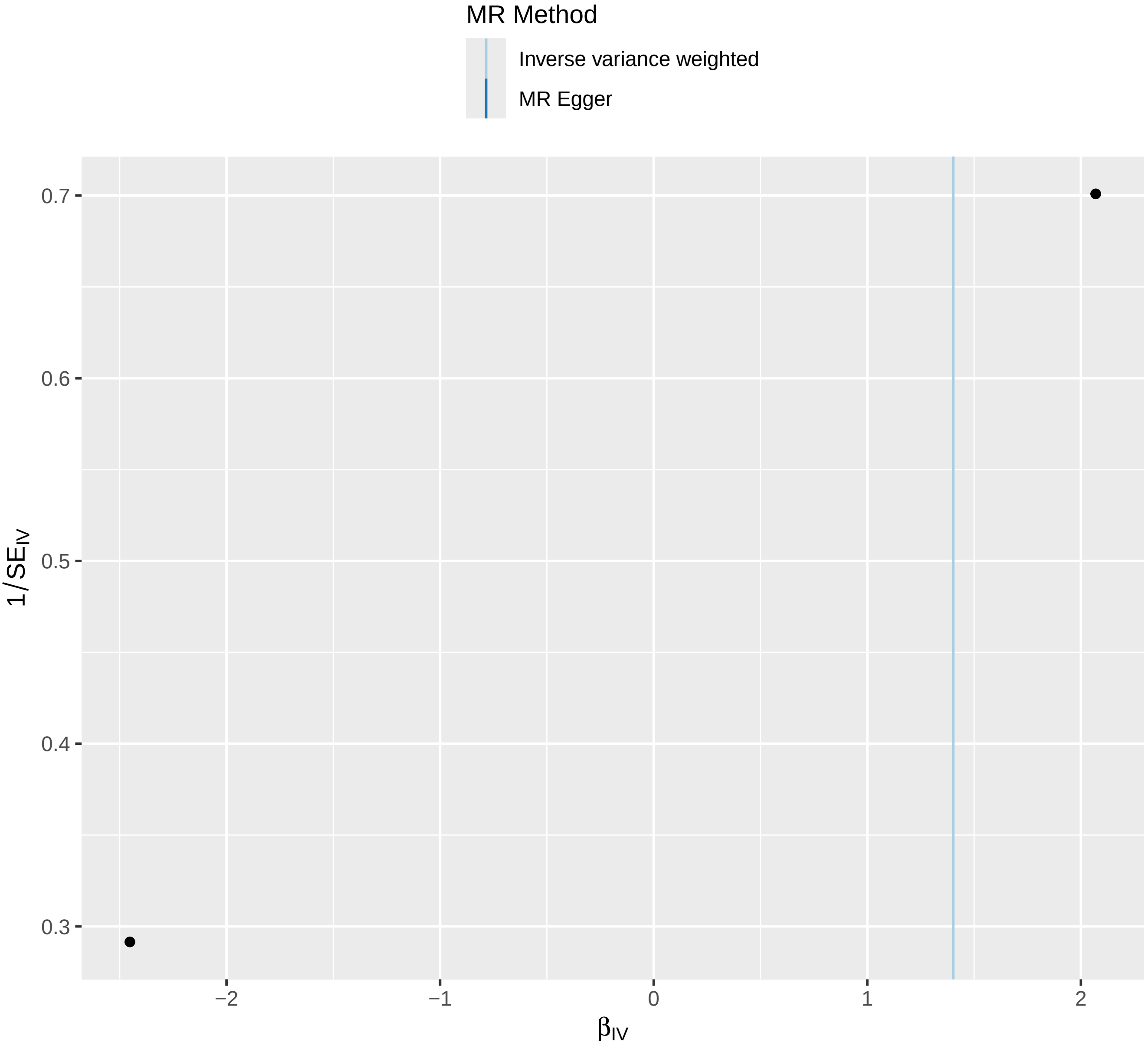

MR Method
Inverse variance weighted
MR Egger
0.7
0.6
0.5
0.4
0.3
1/SE_IV
−2
−1
0
1
2
β_IV

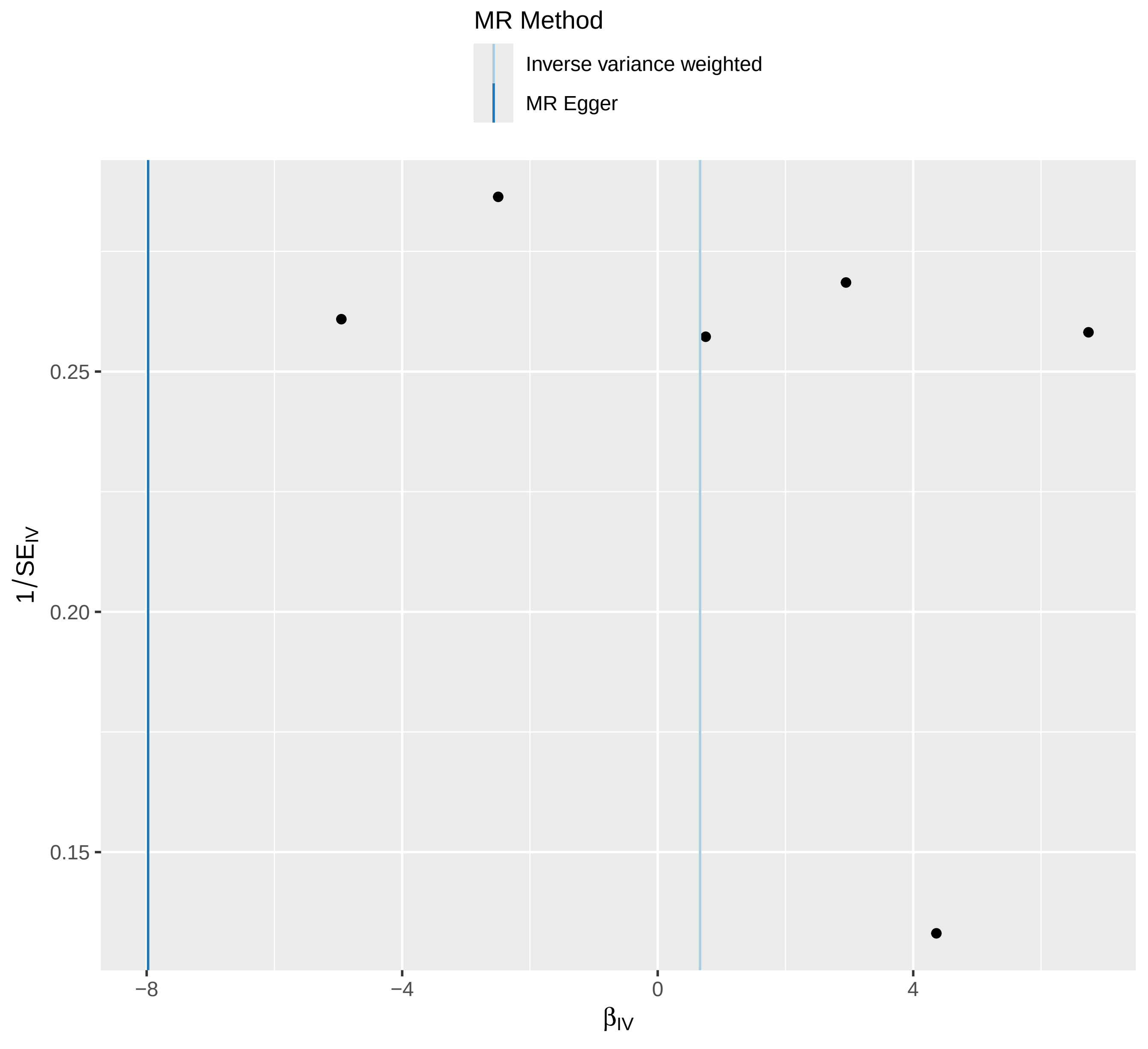
MR Method
Inverse variance weighted
MR Egger
1/SE_IV
0.25
0.20
0.15
−8
−4
0
4
β_IV

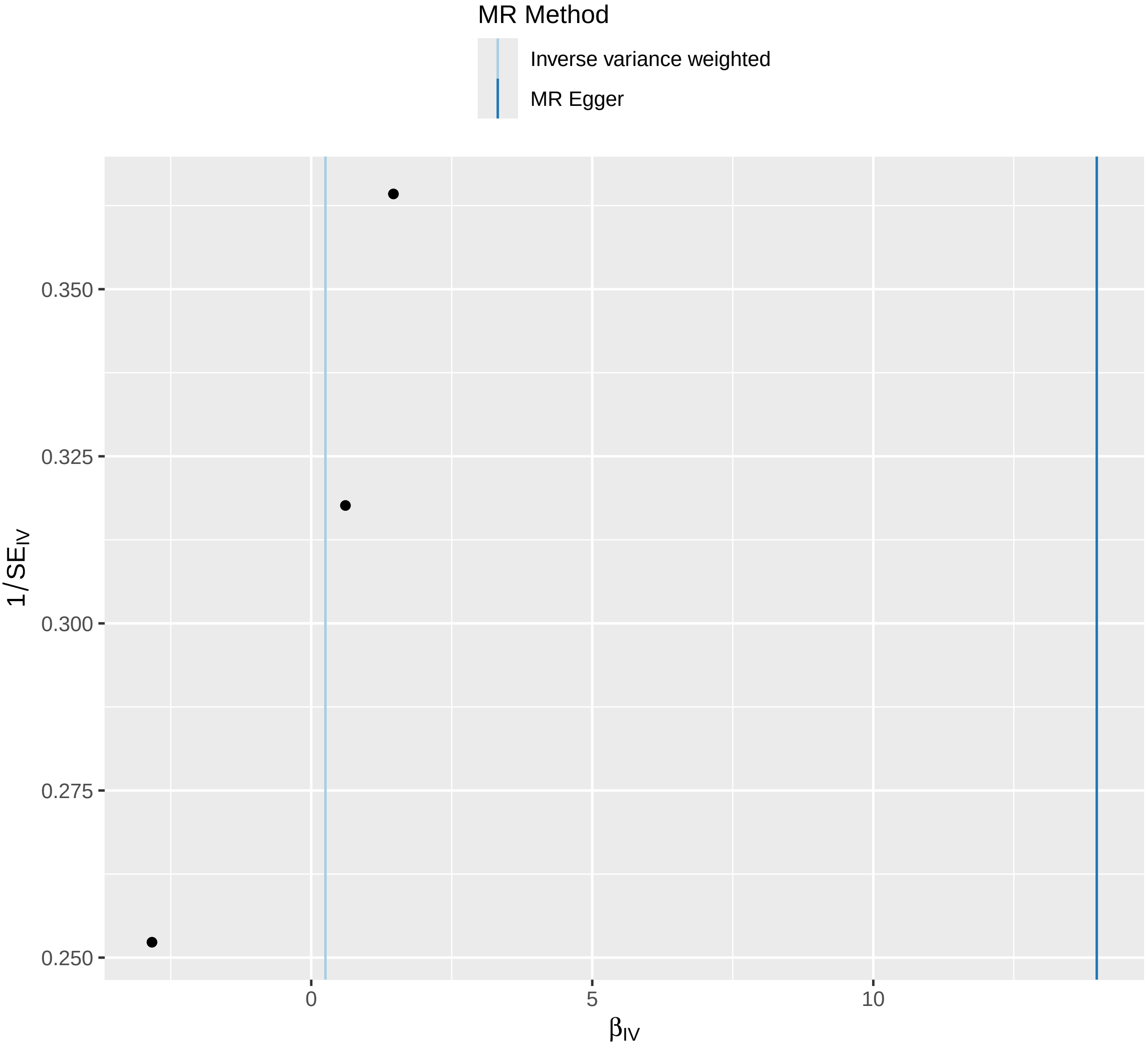
MR Method
Inverse variance weighted
MR Egger
0.350
0.325
0.300
0.275
0.250
1/SE$_{IV}$
0
5
10
β$_{IV}$

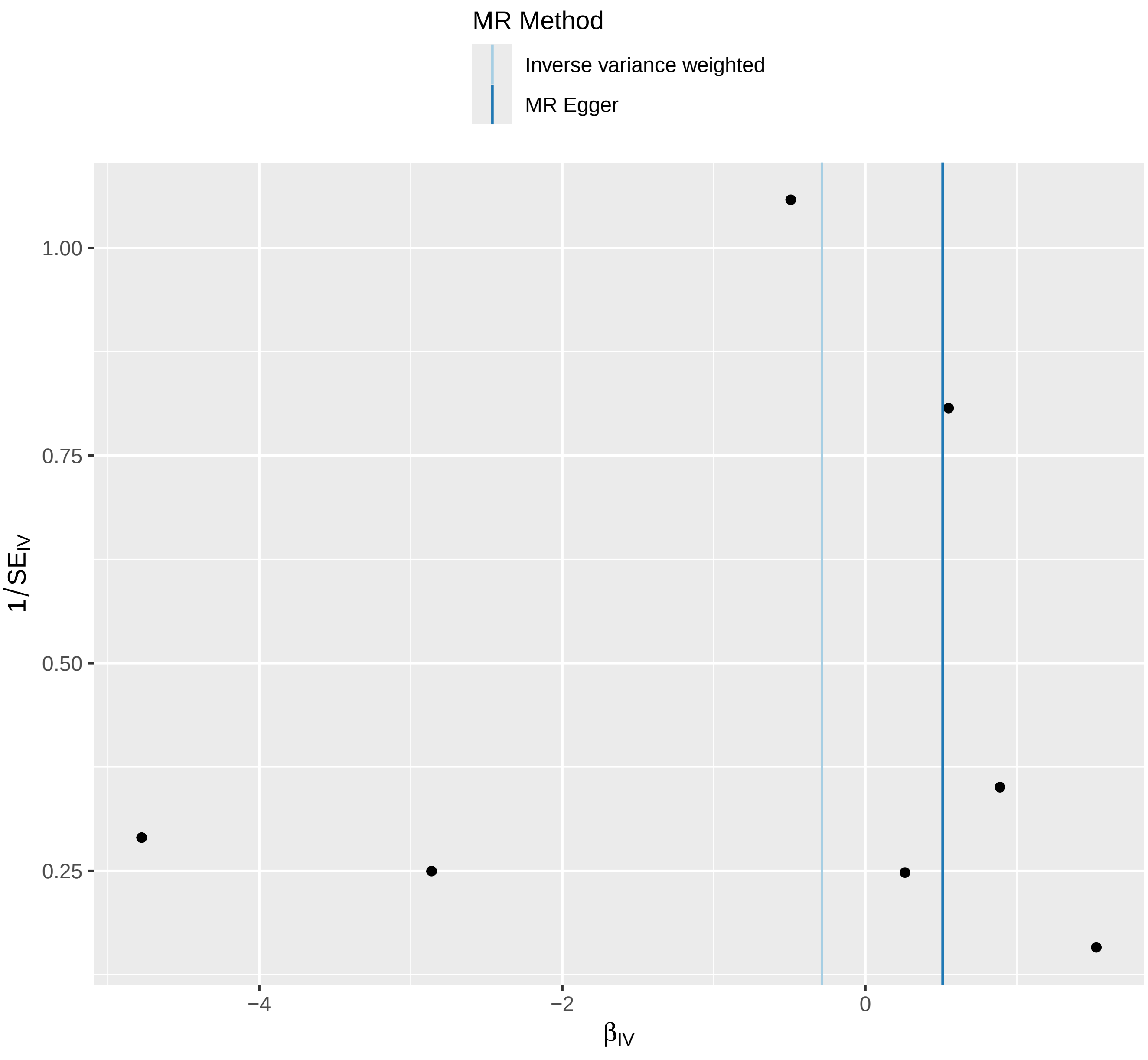
MR Method
Inverse variance weighted
MR Egger
1.00
0.75
0.50
0.25
−4
−2
0
1/SE_IV
β_IV

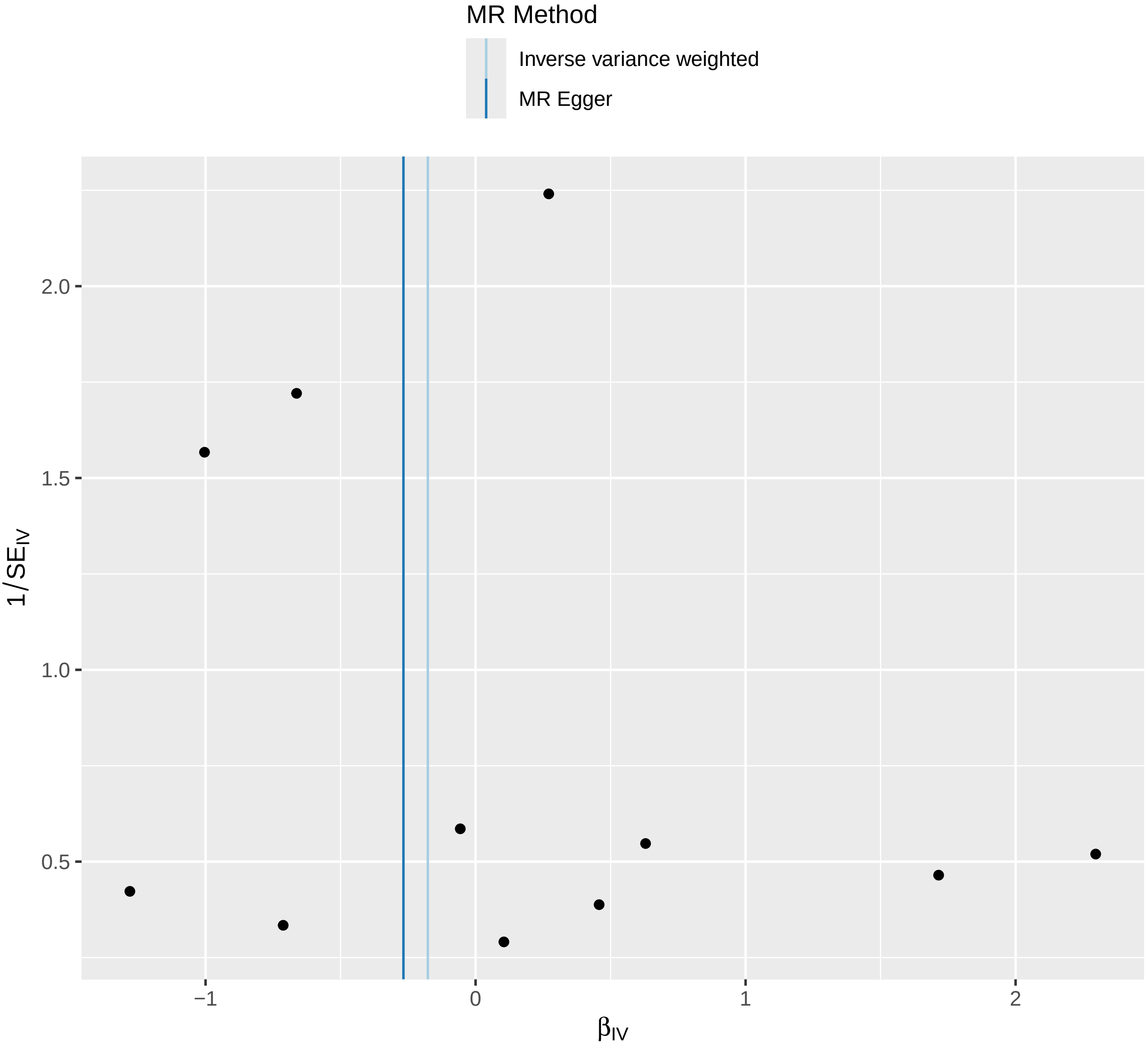
MR Method
Inverse variance weighted
MR Egger
2.0
1.5
1.0
0.5
1/SE_IV
−1
0
1
2
β_IV

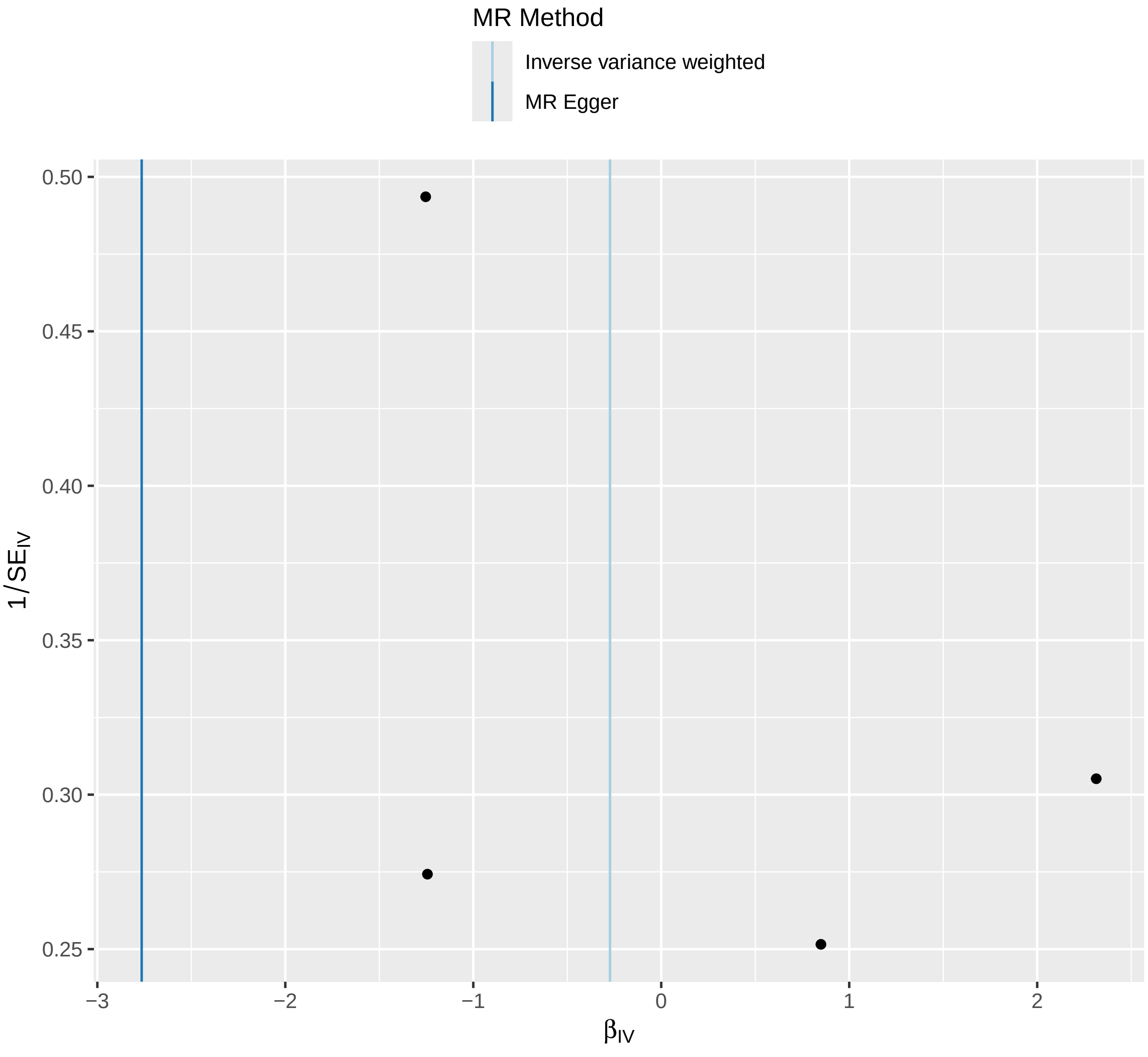
MR Method
Inverse variance weighted
MR Egger
0.50
0.45
0.40
0.35
0.30
0.25
1/SE$_{IV}$
−3
−2
−1
0
1
2
β$_{IV}$

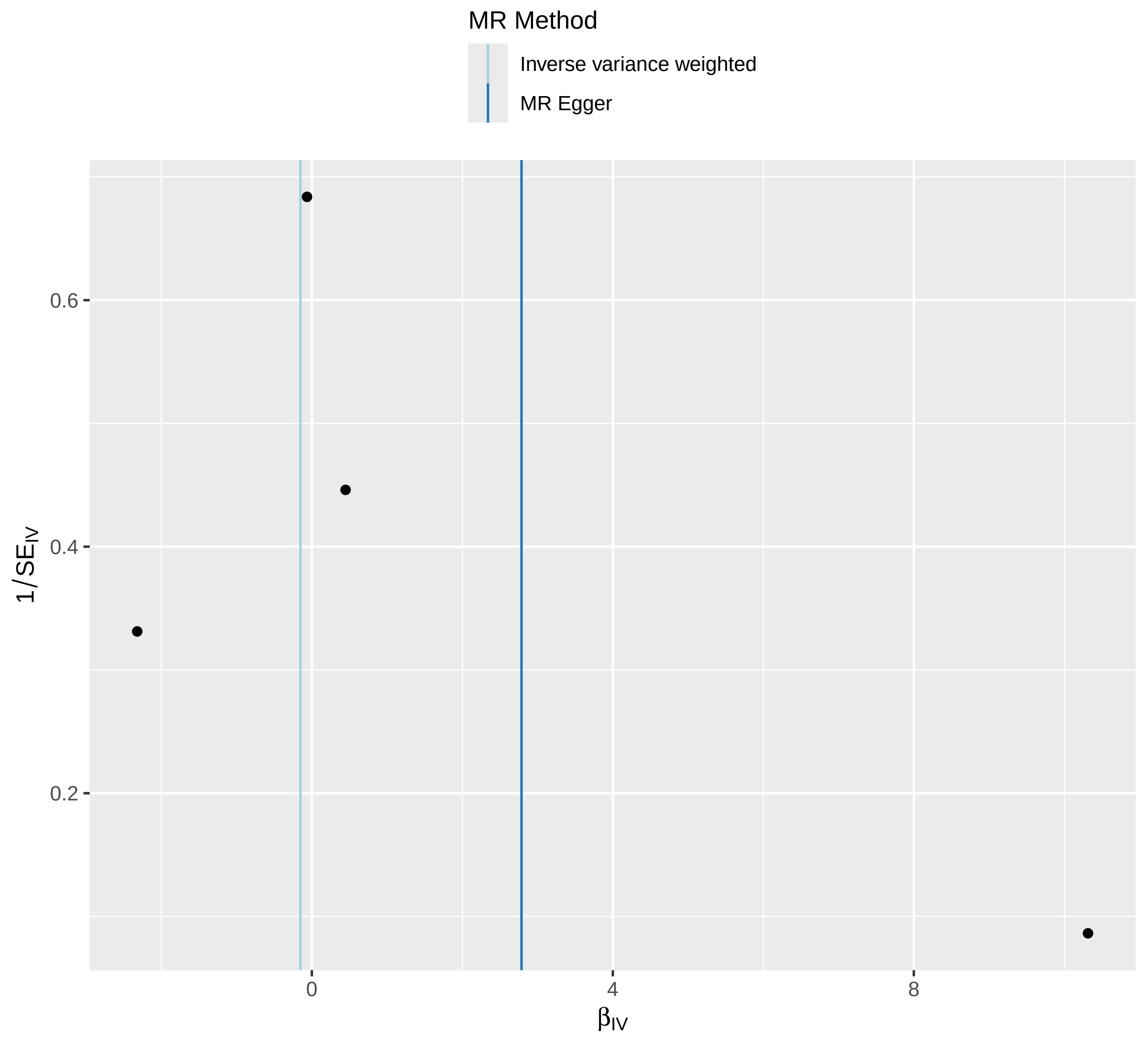
MR Method
Inverse variance weighted
MR Egger
0.6
0.4
0.2
1/SE$_{IV}$
0
4
8
β$_{IV}$

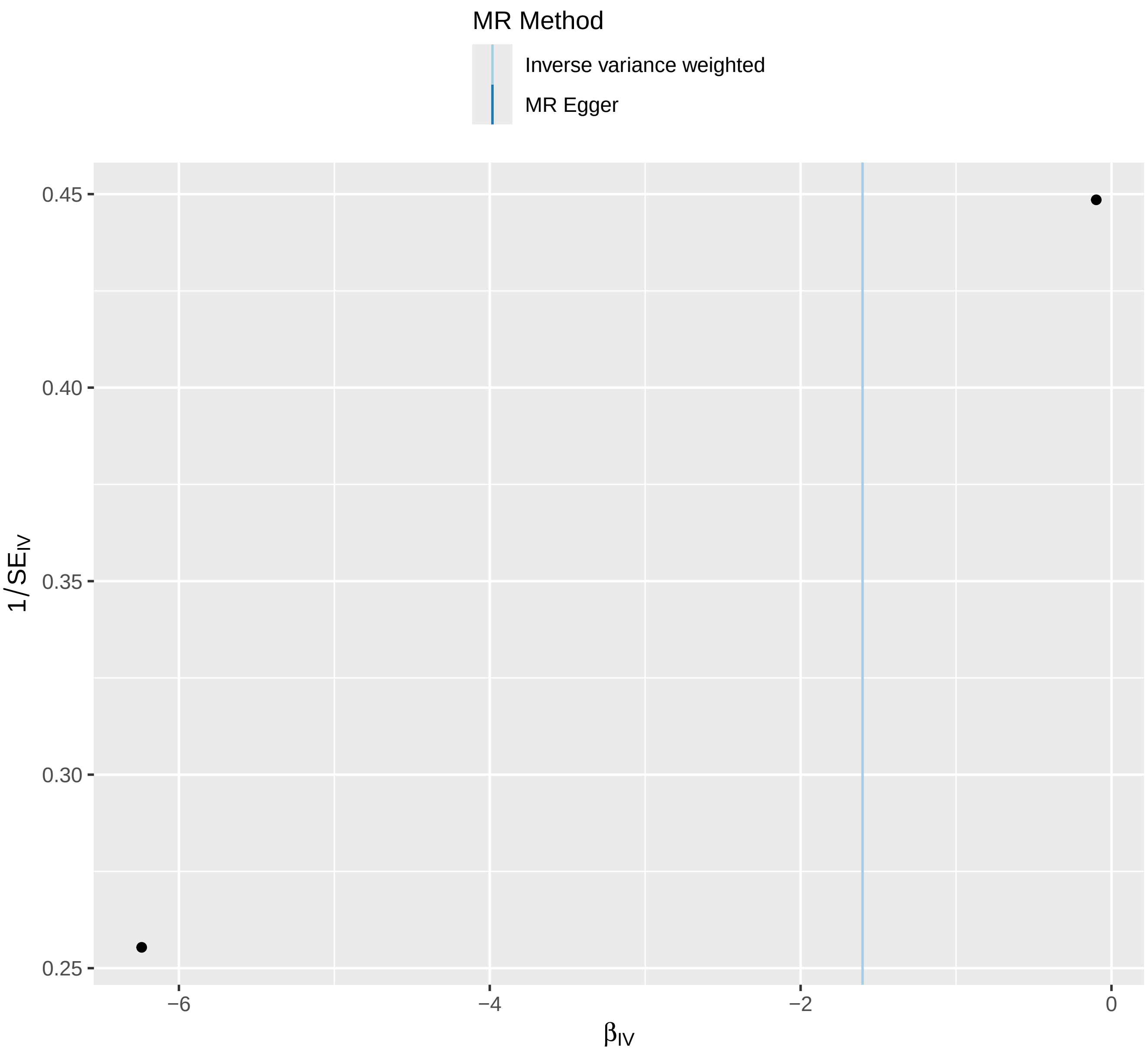
MR Method
Inverse variance weighted
MR Egger
0.45
0.40
0.35
0.30
0.25
1/SE_IV
−6
−4
−2
0
β_IV

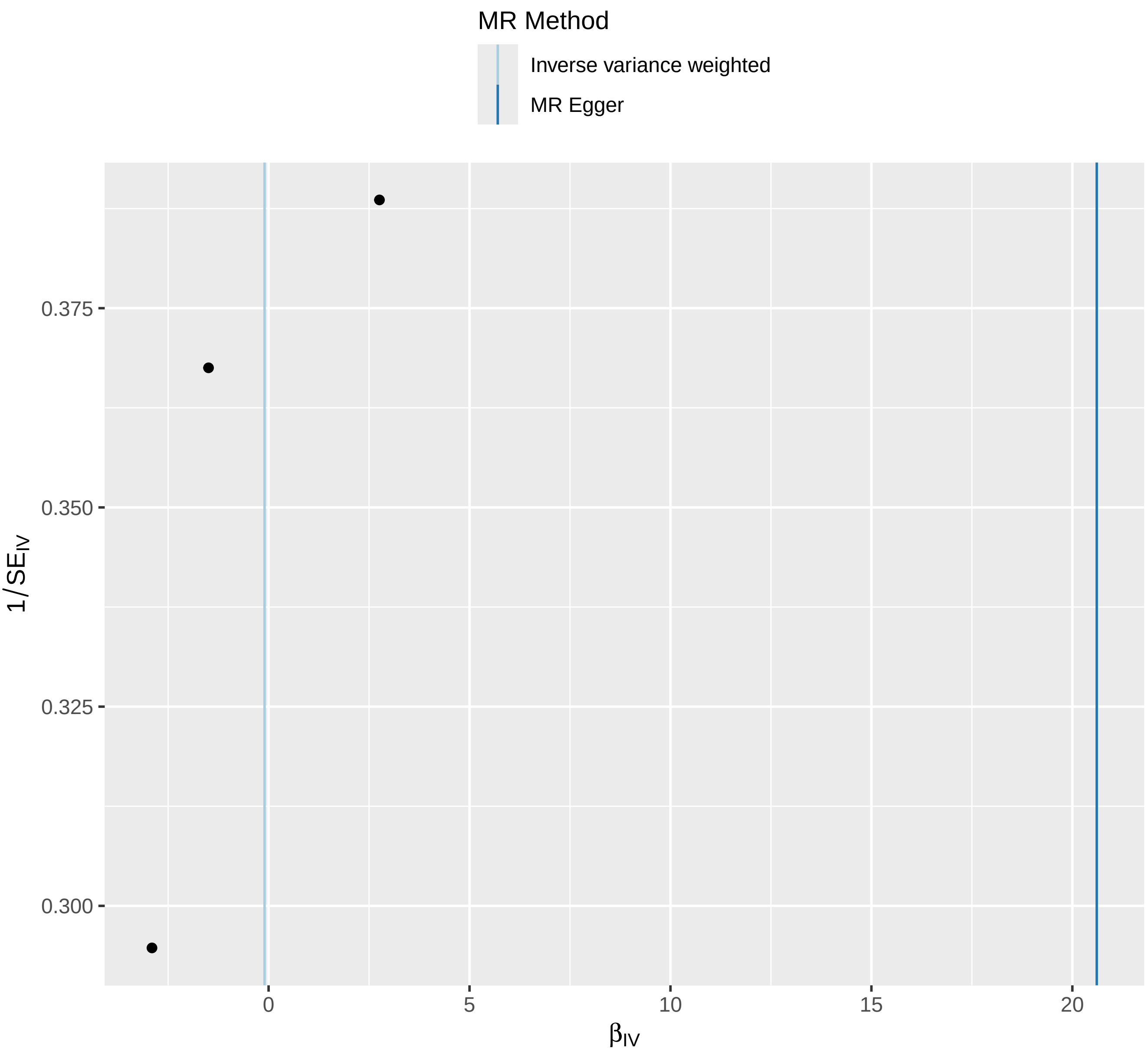
MR Method
Inverse variance weighted
MR Egger
0.375
0.350
0.325
0.300
1/SE_IV
0
5
10
15
20
β_IV

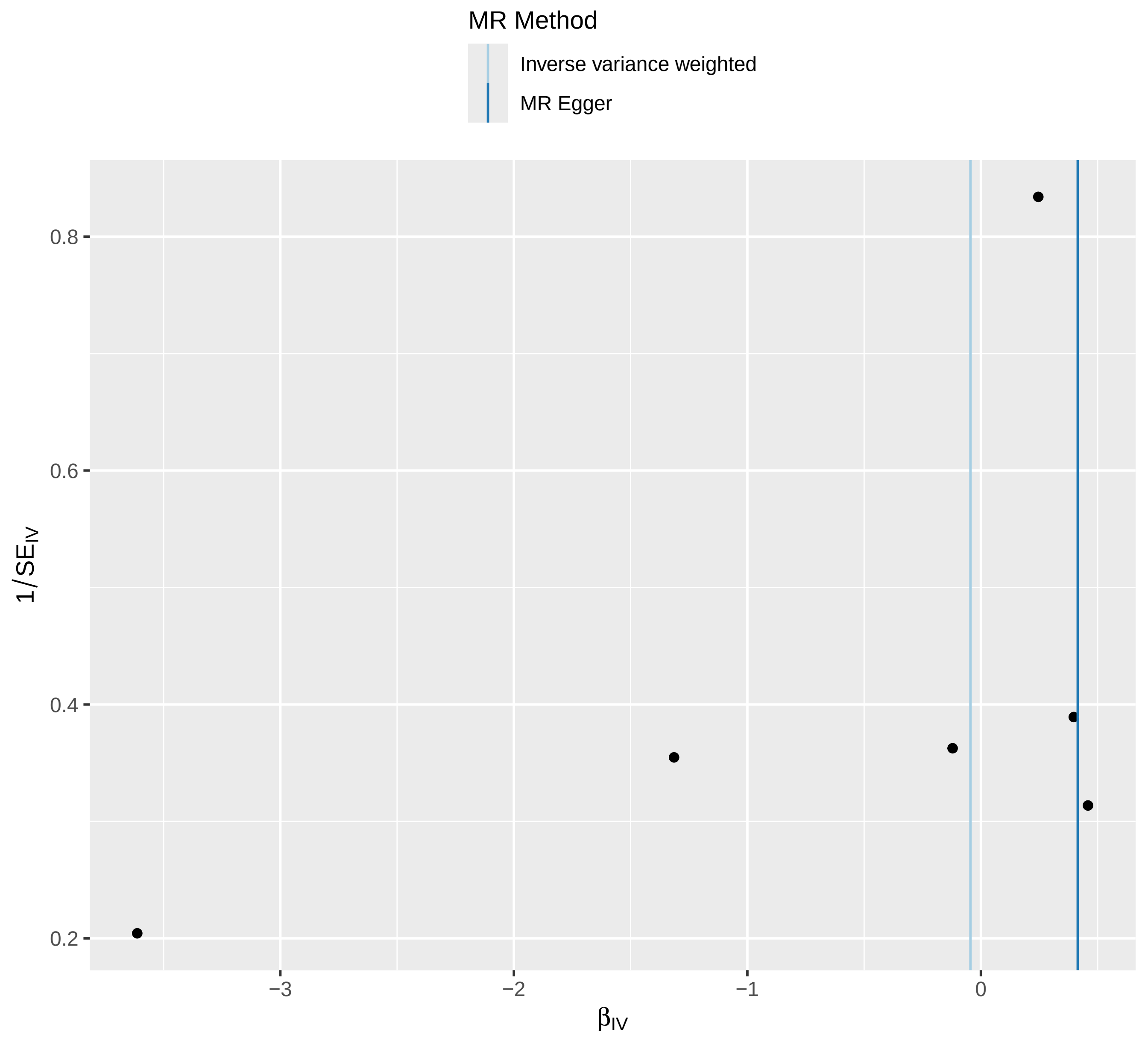
MR Method
Inverse variance weighted
MR Egger
0.8
0.6
0.4
0.2
1/SE_IV
−3
−2
−1
0
β_IV

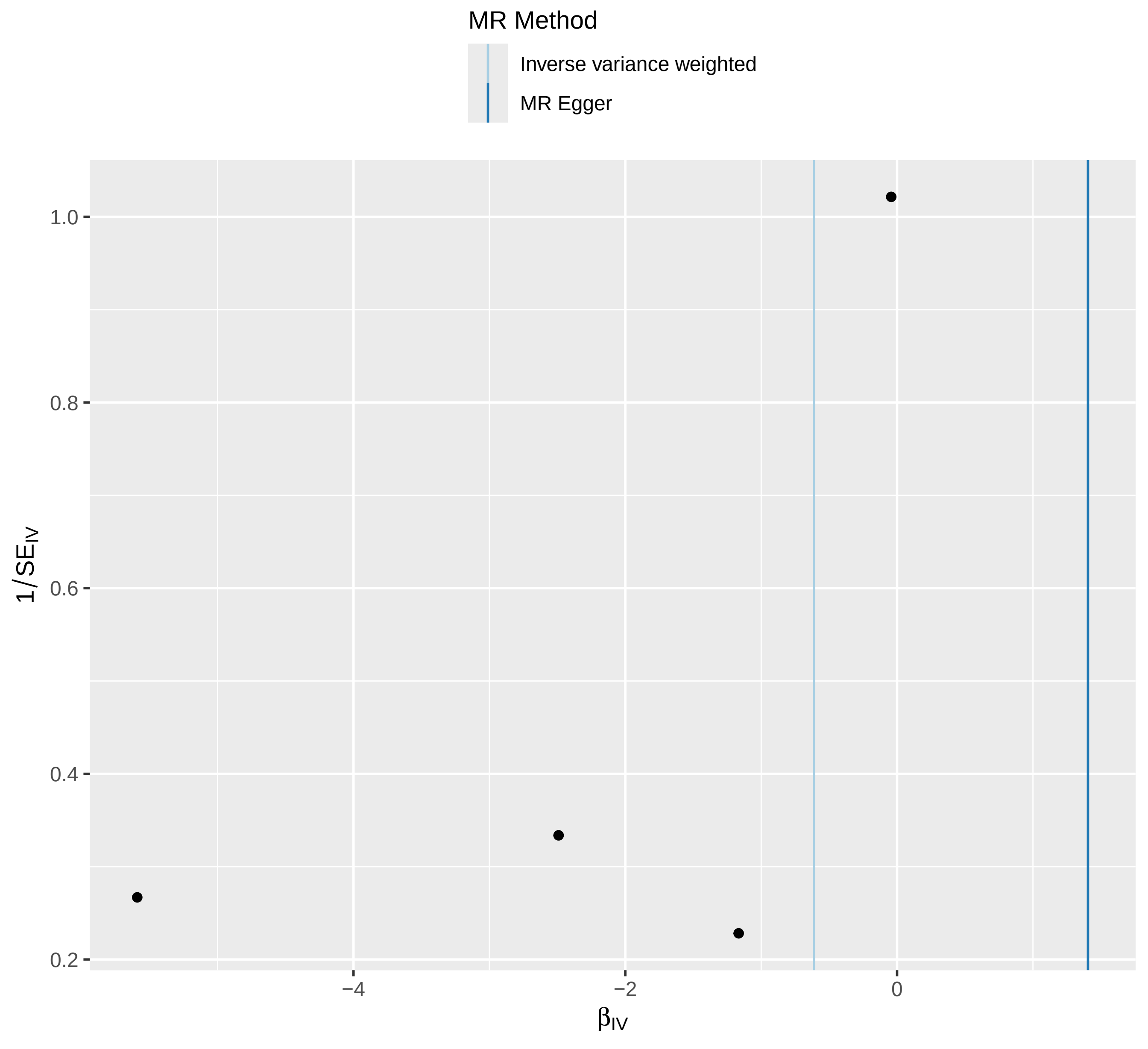
MR Method
Inverse variance weighted
MR Egger
1.0
0.8
0.6
0.4
0.2
1/SE_IV
−4
−2
0
β_IV

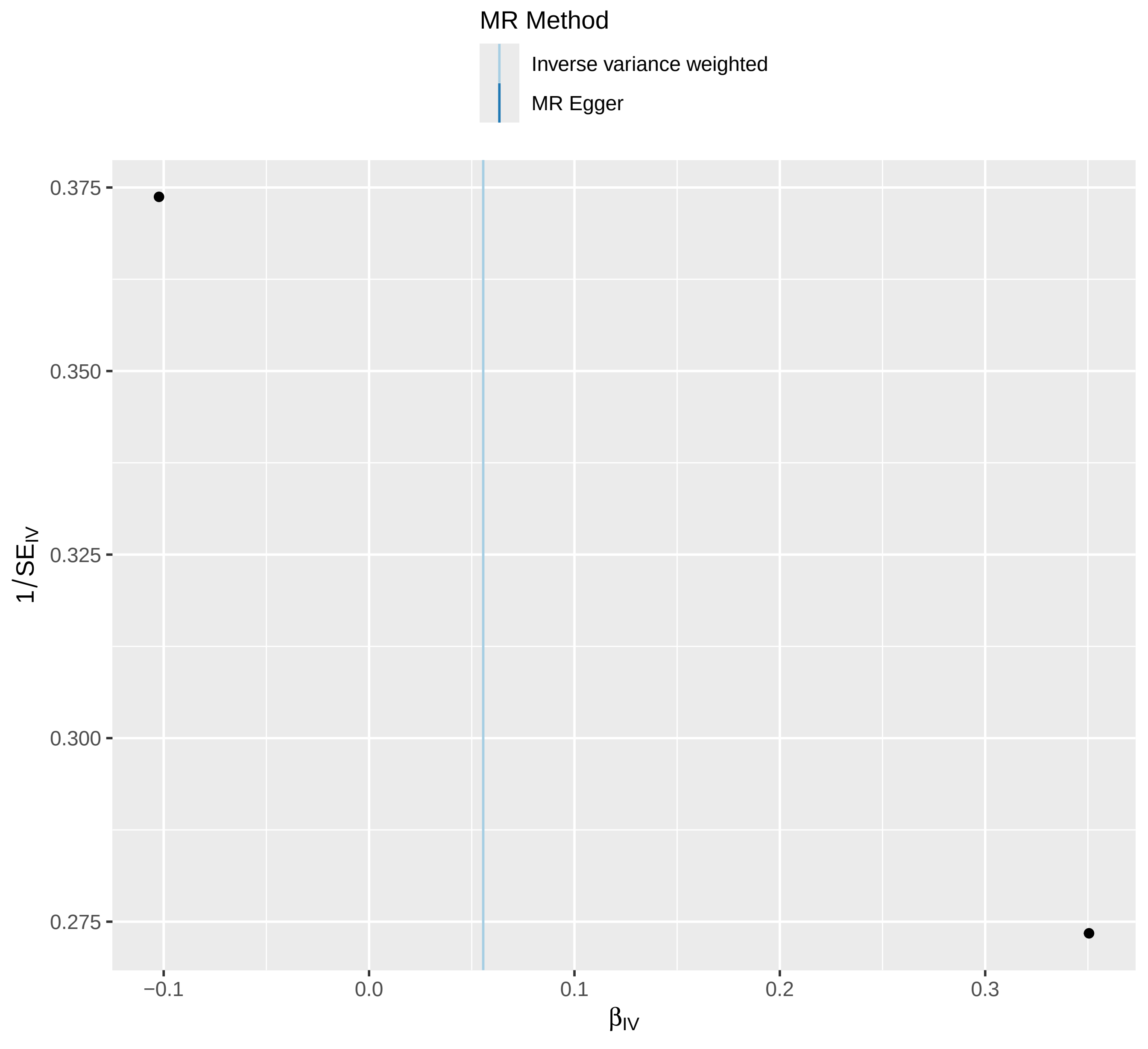
MR Method
Inverse variance weighted
MR Egger
0.375
0.350
0.325
0.300
0.275
1/SE_IV
−0.1
0.0
0.1
0.2
0.3
β_IV

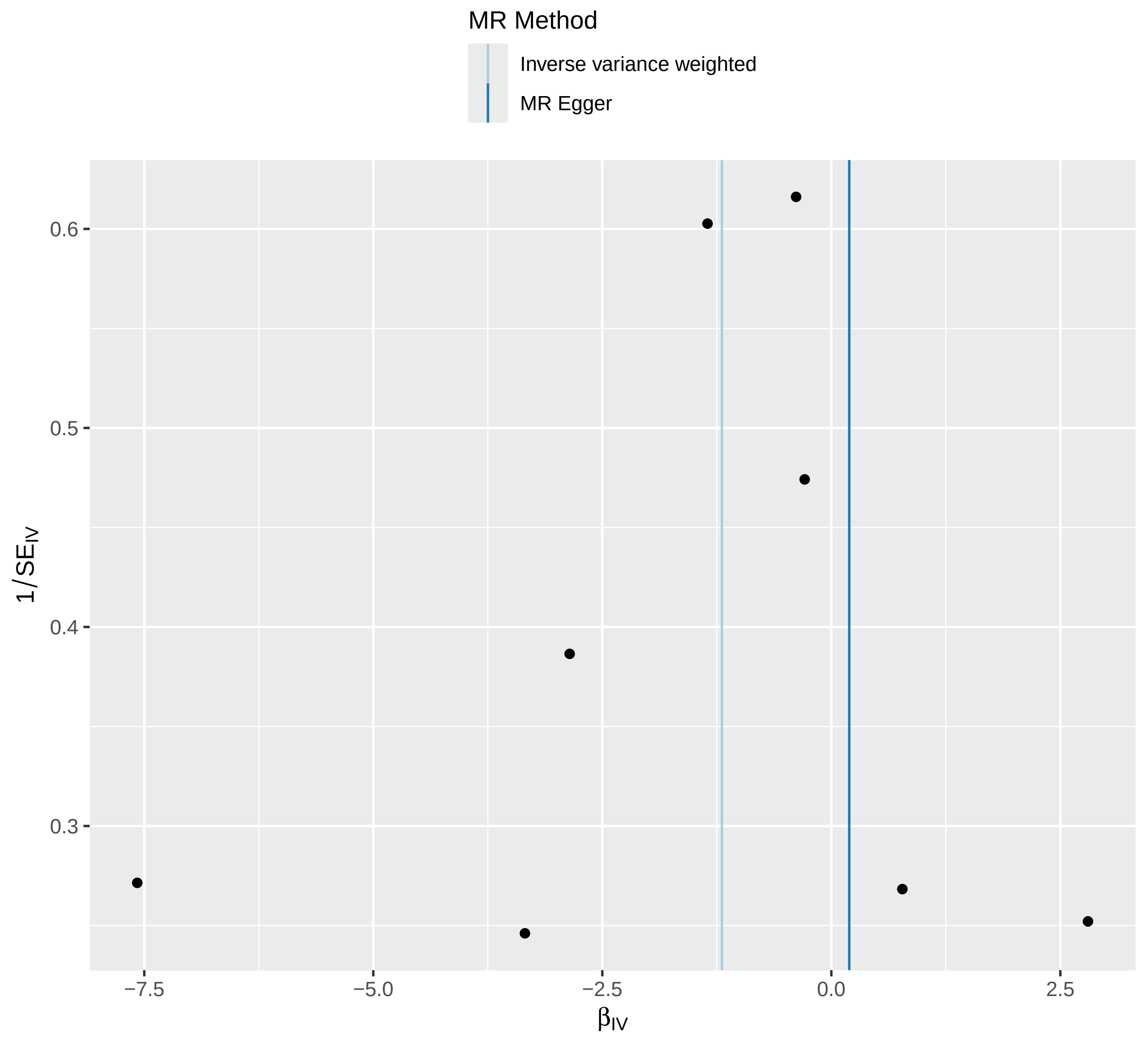
MR Method
Inverse variance weighted
MR Egger
0.6
0.5
0.4
0.3
1/SE_IV
−7.5
−5.0
−2.5
0.0
2.5
β_IV

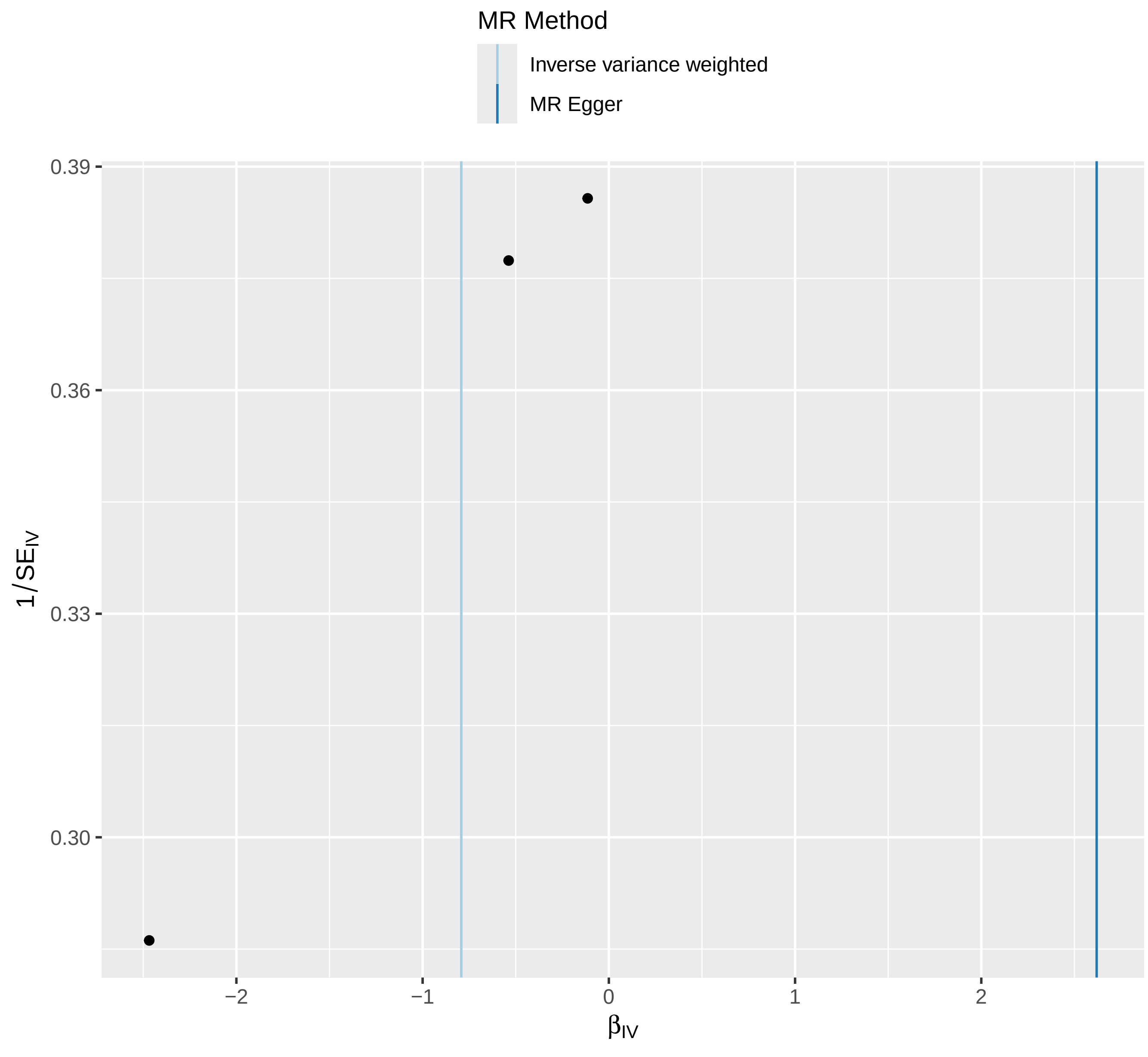
MR Method
Inverse variance weighted
MR Egger
0.39
0.36
0.33
0.30
$1/SE_{IV}$
−2
−1
0
1
2
β_{IV}

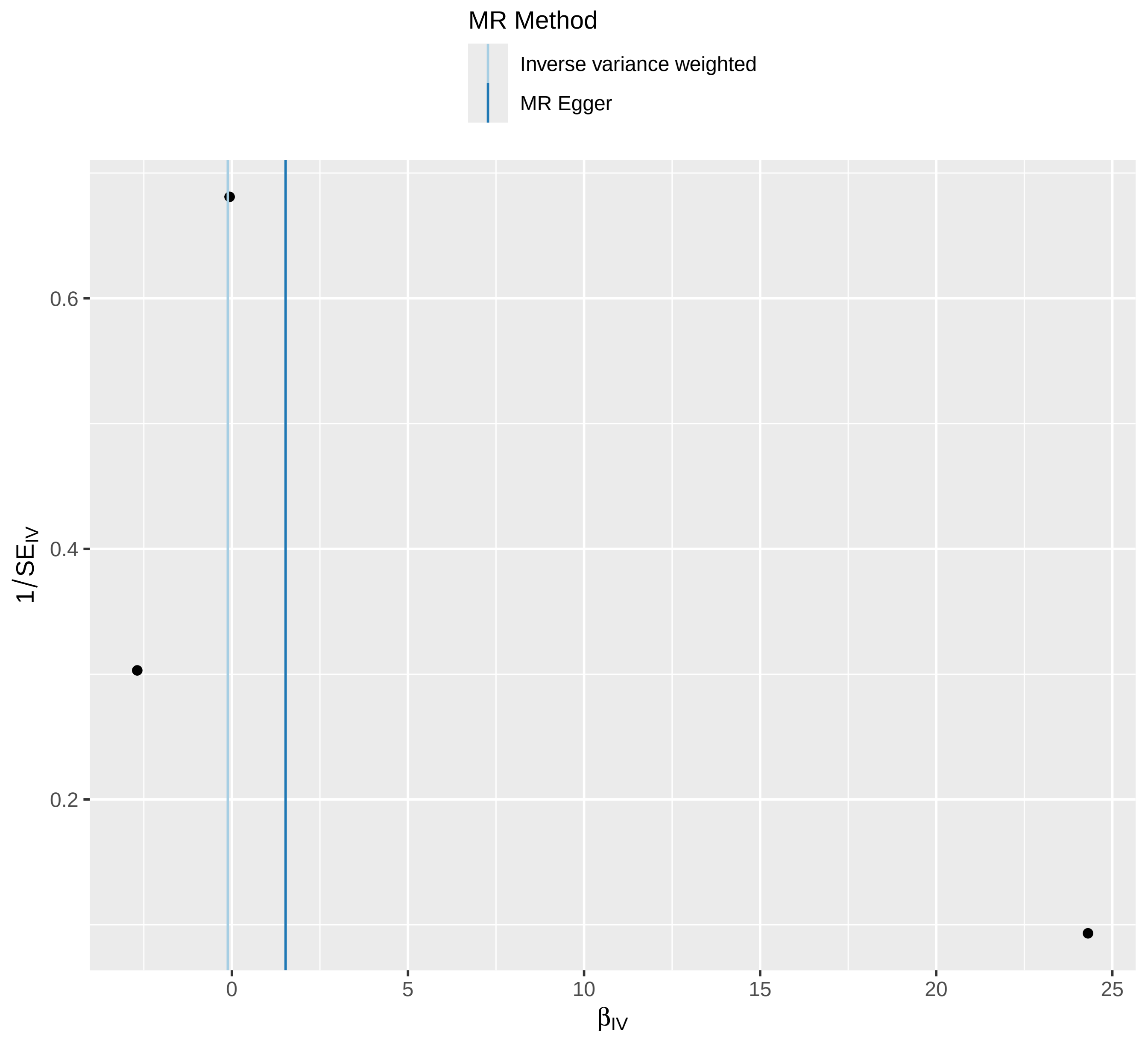
MR Method
Inverse variance weighted
MR Egger
0.6
0.4
0.2
1/SE_IV
0
5
10
15
20
25
β_IV

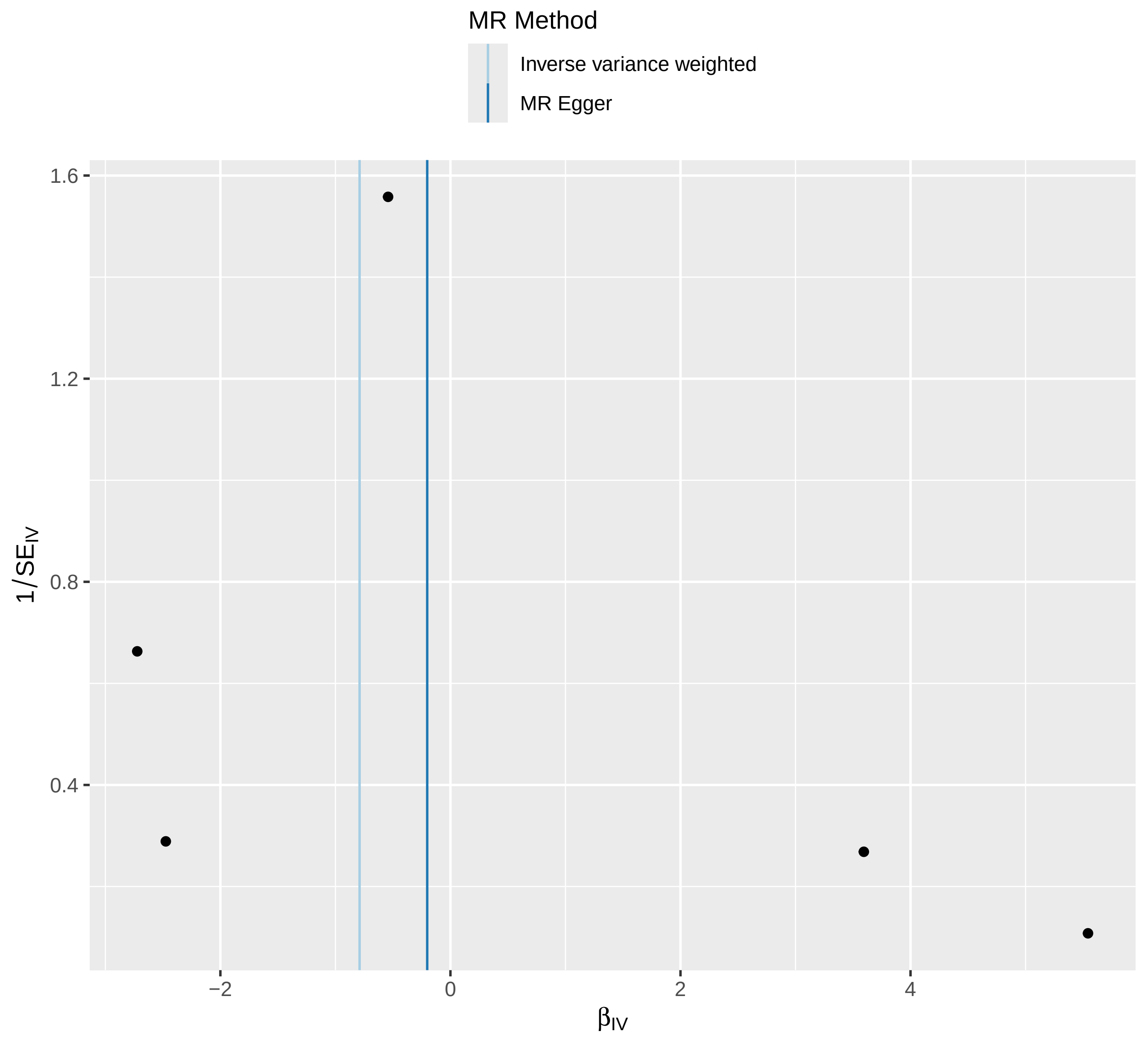
MR Method
Inverse variance weighted
MR Egger
1.6
1.2
0.8
0.4
1/SE_IV
−2
0
2
4
β_IV

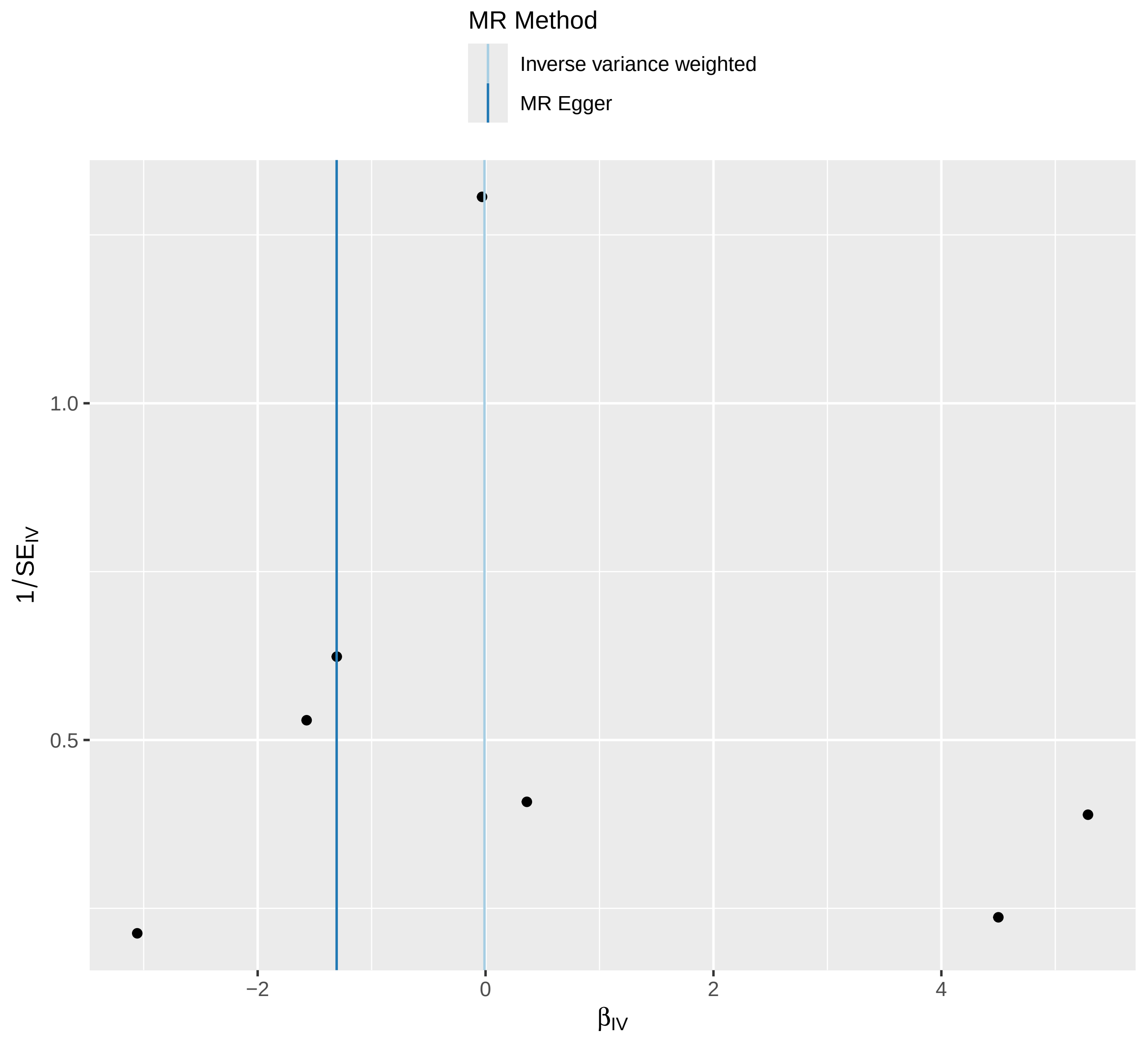

MR Method
Inverse variance weighted
MR Egger
1.0
0.5
1/SE_IV
−2
0
2
4
β_IV

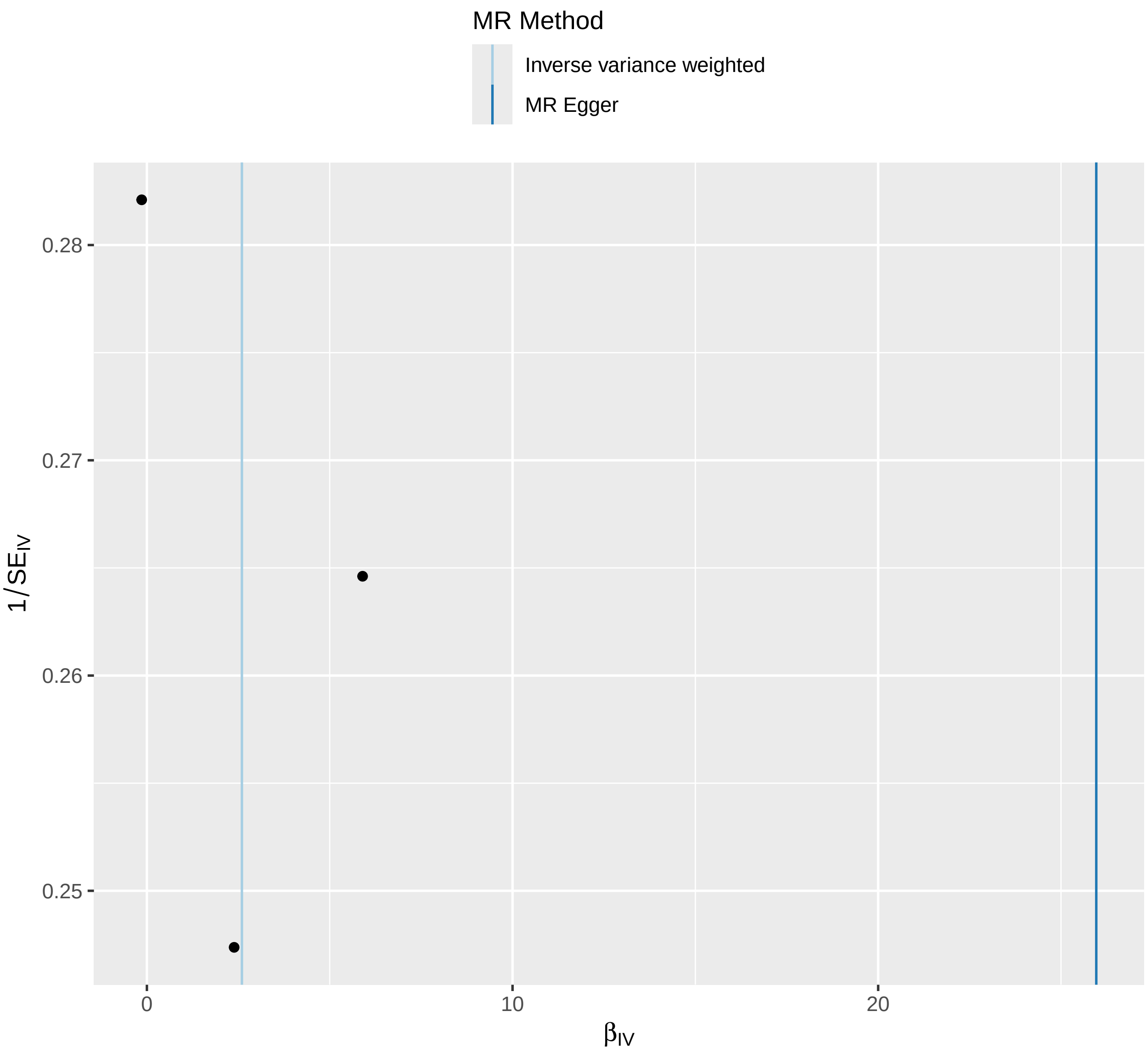
MR Method
Inverse variance weighted
MR Egger
0.28
0.27
0.26
0.25
1/SE$_{IV}$
0
10
20
β$_{IV}$

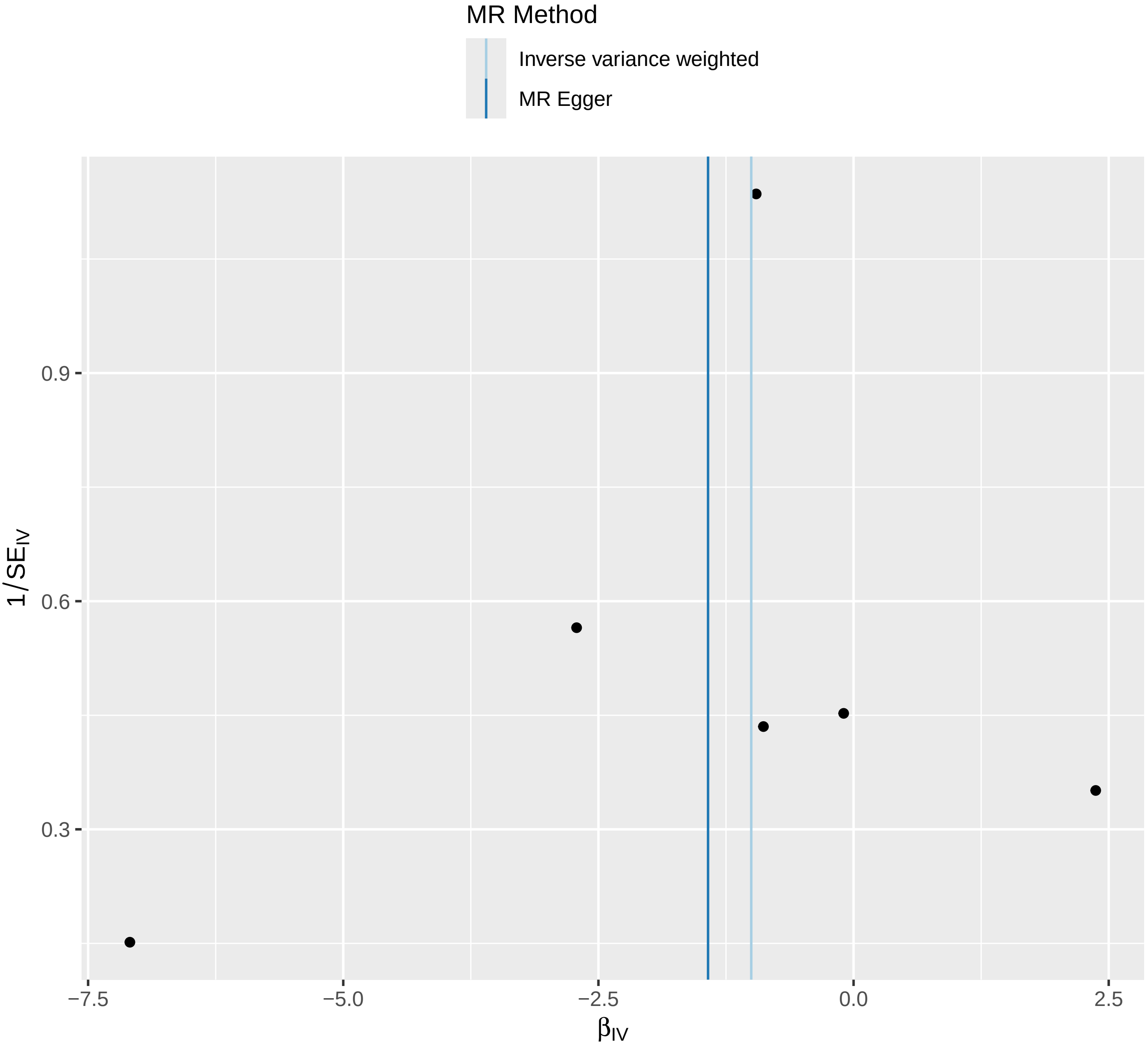
MR Method
Inverse variance weighted
MR Egger
0.9
0.6
0.3
$1/SE_{IV}$
−7.5
−5.0
−2.5
0.0
2.5
β_{IV}

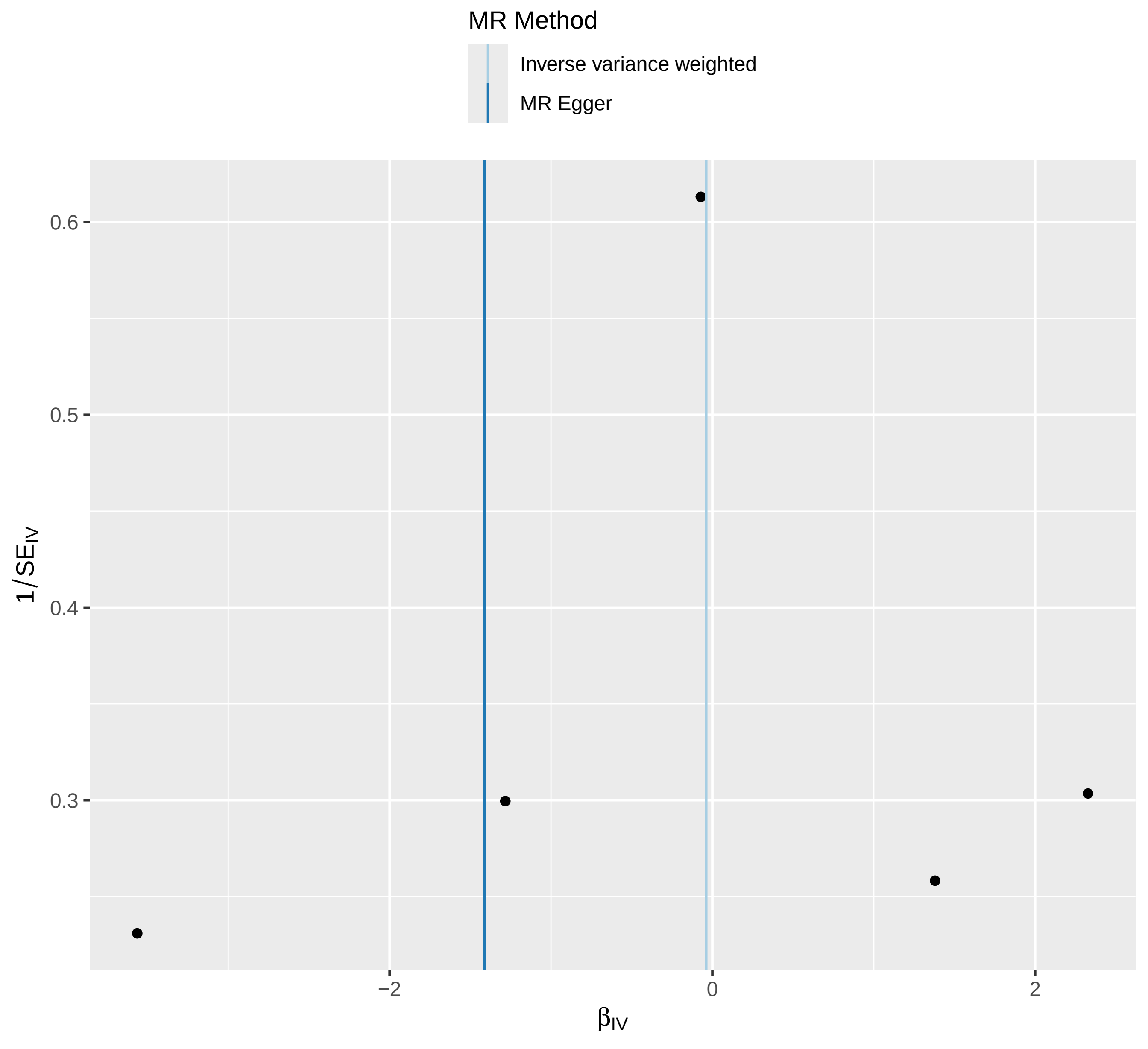

MR Method
Inverse variance weighted
MR Egger
0.6
0.5
0.4
0.3
1/SE_IV
−2
0
2
β_IV

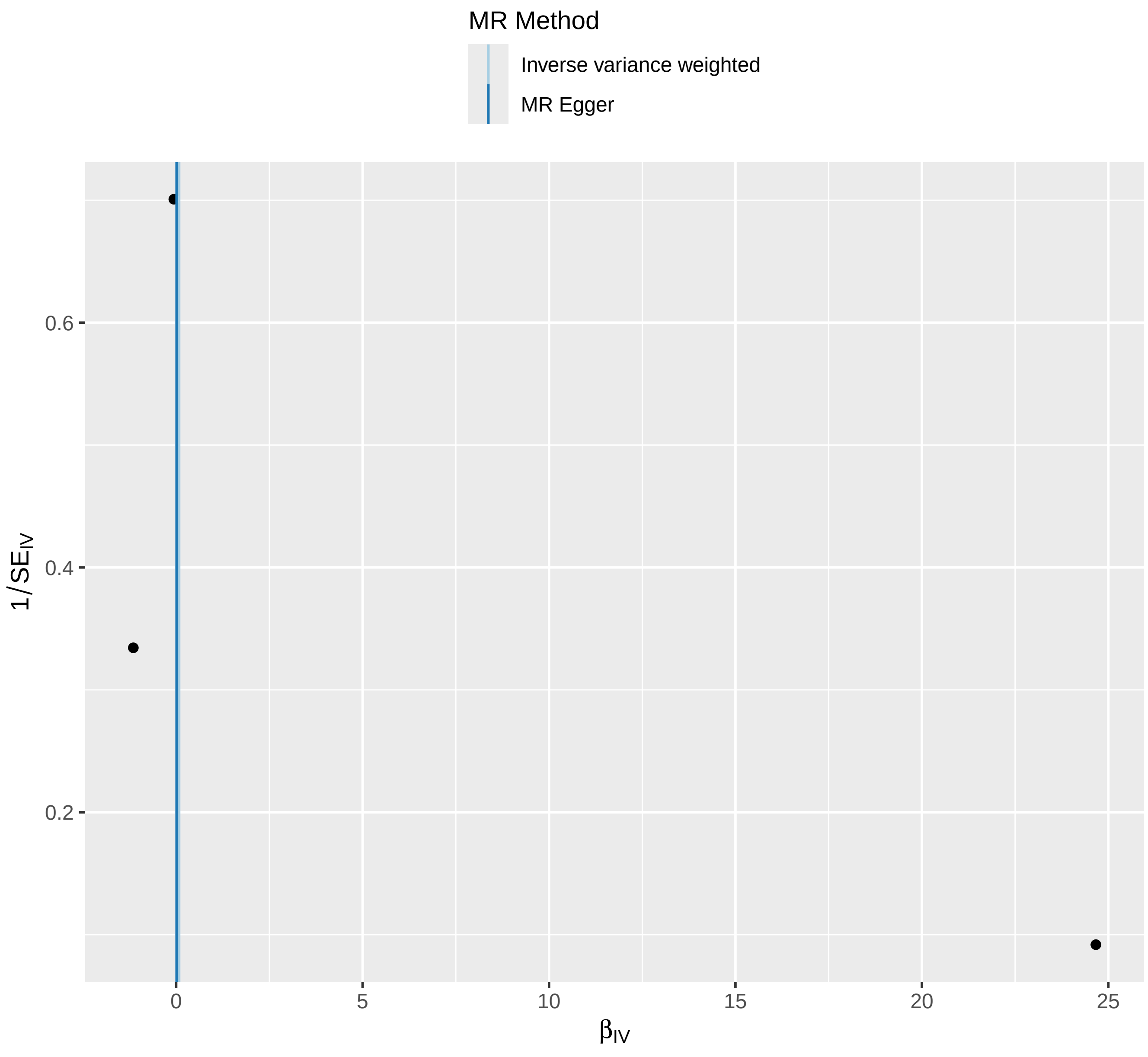
MR Method
Inverse variance weighted
MR Egger
0.6
0.4
0.2
$1/SE_{IV}$
0
5
10
15
20
25
β_{IV}

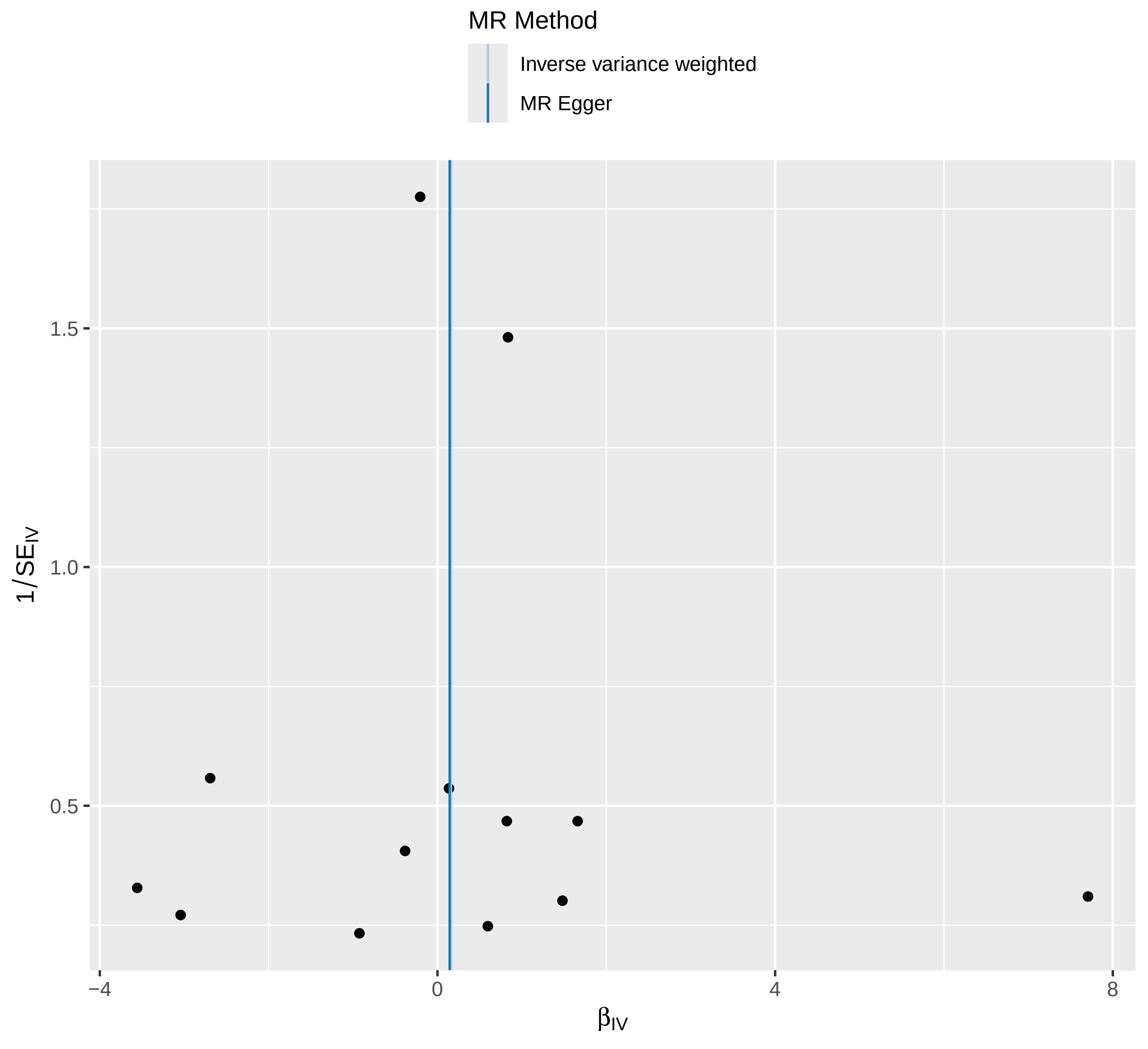
MR Method
Inverse variance weighted
MR Egger
1/SE_IV
1.5
1.0
0.5
−4
0
4
8
β_IV

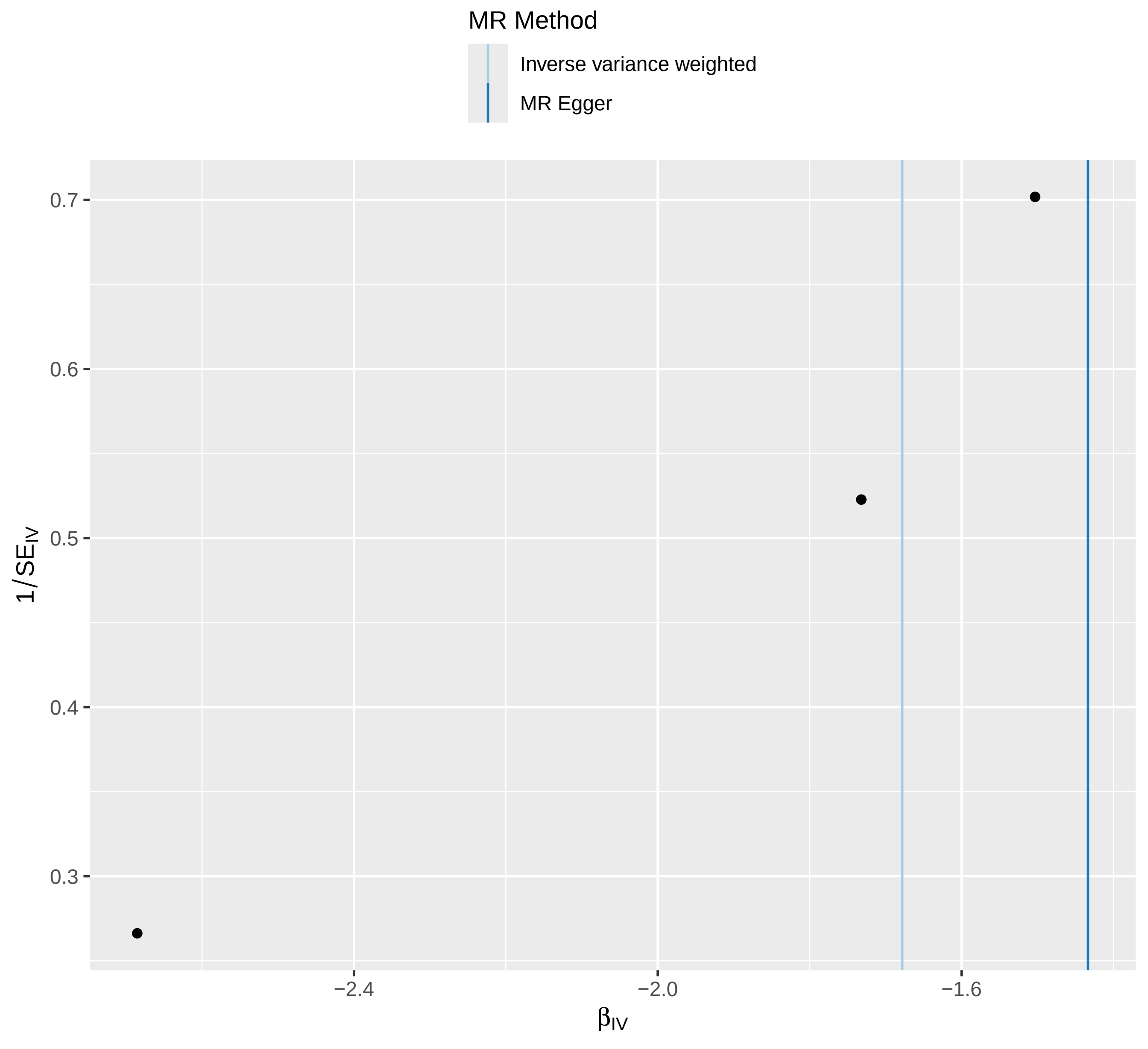
MR Method
Inverse variance weighted
MR Egger
0.7
0.6
0.5
0.4
0.3
1/SE_IV
−2.4
−2.0
−1.6
β_IV

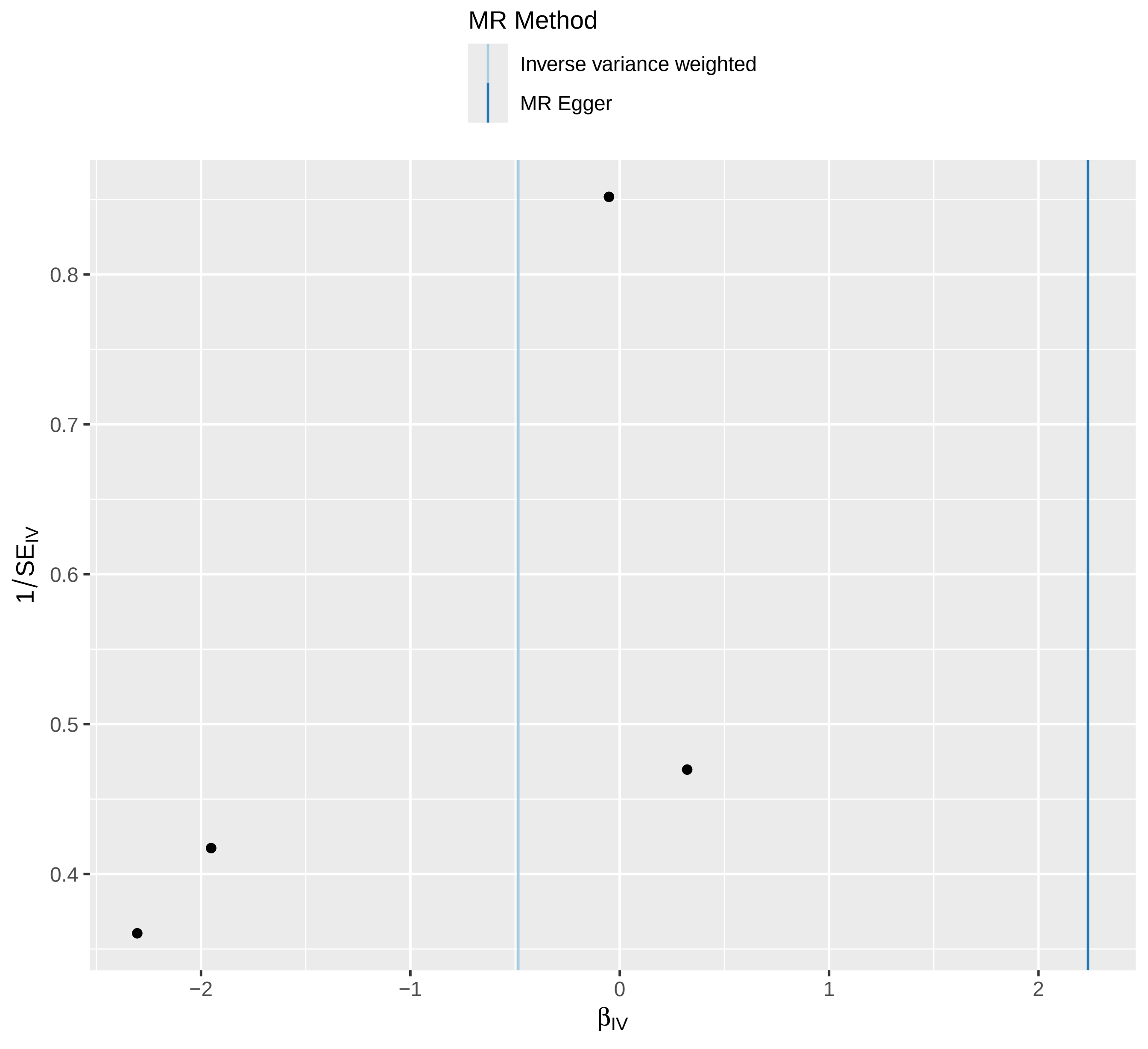
MR Method
Inverse variance weighted
MR Egger
0.8
0.7
0.6
0.5
0.4
1/SE_IV
−2
−1
0
1
2
β_IV

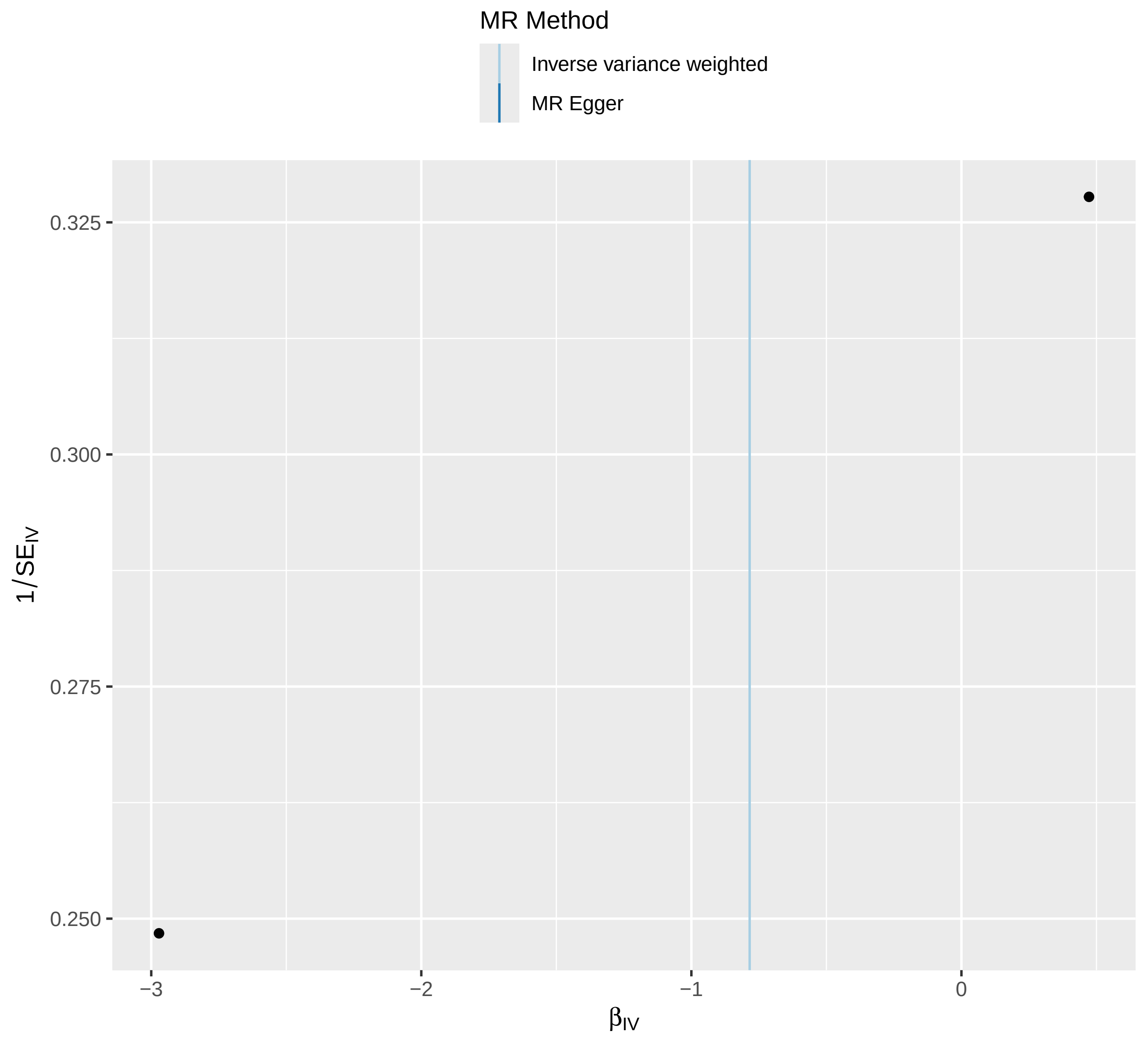
MR Method
Inverse variance weighted
MR Egger
0.325
0.300
0.275
0.250
1/SE_IV
−3
−2
−1
0
β_IV

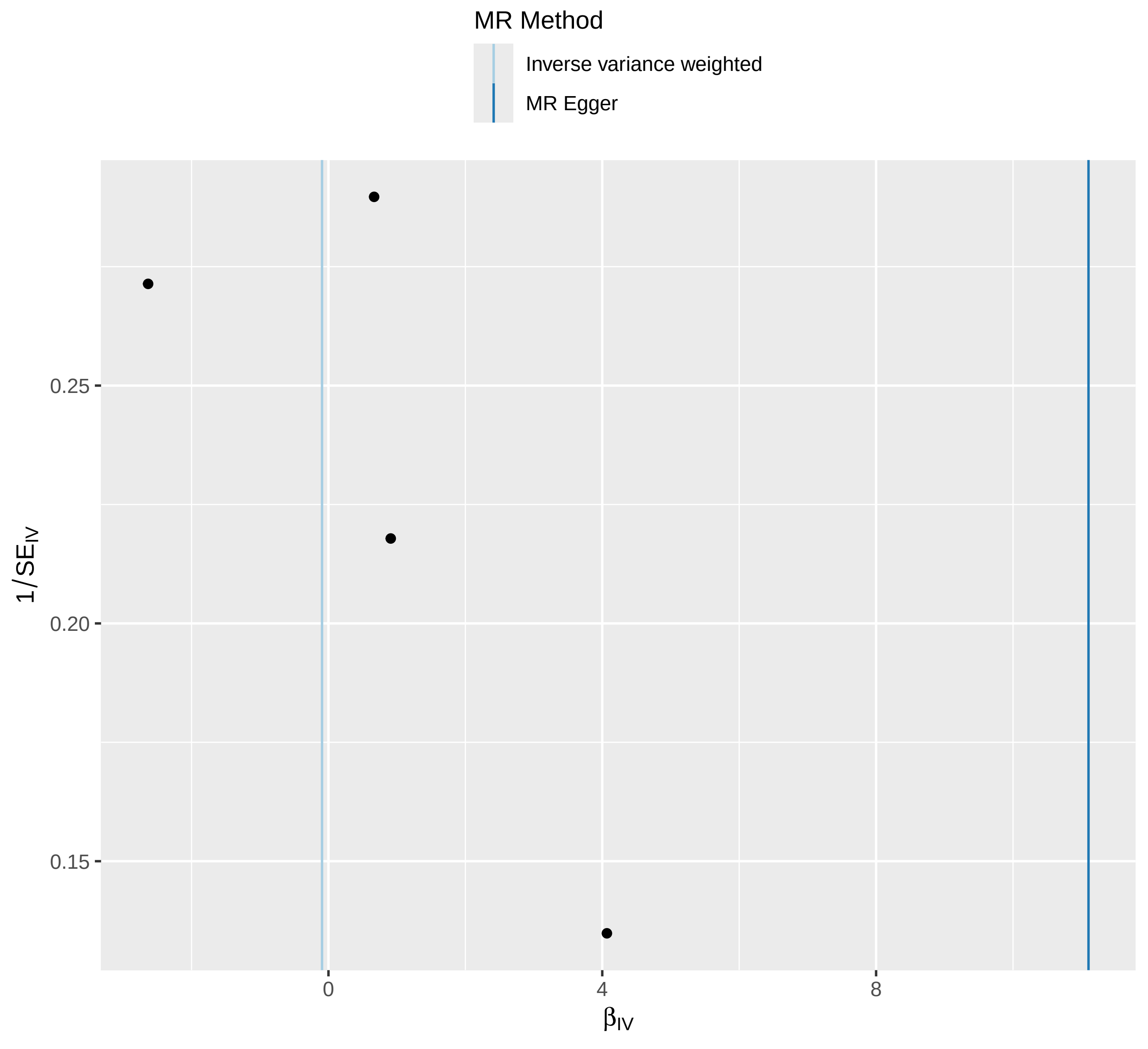
MR Method
Inverse variance weighted
MR Egger
0.25
0.20
0.15
$1/SE_{IV}$
0
4
8
β_{IV}

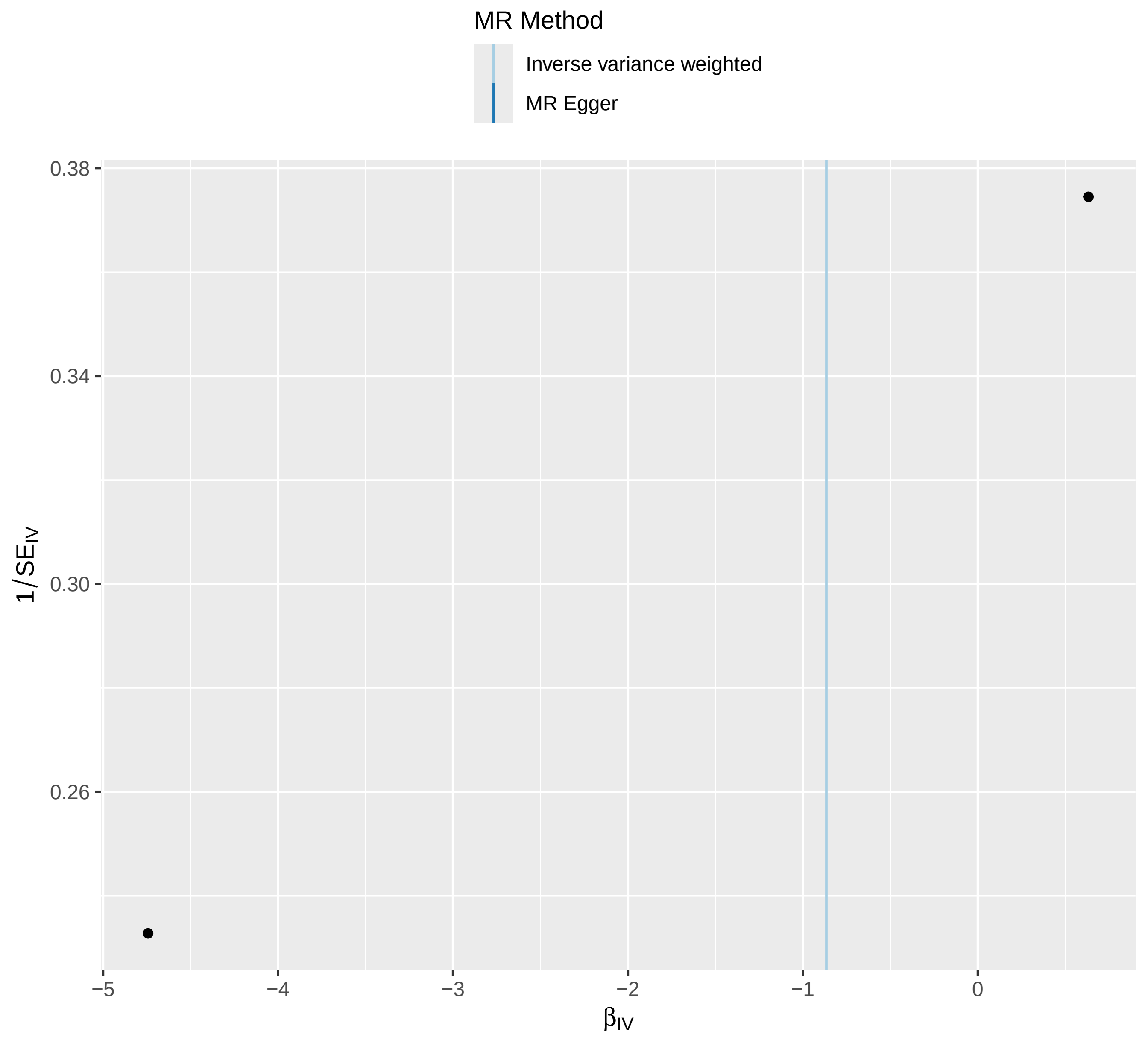
MR Method
Inverse variance weighted
MR Egger
0.38
0.34
0.30
0.26
1/SE_IV
−5
−4
−3
−2
−1
0
β_IV

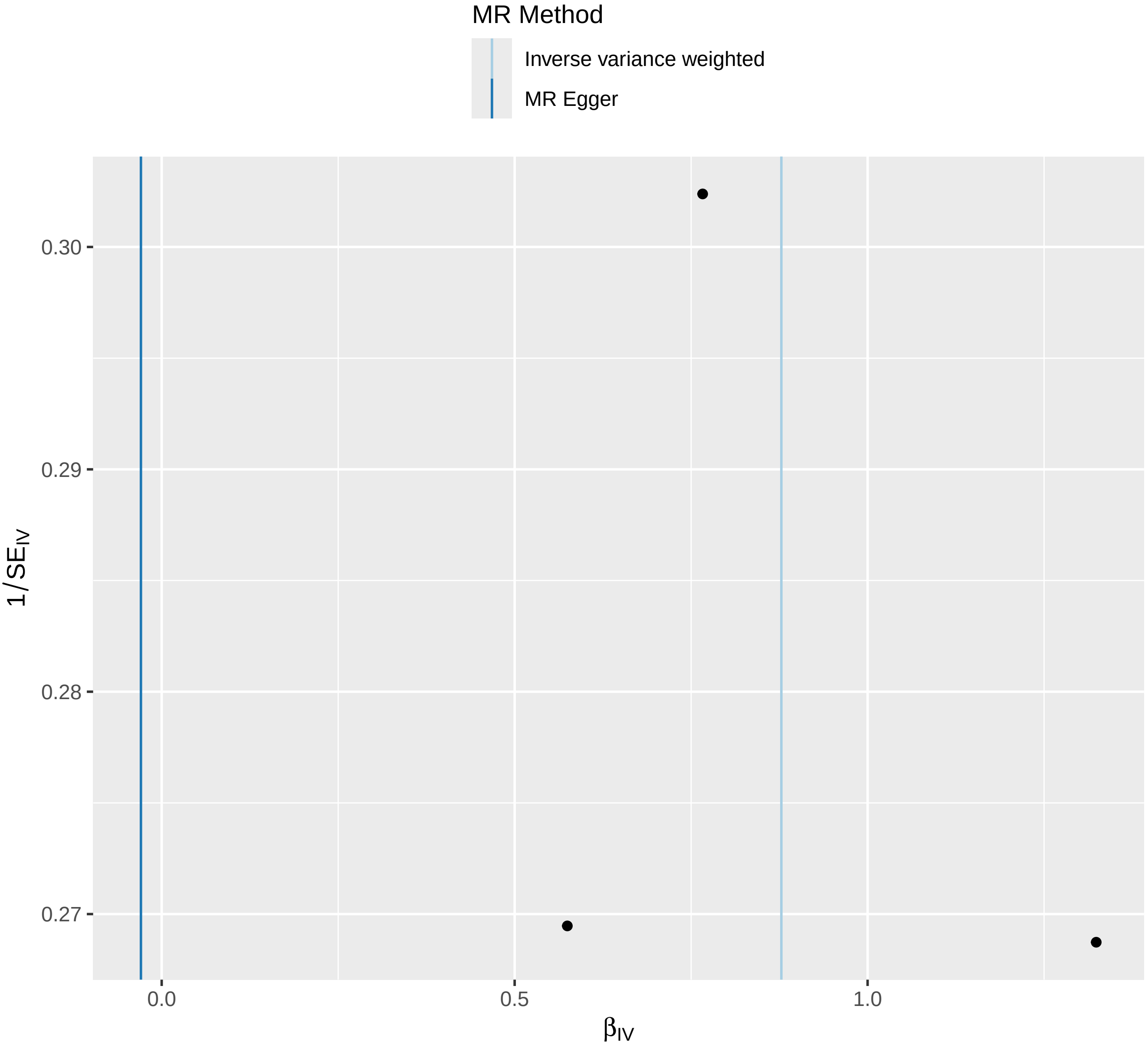
MR Method
Inverse variance weighted
MR Egger
0.30
0.29
0.28
0.27
1/SE_IV
0.0
0.5
1.0
β_IV

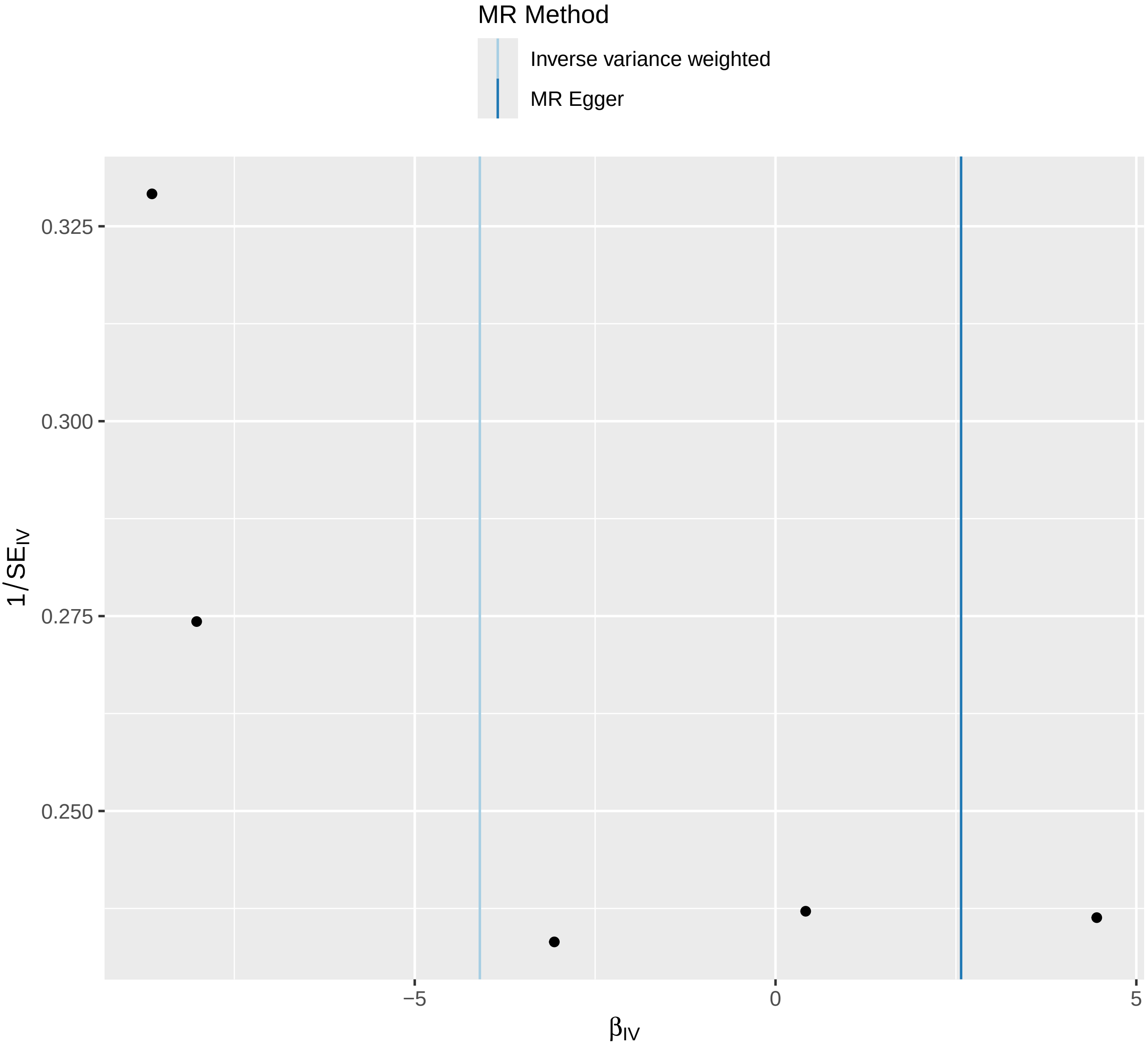
MR Method
Inverse variance weighted
MR Egger
0.325
0.300
0.275
0.250
1/SE_IV
−5
0
5
β_IV

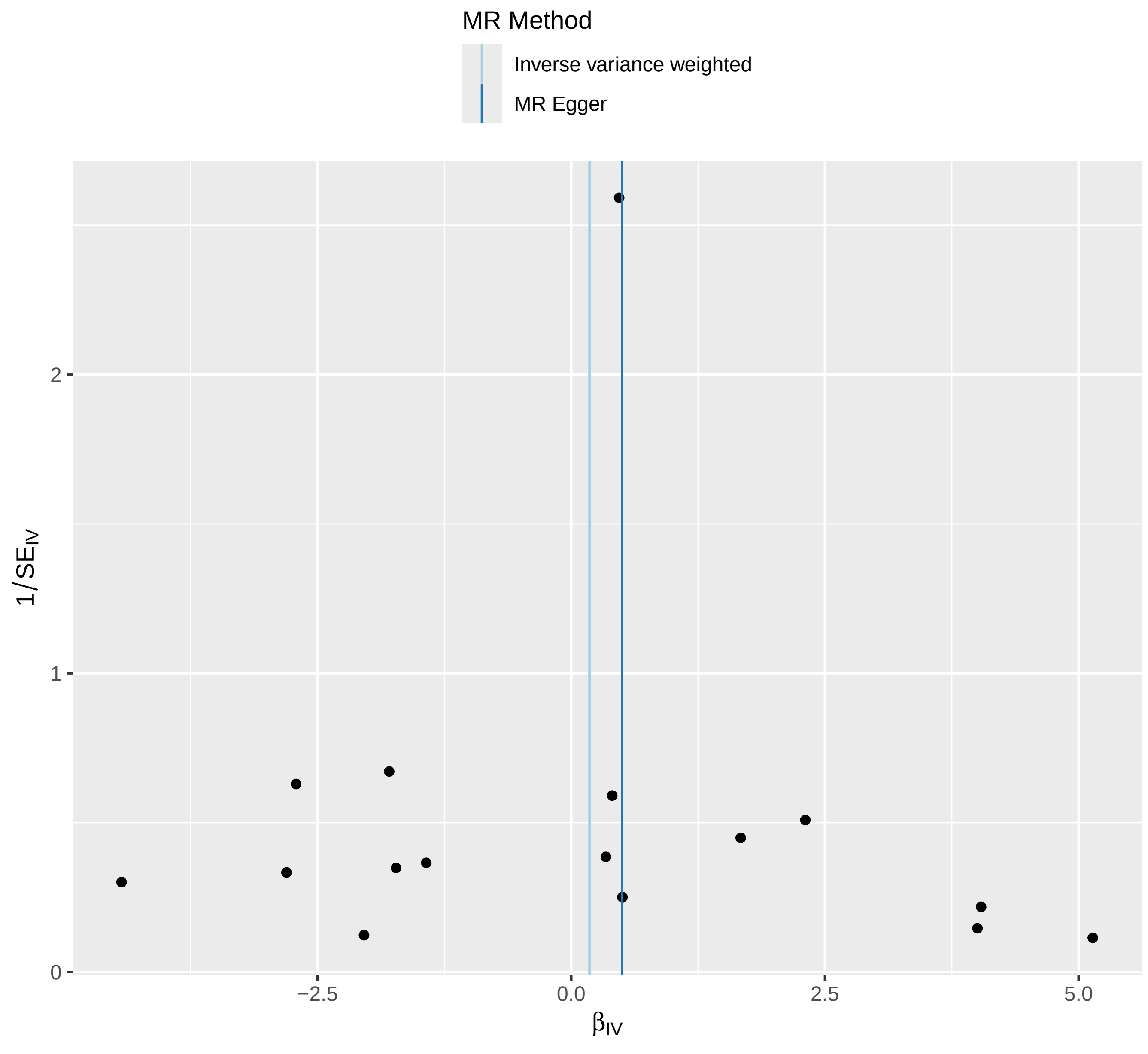
MR Method
Inverse variance weighted
MR Egger
1/SE$_{IV}$
2
1
0
−2.5
0.0
2.5
5.0
β$_{IV}$

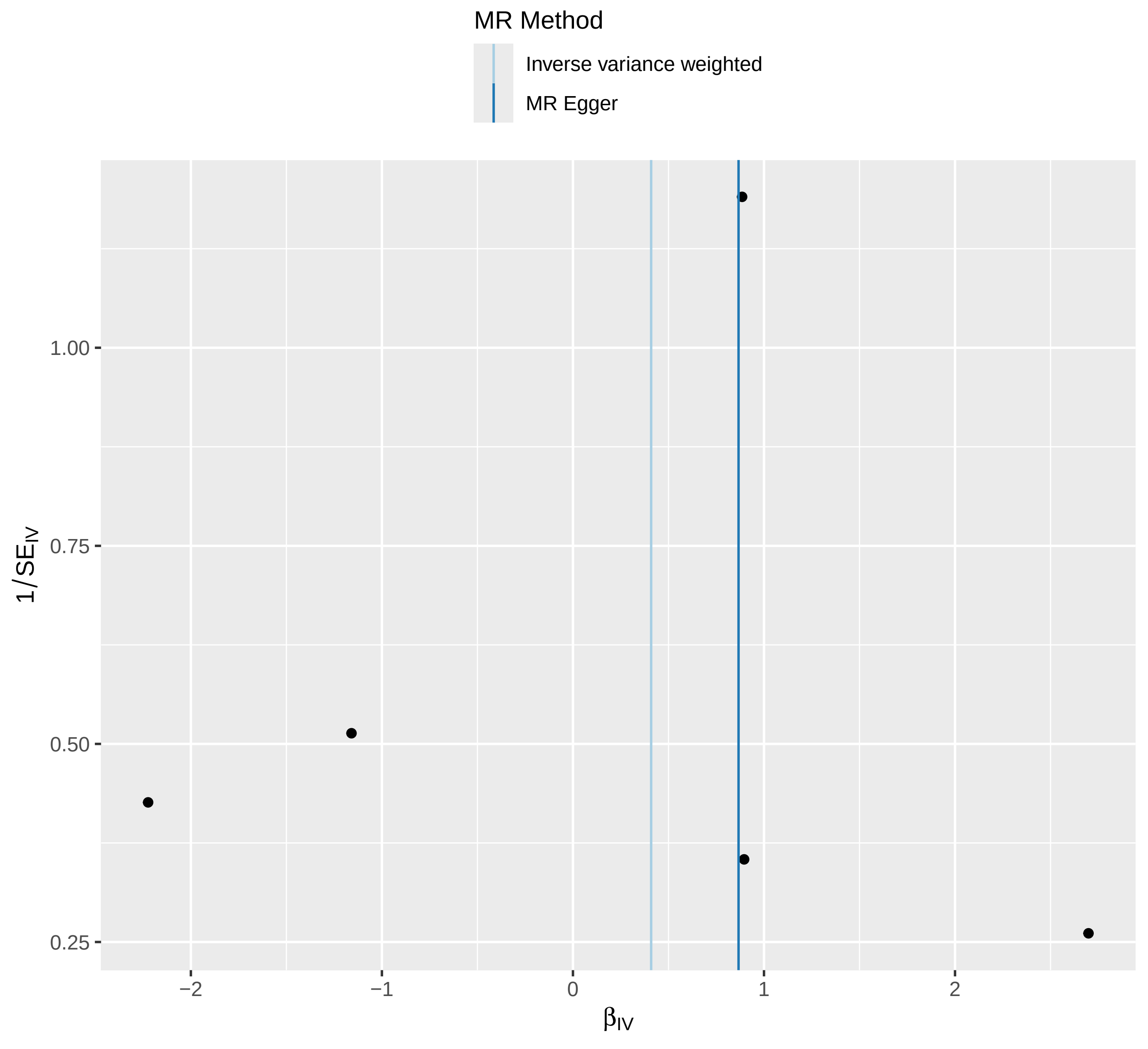
MR Method
Inverse variance weighted
MR Egger
1.00
0.75
0.50
0.25
1/SE$_{IV}$
−2
−1
0
1
2
β$_{IV}$

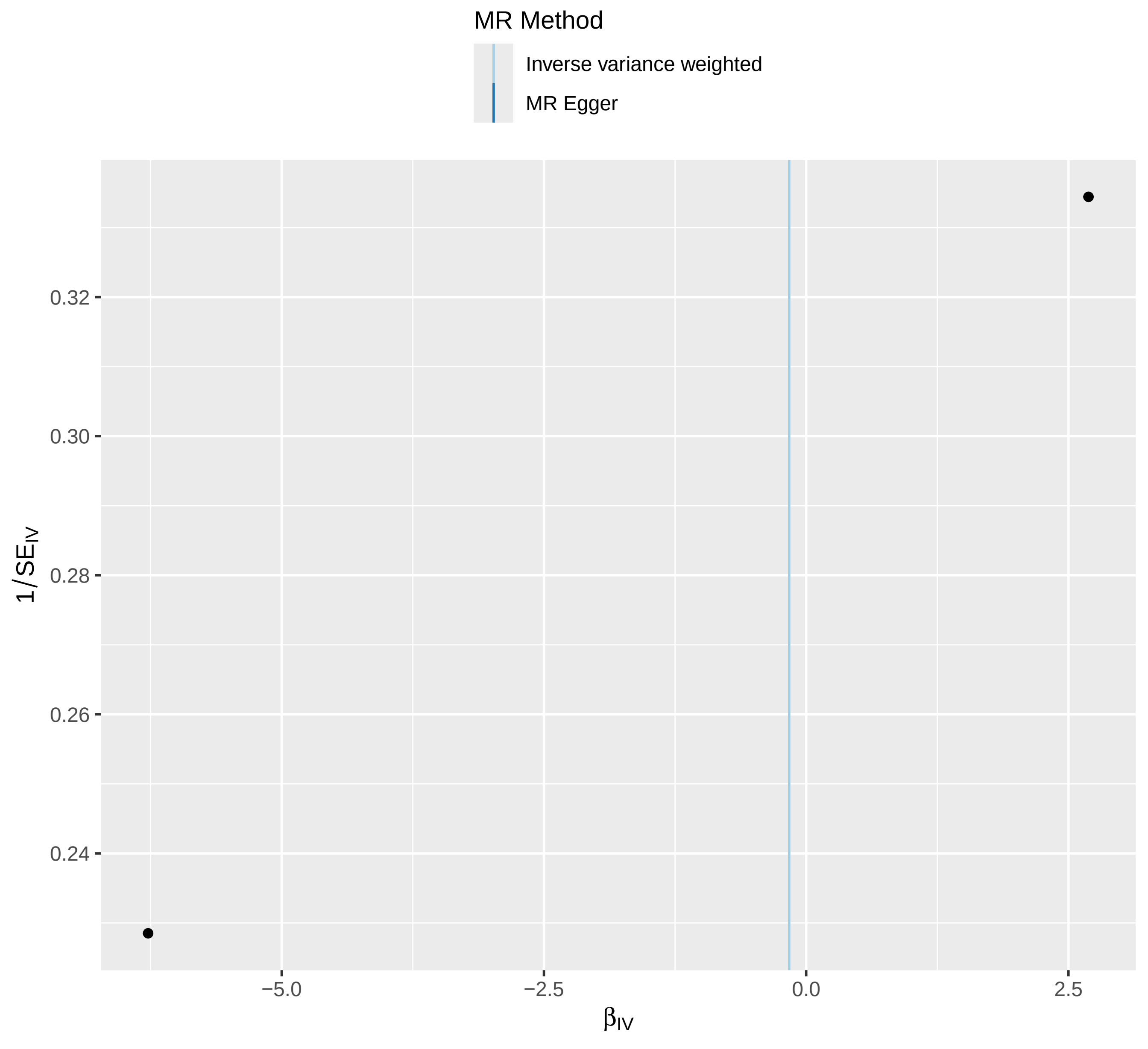

MR Method
Inverse variance weighted
MR Egger
0.32
0.30
0.28
0.26
0.24
1/SE_IV
−5.0
−2.5
0.0
2.5
β_IV

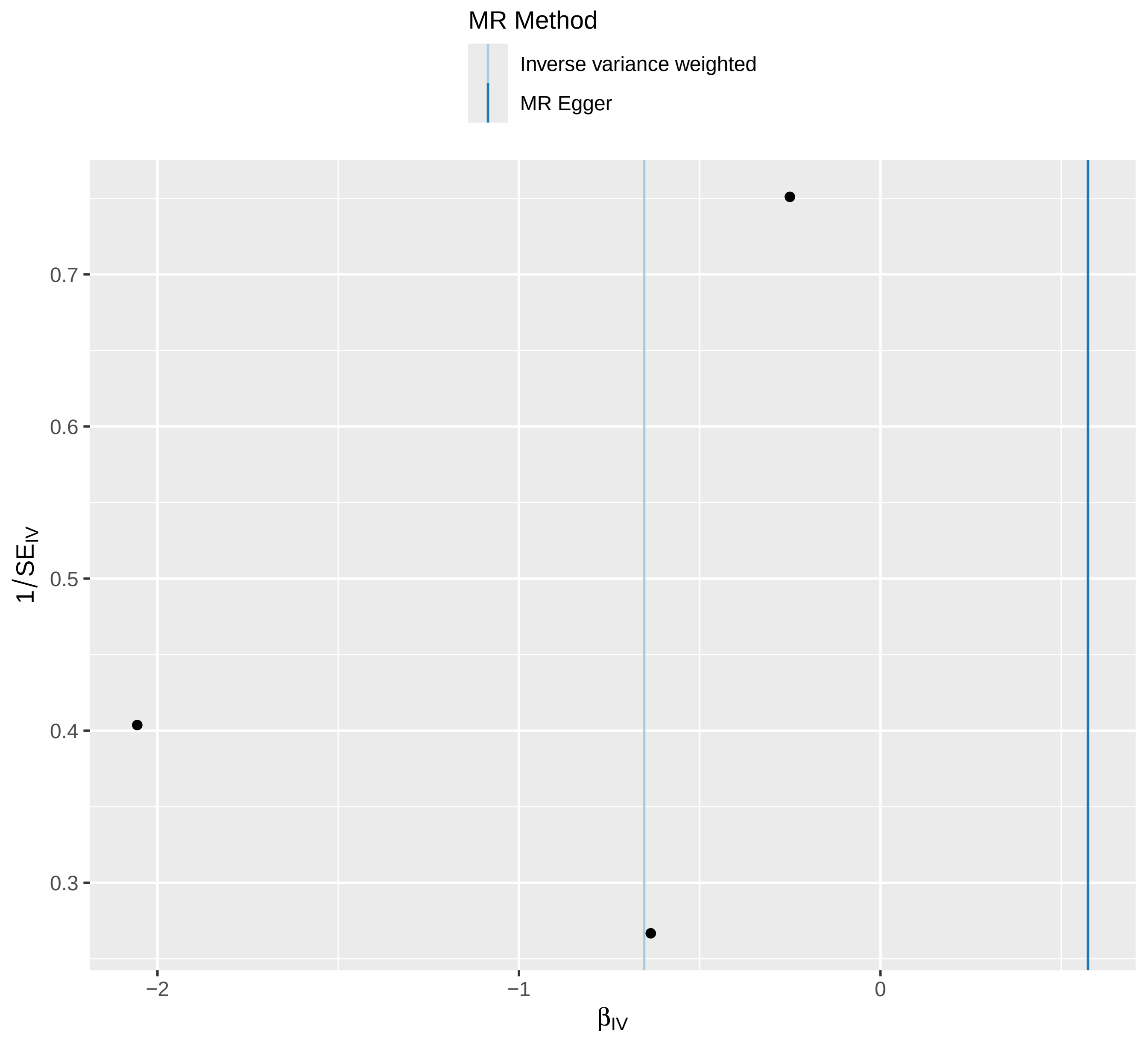
MR Method
Inverse variance weighted
MR Egger
0.7
0.6
0.5
0.4
0.3
1/SE_IV
−2
−1
0
β_IV

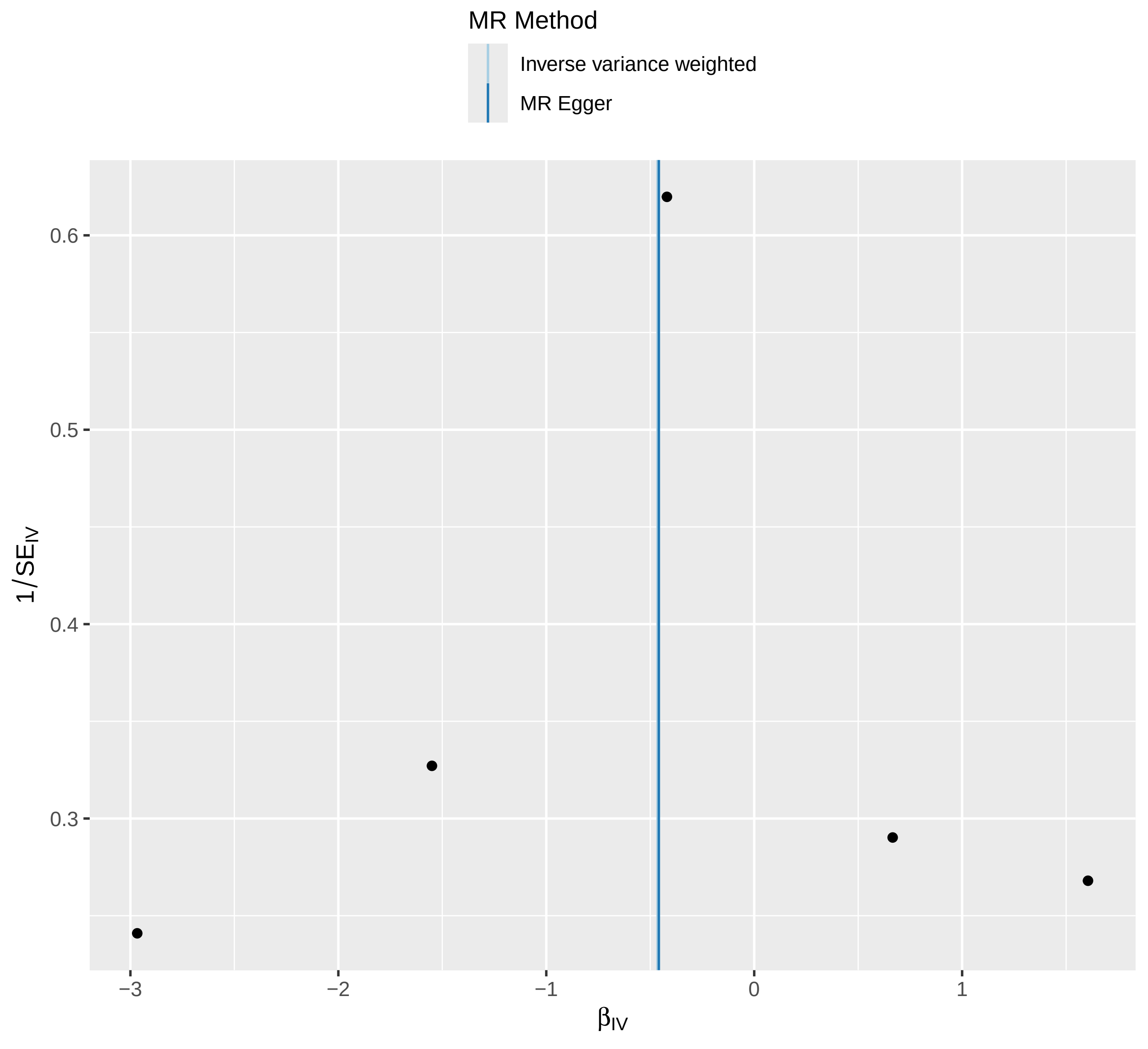
MR Method
Inverse variance weighted
MR Egger
0.6
0.5
0.4
0.3
$1/SE_{IV}$
−3
−2
−1
0
1
β_{IV}

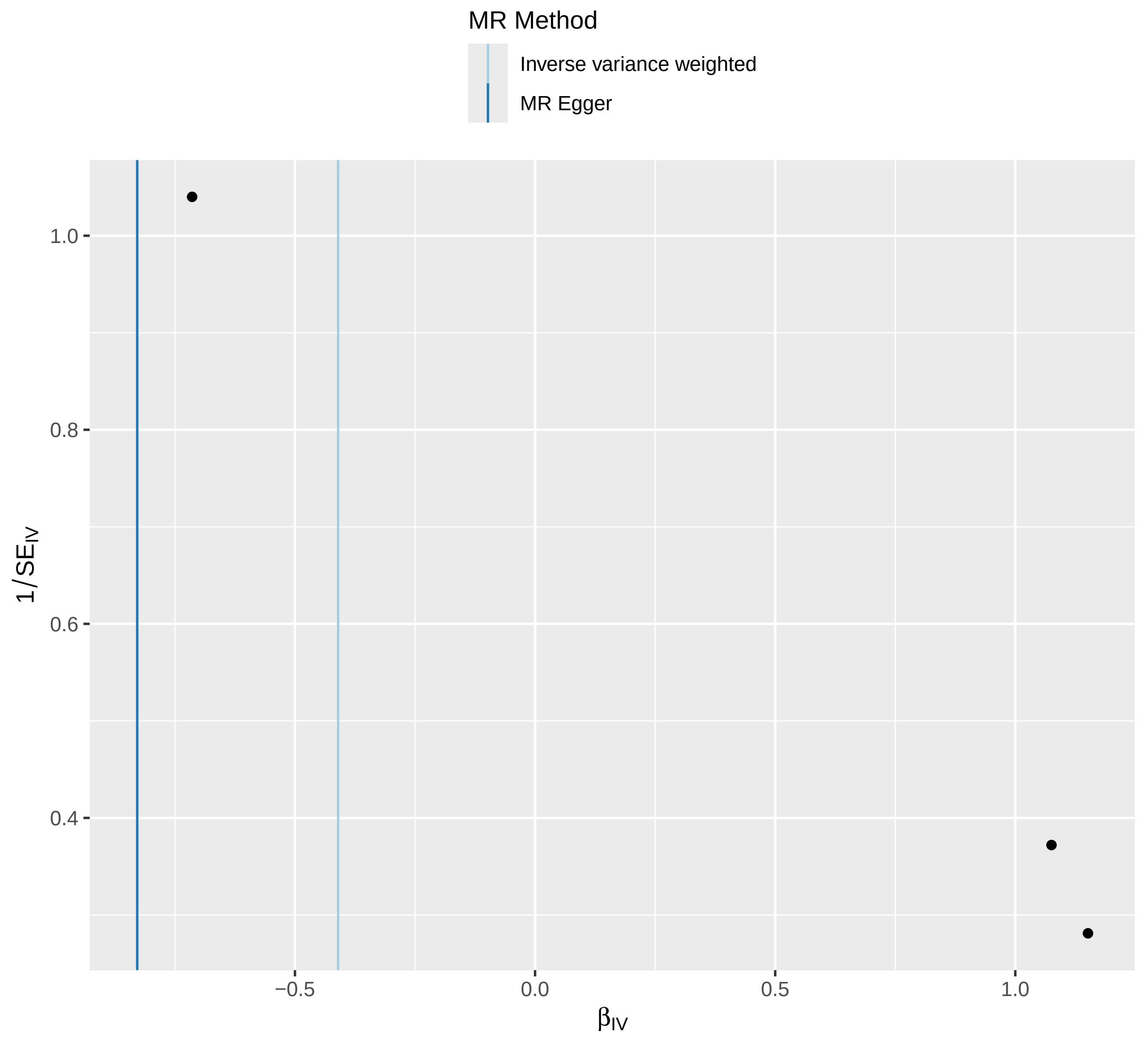
MR Method
Inverse variance weighted
MR Egger
1.0
0.8
0.6
0.4
1/SE_IV
−0.5
0.0
0.5
1.0
β_IV

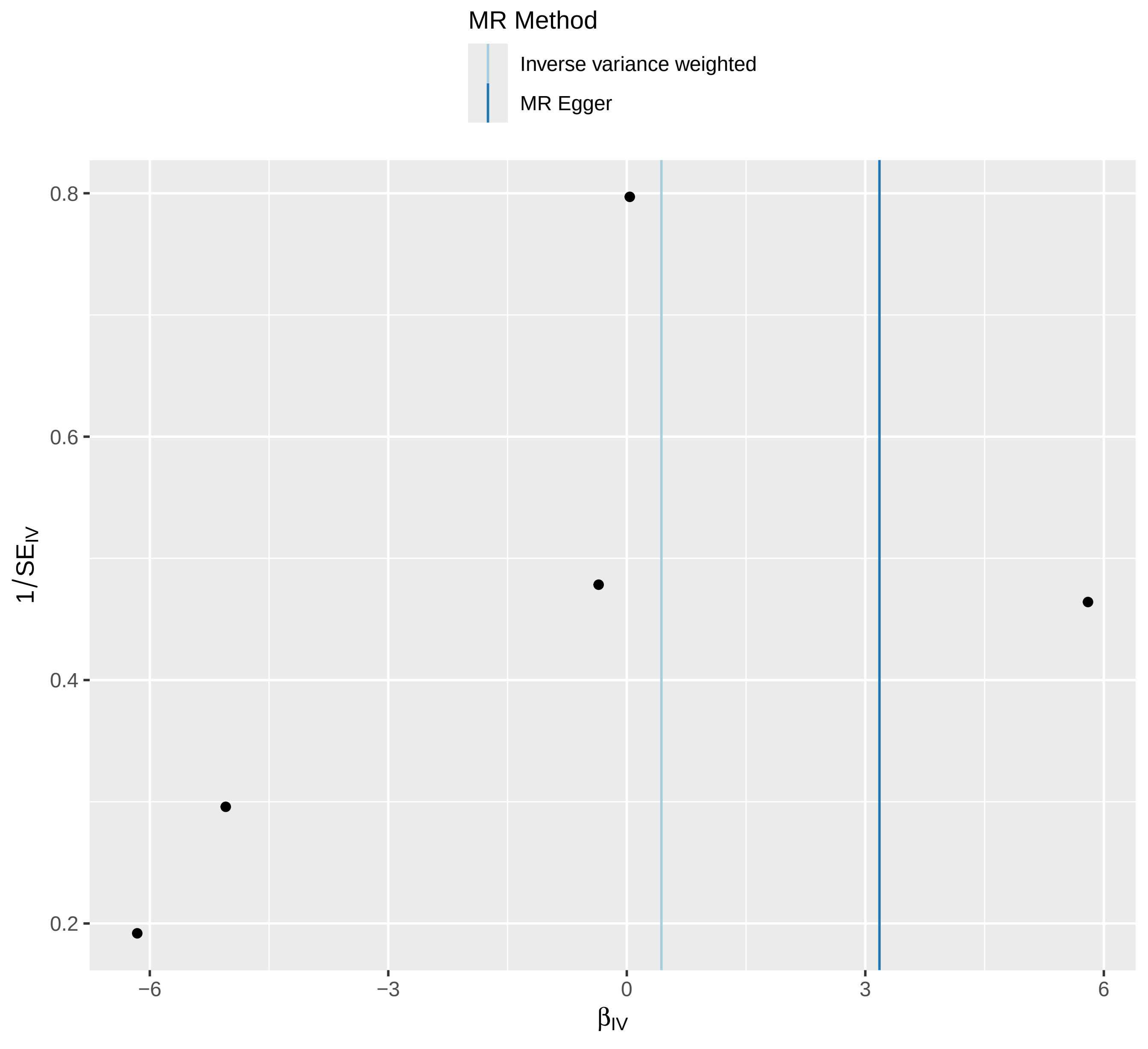
MR Method
Inverse variance weighted
MR Egger
0.8
0.6
0.4
0.2
1/SE$_{IV}$
−6
−3
0
3
6
β$_{IV}$

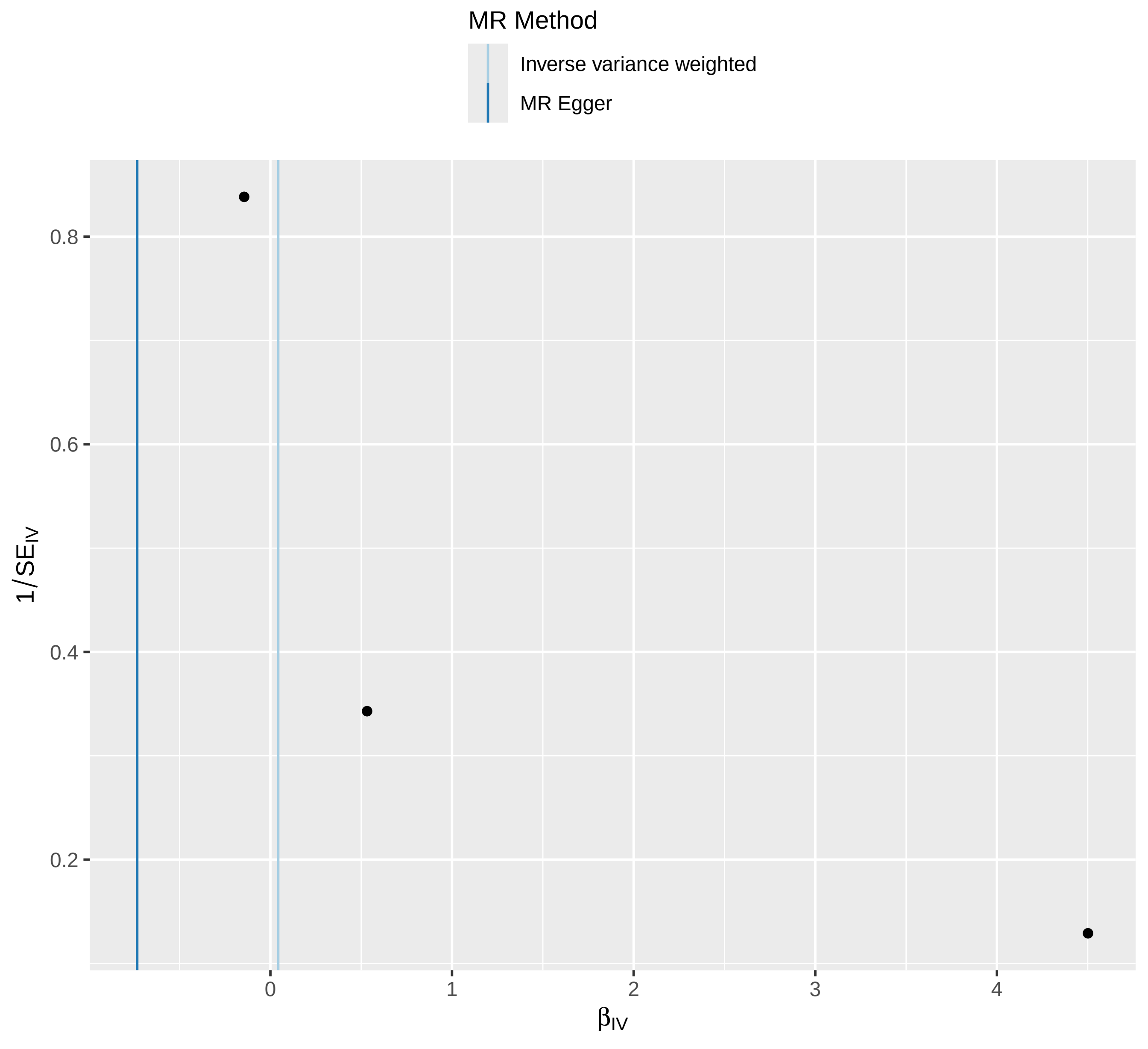
MR Method
Inverse variance weighted
MR Egger
0.8
0.6
0.4
0.2
1/SE_IV
0
1
2
3
4
β_IV

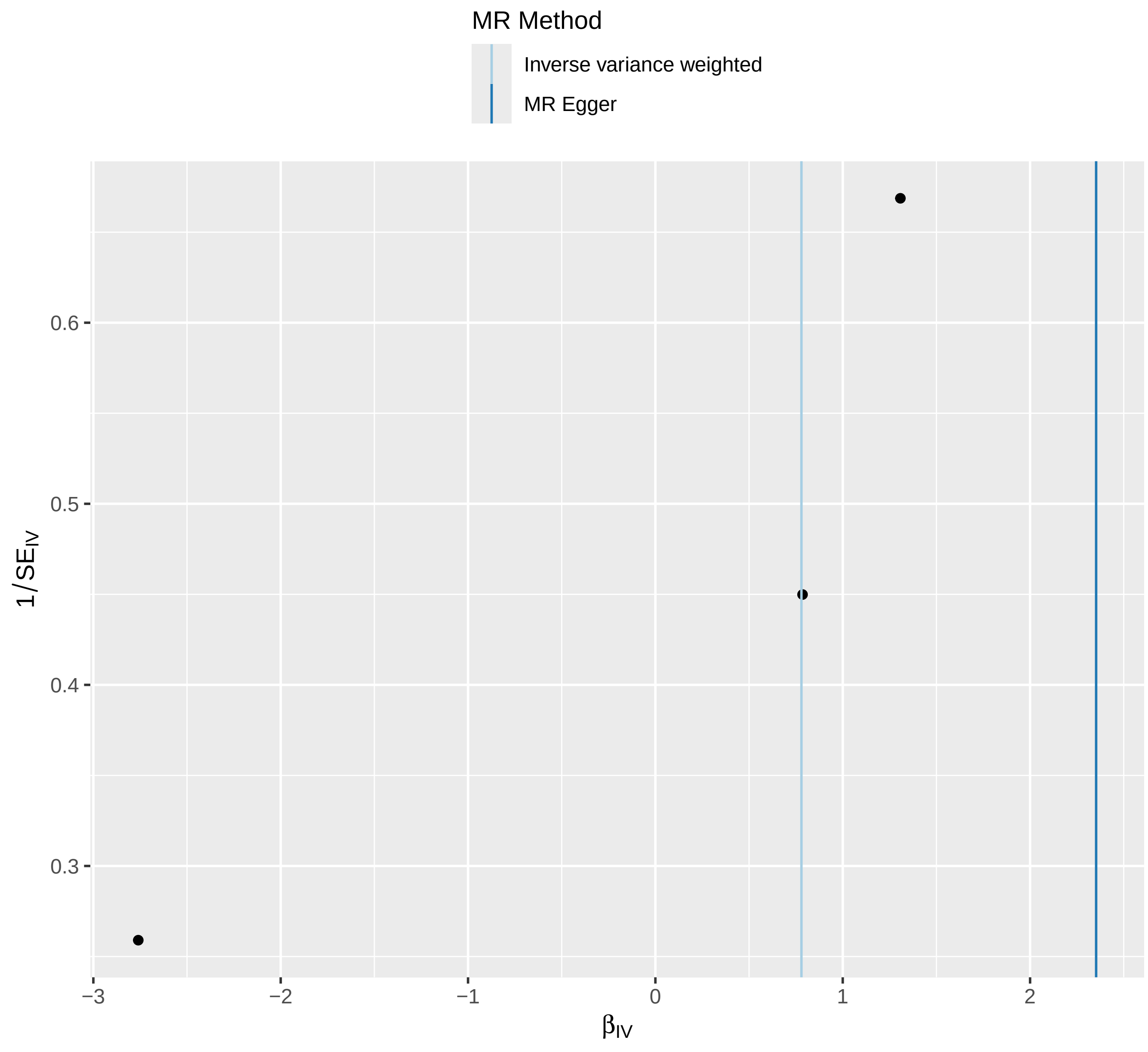
MR Method
Inverse variance weighted
MR Egger
0.6
0.5
0.4
0.3
1/SE$_{IV}$
−3
−2
−1
0
1
2
β_{IV}

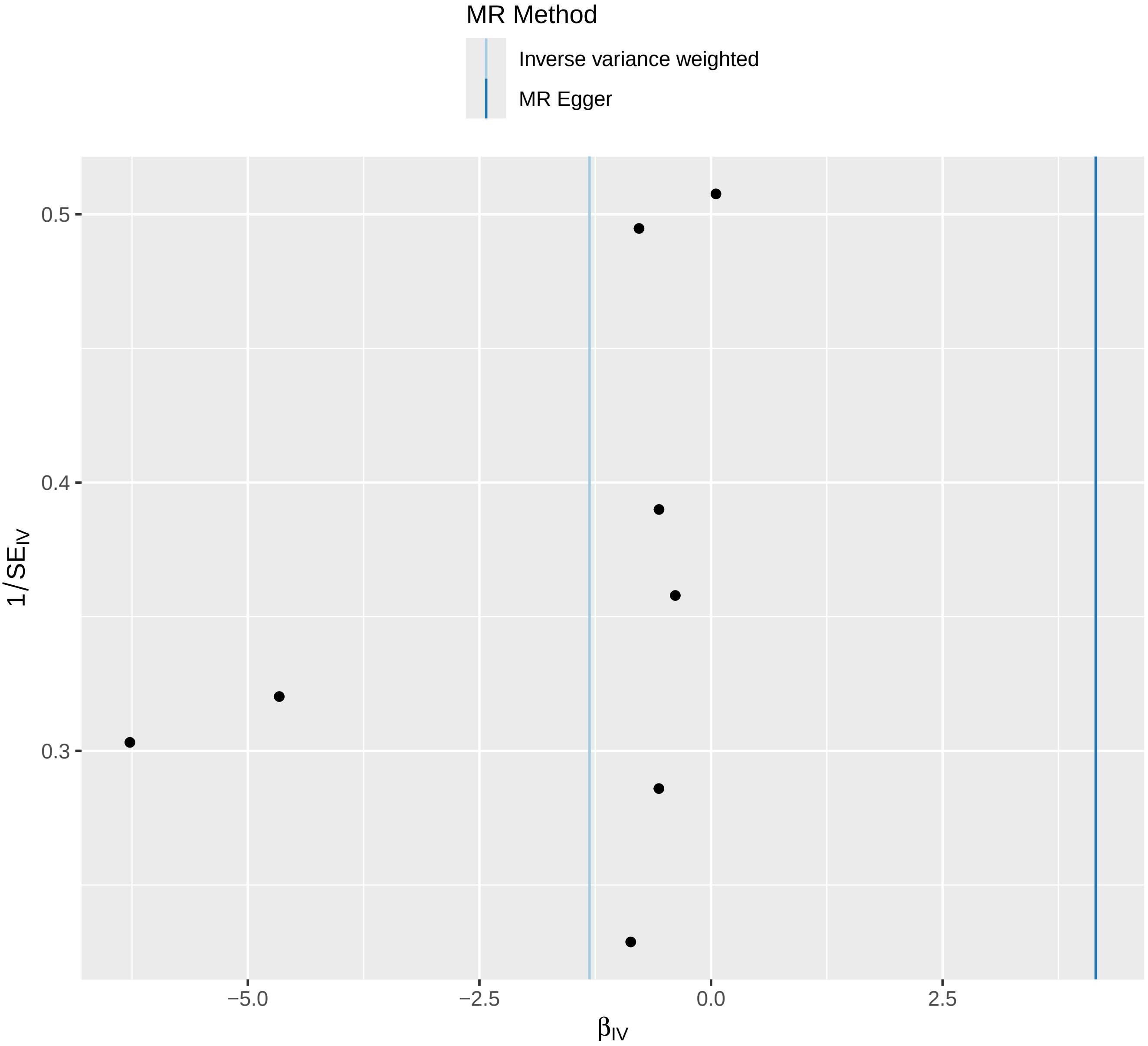
MR Method
Inverse variance weighted
MR Egger
0.5
0.4
0.3
1/SE_IV
−5.0
−2.5
0.0
2.5
β_IV

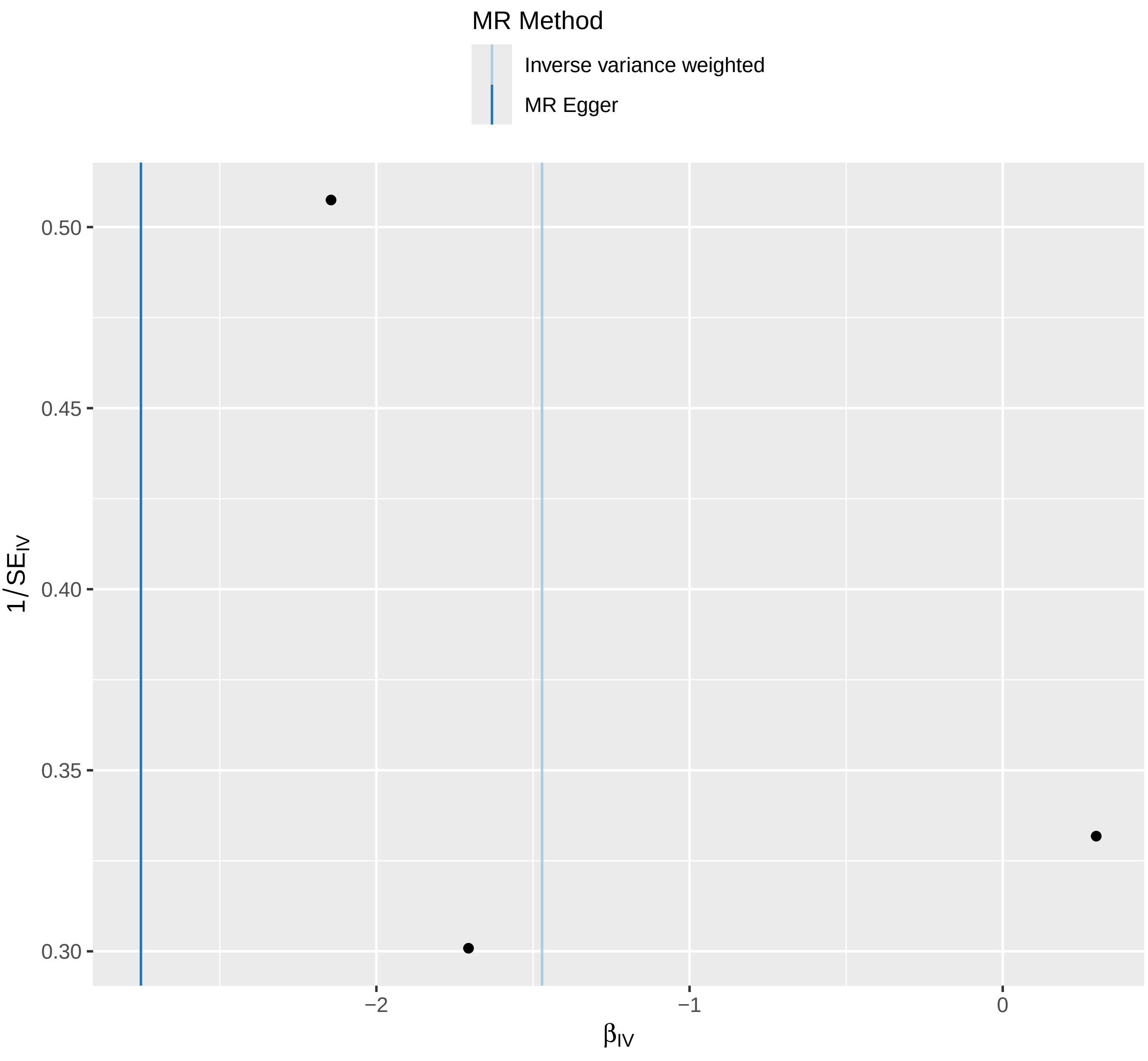
MR Method
Inverse variance weighted
MR Egger
0.50
0.45
0.40
0.35
0.30
$1/SE_{IV}$
−2
−1
0
β_{IV}

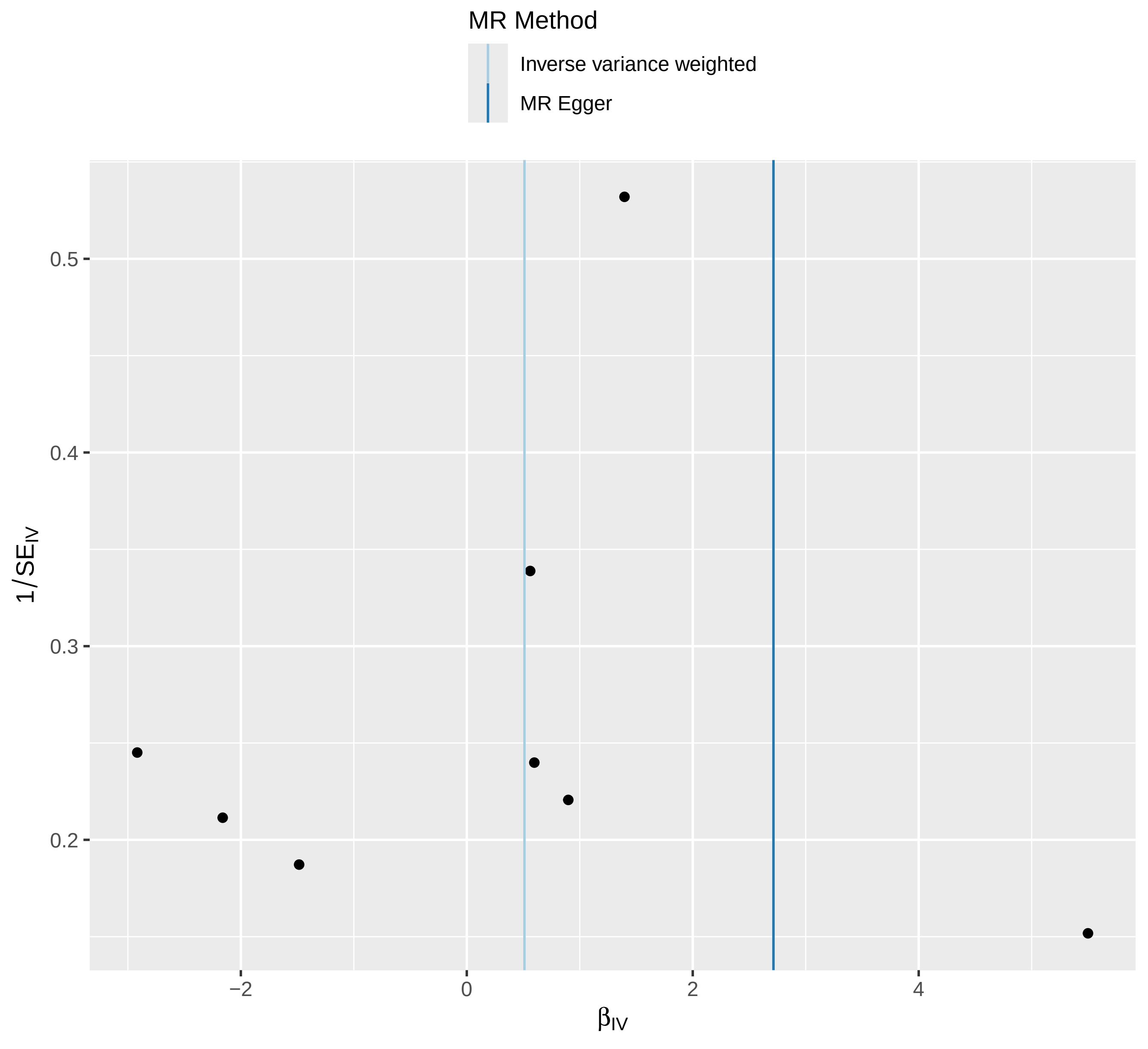

MR Method
Inverse variance weighted
MR Egger
0.5
0.4
0.3
0.2
1/SE$_{IV}$
−2
0
2
4
β_{IV}

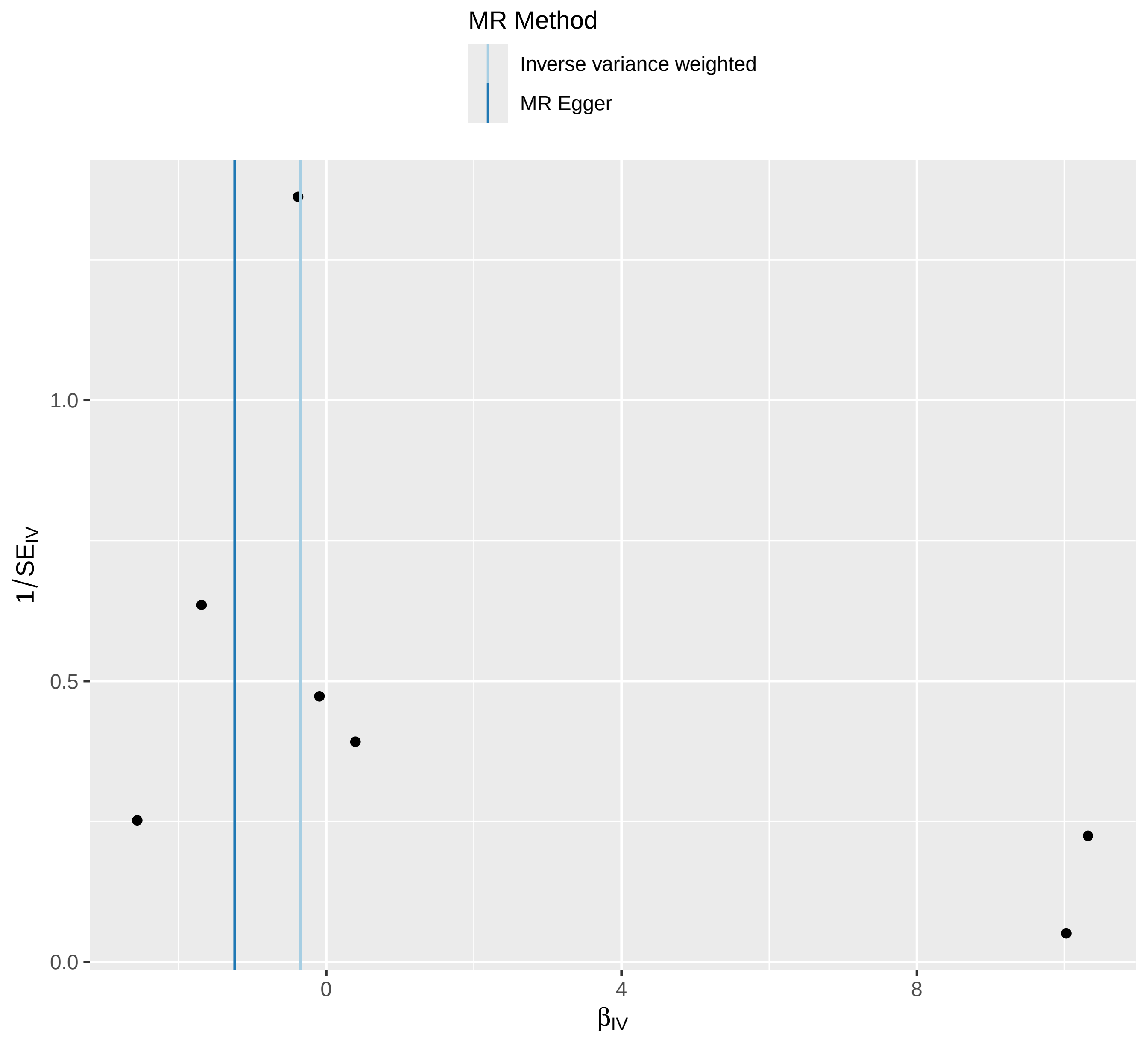
MR Method
Inverse variance weighted
MR Egger
1/SE_IV
1.0
0.5
0.0
0
4
8
β_IV

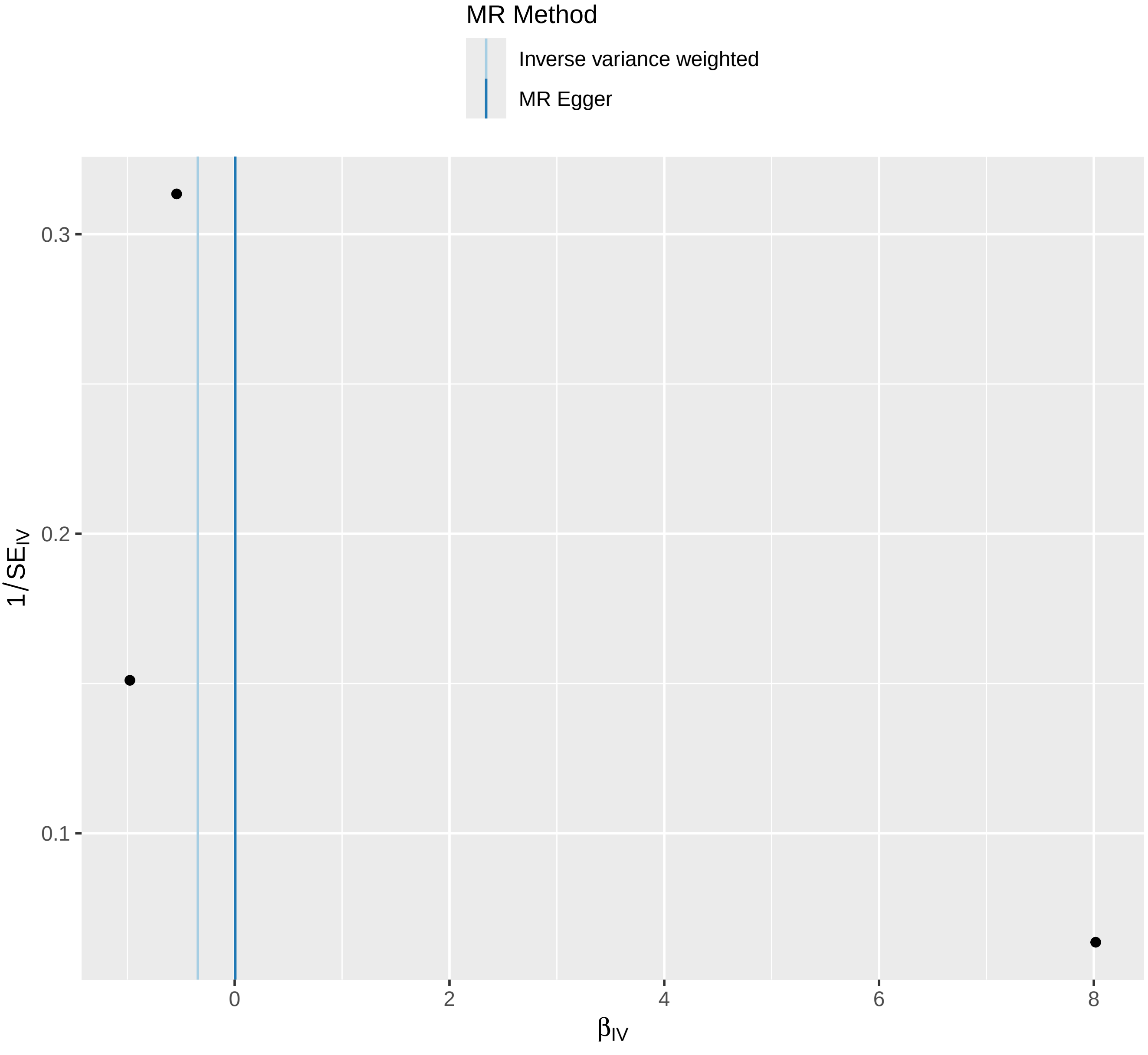

MR Method
Inverse variance weighted
MR Egger
0.3
0.2
0.1
$1/SE_{IV}$
0
2
4
6
8
β_{IV}

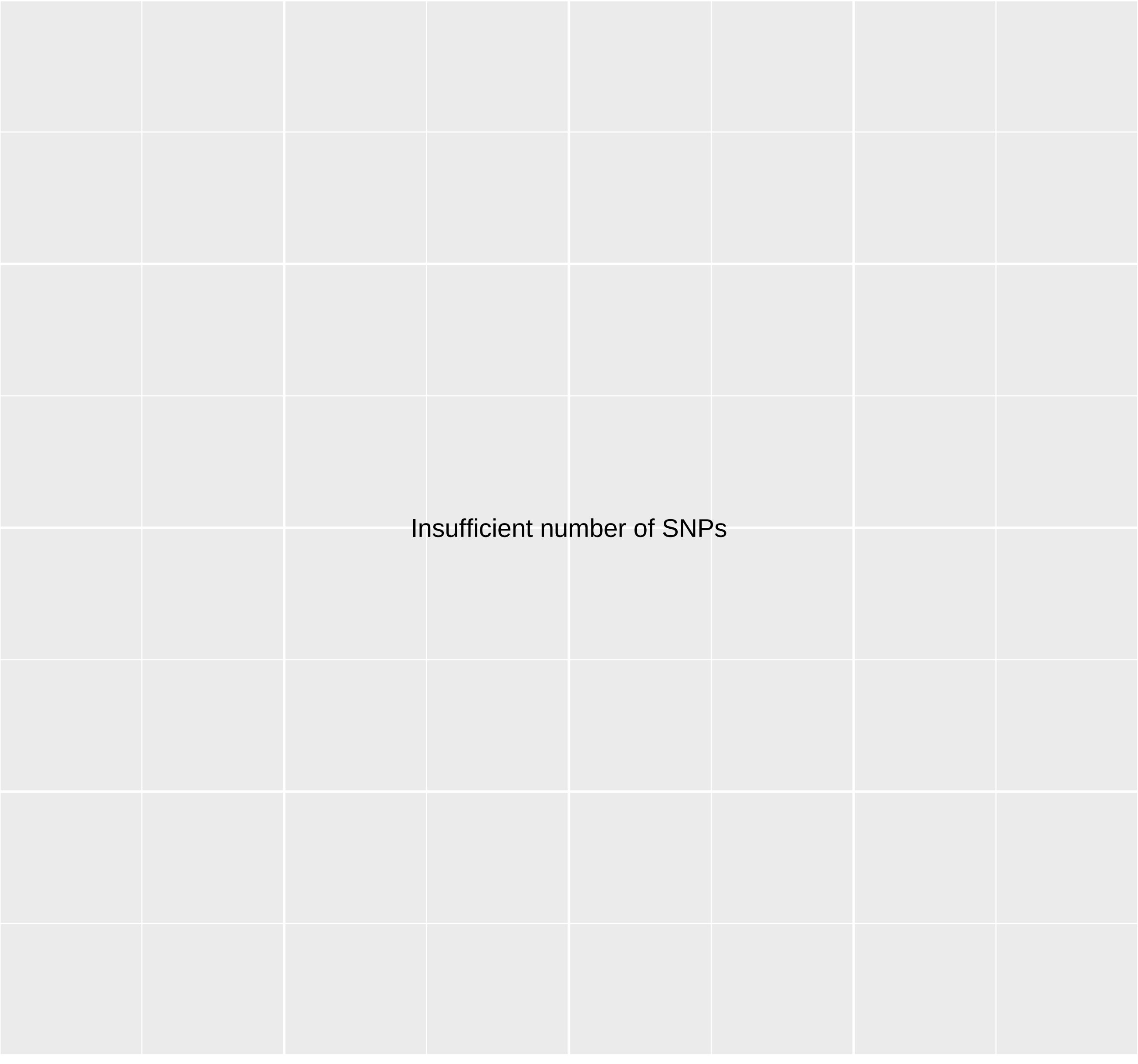
Insufficient number of SNPs

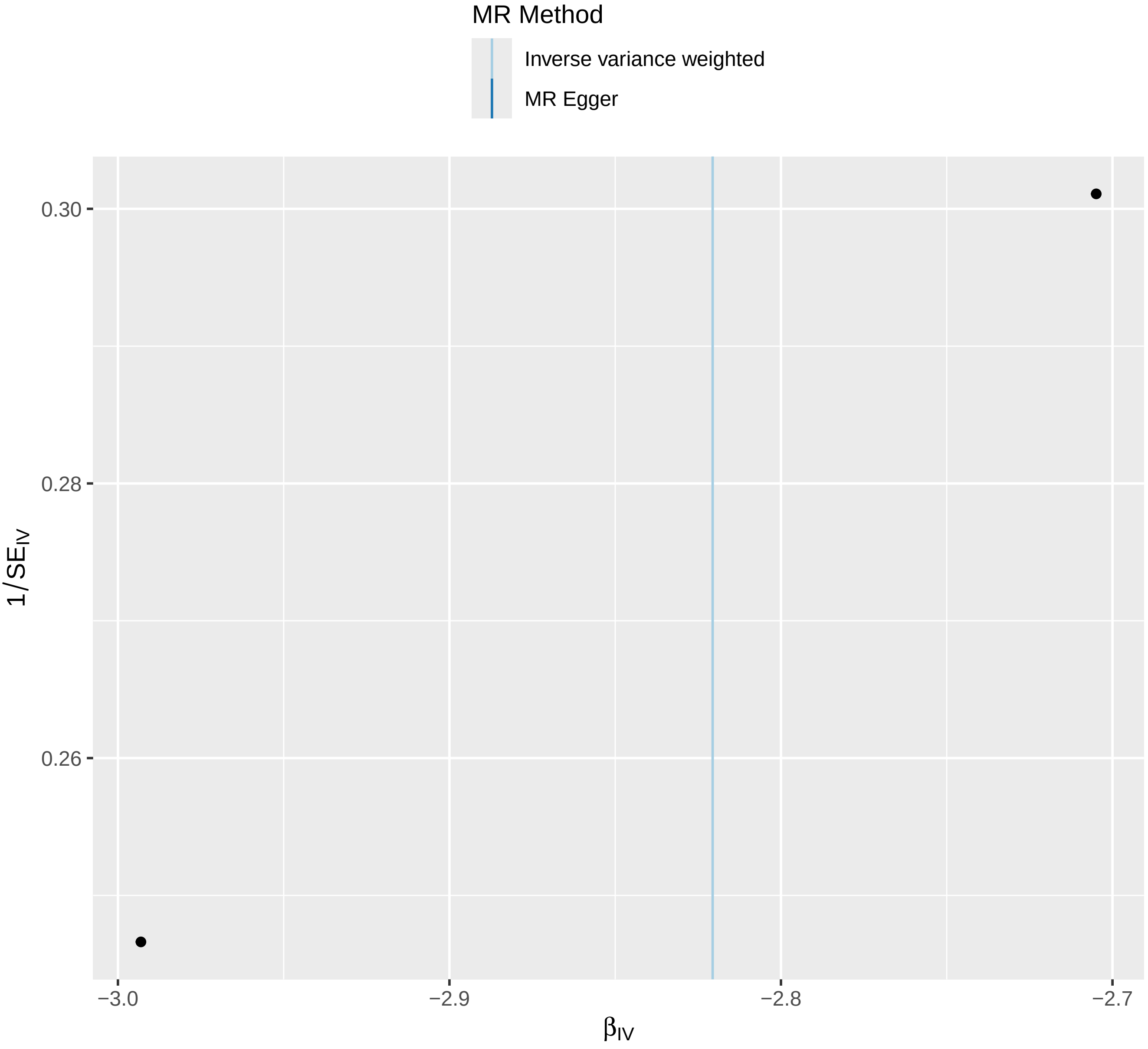
MR Method
Inverse variance weighted
MR Egger
0.30
0.28
0.26
1/SE_IV
−3.0
−2.9
−2.8
−2.7
β_IV

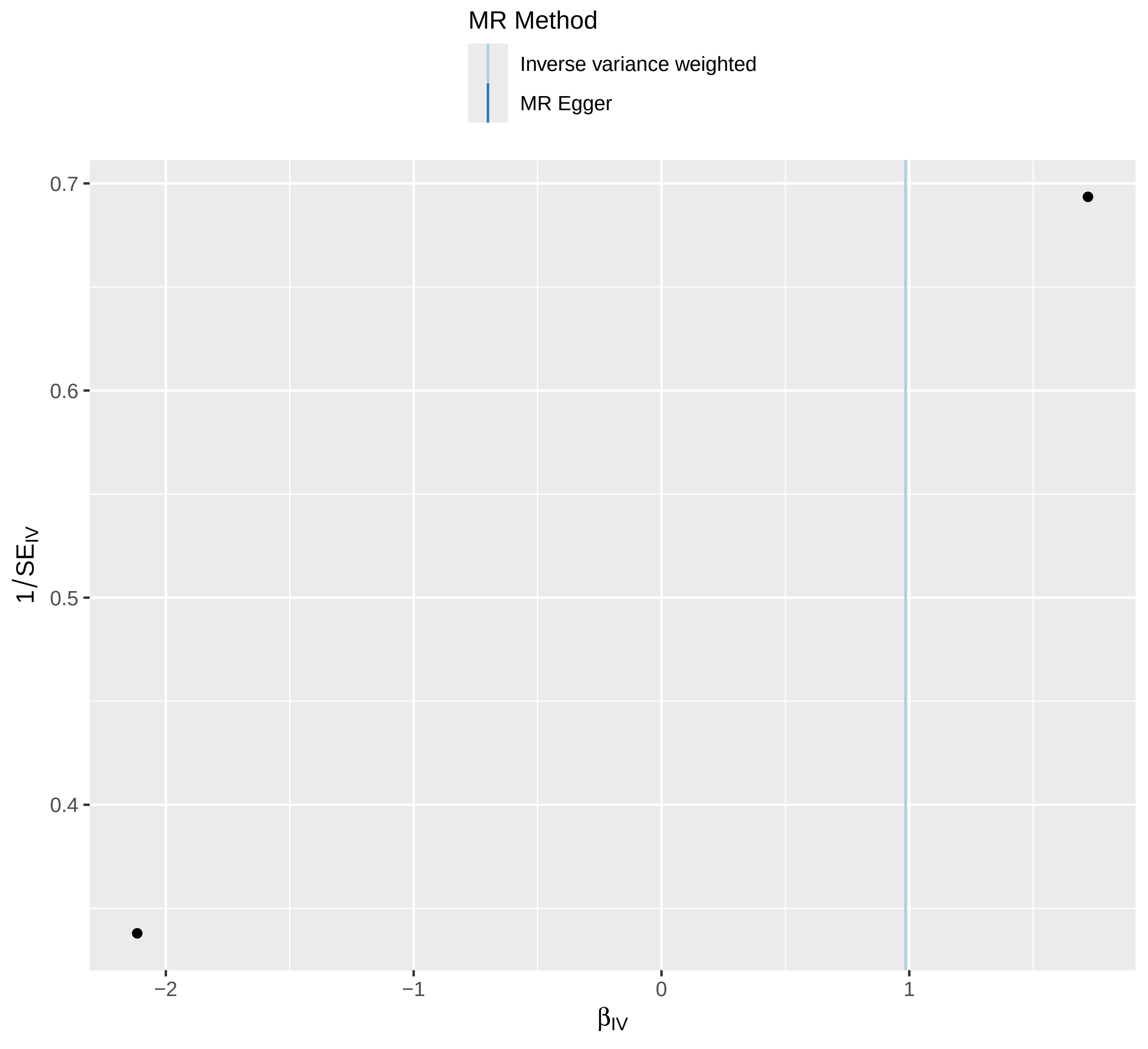
MR Method
Inverse variance weighted
MR Egger
0.7
0.6
0.5
0.4
1/SE_IV
−2
−1
0
1
β_IV

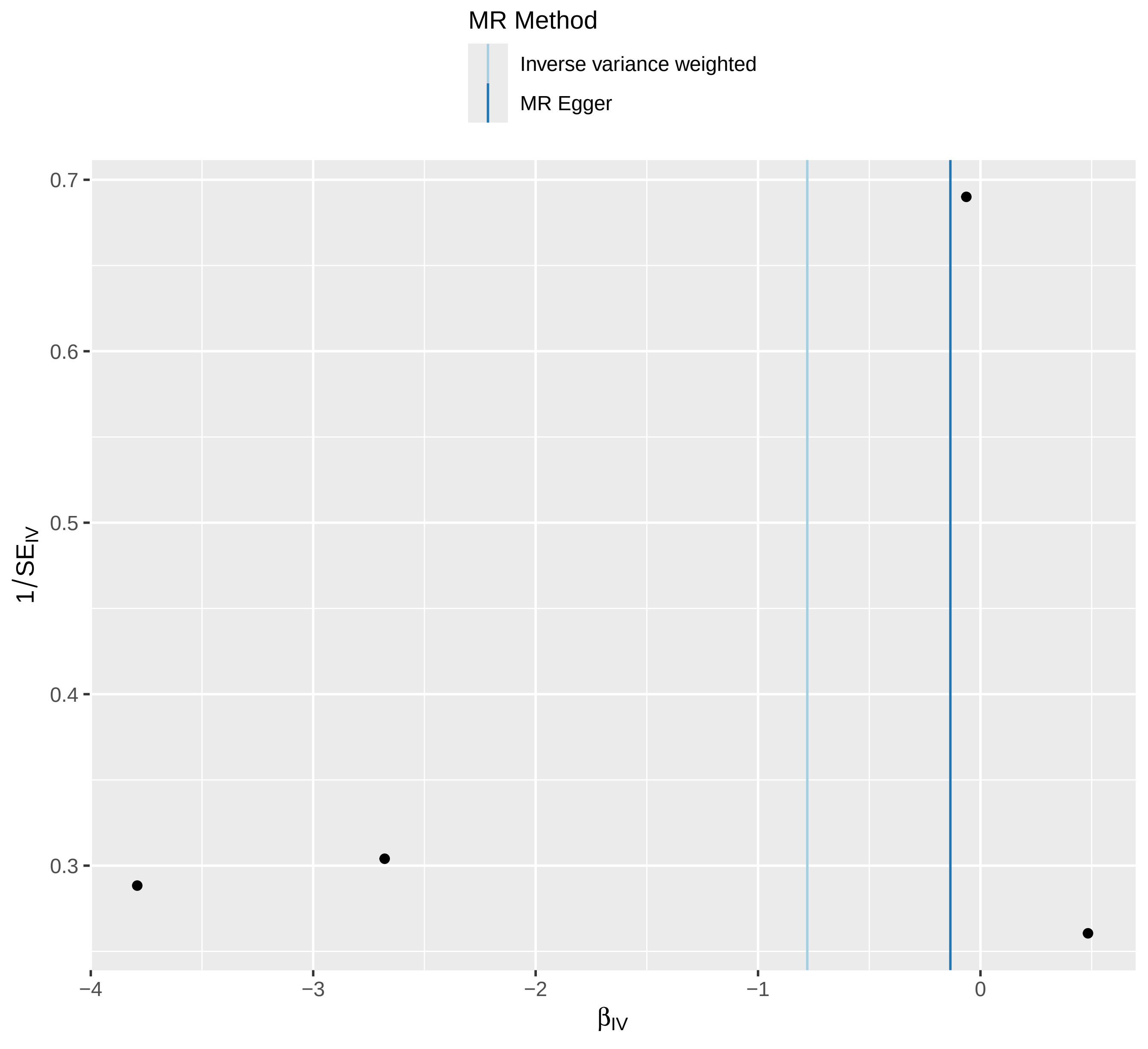
MR Method
Inverse variance weighted
MR Egger
0.7
0.6
0.5
0.4
0.3
1/SE_IV
−4
−3
−2
−1
0
β_IV

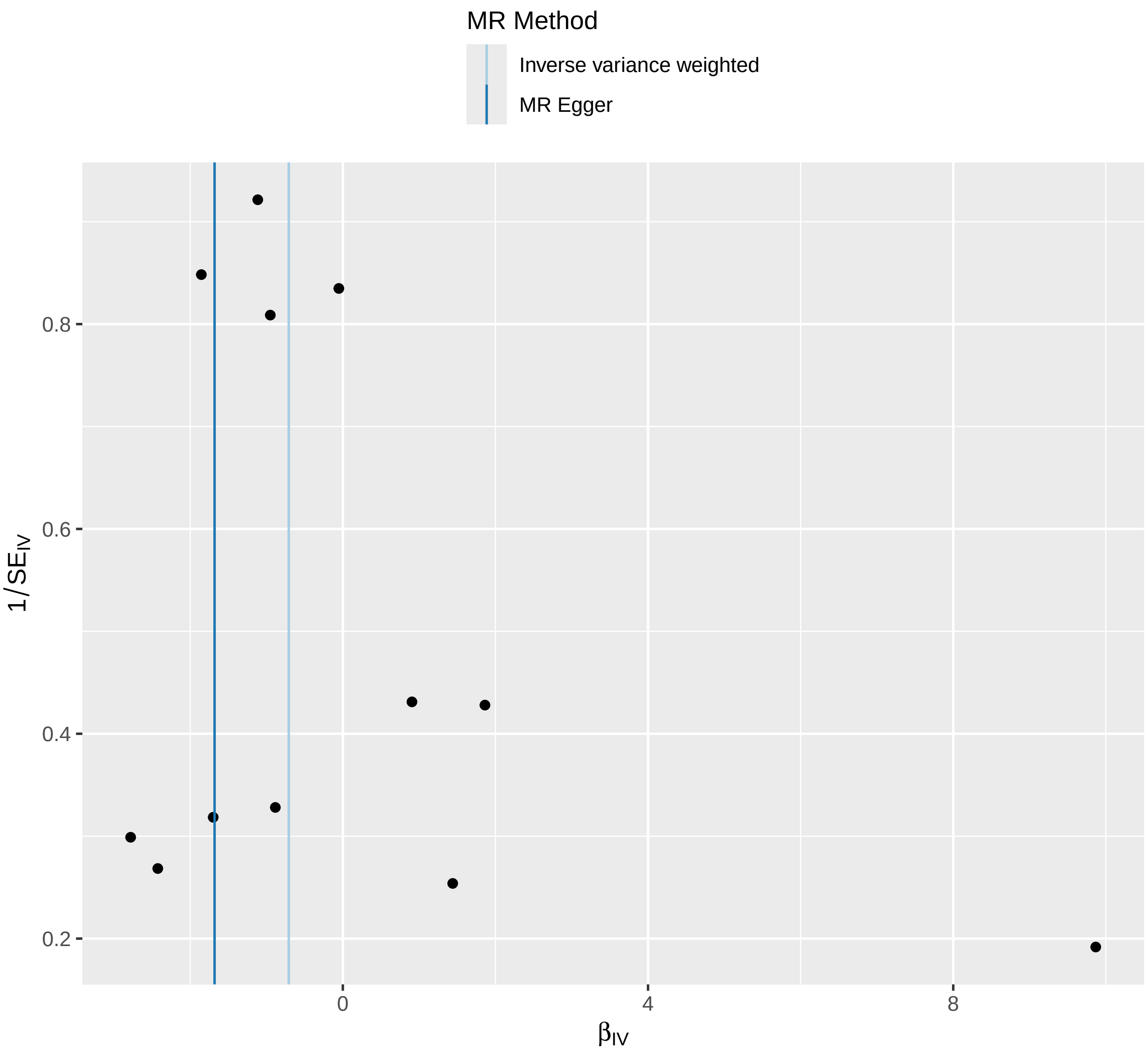
MR Method
Inverse variance weighted
MR Egger
0.8
0.6
0.4
0.2
$1/SE_{IV}$
0
4
8
β_{IV}

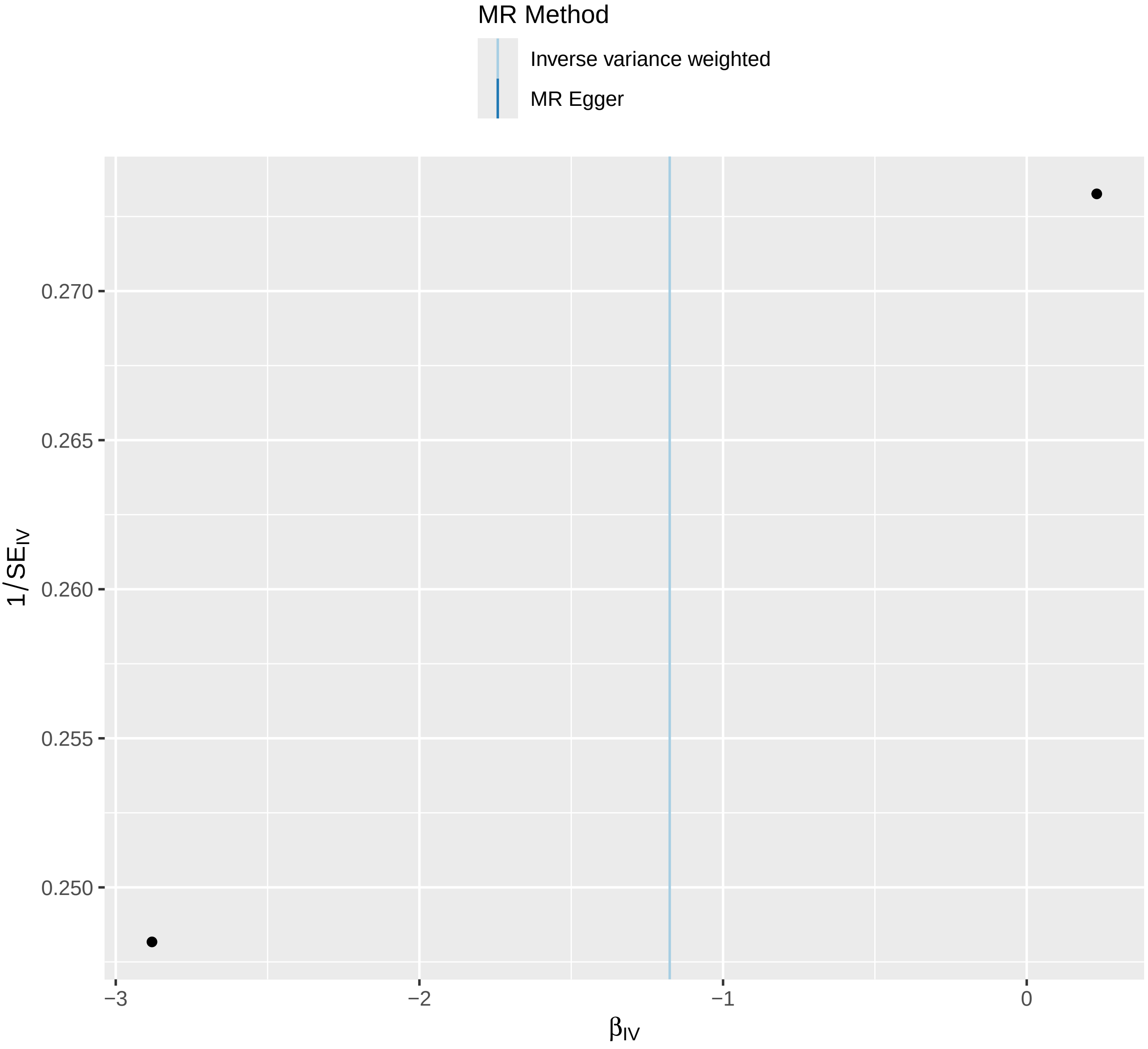

MR Method
Inverse variance weighted
MR Egger
0.270
0.265
0.260
0.255
0.250
1/SE_IV
−3
−2
−1
0
β_IV

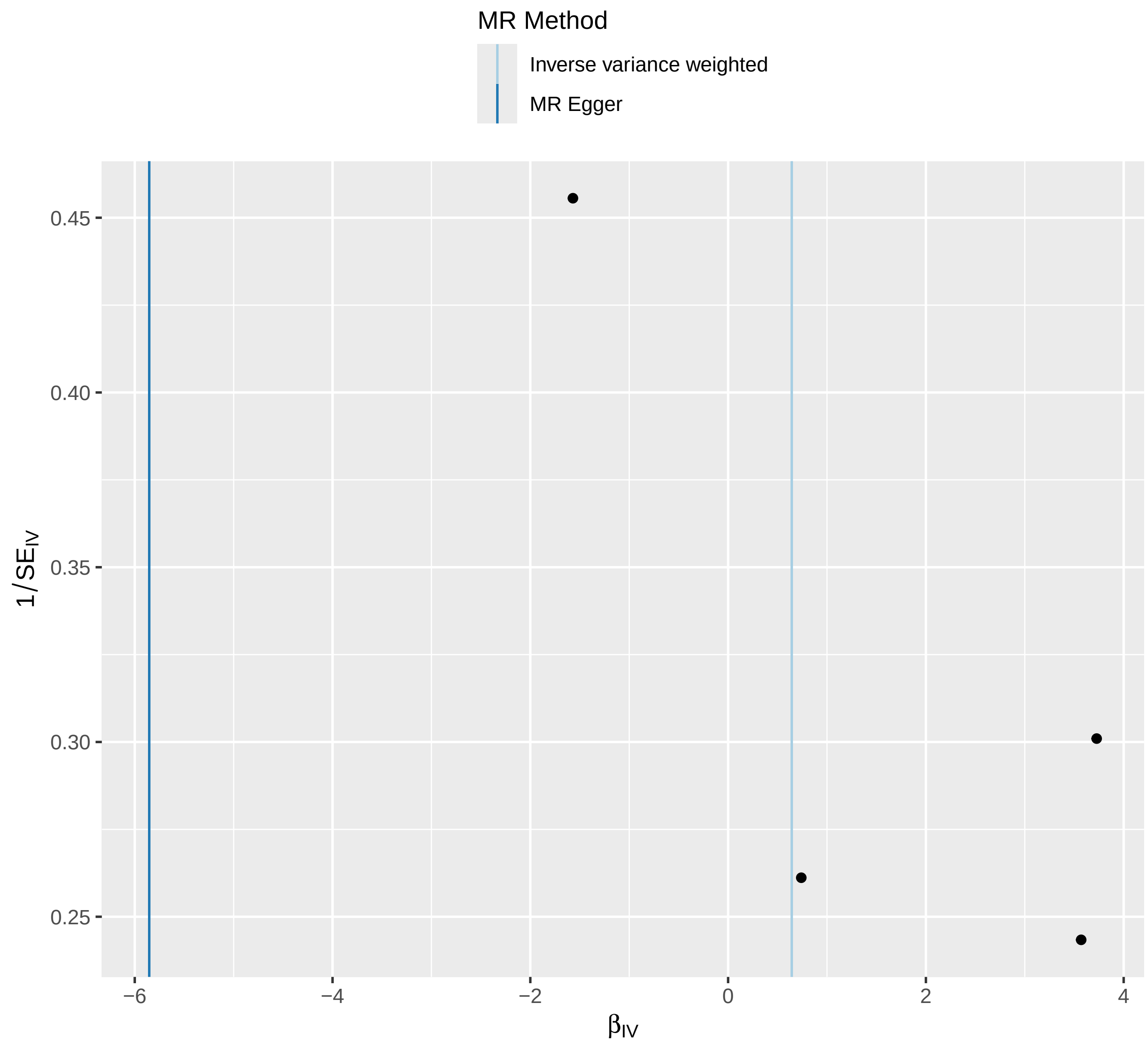

MR Method
Inverse variance weighted
MR Egger
0.45
0.40
0.35
0.30
0.25
1/SE_IV
−6
−4
−2
0
2
4
β_IV

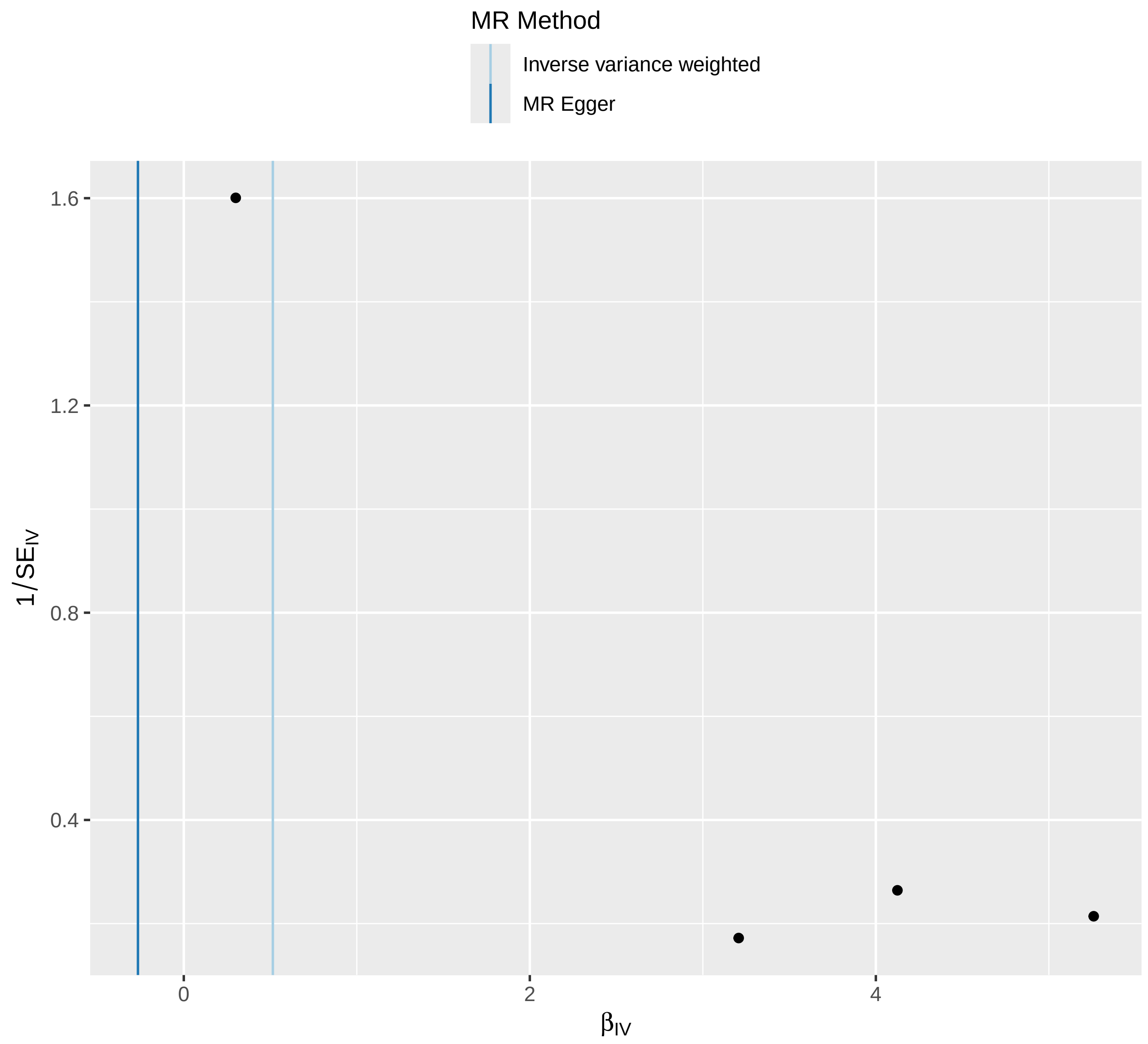
MR Method
Inverse variance weighted
MR Egger
1.6
1.2
0.8
0.4
$1/SE_{IV}$
0
2
4
β_{IV}

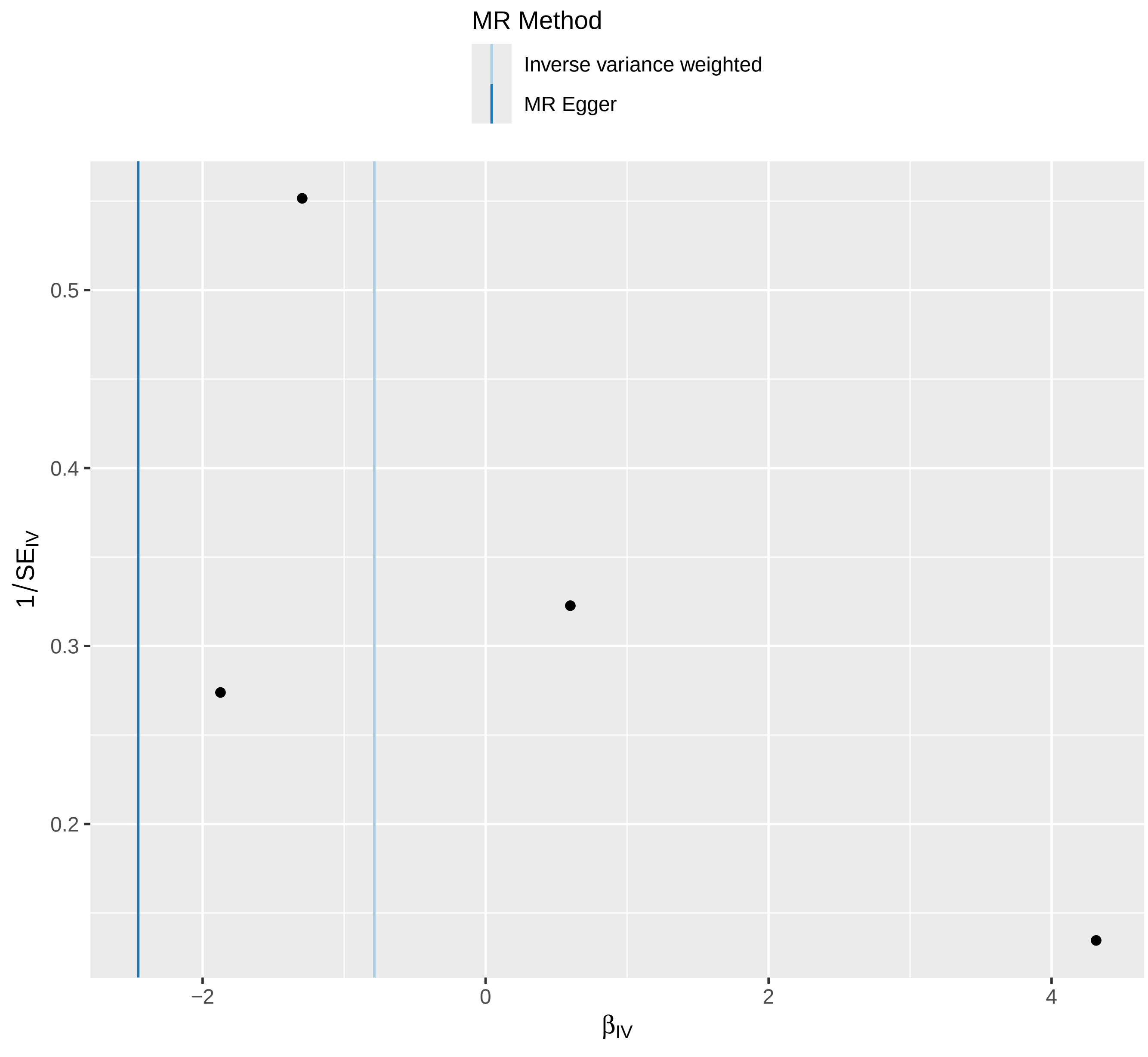
MR Method
Inverse variance weighted
MR Egger
0.5
0.4
0.3
0.2
1/SE_IV
−2
0
2
4
β_IV

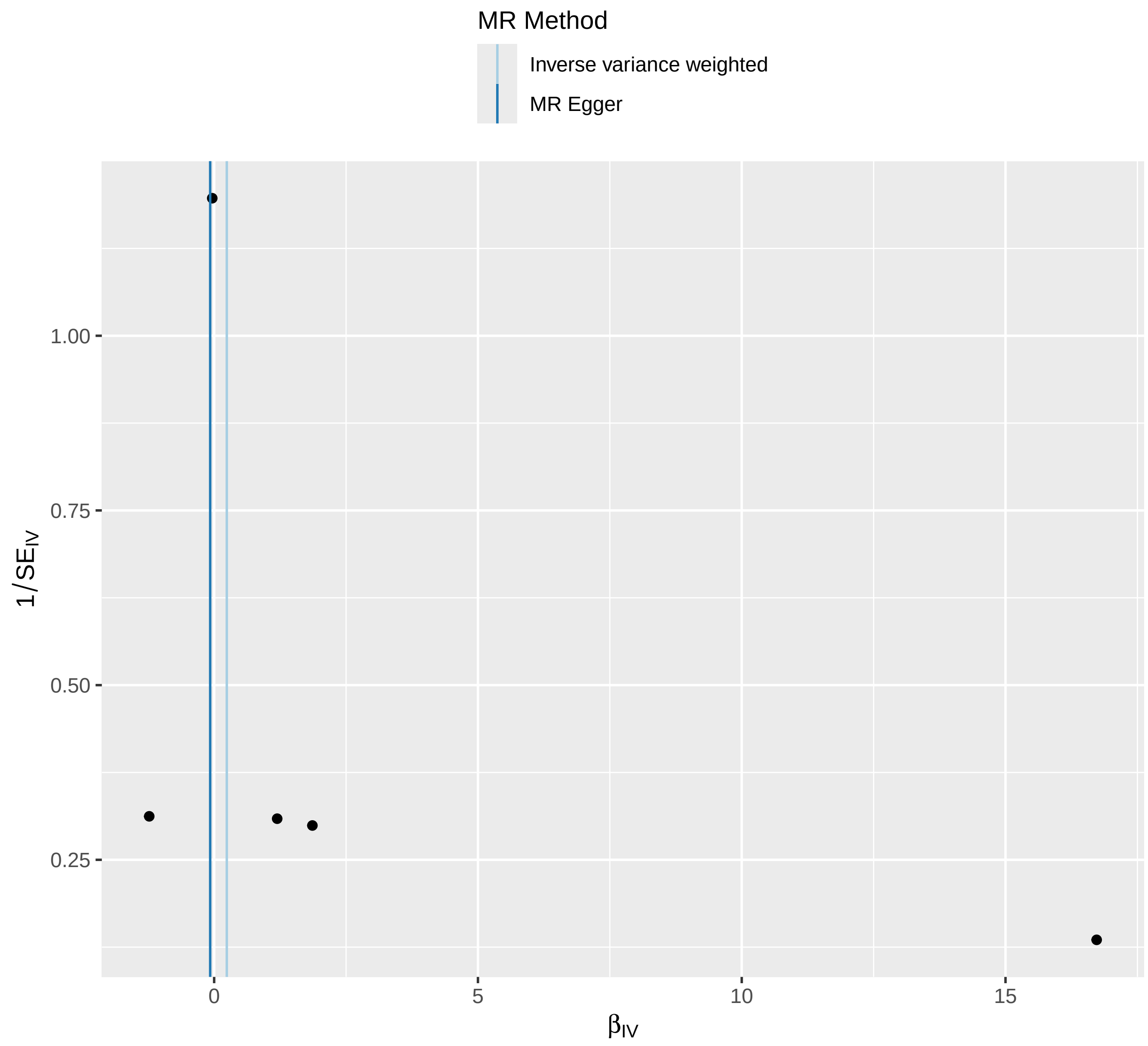
MR Method
Inverse variance weighted
MR Egger
1.00
0.75
0.50
0.25
1/SE_{IV}
0
5
10
15
β_{IV}

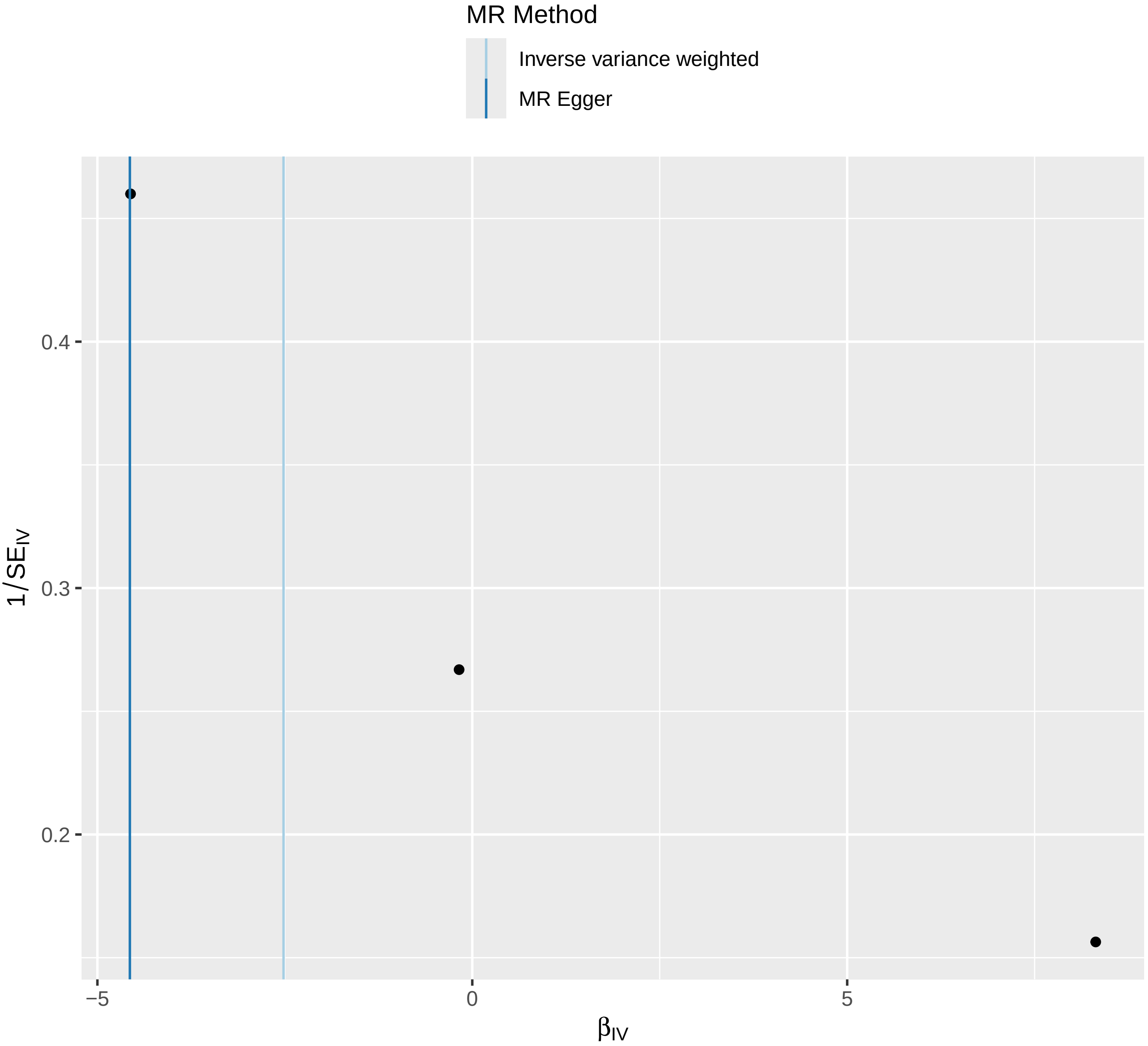

MR Method
Inverse variance weighted
MR Egger
0.4
0.3
0.2
$1/SE_{IV}$
−5
0
5
β_{IV}

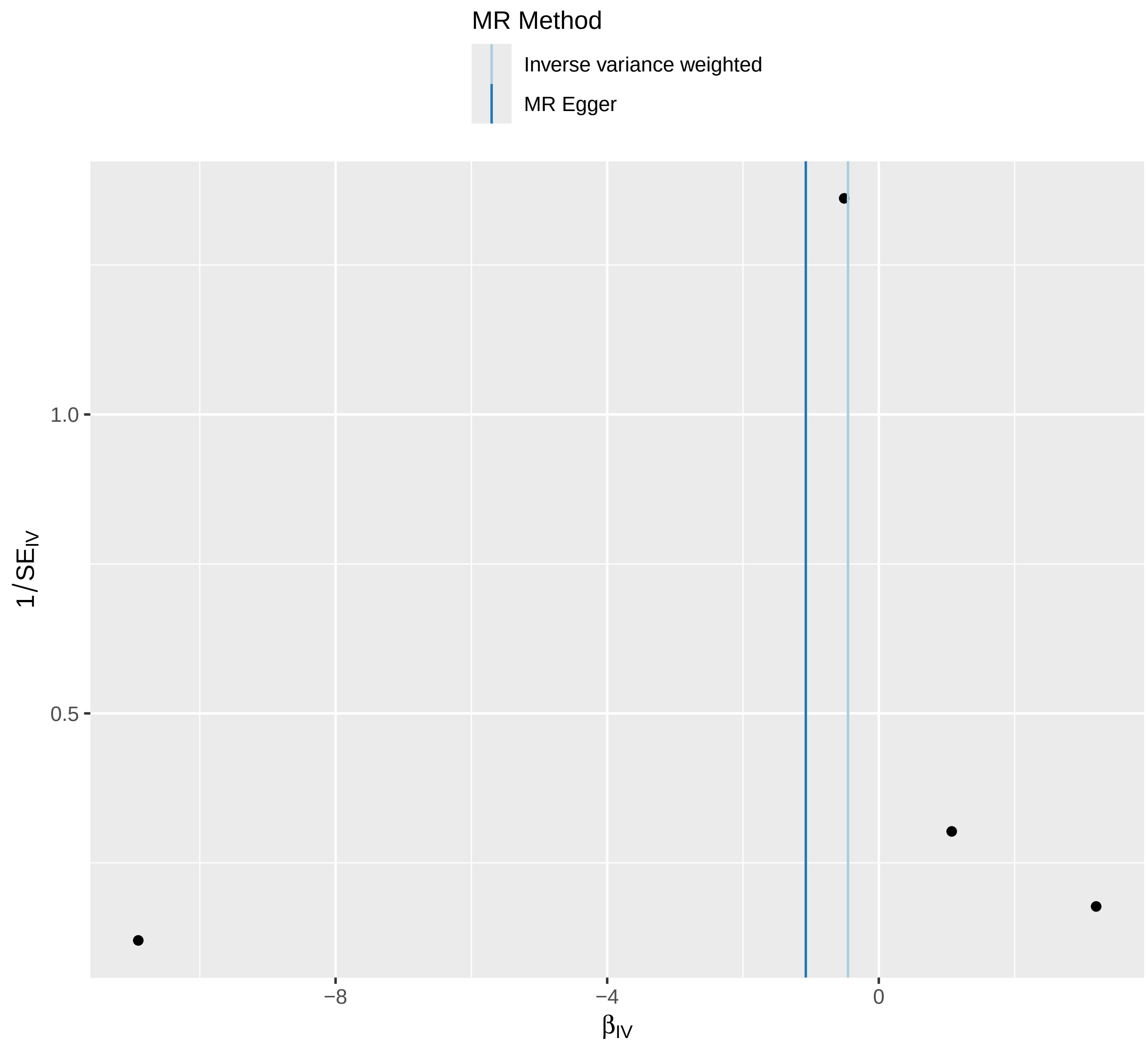
MR Method
Inverse variance weighted
MR Egger
1.0
0.5
−8
−4
0
1/SE_IV
β_IV

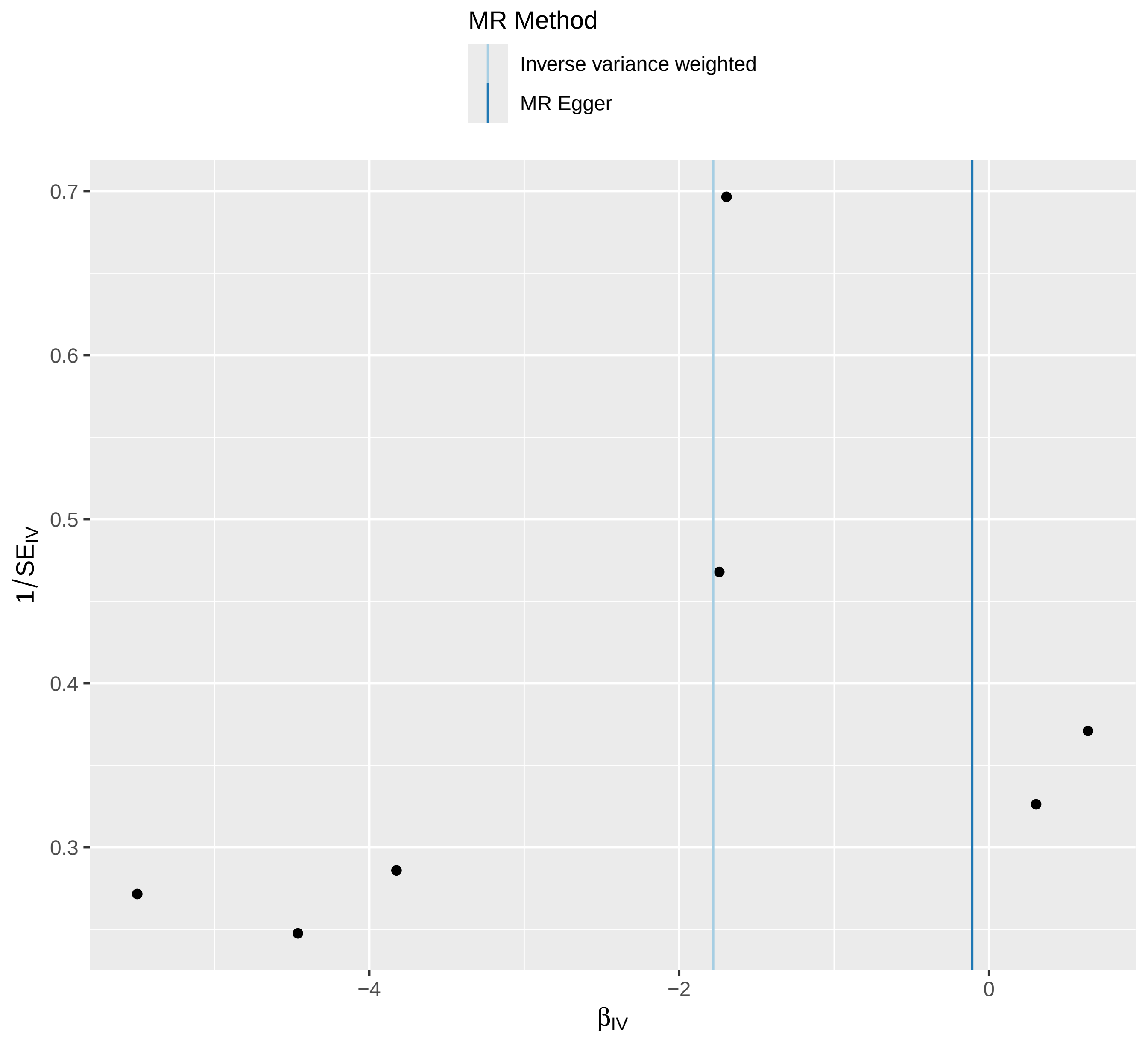
MR Method
Inverse variance weighted
MR Egger
0.7
0.6
0.5
0.4
0.3
$1/SE_{IV}$
−4
−2
0
β_{IV}

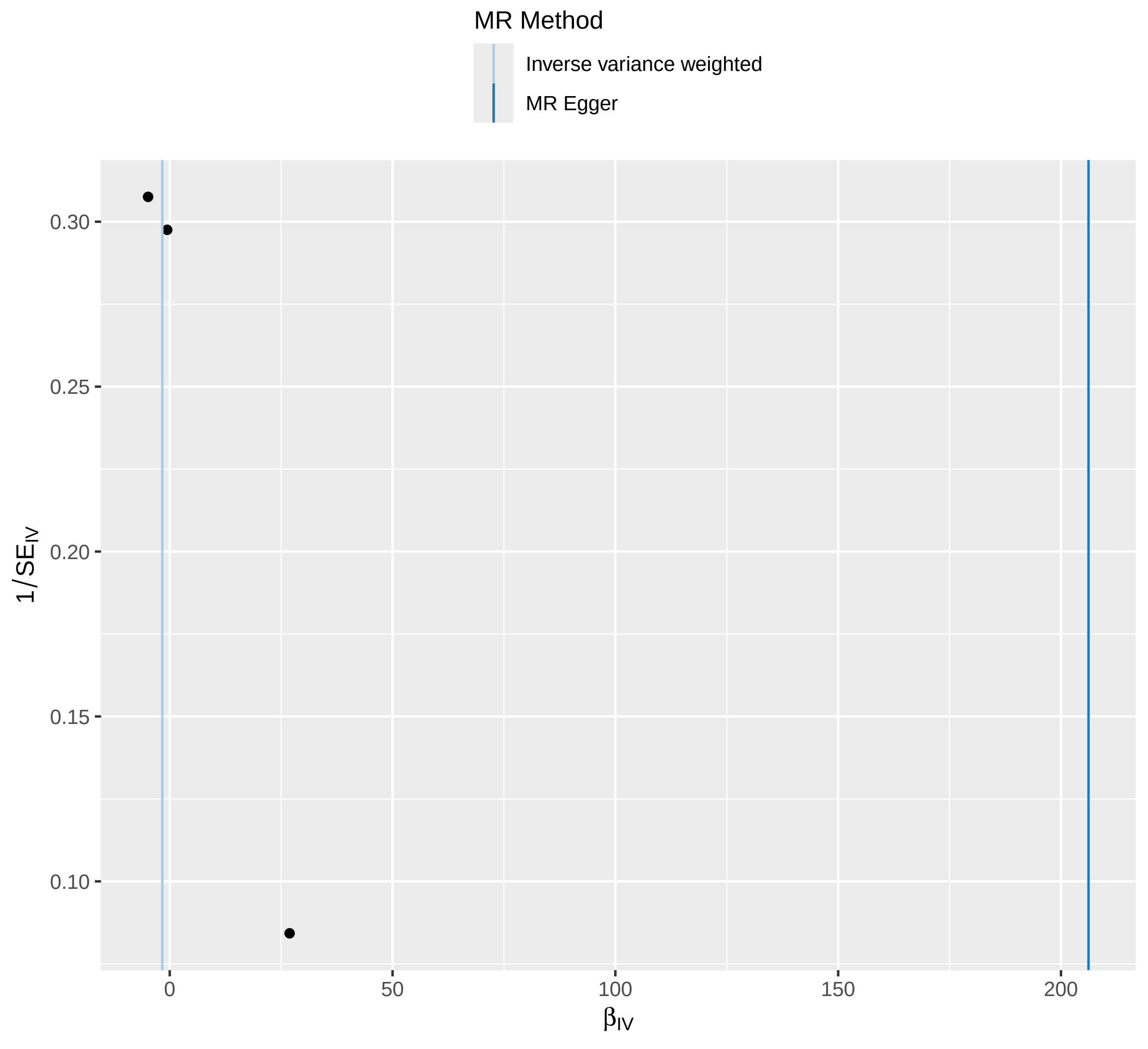
MR Method
Inverse variance weighted
MR Egger
1/SE_IV
0.30
0.25
0.20
0.15
0.10
0
50
100
150
200
β_IV

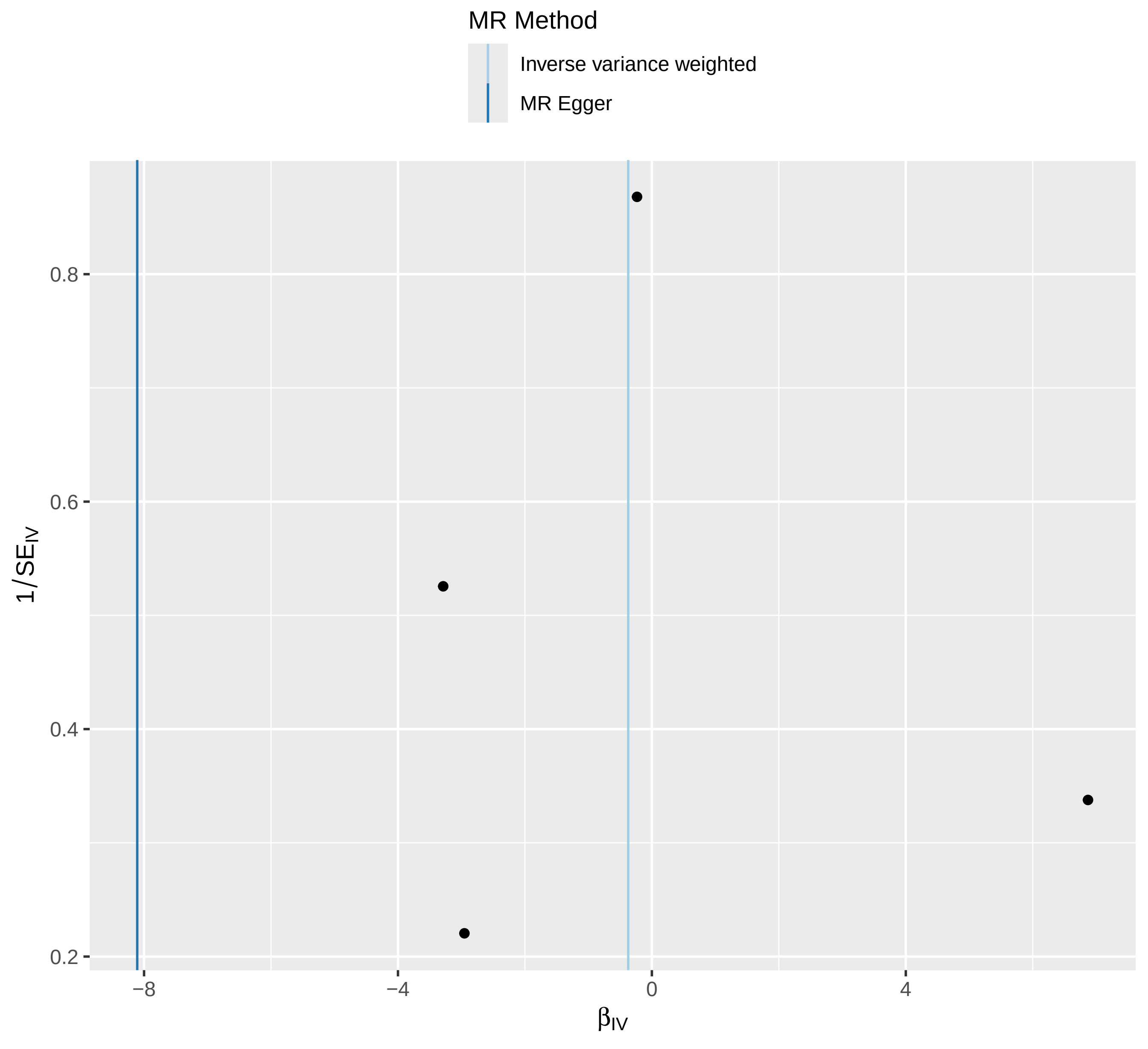
MR Method
Inverse variance weighted
MR Egger
0.8
0.6
0.4
0.2
1/SE_IV
−8
−4
0
4
β_IV

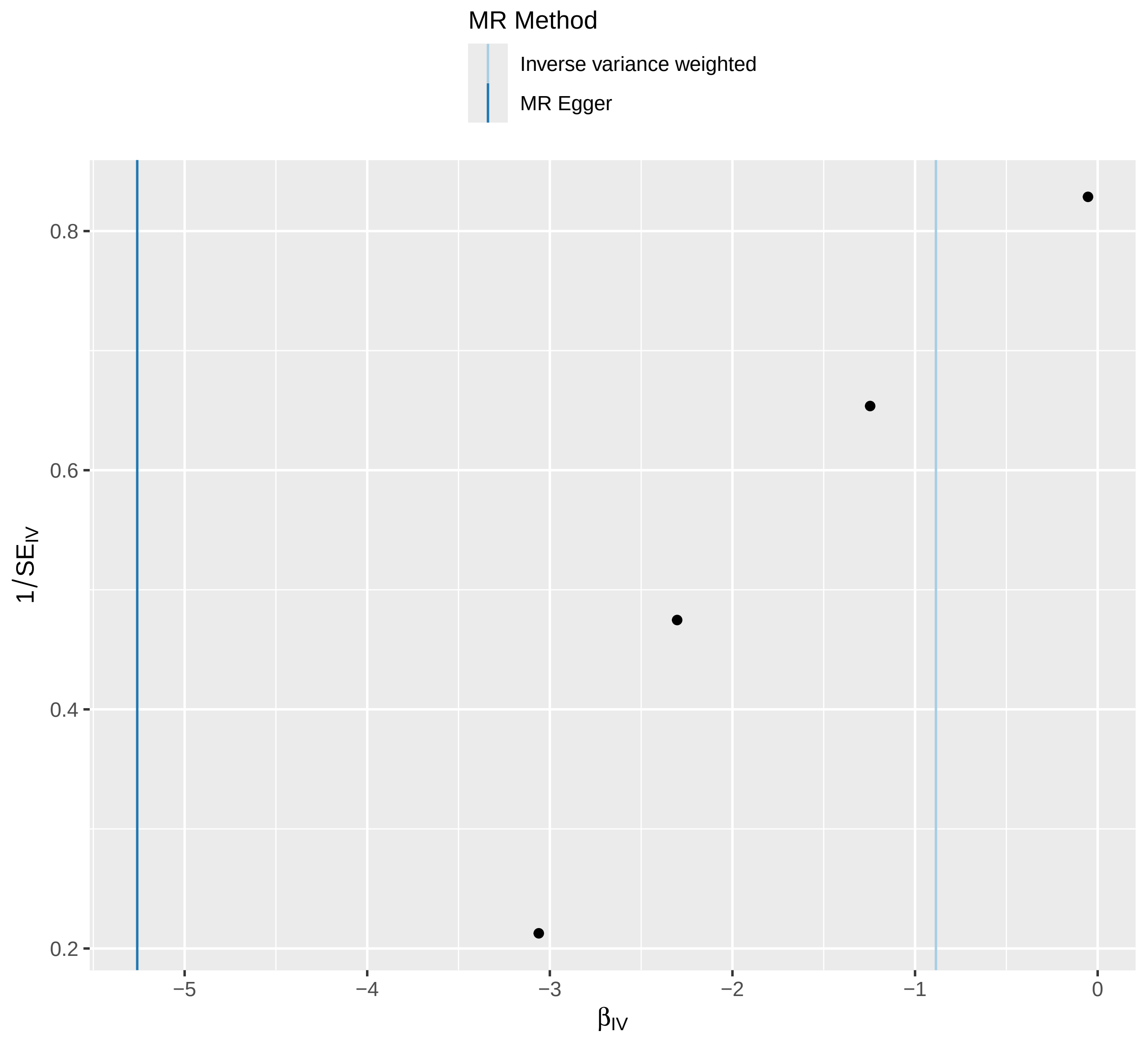

MR Method
Inverse variance weighted
MR Egger
0.8
0.6
0.4
0.2
1/SE_IV
−5
−4
−3
−2
−1
0
β_IV

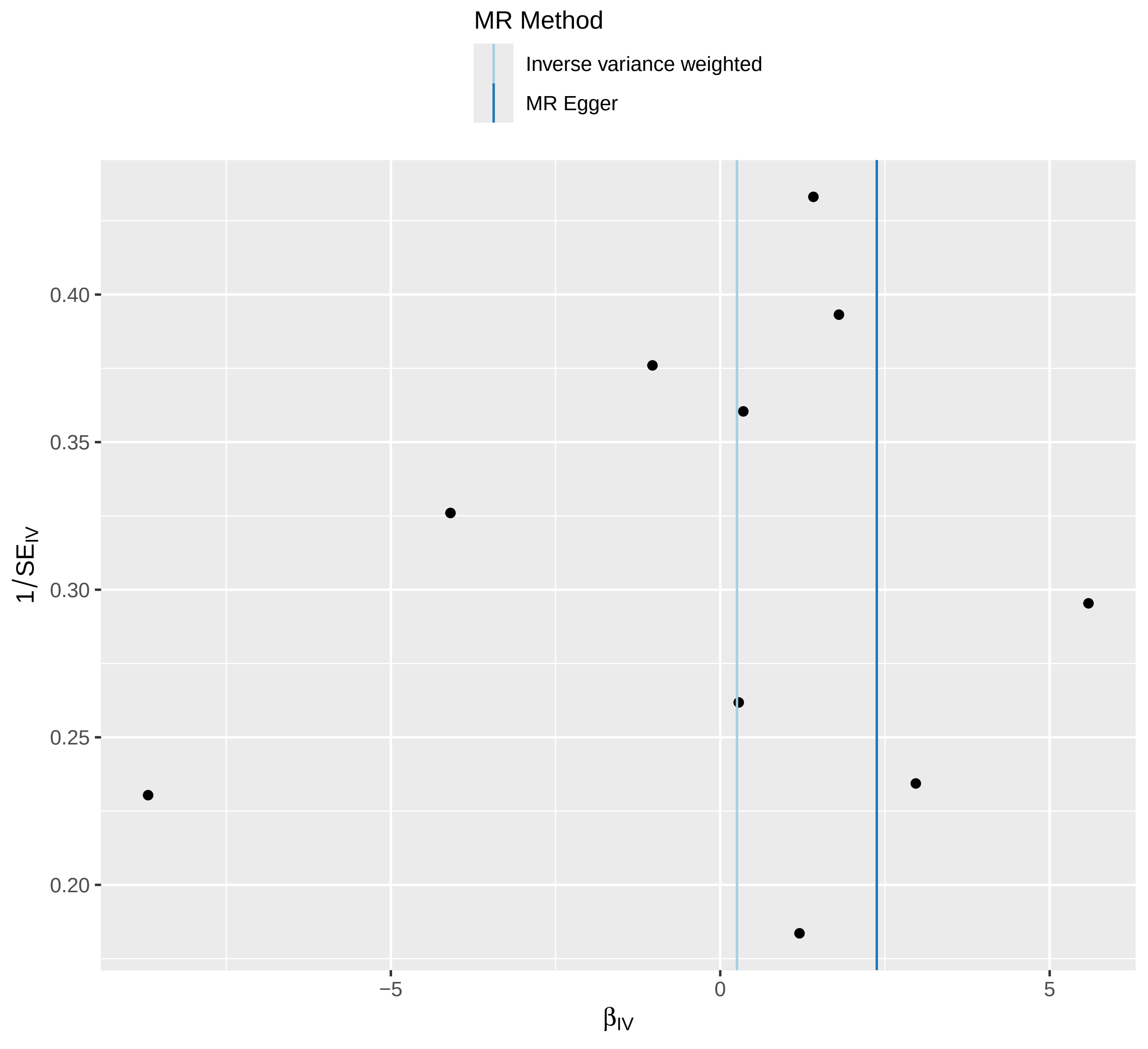
MR Method
Inverse variance weighted
MR Egger
0.40
0.35
0.30
0.25
0.20
1/SE_IV
−5
0
5
β_IV

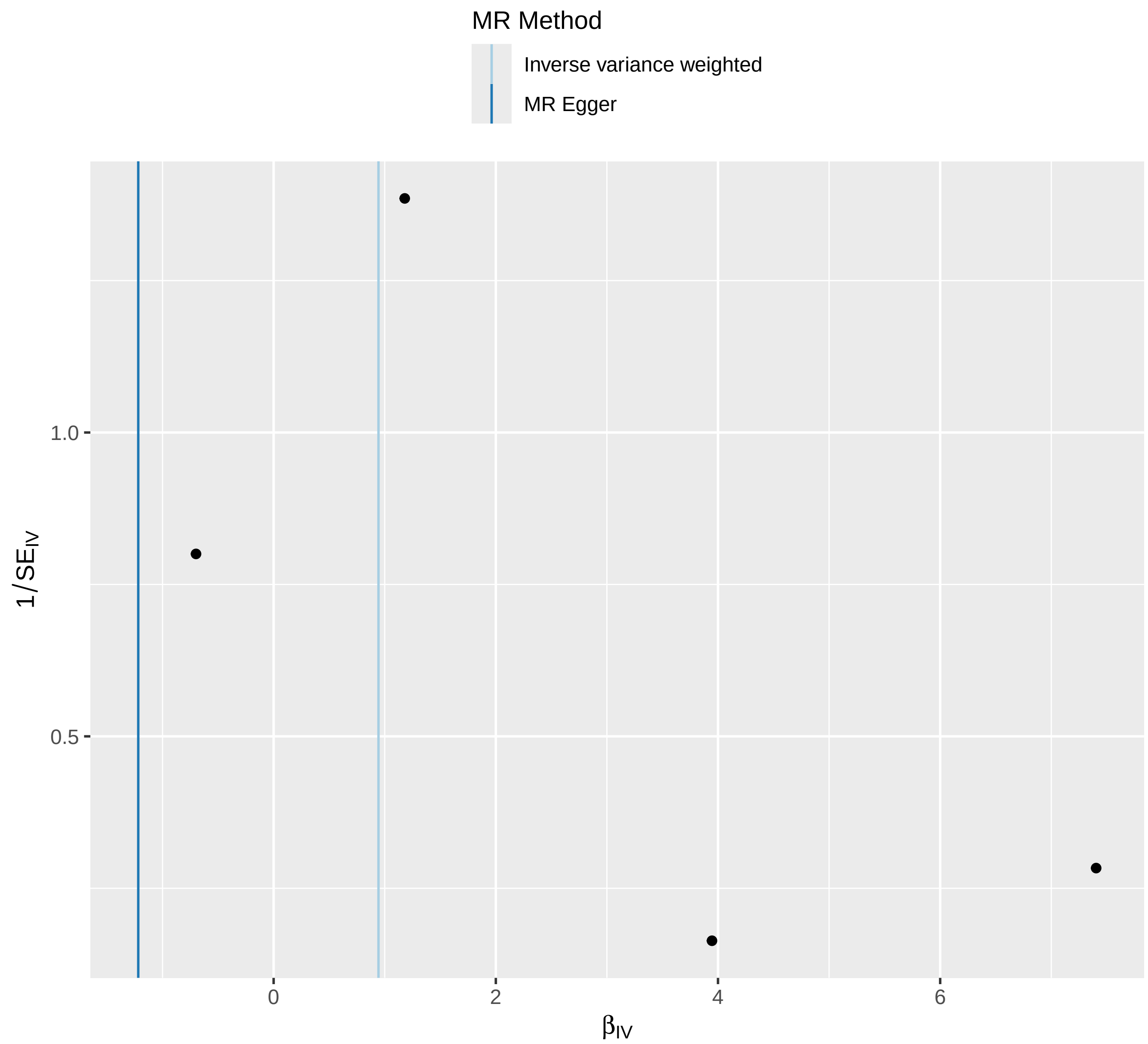
MR Method
Inverse variance weighted
MR Egger
1.0
0.5
0
2
4
6
β_{IV}
$1/SE_{IV}$

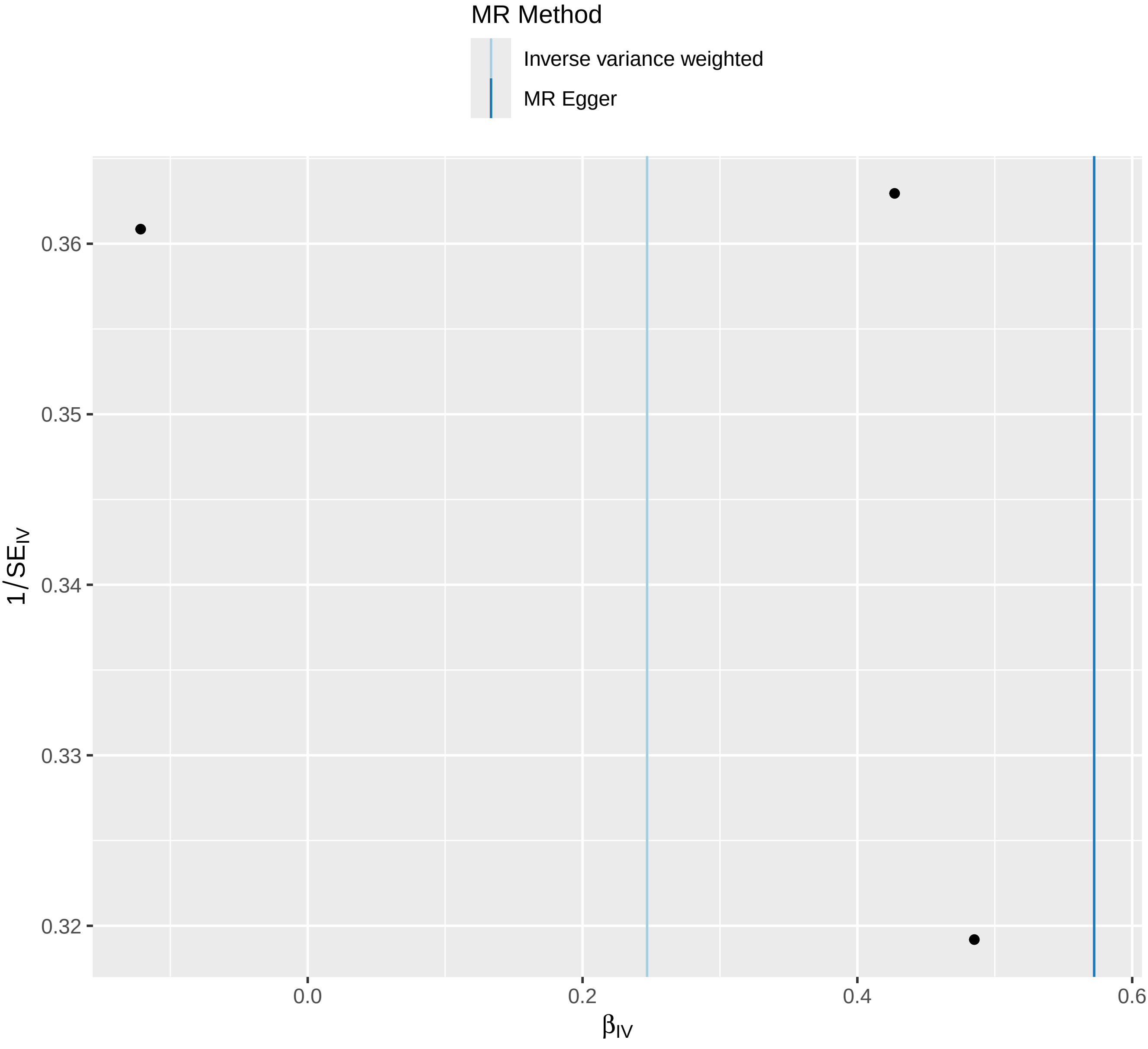

MR Method
Inverse variance weighted
MR Egger
0.36
0.35
0.34
0.33
0.32
$1/SE_{IV}$
0.0
0.2
0.4
0.6
β_{IV}

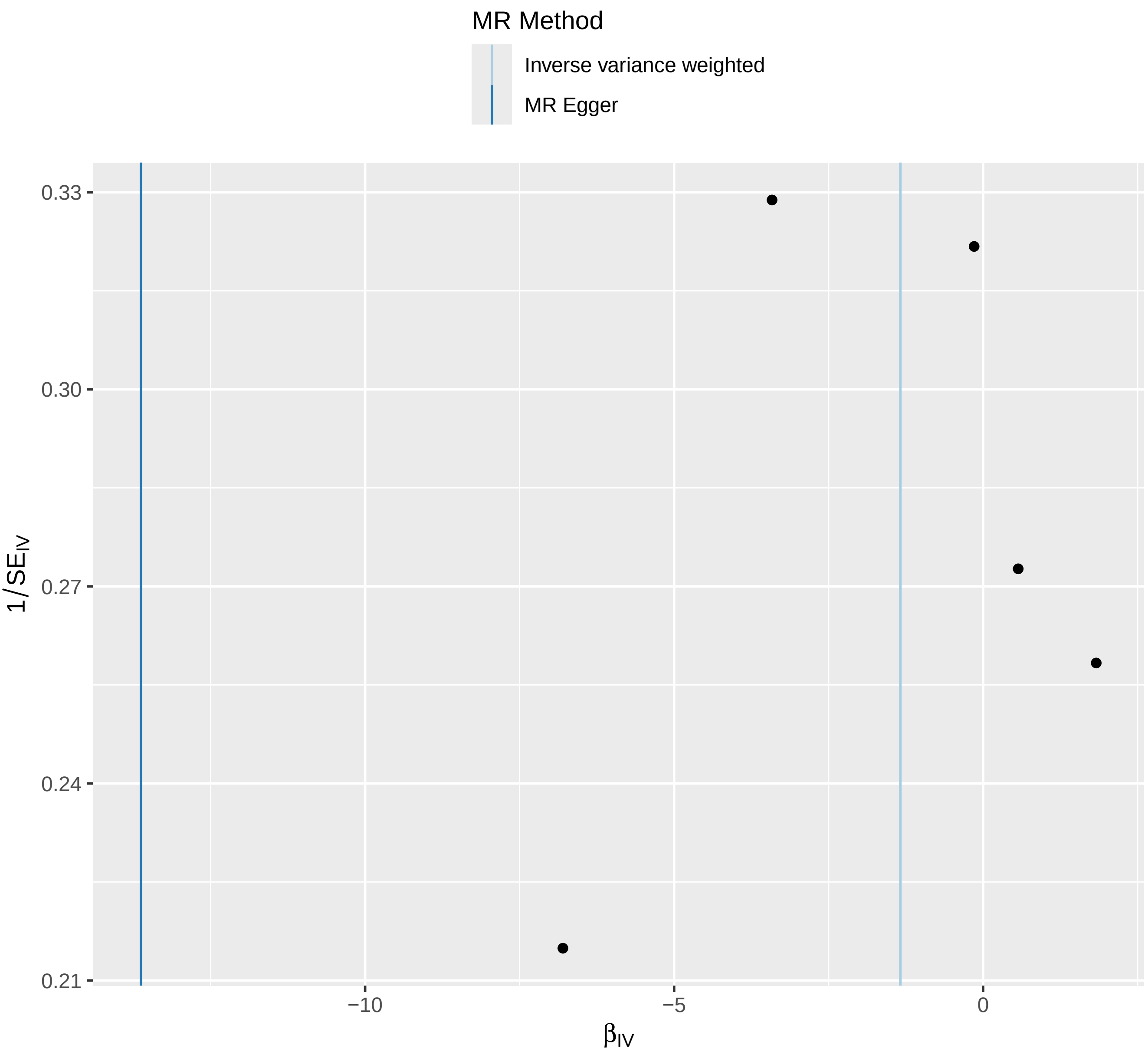
MR Method
Inverse variance weighted
MR Egger
0.33
0.30
0.27
0.24
0.21
1/SE_IV
−10
−5
0
β_IV

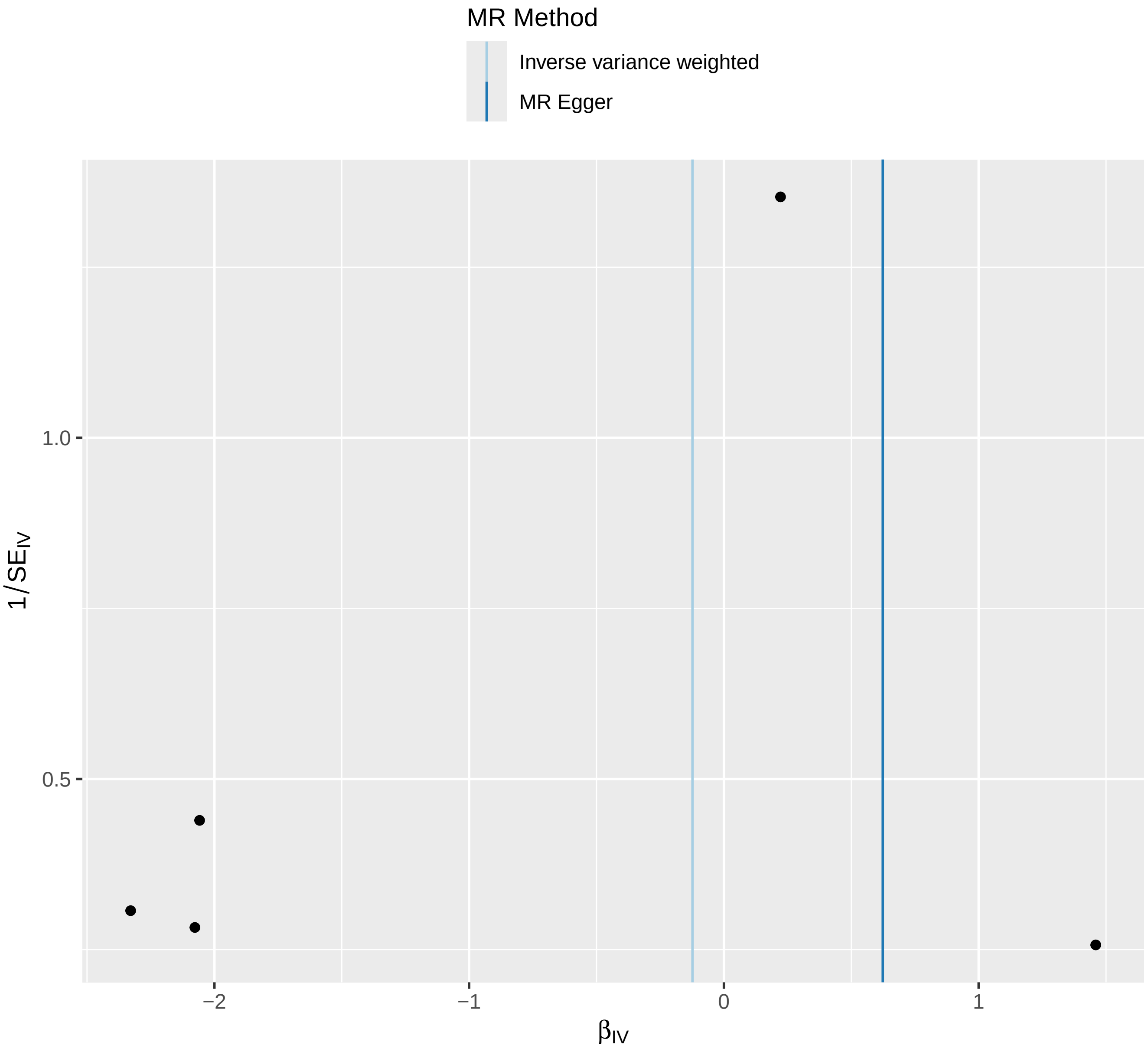

MR Method
Inverse variance weighted
MR Egger
1.0
0.5
1/SE$_{IV}$
−2
−1
0
1
β$_{IV}$

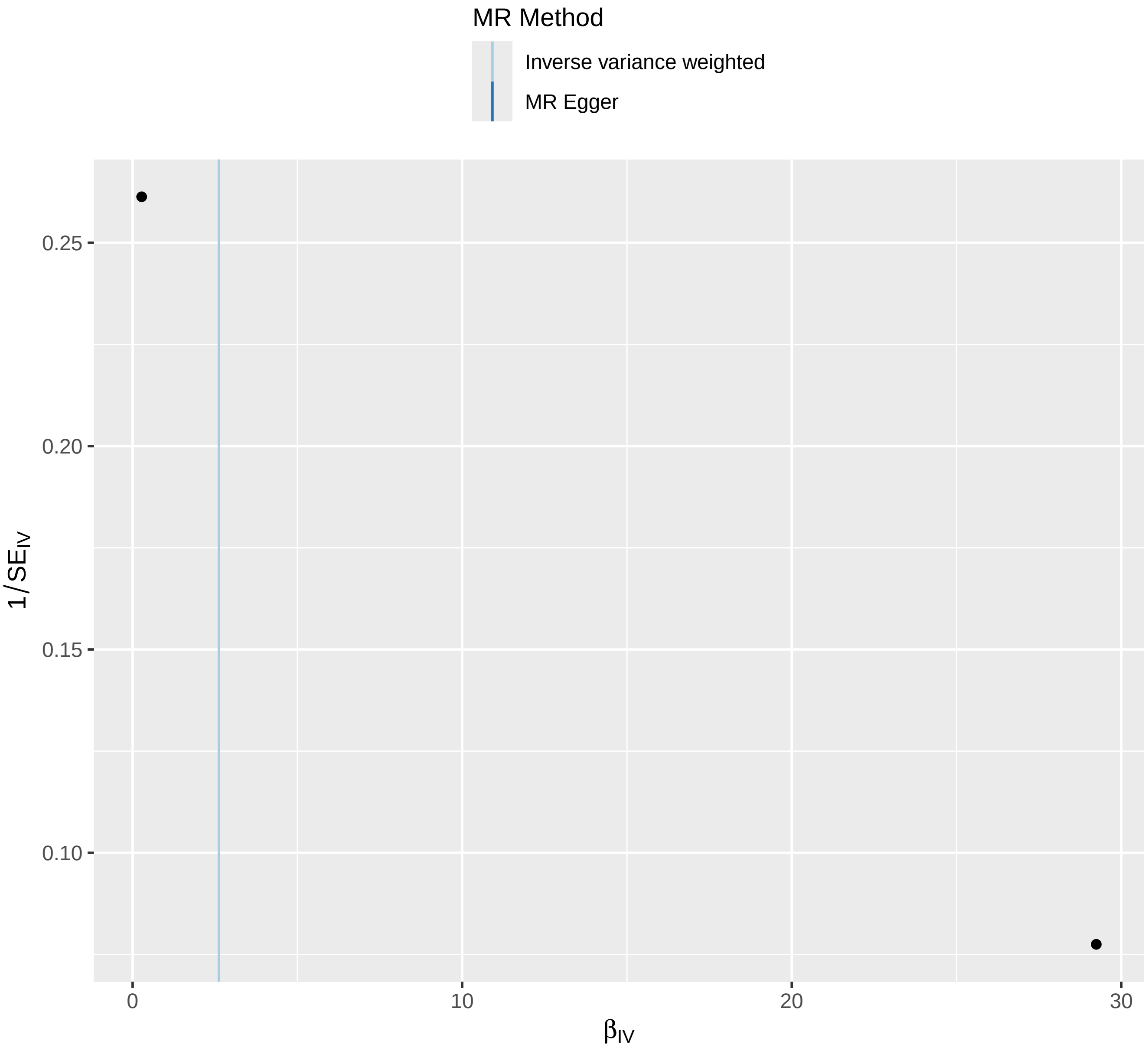
MR Method
Inverse variance weighted
MR Egger
0.25
0.20
0.15
0.10
$1/SE_{IV}$
0
10
20
30
β_{IV}

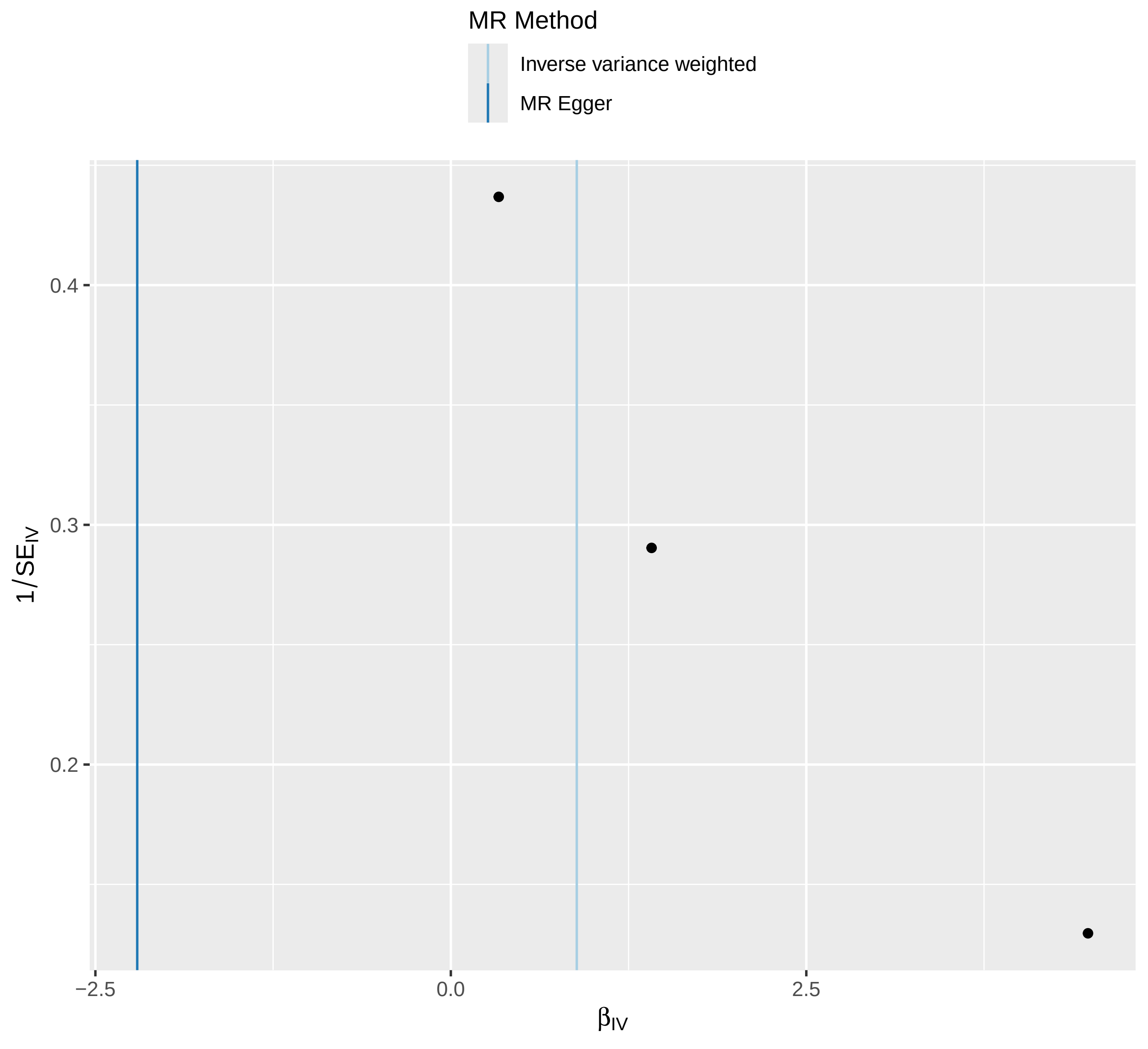

MR Method
Inverse variance weighted
MR Egger
0.4
0.3
0.2
1/SE_IV
−2.5
0.0
2.5
β_IV

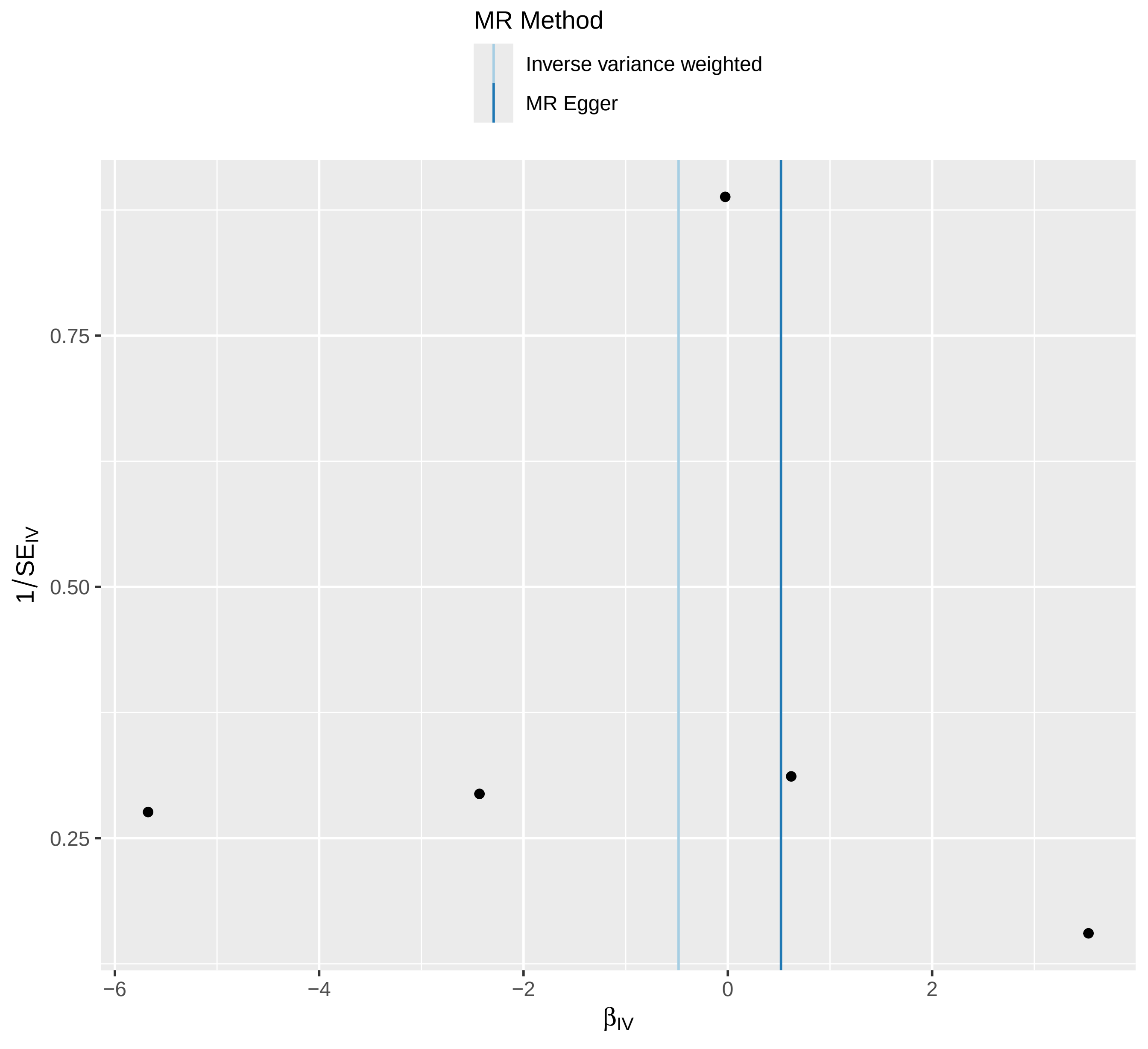
MR Method
Inverse variance weighted
MR Egger
0.75
0.50
0.25
1/SE_IV
−6
−4
−2
0
2
β_IV

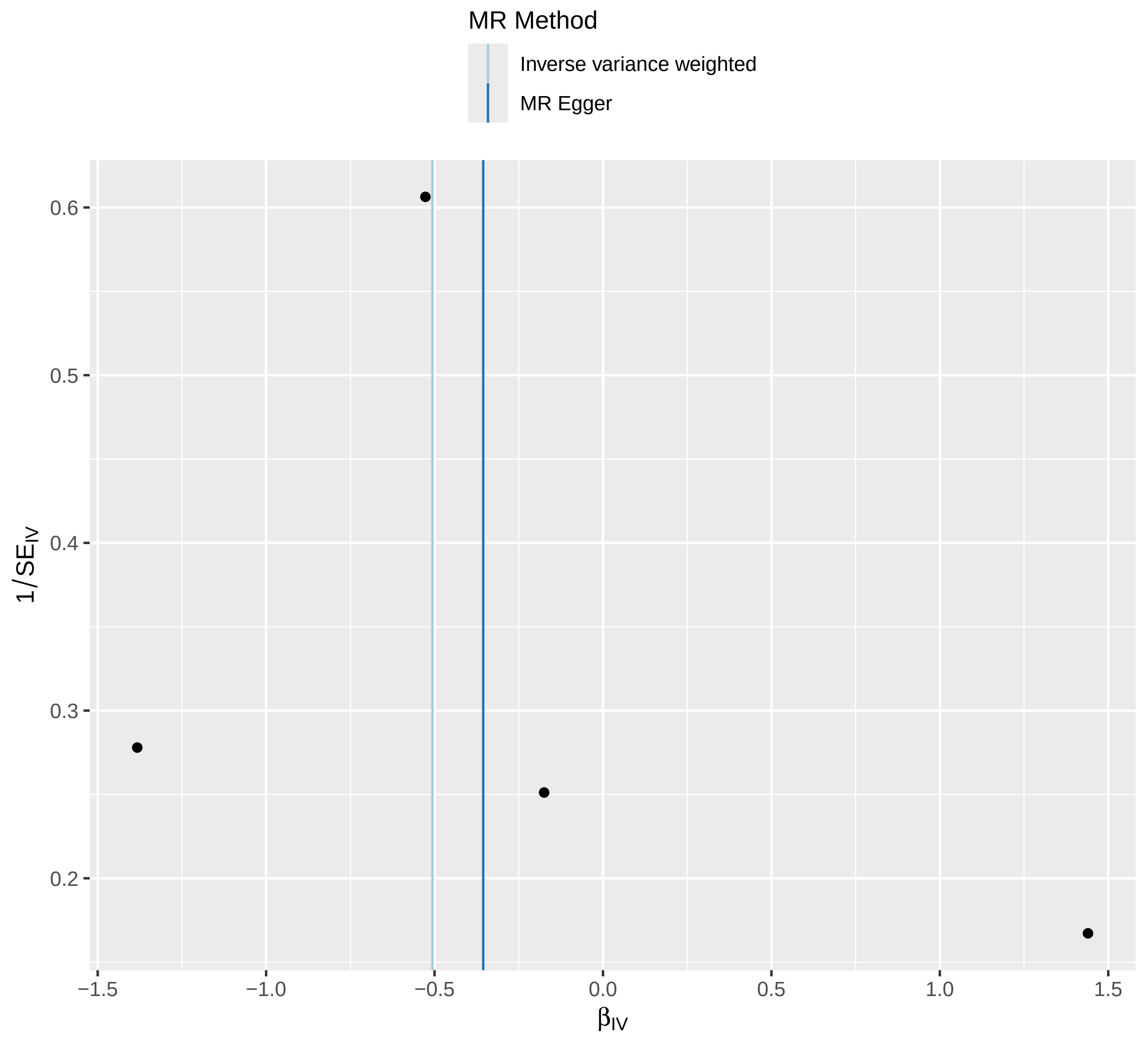
MR Method
Inverse variance weighted
MR Egger
0.6
0.5
0.4
0.3
0.2
1/SE_IV
−1.5
−1.0
−0.5
0.0
0.5
1.0
1.5
β_IV

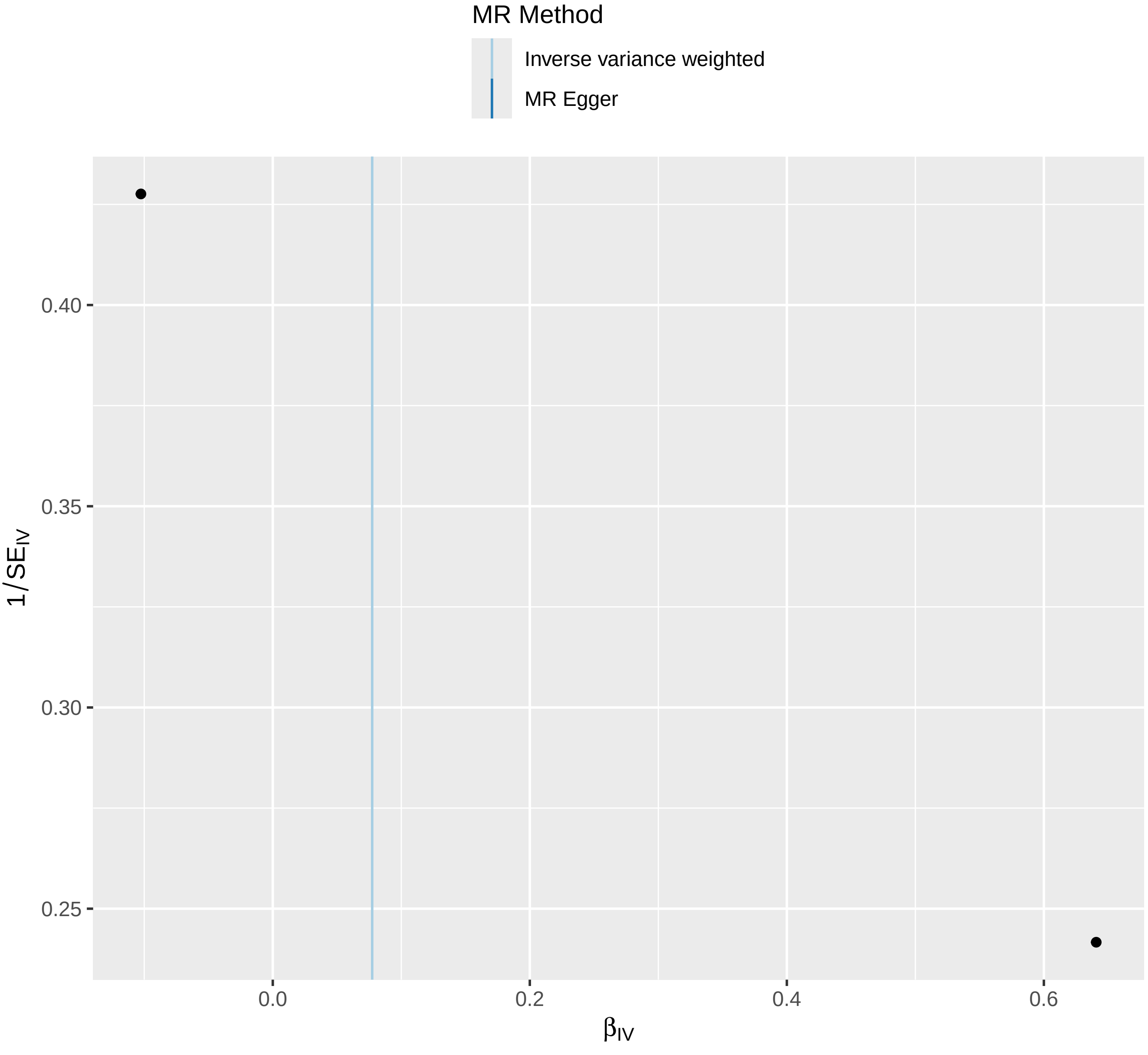

MR Method
Inverse variance weighted
MR Egger
0.40
0.35
0.30
0.25
1/SE$_{IV}$
0.0
0.2
0.4
0.6
β$_{IV}$

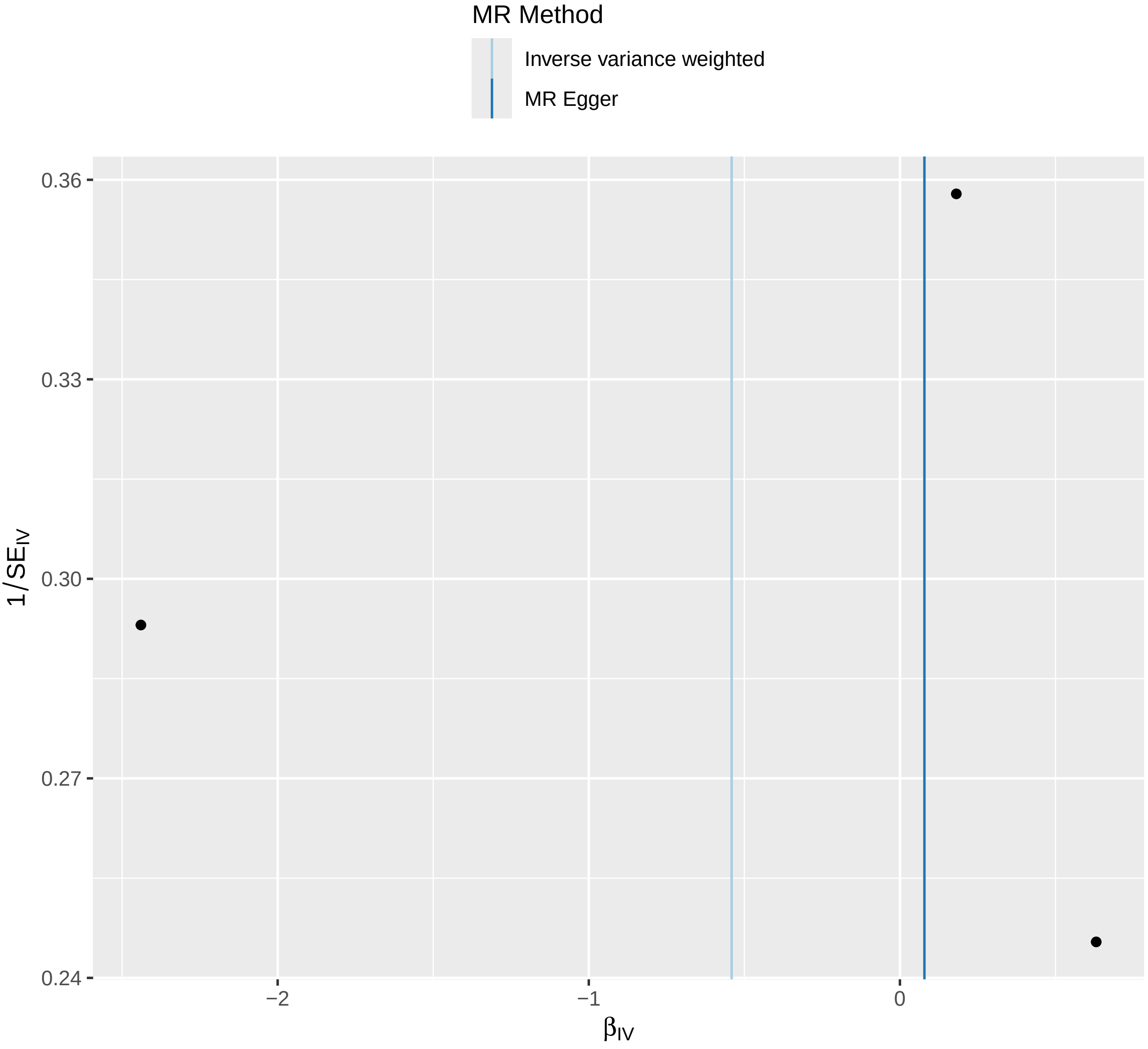
MR Method
Inverse variance weighted
MR Egger
0.36
0.33
0.30
0.27
0.24
1/SE_IV
−2
−1
0
β_IV

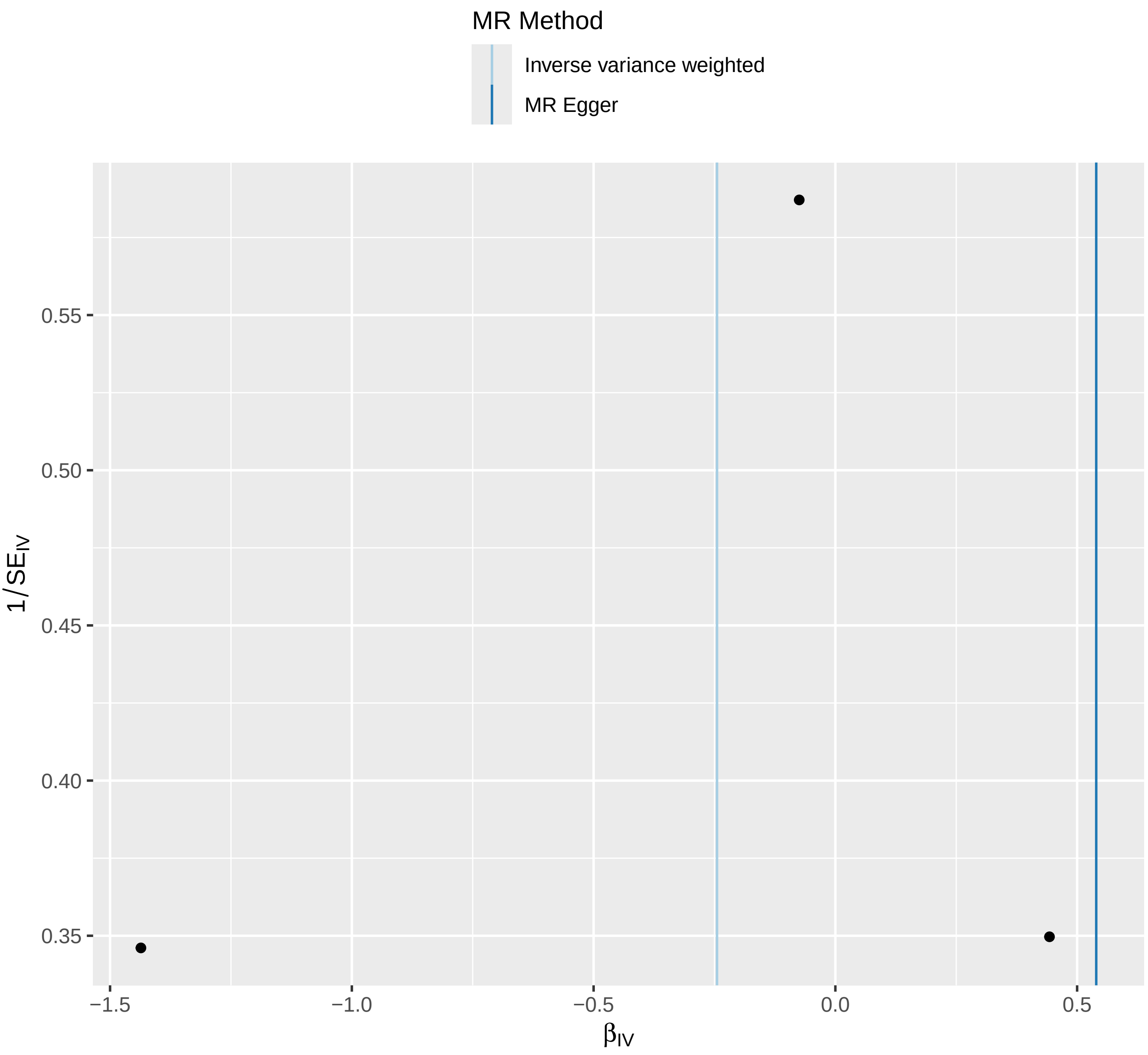
MR Method
Inverse variance weighted
MR Egger
0.55
0.50
0.45
0.40
0.35
1/SE_IV
−1.5
−1.0
−0.5
0.0
0.5
β_IV

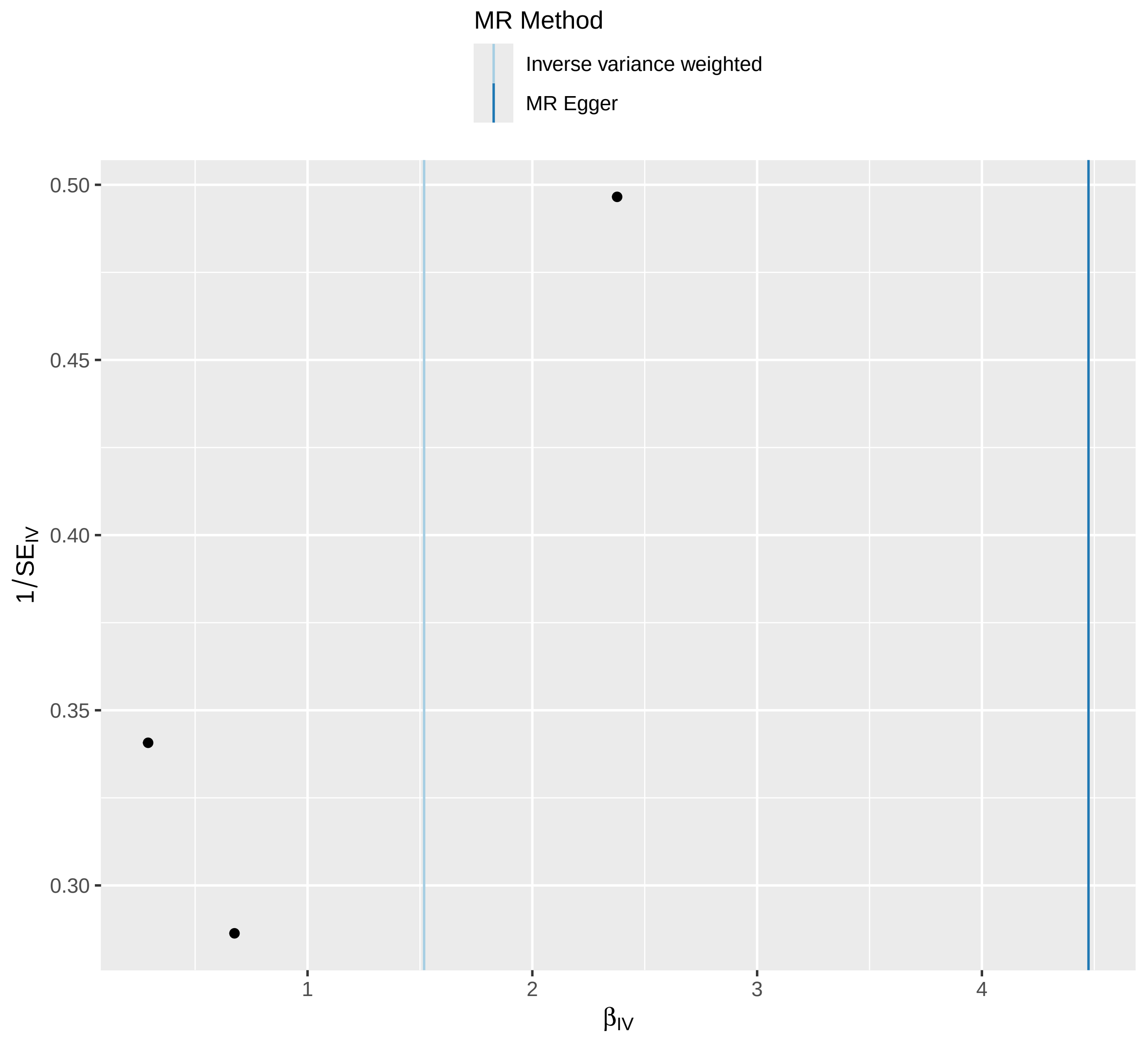
MR Method
Inverse variance weighted
MR Egger
0.50
0.45
0.40
0.35
0.30
1/SE_IV
1
2
3
4
β_IV

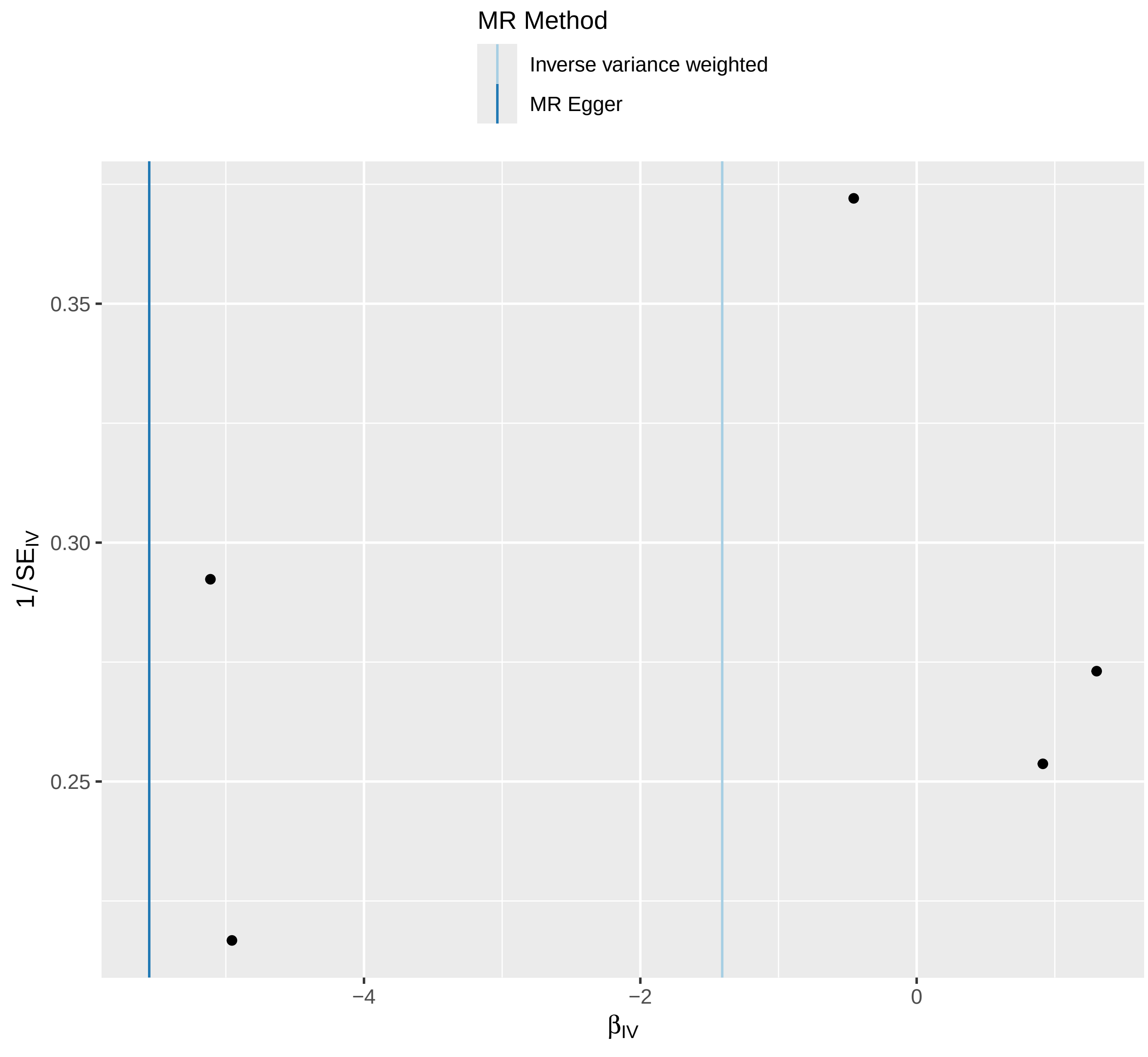

MR Method
Inverse variance weighted
MR Egger
0.35
0.30
0.25
1/SE_IV
−4
−2
0
β_IV

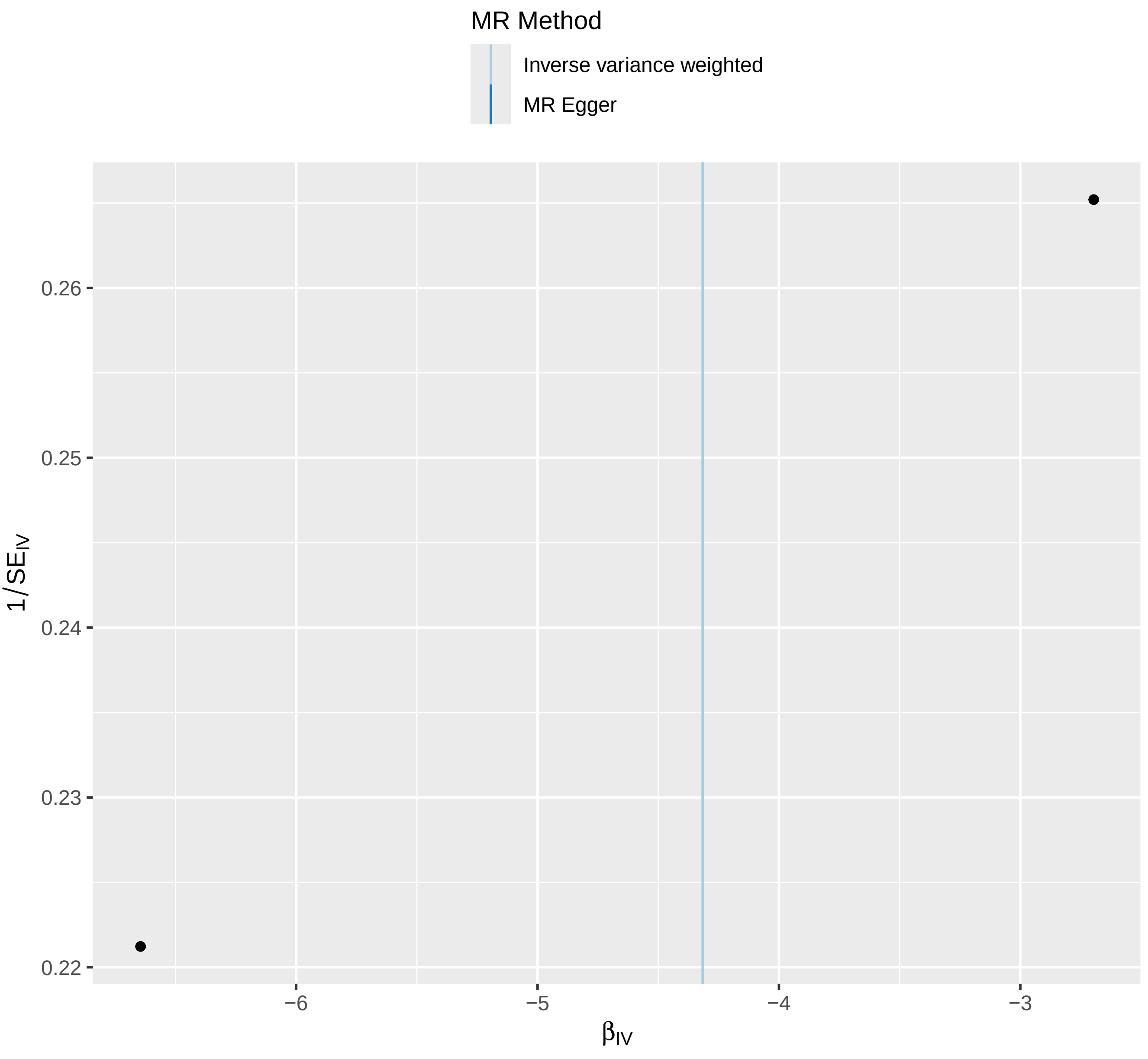
MR Method
Inverse variance weighted
MR Egger
0.26
0.25
0.24
0.23
0.22
1/SE_IV
−6
−5
−4
−3
β_IV

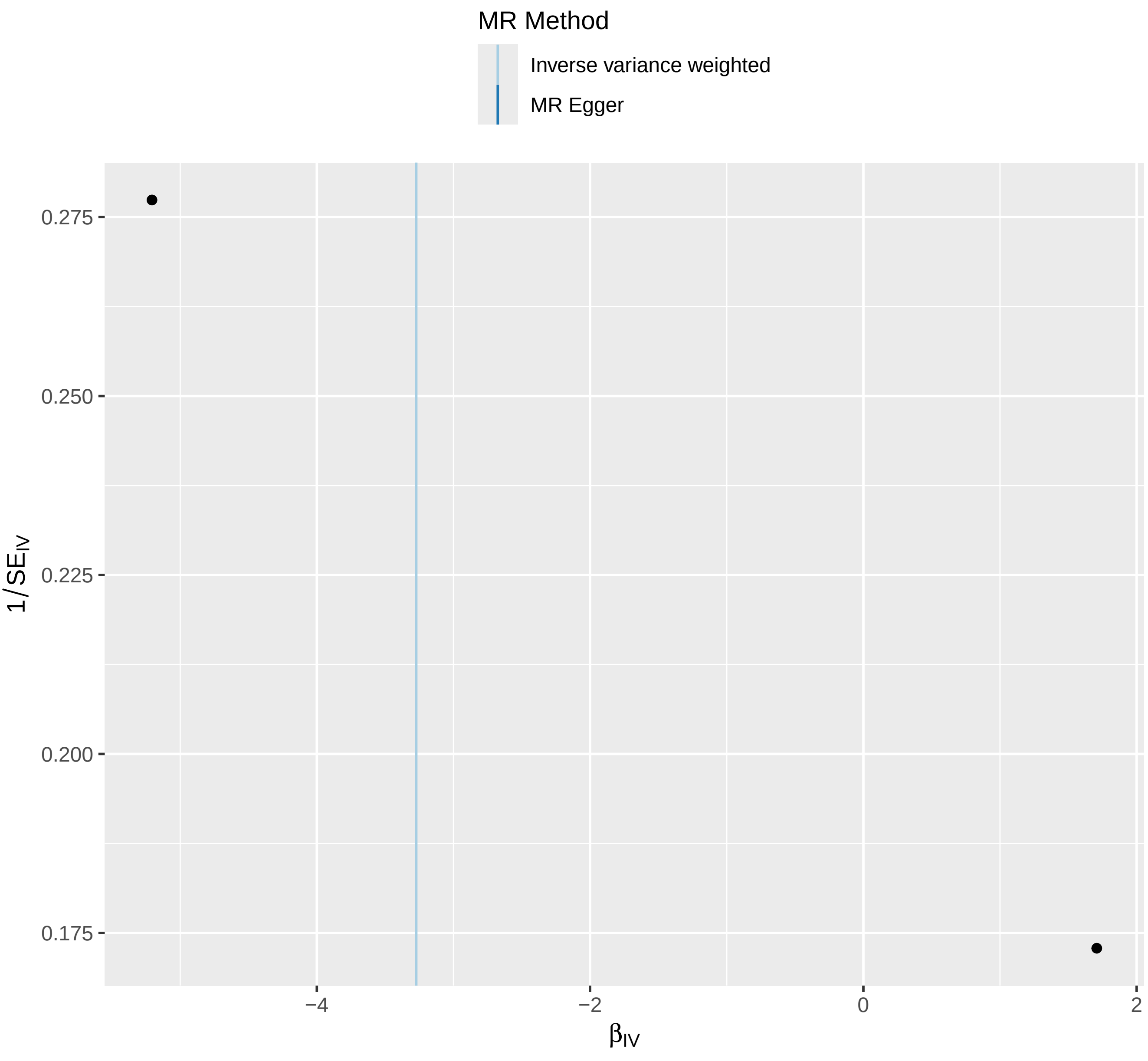
MR Method
Inverse variance weighted
MR Egger
0.275
0.250
0.225
0.200
0.175
1/SE_IV
−4
−2
0
2
β_IV

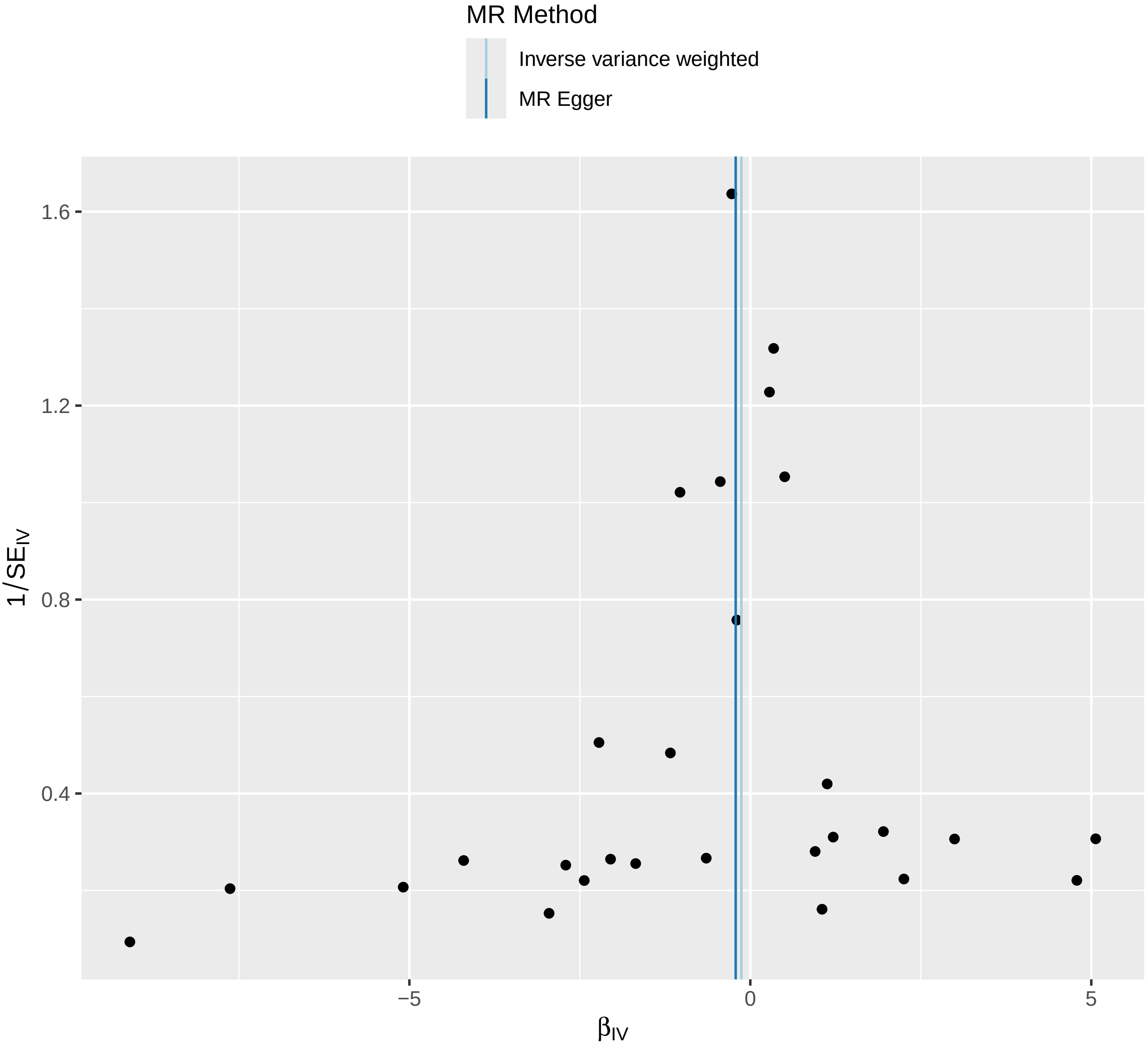

MR Method
Inverse variance weighted
MR Egger
1.6
1.2
0.8
0.4
1/SE_IV
−5
0
5
β_IV

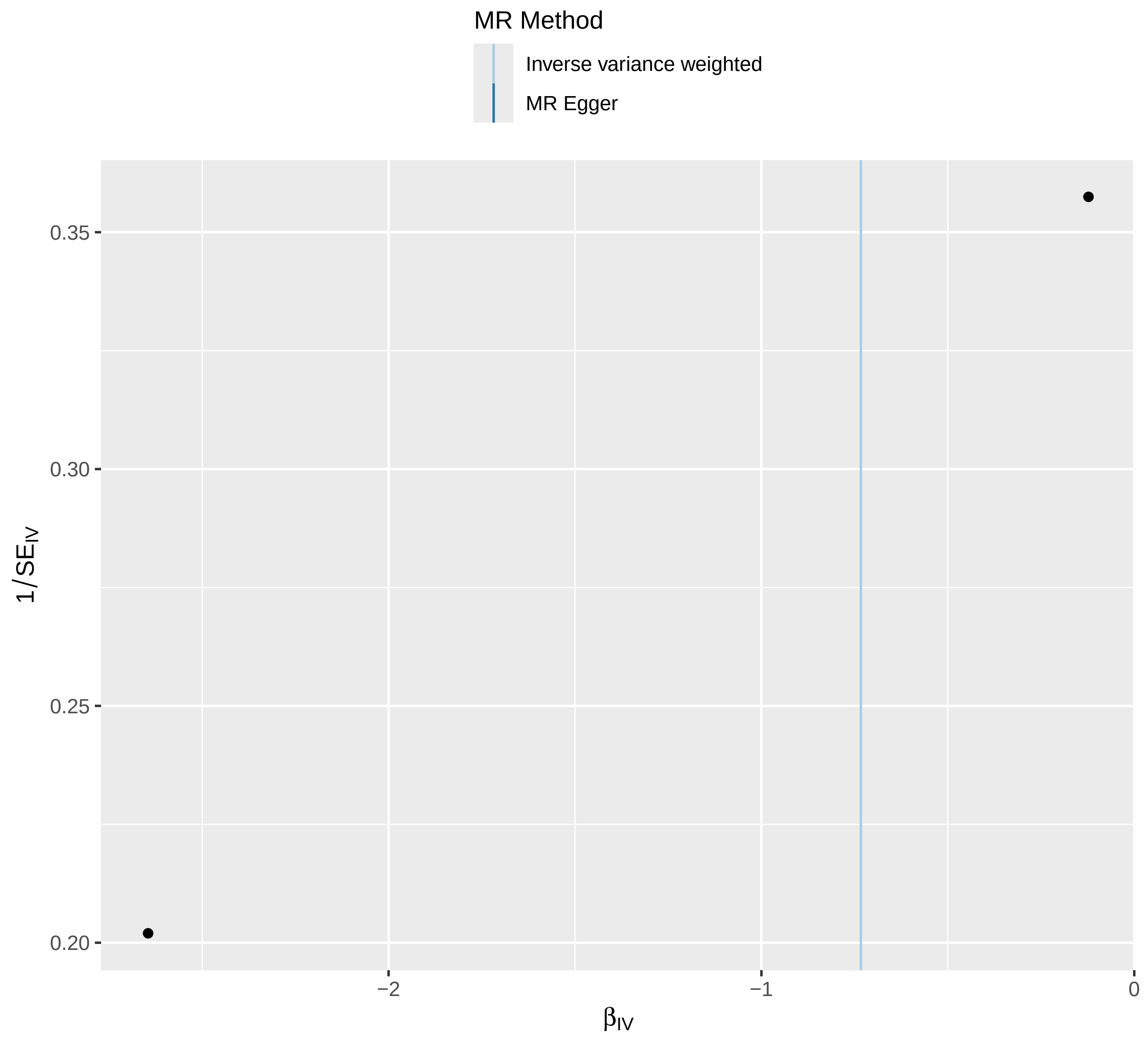
MR Method
Inverse variance weighted
MR Egger
0.35
0.30
0.25
0.20
1/SE_IV
−2
−1
0
β_IV

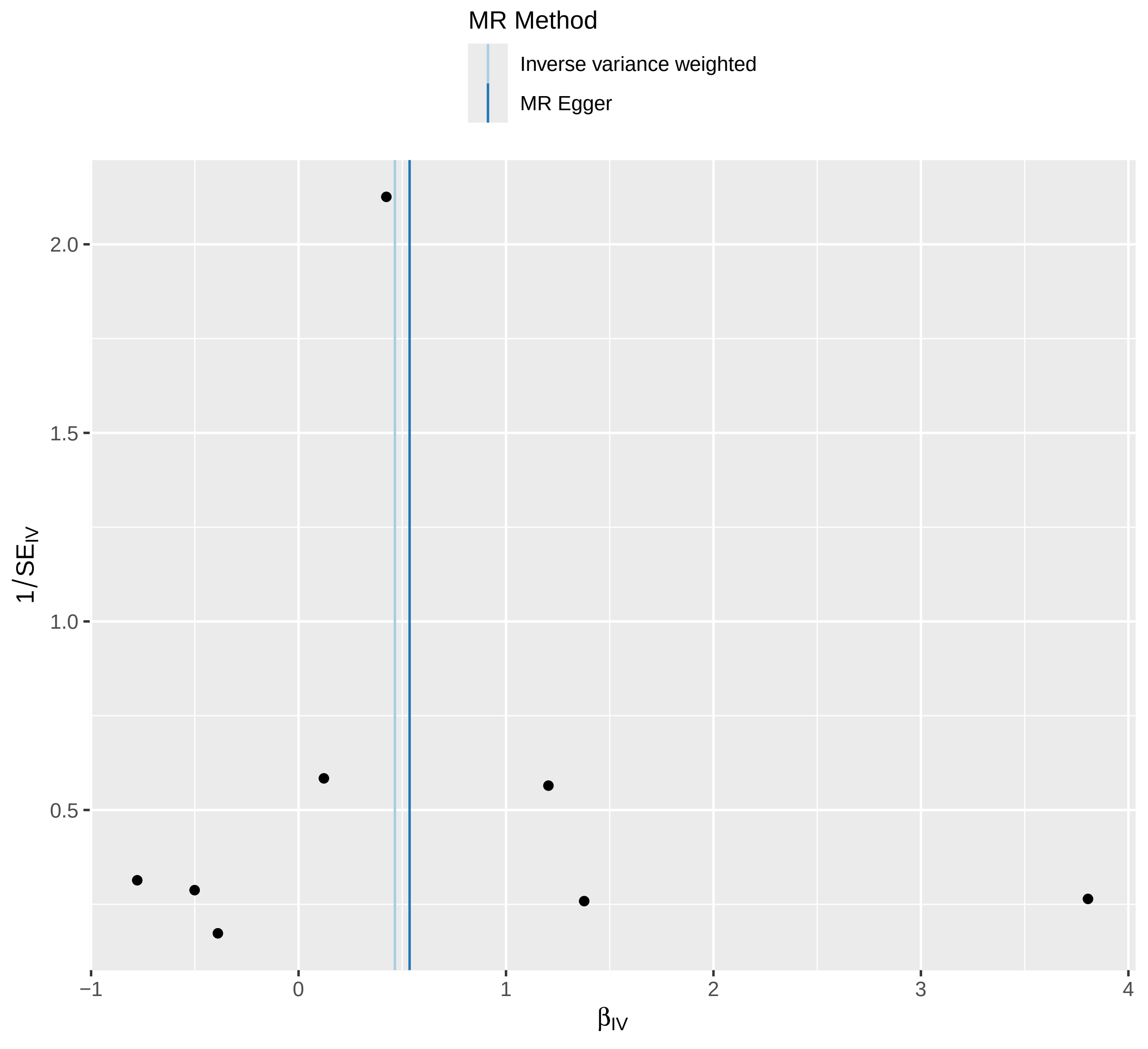
MR Method
Inverse variance weighted
MR Egger
2.0
1.5
1.0
0.5
1/SE_IV
−1
0
1
2
3
4
β_IV

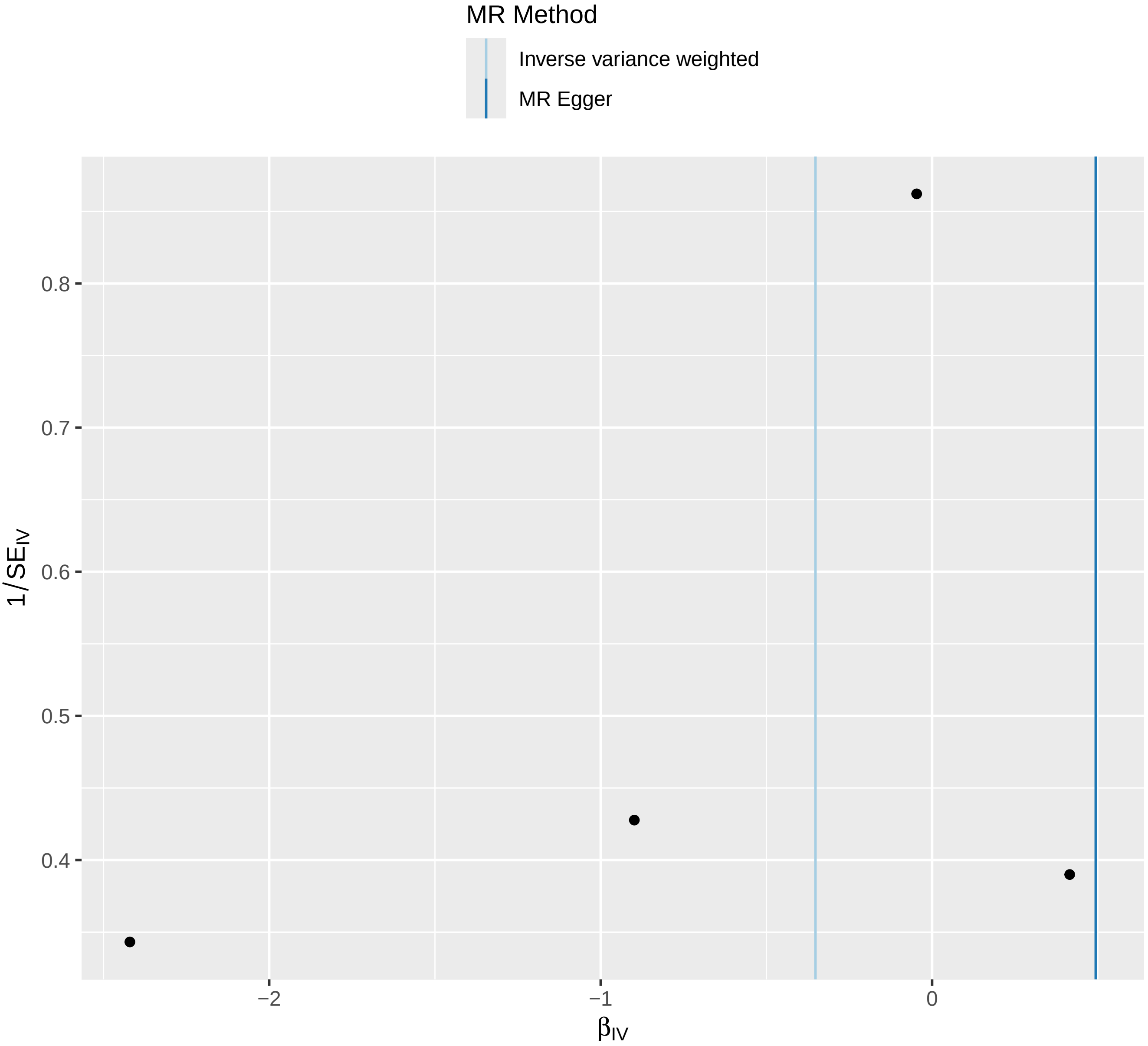

MR Method
Inverse variance weighted
MR Egger
0.8
0.7
0.6
0.5
0.4
1/SE_IV
−2
−1
0
β_IV

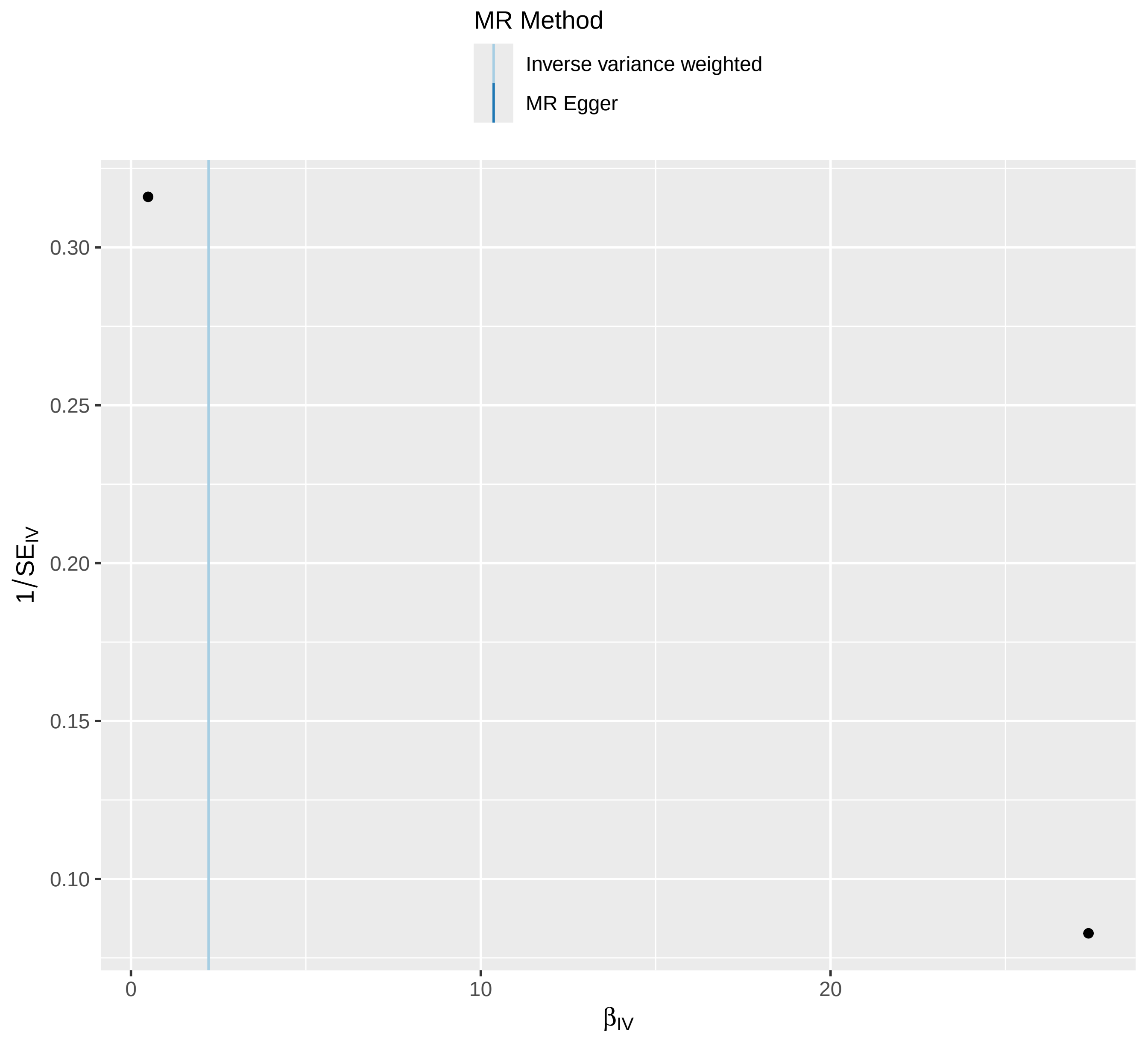
MR Method
Inverse variance weighted
MR Egger
0.30
0.25
0.20
0.15
0.10
$1/SE_{IV}$
0
10
20
β_{IV}

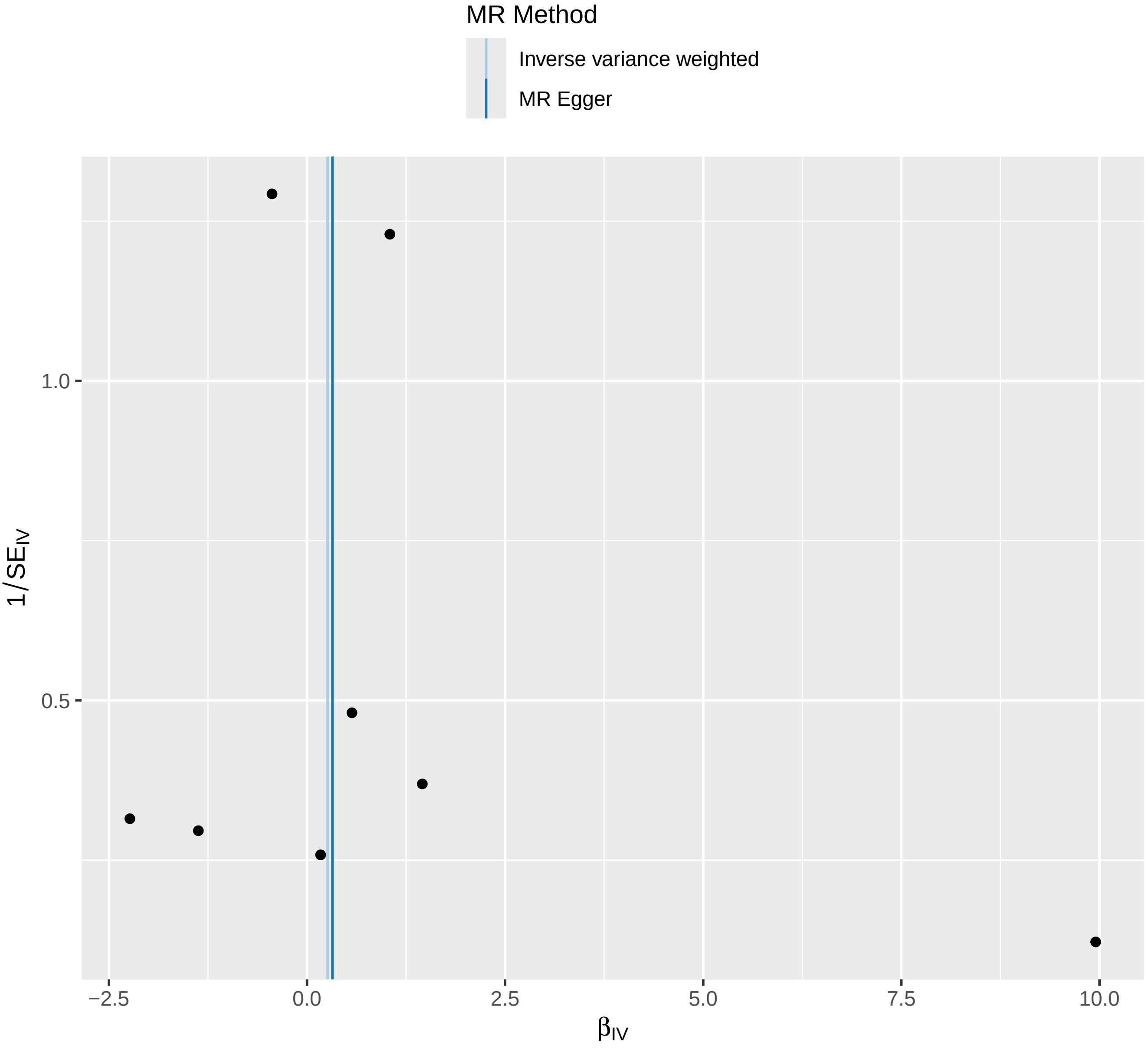
MR Method
Inverse variance weighted
MR Egger
1.0
0.5
1/SE_IV
−2.5
0.0
2.5
5.0
7.5
10.0
β_IV

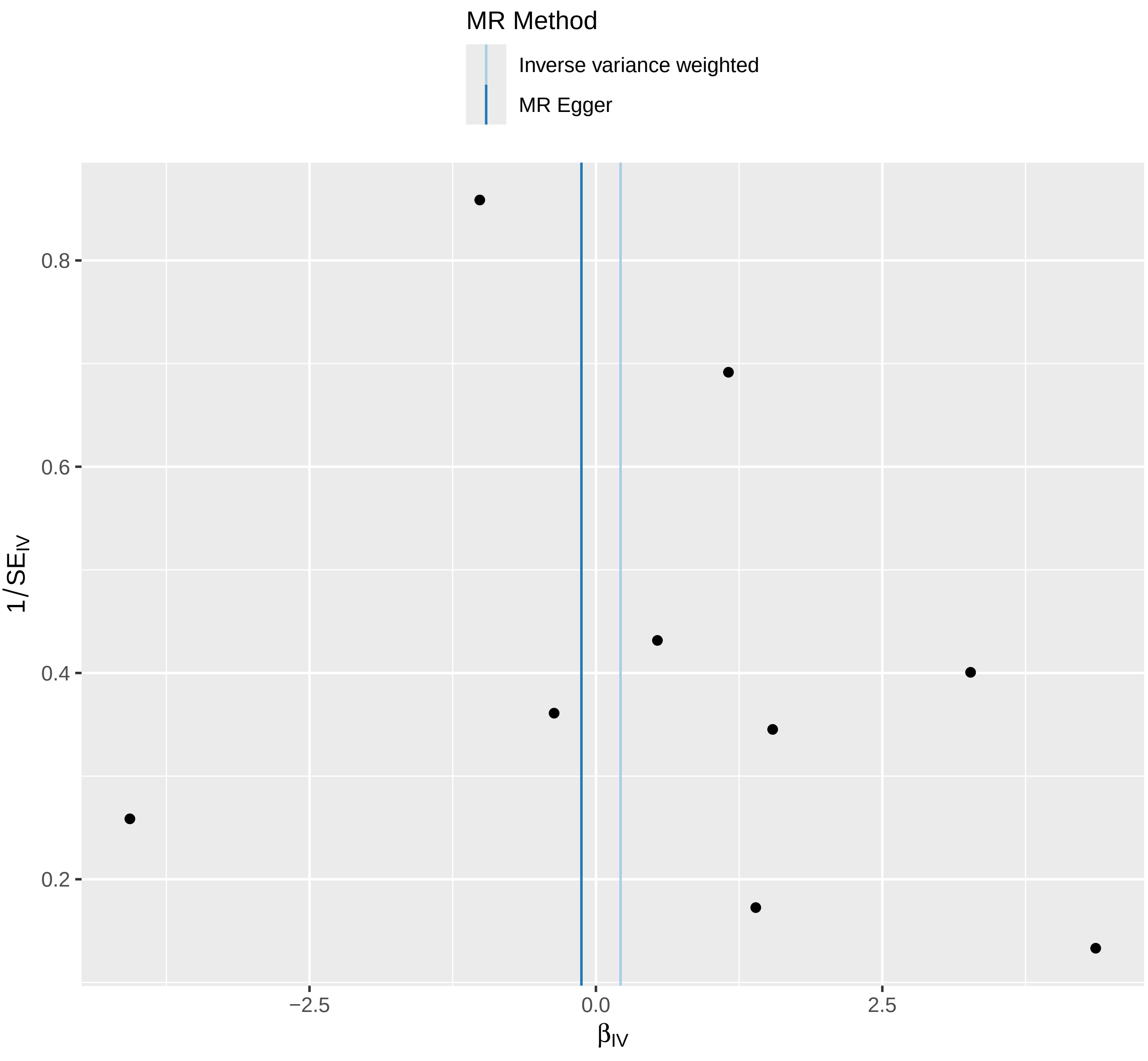
MR Method
Inverse variance weighted
MR Egger
0.8
0.6
0.4
0.2
$1/SE_{IV}$
−2.5
0.0
2.5
β_{IV}

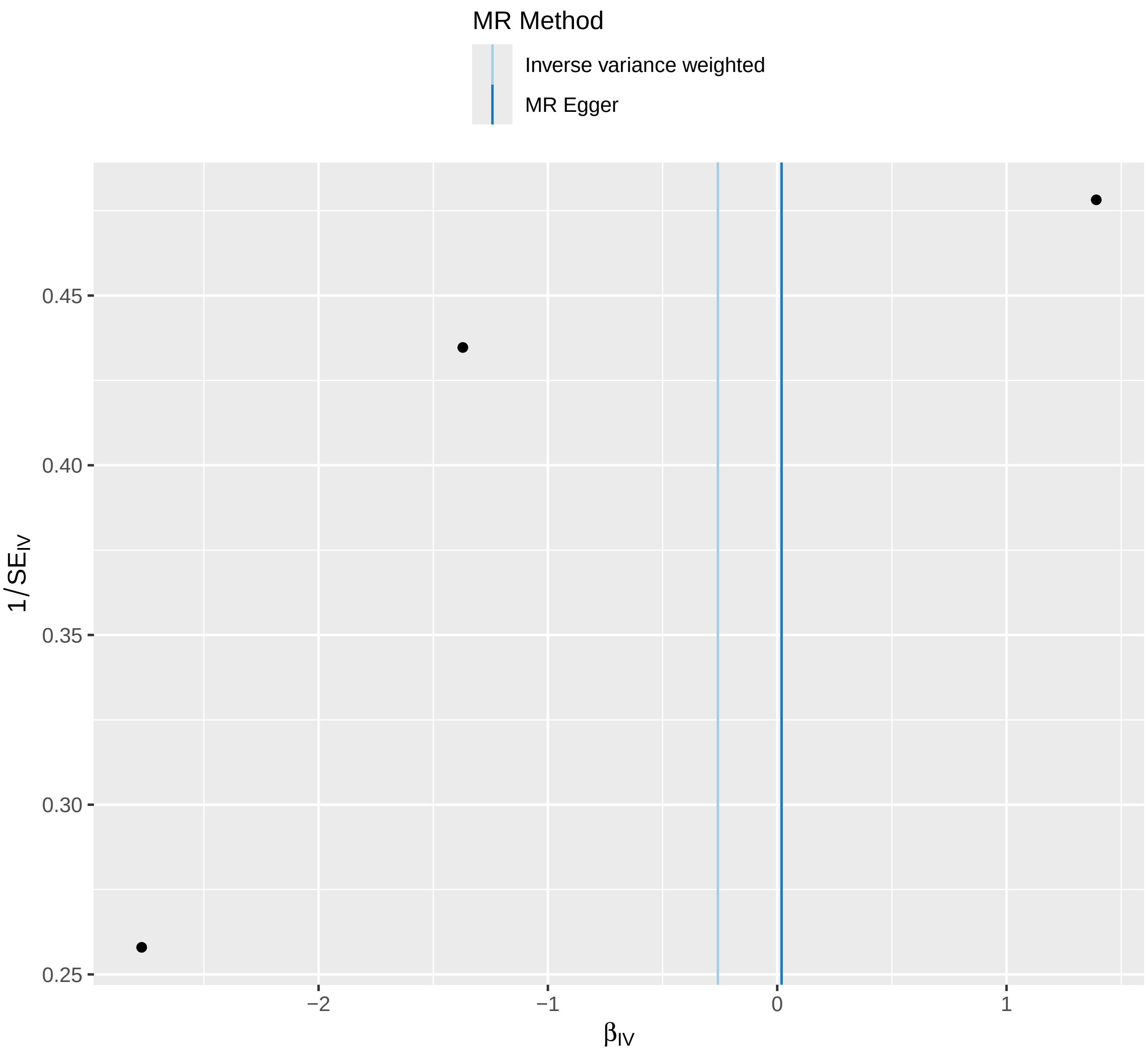
MR Method
Inverse variance weighted
MR Egger
0.45
0.40
0.35
0.30
0.25
1/SE_IV
−2
−1
0
1
β_IV

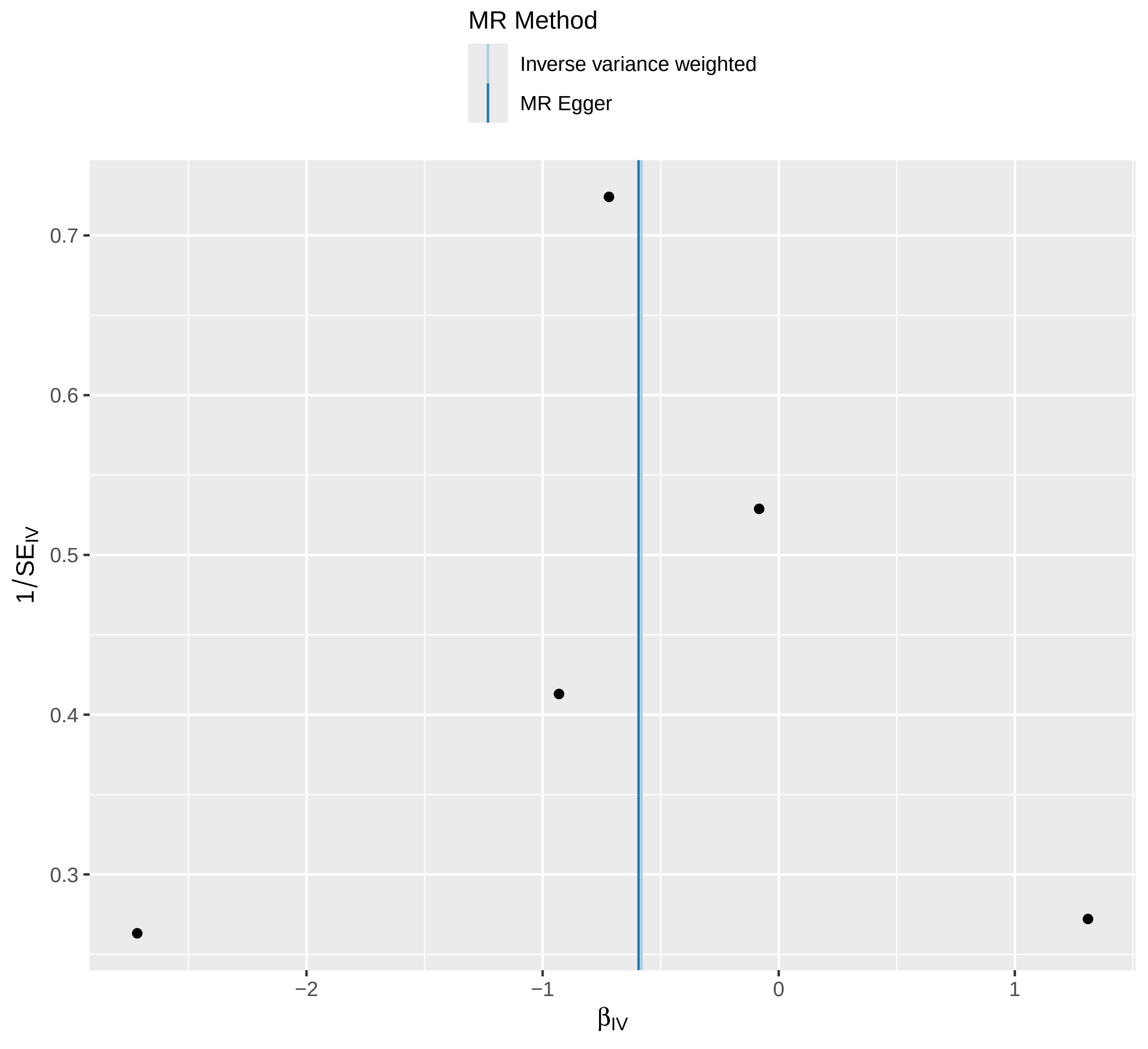

MR Method
Inverse variance weighted
MR Egger
0.7
0.6
0.5
0.4
0.3
1/SE_IV
−2
−1
0
1
β_IV

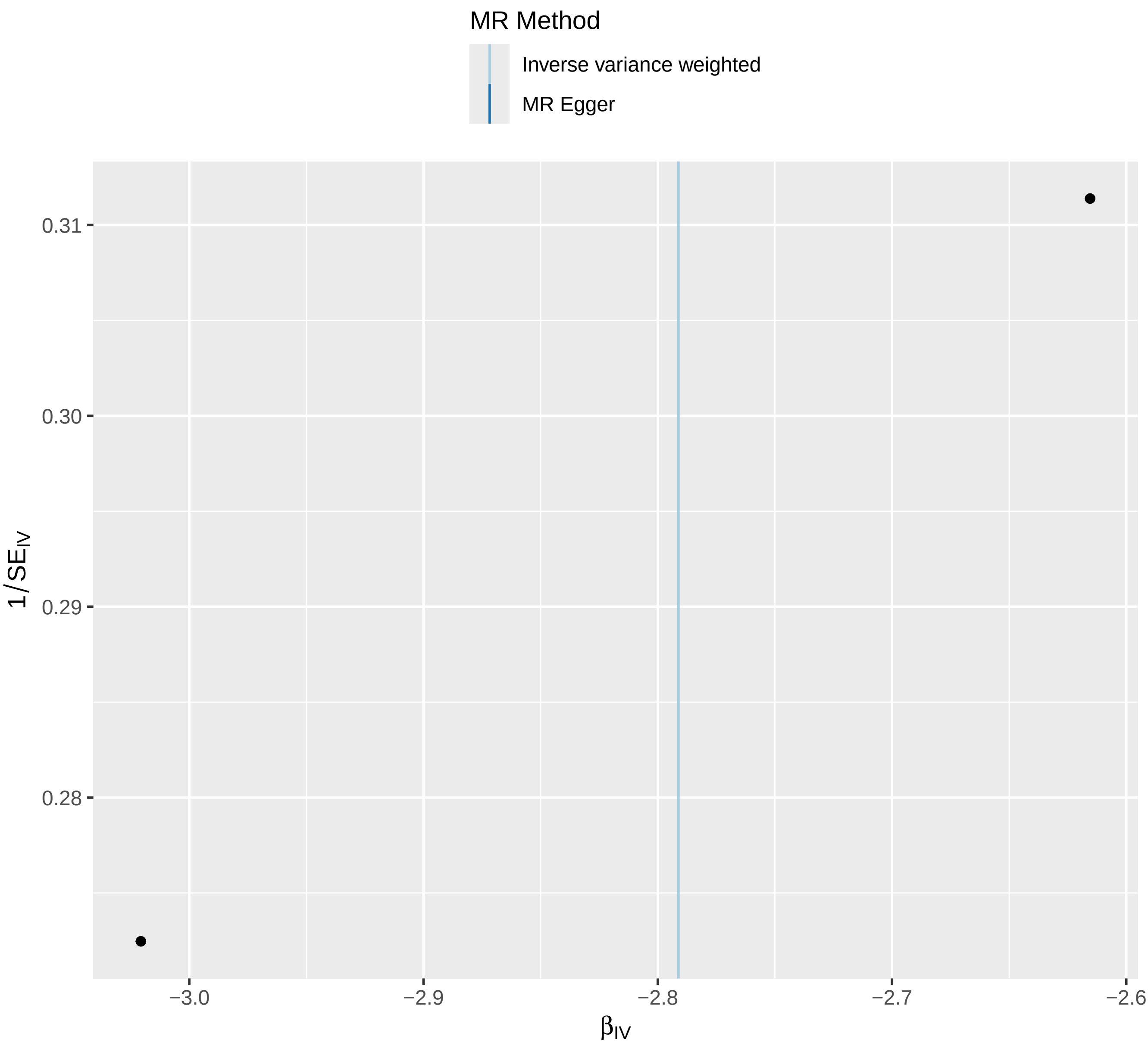
MR Method
Inverse variance weighted
MR Egger
0.31
0.30
0.29
0.28
1/SE_IV
−3.0
−2.9
−2.8
−2.7
−2.6
β_IV

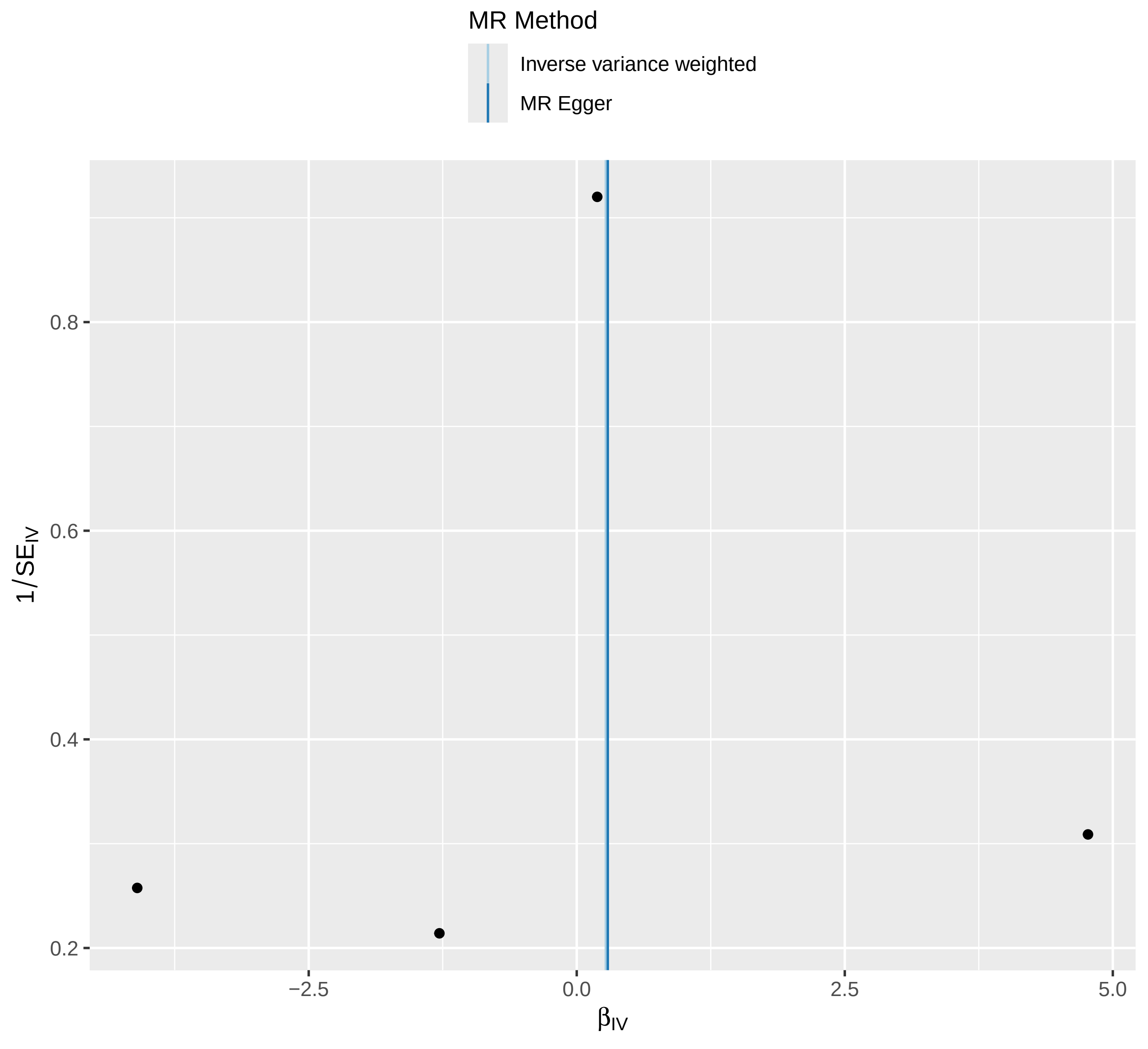
MR Method
Inverse variance weighted
MR Egger
0.8
0.6
0.4
0.2
1/SE_IV
−2.5
0.0
2.5
5.0
β_IV

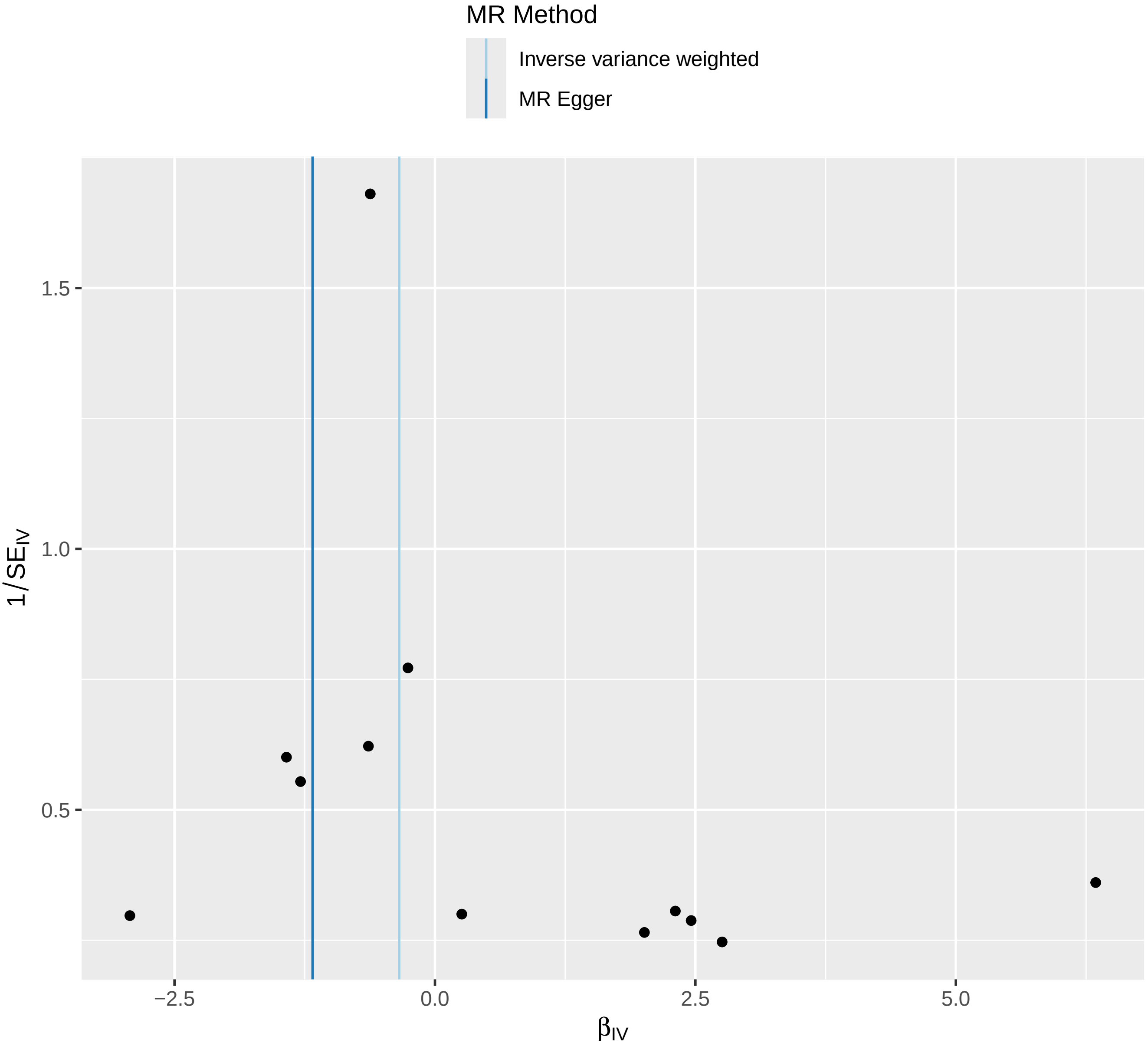

MR Method
Inverse variance weighted
MR Egger
1.5
1.0
0.5
1/SE$_{IV}$
−2.5
0.0
2.5
5.0
β$_{IV}$

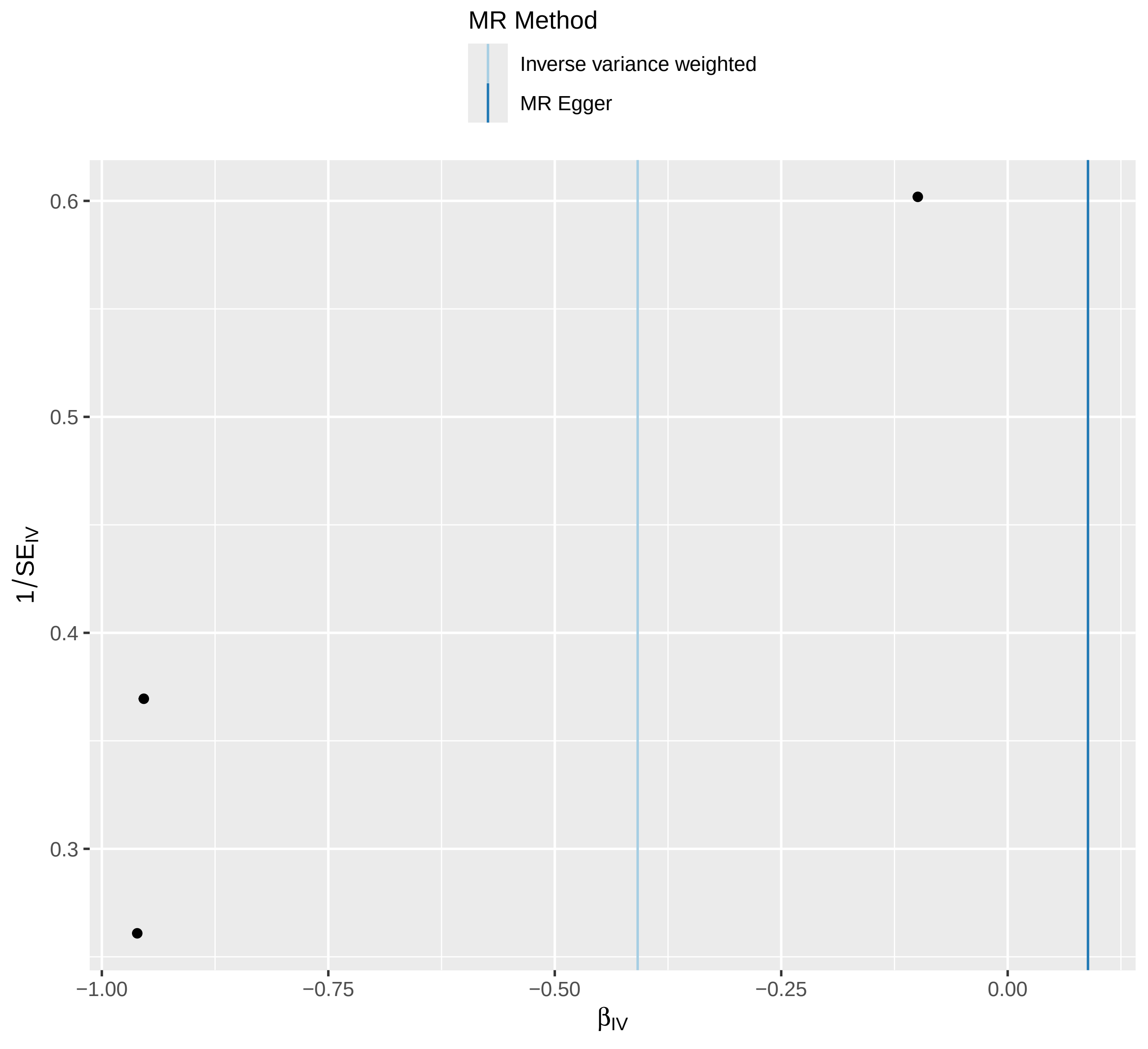

MR Method
Inverse variance weighted
MR Egger
0.6
0.5
0.4
0.3
1/SE_IV
−1.00
−0.75
−0.50
−0.25
0.00
β_IV

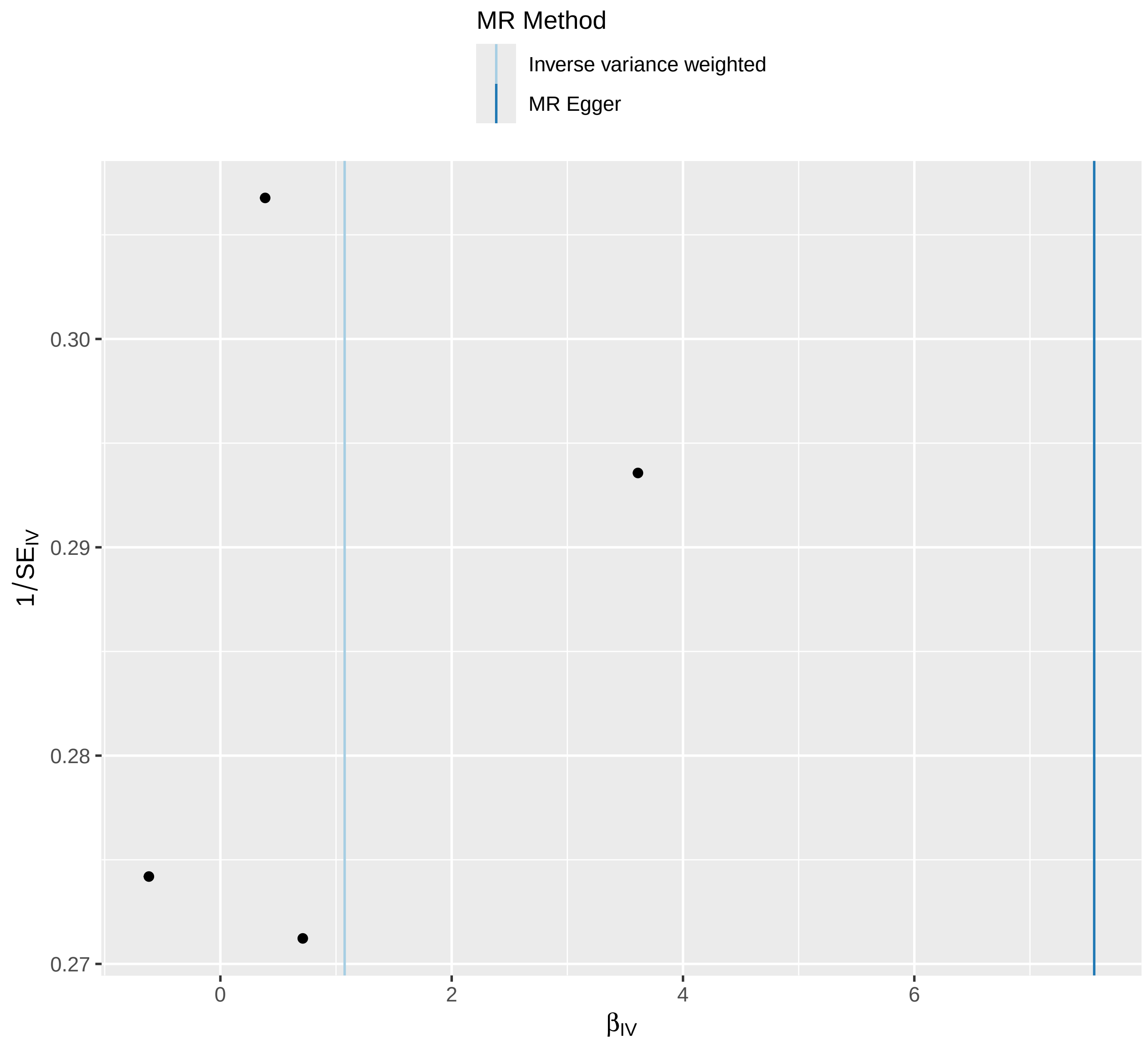
MR Method
Inverse variance weighted
MR Egger
0.30
0.29
0.28
0.27
1/SE$_{IV}$
0
2
4
6
β$_{IV}$

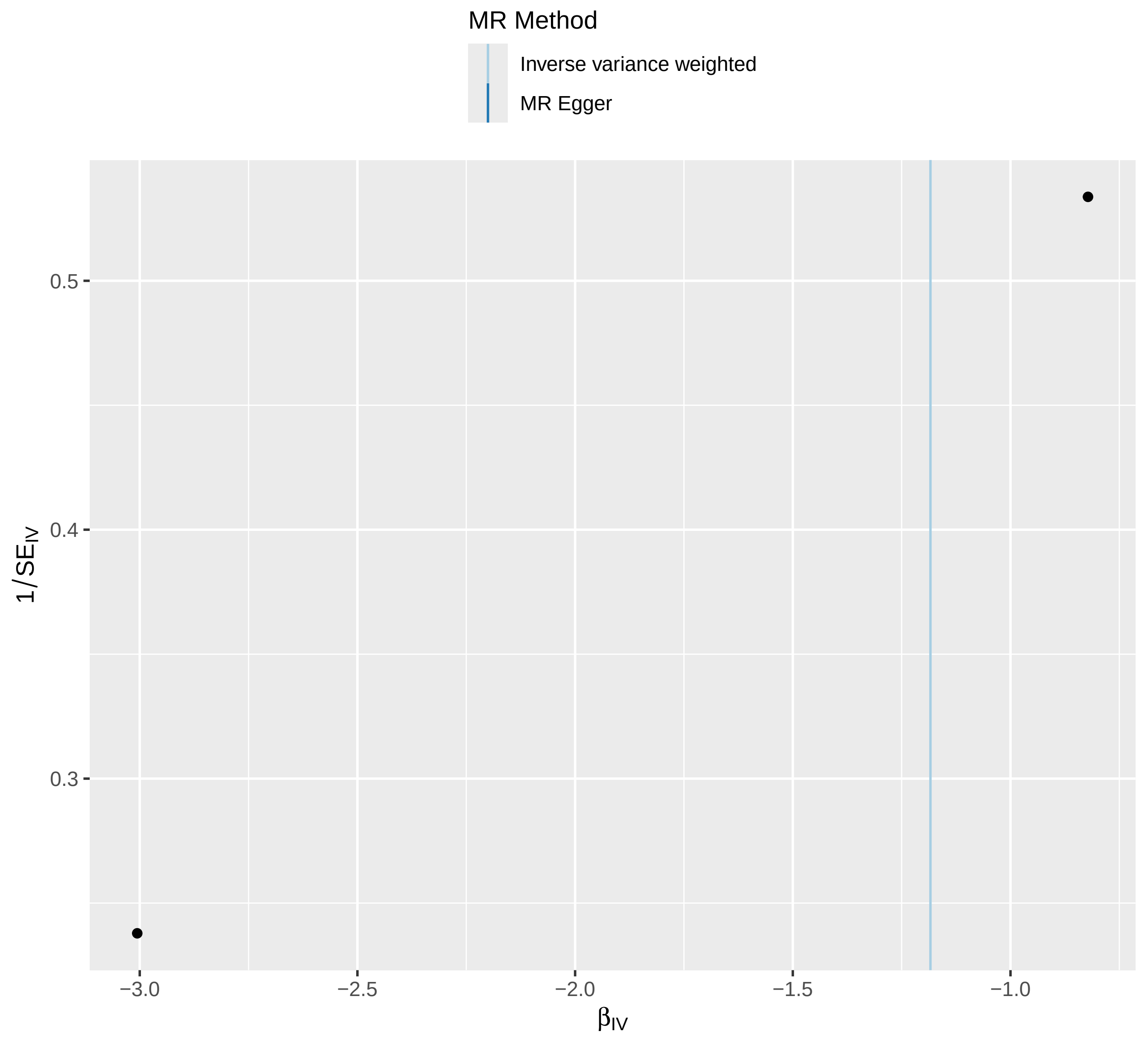

MR Method
Inverse variance weighted
MR Egger
0.5
0.4
0.3
1/SE_IV
−3.0
−2.5
−2.0
−1.5
−1.0
β_IV

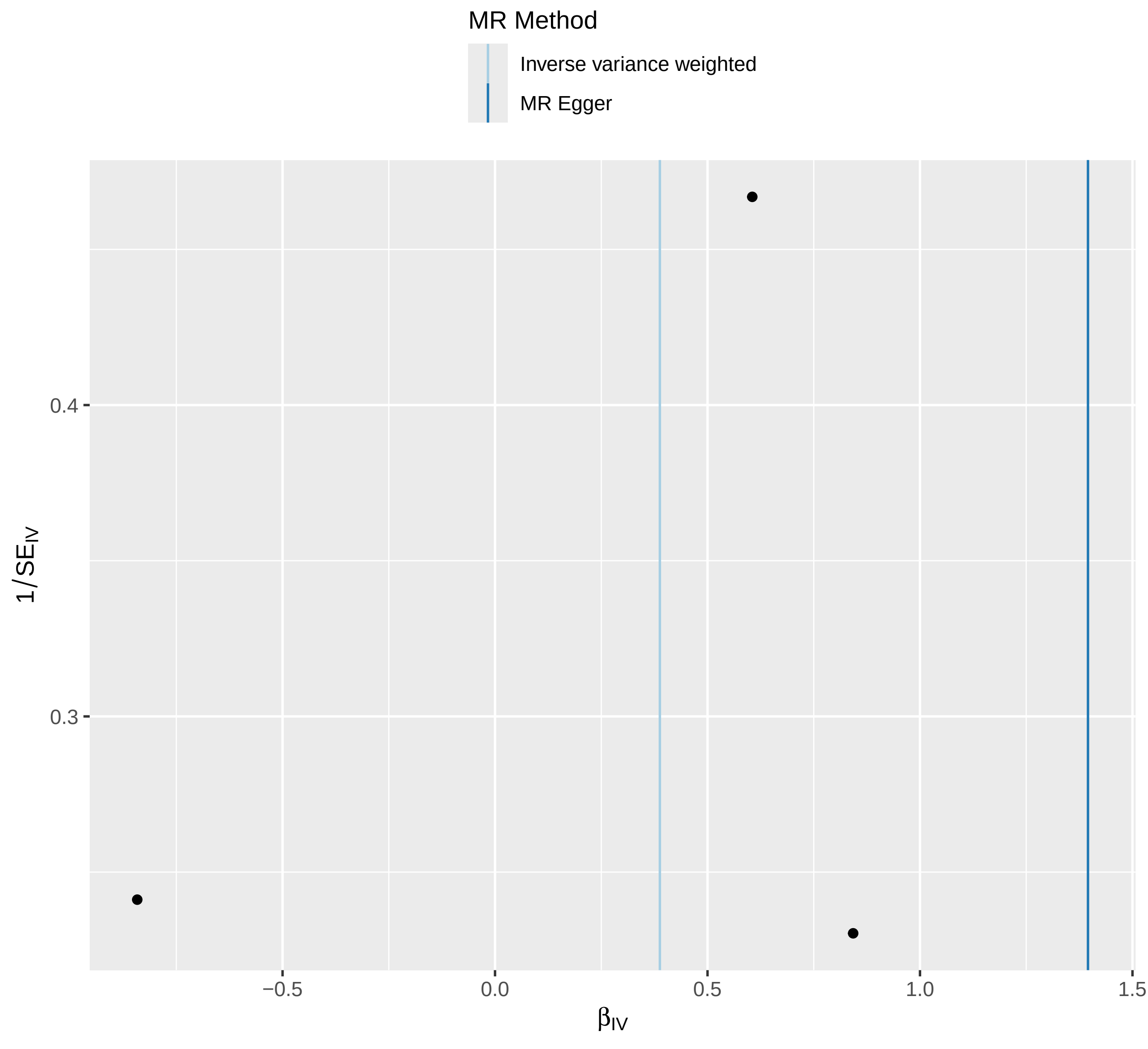

MR Method
Inverse variance weighted
MR Egger
0.4
0.3
1/SE_IV
−0.5
0.0
0.5
1.0
1.5
β_IV

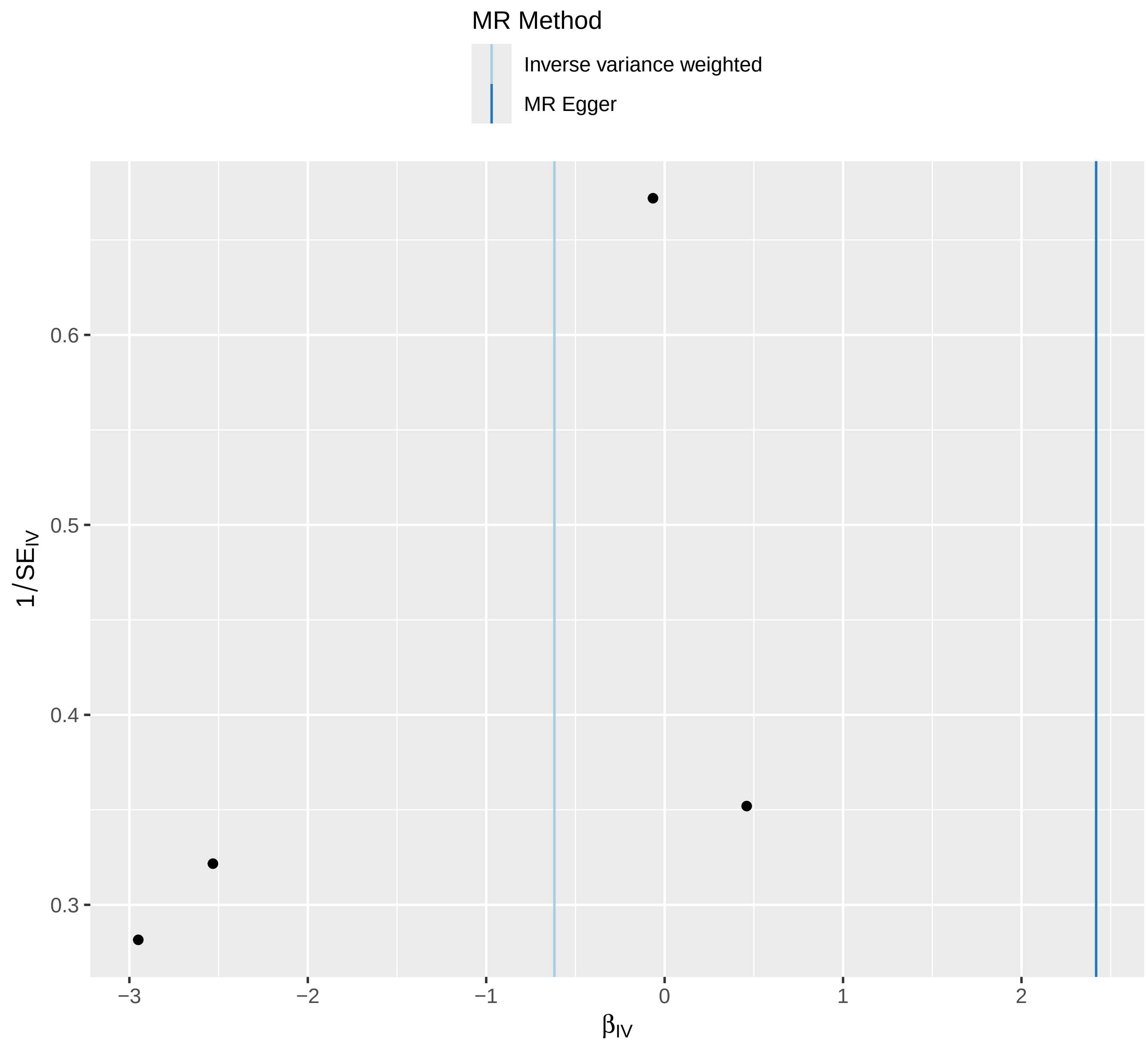
MR Method
Inverse variance weighted
MR Egger
0.6
0.5
0.4
0.3
1/SE_IV
−3
−2
−1
0
1
2
β_IV

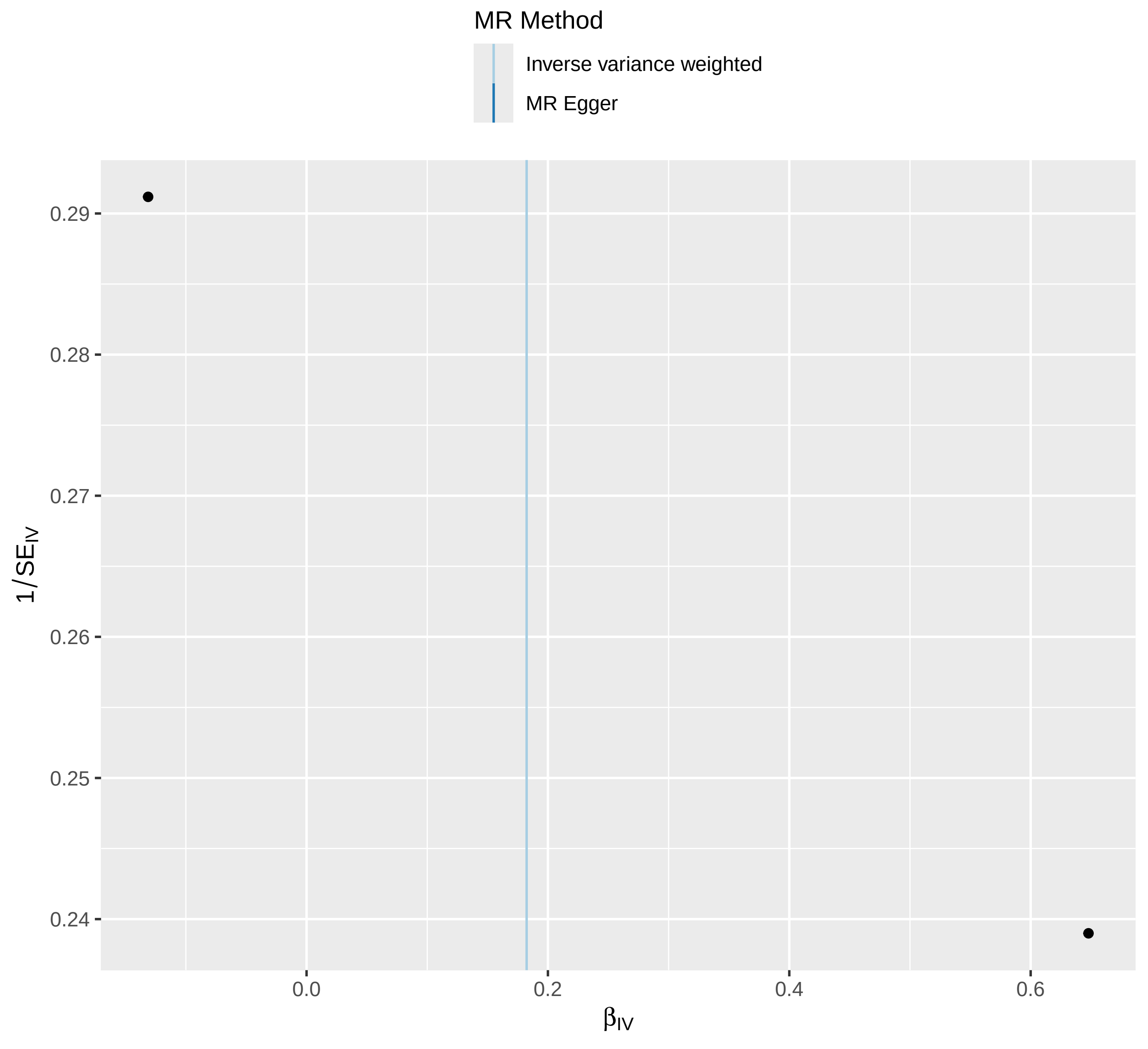
MR Method
Inverse variance weighted
MR Egger
0.29
0.28
0.27
0.26
0.25
0.24
$1/SE_{IV}$
0.0
0.2
0.4
0.6
β_{IV}

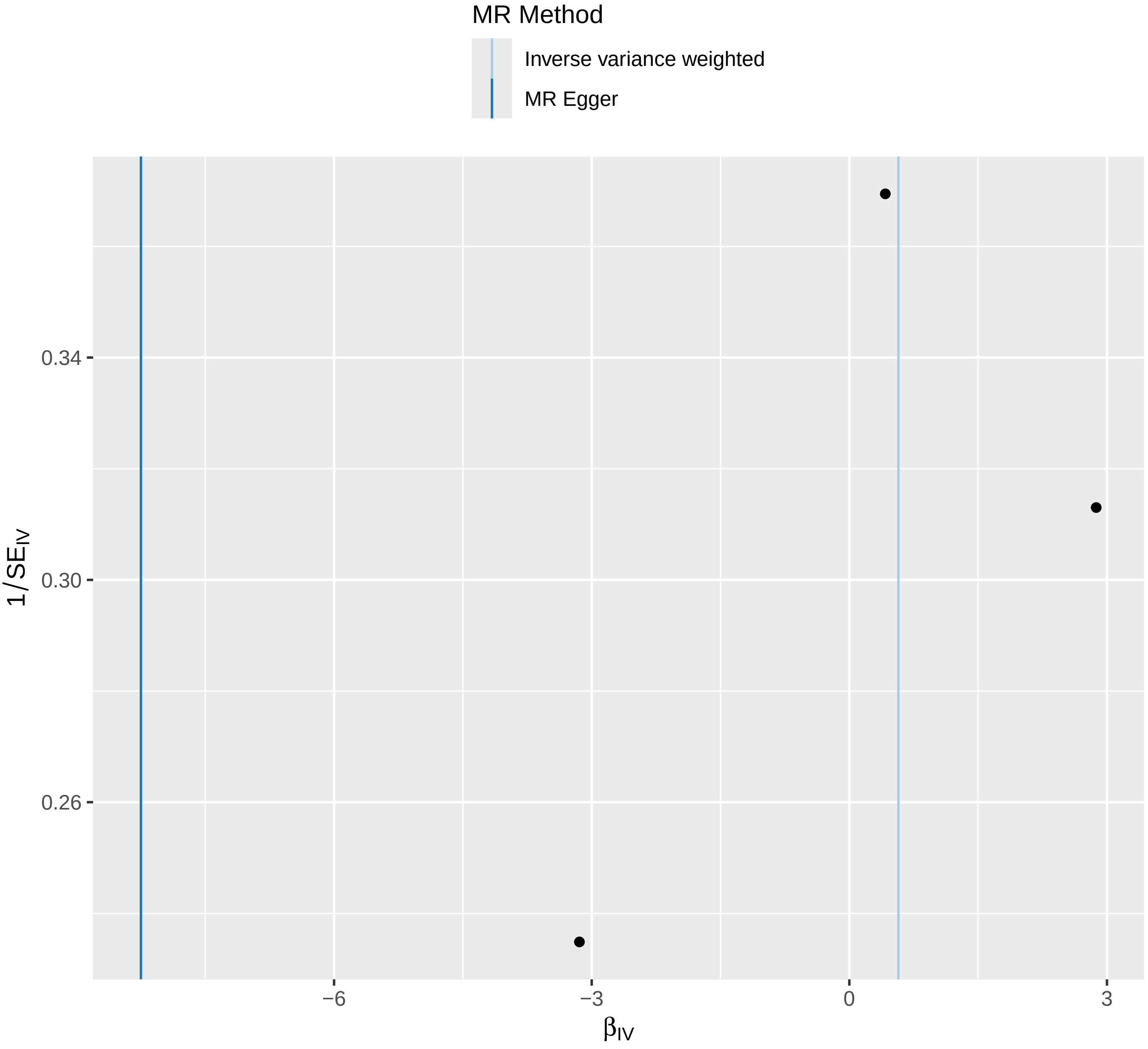
MR Method
Inverse variance weighted
MR Egger
0.34
0.30
0.26
1/SE_IV
−6
−3
0
3
β_IV

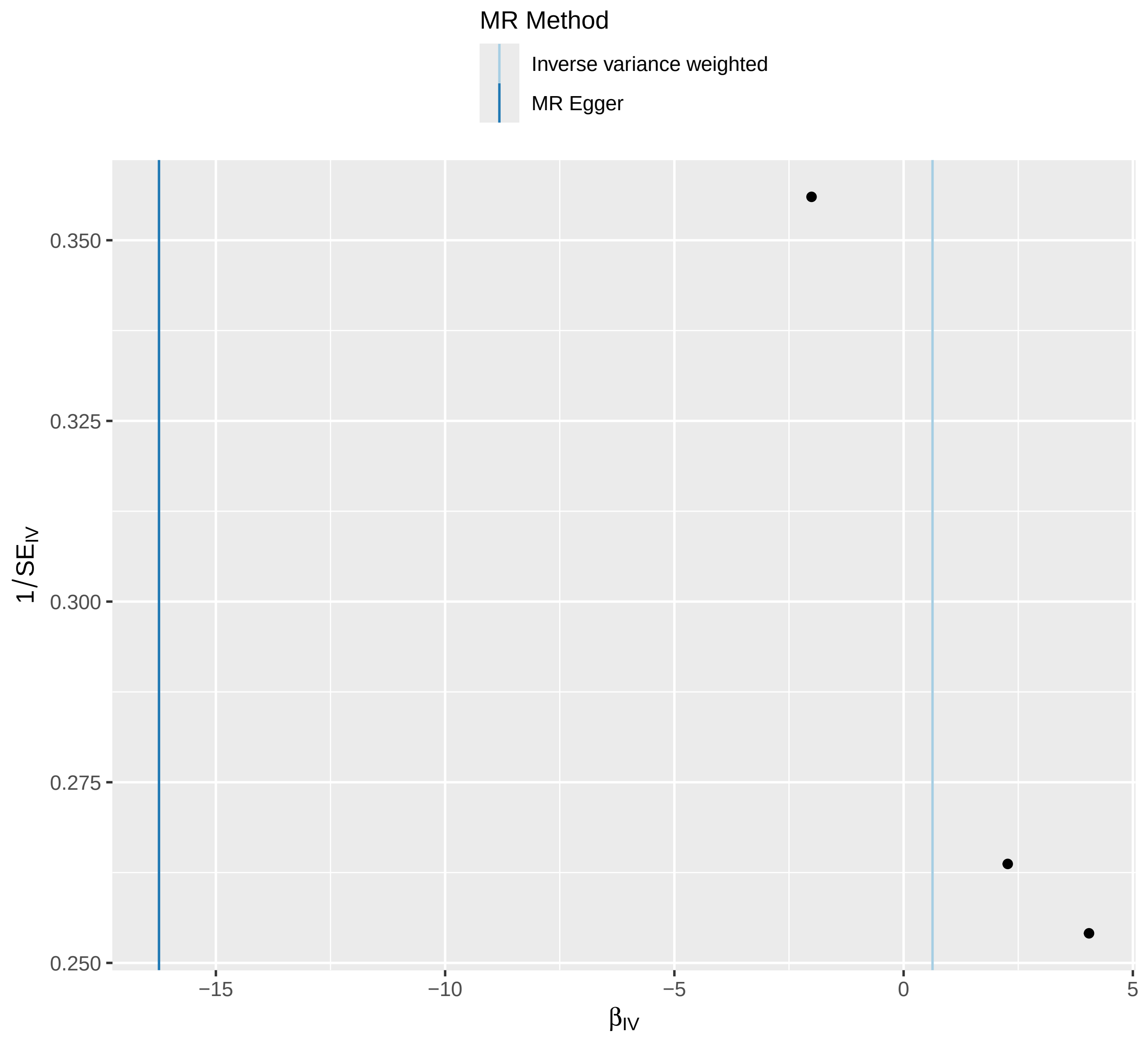

MR Method
Inverse variance weighted
MR Egger
0.350
0.325
0.300
0.275
0.250
$1/SE_{IV}$
−15
−10
−5
0
5
β_{IV}

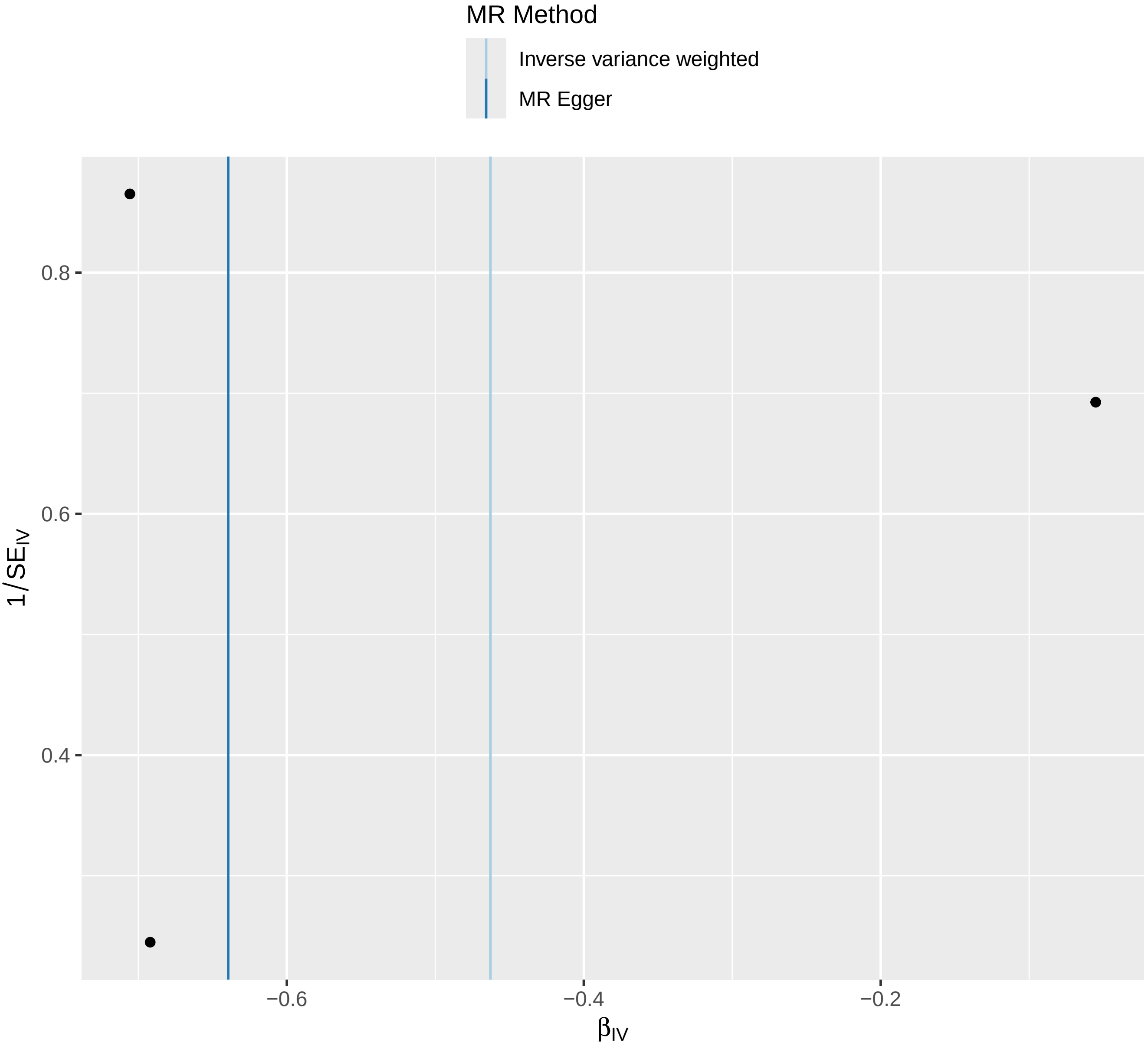
MR Method
Inverse variance weighted
MR Egger
0.8
0.6
0.4
1/SE_IV
−0.6
−0.4
−0.2
β_IV

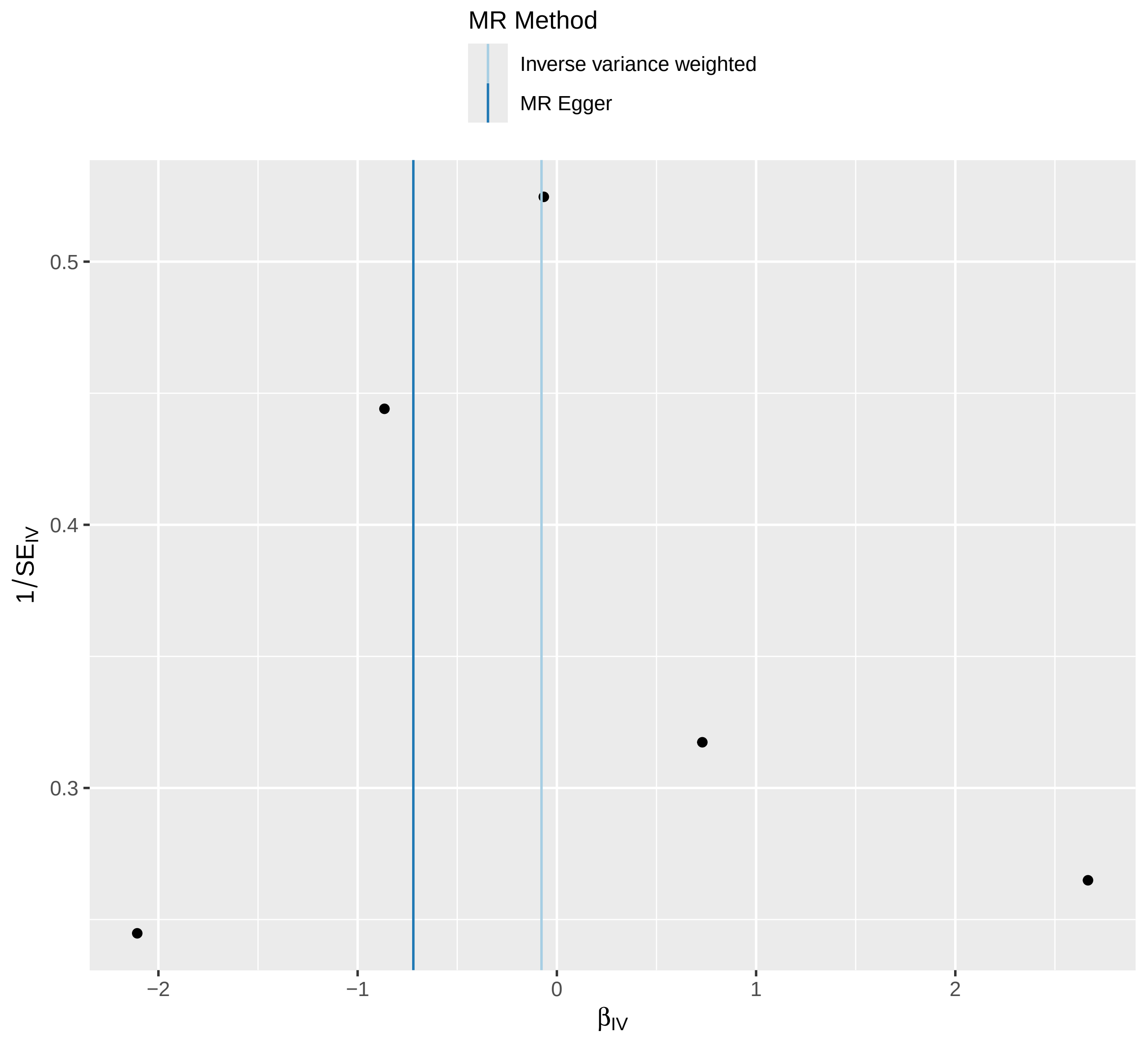
MR Method
Inverse variance weighted
MR Egger
0.5
0.4
0.3
1/SE_IV
−2
−1
0
1
2
β_IV

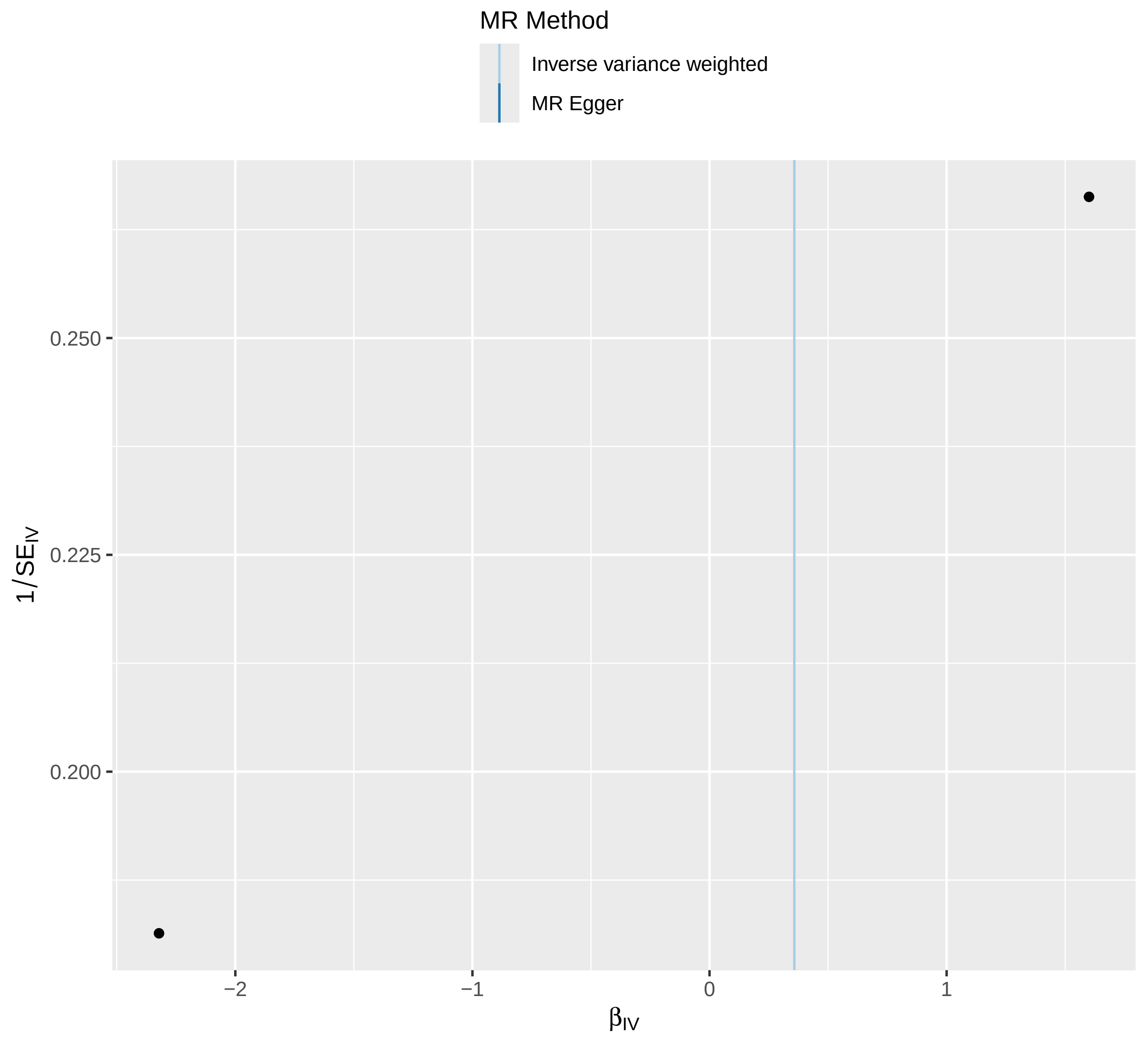
MR Method
Inverse variance weighted
MR Egger
0.250
0.225
0.200
1/SE_IV
−2
−1
0
1
β_IV

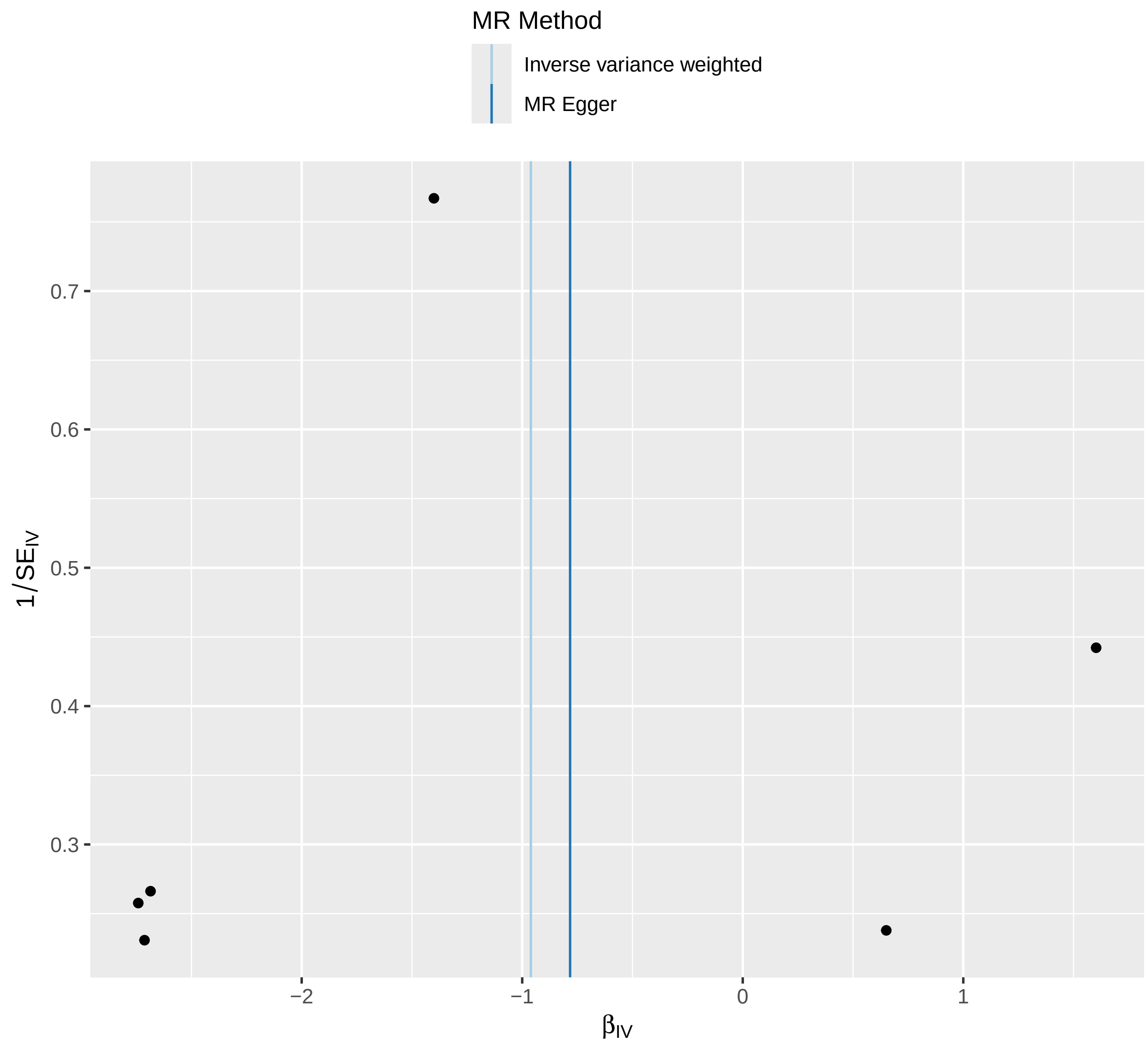
MR Method
Inverse variance weighted
MR Egger
1/SE_IV
0.7
0.6
0.5
0.4
0.3
−2
−1
0
1
β_IV

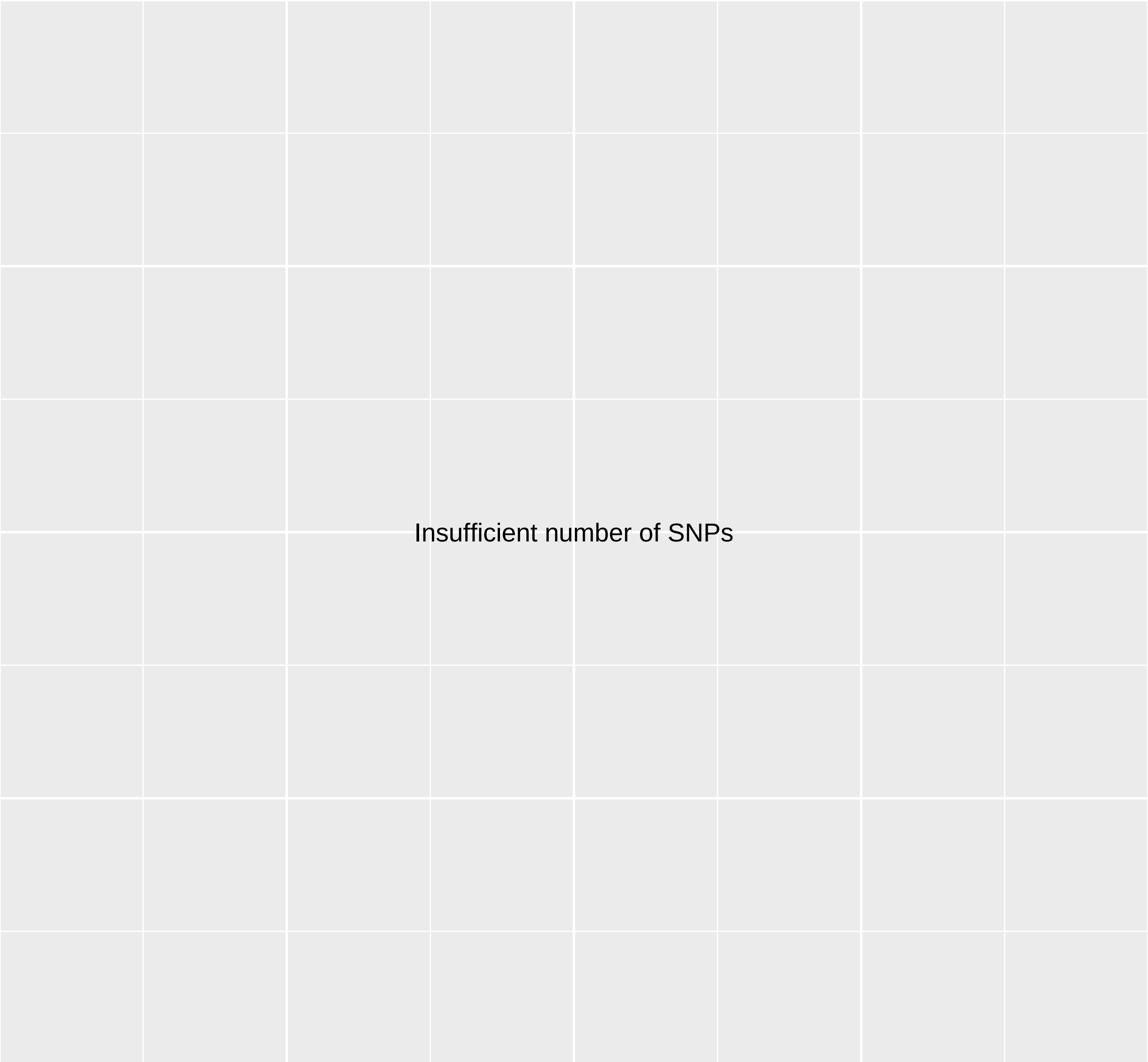
Insufficient number of SNPs

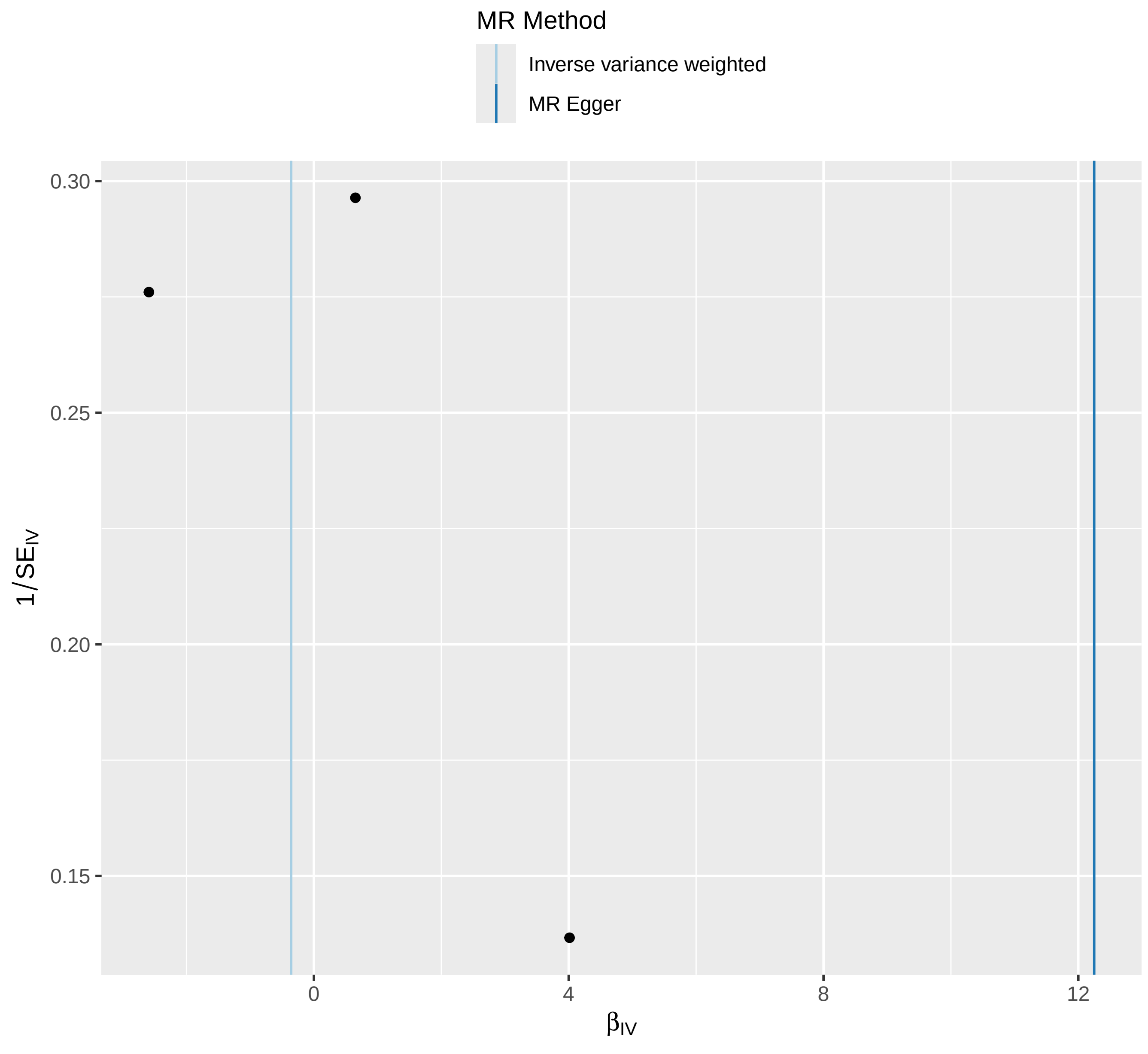
MR Method
Inverse variance weighted
MR Egger
0.30
0.25
0.20
0.15
$1/SE_{IV}$
0
4
8
12
β_{IV}

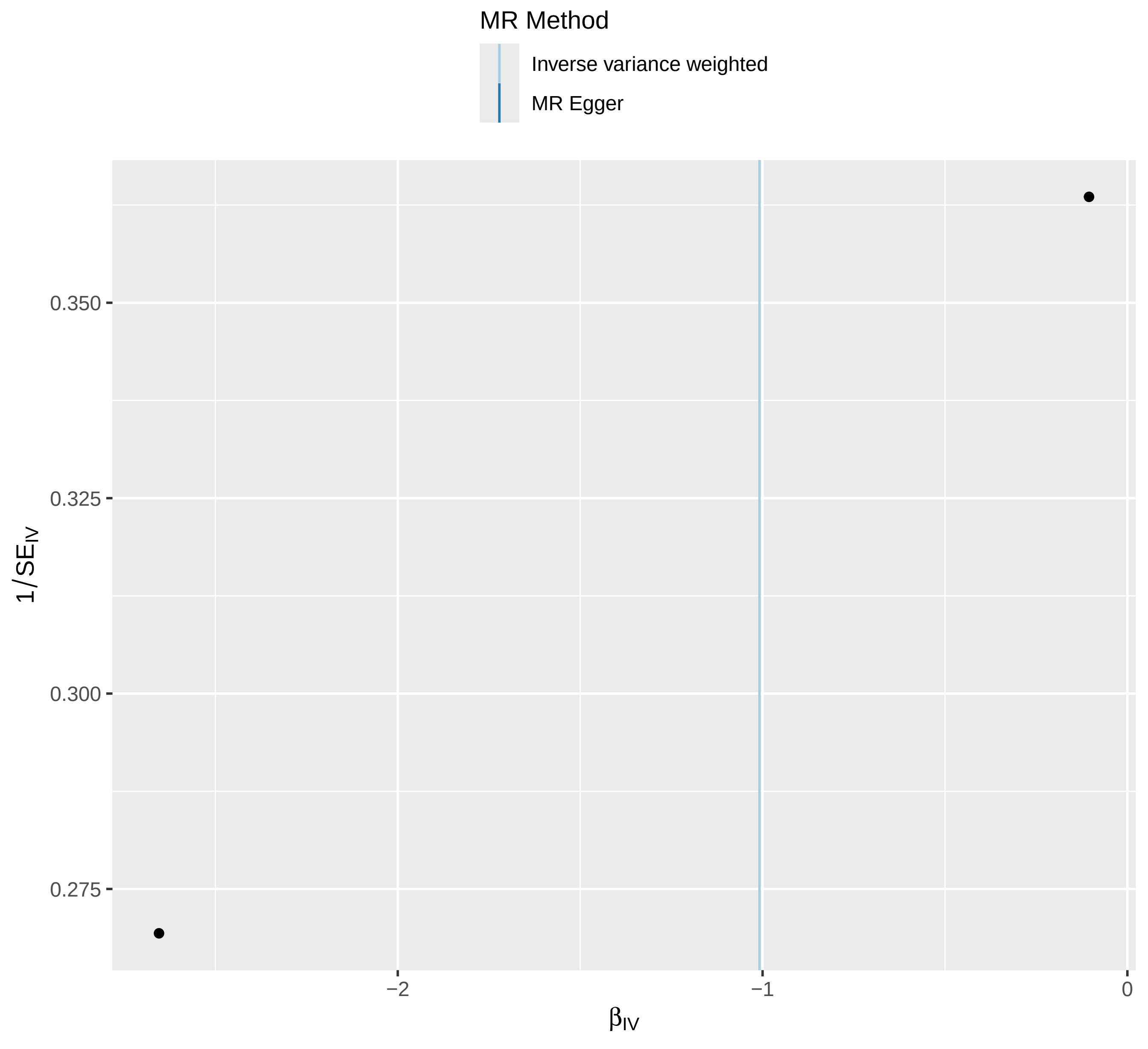

MR Method
Inverse variance weighted
MR Egger
0.350
0.325
0.300
0.275
1/SE$_{IV}$
−2
−1
0
β$_{IV}$

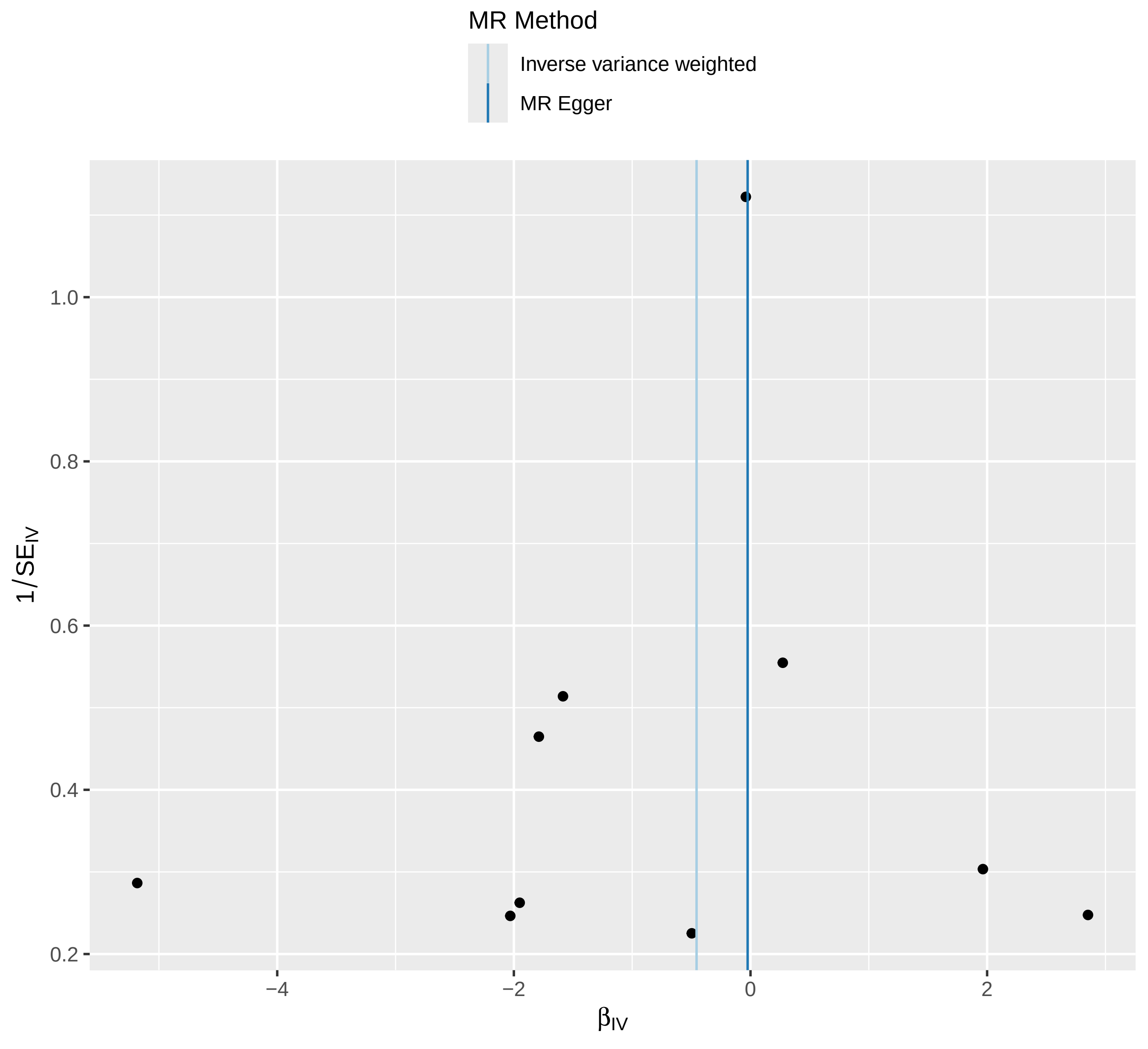
MR Method
Inverse variance weighted
MR Egger
1/SE_IV
1.0
0.8
0.6
0.4
0.2
−4
−2
0
2
β_IV

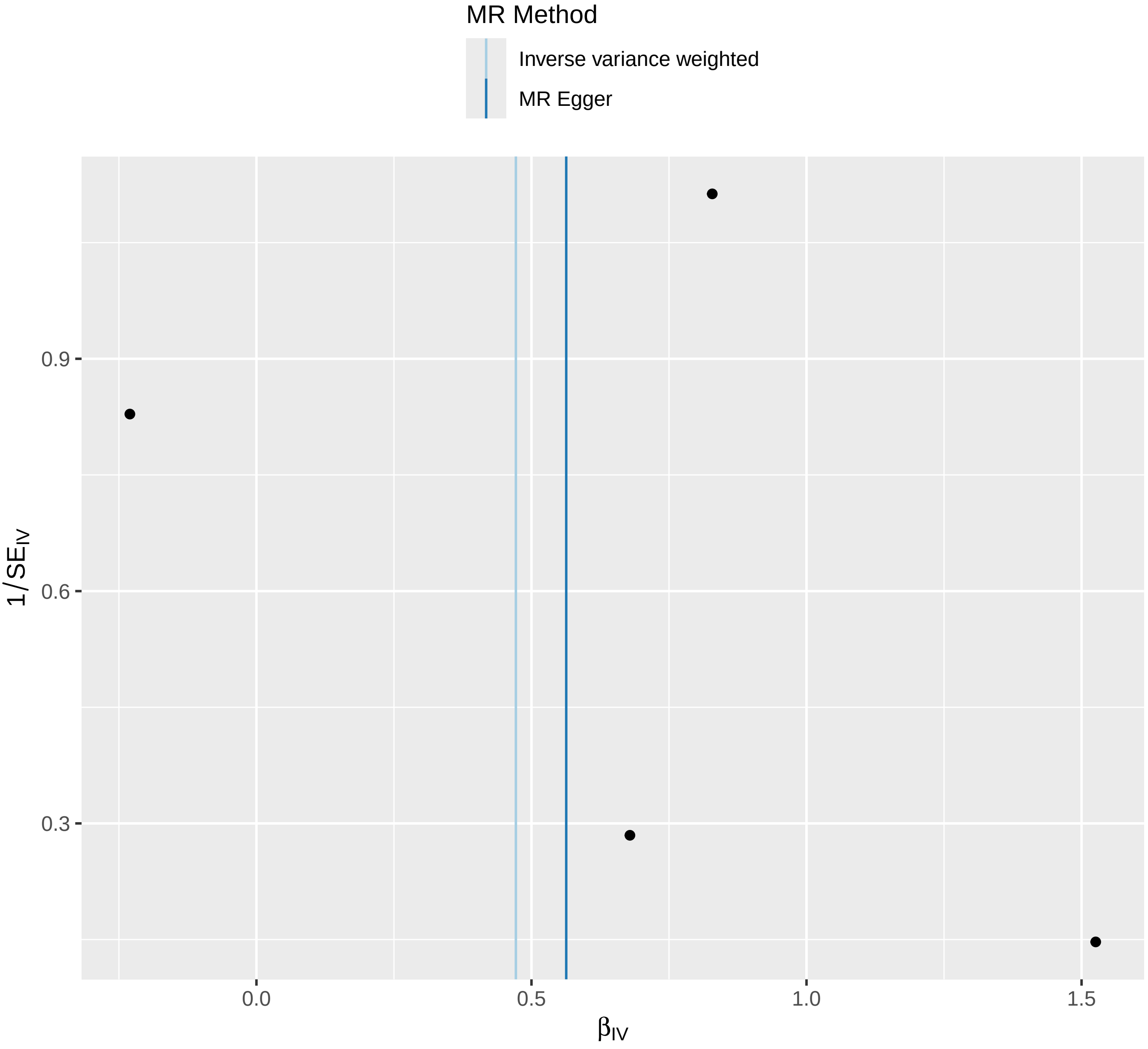
MR Method
Inverse variance weighted
MR Egger
0.9
0.6
0.3
1/SE_IV
0.0
0.5
1.0
1.5
β_IV

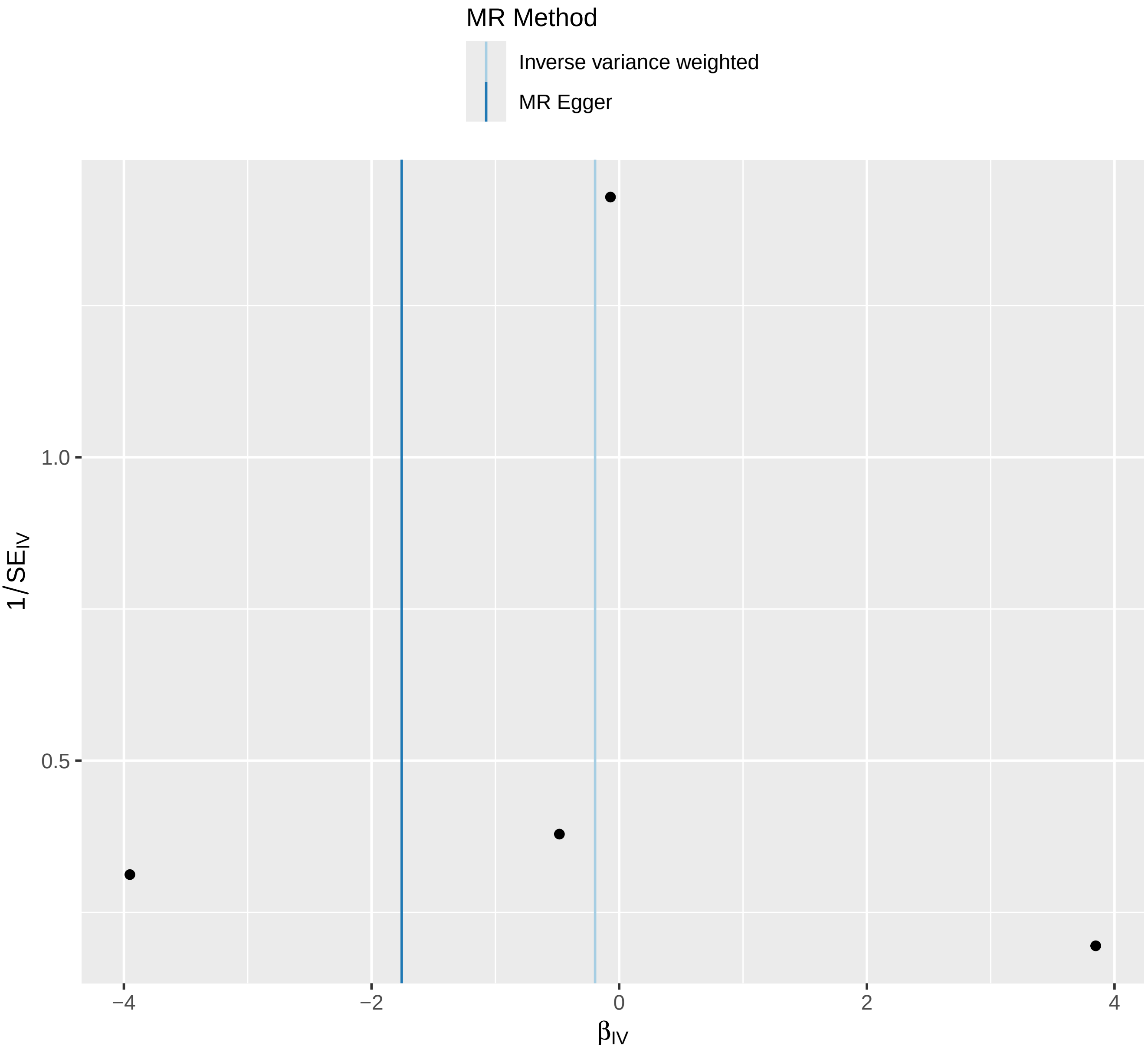
MR Method
Inverse variance weighted
MR Egger
1.0
0.5
1/SE$_{IV}$
−4
−2
0
2
4
β$_{IV}$

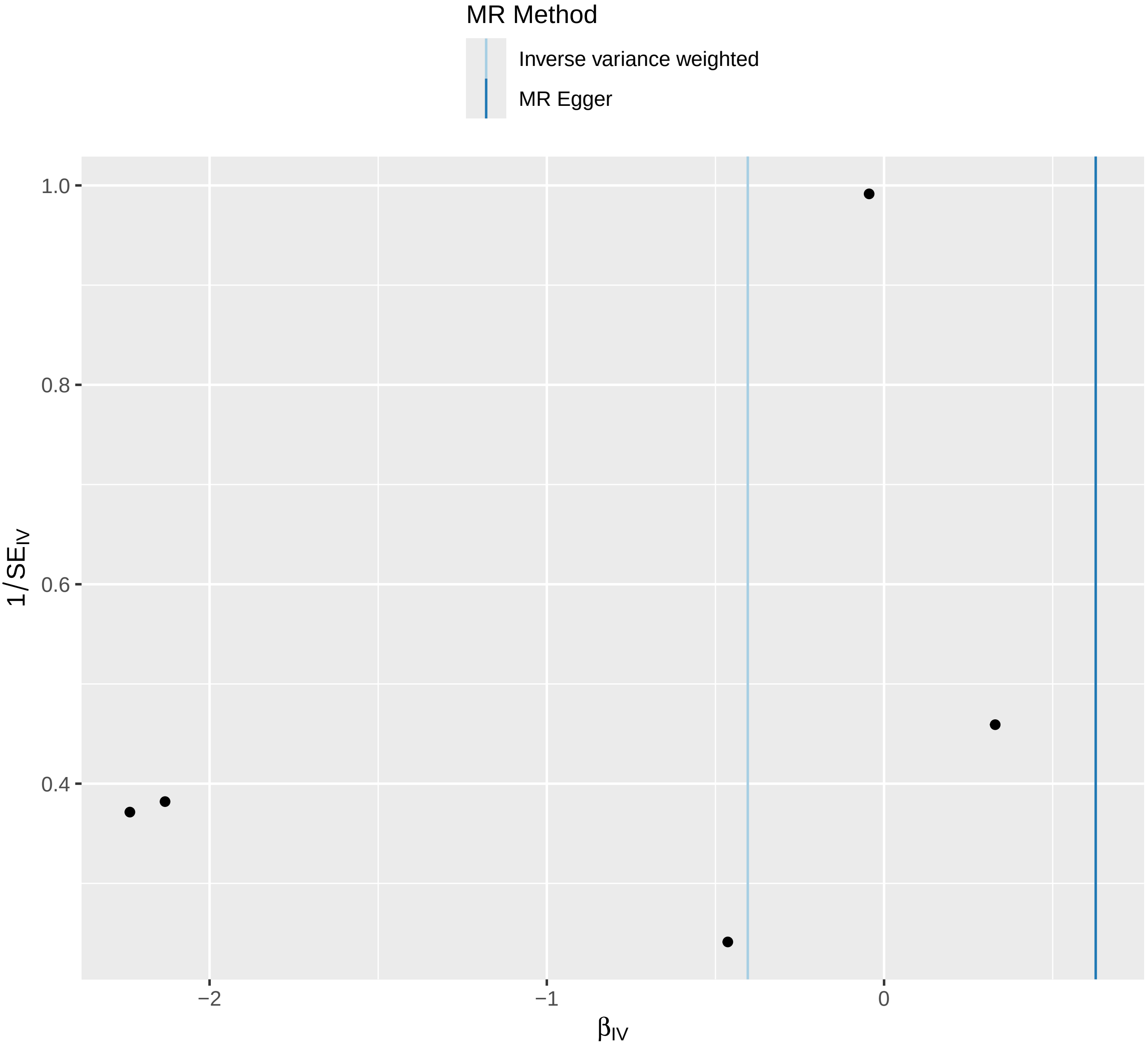

MR Method
Inverse variance weighted
MR Egger
1.0
0.8
0.6
0.4
$1/SE_{IV}$
−2
−1
0
β_{IV}

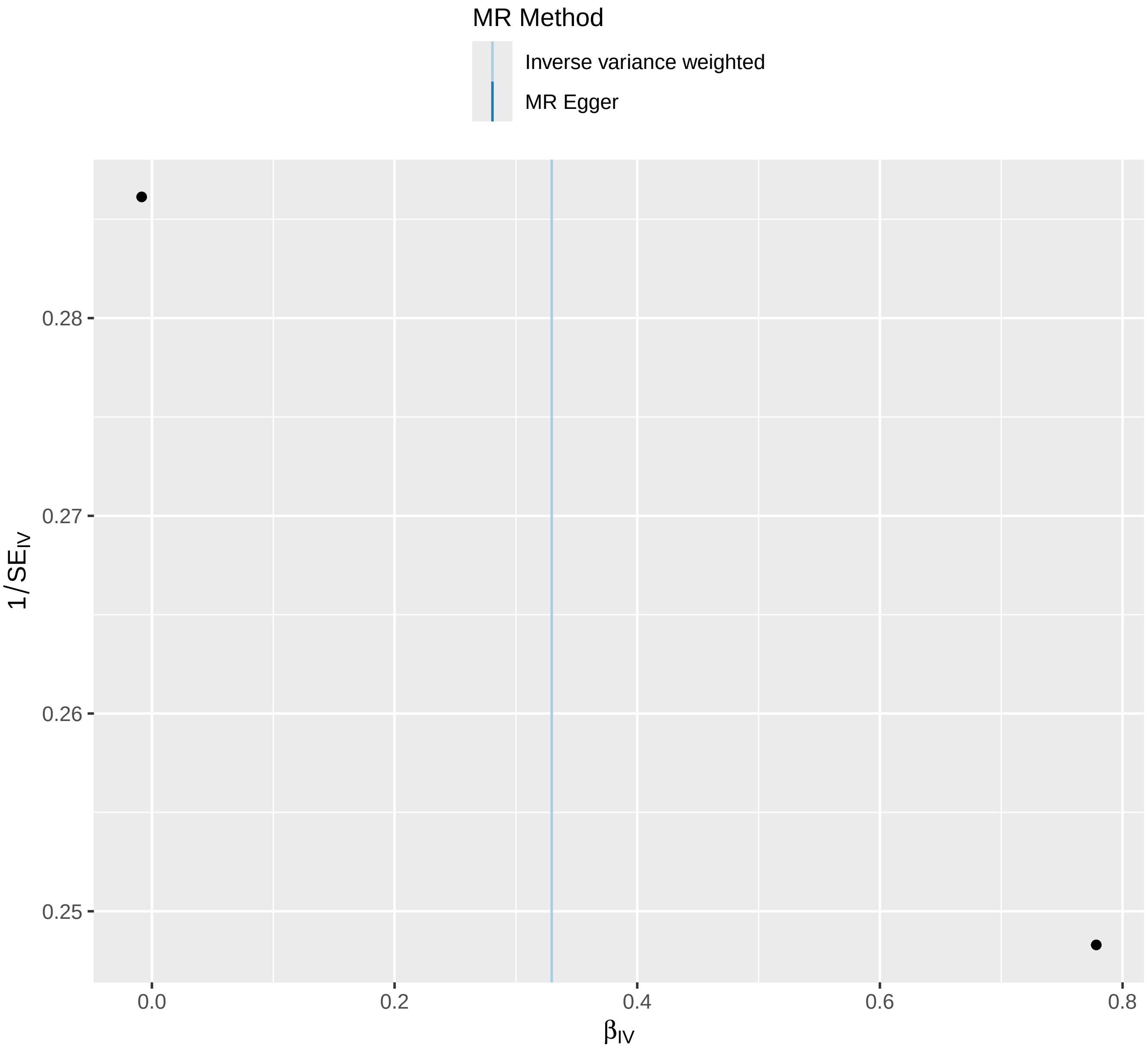

MR Method
Inverse variance weighted
MR Egger
0.28
0.27
0.26
0.25
1/SE_IV
0.0
0.2
0.4
0.6
0.8
β_IV

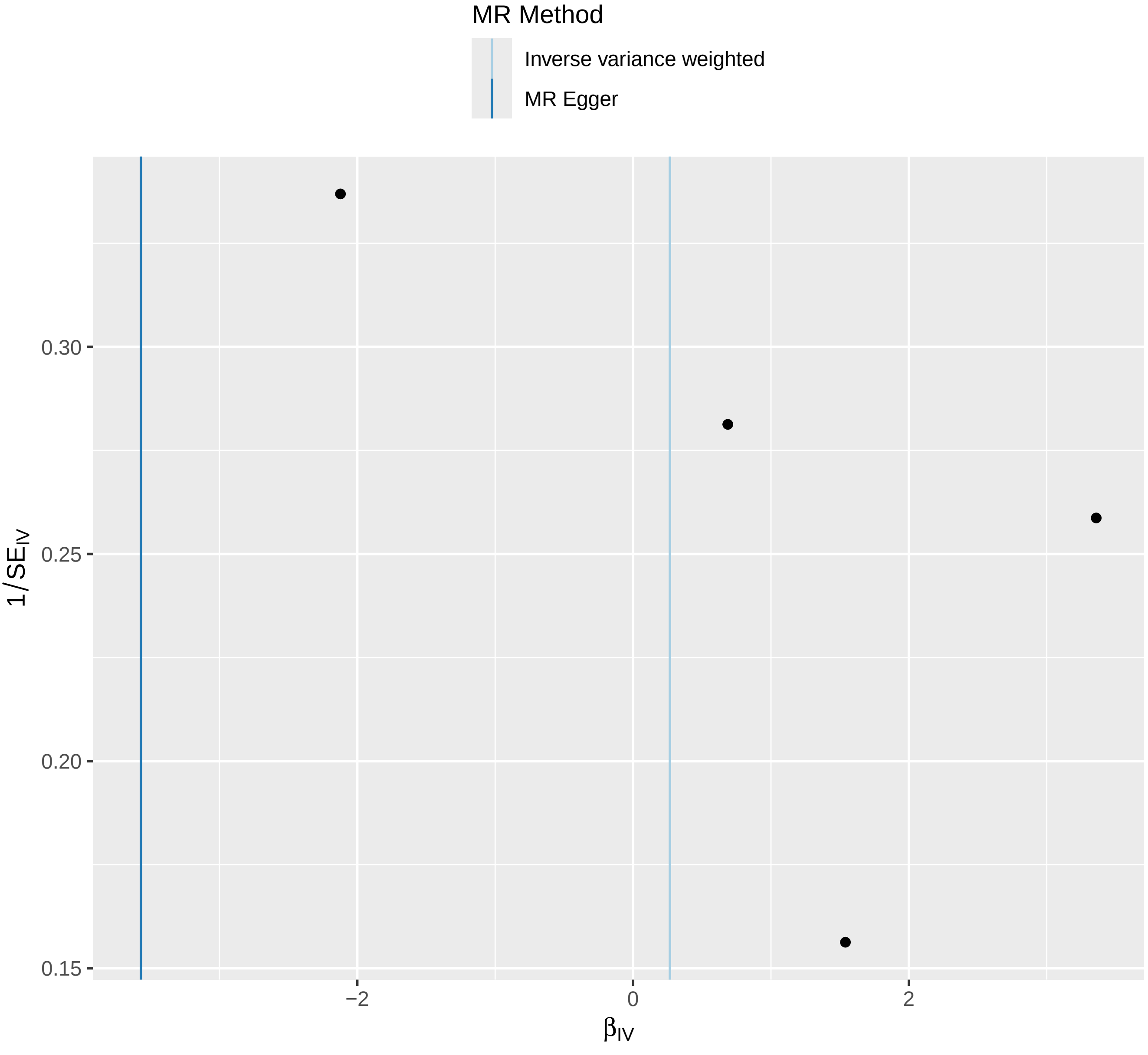
MR Method
Inverse variance weighted
MR Egger
0.30
0.25
0.20
0.15
1/SE$_{IV}$
−2
0
2
β_{IV}

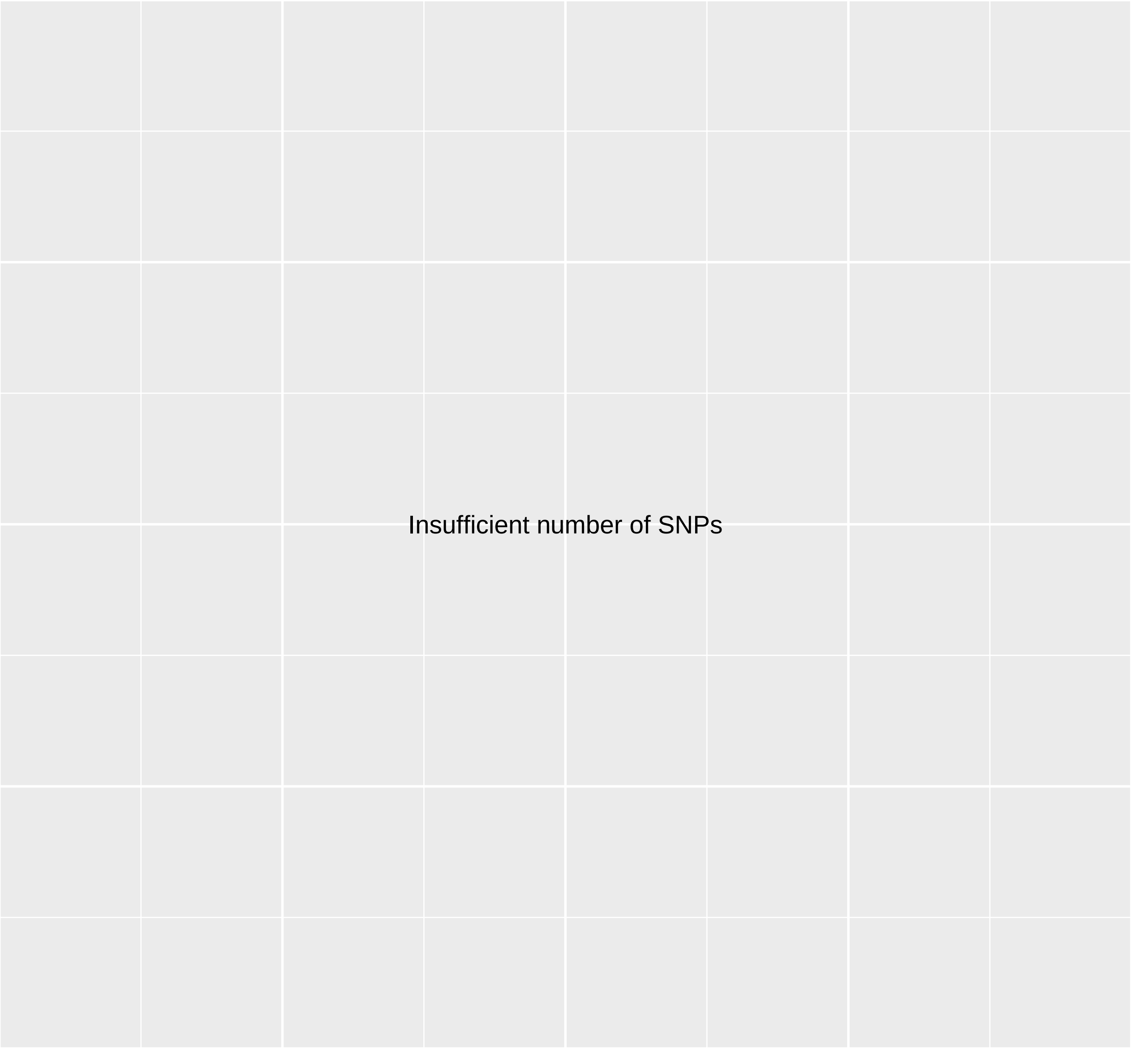
Insufficient number of SNPs

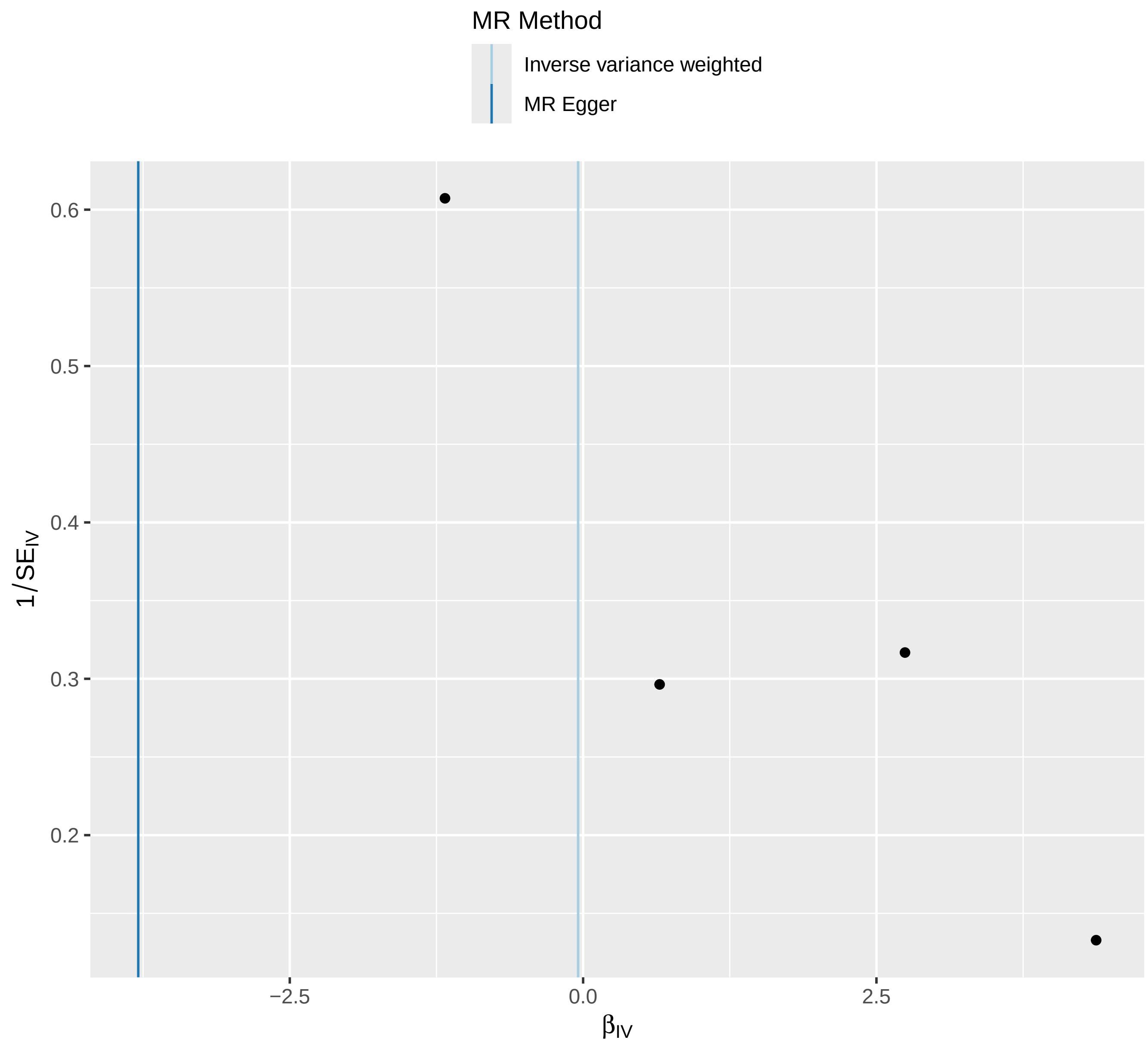
MR Method
Inverse variance weighted
MR Egger
0.6
0.5
0.4
0.3
0.2
1/SE_IV
−2.5
0.0
2.5
β_IV

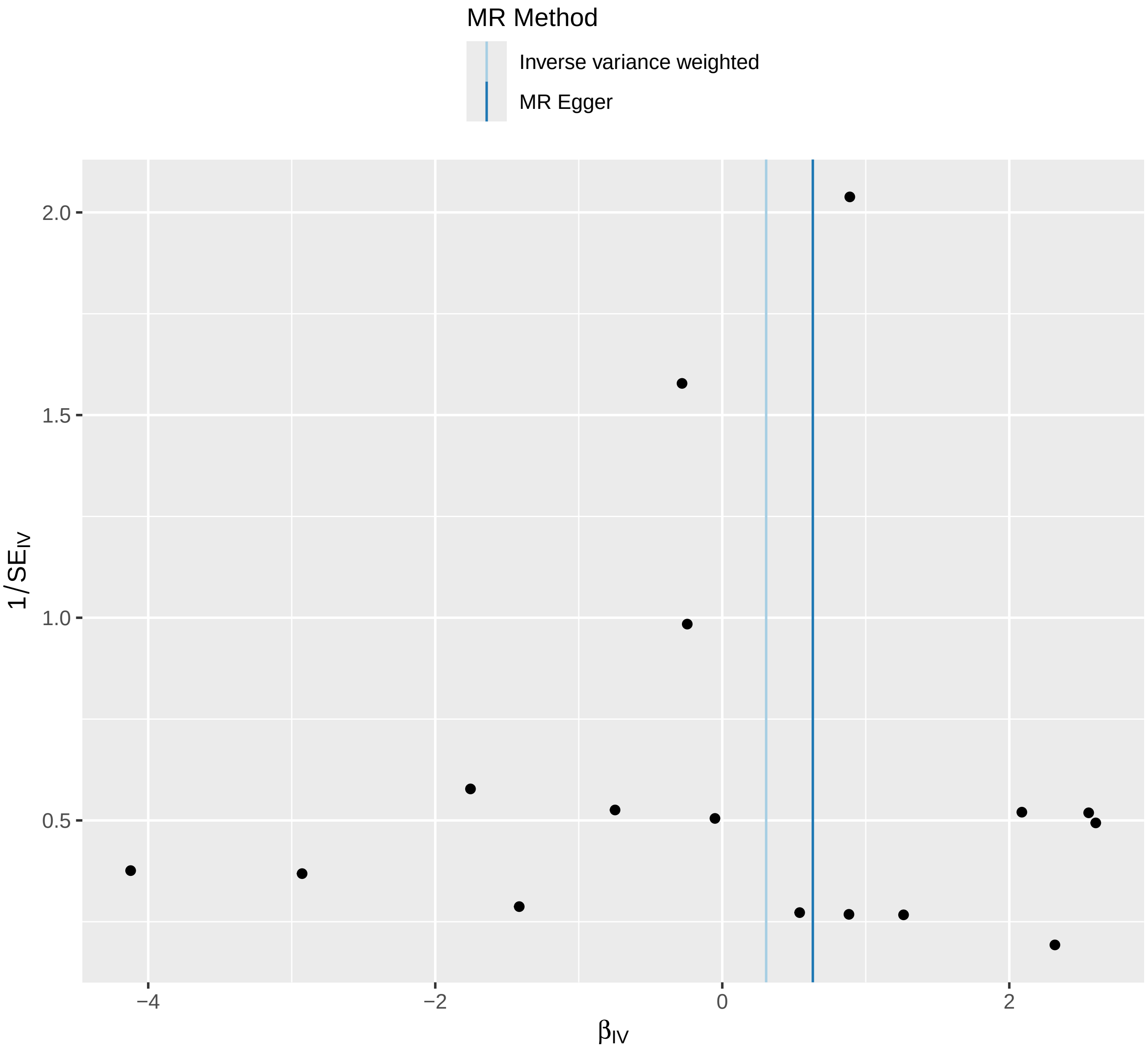
MR Method
Inverse variance weighted
MR Egger
2.0
1.5
1.0
0.5
1/SE_IV
−4
−2
0
2
β_IV

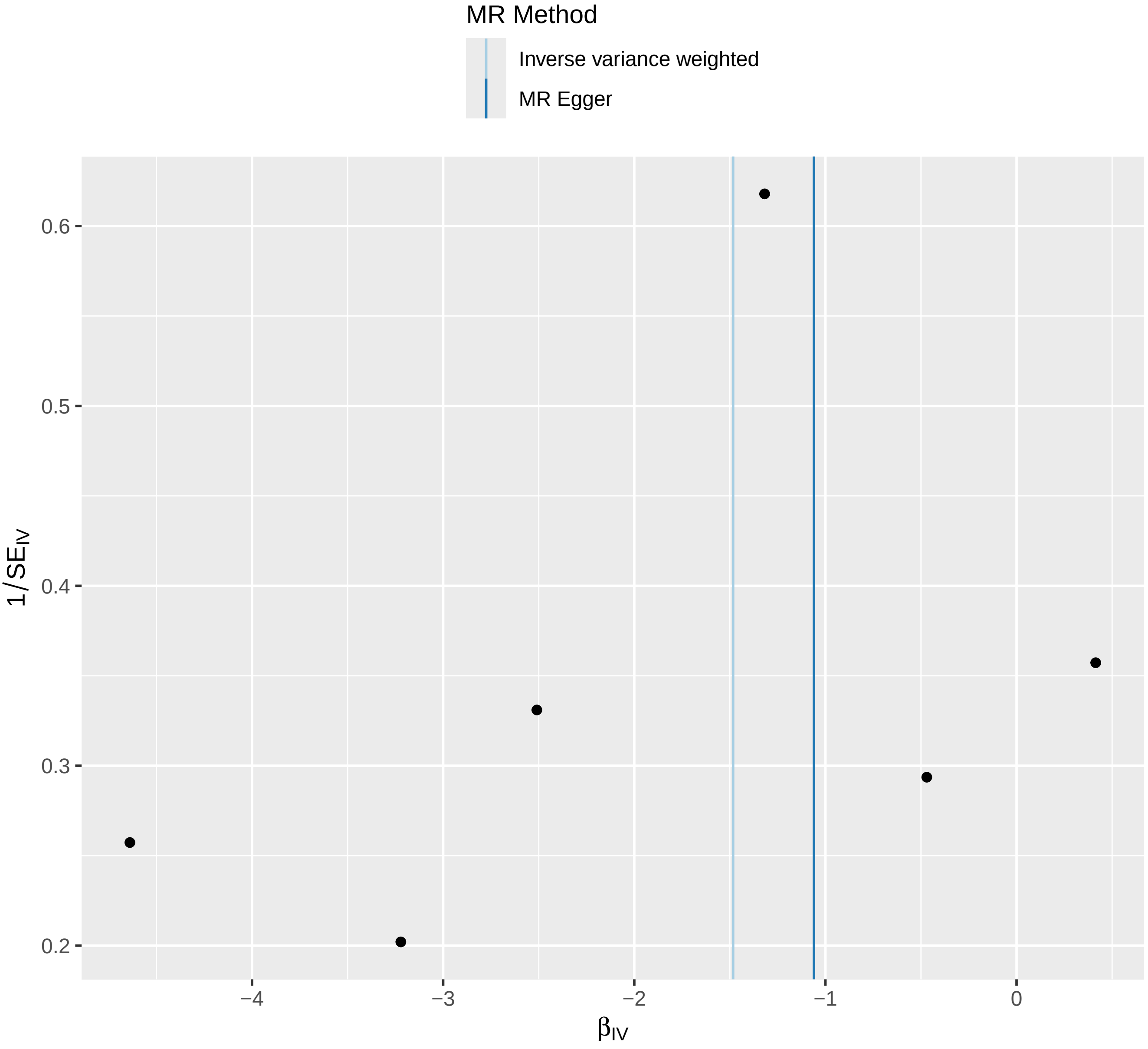

MR Method
Inverse variance weighted
MR Egger
0.6
0.5
0.4
0.3
0.2
$1/SE_{IV}$
−4
−3
−2
−1
0
β_{IV}

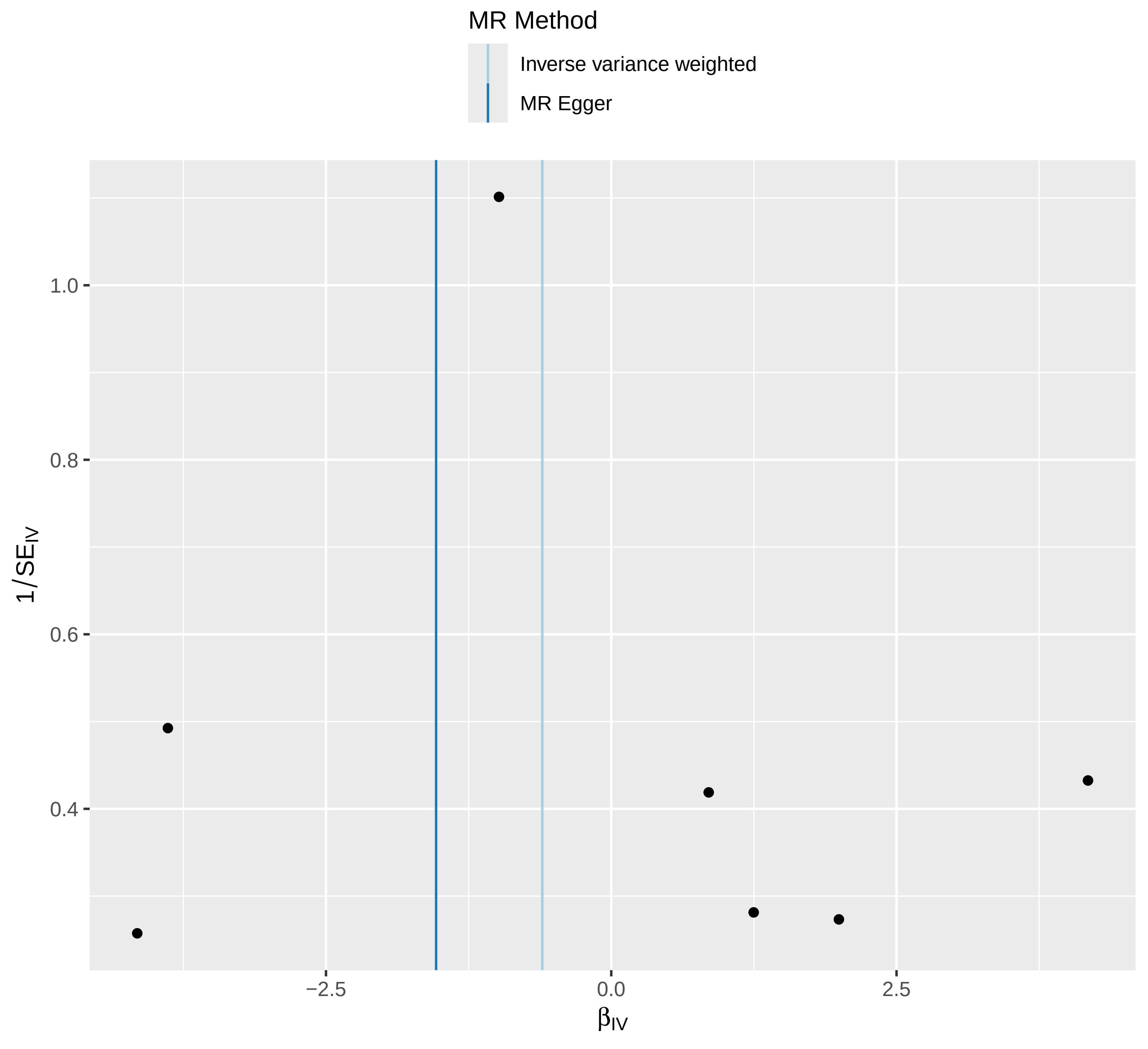

MR Method
Inverse variance weighted
MR Egger
1.0
0.8
0.6
0.4
1/SE$_{IV}$
−2.5
0.0
2.5
β$_{IV}$

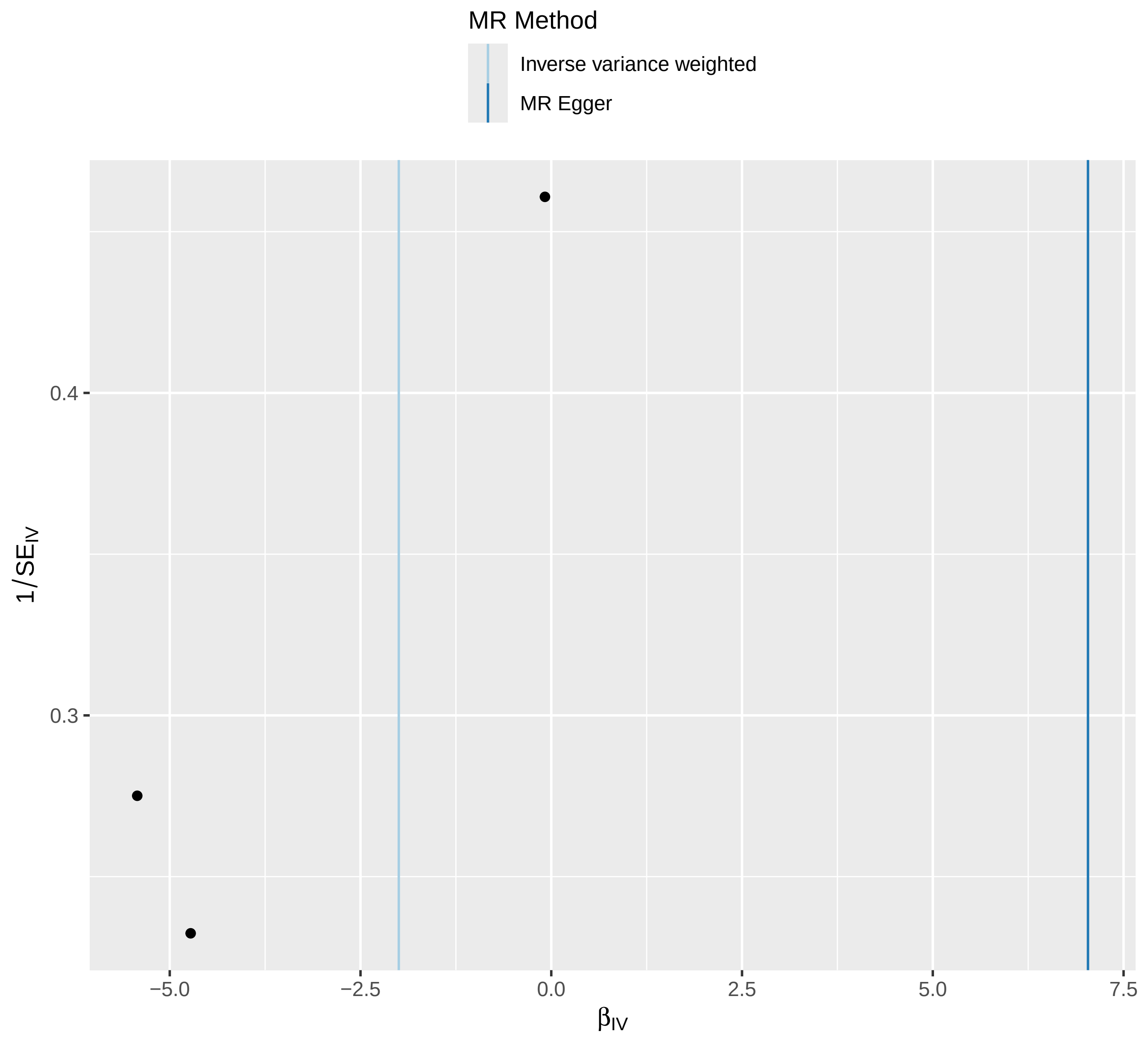
MR Method
Inverse variance weighted
MR Egger
0.4
0.3
1/SE_IV
−5.0
−2.5
0.0
2.5
5.0
7.5
β_IV

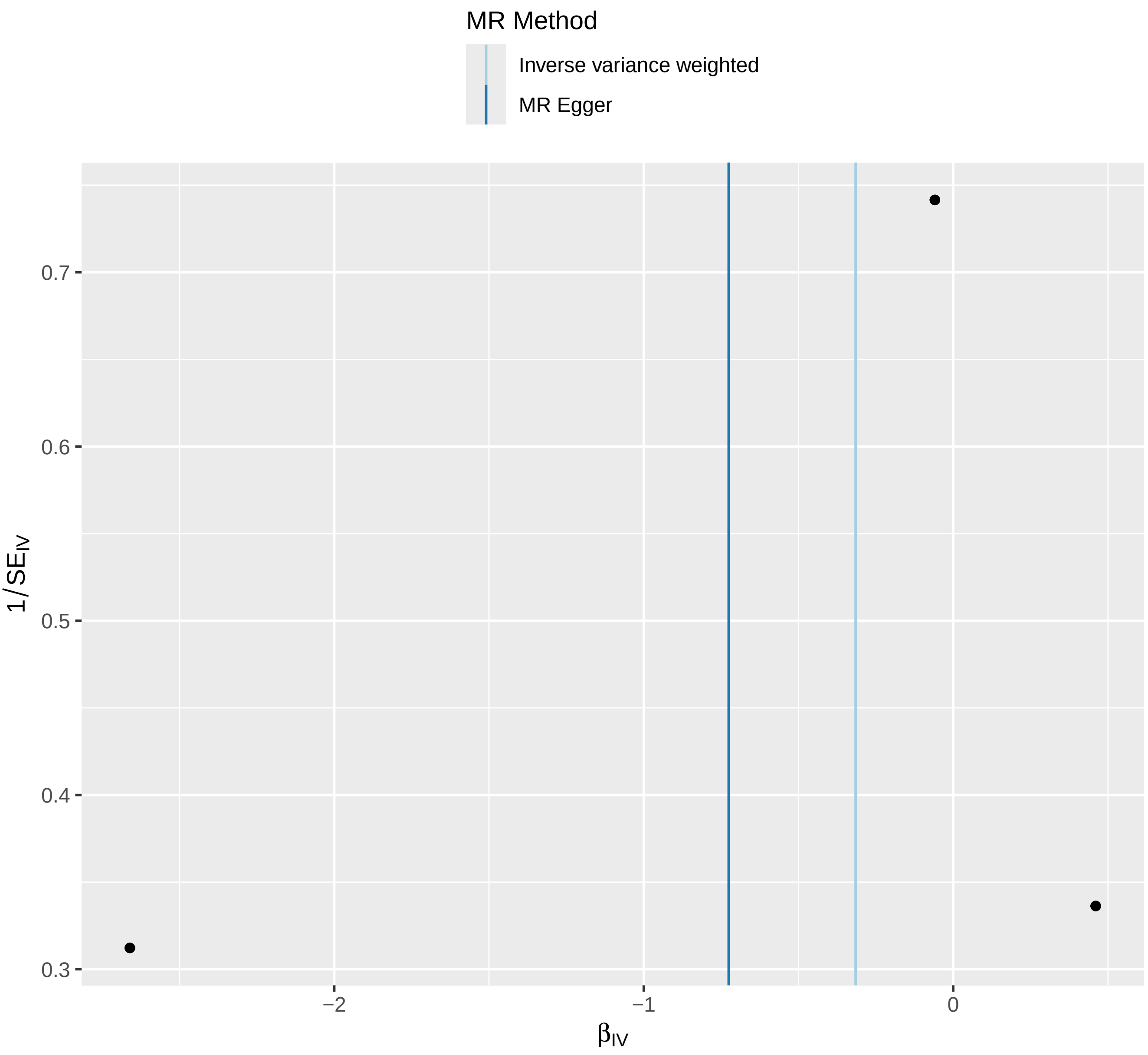

MR Method
Inverse variance weighted
MR Egger
0.7
0.6
0.5
0.4
0.3
1/SE$_{IV}$
−2
−1
0
β$_{IV}$

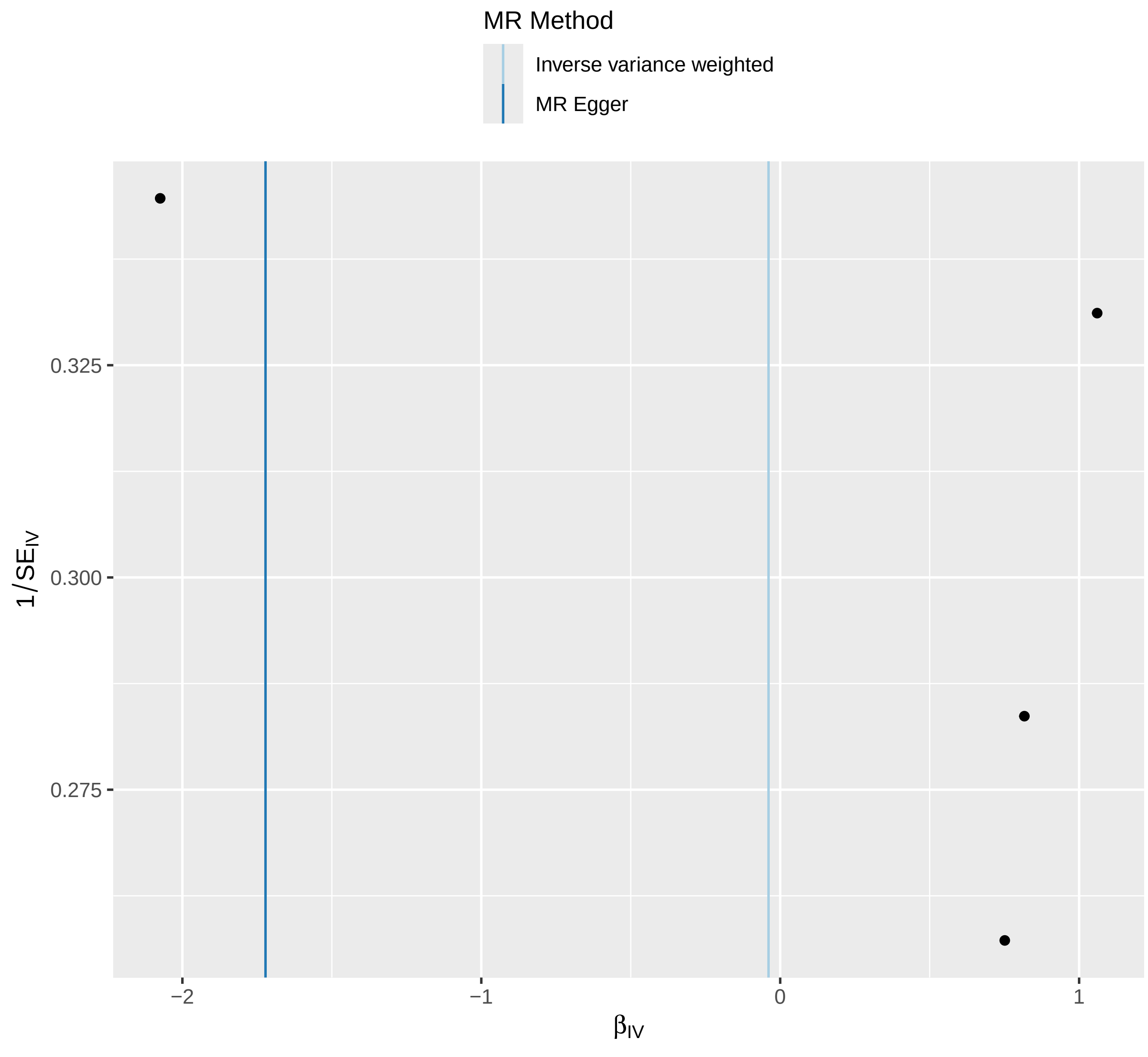
MR Method
Inverse variance weighted
MR Egger
0.325
0.300
0.275
$1/SE_{IV}$
−2
−1
0
1
β_{IV}

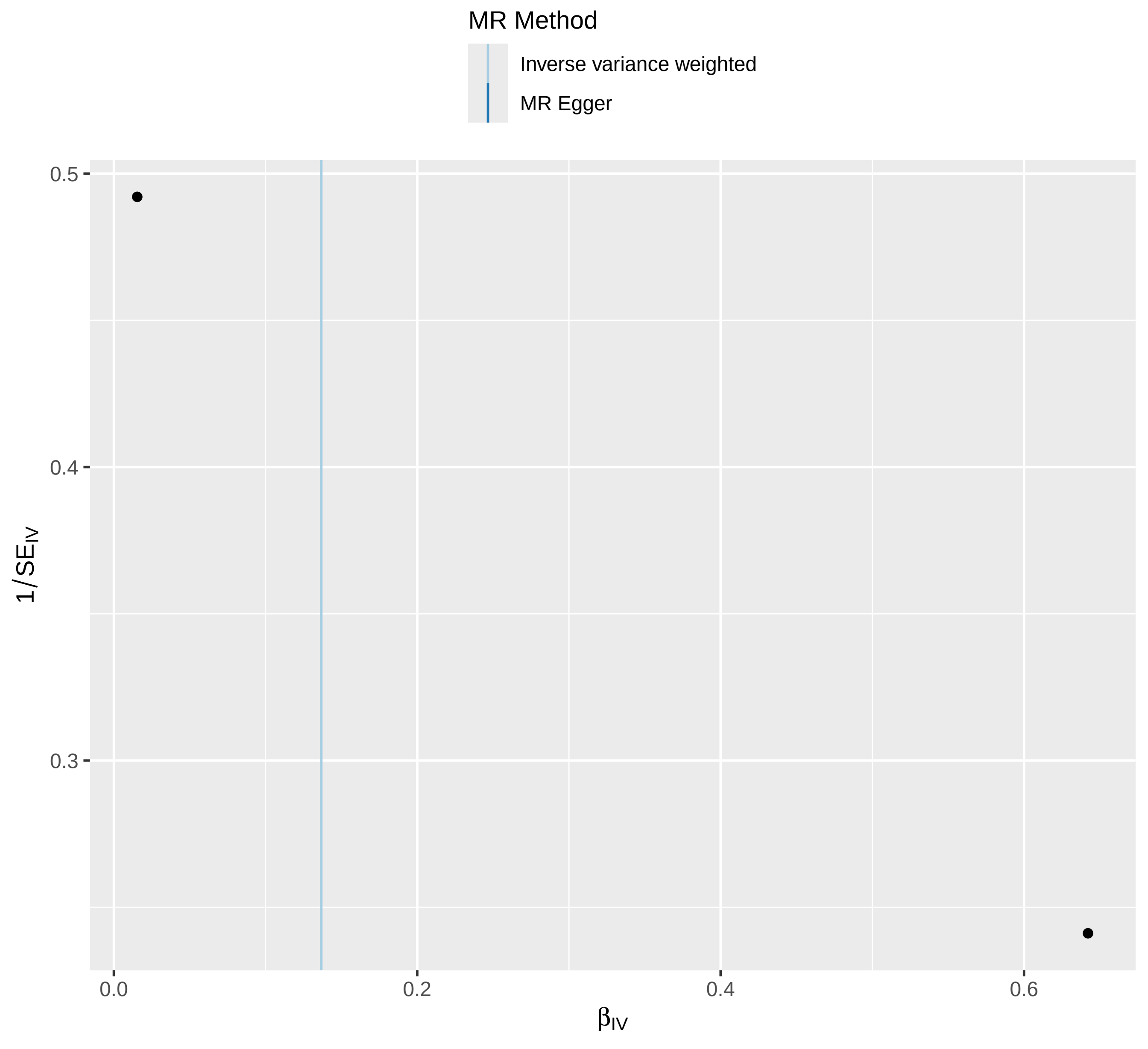
MR Method
Inverse variance weighted
MR Egger
0.5
0.4
0.3
1/SE$_{IV}$
0.0
0.2
0.4
0.6
β$_{IV}$

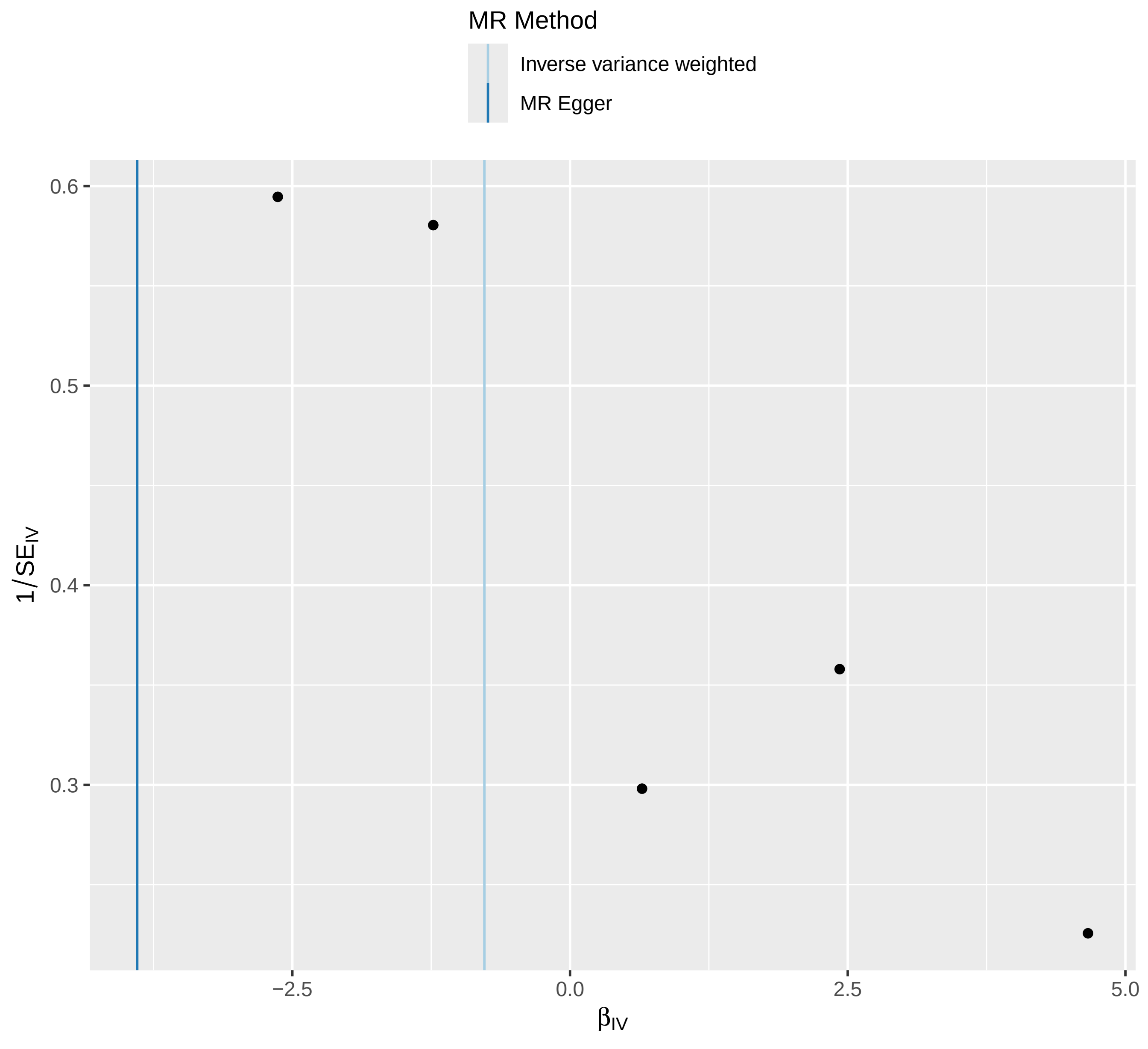
MR Method
Inverse variance weighted
MR Egger
0.6
0.5
0.4
0.3
1/SE_IV
−2.5
0.0
2.5
5.0
β_IV

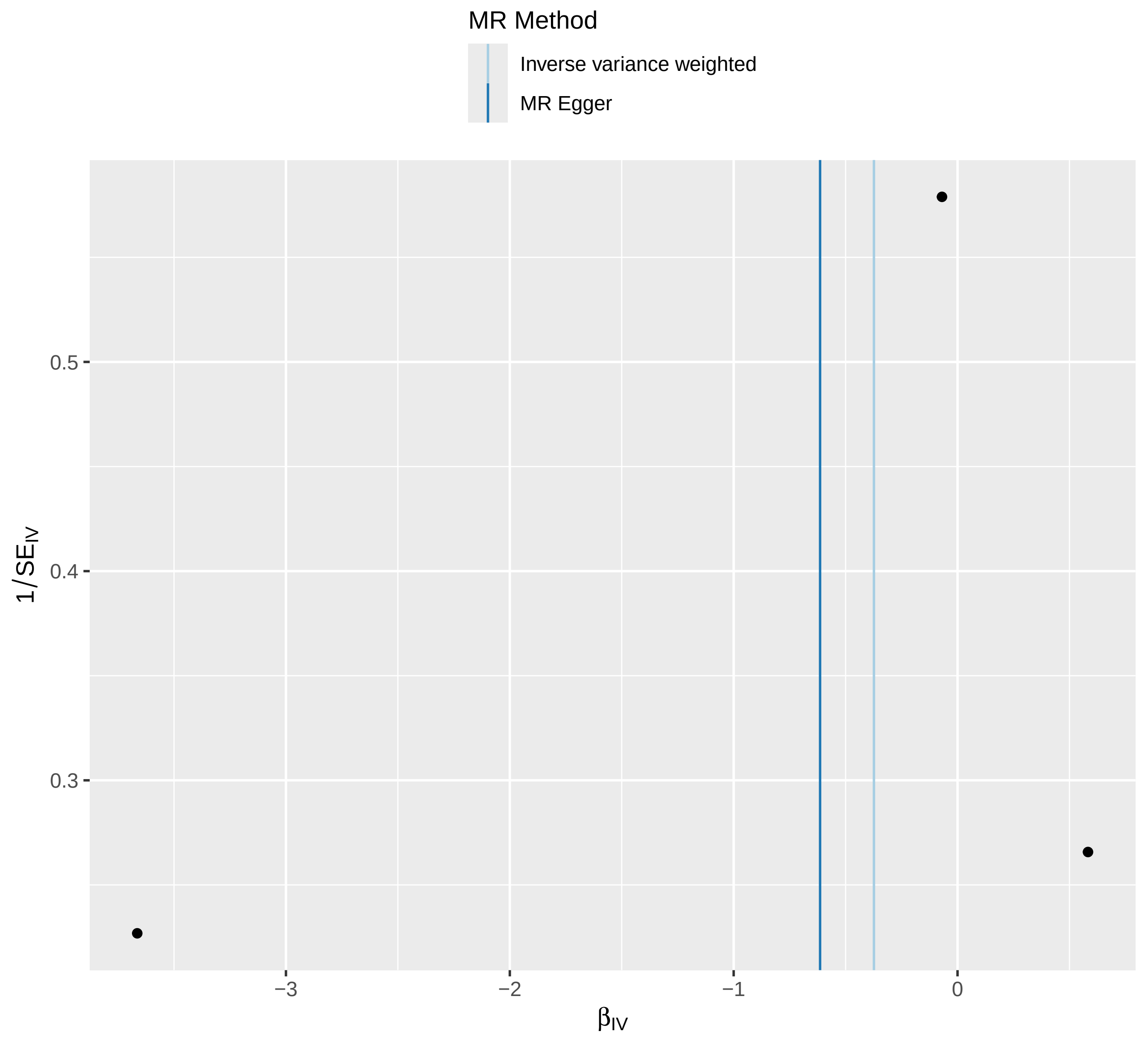
MR Method
Inverse variance weighted
MR Egger
0.5
0.4
0.3
1/SE$_{IV}$
−3
−2
−1
0
β$_{IV}$

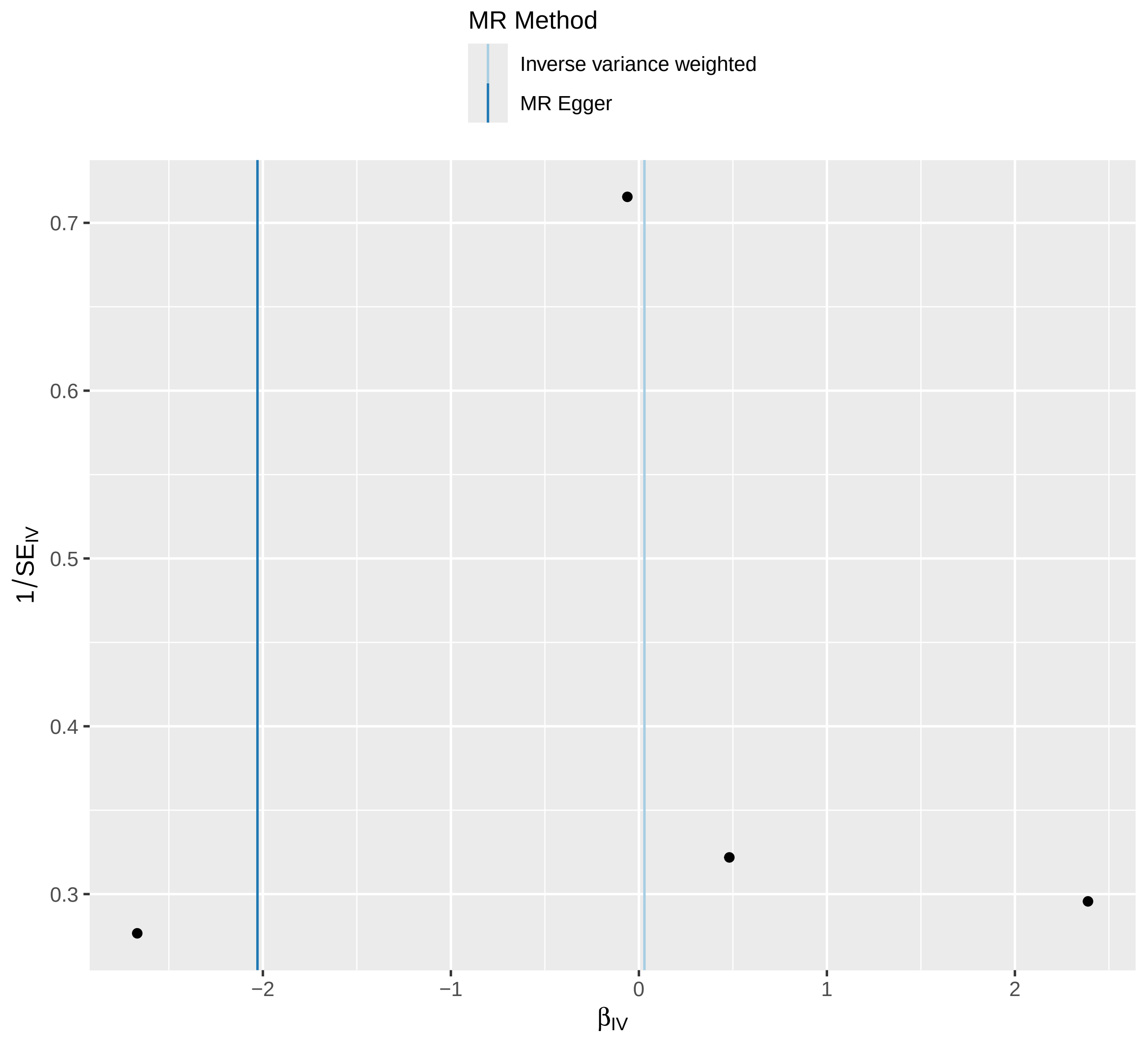
MR Method
Inverse variance weighted
MR Egger
0.7
0.6
0.5
0.4
0.3
1/SE$_{IV}$
−2
−1
0
1
2
β$_{IV}$

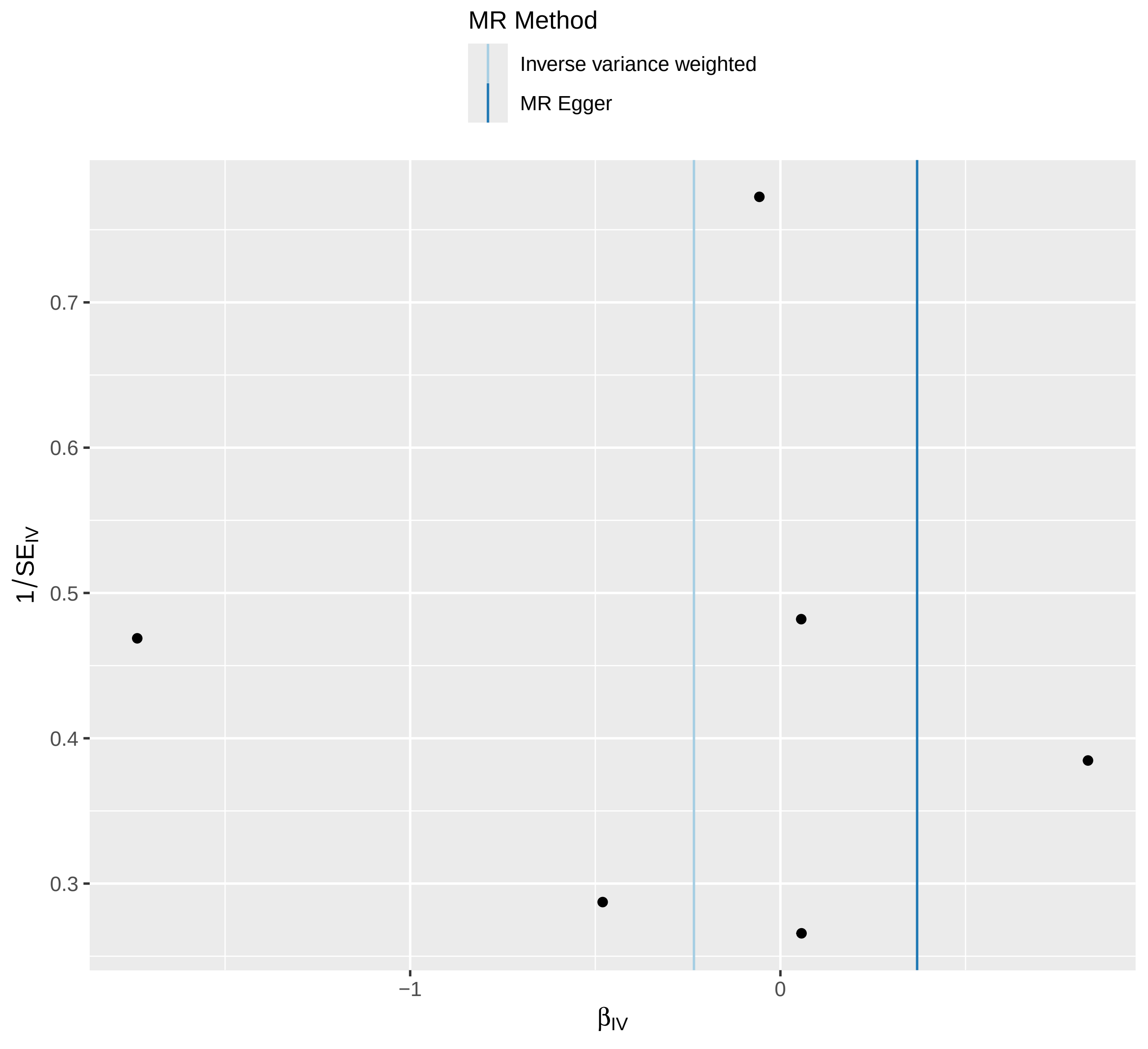
MR Method
Inverse variance weighted
MR Egger
0.7
0.6
0.5
0.4
0.3
1/SE_IV
−1
0
β_IV

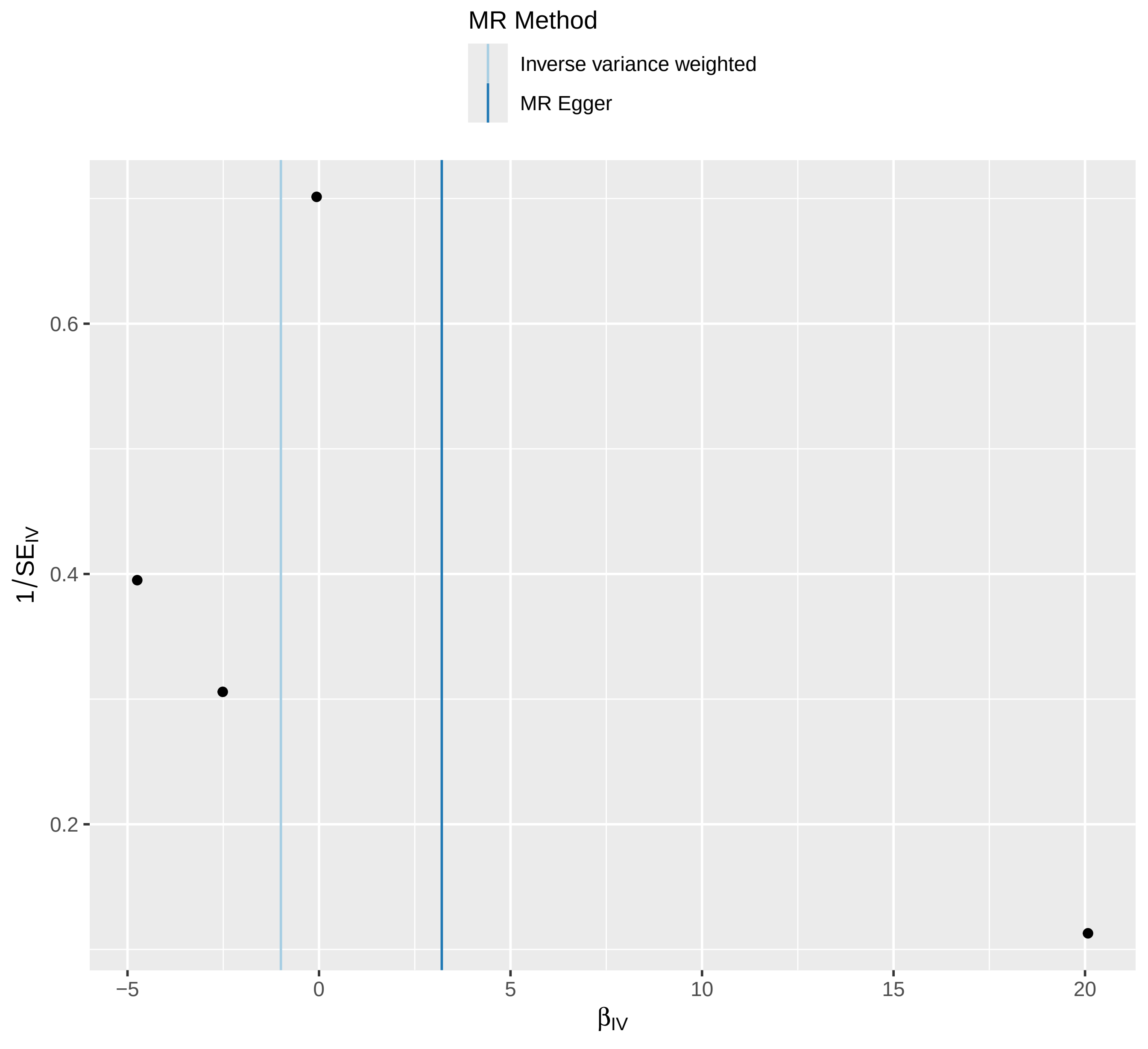
MR Method
Inverse variance weighted
MR Egger
0.6
0.4
0.2
1/SE$_{IV}$
−5
0
5
10
15
20
β$_{IV}$

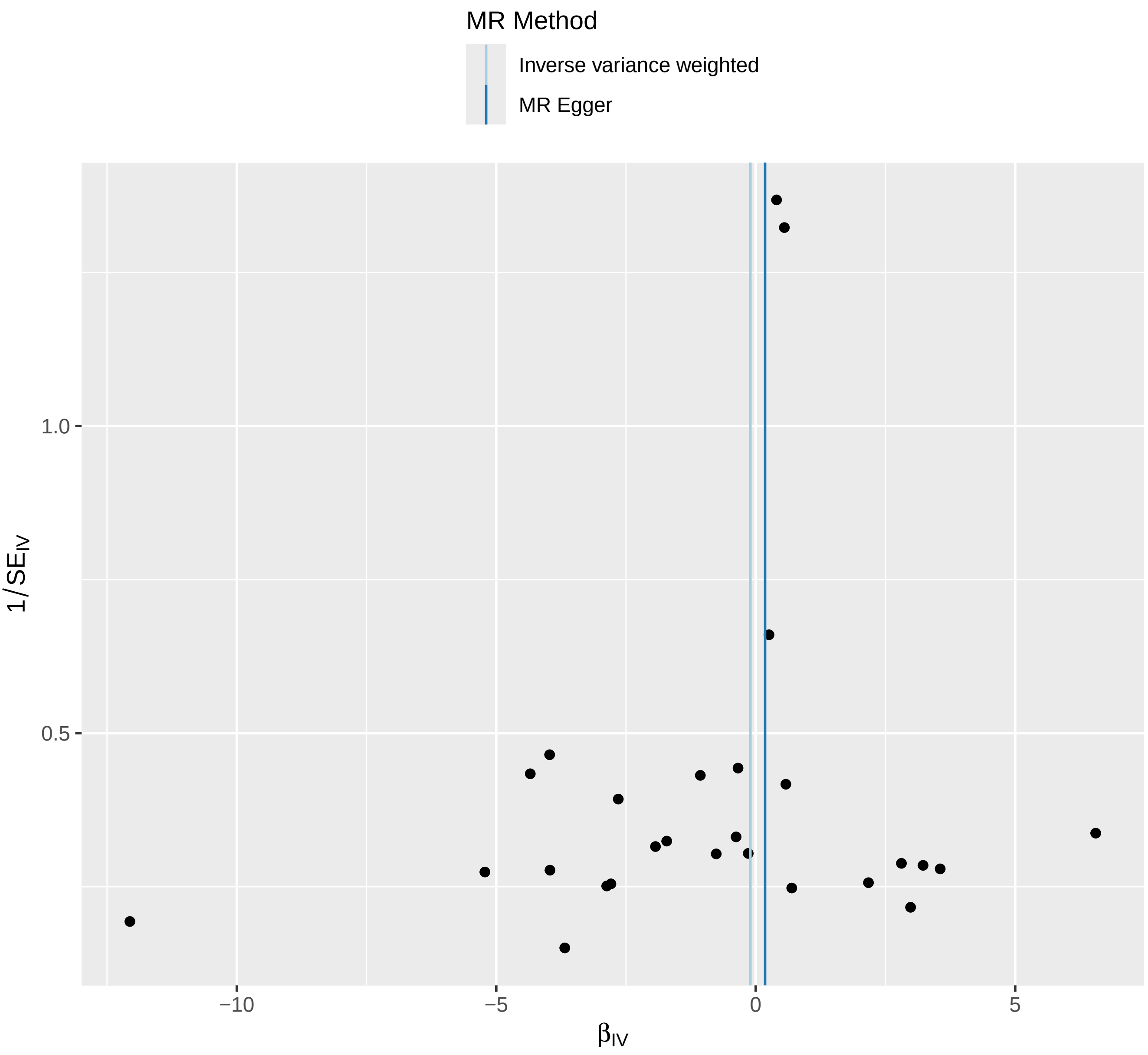
MR Method
Inverse variance weighted
MR Egger
1.0
0.5
1/SE$_{IV}$
−10
−5
0
5
β$_{IV}$

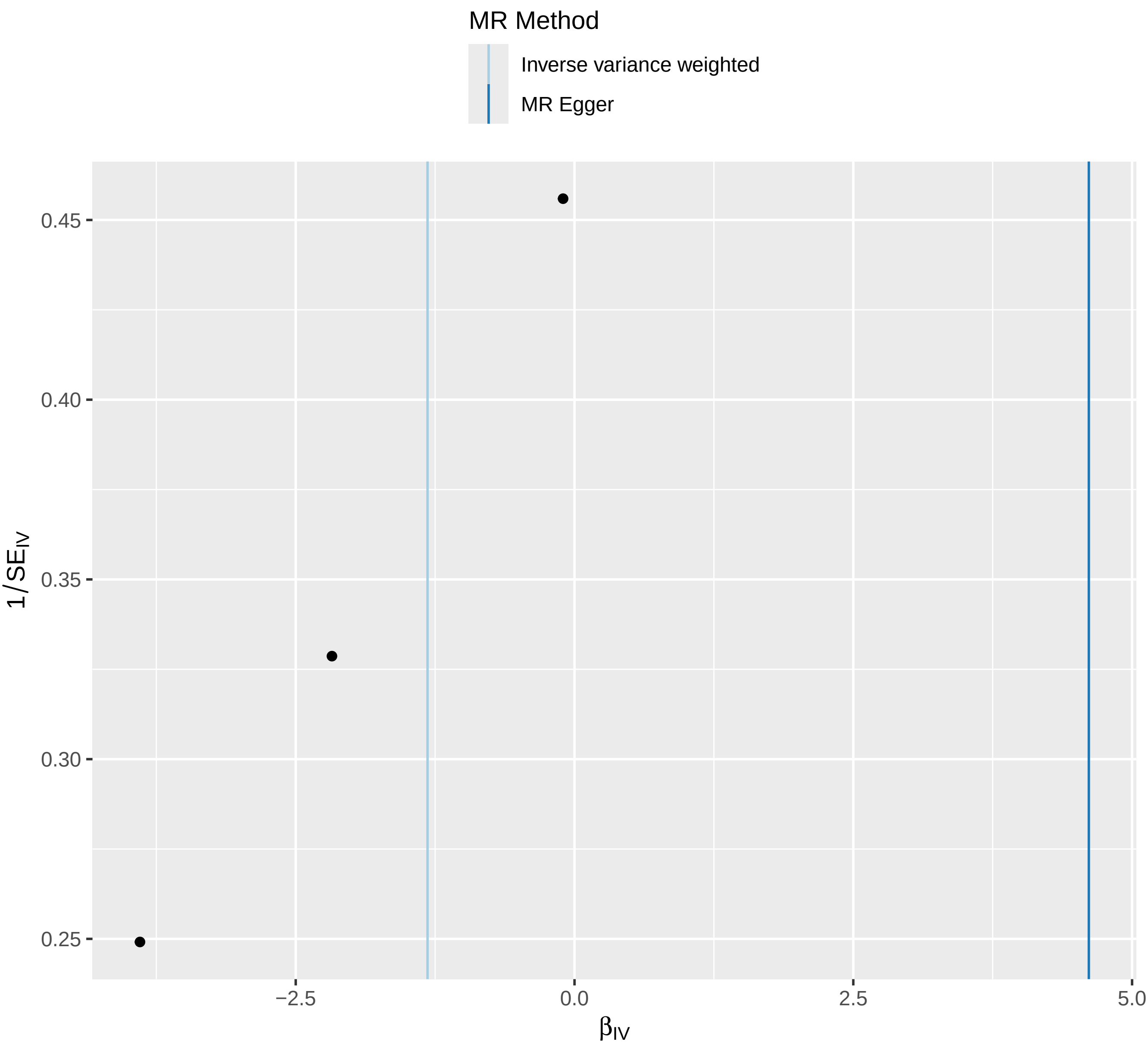
MR Method
Inverse variance weighted
MR Egger
0.45
0.40
0.35
0.30
0.25
1/SE_IV
−2.5
0.0
2.5
5.0
β_IV

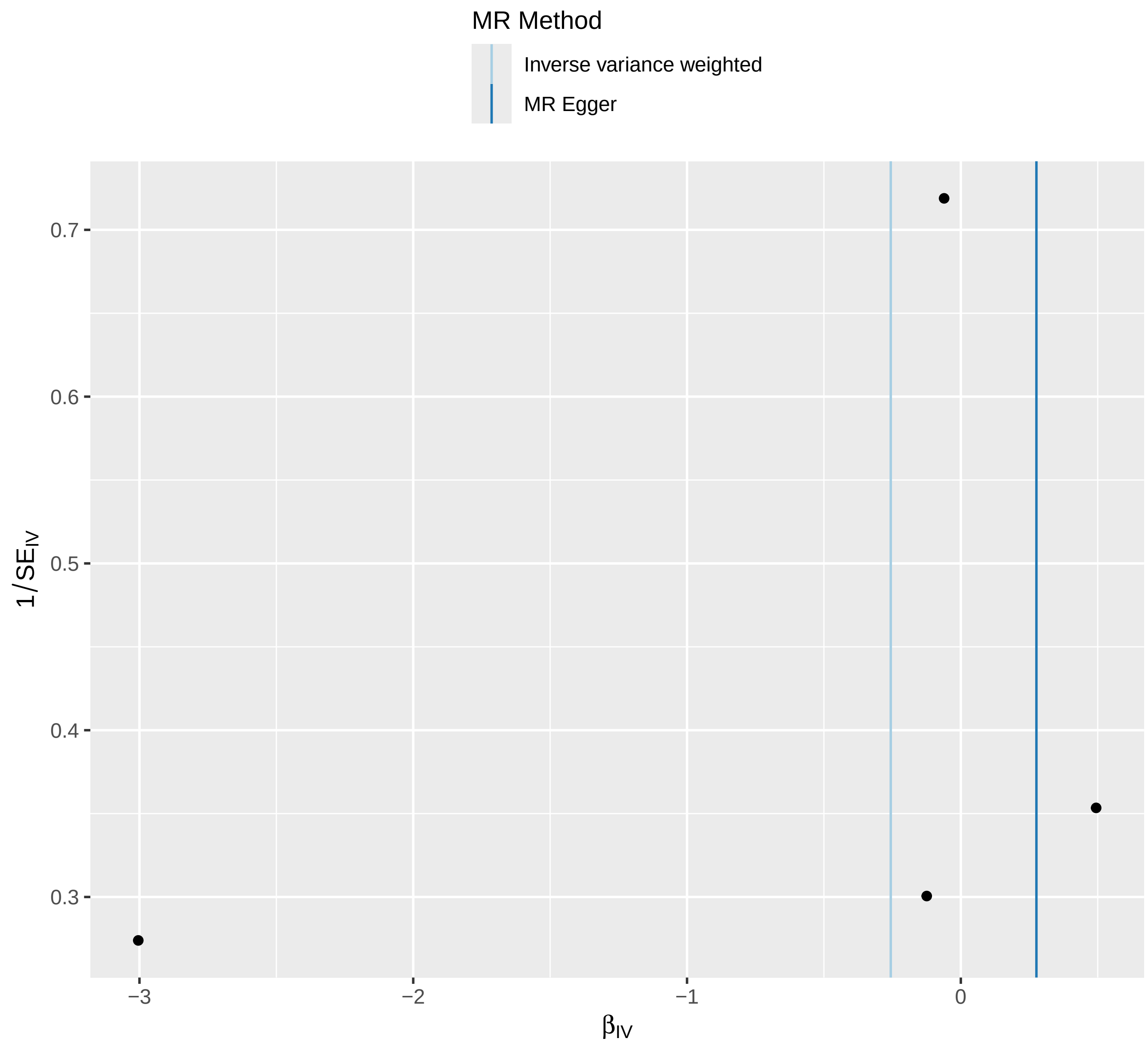
MR Method
Inverse variance weighted
MR Egger
0.7
0.6
0.5
0.4
0.3
1/SE_IV
−3
−2
−1
0
β_IV

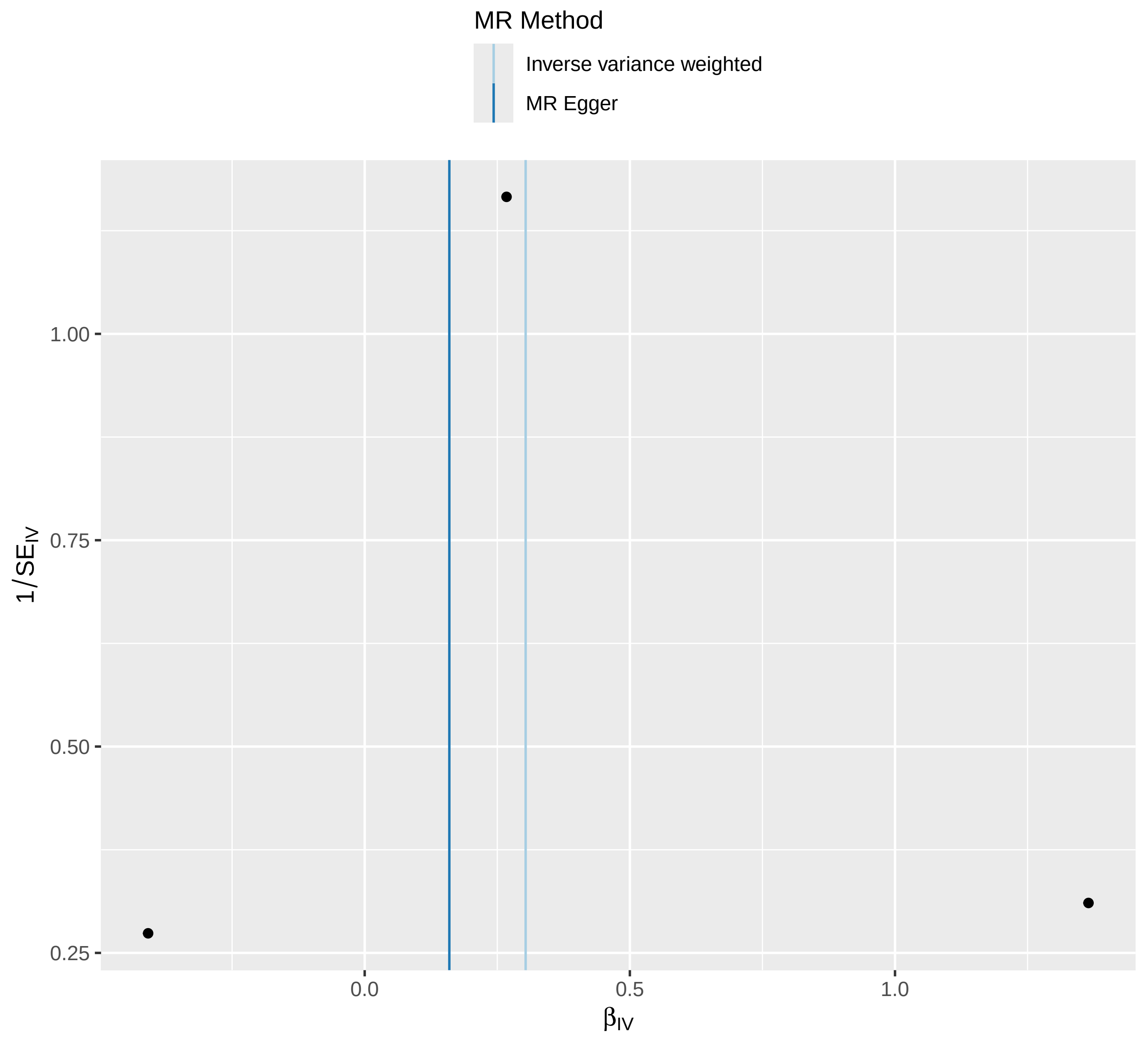
MR Method
Inverse variance weighted
MR Egger
1.00
0.75
0.50
0.25
$1/SE_{IV}$
0.0
0.5
1.0
β_{IV}

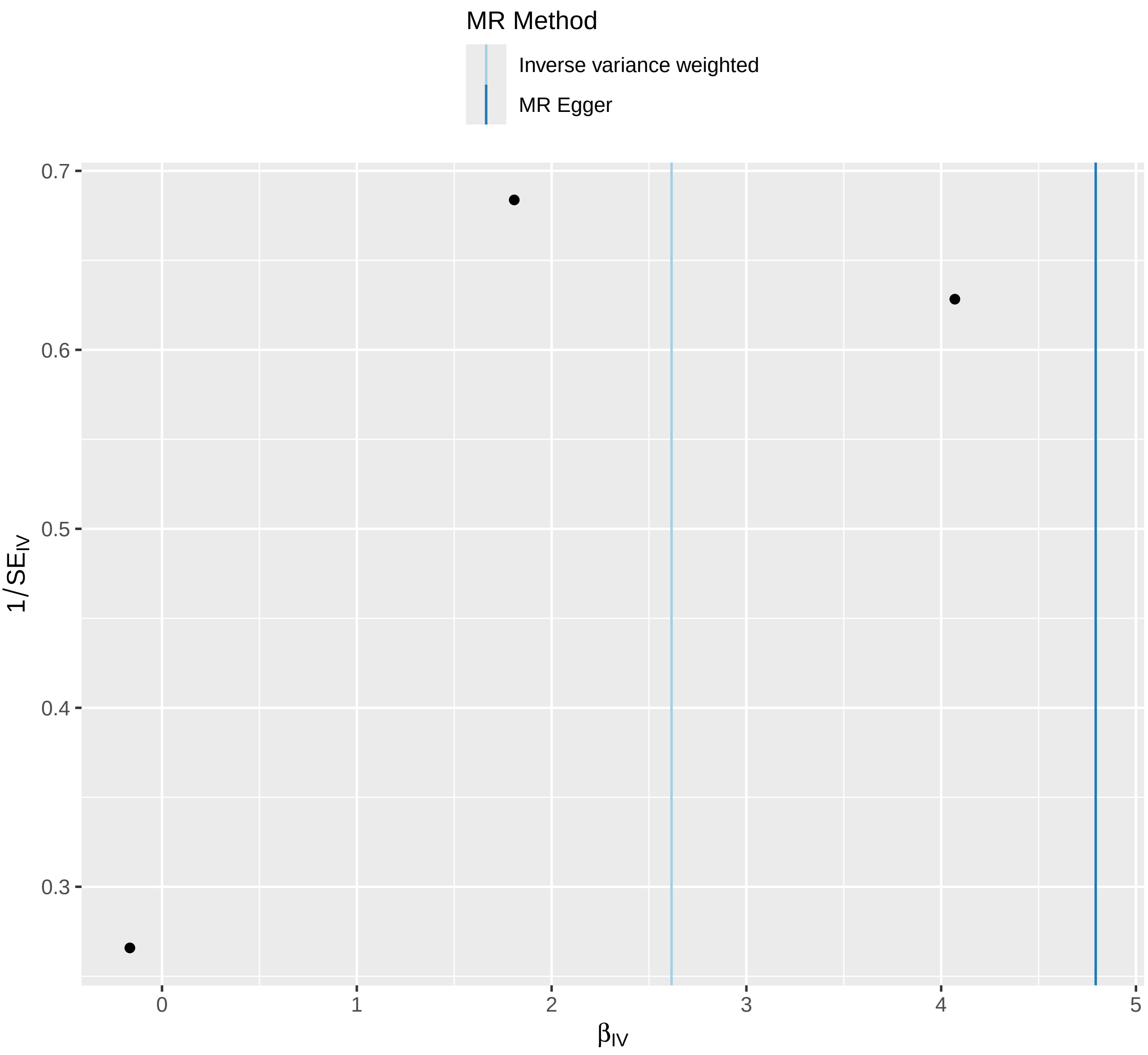
MR Method
Inverse variance weighted
MR Egger
0.7
0.6
0.5
0.4
0.3
1/SE_IV
0
1
2
3
4
5
β_IV

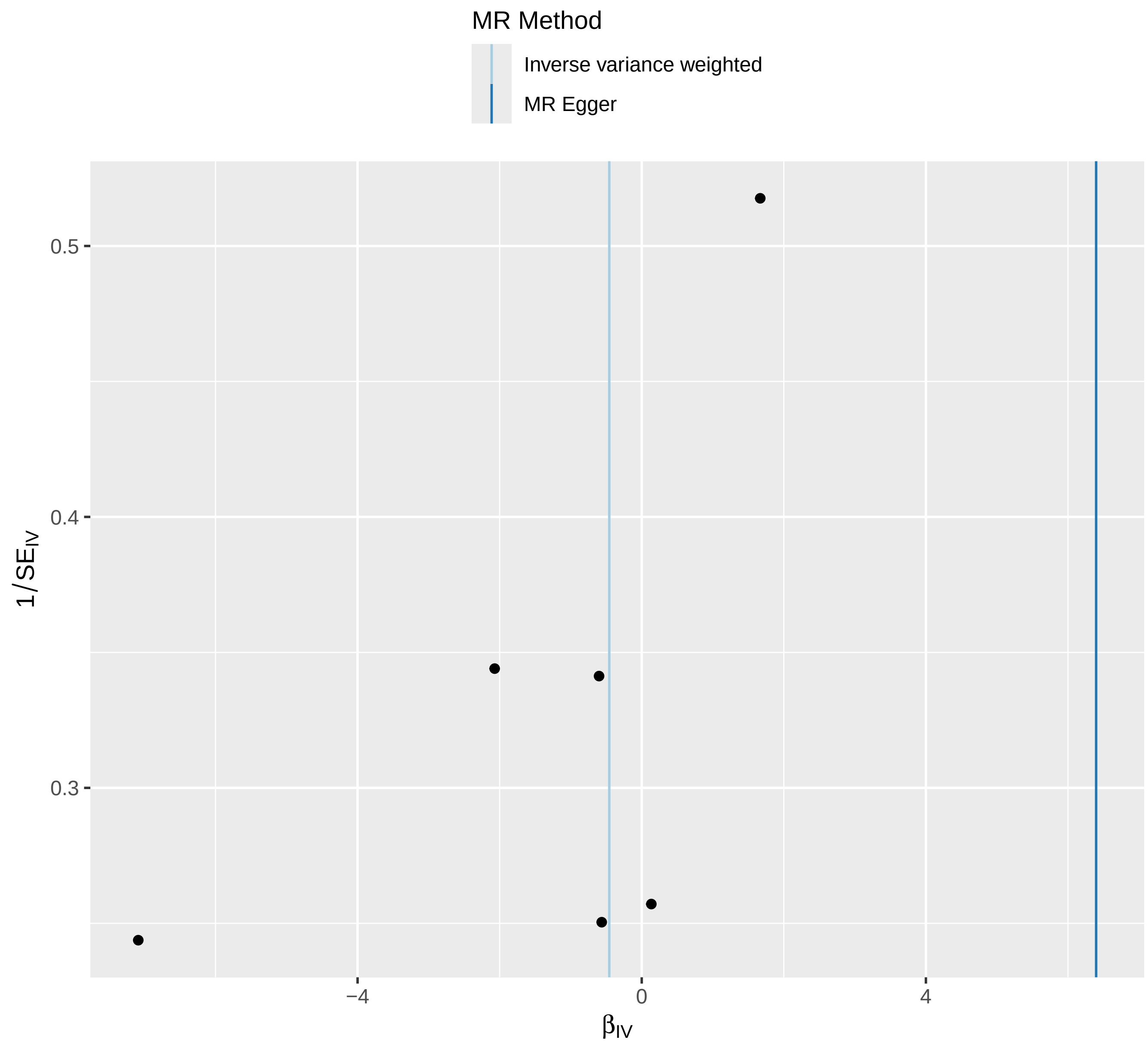
MR Method
Inverse variance weighted
MR Egger
0.5
0.4
0.3
1/SE$_{IV}$
−4
0
4
β$_{IV}$

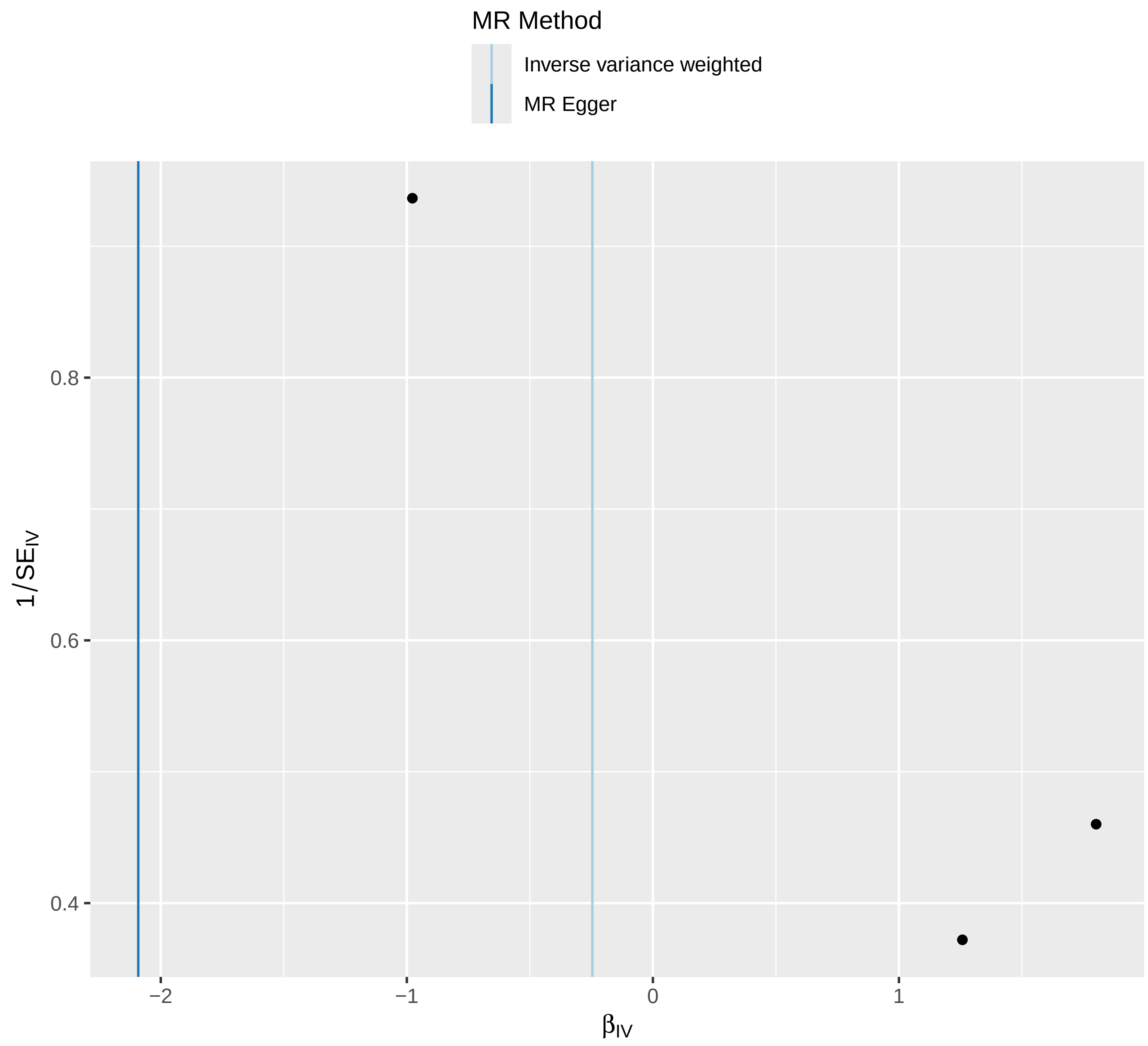

MR Method
Inverse variance weighted
MR Egger
1/SE_IV
0.8
0.6
0.4
−2
−1
0
1
β_IV

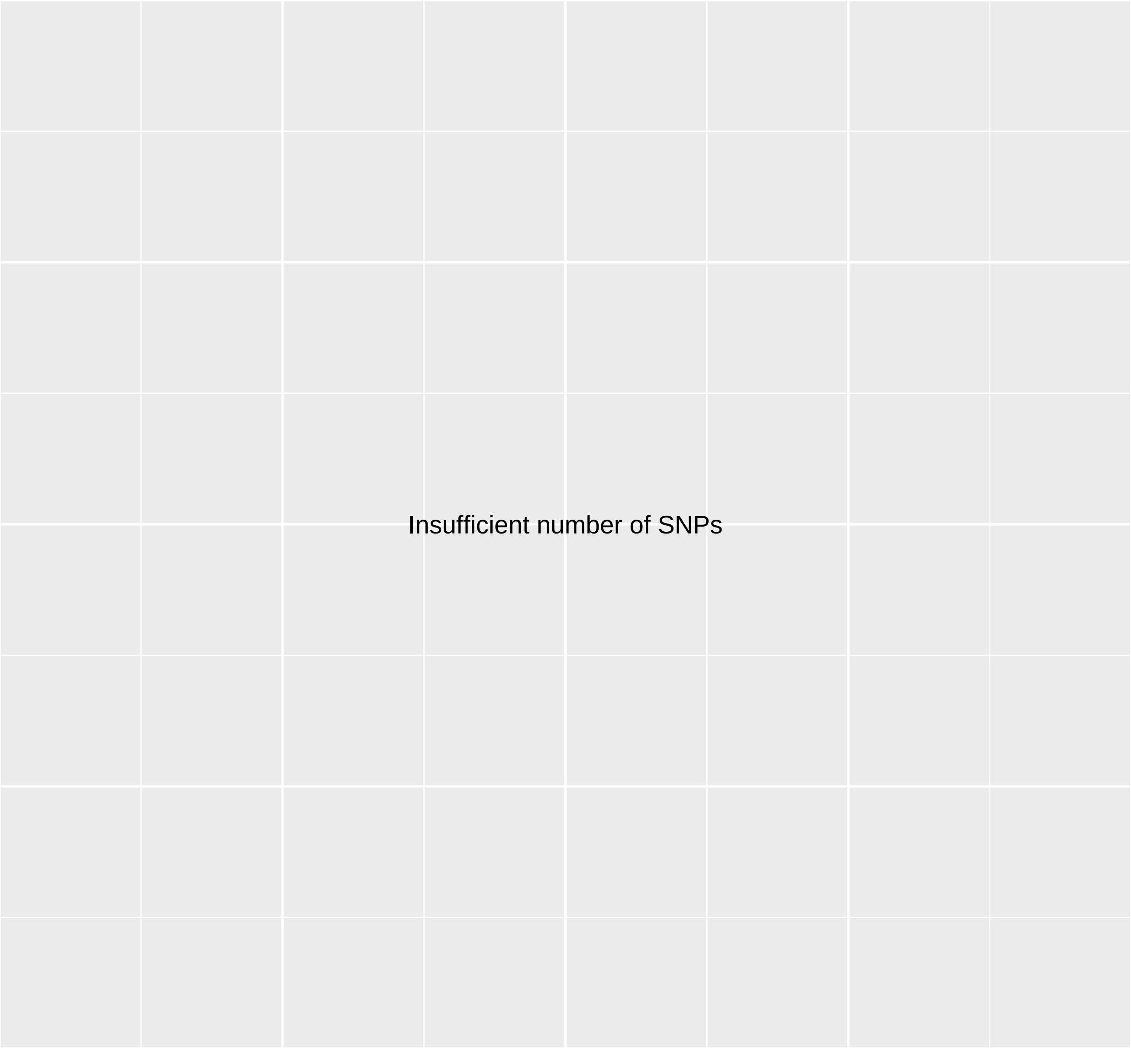
Insufficient number of SNPs

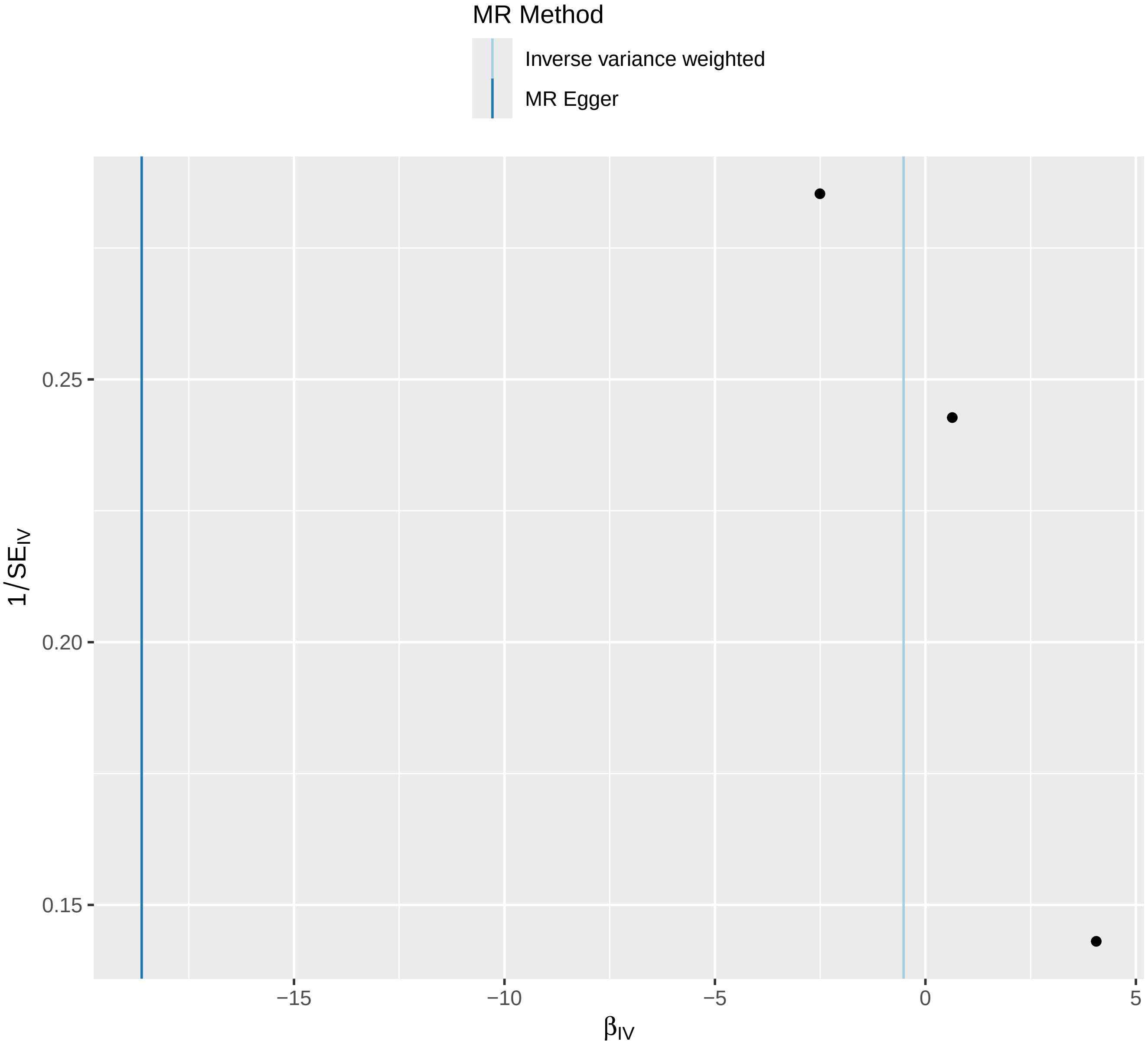
MR Method
Inverse variance weighted
MR Egger
0.25
0.20
0.15
1/SE_IV
−15
−10
−5
0
5
β_IV

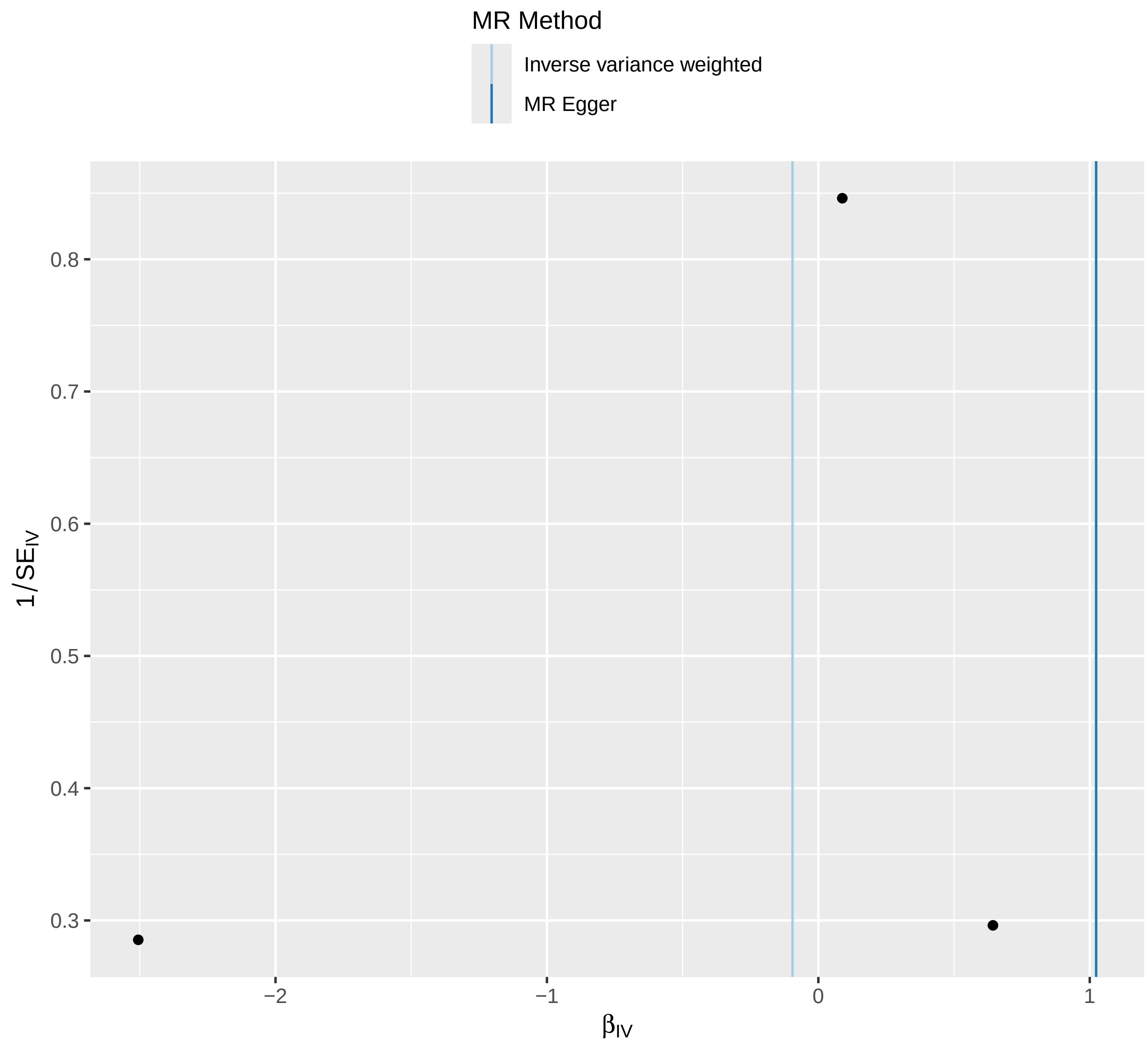
MR Method
Inverse variance weighted
MR Egger
1/SE_IV
0.8
0.7
0.6
0.5
0.4
0.3
−2
−1
0
1
β_IV

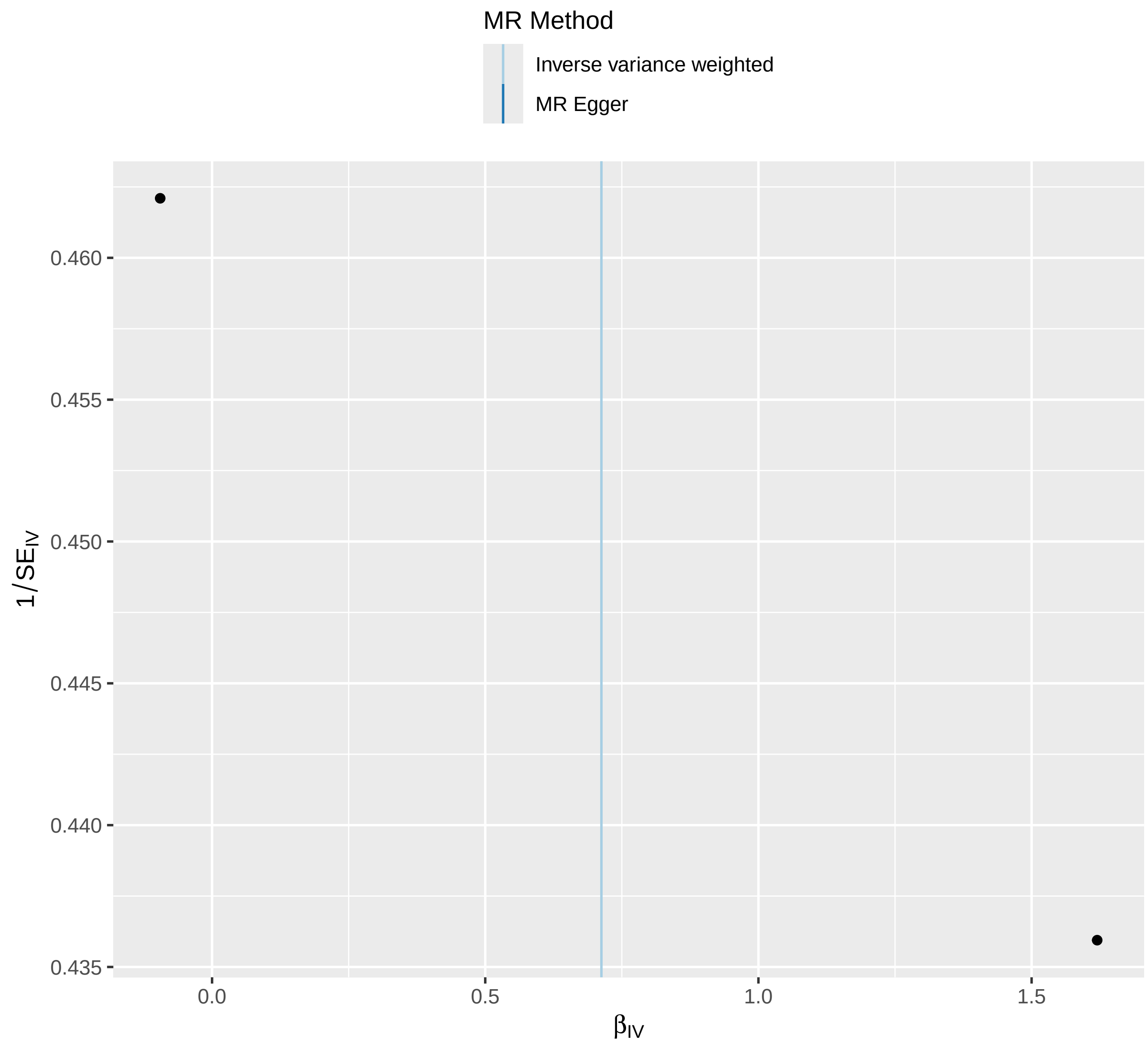
MR Method
Inverse variance weighted
MR Egger
0.460
0.455
0.450
0.445
0.440
0.435
1/SE$_{IV}$
0.0
0.5
1.0
1.5
β$_{IV}$

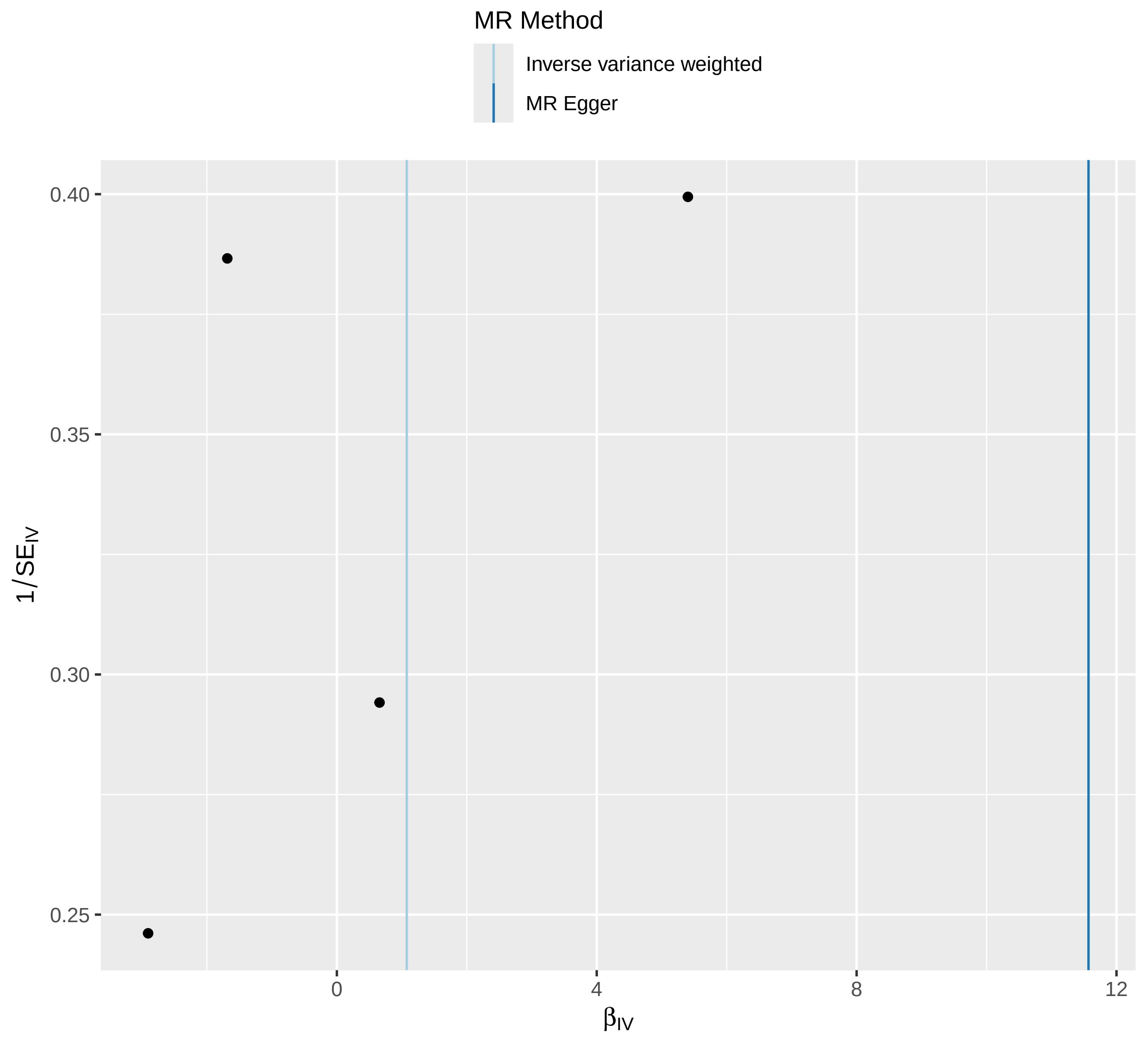
MR Method
Inverse variance weighted
MR Egger
0.40
0.35
0.30
0.25
$1/SE_{IV}$
0
4
8
12
β_{IV}

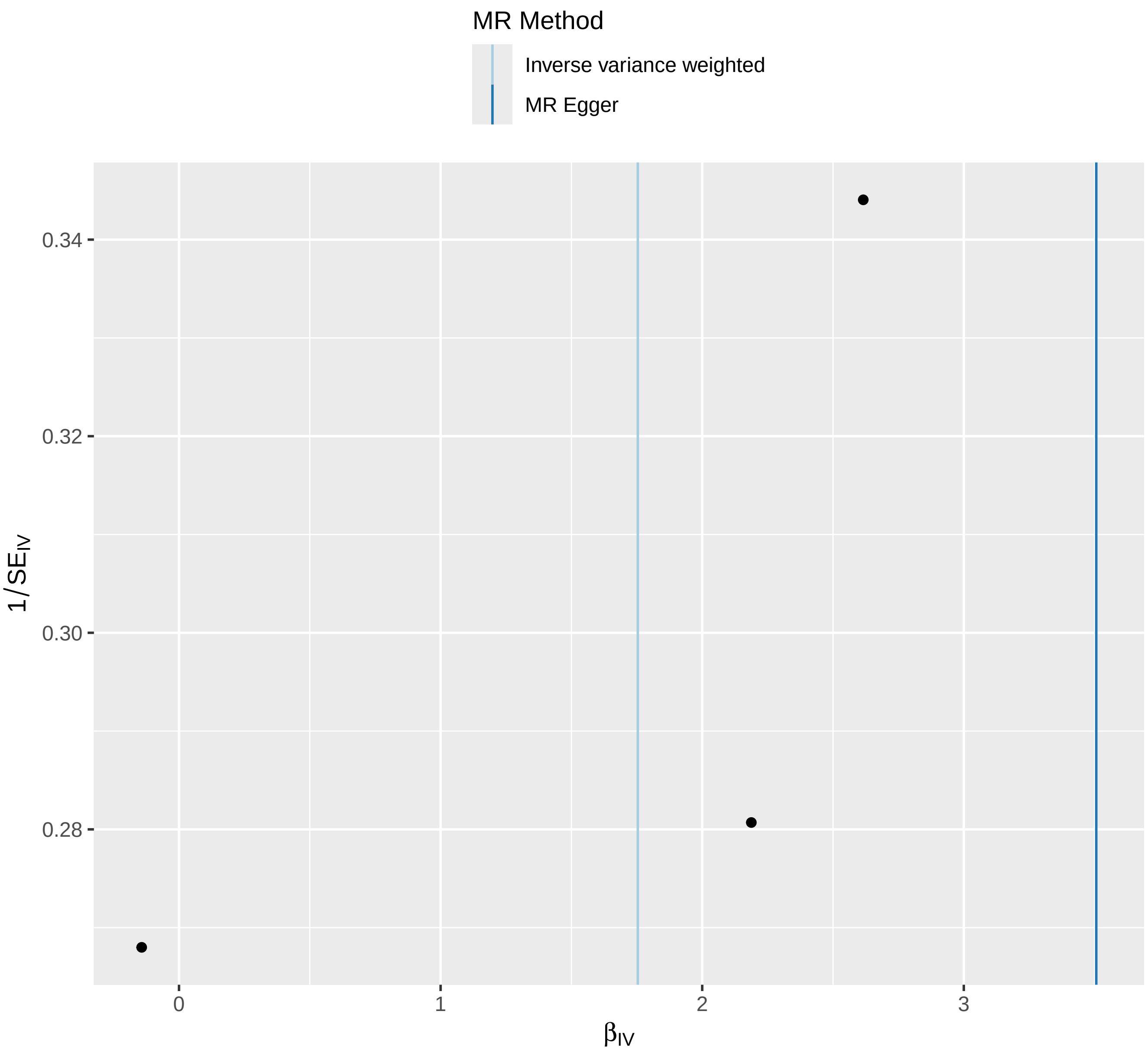
MR Method
Inverse variance weighted
MR Egger
0.34
0.32
0.30
0.28
1/SE_IV
0
1
2
3
β_IV

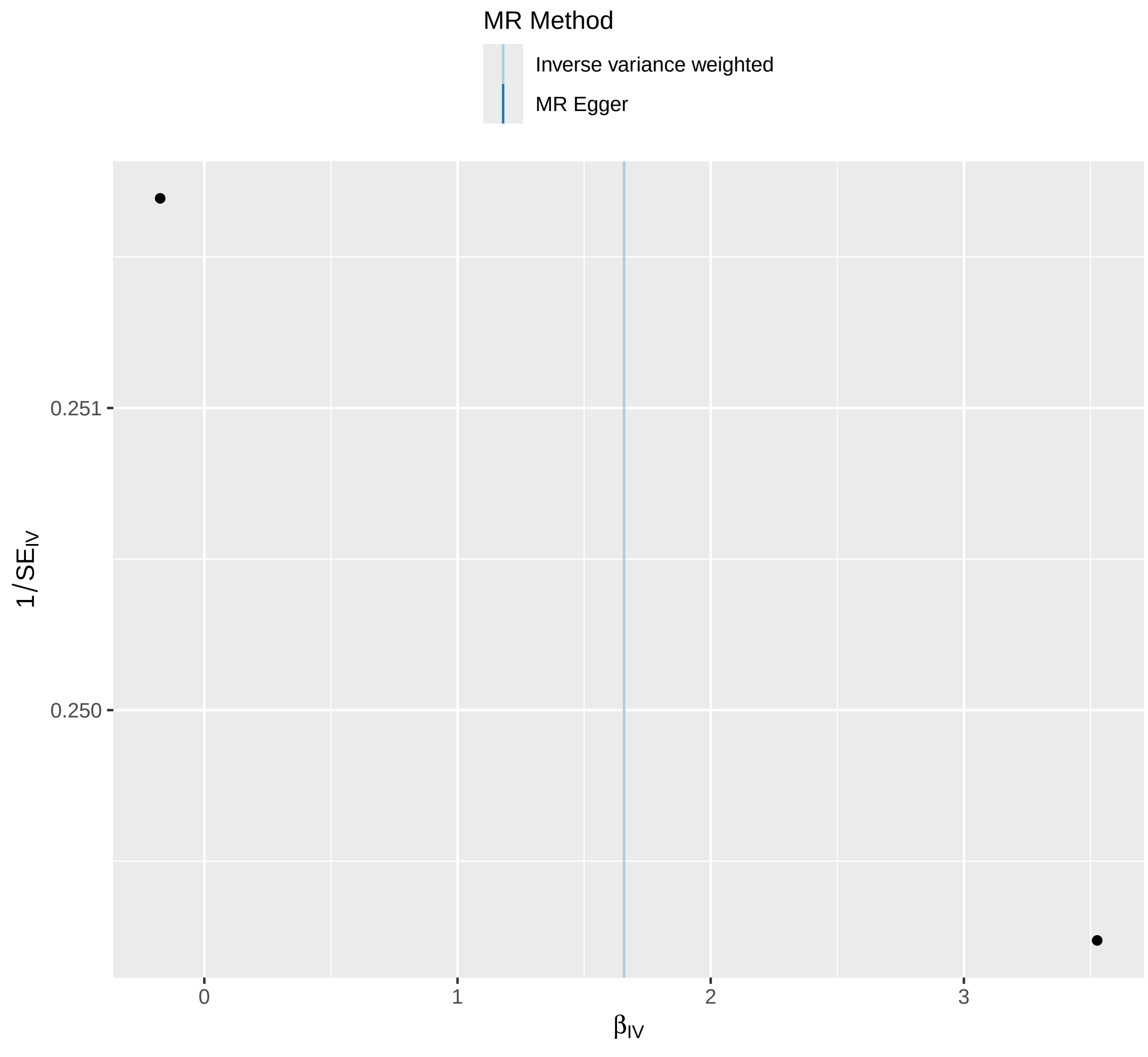
MR Method
Inverse variance weighted
MR Egger
0.251
0.250
1/SE_IV
0
1
2
3
β_IV

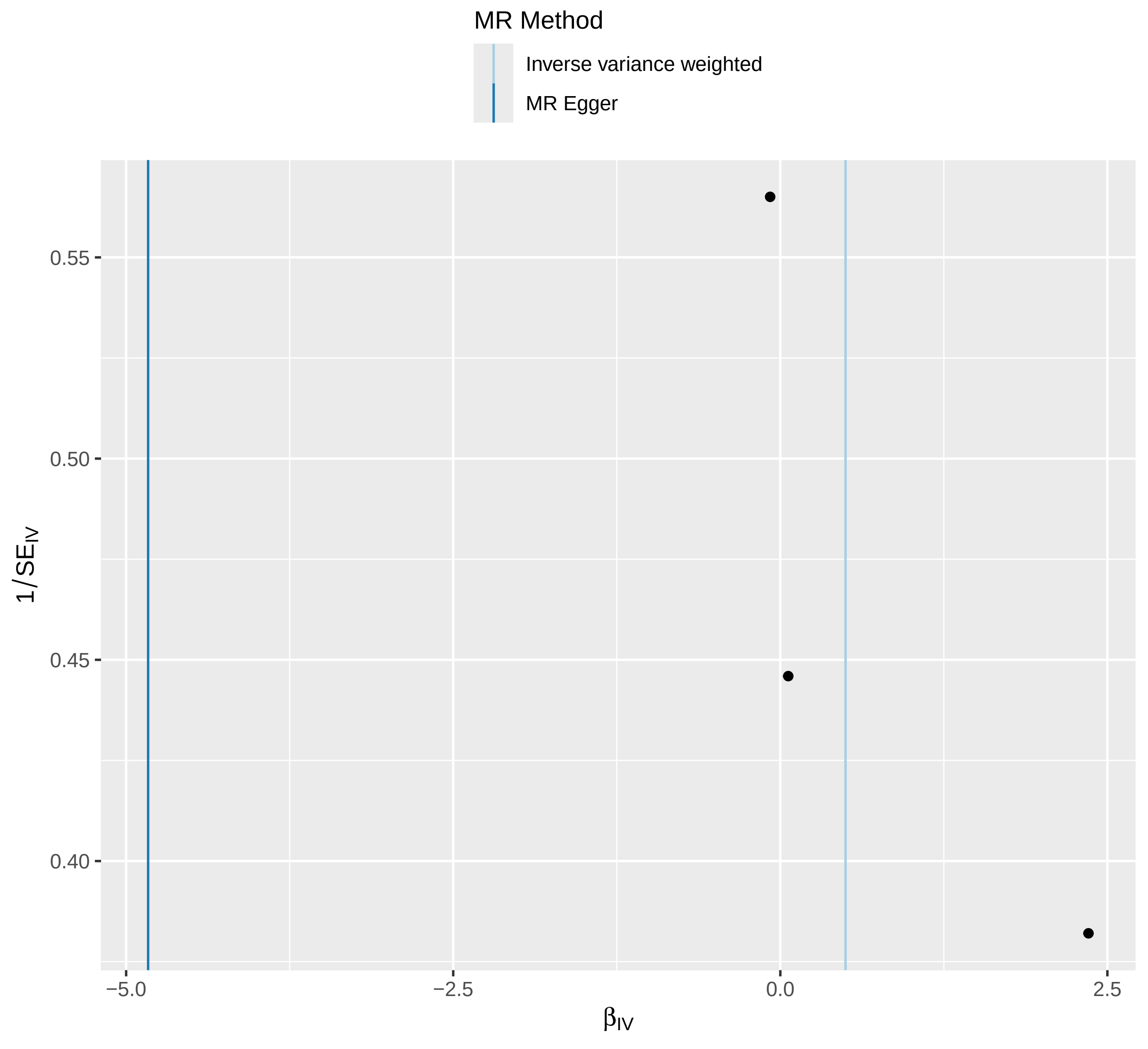

MR Method
Inverse variance weighted
MR Egger
0.55
0.50
0.45
0.40
1/SE_IV
−5.0
−2.5
0.0
2.5
β_IV

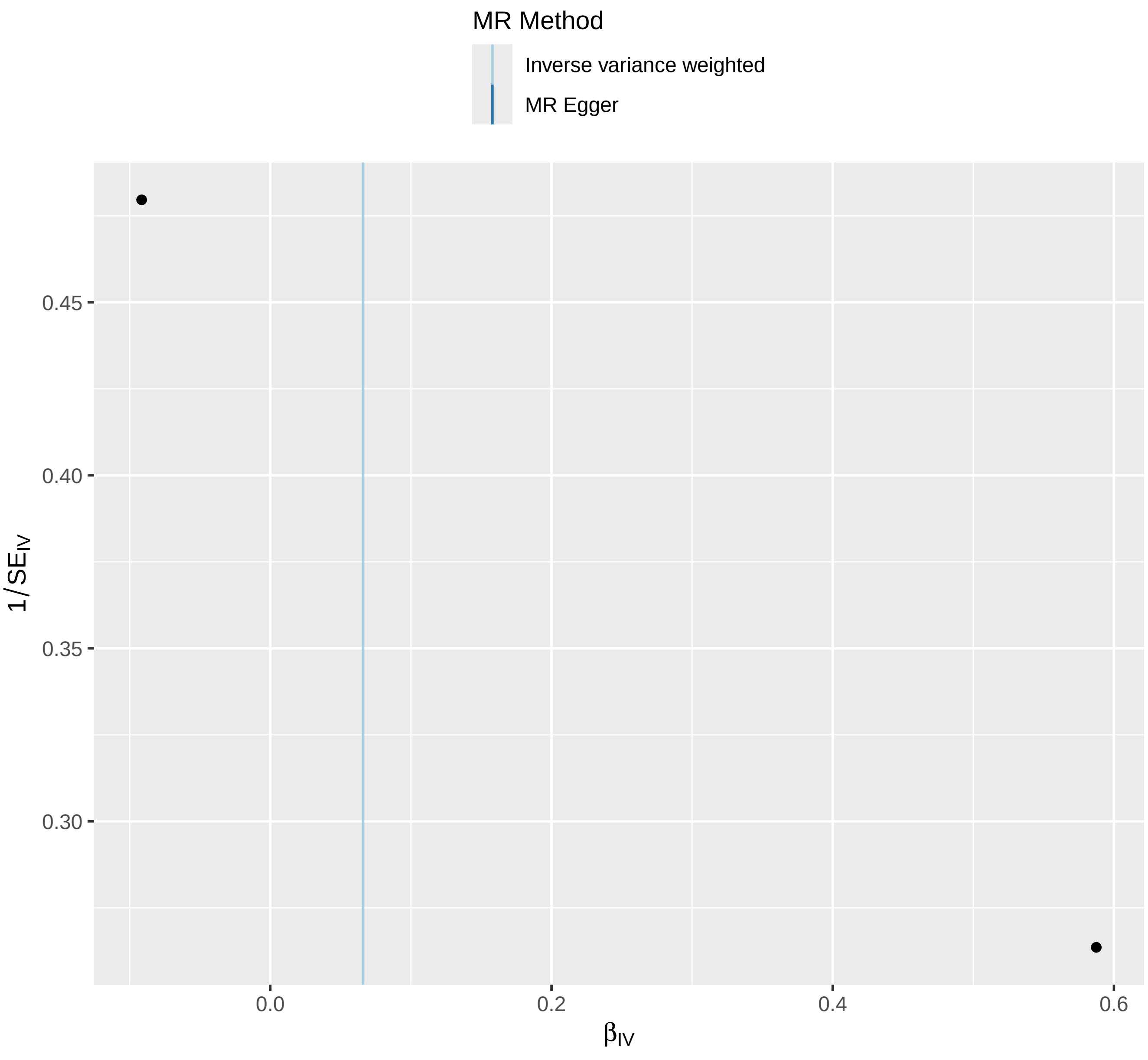
MR Method
Inverse variance weighted
MR Egger
0.45
0.40
0.35
0.30
1/SE_IV
0.0
0.2
0.4
0.6
β_IV

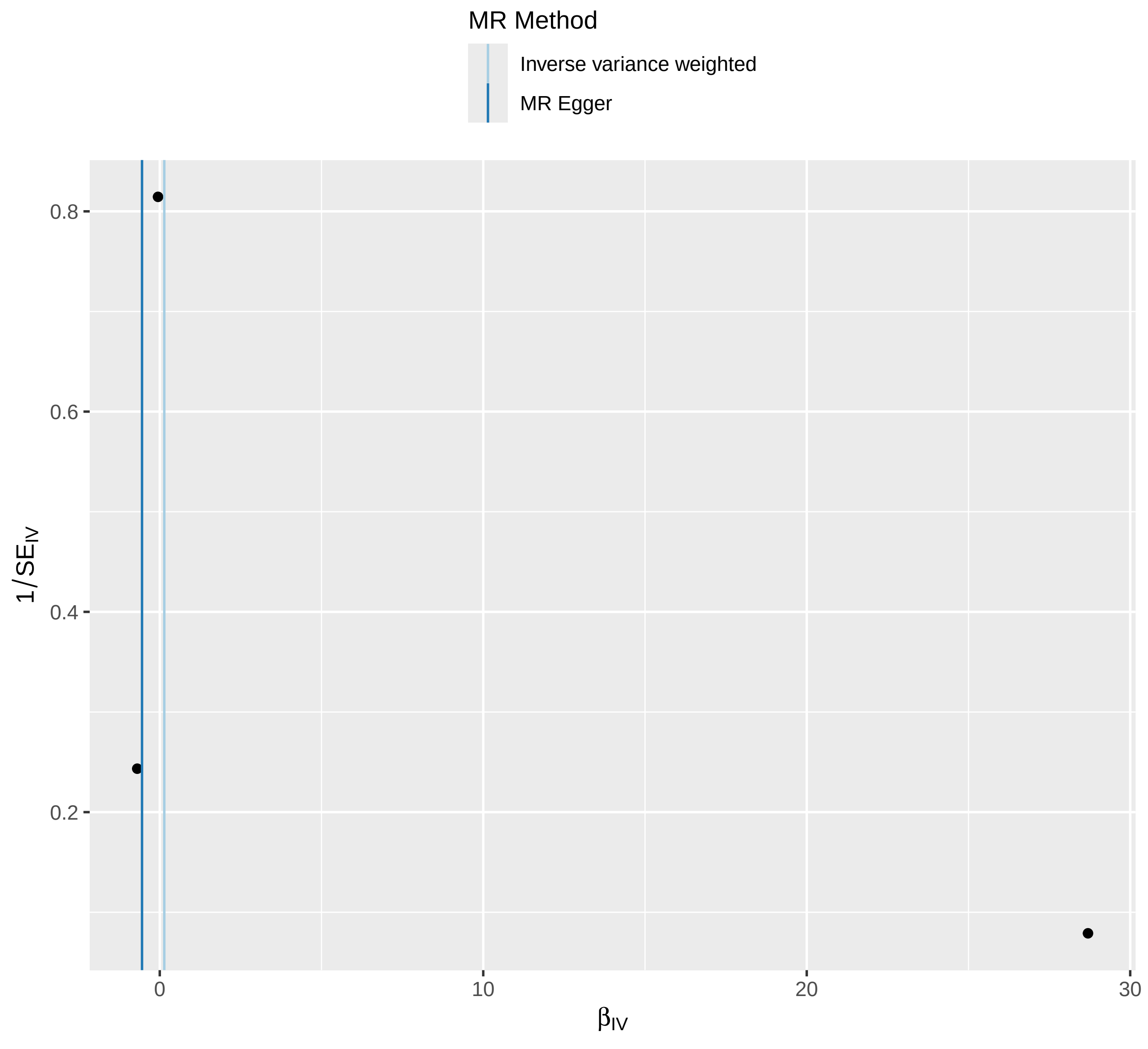
MR Method
Inverse variance weighted
MR Egger
0.8
0.6
0.4
0.2
1/SE_IV
0
10
20
30
β_IV

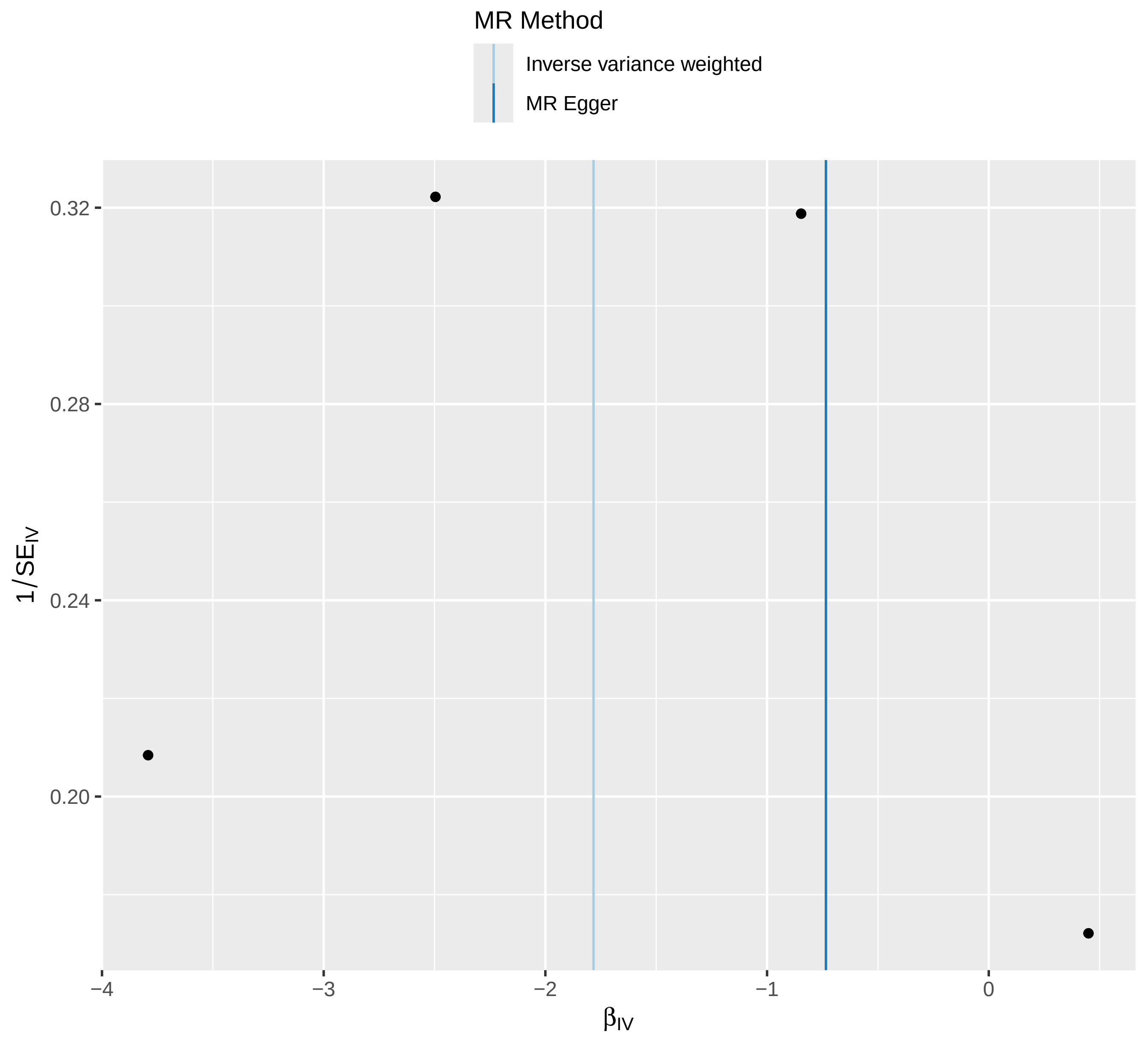
MR Method
Inverse variance weighted
MR Egger
0.32
0.28
0.24
0.20
1/SE_IV
−4
−3
−2
−1
0
β_IV

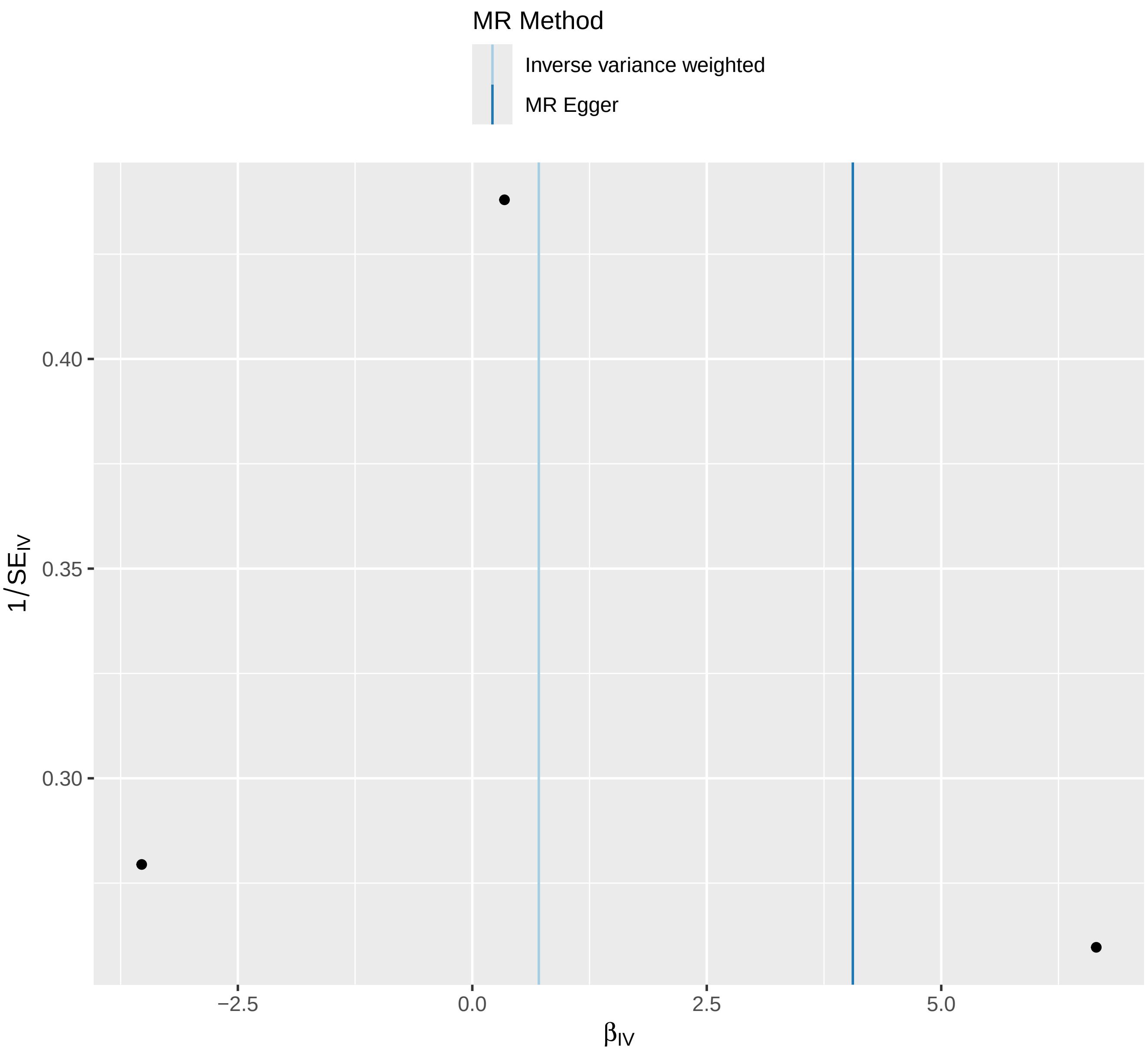
MR Method
Inverse variance weighted
MR Egger
0.40
0.35
0.30
$1/SE_{IV}$
−2.5
0.0
2.5
5.0
β_{IV}

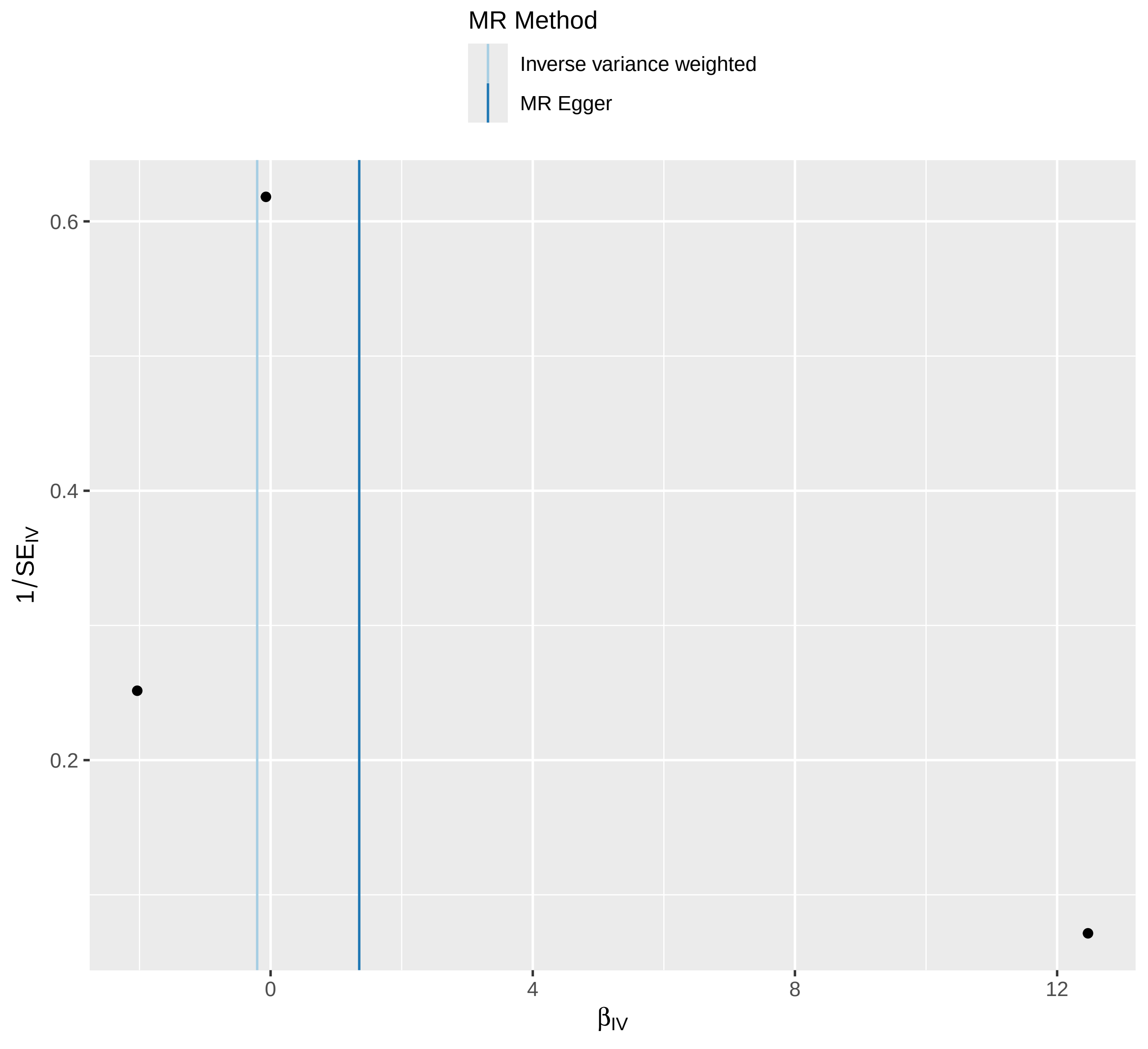
MR Method
Inverse variance weighted
MR Egger
0.6
0.4
0.2
$1/SE_{IV}$
0
4
8
12
β_{IV}

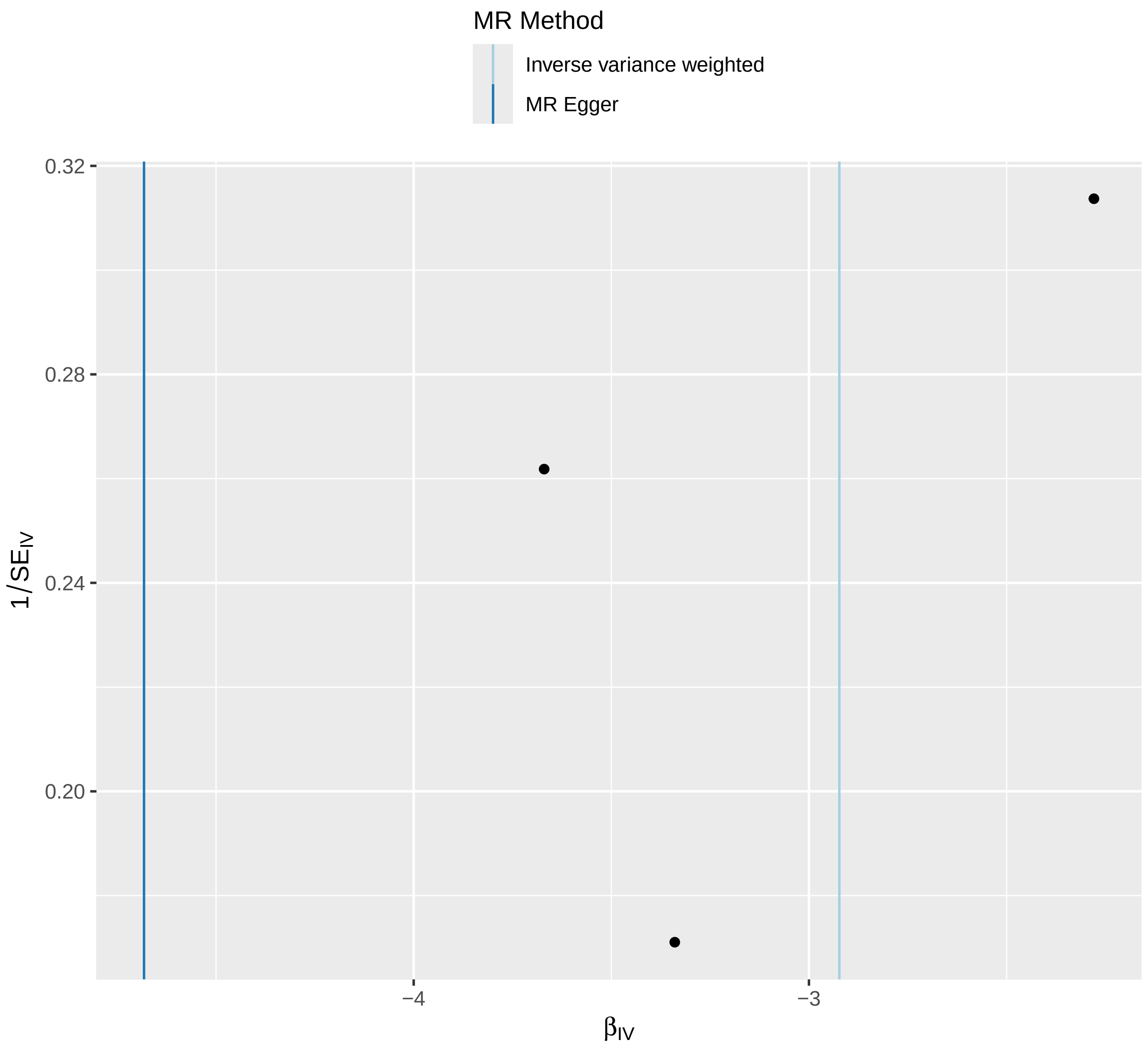
MR Method
Inverse variance weighted
MR Egger
0.32
0.28
0.24
0.20
$1/SE_{IV}$
−4
−3
β_{IV}

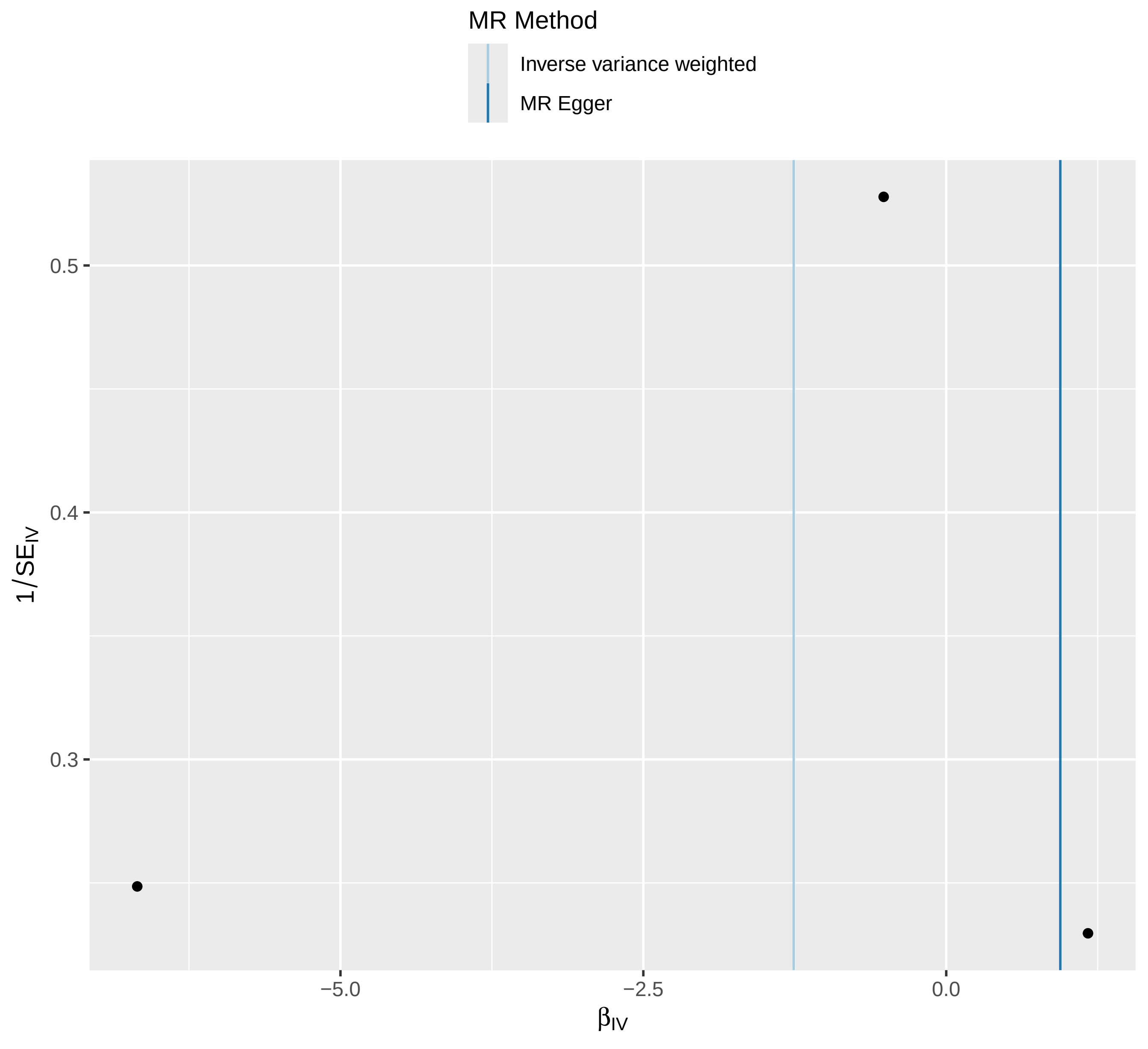
MR Method
Inverse variance weighted
MR Egger
0.5
0.4
0.3
1/SE_IV
−5.0
−2.5
0.0
β_IV

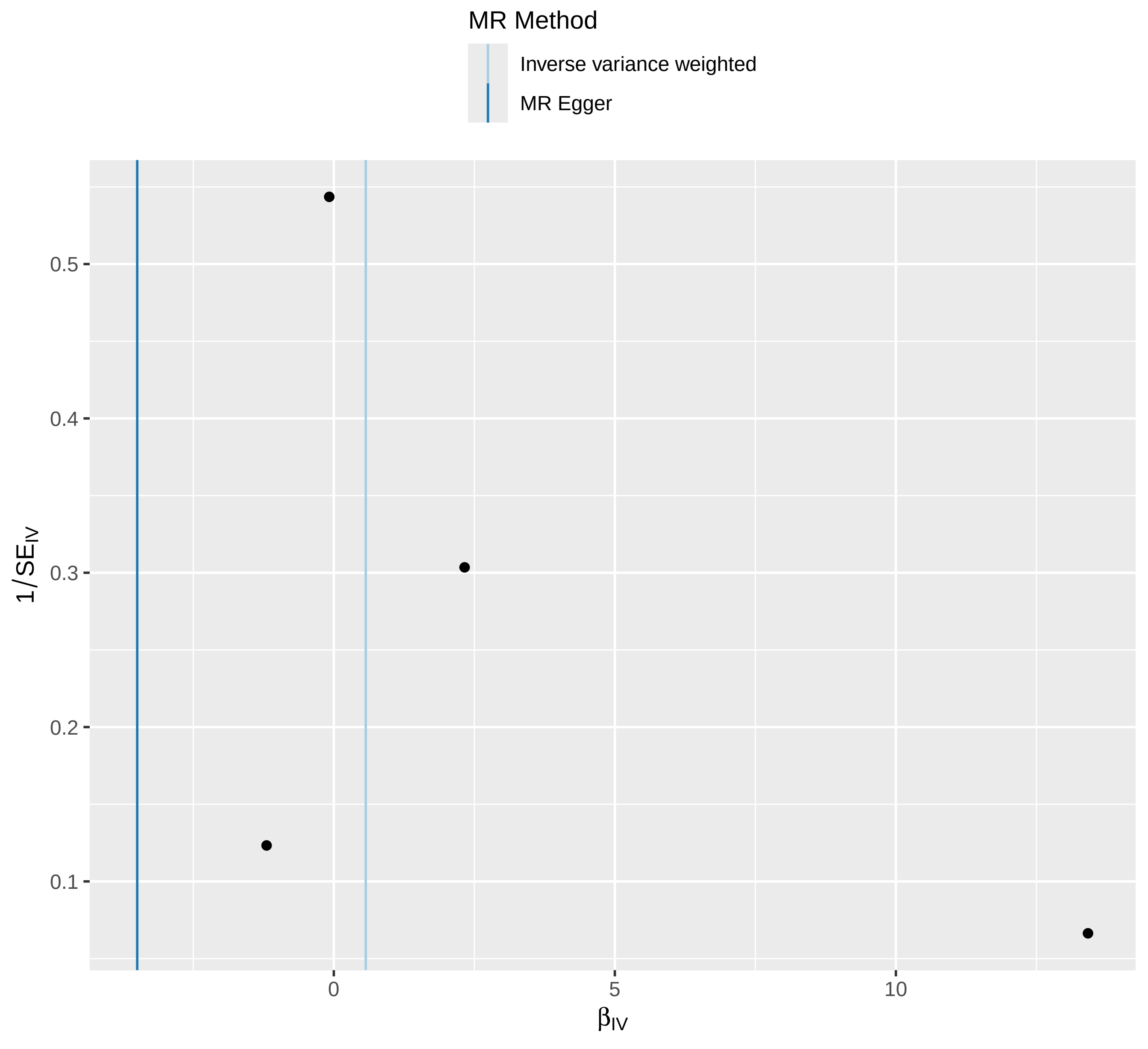

MR Method
Inverse variance weighted
MR Egger
0.5
0.4
0.3
0.2
0.1
1/SE$_{IV}$
0
5
10
β$_{IV}$

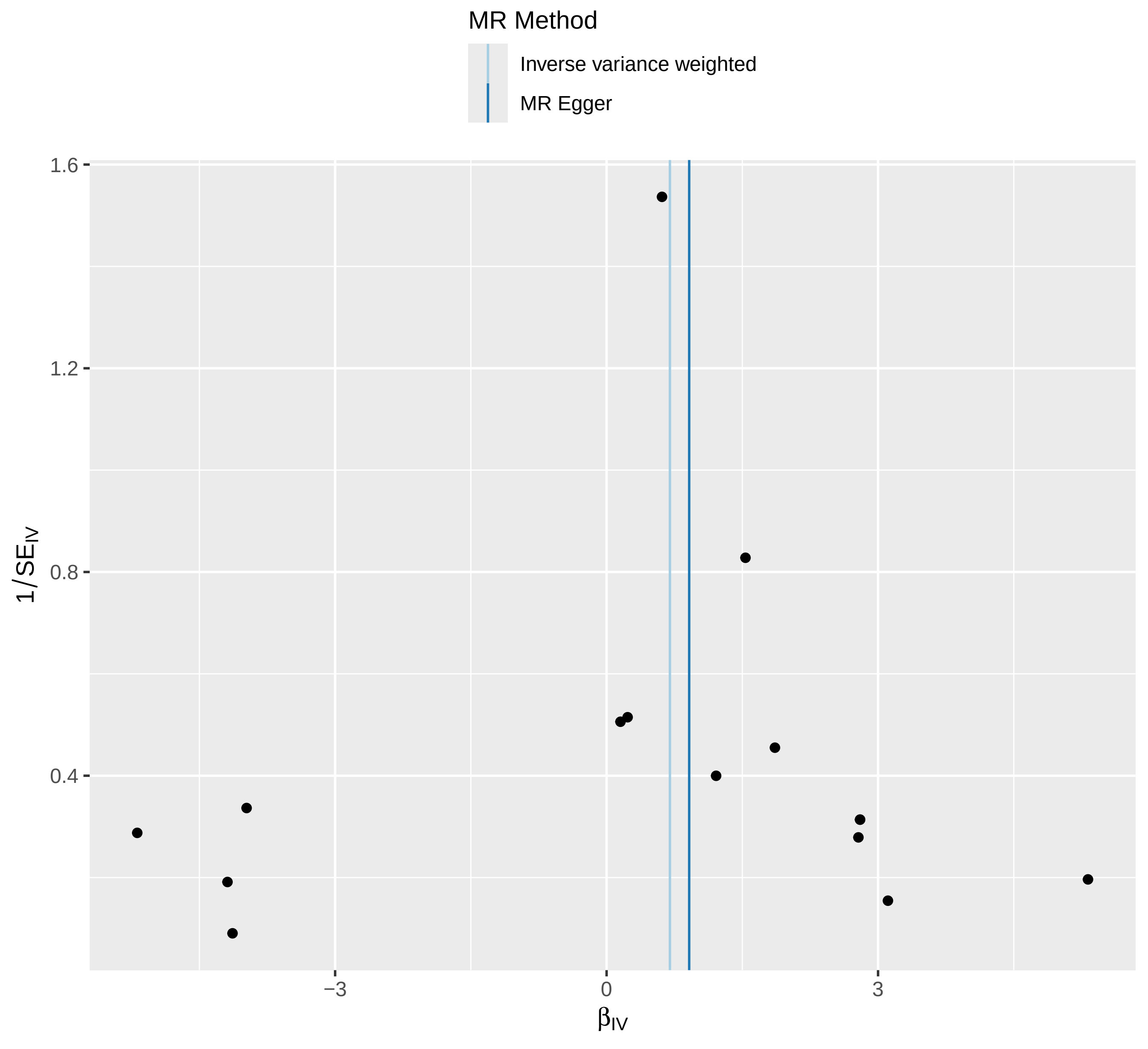
MR Method
Inverse variance weighted
MR Egger
1.6
1.2
0.8
0.4
1/SE_IV
−3
0
3
β_IV

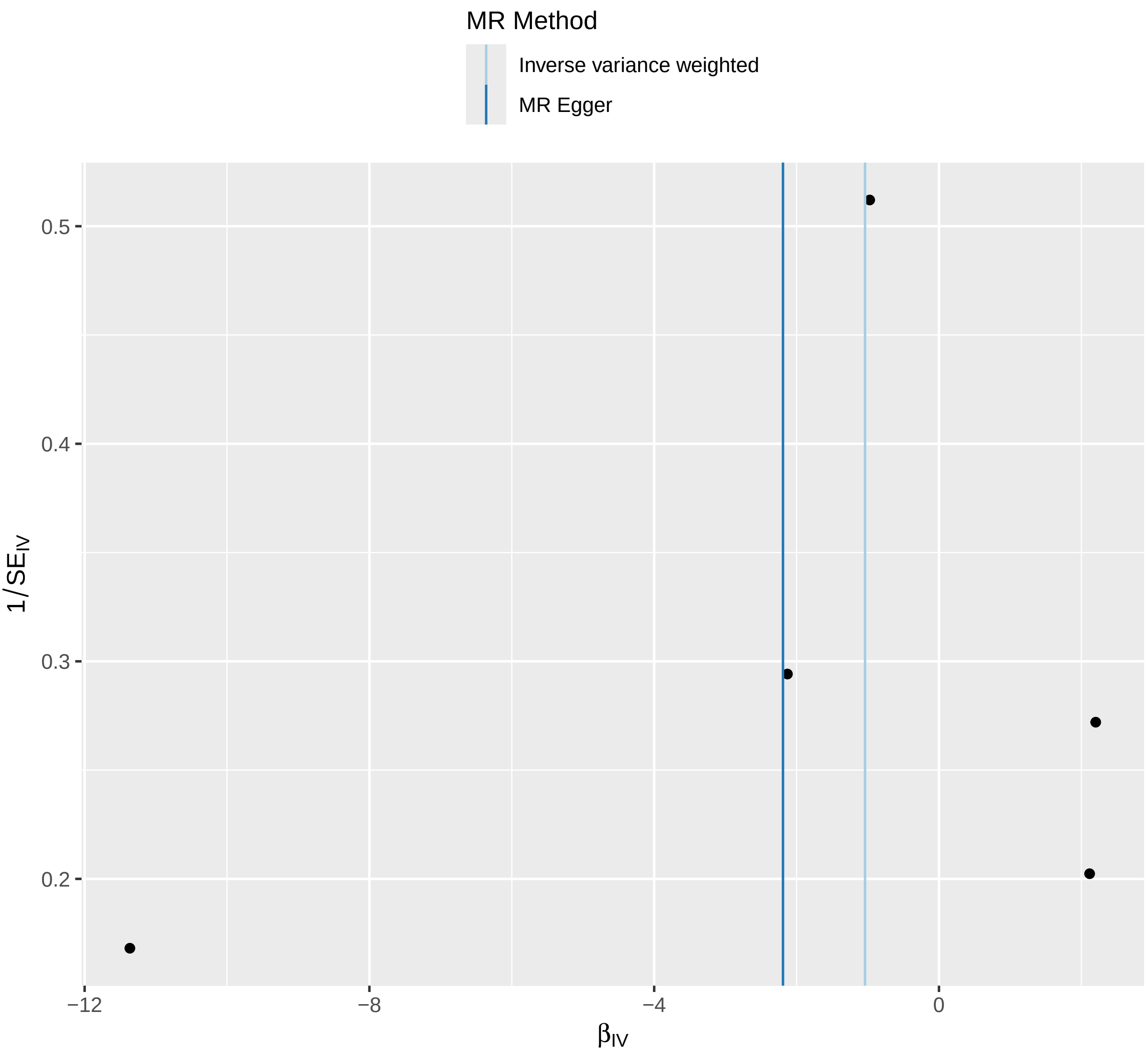
MR Method
Inverse variance weighted
MR Egger
0.5
0.4
0.3
0.2
1/SE$_{IV}$
−12
−8
−4
0
β$_{IV}$

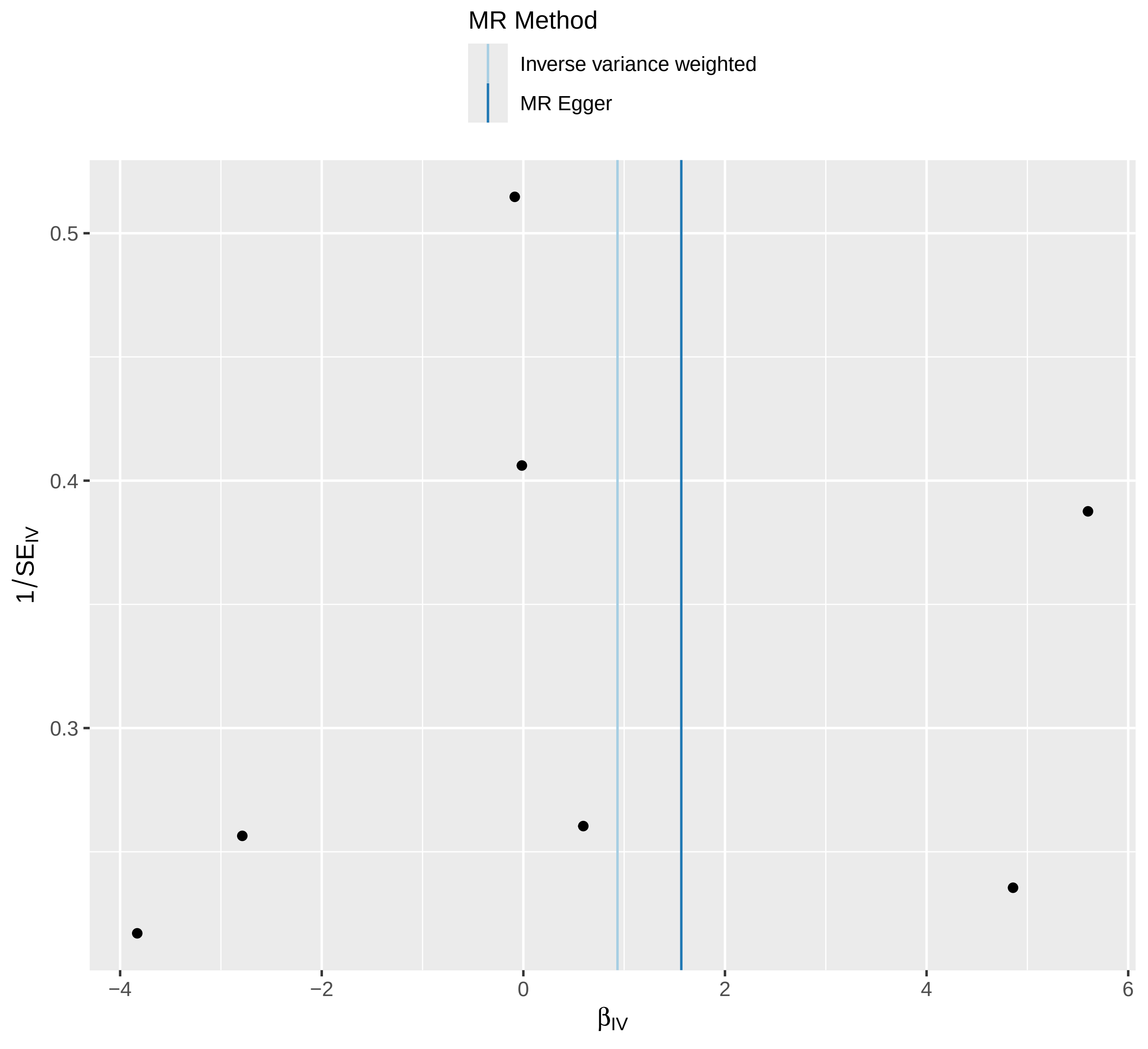

MR Method
Inverse variance weighted
MR Egger
0.5
0.4
0.3
1/SE_IV
−4
−2
0
2
4
6
β_IV

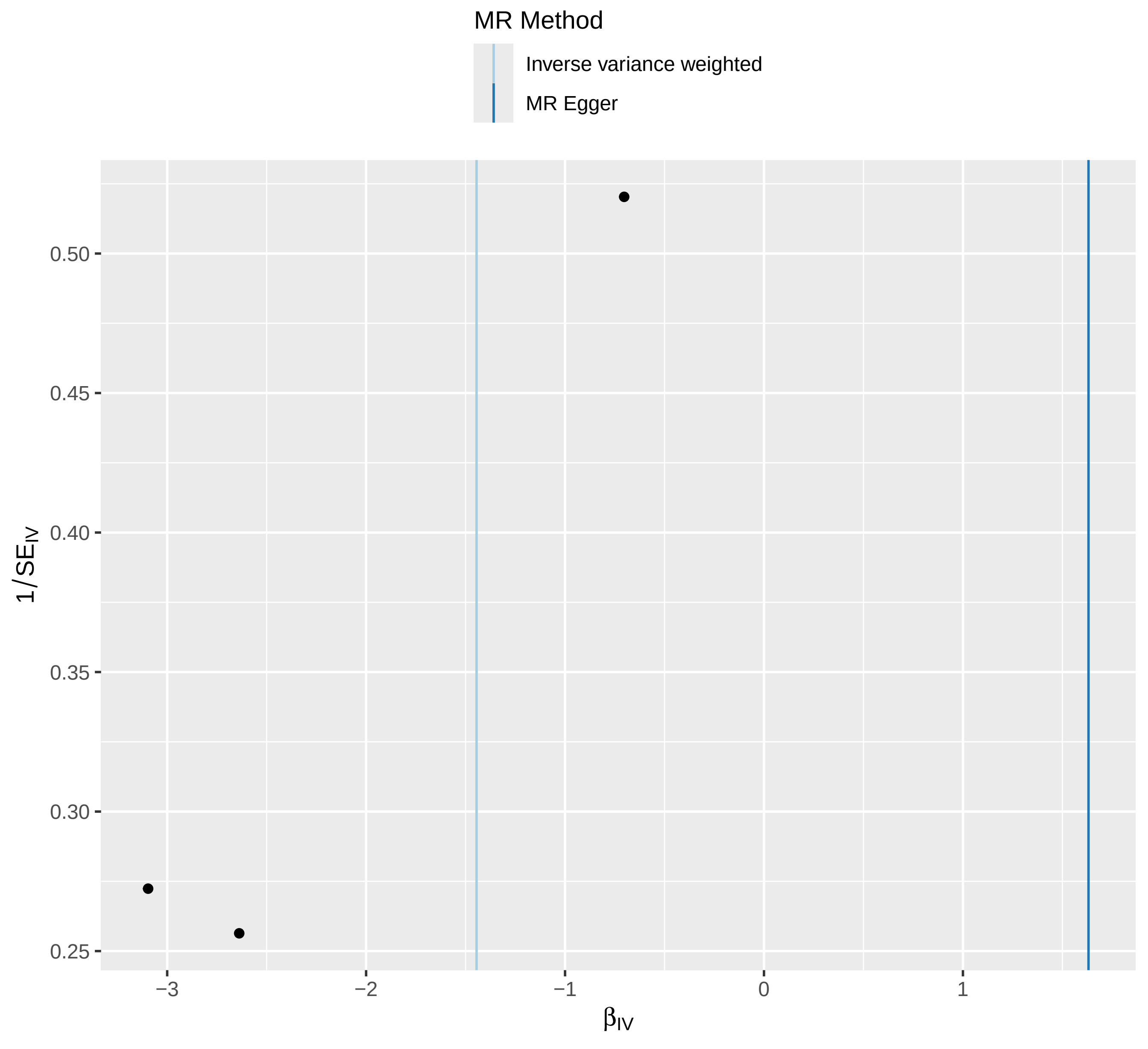

MR Method
Inverse variance weighted
MR Egger
0.50
0.45
0.40
0.35
0.30
0.25
1/SE_IV
−3
−2
−1
0
1
β_IV

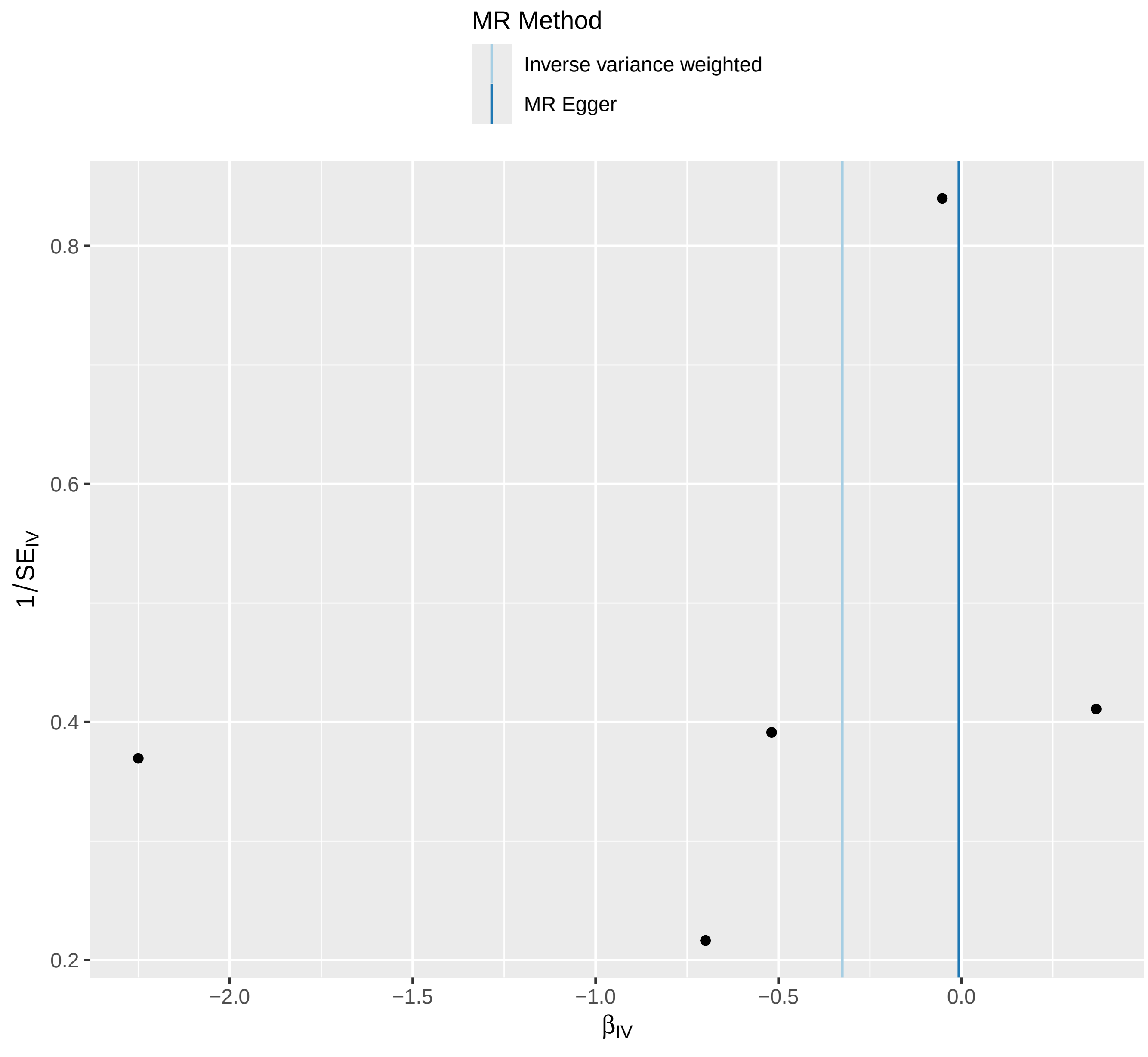
MR Method
Inverse variance weighted
MR Egger
0.8
0.6
0.4
0.2
1/SE_IV
−2.0
−1.5
−1.0
−0.5
0.0
β_IV

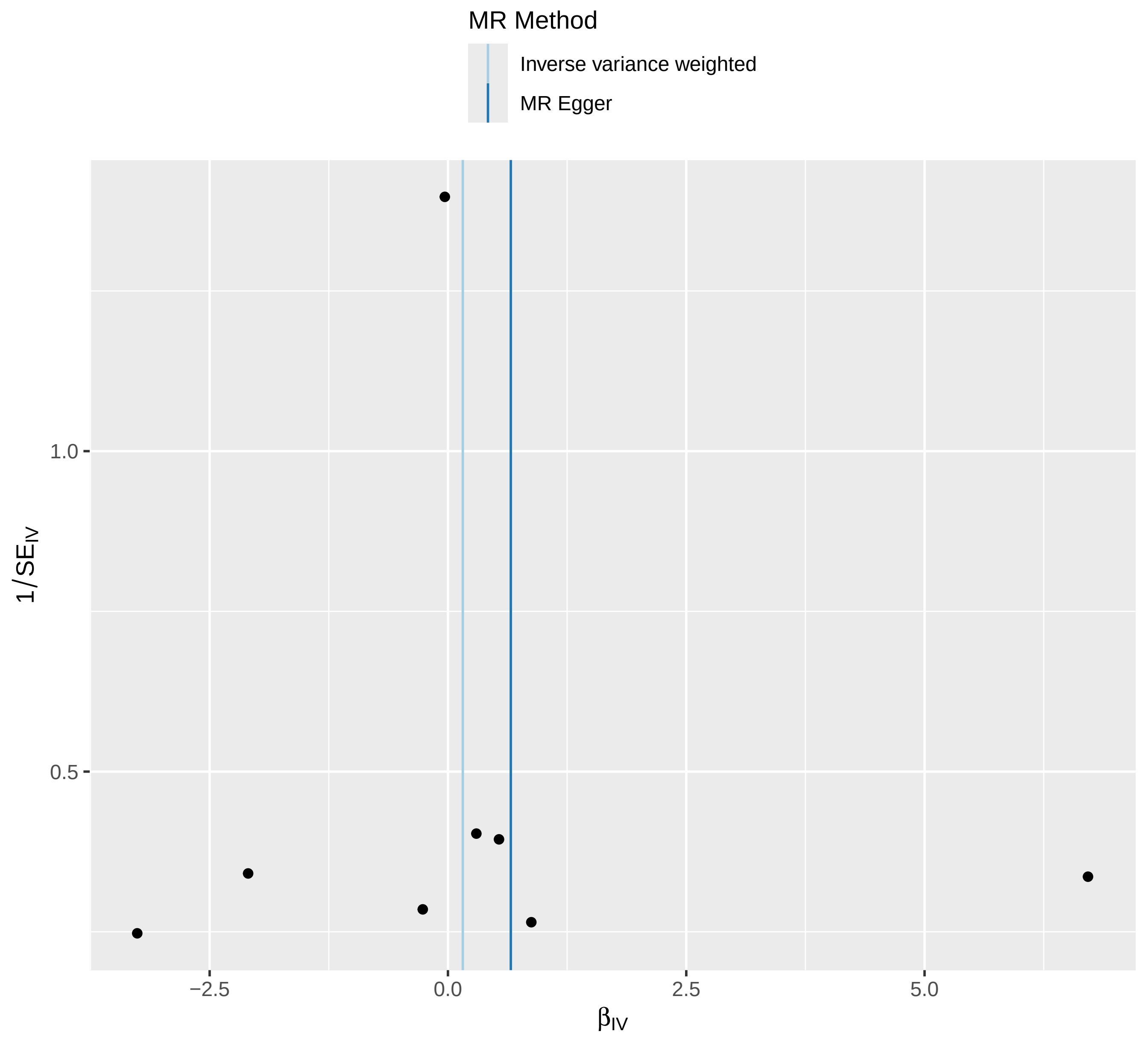
MR Method
Inverse variance weighted
MR Egger
1.0
0.5
1/SE$_{IV}$
−2.5
0.0
2.5
5.0
β$_{IV}$

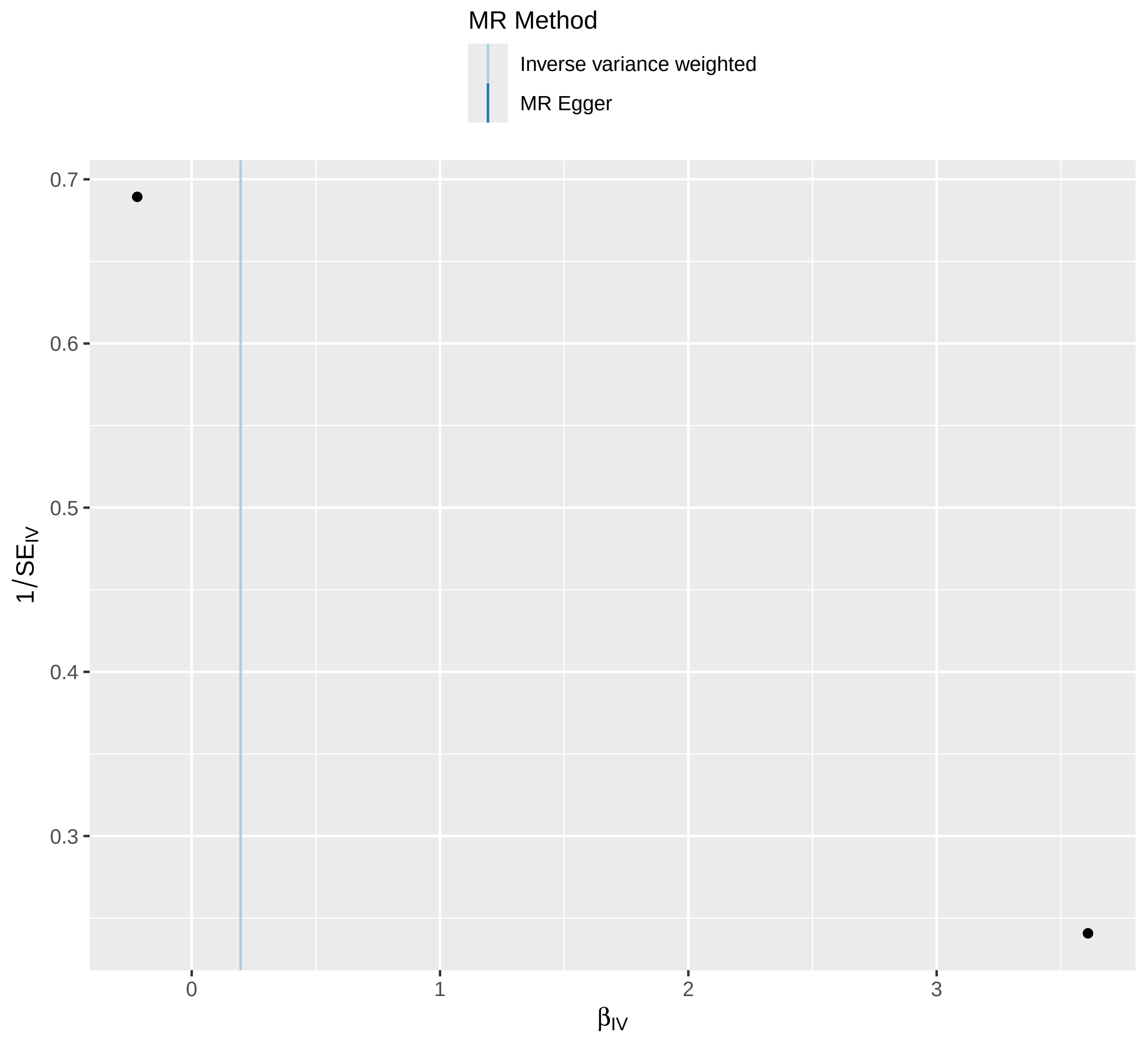
MR Method
Inverse variance weighted
MR Egger
0.7
0.6
0.5
0.4
0.3
0
1
2
3
$1/SE_{IV}$
β_{IV}

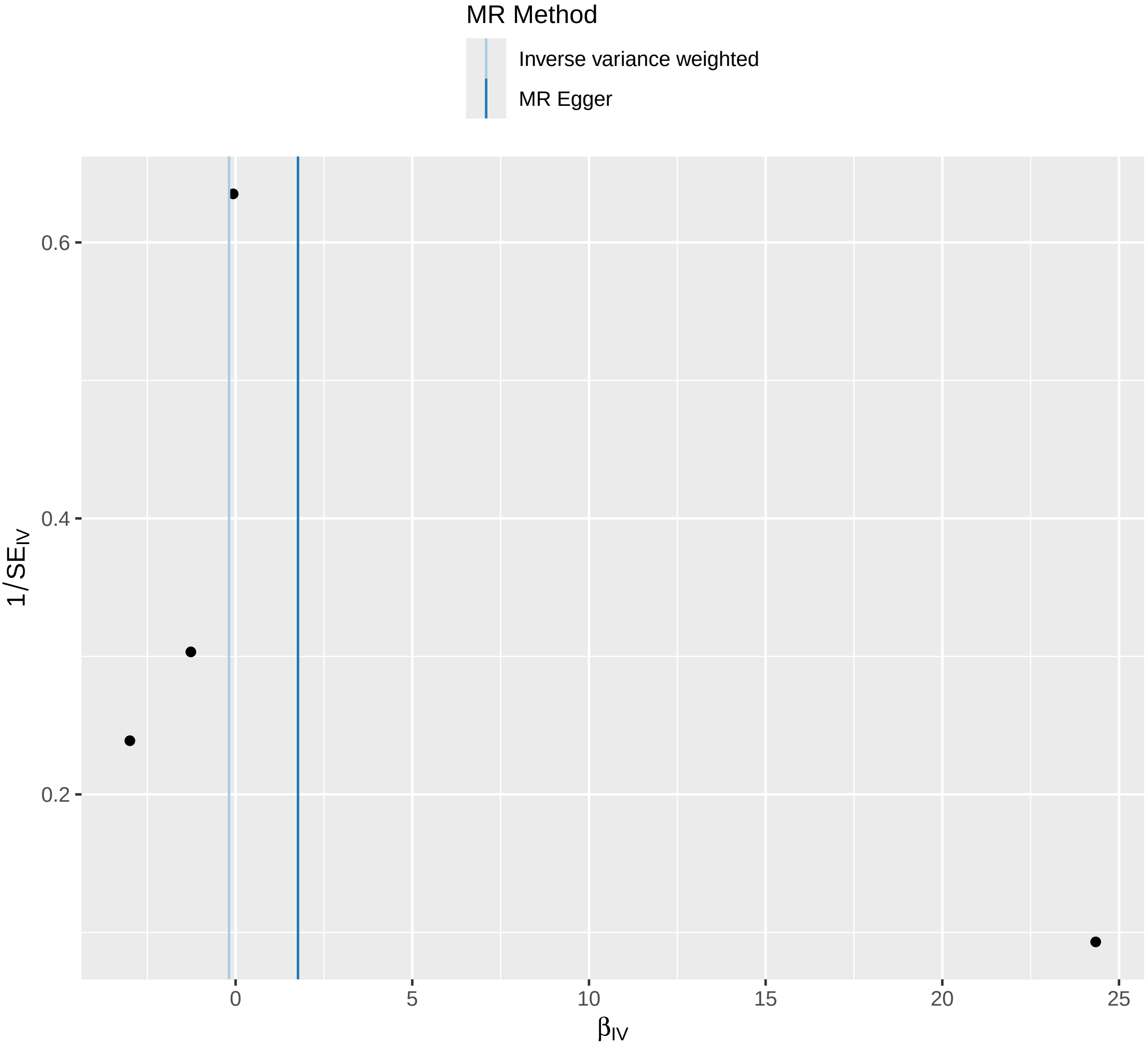
MR Method
Inverse variance weighted
MR Egger
0.6
0.4
0.2
$1/SE_{IV}$
0
5
10
15
20
25
β_{IV}

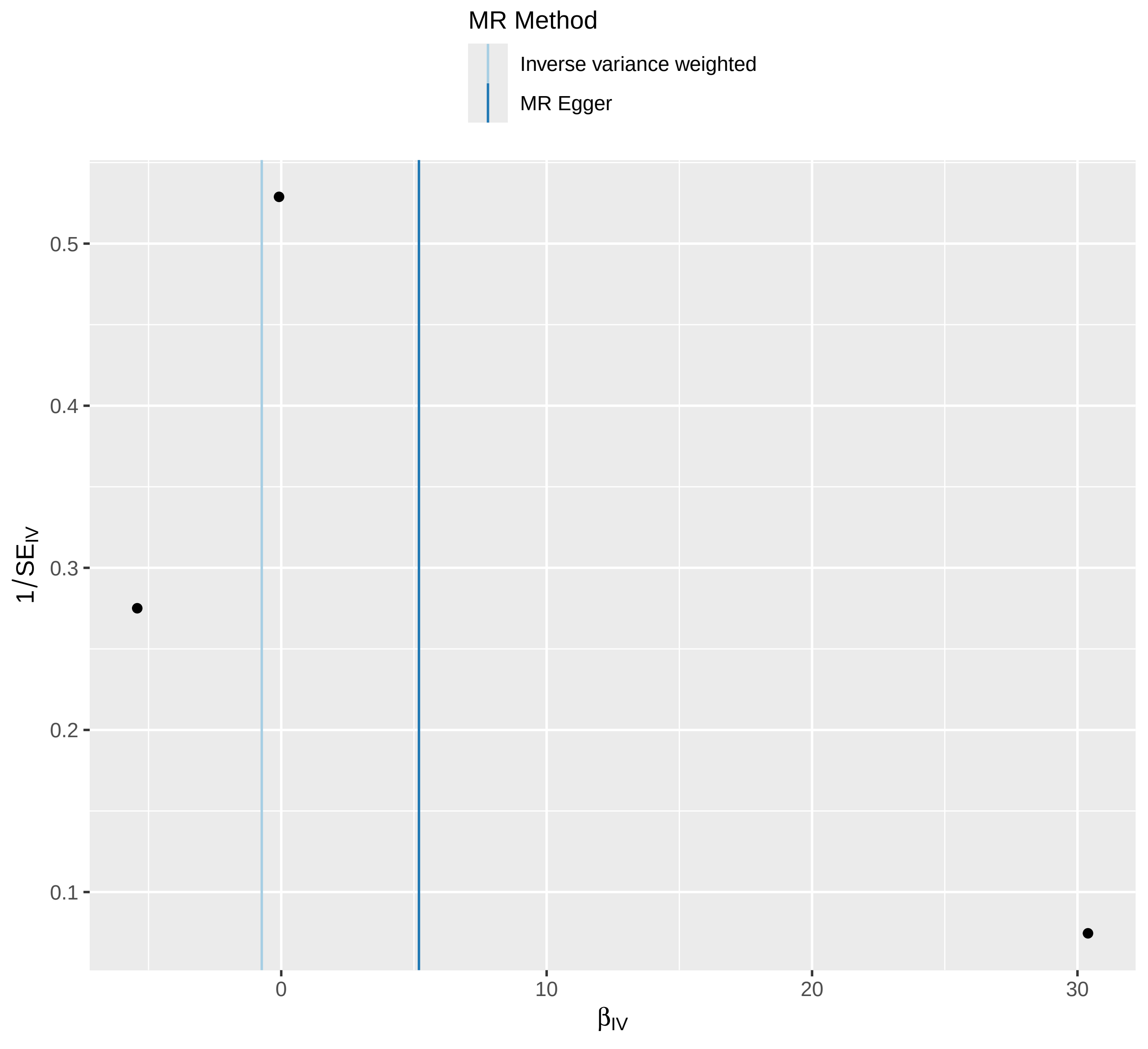
MR Method
Inverse variance weighted
MR Egger
0.5
0.4
0.3
0.2
0.1
1/SE_IV
0
10
20
30
β_IV

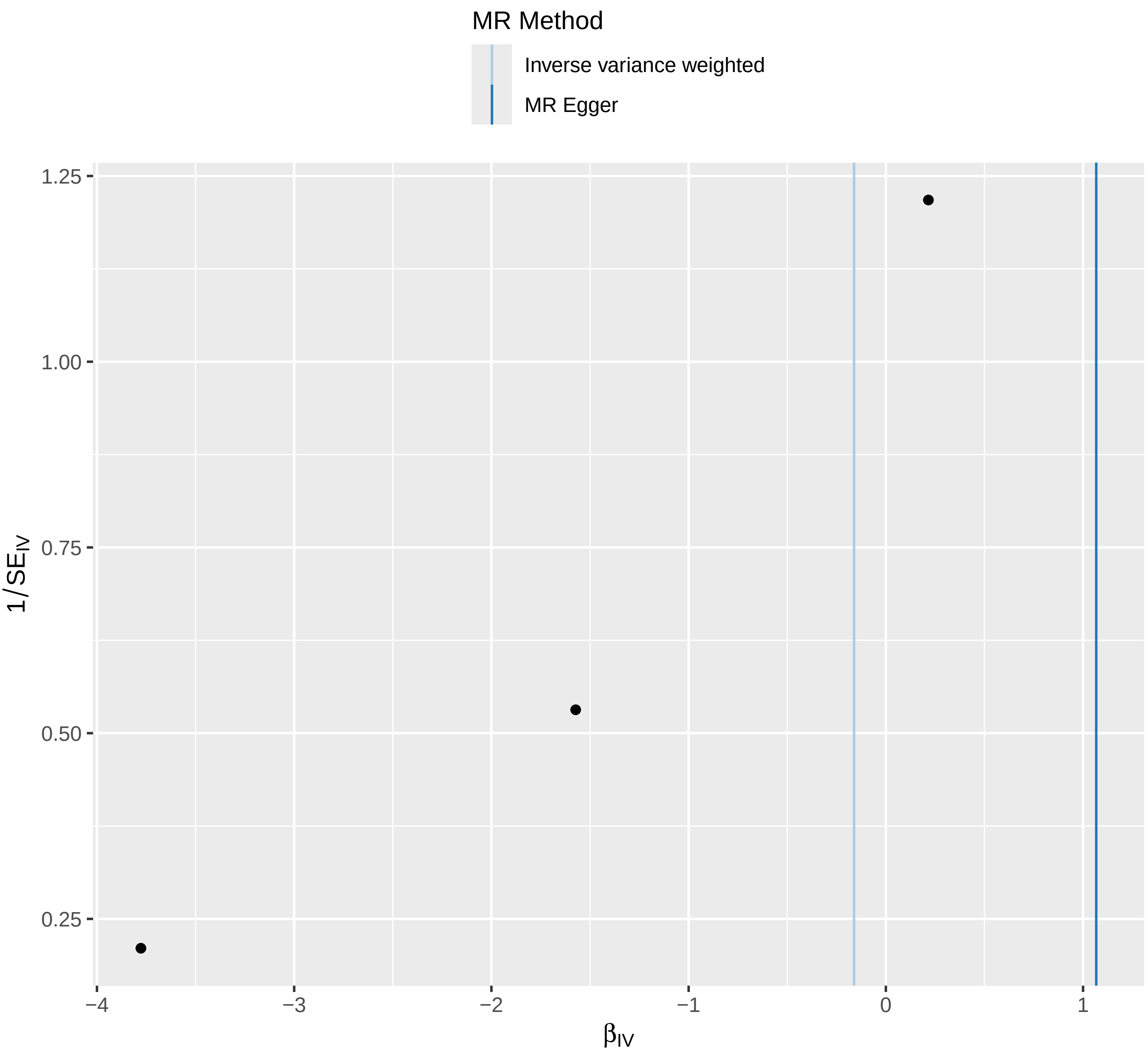
MR Method
Inverse variance weighted
MR Egger
1.25
1.00
0.75
0.50
0.25
1/SE_IV
−4
−3
−2
−1
0
1
β_IV

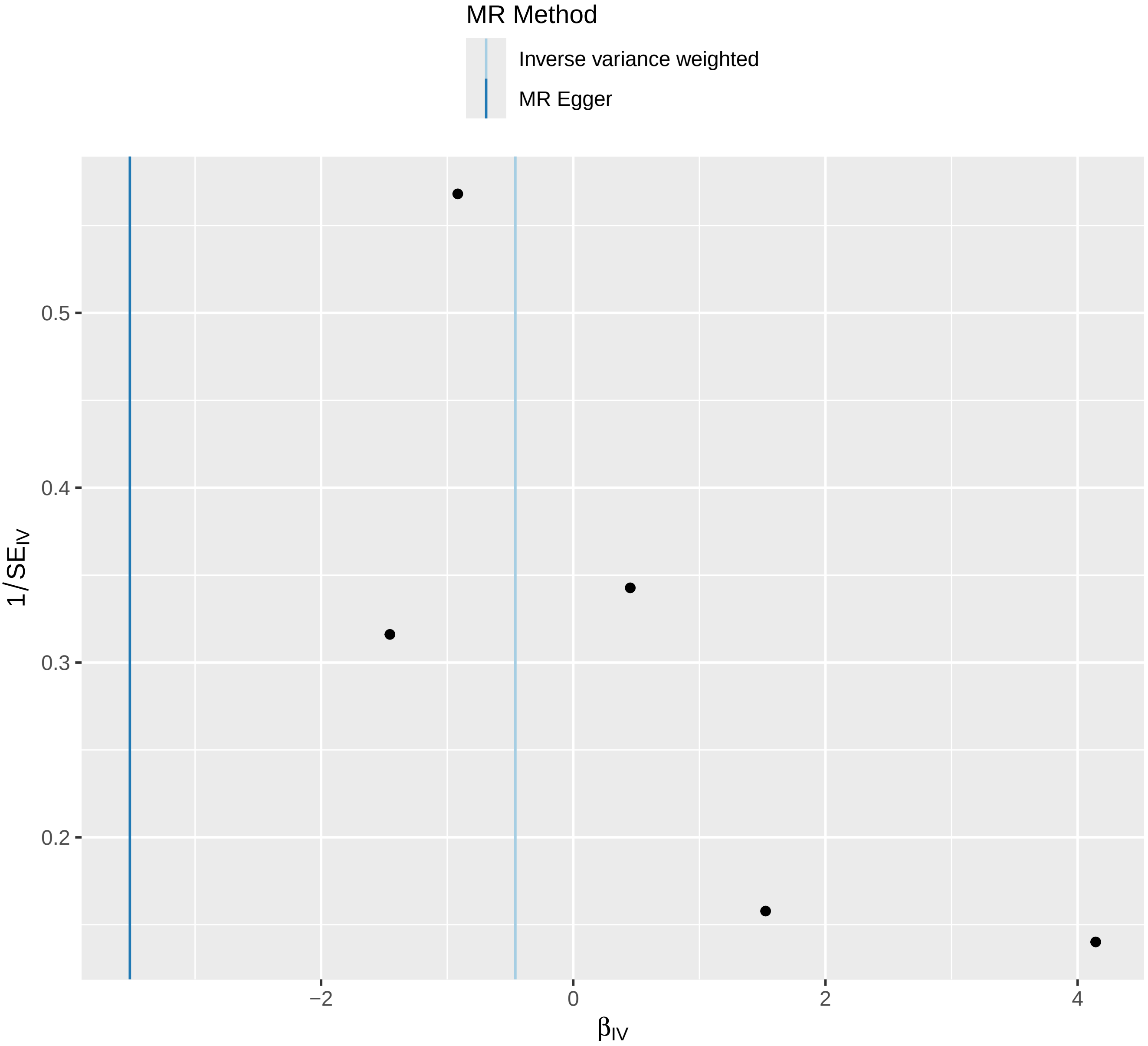
MR Method
Inverse variance weighted
MR Egger
0.5
0.4
0.3
0.2
−2
0
2
4
$1/SE_{IV}$
β_{IV}

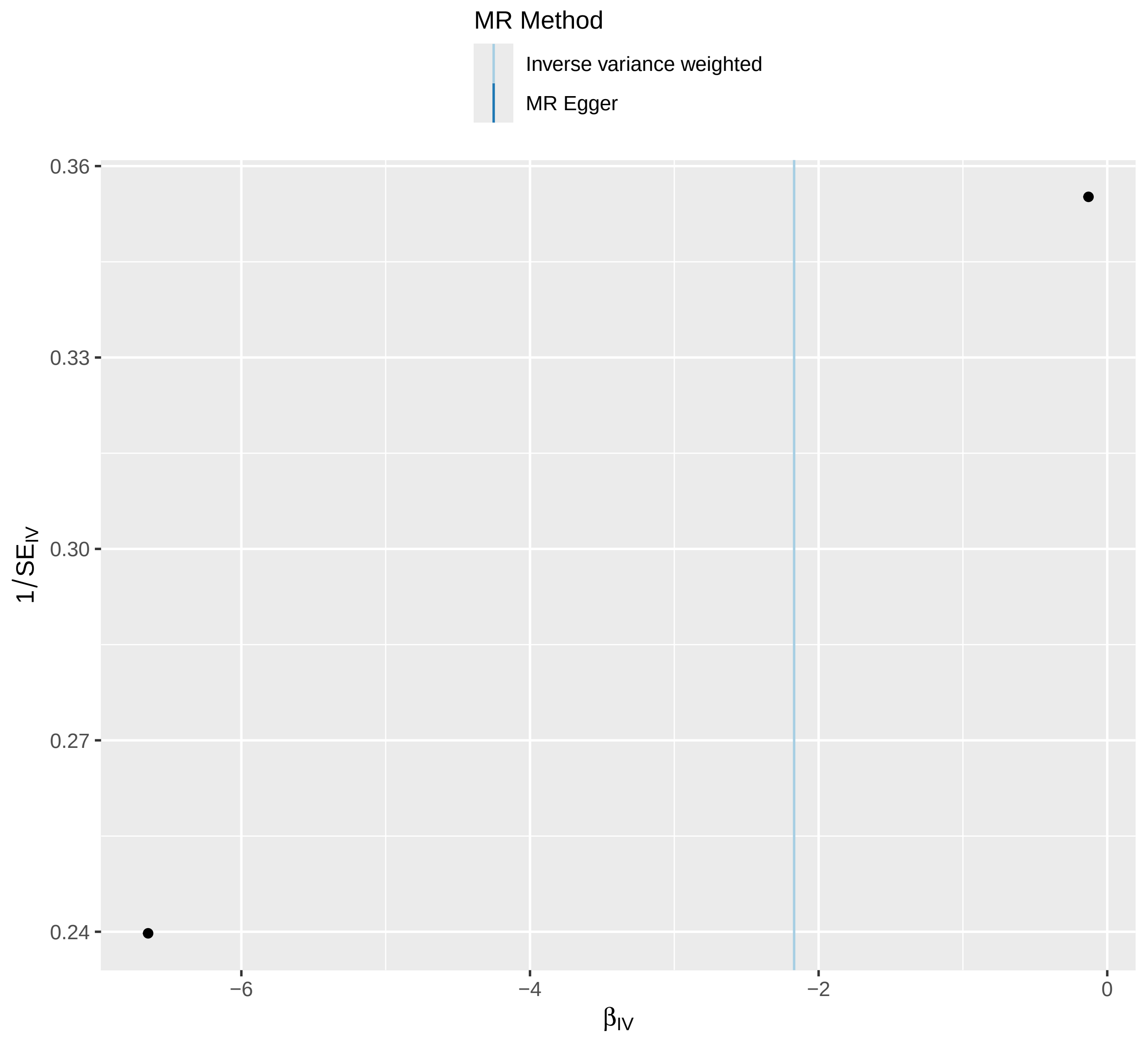
MR Method
Inverse variance weighted
MR Egger
0.36
0.33
0.30
0.27
0.24
1/SE_IV
−6
−4
−2
0
β_IV

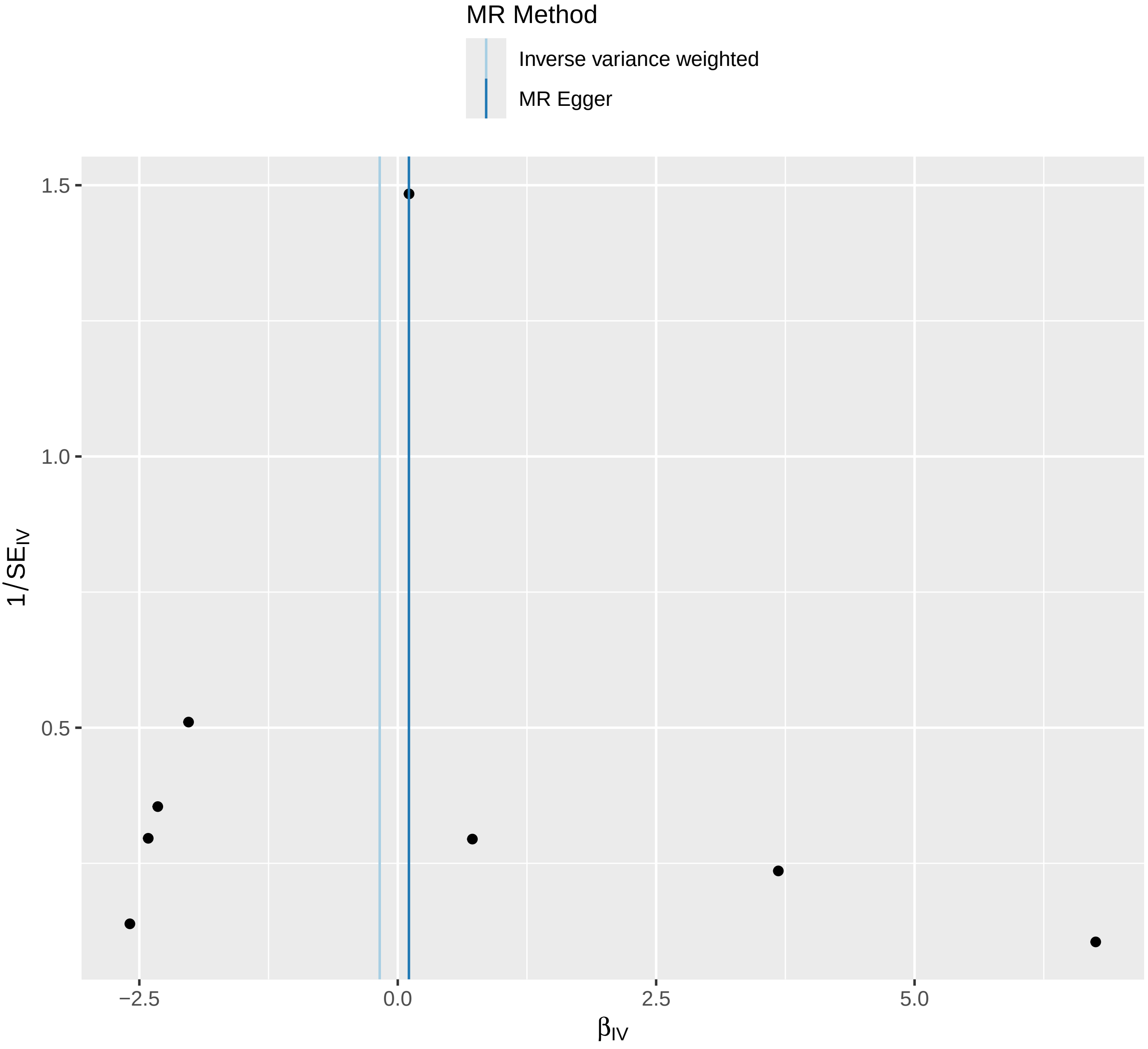

MR Method
Inverse variance weighted
MR Egger
1.5
1.0
0.5
1/SE_IV
−2.5
0.0
2.5
5.0
β_IV

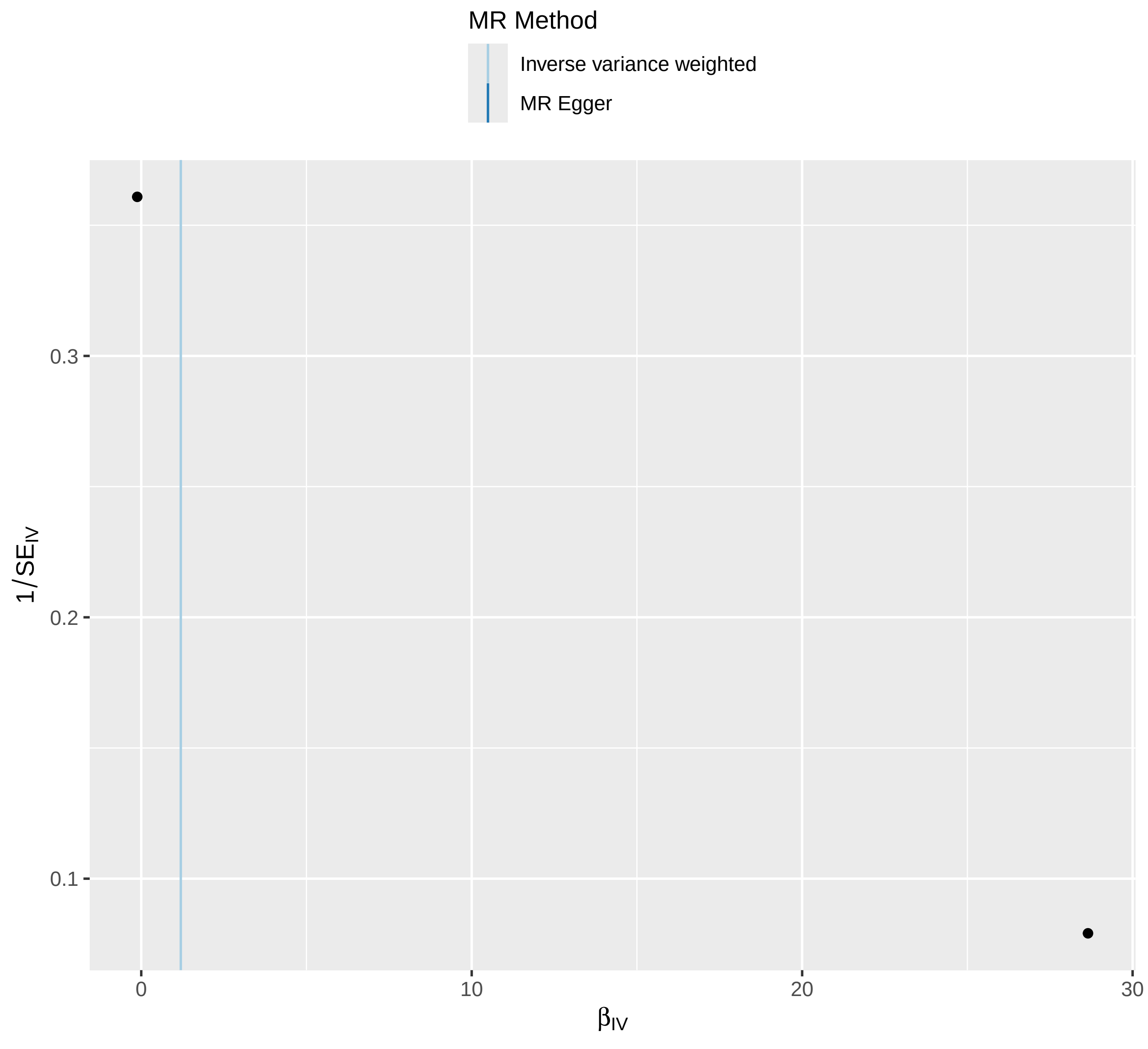
MR Method
Inverse variance weighted
MR Egger
0.3
0.2
0.1
1/SE_IV
0
10
20
30
β_IV

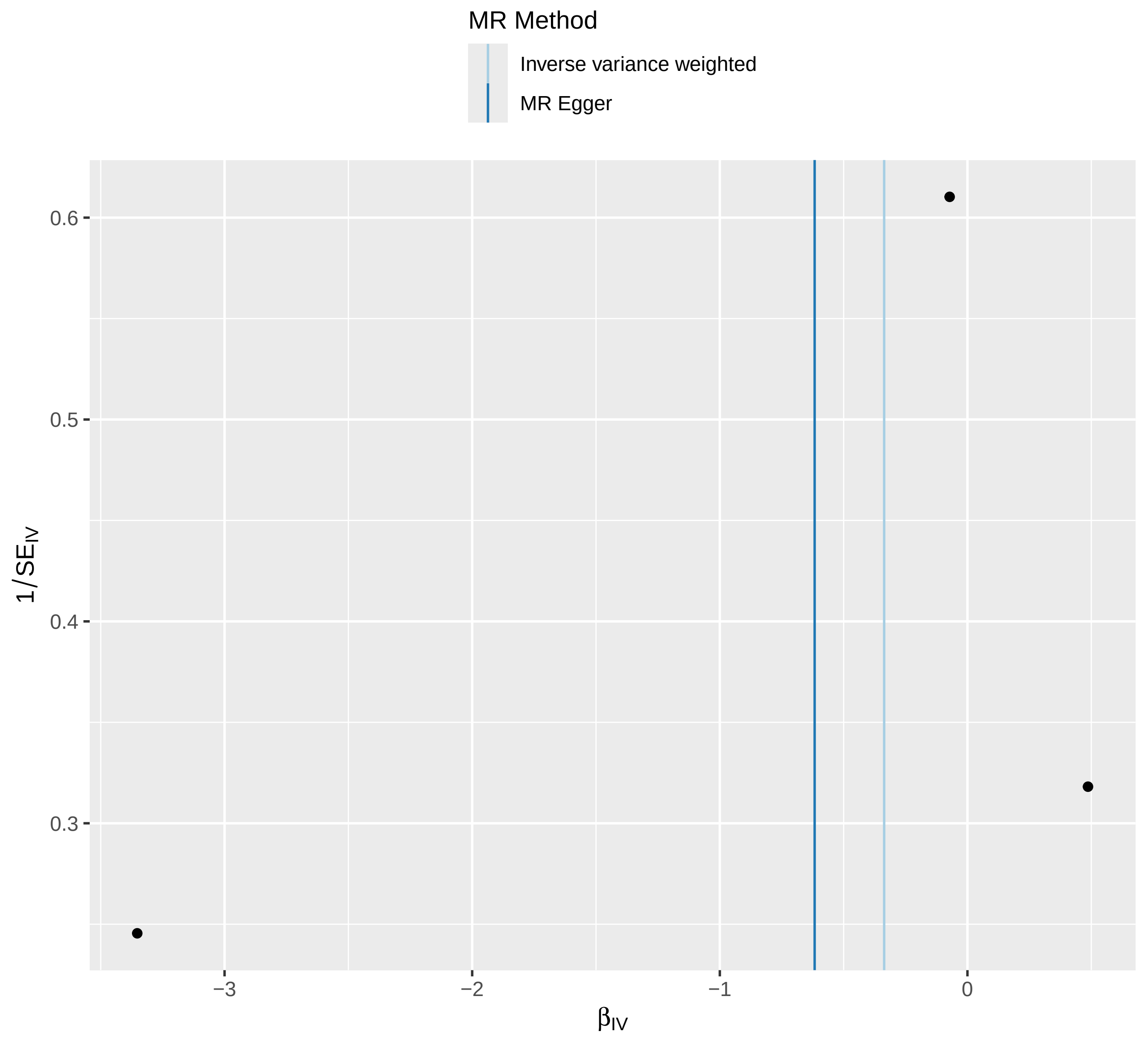
MR Method
Inverse variance weighted
MR Egger
0.6
0.5
0.4
0.3
1/SE_IV
−3
−2
−1
0
β_IV

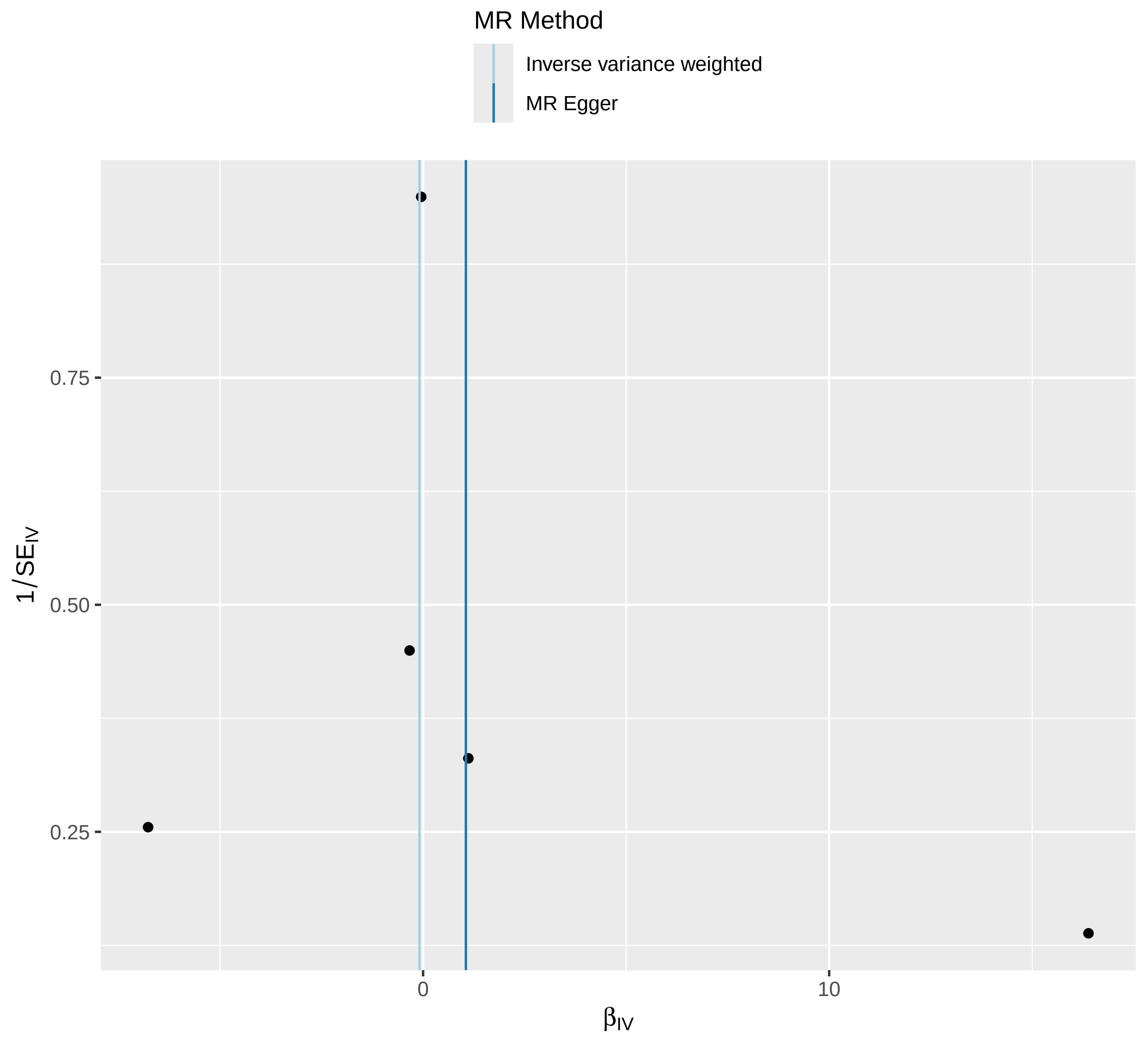
MR Method
Inverse variance weighted
MR Egger
0.75
0.50
0.25
$1/SE_{IV}$
0
10
β_{IV}

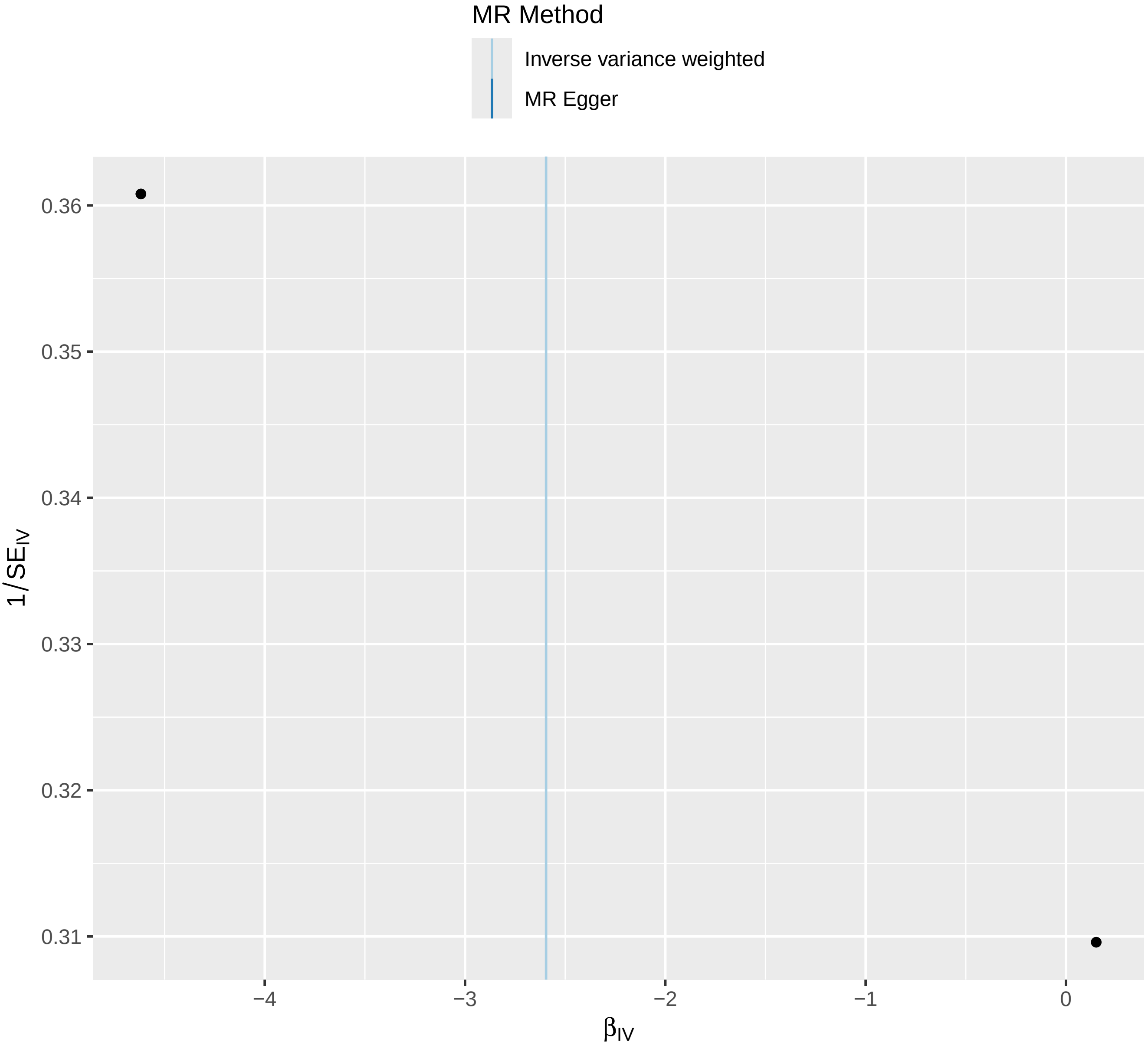
MR Method
Inverse variance weighted
MR Egger
0.36
0.35
0.34
0.33
0.32
0.31
1/SE_IV
−4
−3
−2
−1
0
β_IV

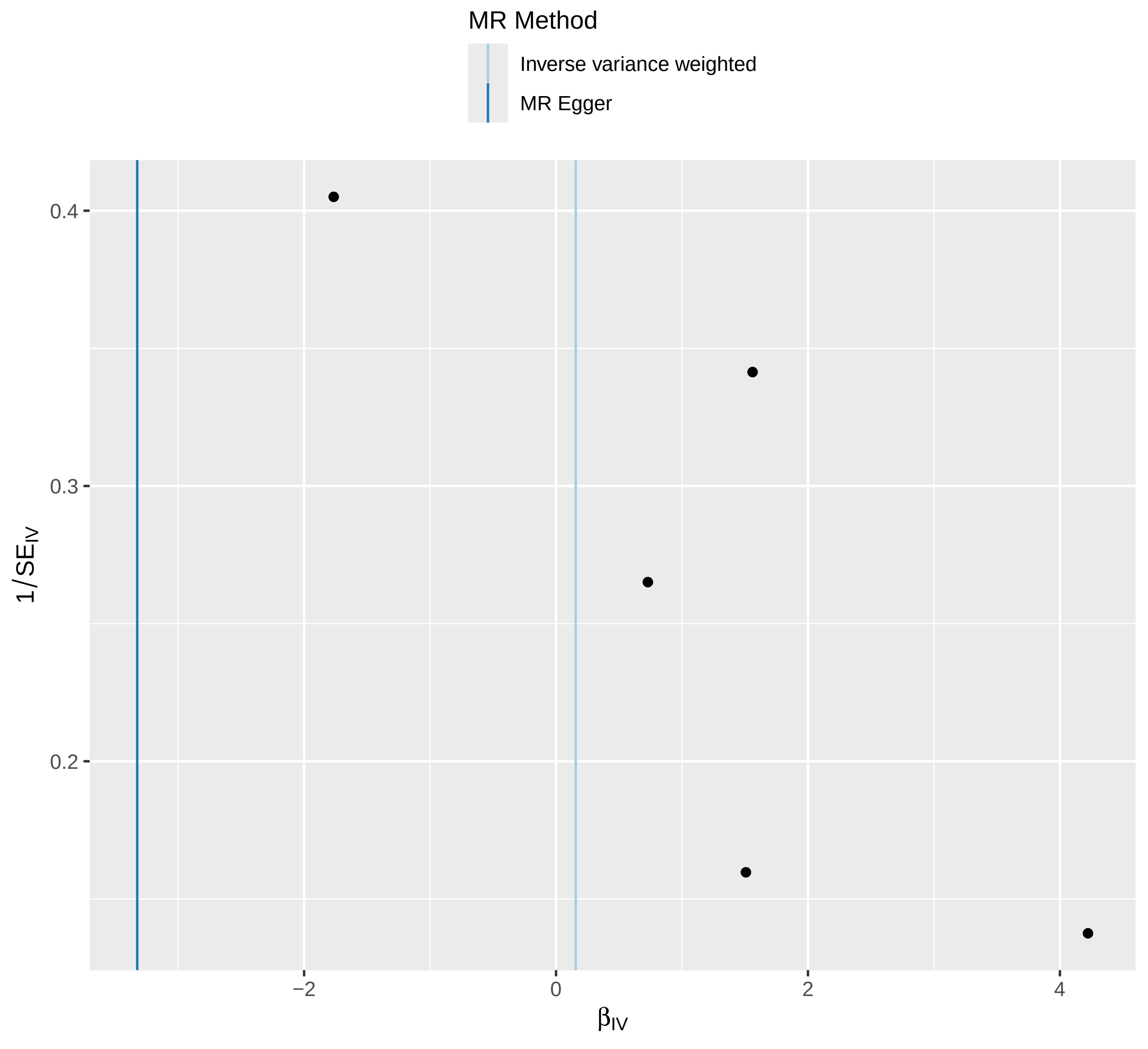
MR Method
Inverse variance weighted
MR Egger
0.4
0.3
0.2
1/SE$_{IV}$
−2
0
2
4
β$_{IV}$

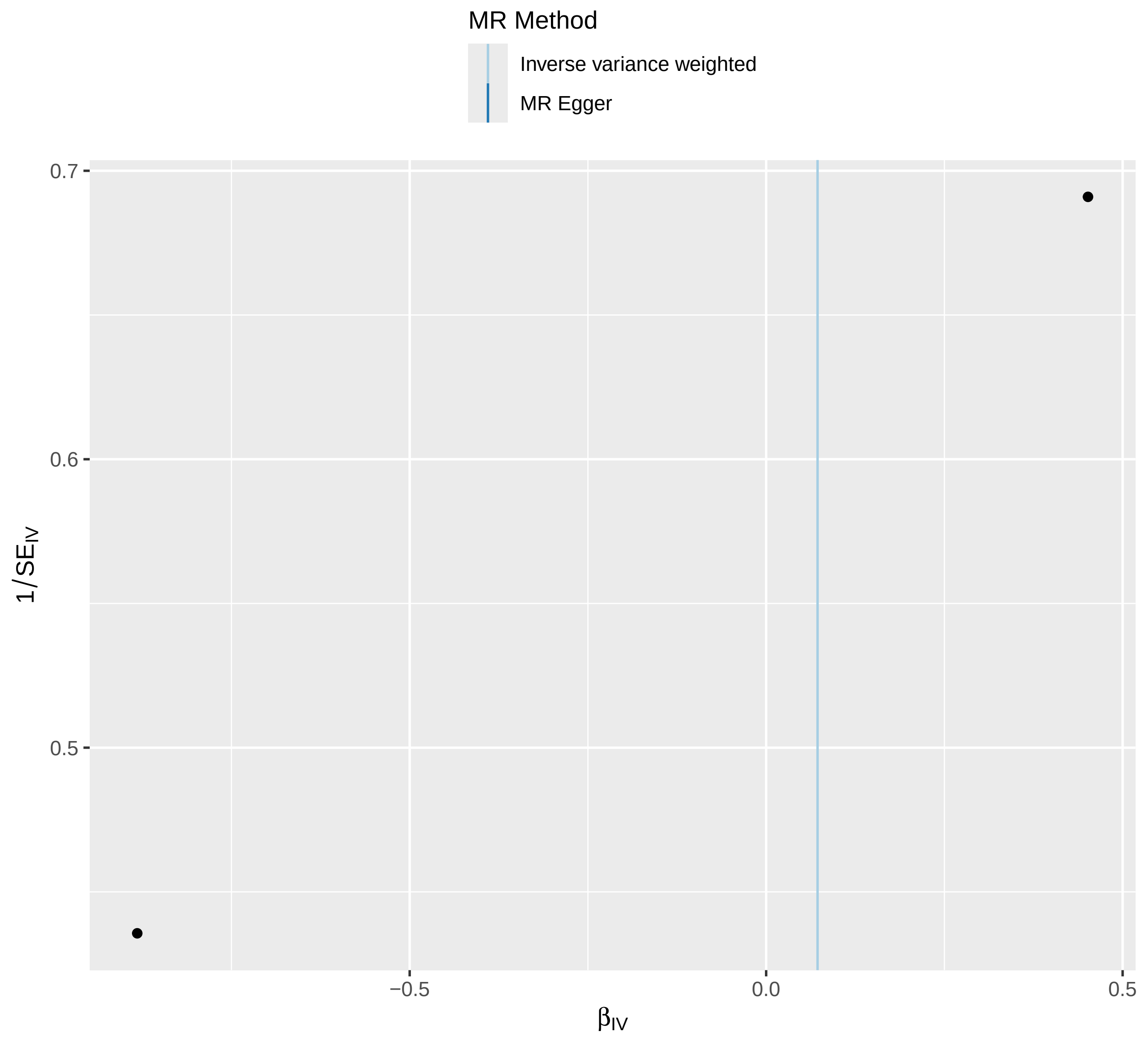

MR Method
Inverse variance weighted
MR Egger
0.7
0.6
0.5
1/SE_IV
−0.5
0.0
0.5
β_IV

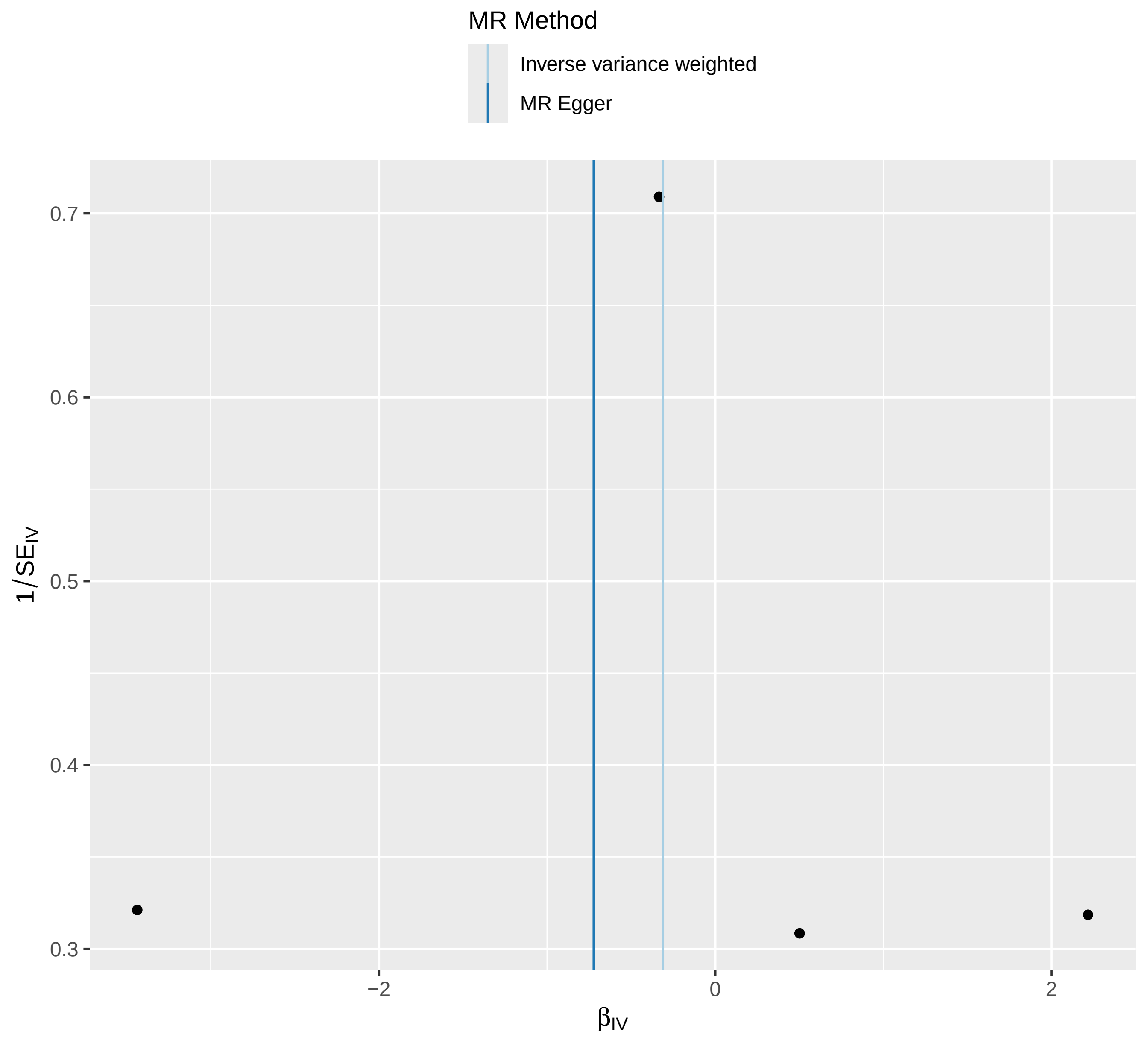
MR Method
Inverse variance weighted
MR Egger
0.7
0.6
0.5
0.4
0.3
−2
0
2
$1/SE_{IV}$
β_{IV}

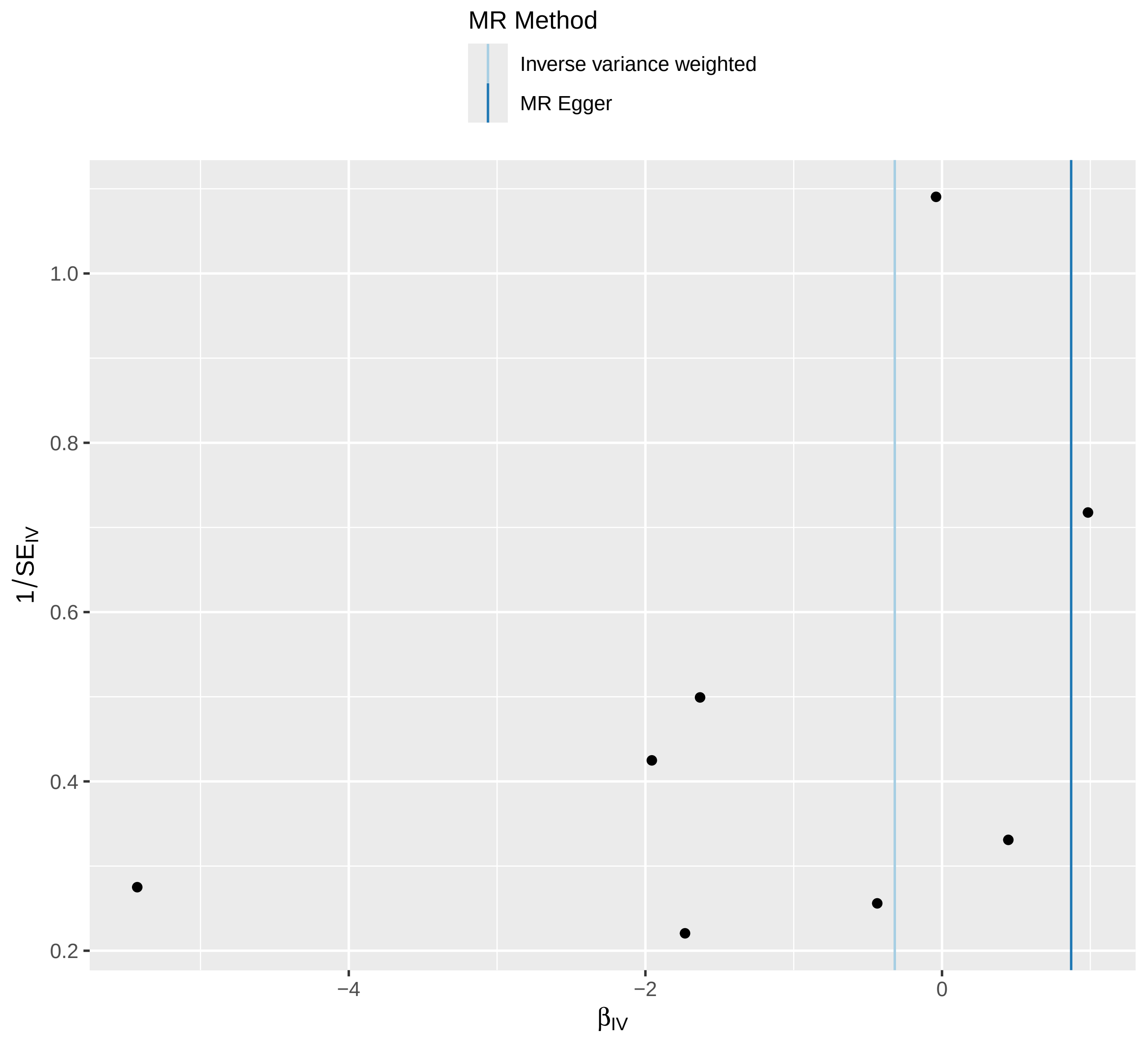
MR Method
Inverse variance weighted
MR Egger
1.0
0.8
0.6
0.4
0.2
1/SE_IV
−4
−2
0
β_IV

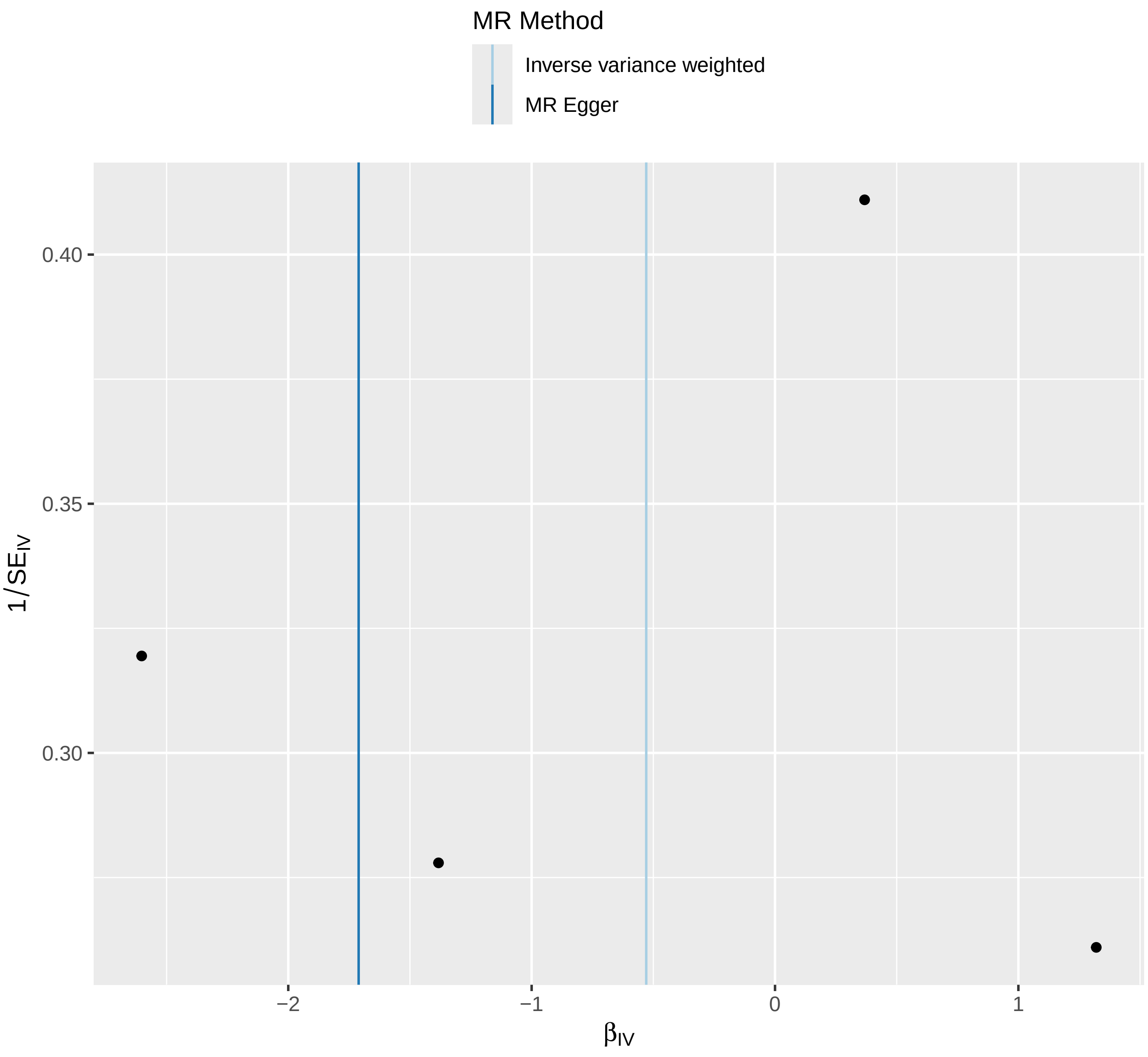
MR Method
Inverse variance weighted
MR Egger
0.40
0.35
0.30
1/SE$_{IV}$
−2
−1
0
1
β$_{IV}$

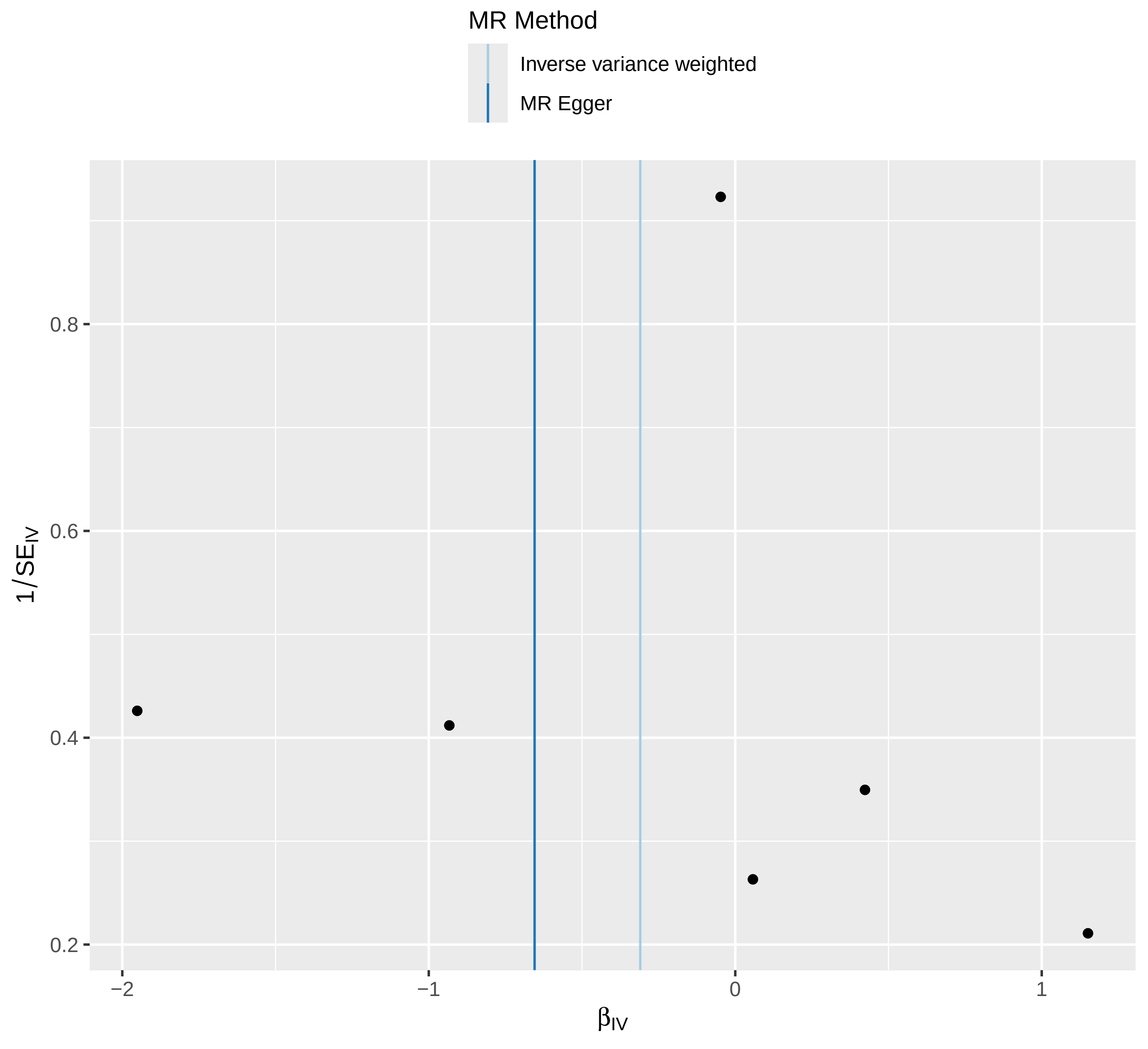
MR Method
Inverse variance weighted
MR Egger
0.8
0.6
0.4
0.2
1/SE$_{IV}$
−2
−1
0
1
β$_{IV}$

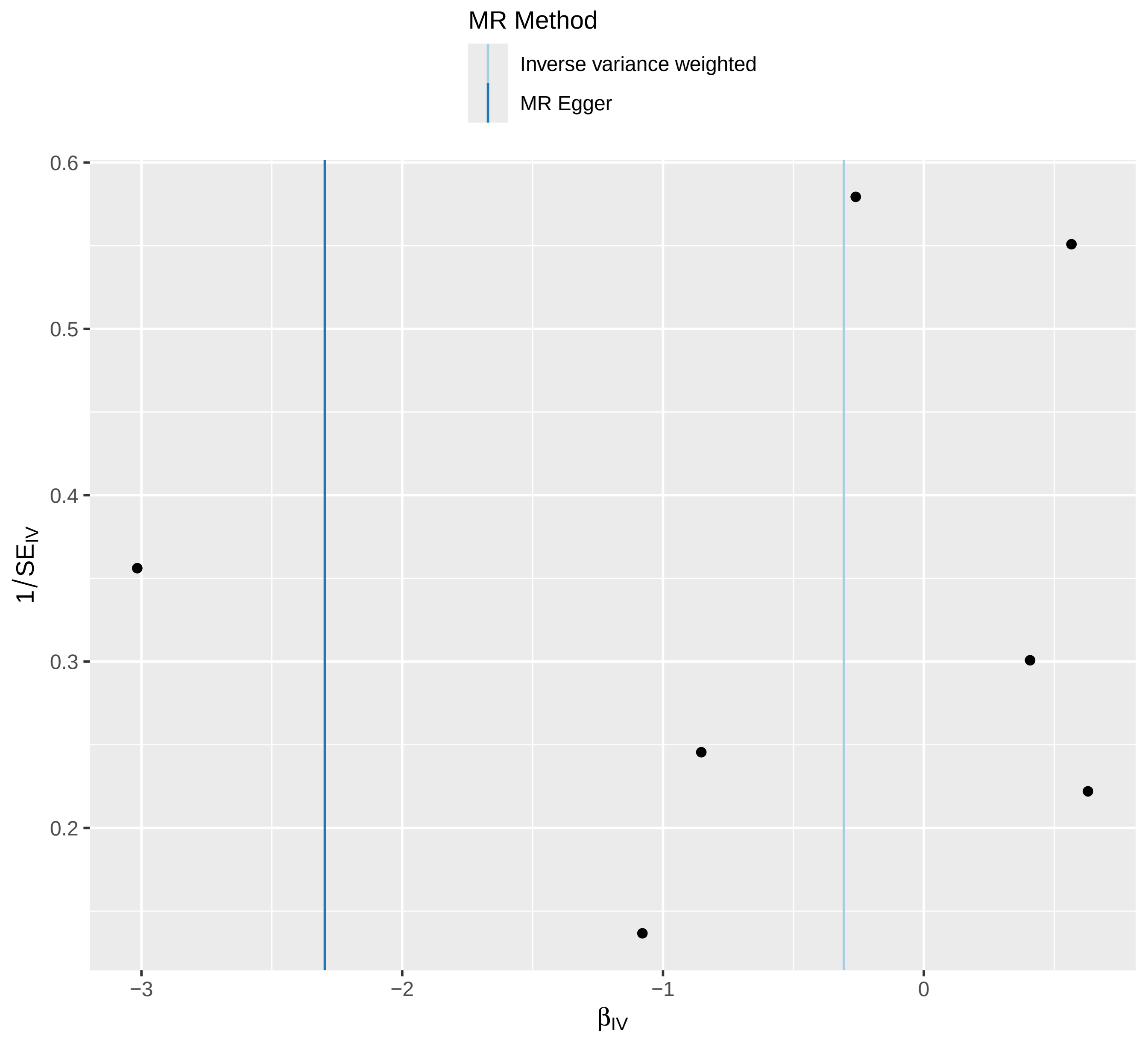
MR Method
Inverse variance weighted
MR Egger
0.6
0.5
0.4
0.3
0.2
1/SE_IV
−3
−2
−1
0
β_IV

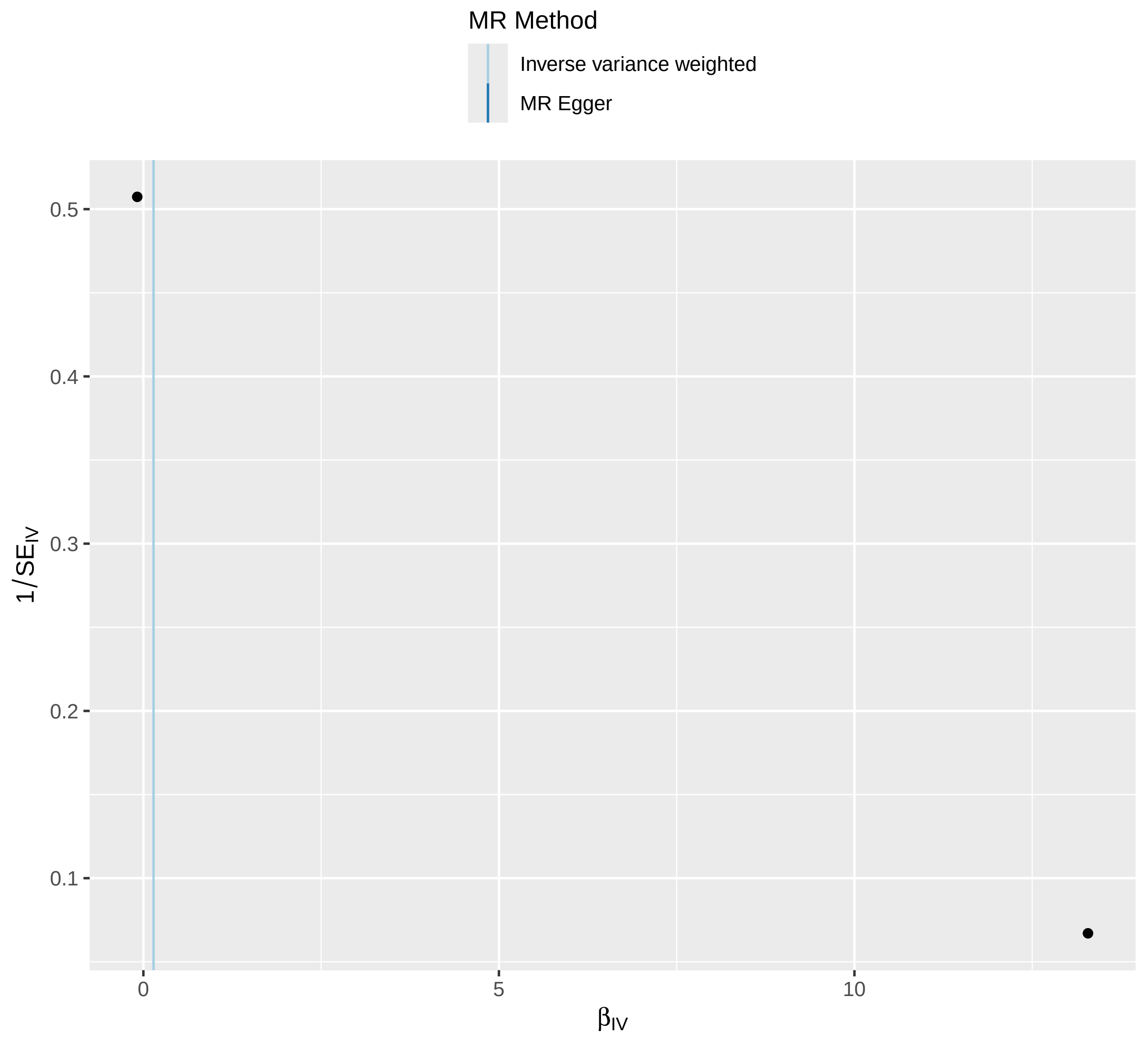
MR Method
Inverse variance weighted
MR Egger
0.5
0.4
0.3
0.2
0.1
0
5
10
1/SE_IV
β_IV

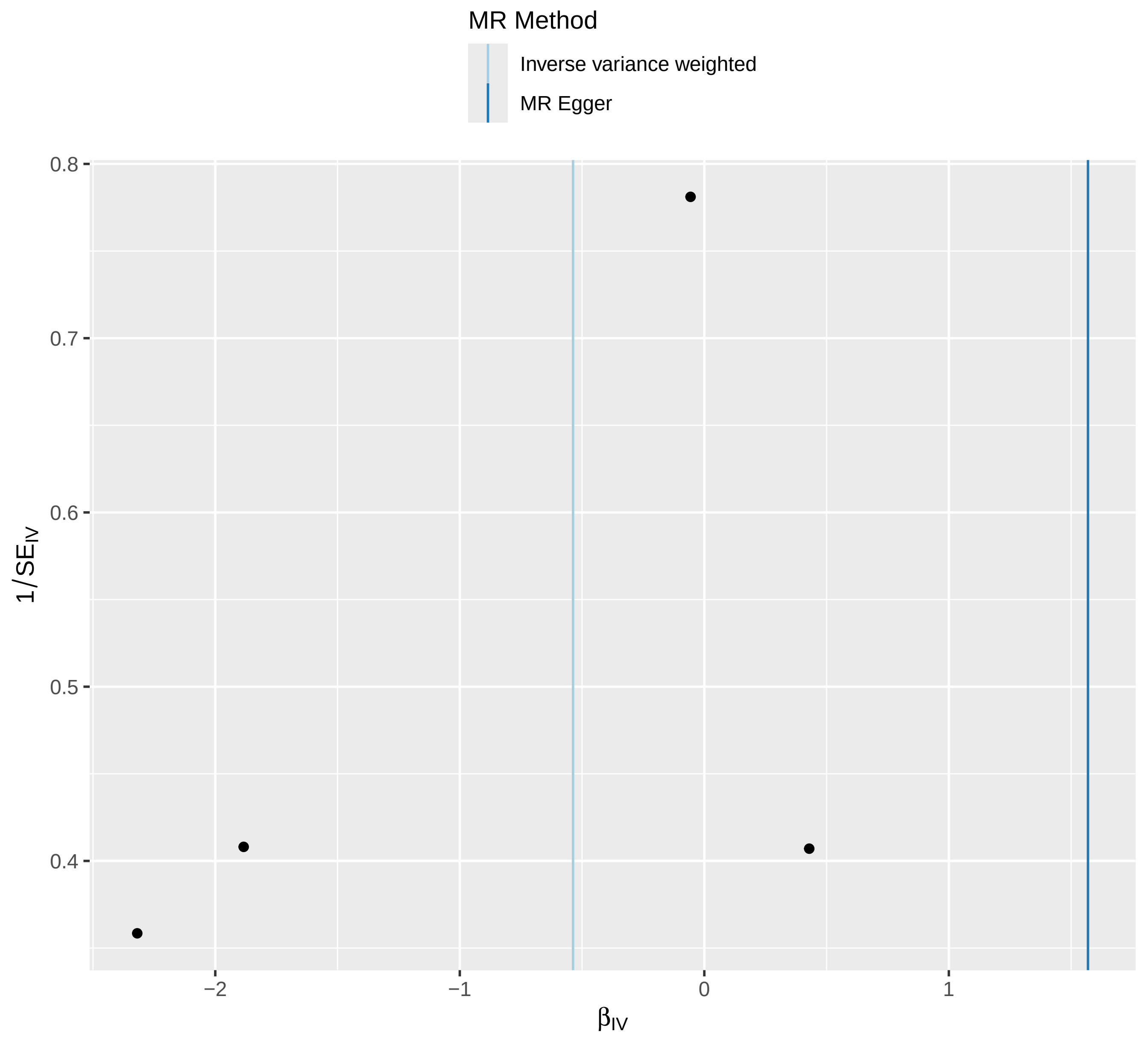
MR Method
Inverse variance weighted
MR Egger
0.8
0.7
0.6
0.5
0.4
1/SE_IV
−2
−1
0
1
β_IV

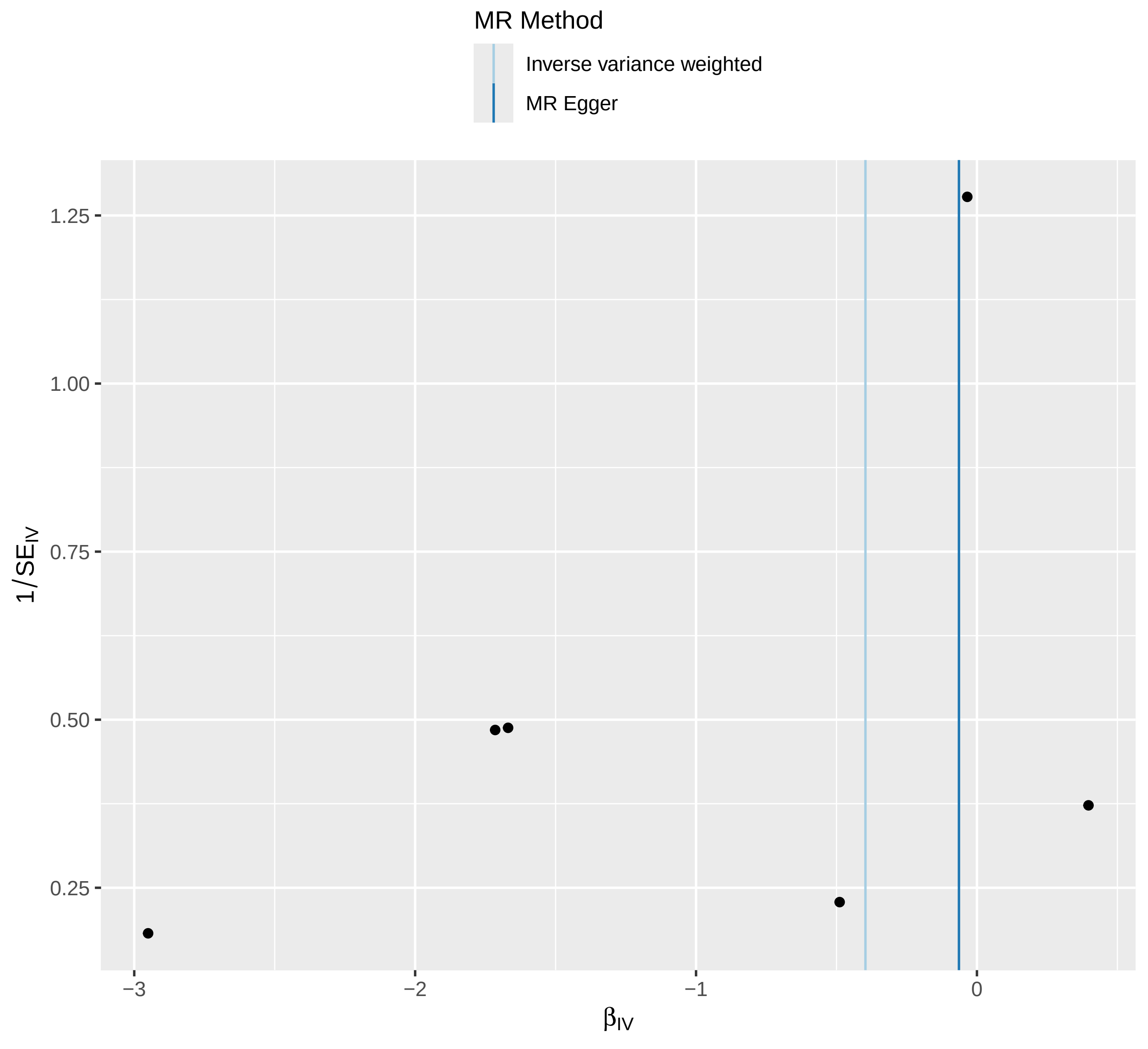

MR Method
Inverse variance weighted
MR Egger
1.25
1.00
0.75
0.50
0.25
1/SE$_{IV}$
−3
−2
−1
0
β$_{IV}$

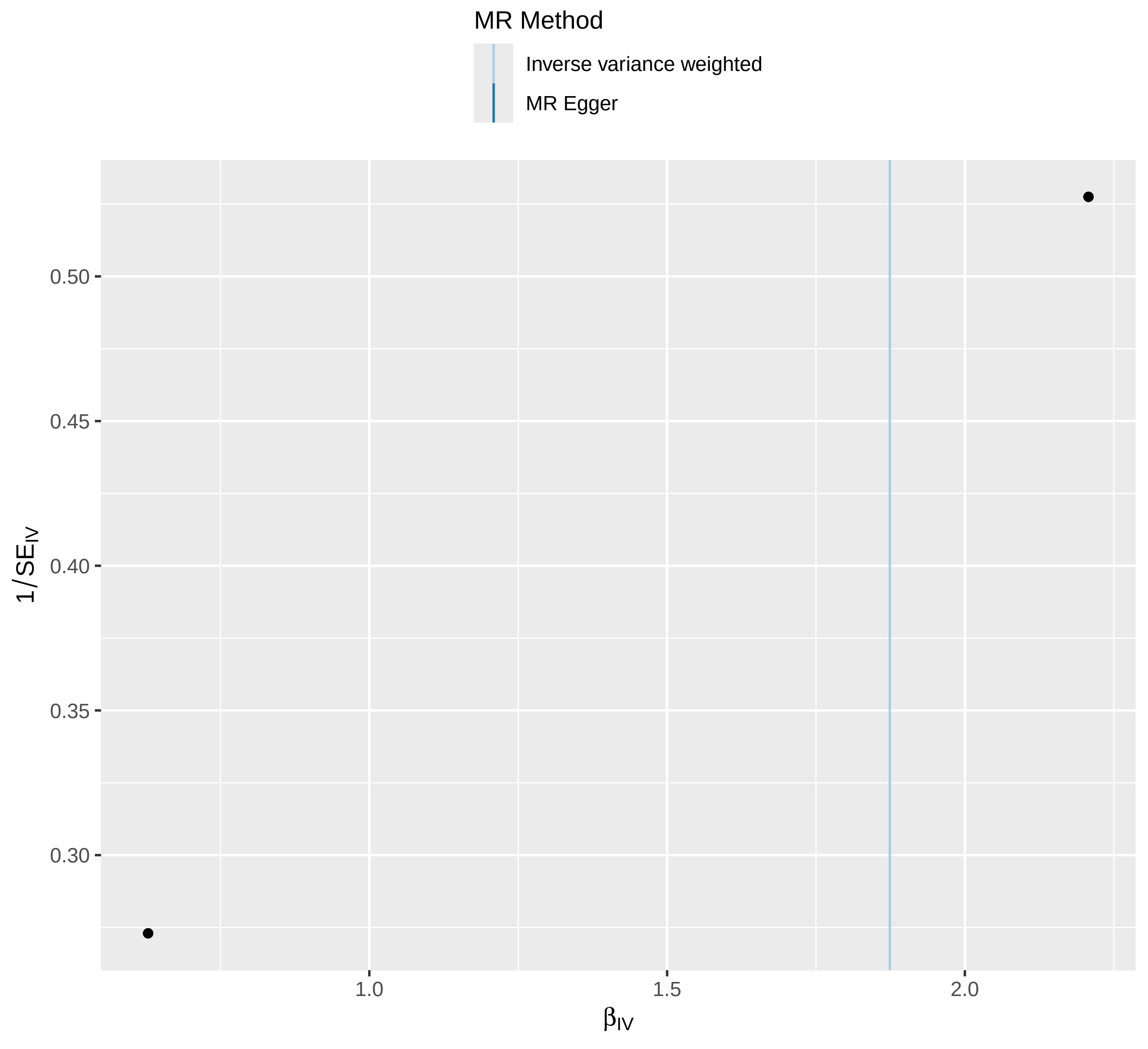
MR Method
Inverse variance weighted
MR Egger
0.50
0.45
0.40
0.35
0.30
1/SE_IV
1.0
1.5
2.0
β_IV

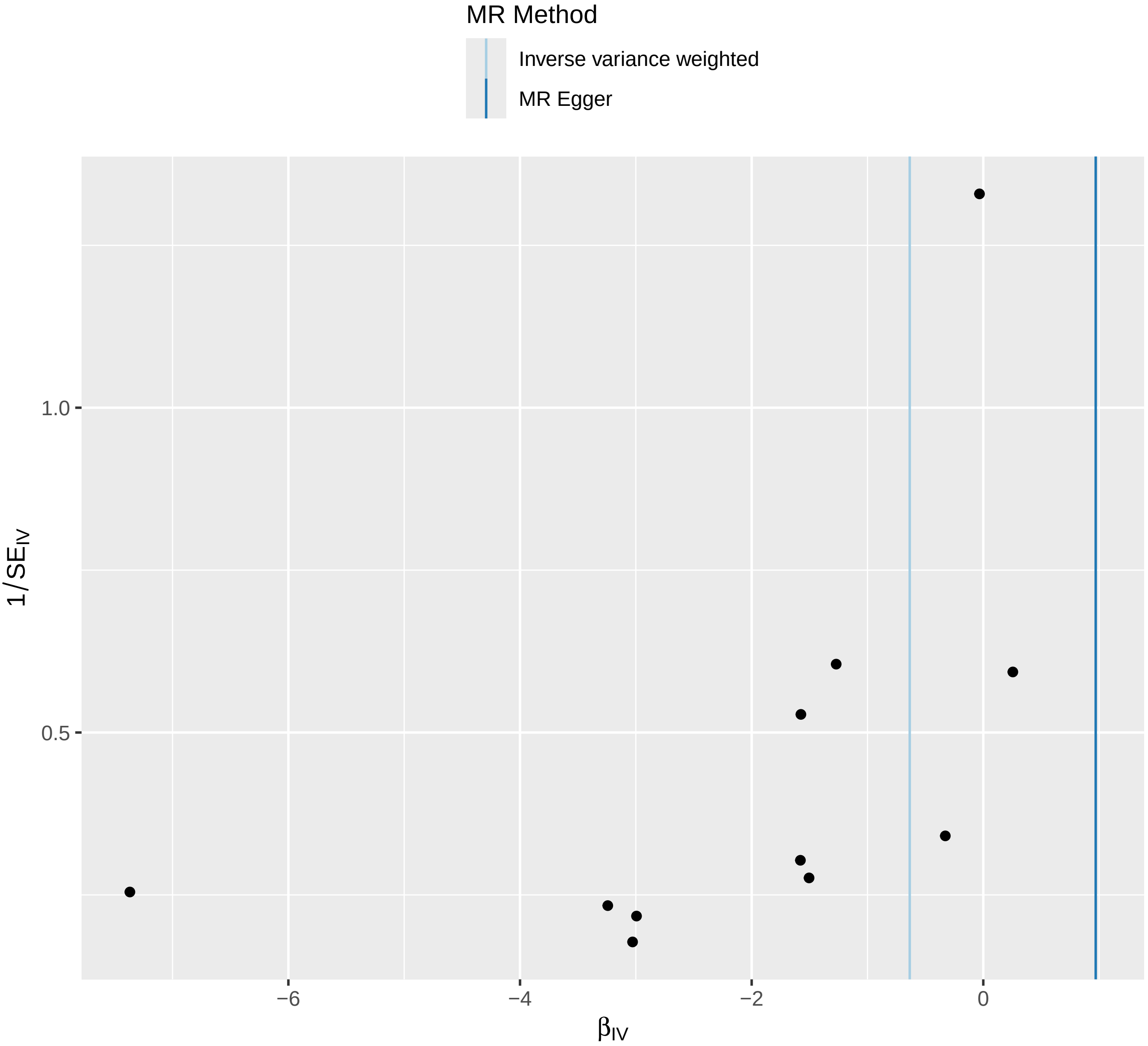
MR Method
Inverse variance weighted
MR Egger
1.0
0.5
1/SE_IV
−6
−4
−2
0
β_IV

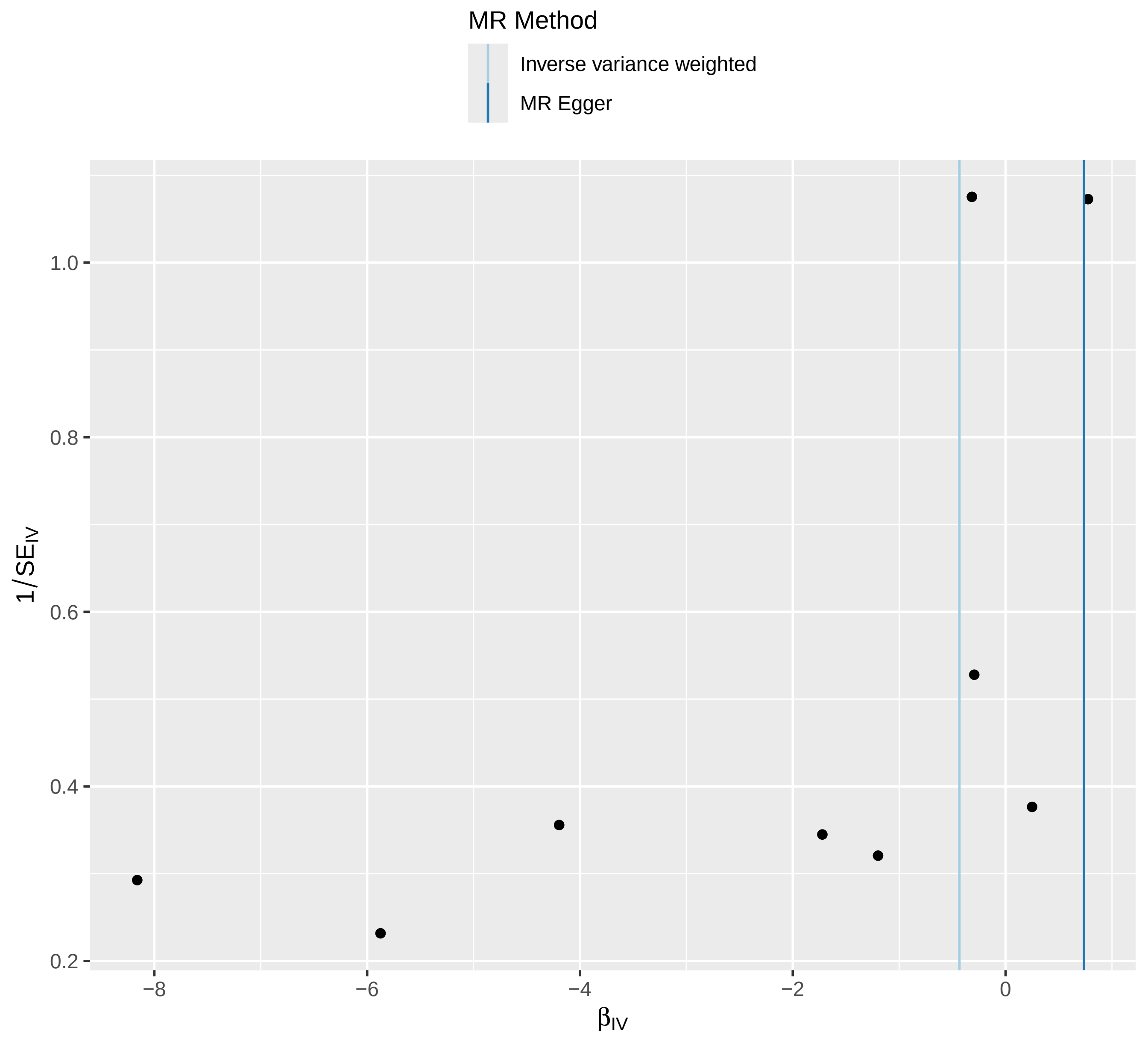
MR Method
Inverse variance weighted
MR Egger
1/SE$_{IV}$
1.0
0.8
0.6
0.4
0.2
−8
−6
−4
−2
0
β$_{IV}$

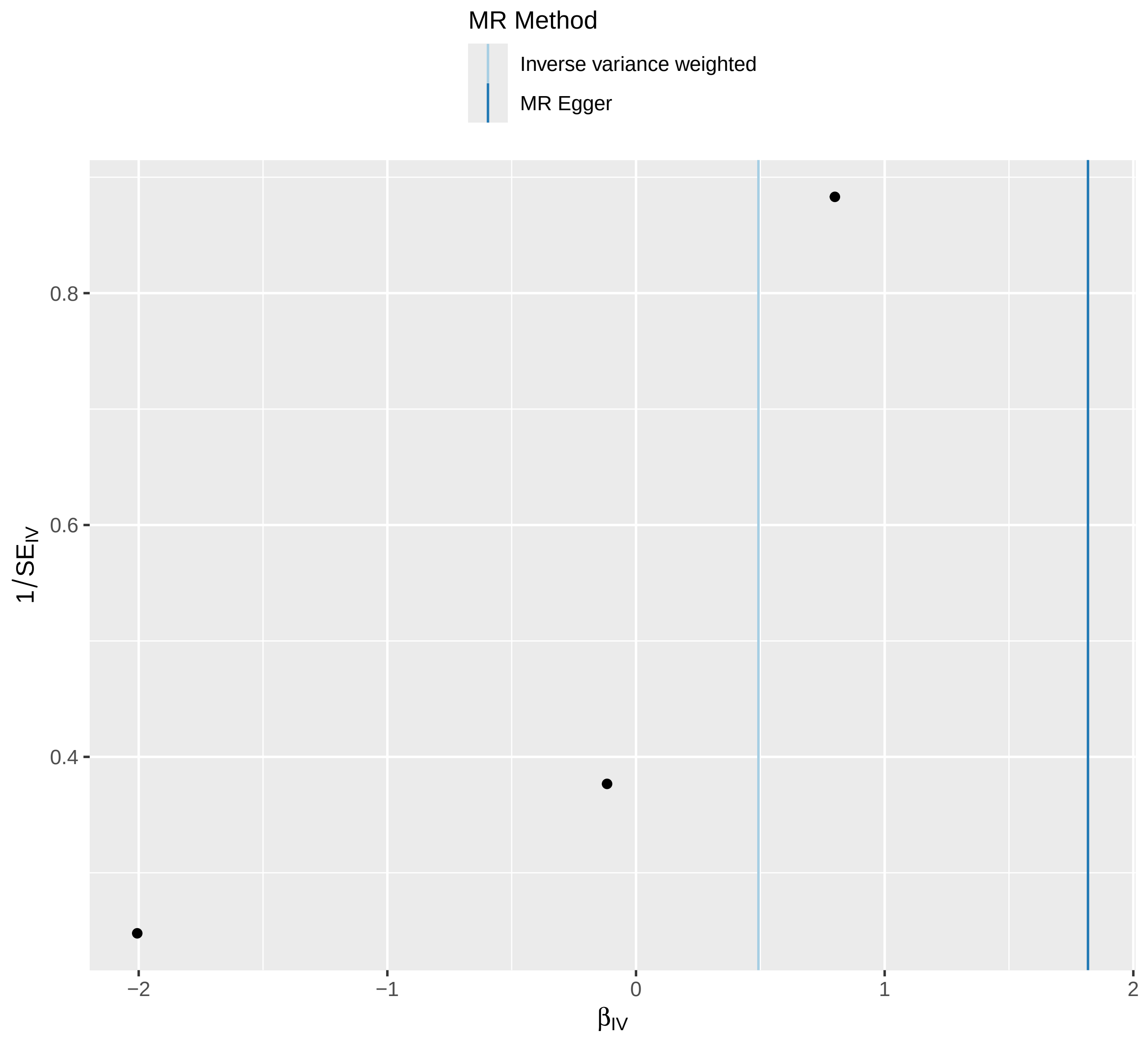
MR Method
Inverse variance weighted
MR Egger
0.8
0.6
0.4
1/SE$_{IV}$
−2
−1
0
1
2
β$_{IV}$

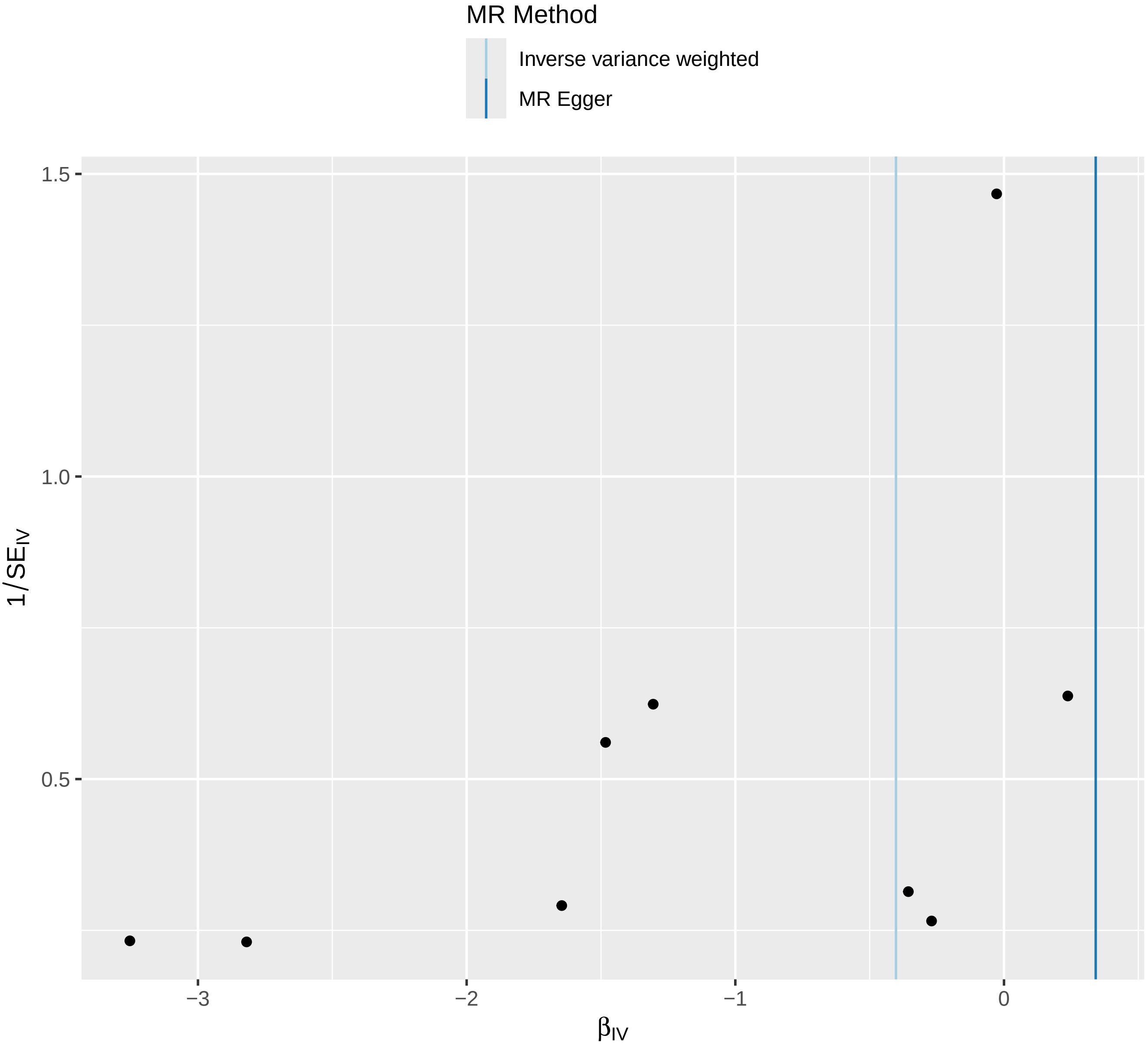
MR Method
Inverse variance weighted
MR Egger
1.5
1.0
0.5
1/SE_IV
−3
−2
−1
0
β_IV

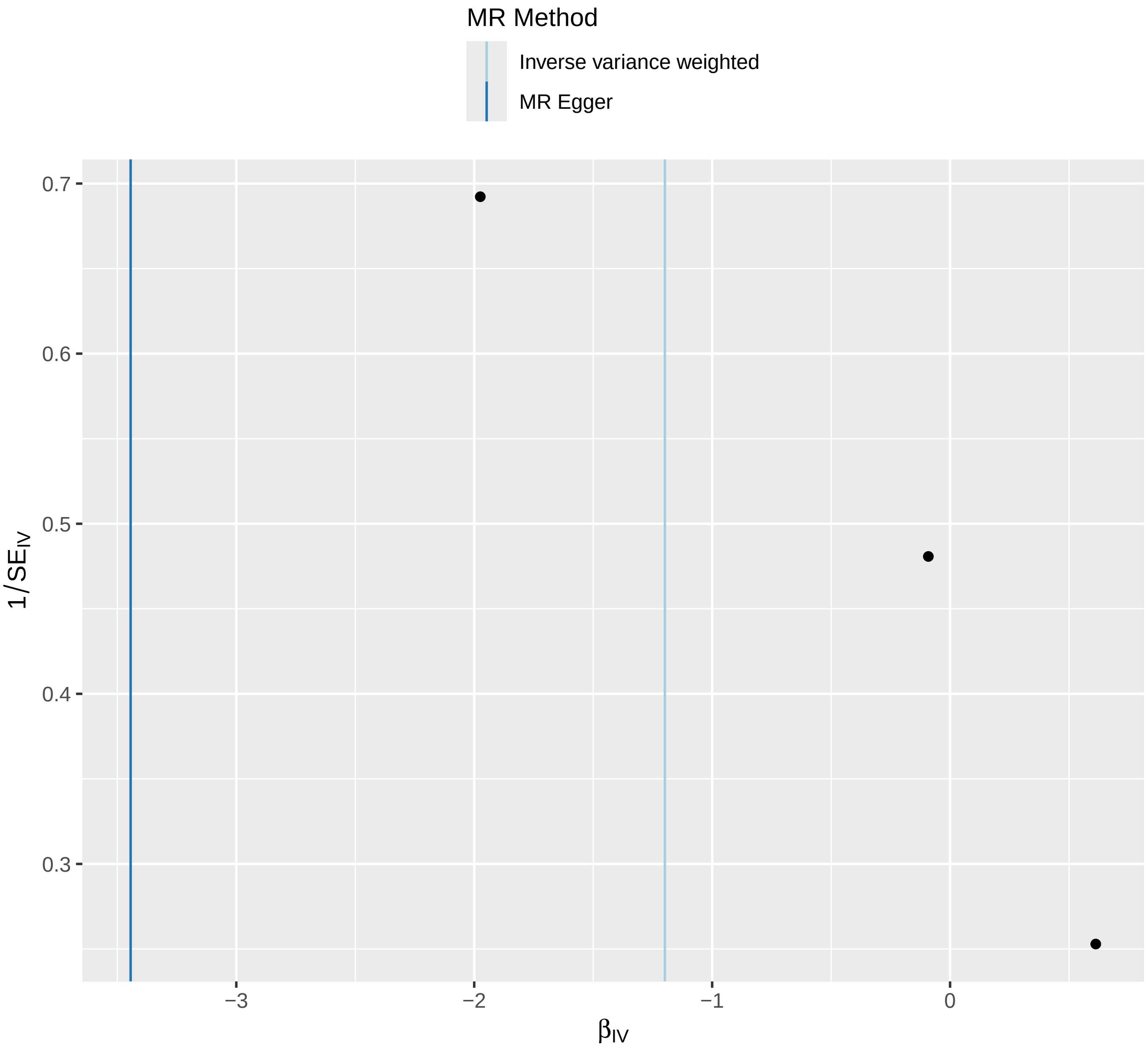
MR Method
Inverse variance weighted
MR Egger
0.7
0.6
0.5
0.4
0.3
1/SE_IV
−3
−2
−1
0
β_IV

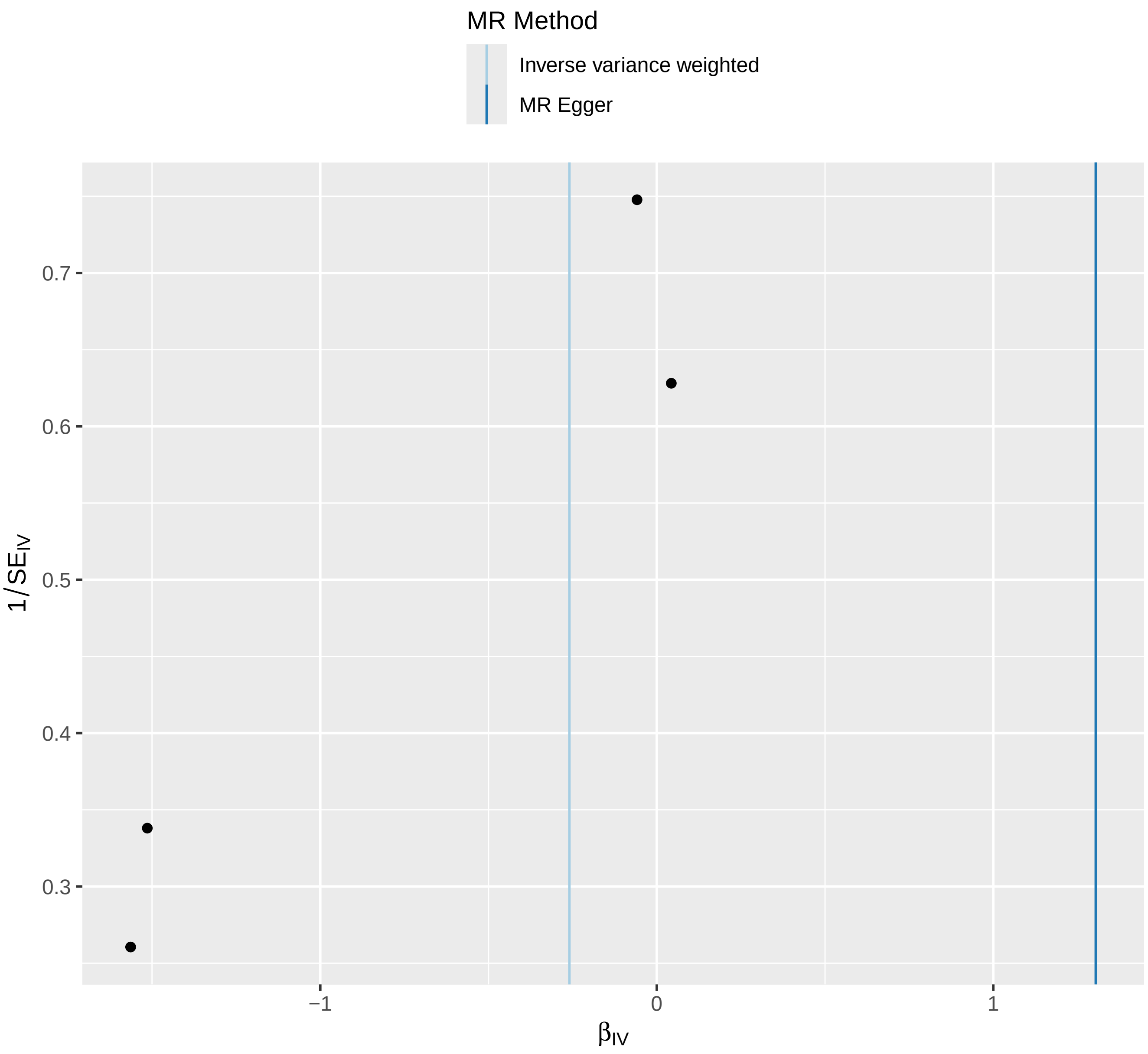
MR Method
Inverse variance weighted
MR Egger
0.7
0.6
0.5
0.4
0.3
1/SE_IV
−1
0
1
β_IV

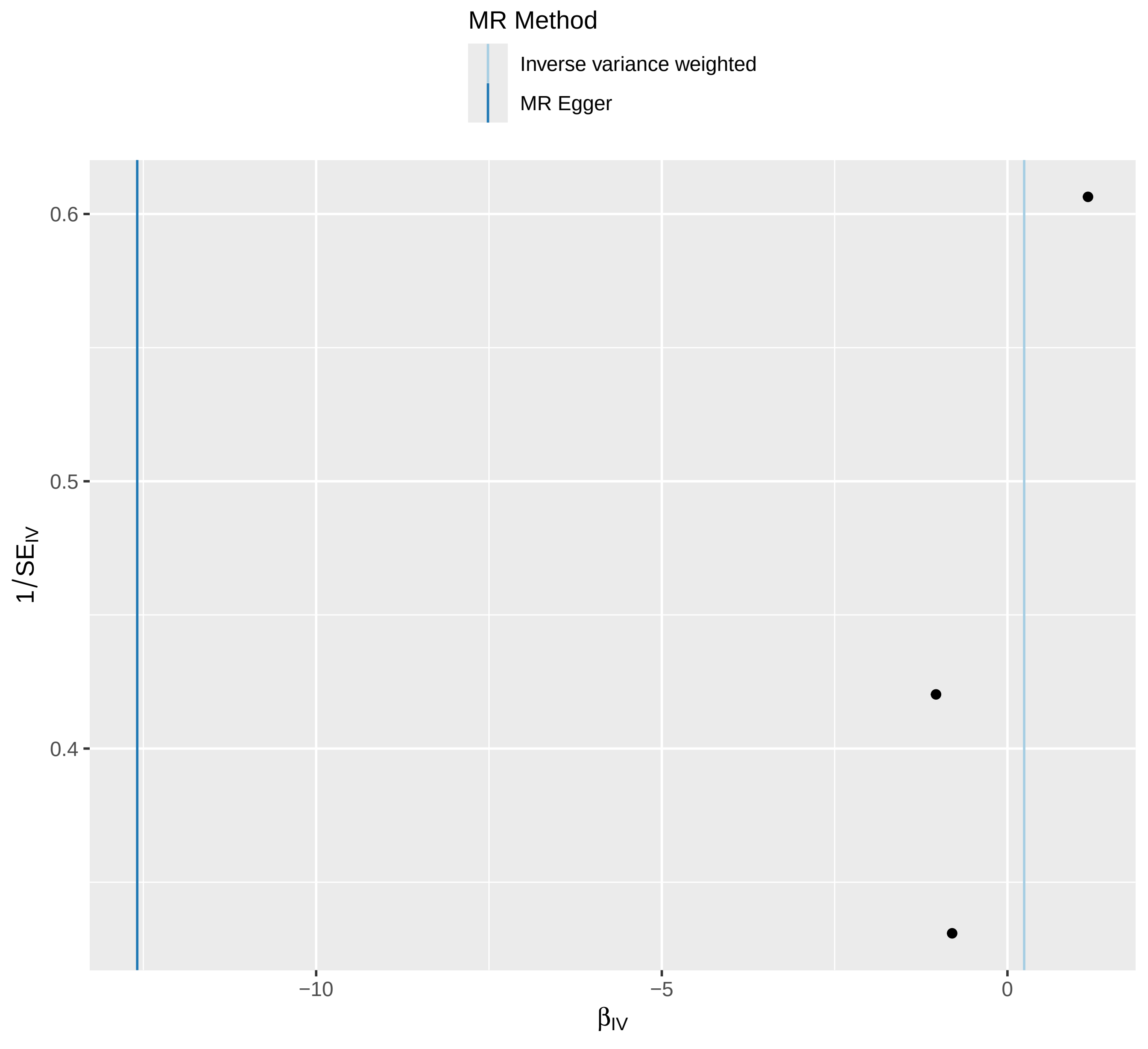
MR Method
Inverse variance weighted
MR Egger
0.6
0.5
0.4
$1/SE_{IV}$
−10
−5
0
β_{IV}

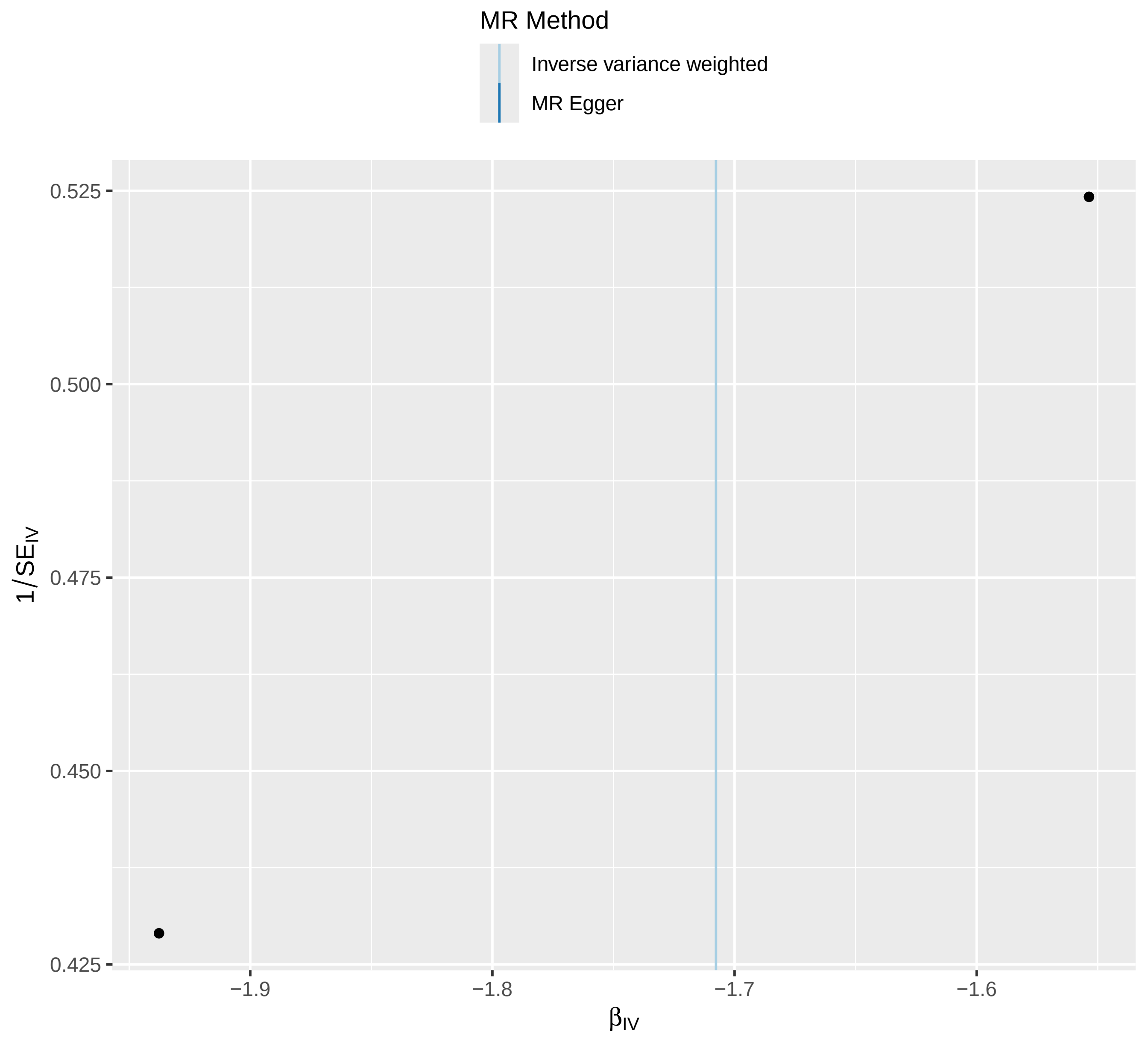
MR Method
Inverse variance weighted
MR Egger
0.525
0.500
0.475
0.450
0.425
1/SEIV
−1.9
−1.8
−1.7
−1.6
βIV

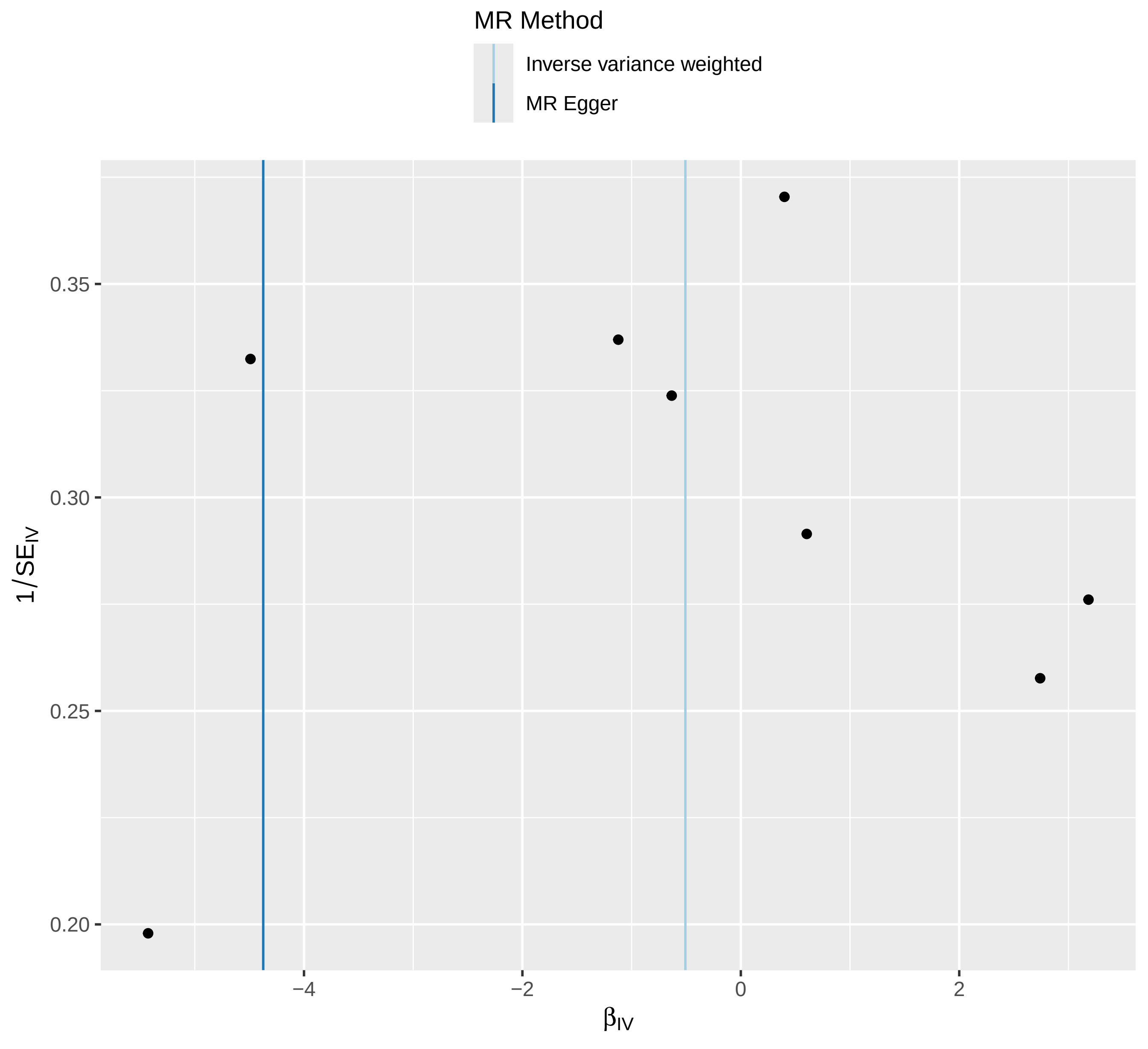
MR Method
Inverse variance weighted
MR Egger
0.35
0.30
0.25
0.20
1/SE$_{IV}$
−4
−2
0
2
β$_{IV}$

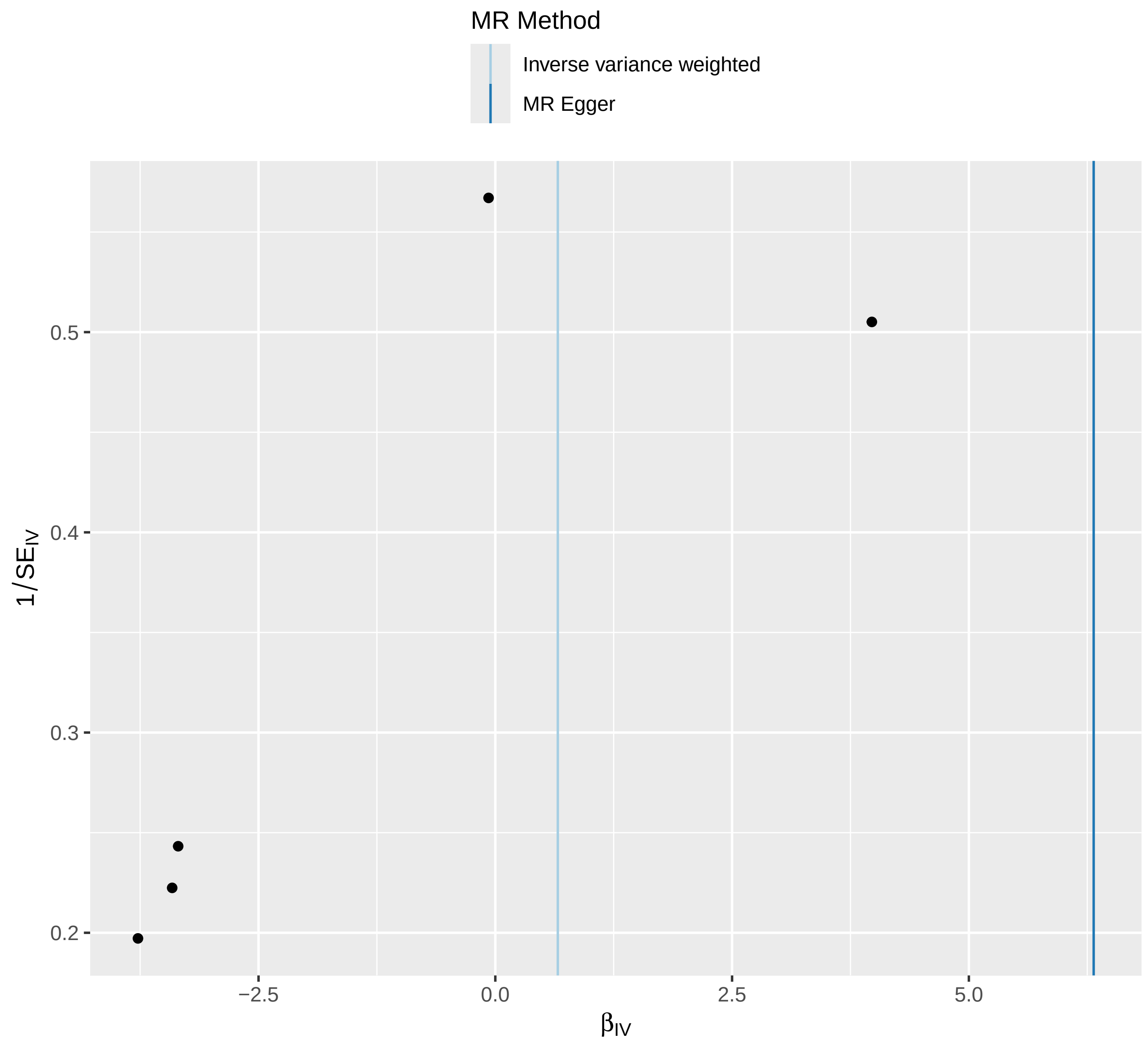
MR Method
Inverse variance weighted
MR Egger
0.5
0.4
0.3
0.2
$1/SE_{IV}$
−2.5
0.0
2.5
5.0
β_{IV}

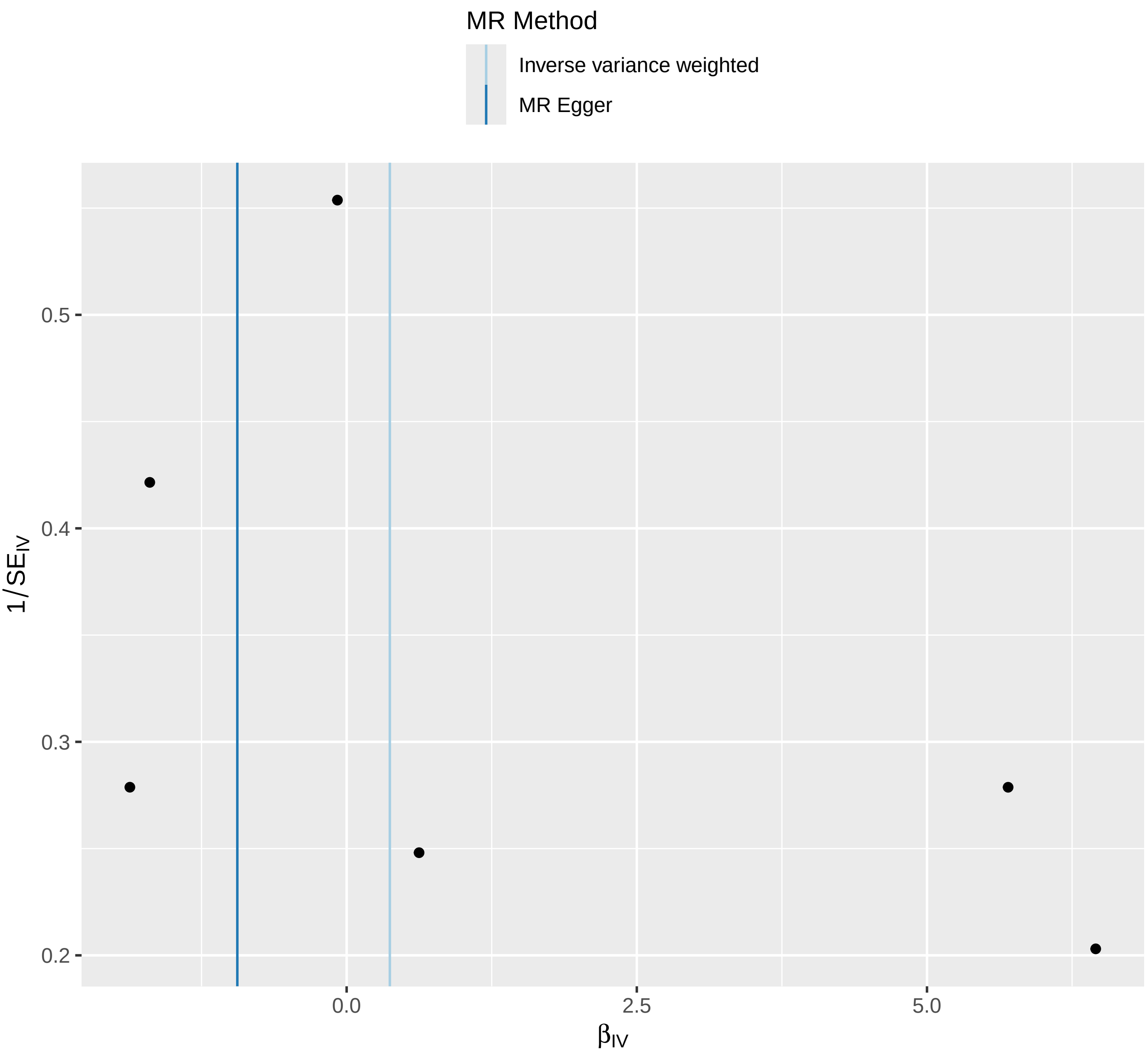
MR Method
Inverse variance weighted
MR Egger
0.5
0.4
0.3
0.2
1/SE_IV
0.0
2.5
5.0
β_IV

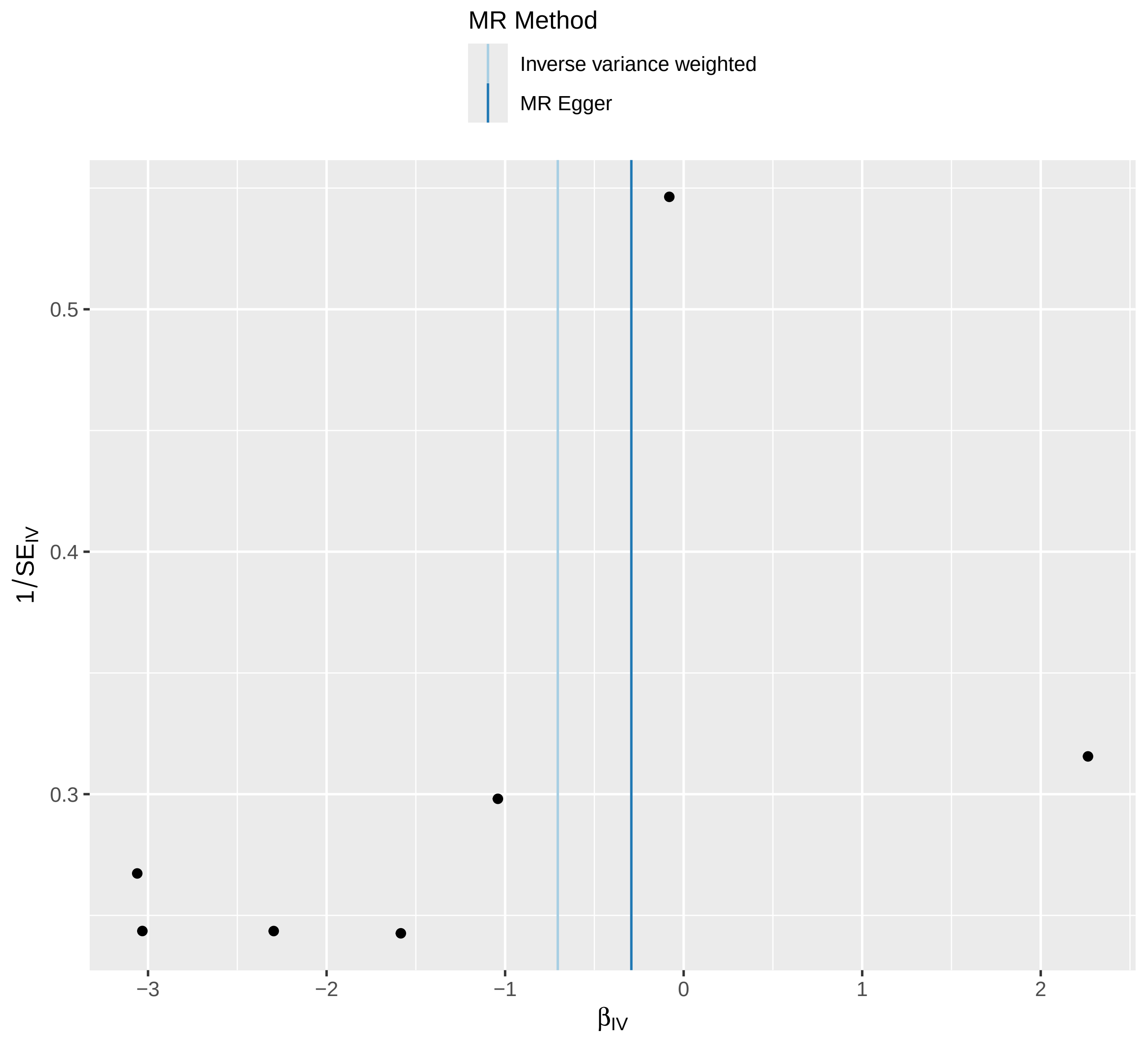
MR Method
Inverse variance weighted
MR Egger
0.5
0.4
0.3
1/SE_IV
−3
−2
−1
0
1
2
β_IV

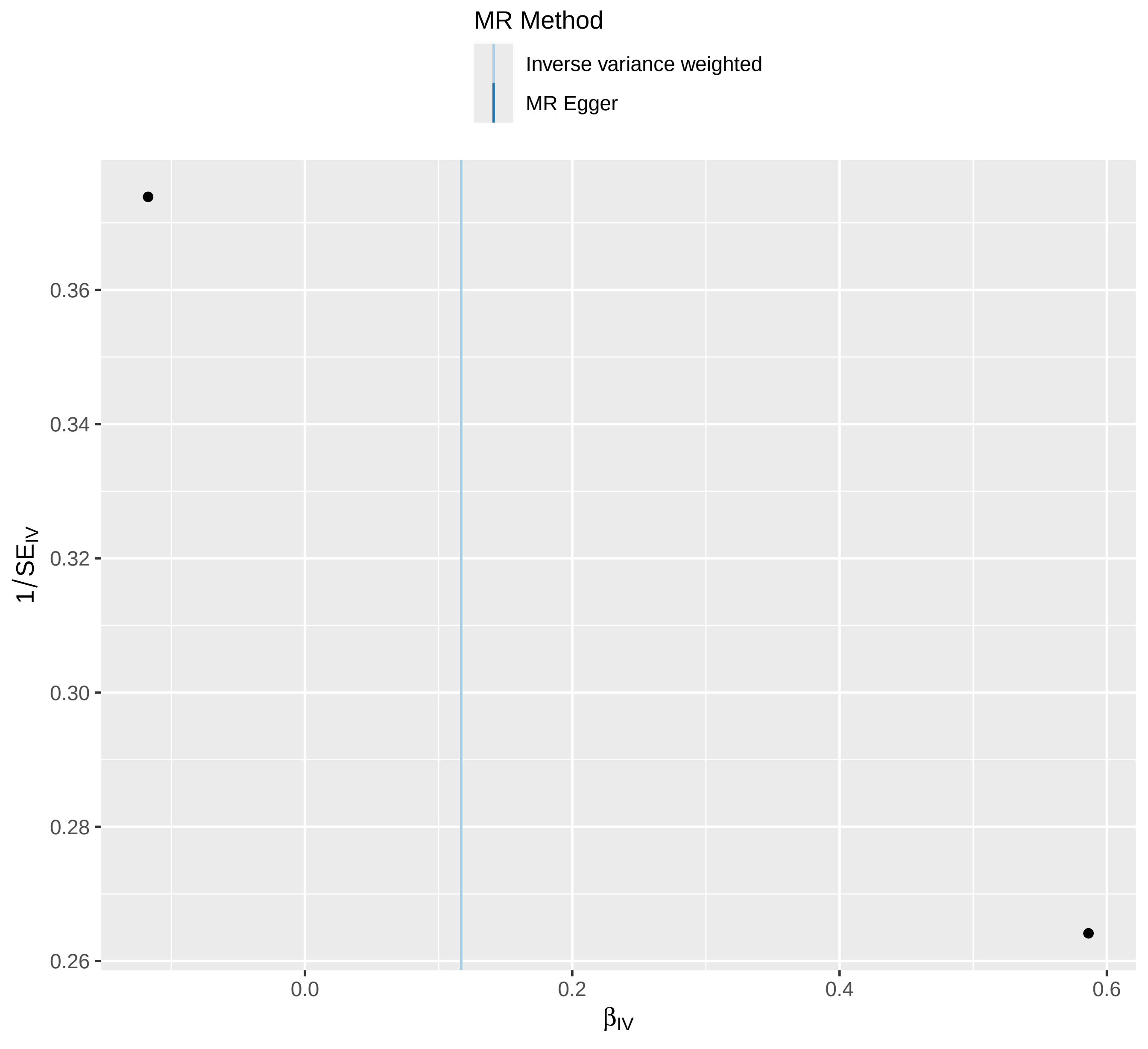
MR Method
Inverse variance weighted
MR Egger
0.36
0.34
0.32
0.30
0.28
0.26
$1/SE_{IV}$
0.0
0.2
0.4
0.6
β_{IV}

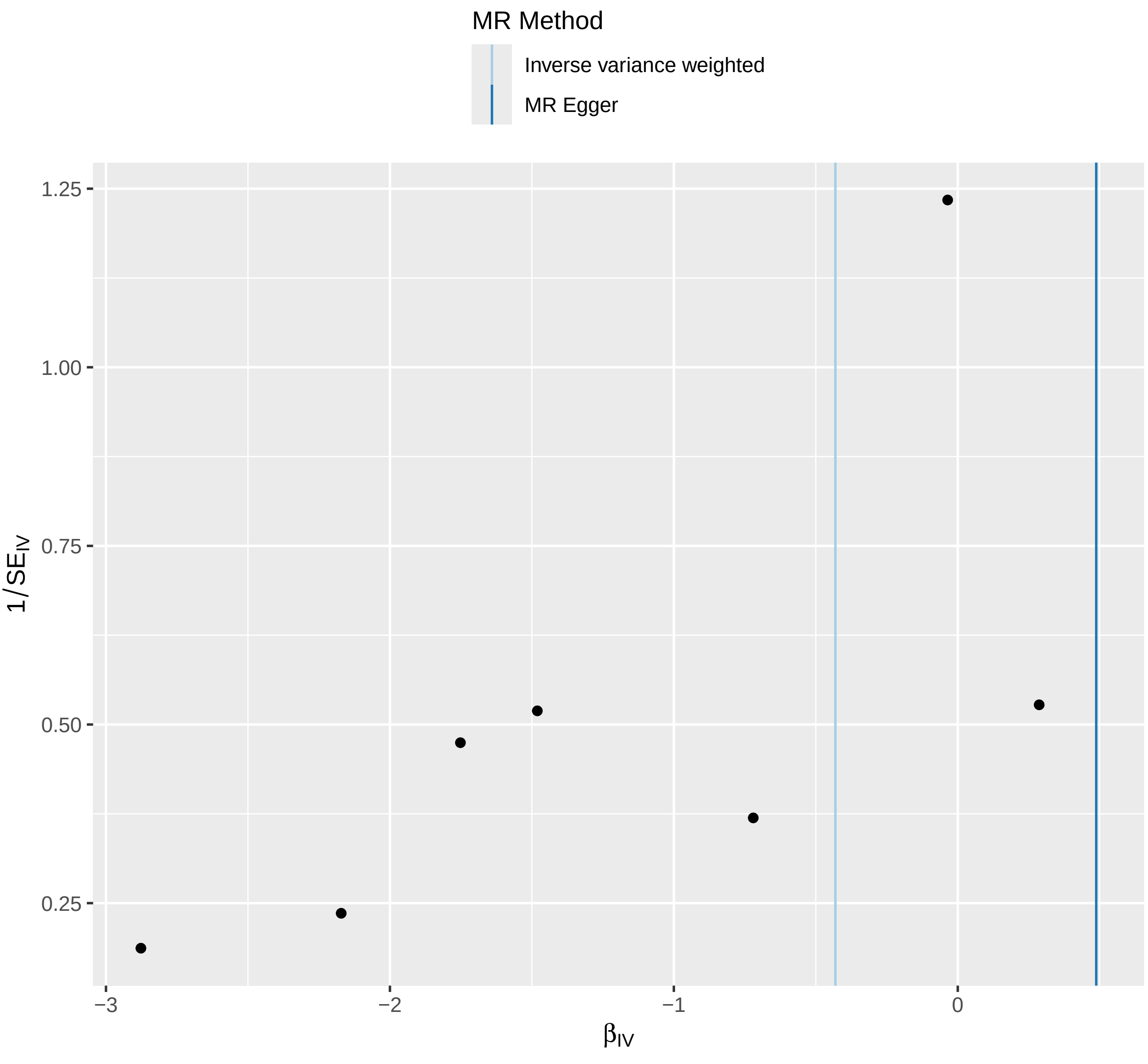
MR Method
Inverse variance weighted
MR Egger
1.25
1.00
0.75
0.50
0.25
1/SE_IV
−3
−2
−1
0
β_IV

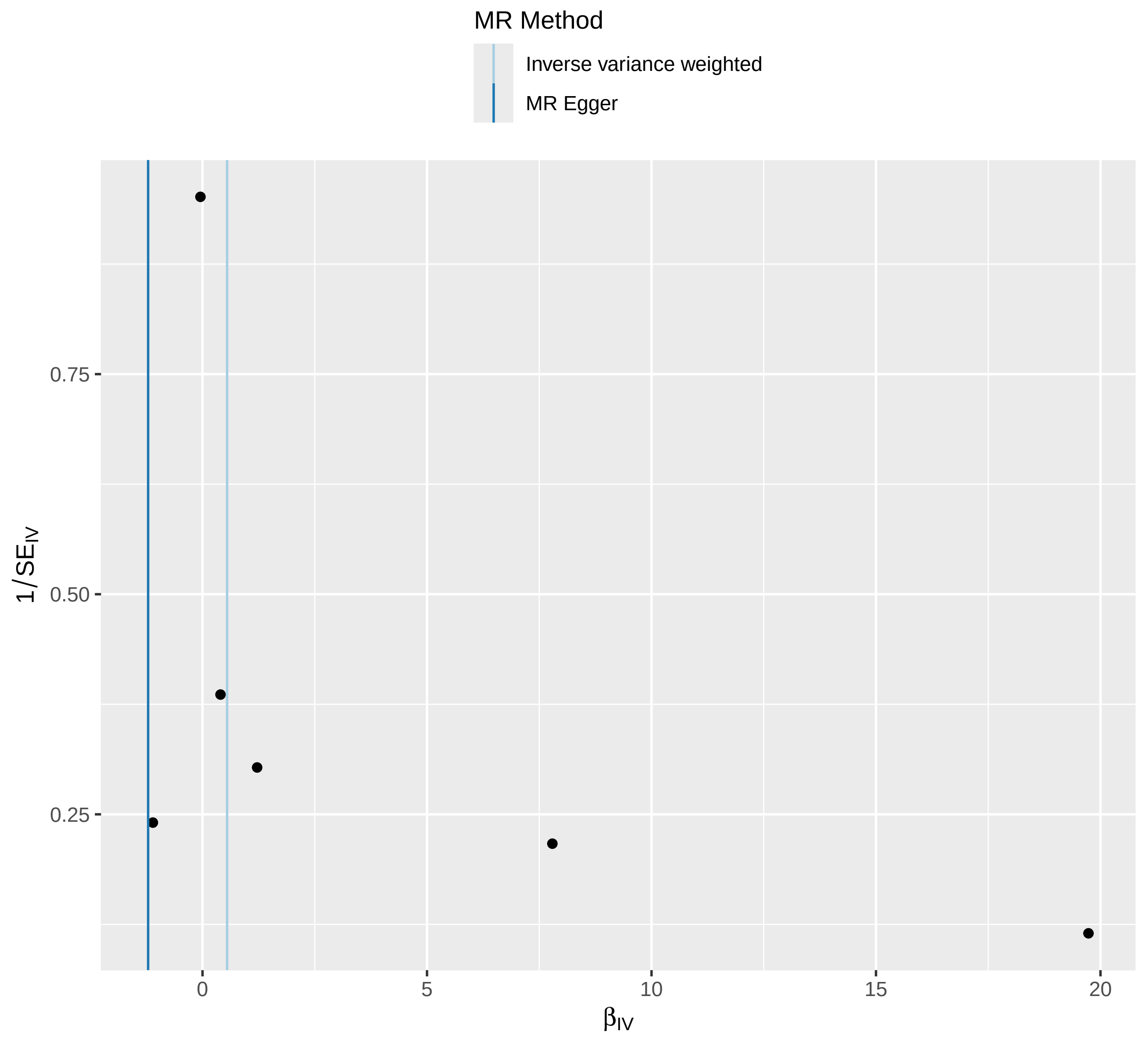
MR Method
Inverse variance weighted
MR Egger
0.75
0.50
0.25
1/SE_IV
0
5
10
15
20
β_IV

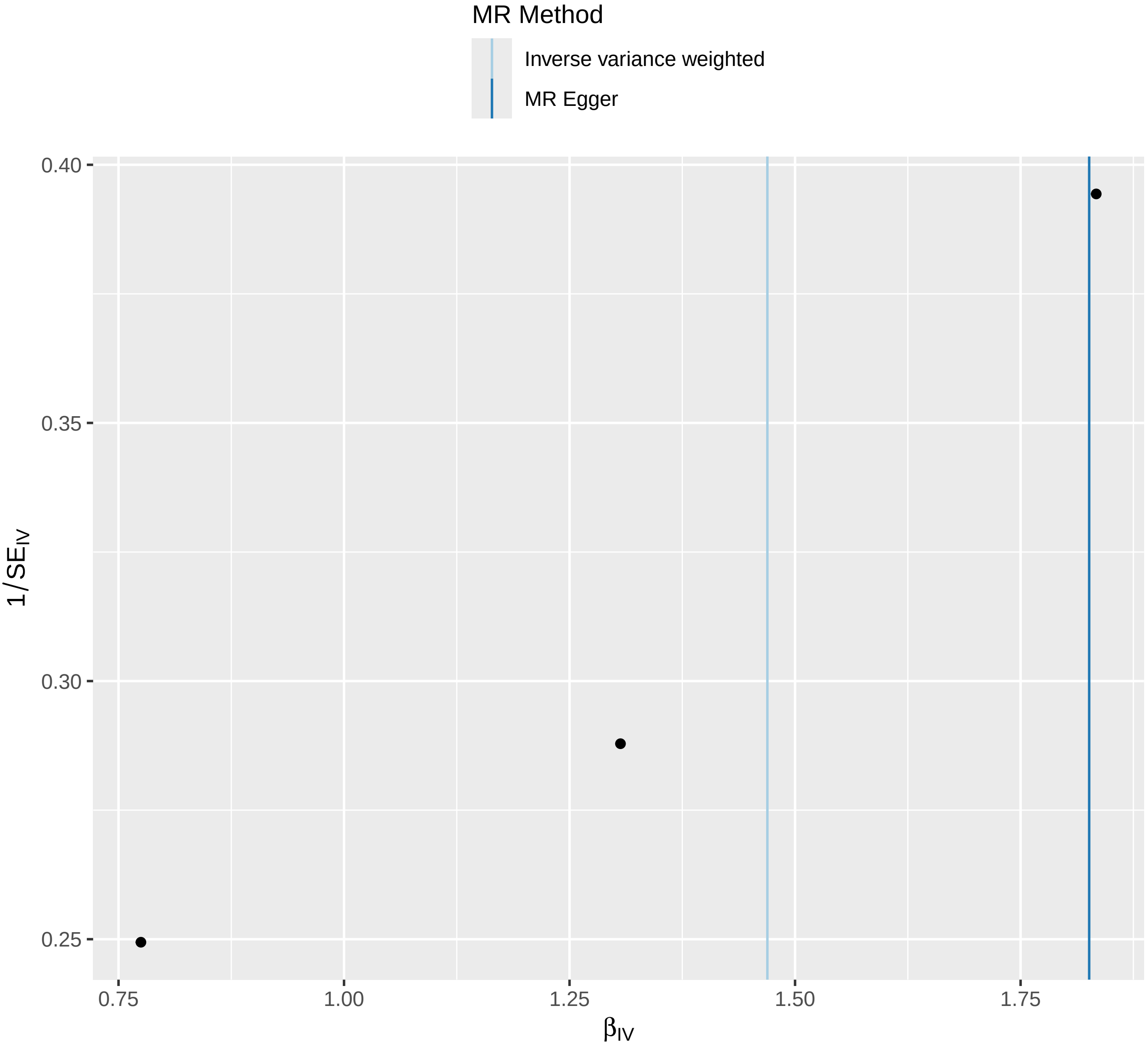
MR Method
Inverse variance weighted
MR Egger
0.40
0.35
0.30
0.25
1/SE_IV
0.75
1.00
1.25
1.50
1.75
β_IV

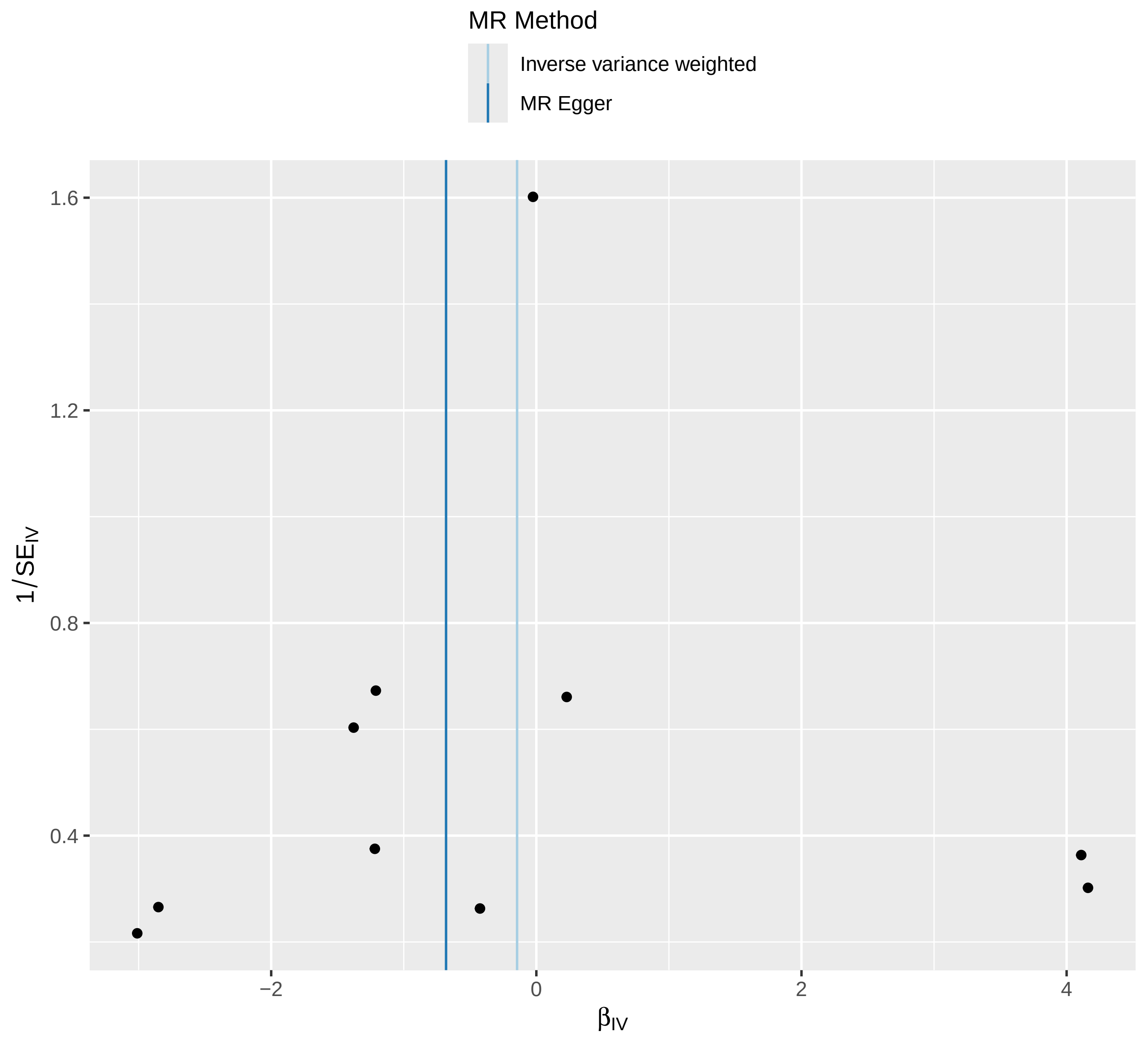
MR Method
Inverse variance weighted
MR Egger
1.6
1.2
0.8
0.4
1/SE$_{IV}$
−2
0
2
4
β_{IV}

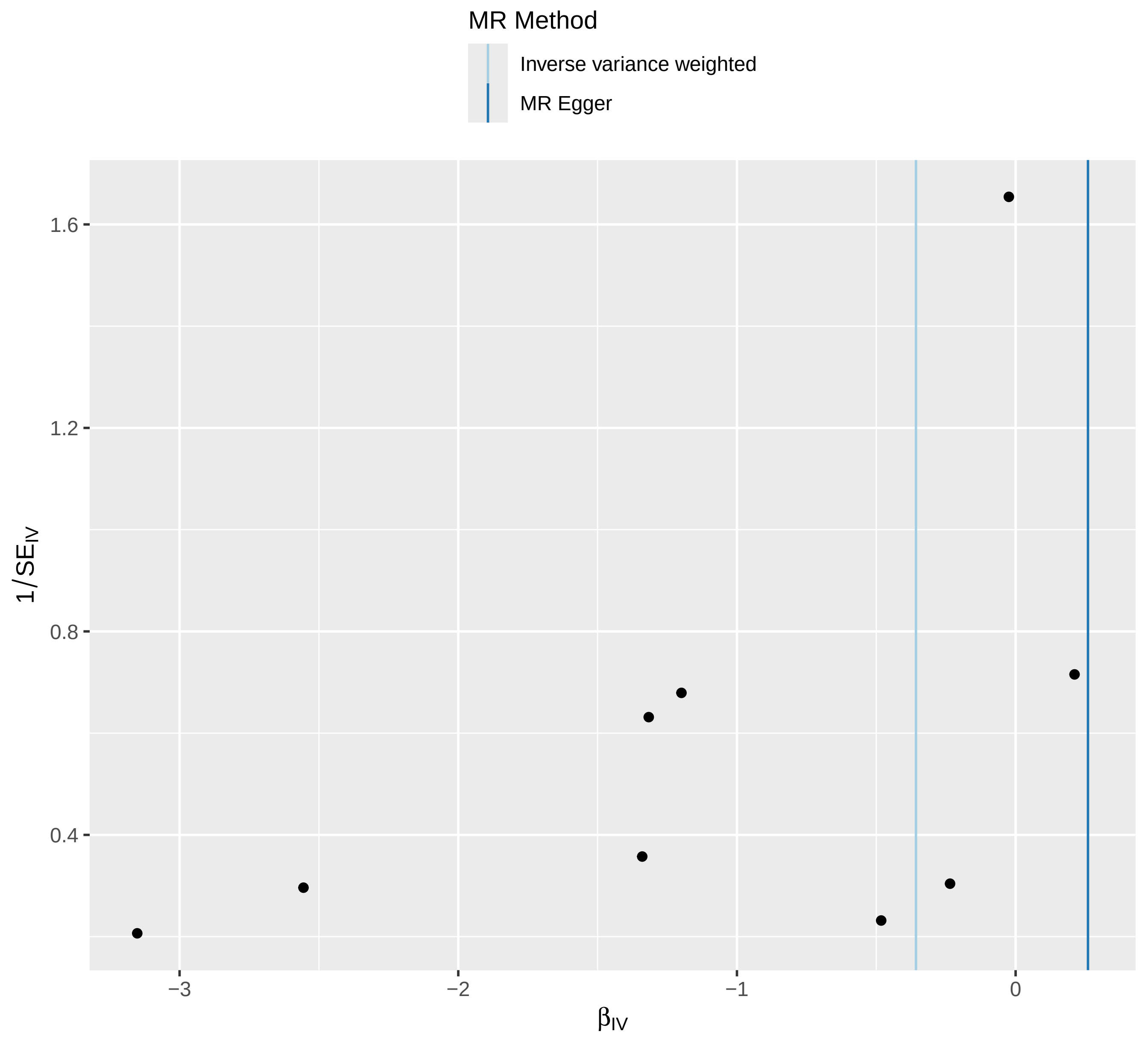
MR Method
Inverse variance weighted
MR Egger
1.6
1.2
0.8
0.4
1/SE_IV
−3
−2
−1
0
β_IV

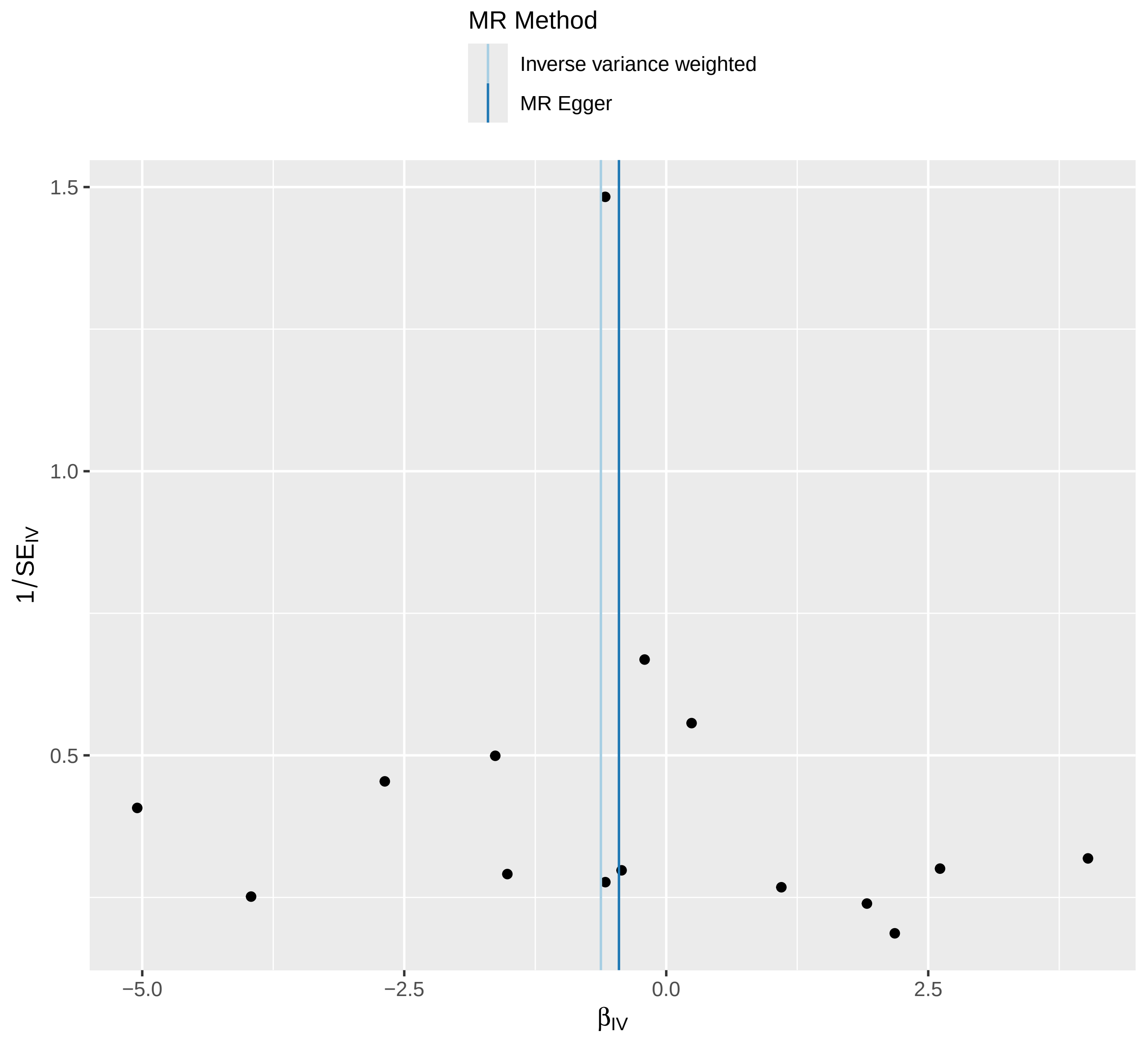
MR Method
Inverse variance weighted
MR Egger
1.5
1.0
0.5
1/SE_IV
−5.0
−2.5
0.0
2.5
β_IV

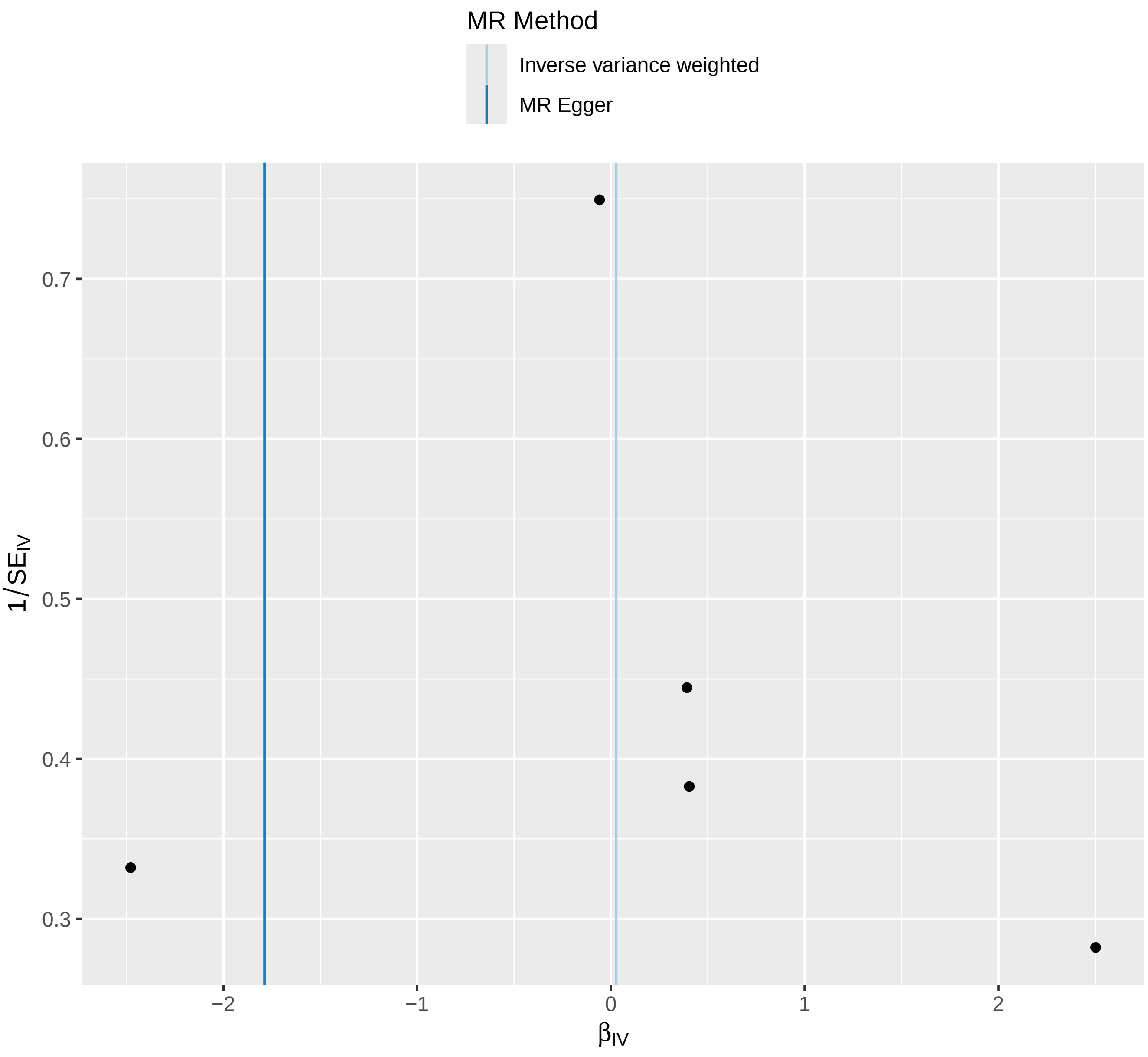
MR Method
Inverse variance weighted
MR Egger
0.7
0.6
0.5
0.4
0.3
$1/SE_{IV}$
−2
−1
0
1
2
β_{IV}

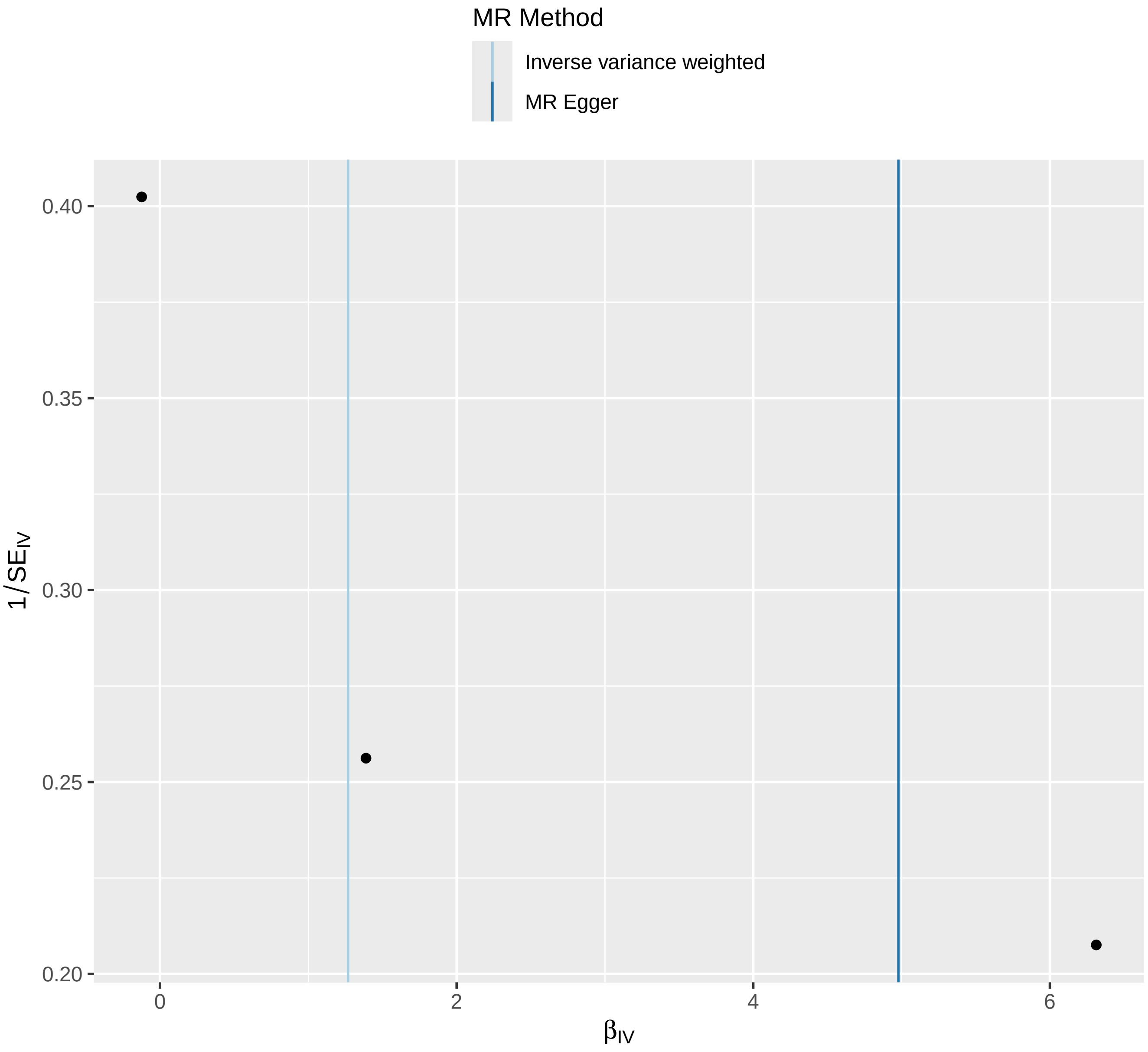
MR Method
Inverse variance weighted
MR Egger
0.40
0.35
0.30
0.25
0.20
1/SE_IV
0
2
4
6
β_IV

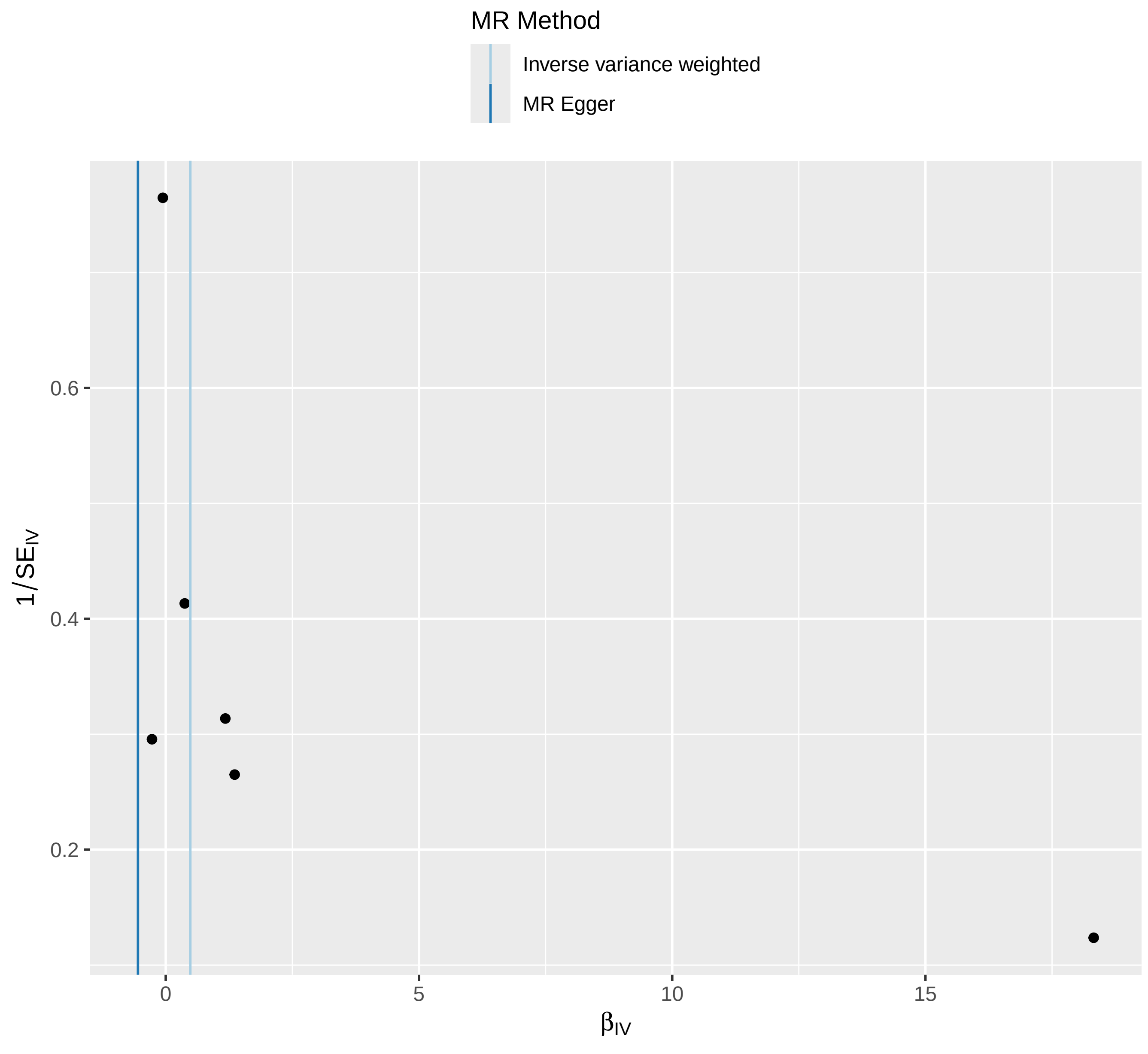
MR Method
Inverse variance weighted
MR Egger
0.6
0.4
0.2
$1/SE_{IV}$
0
5
10
15
β_{IV}

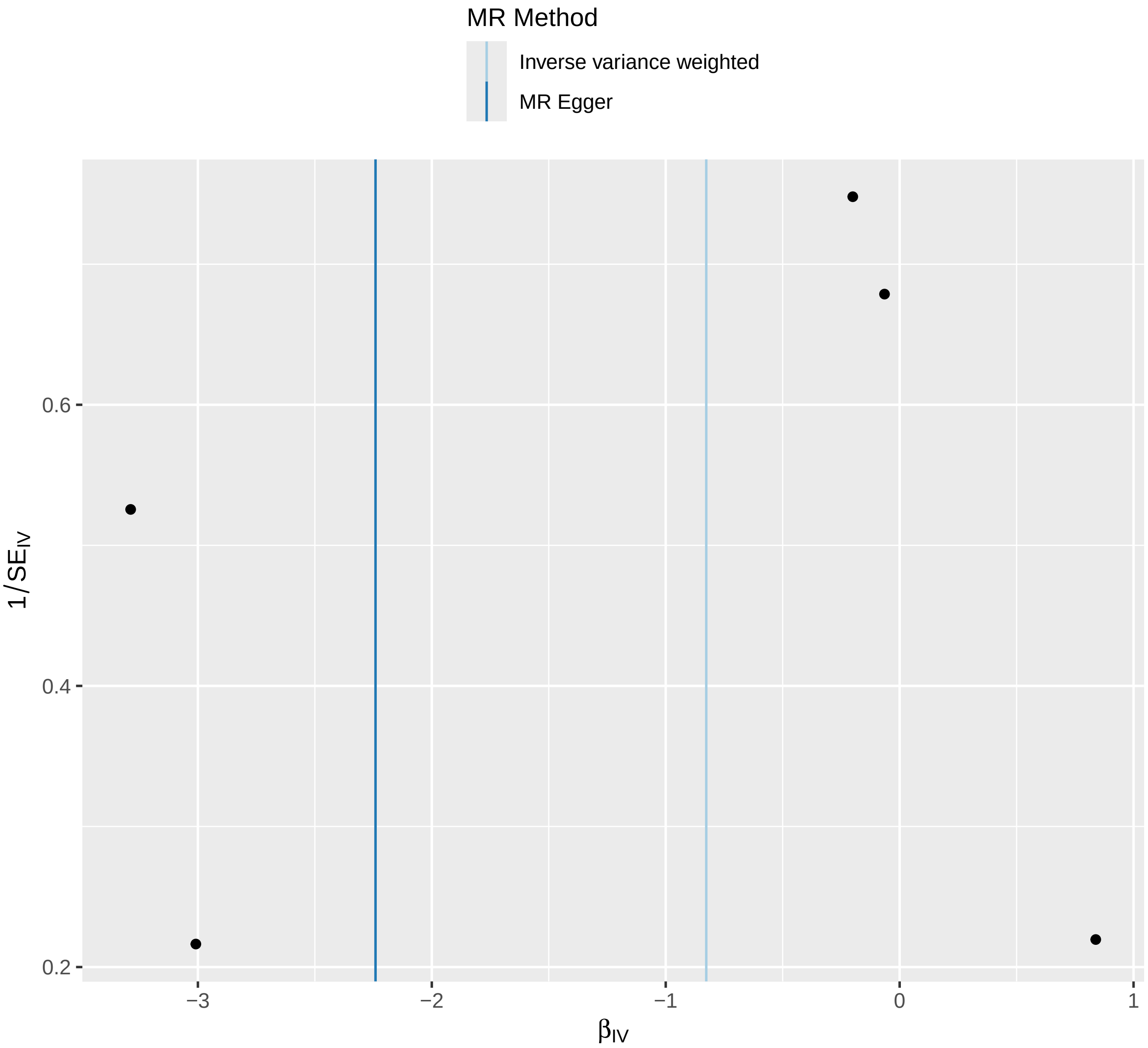
MR Method
Inverse variance weighted
MR Egger
0.6
0.4
0.2
−3
−2
−1
0
1
β_{IV}
$1/SE_{IV}$

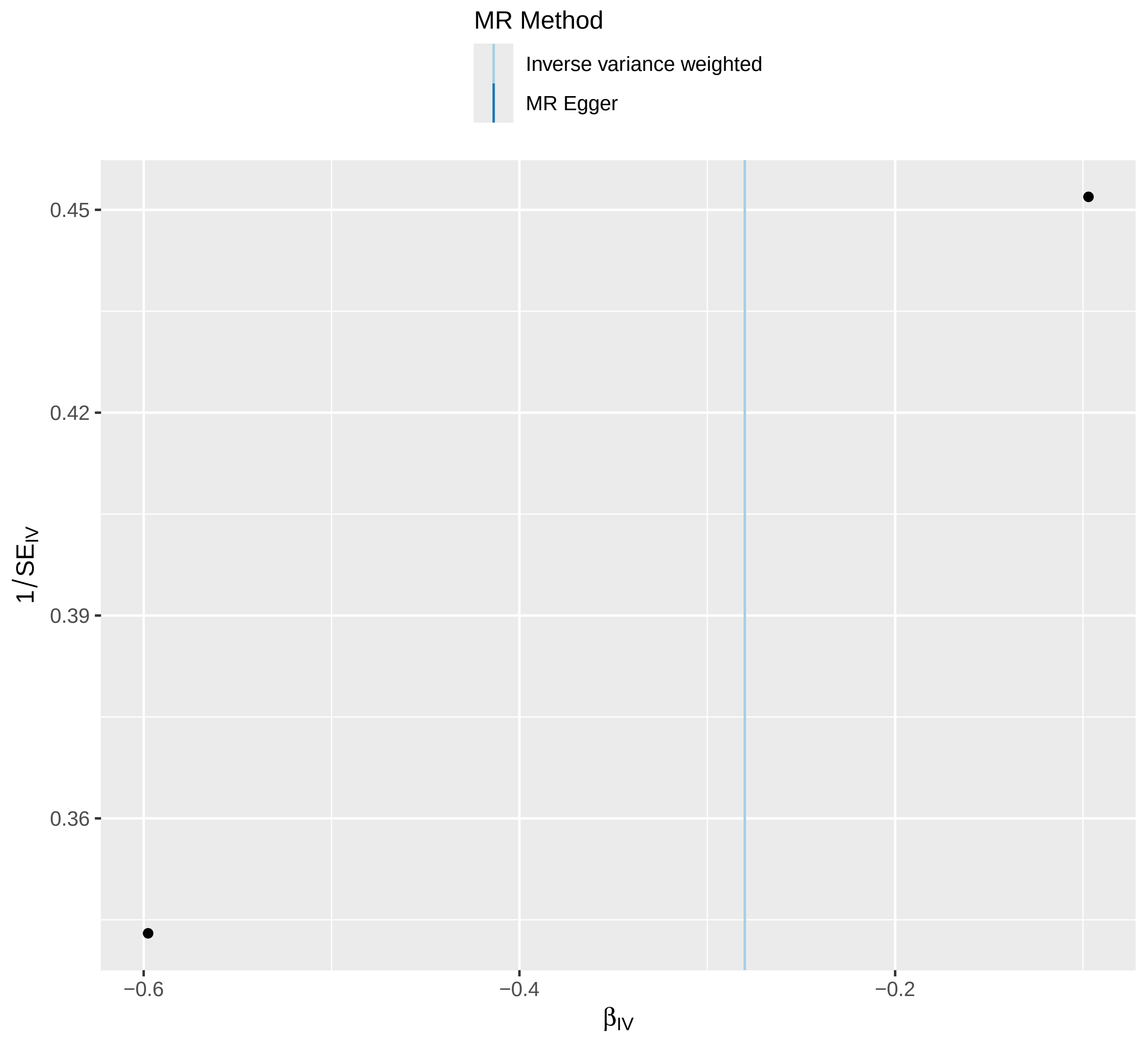
MR Method
Inverse variance weighted
MR Egger
0.45
0.42
0.39
0.36
$1/SE_{IV}$
−0.6
−0.4
−0.2
β_{IV}

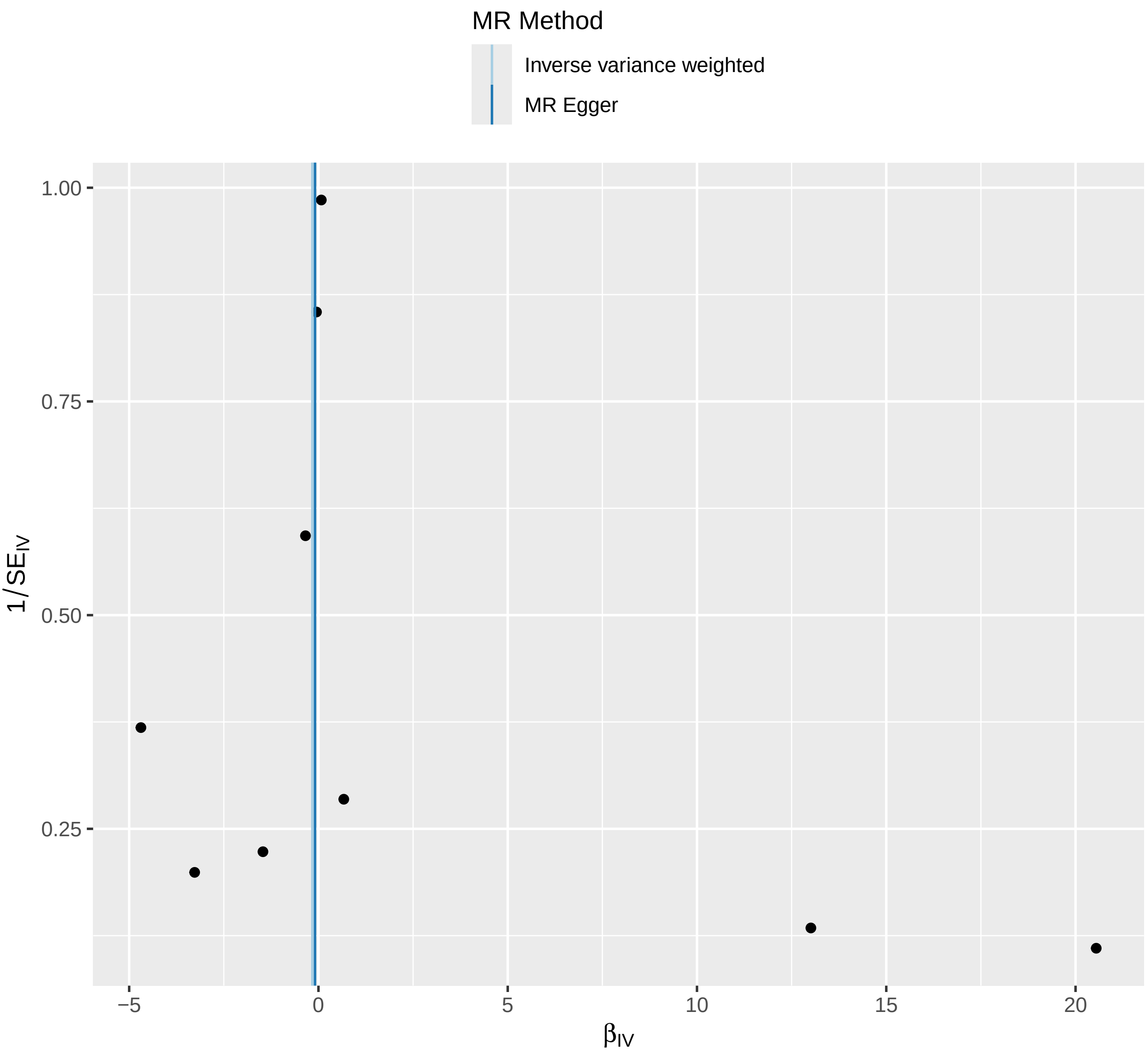
MR Method
Inverse variance weighted
MR Egger
1.00
0.75
0.50
0.25
1/SE_IV
−5
0
5
10
15
20
β_IV

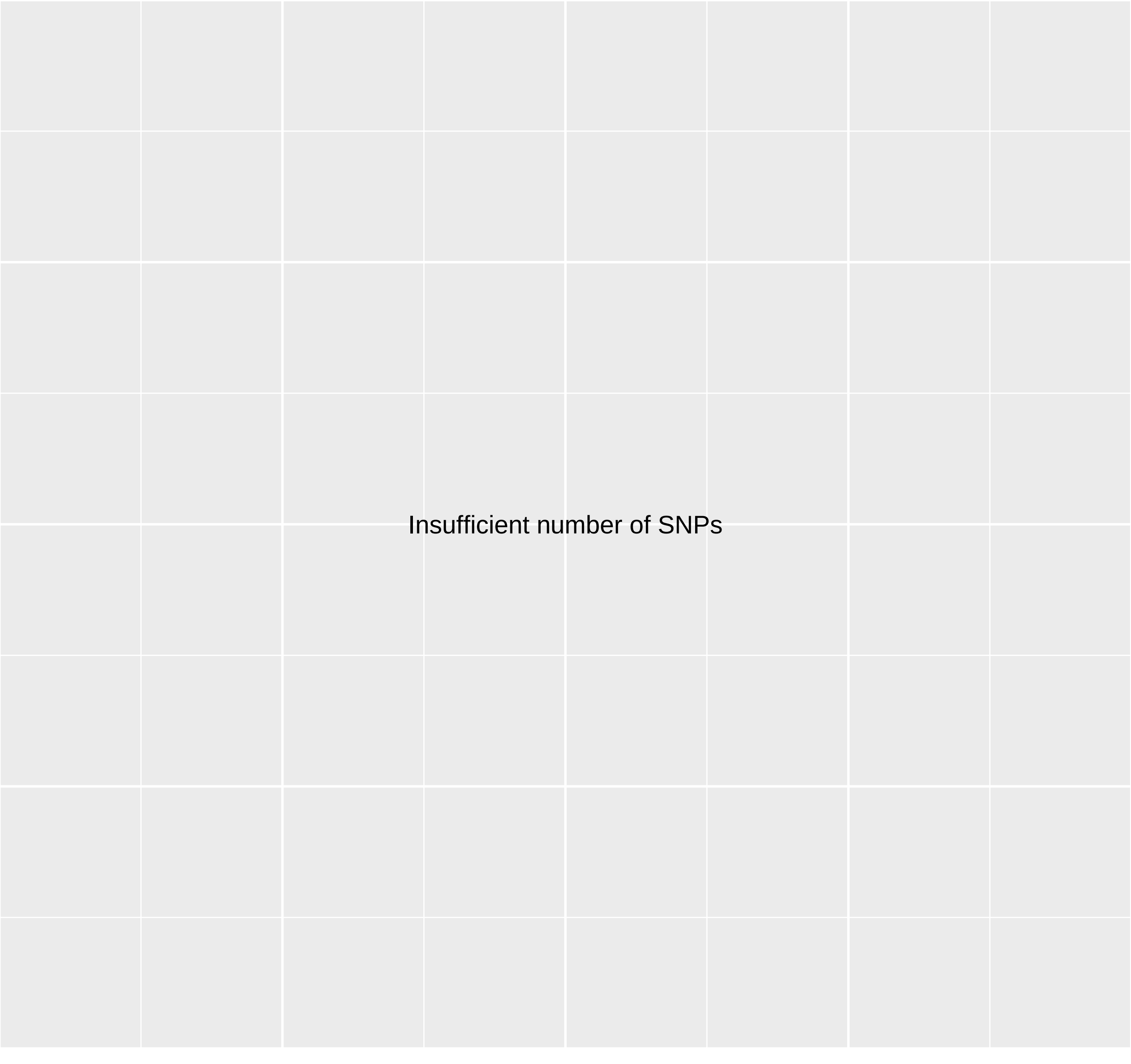
Insufficient number of SNPs

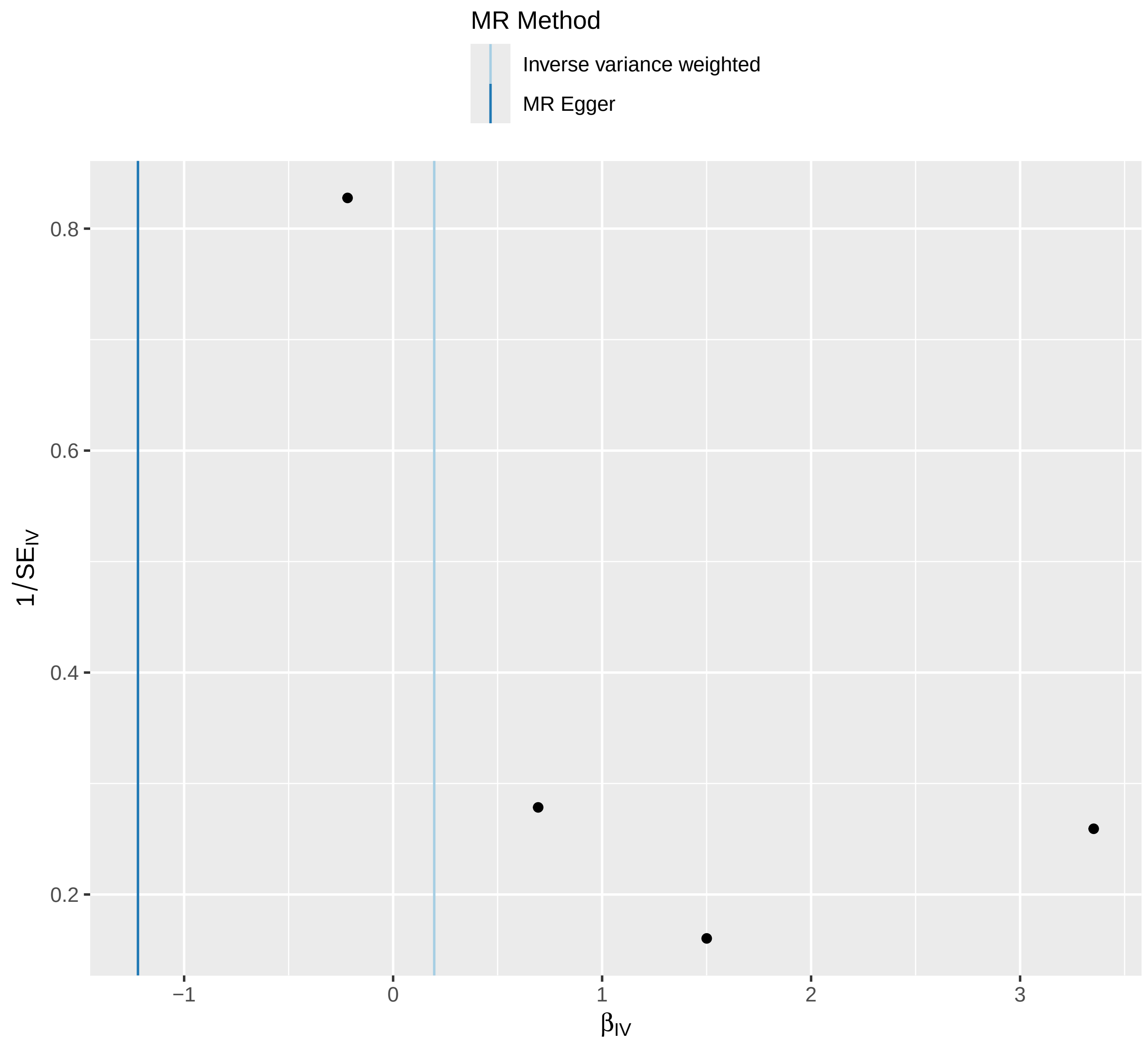
MR Method
Inverse variance weighted
MR Egger
0.8
0.6
0.4
0.2
1/SE_IV
−1
0
1
2
3
β_IV

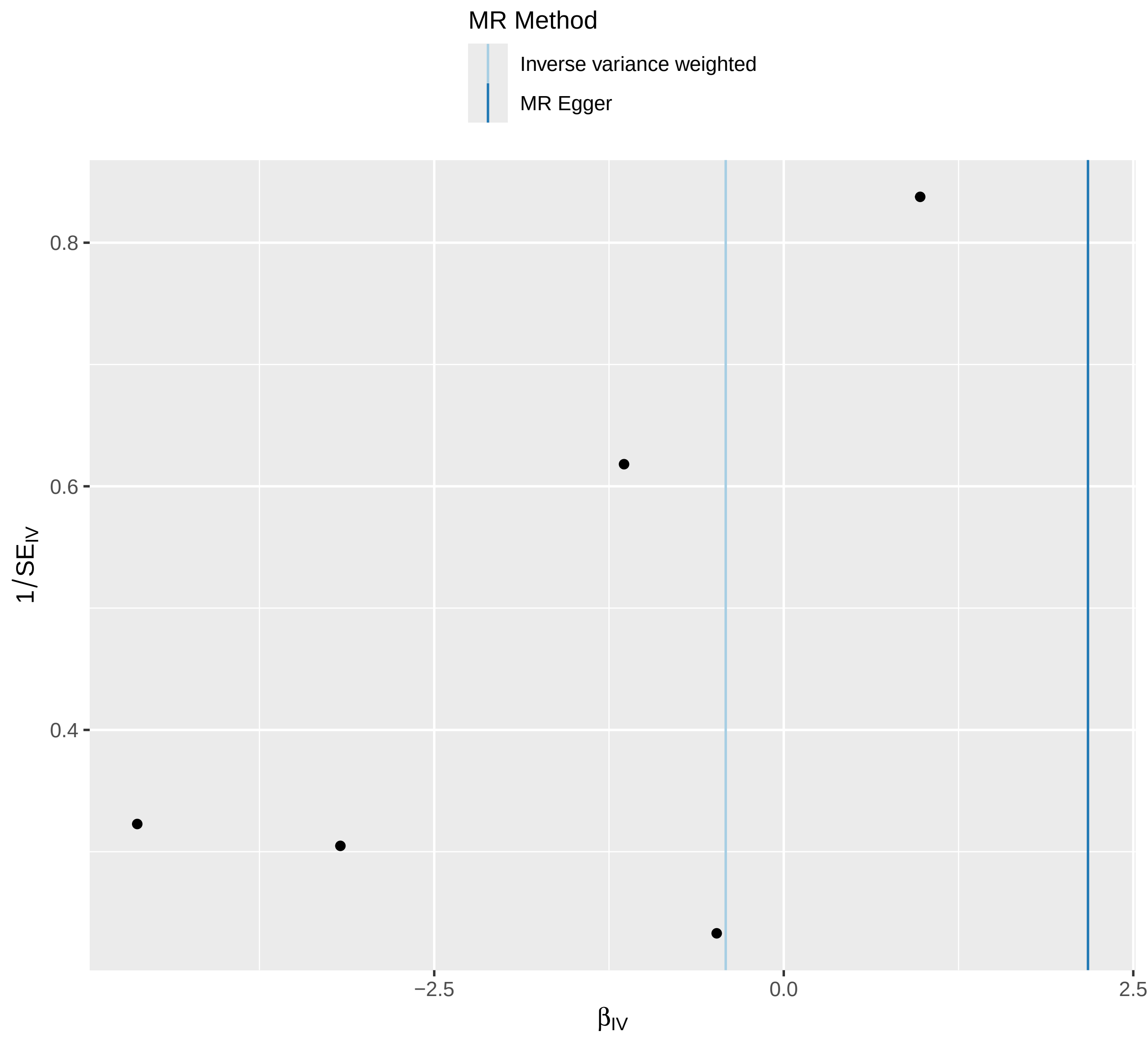
MR Method
Inverse variance weighted
MR Egger
0.8
0.6
0.4
1/SE_IV
−2.5
0.0
2.5
β_IV

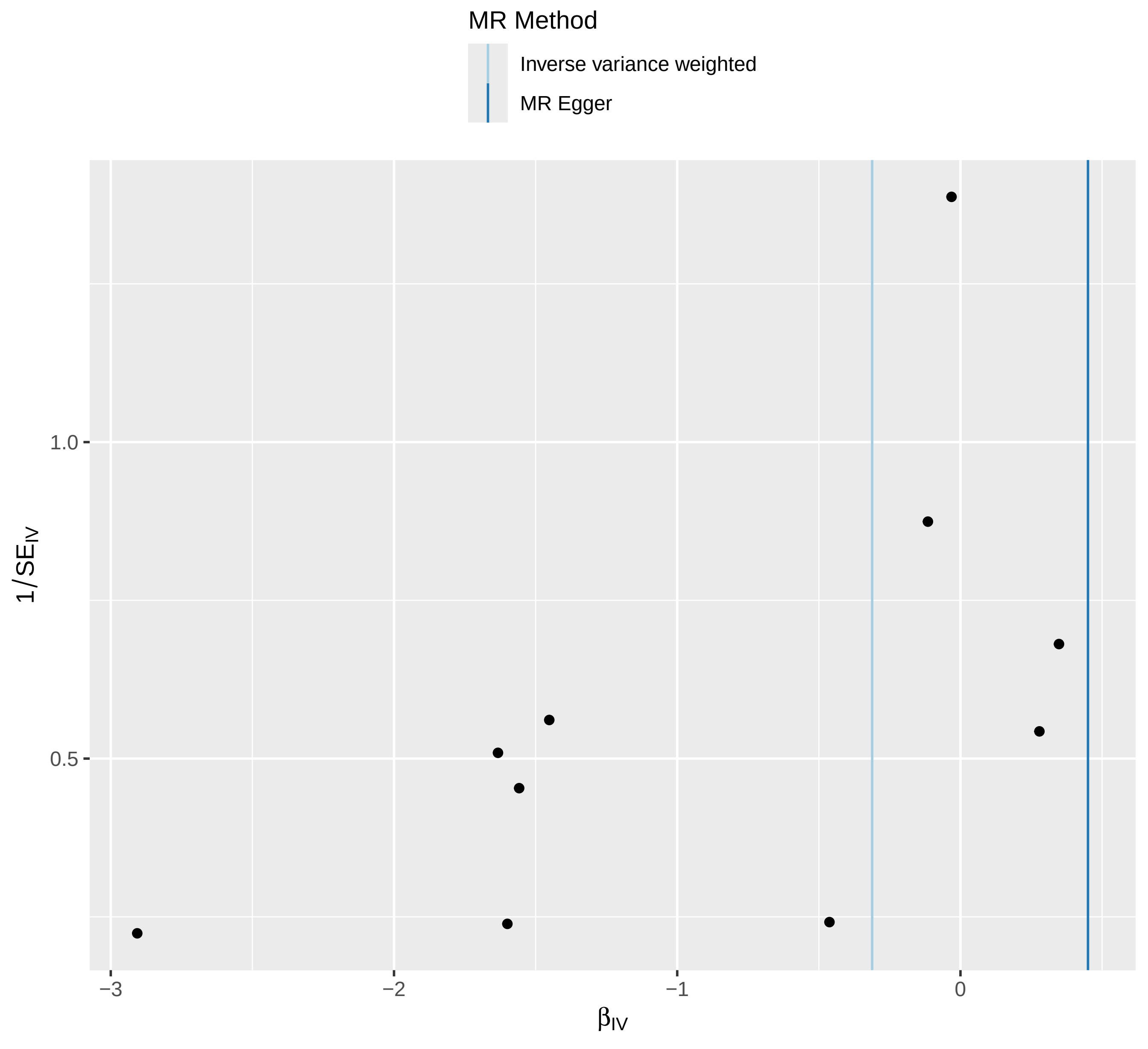
MR Method
Inverse variance weighted
MR Egger
1.0
0.5
1/SE_IV
−3
−2
−1
0
β_IV

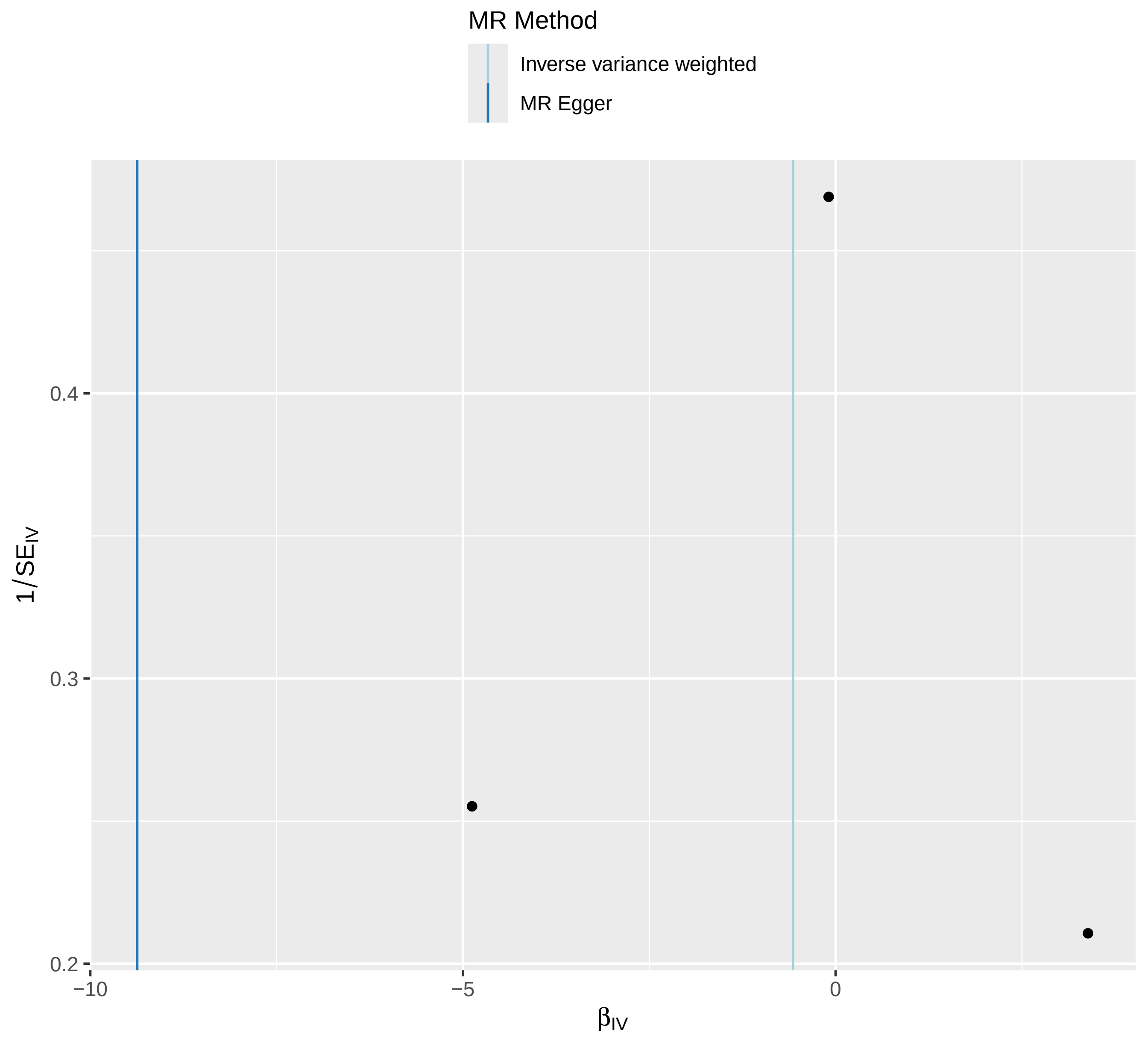
MR Method
Inverse variance weighted
MR Egger
0.4
0.3
0.2
1/SE$_{IV}$
−10
−5
0
β$_{IV}$

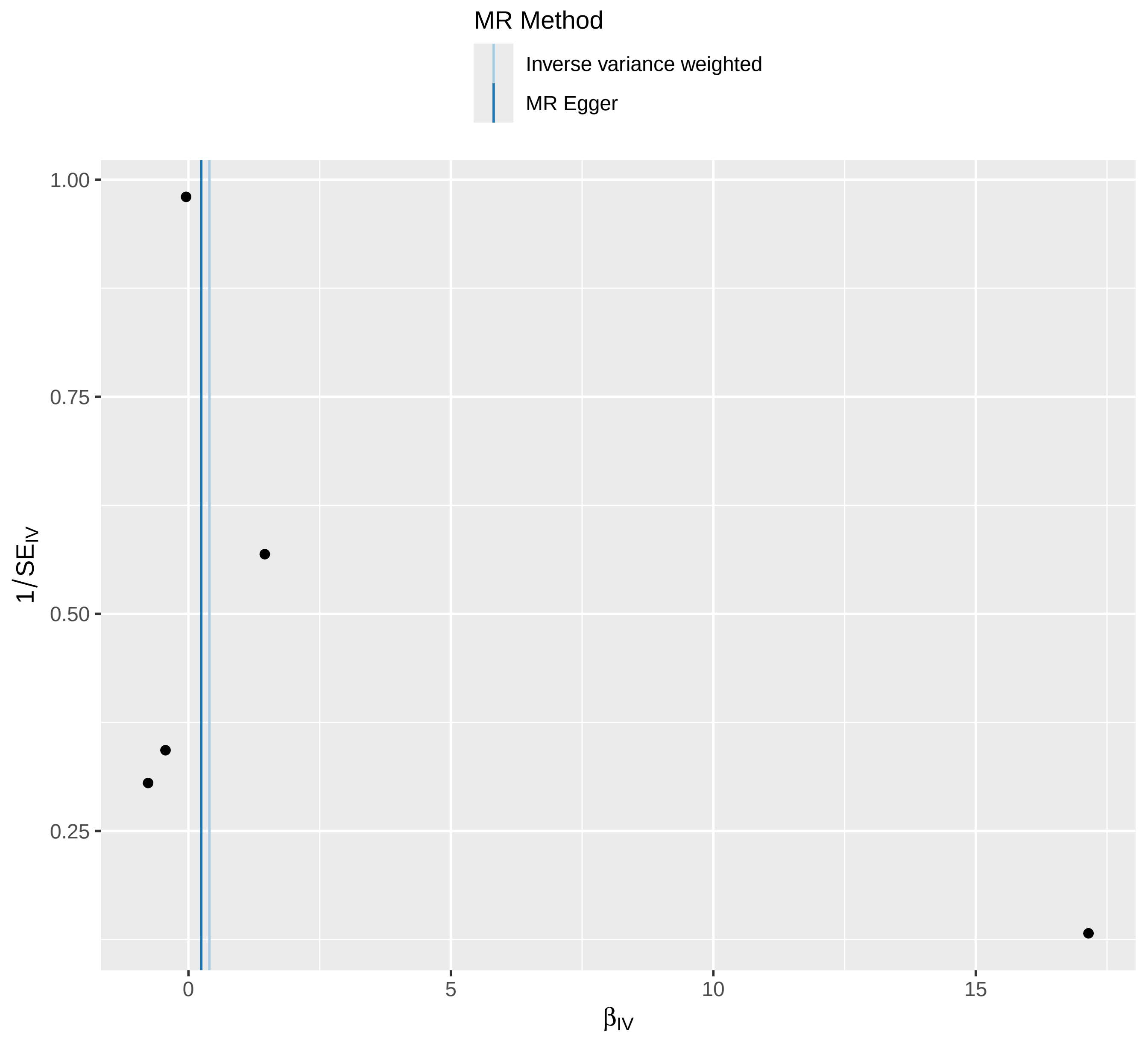
MR Method
Inverse variance weighted
MR Egger
1.00
0.75
0.50
0.25
$1/SE_{IV}$
0
5
10
15
β_{IV}

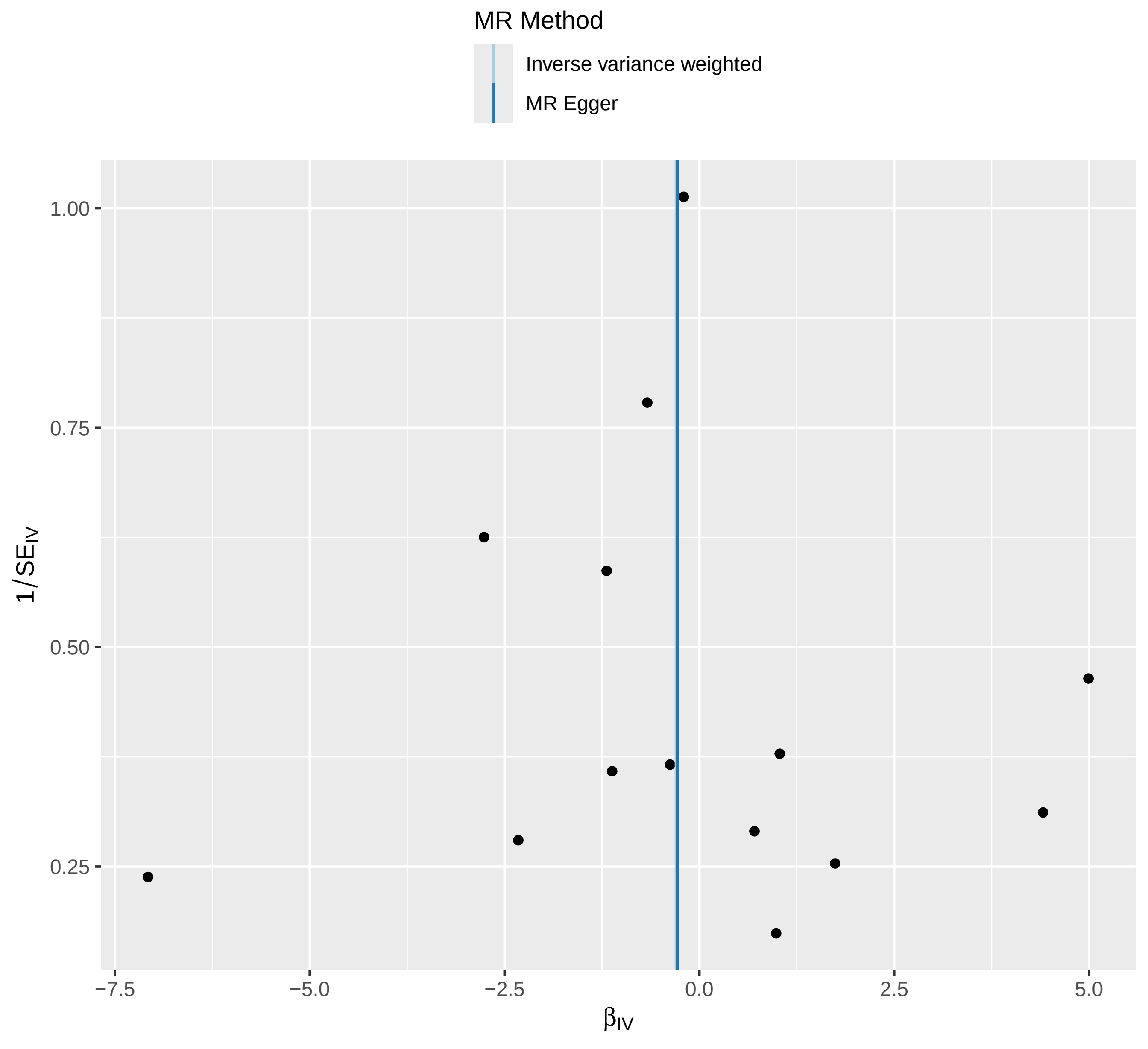

MR Method
Inverse variance weighted
MR Egger
1.00
0.75
0.50
0.25
$1/SE_{IV}$
−7.5
−5.0
−2.5
0.0
2.5
5.0
β_{IV}

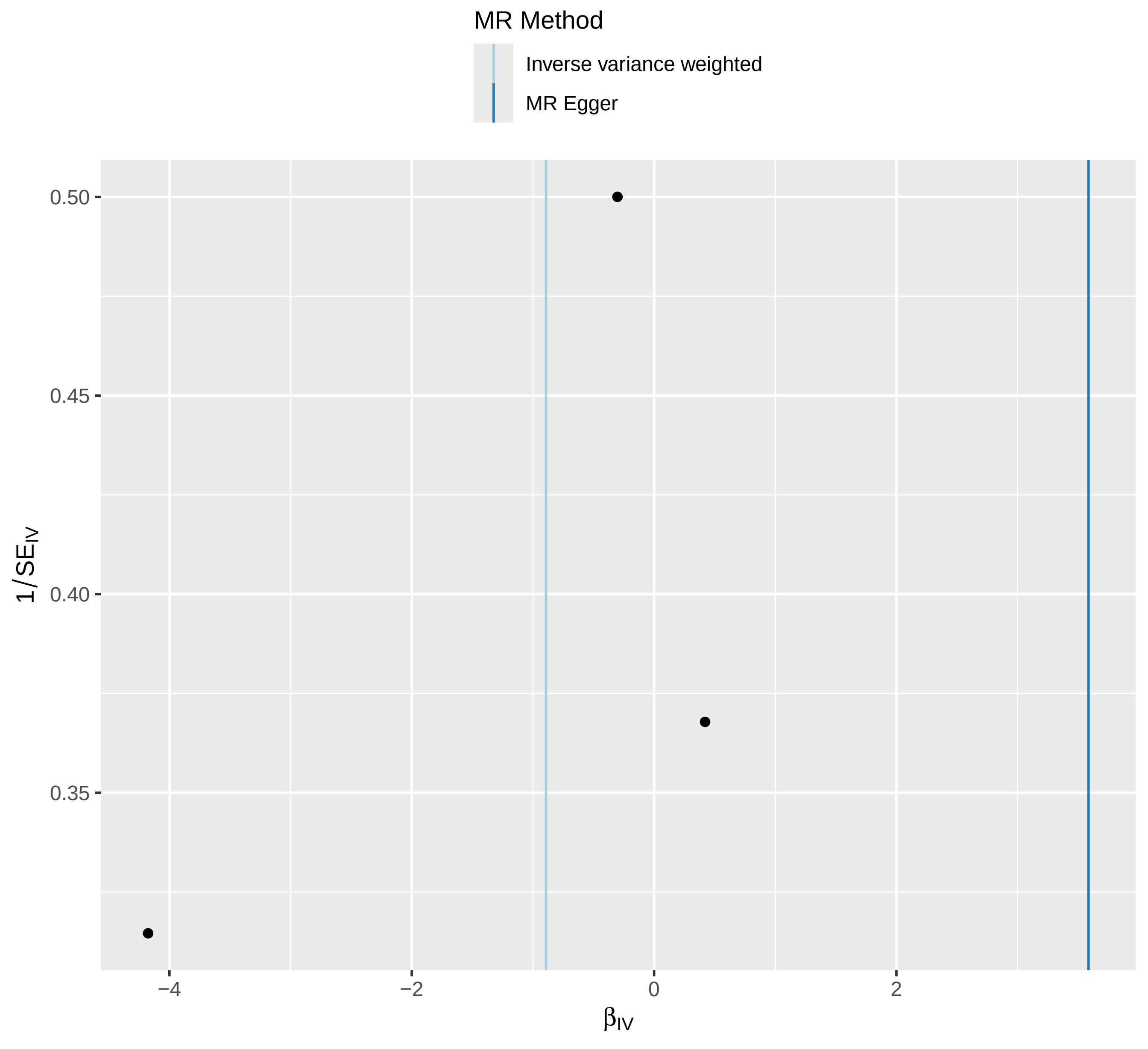
MR Method
Inverse variance weighted
MR Egger
0.50
0.45
0.40
0.35
1/SE_IV
−4
−2
0
2
β_IV

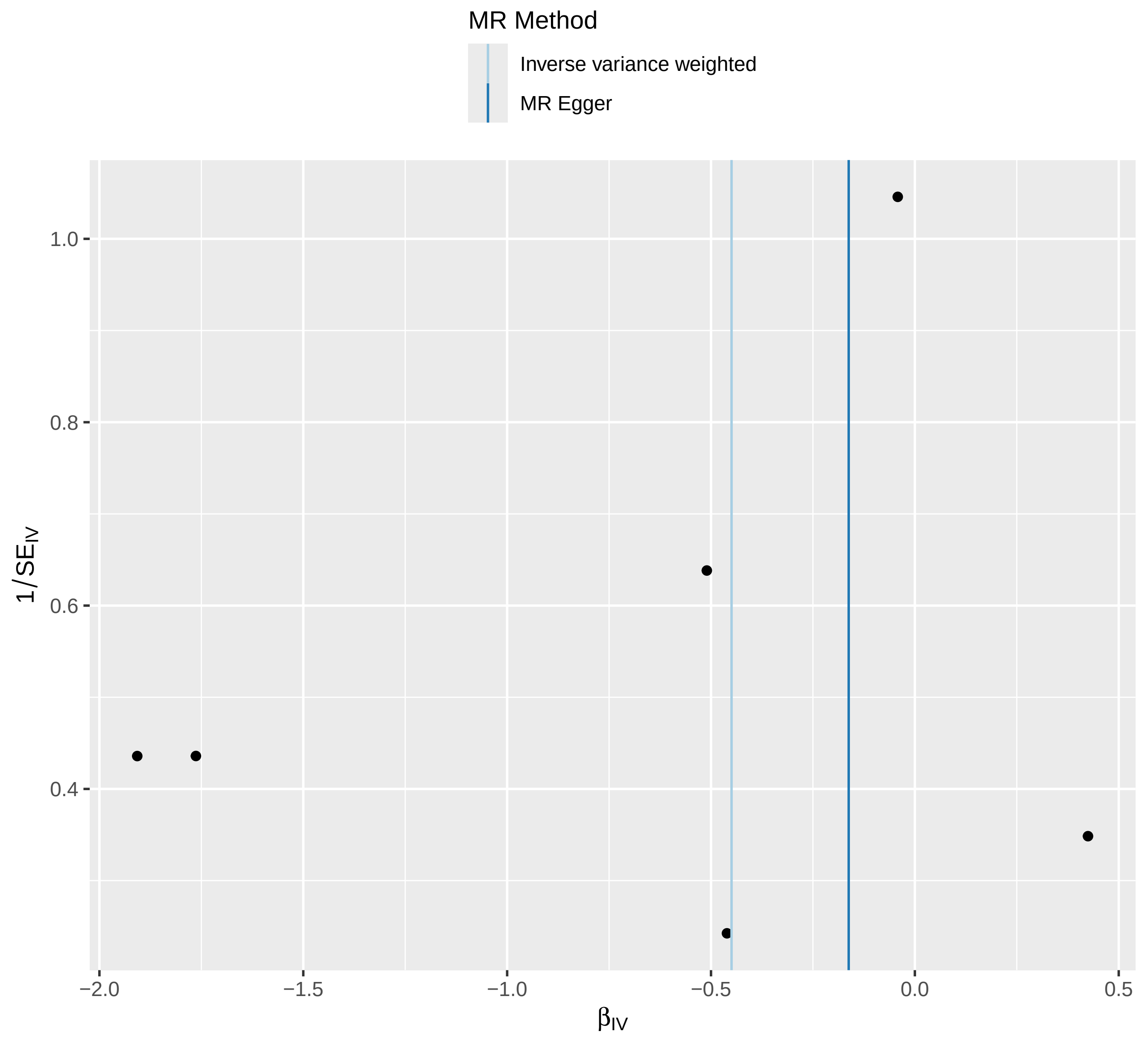
MR Method
Inverse variance weighted
MR Egger
1.0
0.8
0.6
0.4
$1/SE_{IV}$
−2.0
−1.5
−1.0
−0.5
0.0
0.5
β_{IV}

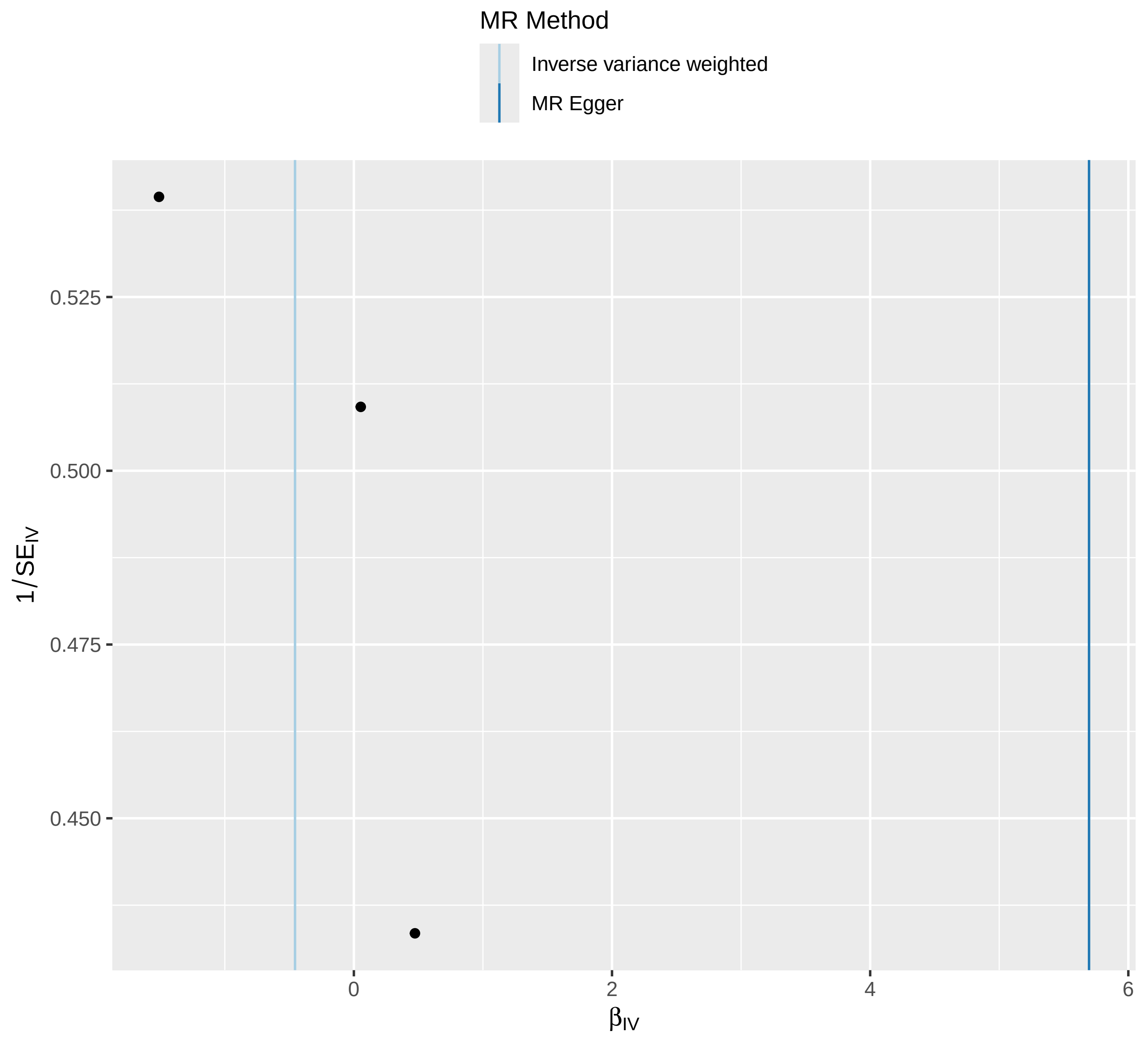
MR Method
Inverse variance weighted
MR Egger
0.525
0.500
0.475
0.450
1/SE_IV
0
2
4
6
β_IV

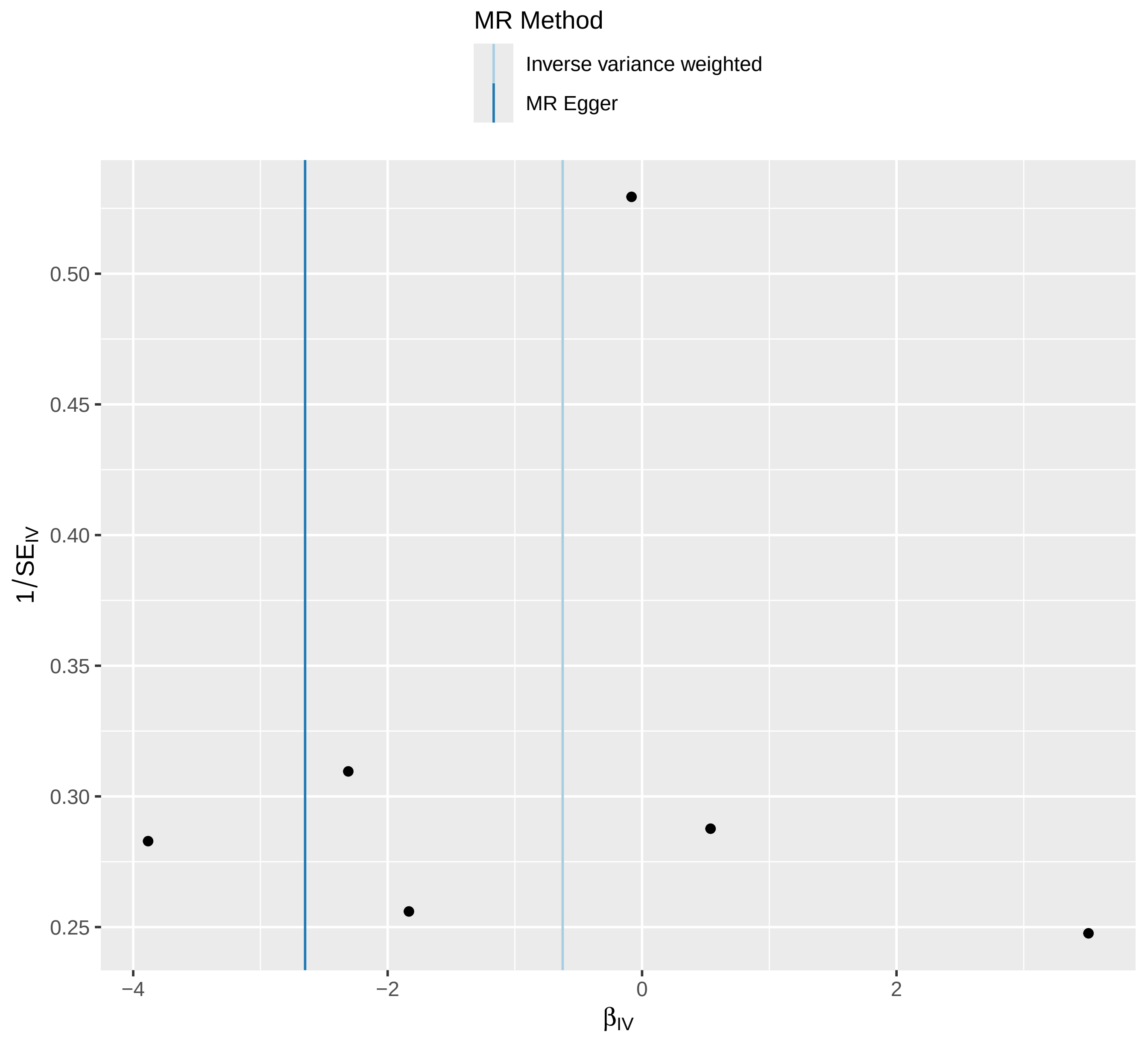
MR Method
Inverse variance weighted
MR Egger
0.50
0.45
0.40
0.35
0.30
0.25
$1/SE_{IV}$
−4
−2
0
2
β_{IV}

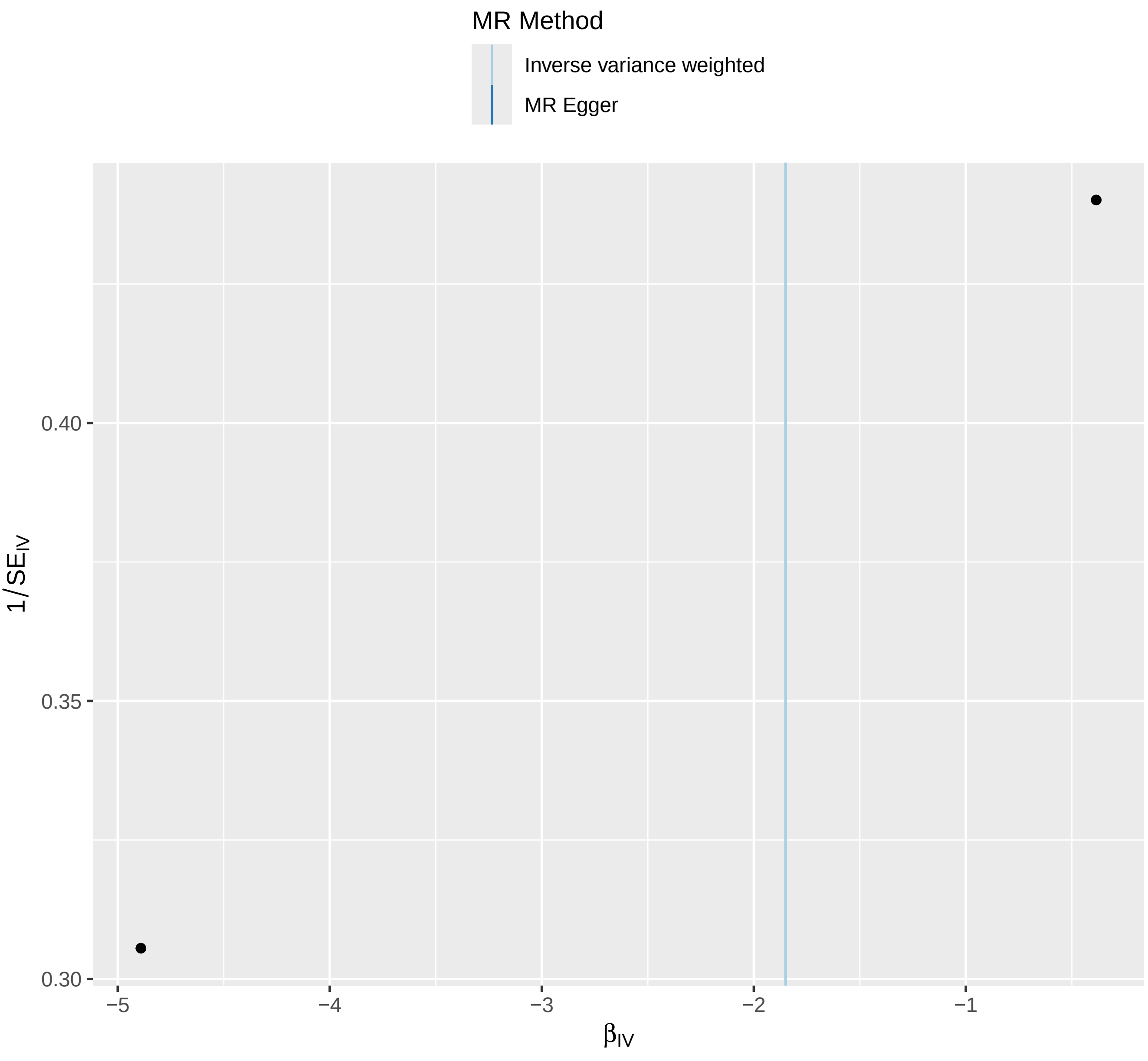

MR Method
Inverse variance weighted
MR Egger
0.40
0.35
0.30
1/SE_IV
−5
−4
−3
−2
−1
β_IV

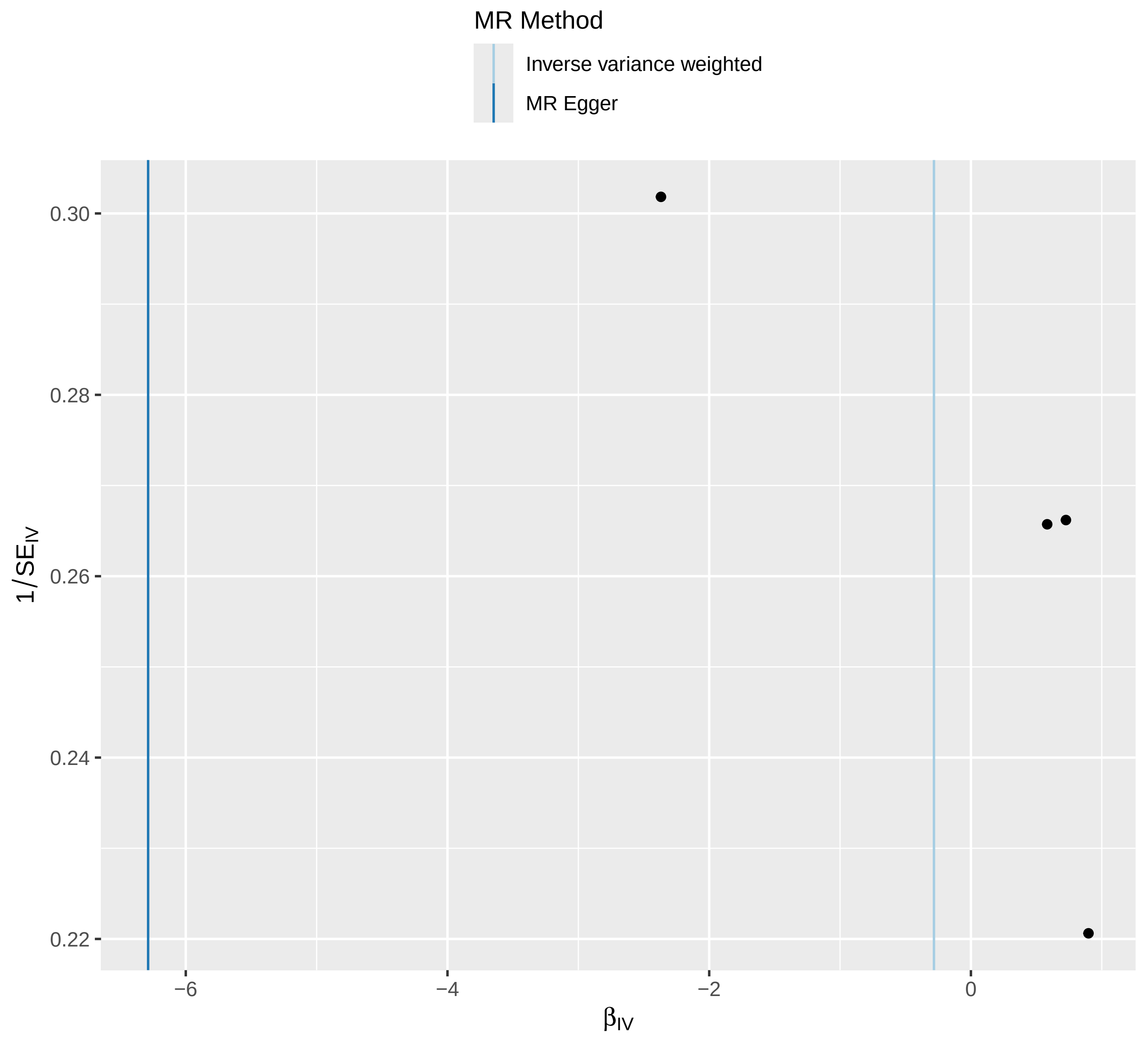
MR Method
Inverse variance weighted
MR Egger
0.30
0.28
0.26
0.24
0.22
1/SE$_{IV}$
−6
−4
−2
0
β$_{IV}$

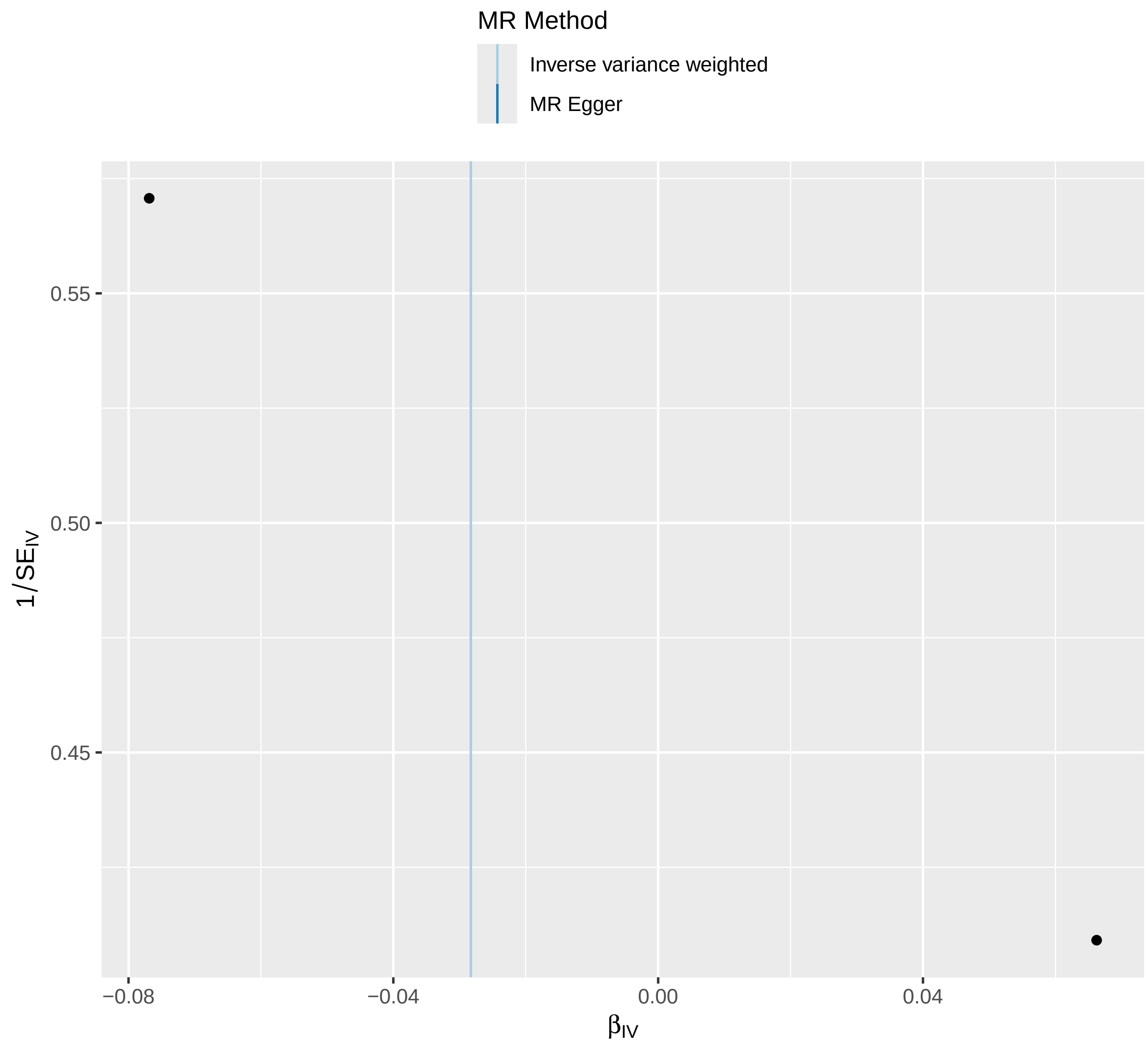

MR Method
Inverse variance weighted
MR Egger
0.55
0.50
0.45
1/SE$_{IV}$
−0.08
−0.04
0.00
0.04
β$_{IV}$

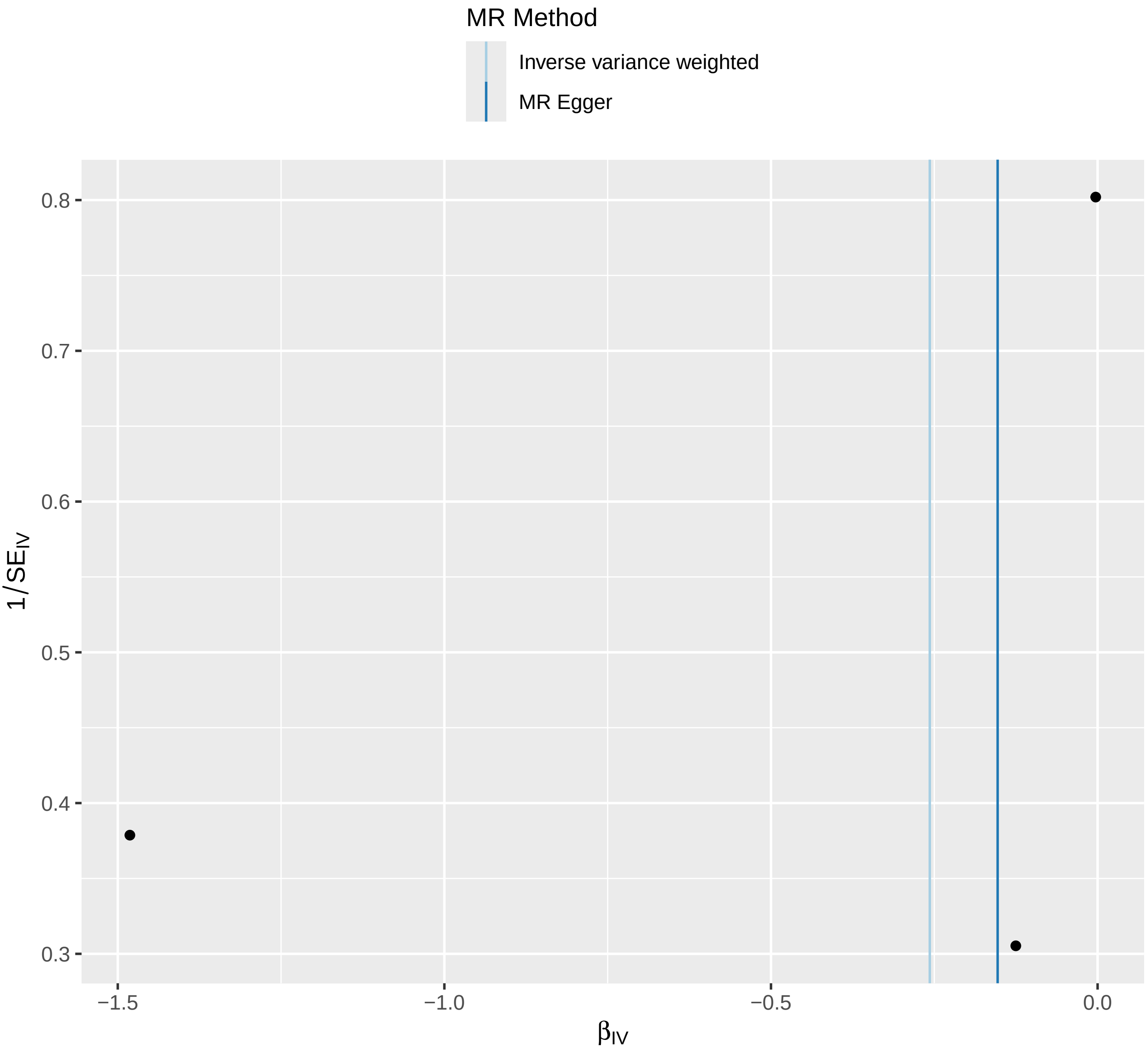
MR Method
Inverse variance weighted
MR Egger
0.8
0.7
0.6
0.5
0.4
0.3
$1/SE_{IV}$
−1.5
−1.0
−0.5
0.0
β_{IV}

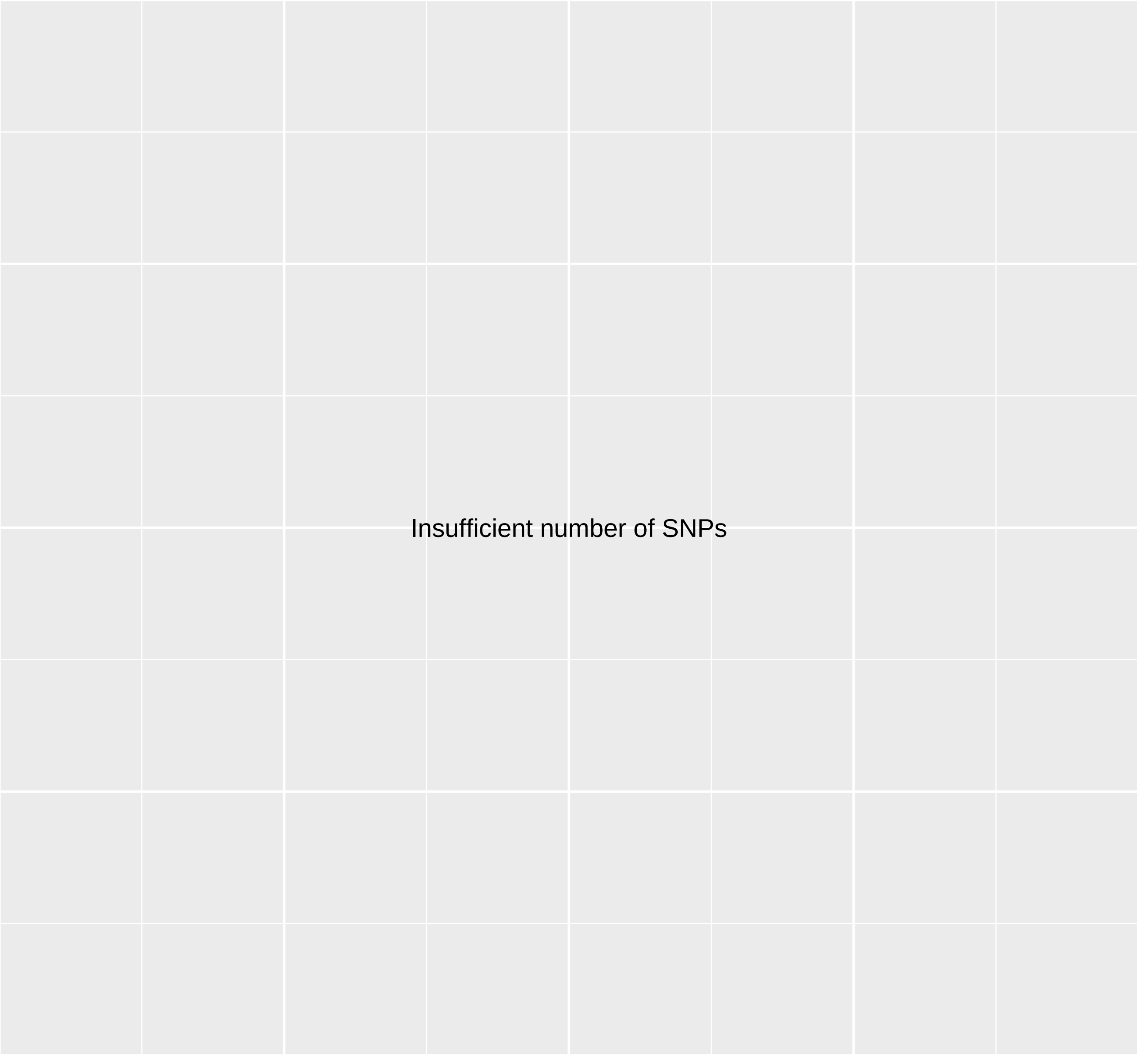
Insufficient number of SNPs

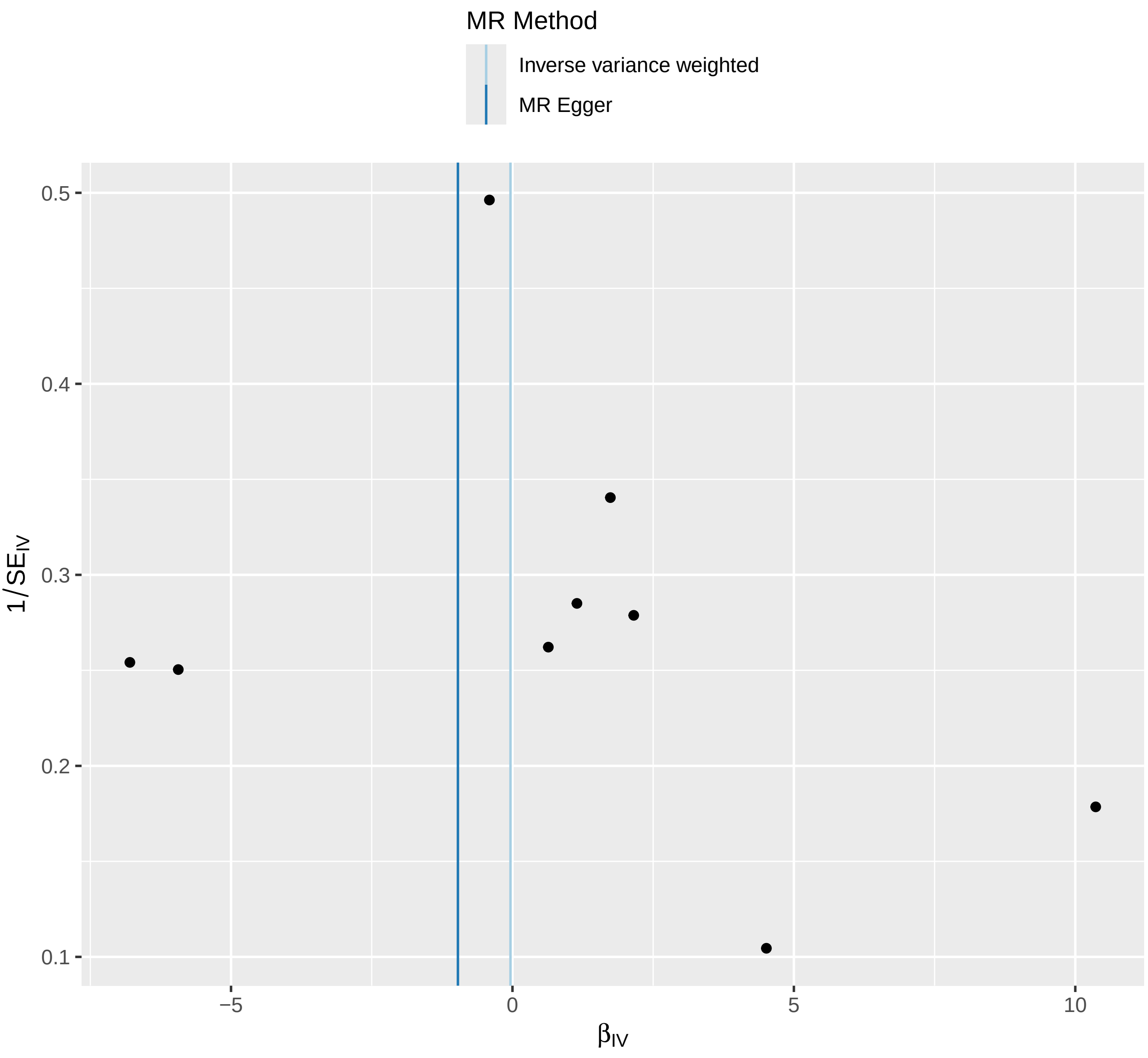

MR Method
Inverse variance weighted
MR Egger
0.5
0.4
0.3
0.2
0.1
1/SE_IV
−5
0
5
10
β_IV

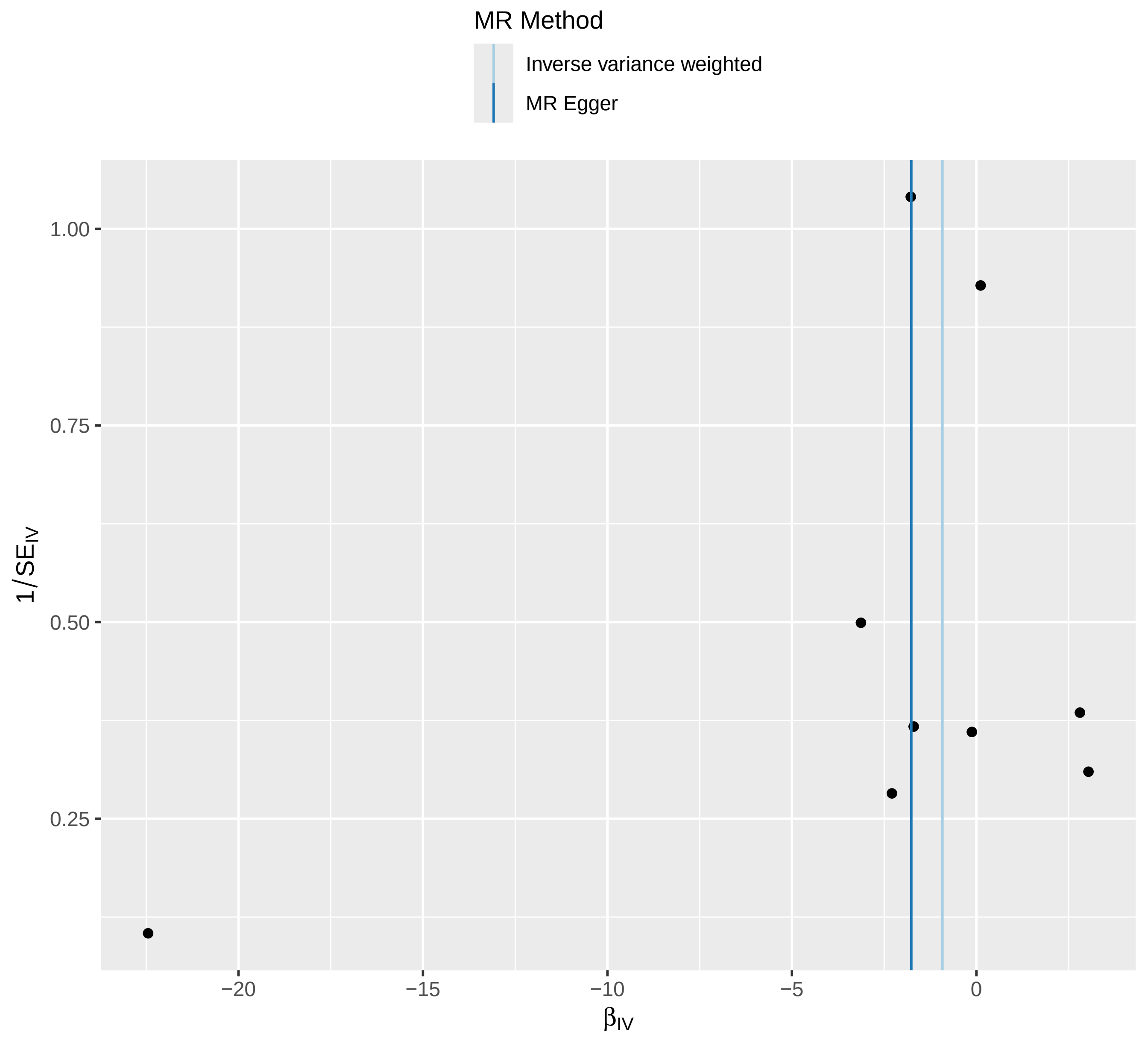
MR Method
Inverse variance weighted
MR Egger
1.00
0.75
0.50
0.25
1/SE$_{IV}$
−20
−15
−10
−5
0
β$_{IV}$

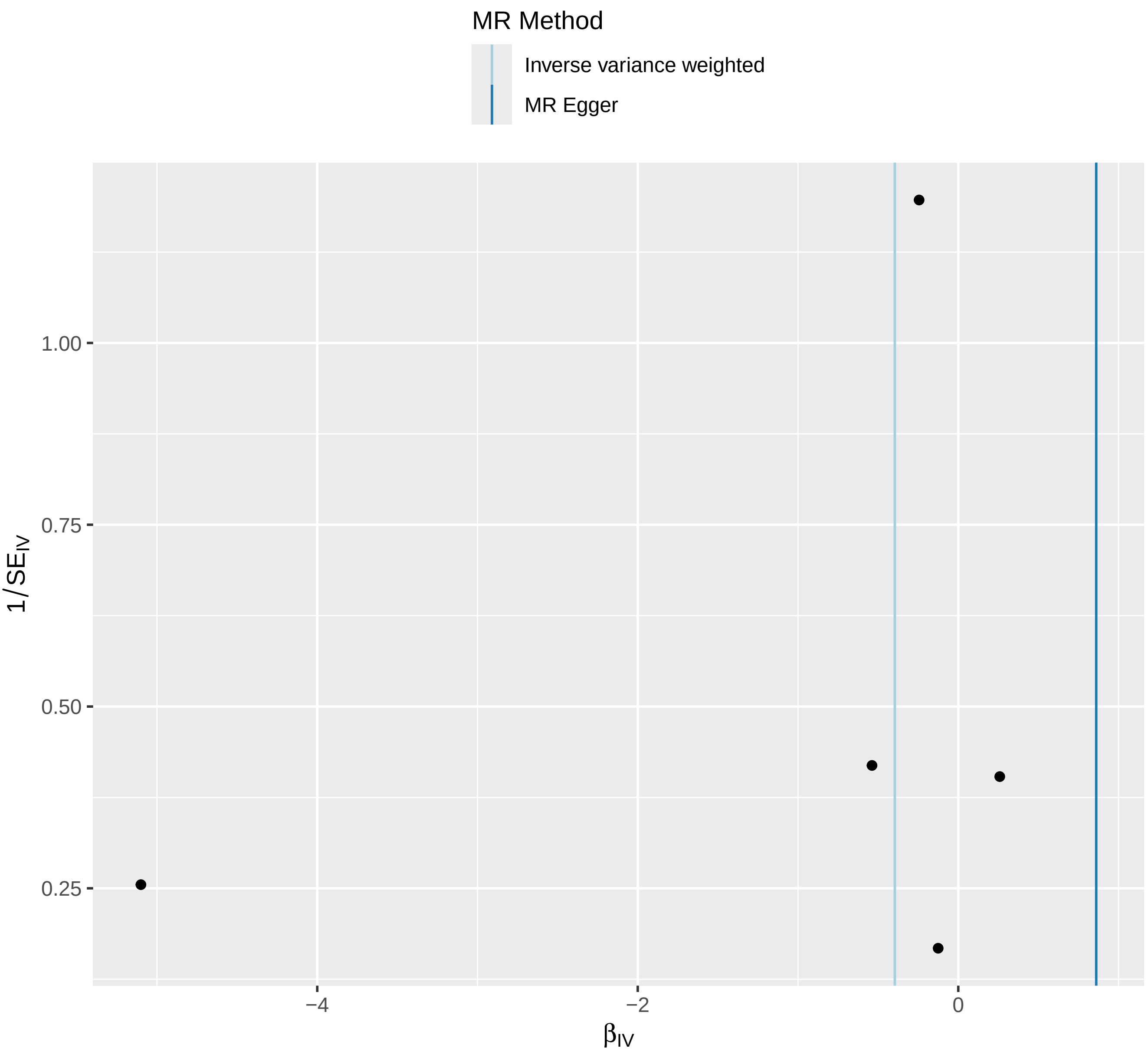
MR Method
Inverse variance weighted
MR Egger
1.00
0.75
0.50
0.25
1/SE_IV
−4
−2
0
β_IV

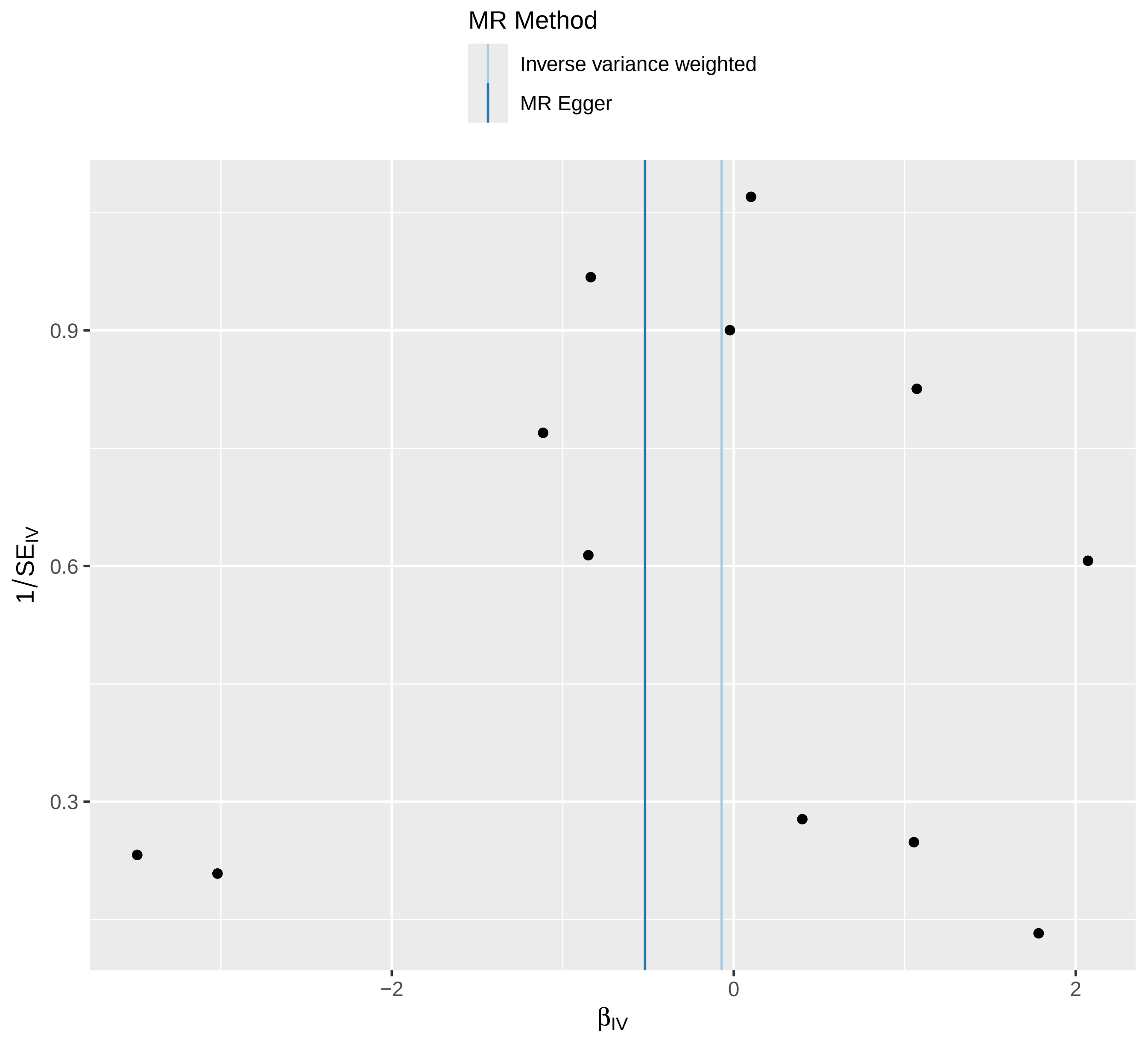
MR Method
Inverse variance weighted
MR Egger
1/SE$_{IV}$
0.9
0.6
0.3
−2
0
2
β$_{IV}$

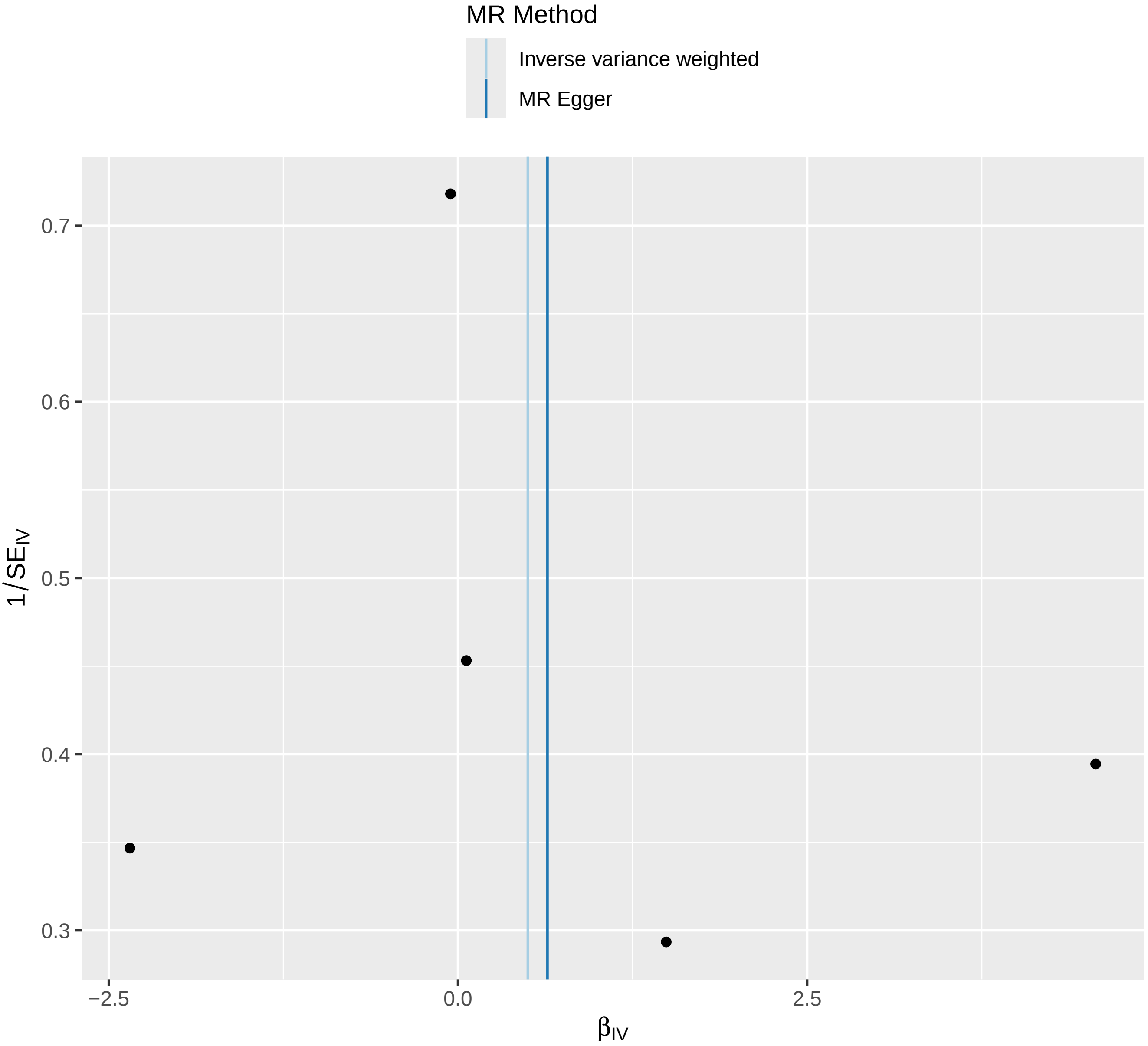
MR Method
Inverse variance weighted
MR Egger
0.7
0.6
0.5
0.4
0.3
1/SE$_{IV}$
−2.5
0.0
2.5
β$_{IV}$

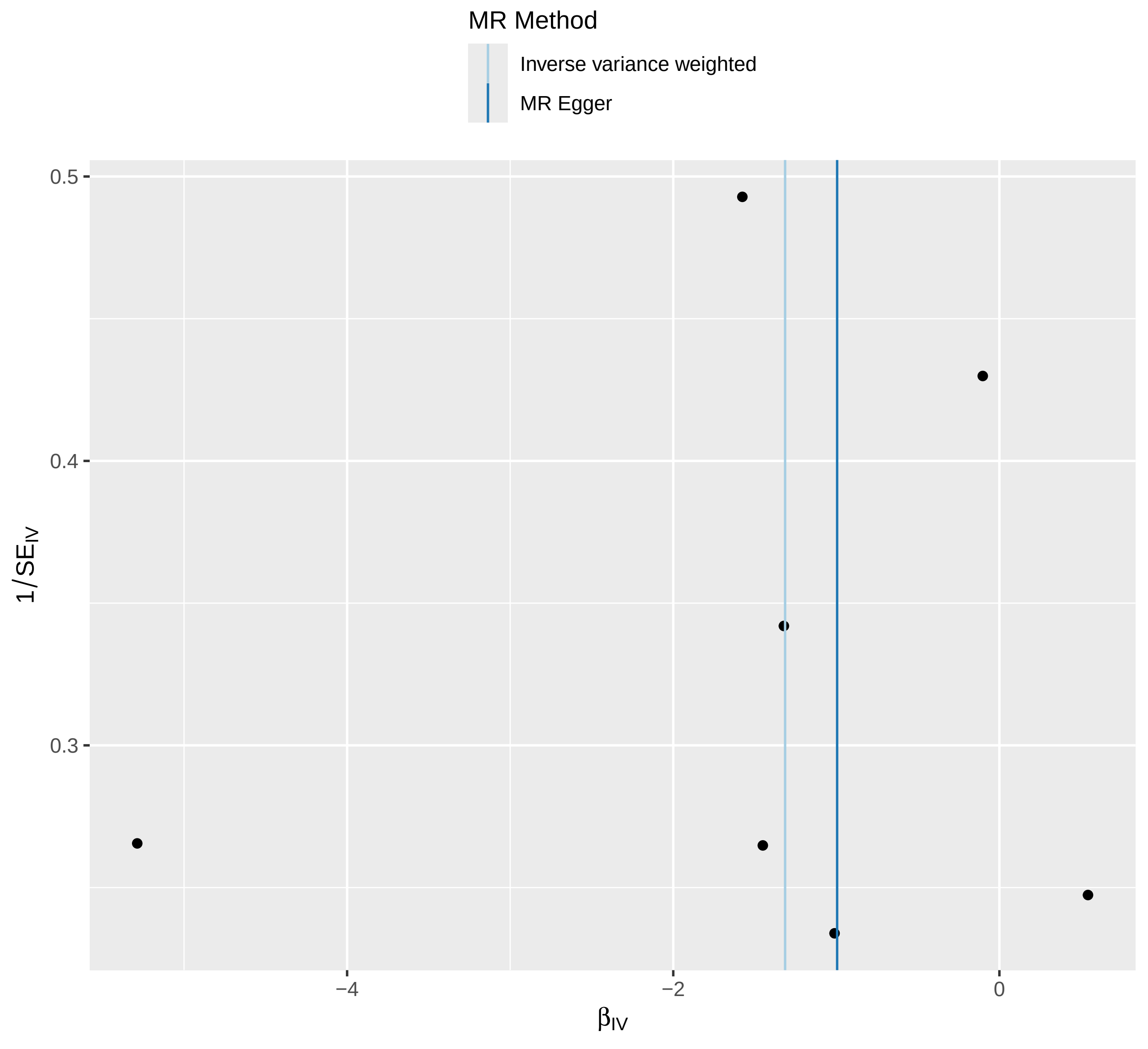

MR Method
Inverse variance weighted
MR Egger
0.5
0.4
0.3
1/SE_IV
−4
−2
0
β_IV

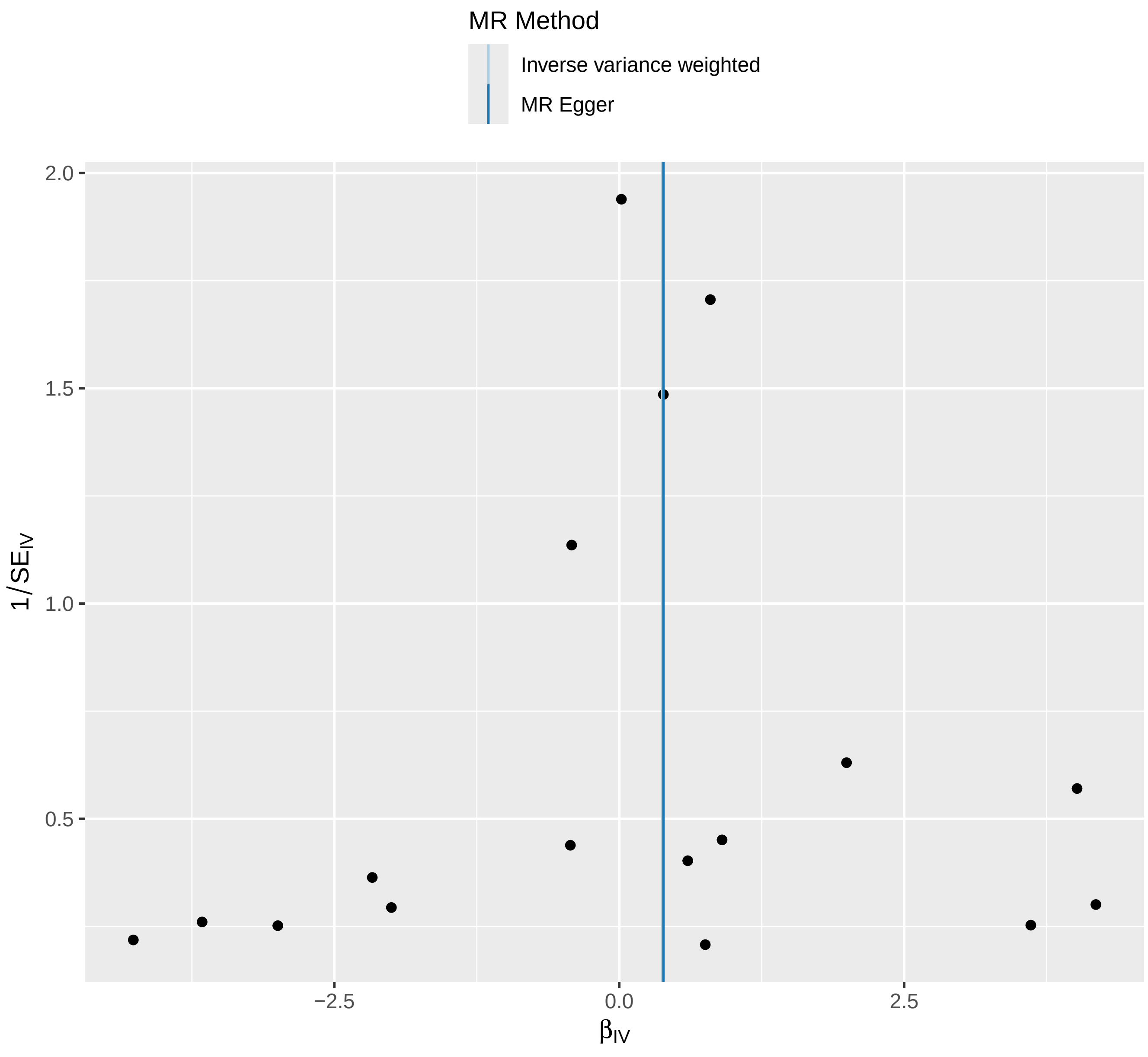
MR Method
Inverse variance weighted
MR Egger
2.0
1.5
1.0
0.5
1/SE_IV
−2.5
0.0
2.5
β_IV

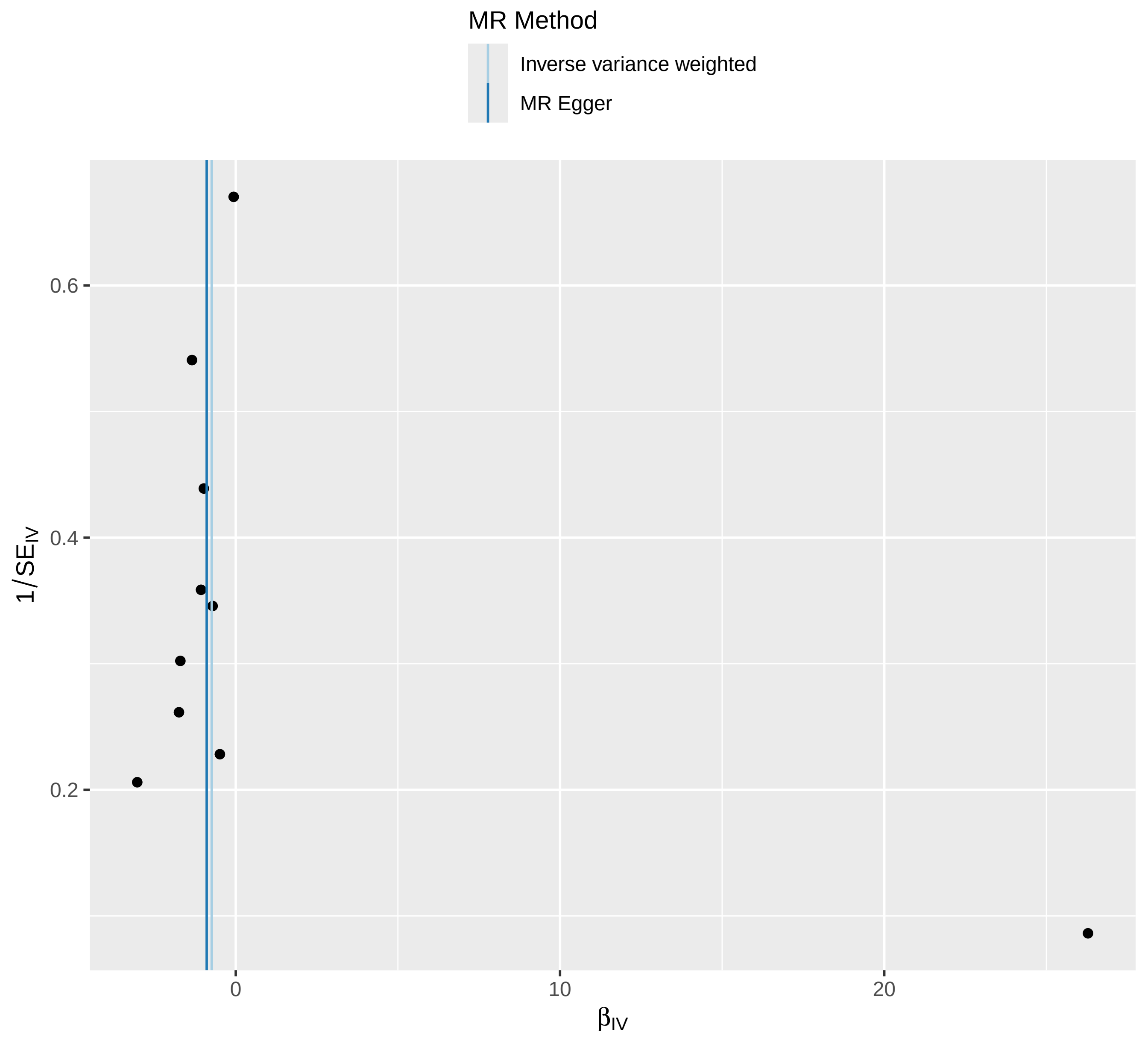

MR Method
Inverse variance weighted
MR Egger
0.6
0.4
0.2
1/SE_IV
0
10
20
β_IV

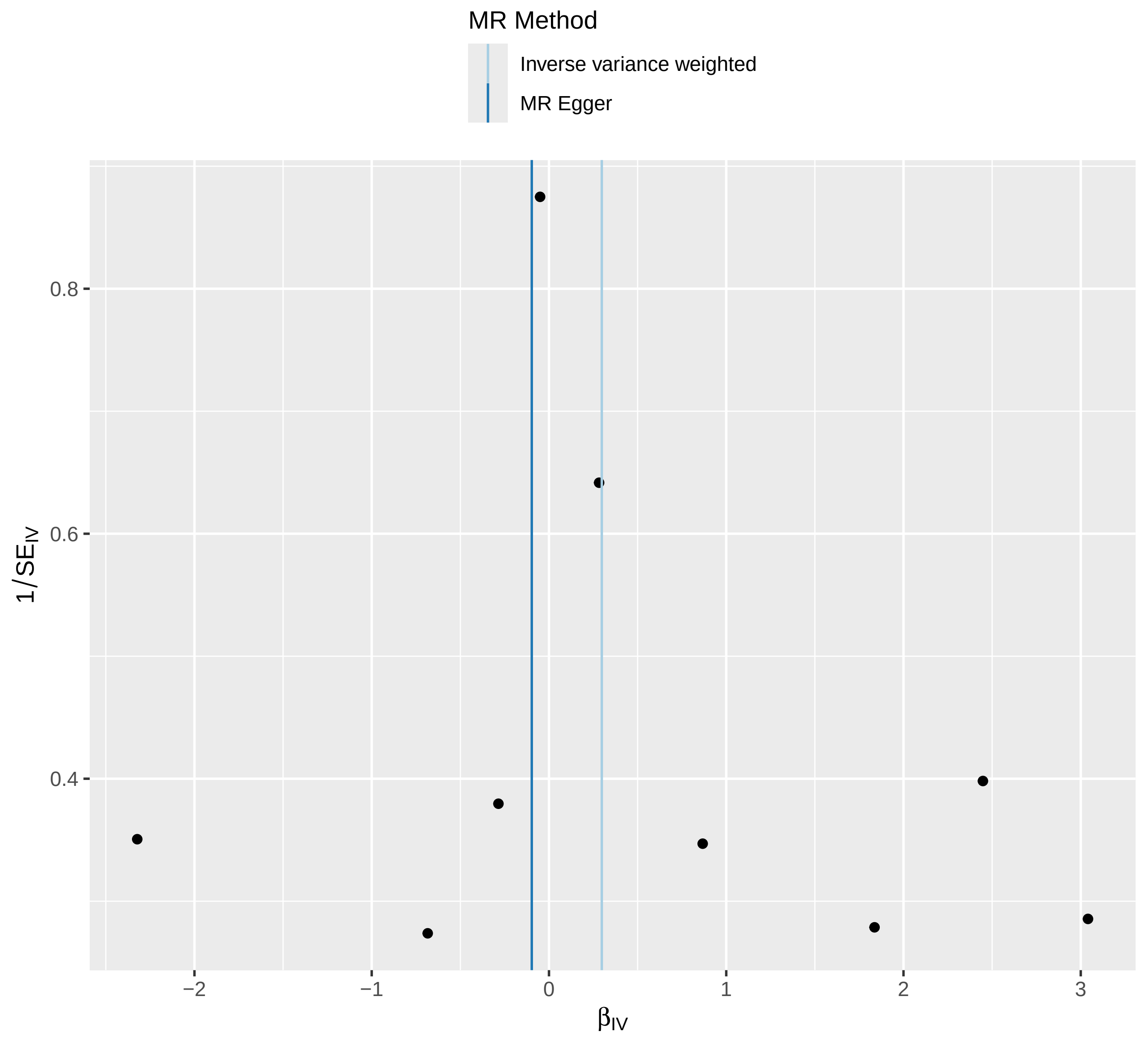
MR Method
Inverse variance weighted
MR Egger
0.8
0.6
0.4
1/SE$_{IV}$
−2
−1
0
1
2
3
β$_{IV}$

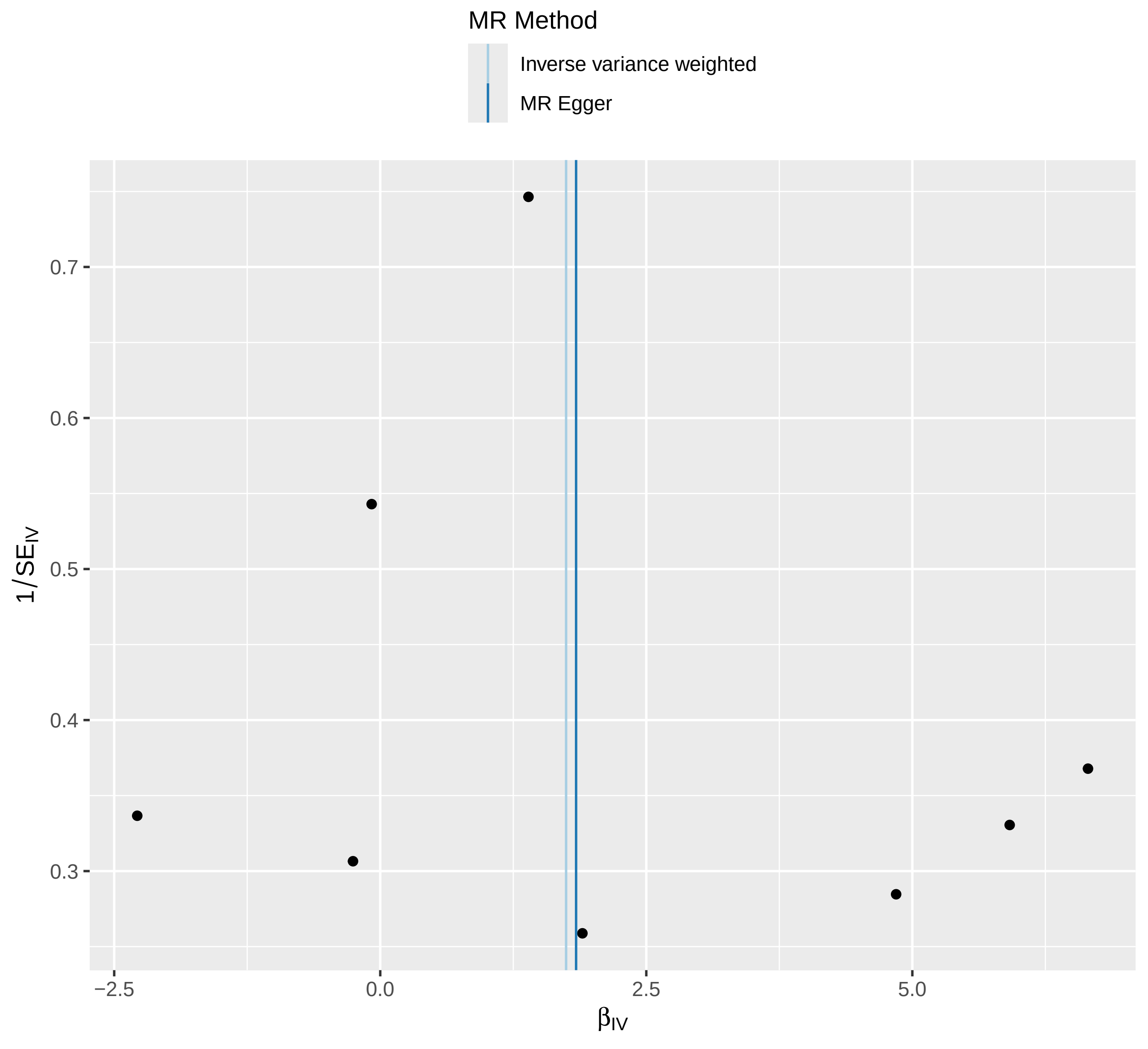
MR Method
Inverse variance weighted
MR Egger
0.7
0.6
0.5
0.4
0.3
$1/SE_{IV}$
−2.5
0.0
2.5
5.0
β_{IV}

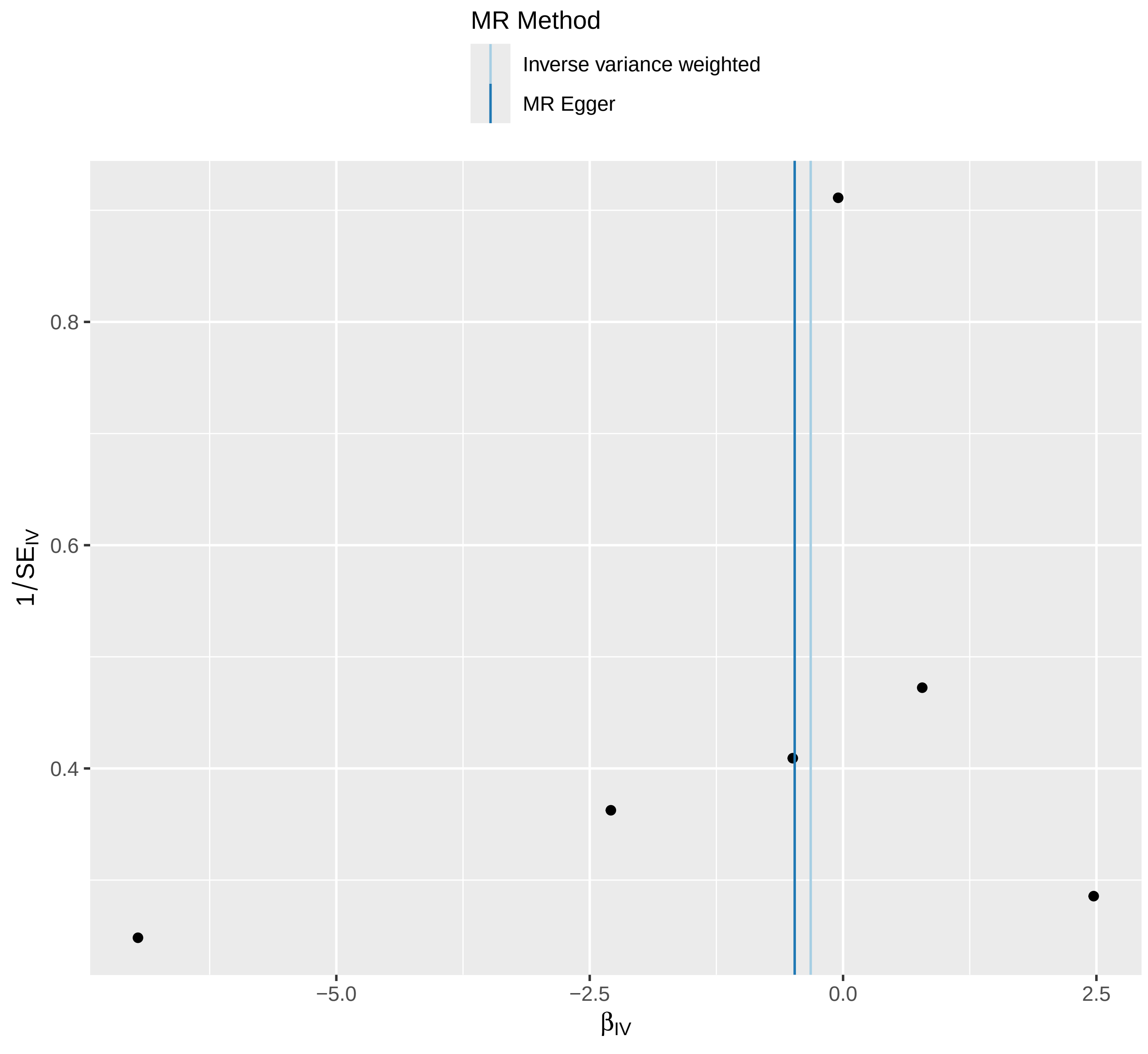
MR Method
Inverse variance weighted
MR Egger
0.8
0.6
0.4
$1/SE_{IV}$
−5.0
−2.5
0.0
2.5
β_{IV}

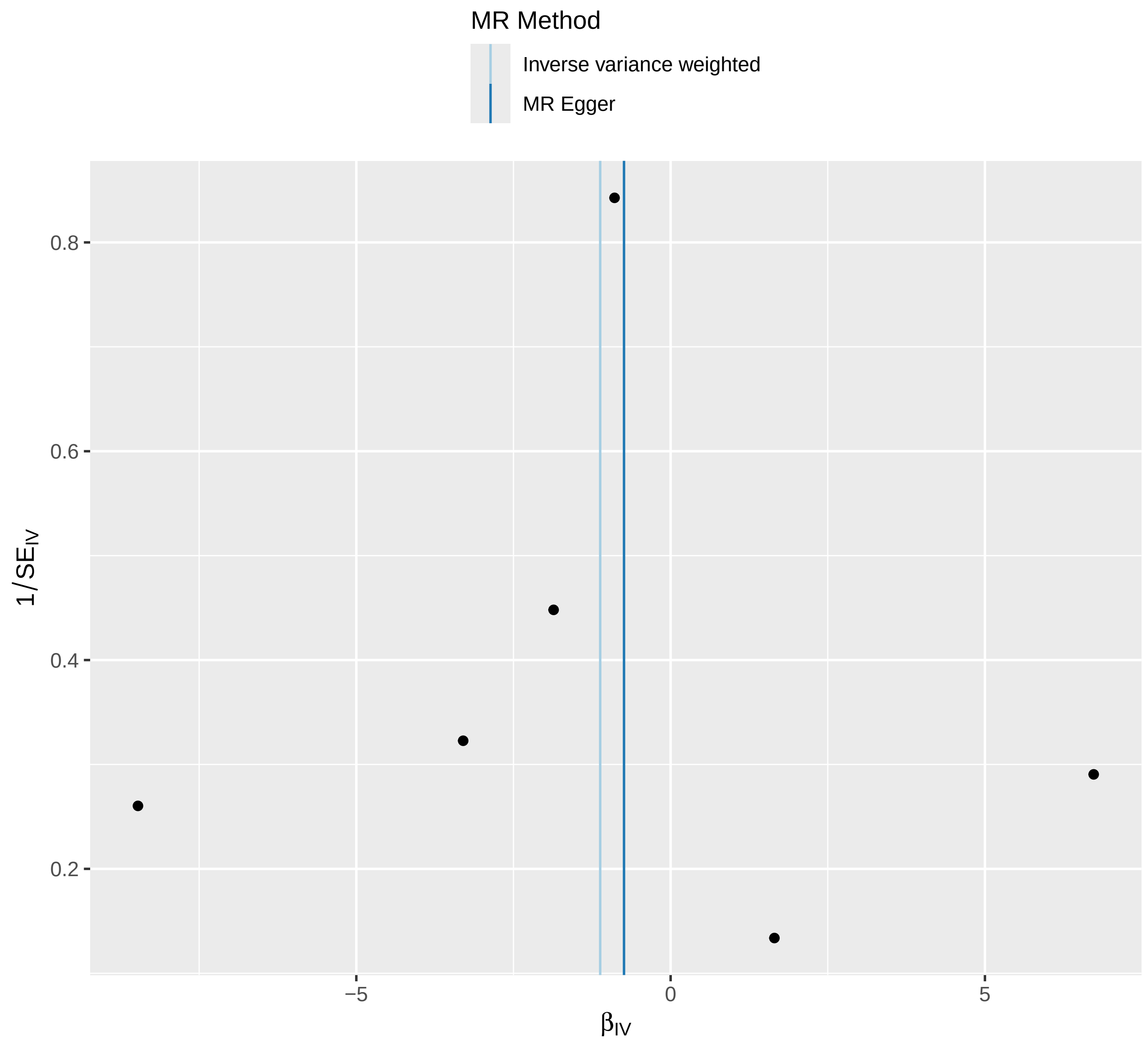

MR Method
Inverse variance weighted
MR Egger
0.8
0.6
0.4
0.2
−5
0
5
$1/SE_{IV}$
β_{IV}

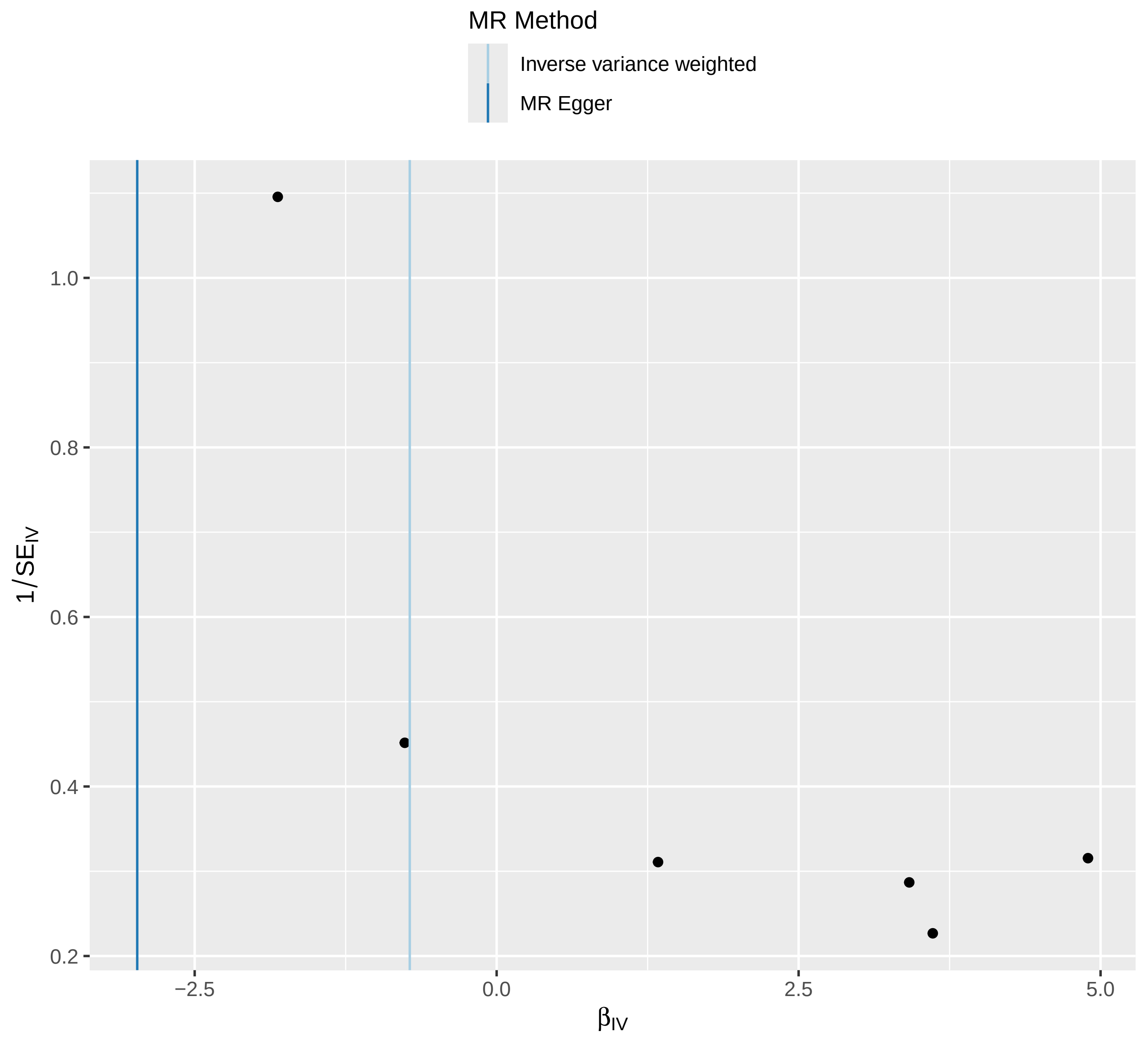
MR Method
Inverse variance weighted
MR Egger
1/SE$_{IV}$
1.0
0.8
0.6
0.4
0.2
−2.5
0.0
2.5
5.0
β$_{IV}$

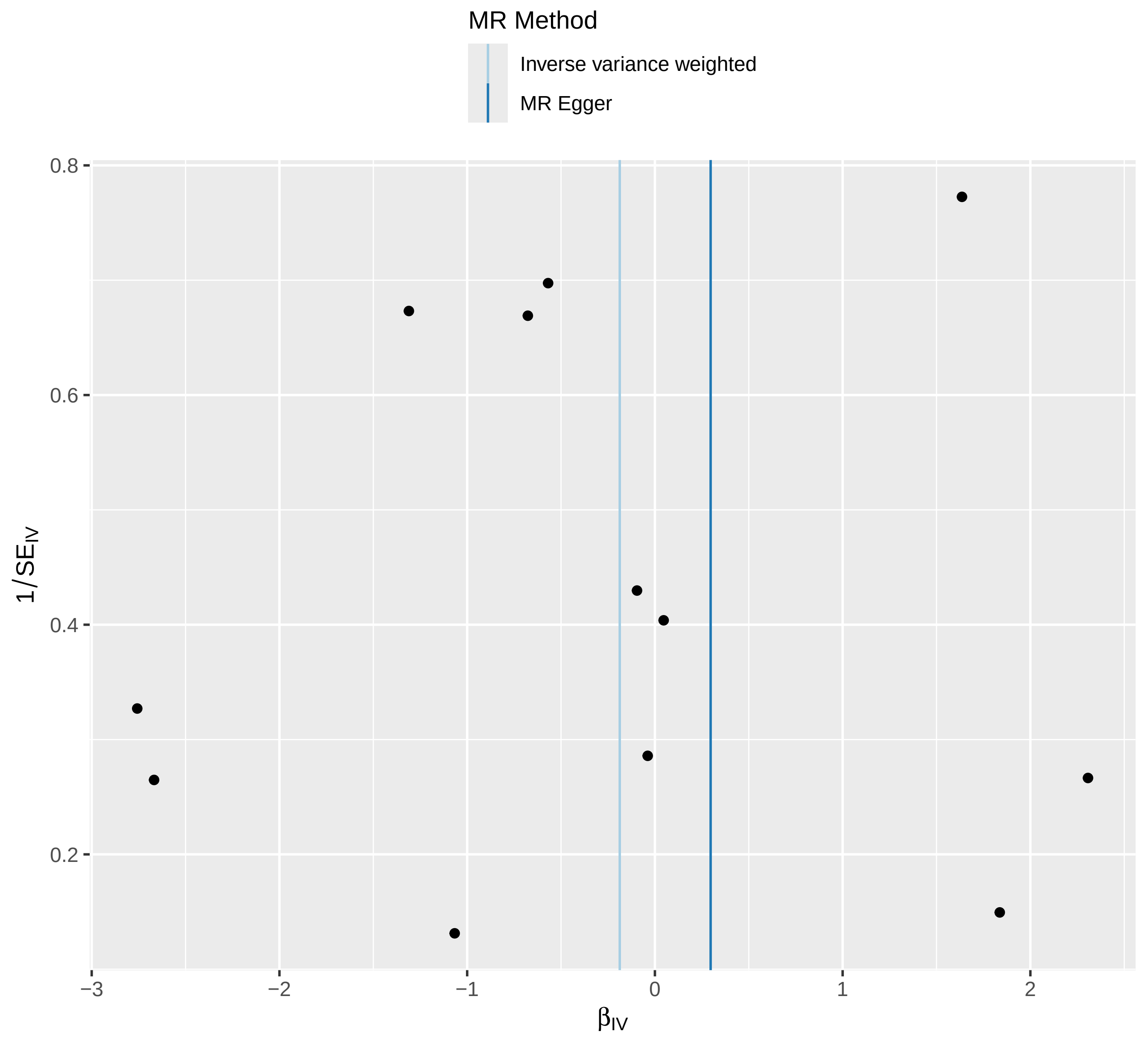

MR Method
Inverse variance weighted
MR Egger
0.8
0.6
0.4
0.2
1/SE$_{IV}$
−3
−2
−1
0
1
2
β$_{IV}$

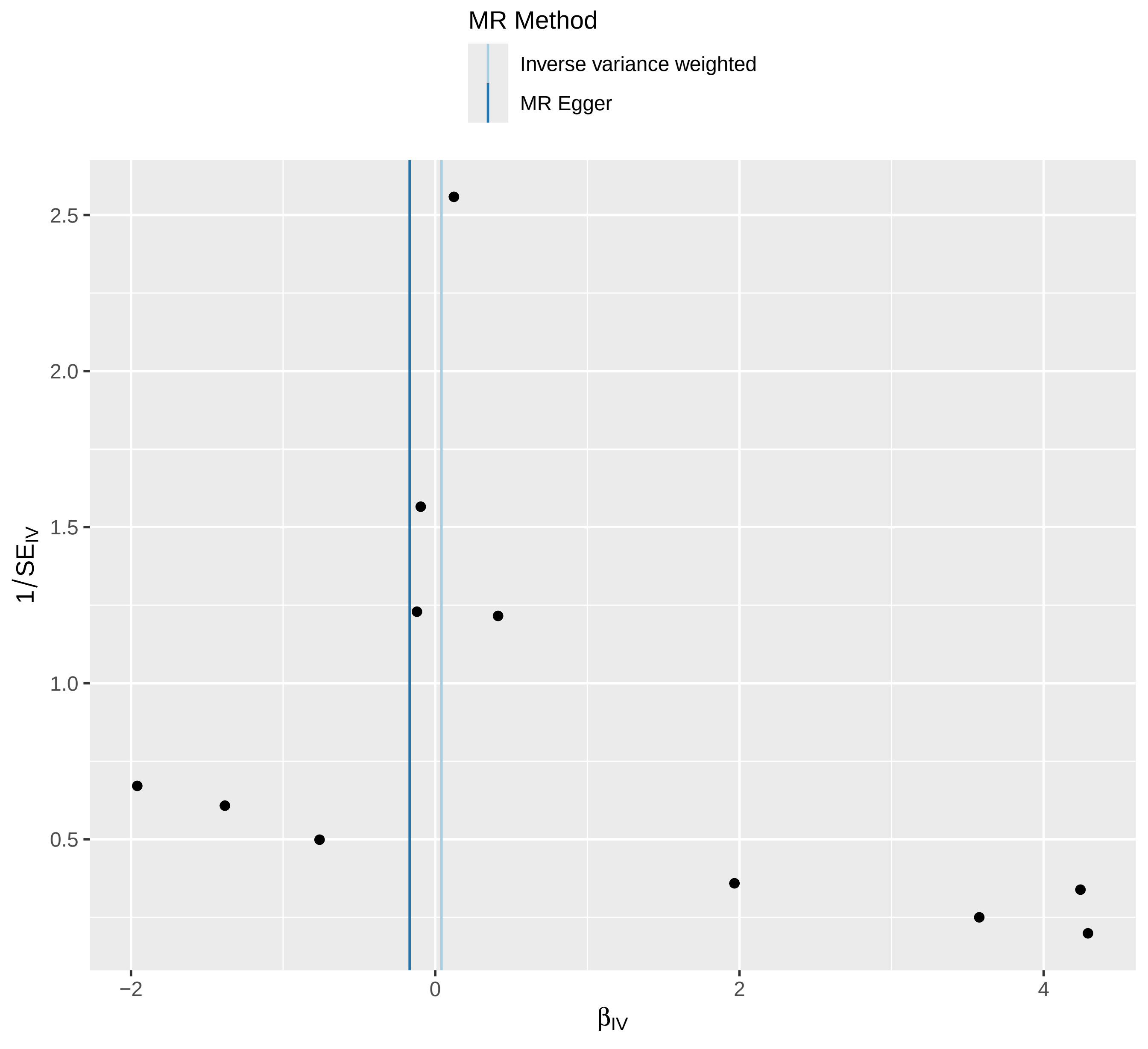
MR Method
Inverse variance weighted
MR Egger
2.5
2.0
1.5
1.0
0.5
1/SE_IV
−2
0
2
4
β_IV

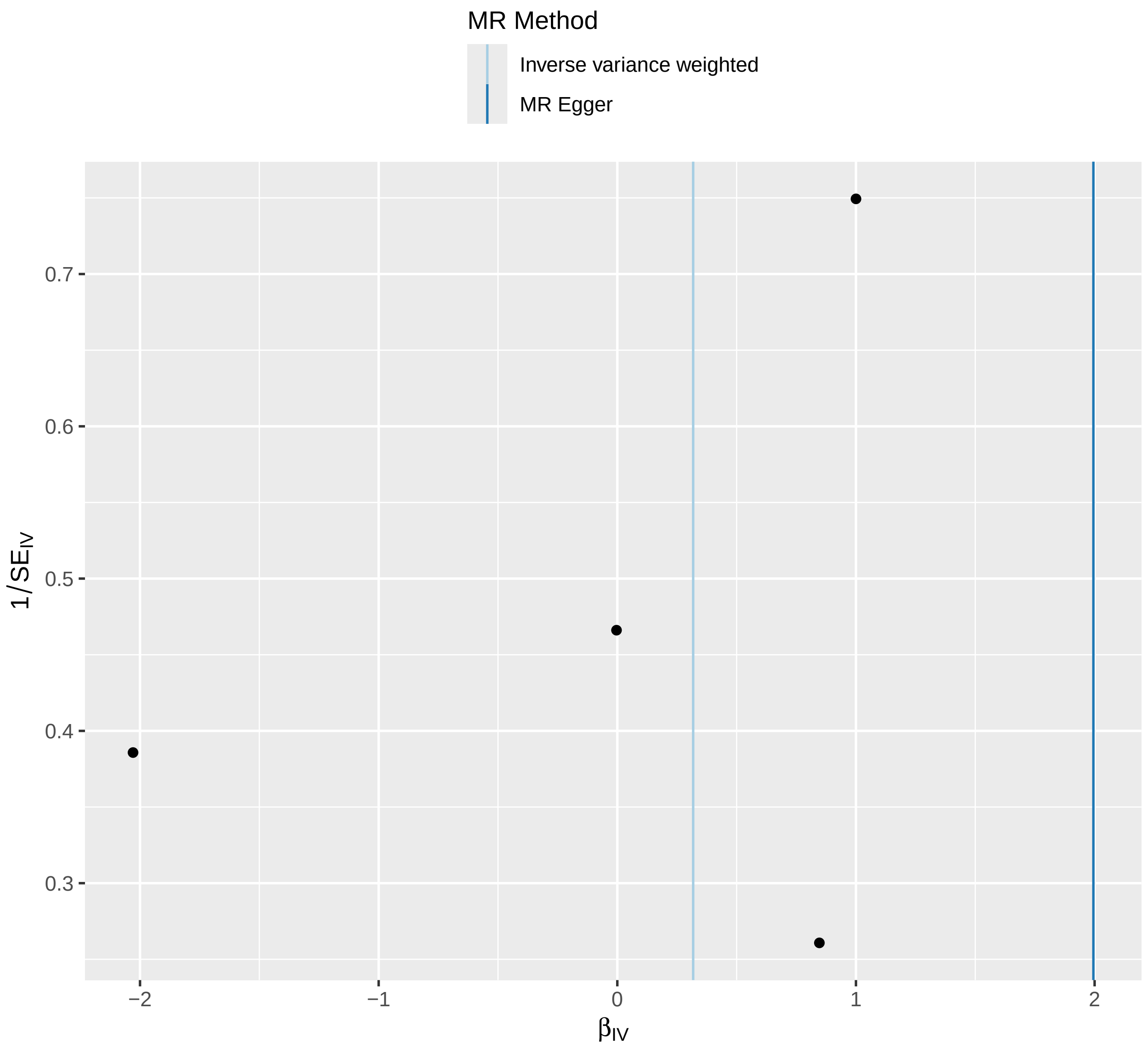
MR Method
Inverse variance weighted
MR Egger
0.7
0.6
0.5
0.4
0.3
1/SE_IV
−2
−1
0
1
2
β_IV

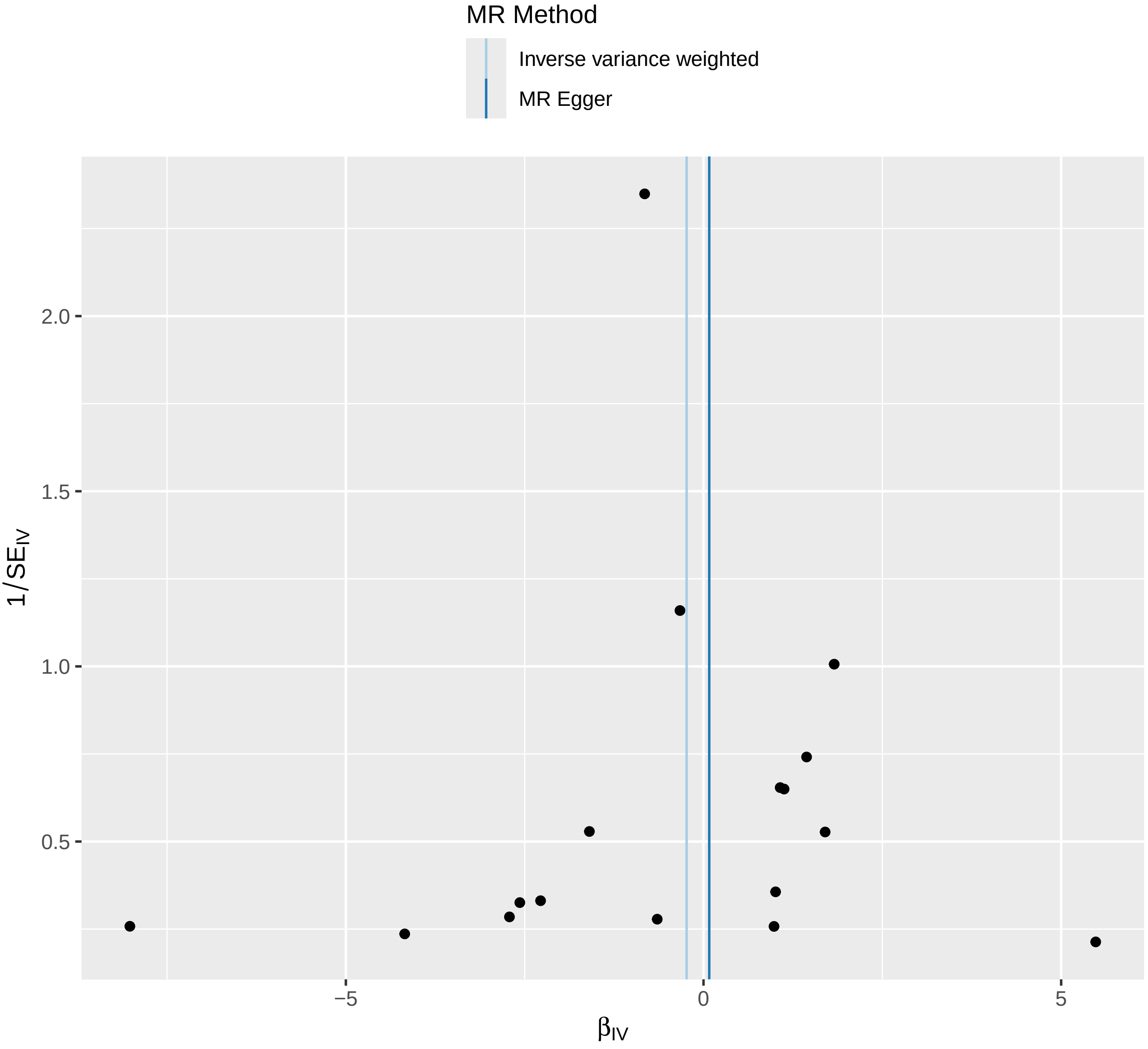
MR Method
Inverse variance weighted
MR Egger
2.0
1.5
1.0
0.5
$1/SE_{IV}$
−5
0
5
β_{IV}

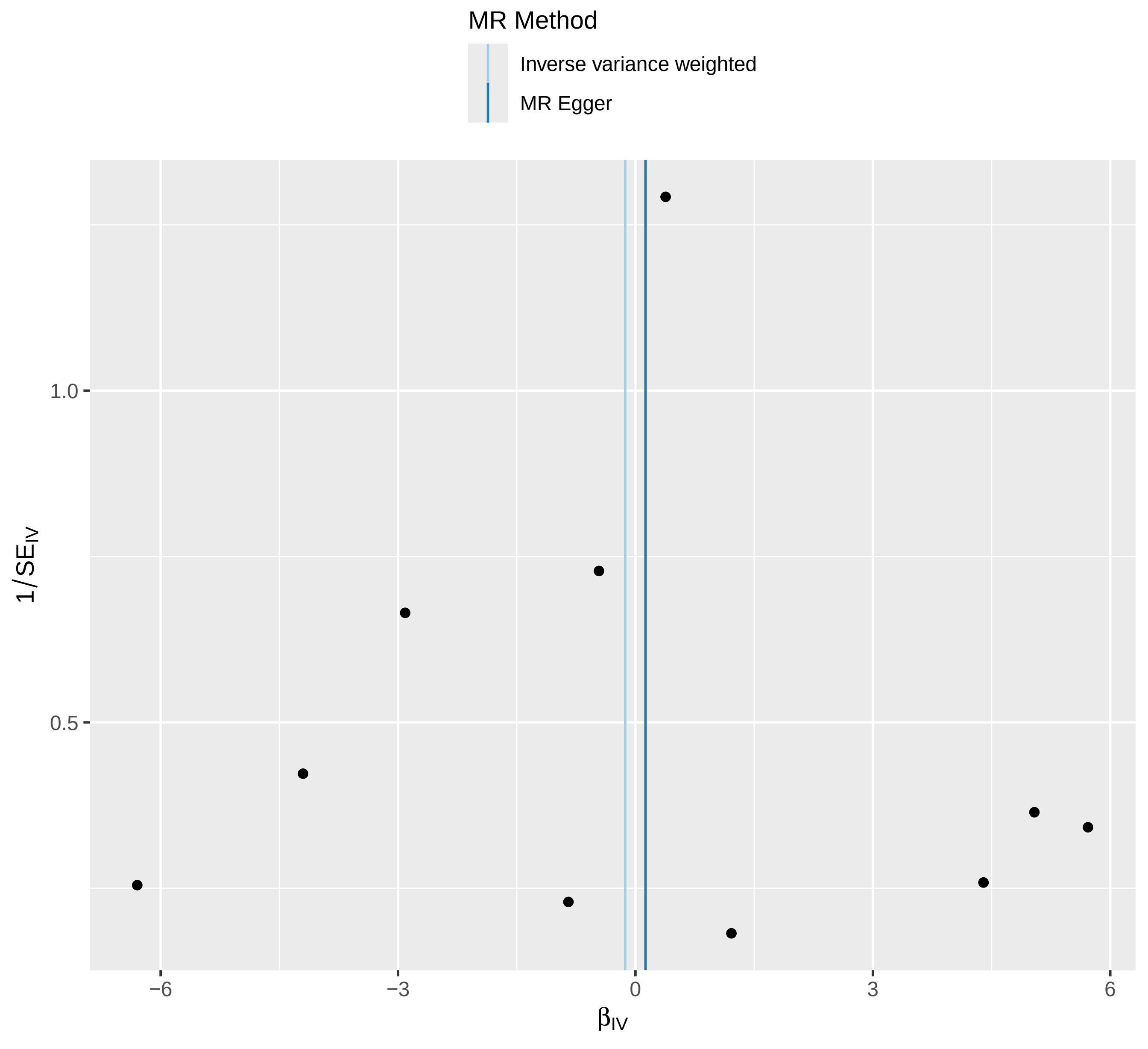

MR Method
Inverse variance weighted
MR Egger
1.0
0.5
1/SE$_{IV}$
−6
−3
0
3
6
β$_{IV}$

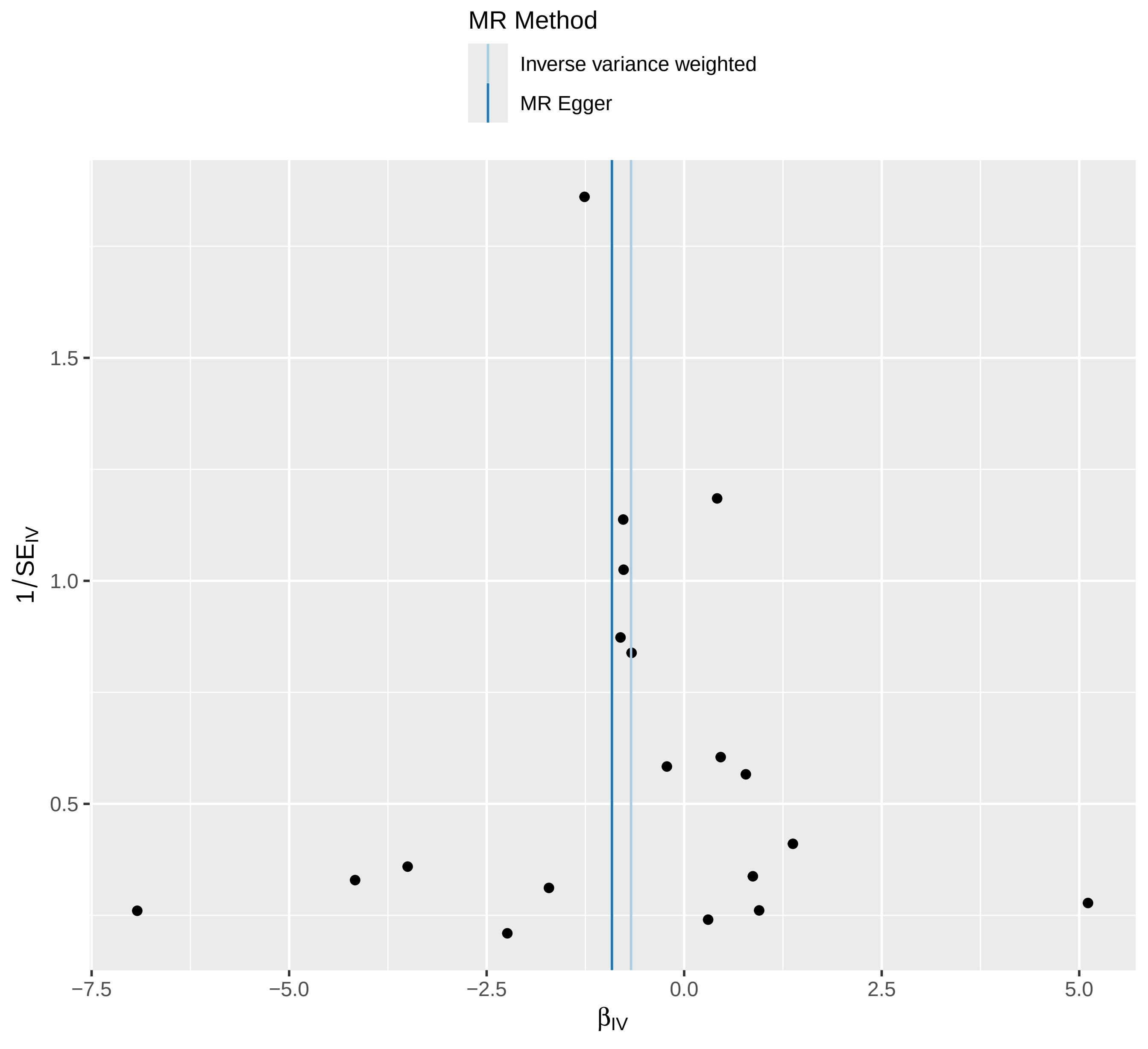

MR Method
Inverse variance weighted
MR Egger
1/SE_IV
1.5
1.0
0.5
−7.5
−5.0
−2.5
0.0
2.5
5.0
β_IV

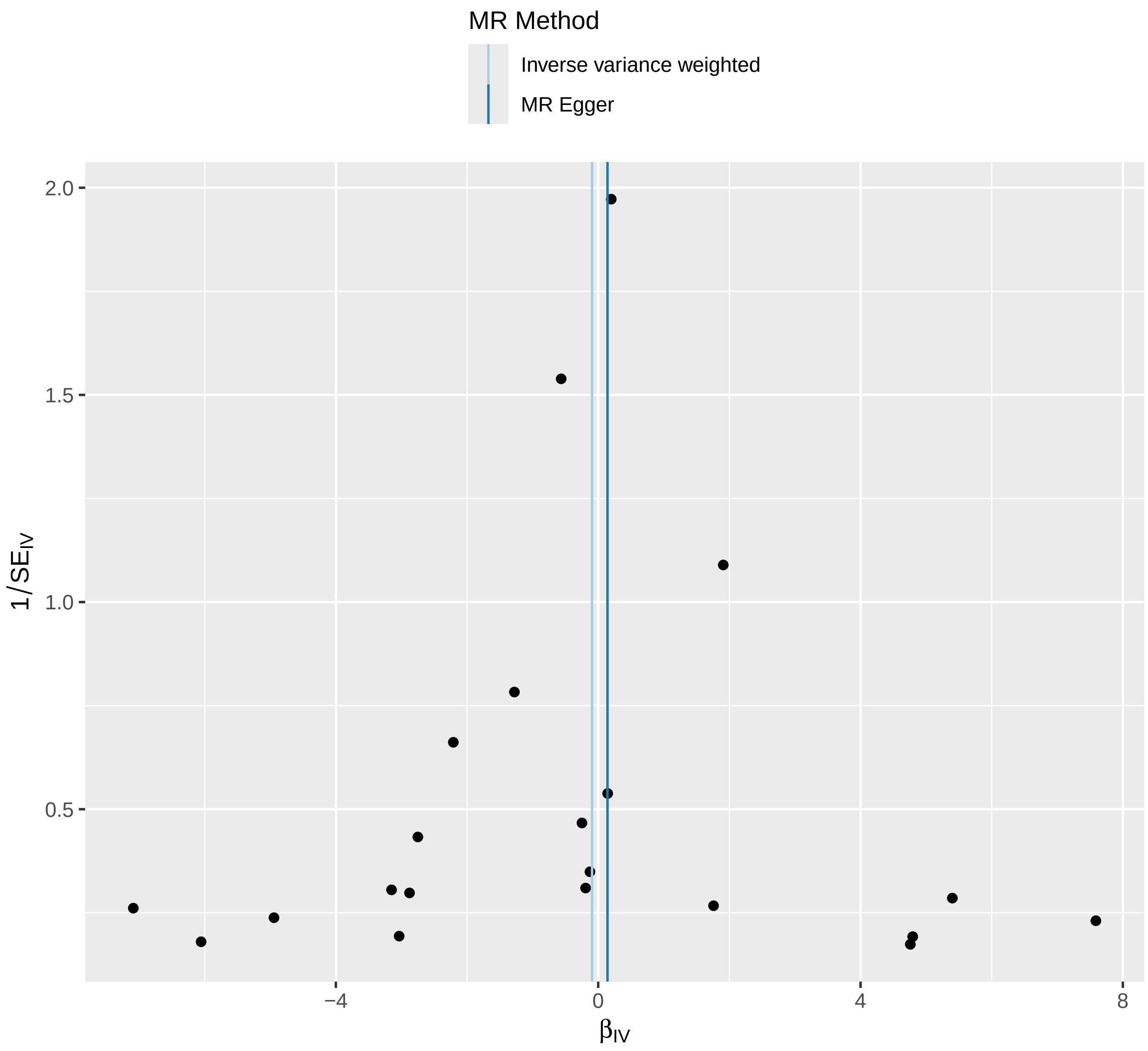
MR Method
Inverse variance weighted
MR Egger
2.0
1.5
1.0
0.5
1/SE$_{IV}$
−4
0
4
8
β$_{IV}$

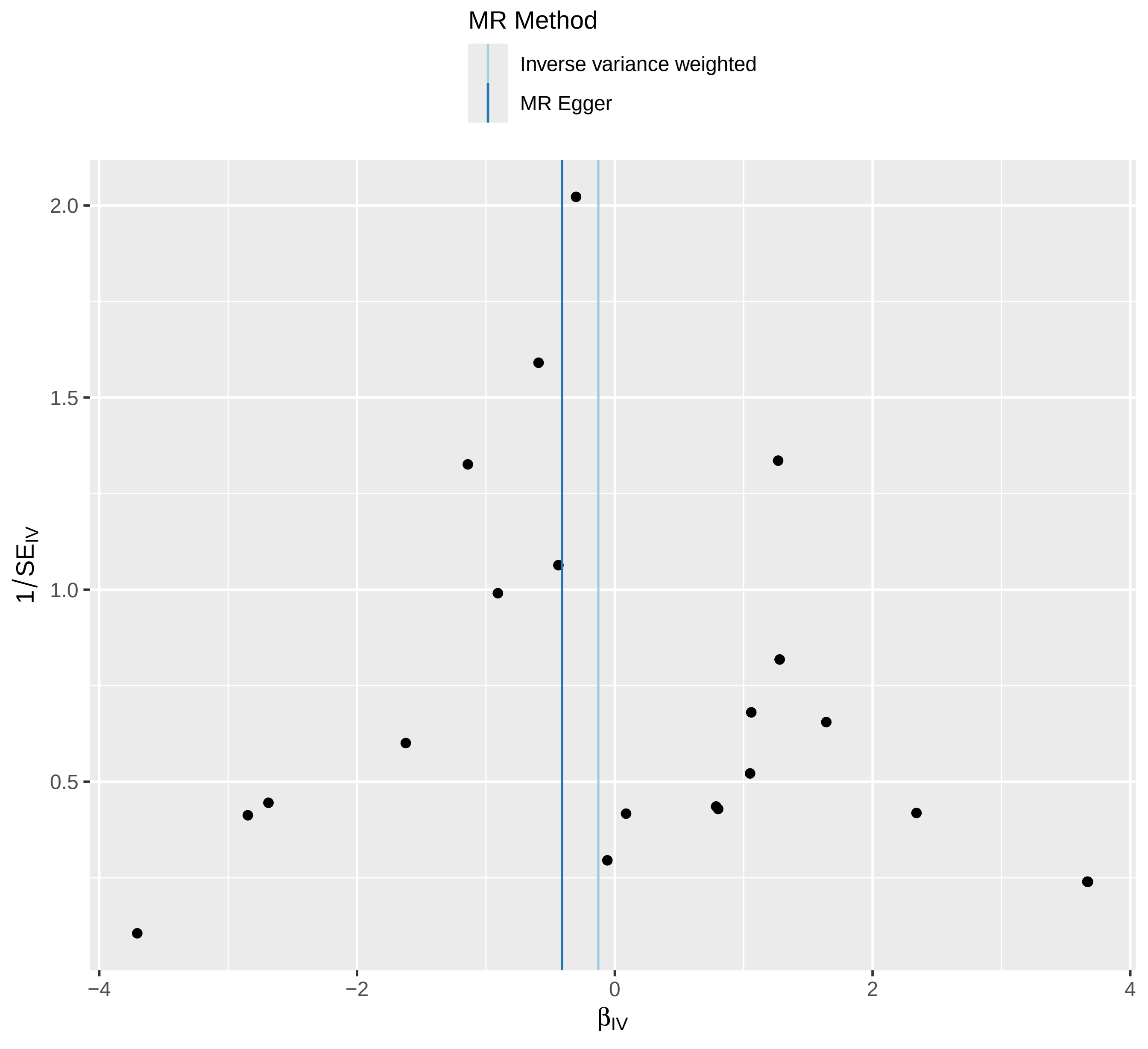

MR Method
Inverse variance weighted
MR Egger
2.0
1.5
1.0
0.5
$1/SE_{IV}$
−4
−2
0
2
4
β_{IV}

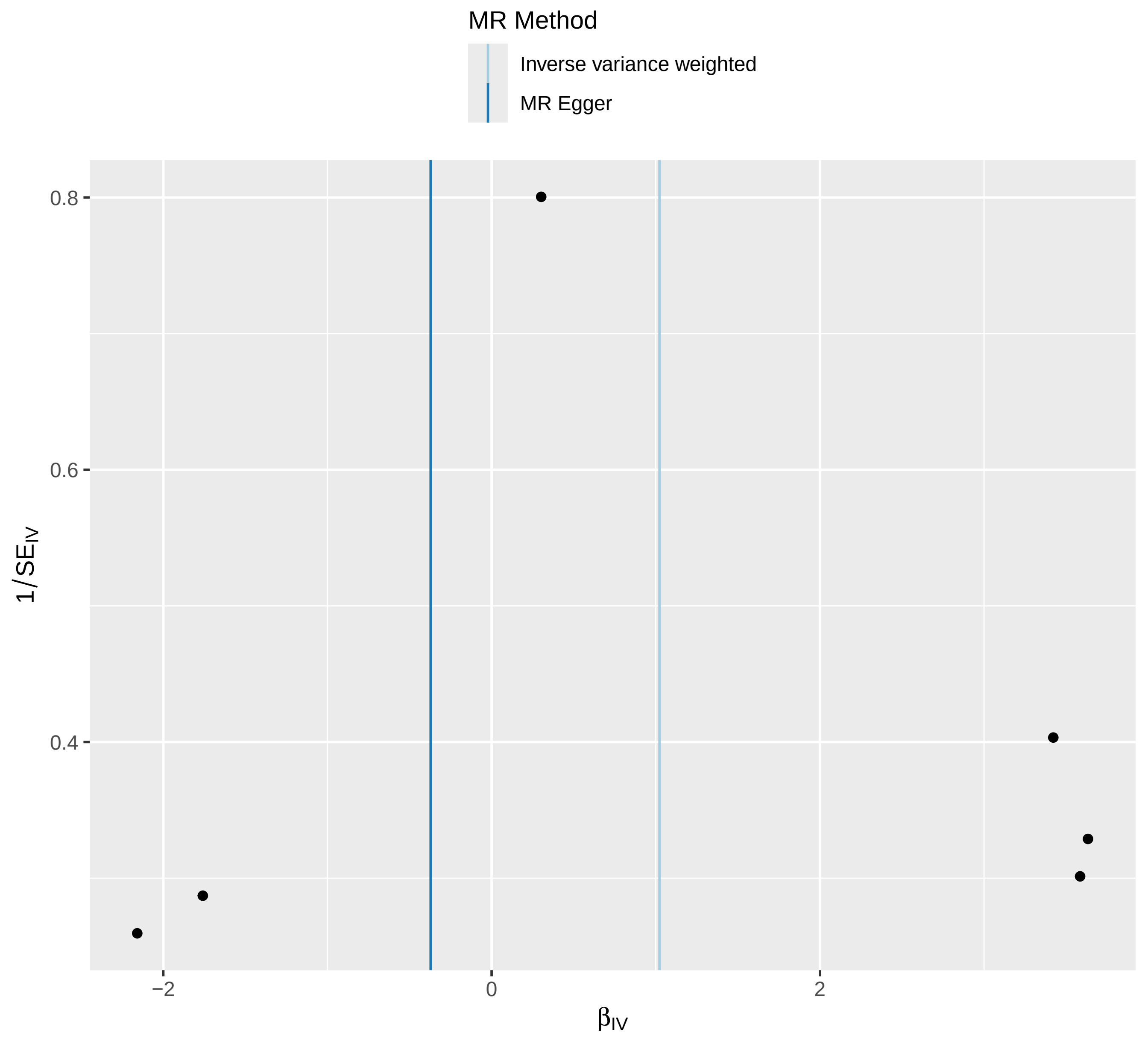
MR Method
Inverse variance weighted
MR Egger
0.8
0.6
0.4
1/SE_IV
−2
0
2
β_IV

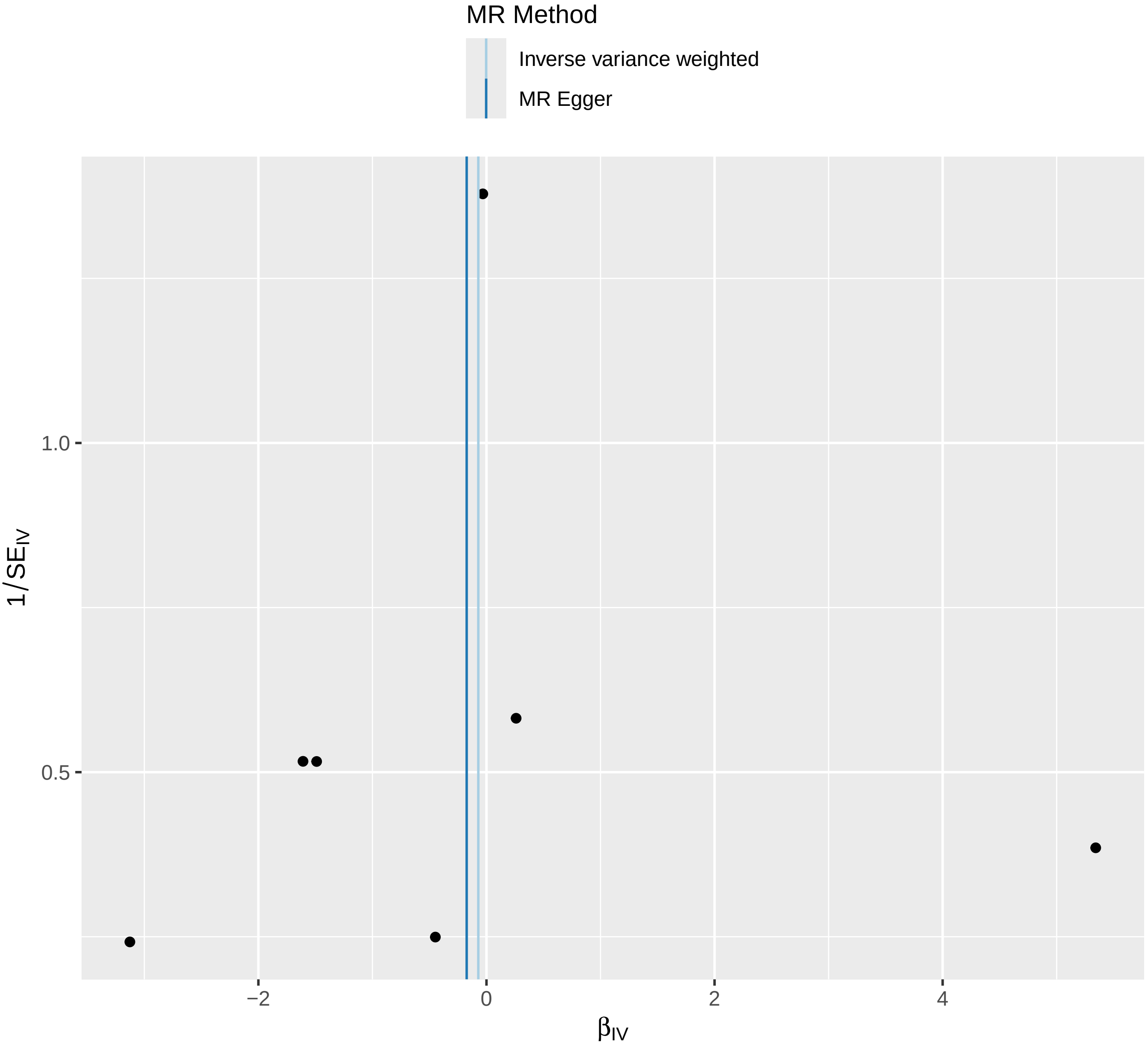
MR Method
Inverse variance weighted
MR Egger
1.0
0.5
$1/SE_{IV}$
−2
0
2
4
β_{IV}

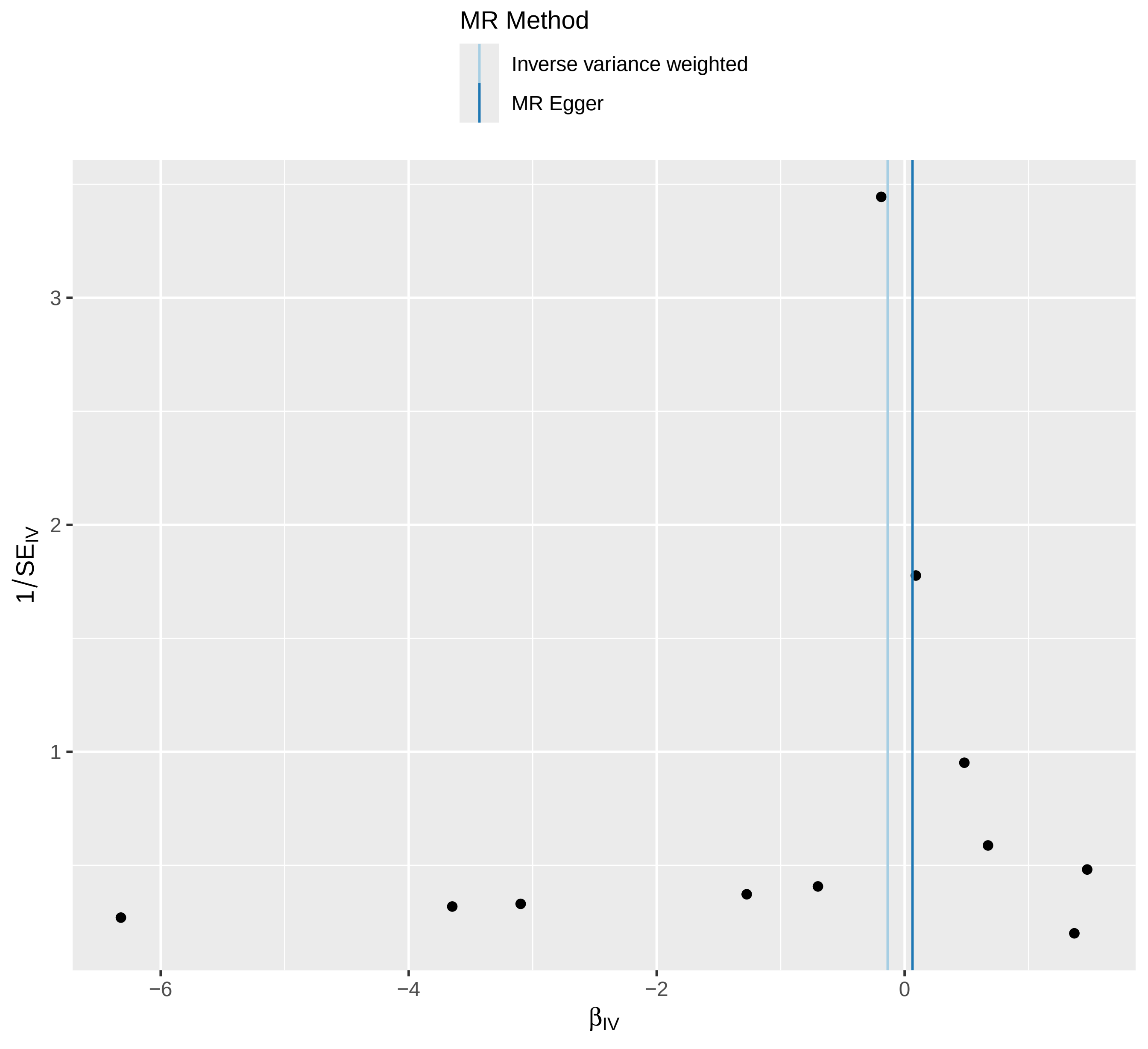
MR Method
Inverse variance weighted
MR Egger
3
2
1
1/SE_IV
−6
−4
−2
0
β_IV

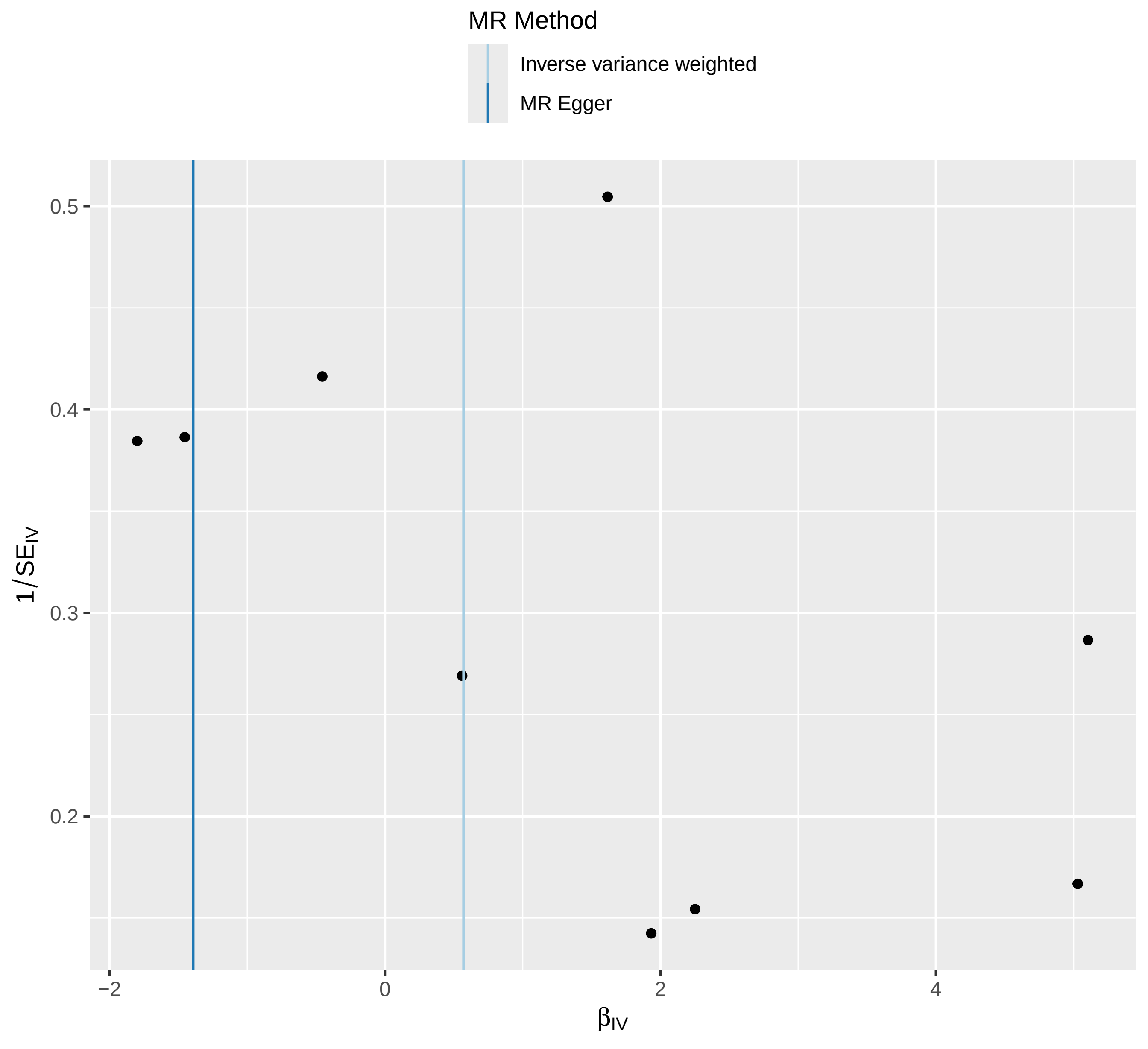

MR Method
Inverse variance weighted
MR Egger
0.5
0.4
0.3
0.2
1/SE_IV
−2
0
2
4
β_IV

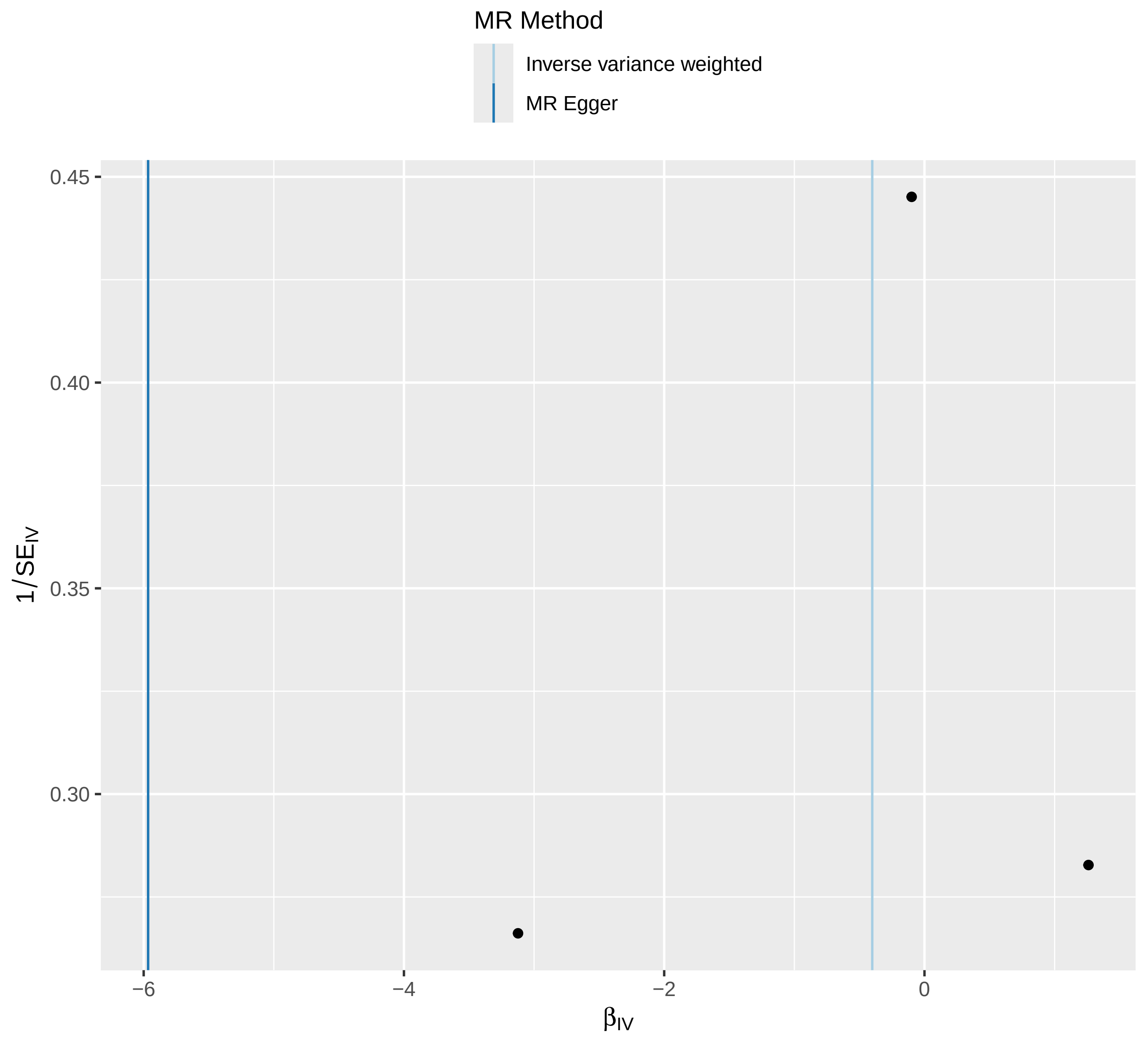

MR Method
Inverse variance weighted
MR Egger
0.45
0.40
0.35
0.30
$1/SE_{IV}$
−6
−4
−2
0
β_{IV}

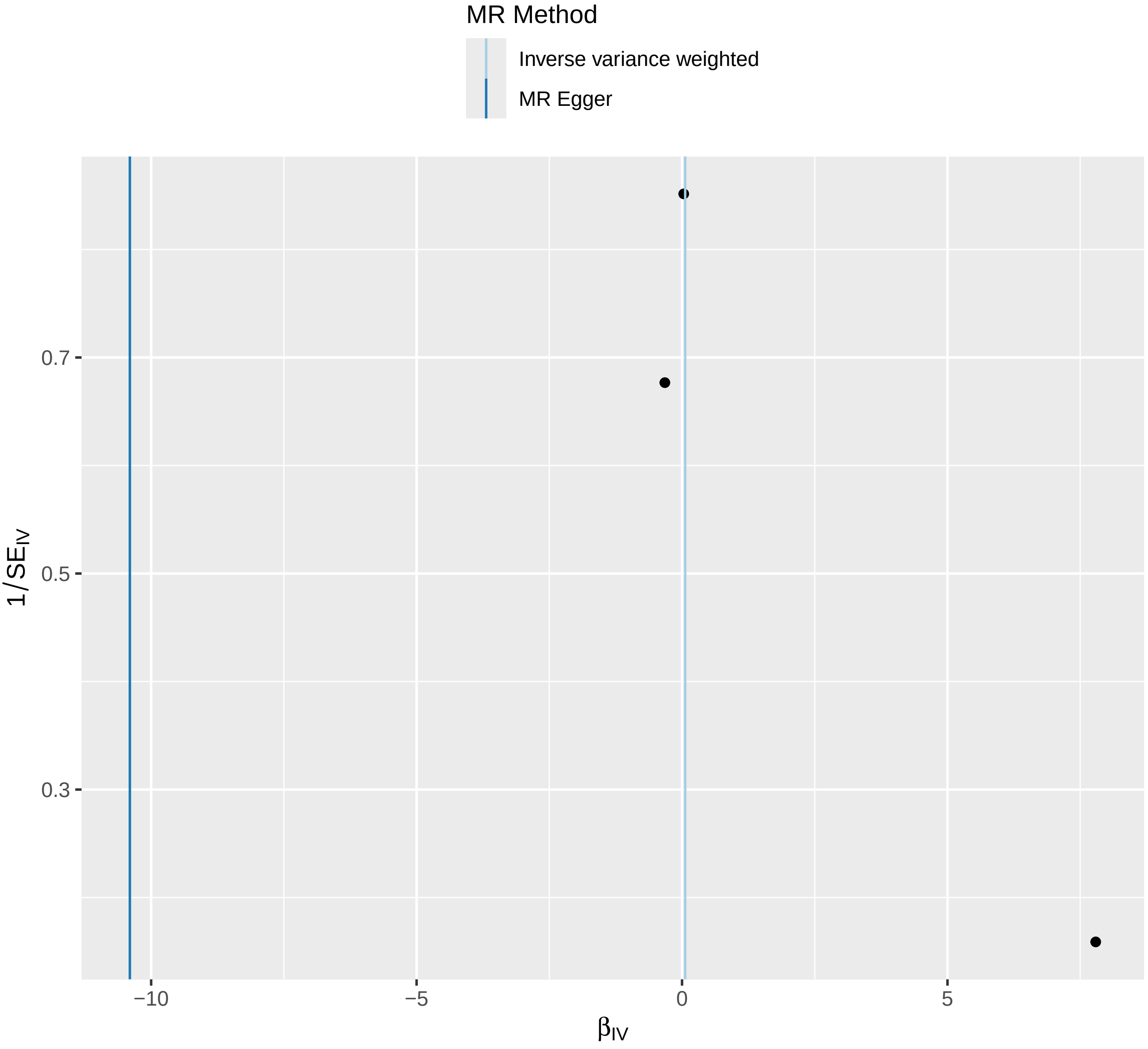
MR Method
Inverse variance weighted
MR Egger
0.7
0.5
0.3
$1/SE_{IV}$
−10
−5
0
5
β_{IV}

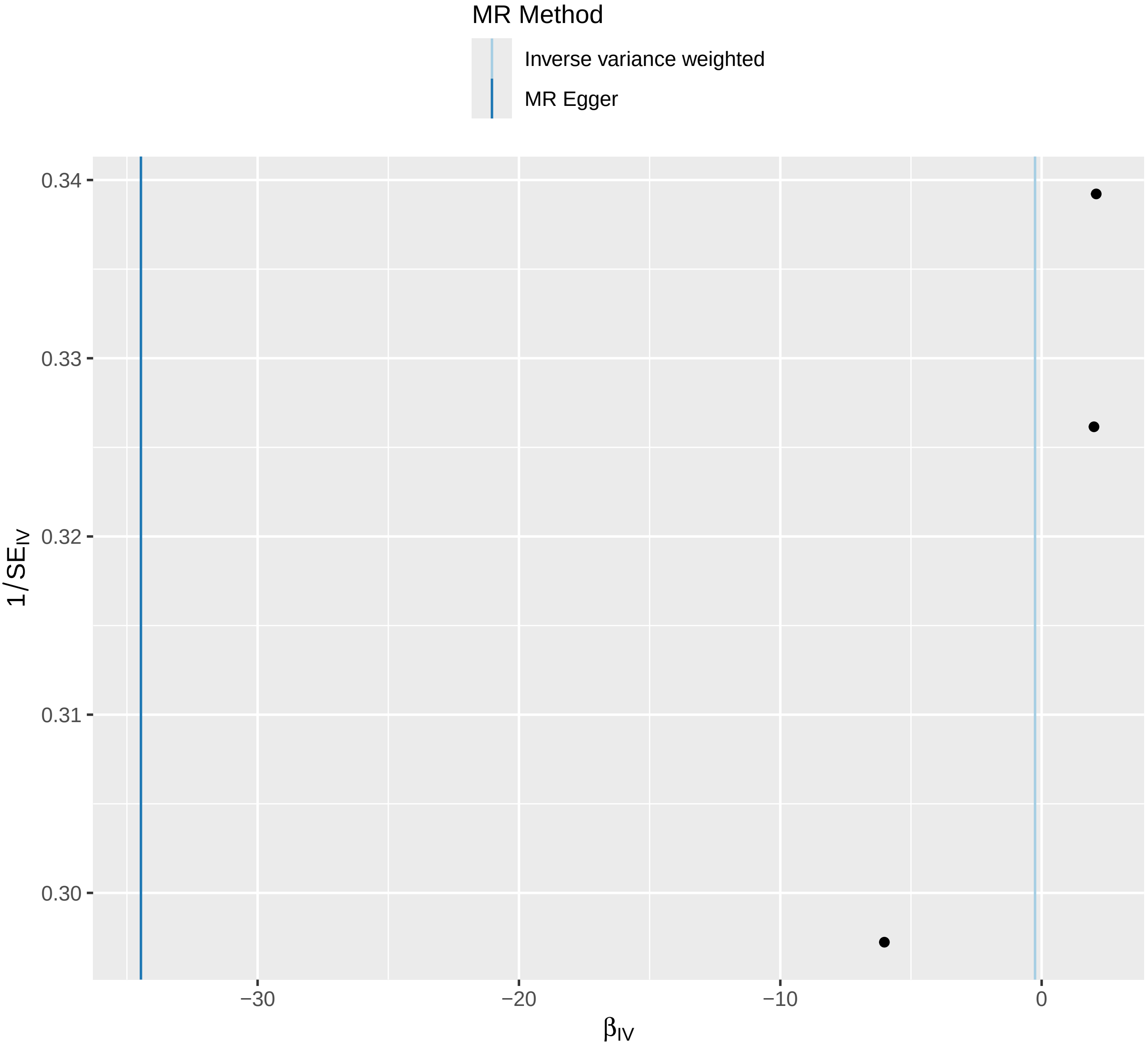

MR Method
Inverse variance weighted
MR Egger
0.34
0.33
0.32
0.31
0.30
1/SE_IV
−30
−20
−10
0
β_IV

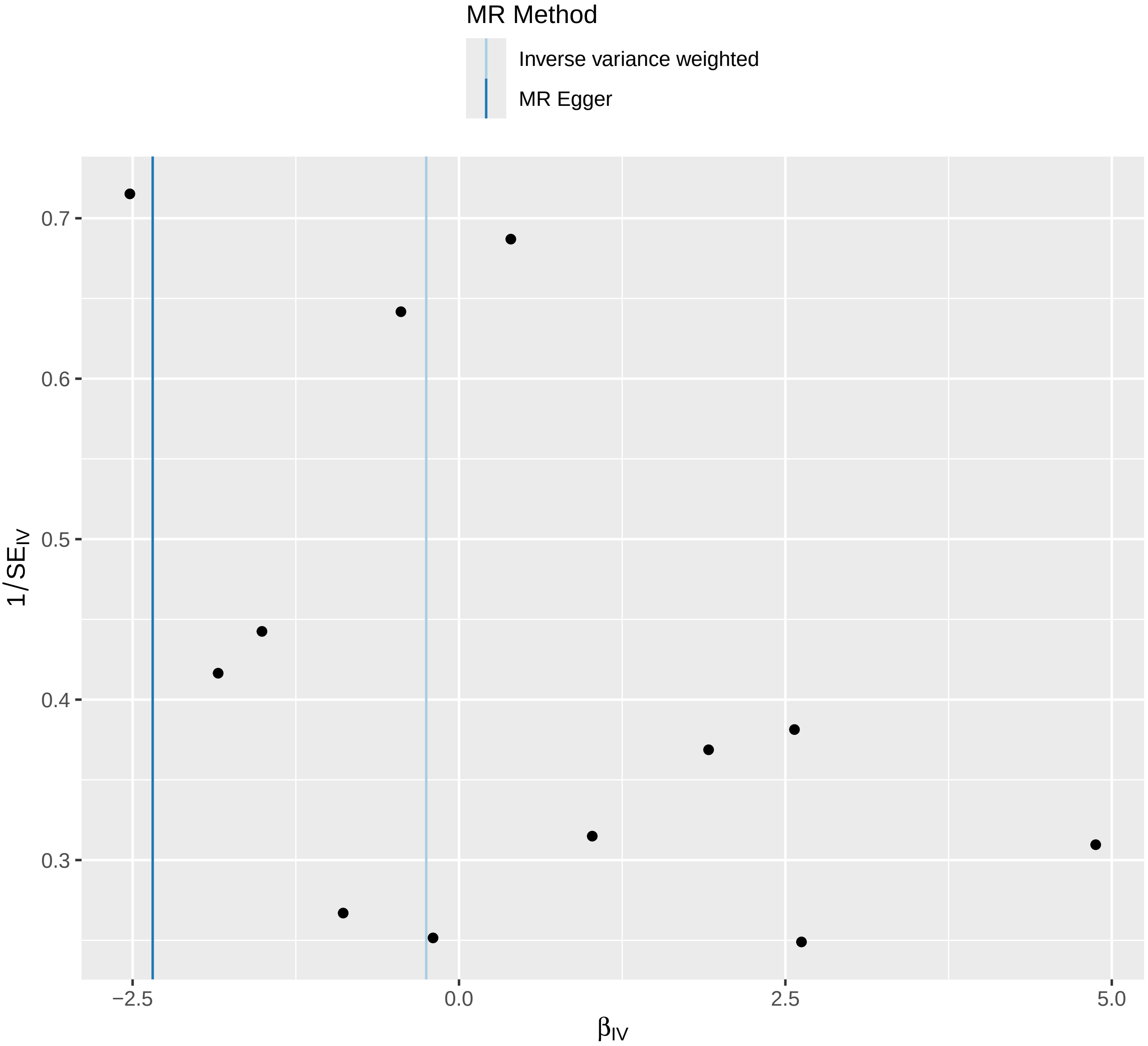
MR Method
Inverse variance weighted
MR Egger
0.7
0.6
0.5
0.4
0.3
1/SE_IV
−2.5
0.0
2.5
5.0
β_IV

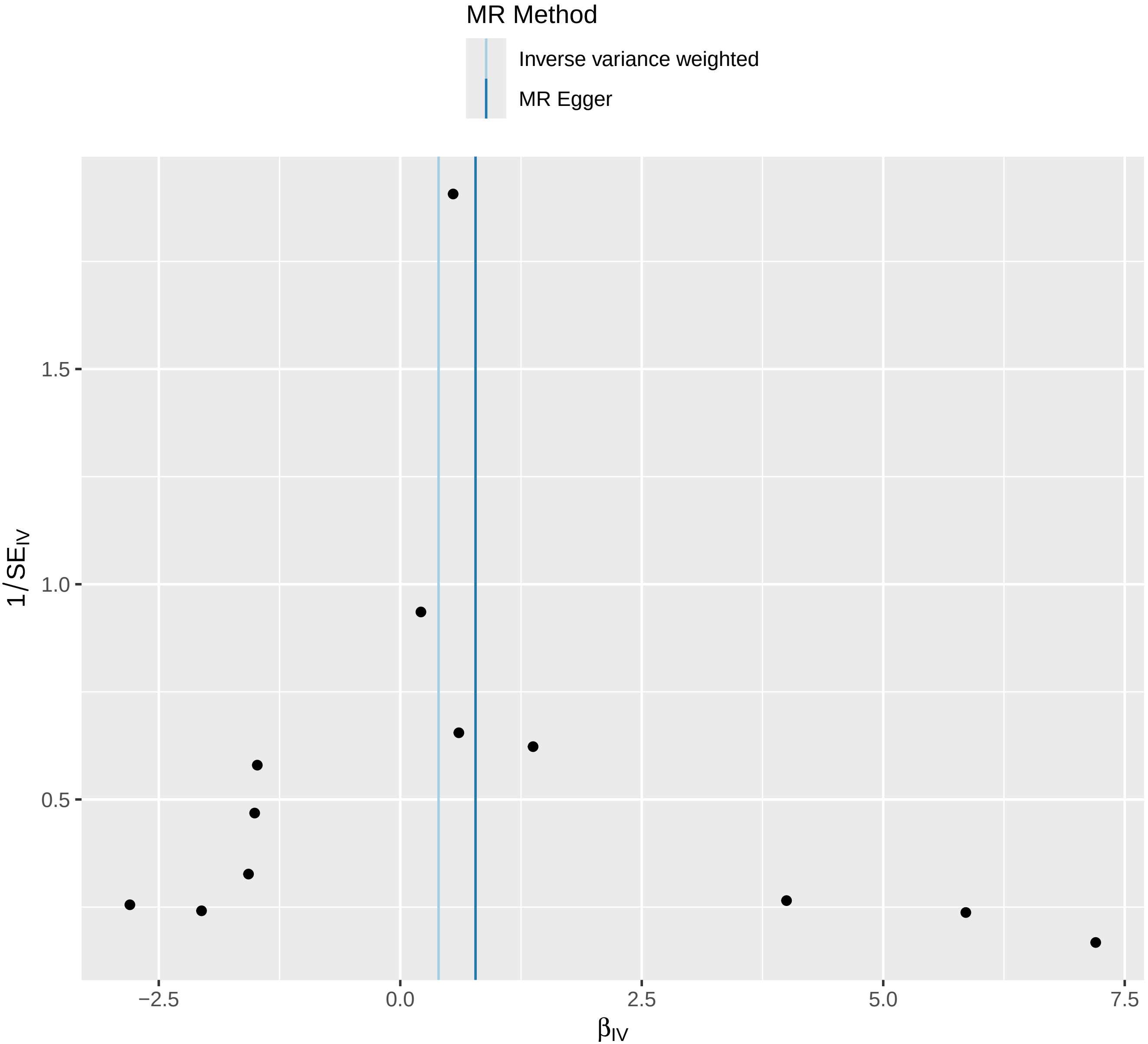
MR Method
Inverse variance weighted
MR Egger
1.5
1.0
0.5
1/SE_IV
−2.5
0.0
2.5
5.0
7.5
β_IV

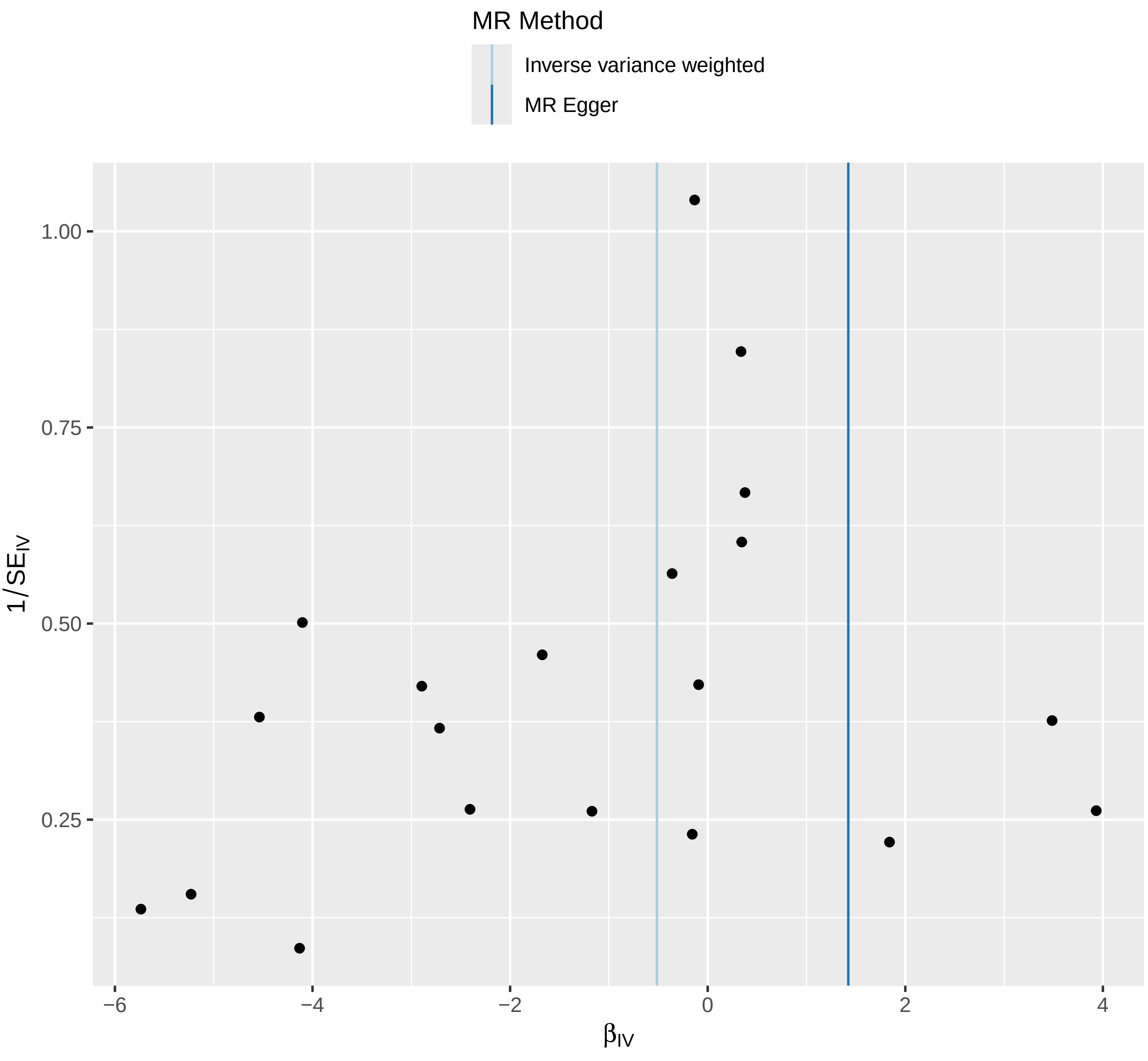
MR Method
Inverse variance weighted
MR Egger
1/SE_IV
1.00
0.75
0.50
0.25
−6
−4
−2
0
2
4
β_IV

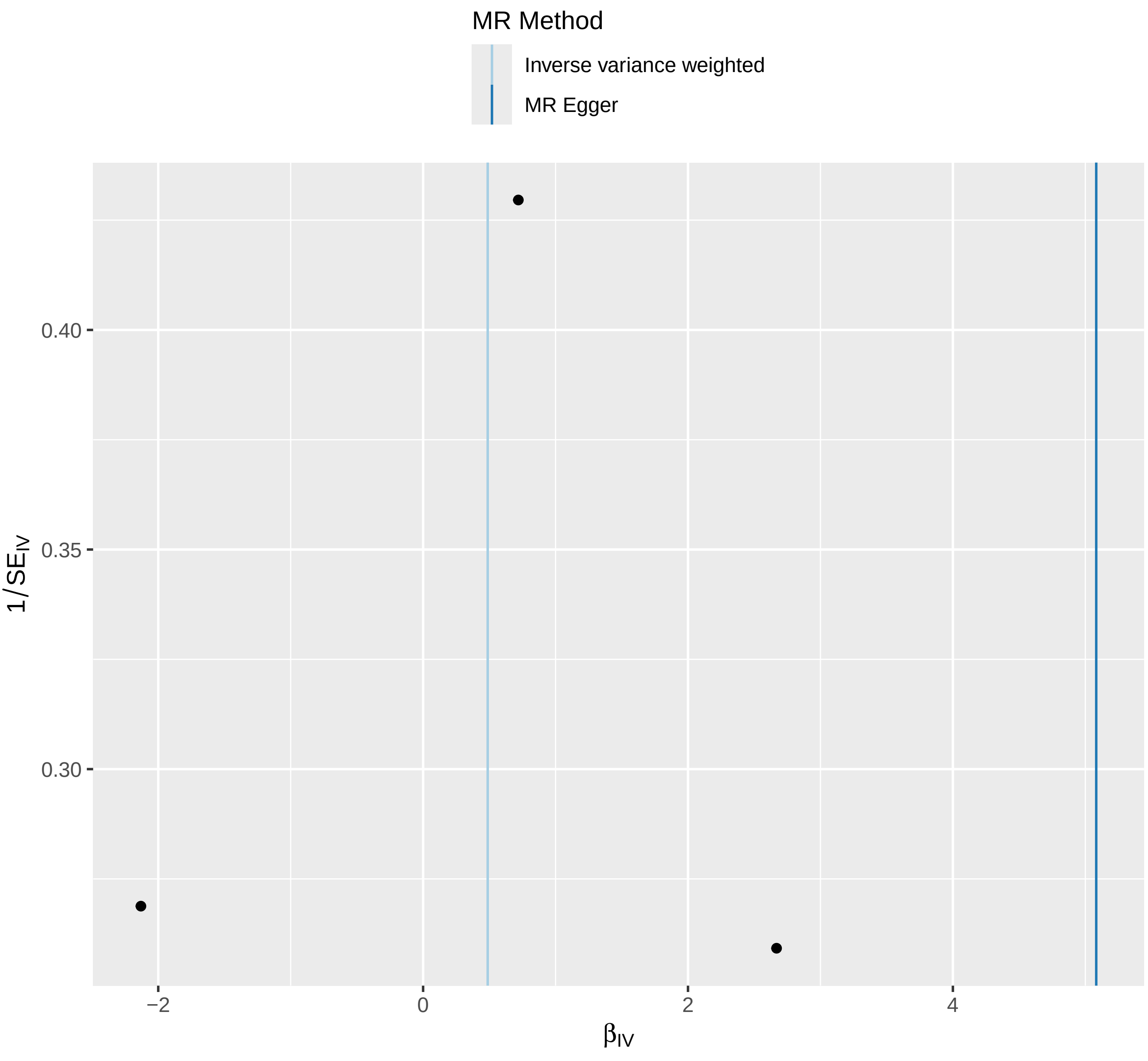
MR Method
Inverse variance weighted
MR Egger
0.40
0.35
0.30
$1/SE_{IV}$
−2
0
2
4
β_{IV}

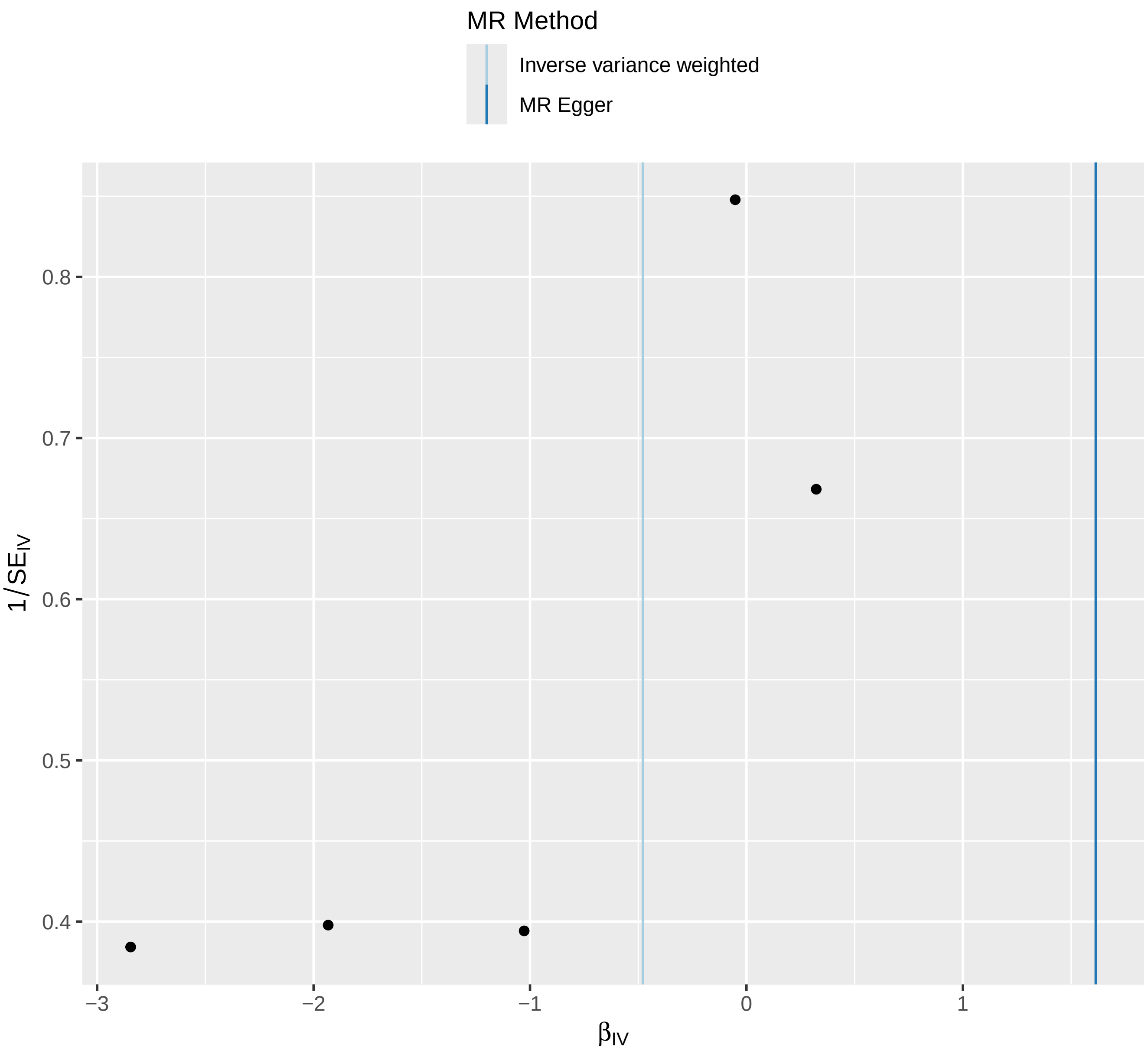
MR Method
Inverse variance weighted
MR Egger
0.8
0.7
0.6
0.5
0.4
1/SE_IV
−3
−2
−1
0
1
β_IV

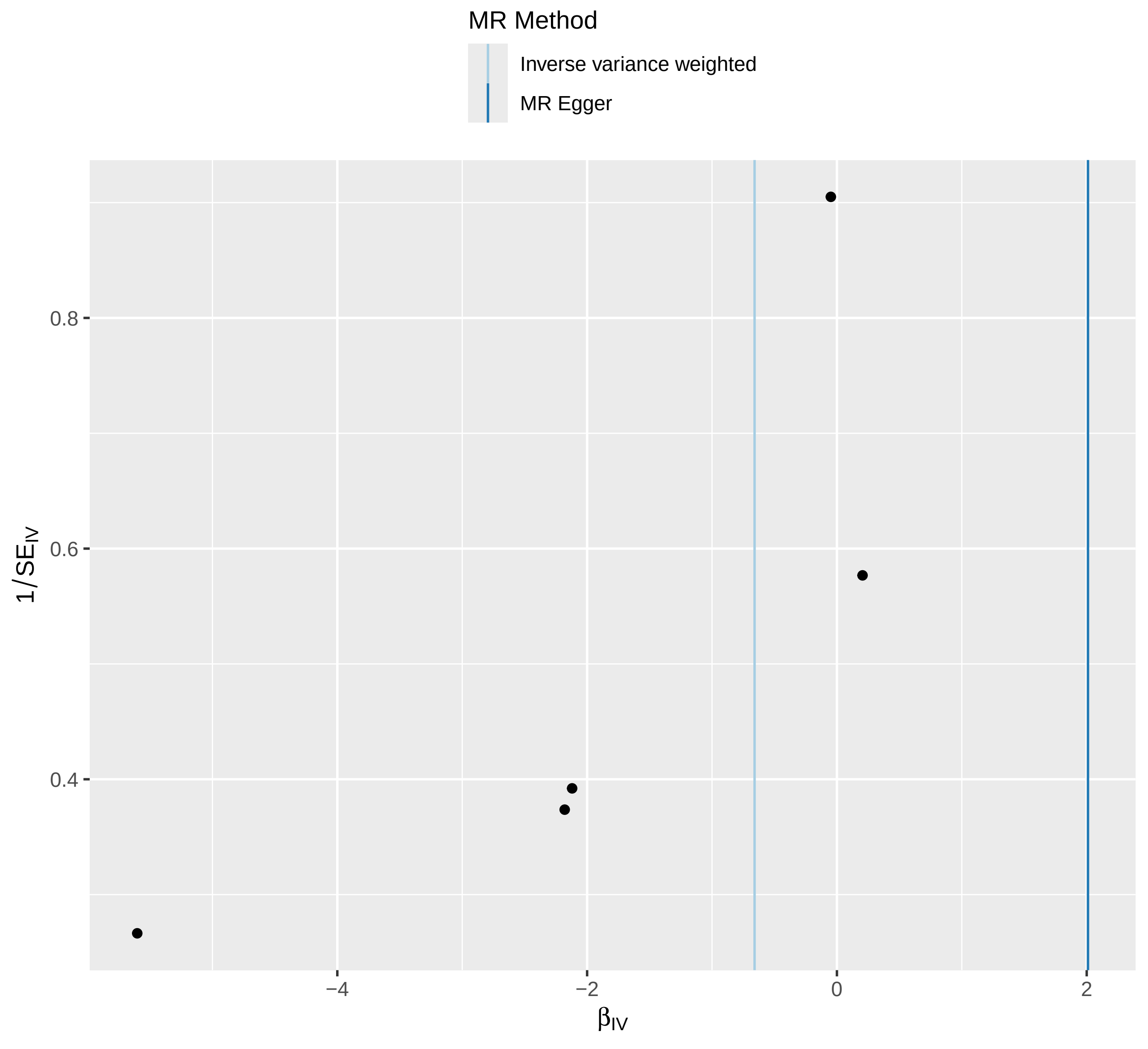
MR Method
Inverse variance weighted
MR Egger
0.8
0.6
0.4
−4
−2
0
2
$1/SE_{IV}$
β_{IV}

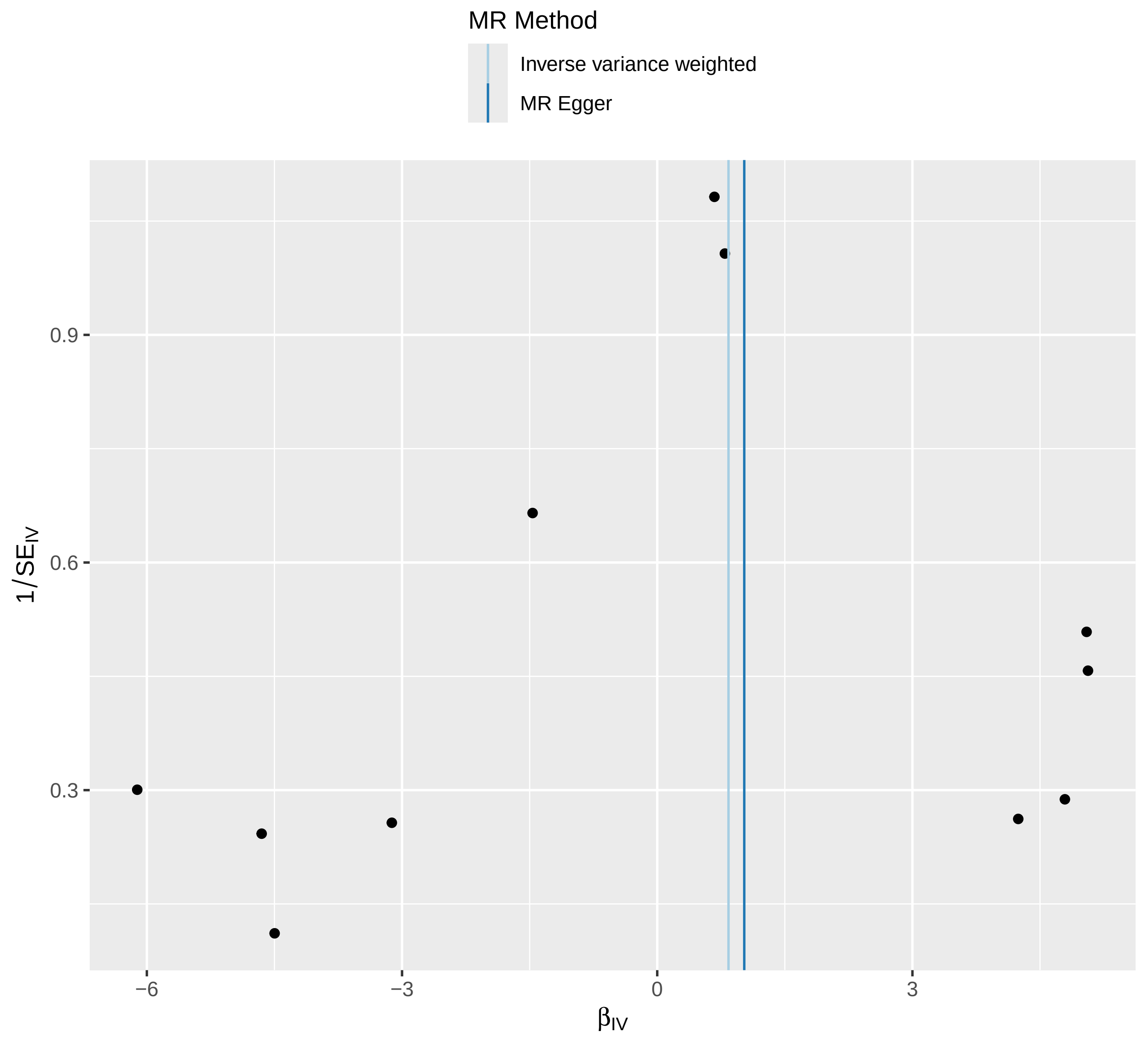
MR Method
Inverse variance weighted
MR Egger
0.9
0.6
0.3
$1/SE_{IV}$
−6
−3
0
3
β_{IV}

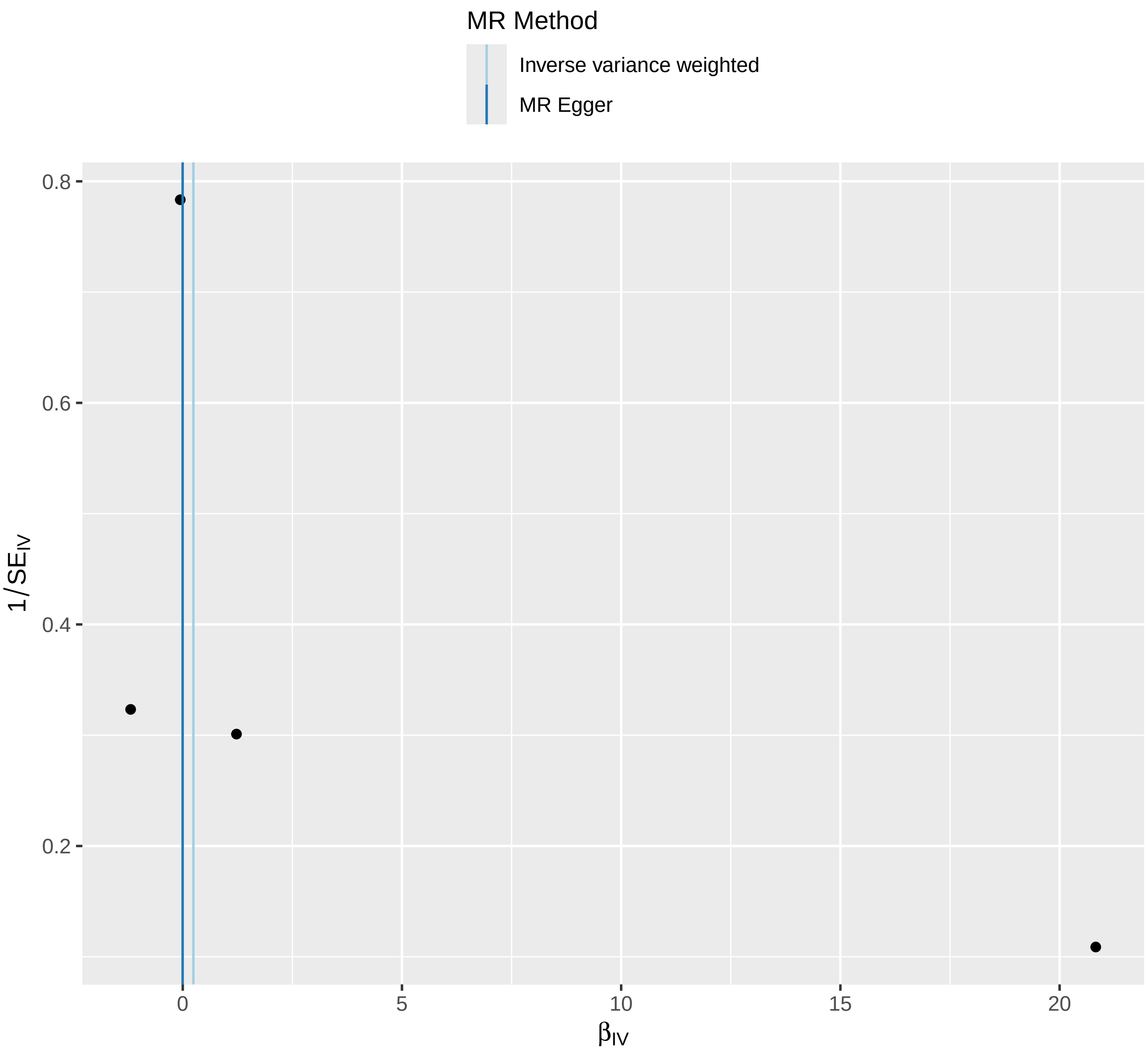
MR Method
Inverse variance weighted
MR Egger
0.8
0.6
0.4
0.2
$1/SE_{IV}$
0
5
10
15
20
β_{IV}

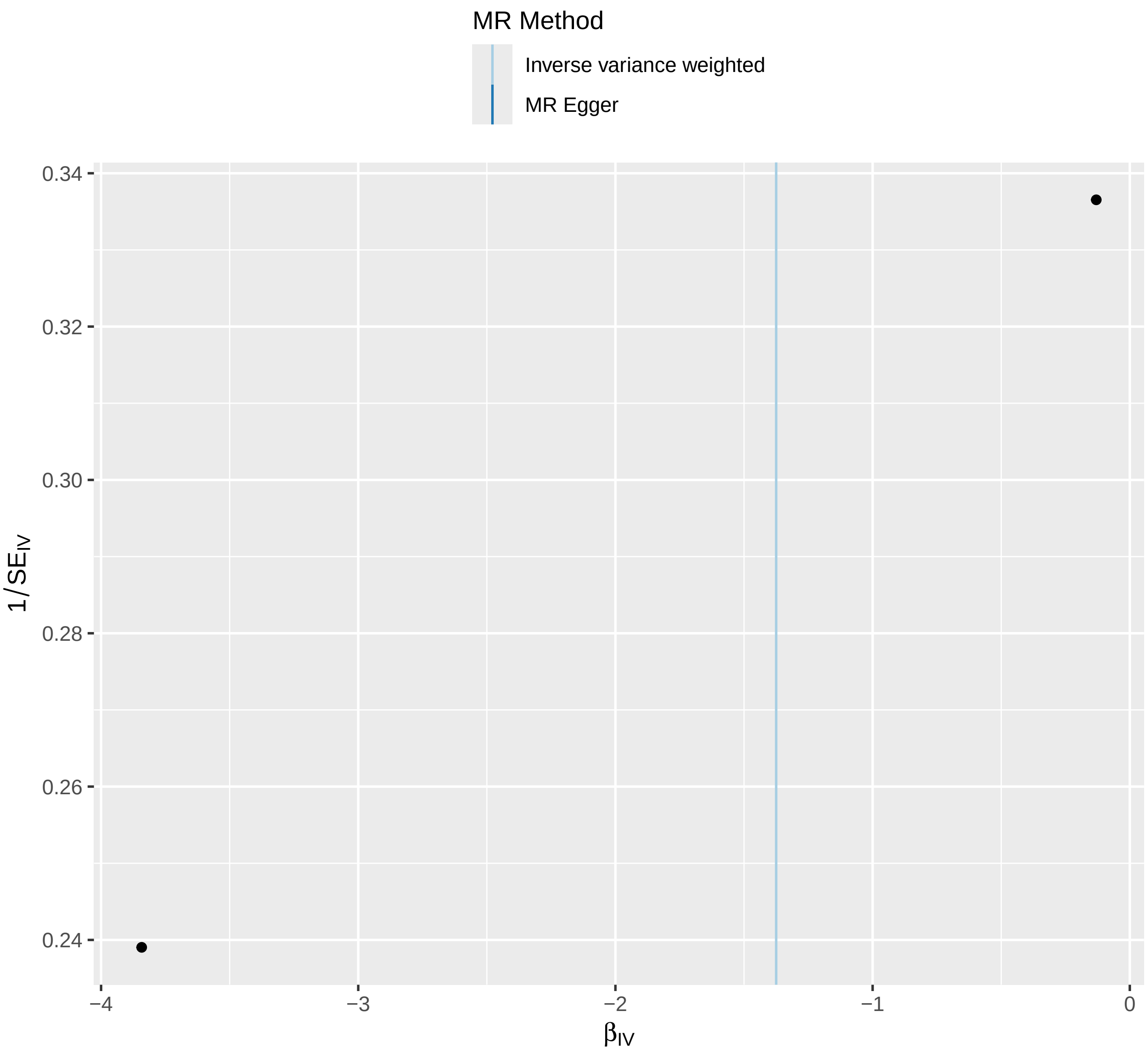

MR Method
Inverse variance weighted
MR Egger
1/SE_IV
β_IV
0.34
0.32
0.30
0.28
0.26
0.24
−4
−3
−2
−1
0

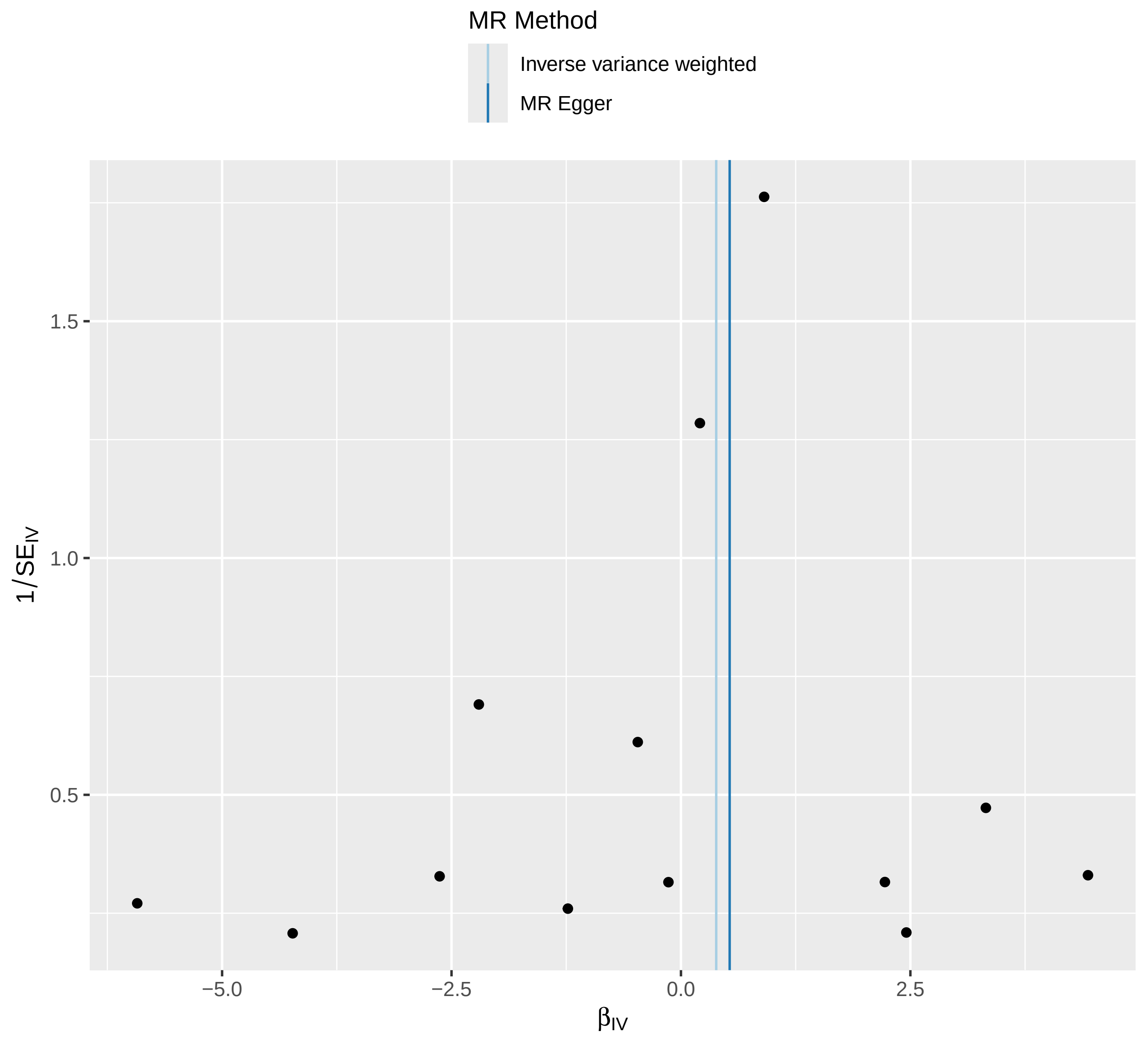
MR Method
Inverse variance weighted
MR Egger
1/SE_IV
1.5
1.0
0.5
−5.0
−2.5
0.0
2.5
β_IV

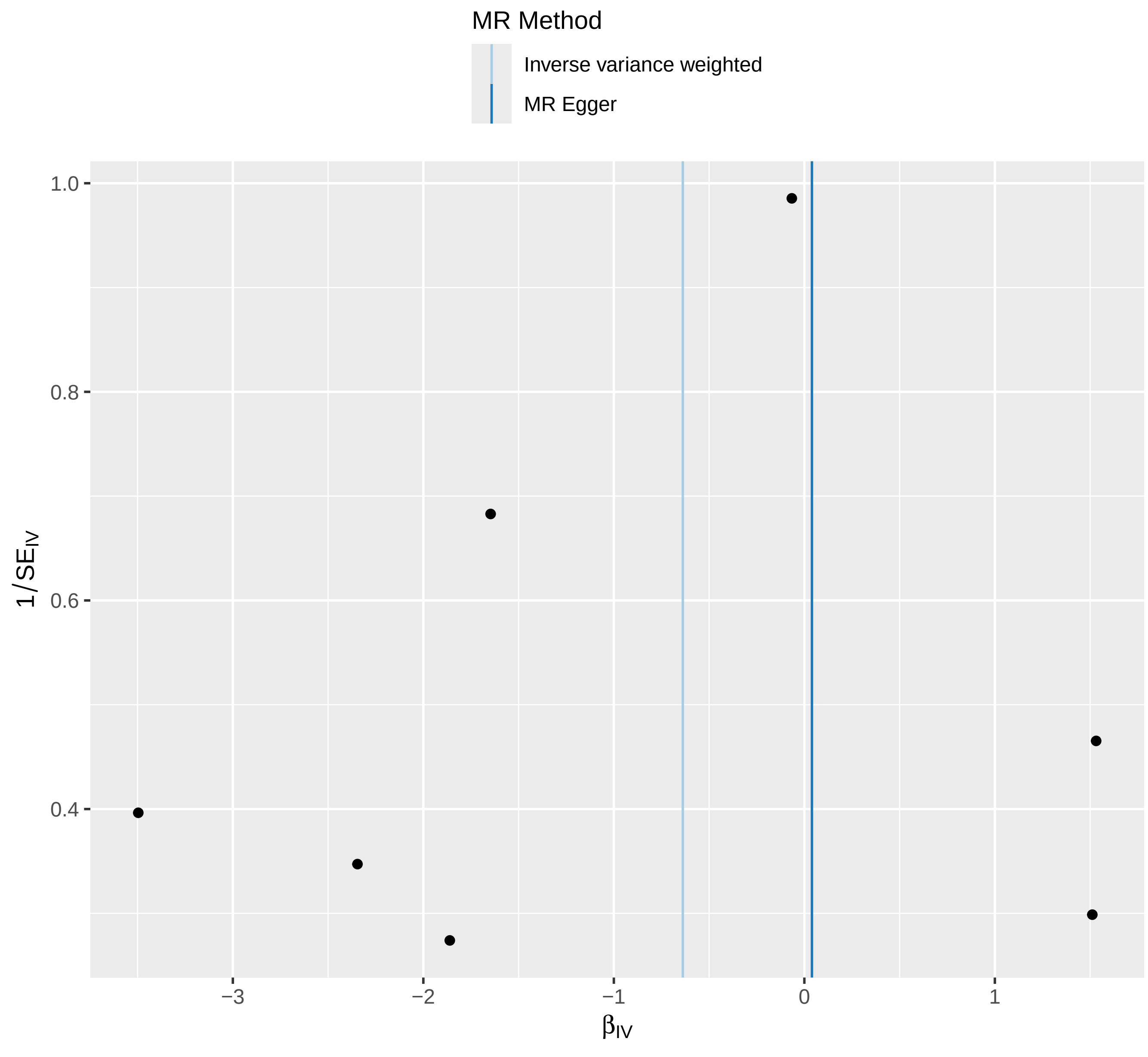

MR Method
Inverse variance weighted
MR Egger
1.0
0.8
0.6
0.4
1/SE_IV
−3
−2
−1
0
1
β_IV

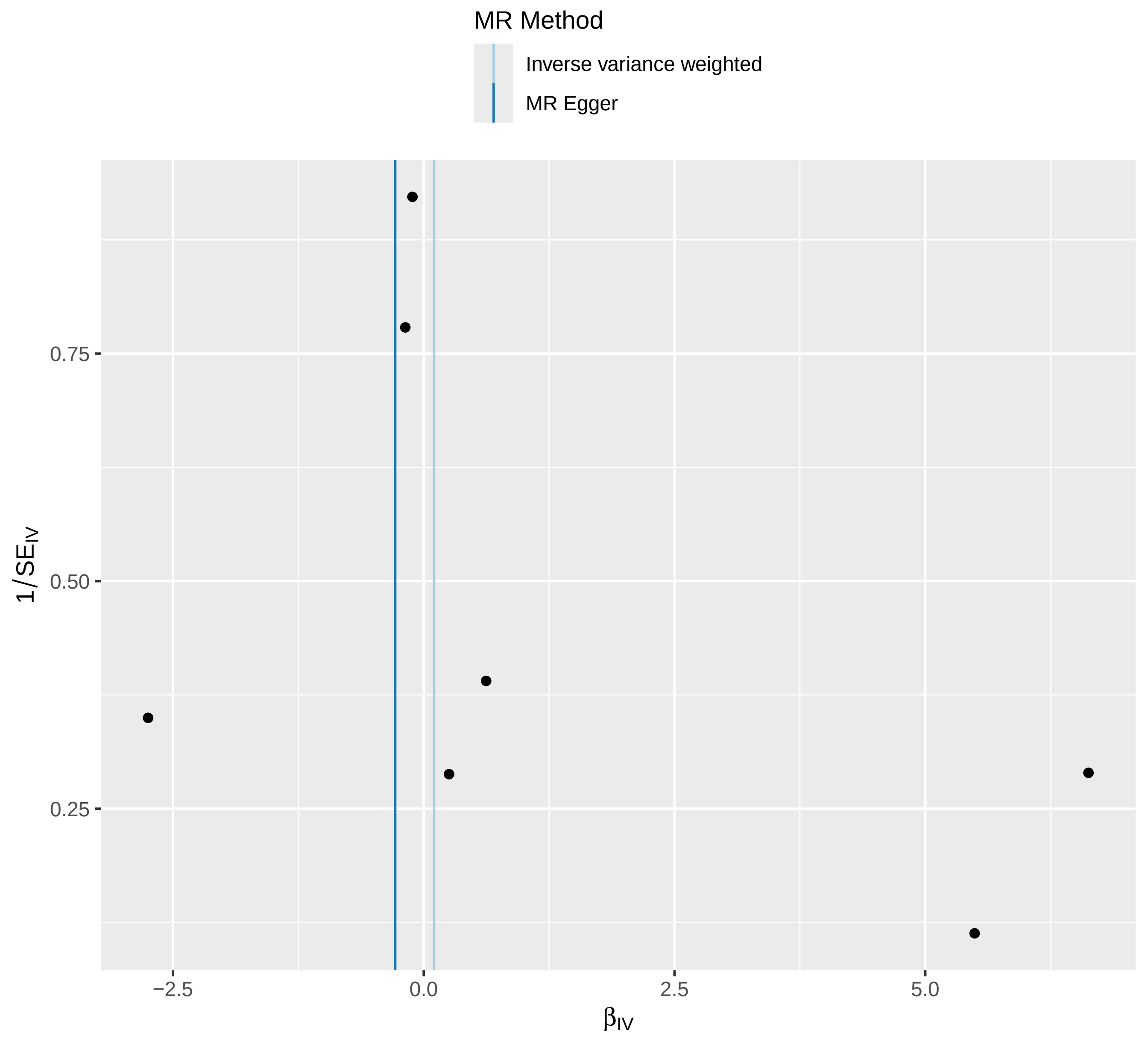
MR Method
Inverse variance weighted
MR Egger
0.75
0.50
0.25
1/SE$_{IV}$
−2.5
0.0
2.5
5.0
β$_{IV}$

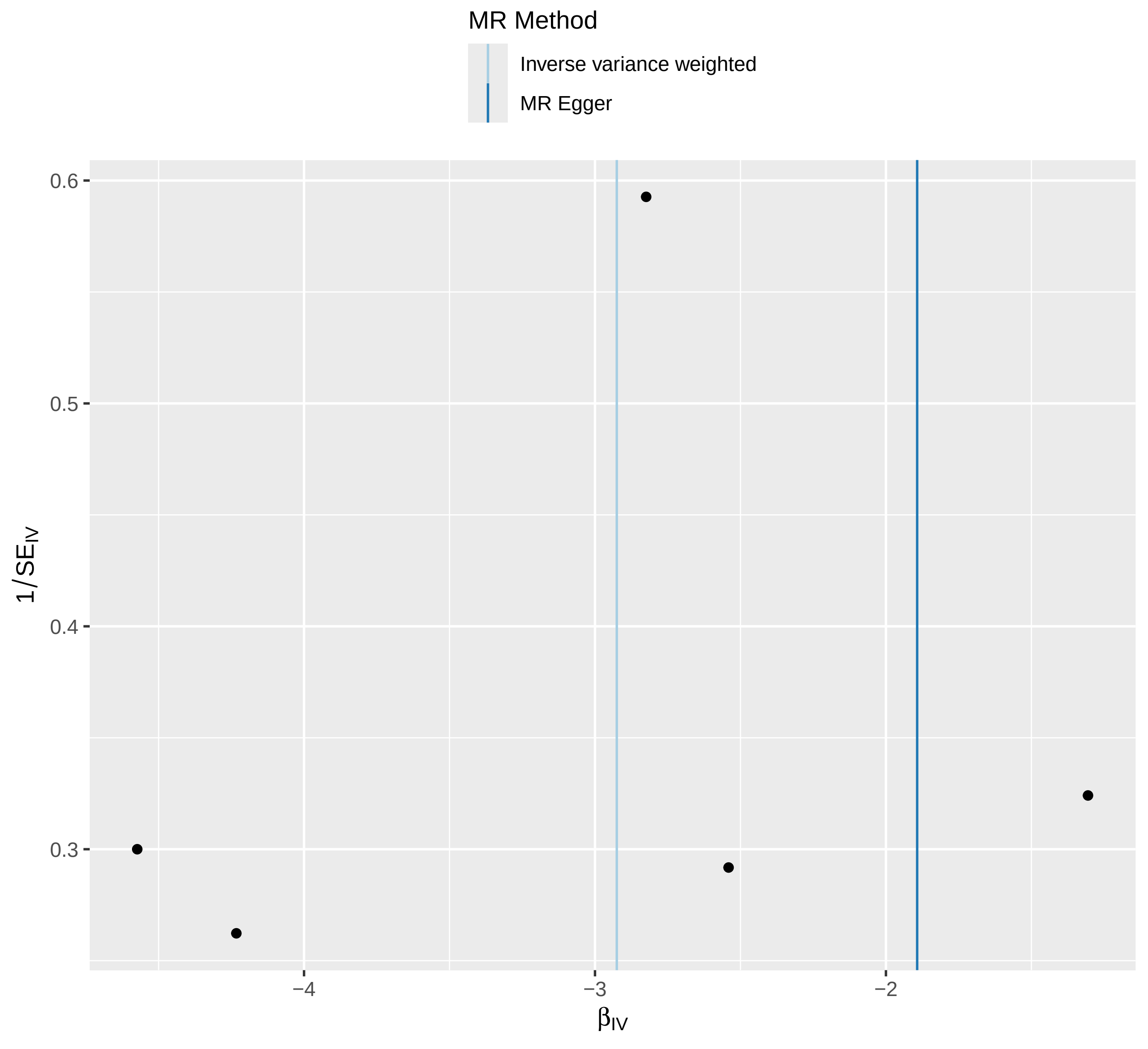
MR Method
Inverse variance weighted
MR Egger
0.6
0.5
0.4
0.3
1/SE_IV
−4
−3
−2
β_IV

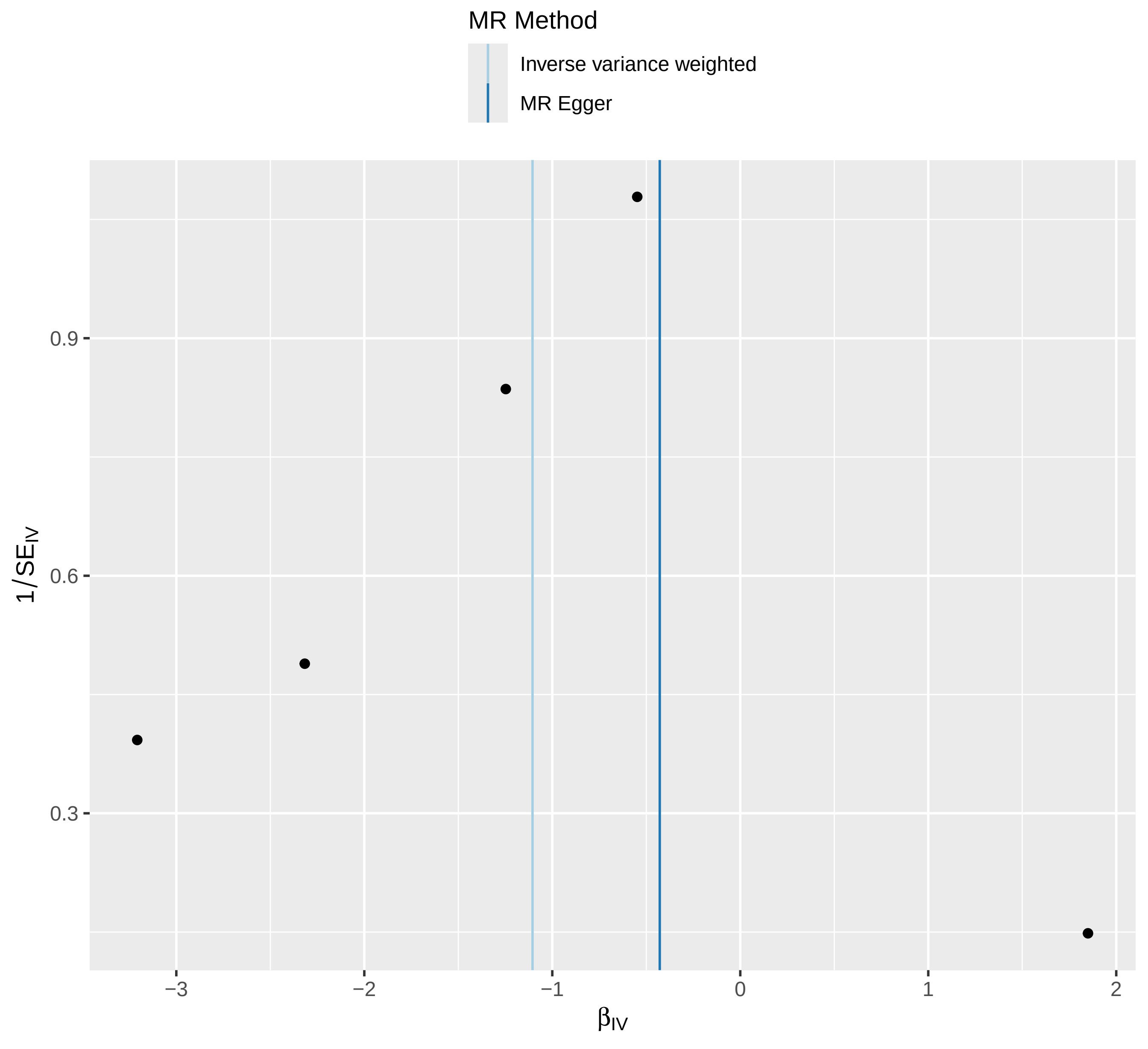
MR Method
Inverse variance weighted
MR Egger
0.9
0.6
0.3
$1/SE_{IV}$
−3
−2
−1
0
1
2
β_{IV}

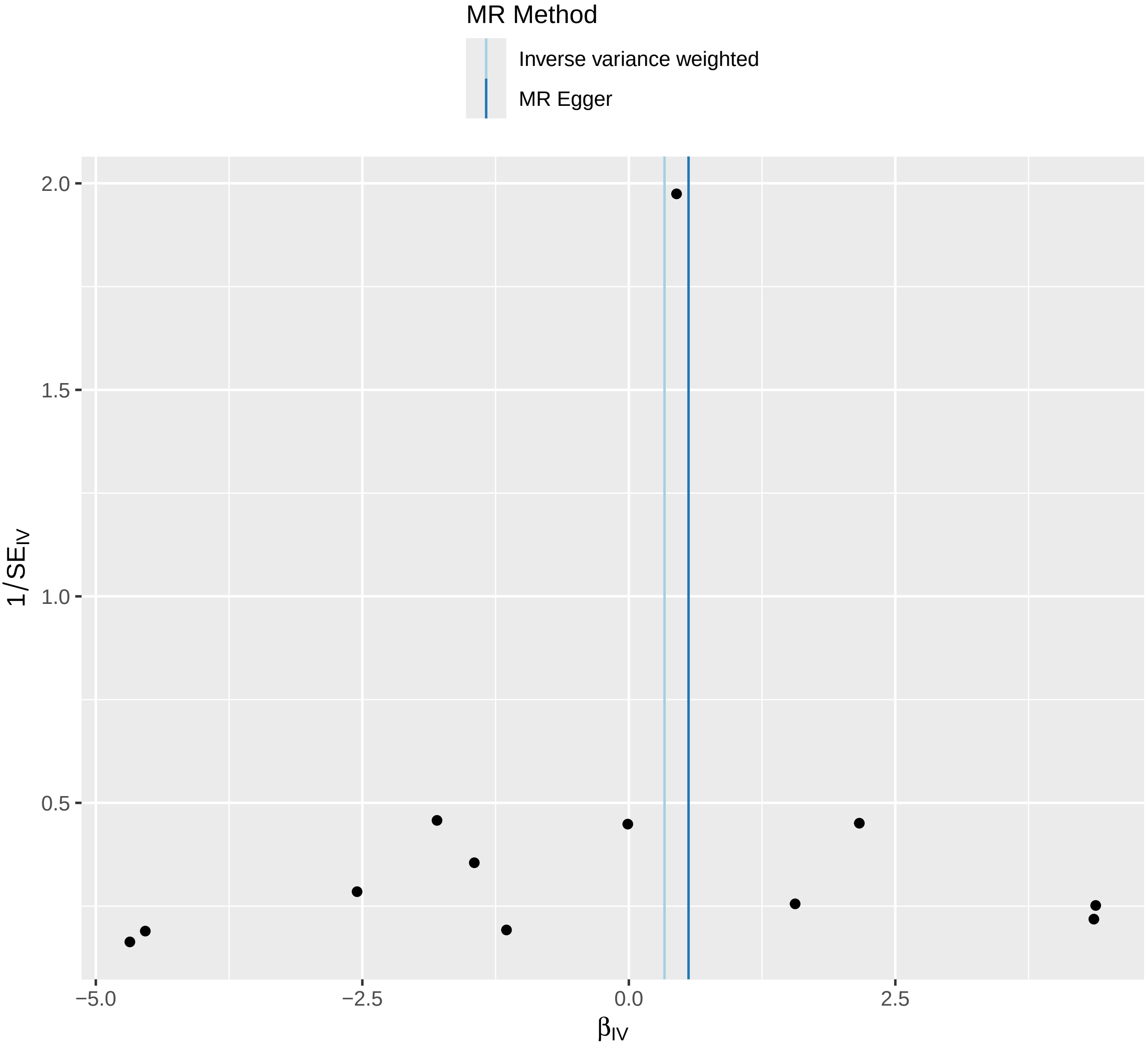
MR Method
Inverse variance weighted
MR Egger
2.0
1.5
1.0
0.5
1/SE_IV
−5.0
−2.5
0.0
2.5
β_IV

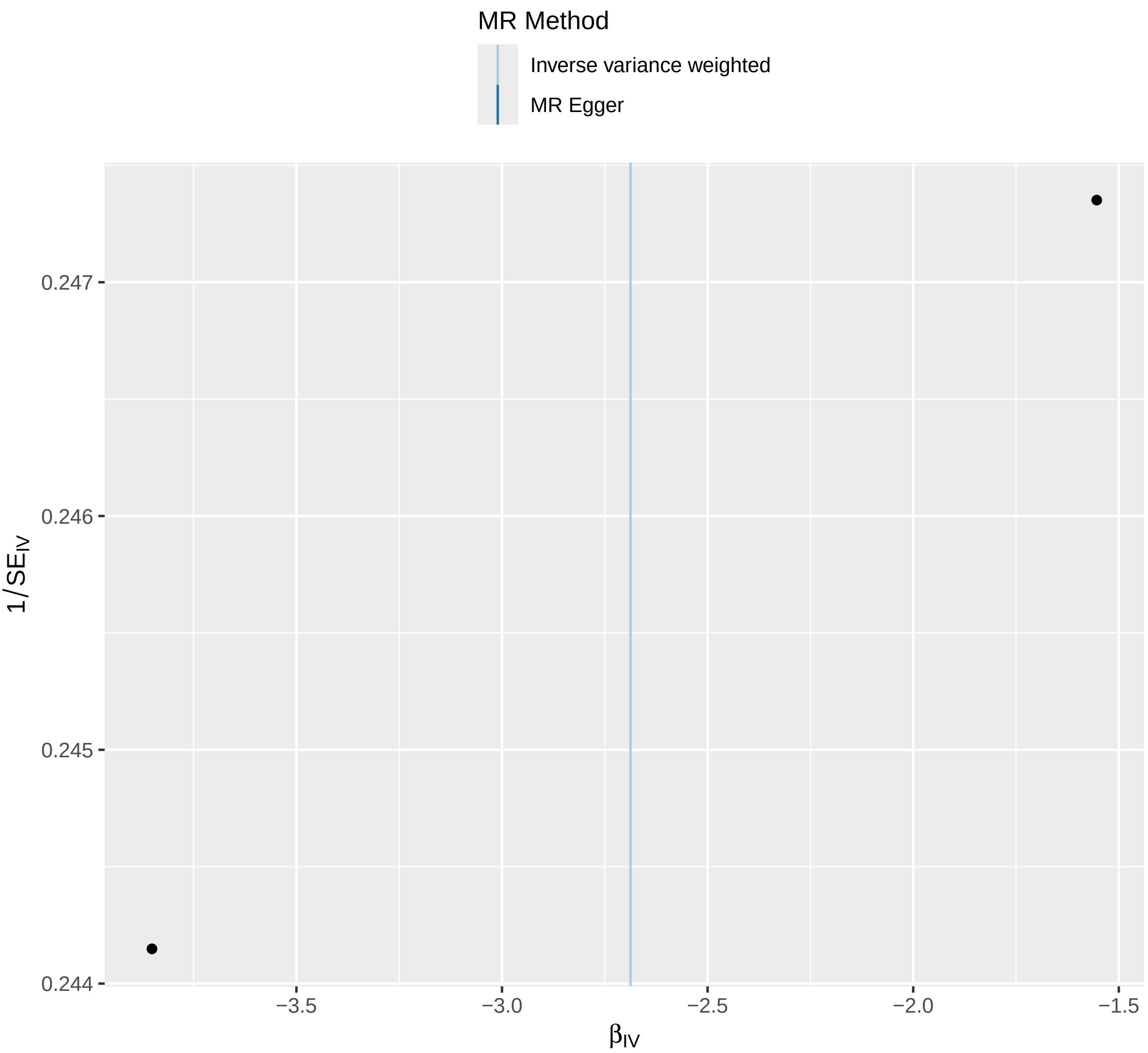
MR Method
Inverse variance weighted
MR Egger
0.247
0.246
0.245
0.244
1/SE_IV
−3.5
−3.0
−2.5
−2.0
−1.5
β_IV

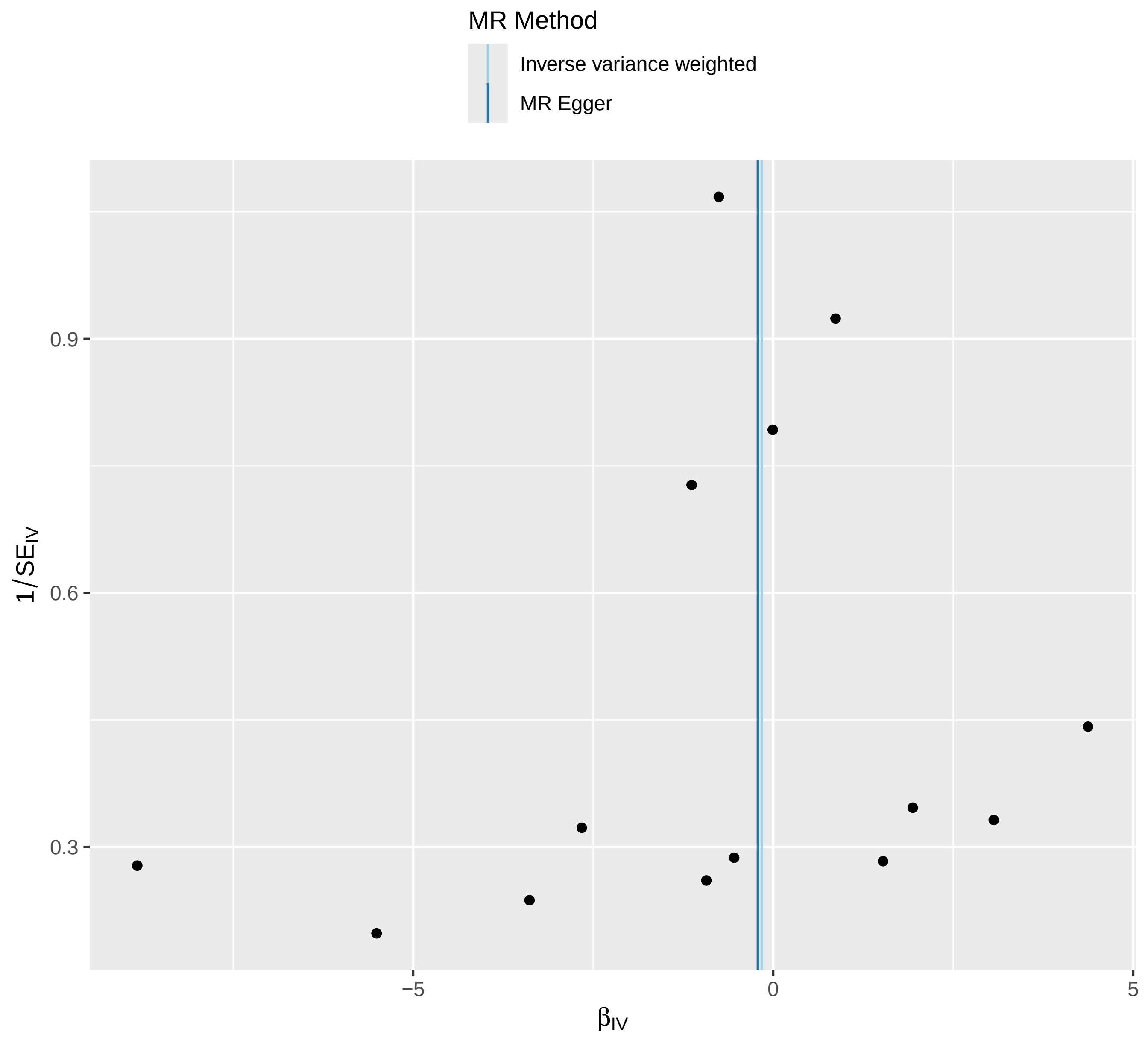
MR Method
Inverse variance weighted
MR Egger
0.9
0.6
0.3
$1/SE_{IV}$
−5
0
5
β_{IV}

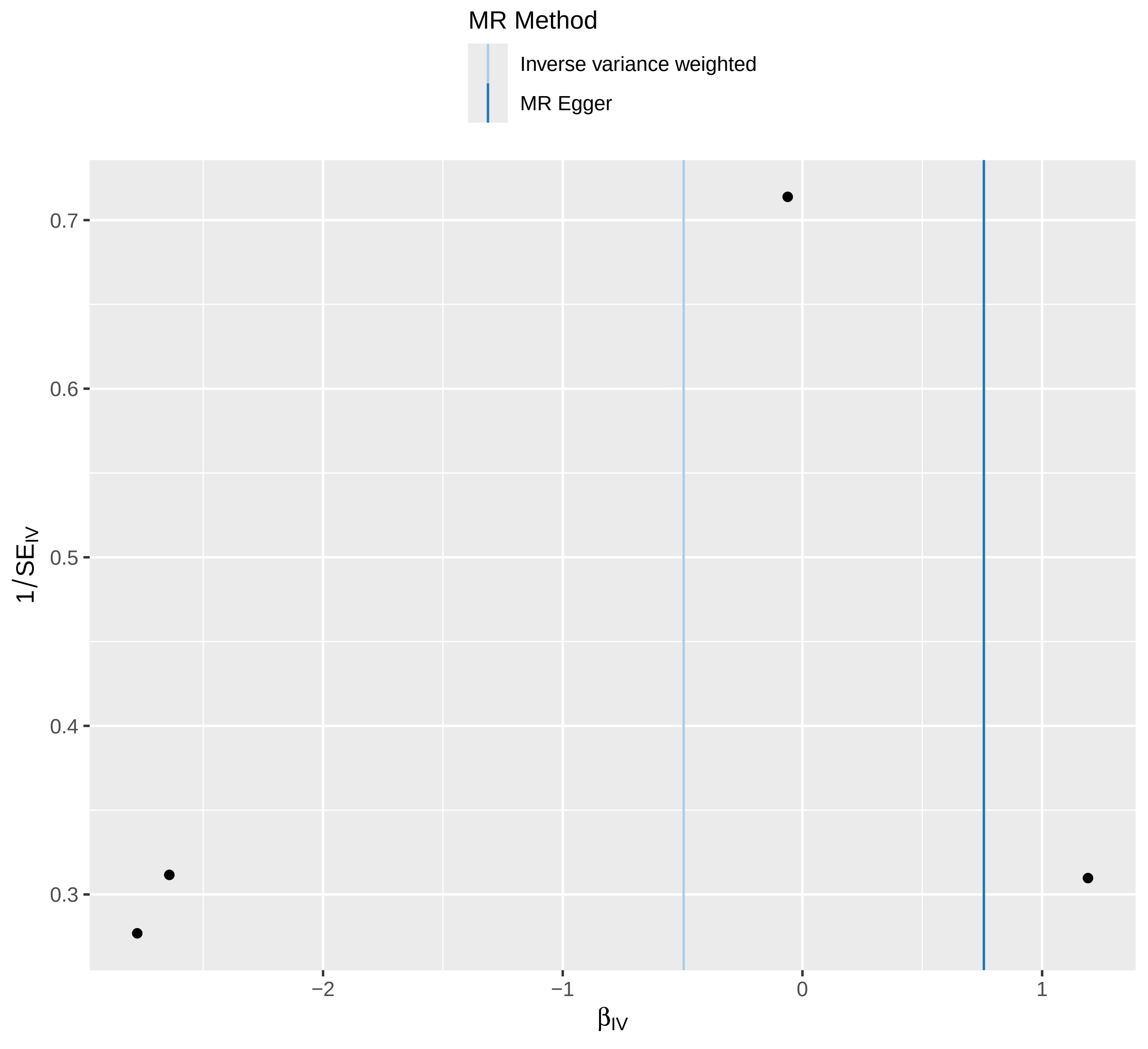

MR Method
Inverse variance weighted
MR Egger
0.7
0.6
0.5
0.4
0.3
1/SE$_{IV}$
−2
−1
0
1
β$_{IV}$

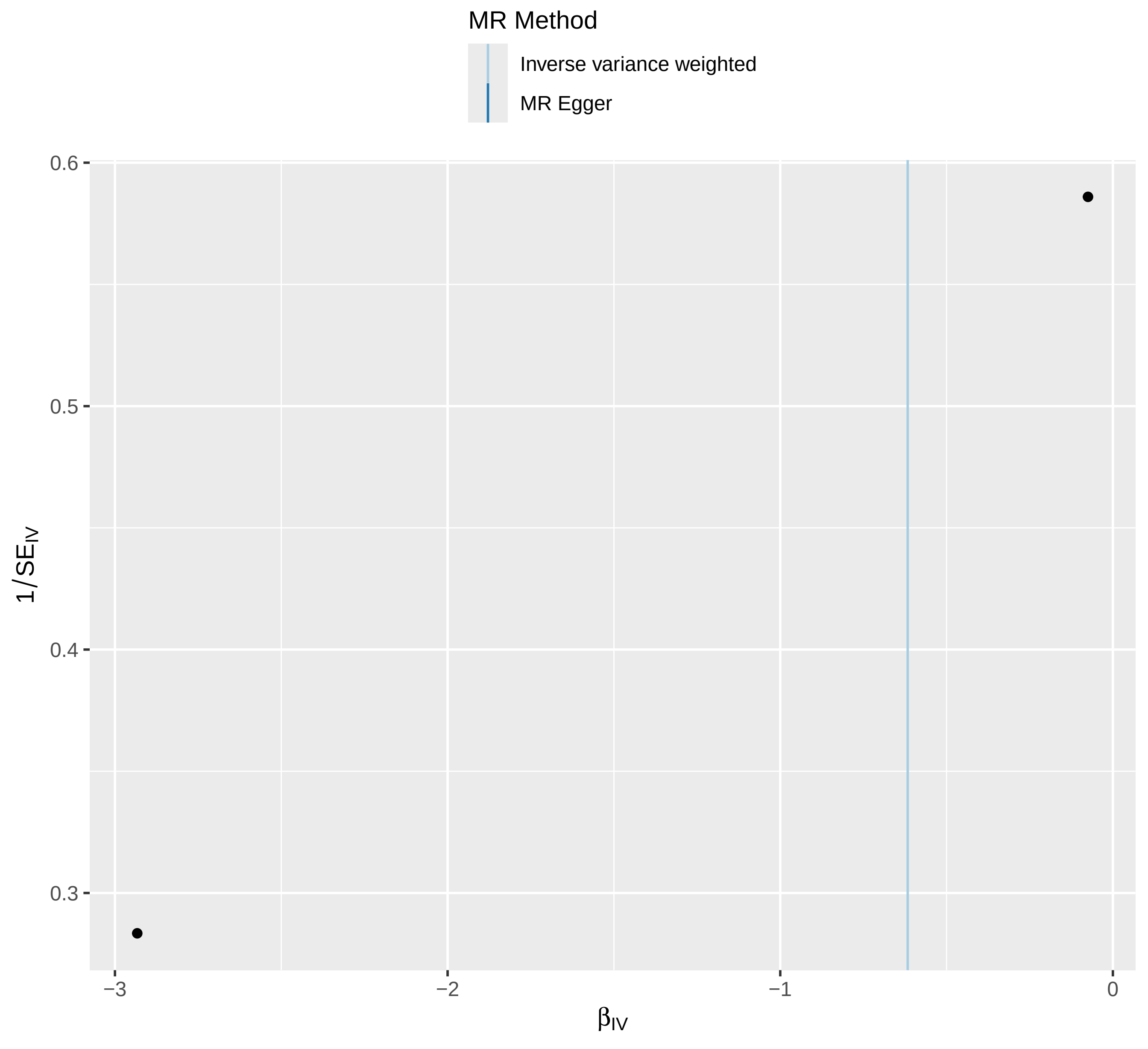
MR Method
Inverse variance weighted
MR Egger
0.6
0.5
0.4
0.3
1/SE_IV
−3
−2
−1
0
β_IV

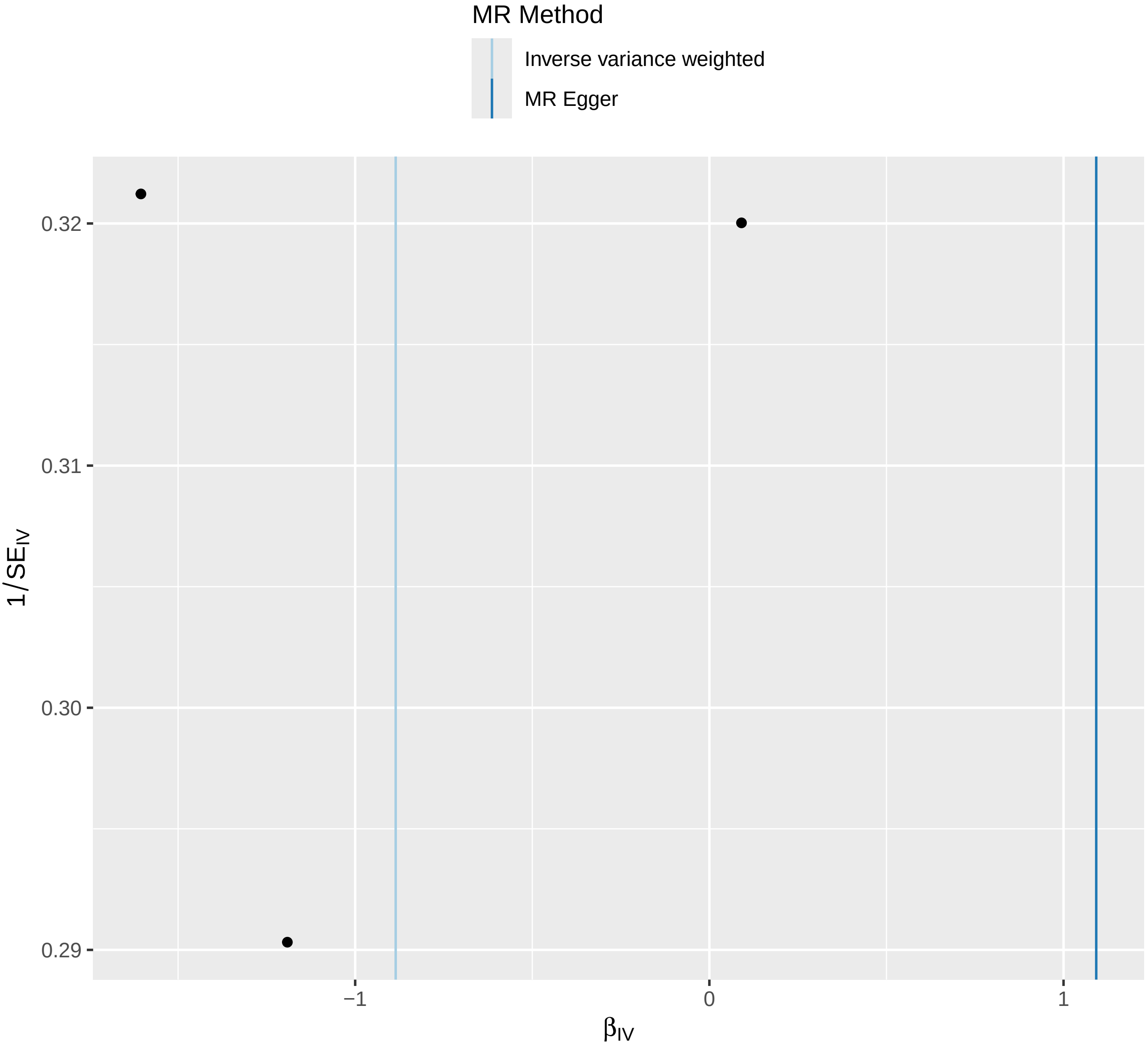
MR Method
Inverse variance weighted
MR Egger
0.32
0.31
0.30
0.29
1/SE_IV
−1
0
1
β_IV

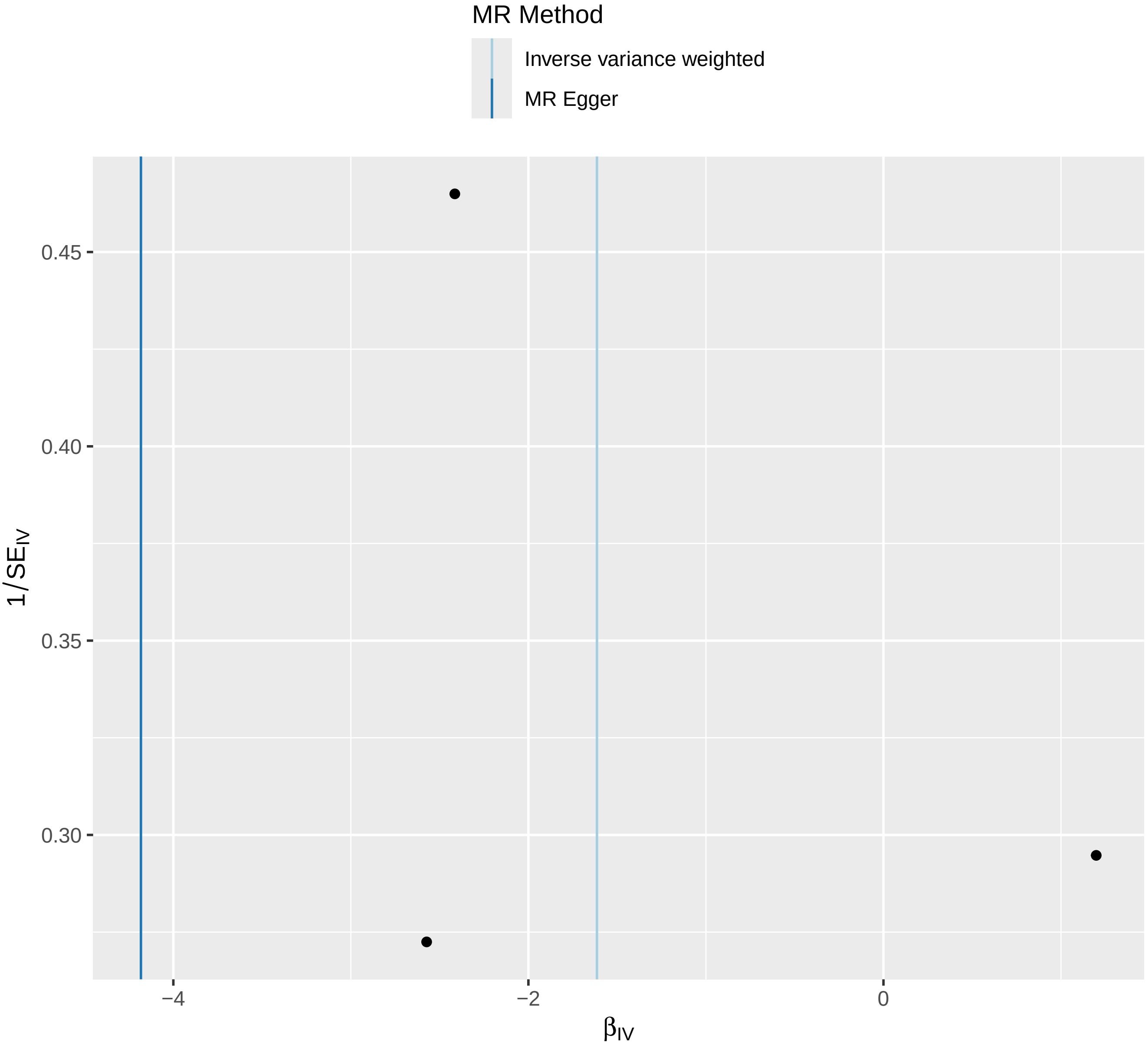
MR Method
Inverse variance weighted
MR Egger
0.45
0.40
0.35
0.30
1/SE_IV
−4
−2
0
β_IV

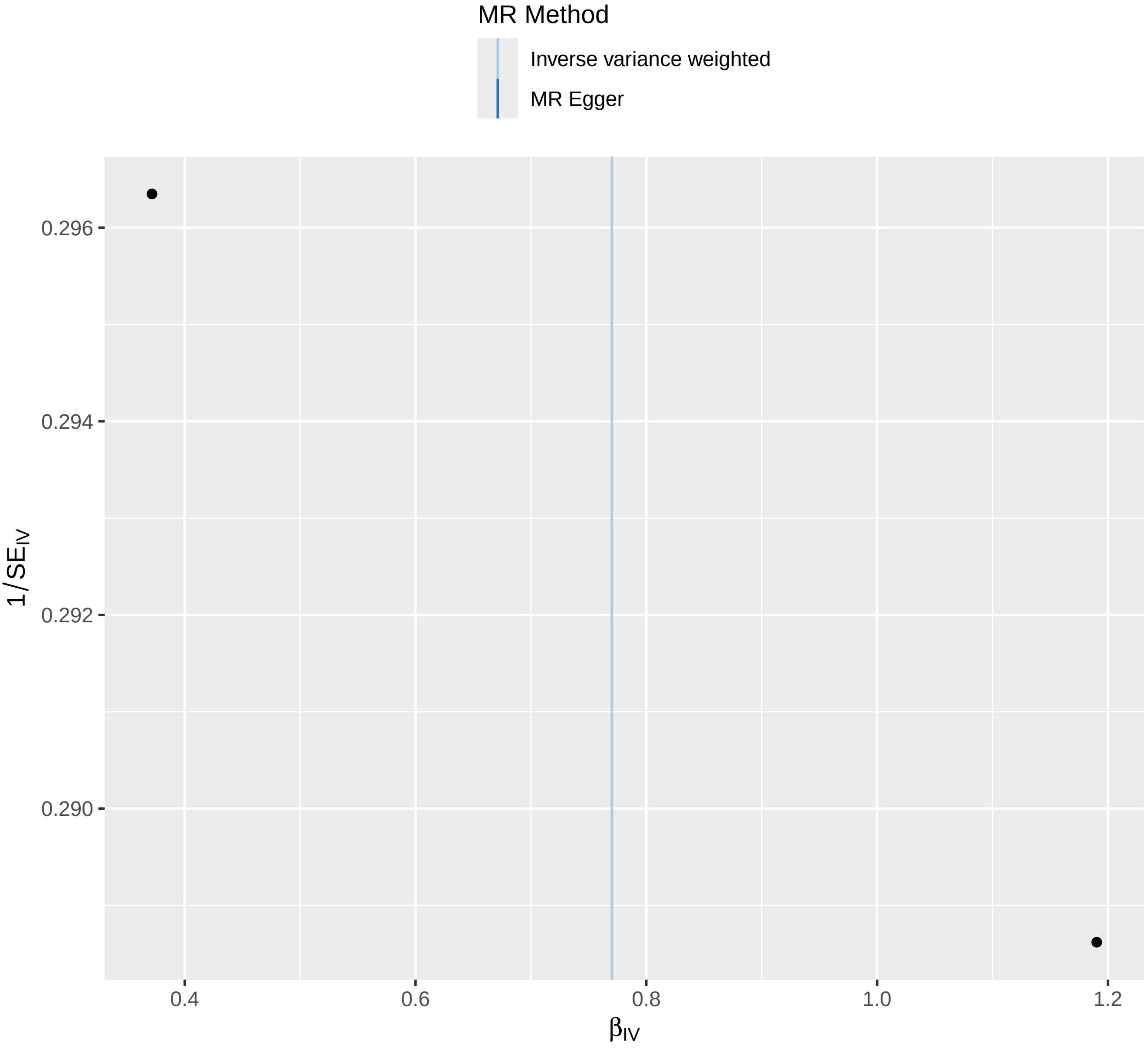
MR Method
Inverse variance weighted
MR Egger
0.296
0.294
0.292
0.290
1/SE_IV
0.4
0.6
0.8
1.0
1.2
β_IV

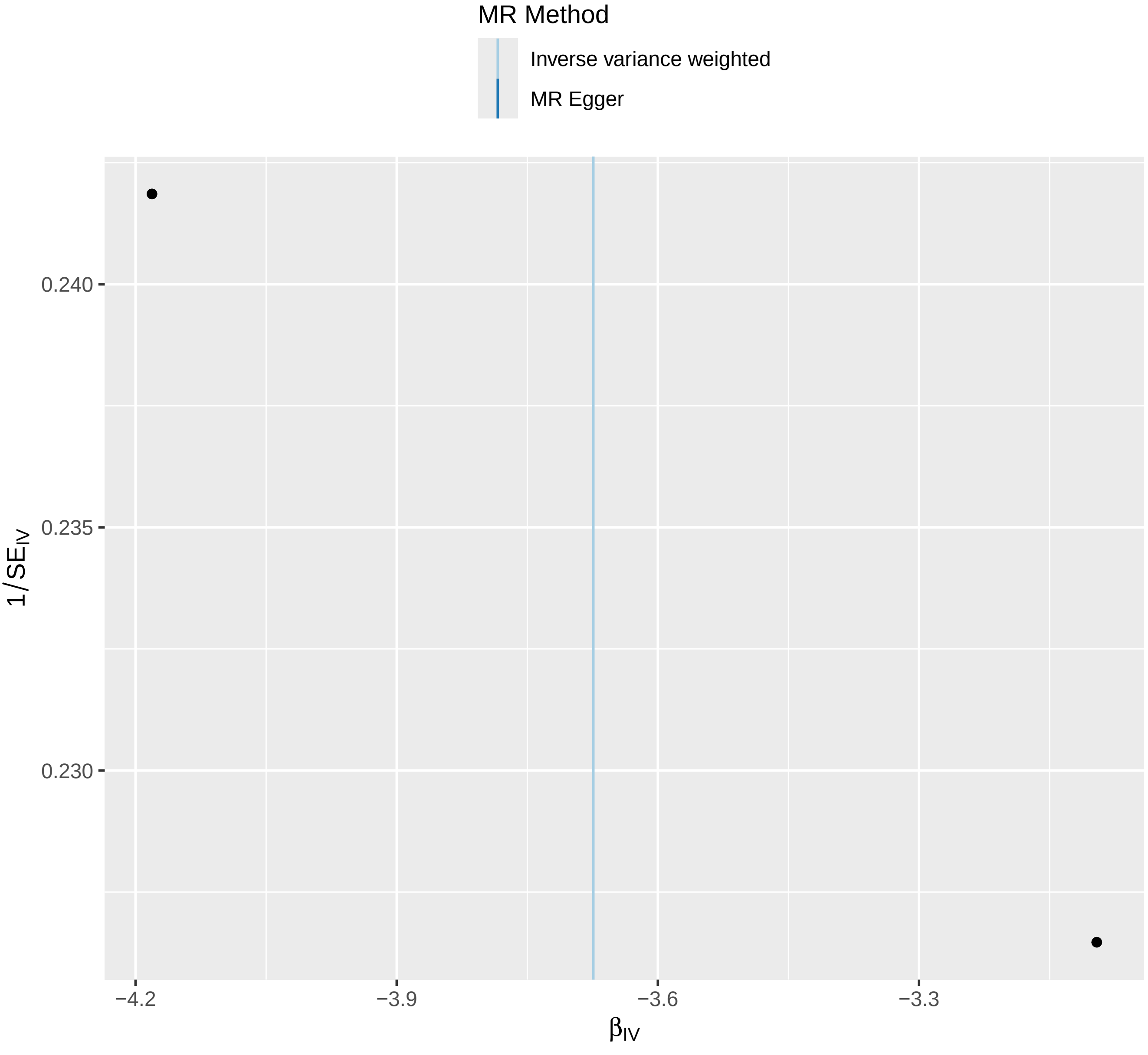
MR Method
Inverse variance weighted
MR Egger
0.240
0.235
0.230
1/SE$_{IV}$
−4.2
−3.9
−3.6
−3.3
β$_{IV}$

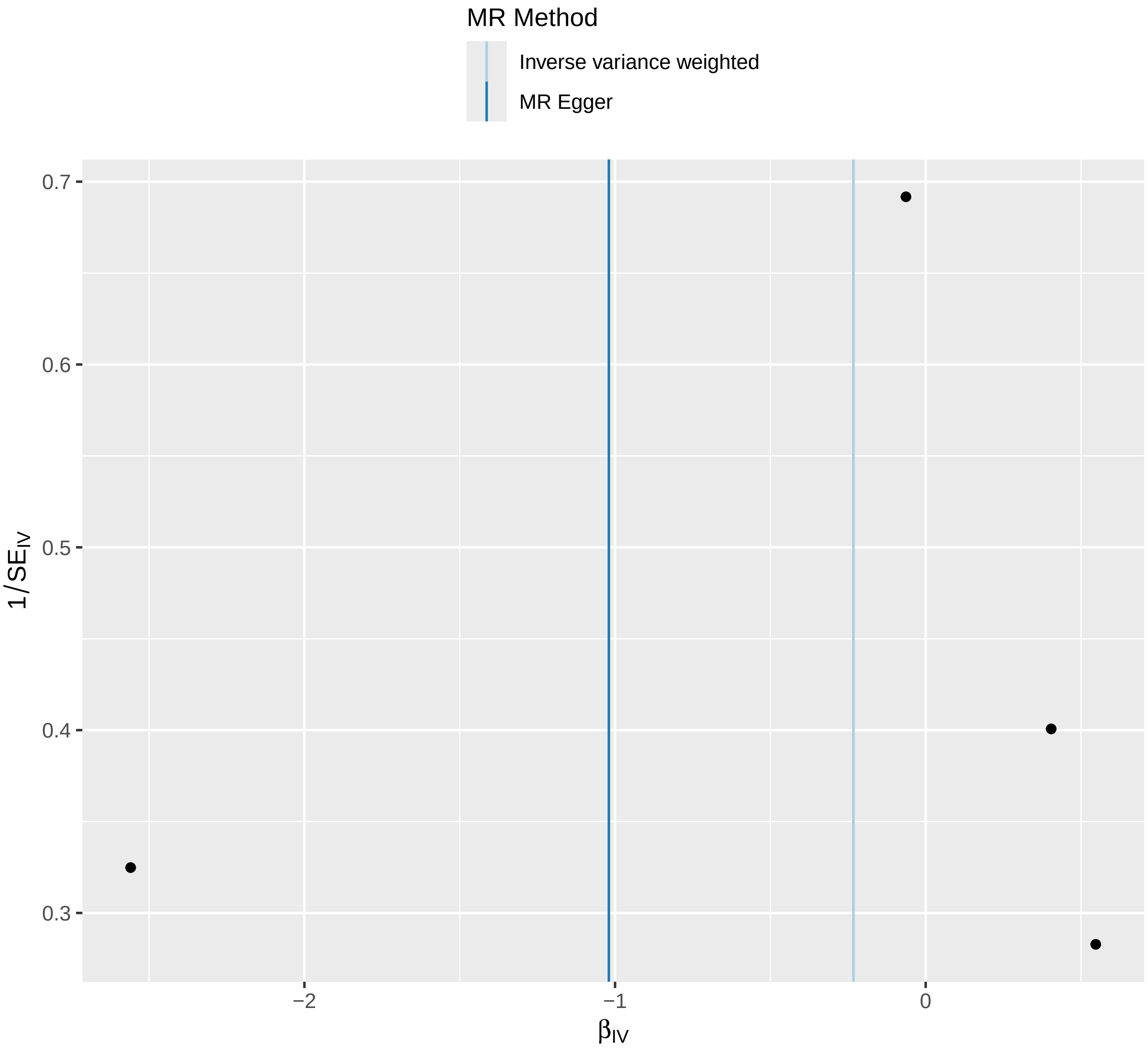
MR Method
Inverse variance weighted
MR Egger
0.7
0.6
0.5
0.4
0.3
1/SE_{IV}
−2
−1
0
β_{IV}

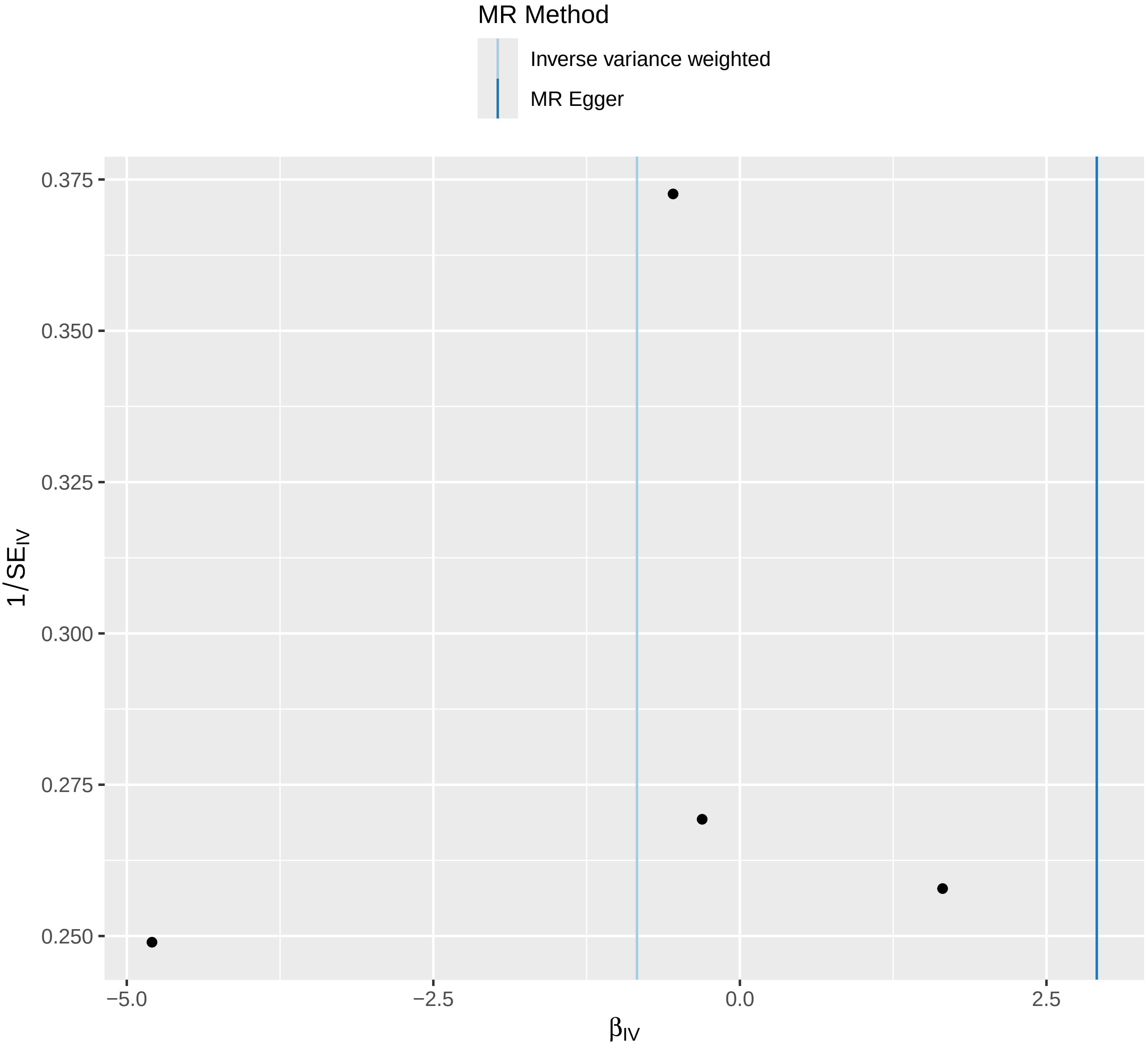
MR Method
Inverse variance weighted
MR Egger
0.375
0.350
0.325
0.300
0.275
0.250
1/SE_IV
−5.0
−2.5
0.0
2.5
β_IV

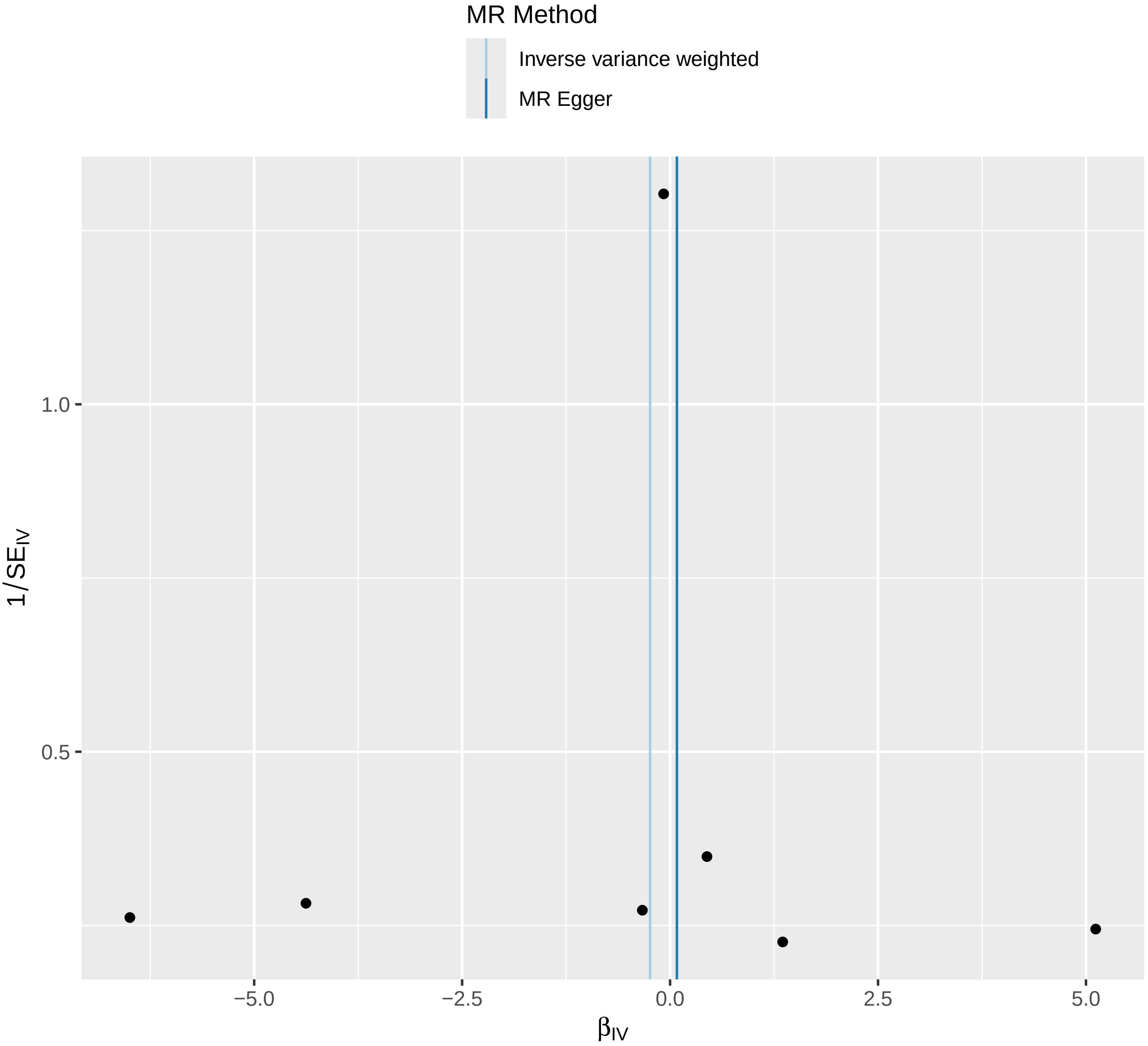
MR Method
Inverse variance weighted
MR Egger
1.0
0.5
1/SE_IV
−5.0
−2.5
0.0
2.5
5.0
β_IV

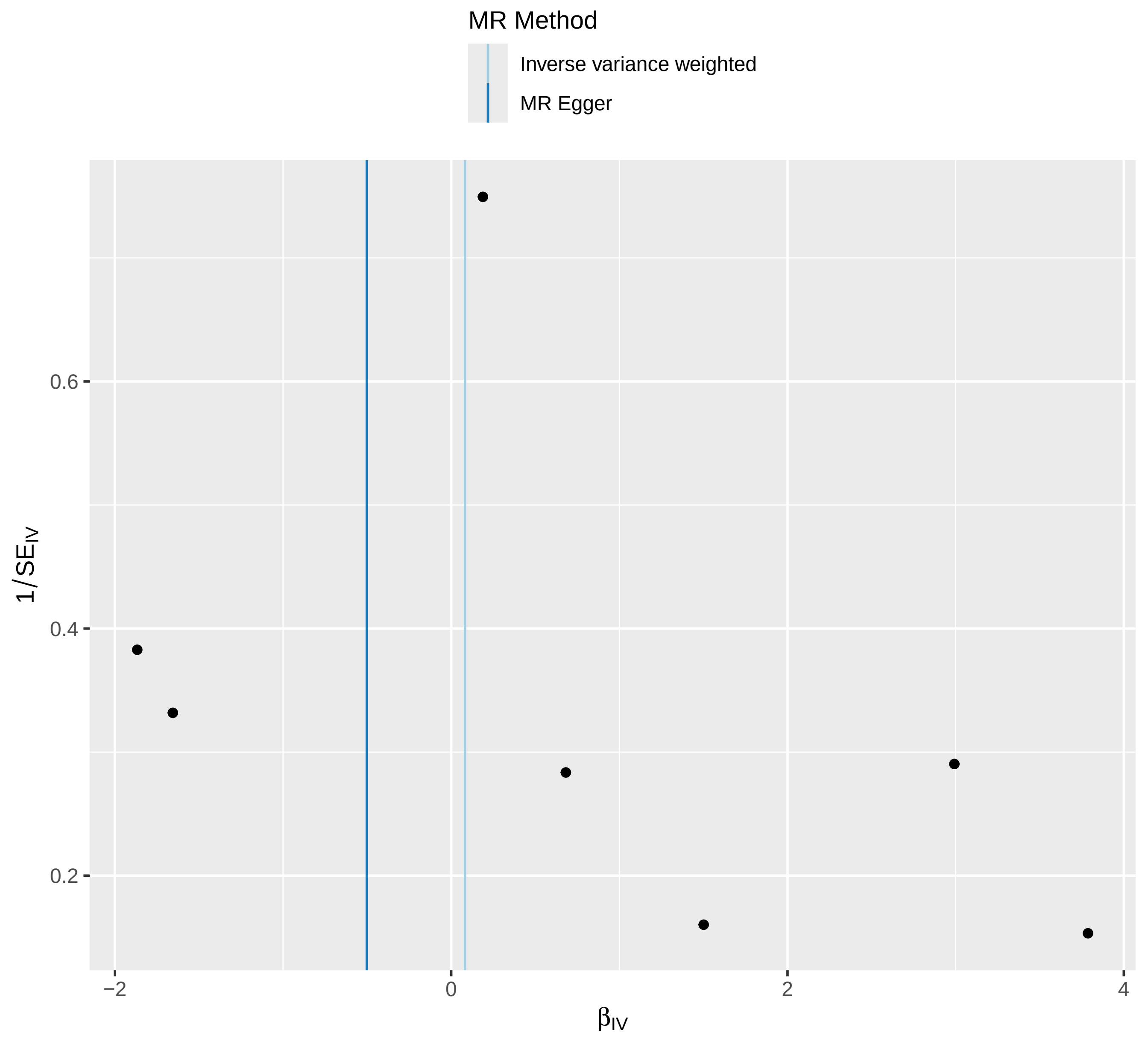

MR Method
Inverse variance weighted
MR Egger
0.6
0.4
0.2
1/SE$_{IV}$
−2
0
2
4
β$_{IV}$

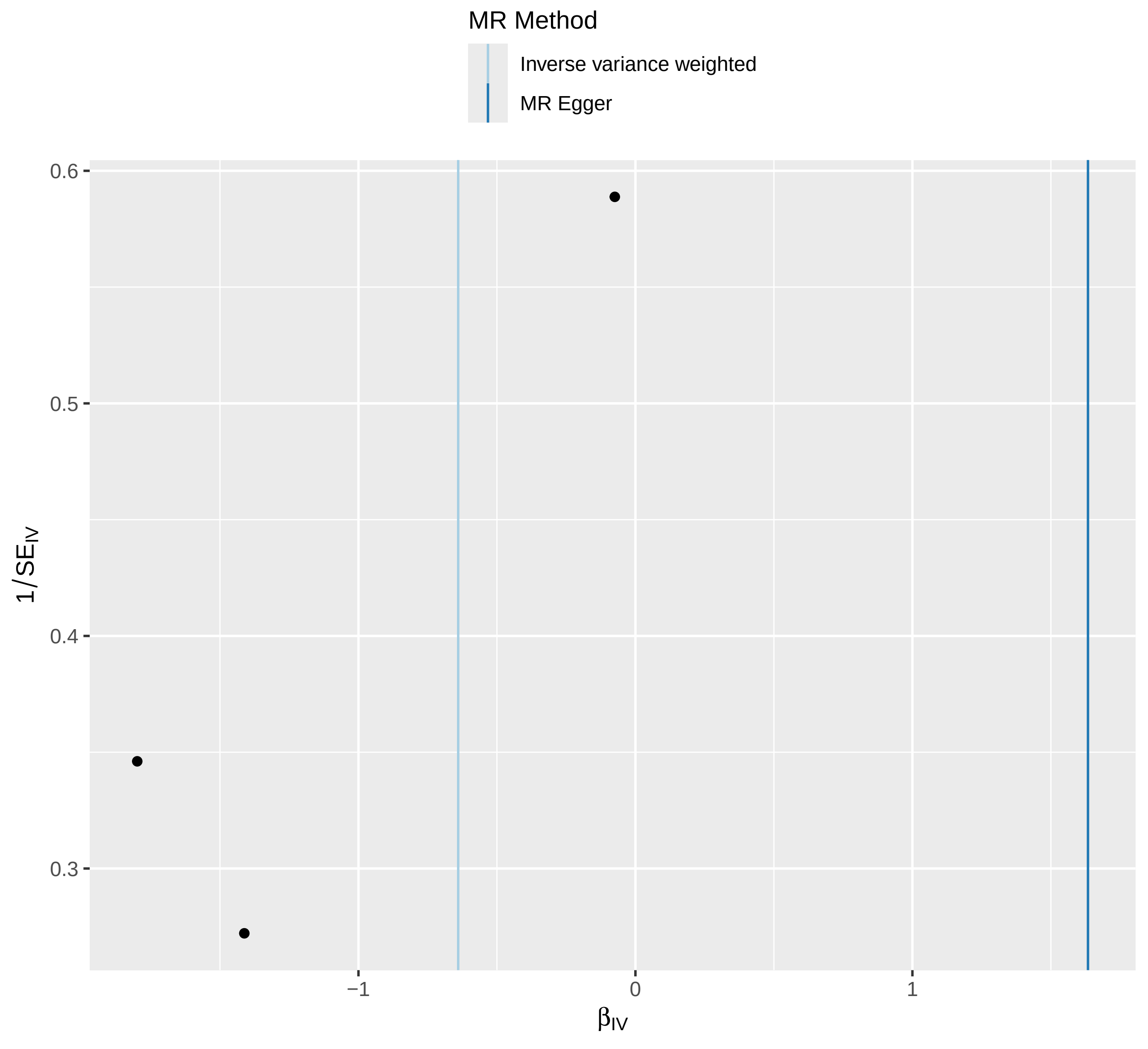
MR Method
Inverse variance weighted
MR Egger
0.6
0.5
0.4
0.3
1/SE_IV
−1
0
1
β_IV

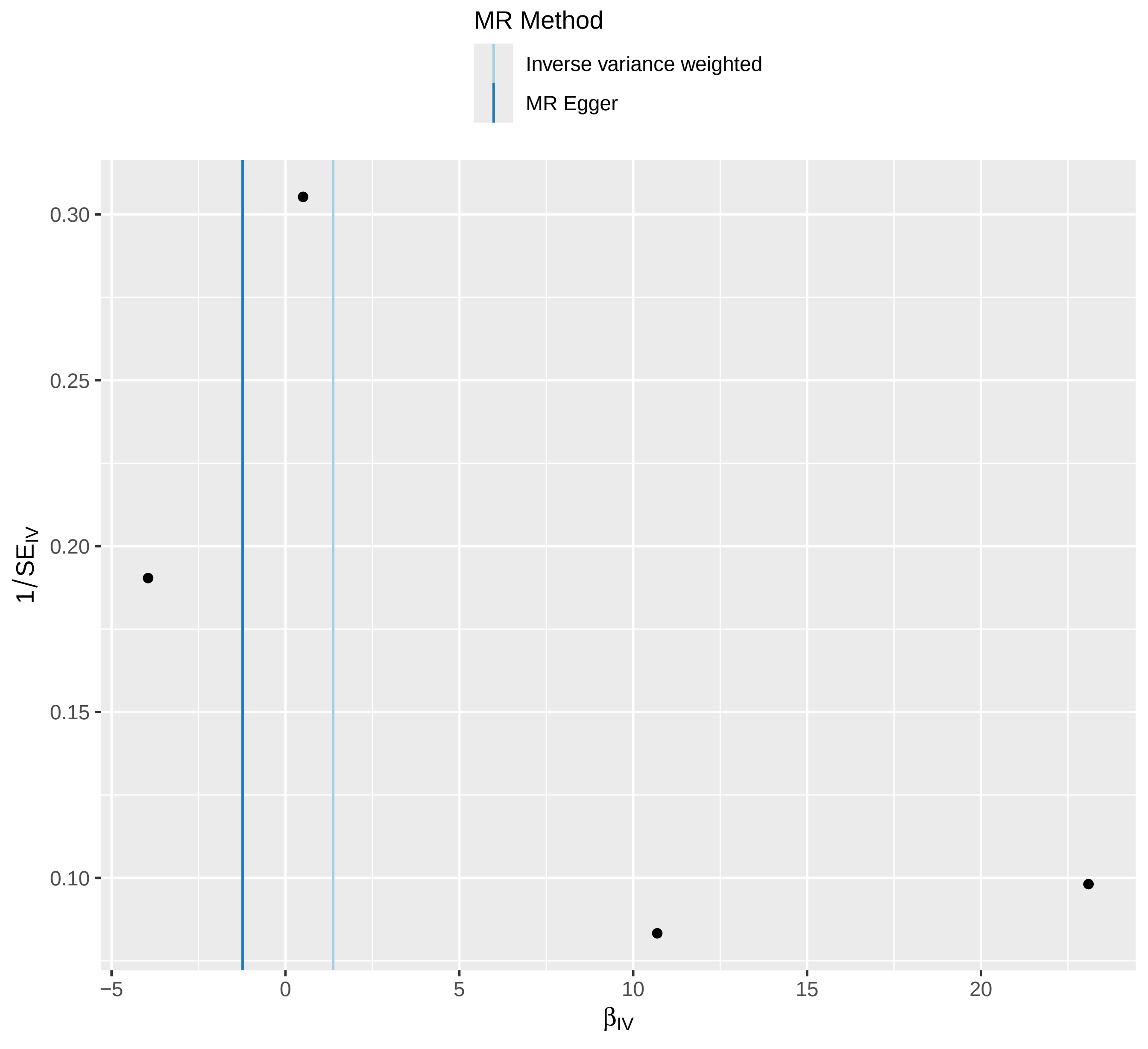
MR Method
Inverse variance weighted
MR Egger
0.30
0.25
0.20
0.15
0.10
1/SE_IV
−5
0
5
10
15
20
β_IV

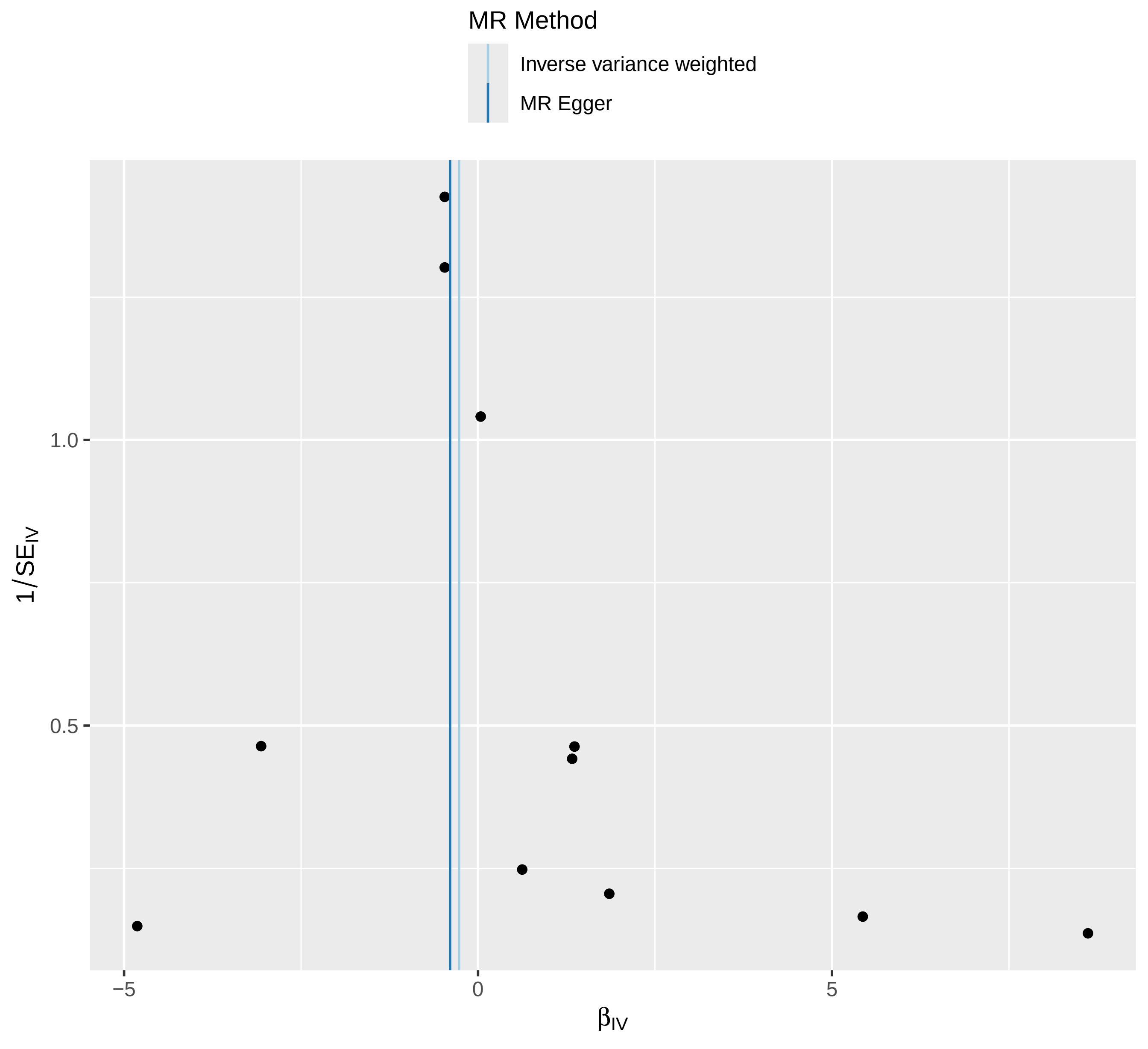
MR Method
Inverse variance weighted
MR Egger
1.0
0.5
−5
0
5
$1/SE_{IV}$
β_{IV}

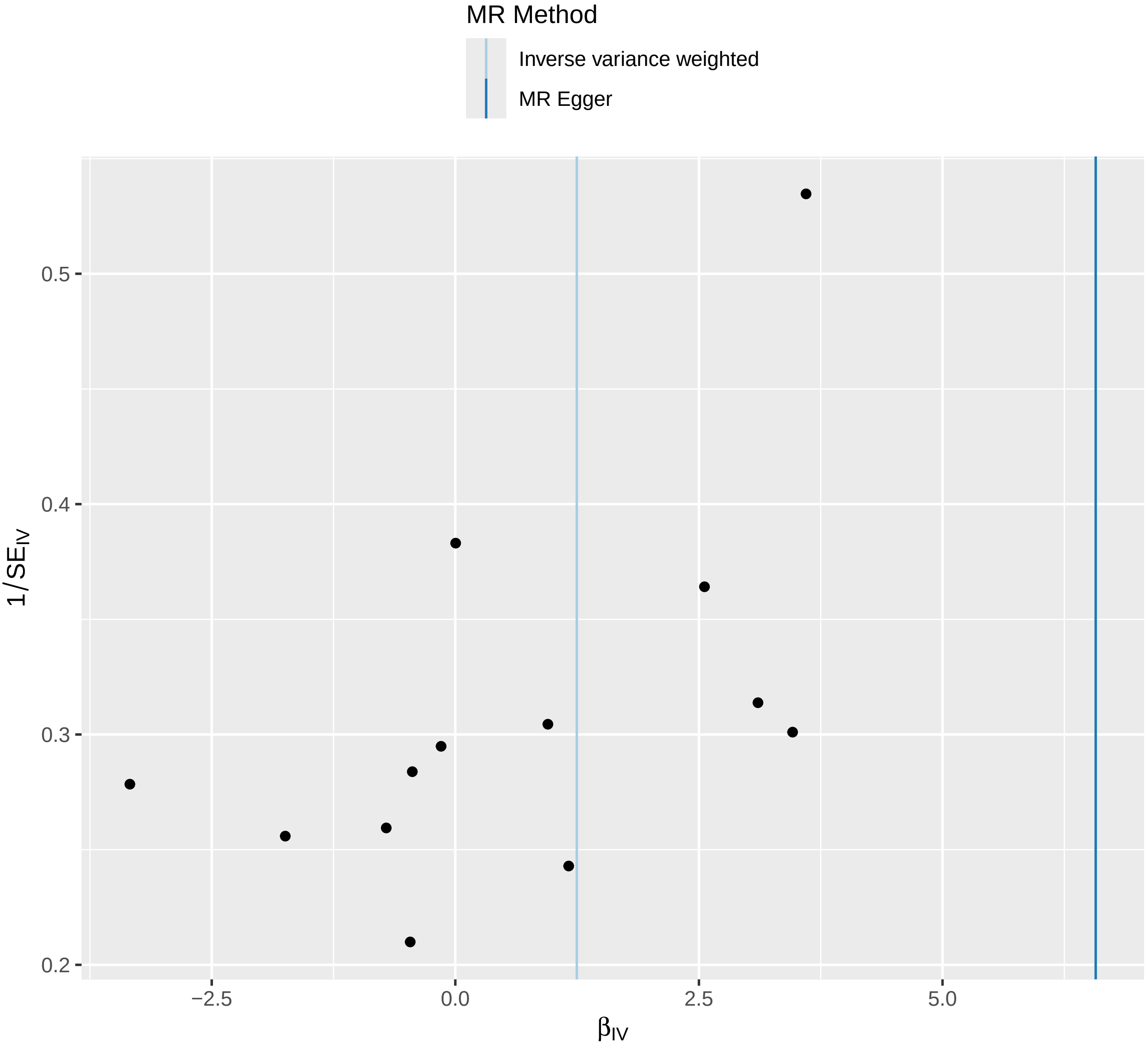
MR Method
Inverse variance weighted
MR Egger
0.5
0.4
0.3
0.2
1/SE_IV
−2.5
0.0
2.5
5.0
β_IV

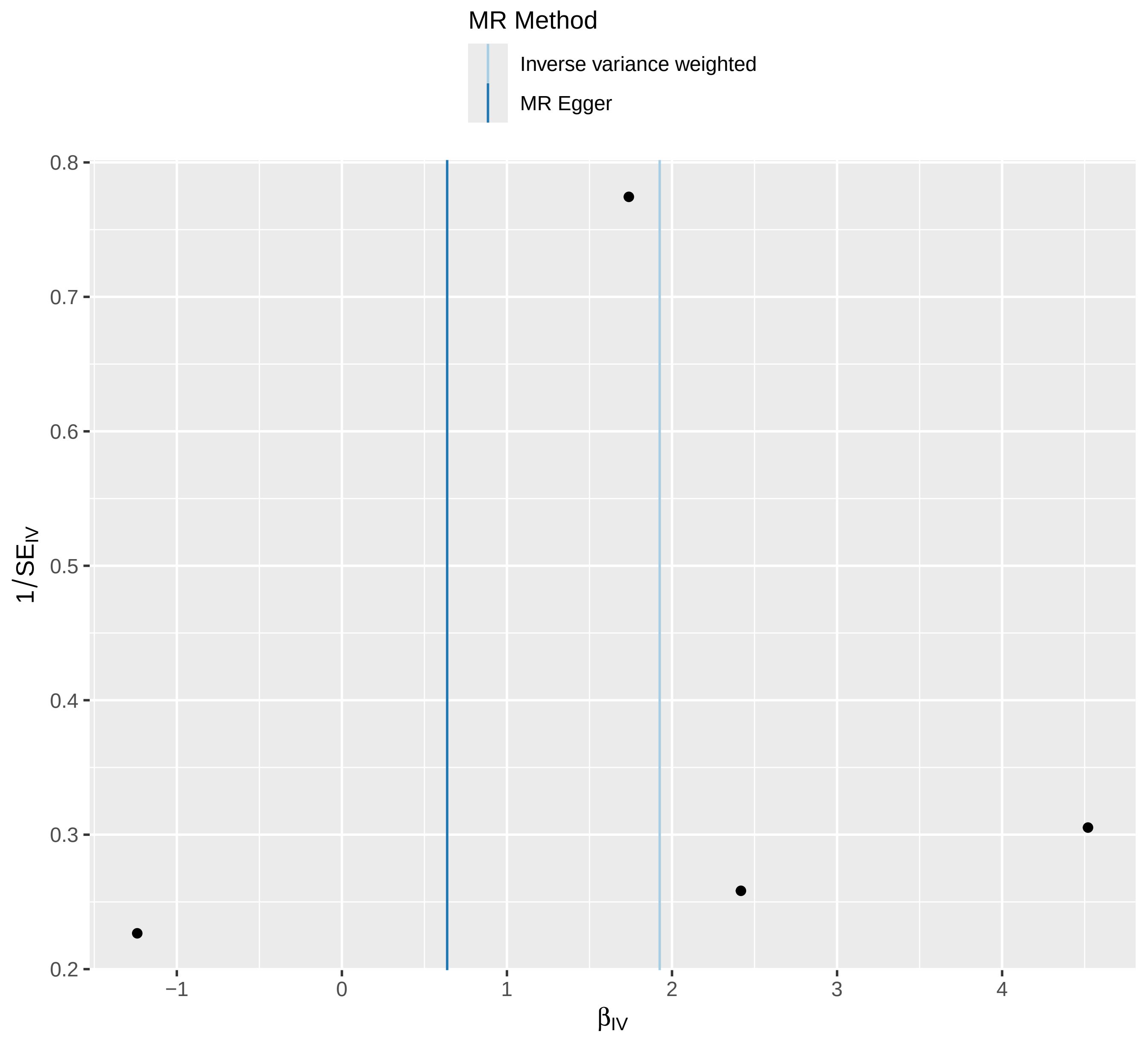
MR Method
Inverse variance weighted
MR Egger
0.8
0.7
0.6
0.5
0.4
0.3
0.2
1/SE_IV
−1
0
1
2
3
4
β_IV

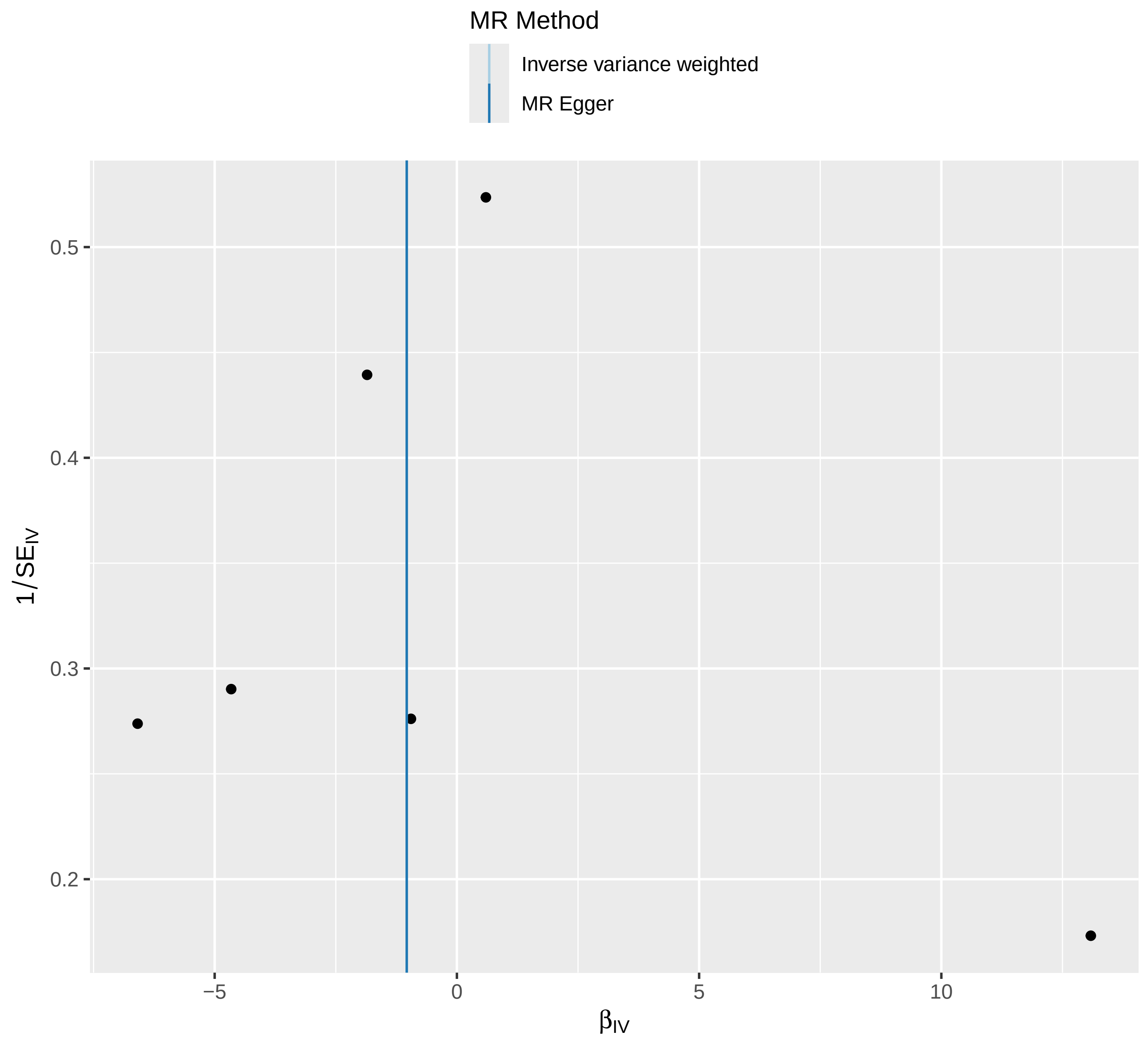

MR Method
Inverse variance weighted
MR Egger
0.5
0.4
0.3
0.2
$1/SE_{IV}$
−5
0
5
10
β_{IV}

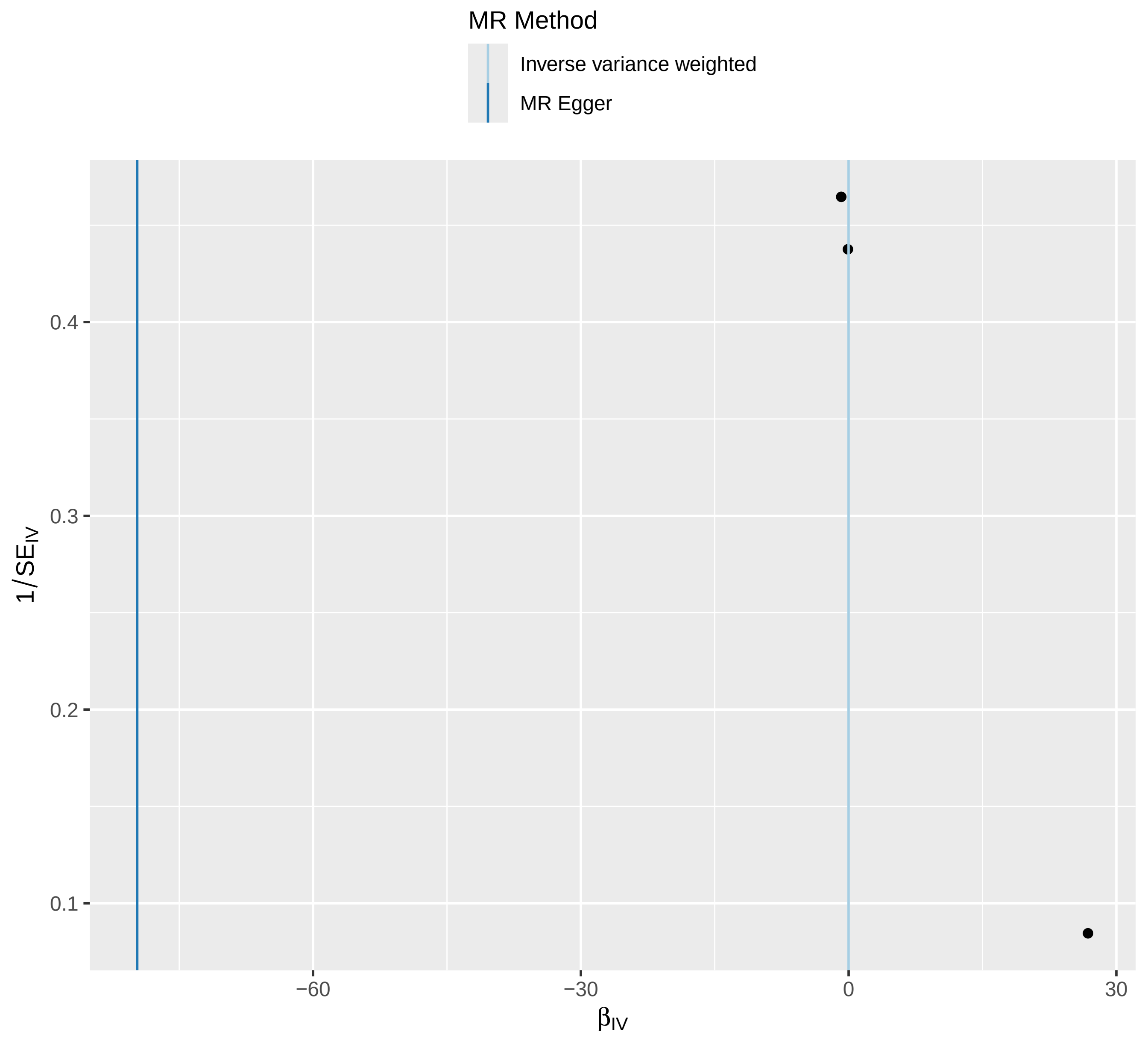
MR Method
Inverse variance weighted
MR Egger
0.4
0.3
0.2
0.1
$1/SE_{IV}$
−60
−30
0
30
β_{IV}

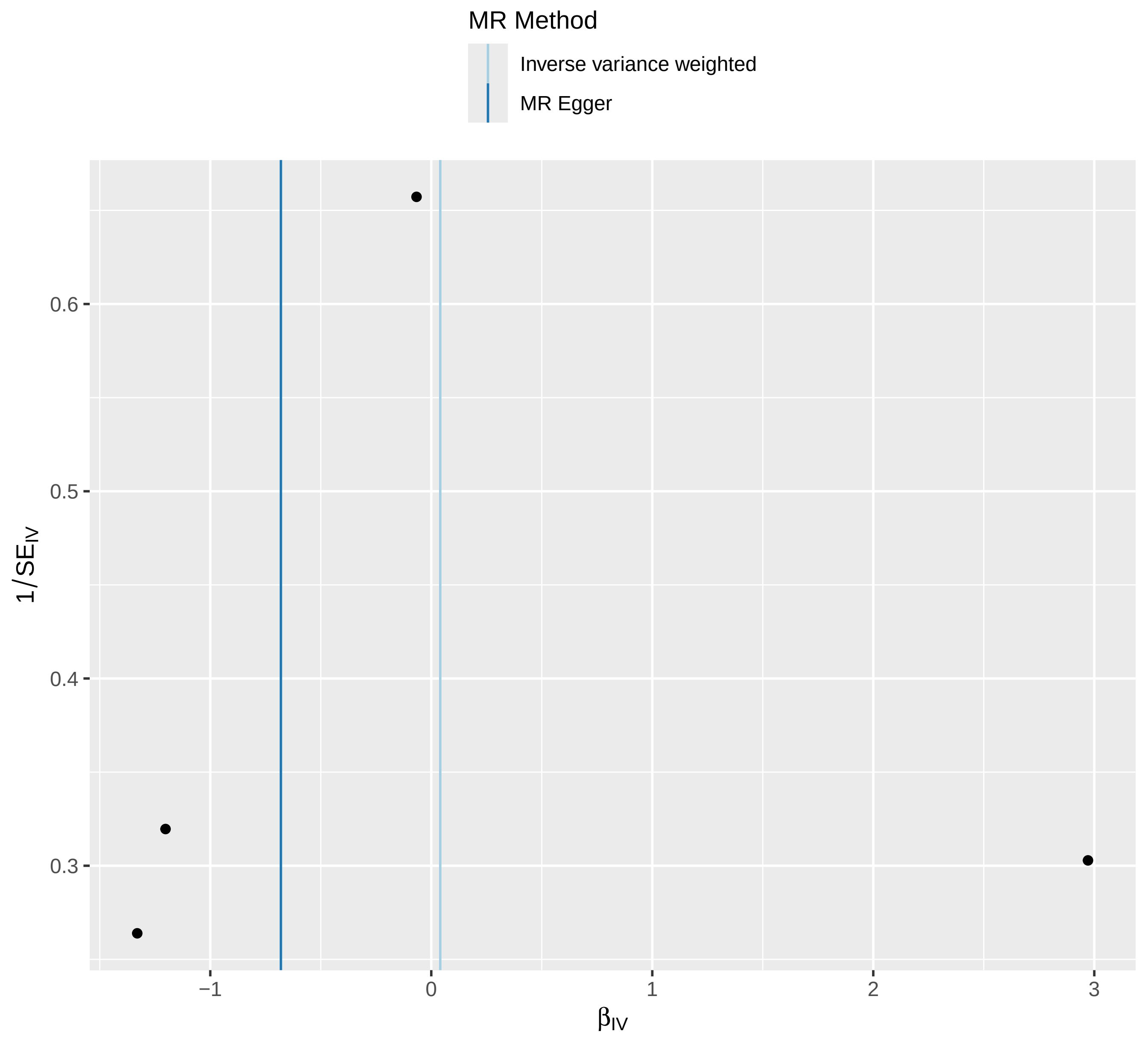

MR Method
Inverse variance weighted
MR Egger
0.6
0.5
0.4
0.3
1/SE_IV
−1
0
1
2
3
β_IV

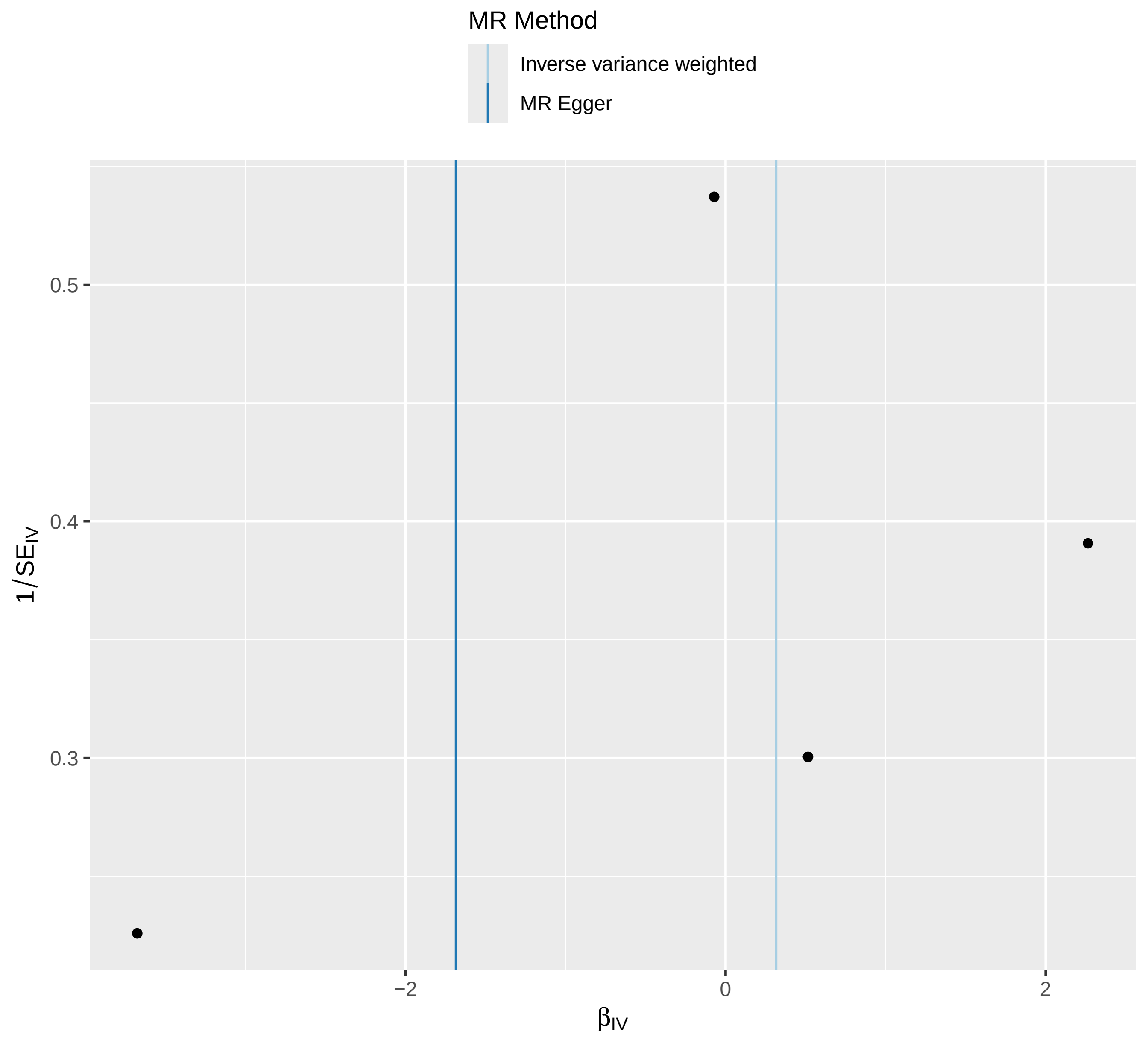
MR Method
Inverse variance weighted
MR Egger
0.5
0.4
0.3
1/SE_IV
−2
0
2
β_IV

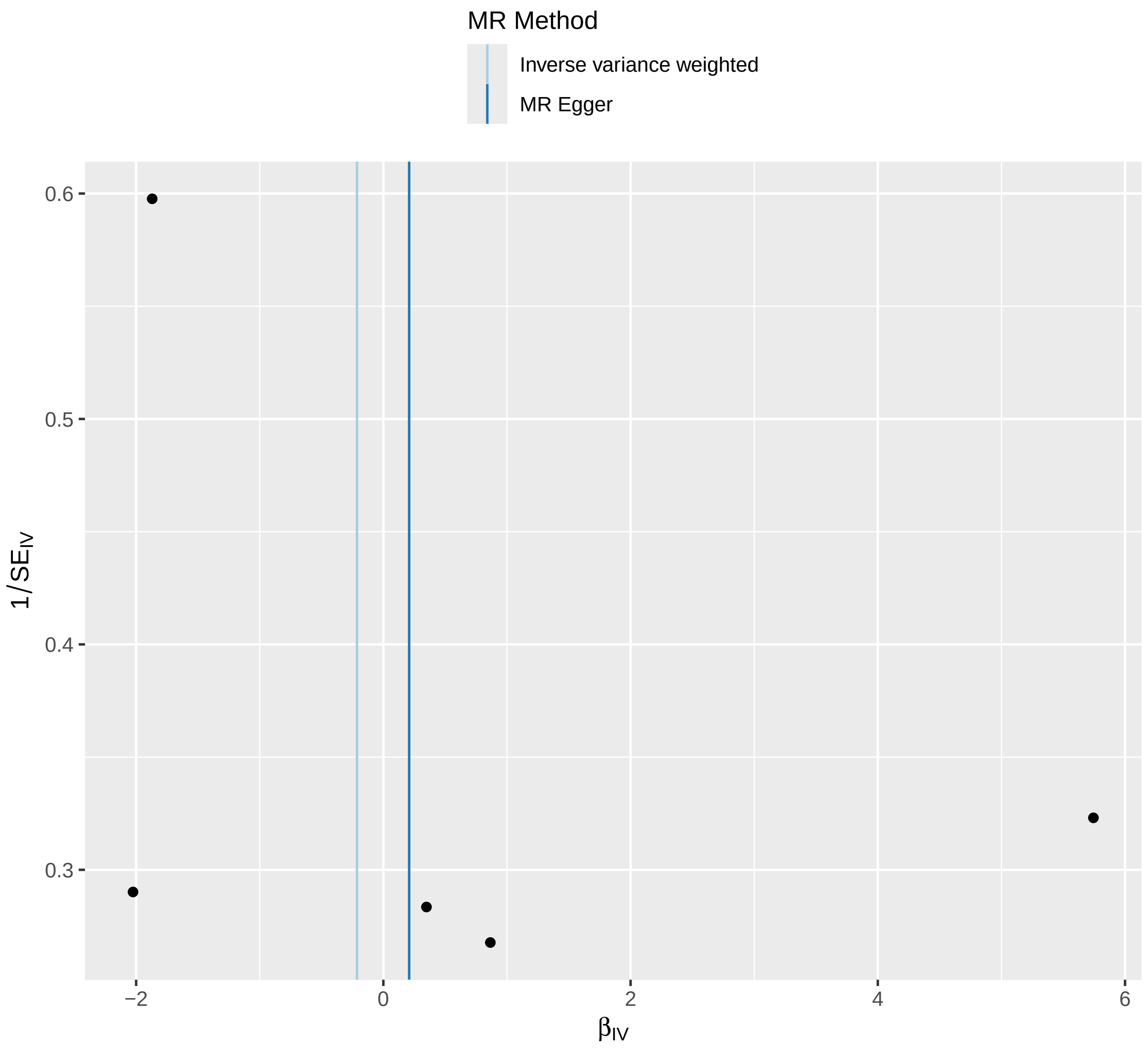
MR Method
Inverse variance weighted
MR Egger
0.6
0.5
0.4
0.3
1/SE_IV
−2
0
2
4
6
β_IV

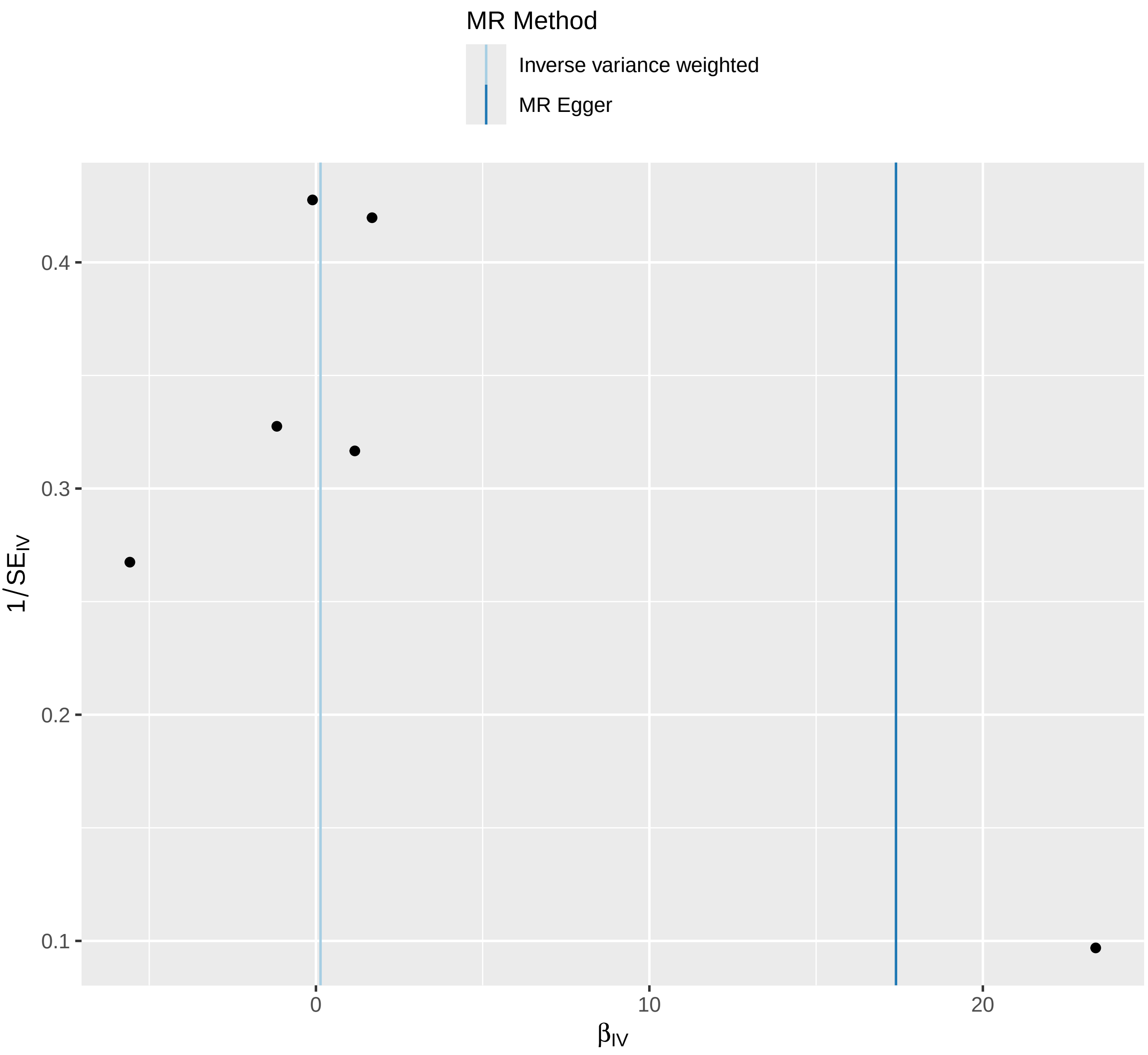
MR Method
Inverse variance weighted
MR Egger
0.4
0.3
0.2
0.1
1/SE_IV
0
10
20
β_IV

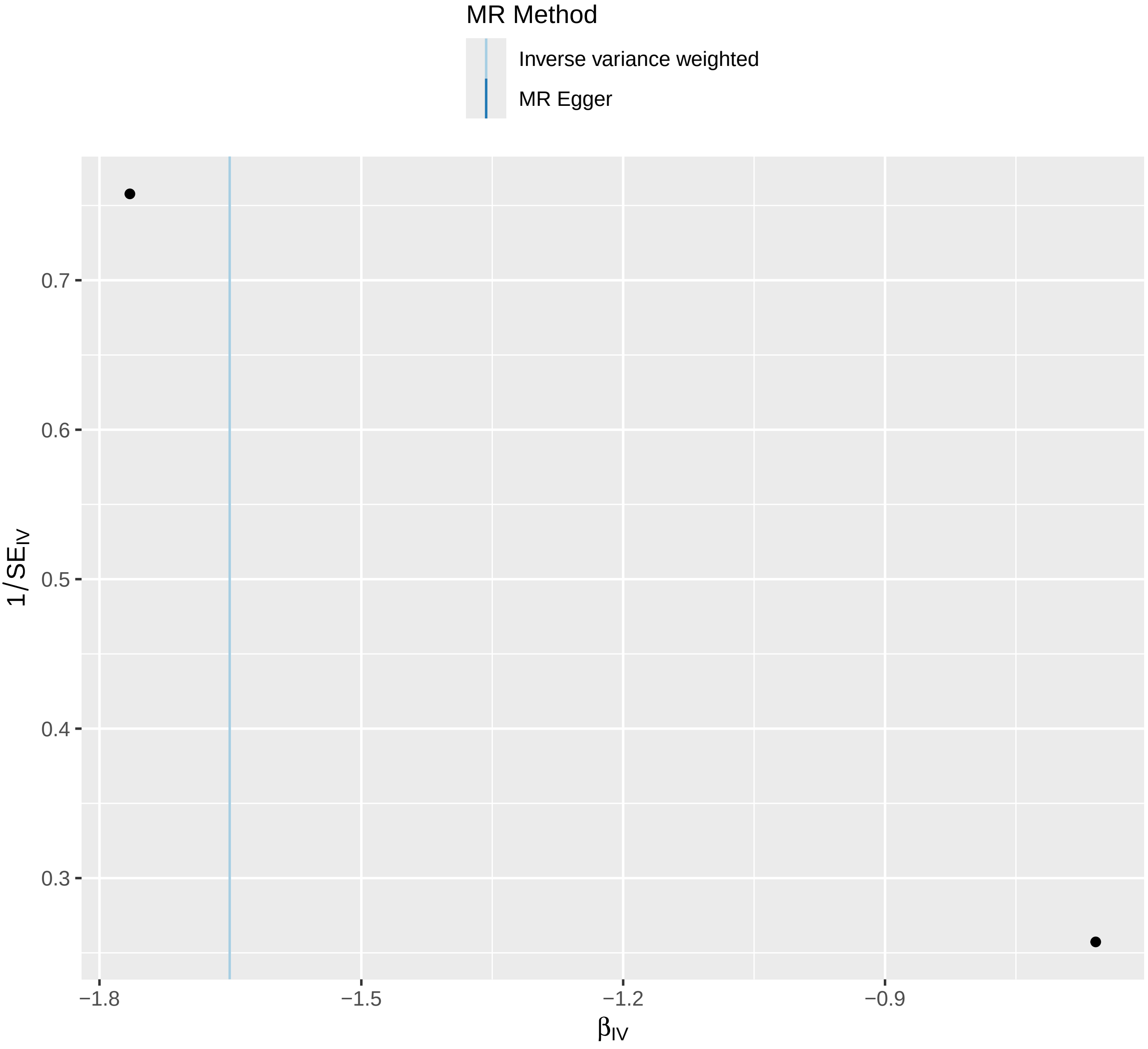

MR Method
Inverse variance weighted
MR Egger
0.7
0.6
0.5
0.4
0.3
1/SE_IV
−1.8
−1.5
−1.2
−0.9
β_IV

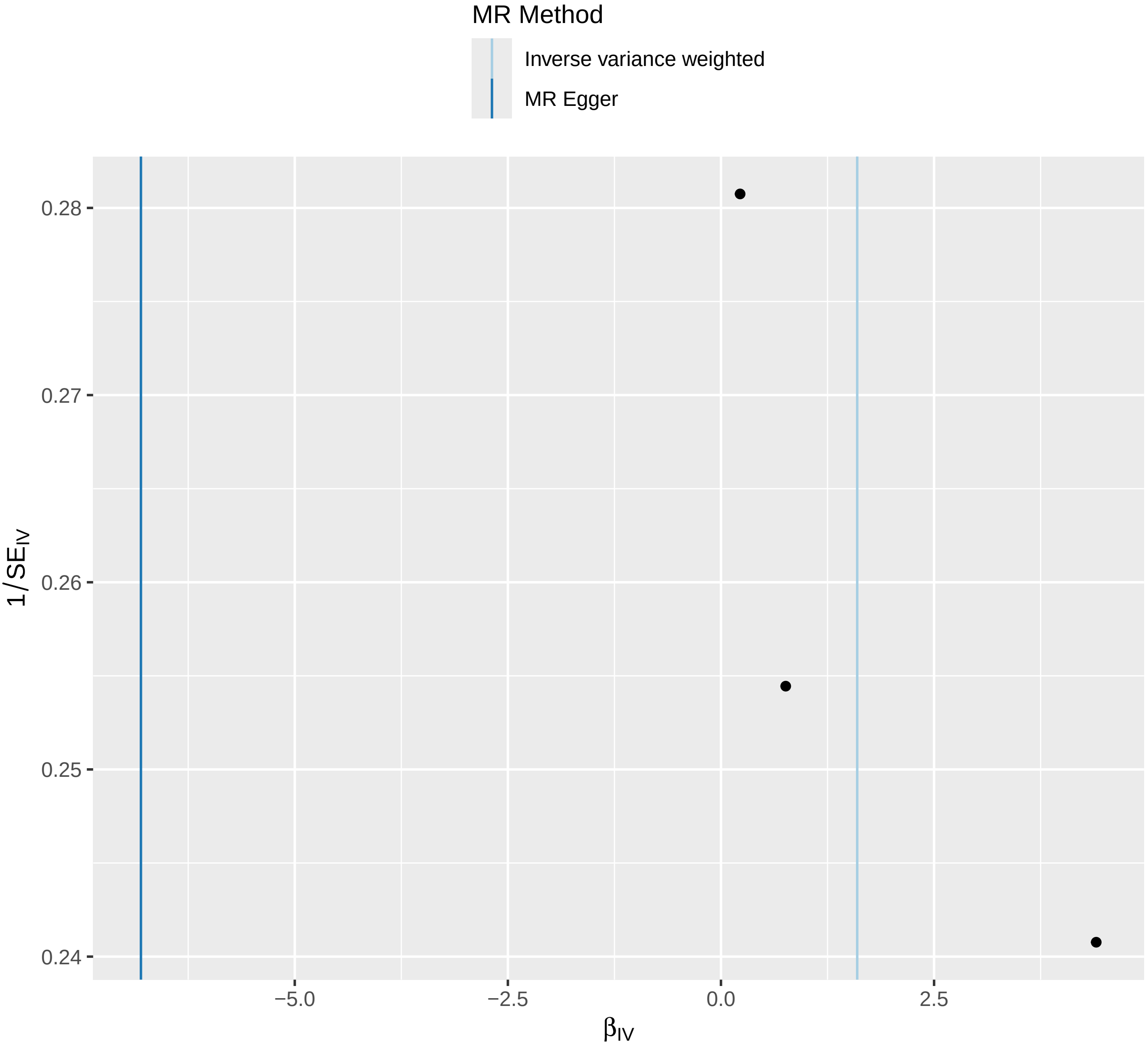

MR Method
Inverse variance weighted
MR Egger
0.28
0.27
0.26
0.25
0.24
1/SE_IV
−5.0
−2.5
0.0
2.5
β_IV

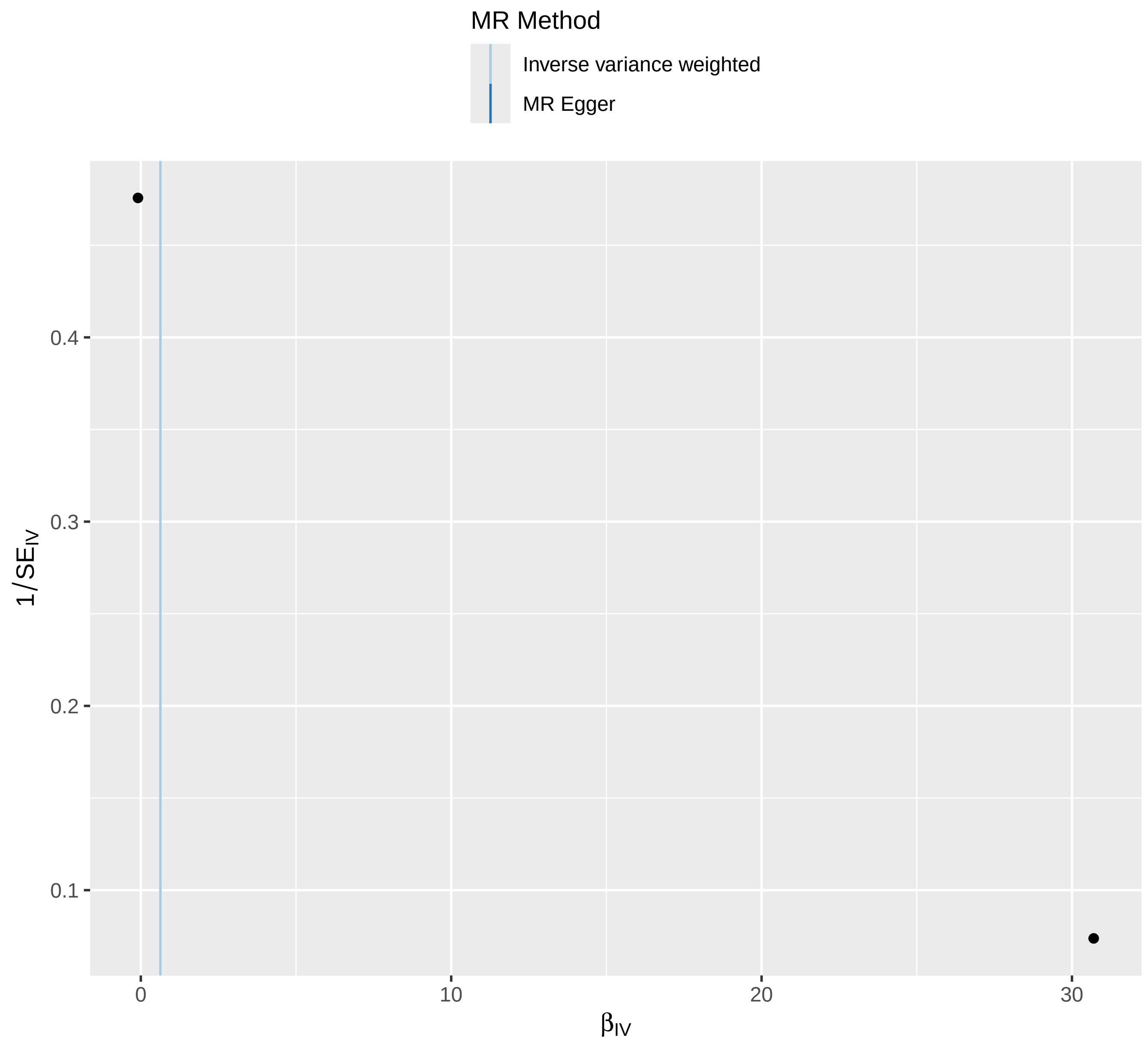
MR Method
Inverse variance weighted
MR Egger
0.4
0.3
0.2
0.1
1/SE_IV
0
10
20
30
β_IV

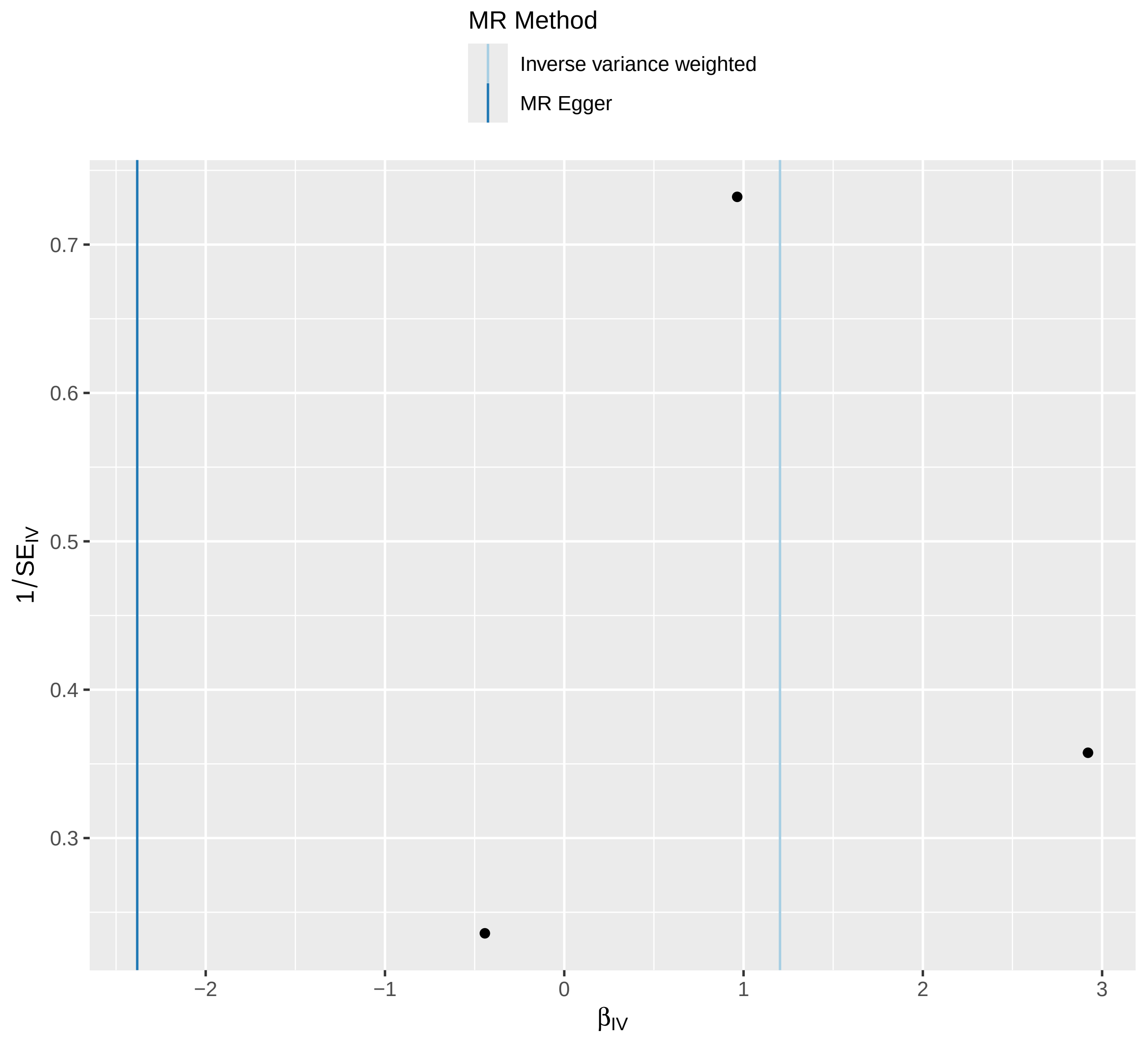
MR Method
Inverse variance weighted
MR Egger
0.7
0.6
0.5
0.4
0.3
1/SE_IV
−2
−1
0
1
2
3
β_IV

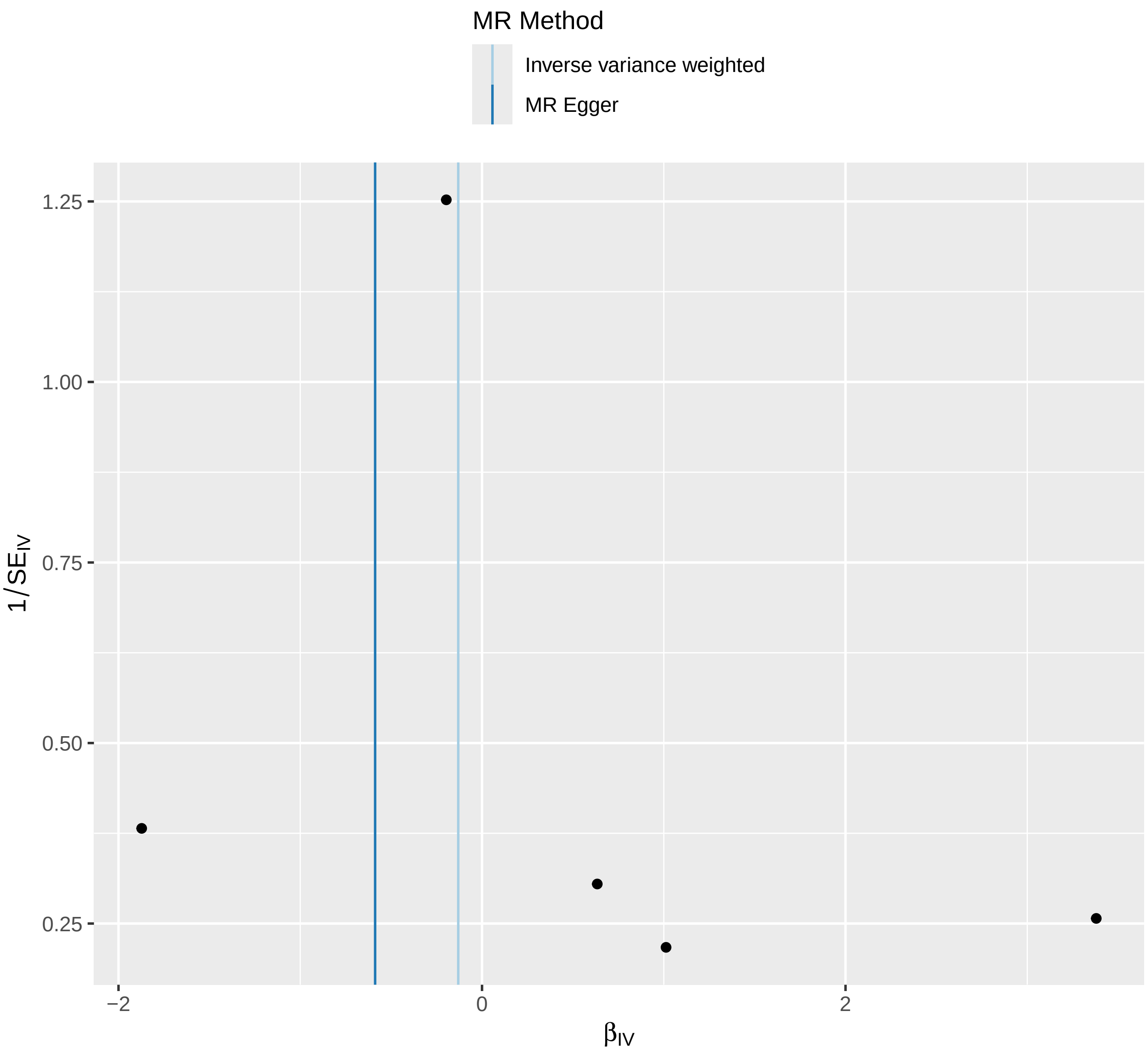
MR Method
Inverse variance weighted
MR Egger
1.25
1.00
0.75
0.50
0.25
1/SE_IV
−2
0
2
β_IV

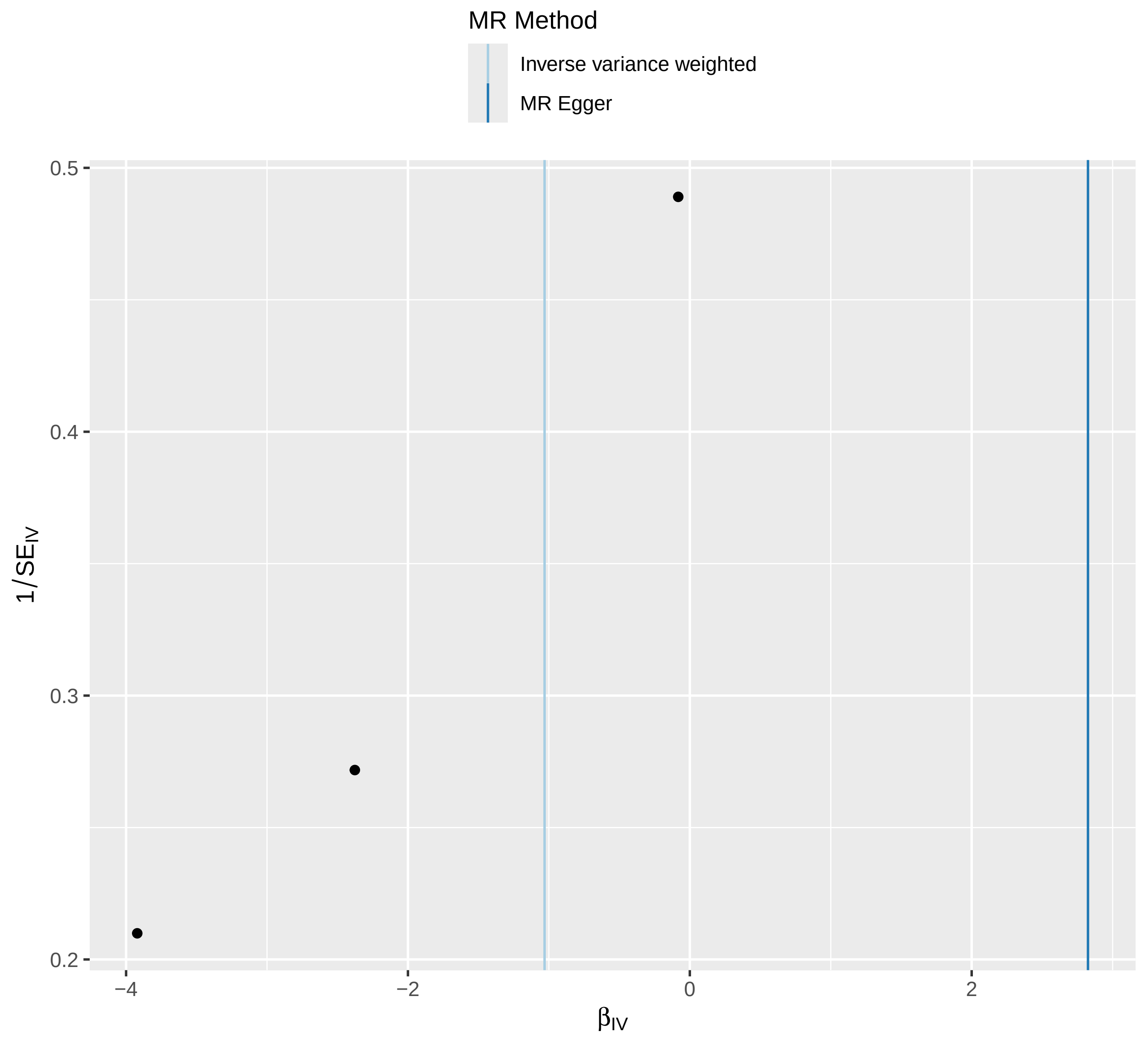
MR Method
Inverse variance weighted
MR Egger
0.5
0.4
0.3
0.2
1/SE$_{IV}$
−4
−2
0
2
β$_{IV}$

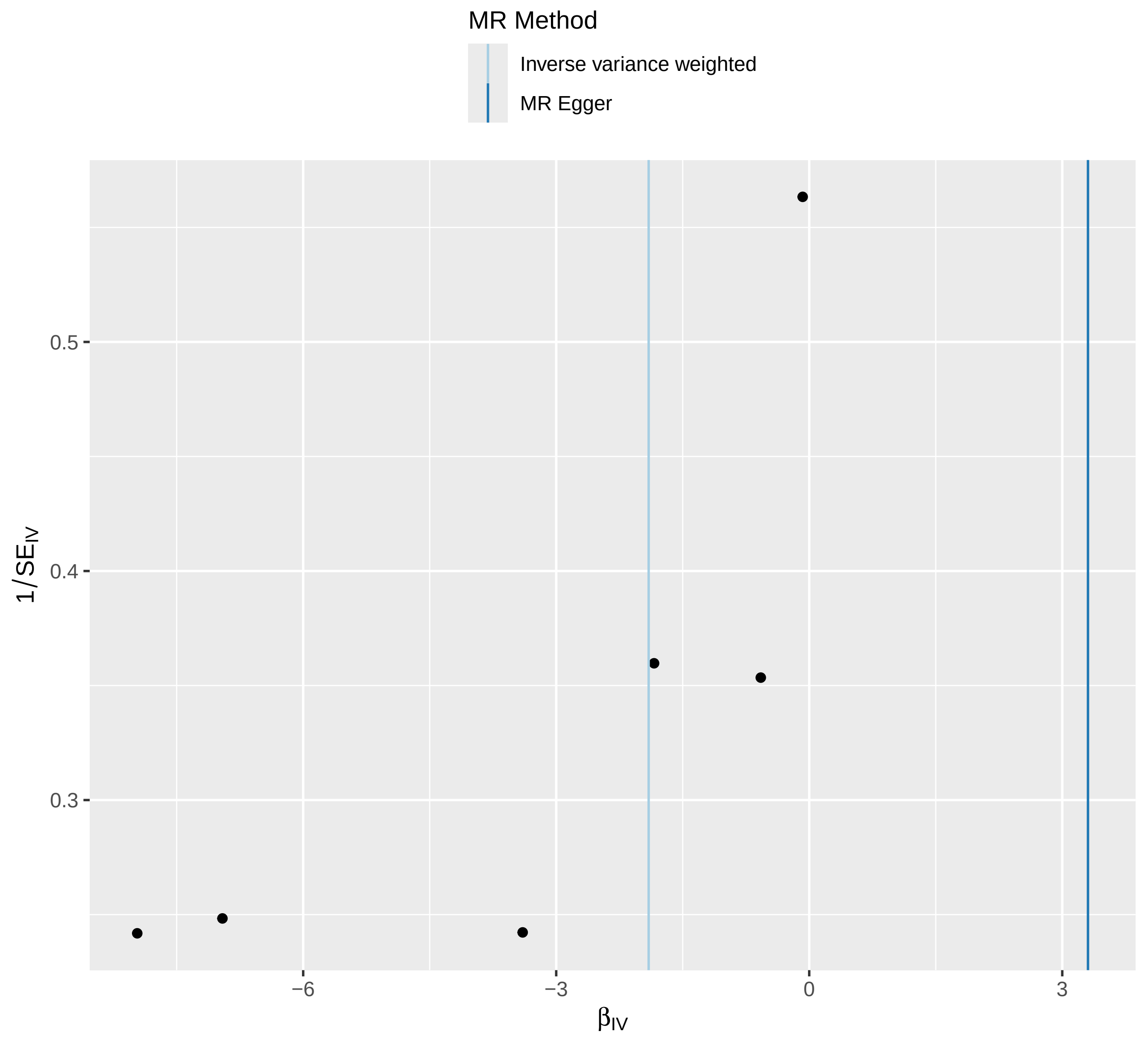
MR Method
Inverse variance weighted
MR Egger
0.5
0.4
0.3
1/SE_IV
−6
−3
0
3
β_IV

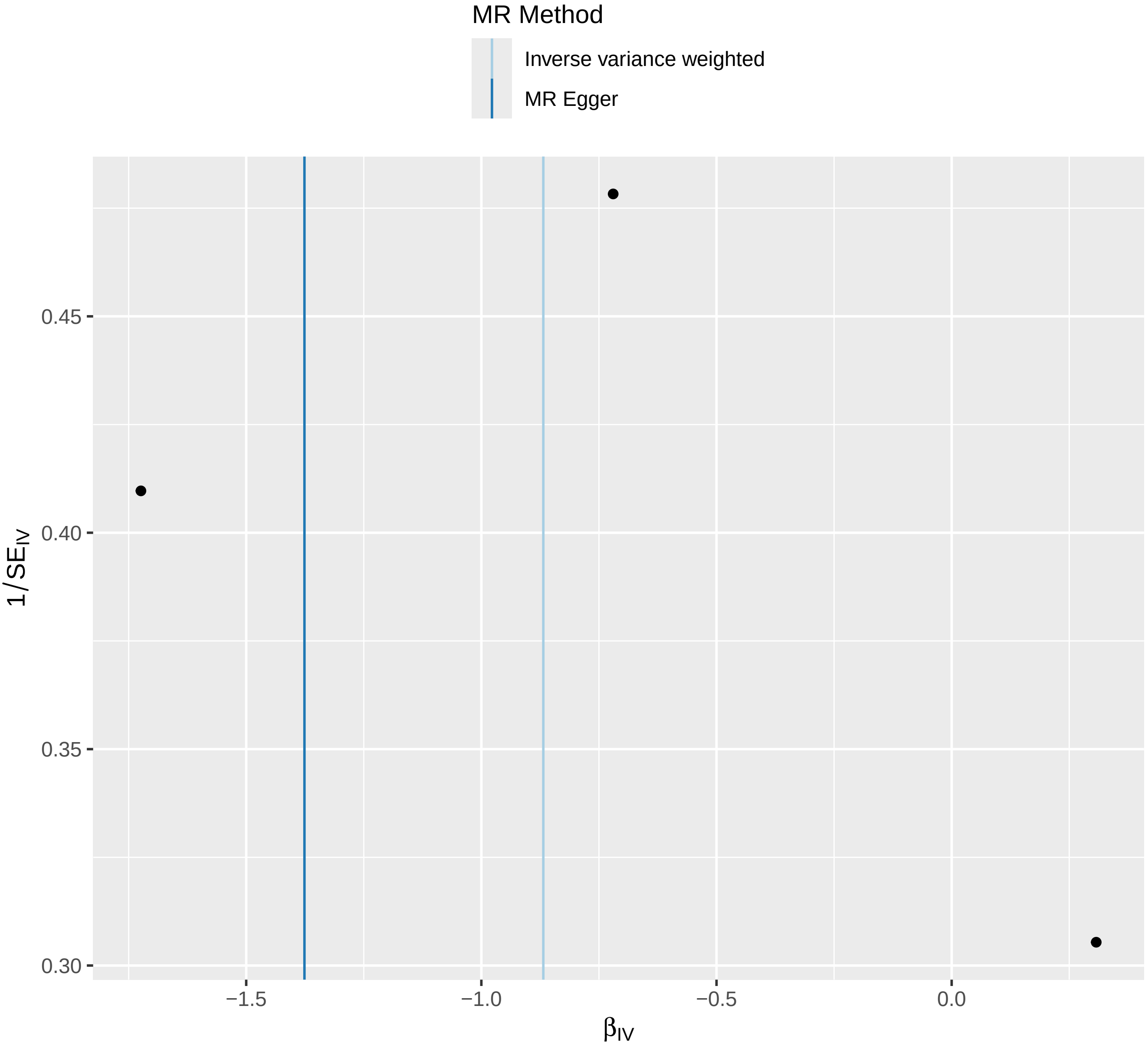
MR Method
Inverse variance weighted
MR Egger
0.45
0.40
0.35
0.30
1/SE$_{IV}$
−1.5
−1.0
−0.5
0.0
β$_{IV}$

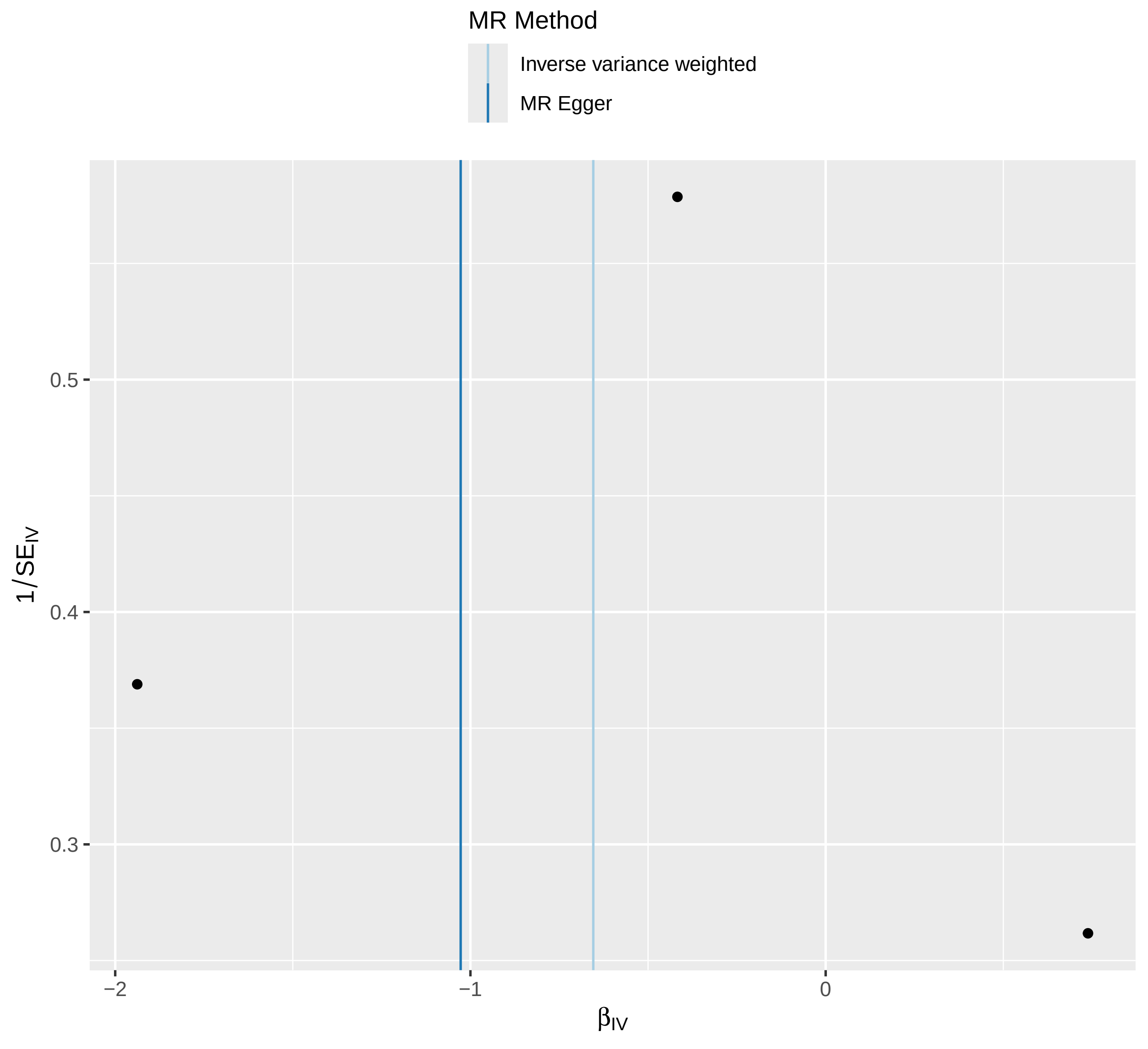
MR Method
Inverse variance weighted
MR Egger
0.5
0.4
0.3
1/SE$_{IV}$
−2
−1
0
β$_{IV}$

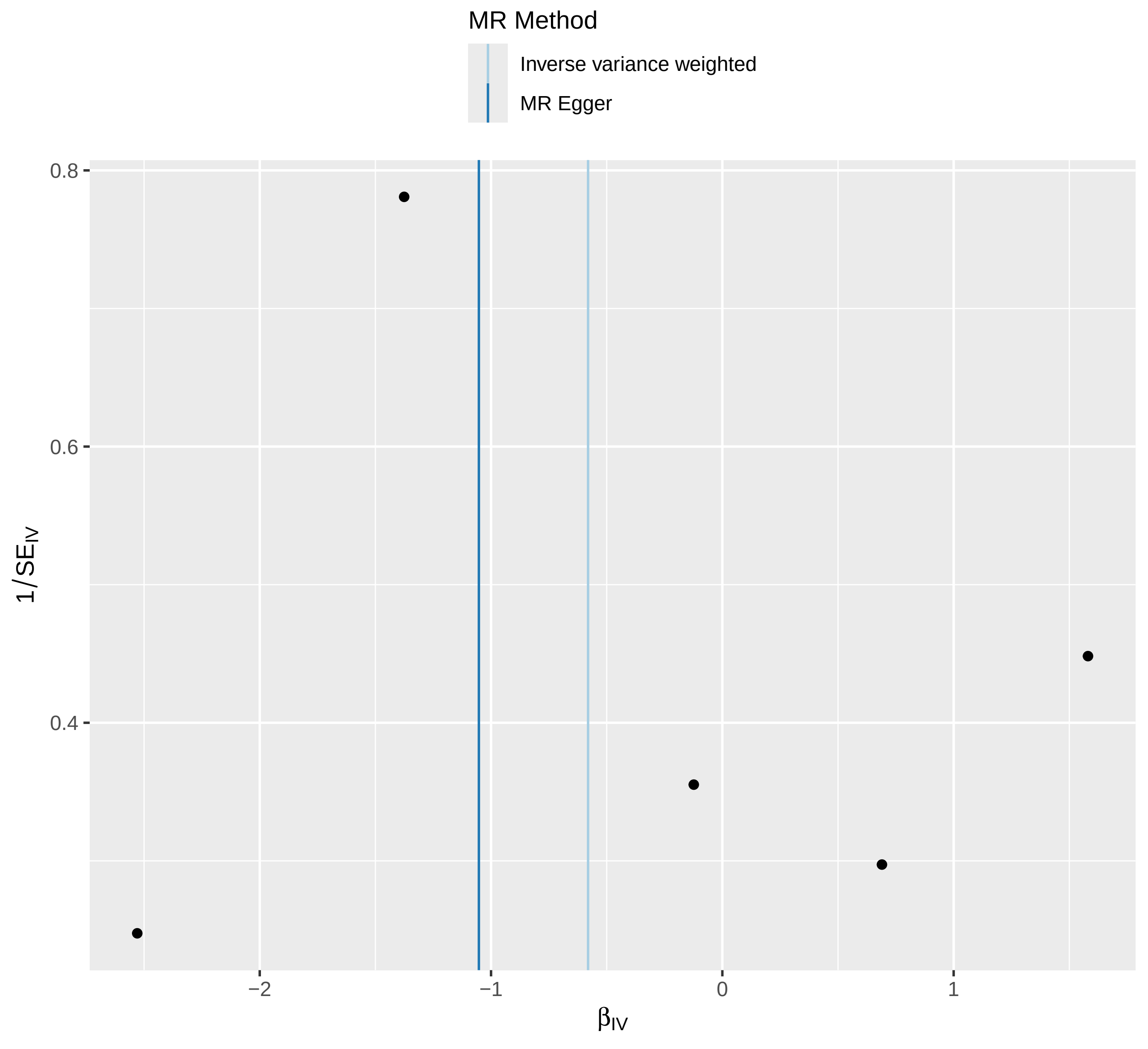
MR Method
Inverse variance weighted
MR Egger
0.8
0.6
0.4
1/SE_IV
−2
−1
0
1
β_IV

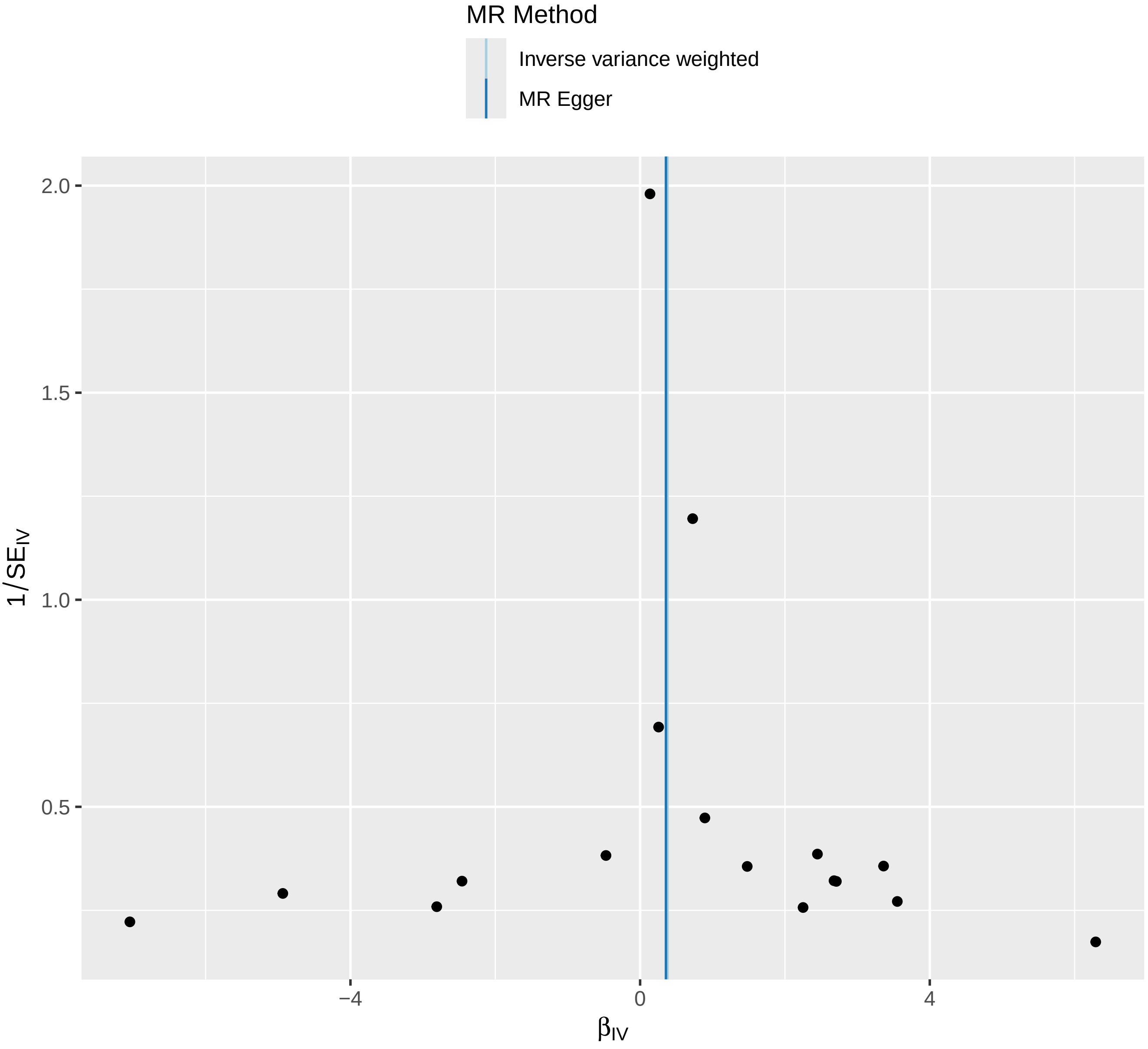

MR Method
Inverse variance weighted
MR Egger
2.0
1.5
1.0
0.5
1/SE_IV
−4
0
4
β_IV

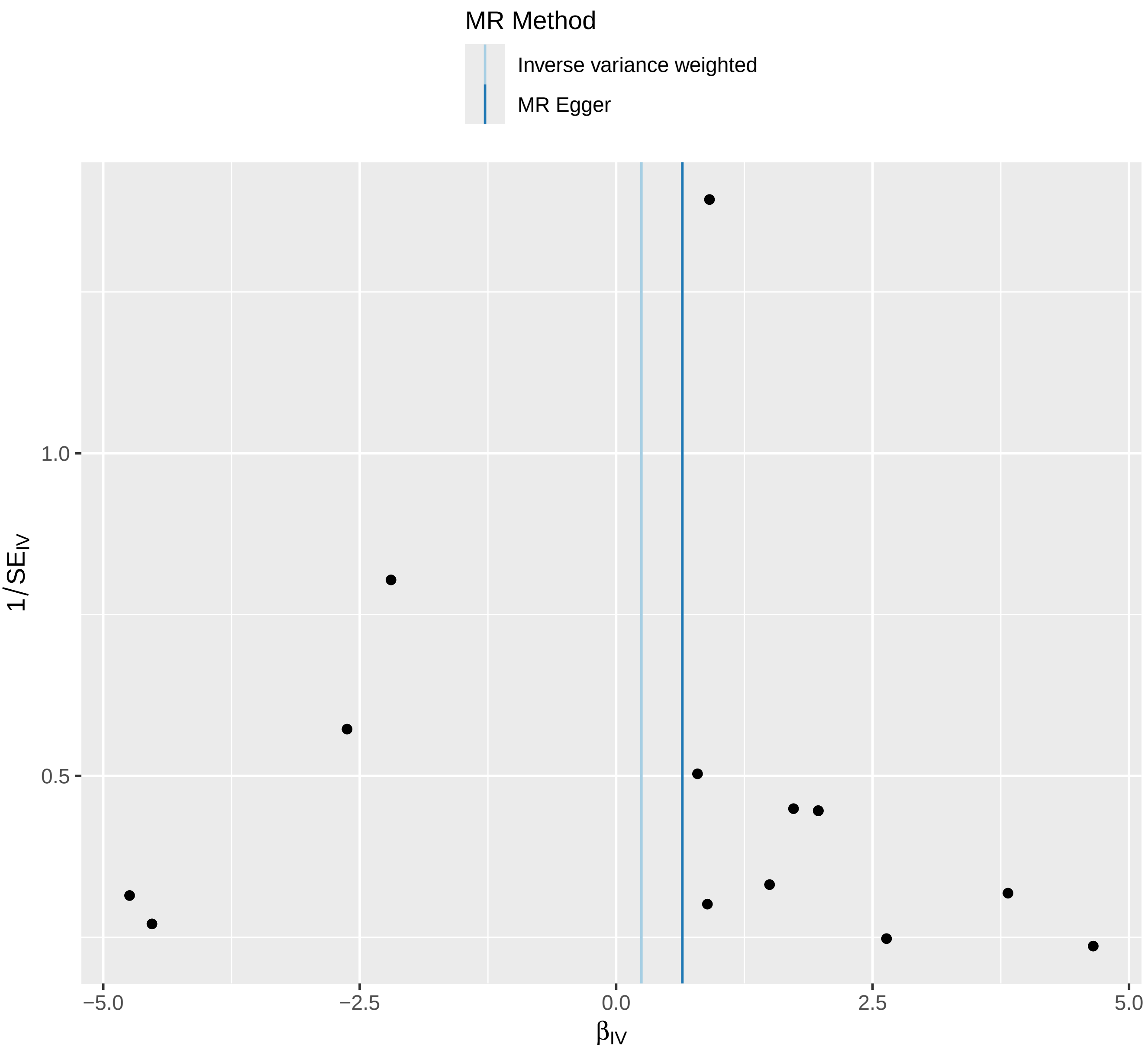
MR Method
Inverse variance weighted
MR Egger
1.0
0.5
1/SE$_{IV}$
−5.0
−2.5
0.0
2.5
5.0
β$_{IV}$

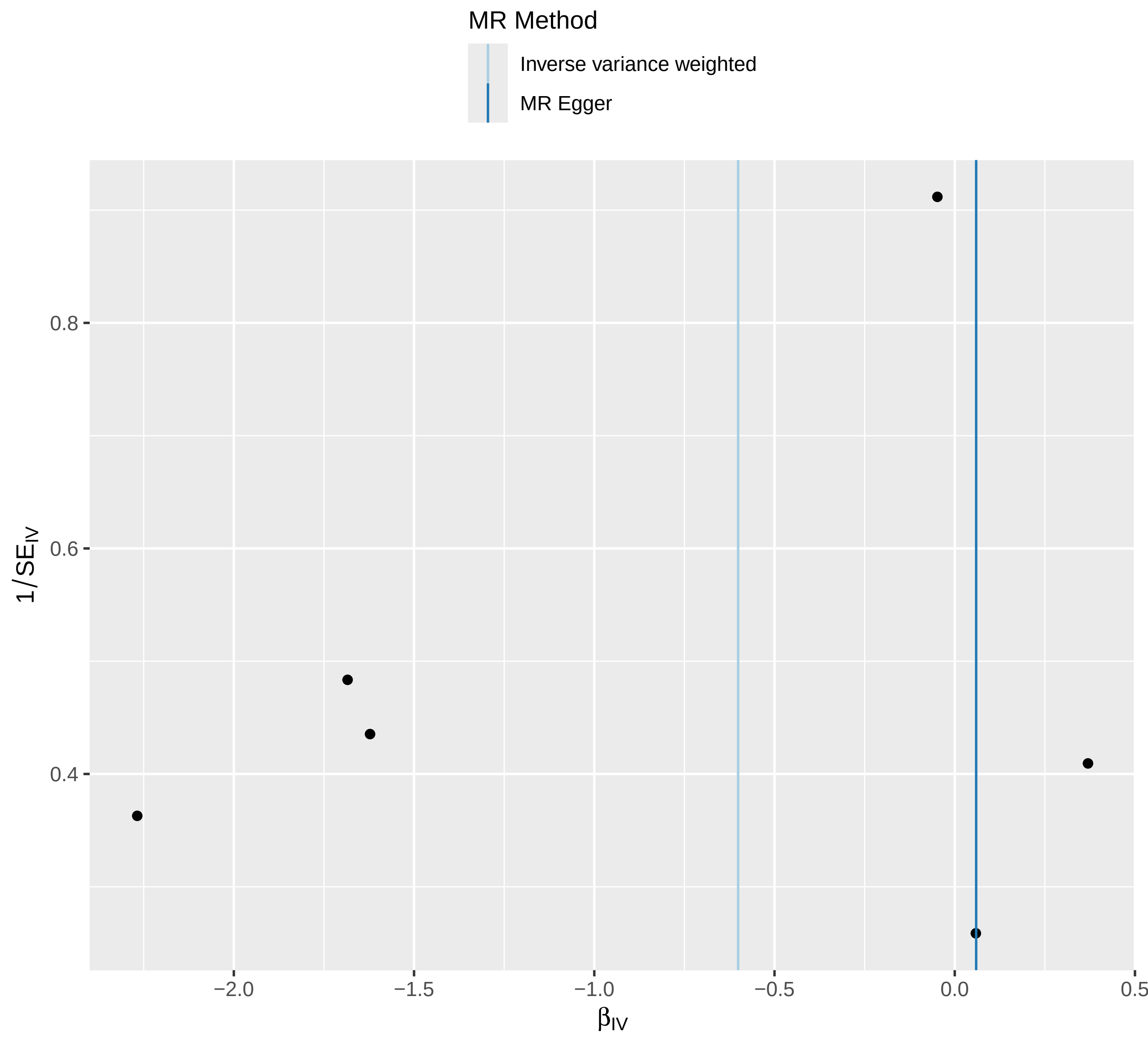
MR Method
Inverse variance weighted
MR Egger
0.8
0.6
0.4
$1/SE_{IV}$
−2.0
−1.5
−1.0
−0.5
0.0
0.5
β_{IV}

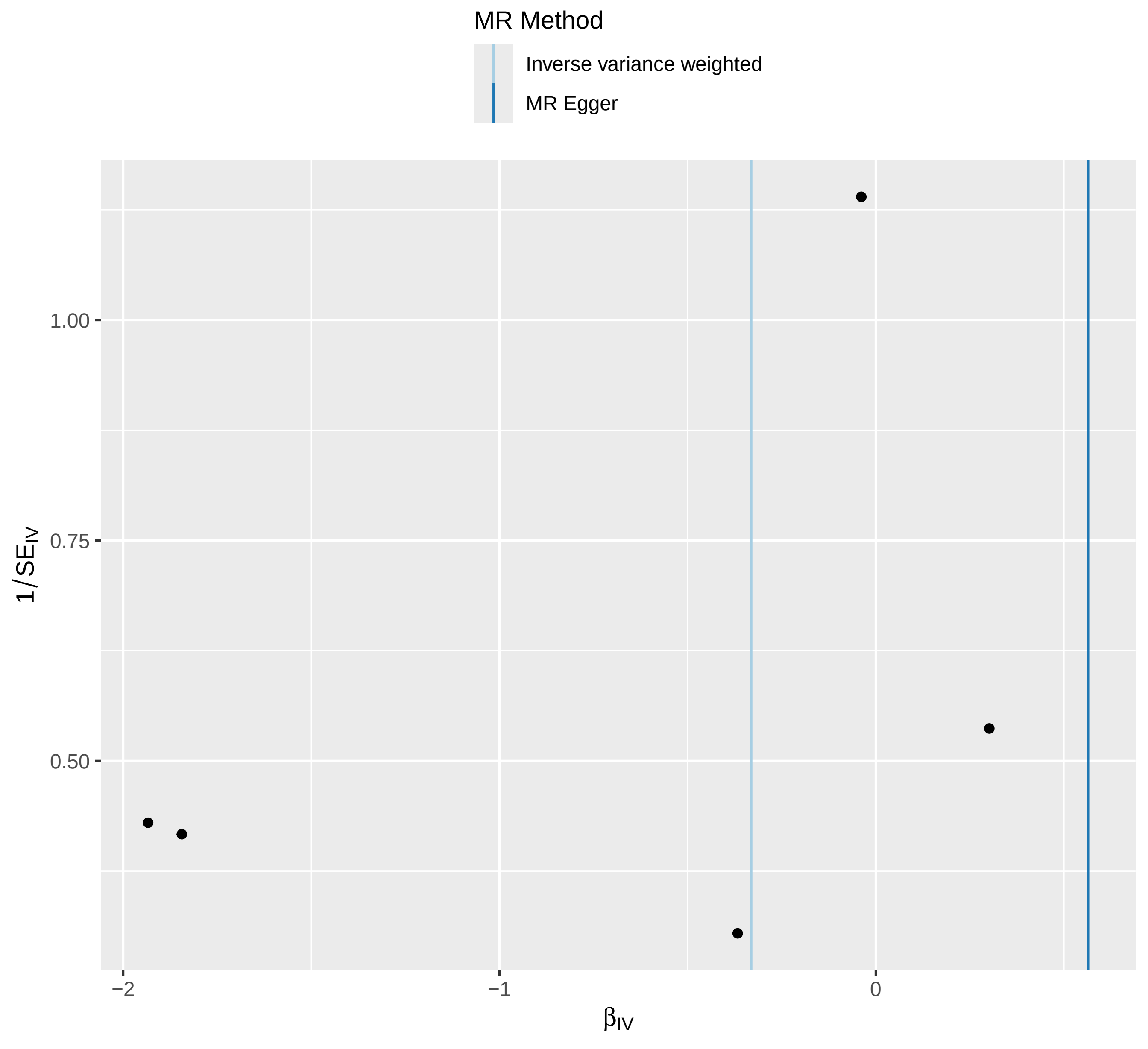
MR Method
Inverse variance weighted
MR Egger
1.00
0.75
0.50
1/SE_IV
−2
−1
0
β_IV

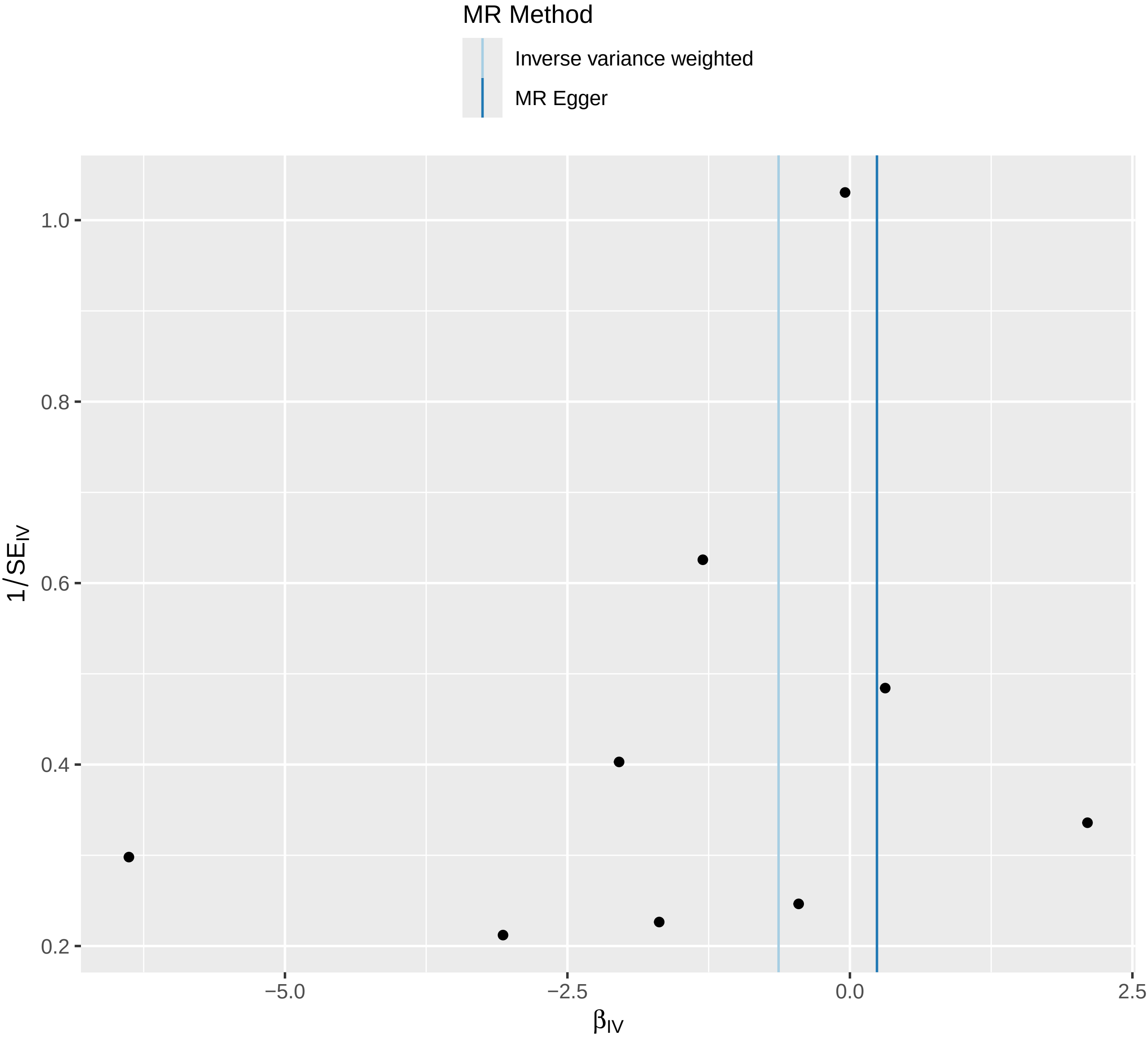
MR Method
Inverse variance weighted
MR Egger
1.0
0.8
0.6
0.4
0.2
$1/SE_{IV}$
−5.0
−2.5
0.0
2.5
β_{IV}

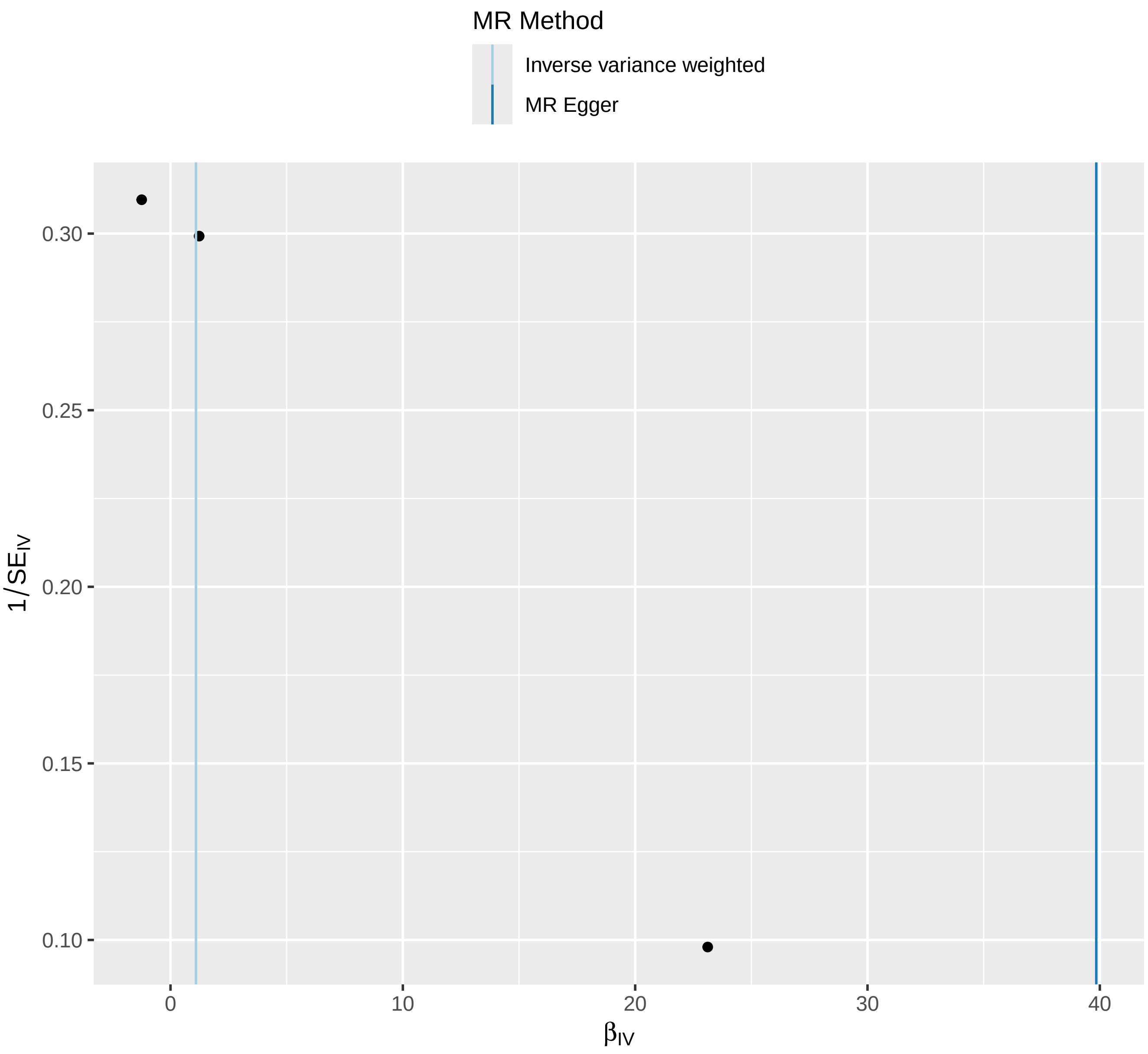
MR Method
Inverse variance weighted
MR Egger
0.30
0.25
0.20
0.15
0.10
1/SE_IV
0
10
20
30
40
β_IV

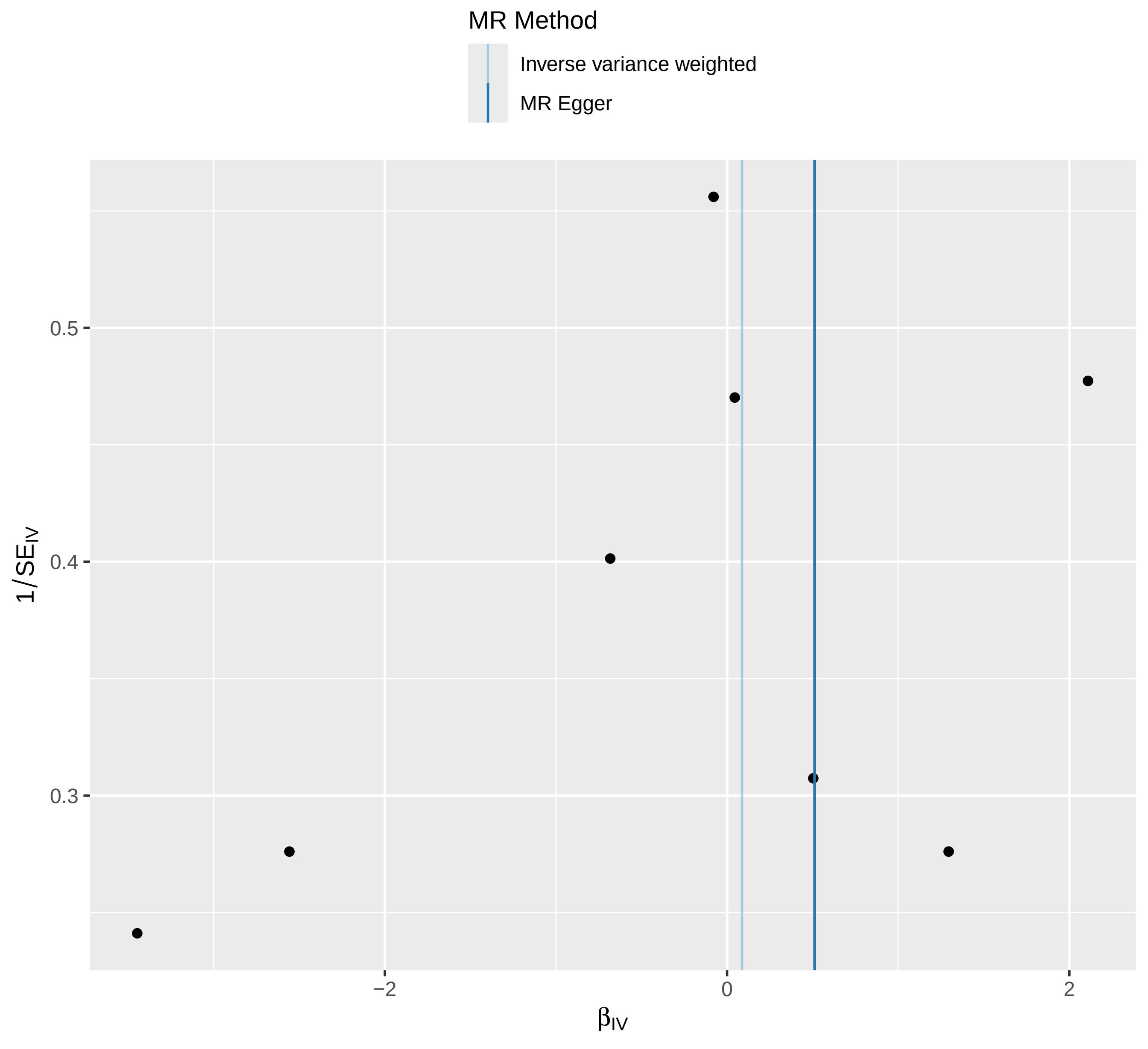
MR Method
Inverse variance weighted
MR Egger
1/SE_IV
0.5
0.4
0.3
−2
0
2
β_IV

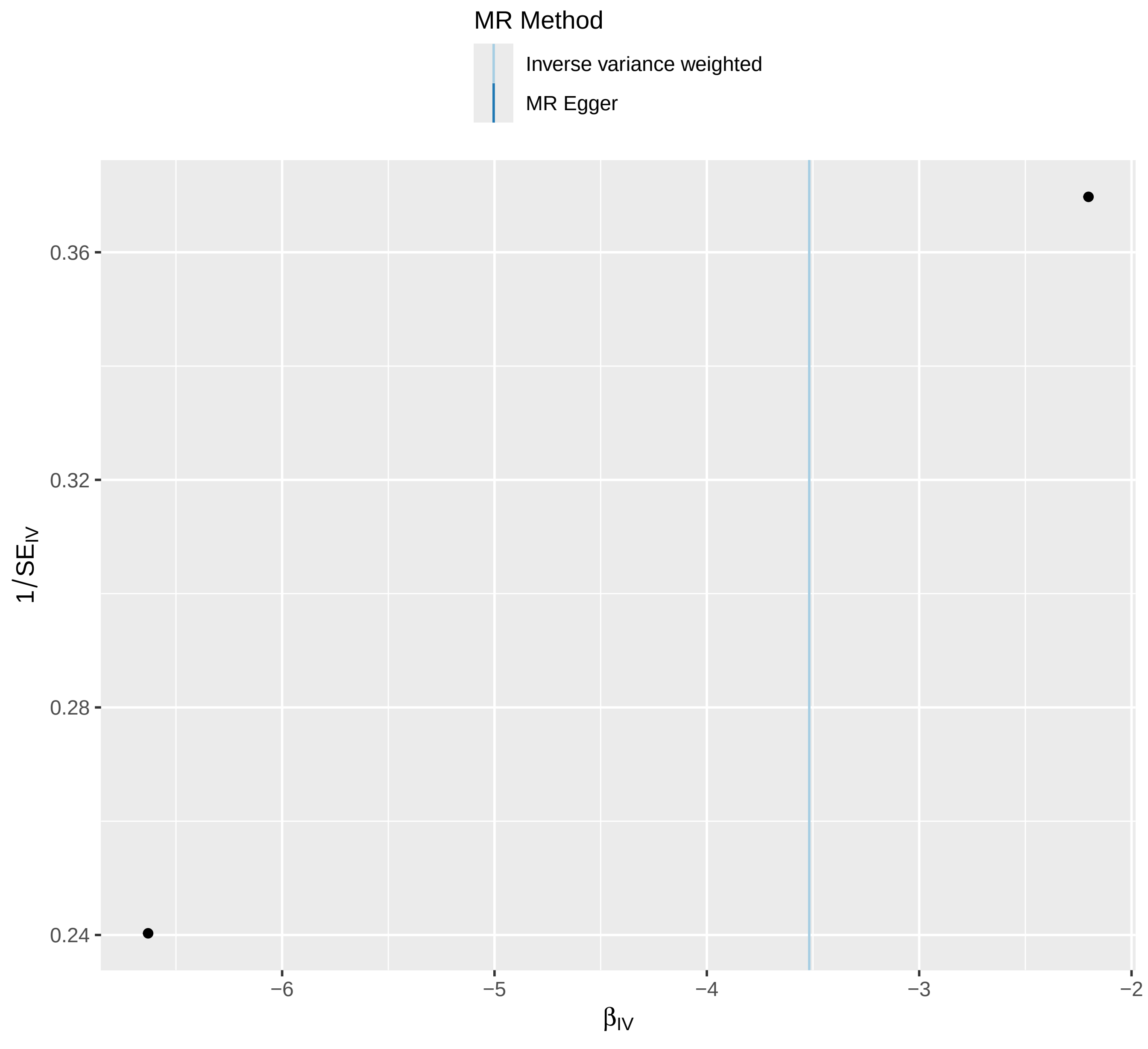

MR Method
Inverse variance weighted
MR Egger
0.36
0.32
0.28
0.24
1/SE_IV
−6
−5
−4
−3
−2
β_IV

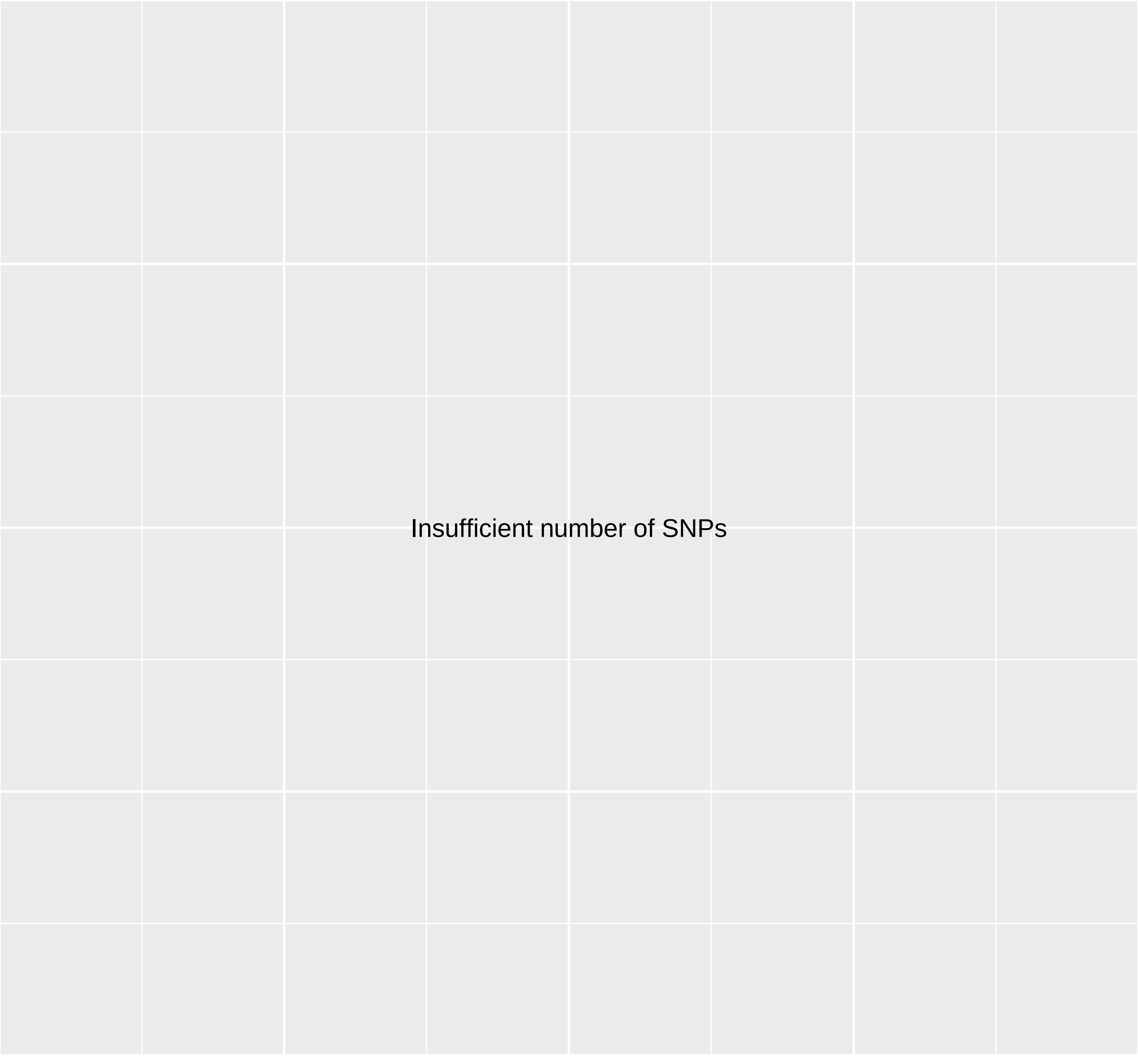
Insufficient number of SNPs

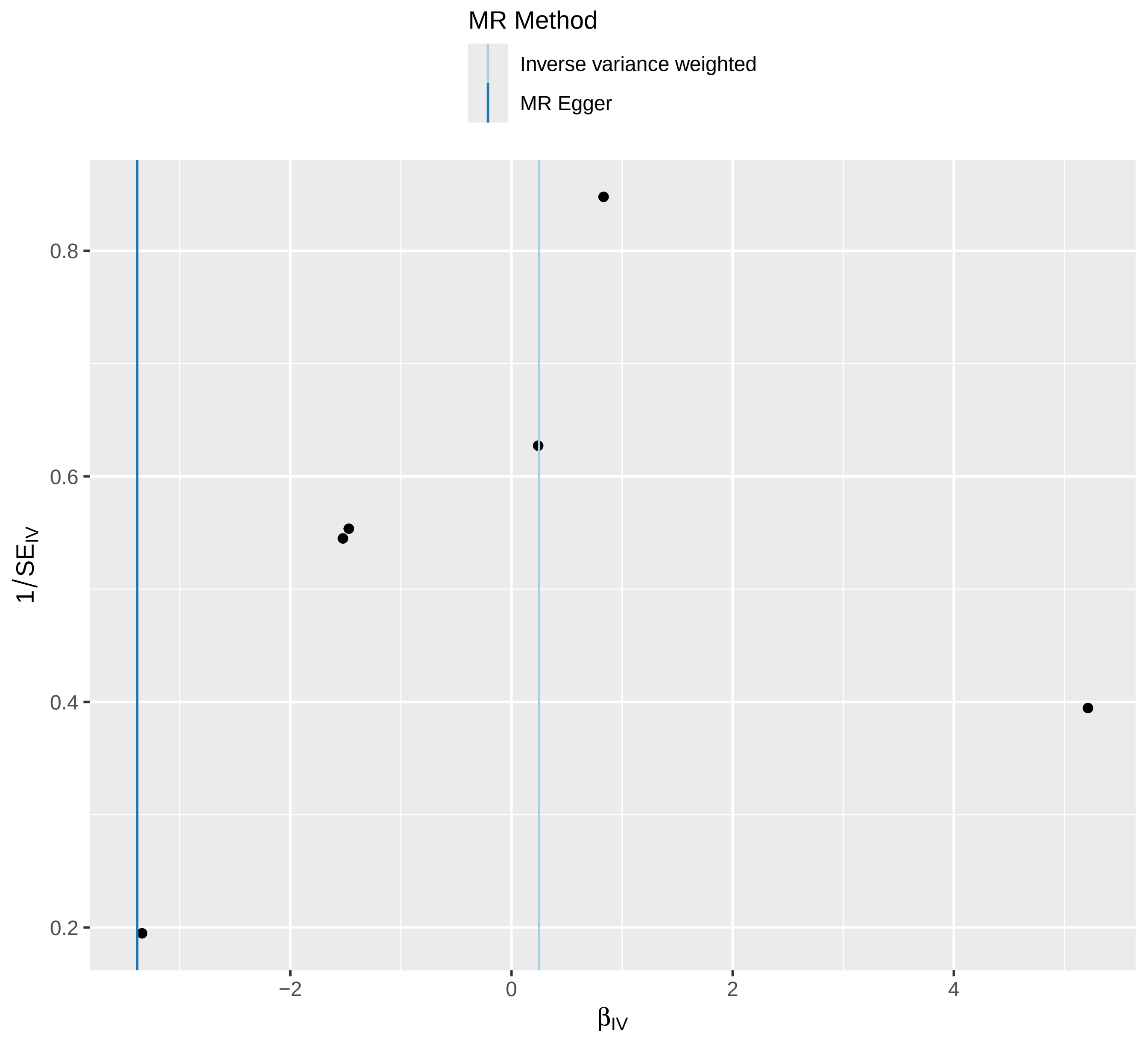
MR Method
Inverse variance weighted
MR Egger
0.8
0.6
0.4
0.2
1/SE_IV
−2
0
2
4
β_IV

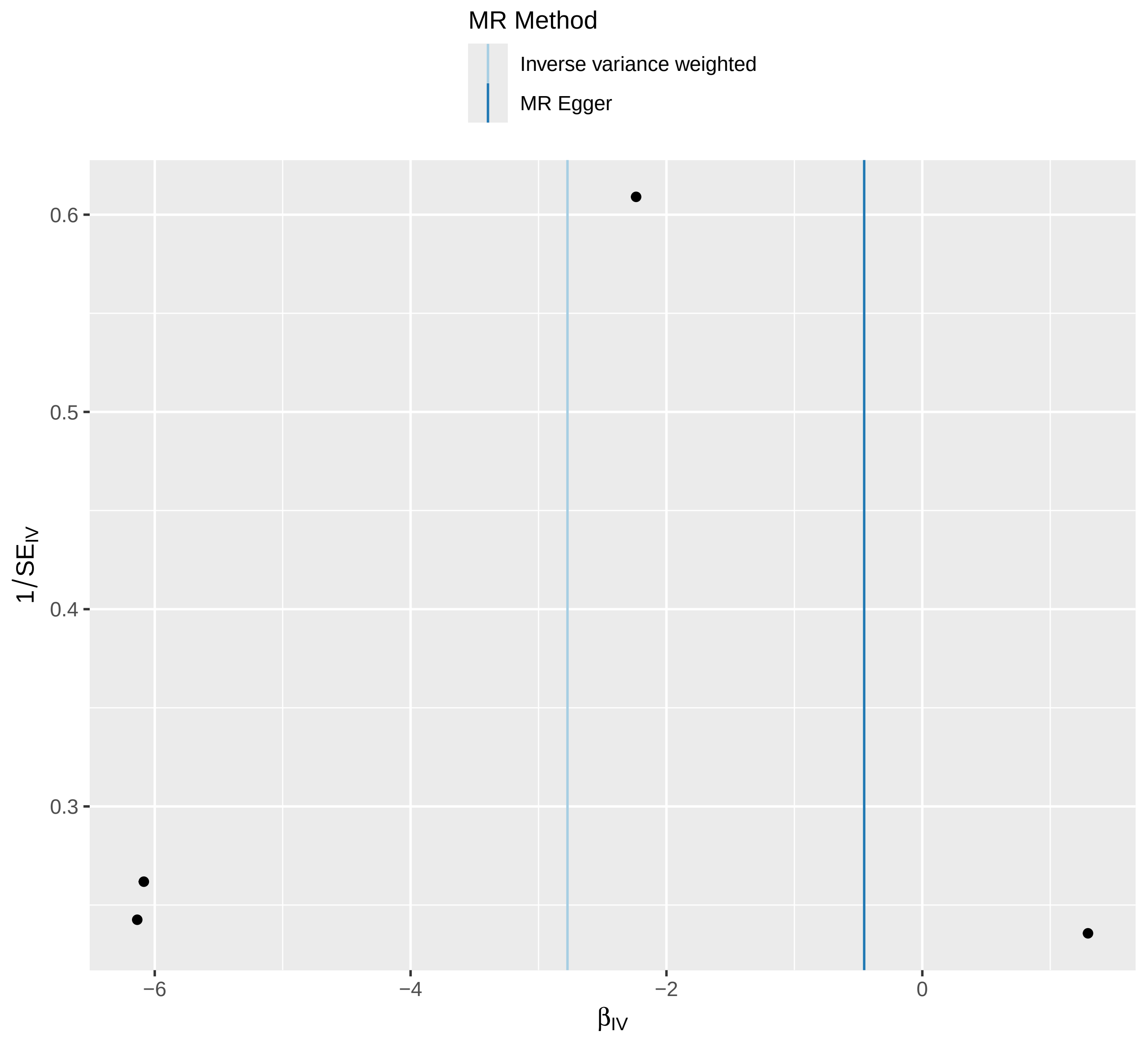
MR Method
Inverse variance weighted
MR Egger
0.6
0.5
0.4
0.3
1/SE_IV
−6
−4
−2
0
β_IV

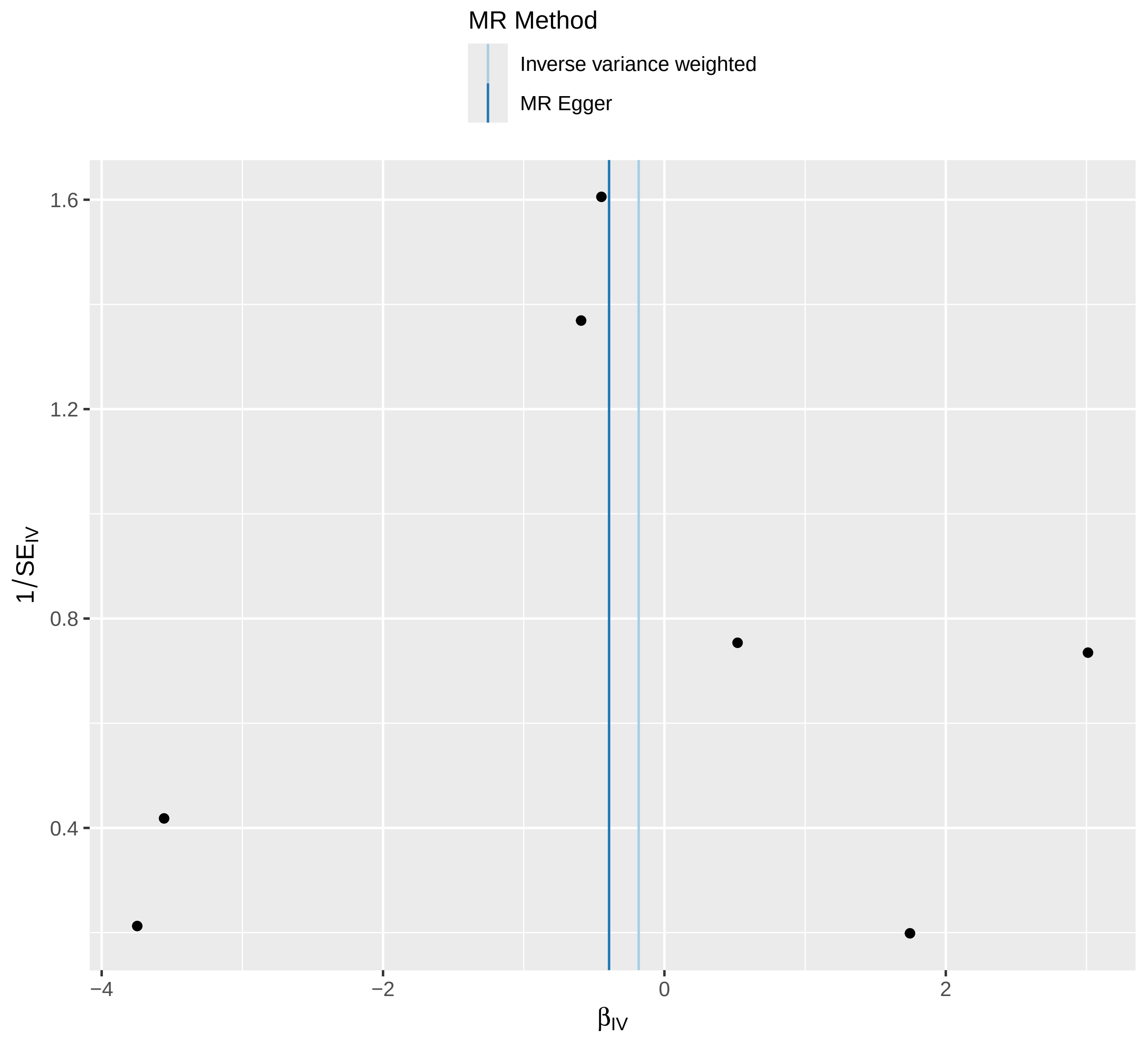
MR Method
Inverse variance weighted
MR Egger
1.6
1.2
0.8
0.4
−4
−2
0
2
β_{IV}
$1/SE_{IV}$

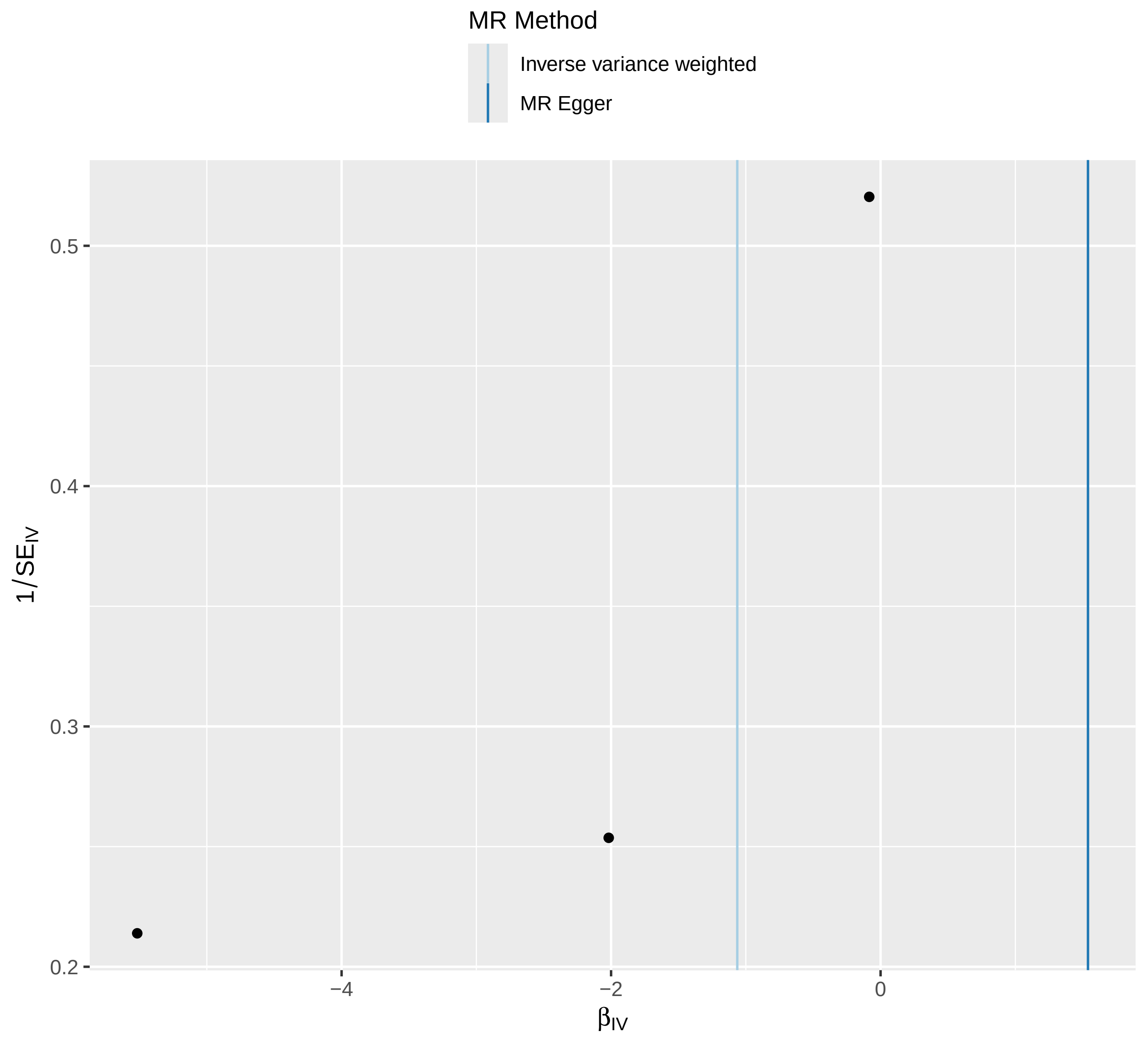
MR Method
Inverse variance weighted
MR Egger
0.5
0.4
0.3
0.2
1/SE_IV
−4
−2
0
β_IV

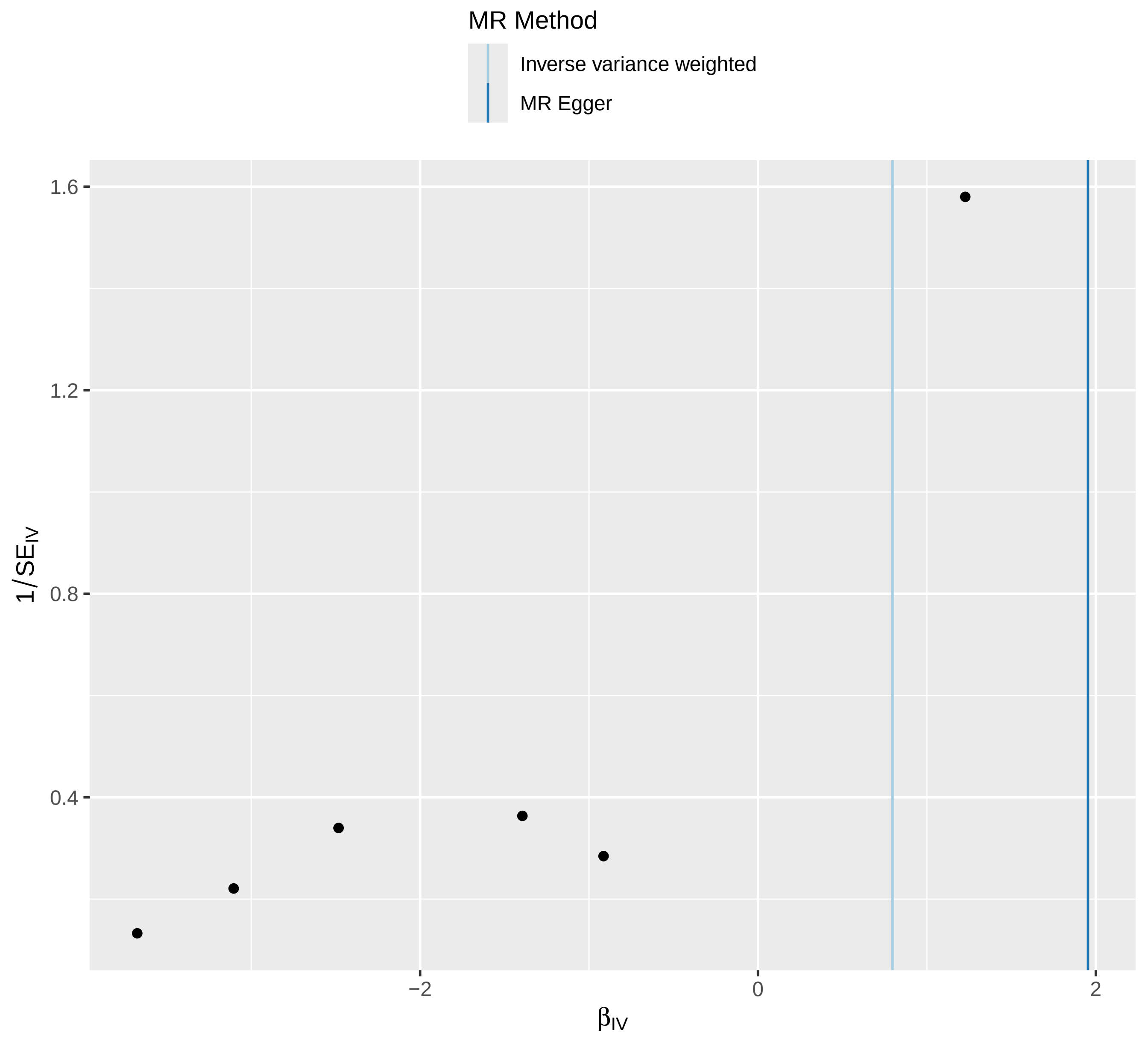

MR Method
Inverse variance weighted
MR Egger
1.6
1.2
0.8
0.4
1/SE_IV
−2
0
2
β_IV

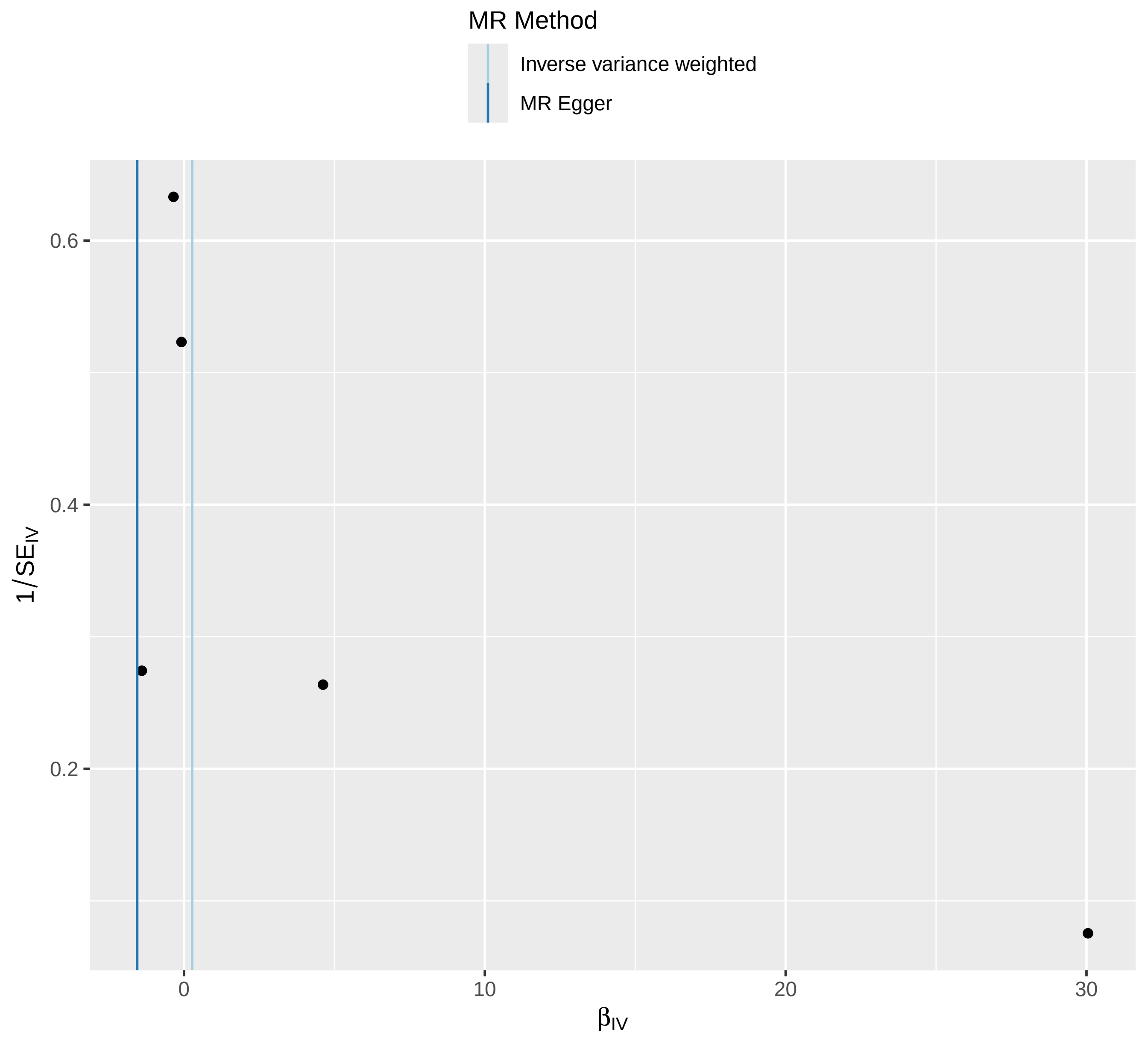
MR Method
Inverse variance weighted
MR Egger
0.6
0.4
0.2
1/SE_IV
0
10
20
30
β_IV

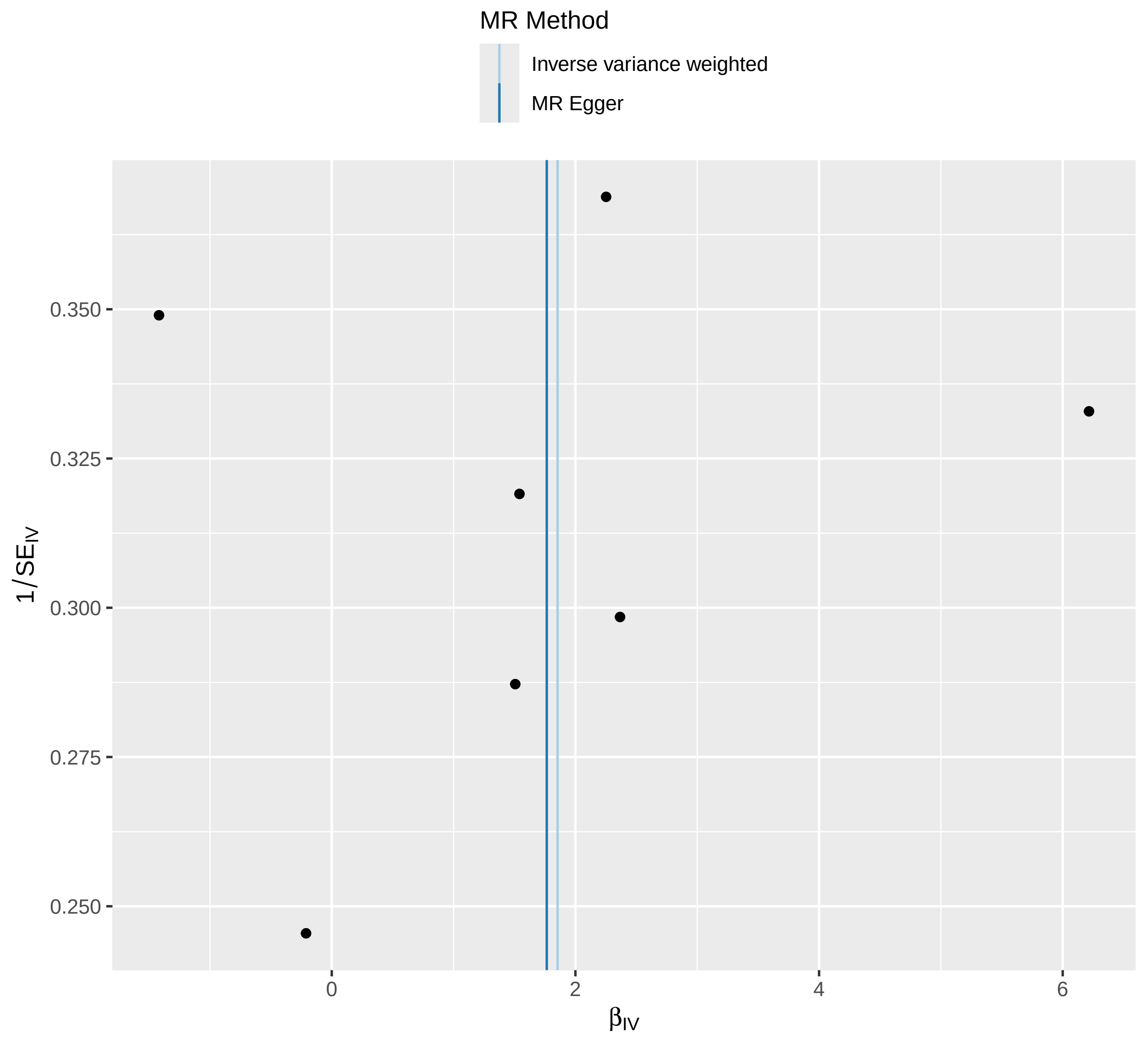
MR Method
Inverse variance weighted
MR Egger
0.350
0.325
0.300
0.275
0.250
$1/SE_{IV}$
0
2
4
6
β_{IV}

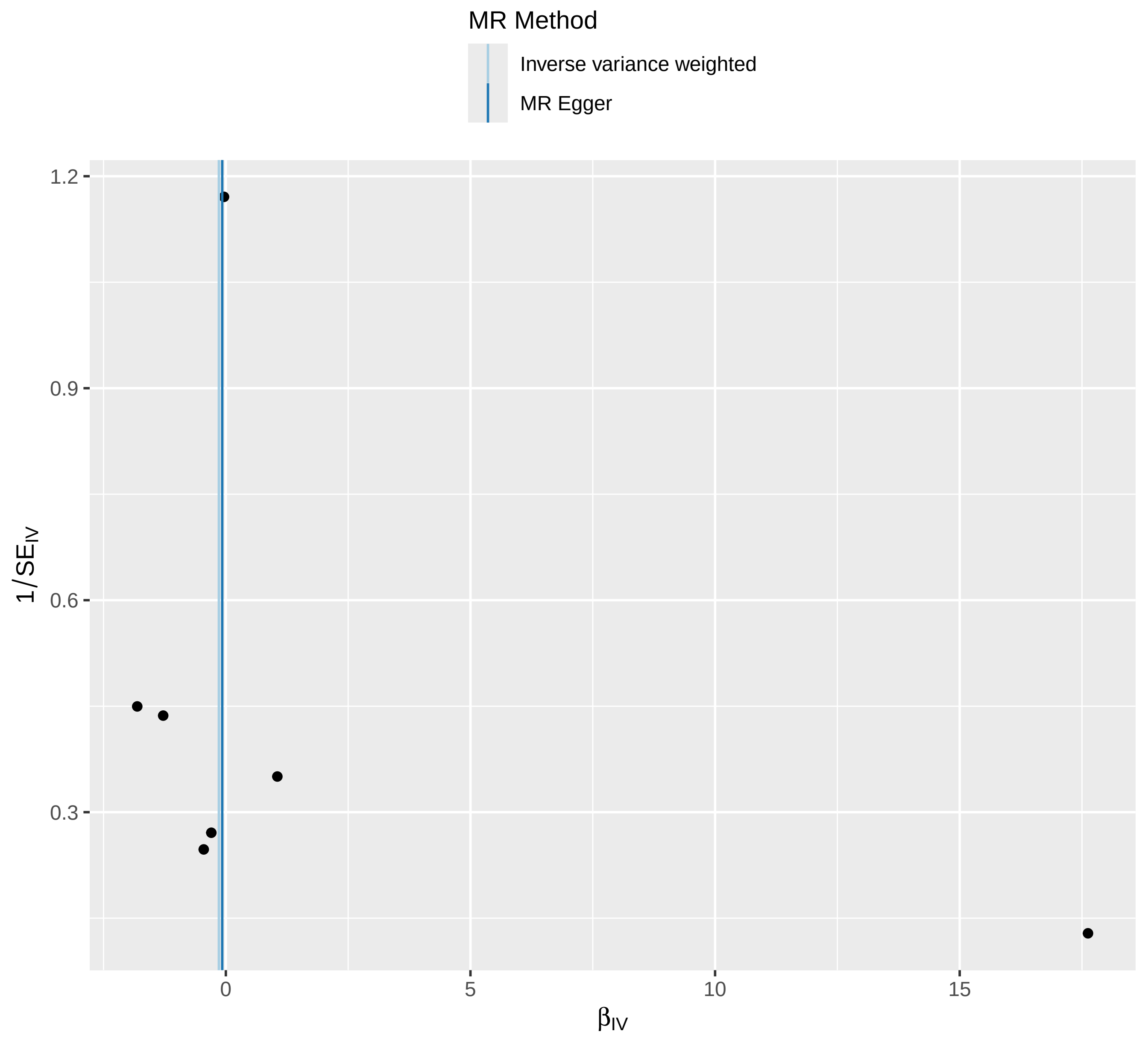

MR Method
Inverse variance weighted
MR Egger
1.2
0.9
0.6
0.3
$1/SE_{IV}$
0
5
10
15
β_{IV}

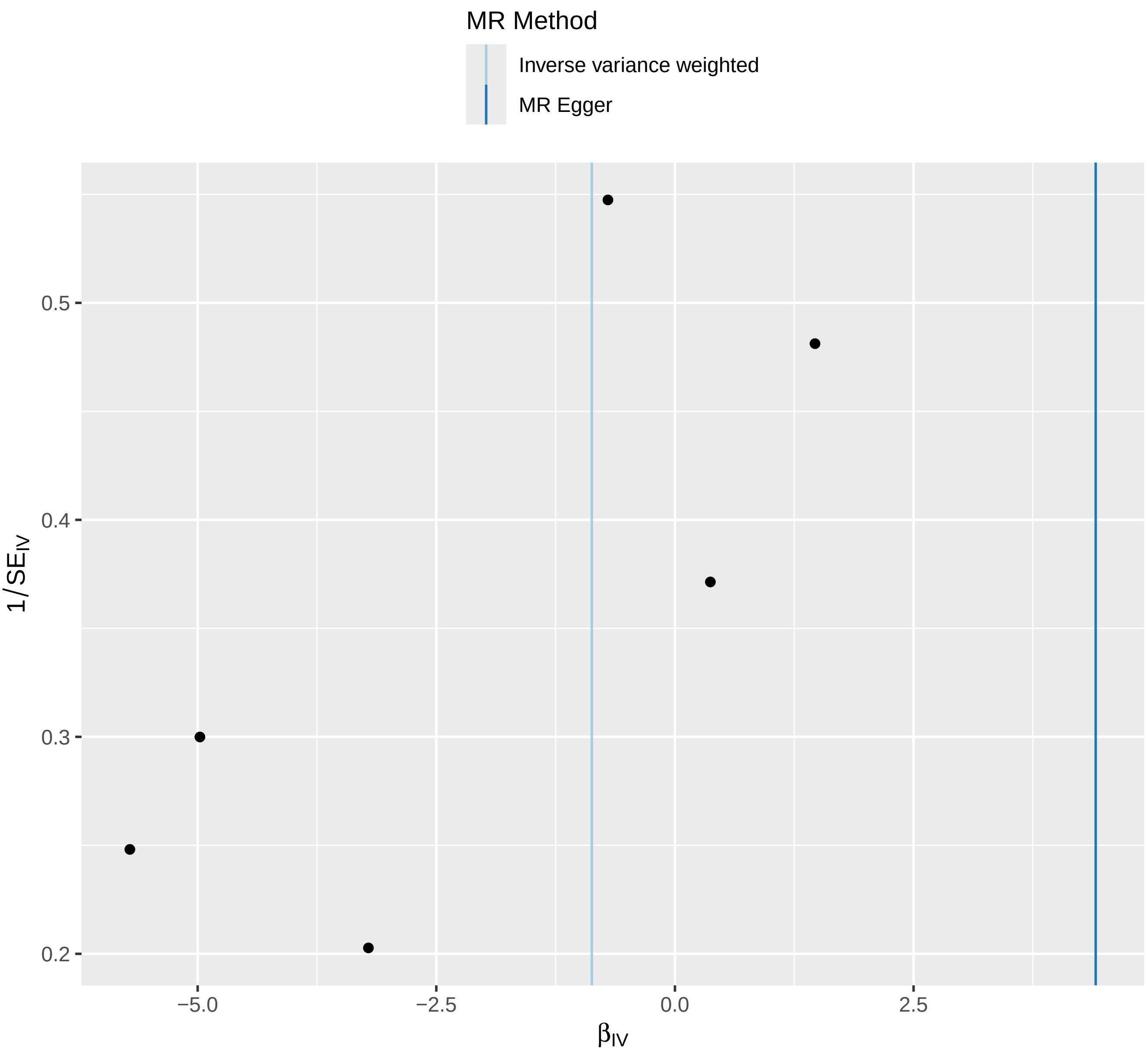
MR Method
Inverse variance weighted
MR Egger
0.5
0.4
0.3
0.2
1/SE$_{IV}$
−5.0
−2.5
0.0
2.5
β$_{IV}$

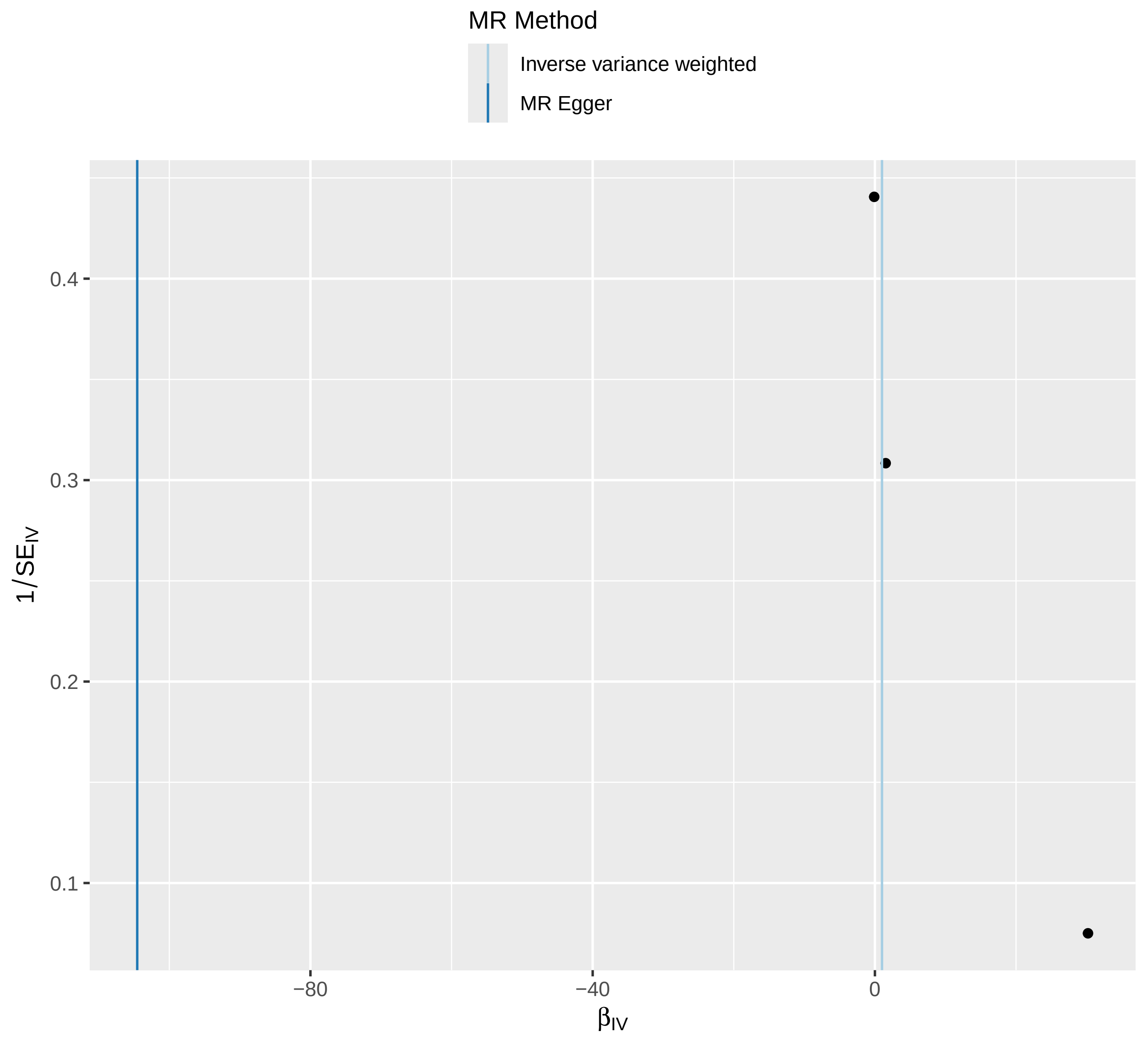
MR Method
Inverse variance weighted
MR Egger
0.4
0.3
0.2
0.1
$1/SE_{IV}$
−80
−40
0
β_{IV}

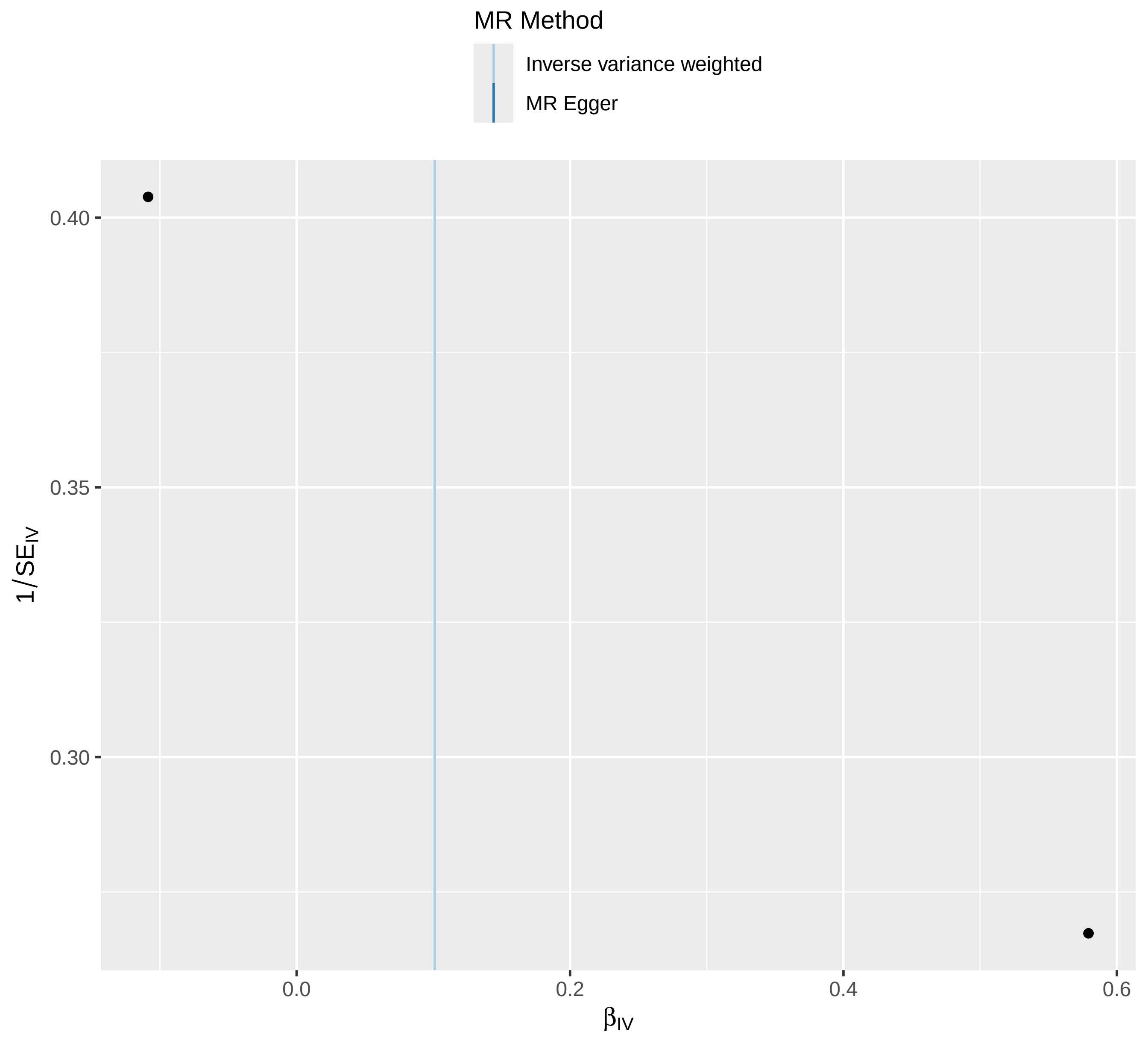
MR Method
Inverse variance weighted
MR Egger
0.40
0.35
0.30
1/SE_IV
0.0
0.2
0.4
0.6
β_IV

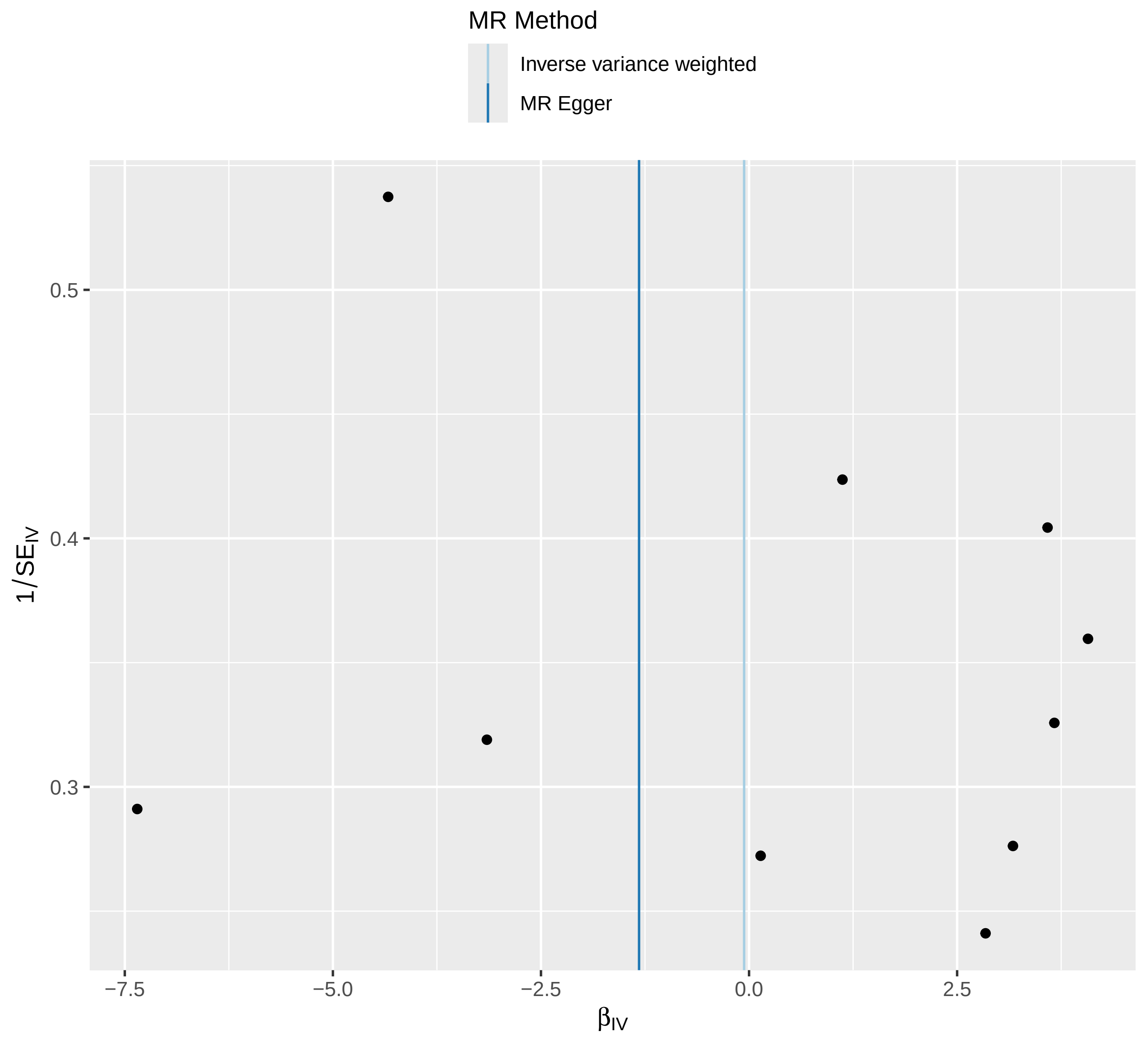
MR Method
Inverse variance weighted
MR Egger
1/SE_IV
0.5
0.4
0.3
−7.5
−5.0
−2.5
0.0
2.5
β_IV

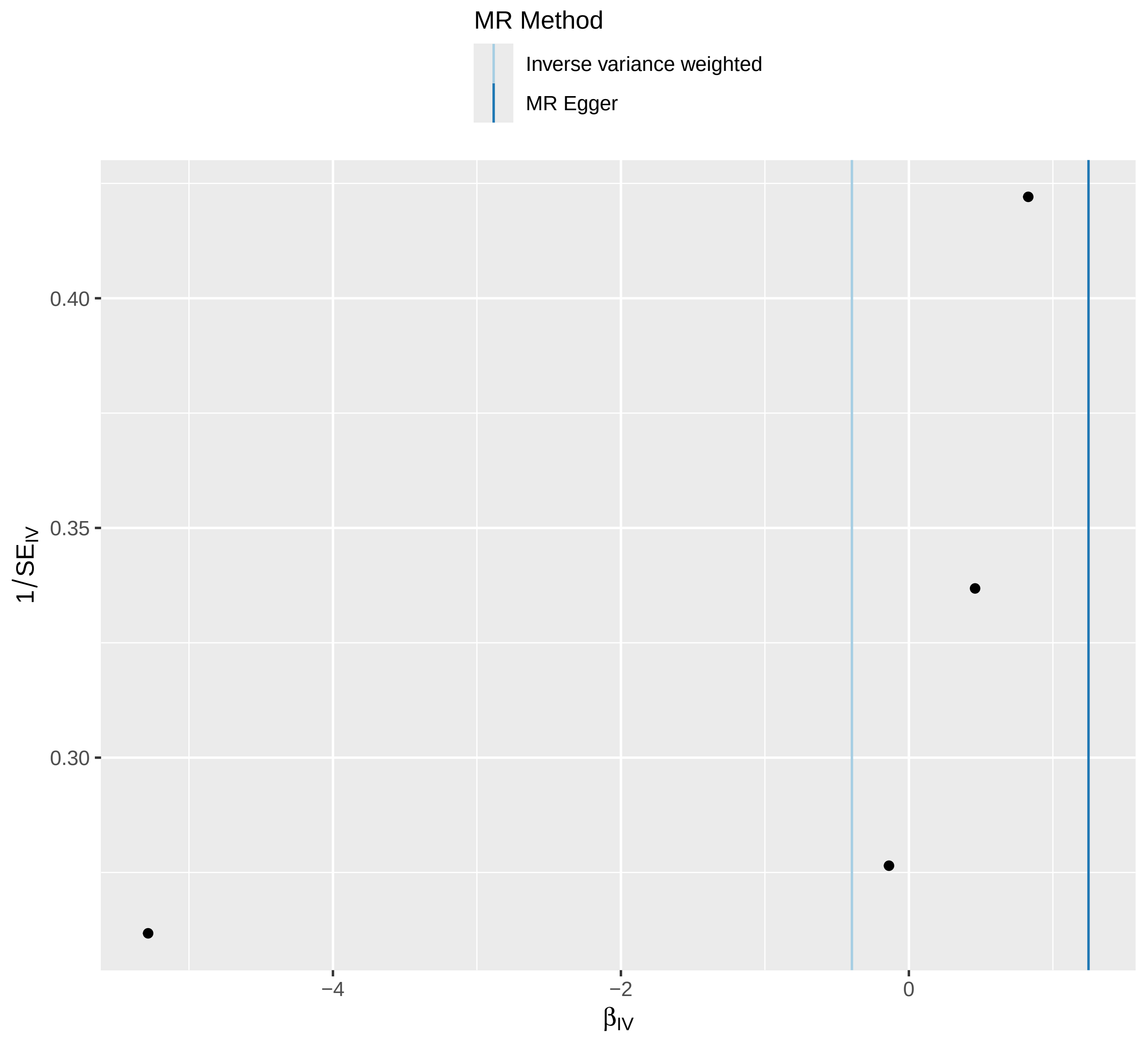
MR Method
Inverse variance weighted
MR Egger
0.40
0.35
0.30
1/SE_IV
−4
−2
0
β_IV

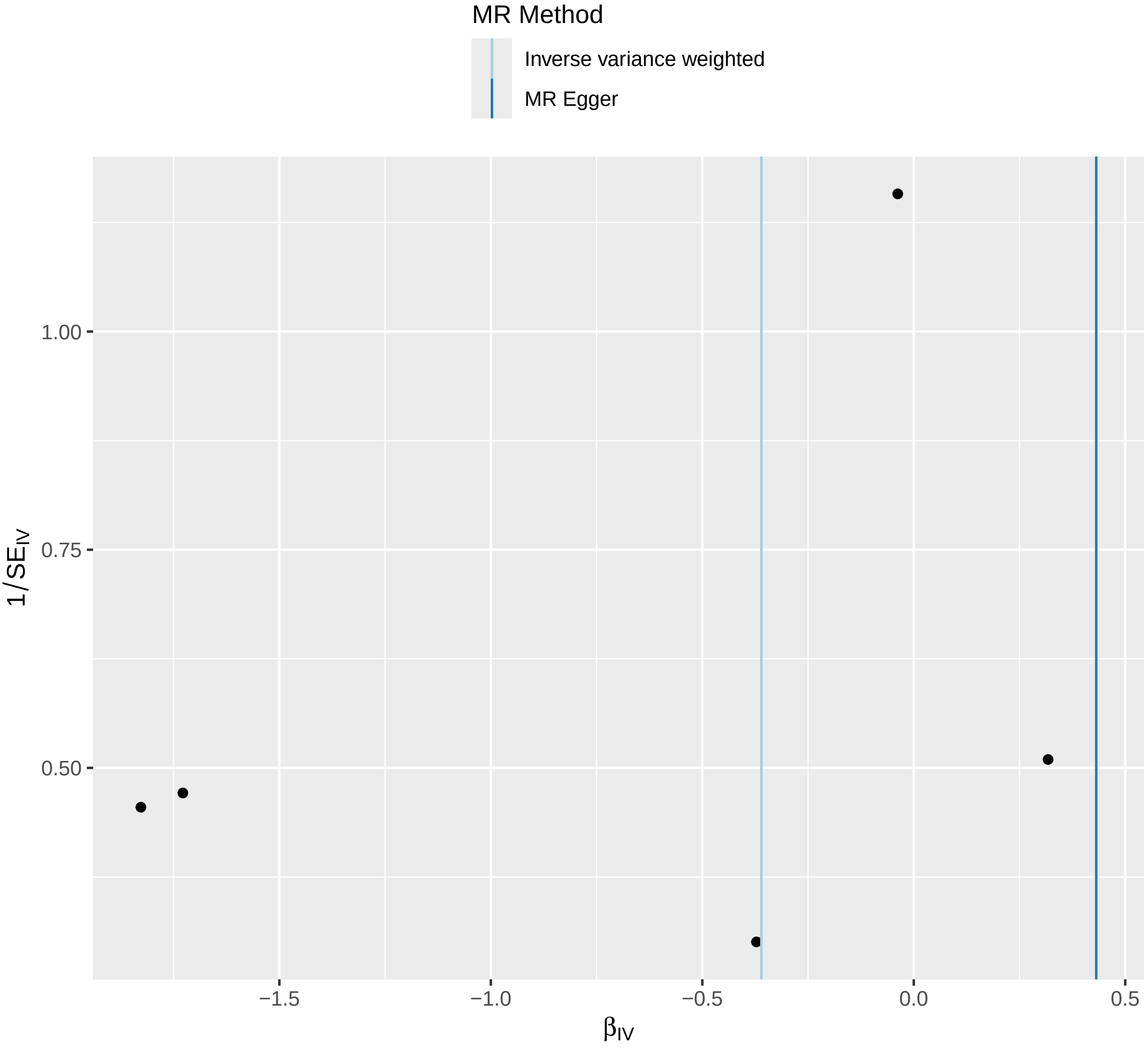
MR Method
Inverse variance weighted
MR Egger
1.00
0.75
0.50
1/SE$_{IV}$
−1.5
−1.0
−0.5
0.0
0.5
β$_{IV}$

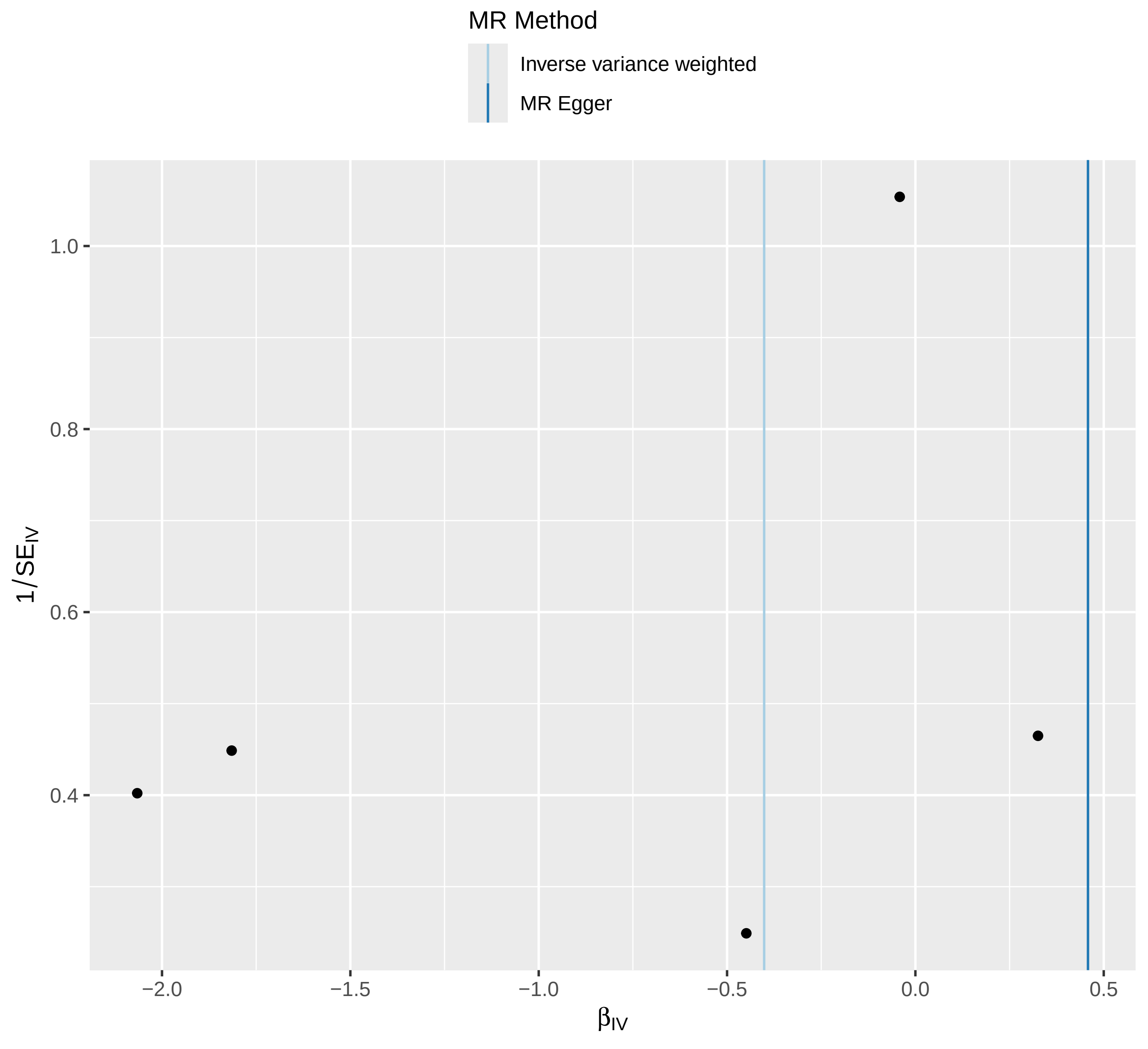
MR Method
Inverse variance weighted
MR Egger
1/SE_IV
1.0
0.8
0.6
0.4
−2.0
−1.5
−1.0
−0.5
0.0
0.5
β_IV

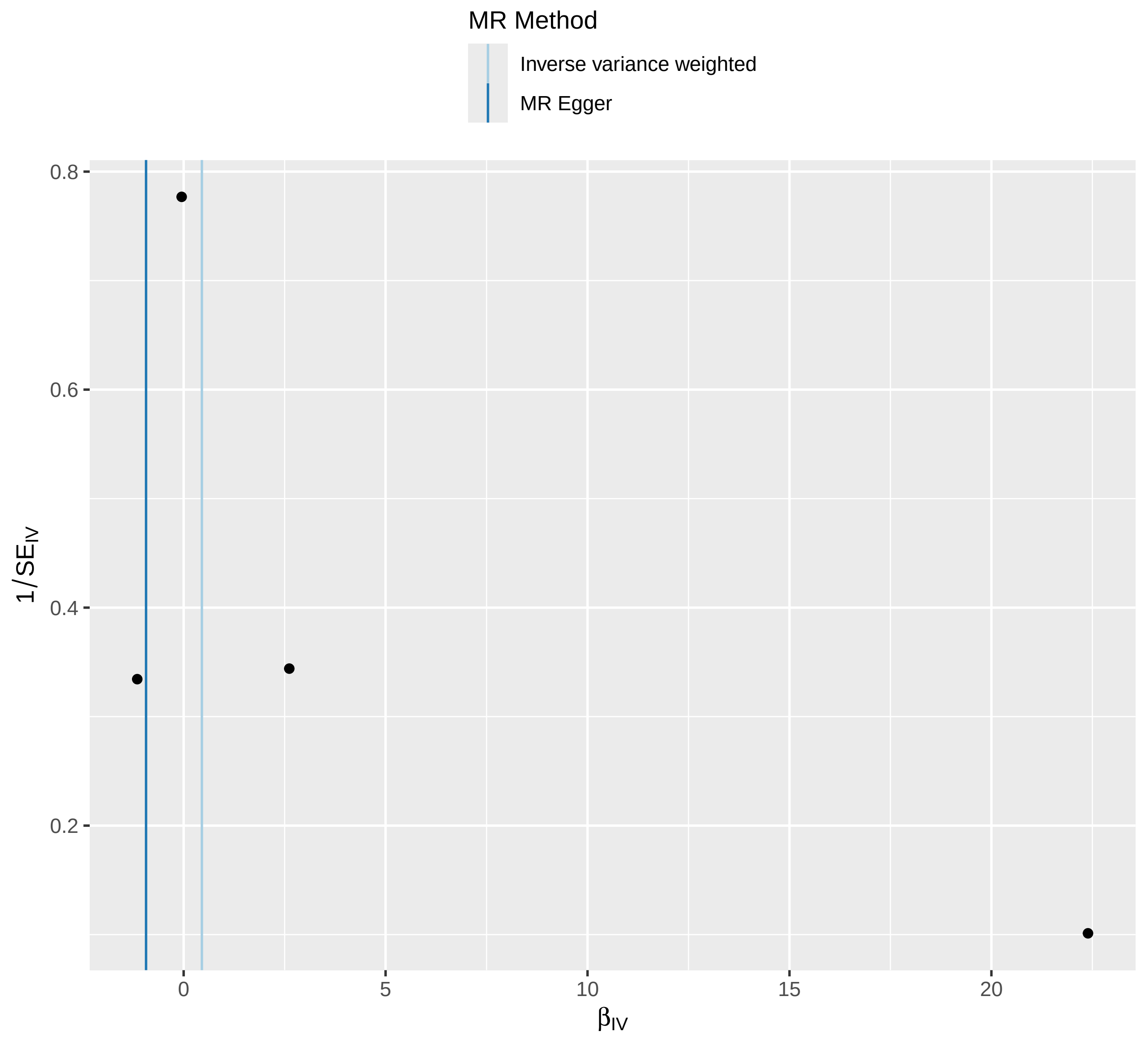

MR Method
Inverse variance weighted
MR Egger
0.8
0.6
0.4
0.2
$1/SE_{IV}$
0
5
10
15
20
β_{IV}

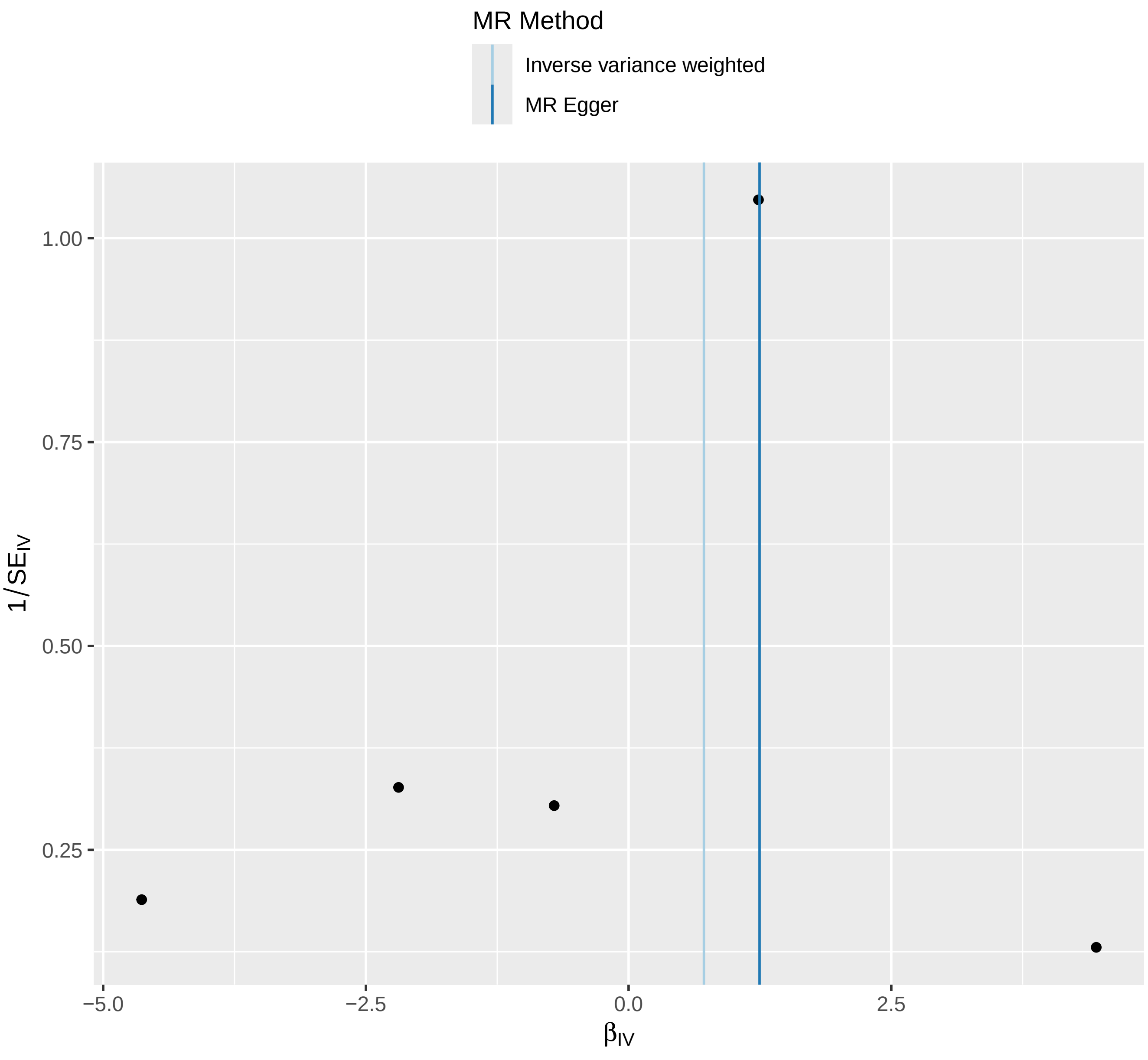
MR Method
Inverse variance weighted
MR Egger
1.00
0.75
0.50
0.25
1/SE$_{IV}$
−5.0
−2.5
0.0
2.5
β$_{IV}$

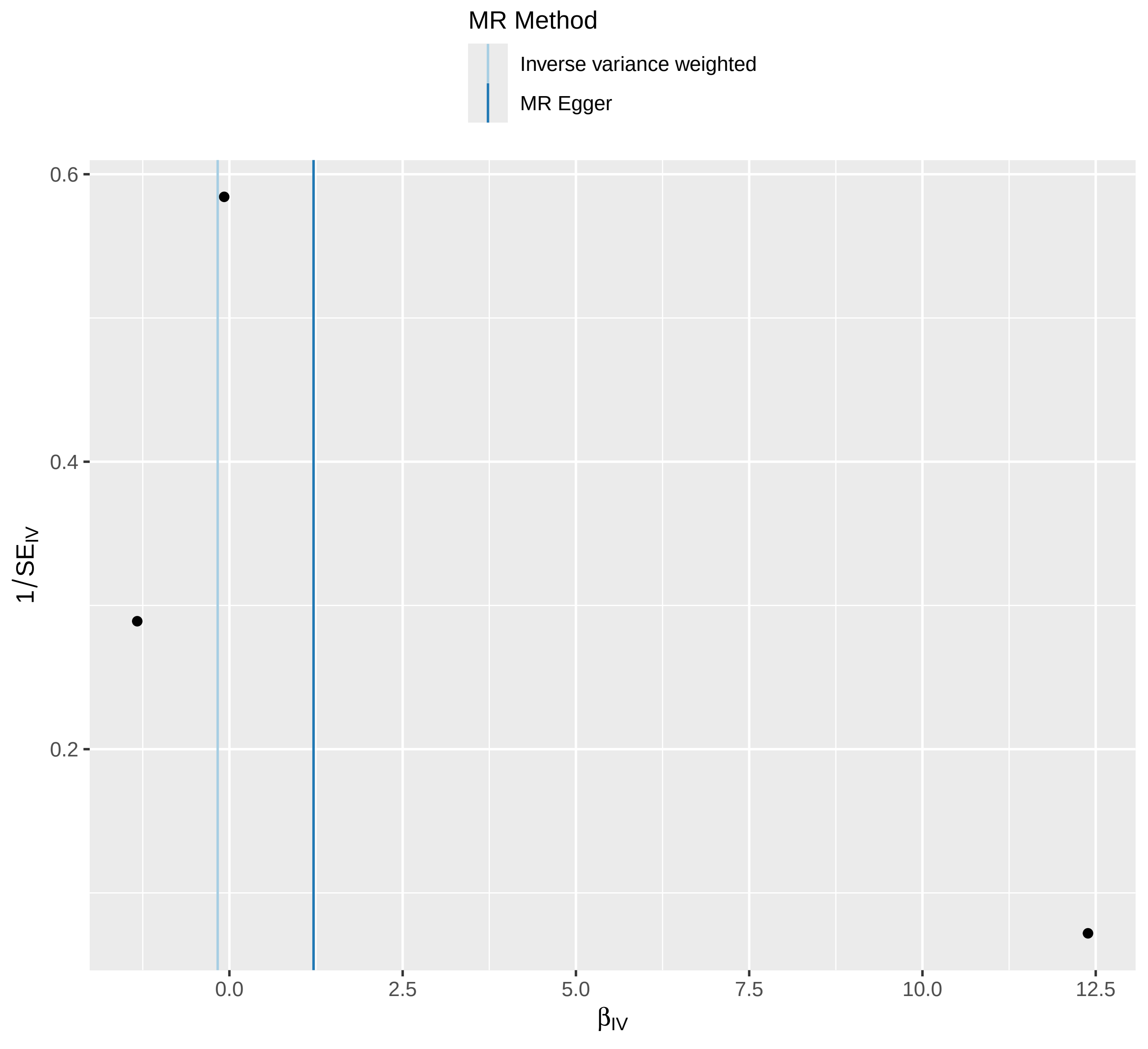

MR Method
Inverse variance weighted
MR Egger
0.6
0.4
0.2
1/SE$_{IV}$
0.0
2.5
5.0
7.5
10.0
12.5
β$_{IV}$

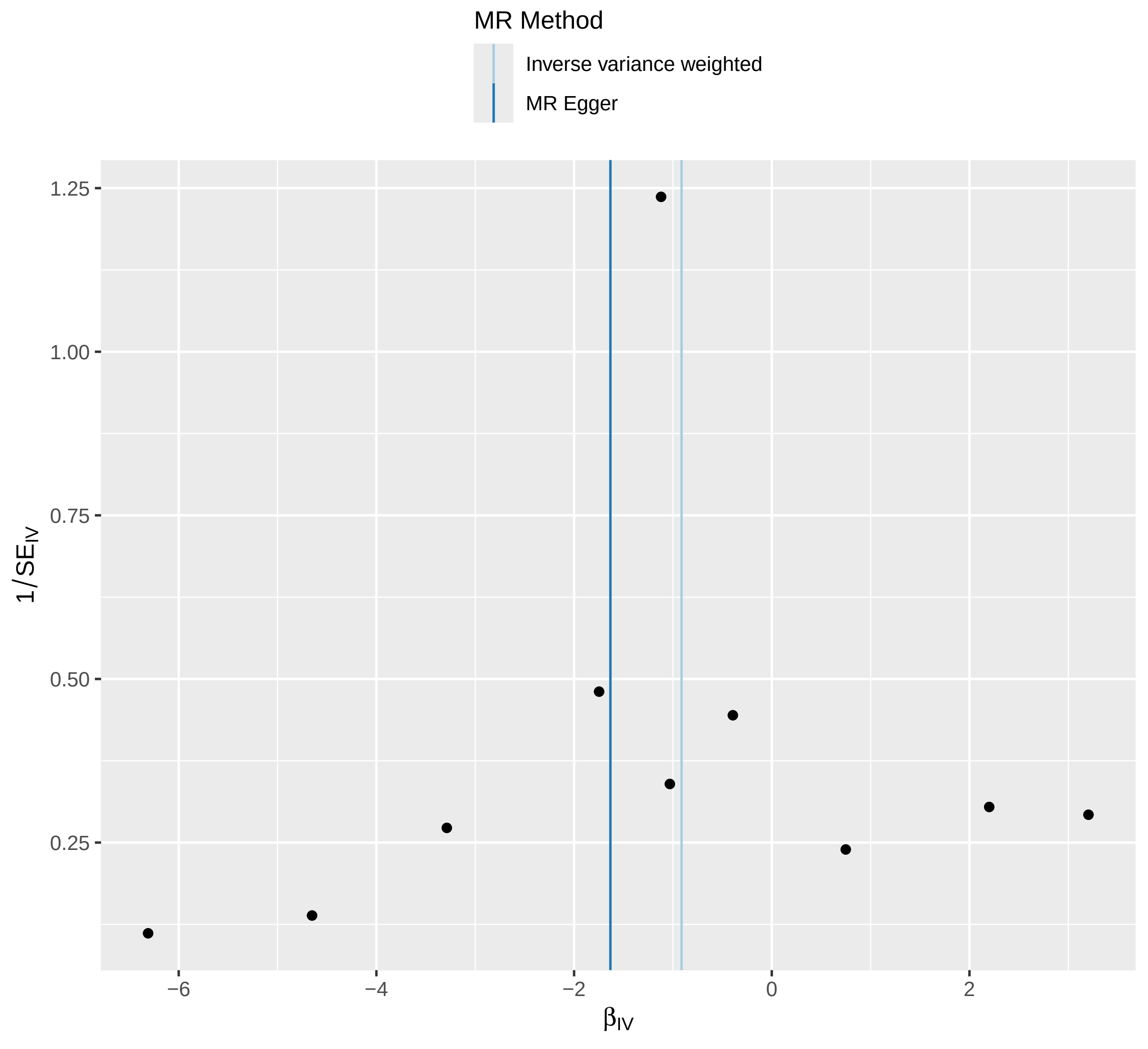

MR Method
Inverse variance weighted
MR Egger
1.25
1.00
0.75
0.50
0.25
$1/SE_{IV}$
−6
−4
−2
0
2
β_{IV}

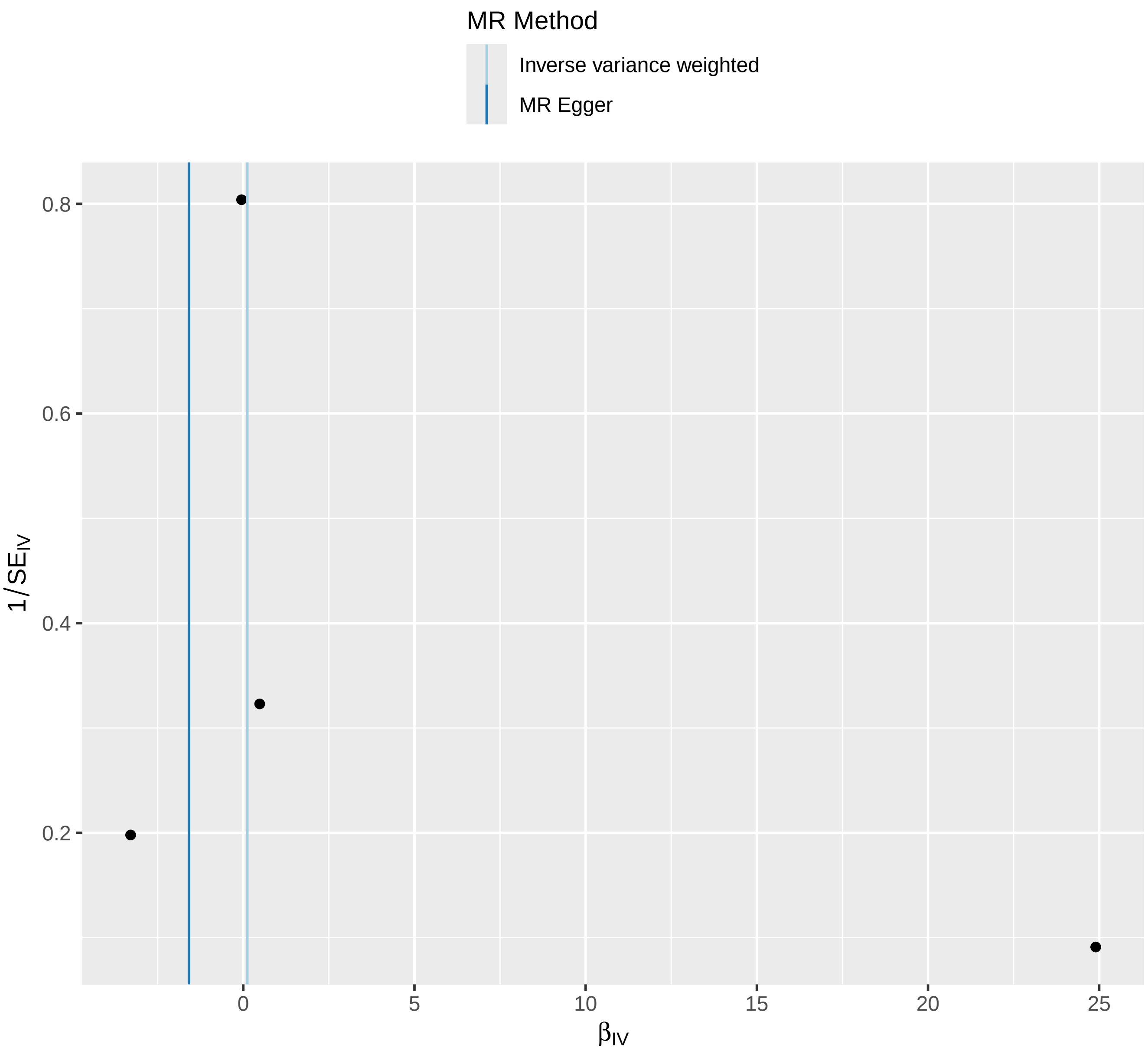
MR Method
Inverse variance weighted
MR Egger
0.8
0.6
0.4
0.2
1/SE_IV
0
5
10
15
20
25
β_IV

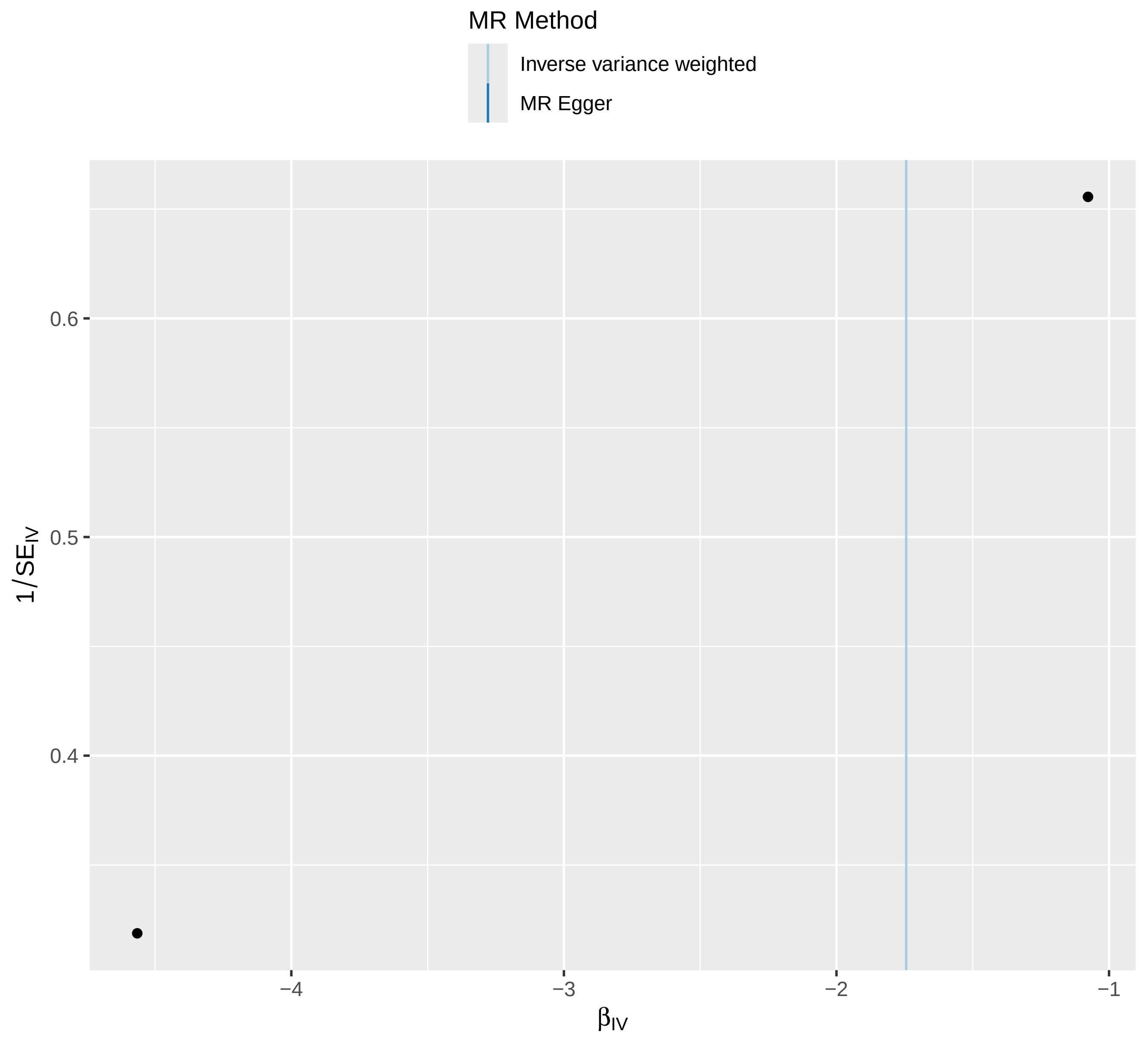
MR Method
Inverse variance weighted
MR Egger
0.6
0.5
0.4
1/SE_IV
−4
−3
−2
−1
β_IV

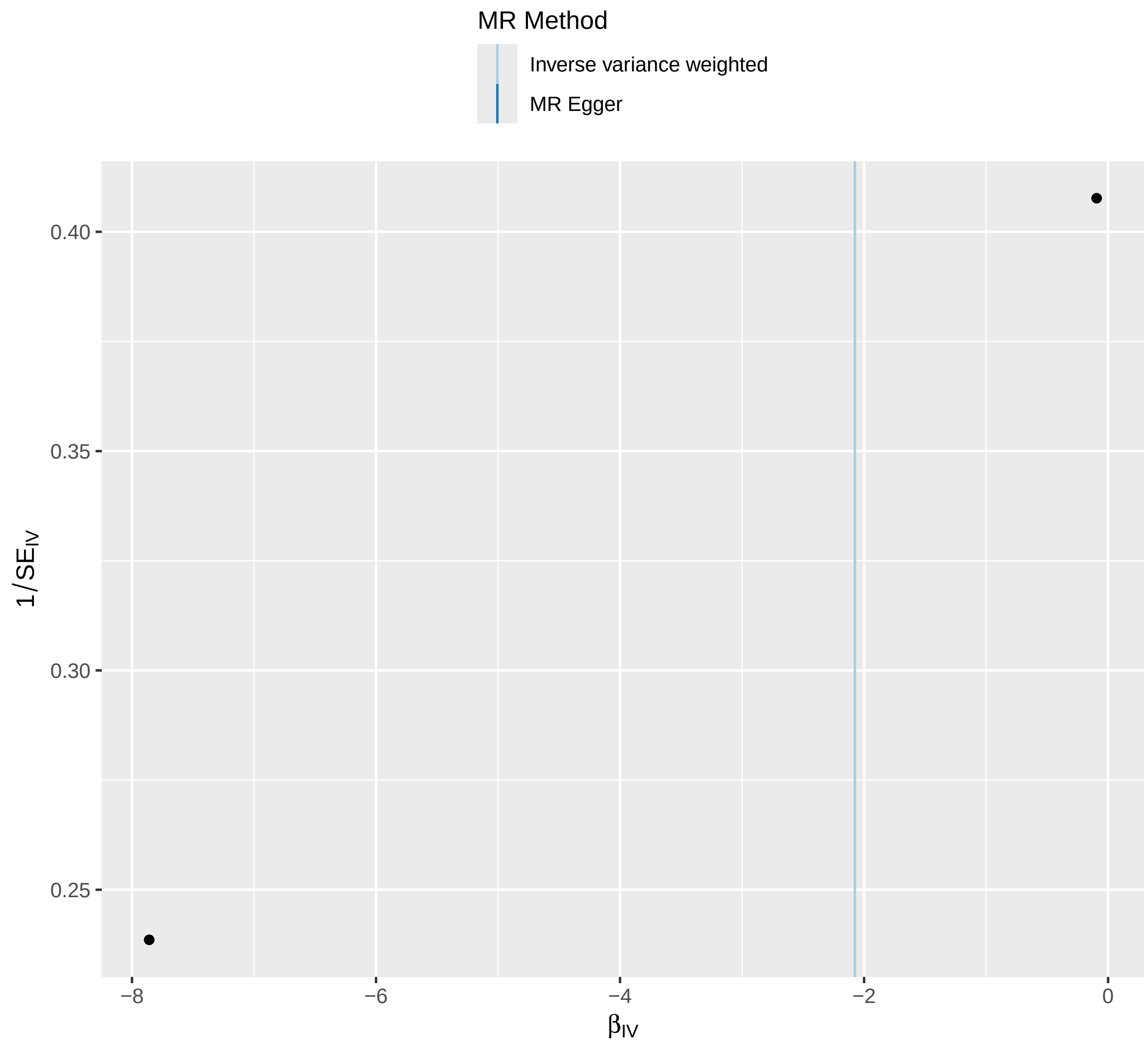
MR Method
Inverse variance weighted
MR Egger
1/SE$_{IV}$
0.40
0.35
0.30
0.25
−8
−6
−4
−2
0
β$_{IV}$

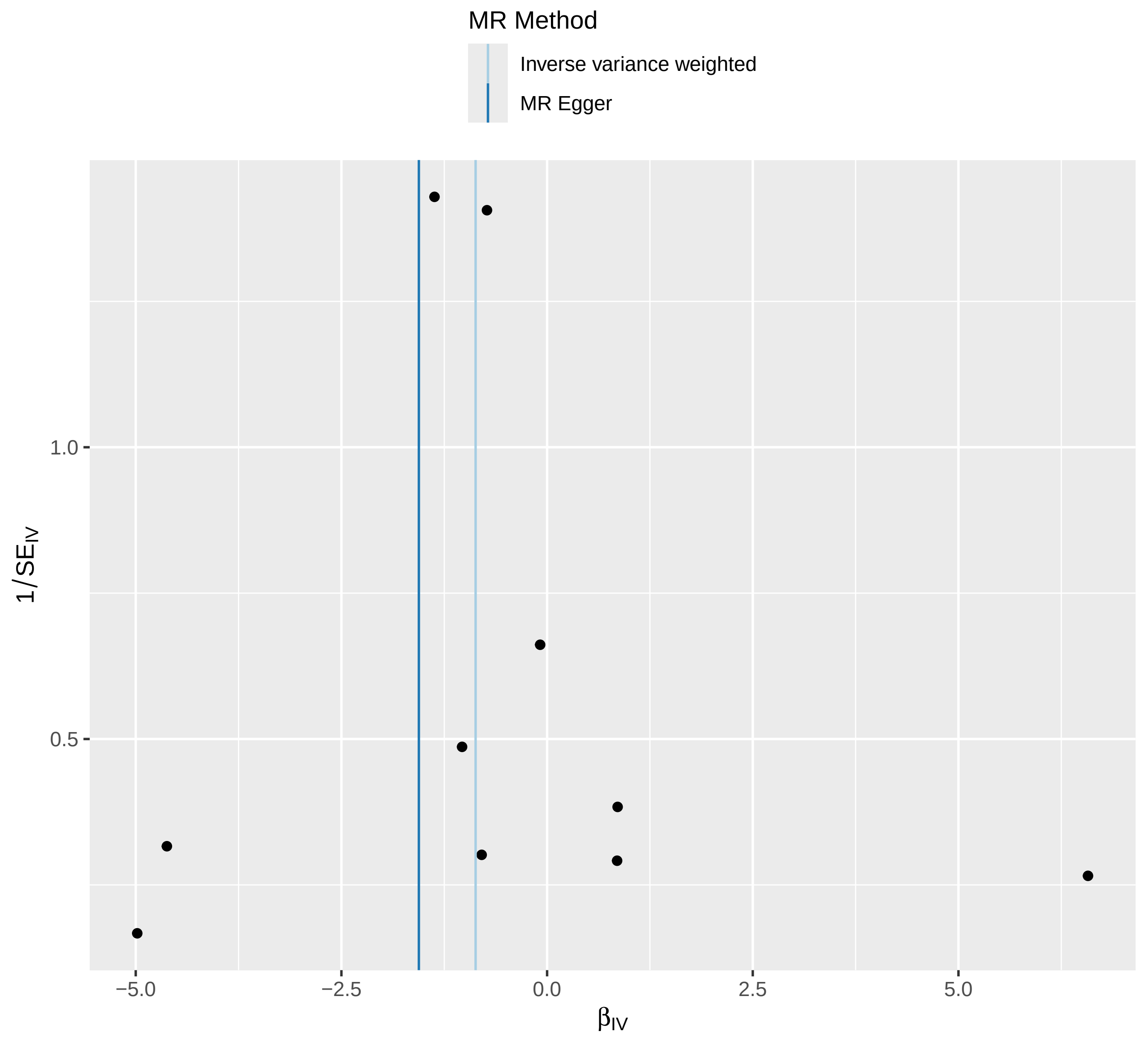
MR Method
Inverse variance weighted
MR Egger
1.0
0.5
−5.0
−2.5
0.0
2.5
5.0
$1/SE_{IV}$
β_{IV}

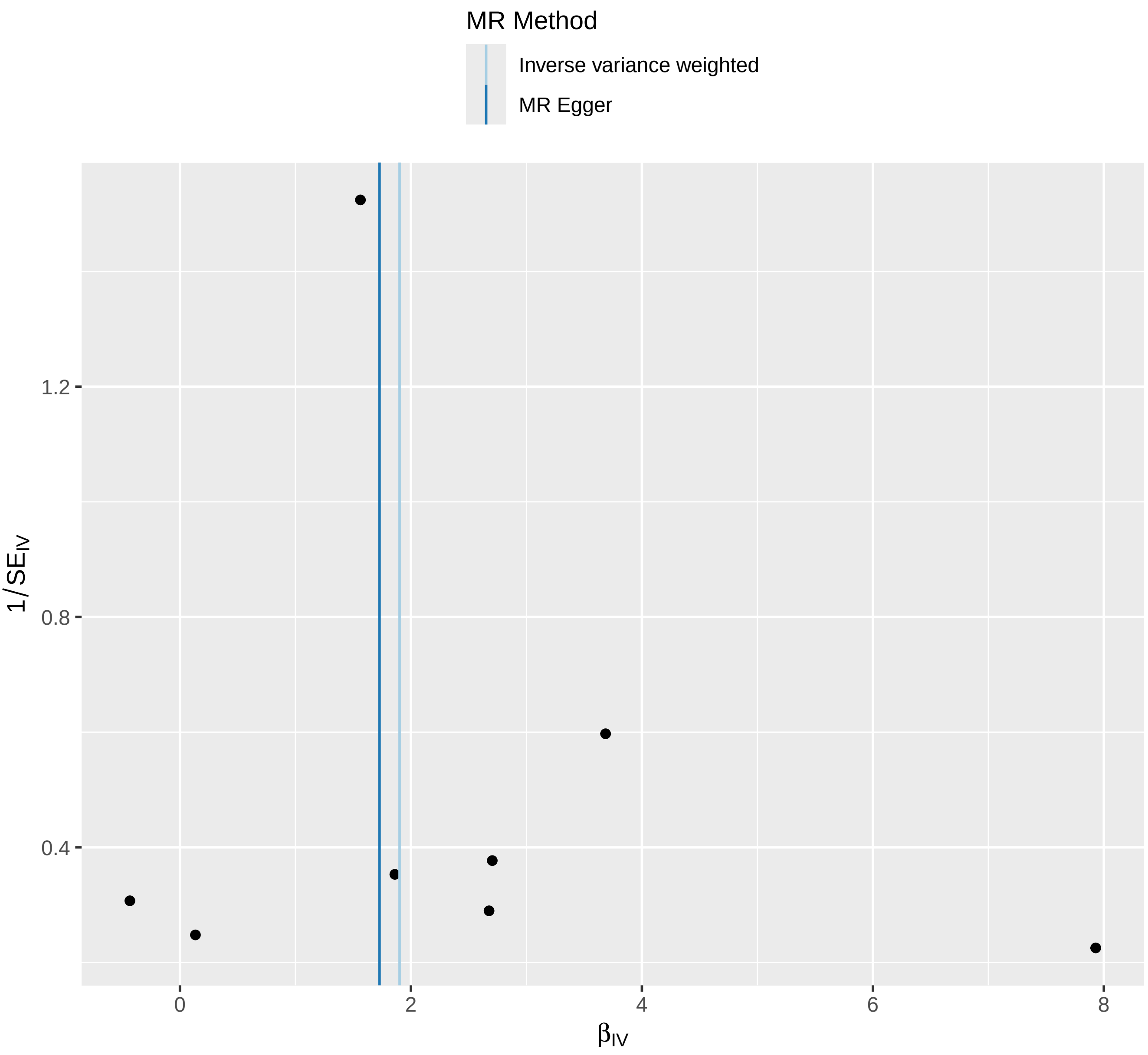
MR Method
Inverse variance weighted
MR Egger
1.2
0.8
0.4
$1/SE_{IV}$
0
2
4
6
8
β_{IV}

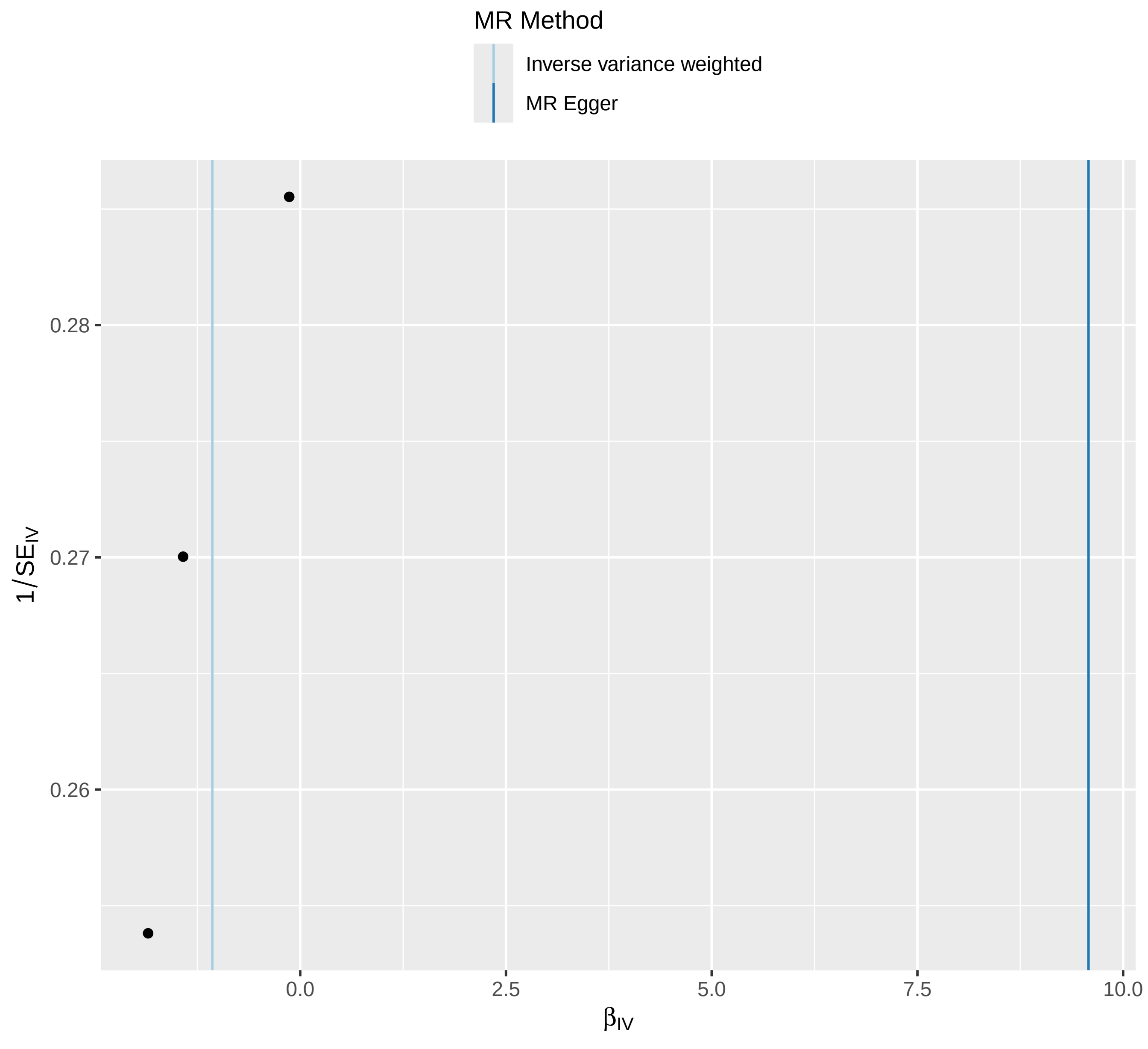
MR Method
Inverse variance weighted
MR Egger
0.28
0.27
0.26
1/SE$_{IV}$
0.0
2.5
5.0
7.5
10.0
β$_{IV}$

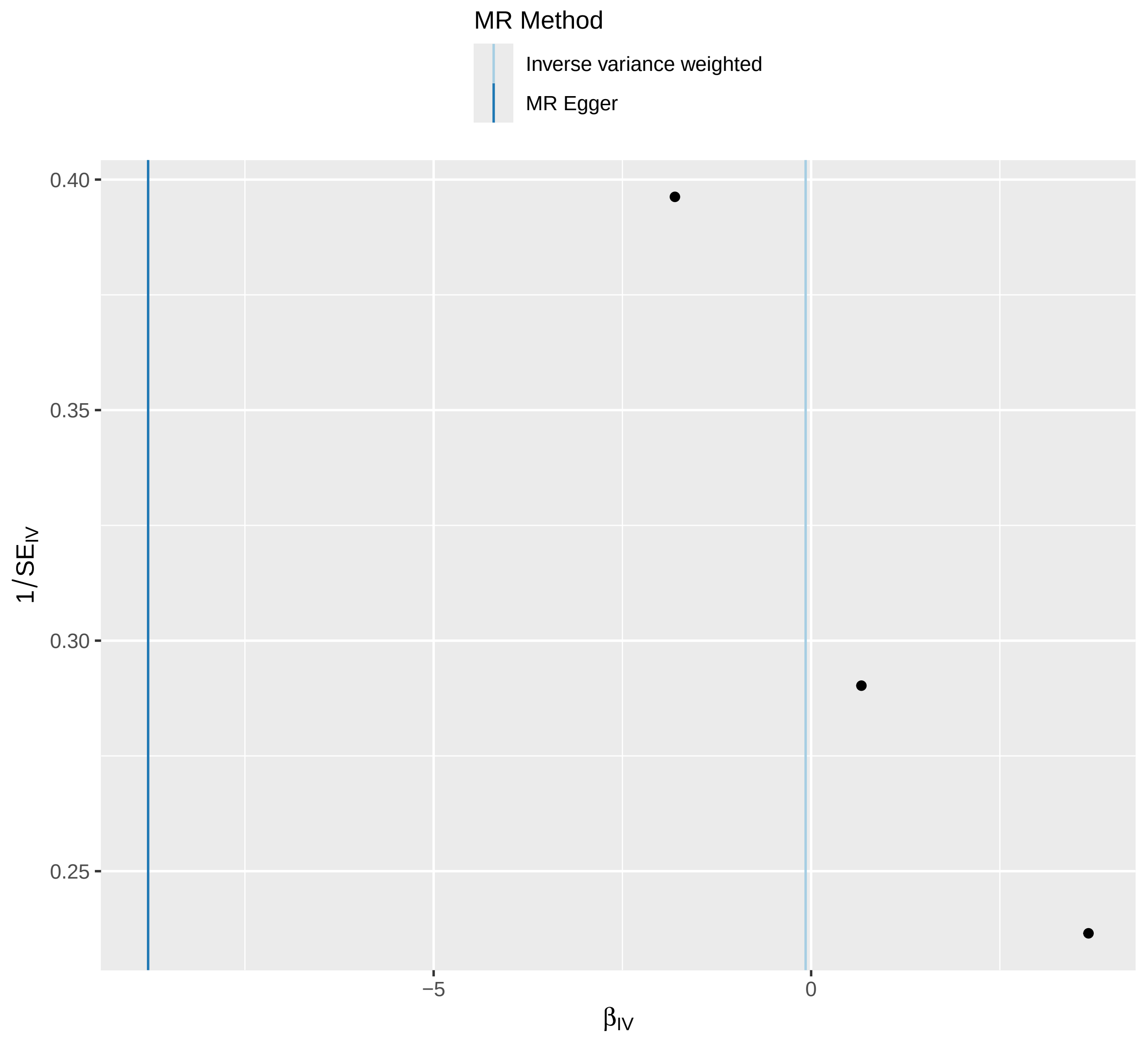

MR Method
Inverse variance weighted
MR Egger
0.40
0.35
0.30
0.25
1/SE_IV
−5
0
β_IV

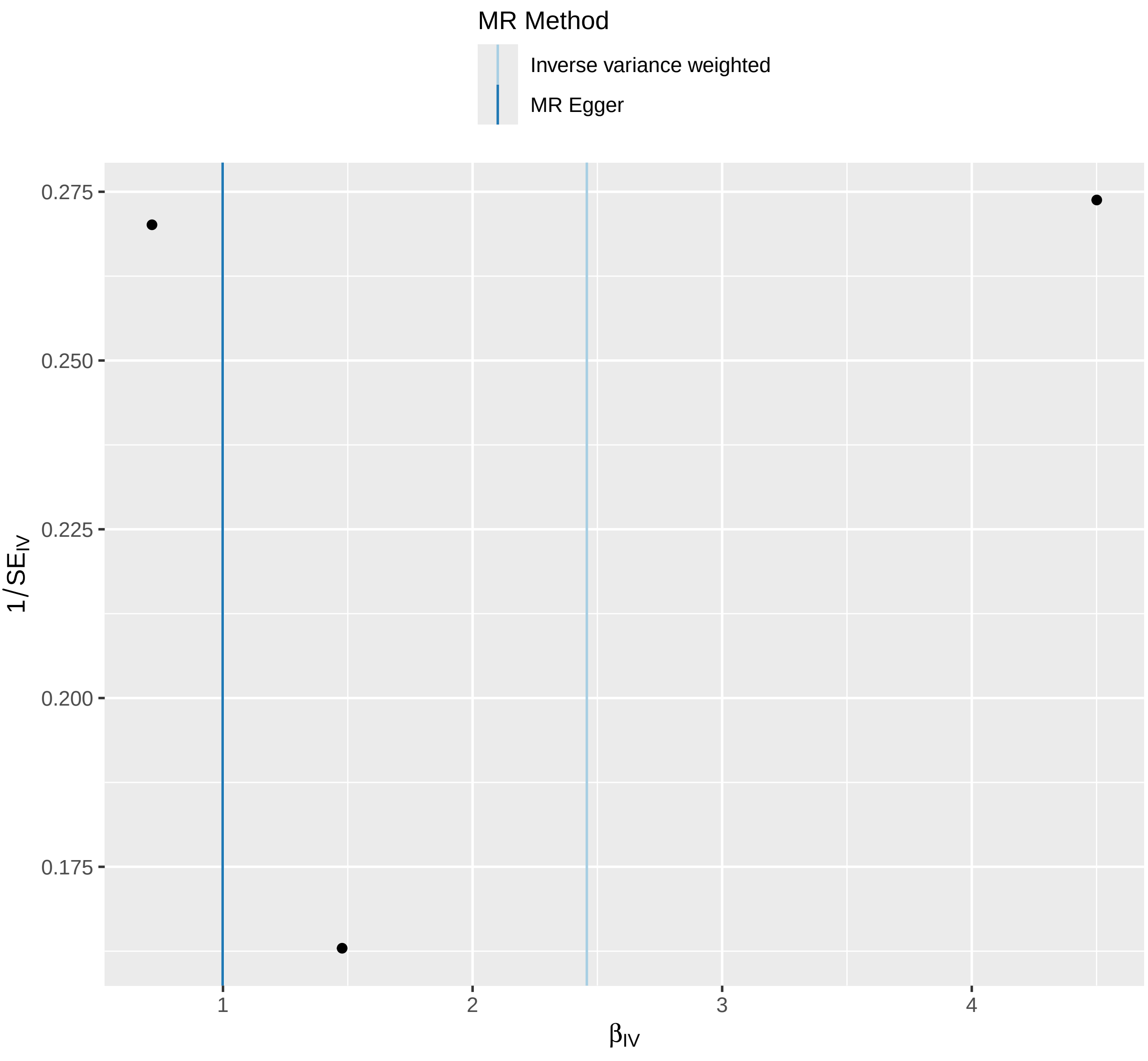

MR Method
Inverse variance weighted
MR Egger
0.275
0.250
0.225
0.200
0.175
1/SE_IV
1
2
3
4
β_IV

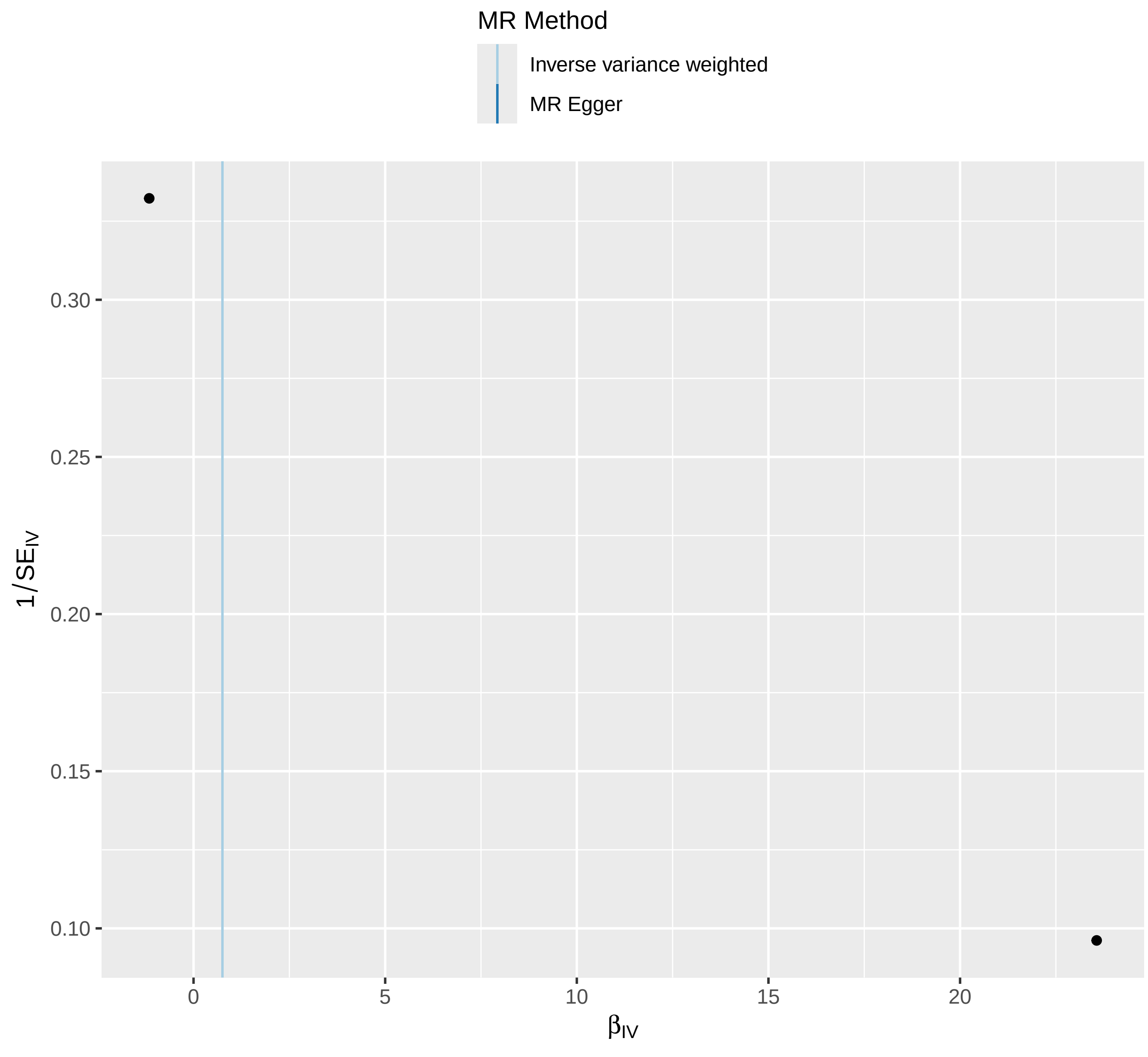
MR Method
Inverse variance weighted
MR Egger
0.30
0.25
0.20
0.15
0.10
$1/SE_{IV}$
0
5
10
15
20
β_{IV}

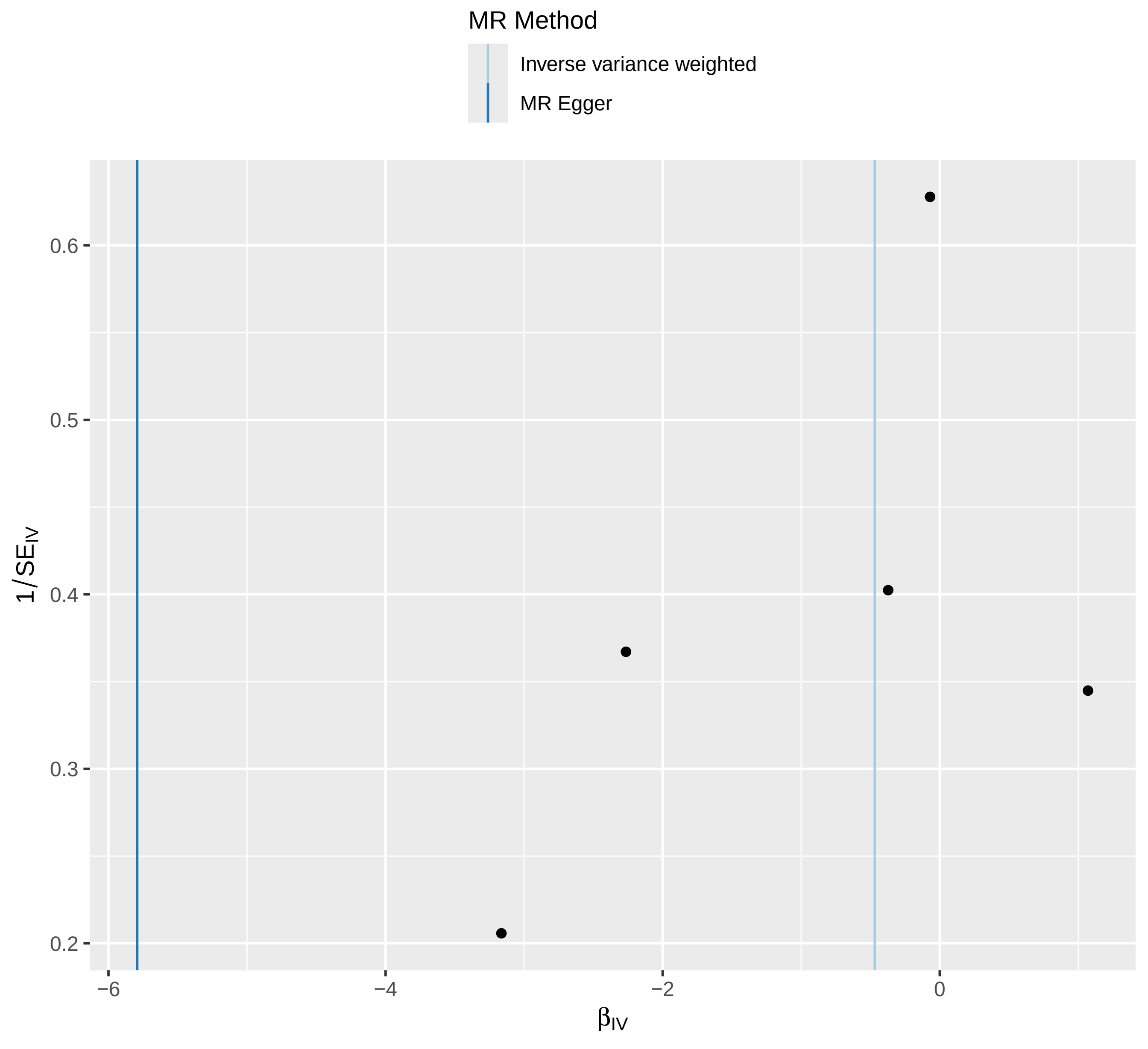
MR Method
Inverse variance weighted
MR Egger
0.6
0.5
0.4
0.3
0.2
1/SE_IV
−6
−4
−2
0
β_IV

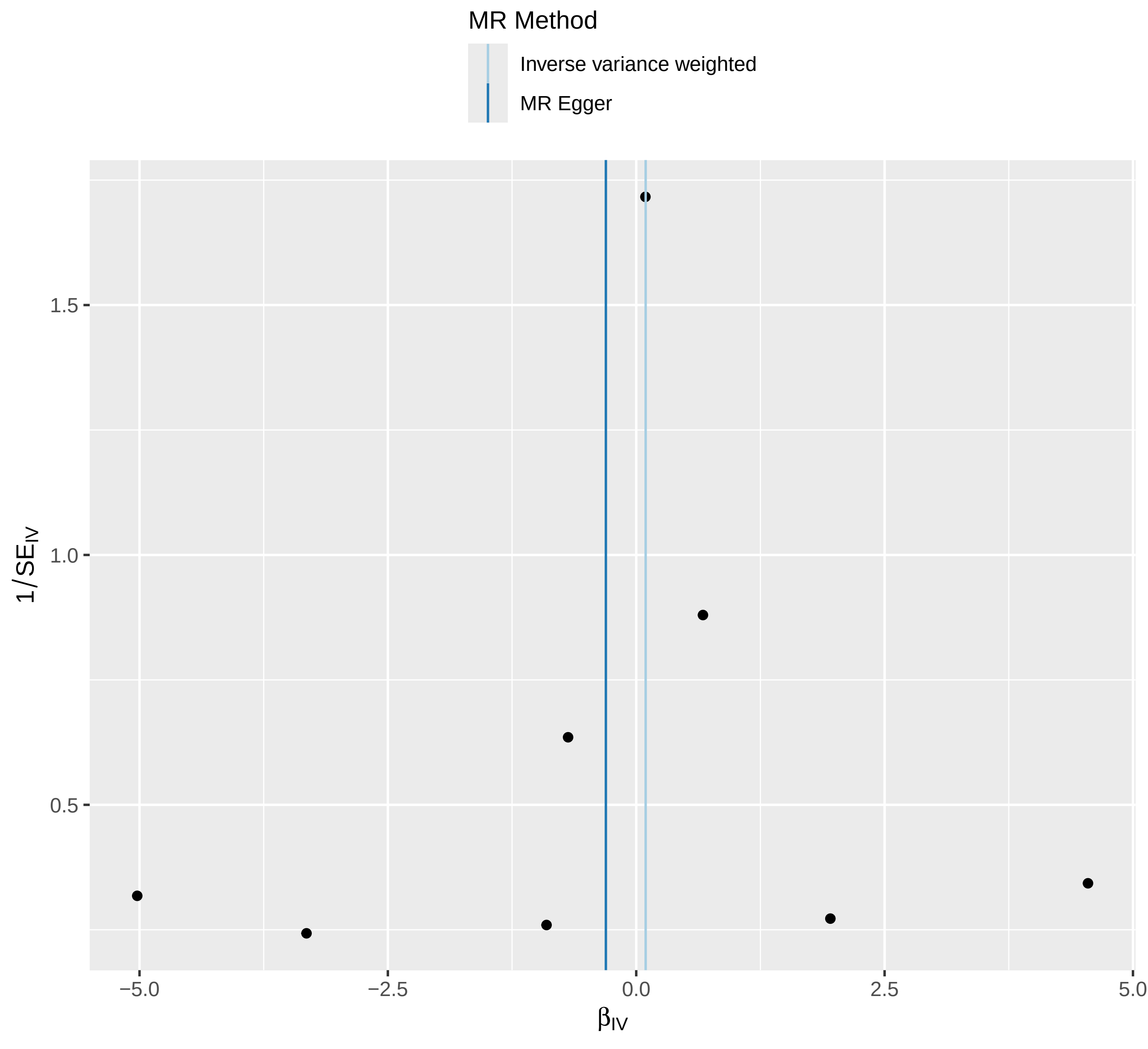

MR Method
Inverse variance weighted
MR Egger
1.5
1.0
0.5
1/SE_IV
−5.0
−2.5
0.0
2.5
5.0
β_IV

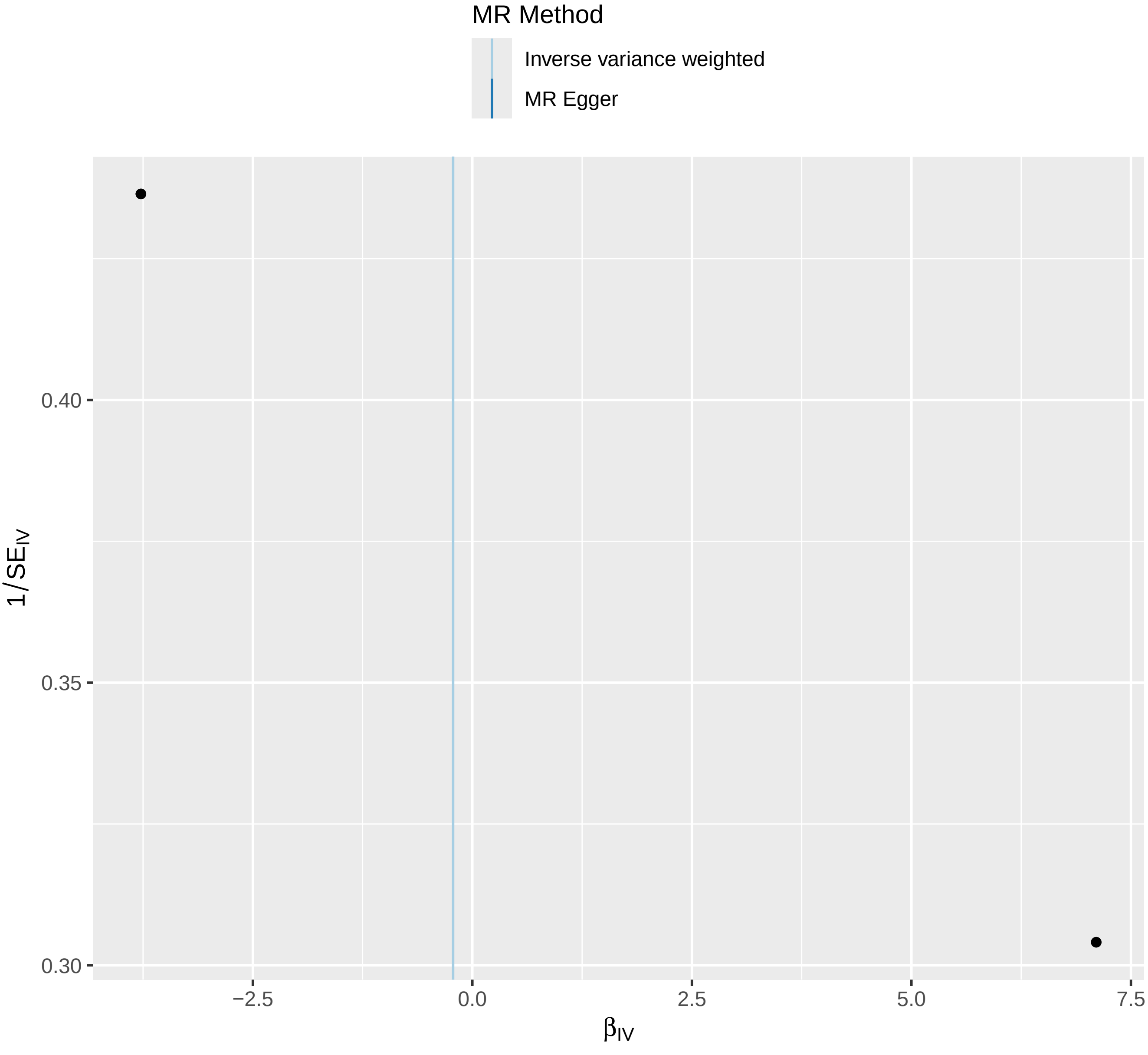
MR Method
Inverse variance weighted
MR Egger
0.40
0.35
0.30
1/SE$_{IV}$
−2.5
0.0
2.5
5.0
7.5
β$_{IV}$

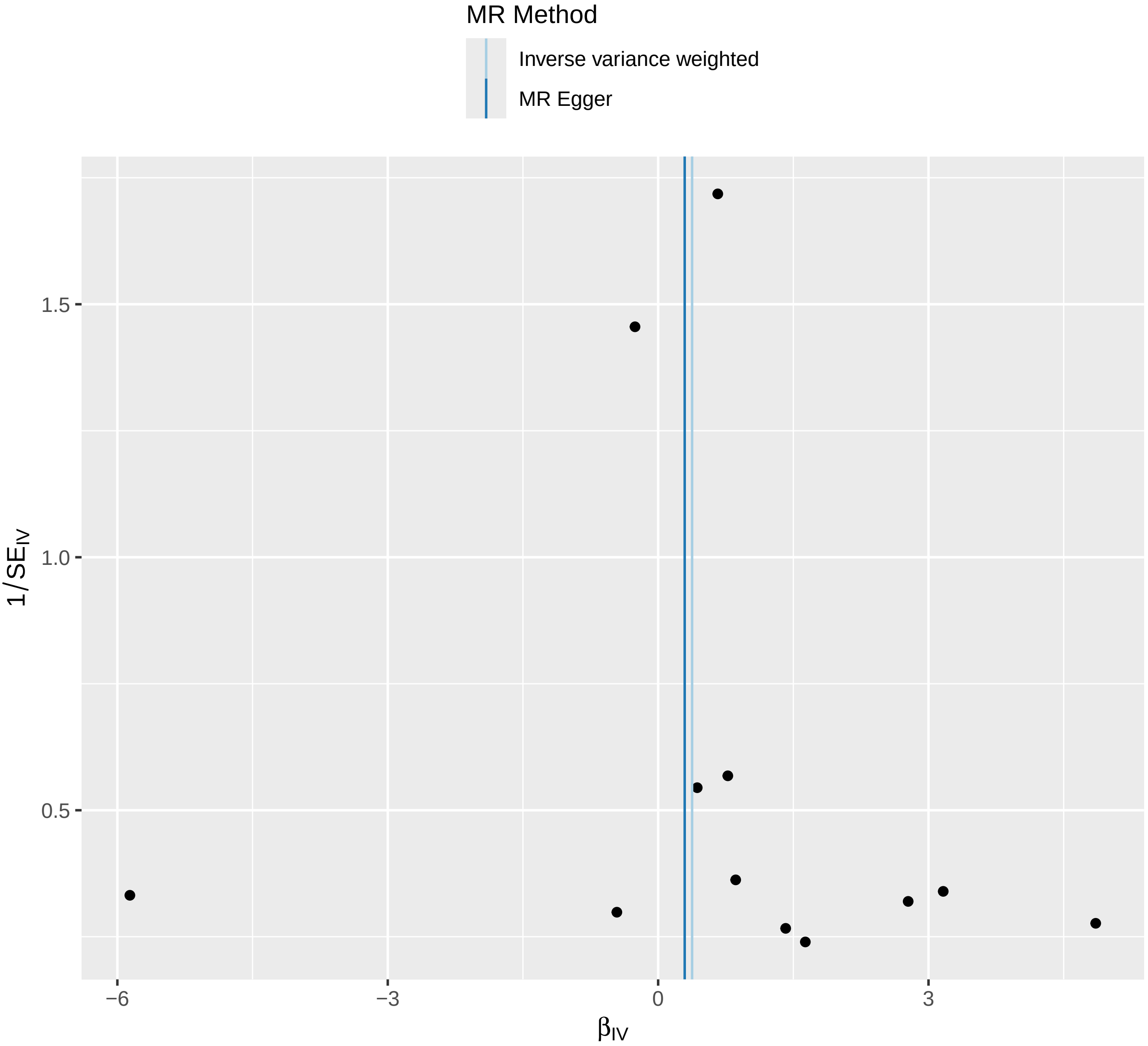

MR Method
Inverse variance weighted
MR Egger
1.5
1.0
0.5
$1/SE_{IV}$
−6
−3
0
3
β_{IV}

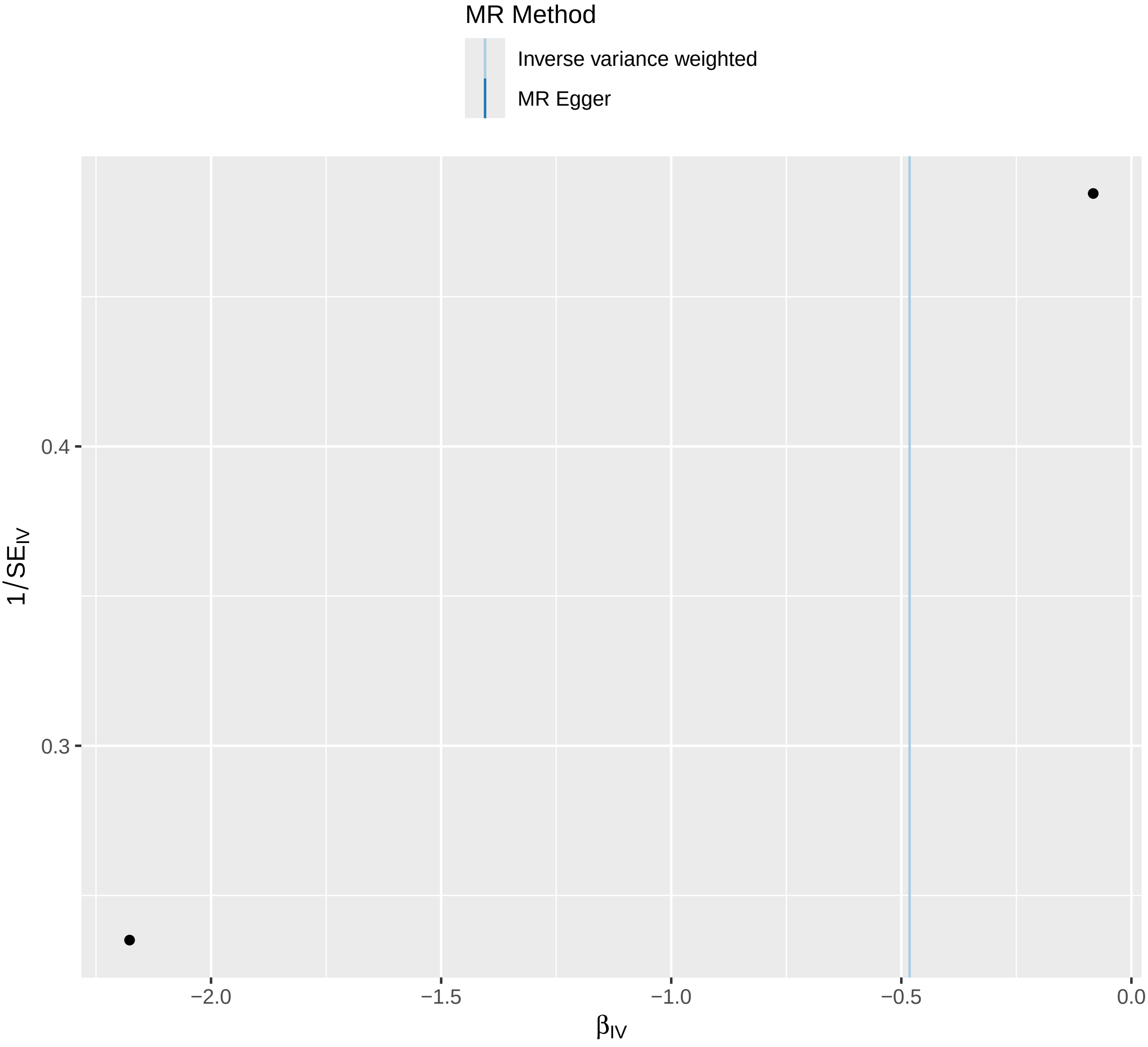
MR Method
Inverse variance weighted
MR Egger
0.4
0.3
1/SE$_{IV}$
−2.0
−1.5
−1.0
−0.5
0.0
β$_{IV}$

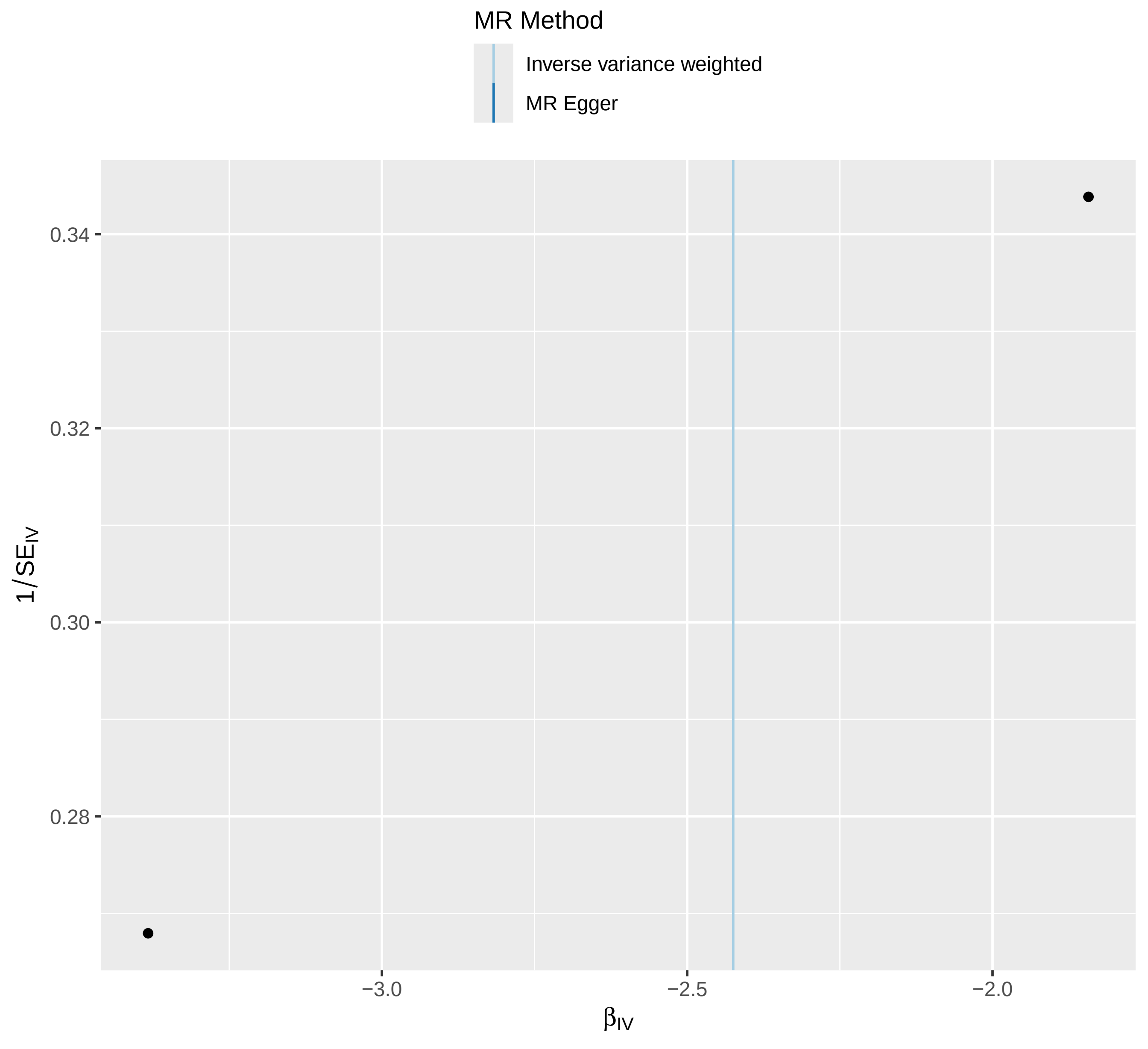

MR Method
Inverse variance weighted
MR Egger
0.34
0.32
0.30
0.28
1/SE_IV
−3.0
−2.5
−2.0
β_IV

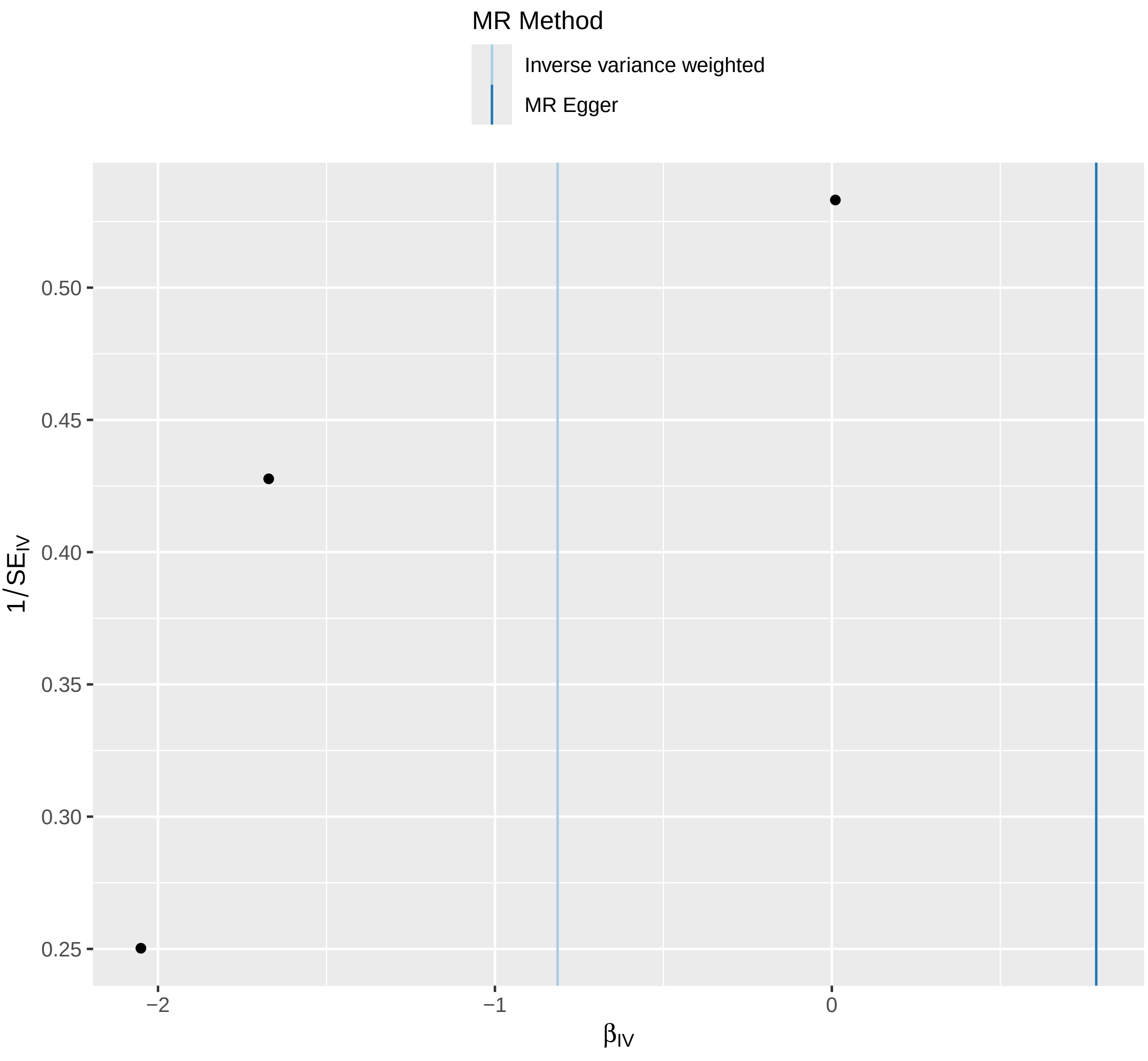
MR Method
Inverse variance weighted
MR Egger
0.50
0.45
0.40
0.35
0.30
0.25
1/SE_IV
−2
−1
0
β_IV

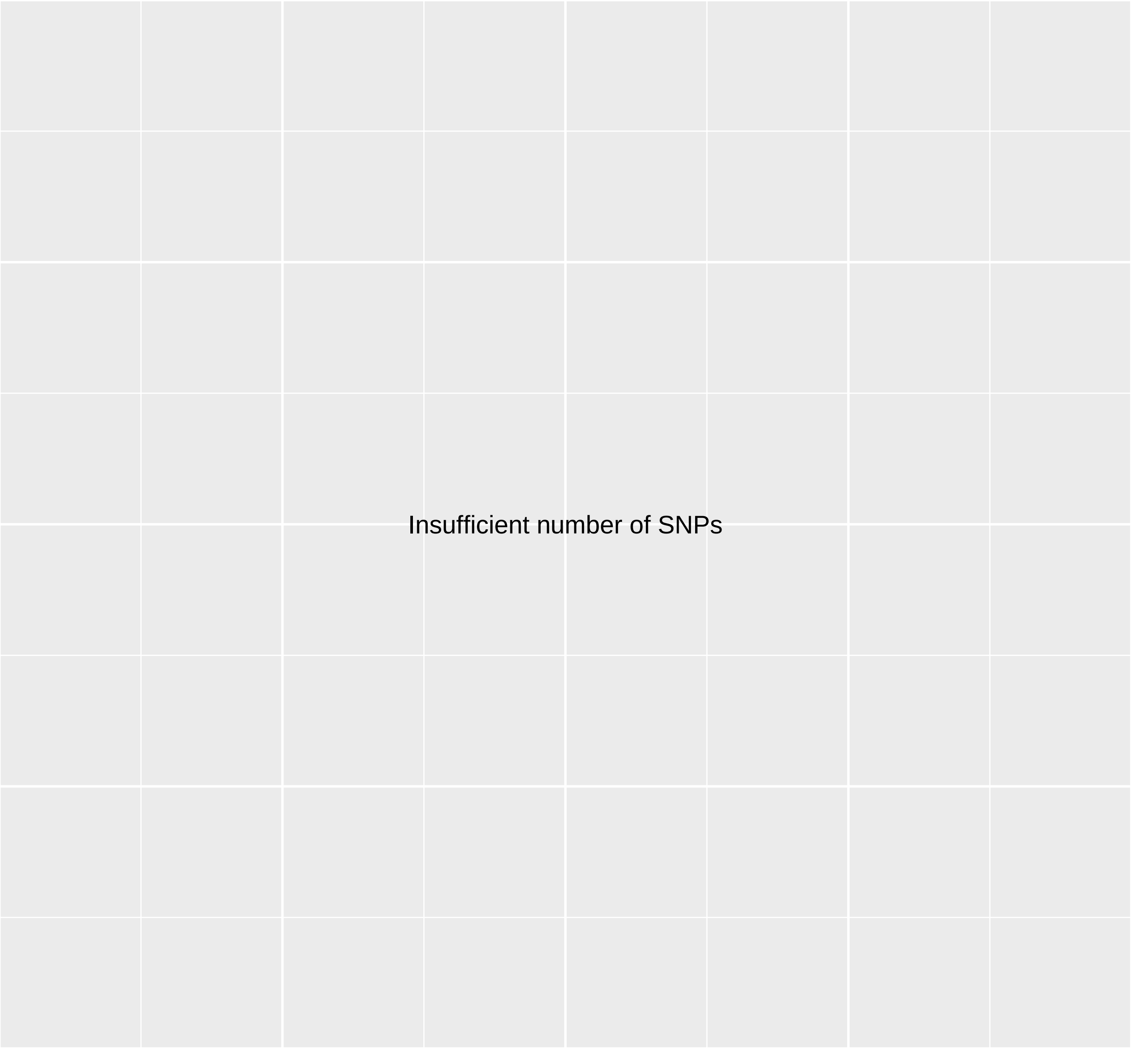
Insufficient number of SNPs

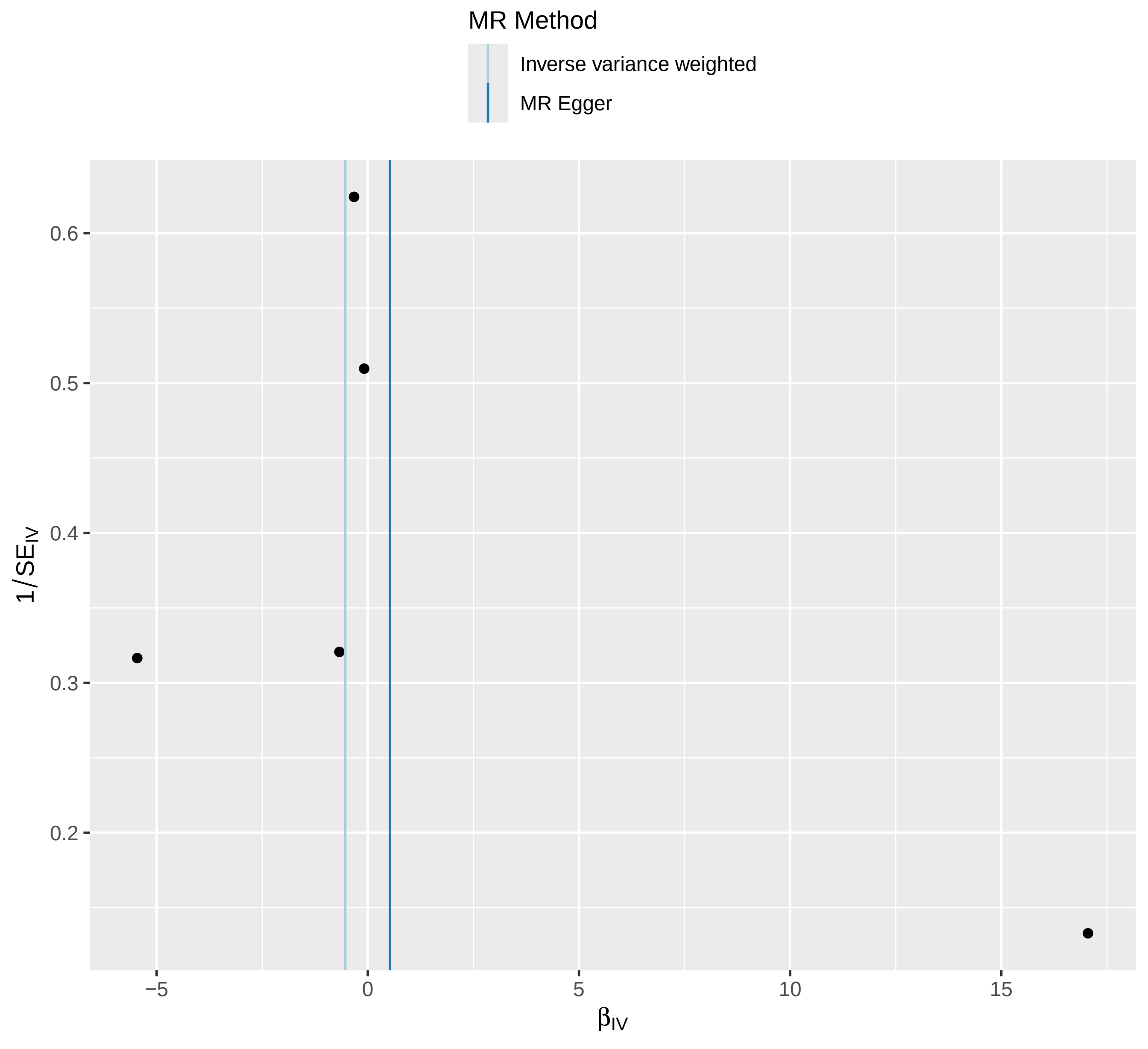

MR Method
Inverse variance weighted
MR Egger
0.6
0.5
0.4
0.3
0.2
1/SE_IV
−5
0
5
10
15
β_IV

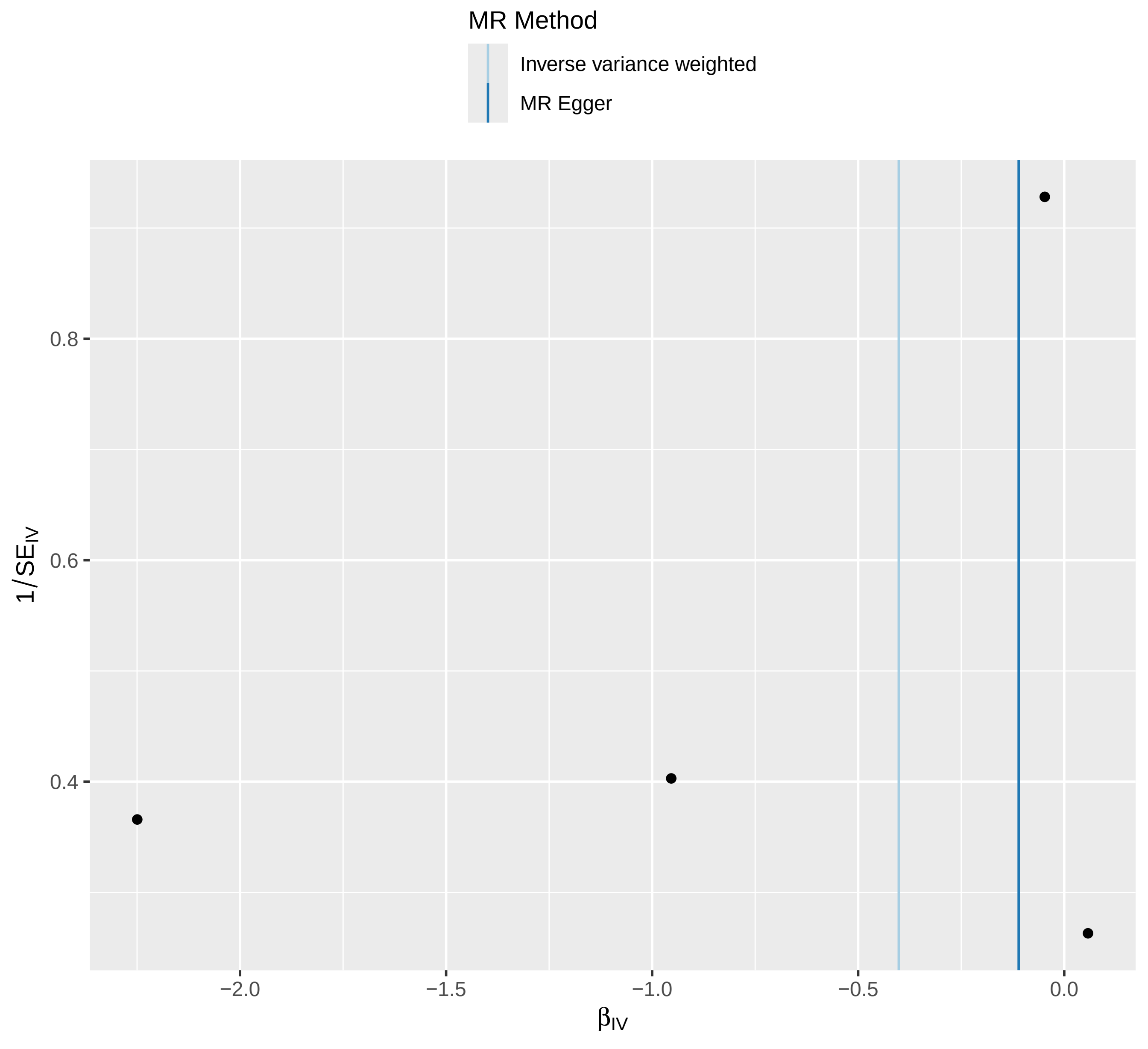
MR Method
Inverse variance weighted
MR Egger
1/SE$_{IV}$
0.8
0.6
0.4
−2.0
−1.5
−1.0
−0.5
0.0
β$_{IV}$

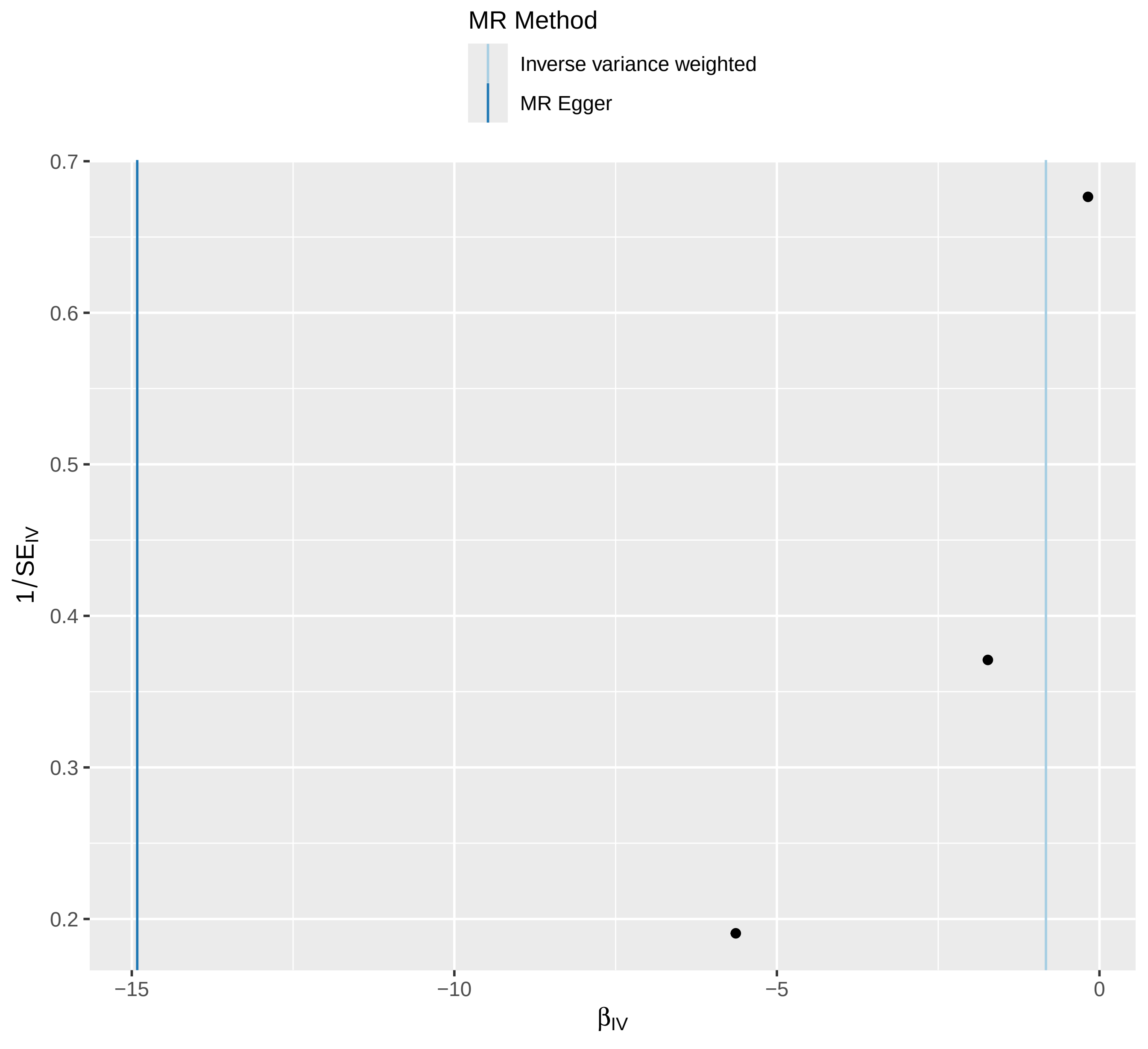
MR Method
Inverse variance weighted
MR Egger
0.7
0.6
0.5
0.4
0.3
0.2
1/SE_IV
−15
−10
−5
0
β_IV

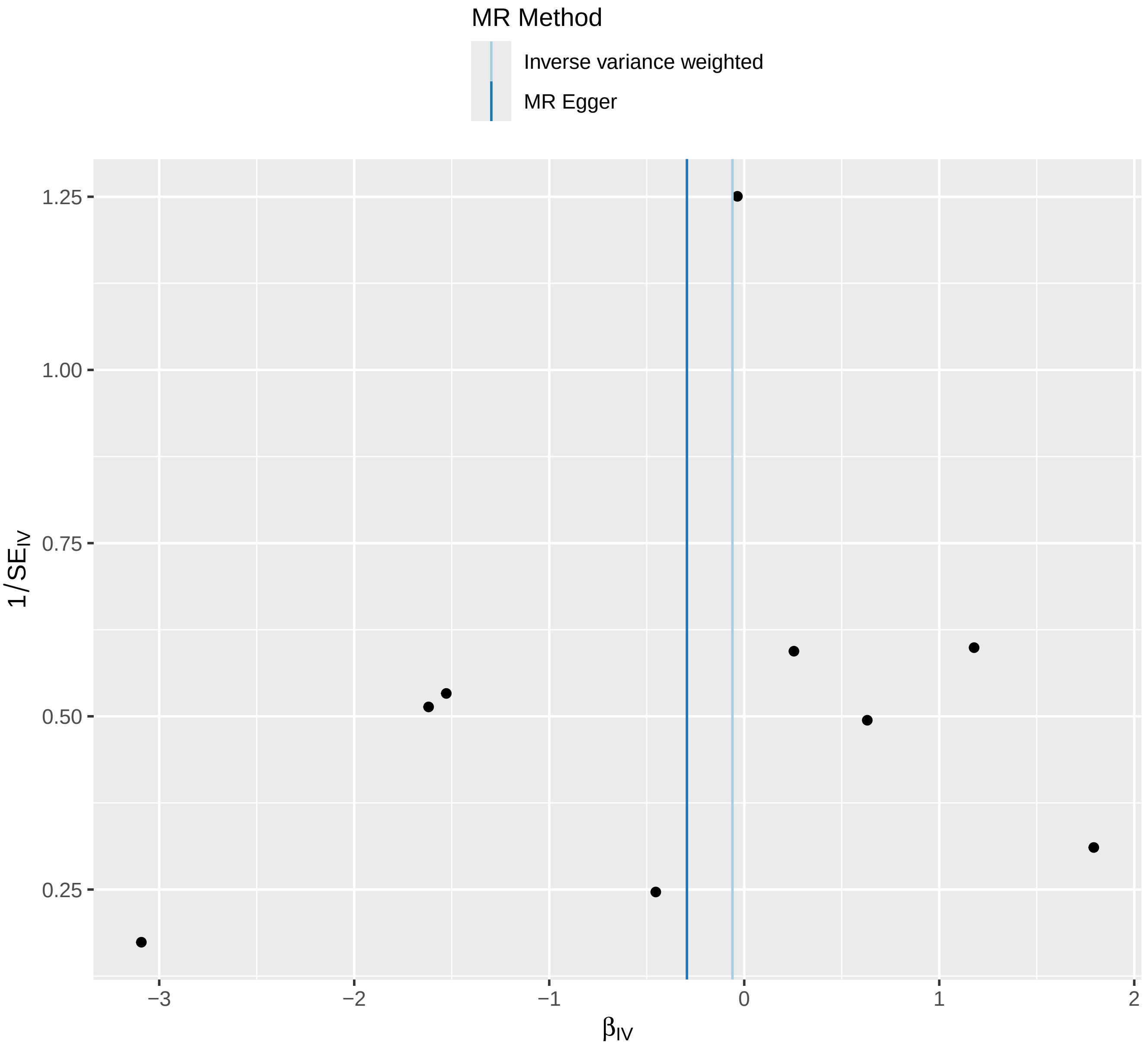

MR Method
Inverse variance weighted
MR Egger
1.25
1.00
0.75
0.50
0.25
1/SE_IV
−3
−2
−1
0
1
2
β_IV

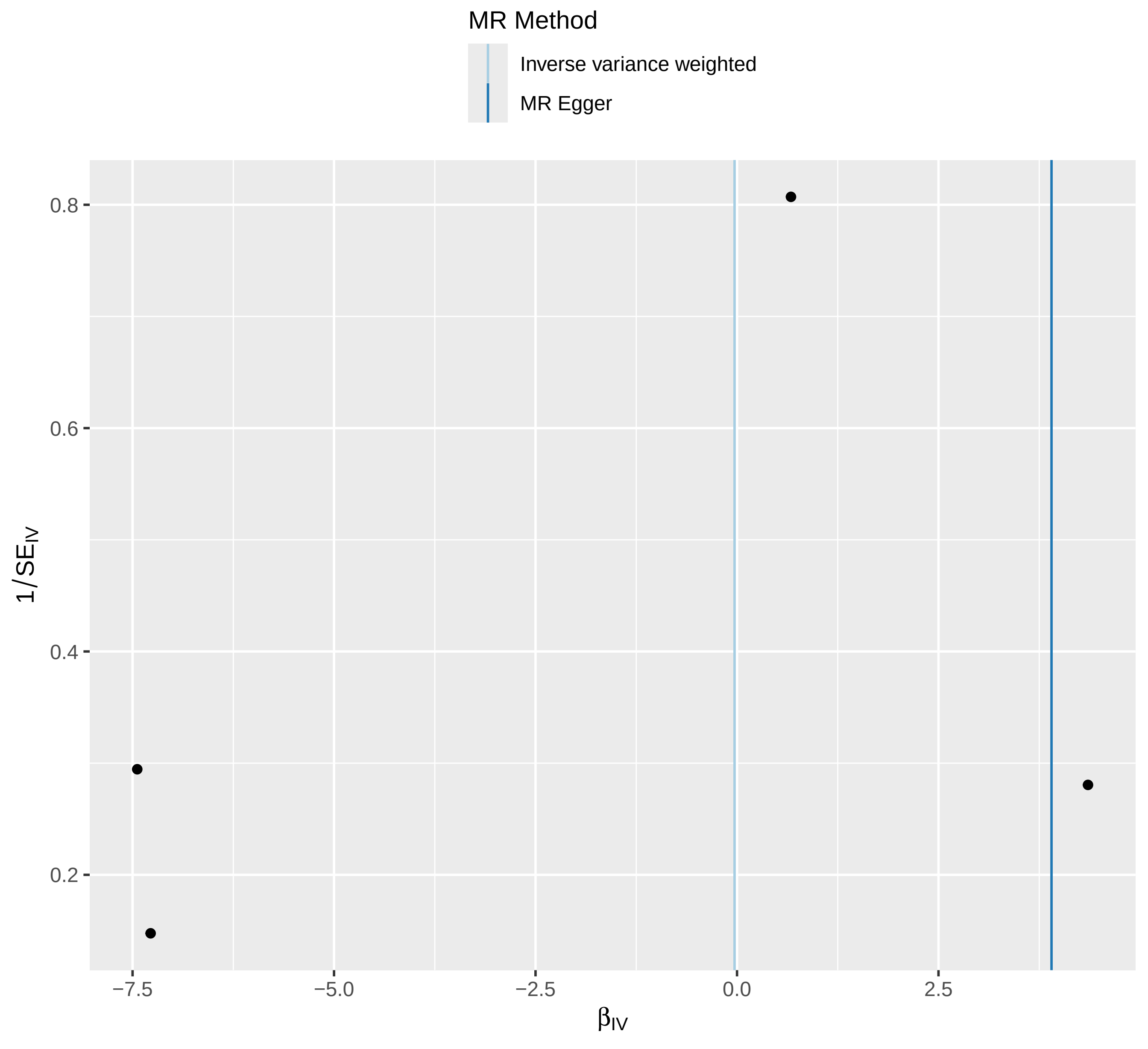

MR Method
Inverse variance weighted
MR Egger
0.8
0.6
0.4
0.2
$1/SE_{IV}$
−7.5
−5.0
−2.5
0.0
2.5
β_{IV}

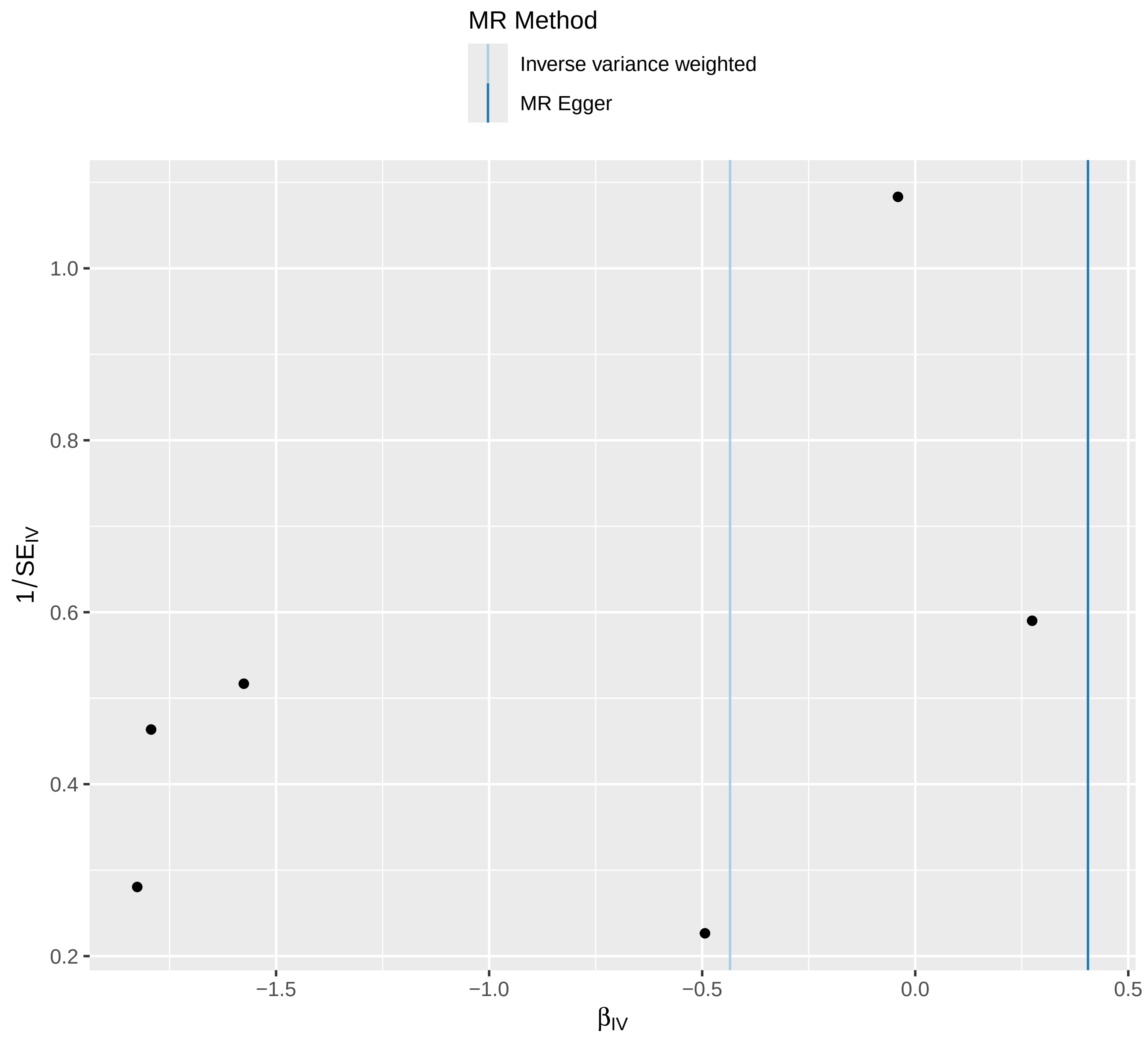
MR Method
Inverse variance weighted
MR Egger
1.0
0.8
0.6
0.4
0.2
−1.5
−1.0
−0.5
0.0
0.5
1/SE_IV
β_IV

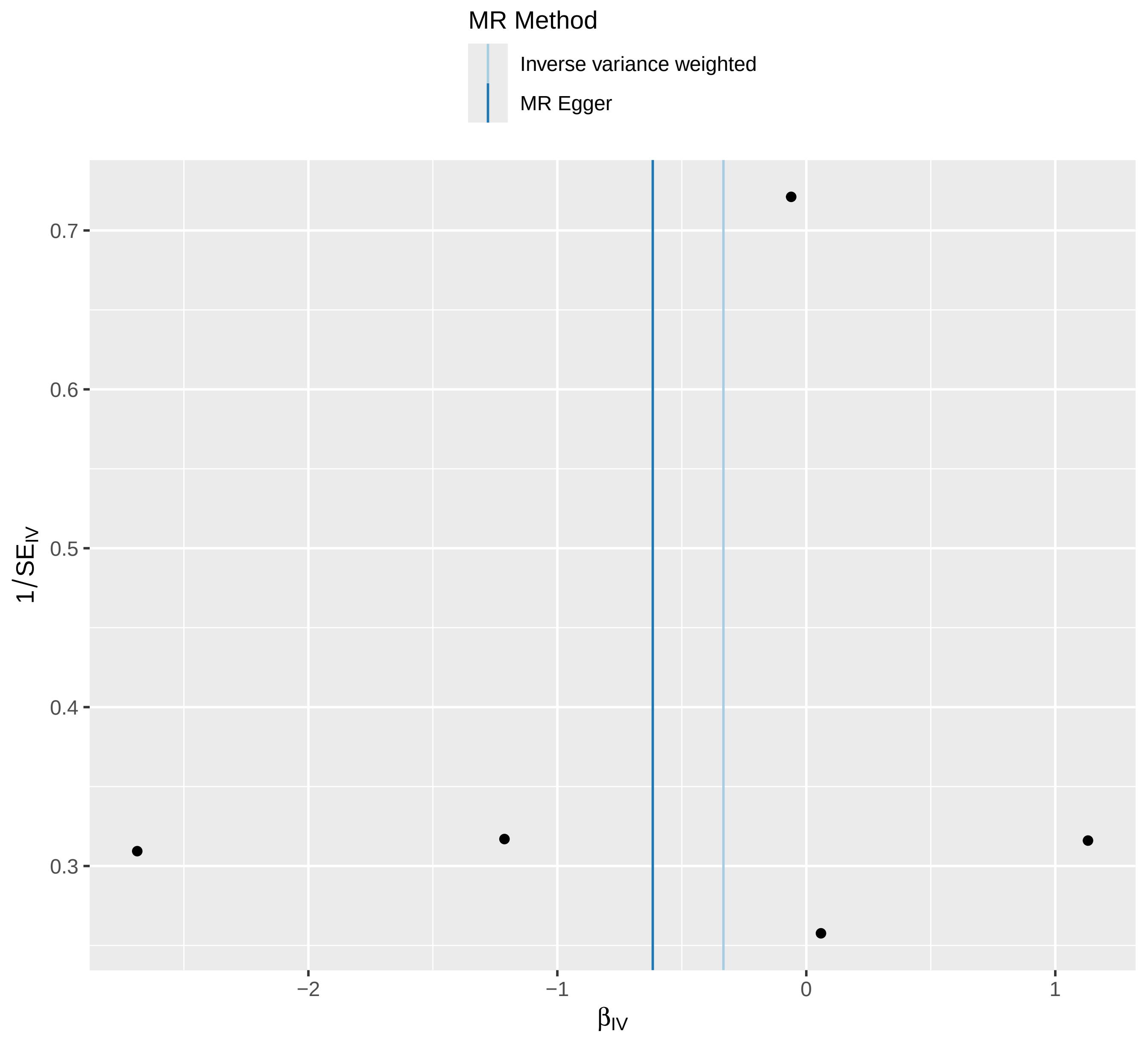
MR Method
Inverse variance weighted
MR Egger
0.7
0.6
0.5
0.4
0.3
1/SE_IV
−2
−1
0
1
β_IV

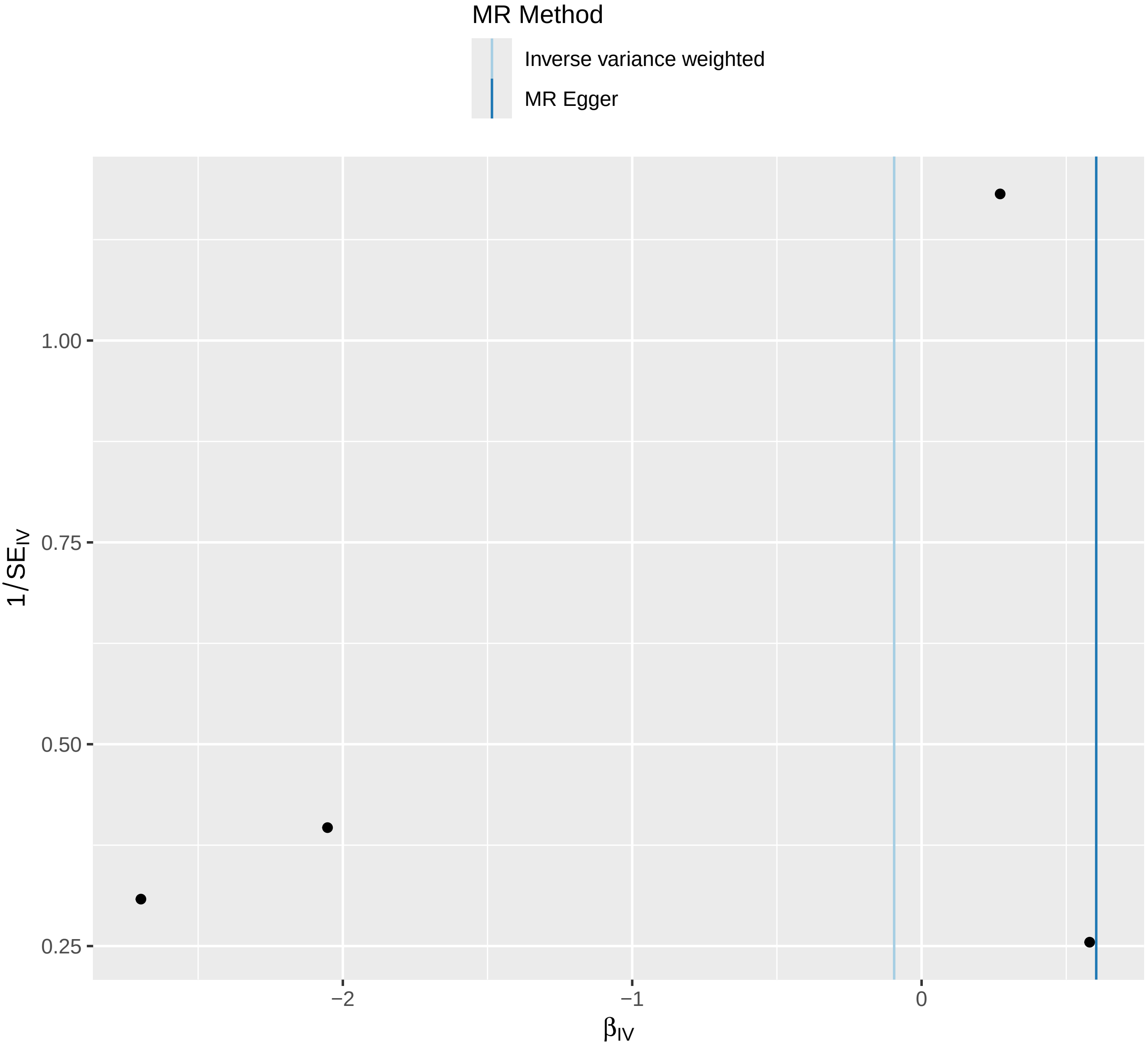
MR Method
Inverse variance weighted
MR Egger
1.00
0.75
0.50
0.25
1/SE$_{IV}$
−2
−1
0
β$_{IV}$

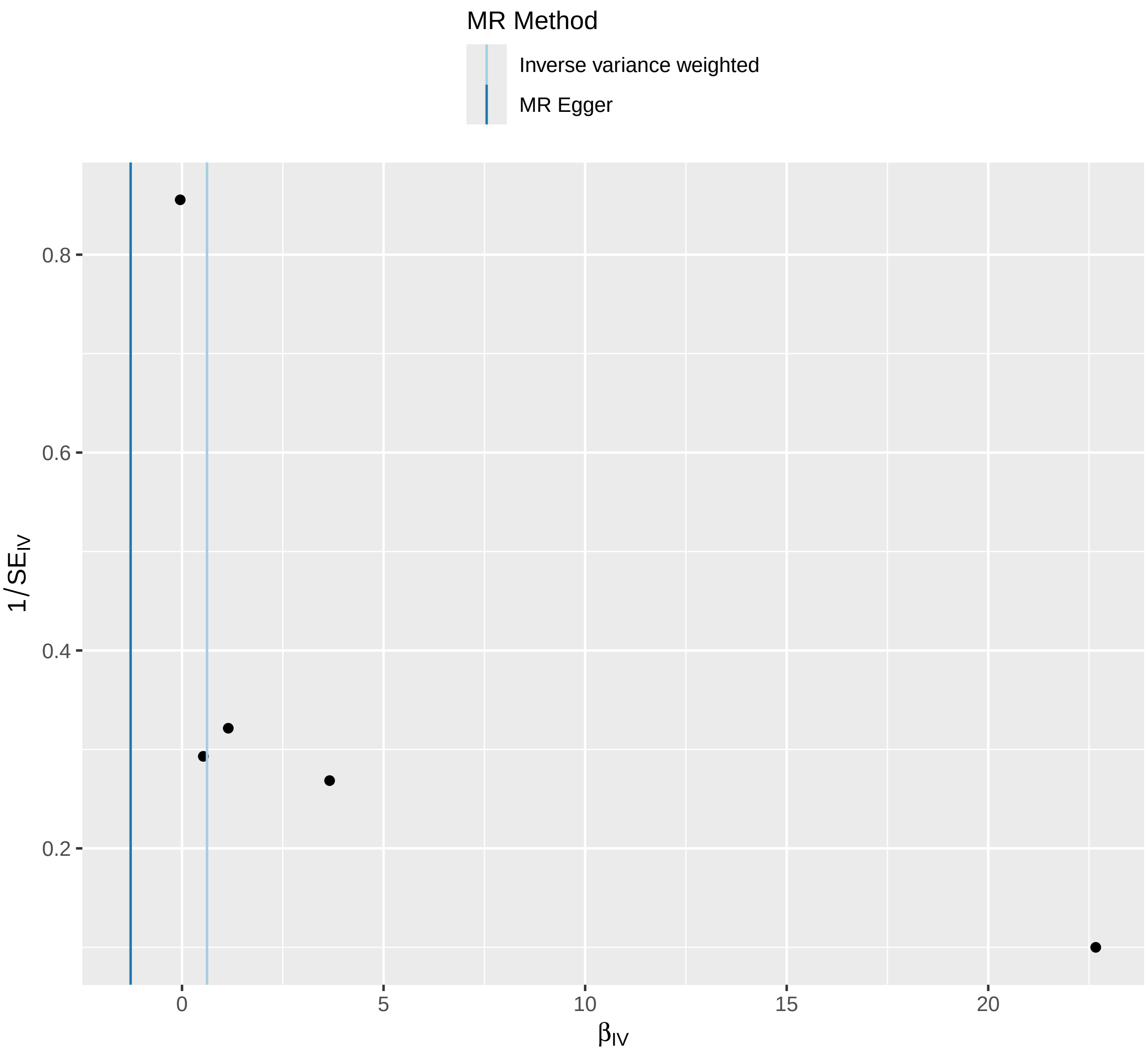
MR Method
Inverse variance weighted
MR Egger
0.8
0.6
0.4
0.2
$1/SE_{IV}$
0
5
10
15
20
β_{IV}

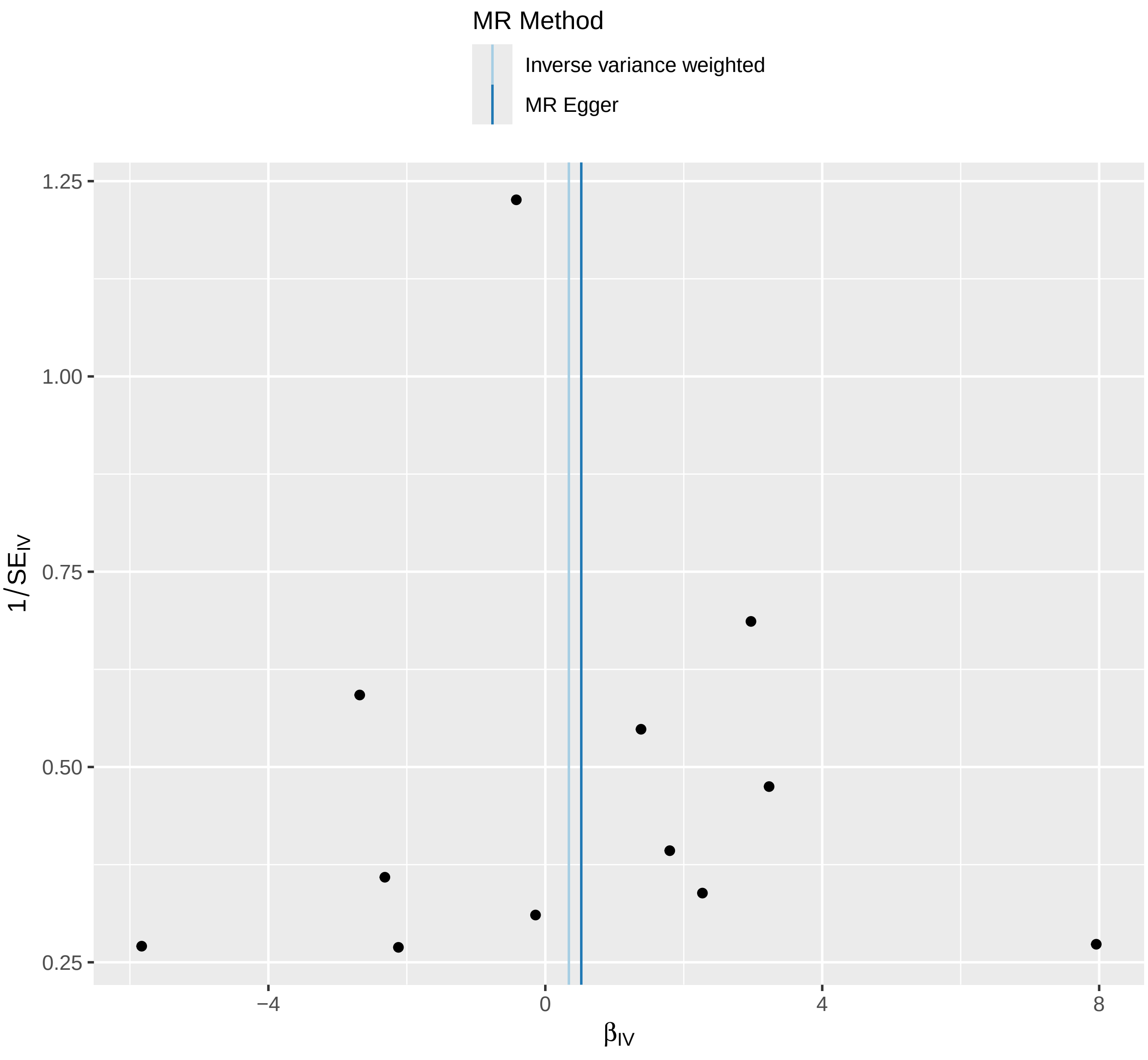
MR Method
Inverse variance weighted
MR Egger
1.25
1.00
0.75
0.50
0.25
1/SE_IV
−4
0
4
8
β_IV

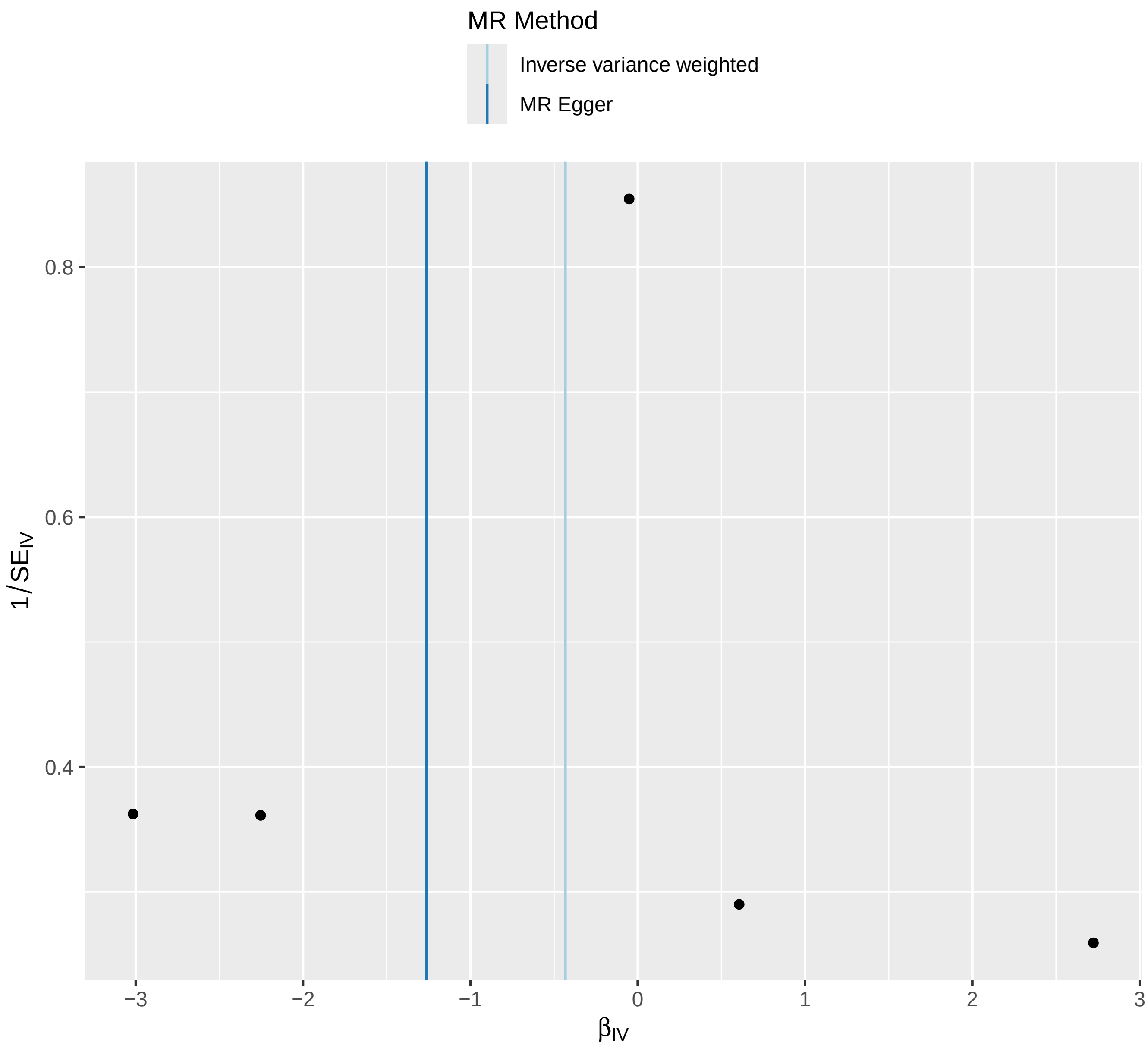

MR Method
Inverse variance weighted
MR Egger
0.8
0.6
0.4
1/SE_IV
−3
−2
−1
0
1
2
3
β_IV

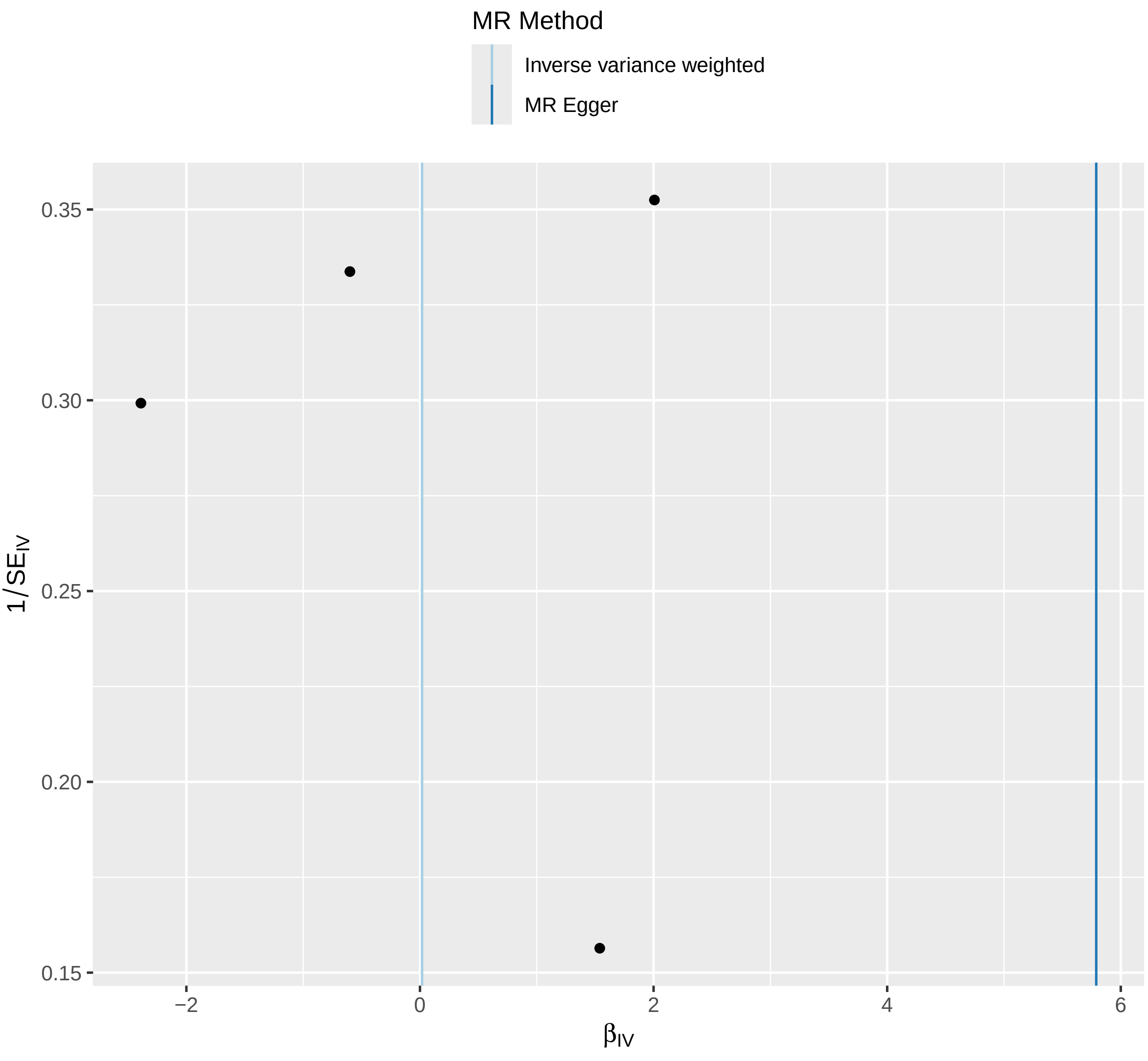
MR Method
Inverse variance weighted
MR Egger
0.35
0.30
0.25
0.20
0.15
1/SE_IV
−2
0
2
4
6
β_IV

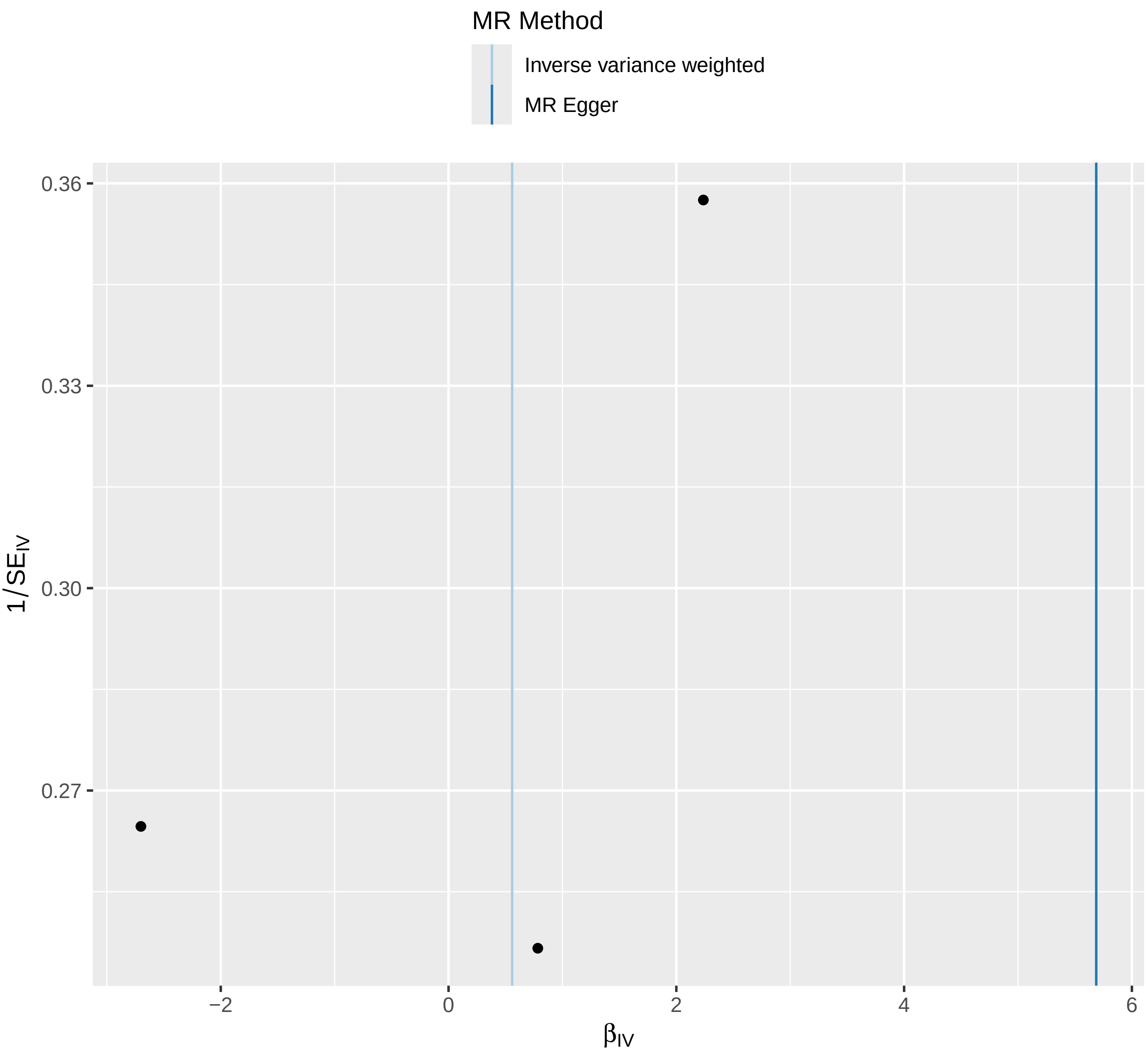

MR Method
Inverse variance weighted
MR Egger
0.36
0.33
0.30
0.27
1/SE_IV
−2
0
2
4
6
β_IV

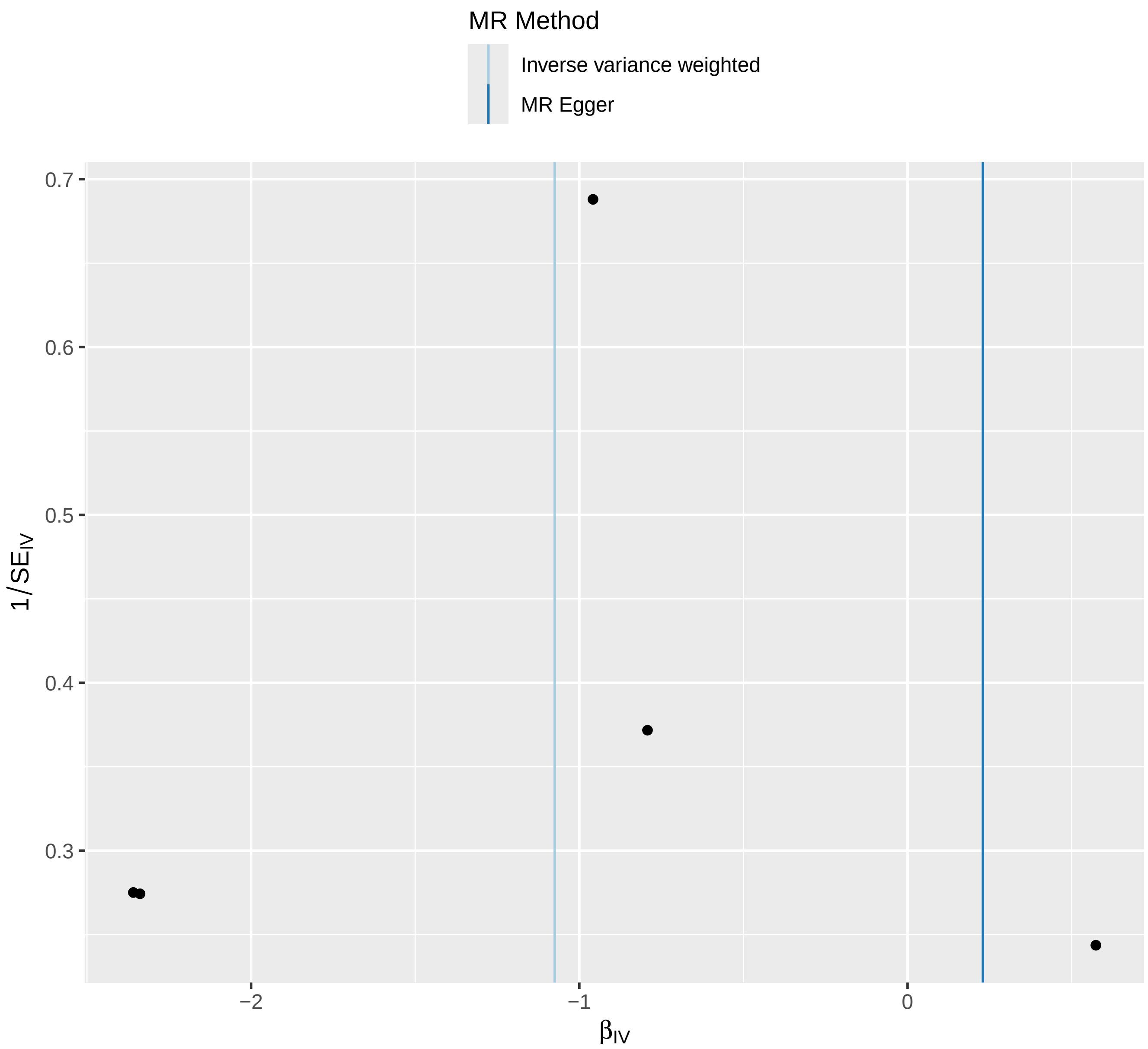

MR Method
Inverse variance weighted
MR Egger
0.7
0.6
0.5
0.4
0.3
1/SE_IV
−2
−1
0
β_IV

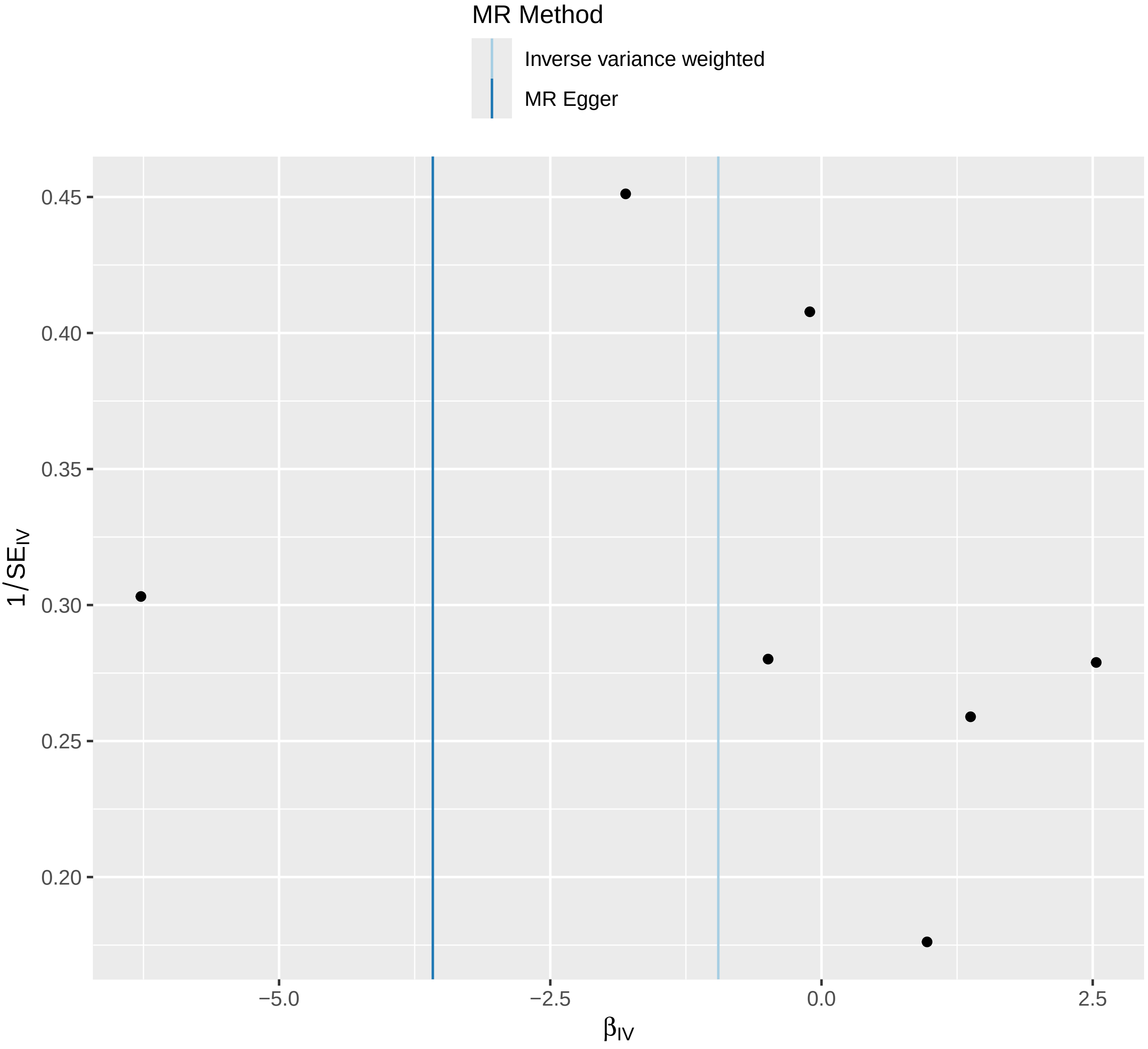
MR Method
Inverse variance weighted
MR Egger
0.45
0.40
0.35
0.30
0.25
0.20
1/SE_IV
−5.0
−2.5
0.0
2.5
β_IV

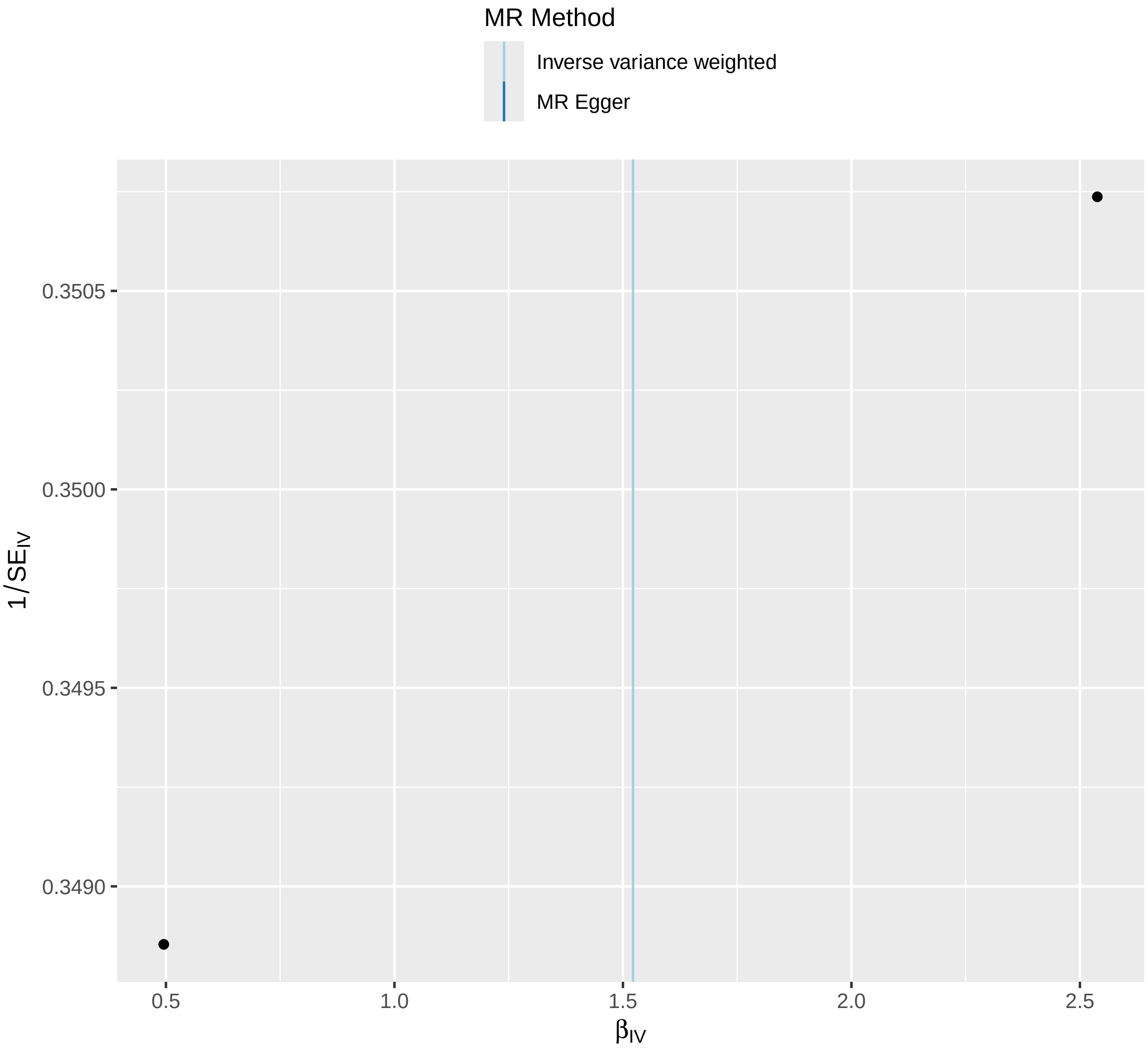

MR Method
Inverse variance weighted
MR Egger
0.3505
0.3500
0.3495
0.3490
$1/SE_{IV}$
0.5
1.0
1.5
2.0
2.5
β_{IV}

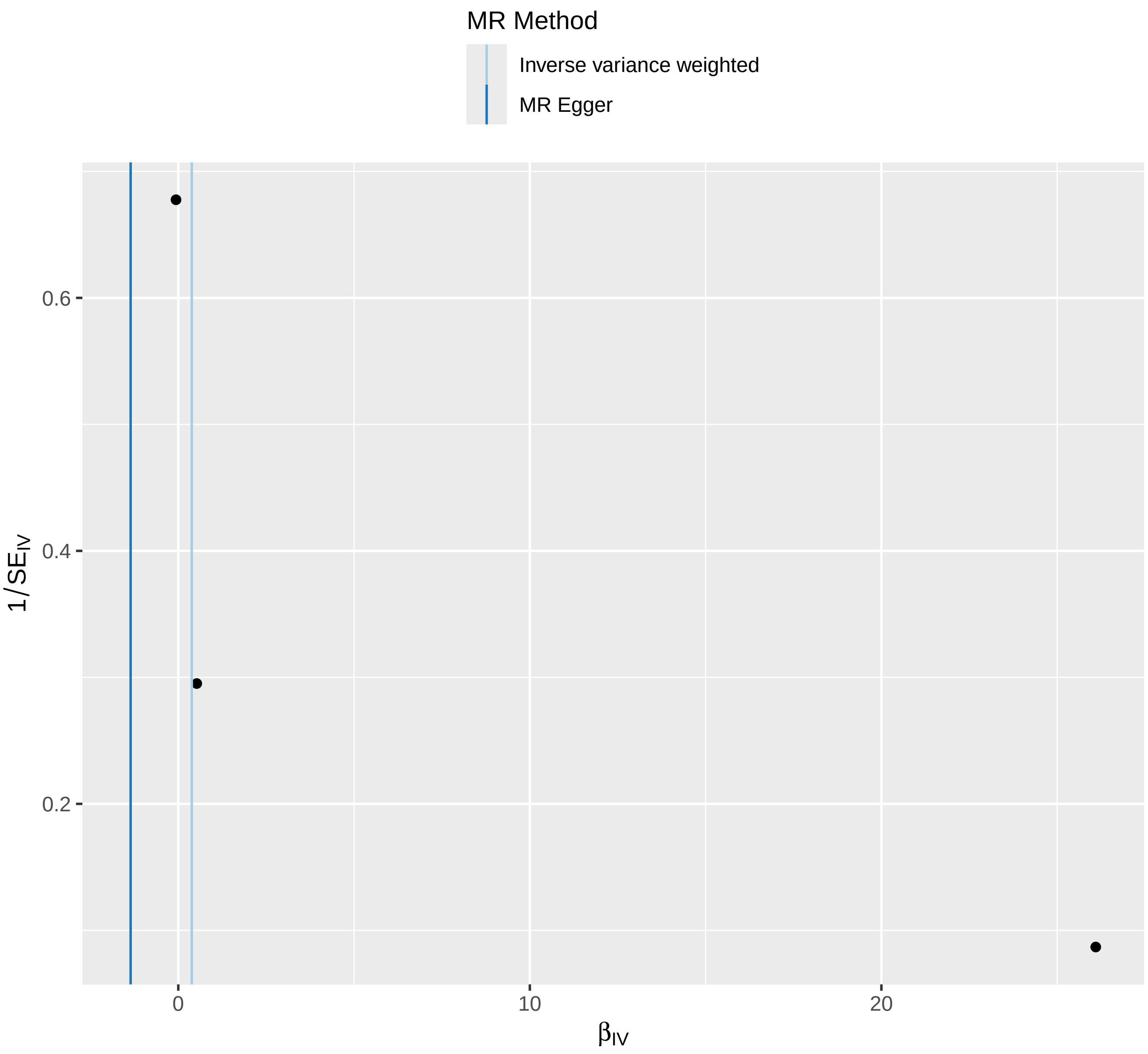
MR Method
Inverse variance weighted
MR Egger
0.6
0.4
0.2
1/SE_IV
0
10
20
β_IV

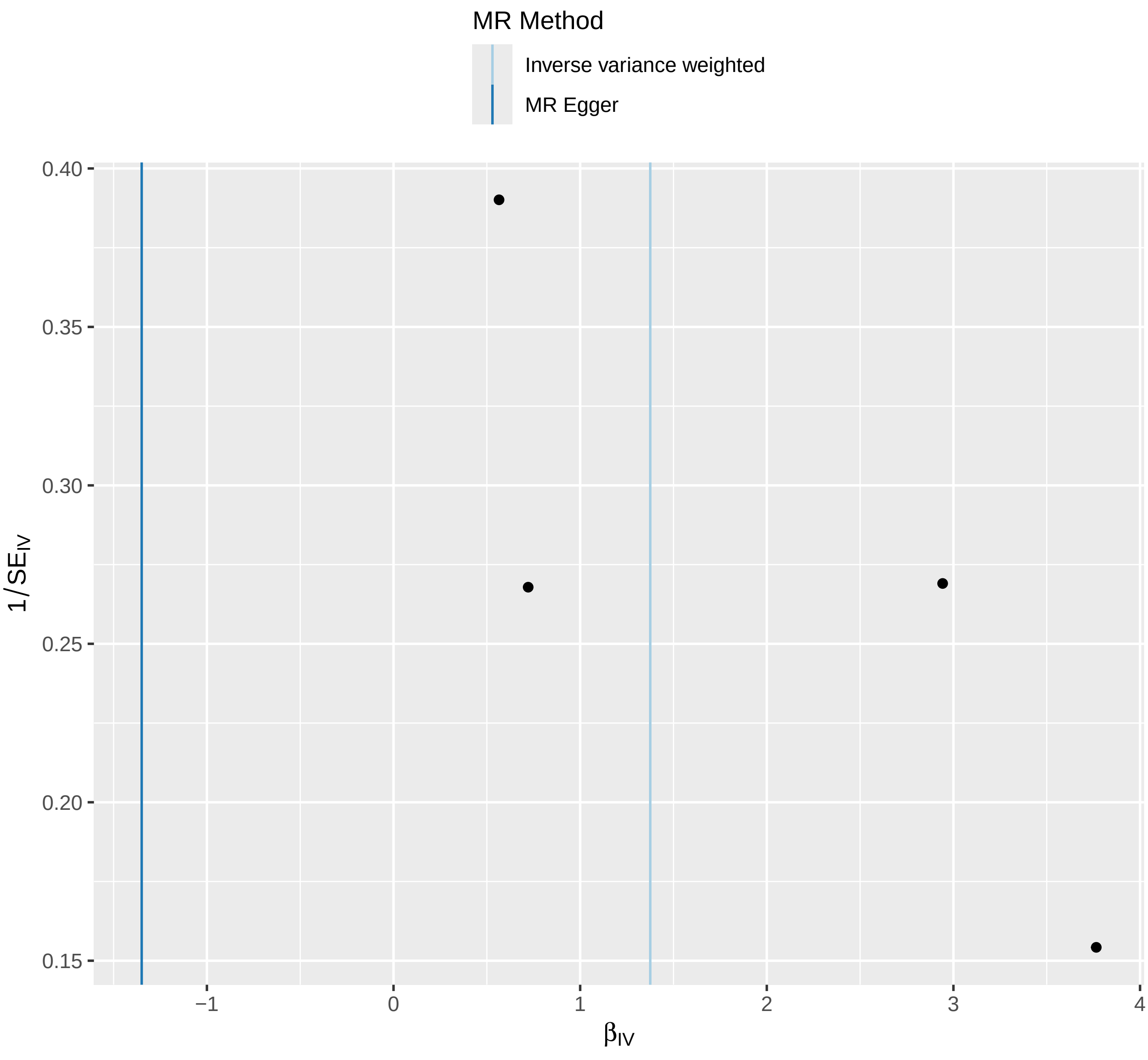
MR Method
Inverse variance weighted
MR Egger
0.40
0.35
0.30
0.25
0.20
0.15
1/SE$_{IV}$
−1
0
1
2
3
4
β$_{IV}$

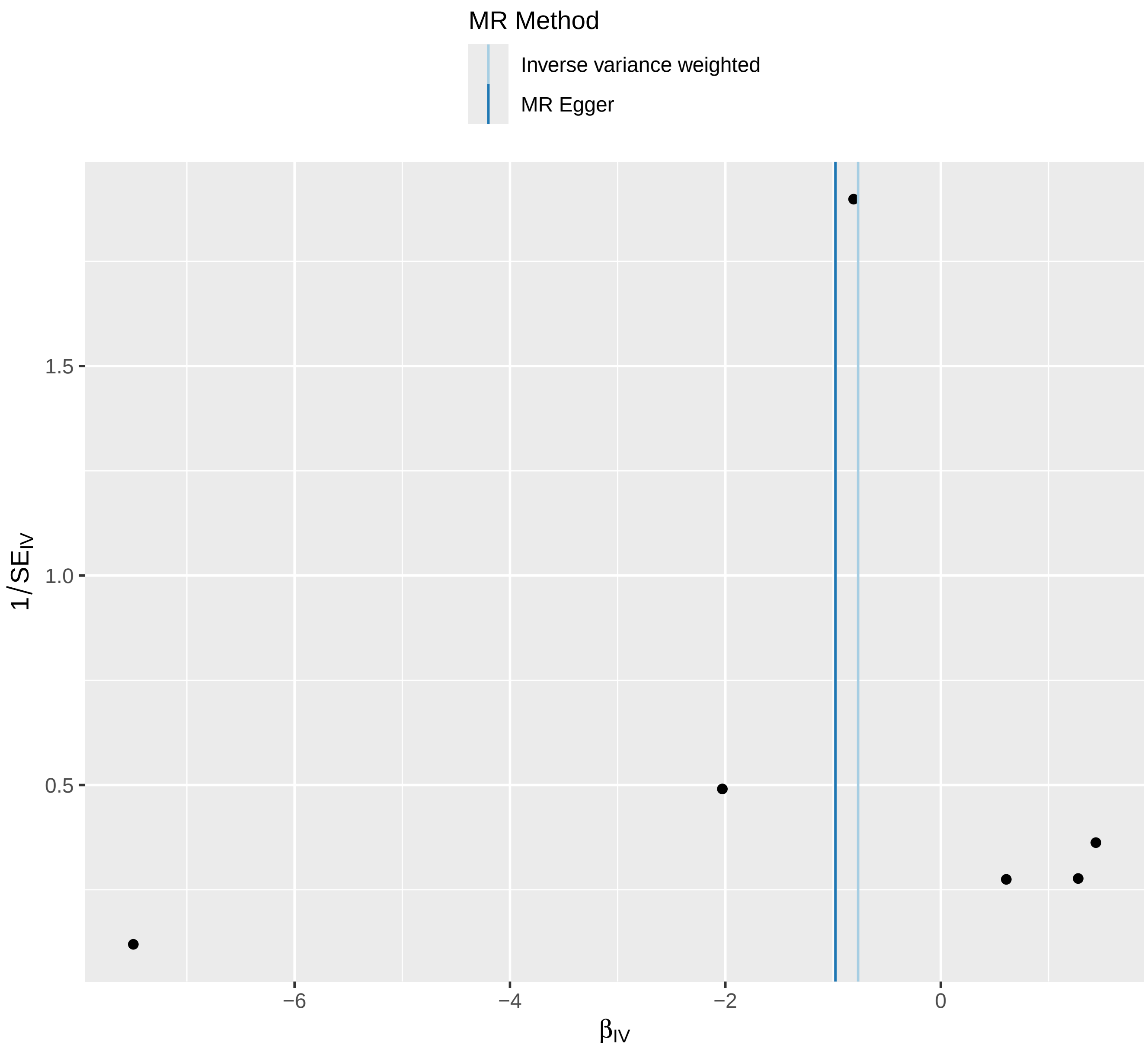
MR Method
Inverse variance weighted
MR Egger
1/SE_IV
1.5
1.0
0.5
−6
−4
−2
0
β_IV

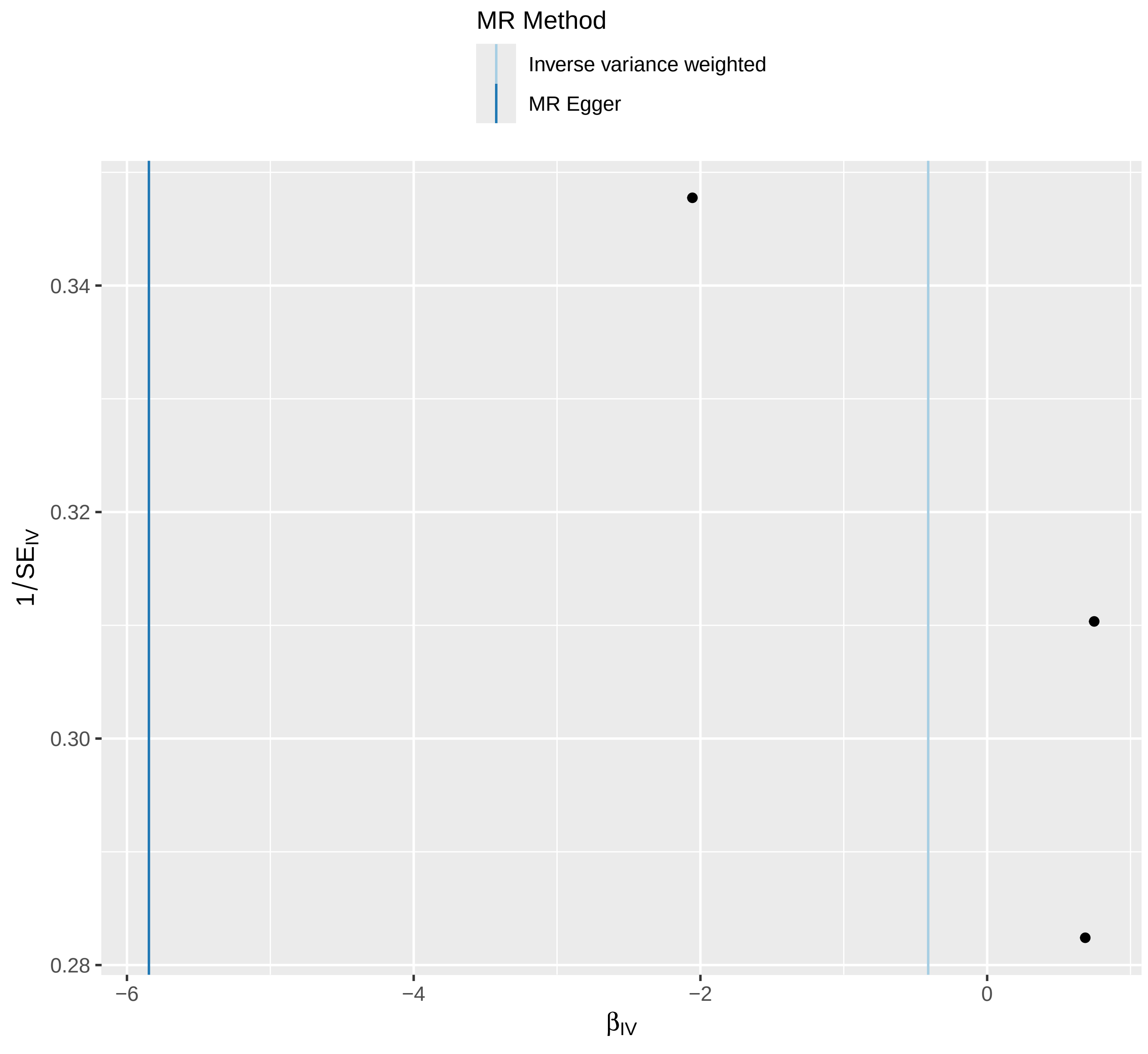
MR Method
Inverse variance weighted
MR Egger
0.34
0.32
0.30
0.28
$1/SE_{IV}$
−6
−4
−2
0
β_{IV}

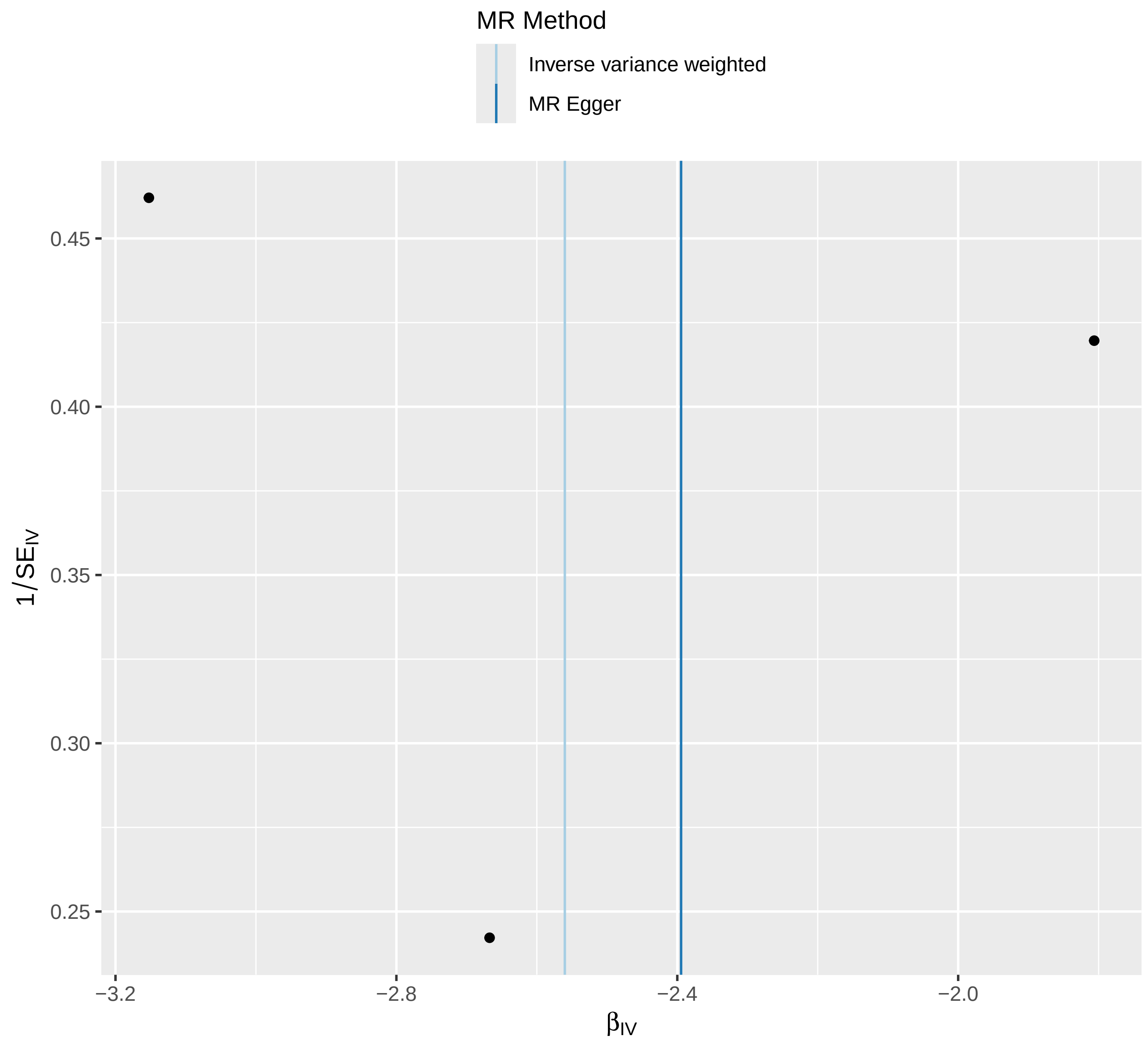
MR Method
Inverse variance weighted
MR Egger
0.45
0.40
0.35
0.30
0.25
1/SE_IV
−3.2
−2.8
−2.4
−2.0
β_IV

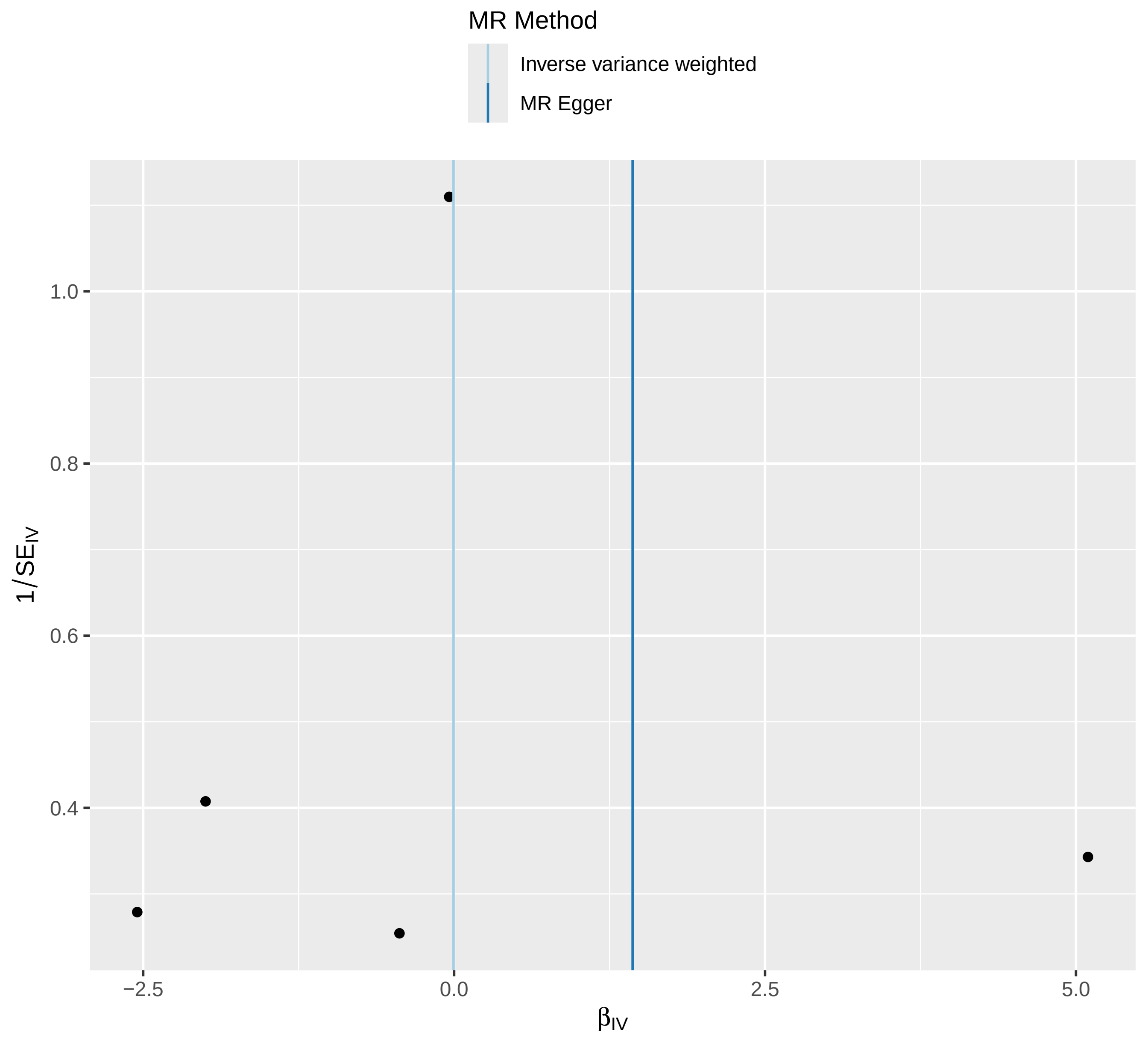
MR Method
Inverse variance weighted
MR Egger
1.0
0.8
0.6
0.4
$1/SE_{IV}$
−2.5
0.0
2.5
5.0
β_{IV}

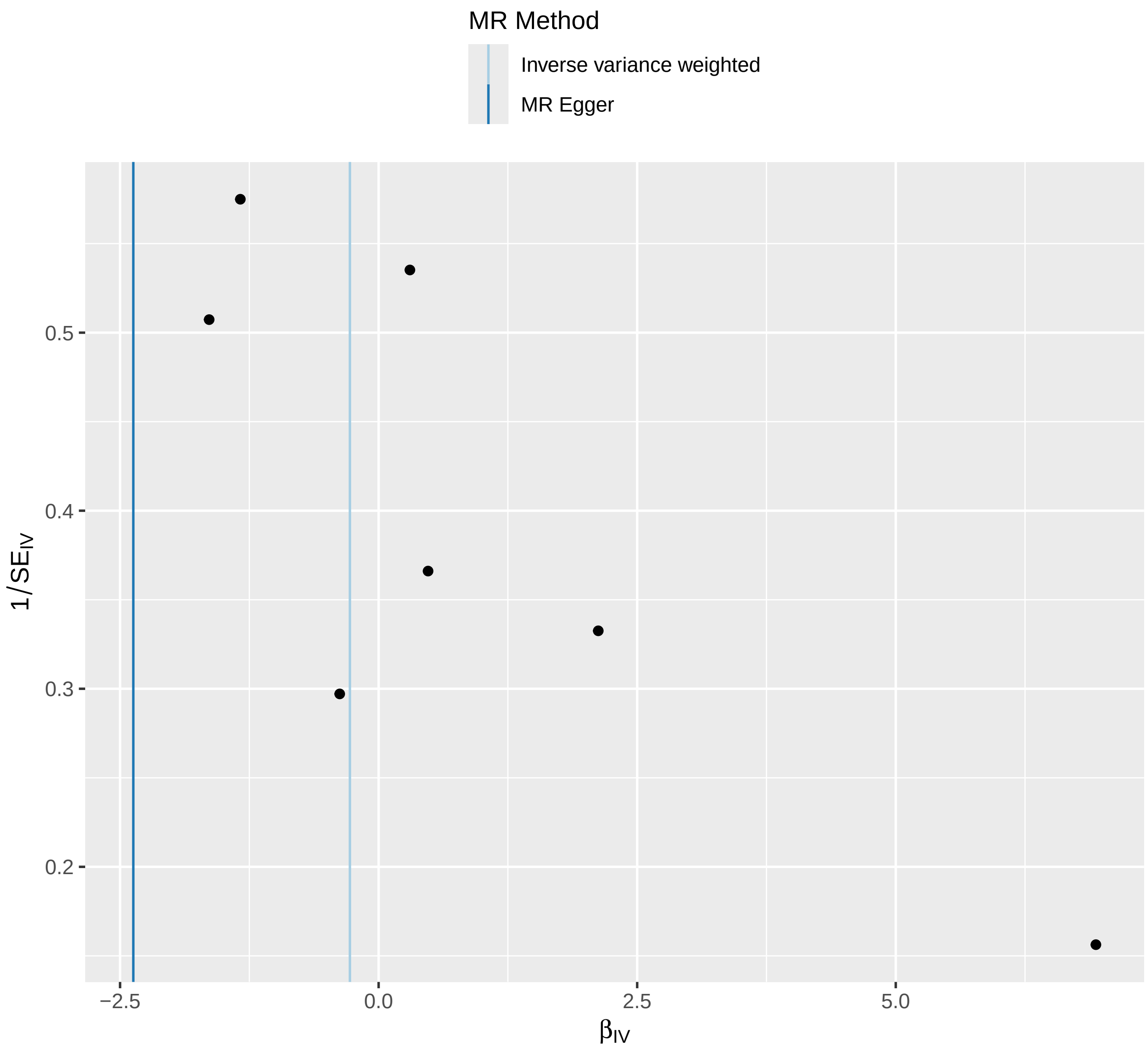
MR Method
Inverse variance weighted
MR Egger
1/SE_IV
β_IV
0.5
0.4
0.3
0.2
−2.5
0.0
2.5
5.0

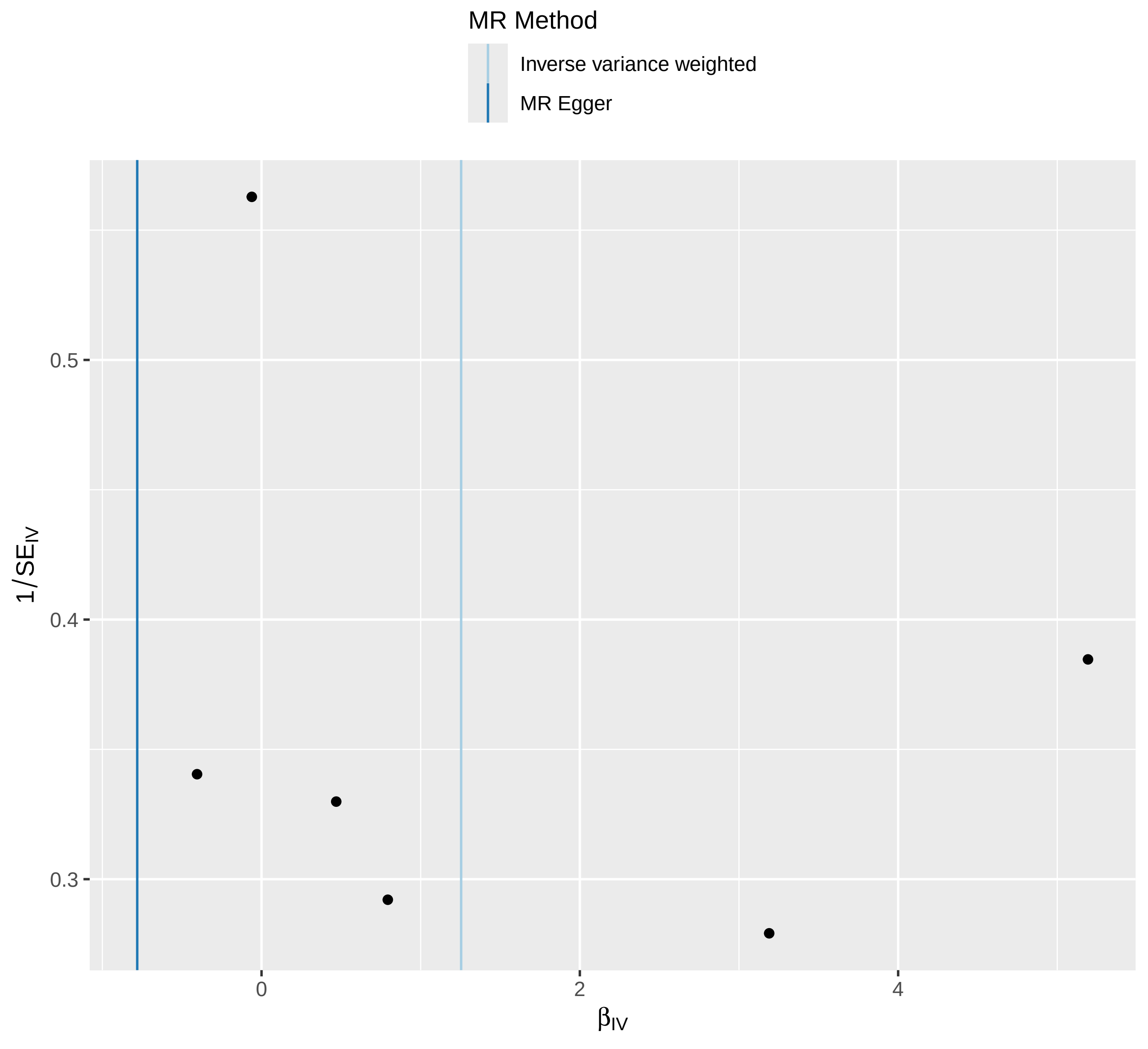
MR Method
Inverse variance weighted
MR Egger
0.5
0.4
0.3
$1/SE_{IV}$
0
2
4
β_{IV}

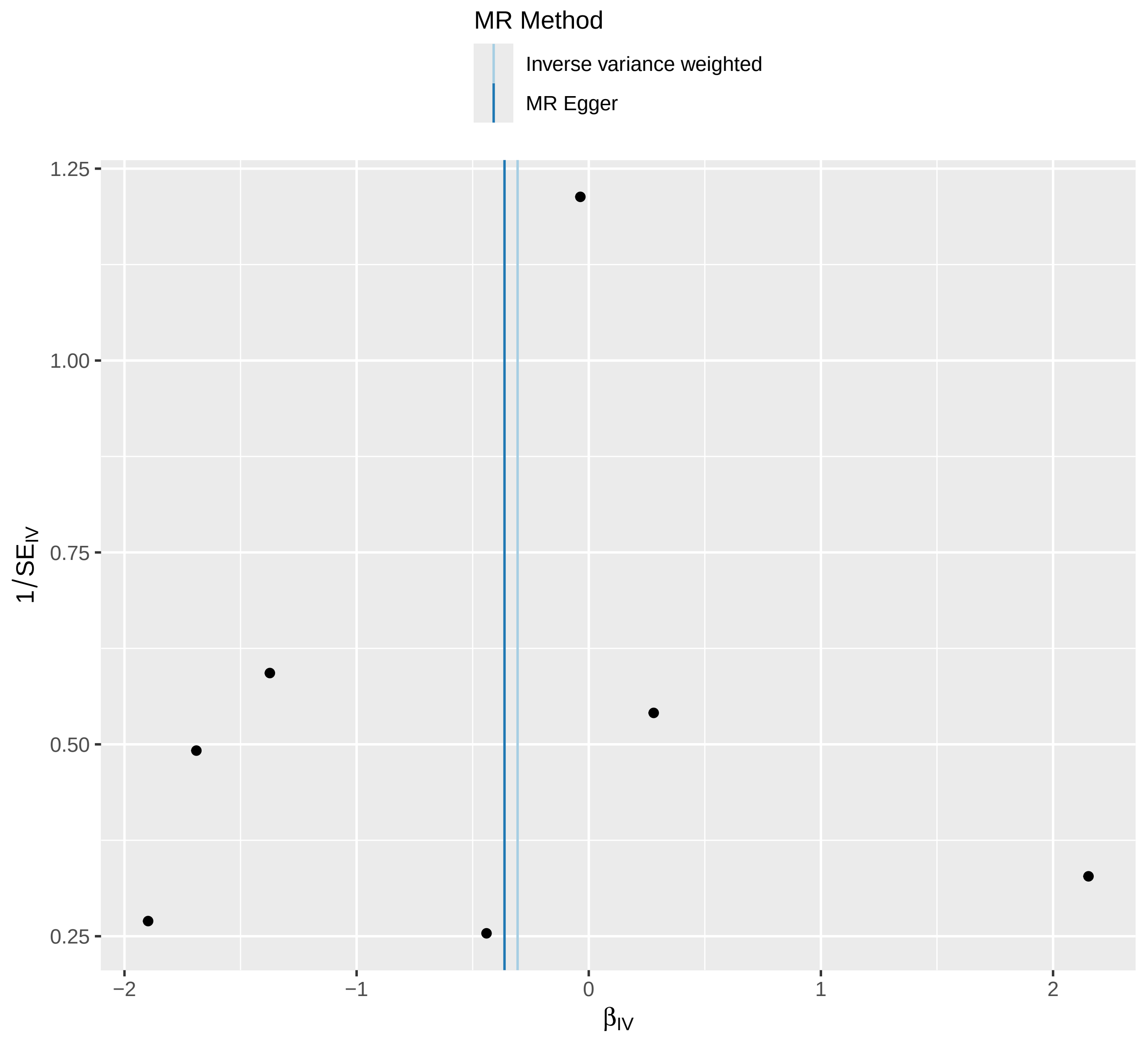
MR Method
Inverse variance weighted
MR Egger
1.25
1.00
0.75
0.50
0.25
1/SE_IV
−2
−1
0
1
2
β_IV

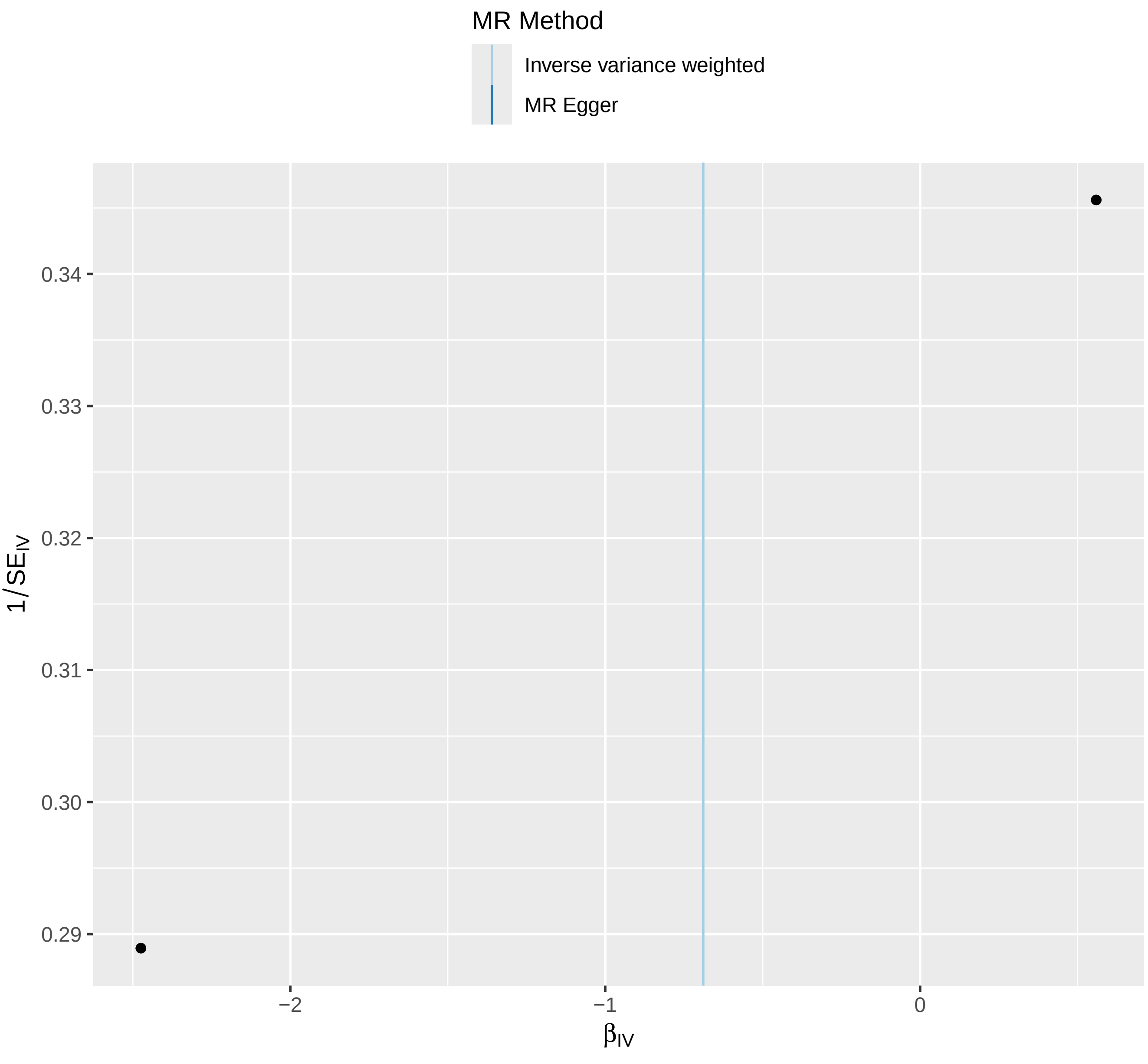
MR Method
Inverse variance weighted
MR Egger
0.34
0.33
0.32
0.31
0.30
0.29
1/SE$_{IV}$
−2
−1
0
β$_{IV}$

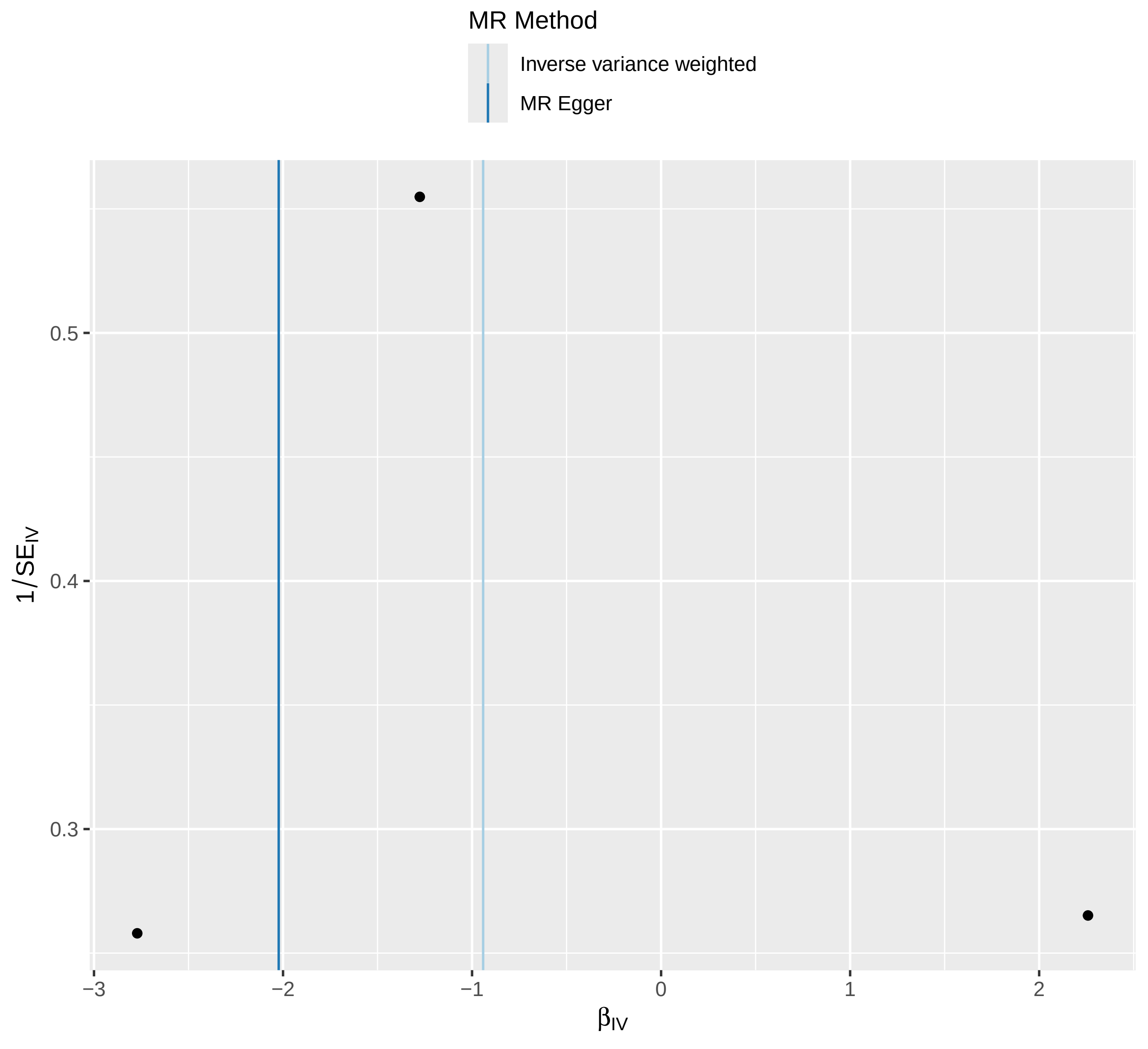

MR Method
Inverse variance weighted
MR Egger
0.5
0.4
0.3
1/SE$_{IV}$
−3
−2
−1
0
1
2
β_{IV}

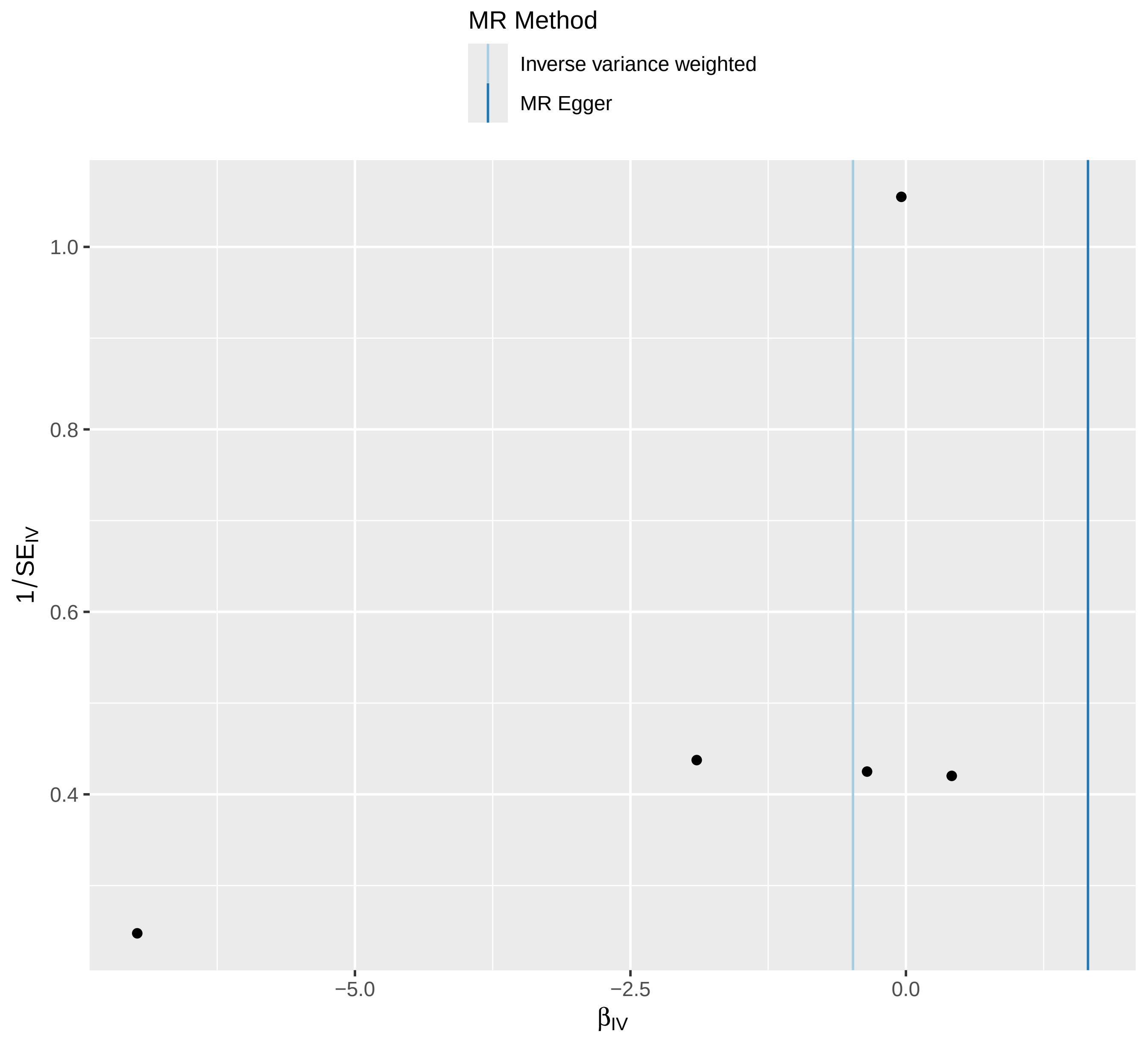

MR Method
Inverse variance weighted
MR Egger
1.0
0.8
0.6
0.4
1/SE_IV
−5.0
−2.5
0.0
β_IV

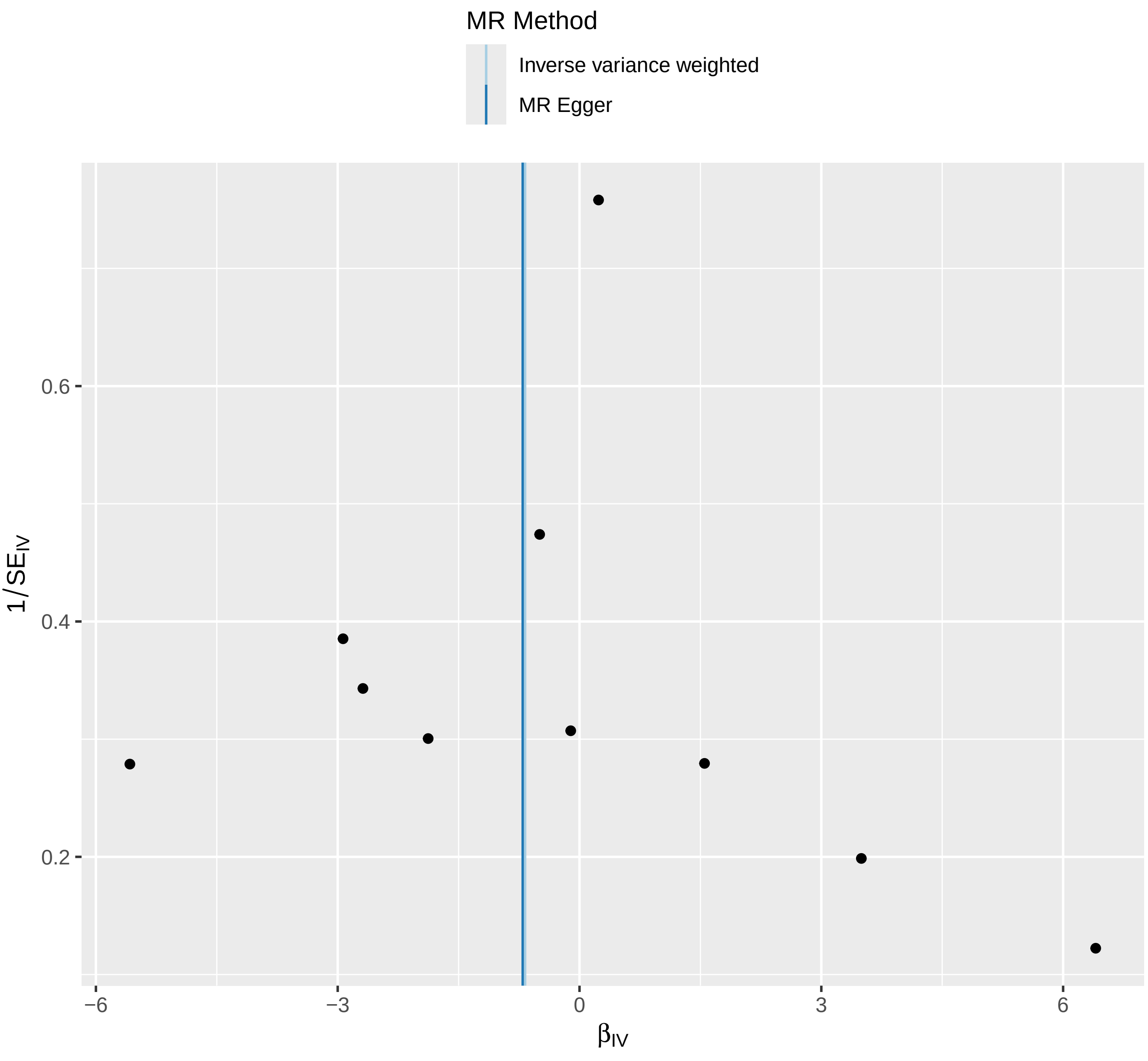
MR Method
Inverse variance weighted
MR Egger
0.6
0.4
0.2
1/SE_{IV}
−6
−3
0
3
6
β_{IV}

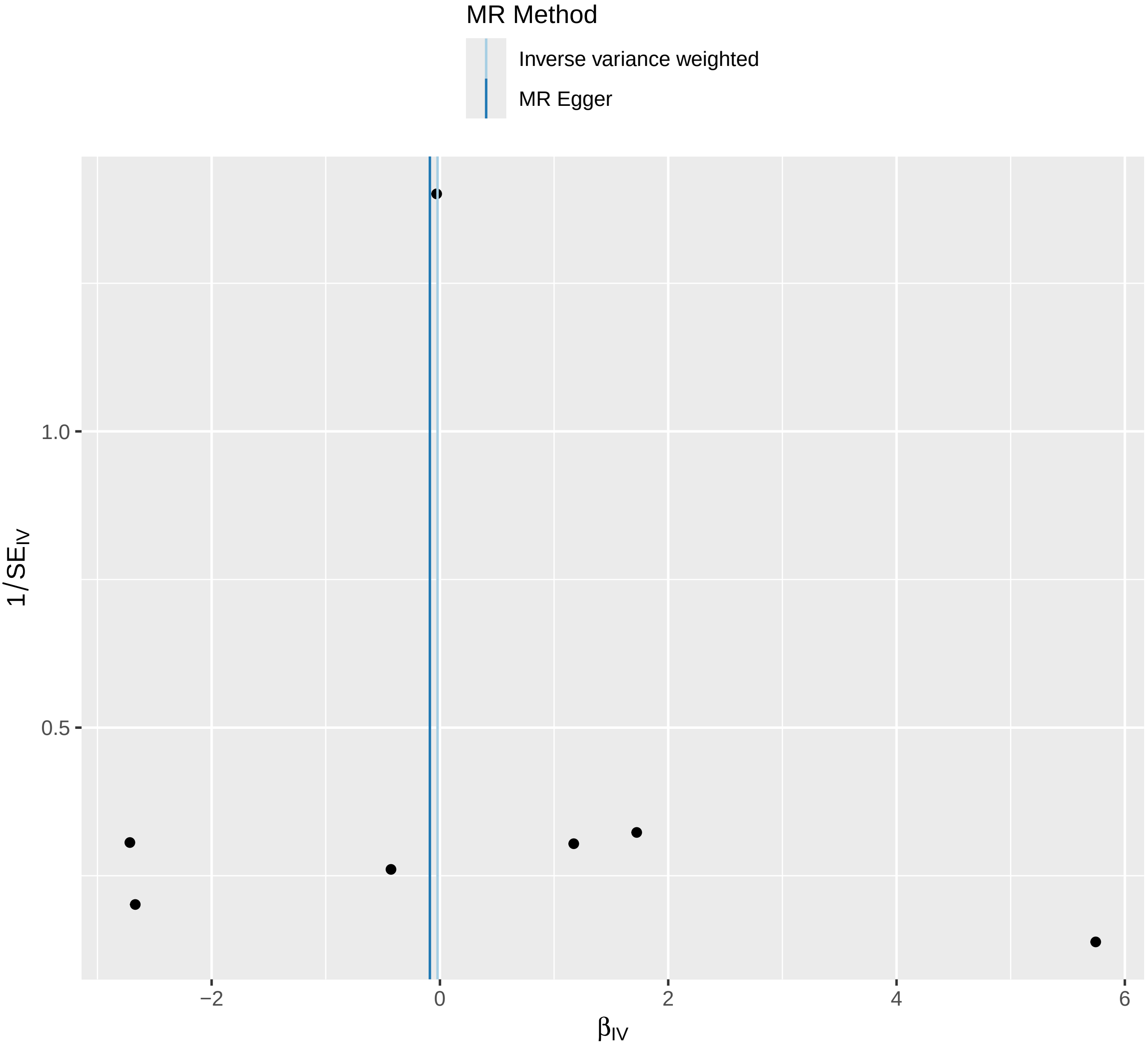
MR Method
Inverse variance weighted
MR Egger
1.0
0.5
1/SE_IV
−2
0
2
4
6
β_IV

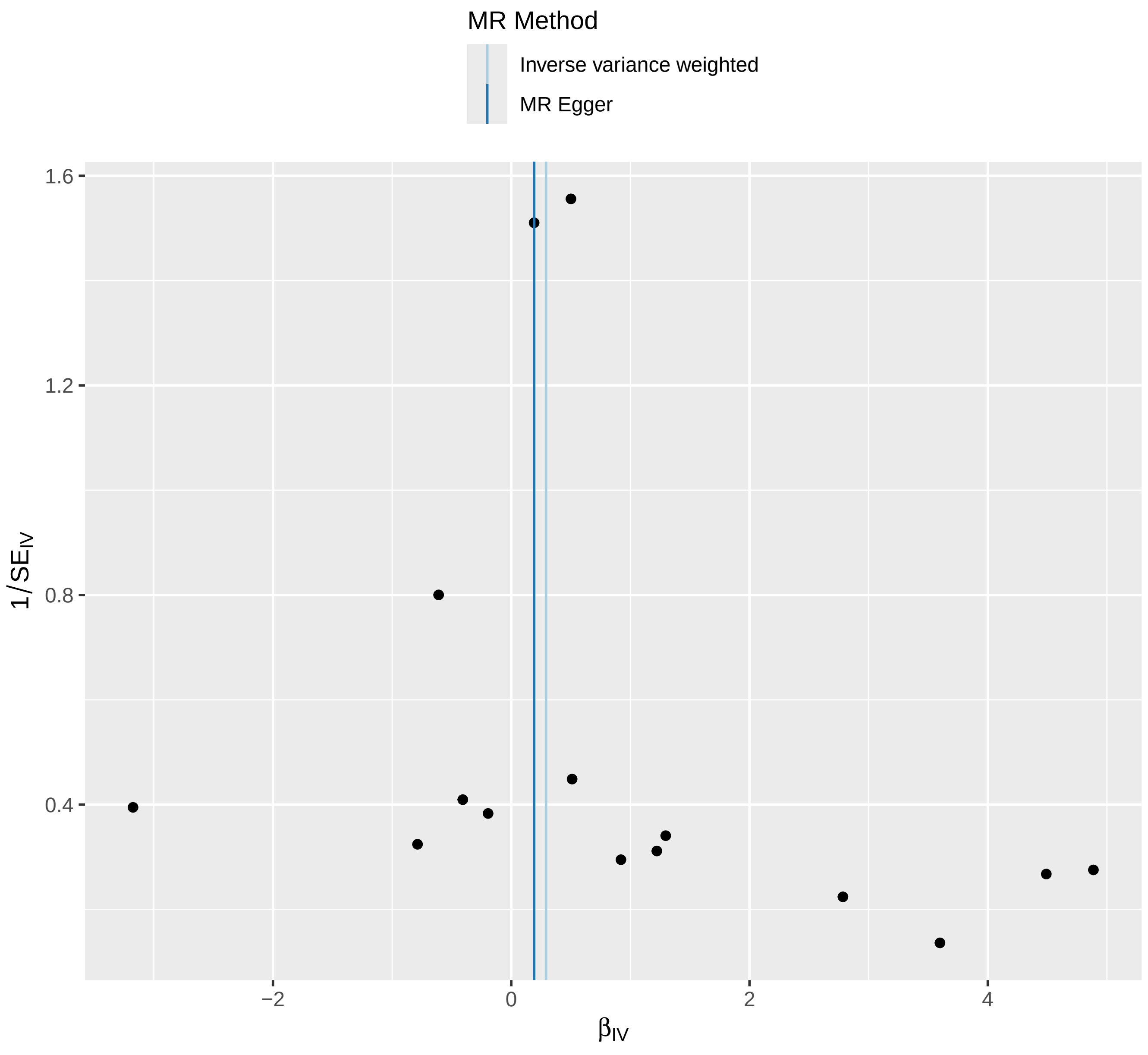
MR Method
Inverse variance weighted
MR Egger
1.6
1.2
0.8
0.4
1/SE_IV
−2
0
2
4
β_IV

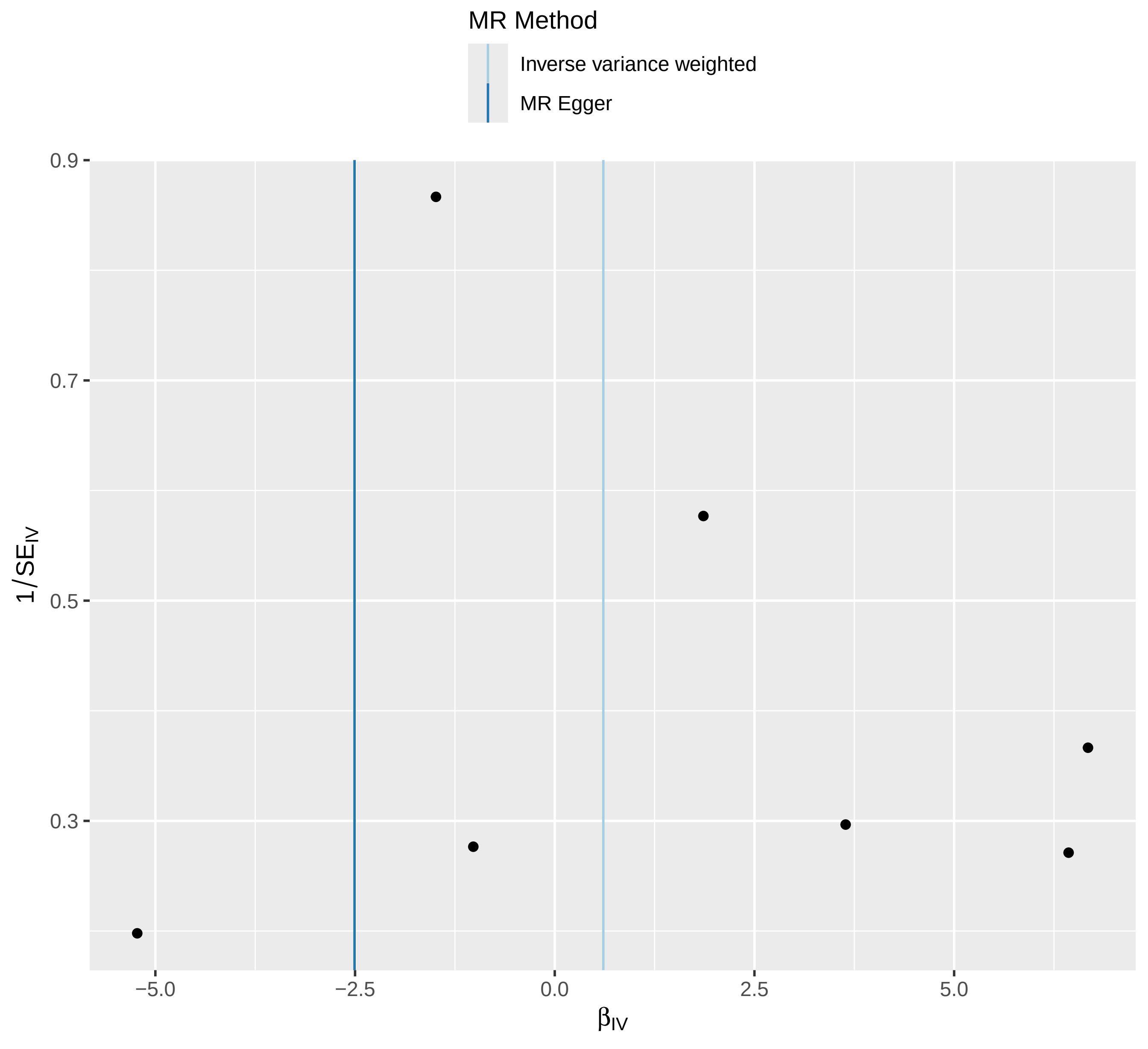
MR Method
Inverse variance weighted
MR Egger
0.9
0.7
0.5
0.3
1/SE_IV
−5.0
−2.5
0.0
2.5
5.0
β_IV

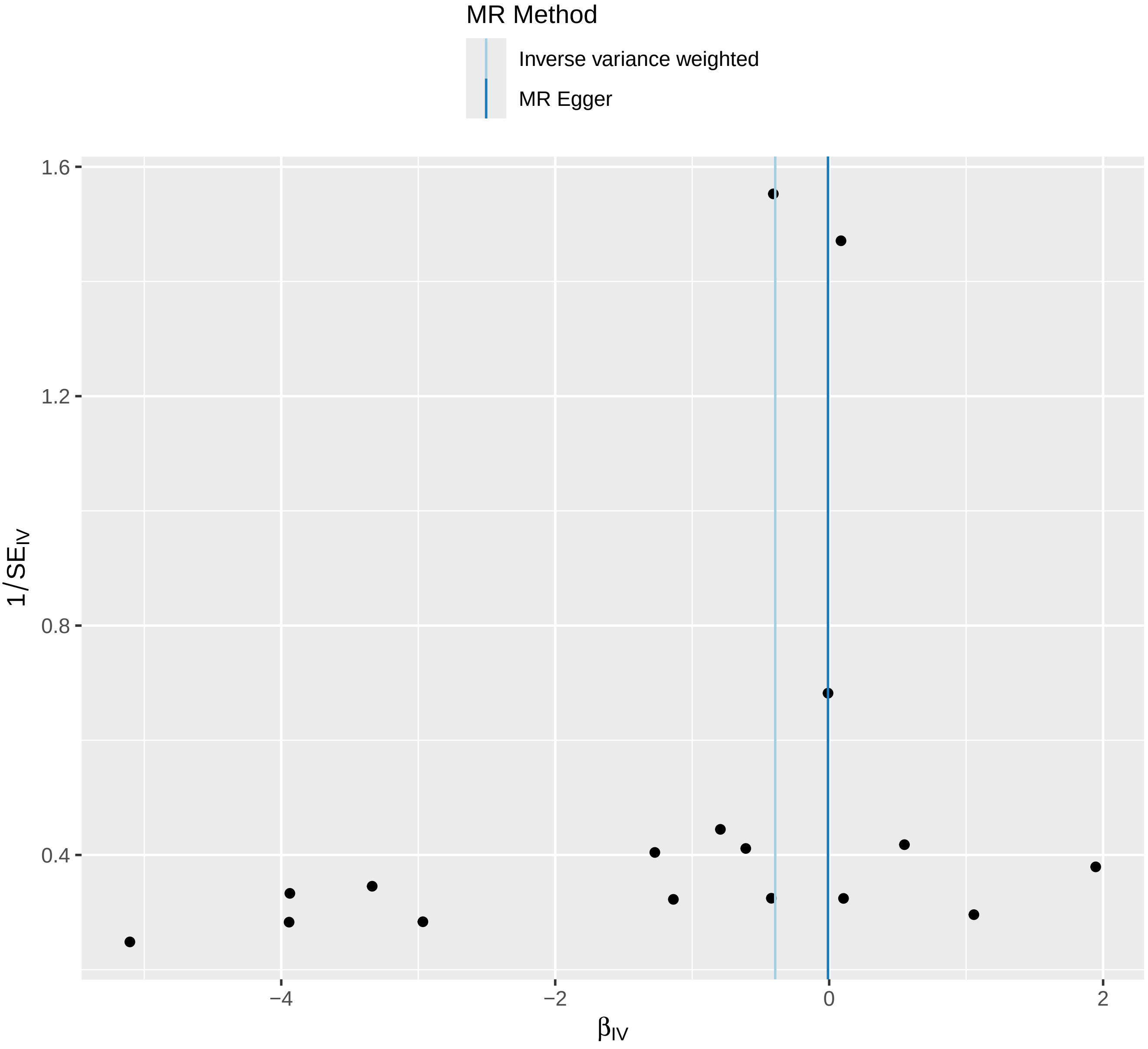

MR Method
Inverse variance weighted
MR Egger
1.6
1.2
0.8
0.4
1/SE$_{IV}$
−4
−2
0
2
β$_{IV}$

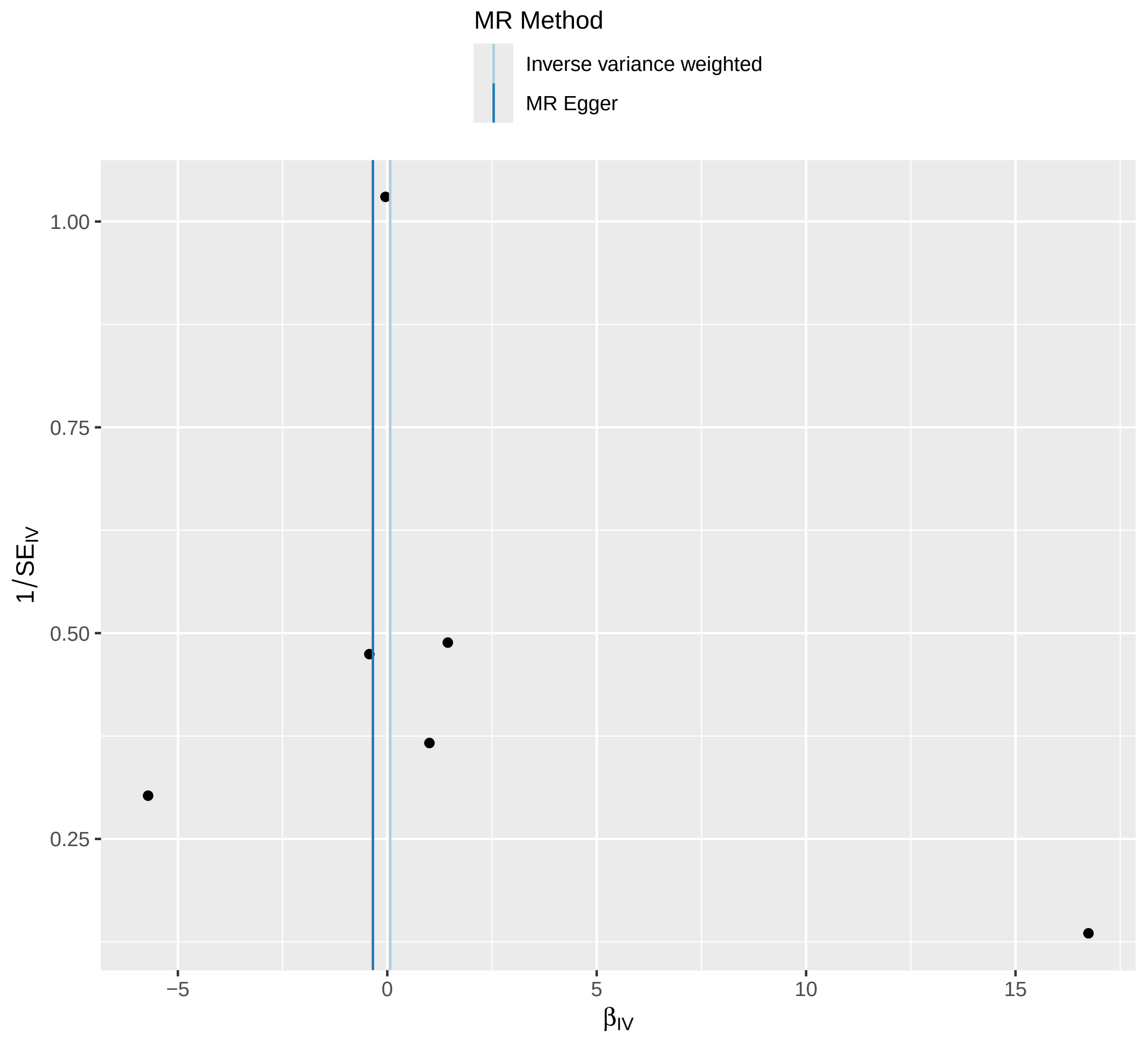

MR Method
Inverse variance weighted
MR Egger
1.00
0.75
0.50
0.25
1/SE$_{IV}$
−5
0
5
10
15
β$_{IV}$

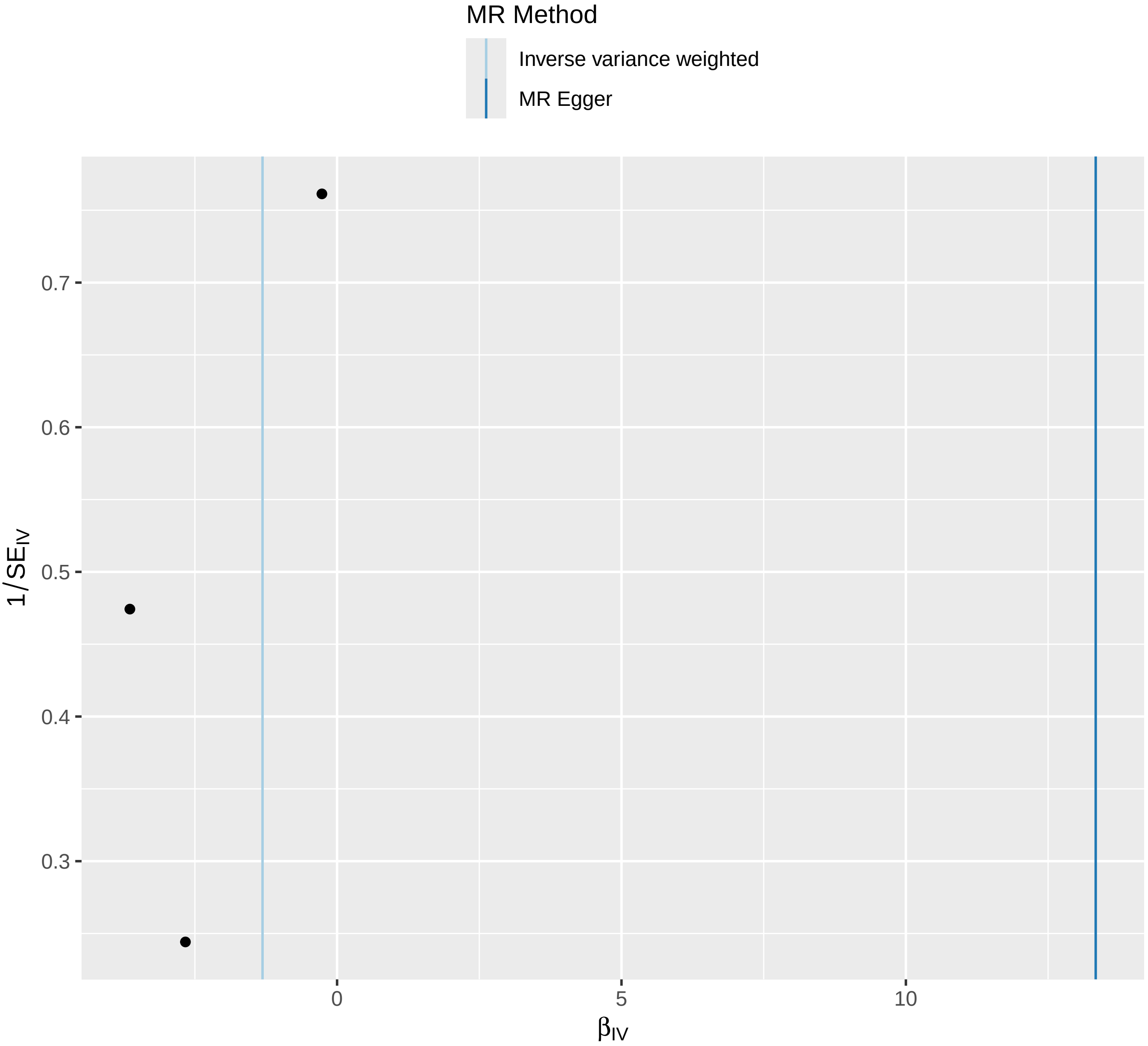
MR Method
Inverse variance weighted
MR Egger
0.7
0.6
0.5
0.4
0.3
1/SE$_{IV}$
0
5
10
β$_{IV}$

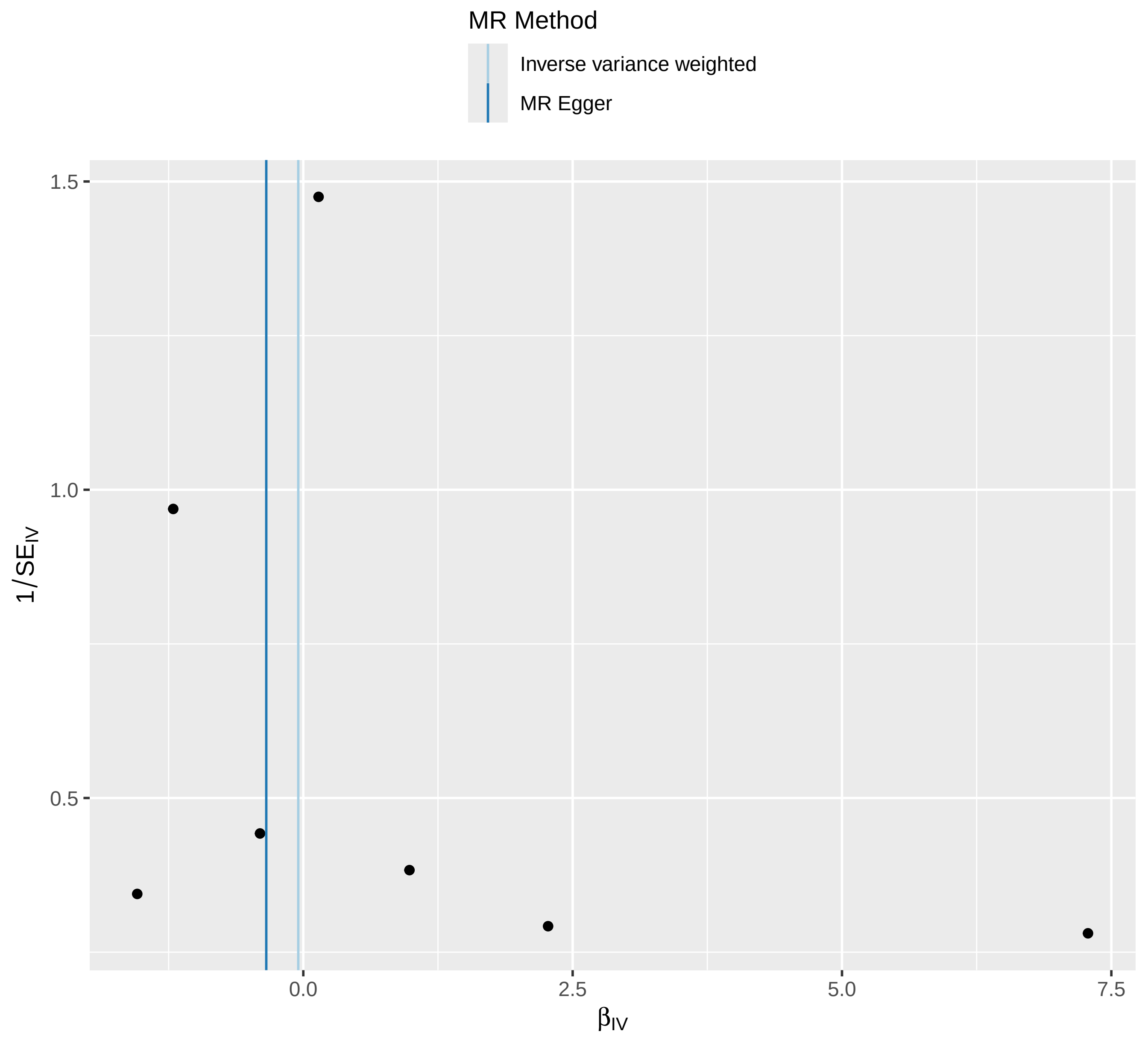
MR Method
Inverse variance weighted
MR Egger
1.5
1.0
0.5
0.0
2.5
5.0
7.5
1/SE_IV
β_IV

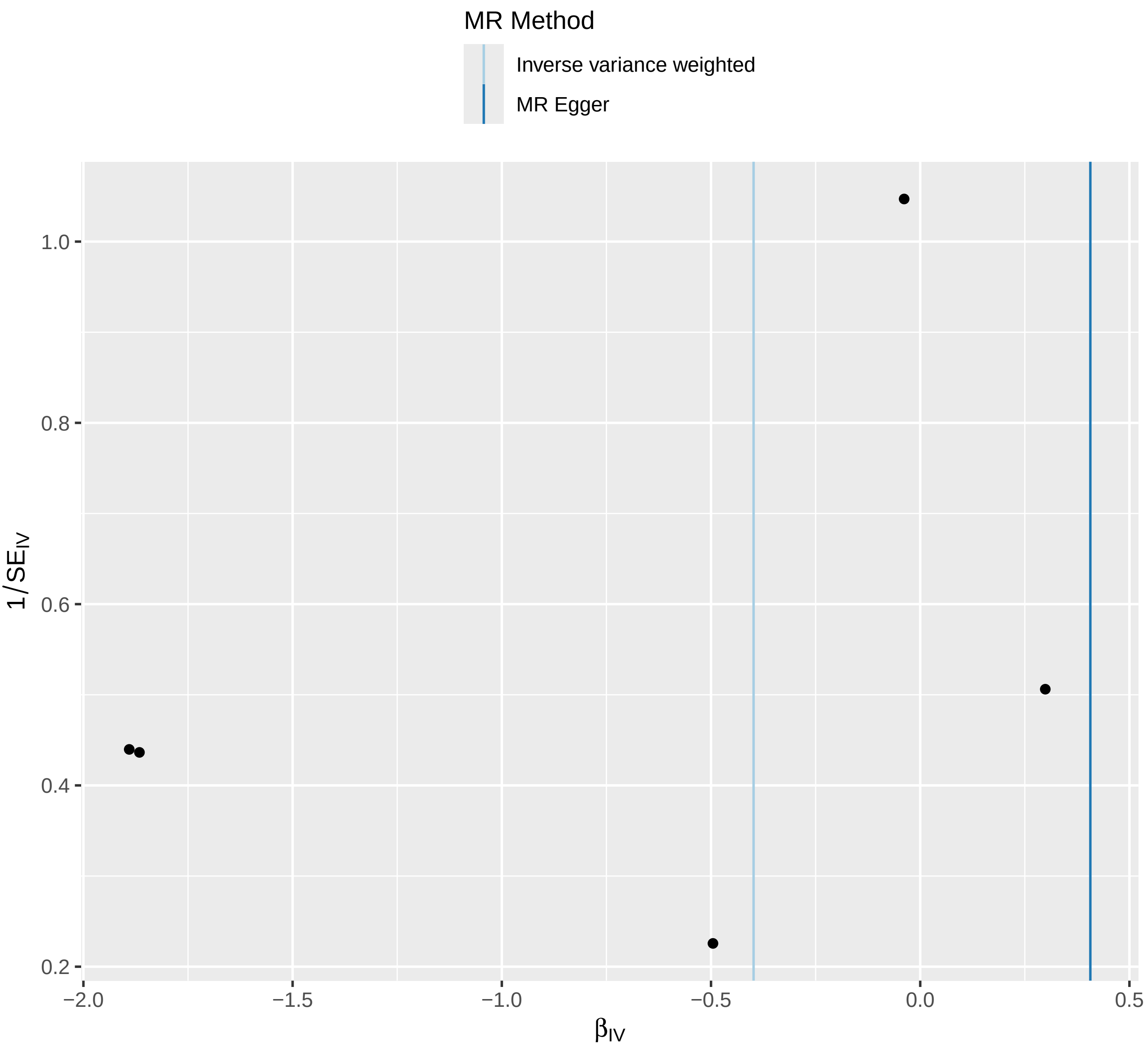
MR Method
Inverse variance weighted
MR Egger
1.0
0.8
0.6
0.4
0.2
1/SE$_{IV}$
−2.0
−1.5
−1.0
−0.5
0.0
0.5
β$_{IV}$

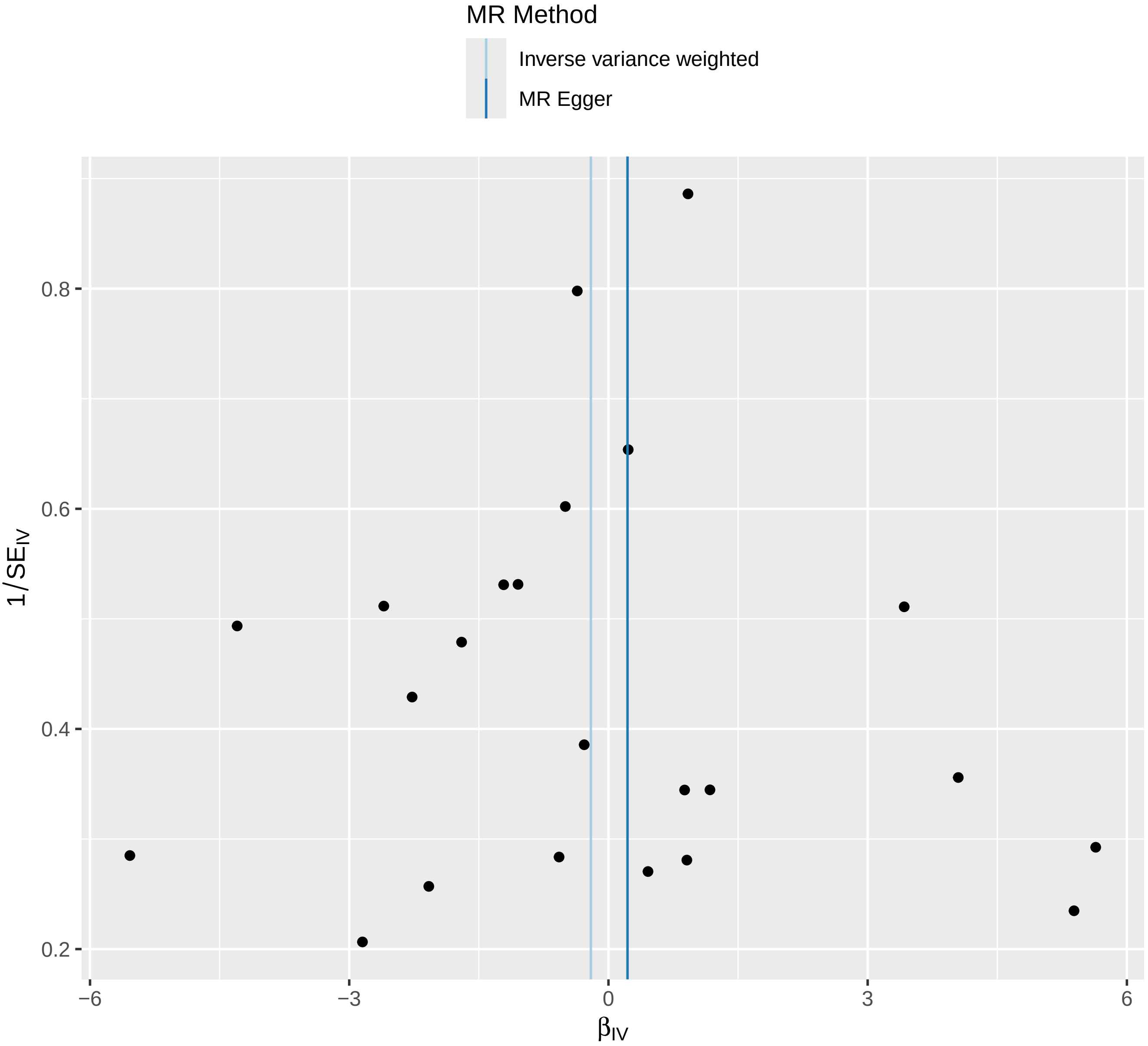
MR Method
Inverse variance weighted
MR Egger
1/SE_IV
0.8
0.6
0.4
0.2
−6
−3
0
3
6
β_IV

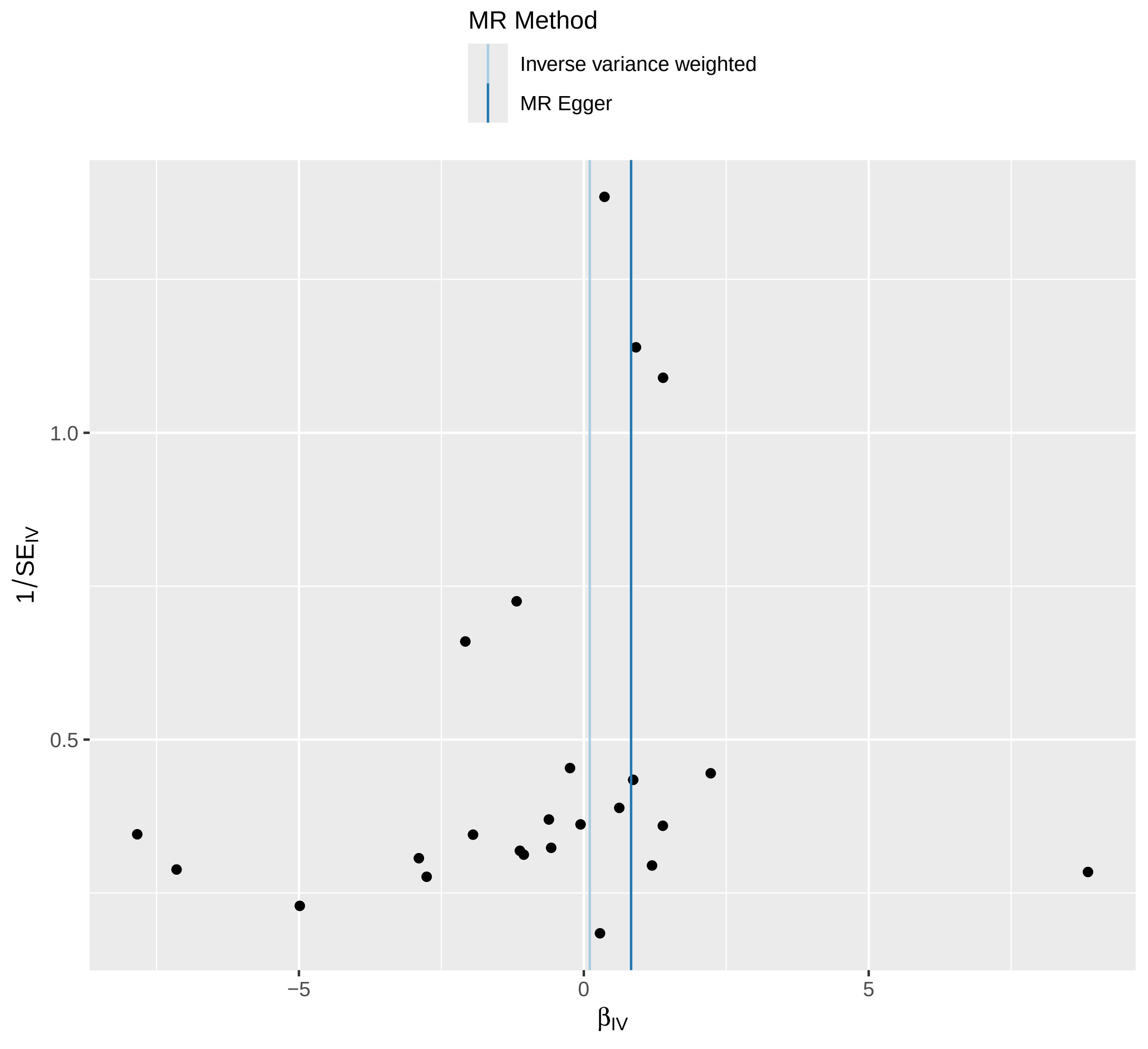
MR Method
Inverse variance weighted
MR Egger
1/SE_{IV}
1.0
0.5
−5
0
5
β_{IV}

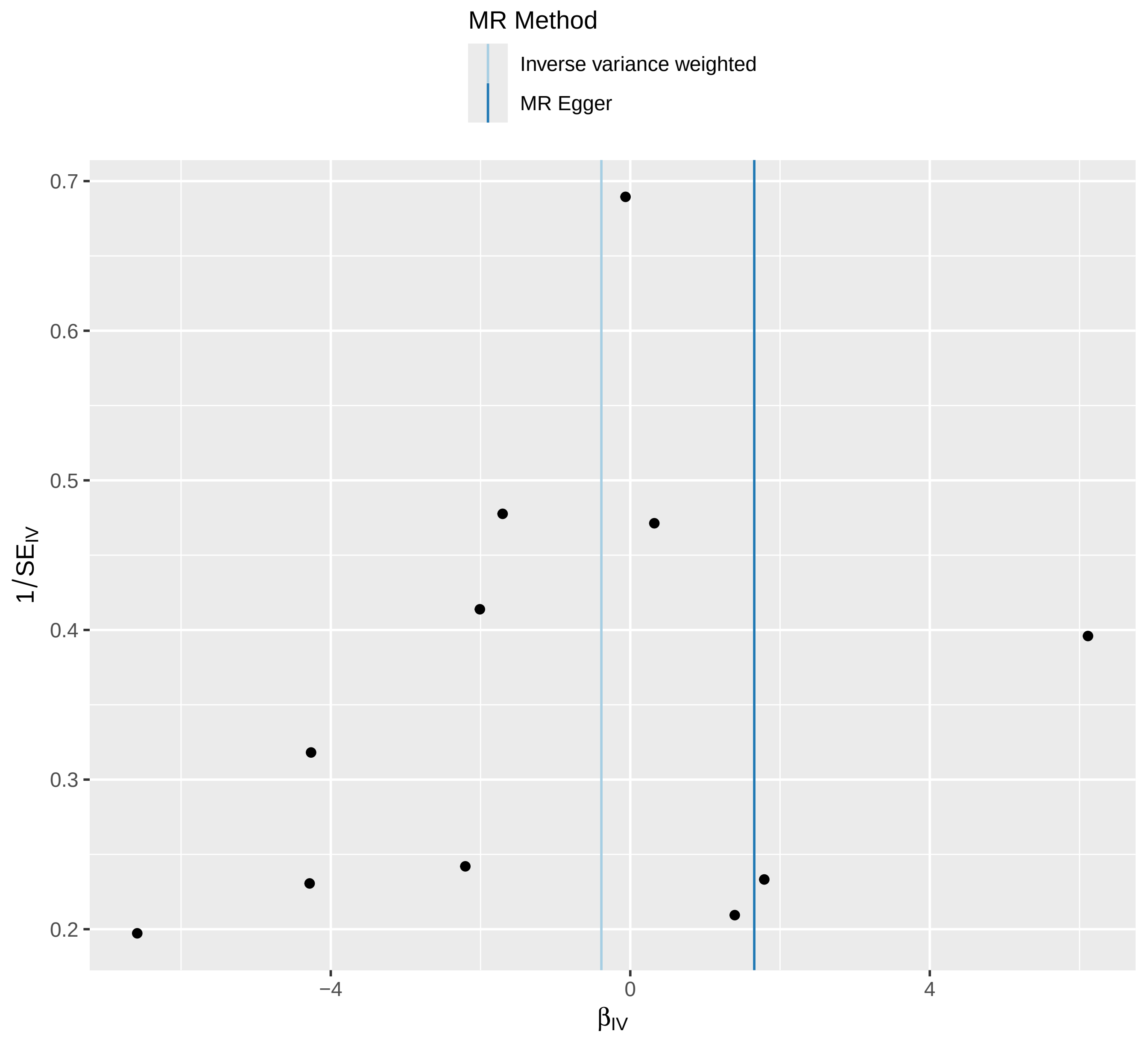
MR Method
Inverse variance weighted
MR Egger
0.7
0.6
0.5
0.4
0.3
0.2
1/SE$_{IV}$
−4
0
4
β$_{IV}$

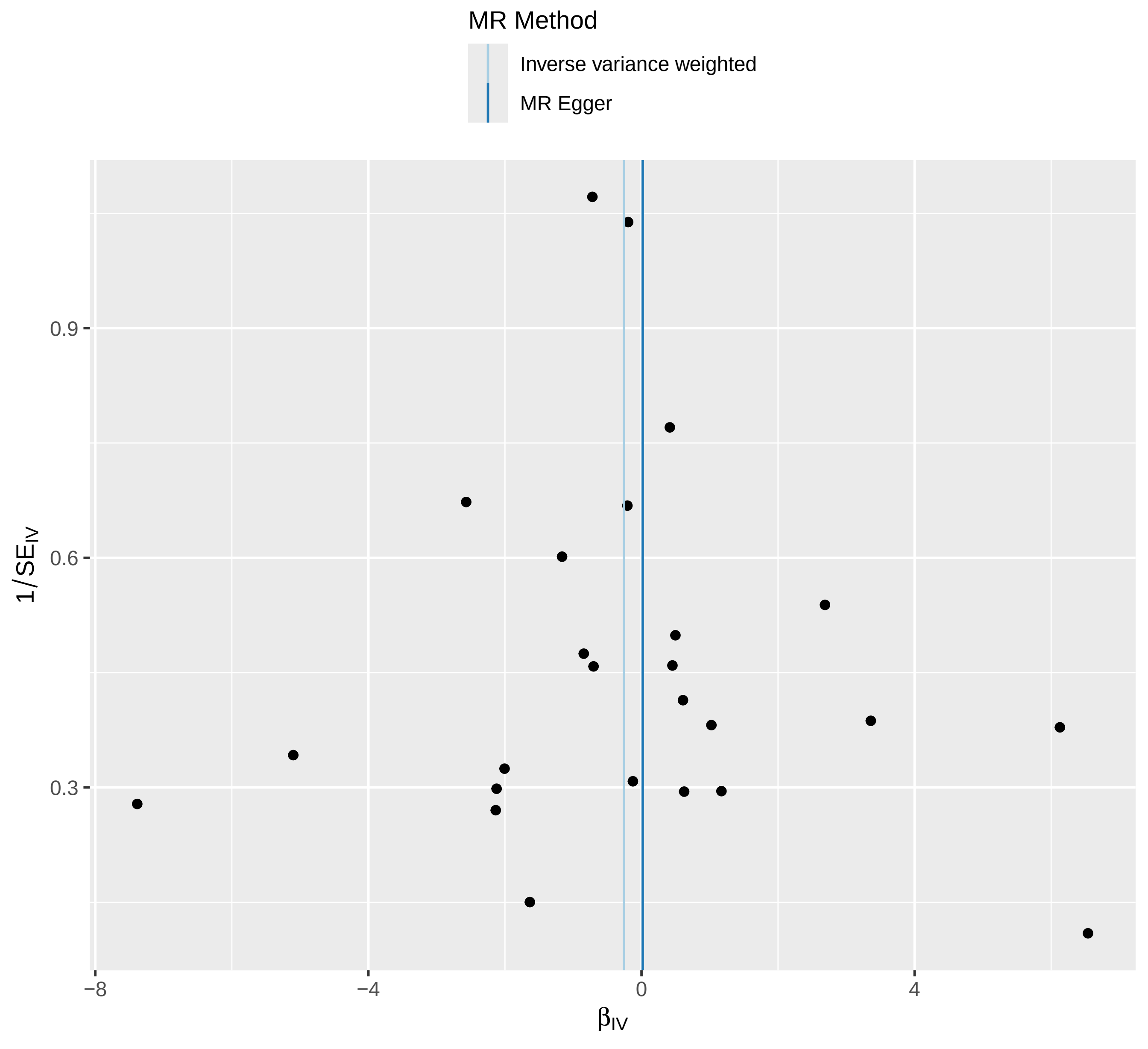

MR Method
Inverse variance weighted
MR Egger
1/SE$_{IV}$
0.9
0.6
0.3
−8
−4
0
4
β$_{IV}$

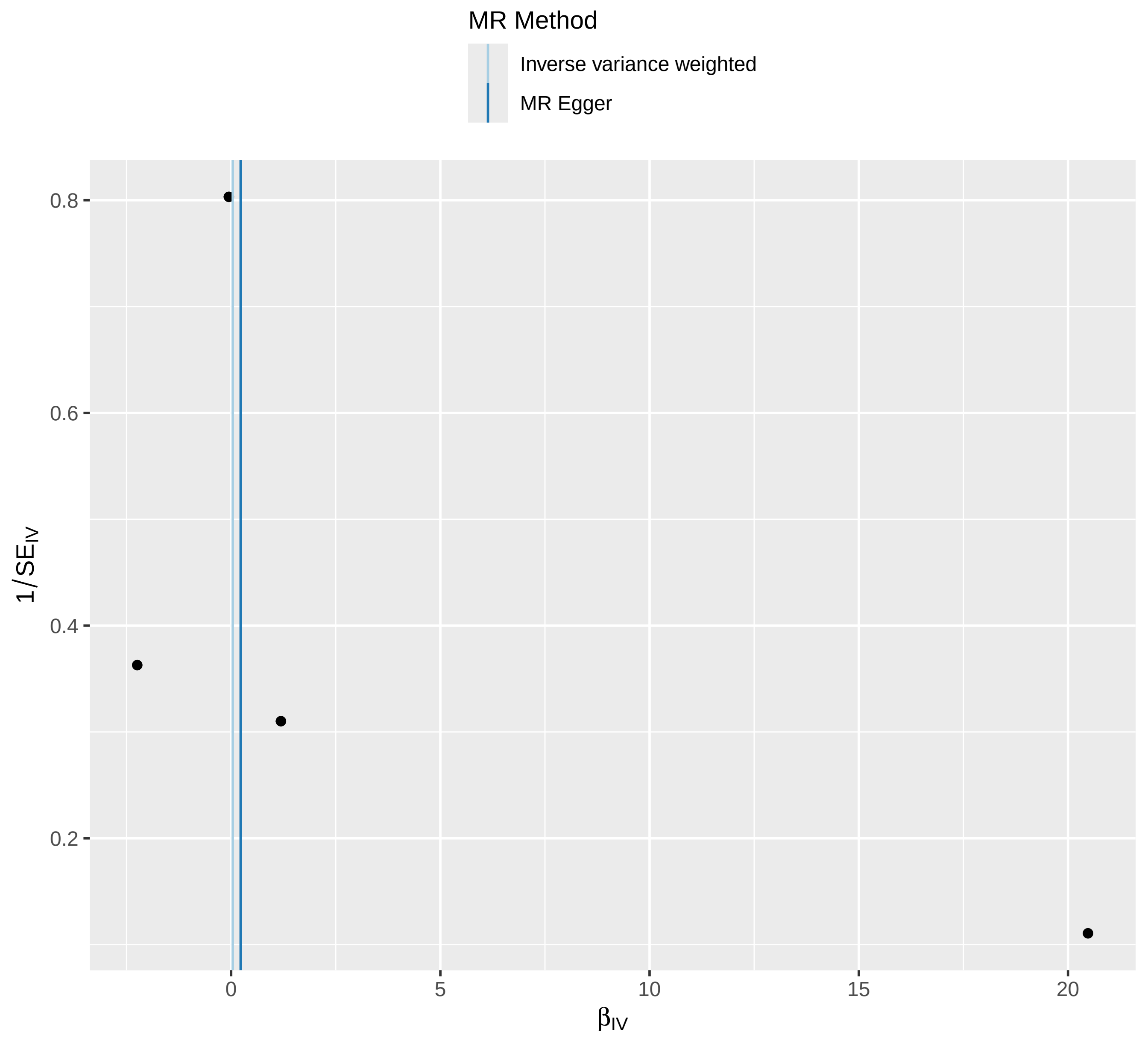

MR Method
Inverse variance weighted
MR Egger
0.8
0.6
0.4
0.2
$1/SE_{IV}$
0
5
10
15
20
β_{IV}

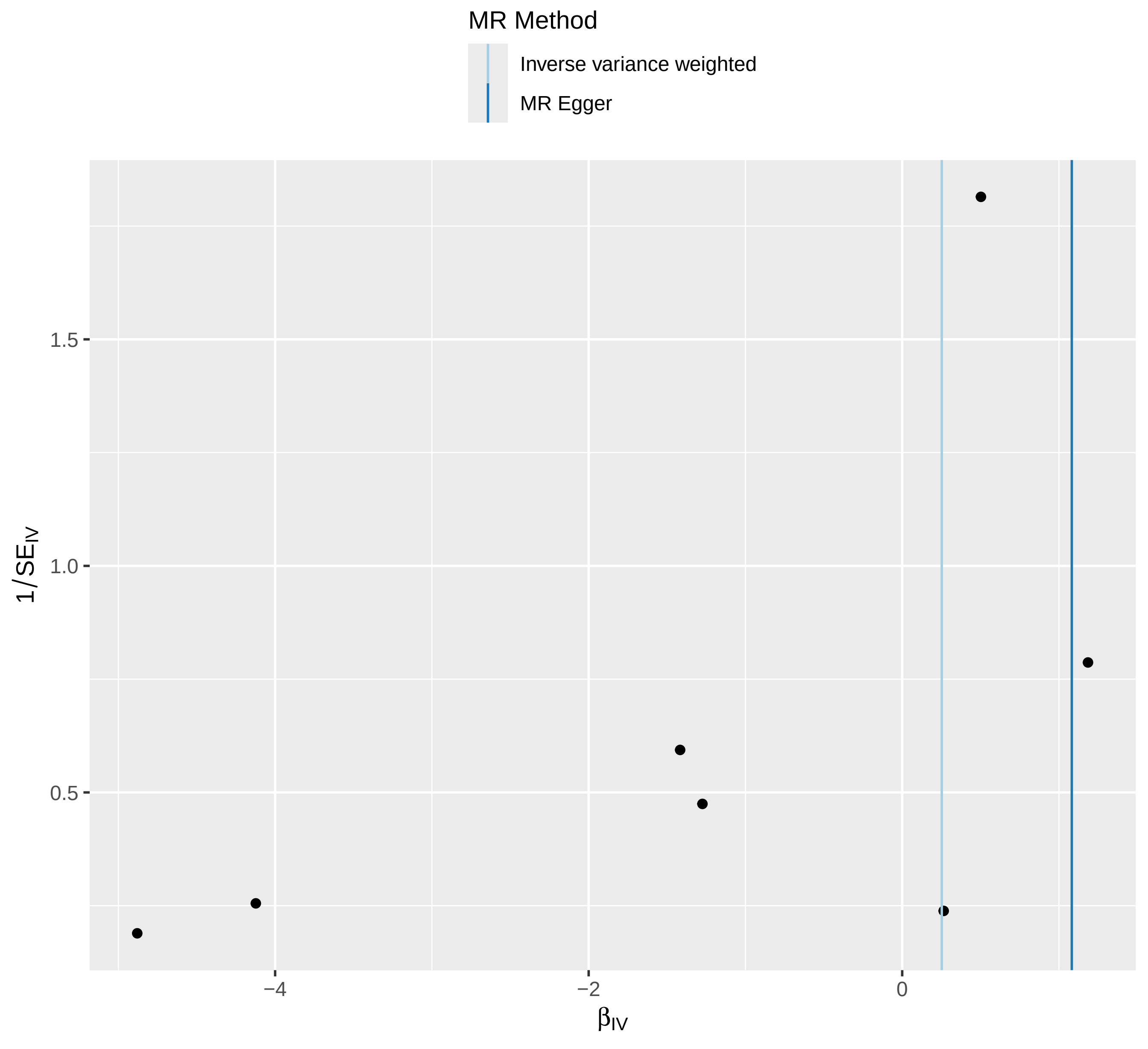
MR Method
Inverse variance weighted
MR Egger
1.5
1.0
0.5
$1/SE_{IV}$
−4
−2
0
β_{IV}

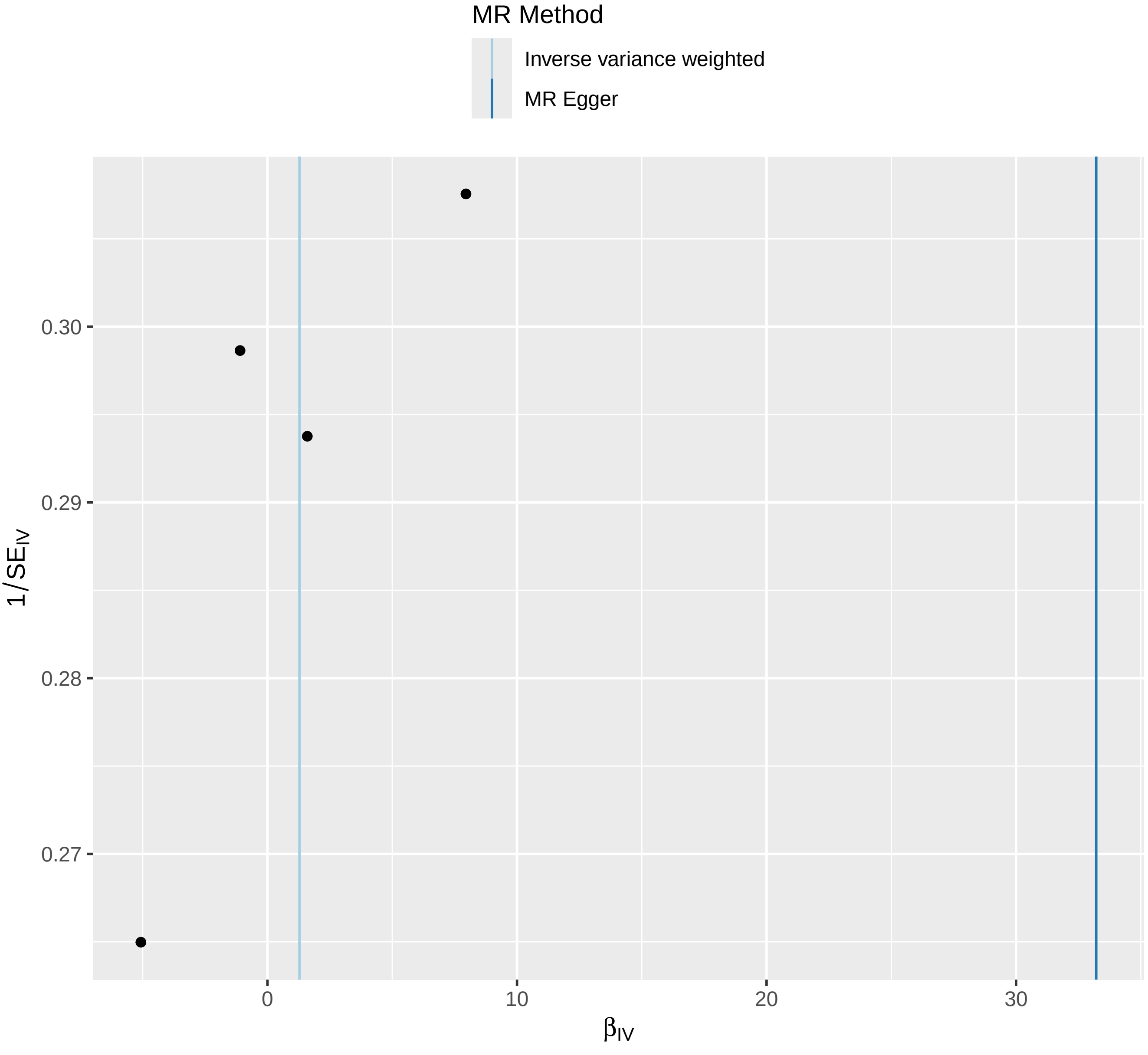
MR Method
Inverse variance weighted
MR Egger
0.30
0.29
0.28
0.27
1/SE_IV
0
10
20
30
β_IV

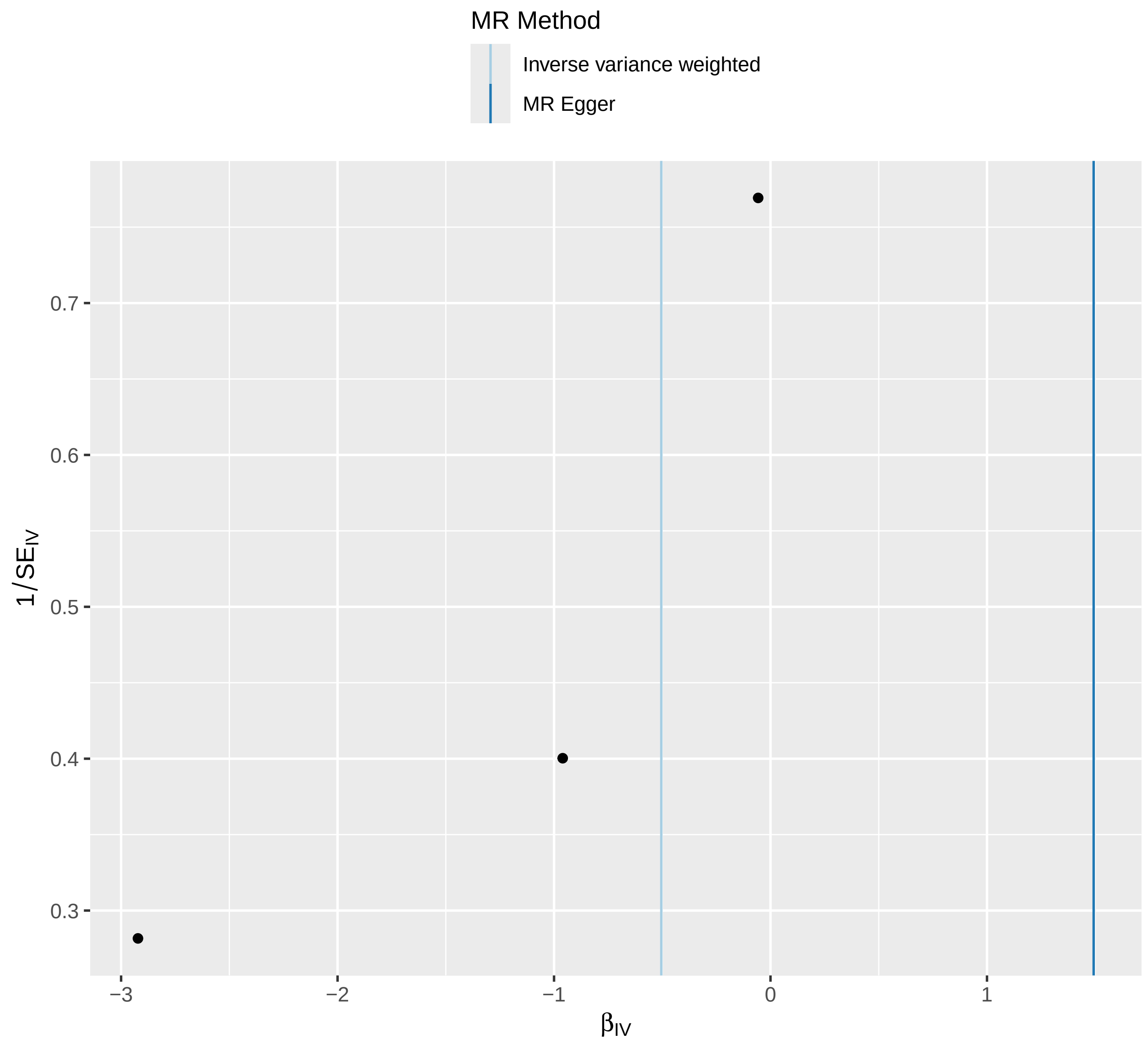
MR Method
Inverse variance weighted
MR Egger
0.7
0.6
0.5
0.4
0.3
1/SE_IV
−3
−2
−1
0
1
β_IV

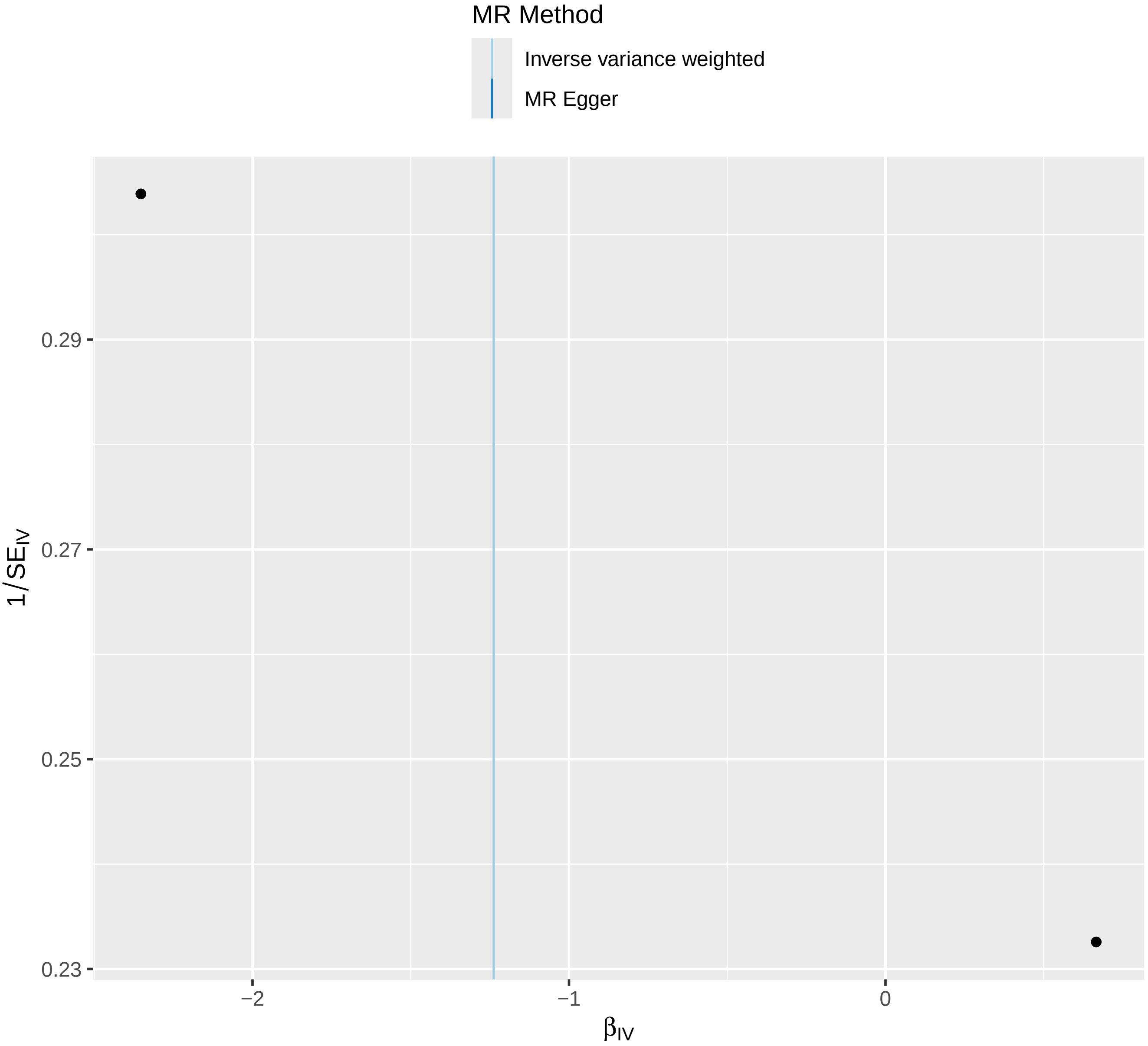

MR Method
Inverse variance weighted
MR Egger
0.29
0.27
0.25
0.23
1/SE_IV
−2
−1
0
β_IV

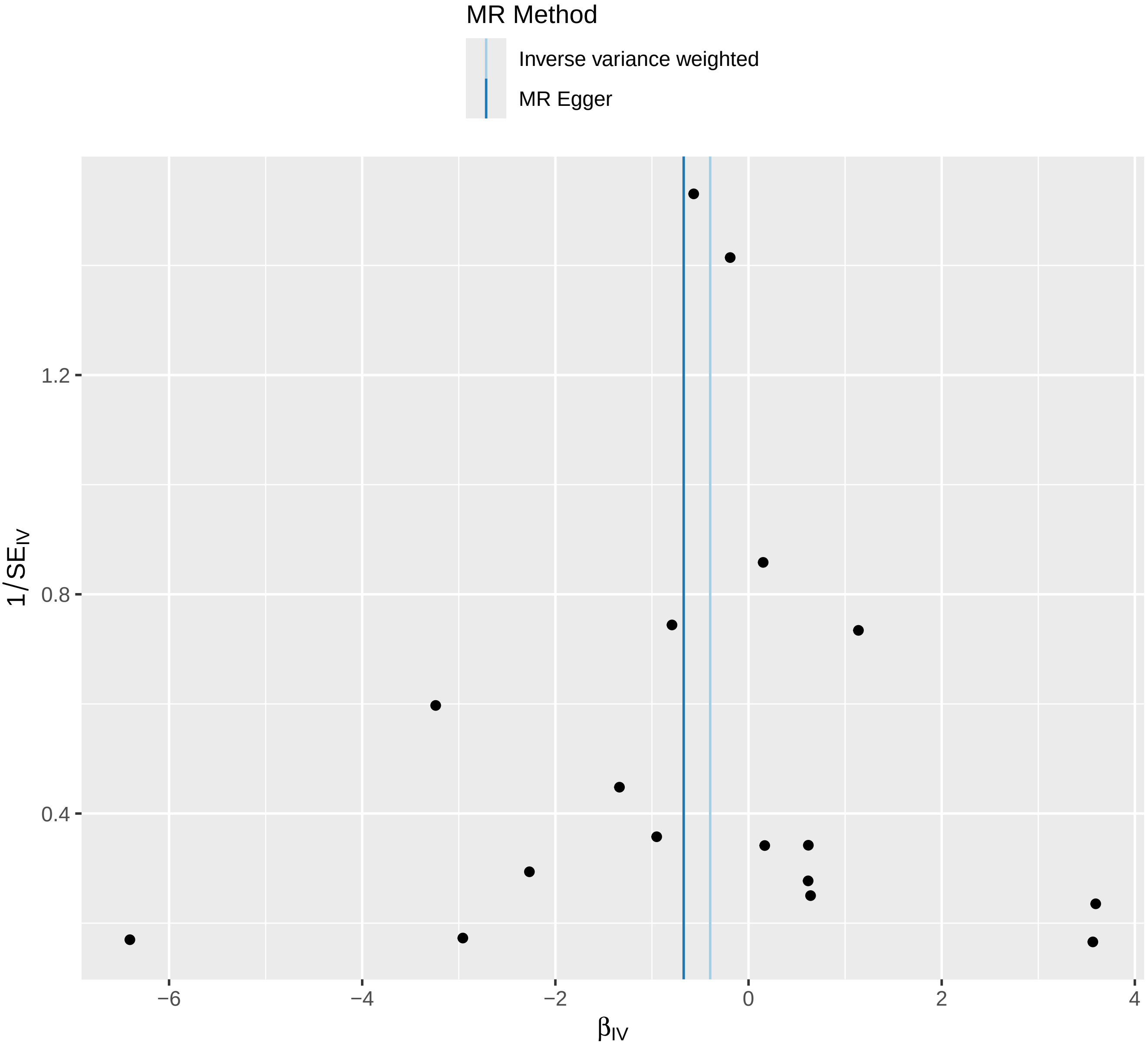

MR Method
Inverse variance weighted
MR Egger
1.2
0.8
0.4
$1/SE_{IV}$
−6
−4
−2
0
2
4
β_{IV}

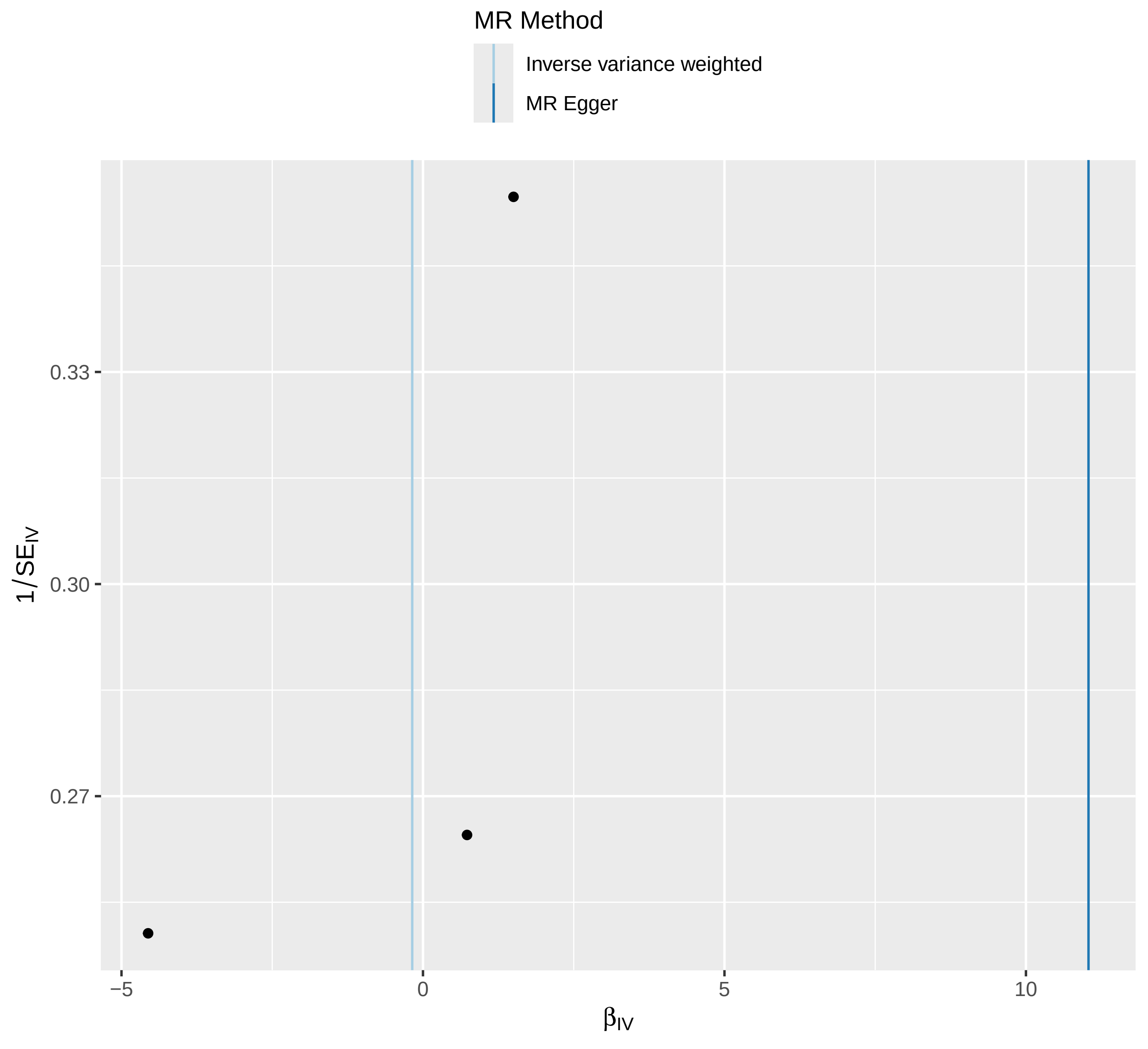
MR Method
Inverse variance weighted
MR Egger
0.33
0.30
0.27
1/SE_IV
−5
0
5
10
β_IV

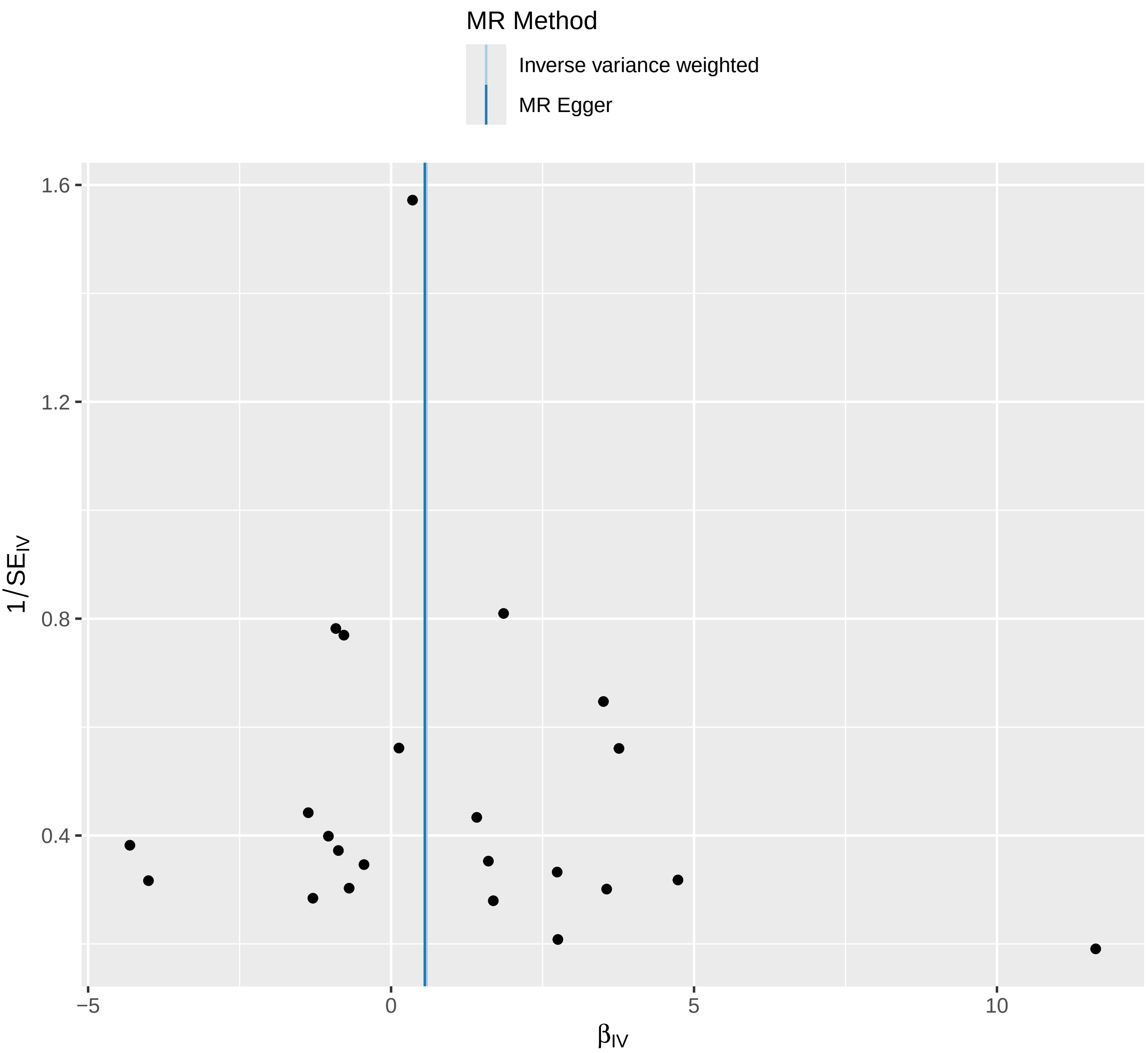
MR Method
Inverse variance weighted
MR Egger
1.6
1.2
0.8
0.4
1/SE_IV
−5
0
5
10
β_IV

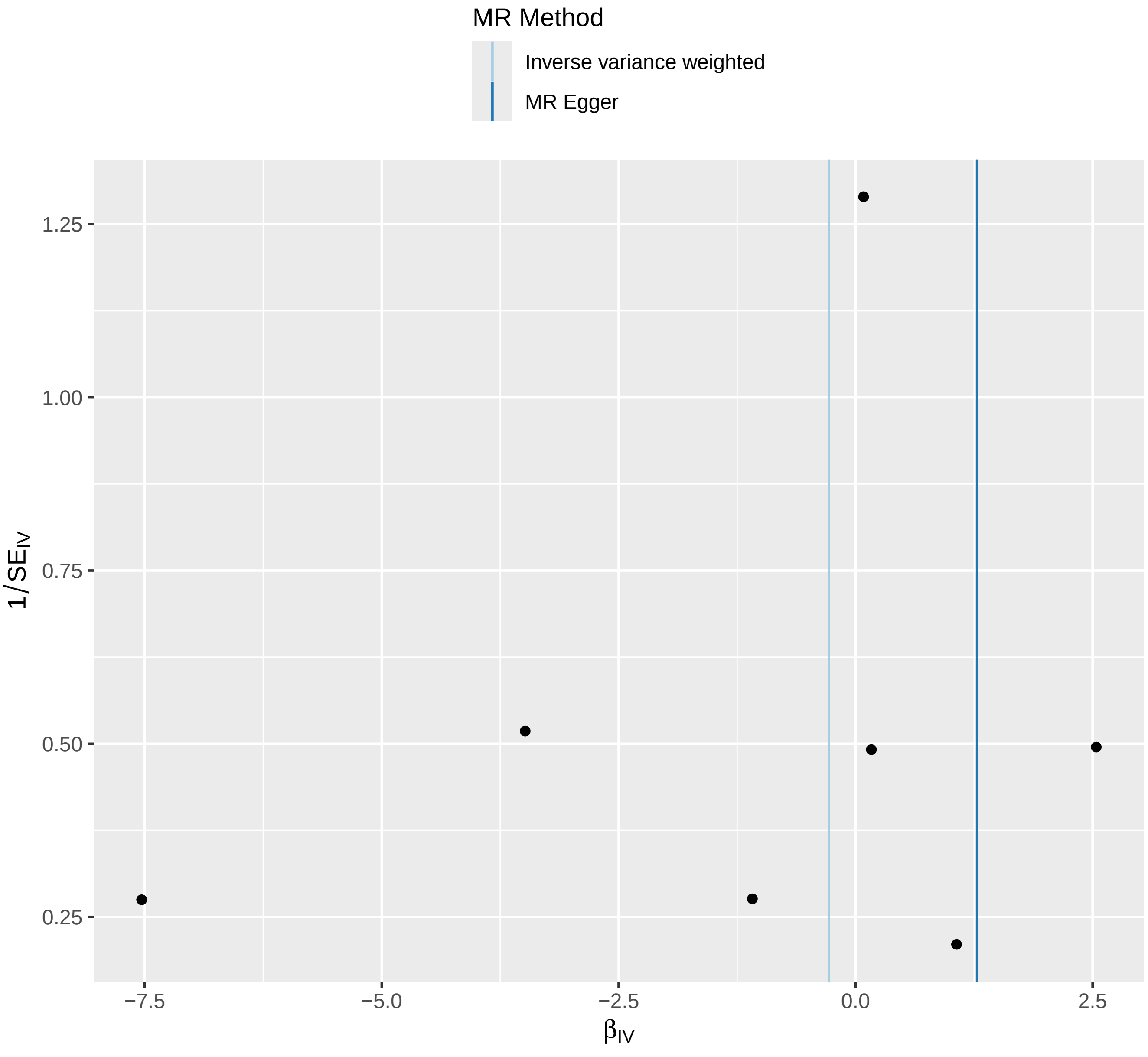

MR Method
Inverse variance weighted
MR Egger
1.25
1.00
0.75
0.50
0.25
1/SE_IV
−7.5
−5.0
−2.5
0.0
2.5
β_IV

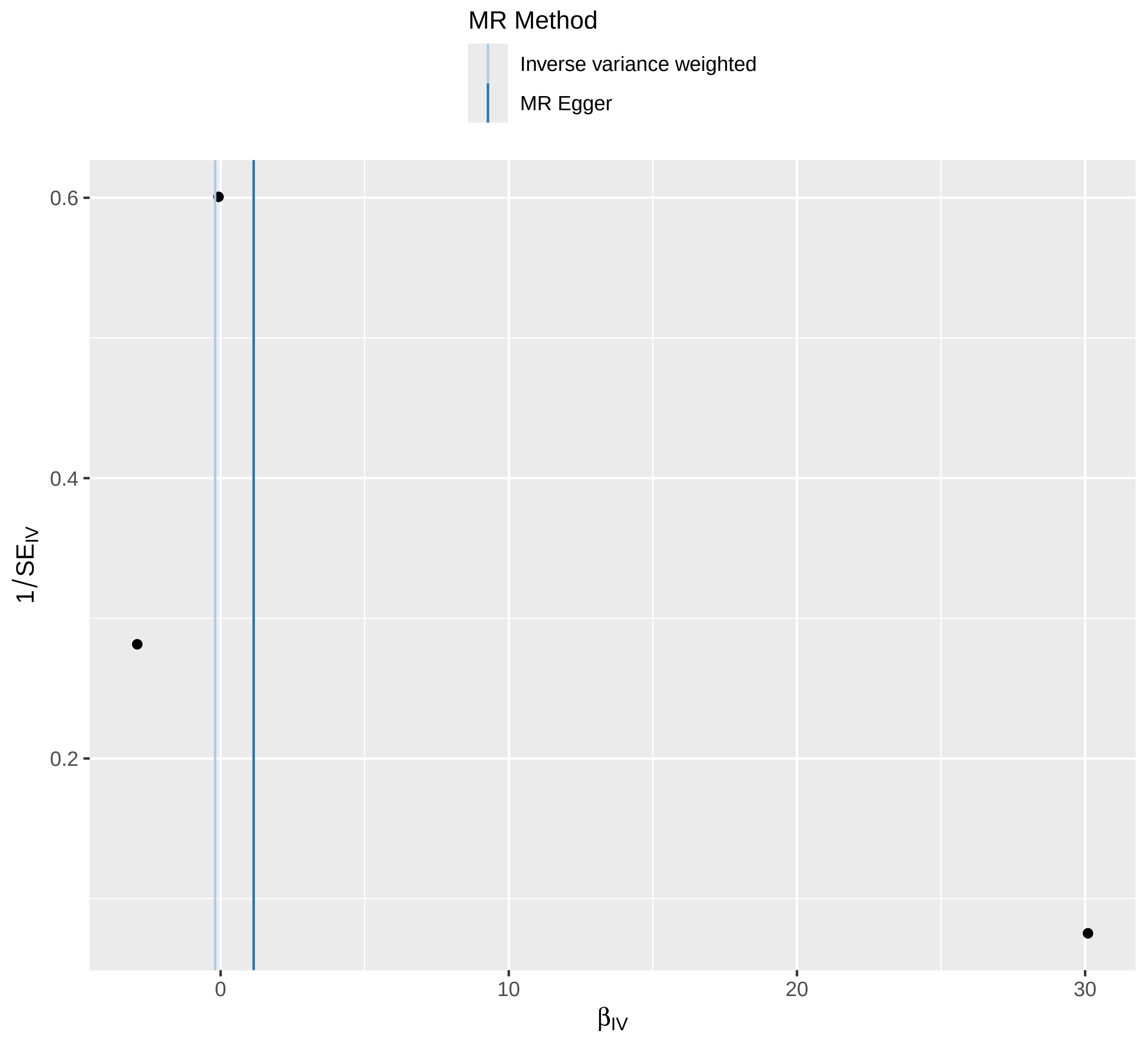
MR Method
Inverse variance weighted
MR Egger
0.6
0.4
0.2
1/SE_IV
0
10
20
30
β_IV

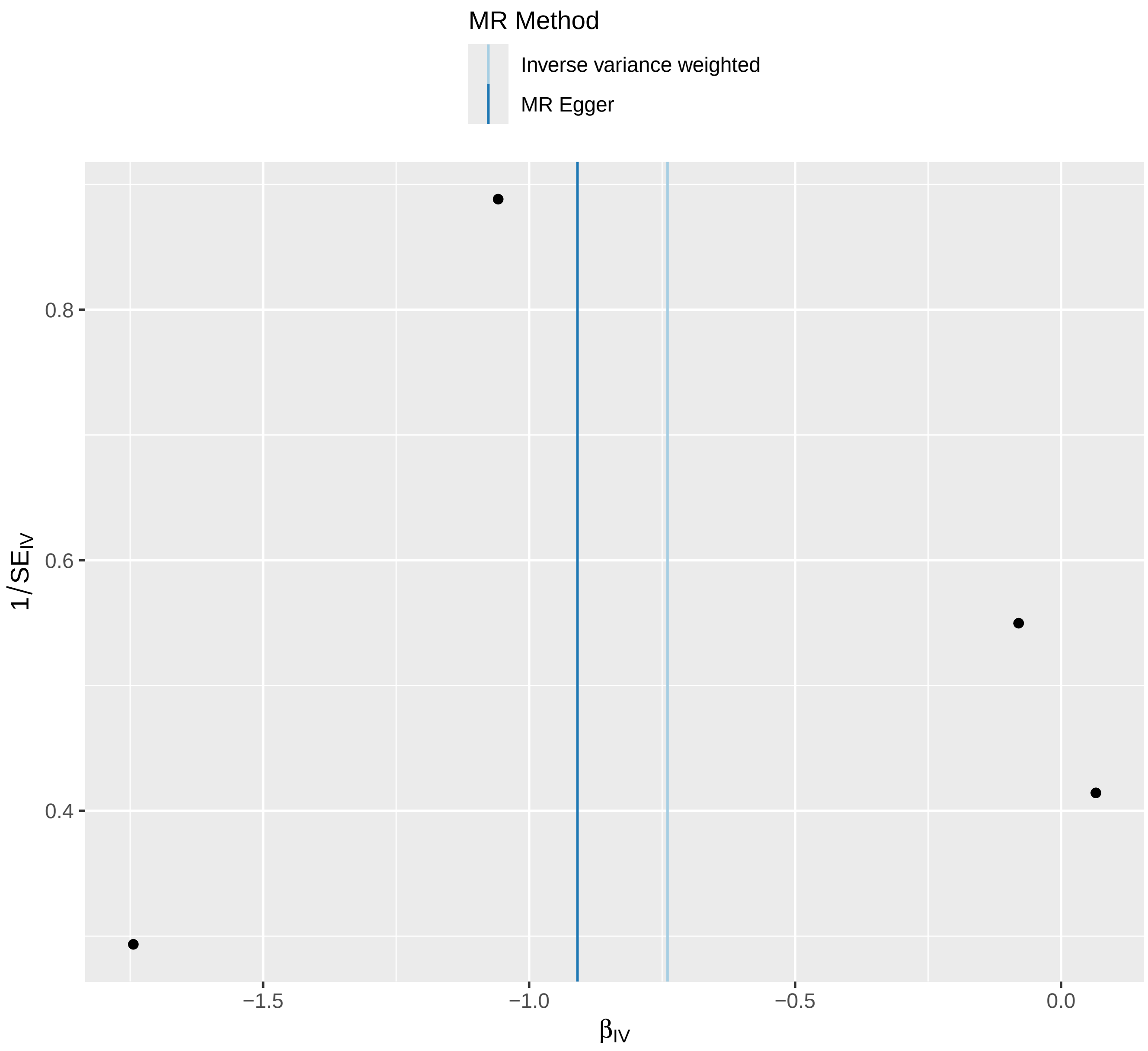
MR Method
Inverse variance weighted
MR Egger
0.8
0.6
0.4
1/SE$_{IV}$
−1.5
−1.0
−0.5
0.0
β$_{IV}$

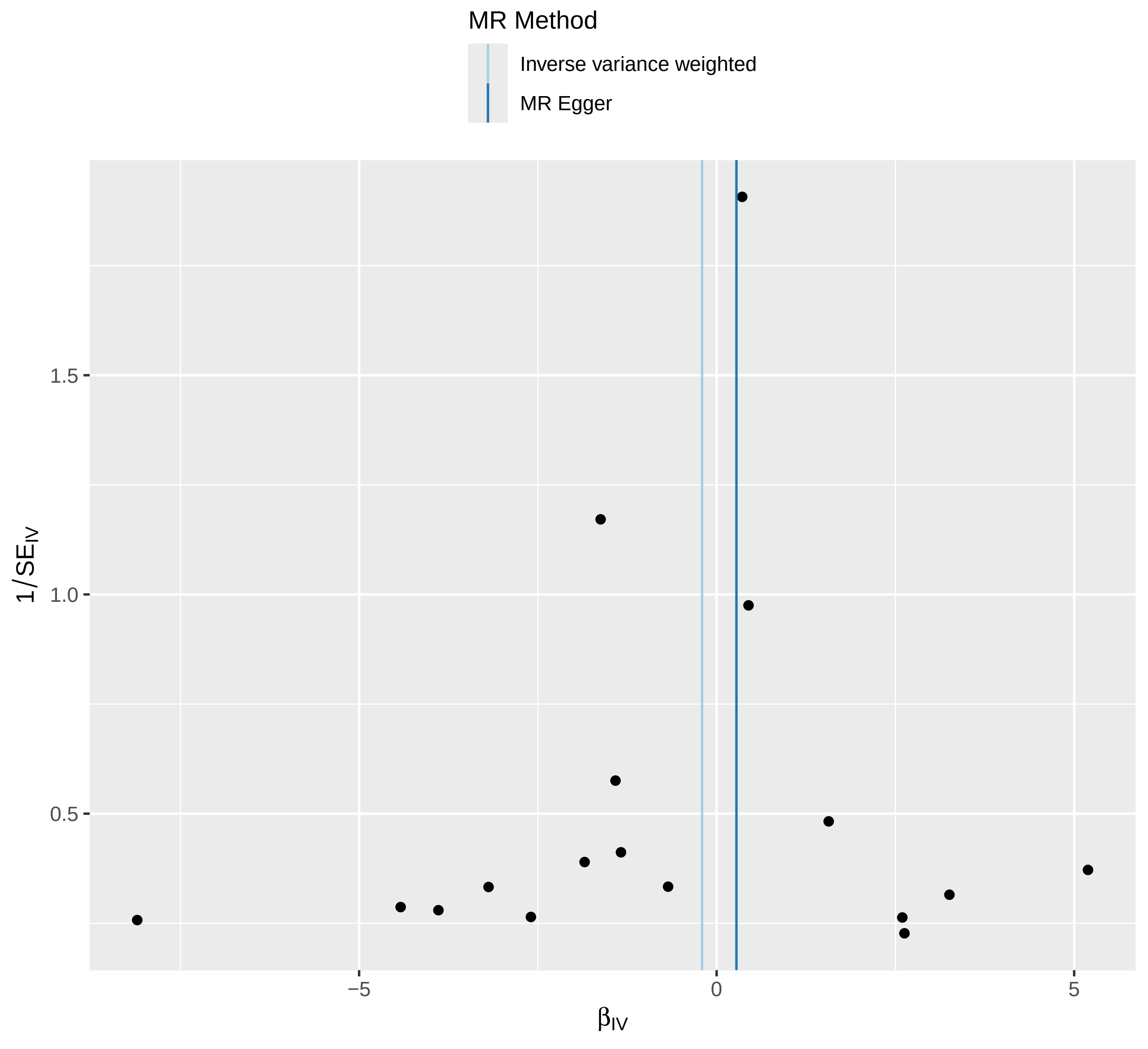

MR Method
Inverse variance weighted
MR Egger
1.5
1.0
0.5
1/SE_IV
−5
0
5
β_IV

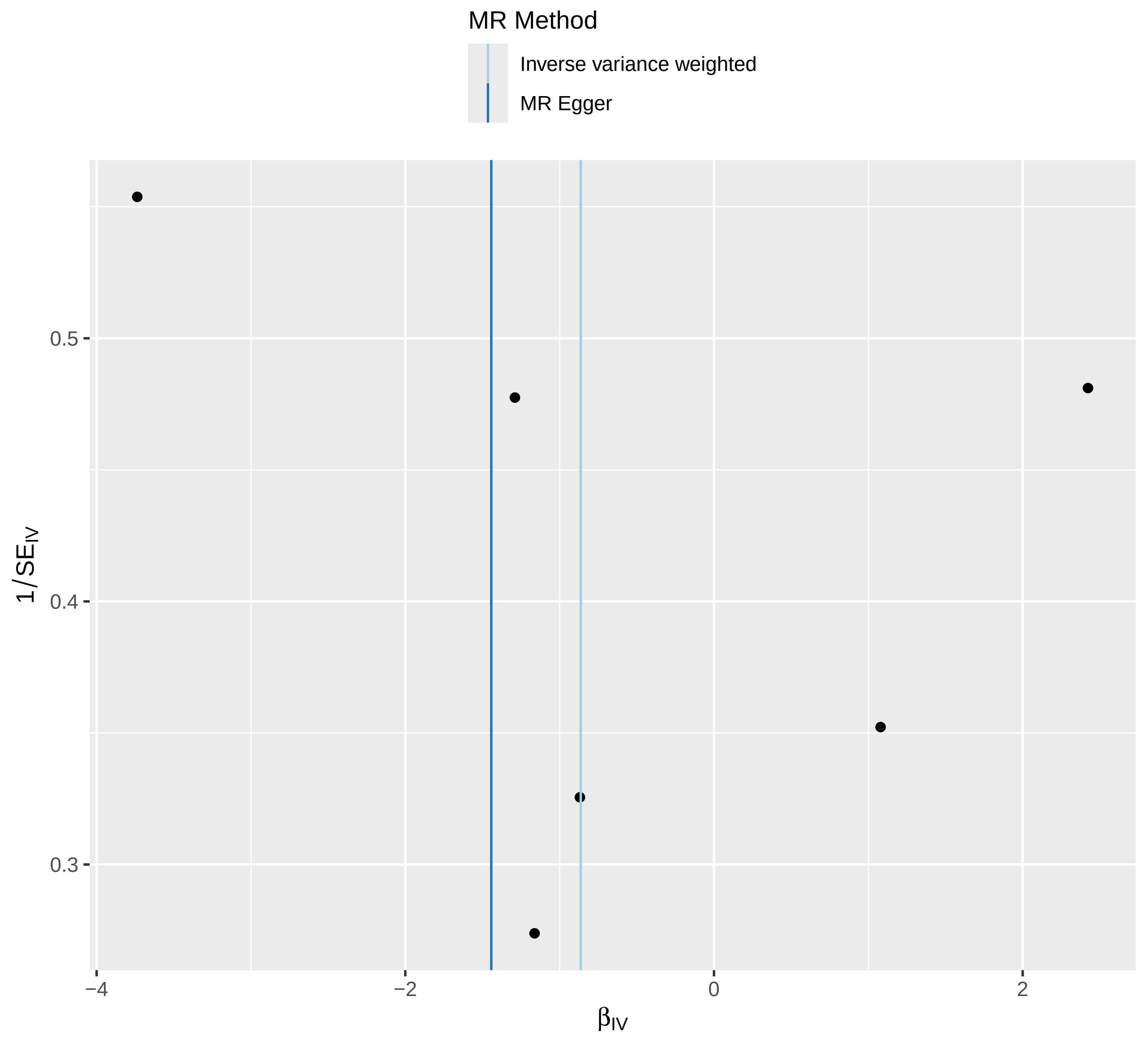

MR Method
Inverse variance weighted
MR Egger
0.5
0.4
0.3
1/SE_IV
−4
−2
0
2
β_IV

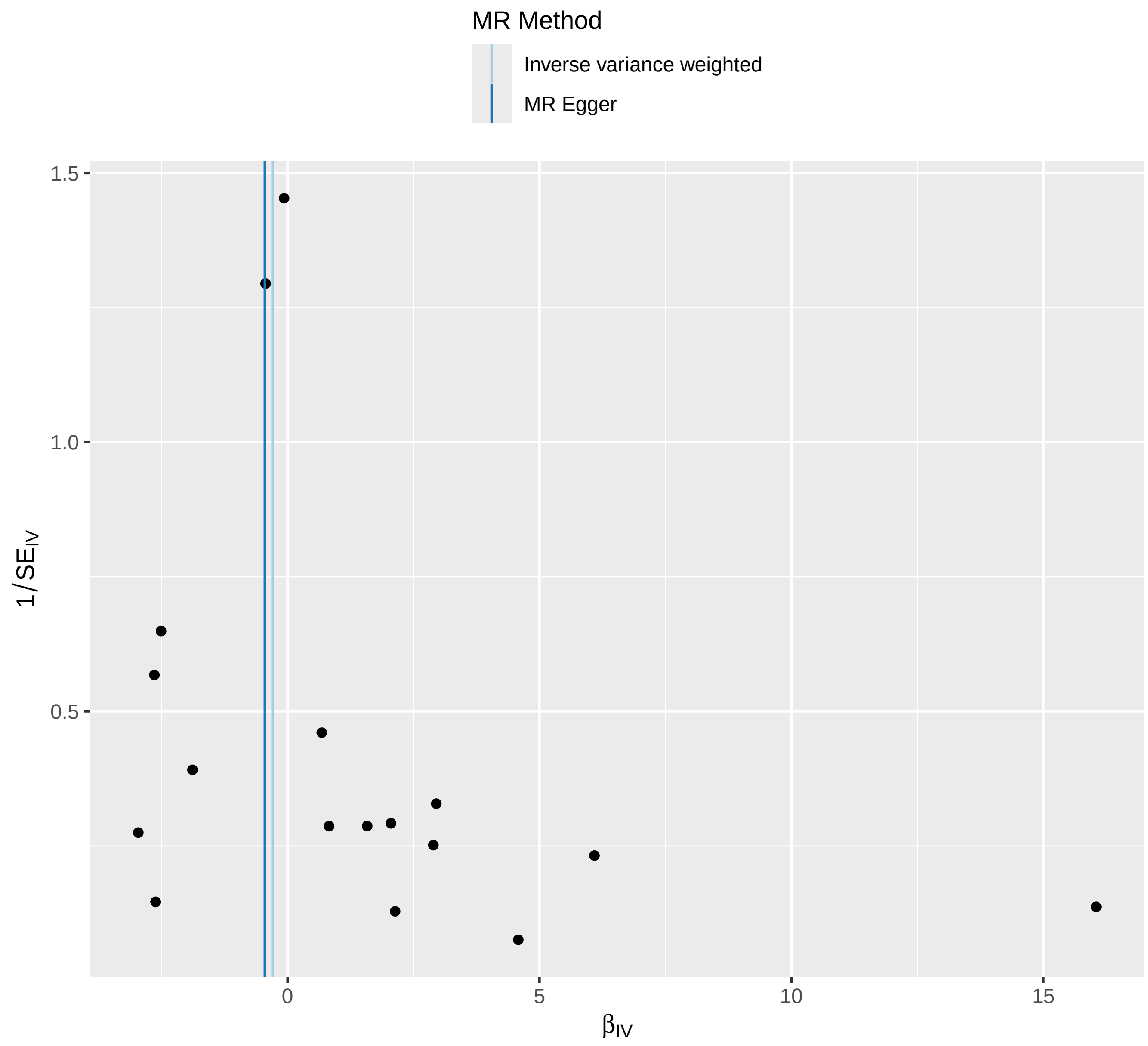
MR Method
Inverse variance weighted
MR Egger
1.5
1.0
0.5
1/SE_IV
0
5
10
15
β_IV

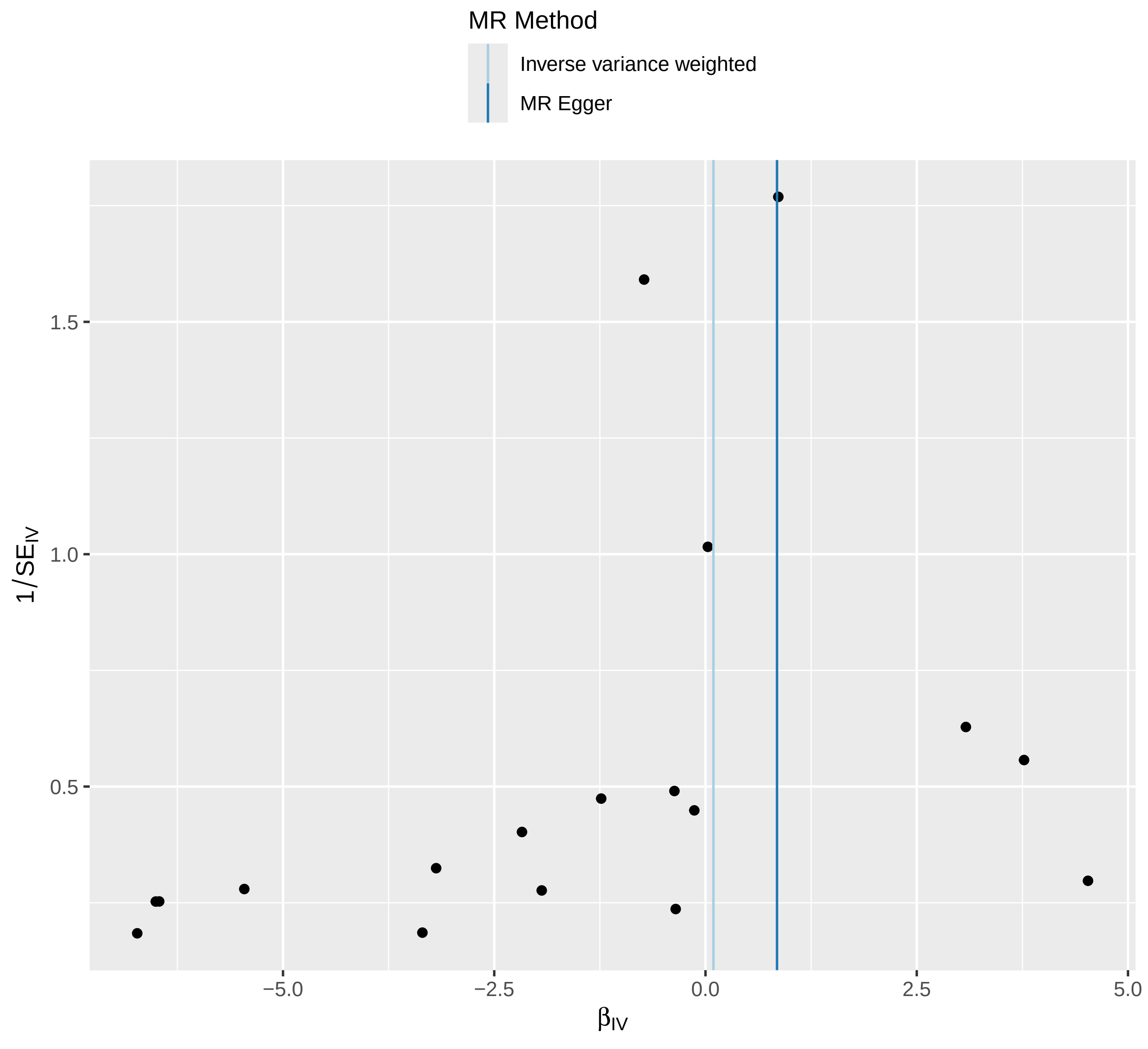
MR Method
Inverse variance weighted
MR Egger
1.5
1.0
0.5
1/SE_IV
−5.0
−2.5
0.0
2.5
5.0
β_IV

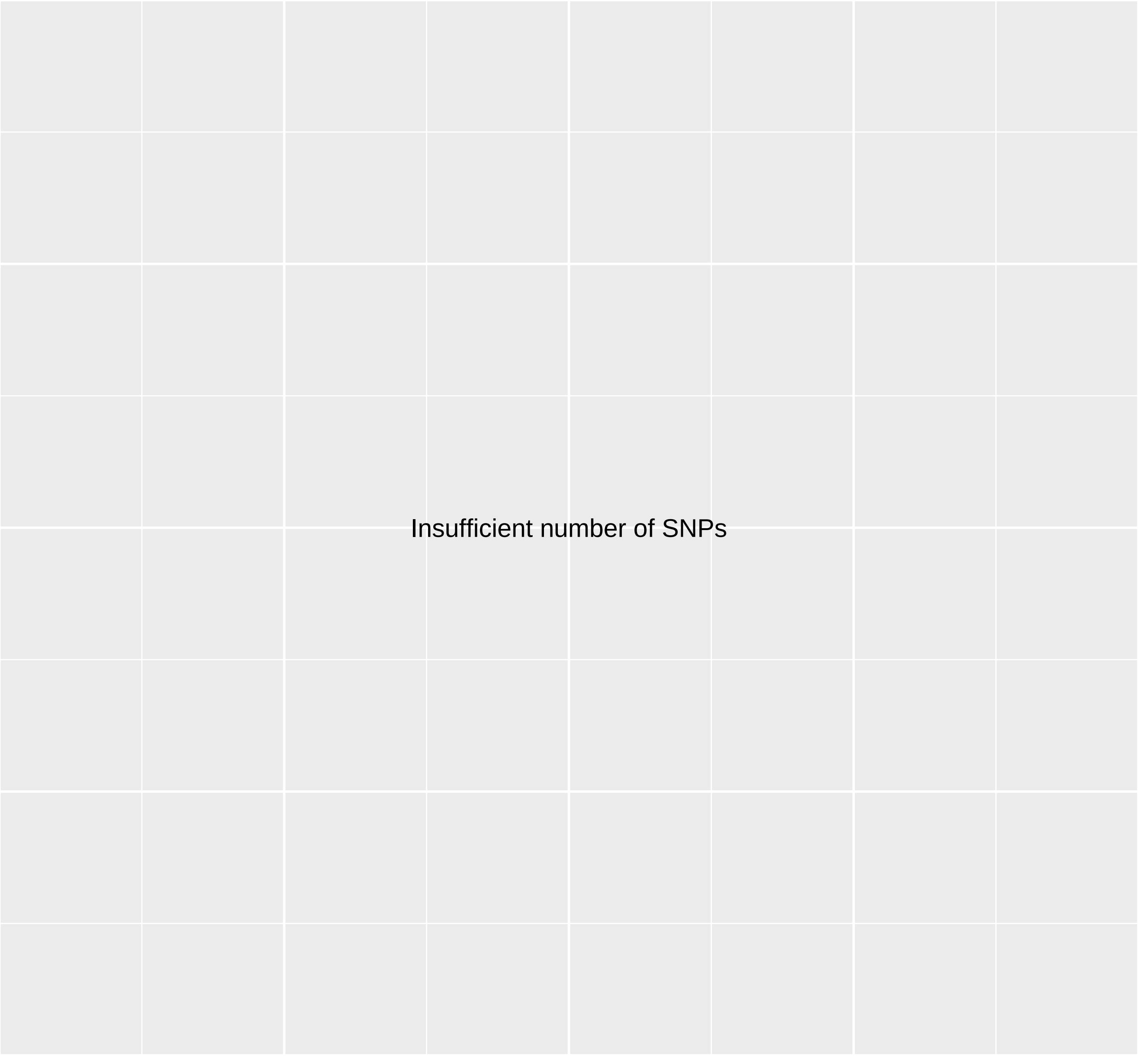
Insufficient number of SNPs

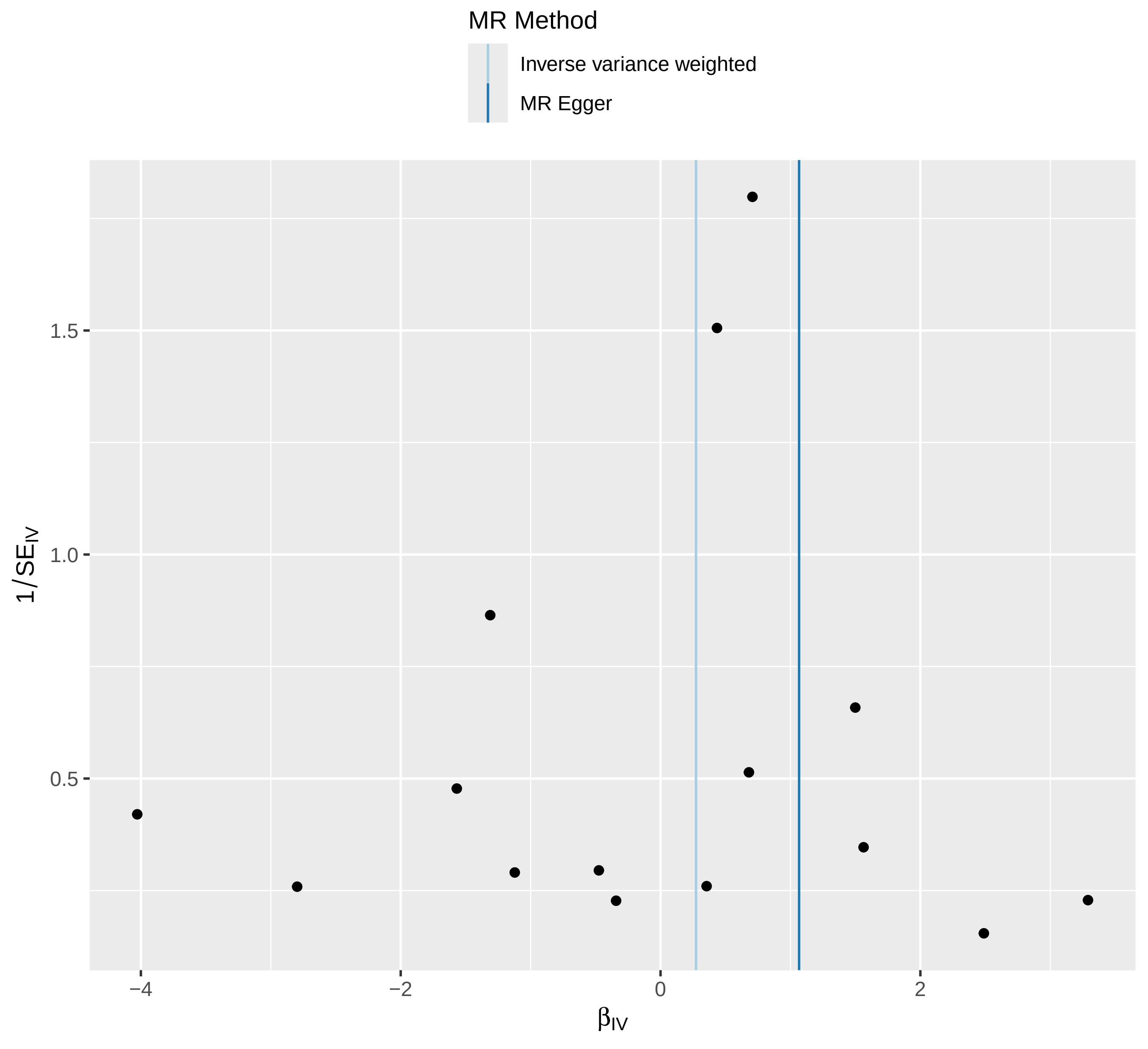
MR Method
Inverse variance weighted
MR Egger
1.5
1.0
0.5
$1/SE_{IV}$
−4
−2
0
2
β_{IV}

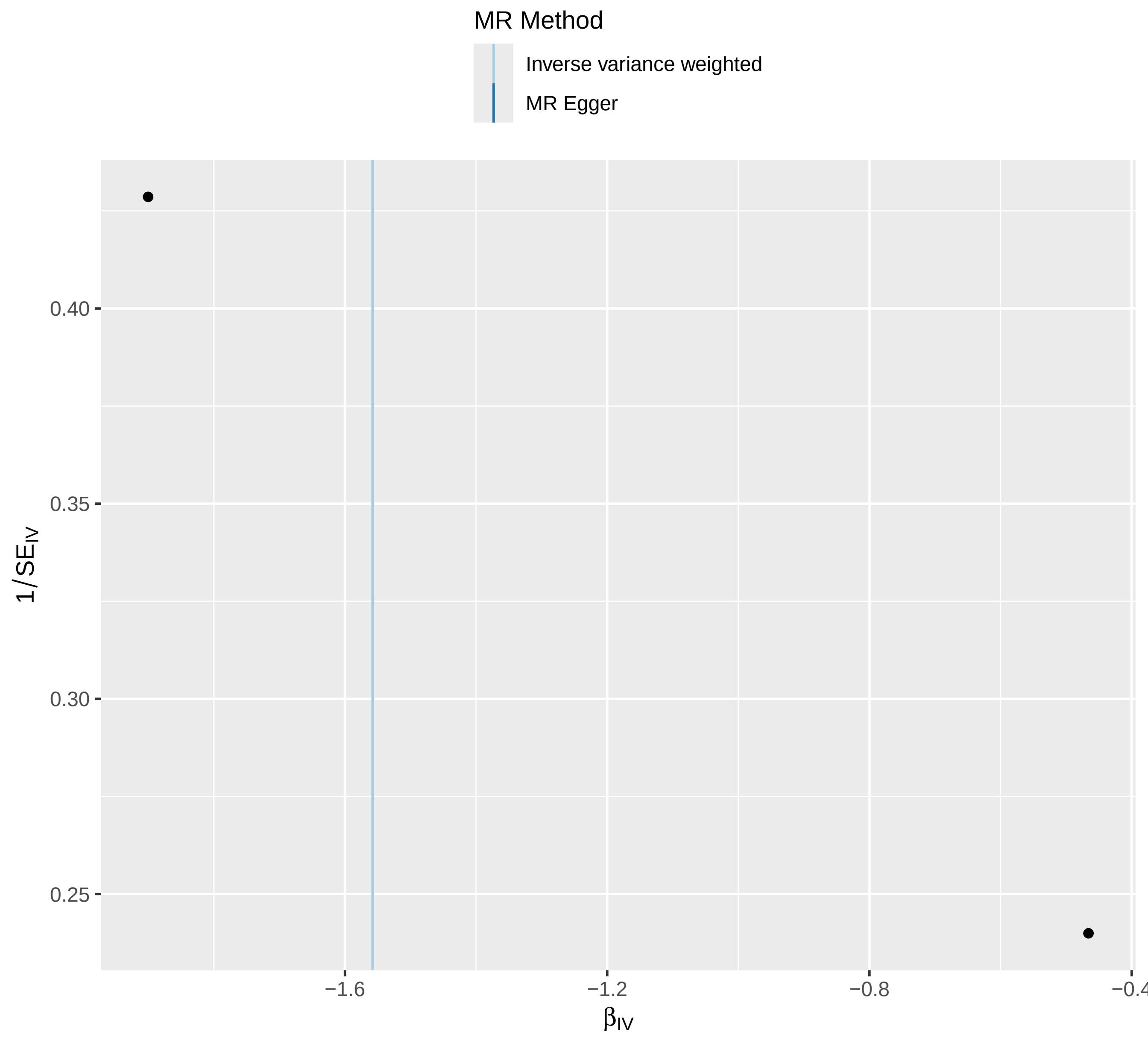
MR Method
Inverse variance weighted
MR Egger
0.40
0.35
0.30
0.25
1/SE_IV
−1.6
−1.2
−0.8
−0.4
β_IV

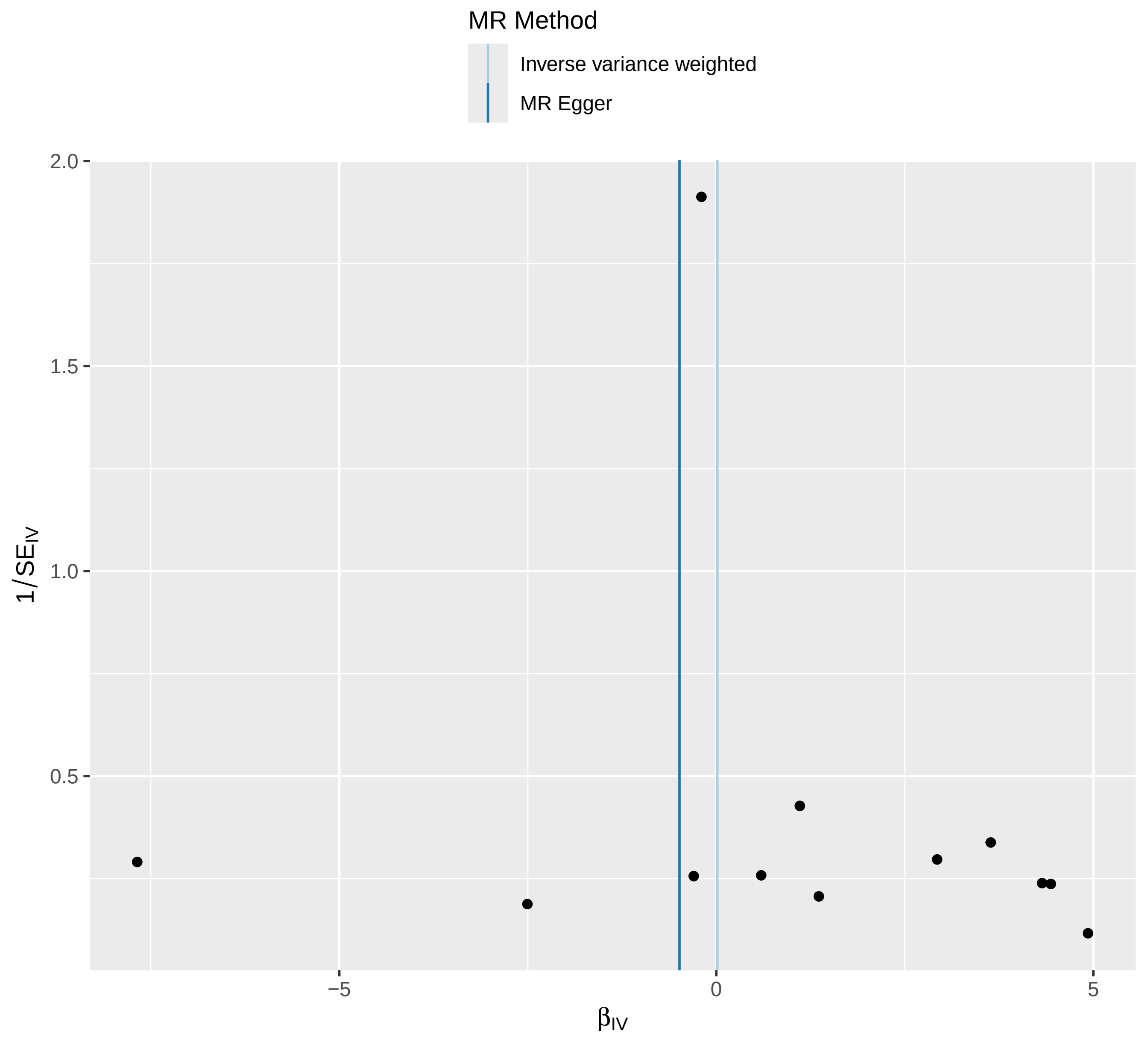
MR Method
Inverse variance weighted
MR Egger
2.0
1.5
1.0
0.5
1/SE_IV
−5
0
5
β_IV

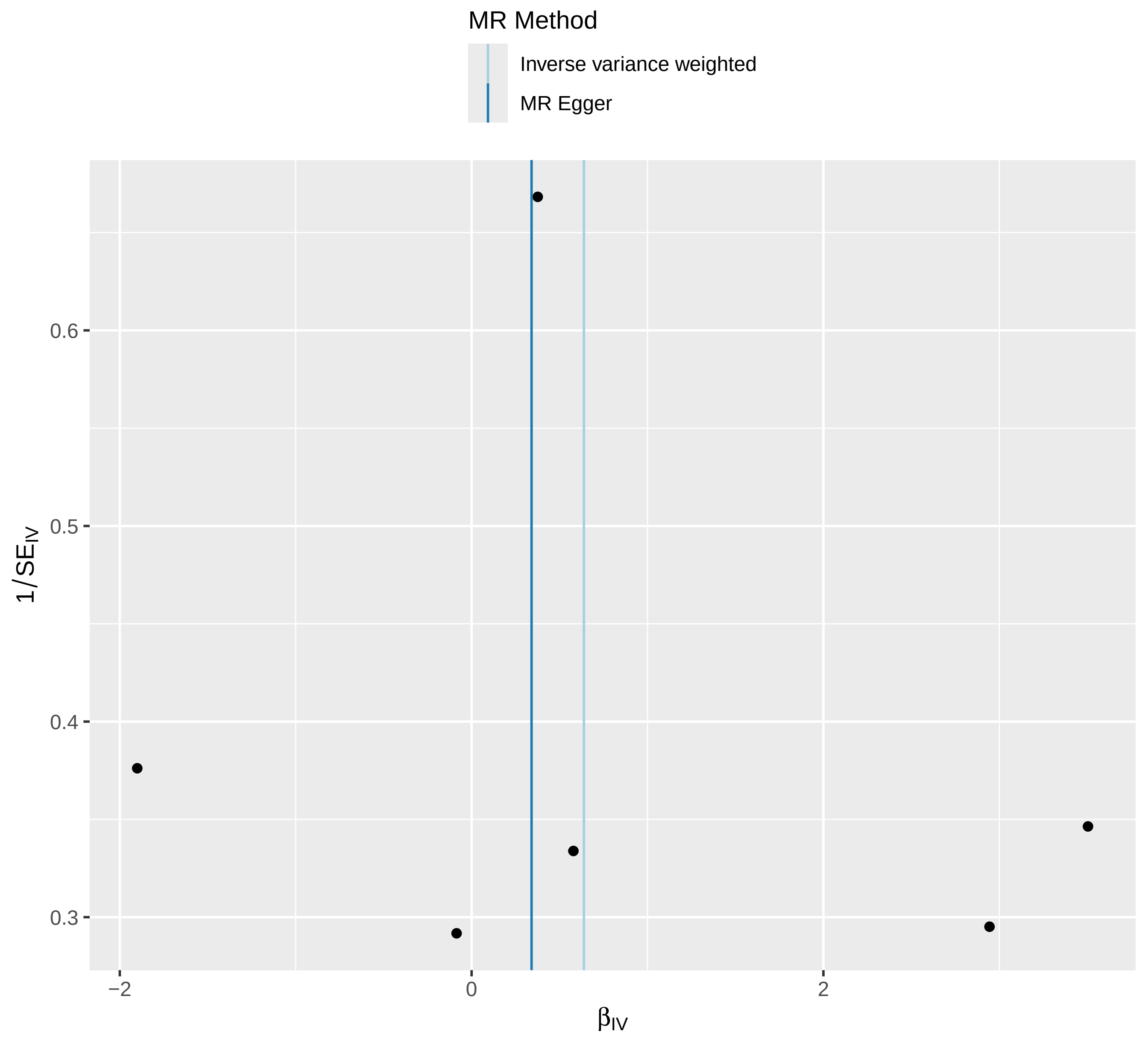
MR Method
Inverse variance weighted
MR Egger
0.6
0.5
0.4
0.3
1/SE_IV
−2
0
2
β_IV

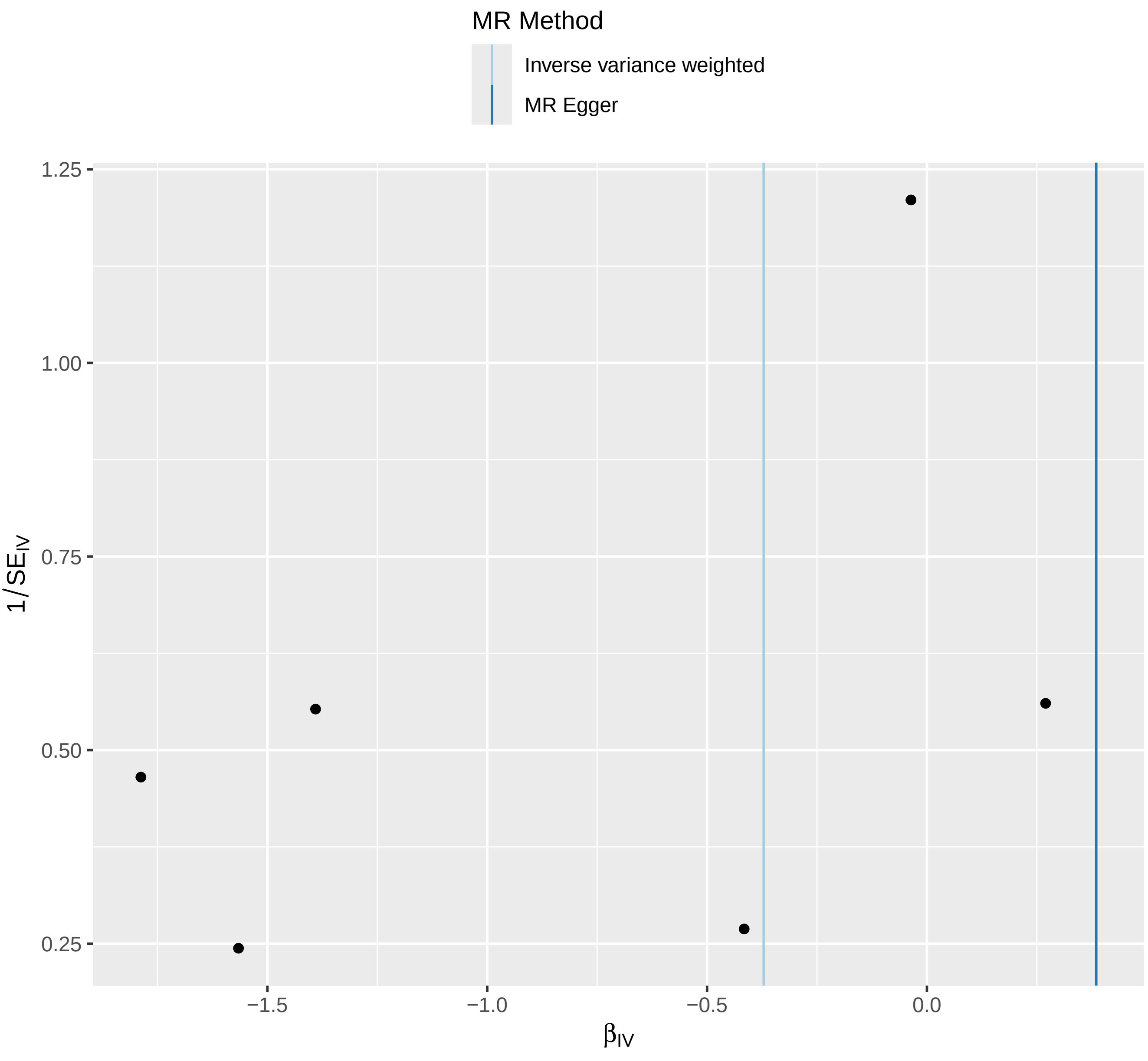
MR Method
Inverse variance weighted
MR Egger
1.25
1.00
0.75
0.50
0.25
1/SE_IV
−1.5
−1.0
−0.5
0.0
β_IV

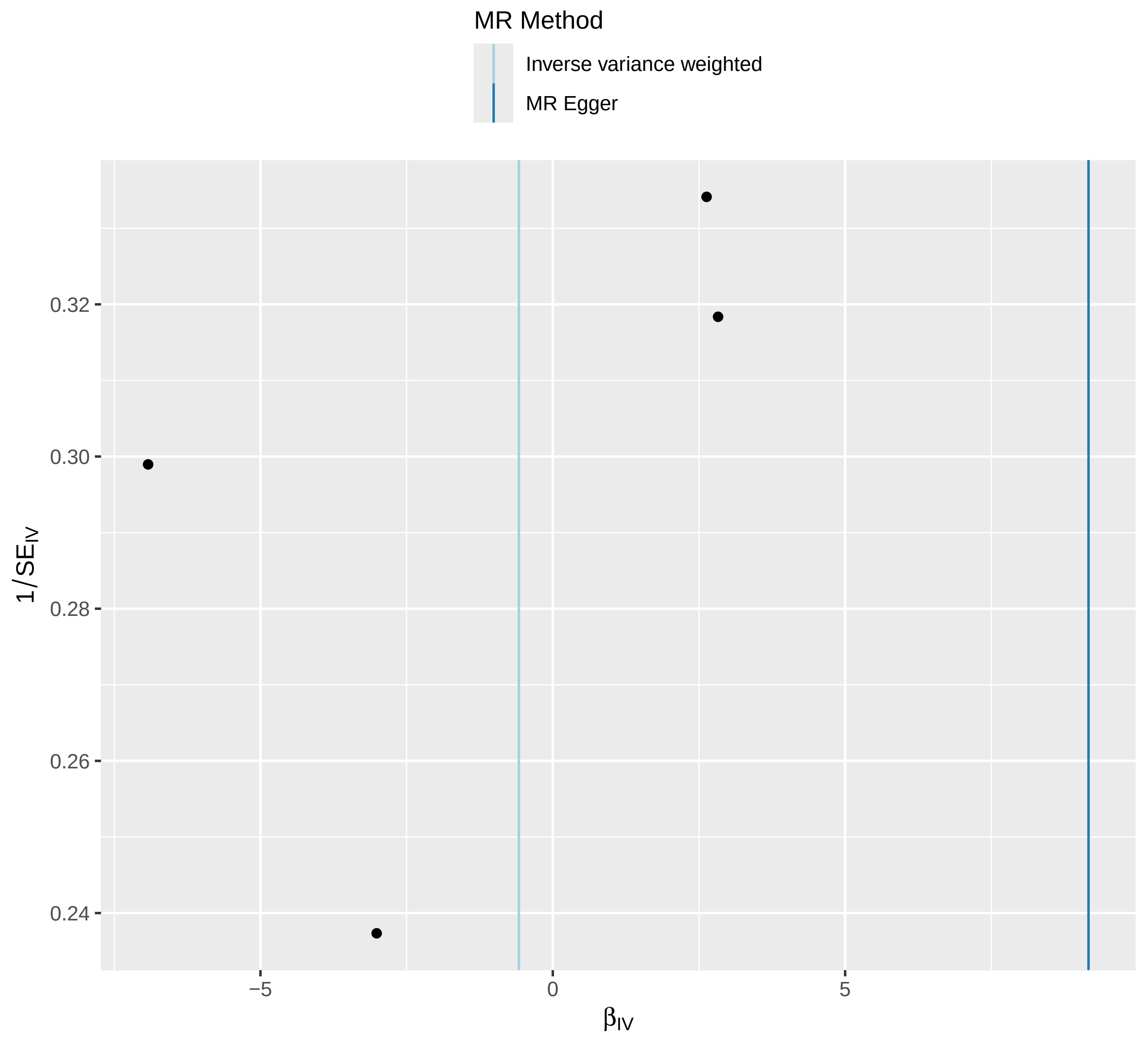
MR Method
Inverse variance weighted
MR Egger
0.32
0.30
0.28
0.26
0.24
1/SE_IV
−5
0
5
β_IV

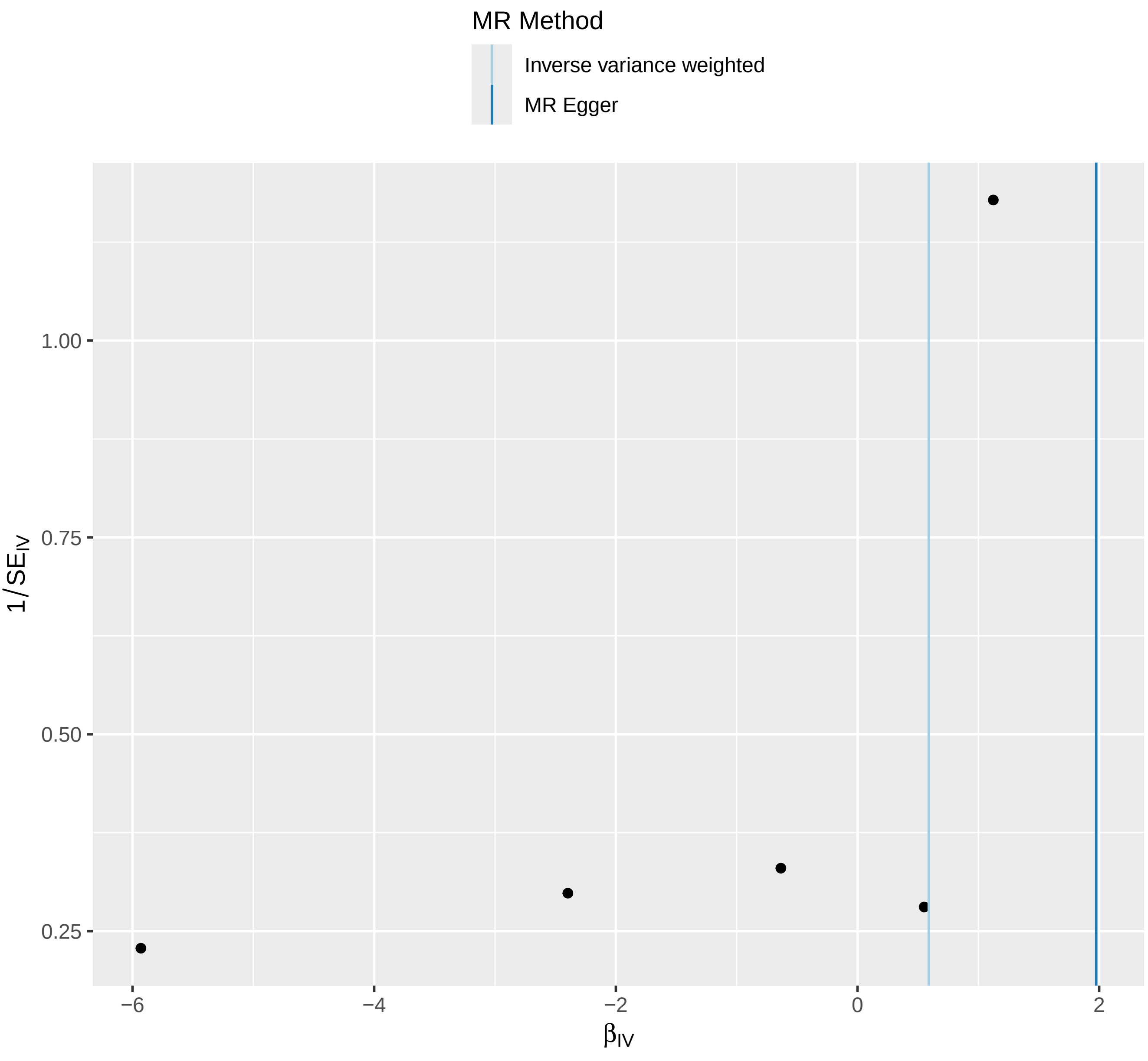
MR Method
Inverse variance weighted
MR Egger
1.00
0.75
0.50
0.25
1/SE$_{IV}$
−6
−4
−2
0
2
β$_{IV}$

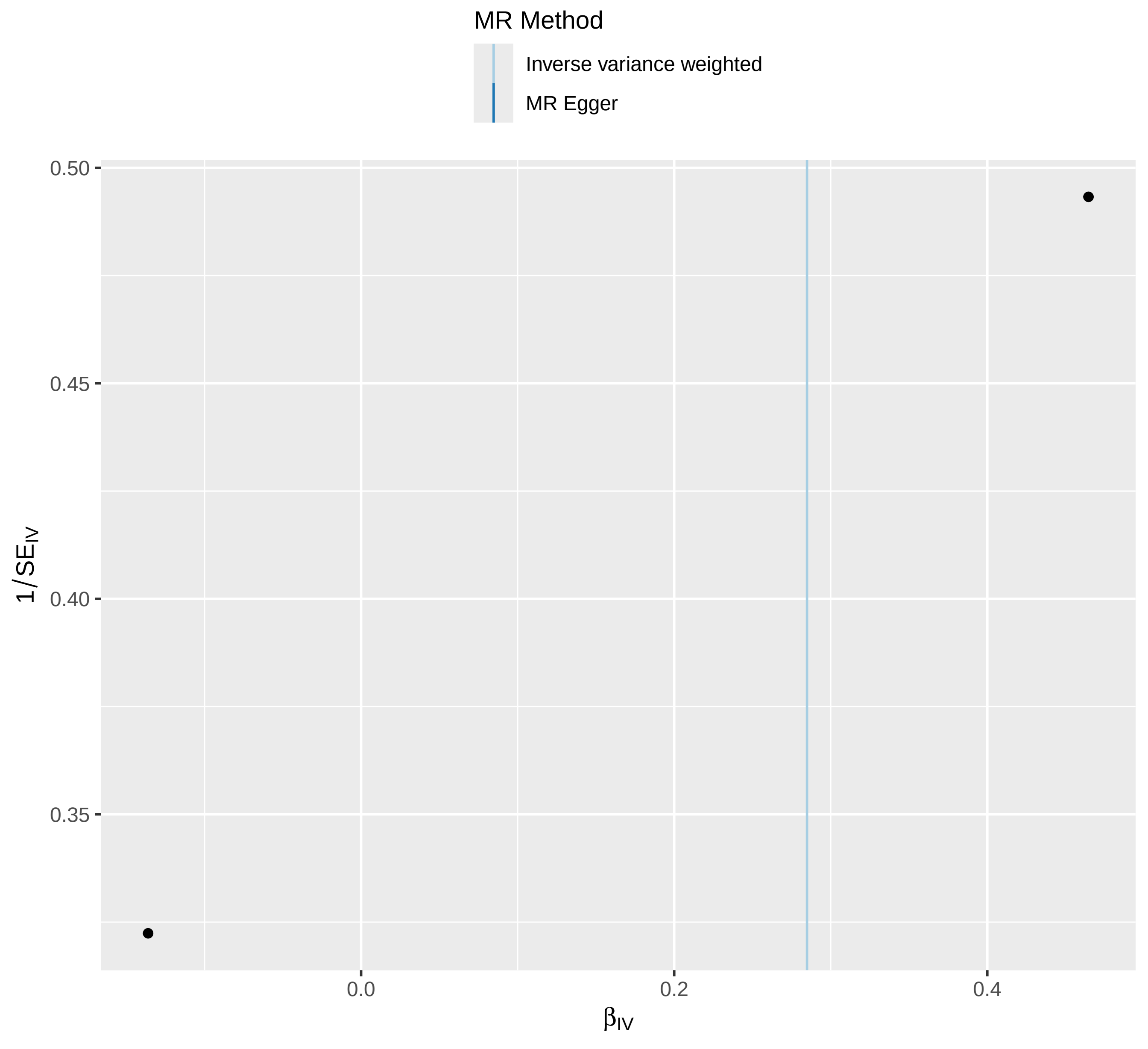
MR Method
Inverse variance weighted
MR Egger
0.50
0.45
0.40
0.35
1/SE_IV
0.0
0.2
0.4
β_IV

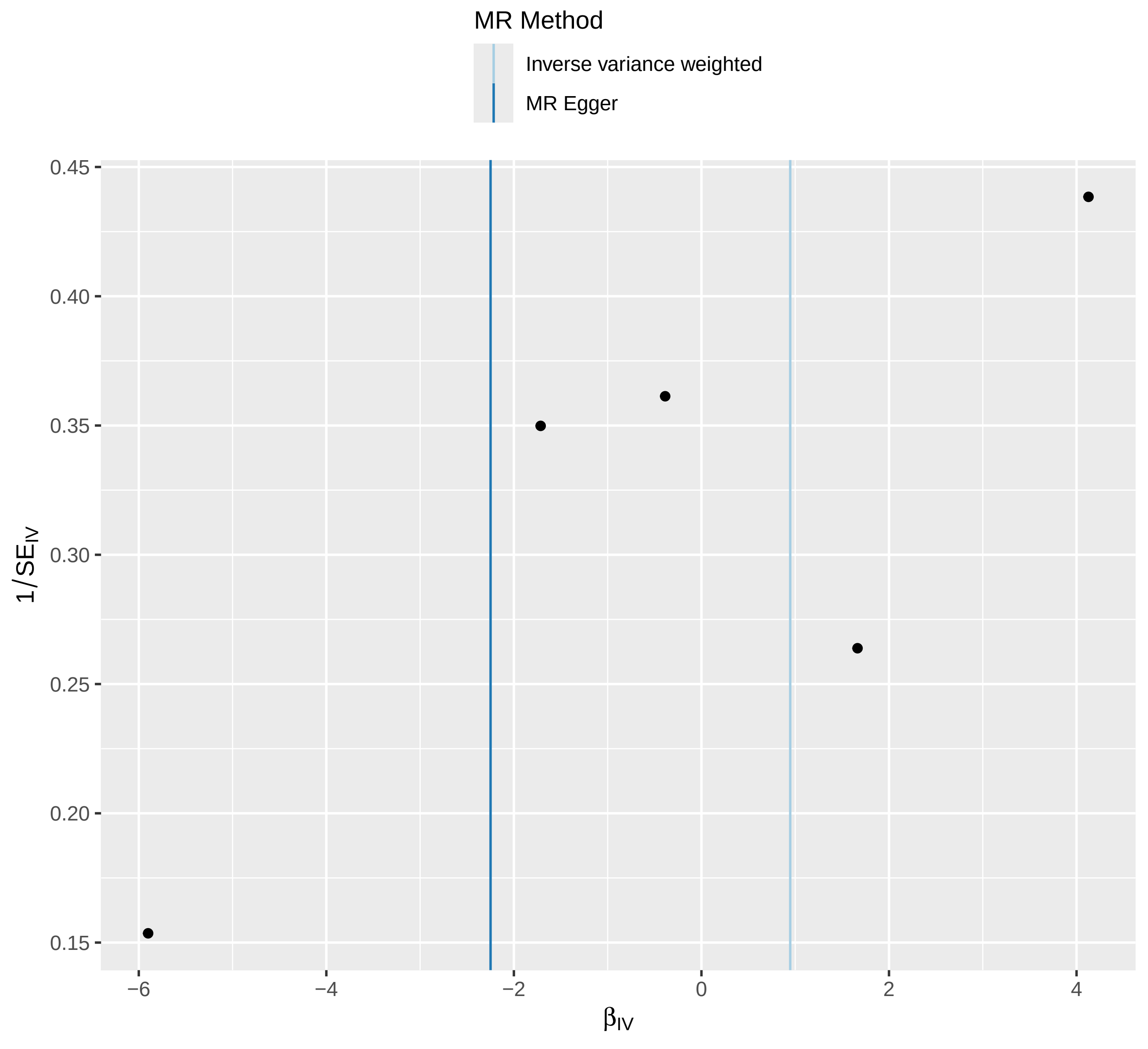
MR Method
Inverse variance weighted
MR Egger
0.45
0.40
0.35
0.30
0.25
0.20
0.15
1/SE$_{IV}$
−6
−4
−2
0
2
4
β$_{IV}$

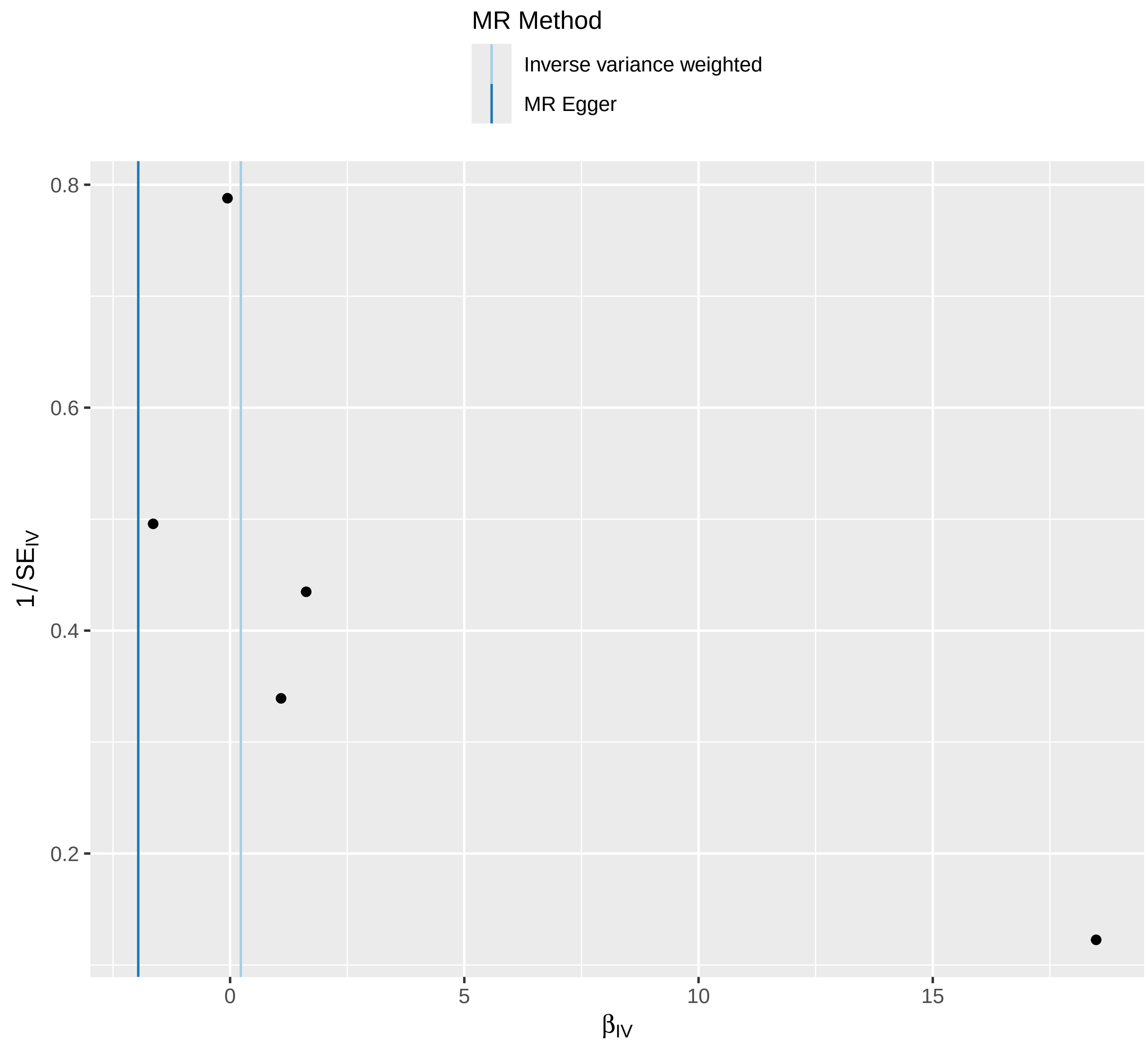
MR Method
Inverse variance weighted
MR Egger
0.8
0.6
0.4
0.2
$1/SE_{IV}$
0
5
10
15
β_{IV}

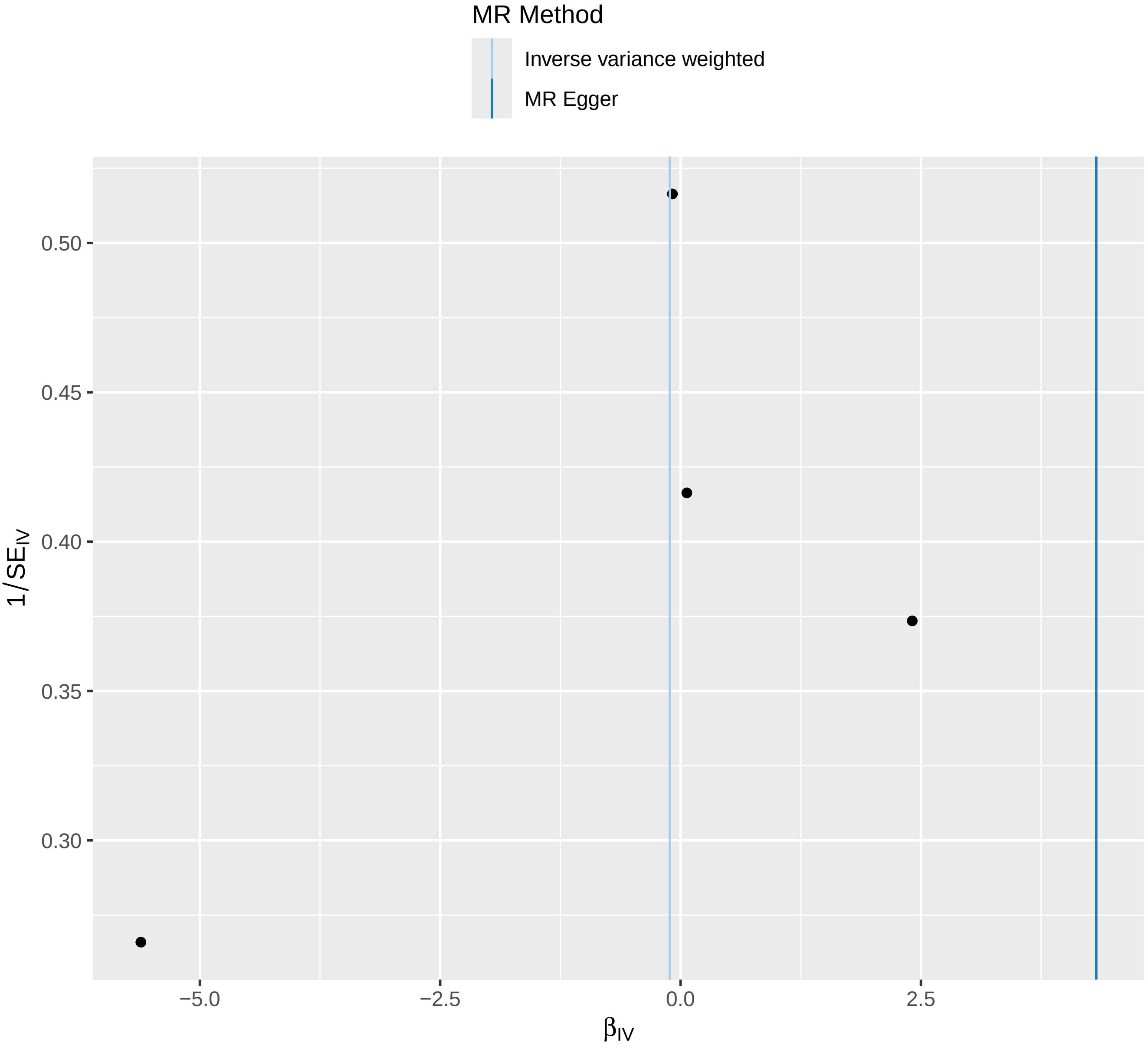
MR Method
Inverse variance weighted
MR Egger
0.50
0.45
0.40
0.35
0.30
$1/SE_{IV}$
−5.0
−2.5
0.0
2.5
β_{IV}

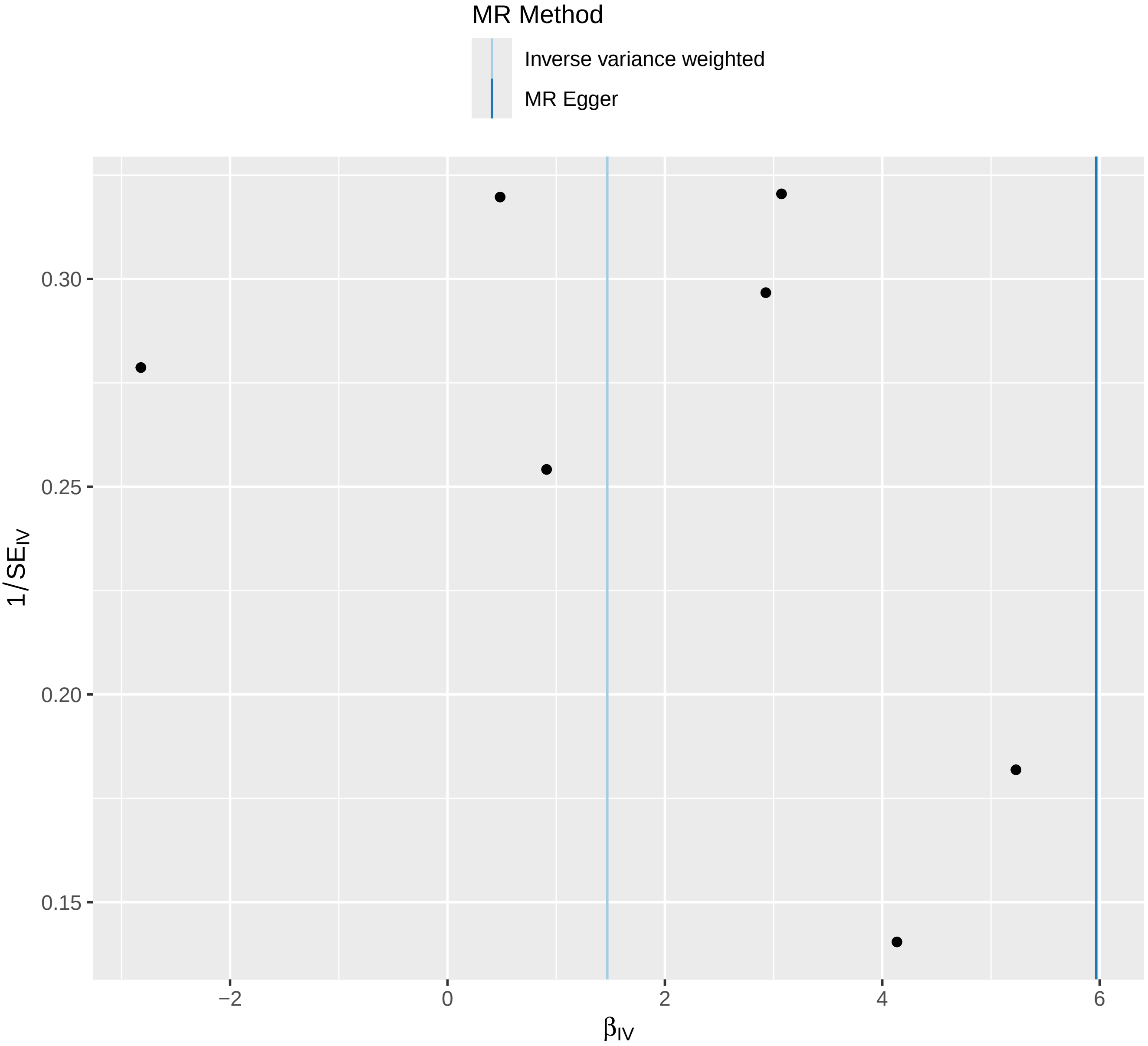

MR Method
Inverse variance weighted
MR Egger
0.30
0.25
0.20
0.15
1/SE_IV
−2
0
2
4
6
β_IV

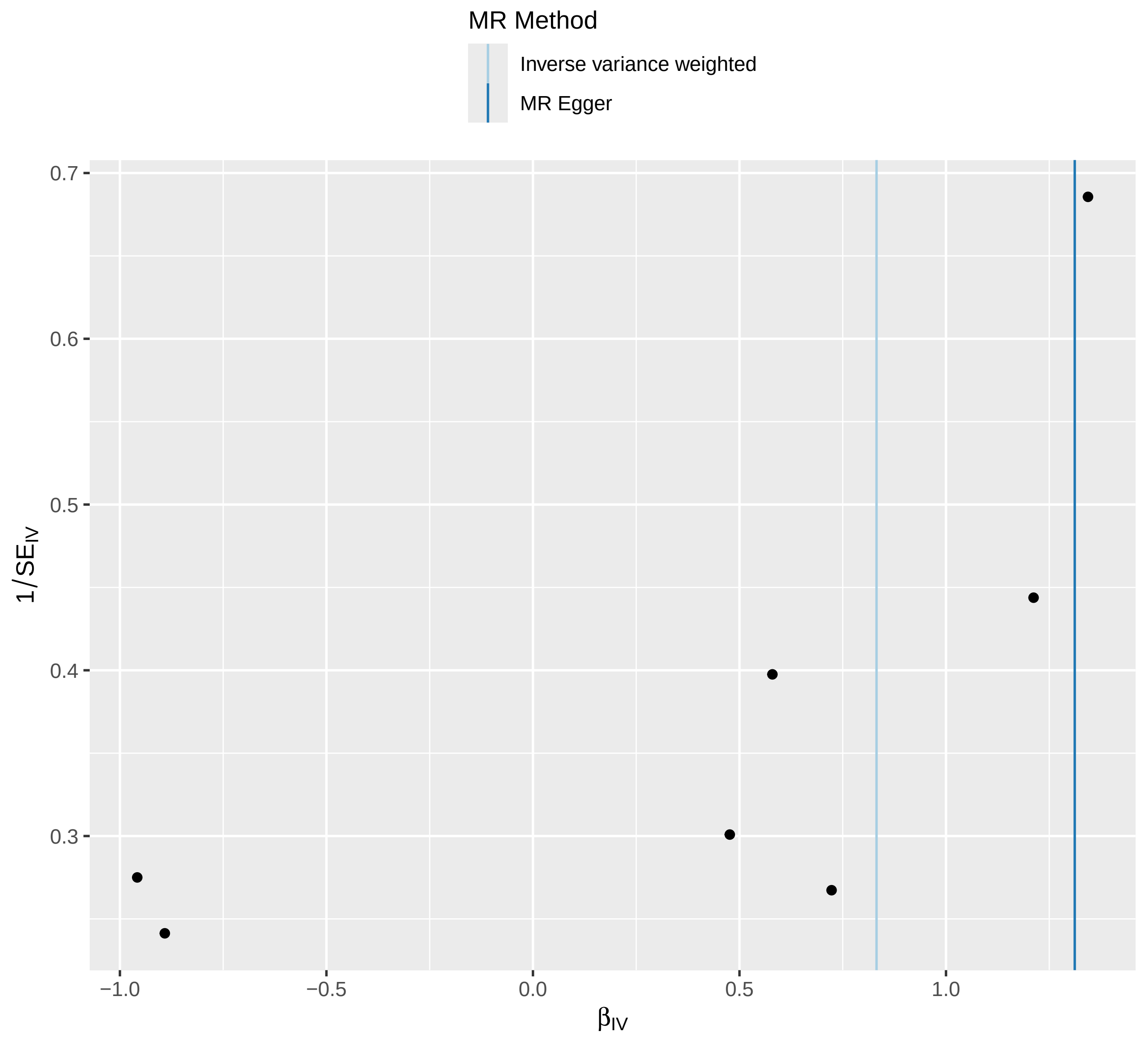
MR Method
Inverse variance weighted
MR Egger
0.7
0.6
0.5
0.4
0.3
1/SE_IV
−1.0
−0.5
0.0
0.5
1.0
β_IV

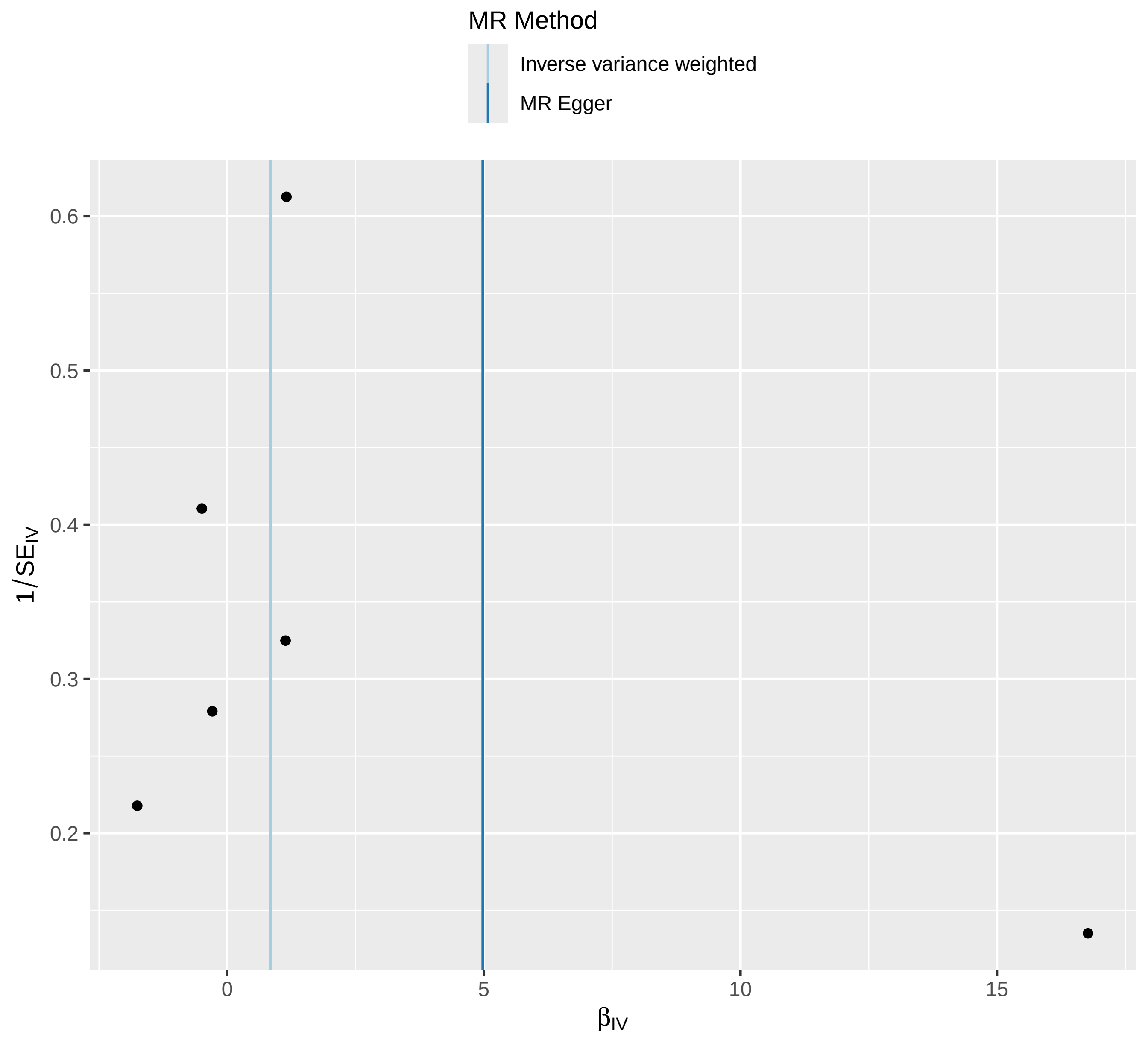
MR Method
Inverse variance weighted
MR Egger
0.6
0.5
0.4
0.3
0.2
1/SE$_{IV}$
0
5
10
15
β$_{IV}$

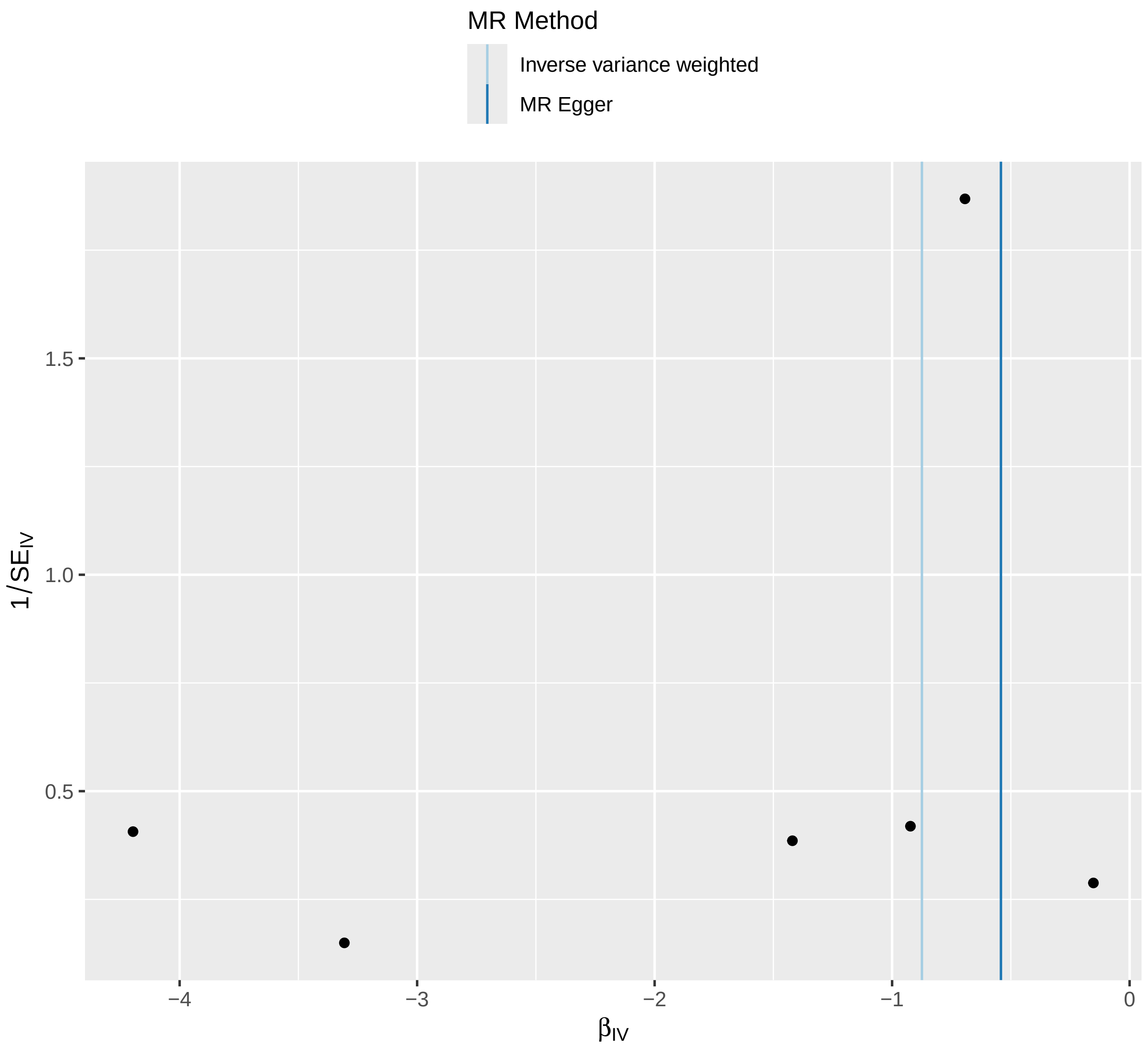
MR Method
Inverse variance weighted
MR Egger
1/SE_IV
1.5
1.0
0.5
−4
−3
−2
−1
0
β_IV

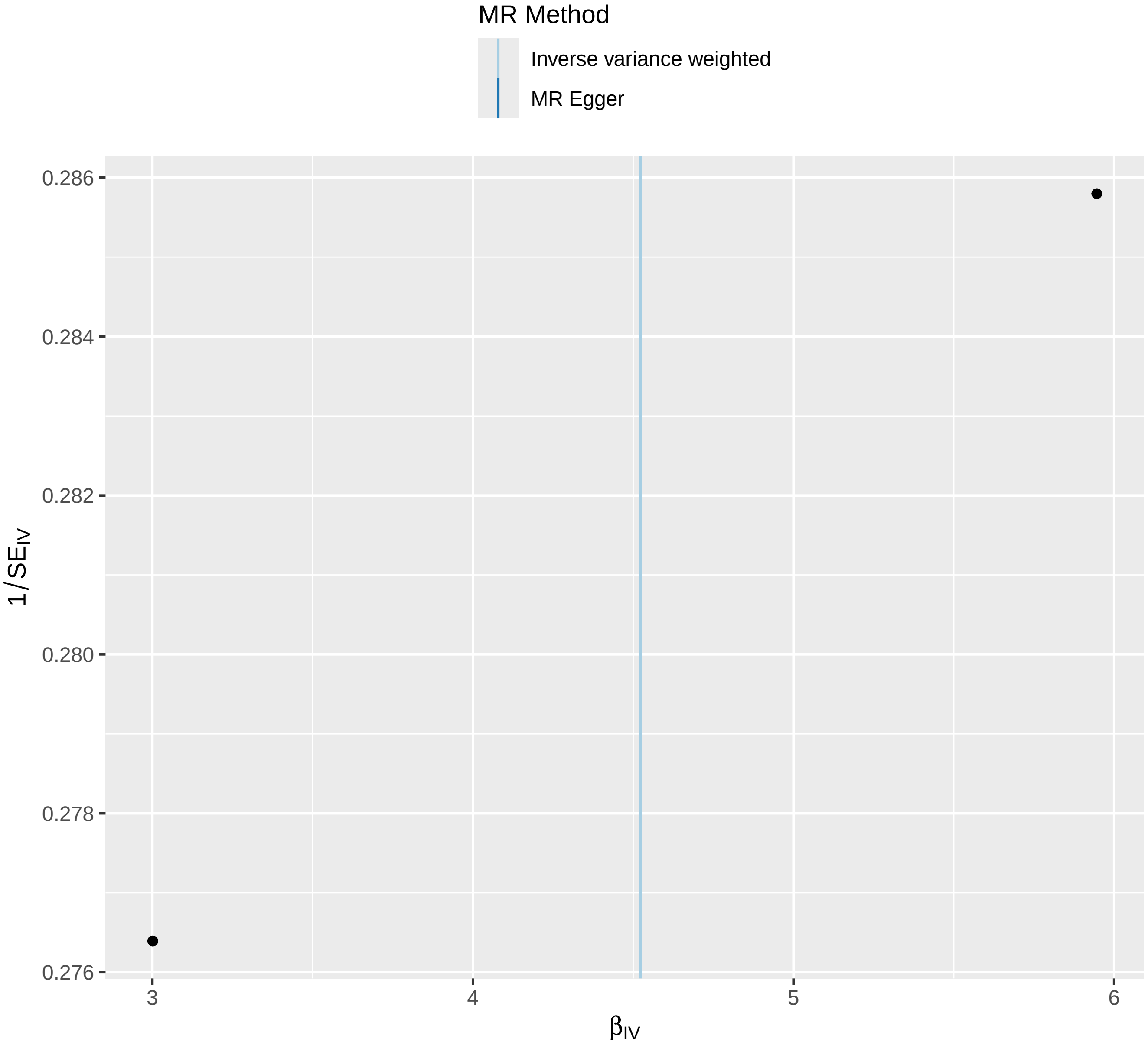
MR Method
Inverse variance weighted
MR Egger
0.286
0.284
0.282
0.280
0.278
0.276
1/SE_IV
3
4
5
6
β_IV

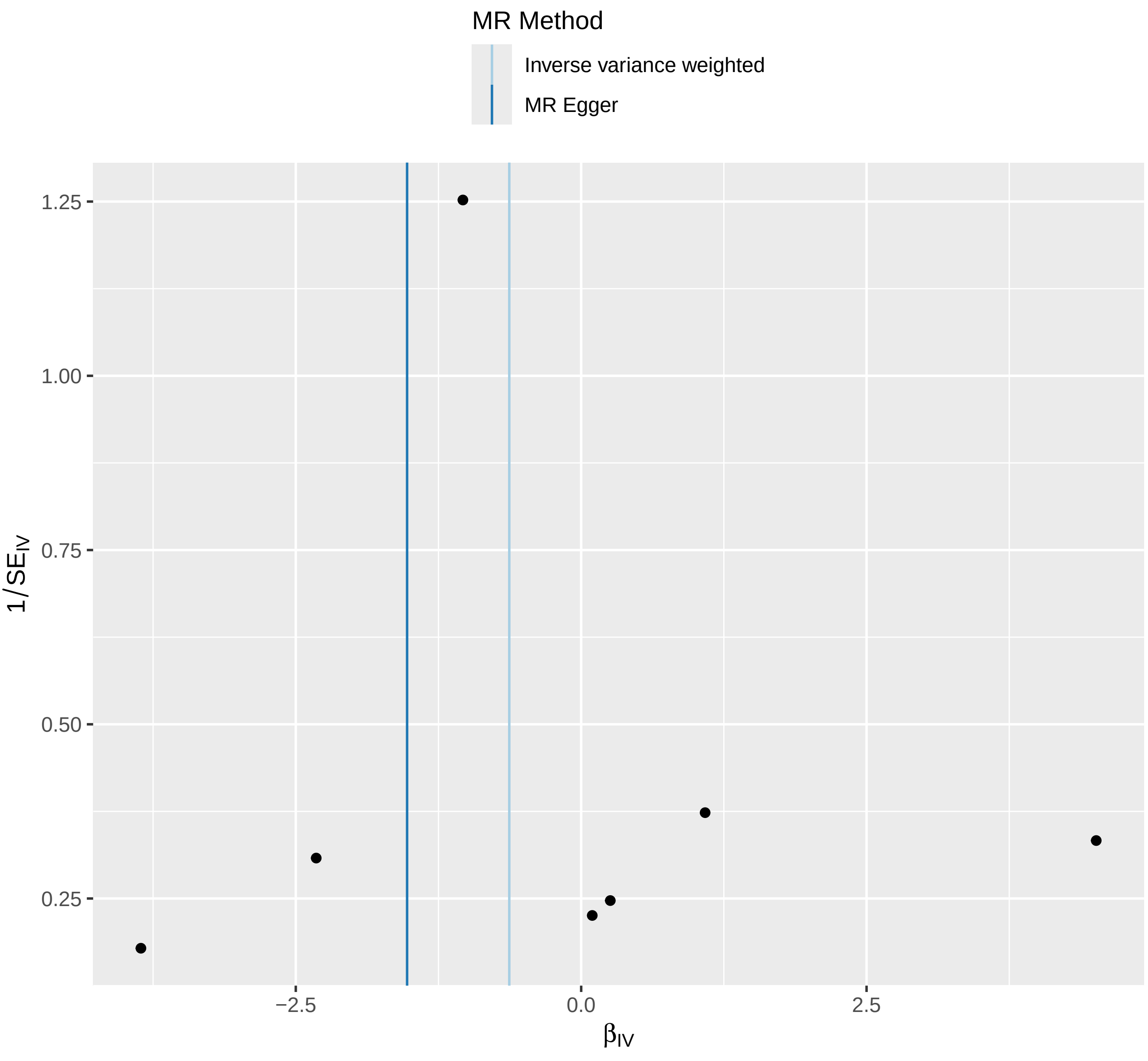

MR Method
Inverse variance weighted
MR Egger
1.25
1.00
0.75
0.50
0.25
−2.5
0.0
2.5
$1/SE_{IV}$
β_{IV}

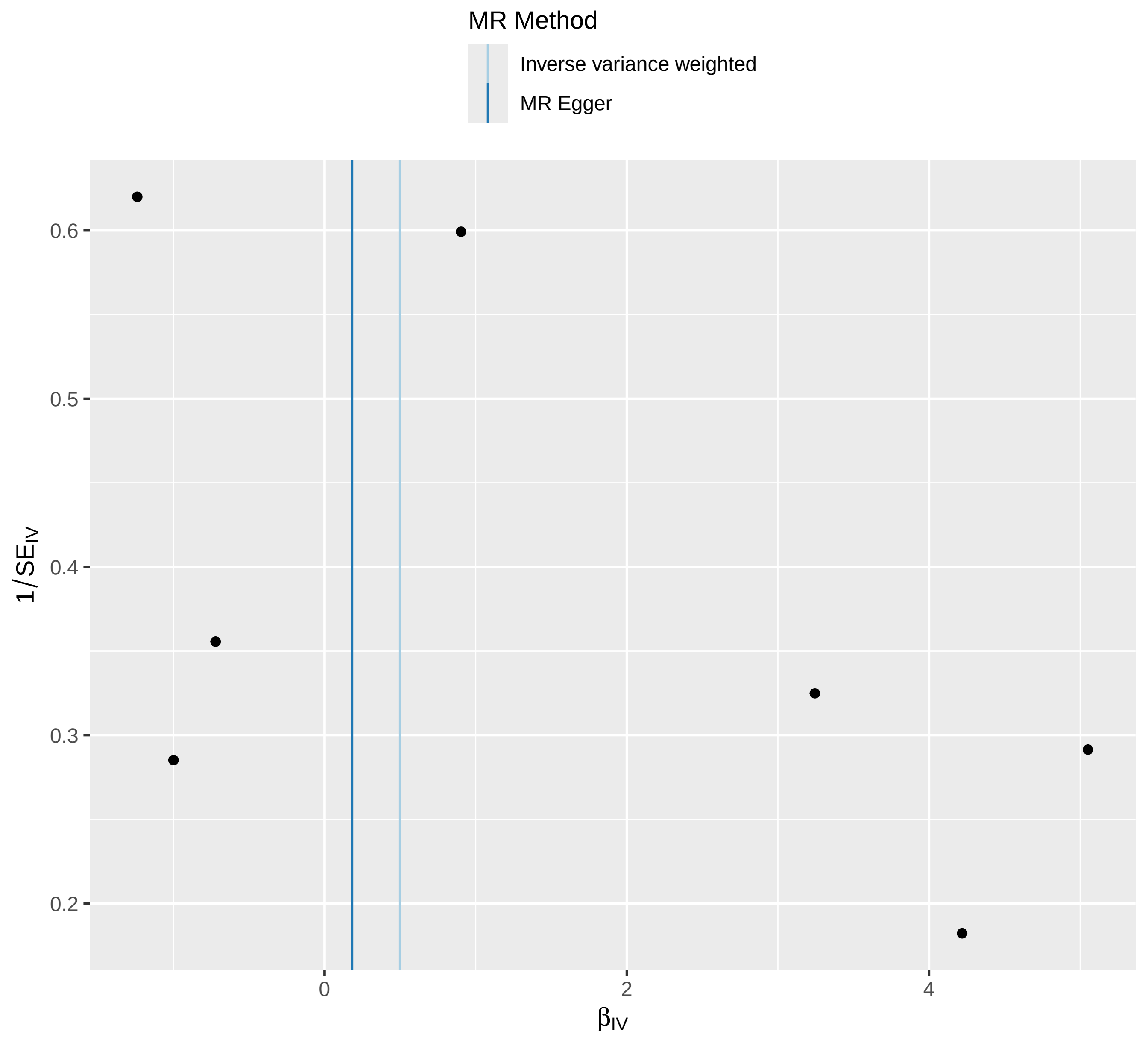

MR Method
Inverse variance weighted
MR Egger
0.6
0.5
0.4
0.3
0.2
1/SE_IV
0
2
4
β_IV

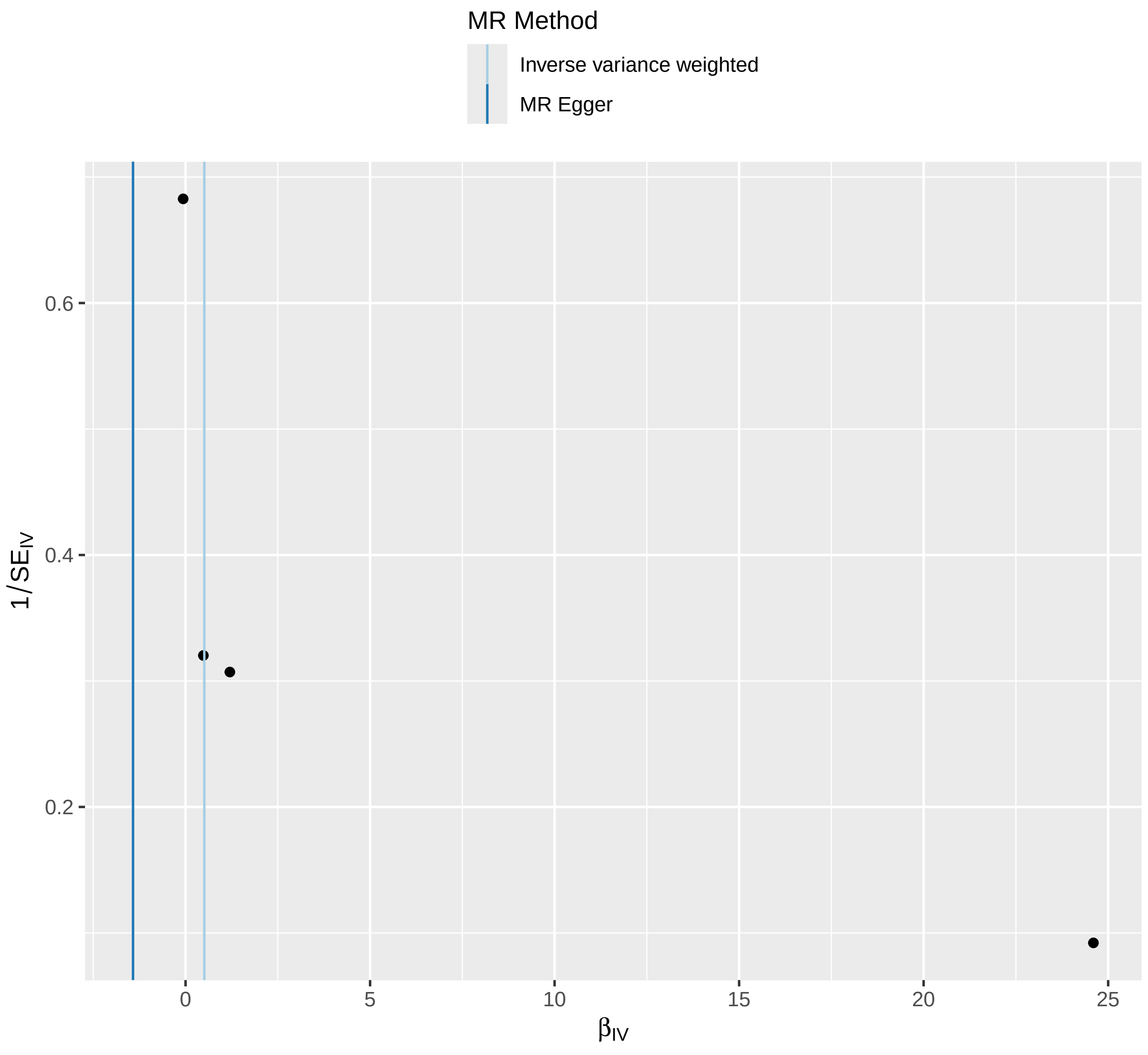

MR Method
Inverse variance weighted
MR Egger
0.6
0.4
0.2
1/SE_IV
0
5
10
15
20
25
β_IV

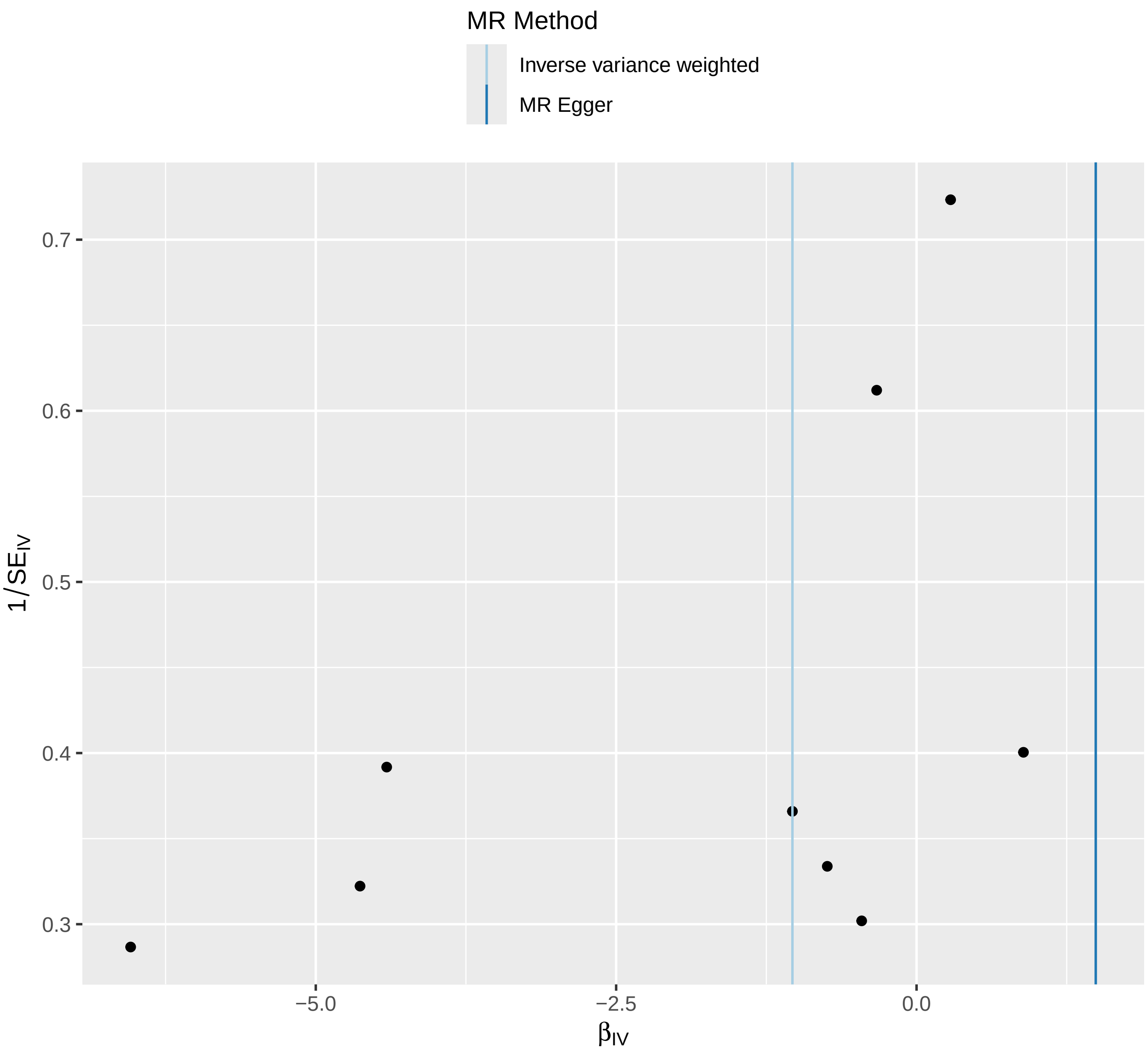
MR Method
Inverse variance weighted
MR Egger
0.7
0.6
0.5
0.4
0.3
1/SE$_{IV}$
−5.0
−2.5
0.0
β$_{IV}$

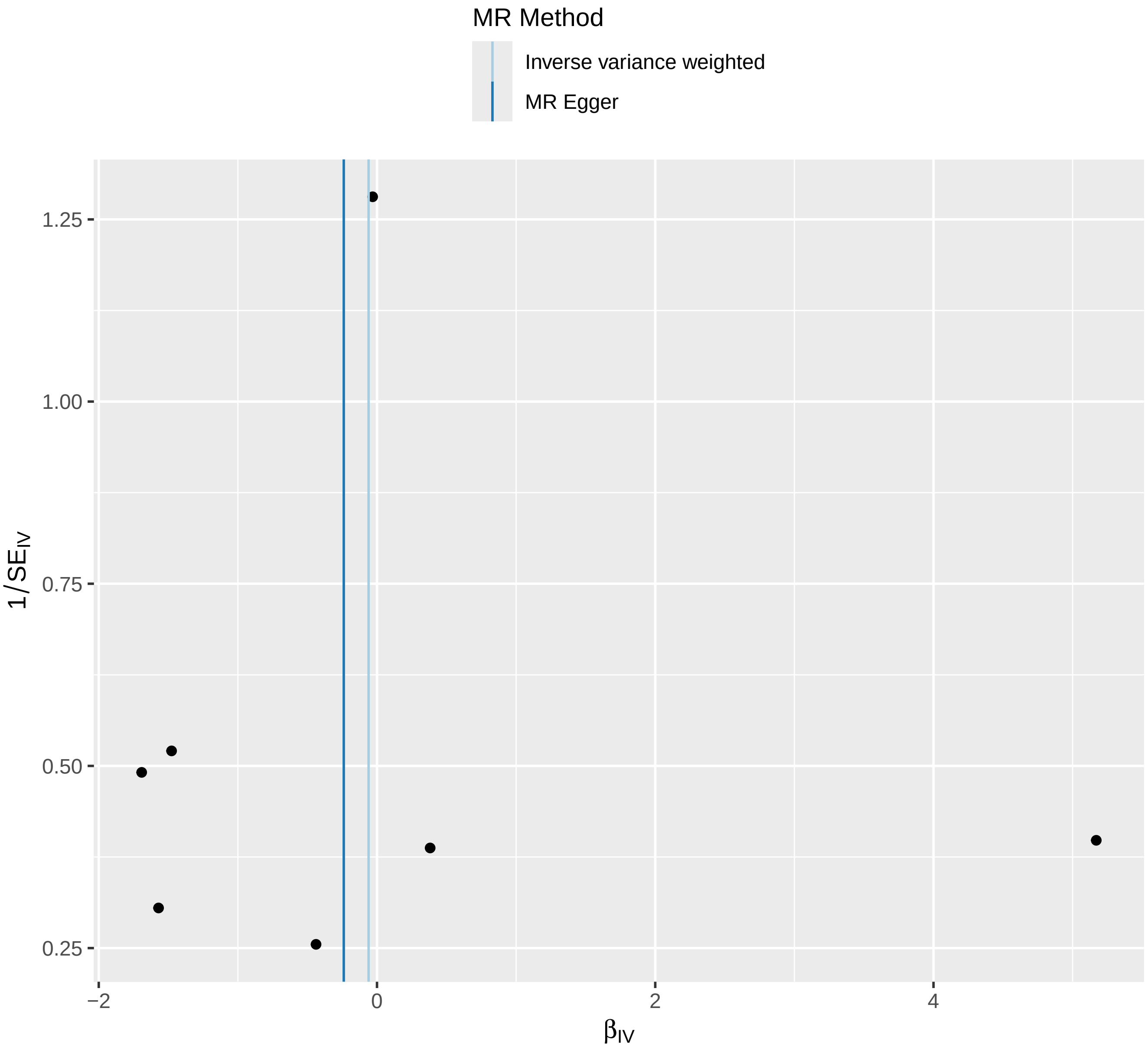
MR Method
Inverse variance weighted
MR Egger
1.25
1.00
0.75
0.50
0.25
1/SE_IV
−2
0
2
4
β_IV

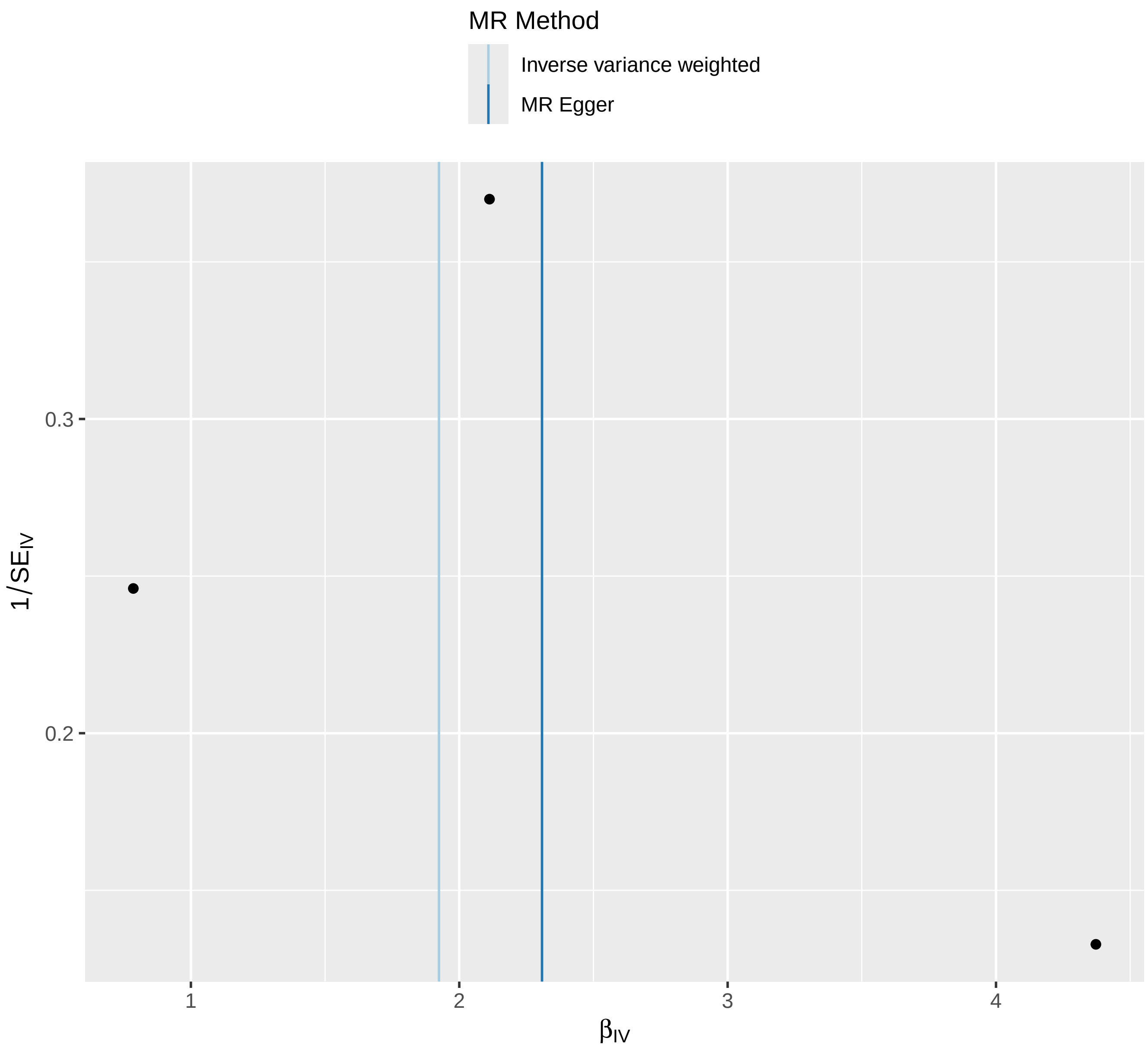

MR Method
Inverse variance weighted
MR Egger
0.3
0.2
1/SE_IV
1
2
3
4
β_IV

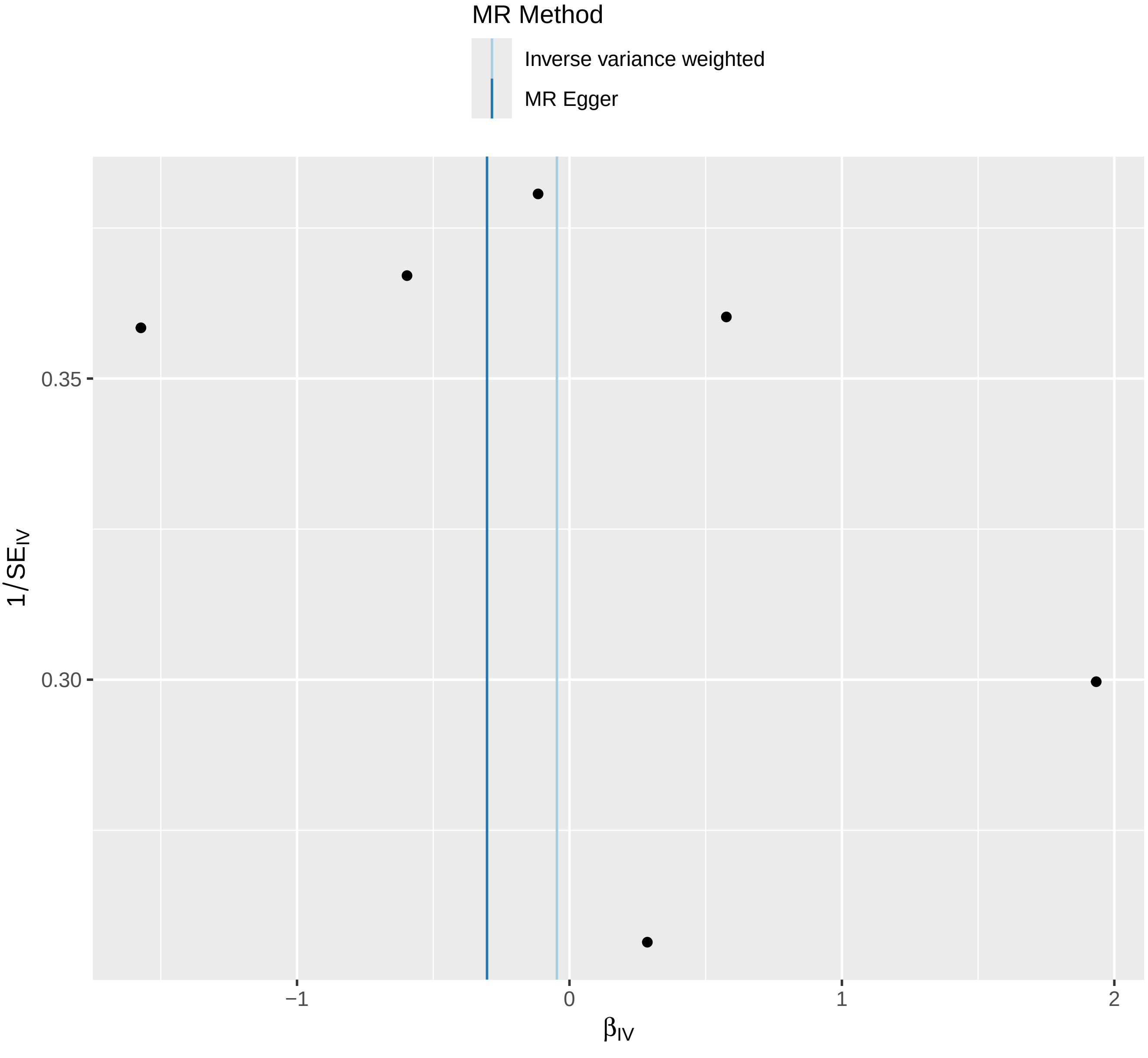

MR Method
Inverse variance weighted
MR Egger
0.35
0.30
1/SE_IV
−1
0
1
2
β_IV

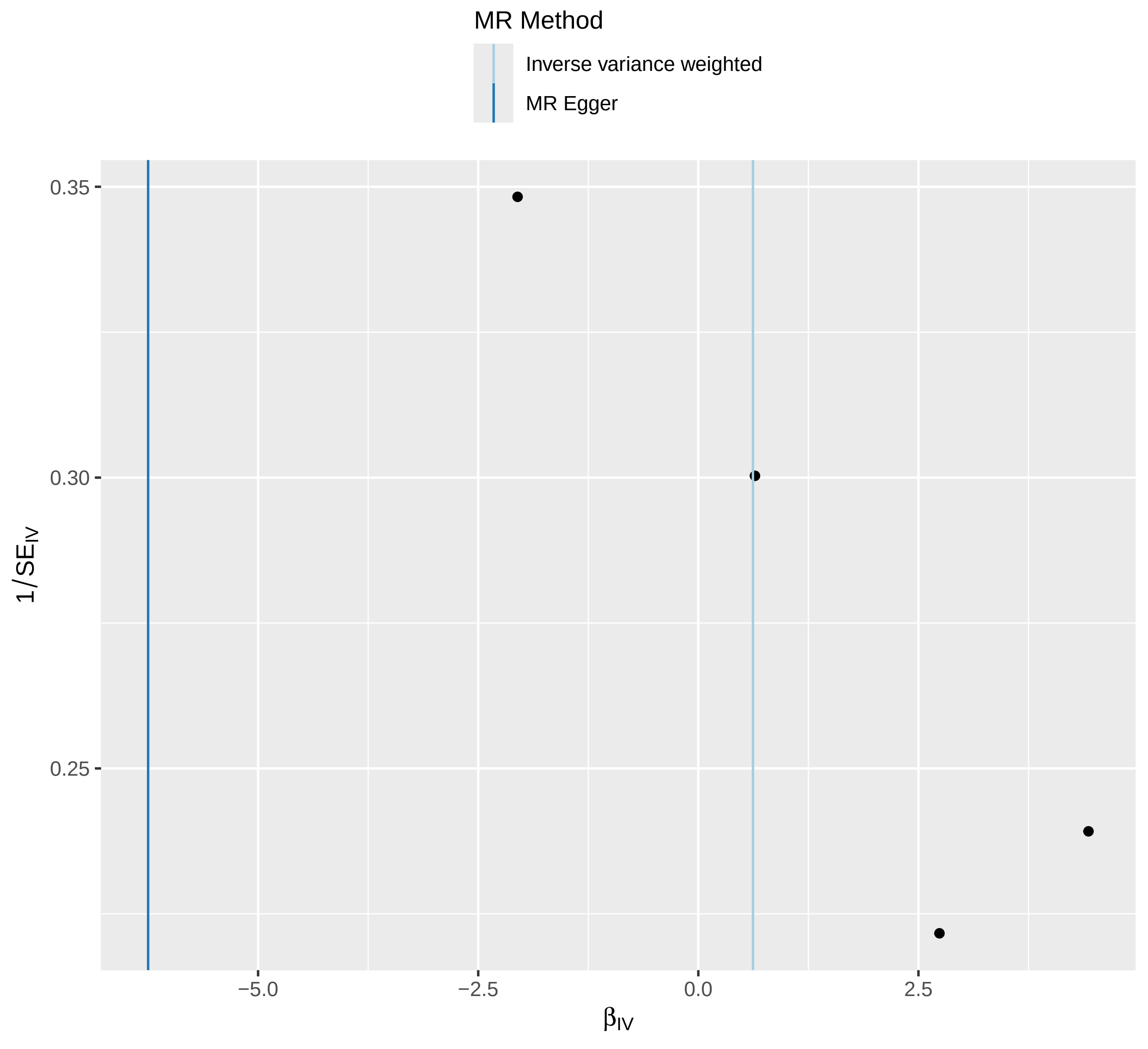

MR Method
Inverse variance weighted
MR Egger
0.35
0.30
0.25
1/SE$_{IV}$
−5.0
−2.5
0.0
2.5
β$_{IV}$

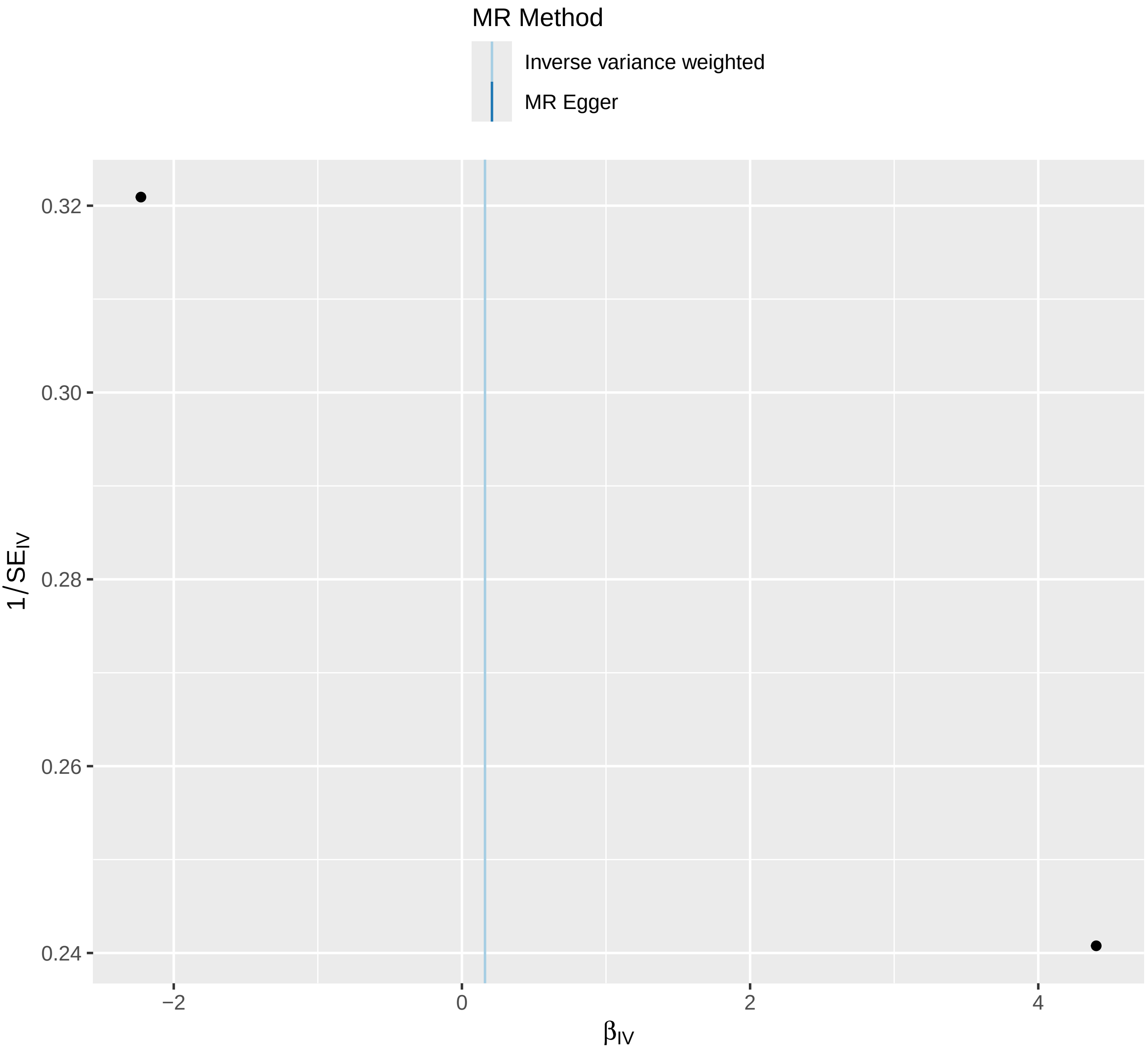
MR Method
Inverse variance weighted
MR Egger
0.32
0.30
0.28
0.26
0.24
$1/SE_{IV}$
−2
0
2
4
β_{IV}

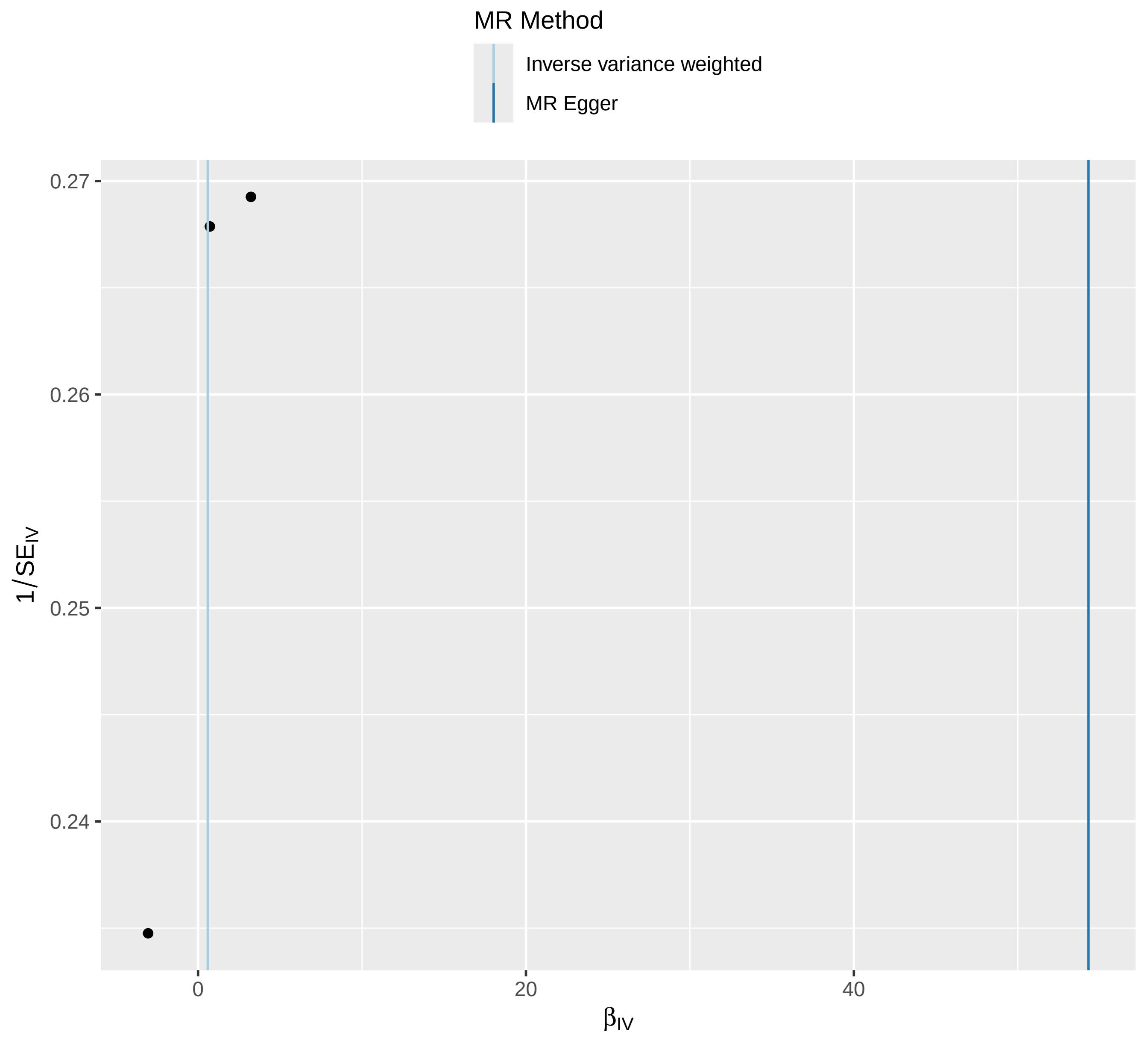

MR Method
Inverse variance weighted
MR Egger
0.27
0.26
0.25
0.24
0
20
40
$1/SE_{IV}$
β_{IV}

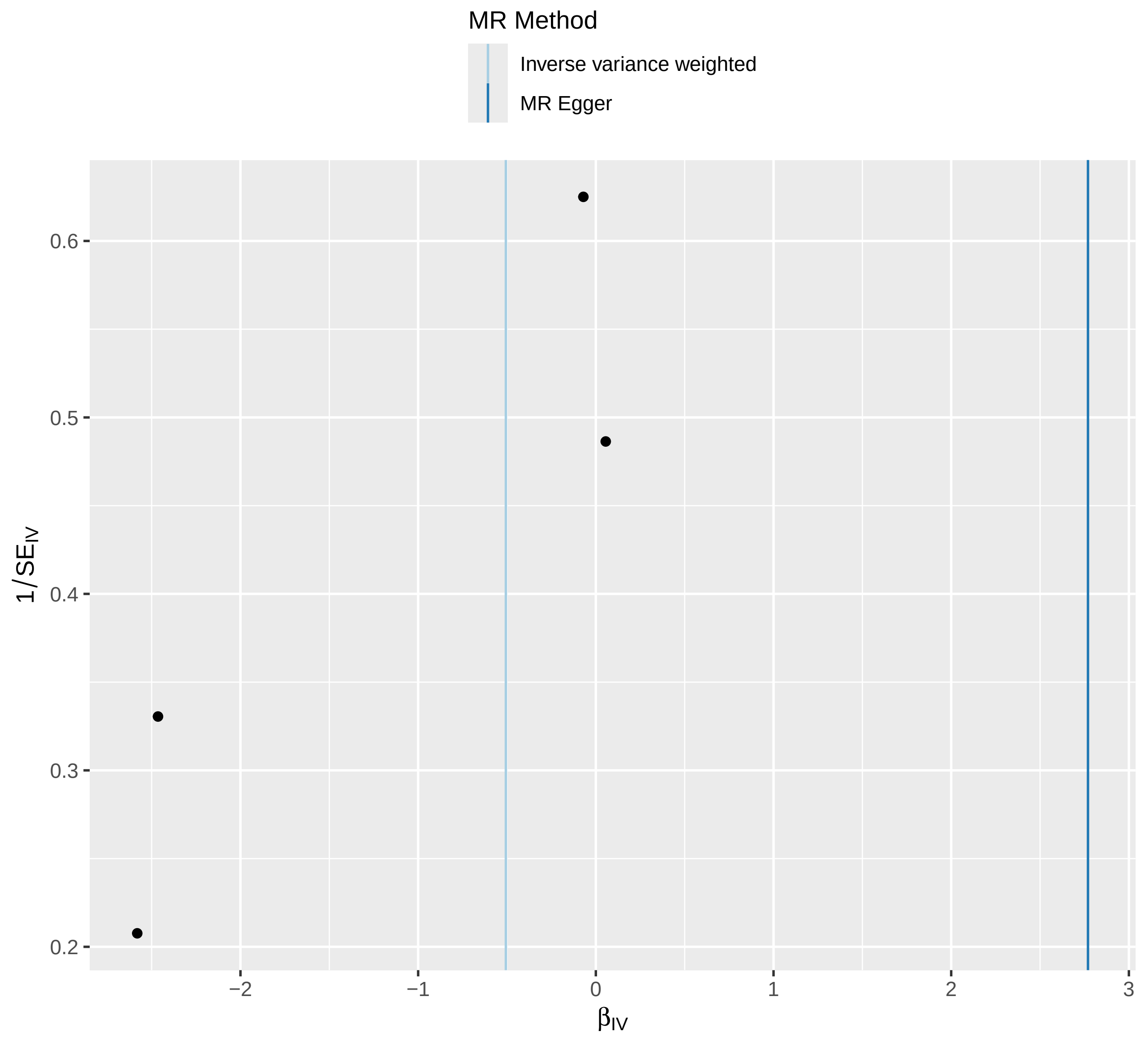

MR Method
Inverse variance weighted
MR Egger
0.6
0.5
0.4
0.3
0.2
1/SE_IV
−2
−1
0
1
2
3
β_IV

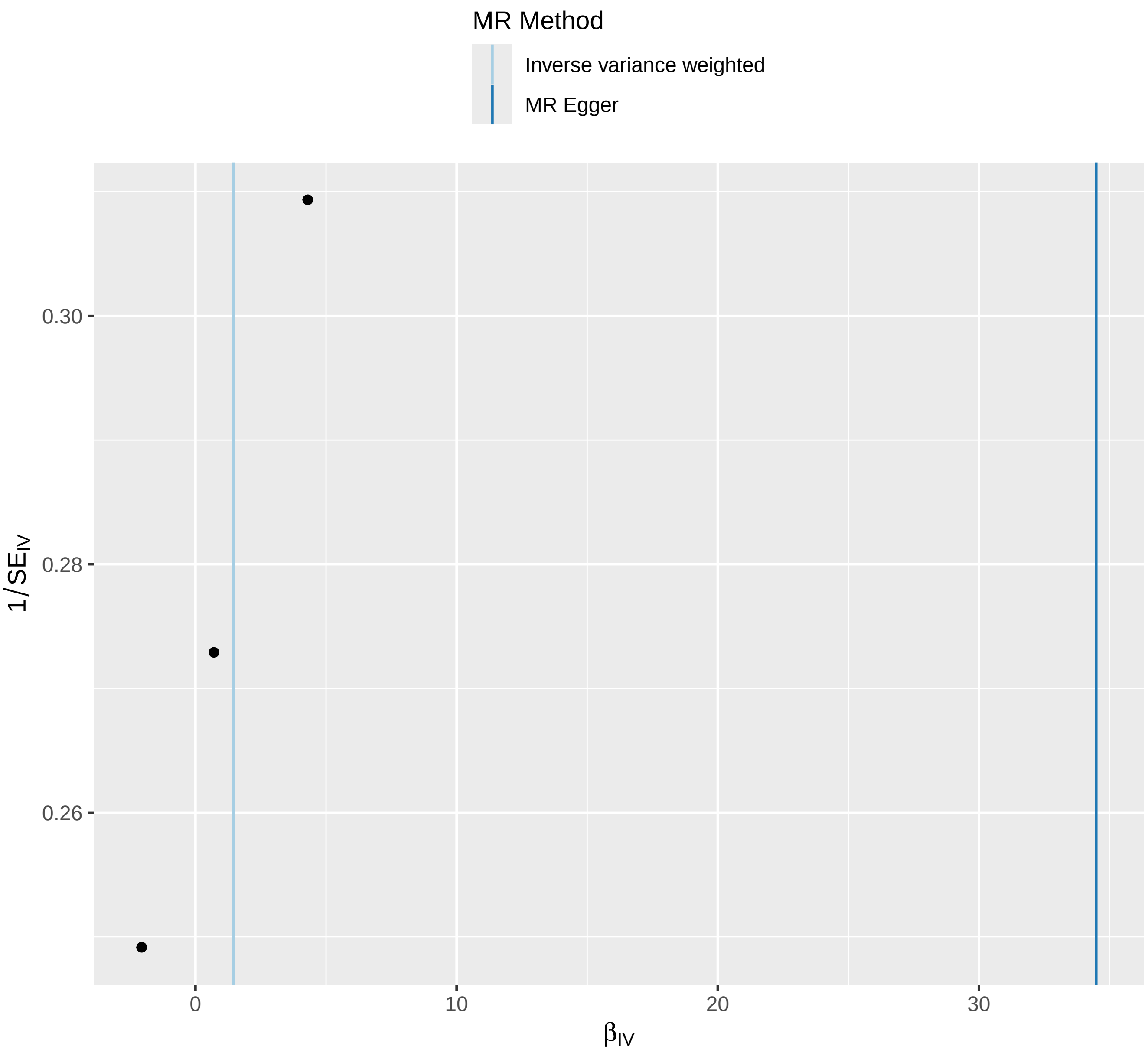

MR Method
Inverse variance weighted
MR Egger
0.30
0.28
0.26
0
10
20
30
$1/SE_{IV}$
β_{IV}

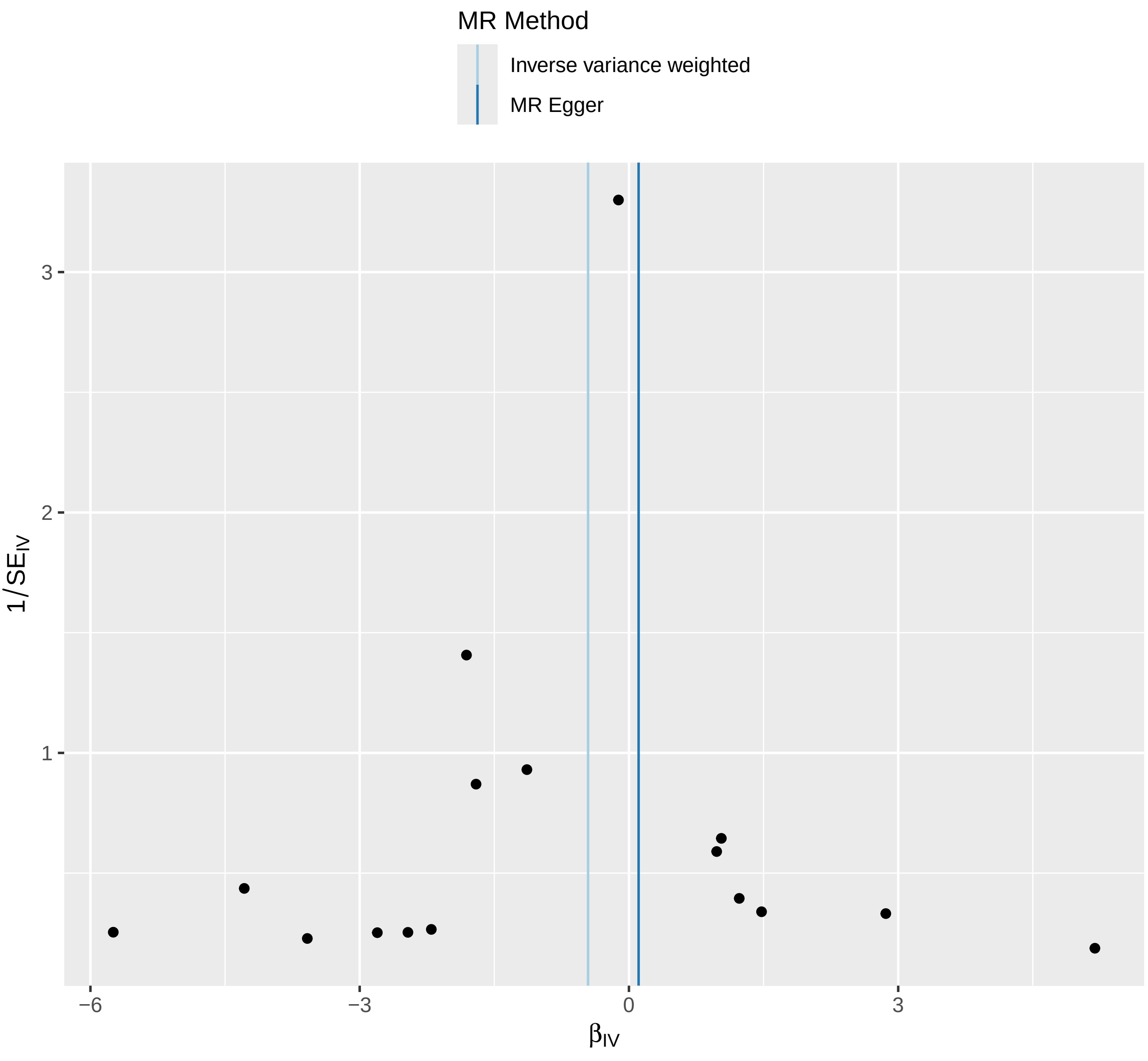
MR Method
Inverse variance weighted
MR Egger
3
2
1
1/SE_IV
−6
−3
0
3
β_IV

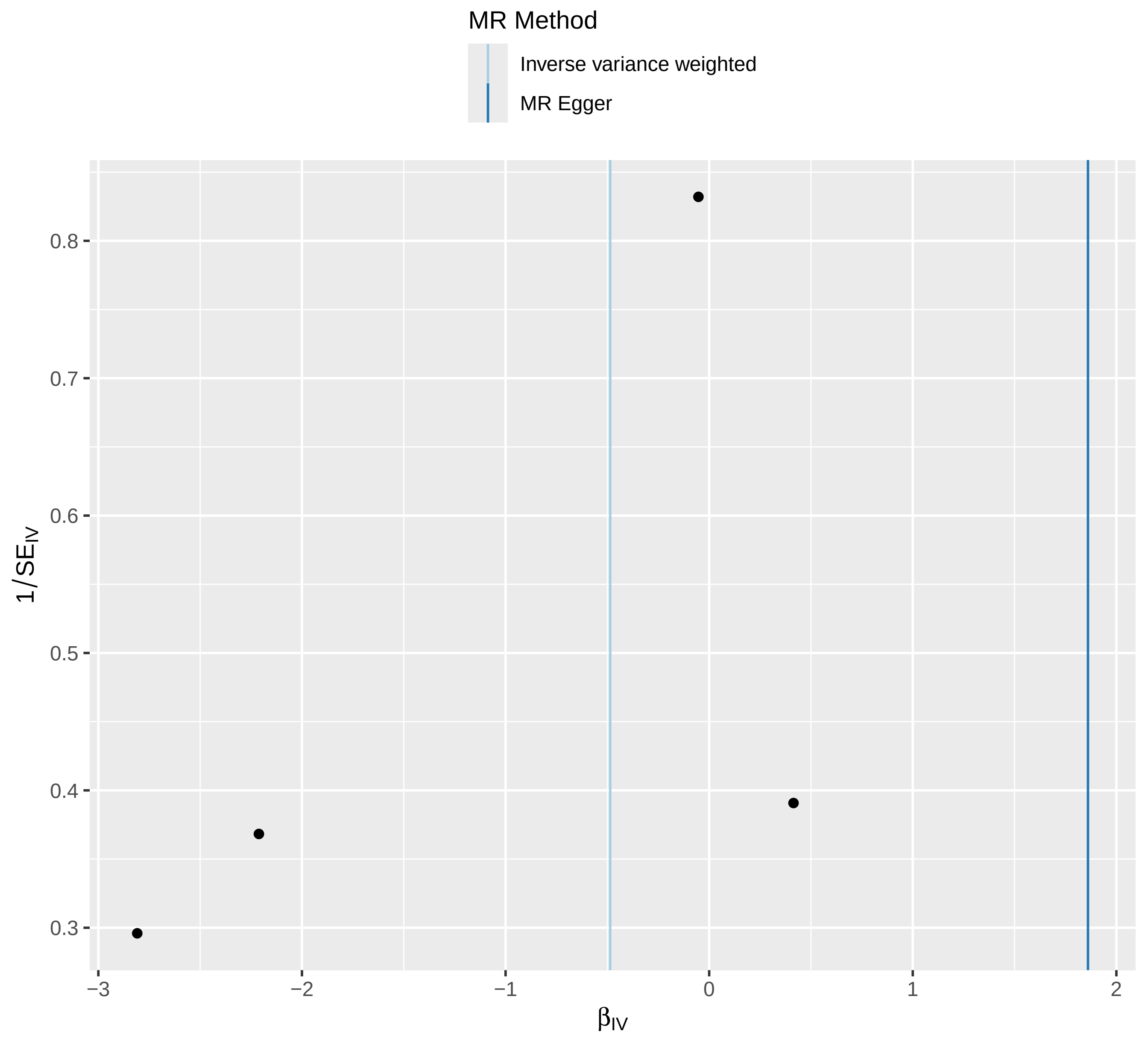
MR Method
Inverse variance weighted
MR Egger
0.8
0.7
0.6
0.5
0.4
0.3
1/SE_IV
−3
−2
−1
0
1
2
β_IV

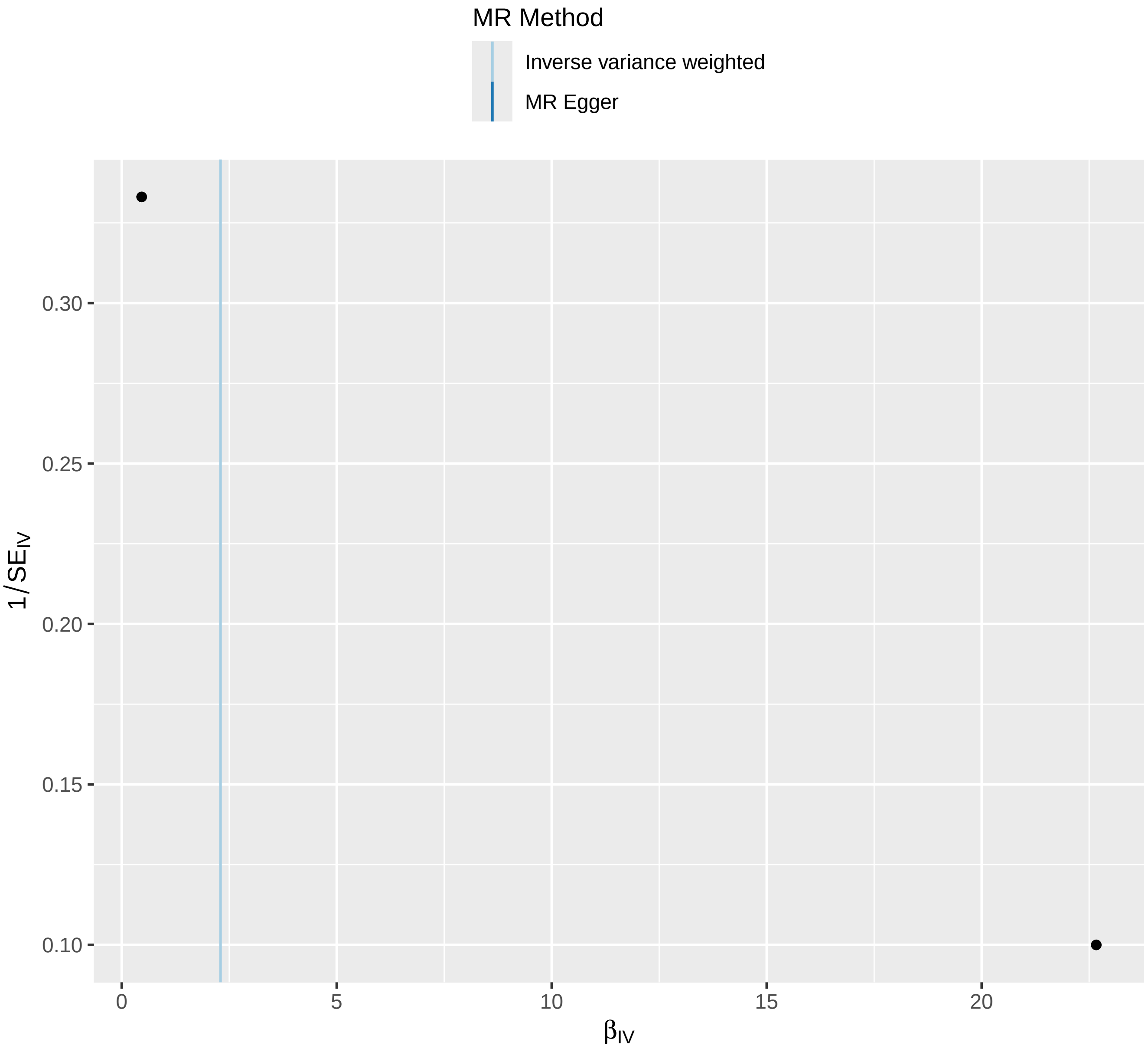
MR Method
Inverse variance weighted
MR Egger
0.30
0.25
0.20
0.15
0.10
$1/SE_{IV}$
0
5
10
15
20
β_{IV}

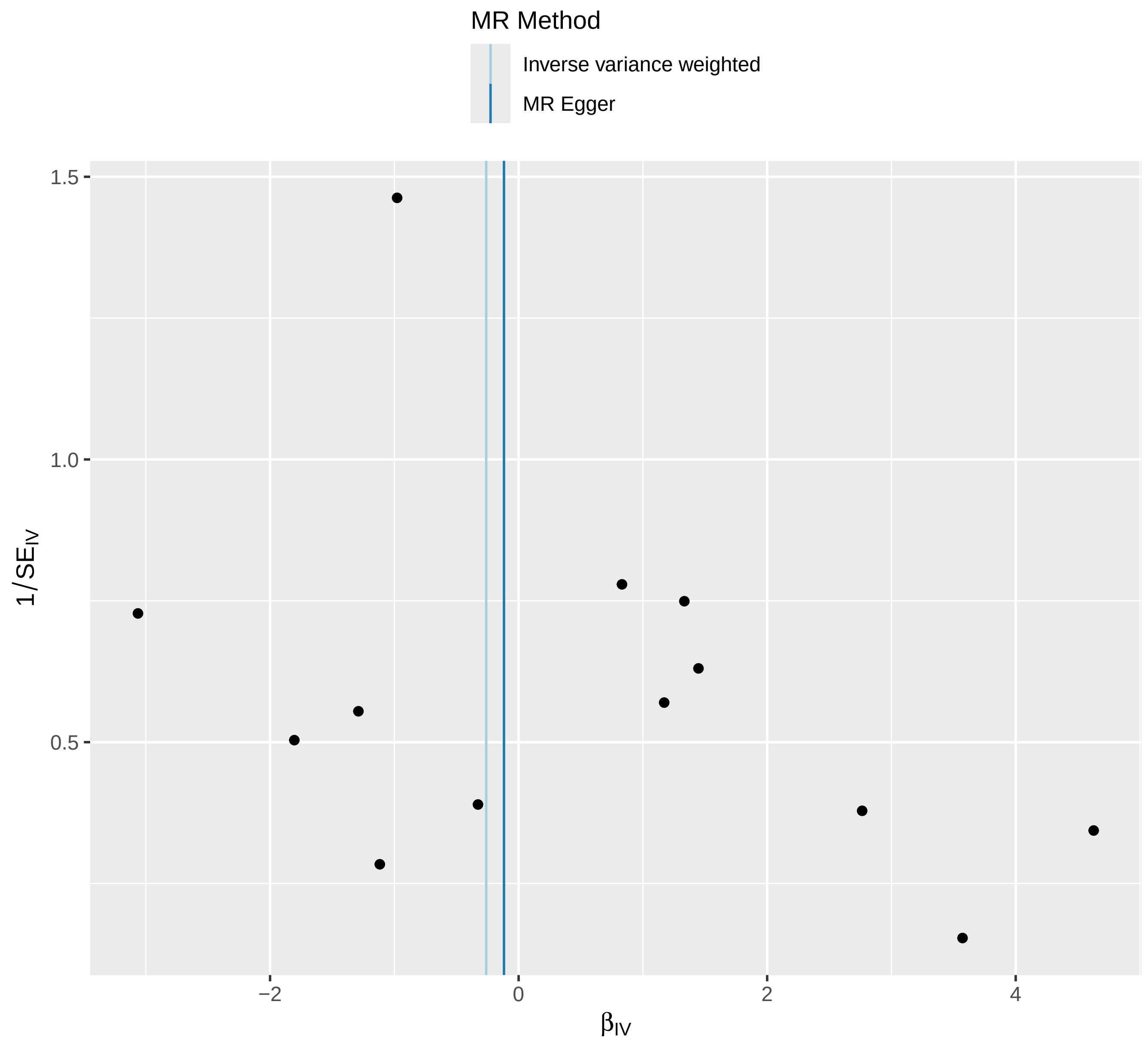
MR Method
Inverse variance weighted
MR Egger
1.5
1.0
0.5
−2
0
2
4
$1/SE_{IV}$
β_{IV}

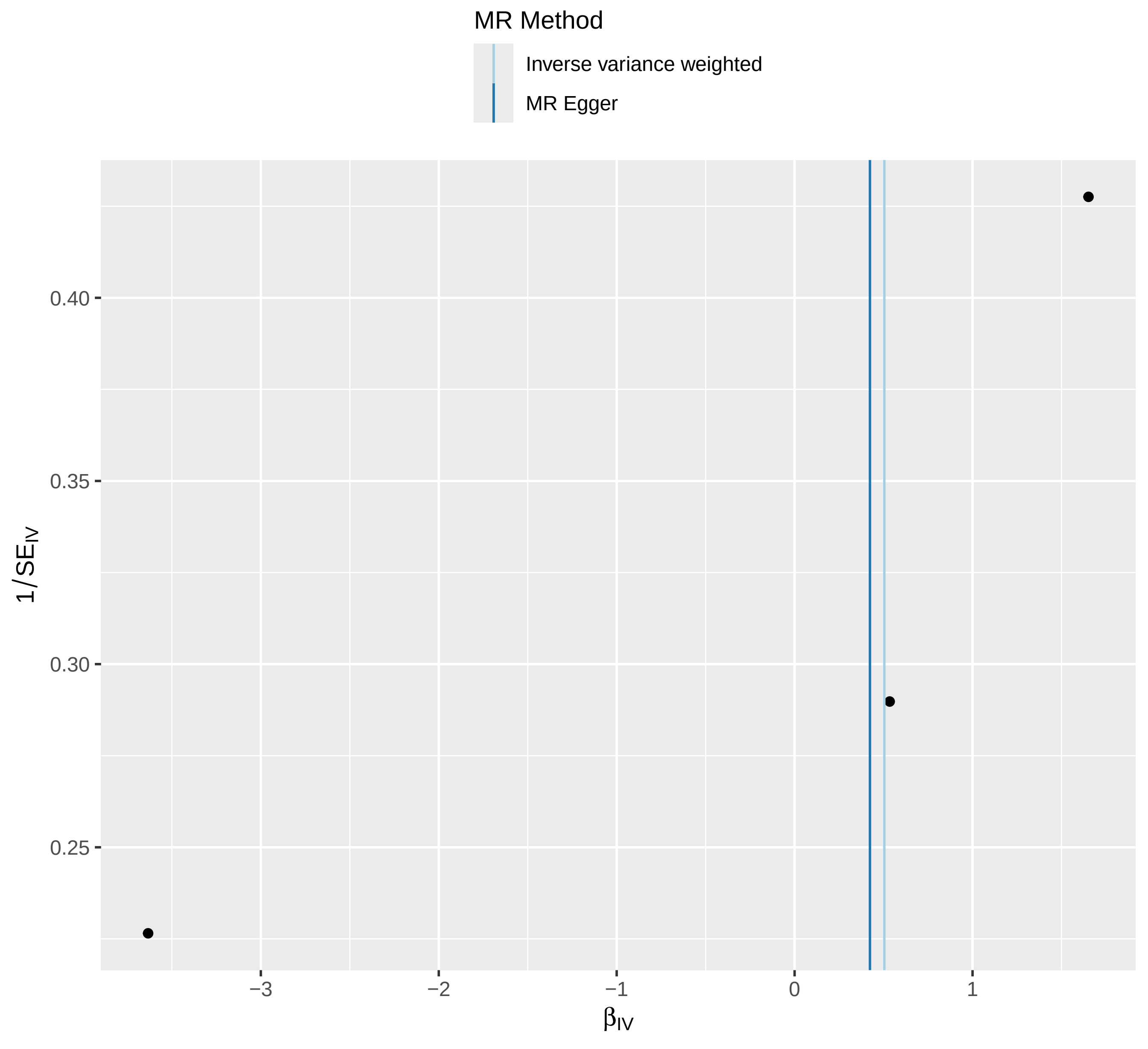
MR Method
Inverse variance weighted
MR Egger
0.40
0.35
0.30
0.25
1/SE_IV
−3
−2
−1
0
1
β_IV

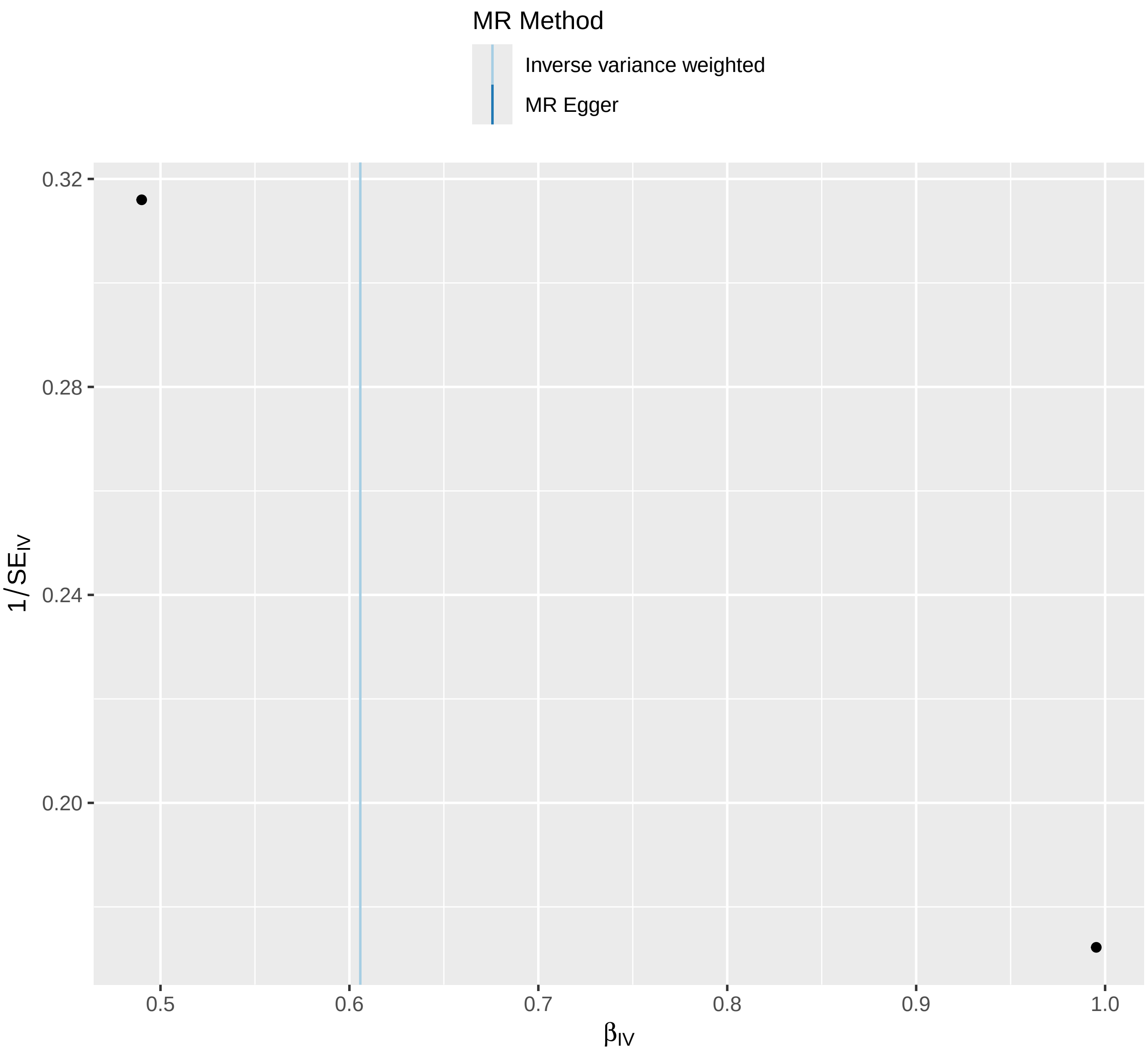
MR Method
Inverse variance weighted
MR Egger
0.32
0.28
0.24
0.20
0.5
0.6
0.7
0.8
0.9
1.0
$1/SE_{IV}$
β_{IV}

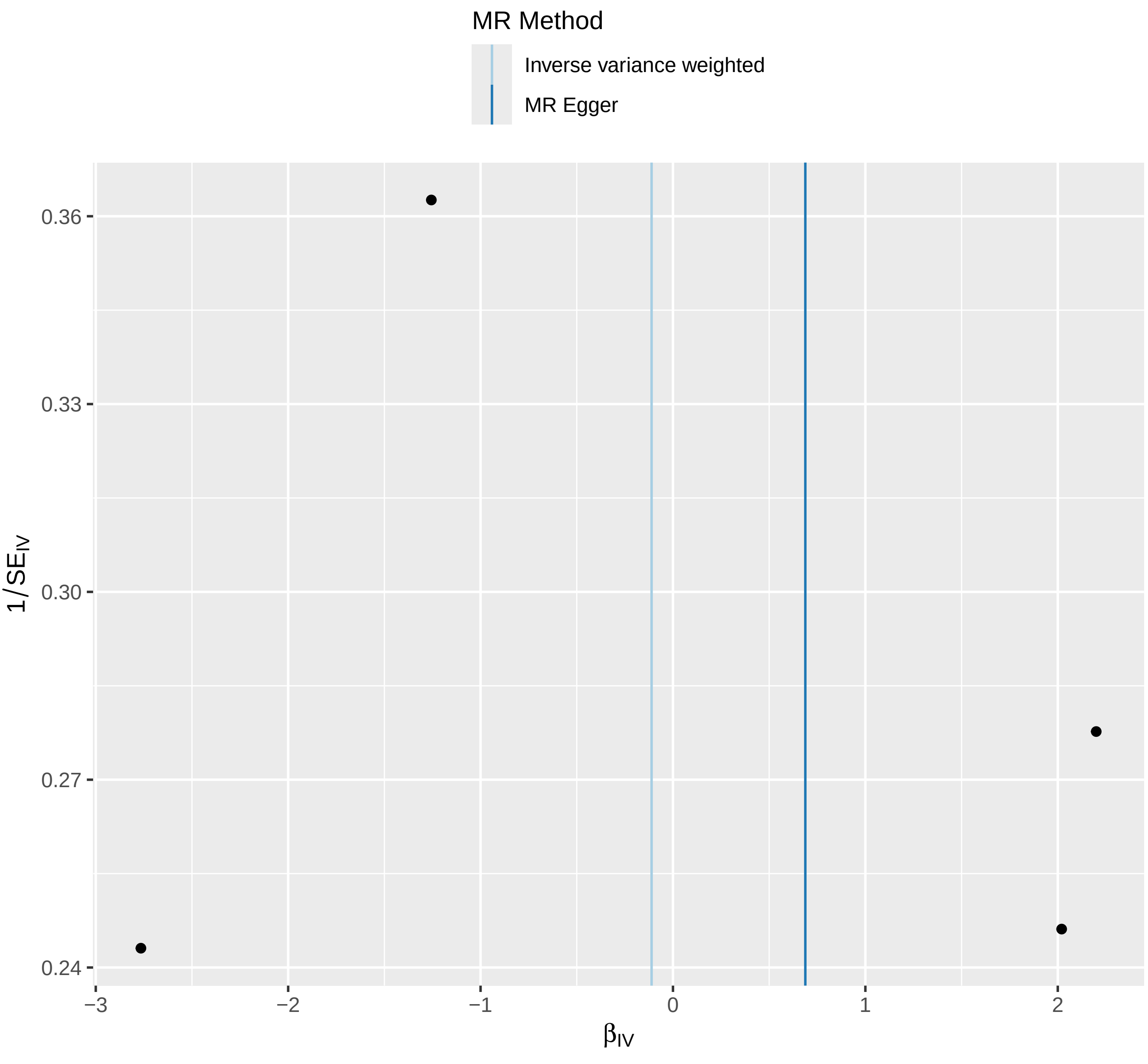
MR Method
Inverse variance weighted
MR Egger
0.36
0.33
0.30
0.27
0.24
1/SE_IV
−3
−2
−1
0
1
2
β_IV

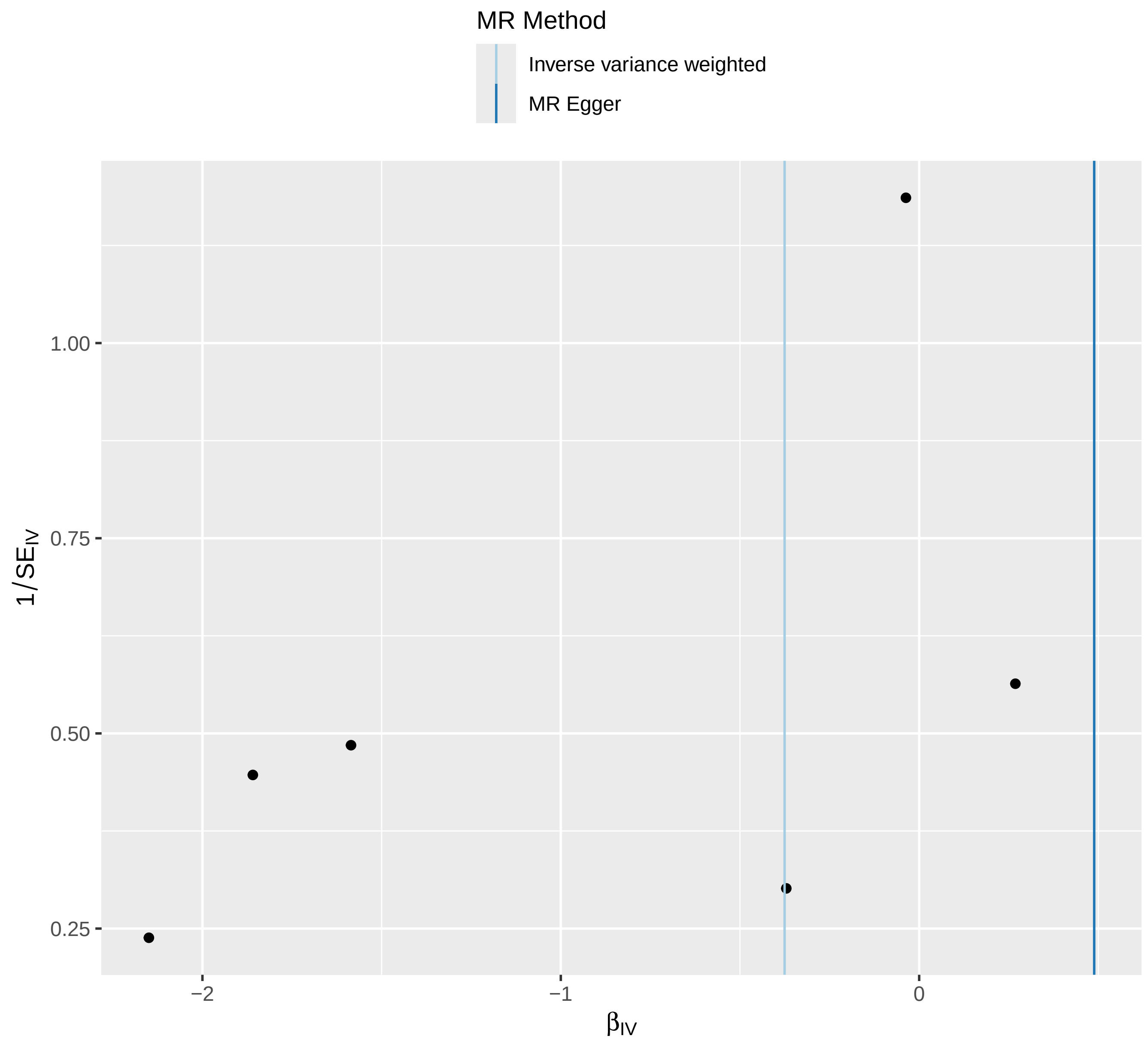
MR Method
Inverse variance weighted
MR Egger
1.00
0.75
0.50
0.25
1/SE_IV
−2
−1
0
β_IV

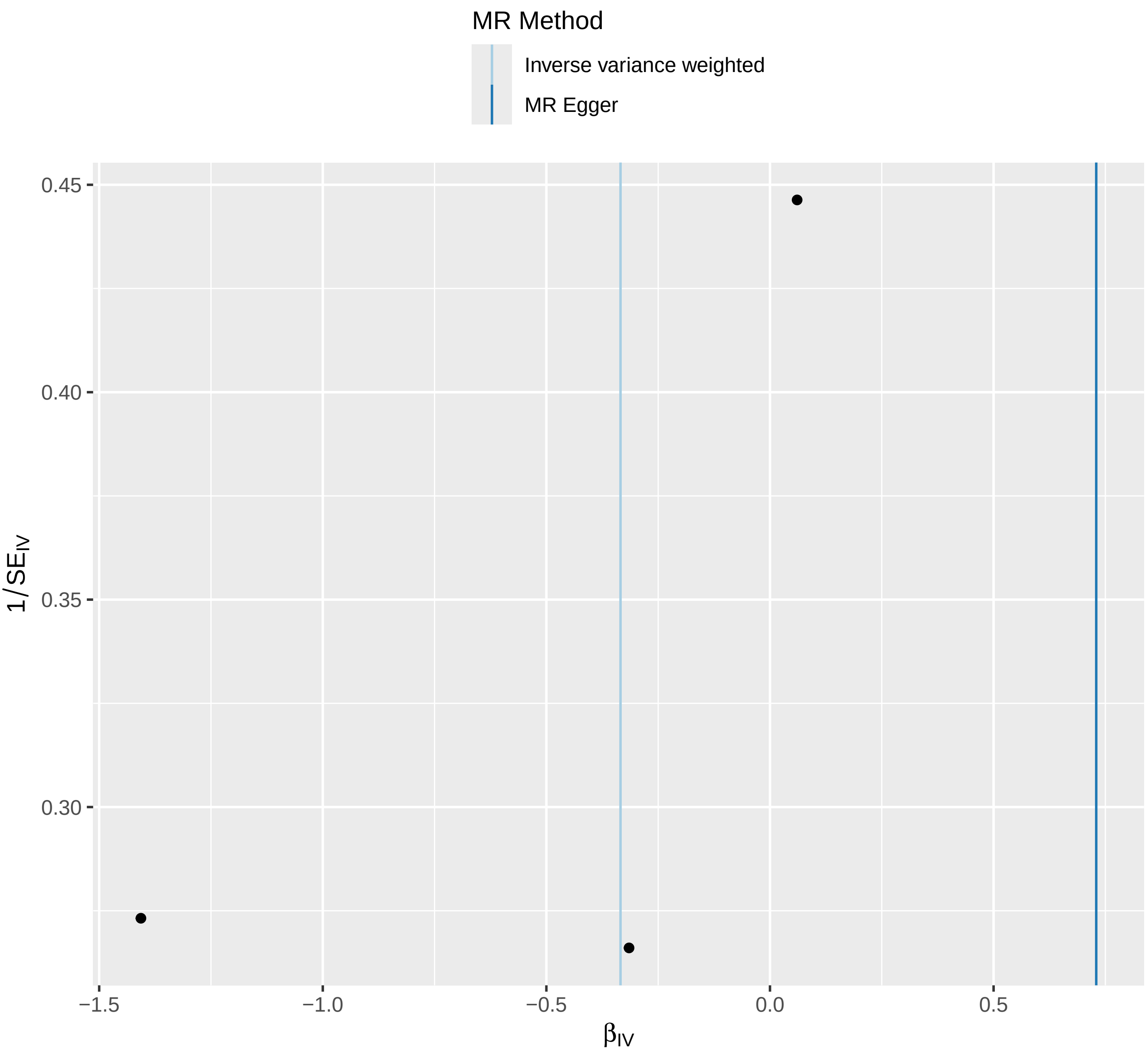
MR Method
Inverse variance weighted
MR Egger
0.45
0.40
0.35
0.30
1/SE_IV
−1.5
−1.0
−0.5
0.0
0.5
β_IV

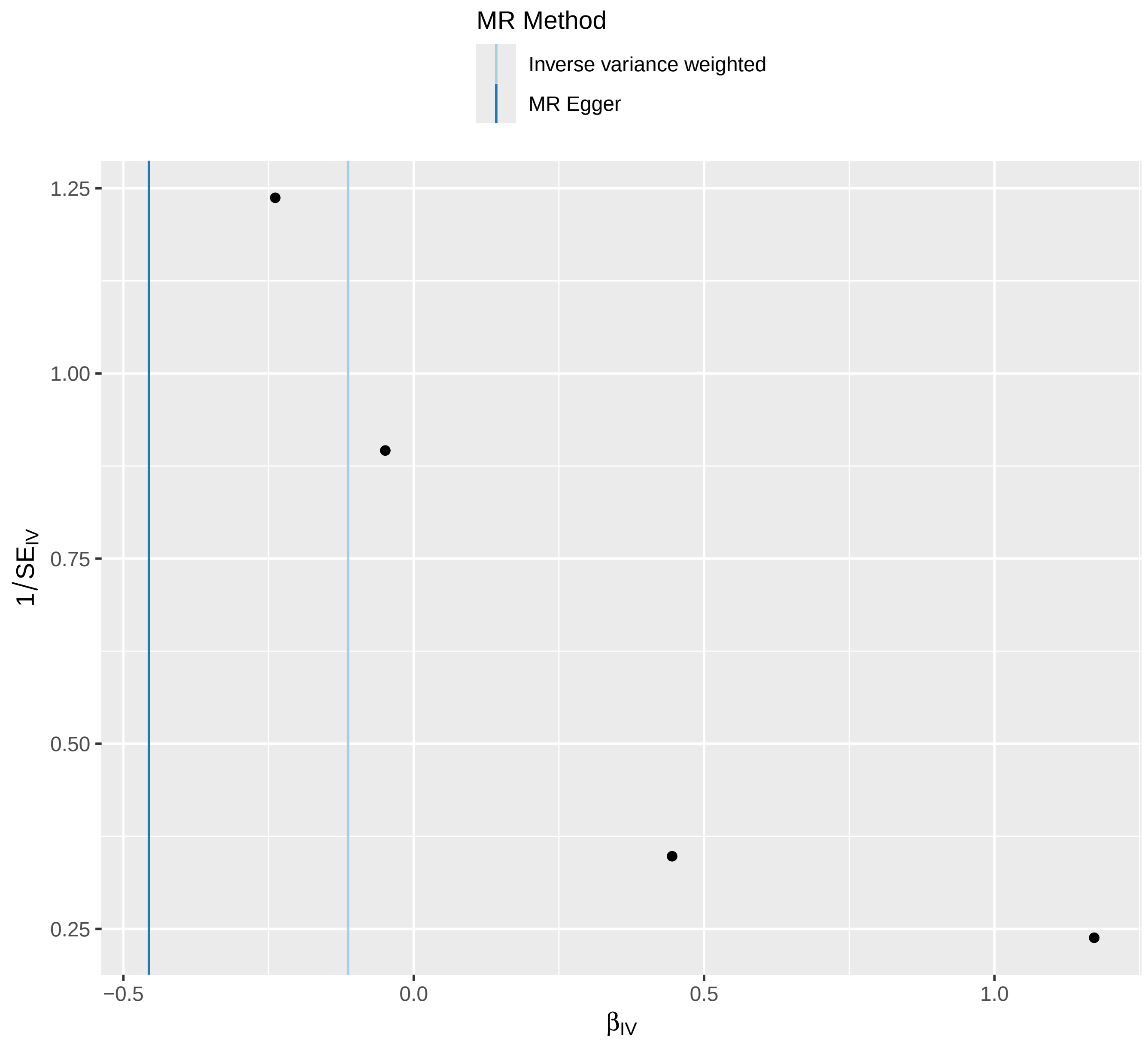
MR Method
Inverse variance weighted
MR Egger
1.25
1.00
0.75
0.50
0.25
1/SE_IV
−0.5
0.0
0.5
1.0
β_IV

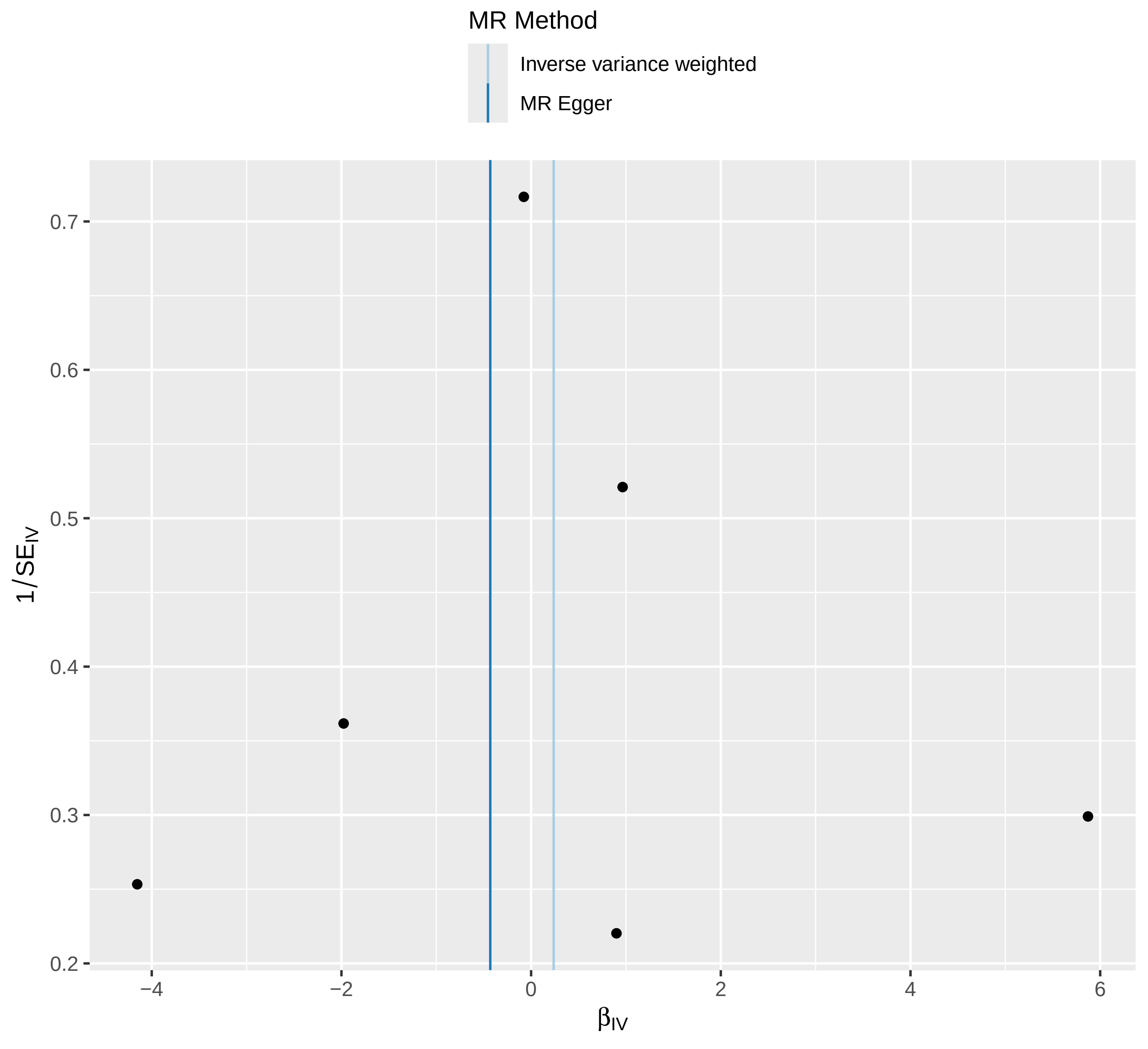
MR Method
Inverse variance weighted
MR Egger
0.7
0.6
0.5
0.4
0.3
0.2
1/SE$_{IV}$
−4
−2
0
2
4
6
β_{IV}

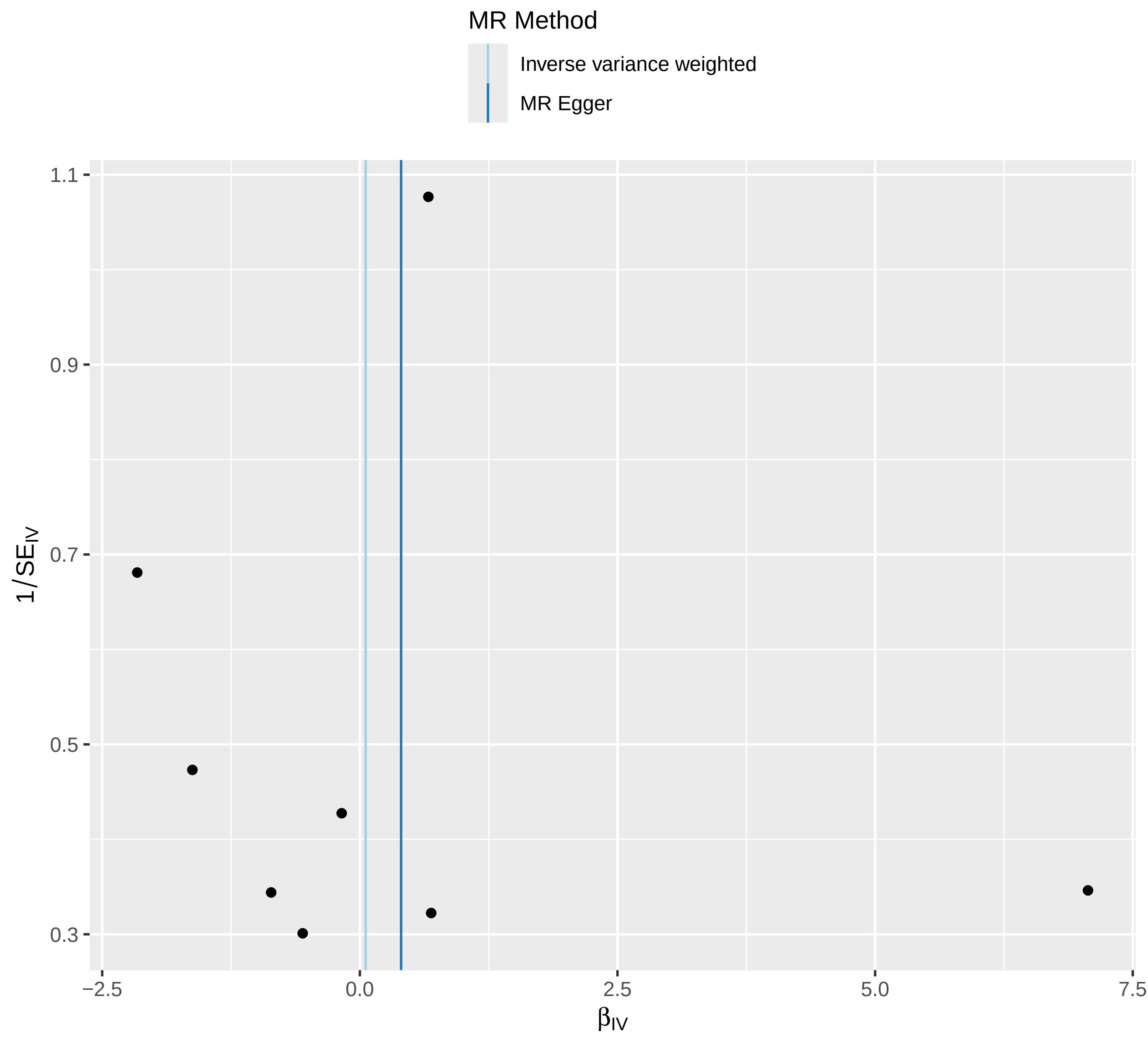

MR Method
Inverse variance weighted
MR Egger
1/SE_IV
1.1
0.9
0.7
0.5
0.3
−2.5
0.0
2.5
5.0
7.5
β_IV

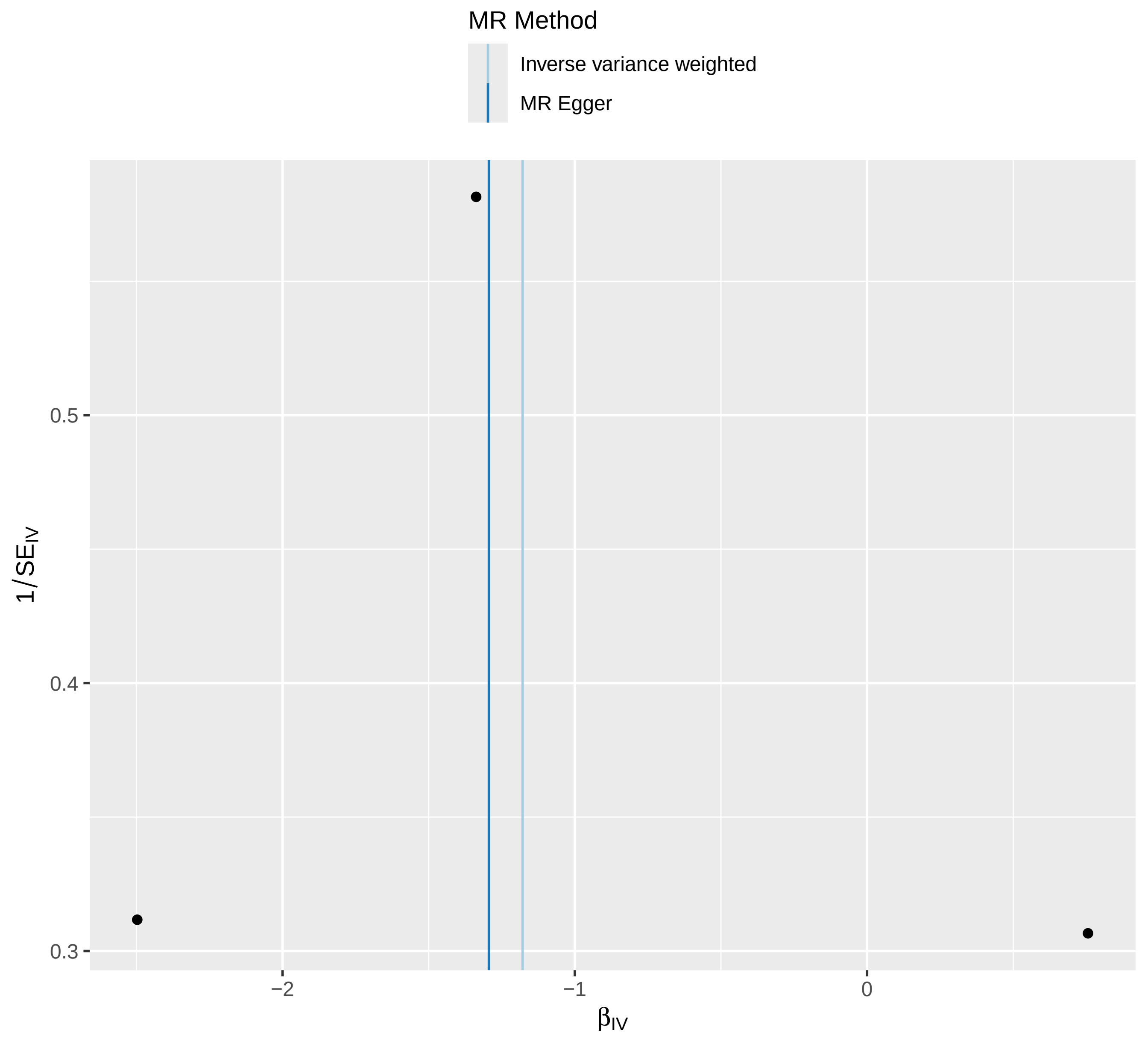
MR Method
Inverse variance weighted
MR Egger
0.5
0.4
0.3
−2
−1
0
1/SE_IV
β_IV

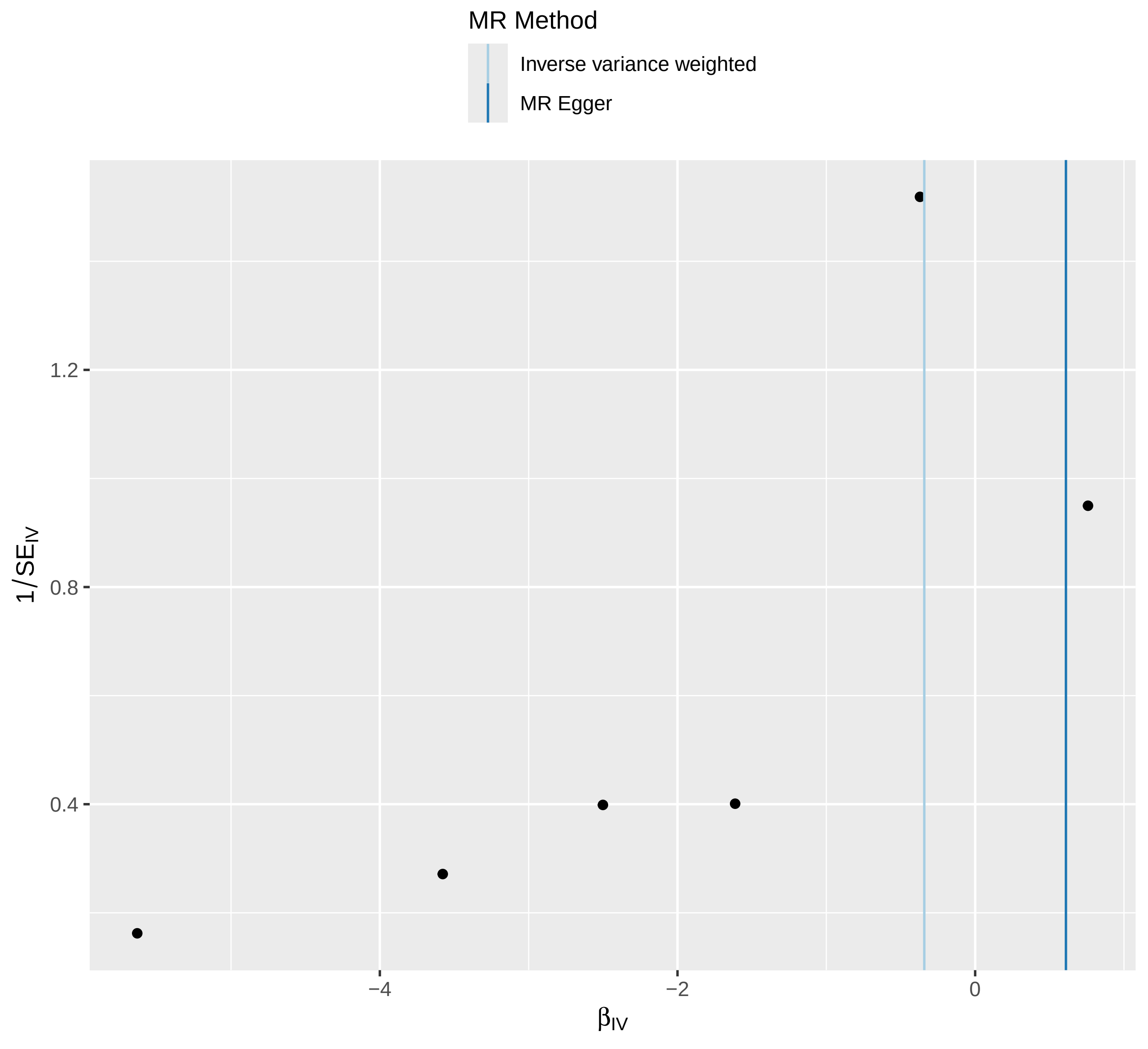
MR Method
Inverse variance weighted
MR Egger
1.2
0.8
0.4
−4
−2
0
β_{IV}
$1/SE_{IV}$

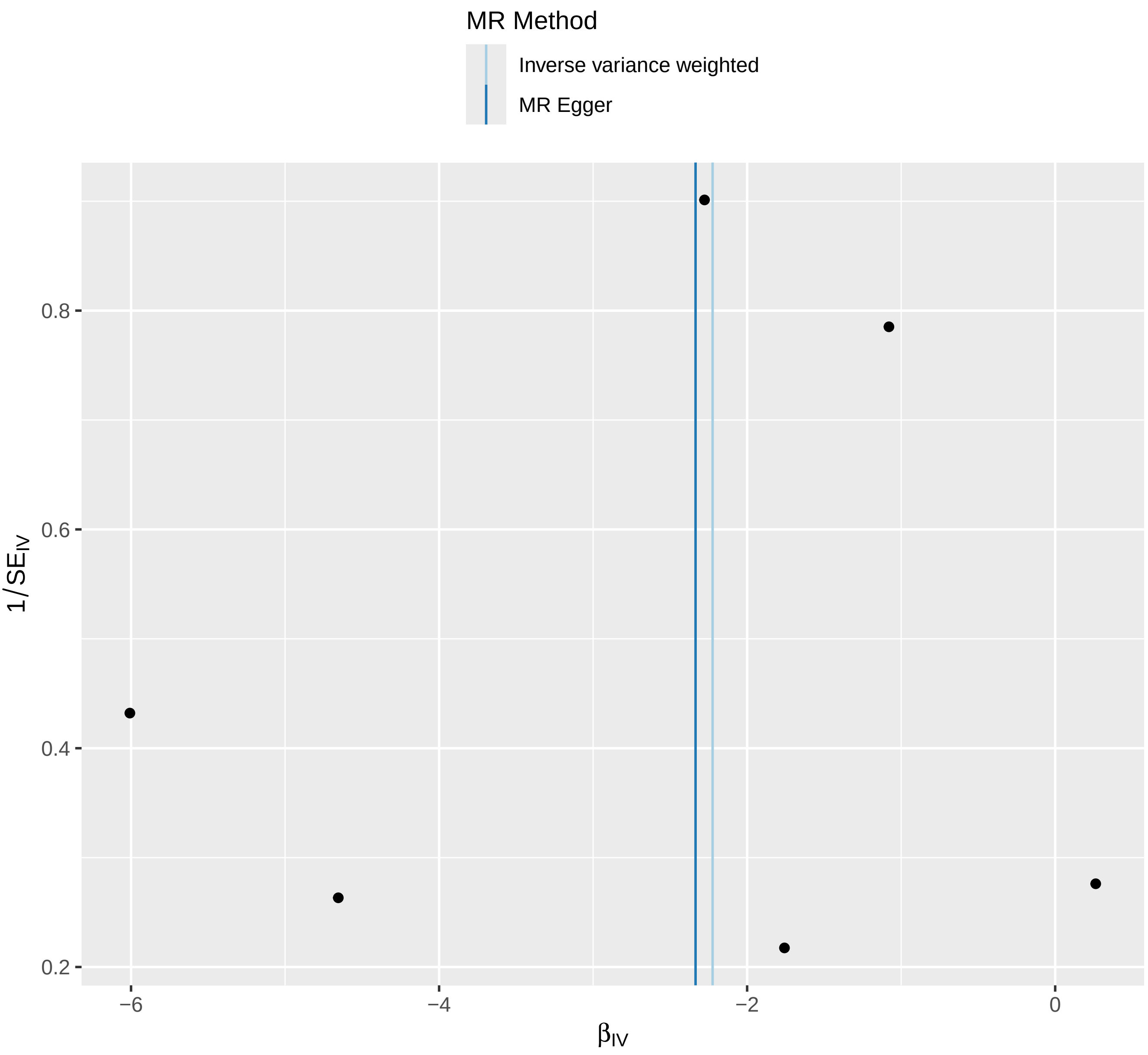
MR Method
Inverse variance weighted
MR Egger
0.8
0.6
0.4
0.2
1/SE$_{IV}$
−6
−4
−2
0
β$_{IV}$

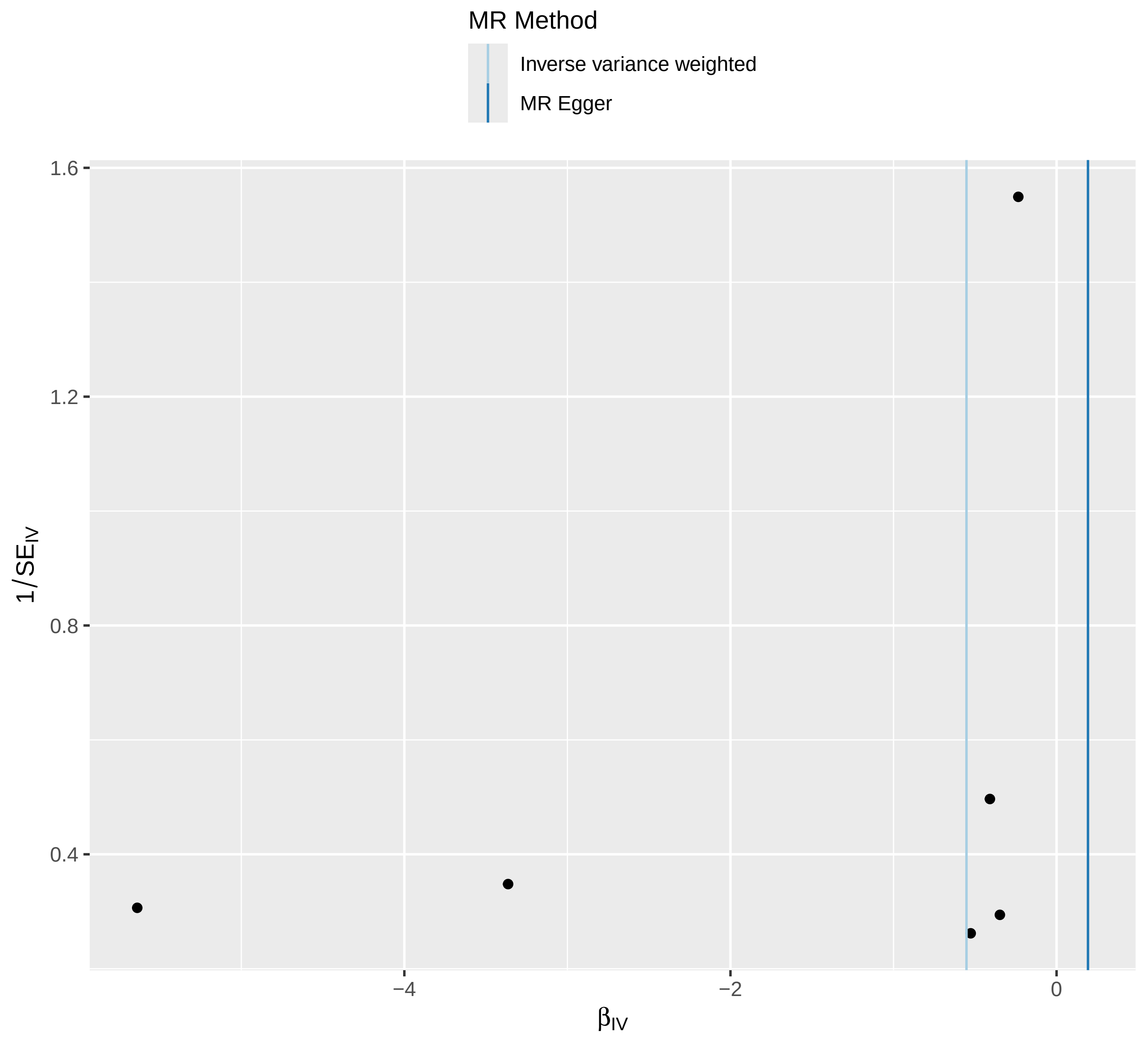

MR Method
Inverse variance weighted
MR Egger
1.6
1.2
0.8
0.4
1/SE_IV
−4
−2
0
β_IV

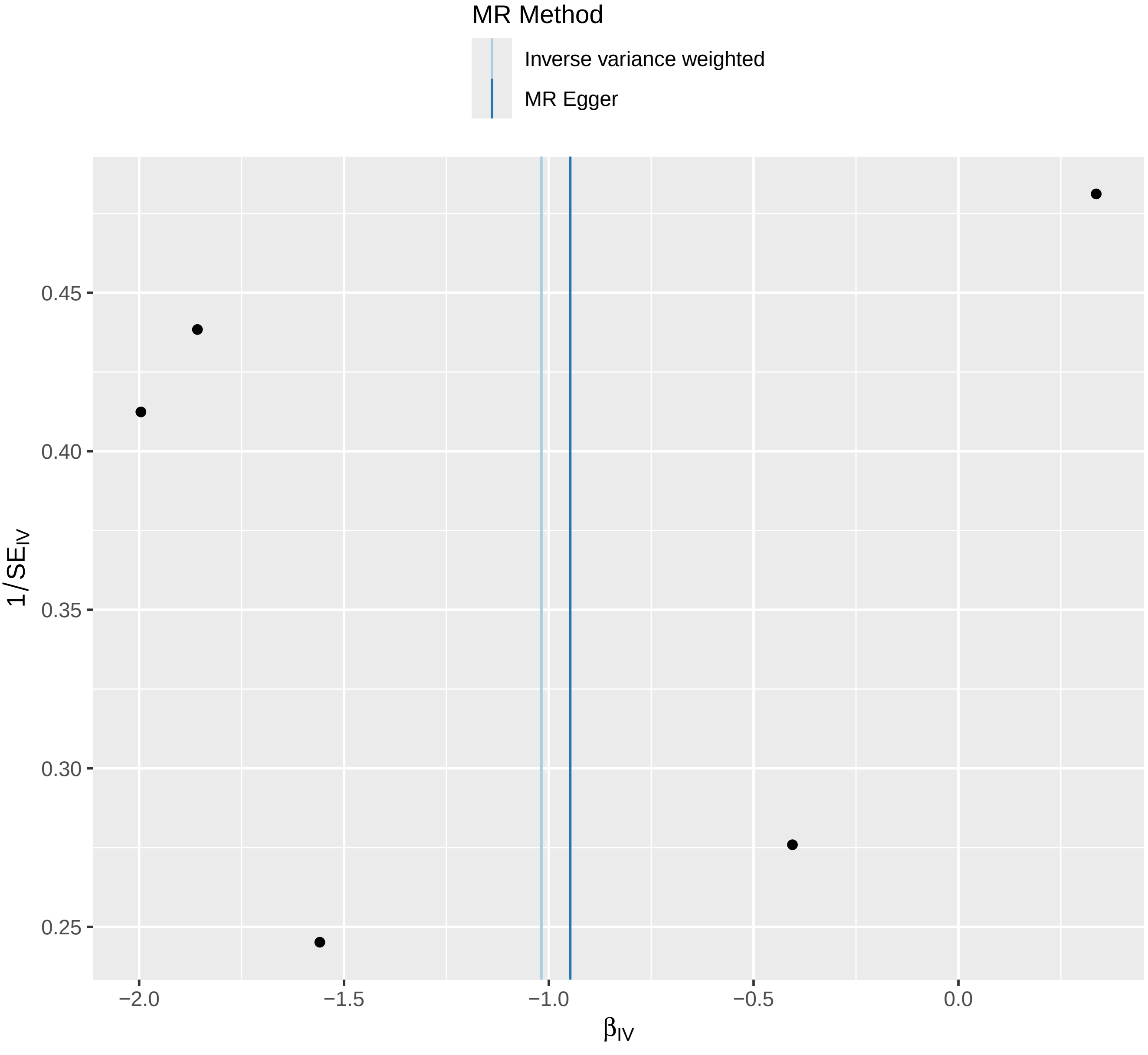
MR Method
Inverse variance weighted
MR Egger
0.45
0.40
0.35
0.30
0.25
1/SE_IV
−2.0
−1.5
−1.0
−0.5
0.0
β_IV

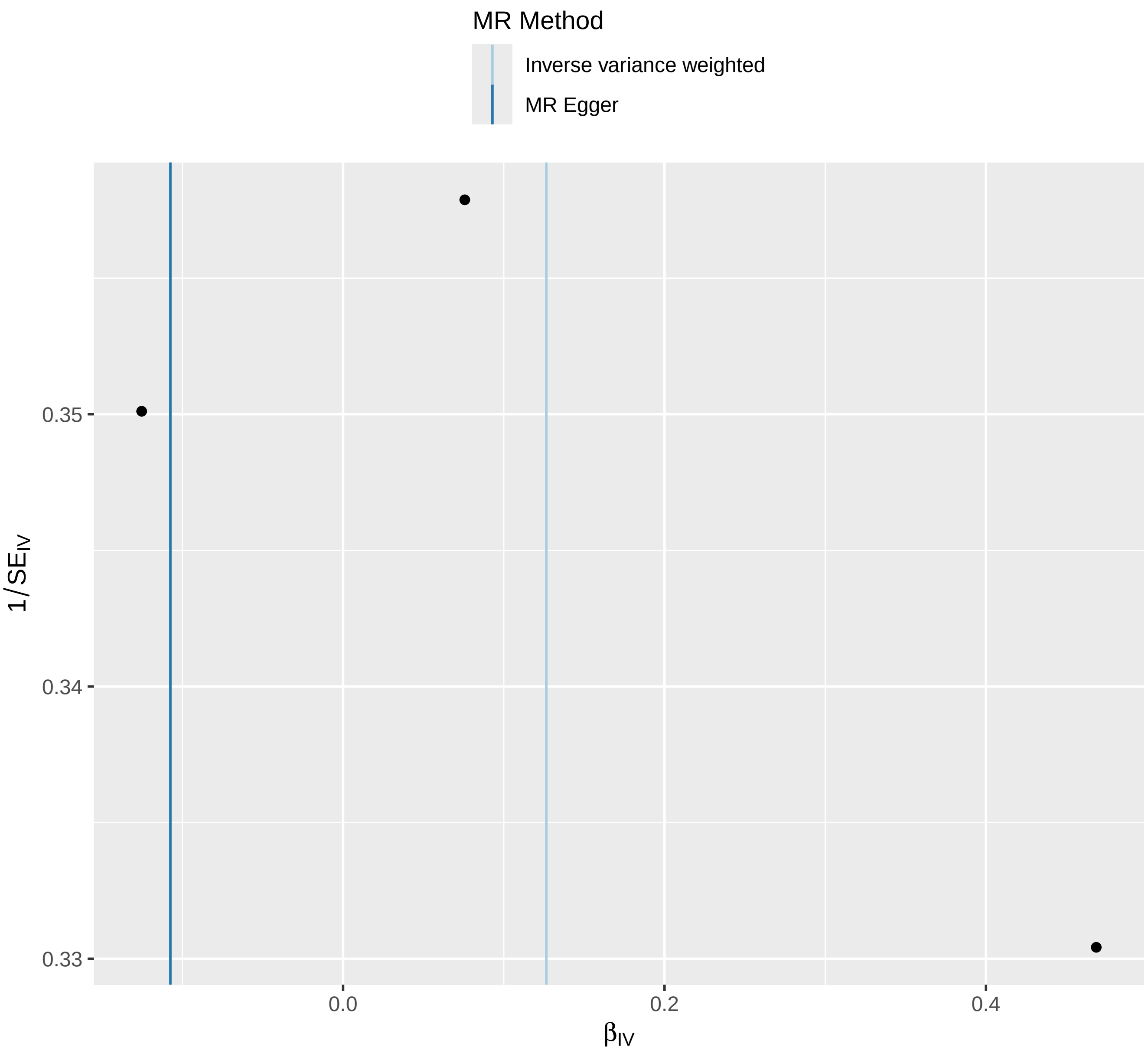
MR Method
Inverse variance weighted
MR Egger
0.35
0.34
0.33
1/SE_IV
0.0
0.2
0.4
β_IV

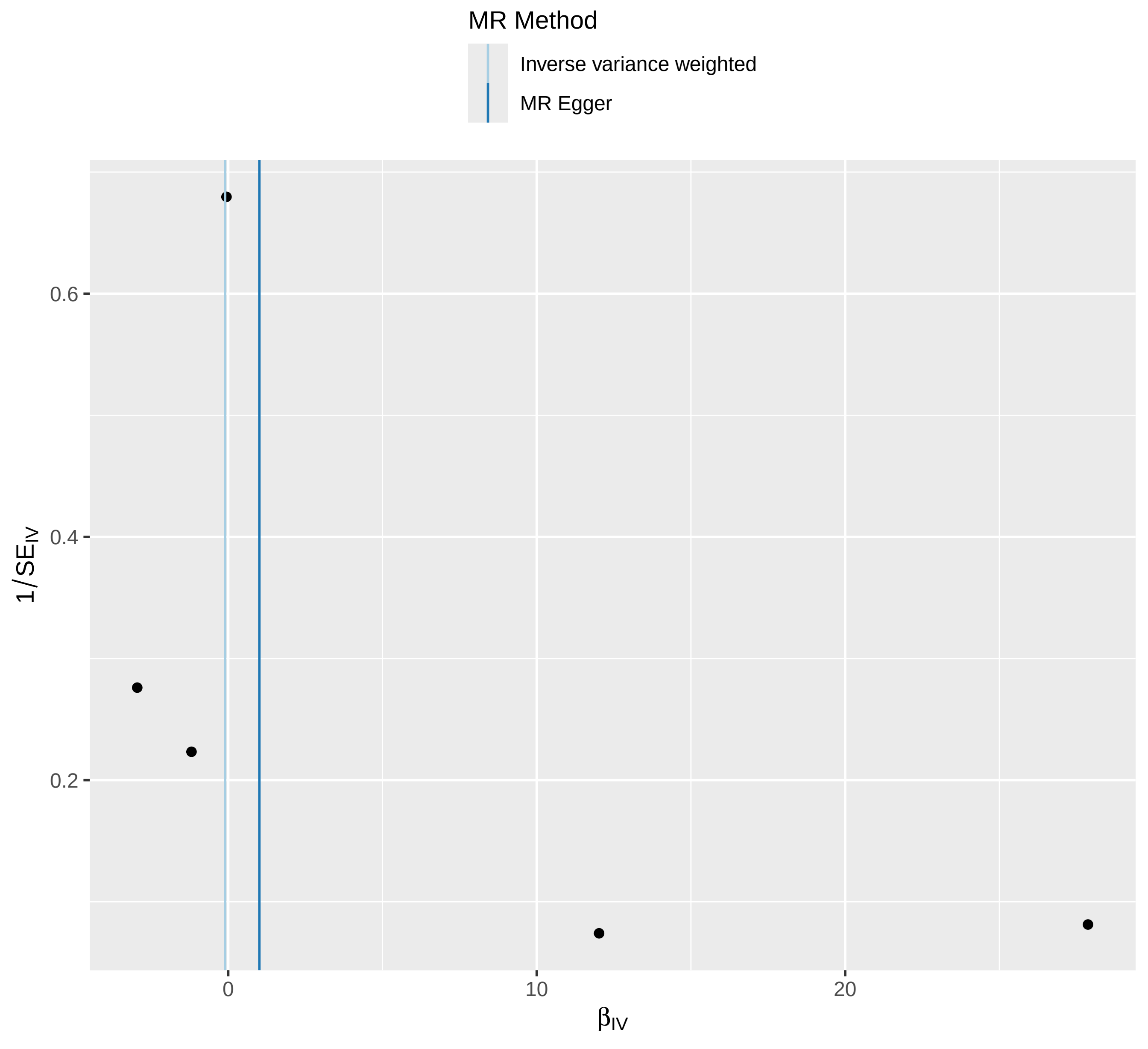

MR Method
Inverse variance weighted
MR Egger
0.6
0.4
0.2
1/SE_IV
0
10
20
β_IV

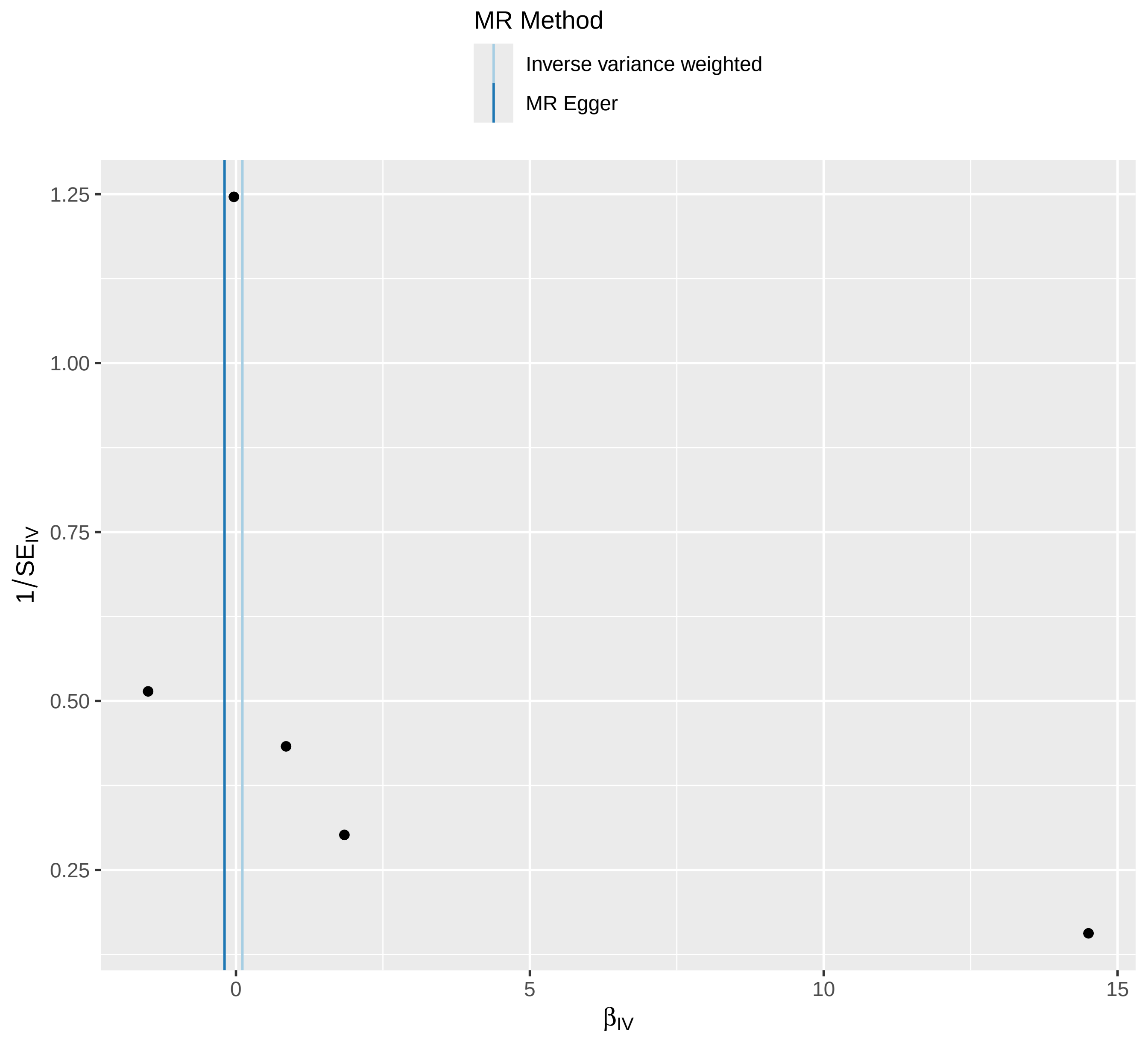
MR Method
Inverse variance weighted
MR Egger
1.25
1.00
0.75
0.50
0.25
1/SE_IV
0
5
10
15
β_IV

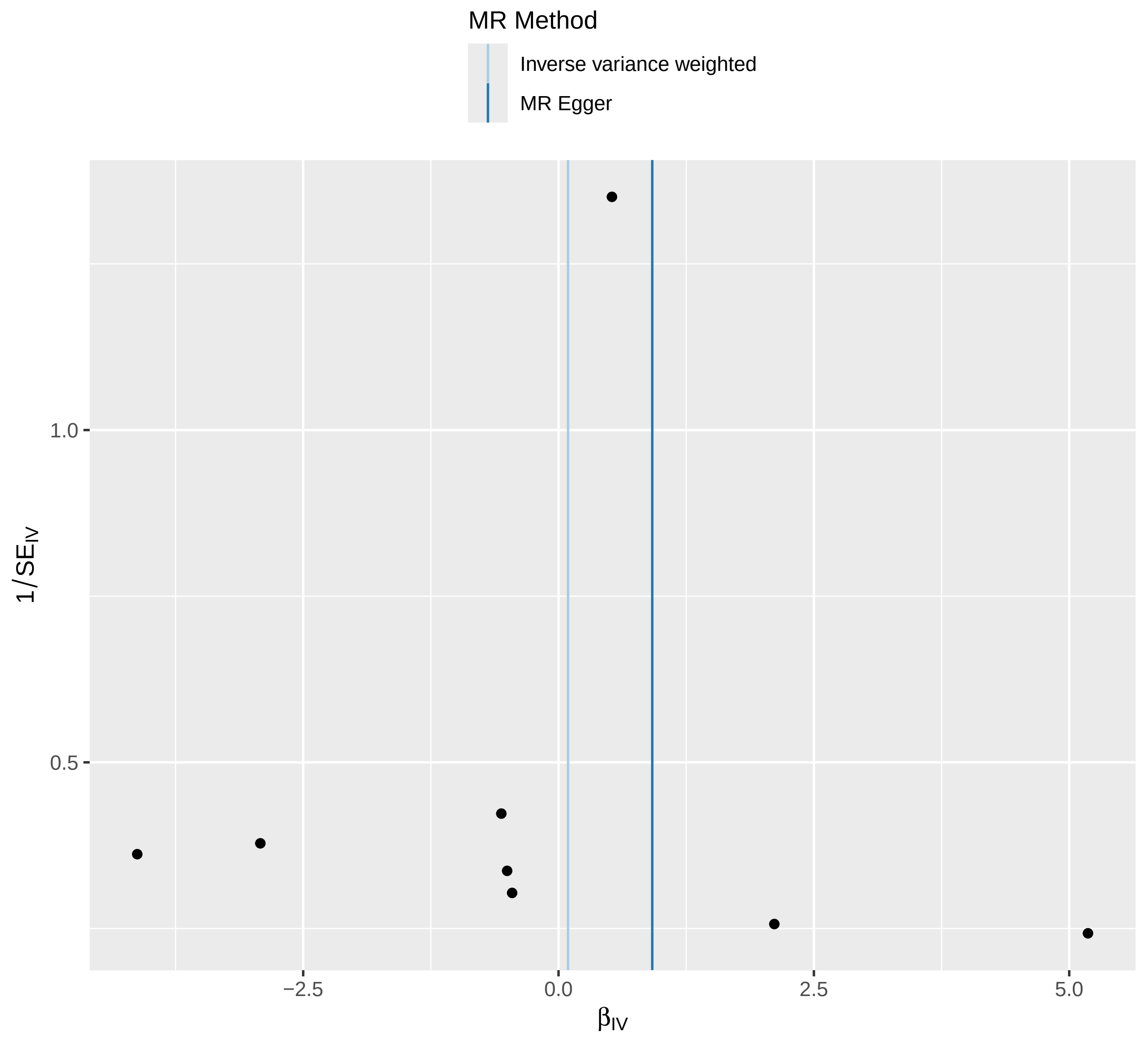
MR Method
Inverse variance weighted
MR Egger
1.0
0.5
1/SE$_{IV}$
−2.5
0.0
2.5
5.0
β$_{IV}$

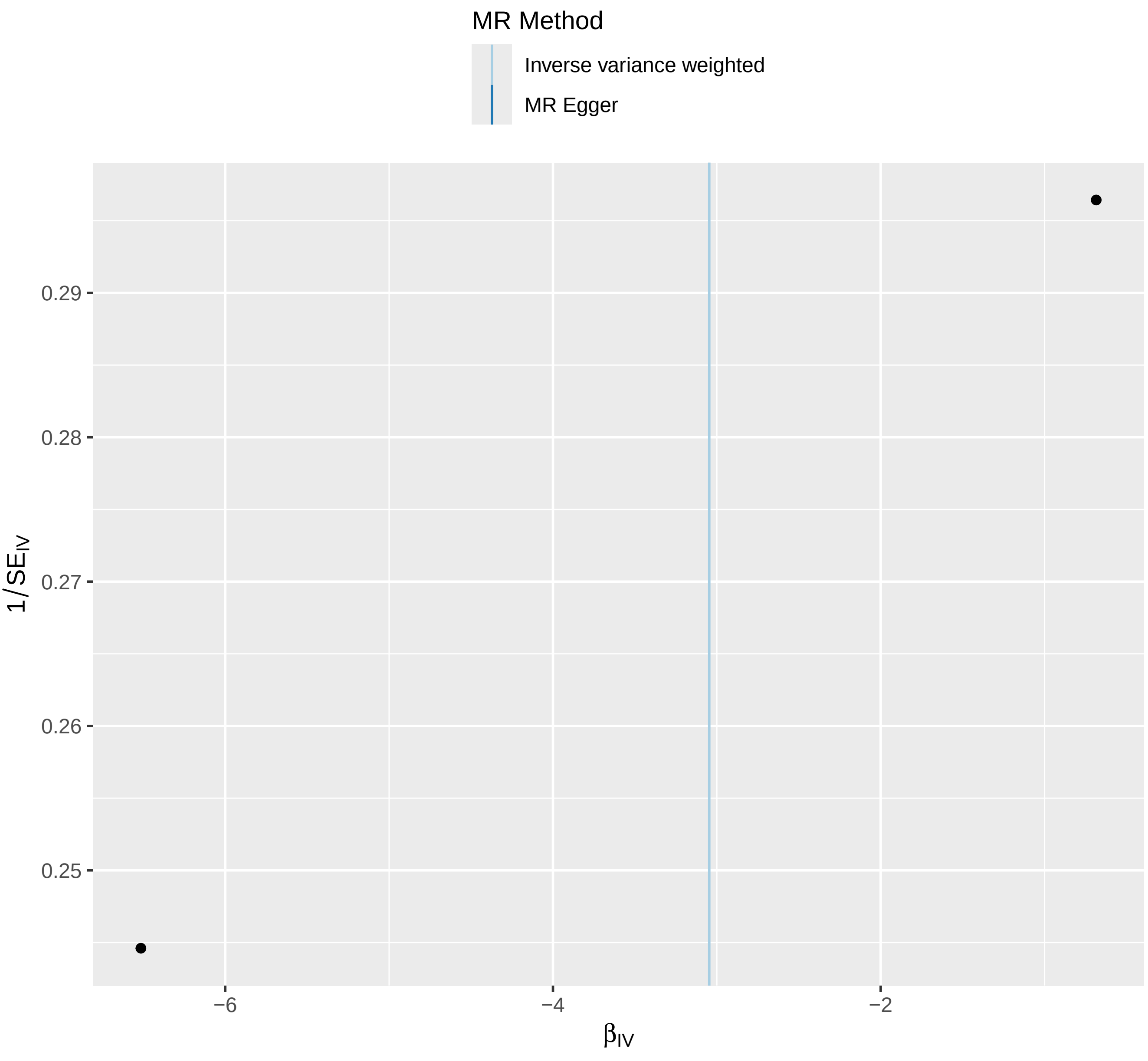
MR Method
Inverse variance weighted
MR Egger
0.29
0.28
0.27
0.26
0.25
1/SE_IV
−6
−4
−2
β_IV

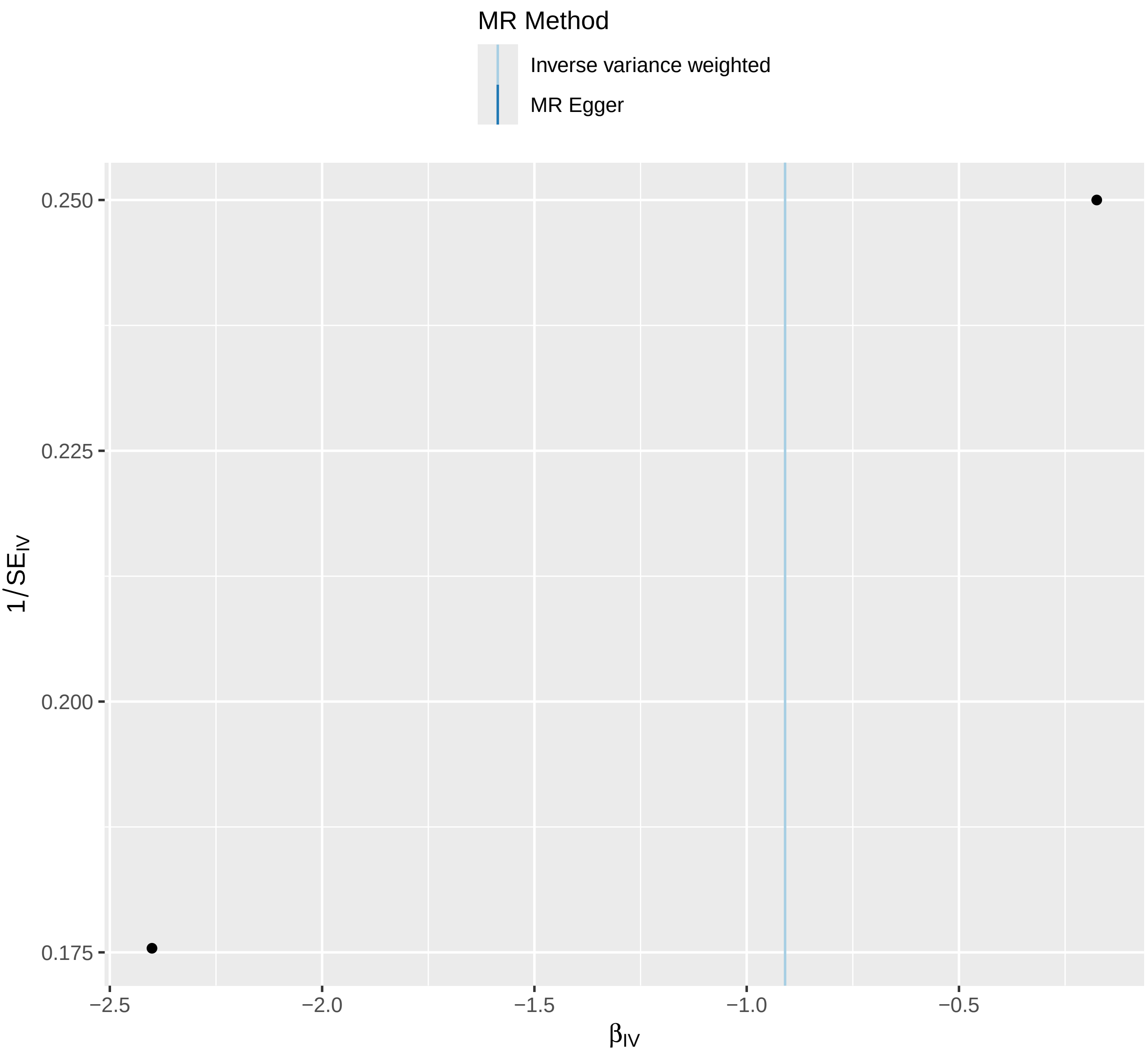
MR Method
Inverse variance weighted
MR Egger
0.250
0.225
0.200
0.175
1/SE$_{IV}$
−2.5
−2.0
−1.5
−1.0
−0.5
β$_{IV}$

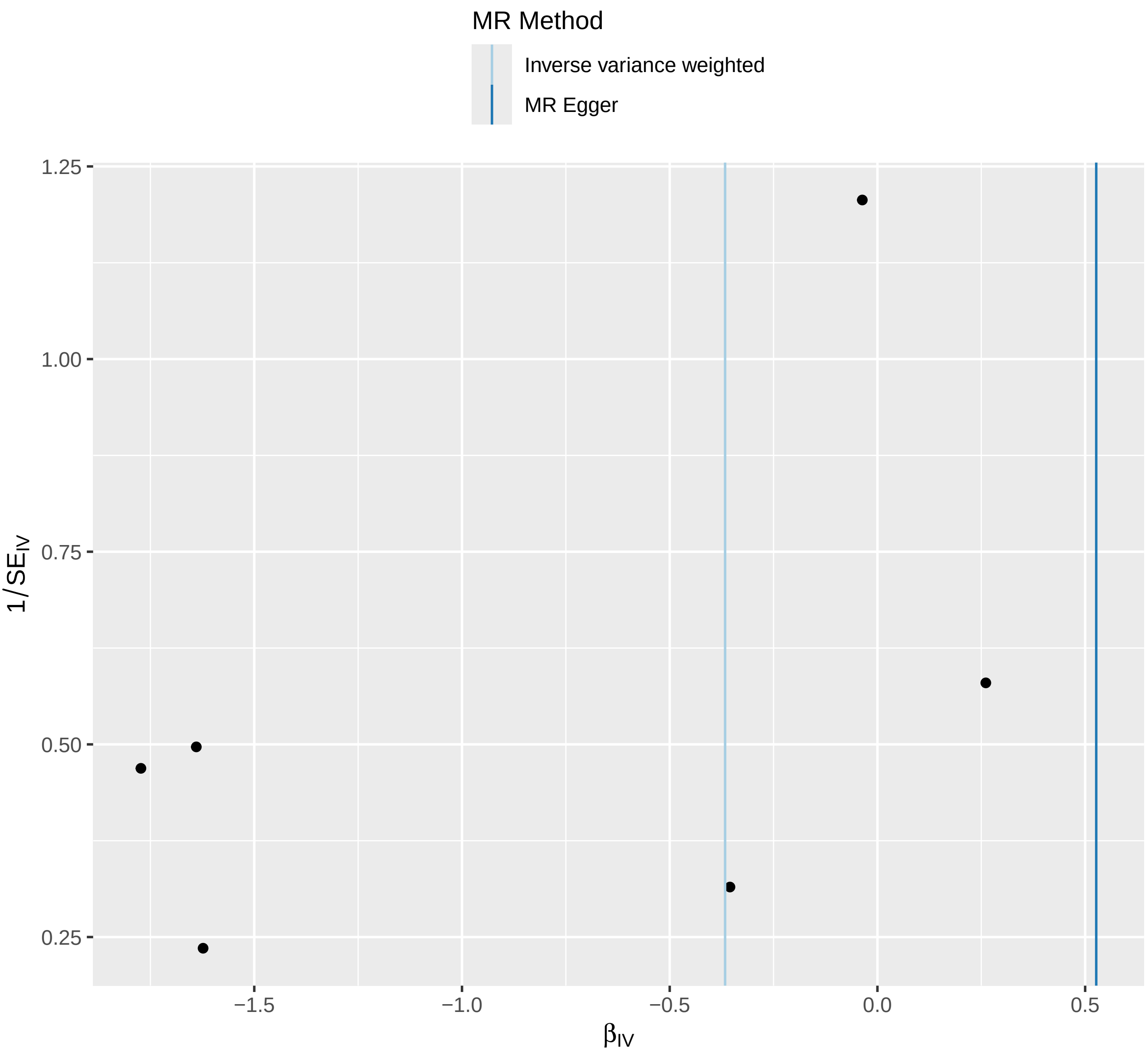
MR Method
Inverse variance weighted
MR Egger
1.25
1.00
0.75
0.50
0.25
1/SE_IV
−1.5
−1.0
−0.5
0.0
0.5
β_IV

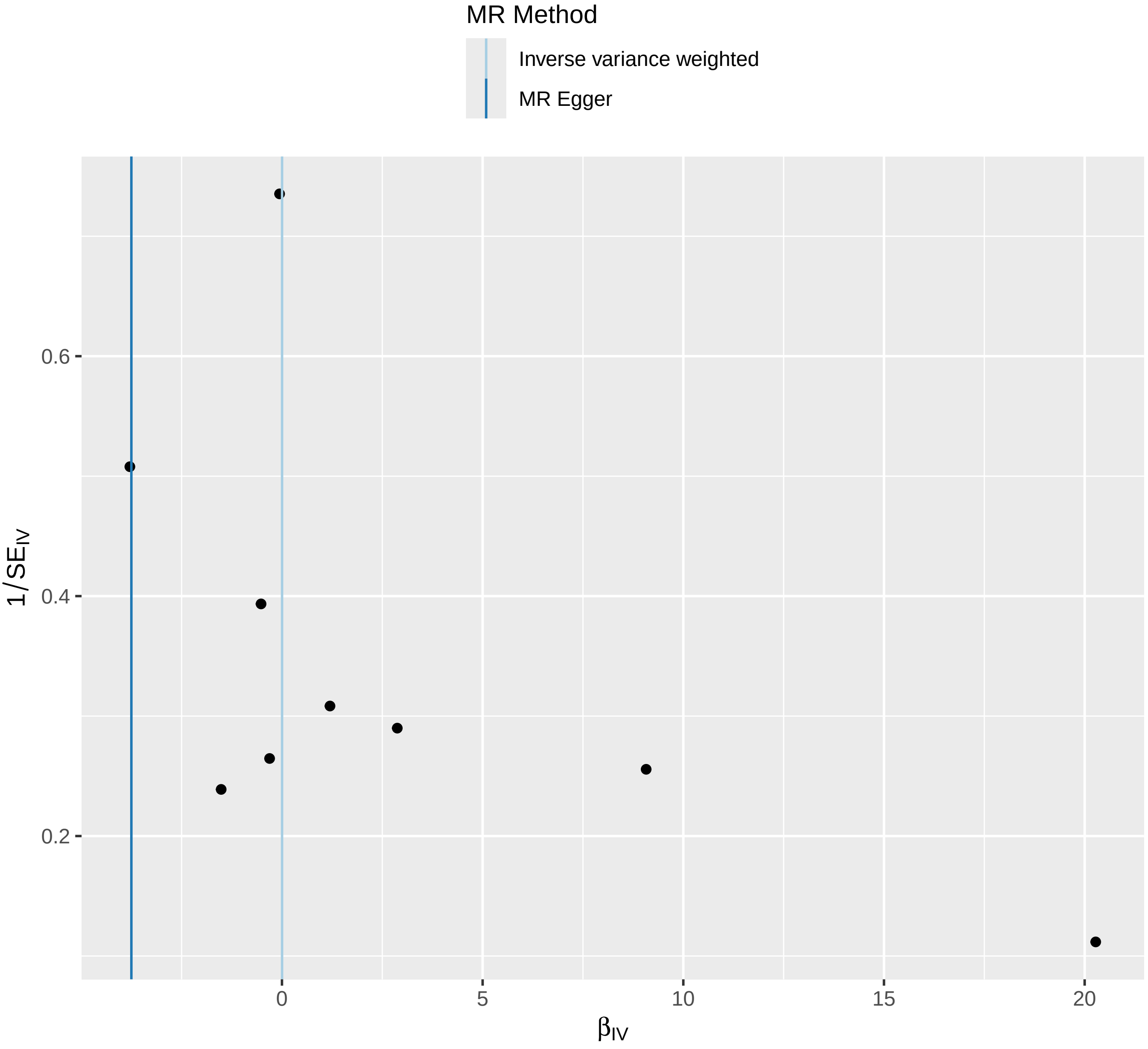
MR Method
Inverse variance weighted
MR Egger
0.6
0.4
0.2
1/SE_IV
0
5
10
15
20
β_IV

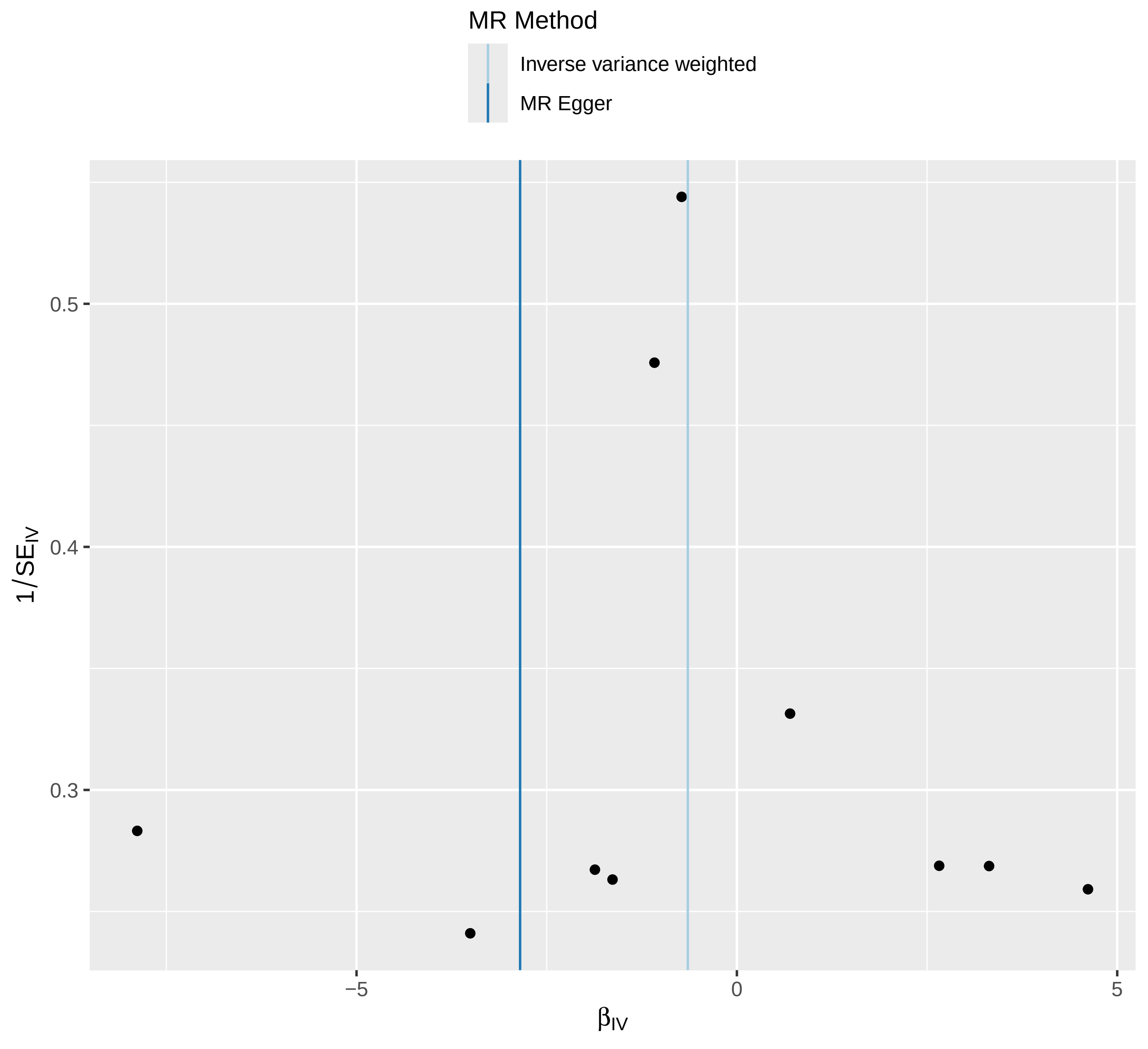
MR Method
Inverse variance weighted
MR Egger
0.5
0.4
0.3
1/SE_IV
−5
0
5
β_IV

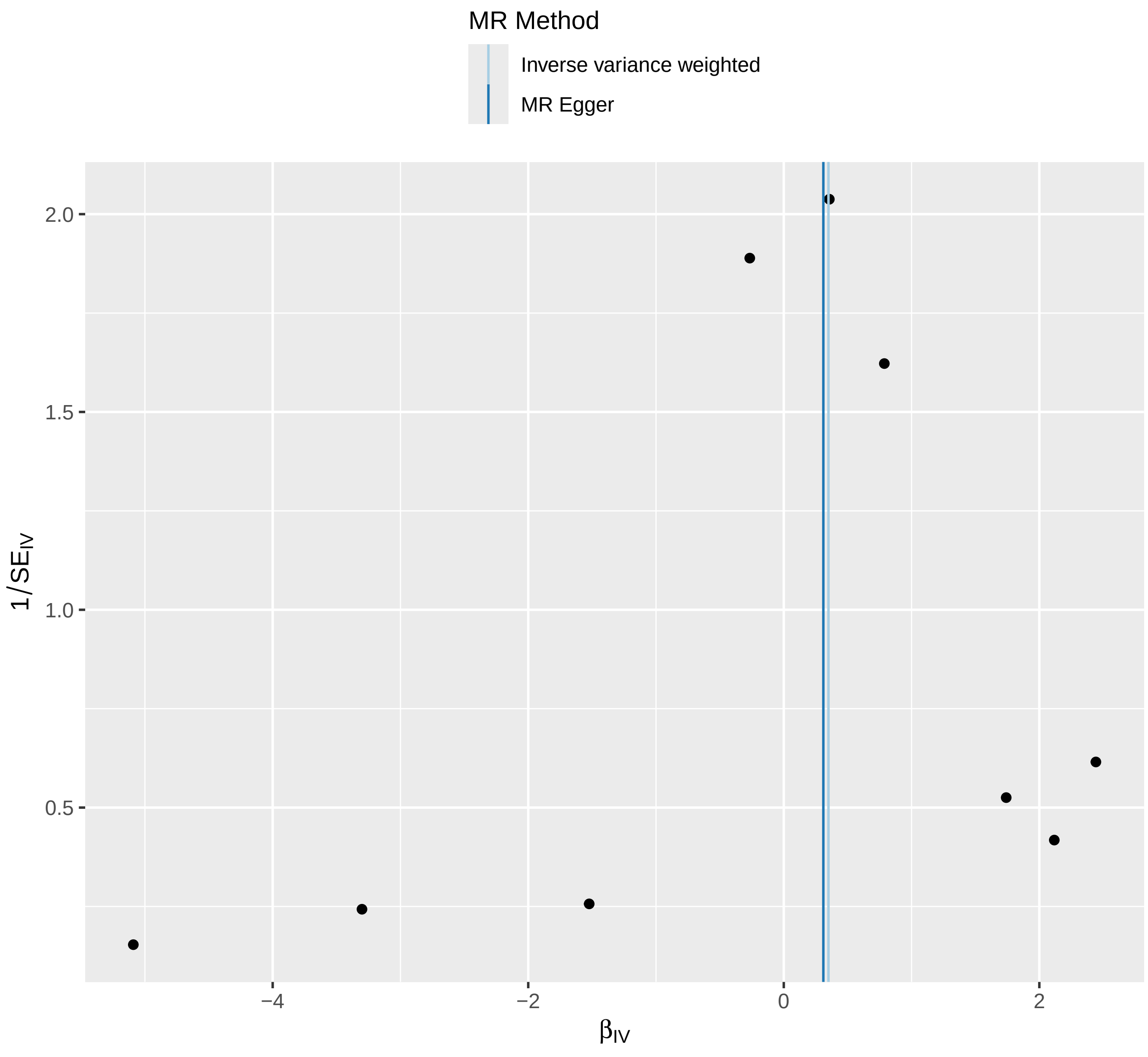

MR Method
Inverse variance weighted
MR Egger
2.0
1.5
1.0
0.5
1/SE$_{IV}$
−4
−2
0
2
β$_{IV}$

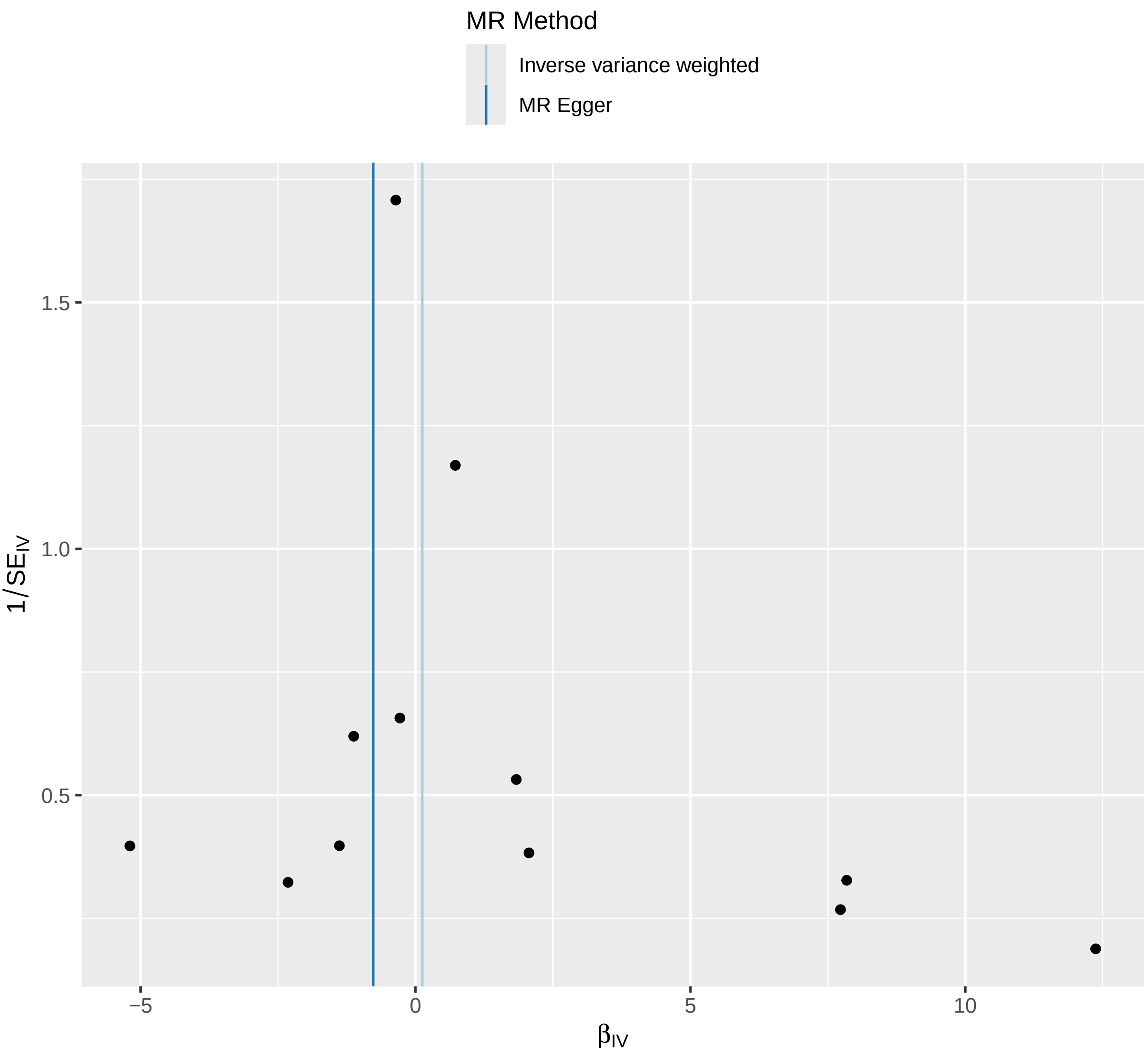

MR Method
Inverse variance weighted
MR Egger
1.5
1.0
0.5
$1/SE_{IV}$
−5
0
5
10
β_{IV}

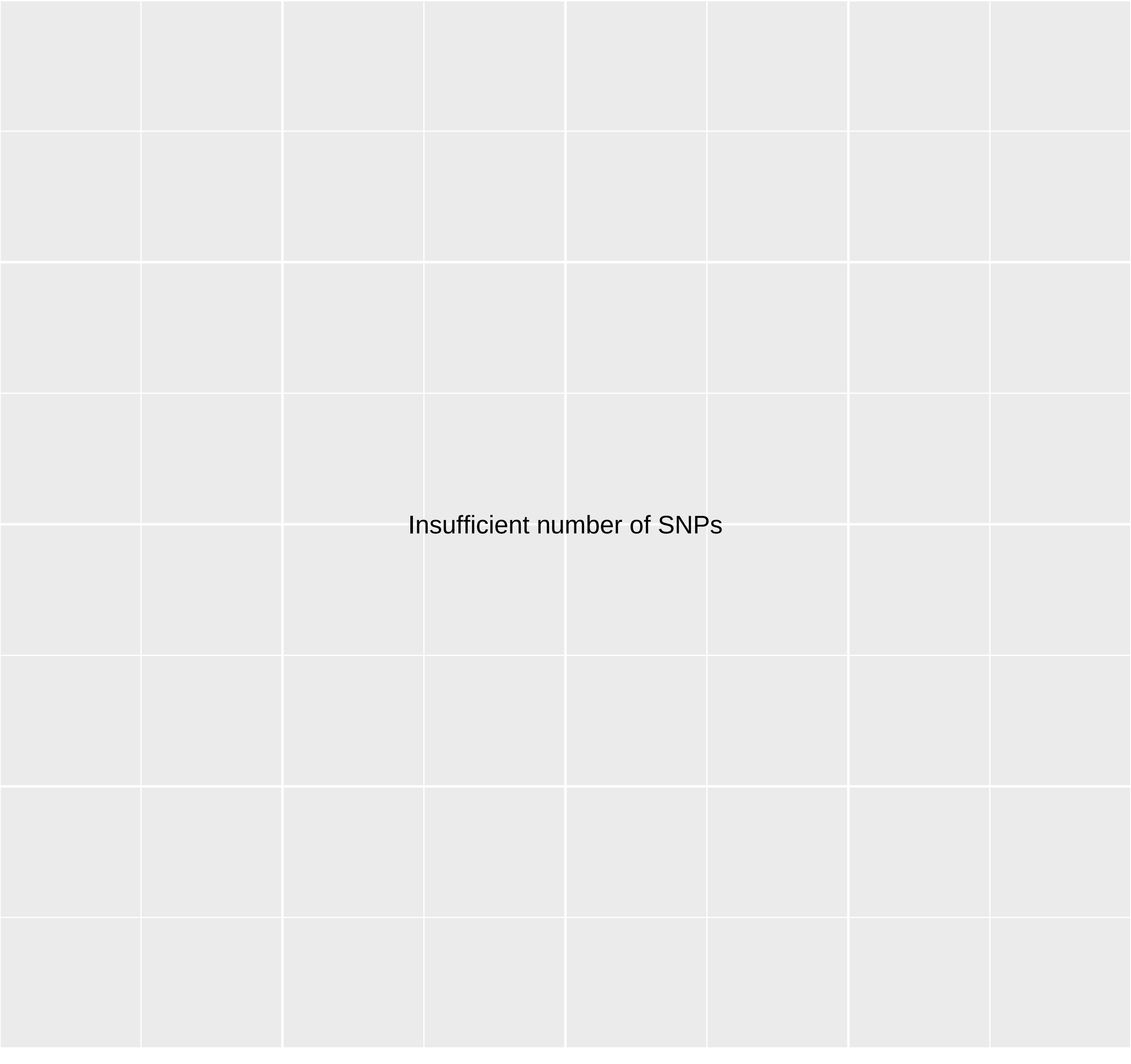
Insufficient number of SNPs

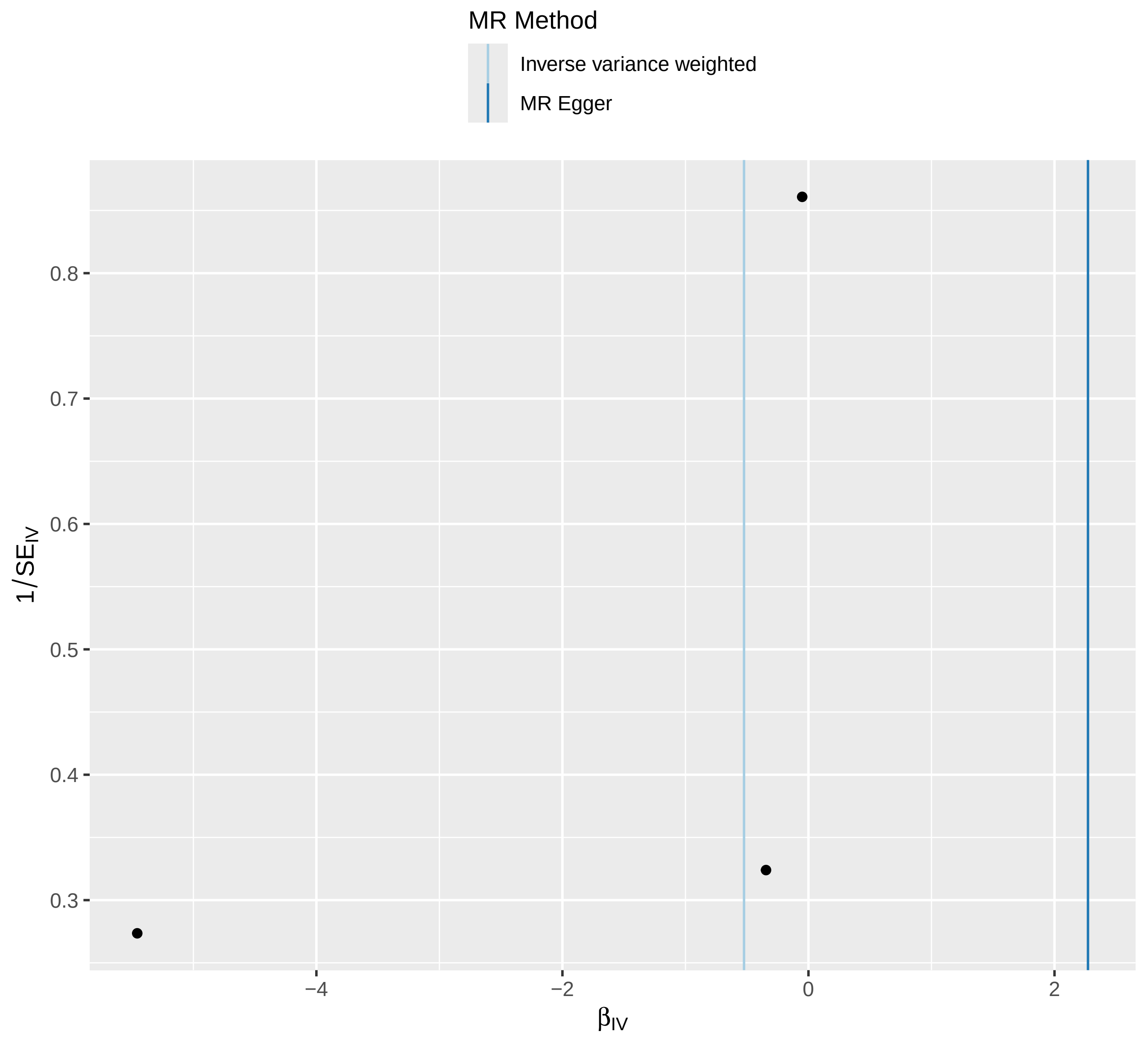
MR Method
Inverse variance weighted
MR Egger
0.8
0.7
0.6
0.5
0.4
0.3
$1/SE_{IV}$
−4
−2
0
2
β_{IV}

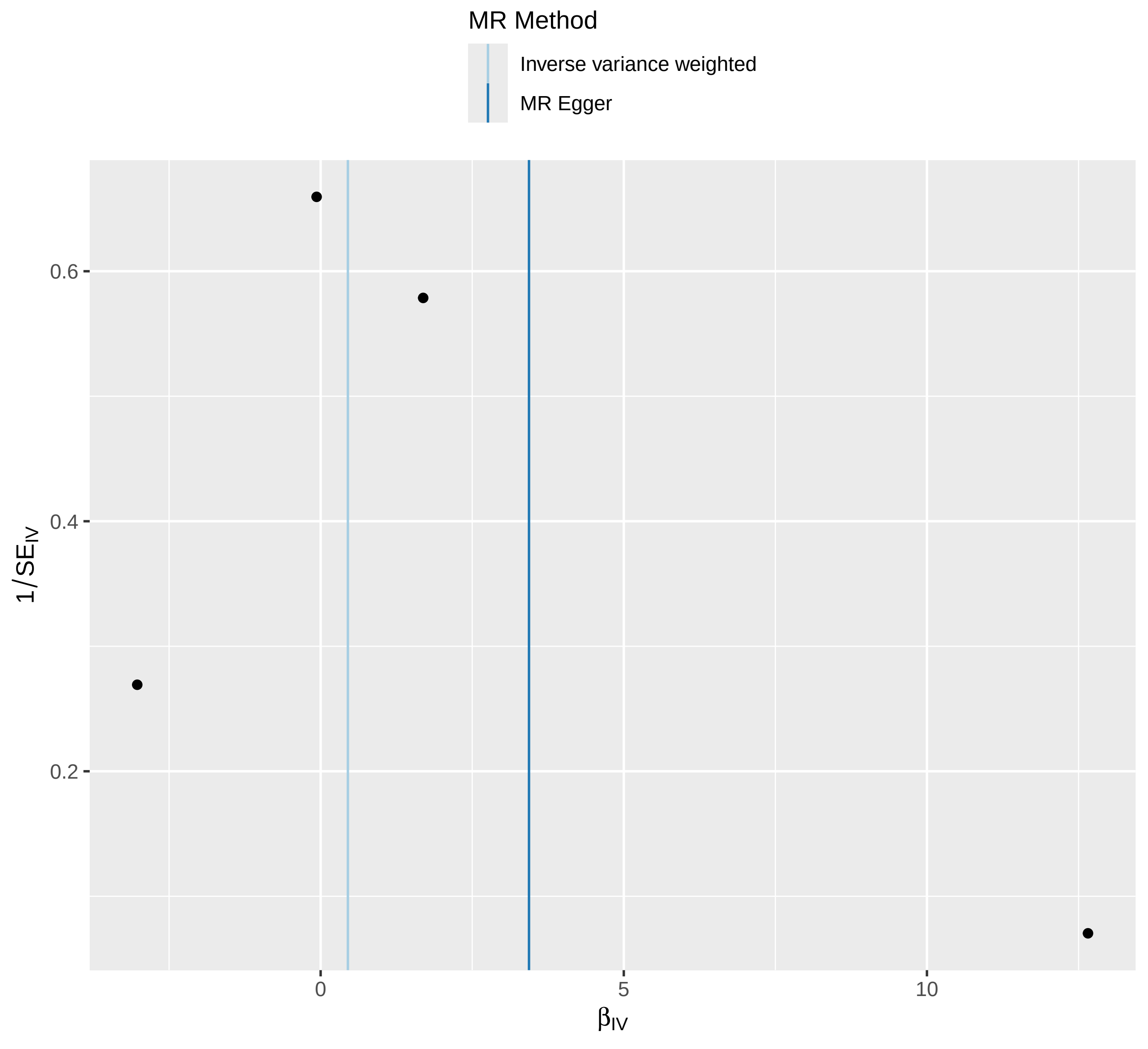

MR Method
Inverse variance weighted
MR Egger
0.6
0.4
0.2
$1/SE_{IV}$
0
5
10
β_{IV}

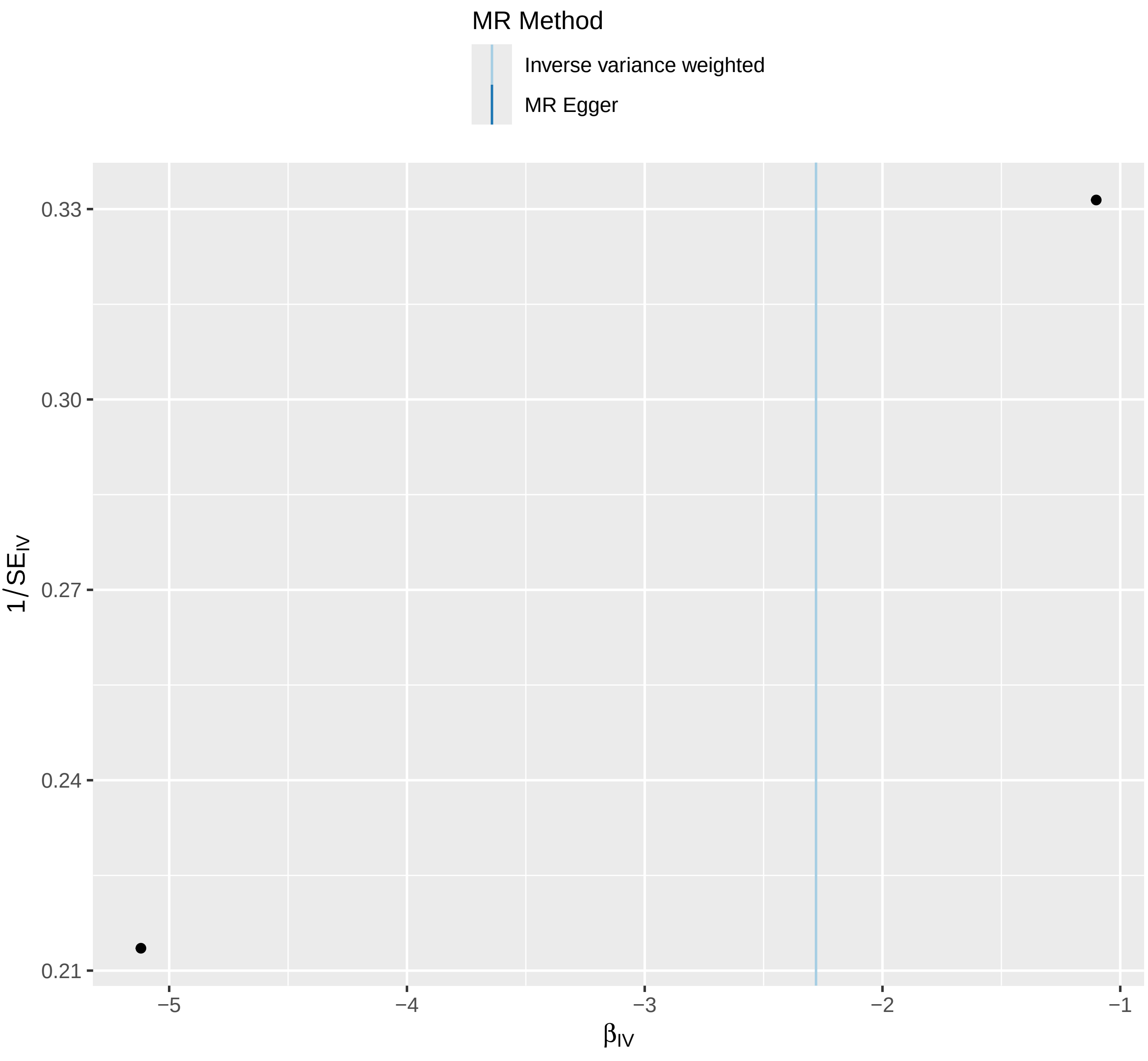
MR Method
Inverse variance weighted
MR Egger
0.33
0.30
0.27
0.24
0.21
$1/SE_{IV}$
−5
−4
−3
−2
−1
β_{IV}

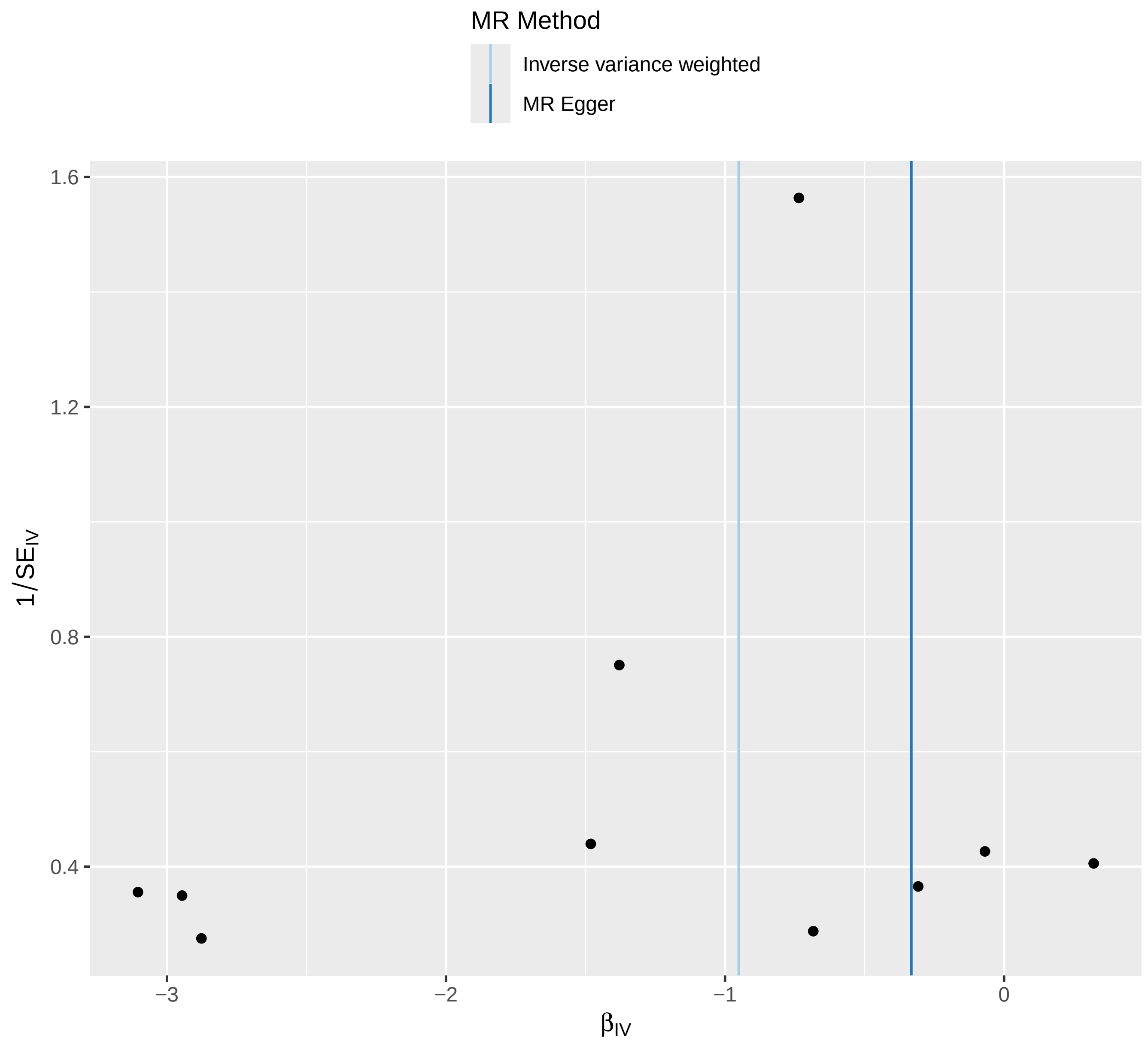

MR Method
Inverse variance weighted
MR Egger
1.6
1.2
0.8
0.4
−3
−2
−1
0
$1/SE_{IV}$
β_{IV}

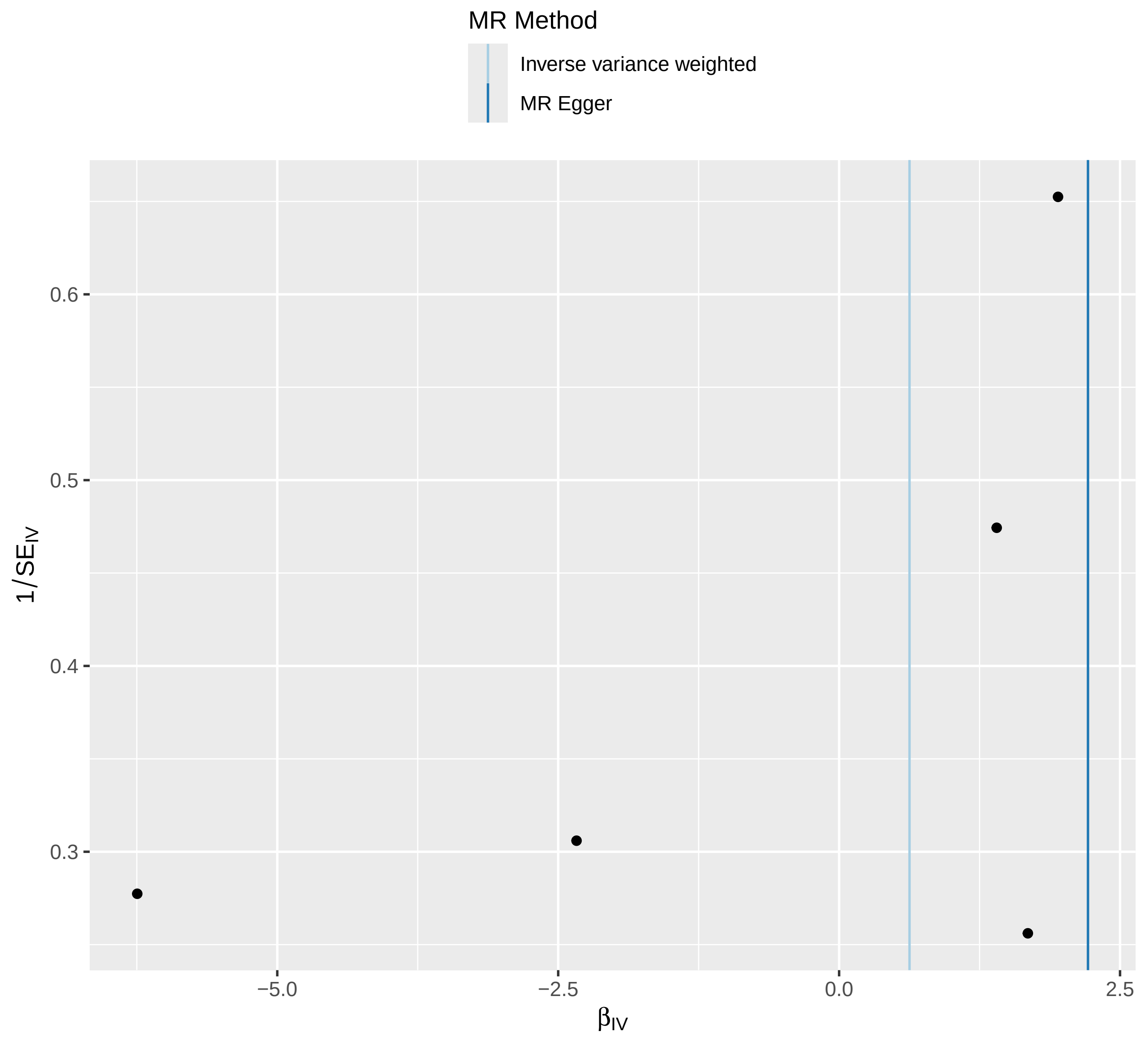
MR Method
Inverse variance weighted
MR Egger
1/SE$_{IV}$
0.6
0.5
0.4
0.3
−5.0
−2.5
0.0
2.5
β$_{IV}$

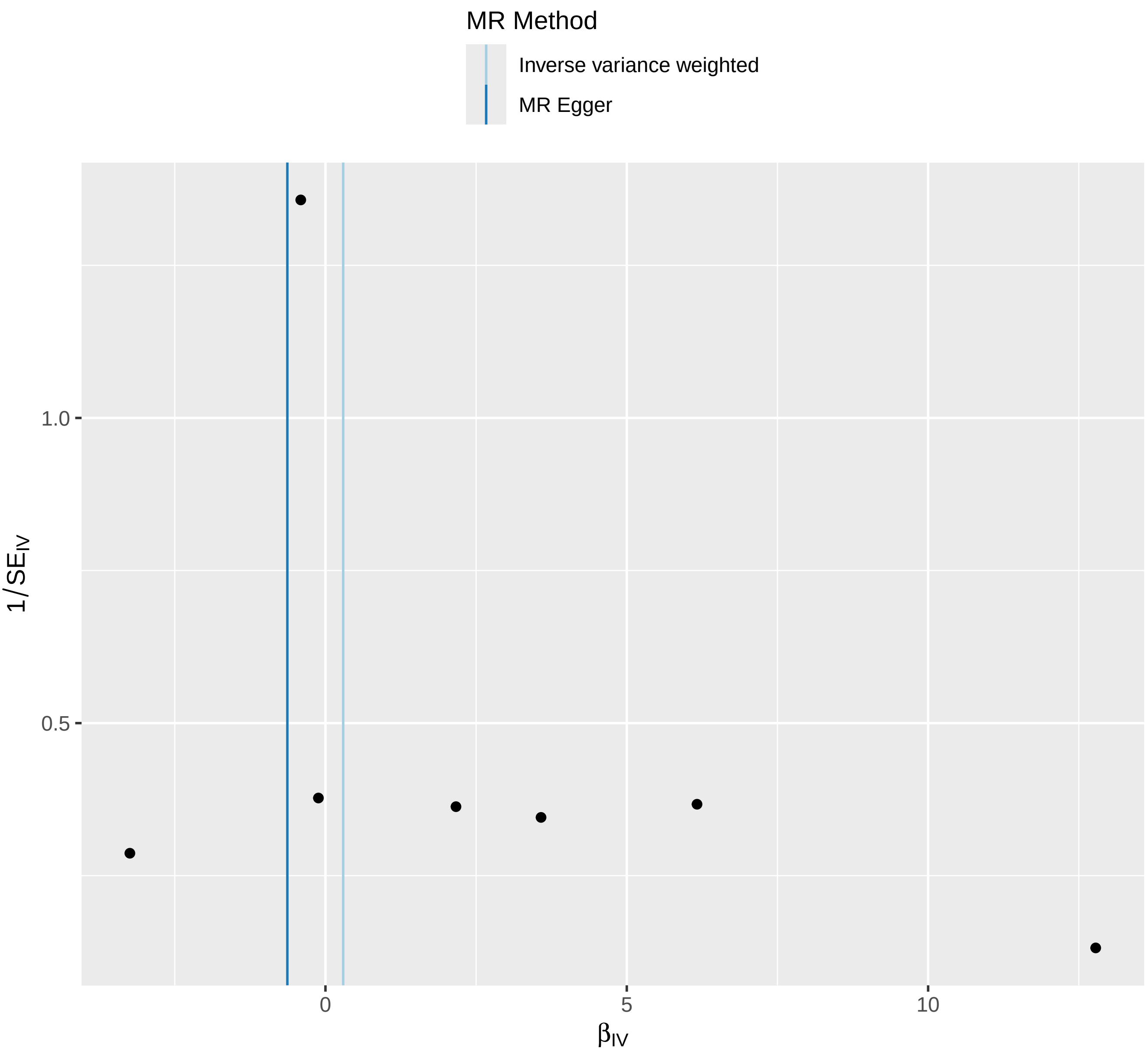

MR Method
Inverse variance weighted
MR Egger
1.0
0.5
1/SE_IV
0
5
10
β_IV

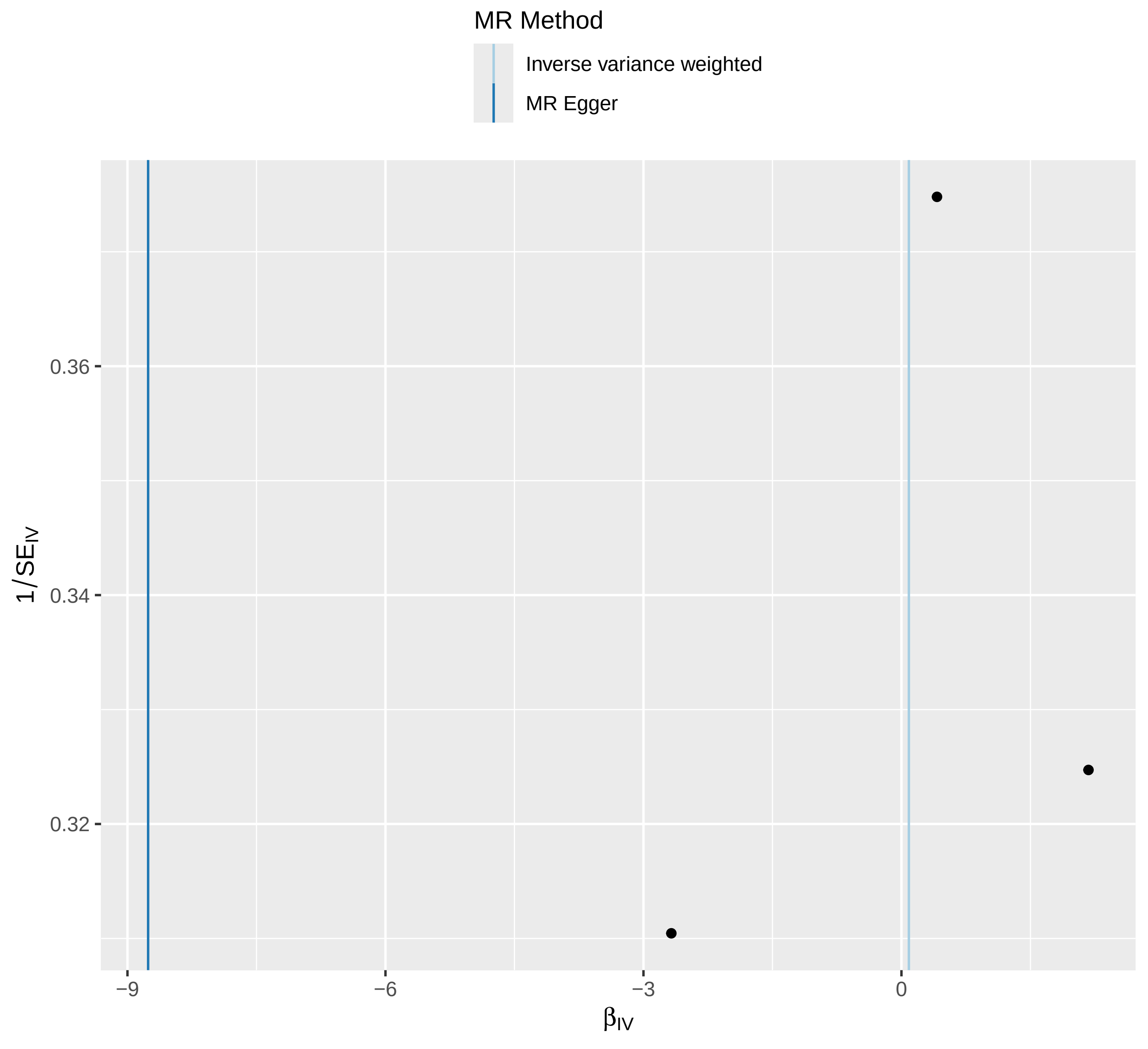
MR Method
Inverse variance weighted
MR Egger
0.36
0.34
0.32
1/SE_IV
−9
−6
−3
0
β_IV

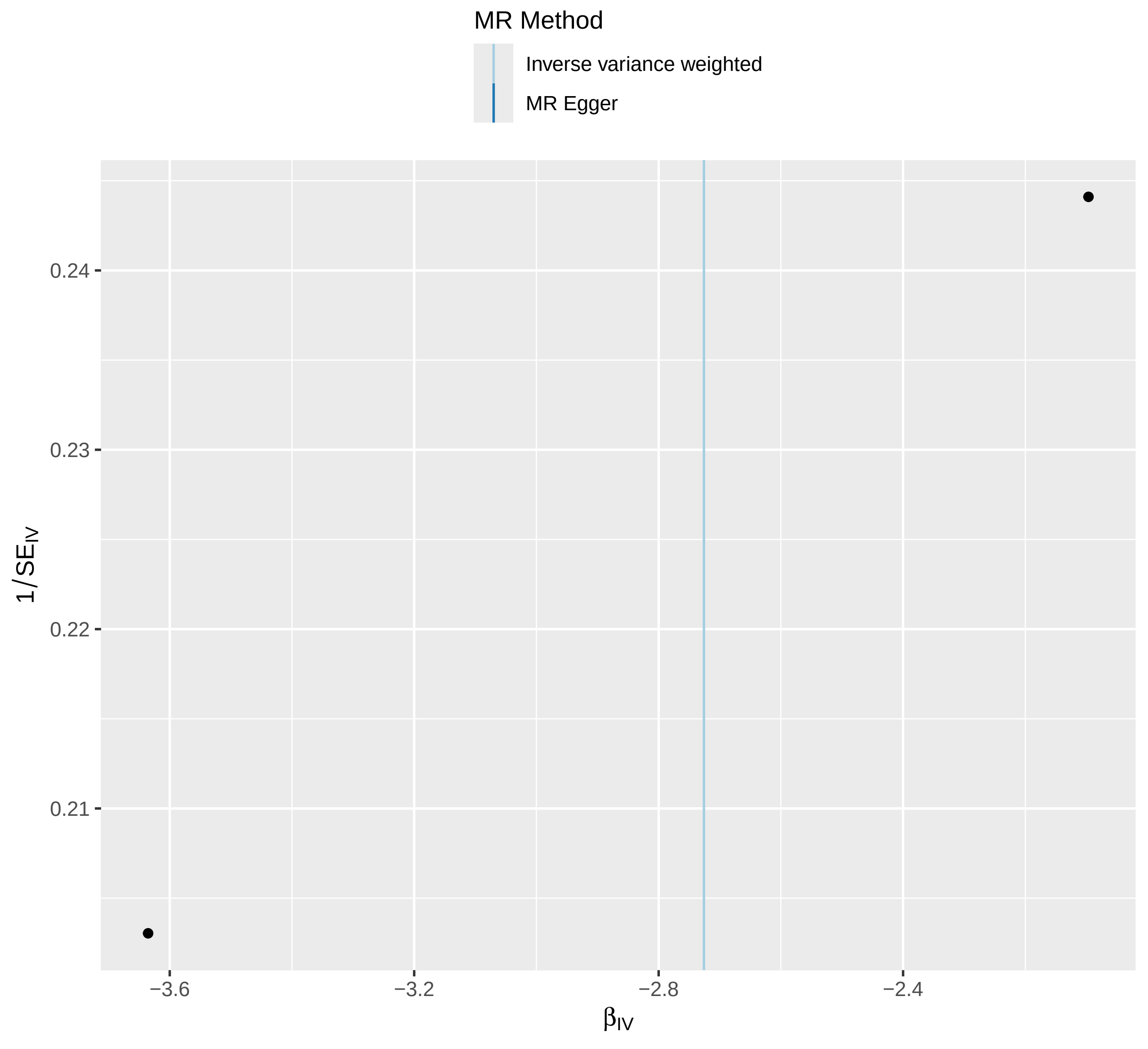
MR Method
Inverse variance weighted
MR Egger
0.24
0.23
0.22
0.21
1/SE$_{IV}$
−3.6
−3.2
−2.8
−2.4
β$_{IV}$

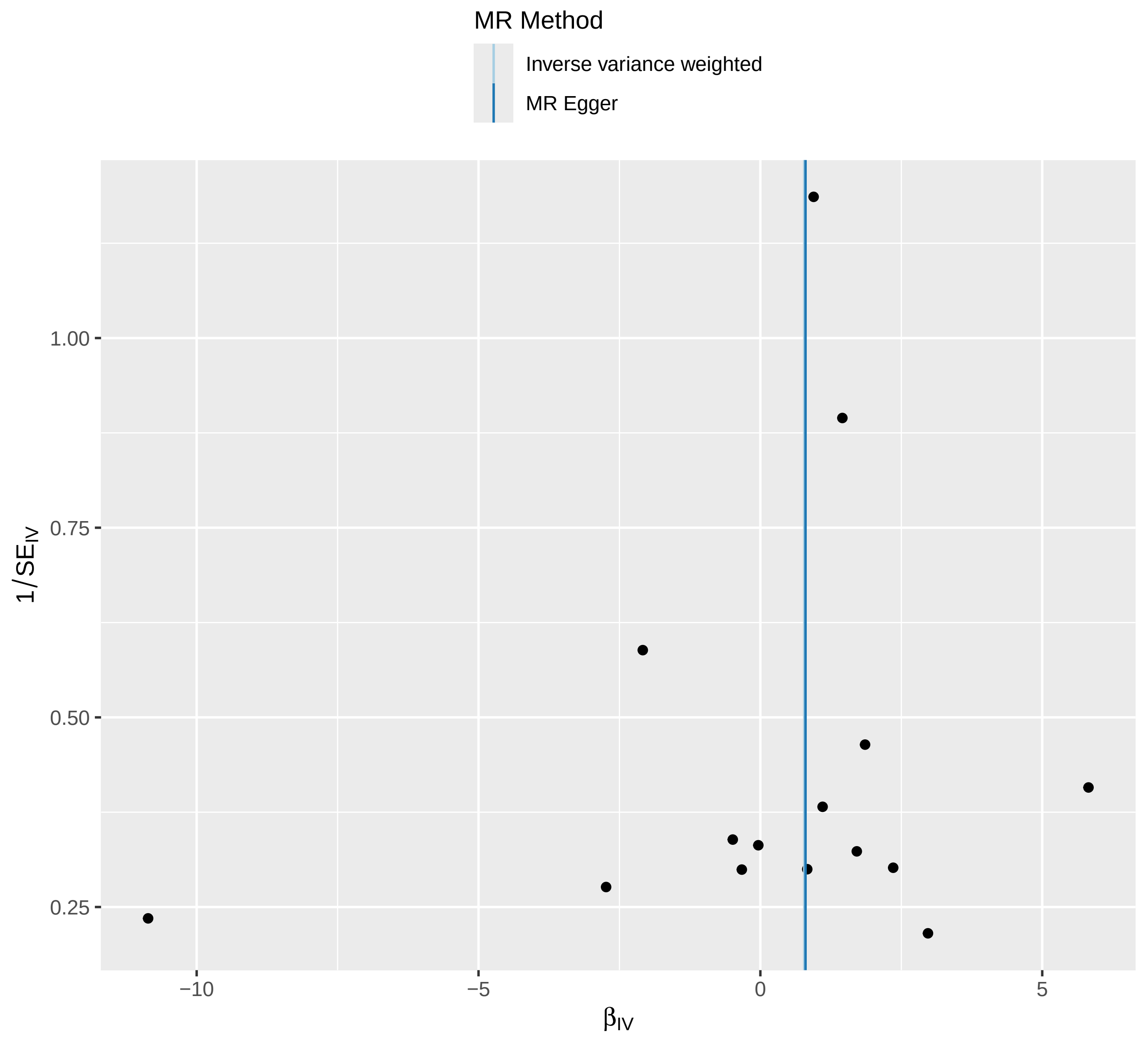
MR Method
Inverse variance weighted
MR Egger
1.00
0.75
0.50
0.25
1/SE$_{IV}$
−10
−5
0
5
β$_{IV}$

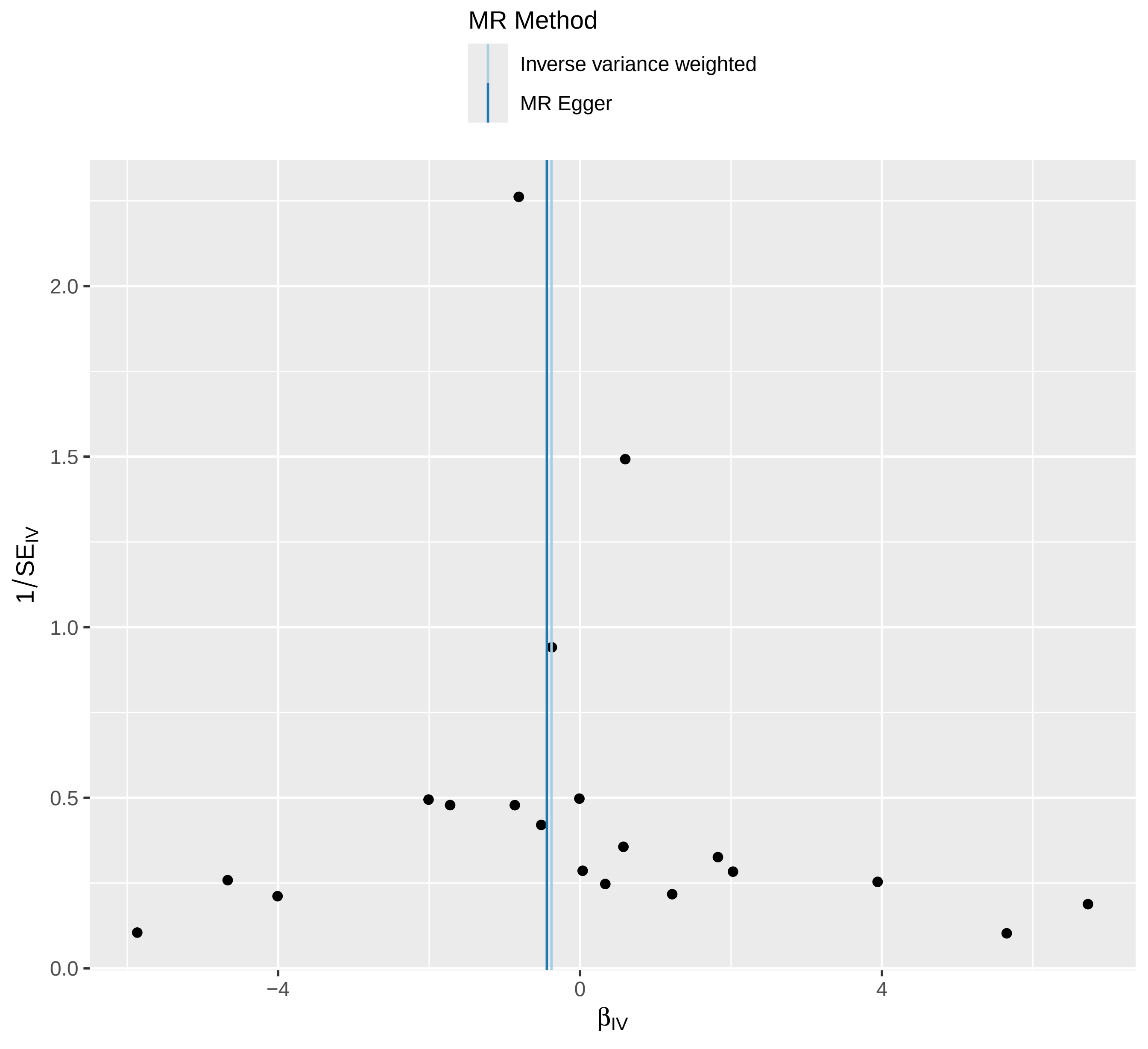

MR Method
Inverse variance weighted
MR Egger
2.0
1.5
1.0
0.5
0.0
−4
0
4
$1/SE_{IV}$
β_{IV}

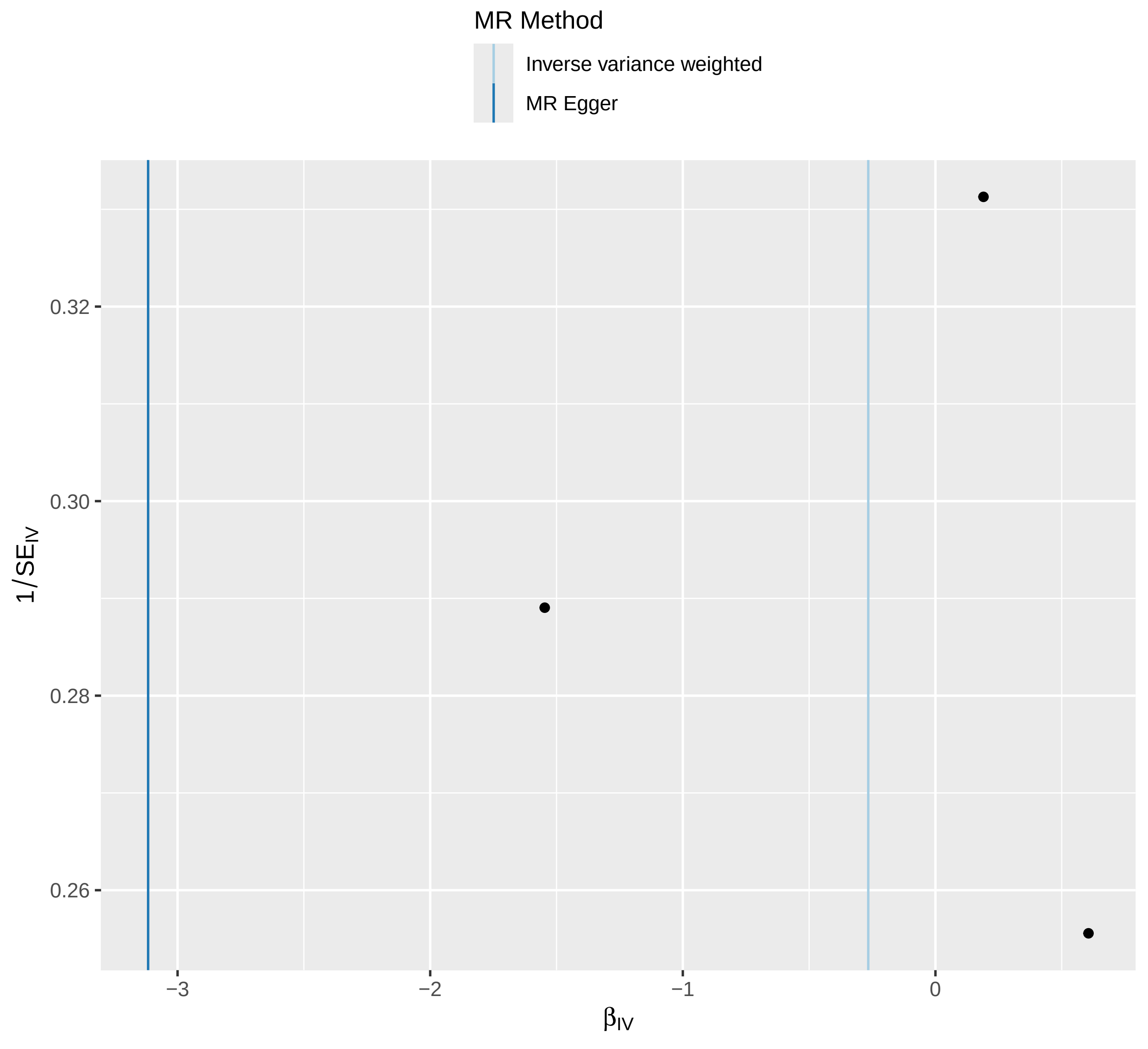

MR Method
Inverse variance weighted
MR Egger
0.32
0.30
0.28
0.26
1/SE$_{IV}$
−3
−2
−1
0
β$_{IV}$

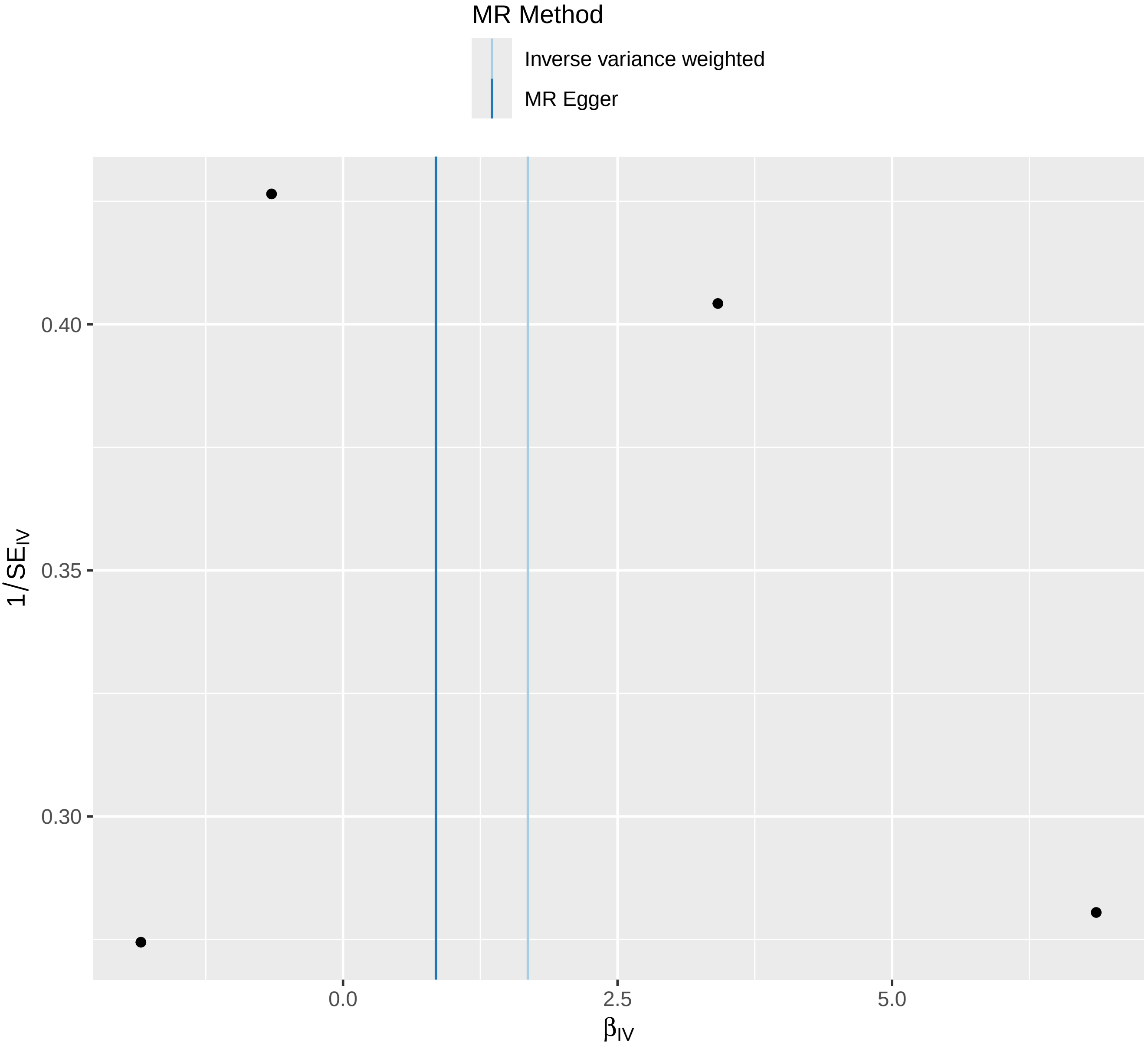

MR Method
Inverse variance weighted
MR Egger
0.40
0.35
0.30
1/SE_IV
0.0
2.5
5.0
β_IV

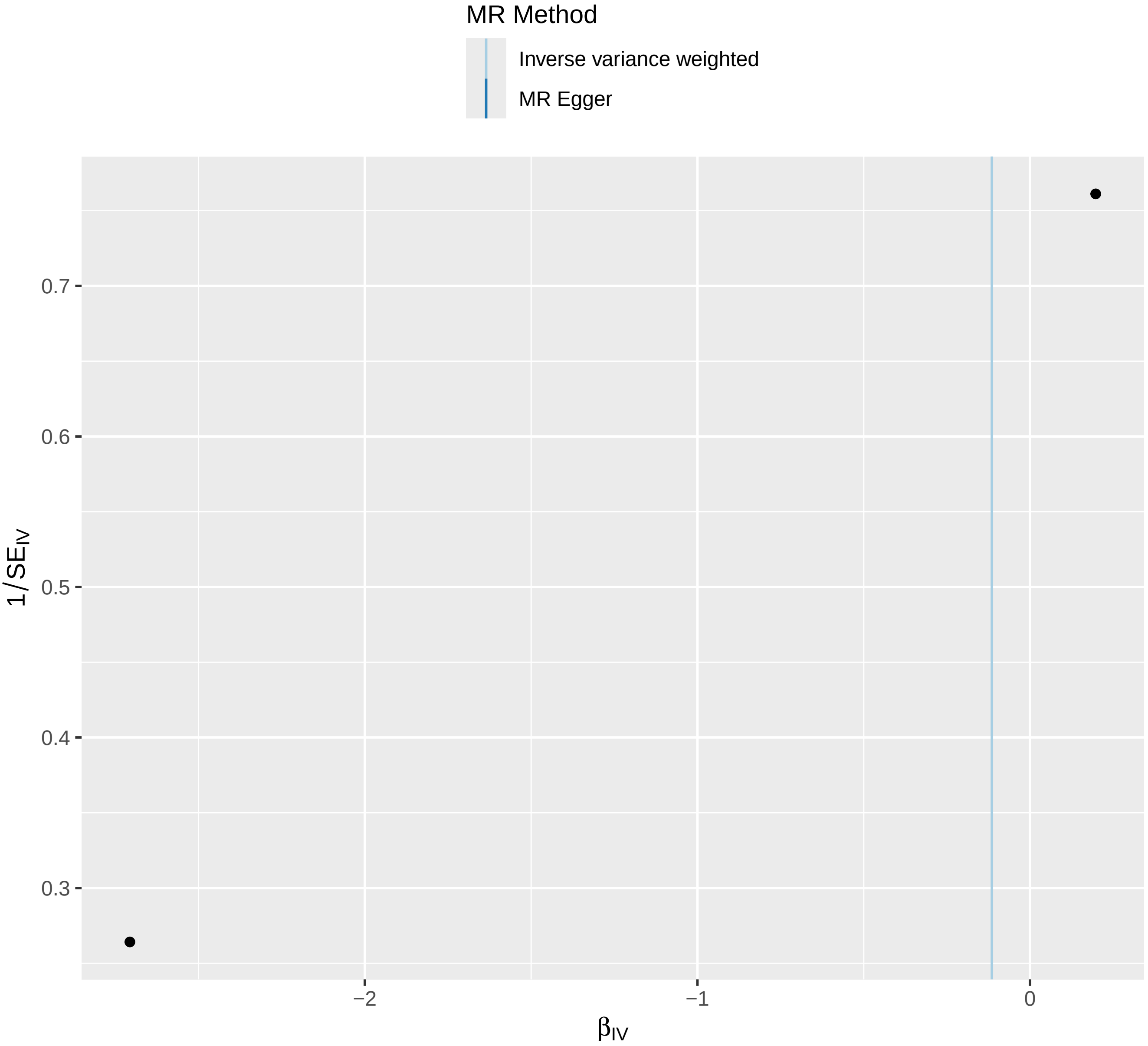
MR Method
Inverse variance weighted
MR Egger
0.7
0.6
0.5
0.4
0.3
1/SE_IV
−2
−1
0
β_IV

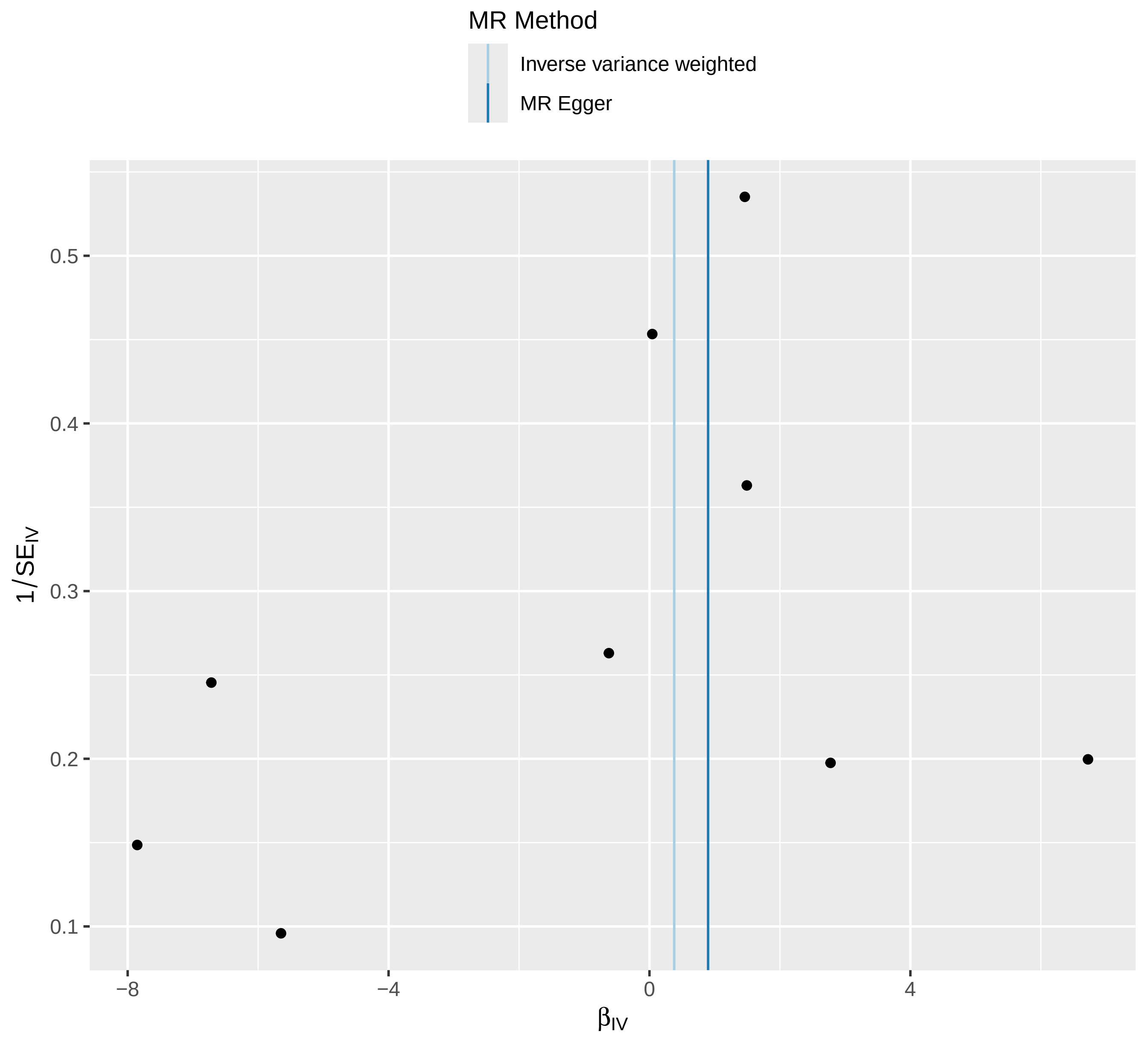

MR Method
Inverse variance weighted
MR Egger
0.5
0.4
0.3
0.2
0.1
$1/SE_{IV}$
−8
−4
0
4
β_{IV}

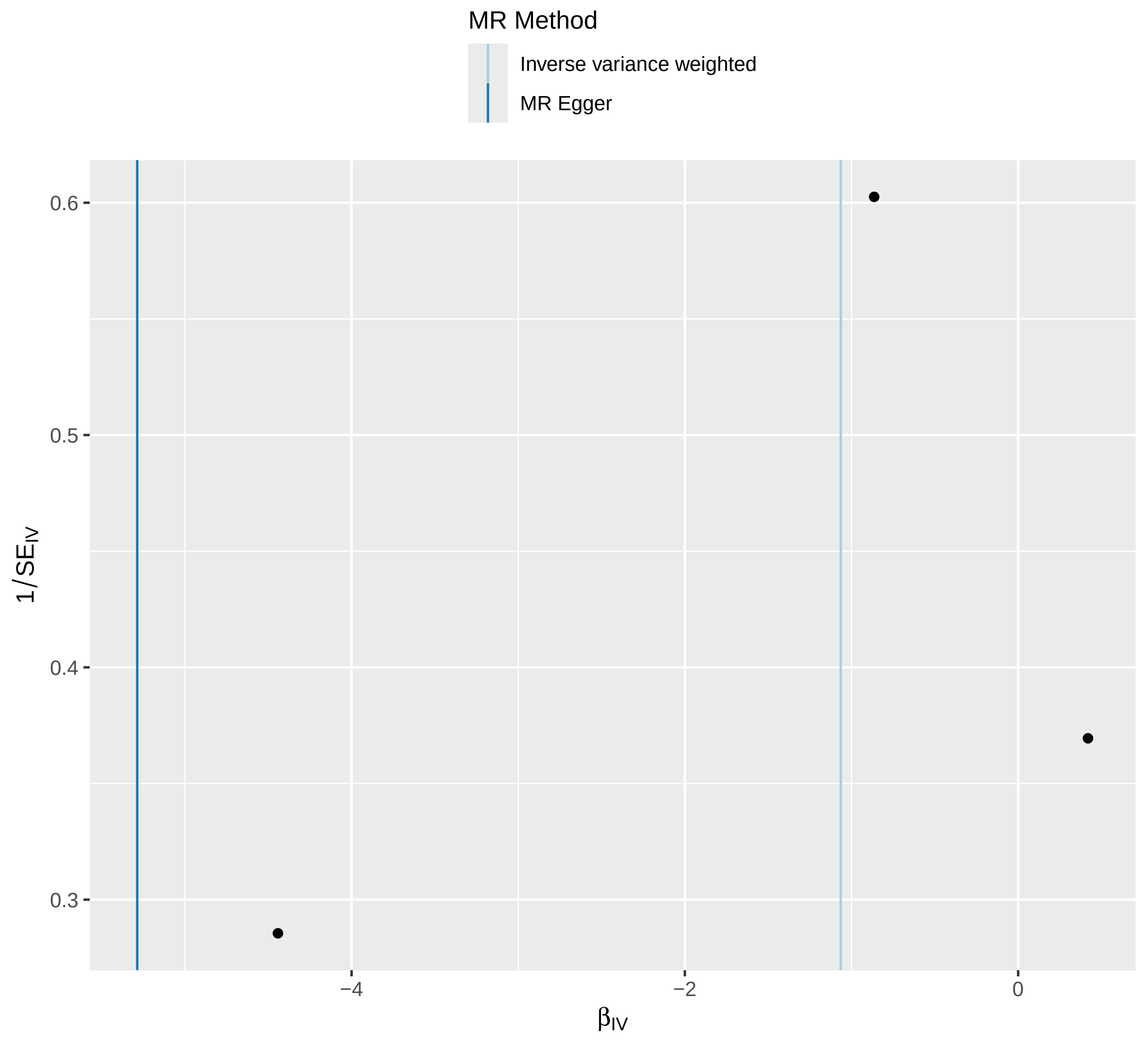
MR Method
Inverse variance weighted
MR Egger
0.6
0.5
0.4
0.3
−4
−2
0
1/SE_IV
β_IV

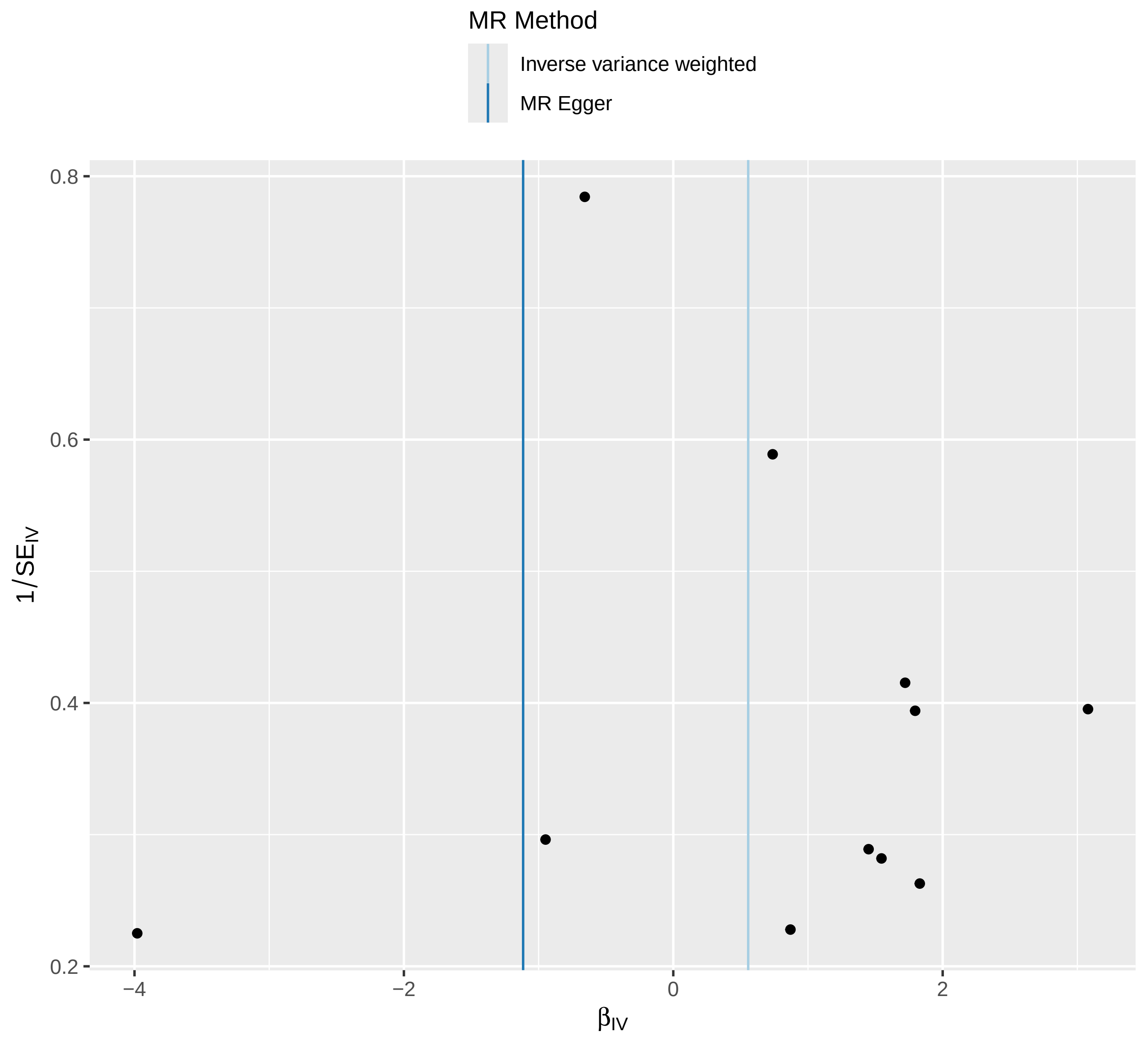
MR Method
Inverse variance weighted
MR Egger
0.8
0.6
0.4
0.2
1/SE$_{IV}$
−4
−2
0
2
β$_{IV}$

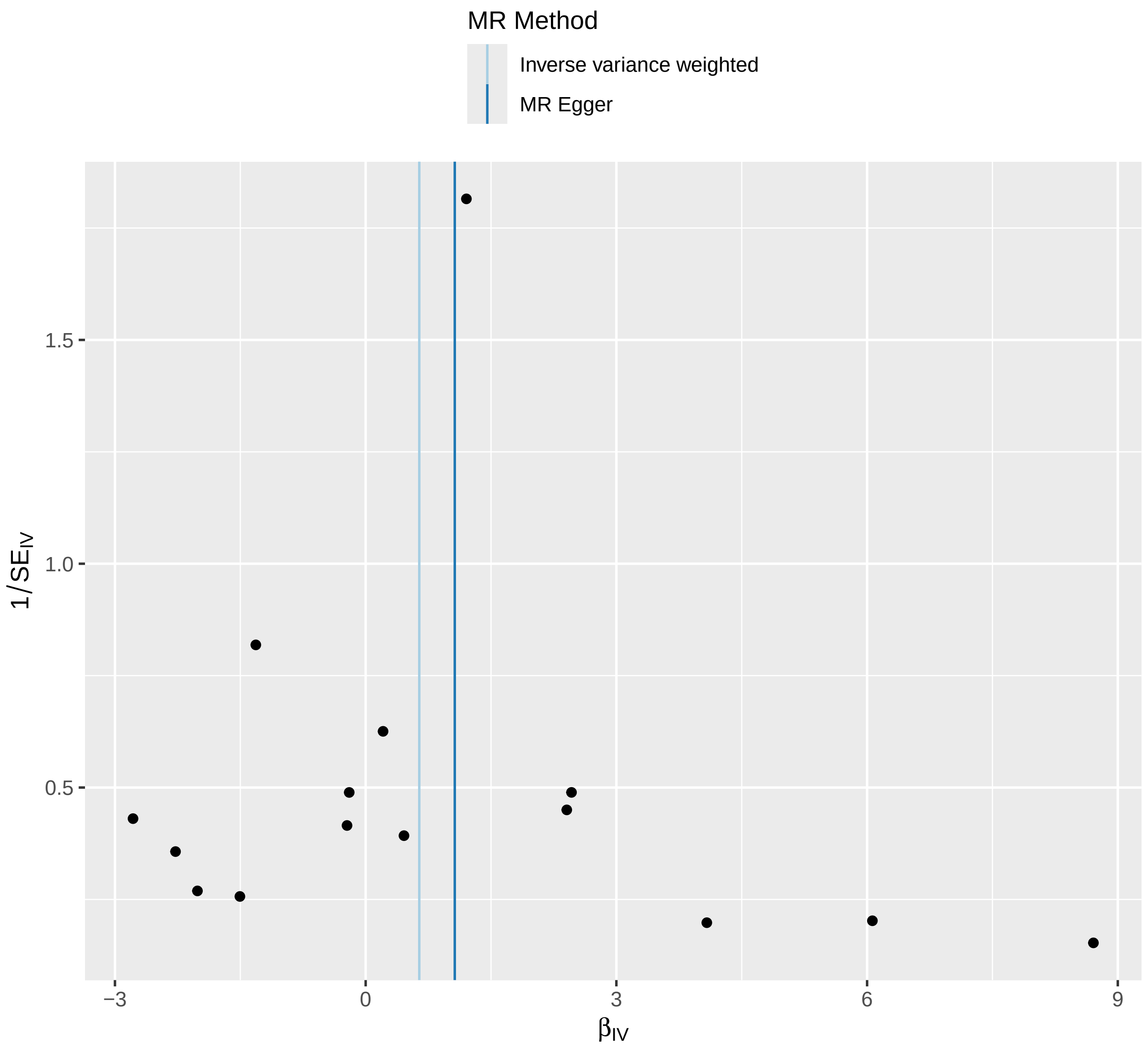
MR Method
Inverse variance weighted
MR Egger
1.5
1.0
0.5
$1/SE_{IV}$
−3
0
3
6
9
β_{IV}

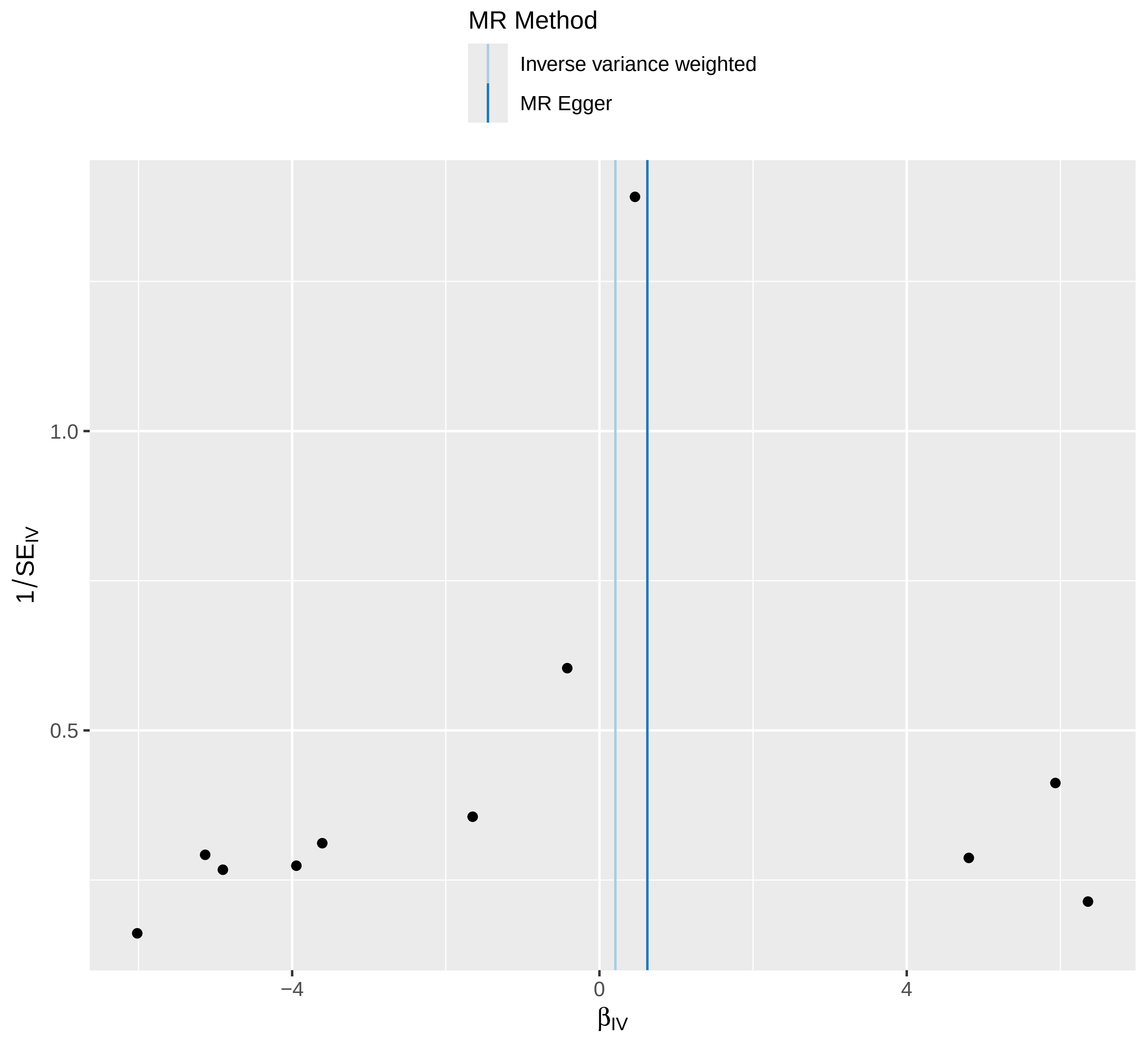
MR Method
Inverse variance weighted
MR Egger
1.0
0.5
1/SE_IV
−4
0
4
β_IV

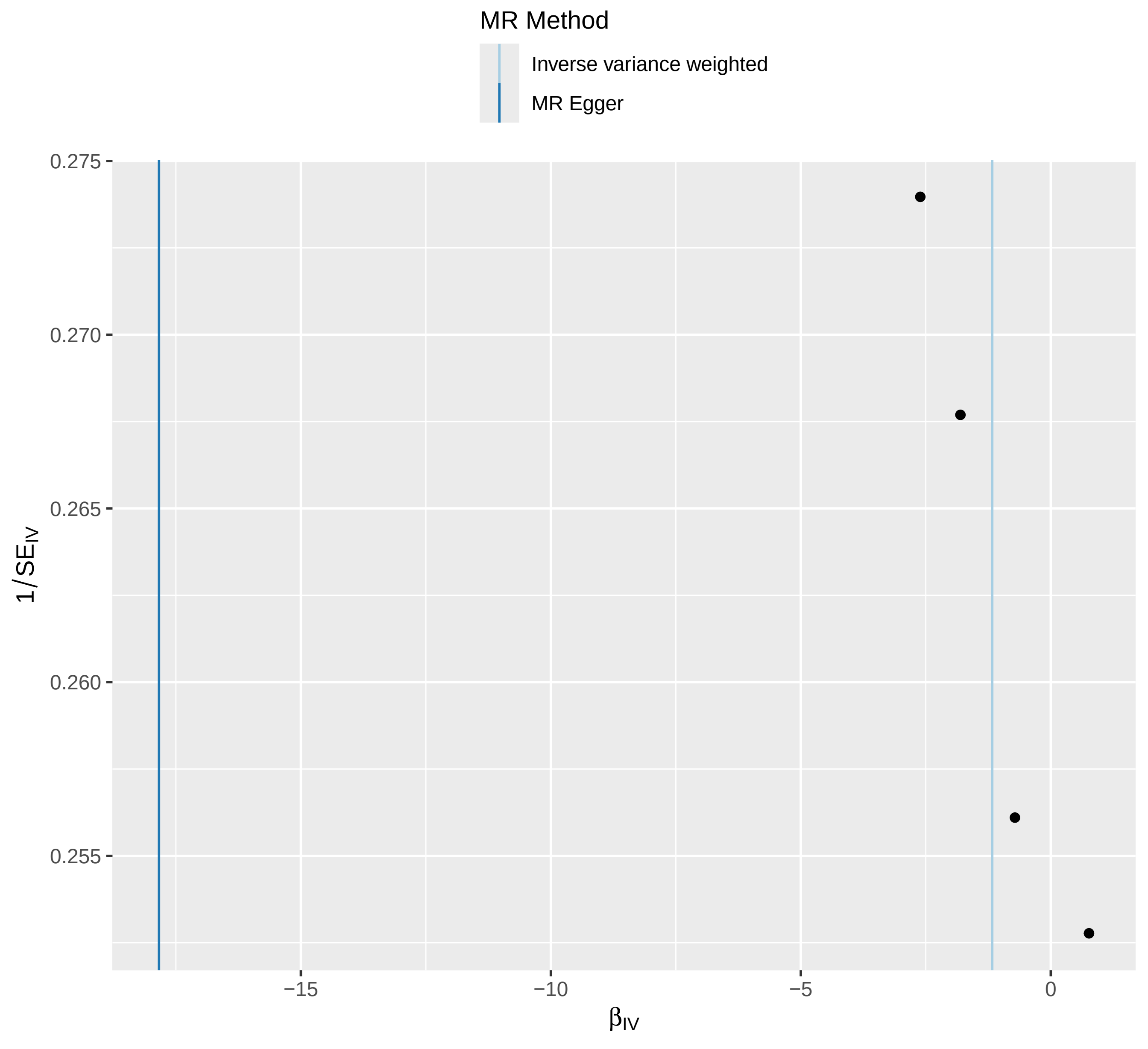
MR Method
Inverse variance weighted
MR Egger
0.275
0.270
0.265
0.260
0.255
1/SE_IV
−15
−10
−5
0
β_IV

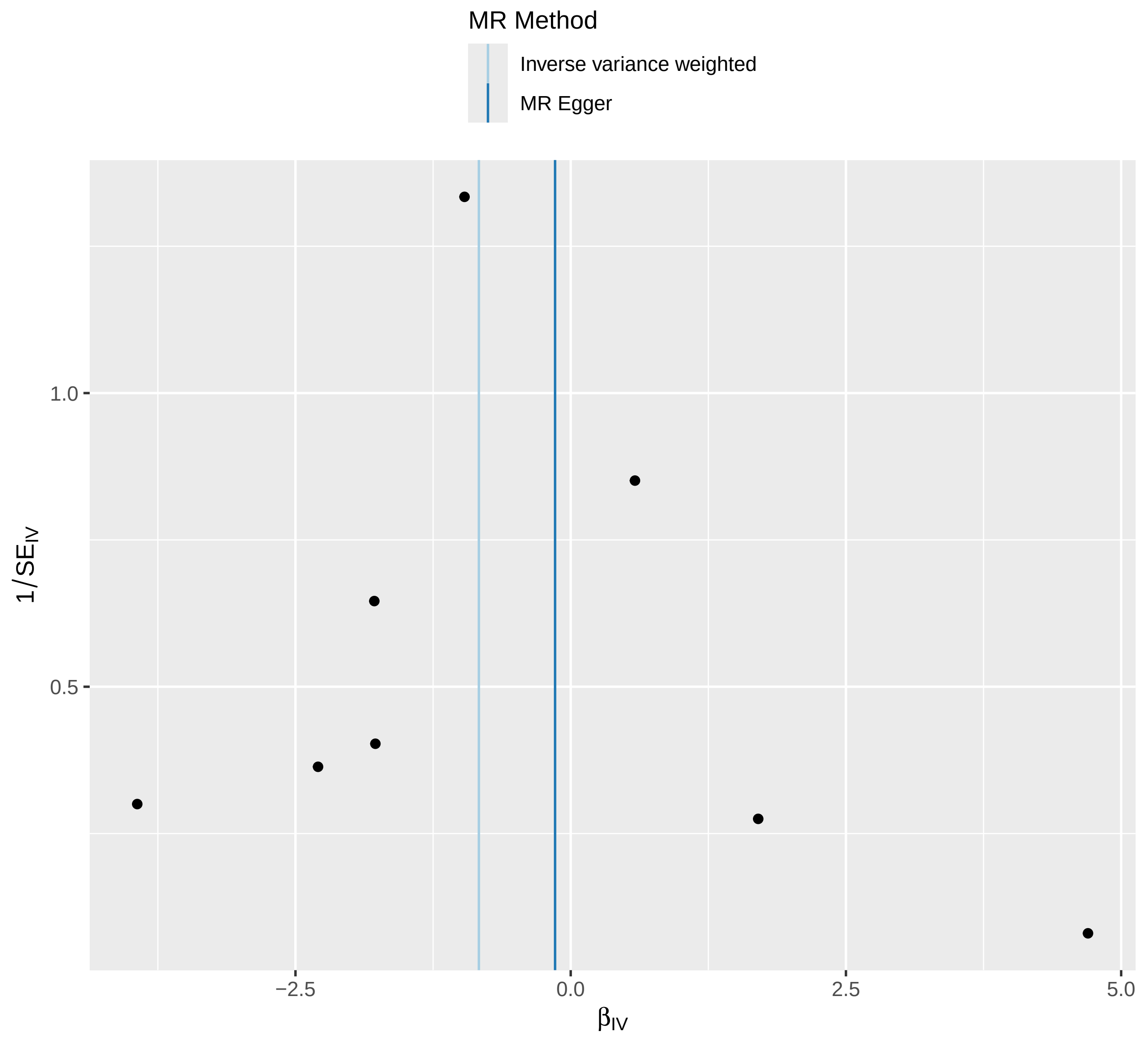

MR Method
Inverse variance weighted
MR Egger
1.0
0.5
1/SE$_{IV}$
−2.5
0.0
2.5
5.0
β$_{IV}$

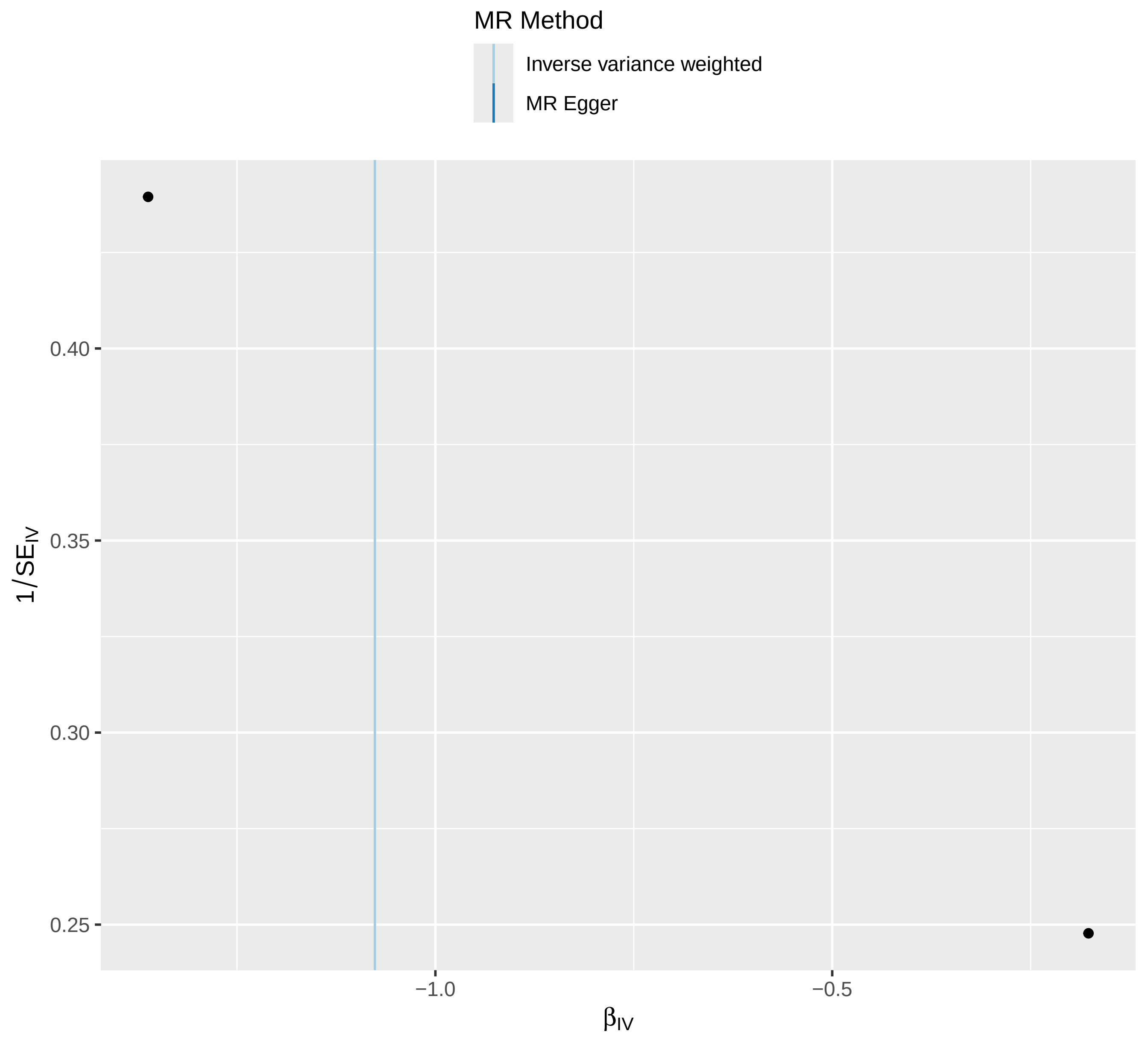
MR Method
Inverse variance weighted
MR Egger
0.40
0.35
0.30
0.25
1/SE$_{IV}$
−1.0
−0.5
β_{IV}

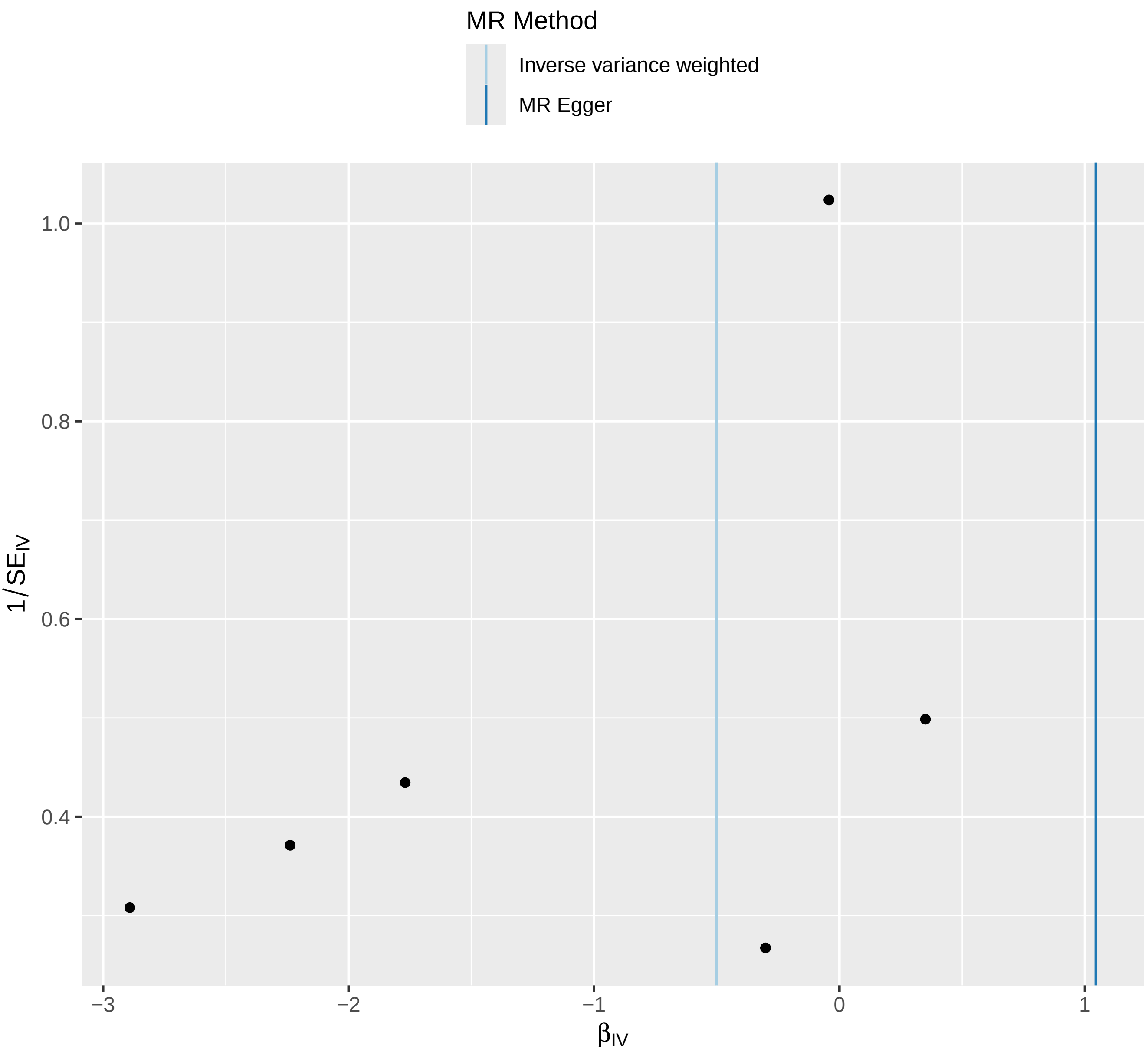

MR Method
Inverse variance weighted
MR Egger
1.0
0.8
0.6
0.4
1/SE_IV
−3
−2
−1
0
1
β_IV

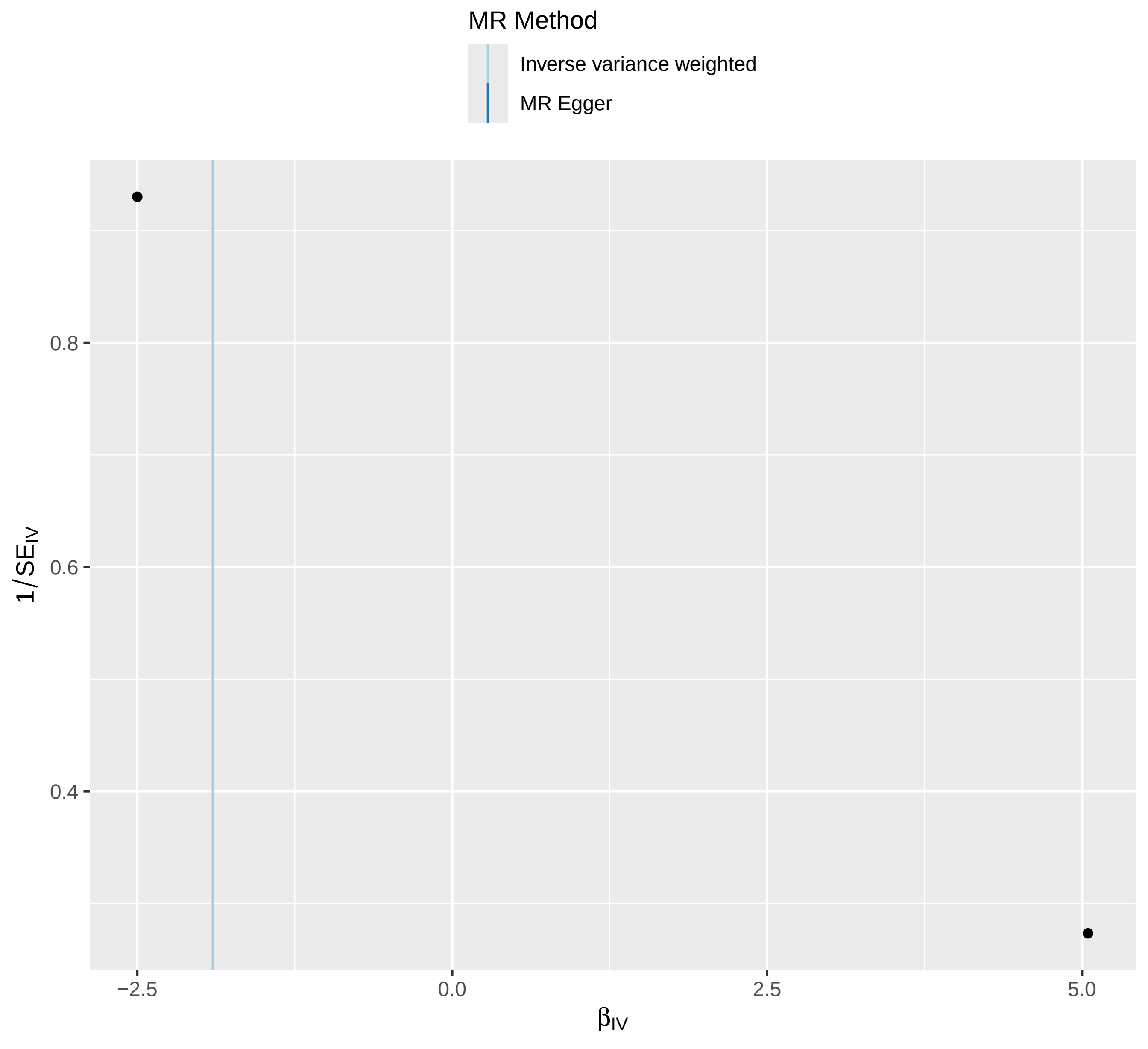
MR Method
Inverse variance weighted
MR Egger
1/SE$_{IV}$
0.8
0.6
0.4
−2.5
0.0
2.5
5.0
β$_{IV}$

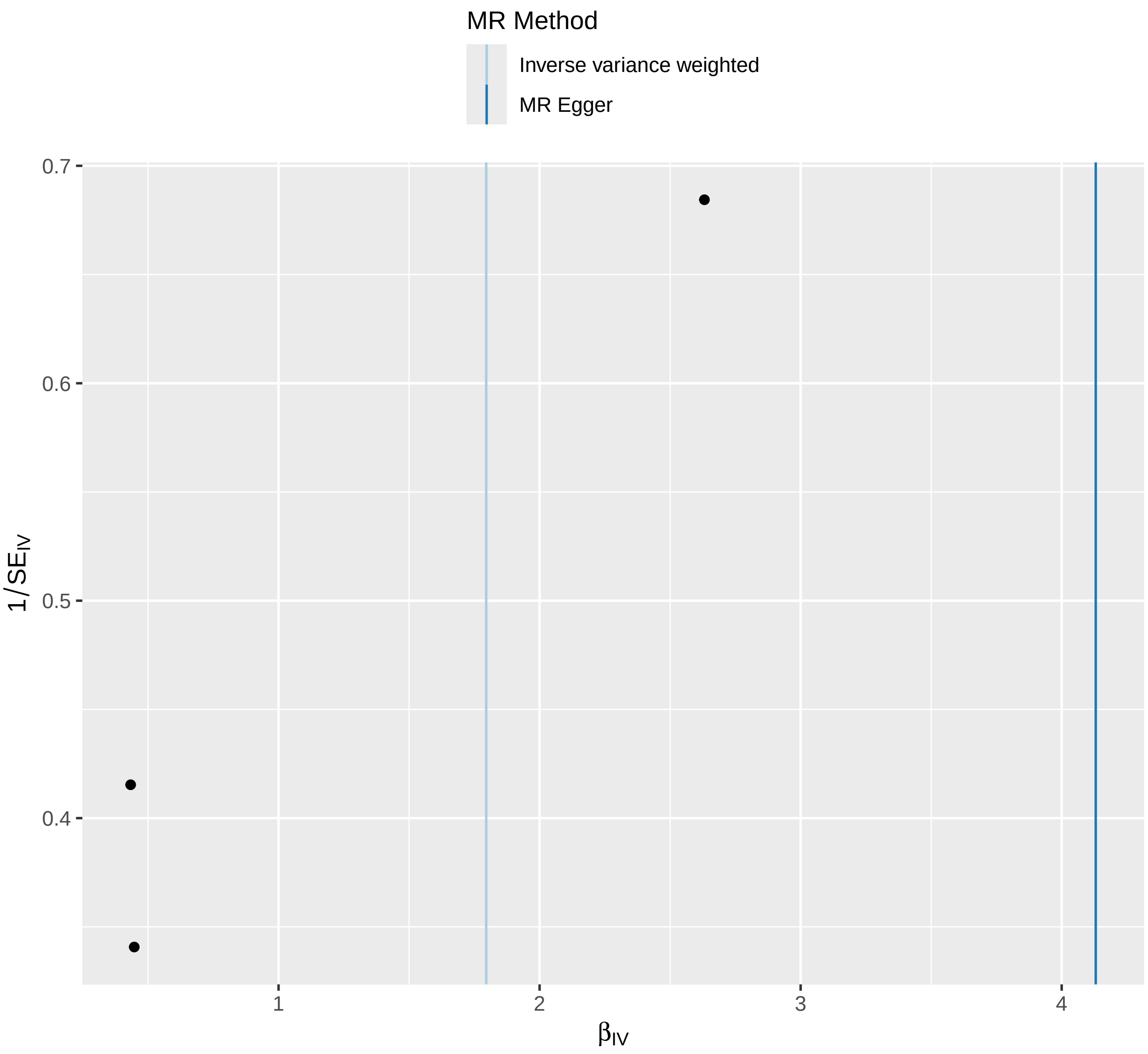
MR Method
Inverse variance weighted
MR Egger
0.7
0.6
0.5
0.4
1/SE_IV
1
2
3
4
β_IV

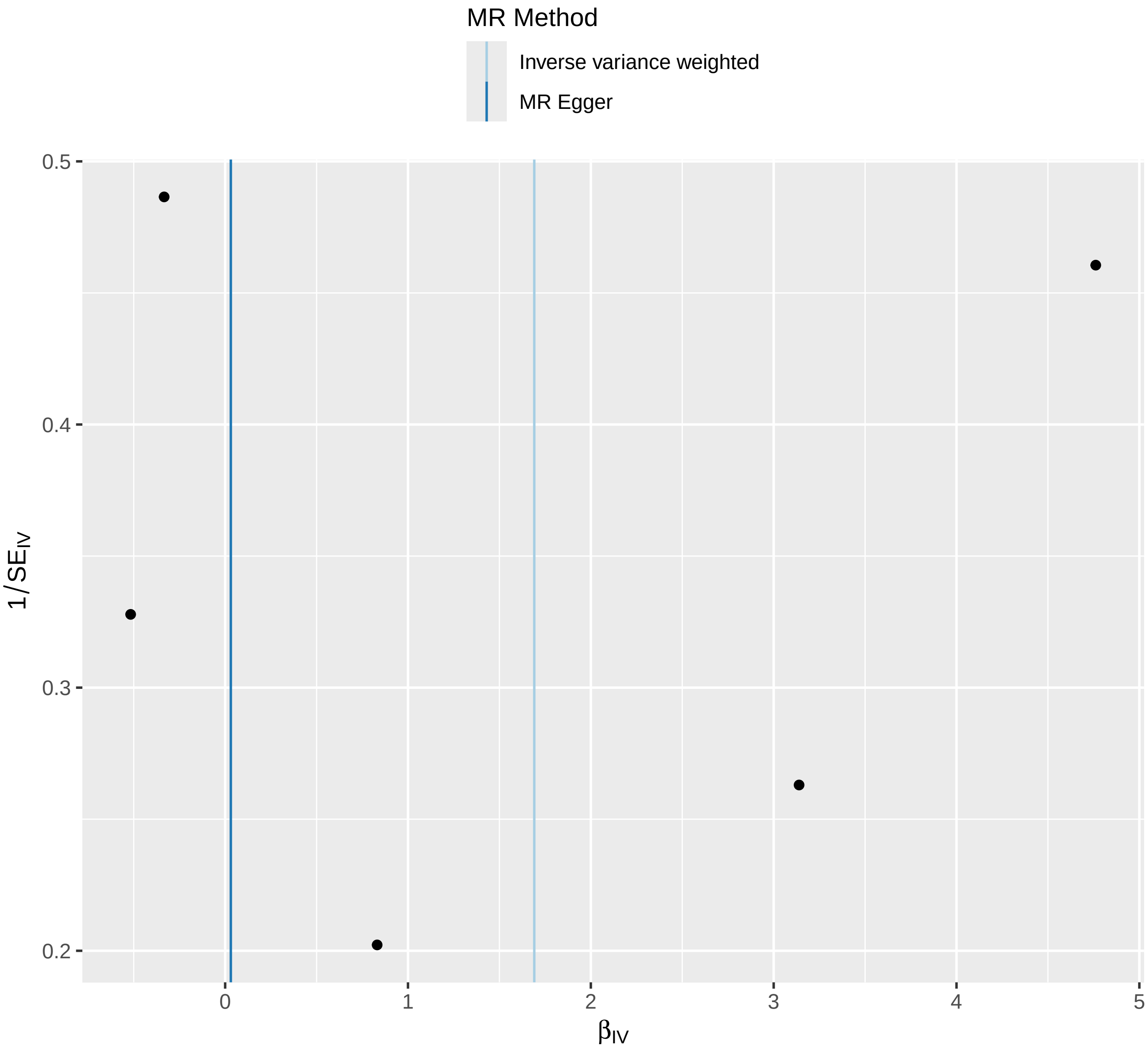
MR Method
Inverse variance weighted
MR Egger
0.5
0.4
0.3
0.2
$1/SE_{IV}$
0
1
2
3
4
5
β_{IV}

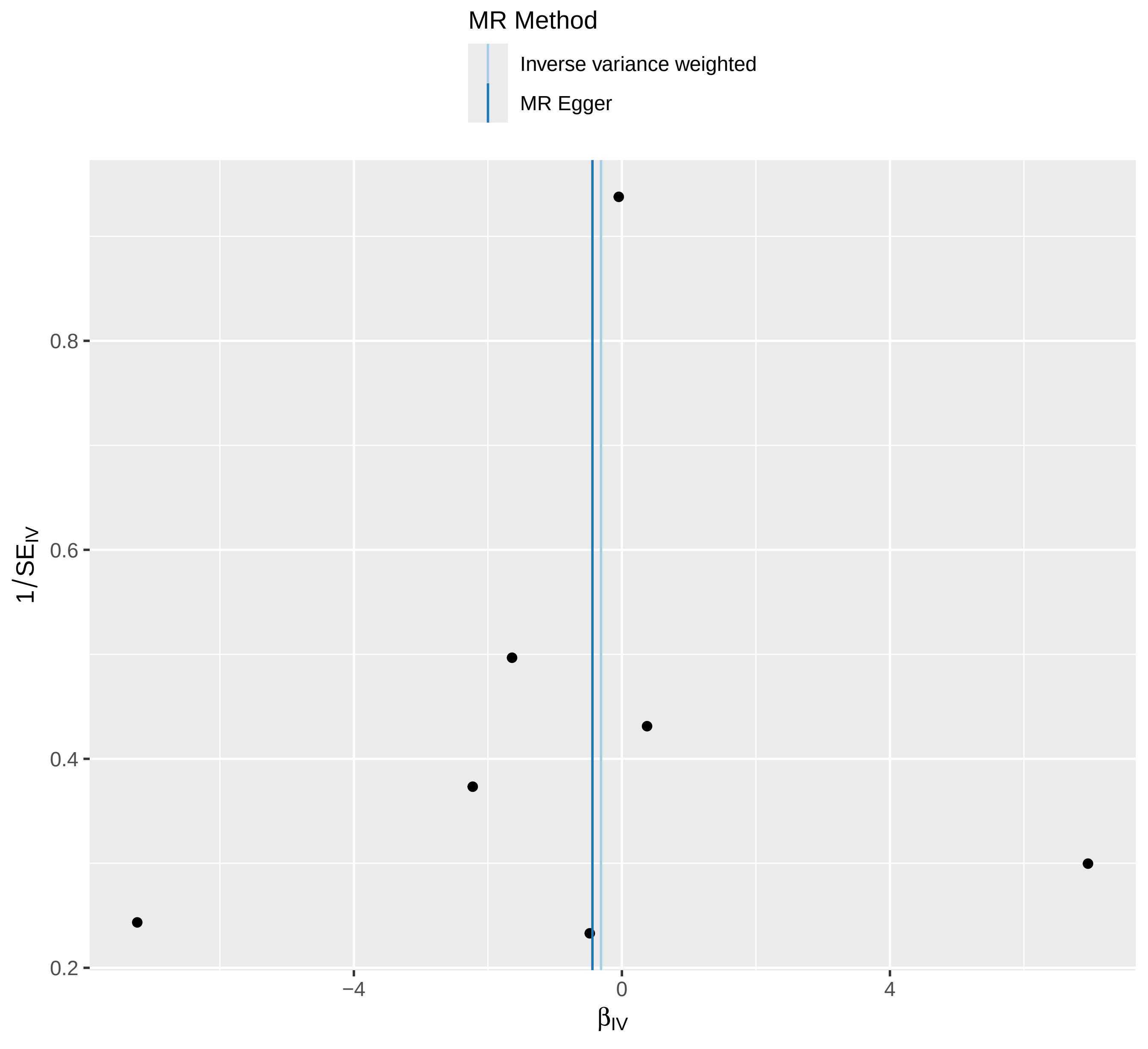

MR Method
Inverse variance weighted
MR Egger
0.8
0.6
0.4
0.2
−4
0
4
$1/SE_{IV}$
β_{IV}

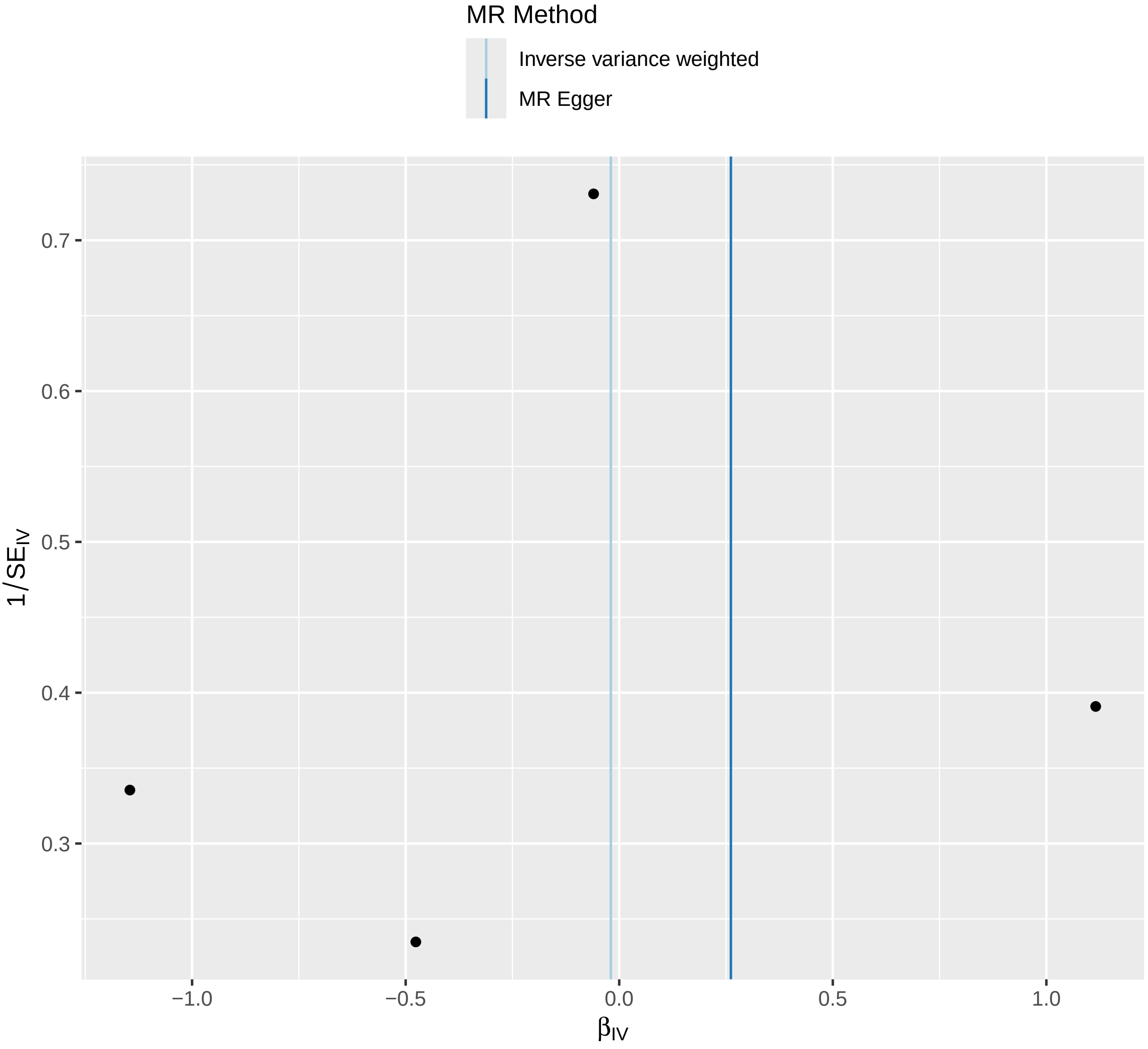

MR Method
Inverse variance weighted
MR Egger
1/SE_IV
0.7
0.6
0.5
0.4
0.3
−1.0
−0.5
0.0
0.5
1.0
β_IV

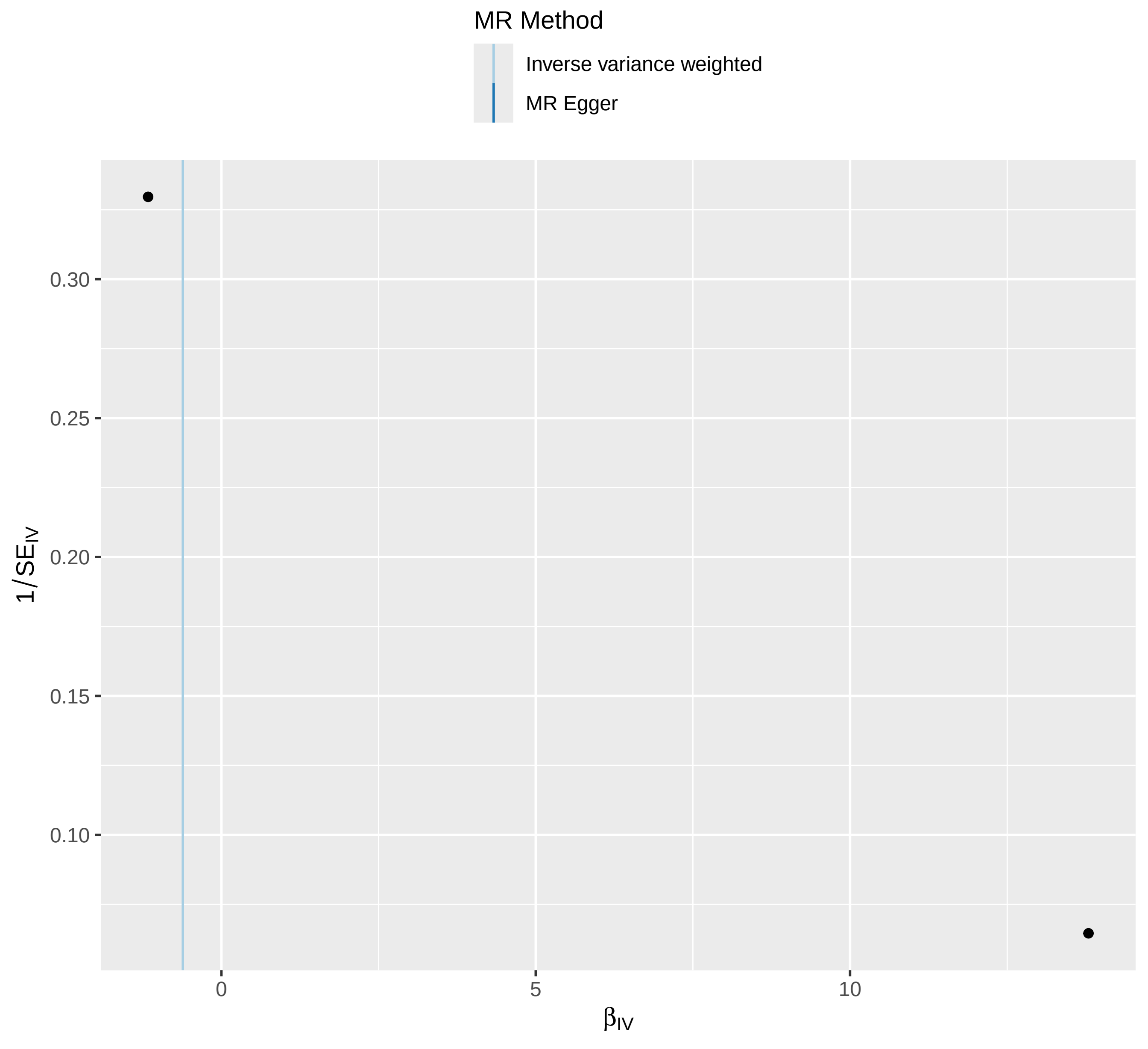
MR Method
Inverse variance weighted
MR Egger
0.30
0.25
0.20
0.15
0.10
$1/SE_{IV}$
0
5
10
β_{IV}

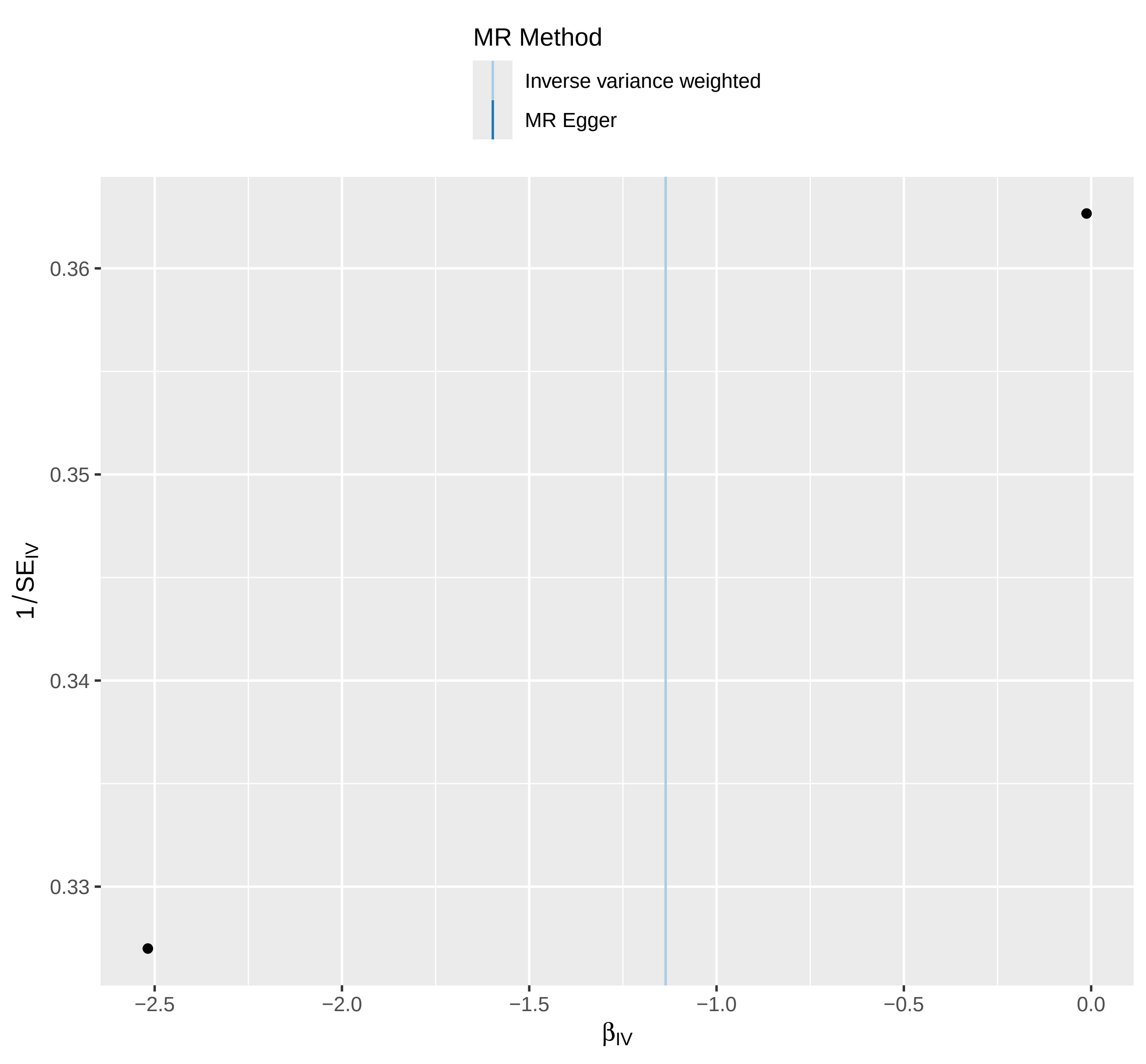
MR Method
Inverse variance weighted
MR Egger
0.36
0.35
0.34
0.33
1/SE_IV
−2.5
−2.0
−1.5
−1.0
−0.5
0.0
β_IV

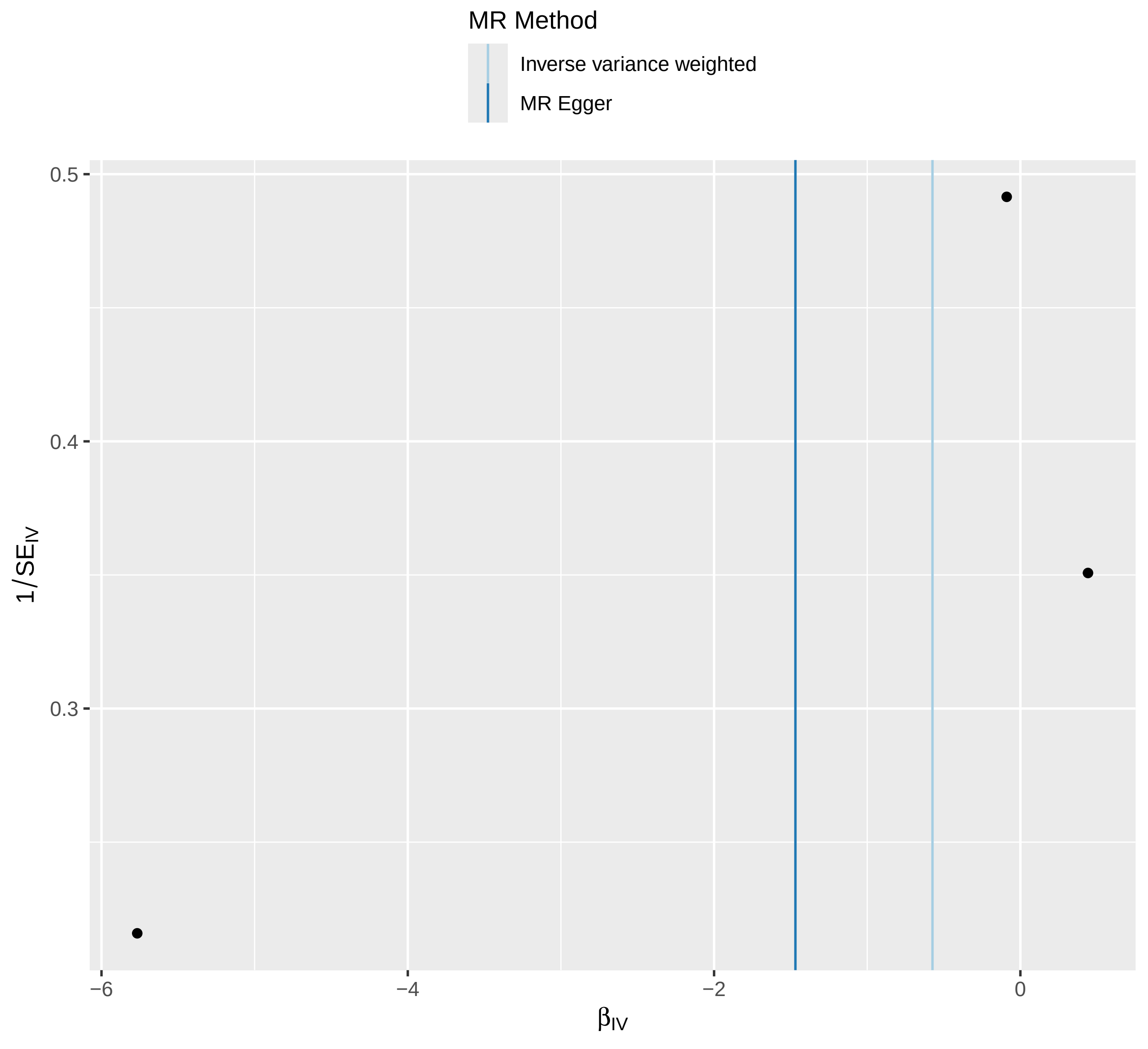
MR Method
Inverse variance weighted
MR Egger
0.5
0.4
0.3
1/SE_IV
−6
−4
−2
0
β_IV

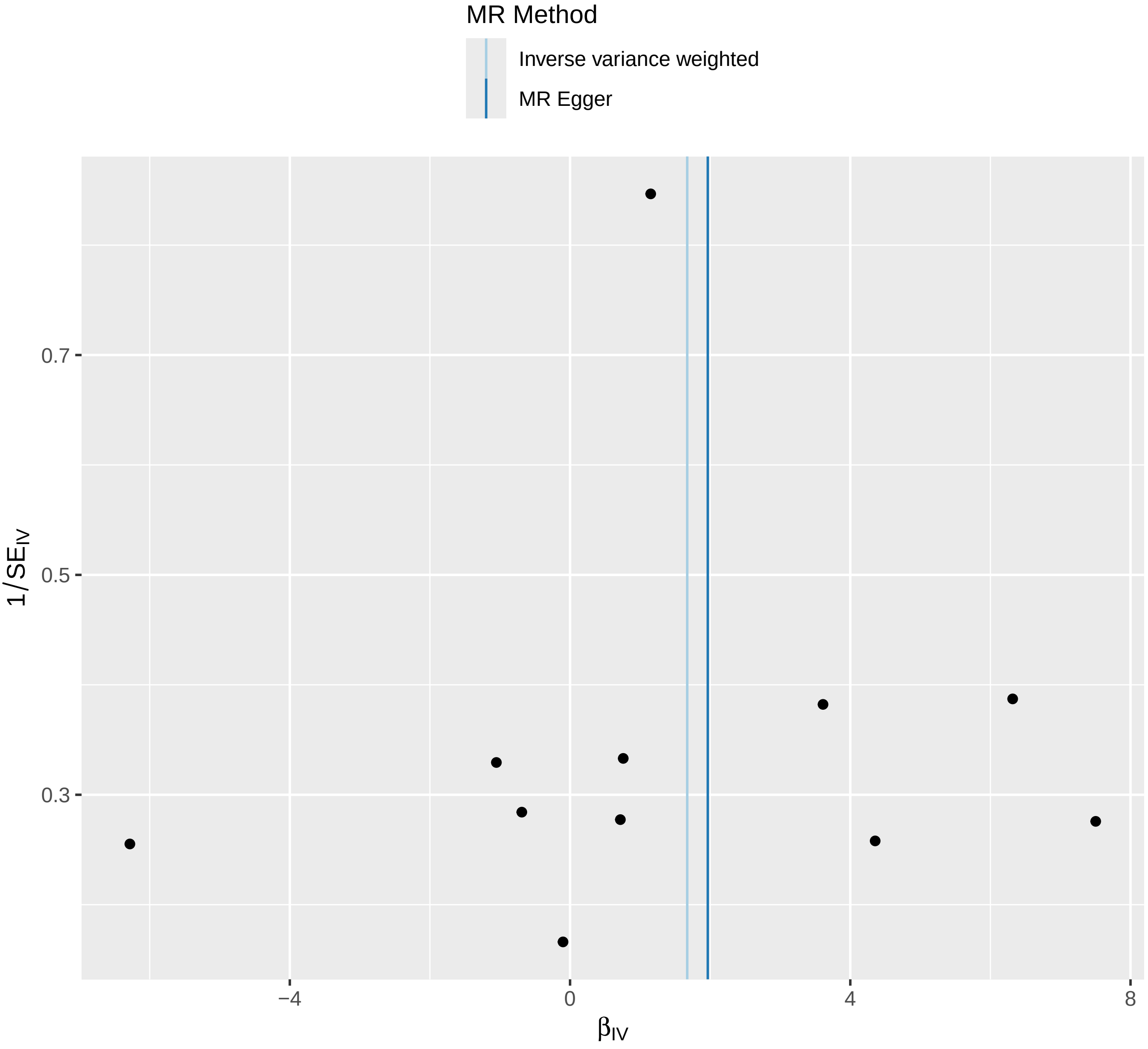

MR Method
Inverse variance weighted
MR Egger
0.7
0.5
0.3
1/SE$_{IV}$
−4
0
4
8
β$_{IV}$

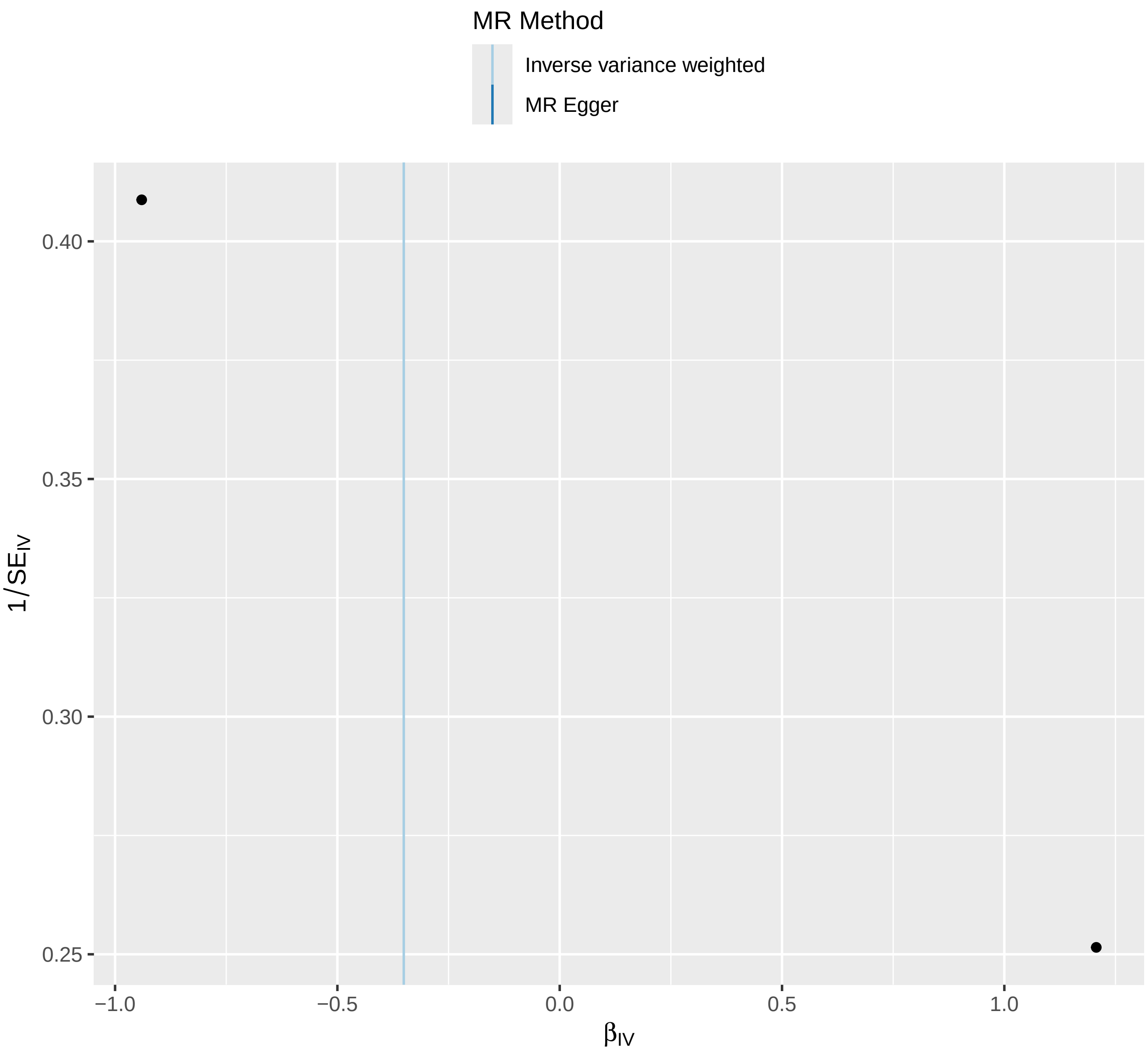
MR Method
Inverse variance weighted
MR Egger
0.40
0.35
0.30
0.25
1/SE_IV
−1.0
−0.5
0.0
0.5
1.0
β_IV

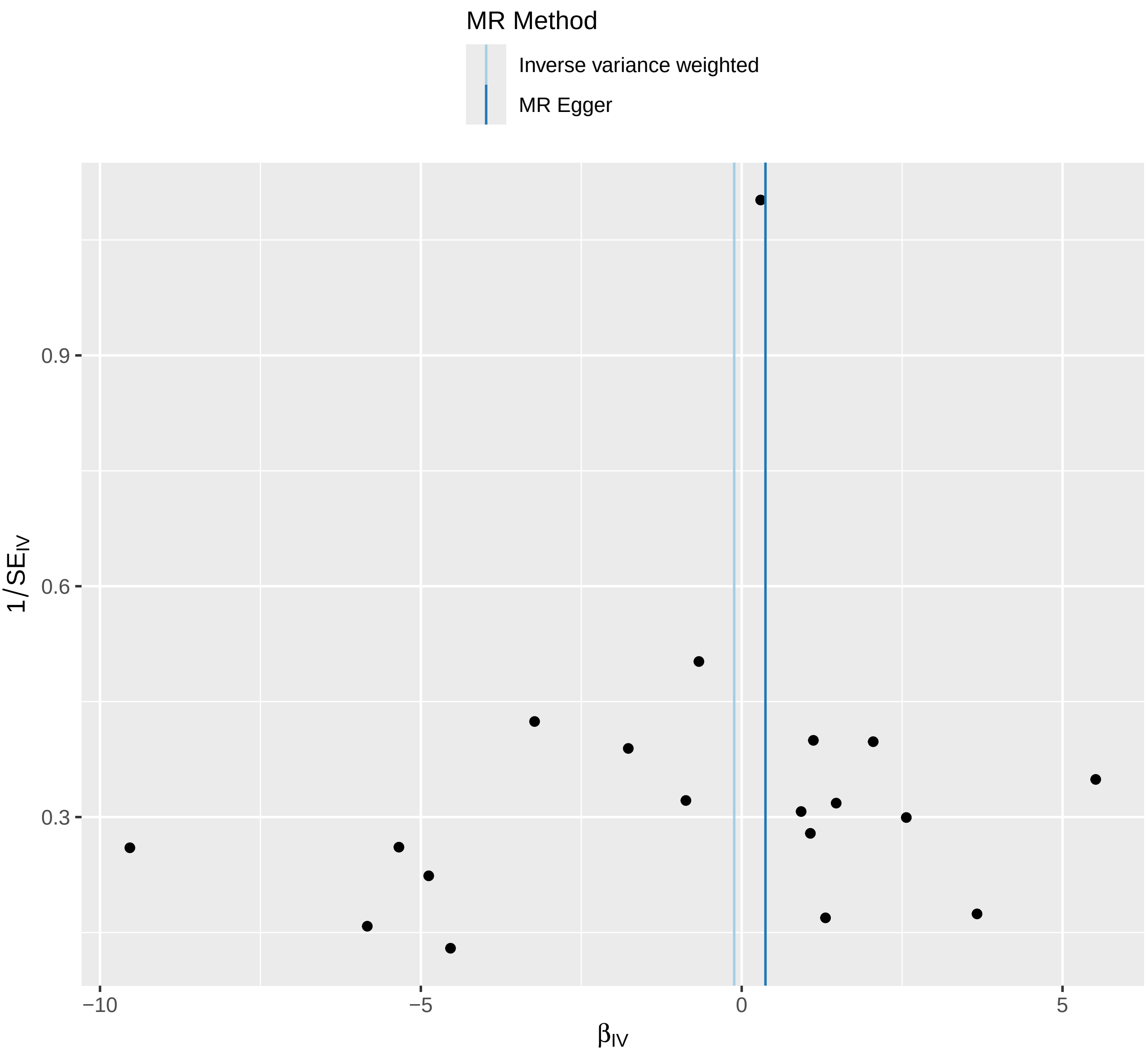
MR Method
Inverse variance weighted
MR Egger
0.9
0.6
0.3
1/SE_IV
−10
−5
0
5
β_IV

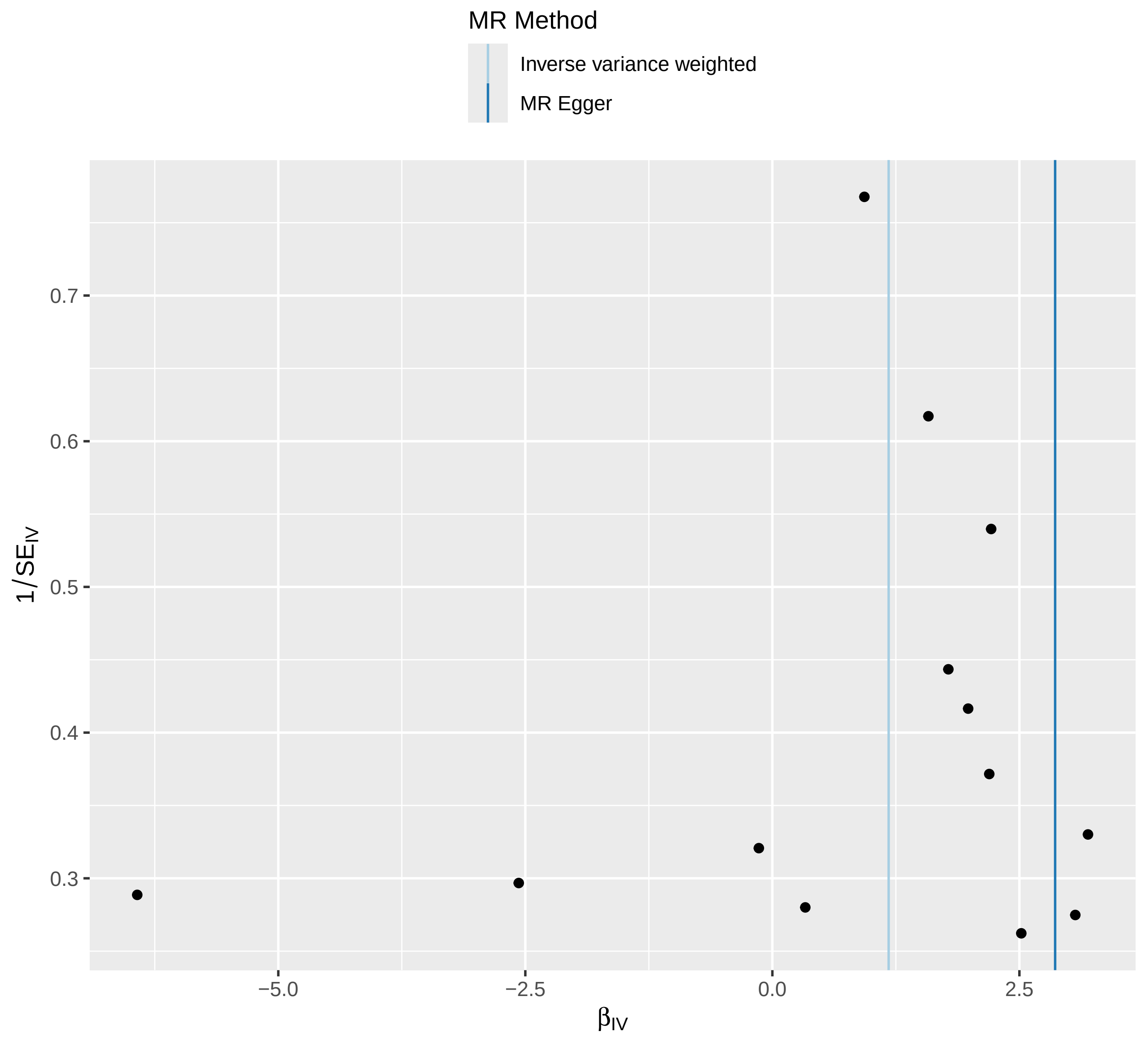
MR Method
Inverse variance weighted
MR Egger
0.7
0.6
0.5
0.4
0.3
$1/SE_{IV}$
−5.0
−2.5
0.0
2.5
β_{IV}

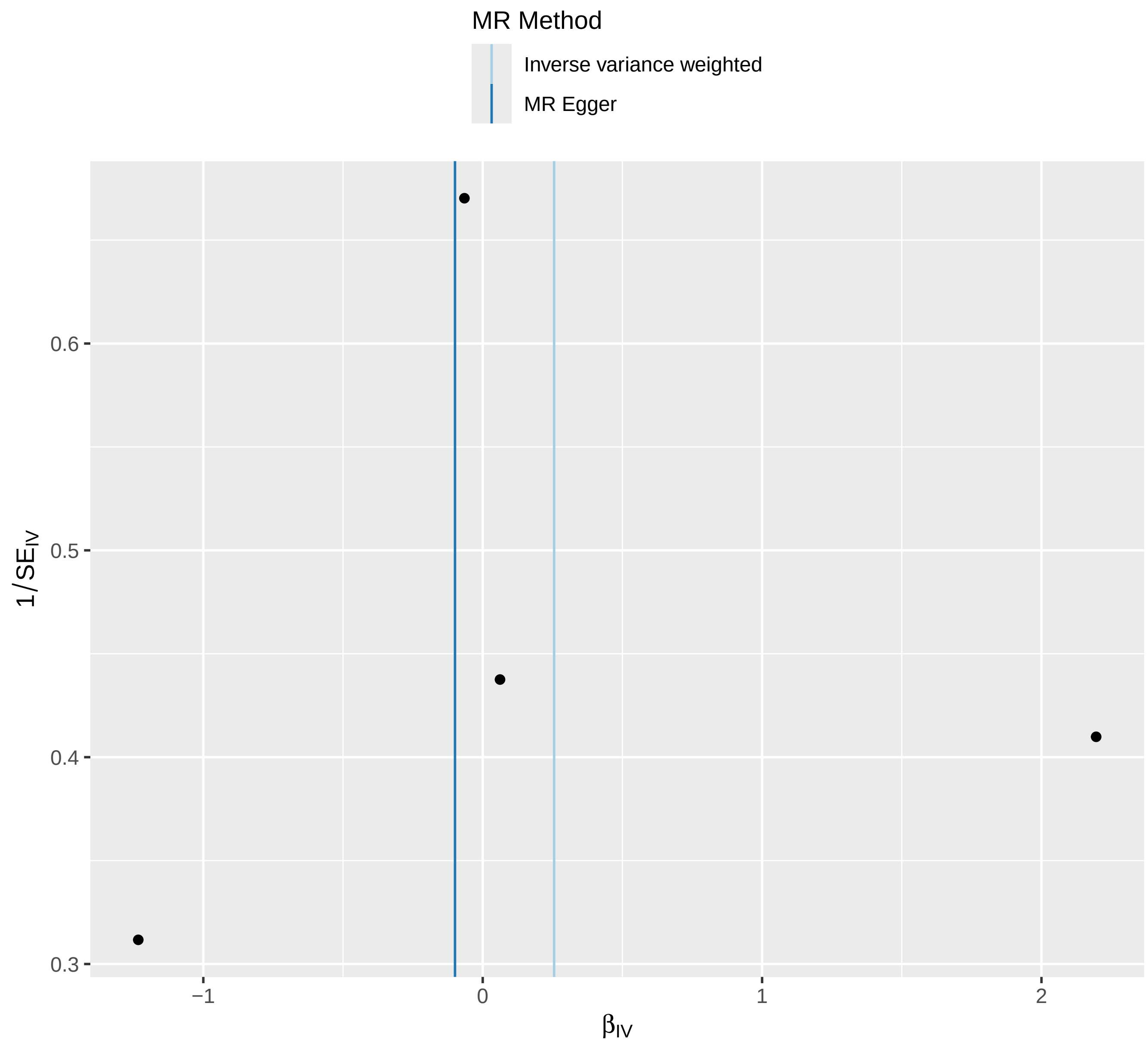
MR Method
Inverse variance weighted
MR Egger
0.6
0.5
0.4
0.3
−1
0
1
2
$1/SE_{IV}$
β_{IV}

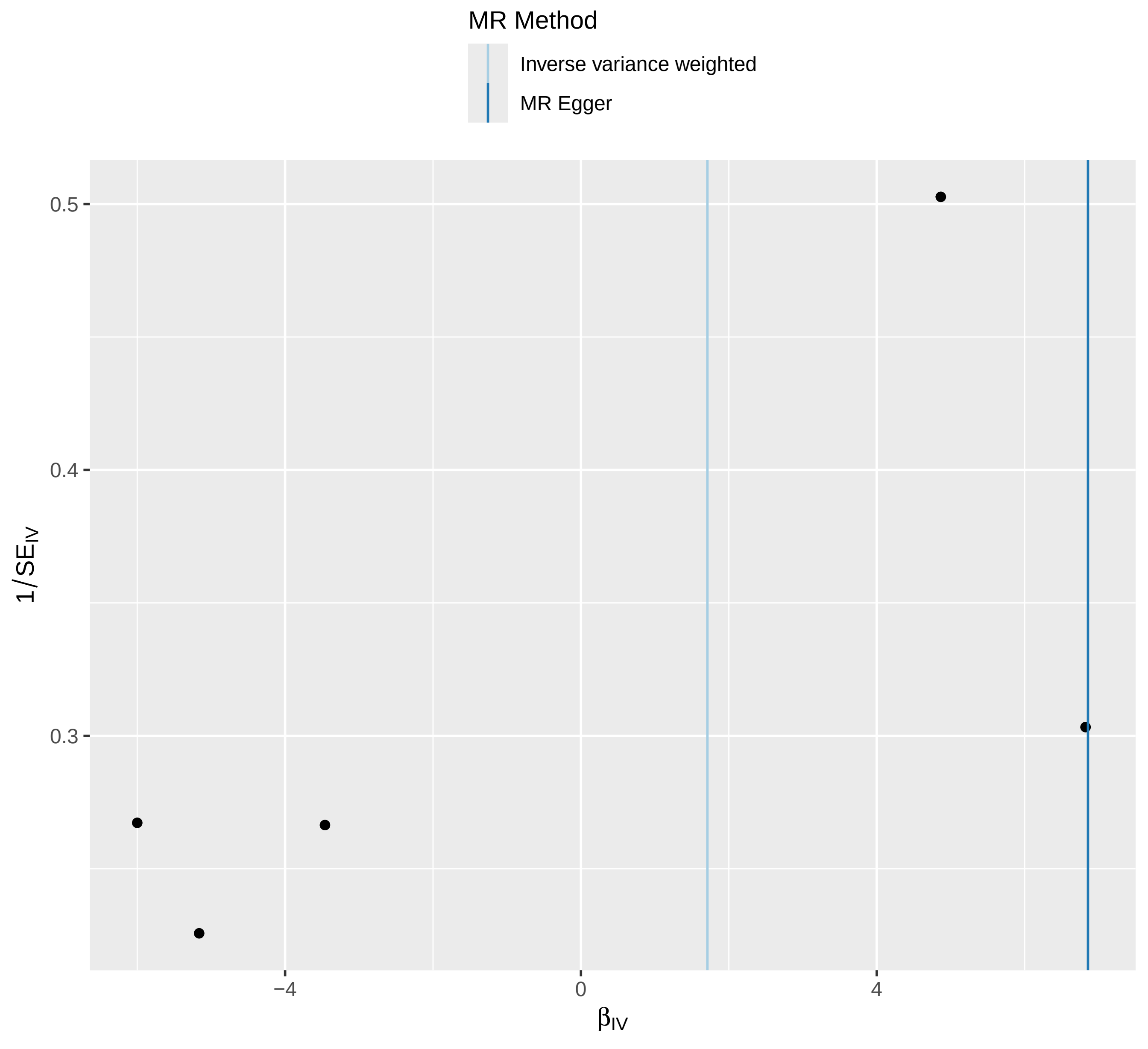
MR Method
Inverse variance weighted
MR Egger
0.5
0.4
0.3
1/SE_IV
−4
0
4
β_IV

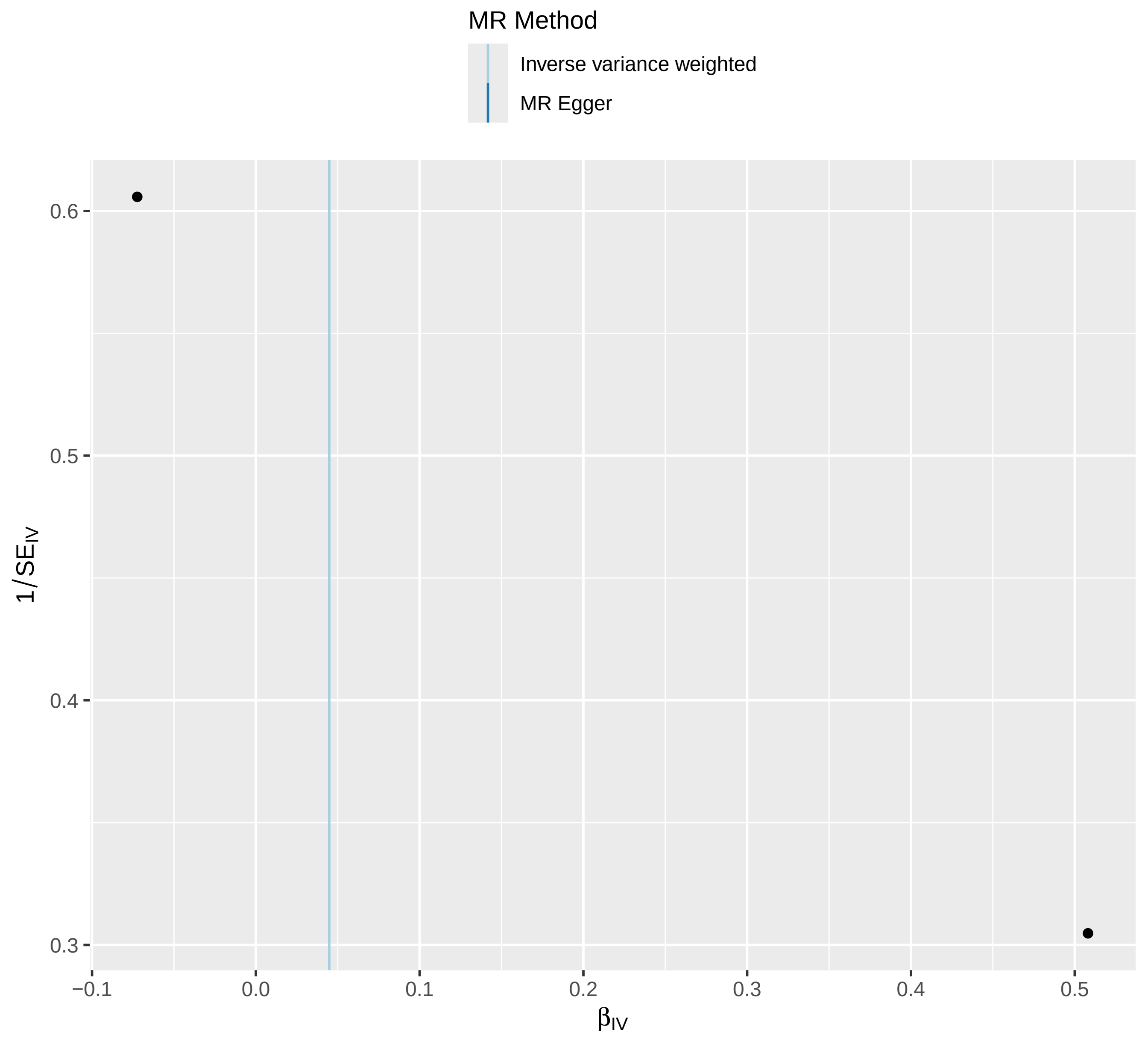

MR Method
Inverse variance weighted
MR Egger
0.6
0.5
0.4
0.3
1/SE_IV
−0.1
0.0
0.1
0.2
0.3
0.4
0.5
β_IV

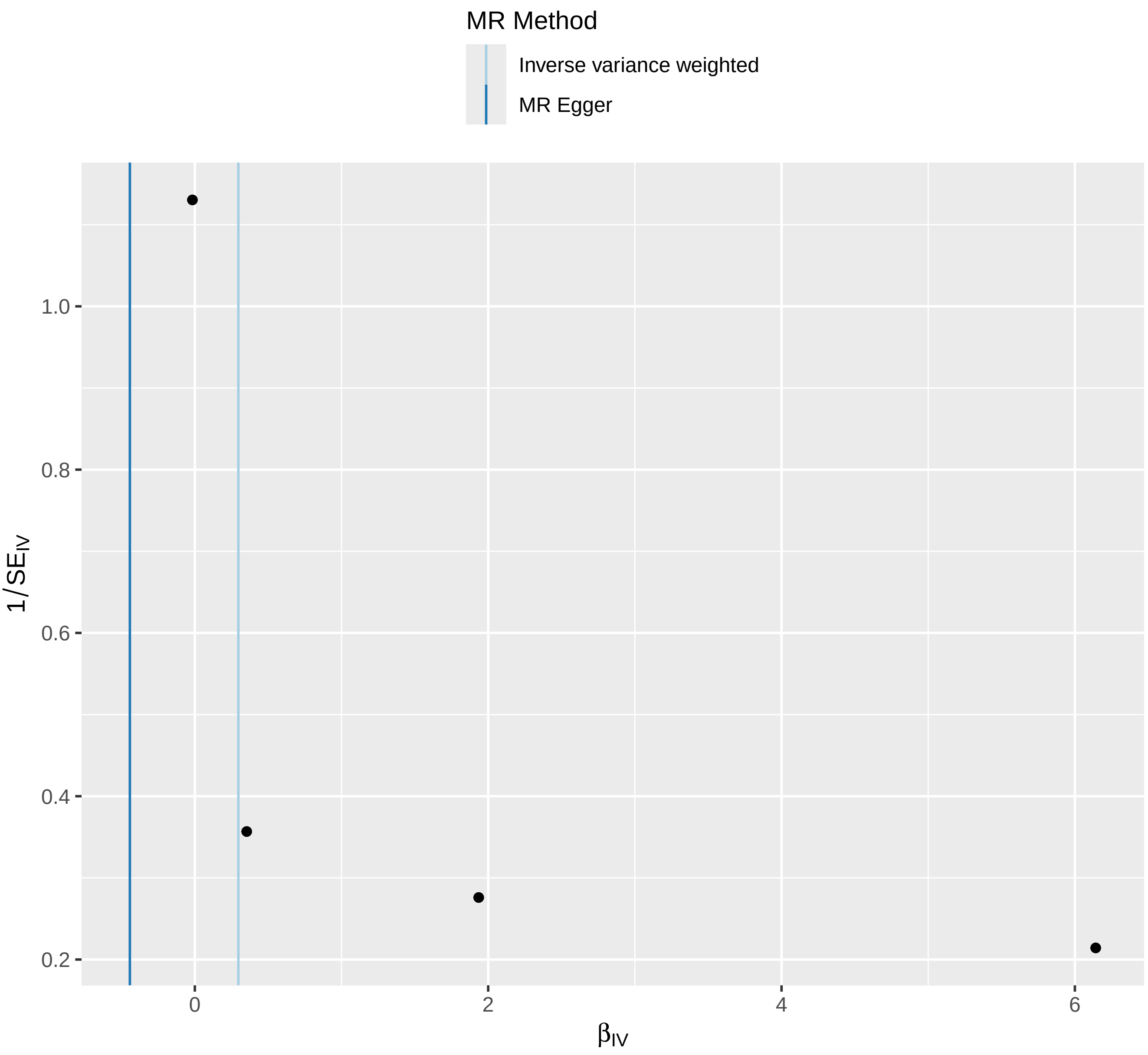
MR Method
Inverse variance weighted
MR Egger
1.0
0.8
0.6
0.4
0.2
1/SE_IV
0
2
4
6
β_IV

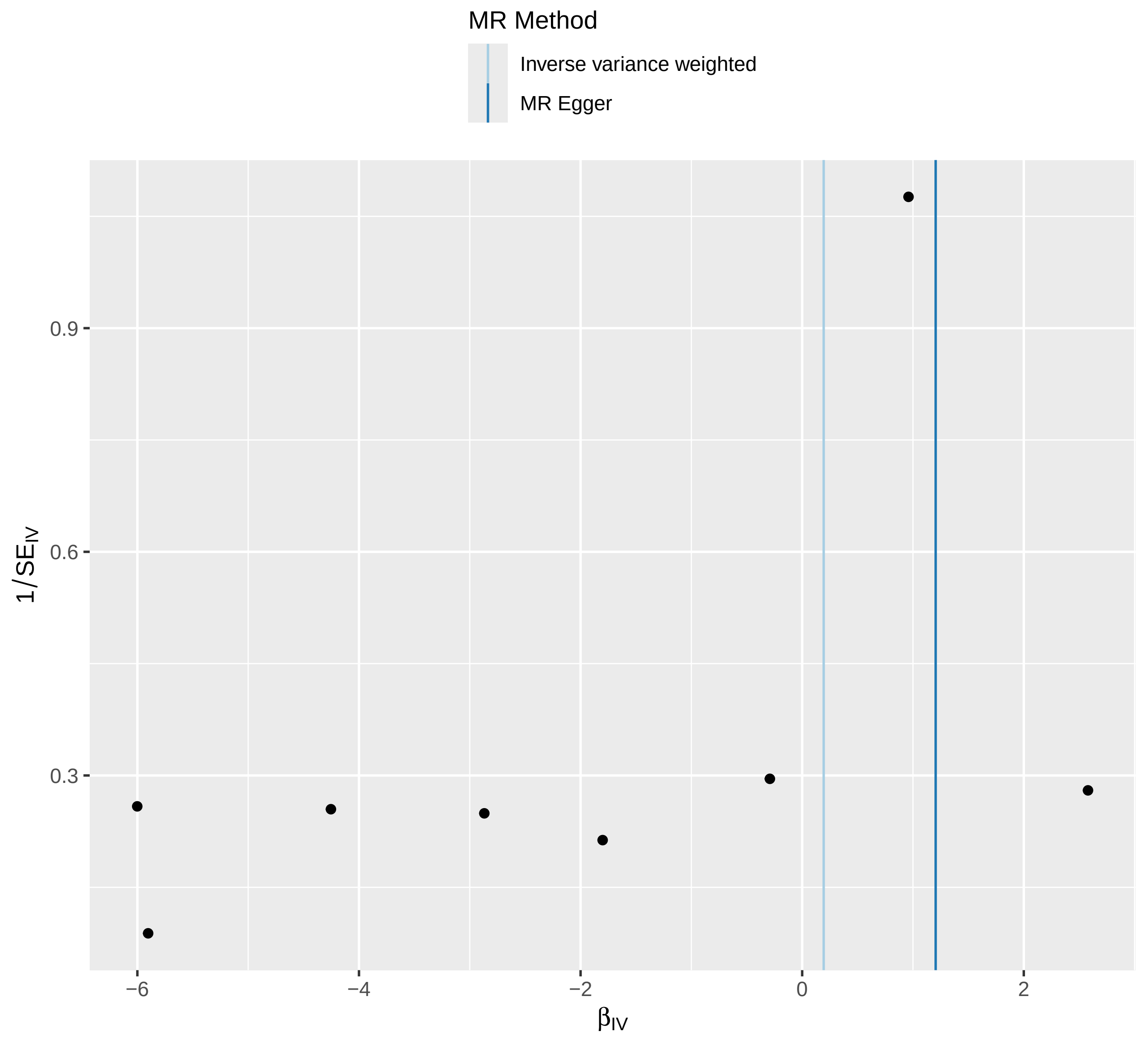
MR Method
Inverse variance weighted
MR Egger
0.9
0.6
0.3
1/SE_IV
−6
−4
−2
0
2
β_IV

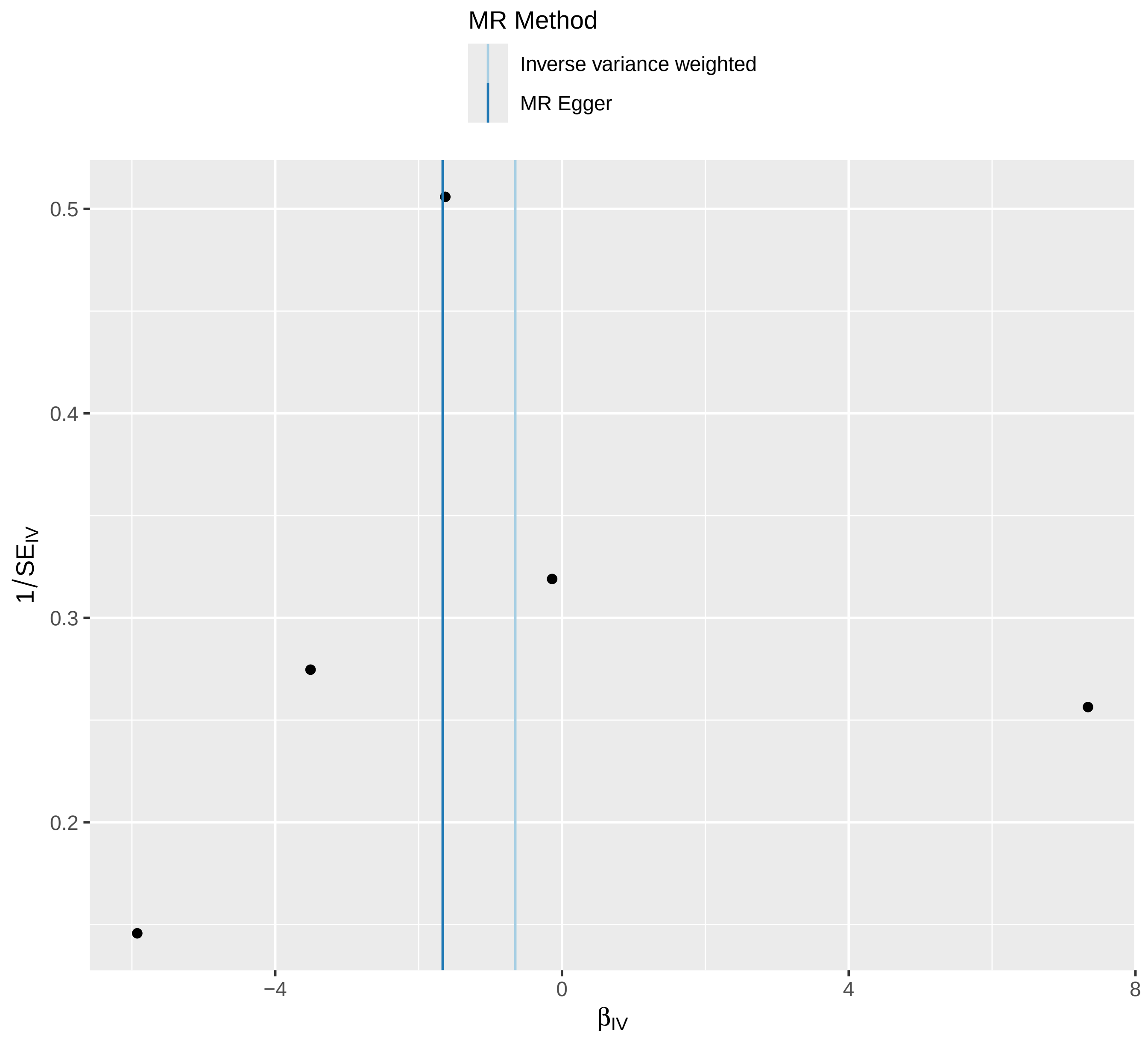
MR Method
Inverse variance weighted
MR Egger
1/SE_IV
0.5
0.4
0.3
0.2
−4
0
4
8
β_IV

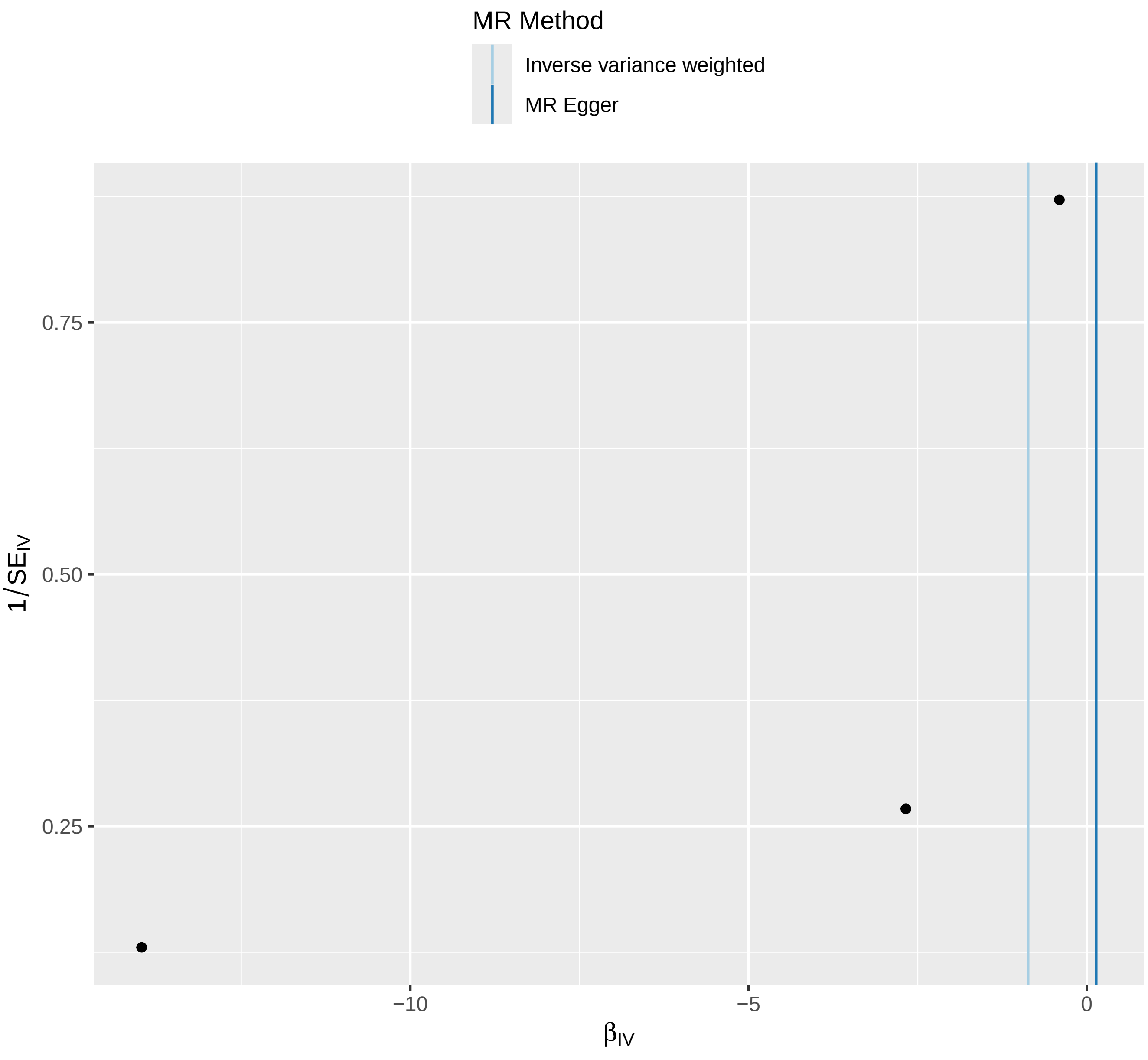
MR Method
Inverse variance weighted
MR Egger
0.75
0.50
0.25
1/SE_IV
−10
−5
0
β_IV

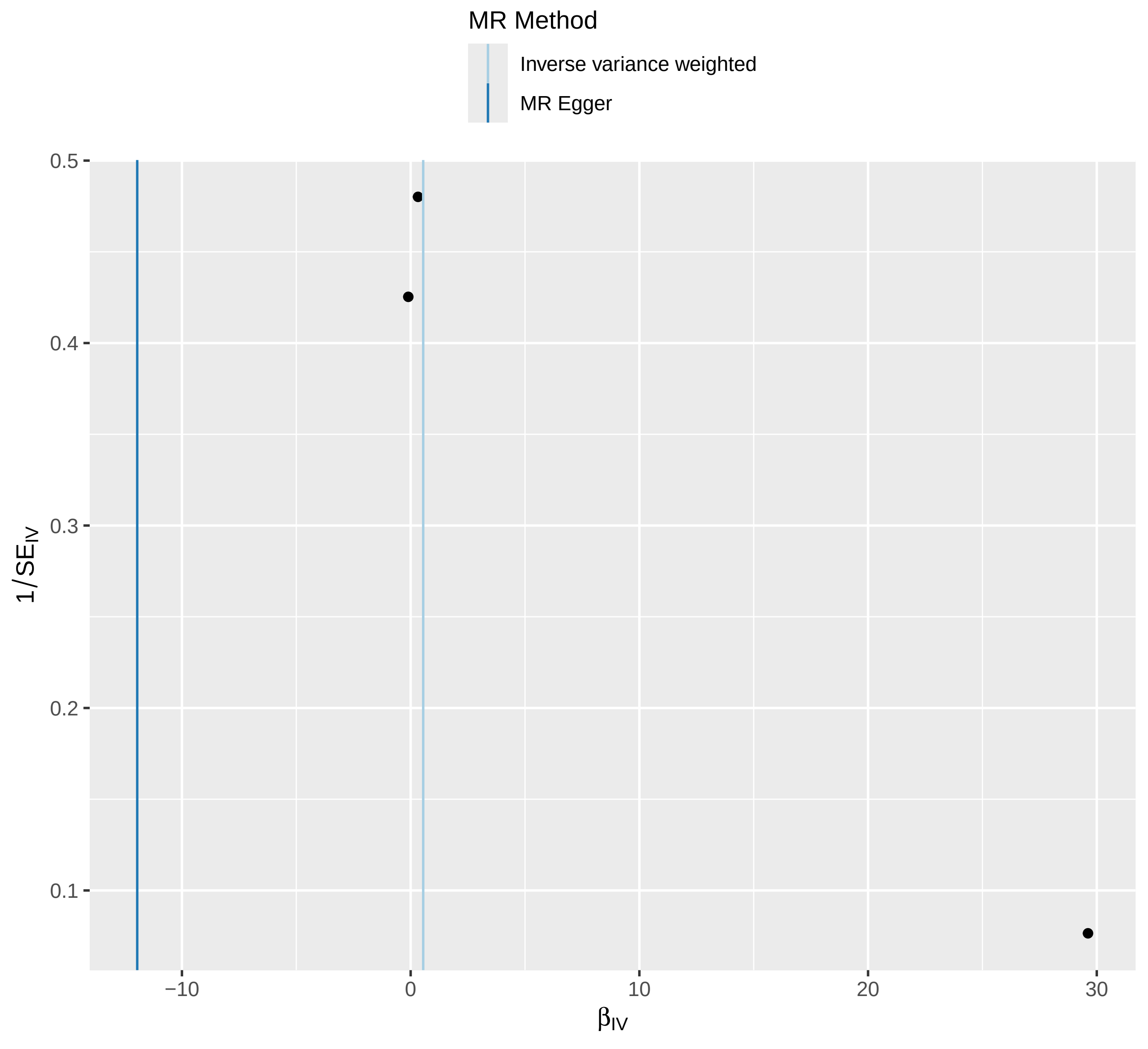
MR Method
Inverse variance weighted
MR Egger
0.5
0.4
0.3
0.2
0.1
1/SE_IV
−10
0
10
20
30
β_IV

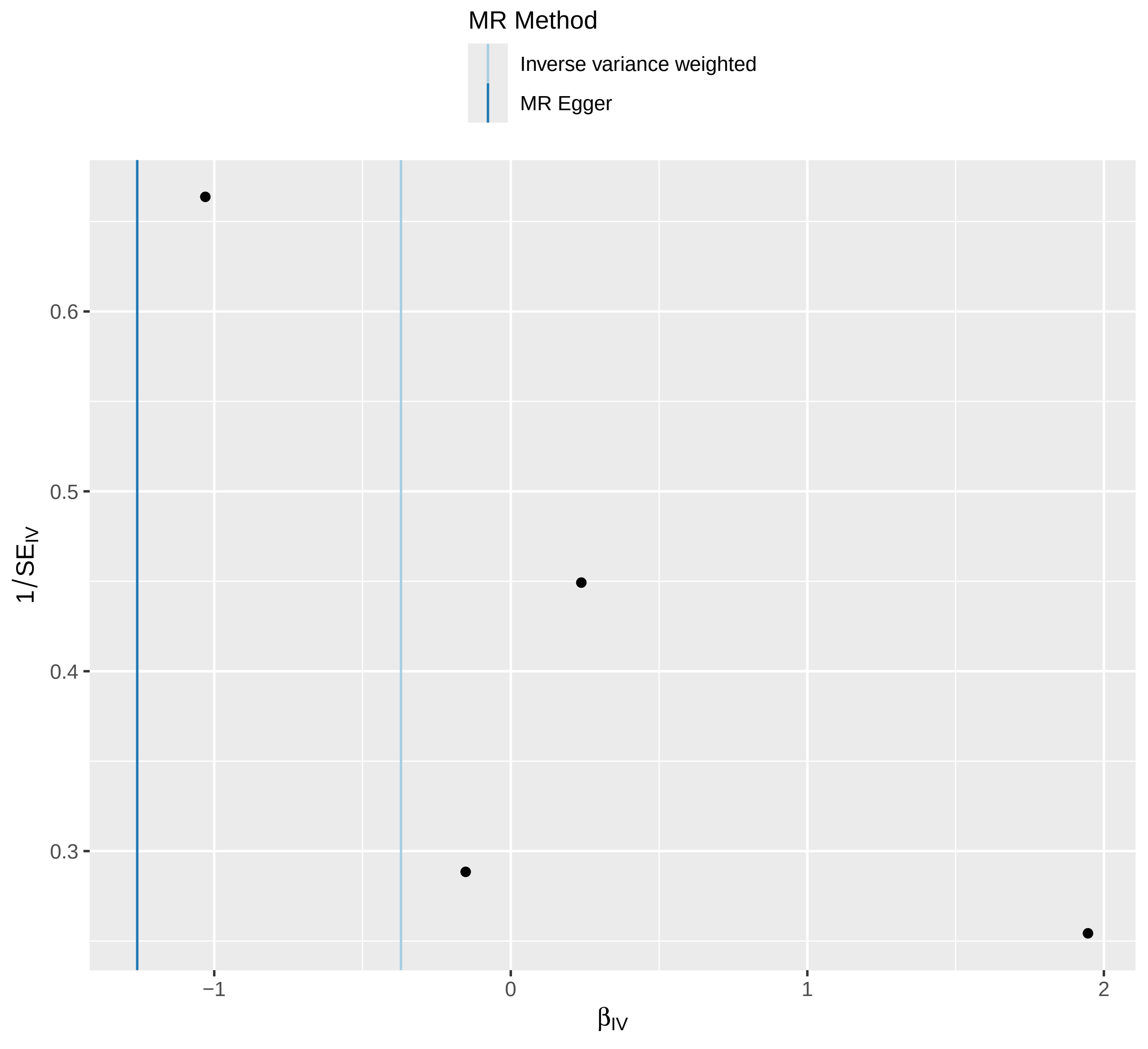
MR Method
Inverse variance weighted
MR Egger
0.6
0.5
0.4
0.3
1/SE_IV
−1
0
1
2
β_IV

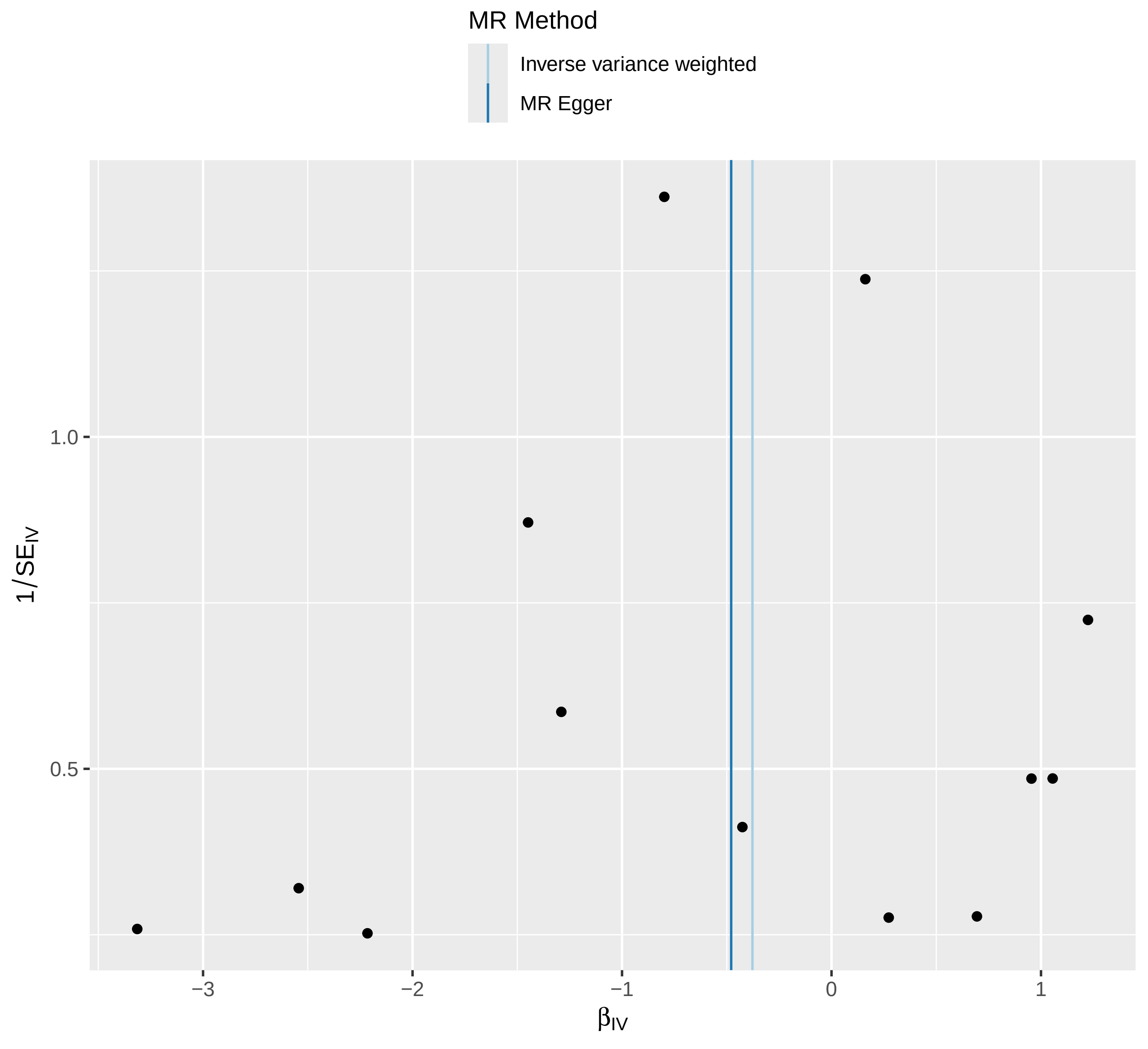

MR Method
Inverse variance weighted
MR Egger
1.0
0.5
1/SE$_{IV}$
−3
−2
−1
0
1
β$_{IV}$

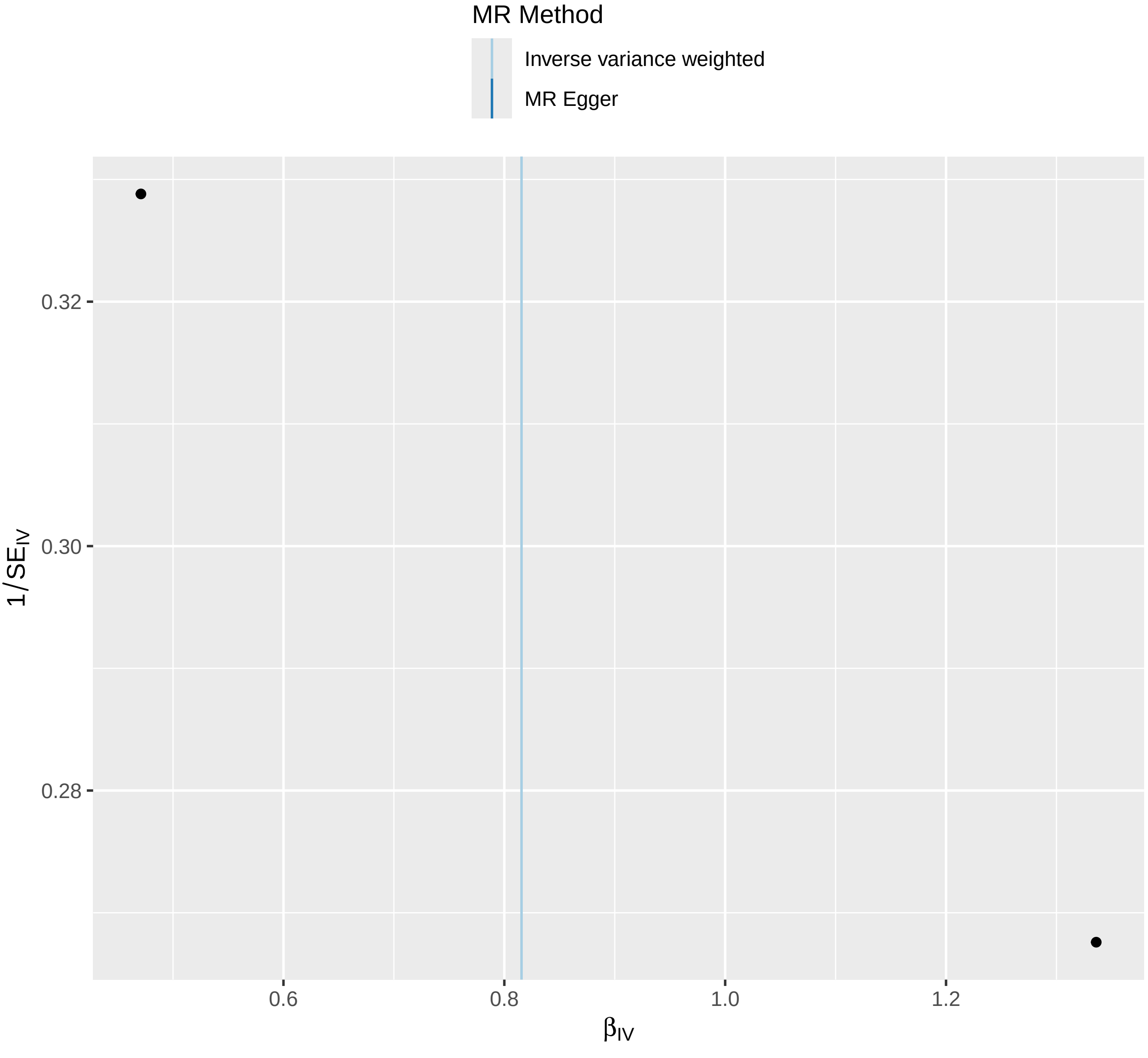

MR Method
Inverse variance weighted
MR Egger
0.32
0.30
0.28
$1/SE_{IV}$
0.6
0.8
1.0
1.2
β_{IV}

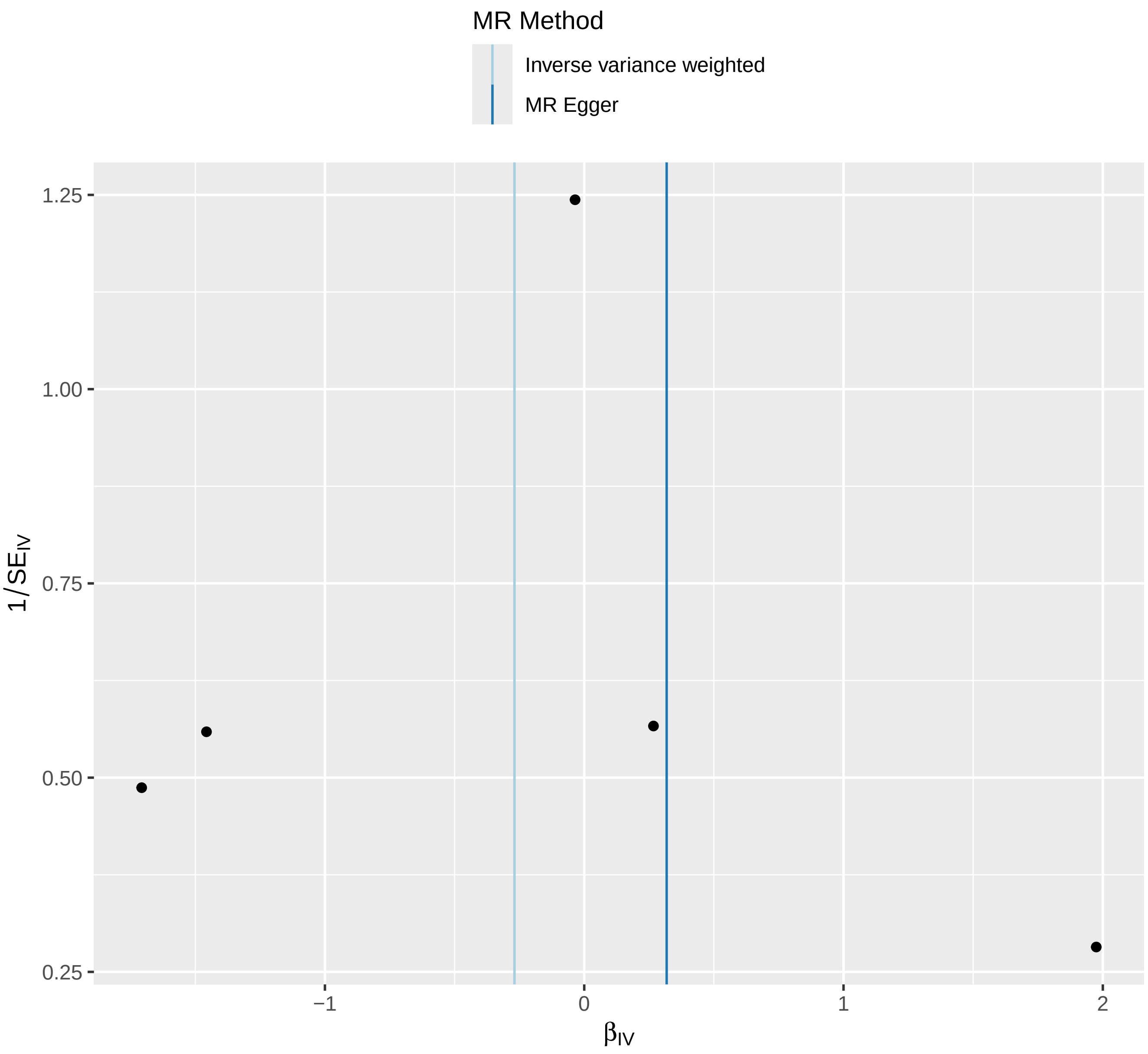
MR Method
Inverse variance weighted
MR Egger
1.25
1.00
0.75
0.50
0.25
1/SE_IV
−1
0
1
2
β_IV

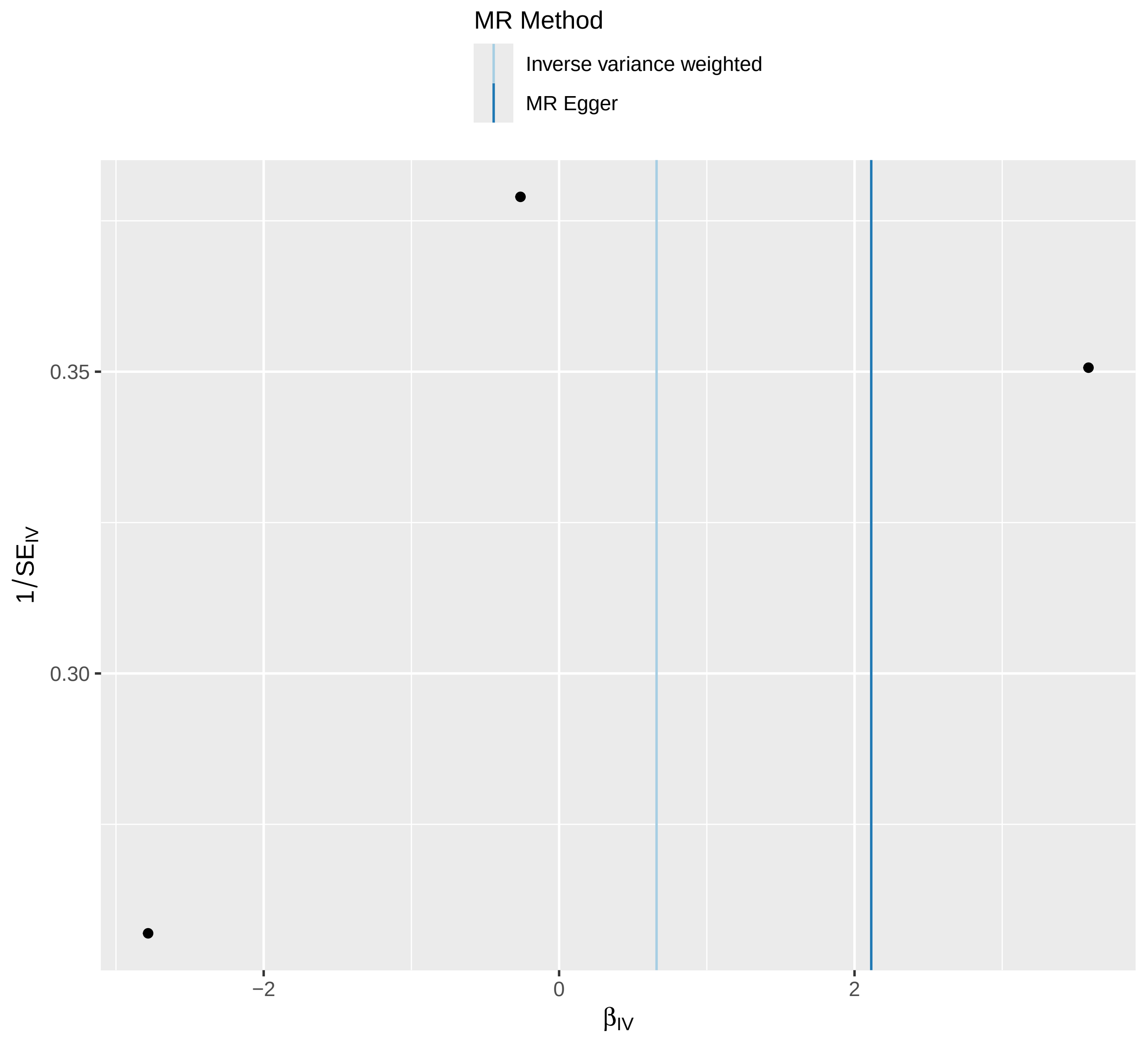

MR Method
Inverse variance weighted
MR Egger
0.35
0.30
1/SE_IV
−2
0
2
β_IV

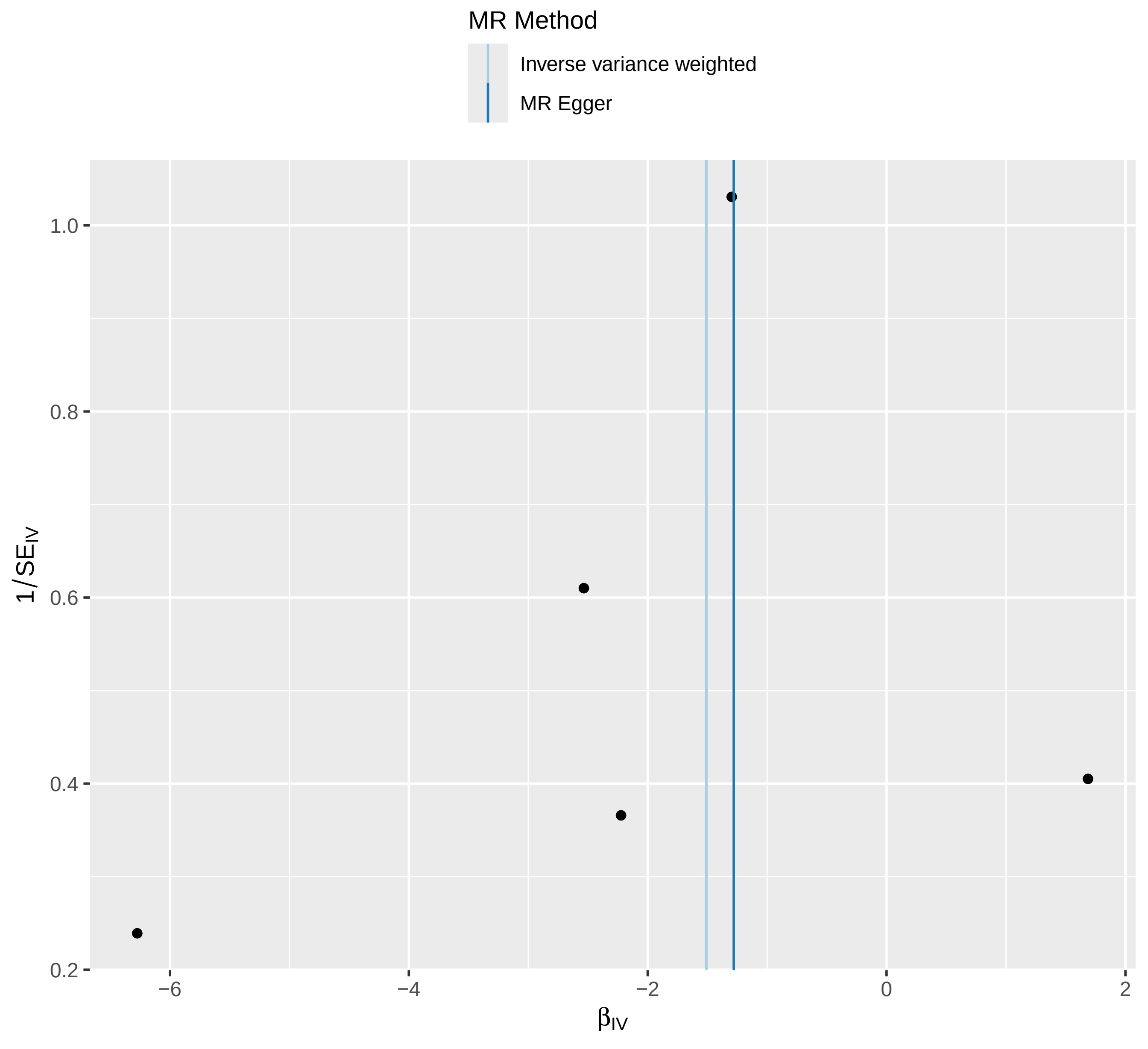
MR Method
Inverse variance weighted
MR Egger
1.0
0.8
0.6
0.4
0.2
1/SE_IV
−6
−4
−2
0
2
β_IV

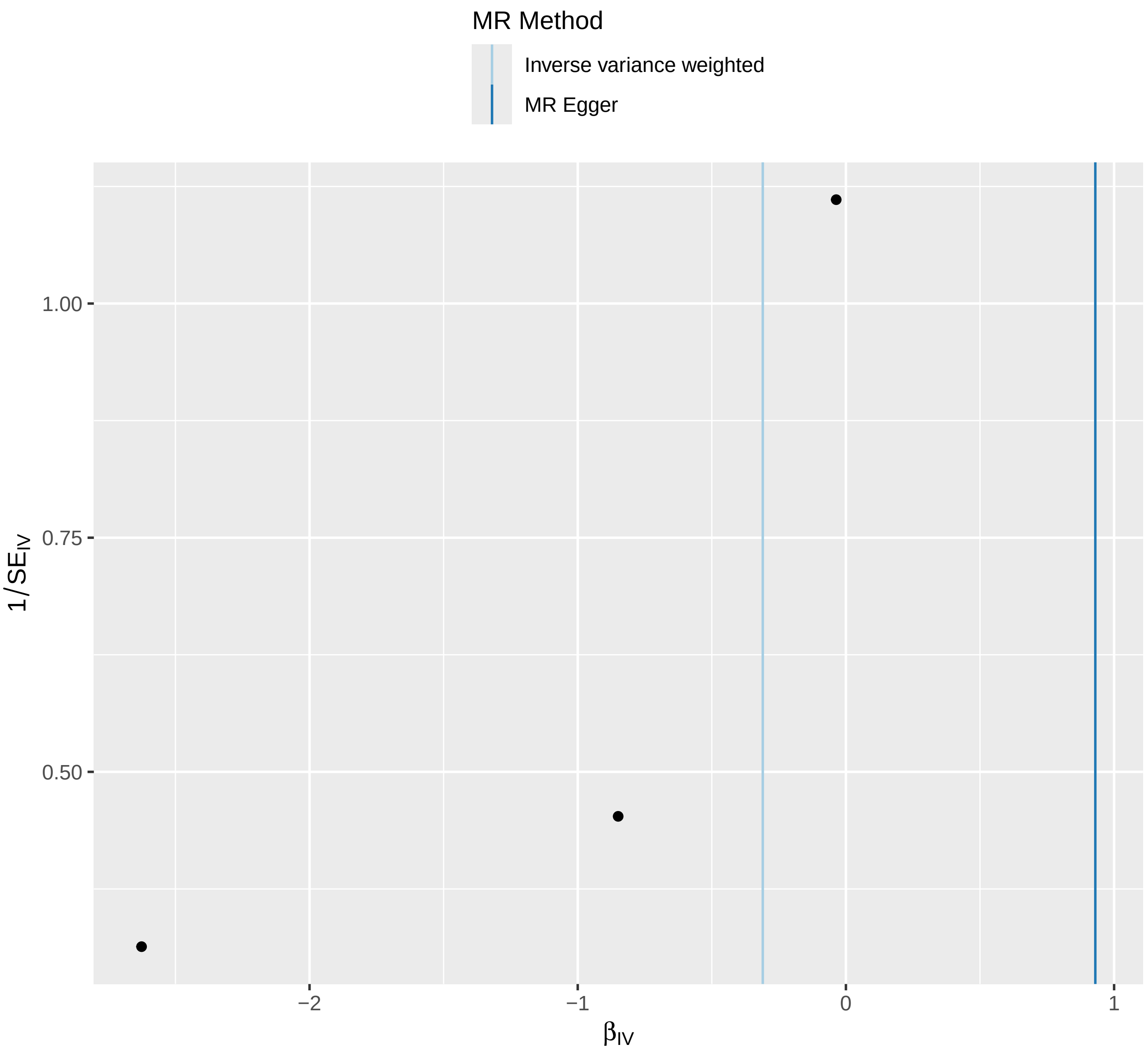
MR Method
Inverse variance weighted
MR Egger
1.00
0.75
0.50
1/SE_{IV}
−2
−1
0
1
β_{IV}

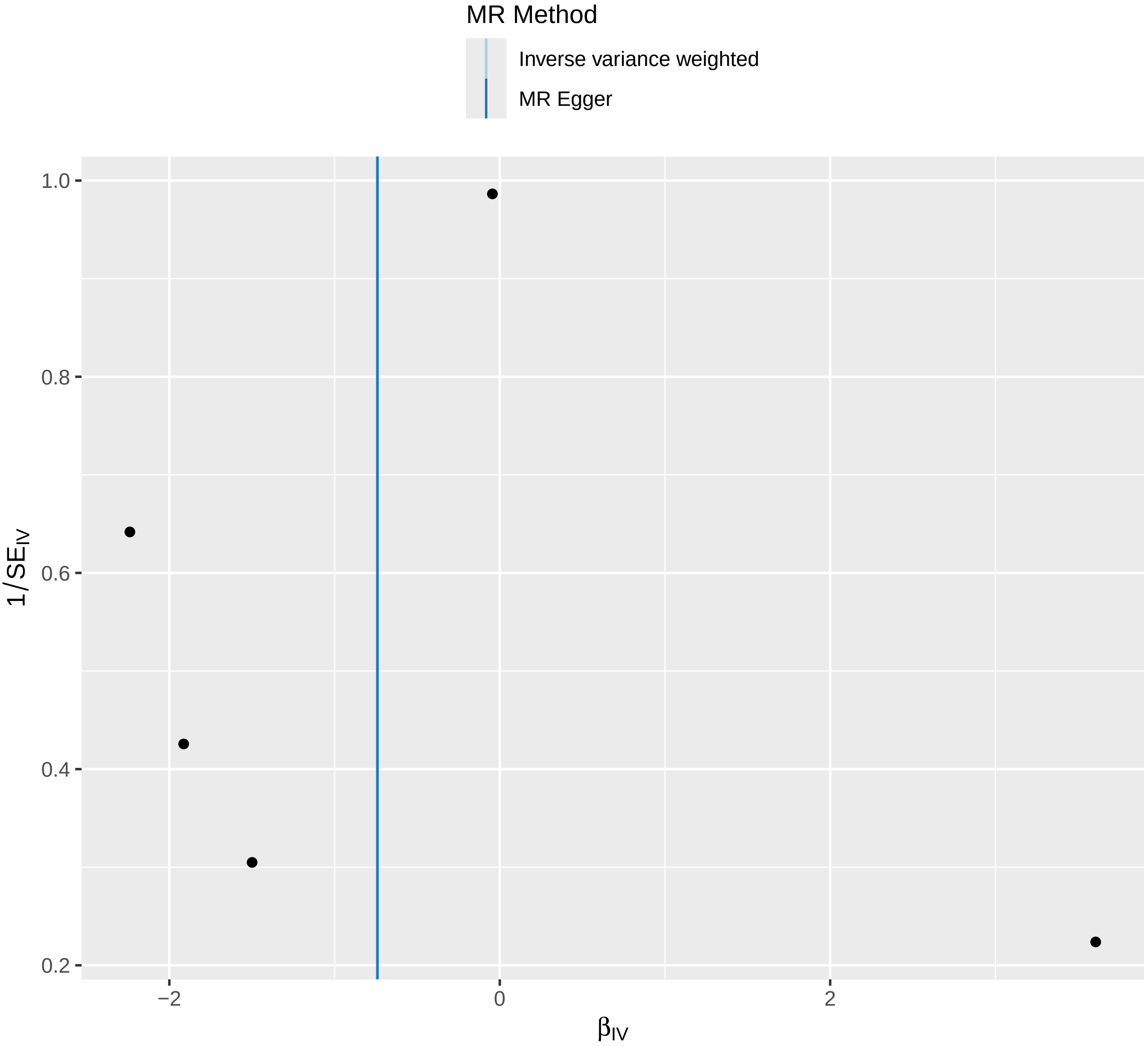
MR Method
Inverse variance weighted
MR Egger
1.0
0.8
0.6
0.4
0.2
−2
0
2
1/SE_IV
β_IV

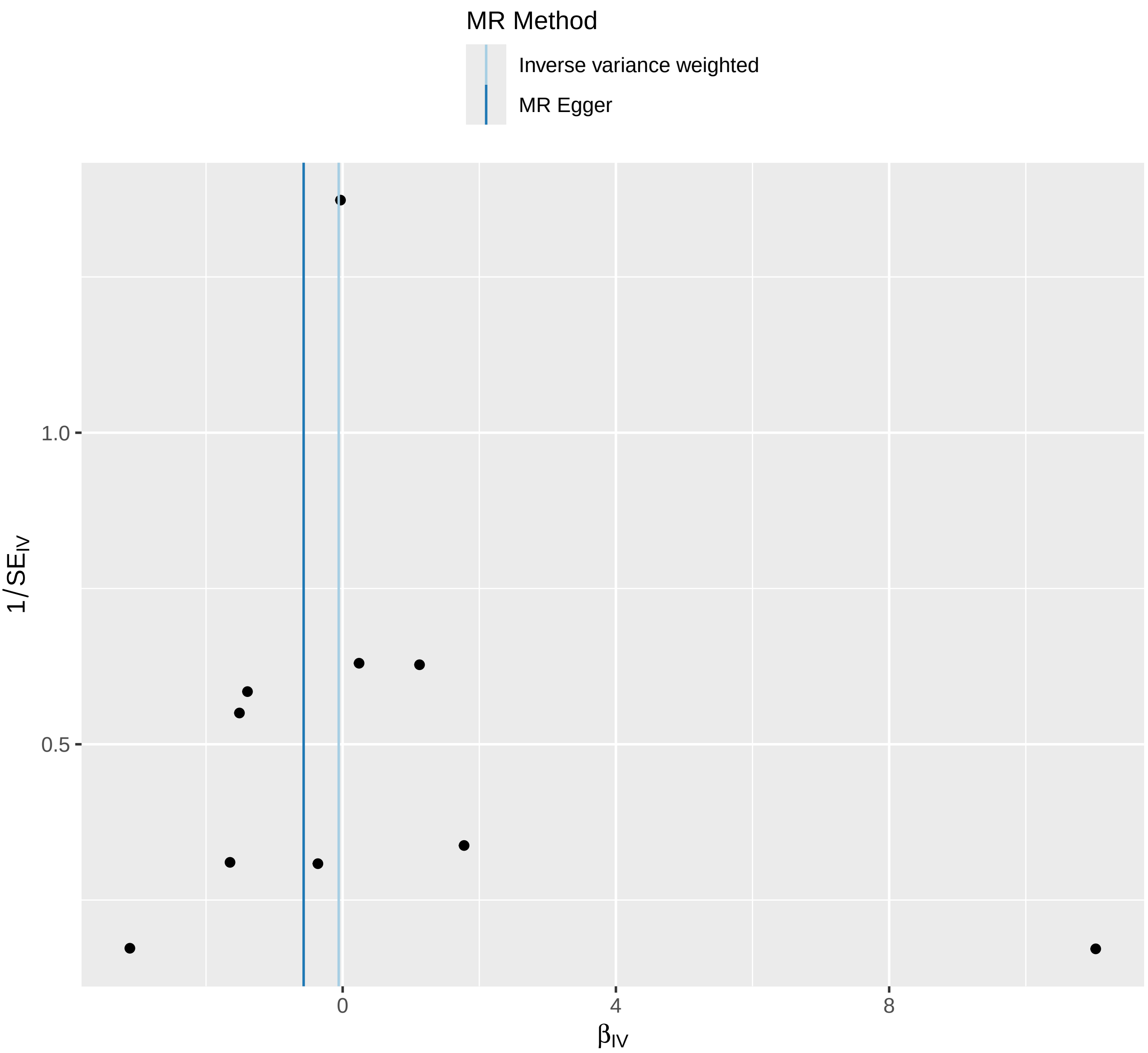
MR Method
Inverse variance weighted
MR Egger
1.0
0.5
$1/SE_{IV}$
0
4
8
β_{IV}

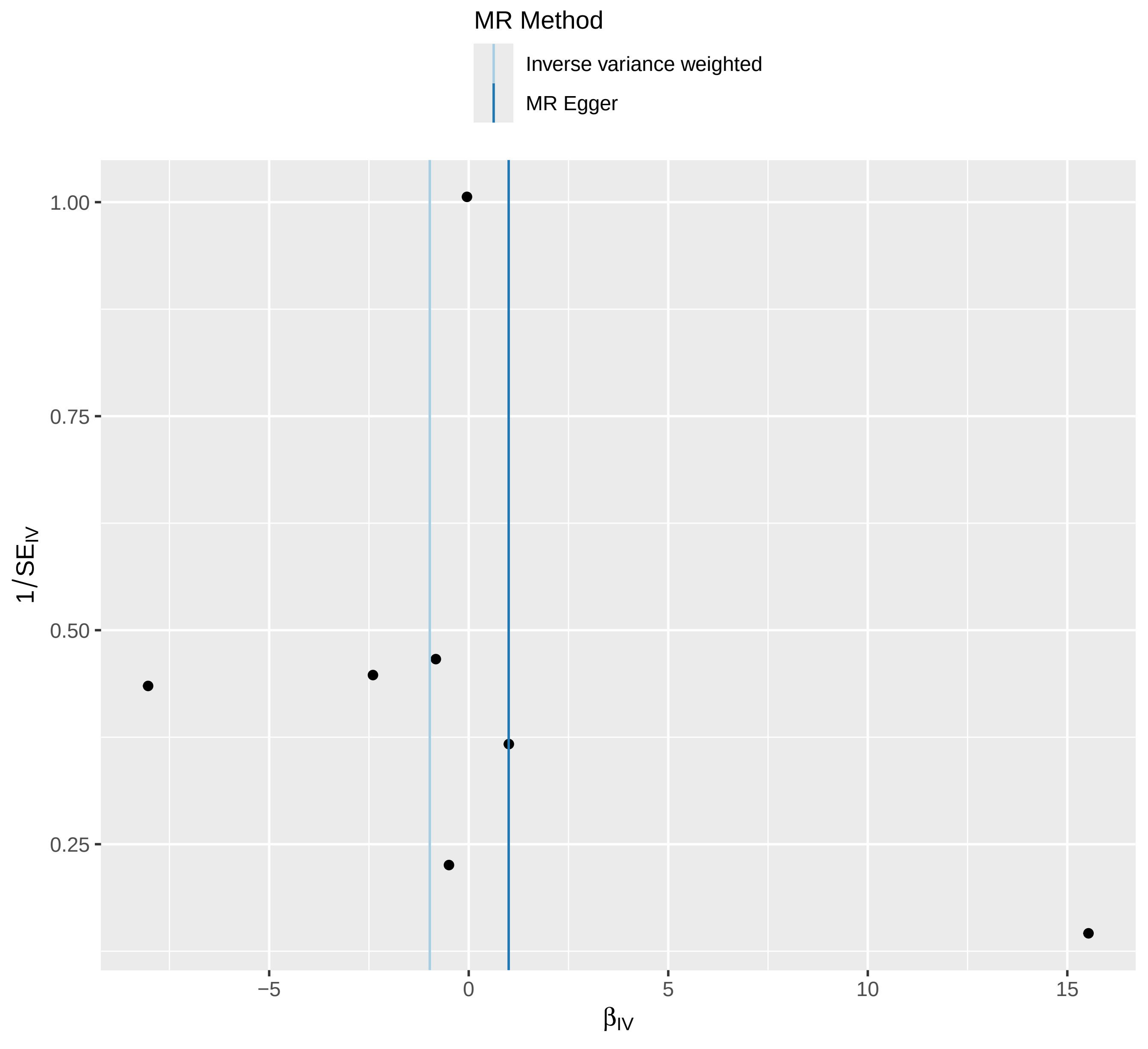
MR Method
Inverse variance weighted
MR Egger
1.00
0.75
0.50
0.25
$1/SE_{IV}$
−5
0
5
10
15
β_{IV}

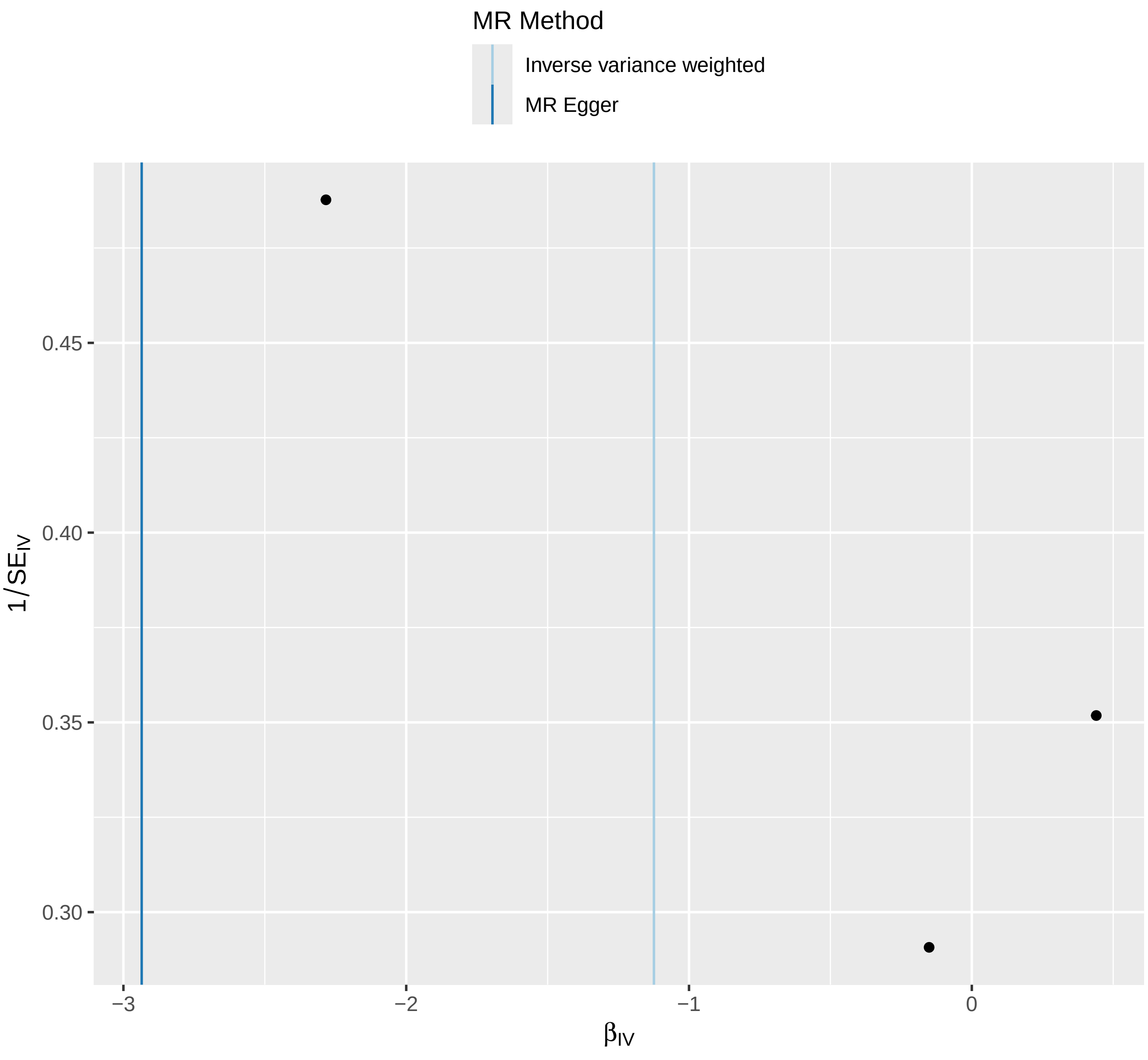

MR Method
Inverse variance weighted
MR Egger
0.45
0.40
0.35
0.30
1/SE$_{IV}$
−3
−2
−1
0
β$_{IV}$

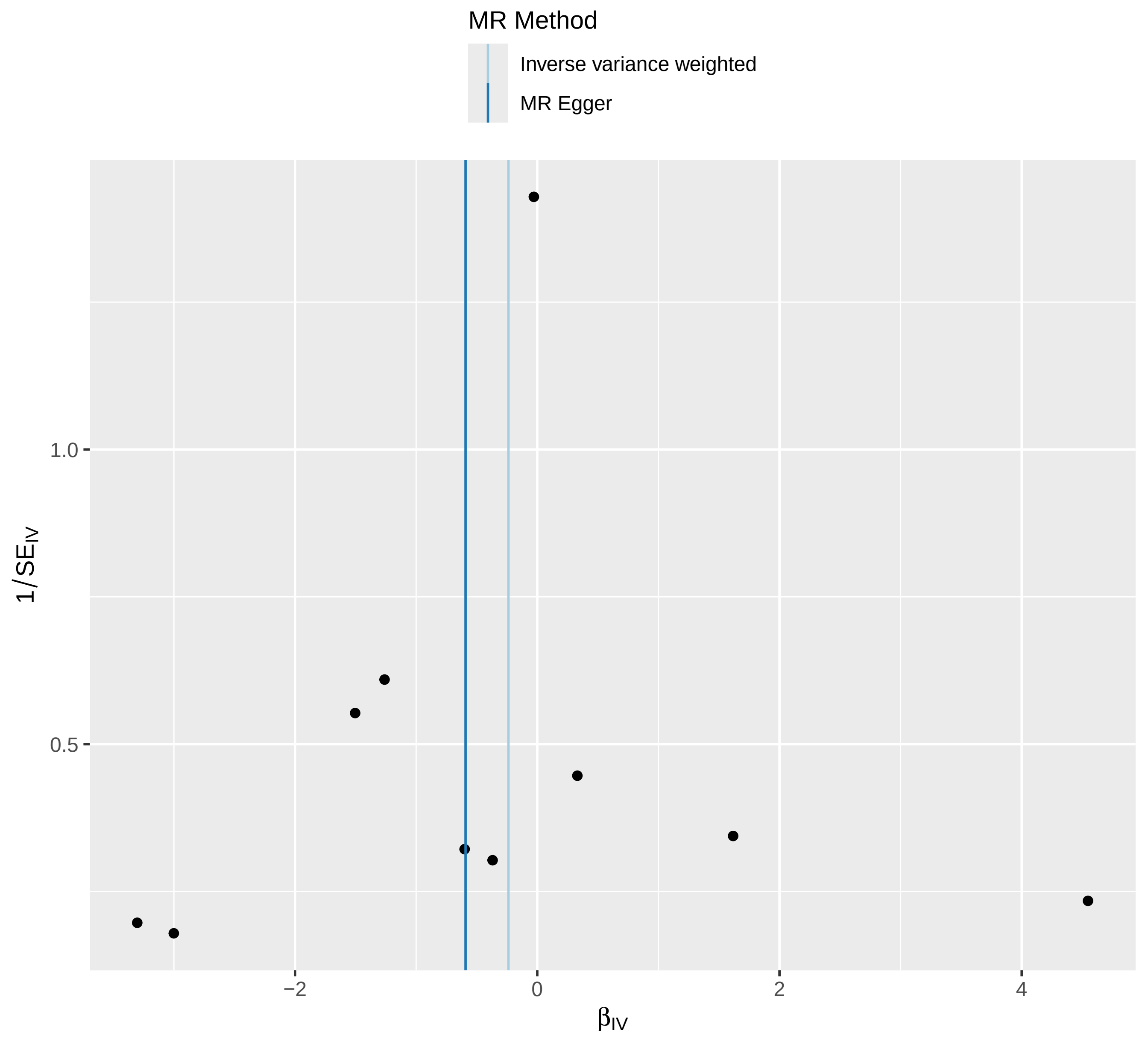
MR Method
Inverse variance weighted
MR Egger
1.0
0.5
1/SE_IV
−2
0
2
4
β_IV

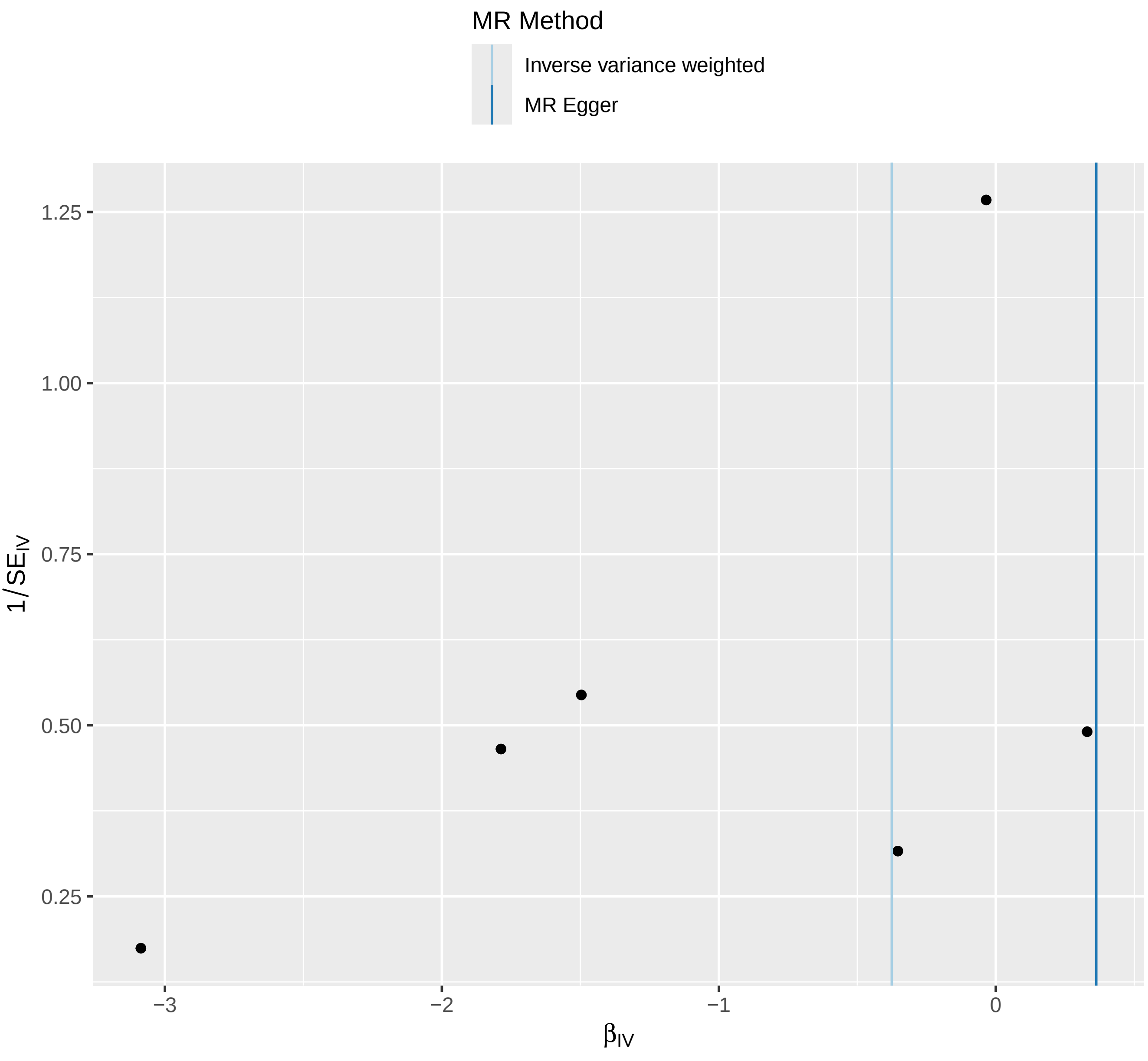
MR Method
Inverse variance weighted
MR Egger
1.25
1.00
0.75
0.50
0.25
1/SE_IV
−3
−2
−1
0
β_IV

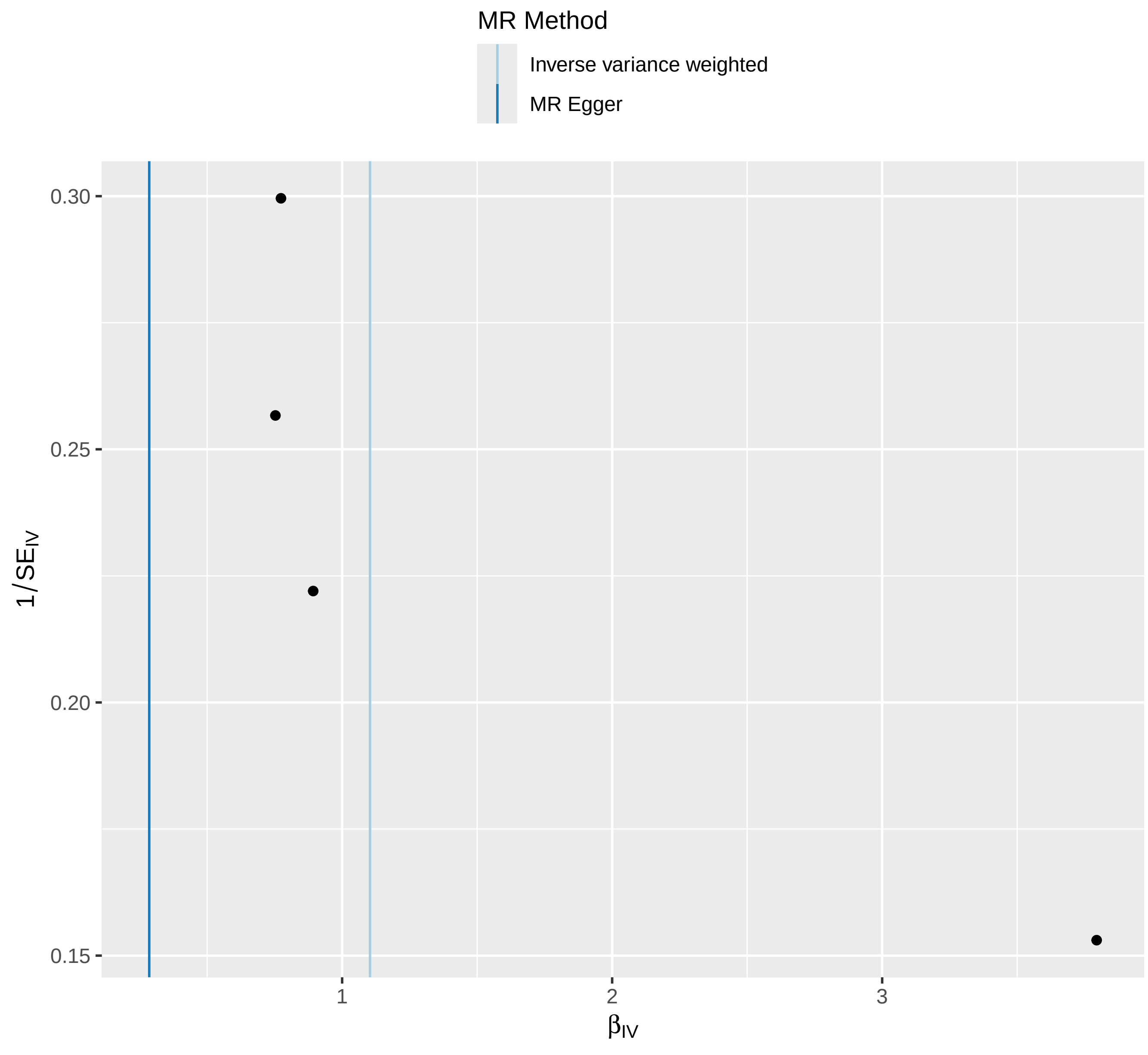
MR Method
Inverse variance weighted
MR Egger
0.30
0.25
0.20
0.15
1/SE_IV
1
2
3
β_IV

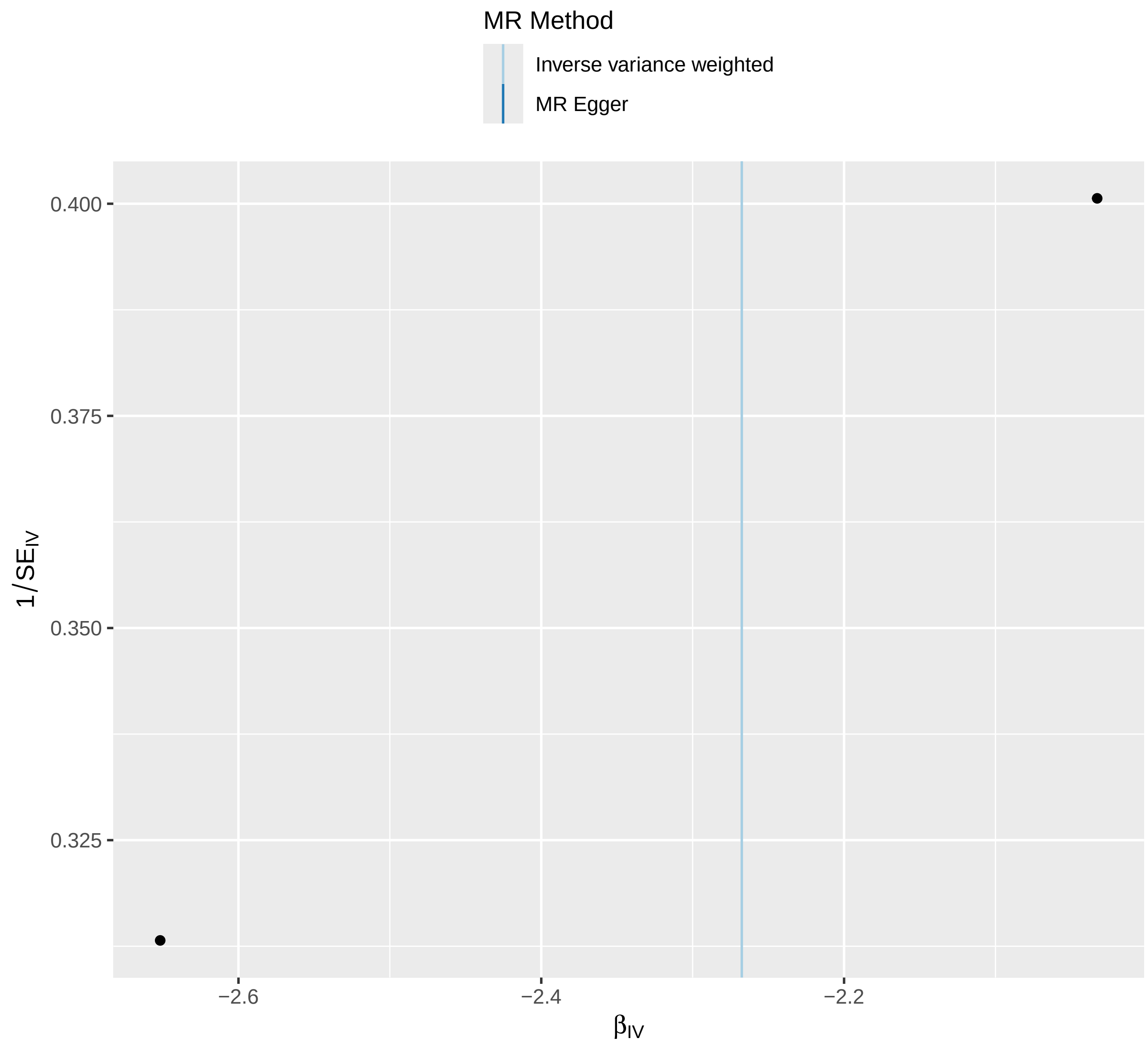

MR Method
Inverse variance weighted
MR Egger
0.400
0.375
0.350
0.325
1/SE_IV
−2.6
−2.4
−2.2
β_IV

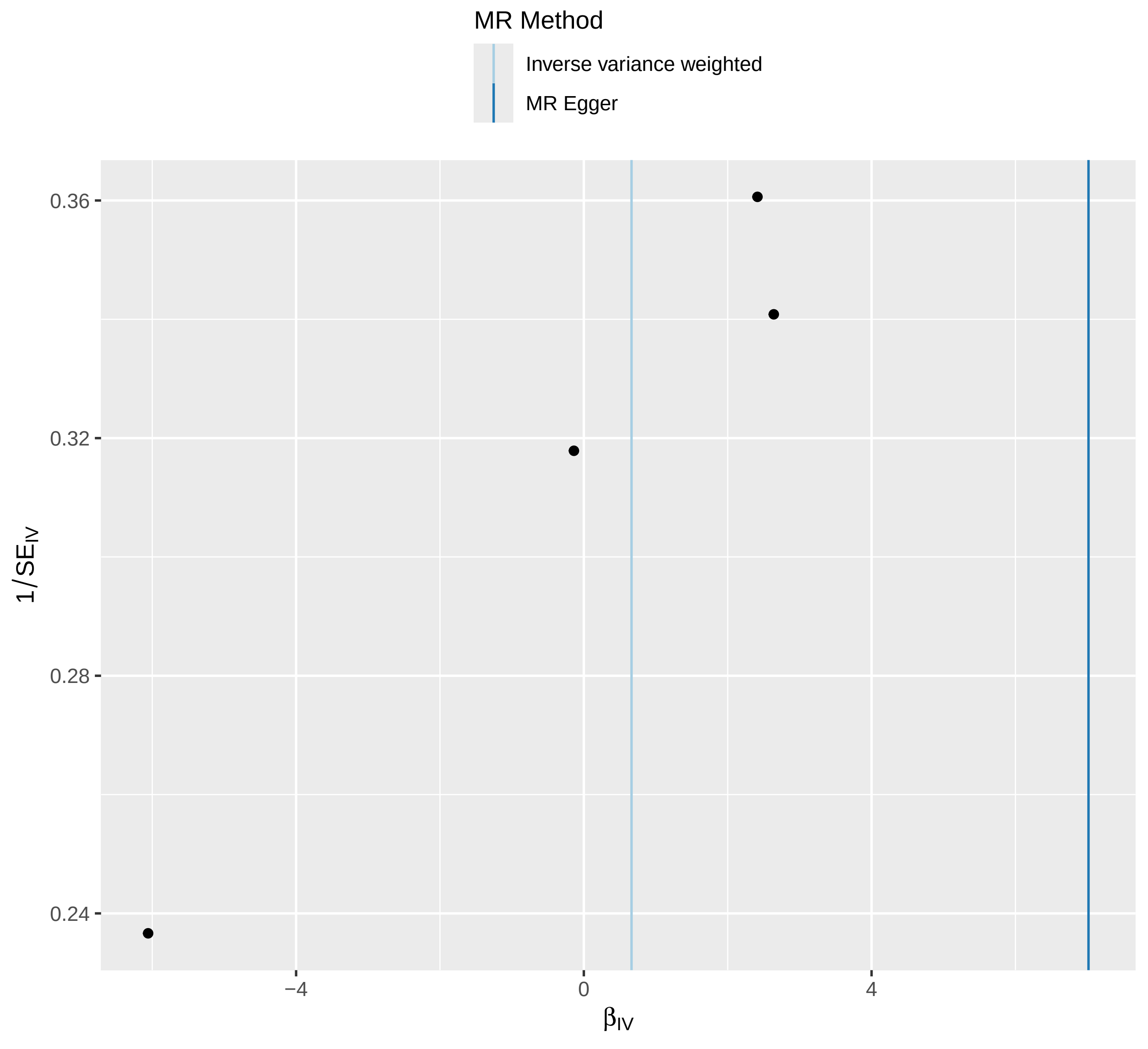
MR Method
Inverse variance weighted
MR Egger
0.36
0.32
0.28
0.24
−4
0
4
1/SE$_{IV}$
β$_{IV}$

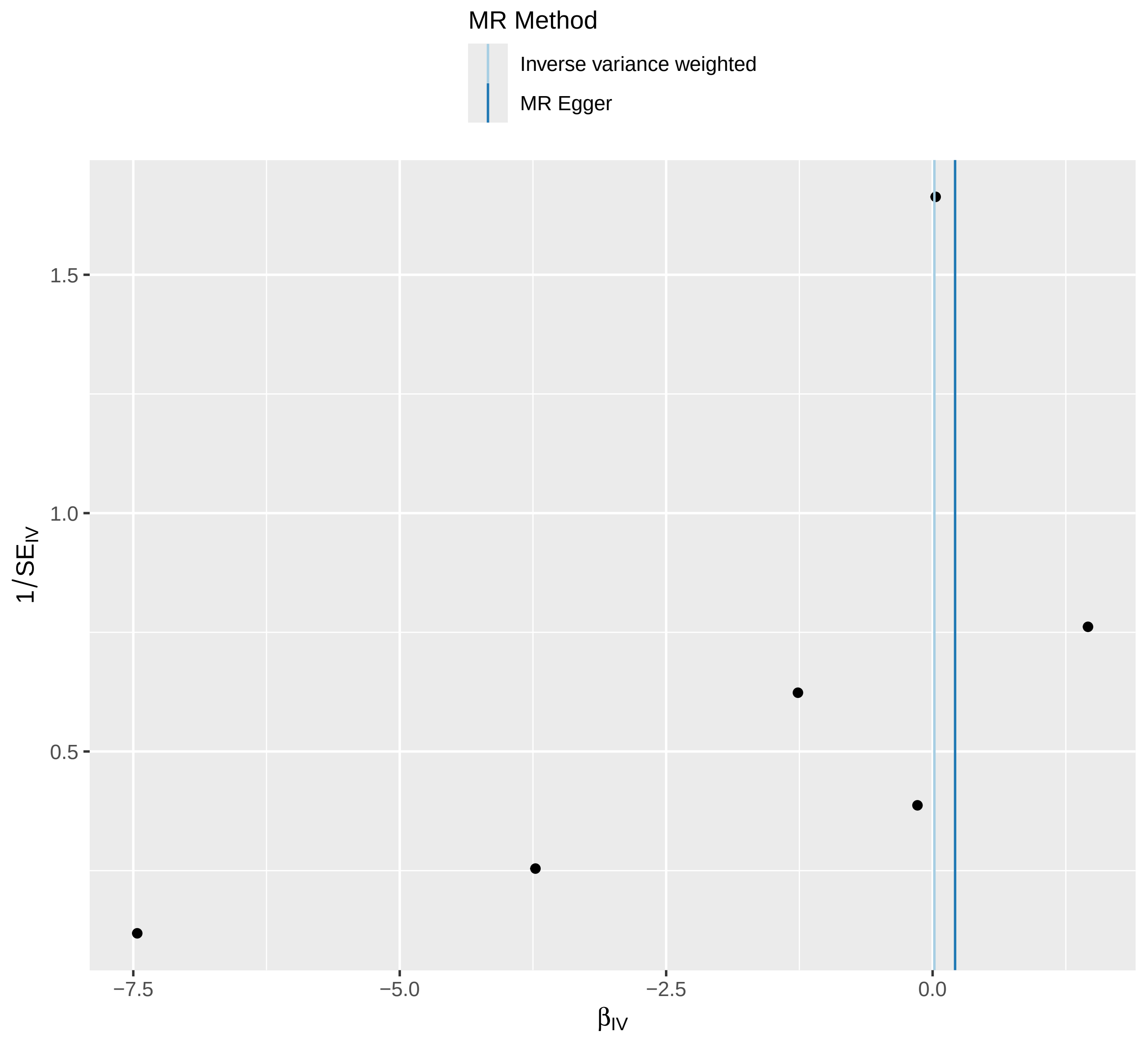
MR Method
Inverse variance weighted
MR Egger
1.5
1.0
0.5
−7.5
−5.0
−2.5
0.0
1/SE_IV
β_IV

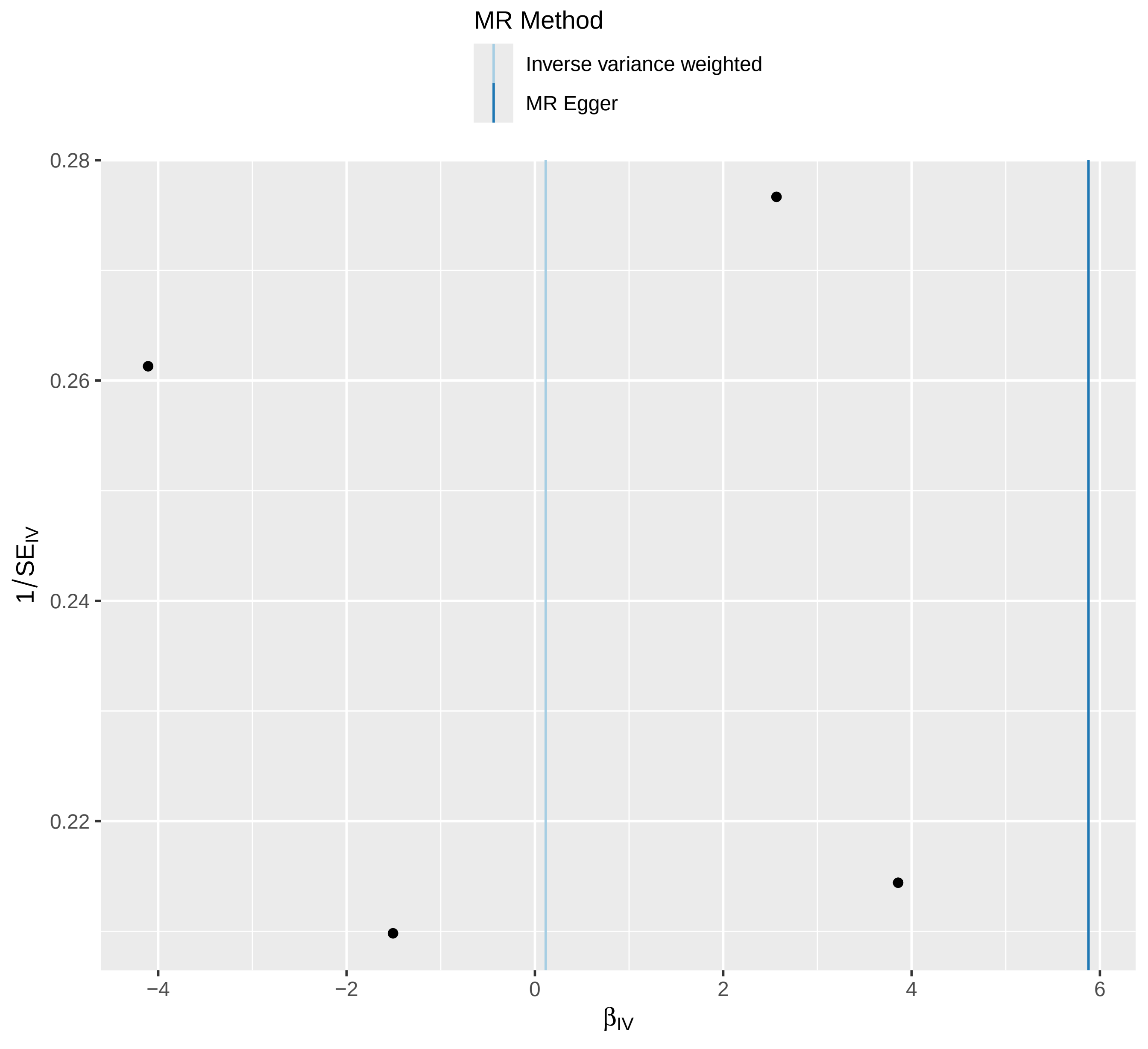

MR Method
Inverse variance weighted
MR Egger
0.28
0.26
0.24
0.22
1/SE_IV
−4
−2
0
2
4
6
β_IV

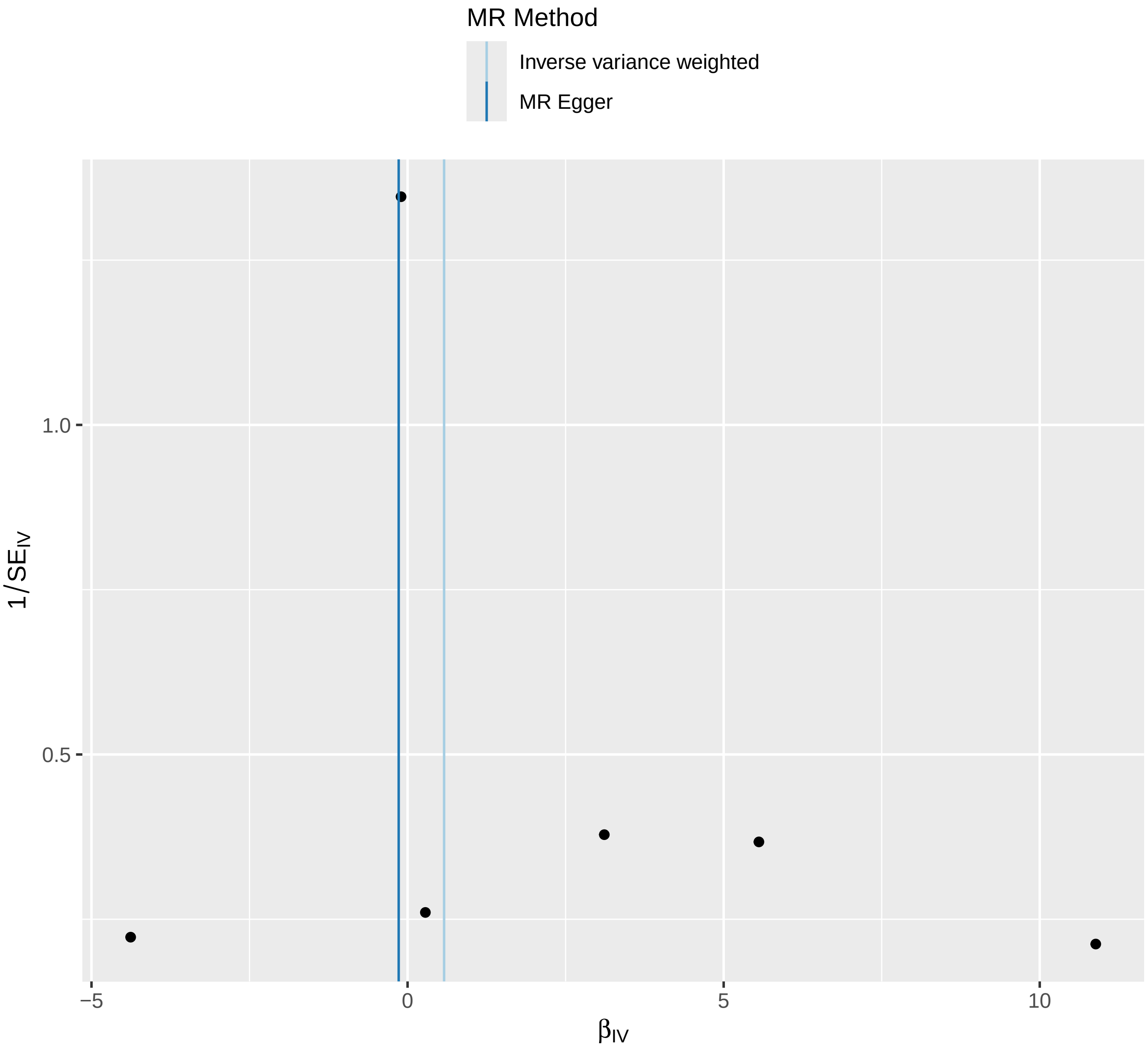

MR Method
Inverse variance weighted
MR Egger
1.0
0.5
1/SE_IV
−5
0
5
10
β_IV

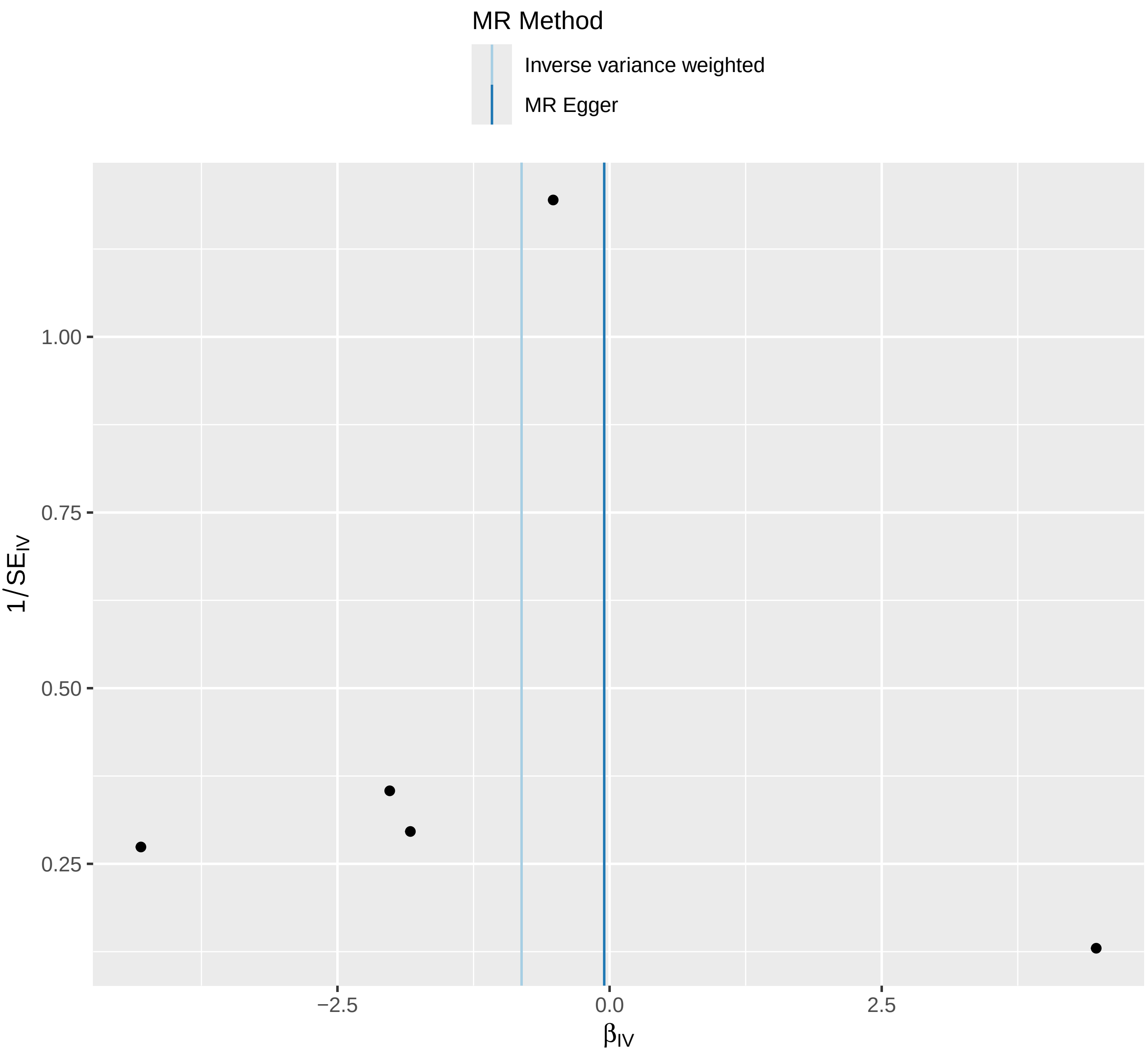
MR Method
Inverse variance weighted
MR Egger
1.00
0.75
0.50
0.25
1/SE$_{IV}$
−2.5
0.0
2.5
β_{IV}

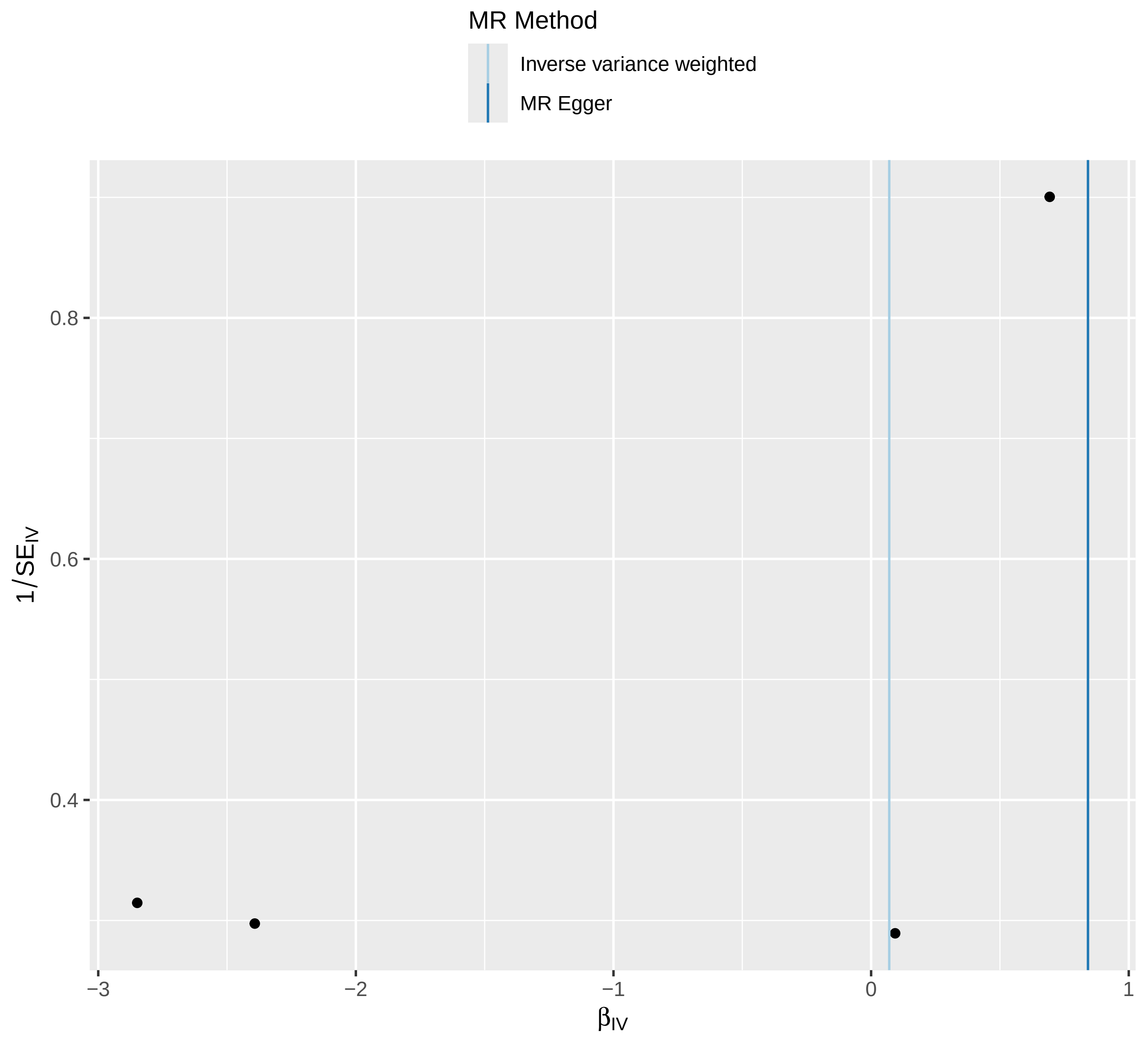
MR Method
Inverse variance weighted
MR Egger
0.8
0.6
0.4
1/SE_IV
−3
−2
−1
0
1
β_IV

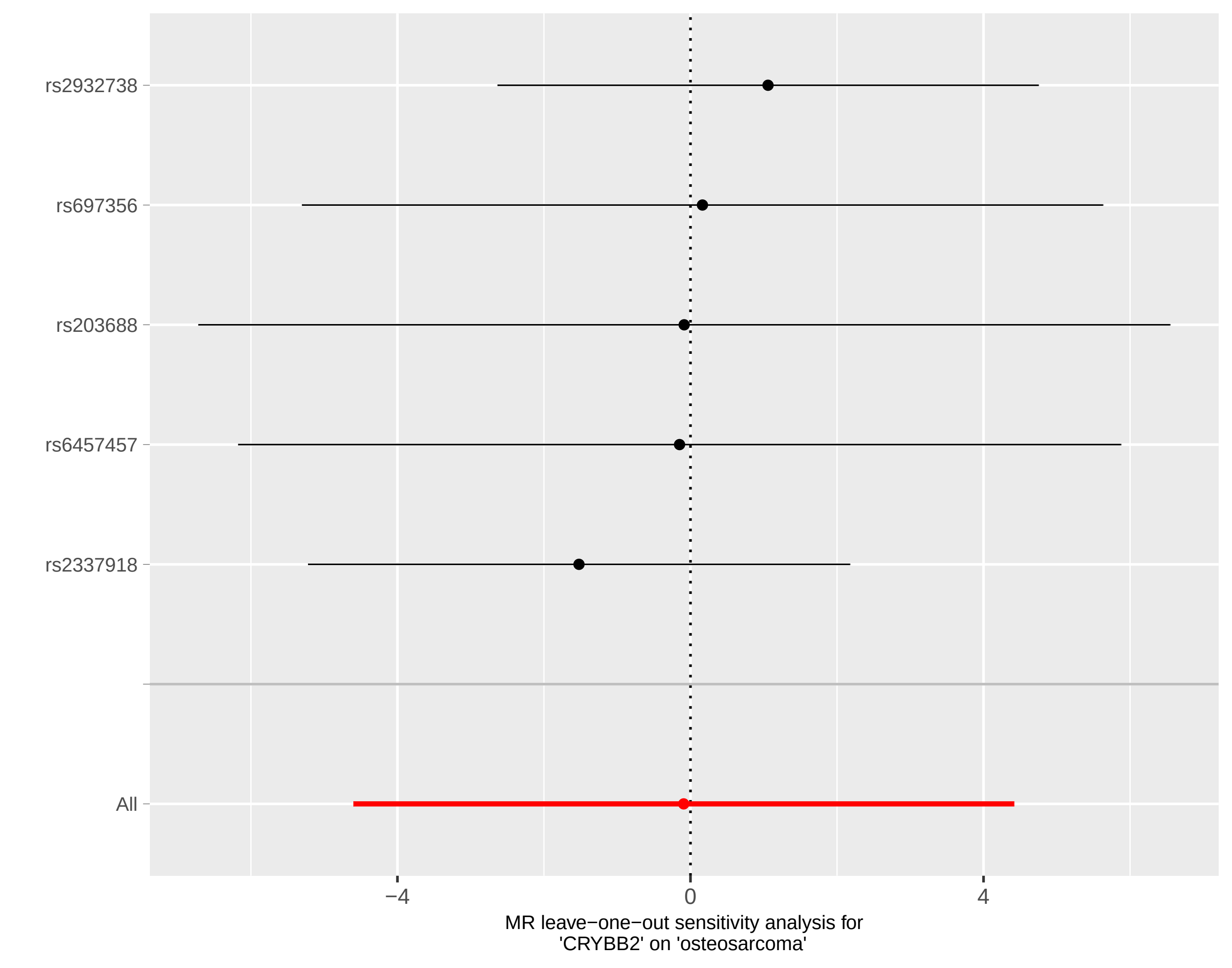
rs2932738
rs697356
rs203688
rs6457457
rs2337918
All
−4
0
4
MR leave−one−out sensitivity analysis for
'CRYBB2' on 'osteosarcoma'

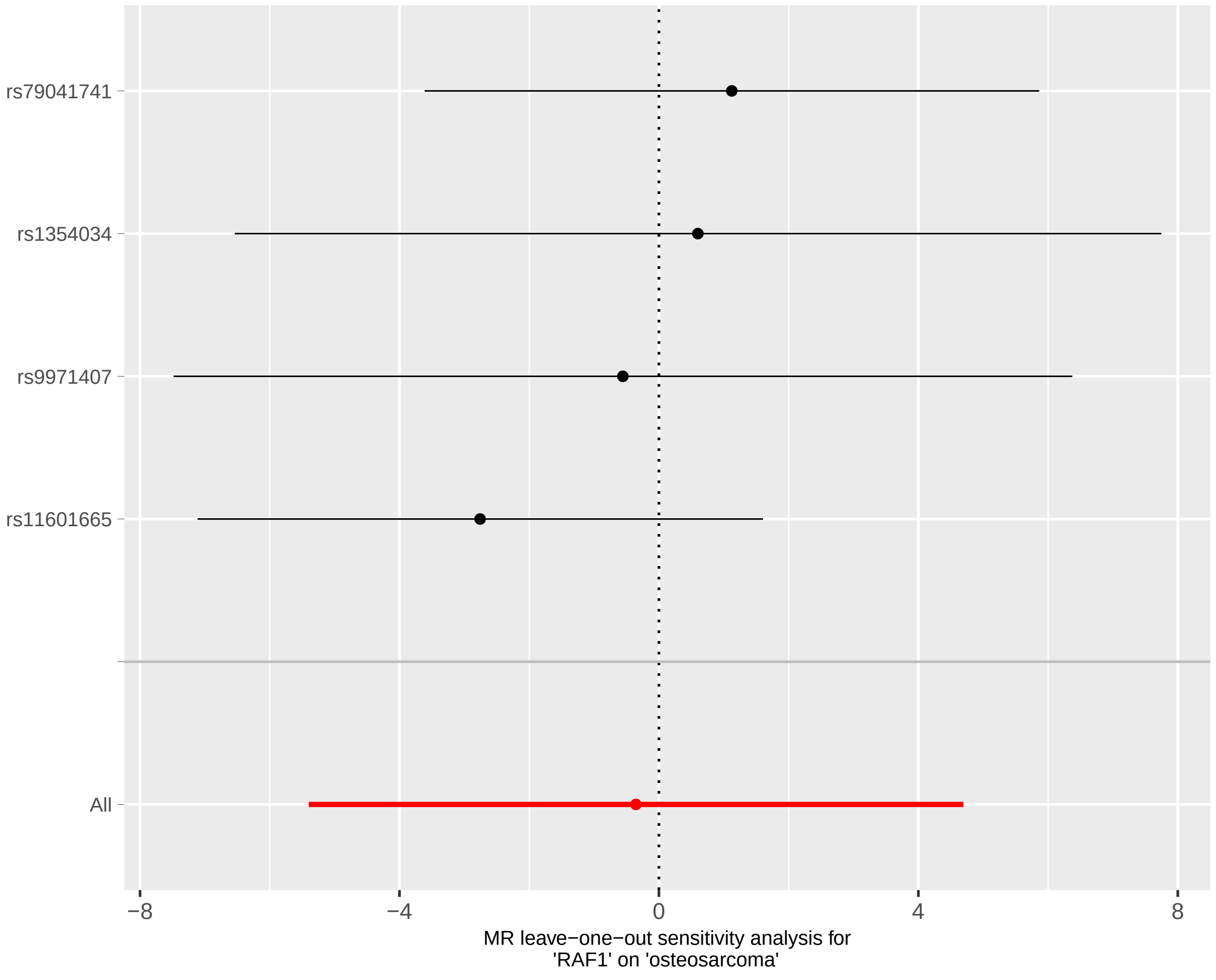

rs79041741
rs1354034
rs9971407
rs11601665
All
−8
−4
0
4
8
MR leave−one−out sensitivity analysis for
'RAF1' on 'osteosarcoma'

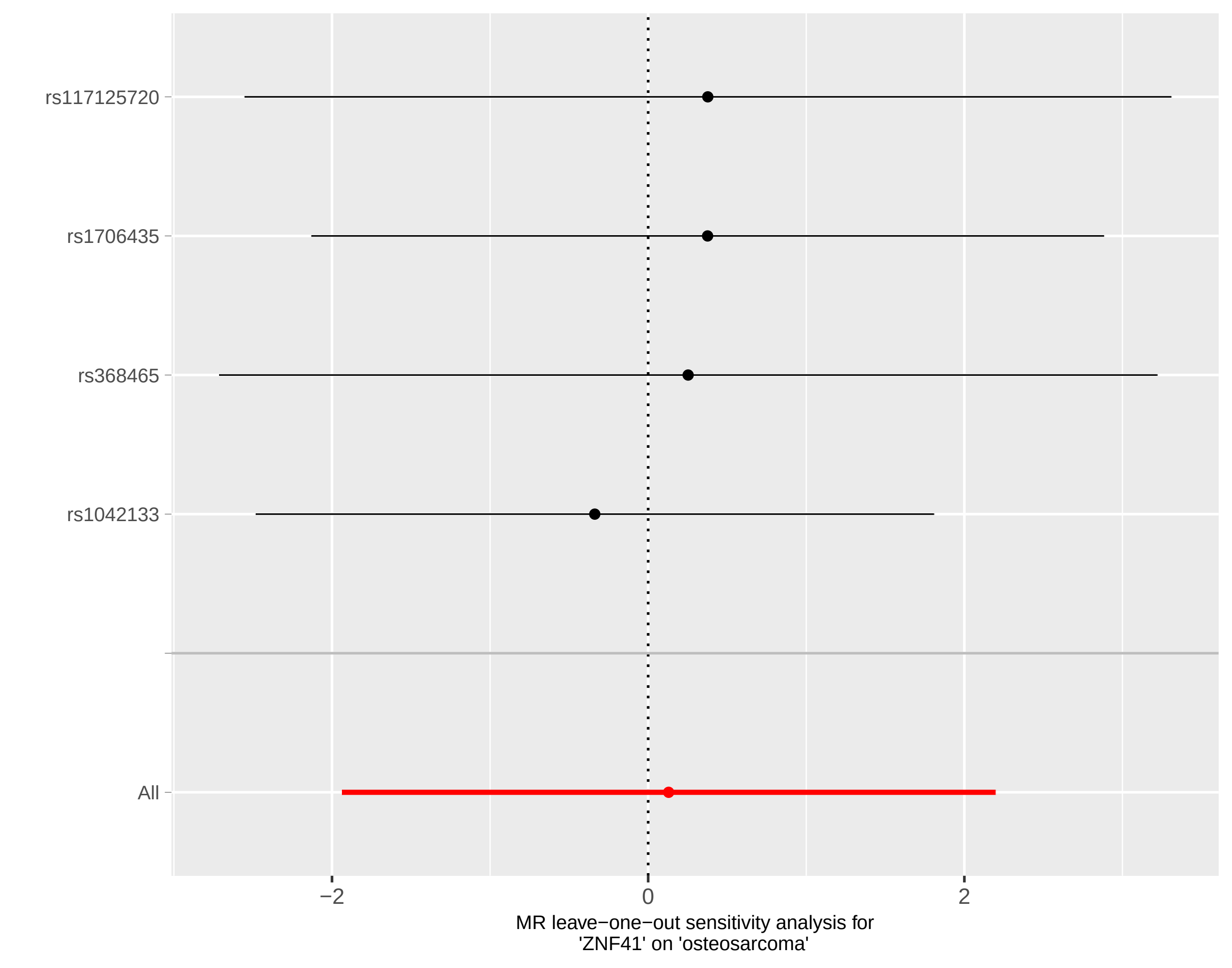
rs117125720
rs1706435
rs368465
rs1042133
All
−2
0
2
MR leave−one−out sensitivity analysis for
'ZNF41' on 'osteosarcoma'

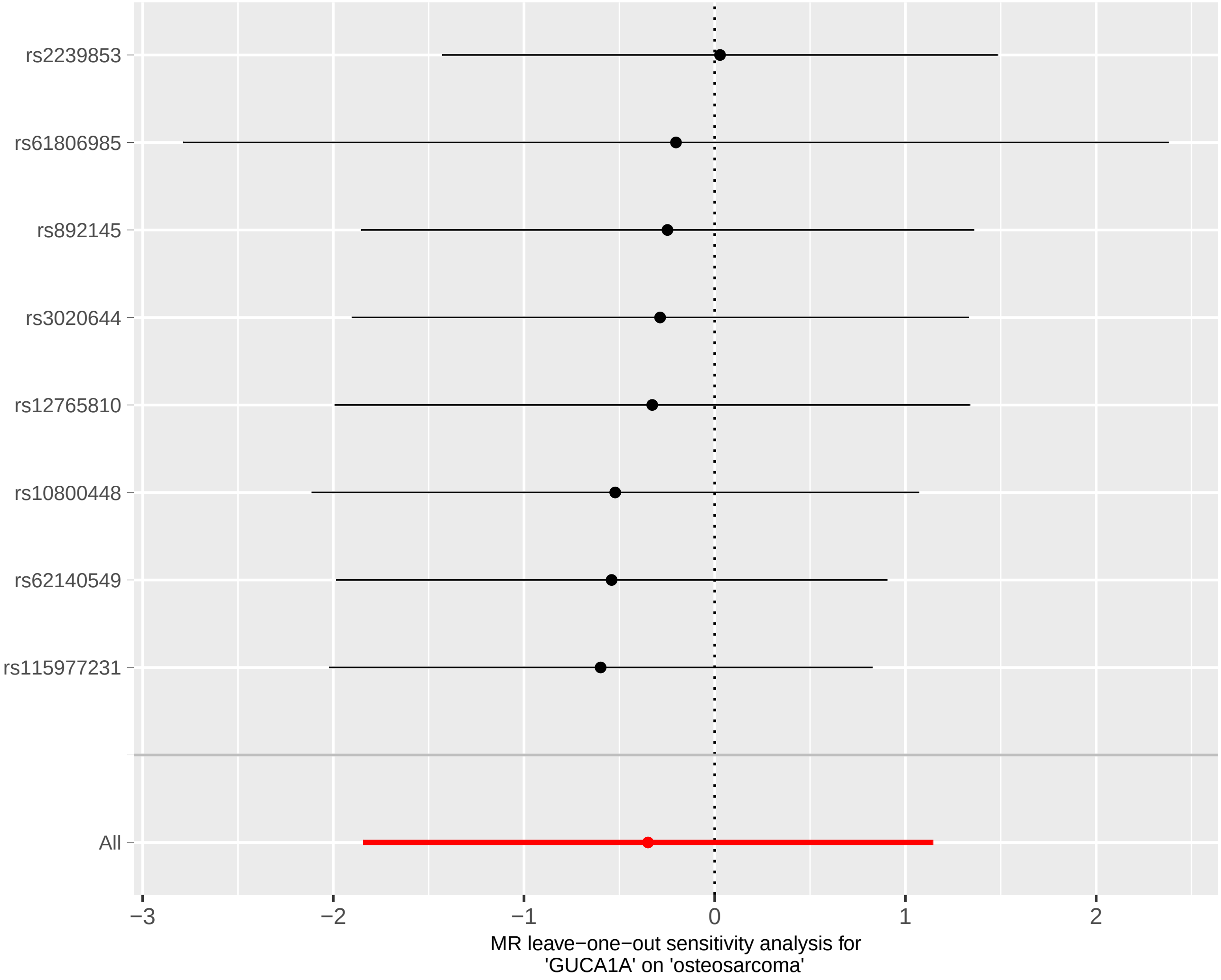

rs2239853
rs61806985
rs892145
rs3020644
rs12765810
rs10800448
rs62140549
rs115977231
All
−3
−2
−1
0
1
2
MR leave−one−out sensitivity analysis for
'GUCA1A' on 'osteosarcoma'

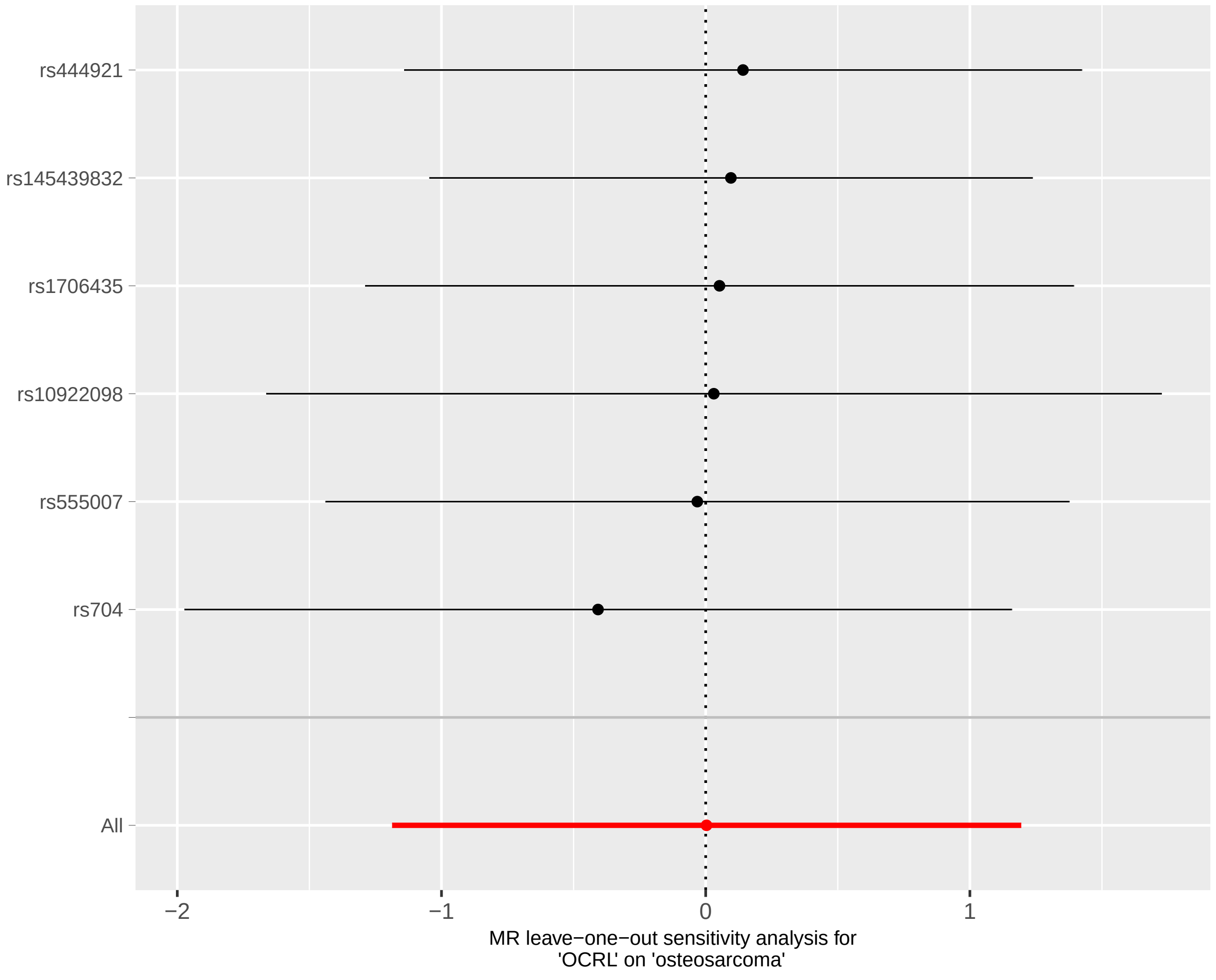
rs444921
rs145439832
rs1706435
rs10922098
rs555007
rs704
All
−2
−1
0
1
MR leave−one−out sensitivity analysis for
'OCRL' on 'osteosarcoma'

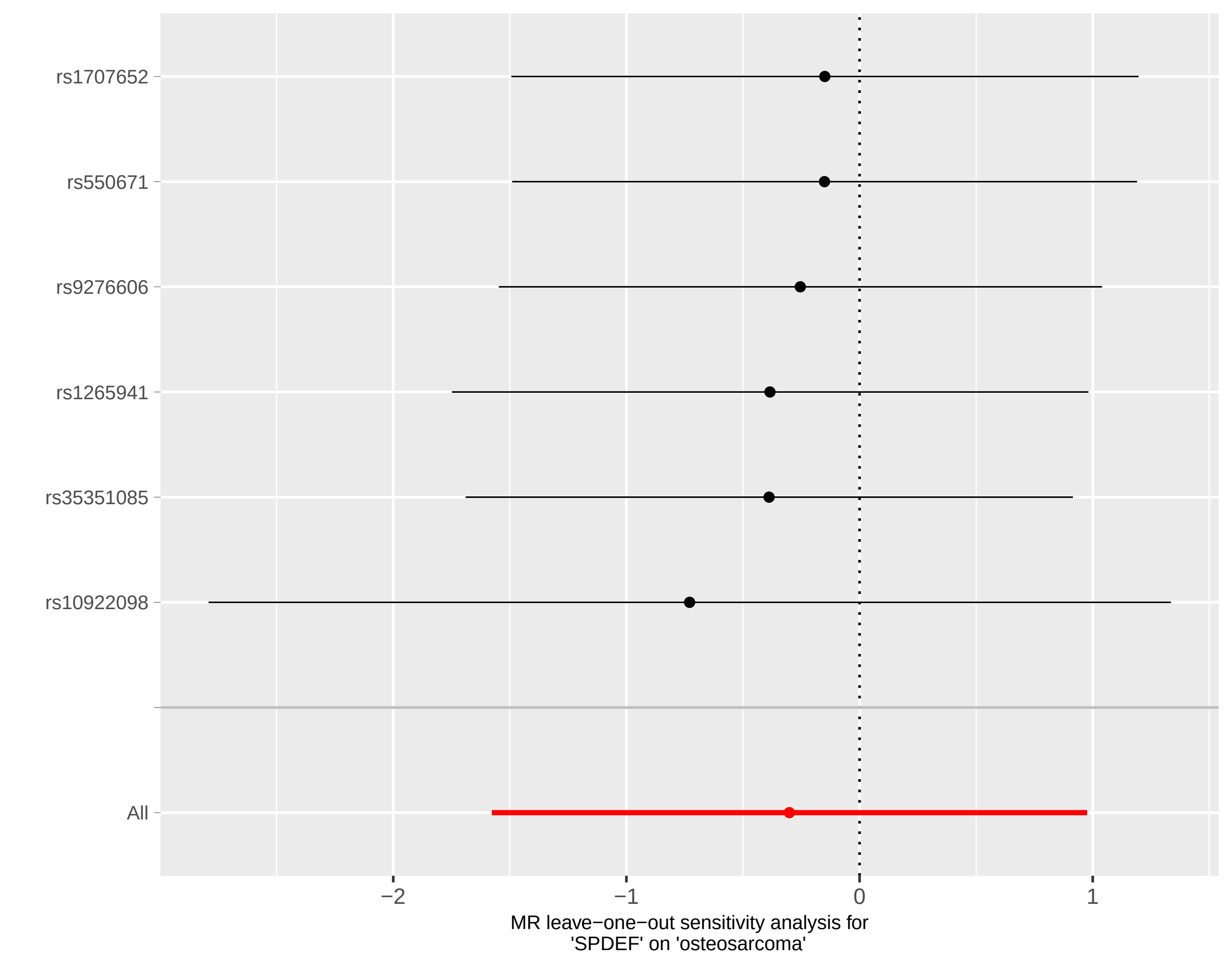

rs1707652
rs550671
rs9276606
rs1265941
rs35351085
rs10922098
All
−2
−1
0
1
MR leave−one−out sensitivity analysis for
'SPDEF' on 'osteosarcoma'

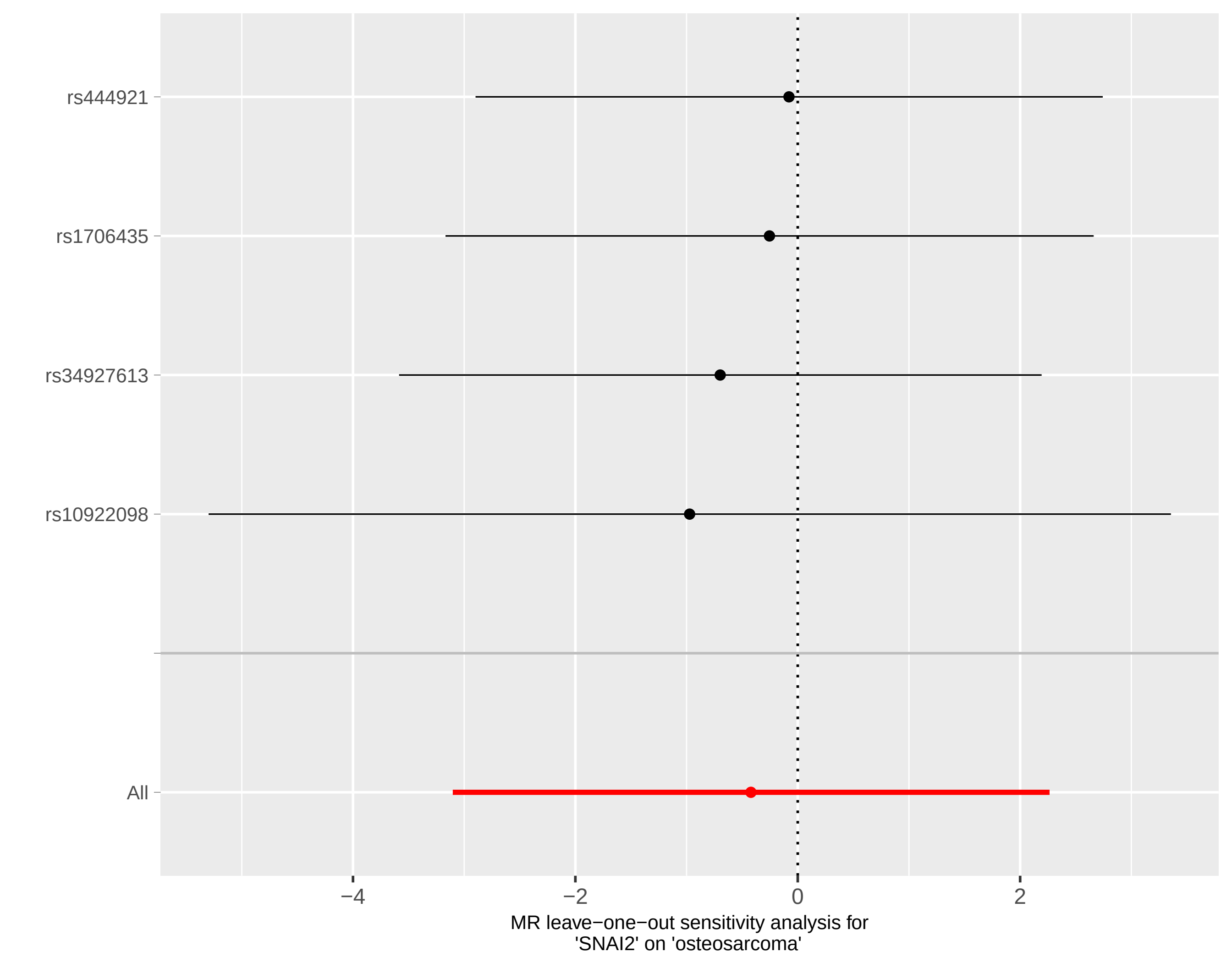
rs444921
rs1706435
rs34927613
rs10922098
All
−4
−2
0
2
MR leave−one−out sensitivity analysis for
'SNAI2' on 'osteosarcoma'

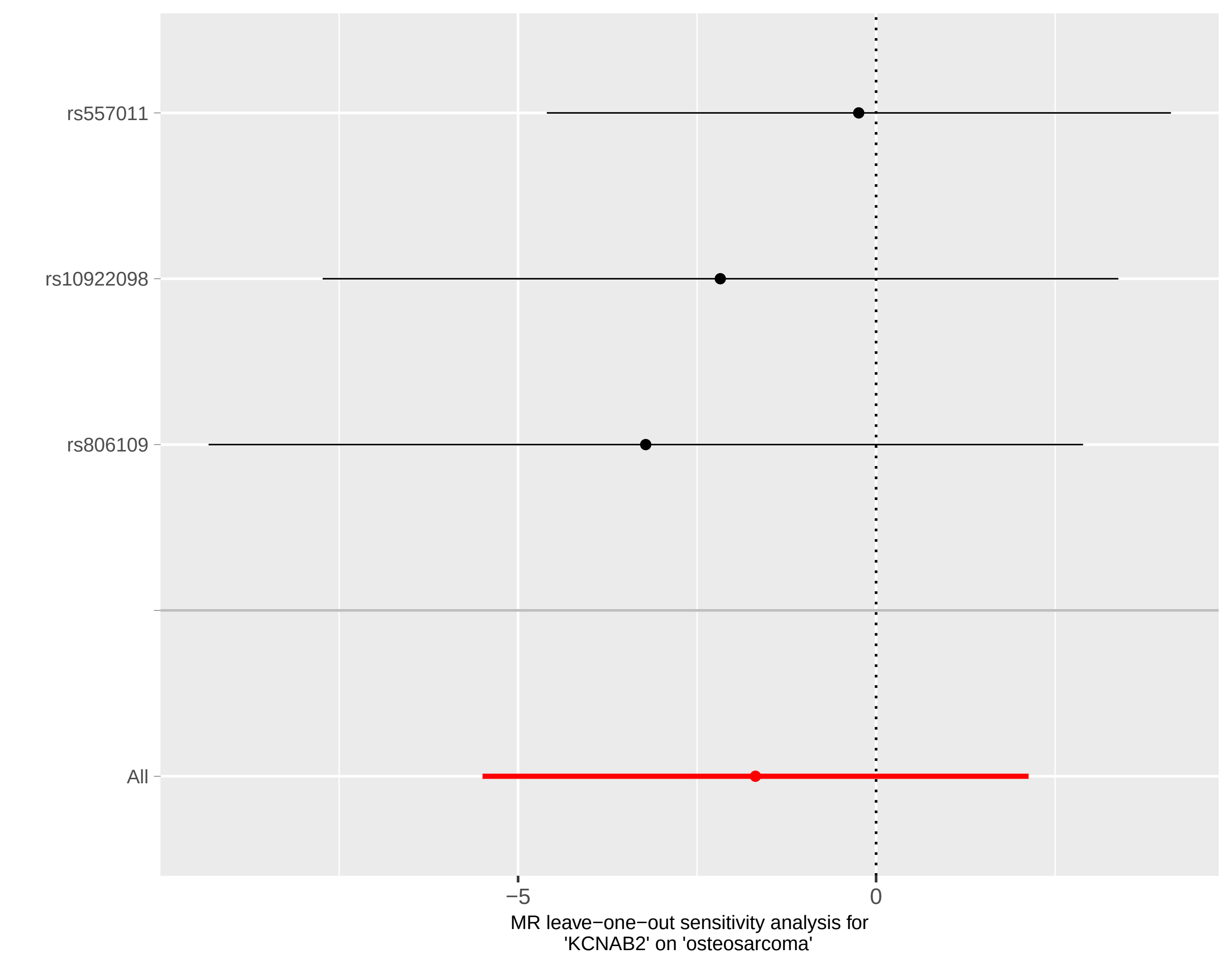
rs557011
rs10922098
rs806109
All
−5
0
MR leave−one−out sensitivity analysis for
'KCNAB2' on 'osteosarcoma'

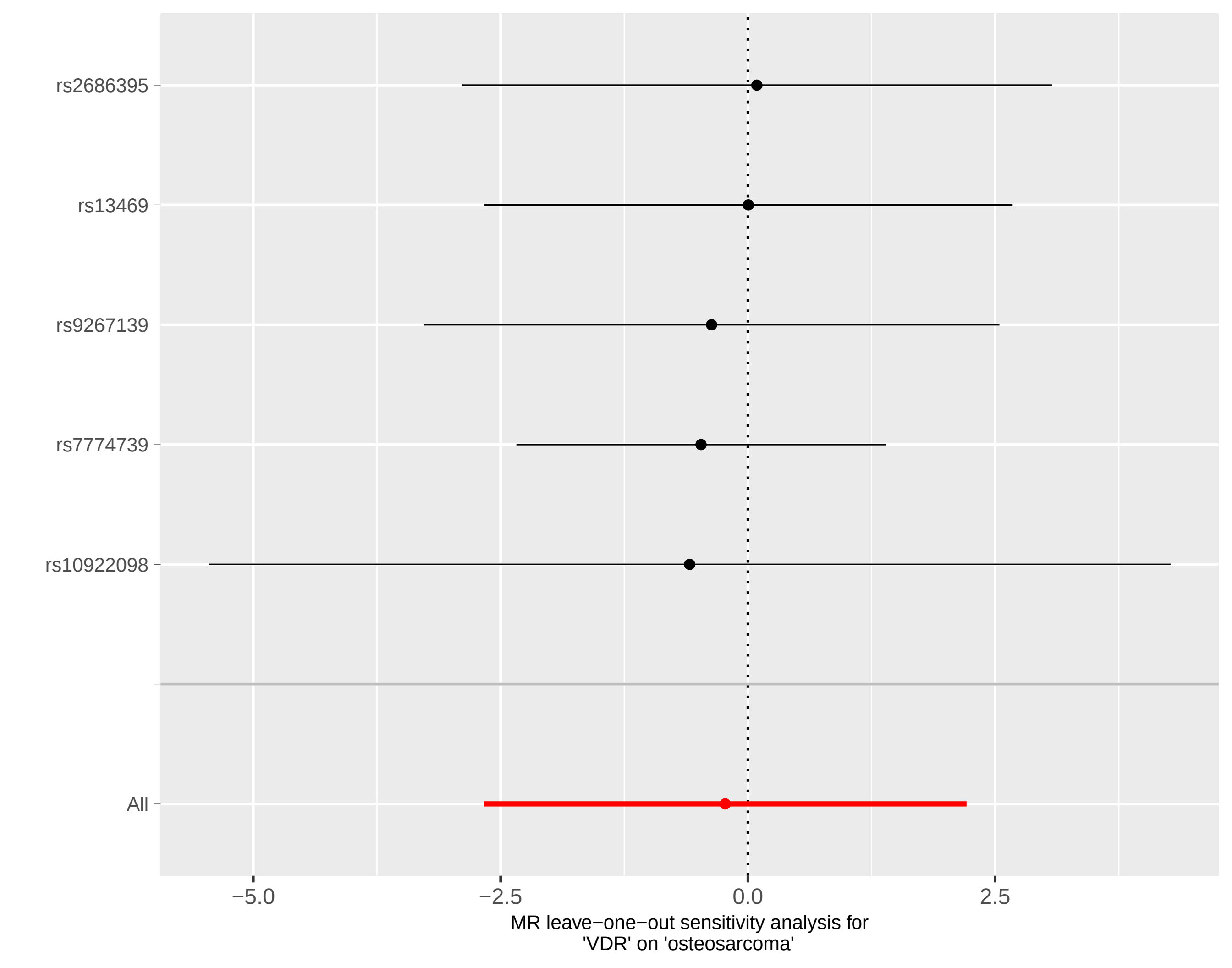

rs2686395
rs13469
rs9267139
rs7774739
rs10922098
All
−5.0
−2.5
0.0
2.5
MR leave−one−out sensitivity analysis for
'VDR' on 'osteosarcoma'

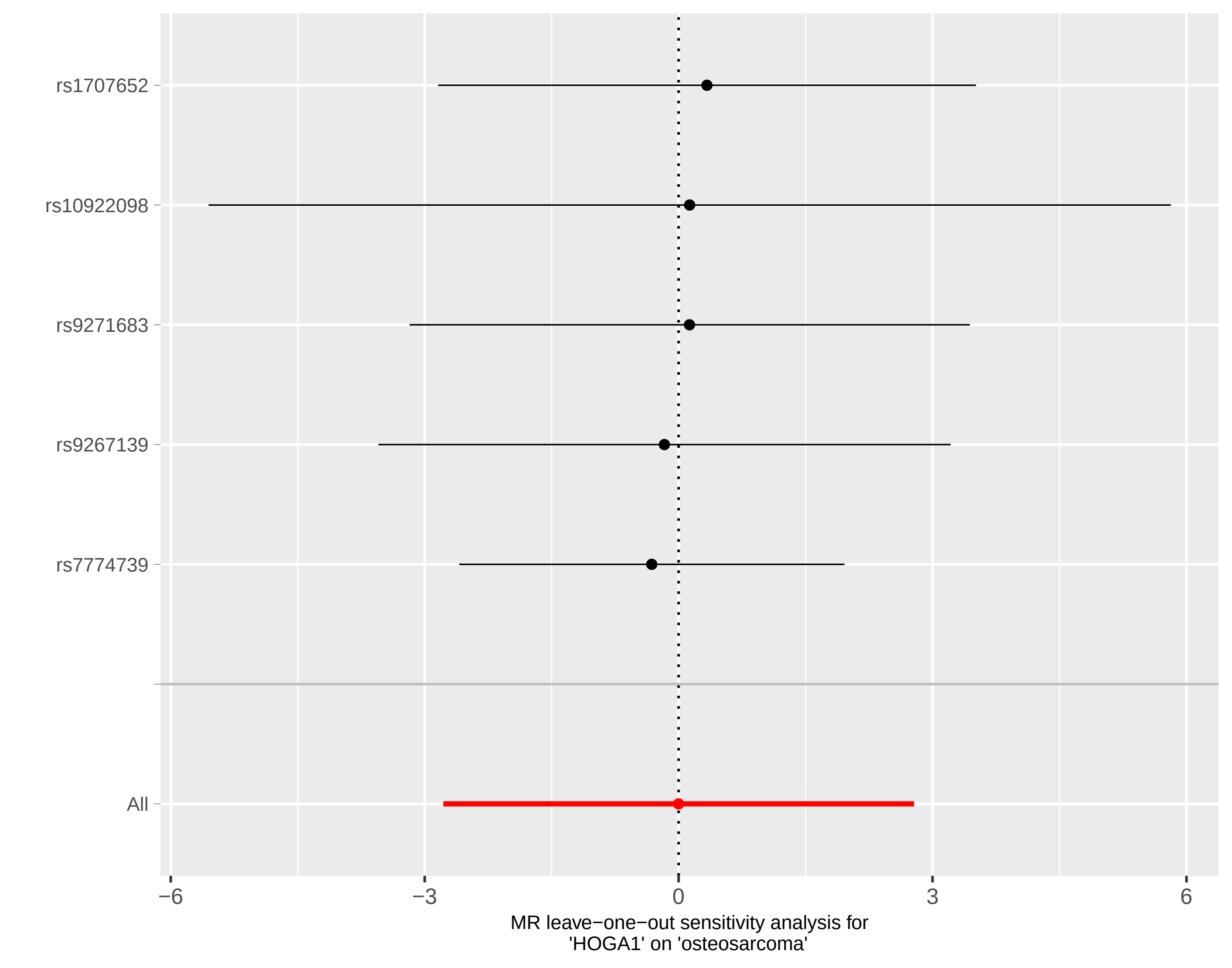

rs1707652
rs10922098
rs9271683
rs9267139
rs7774739
All
−6
−3
0
3
6
MR leave−one−out sensitivity analysis for
'HOGA1' on 'osteosarcoma'

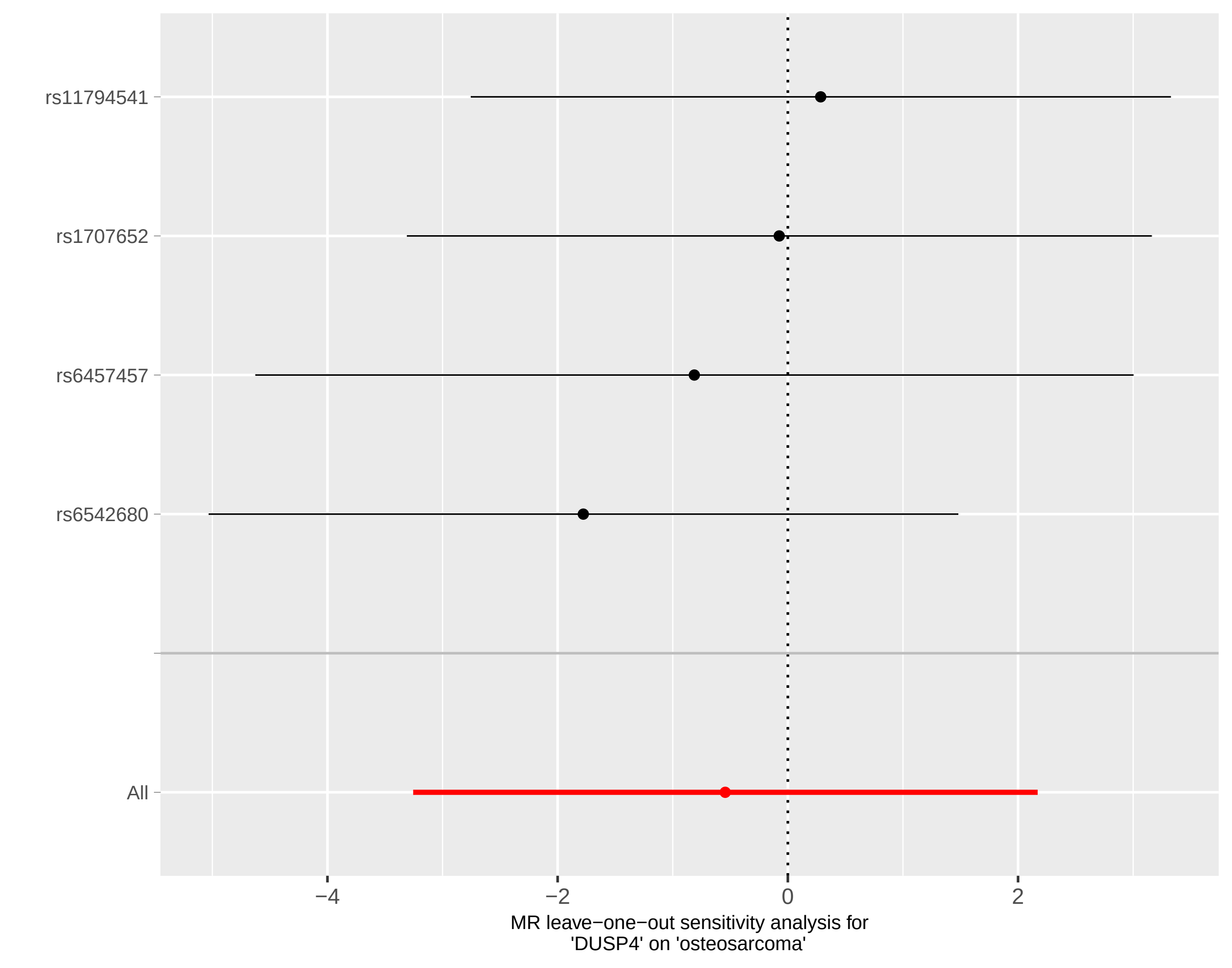
rs11794541
rs1707652
rs6457457
rs6542680
All
−4
−2
0
2
MR leave−one−out sensitivity analysis for
'DUSP4' on 'osteosarcoma'

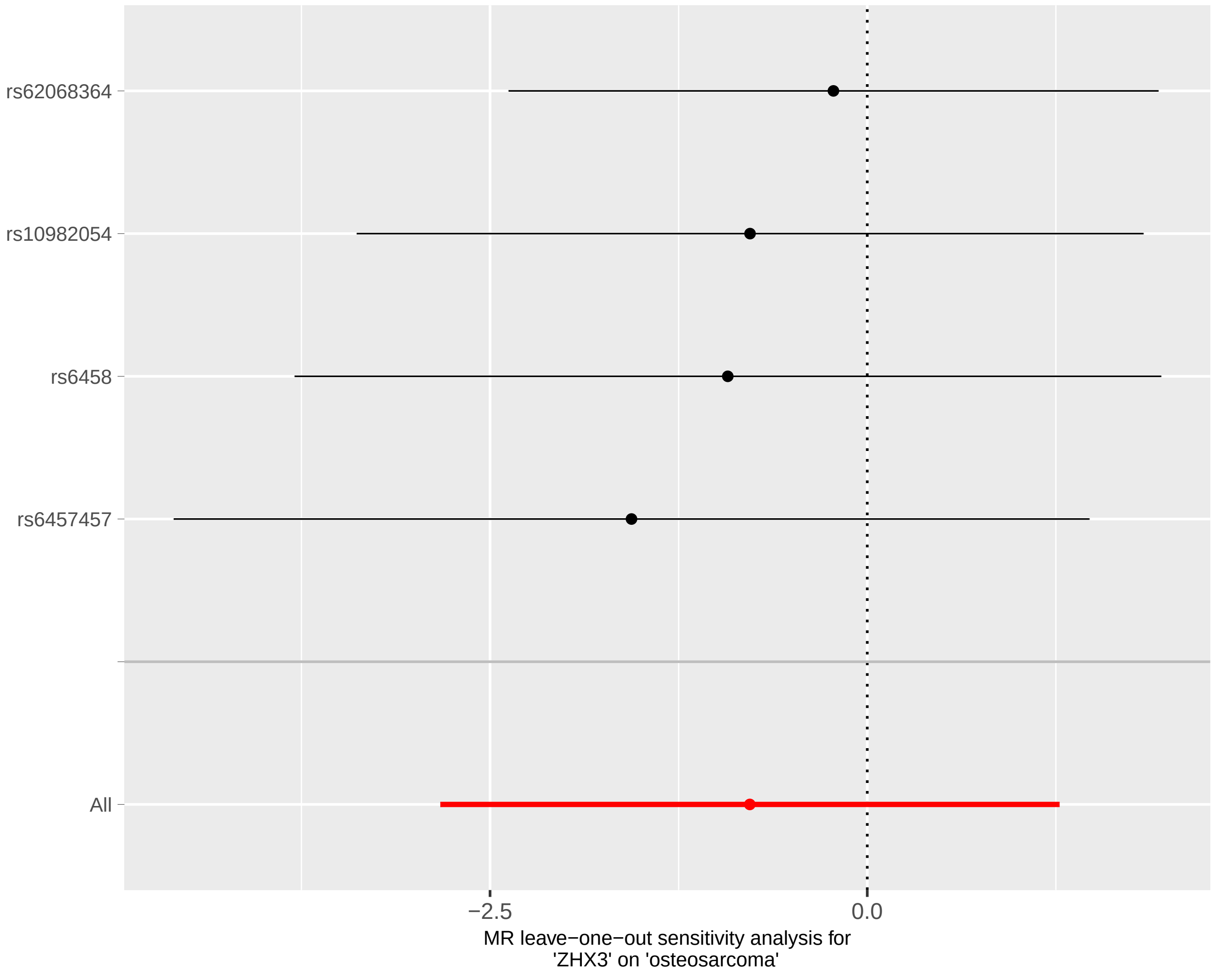
rs62068364
rs10982054
rs6458
rs6457457
All
−2.5
0.0
MR leave−one−out sensitivity analysis for
'ZHX3' on 'osteosarcoma'

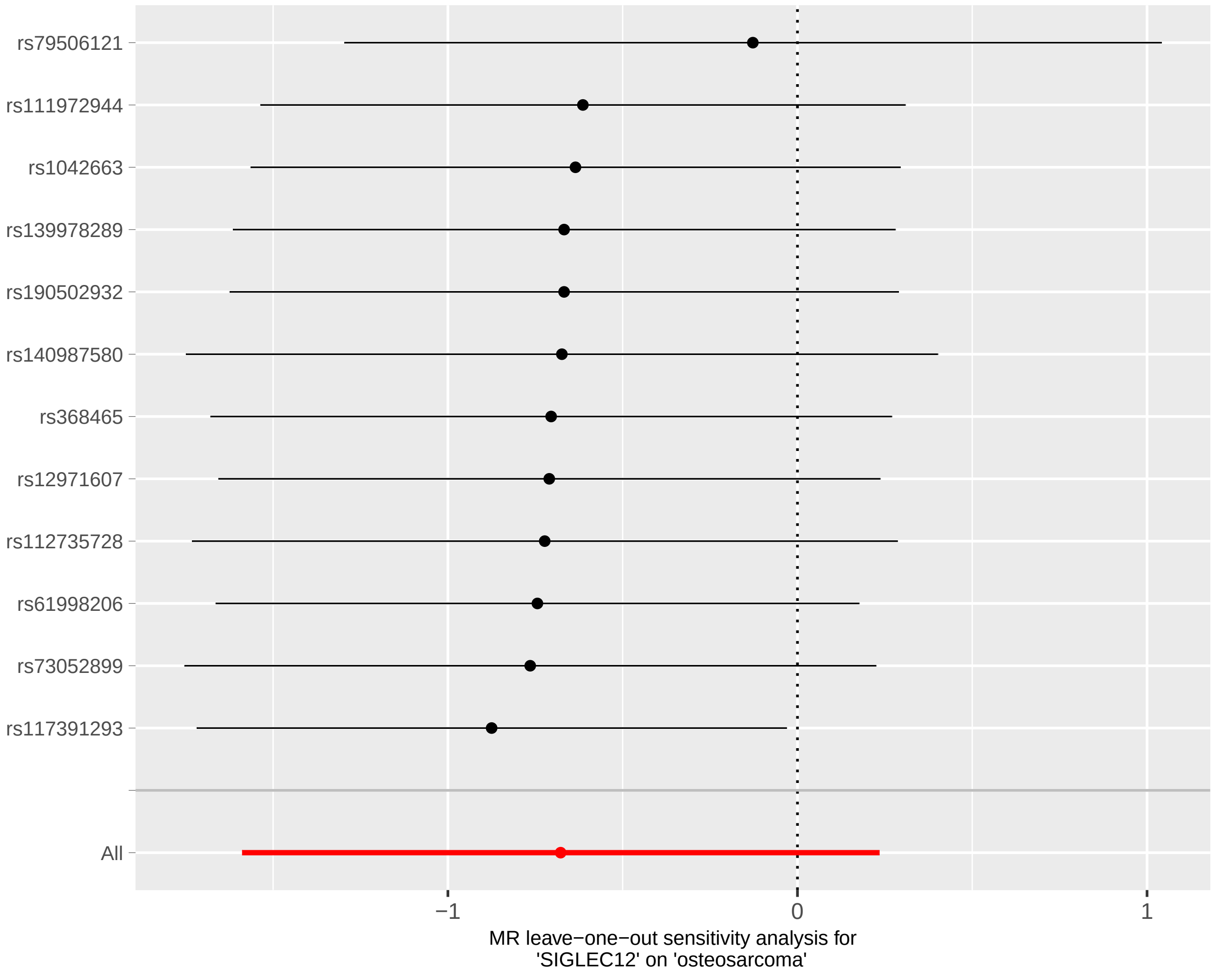
rs79506121
rs111972944
rs1042663
rs139978289
rs190502932
rs140987580
rs368465
rs12971607
rs112735728
rs61998206
rs73052899
rs117391293
All
−1
0
1
MR leave−one−out sensitivity analysis for
'SIGLEC12' on 'osteosarcoma'

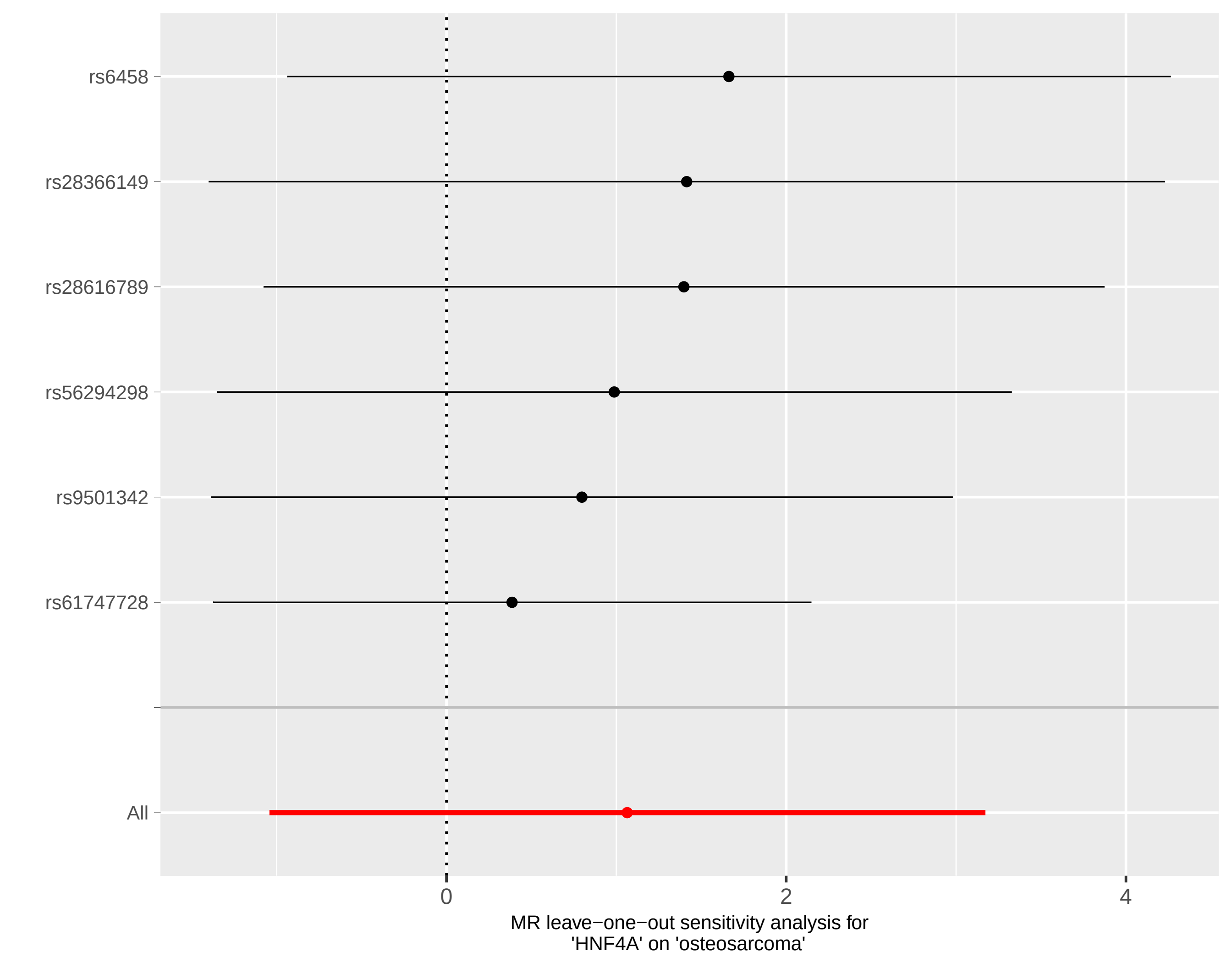

rs6458
rs28366149
rs28616789
rs56294298
rs9501342
rs61747728
All
0
2
4
MR leave−one−out sensitivity analysis for
'HNF4A' on 'osteosarcoma'

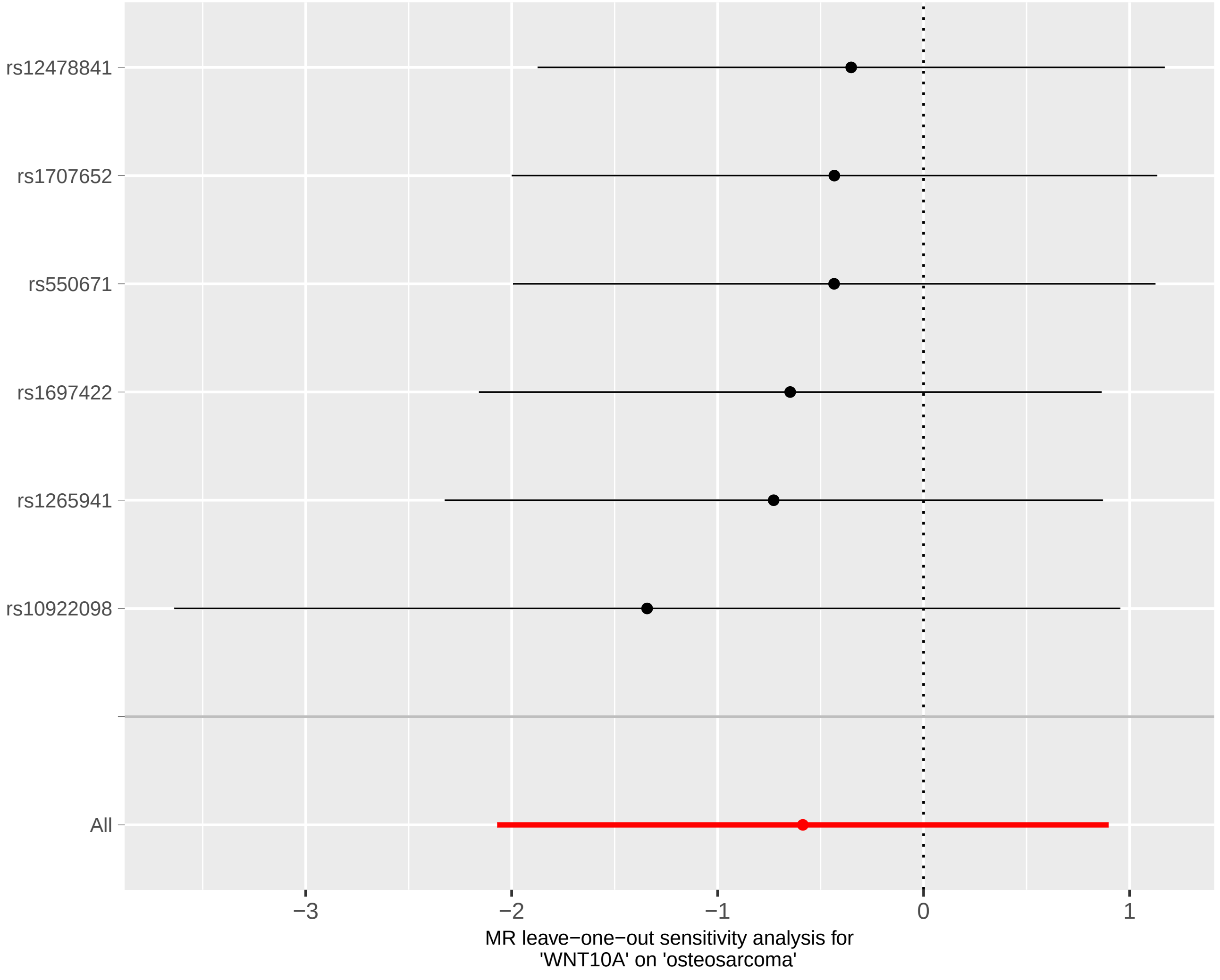
rs12478841
rs1707652
rs550671
rs1697422
rs1265941
rs10922098
All
−3
−2
−1
0
1
MR leave−one−out sensitivity analysis for
'WNT10A' on 'osteosarcoma'

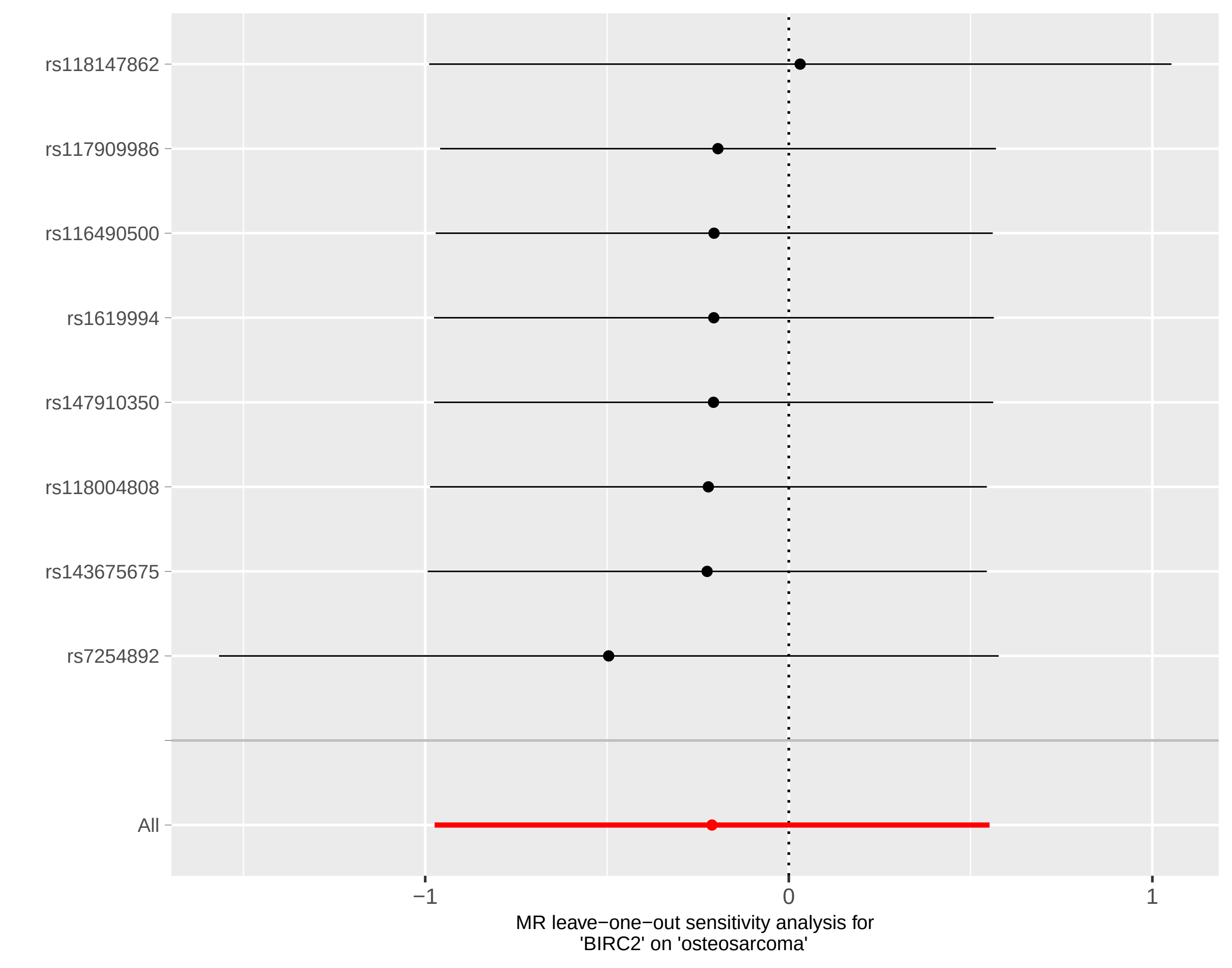
rs118147862
rs117909986
rs116490500
rs1619994
rs147910350
rs118004808
rs143675675
rs7254892
All
−1
0
1
MR leave−one−out sensitivity analysis for
'BIRC2' on 'osteosarcoma'

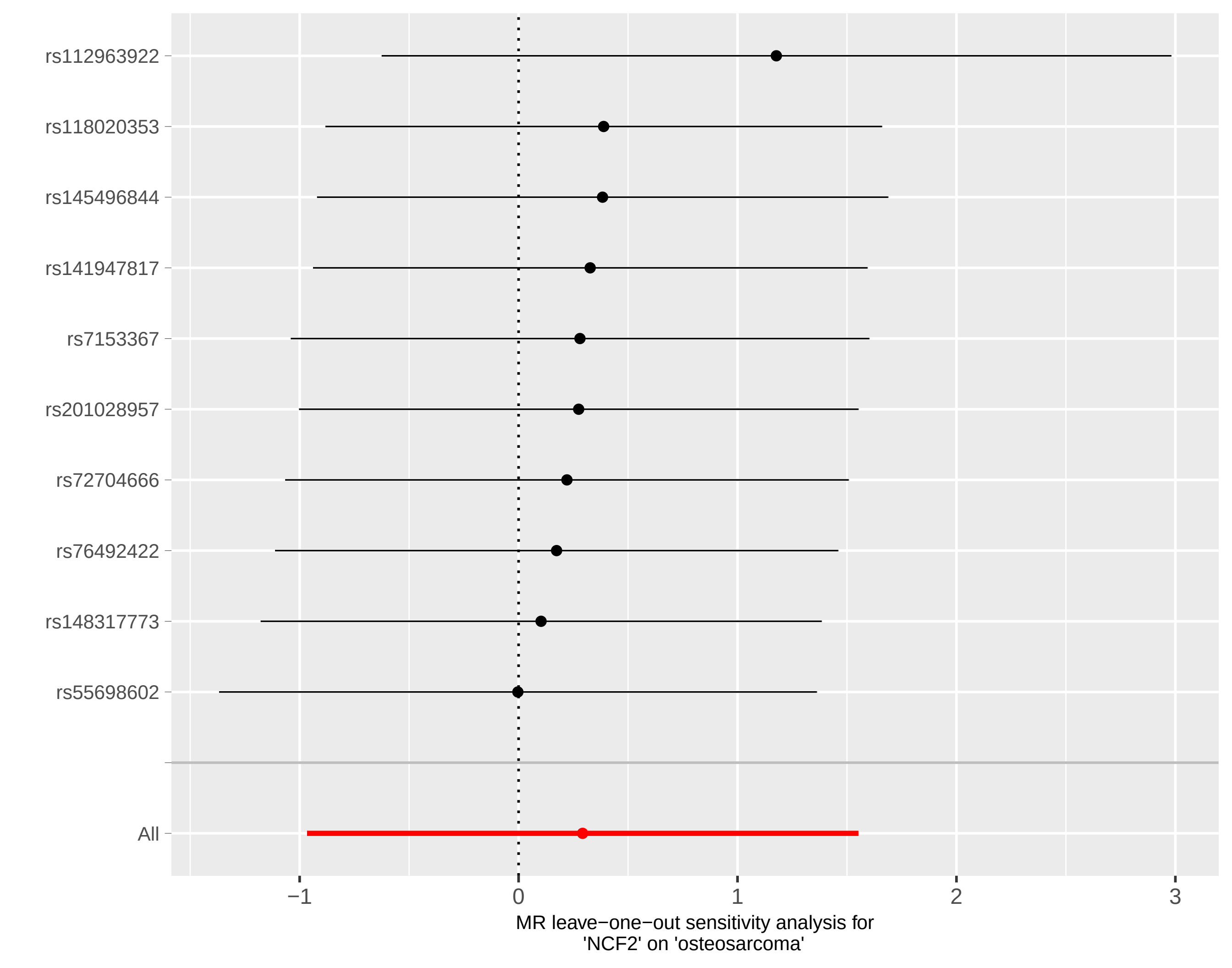

rs112963922
rs118020353
rs145496844
rs141947817
rs7153367
rs201028957
rs72704666
rs76492422
rs148317773
rs55698602
All
−1
0
1
2
3
MR leave−one−out sensitivity analysis for
'NCF2' on 'osteosarcoma'

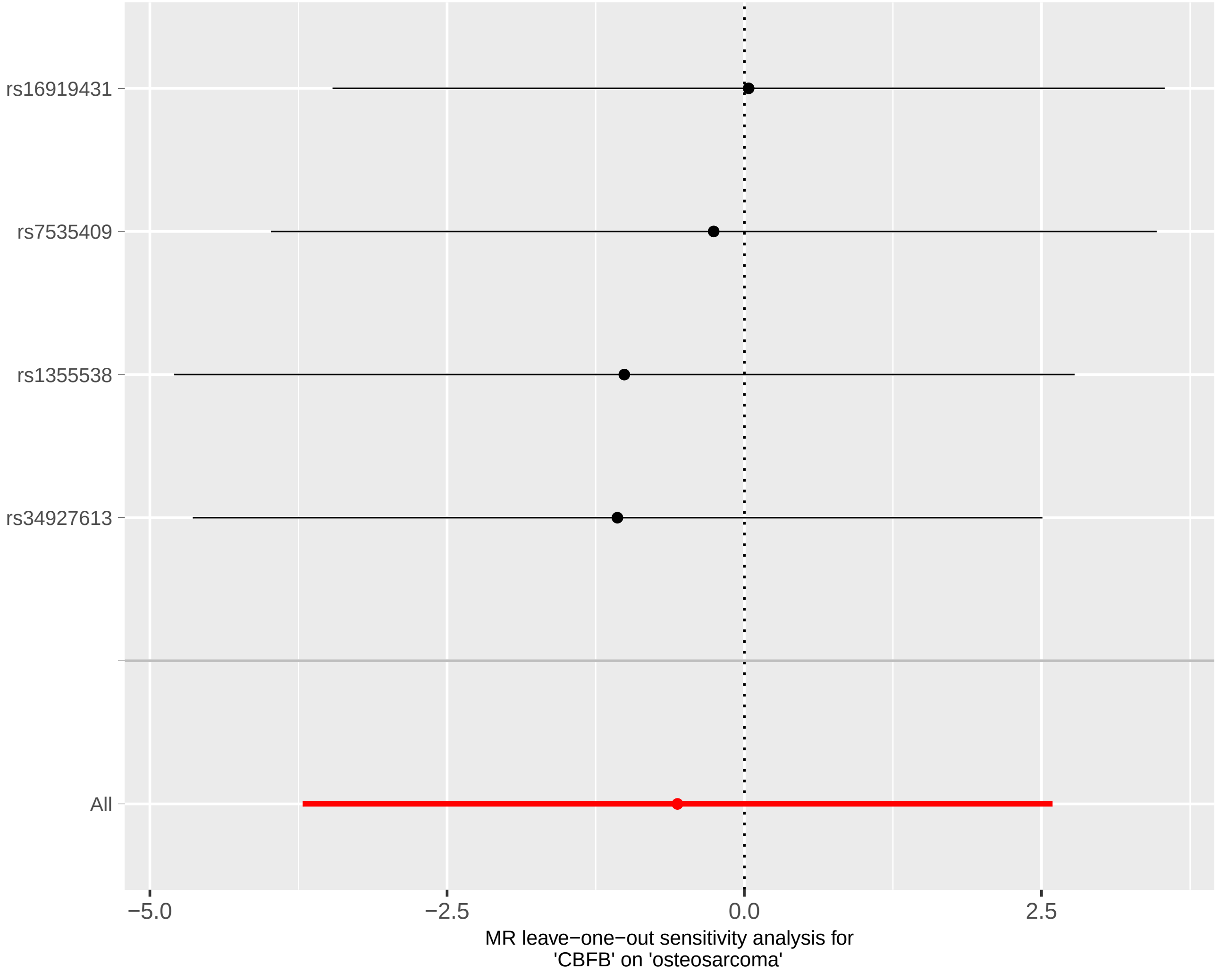
rs16919431
rs7535409
rs1355538
rs34927613
All
−5.0
−2.5
0.0
2.5
MR leave−one−out sensitivity analysis for
'CBFB' on 'osteosarcoma'

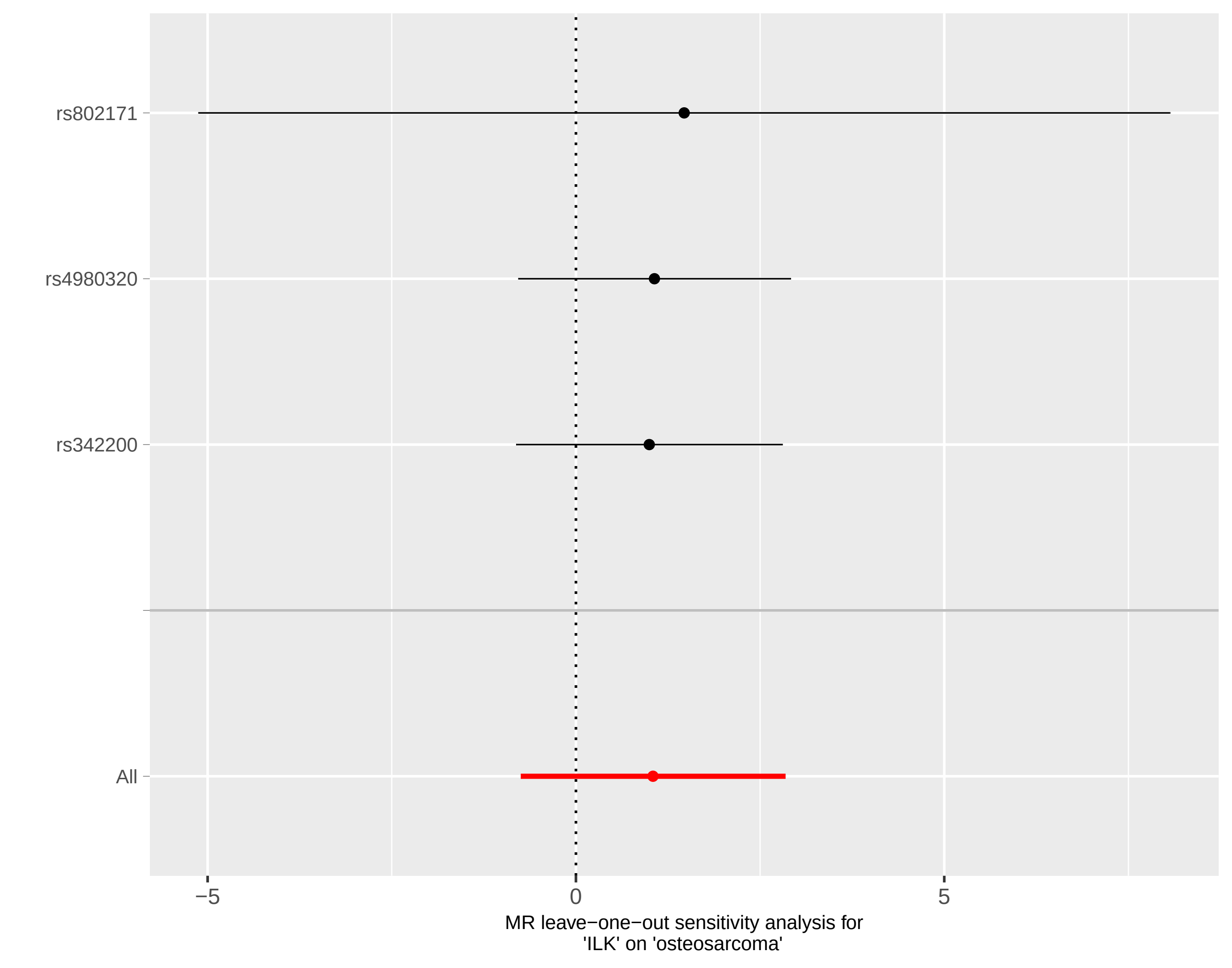
rs802171
rs4980320
rs342200
All
−5
0
5
MR leave−one−out sensitivity analysis for
'ILK' on 'osteosarcoma'

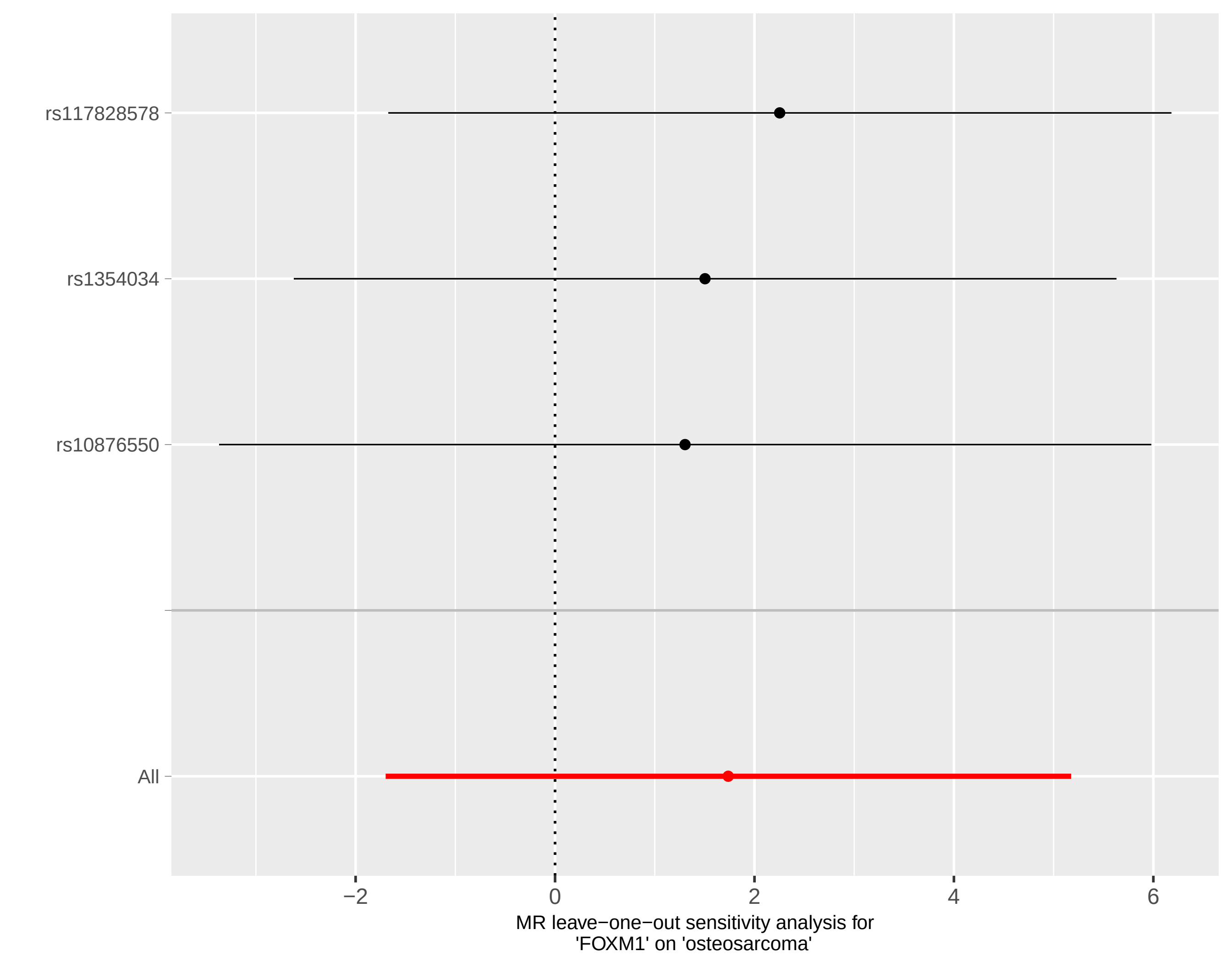

rs117828578
rs1354034
rs10876550
All
−2
0
2
4
6
MR leave−one−out sensitivity analysis for
'FOXM1' on 'osteosarcoma'

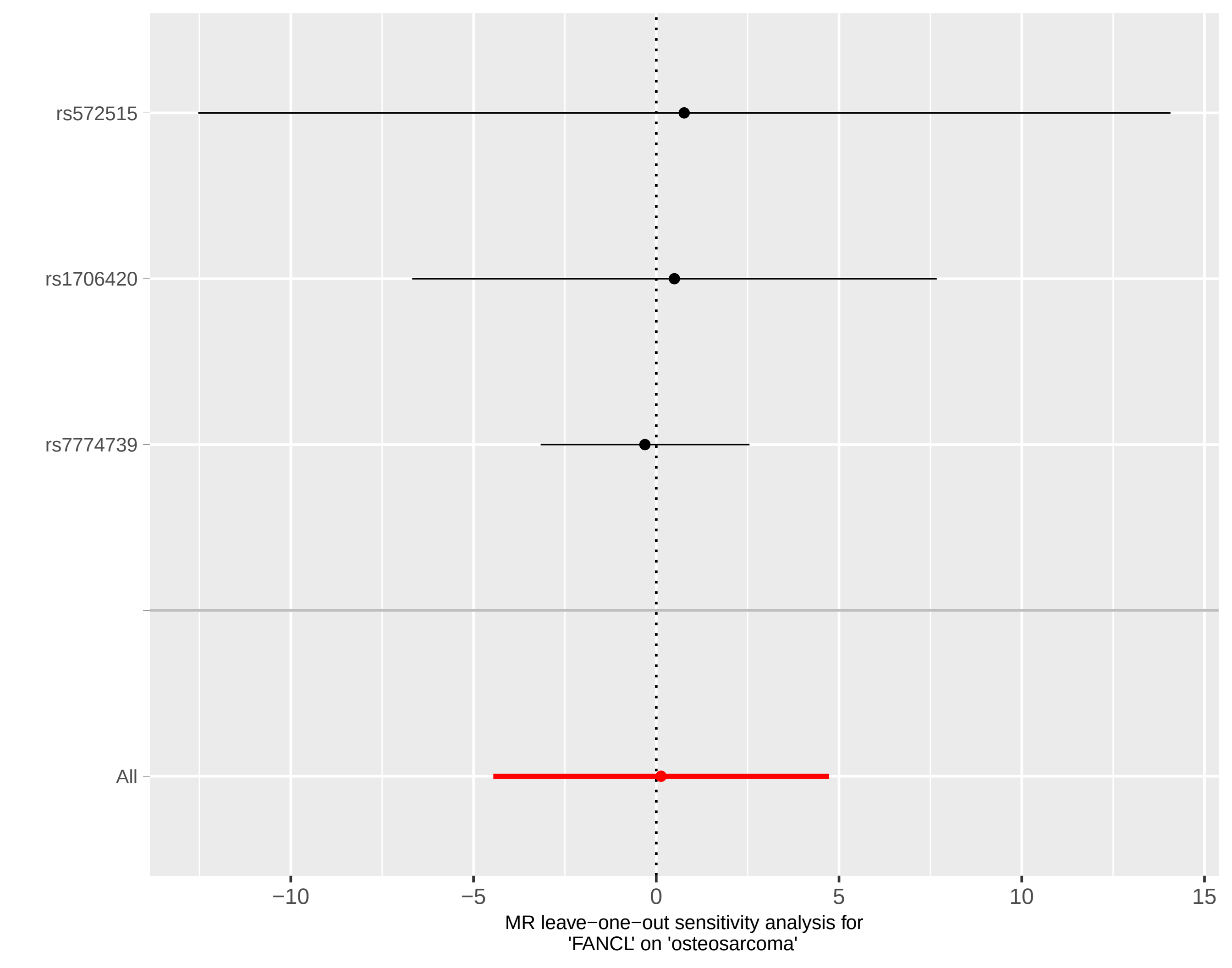
rs572515
rs1706420
rs7774739
All
−10
−5
0
5
10
15
MR leave−one−out sensitivity analysis for
'FANCL' on 'osteosarcoma'

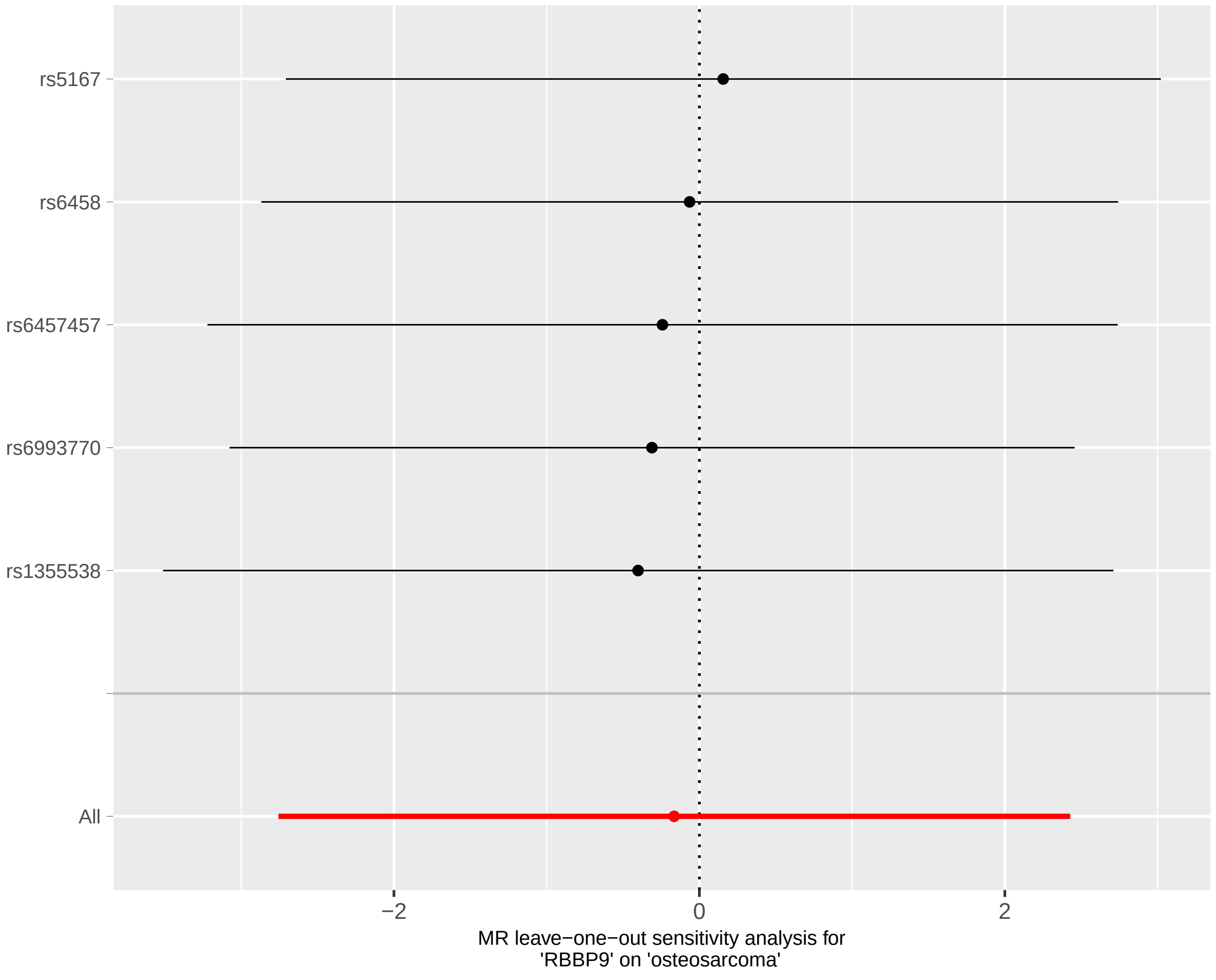
rs5167
rs6458
rs6457457
rs6993770
rs1355538
All
−2
0
2
MR leave−one−out sensitivity analysis for
'RBBP9' on 'osteosarcoma'

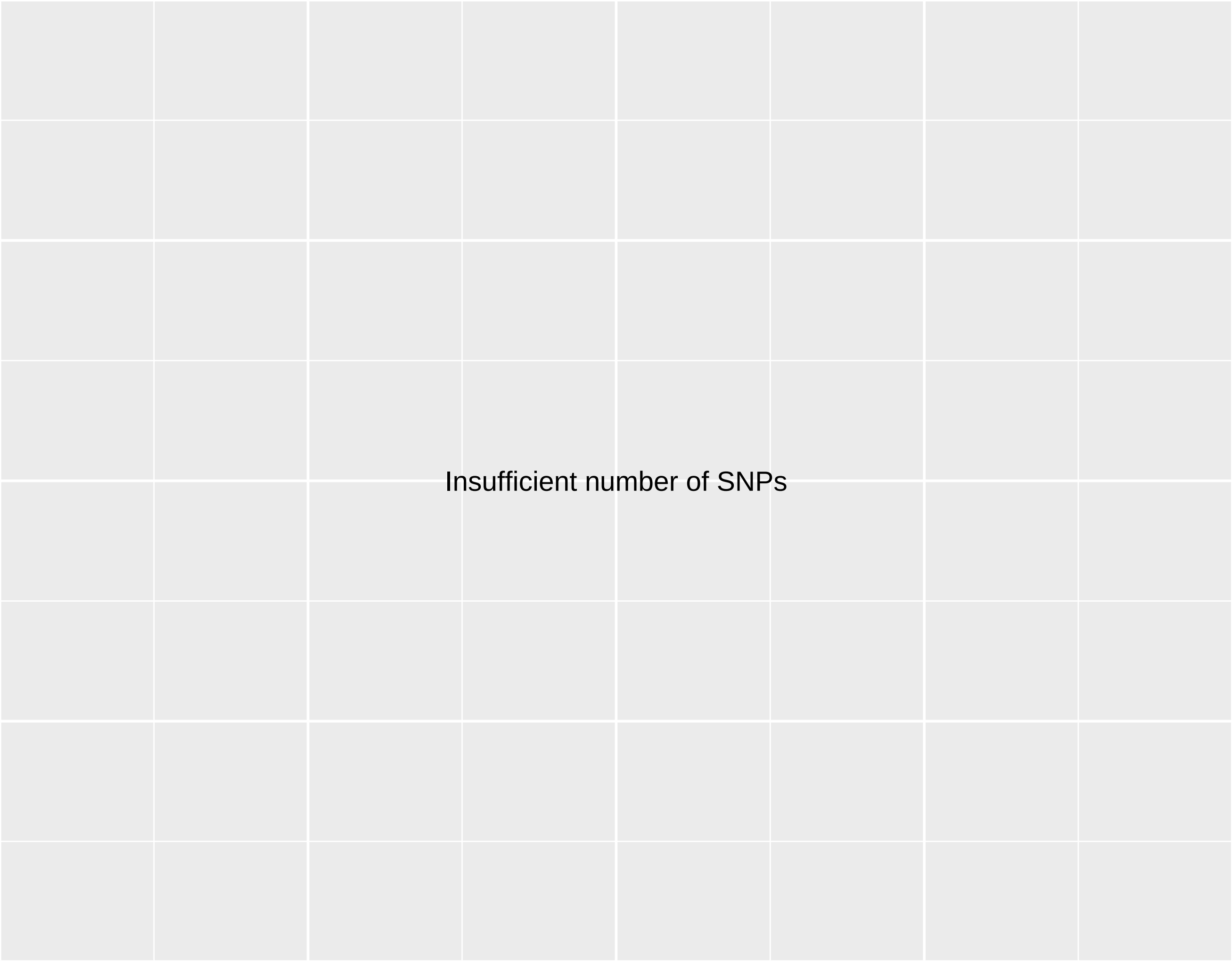
Insufficient number of SNPs

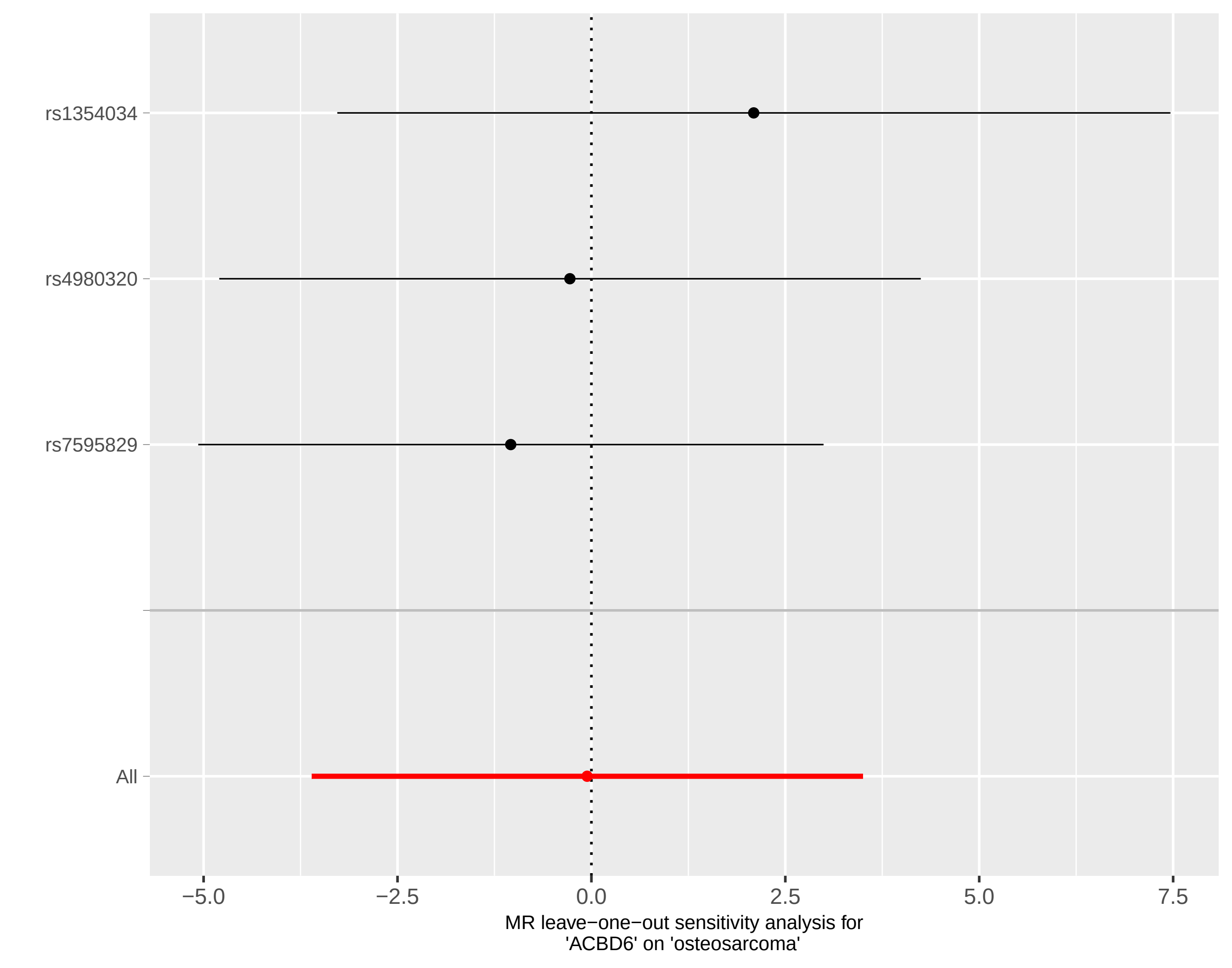

rs1354034
rs4980320
rs7595829
All
−5.0
−2.5
0.0
2.5
5.0
7.5
MR leave−one−out sensitivity analysis for
'ACBD6' on 'osteosarcoma'

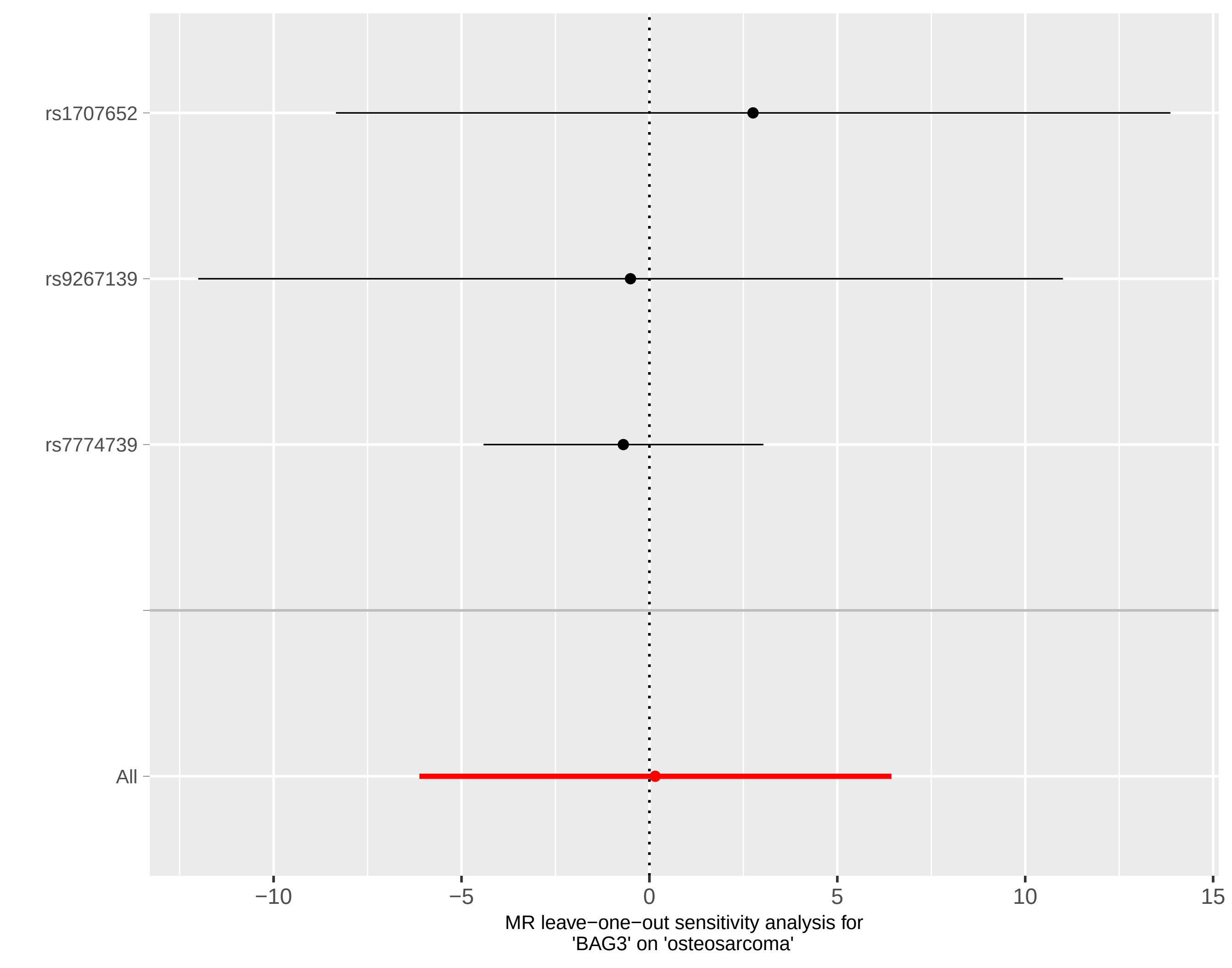
rs1707652
rs9267139
rs7774739
All
−10
−5
0
5
10
15
MR leave−one−out sensitivity analysis for
'BAG3' on 'osteosarcoma'

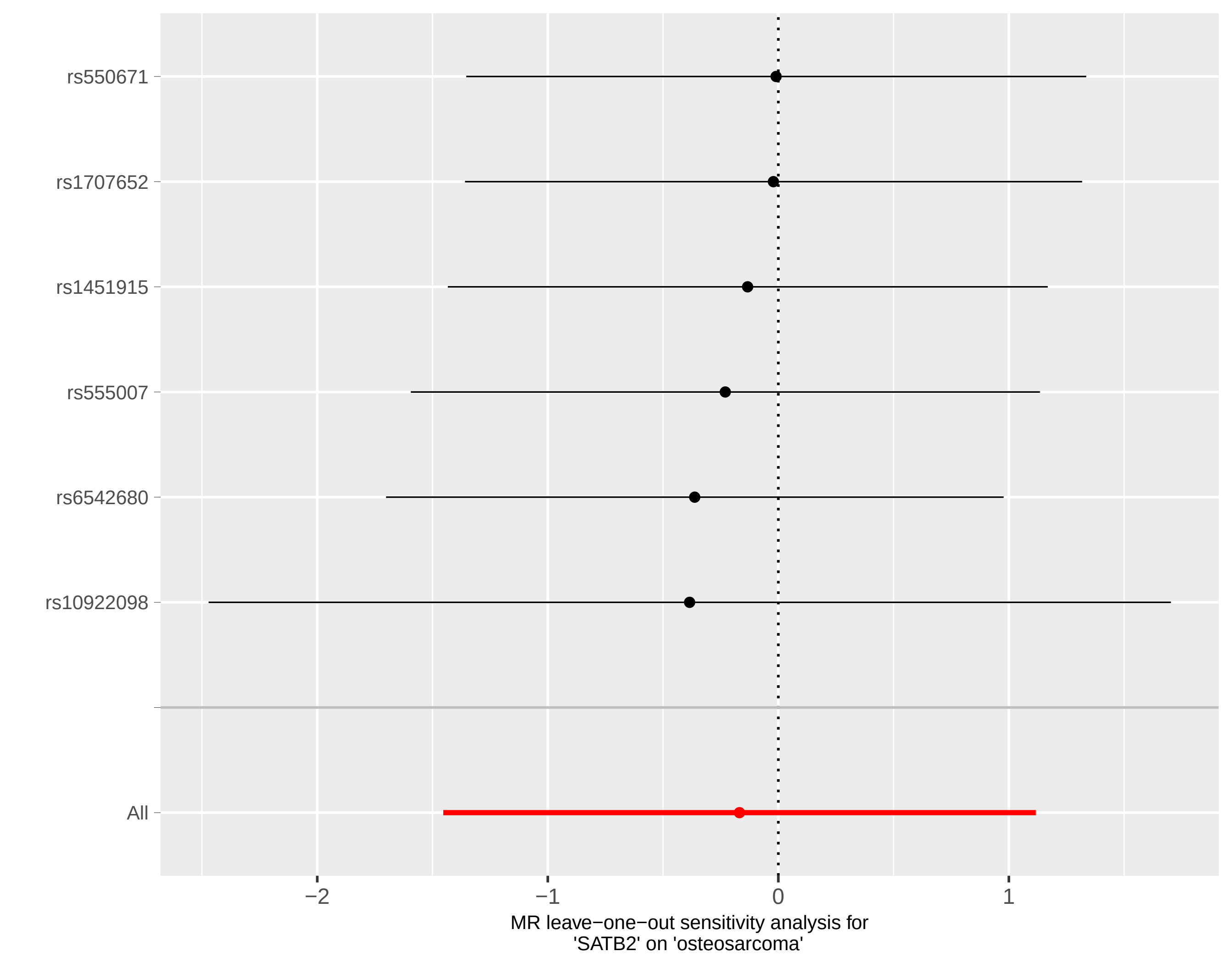
rs550671
rs1707652
rs1451915
rs555007
rs6542680
rs10922098
All
−2
−1
0
1
MR leave−one−out sensitivity analysis for
'SATB2' on 'osteosarcoma'

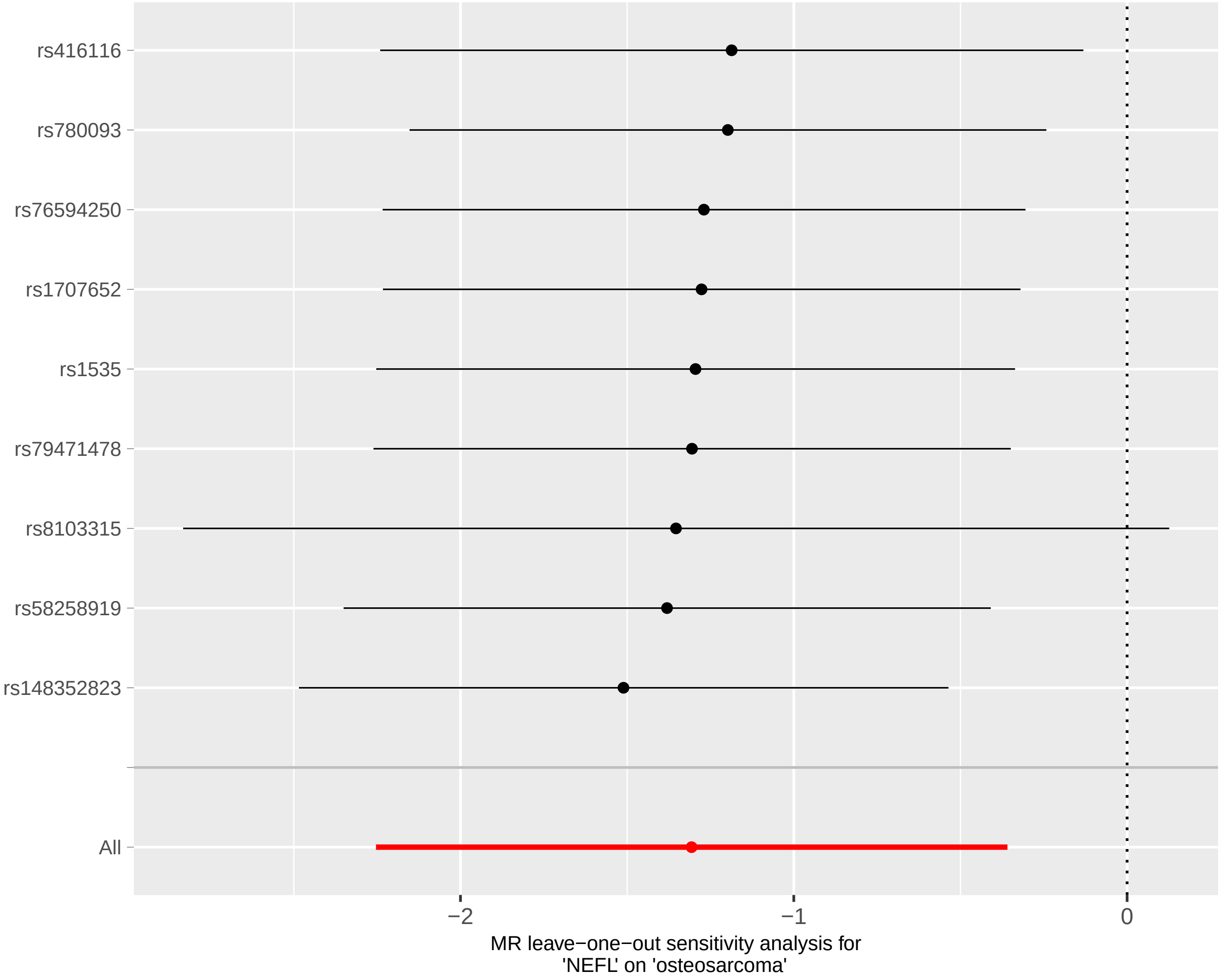

rs416116
rs780093
rs76594250
rs1707652
rs1535
rs79471478
rs8103315
rs58258919
rs148352823
All
−2
−1
0
MR leave−one−out sensitivity analysis for
'NEFL' on 'osteosarcoma'

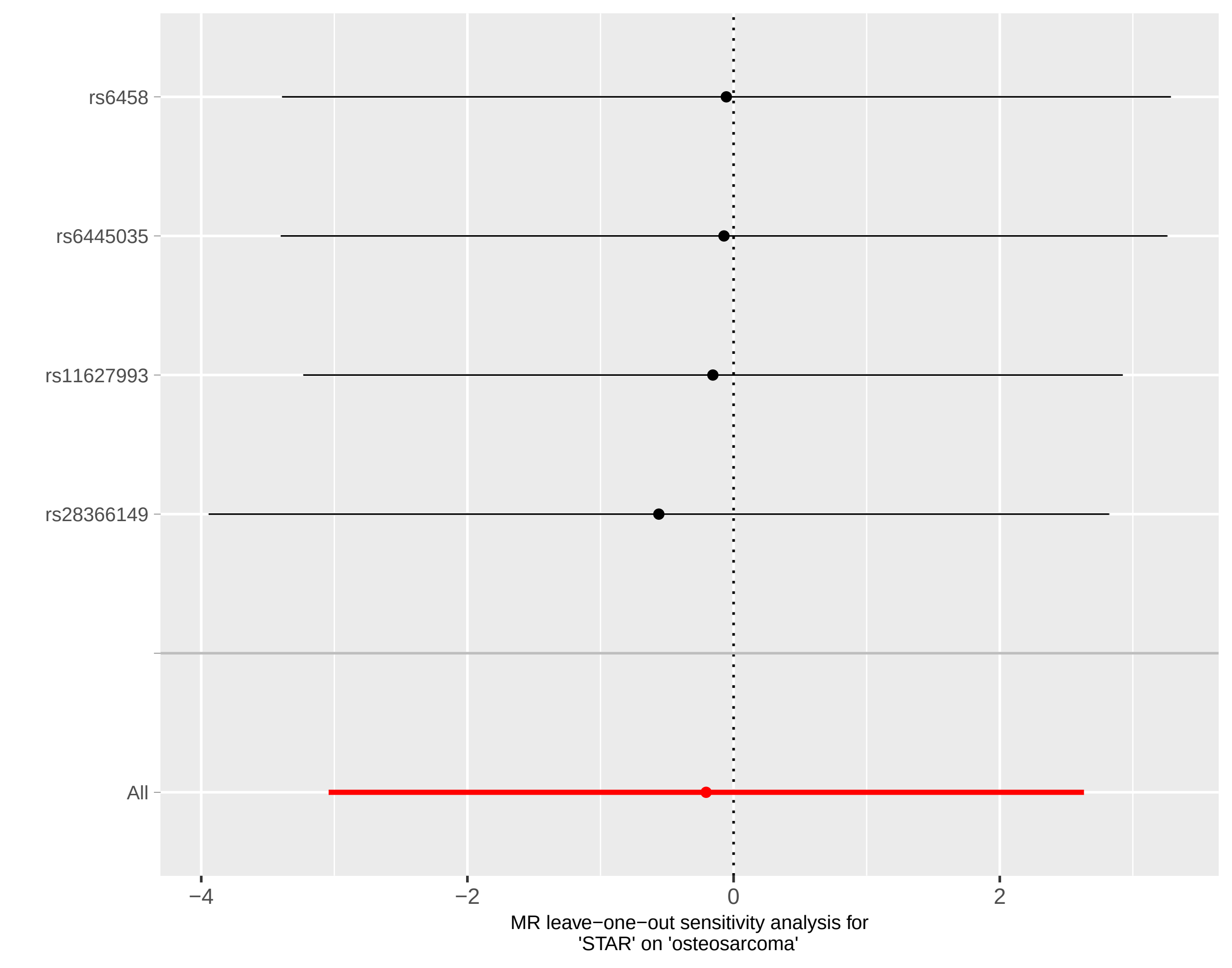
rs6458
rs6445035
rs11627993
rs28366149
All
−4
−2
0
2
MR leave−one−out sensitivity analysis for
'STAR' on 'osteosarcoma'

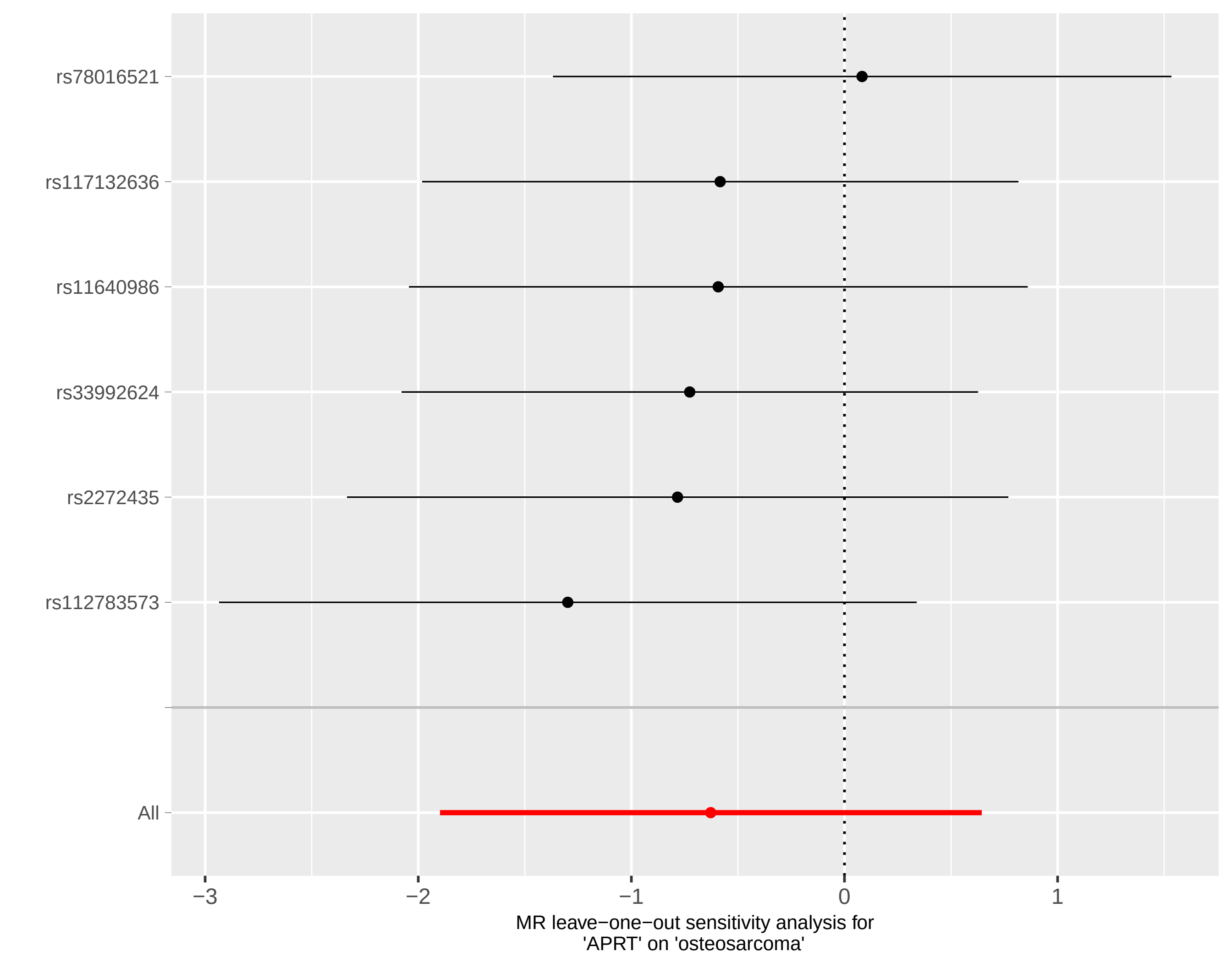

rs78016521
rs117132636
rs11640986
rs33992624
rs2272435
rs112783573
All
−3
−2
−1
0
1
MR leave−one−out sensitivity analysis for
'APRT' on 'osteosarcoma'

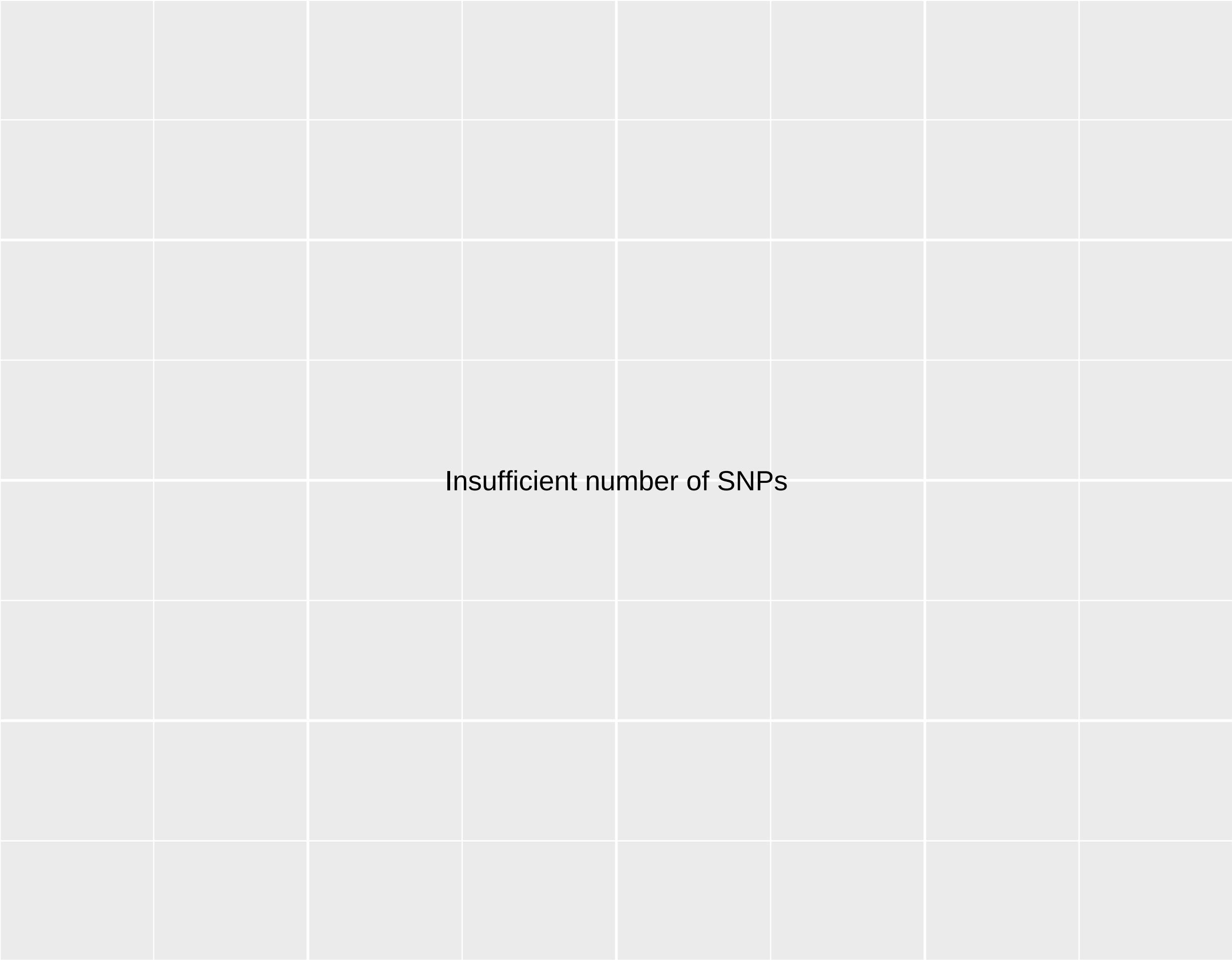
Insufficient number of SNPs

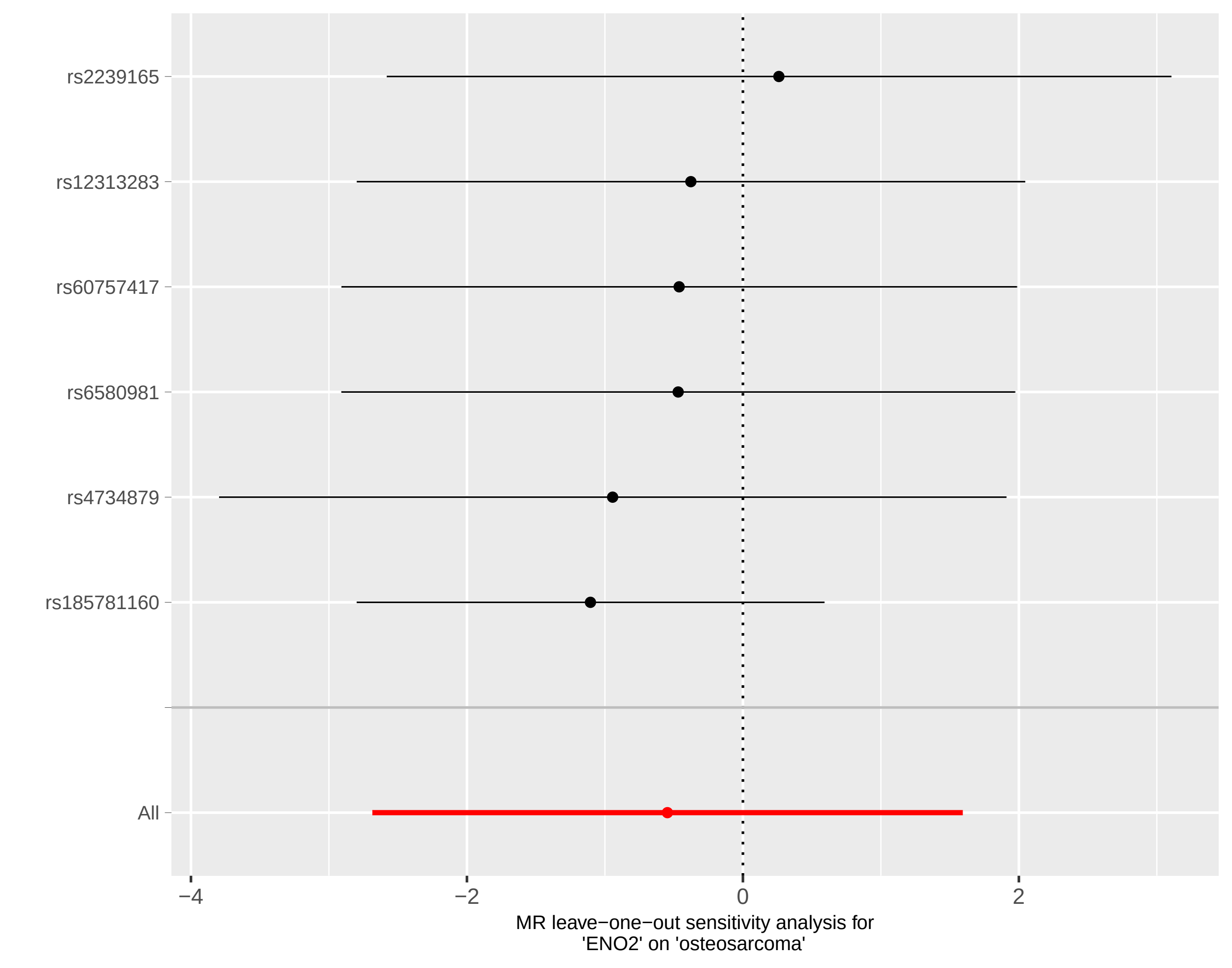
rs2239165
rs12313283
rs60757417
rs6580981
rs4734879
rs185781160
All
−4
−2
0
2
MR leave−one−out sensitivity analysis for
'ENO2' on 'osteosarcoma'

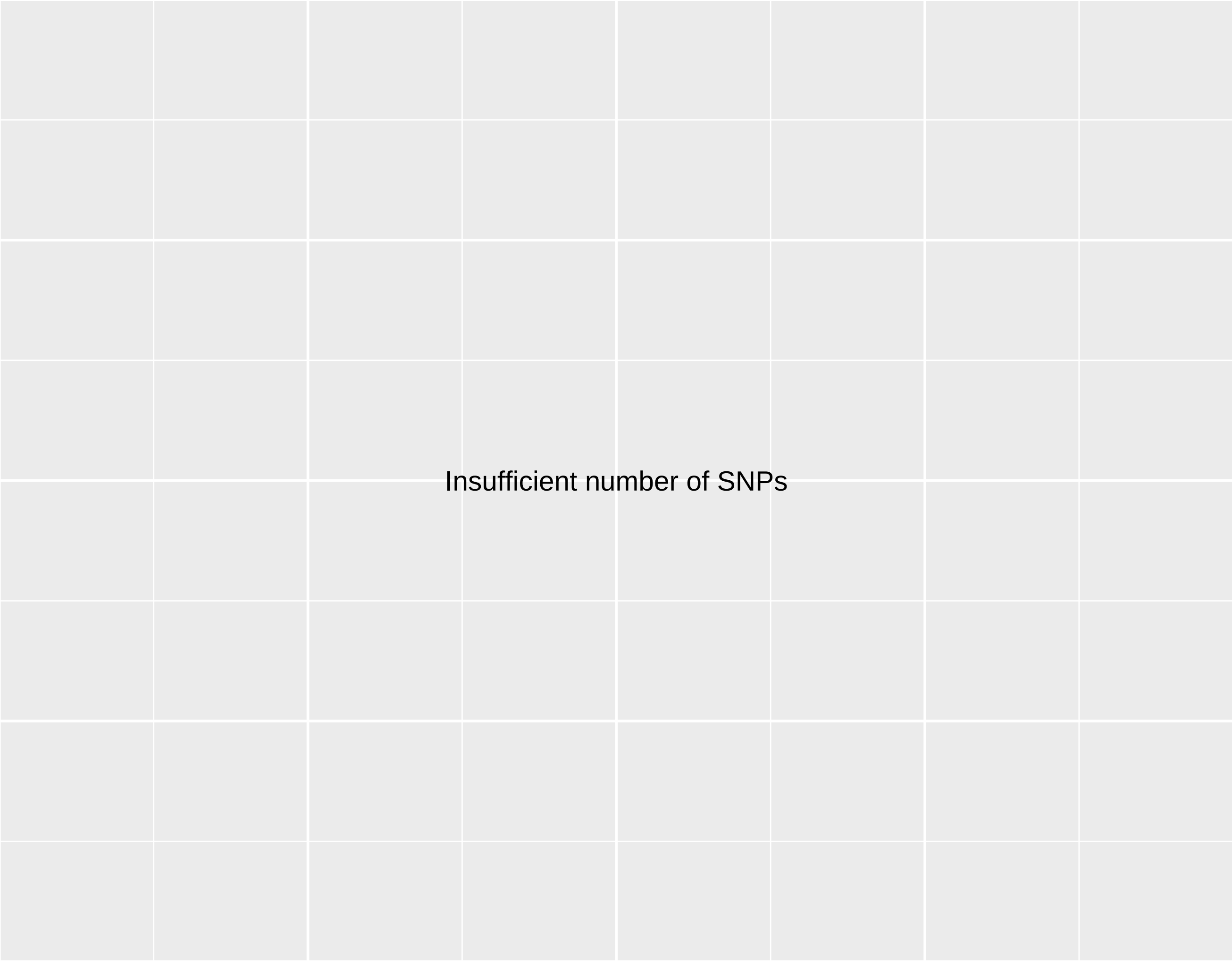
Insufficient number of SNPs

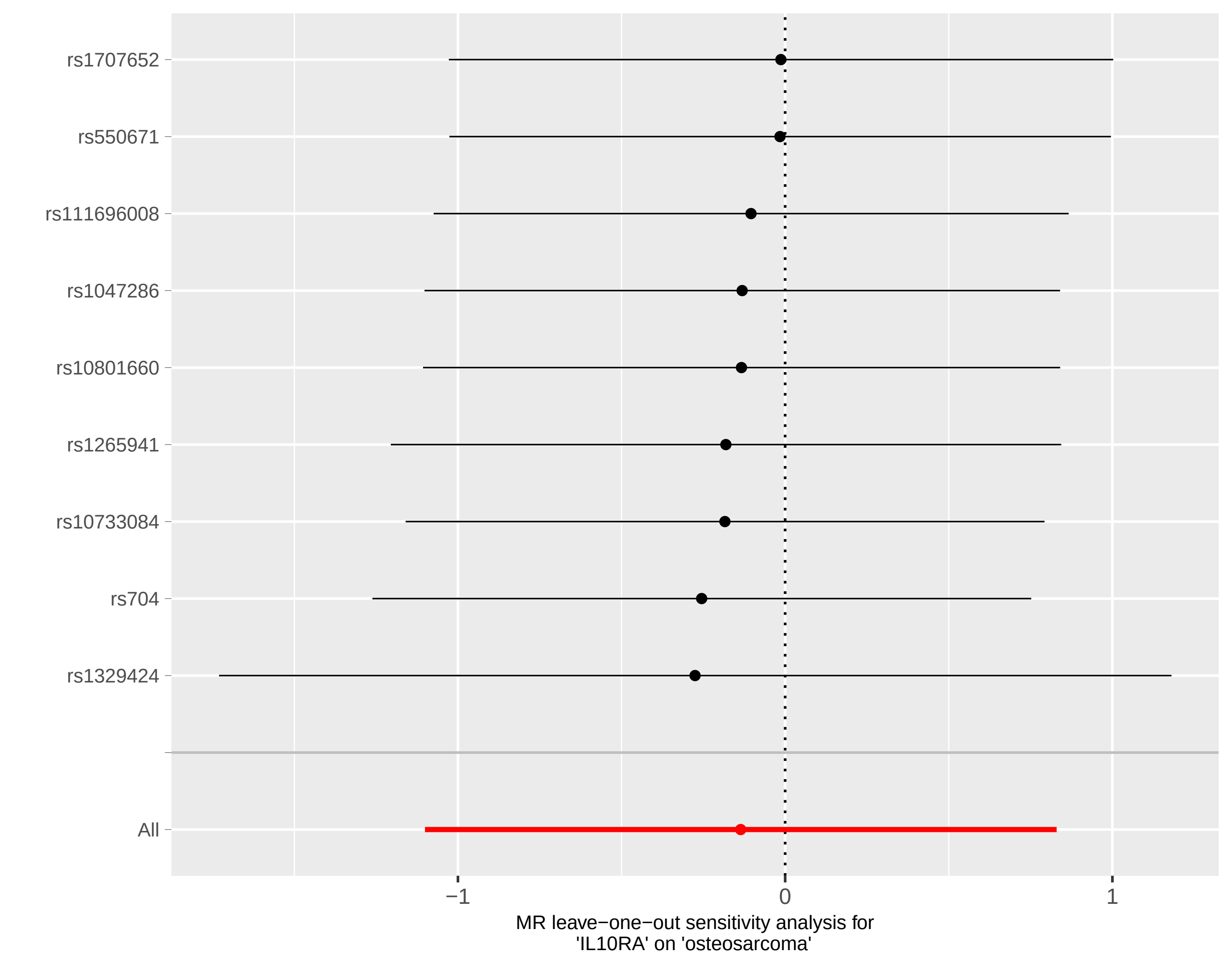
rs1707652
rs550671
rs111696008
rs1047286
rs10801660
rs1265941
rs10733084
rs704
rs1329424
All
−1
0
1
MR leave−one−out sensitivity analysis for
'IL10RA' on 'osteosarcoma'

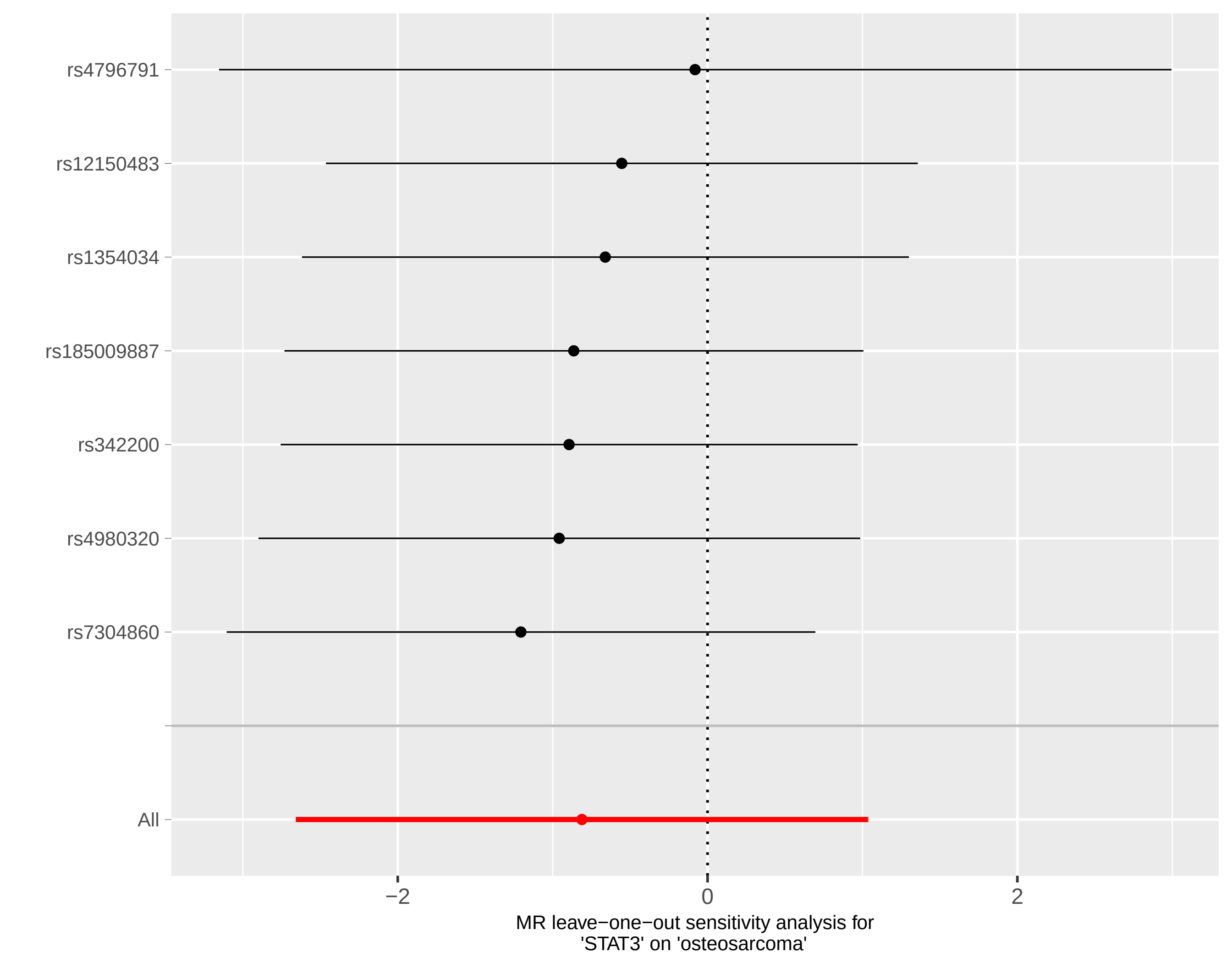
rs4796791
rs12150483
rs1354034
rs185009887
rs342200
rs4980320
rs7304860
All
−2
0
2
MR leave−one−out sensitivity analysis for
'STAT3' on 'osteosarcoma'

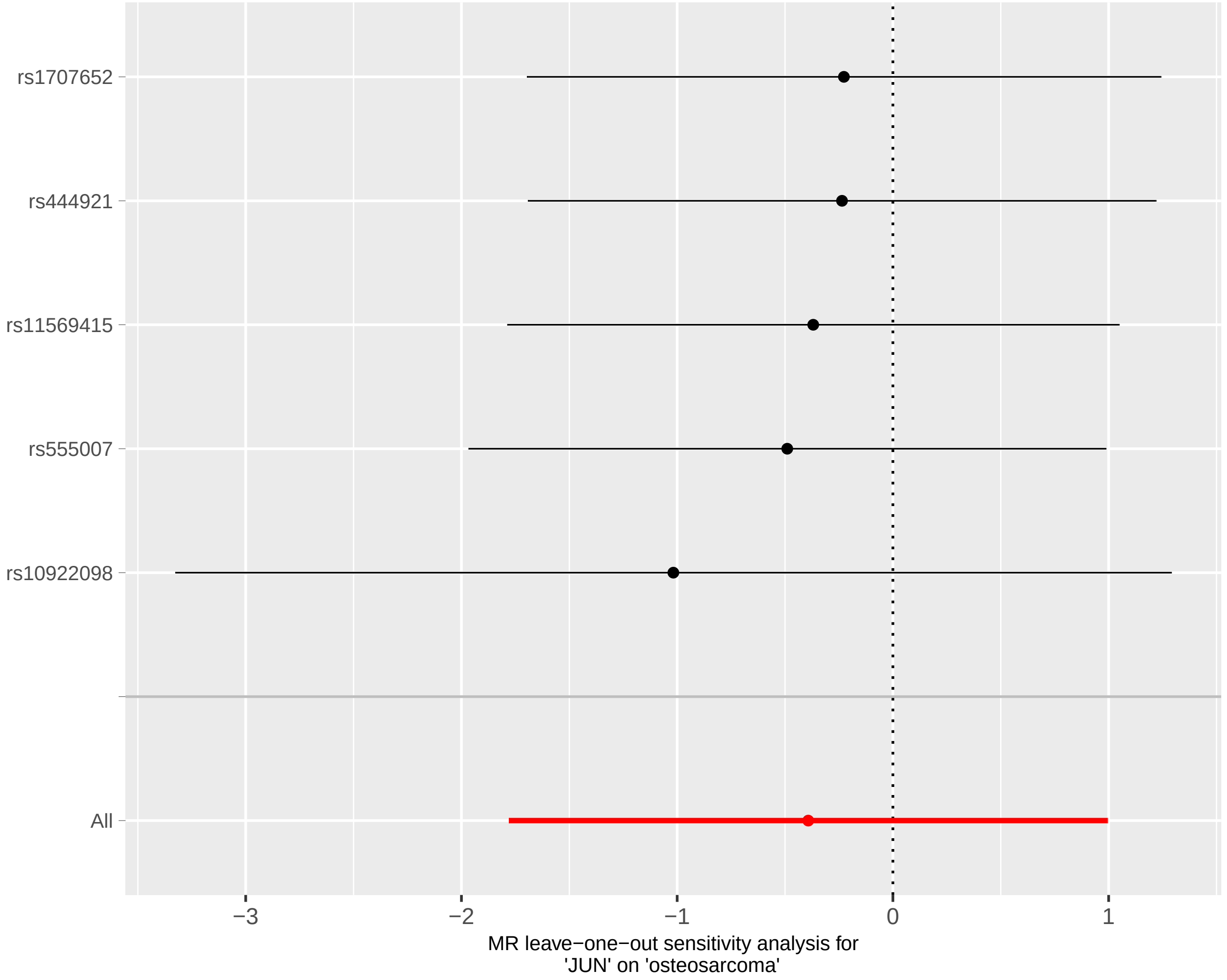

rs1707652
rs444921
rs11569415
rs555007
rs10922098
All
−3
−2
−1
0
1
MR leave−one−out sensitivity analysis for
'JUN' on 'osteosarcoma'

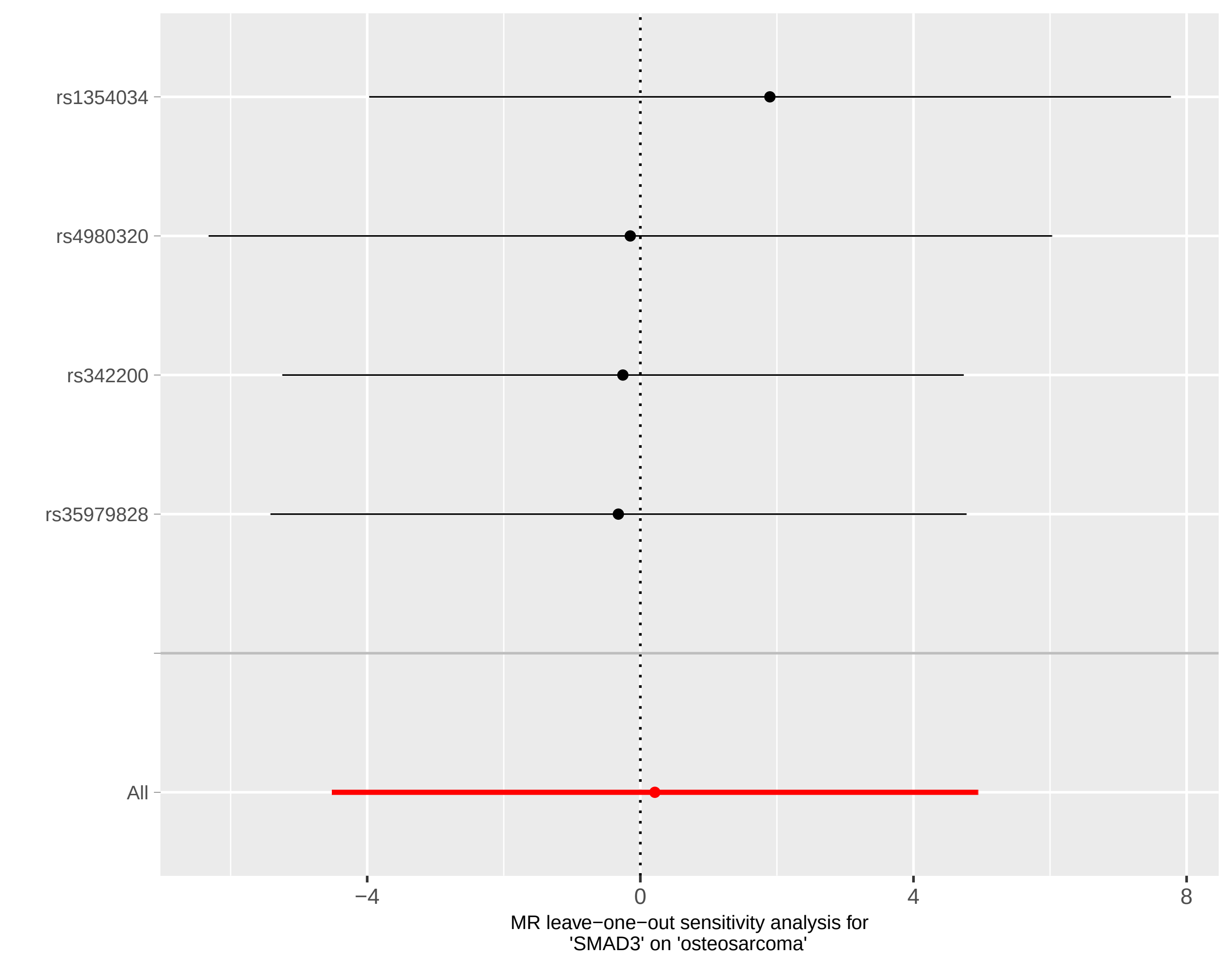
rs1354034
rs4980320
rs342200
rs35979828
All
−4
0
4
8
MR leave−one−out sensitivity analysis for
'SMAD3' on 'osteosarcoma'

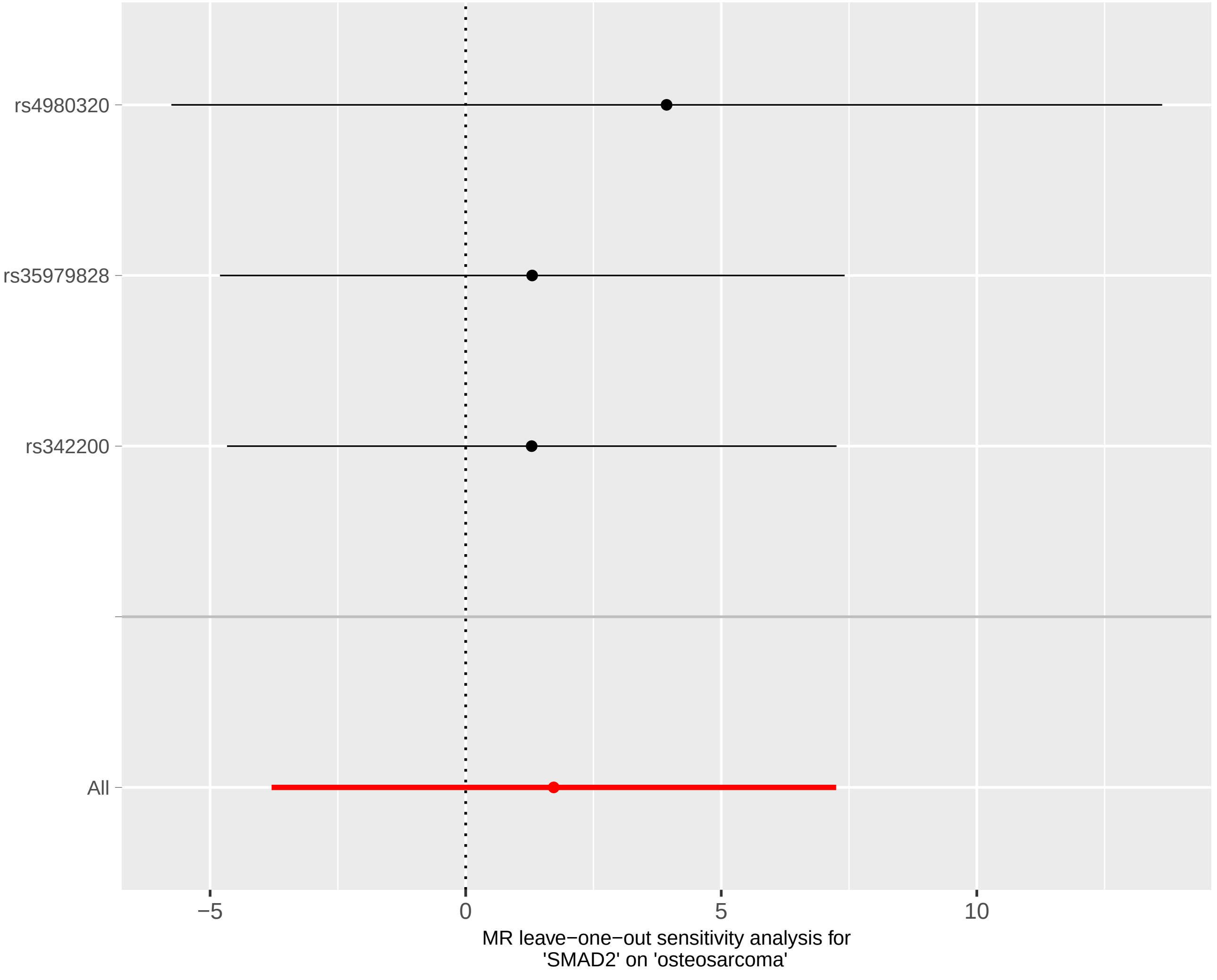
rs4980320
rs35979828
rs342200
All
−5
0
5
10
MR leave−one−out sensitivity analysis for
'SMAD2' on 'osteosarcoma'

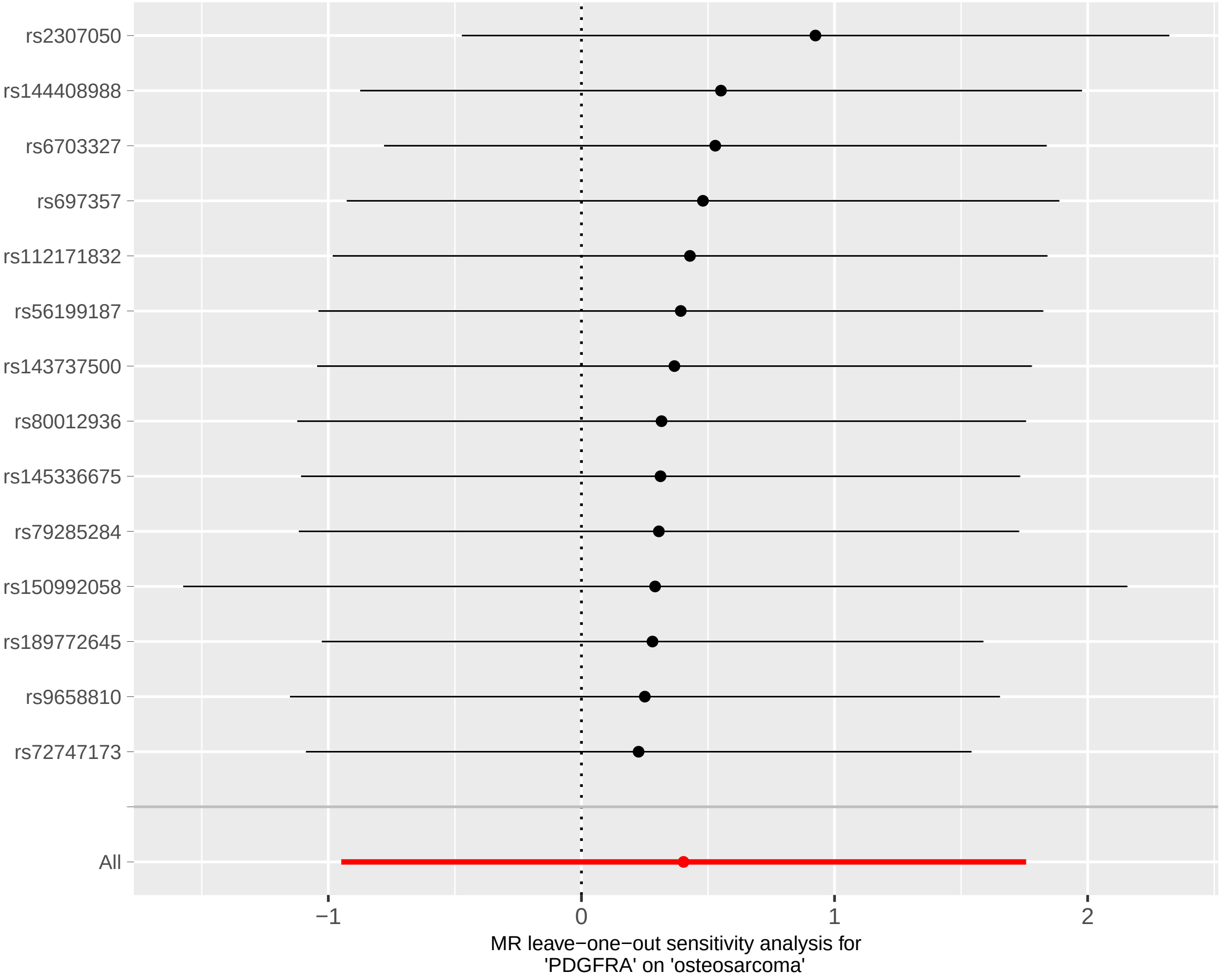
rs2307050
rs144408988
rs6703327
rs697357
rs112171832
rs56199187
rs143737500
rs80012936
rs145336675
rs79285284
rs150992058
rs189772645
rs9658810
rs72747173
All
−1
0
1
2
MR leave−one−out sensitivity analysis for
'PDGFRA' on 'osteosarcoma'

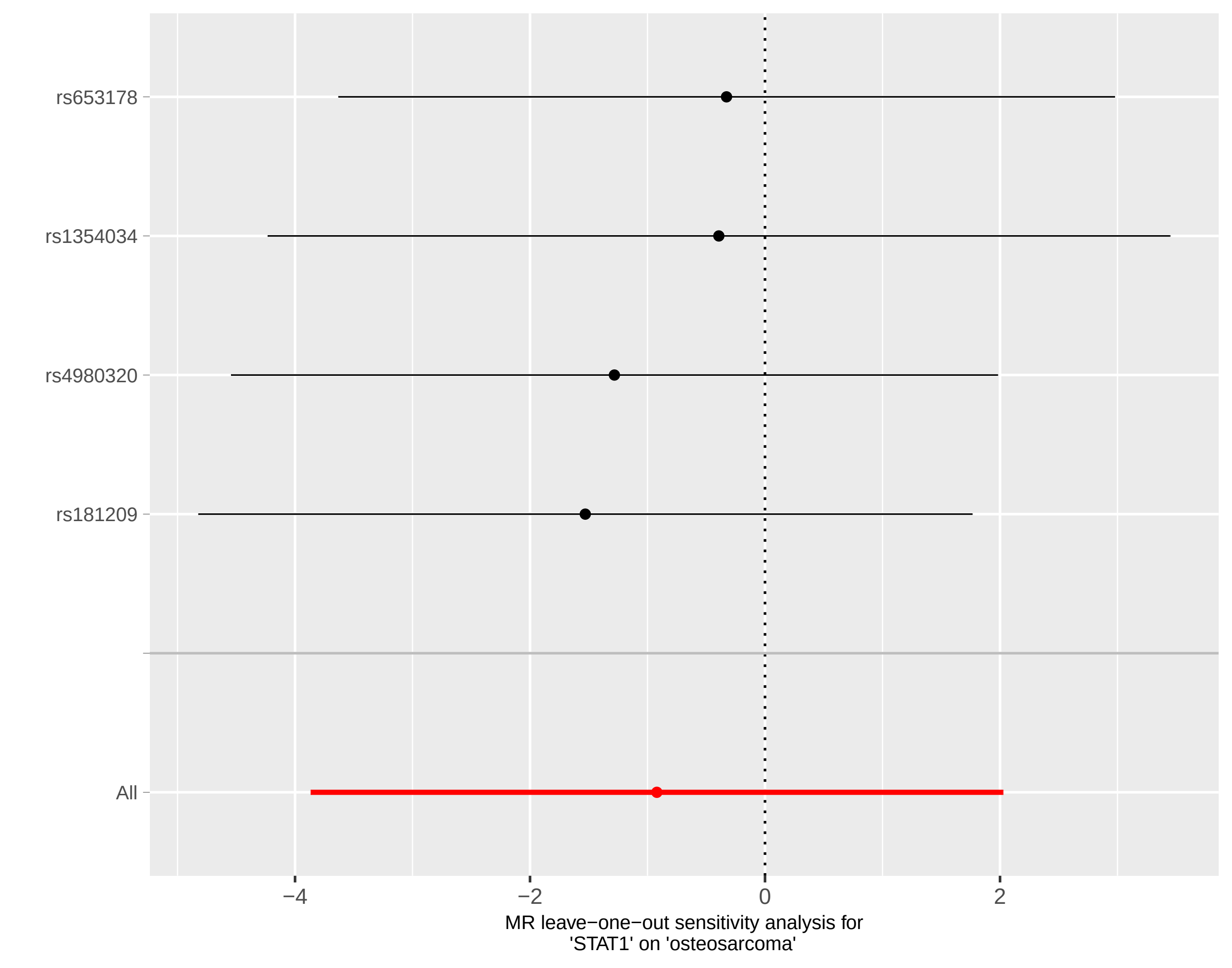

rs653178
rs1354034
rs4980320
rs181209
All
−4
−2
0
2
MR leave−one−out sensitivity analysis for
'STAT1' on 'osteosarcoma'

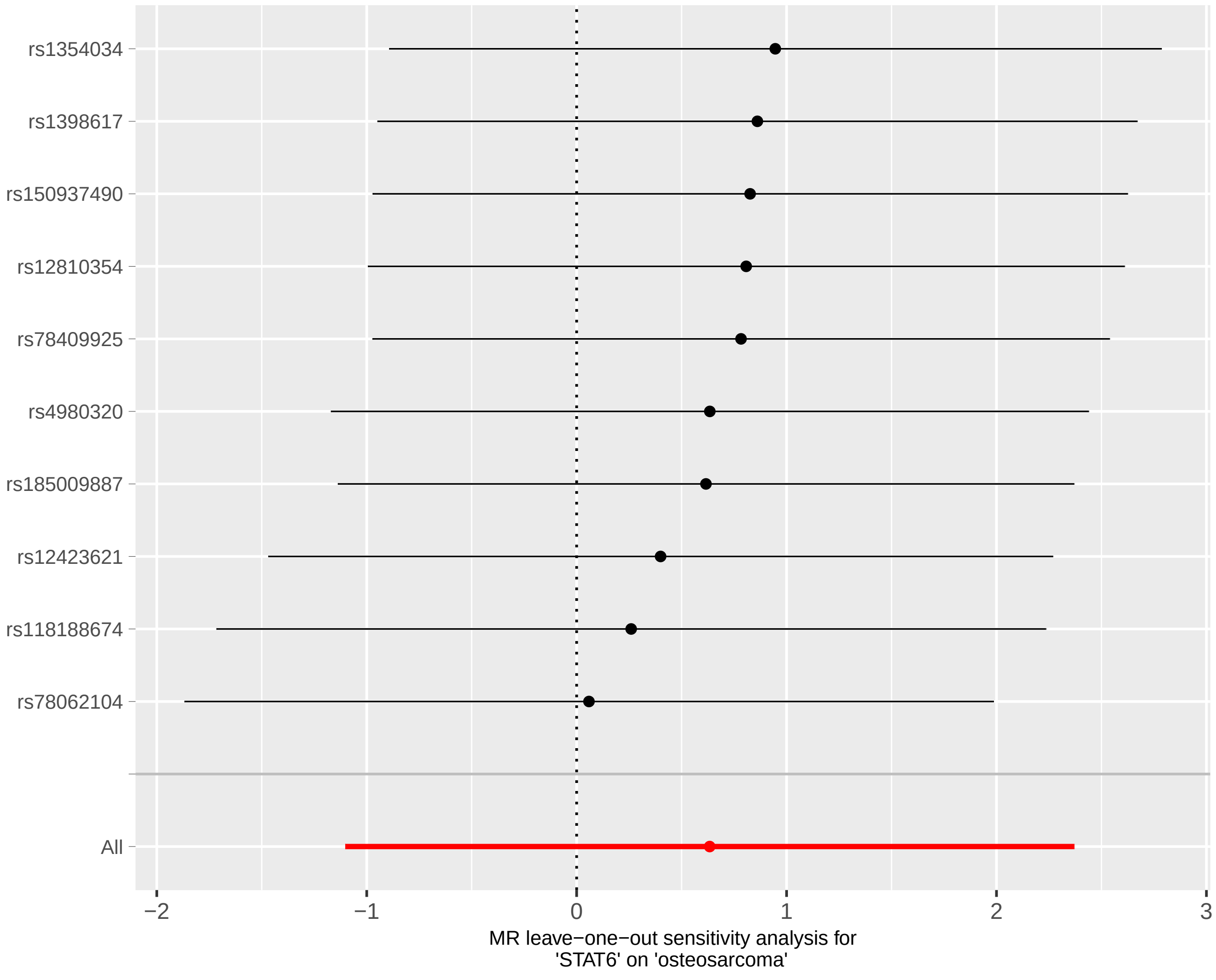
rs1354034
rs1398617
rs150937490
rs12810354
rs78409925
rs4980320
rs185009887
rs12423621
rs118188674
rs78062104
All
−2
−1
0
1
2
3
MR leave−one−out sensitivity analysis for
'STAT6' on 'osteosarcoma'

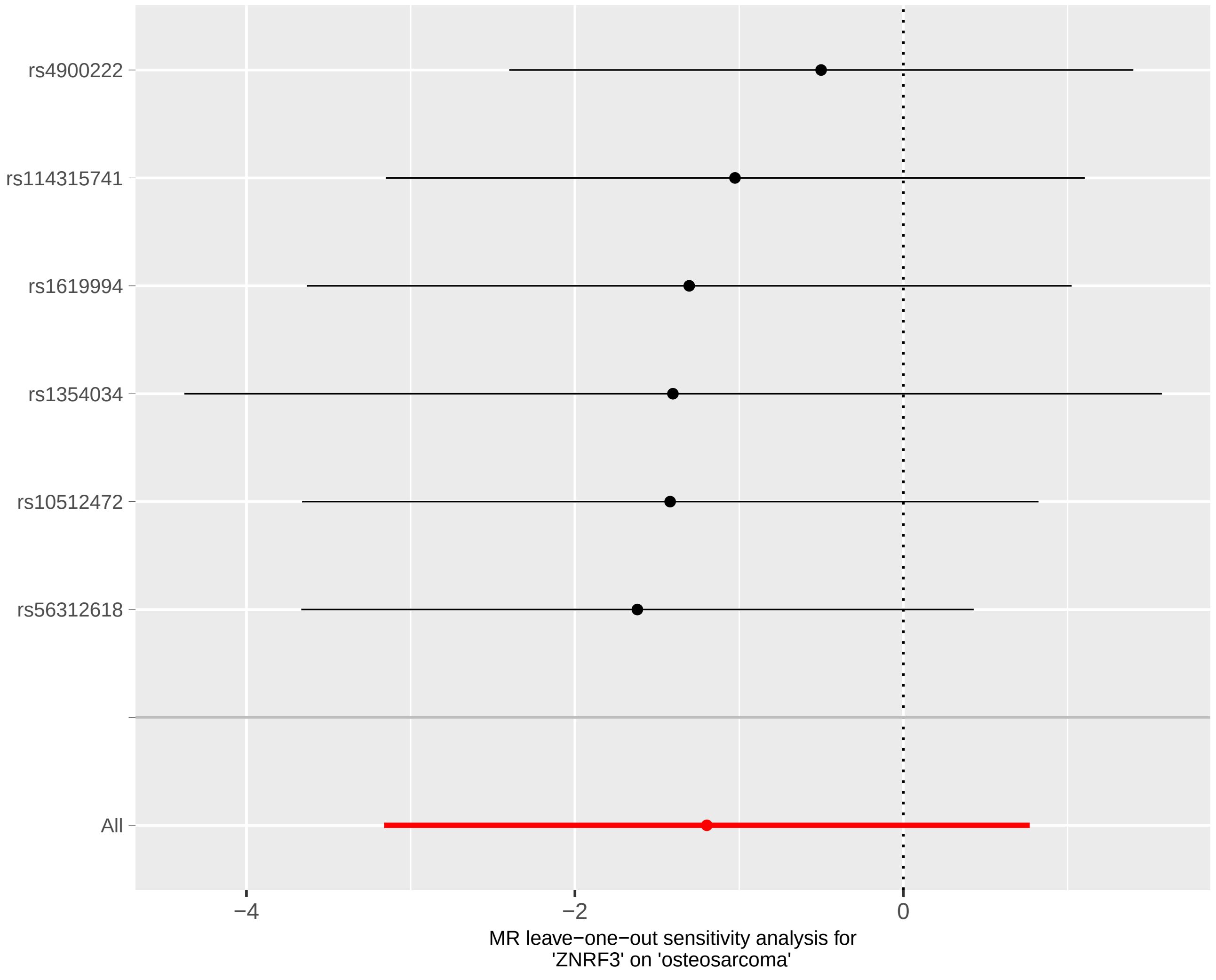
rs4900222
rs114315741
rs1619994
rs1354034
rs10512472
rs56312618
All
−4
−2
0
MR leave−one−out sensitivity analysis for
'ZNRF3' on 'osteosarcoma'

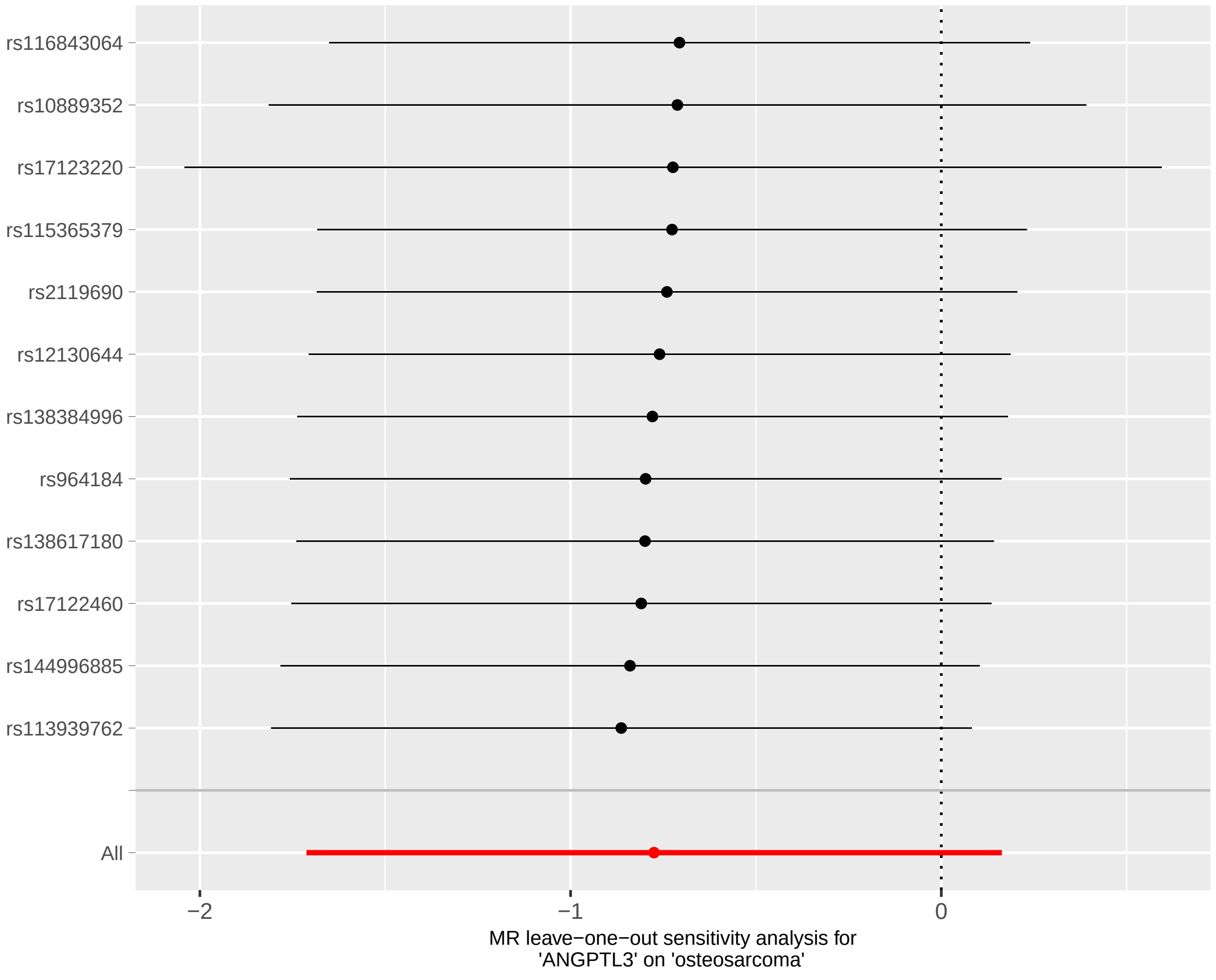

rs116843064
rs10889352
rs17123220
rs115365379
rs2119690
rs12130644
rs138384996
rs964184
rs138617180
rs17122460
rs144996885
rs113939762
All
−2
−1
0
MR leave−one−out sensitivity analysis for
'ANGPTL3' on 'osteosarcoma'

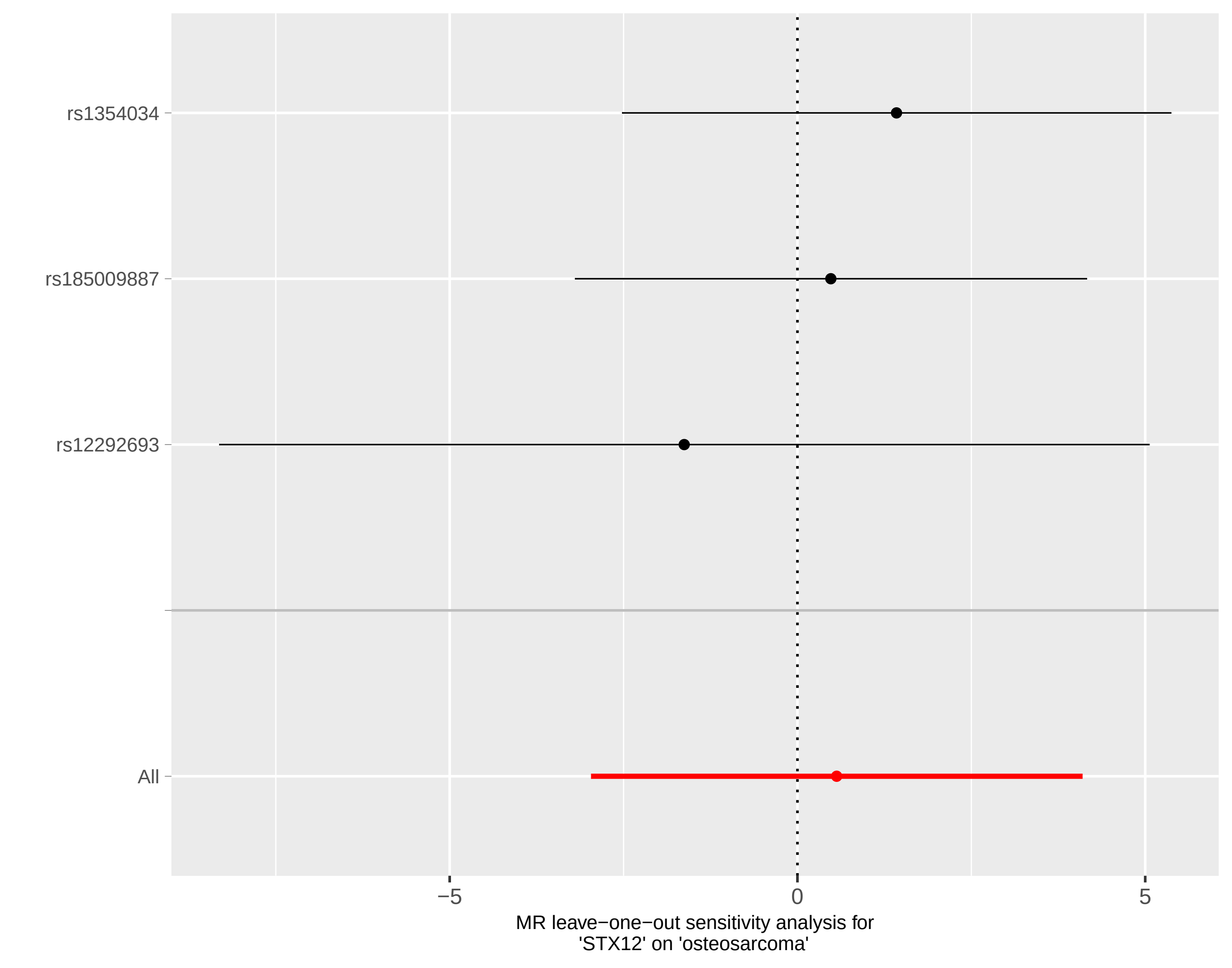
rs1354034
rs185009887
rs12292693
All
−5
0
5
MR leave−one−out sensitivity analysis for
'STX12' on 'osteosarcoma'

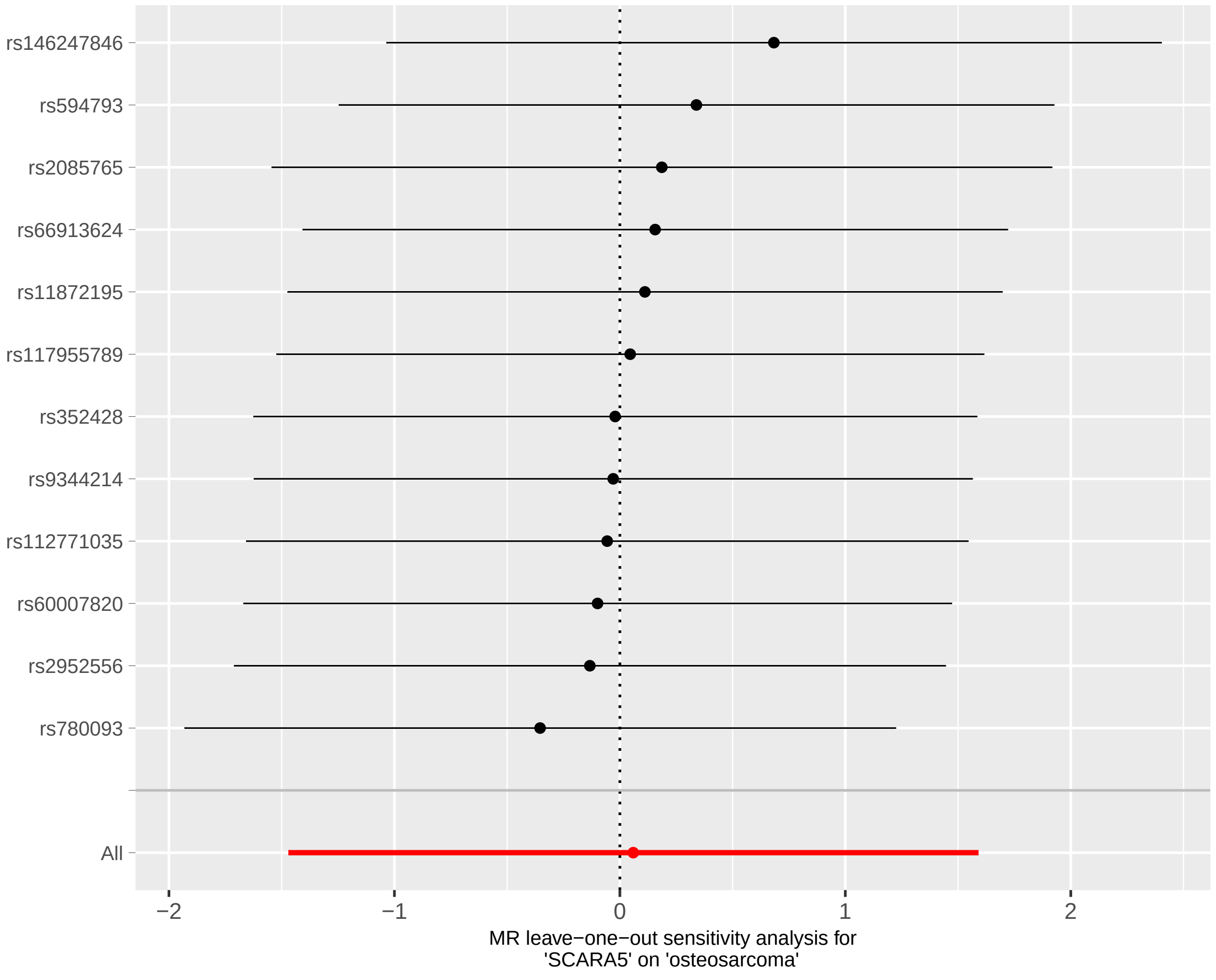

rs146247846
rs594793
rs2085765
rs66913624
rs11872195
rs117955789
rs352428
rs9344214
rs112771035
rs60007820
rs2952556
rs780093
All
−2
−1
0
1
2
MR leave−one−out sensitivity analysis for
'SCARA5' on 'osteosarcoma'

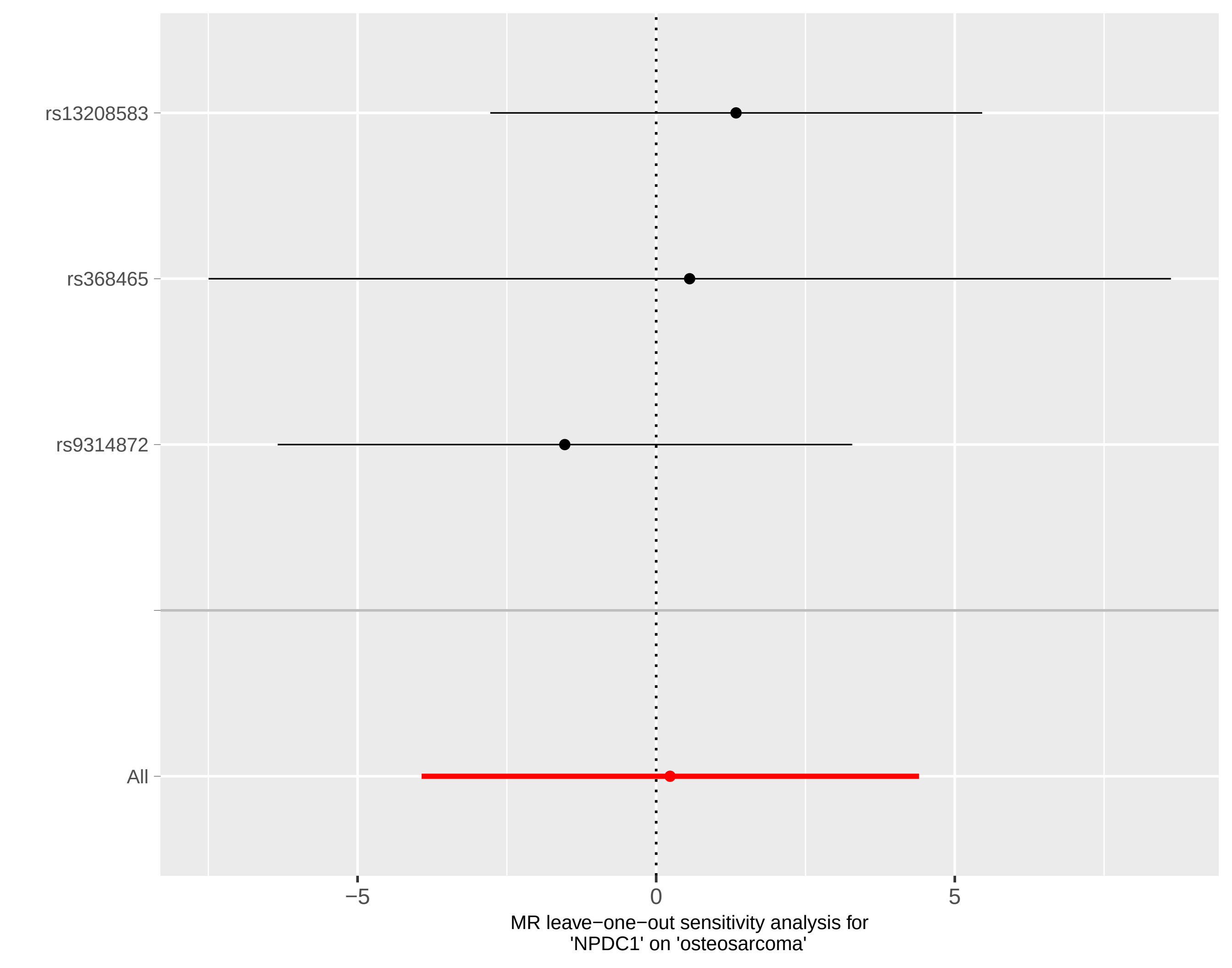
rs13208583
rs368465
rs9314872
All
−5
0
5
MR leave−one−out sensitivity analysis for
'NPDC1' on 'osteosarcoma'

Insufficient number of SNPs

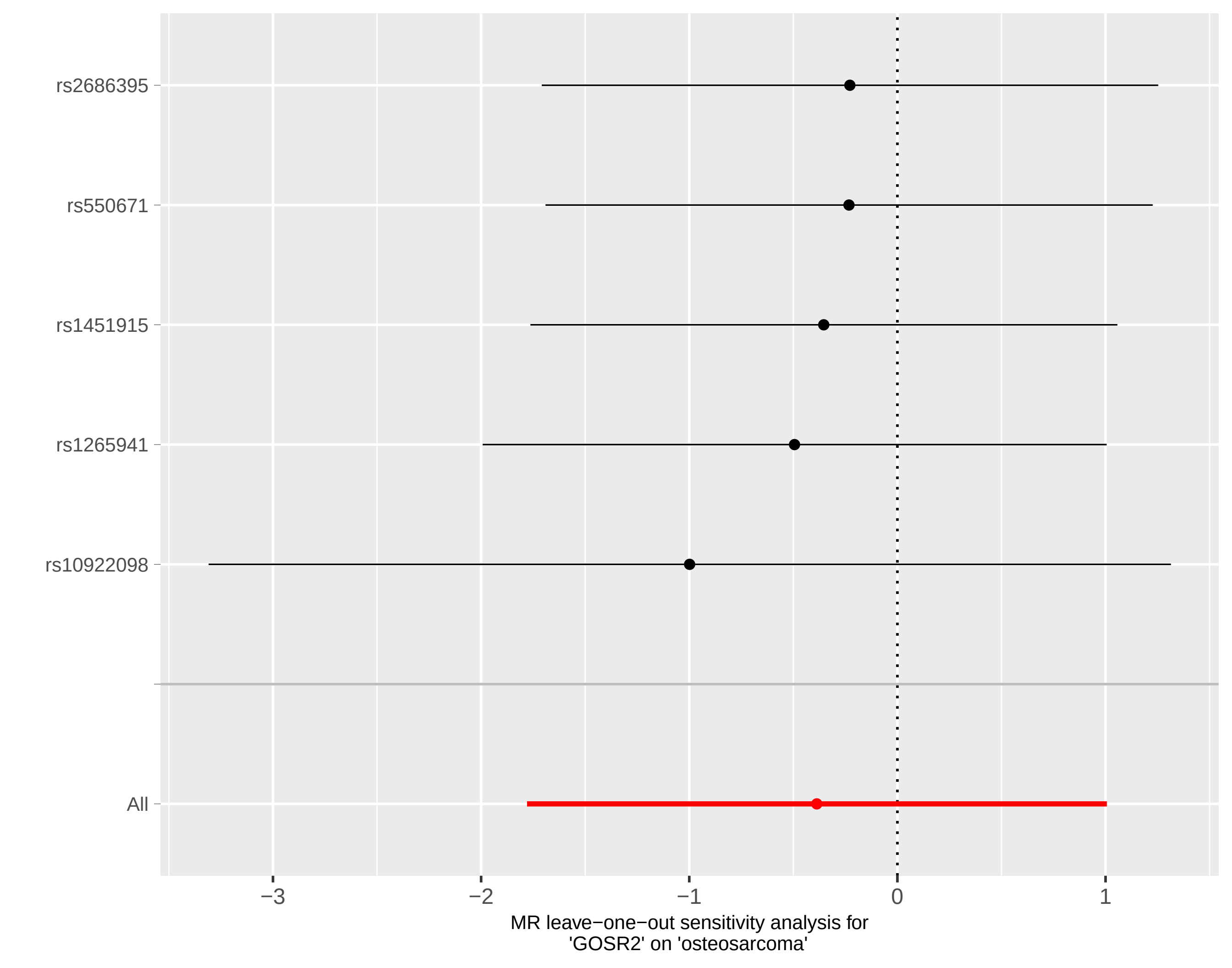
rs2686395
rs550671
rs1451915
rs1265941
rs10922098
All
−3
−2
−1
0
1
MR leave−one−out sensitivity analysis for
'GOSR2' on 'osteosarcoma'

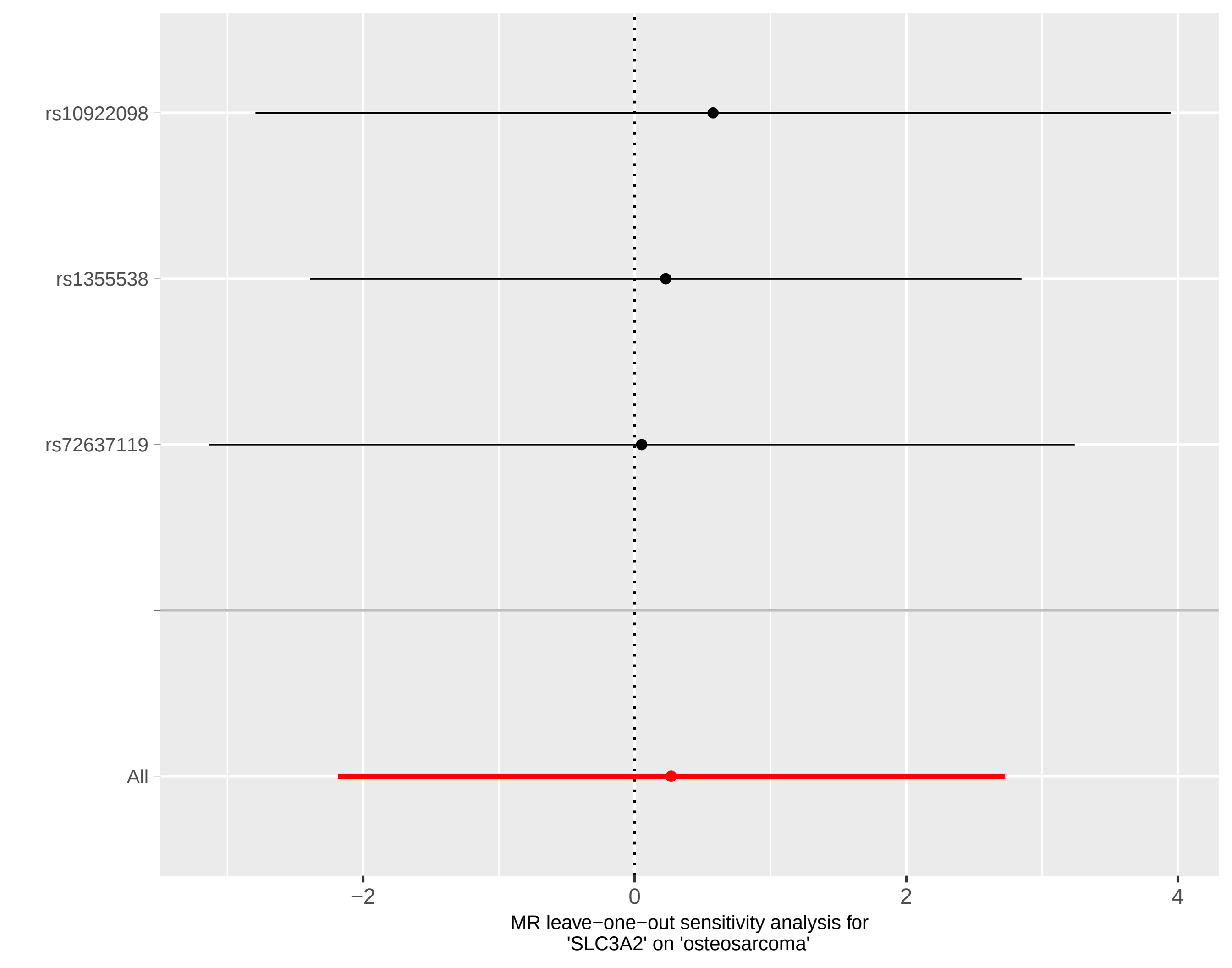
rs10922098
rs1355538
rs72637119
All
−2
0
2
4
MR leave−one−out sensitivity analysis for
'SLC3A2' on 'osteosarcoma'

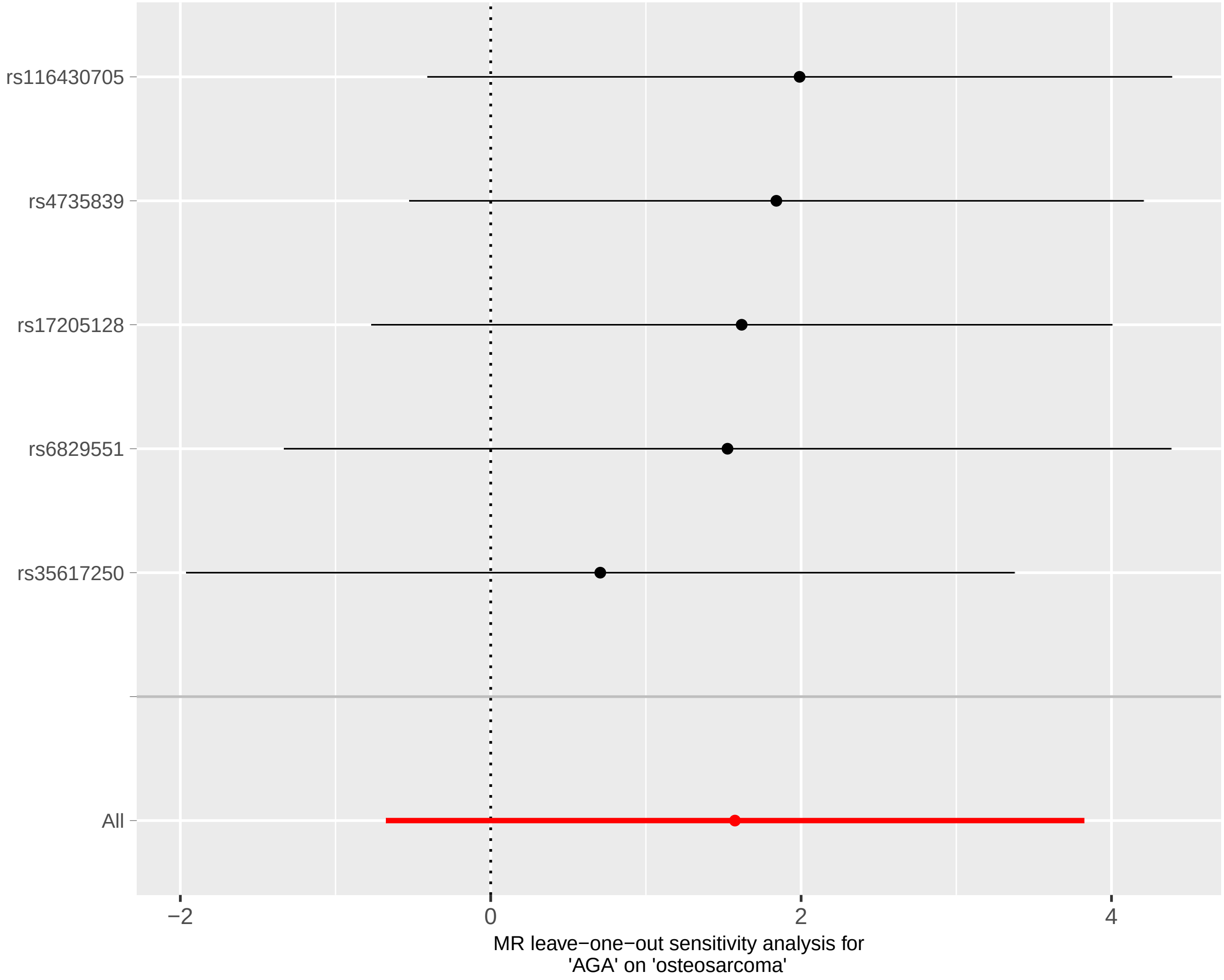

rs116430705
rs4735839
rs17205128
rs6829551
rs35617250
All
−2
0
2
4
MR leave−one−out sensitivity analysis for
'AGA' on 'osteosarcoma'

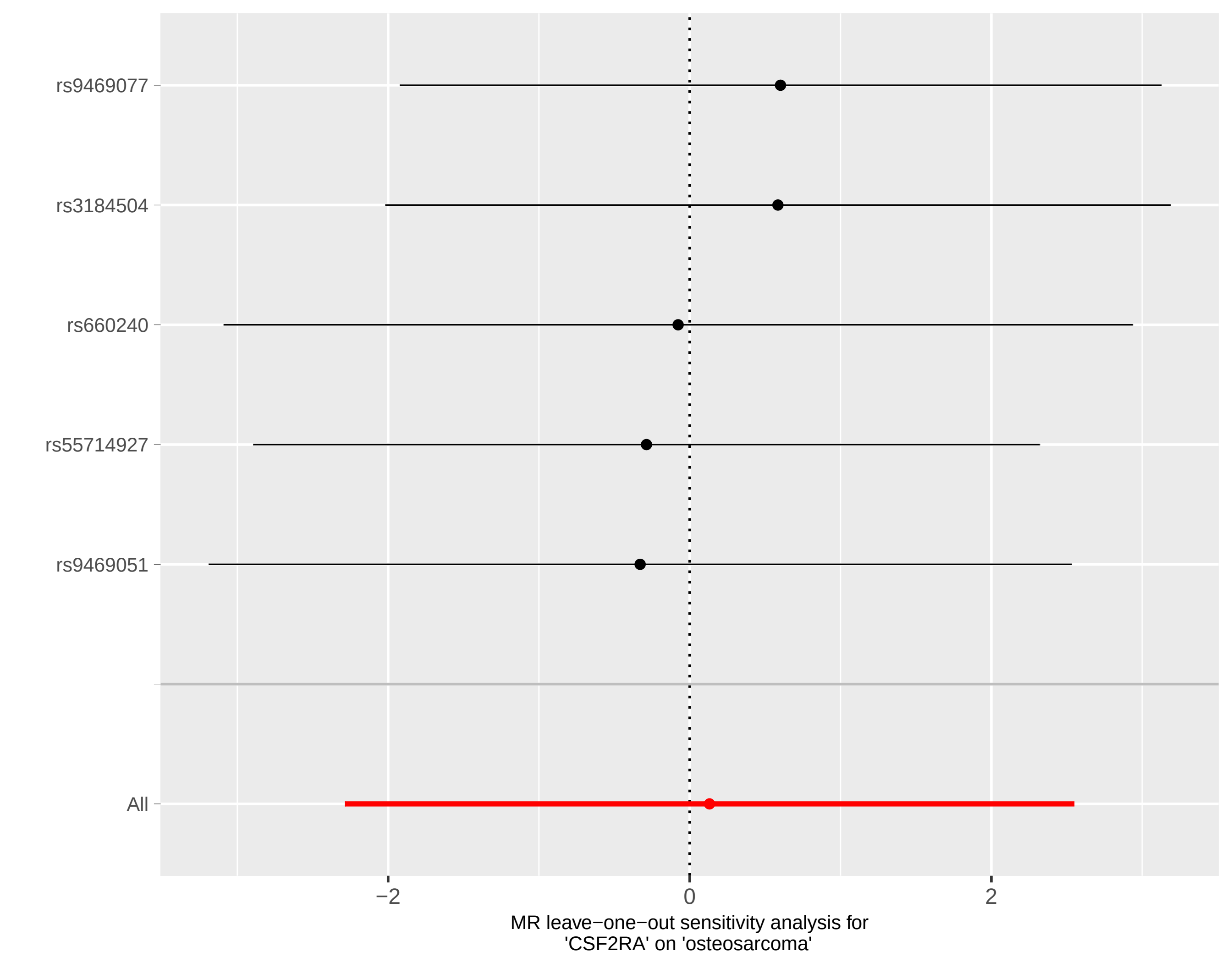
rs9469077
rs3184504
rs660240
rs55714927
rs9469051
All
−2
0
2
MR leave−one−out sensitivity analysis for
'CSF2RA' on 'osteosarcoma'

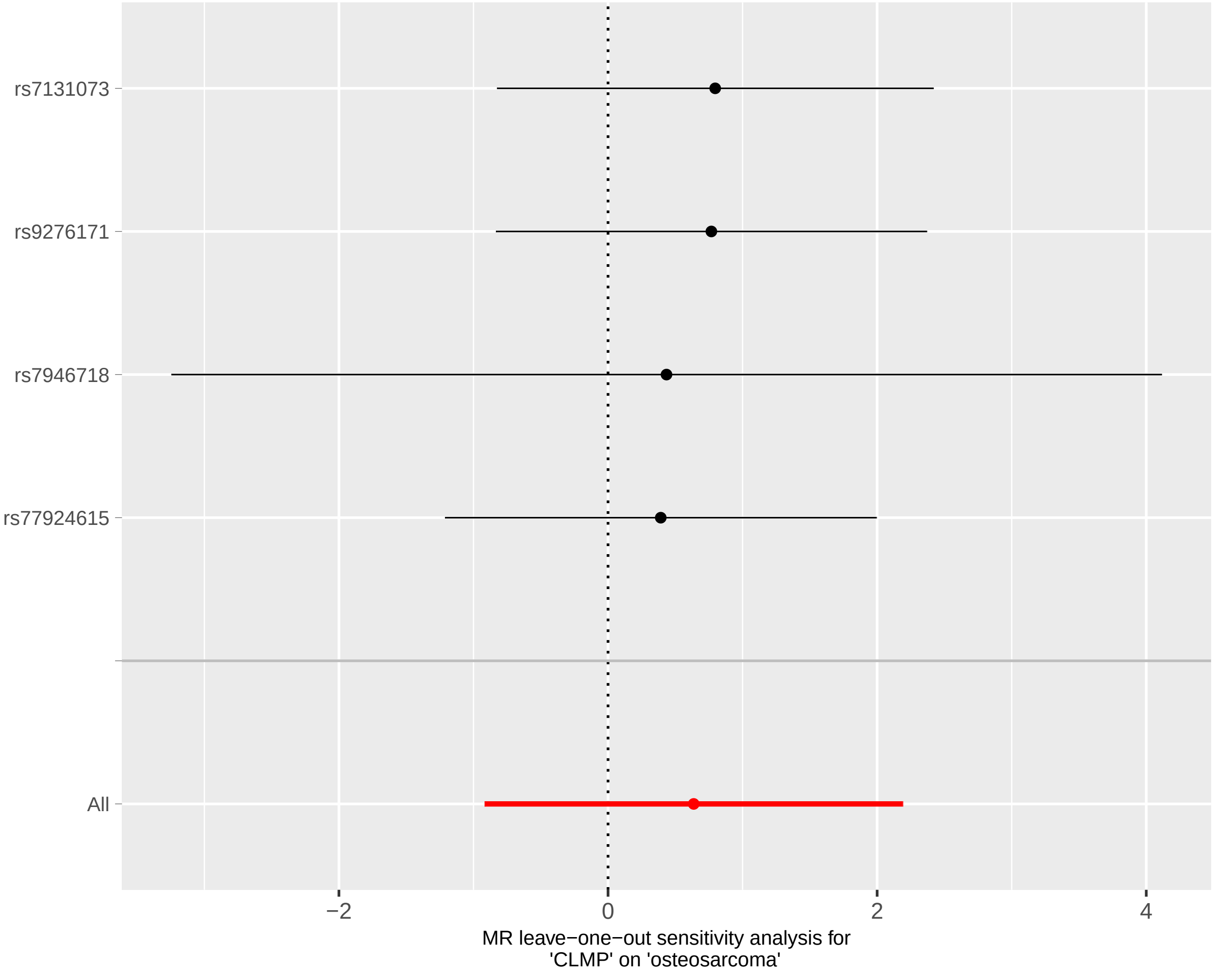
rs7131073
rs9276171
rs7946718
rs77924615
All
−2
0
2
4
MR leave−one−out sensitivity analysis for
'CLMP' on 'osteosarcoma'

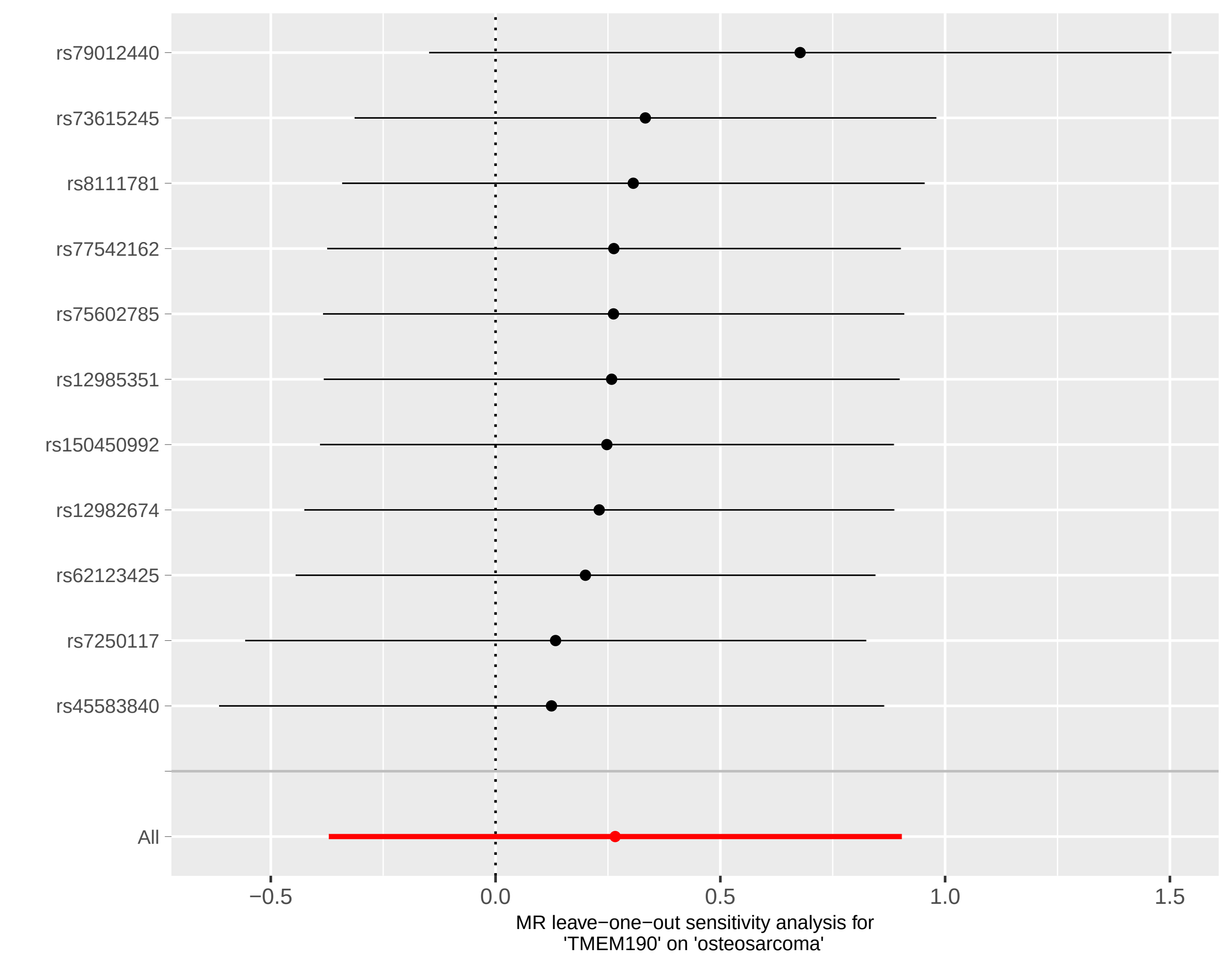
rs79012440
rs73615245
rs8111781
rs77542162
rs75602785
rs12985351
rs150450992
rs12982674
rs62123425
rs7250117
rs45583840
All
−0.5
0.0
0.5
1.0
1.5
MR leave−one−out sensitivity analysis for
'TMEM190' on 'osteosarcoma'

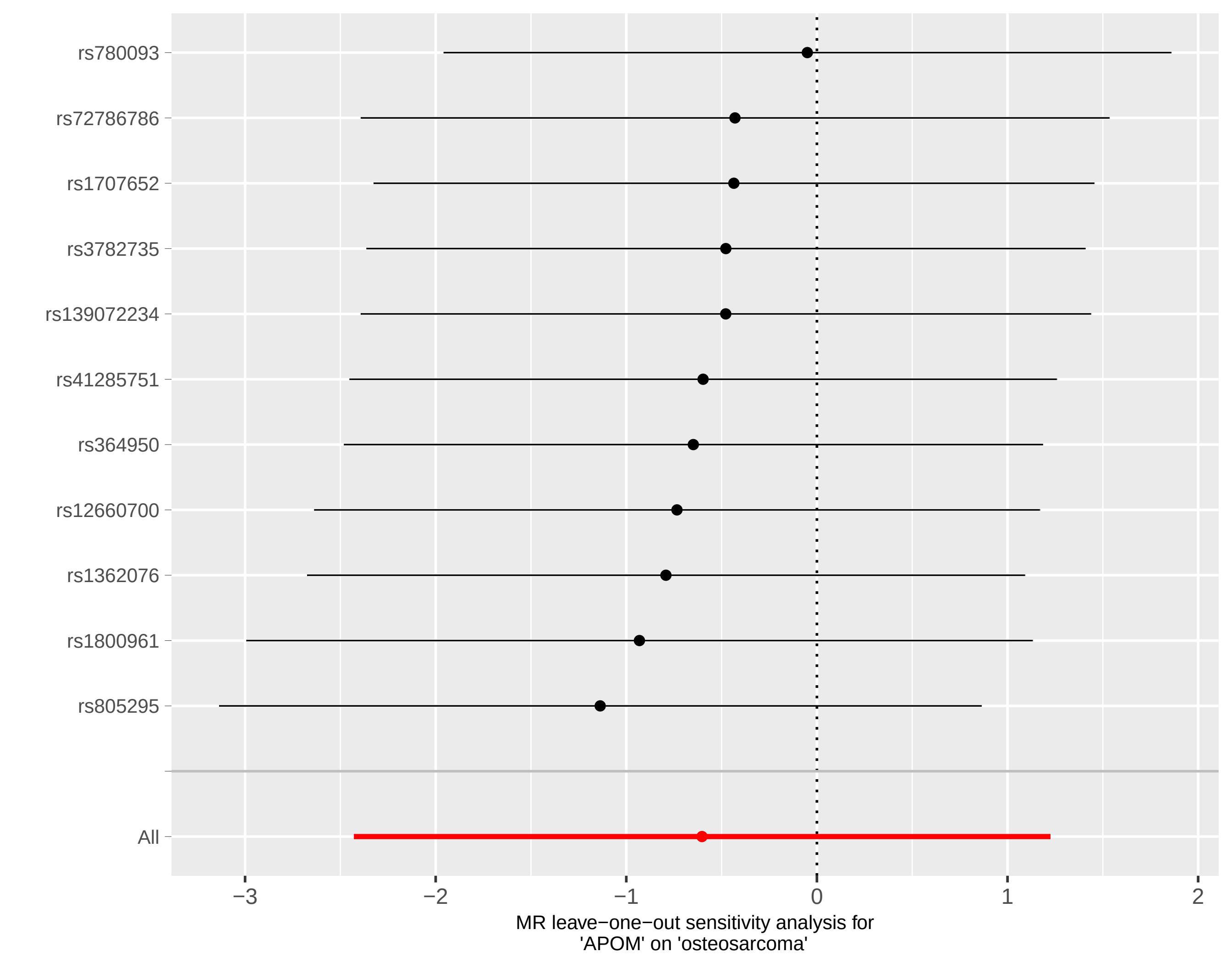
rs780093
rs72786786
rs1707652
rs3782735
rs139072234
rs41285751
rs364950
rs12660700
rs1362076
rs1800961
rs805295
All
−3
−2
−1
0
1
2
MR leave−one−out sensitivity analysis for
'APOM' on 'osteosarcoma'

Insufficient number of SNPs

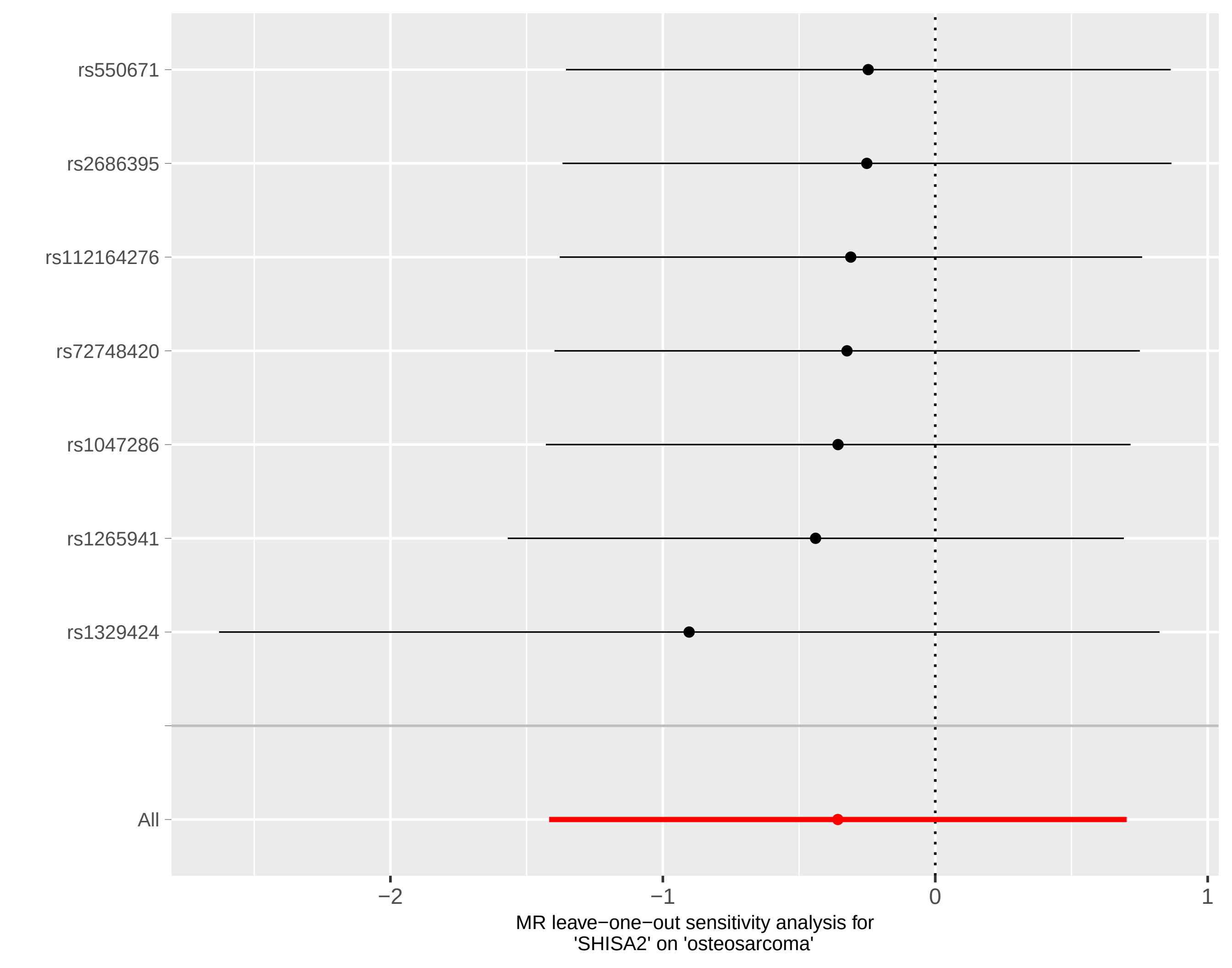

rs550671
rs2686395
rs112164276
rs72748420
rs1047286
rs1265941
rs1329424
All
−2
−1
0
1
MR leave−one−out sensitivity analysis for
'SHISA2' on 'osteosarcoma'

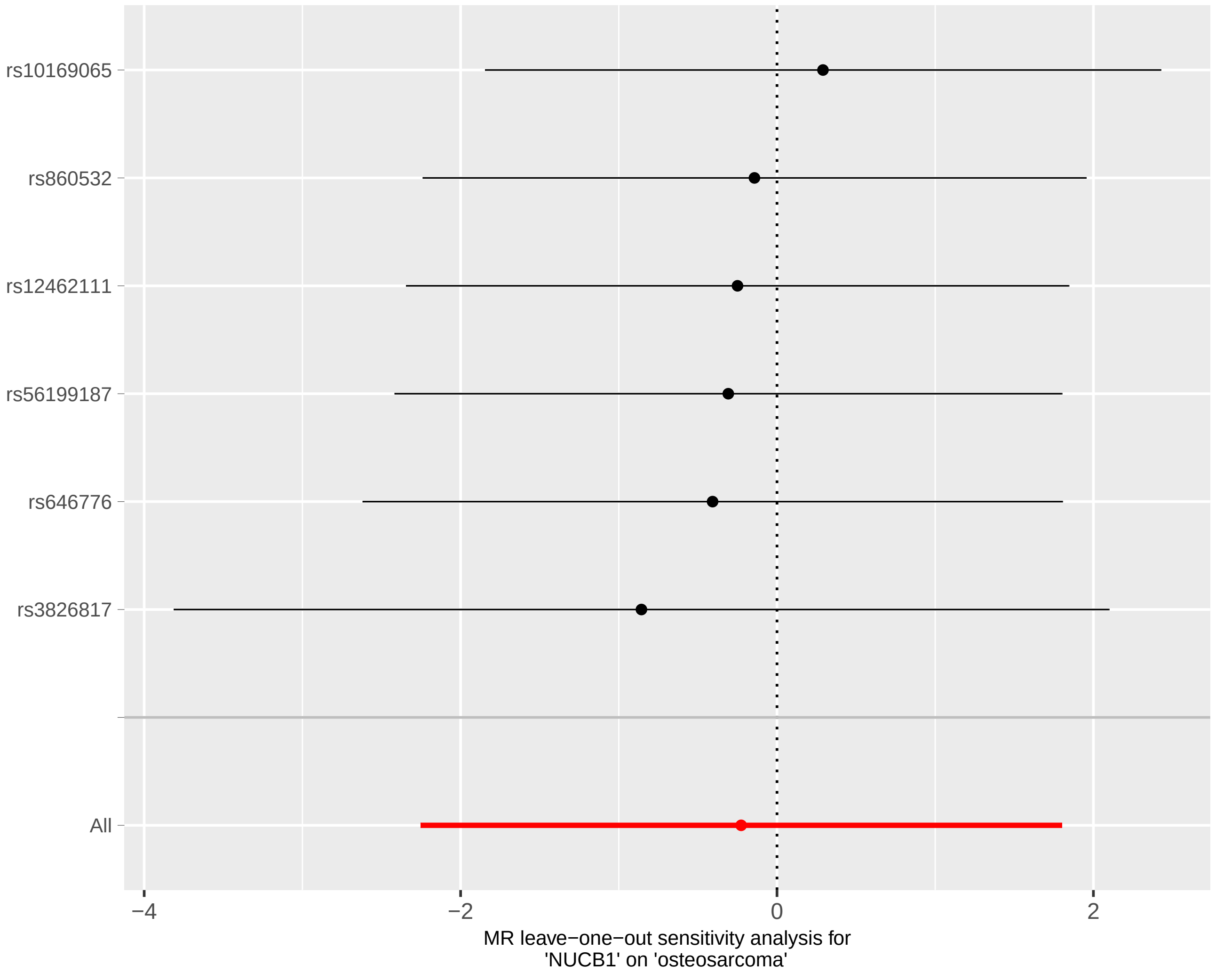

rs10169065
rs860532
rs12462111
rs56199187
rs646776
rs3826817
All
−4
−2
0
2
MR leave−one−out sensitivity analysis for
'NUCB1' on 'osteosarcoma'

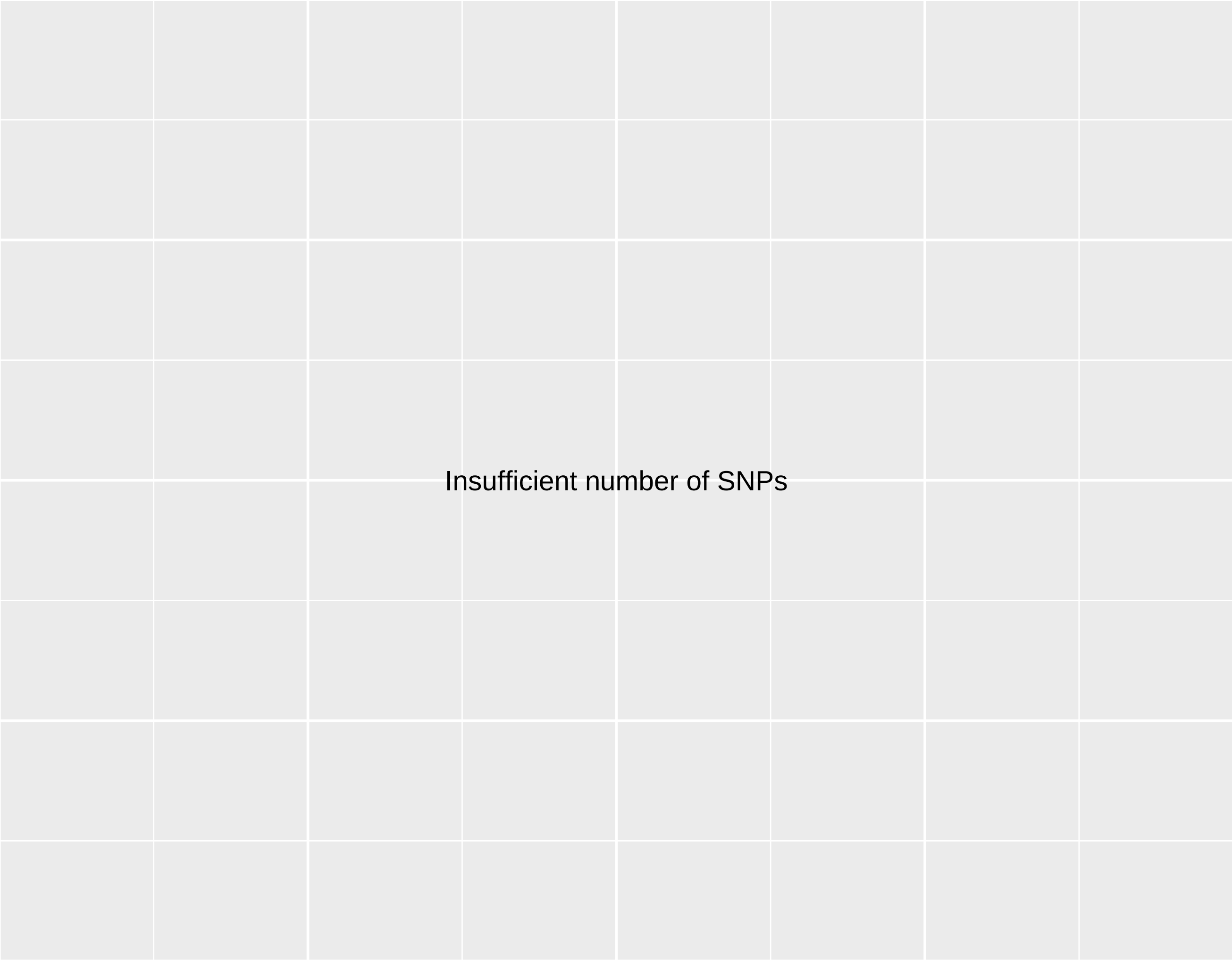
Insufficient number of SNPs

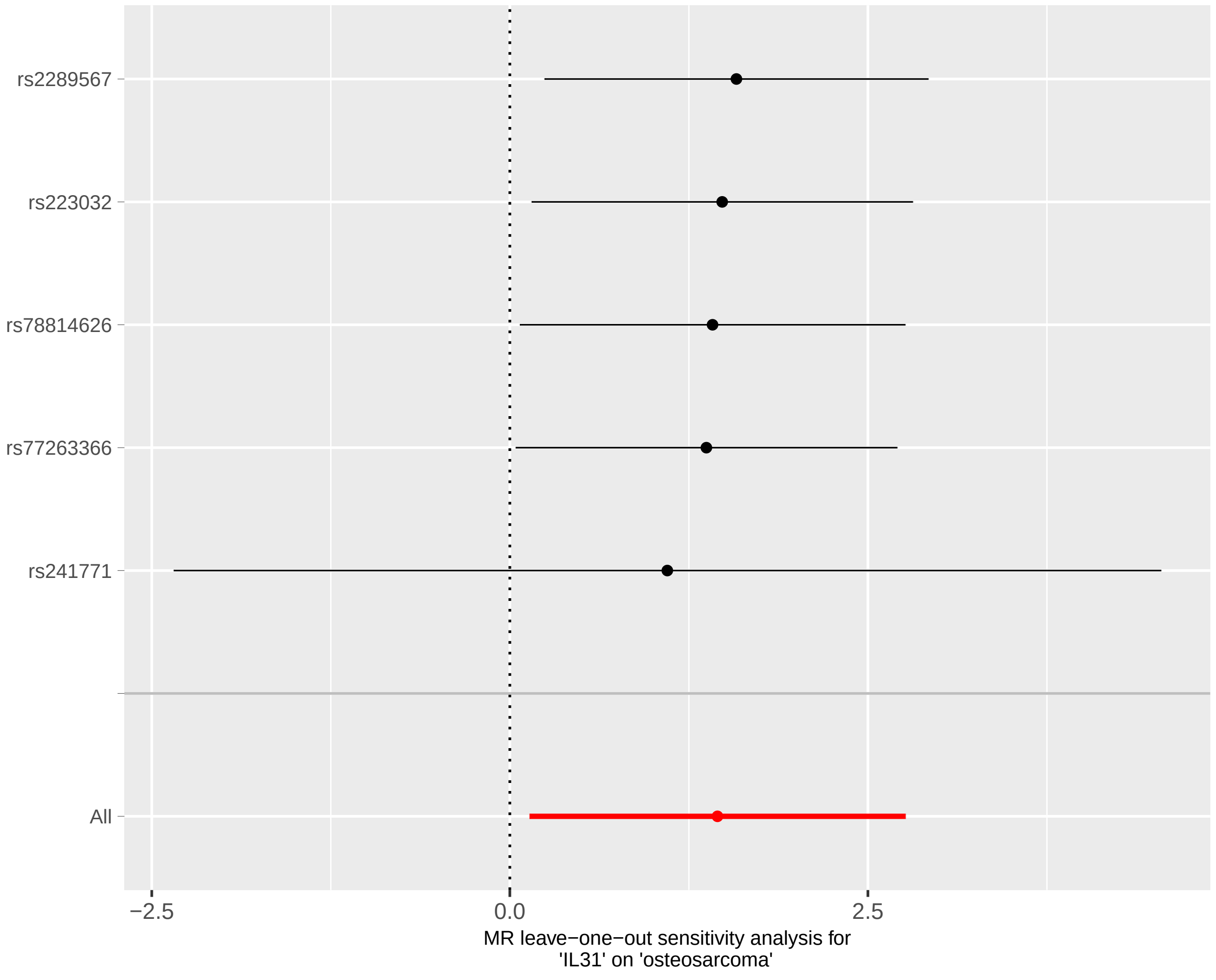
rs2289567
rs223032
rs78814626
rs77263366
rs241771
All
−2.5
0.0
2.5
MR leave−one−out sensitivity analysis for
'IL31' on 'osteosarcoma'

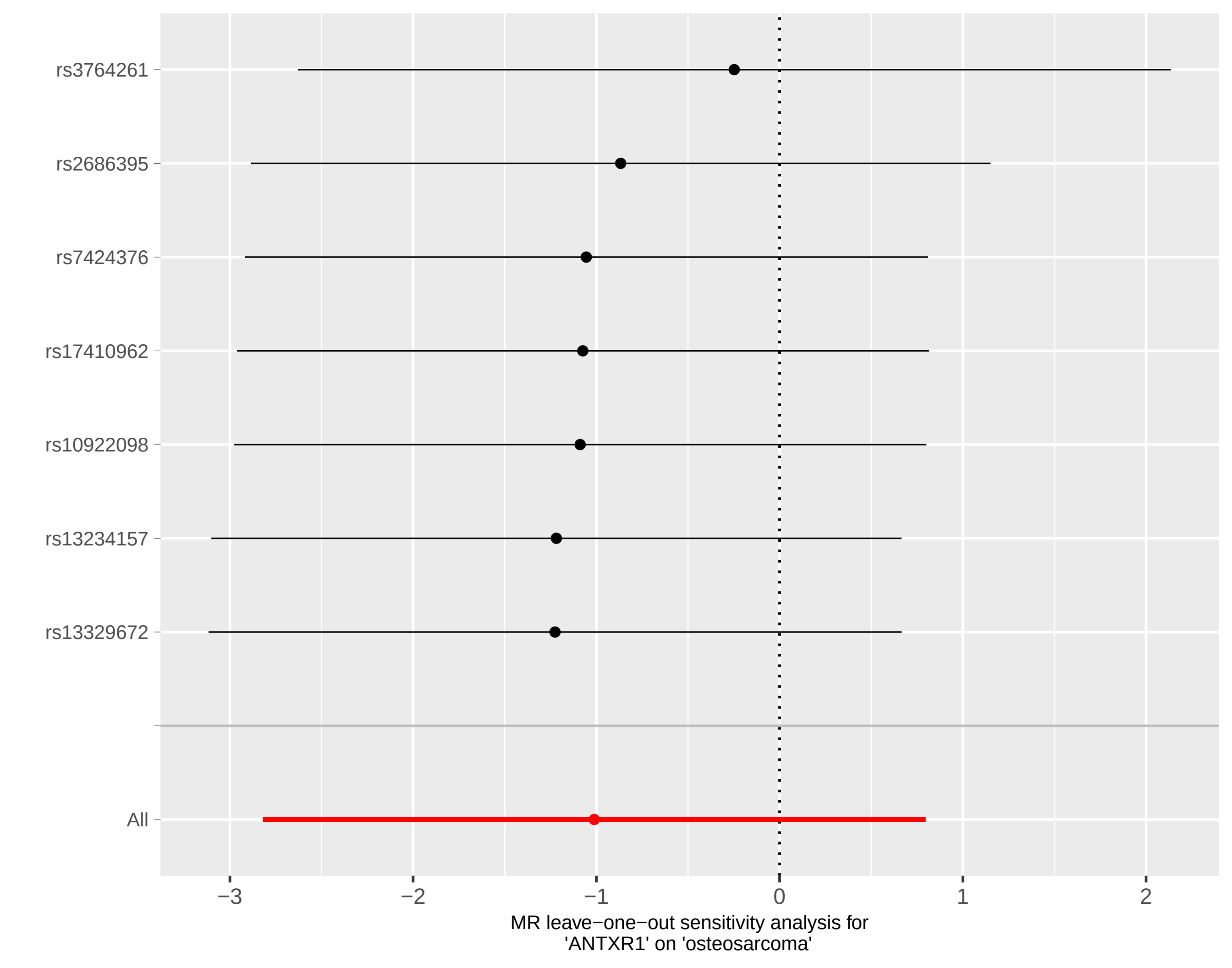
rs3764261
rs2686395
rs7424376
rs17410962
rs10922098
rs13234157
rs13329672
All
−3
−2
−1
0
1
2
MR leave−one−out sensitivity analysis for
'ANTXR1' on 'osteosarcoma'

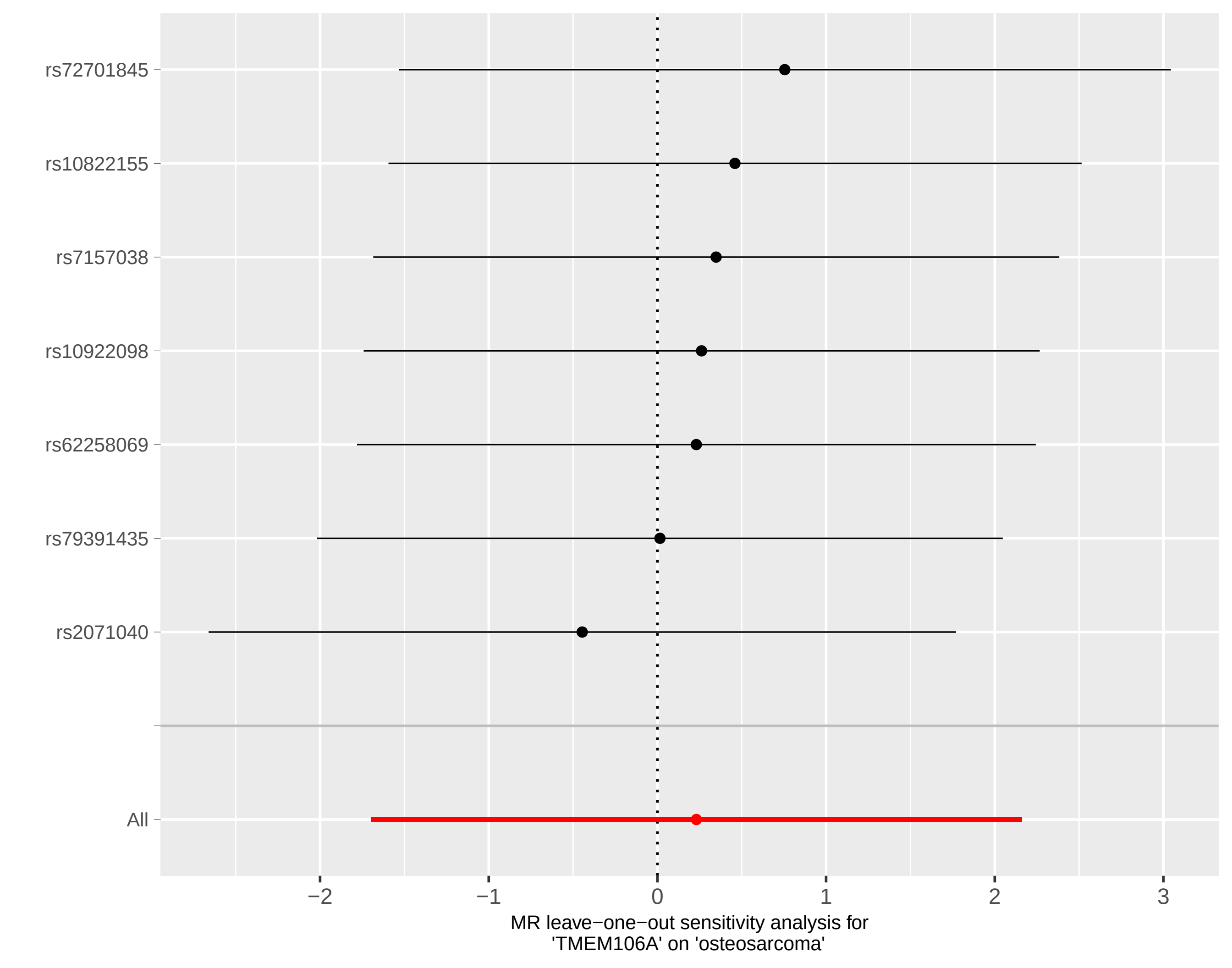

rs72701845
rs10822155
rs7157038
rs10922098
rs62258069
rs79391435
rs2071040
All
−2
−1
0
1
2
3
MR leave−one−out sensitivity analysis for
'TMEM106A' on 'osteosarcoma'

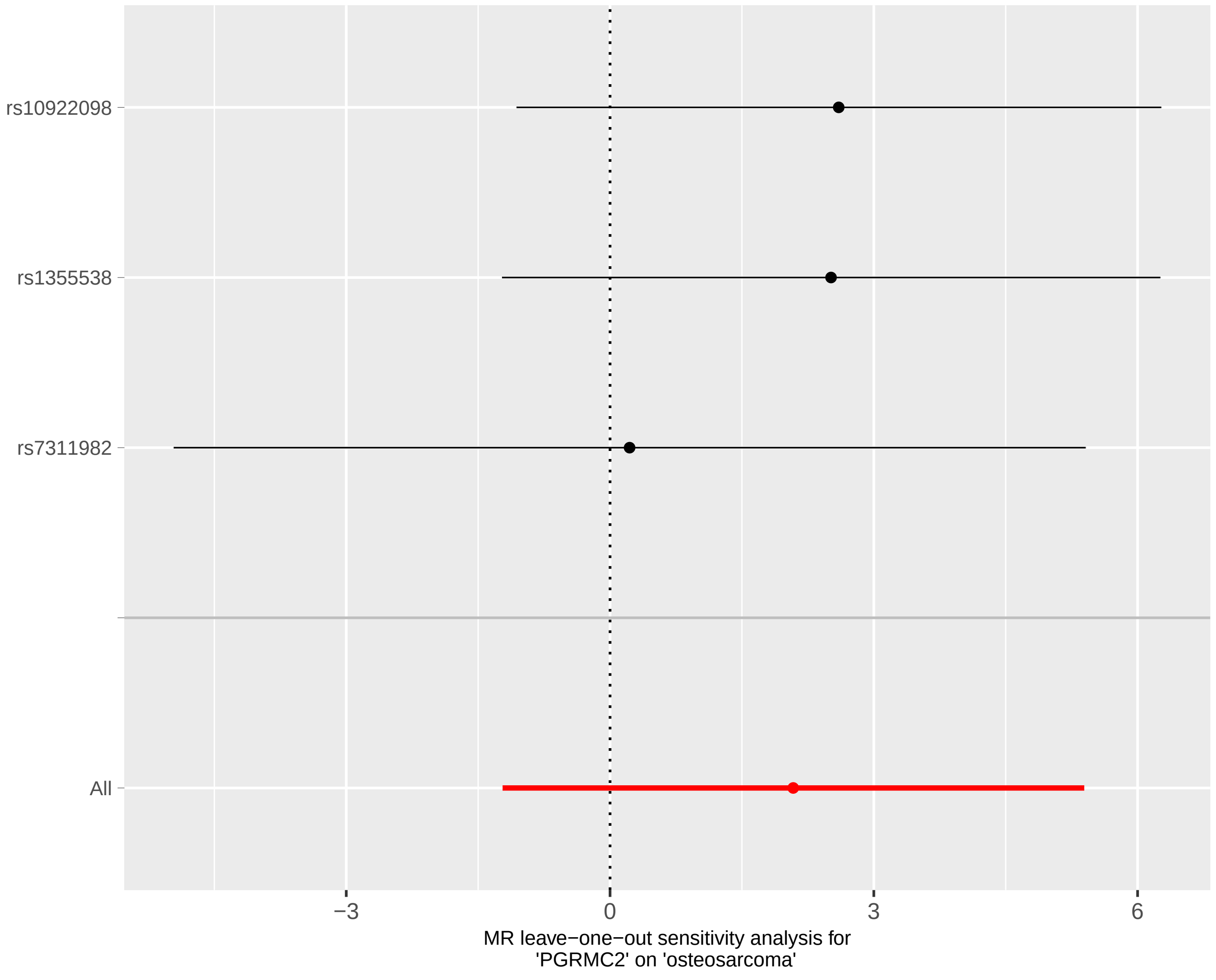
rs10922098
rs1355538
rs7311982
All
−3
0
3
6
MR leave−one−out sensitivity analysis for
'PGRMC2' on 'osteosarcoma'

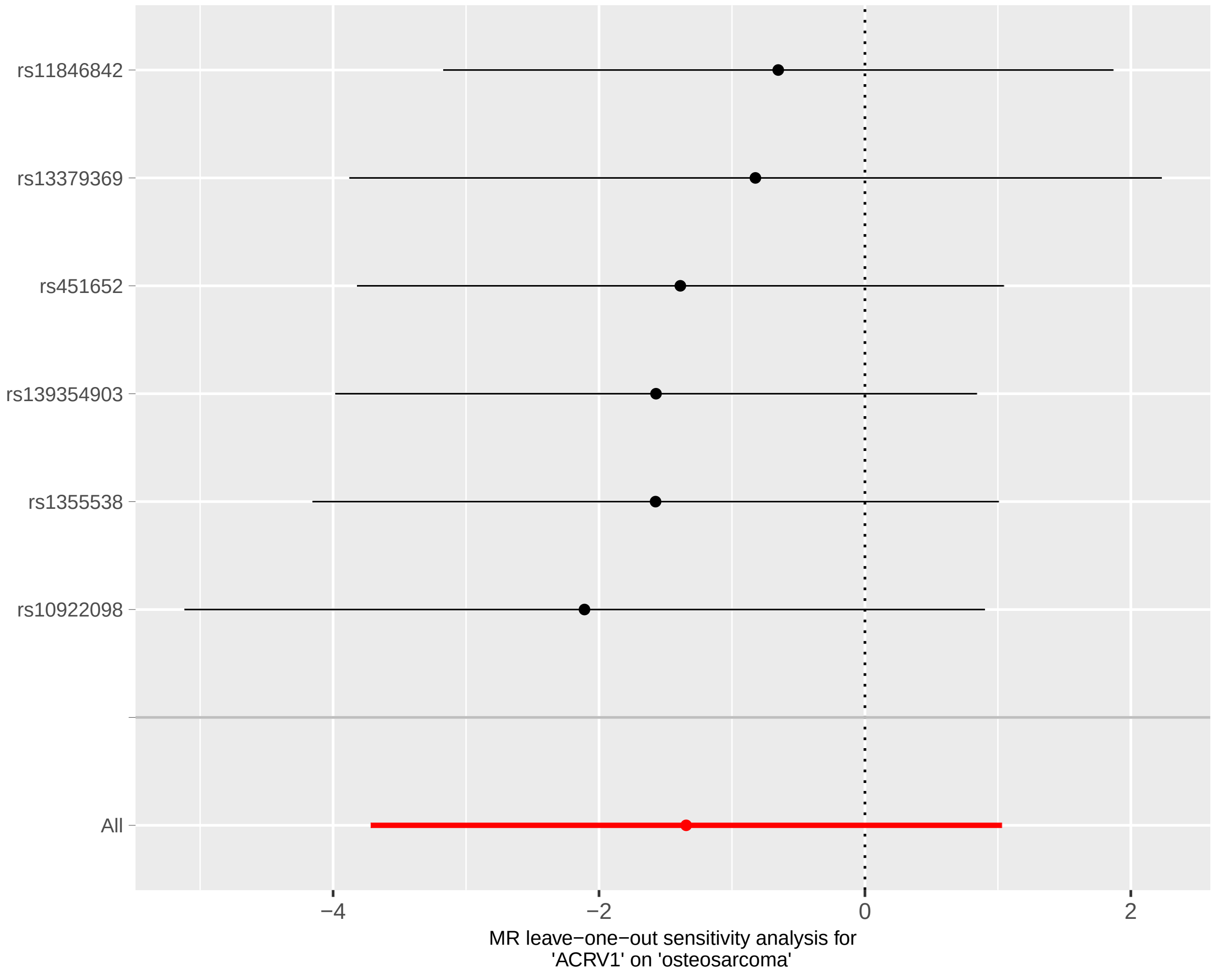

rs11846842
rs13379369
rs451652
rs139354903
rs1355538
rs10922098
All
−4
−2
0
2
MR leave−one−out sensitivity analysis for
'ACRV1' on 'osteosarcoma'

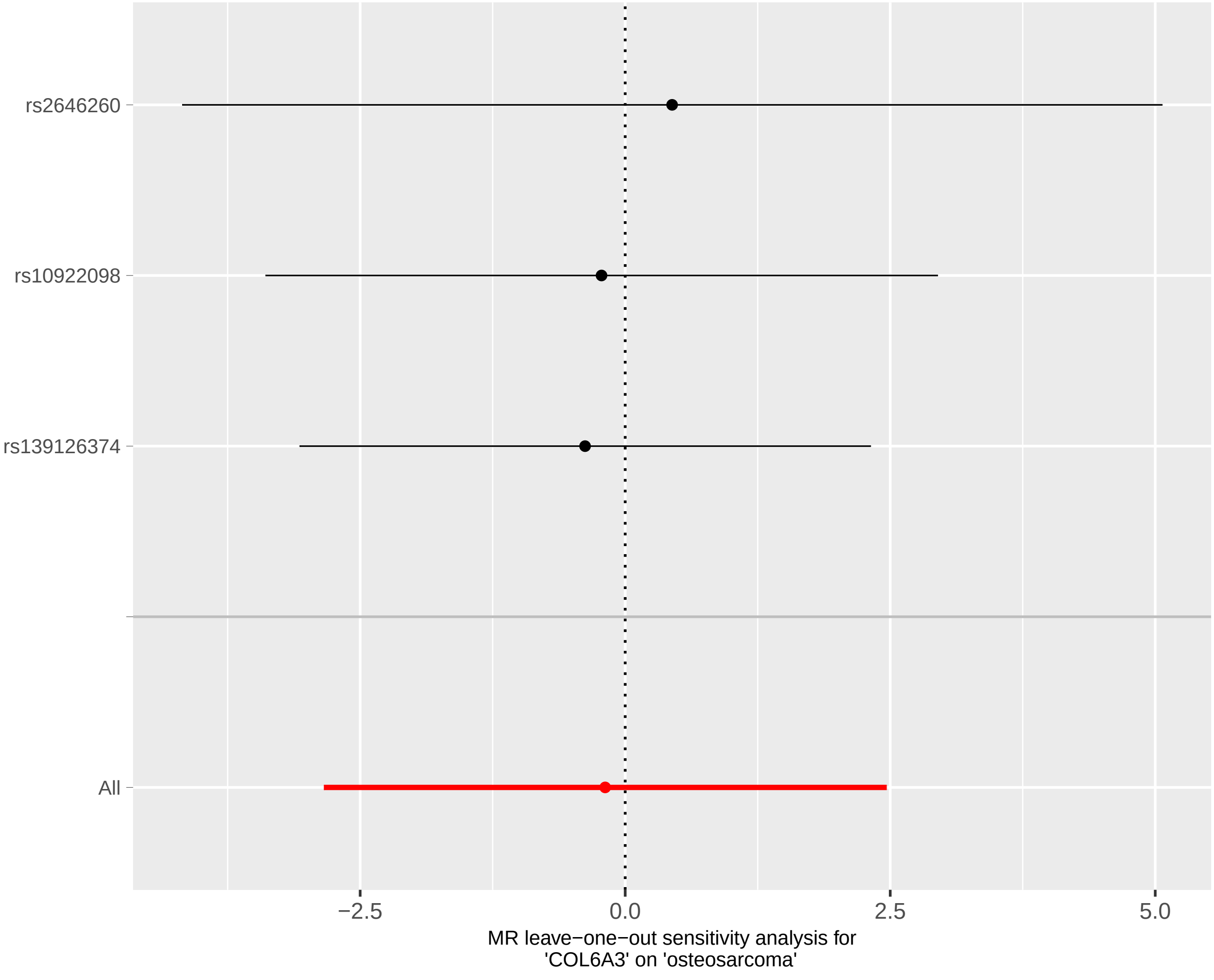
rs2646260
rs10922098
rs139126374
All
−2.5
0.0
2.5
5.0
MR leave−one−out sensitivity analysis for
'COL6A3' on 'osteosarcoma'

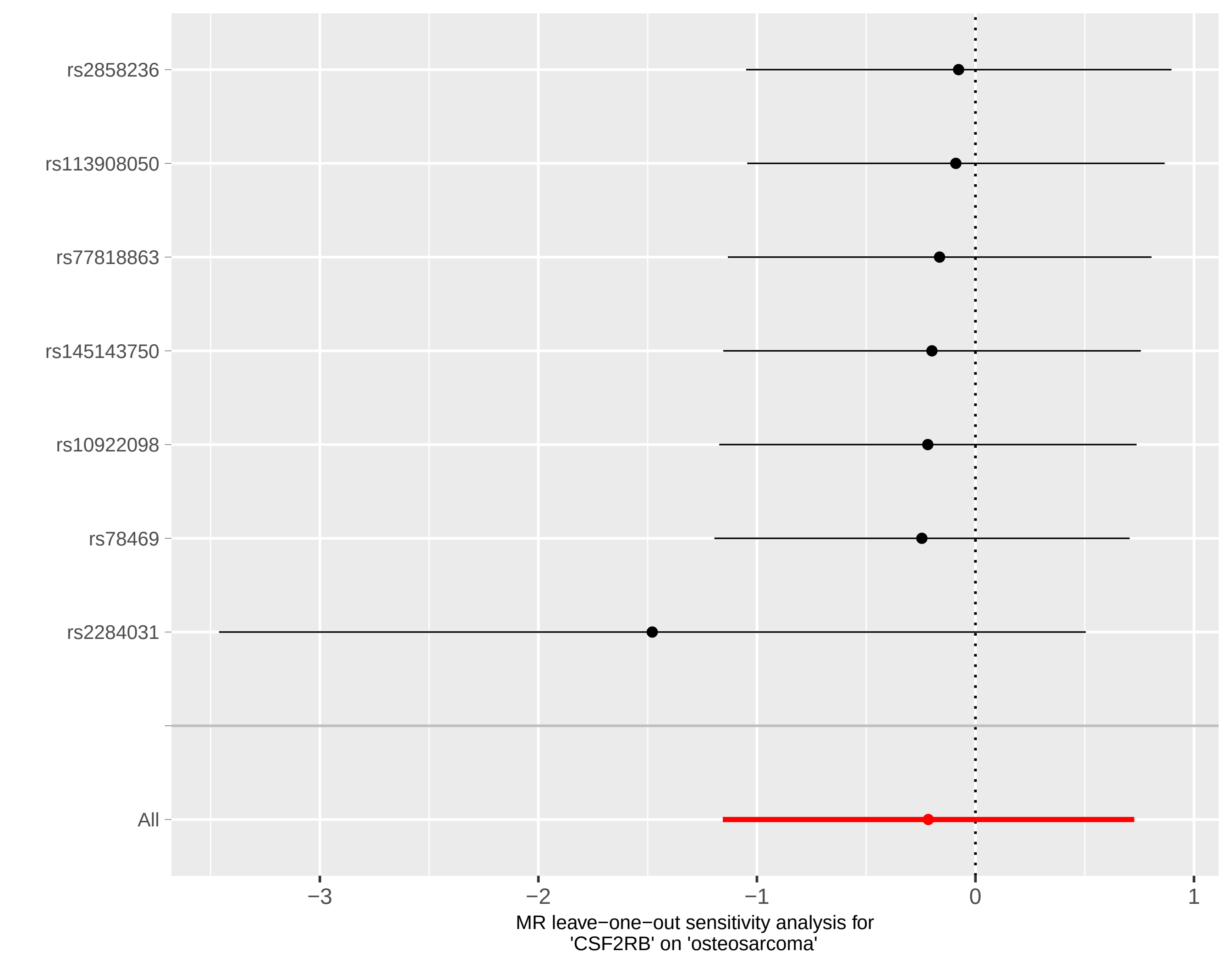
rs2858236
rs113908050
rs77818863
rs145143750
rs10922098
rs78469
rs2284031
All
−3
−2
−1
0
1
MR leave−one−out sensitivity analysis for
'CSF2RB' on 'osteosarcoma'

Insufficient number of SNPs

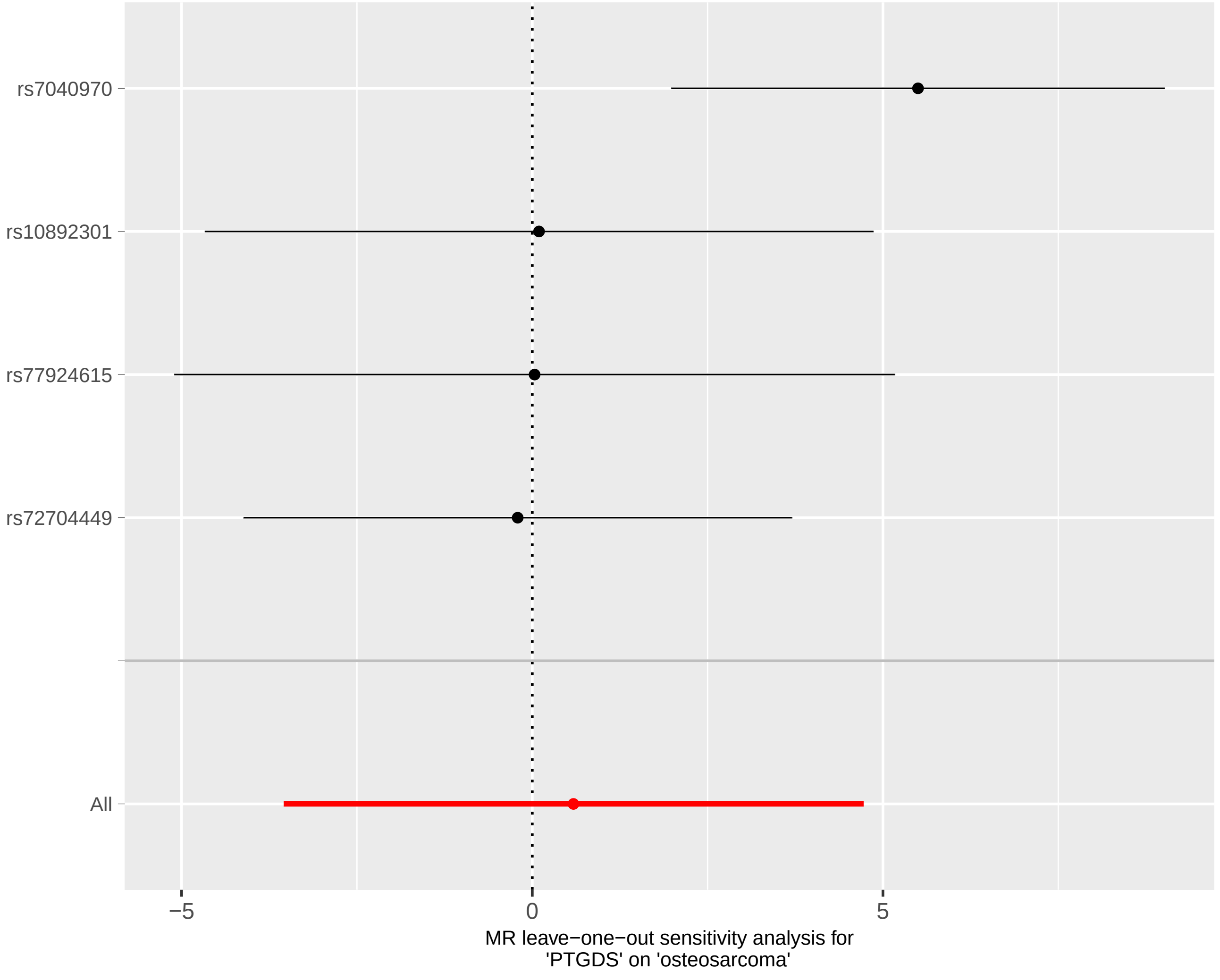
rs7040970
rs10892301
rs77924615
rs72704449
All
−5
0
5
MR leave−one−out sensitivity analysis for
'PTGDS' on 'osteosarcoma'

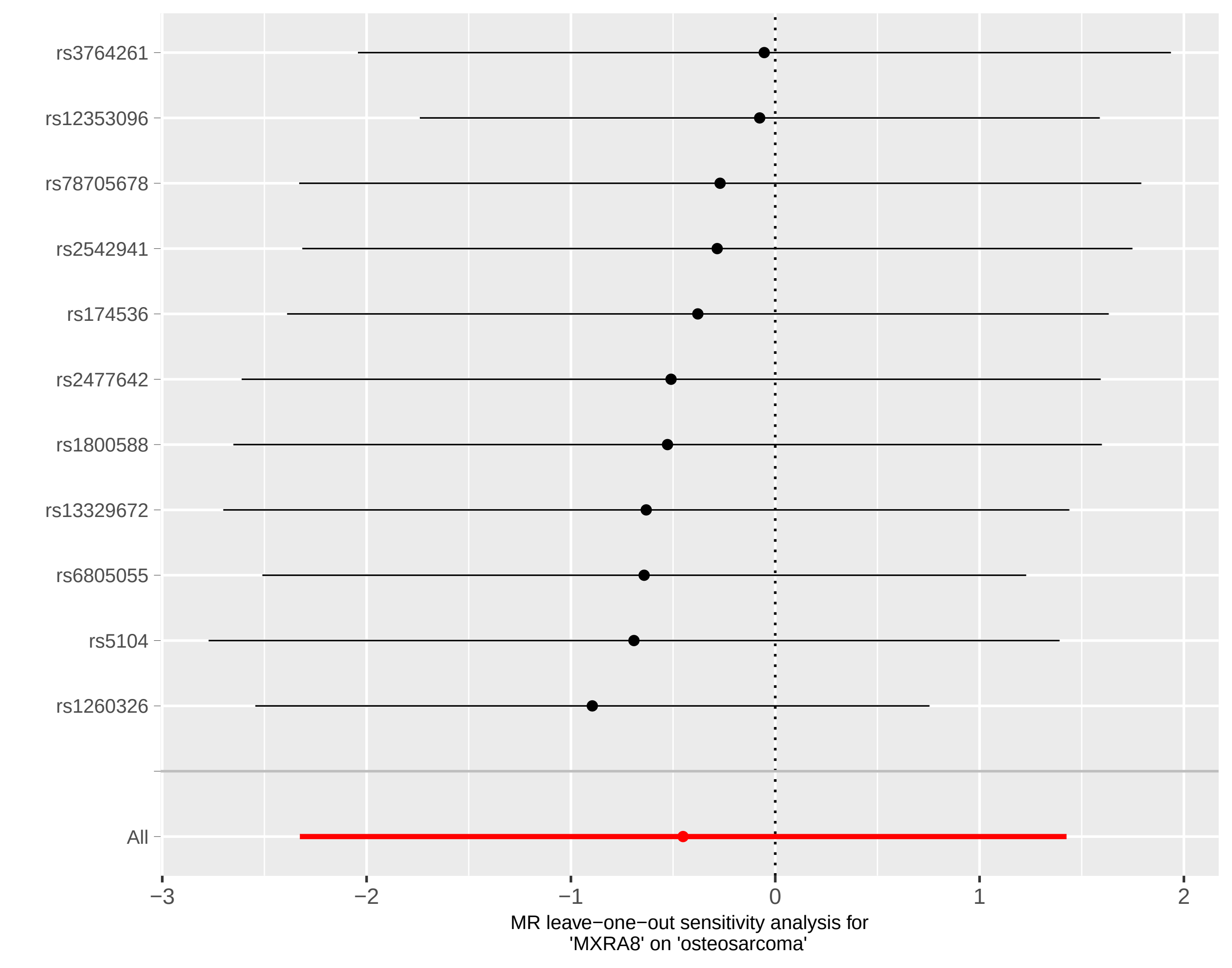
rs3764261
rs12353096
rs78705678
rs2542941
rs174536
rs2477642
rs1800588
rs13329672
rs6805055
rs5104
rs1260326
All
−3
−2
−1
0
1
2
MR leave−one−out sensitivity analysis for
'MXRA8' on 'osteosarcoma'

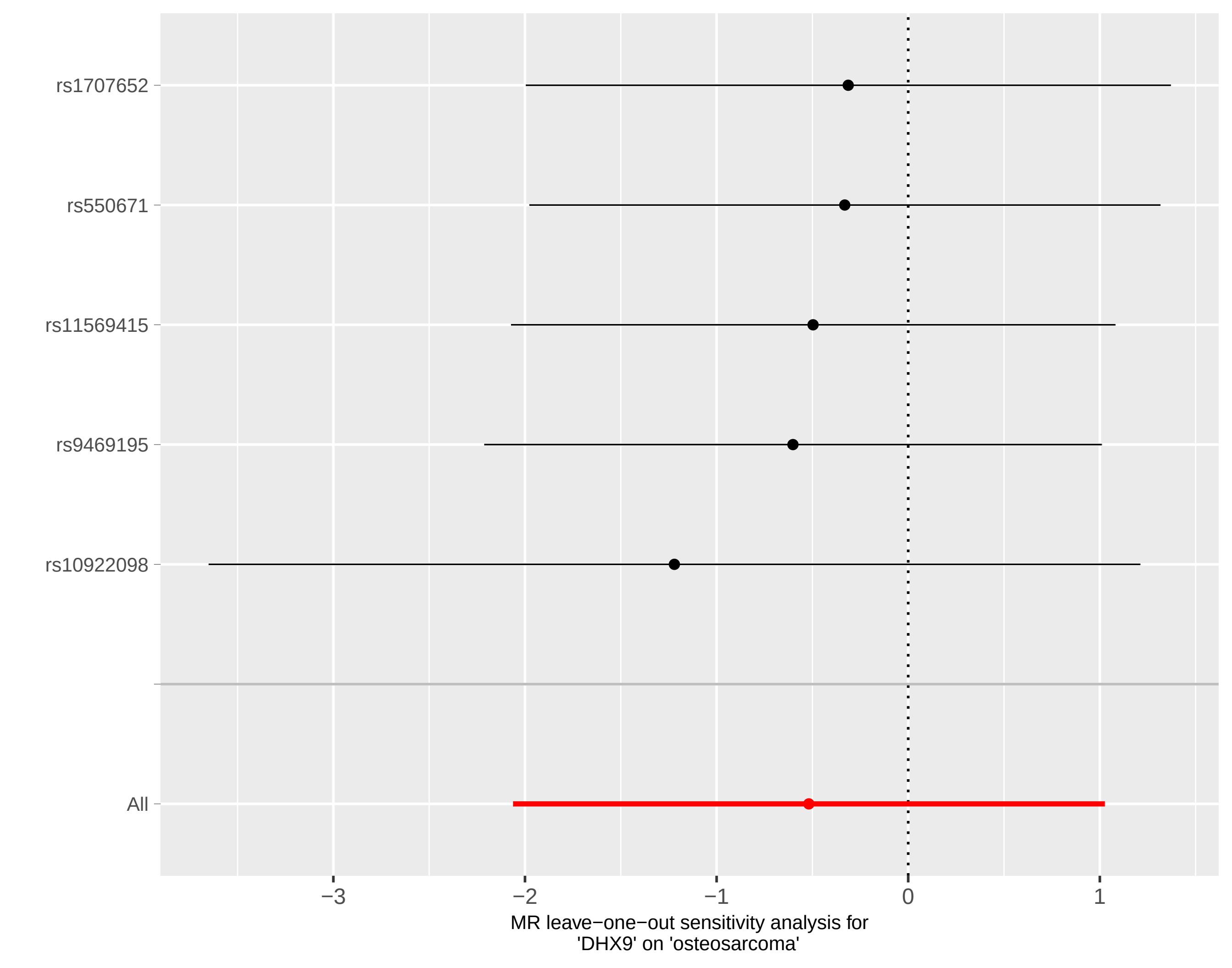
rs1707652
rs550671
rs11569415
rs9469195
rs10922098
All
−3
−2
−1
0
1
MR leave−one−out sensitivity analysis for
'DHX9' on 'osteosarcoma'

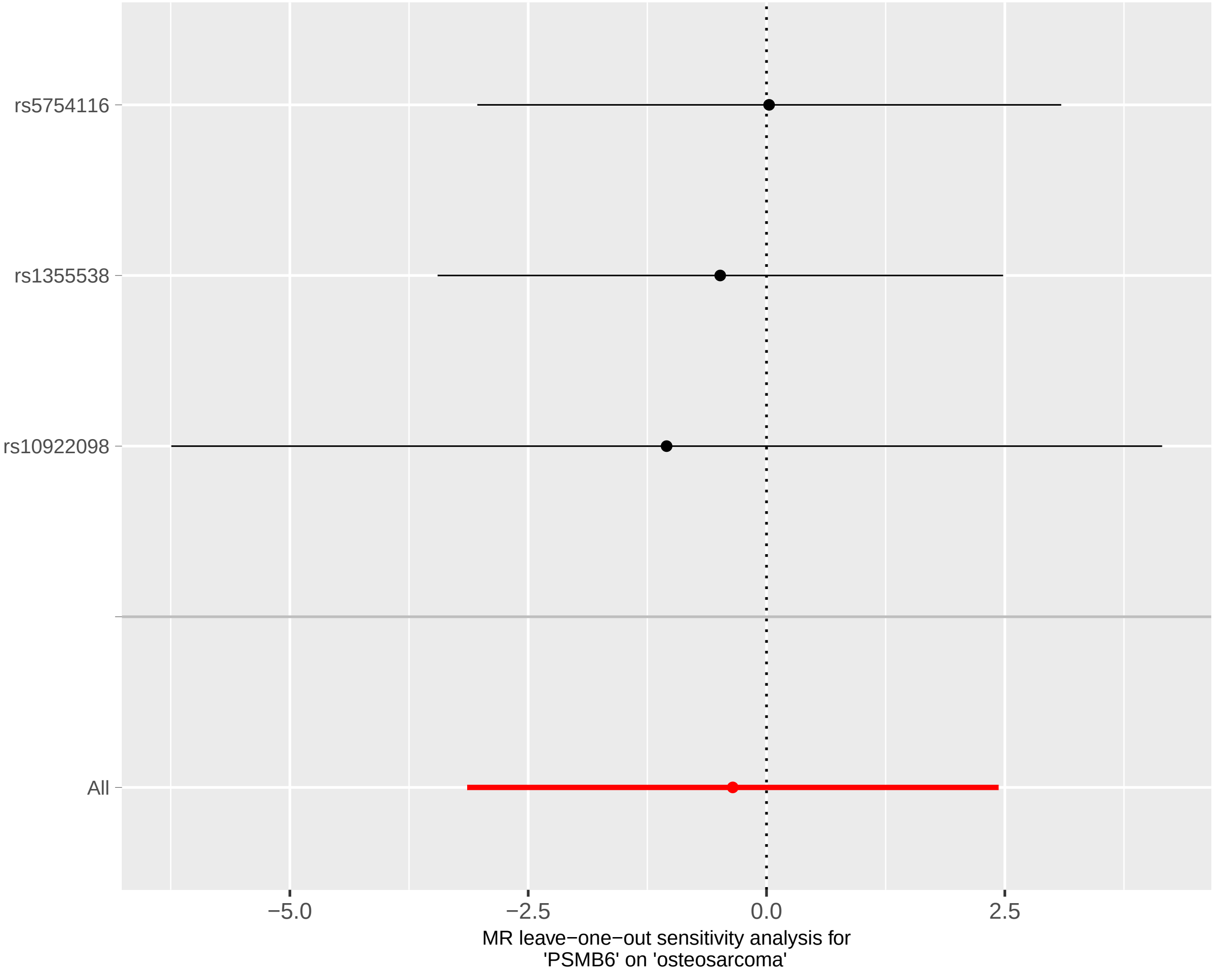

rs5754116
rs1355538
rs10922098
All
−5.0
−2.5
0.0
2.5
MR leave−one−out sensitivity analysis for
'PSMB6' on 'osteosarcoma'

Insufficient number of SNPs

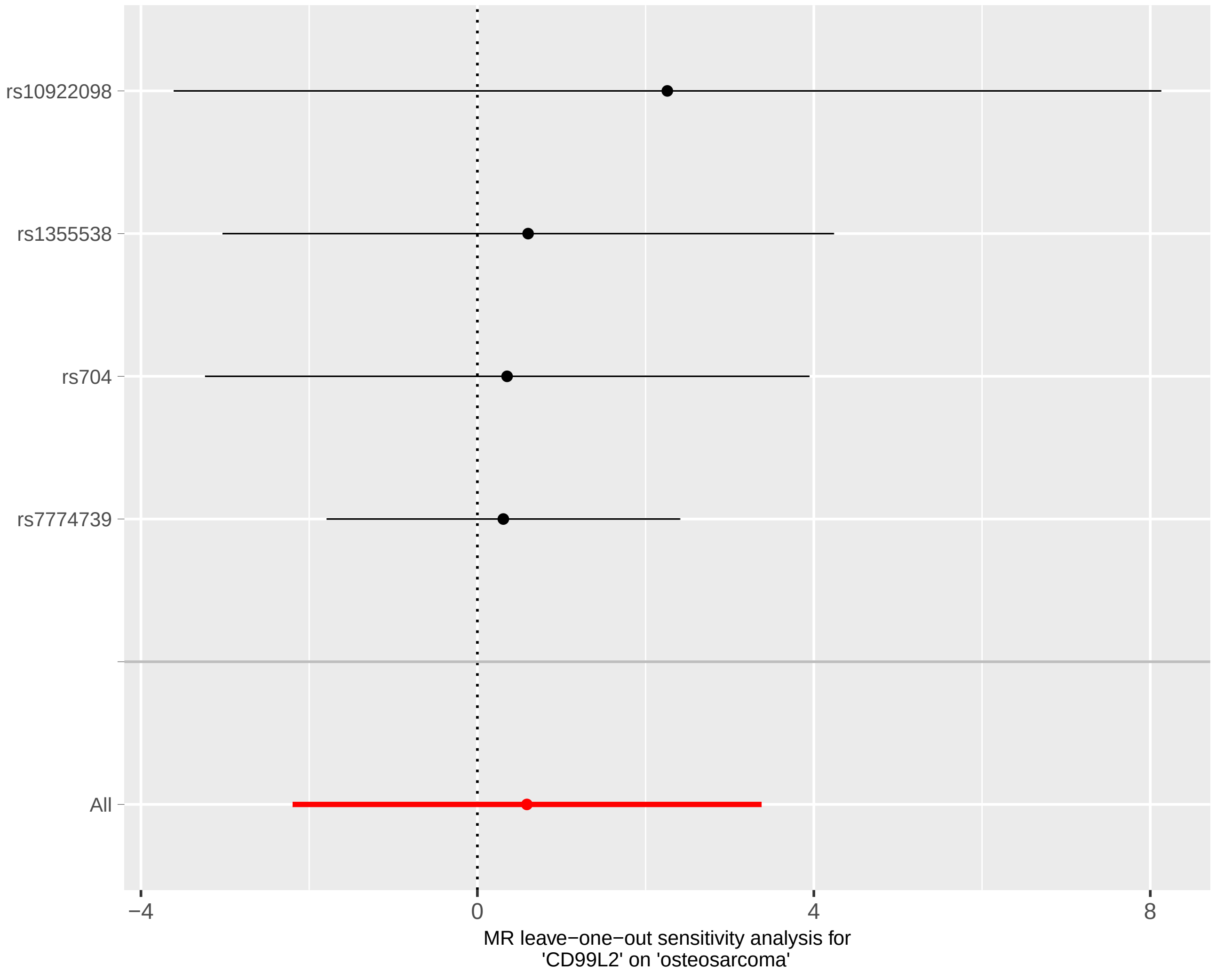
rs10922098
rs1355538
rs704
rs7774739
All
−4
0
4
8
MR leave−one−out sensitivity analysis for
'CD99L2' on 'osteosarcoma'

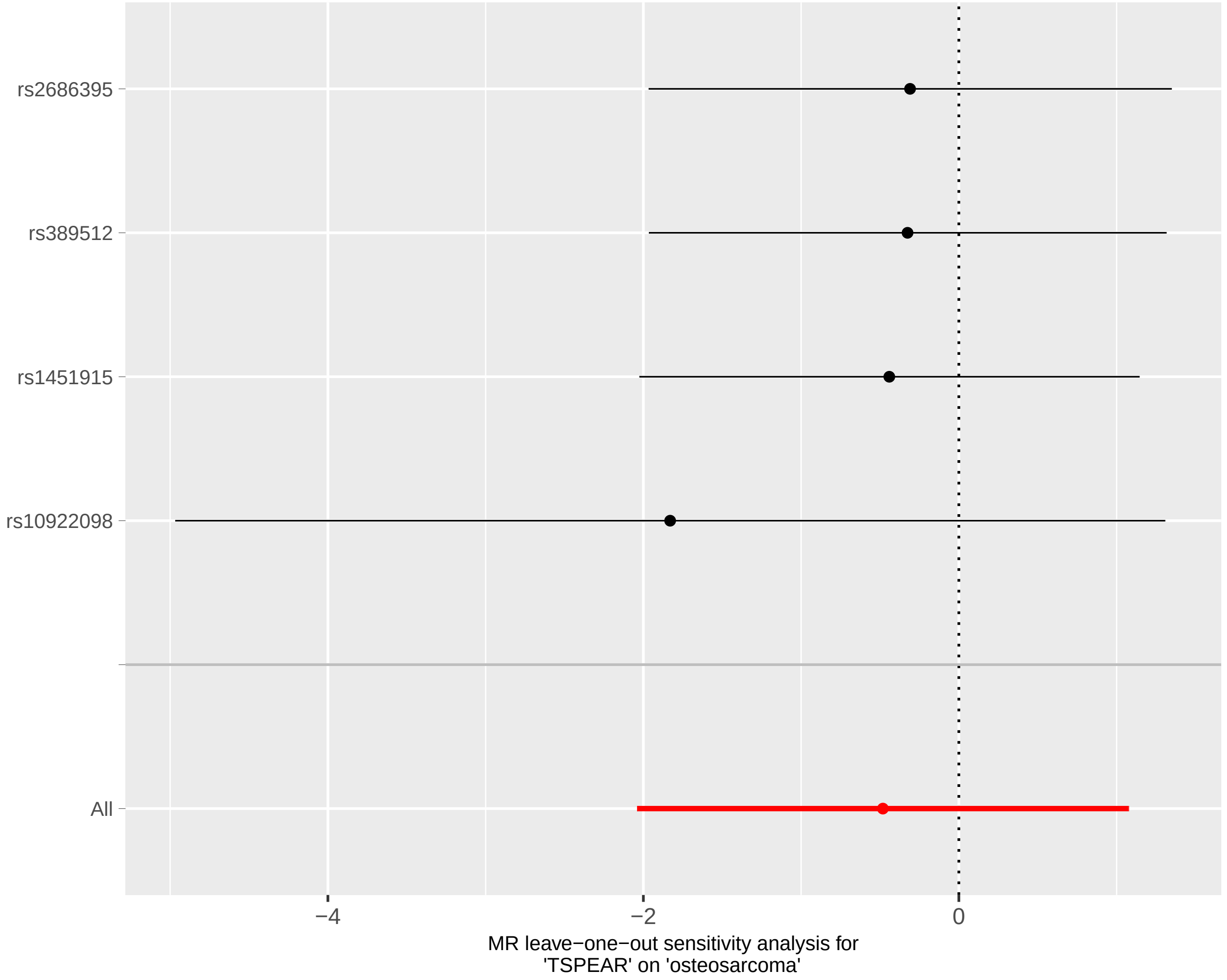

rs2686395
rs389512
rs1451915
rs10922098
All
−4
−2
0
MR leave−one−out sensitivity analysis for
'TSPEAR' on 'osteosarcoma'

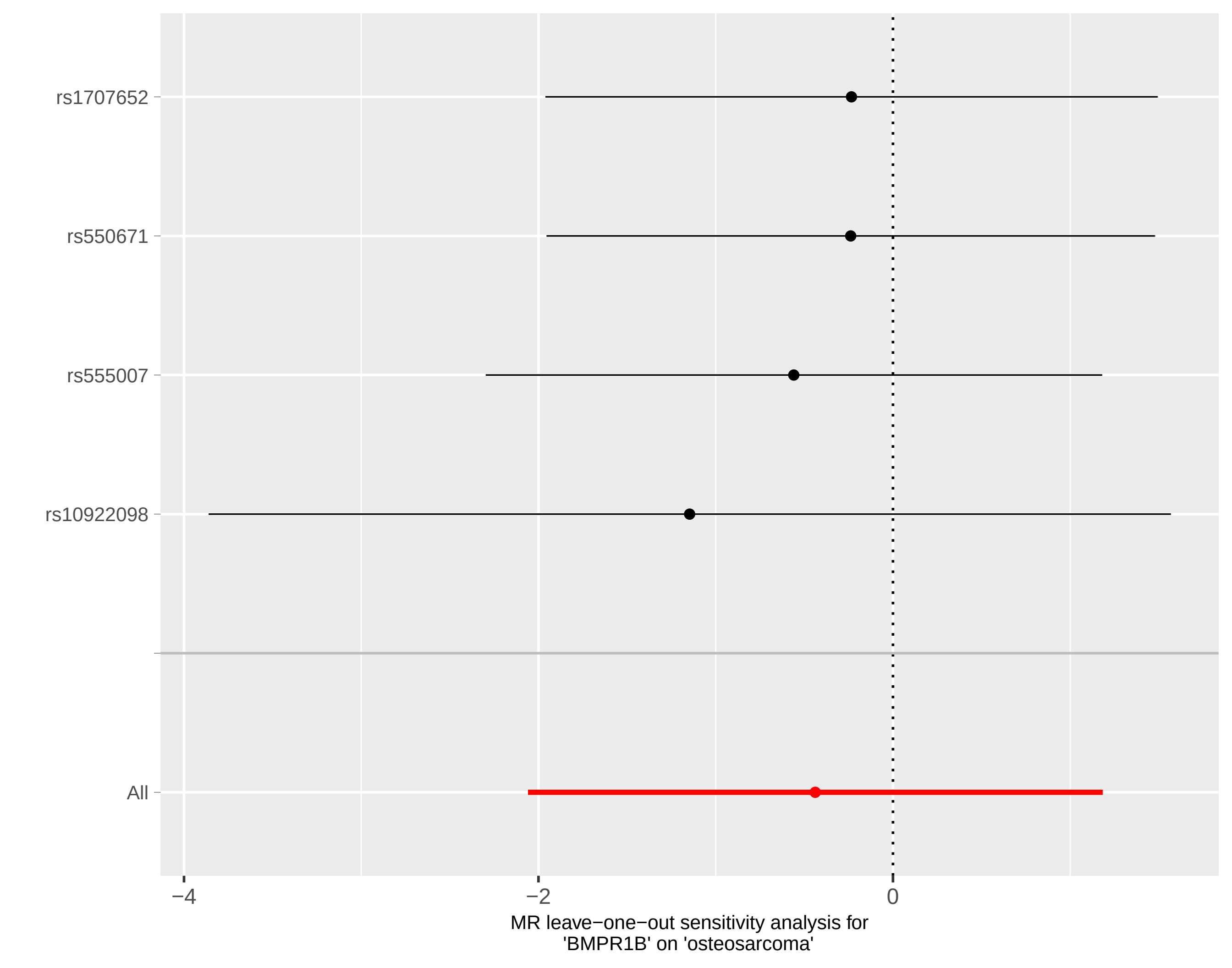

rs1707652
rs550671
rs555007
rs10922098
All
−4
−2
0
MR leave−one−out sensitivity analysis for
'BMPR1B' on 'osteosarcoma'

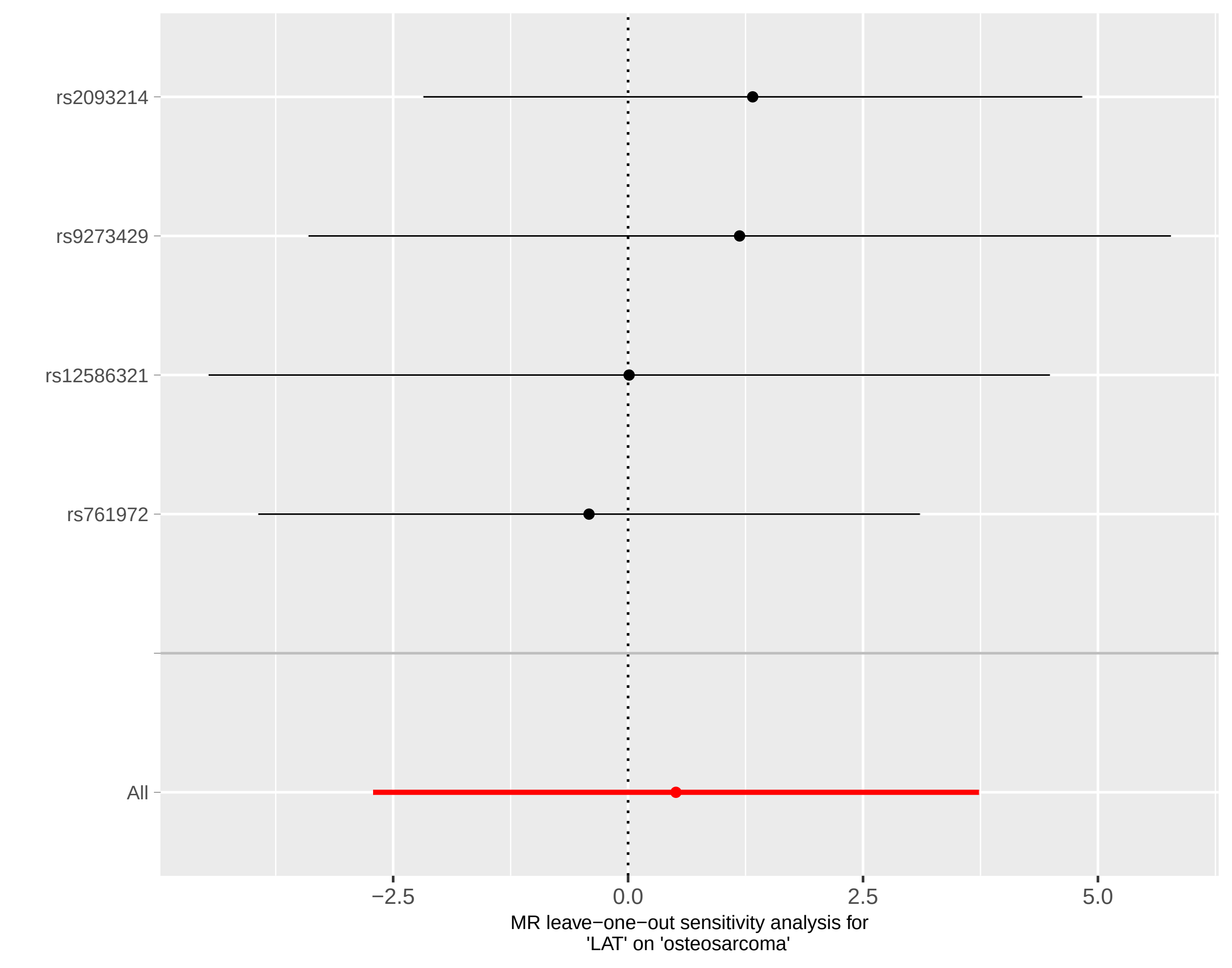
rs2093214
rs9273429
rs12586321
rs761972
All
−2.5
0.0
2.5
5.0
MR leave−one−out sensitivity analysis for
'LAT' on 'osteosarcoma'

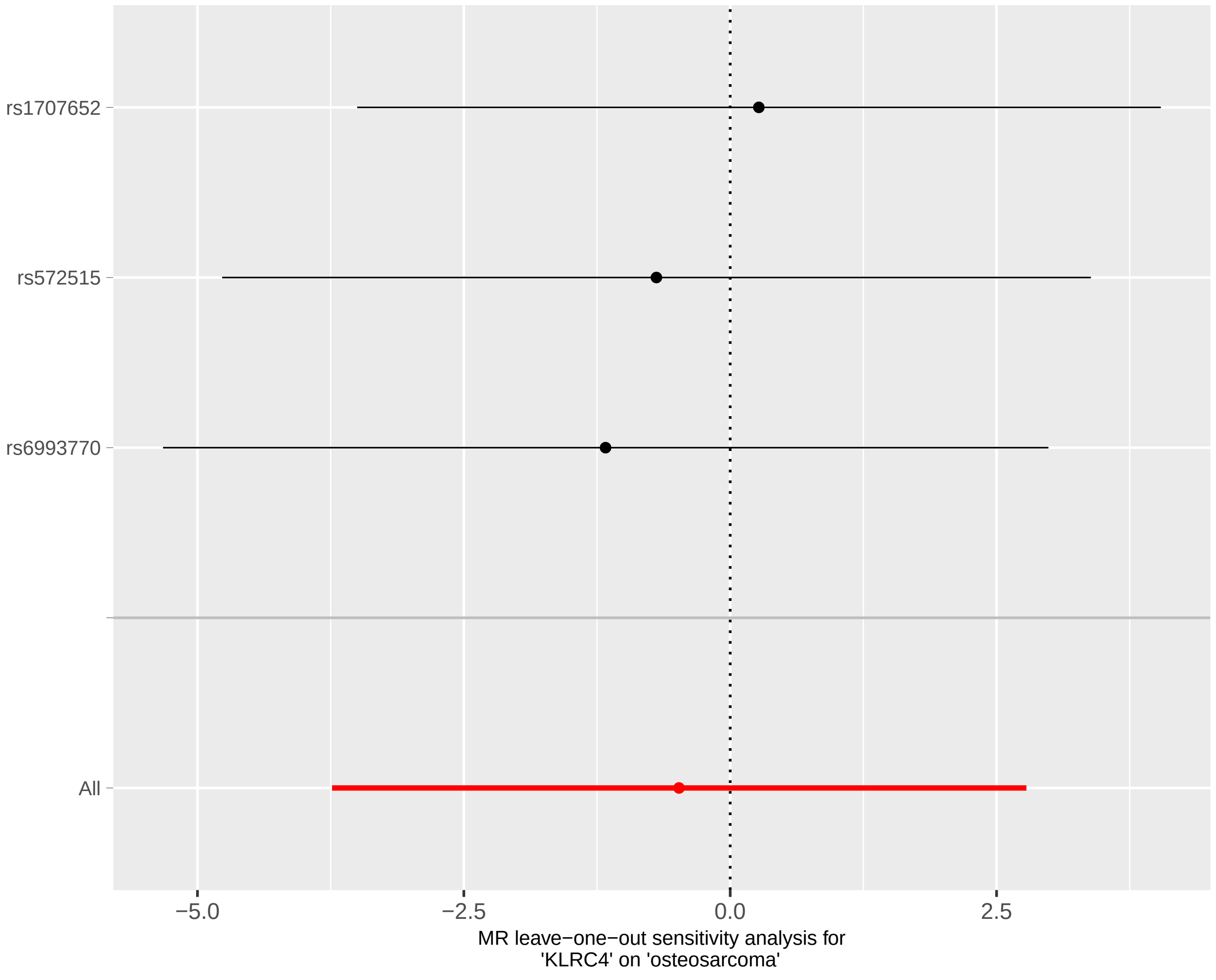
rs1707652
rs572515
rs6993770
All
−5.0
−2.5
0.0
2.5
MR leave−one−out sensitivity analysis for
'KLRC4' on 'osteosarcoma'

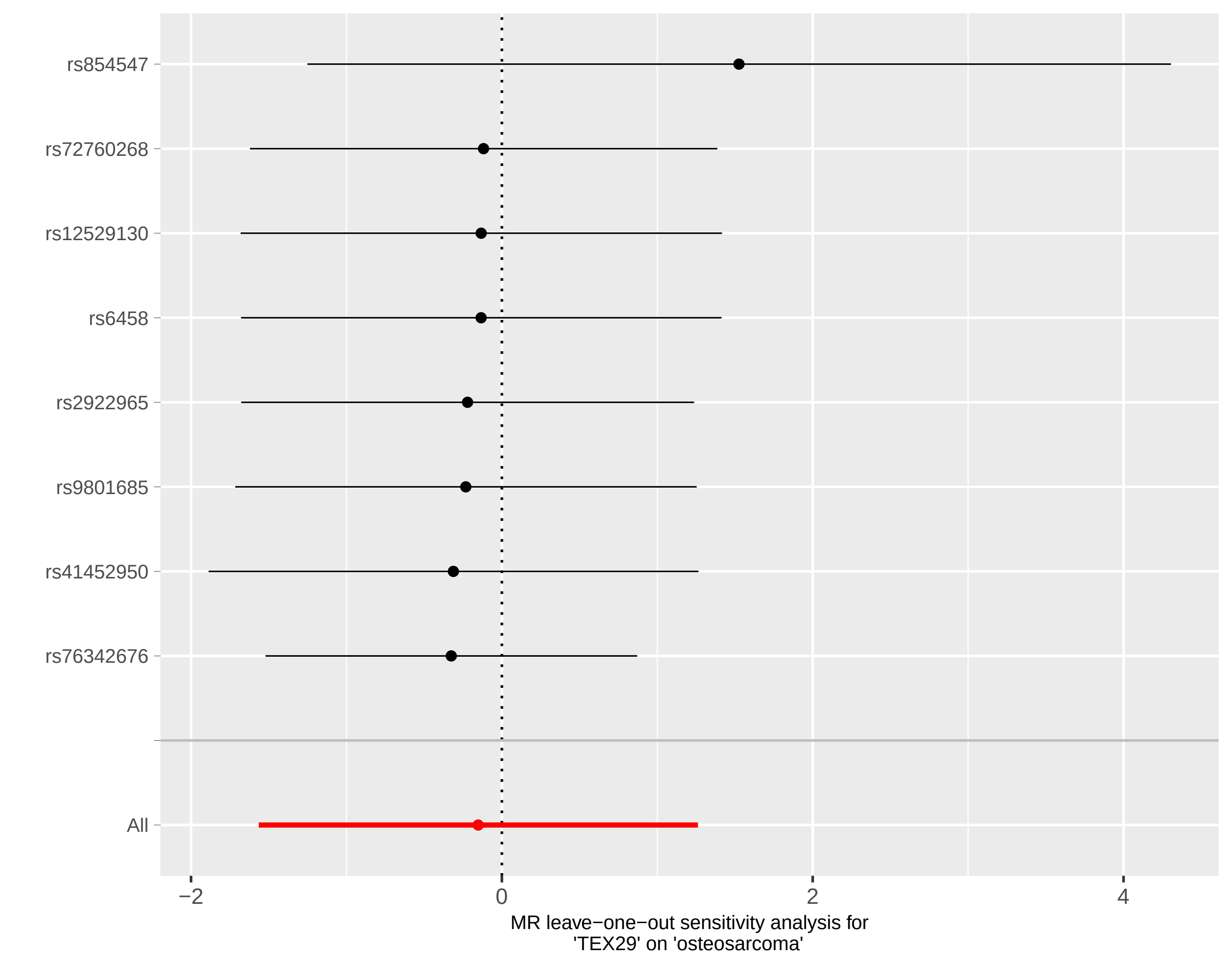
rs854547
rs72760268
rs12529130
rs6458
rs2922965
rs9801685
rs41452950
rs76342676
All
−2
0
2
4
MR leave−one−out sensitivity analysis for
'TEX29' on 'osteosarcoma'

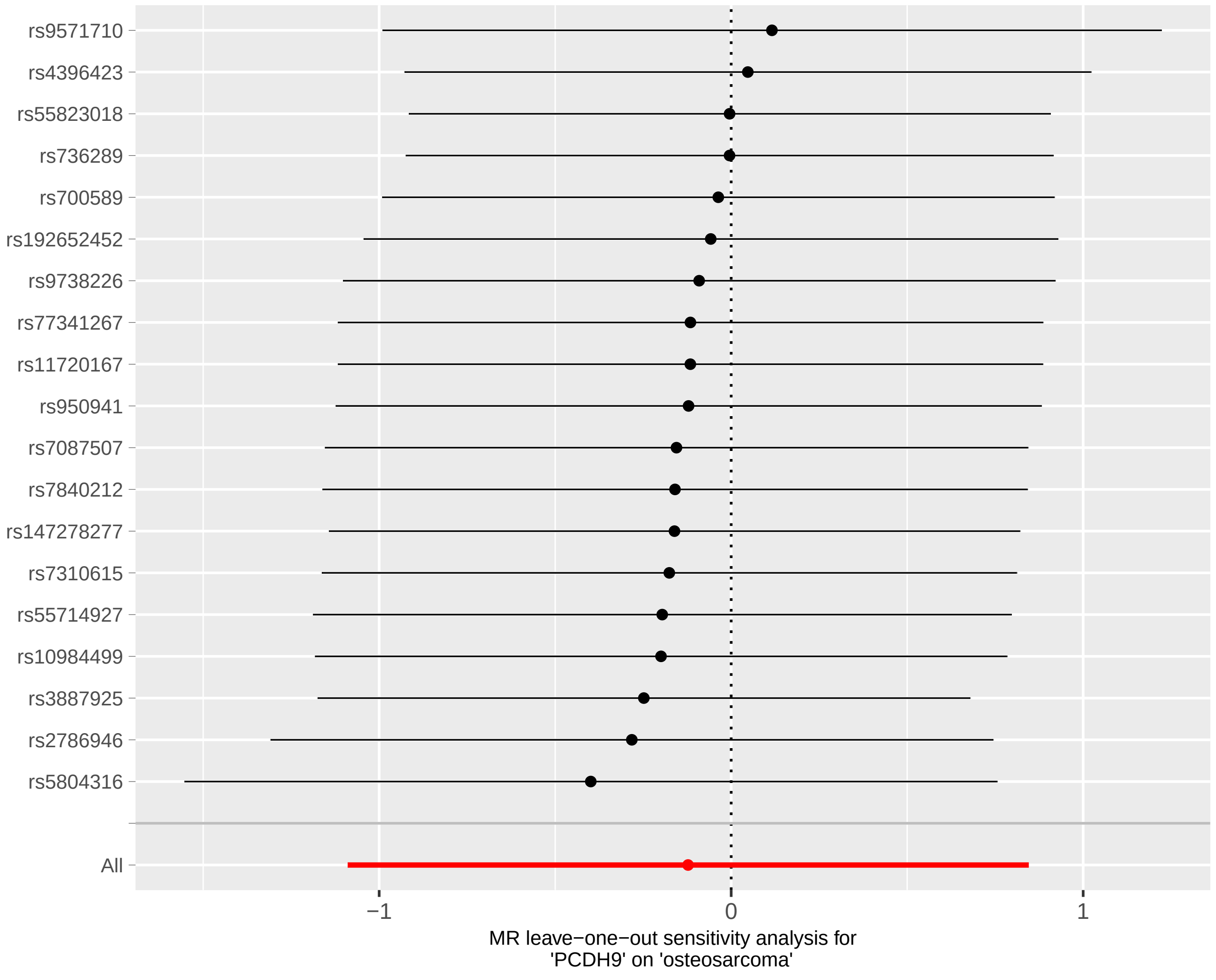

rs9571710
rs4396423
rs55823018
rs736289
rs700589
rs192652452
rs9738226
rs77341267
rs11720167
rs950941
rs7087507
rs7840212
rs147278277
rs7310615
rs55714927
rs10984499
rs3887925
rs2786946
rs5804316
All
−1
0
1
MR leave−one−out sensitivity analysis for
'PCDH9' on 'osteosarcoma'

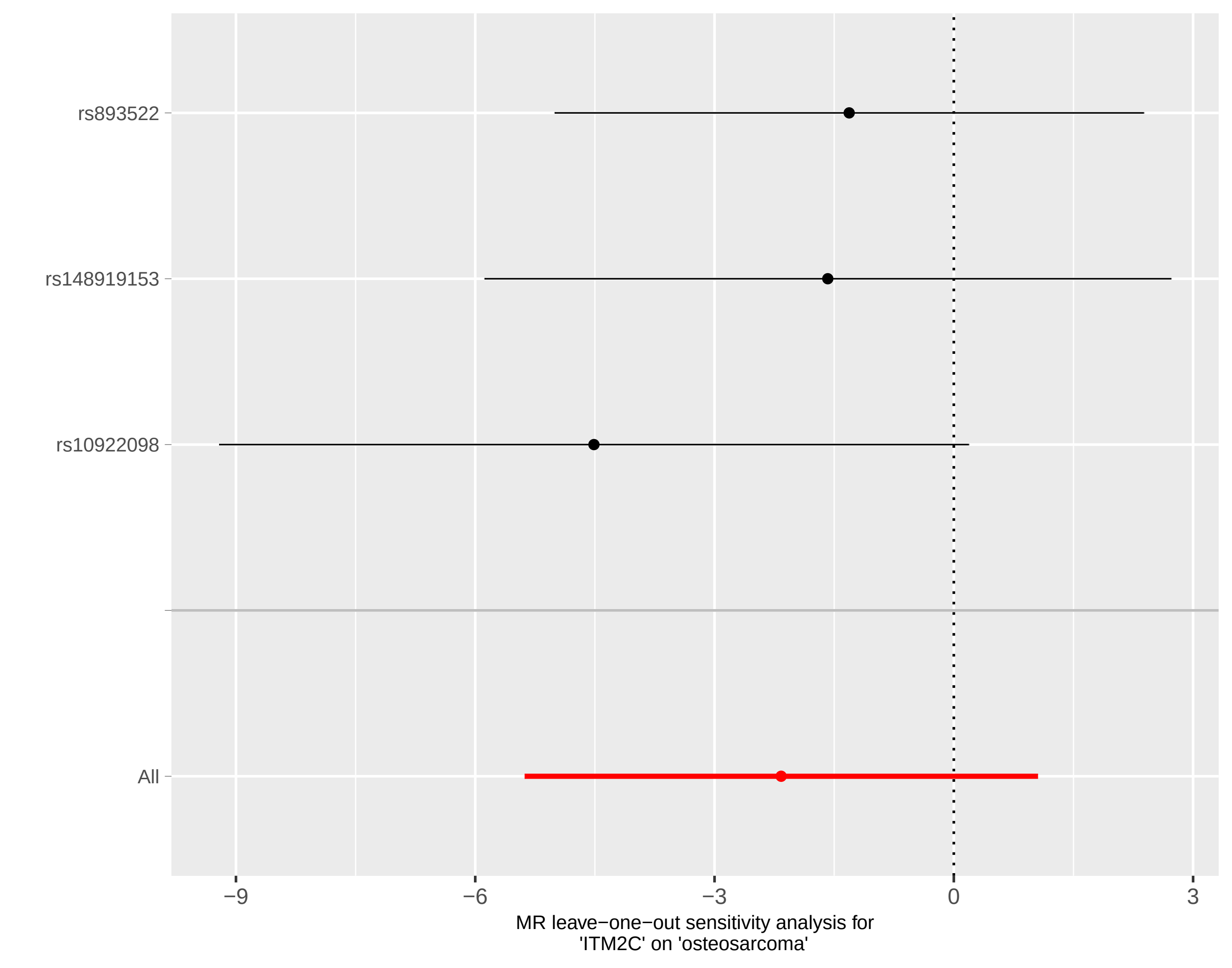
rs893522
rs148919153
rs10922098
All
−9
−6
−3
0
3
MR leave−one−out sensitivity analysis for
'ITM2C' on 'osteosarcoma'

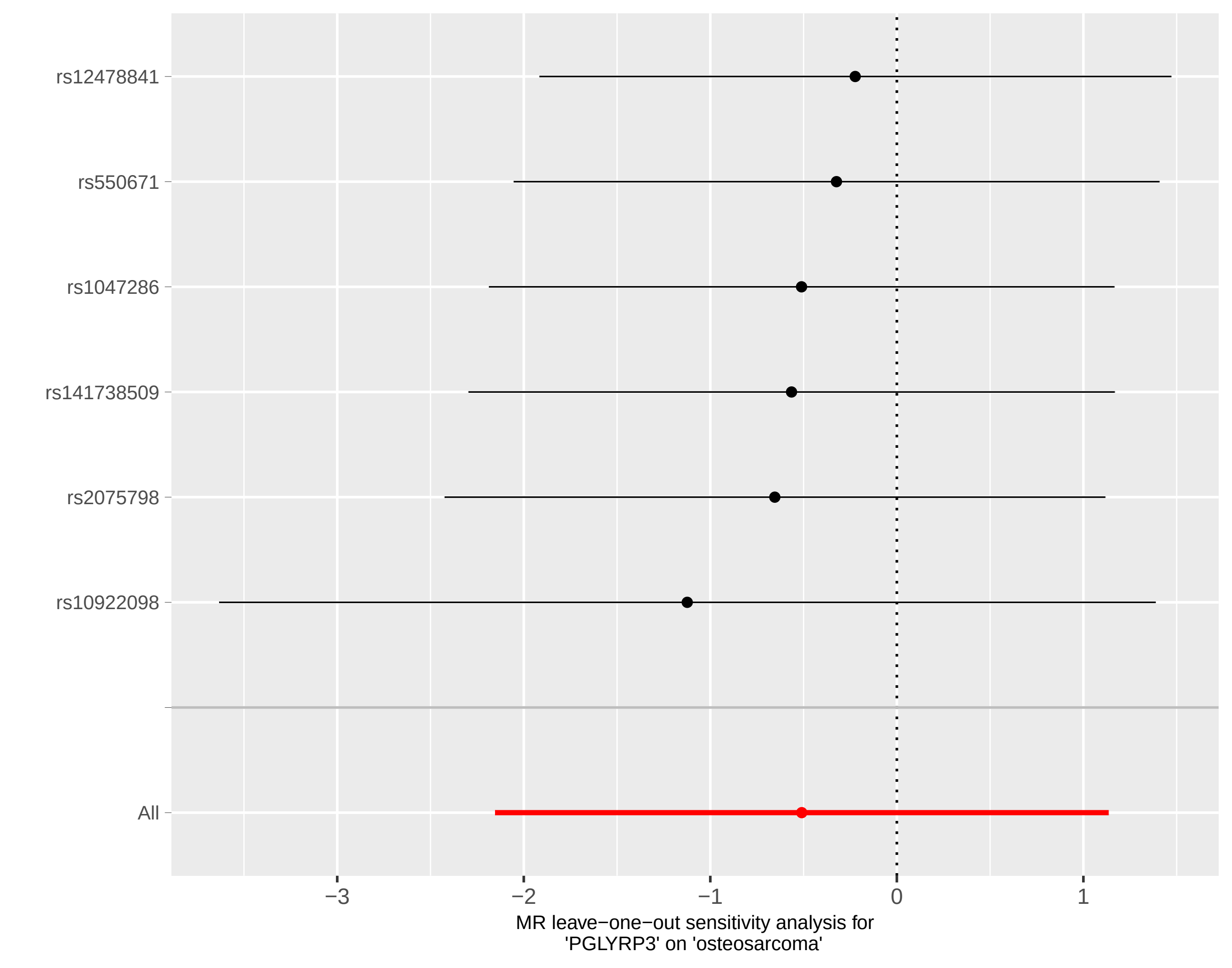

rs12478841
rs550671
rs1047286
rs141738509
rs2075798
rs10922098
All
−3
−2
−1
0
1
MR leave−one−out sensitivity analysis for
'PGLYRP3' on 'osteosarcoma'

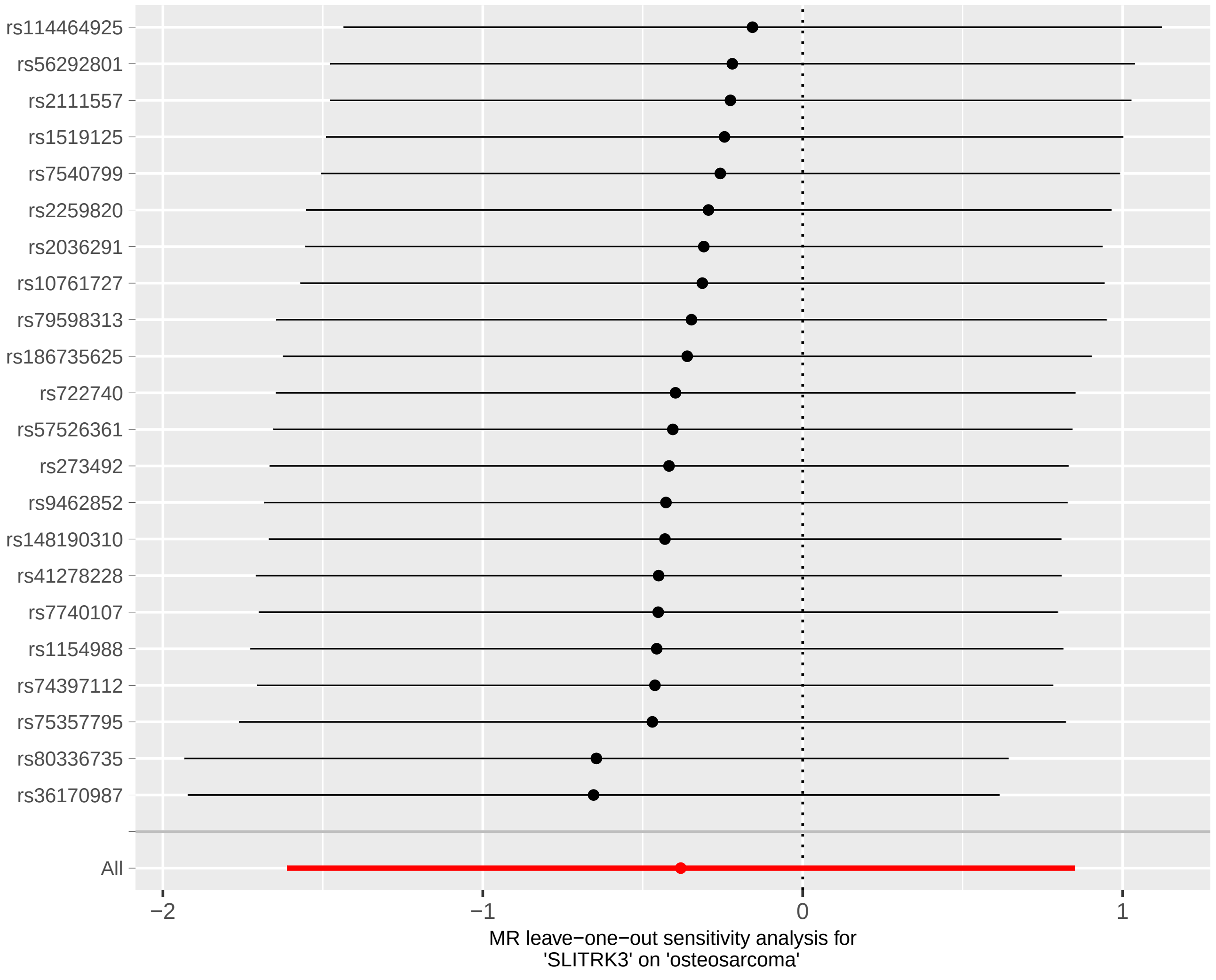
rs114464925
rs56292801
rs2111557
rs1519125
rs7540799
rs2259820
rs2036291
rs10761727
rs79598313
rs186735625
rs722740
rs57526361
rs273492
rs9462852
rs148190310
rs41278228
rs7740107
rs1154988
rs74397112
rs75357795
rs80336735
rs36170987
All
−2
−1
0
1
MR leave−one−out sensitivity analysis for
'SLITRK3' on 'osteosarcoma'

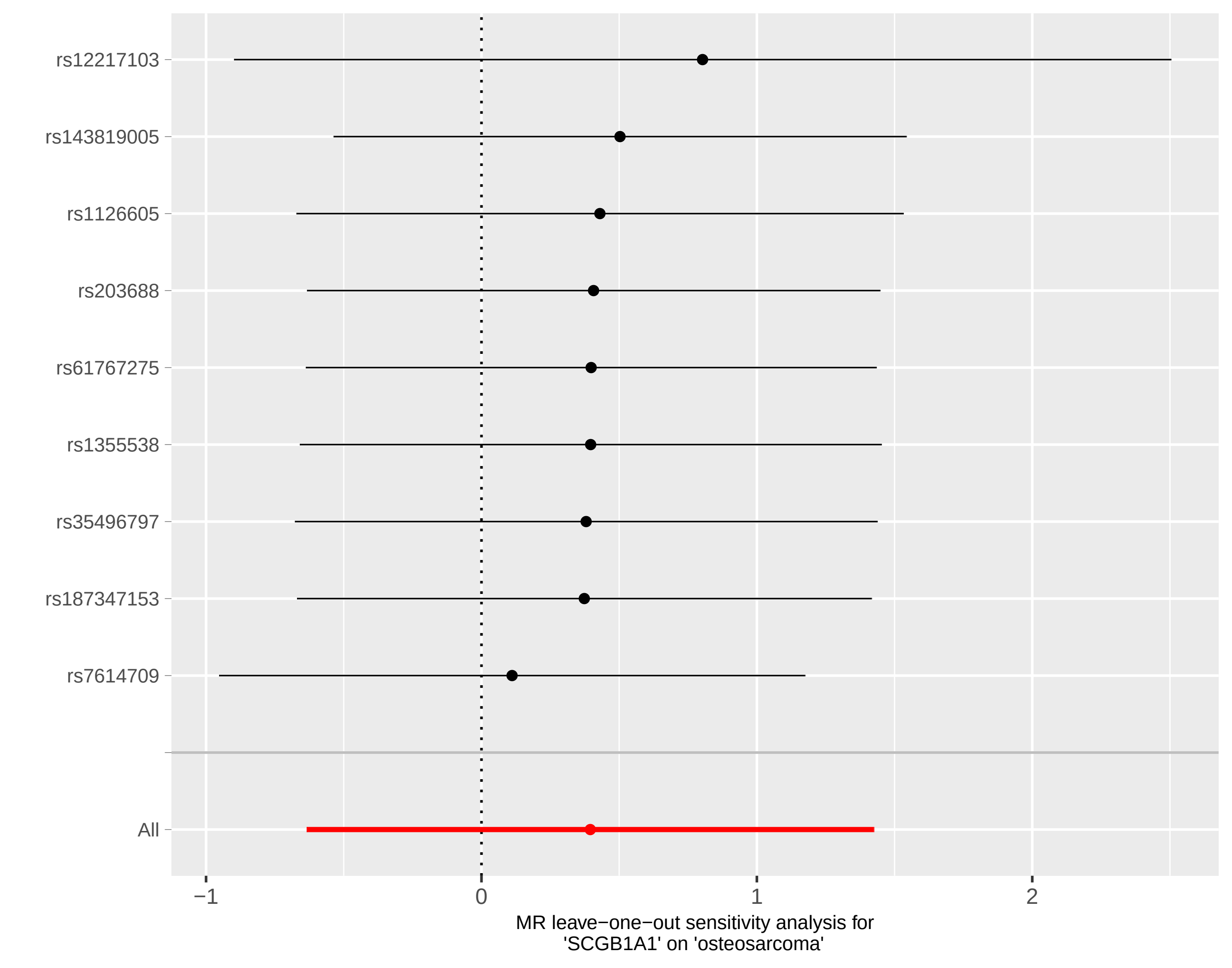

rs12217103
rs143819005
rs1126605
rs203688
rs61767275
rs1355538
rs35496797
rs187347153
rs7614709
All
−1
0
1
2
MR leave−one−out sensitivity analysis for
'SCGB1A1' on 'osteosarcoma'

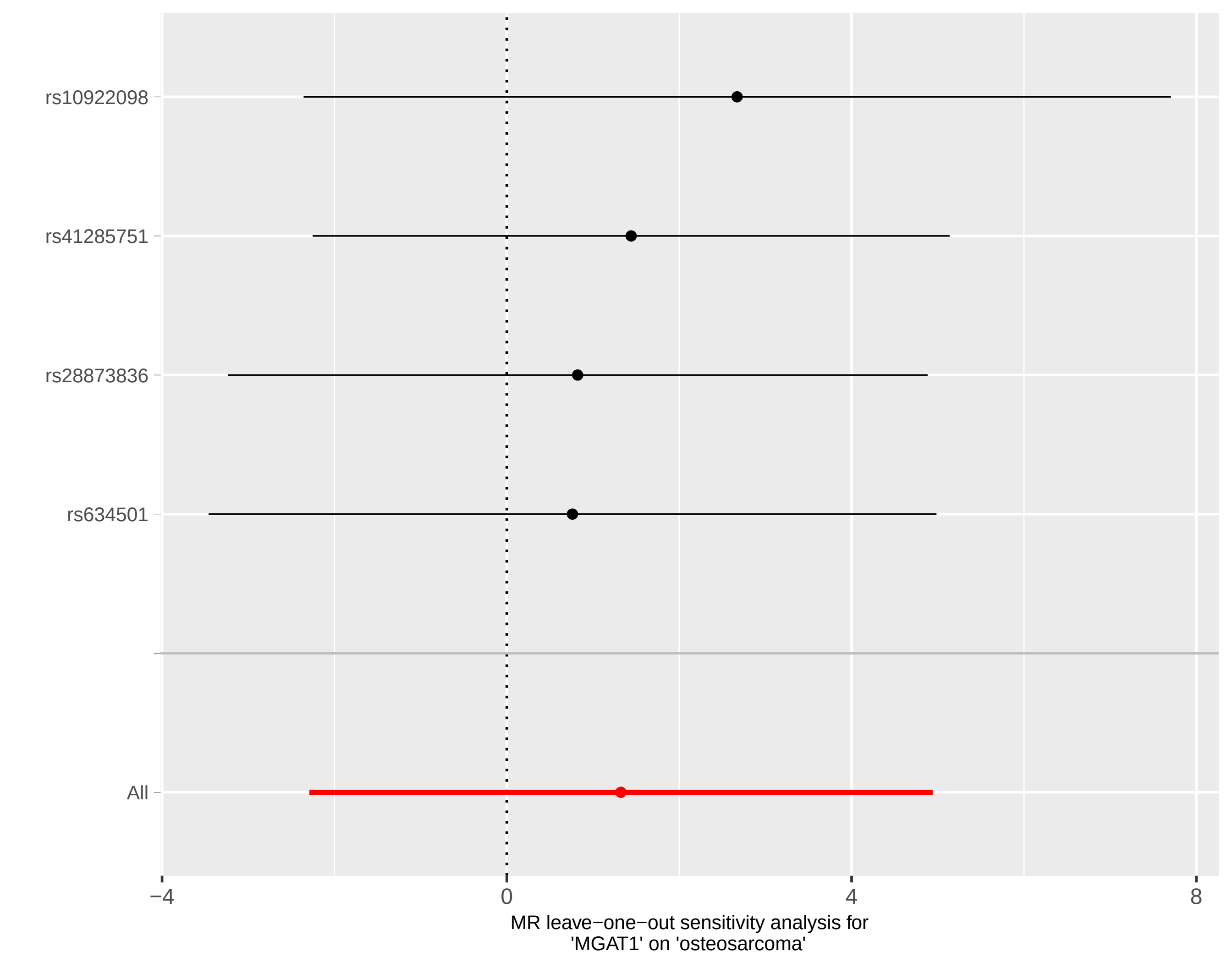
rs10922098
rs41285751
rs28873836
rs634501
All
−4
0
4
8
MR leave−one−out sensitivity analysis for
'MGAT1' on 'osteosarcoma'

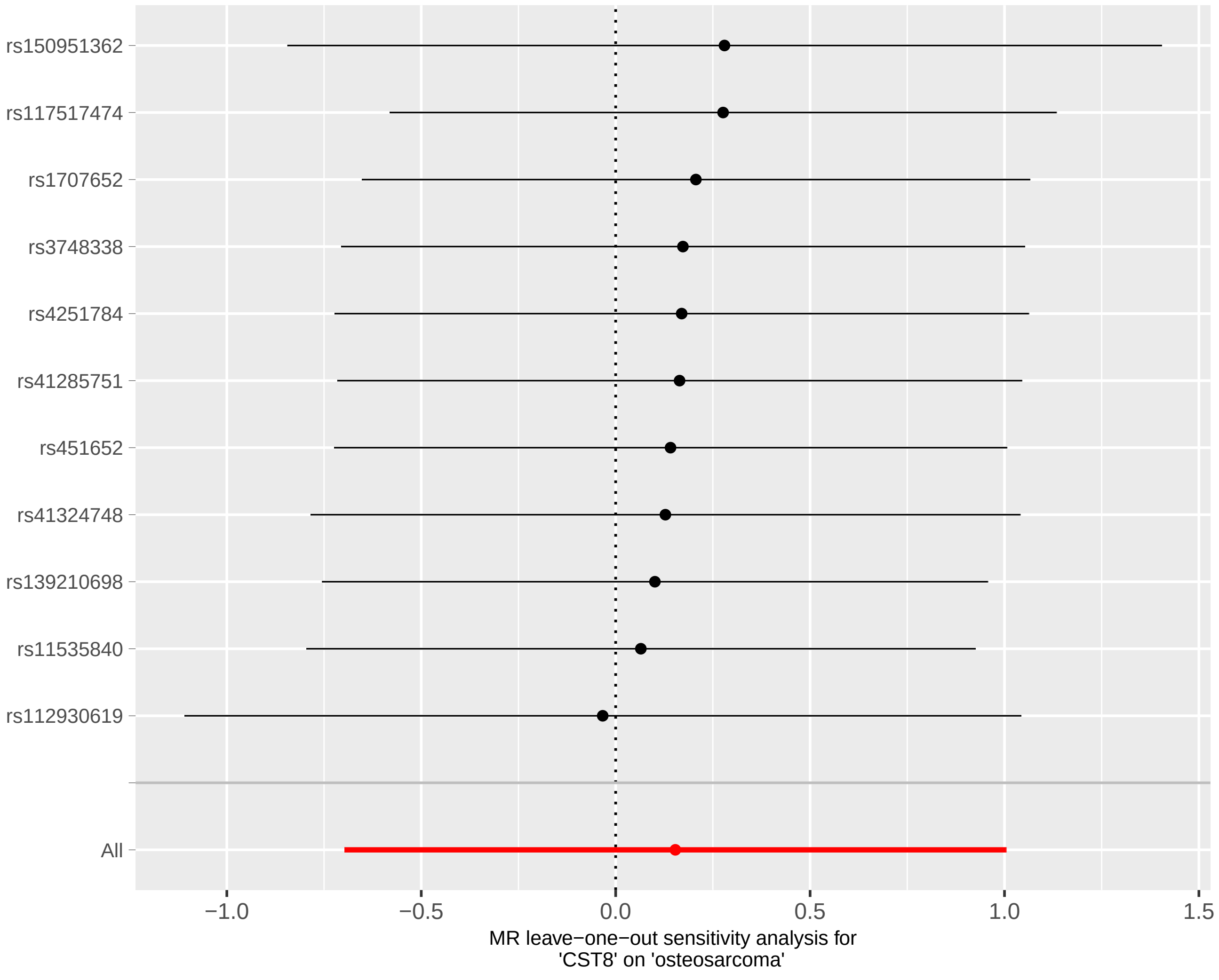

rs150951362
rs117517474
rs1707652
rs3748338
rs4251784
rs41285751
rs451652
rs41324748
rs139210698
rs11535840
rs112930619
All
−1.0
−0.5
0.0
0.5
1.0
1.5
MR leave−one−out sensitivity analysis for
'CST8' on 'osteosarcoma'

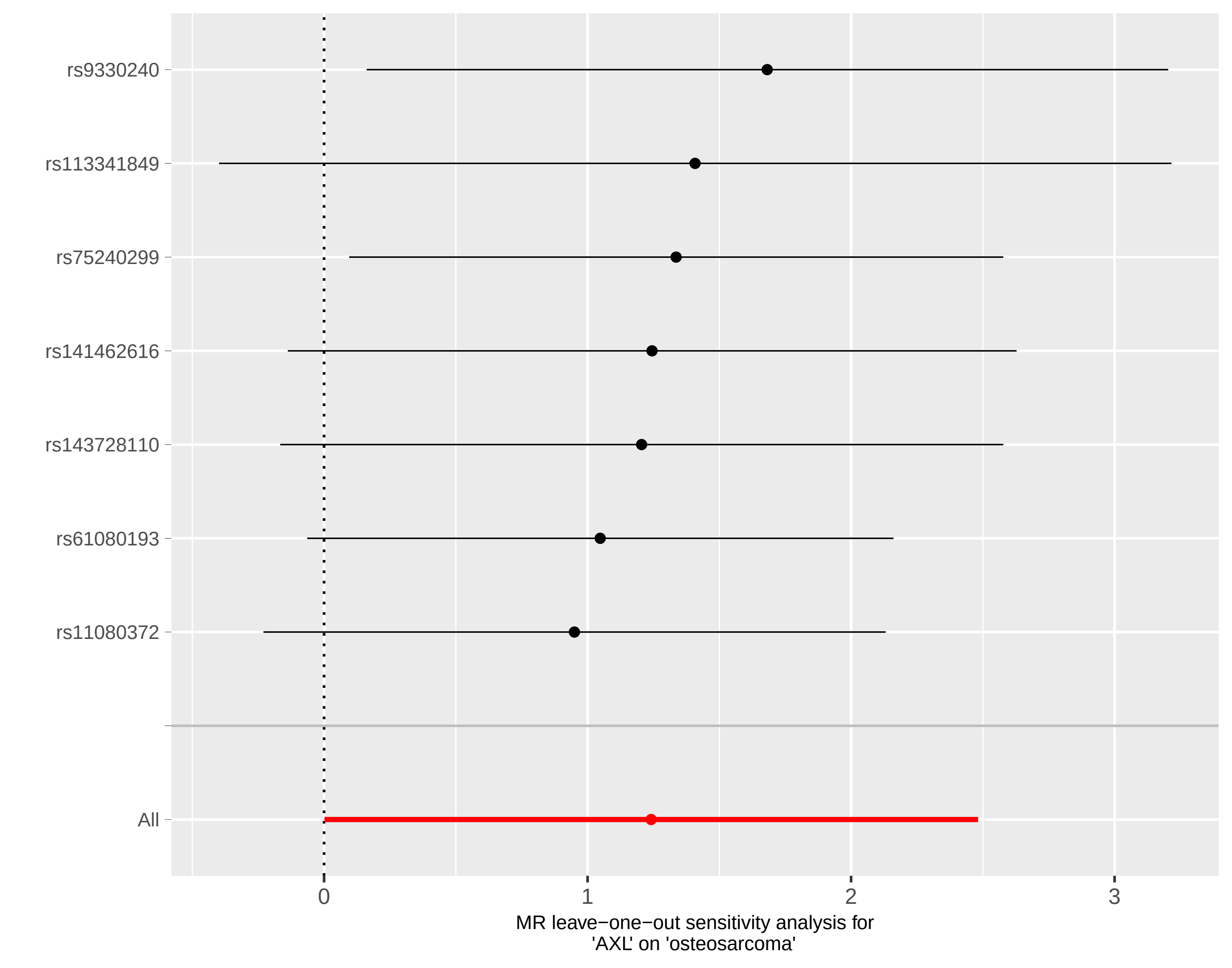
rs9330240
rs113341849
rs75240299
rs141462616
rs143728110
rs61080193
rs11080372
All
0
1
2
3
MR leave−one−out sensitivity analysis for
'AXL' on 'osteosarcoma'

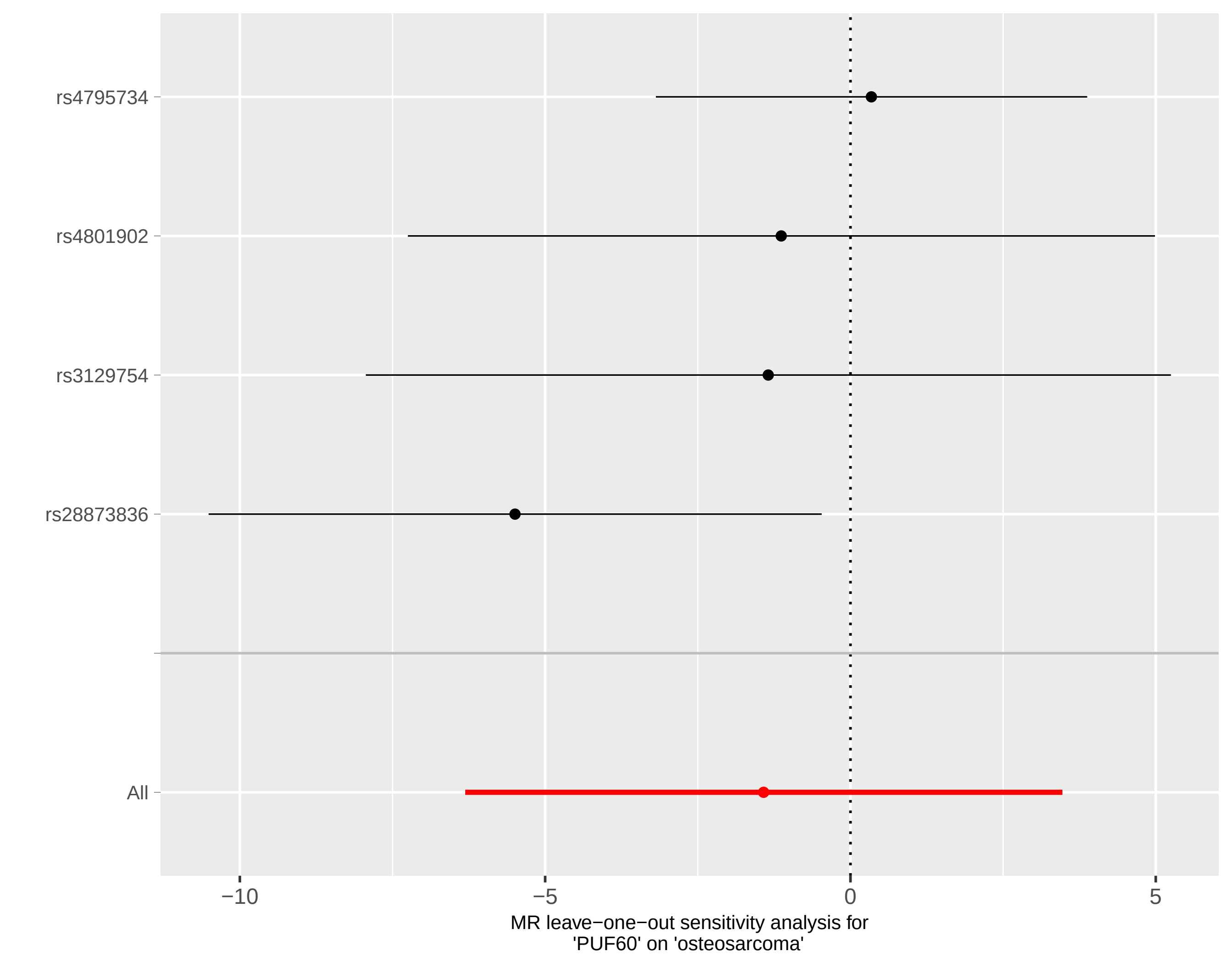

rs4795734
rs4801902
rs3129754
rs28873836
All
−10
−5
0
5
MR leave−one−out sensitivity analysis for
'PUF60' on 'osteosarcoma'

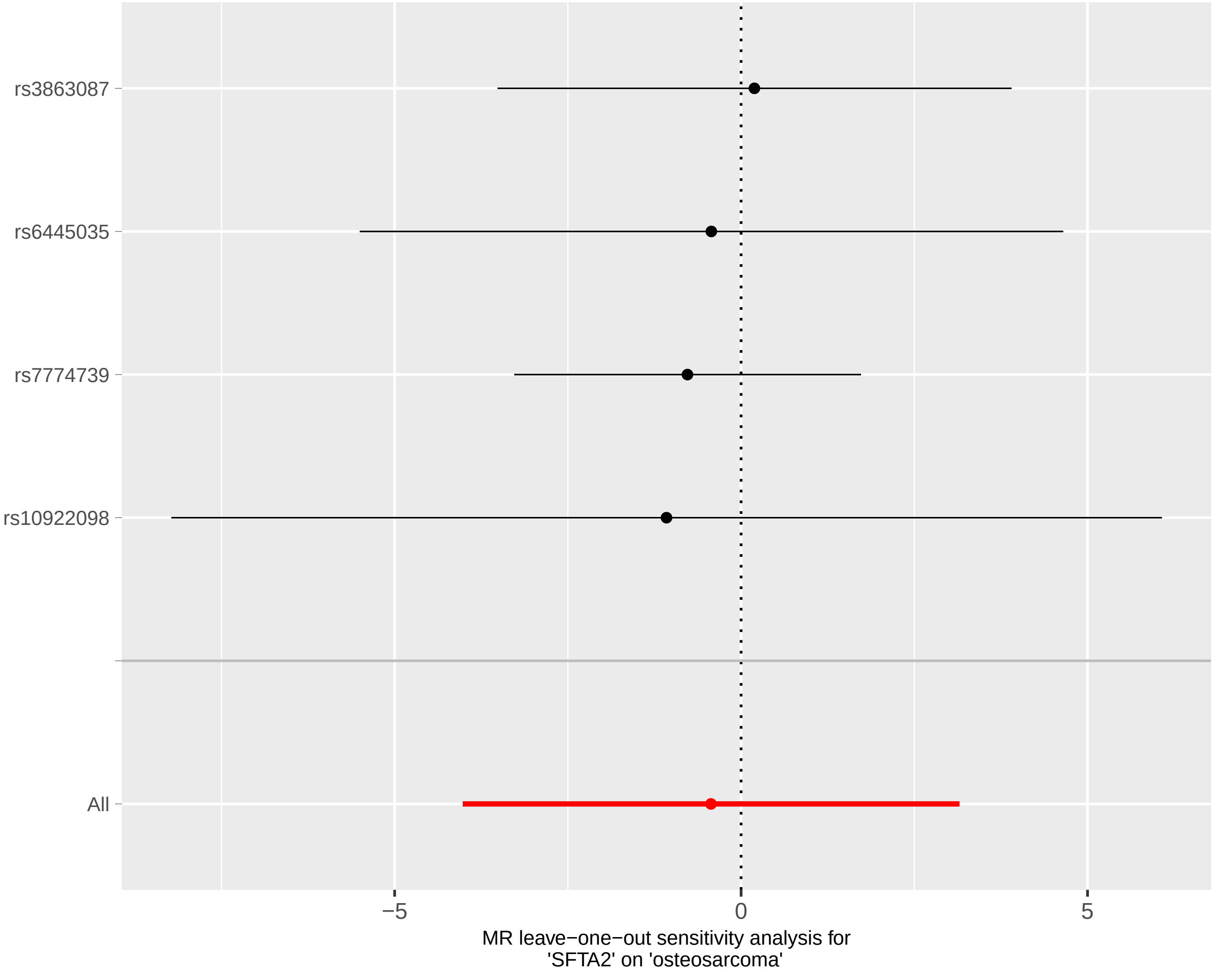

rs3863087
rs6445035
rs7774739
rs10922098
All
−5
0
5
MR leave−one−out sensitivity analysis for
'SFTA2' on 'osteosarcoma'

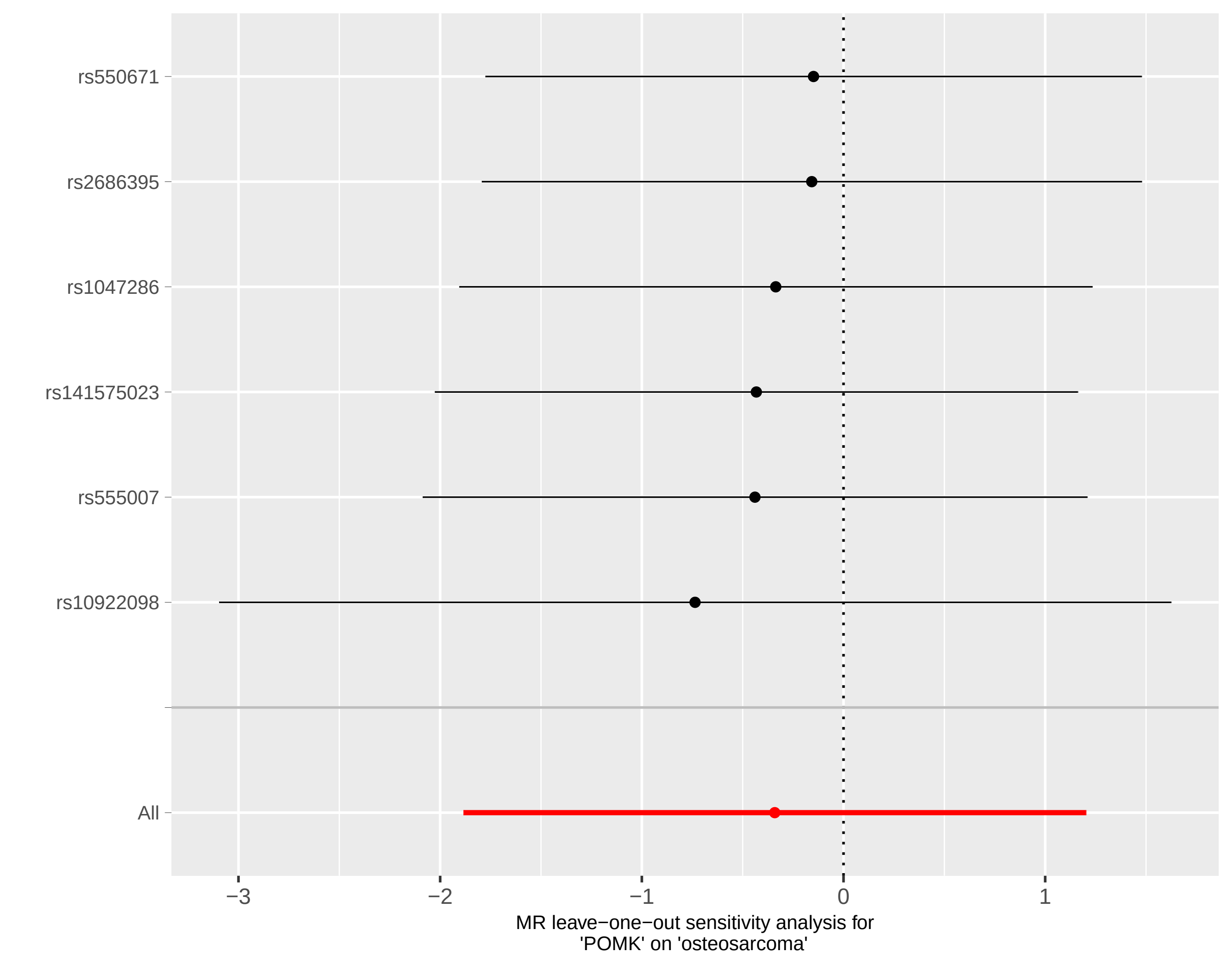

rs550671
rs2686395
rs1047286
rs141575023
rs555007
rs10922098
All
−3
−2
−1
0
1
MR leave−one−out sensitivity analysis for
'POMK' on 'osteosarcoma'

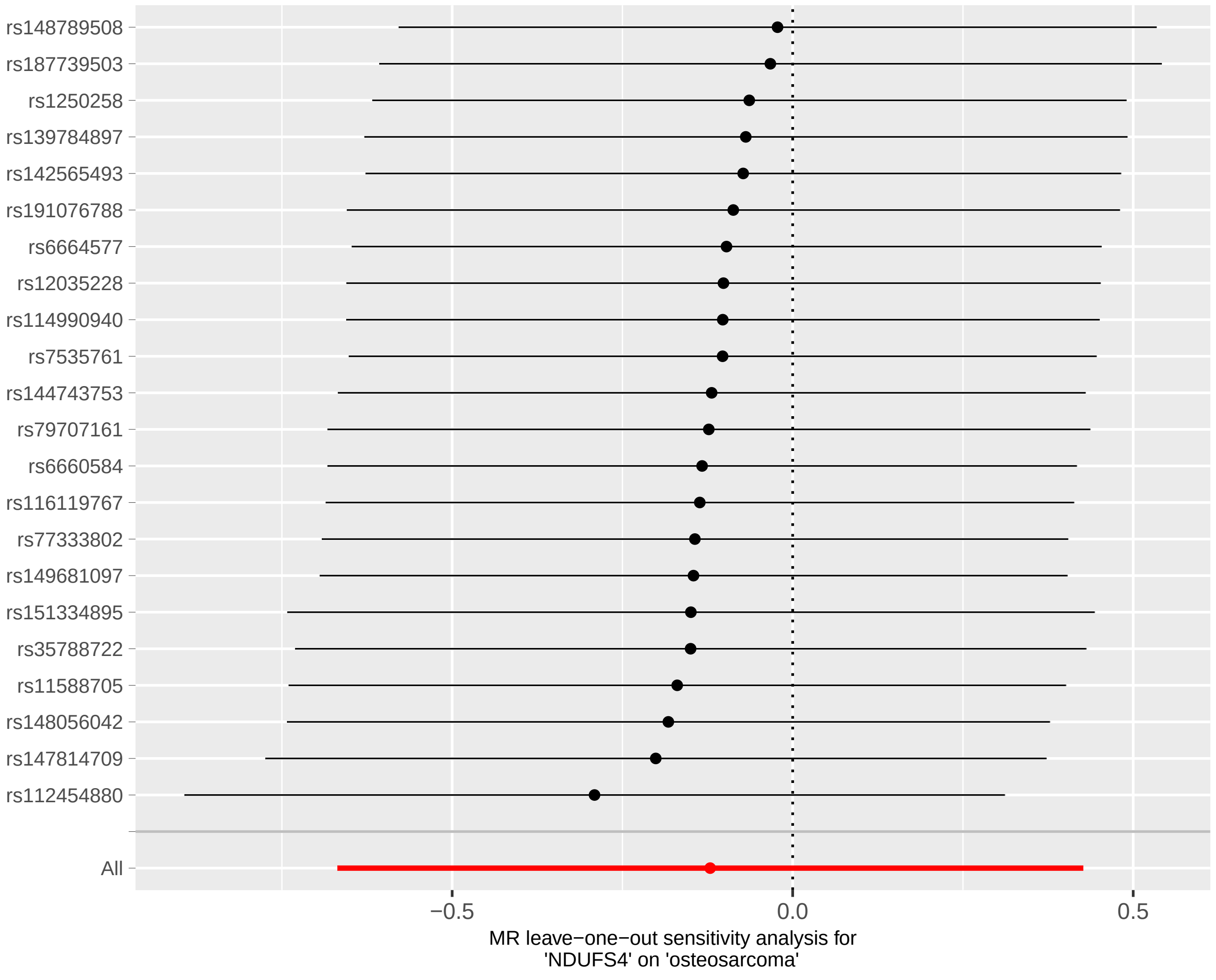

rs148789508
rs187739503
rs1250258
rs139784897
rs142565493
rs191076788
rs6664577
rs12035228
rs114990940
rs7535761
rs144743753
rs79707161
rs6660584
rs116119767
rs77333802
rs149681097
rs151334895
rs35788722
rs11588705
rs148056042
rs147814709
rs112454880
All
−0.5
0.0
0.5
MR leave−one−out sensitivity analysis for
'NDUFS4' on 'osteosarcoma'

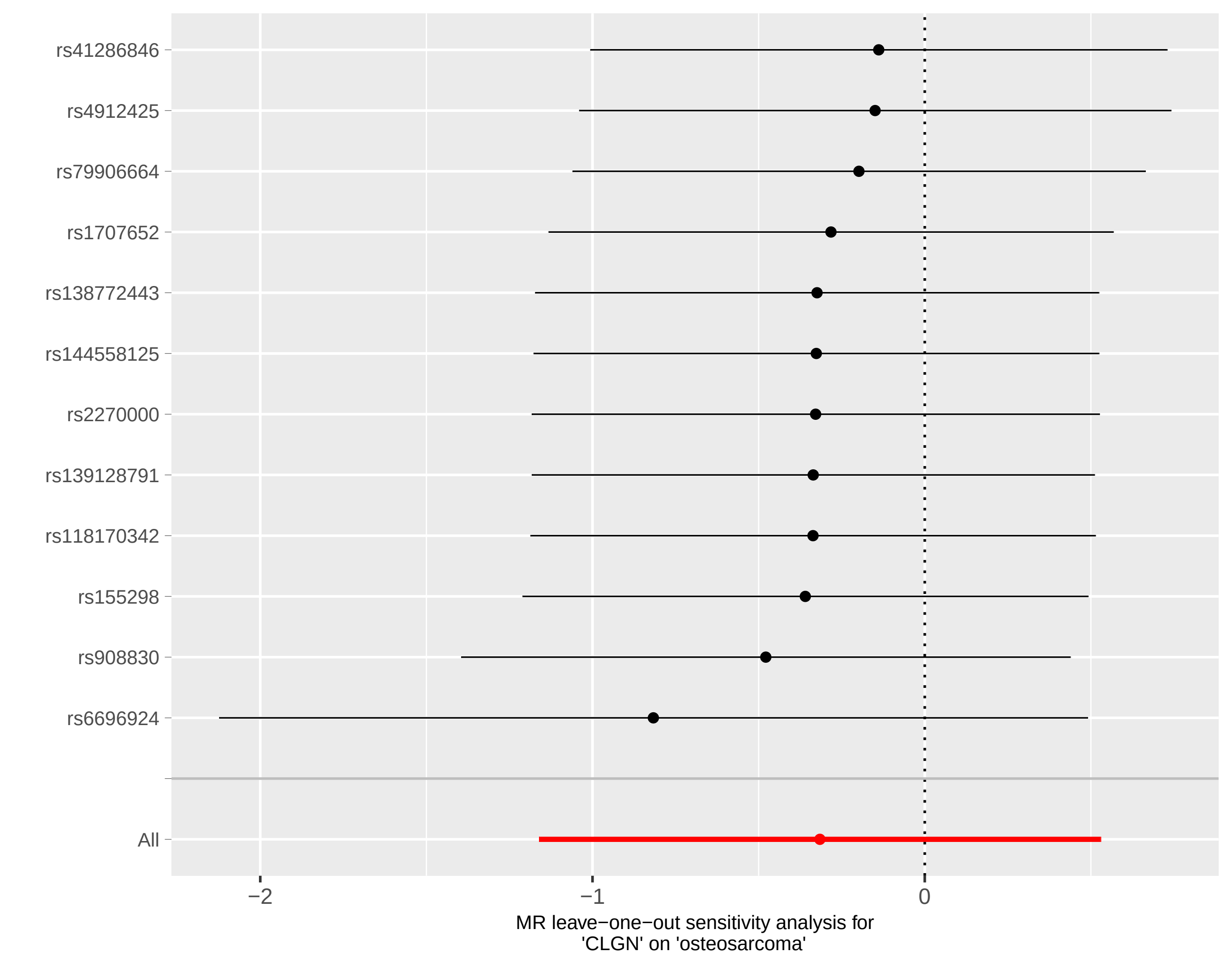
rs41286846
rs4912425
rs79906664
rs1707652
rs138772443
rs144558125
rs2270000
rs139128791
rs118170342
rs155298
rs908830
rs6696924
All
−2
−1
0
MR leave−one−out sensitivity analysis for
'CLGN' on 'osteosarcoma'

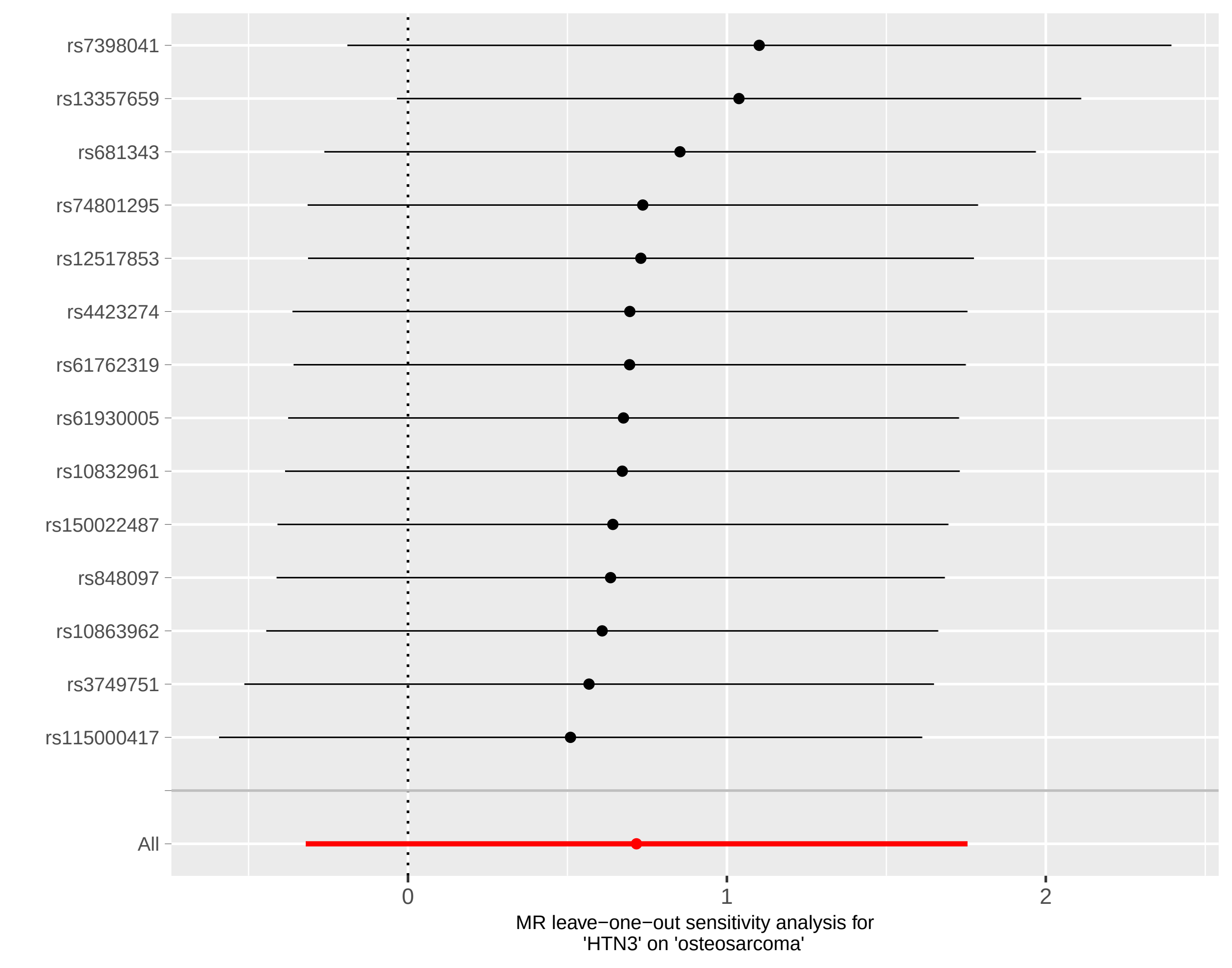

rs7398041
rs13357659
rs681343
rs74801295
rs12517853
rs4423274
rs61762319
rs61930005
rs10832961
rs150022487
rs848097
rs10863962
rs3749751
rs115000417
All
0
1
2
MR leave−one−out sensitivity analysis for
'HTN3' on 'osteosarcoma'

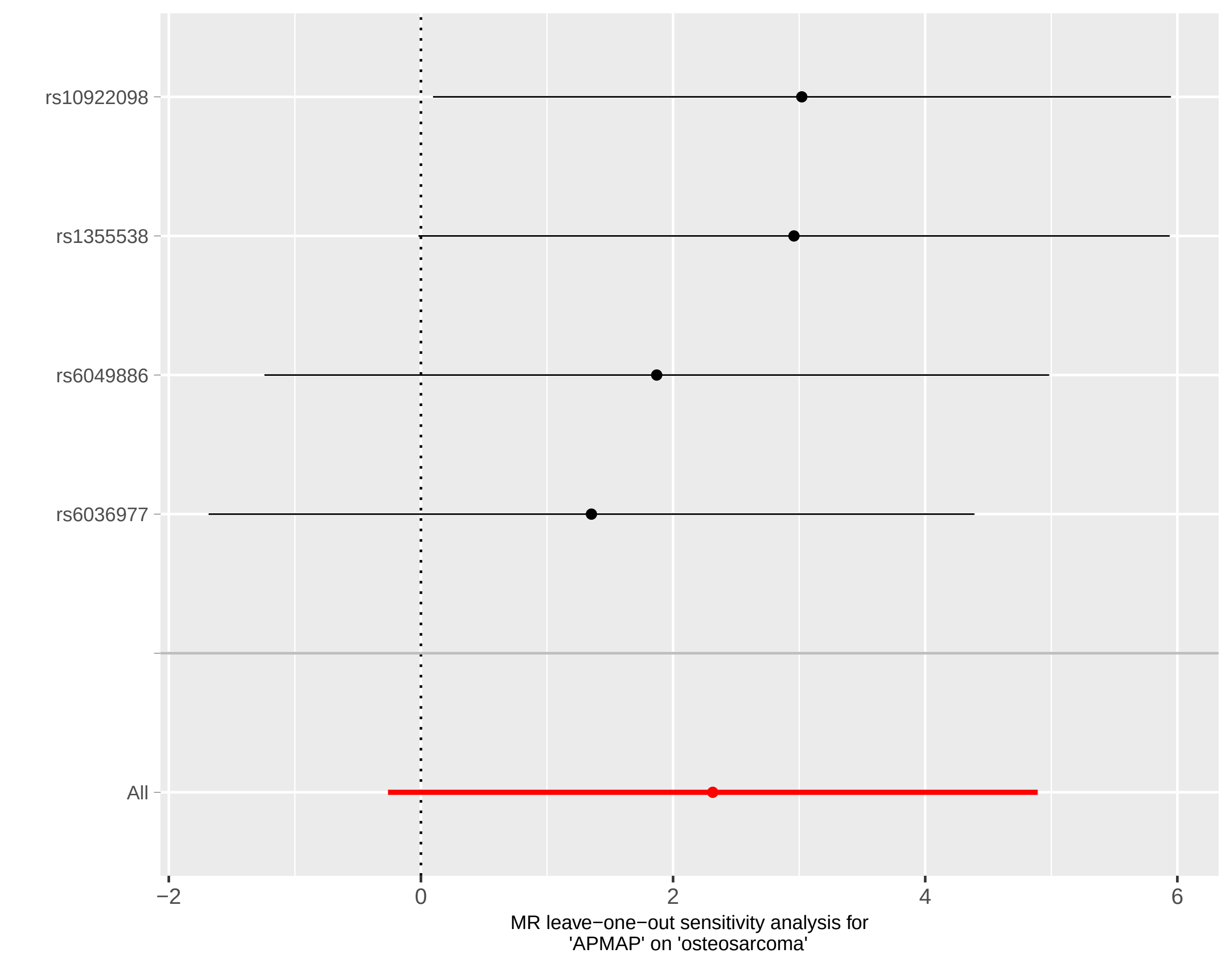
rs10922098
rs1355538
rs6049886
rs6036977
All
−2
0
2
4
6
MR leave−one−out sensitivity analysis for
'APMAP' on 'osteosarcoma'

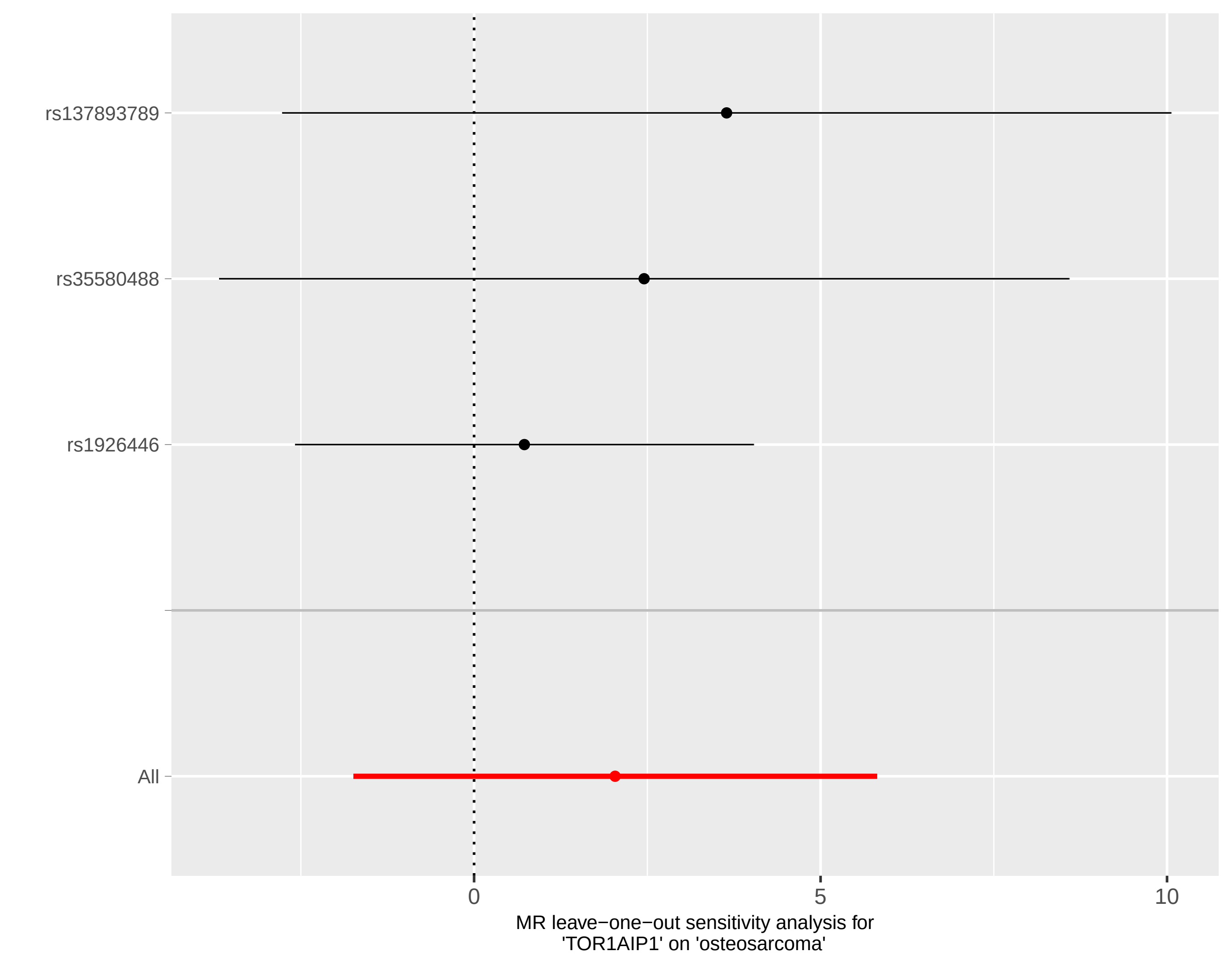
rs137893789
rs35580488
rs1926446
All
0
5
10
MR leave−one−out sensitivity analysis for
'TOR1AIP1' on 'osteosarcoma'

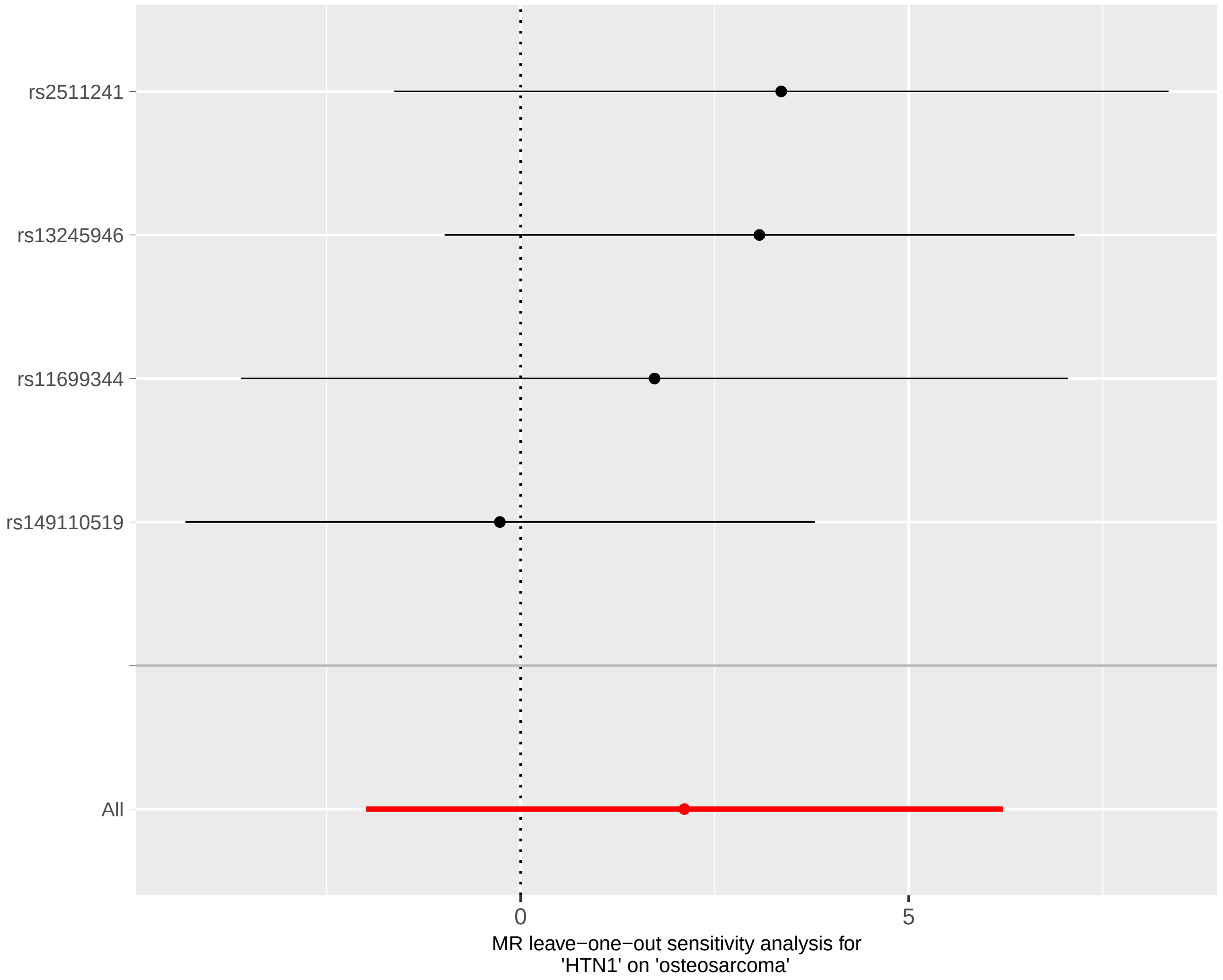

rs2511241
rs13245946
rs11699344
rs149110519
All
0
5
MR leave−one−out sensitivity analysis for
'HTN1' on 'osteosarcoma'

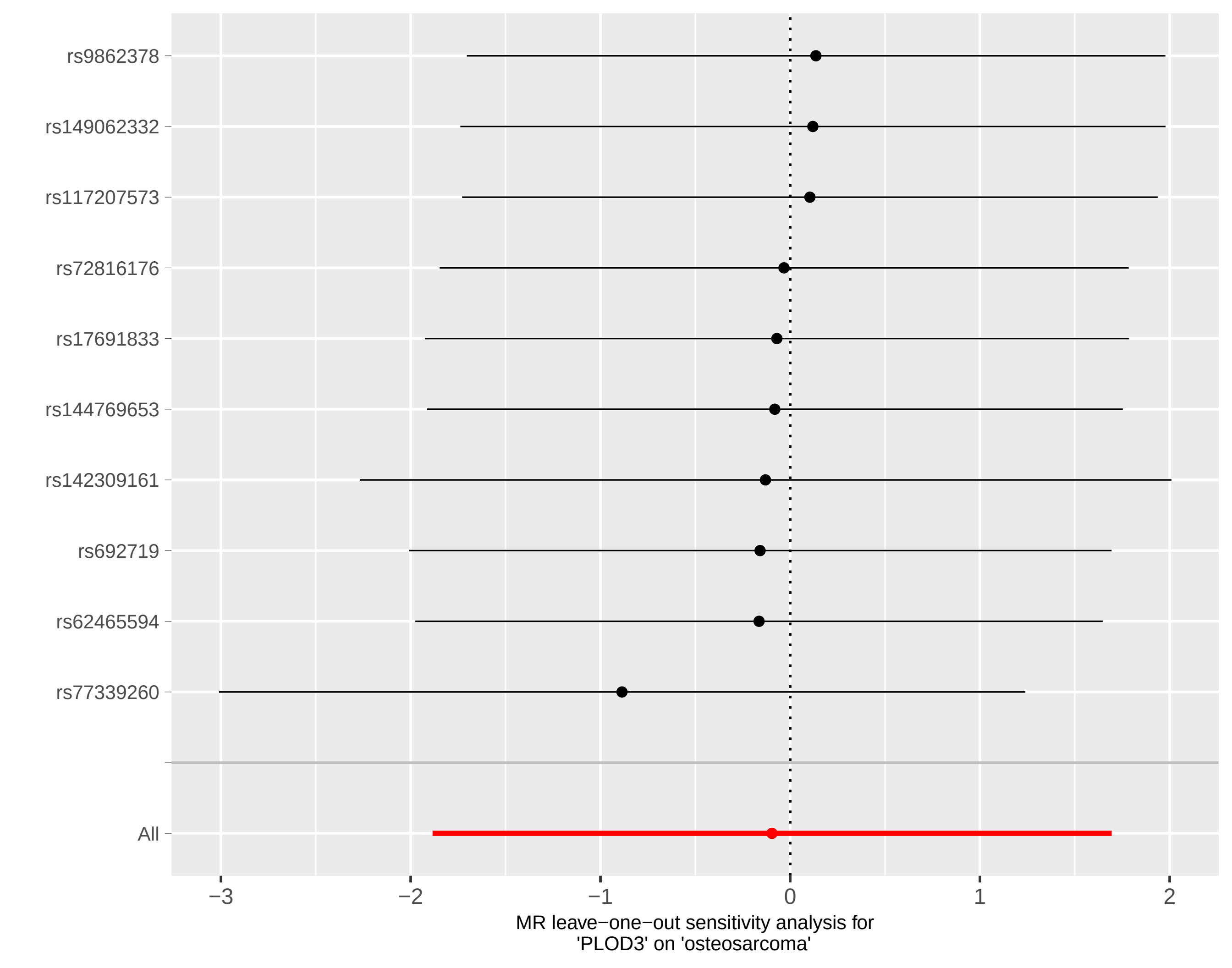

rs9862378
rs149062332
rs117207573
rs72816176
rs17691833
rs144769653
rs142309161
rs692719
rs62465594
rs77339260
All
−3
−2
−1
0
1
2
MR leave−one−out sensitivity analysis for
'PLOD3' on 'osteosarcoma'

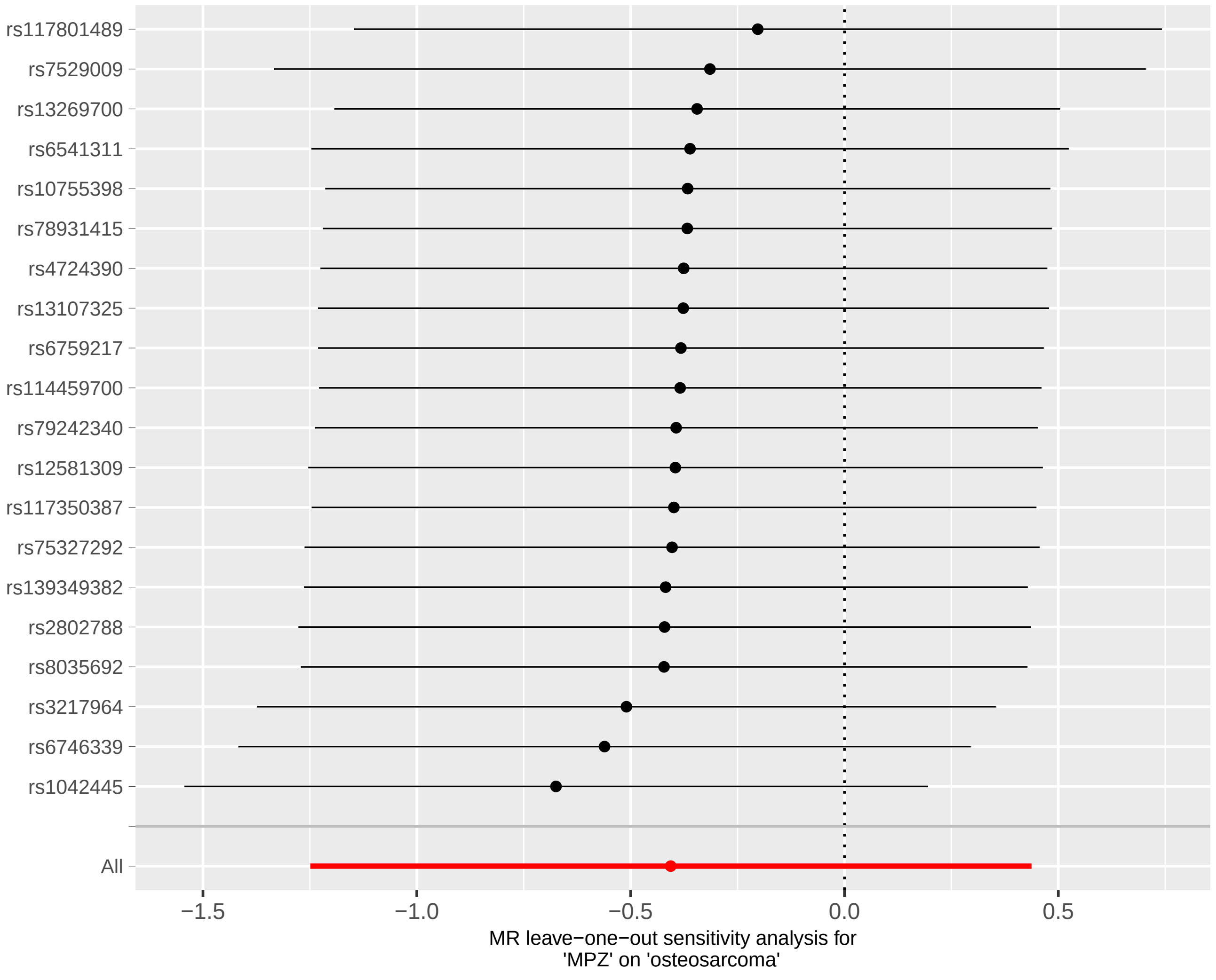

rs117801489
rs7529009
rs13269700
rs6541311
rs10755398
rs78931415
rs4724390
rs13107325
rs6759217
rs114459700
rs79242340
rs12581309
rs117350387
rs75327292
rs139349382
rs2802788
rs8035692
rs3217964
rs6746339
rs1042445
All
−1.5
−1.0
−0.5
0.0
0.5
MR leave−one−out sensitivity analysis for
'MPZ' on 'osteosarcoma'

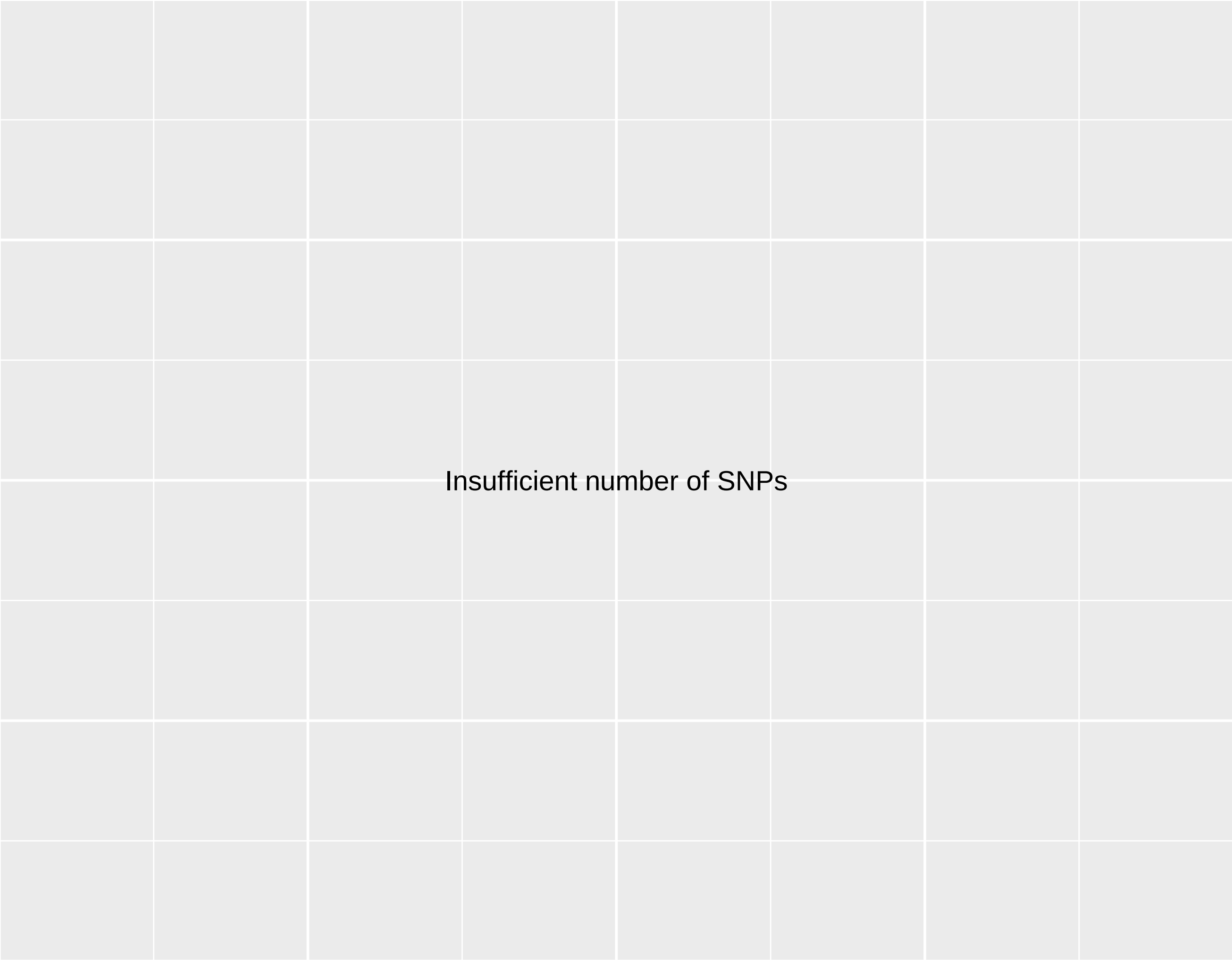
Insufficient number of SNPs

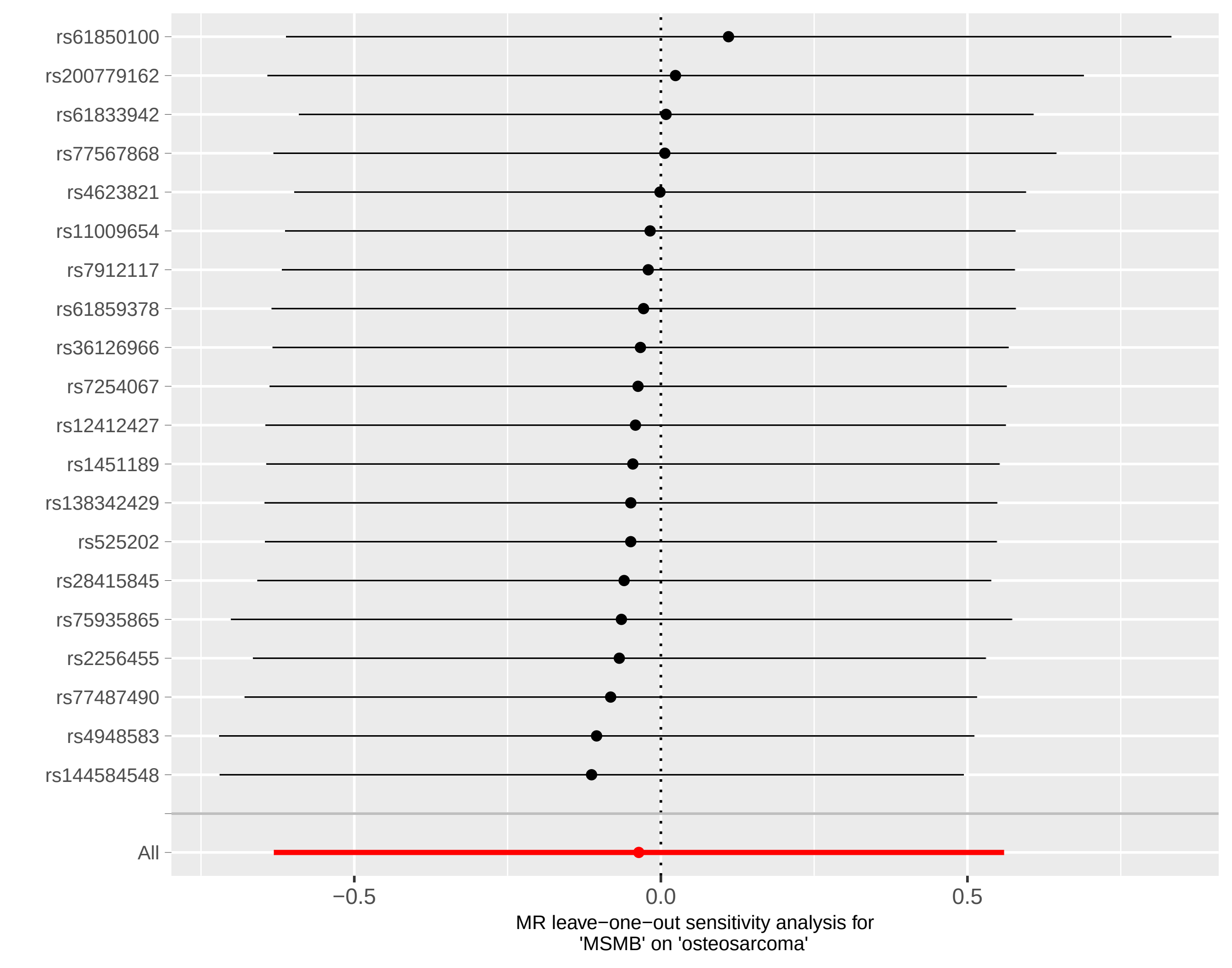
rs61850100
rs200779162
rs61833942
rs77567868
rs4623821
rs11009654
rs7912117
rs61859378
rs36126966
rs7254067
rs12412427
rs1451189
rs138342429
rs525202
rs28415845
rs75935865
rs2256455
rs77487490
rs4948583
rs144584548
All
−0.5
0.0
0.5
MR leave−one−out sensitivity analysis for
'MSMB' on 'osteosarcoma'

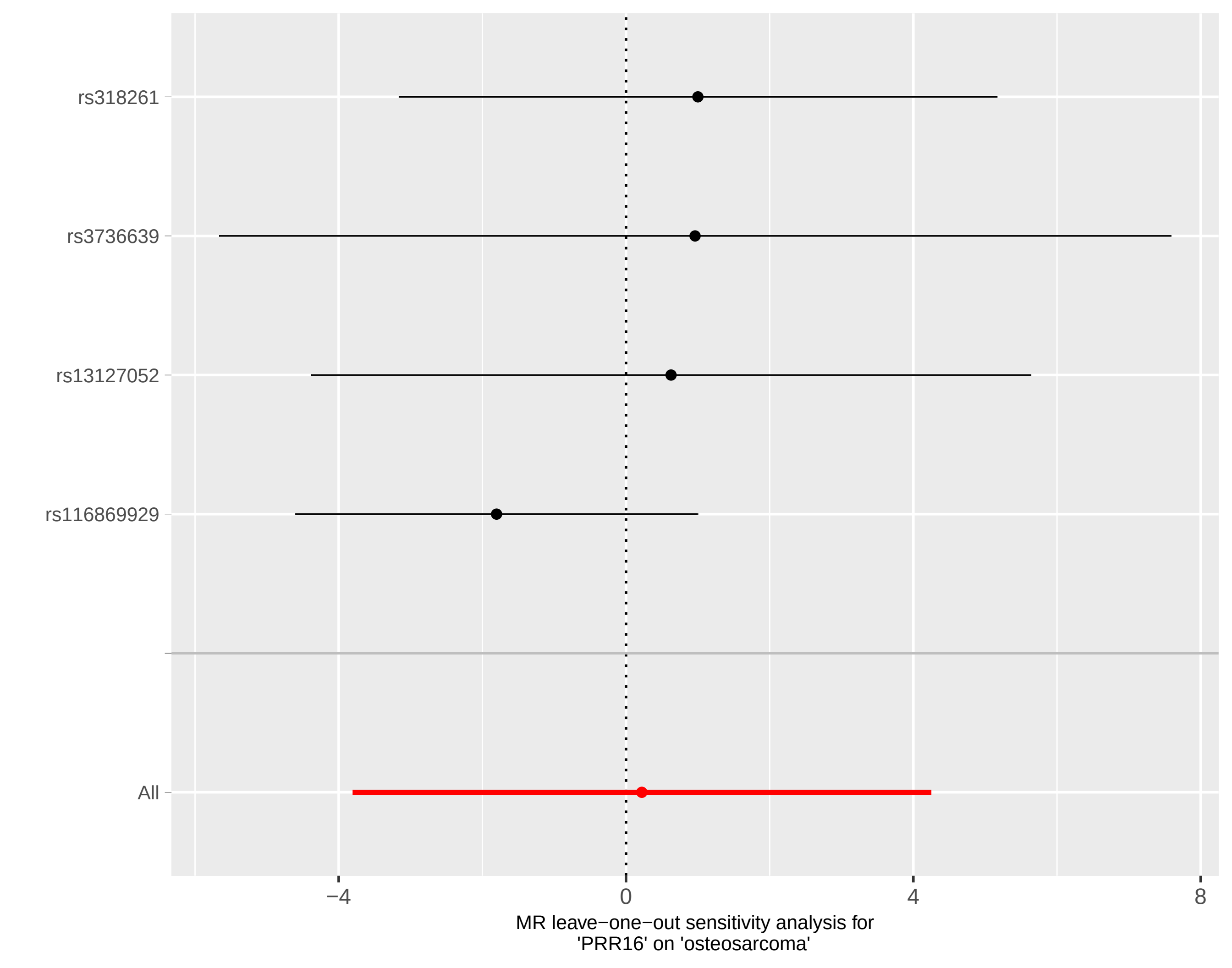

rs318261
rs3736639
rs13127052
rs116869929
All
−4
0
4
8
MR leave−one−out sensitivity analysis for
'PRR16' on 'osteosarcoma'

Insufficient number of SNPs

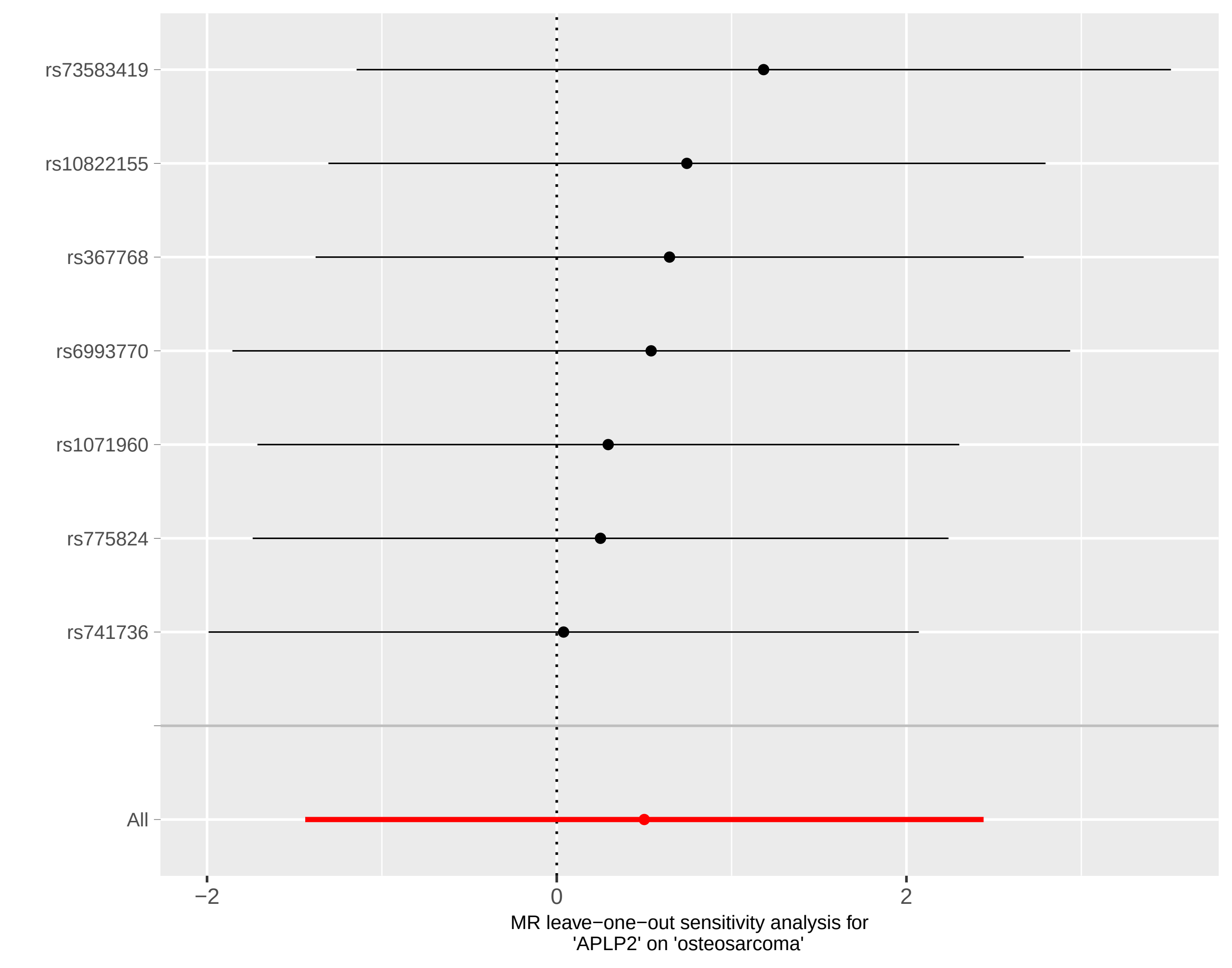
rs73583419
rs10822155
rs367768
rs6993770
rs1071960
rs775824
rs741736
All
−2
0
2
MR leave−one−out sensitivity analysis for
'APLP2' on 'osteosarcoma'

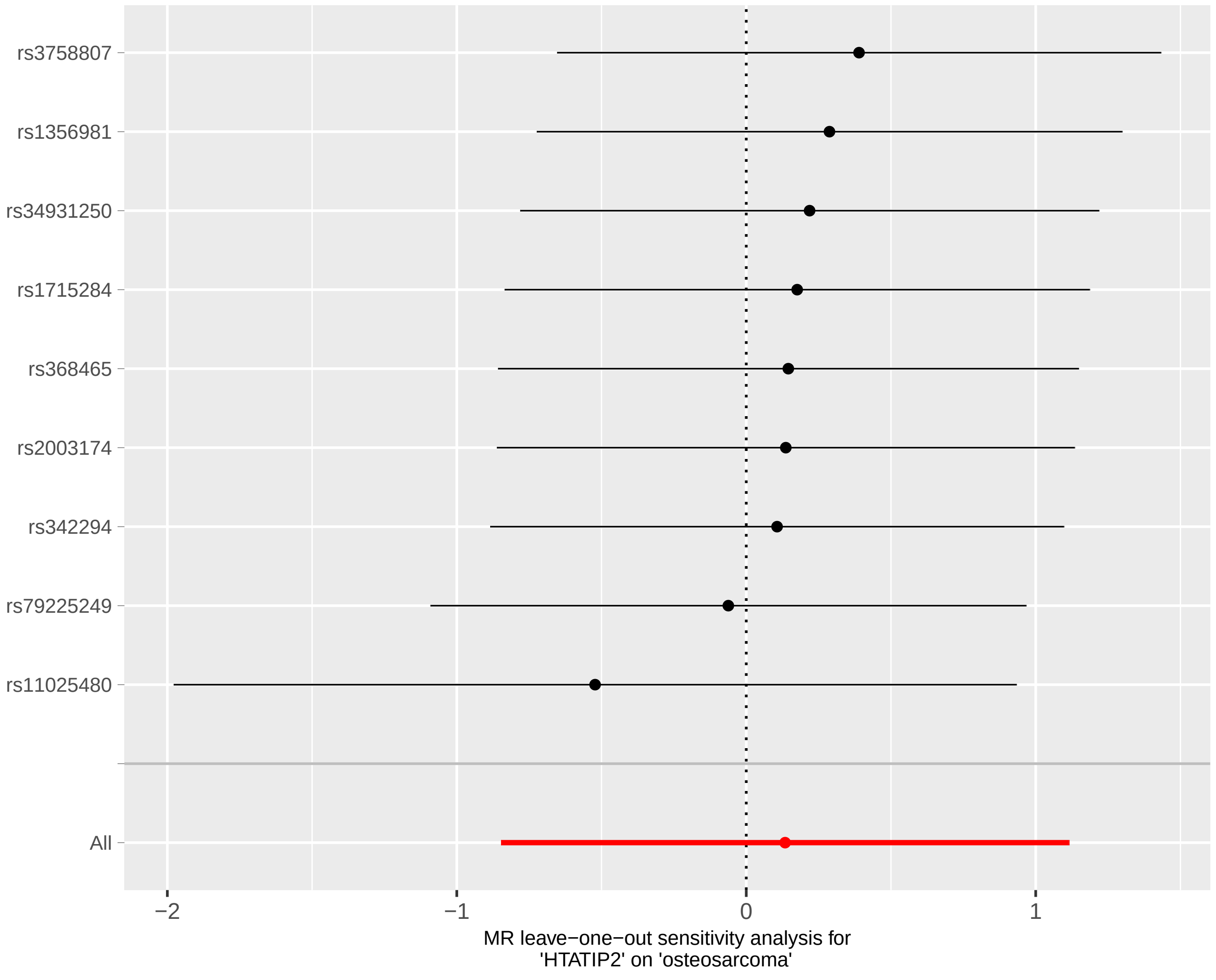
rs3758807
rs1356981
rs34931250
rs1715284
rs368465
rs2003174
rs342294
rs79225249
rs11025480
All
−2
−1
0
1
MR leave−one−out sensitivity analysis for
'HTATIP2' on 'osteosarcoma'

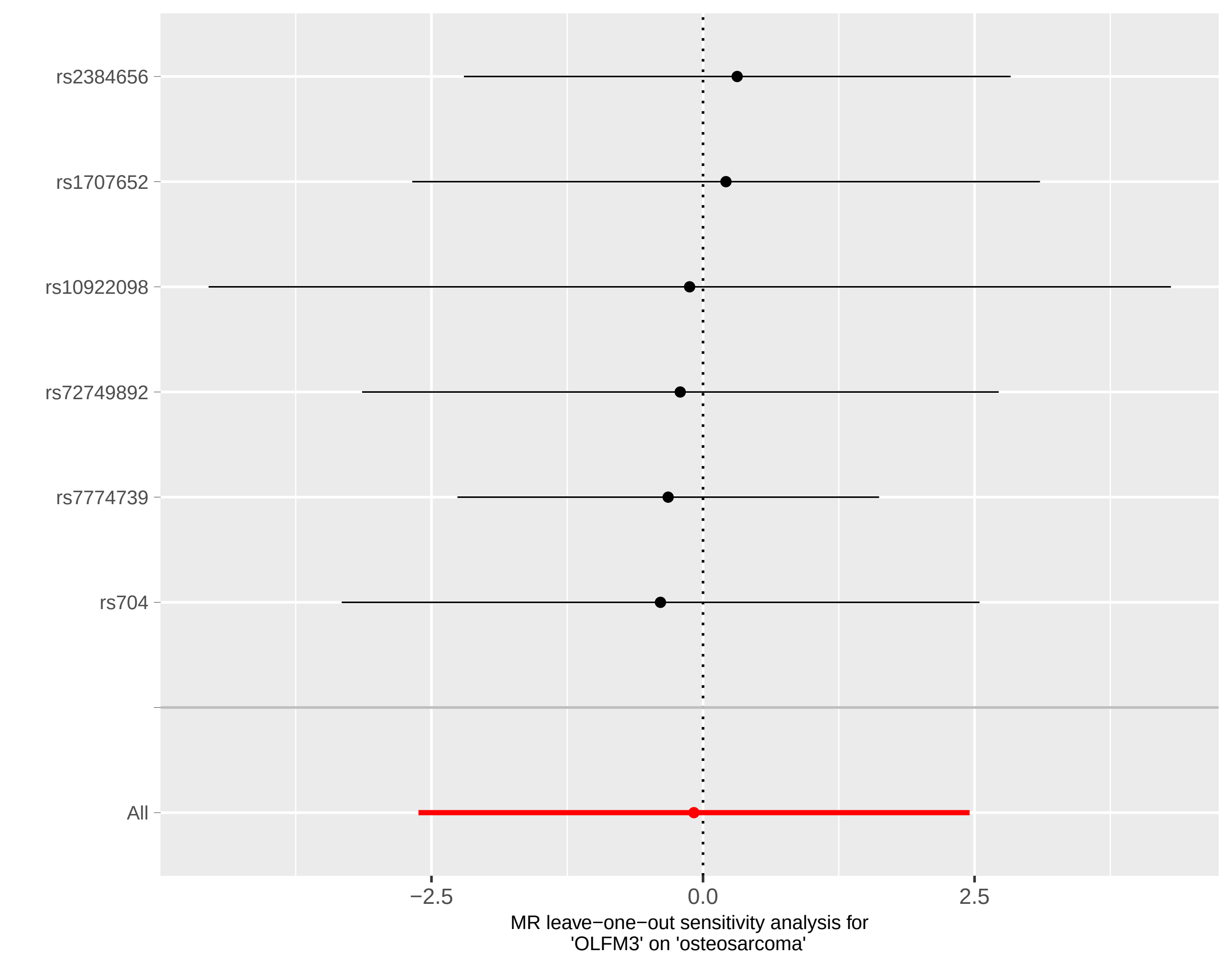
rs2384656
rs1707652
rs10922098
rs72749892
rs7774739
rs704
All
−2.5
0.0
2.5
MR leave−one−out sensitivity analysis for
'OLFM3' on 'osteosarcoma'

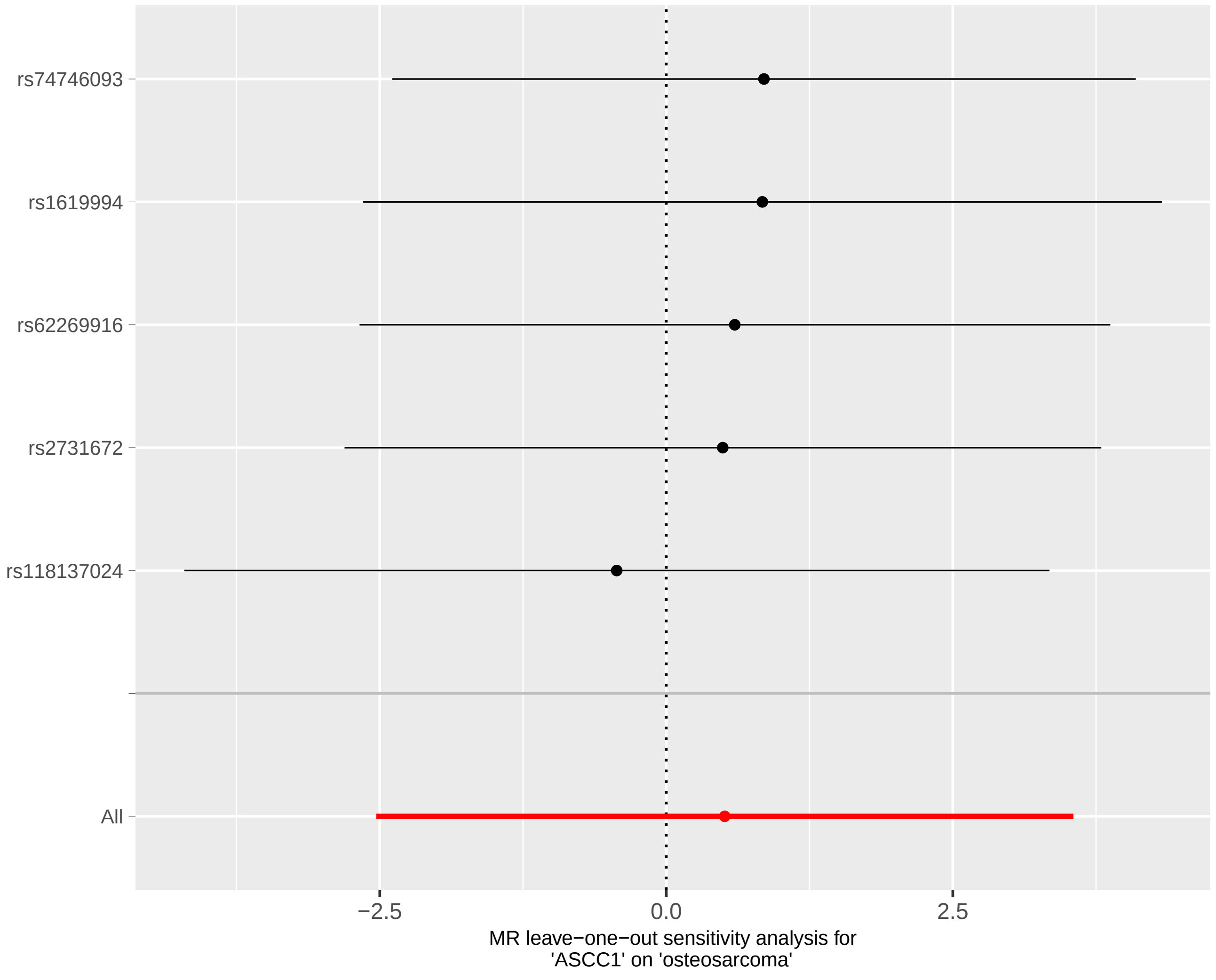
rs74746093
rs1619994
rs62269916
rs2731672
rs118137024
All
−2.5
0.0
2.5
MR leave−one−out sensitivity analysis for
'ASCC1' on 'osteosarcoma'

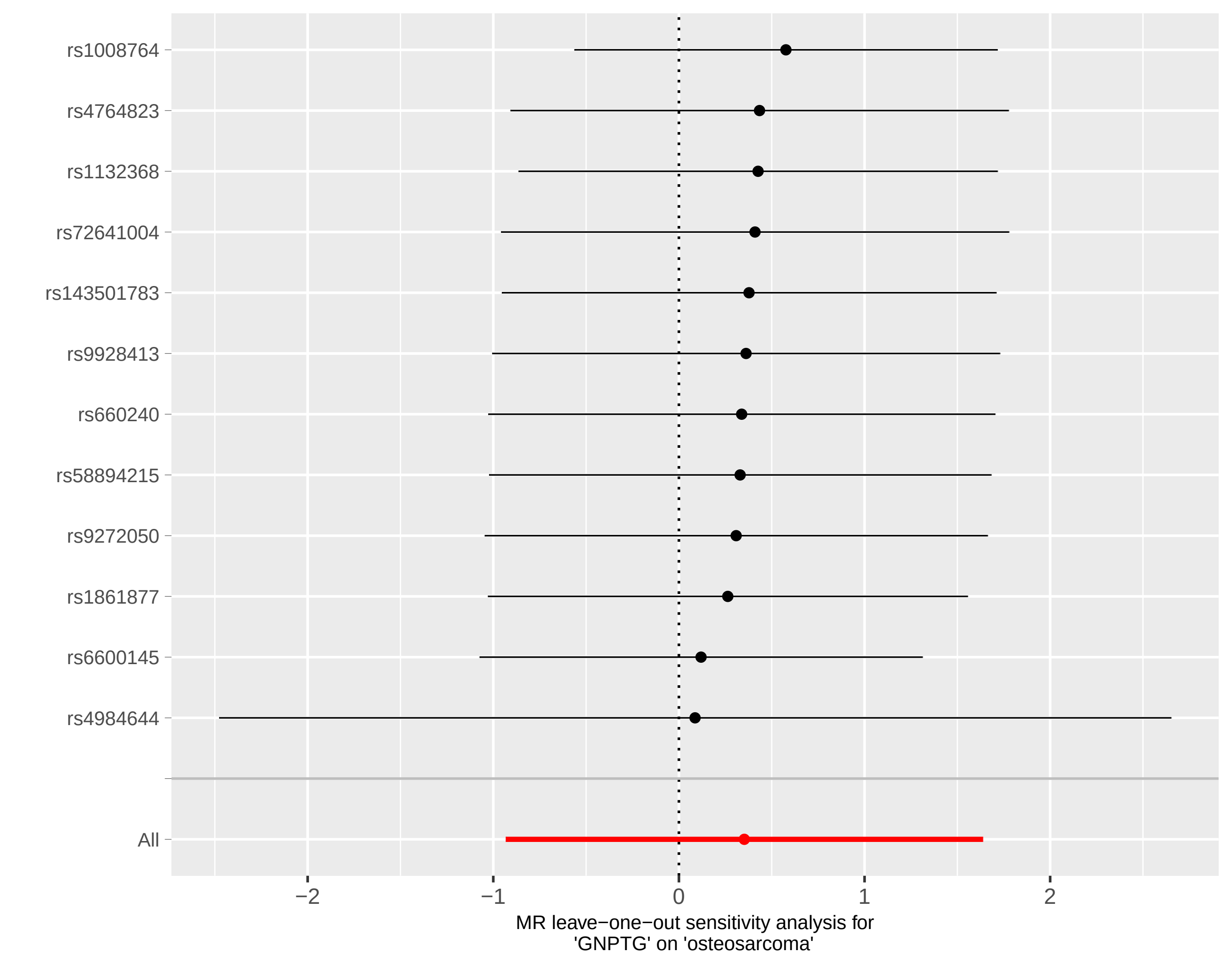

rs1008764
rs4764823
rs1132368
rs72641004
rs143501783
rs9928413
rs660240
rs58894215
rs9272050
rs1861877
rs6600145
rs4984644
All
−2
−1
0
1
2
MR leave−one−out sensitivity analysis for
'GNPTG' on 'osteosarcoma'

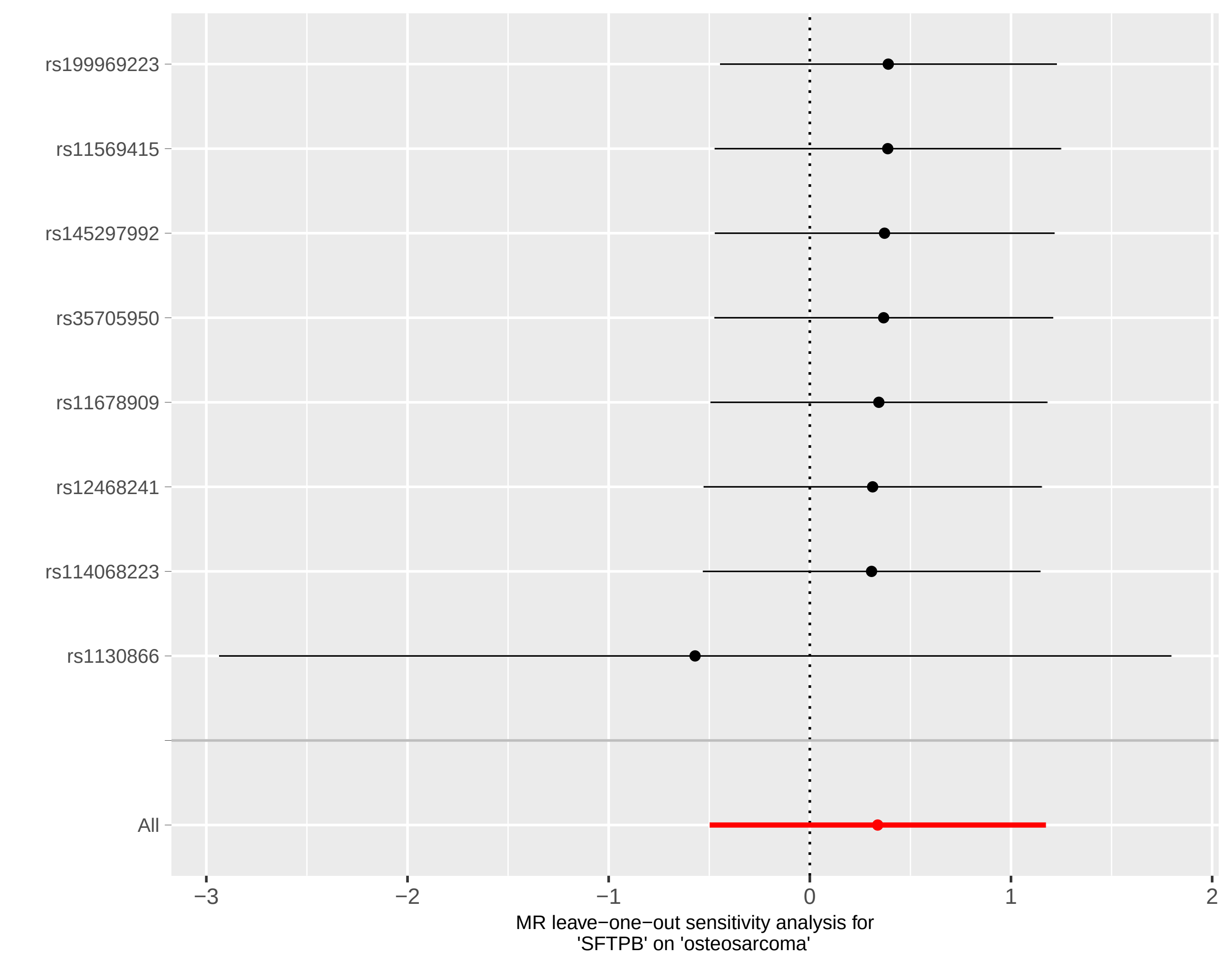
rs199969223
rs11569415
rs145297992
rs35705950
rs11678909
rs12468241
rs114068223
rs1130866
All
−3
−2
−1
0
1
2
MR leave−one−out sensitivity analysis for
'SFTPB' on 'osteosarcoma'

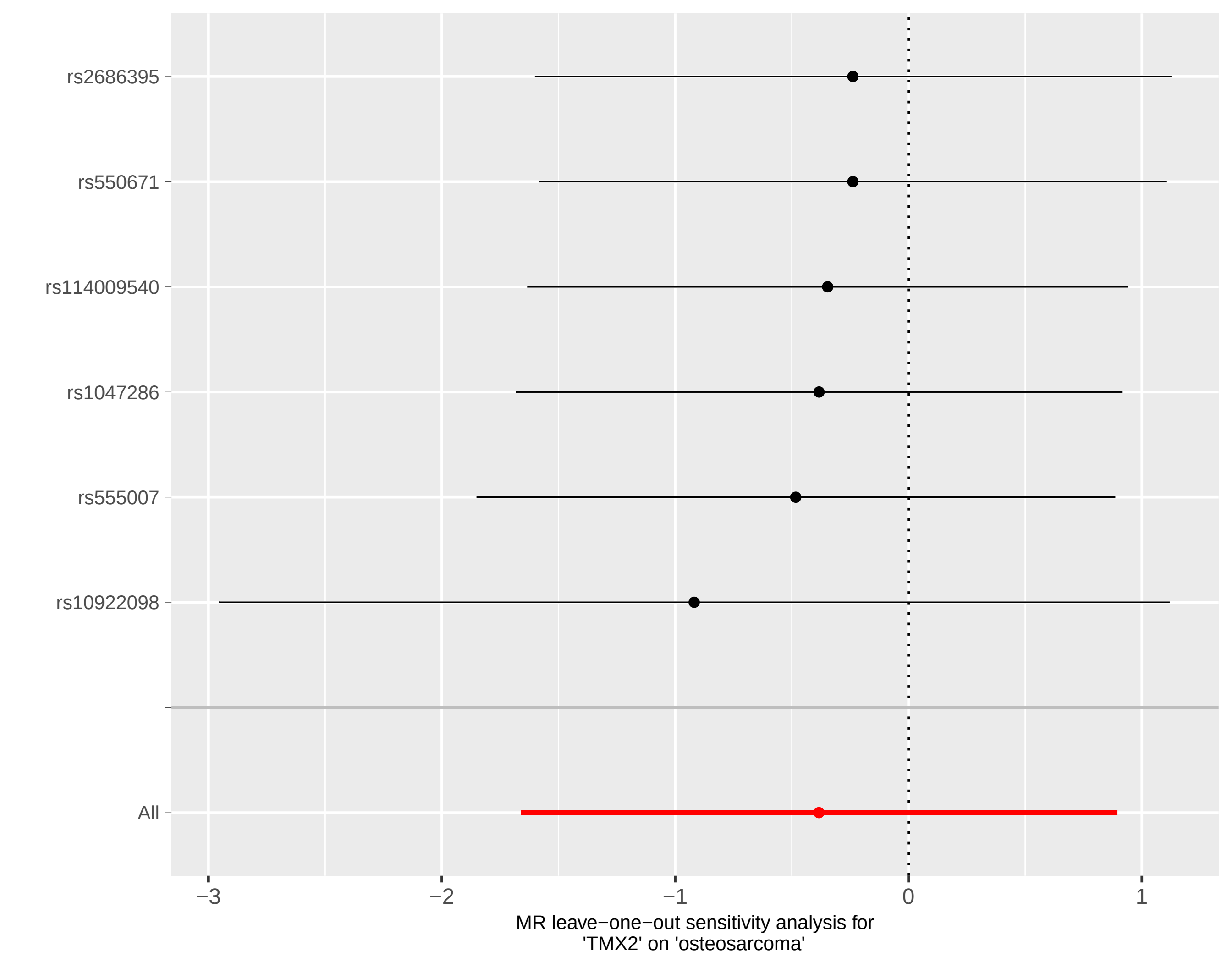

rs2686395
rs550671
rs114009540
rs1047286
rs555007
rs10922098
All
−3
−2
−1
0
1
MR leave−one−out sensitivity analysis for
'TMX2' on 'osteosarcoma'

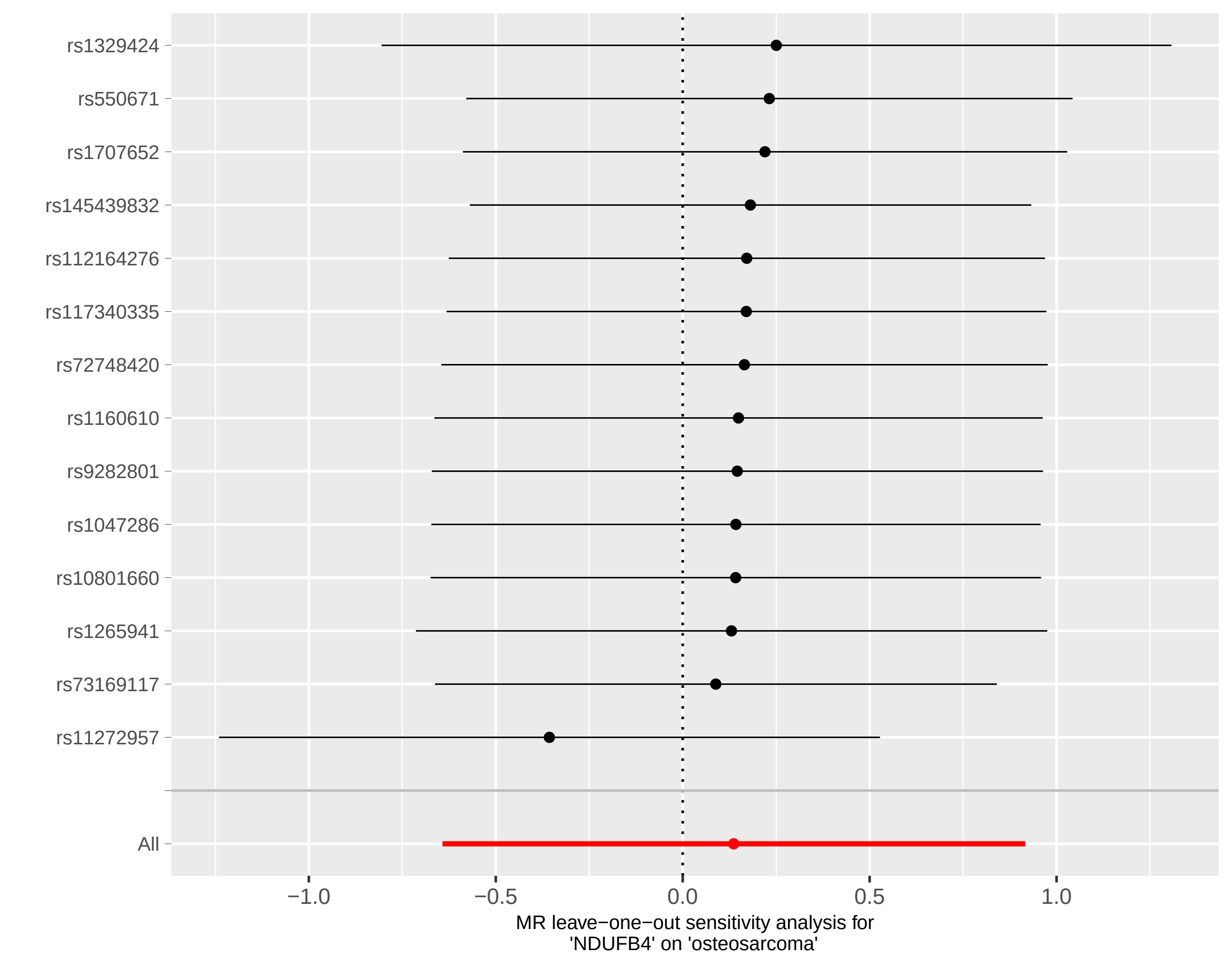
rs1329424
rs550671
rs1707652
rs145439832
rs112164276
rs117340335
rs72748420
rs1160610
rs9282801
rs1047286
rs10801660
rs1265941
rs73169117
rs11272957
All
−1.0
−0.5
0.0
0.5
1.0
MR leave−one−out sensitivity analysis for
'NDUFB4' on 'osteosarcoma'

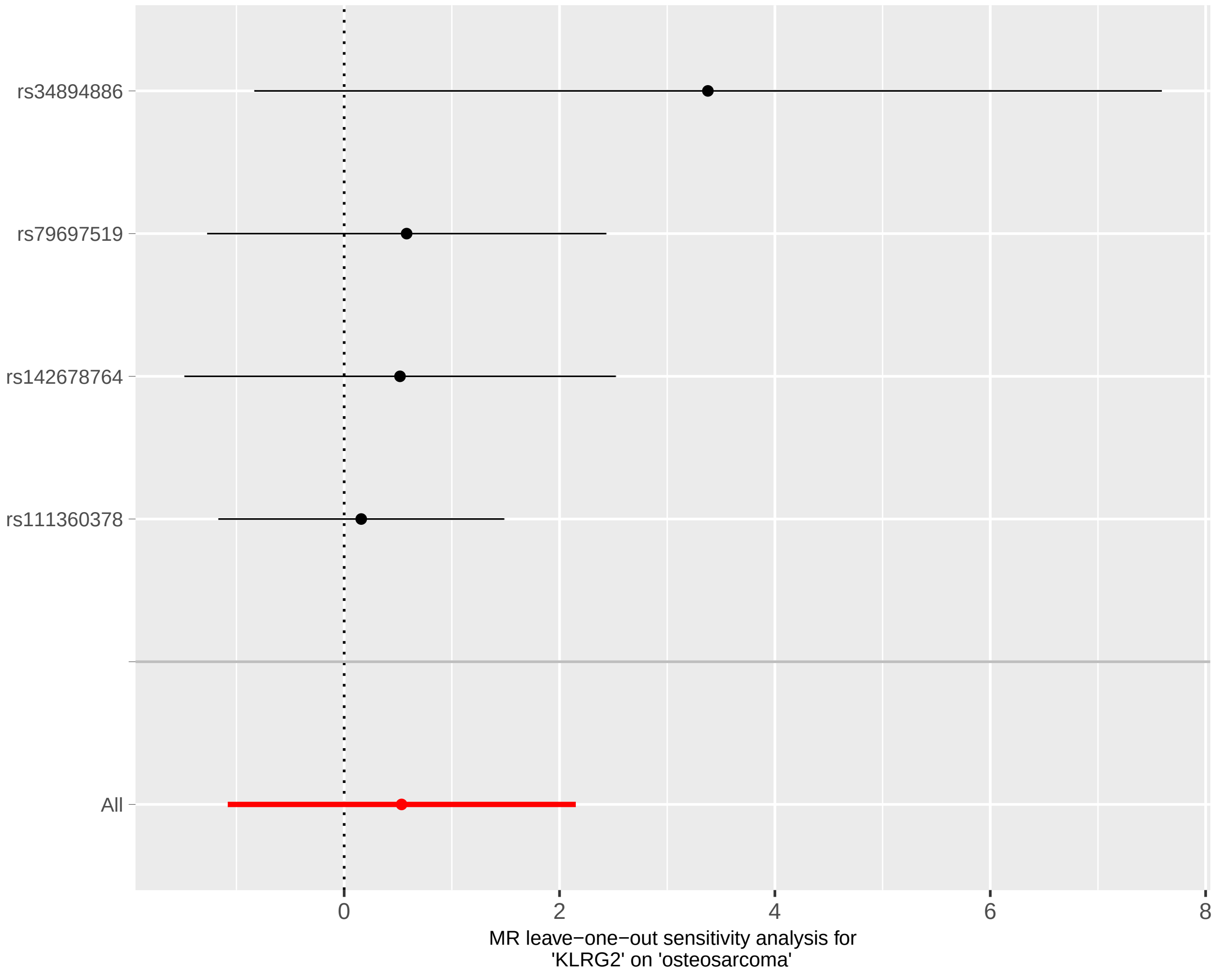
rs34894886
rs79697519
rs142678764
rs111360378
All
0
2
4
6
8
MR leave−one−out sensitivity analysis for
'KLRG2' on 'osteosarcoma'

Insufficient number of SNPs

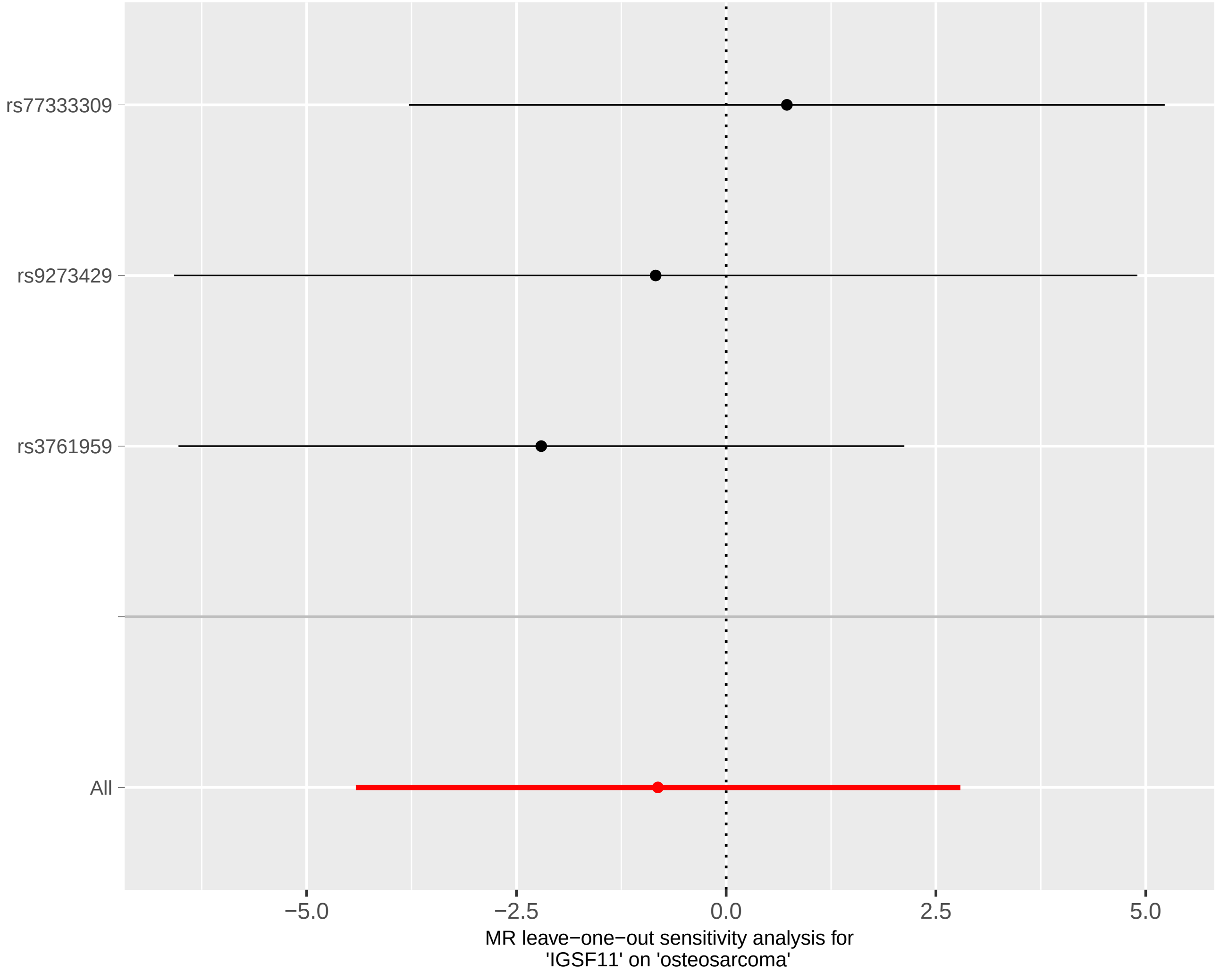
rs77333309
rs9273429
rs3761959
All
−5.0
−2.5
0.0
2.5
5.0
MR leave−one−out sensitivity analysis for
'IGSF11' on 'osteosarcoma'

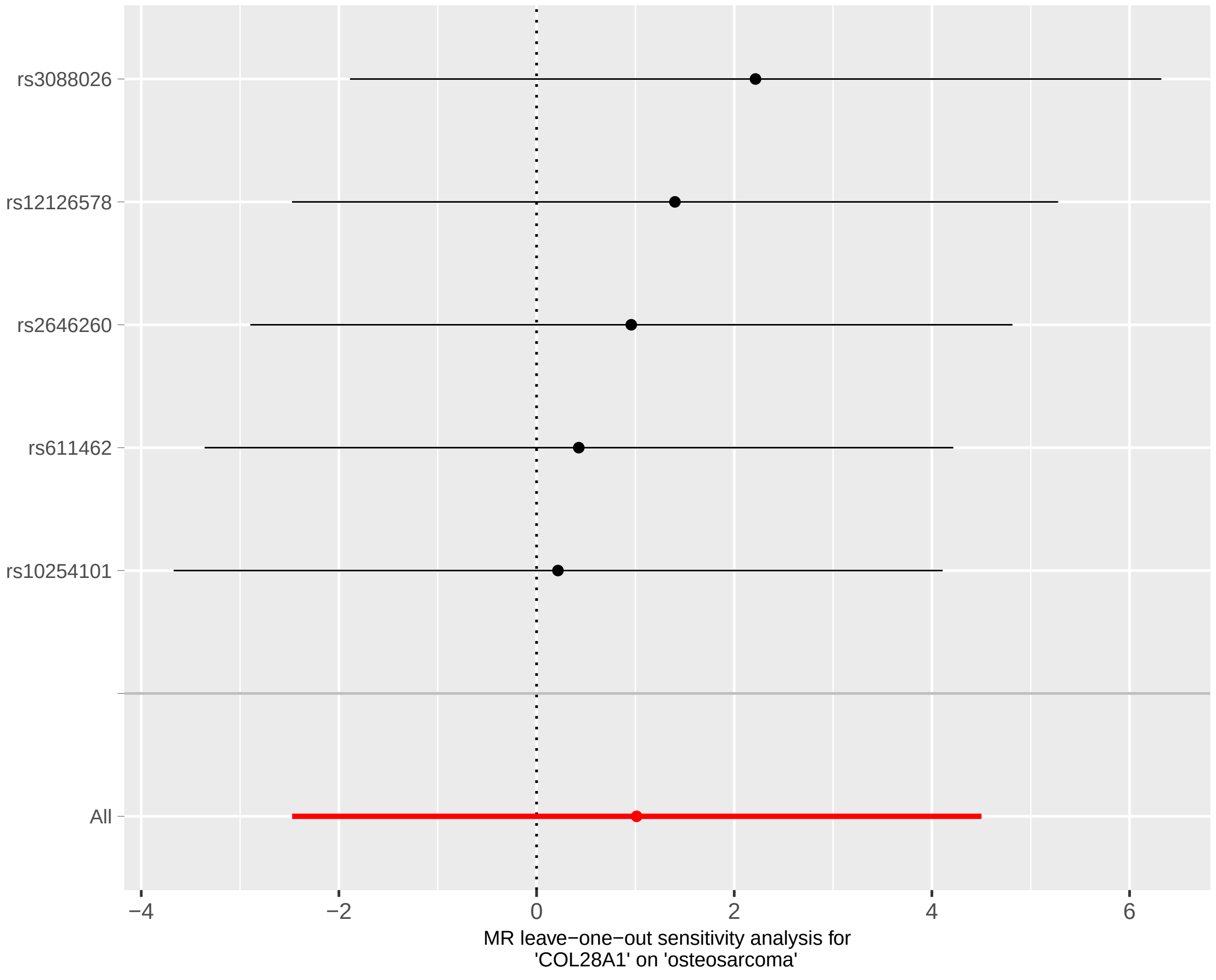

rs3088026
rs12126578
rs2646260
rs611462
rs10254101
All
−4
−2
0
2
4
6
MR leave−one−out sensitivity analysis for
'COL28A1' on 'osteosarcoma'

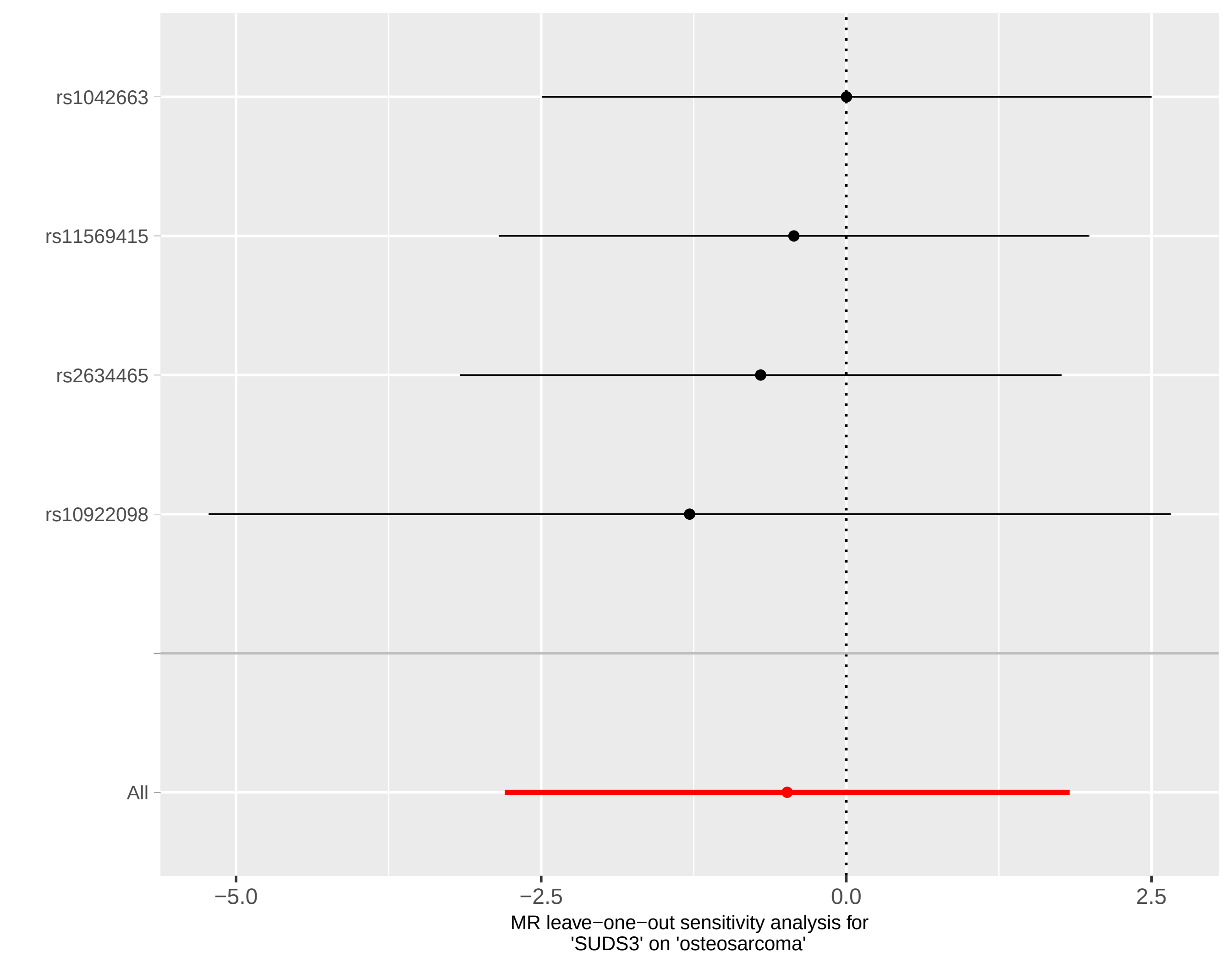

rs1042663
rs11569415
rs2634465
rs10922098
All
−5.0
−2.5
0.0
2.5
MR leave−one−out sensitivity analysis for
'SUDS3' on 'osteosarcoma'

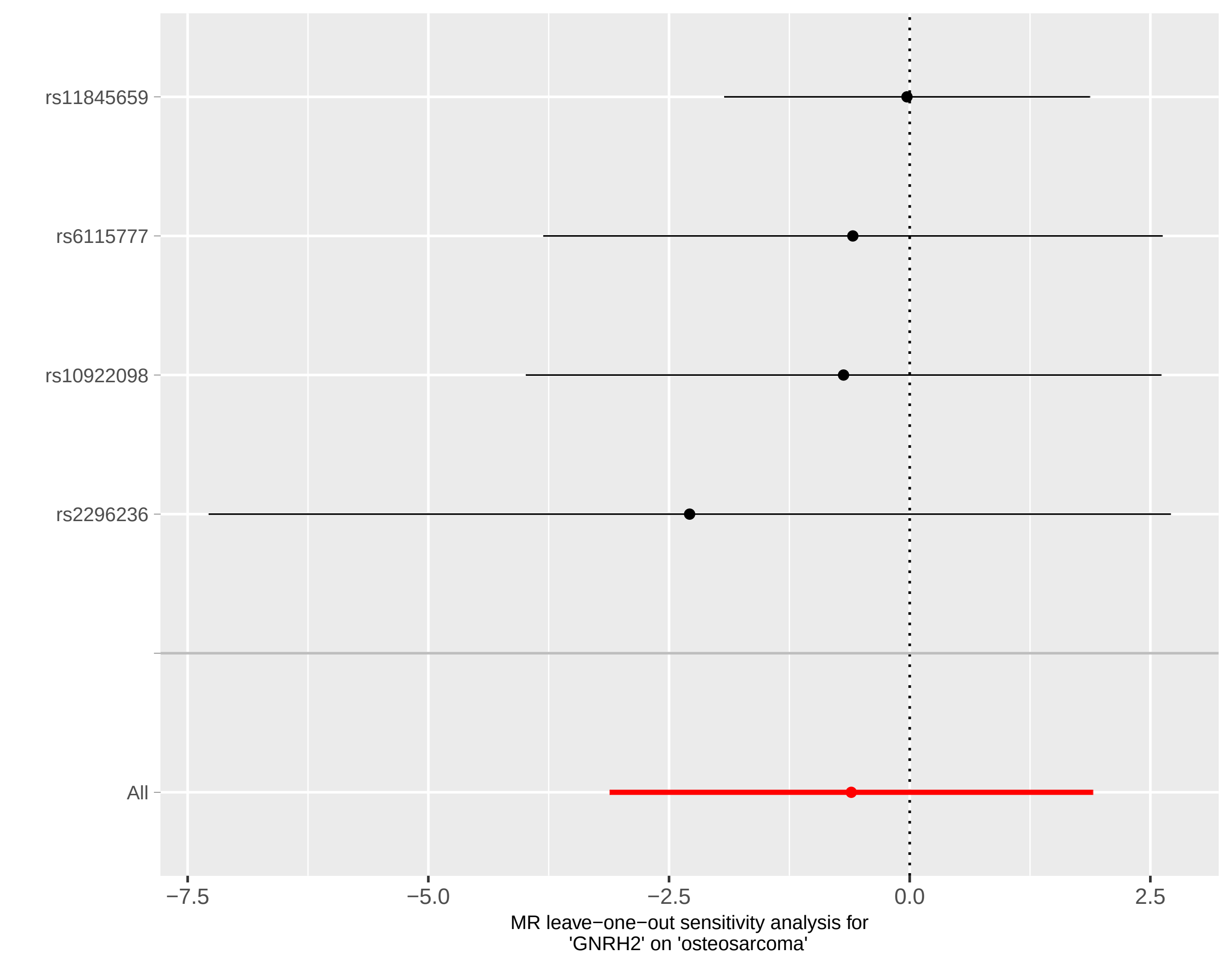

rs11845659
rs6115777
rs10922098
rs2296236
All
−7.5
−5.0
−2.5
0.0
2.5
MR leave−one−out sensitivity analysis for
'GNRH2' on 'osteosarcoma'

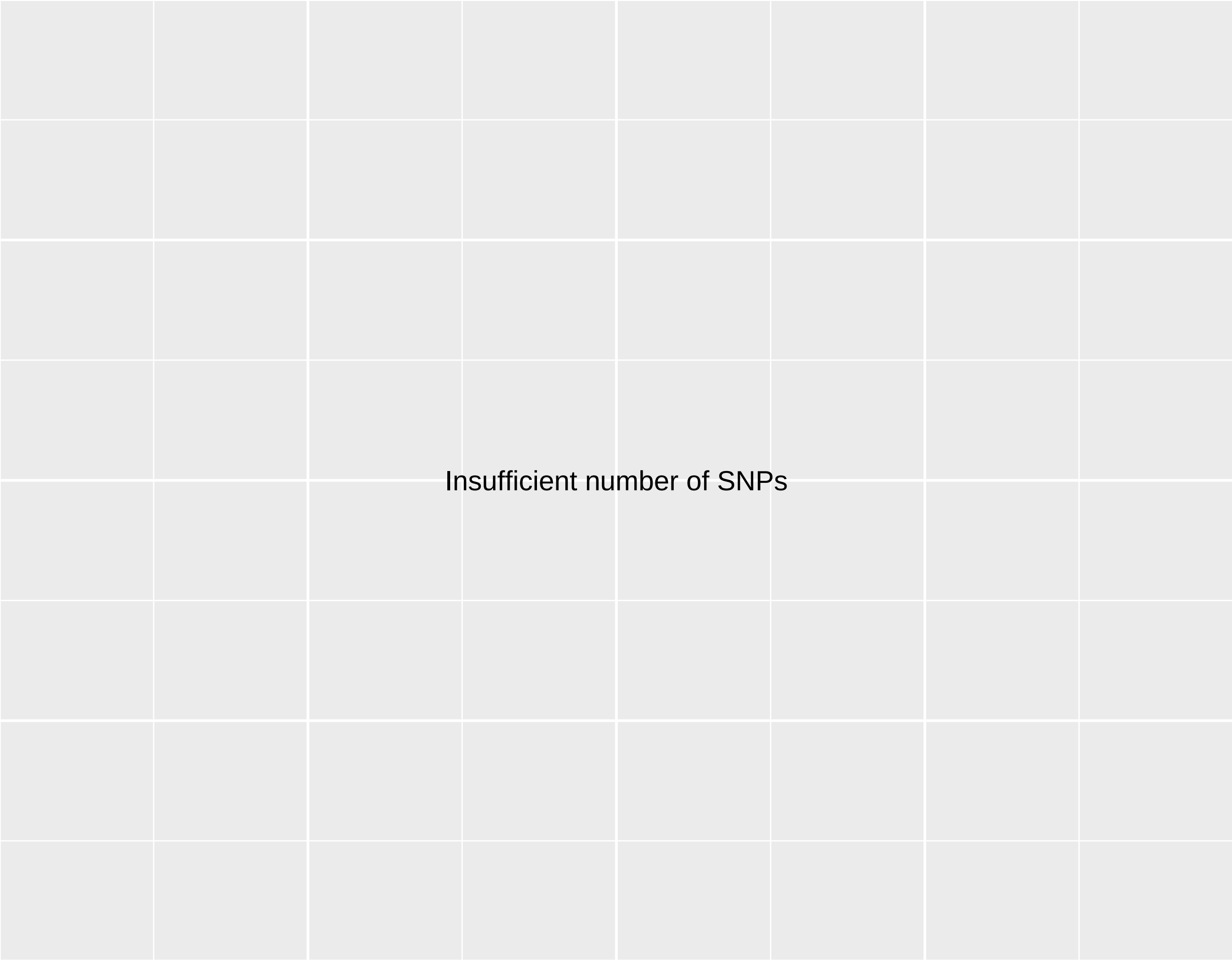
Insufficient number of SNPs

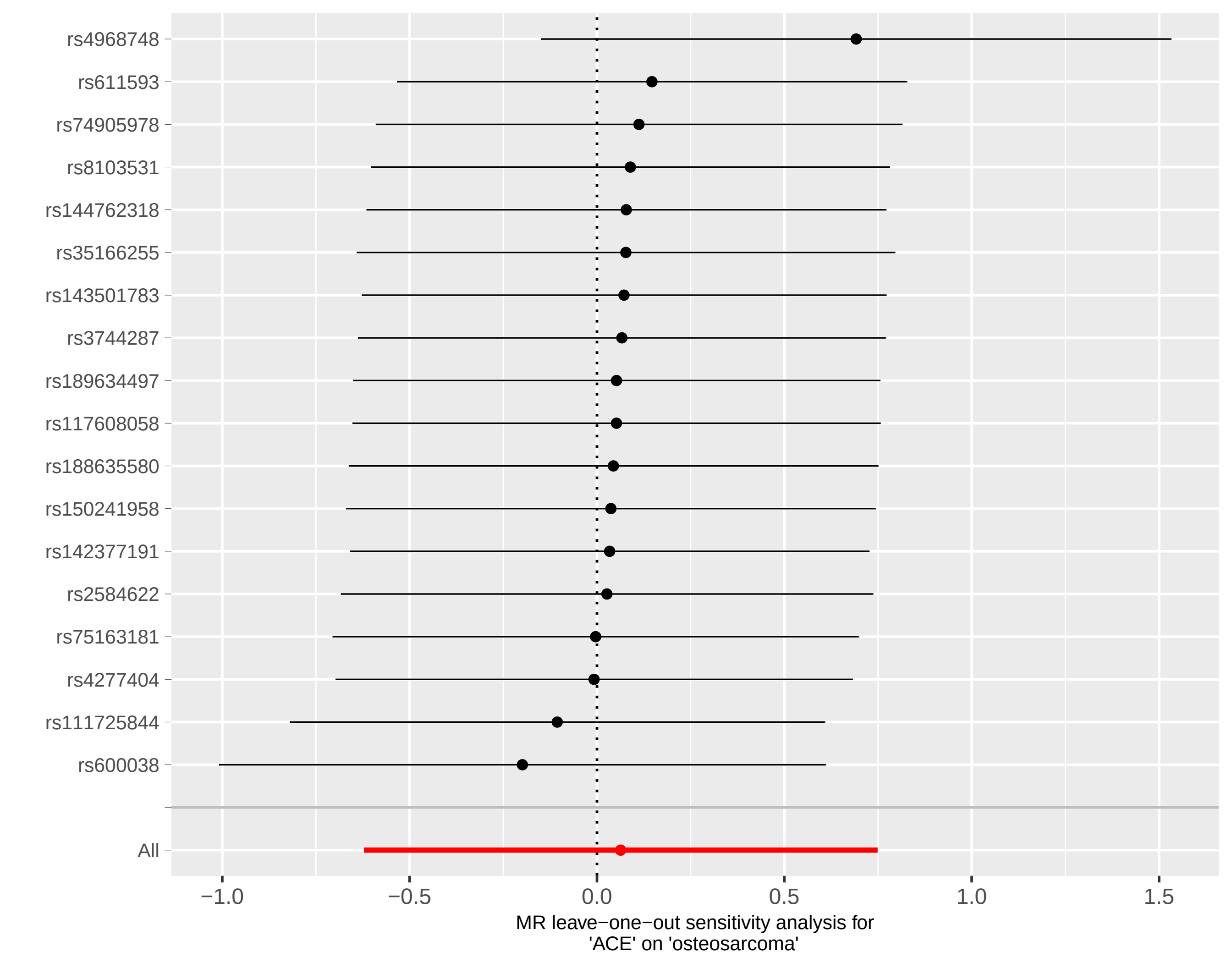
rs4968748
rs611593
rs74905978
rs8103531
rs144762318
rs35166255
rs143501783
rs3744287
rs189634497
rs117608058
rs188635580
rs150241958
rs142377191
rs2584622
rs75163181
rs4277404
rs111725844
rs600038
All
−1.0
−0.5
0.0
0.5
1.0
1.5
MR leave−one−out sensitivity analysis for
'ACE' on 'osteosarcoma'

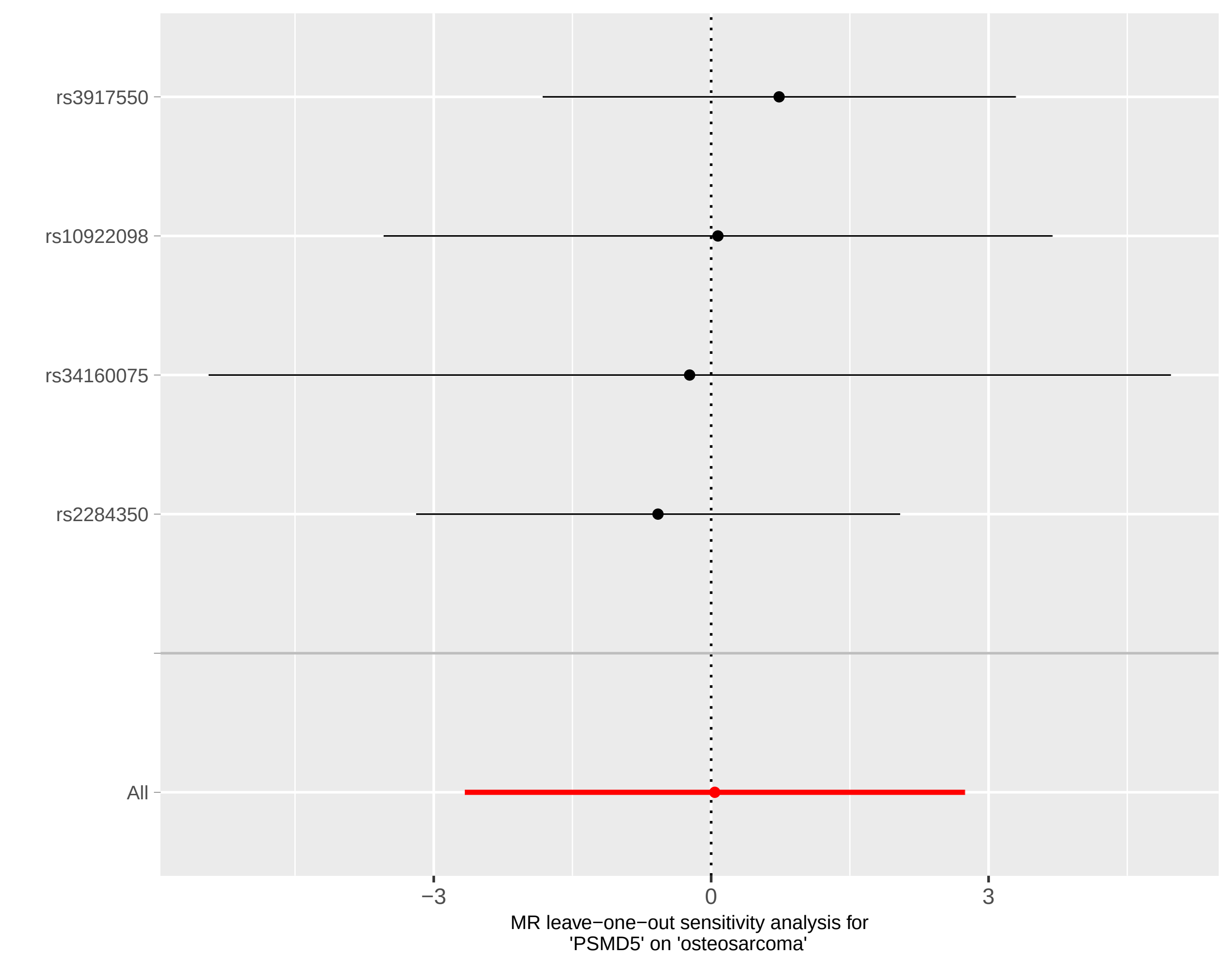

rs3917550
rs10922098
rs34160075
rs2284350
All
−3
0
3
MR leave−one−out sensitivity analysis for
'PSMD5' on 'osteosarcoma'

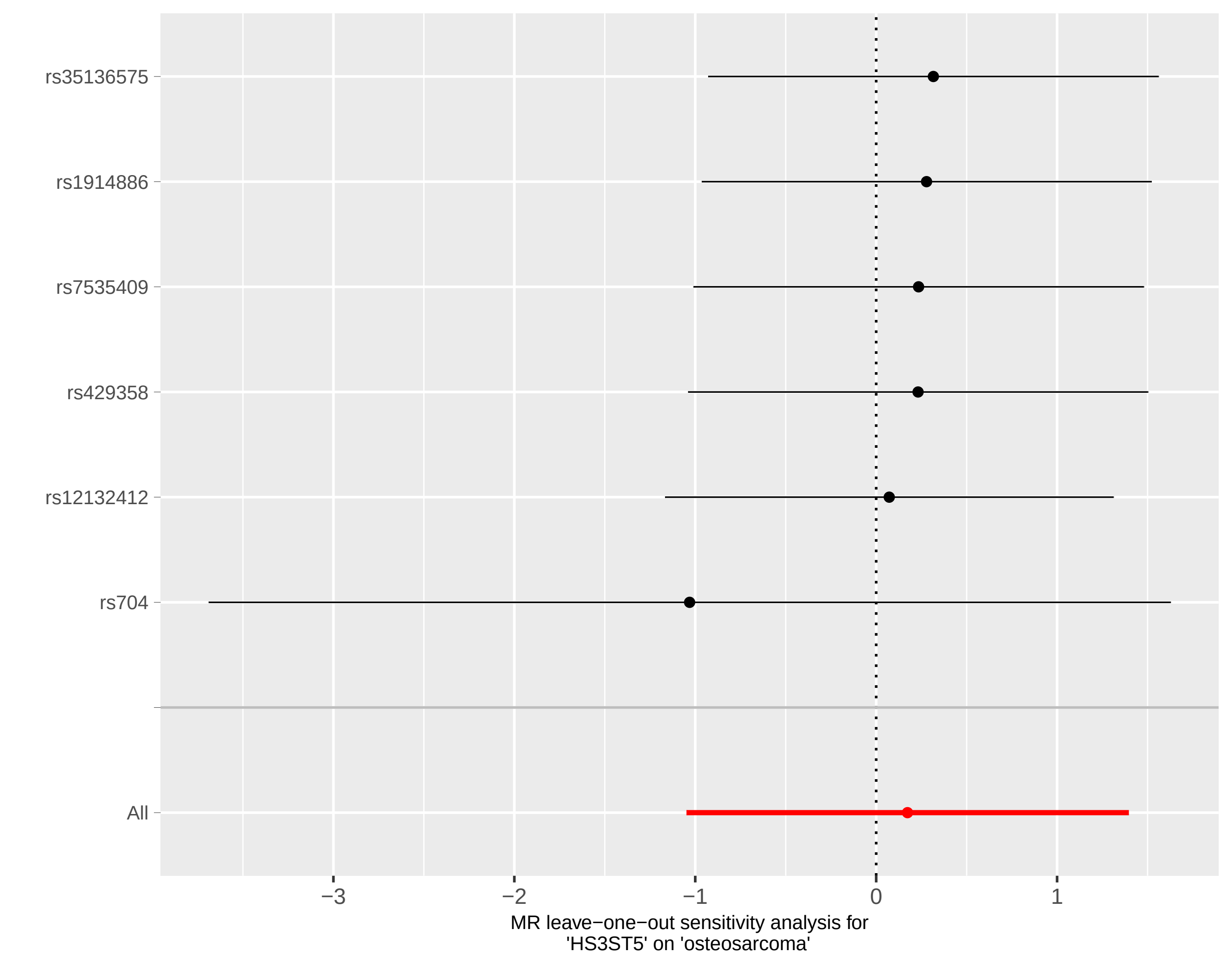
rs35136575
rs1914886
rs7535409
rs429358
rs12132412
rs704
All
−3
−2
−1
0
1
MR leave−one−out sensitivity analysis for
'HS3ST5' on 'osteosarcoma'

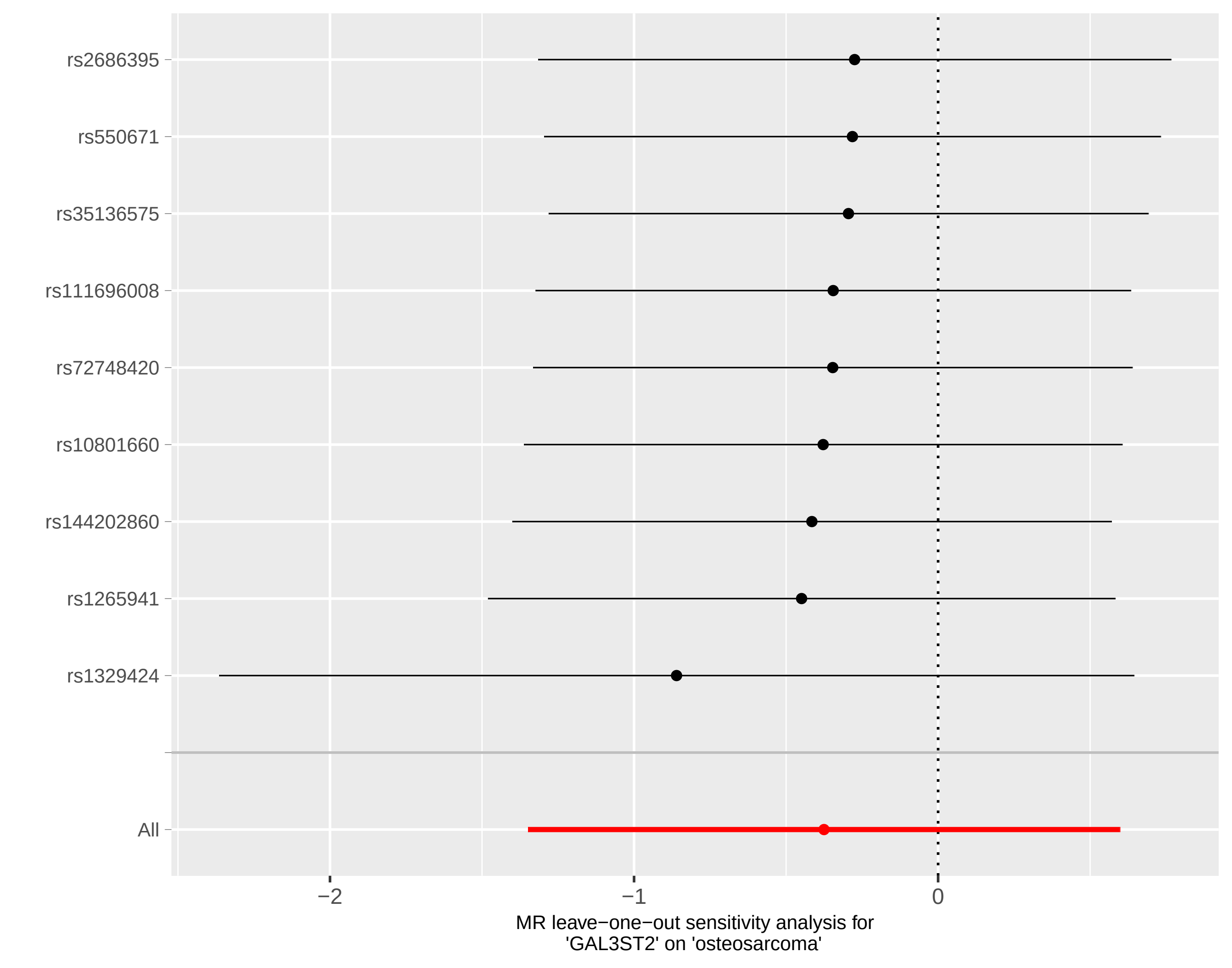
rs2686395
rs550671
rs35136575
rs111696008
rs72748420
rs10801660
rs144202860
rs1265941
rs1329424
All
−2
−1
0
MR leave−one−out sensitivity analysis for
'GAL3ST2' on 'osteosarcoma'

Insufficient number of SNPs

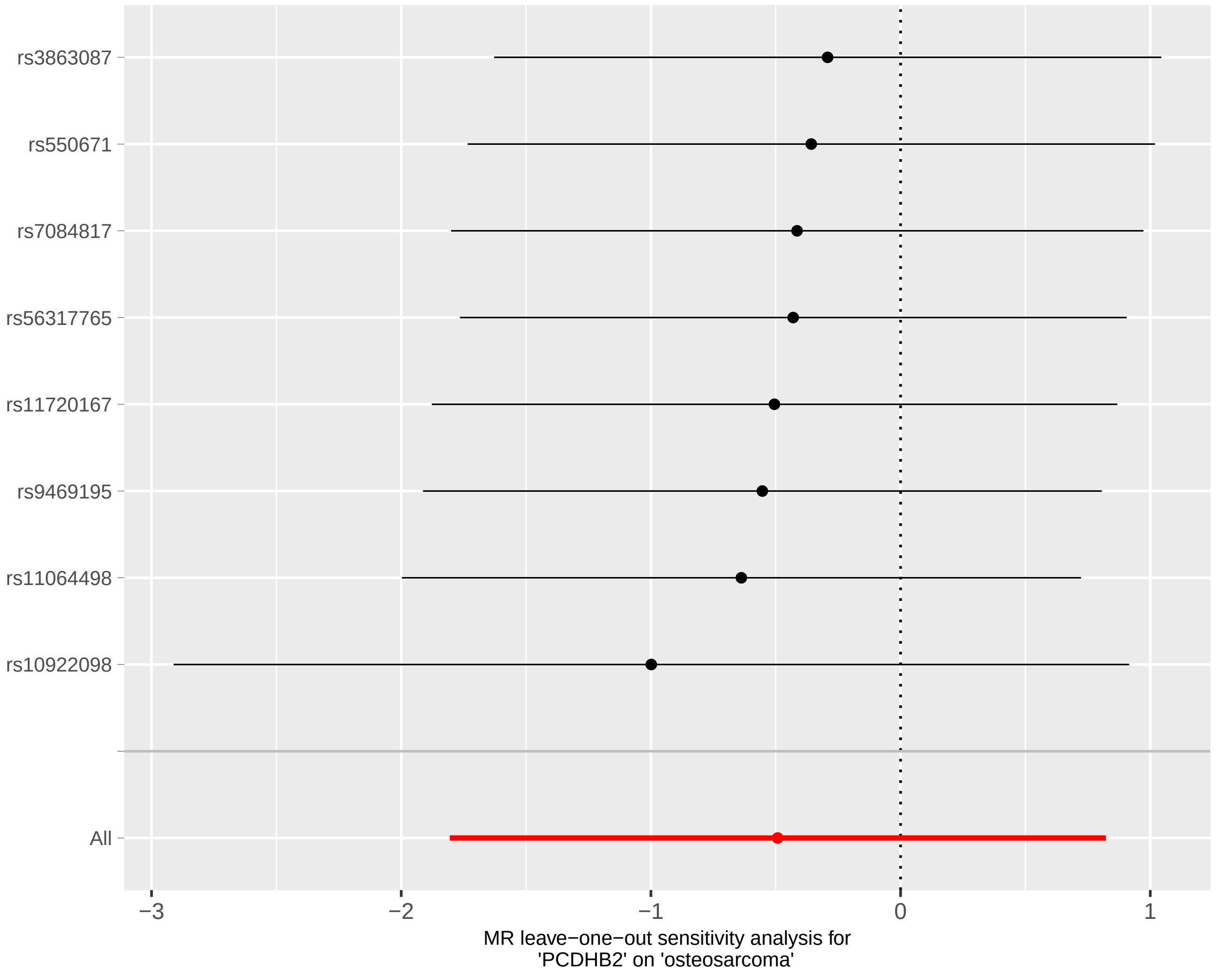
rs3863087
rs550671
rs7084817
rs56317765
rs11720167
rs9469195
rs11064498
rs10922098
All
−3
−2
−1
0
1
MR leave−one−out sensitivity analysis for
'PCDHB2' on 'osteosarcoma'

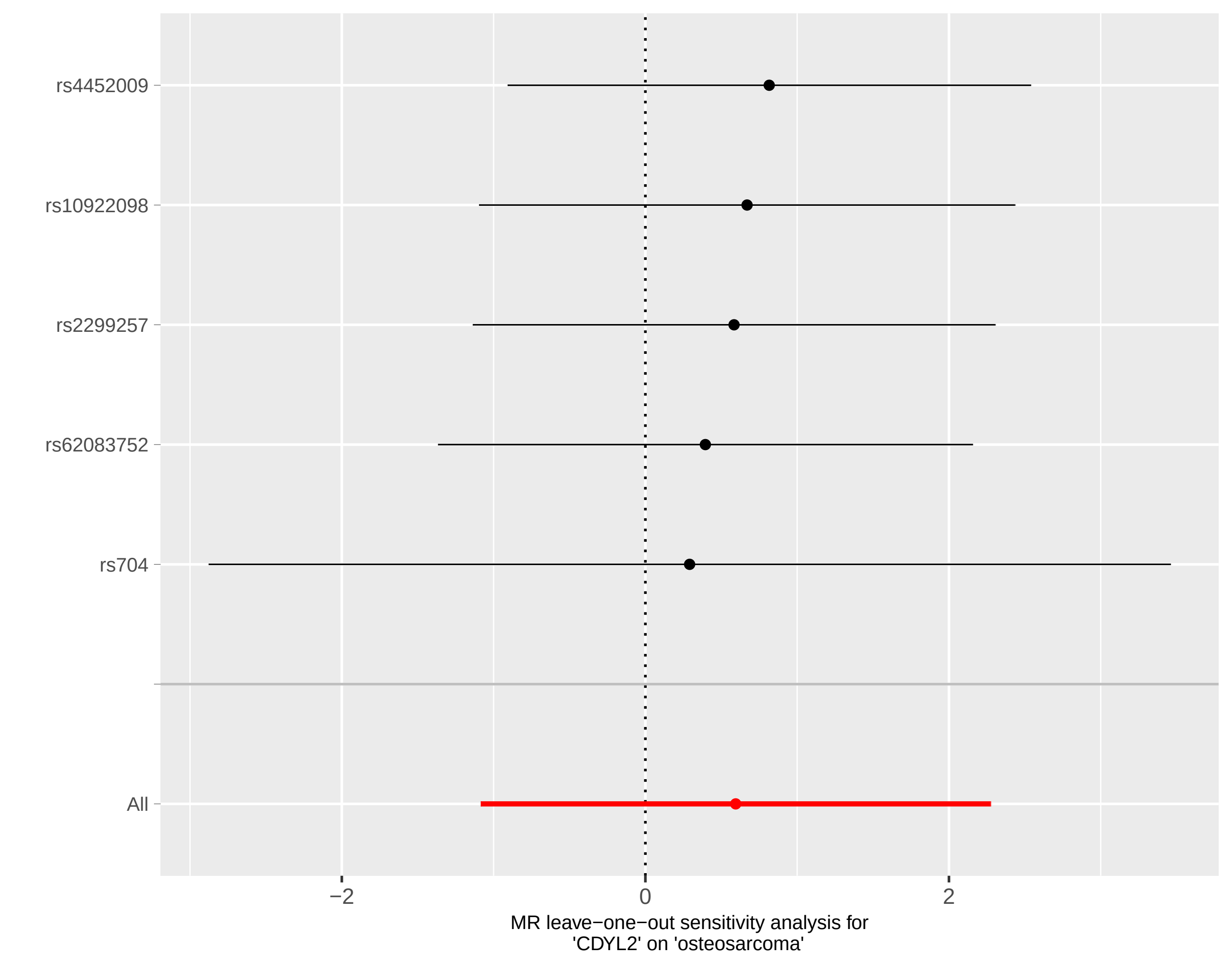

rs4452009
rs10922098
rs2299257
rs62083752
rs704
All
−2
0
2
MR leave−one−out sensitivity analysis for
'CDYL2' on 'osteosarcoma'

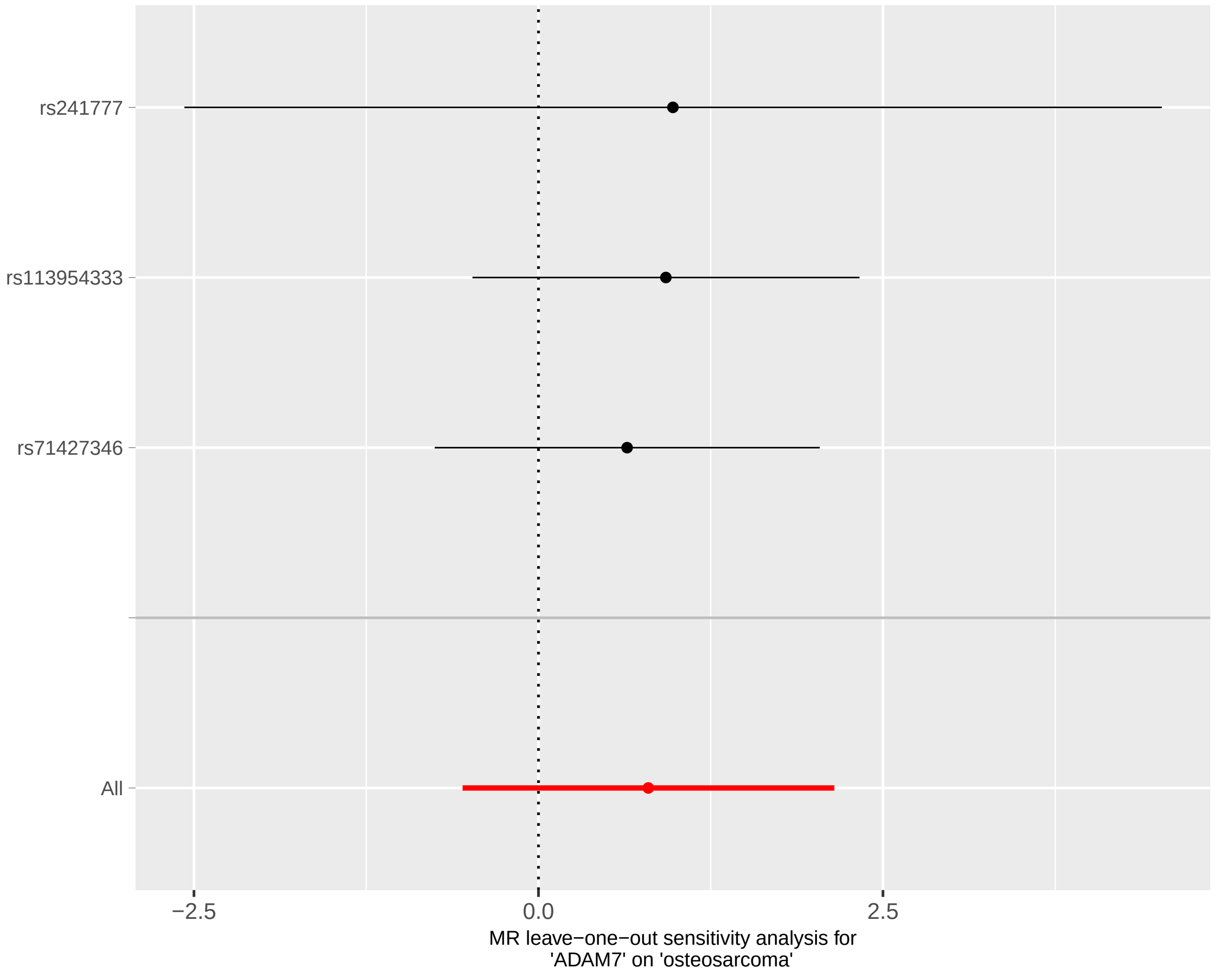
rs241777
rs113954333
rs71427346
All
−2.5
0.0
2.5
MR leave−one−out sensitivity analysis for
'ADAM7' on 'osteosarcoma'

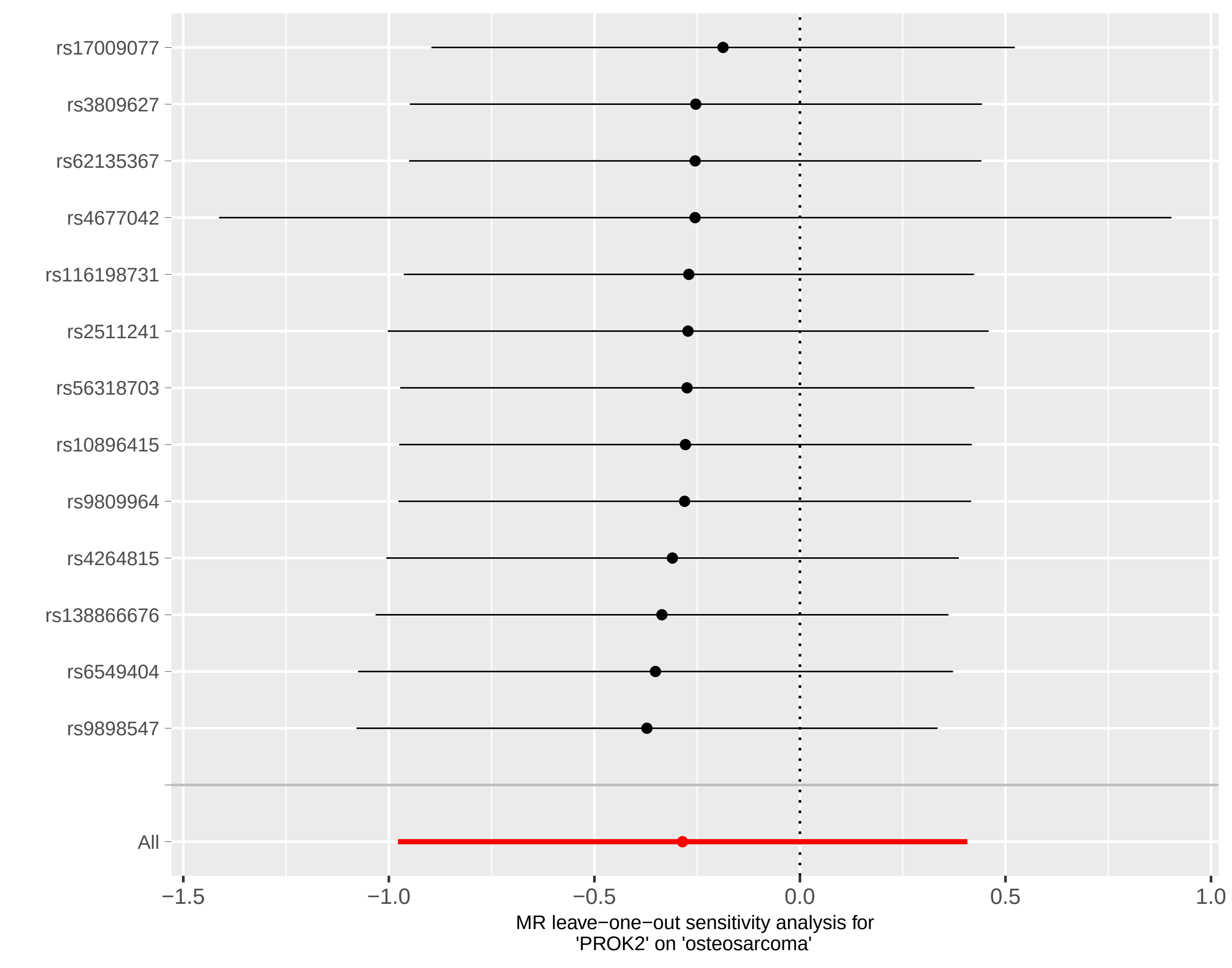

rs17009077
rs3809627
rs62135367
rs4677042
rs116198731
rs2511241
rs56318703
rs10896415
rs9809964
rs4264815
rs138866676
rs6549404
rs9898547
All
−1.5
−1.0
−0.5
0.0
0.5
1.0
MR leave−one−out sensitivity analysis for
'PROK2' on 'osteosarcoma'

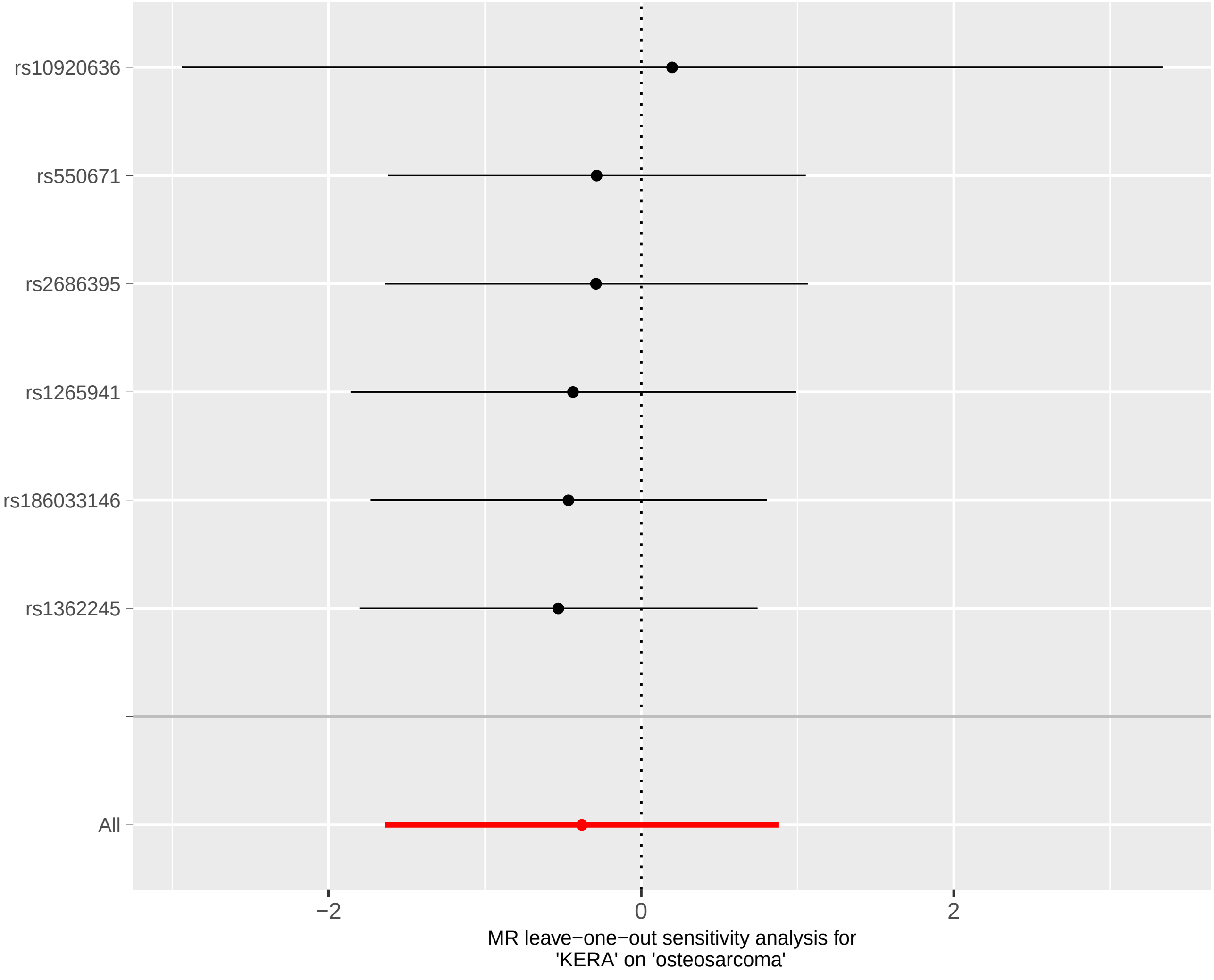
rs10920636
rs550671
rs2686395
rs1265941
rs186033146
rs1362245
All
−2
0
2
MR leave−one−out sensitivity analysis for
'KERA' on 'osteosarcoma'

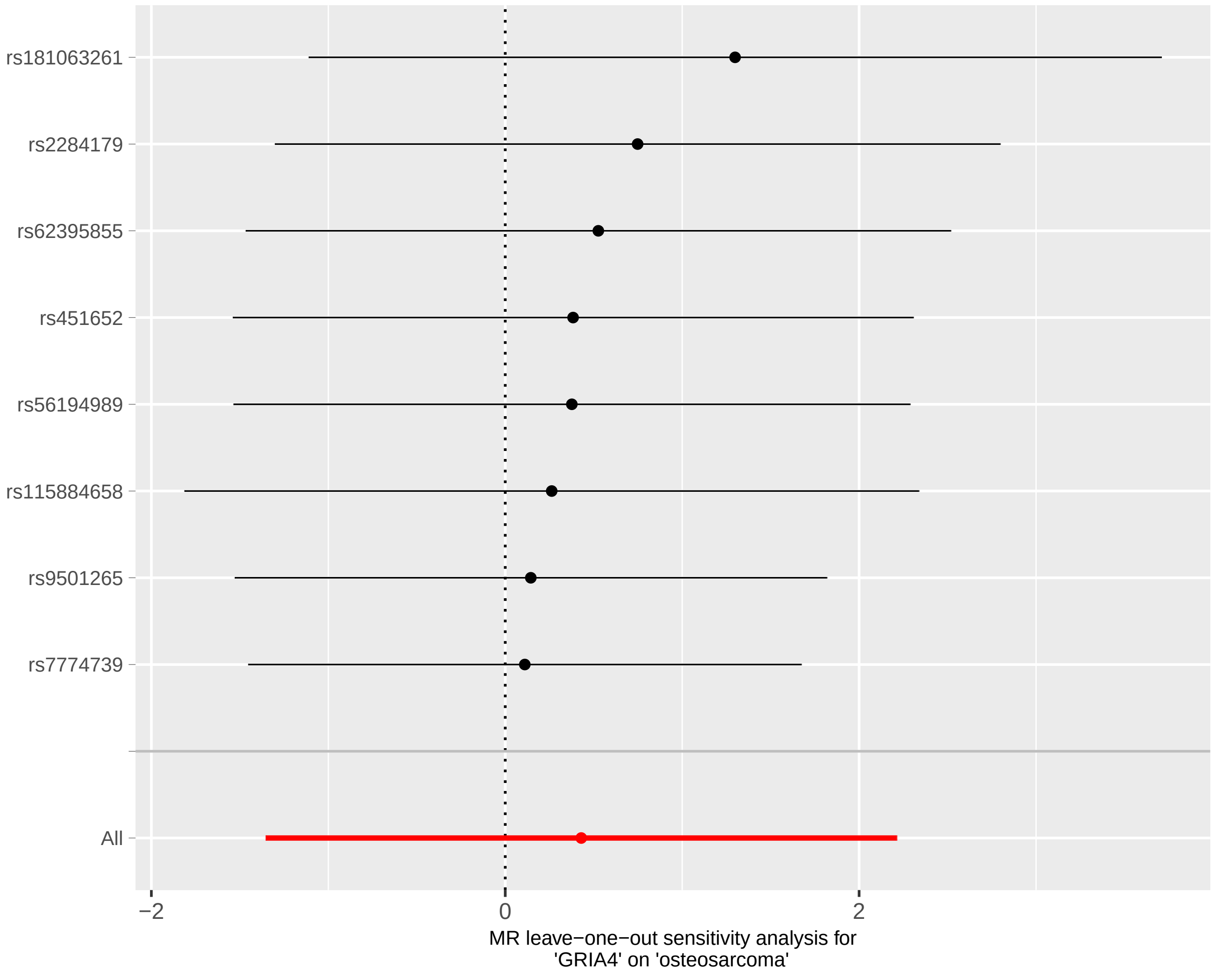

rs181063261
rs2284179
rs62395855
rs451652
rs56194989
rs115884658
rs9501265
rs7774739
All
−2
0
2
MR leave−one−out sensitivity analysis for
'GRIA4' on 'osteosarcoma'

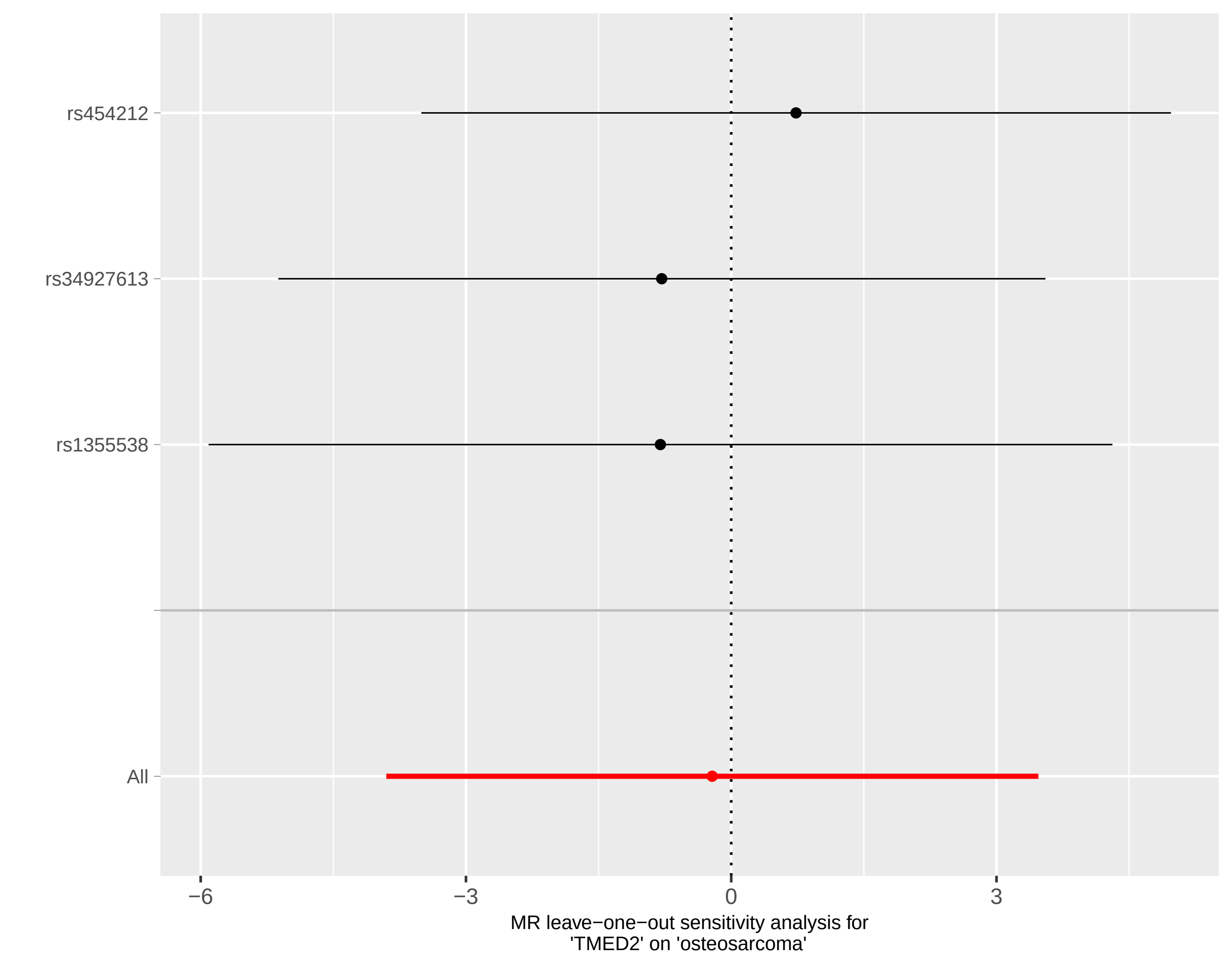
rs454212
rs34927613
rs1355538
All
−6
−3
0
3
MR leave−one−out sensitivity analysis for
'TMED2' on 'osteosarcoma'

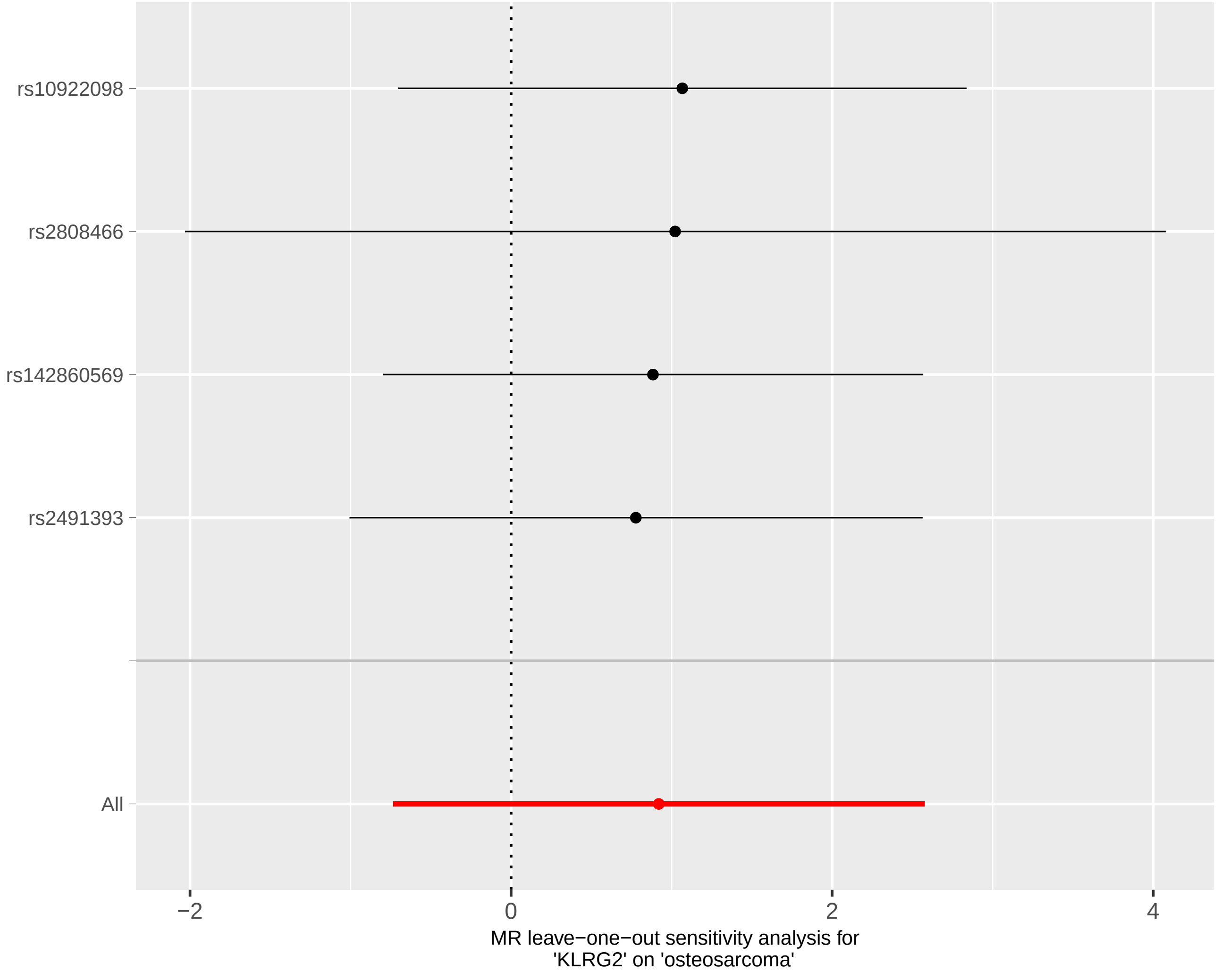
rs10922098
rs2808466
rs142860569
rs2491393
All
−2
0
2
4
MR leave−one−out sensitivity analysis for
'KLRG2' on 'osteosarcoma'

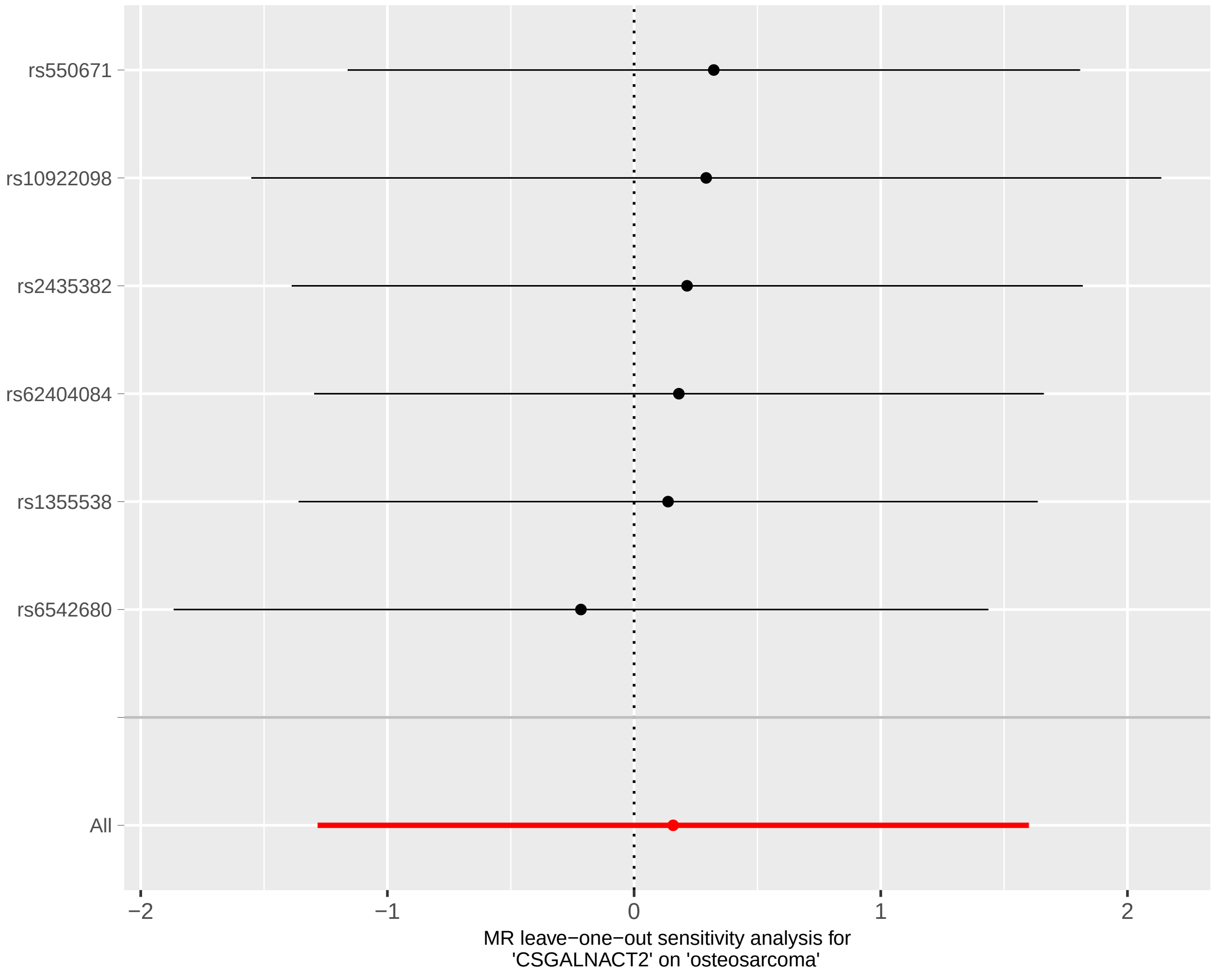
rs550671
rs10922098
rs2435382
rs62404084
rs1355538
rs6542680
All
−2
−1
0
1
2
MR leave−one−out sensitivity analysis for
'CSGALNACT2' on 'osteosarcoma'

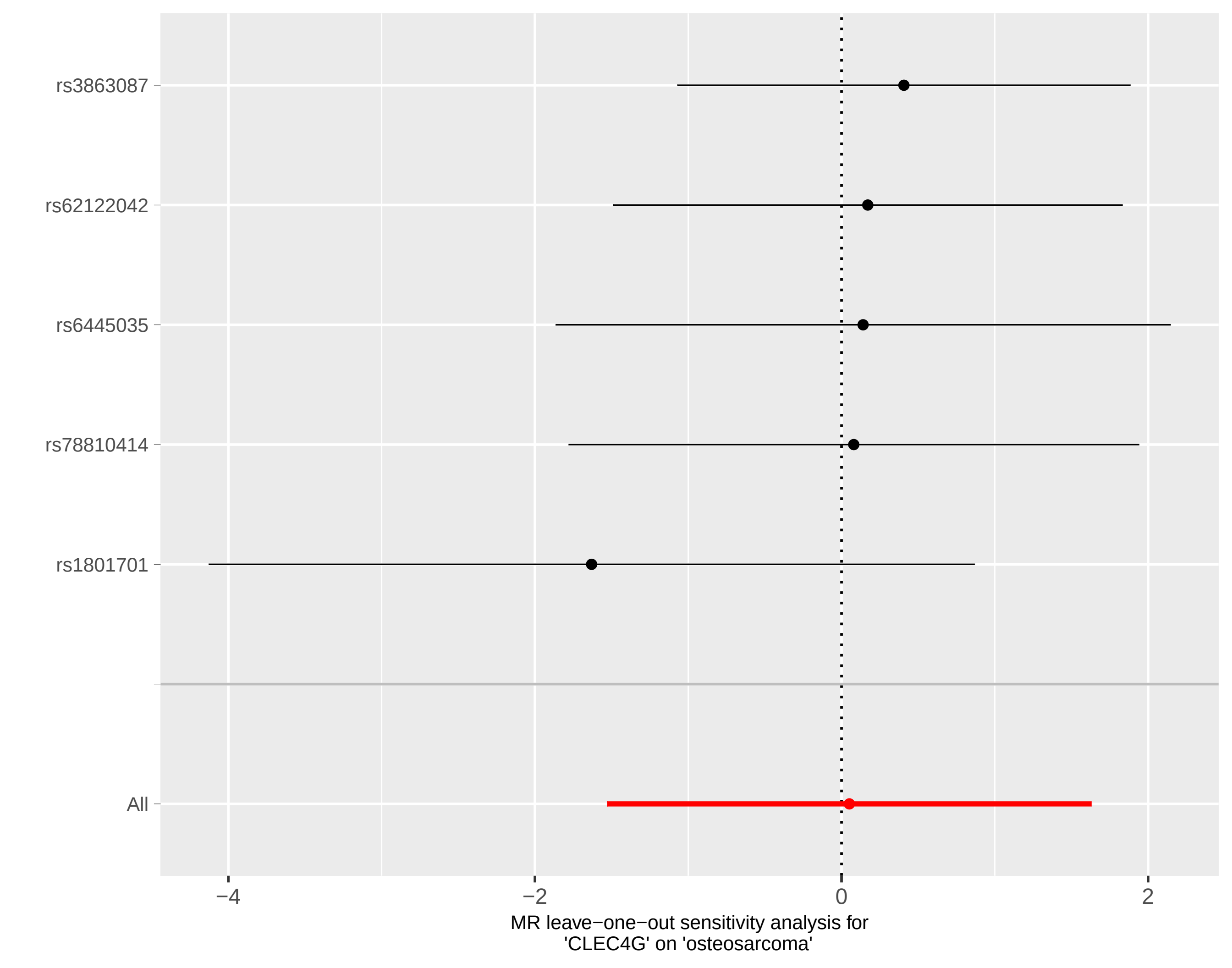
rs3863087
rs62122042
rs6445035
rs78810414
rs1801701
All
−4
−2
0
2
MR leave−one−out sensitivity analysis for
'CLEC4G' on 'osteosarcoma'

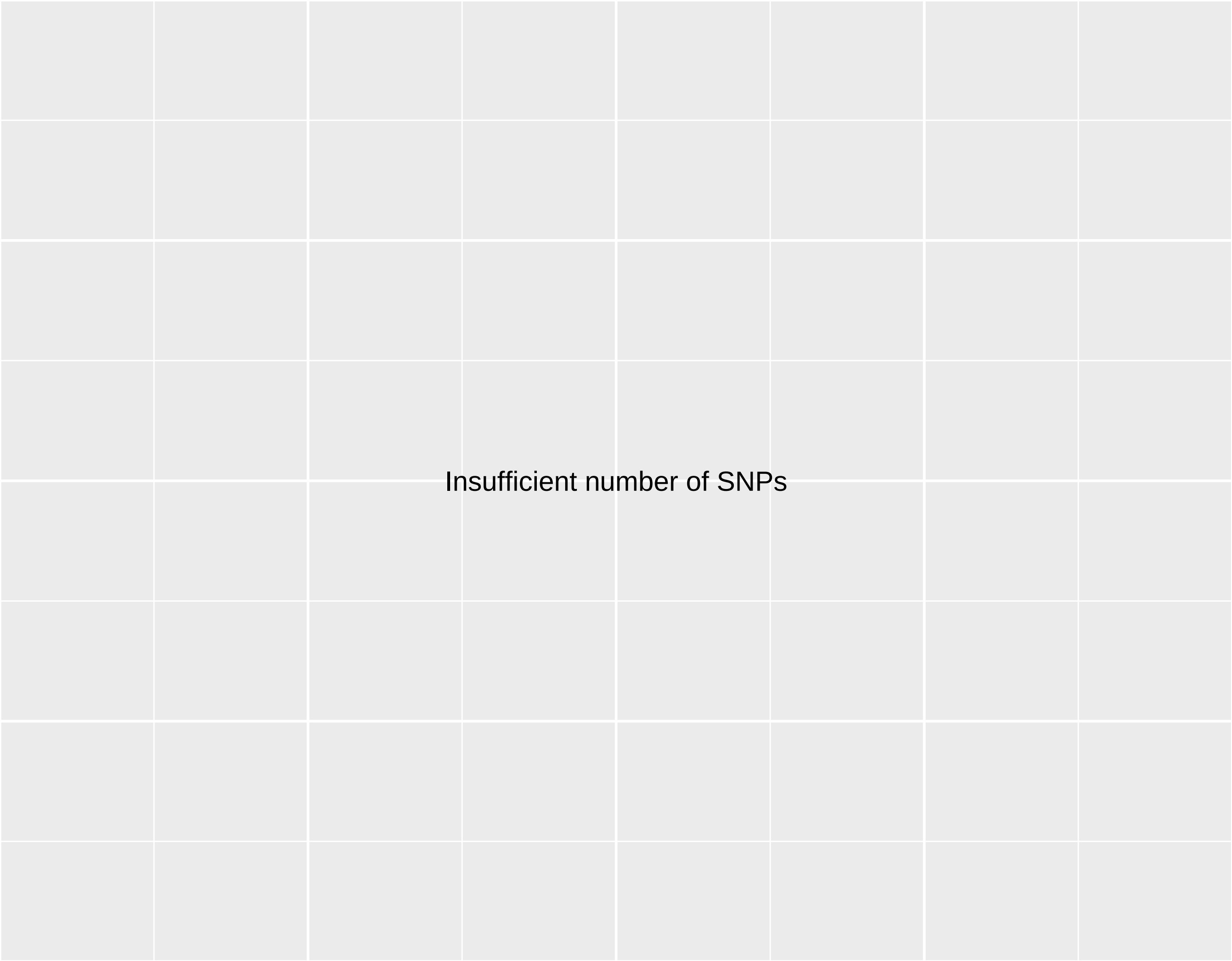
Insufficient number of SNPs

Insufficient number of SNPs

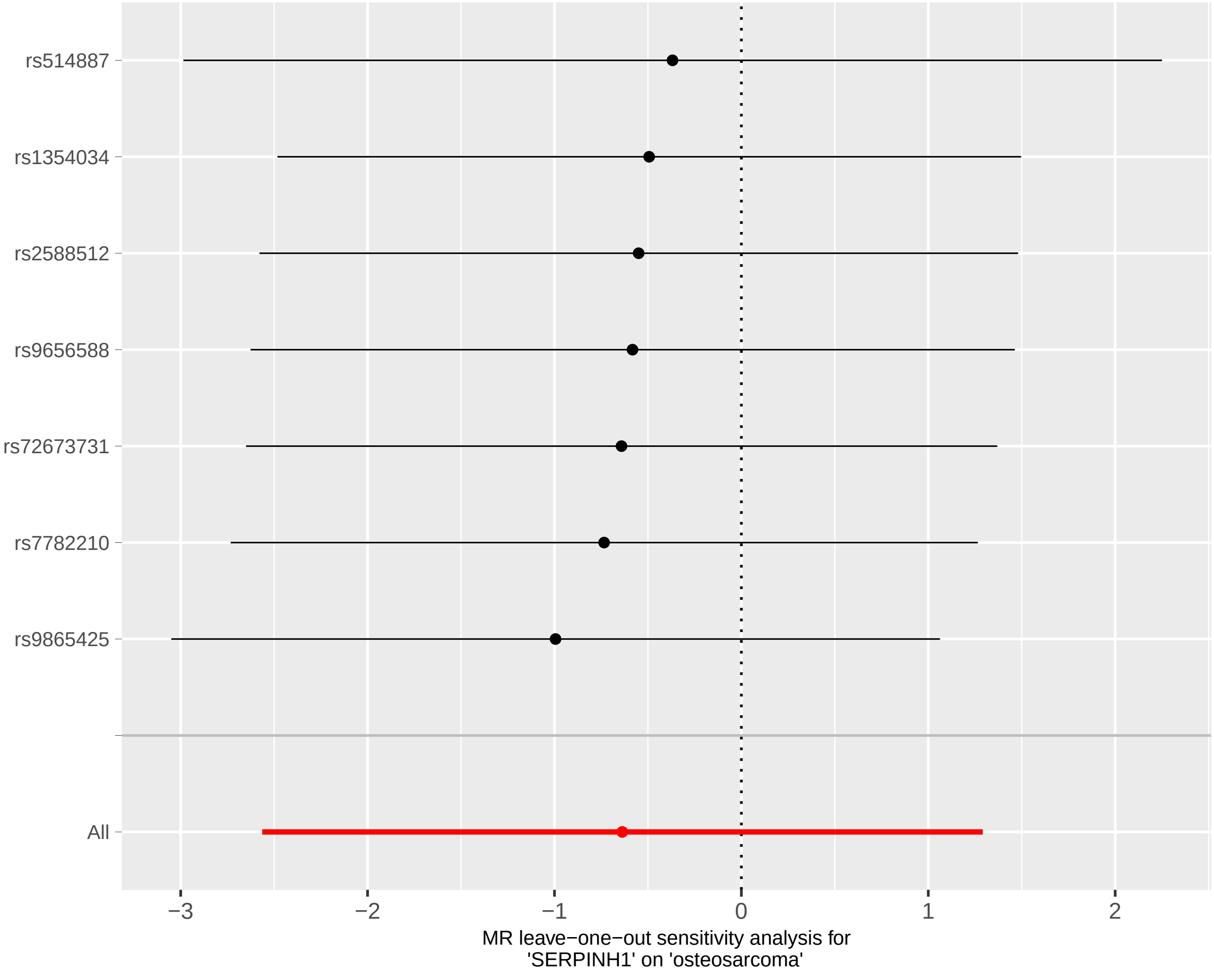
rs514887
rs1354034
rs2588512
rs9656588
rs72673731
rs7782210
rs9865425
All
−3
−2
−1
0
1
2
MR leave−one−out sensitivity analysis for
'SERPINH1' on 'osteosarcoma'

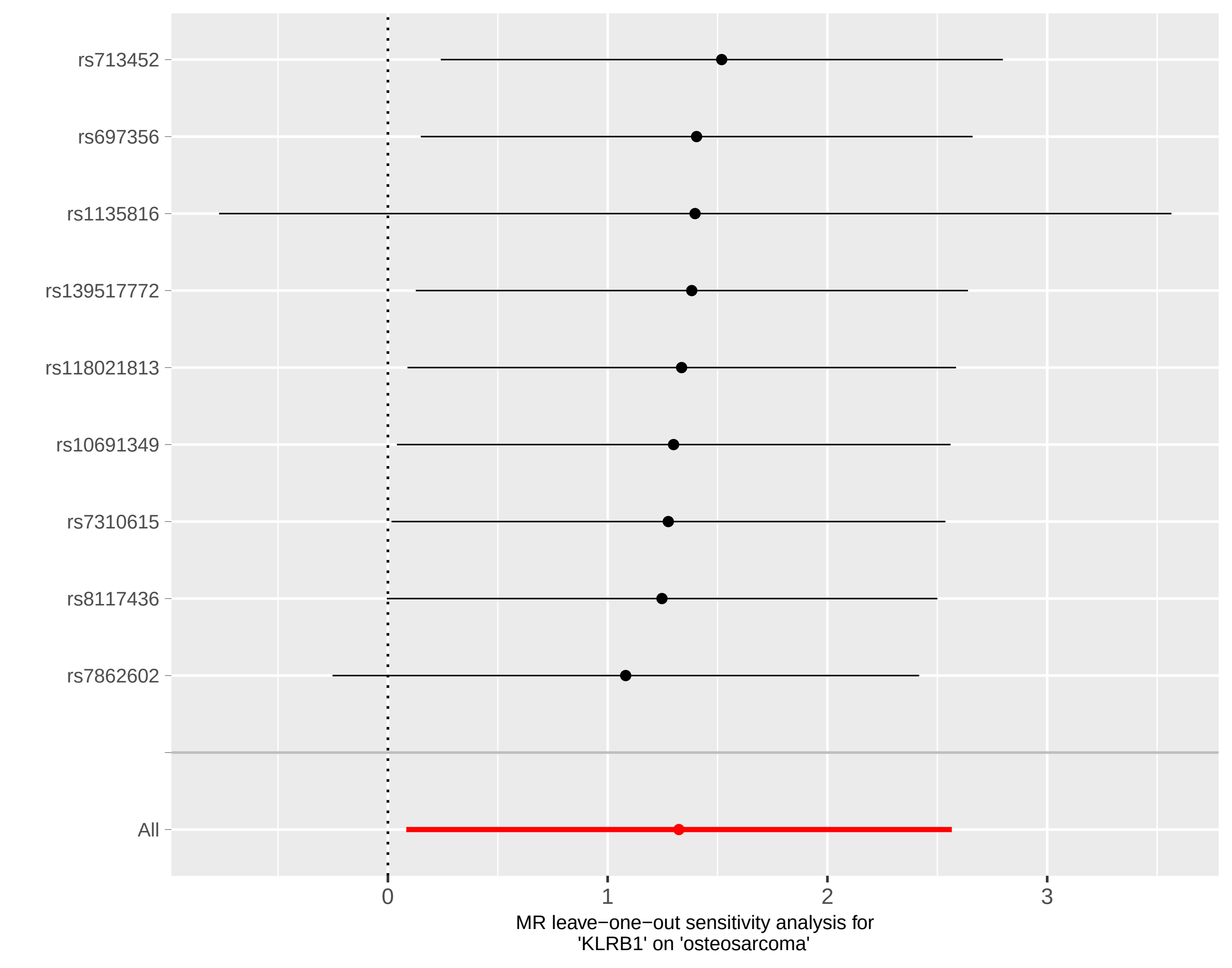

rs713452
rs697356
rs1135816
rs139517772
rs118021813
rs10691349
rs7310615
rs8117436
rs7862602
All
0
1
2
3
MR leave−one−out sensitivity analysis for
'KLRB1' on 'osteosarcoma'

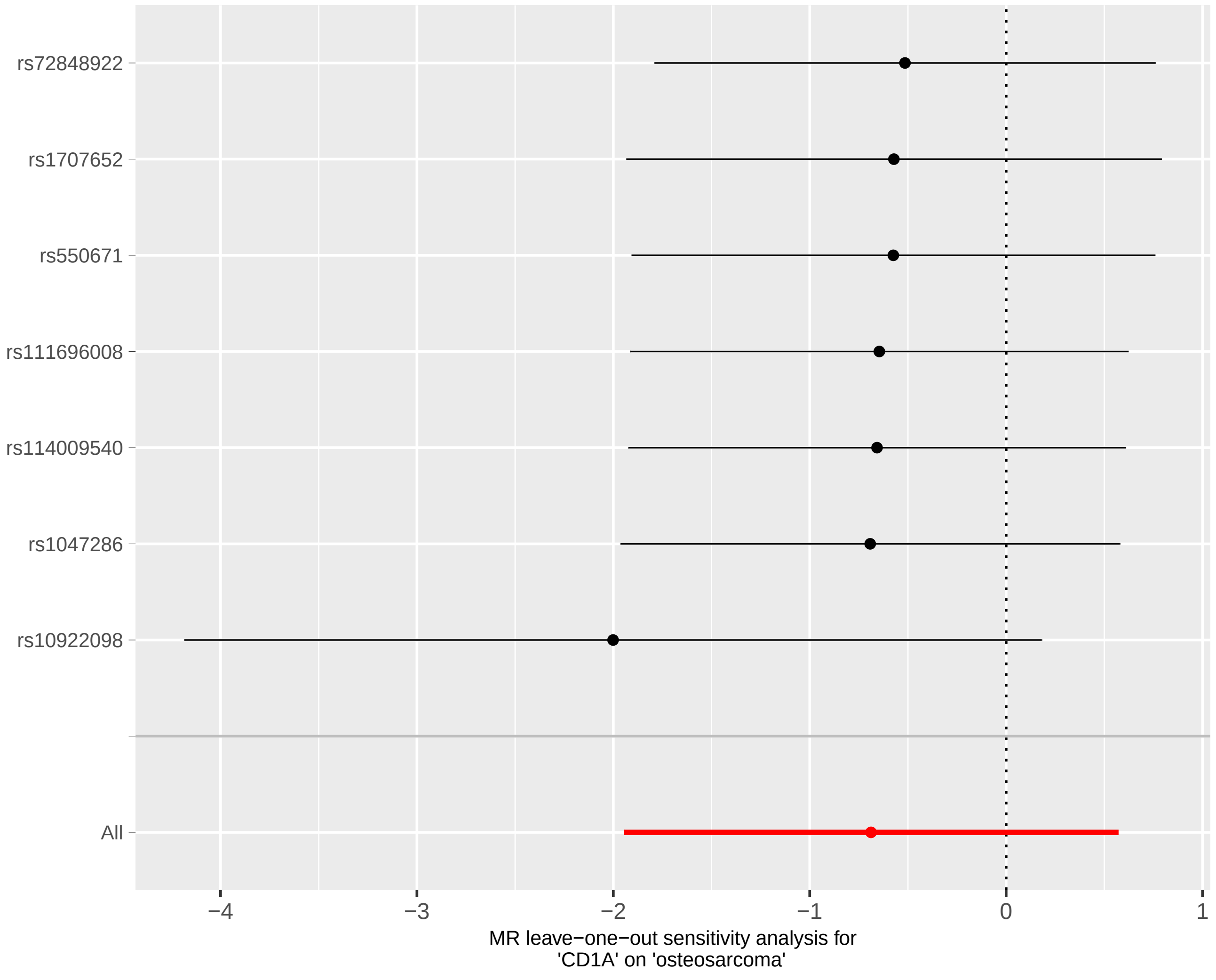

rs72848922
rs1707652
rs550671
rs111696008
rs114009540
rs1047286
rs10922098
All
−4
−3
−2
−1
0
1
MR leave−one−out sensitivity analysis for
'CD1A' on 'osteosarcoma'

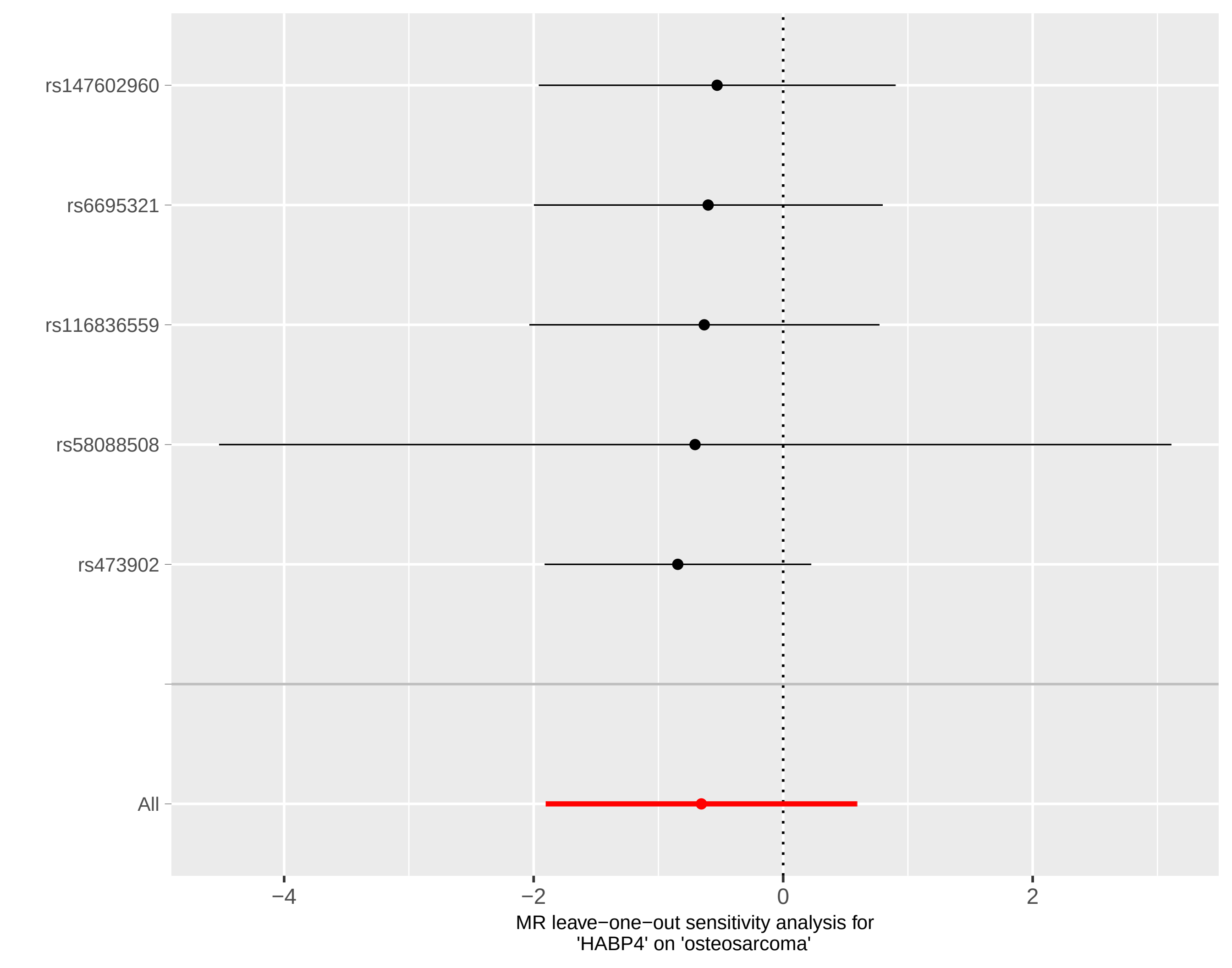
rs147602960
rs6695321
rs116836559
rs58088508
rs473902
All
−4
−2
0
2
MR leave−one−out sensitivity analysis for
'HABP4' on 'osteosarcoma'

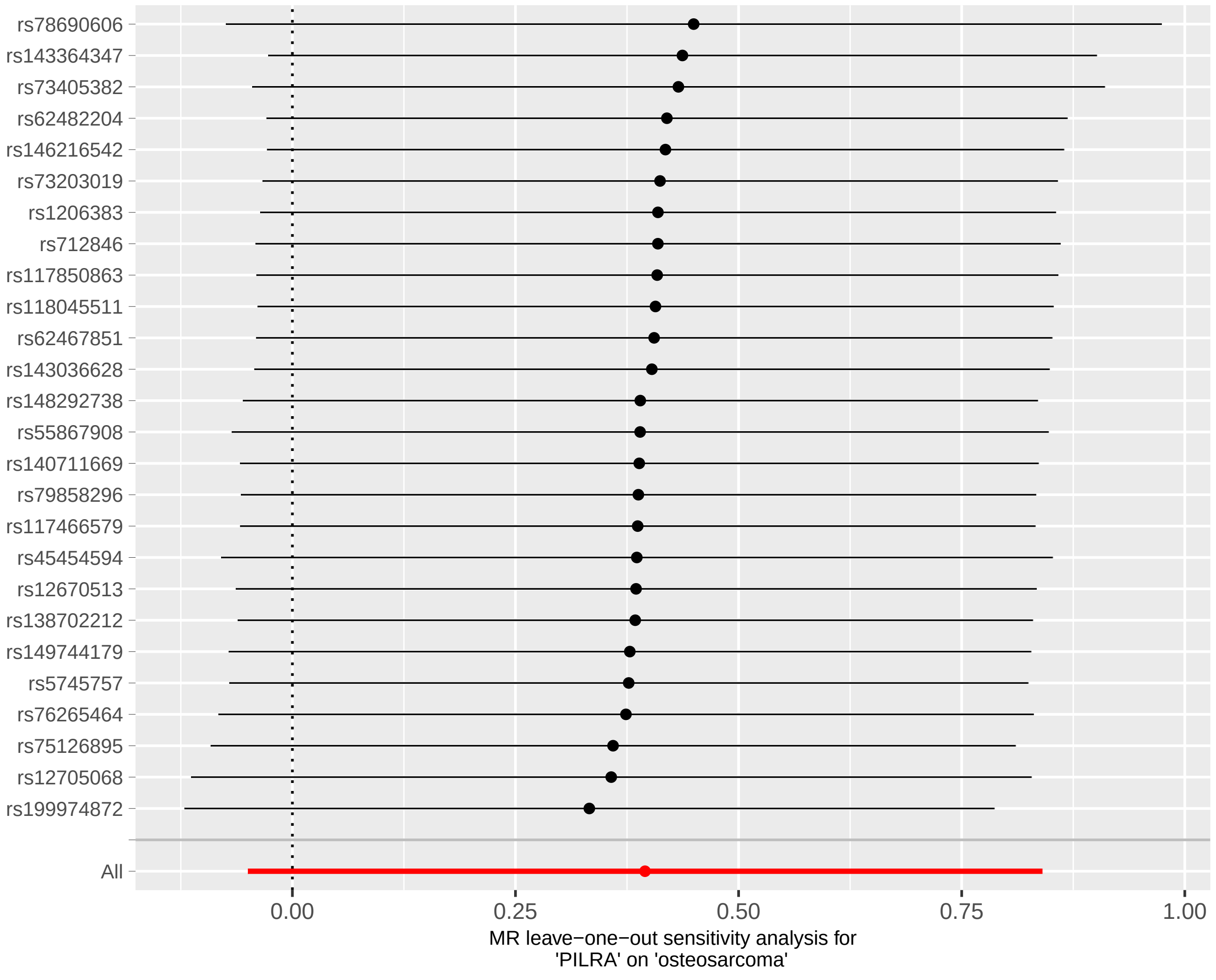

rs78690606
rs143364347
rs73405382
rs62482204
rs146216542
rs73203019
rs1206383
rs712846
rs117850863
rs118045511
rs62467851
rs143036628
rs148292738
rs55867908
rs140711669
rs79858296
rs117466579
rs45454594
rs12670513
rs138702212
rs149744179
rs5745757
rs76265464
rs75126895
rs12705068
rs199974872
All
0.00
0.25
0.50
0.75
1.00
MR leave−one−out sensitivity analysis for
'PILRA' on 'osteosarcoma'

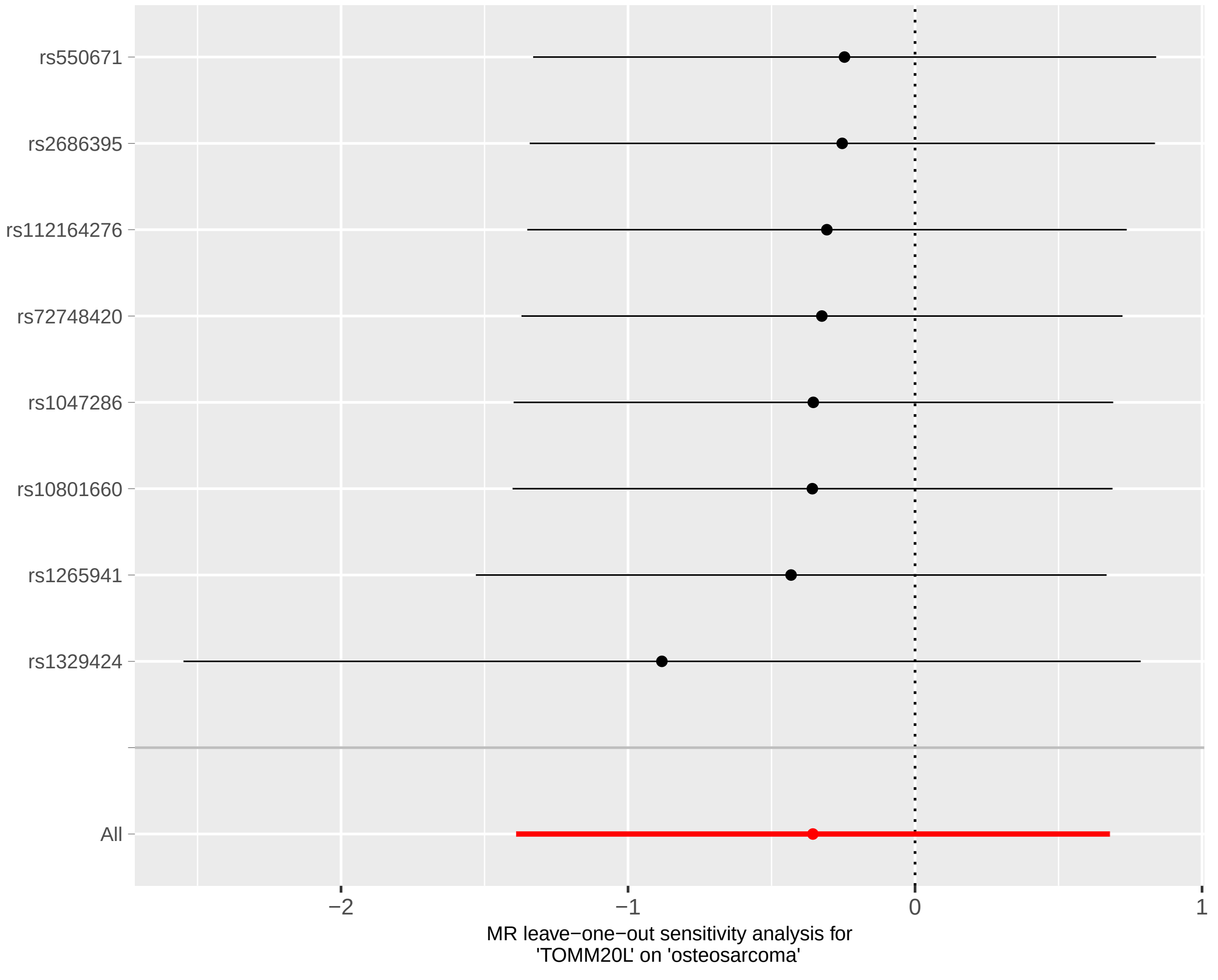

rs550671
rs2686395
rs112164276
rs72748420
rs1047286
rs10801660
rs1265941
rs1329424
All
−2
−1
0
1
MR leave−one−out sensitivity analysis for
'TOMM20L' on 'osteosarcoma'

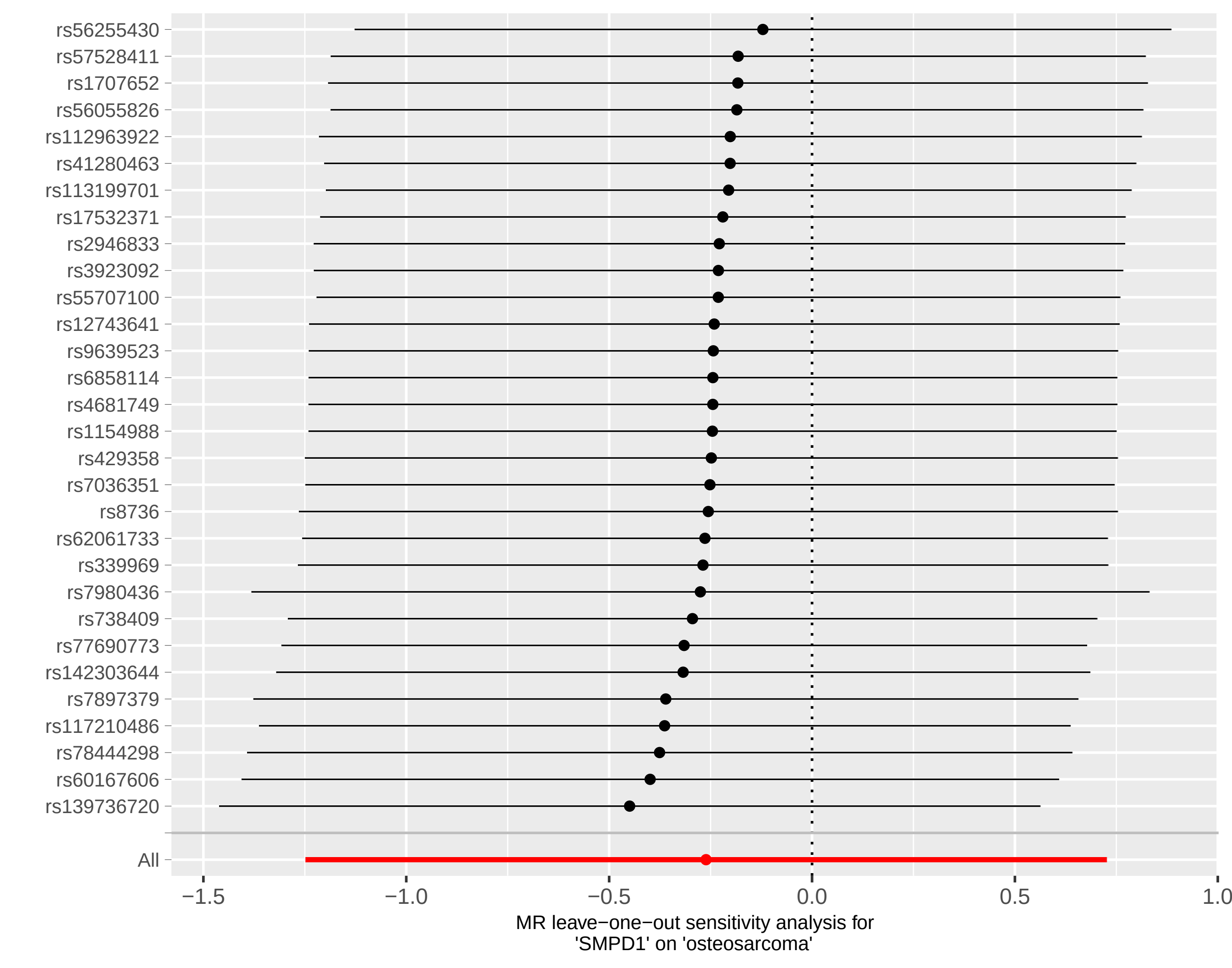

rs56255430
rs57528411
rs1707652
rs56055826
rs112963922
rs41280463
rs113199701
rs17532371
rs2946833
rs3923092
rs55707100
rs12743641
rs9639523
rs6858114
rs4681749
rs1154988
rs429358
rs7036351
rs8736
rs62061733
rs339969
rs7980436
rs738409
rs77690773
rs142303644
rs7897379
rs117210486
rs78444298
rs60167606
rs139736720
All
−1.5
−1.0
−0.5
0.0
0.5
1.0
MR leave−one−out sensitivity analysis for
'SMPD1' on 'osteosarcoma'

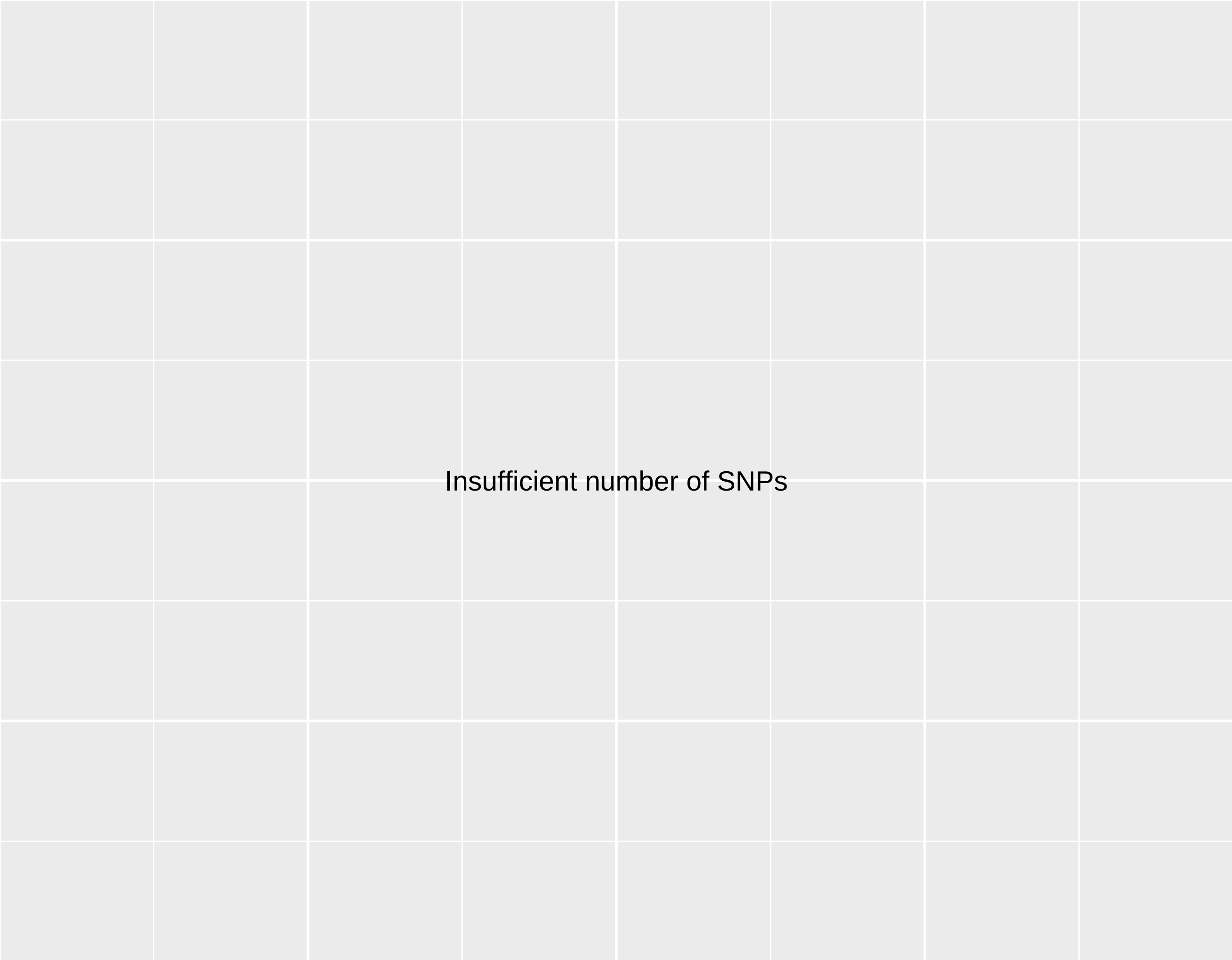
Insufficient number of SNPs

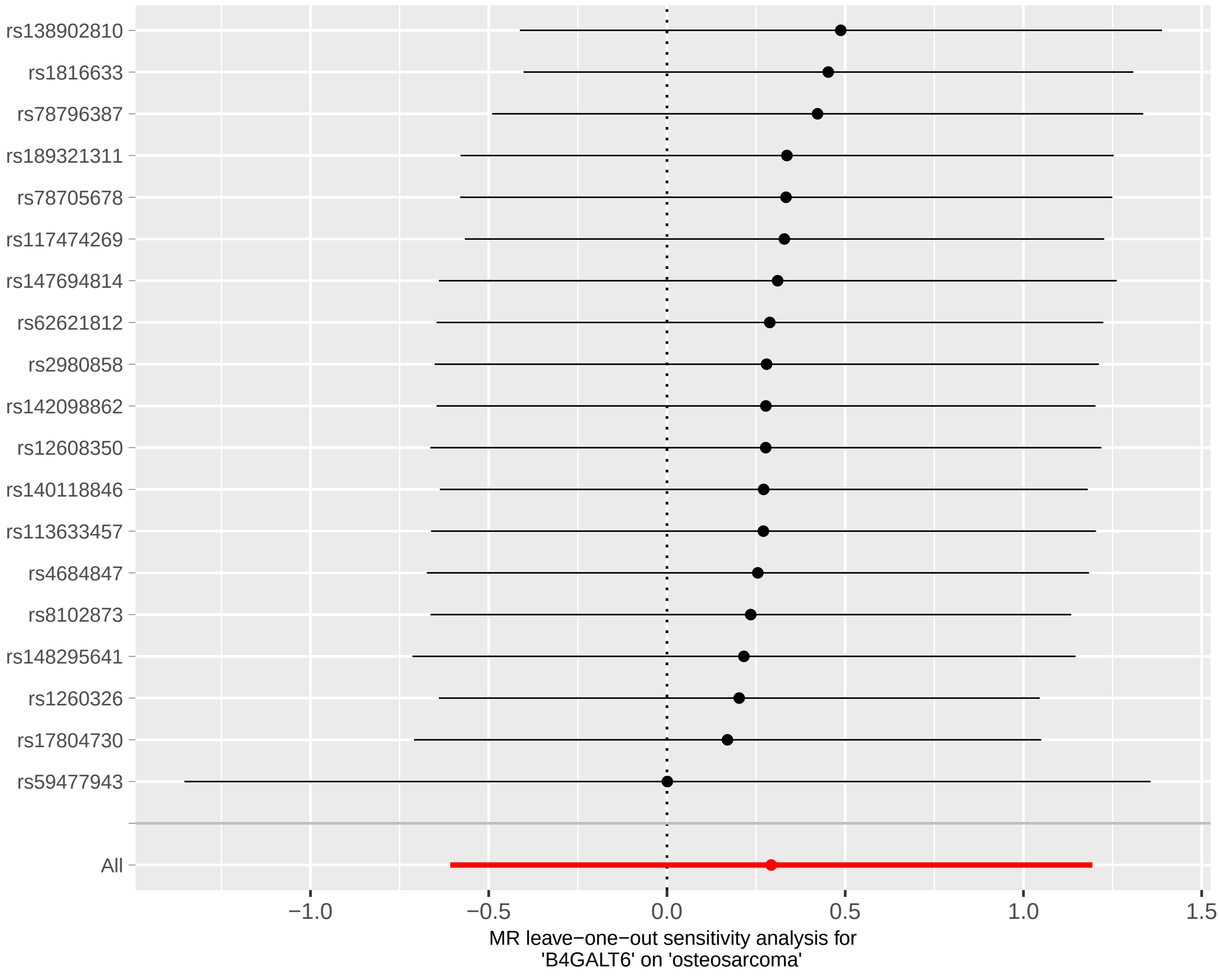
rs138902810
rs1816633
rs78796387
rs189321311
rs78705678
rs117474269
rs147694814
rs62621812
rs2980858
rs142098862
rs12608350
rs140118846
rs113633457
rs4684847
rs8102873
rs148295641
rs1260326
rs17804730
rs59477943
All
−1.0
−0.5
0.0
0.5
1.0
1.5
MR leave−one−out sensitivity analysis for
'B4GALT6' on 'osteosarcoma'

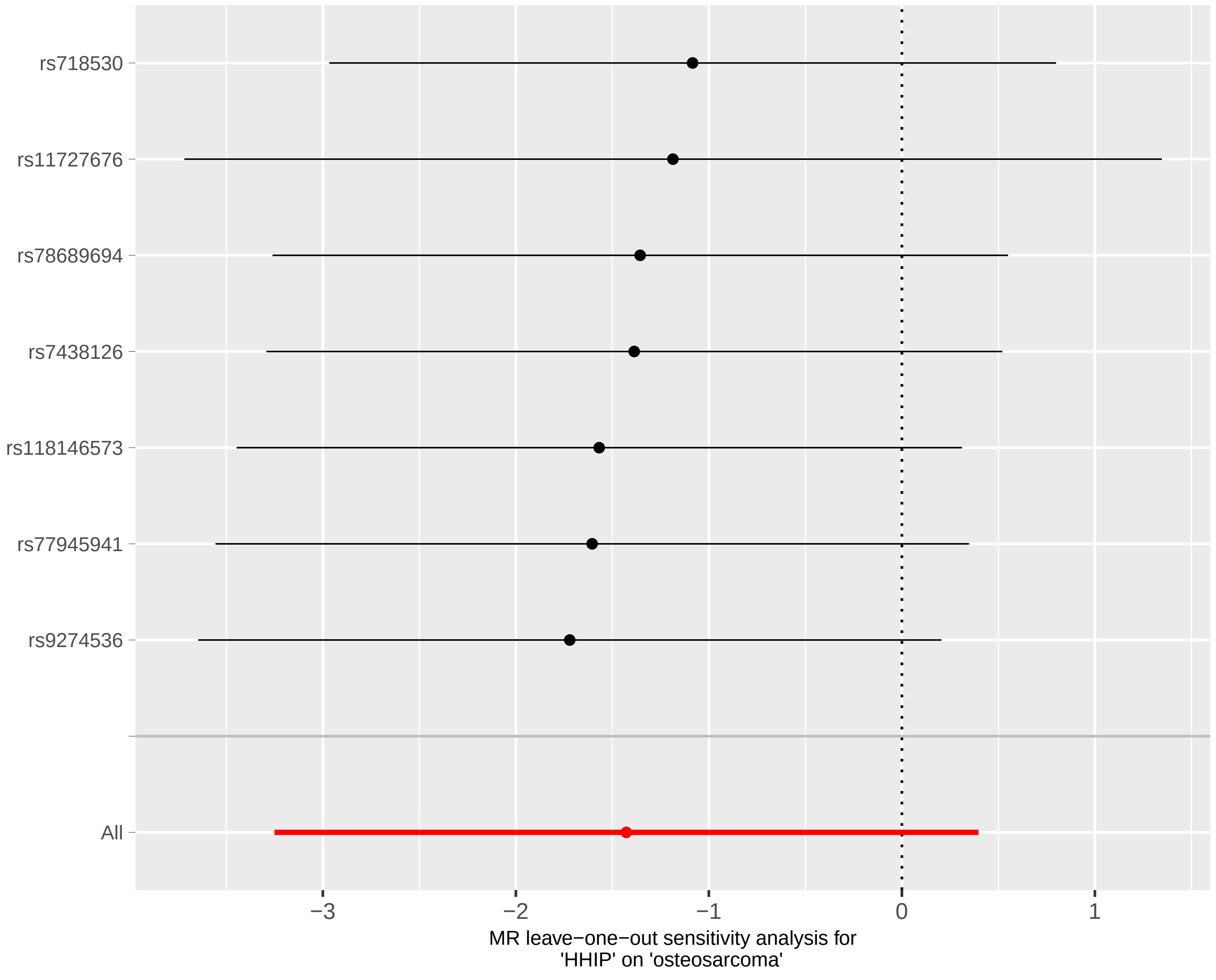

rs718530
rs11727676
rs78689694
rs7438126
rs118146573
rs77945941
rs9274536
All
−3
−2
−1
0
1
MR leave−one−out sensitivity analysis for
'HHIP' on 'osteosarcoma'

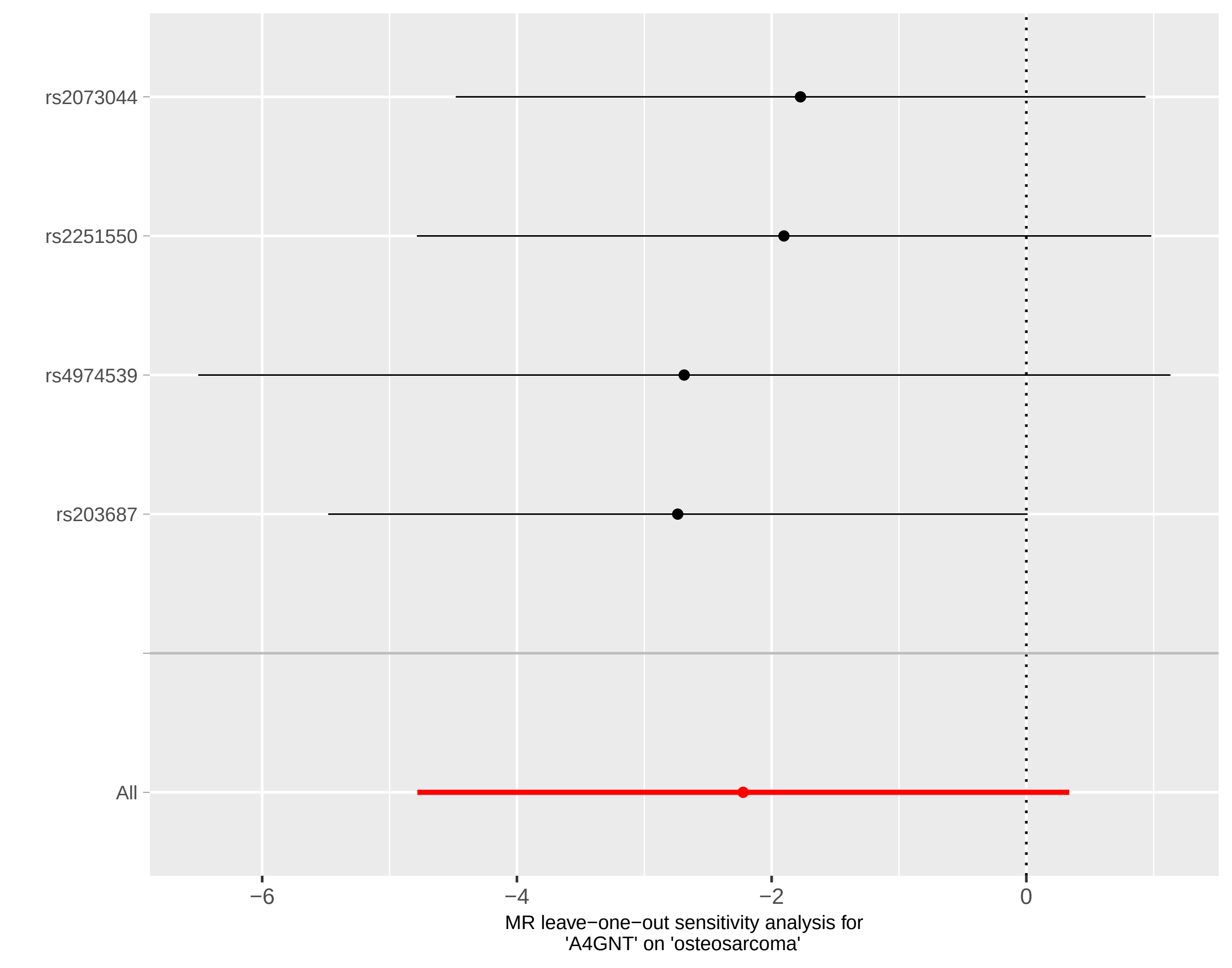
rs2073044
rs2251550
rs4974539
rs203687
All
−6
−4
−2
0
MR leave−one−out sensitivity analysis for
'A4GNT' on 'osteosarcoma'

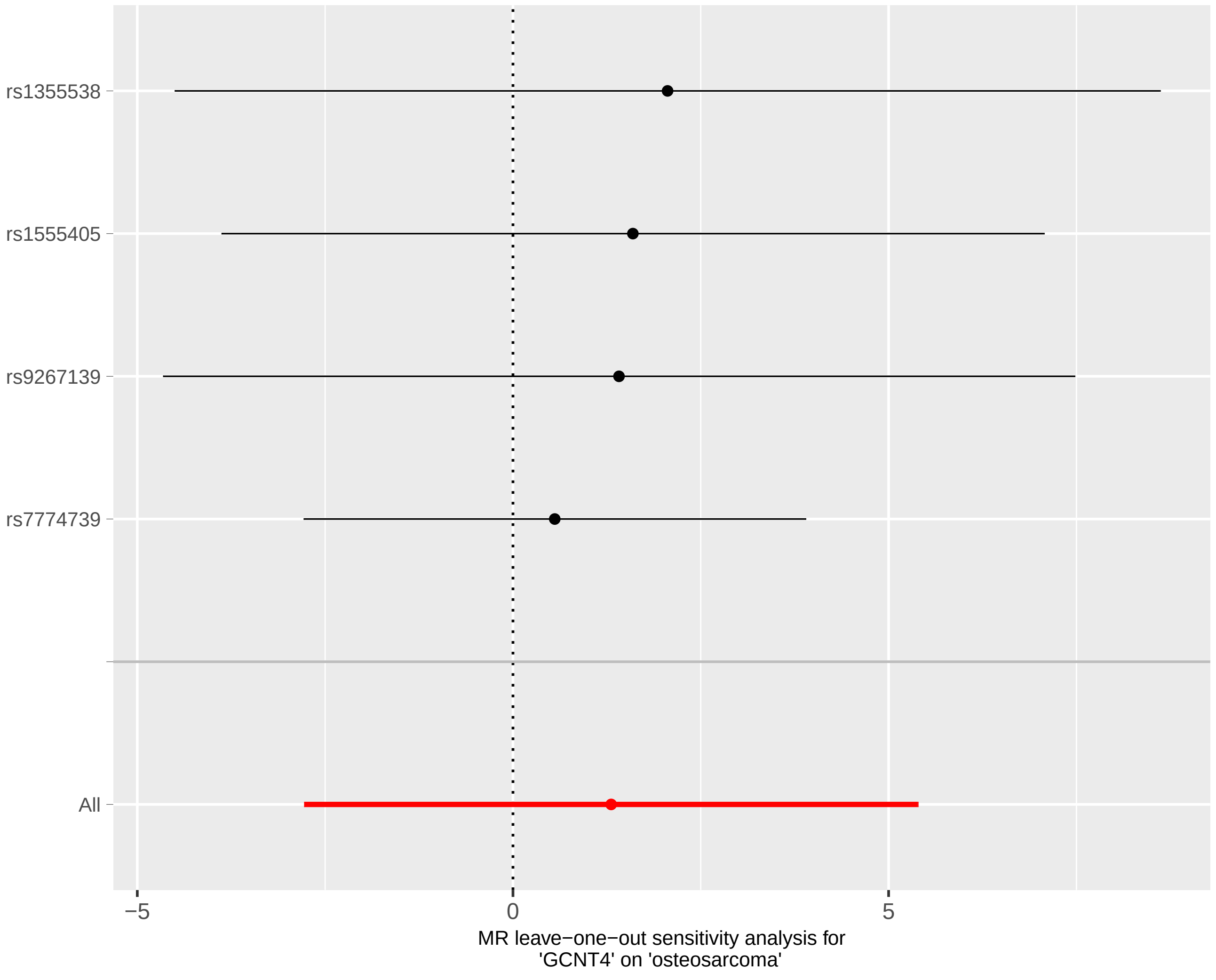

rs1355538
rs1555405
rs9267139
rs7774739
All
−5
0
5
MR leave−one−out sensitivity analysis for
'GCNT4' on 'osteosarcoma'

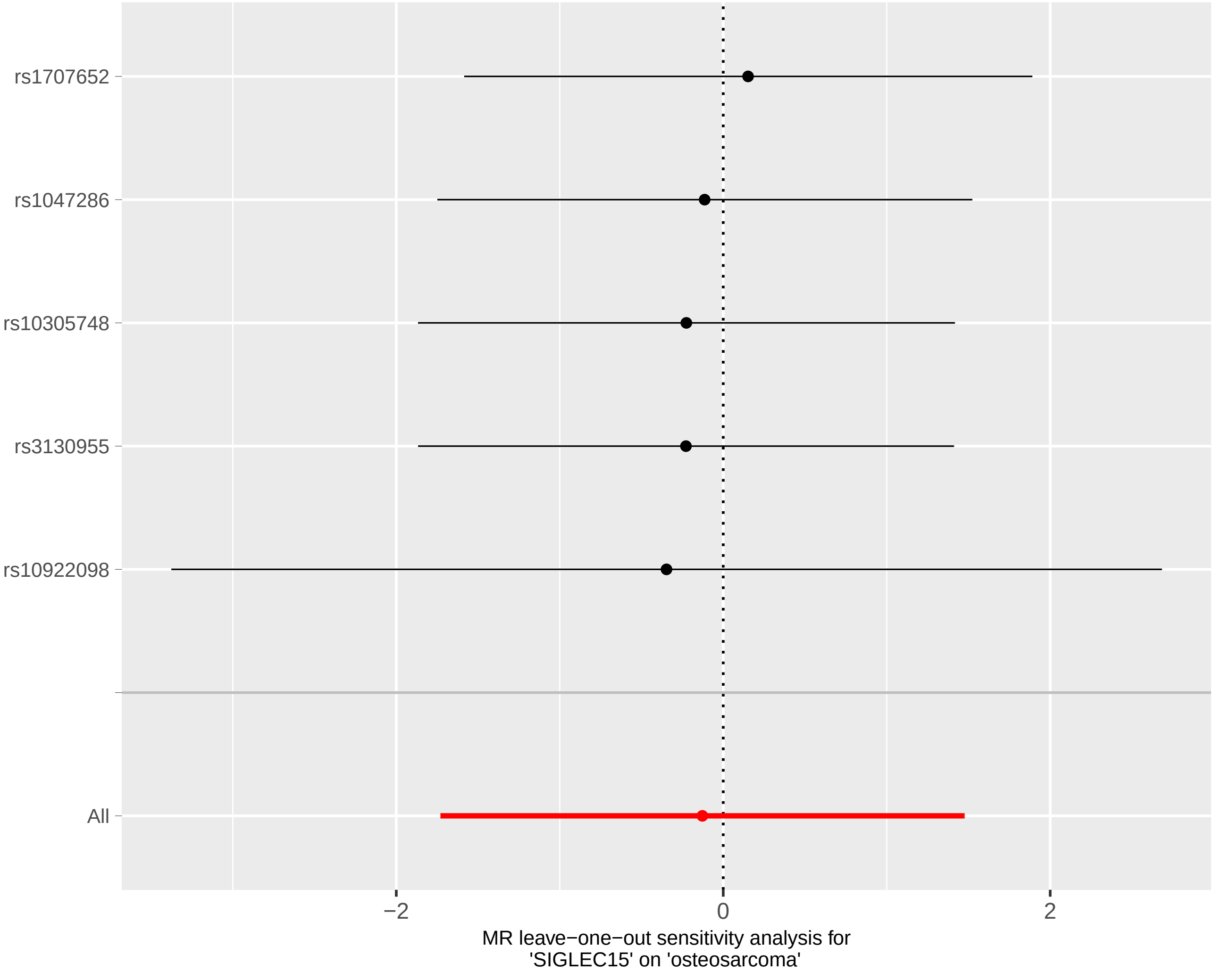

rs1707652
rs1047286
rs10305748
rs3130955
rs10922098
All
−2
0
2
MR leave−one−out sensitivity analysis for
'SIGLEC15' on 'osteosarcoma'

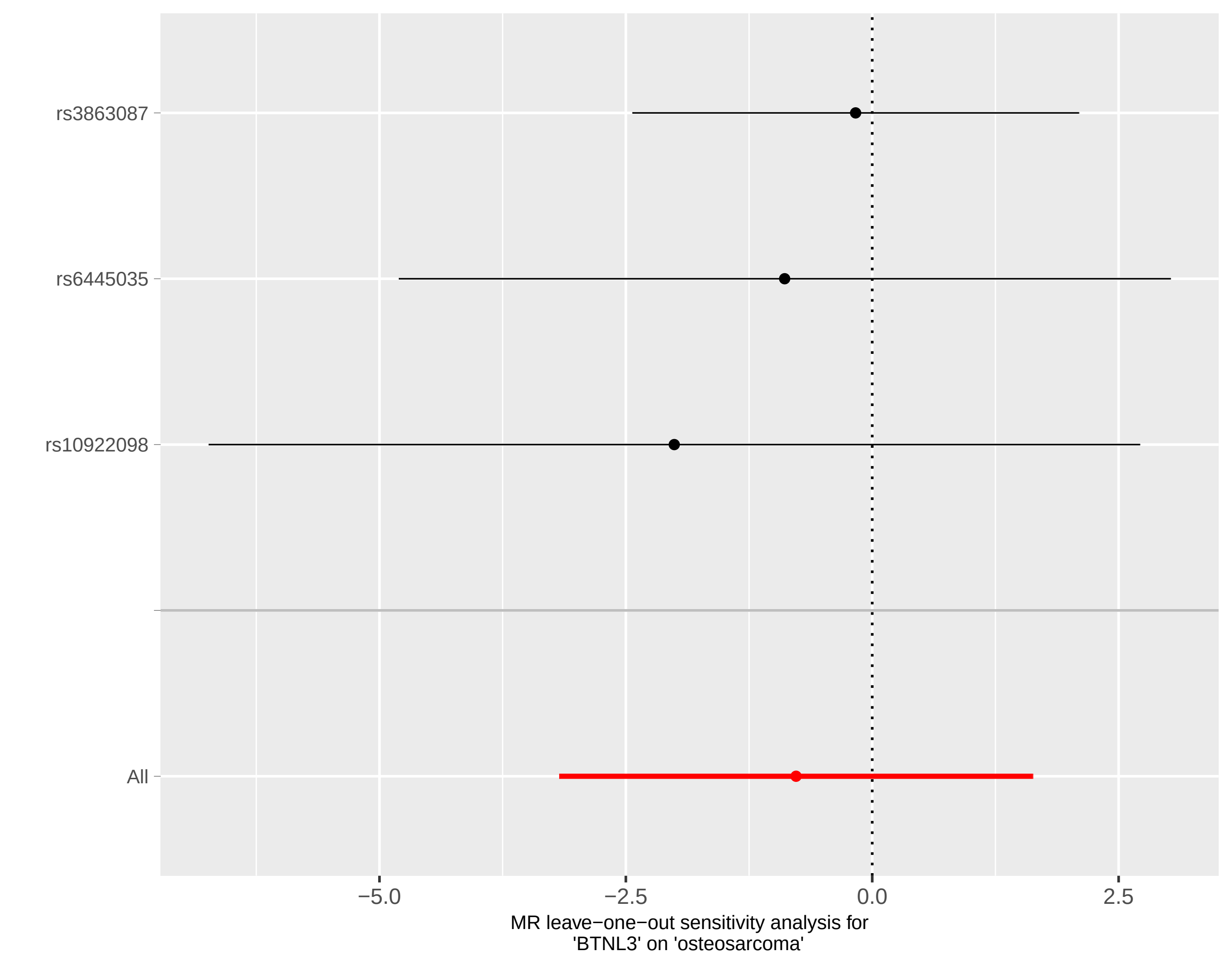
rs3863087
rs6445035
rs10922098
All
−5.0
−2.5
0.0
2.5
MR leave−one−out sensitivity analysis for
'BTNL3' on 'osteosarcoma'

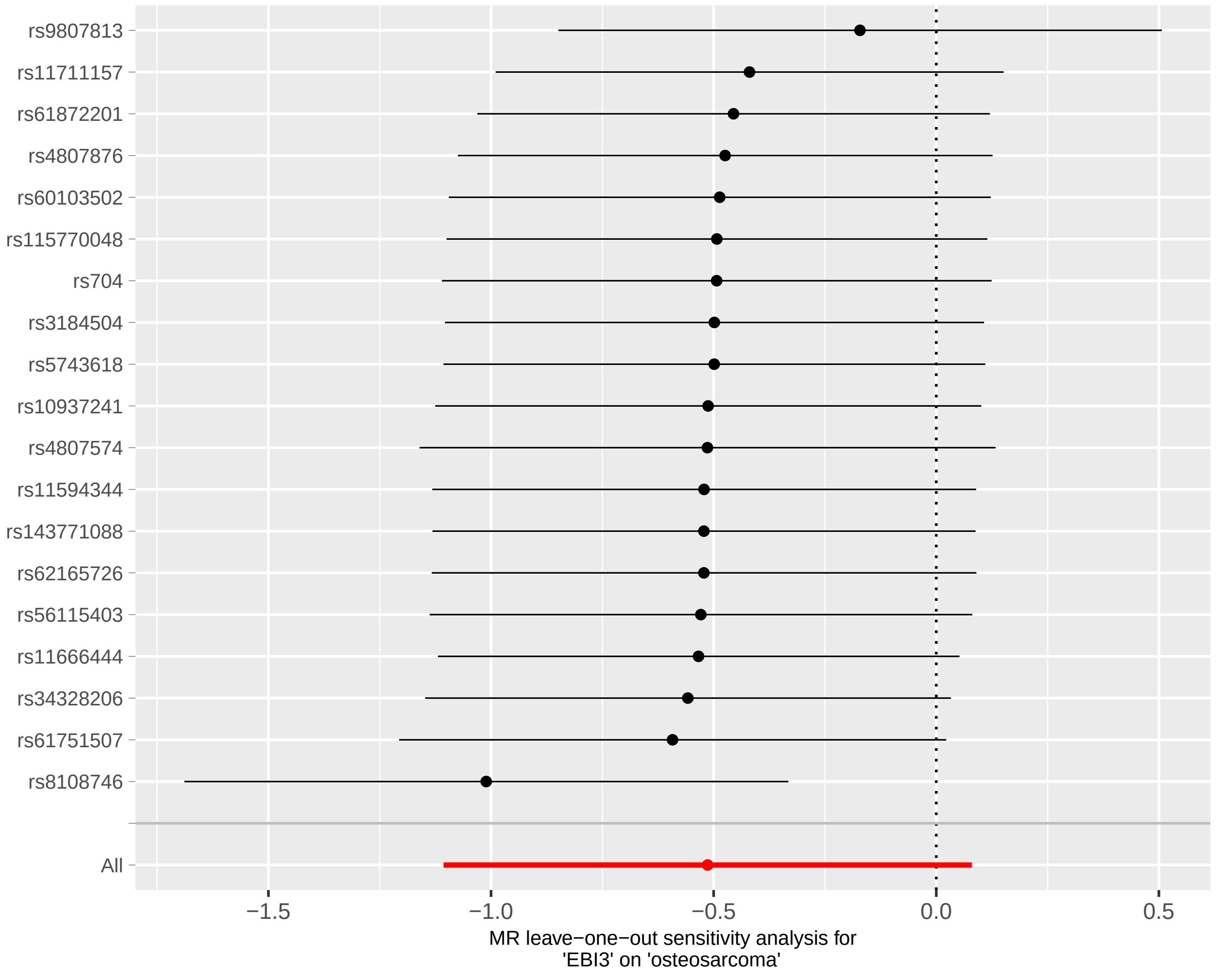

rs9807813
rs11711157
rs61872201
rs4807876
rs60103502
rs115770048
rs704
rs3184504
rs5743618
rs10937241
rs4807574
rs11594344
rs143771088
rs62165726
rs56115403
rs11666444
rs34328206
rs61751507
rs8108746
All
−1.5
−1.0
−0.5
0.0
0.5
MR leave−one−out sensitivity analysis for
'EBI3' on 'osteosarcoma'

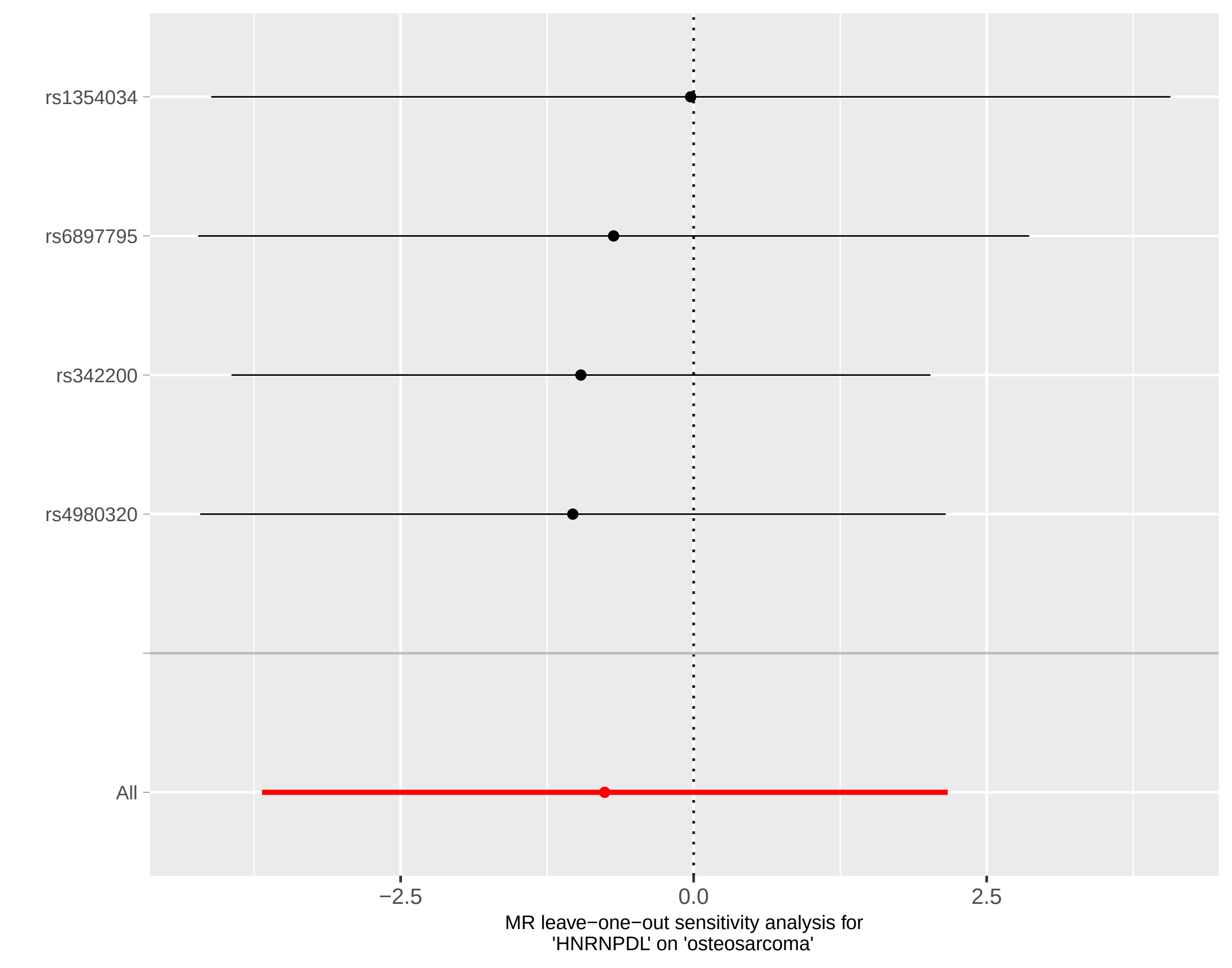

rs1354034
rs6897795
rs342200
rs4980320
All
−2.5
0.0
2.5
MR leave−one−out sensitivity analysis for
'HNRNPDL' on 'osteosarcoma'

Insufficient number of SNPs

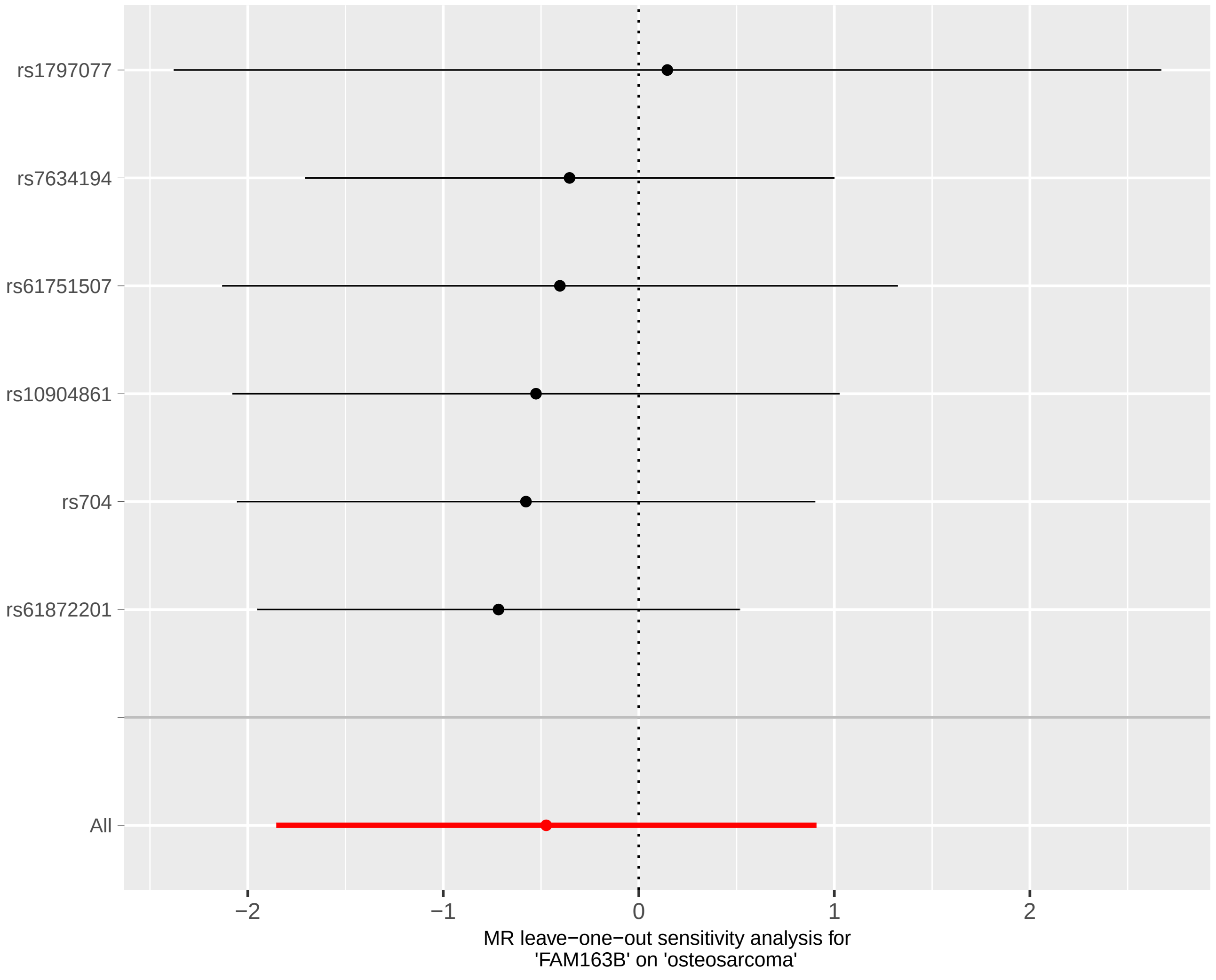

rs1797077
rs7634194
rs61751507
rs10904861
rs704
rs61872201
All
−2
−1
0
1
2
MR leave−one−out sensitivity analysis for
'FAM163B' on 'osteosarcoma'

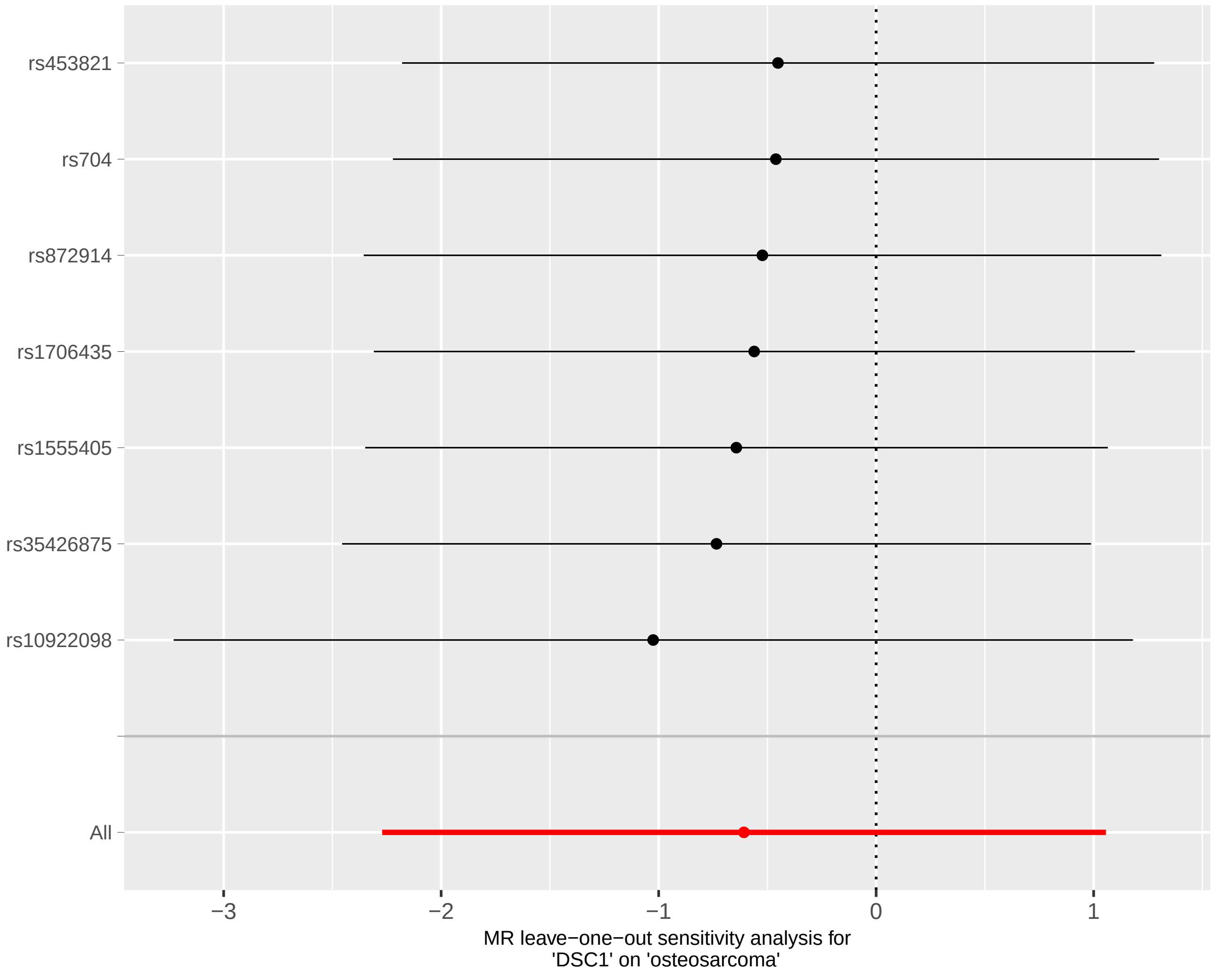
rs453821
rs704
rs872914
rs1706435
rs1555405
rs35426875
rs10922098
All
−3
−2
−1
0
1
MR leave−one−out sensitivity analysis for
'DSC1' on 'osteosarcoma'

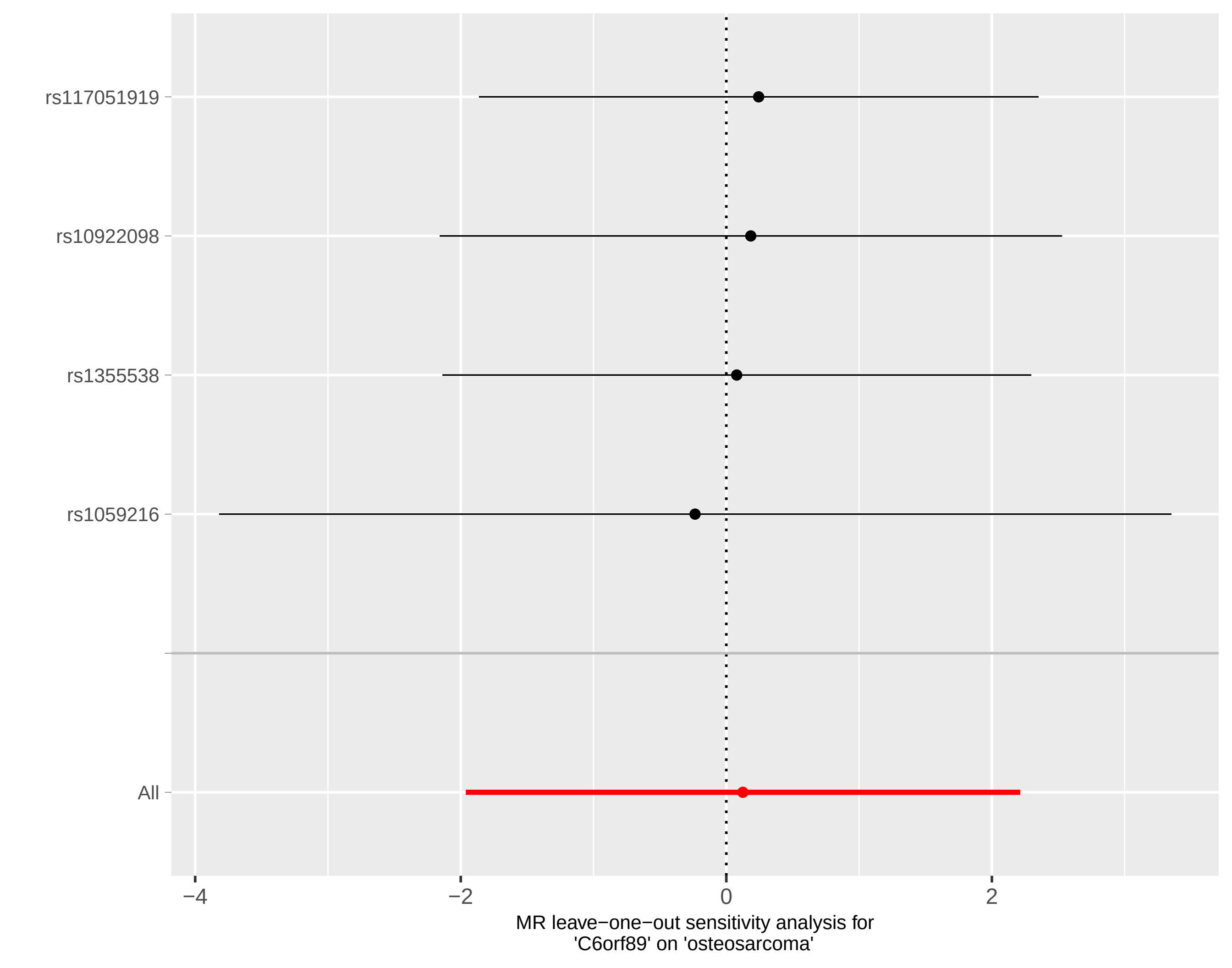

rs117051919
rs10922098
rs1355538
rs1059216
All
−4
−2
0
2
MR leave−one−out sensitivity analysis for
'C6orf89' on 'osteosarcoma'

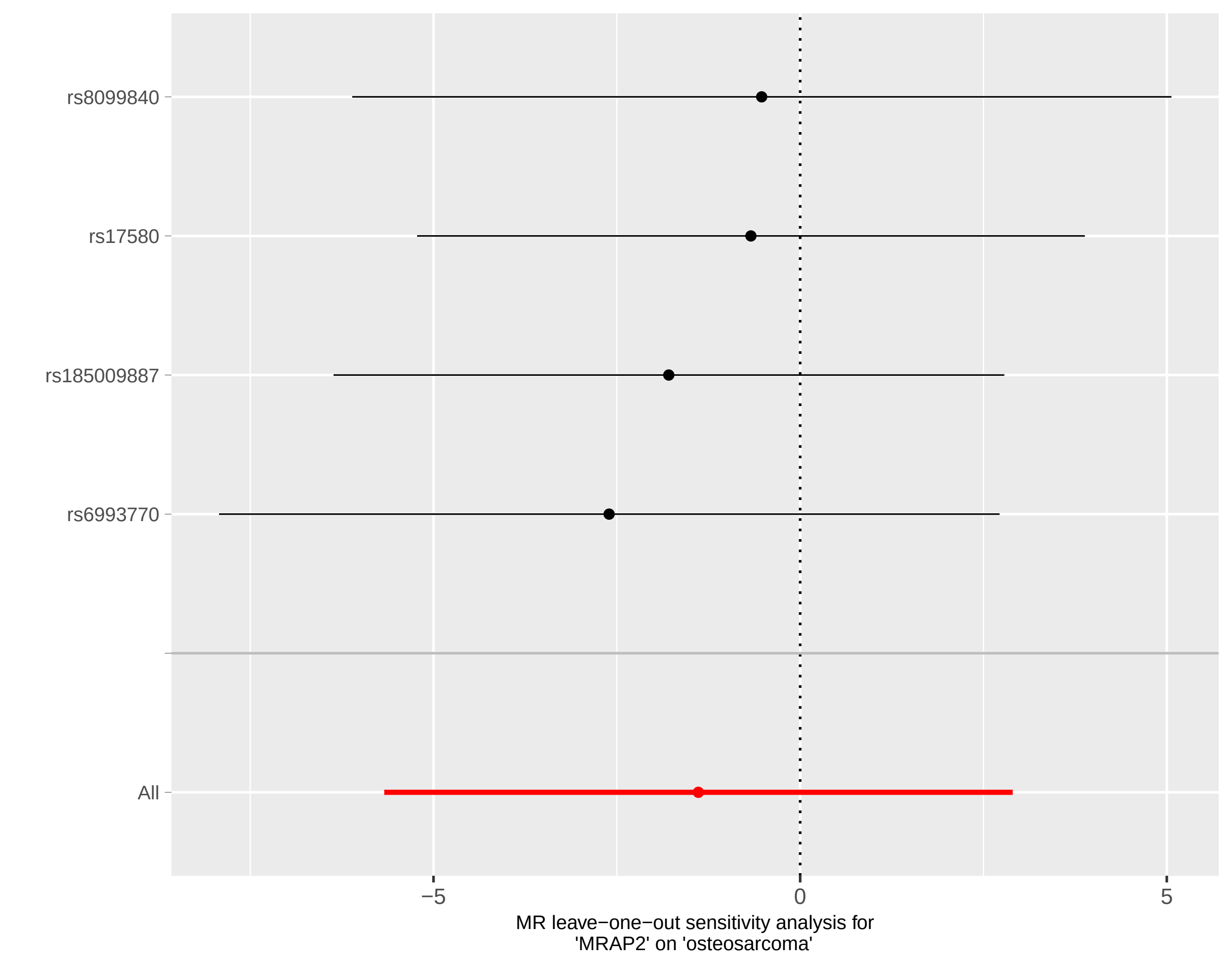
rs8099840
rs17580
rs185009887
rs6993770
All
−5
0
5
MR leave−one−out sensitivity analysis for
'MRAP2' on 'osteosarcoma'

Insufficient number of SNPs

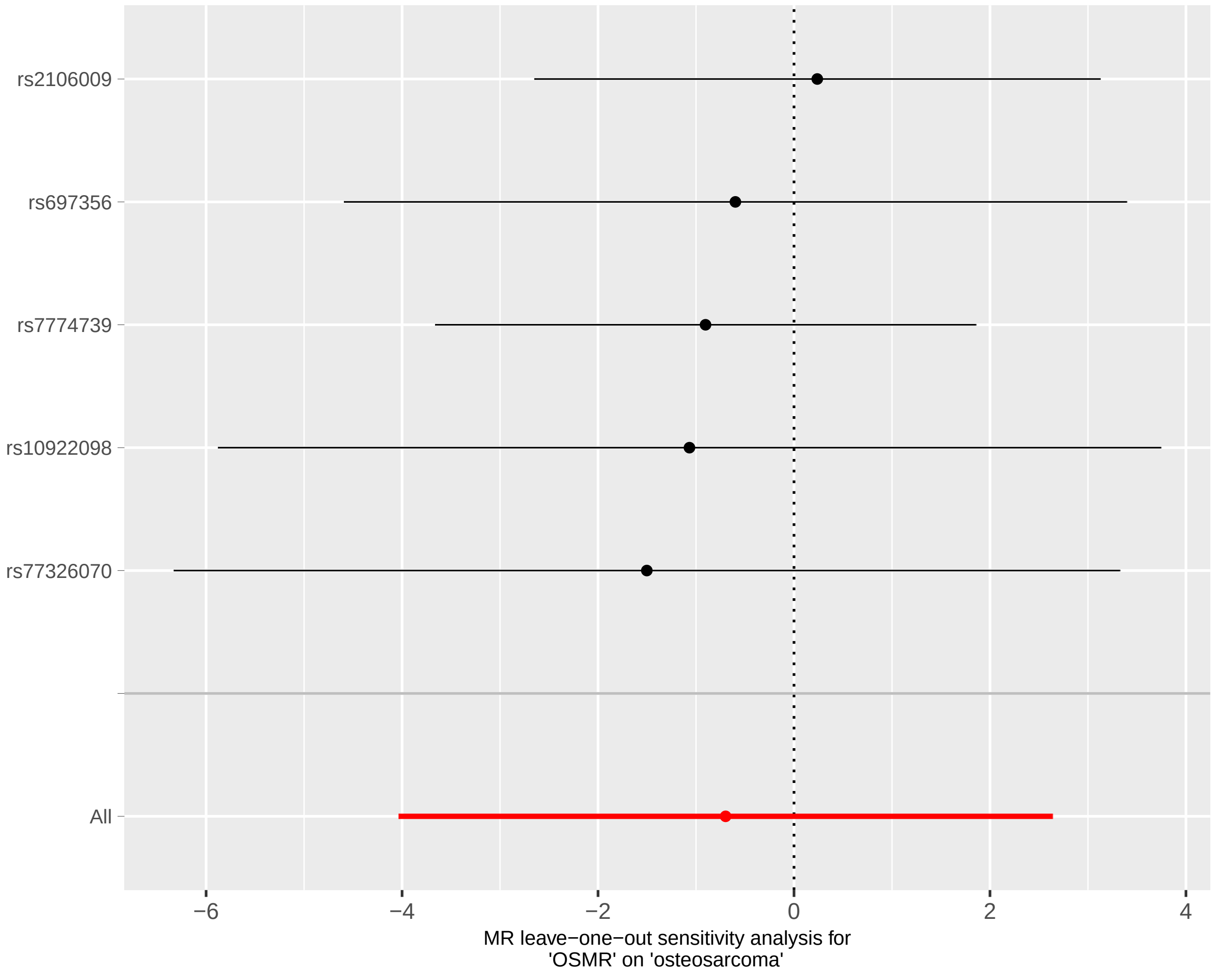
rs2106009
rs697356
rs7774739
rs10922098
rs77326070
All
−6
−4
−2
0
2
4
MR leave−one−out sensitivity analysis for
'OSMR' on 'osteosarcoma'

Insufficient number of SNPs

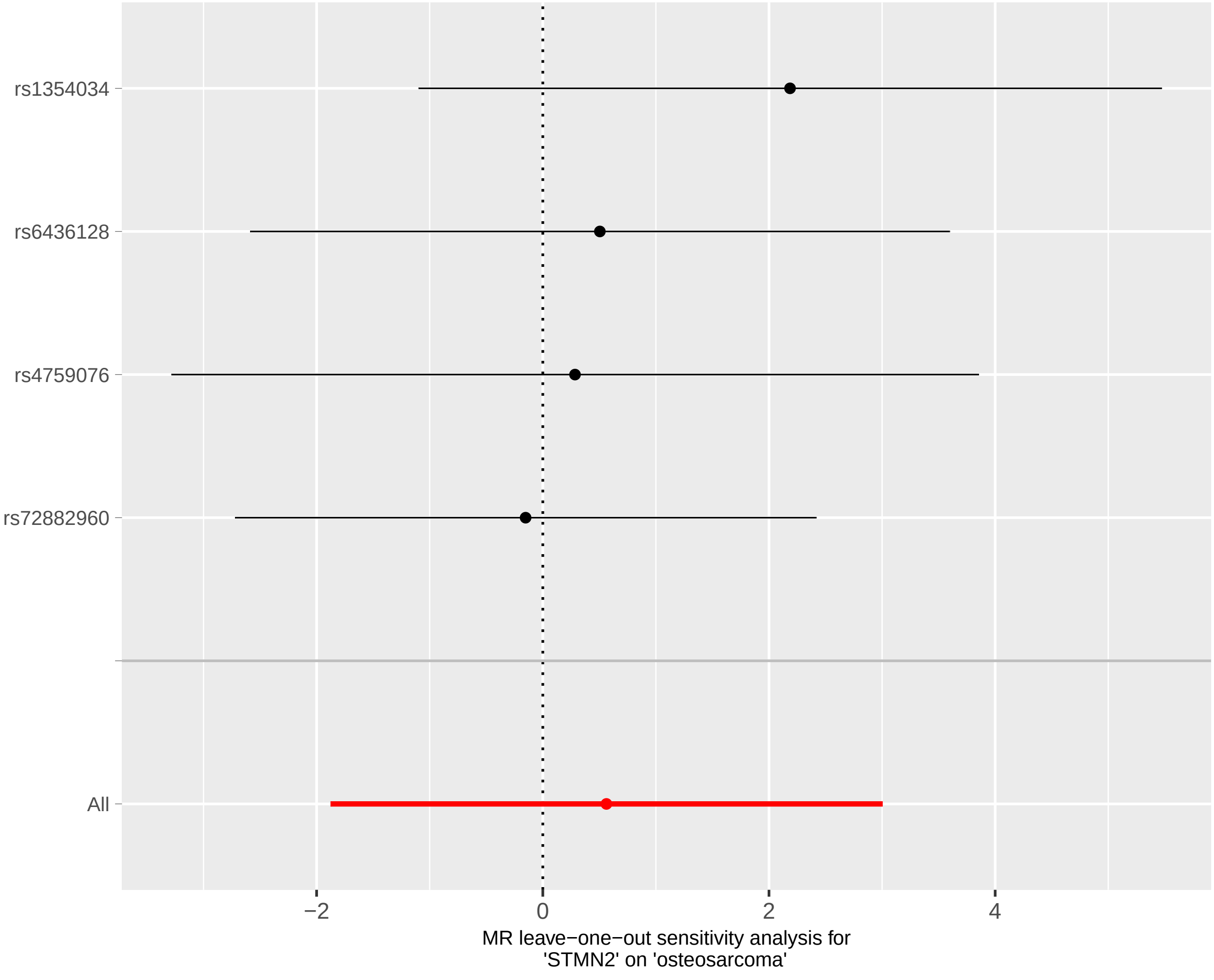
rs1354034
rs6436128
rs4759076
rs72882960
All
−2
0
2
4
MR leave−one−out sensitivity analysis for
'STMN2' on 'osteosarcoma'

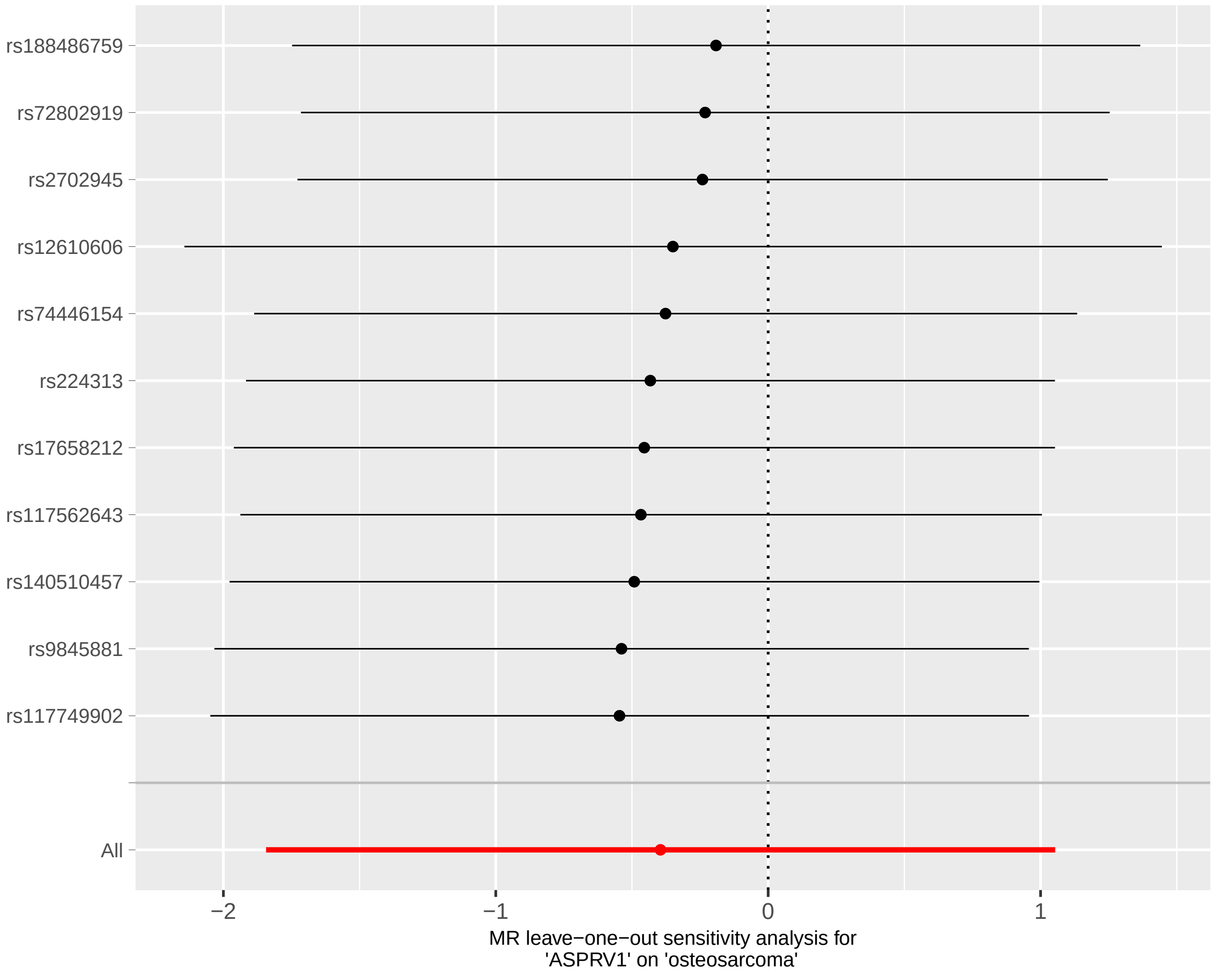
rs188486759
rs72802919
rs2702945
rs12610606
rs74446154
rs224313
rs17658212
rs117562643
rs140510457
rs9845881
rs117749902
All
−2
−1
0
1
MR leave−one−out sensitivity analysis for
'ASPRV1' on 'osteosarcoma'

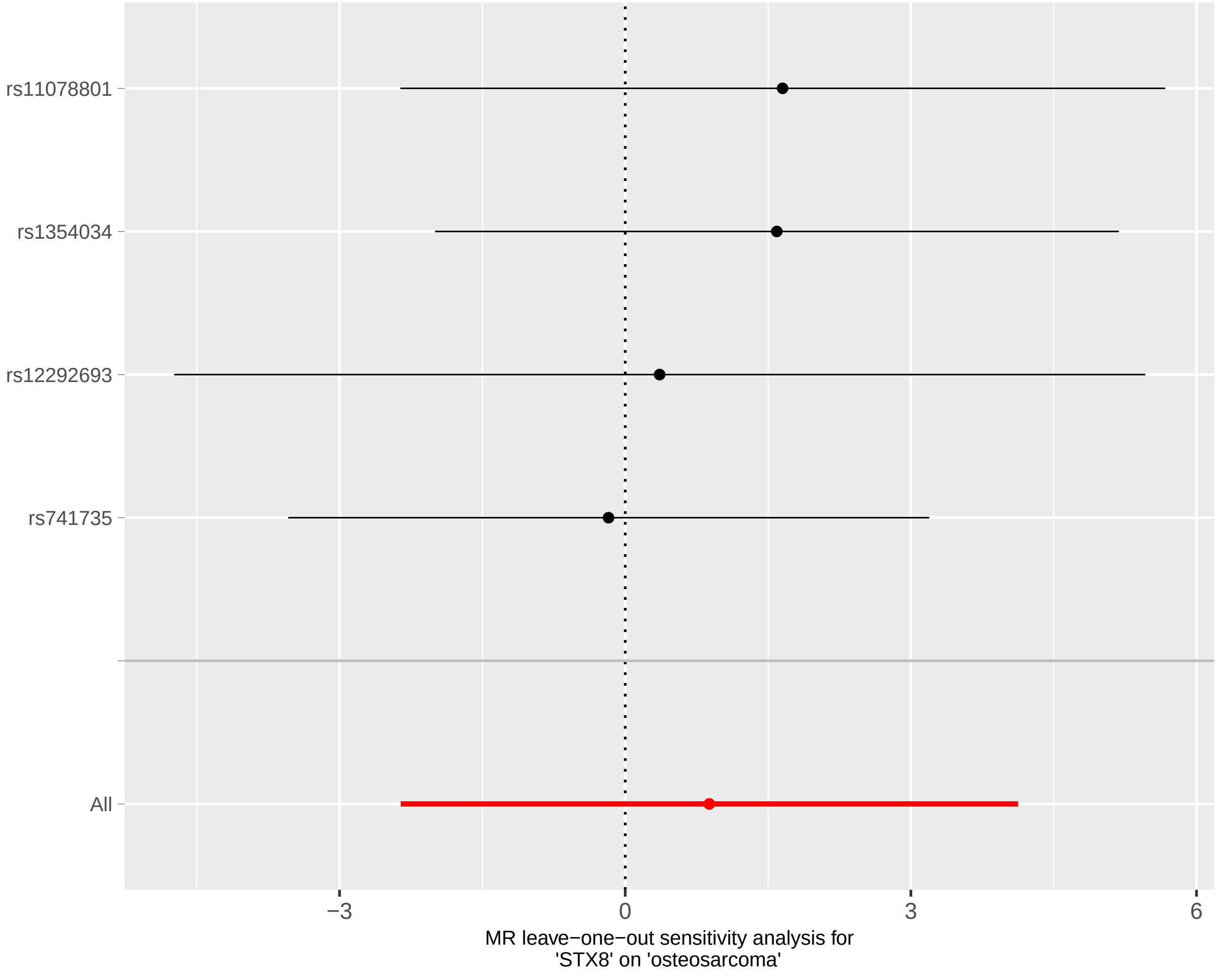
rs11078801
rs1354034
rs12292693
rs741735
All
−3
0
3
6
MR leave−one−out sensitivity analysis for
'STX8' on 'osteosarcoma'

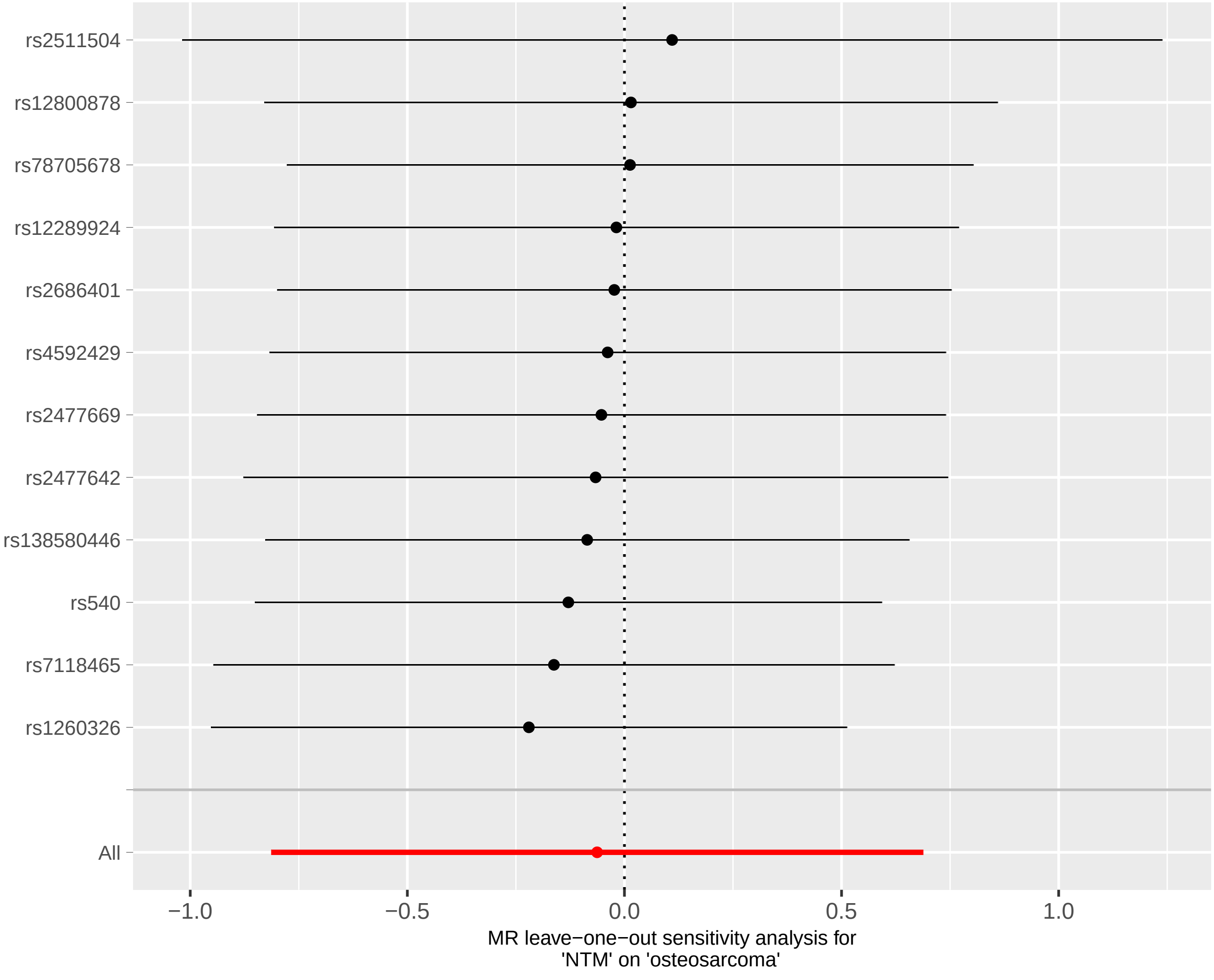
rs2511504
rs12800878
rs78705678
rs12289924
rs2686401
rs4592429
rs2477669
rs2477642
rs138580446
rs540
rs7118465
rs1260326
All
−1.0
−0.5
0.0
0.5
1.0
MR leave−one−out sensitivity analysis for
'NTM' on 'osteosarcoma'

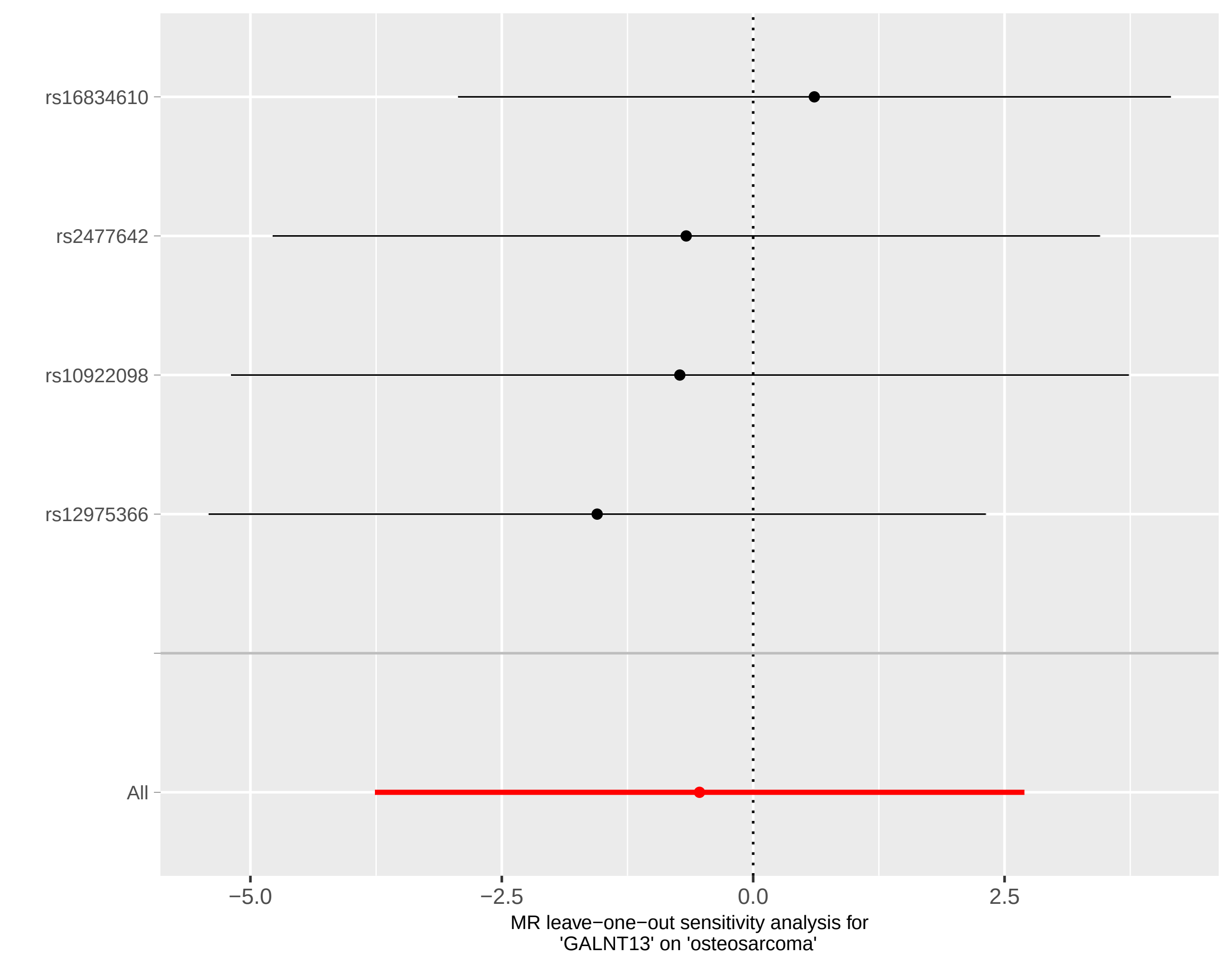

rs16834610
rs2477642
rs10922098
rs12975366
All
−5.0
−2.5
0.0
2.5
MR leave−one−out sensitivity analysis for
'GALNT13' on 'osteosarcoma'

Insufficient number of SNPs

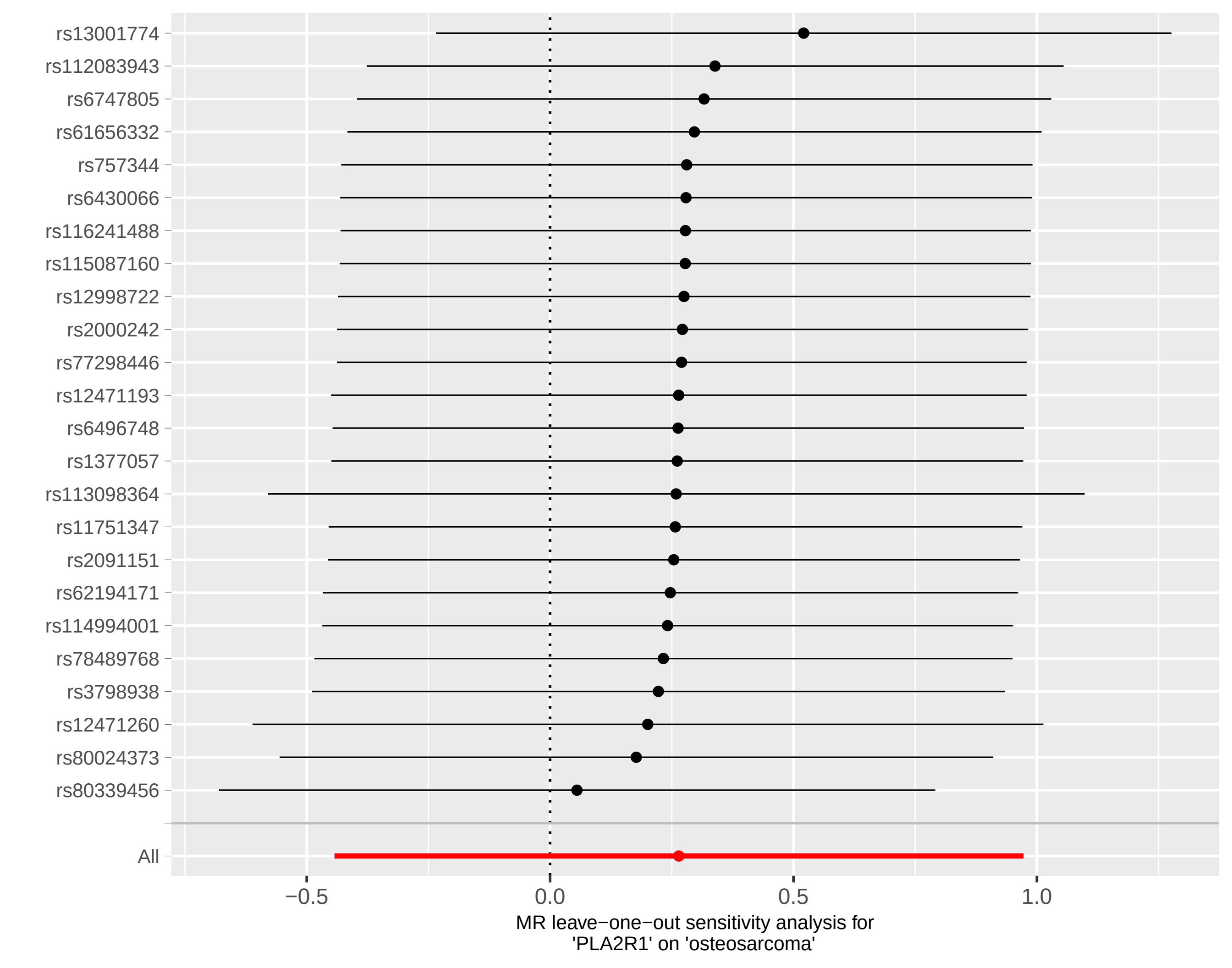

rs13001774
rs112083943
rs6747805
rs61656332
rs757344
rs6430066
rs116241488
rs115087160
rs12998722
rs2000242
rs77298446
rs12471193
rs6496748
rs1377057
rs113098364
rs11751347
rs2091151
rs62194171
rs114994001
rs78489768
rs3798938
rs12471260
rs80024373
rs80339456
All
−0.5
0.0
0.5
1.0
MR leave−one−out sensitivity analysis for
'PLA2R1' on 'osteosarcoma'

Insufficient number of SNPs

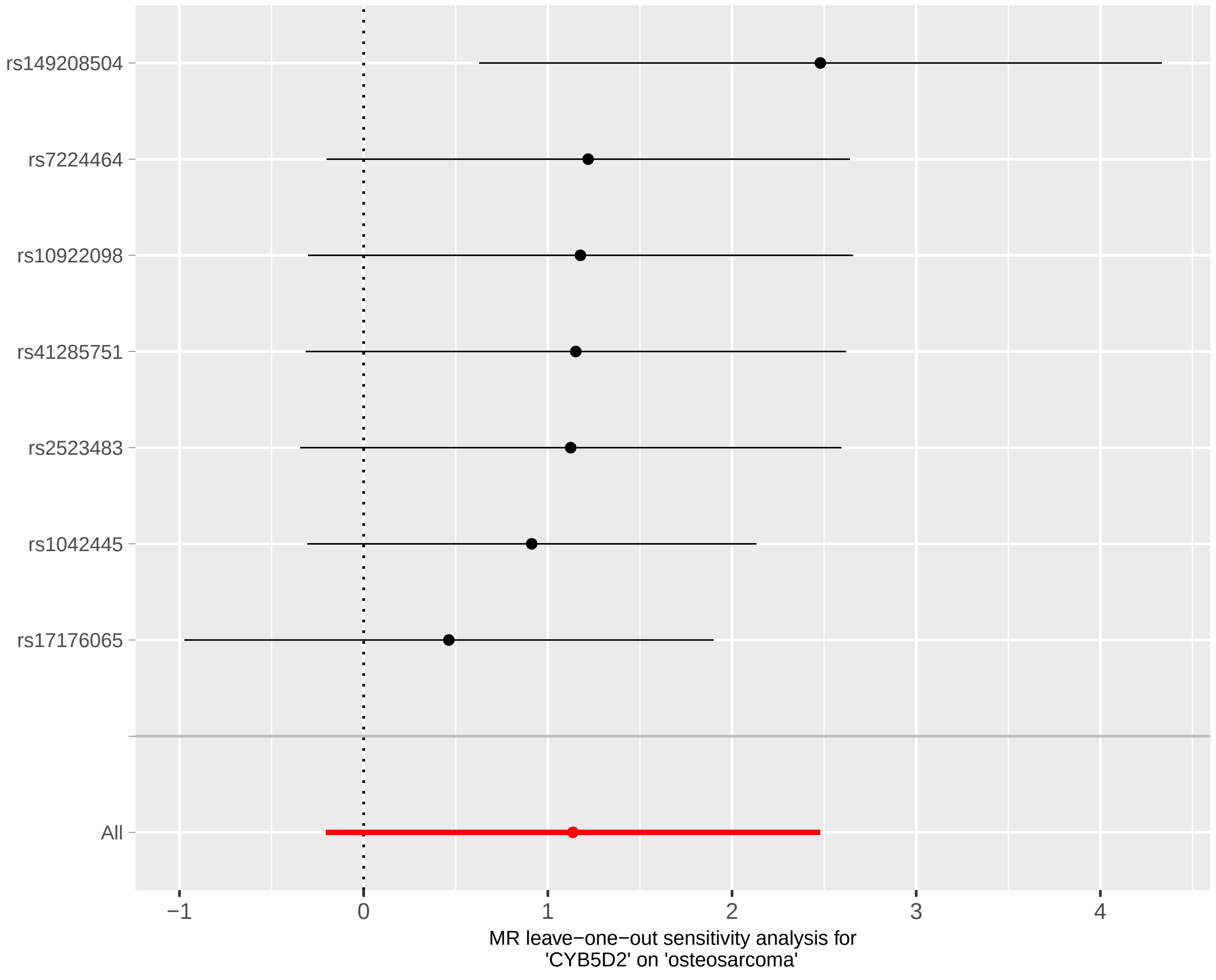

rs149208504
rs7224464
rs10922098
rs41285751
rs2523483
rs1042445
rs17176065
All
−1
0
1
2
3
4
MR leave−one−out sensitivity analysis for
'CYB5D2' on 'osteosarcoma'

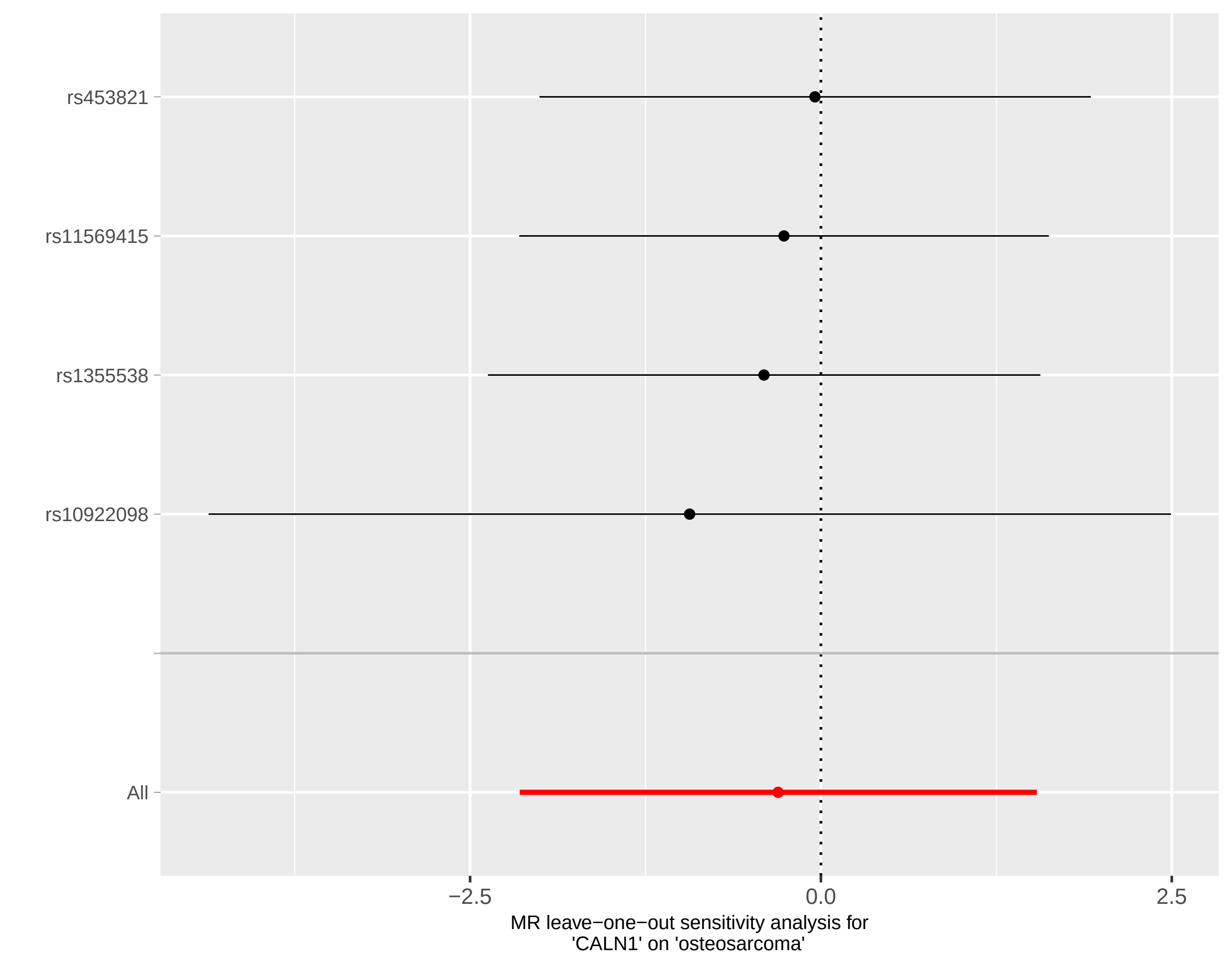
rs453821
rs11569415
rs1355538
rs10922098
All
−2.5
0.0
2.5
MR leave−one−out sensitivity analysis for
'CALN1' on 'osteosarcoma'

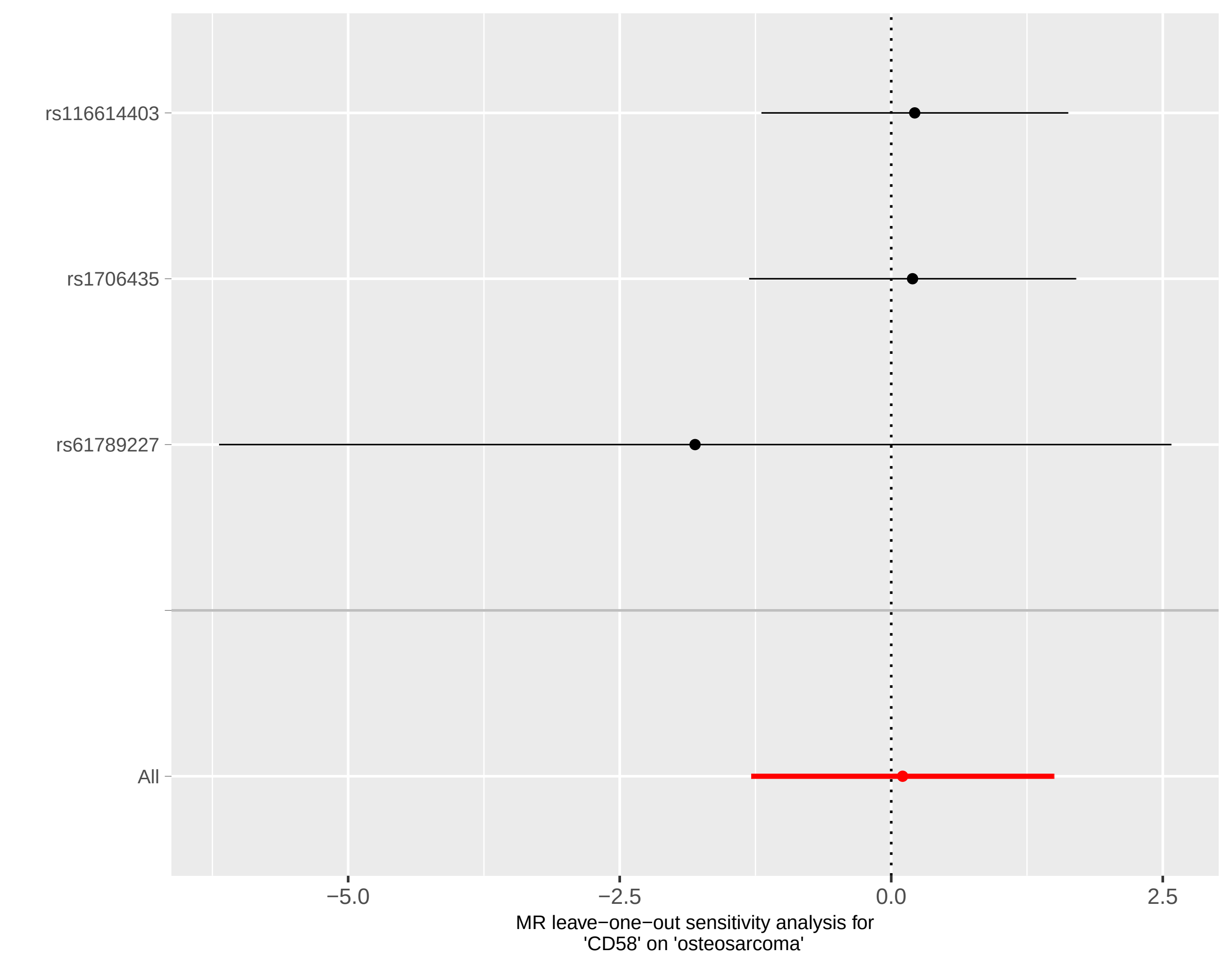

rs116614403
rs1706435
rs61789227
All
−5.0
−2.5
0.0
2.5
MR leave−one−out sensitivity analysis for
'CD58' on 'osteosarcoma'

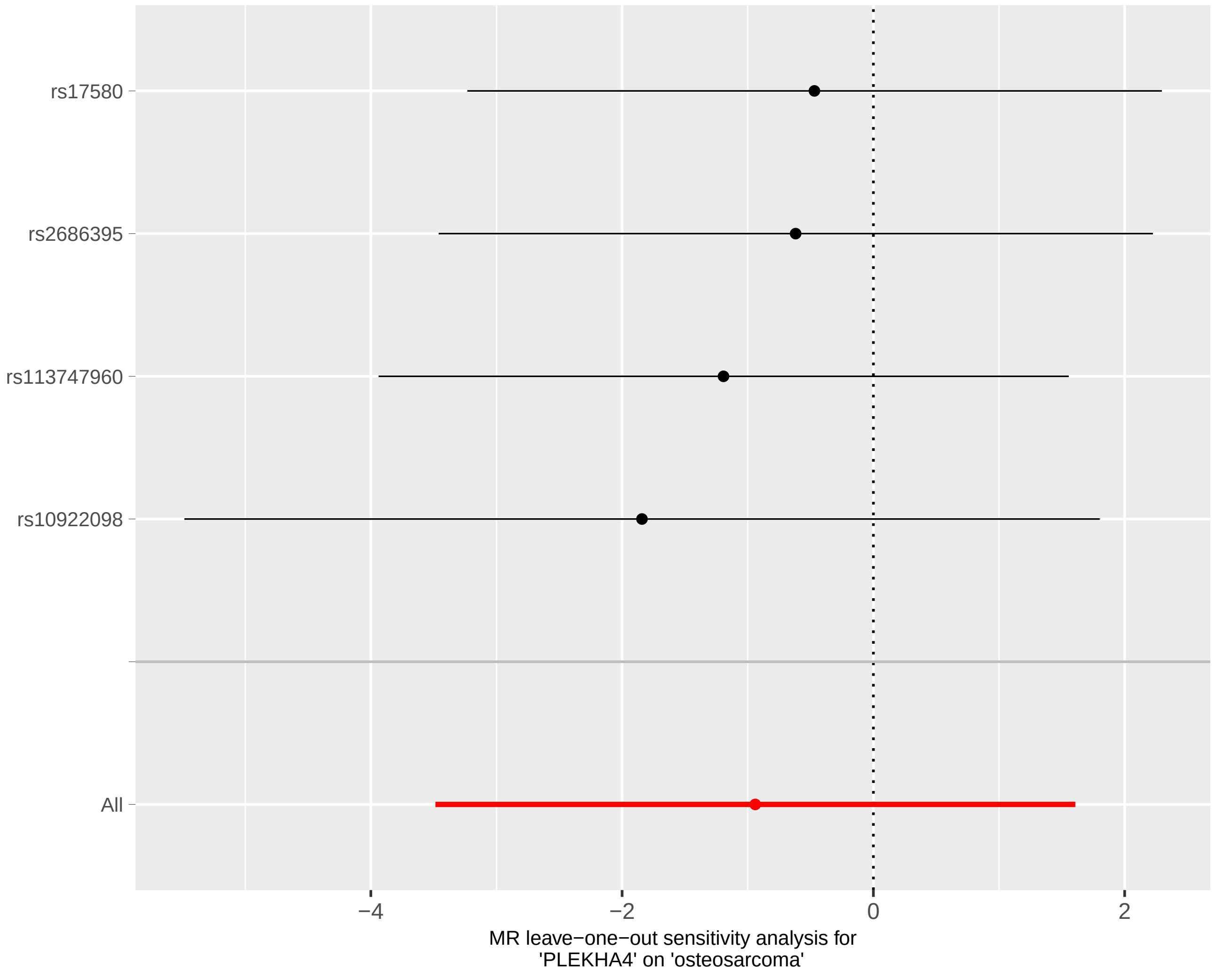
rs17580
rs2686395
rs113747960
rs10922098
All
−4
−2
0
2
MR leave−one−out sensitivity analysis for
'PLEKHA4' on 'osteosarcoma'

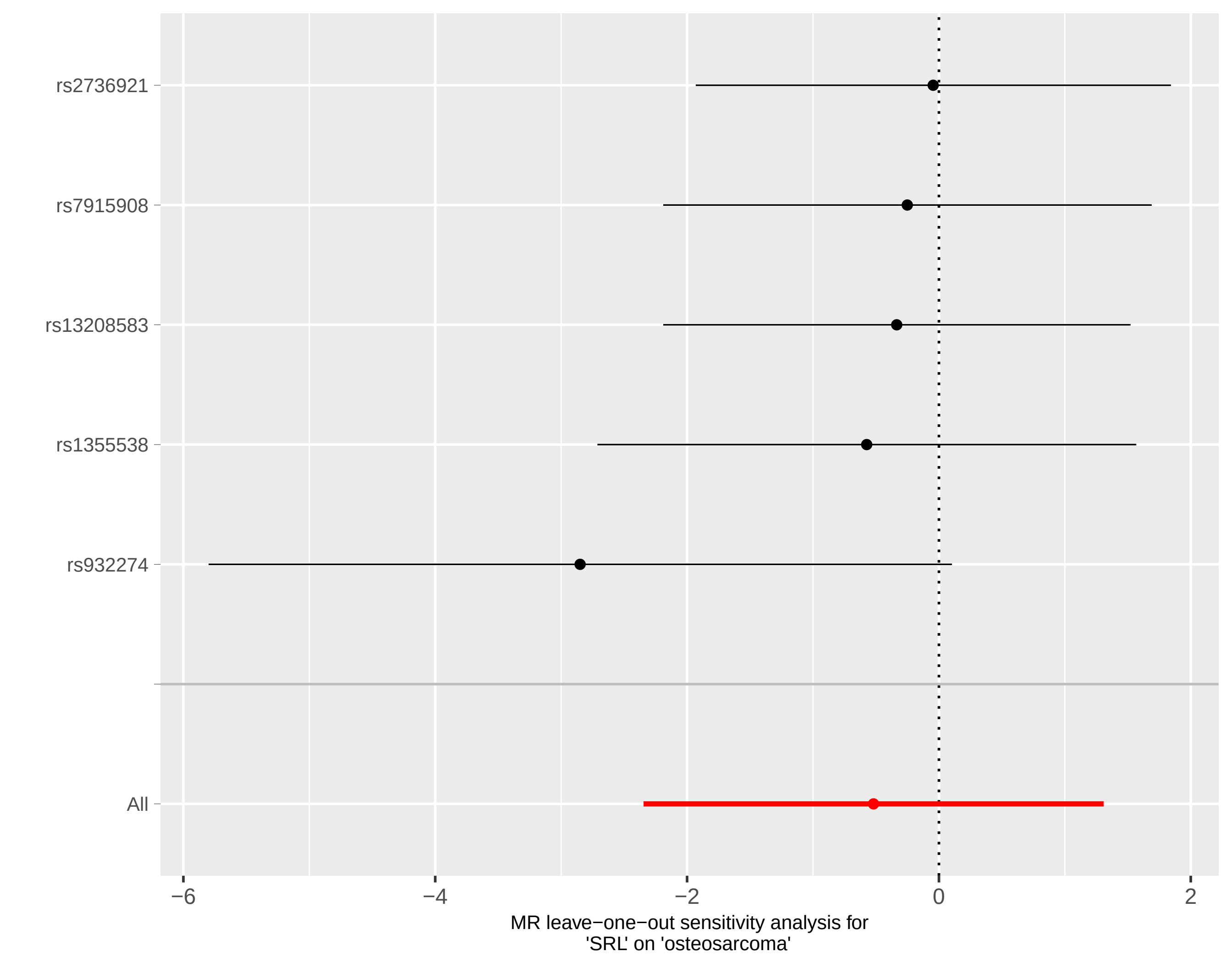
rs2736921
rs7915908
rs13208583
rs1355538
rs932274
All
−6
−4
−2
0
2
MR leave−one−out sensitivity analysis for
'SRL' on 'osteosarcoma'

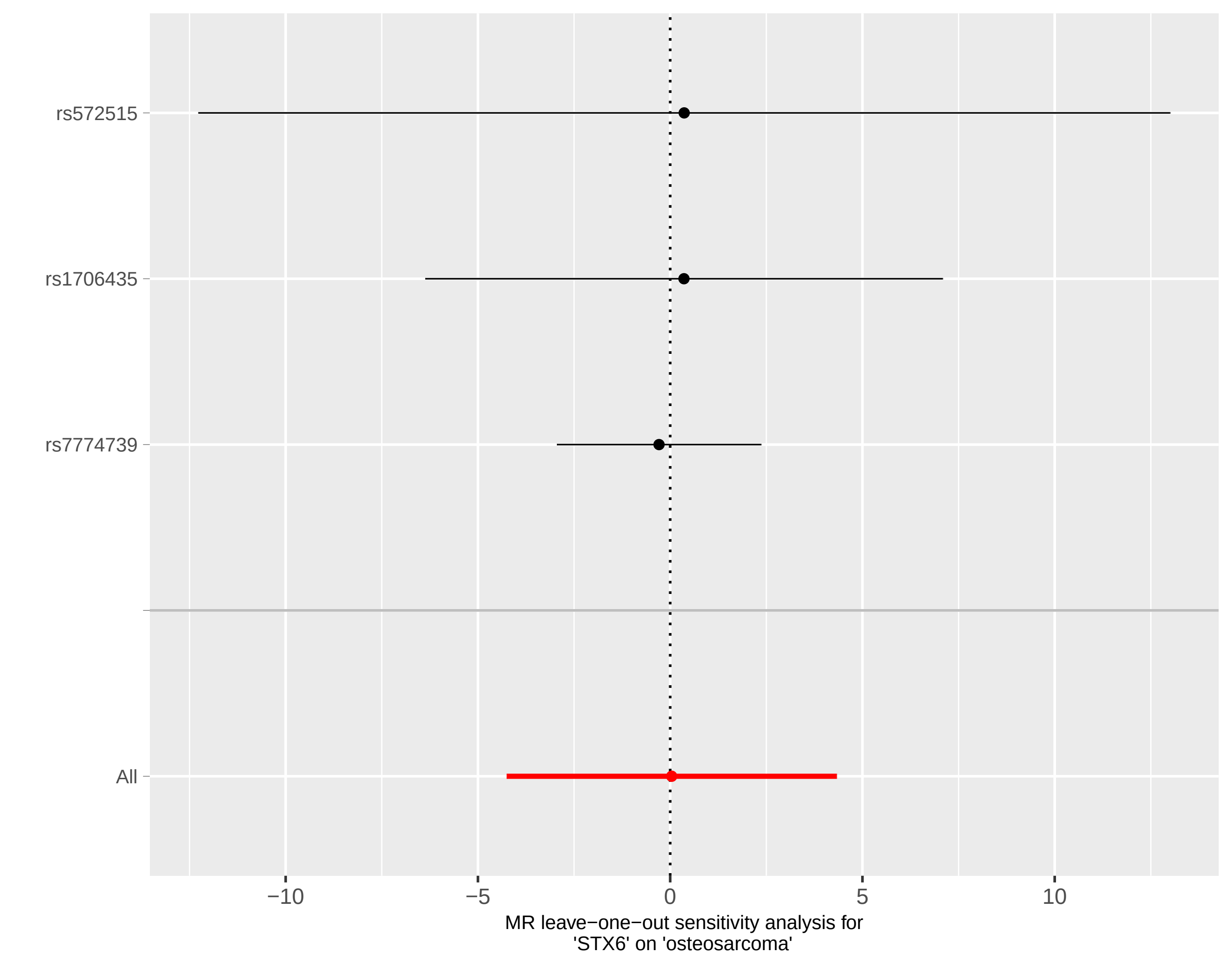
rs572515
rs1706435
rs7774739
All
−10
−5
0
5
10
MR leave−one−out sensitivity analysis for
'STX6' on 'osteosarcoma'

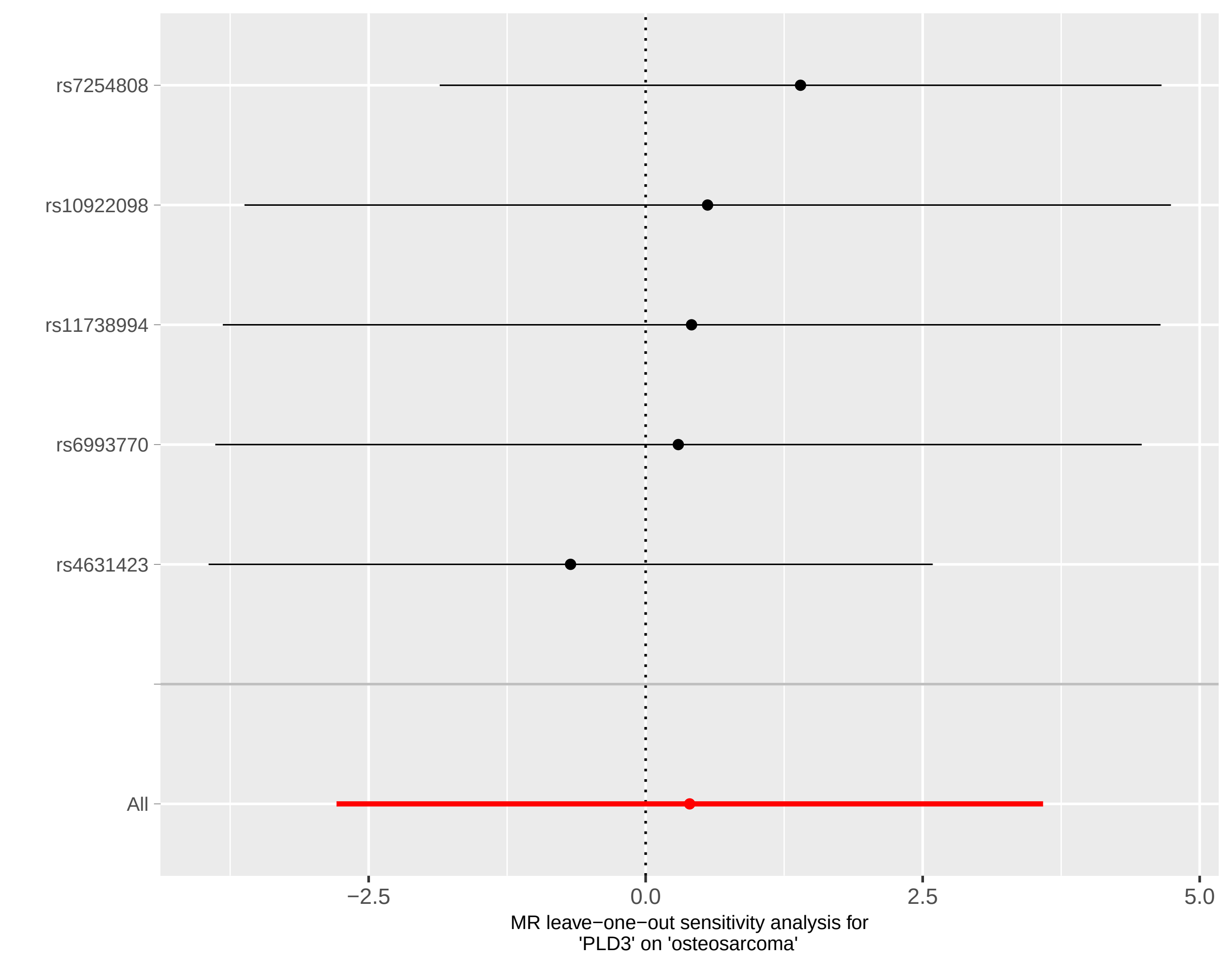
rs7254808
rs10922098
rs11738994
rs6993770
rs4631423
All
−2.5
0.0
2.5
5.0
MR leave−one−out sensitivity analysis for
'PLD3' on 'osteosarcoma'

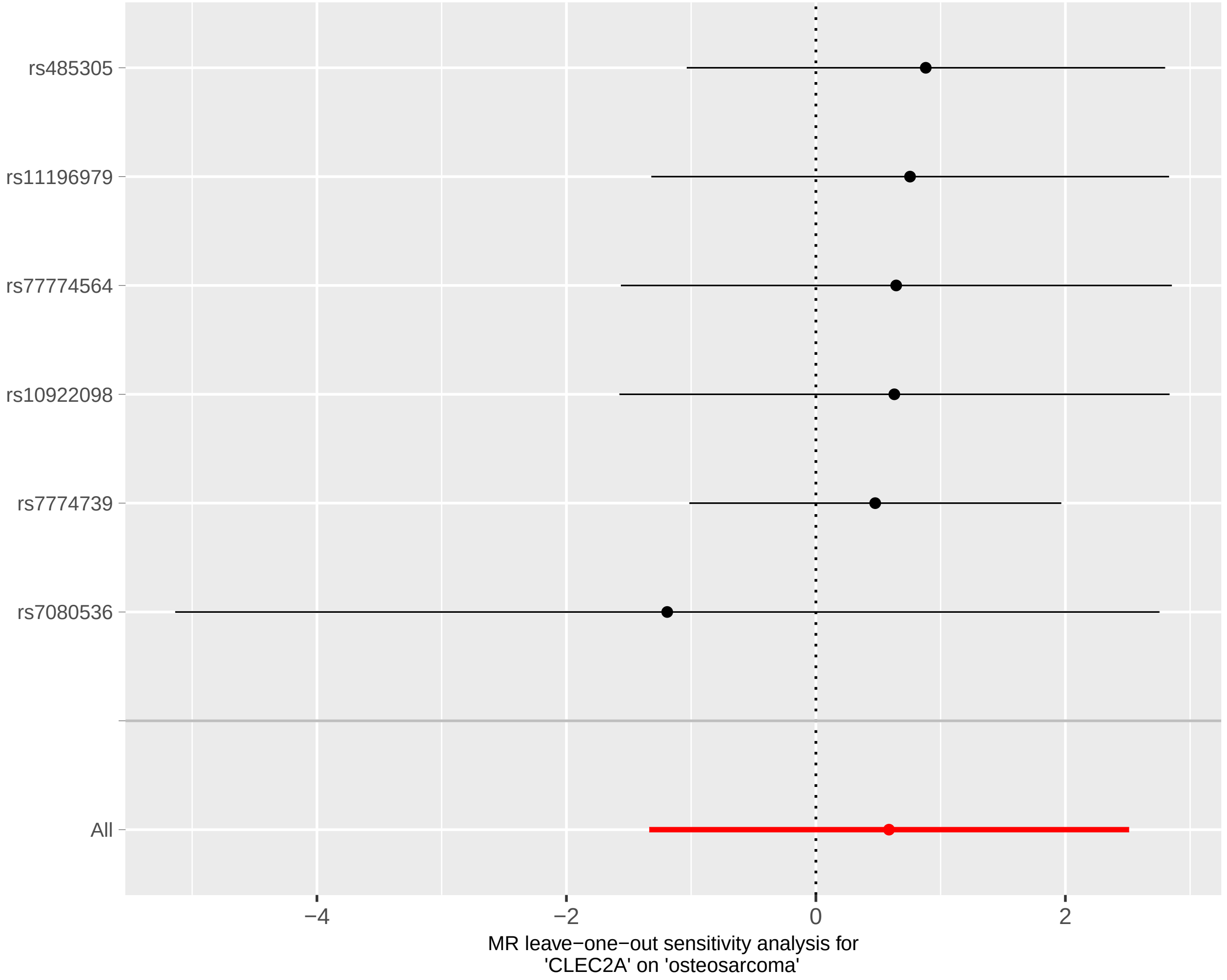
rs485305
rs11196979
rs77774564
rs10922098
rs7774739
rs7080536
All
−4
−2
0
2
MR leave−one−out sensitivity analysis for
'CLEC2A' on 'osteosarcoma'

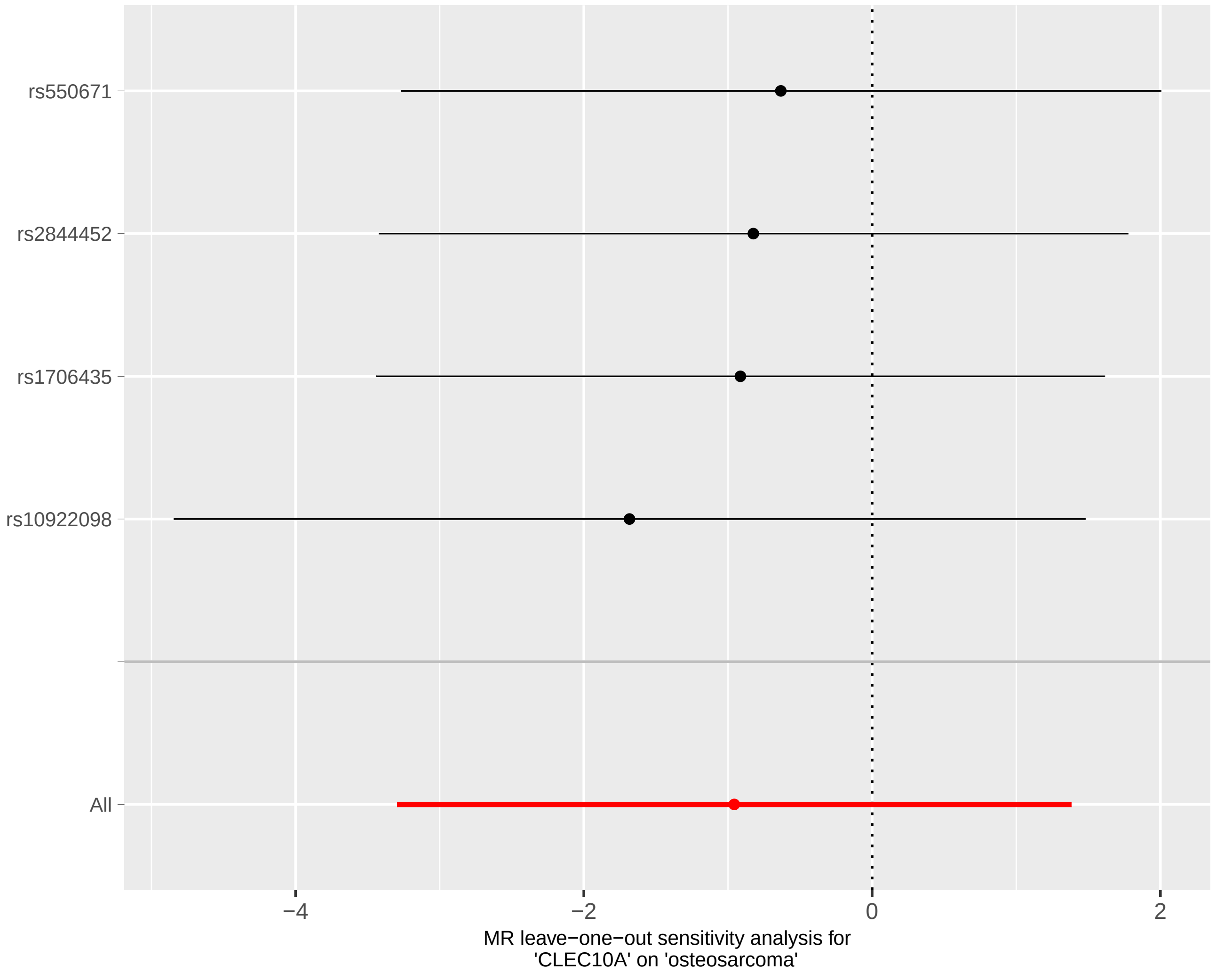
rs550671
rs2844452
rs1706435
rs10922098
All
−4
−2
0
2
MR leave−one−out sensitivity analysis for
'CLEC10A' on 'osteosarcoma'

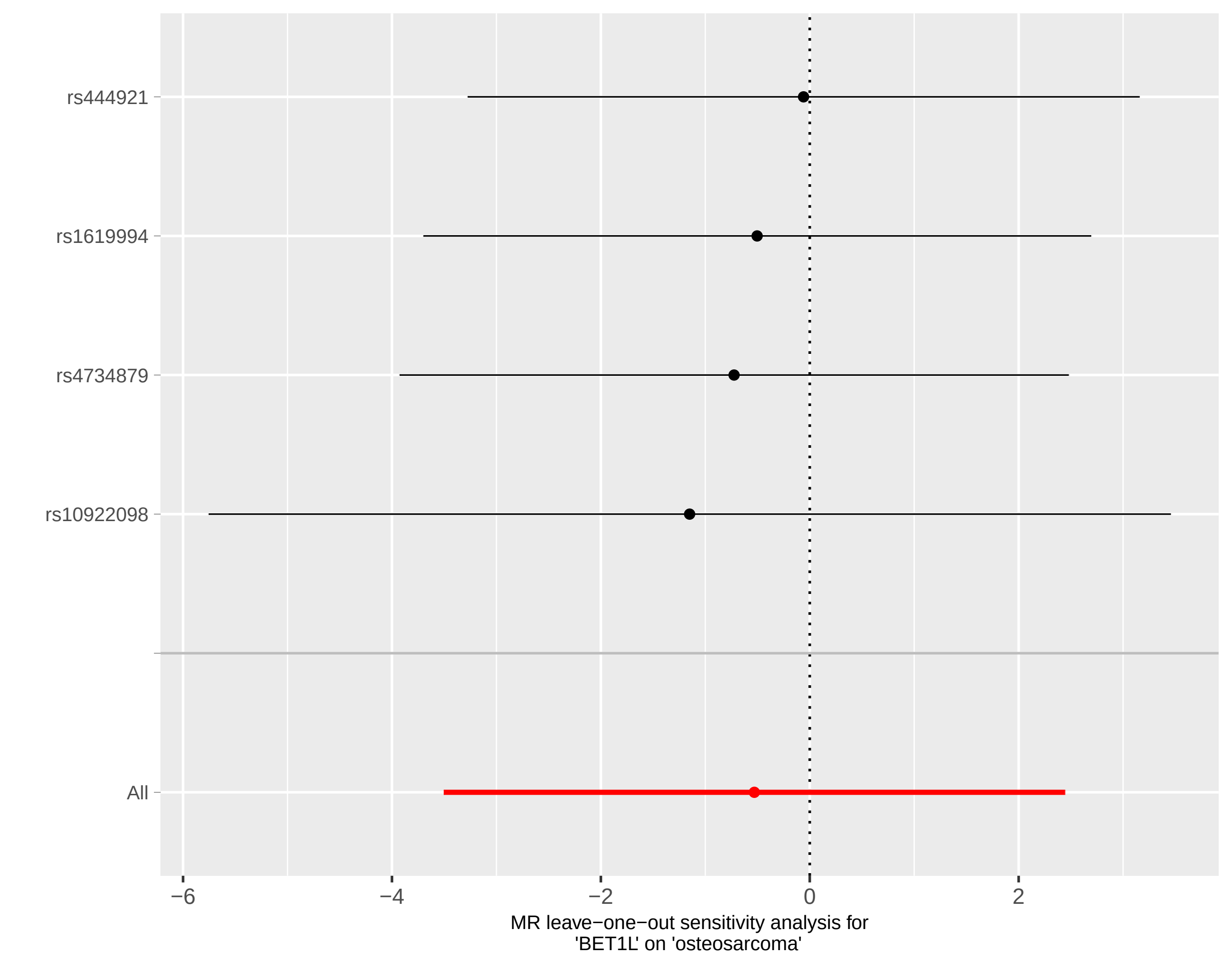

rs444921
rs1619994
rs4734879
rs10922098
All
−6
−4
−2
0
2
MR leave−one−out sensitivity analysis for
'BET1L' on 'osteosarcoma'

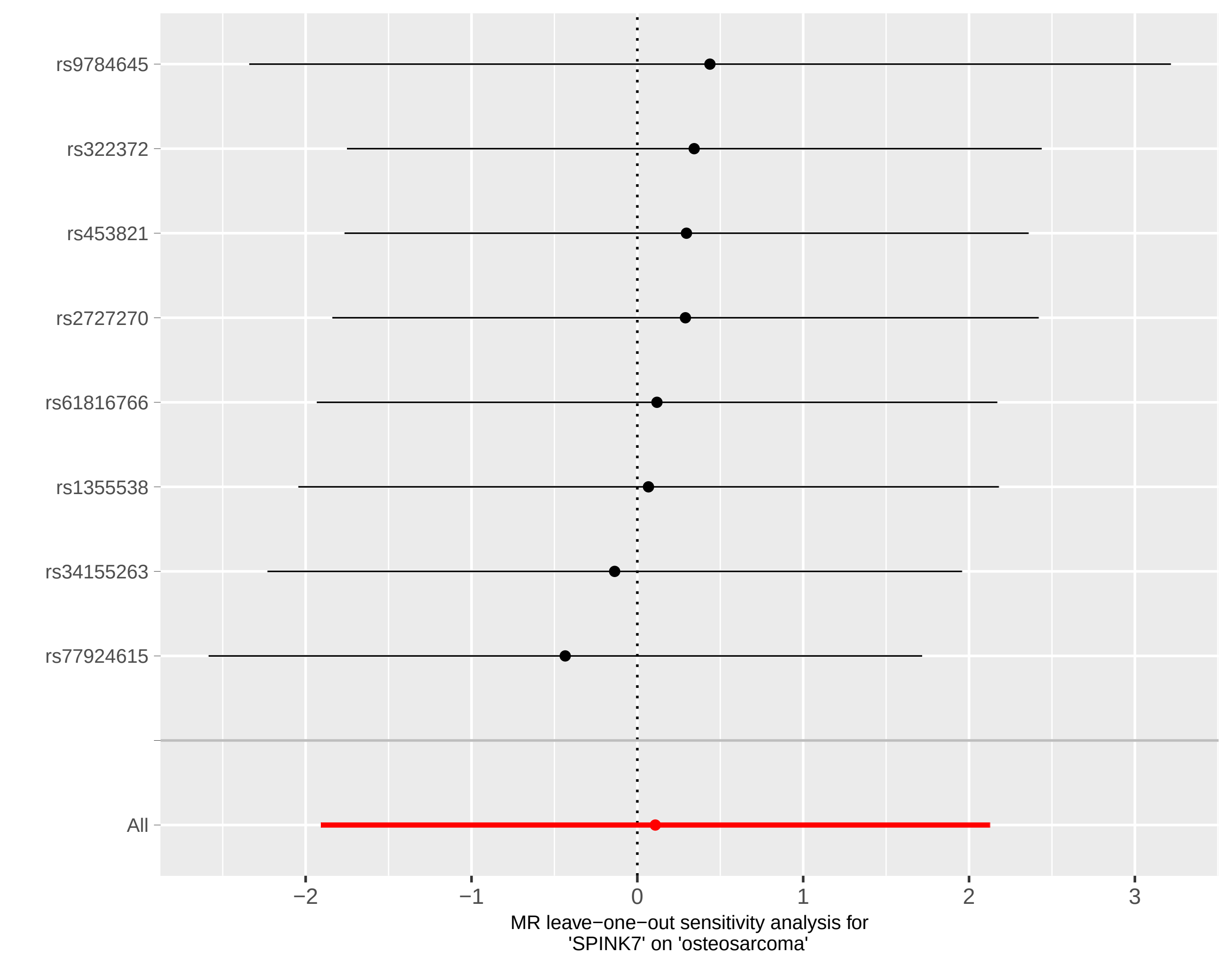

rs9784645
rs322372
rs453821
rs2727270
rs61816766
rs1355538
rs34155263
rs77924615
All
−2
−1
0
1
2
3
MR leave−one−out sensitivity analysis for
'SPINK7' on 'osteosarcoma'

Insufficient number of SNPs

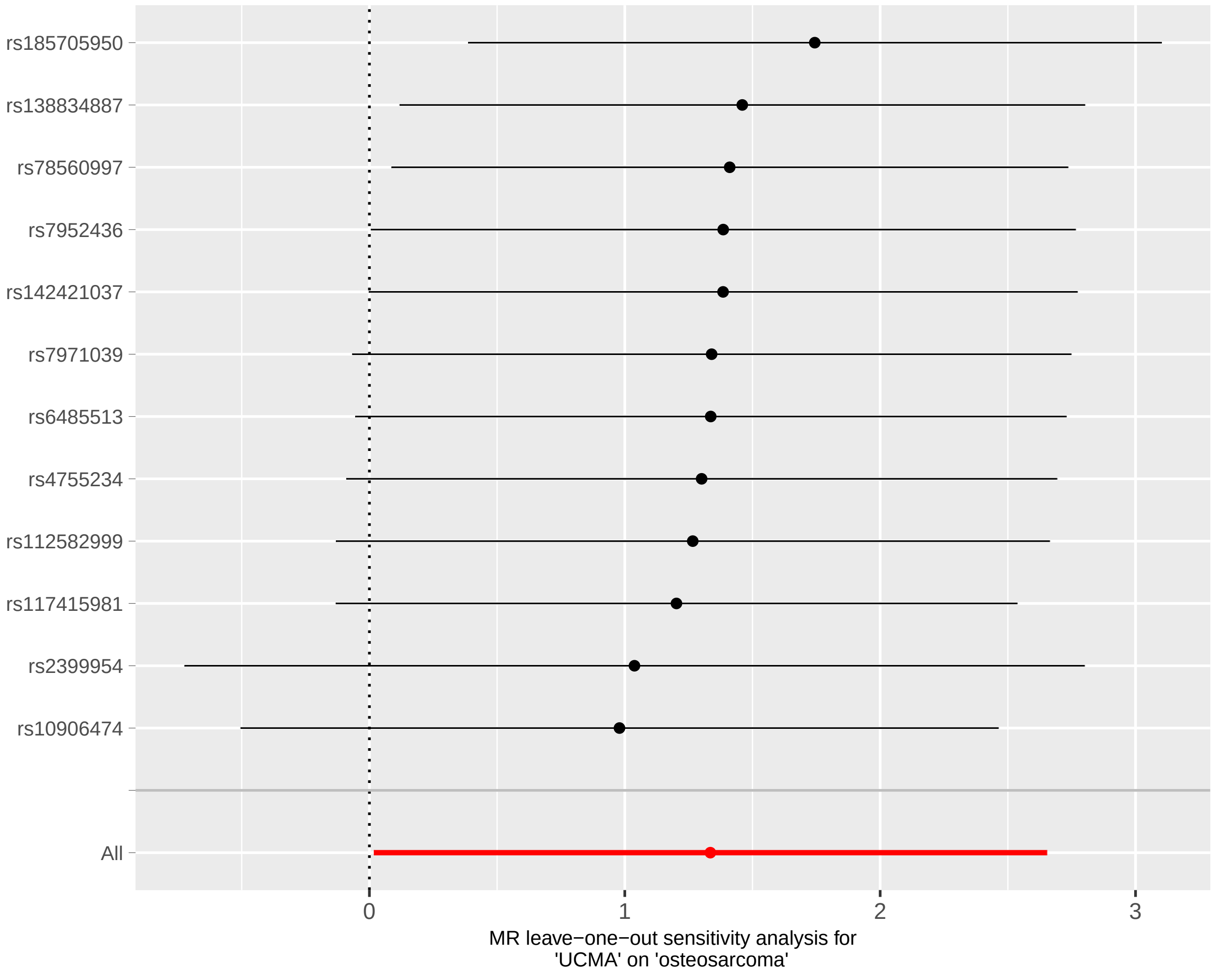
rs185705950
rs138834887
rs78560997
rs7952436
rs142421037
rs7971039
rs6485513
rs4755234
rs112582999
rs117415981
rs2399954
rs10906474
All
0
1
2
3
MR leave−one−out sensitivity analysis for
'UCMA' on 'osteosarcoma'

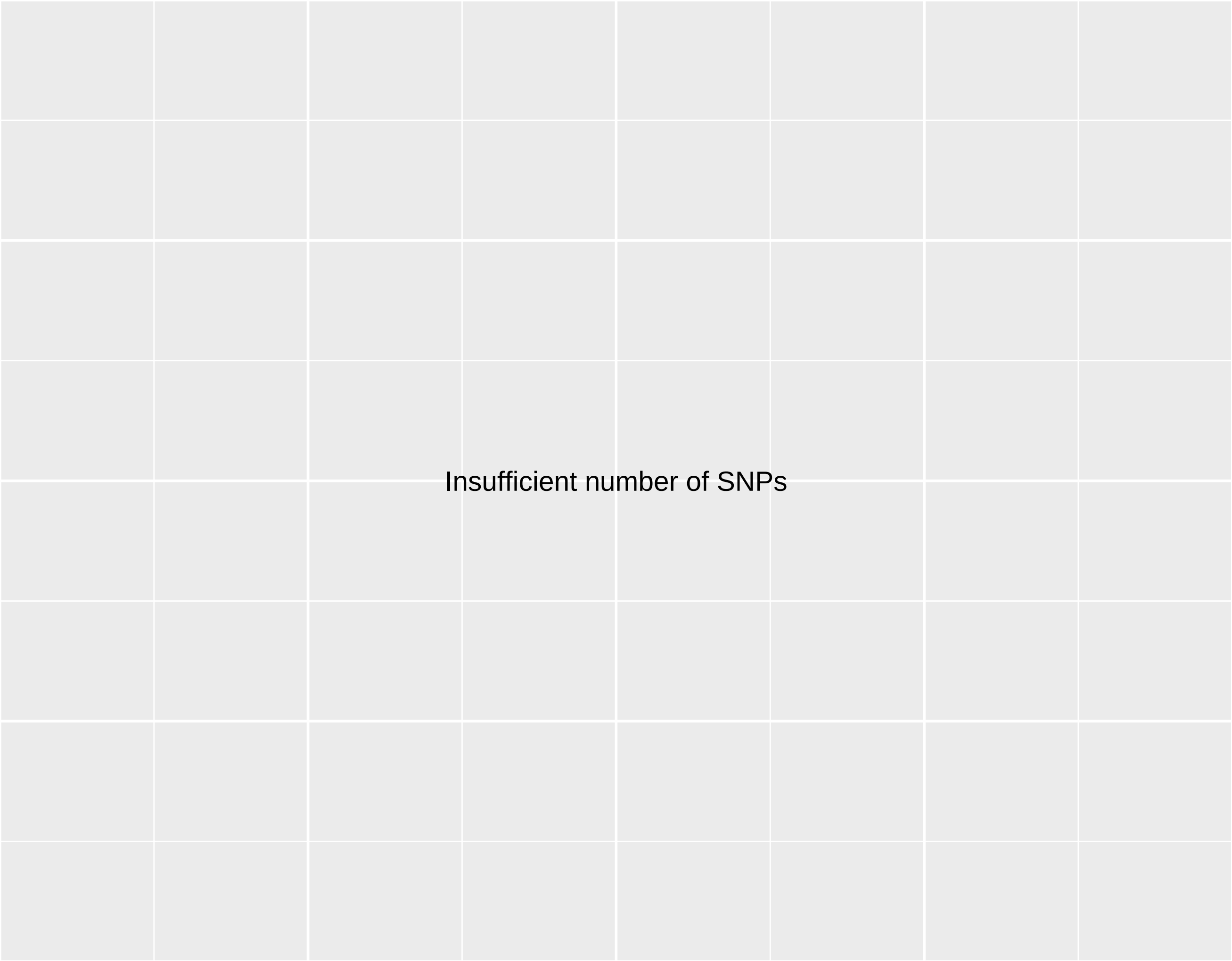
Insufficient number of SNPs

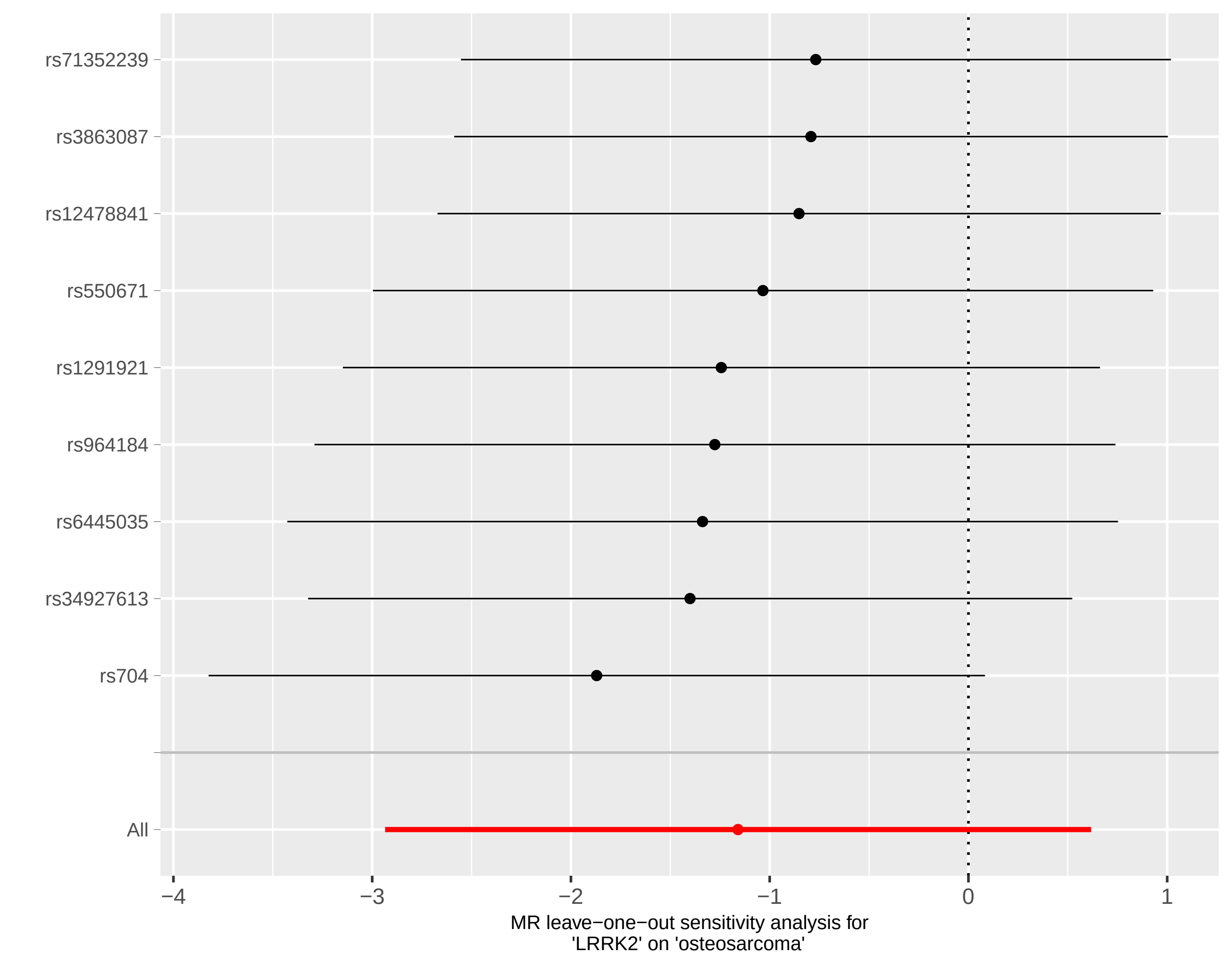

rs71352239
rs3863087
rs12478841
rs550671
rs1291921
rs964184
rs6445035
rs34927613
rs704
All
−4
−3
−2
−1
0
1
MR leave−one−out sensitivity analysis for
'LRRK2' on 'osteosarcoma'

Insufficient number of SNPs

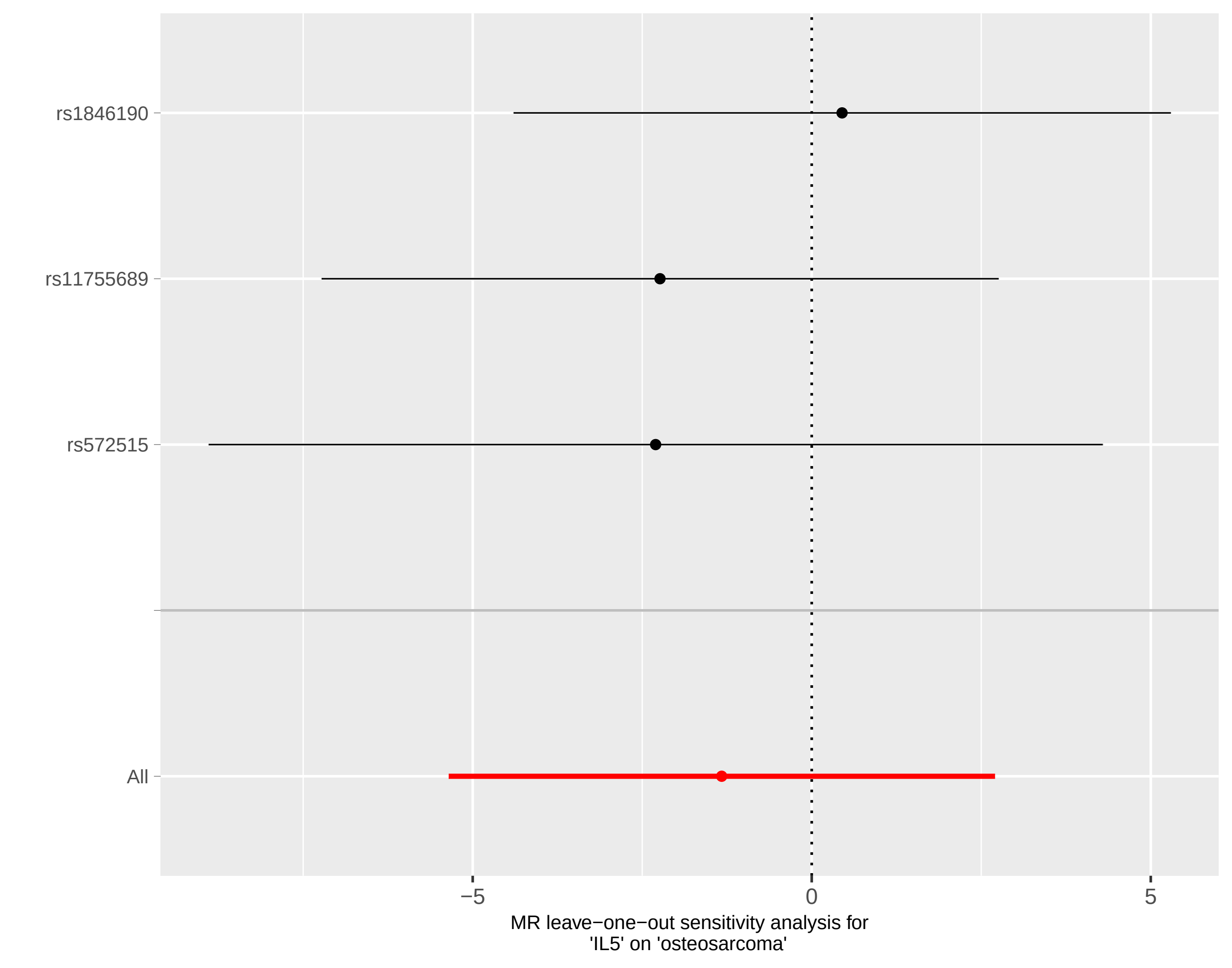

rs1846190
rs11755689
rs572515
All
−5
0
5
MR leave−one−out sensitivity analysis for
'IL5' on 'osteosarcoma'

Insufficient number of SNPs

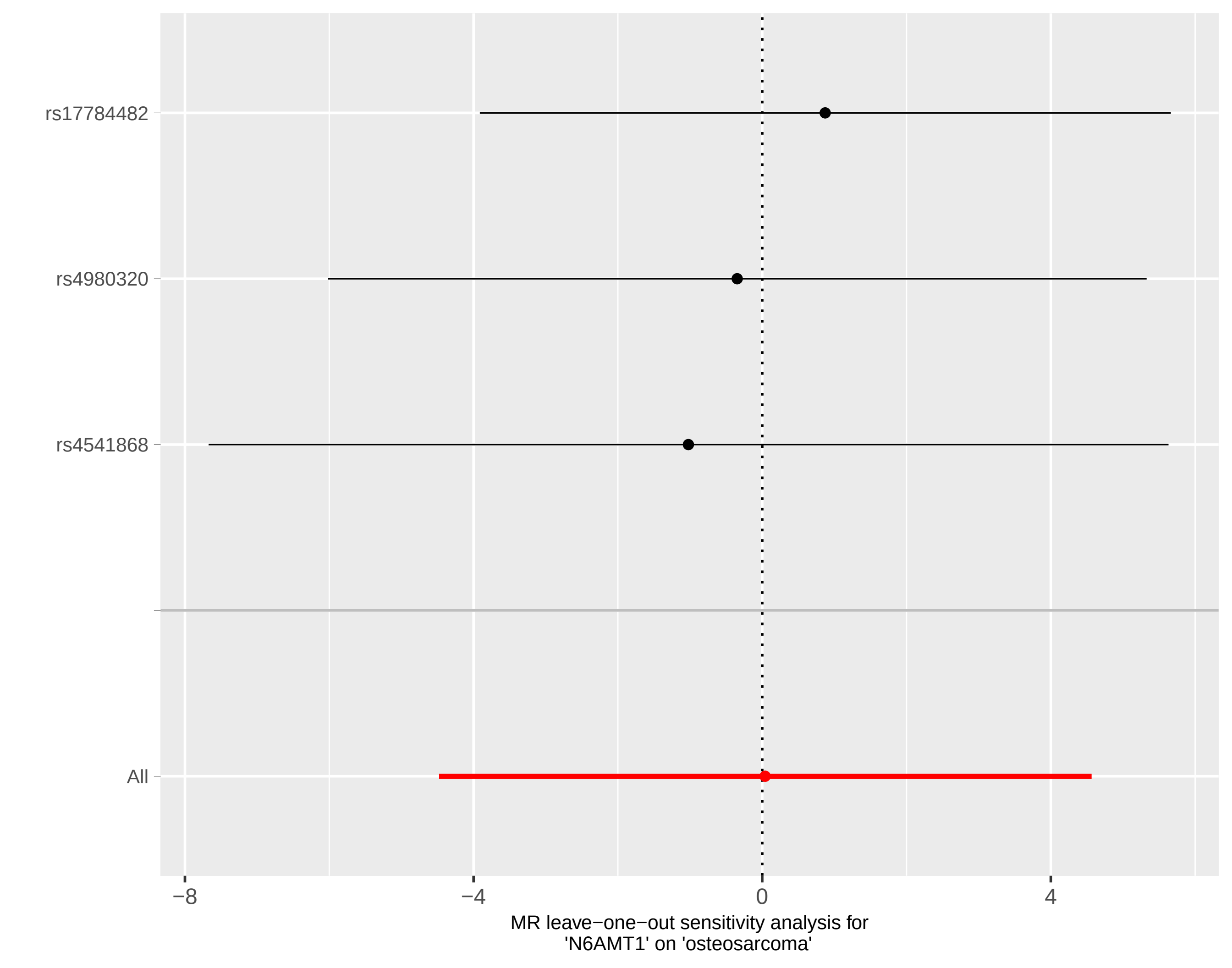
rs17784482
rs4980320
rs4541868
All
−8
−4
0
4
MR leave−one−out sensitivity analysis for
'N6AMT1' on 'osteosarcoma'

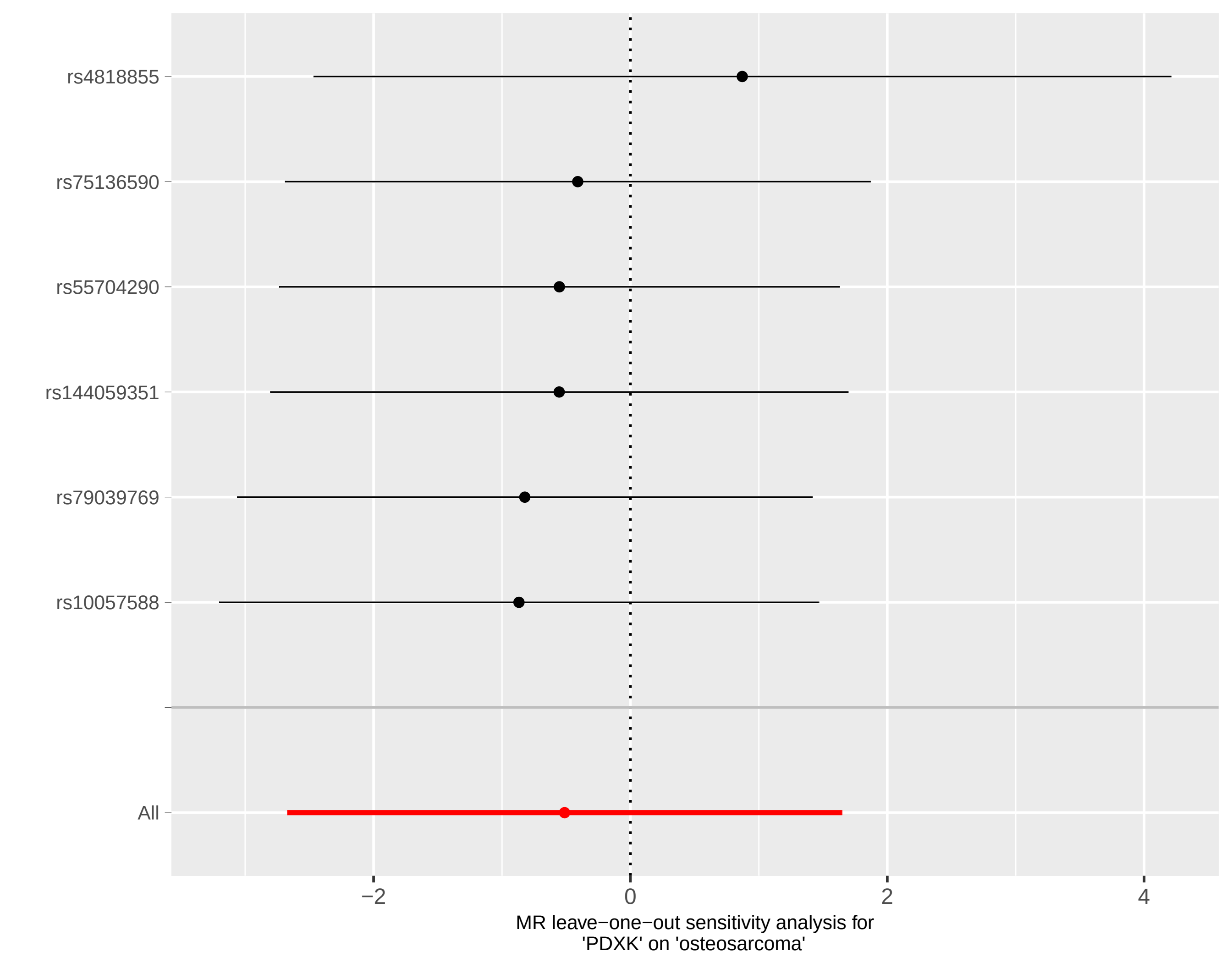
rs4818855
rs75136590
rs55704290
rs144059351
rs79039769
rs10057588
All
−2
0
2
4
MR leave−one−out sensitivity analysis for
'PDXK' on 'osteosarcoma'

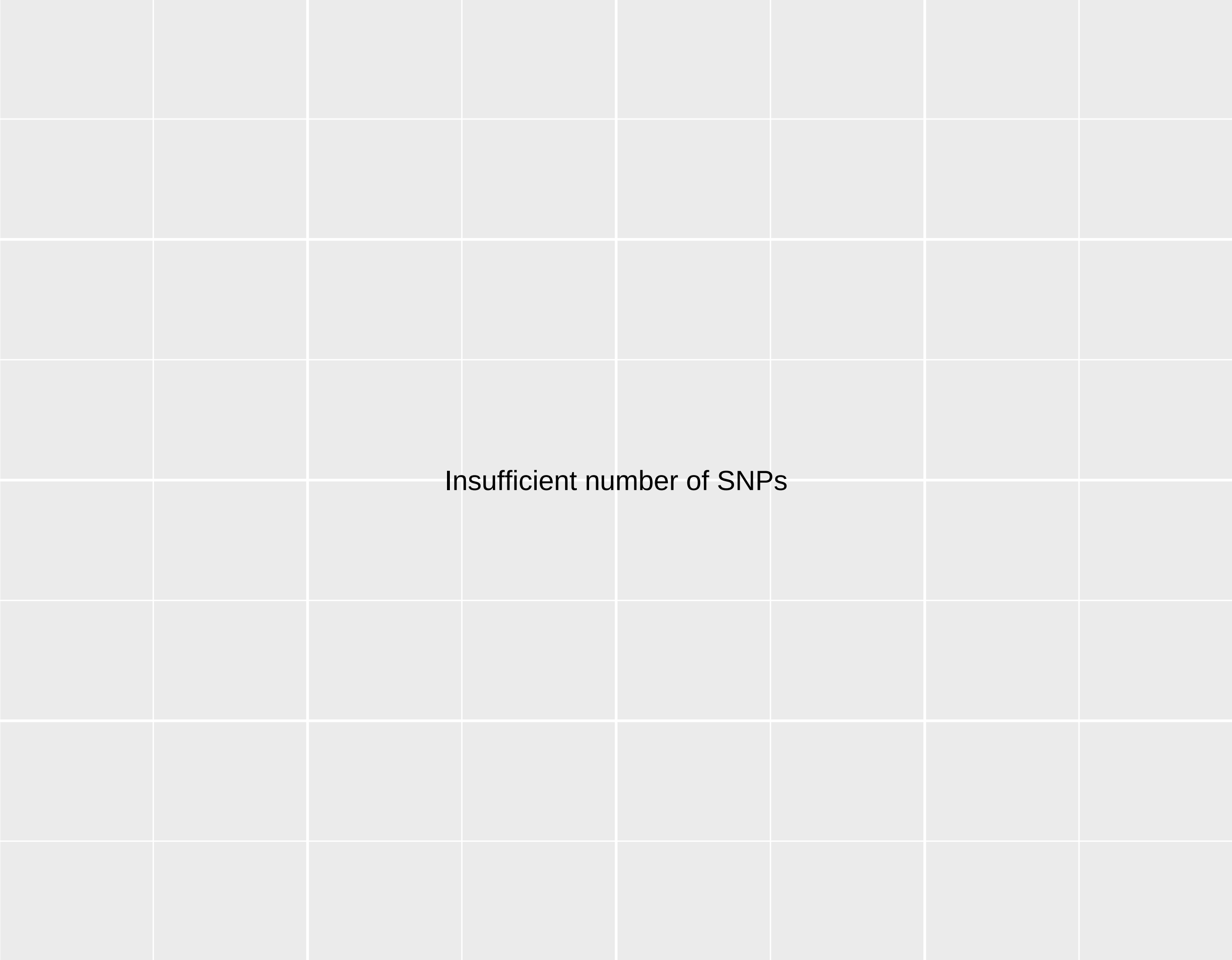
Insufficient number of SNPs

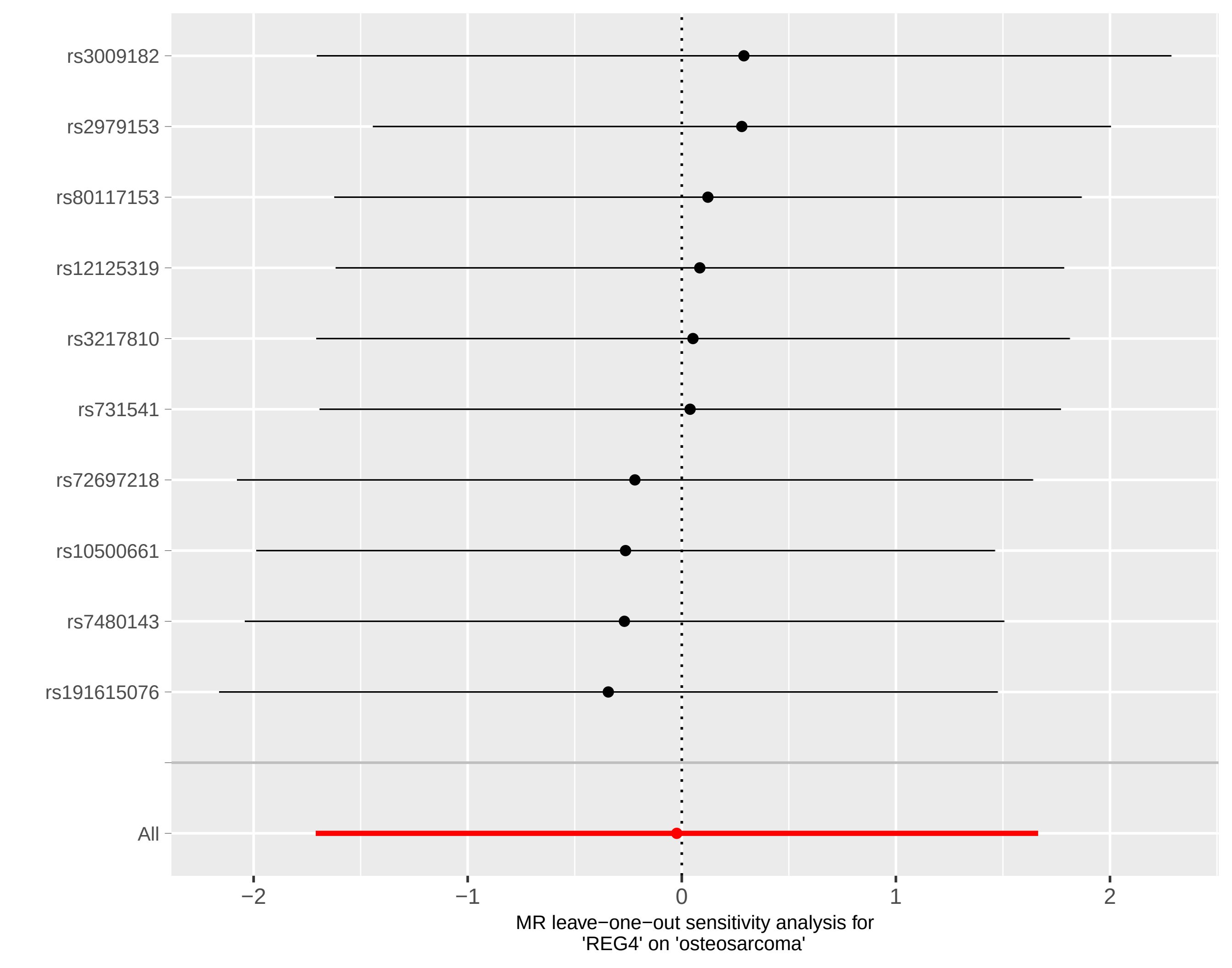

rs3009182
rs2979153
rs80117153
rs12125319
rs3217810
rs731541
rs72697218
rs10500661
rs7480143
rs191615076
All
−2
−1
0
1
2
MR leave−one−out sensitivity analysis for
'REG4' on 'osteosarcoma'

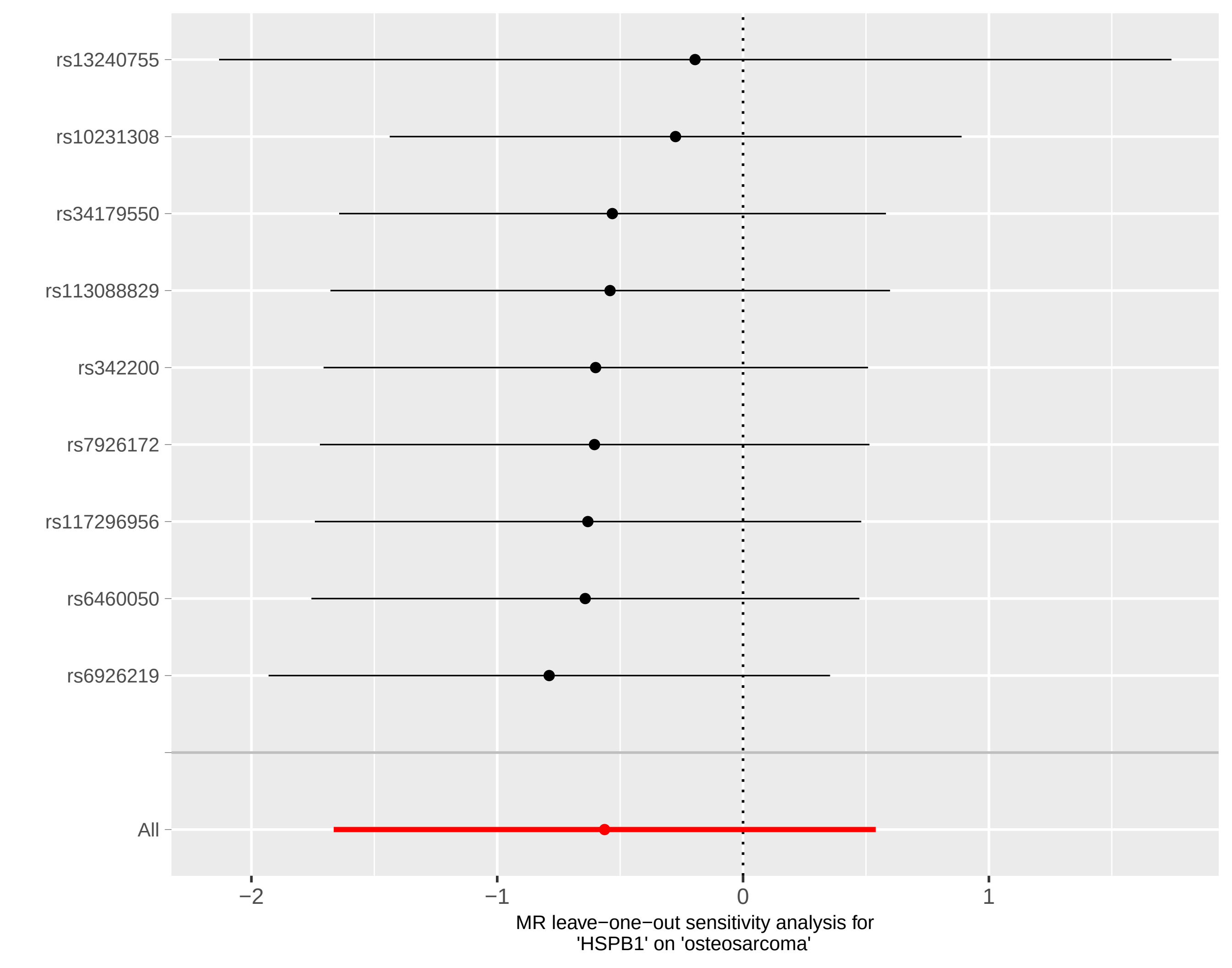

rs13240755
rs10231308
rs34179550
rs113088829
rs342200
rs7926172
rs117296956
rs6460050
rs6926219
All
−2
−1
0
1
MR leave−one−out sensitivity analysis for
'HSPB1' on 'osteosarcoma'

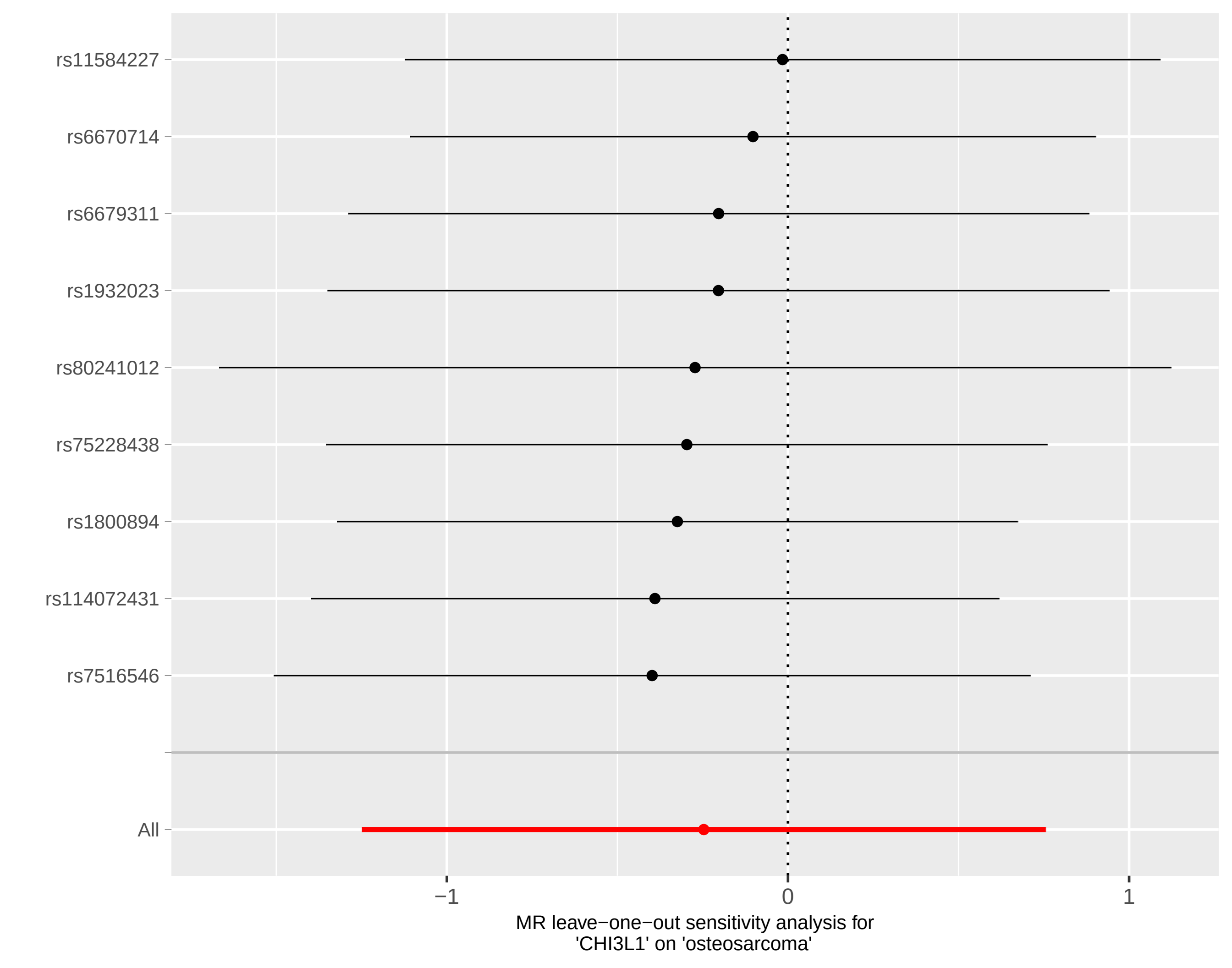

rs11584227
rs6670714
rs6679311
rs1932023
rs80241012
rs75228438
rs1800894
rs114072431
rs7516546
All
−1
0
1
MR leave−one−out sensitivity analysis for
'CHI3L1' on 'osteosarcoma'

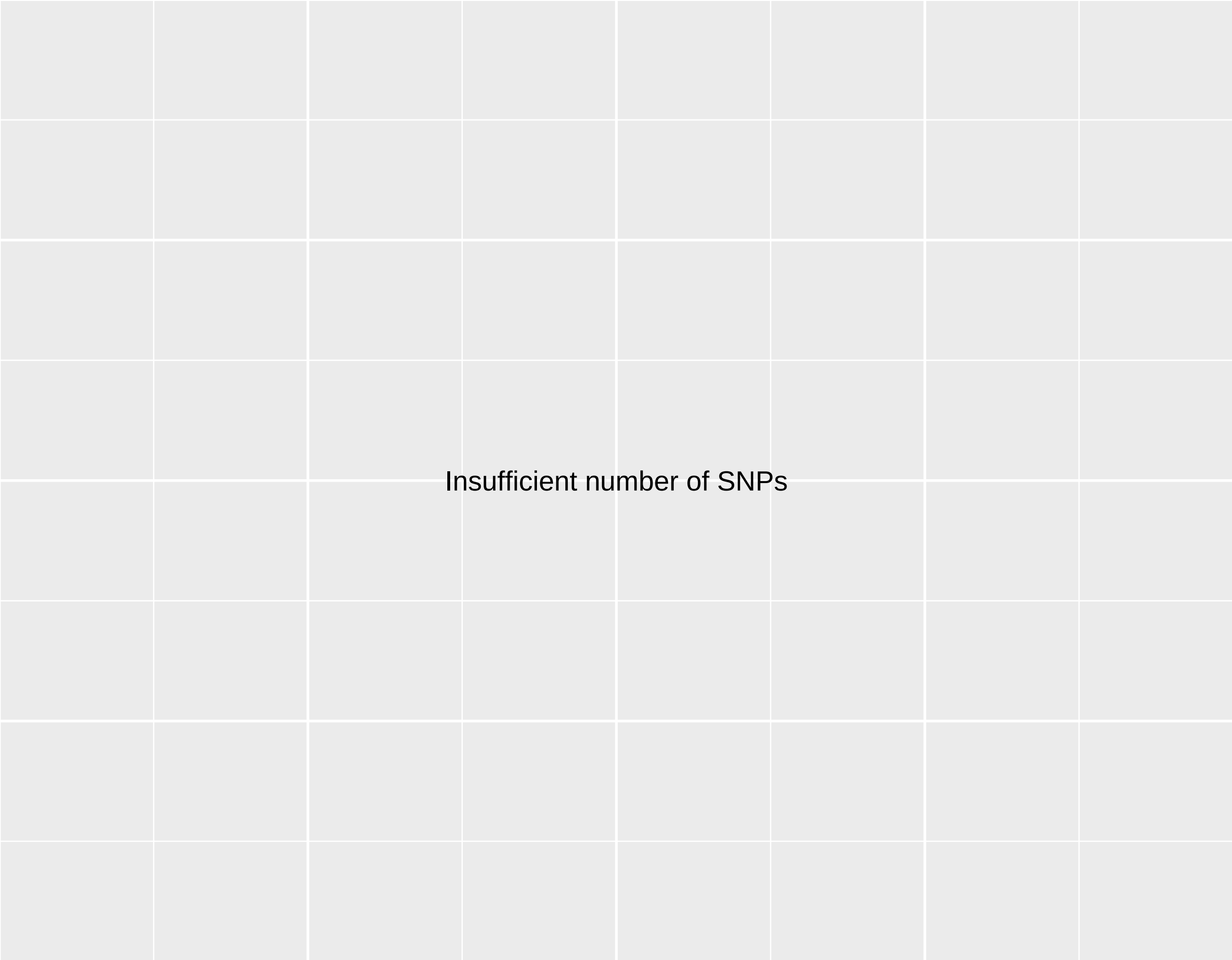
Insufficient number of SNPs

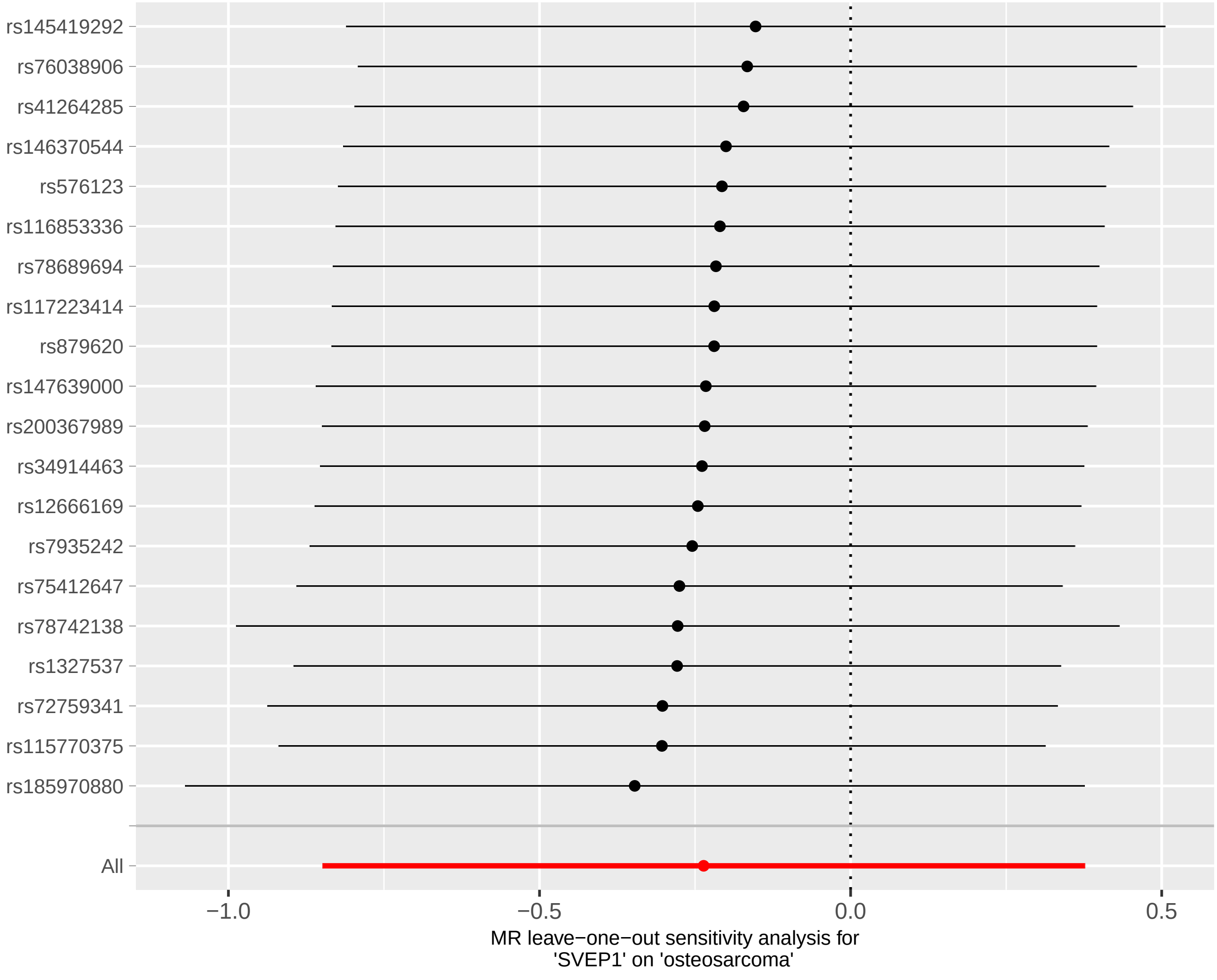

rs145419292
rs76038906
rs41264285
rs146370544
rs576123
rs116853336
rs78689694
rs117223414
rs879620
rs147639000
rs200367989
rs34914463
rs12666169
rs7935242
rs75412647
rs78742138
rs1327537
rs72759341
rs115770375
rs185970880
All
−1.0
−0.5
0.0
0.5
MR leave−one−out sensitivity analysis for
'SVEP1' on 'osteosarcoma'

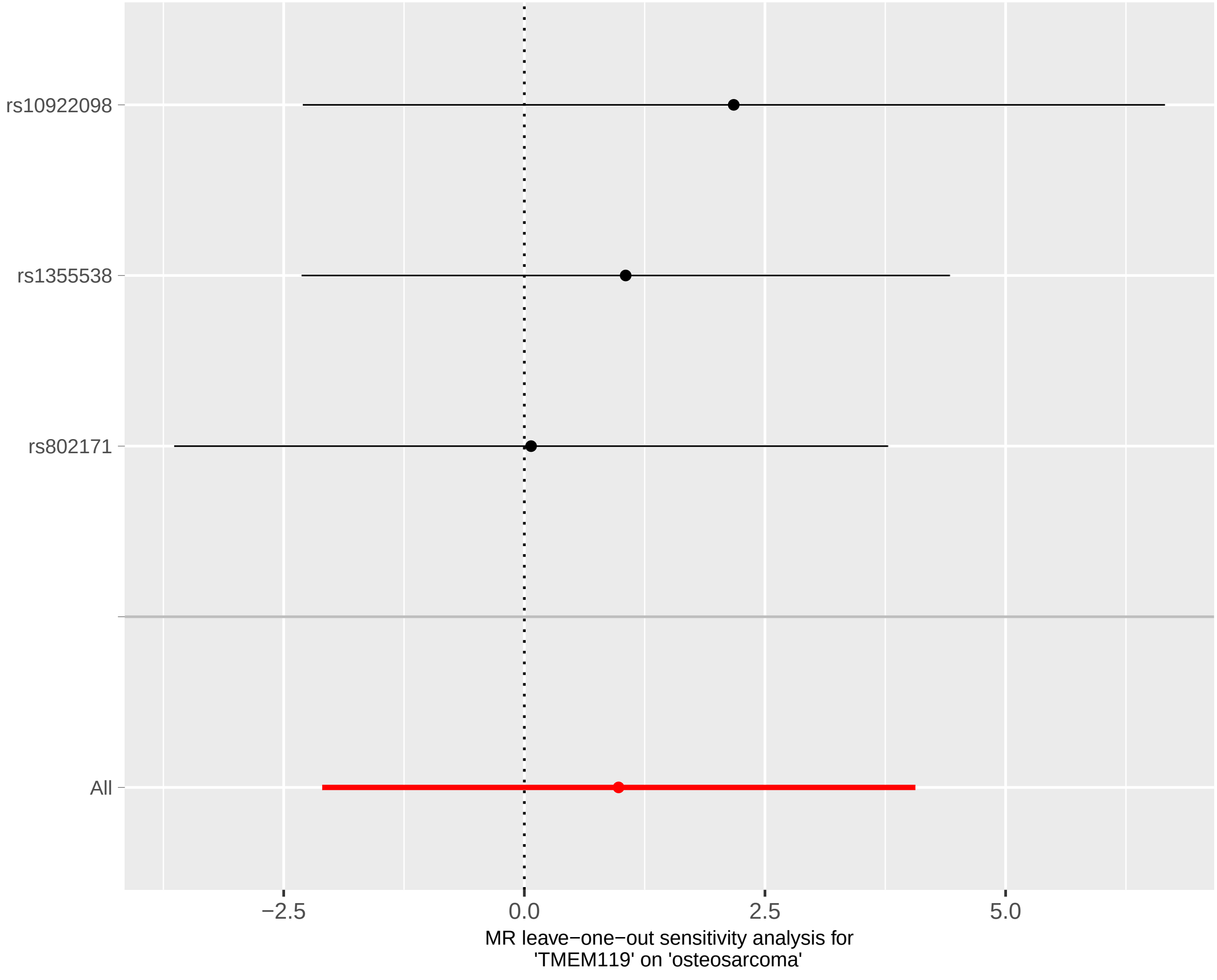
rs10922098
rs1355538
rs802171
All
−2.5
0.0
2.5
5.0
MR leave−one−out sensitivity analysis for
'TMEM119' on 'osteosarcoma'

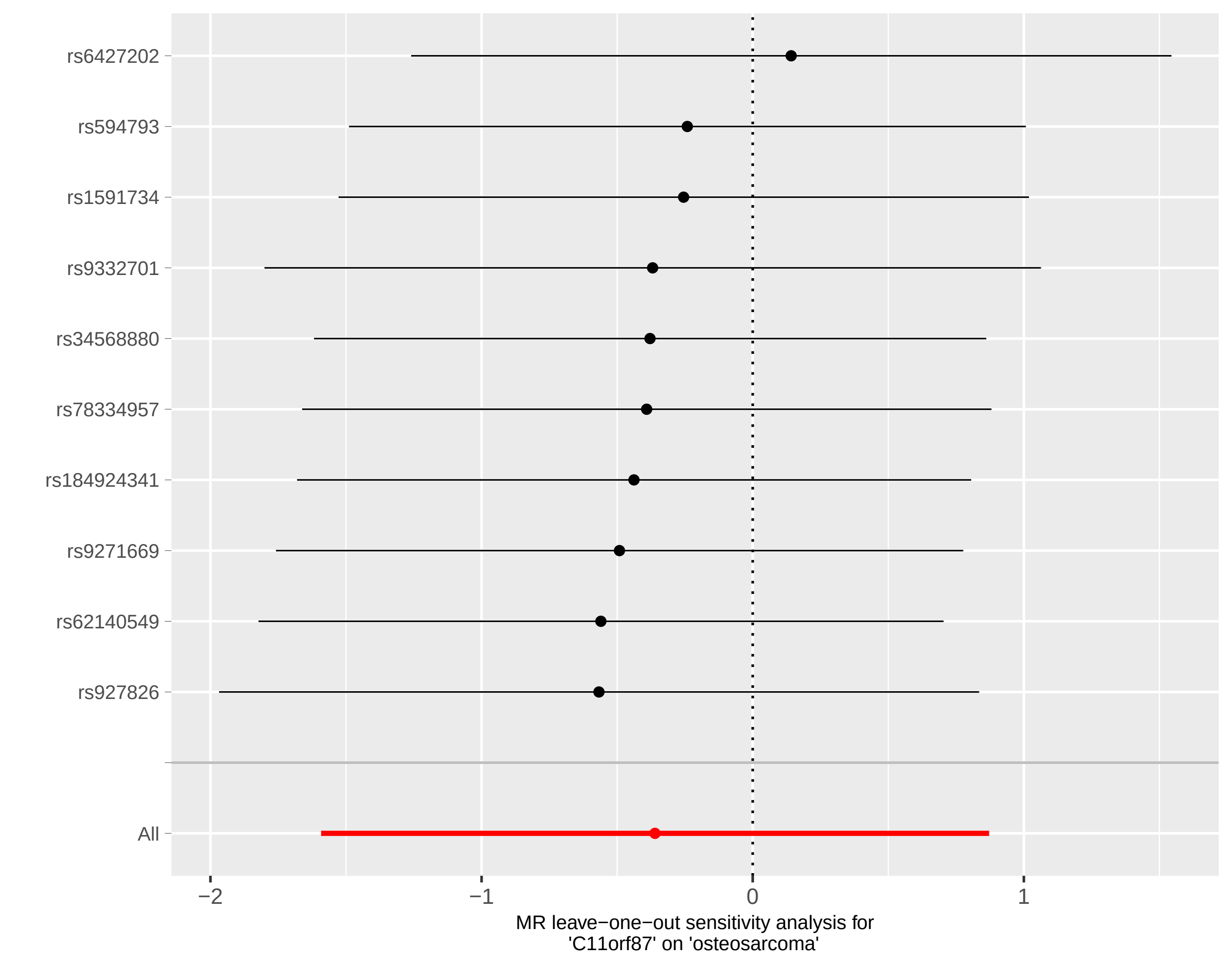
rs6427202
rs594793
rs1591734
rs9332701
rs34568880
rs78334957
rs184924341
rs9271669
rs62140549
rs927826
All
−2
−1
0
1
MR leave−one−out sensitivity analysis for
'C11orf87' on 'osteosarcoma'

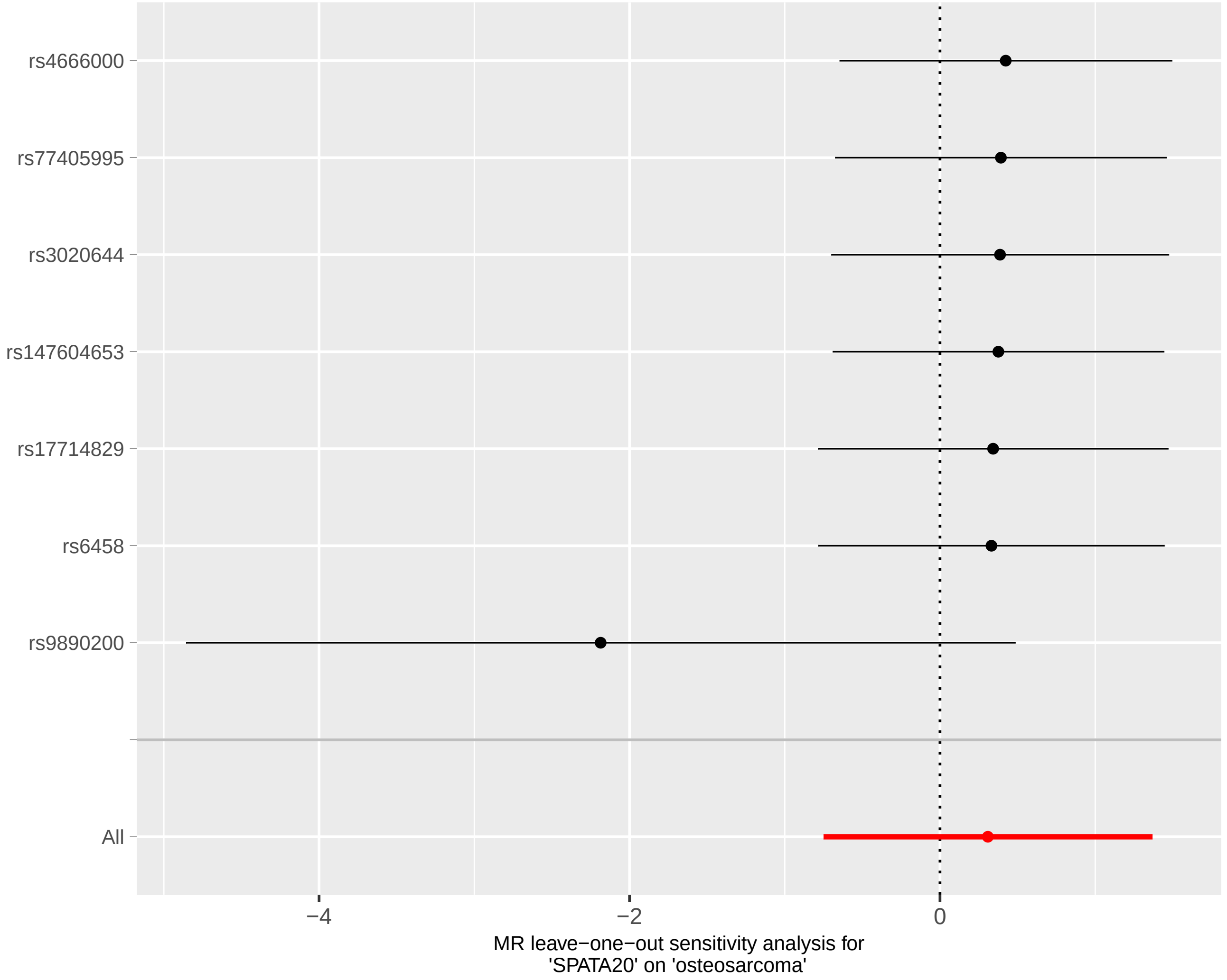

rs4666000
rs77405995
rs3020644
rs147604653
rs17714829
rs6458
rs9890200
All
−4
−2
0
MR leave−one−out sensitivity analysis for
'SPATA20' on 'osteosarcoma'

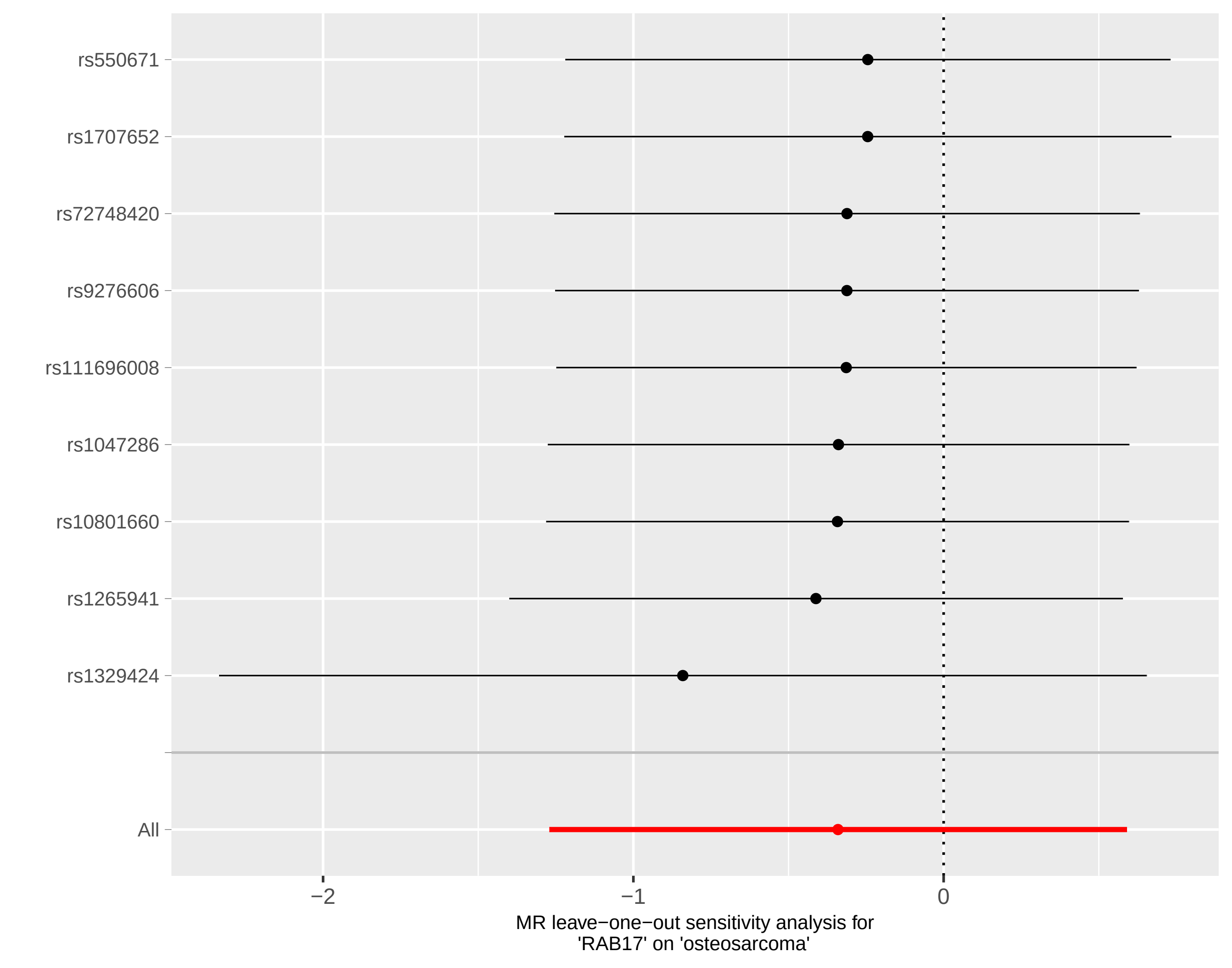

rs550671
rs1707652
rs72748420
rs9276606
rs111696008
rs1047286
rs10801660
rs1265941
rs1329424
All
−2
−1
0
MR leave−one−out sensitivity analysis for
'RAB17' on 'osteosarcoma'

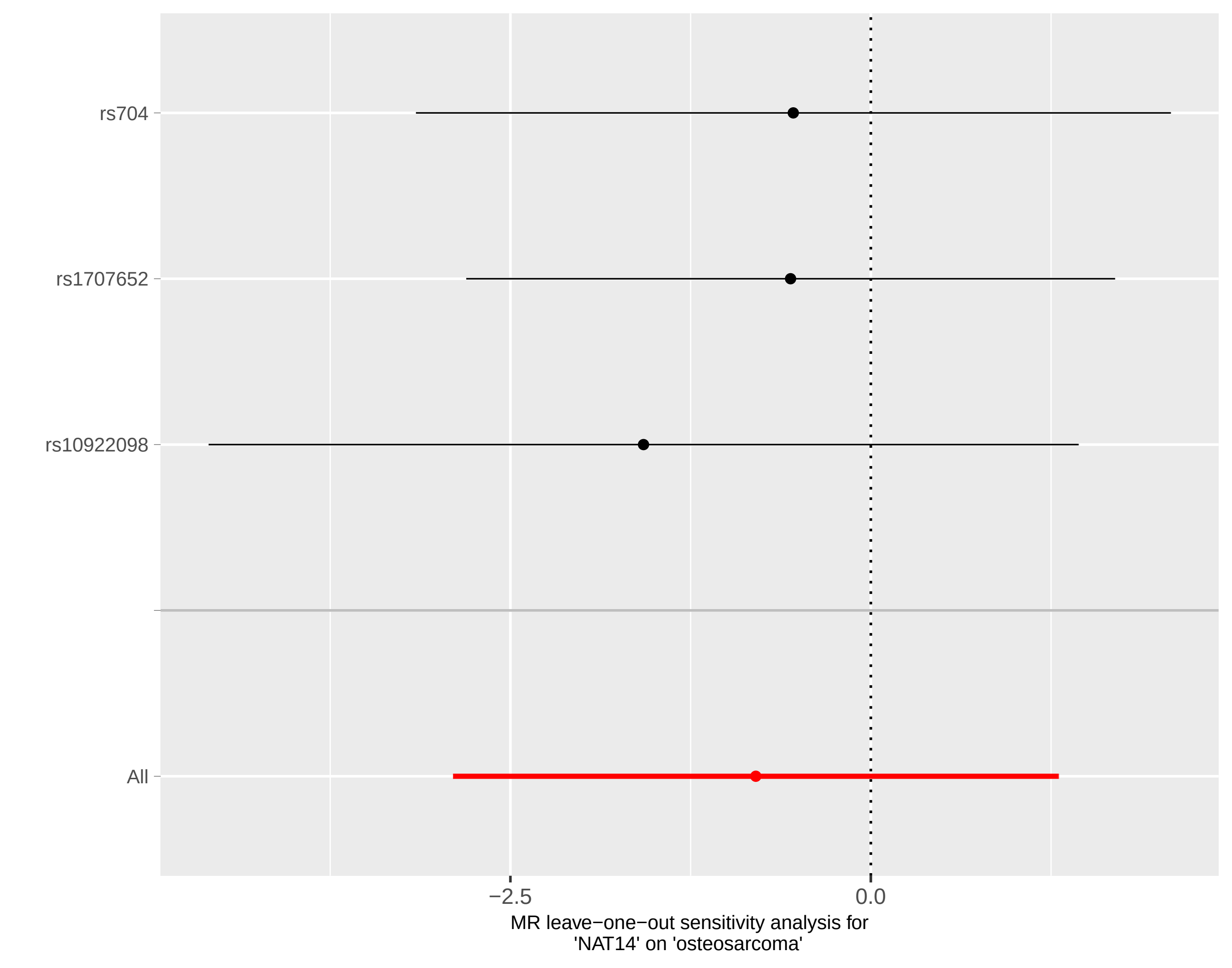
rs704
rs1707652
rs10922098
All
−2.5
0.0
MR leave−one−out sensitivity analysis for
'NAT14' on 'osteosarcoma'

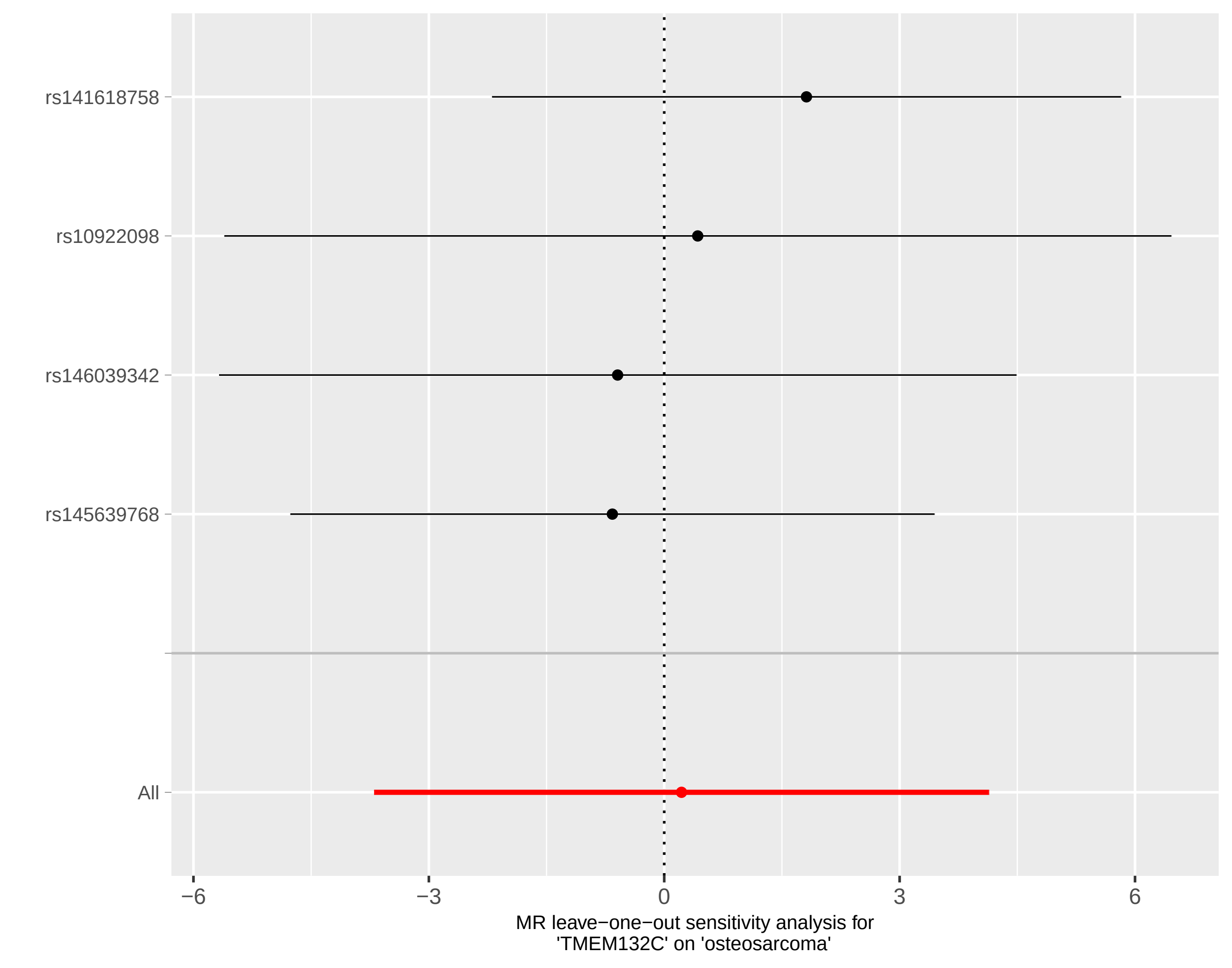
rs141618758
rs10922098
rs146039342
rs145639768
All
−6
−3
0
3
6
MR leave−one−out sensitivity analysis for
'TMEM132C' on 'osteosarcoma'

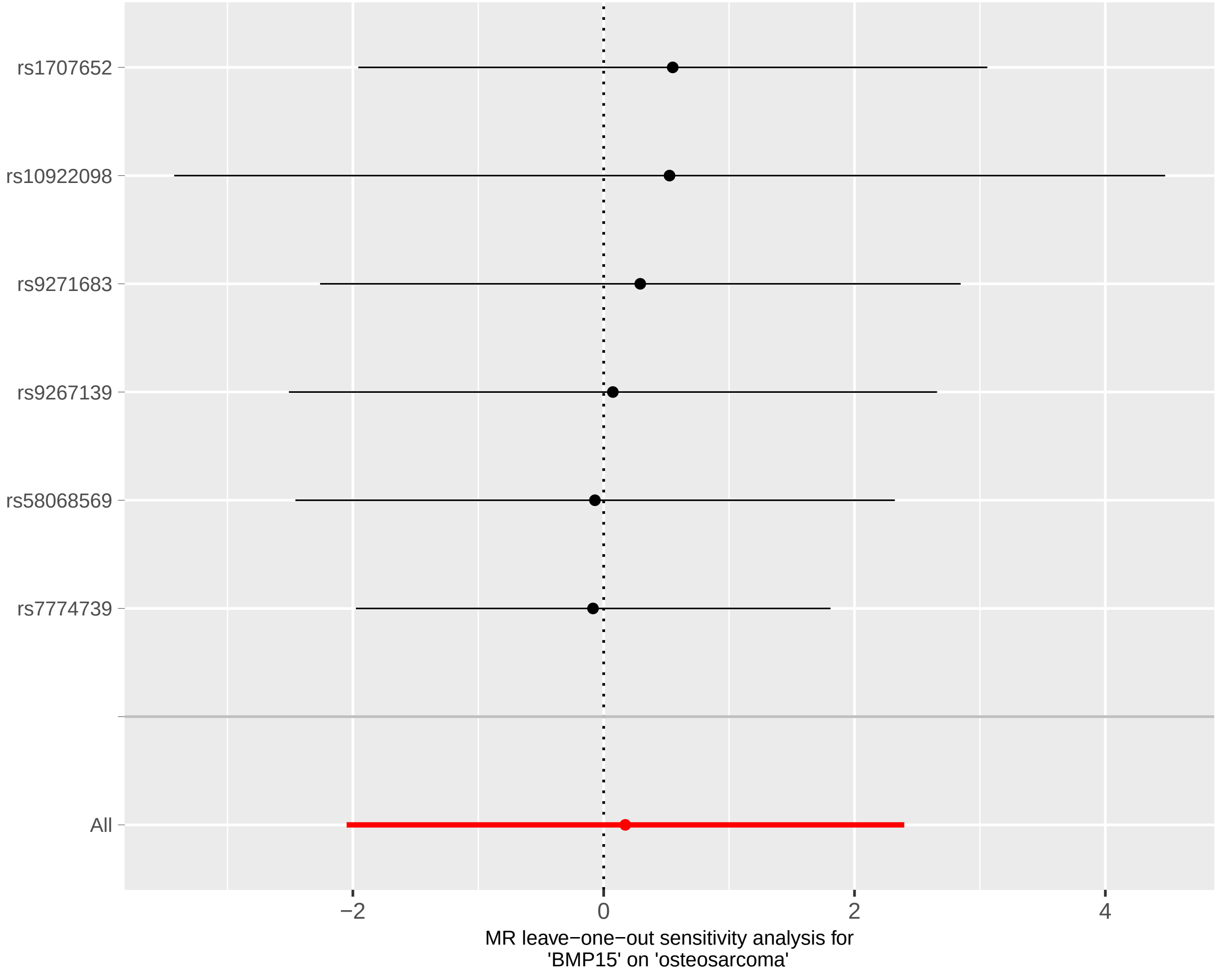
rs1707652
rs10922098
rs9271683
rs9267139
rs58068569
rs7774739
All
−2
0
2
4
MR leave−one−out sensitivity analysis for
'BMP15' on 'osteosarcoma'

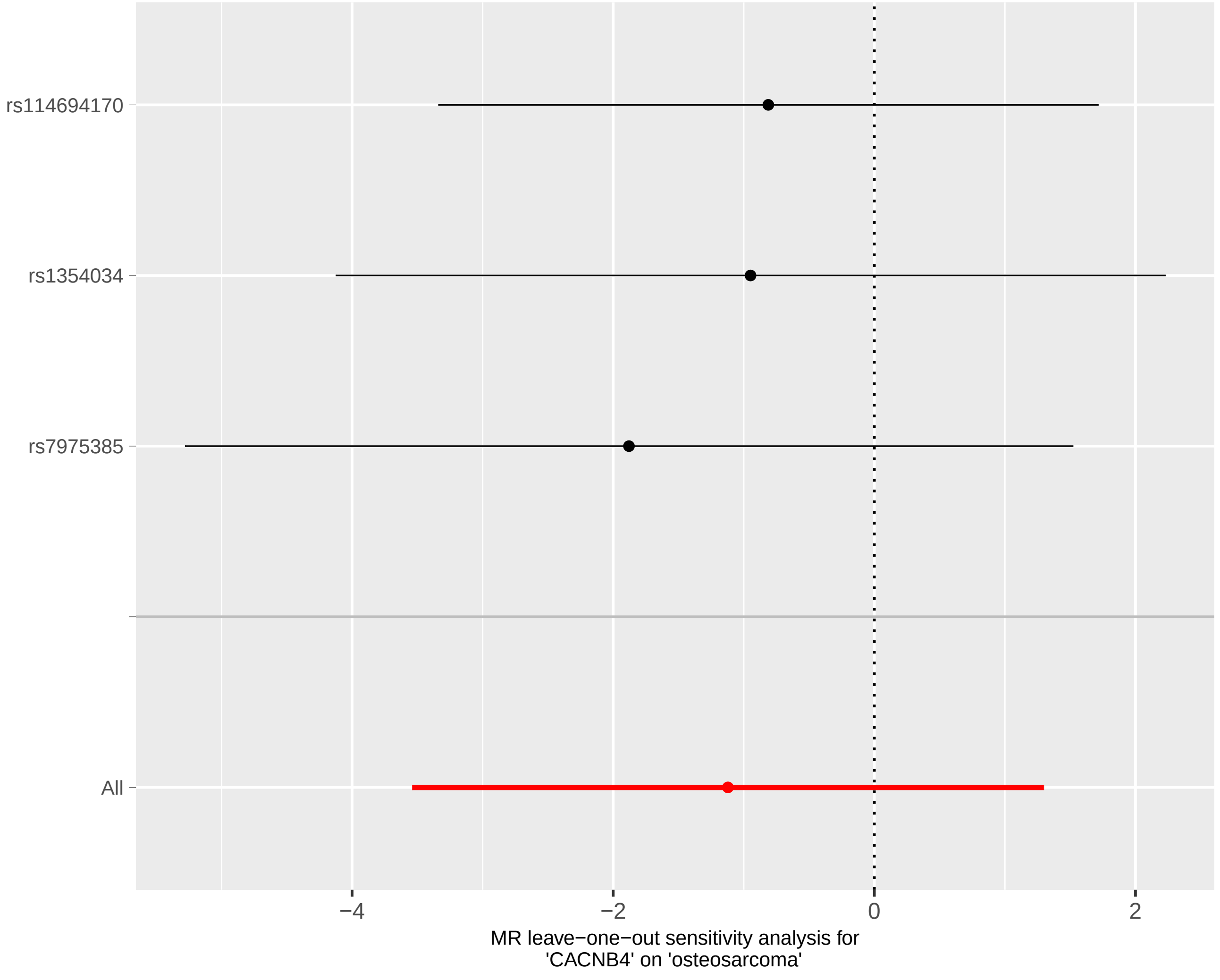

rs114694170
rs1354034
rs7975385
All
−4
−2
0
2
MR leave−one−out sensitivity analysis for
'CACNB4' on 'osteosarcoma'

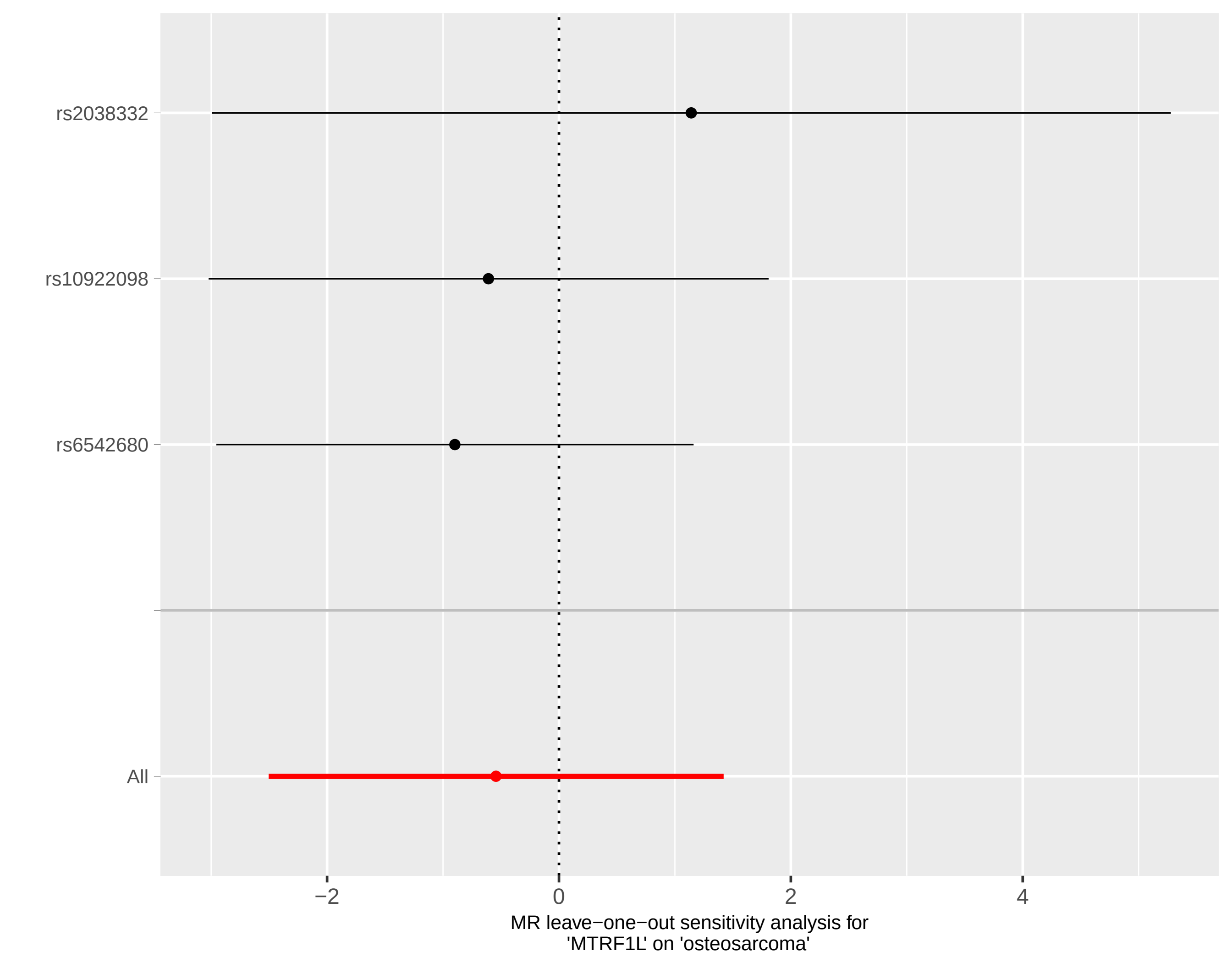

rs2038332
rs10922098
rs6542680
All
−2
0
2
4
MR leave−one−out sensitivity analysis for
'MTRF1L' on 'osteosarcoma'

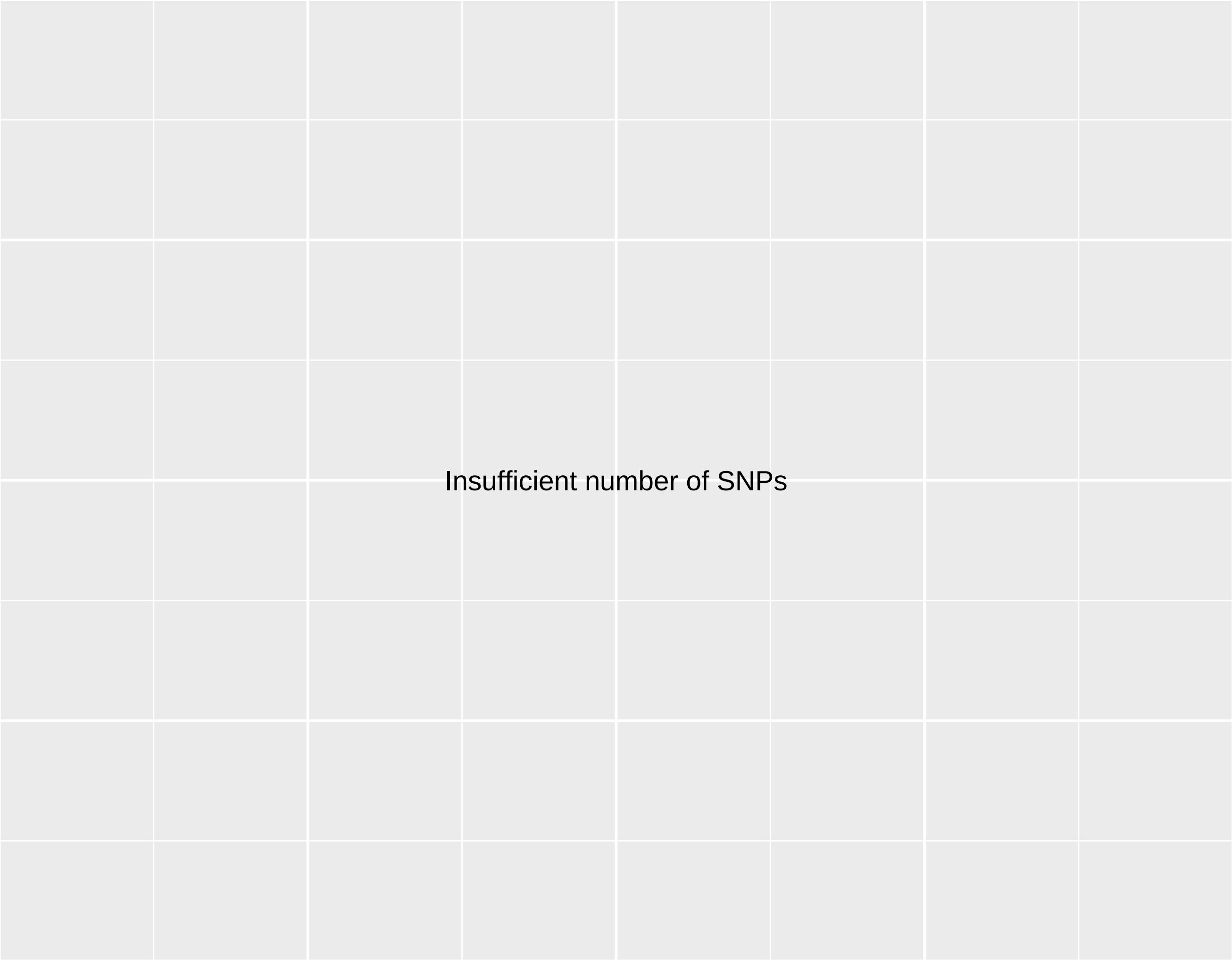
Insufficient number of SNPs

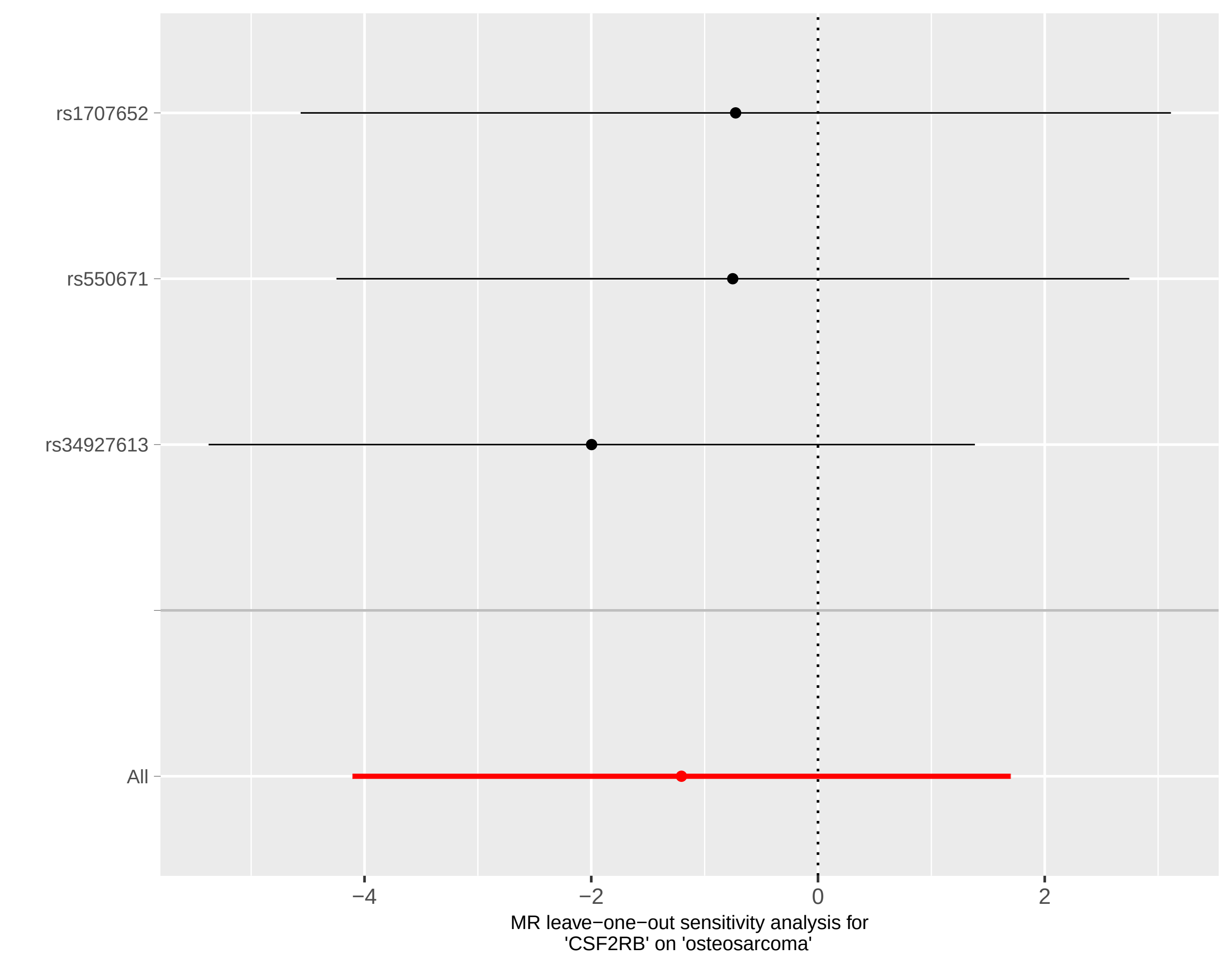
rs1707652
rs550671
rs34927613
All
−4
−2
0
2
MR leave−one−out sensitivity analysis for
'CSF2RB' on 'osteosarcoma'

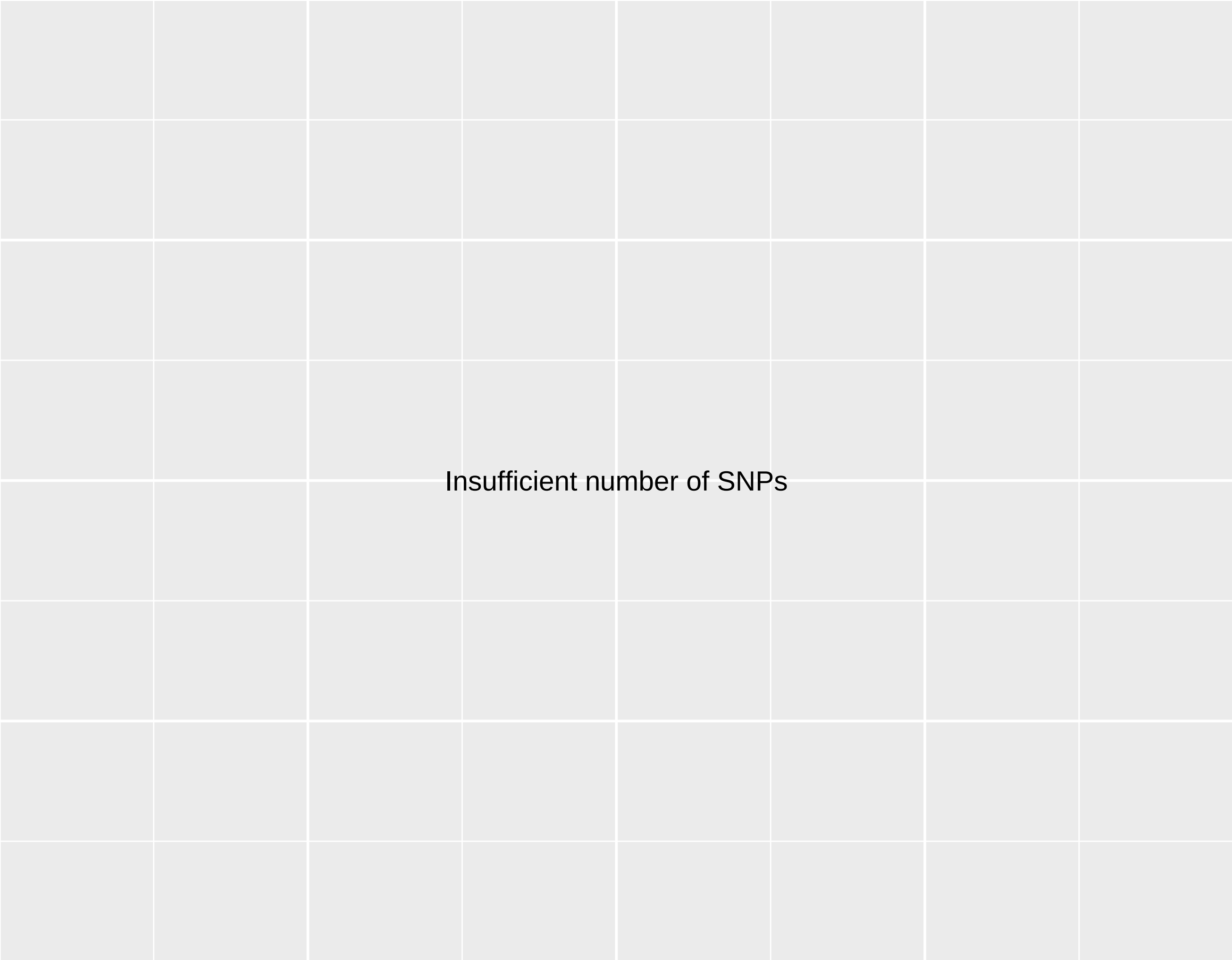
Insufficient number of SNPs

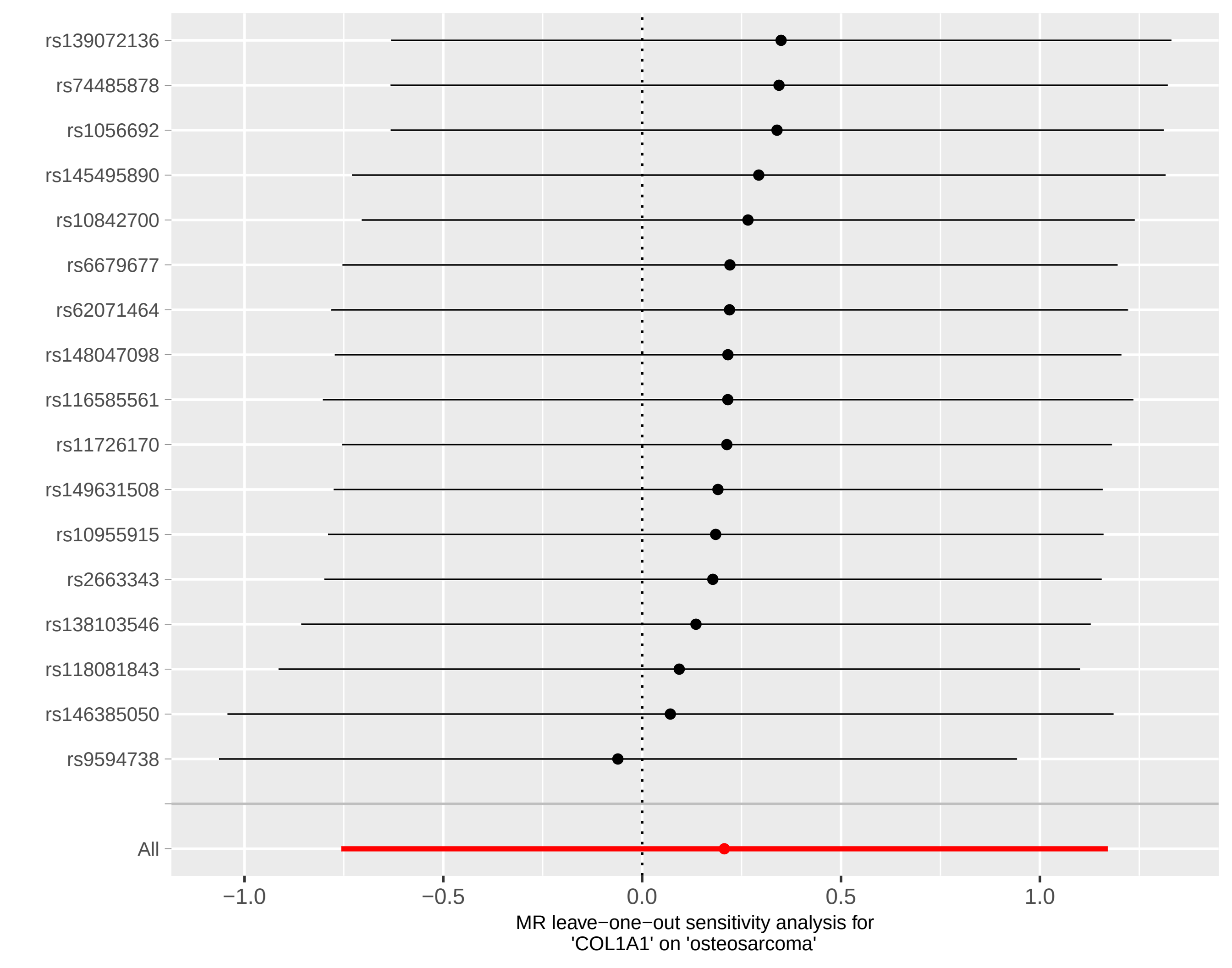
rs139072136
rs74485878
rs1056692
rs145495890
rs10842700
rs6679677
rs62071464
rs148047098
rs116585561
rs11726170
rs149631508
rs10955915
rs2663343
rs138103546
rs118081843
rs146385050
rs9594738
All
−1.0
−0.5
0.0
0.5
1.0
MR leave−one−out sensitivity analysis for
'COL1A1' on 'osteosarcoma'

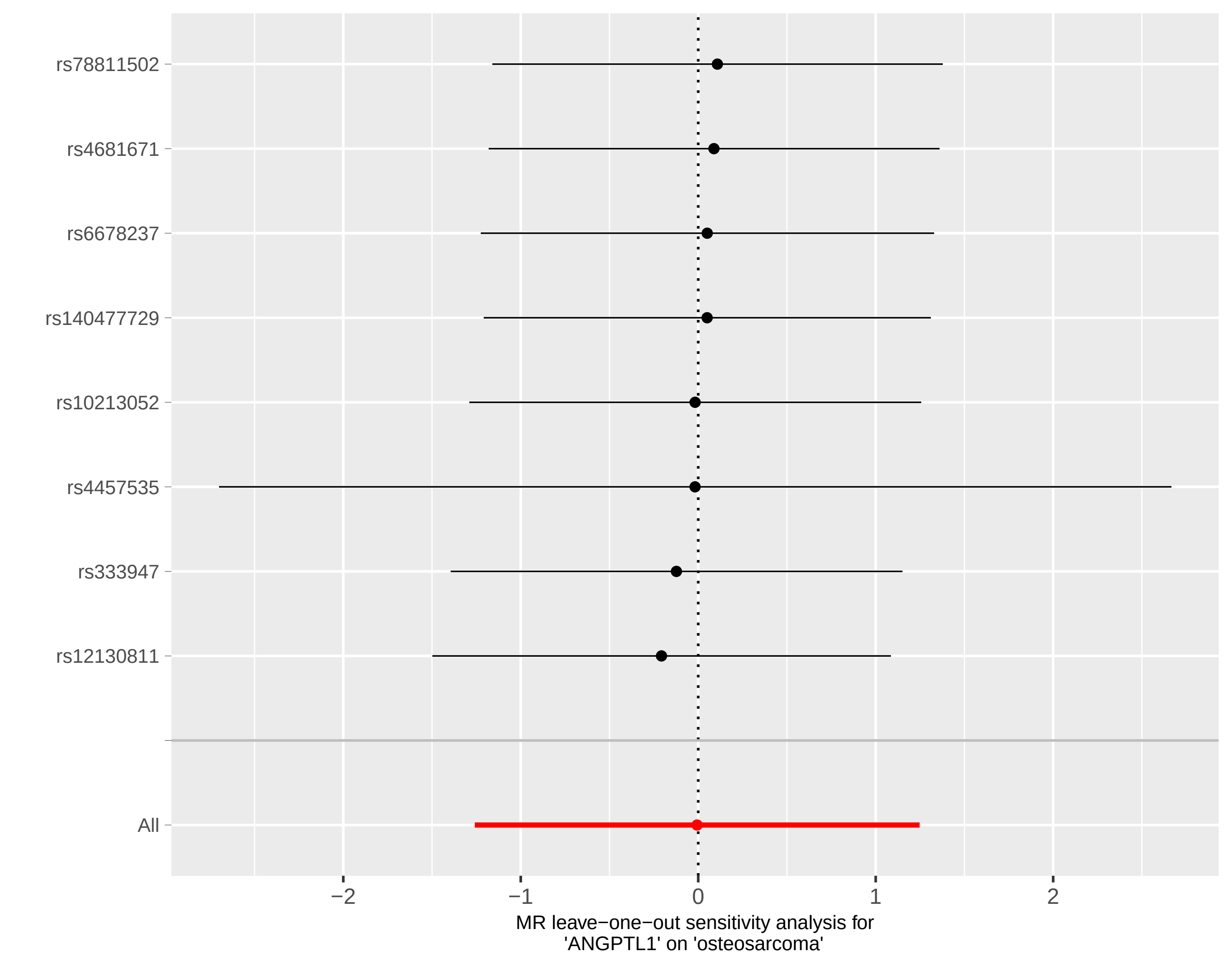

rs78811502
rs4681671
rs6678237
rs140477729
rs10213052
rs4457535
rs333947
rs12130811
All
−2
−1
0
1
2
MR leave−one−out sensitivity analysis for
'ANGPTL1' on 'osteosarcoma'

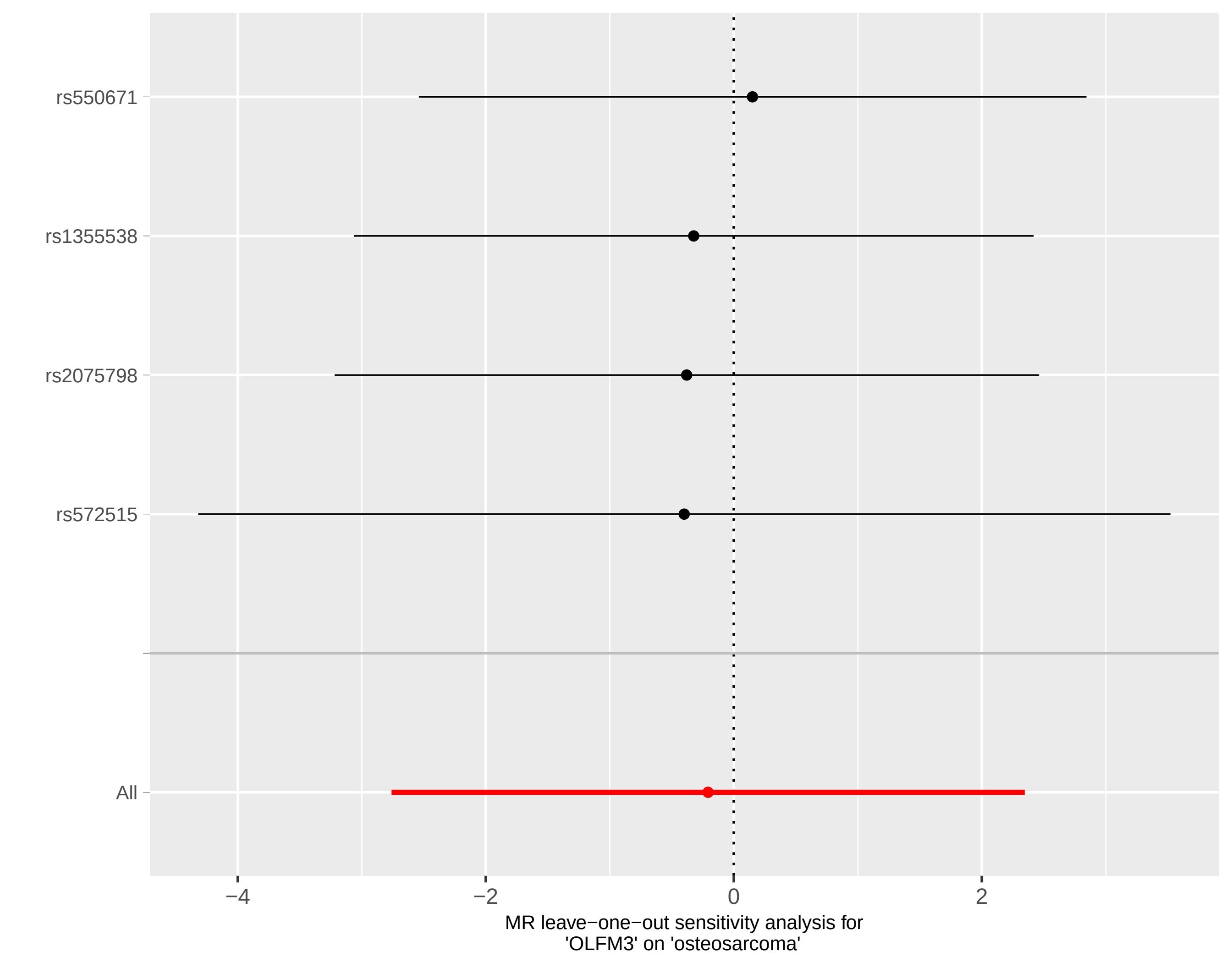

rs550671
rs1355538
rs2075798
rs572515
All
−4
−2
0
2
MR leave−one−out sensitivity analysis for
'OLFM3' on 'osteosarcoma'

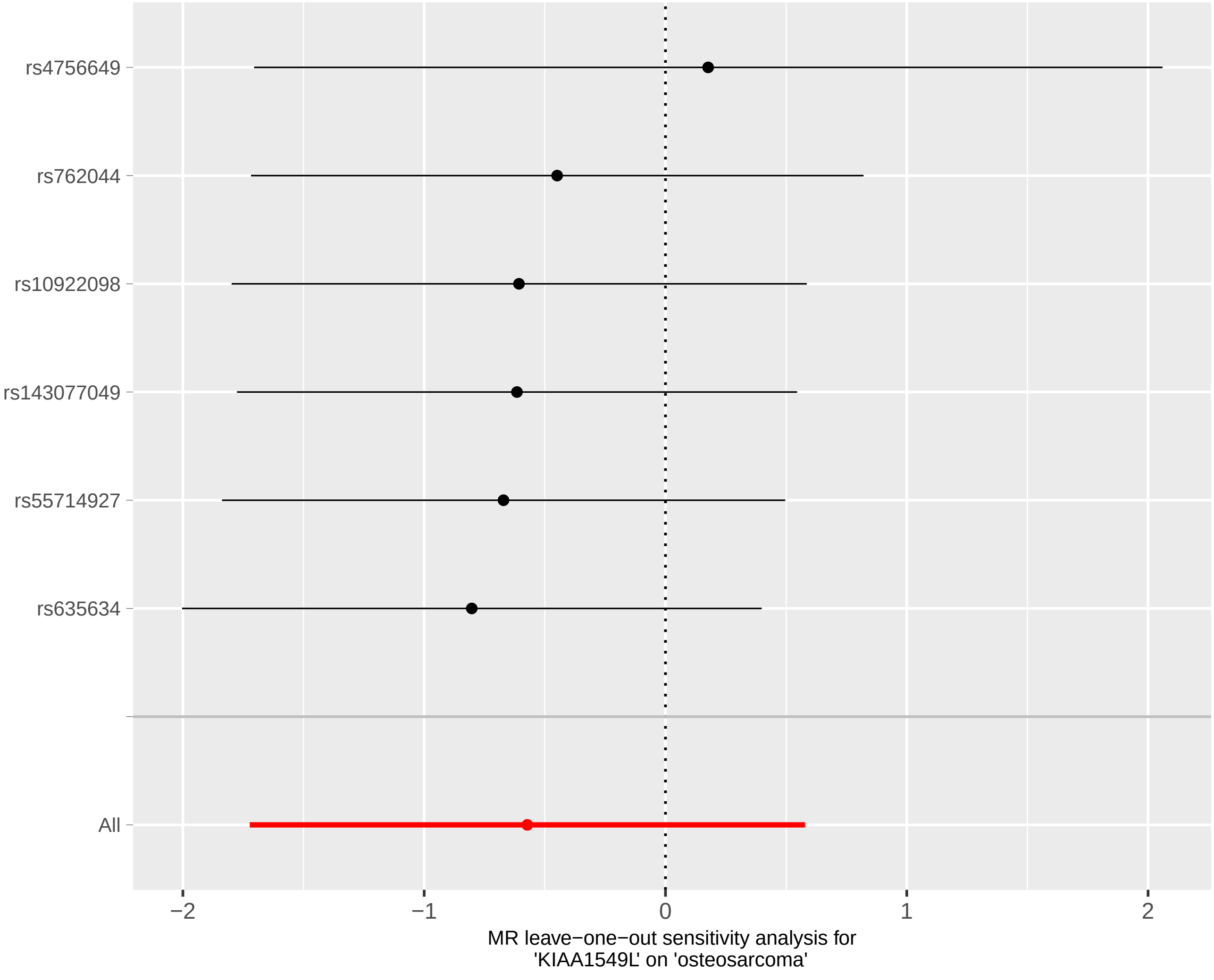
rs4756649
rs762044
rs10922098
rs143077049
rs55714927
rs635634
All
−2
−1
0
1
2
MR leave−one−out sensitivity analysis for
'KIAA1549L' on 'osteosarcoma'

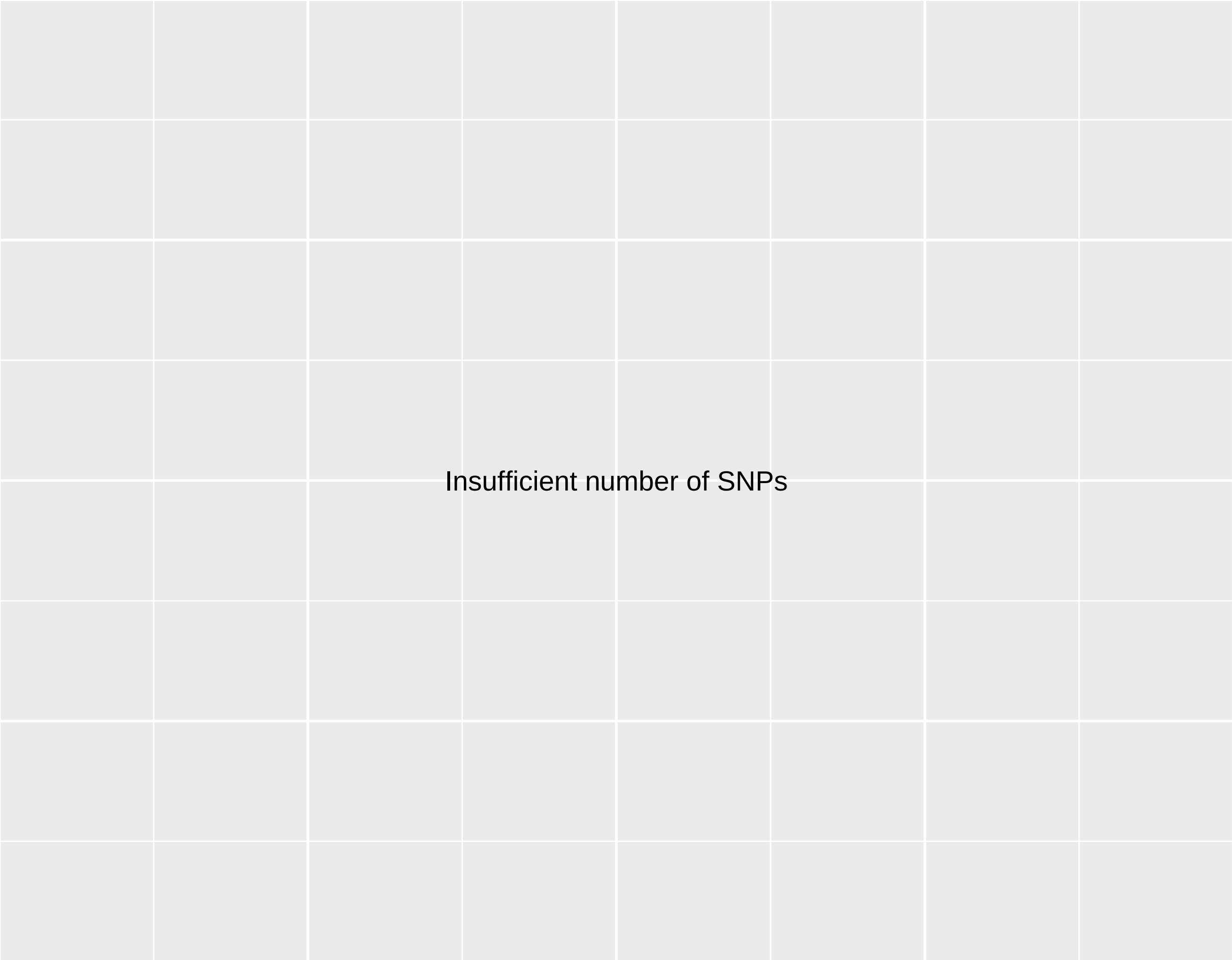
Insufficient number of SNPs

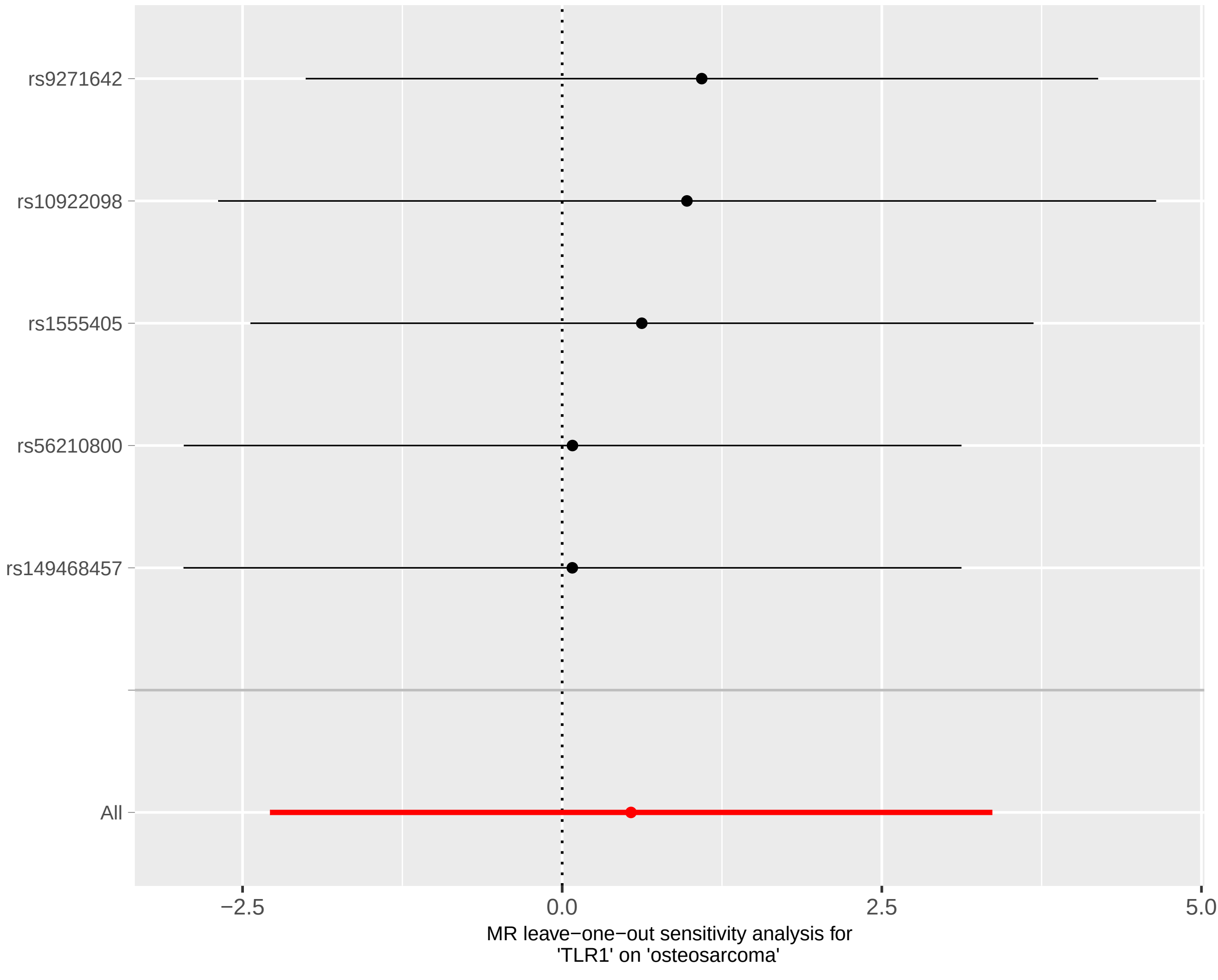
rs9271642
rs10922098
rs1555405
rs56210800
rs149468457
All
−2.5
0.0
2.5
5.0
MR leave−one−out sensitivity analysis for
'TLR1' on 'osteosarcoma'

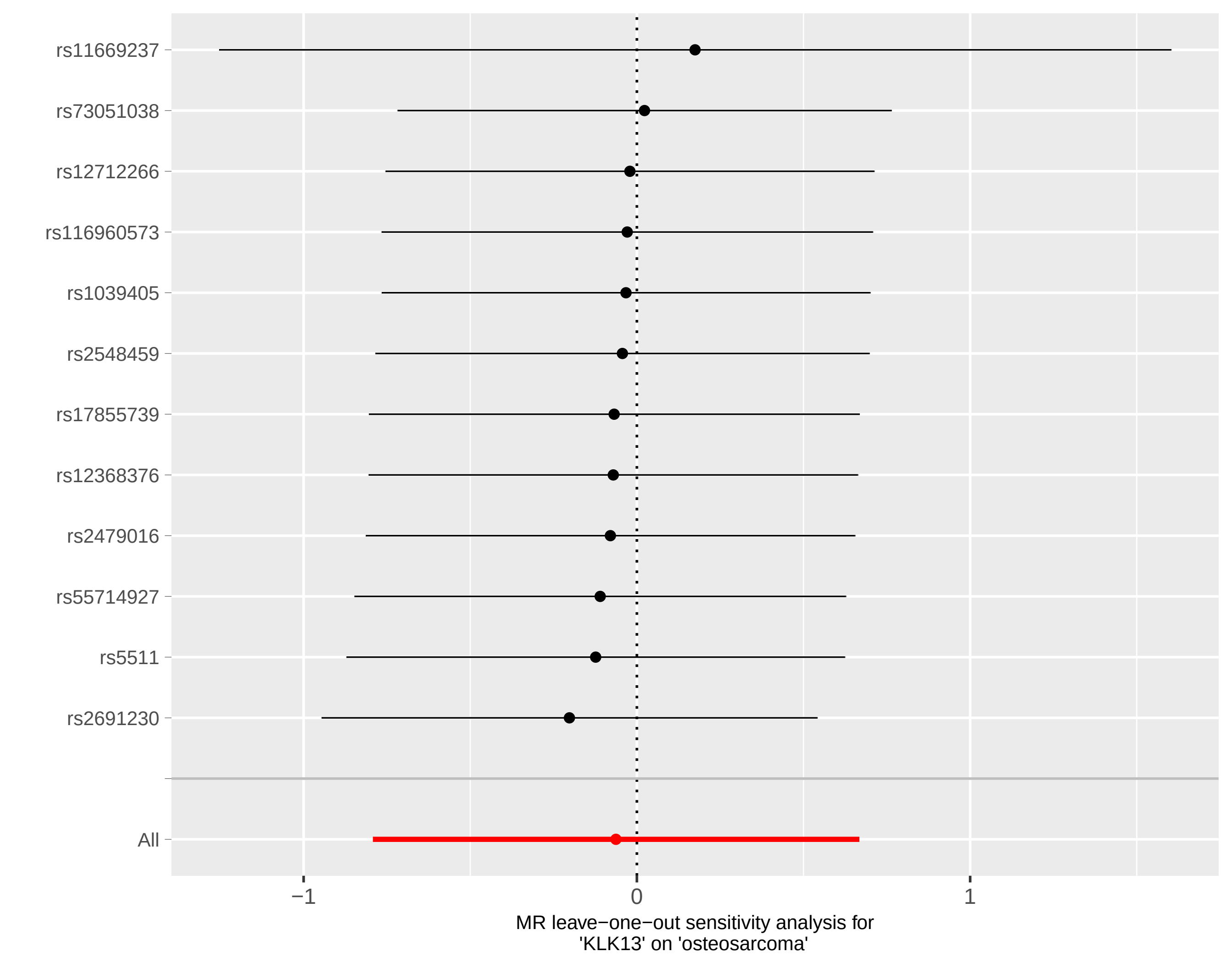
rs11669237
rs73051038
rs12712266
rs116960573
rs1039405
rs2548459
rs17855739
rs12368376
rs2479016
rs55714927
rs5511
rs2691230
All
−1
0
1
MR leave−one−out sensitivity analysis for
'KLK13' on 'osteosarcoma'

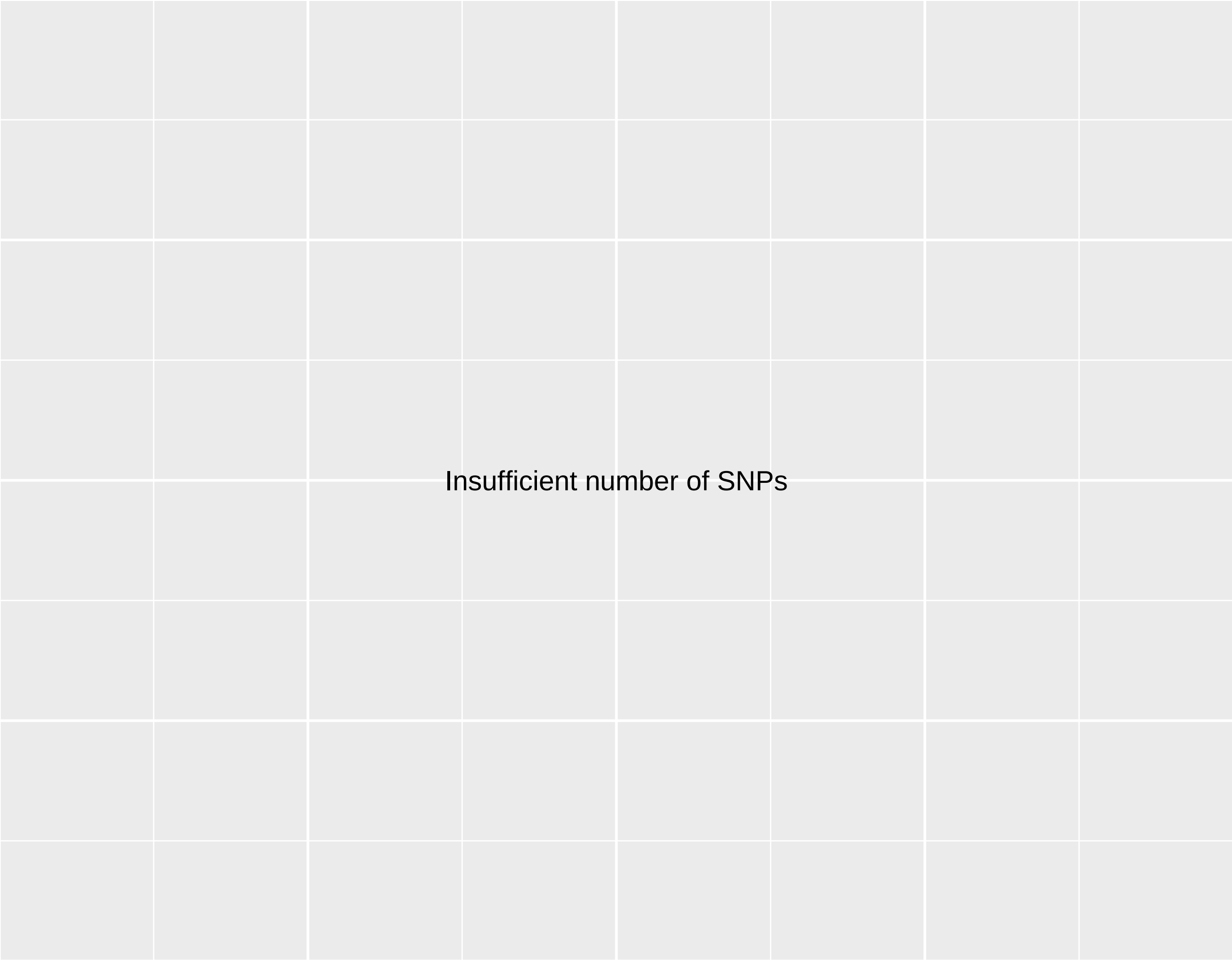
Insufficient number of SNPs

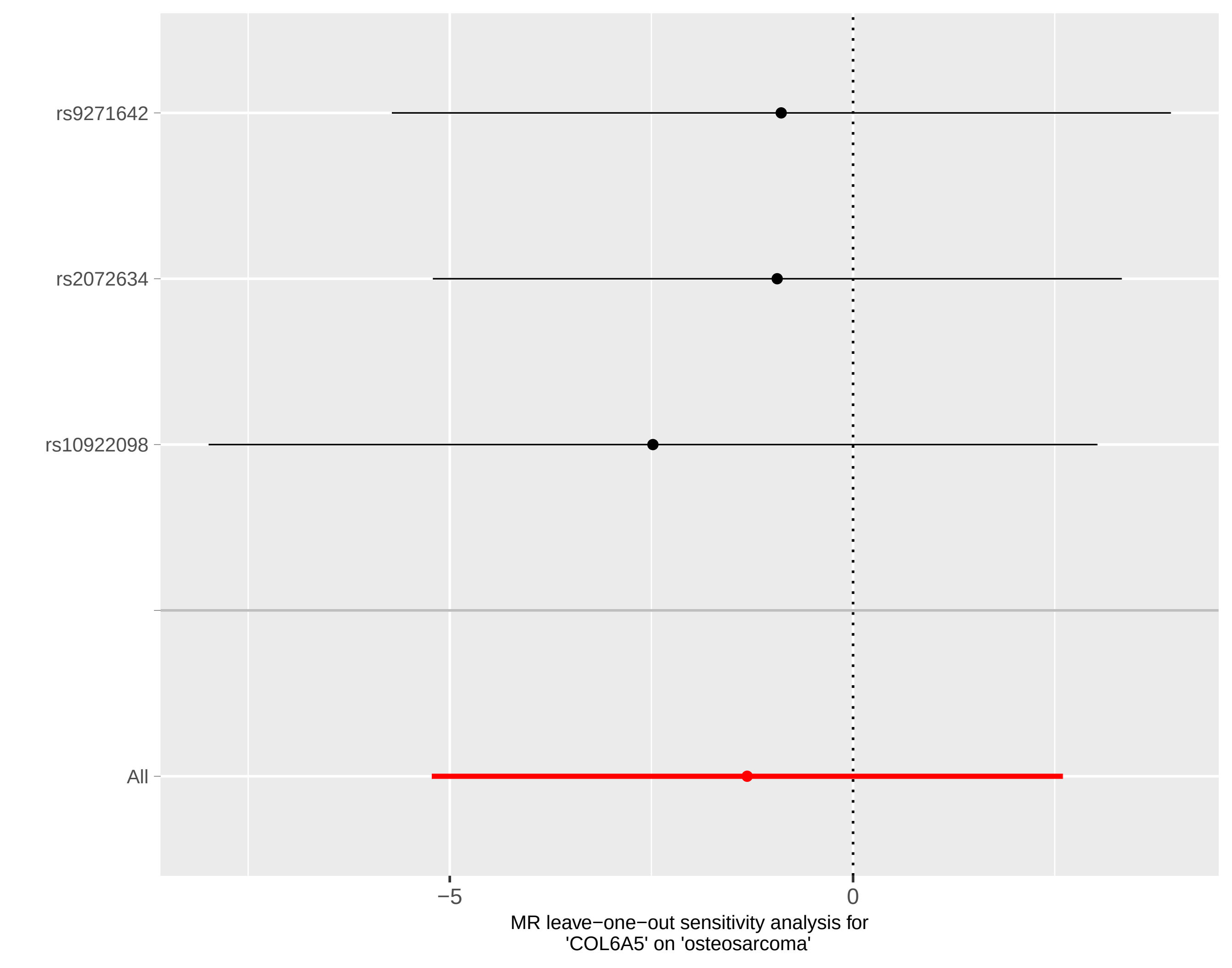
rs9271642
rs2072634
rs10922098
All
−5
0
MR leave−one−out sensitivity analysis for
'COL6A5' on 'osteosarcoma'

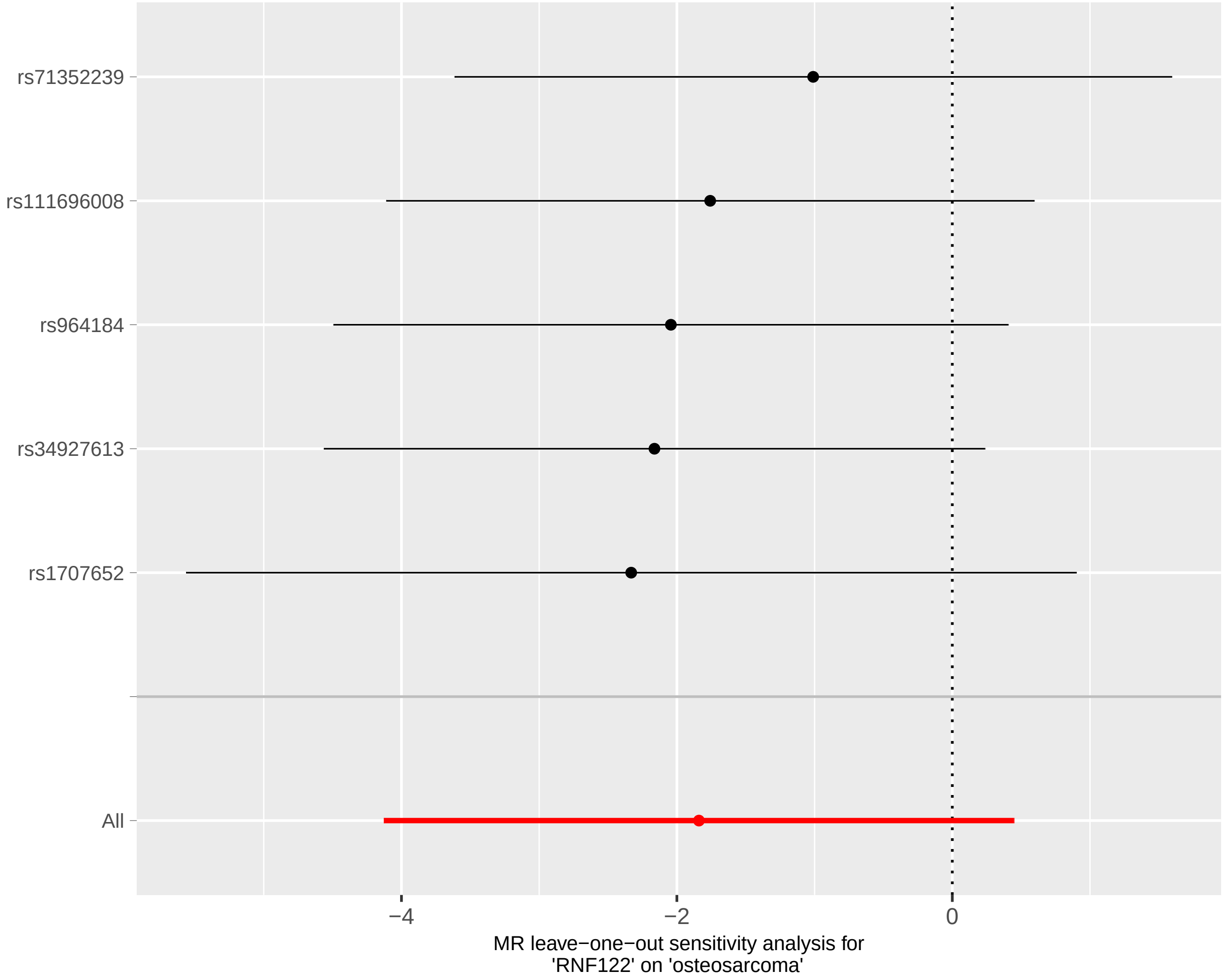
rs71352239
rs111696008
rs964184
rs34927613
rs1707652
All
−4
−2
0
MR leave−one−out sensitivity analysis for
'RNF122' on 'osteosarcoma'

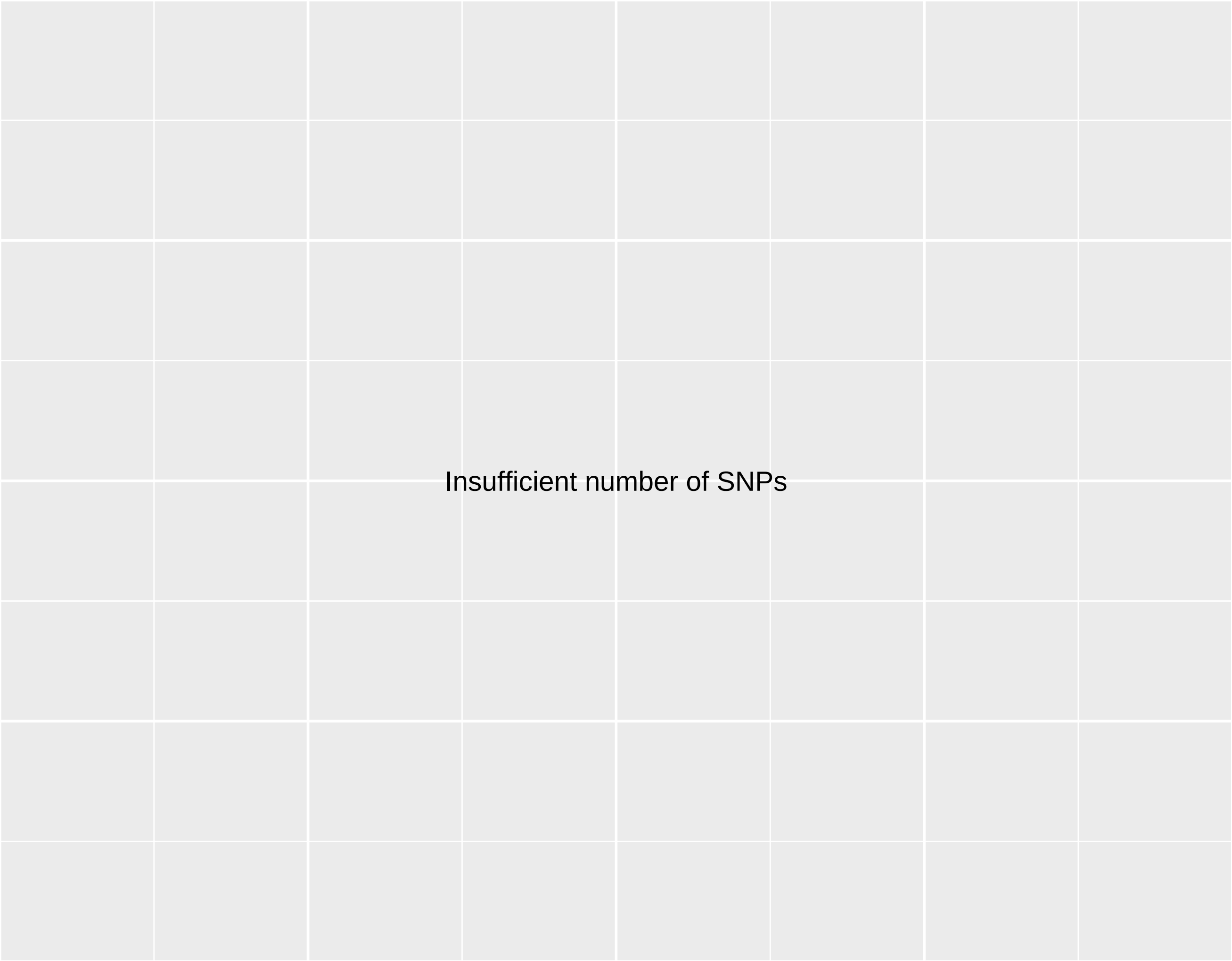
Insufficient number of SNPs

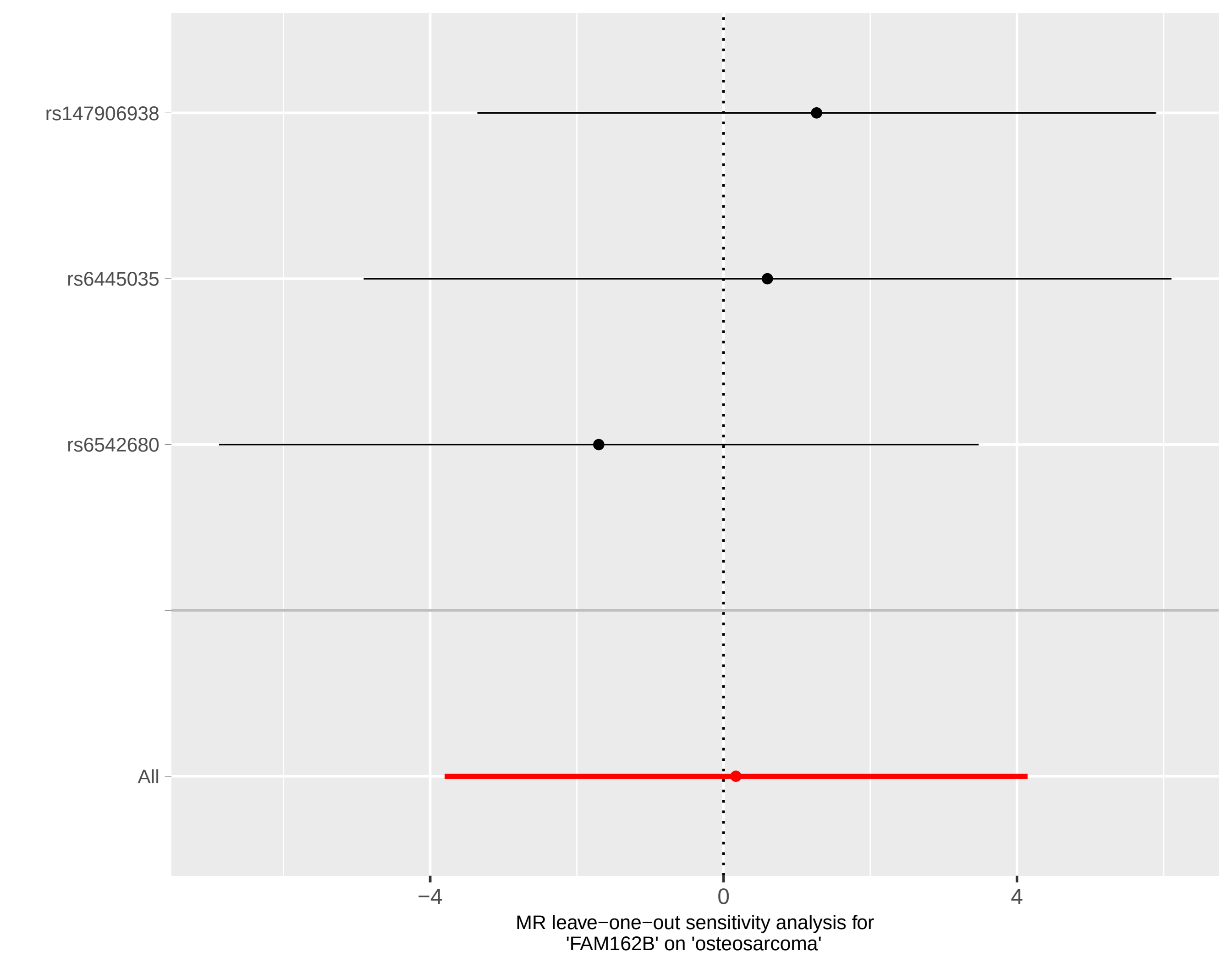
rs147906938
rs6445035
rs6542680
All
−4
0
4
MR leave−one−out sensitivity analysis for
'FAM162B' on 'osteosarcoma'

Insufficient number of SNPs

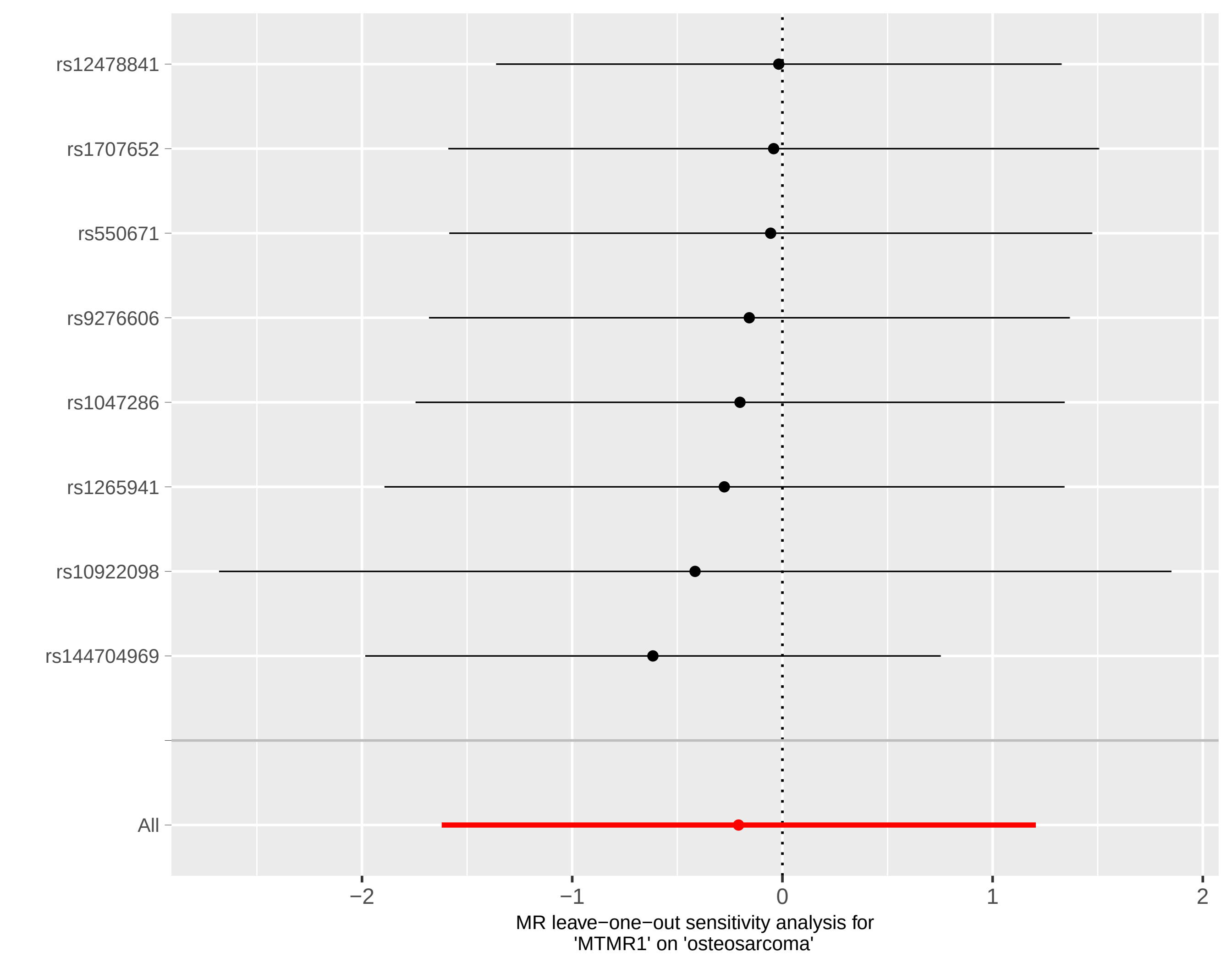

rs12478841
rs1707652
rs550671
rs9276606
rs1047286
rs1265941
rs10922098
rs144704969
All
−2
−1
0
1
2
MR leave−one−out sensitivity analysis for
'MTMR1' on 'osteosarcoma'

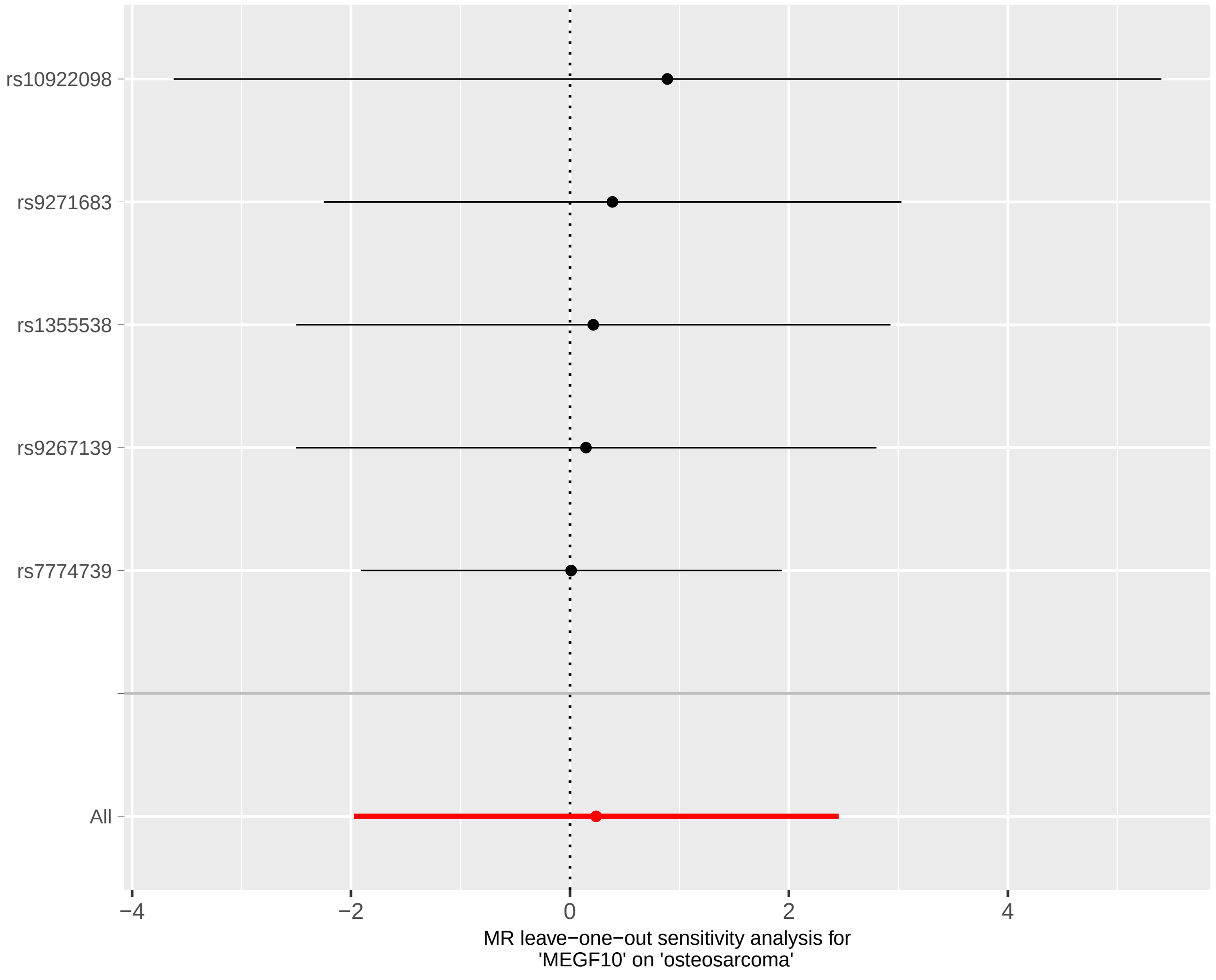
rs10922098
rs9271683
rs1355538
rs9267139
rs7774739
All
−4
−2
0
2
4
MR leave−one−out sensitivity analysis for
'MEGF10' on 'osteosarcoma'

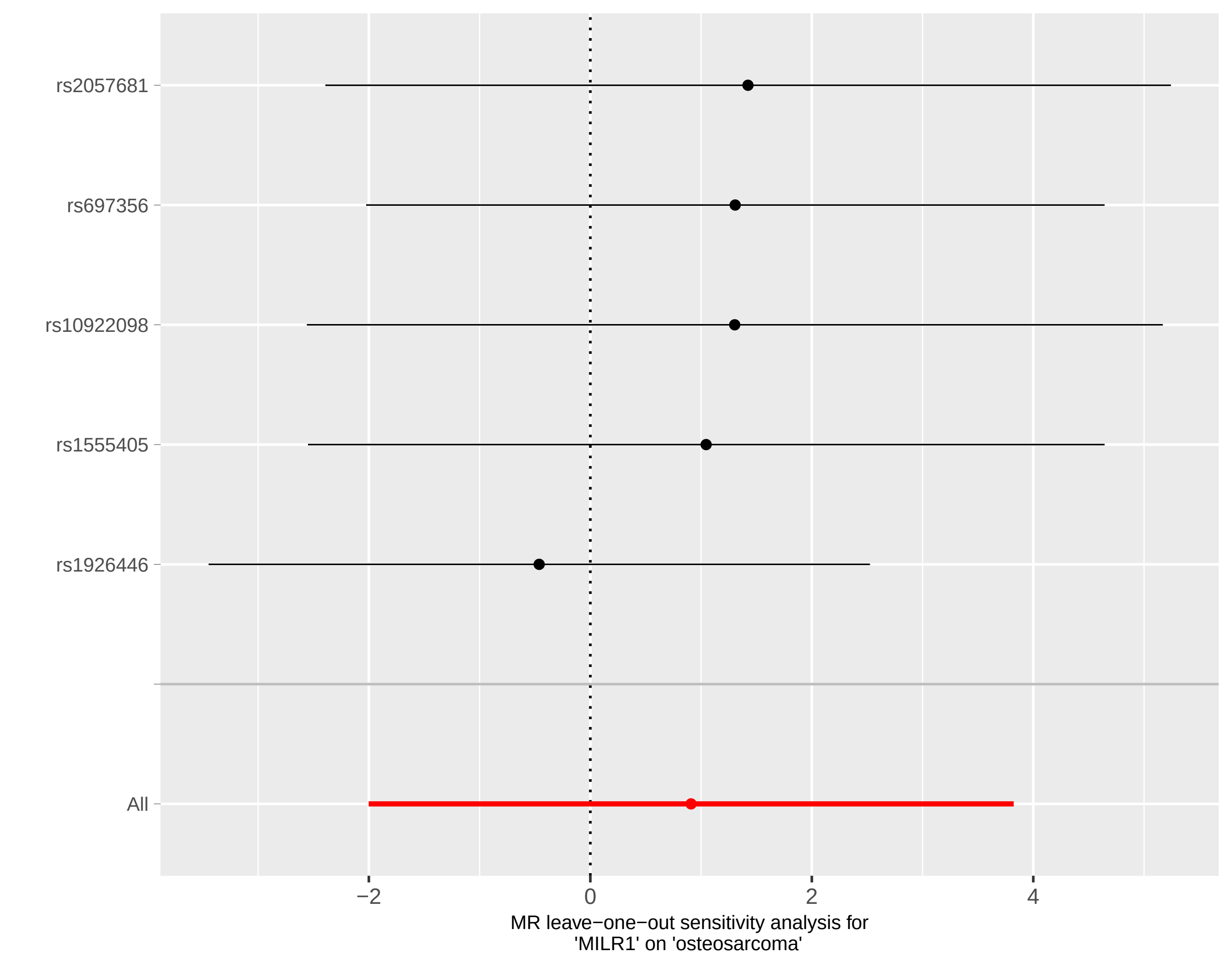
rs2057681
rs697356
rs10922098
rs1555405
rs1926446
All
−2
0
2
4
MR leave−one−out sensitivity analysis for
'MILR1' on 'osteosarcoma'

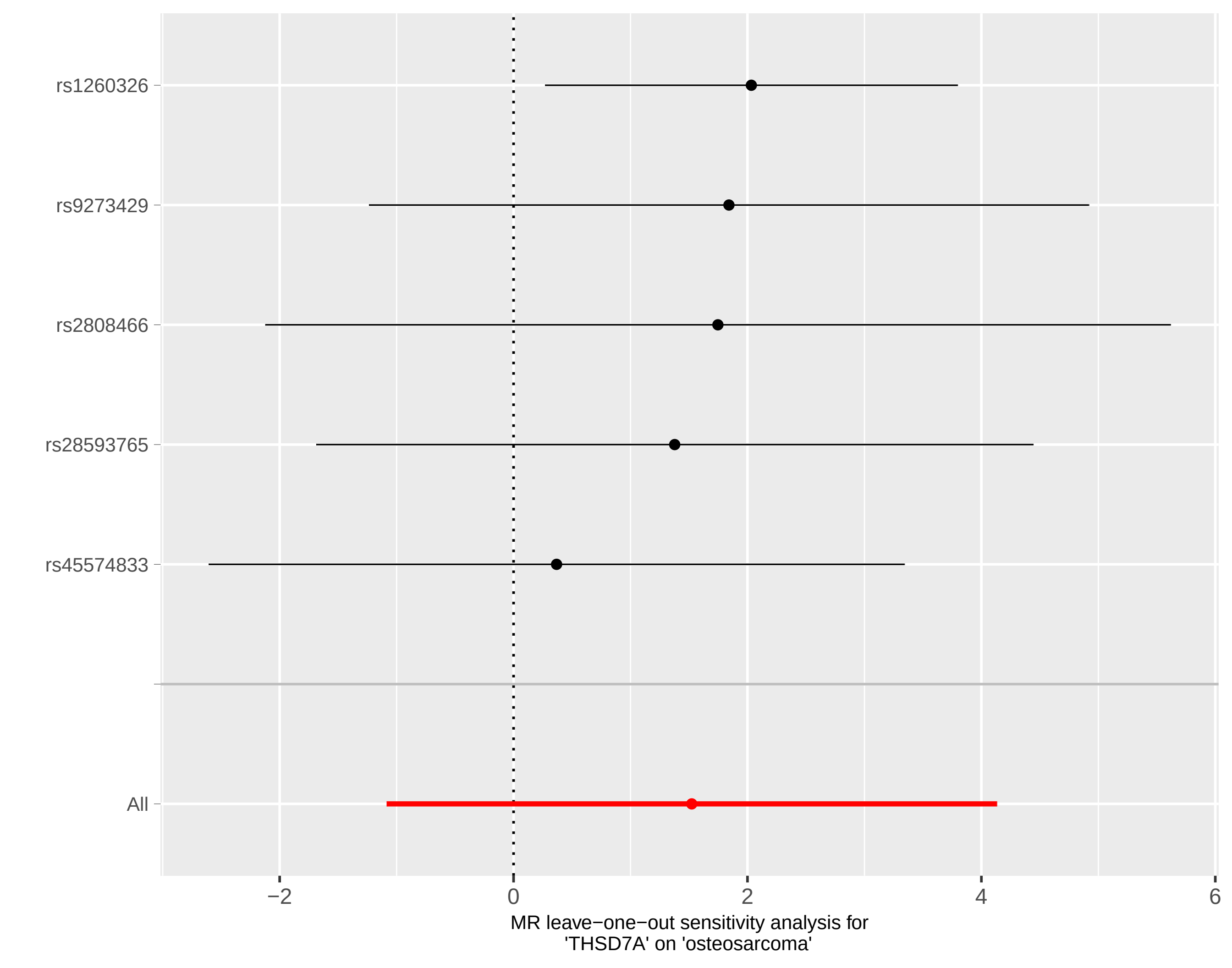
rs1260326
rs9273429
rs2808466
rs28593765
rs45574833
All
−2
0
2
4
6
MR leave−one−out sensitivity analysis for
'THSD7A' on 'osteosarcoma'

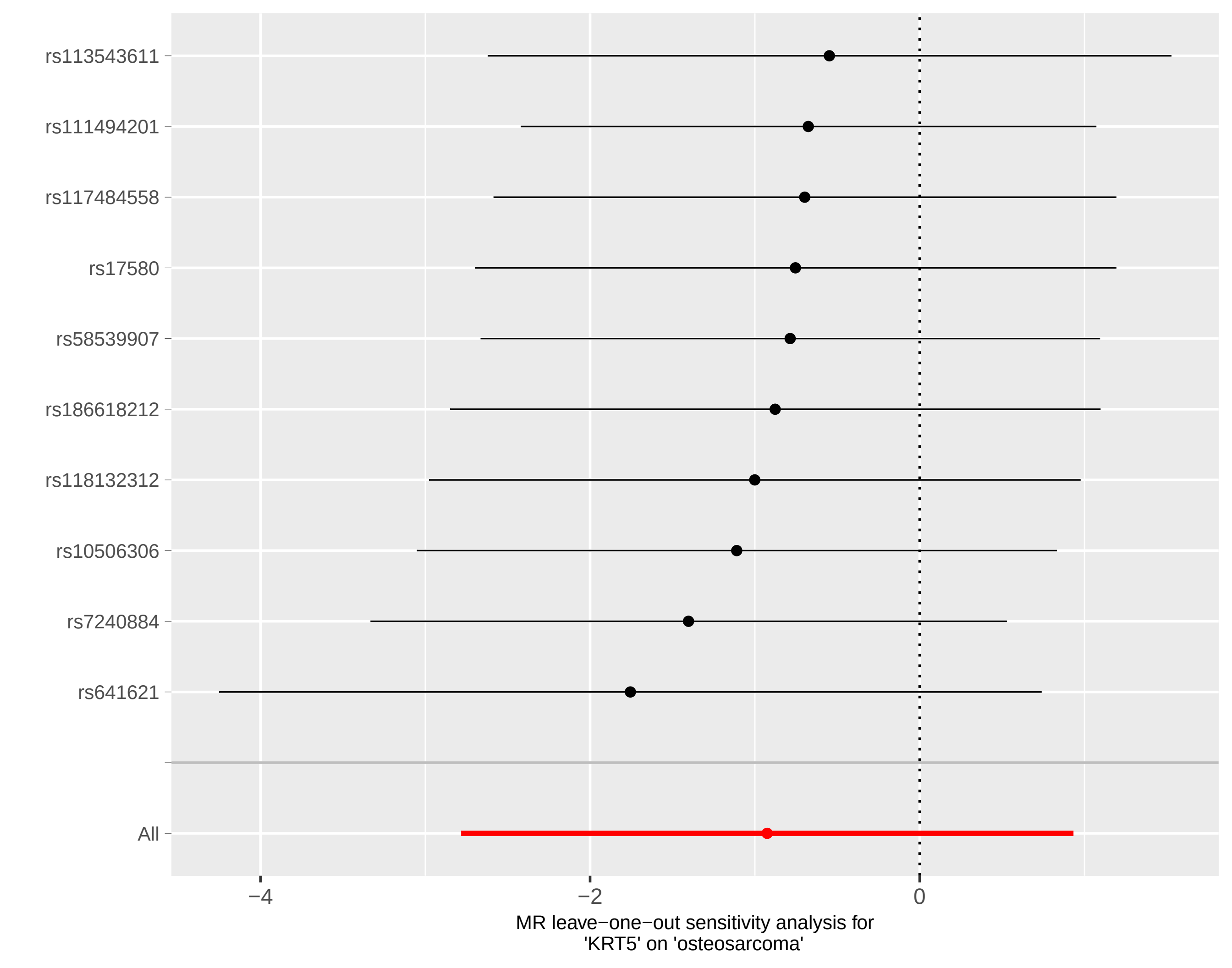

rs113543611
rs111494201
rs117484558
rs17580
rs58539907
rs186618212
rs118132312
rs10506306
rs7240884
rs641621
All
−4
−2
0
MR leave−one−out sensitivity analysis for
'KRT5' on 'osteosarcoma'

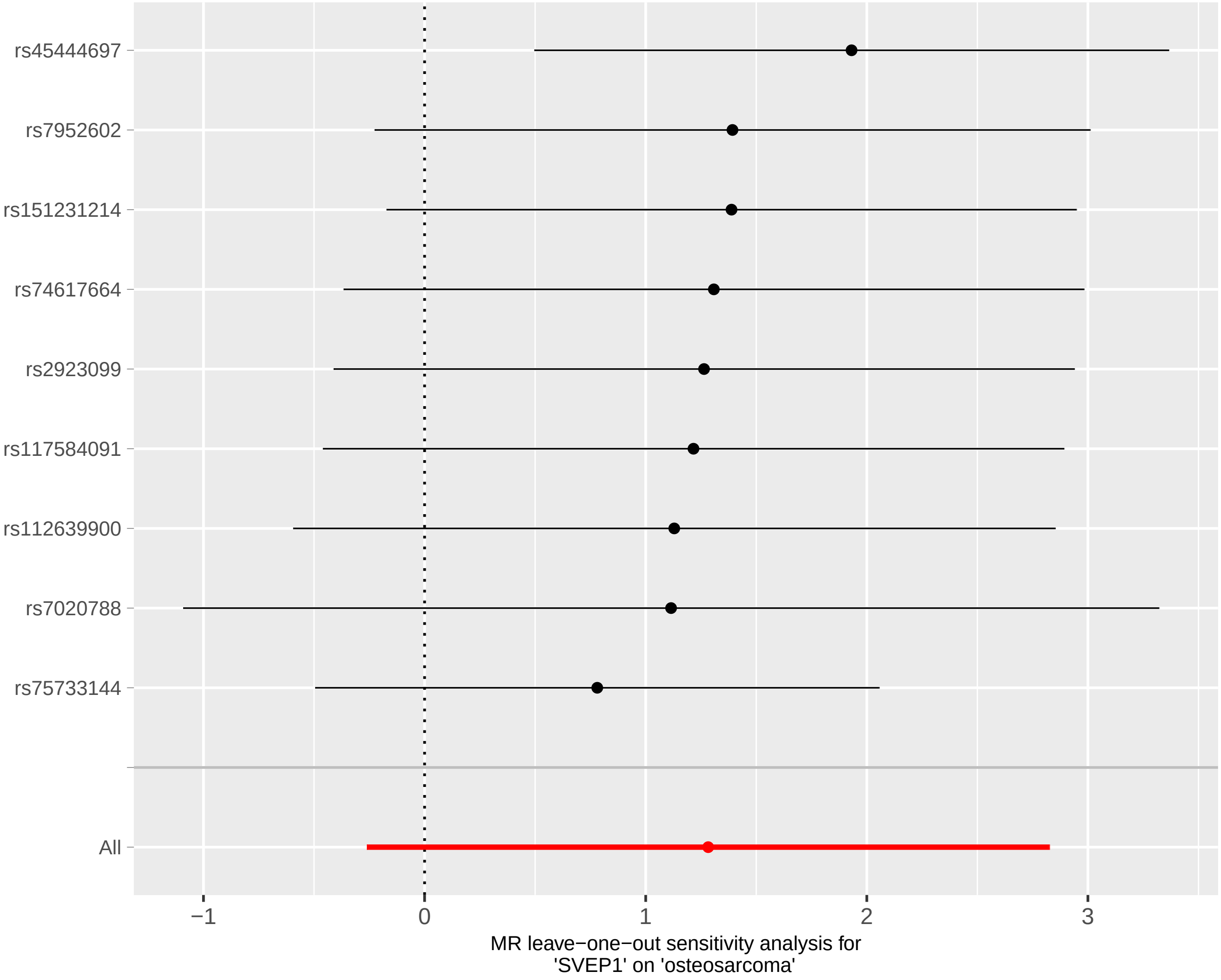
rs45444697
rs7952602
rs151231214
rs74617664
rs2923099
rs117584091
rs112639900
rs7020788
rs75733144
All
−1
0
1
2
3
MR leave−one−out sensitivity analysis for
'SVEP1' on 'osteosarcoma'

Insufficient number of SNPs

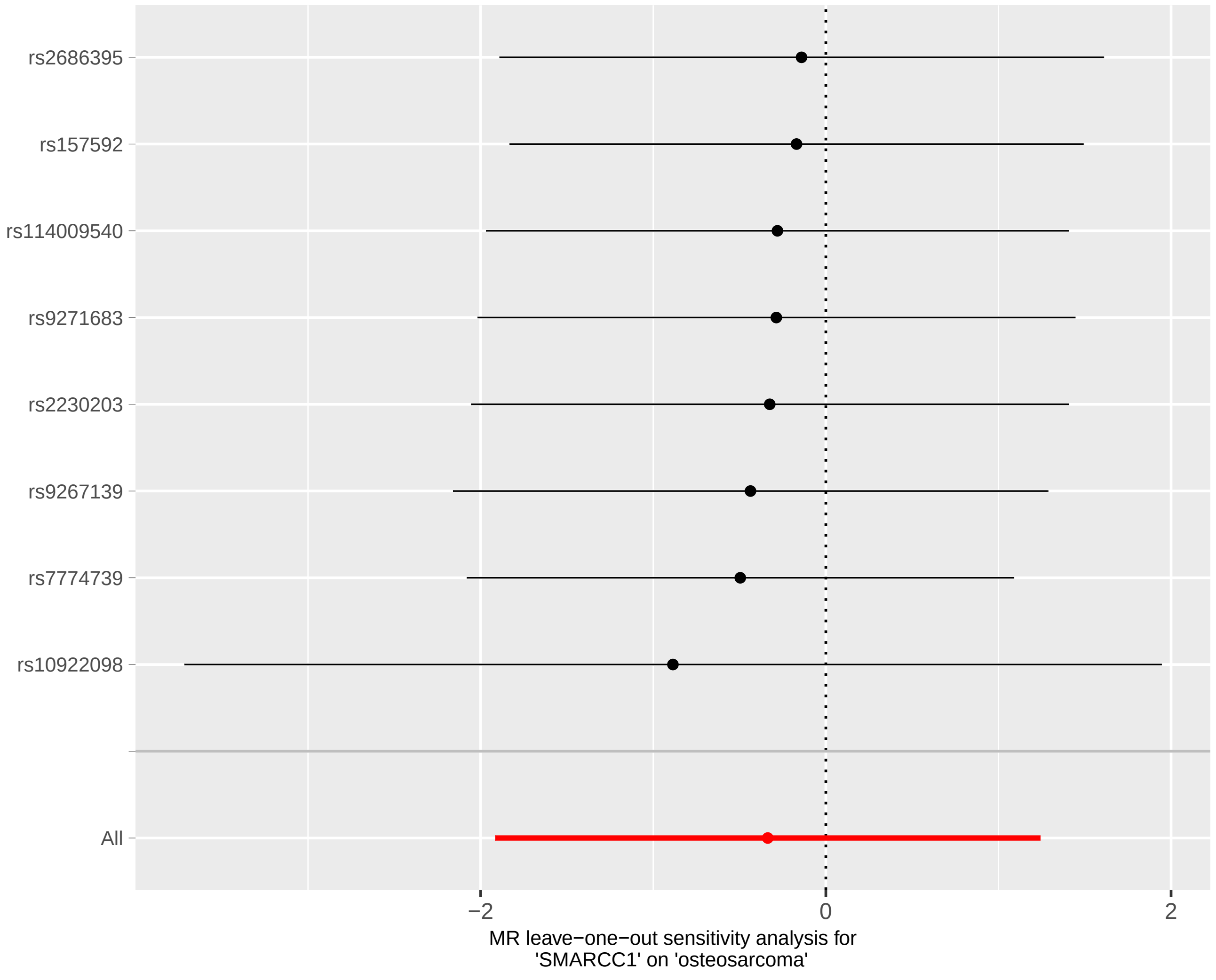
rs2686395
rs157592
rs114009540
rs9271683
rs2230203
rs9267139
rs7774739
rs10922098
All
−2
0
2
MR leave−one−out sensitivity analysis for
'SMARCC1' on 'osteosarcoma'

Insufficient number of SNPs

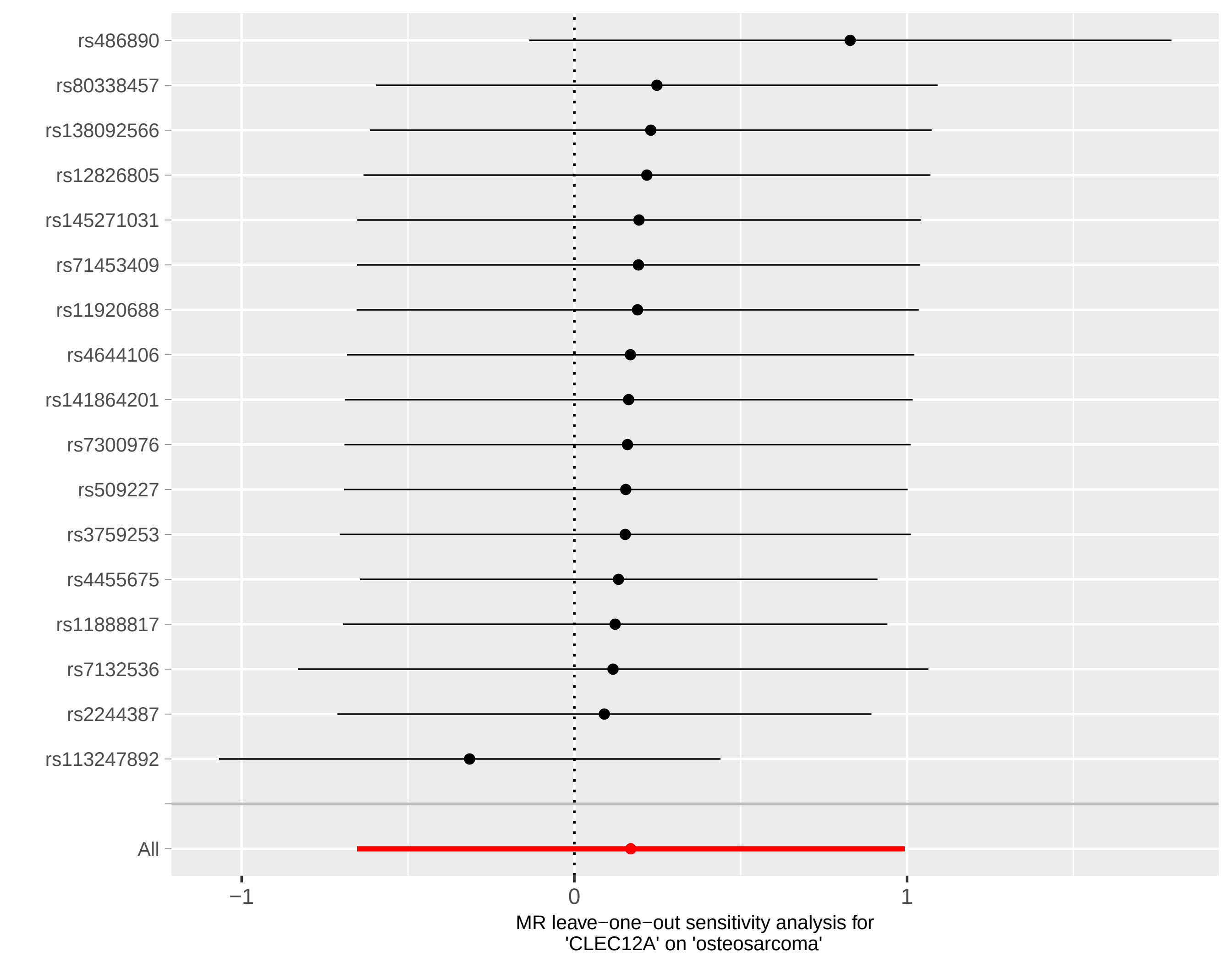
rs486890
rs80338457
rs138092566
rs12826805
rs145271031
rs71453409
rs11920688
rs4644106
rs141864201
rs7300976
rs509227
rs3759253
rs4455675
rs11888817
rs7132536
rs2244387
rs113247892
All
−1
0
1
MR leave−one−out sensitivity analysis for
'CLEC12A' on 'osteosarcoma'

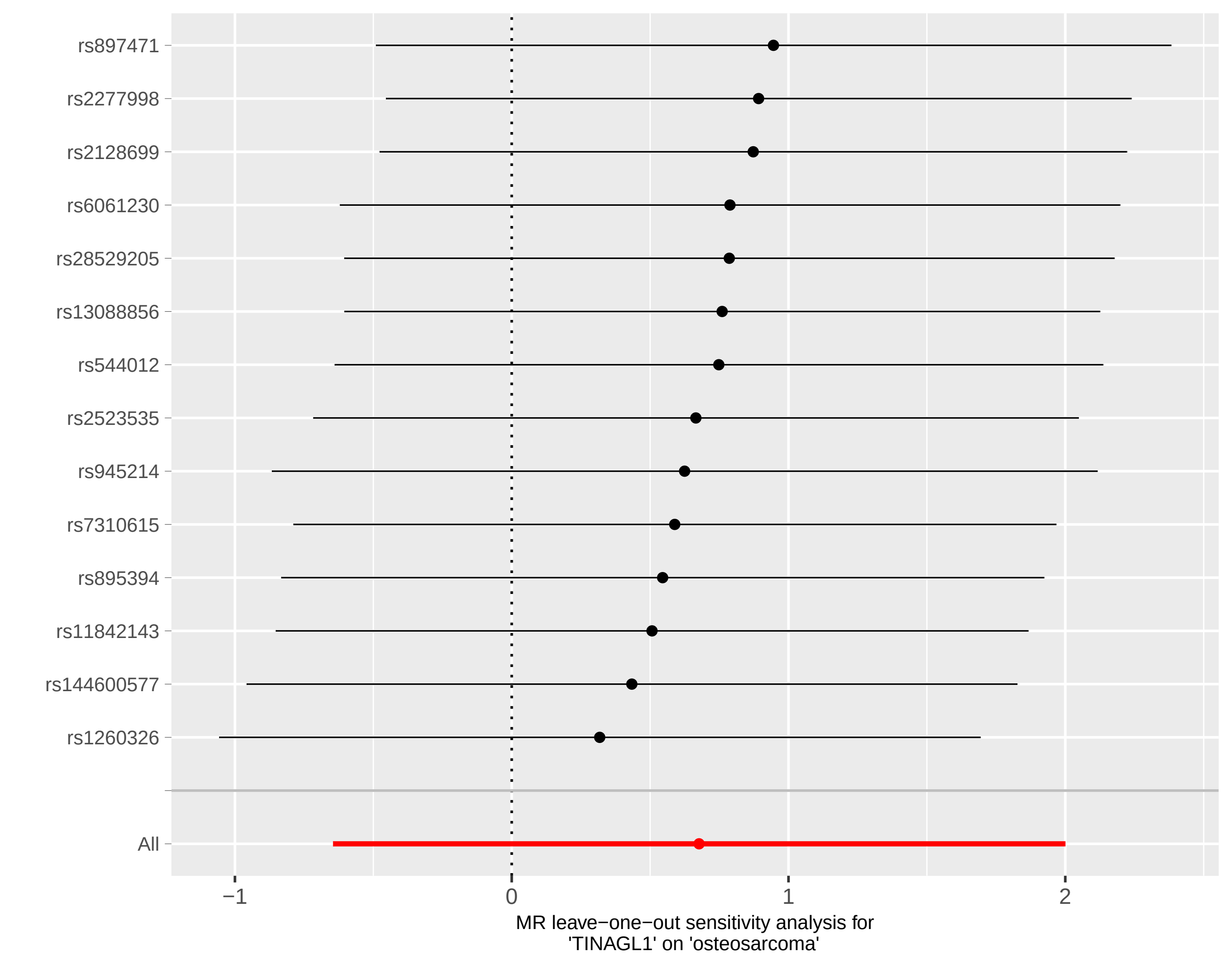
rs897471
rs2277998
rs2128699
rs6061230
rs28529205
rs13088856
rs544012
rs2523535
rs945214
rs7310615
rs895394
rs11842143
rs144600577
rs1260326
All
−1
0
1
2
MR leave−one−out sensitivity analysis for
'TINAGL1' on 'osteosarcoma'

Insufficient number of SNPs

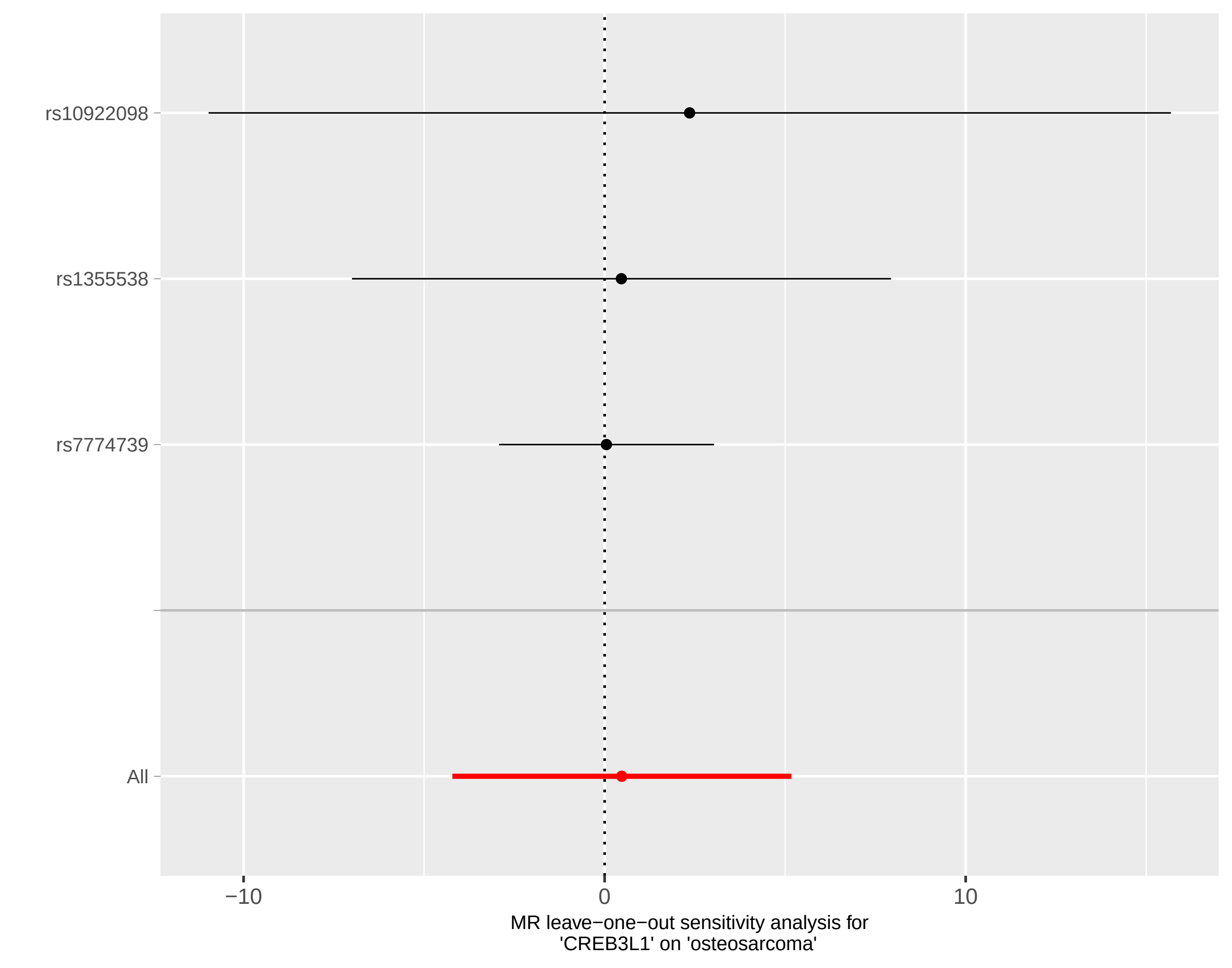

rs10922098
rs1355538
rs7774739
All
−10
0
10
MR leave−one−out sensitivity analysis for
'CREB3L1' on 'osteosarcoma'

Insufficient number of SNPs

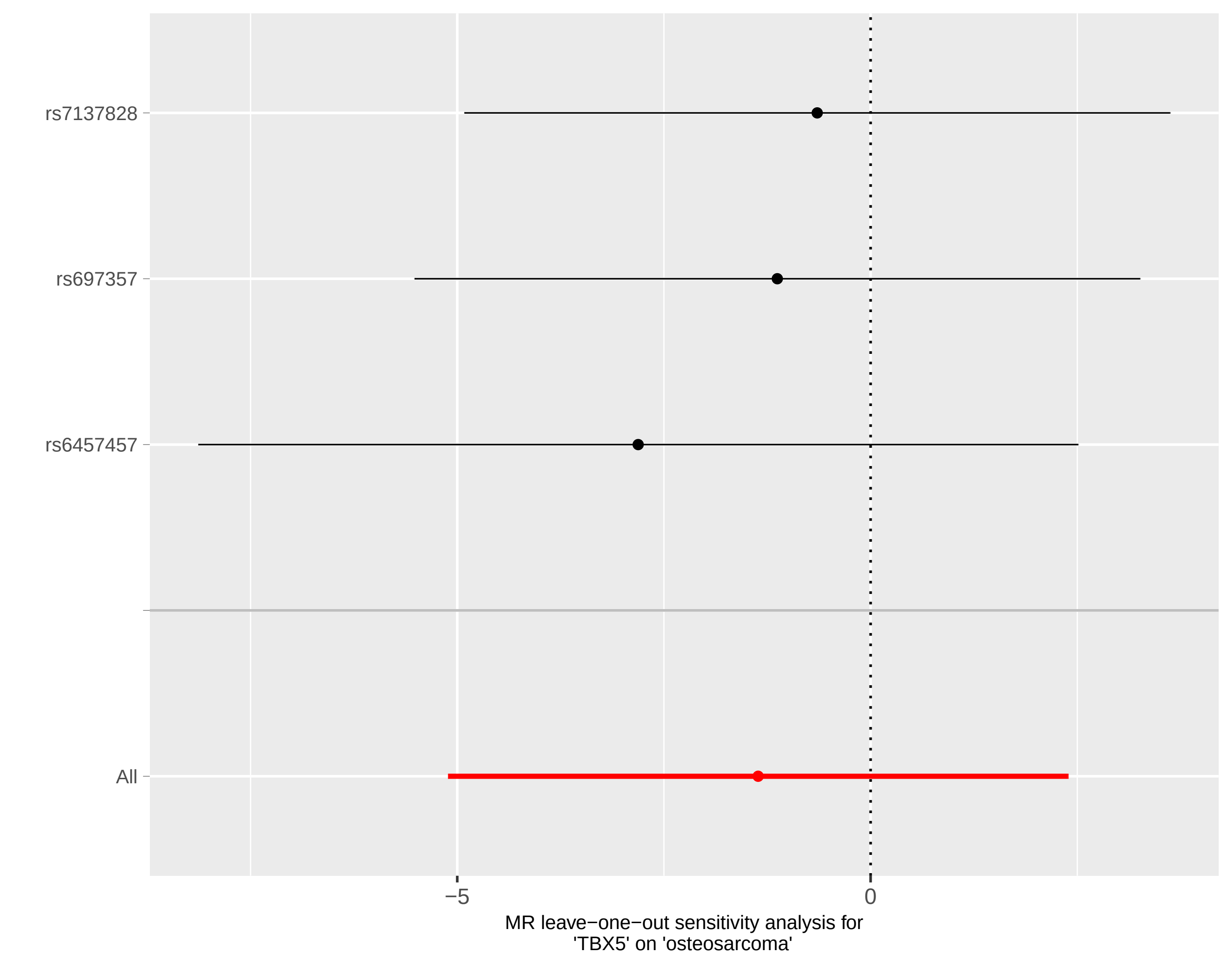

rs7137828
rs697357
rs6457457
All
−5
0
MR leave−one−out sensitivity analysis for
'TBX5' on 'osteosarcoma'

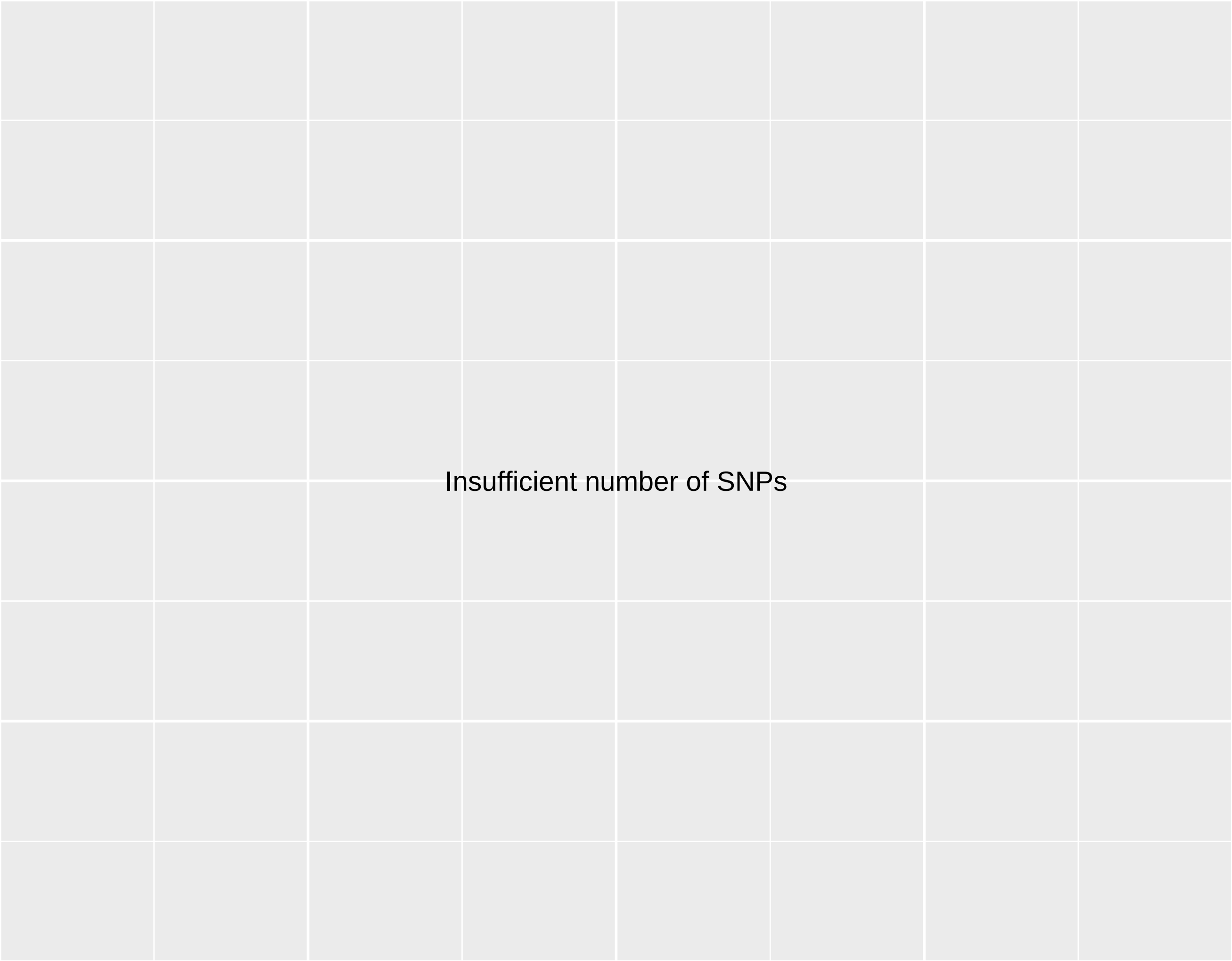
Insufficient number of SNPs

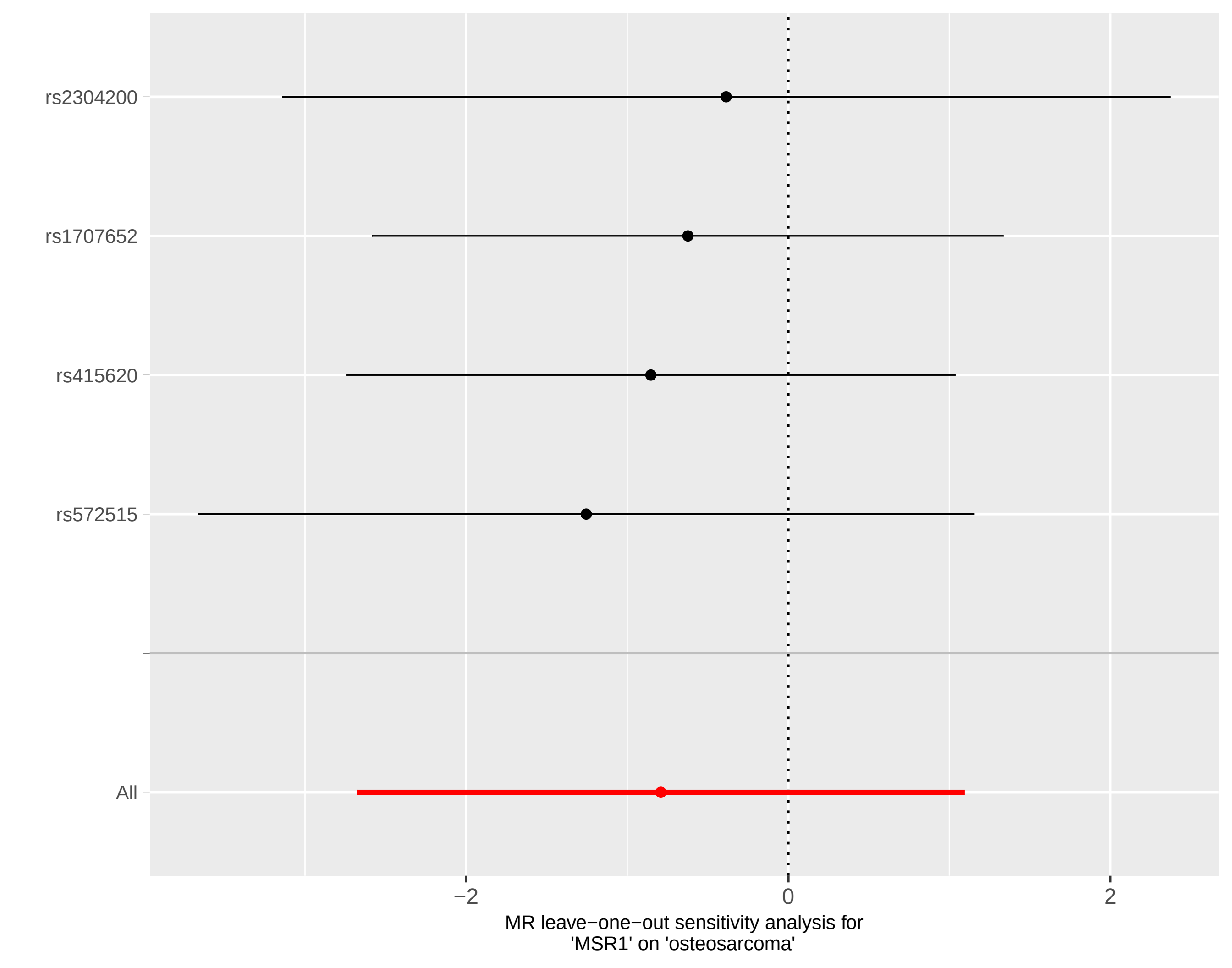
rs2304200
rs1707652
rs415620
rs572515
All
−2
0
2
MR leave−one−out sensitivity analysis for
'MSR1' on 'osteosarcoma'

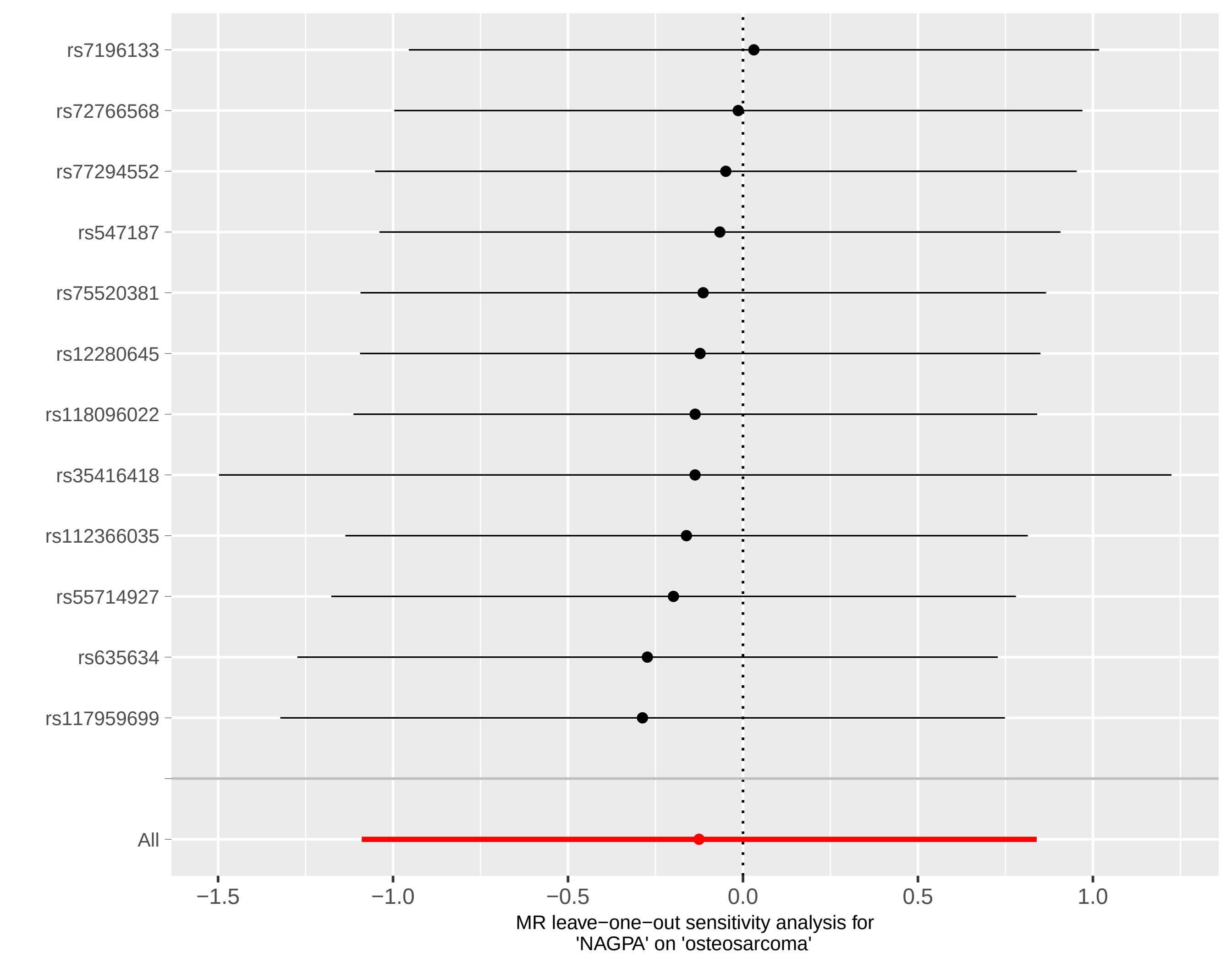
rs7196133
rs72766568
rs77294552
rs547187
rs75520381
rs12280645
rs118096022
rs35416418
rs112366035
rs55714927
rs635634
rs117959699
All
−1.5
−1.0
−0.5
0.0
0.5
1.0
MR leave−one−out sensitivity analysis for
'NAGPA' on 'osteosarcoma'

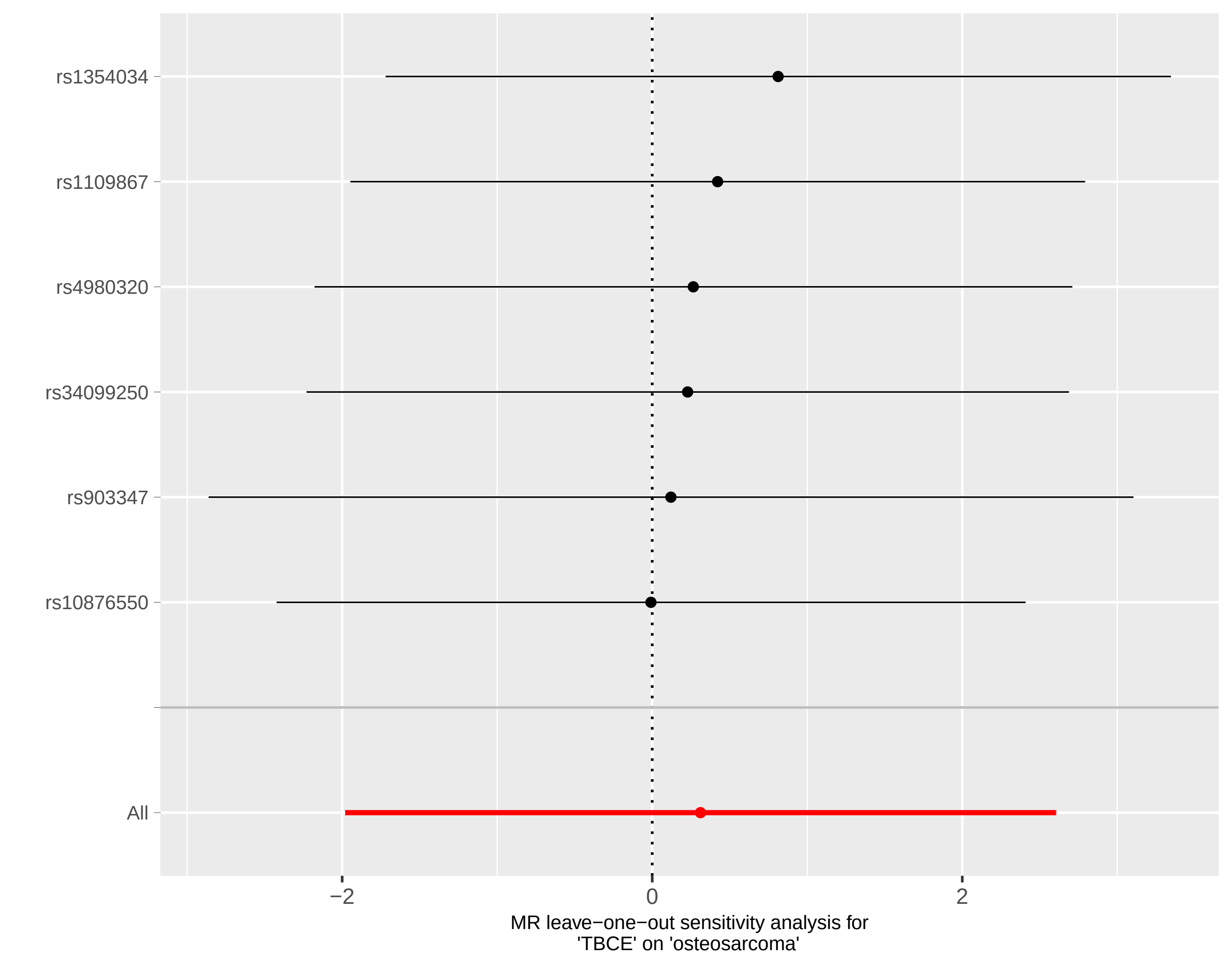
rs1354034
rs1109867
rs4980320
rs34099250
rs903347
rs10876550
All
−2
0
2
MR leave−one−out sensitivity analysis for
'TBCE' on 'osteosarcoma'

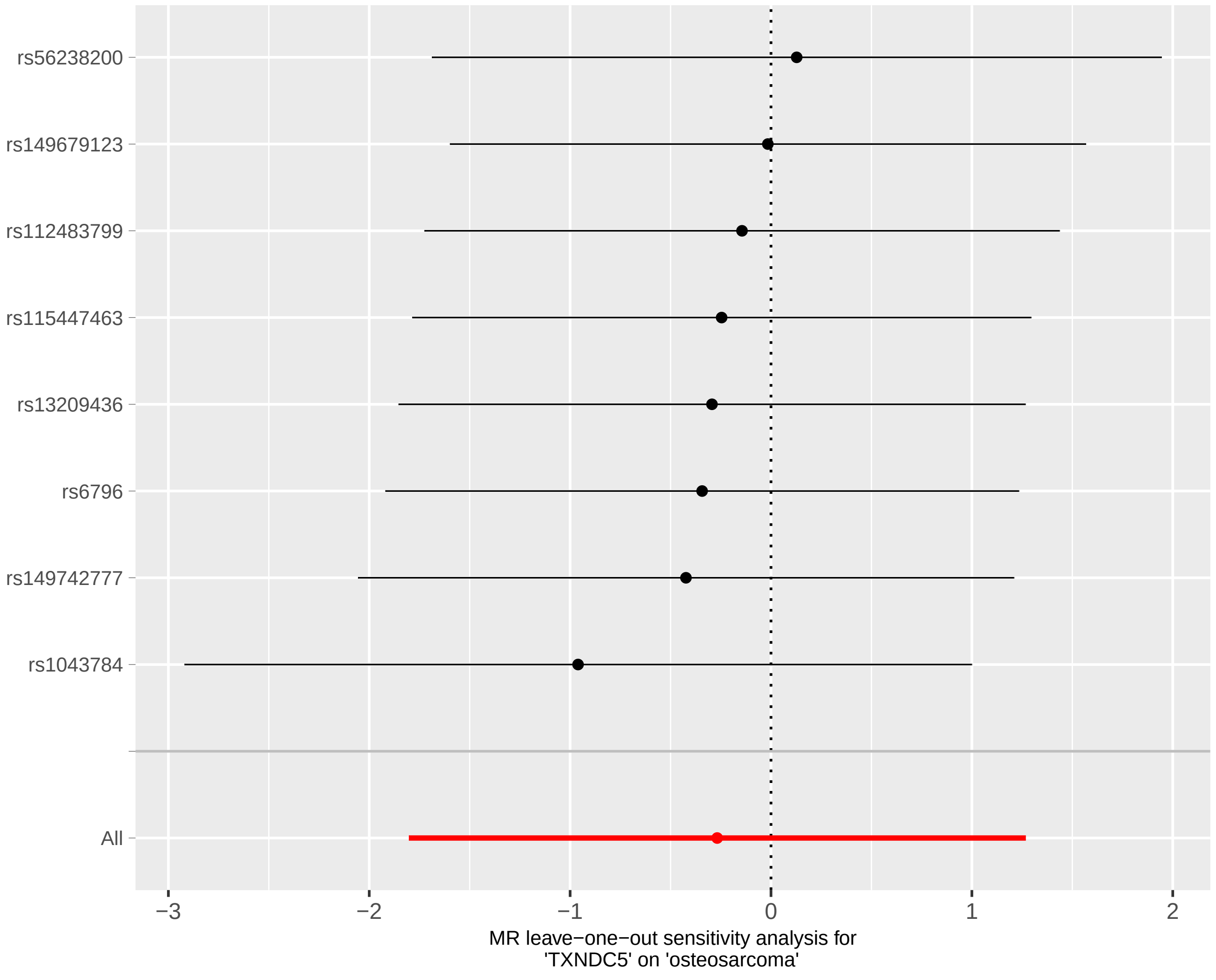

rs56238200
rs149679123
rs112483799
rs115447463
rs13209436
rs6796
rs149742777
rs1043784
All
−3
−2
−1
0
1
2
MR leave−one−out sensitivity analysis for
'TXNDC5' on 'osteosarcoma'

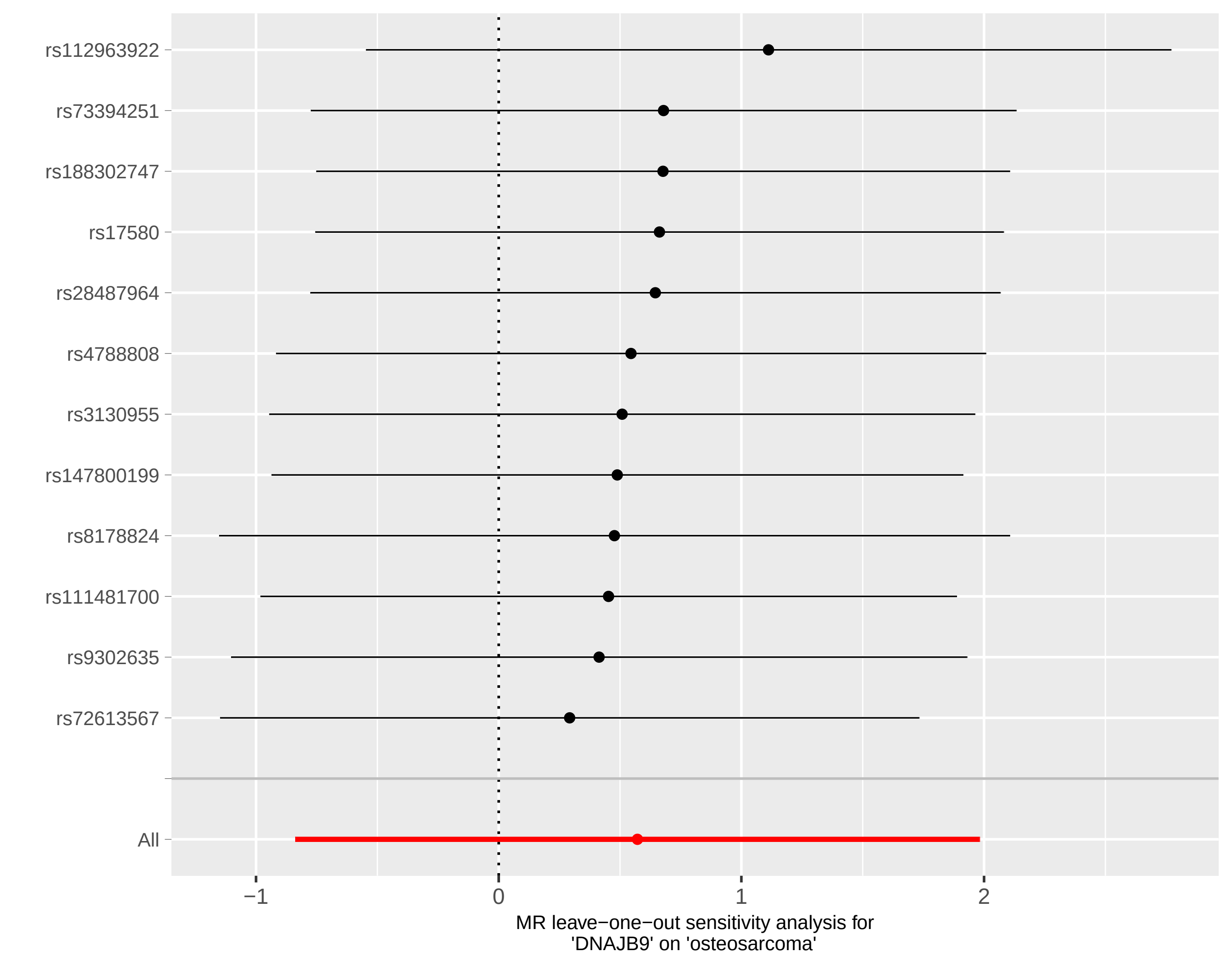

rs112963922
rs73394251
rs188302747
rs17580
rs28487964
rs4788808
rs3130955
rs147800199
rs8178824
rs111481700
rs9302635
rs72613567
All
−1
0
1
2
MR leave−one−out sensitivity analysis for
'DNAJB9' on 'osteosarcoma'

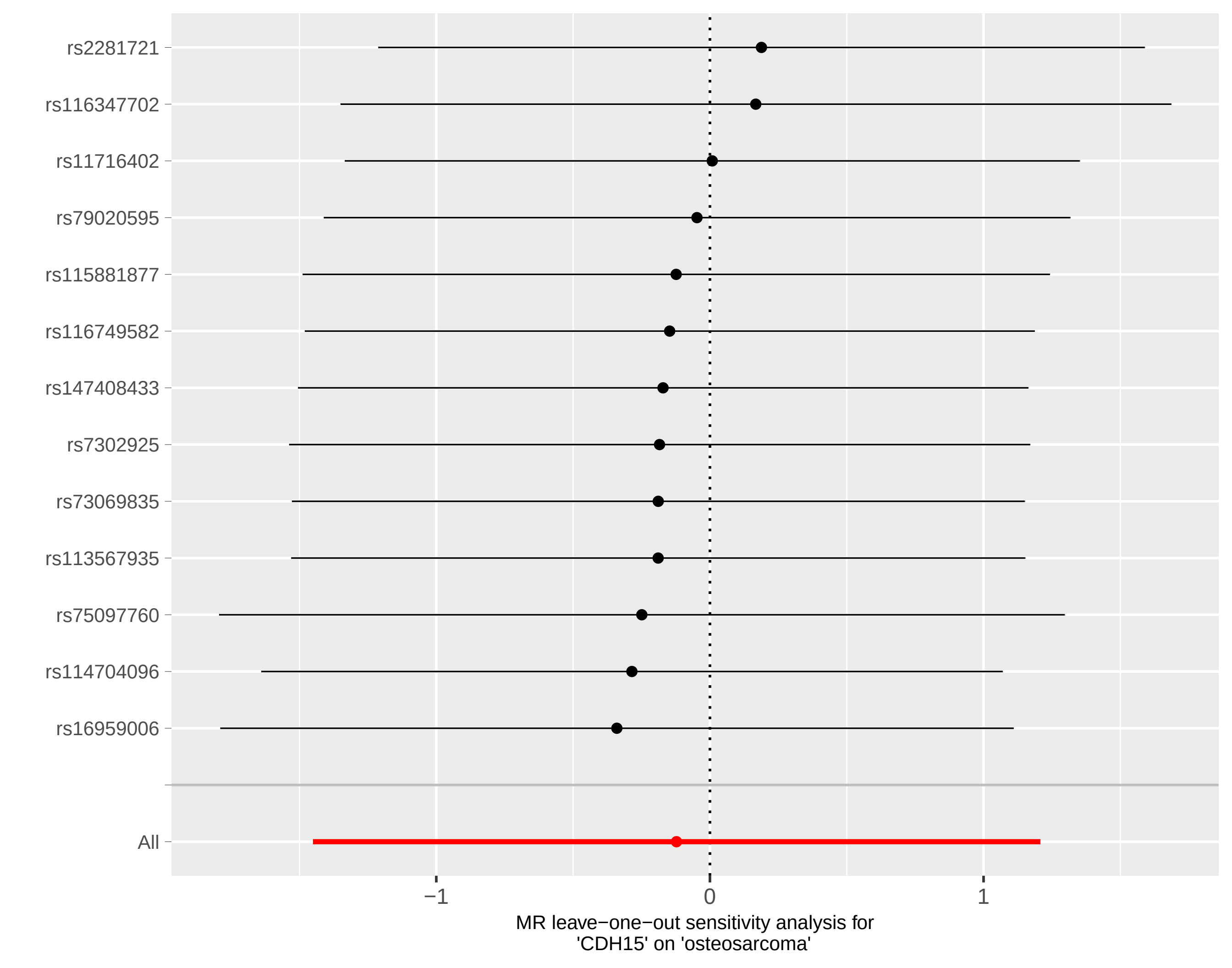
rs2281721
rs116347702
rs11716402
rs79020595
rs115881877
rs116749582
rs147408433
rs7302925
rs73069835
rs113567935
rs75097760
rs114704096
rs16959006
All
−1
0
1
MR leave−one−out sensitivity analysis for
'CDH15' on 'osteosarcoma'

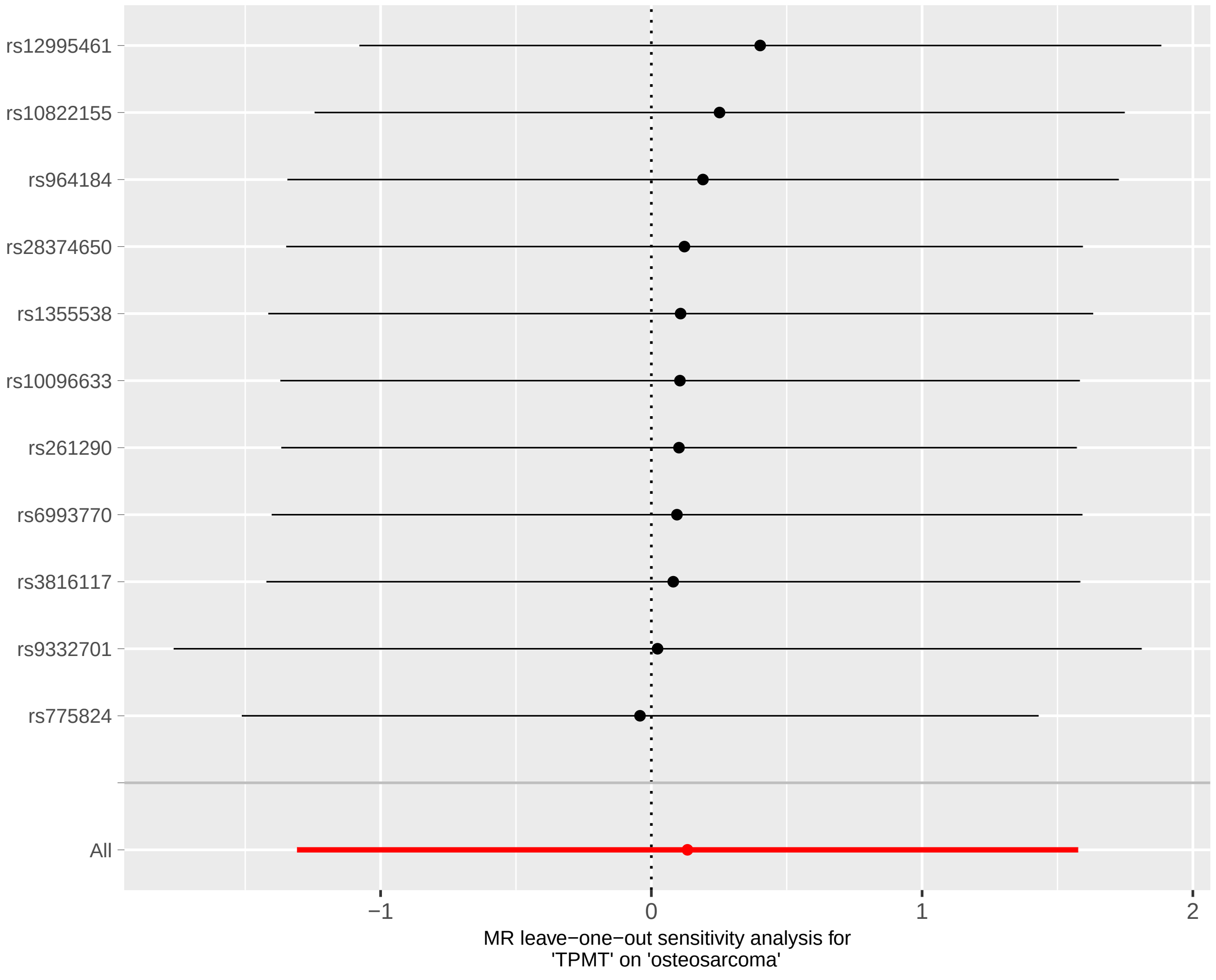
rs12995461
rs10822155
rs964184
rs28374650
rs1355538
rs10096633
rs261290
rs6993770
rs3816117
rs9332701
rs775824
All
−1
0
1
2
MR leave−one−out sensitivity analysis for
'TPMT' on 'osteosarcoma'

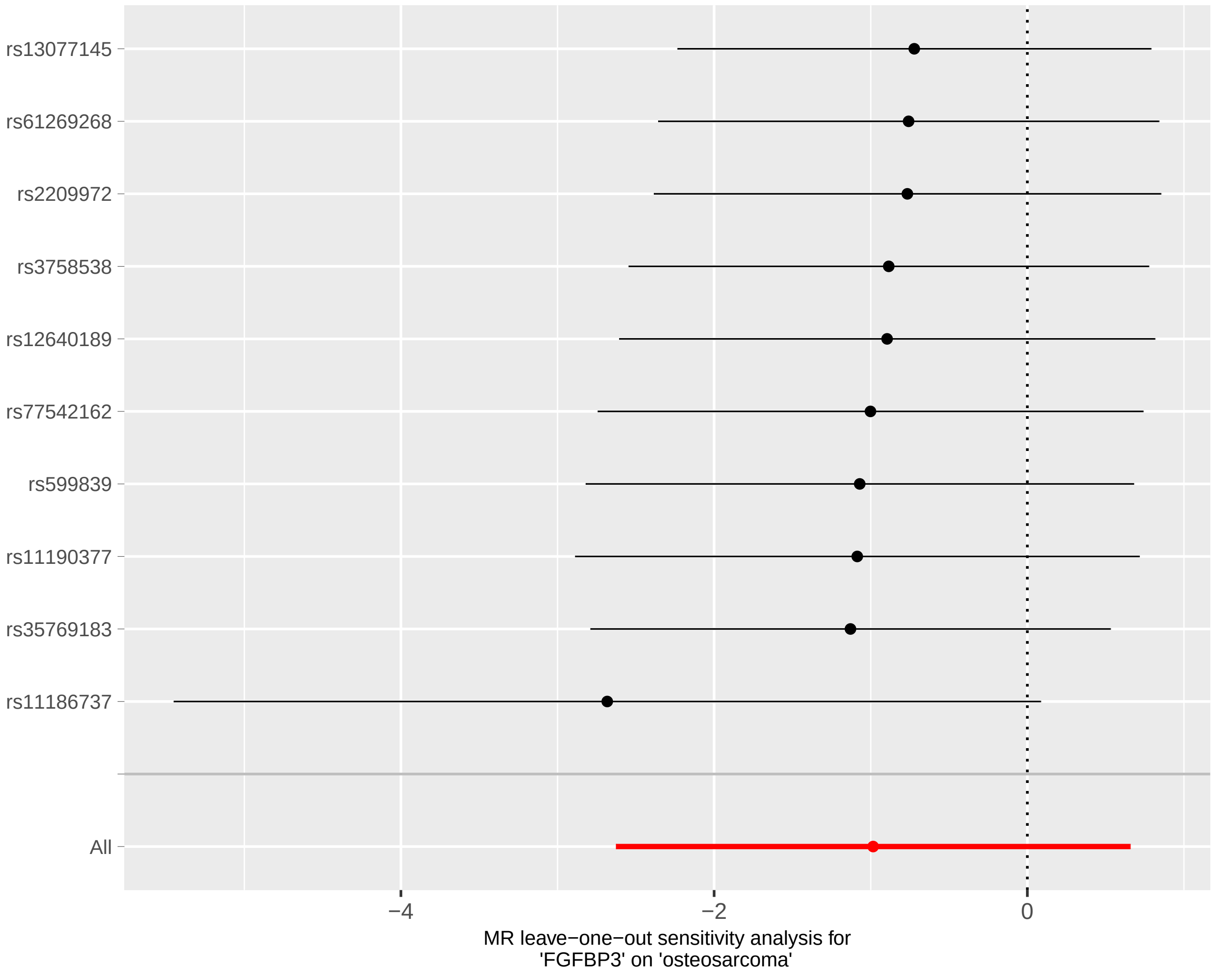
rs13077145
rs61269268
rs2209972
rs3758538
rs12640189
rs77542162
rs599839
rs11190377
rs35769183
rs11186737
All
−4
−2
0
MR leave−one−out sensitivity analysis for
'FGFBP3' on 'osteosarcoma'

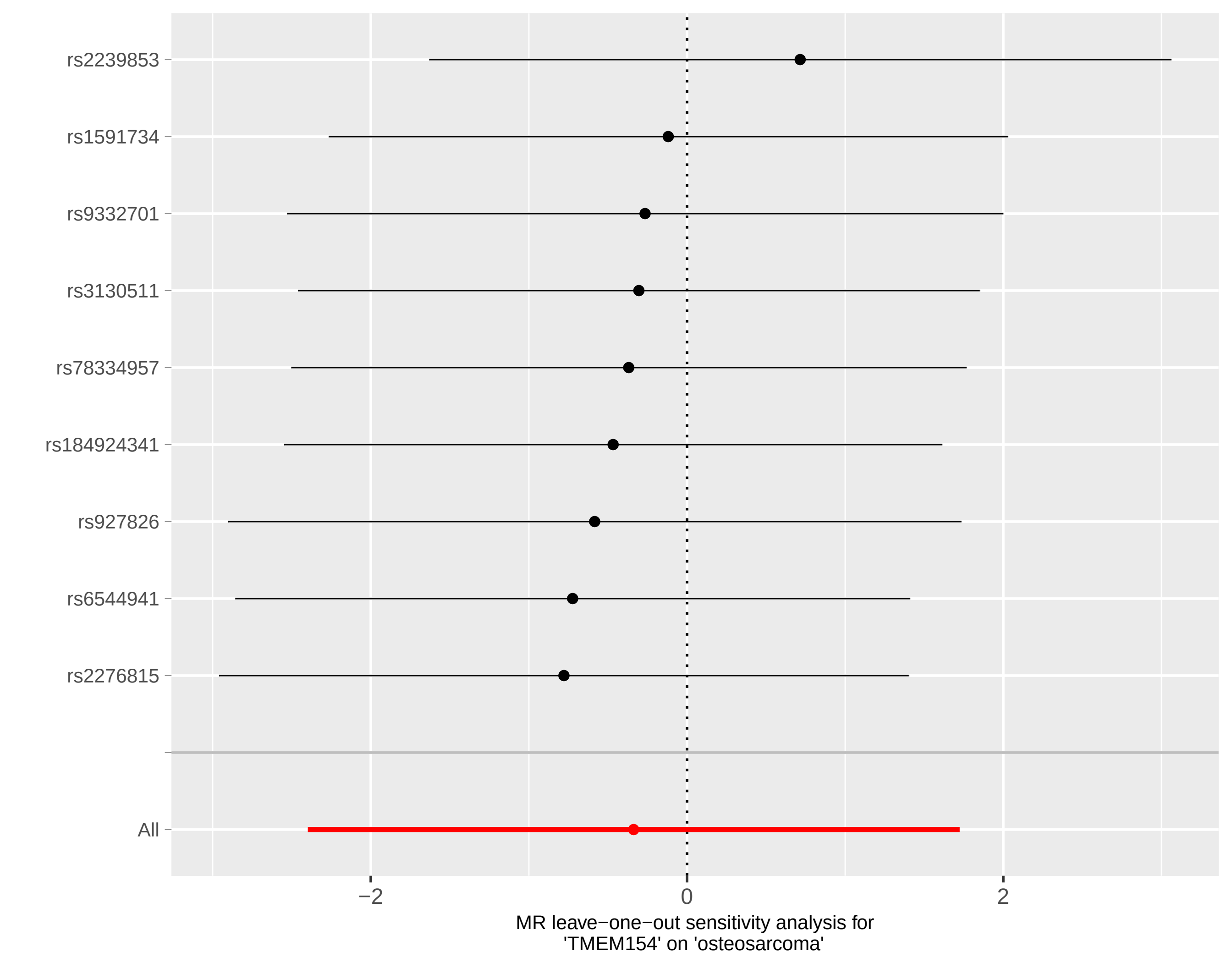
rs2239853
rs1591734
rs9332701
rs3130511
rs78334957
rs184924341
rs927826
rs6544941
rs2276815
All
−2
0
2
MR leave−one−out sensitivity analysis for
'TMEM154' on 'osteosarcoma'

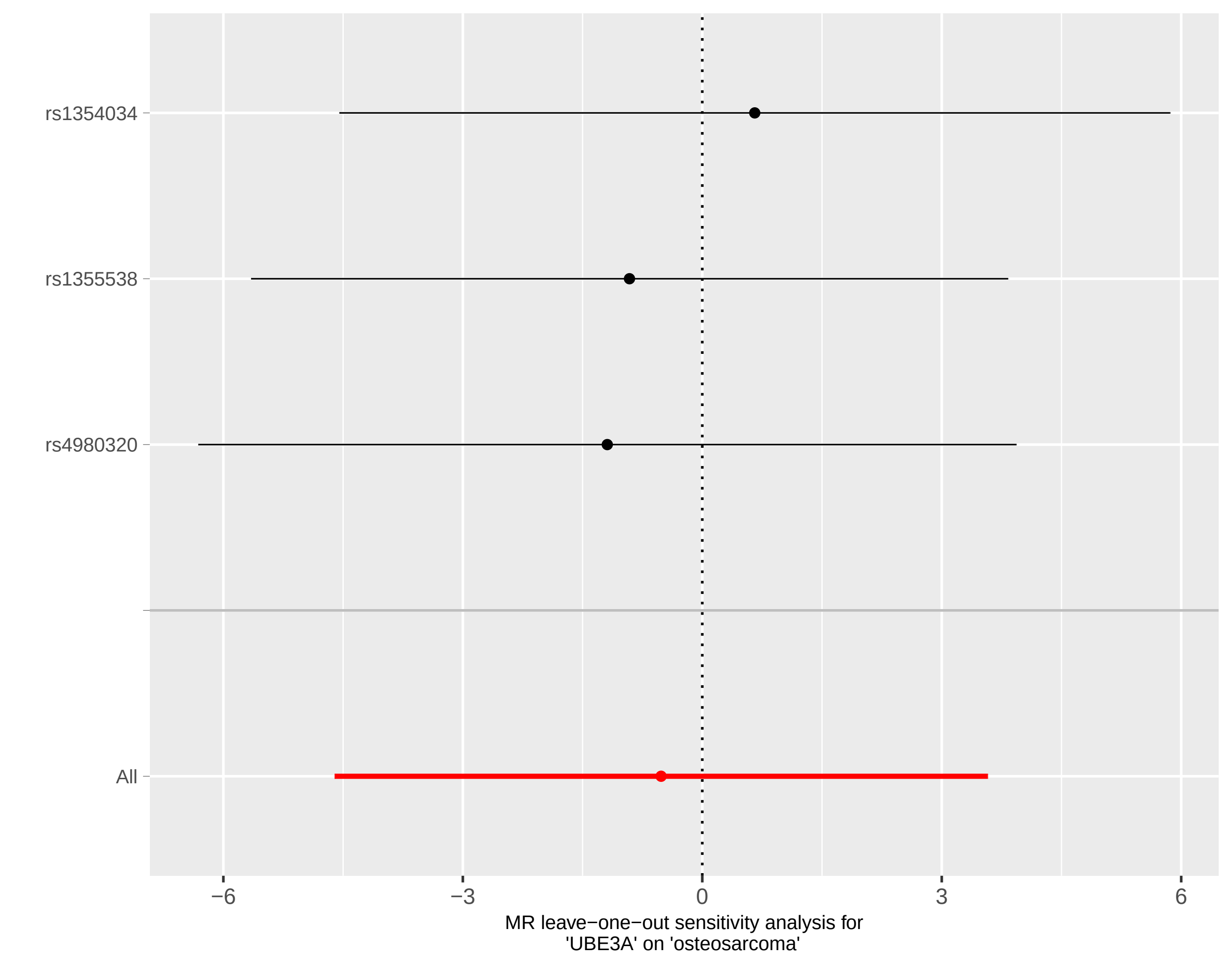
rs1354034
rs1355538
rs4980320
All
−6
−3
0
3
6
MR leave−one−out sensitivity analysis for
'UBE3A' on 'osteosarcoma'

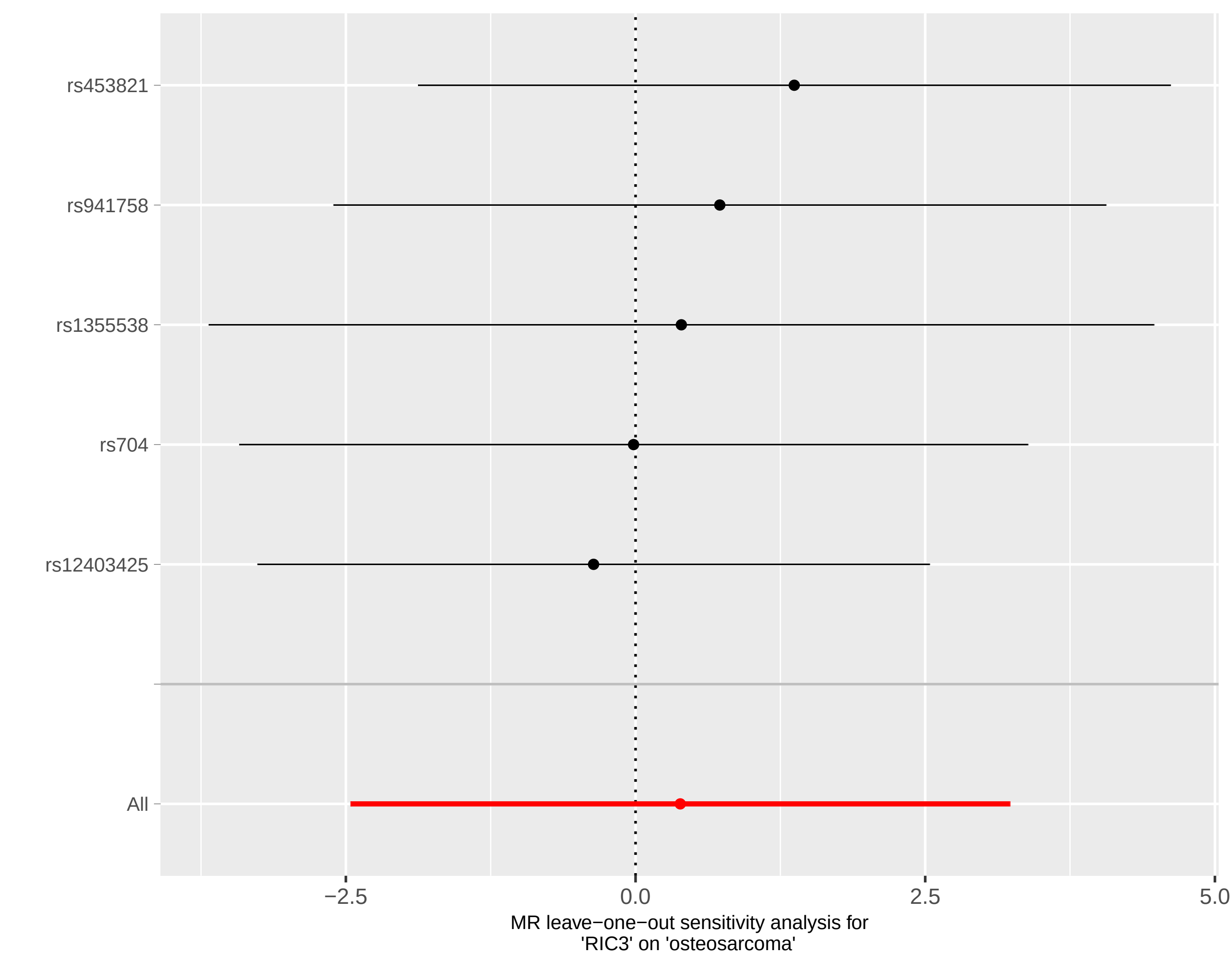
rs453821
rs941758
rs1355538
rs704
rs12403425
All
−2.5
0.0
2.5
5.0
MR leave−one−out sensitivity analysis for
'RIC3' on 'osteosarcoma'

Insufficient number of SNPs

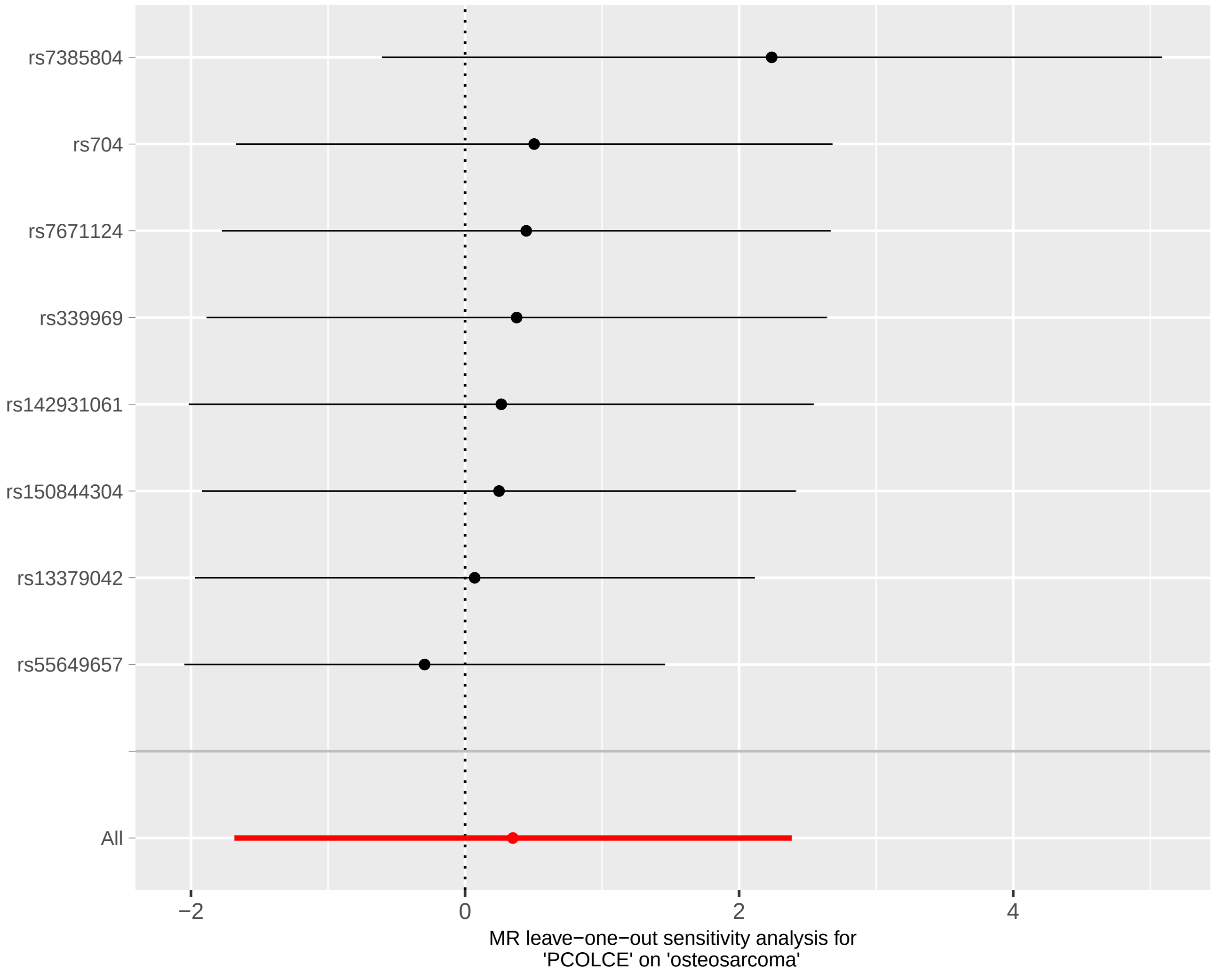

rs7385804
rs704
rs7671124
rs339969
rs142931061
rs150844304
rs13379042
rs55649657
All
−2
0
2
4
MR leave−one−out sensitivity analysis for
'PCOLCE' on 'osteosarcoma'

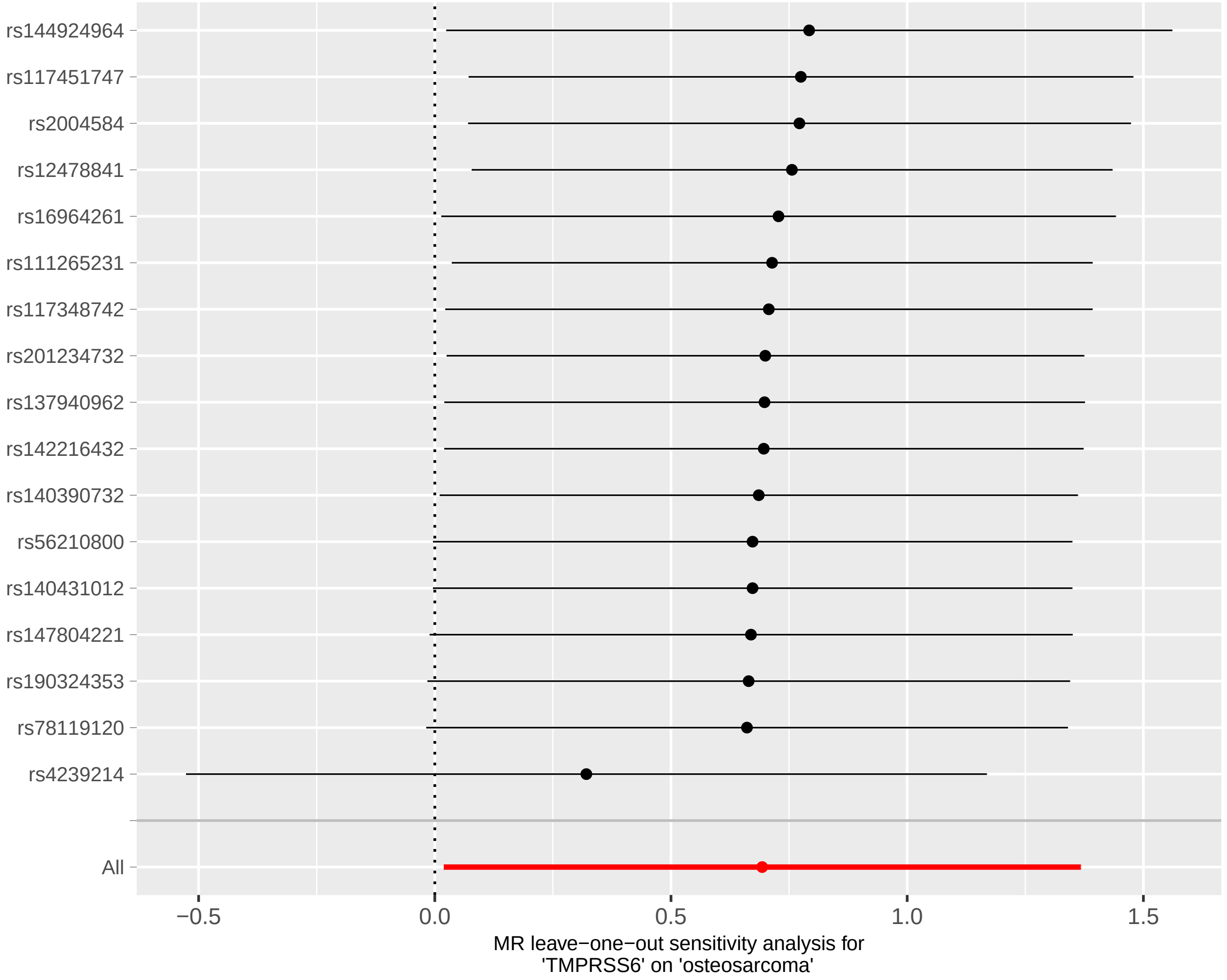
rs144924964
rs117451747
rs2004584
rs12478841
rs16964261
rs111265231
rs117348742
rs201234732
rs137940962
rs142216432
rs140390732
rs56210800
rs140431012
rs147804221
rs190324353
rs78119120
rs4239214
All
−0.5
0.0
0.5
1.0
1.5
MR leave−one−out sensitivity analysis for
'TMPRSS6' on 'osteosarcoma'

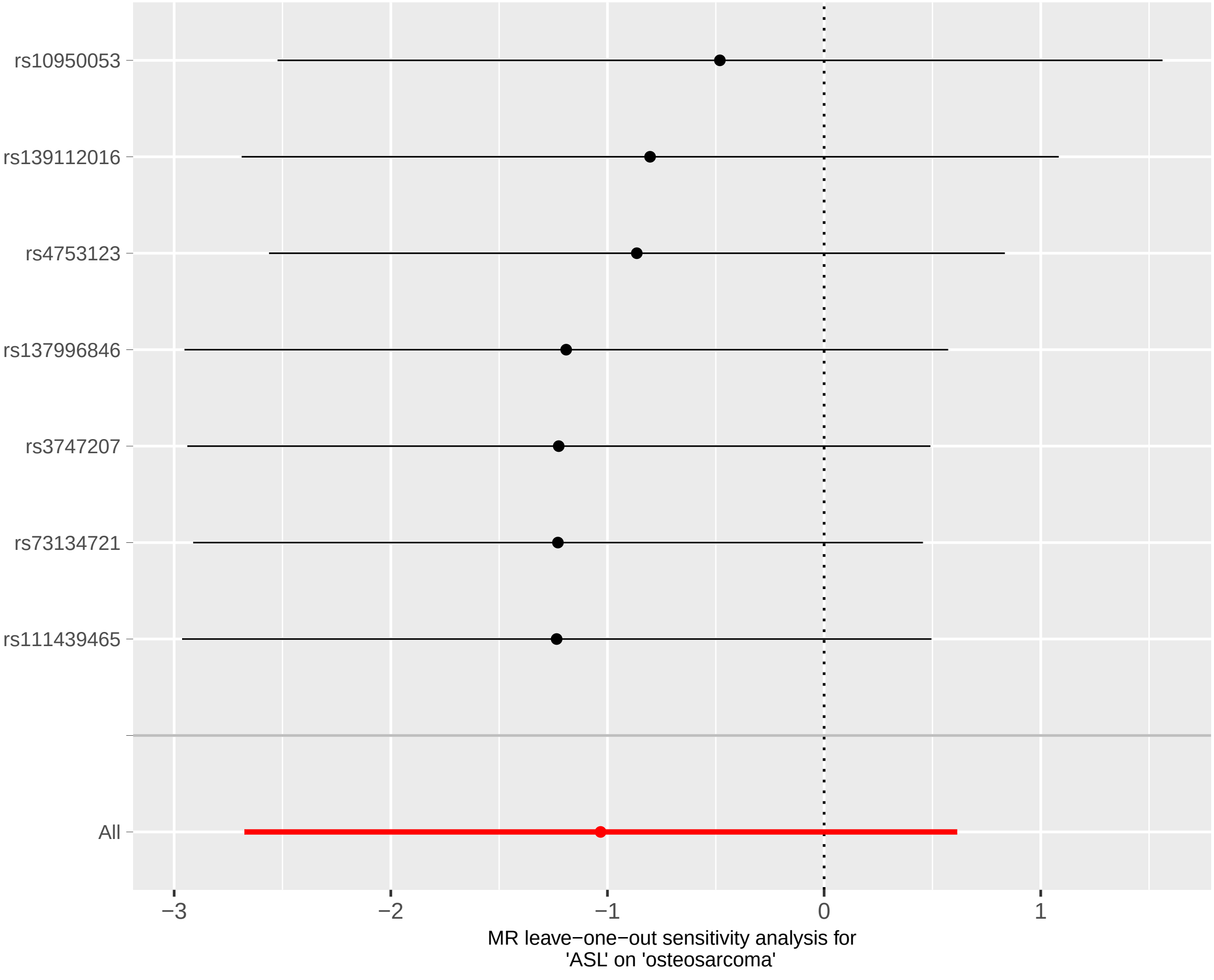
rs10950053
rs139112016
rs4753123
rs137996846
rs3747207
rs73134721
rs111439465
All
−3
−2
−1
0
1
MR leave−one−out sensitivity analysis for
'ASL' on 'osteosarcoma'

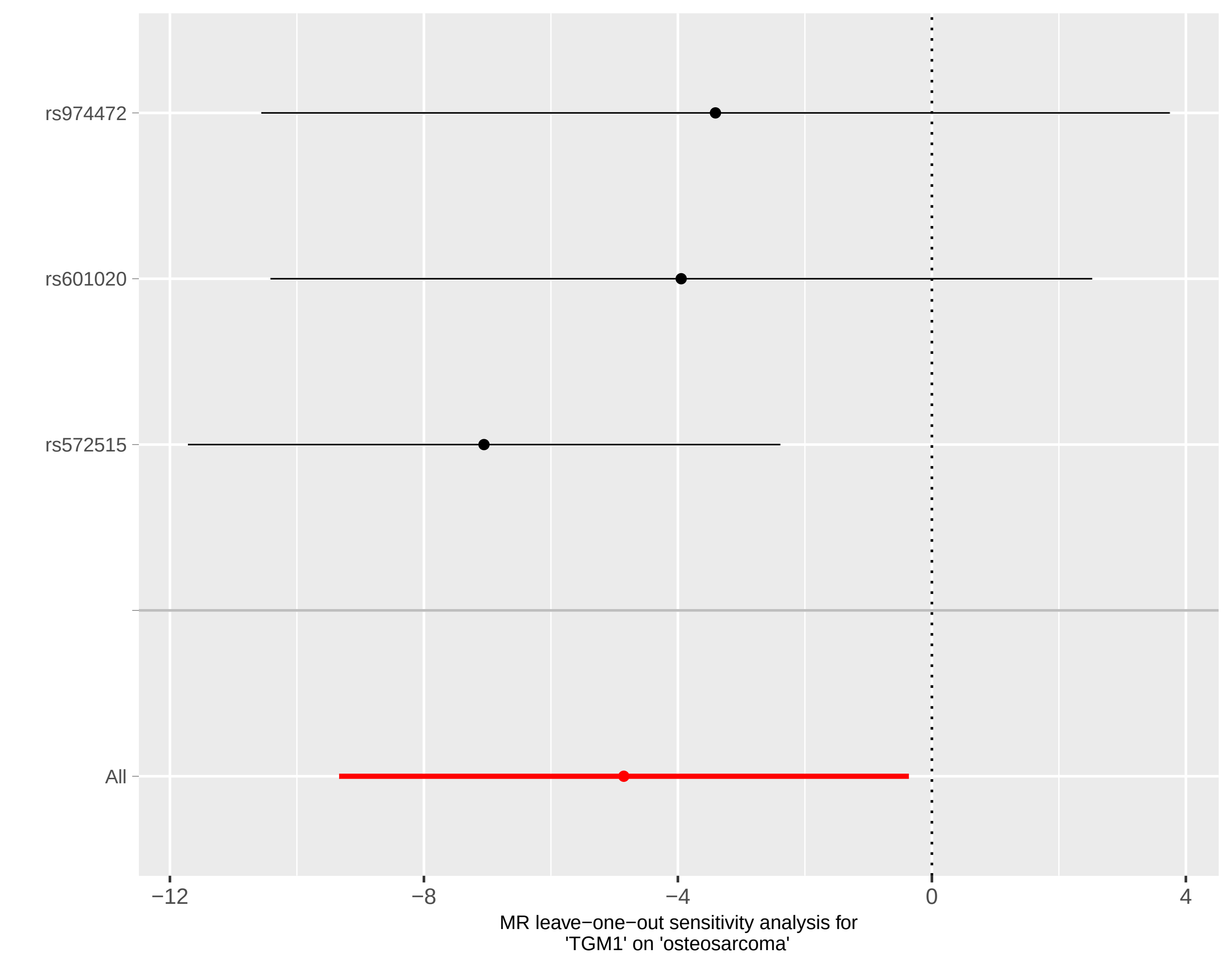
rs974472
rs601020
rs572515
All
−12
−8
−4
0
4
MR leave−one−out sensitivity analysis for
'TGM1' on 'osteosarcoma'

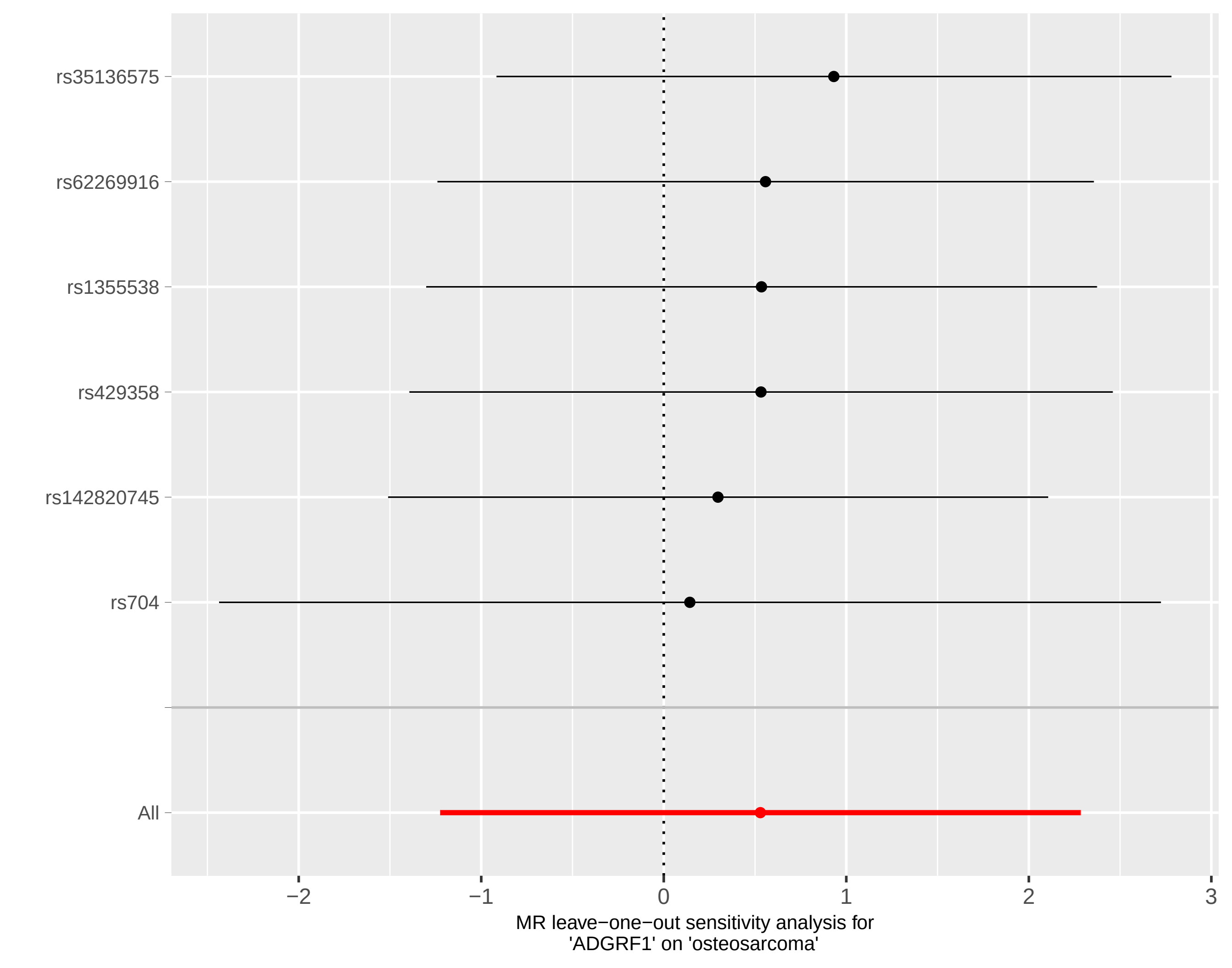

rs35136575
rs62269916
rs1355538
rs429358
rs142820745
rs704
All
−2
−1
0
1
2
3
MR leave−one−out sensitivity analysis for
'ADGRF1' on 'osteosarcoma'

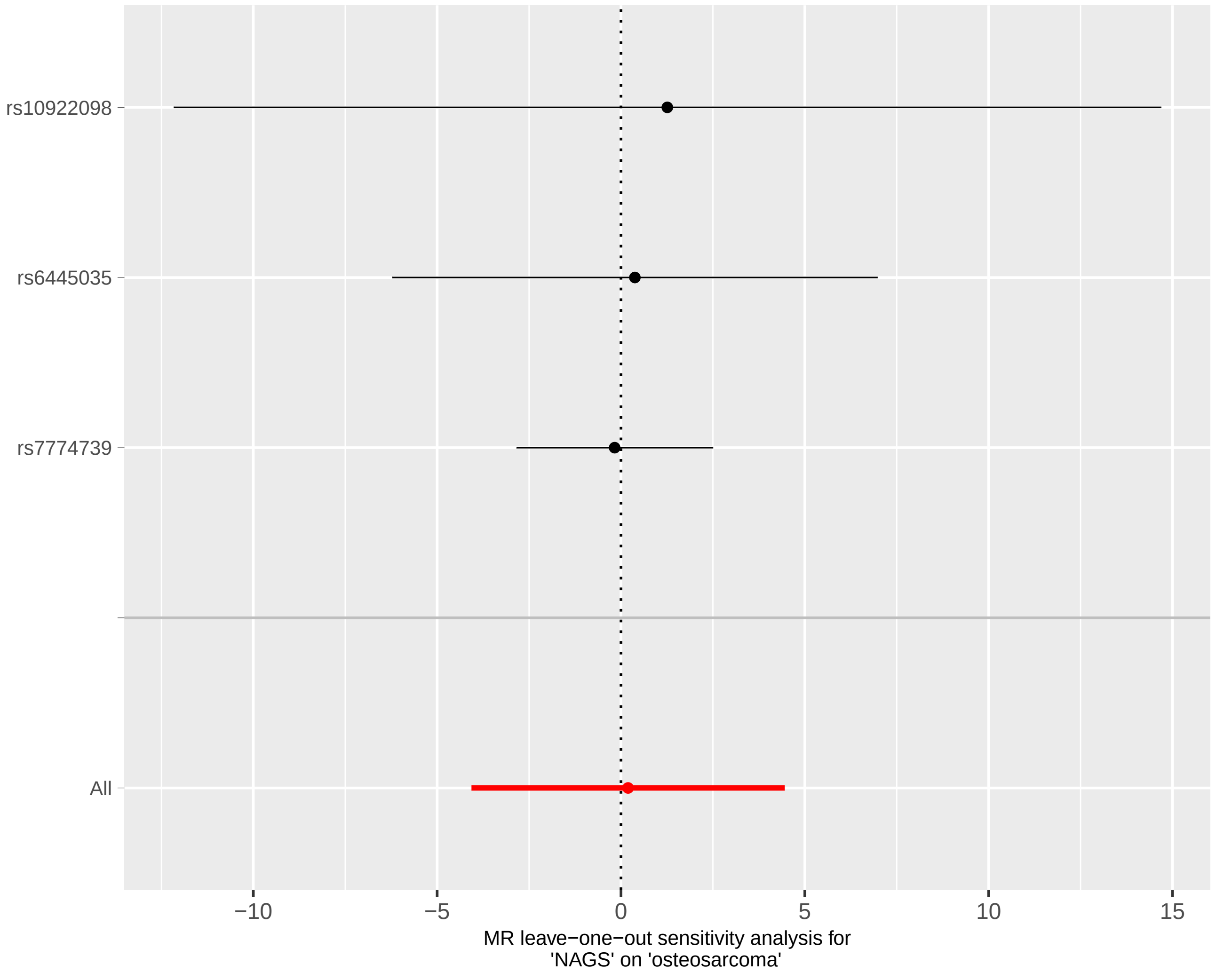

rs10922098
rs6445035
rs7774739
All
−10
−5
0
5
10
15
MR leave−one−out sensitivity analysis for
'NAGS' on 'osteosarcoma'

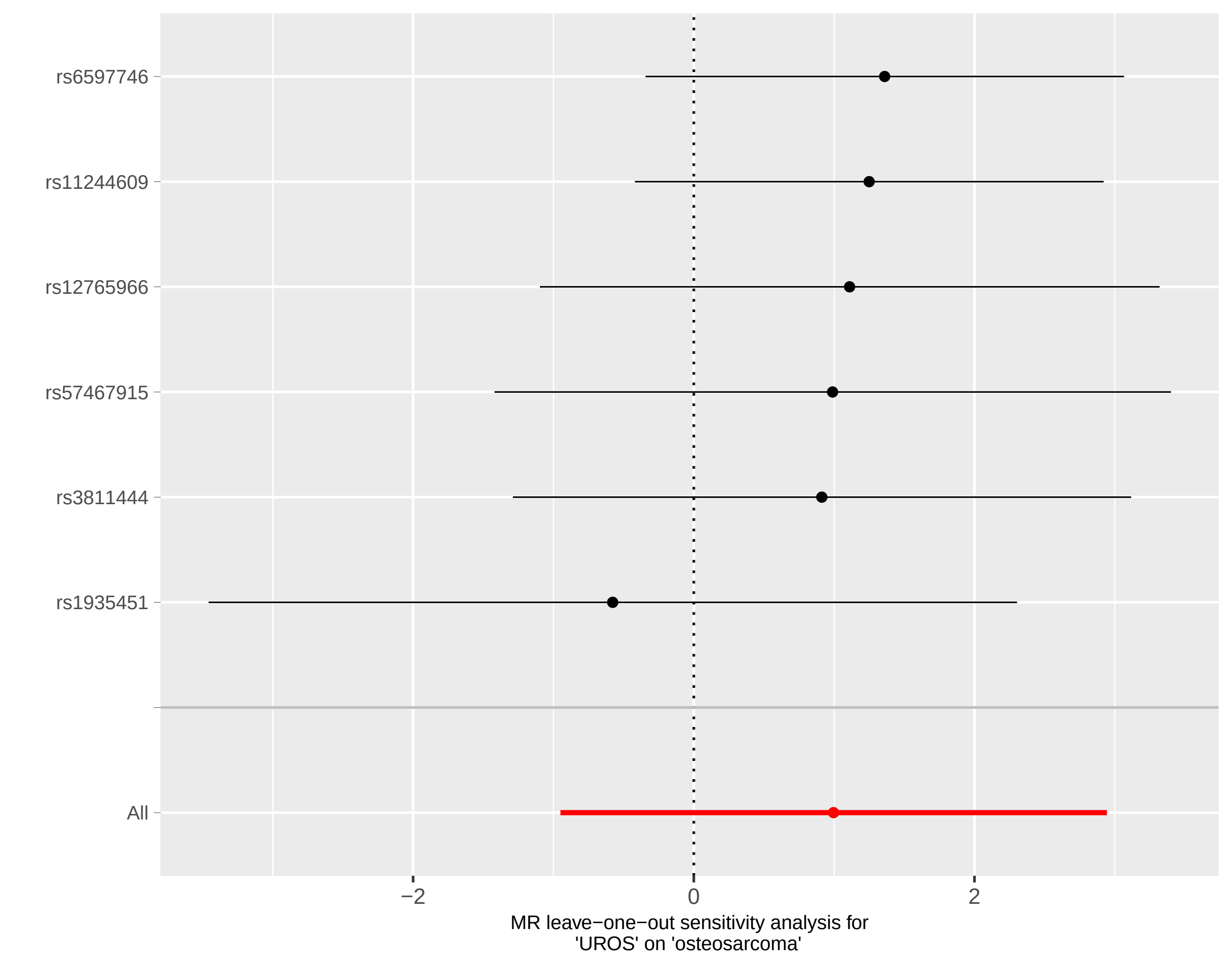

rs6597746
rs11244609
rs12765966
rs57467915
rs3811444
rs1935451
All
−2
0
2
MR leave−one−out sensitivity analysis for
'UROS' on 'osteosarcoma'

Insufficient number of SNPs

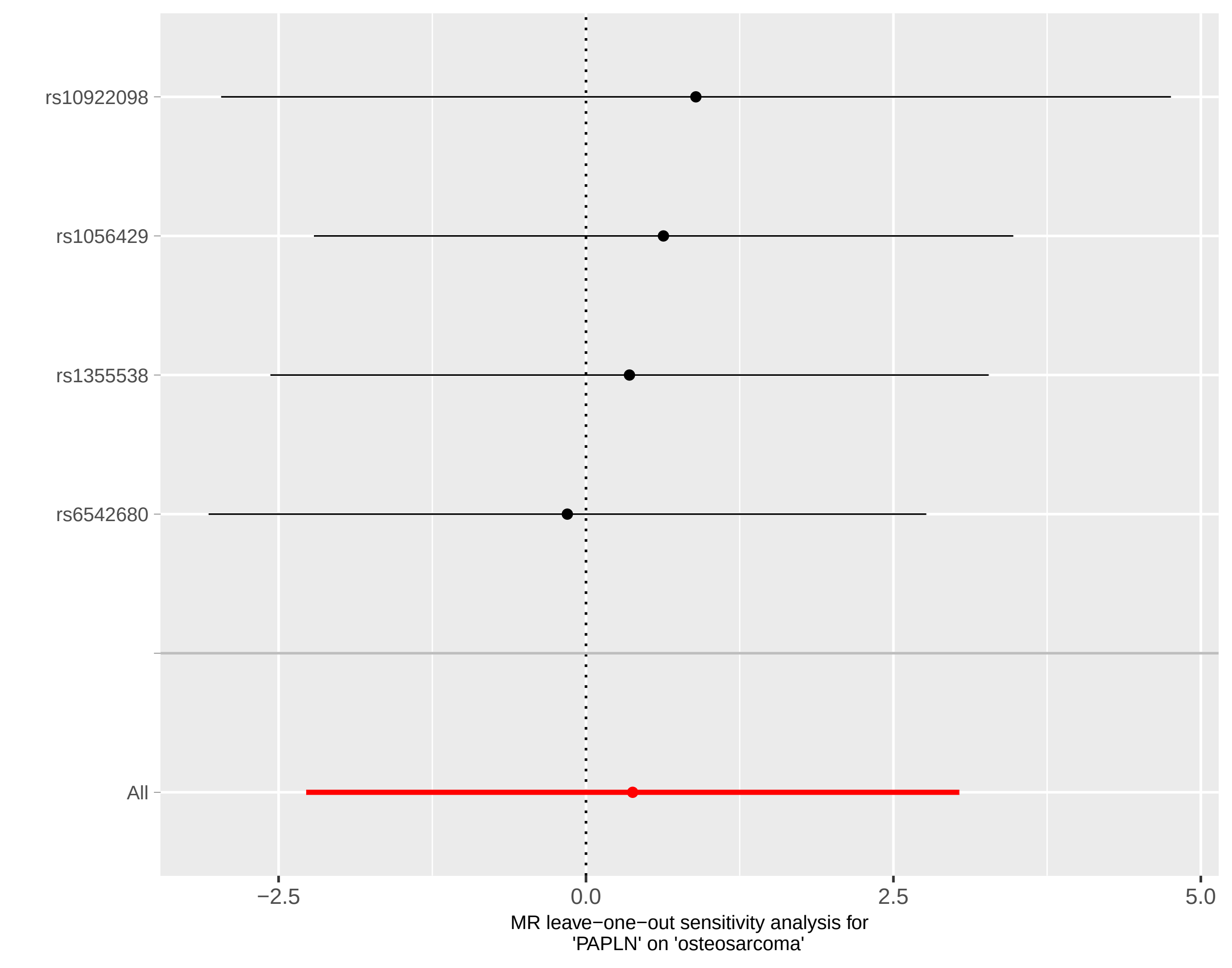
rs10922098
rs1056429
rs1355538
rs6542680
All
−2.5
0.0
2.5
5.0
MR leave−one−out sensitivity analysis for
'PAPLN' on 'osteosarcoma'

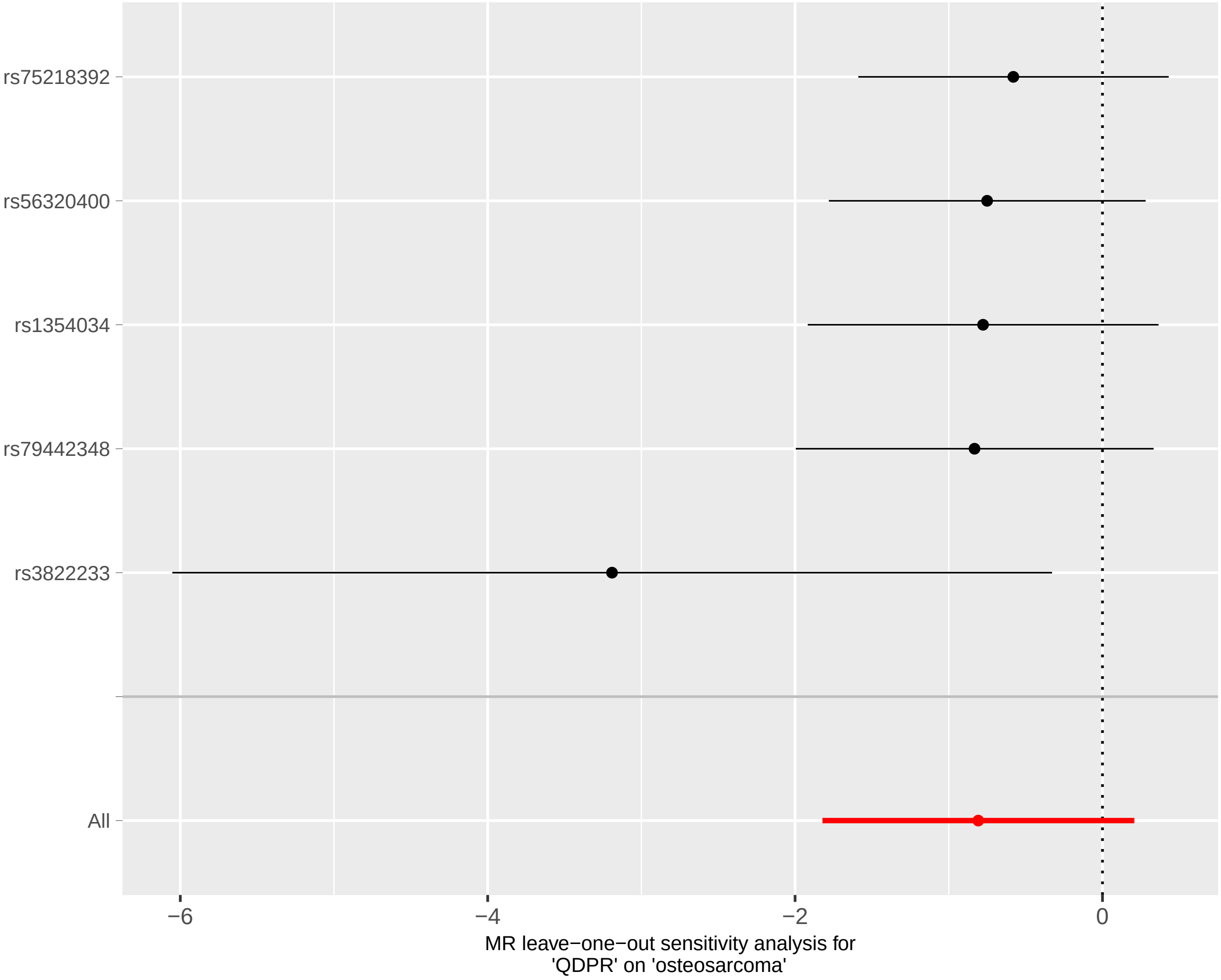

rs75218392
rs56320400
rs1354034
rs79442348
rs3822233
All
−6
−4
−2
0
MR leave−one−out sensitivity analysis for
'QDPR' on 'osteosarcoma'

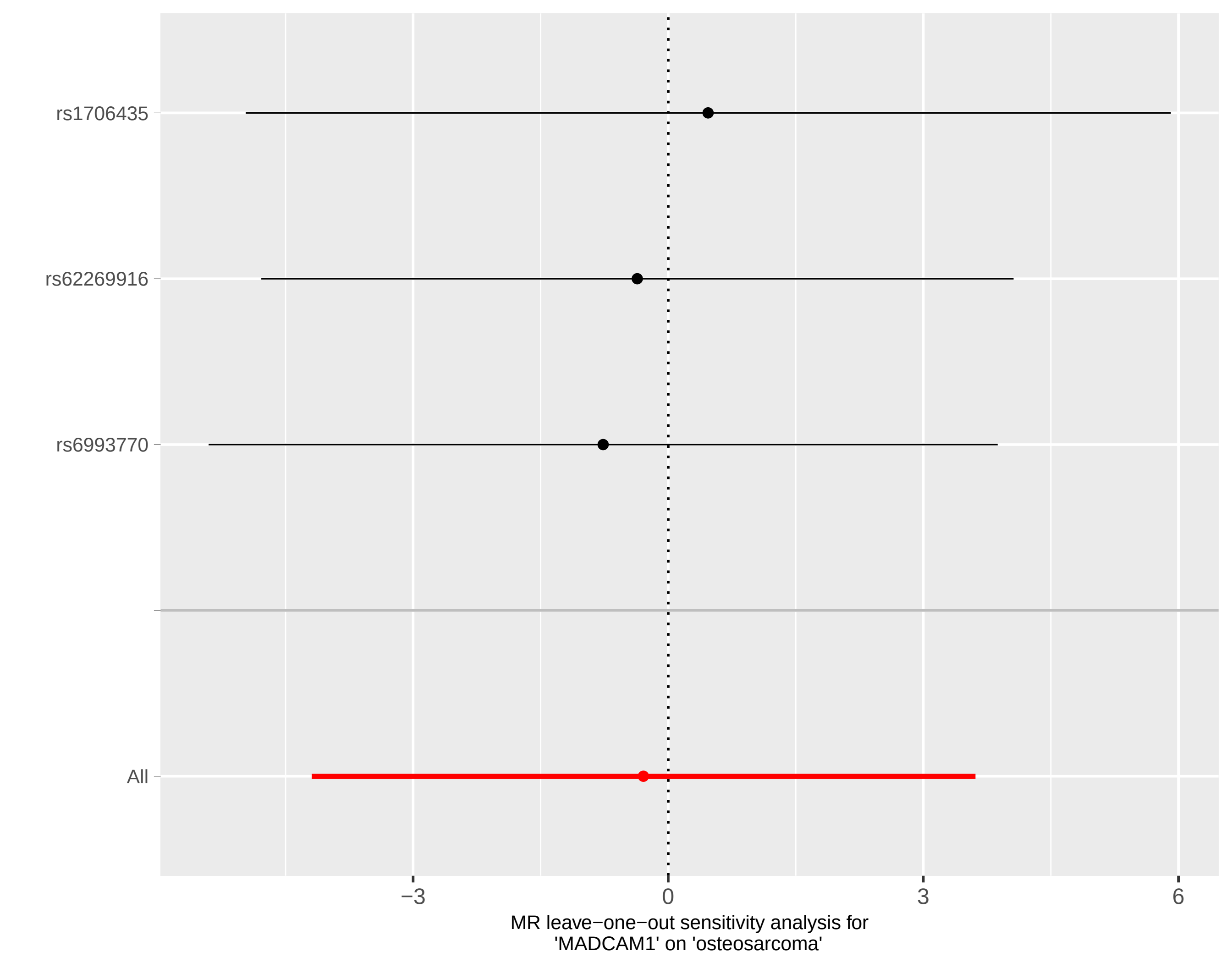
rs1706435
rs62269916
rs6993770
All
−3
0
3
6
MR leave−one−out sensitivity analysis for
'MADCAM1' on 'osteosarcoma'

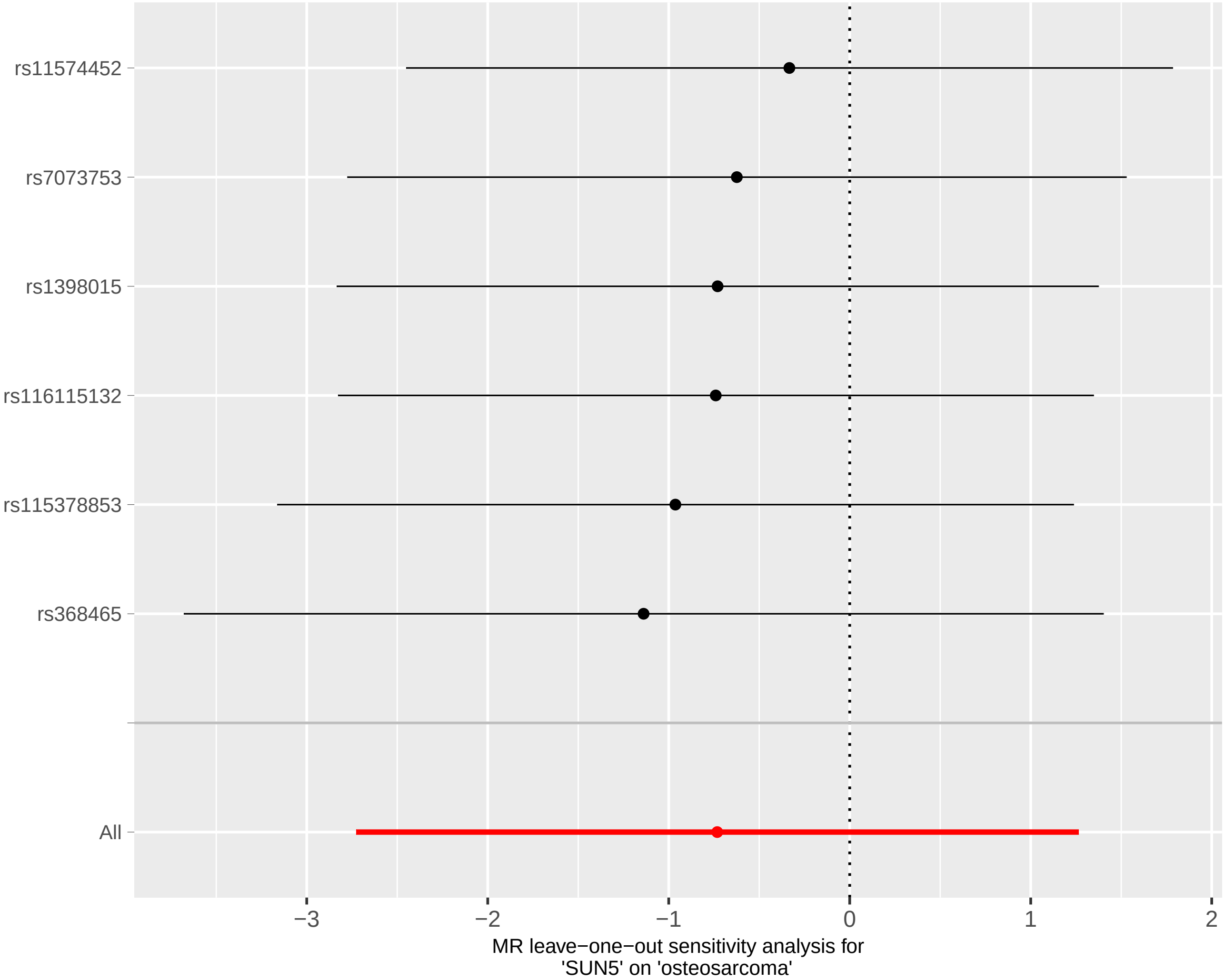
rs11574452
rs7073753
rs1398015
rs116115132
rs115378853
rs368465
All
−3
−2
−1
0
1
2
MR leave−one−out sensitivity analysis for
'SUN5' on 'osteosarcoma'

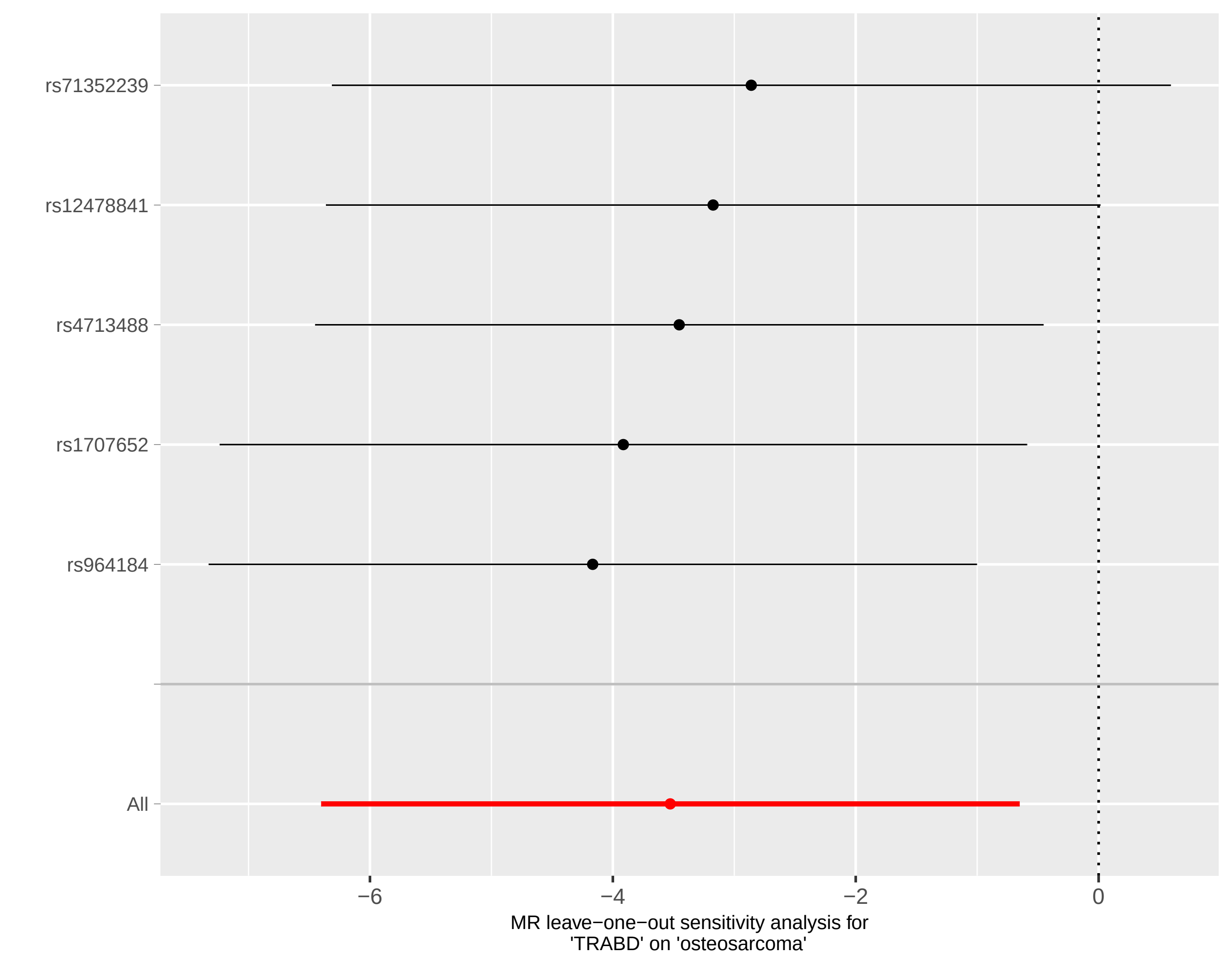
rs71352239
rs12478841
rs4713488
rs1707652
rs964184
All
−6
−4
−2
0
MR leave−one−out sensitivity analysis for
'TRABD' on 'osteosarcoma'

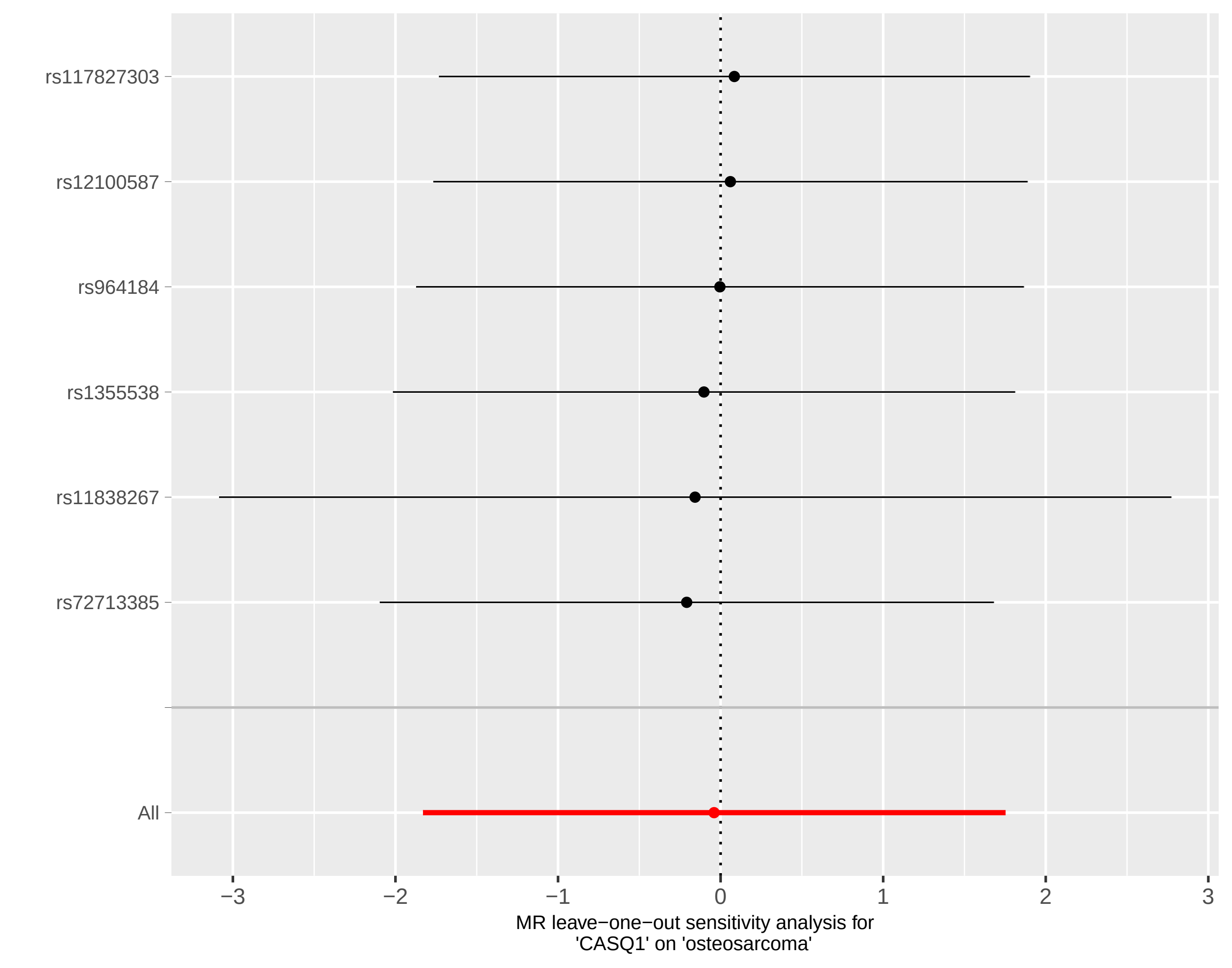
rs117827303
rs12100587
rs964184
rs1355538
rs11838267
rs72713385
All
−3
−2
−1
0
1
2
3
MR leave−one−out sensitivity analysis for
'CASQ1' on 'osteosarcoma'

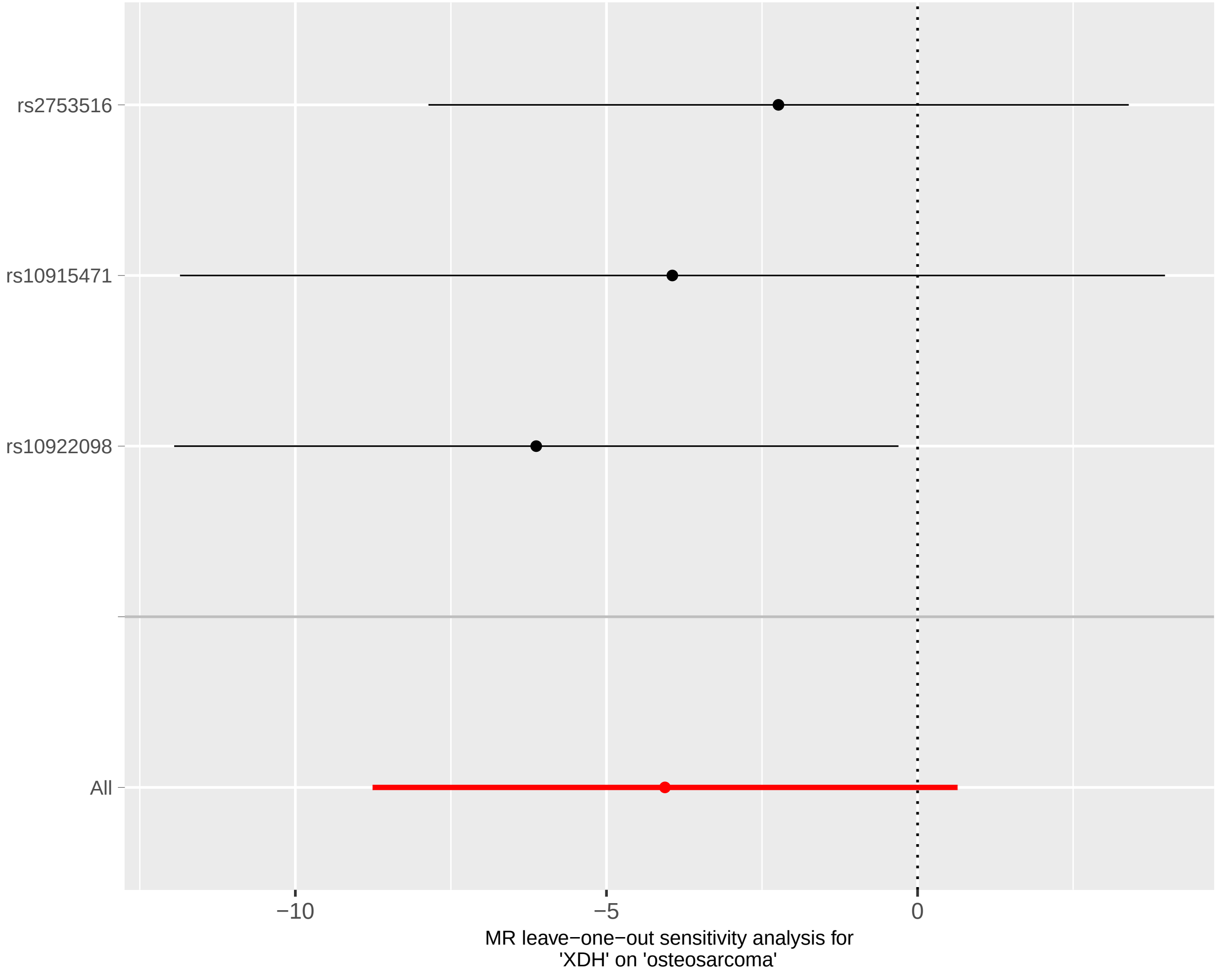

rs2753516
rs10915471
rs10922098
All
−10
−5
0
MR leave−one−out sensitivity analysis for
'XDH' on 'osteosarcoma'

Insufficient number of SNPs

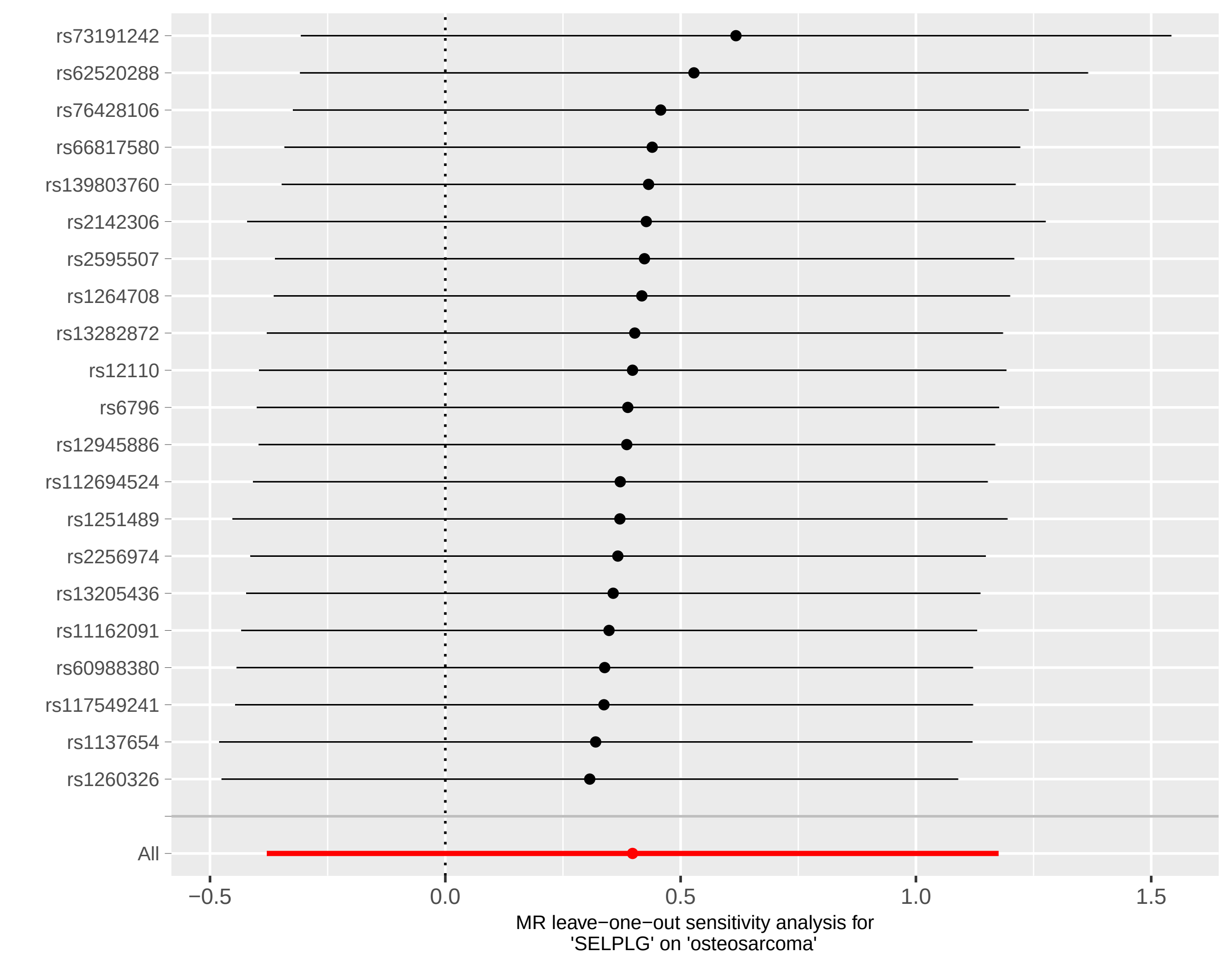

rs73191242
rs62520288
rs76428106
rs66817580
rs139803760
rs2142306
rs2595507
rs1264708
rs13282872
rs12110
rs6796
rs12945886
rs112694524
rs1251489
rs2256974
rs13205436
rs11162091
rs60988380
rs117549241
rs1137654
rs1260326
All
−0.5
0.0
0.5
1.0
1.5
MR leave−one−out sensitivity analysis for
'SELPLG' on 'osteosarcoma'

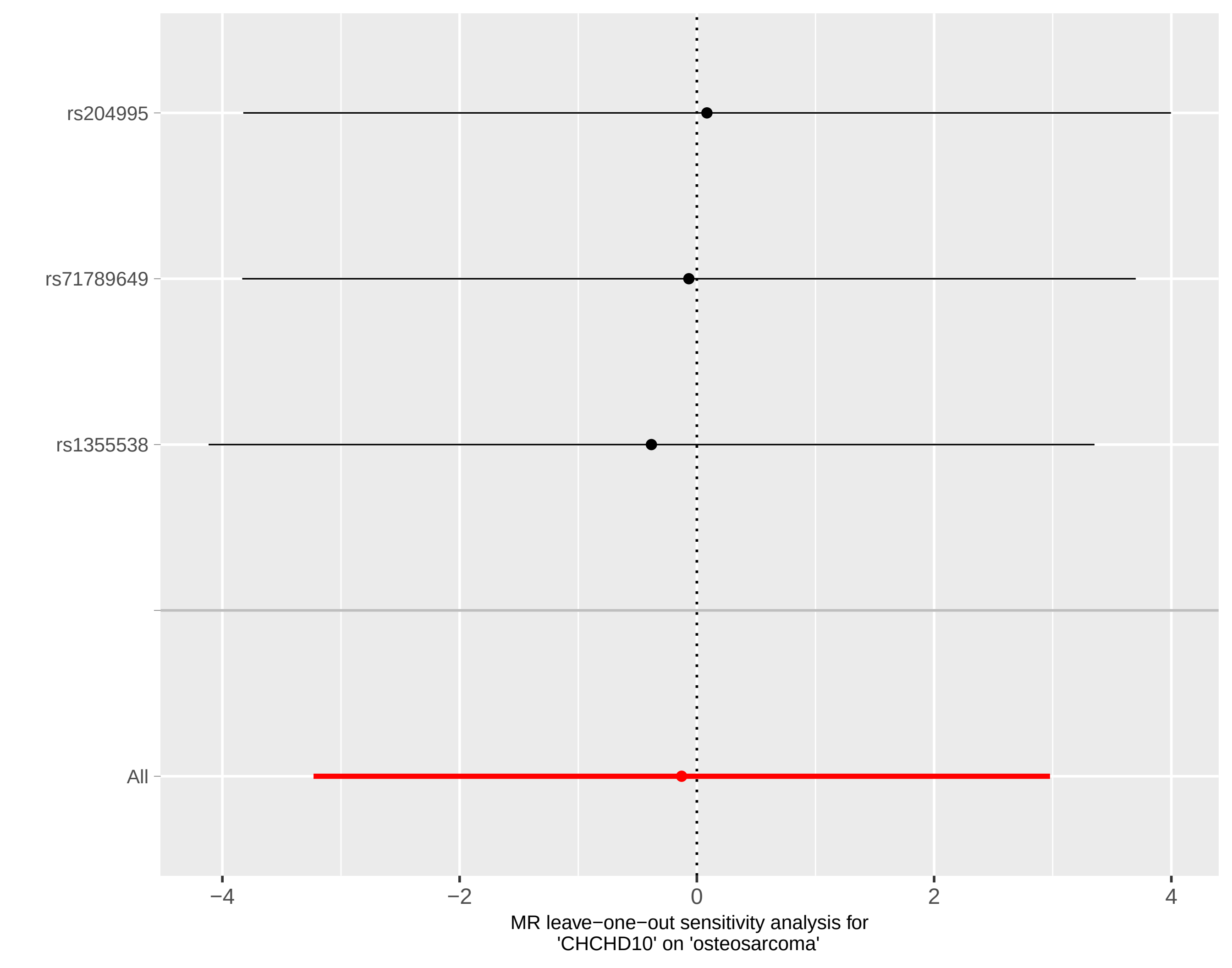

rs204995
rs71789649
rs1355538
All
−4
−2
0
2
4
MR leave−one−out sensitivity analysis for
'CHCHD10' on 'osteosarcoma'

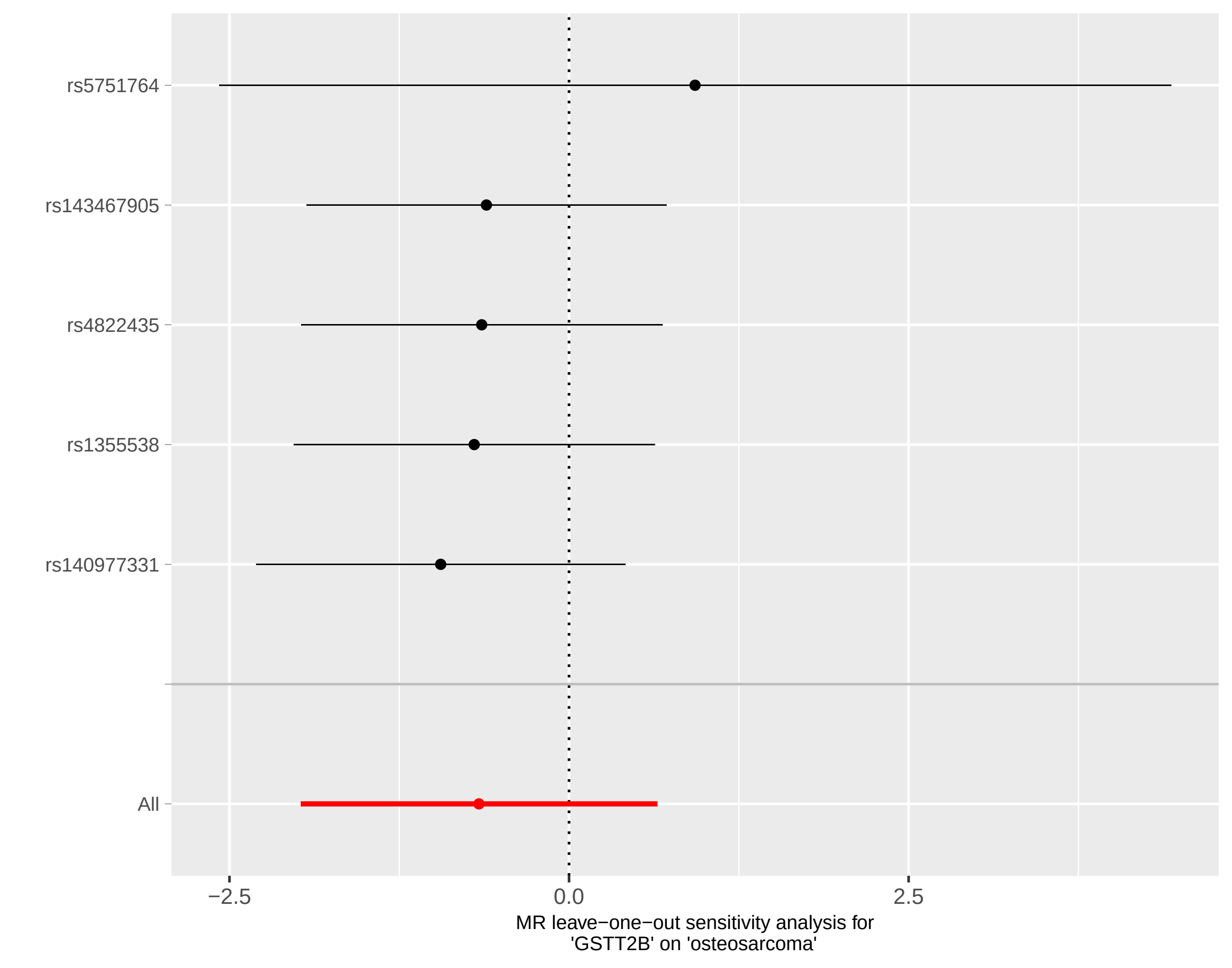
rs5751764
rs143467905
rs4822435
rs1355538
rs140977331
All
−2.5
0.0
2.5
MR leave−one−out sensitivity analysis for
'GSTT2B' on 'osteosarcoma'

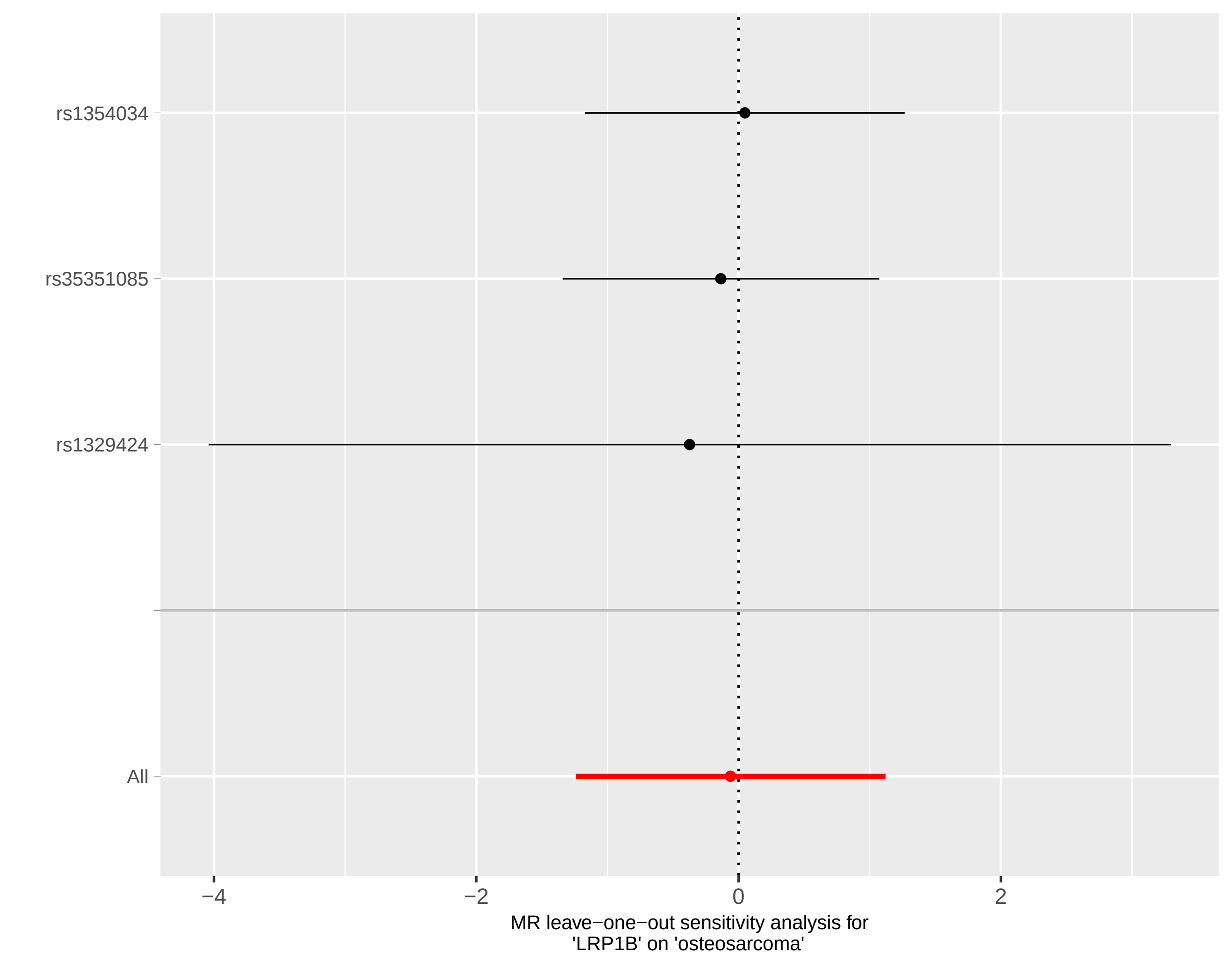

rs1354034
rs35351085
rs1329424
All
−4
−2
0
2
MR leave−one−out sensitivity analysis for
'LRP1B' on 'osteosarcoma'

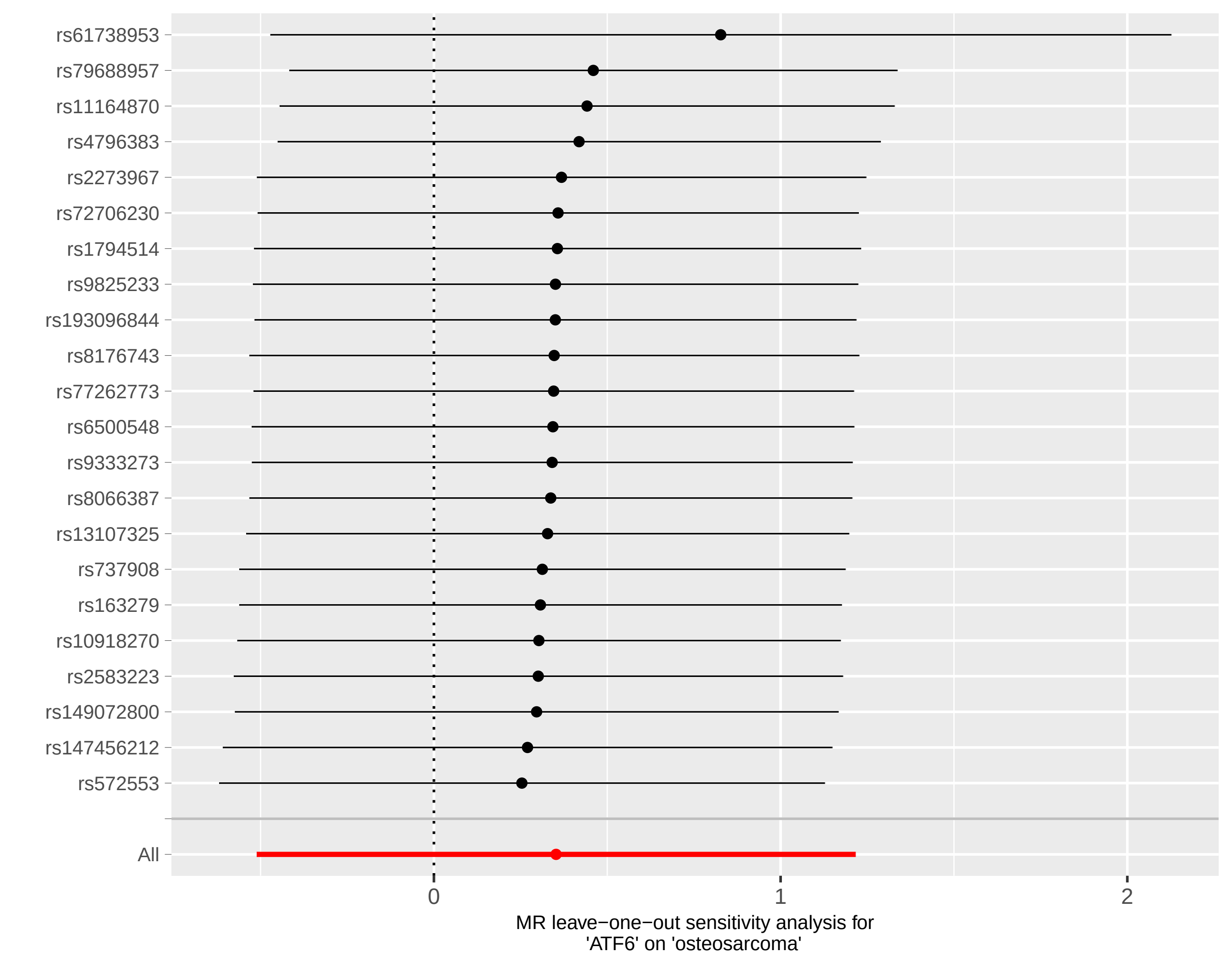
rs61738953
rs79688957
rs11164870
rs4796383
rs2273967
rs72706230
rs1794514
rs9825233
rs193096844
rs8176743
rs77262773
rs6500548
rs9333273
rs8066387
rs13107325
rs737908
rs163279
rs10918270
rs2583223
rs149072800
rs147456212
rs572553
All
0
1
2
MR leave−one−out sensitivity analysis for
'ATF6' on 'osteosarcoma'

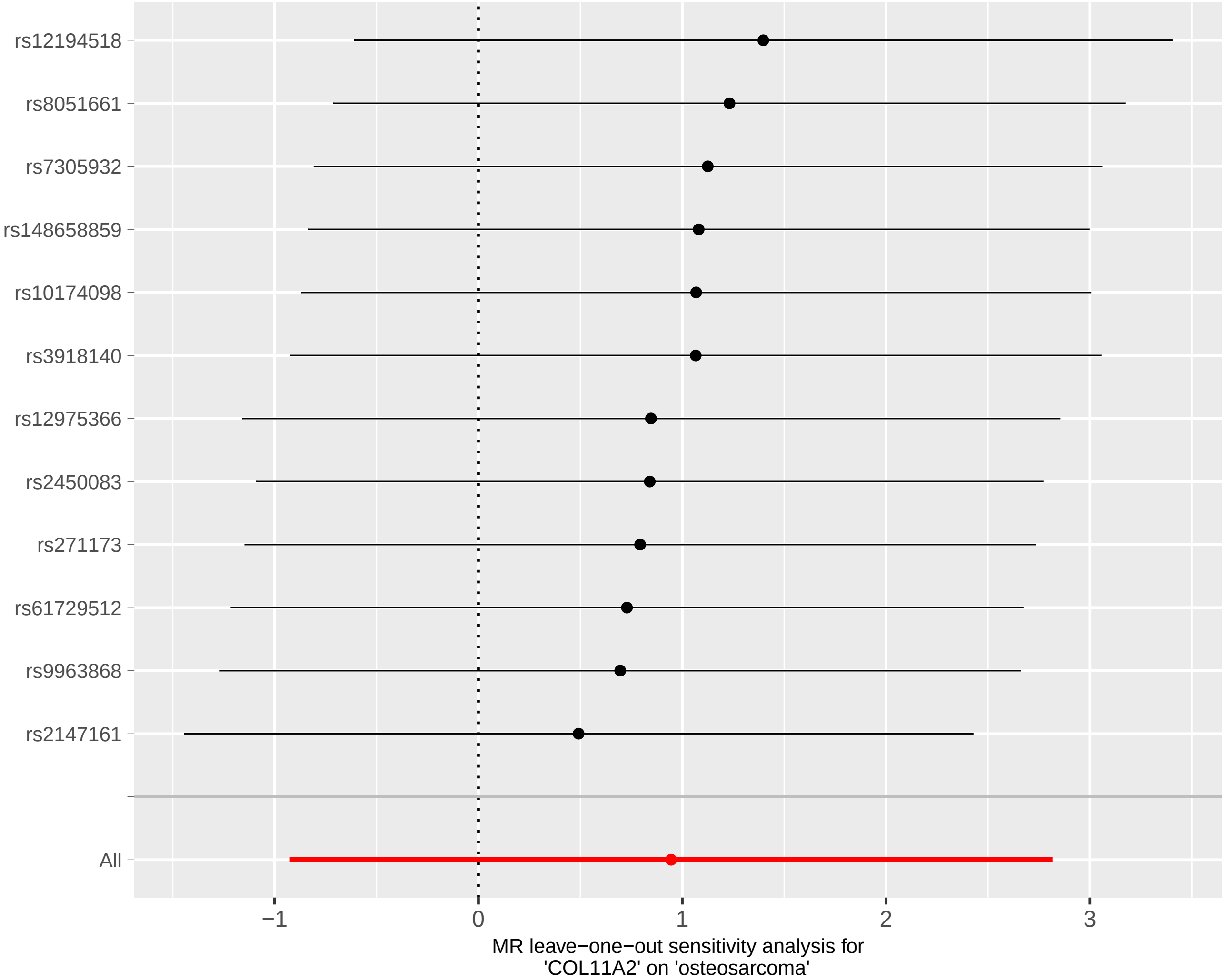
rs12194518
rs8051661
rs7305932
rs148658859
rs10174098
rs3918140
rs12975366
rs2450083
rs271173
rs61729512
rs9963868
rs2147161
All
−1
0
1
2
3
MR leave−one−out sensitivity analysis for
'COL11A2' on 'osteosarcoma'

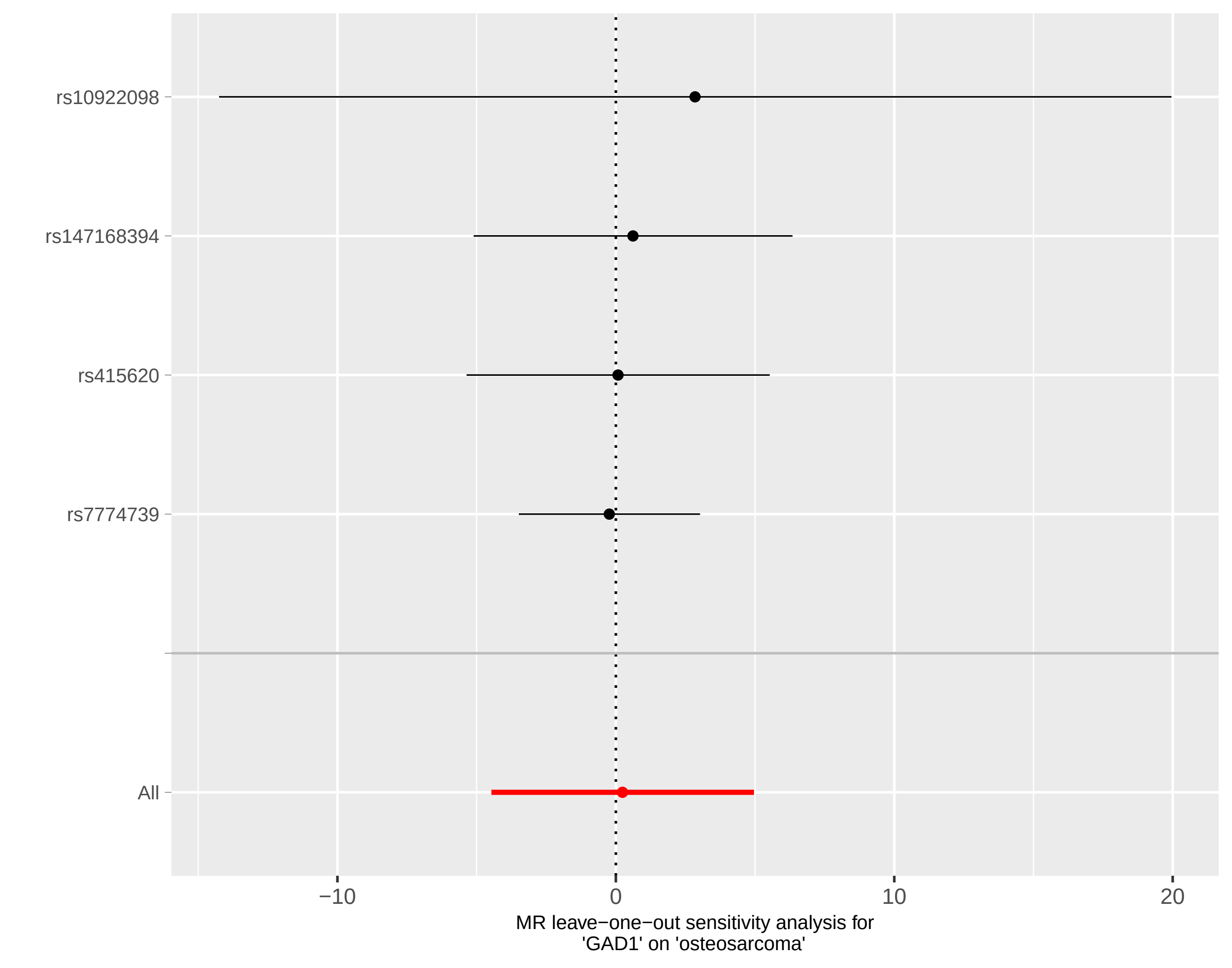
rs10922098
rs147168394
rs415620
rs7774739
All
−10
0
10
20
MR leave−one−out sensitivity analysis for
'GAD1' on 'osteosarcoma'

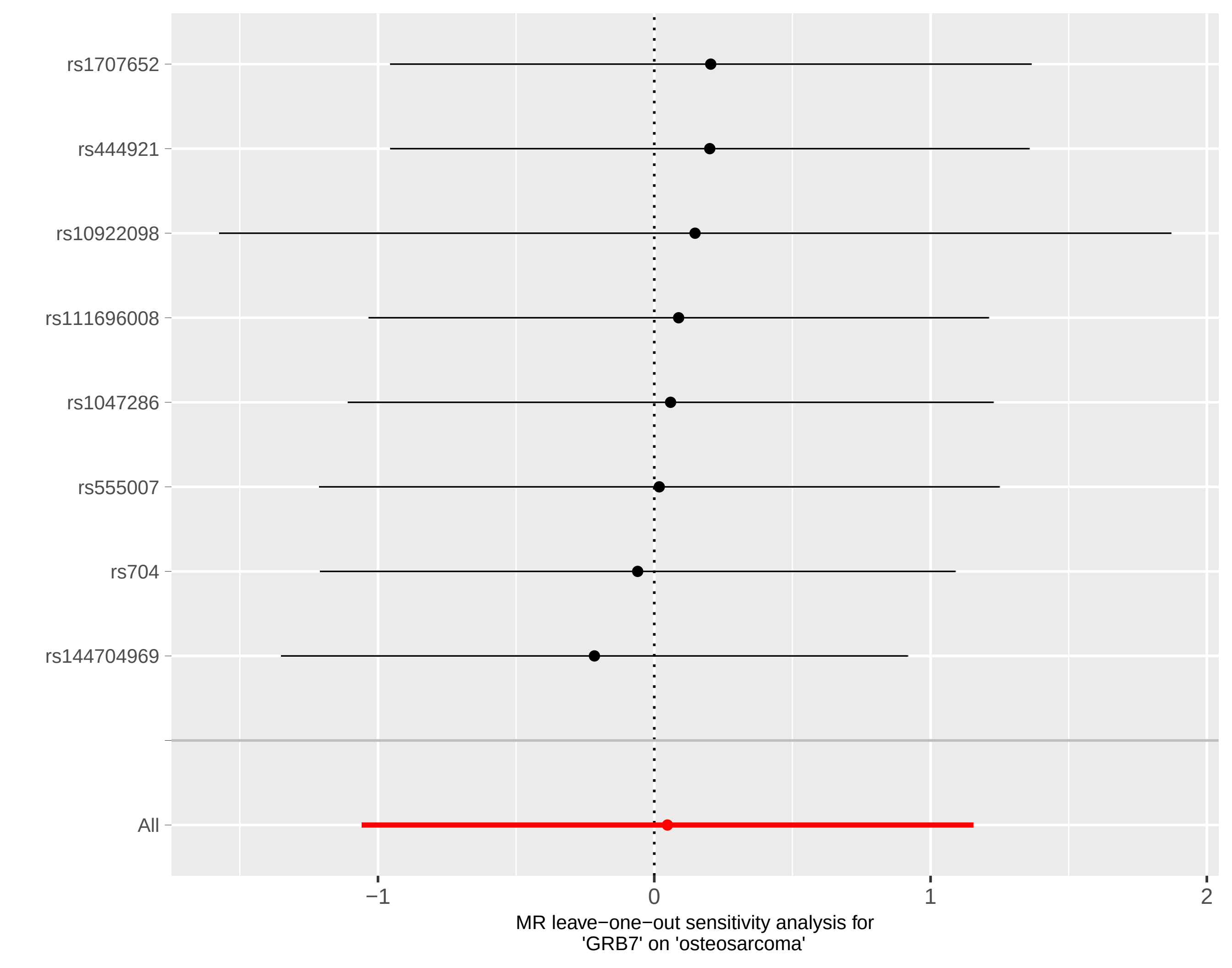
rs1707652
rs444921
rs10922098
rs111696008
rs1047286
rs555007
rs704
rs144704969
All
−1
0
1
2
MR leave−one−out sensitivity analysis for
'GRB7' on 'osteosarcoma'

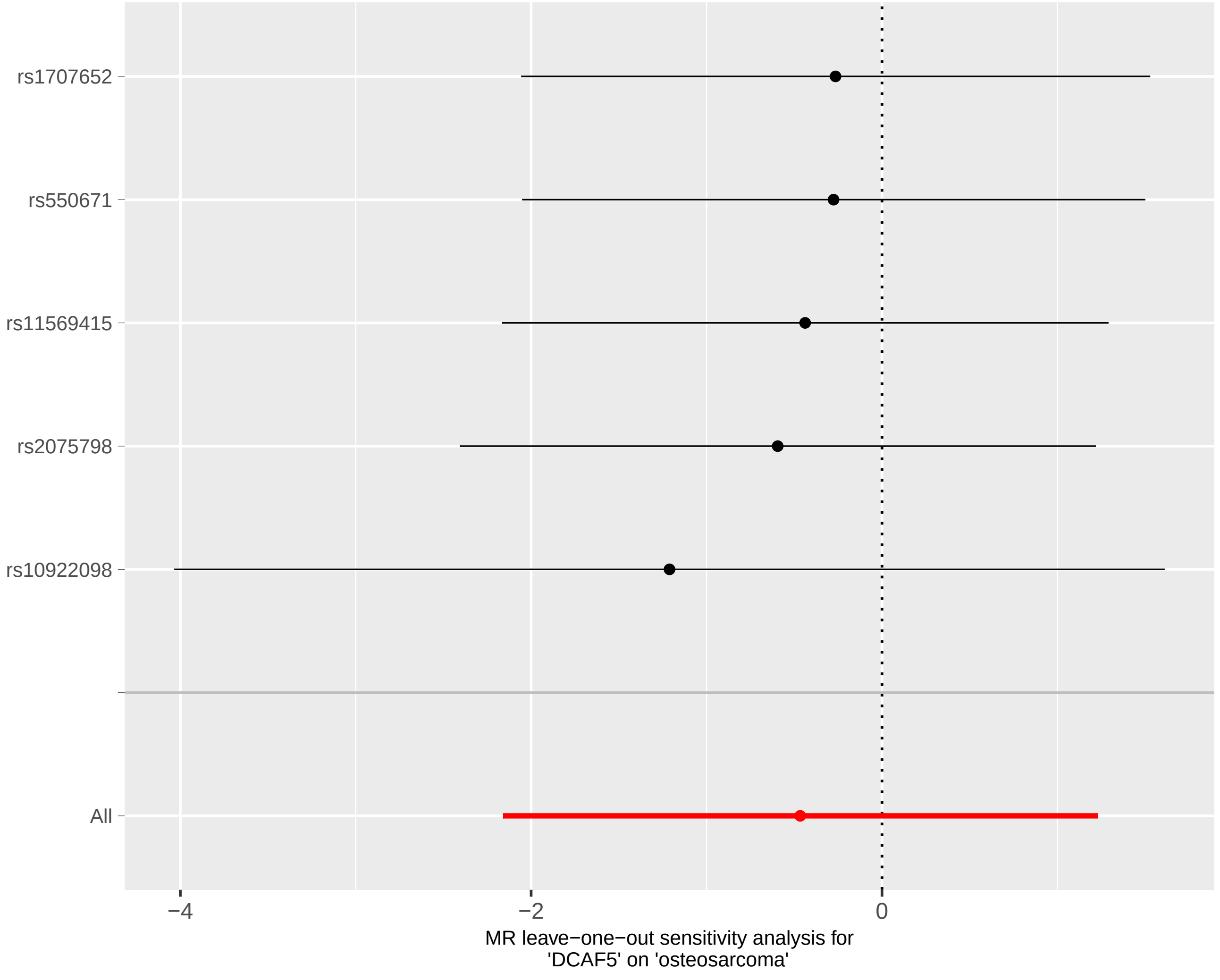

rs1707652
rs550671
rs11569415
rs2075798
rs10922098
All
−4
−2
0
MR leave−one−out sensitivity analysis for
'DCAF5' on 'osteosarcoma'

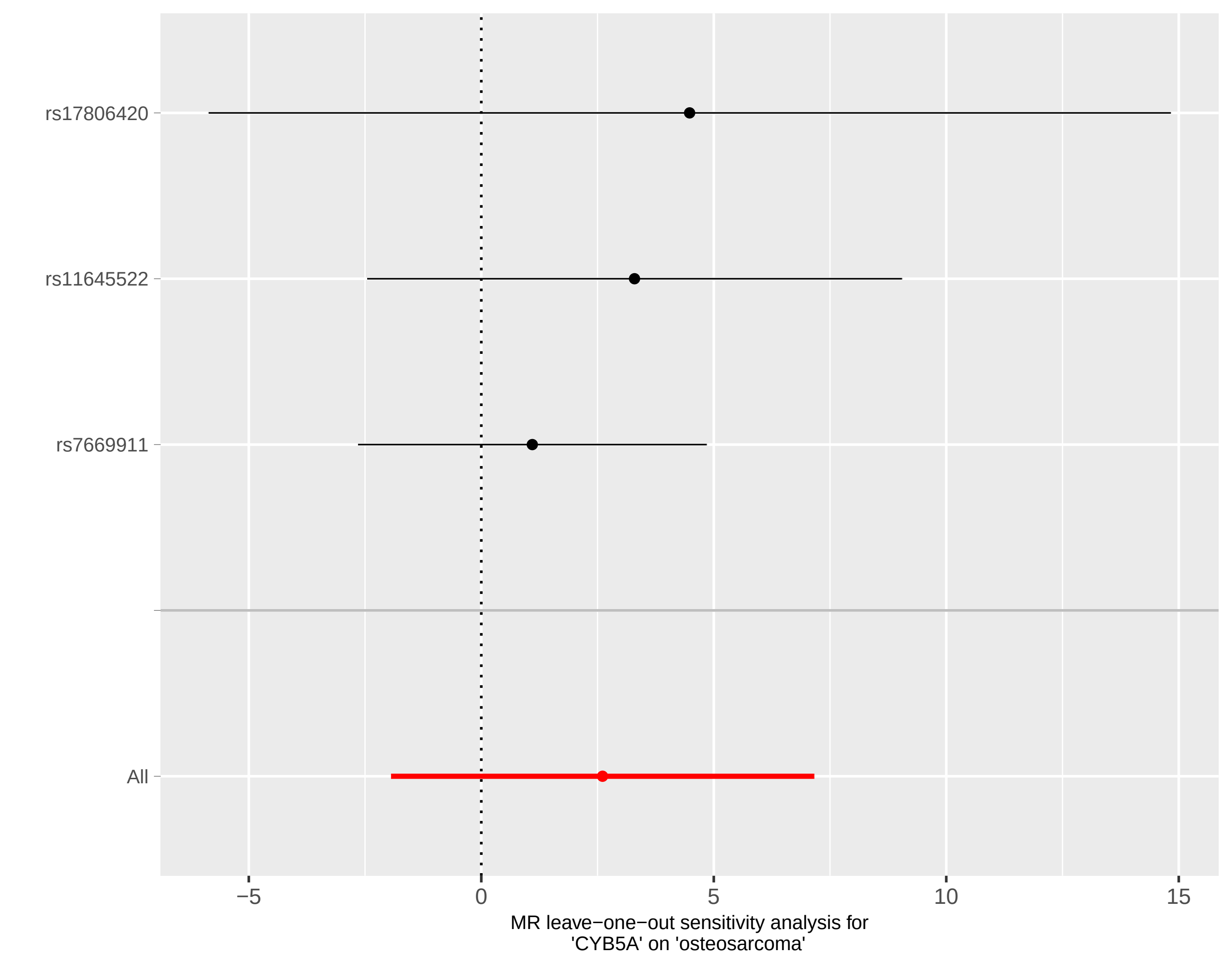

rs17806420
rs11645522
rs7669911
All
−5
0
5
10
15
MR leave−one−out sensitivity analysis for
'CYB5A' on 'osteosarcoma'

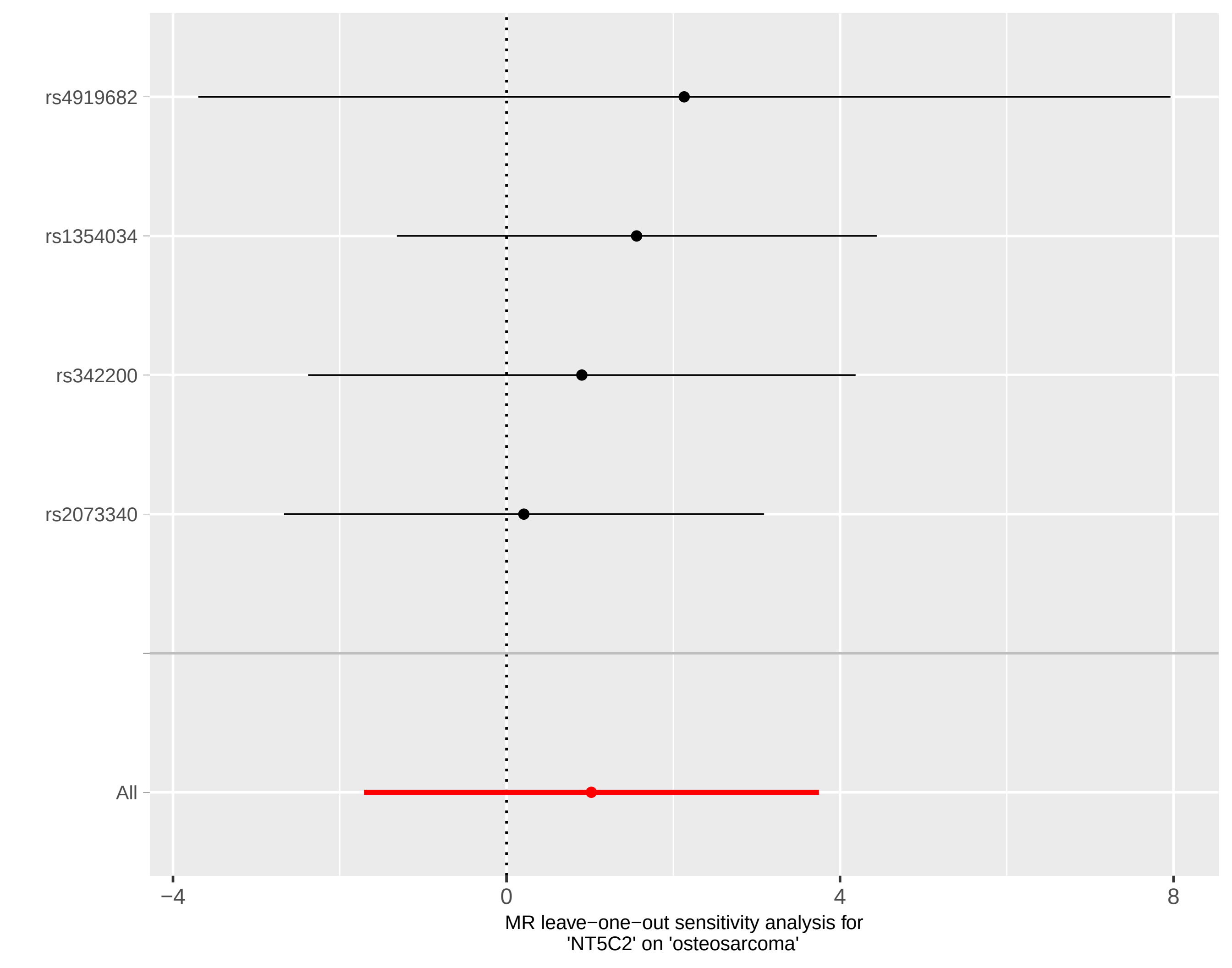
rs4919682
rs1354034
rs342200
rs2073340
All
−4
0
4
8
MR leave−one−out sensitivity analysis for
'NT5C2' on 'osteosarcoma'

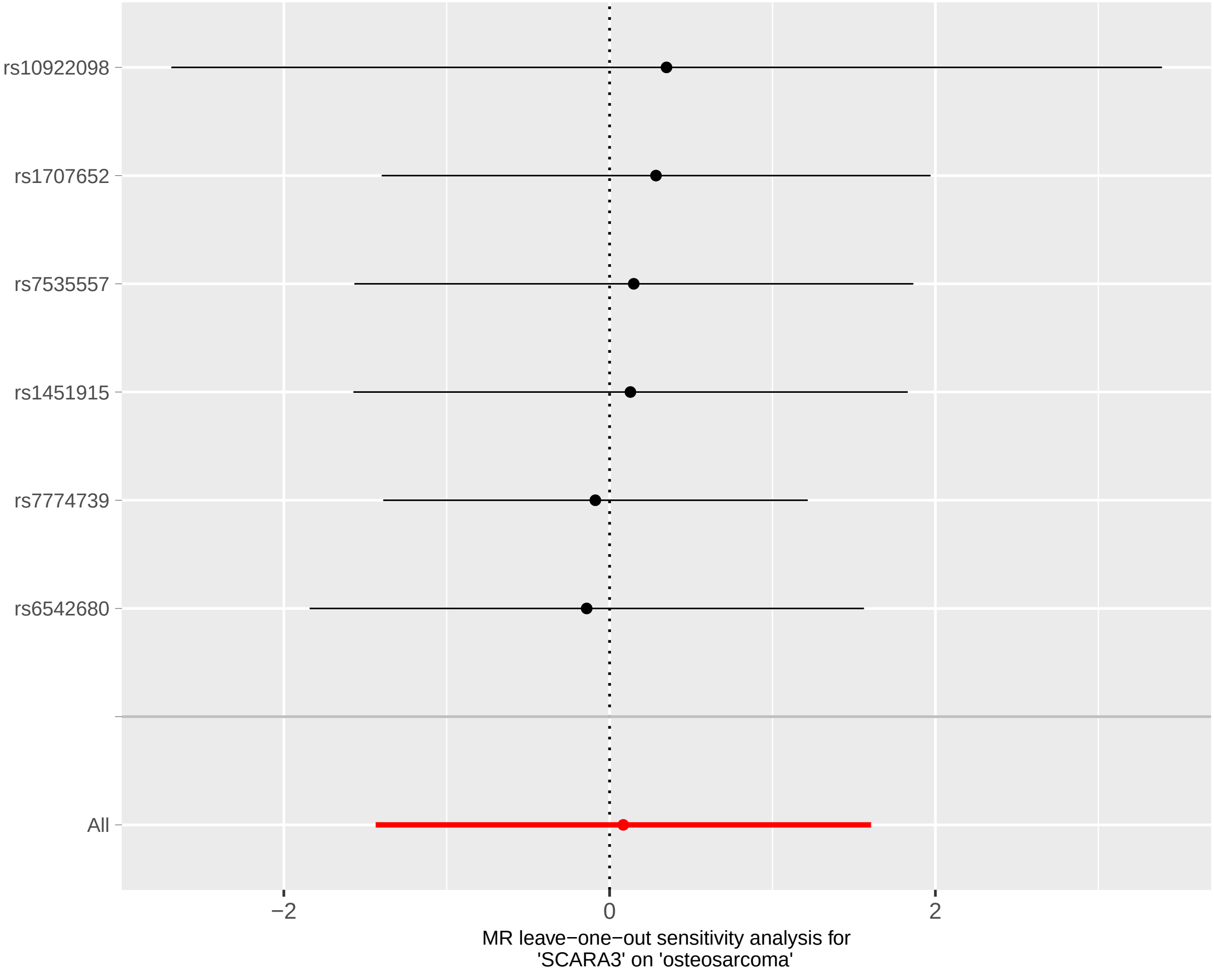
rs10922098
rs1707652
rs7535557
rs1451915
rs7774739
rs6542680
All
−2
0
2
MR leave−one−out sensitivity analysis for
'SCARA3' on 'osteosarcoma'

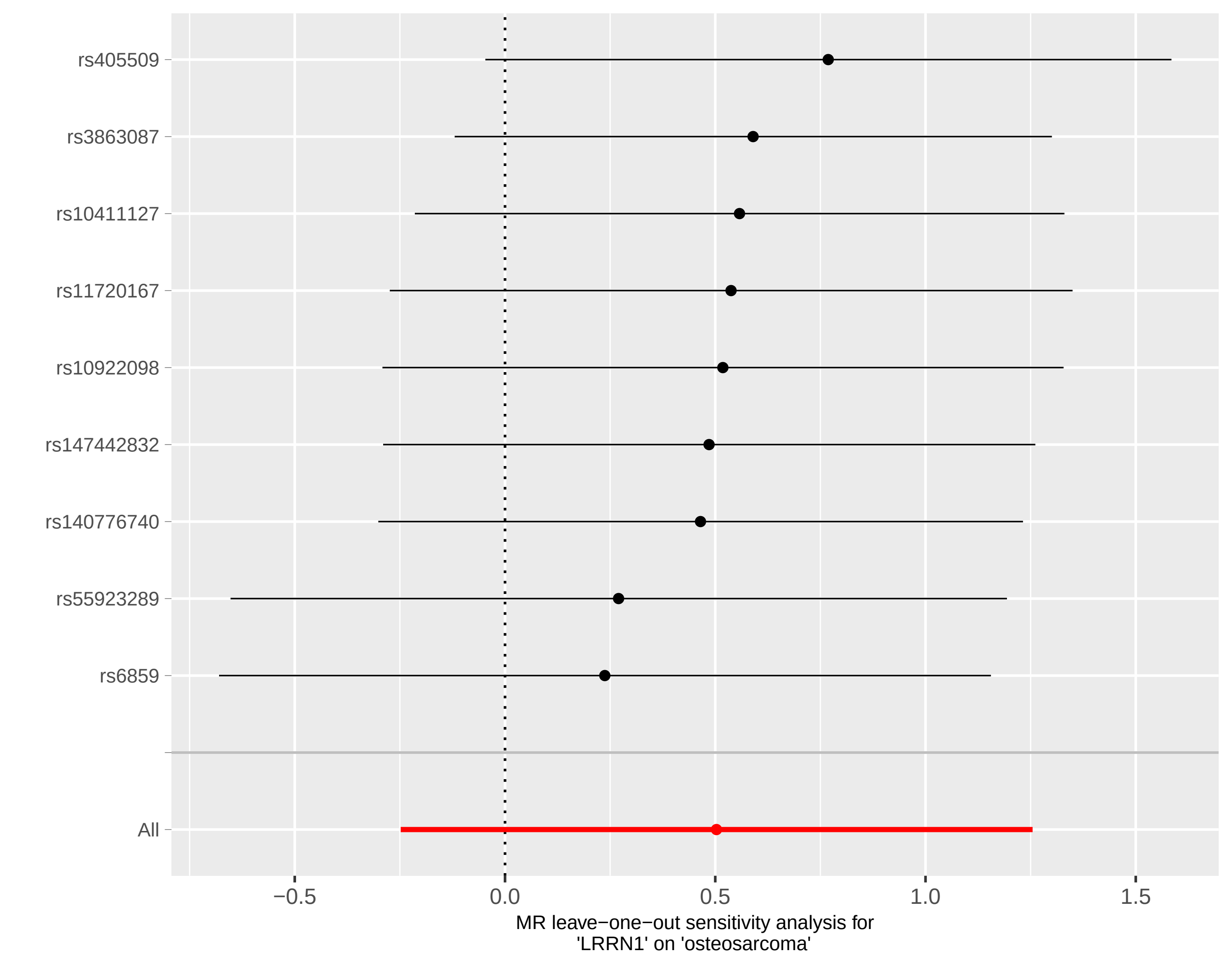

rs405509
rs3863087
rs10411127
rs11720167
rs10922098
rs147442832
rs140776740
rs55923289
rs6859
All
−0.5
0.0
0.5
1.0
1.5
MR leave−one−out sensitivity analysis for
'LRRN1' on 'osteosarcoma'

Insufficient number of SNPs

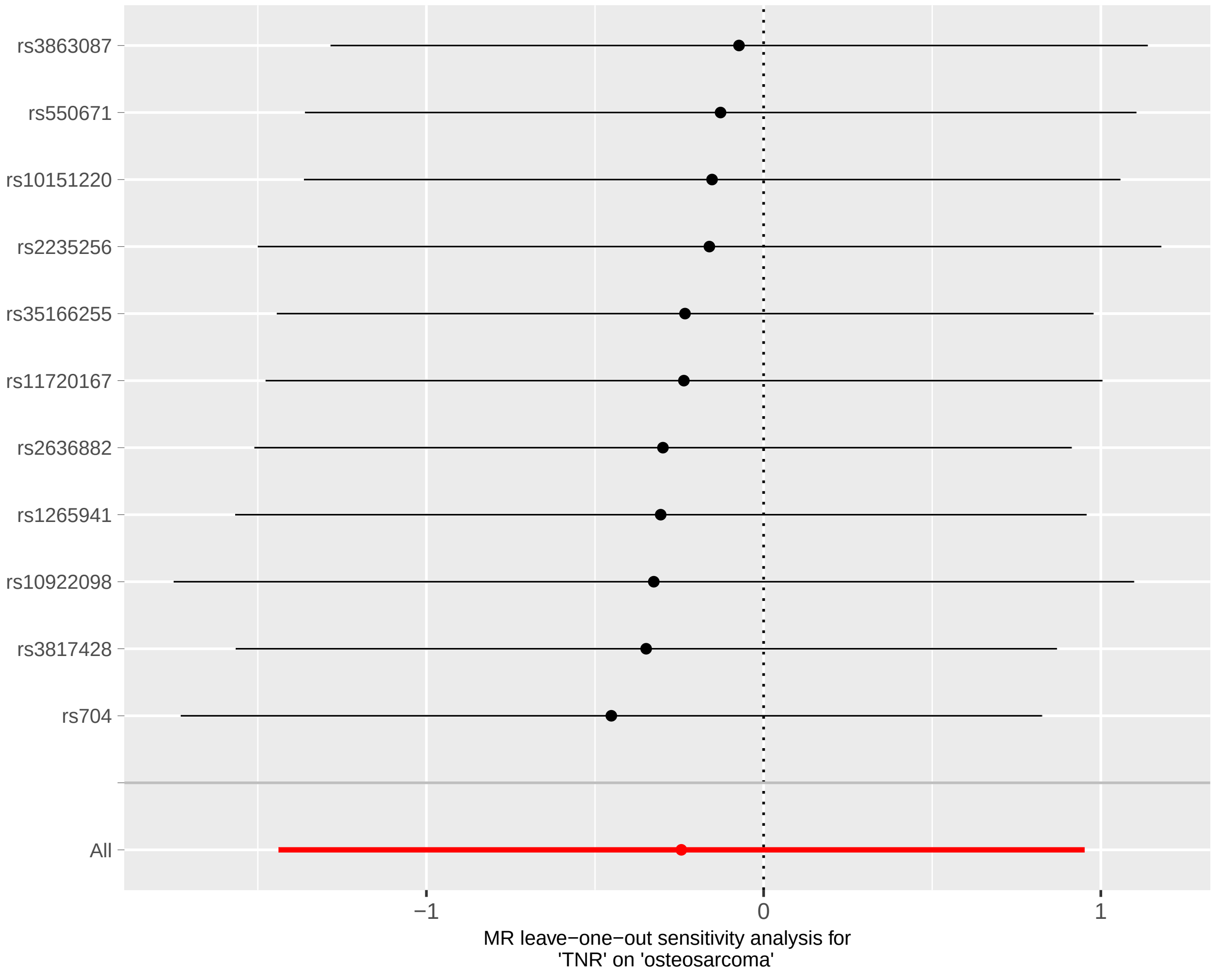
rs3863087
rs550671
rs10151220
rs2235256
rs35166255
rs11720167
rs2636882
rs1265941
rs10922098
rs3817428
rs704
All
−1
0
1
MR leave−one−out sensitivity analysis for
'TNR' on 'osteosarcoma'

Insufficient number of SNPs

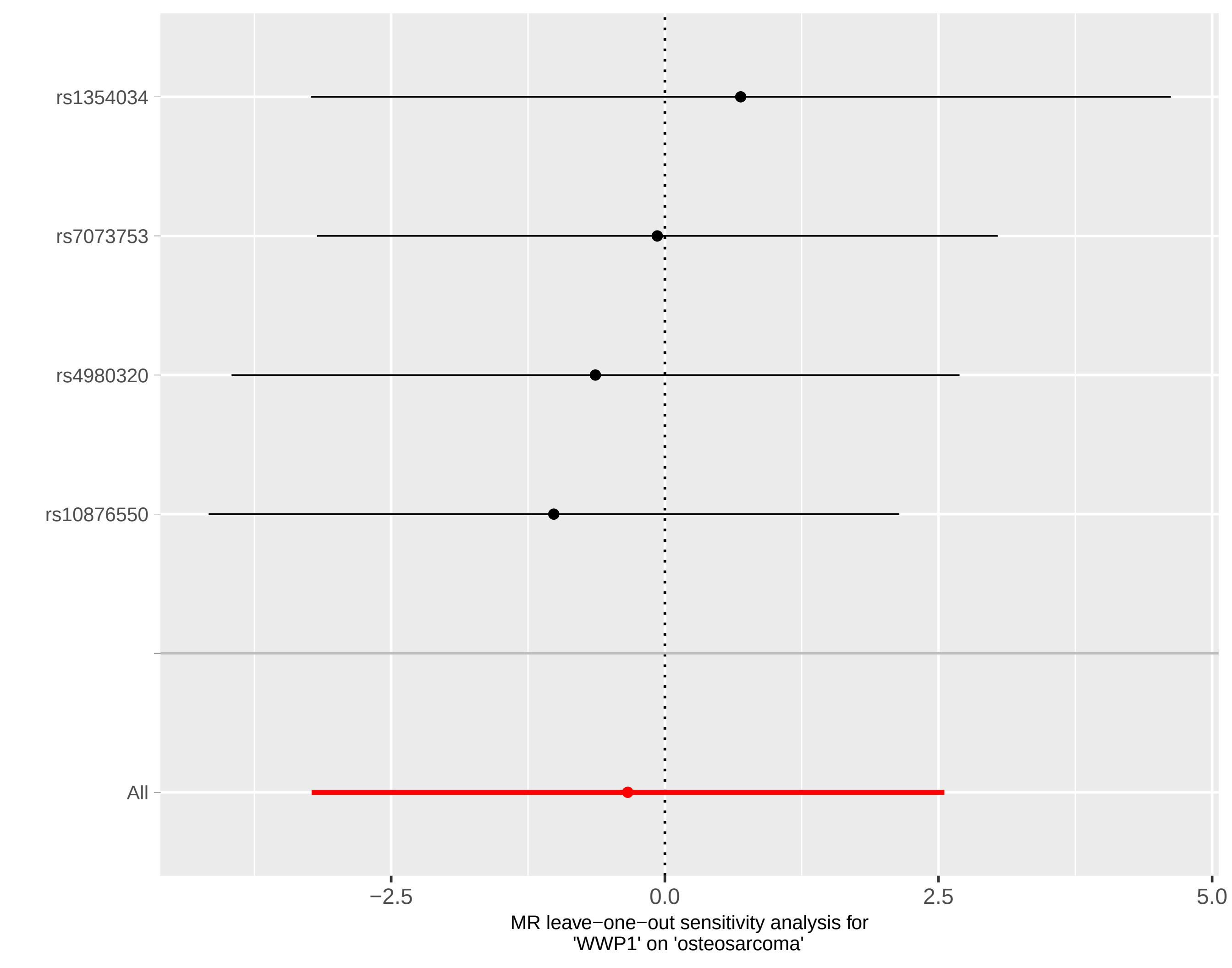
rs1354034
rs7073753
rs4980320
rs10876550
All
−2.5
0.0
2.5
5.0
MR leave−one−out sensitivity analysis for
'WWP1' on 'osteosarcoma'

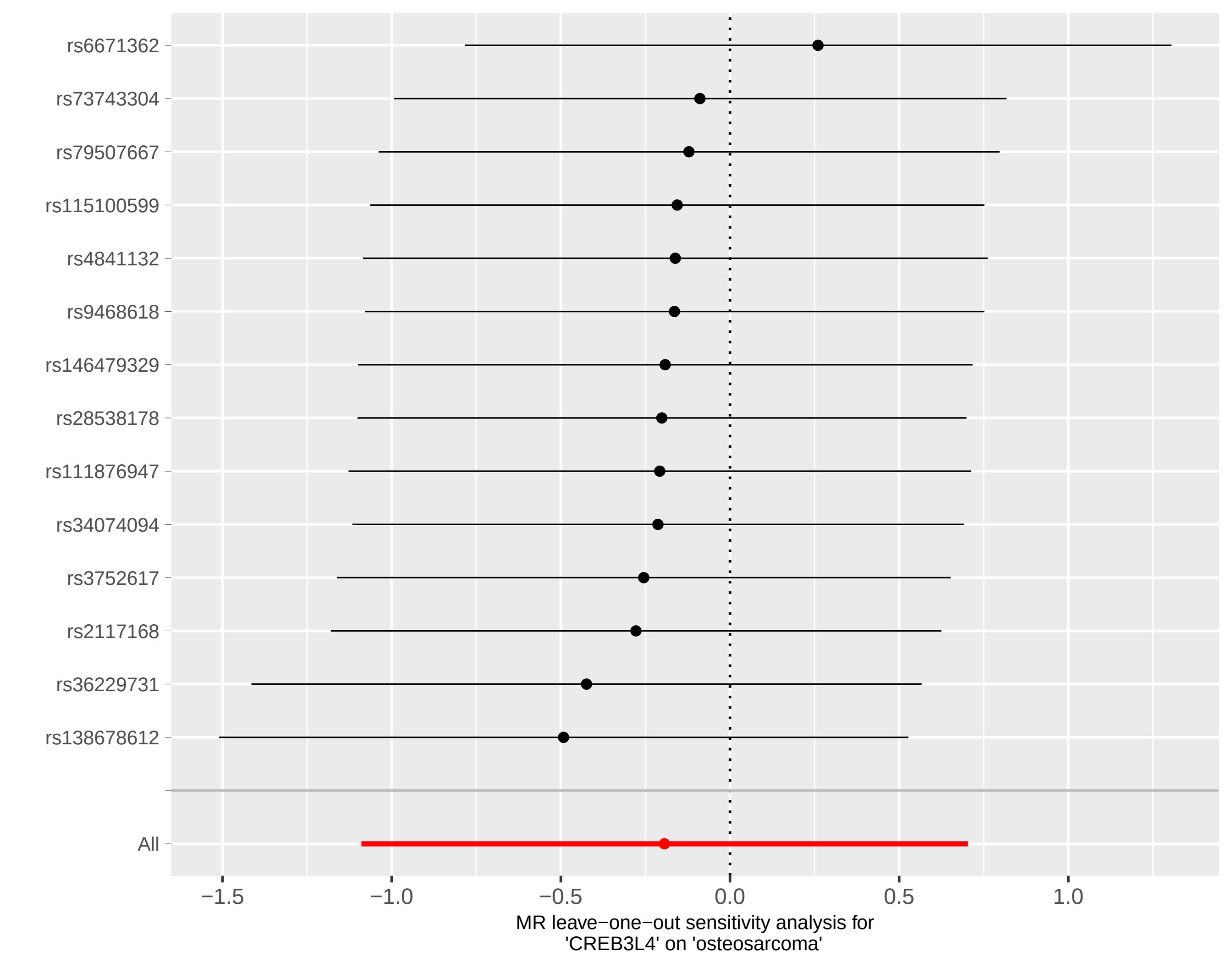

rs6671362
rs73743304
rs79507667
rs115100599
rs4841132
rs9468618
rs146479329
rs28538178
rs111876947
rs34074094
rs3752617
rs2117168
rs36229731
rs138678612
All
−1.5
−1.0
−0.5
0.0
0.5
1.0
MR leave−one−out sensitivity analysis for
'CREB3L4' on 'osteosarcoma'

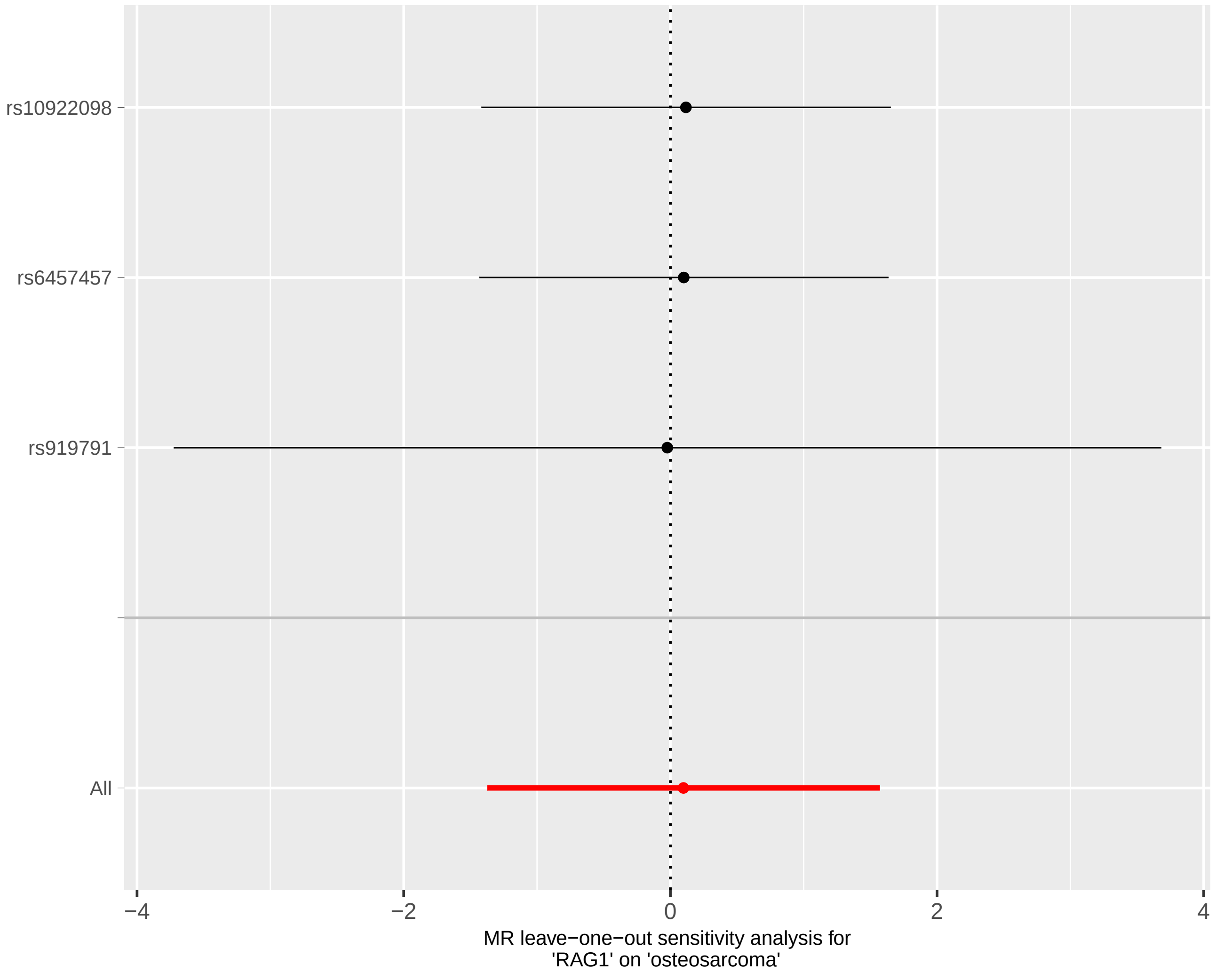
rs10922098
rs6457457
rs919791
All
−4
−2
0
2
4
MR leave−one−out sensitivity analysis for
'RAG1' on 'osteosarcoma'

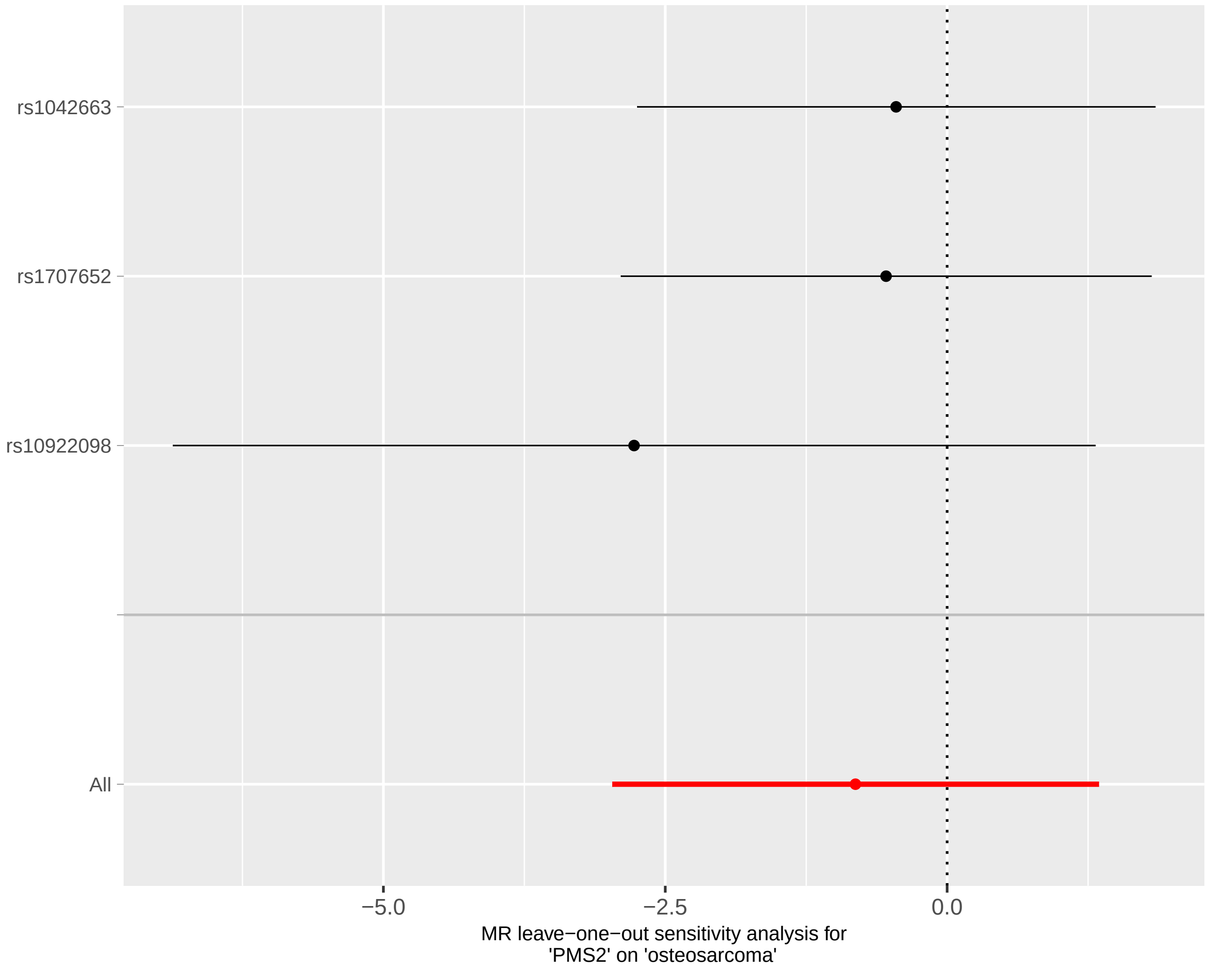
rs1042663
rs1707652
rs10922098
All
−5.0
−2.5
0.0
MR leave−one−out sensitivity analysis for
'PMS2' on 'osteosarcoma'

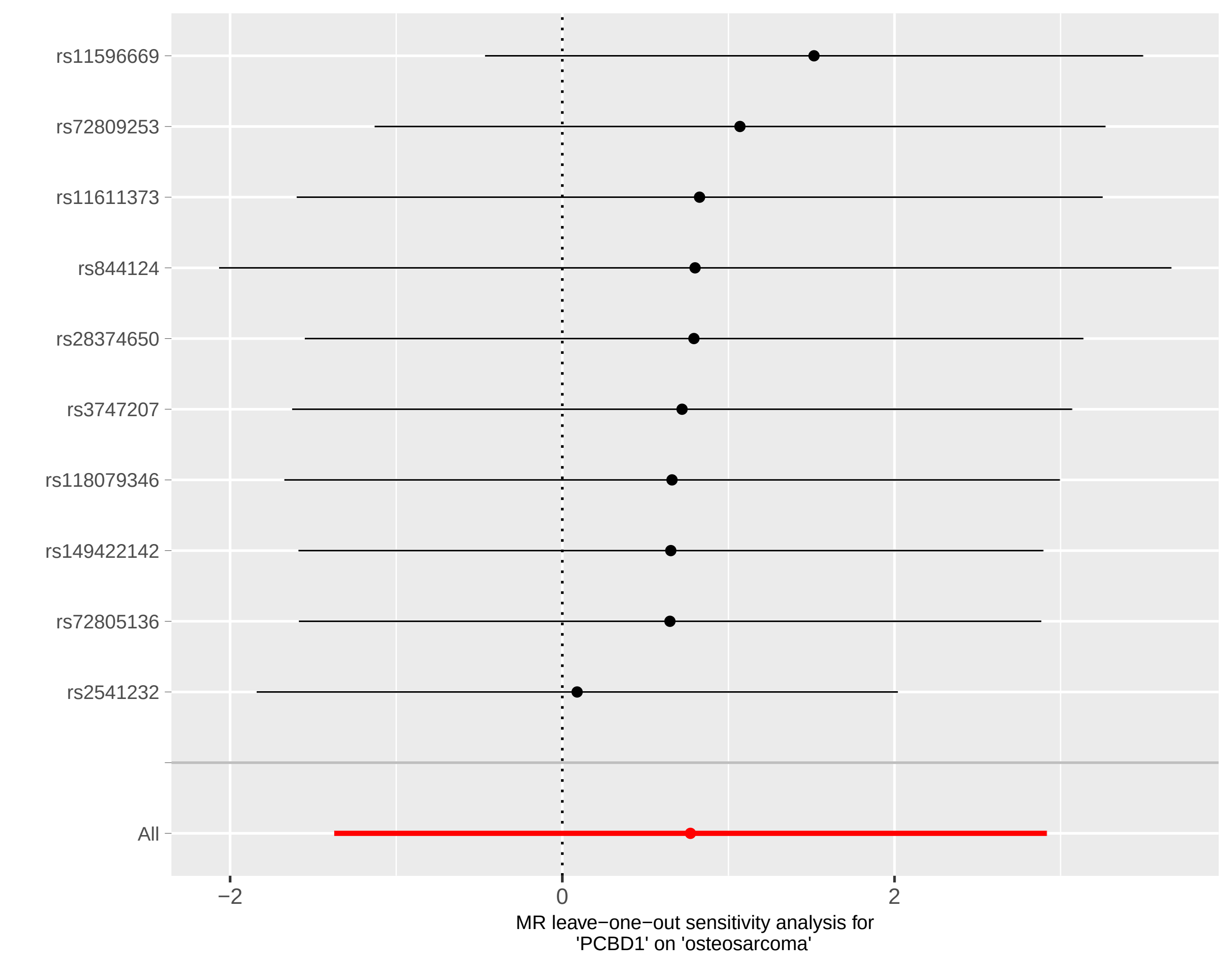

rs11596669
rs72809253
rs11611373
rs844124
rs28374650
rs3747207
rs118079346
rs149422142
rs72805136
rs2541232
All
−2
0
2
MR leave−one−out sensitivity analysis for
'PCBD1' on 'osteosarcoma'

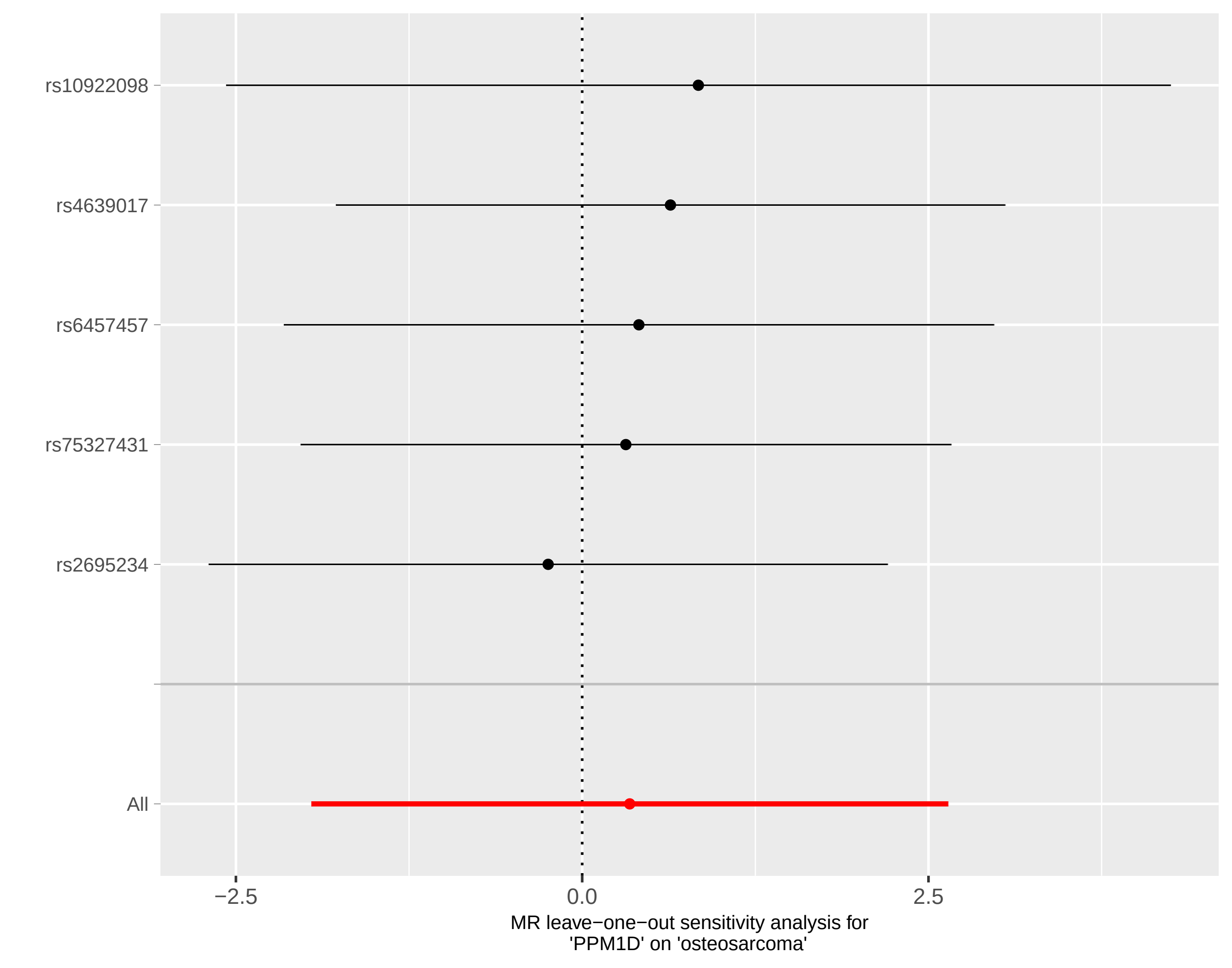
rs10922098
rs4639017
rs6457457
rs75327431
rs2695234
All
−2.5
0.0
2.5
MR leave−one−out sensitivity analysis for
'PPM1D' on 'osteosarcoma'

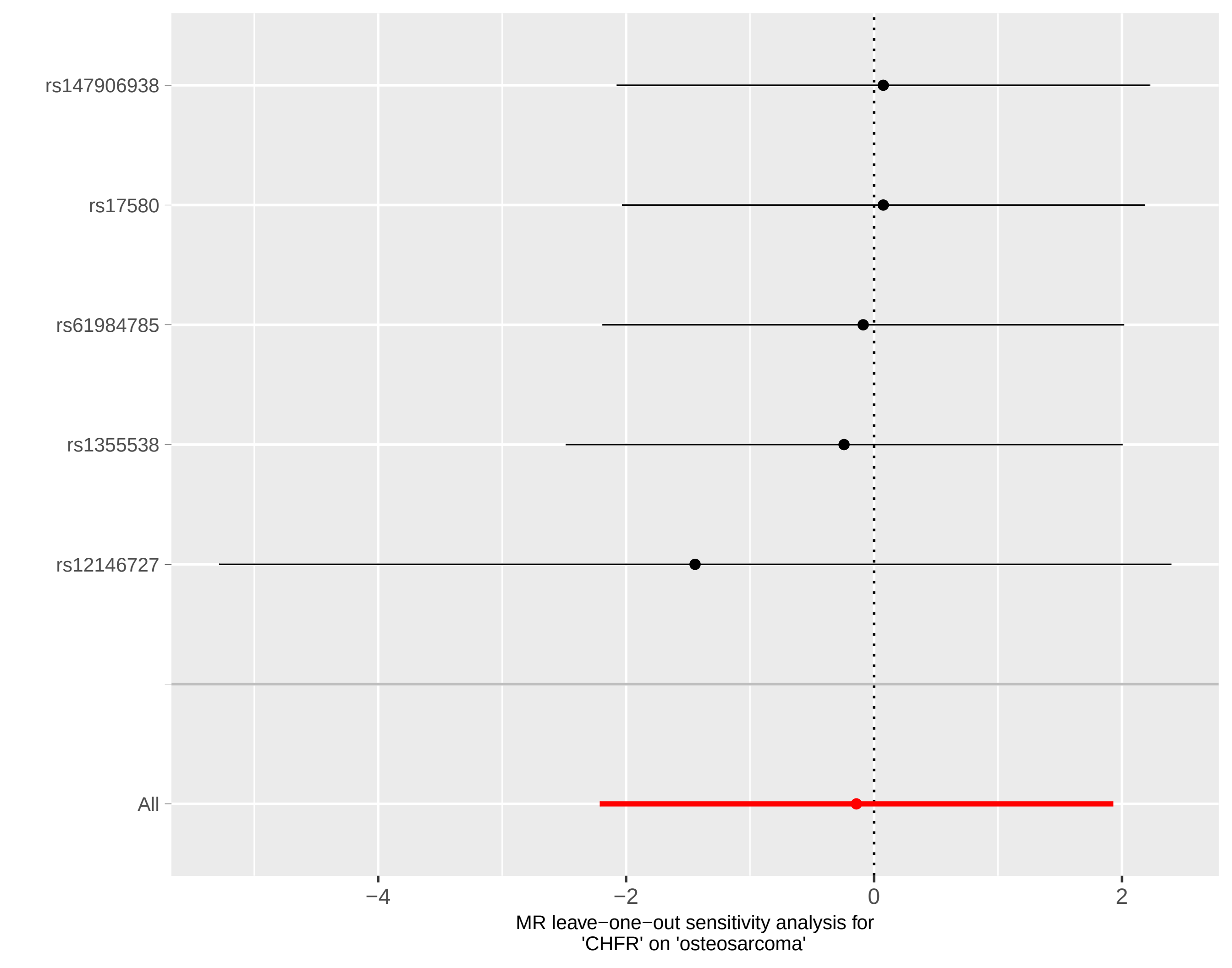
rs147906938
rs17580
rs61984785
rs1355538
rs12146727
All
−4
−2
0
2
MR leave−one−out sensitivity analysis for
'CHFR' on 'osteosarcoma'

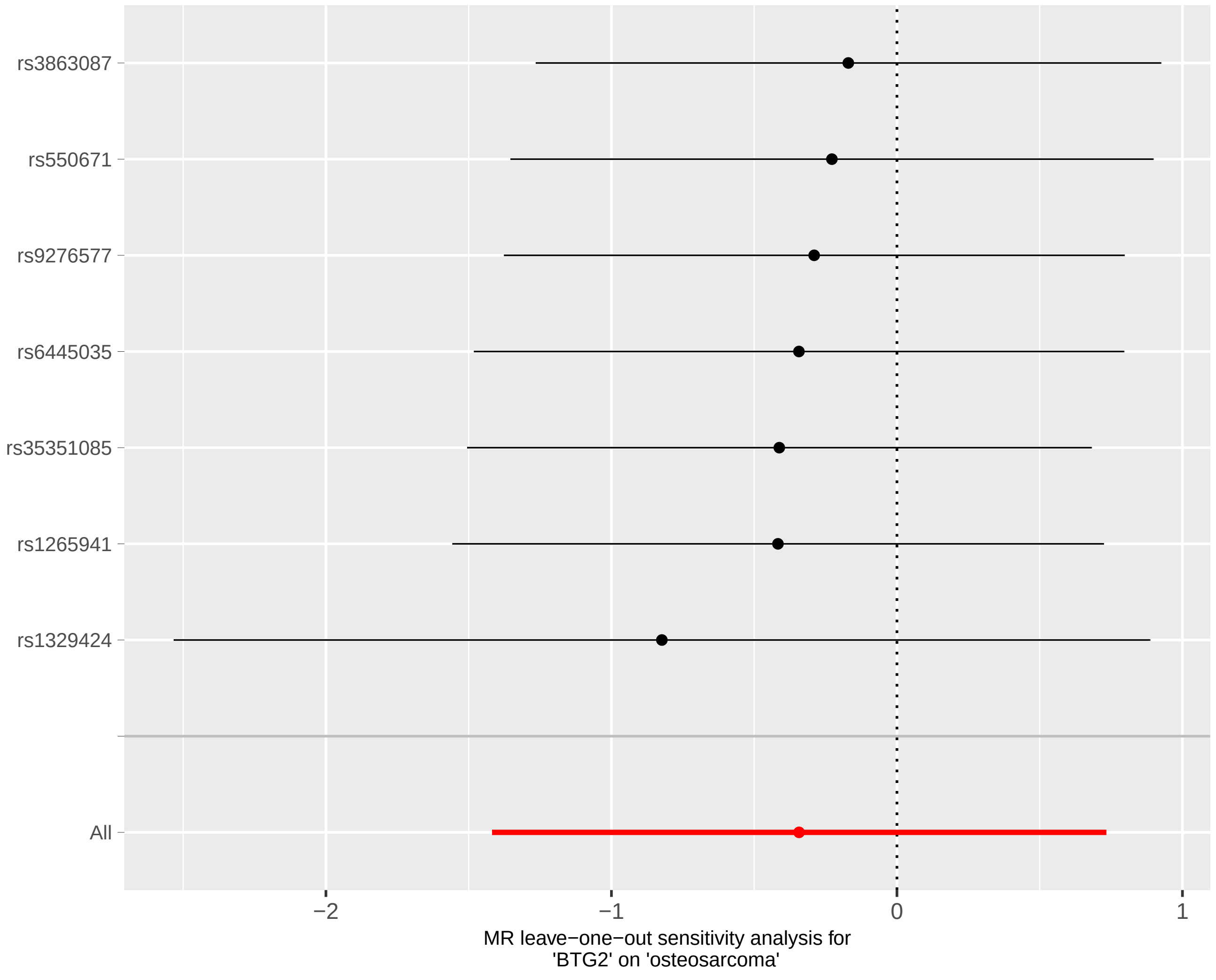

rs3863087
rs550671
rs9276577
rs6445035
rs35351085
rs1265941
rs1329424
All
−2
−1
0
1
MR leave−one−out sensitivity analysis for
'BTG2' on 'osteosarcoma'

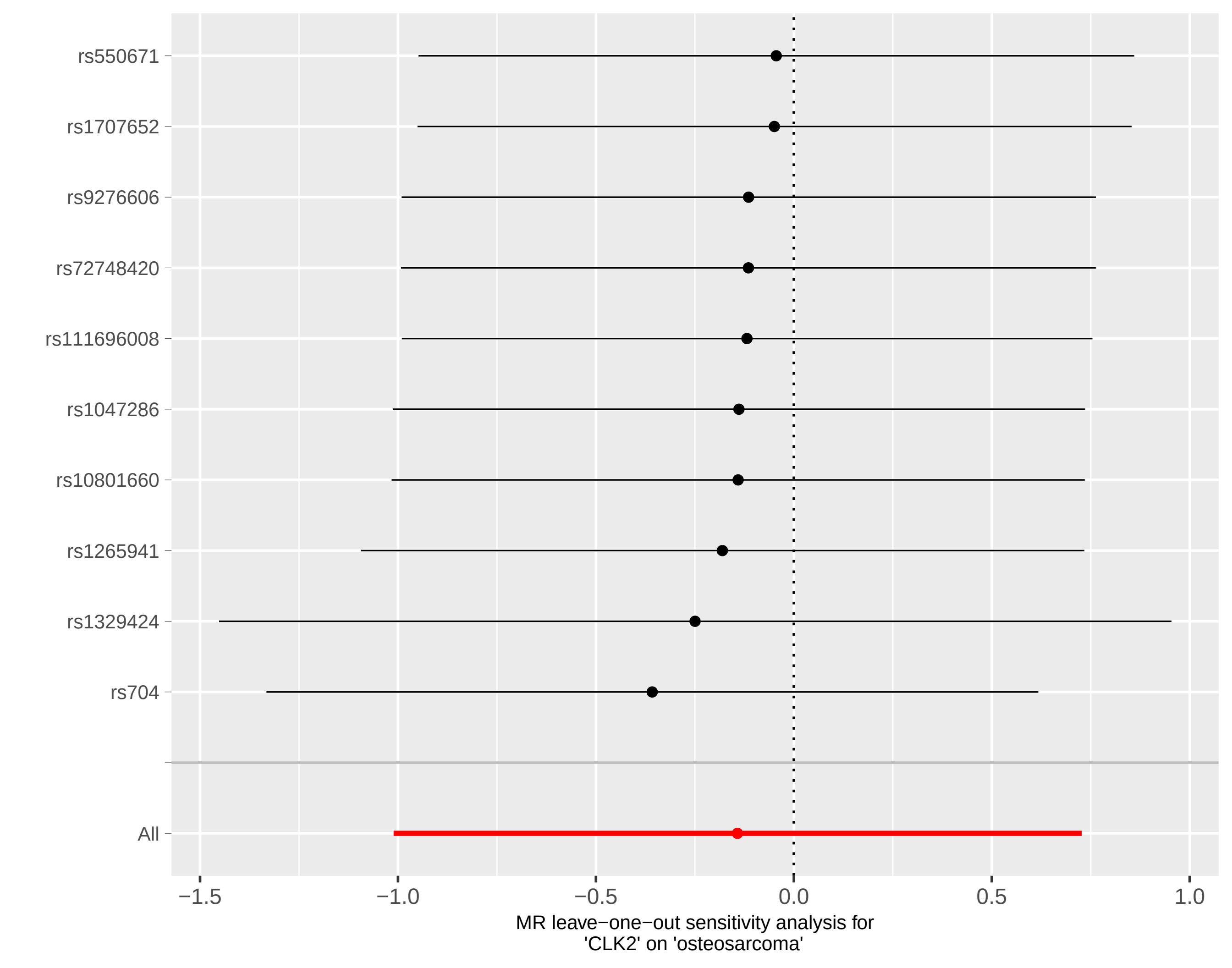

rs550671
rs1707652
rs9276606
rs72748420
rs111696008
rs1047286
rs10801660
rs1265941
rs1329424
rs704
All
−1.5
−1.0
−0.5
0.0
0.5
1.0
MR leave−one−out sensitivity analysis for
'CLK2' on 'osteosarcoma'

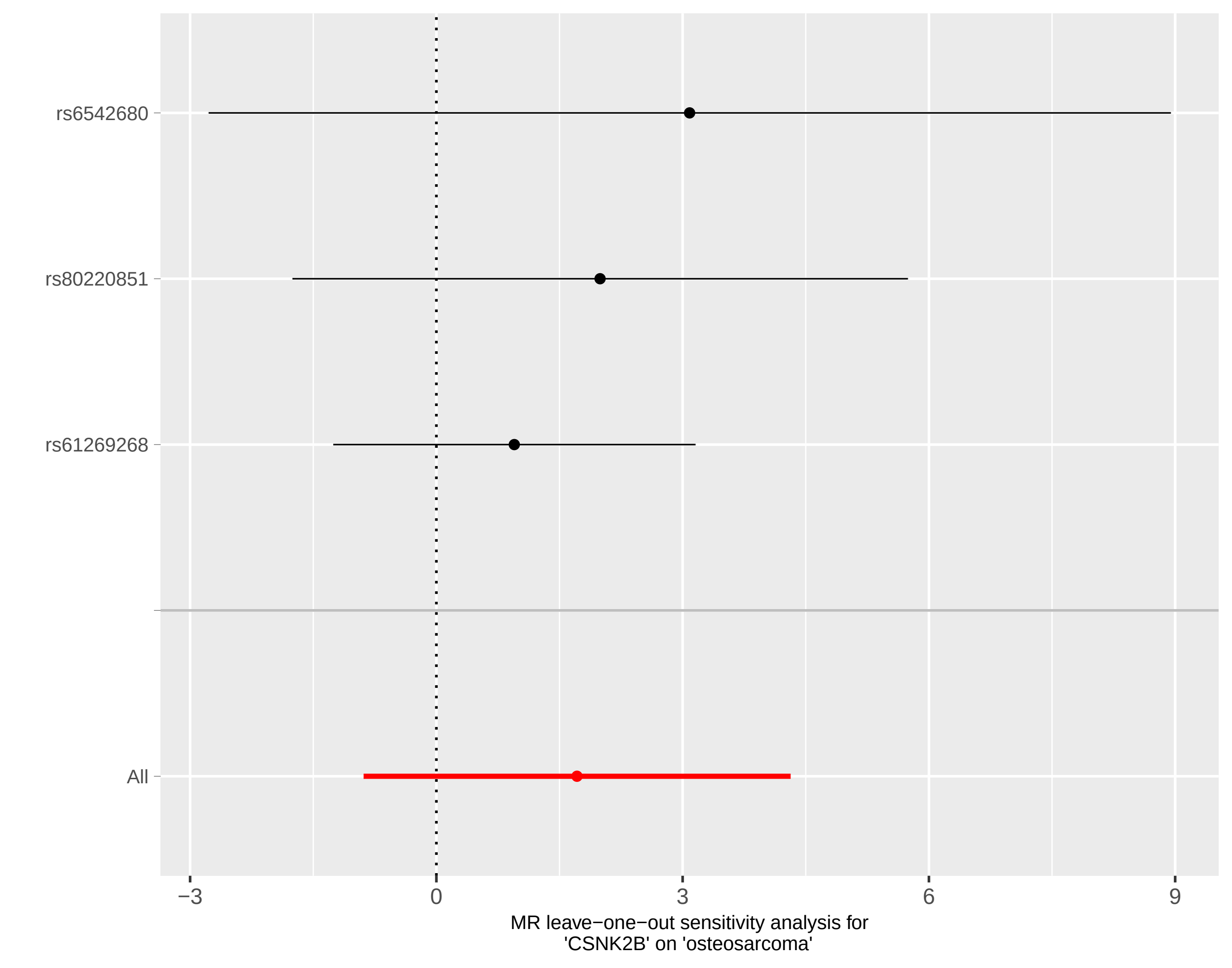

rs6542680
rs80220851
rs61269268
All
−3
0
3
6
9
MR leave−one−out sensitivity analysis for
'CSNK2B' on 'osteosarcoma'

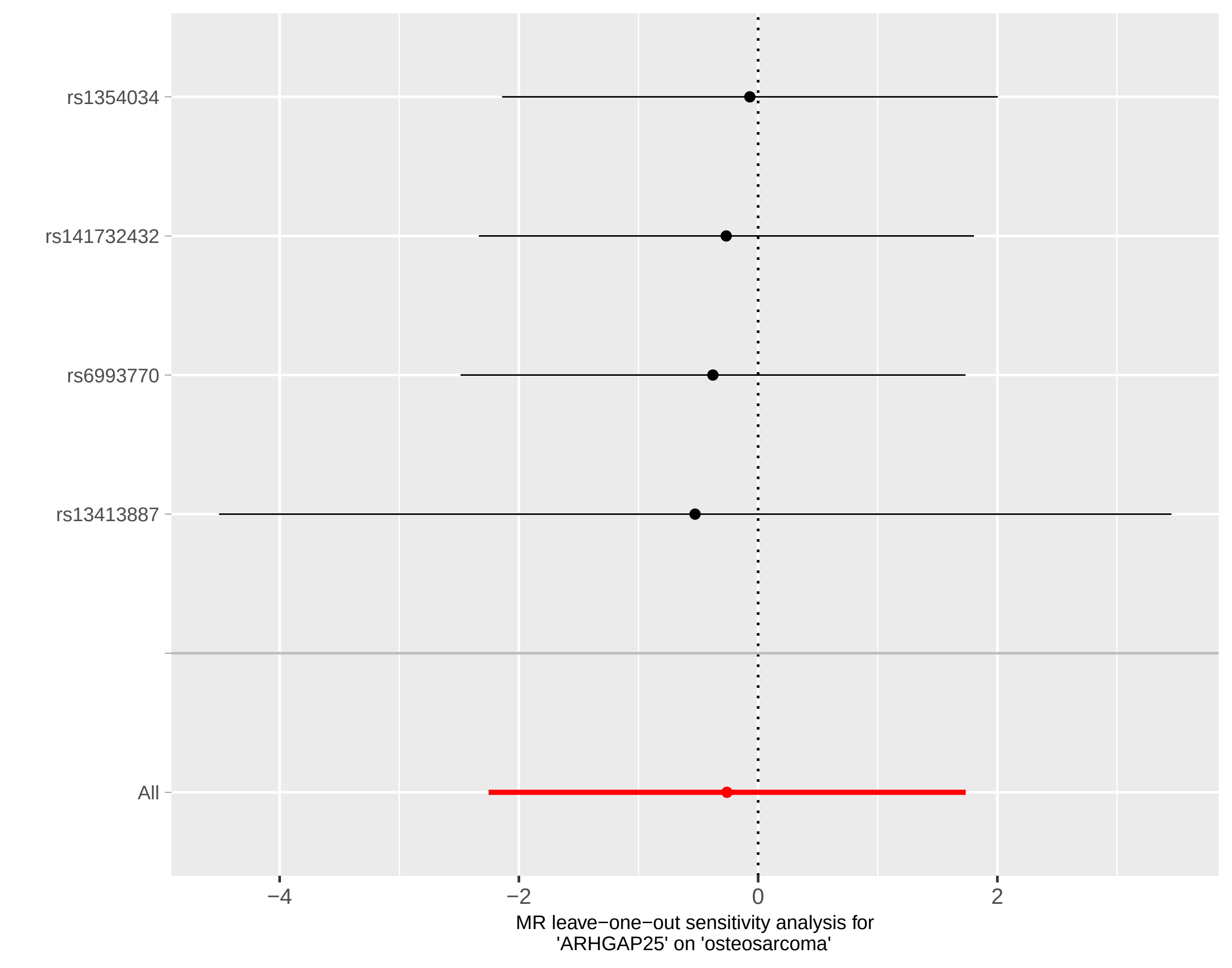
rs1354034
rs141732432
rs6993770
rs13413887
All
−4
−2
0
2
MR leave−one−out sensitivity analysis for
'ARHGAP25' on 'osteosarcoma'

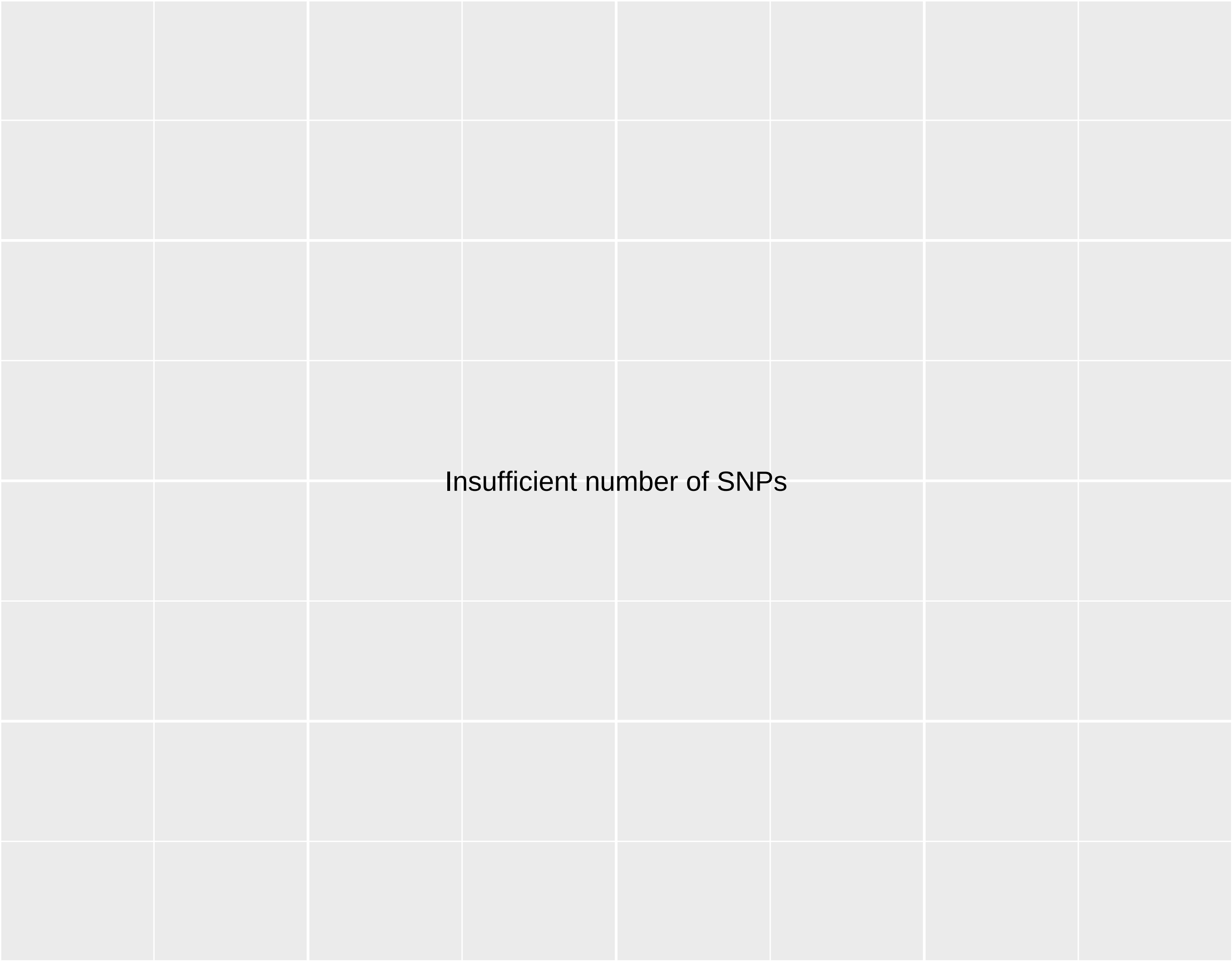
Insufficient number of SNPs

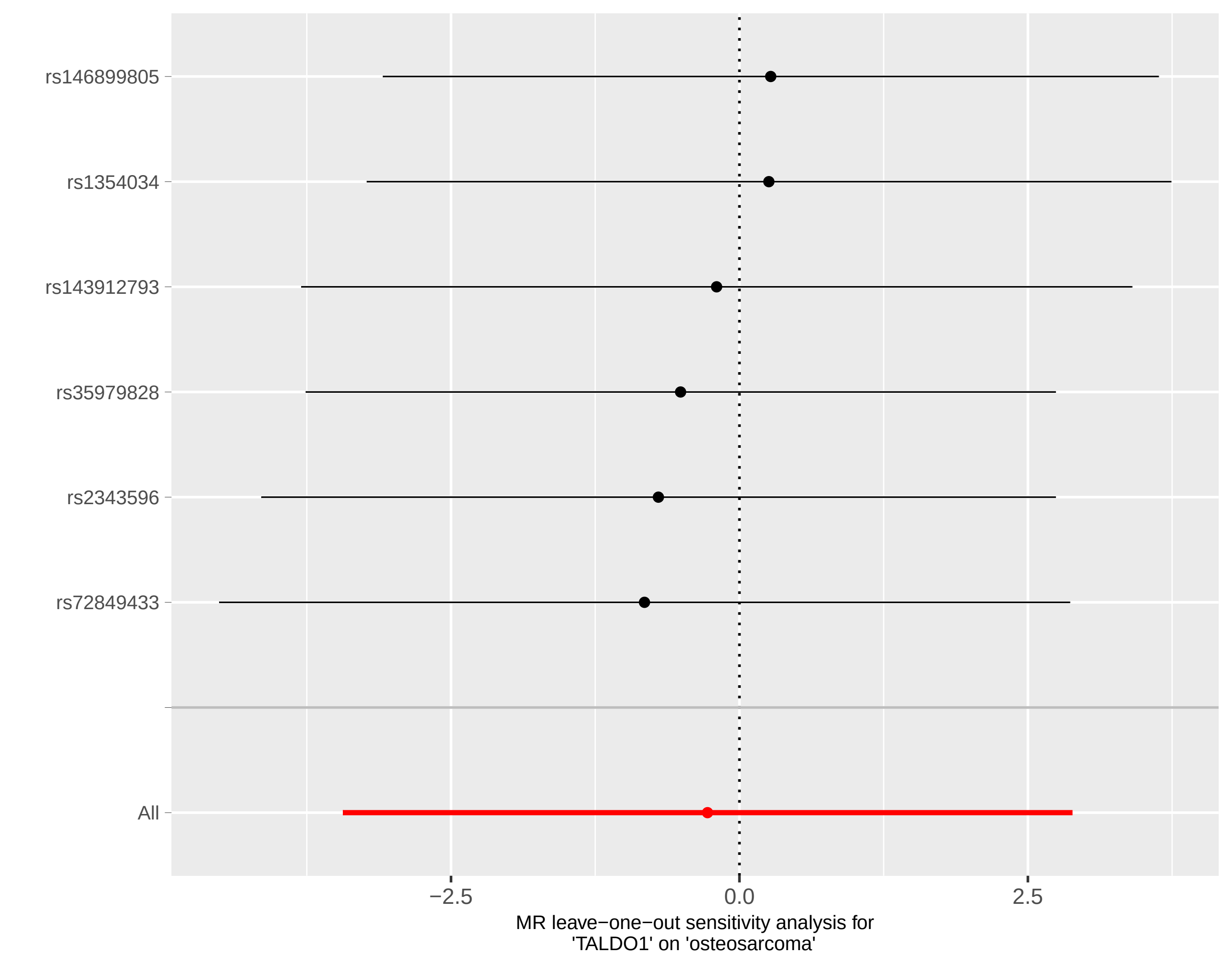

rs146899805
rs1354034
rs143912793
rs35979828
rs2343596
rs72849433
All
−2.5
0.0
2.5
MR leave−one−out sensitivity analysis for
'TALDO1' on 'osteosarcoma'

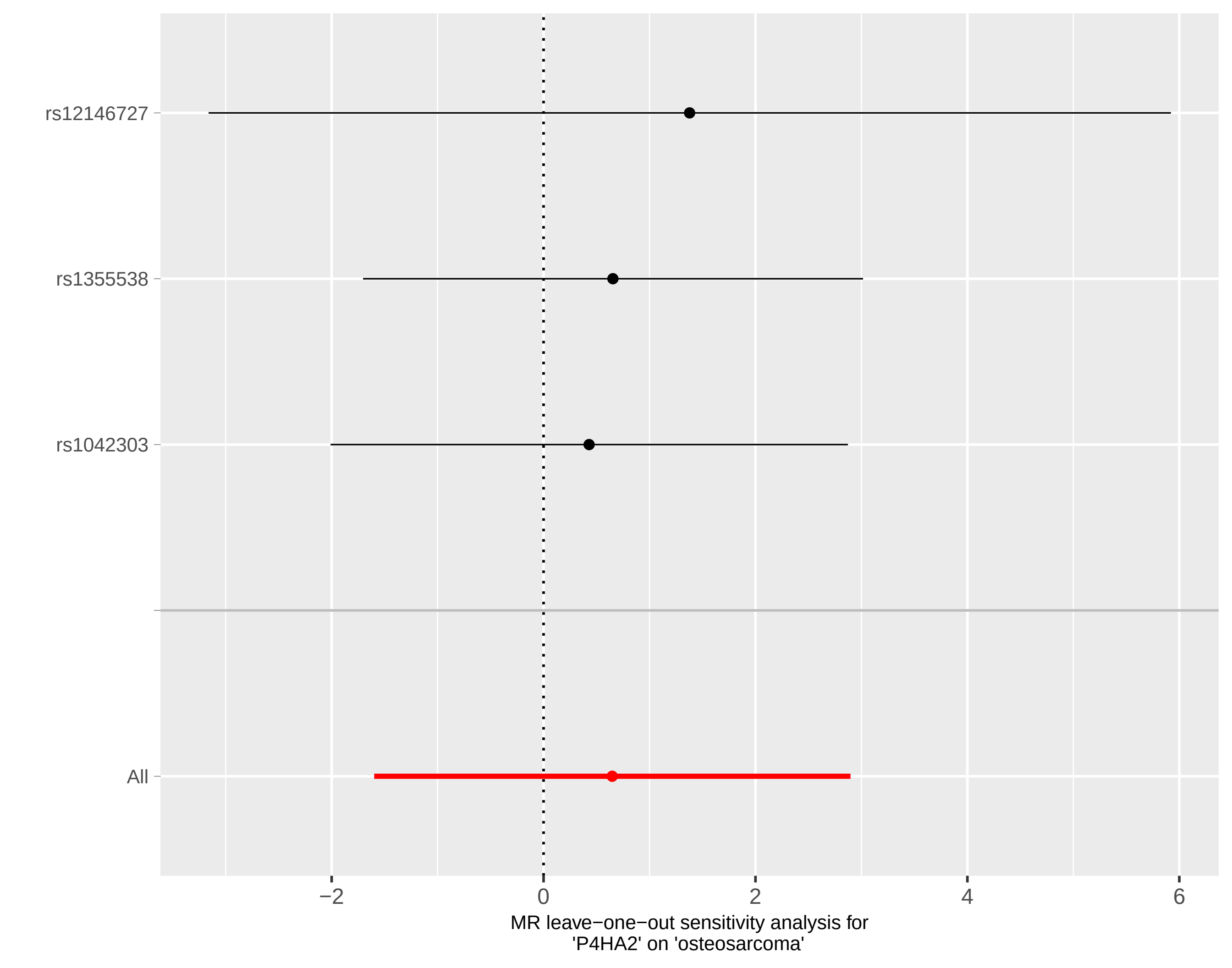

rs12146727
rs1355538
rs1042303
All
−2
0
2
4
6
MR leave−one−out sensitivity analysis for
'P4HA2' on 'osteosarcoma'

Insufficient number of SNPs

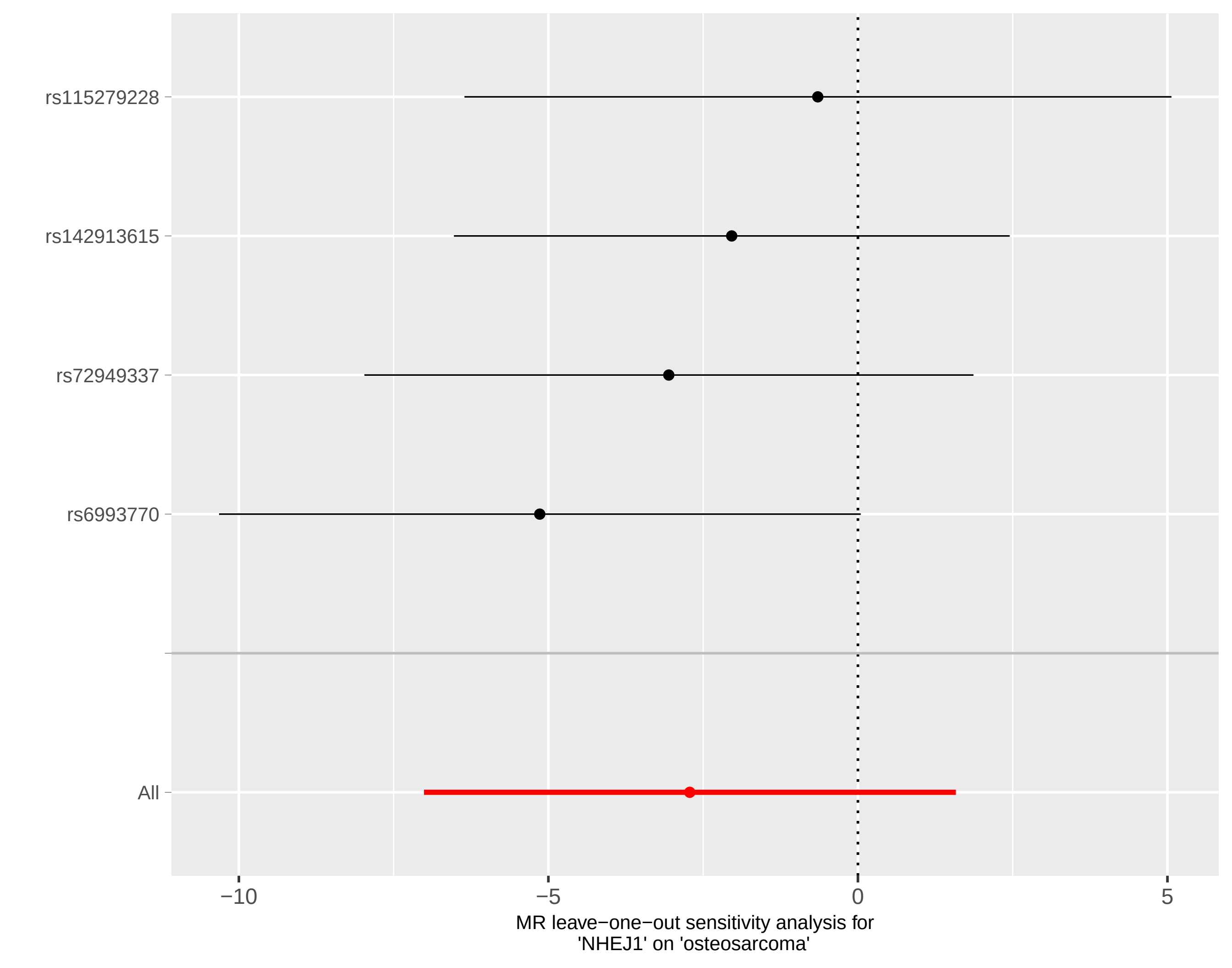
rs115279228
rs142913615
rs72949337
rs6993770
All
−10
−5
0
5
MR leave−one−out sensitivity analysis for
'NHEJ1' on 'osteosarcoma'

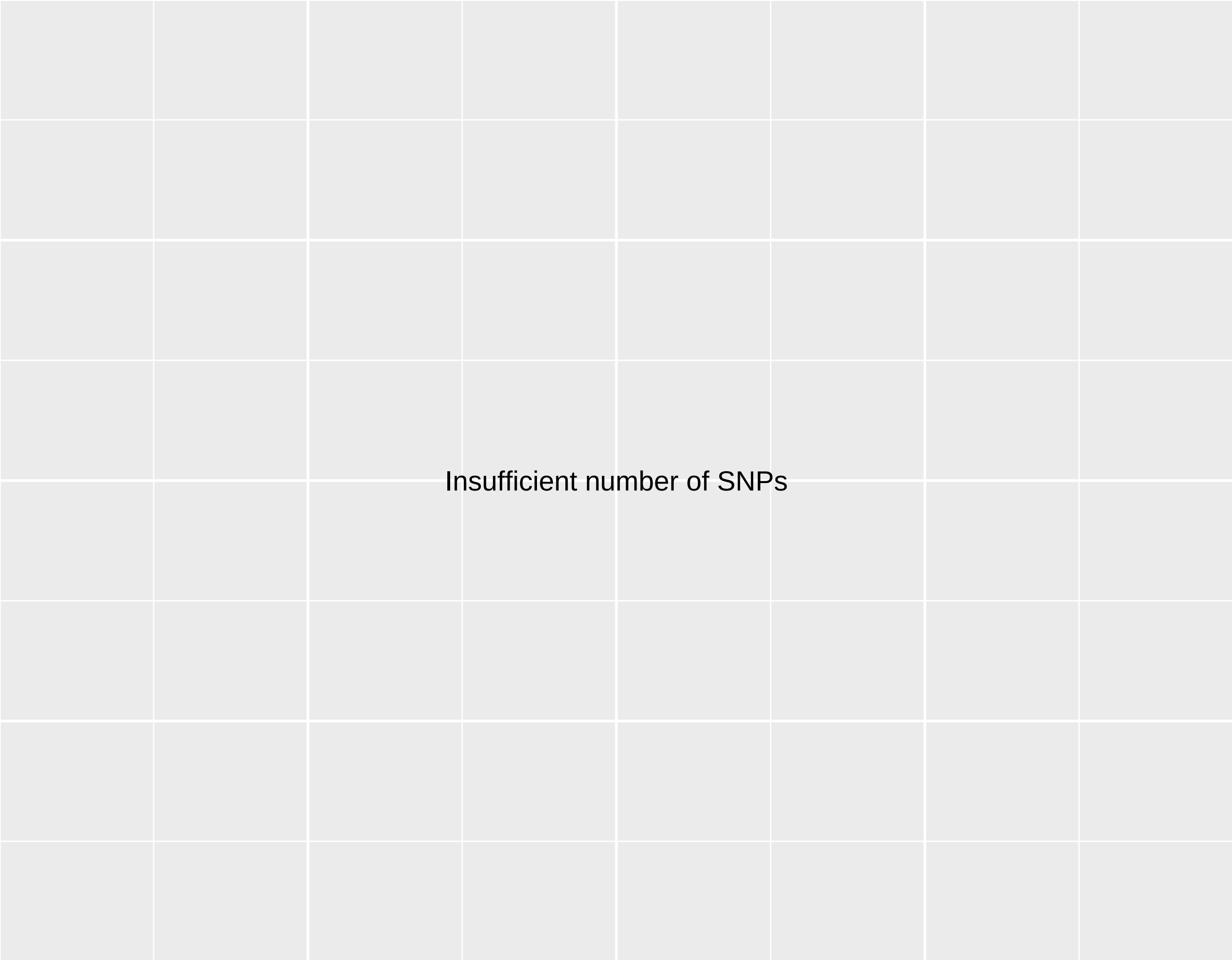
Insufficient number of SNPs

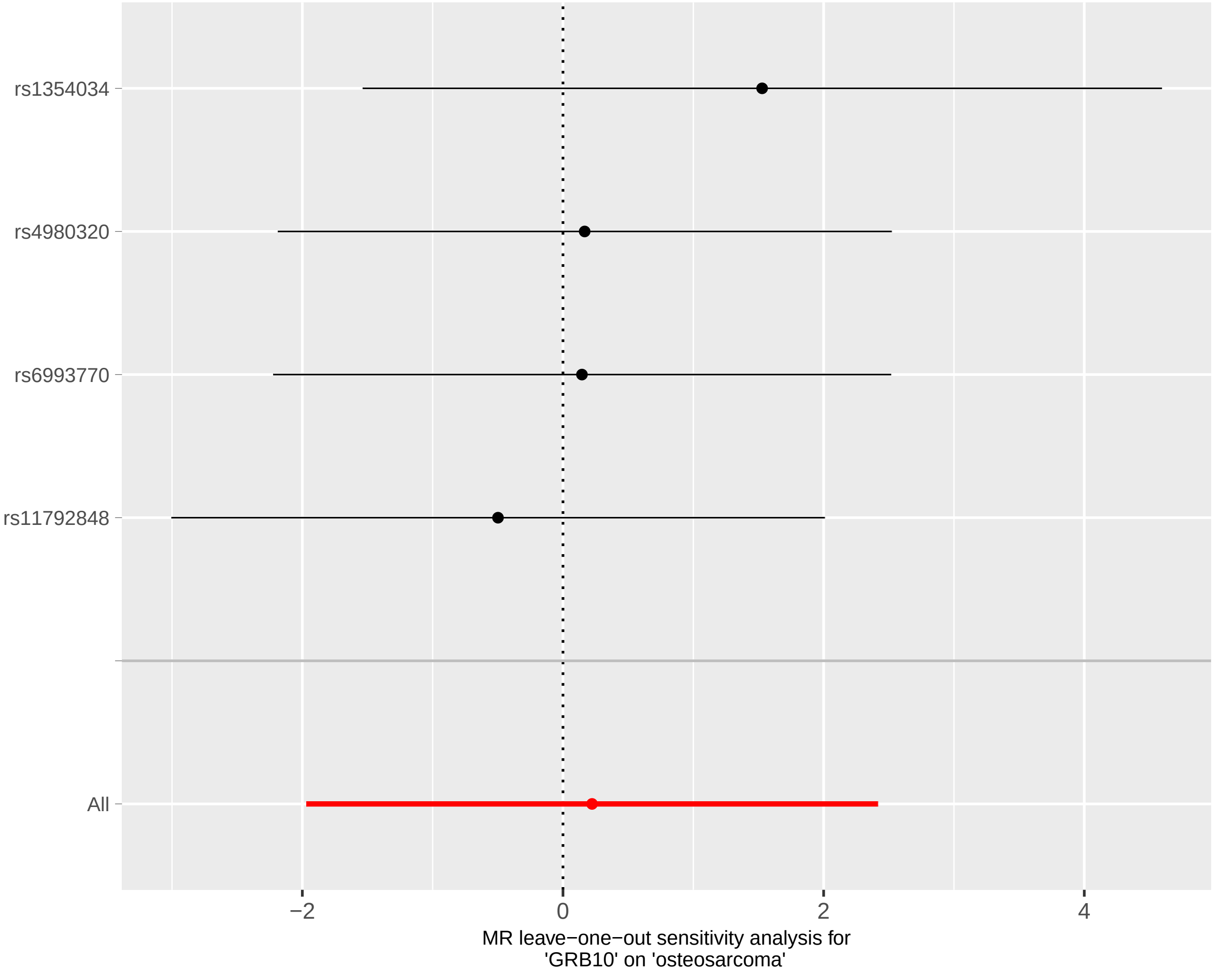
rs1354034
rs4980320
rs6993770
rs11792848
All
−2
0
2
4
MR leave−one−out sensitivity analysis for
'GRB10' on 'osteosarcoma'

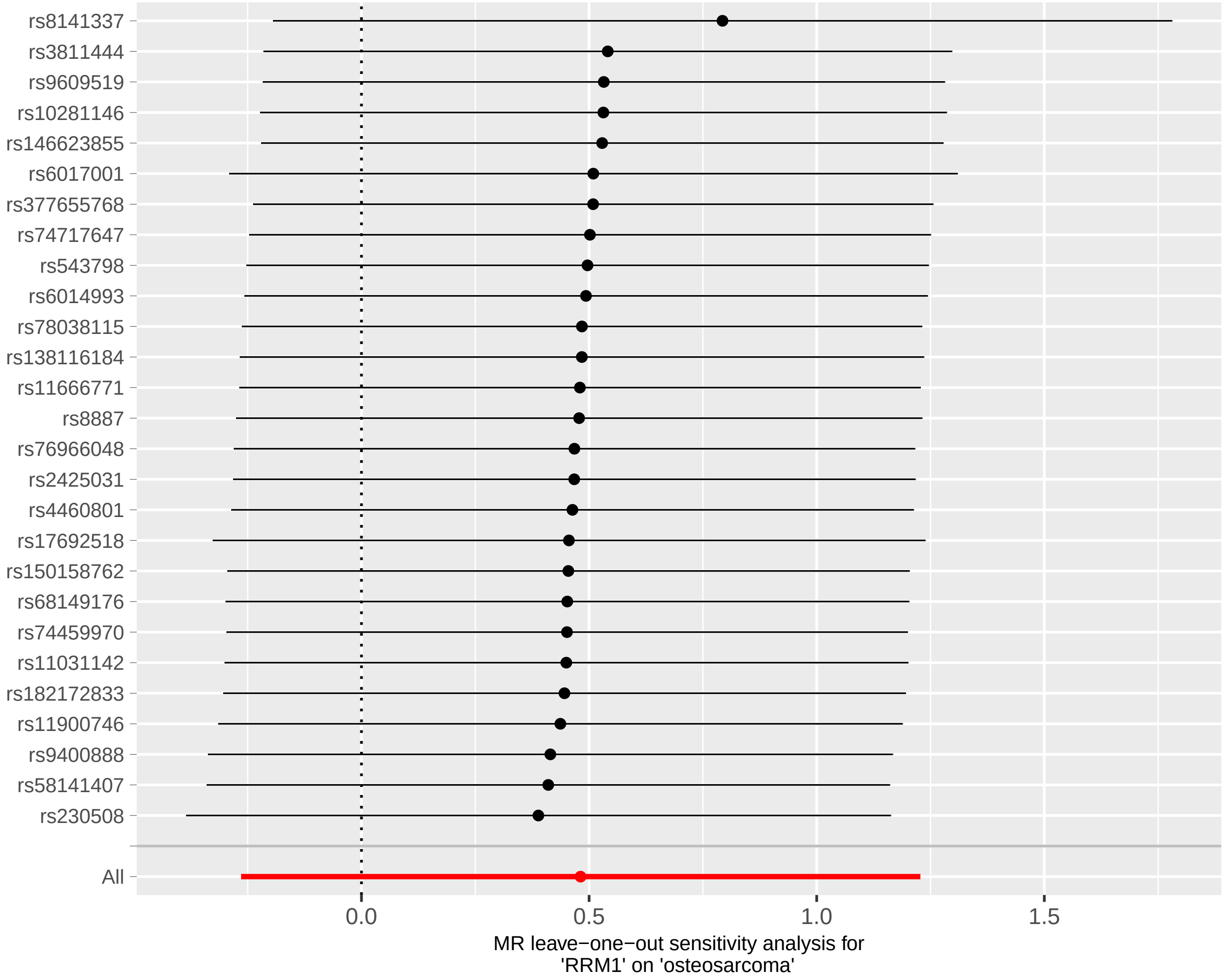

rs8141337
rs3811444
rs9609519
rs10281146
rs146623855
rs6017001
rs377655768
rs74717647
rs543798
rs6014993
rs78038115
rs138116184
rs11666771
rs8887
rs76966048
rs2425031
rs4460801
rs17692518
rs150158762
rs68149176
rs74459970
rs11031142
rs182172833
rs11900746
rs9400888
rs58141407
rs230508
All
0.0
0.5
1.0
1.5
MR leave−one−out sensitivity analysis for
'RRM1' on 'osteosarcoma'

Insufficient number of SNPs

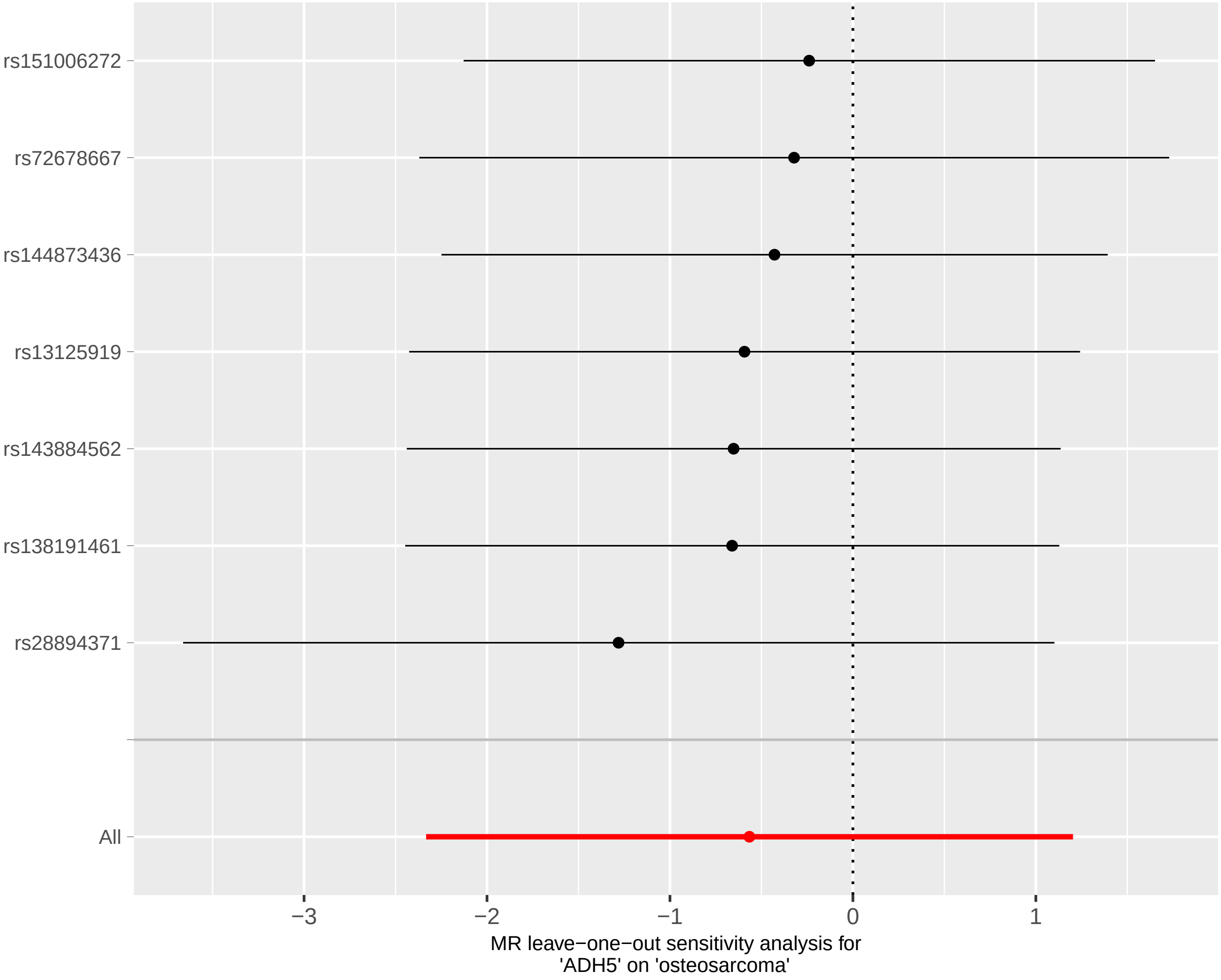

rs151006272
rs72678667
rs144873436
rs13125919
rs143884562
rs138191461
rs28894371
All
−3
−2
−1
0
1
MR leave−one−out sensitivity analysis for
'ADH5' on 'osteosarcoma'

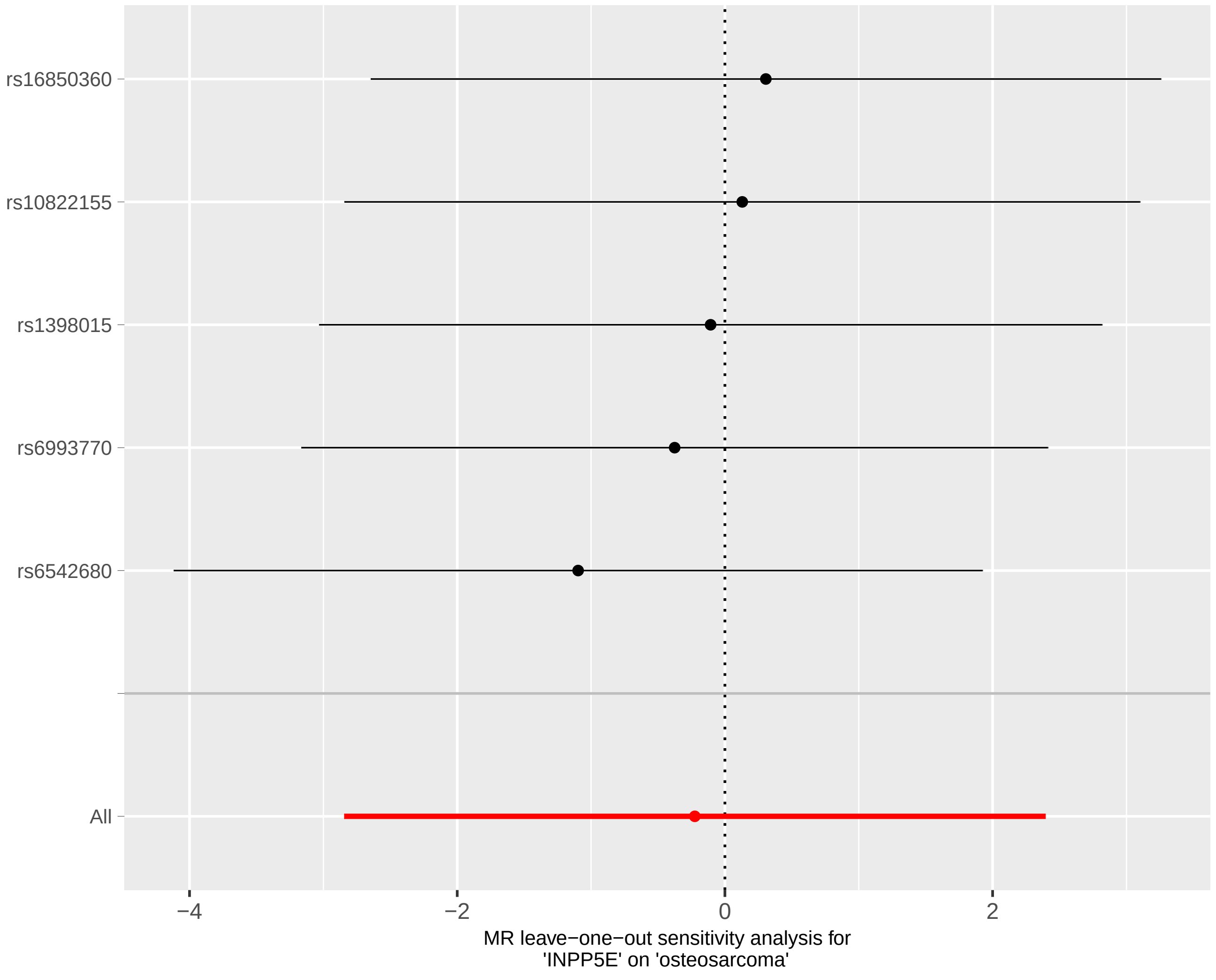

rs16850360
rs10822155
rs1398015
rs6993770
rs6542680
All
−4
−2
0
2
MR leave−one−out sensitivity analysis for
'INPP5E' on 'osteosarcoma'

Insufficient number of SNPs

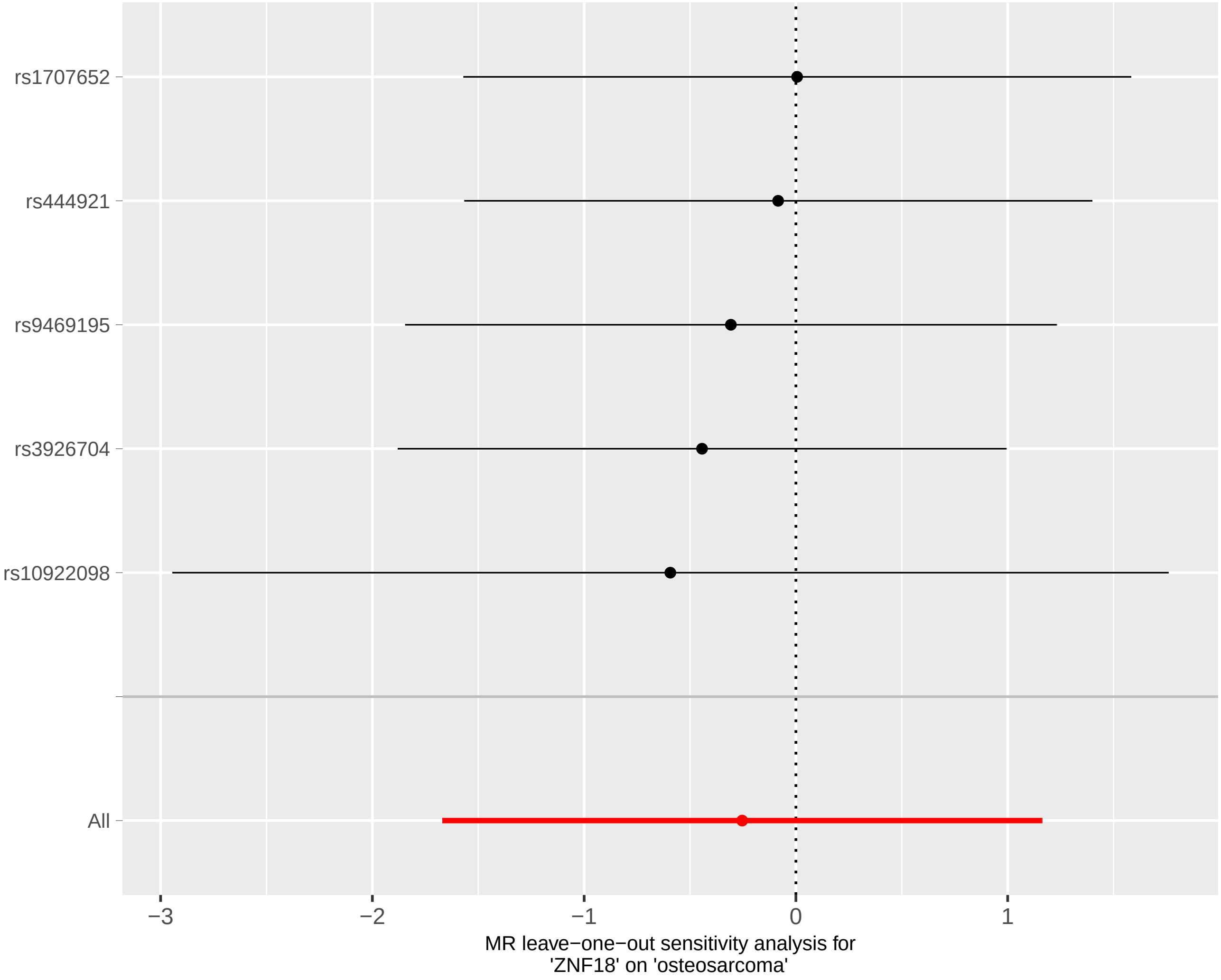
rs1707652
rs444921
rs9469195
rs3926704
rs10922098
All
−3
−2
−1
0
1
MR leave−one−out sensitivity analysis for
'ZNF18' on 'osteosarcoma'

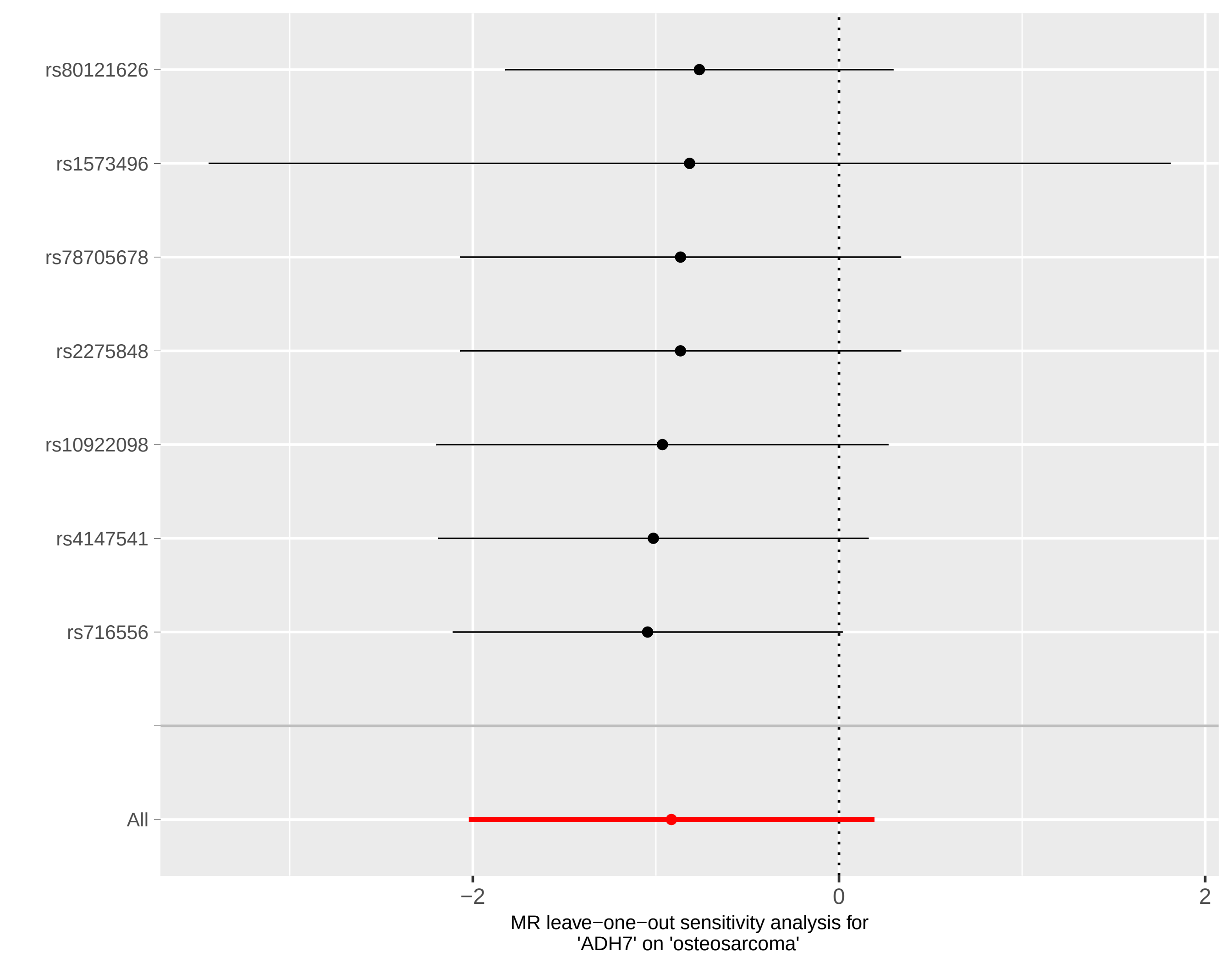
rs80121626
rs1573496
rs78705678
rs2275848
rs10922098
rs4147541
rs716556
All
−2
0
2
MR leave−one−out sensitivity analysis for
'ADH7' on 'osteosarcoma'

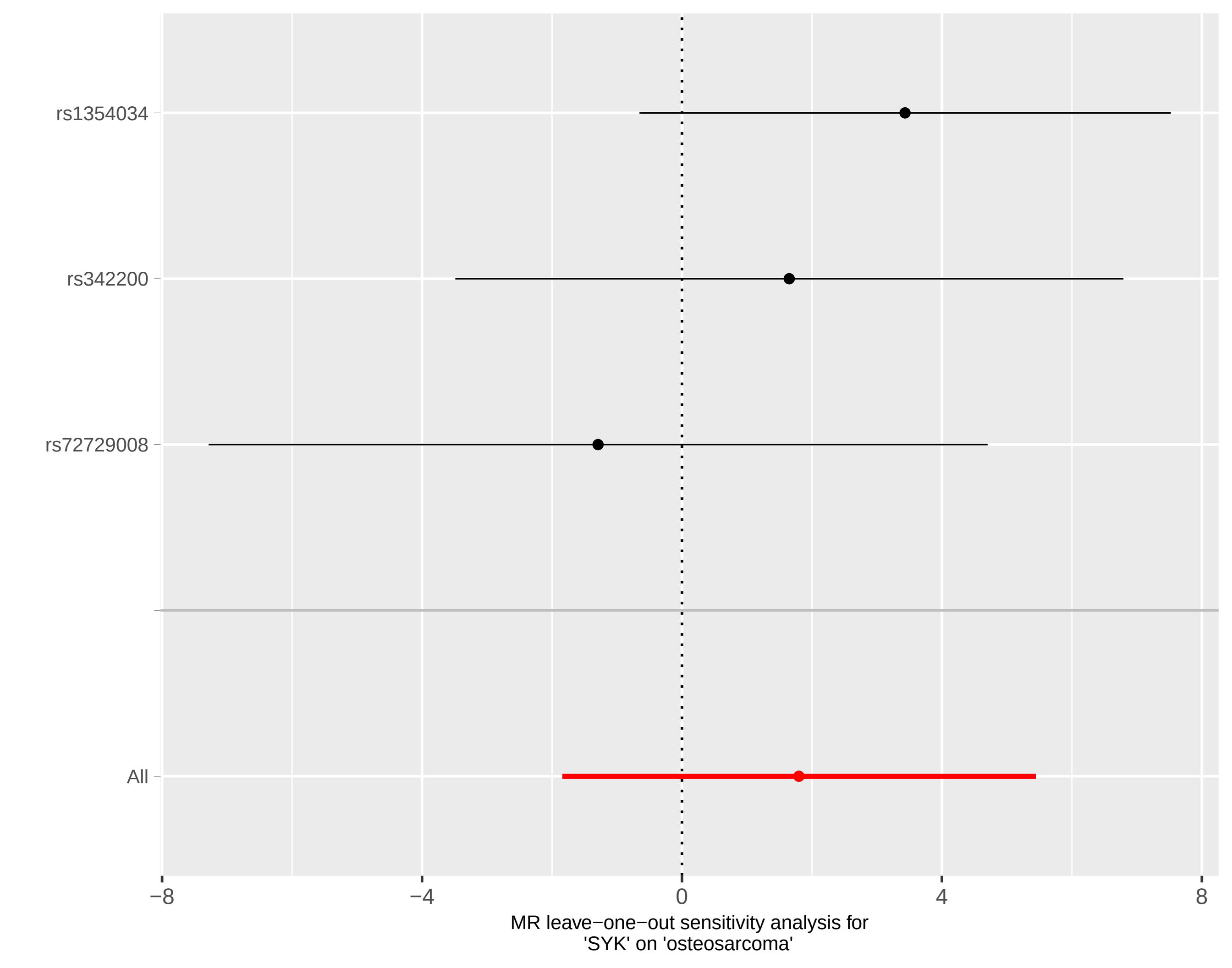
rs1354034
rs342200
rs72729008
All
−8
−4
0
4
8
MR leave−one−out sensitivity analysis for
'SYK' on 'osteosarcoma'

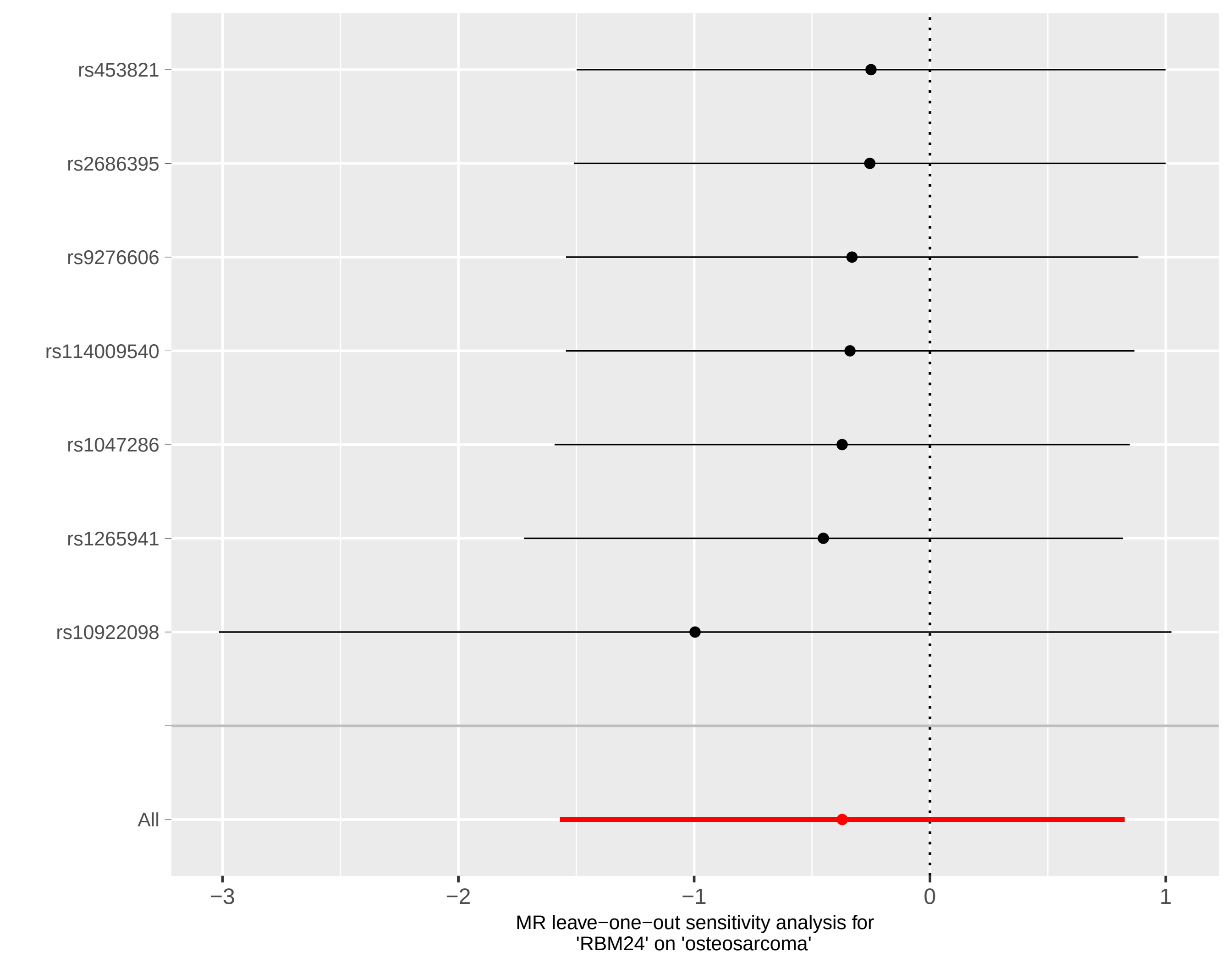

rs453821
rs2686395
rs9276606
rs114009540
rs1047286
rs1265941
rs10922098
All
−3
−2
−1
0
1
MR leave−one−out sensitivity analysis for
'RBM24' on 'osteosarcoma'

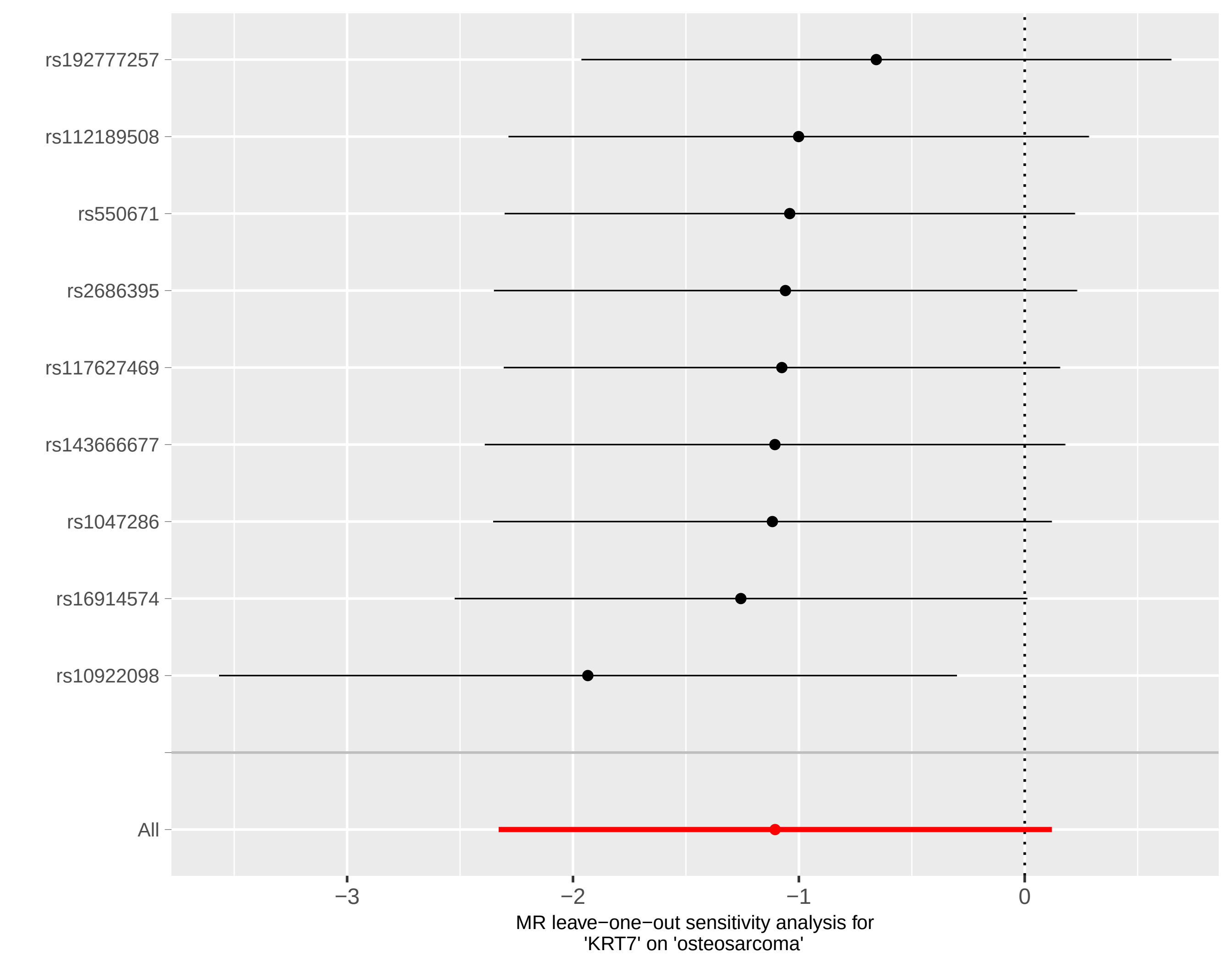

rs192777257
rs112189508
rs550671
rs2686395
rs117627469
rs143666677
rs1047286
rs16914574
rs10922098
All
−3
−2
−1
0
MR leave−one−out sensitivity analysis for
'KRT7' on 'osteosarcoma'

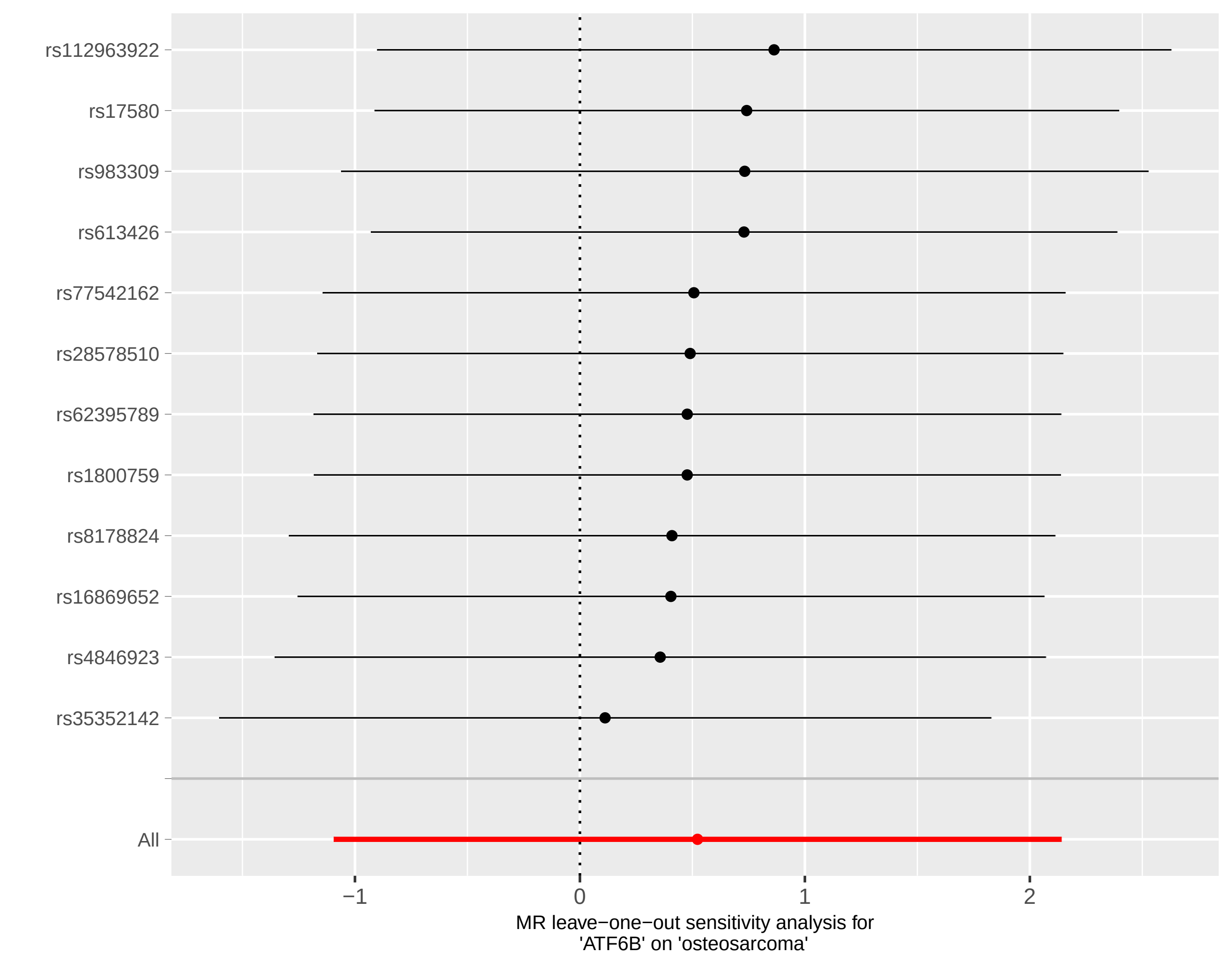
rs112963922
rs17580
rs983309
rs613426
rs77542162
rs28578510
rs62395789
rs1800759
rs8178824
rs16869652
rs4846923
rs35352142
All
−1
0
1
2
MR leave−one−out sensitivity analysis for
'ATF6B' on 'osteosarcoma'

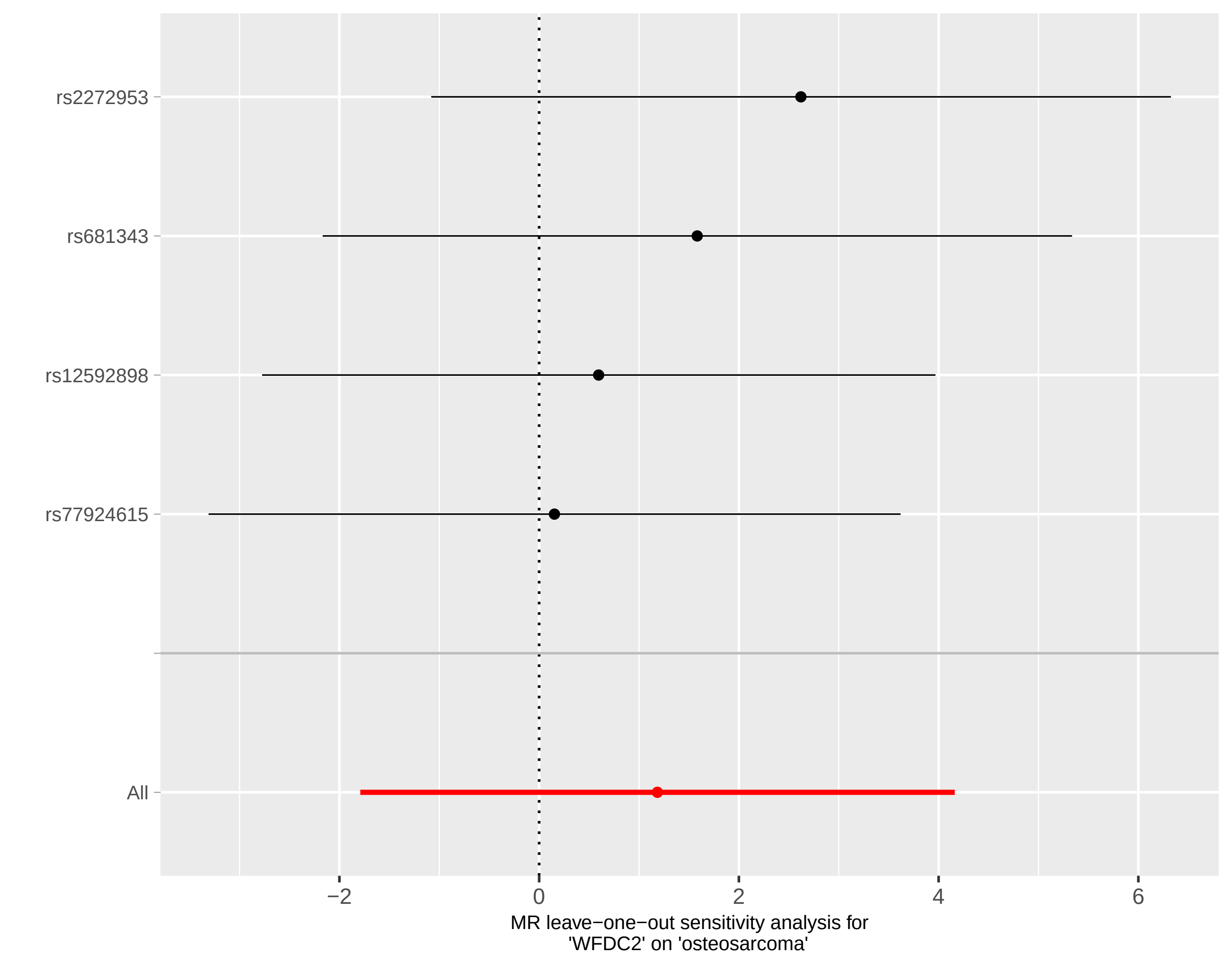
rs2272953
rs681343
rs12592898
rs77924615
All
−2
0
2
4
6
MR leave−one−out sensitivity analysis for
'WFDC2' on 'osteosarcoma'

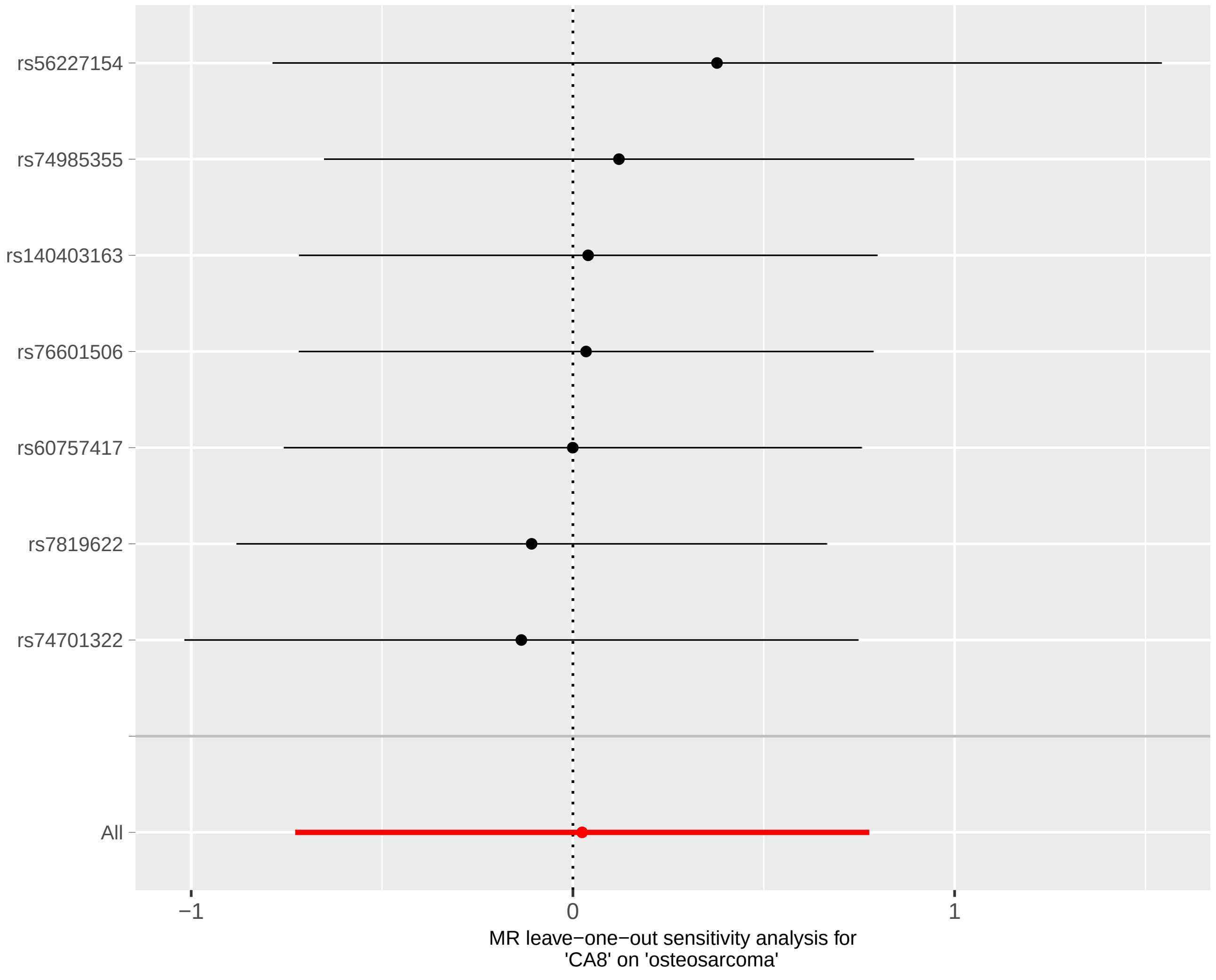

rs56227154
rs74985355
rs140403163
rs76601506
rs60757417
rs7819622
rs74701322
All
−1
0
1
MR leave−one−out sensitivity analysis for
'CA8' on 'osteosarcoma'

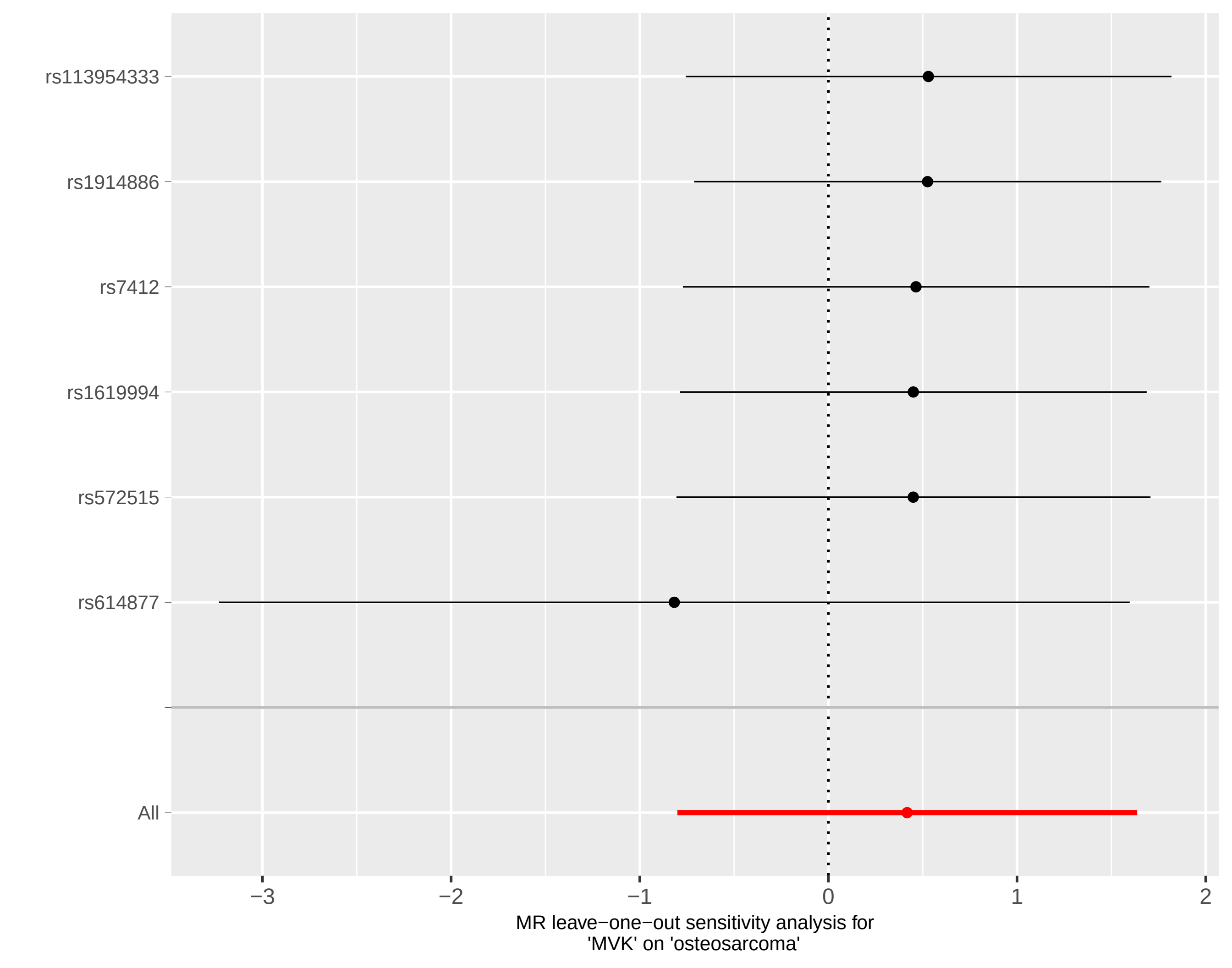

rs113954333
rs1914886
rs7412
rs1619994
rs572515
rs614877
All
−3
−2
−1
0
1
2
MR leave−one−out sensitivity analysis for
'MVK' on 'osteosarcoma'

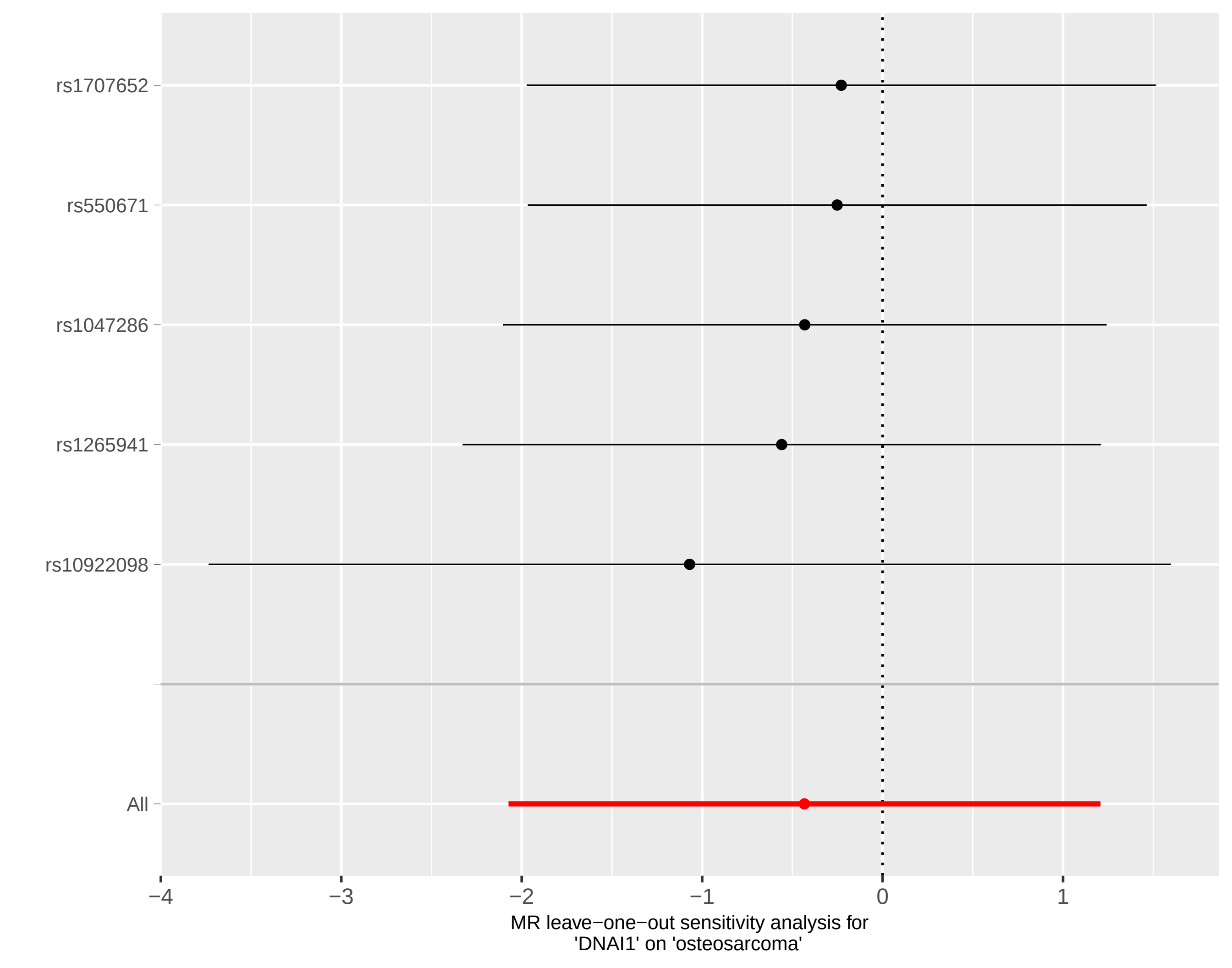
rs1707652
rs550671
rs1047286
rs1265941
rs10922098
All
−4
−3
−2
−1
0
1
MR leave−one−out sensitivity analysis for
'DNAI1' on 'osteosarcoma'

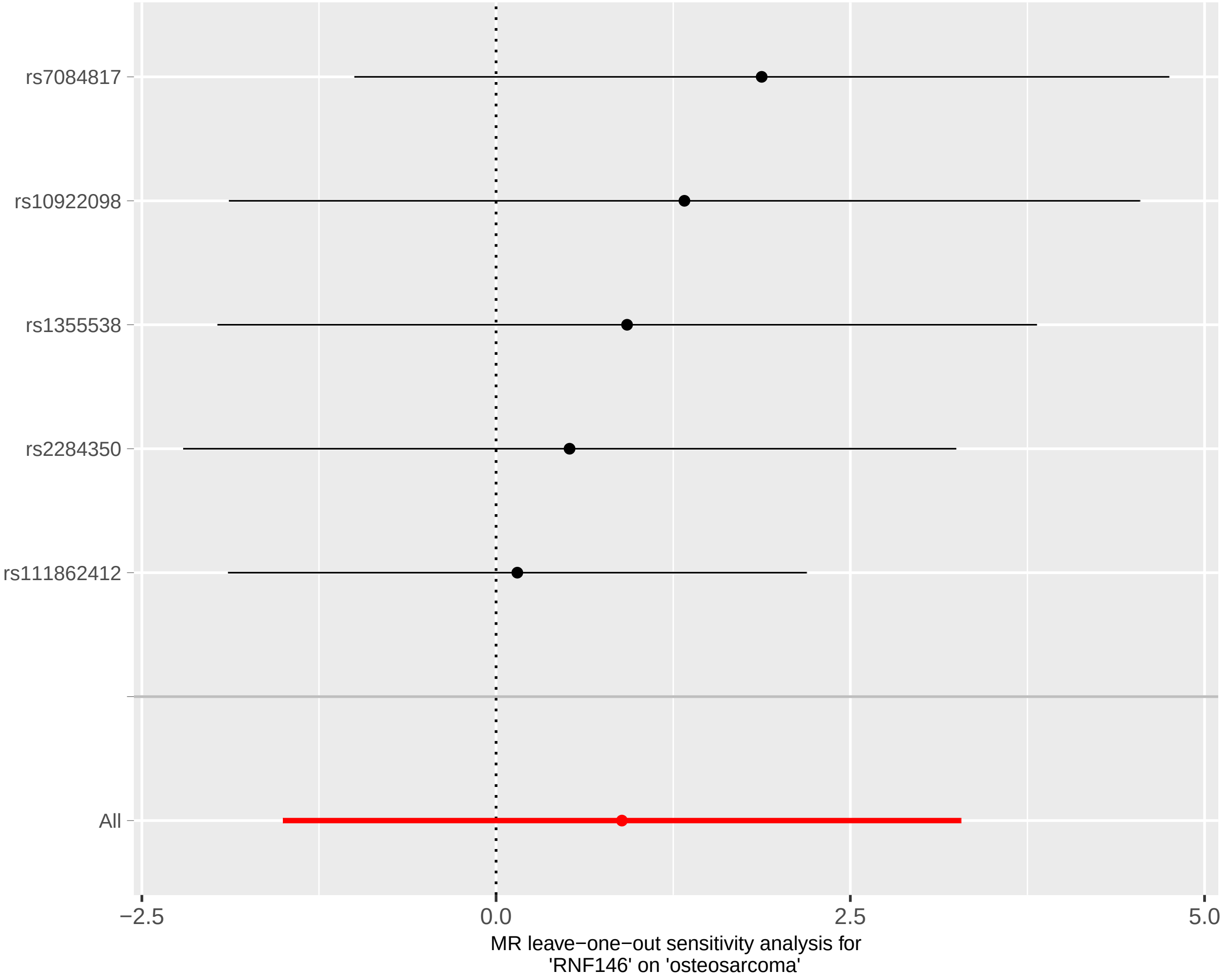
rs7084817
rs10922098
rs1355538
rs2284350
rs111862412
All
−2.5
0.0
2.5
5.0
MR leave−one−out sensitivity analysis for
'RNF146' on 'osteosarcoma'

Insufficient number of SNPs

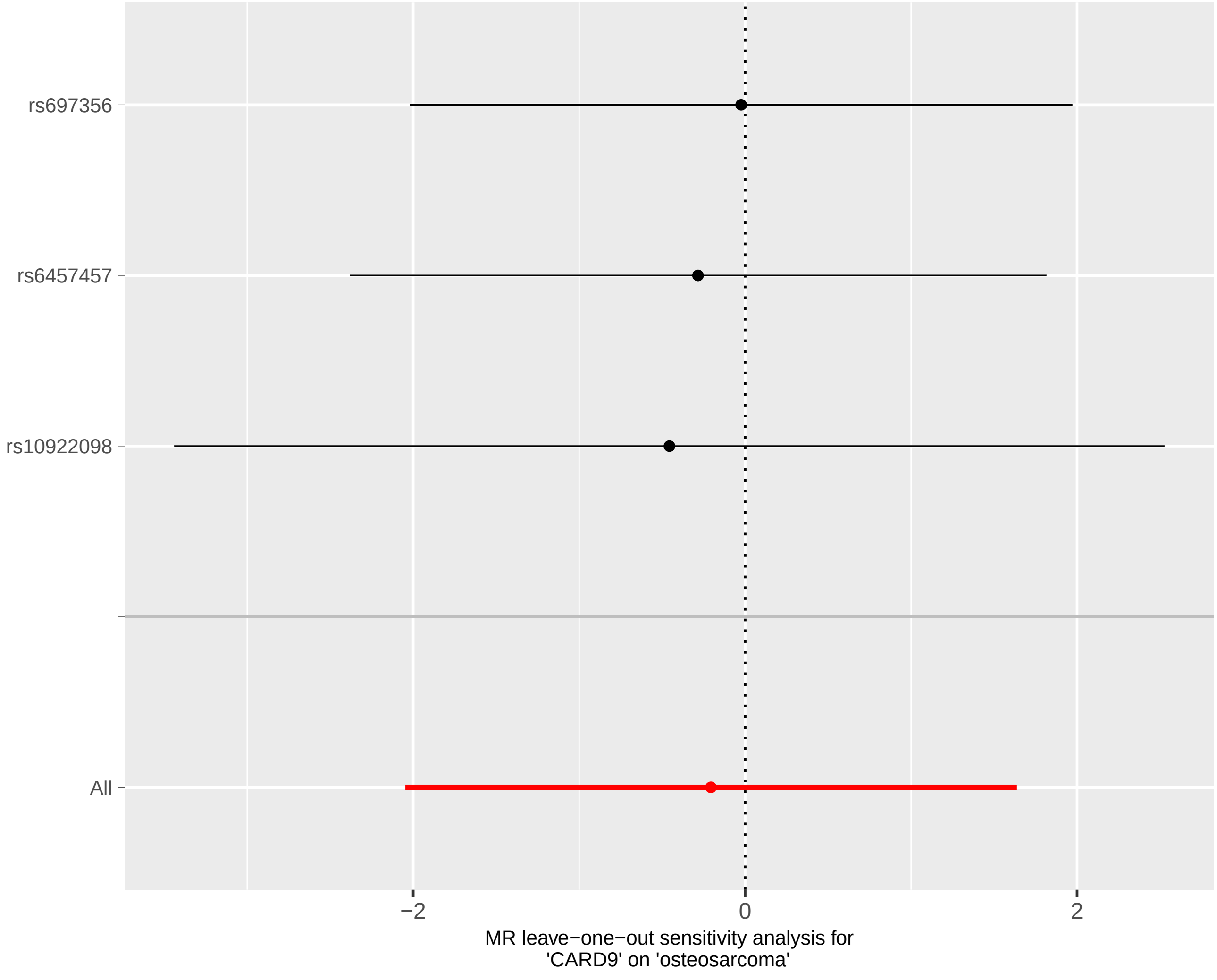

rs697356
rs6457457
rs10922098
All
−2
0
2
MR leave−one−out sensitivity analysis for
'CARD9' on 'osteosarcoma'

Insufficient number of SNPs

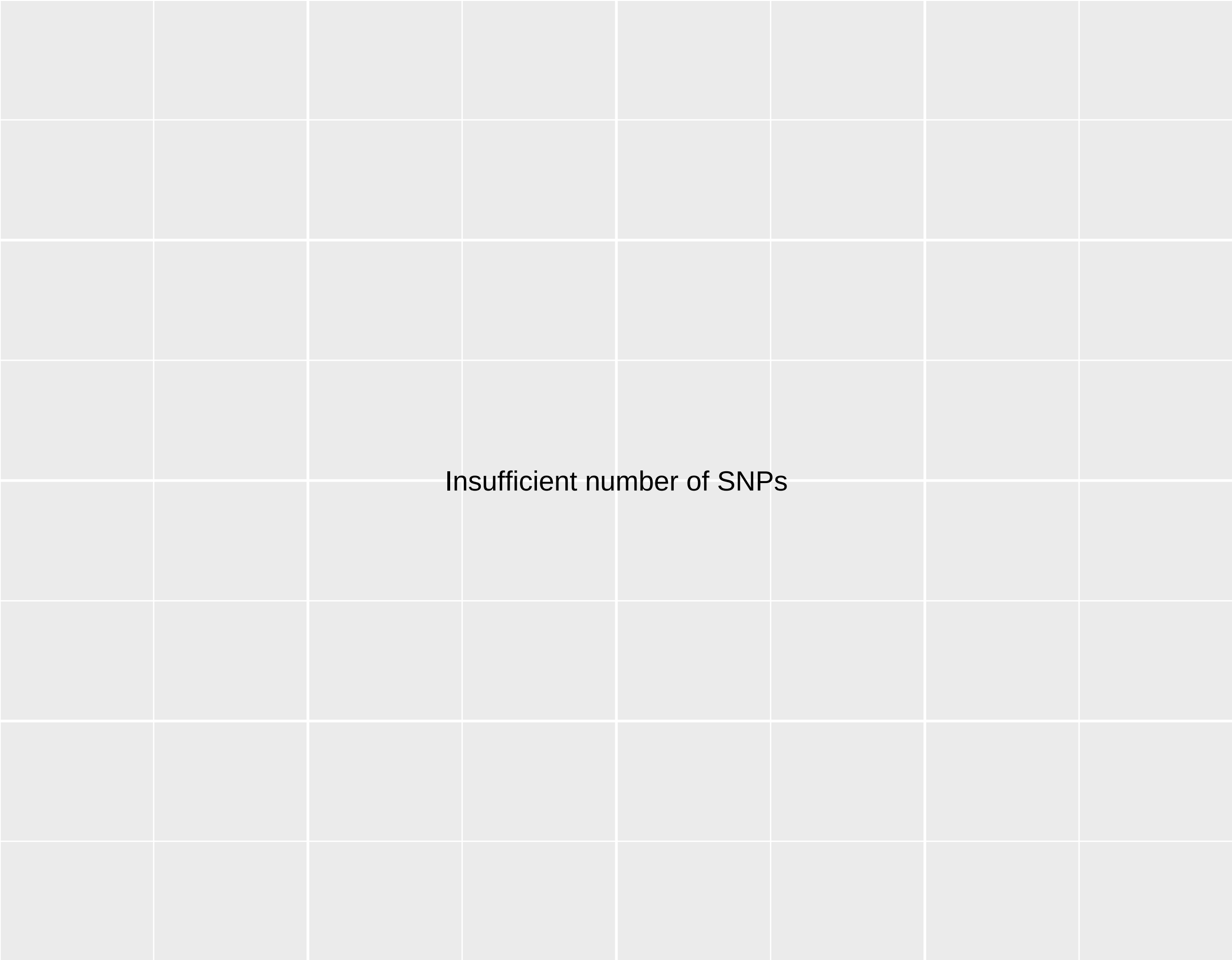
Insufficient number of SNPs

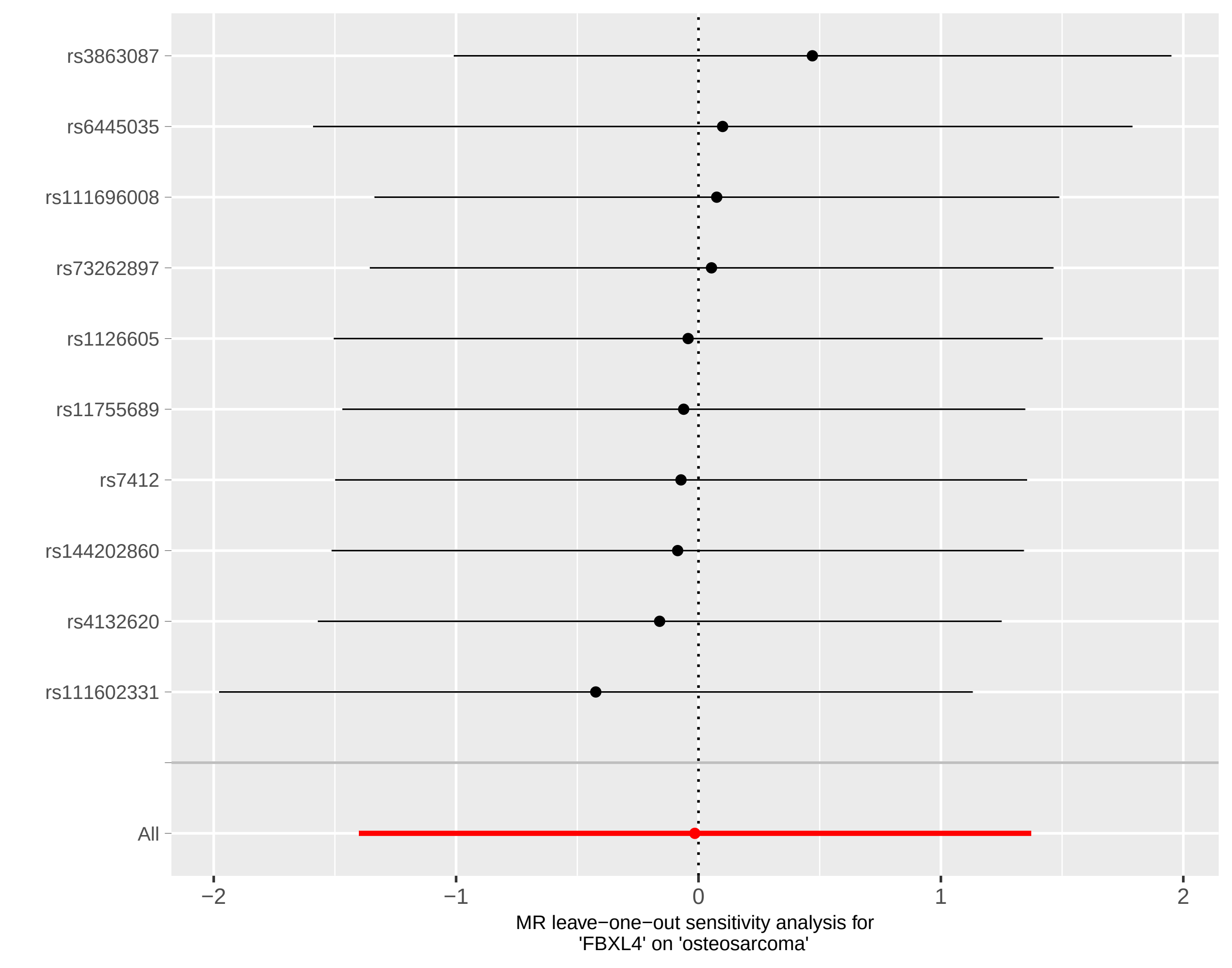
rs3863087
rs6445035
rs111696008
rs73262897
rs1126605
rs11755689
rs7412
rs144202860
rs4132620
rs111602331
All
−2
−1
0
1
2
MR leave−one−out sensitivity analysis for
'FBXL4' on 'osteosarcoma'

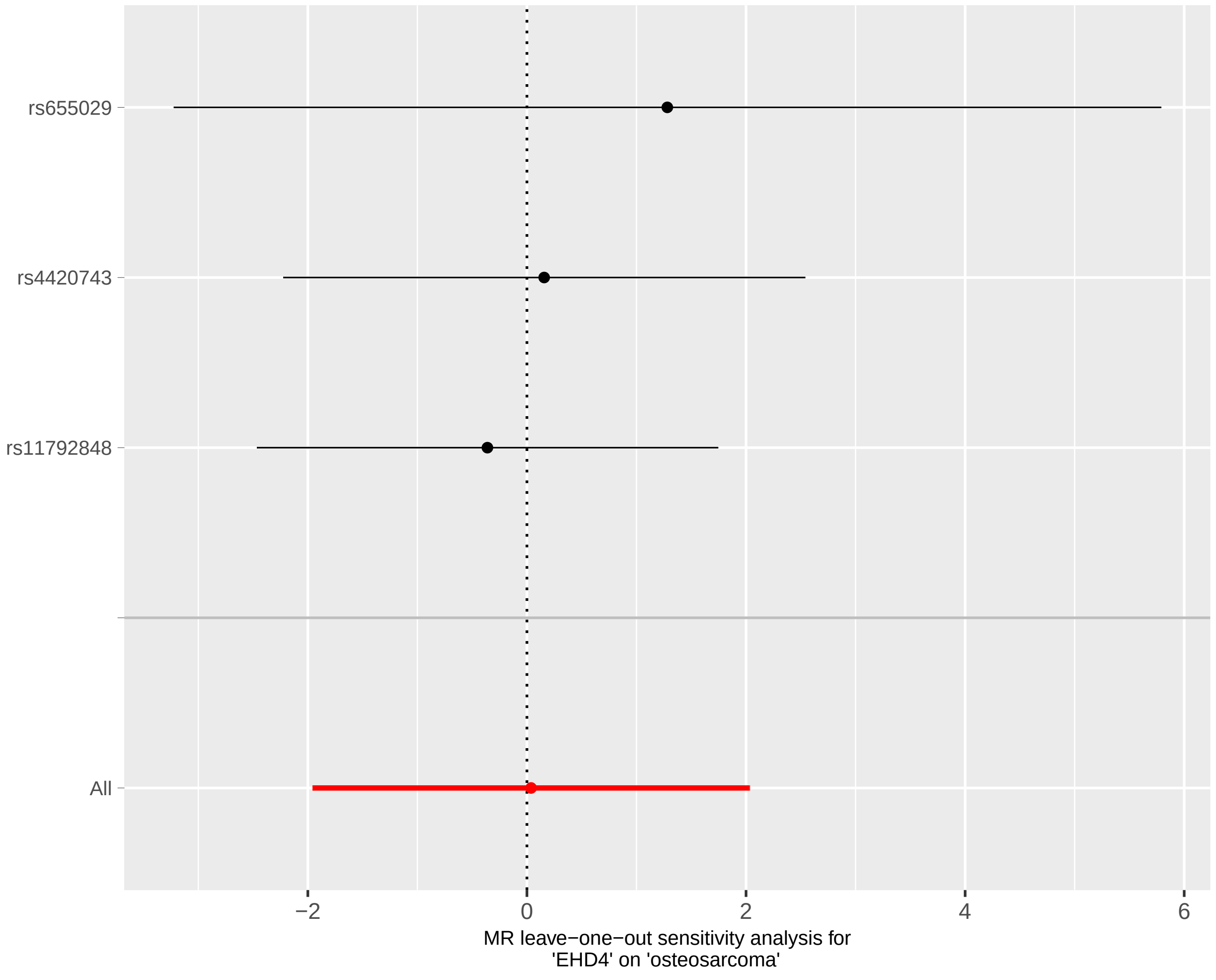
rs655029
rs4420743
rs11792848
All
−2
0
2
4
6
MR leave−one−out sensitivity analysis for
'EHD4' on 'osteosarcoma'

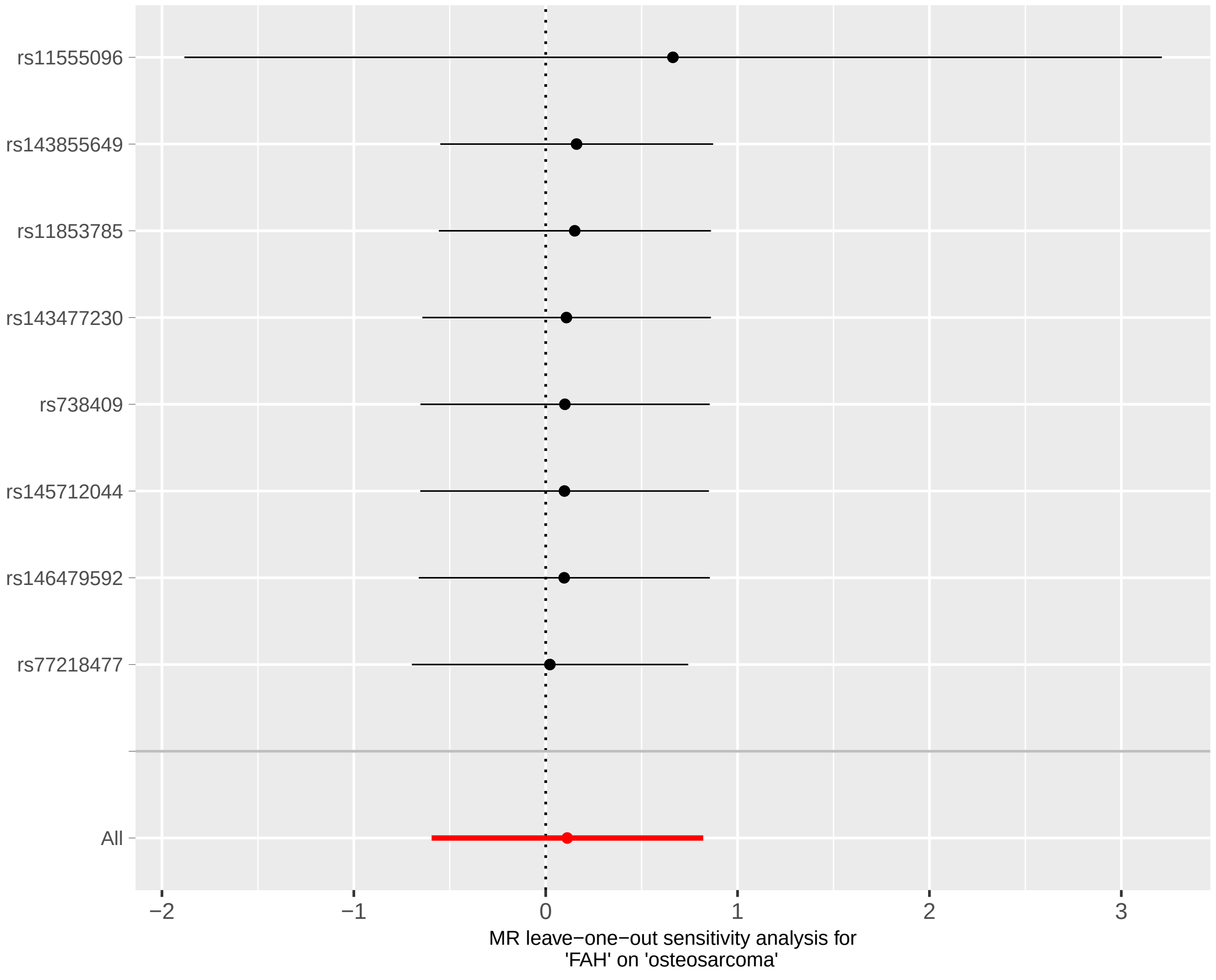
rs11555096
rs143855649
rs11853785
rs143477230
rs738409
rs145712044
rs146479592
rs77218477
All
−2
−1
0
1
2
3
MR leave−one−out sensitivity analysis for
'FAH' on 'osteosarcoma'

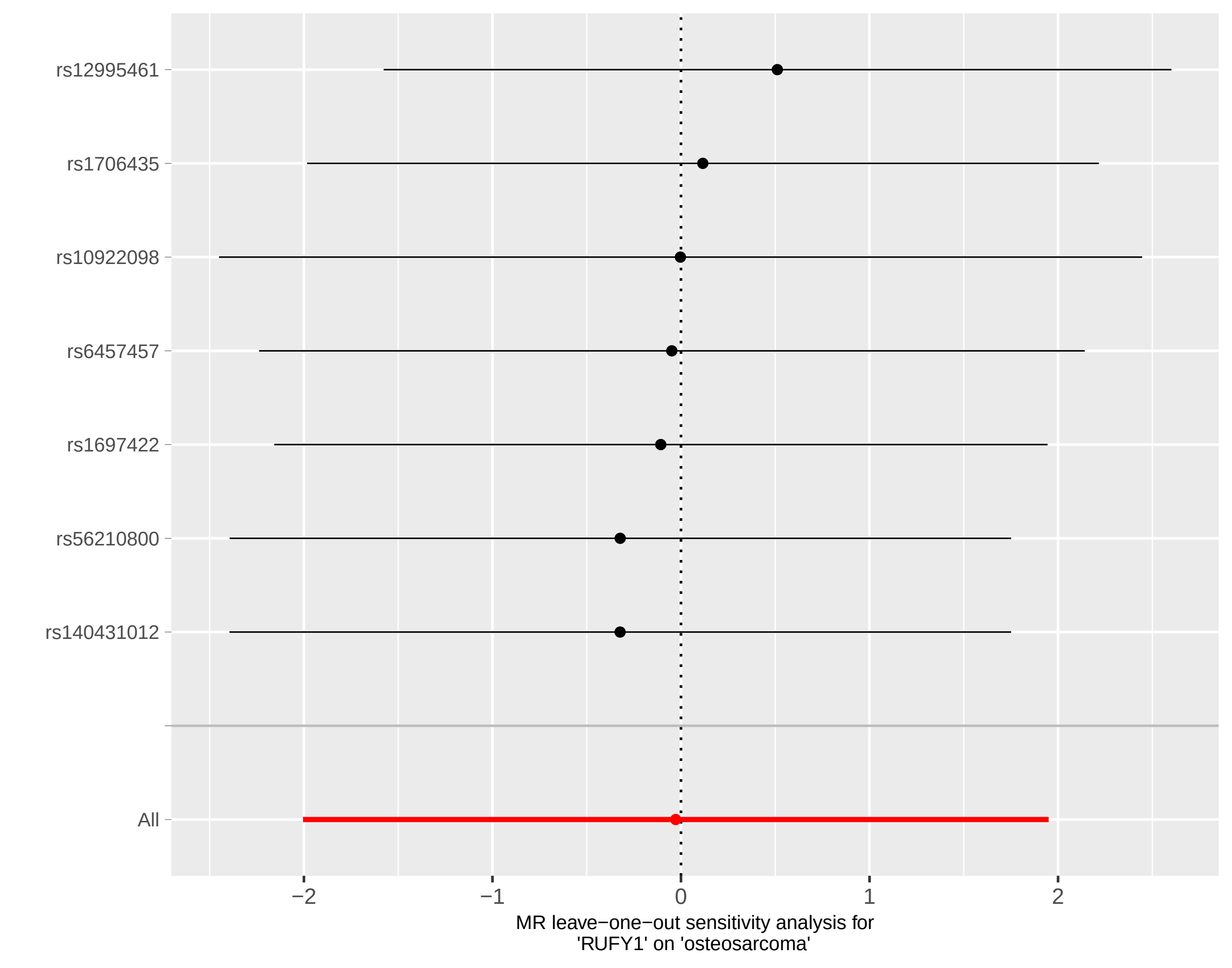
rs12995461
rs1706435
rs10922098
rs6457457
rs1697422
rs56210800
rs140431012
All
−2
−1
0
1
2
MR leave−one−out sensitivity analysis for
'RUFY1' on 'osteosarcoma'

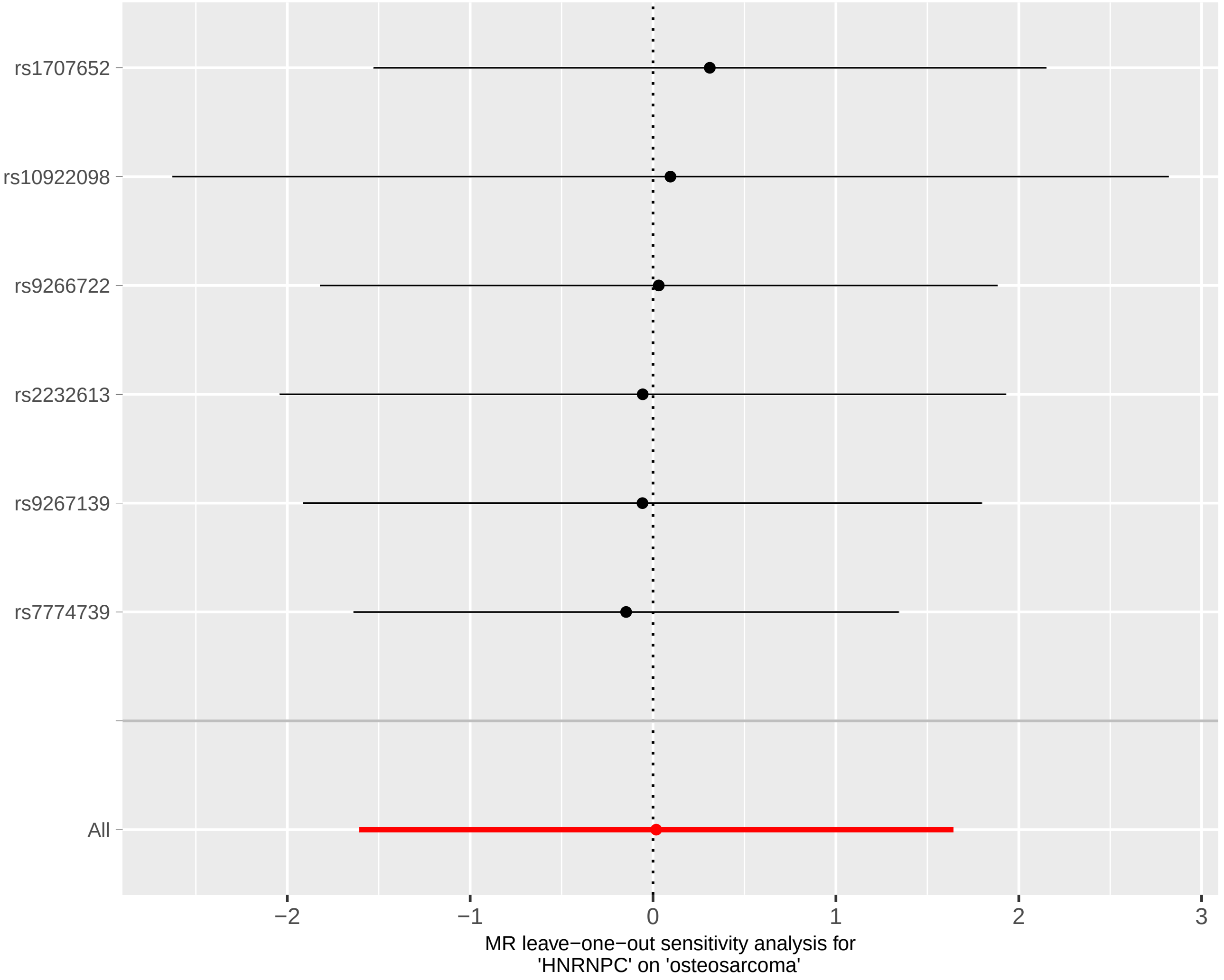

rs1707652
rs10922098
rs9266722
rs2232613
rs9267139
rs7774739
All
−2
−1
0
1
2
3
MR leave−one−out sensitivity analysis for
'HNRNPC' on 'osteosarcoma'

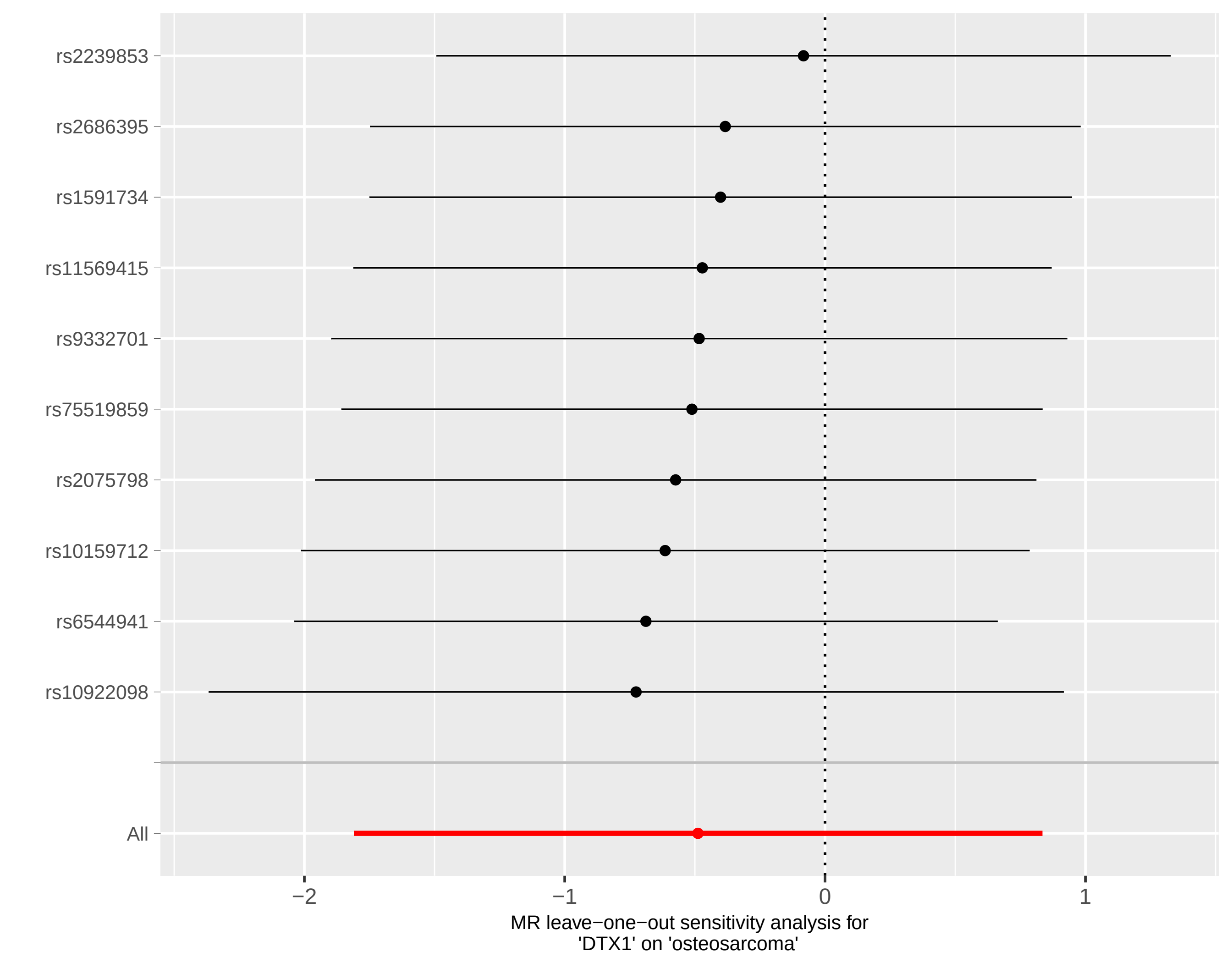
rs2239853
rs2686395
rs1591734
rs11569415
rs9332701
rs75519859
rs2075798
rs10159712
rs6544941
rs10922098
All
−2
−1
0
1
MR leave−one−out sensitivity analysis for
'DTX1' on 'osteosarcoma'

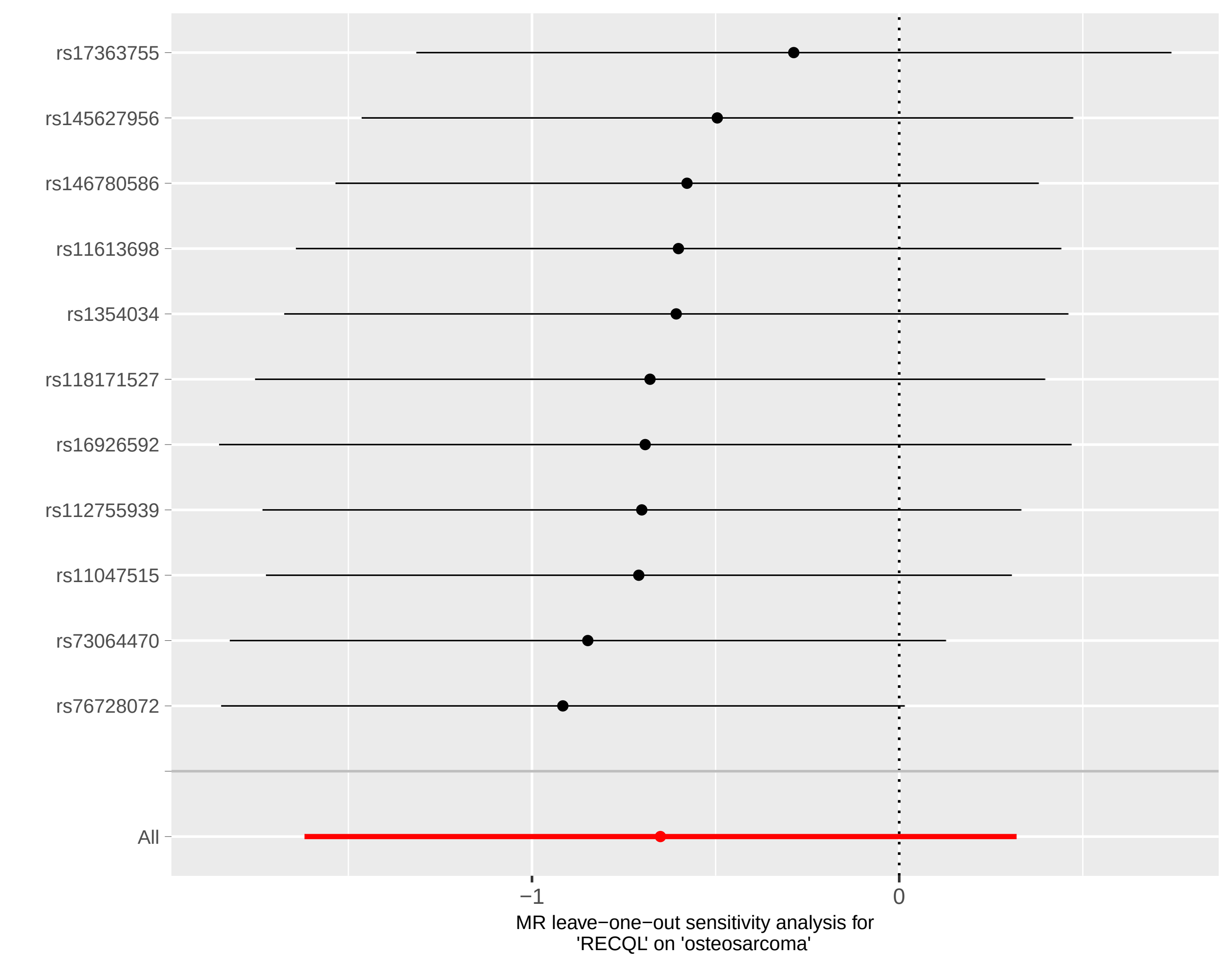

rs17363755
rs145627956
rs146780586
rs11613698
rs1354034
rs118171527
rs16926592
rs112755939
rs11047515
rs73064470
rs76728072
All
−1
0
MR leave−one−out sensitivity analysis for
'RECQL' on 'osteosarcoma'

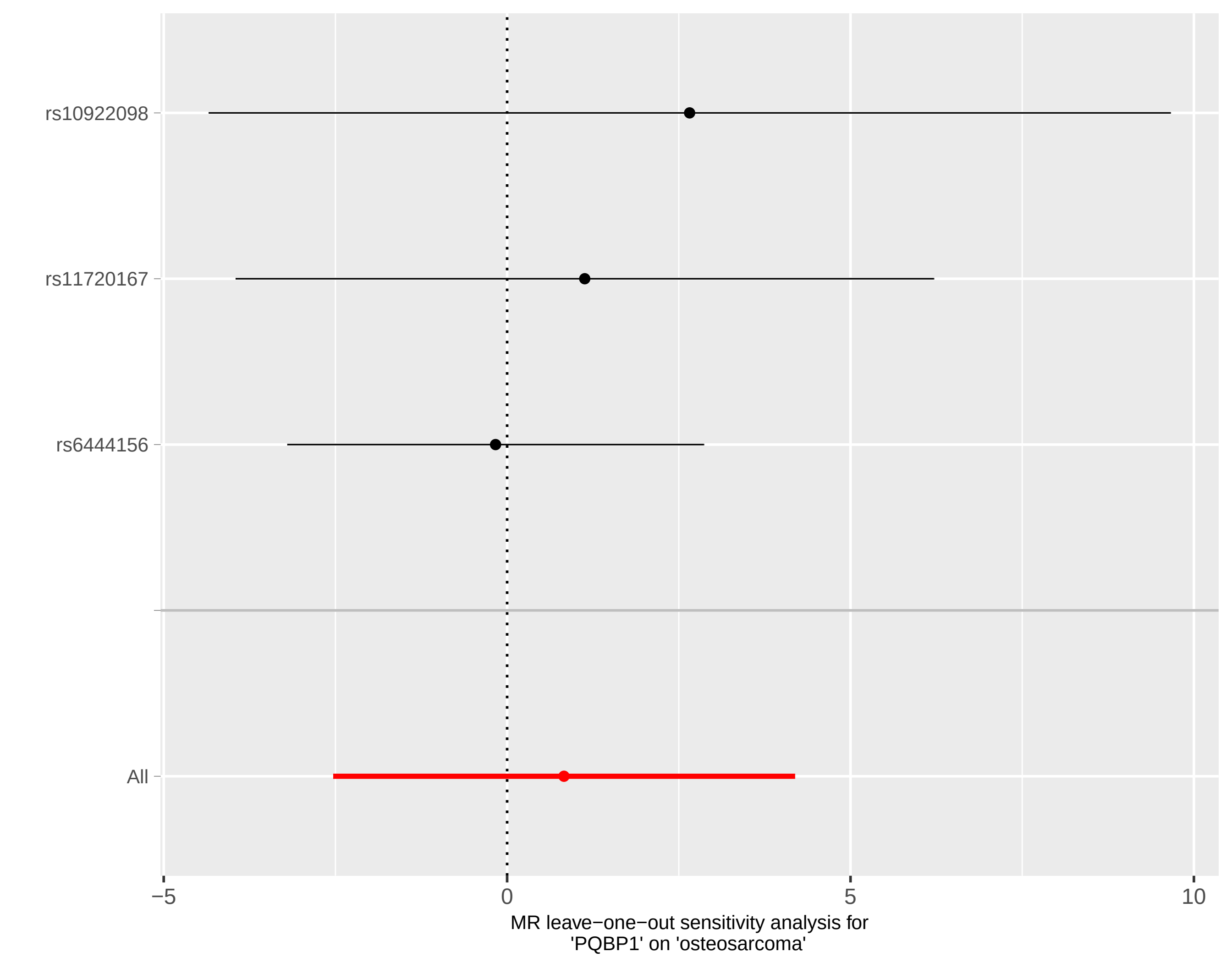
rs10922098
rs11720167
rs6444156
All
−5
0
5
10
MR leave−one−out sensitivity analysis for
'PQBP1' on 'osteosarcoma'

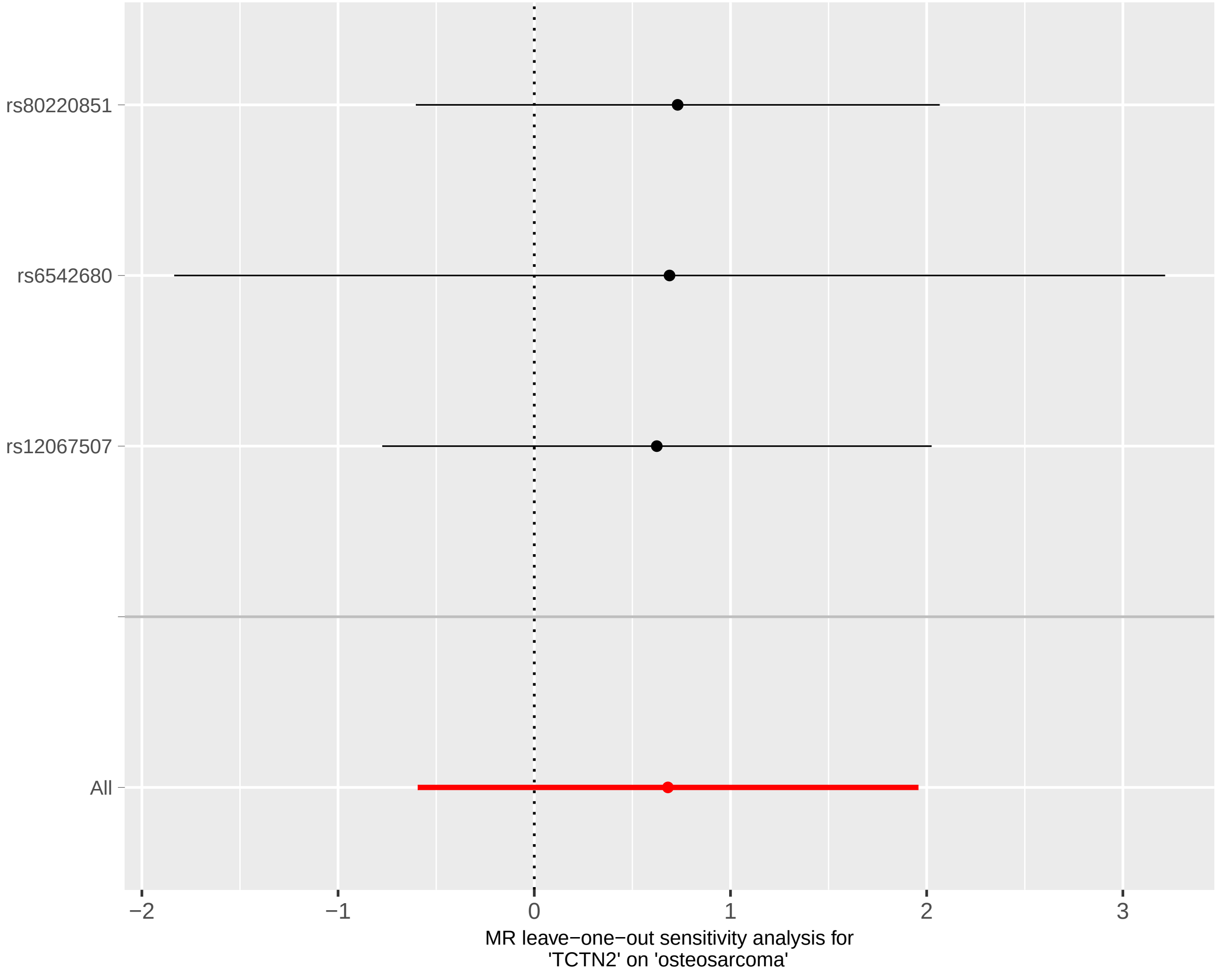
rs80220851
rs6542680
rs12067507
All
−2
−1
0
1
2
3
MR leave−one−out sensitivity analysis for
'TCTN2' on 'osteosarcoma'

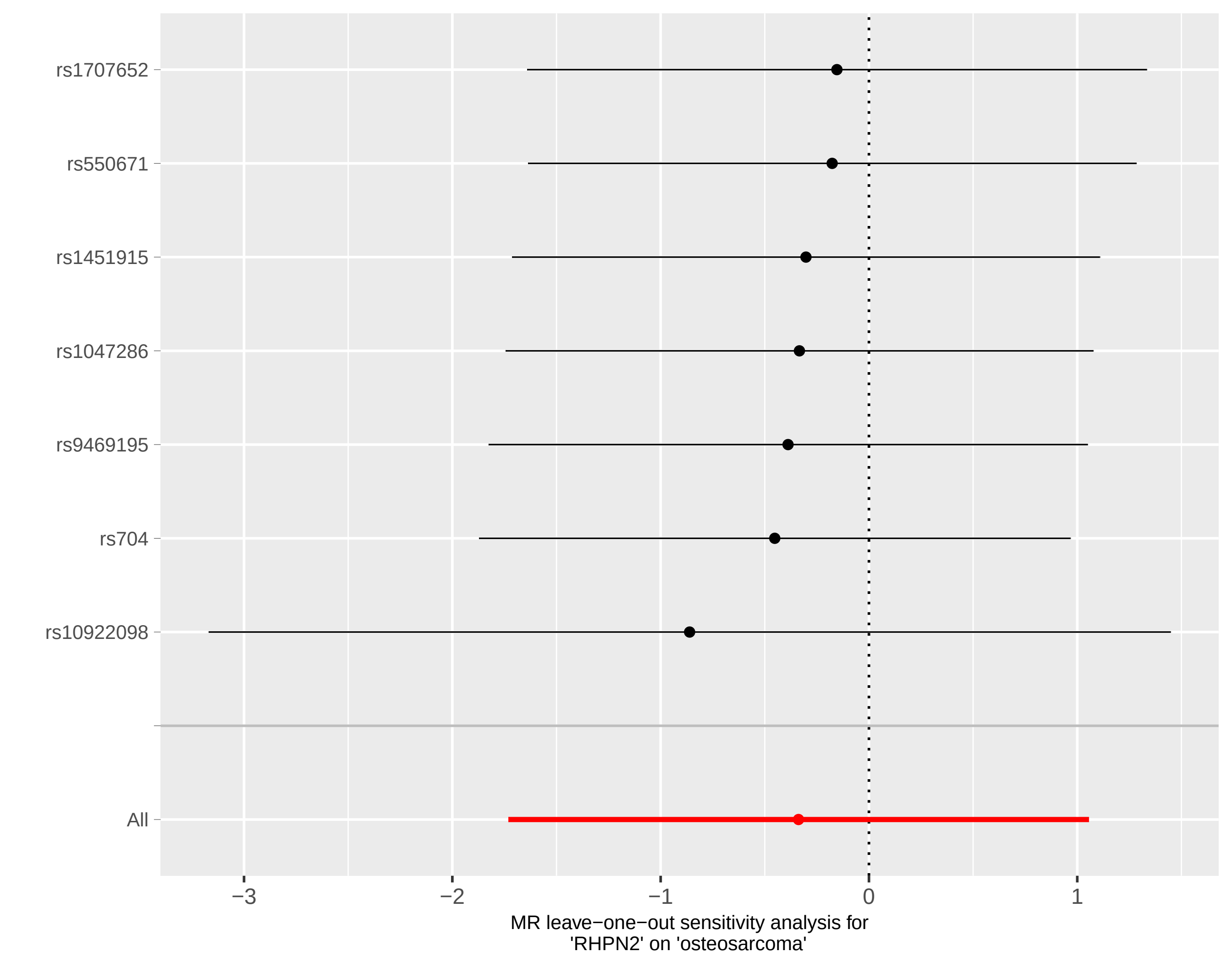
rs1707652
rs550671
rs1451915
rs1047286
rs9469195
rs704
rs10922098
All
−3
−2
−1
0
1
MR leave−one−out sensitivity analysis for
'RHPN2' on 'osteosarcoma'

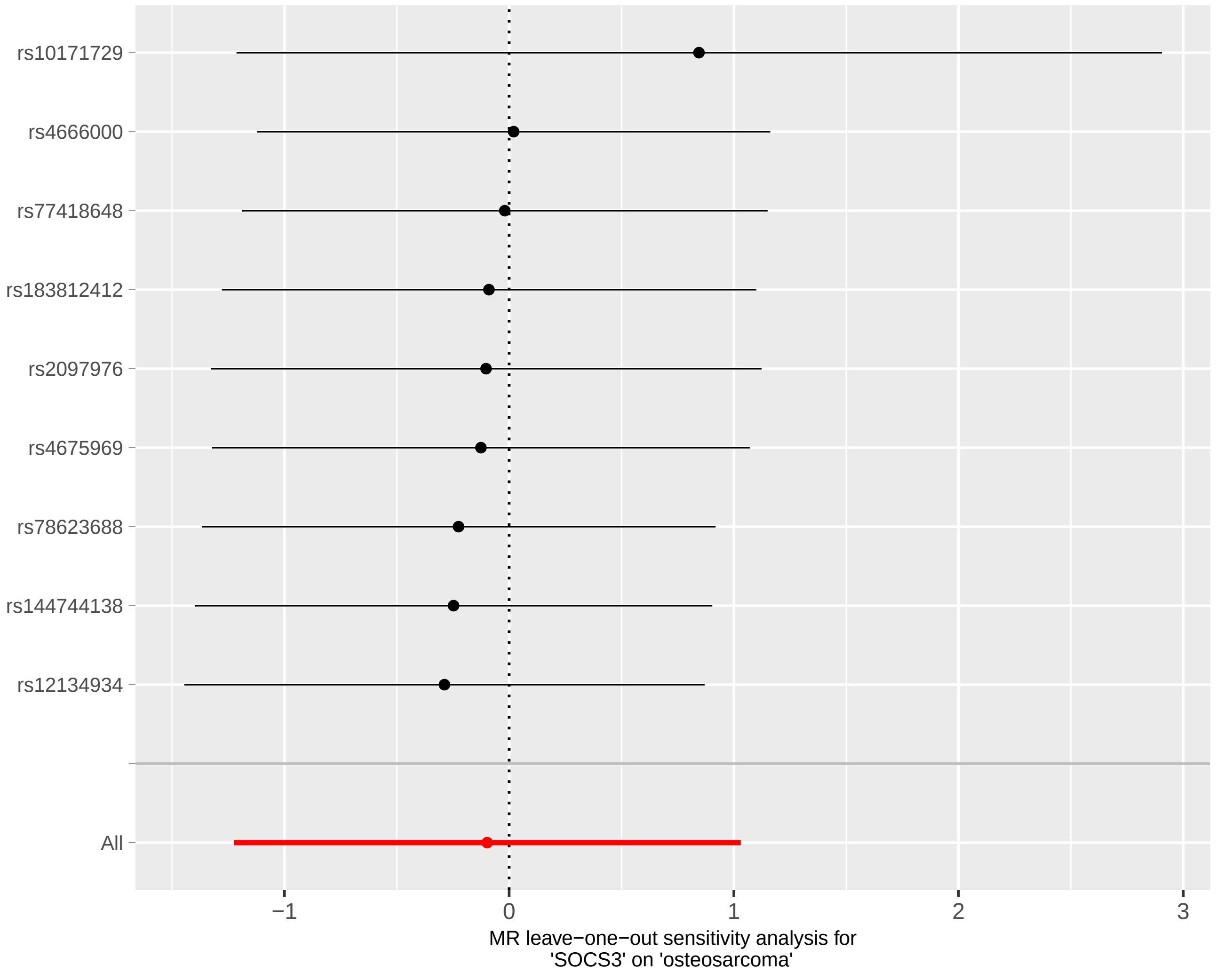
rs10171729
rs4666000
rs77418648
rs183812412
rs2097976
rs4675969
rs78623688
rs144744138
rs12134934
All
−1
0
1
2
3
MR leave−one−out sensitivity analysis for
'SOCS3' on 'osteosarcoma'

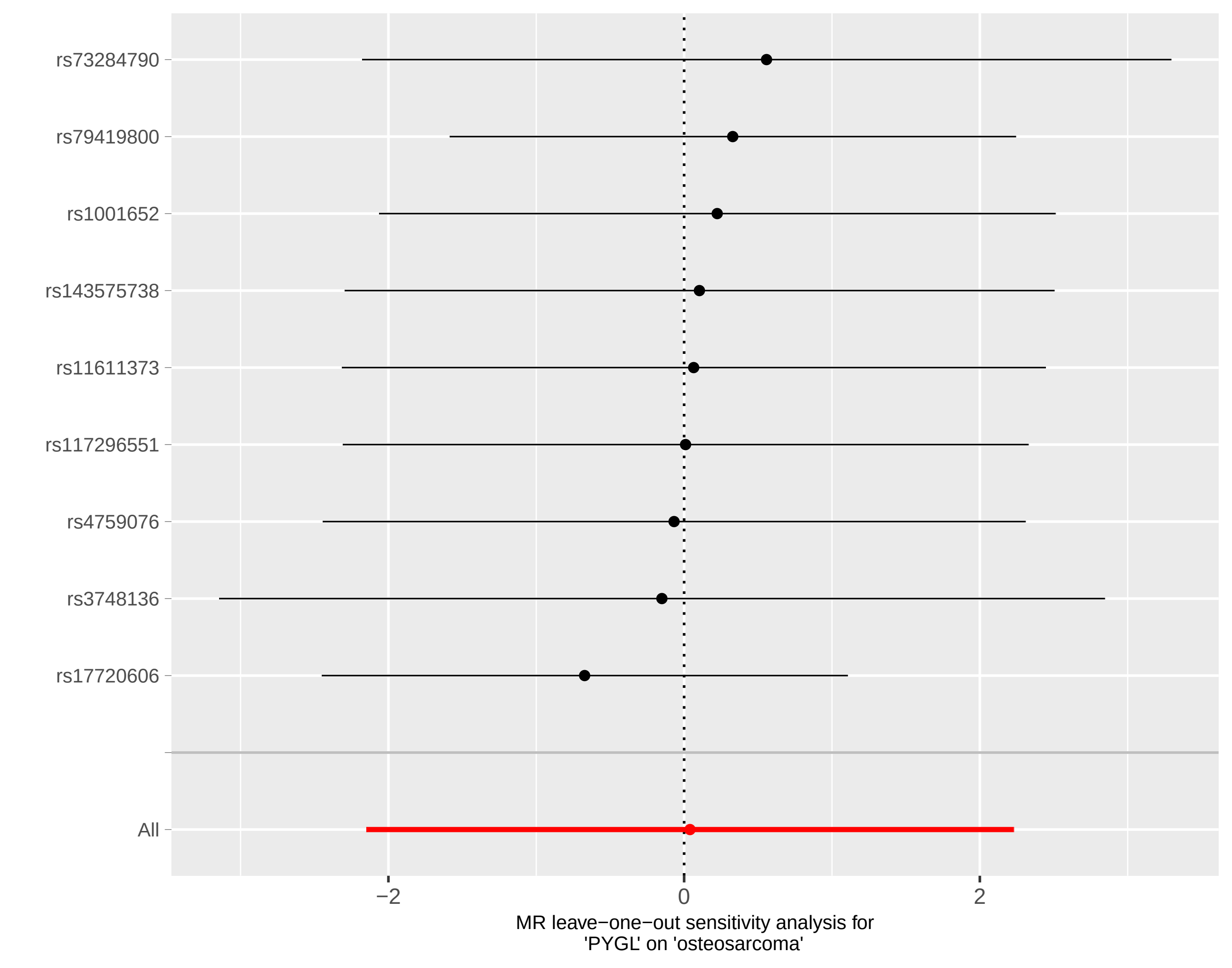

rs73284790
rs79419800
rs1001652
rs143575738
rs11611373
rs117296551
rs4759076
rs3748136
rs17720606
All
−2
0
2
MR leave−one−out sensitivity analysis for
'PYGL' on 'osteosarcoma'

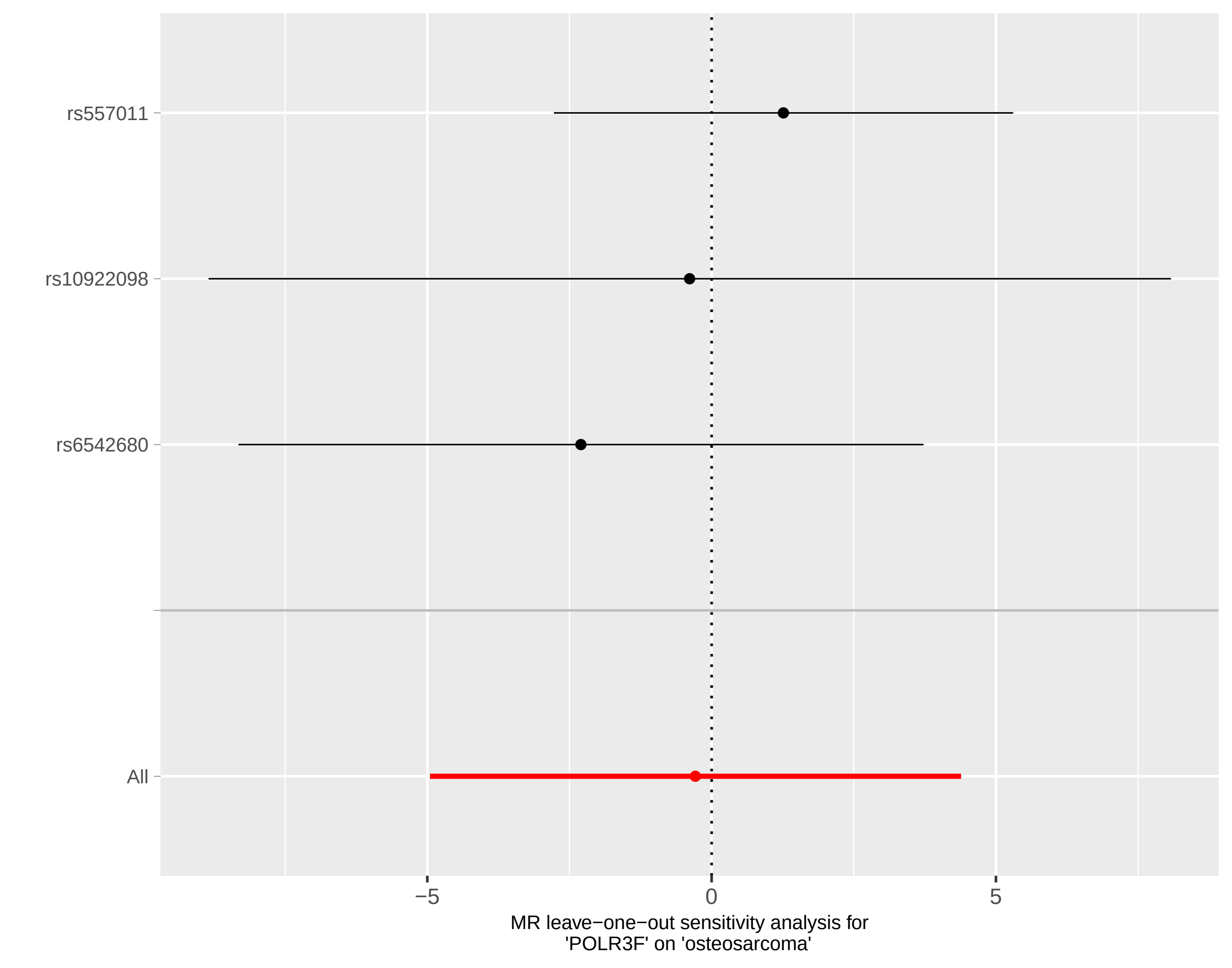

rs557011
rs10922098
rs6542680
All
−5
0
5
MR leave−one−out sensitivity analysis for
'POLR3F' on 'osteosarcoma'

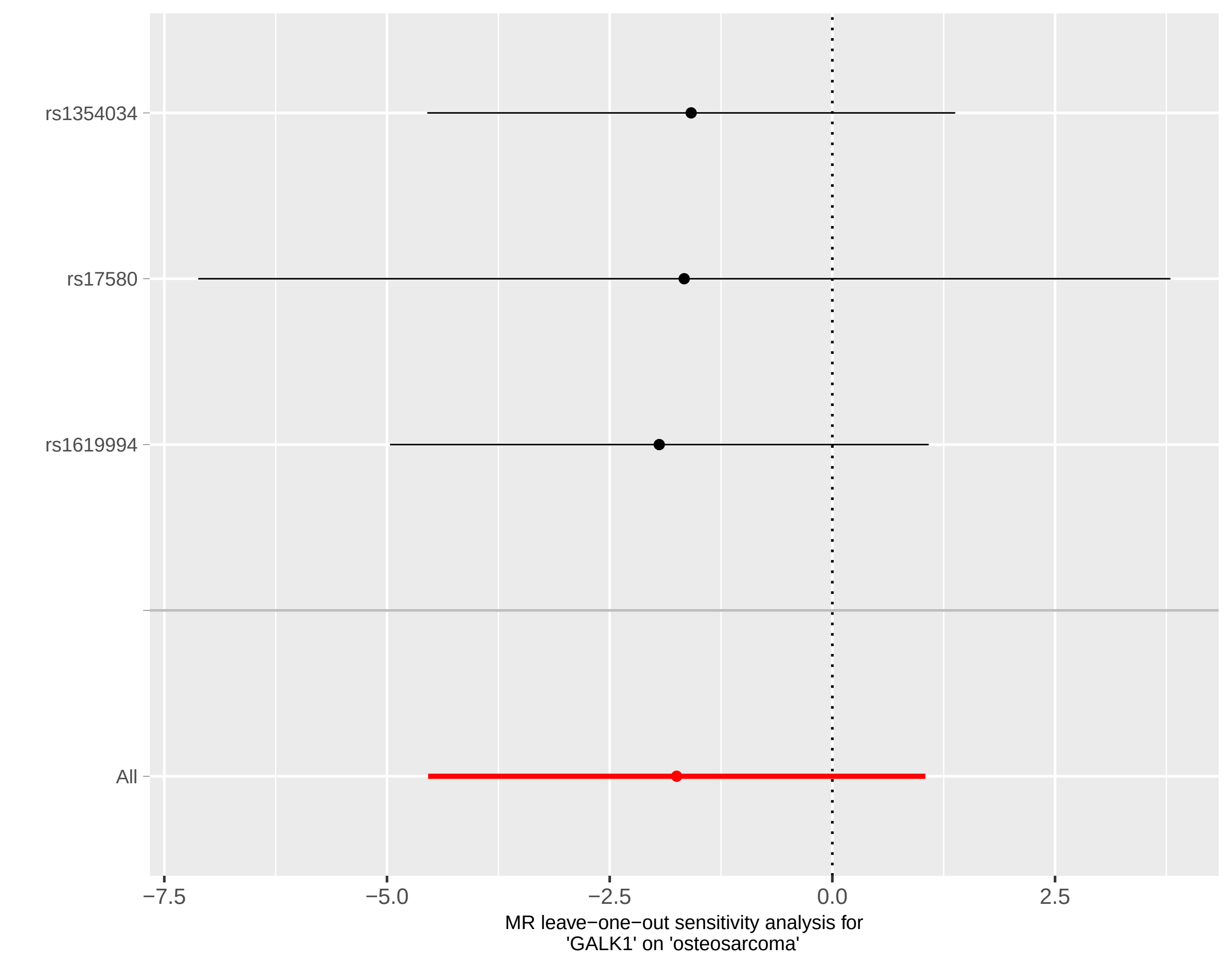
rs1354034
rs17580
rs1619994
All
−7.5
−5.0
−2.5
0.0
2.5
MR leave−one−out sensitivity analysis for
'GALK1' on 'osteosarcoma'

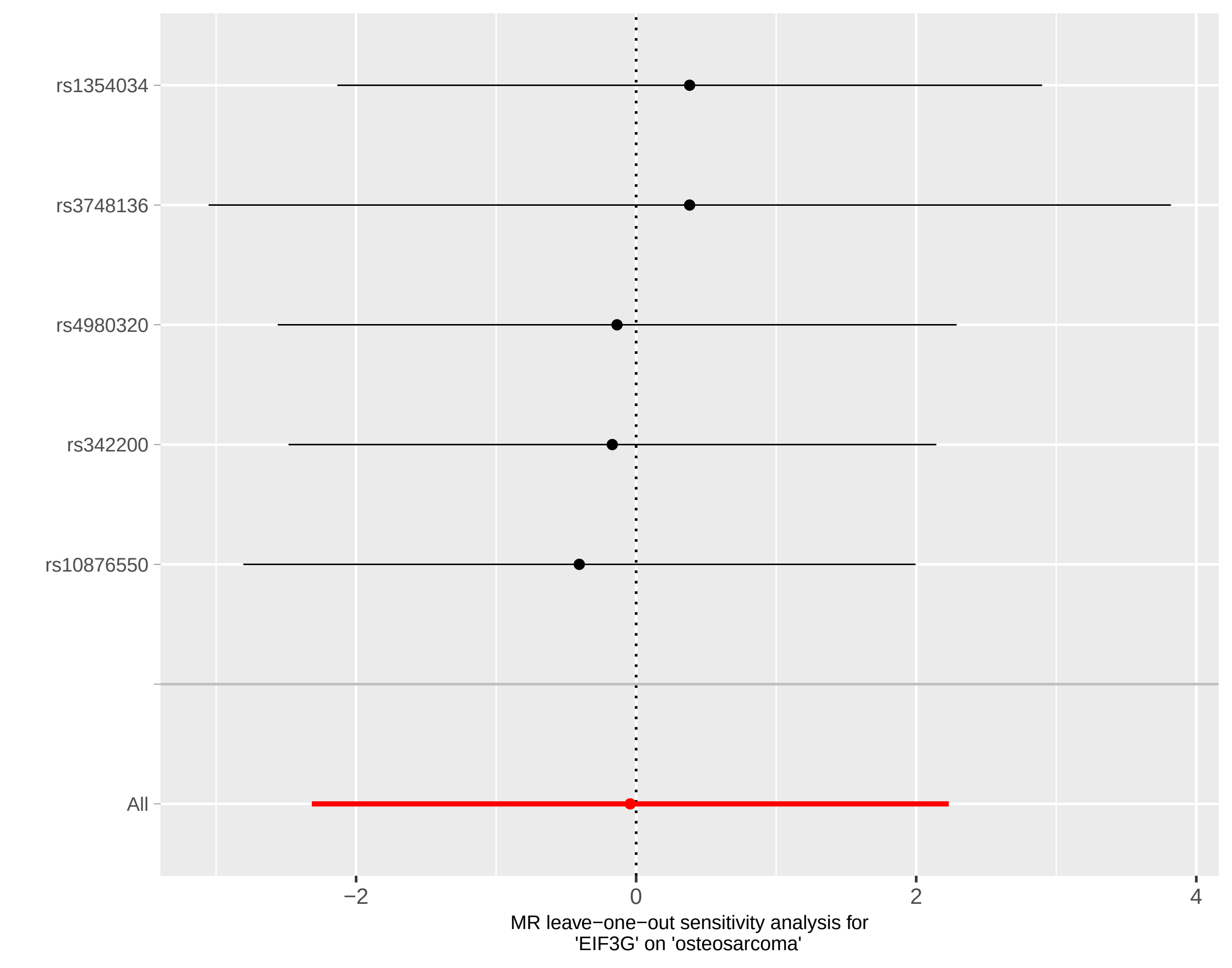

rs1354034
rs3748136
rs4980320
rs342200
rs10876550
All
−2
0
2
4
MR leave−one−out sensitivity analysis for
'EIF3G' on 'osteosarcoma'

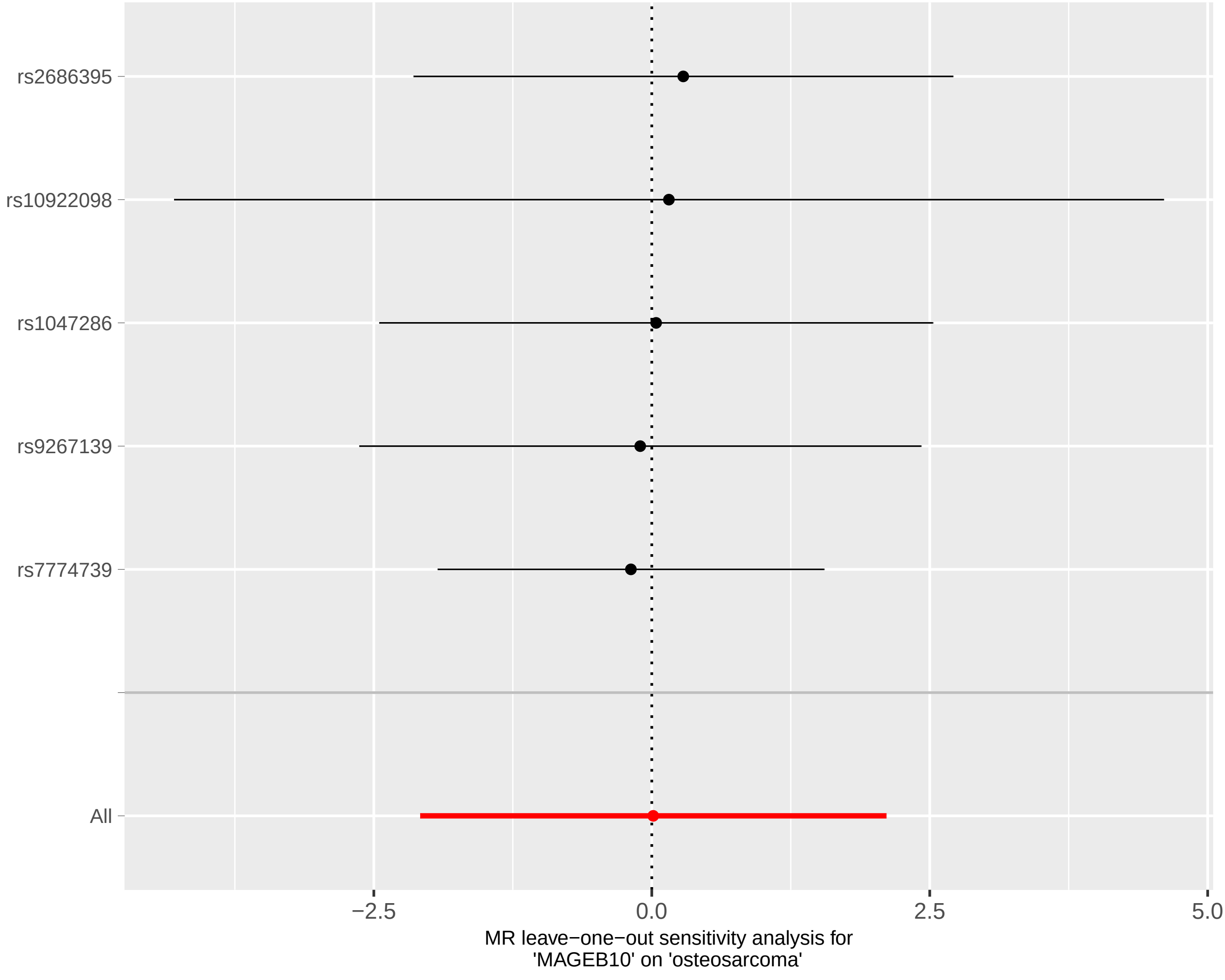
rs2686395
rs10922098
rs1047286
rs9267139
rs7774739
All
−2.5
0.0
2.5
5.0
MR leave−one−out sensitivity analysis for
'MAGEB10' on 'osteosarcoma'

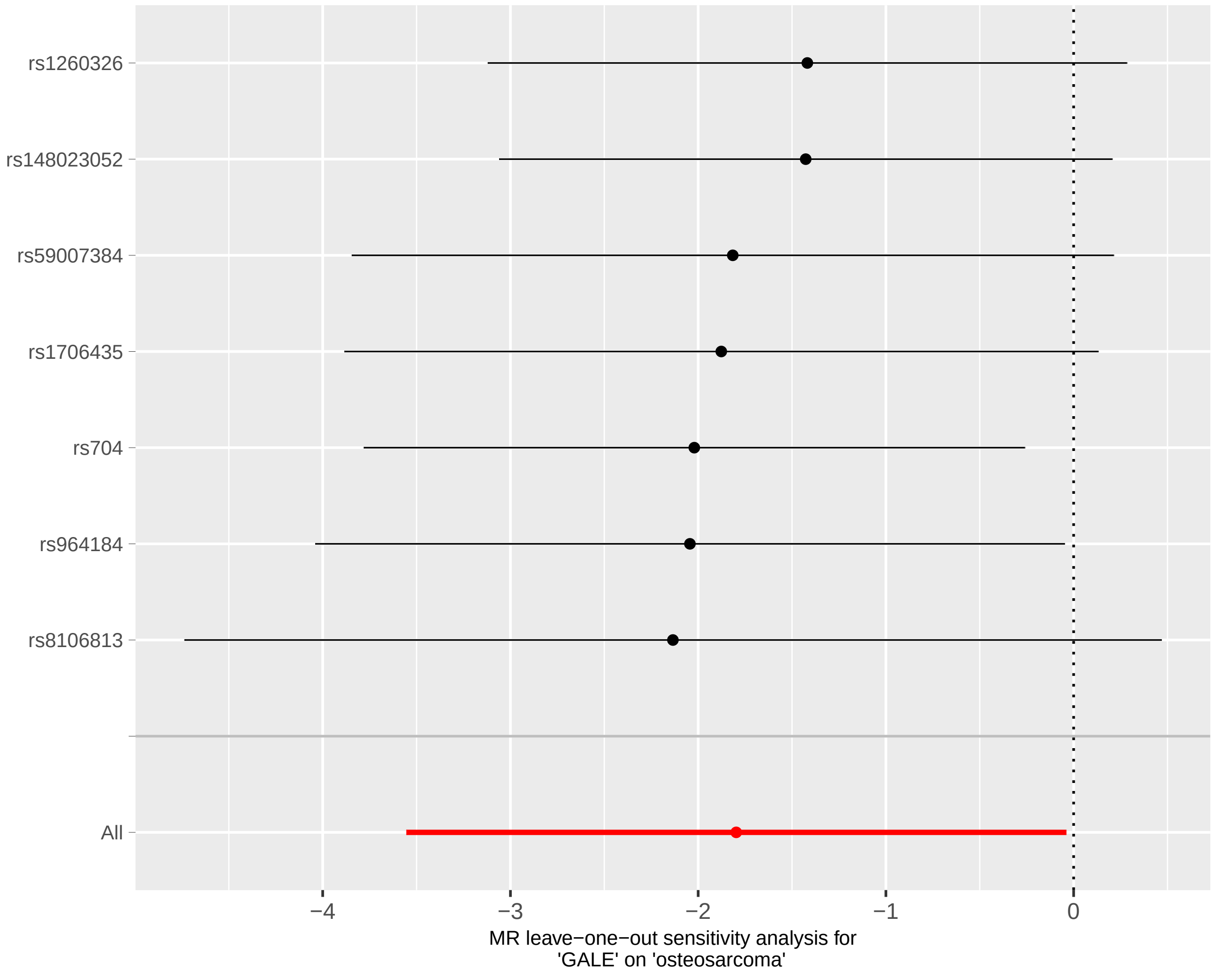

rs1260326
rs148023052
rs59007384
rs1706435
rs704
rs964184
rs8106813
All
−4
−3
−2
−1
0
MR leave−one−out sensitivity analysis for
'GALE' on 'osteosarcoma'

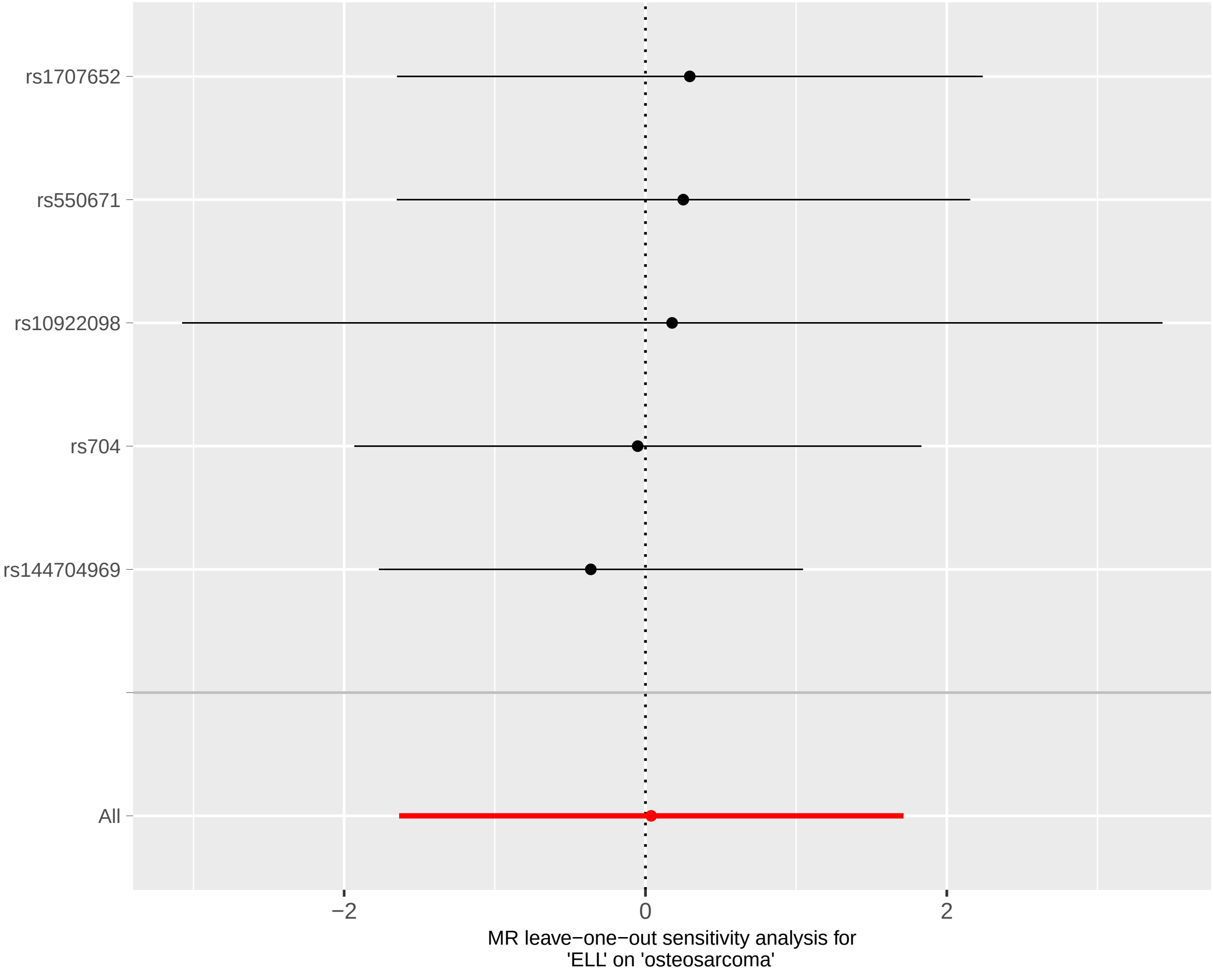
rs1707652
rs550671
rs10922098
rs704
rs144704969
All
−2
0
2
MR leave−one−out sensitivity analysis for
'ELL' on 'osteosarcoma'

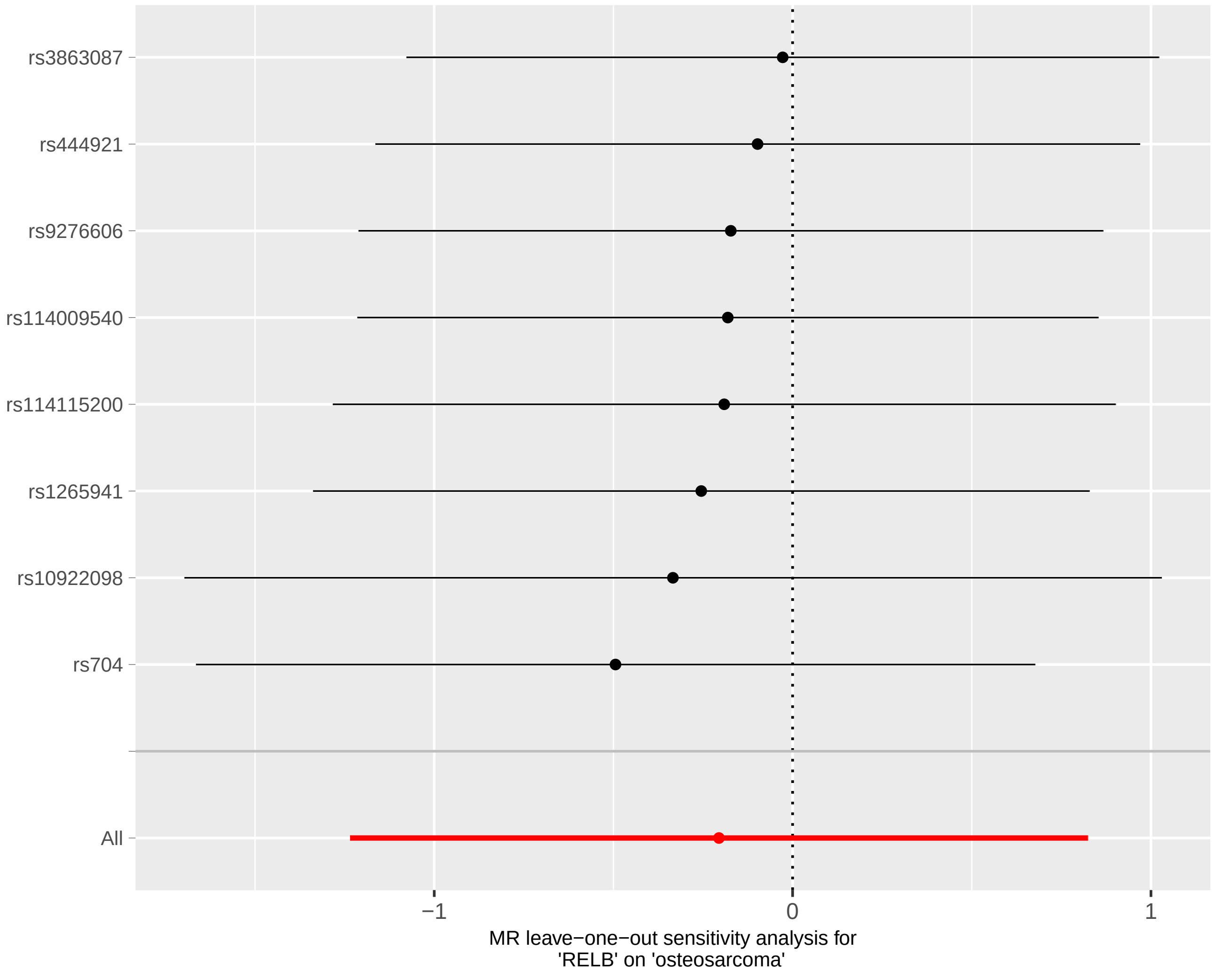
rs3863087
rs444921
rs9276606
rs114009540
rs114115200
rs1265941
rs10922098
rs704
All
−1
0
1
MR leave−one−out sensitivity analysis for
'RELB' on 'osteosarcoma'

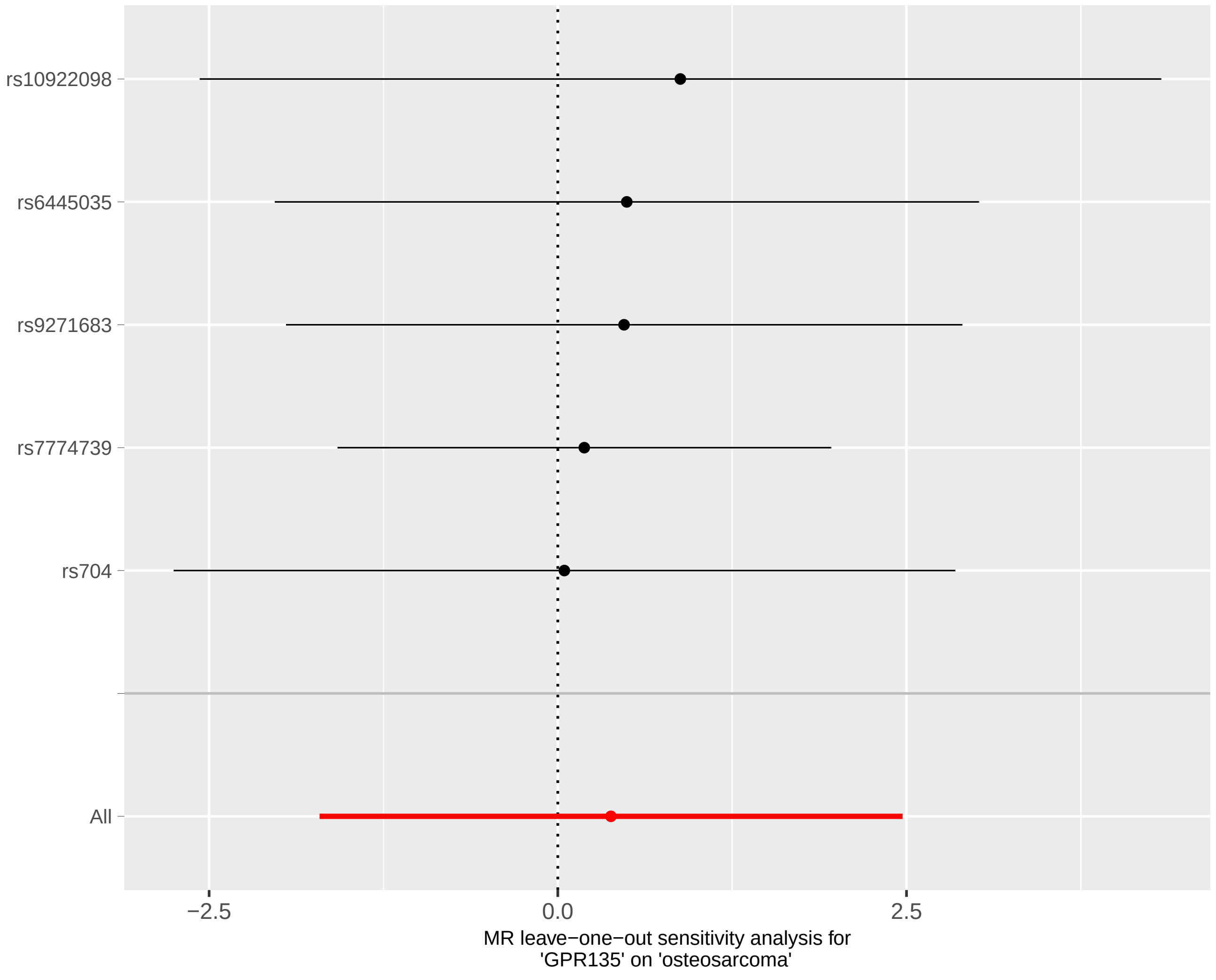
rs10922098
rs6445035
rs9271683
rs7774739
rs704
All
−2.5
0.0
2.5
MR leave−one−out sensitivity analysis for
'GPR135' on 'osteosarcoma'

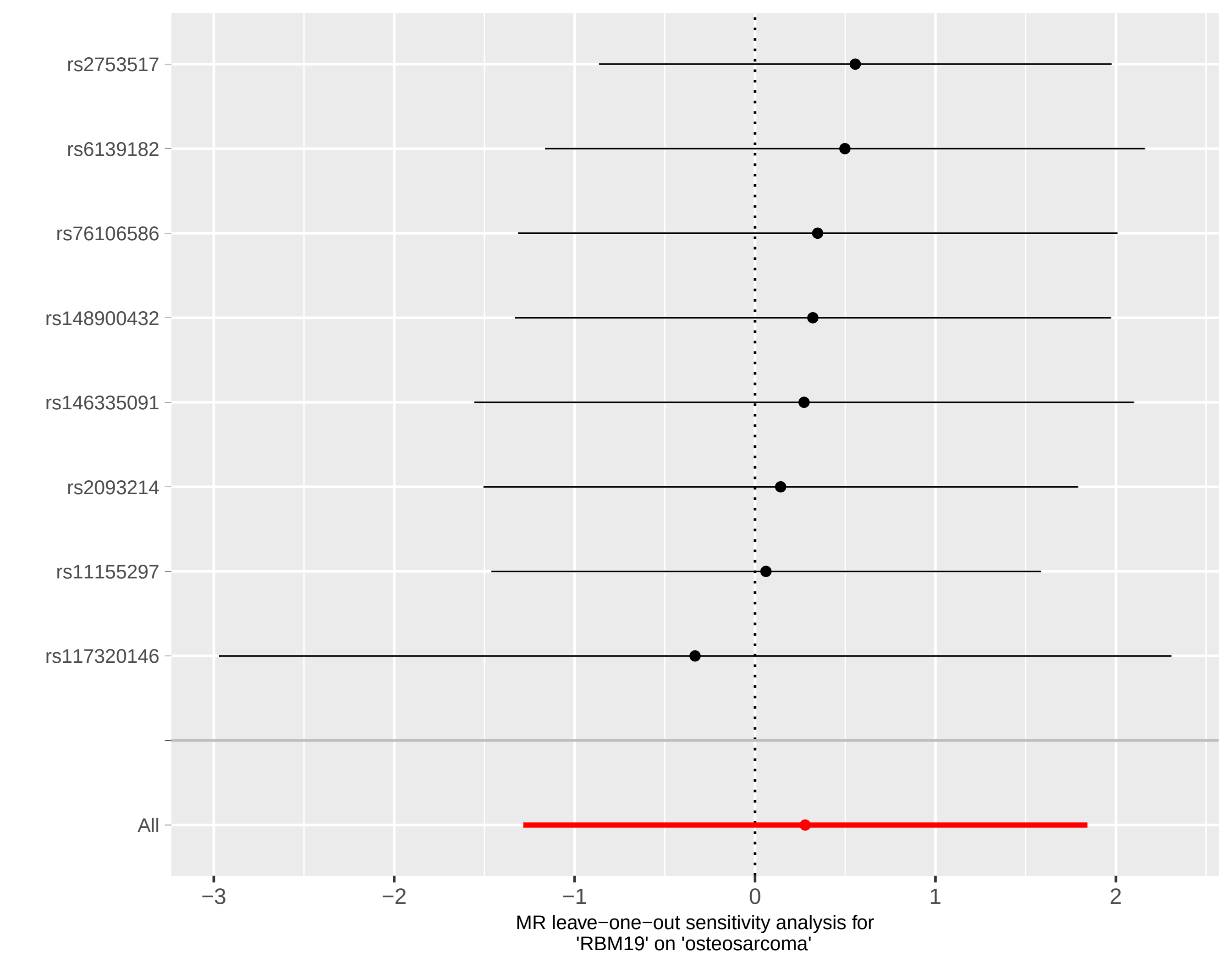

rs2753517
rs6139182
rs76106586
rs148900432
rs146335091
rs2093214
rs11155297
rs117320146
All
−3
−2
−1
0
1
2
MR leave−one−out sensitivity analysis for
'RBM19' on 'osteosarcoma'

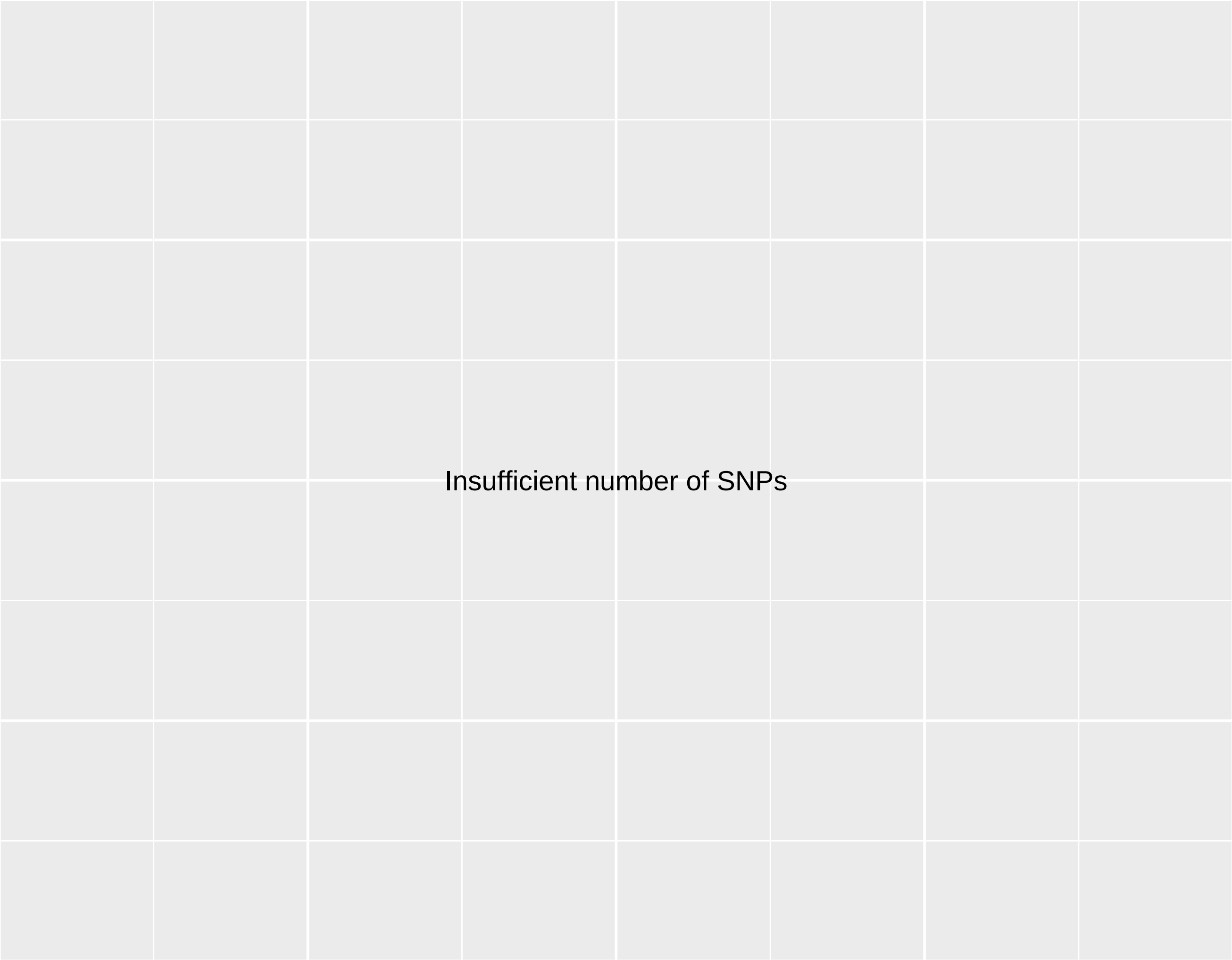
Insufficient number of SNPs

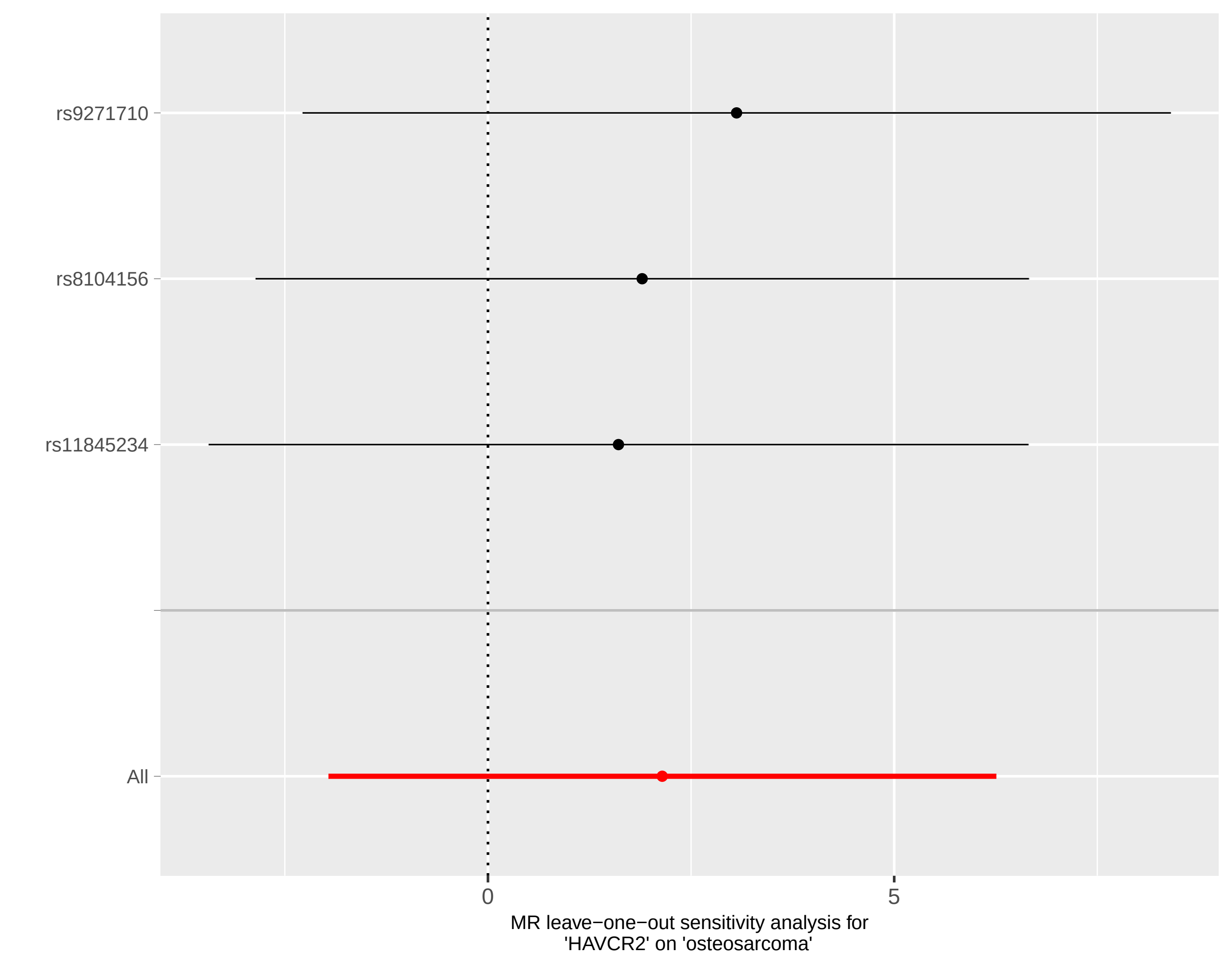
rs9271710
rs8104156
rs11845234
All
0
5
MR leave−one−out sensitivity analysis for
'HAVCR2' on 'osteosarcoma'

Insufficient number of SNPs

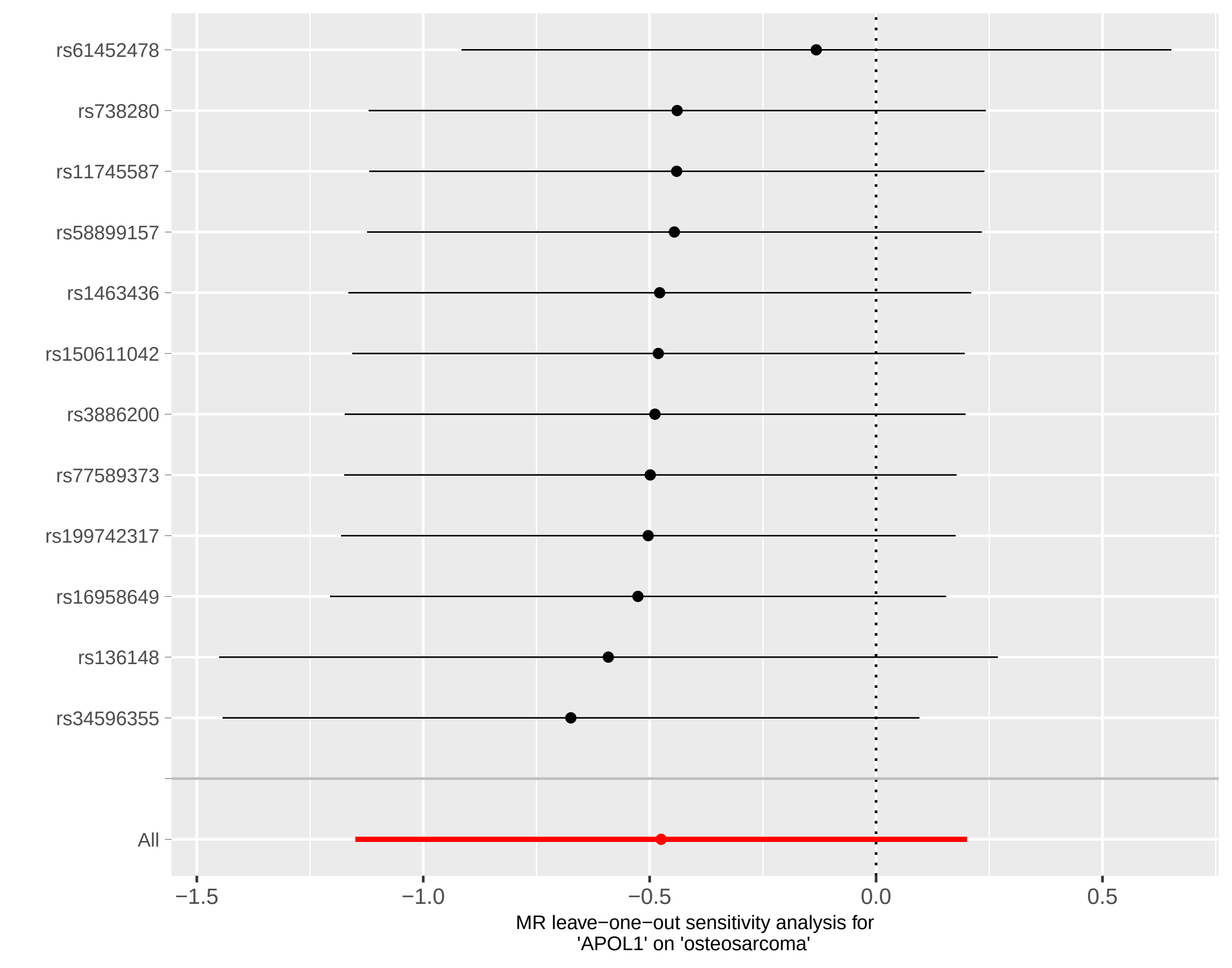
rs61452478
rs738280
rs11745587
rs58899157
rs1463436
rs150611042
rs3886200
rs77589373
rs199742317
rs16958649
rs136148
rs34596355
All
−1.5
−1.0
−0.5
0.0
0.5
MR leave−one−out sensitivity analysis for
'APOL1' on 'osteosarcoma'

Insufficient number of SNPs

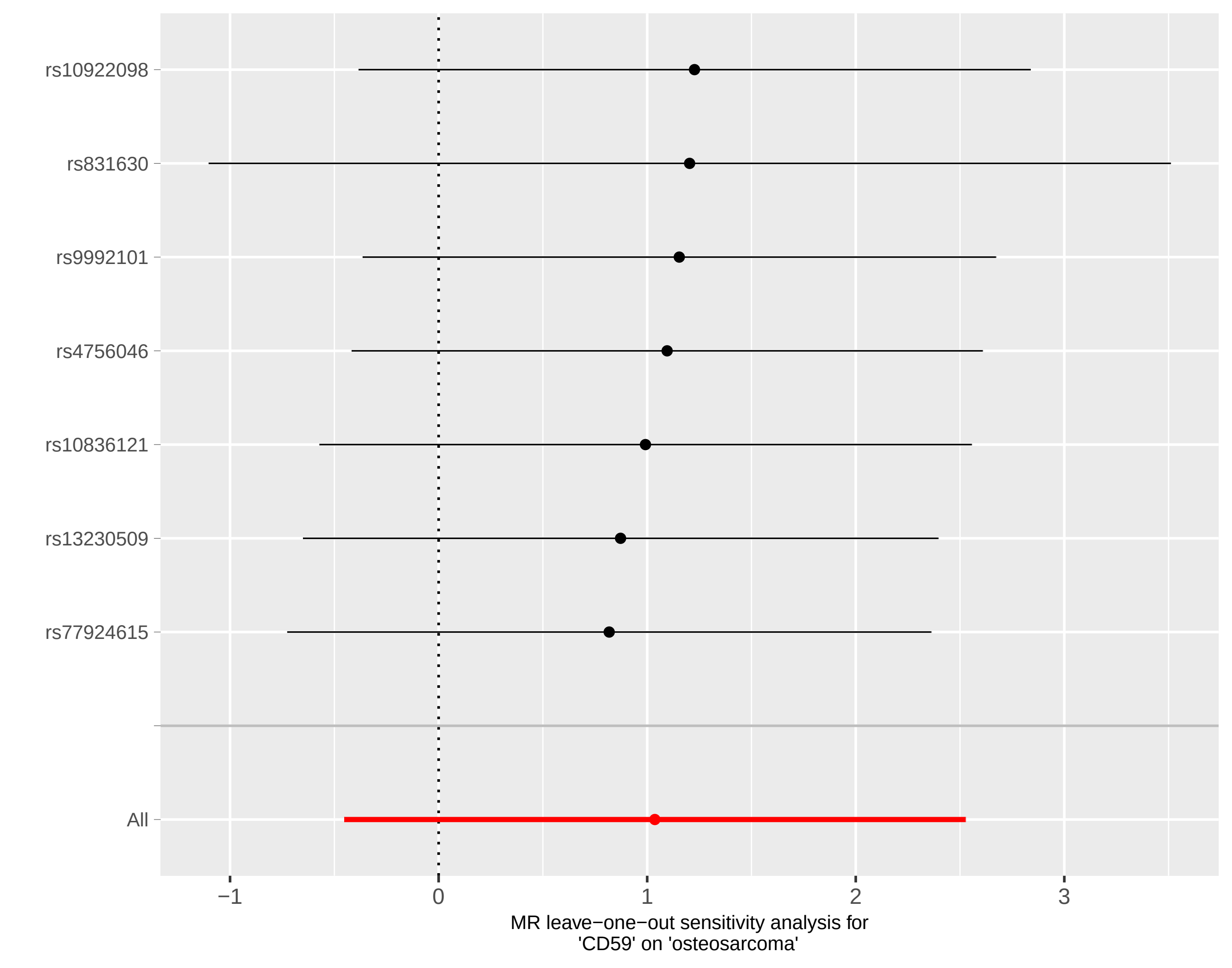
rs10922098
rs831630
rs9992101
rs4756046
rs10836121
rs13230509
rs77924615
All
−1
0
1
2
3
MR leave−one−out sensitivity analysis for
'CD59' on 'osteosarcoma'

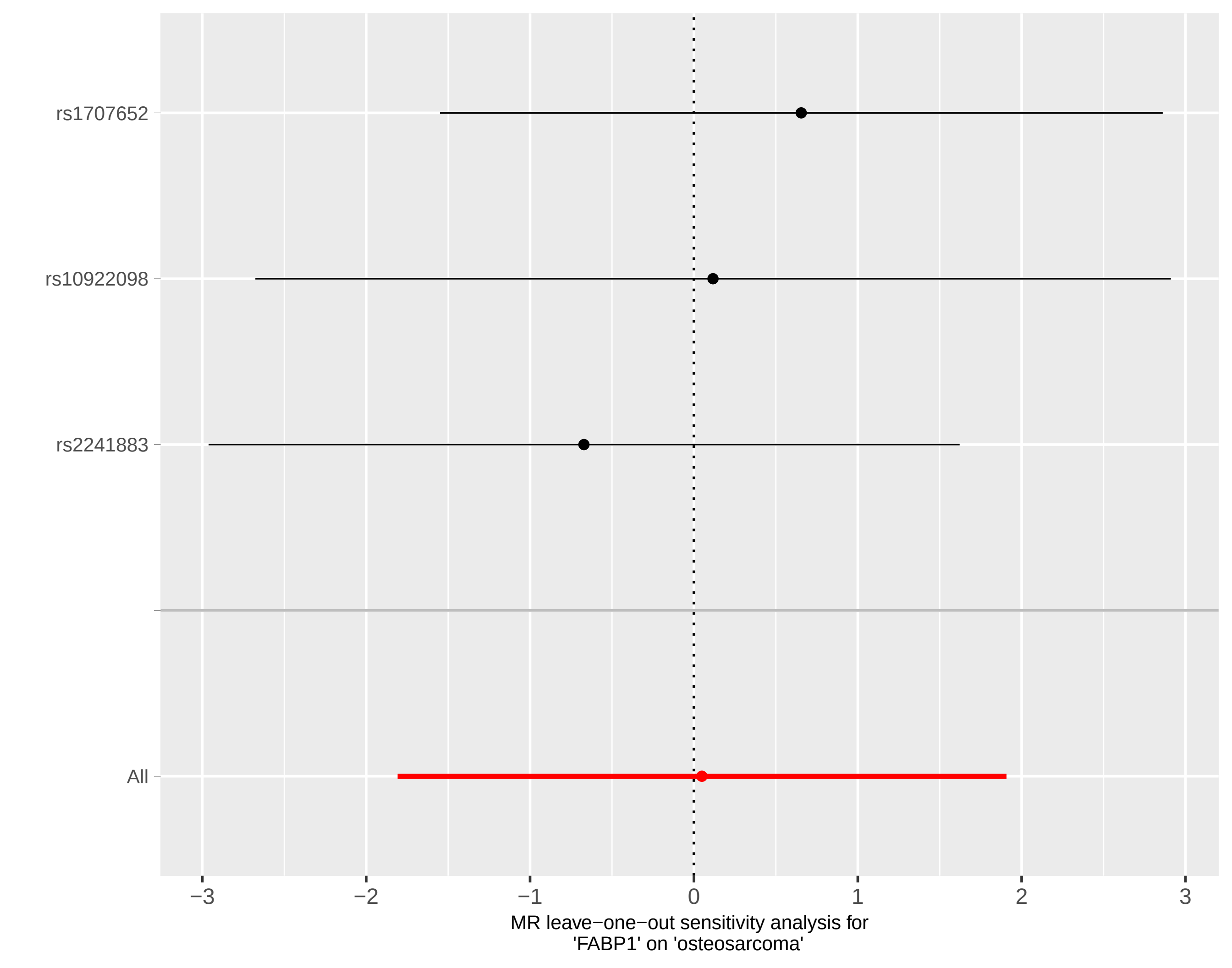
rs1707652
rs10922098
rs2241883
All
−3
−2
−1
0
1
2
3
MR leave−one−out sensitivity analysis for
'FABP1' on 'osteosarcoma'

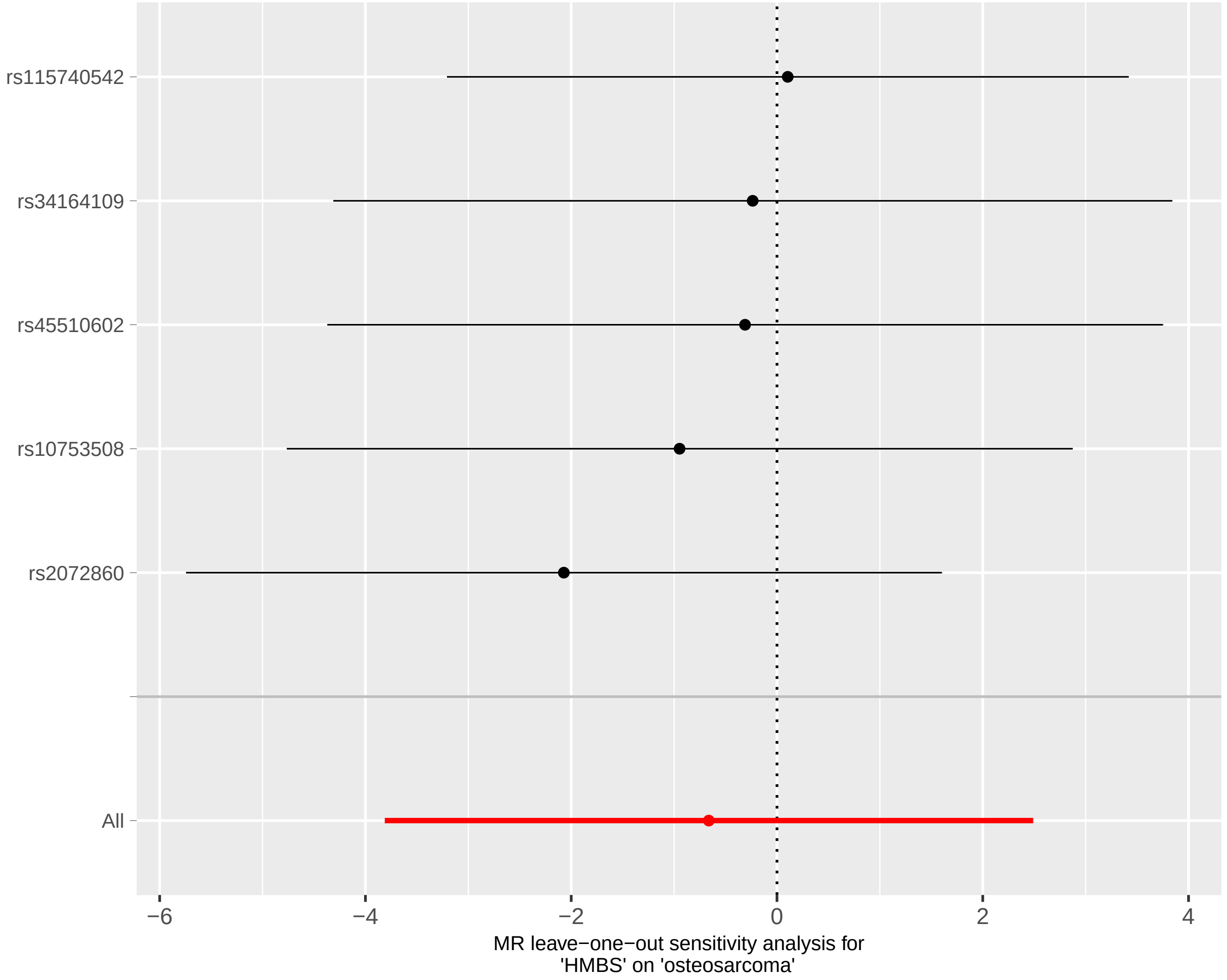

rs115740542
rs34164109
rs45510602
rs10753508
rs2072860
All
−6
−4
−2
0
2
4
MR leave−one−out sensitivity analysis for
'HMBS' on 'osteosarcoma'

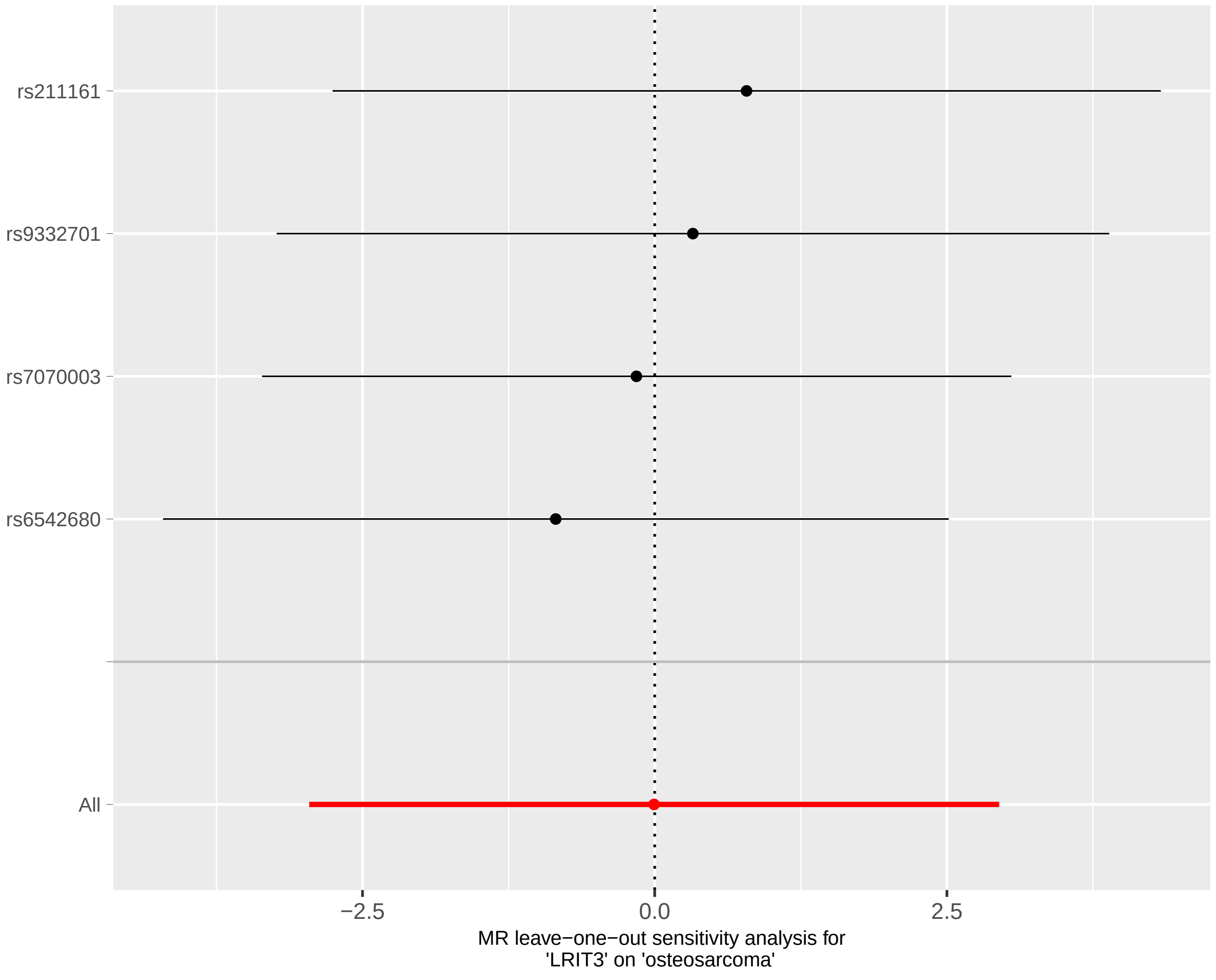
rs211161
rs9332701
rs7070003
rs6542680
All
−2.5
0.0
2.5
MR leave−one−out sensitivity analysis for
'LRIT3' on 'osteosarcoma'

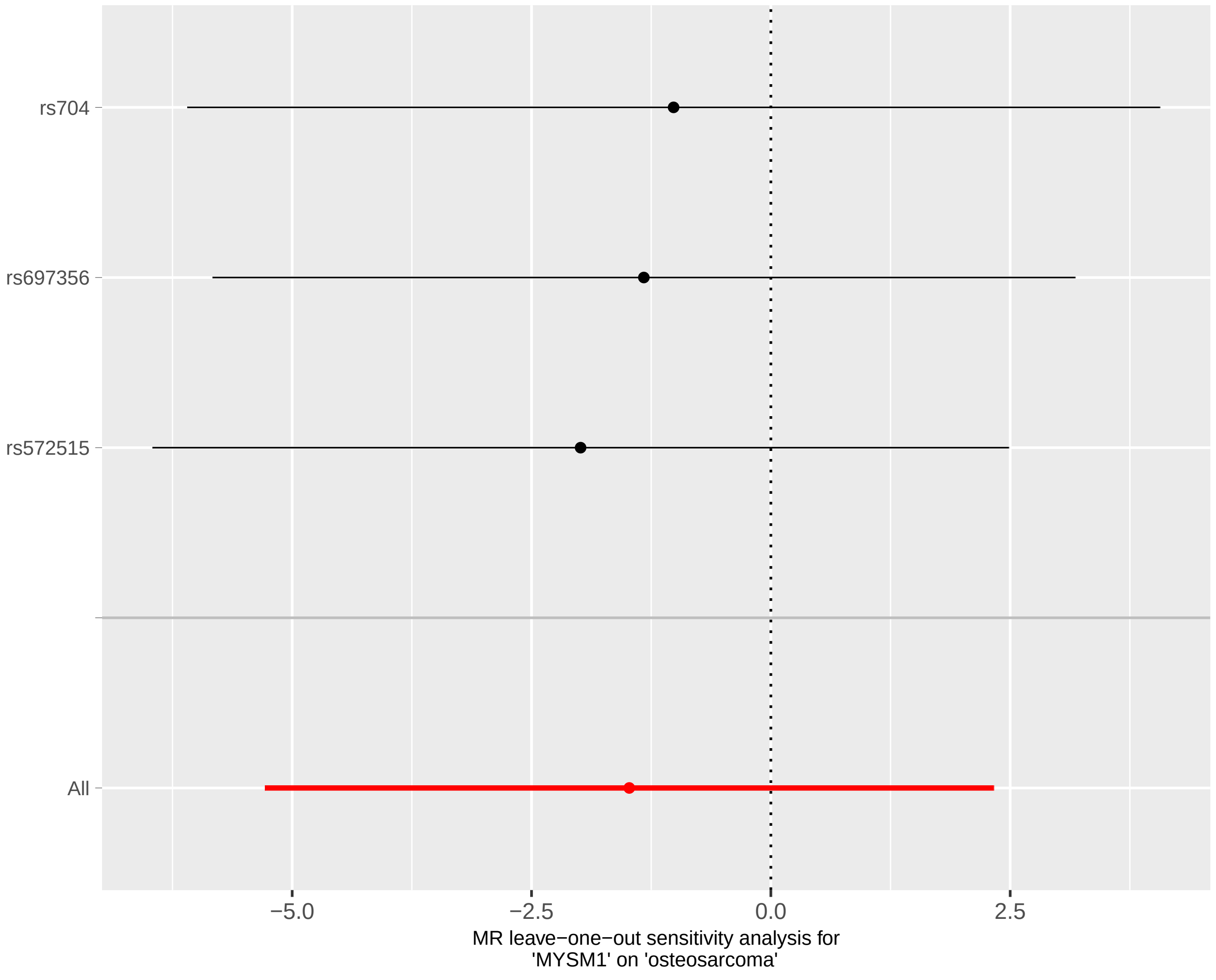
rs704
rs697356
rs572515
All
−5.0
−2.5
0.0
2.5
MR leave−one−out sensitivity analysis for
'MYSM1' on 'osteosarcoma'

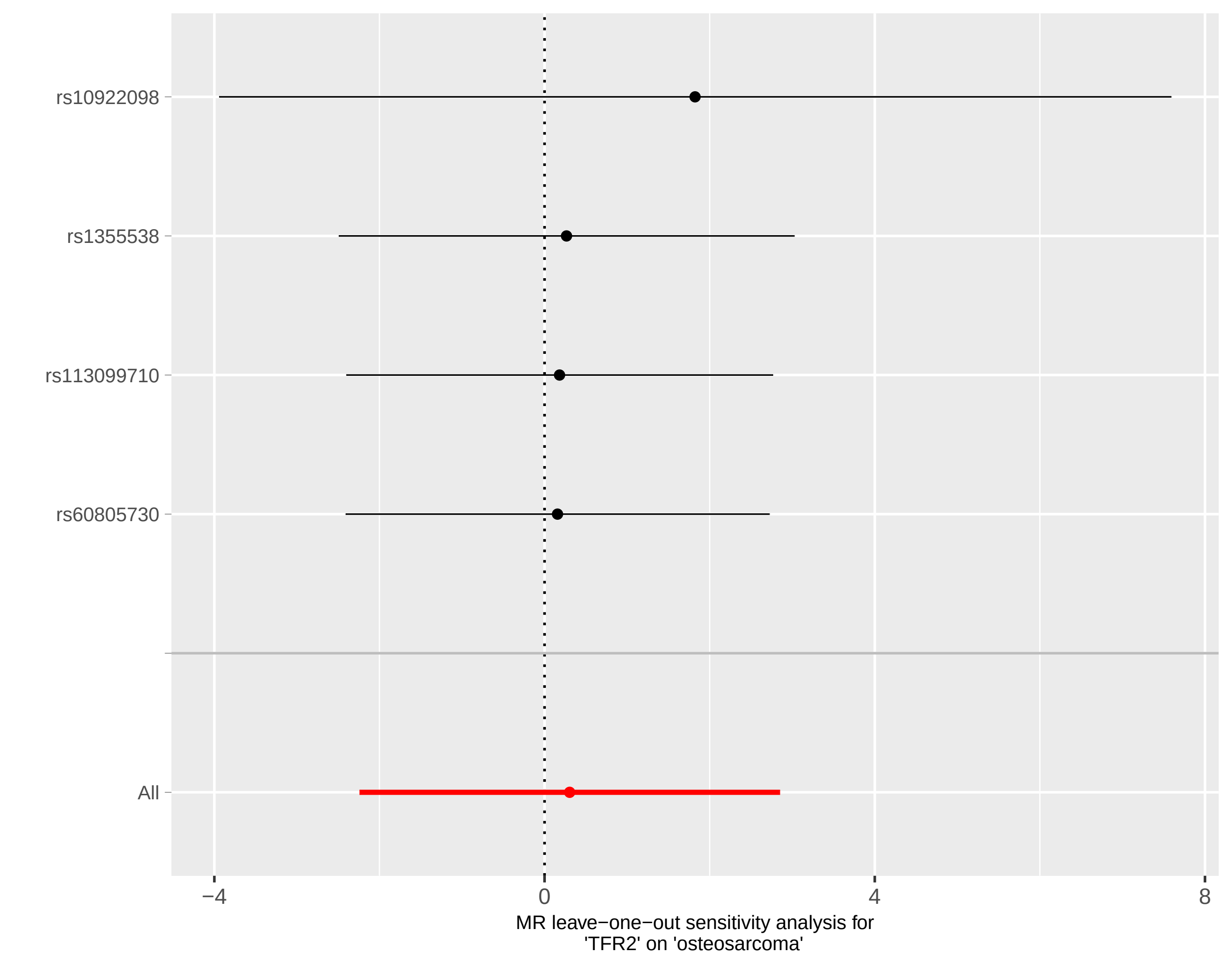
rs10922098
rs1355538
rs113099710
rs60805730
All
−4
0
4
8
MR leave−one−out sensitivity analysis for
'TFR2' on 'osteosarcoma'

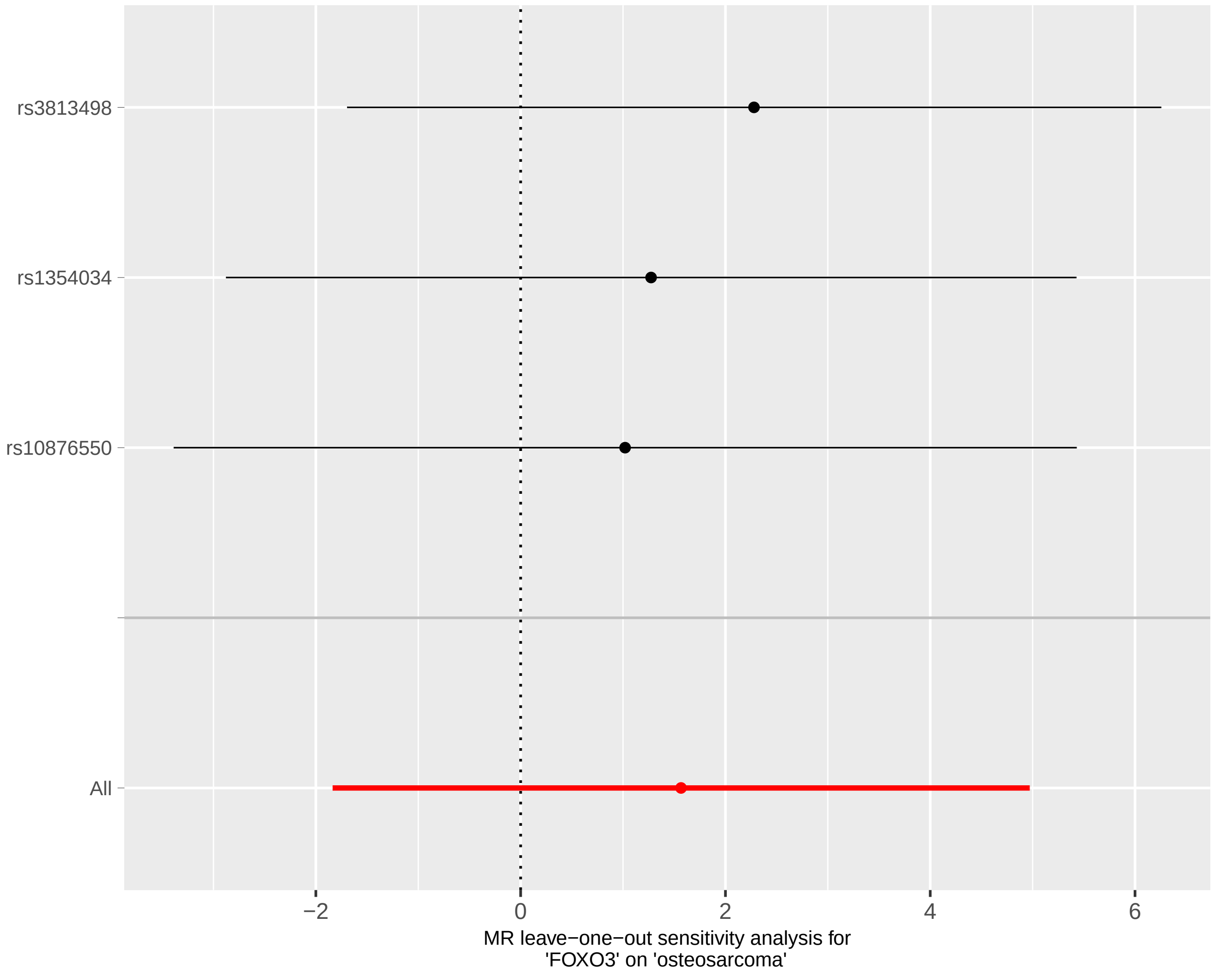

rs3813498
rs1354034
rs10876550
All
−2
0
2
4
6
MR leave−one−out sensitivity analysis for
'FOXO3' on 'osteosarcoma'

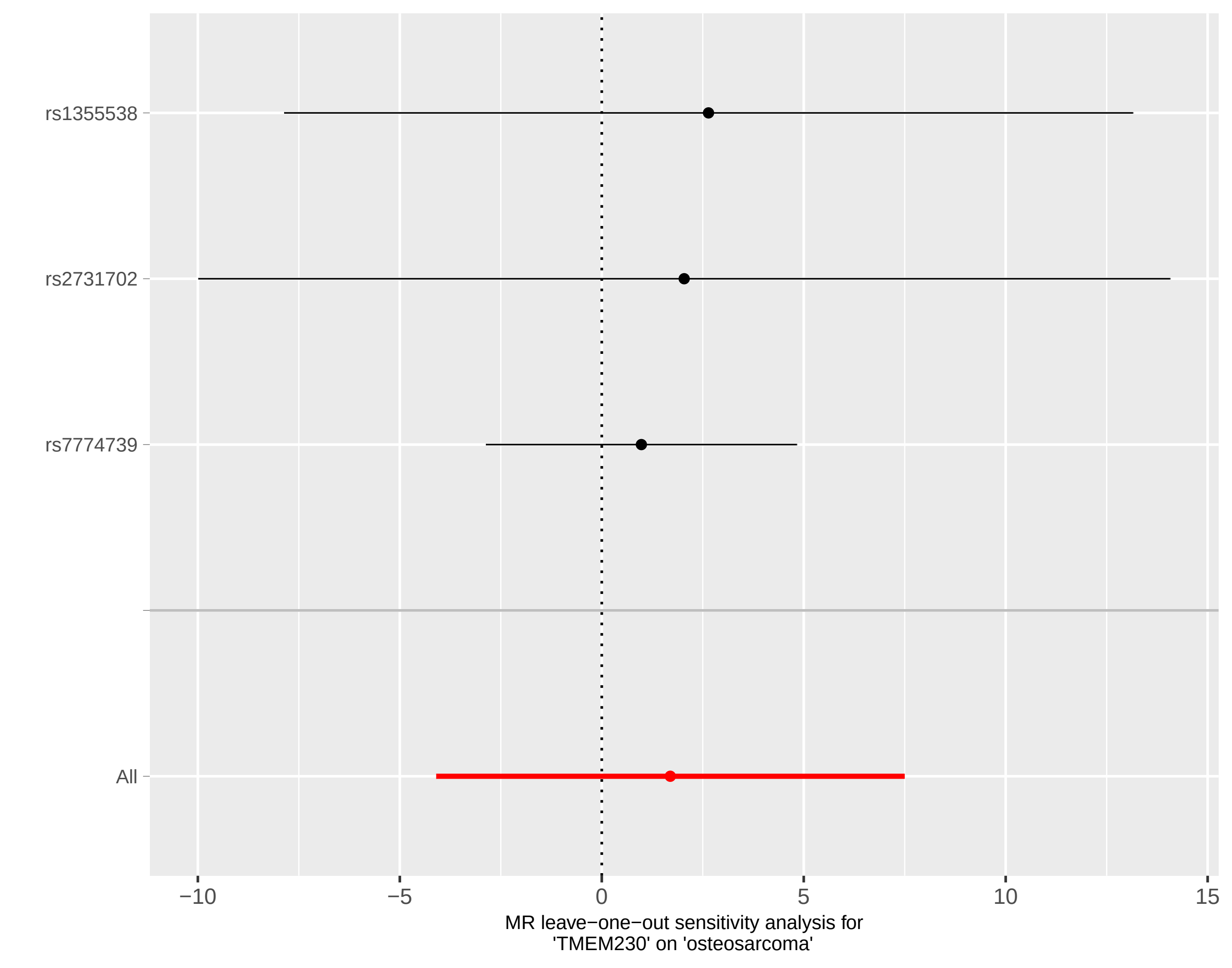
rs1355538
rs2731702
rs7774739
All
−10
−5
0
5
10
15
MR leave−one−out sensitivity analysis for
'TMEM230' on 'osteosarcoma'

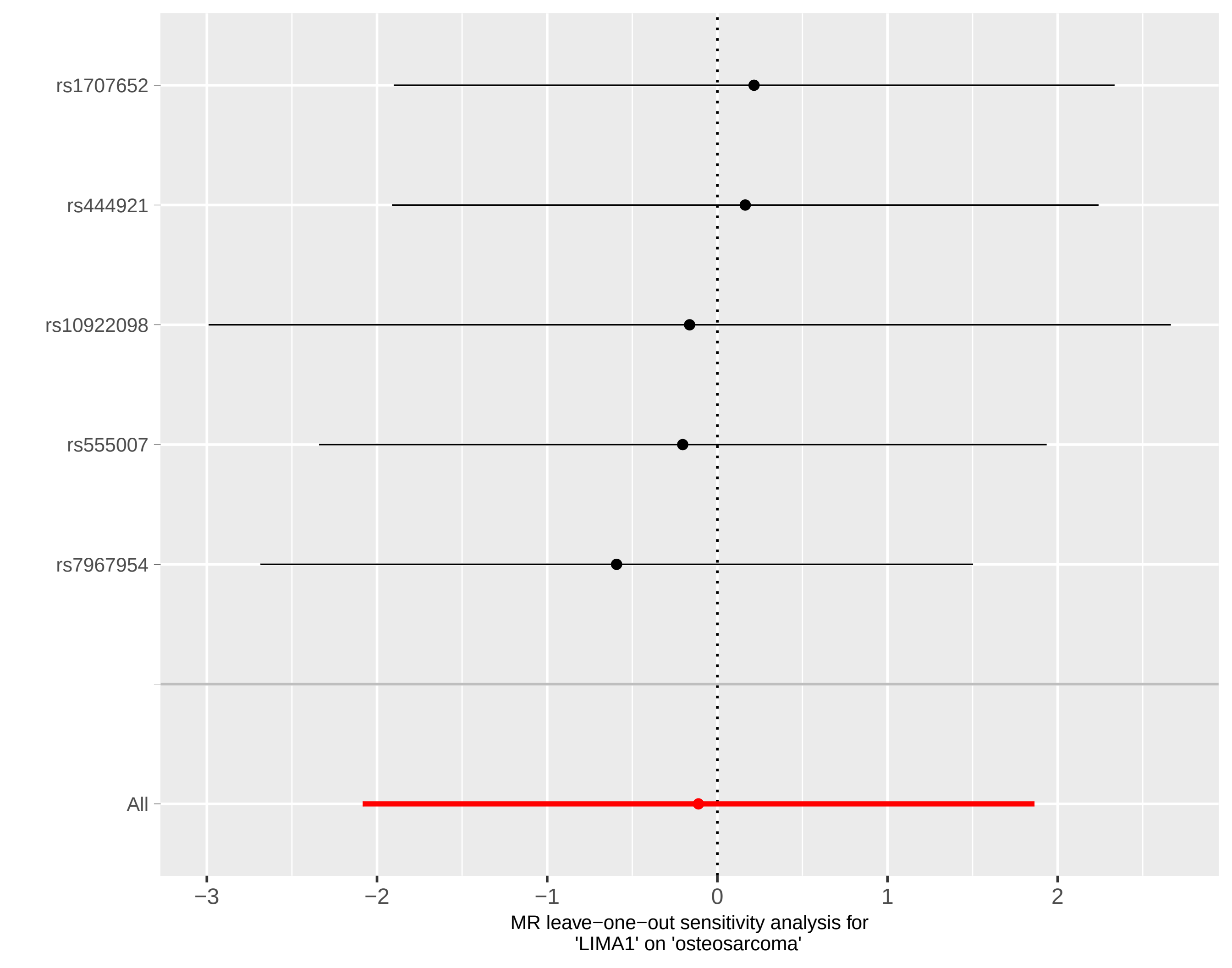

rs1707652
rs444921
rs10922098
rs555007
rs7967954
All
−3
−2
−1
0
1
2
MR leave−one−out sensitivity analysis for
'LIMA1' on 'osteosarcoma'

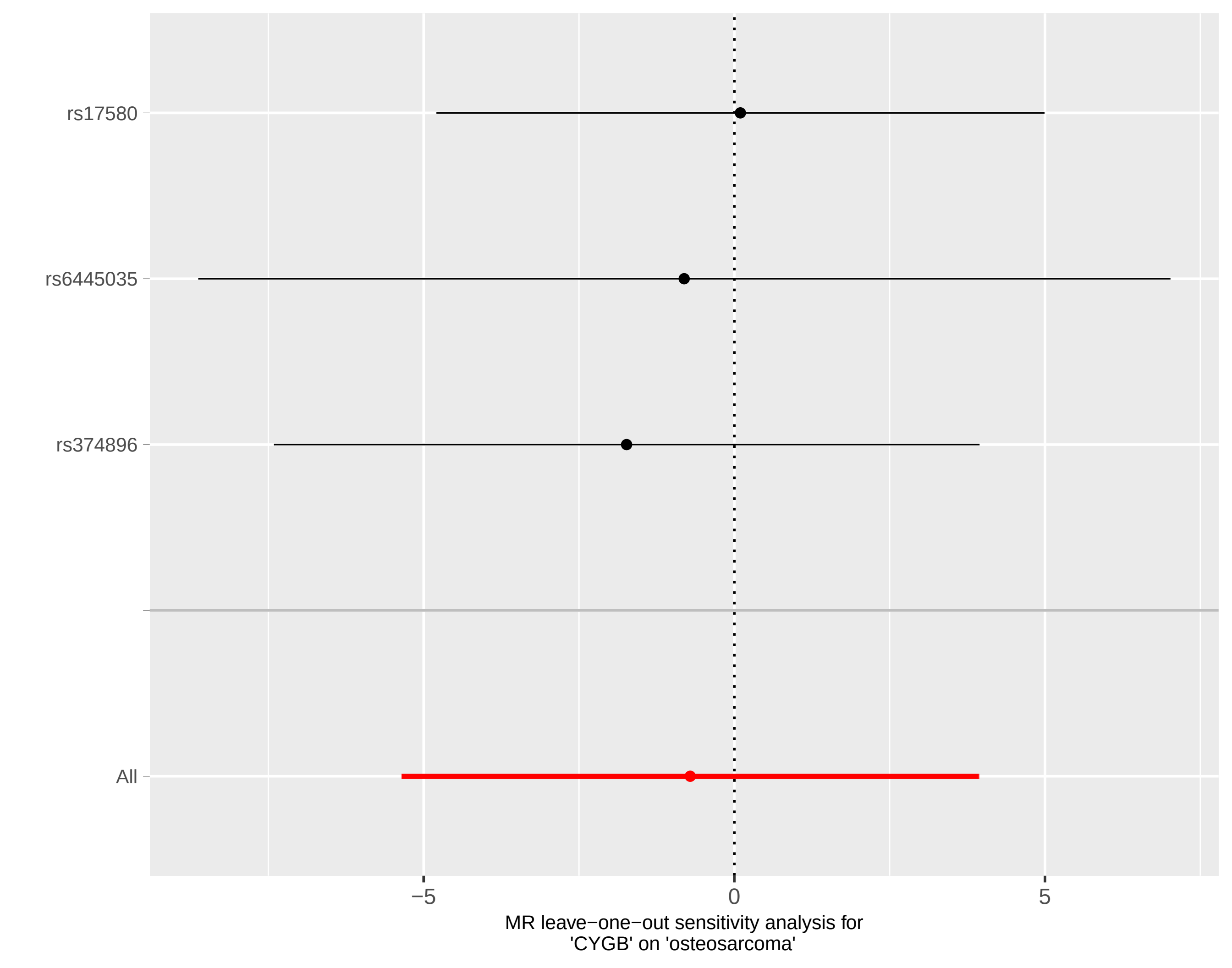

rs17580
rs6445035
rs374896
All
−5
0
5
MR leave−one−out sensitivity analysis for
'CYGB' on 'osteosarcoma'

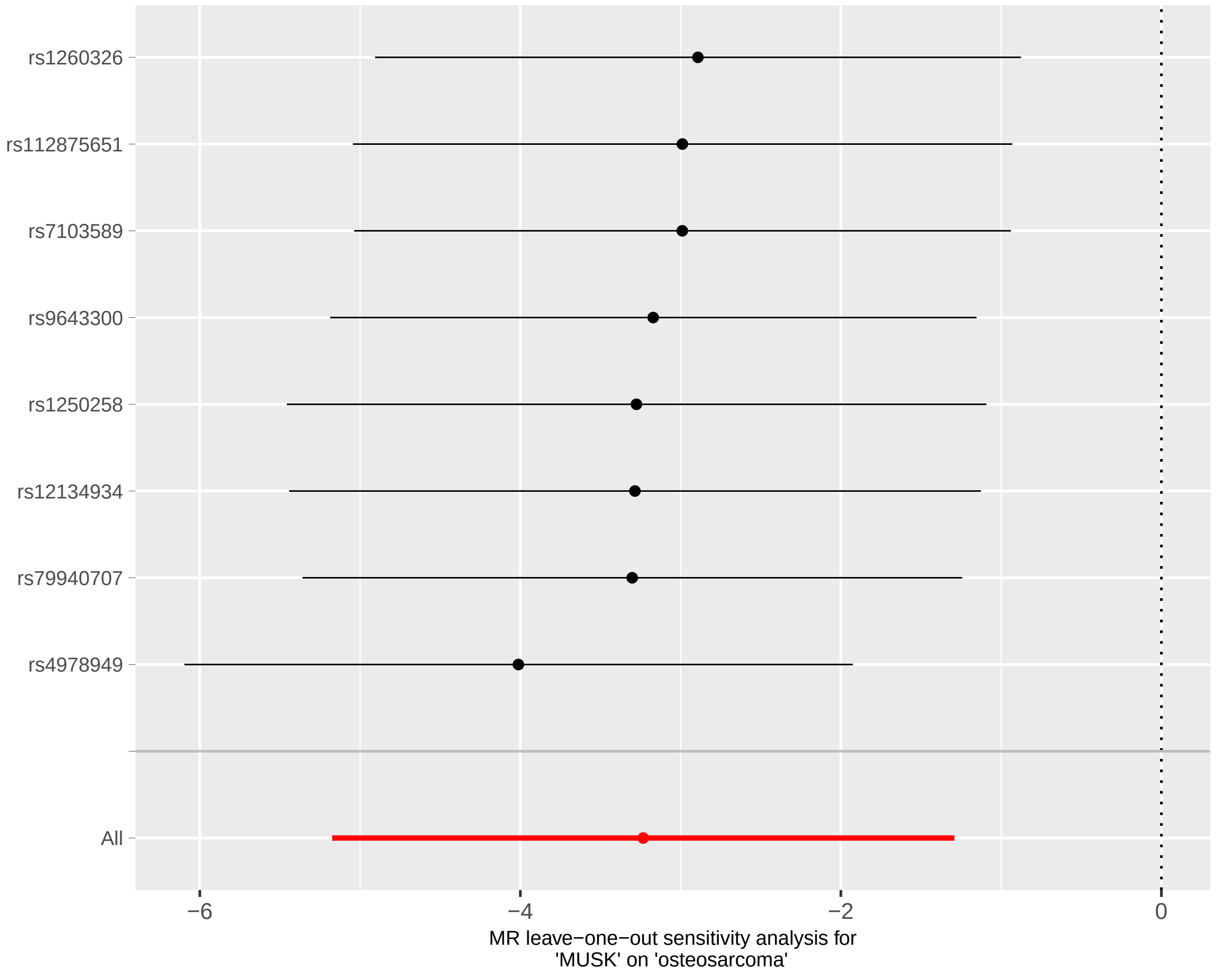
rs1260326
rs112875651
rs7103589
rs9643300
rs1250258
rs12134934
rs79940707
rs4978949
All
−6
−4
−2
0
MR leave−one−out sensitivity analysis for
'MUSK' on 'osteosarcoma'

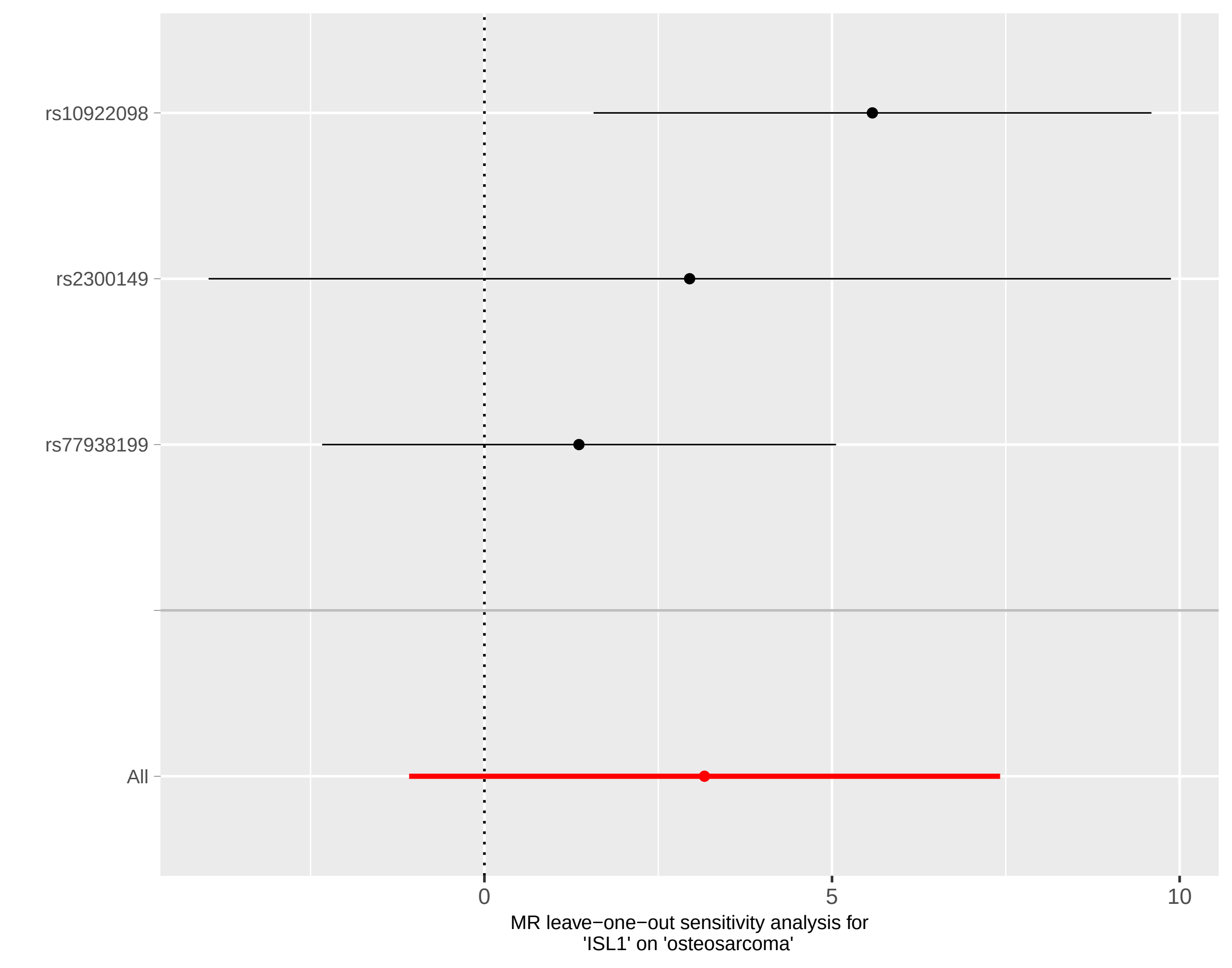

rs10922098
rs2300149
rs77938199
All
0
5
10
MR leave−one−out sensitivity analysis for
'ISL1' on 'osteosarcoma'

Insufficient number of SNPs

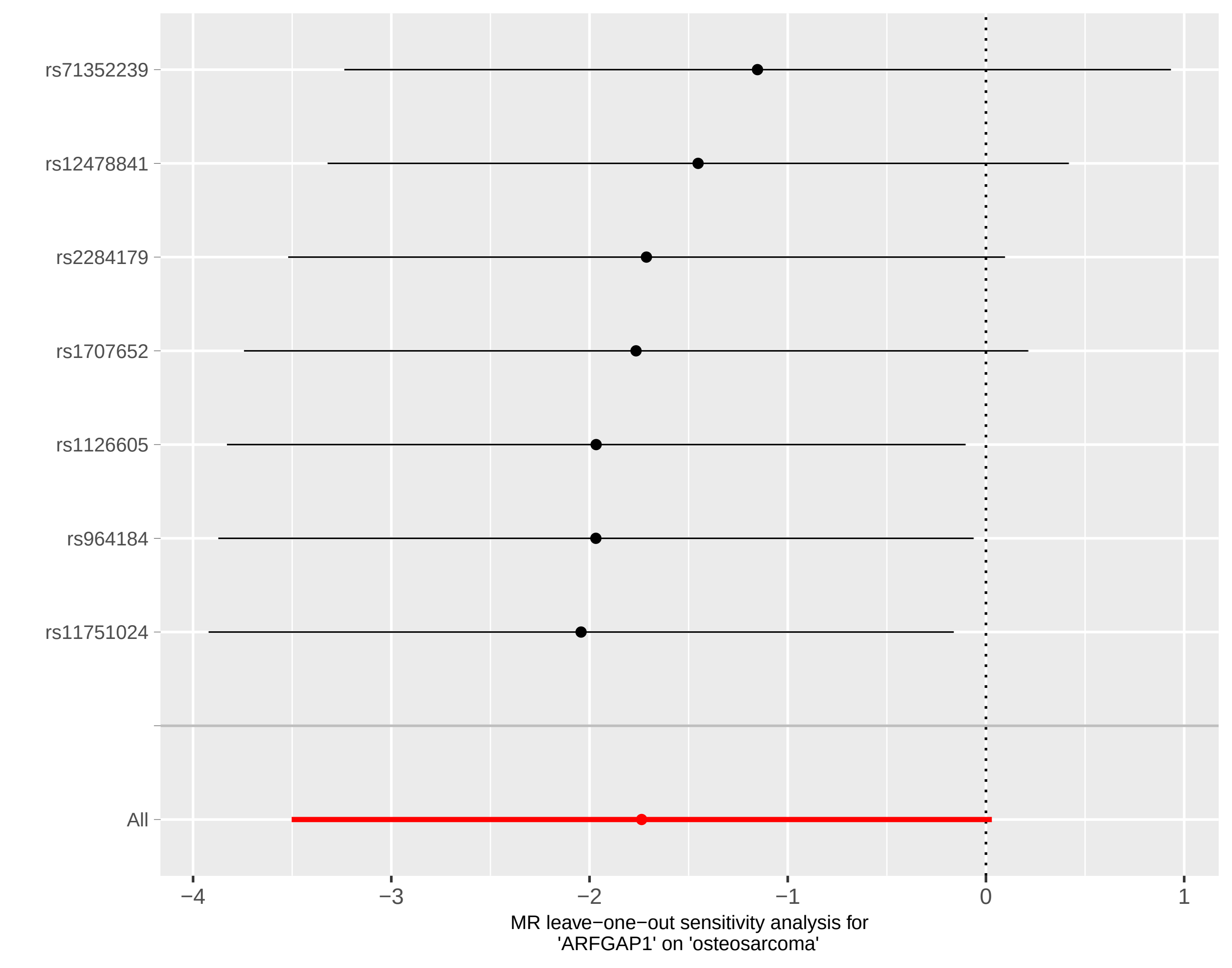
rs71352239
rs12478841
rs2284179
rs1707652
rs1126605
rs964184
rs11751024
All
−4
−3
−2
−1
0
1
MR leave−one−out sensitivity analysis for
'ARFGAP1' on 'osteosarcoma'

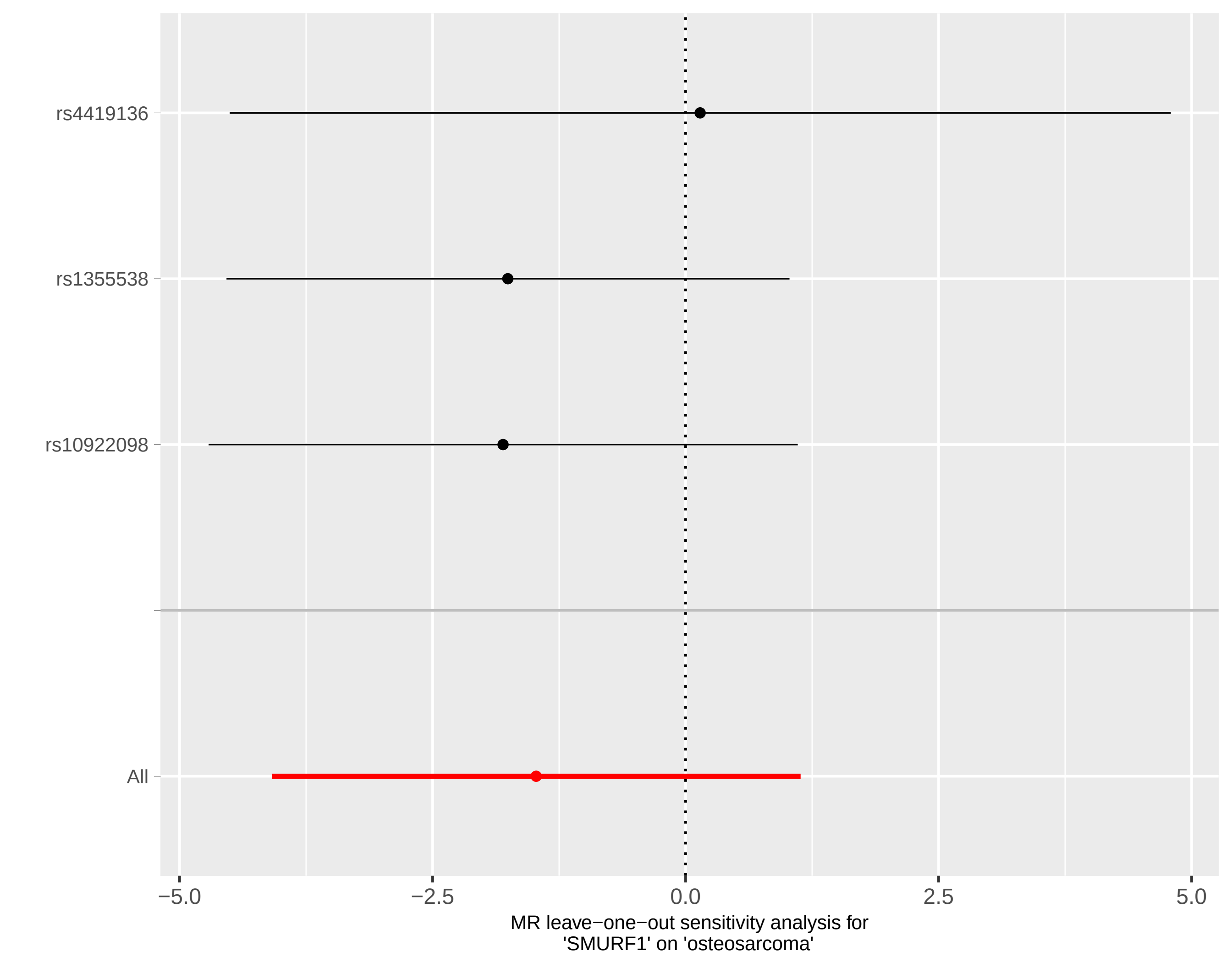
rs4419136
rs1355538
rs10922098
All
−5.0
−2.5
0.0
2.5
5.0
MR leave−one−out sensitivity analysis for
'SMURF1' on 'osteosarcoma'

Insufficient number of SNPs

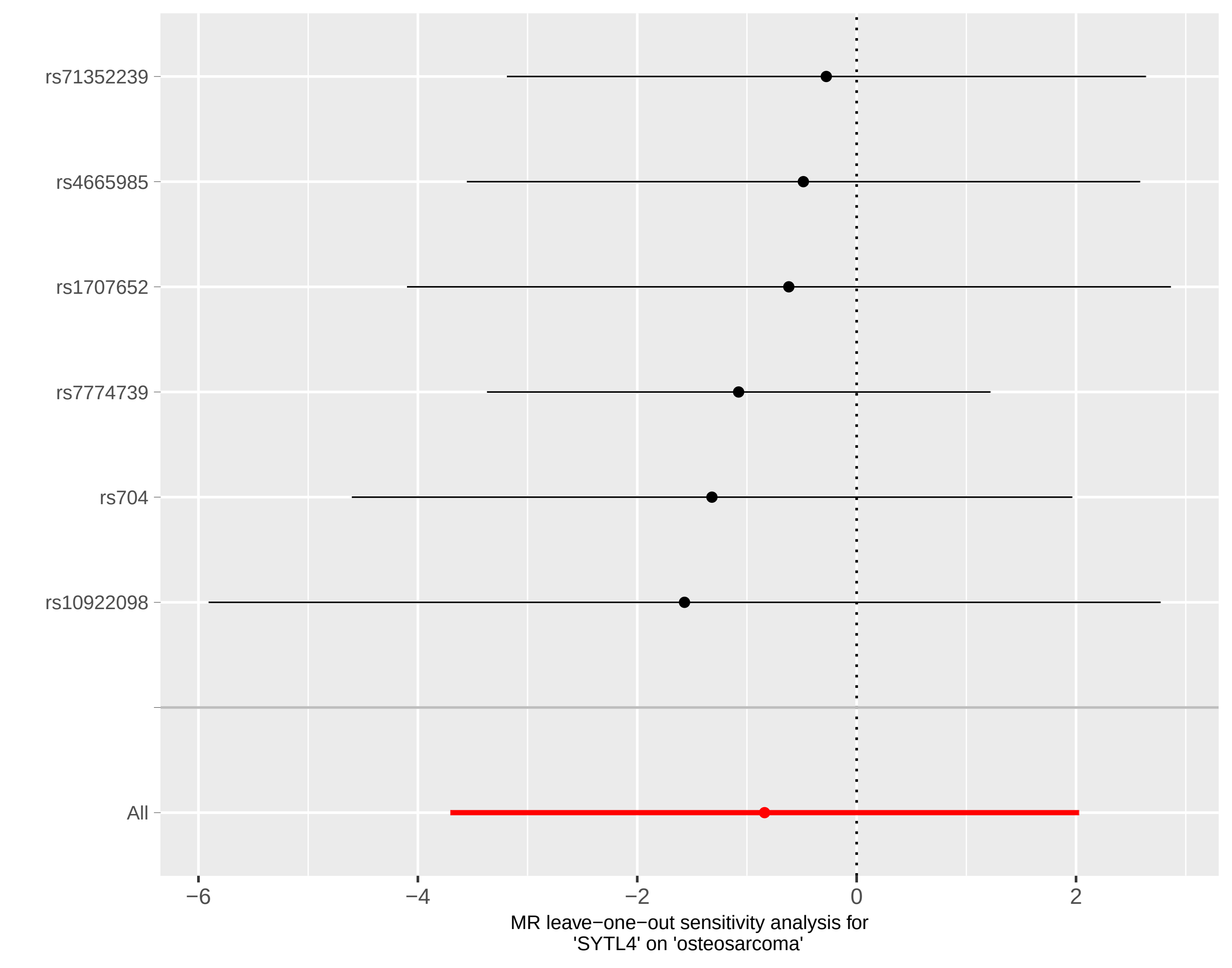

rs71352239
rs4665985
rs1707652
rs7774739
rs704
rs10922098
All
−6
−4
−2
0
2
MR leave−one−out sensitivity analysis for
'SYTL4' on 'osteosarcoma'

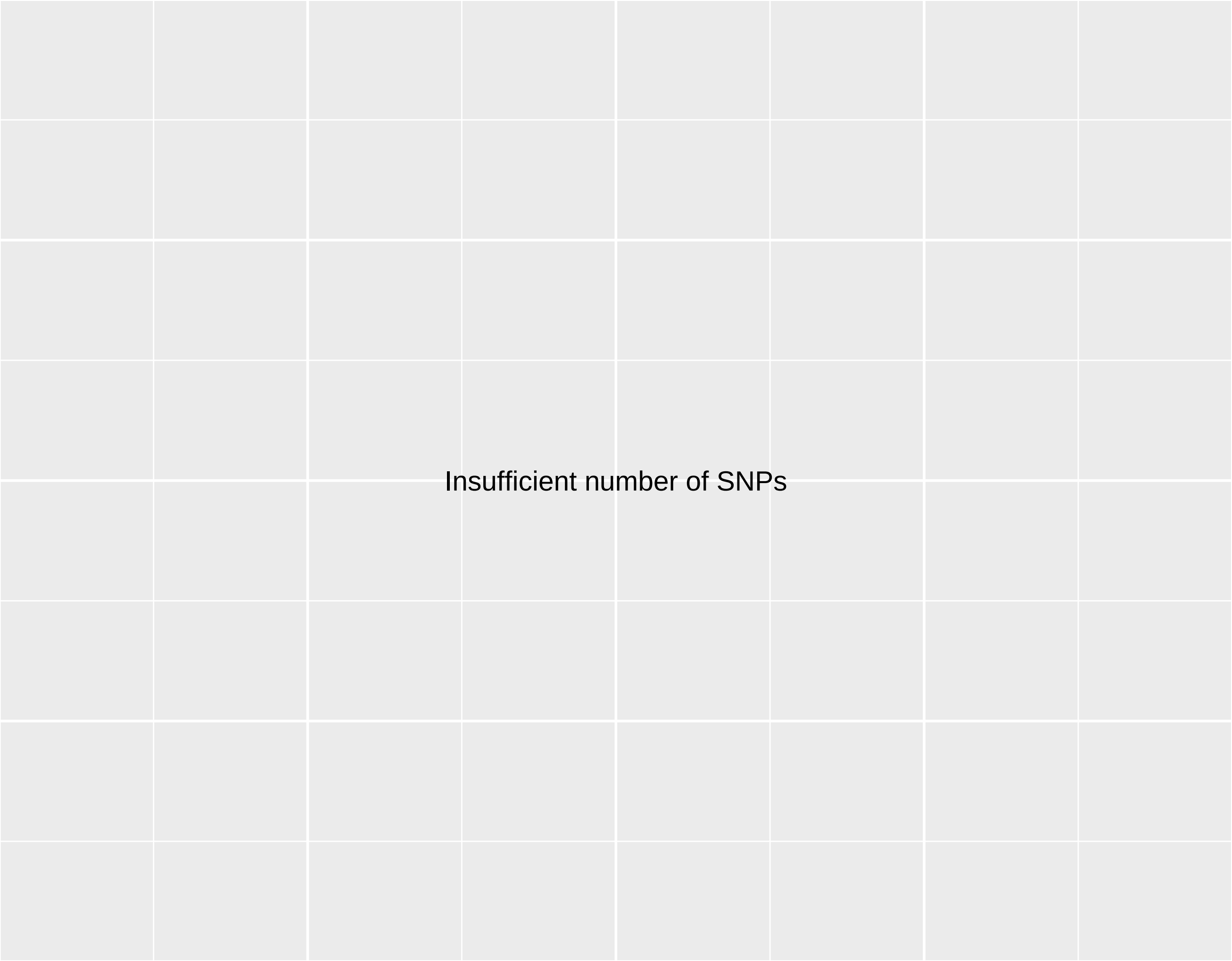
Insufficient number of SNPs

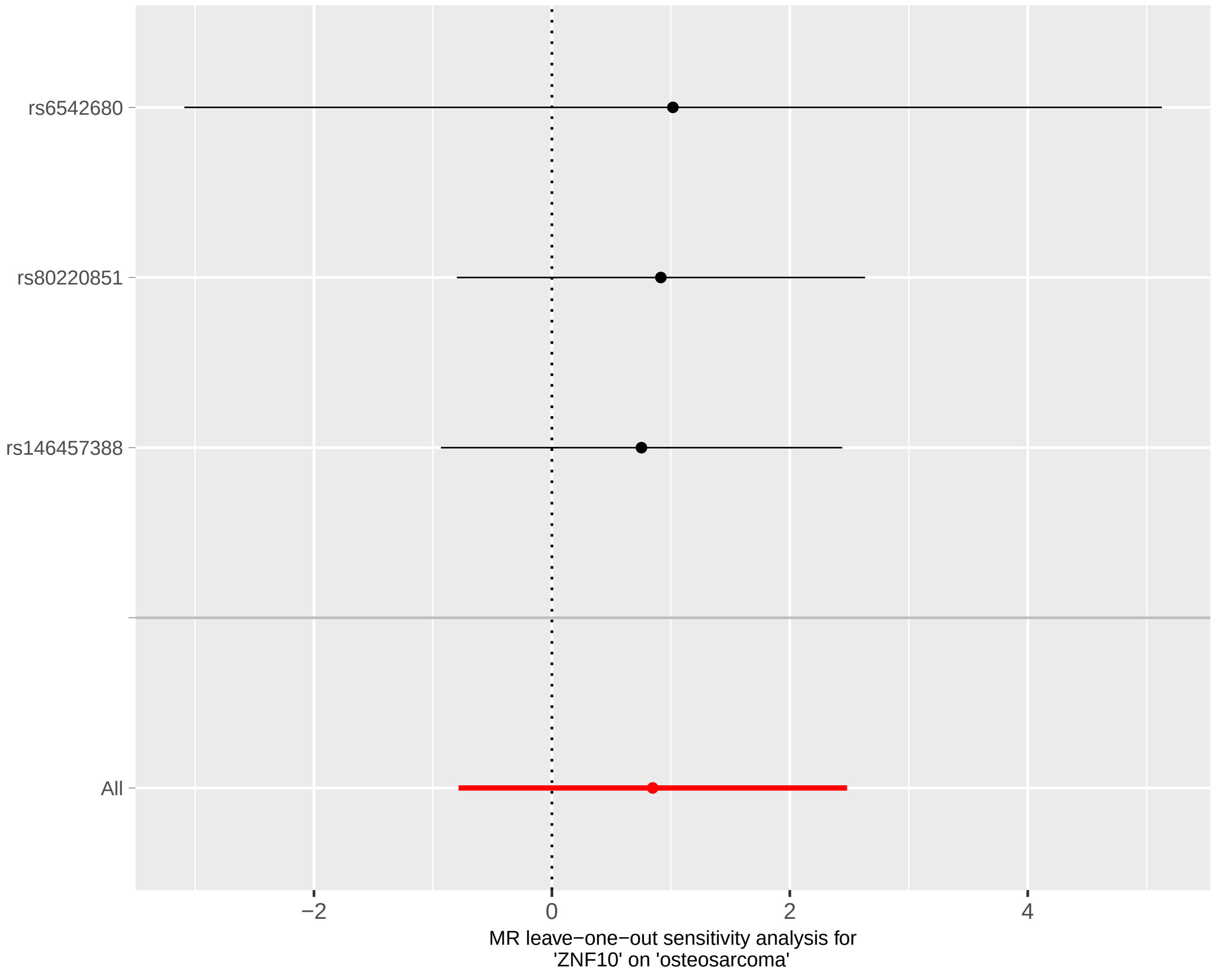
rs6542680
rs80220851
rs146457388
All
−2
0
2
4
MR leave−one−out sensitivity analysis for
'ZNF10' on 'osteosarcoma'

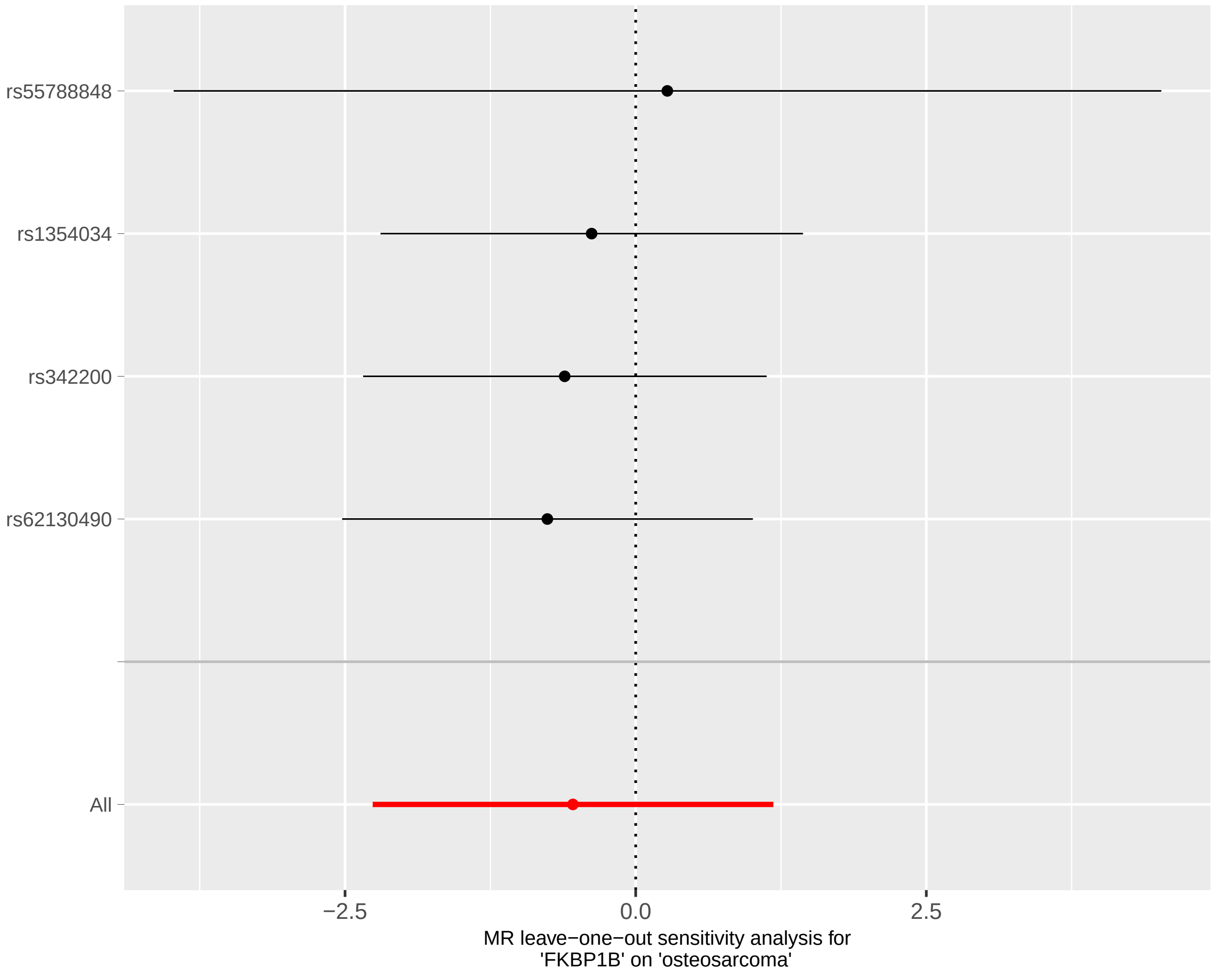
rs55788848
rs1354034
rs342200
rs62130490
All
−2.5
0.0
2.5
MR leave−one−out sensitivity analysis for
'FKBP1B' on 'osteosarcoma'

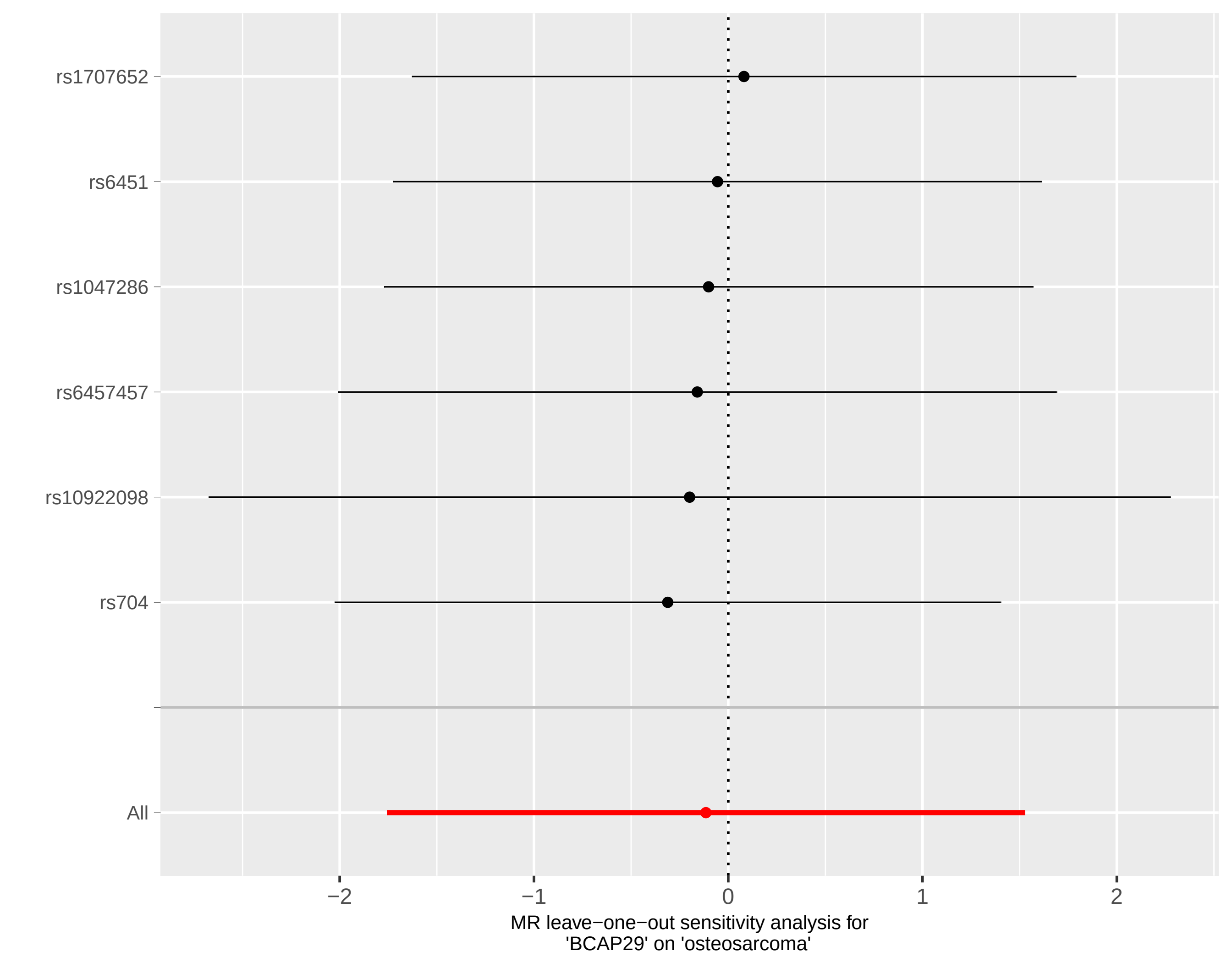
rs1707652
rs6451
rs1047286
rs6457457
rs10922098
rs704
All
−2
−1
0
1
2
MR leave−one−out sensitivity analysis for
'BCAP29' on 'osteosarcoma'

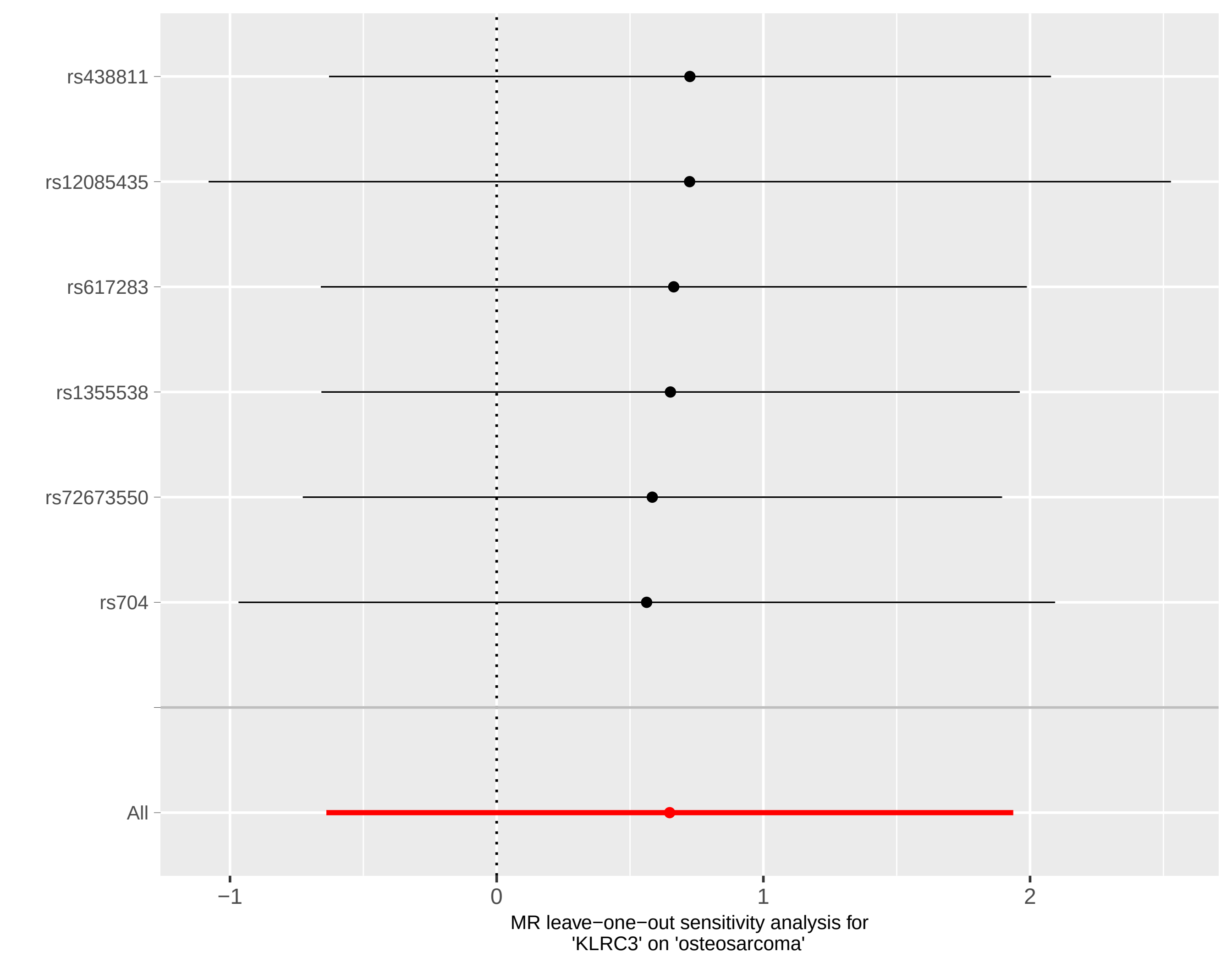
rs438811
rs12085435
rs617283
rs1355538
rs72673550
rs704
All
−1
0
1
2
MR leave−one−out sensitivity analysis for
'KLRC3' on 'osteosarcoma'

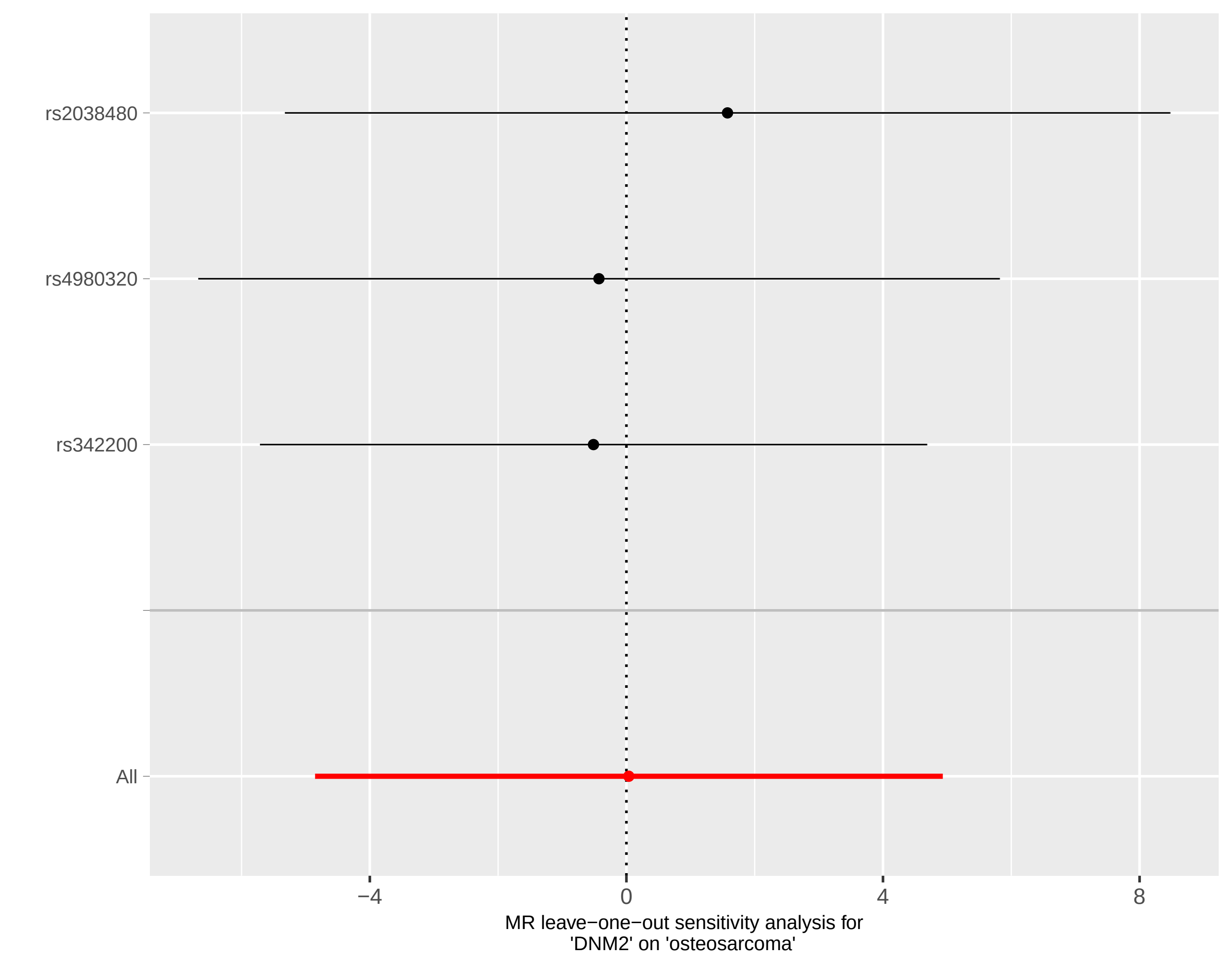
rs2038480
rs4980320
rs342200
All
−4
0
4
8
MR leave−one−out sensitivity analysis for
'DNM2' on 'osteosarcoma'

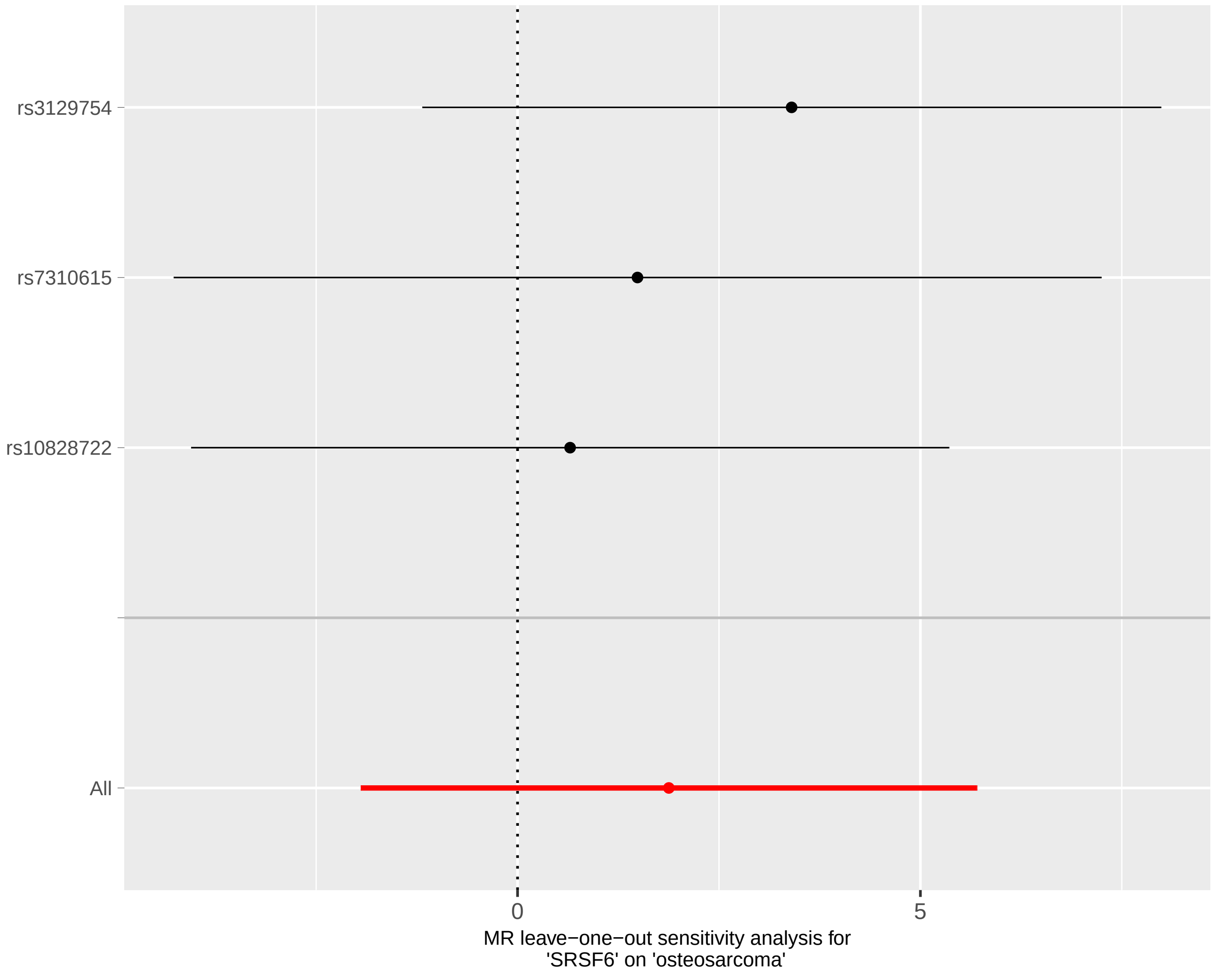
rs3129754
rs7310615
rs10828722
All
0
5
MR leave−one−out sensitivity analysis for
'SRSF6' on 'osteosarcoma'

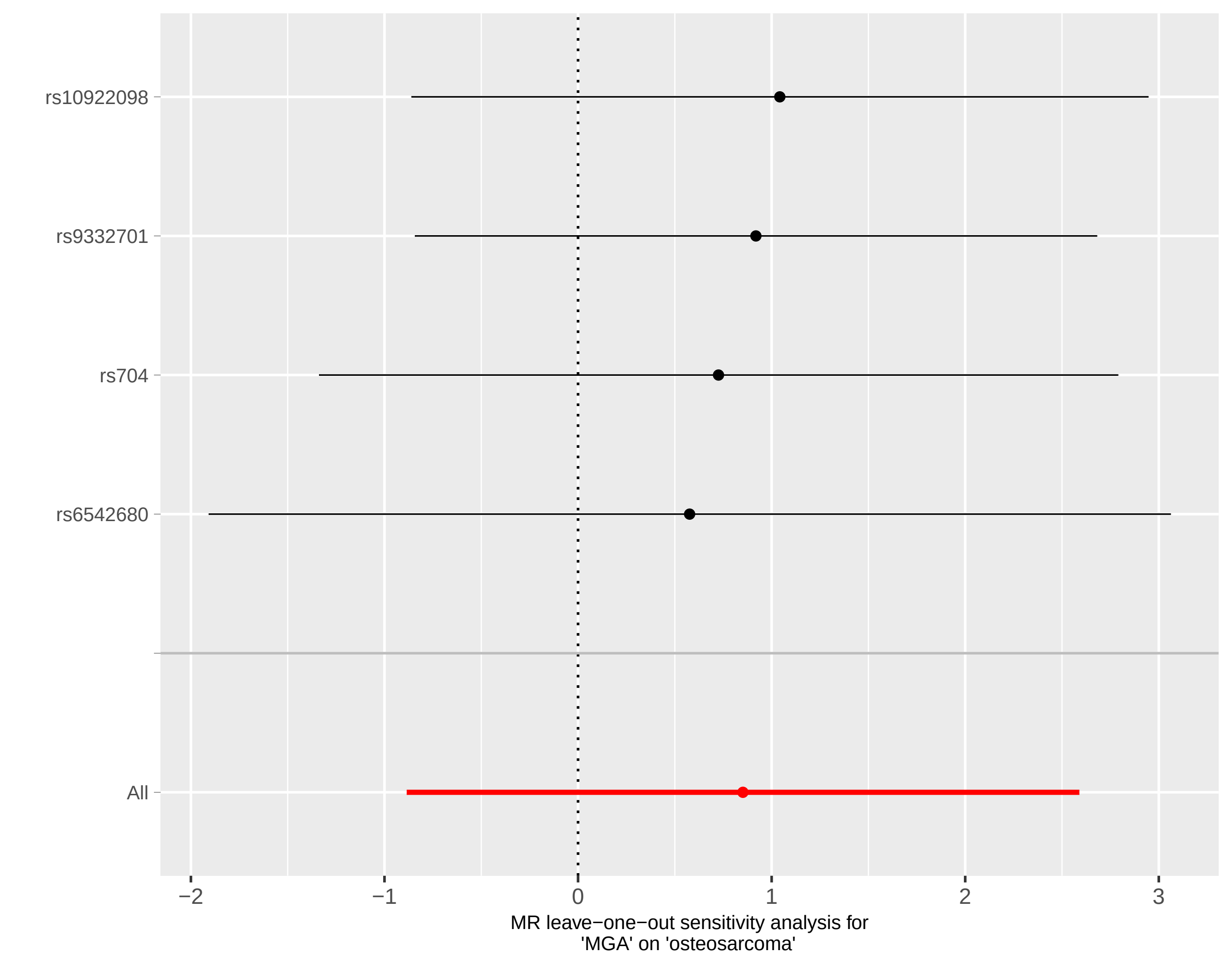
rs10922098
rs9332701
rs704
rs6542680
All
−2
−1
0
1
2
3
MR leave−one−out sensitivity analysis for
'MGA' on 'osteosarcoma'

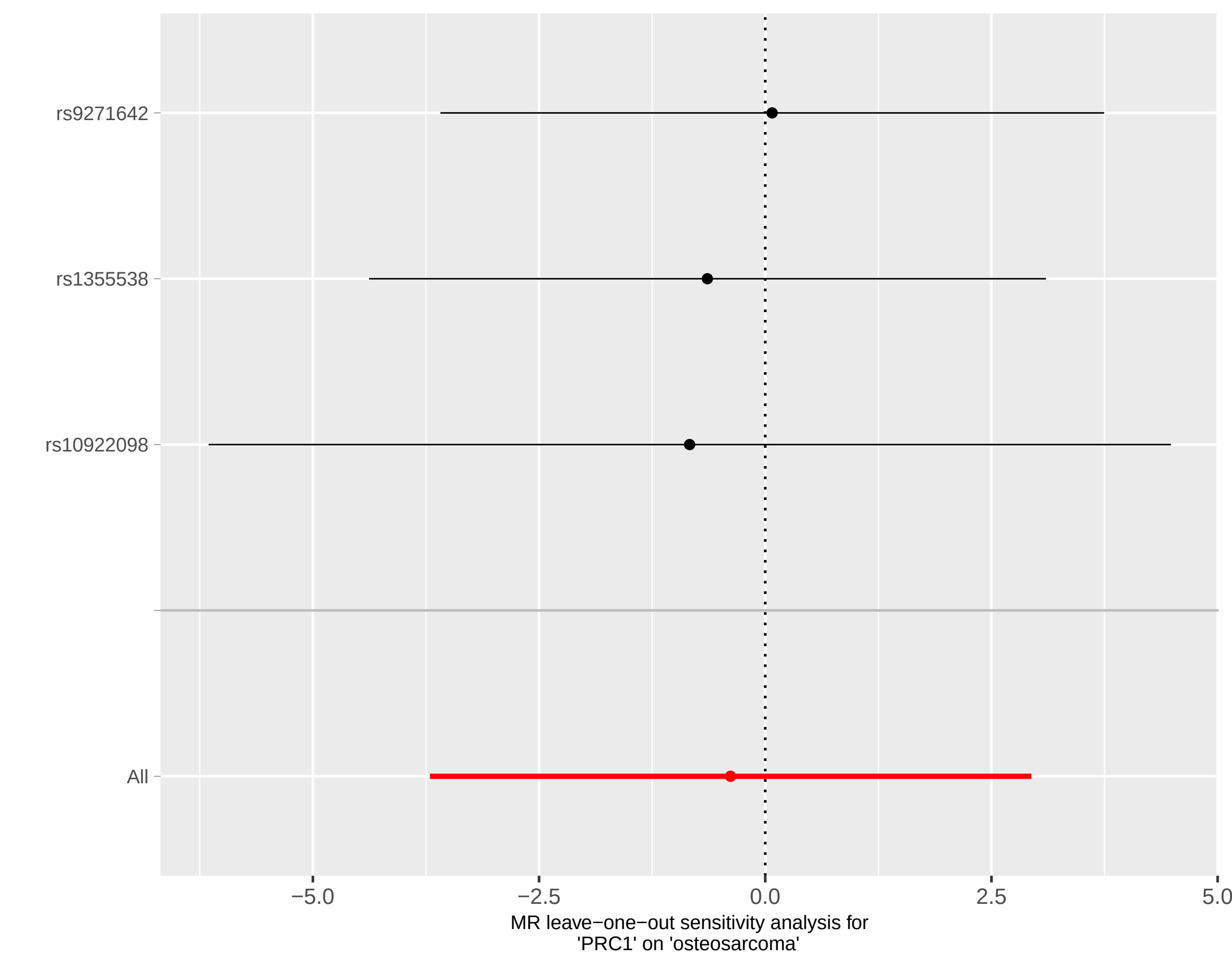
rs9271642
rs1355538
rs10922098
All
−5.0
−2.5
0.0
2.5
5.0
MR leave−one−out sensitivity analysis for
'PRC1' on 'osteosarcoma'

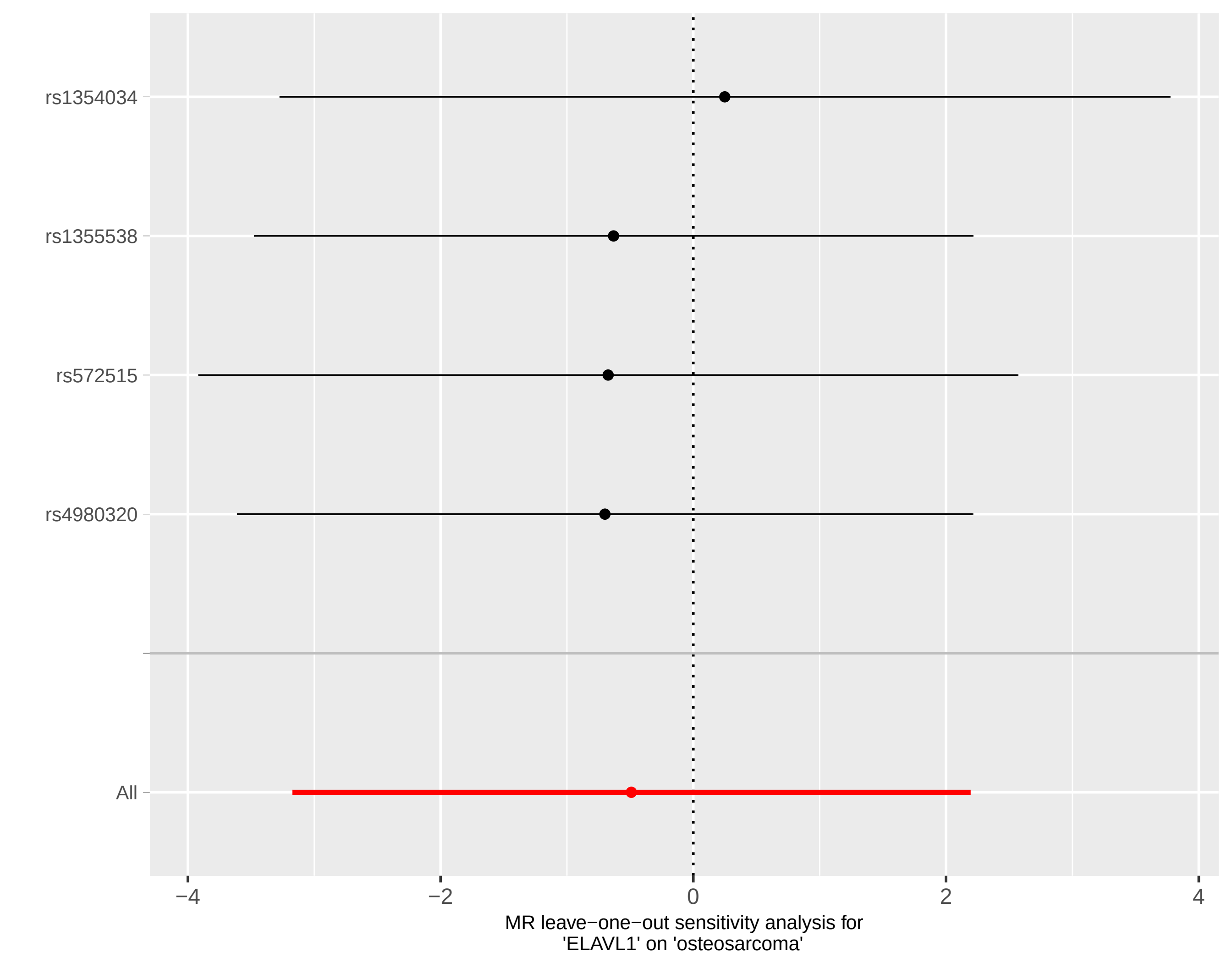
rs1354034
rs1355538
rs572515
rs4980320
All
−4
−2
0
2
4
MR leave−one−out sensitivity analysis for
'ELAVL1' on 'osteosarcoma'

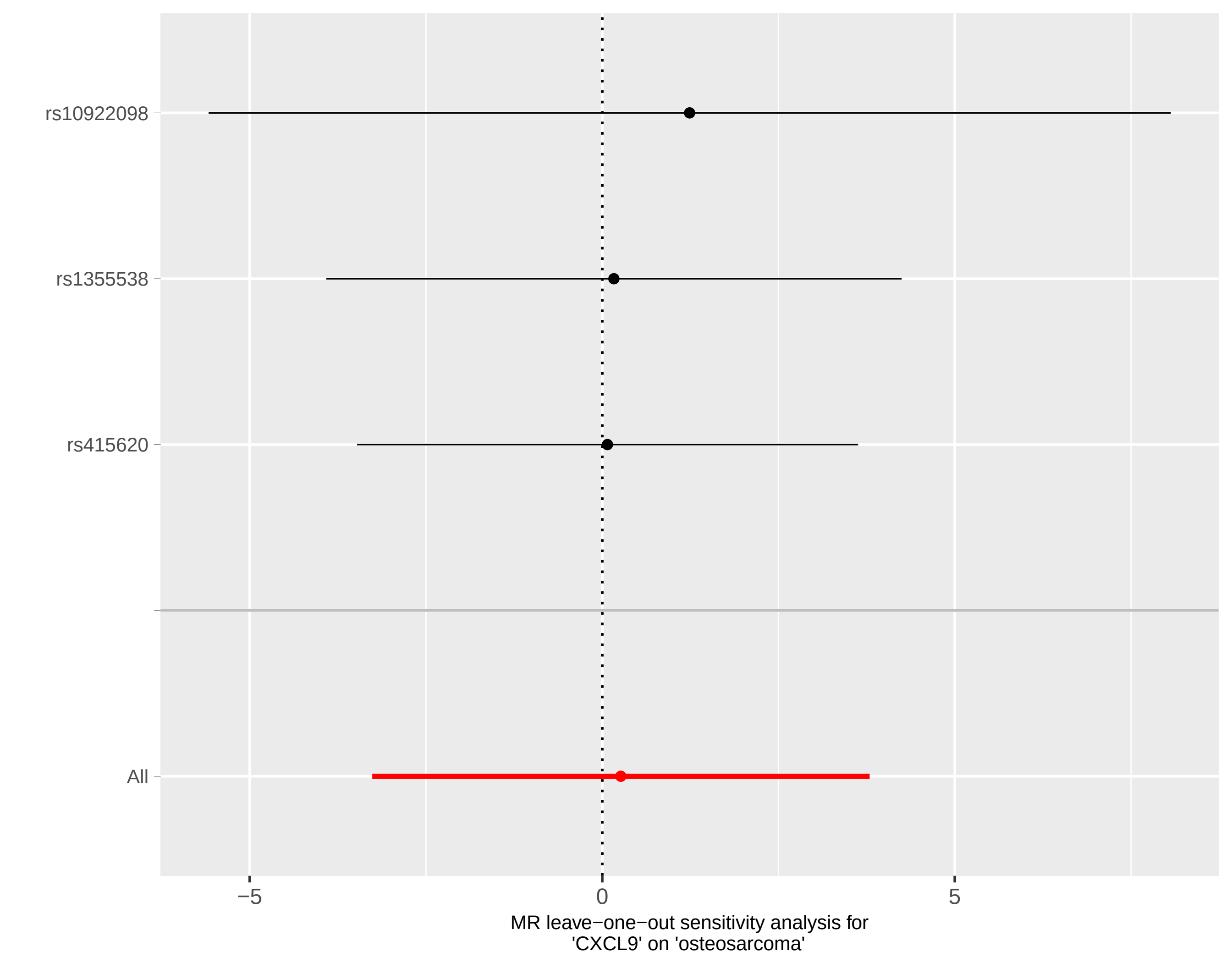
rs10922098
rs1355538
rs415620
All
−5
0
5
MR leave−one−out sensitivity analysis for
'CXCL9' on 'osteosarcoma'

Insufficient number of SNPs

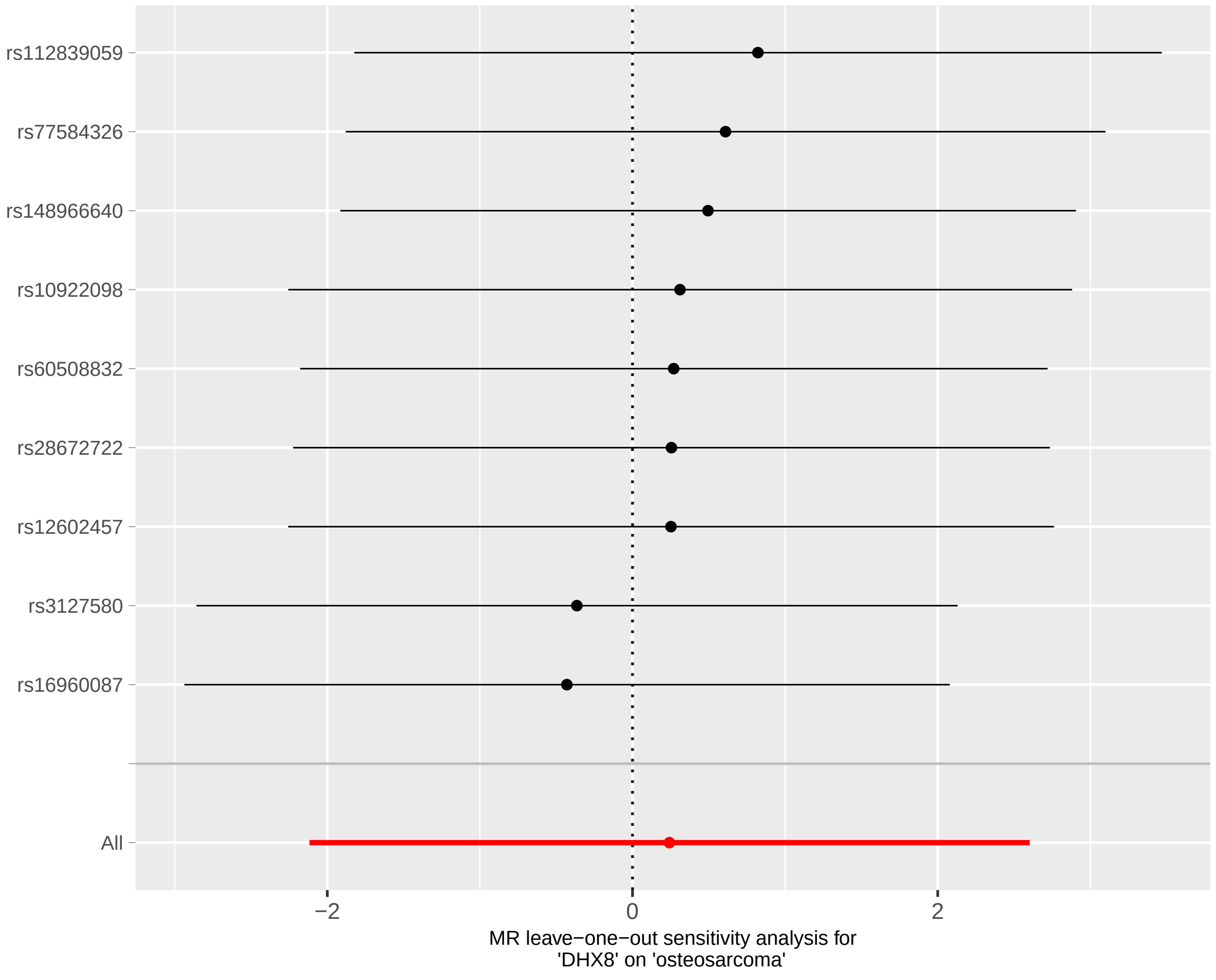
rs112839059
rs77584326
rs148966640
rs10922098
rs60508832
rs28672722
rs12602457
rs3127580
rs16960087
All
−2
0
2
MR leave−one−out sensitivity analysis for
'DHX8' on 'osteosarcoma'

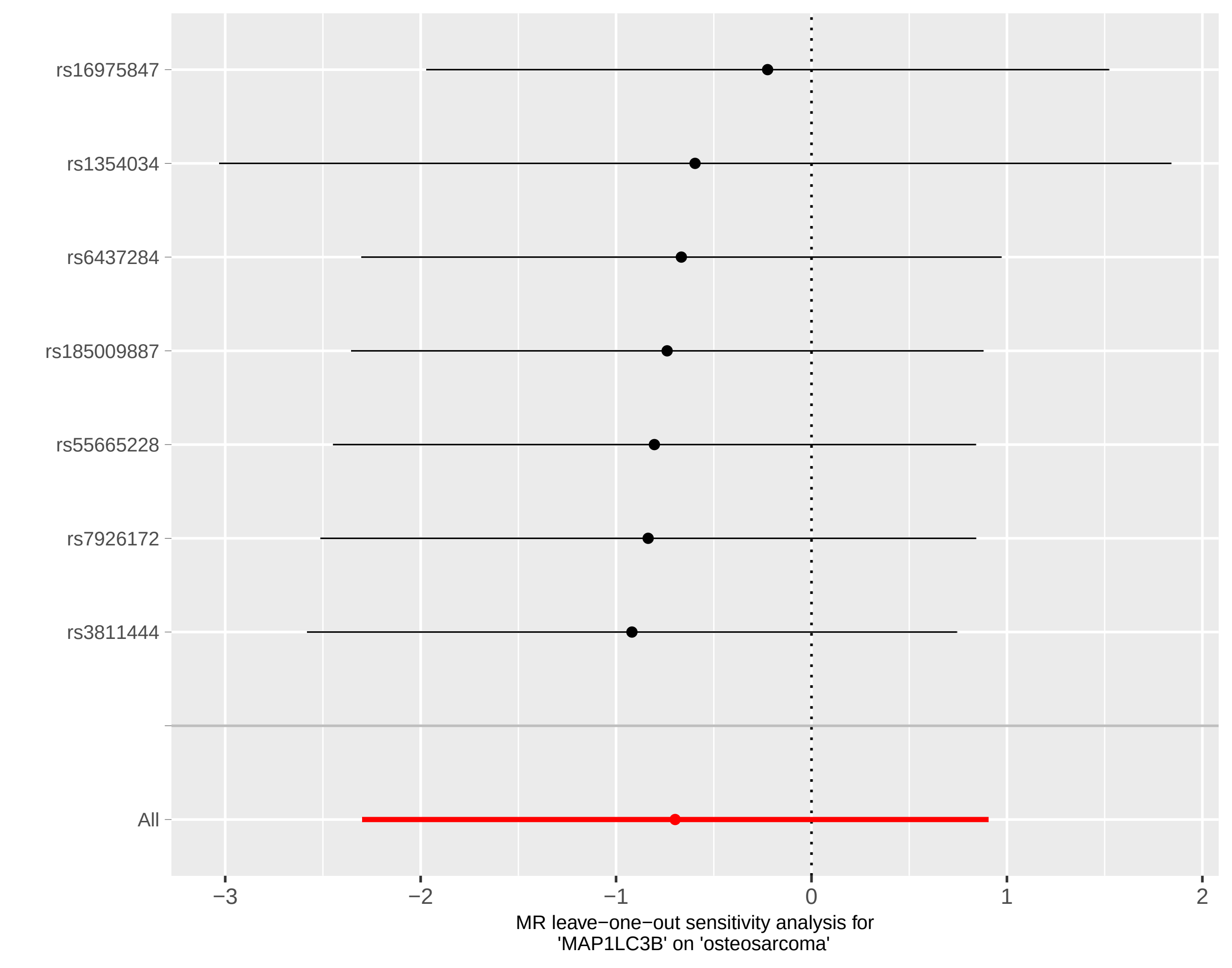
rs16975847
rs1354034
rs6437284
rs185009887
rs55665228
rs7926172
rs3811444
All
−3
−2
−1
0
1
2
MR leave−one−out sensitivity analysis for
'MAP1LC3B' on 'osteosarcoma'

Insufficient number of SNPs

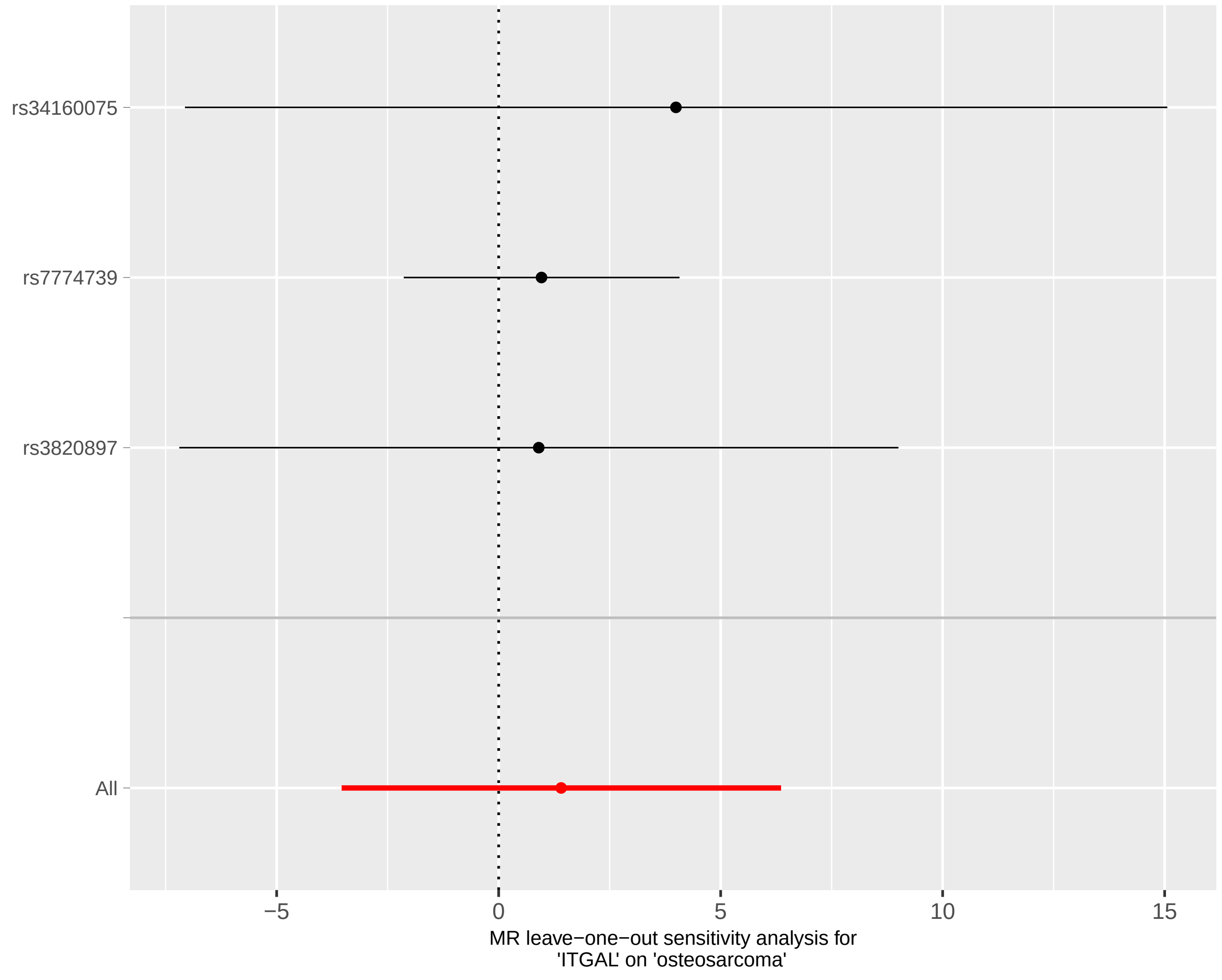
rs34160075
rs7774739
rs3820897
All
−5
0
5
10
15
MR leave−one−out sensitivity analysis for
'ITGAL' on 'osteosarcoma'

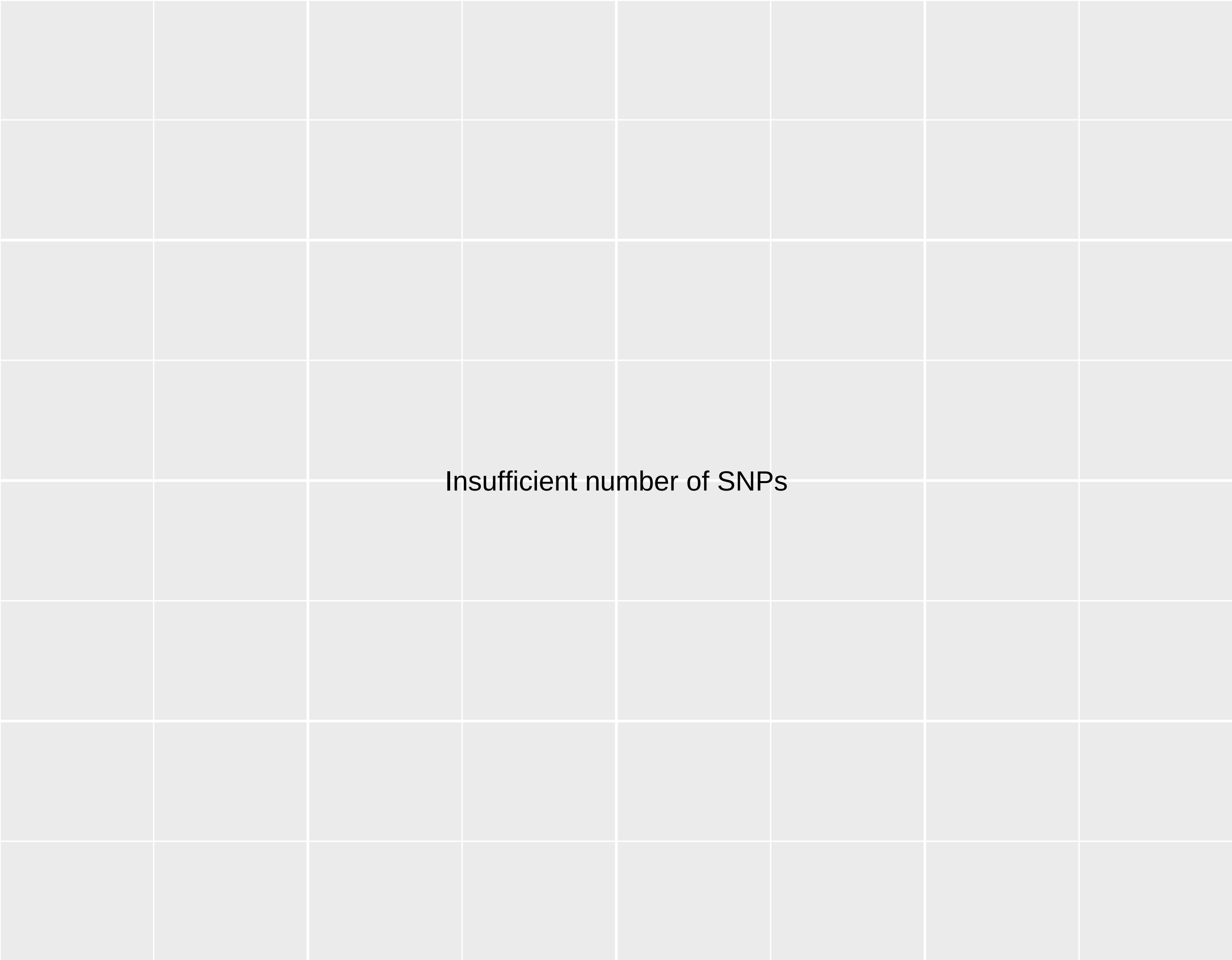
Insufficient number of SNPs

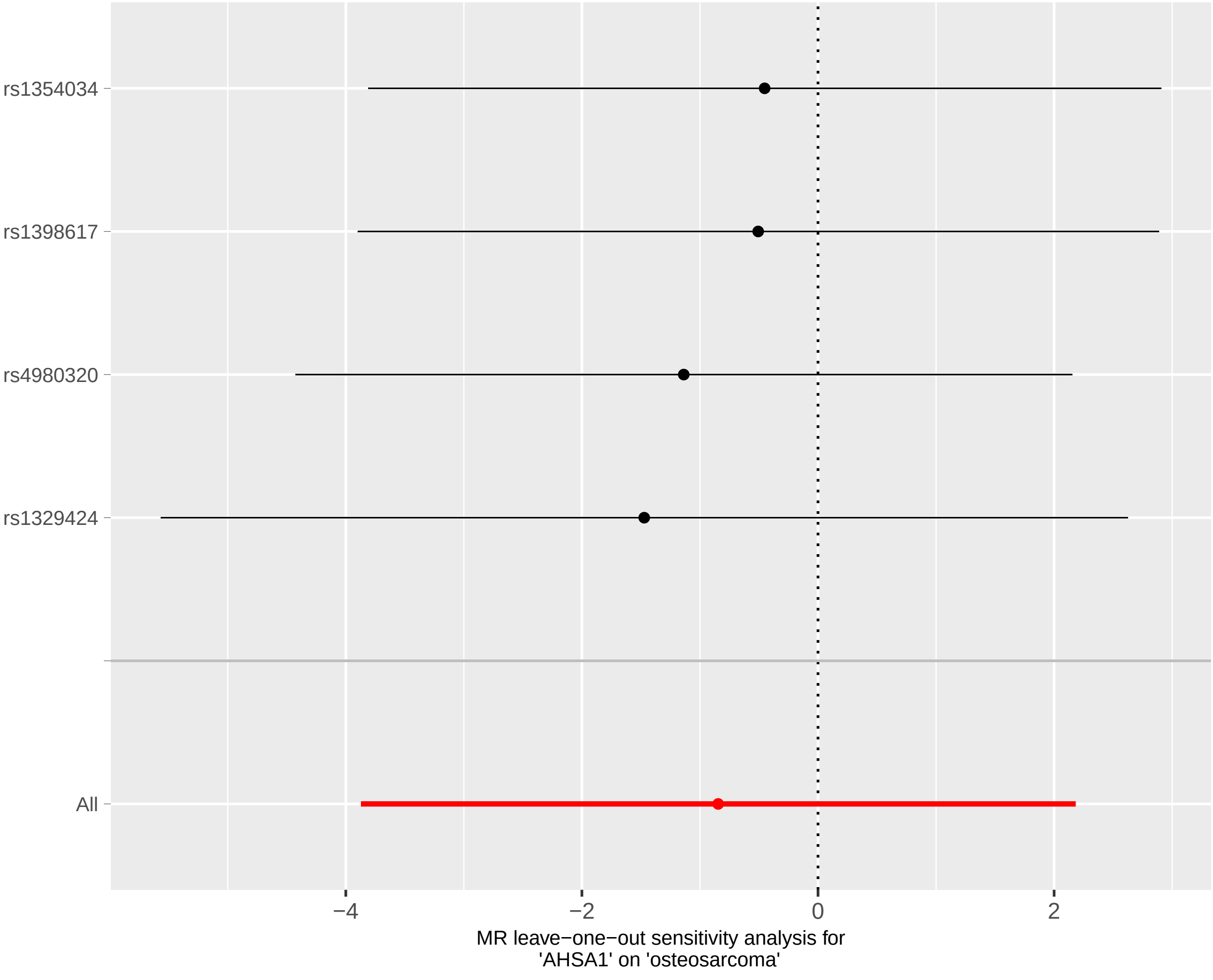

rs1354034
rs1398617
rs4980320
rs1329424
All
−4
−2
0
2
MR leave−one−out sensitivity analysis for
'AHSA1' on 'osteosarcoma'

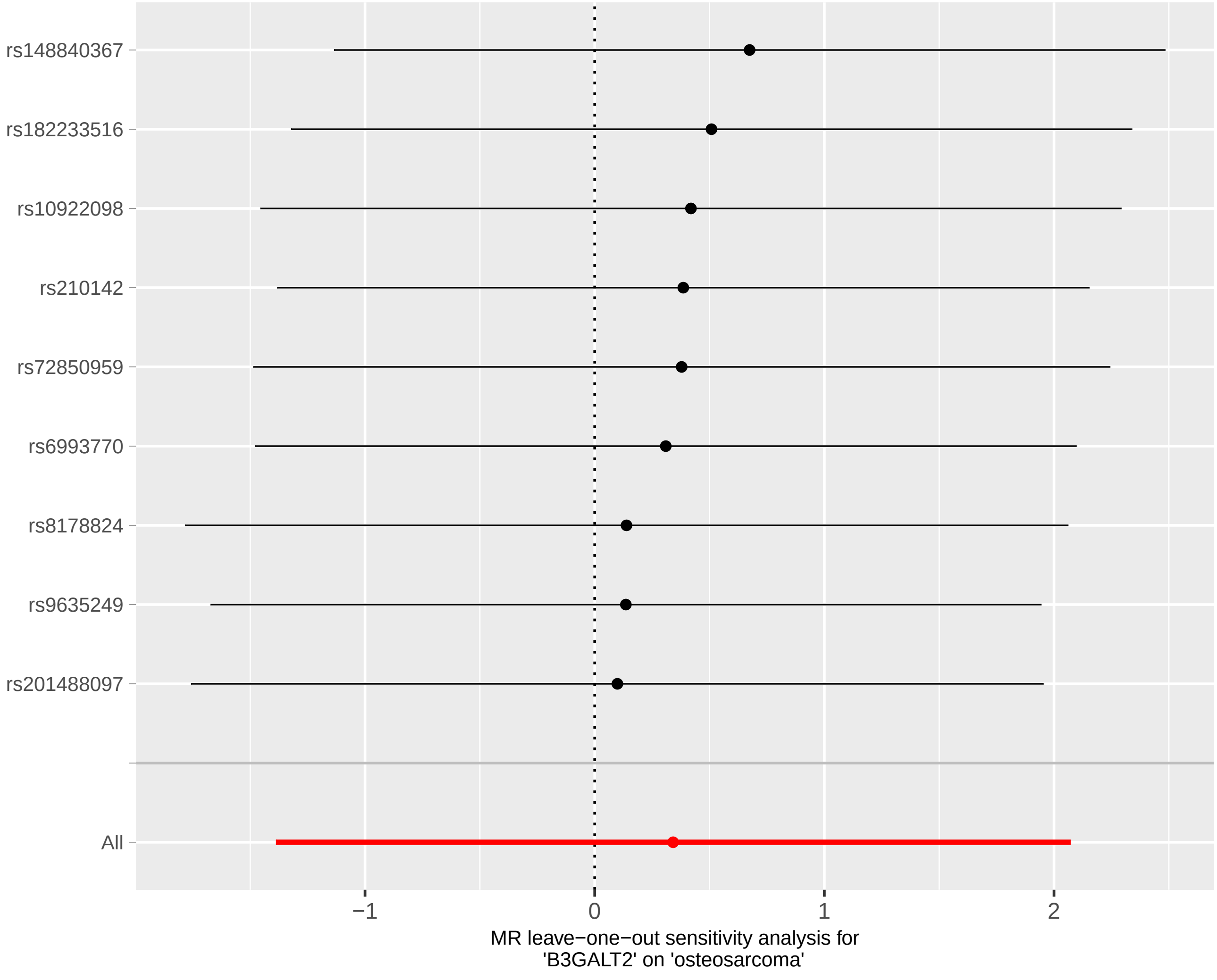

rs148840367
rs182233516
rs10922098
rs210142
rs72850959
rs6993770
rs8178824
rs9635249
rs201488097
All
−1
0
1
2
MR leave−one−out sensitivity analysis for
'B3GALT2' on 'osteosarcoma'

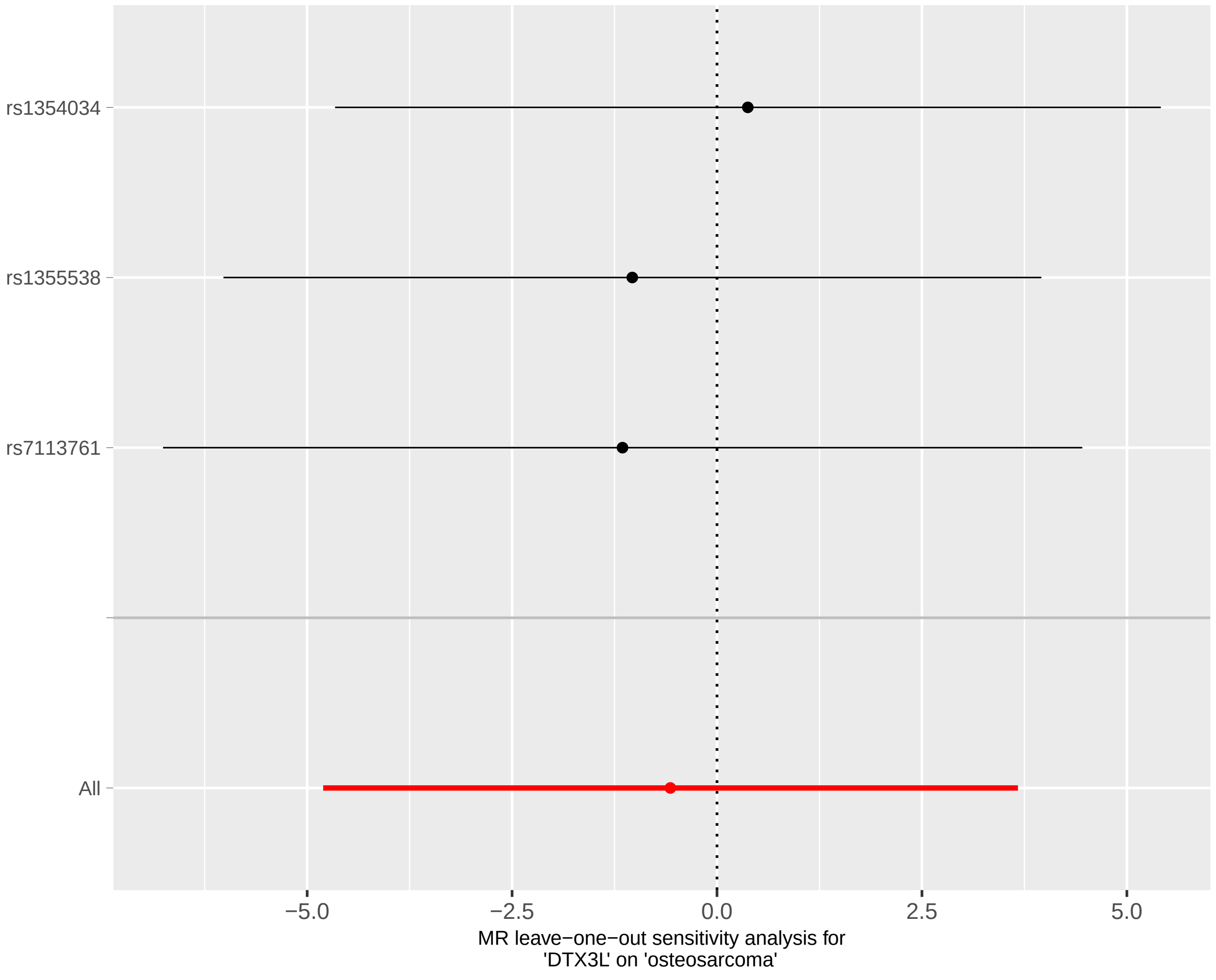

rs1354034
rs1355538
rs7113761
All
−5.0
−2.5
0.0
2.5
5.0
MR leave−one−out sensitivity analysis for
'DTX3L' on 'osteosarcoma'

Insufficient number of SNPs

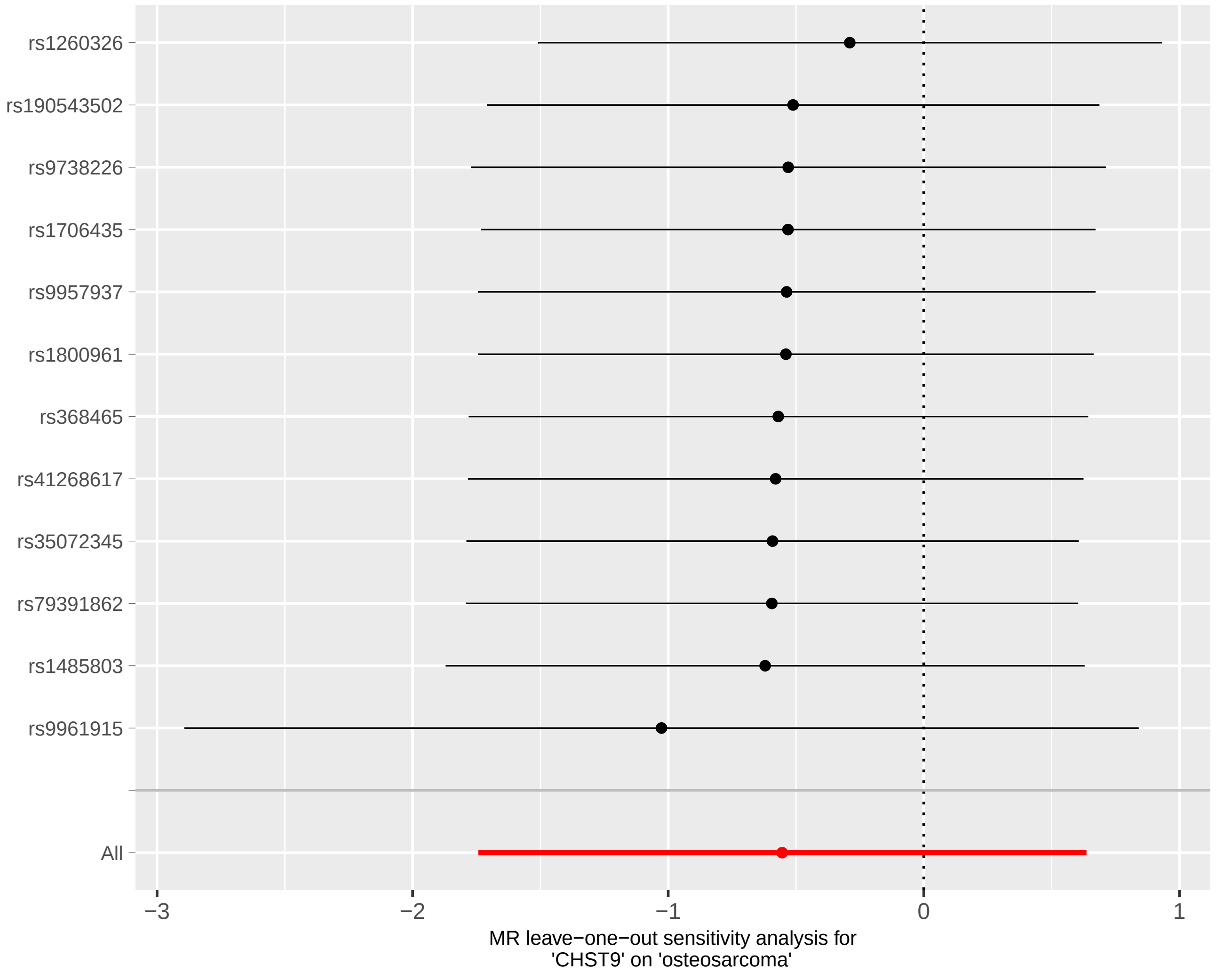
rs1260326
rs190543502
rs9738226
rs1706435
rs9957937
rs1800961
rs368465
rs41268617
rs35072345
rs79391862
rs1485803
rs9961915
All
−3
−2
−1
0
1
MR leave−one−out sensitivity analysis for
'CHST9' on 'osteosarcoma'

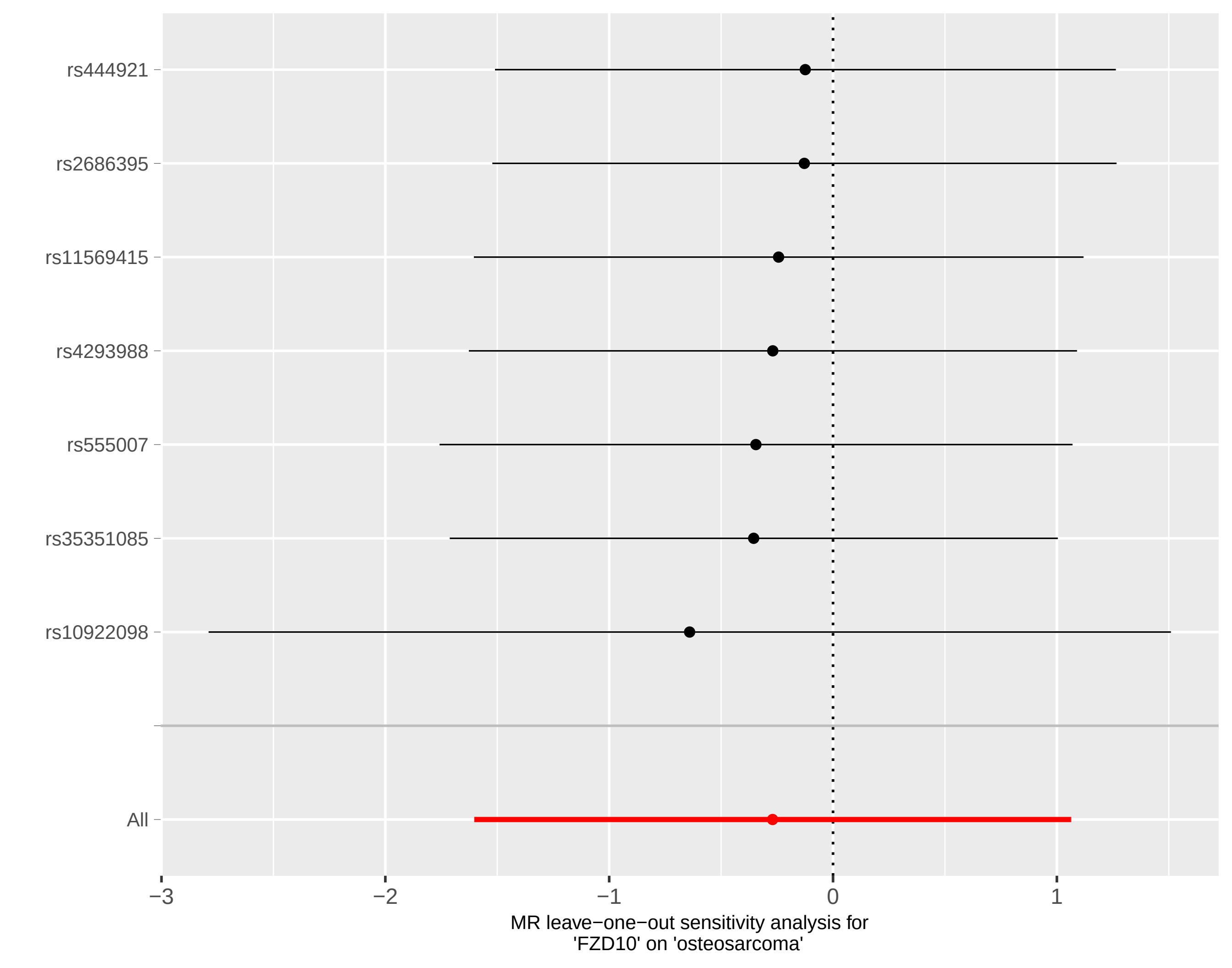
rs444921
rs2686395
rs11569415
rs4293988
rs555007
rs35351085
rs10922098
All
−3
−2
−1
0
1
MR leave−one−out sensitivity analysis for
'FZD10' on 'osteosarcoma'

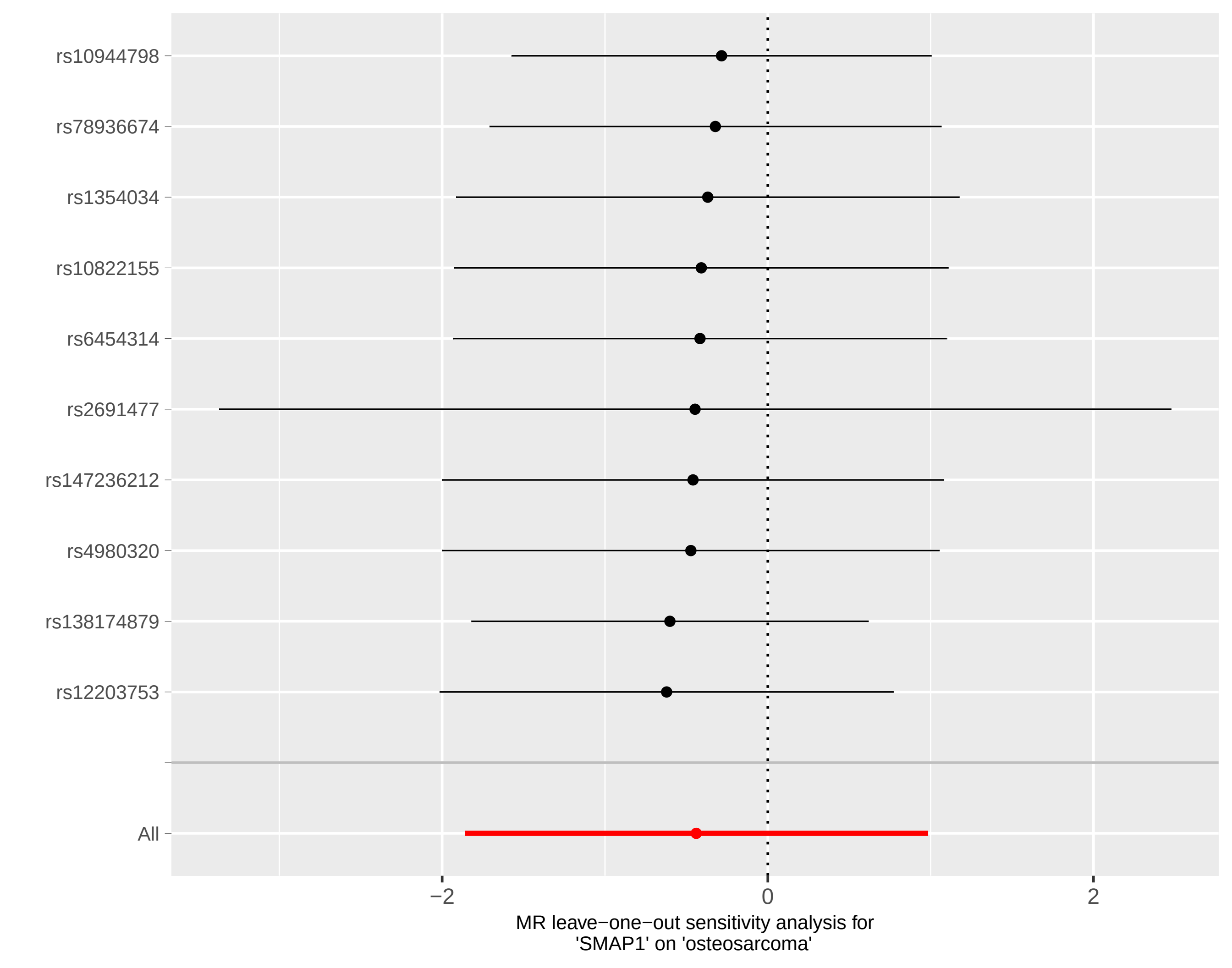
rs10944798
rs78936674
rs1354034
rs10822155
rs6454314
rs2691477
rs147236212
rs4980320
rs138174879
rs12203753
All
−2
0
2
MR leave−one−out sensitivity analysis for
'SMAP1' on 'osteosarcoma'

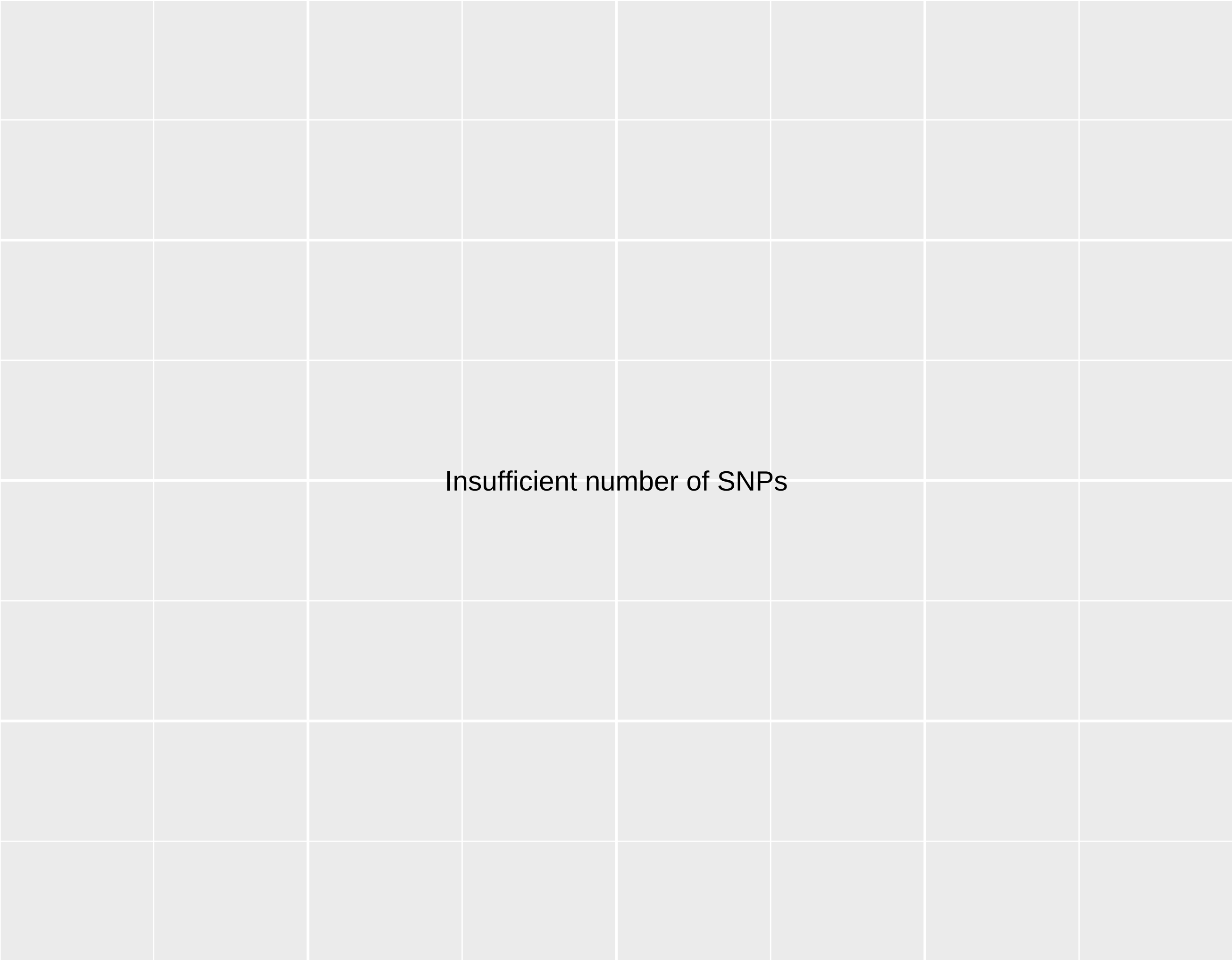
Insufficient number of SNPs

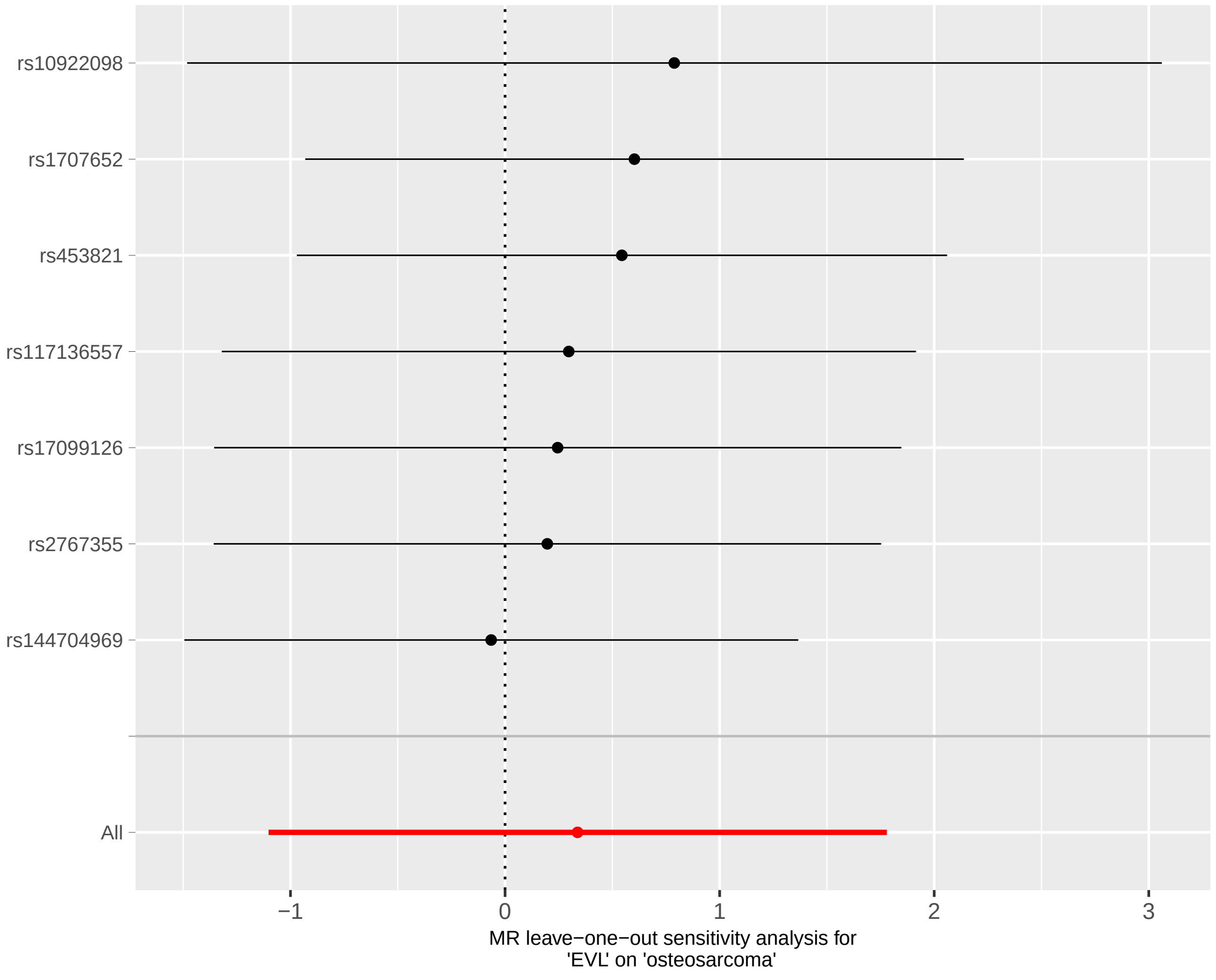
rs10922098
rs1707652
rs453821
rs117136557
rs17099126
rs2767355
rs144704969
All
−1
0
1
2
3
MR leave−one−out sensitivity analysis for
'EVL' on 'osteosarcoma'

Insufficient number of SNPs

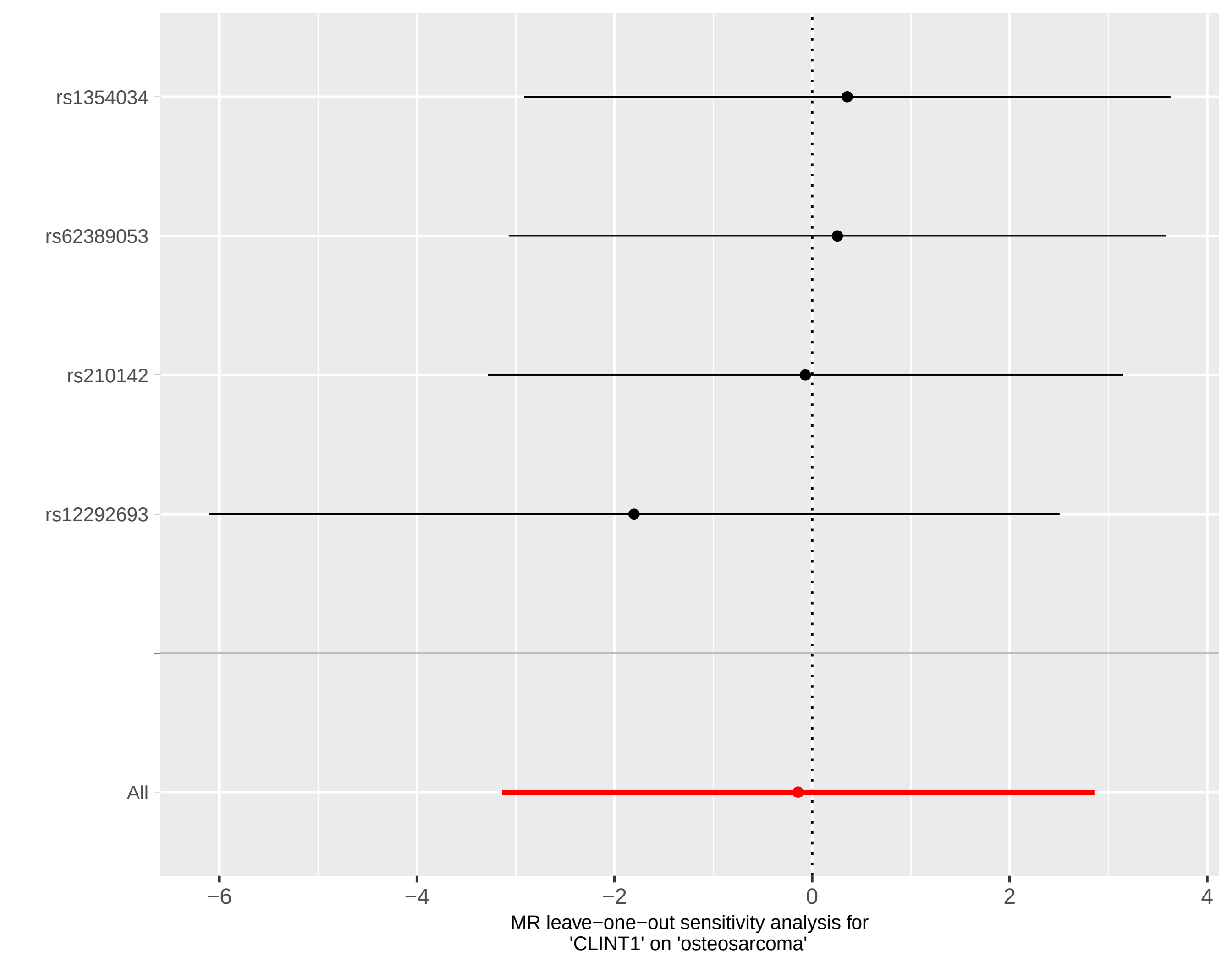
rs1354034
rs62389053
rs210142
rs12292693
All
−6
−4
−2
0
2
4
MR leave−one−out sensitivity analysis for
'CLINT1' on 'osteosarcoma'

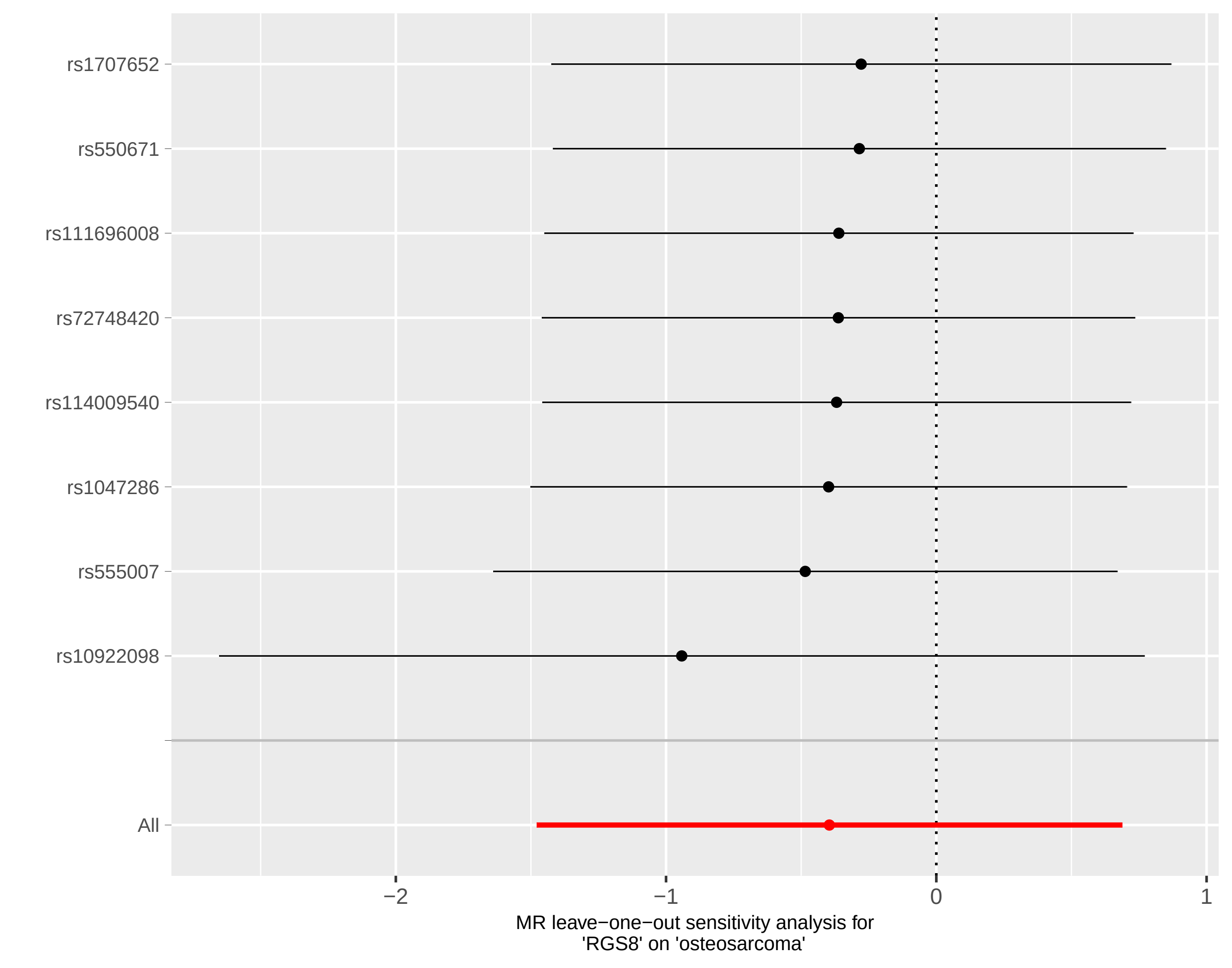
rs1707652
rs550671
rs111696008
rs72748420
rs114009540
rs1047286
rs555007
rs10922098
All
−2
−1
0
1
MR leave−one−out sensitivity analysis for
'RGS8' on 'osteosarcoma'

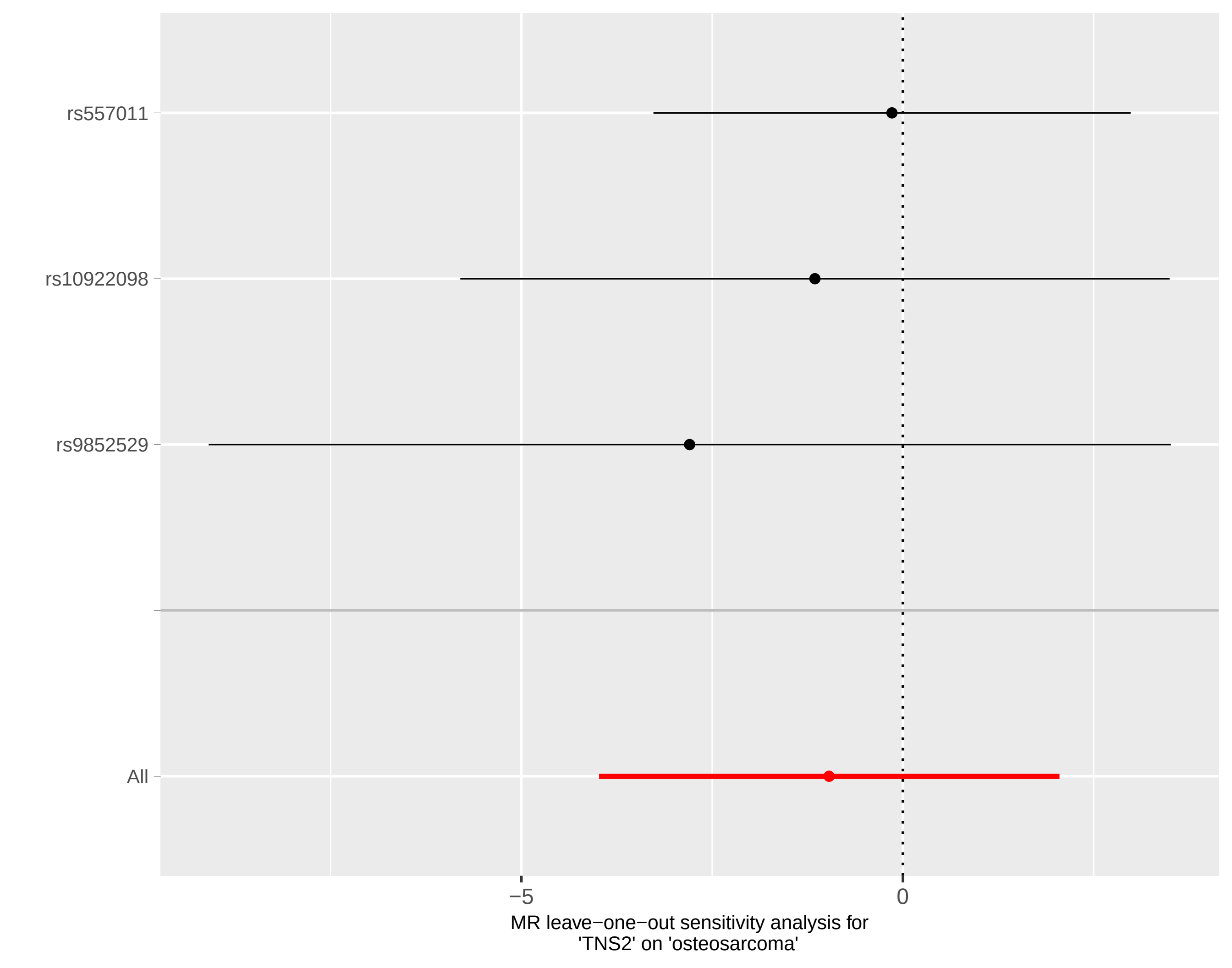
rs557011
rs10922098
rs9852529
All
−5
0
MR leave−one−out sensitivity analysis for
'TNS2' on 'osteosarcoma'

Insufficient number of SNPs

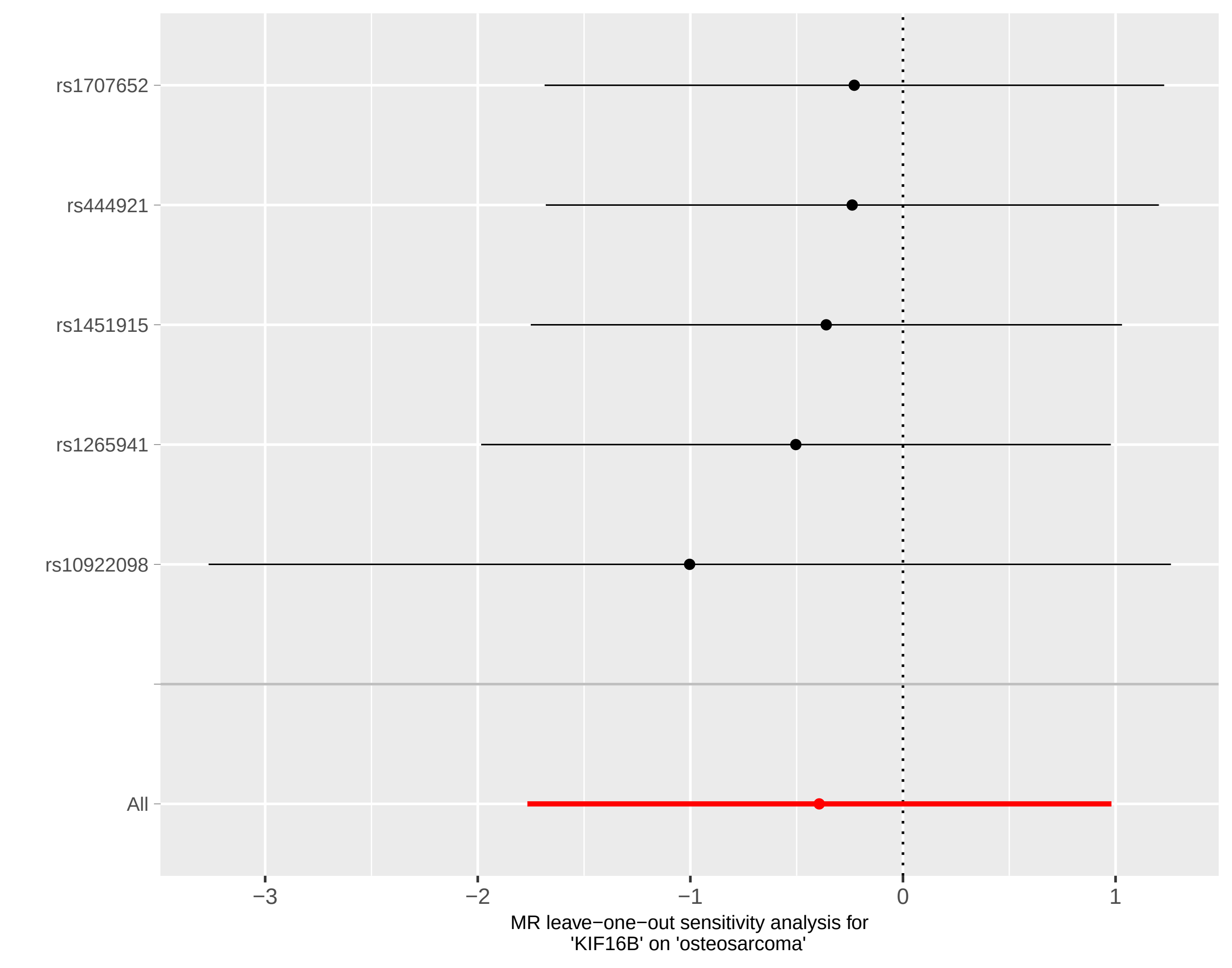
rs1707652
rs444921
rs1451915
rs1265941
rs10922098
All
−3
−2
−1
0
1
MR leave−one−out sensitivity analysis for
'KIF16B' on 'osteosarcoma'

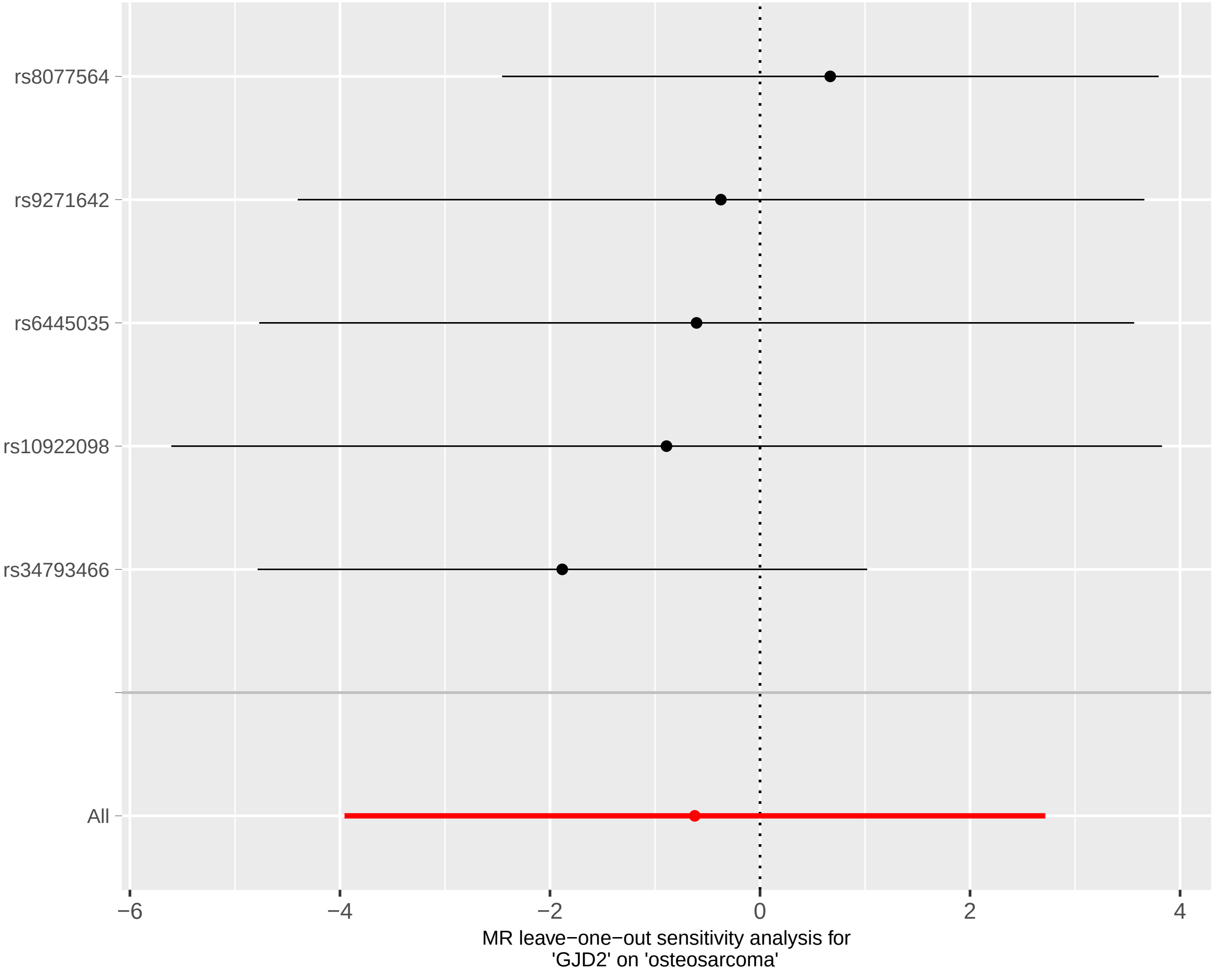

rs8077564
rs9271642
rs6445035
rs10922098
rs34793466
All
−6
−4
−2
0
2
4
MR leave−one−out sensitivity analysis for
'GJD2' on 'osteosarcoma'

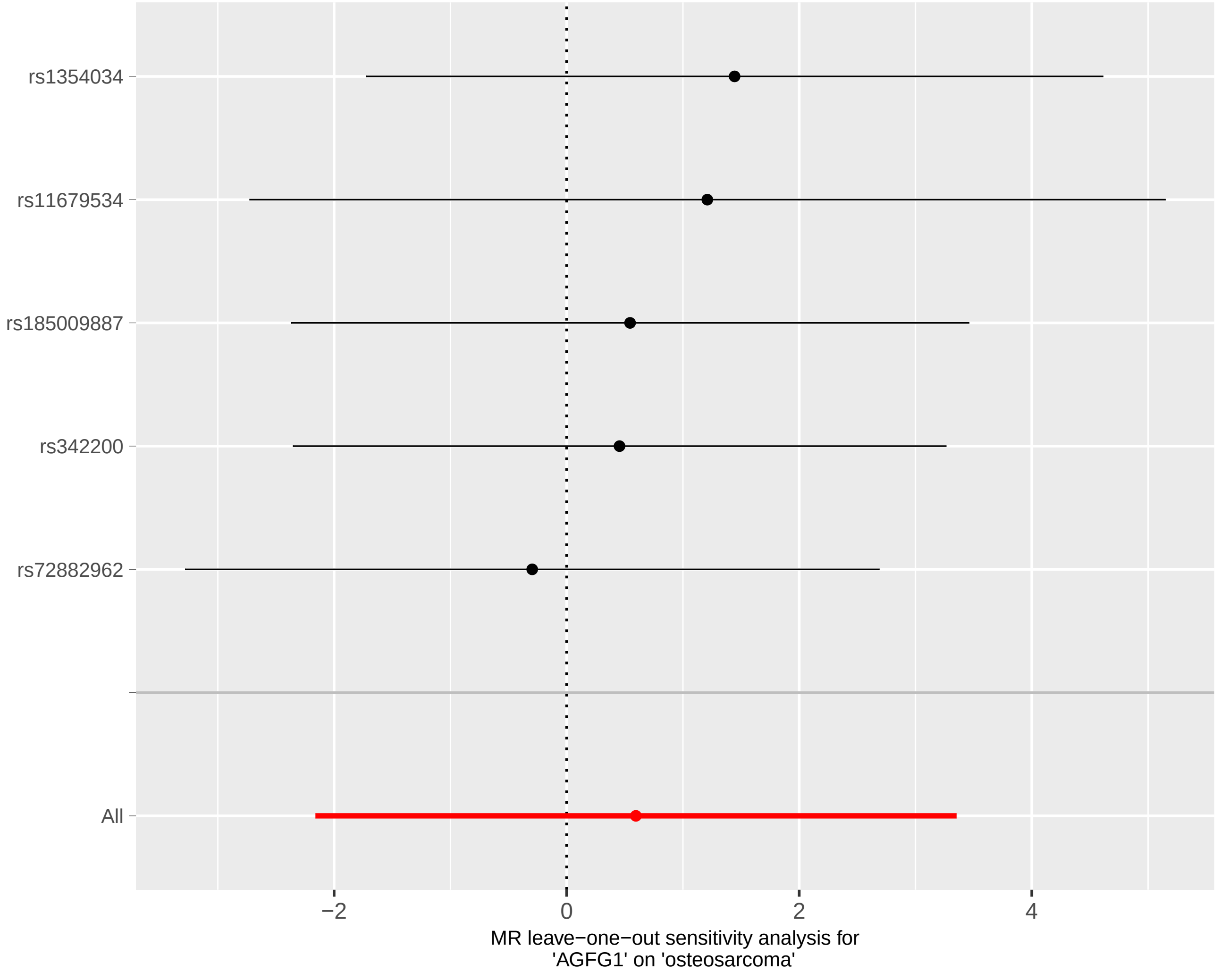
rs1354034
rs11679534
rs185009887
rs342200
rs72882962
All
−2
0
2
4
MR leave−one−out sensitivity analysis for
'AGFG1' on 'osteosarcoma'

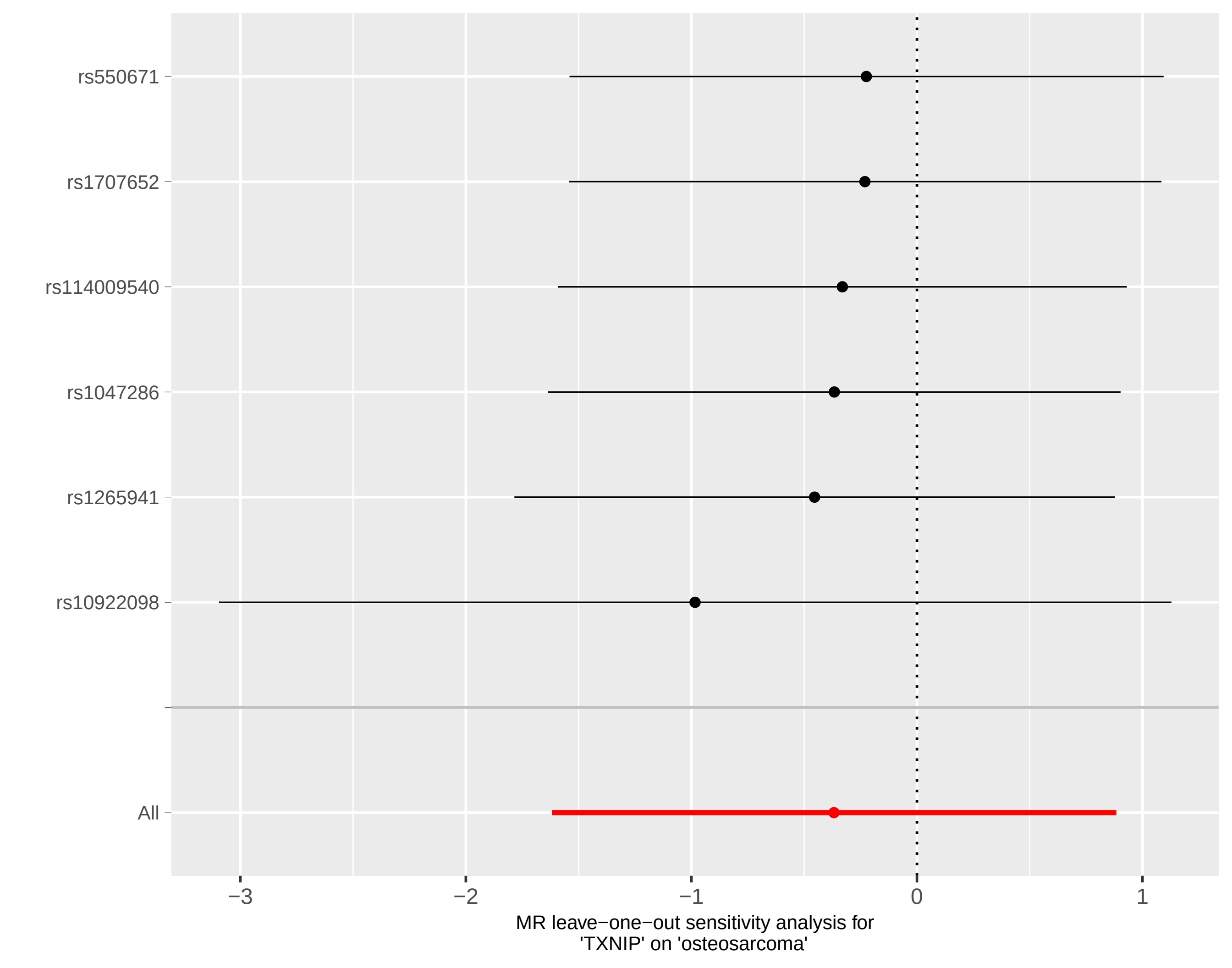

rs550671
rs1707652
rs114009540
rs1047286
rs1265941
rs10922098
All
−3
−2
−1
0
1
MR leave−one−out sensitivity analysis for
'TXNIP' on 'osteosarcoma'

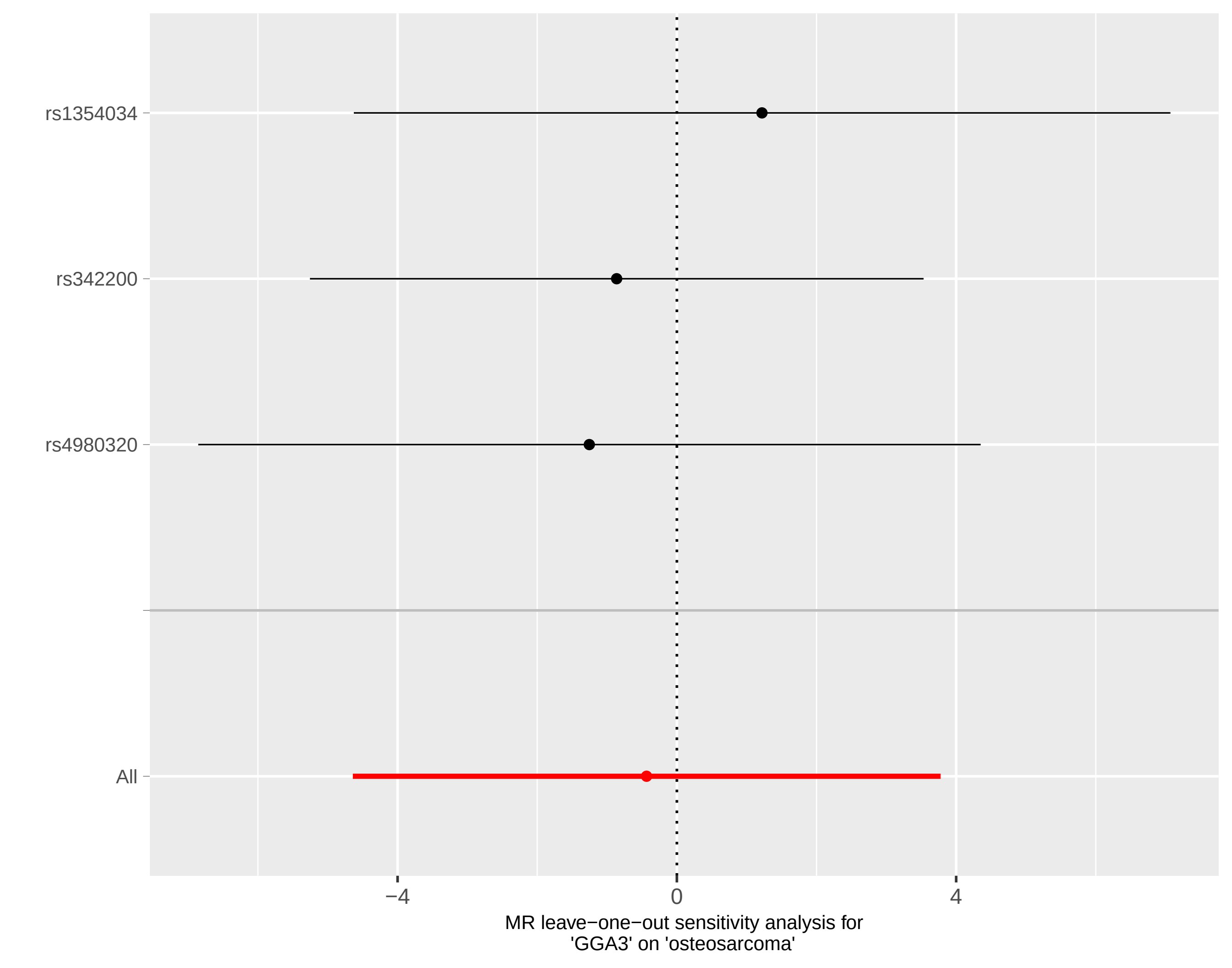
rs1354034
rs342200
rs4980320
All
−4
0
4
MR leave−one−out sensitivity analysis for
'GGA3' on 'osteosarcoma'

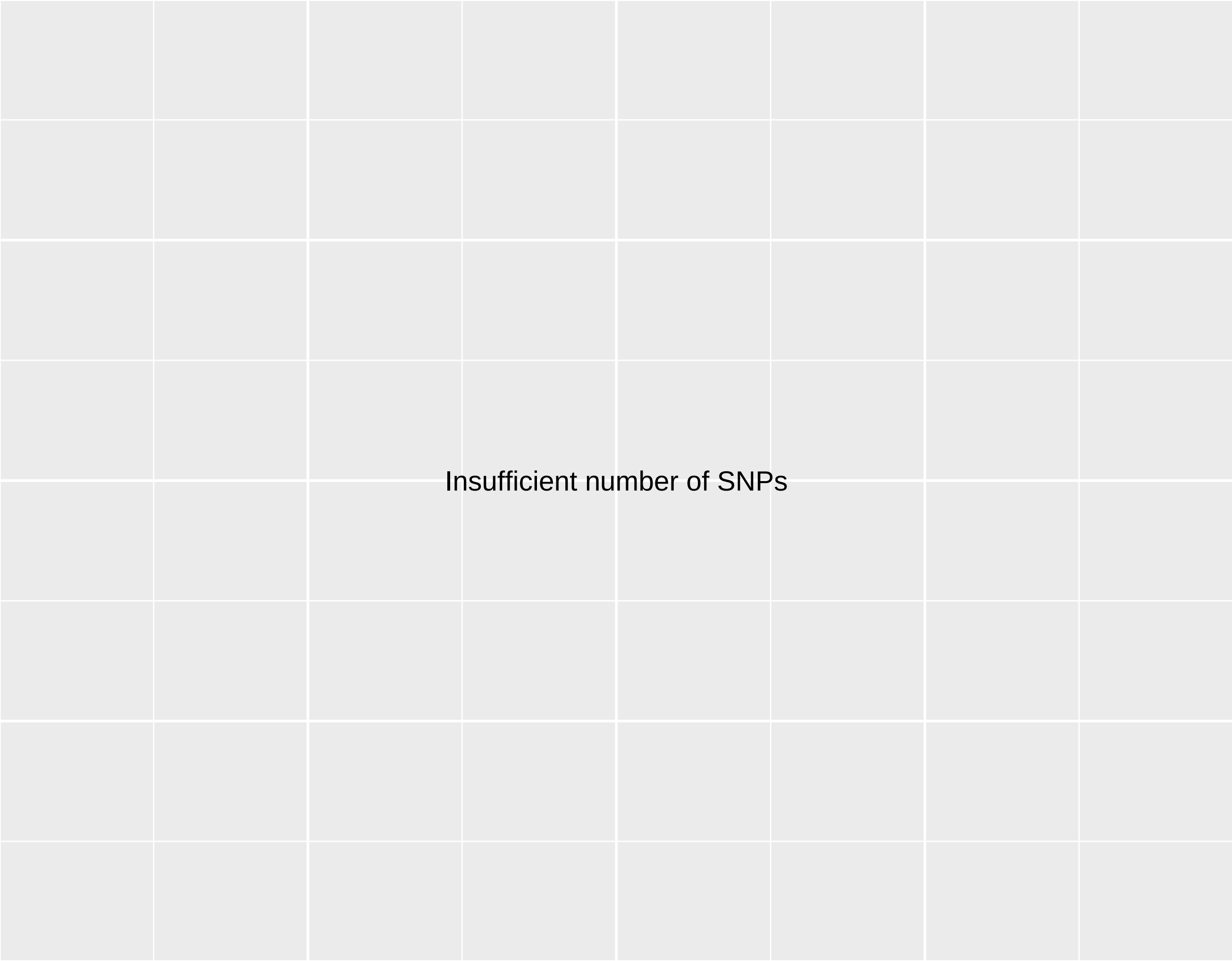
Insufficient number of SNPs

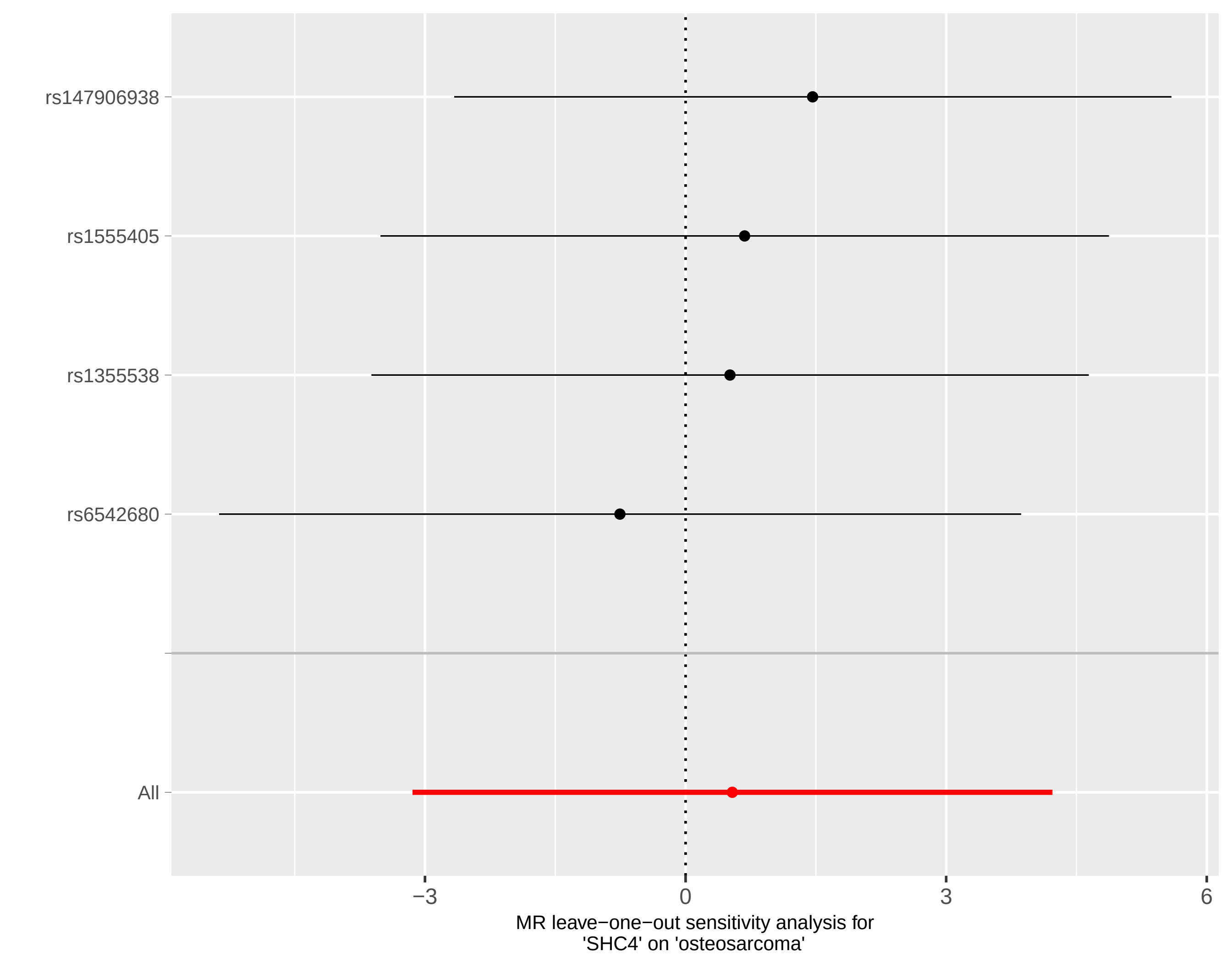
rs147906938
rs1555405
rs1355538
rs6542680
All
−3
0
3
6
MR leave−one−out sensitivity analysis for
'SHC4' on 'osteosarcoma'

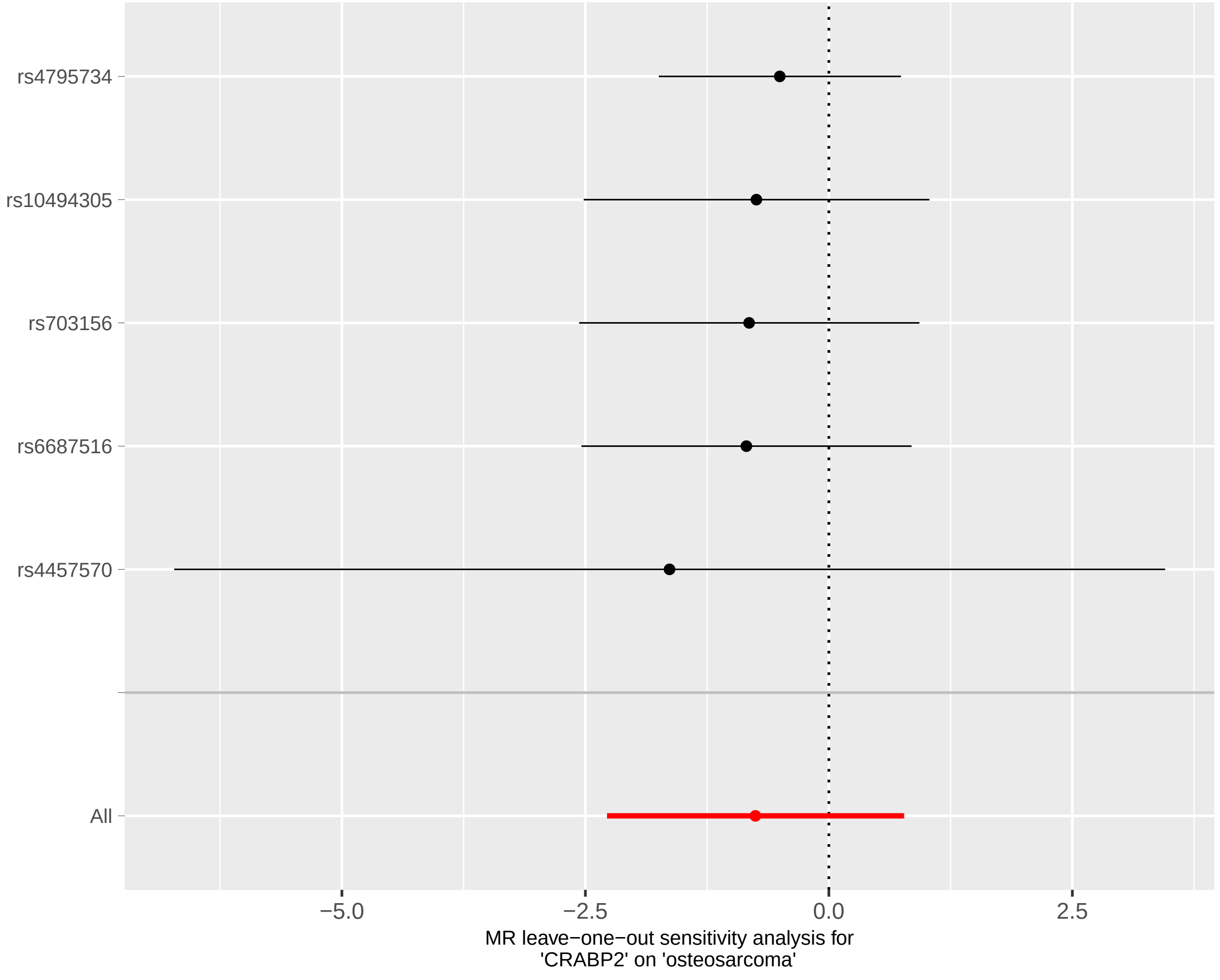

rs4795734
rs10494305
rs703156
rs6687516
rs4457570
All
−5.0
−2.5
0.0
2.5
MR leave−one−out sensitivity analysis for
'CRABP2' on 'osteosarcoma'

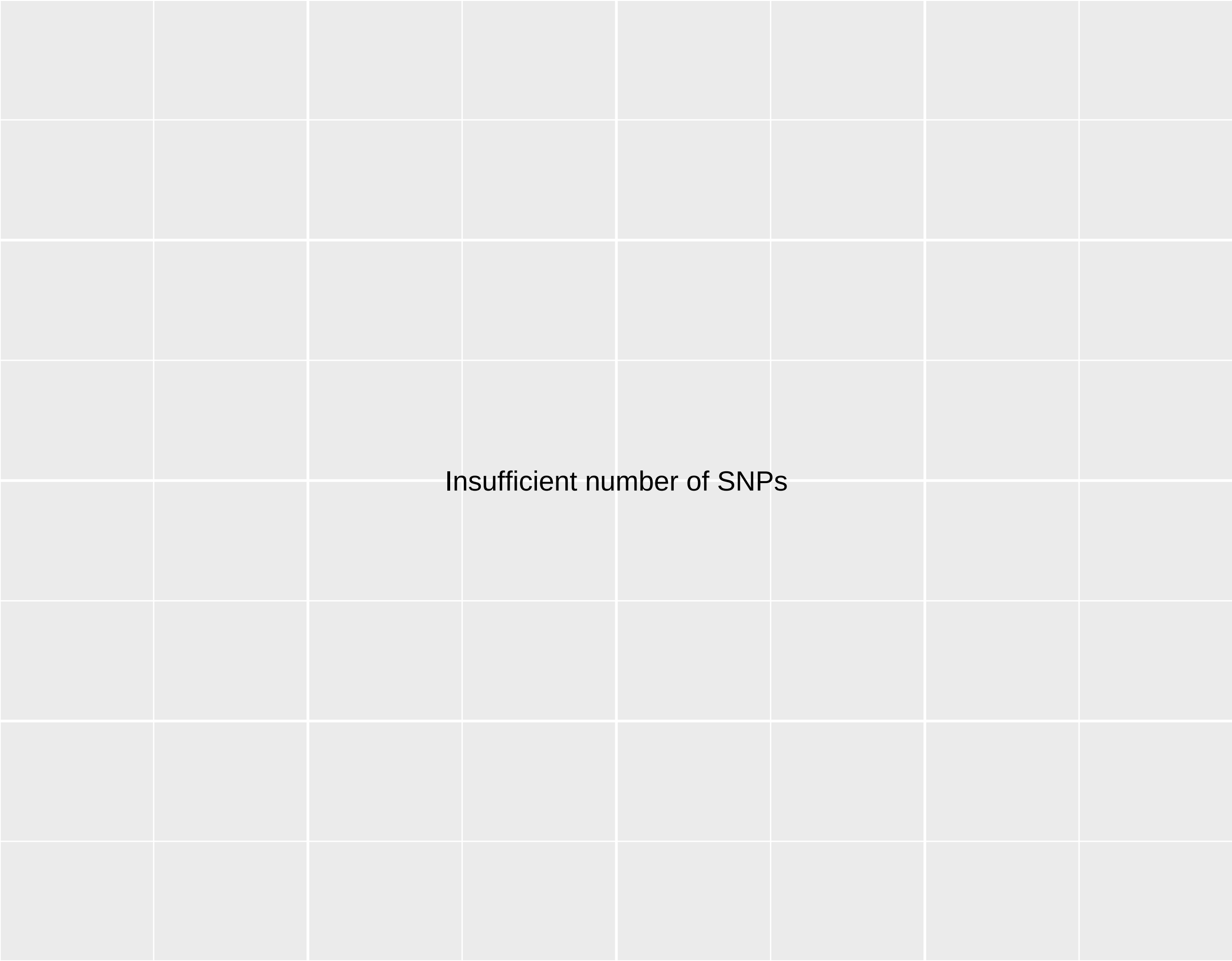
Insufficient number of SNPs

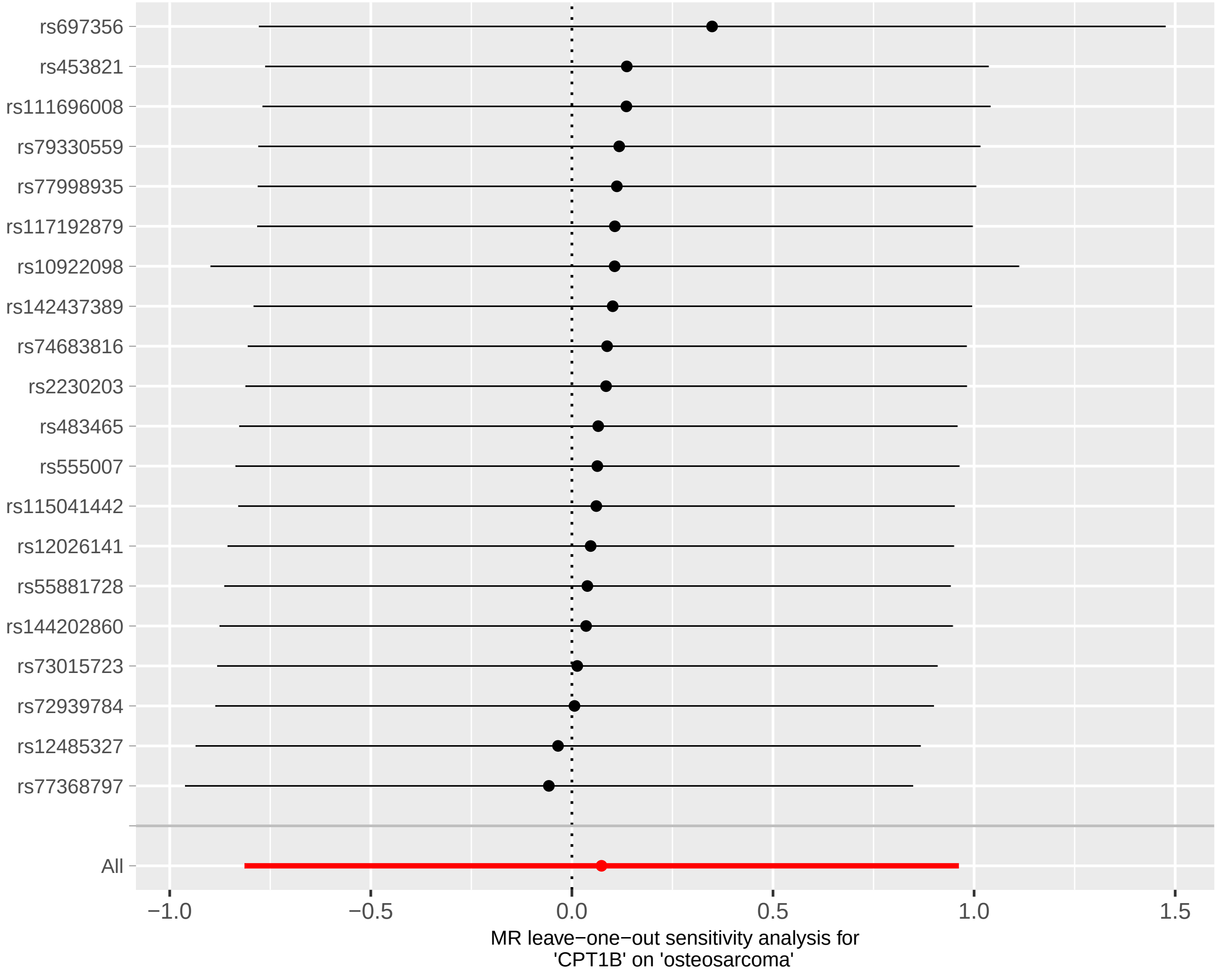
rs697356
rs453821
rs111696008
rs79330559
rs77998935
rs117192879
rs10922098
rs142437389
rs74683816
rs2230203
rs483465
rs555007
rs115041442
rs12026141
rs55881728
rs144202860
rs73015723
rs72939784
rs12485327
rs77368797
All
−1.0
−0.5
0.0
0.5
1.0
1.5
MR leave−one−out sensitivity analysis for
'CPT1B' on 'osteosarcoma'

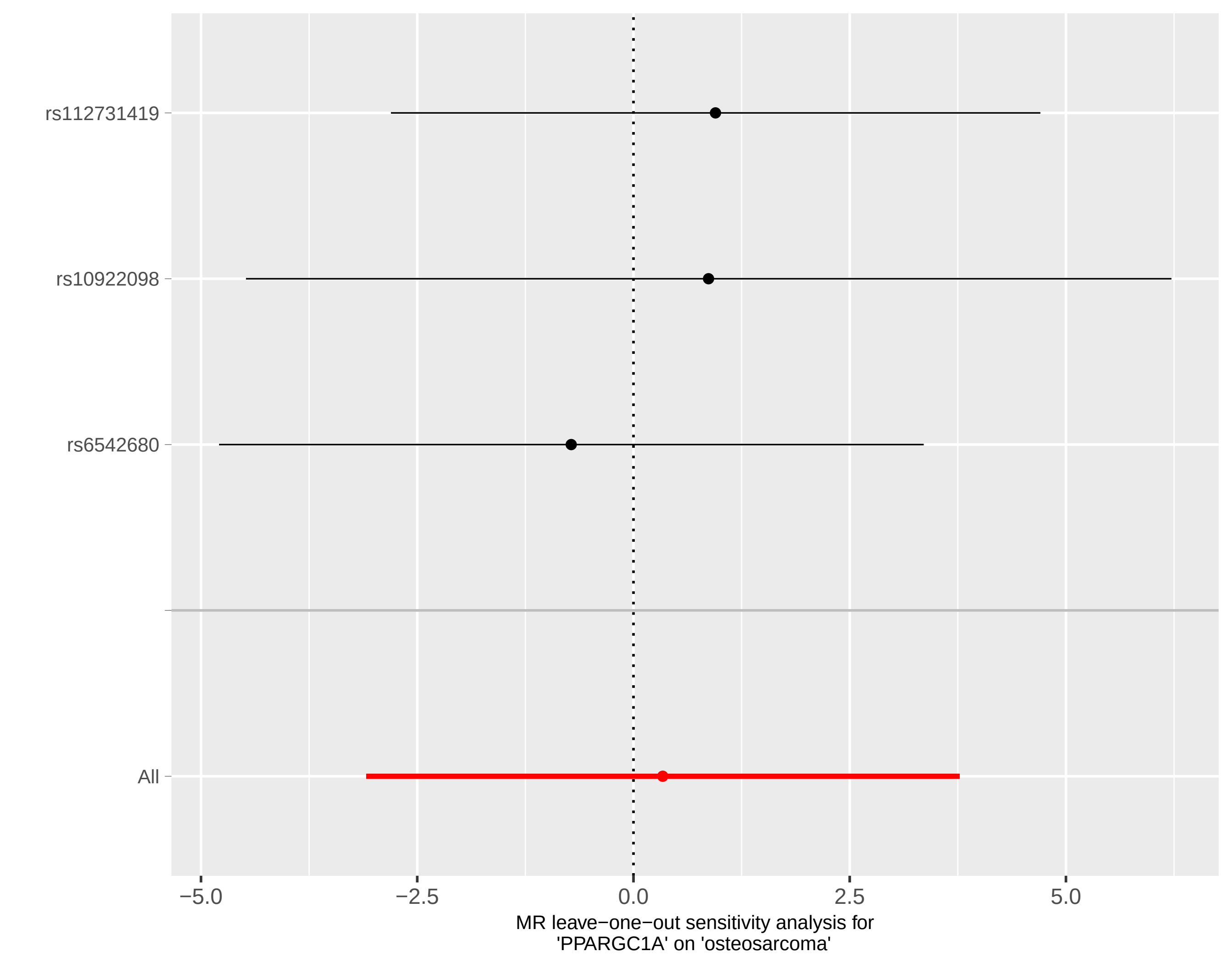
rs112731419
rs10922098
rs6542680
All
−5.0
−2.5
0.0
2.5
5.0
MR leave−one−out sensitivity analysis for
'PPARGC1A' on 'osteosarcoma'

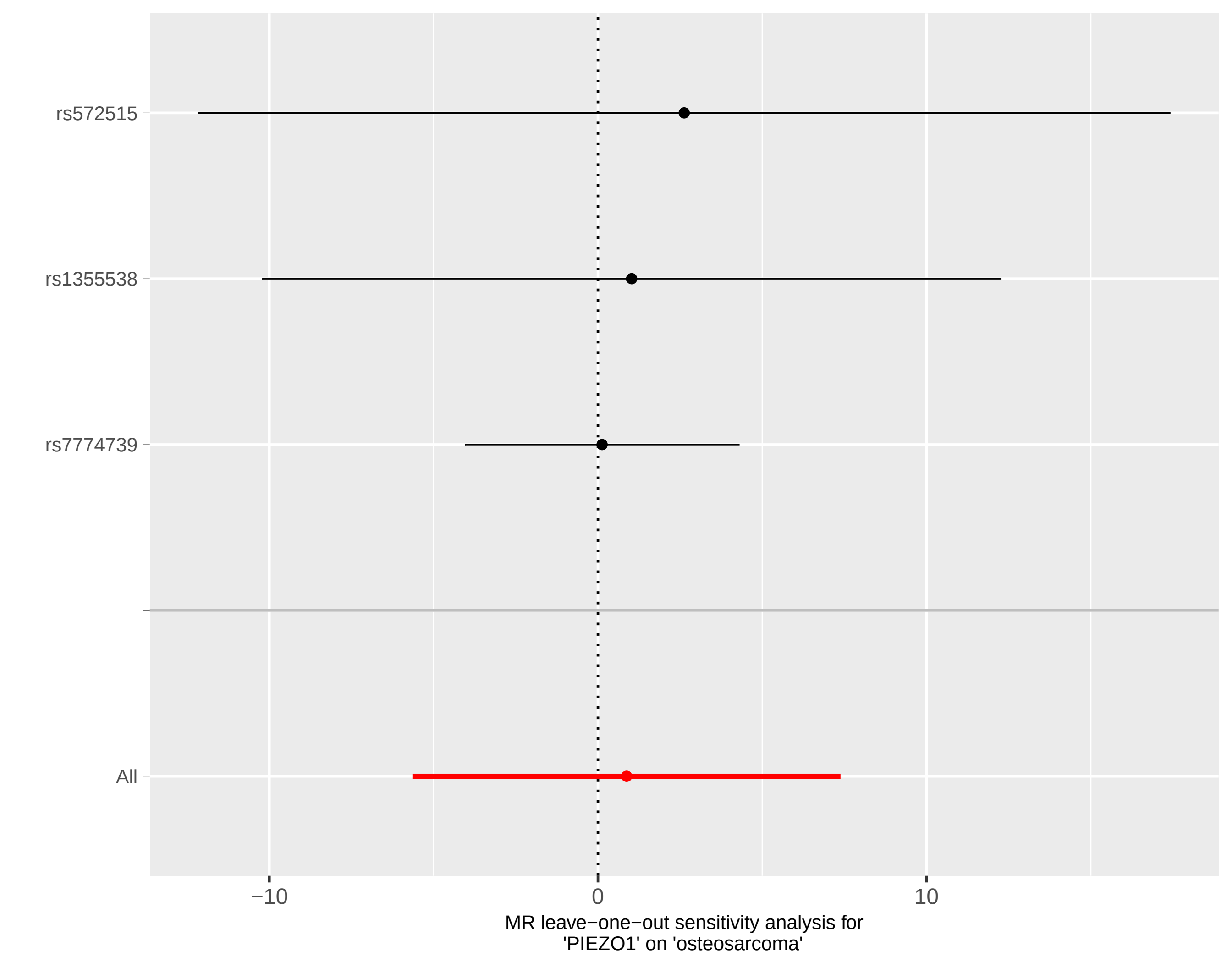
rs572515
rs1355538
rs7774739
All
−10
0
10
MR leave−one−out sensitivity analysis for
'PIEZO1' on 'osteosarcoma'

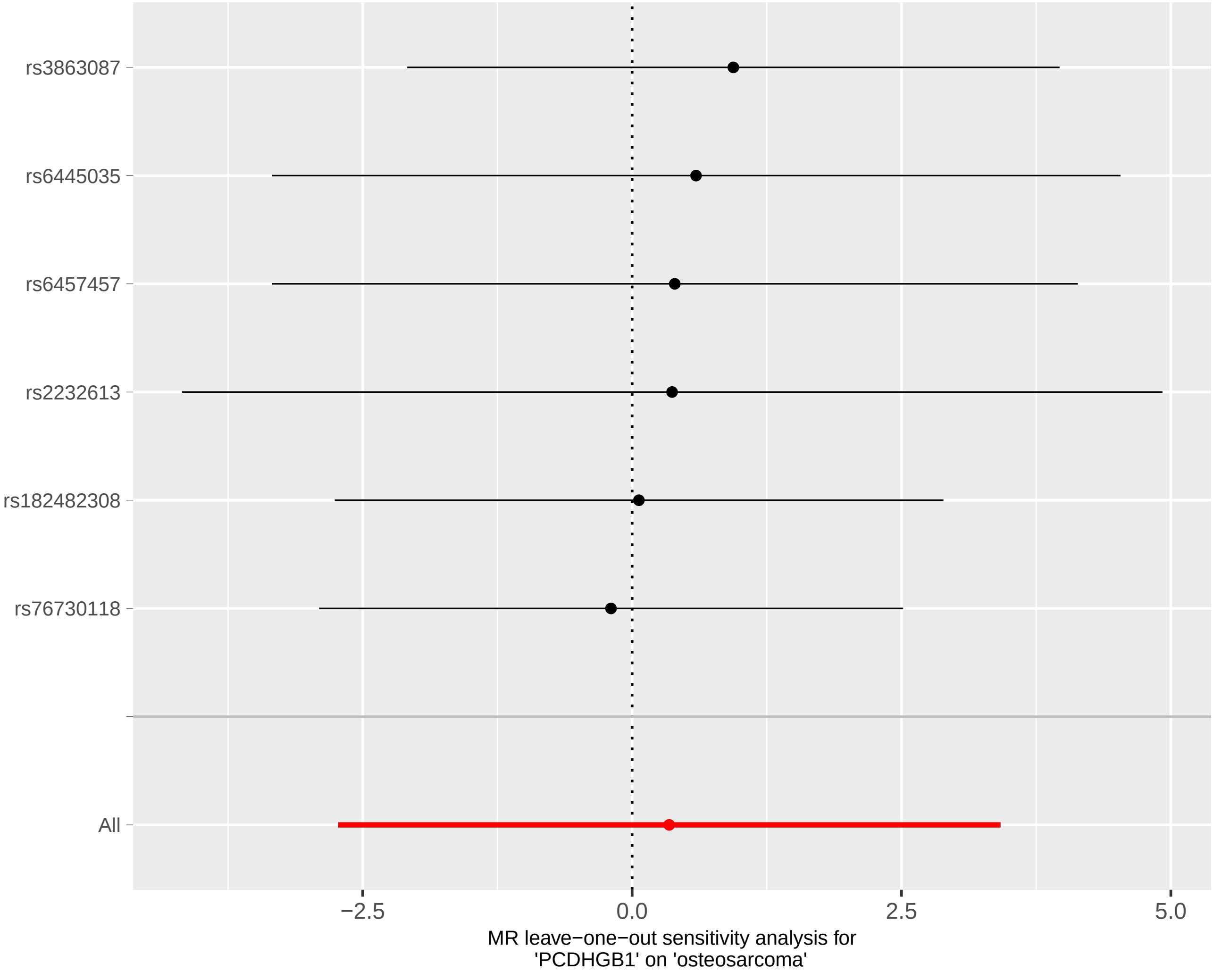
rs3863087
rs6445035
rs6457457
rs2232613
rs182482308
rs76730118
All
−2.5
0.0
2.5
5.0
MR leave−one−out sensitivity analysis for
'PCDHGB1' on 'osteosarcoma'

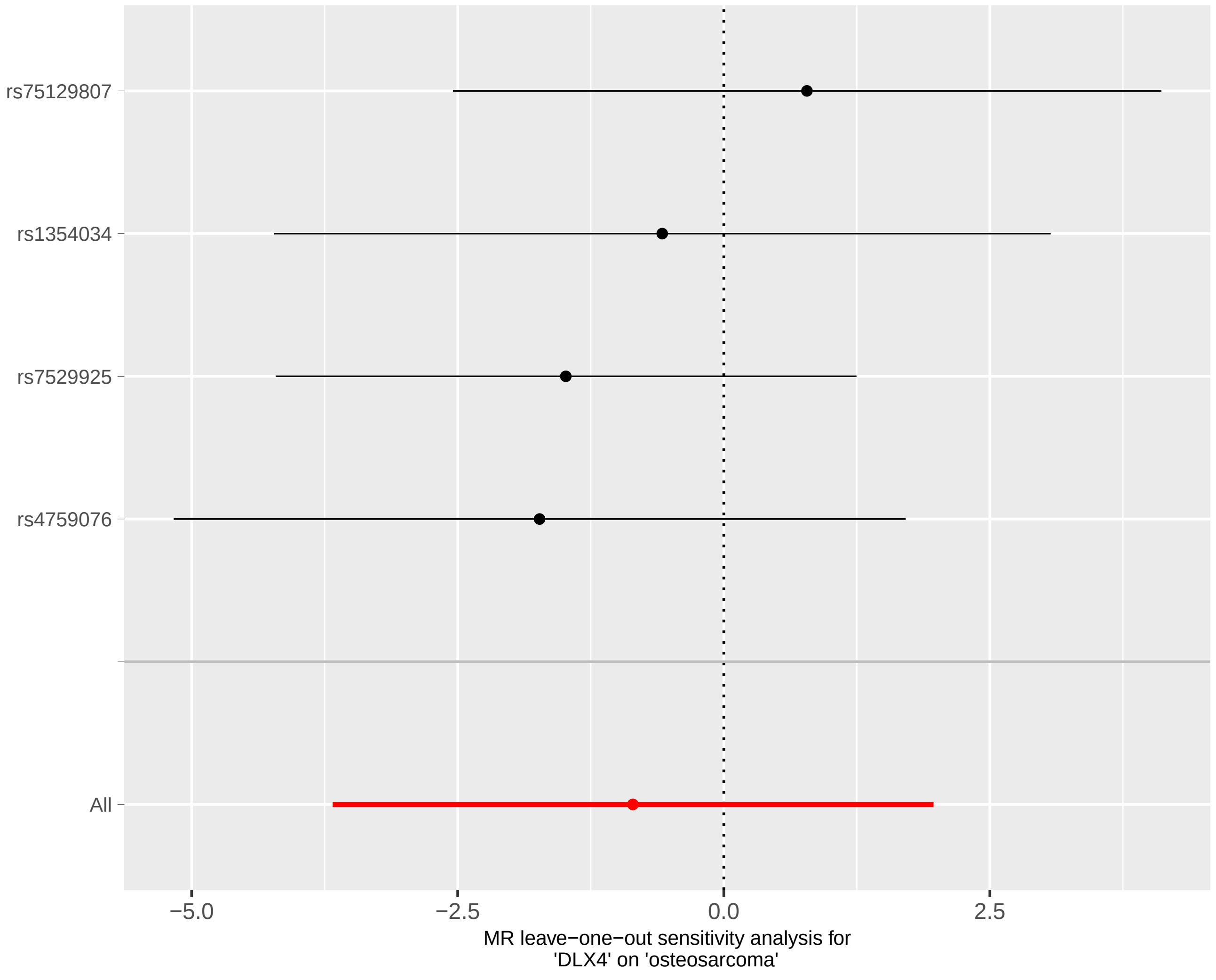
rs75129807
rs1354034
rs7529925
rs4759076
All
−5.0
−2.5
0.0
2.5
MR leave−one−out sensitivity analysis for
'DLX4' on 'osteosarcoma'

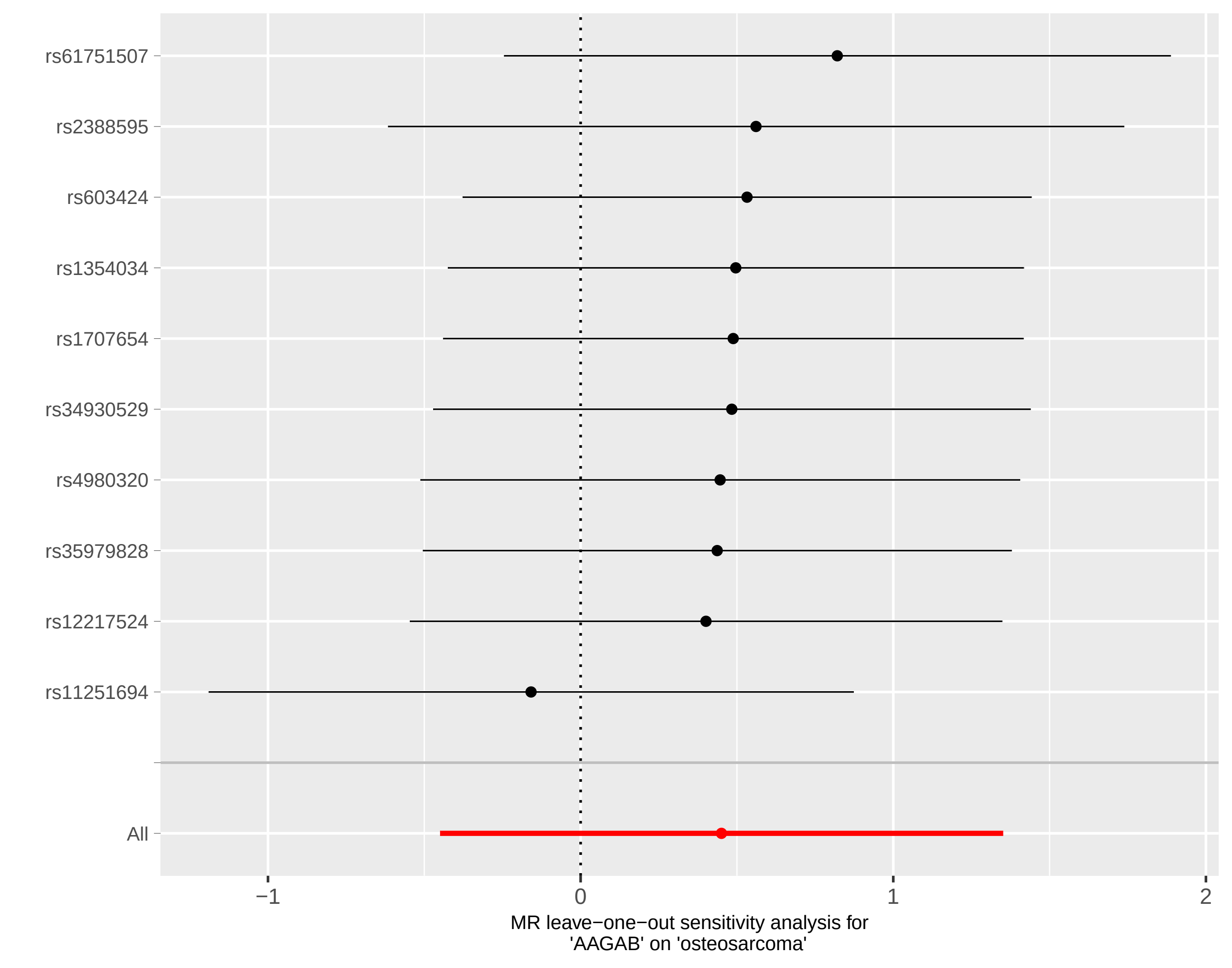

rs61751507
rs2388595
rs603424
rs1354034
rs1707654
rs34930529
rs4980320
rs35979828
rs12217524
rs11251694
All
−1
0
1
2
MR leave−one−out sensitivity analysis for
'AAGAB' on 'osteosarcoma'

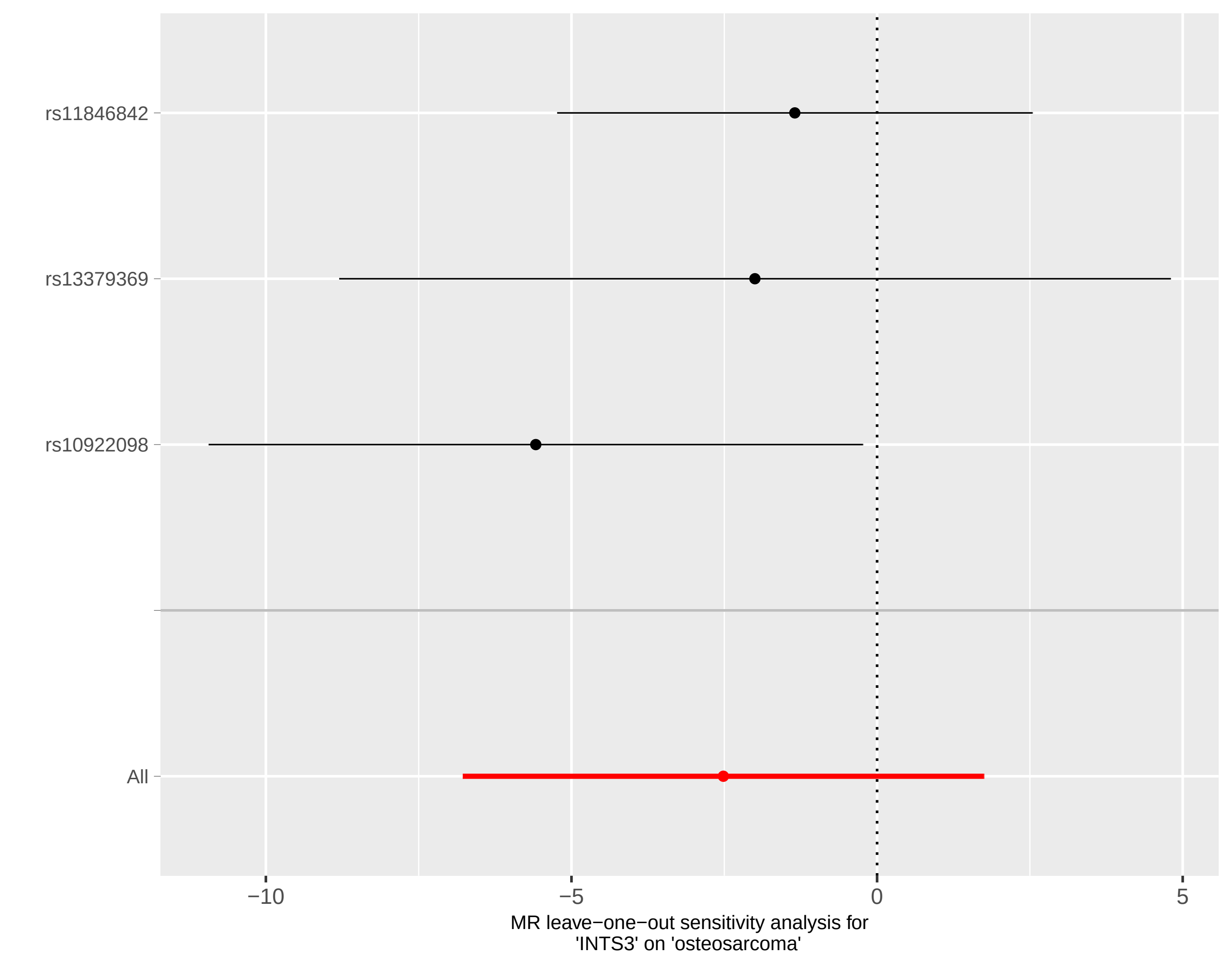
rs11846842
rs13379369
rs10922098
All
−10
−5
0
5
MR leave−one−out sensitivity analysis for
'INTS3' on 'osteosarcoma'

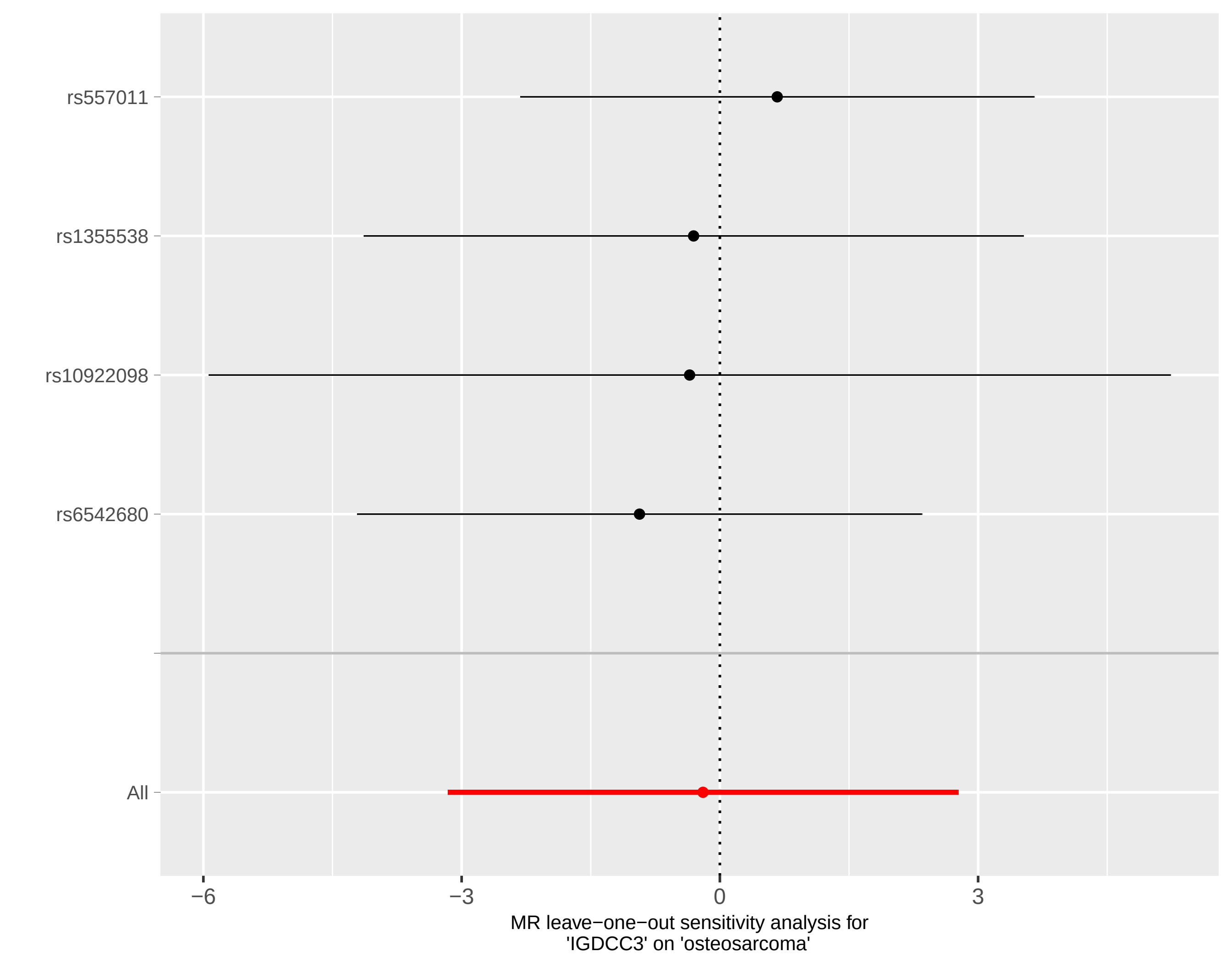

rs557011
rs1355538
rs10922098
rs6542680
All
−6
−3
0
3
MR leave−one−out sensitivity analysis for
'IGDCC3' on 'osteosarcoma'

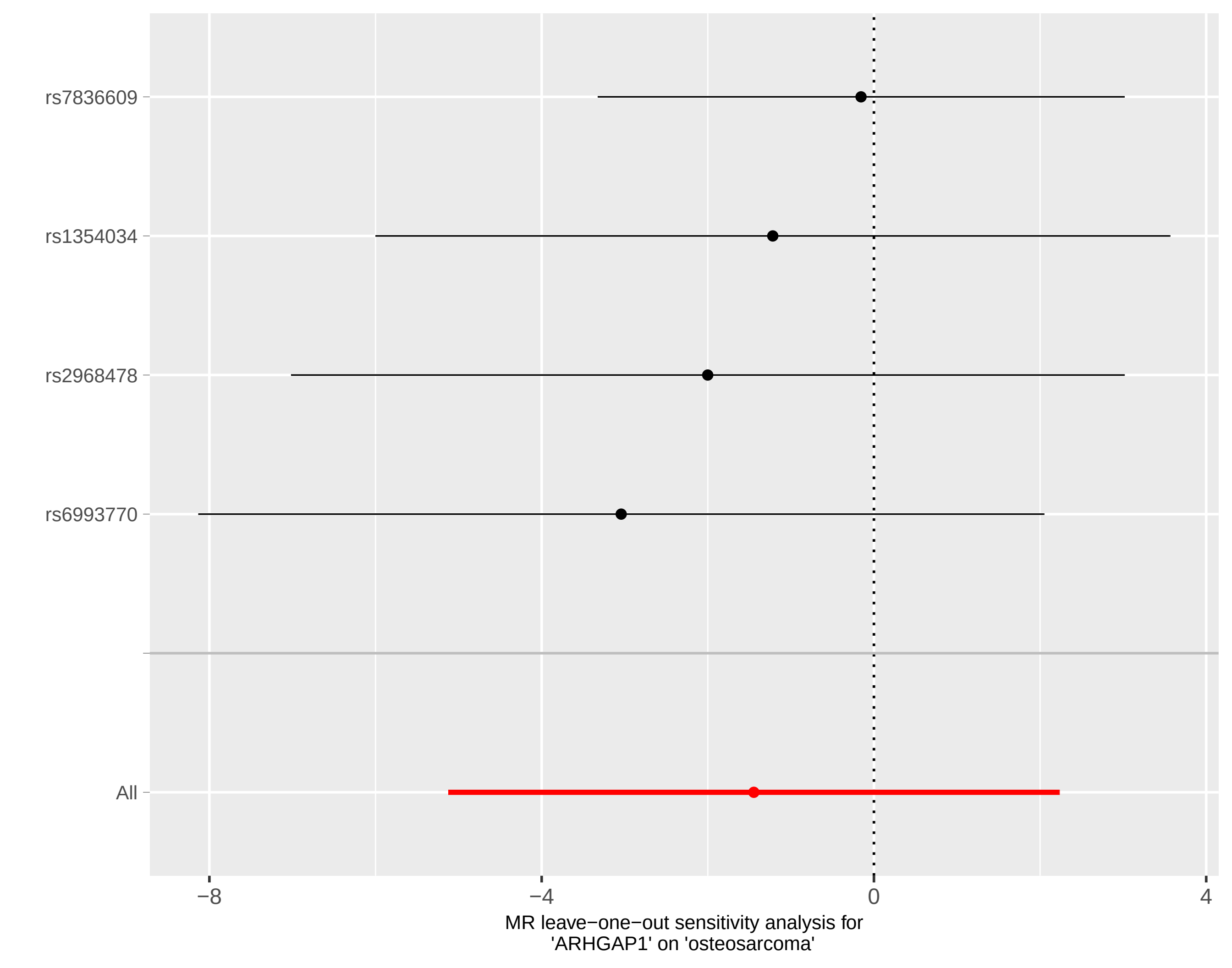
rs7836609
rs1354034
rs2968478
rs6993770
All
−8
−4
0
4
MR leave−one−out sensitivity analysis for
'ARHGAP1' on 'osteosarcoma'

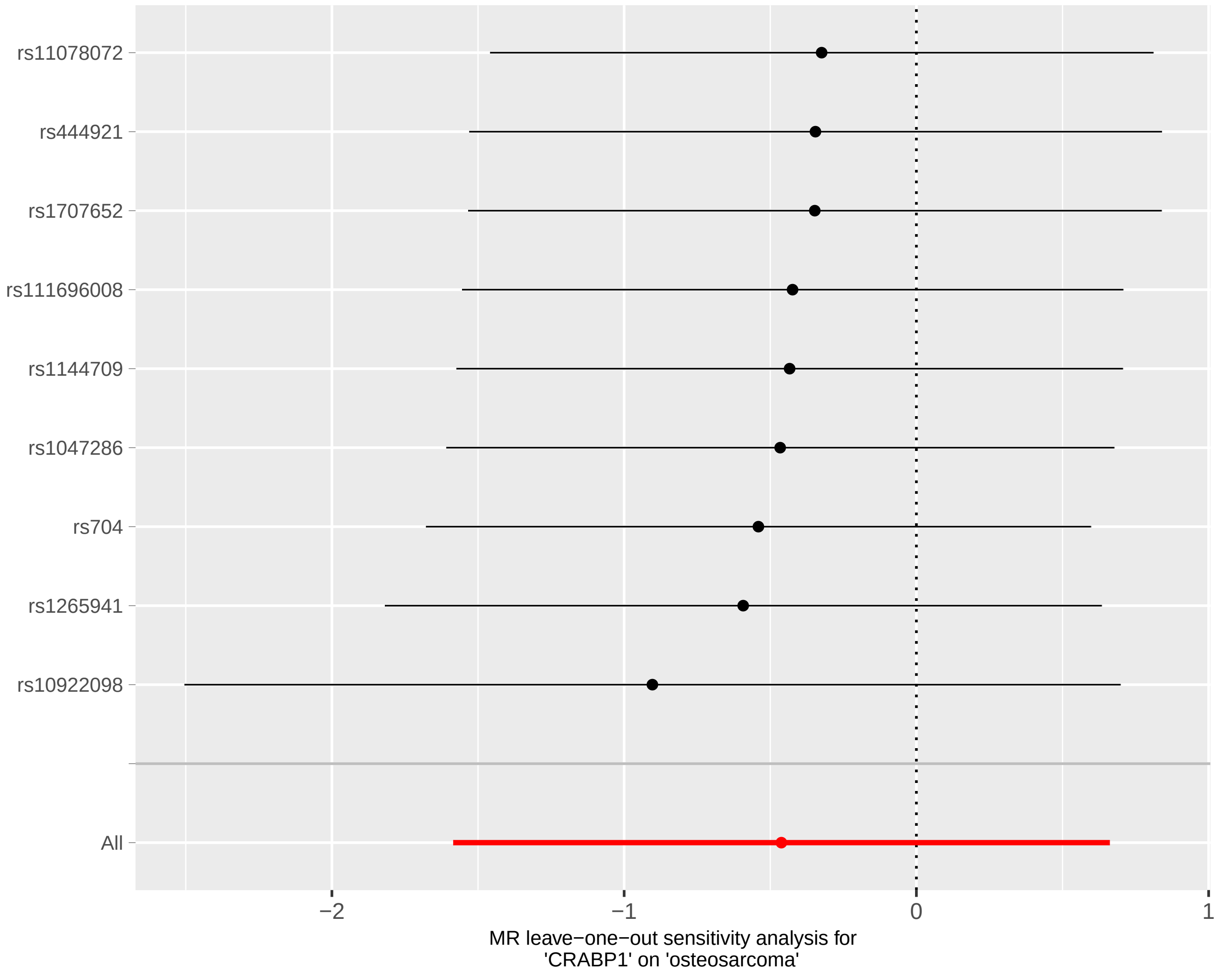
rs11078072
rs444921
rs1707652
rs111696008
rs1144709
rs1047286
rs704
rs1265941
rs10922098
All
−2
−1
0
1
MR leave−one−out sensitivity analysis for
'CRABP1' on 'osteosarcoma'

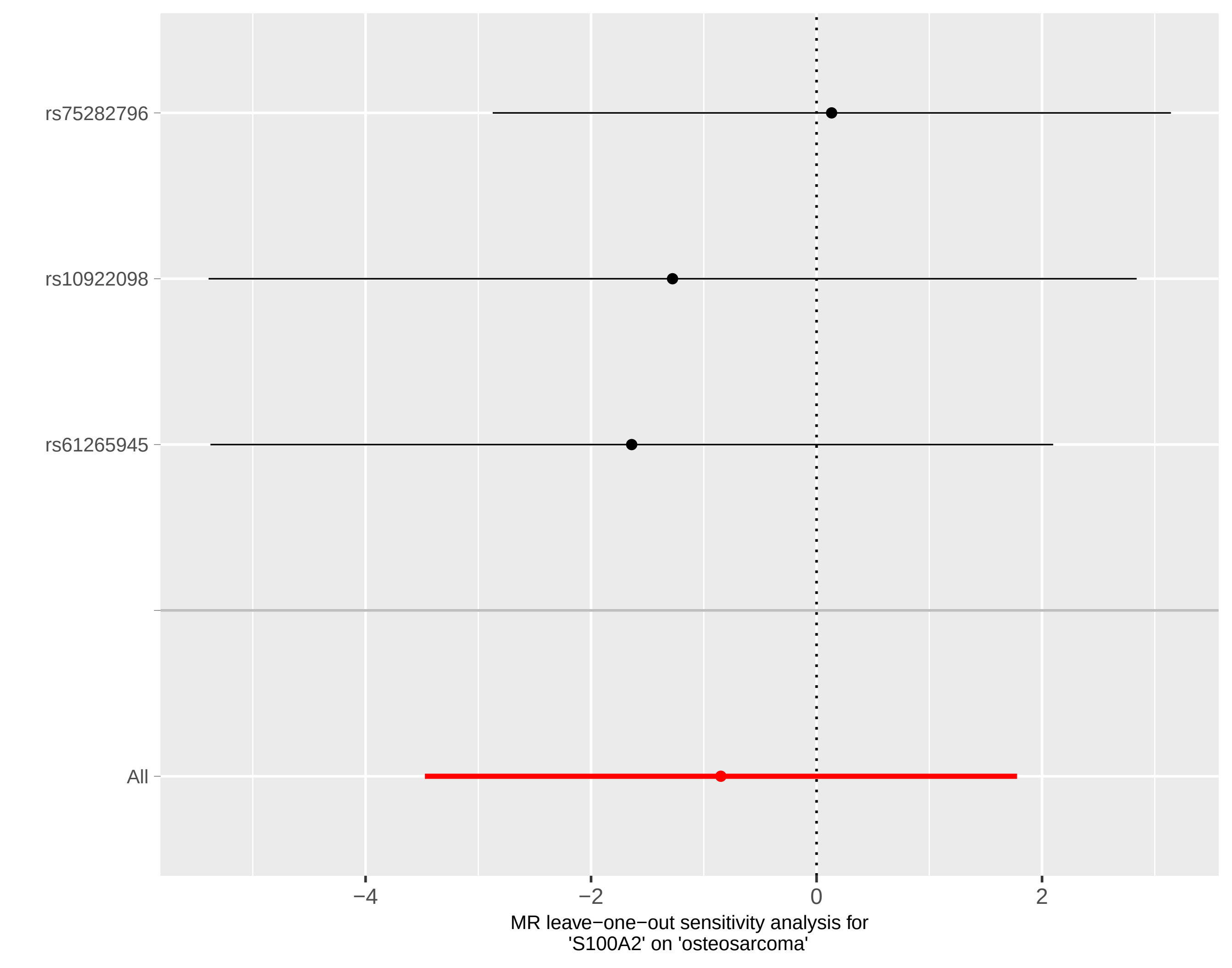

rs75282796
rs10922098
rs61265945
All
−4
−2
0
2
MR leave−one−out sensitivity analysis for
'S100A2' on 'osteosarcoma'

Insufficient number of SNPs

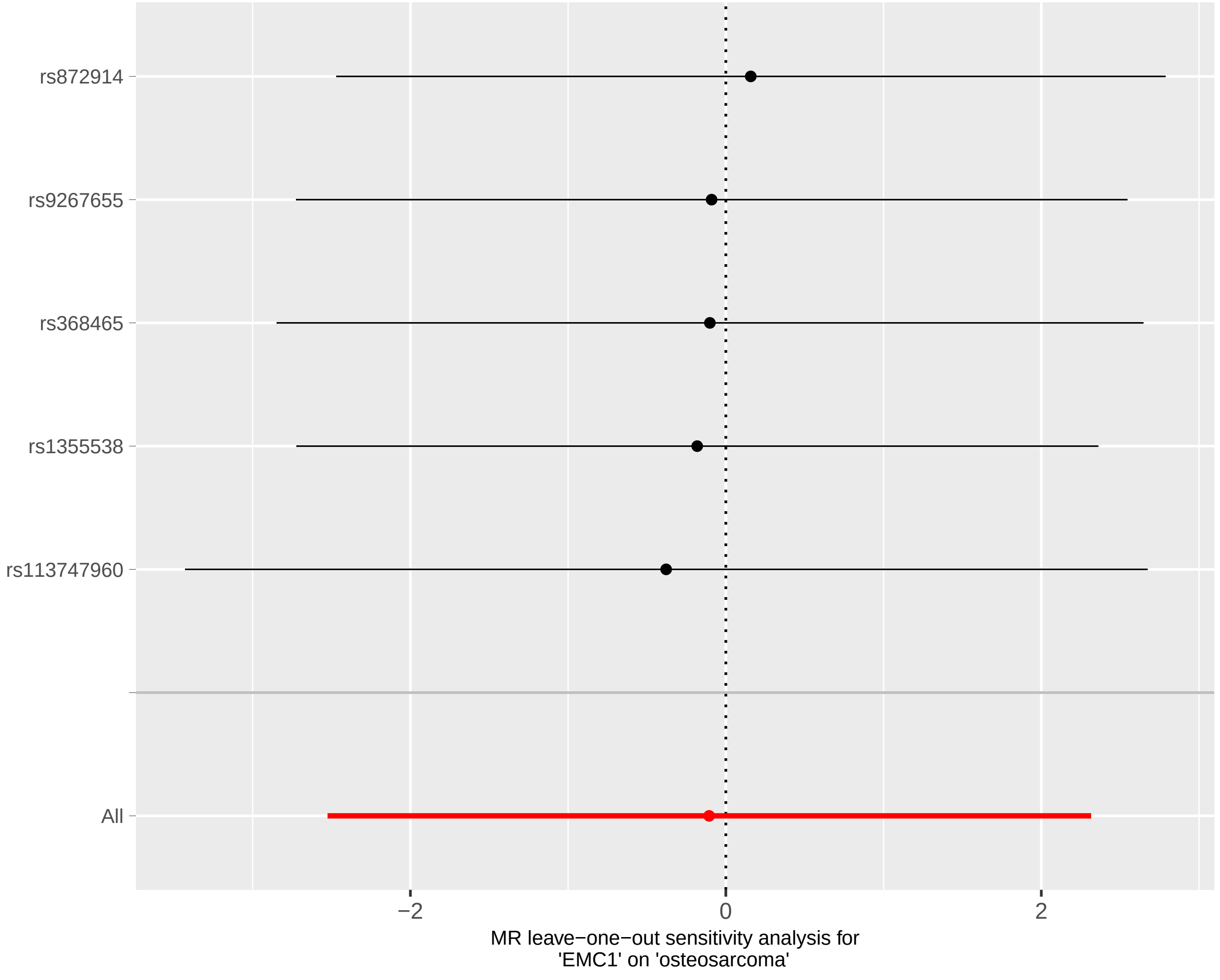
rs872914
rs9267655
rs368465
rs1355538
rs113747960
All
−2
0
2
MR leave−one−out sensitivity analysis for
'EMC1' on 'osteosarcoma'

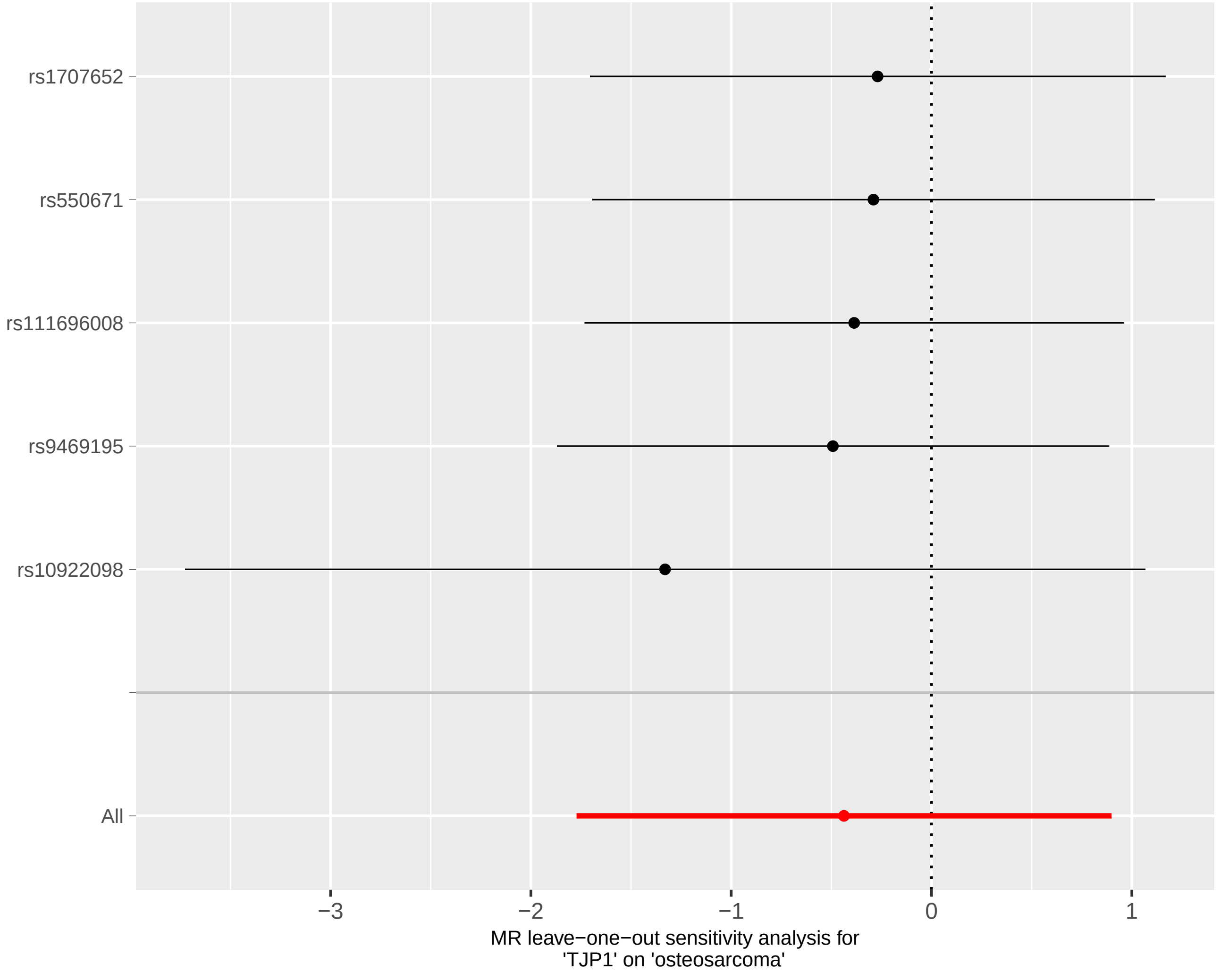
rs1707652
rs550671
rs111696008
rs9469195
rs10922098
All
−3
−2
−1
0
1
MR leave−one−out sensitivity analysis for
'TJP1' on 'osteosarcoma'

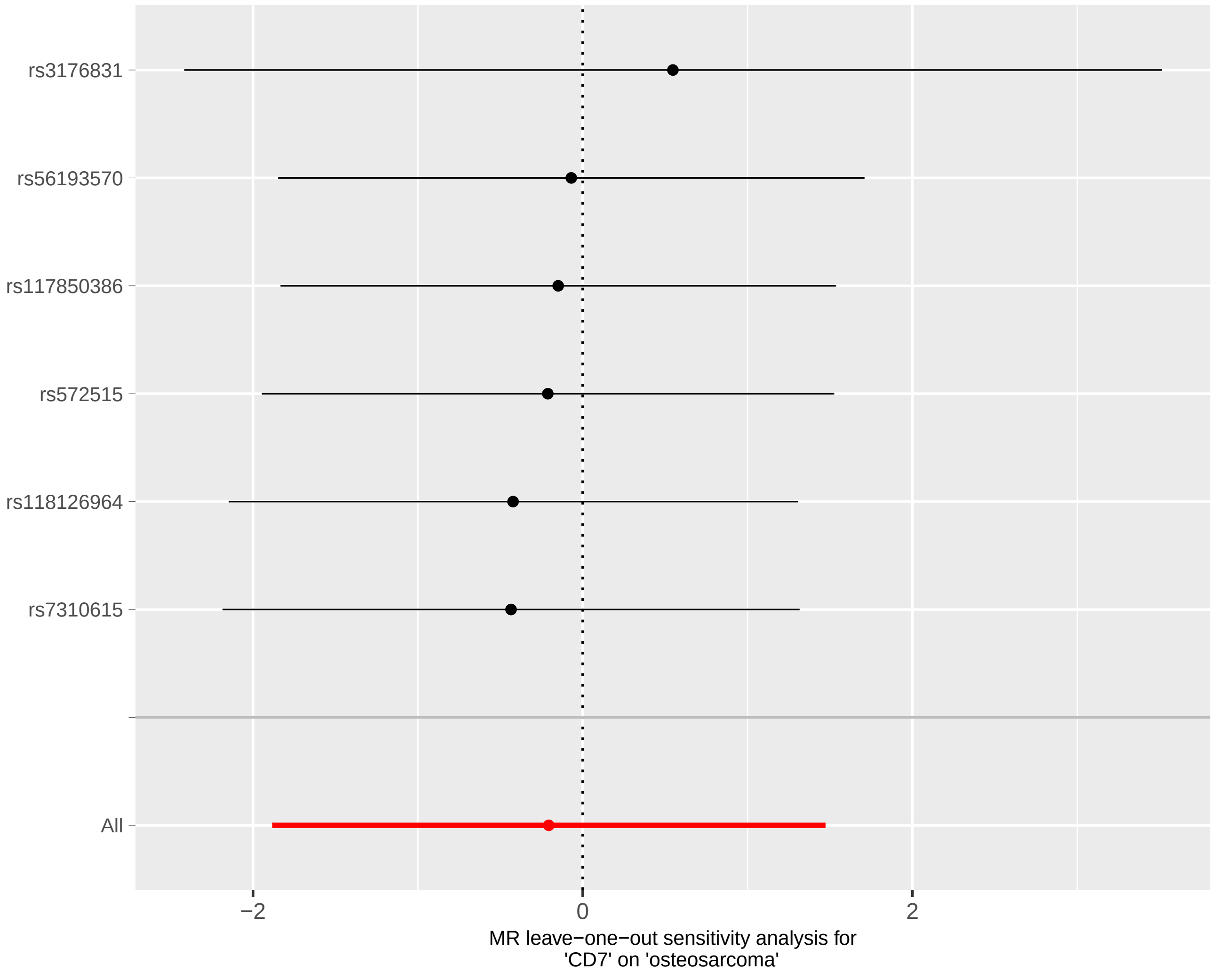

rs3176831
rs56193570
rs117850386
rs572515
rs118126964
rs7310615
All
−2
0
2
MR leave−one−out sensitivity analysis for
'CD7' on 'osteosarcoma'

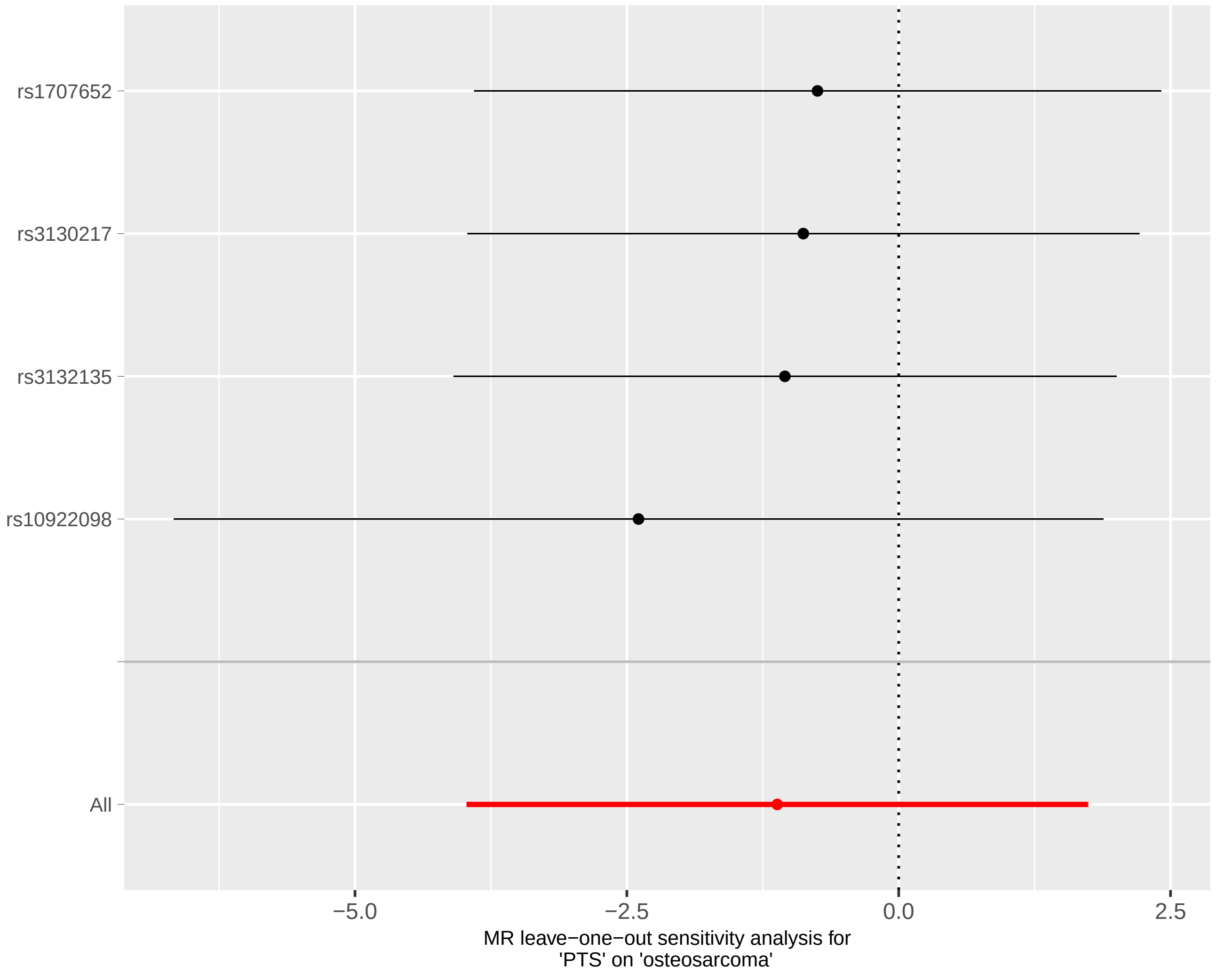

rs1707652
rs3130217
rs3132135
rs10922098
All
−5.0
−2.5
0.0
2.5
MR leave−one−out sensitivity analysis for
'PTS' on 'osteosarcoma'

Insufficient number of SNPs

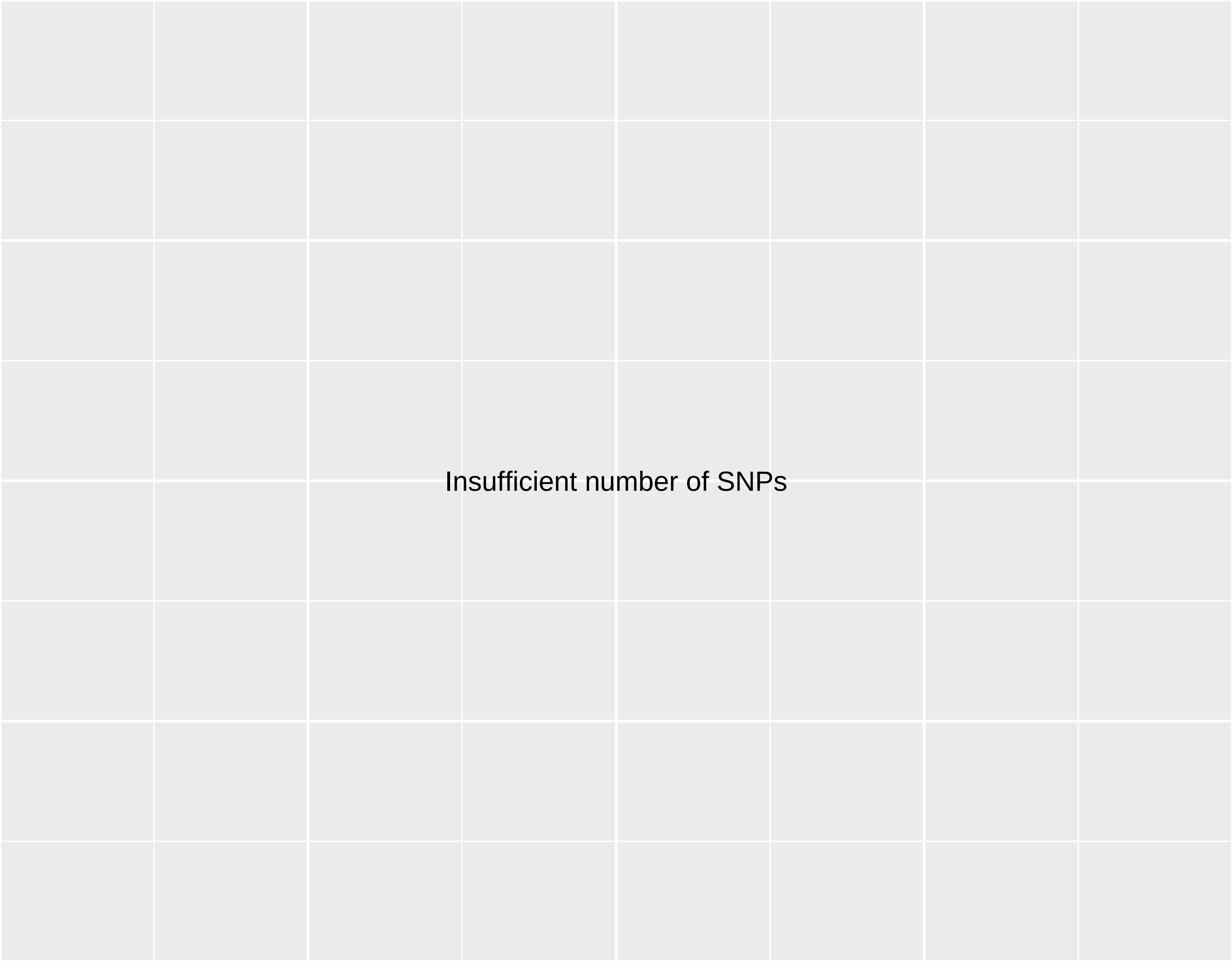
Insufficient number of SNPs

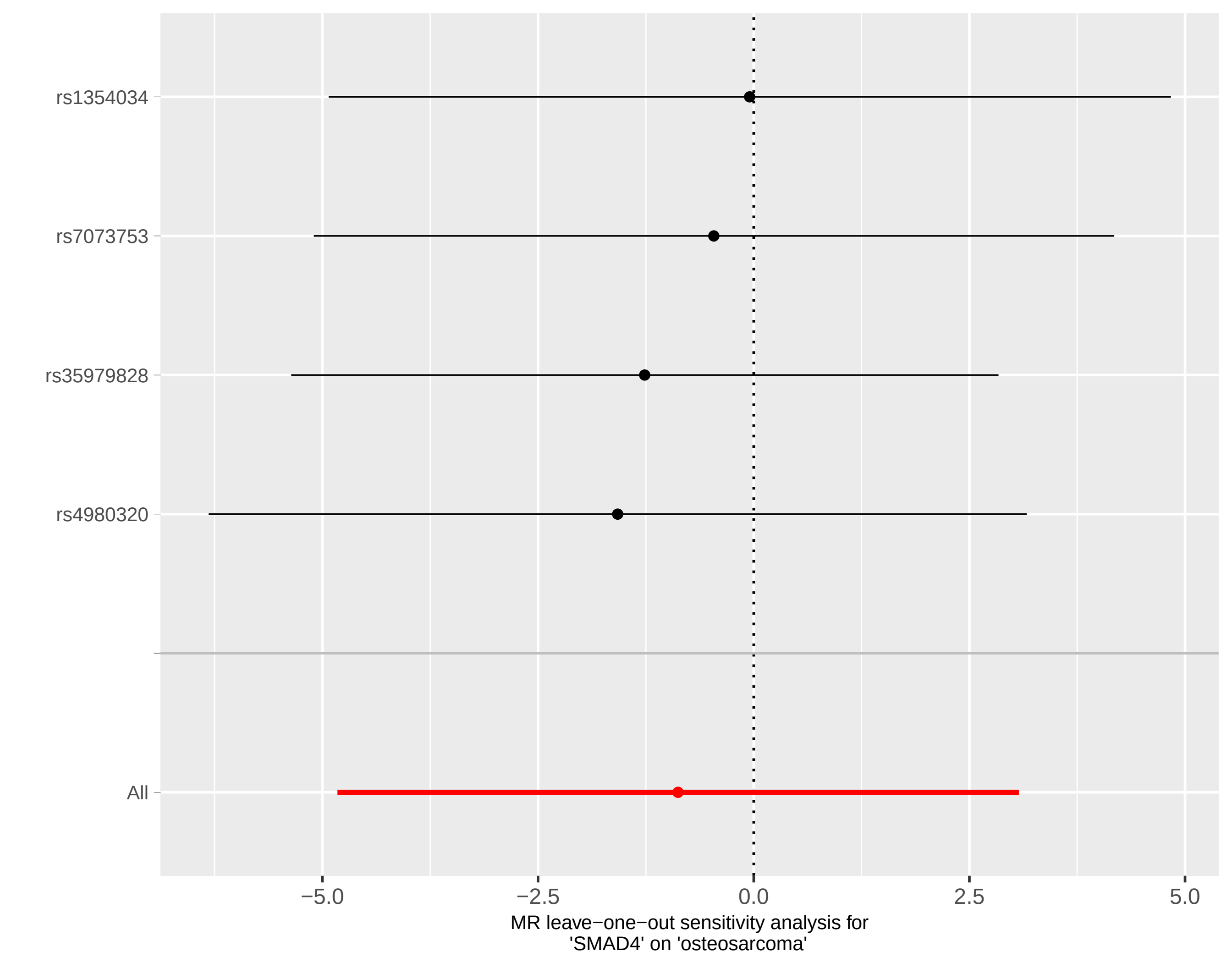

rs1354034
rs7073753
rs35979828
rs4980320
All
−5.0
−2.5
0.0
2.5
5.0
MR leave−one−out sensitivity analysis for
'SMAD4' on 'osteosarcoma'

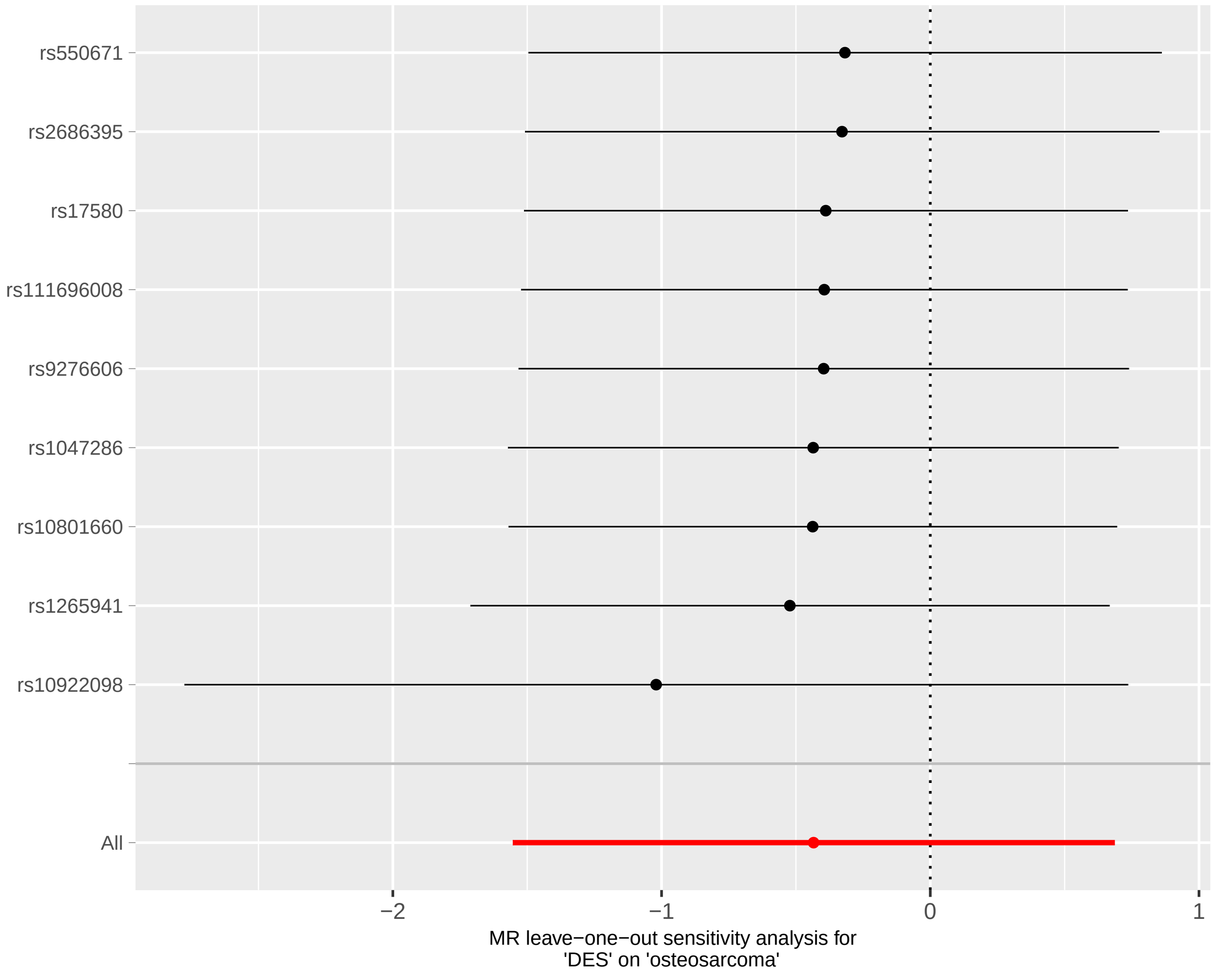
rs550671
rs2686395
rs17580
rs111696008
rs9276606
rs1047286
rs10801660
rs1265941
rs10922098
All
−2
−1
0
1
MR leave−one−out sensitivity analysis for
'DES' on 'osteosarcoma'

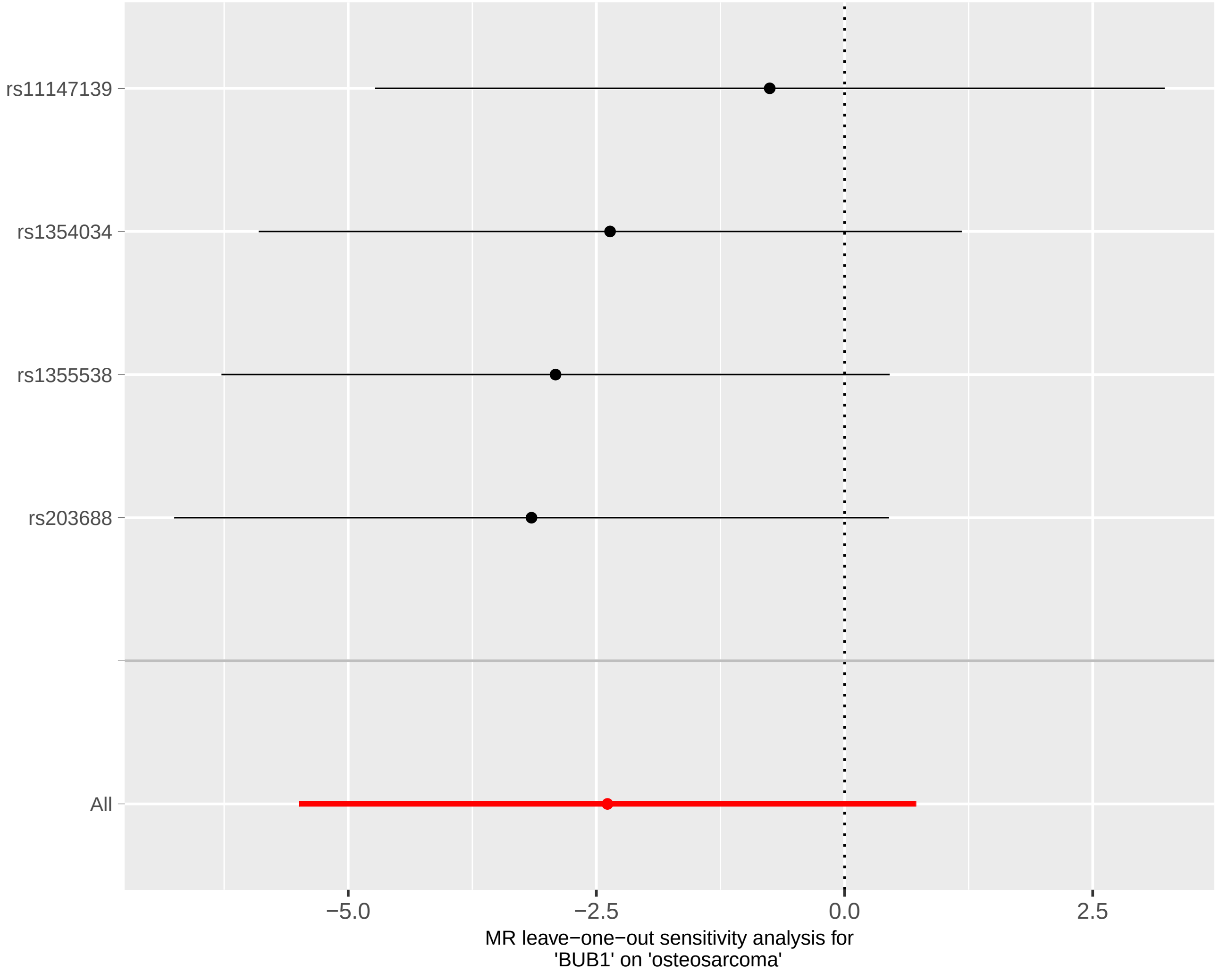

rs11147139
rs1354034
rs1355538
rs203688
All
−5.0
−2.5
0.0
2.5
MR leave−one−out sensitivity analysis for
'BUB1' on 'osteosarcoma'

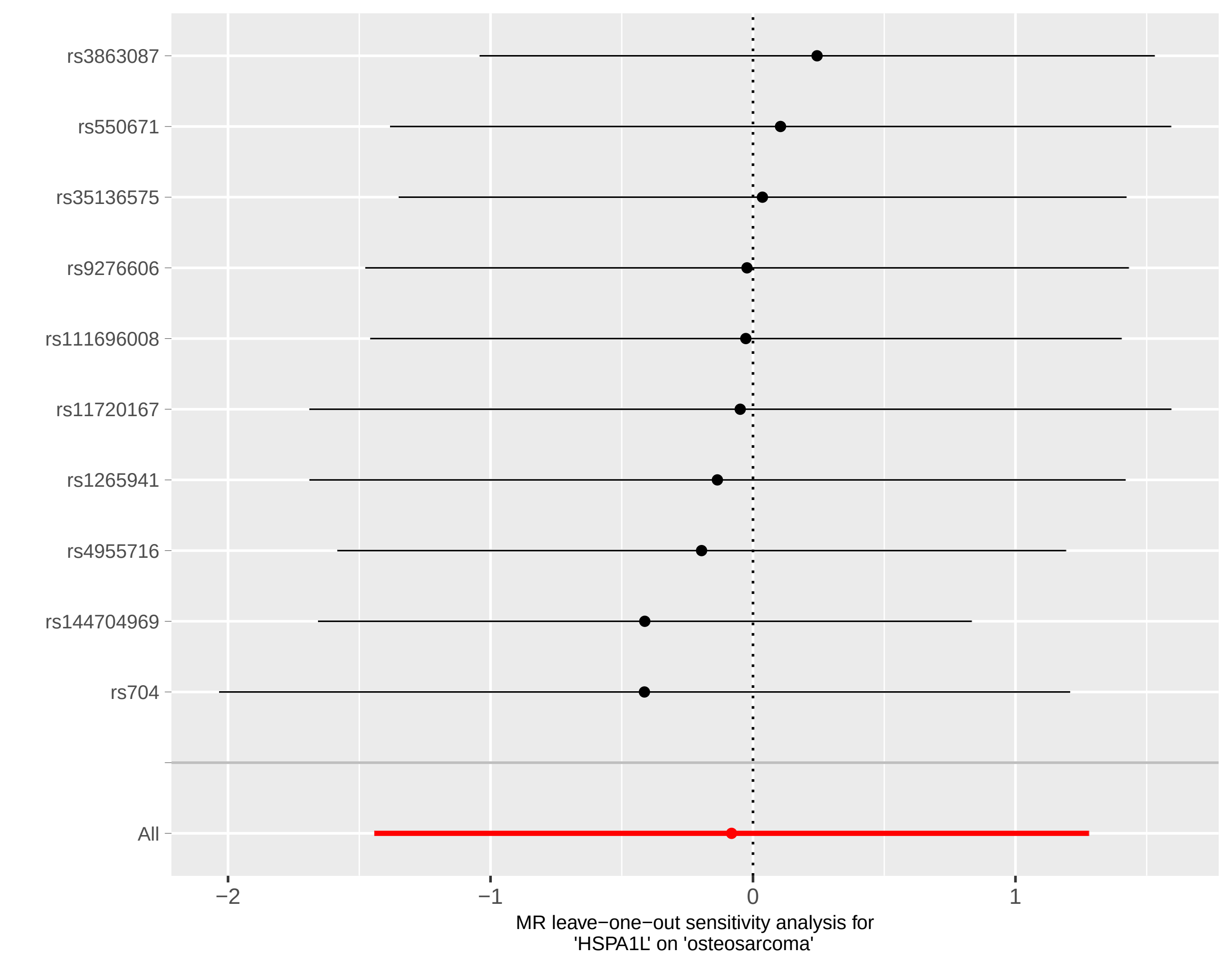

rs3863087
rs550671
rs35136575
rs9276606
rs111696008
rs11720167
rs1265941
rs4955716
rs144704969
rs704
All
−2
−1
0
1
MR leave−one−out sensitivity analysis for
'HSPA1L' on 'osteosarcoma'

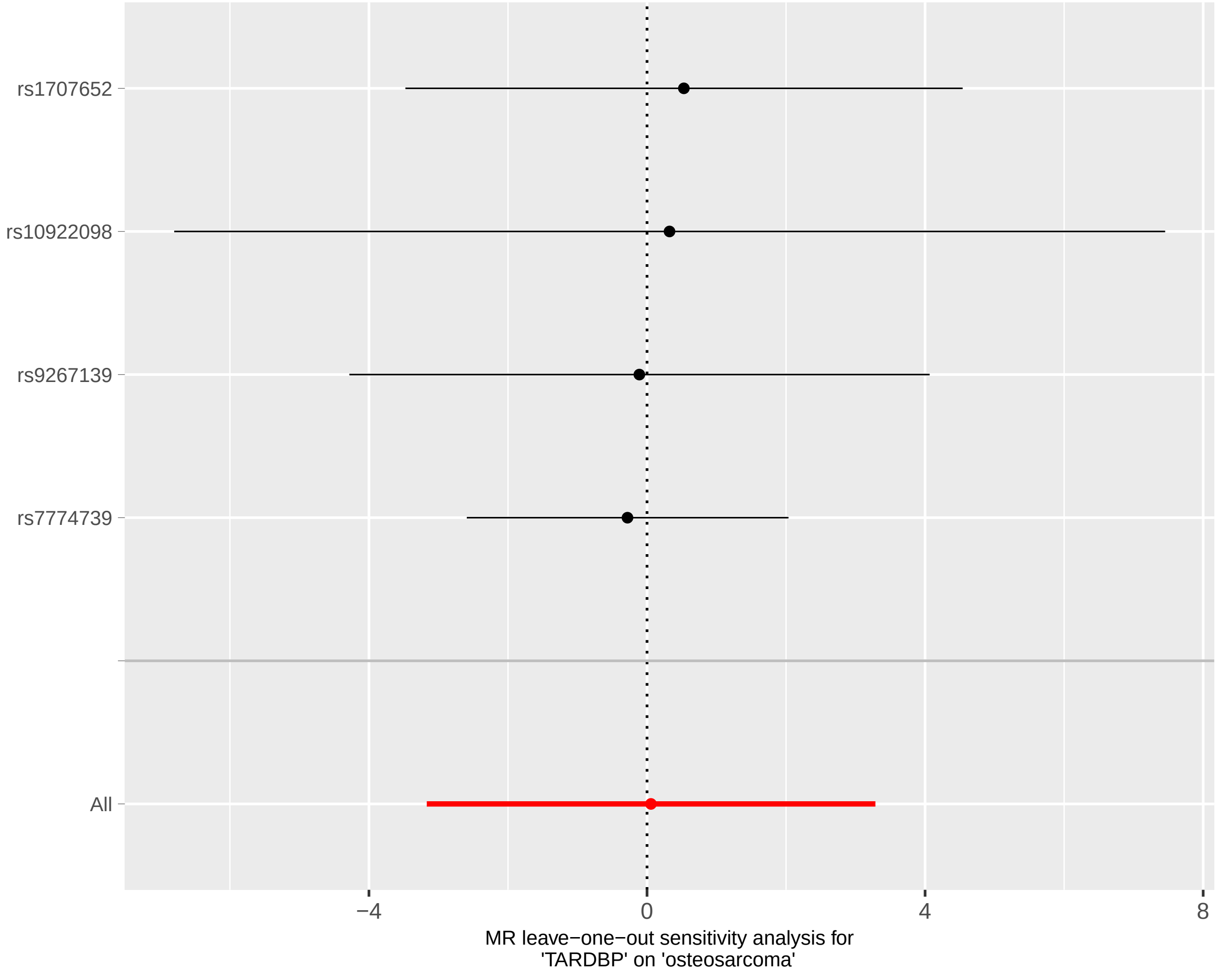

rs1707652
rs10922098
rs9267139
rs7774739
All
−4
0
4
8
MR leave−one−out sensitivity analysis for
'TARDBP' on 'osteosarcoma'

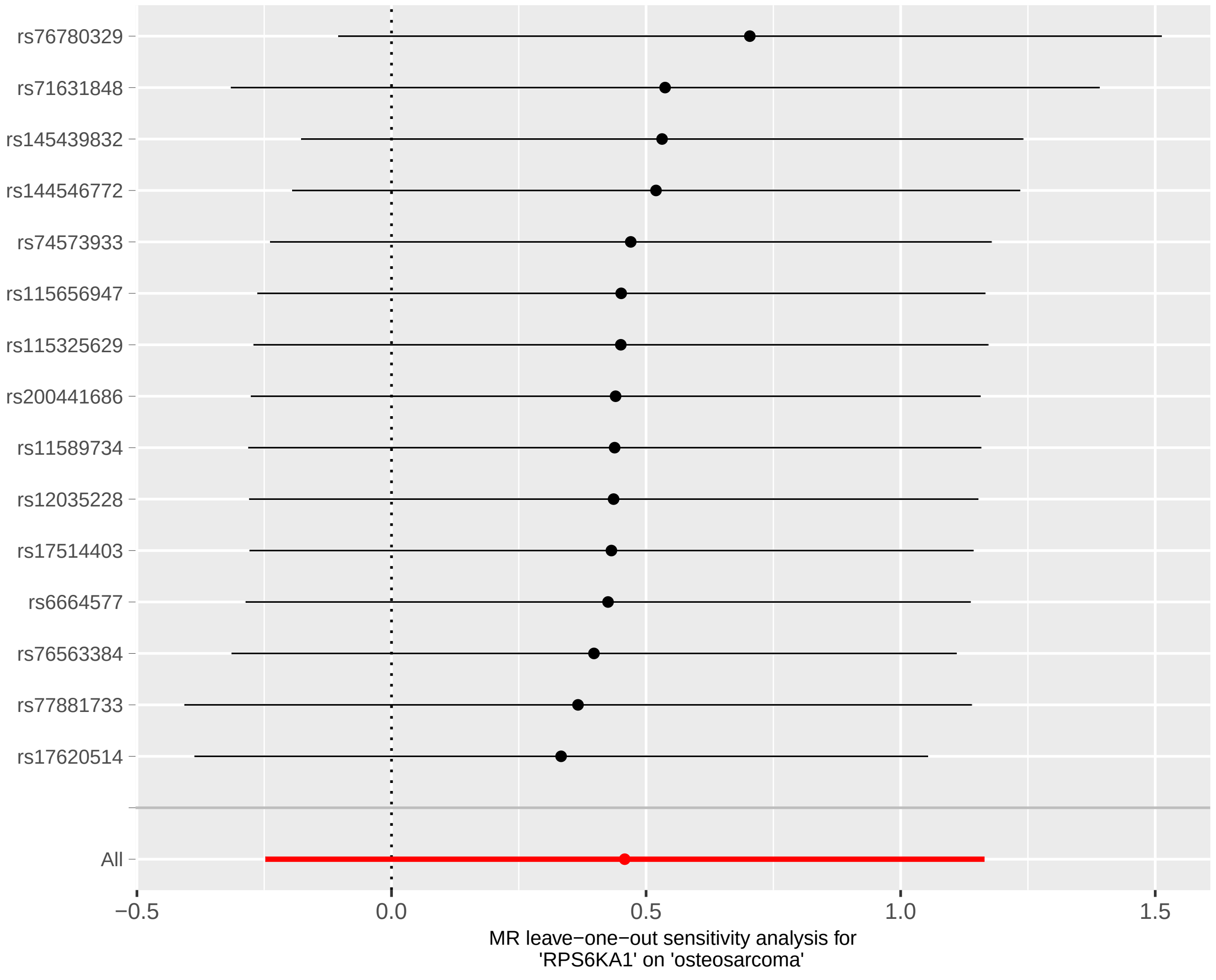

rs76780329
rs71631848
rs145439832
rs144546772
rs74573933
rs115656947
rs115325629
rs200441686
rs11589734
rs12035228
rs17514403
rs6664577
rs76563384
rs77881733
rs17620514
All
−0.5
0.0
0.5
1.0
1.5
MR leave−one−out sensitivity analysis for
'RPS6KA1' on 'osteosarcoma'

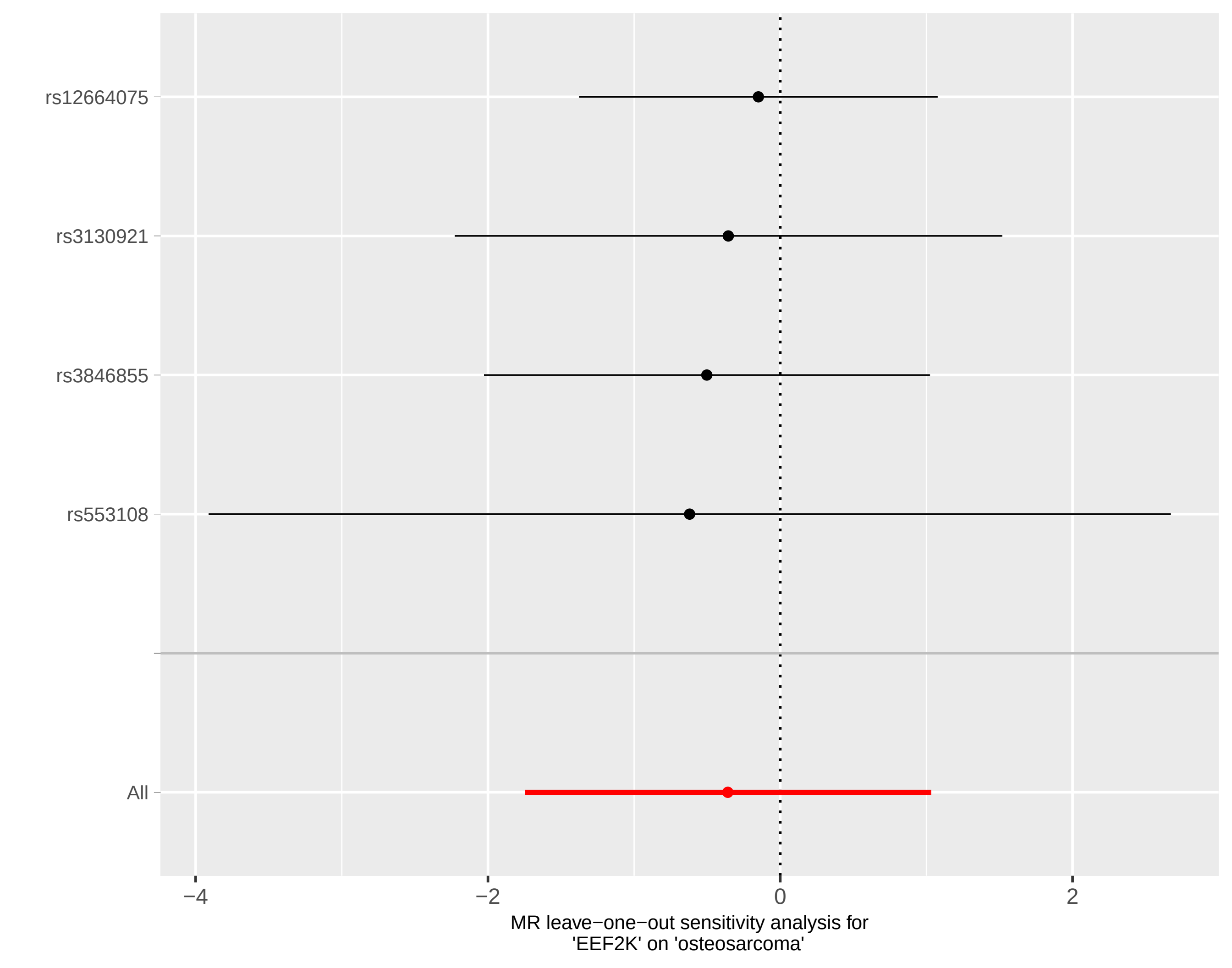
rs12664075
rs3130921
rs3846855
rs553108
All
−4
−2
0
2
MR leave−one−out sensitivity analysis for
'EEF2K' on 'osteosarcoma'

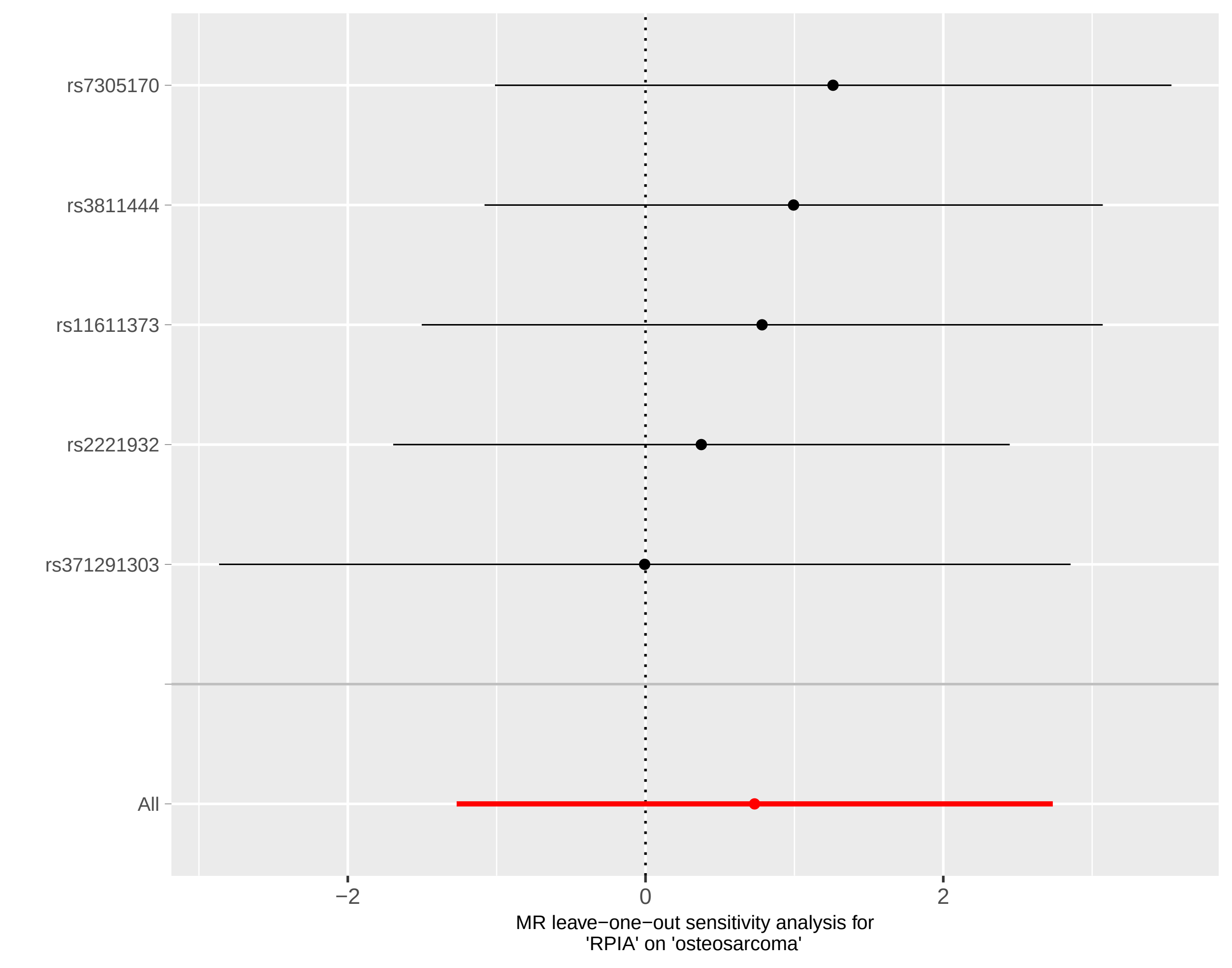
rs7305170
rs3811444
rs11611373
rs2221932
rs371291303
All
−2
0
2
MR leave−one−out sensitivity analysis for
'RPIA' on 'osteosarcoma'

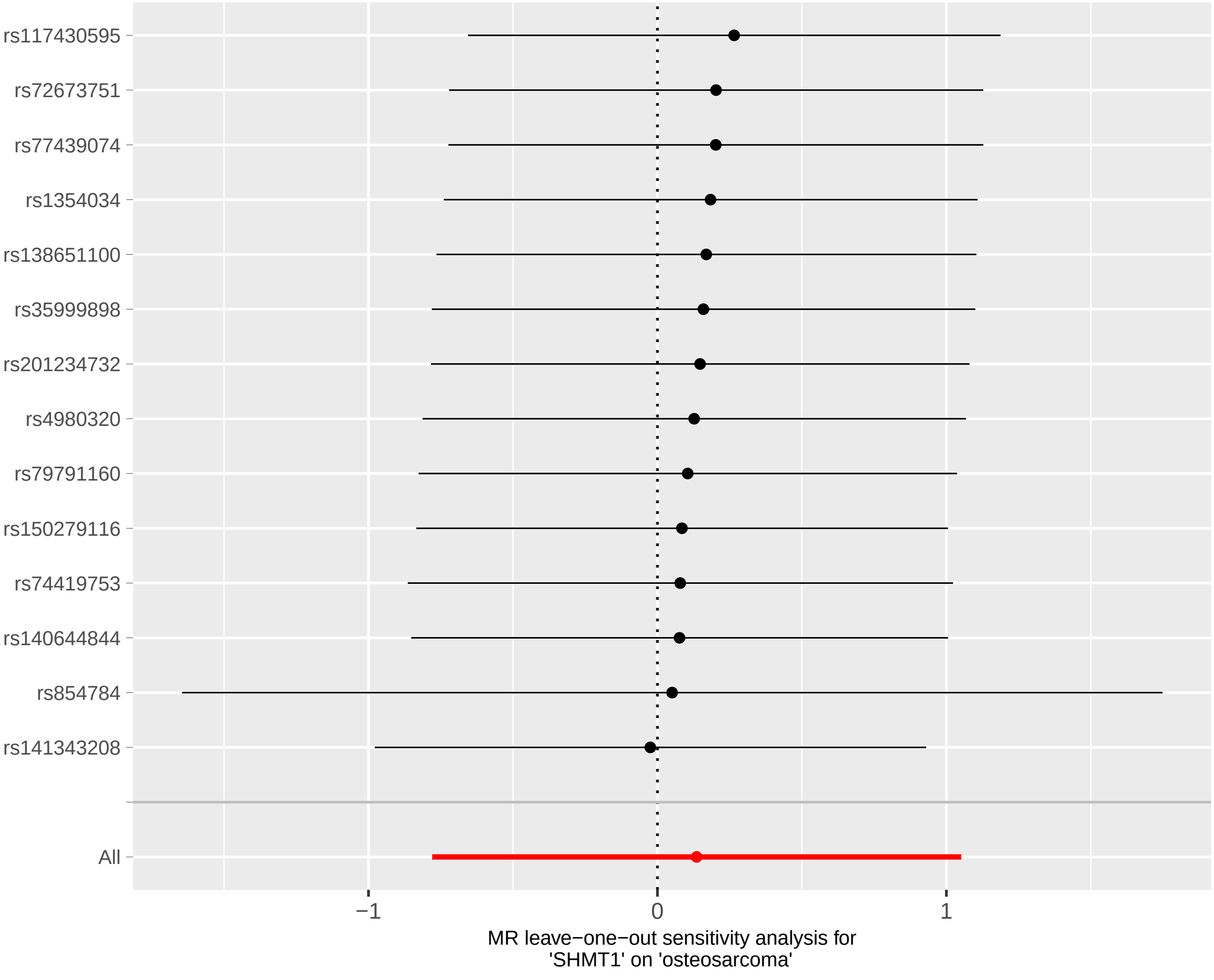
rs117430595
rs72673751
rs77439074
rs1354034
rs138651100
rs35999898
rs201234732
rs4980320
rs79791160
rs150279116
rs74419753
rs140644844
rs854784
rs141343208
All
−1
0
1
MR leave−one−out sensitivity analysis for
'SHMT1' on 'osteosarcoma'

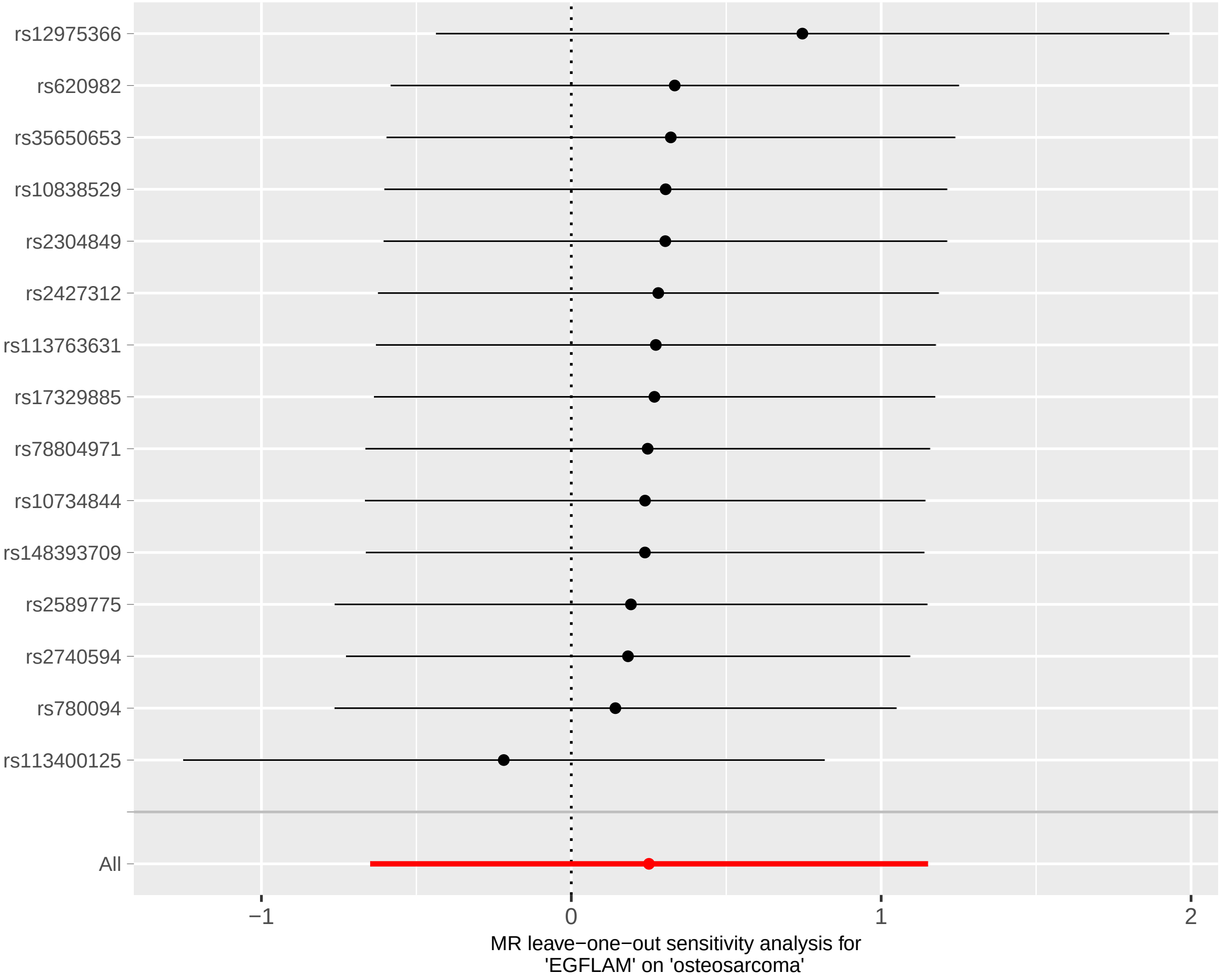

rs12975366
rs620982
rs35650653
rs10838529
rs2304849
rs2427312
rs113763631
rs17329885
rs78804971
rs10734844
rs148393709
rs2589775
rs2740594
rs780094
rs113400125
All
−1
0
1
2
MR leave−one−out sensitivity analysis for
'EGFLAM' on 'osteosarcoma'

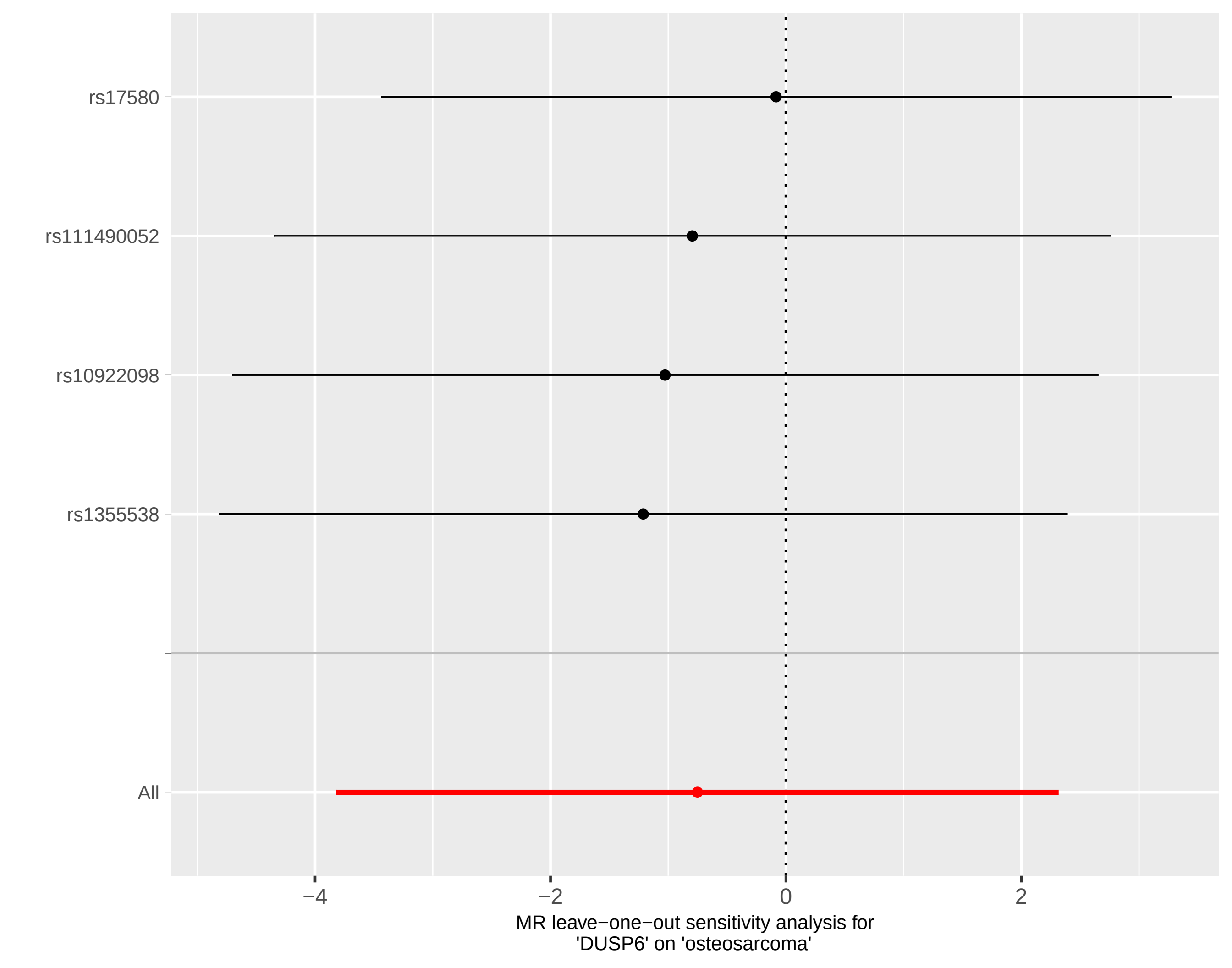
rs17580
rs111490052
rs10922098
rs1355538
All
−4
−2
0
2
MR leave−one−out sensitivity analysis for
'DUSP6' on 'osteosarcoma'

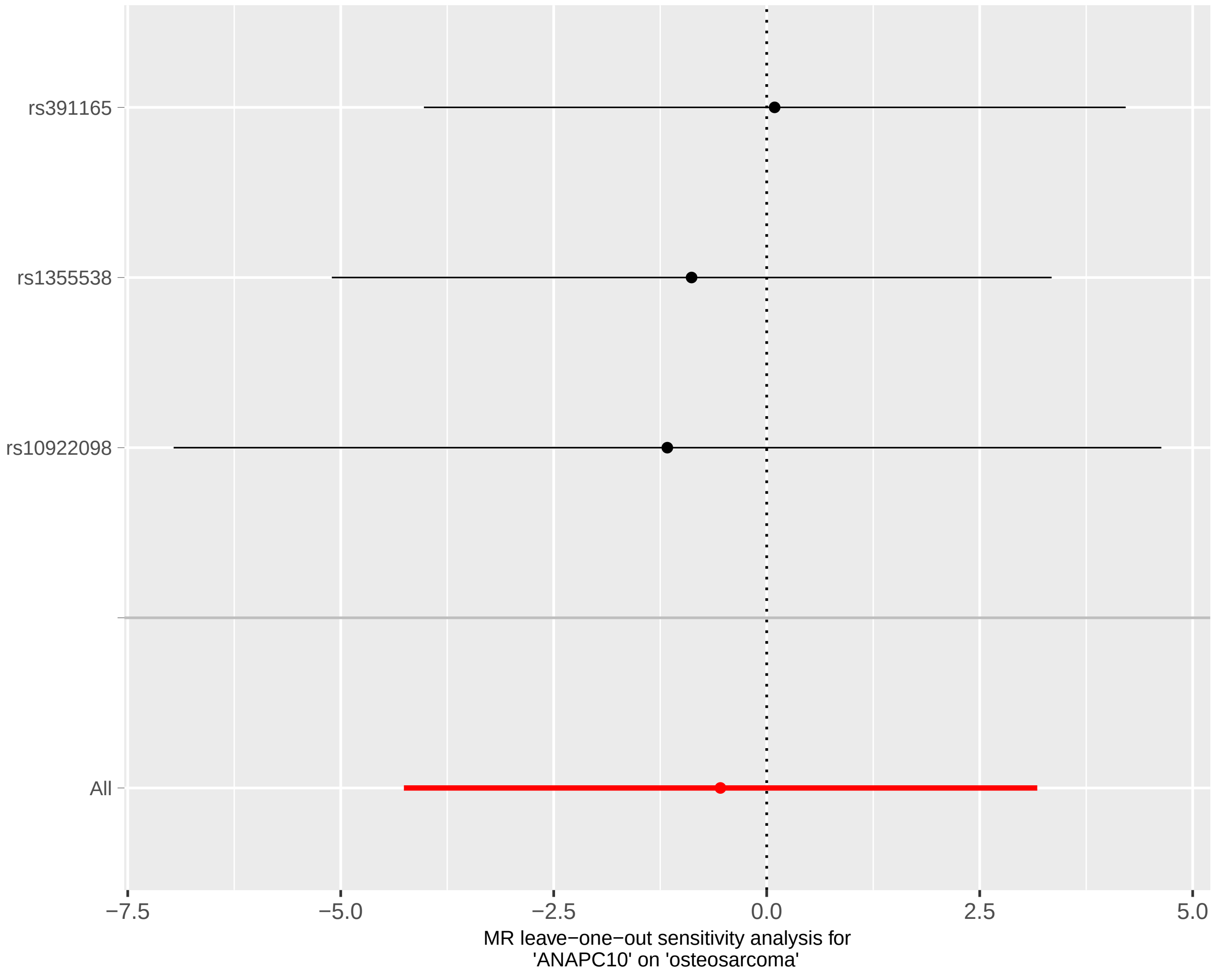
rs391165
rs1355538
rs10922098
All
−7.5
−5.0
−2.5
0.0
2.5
5.0
MR leave−one−out sensitivity analysis for
'ANAPC10' on 'osteosarcoma'

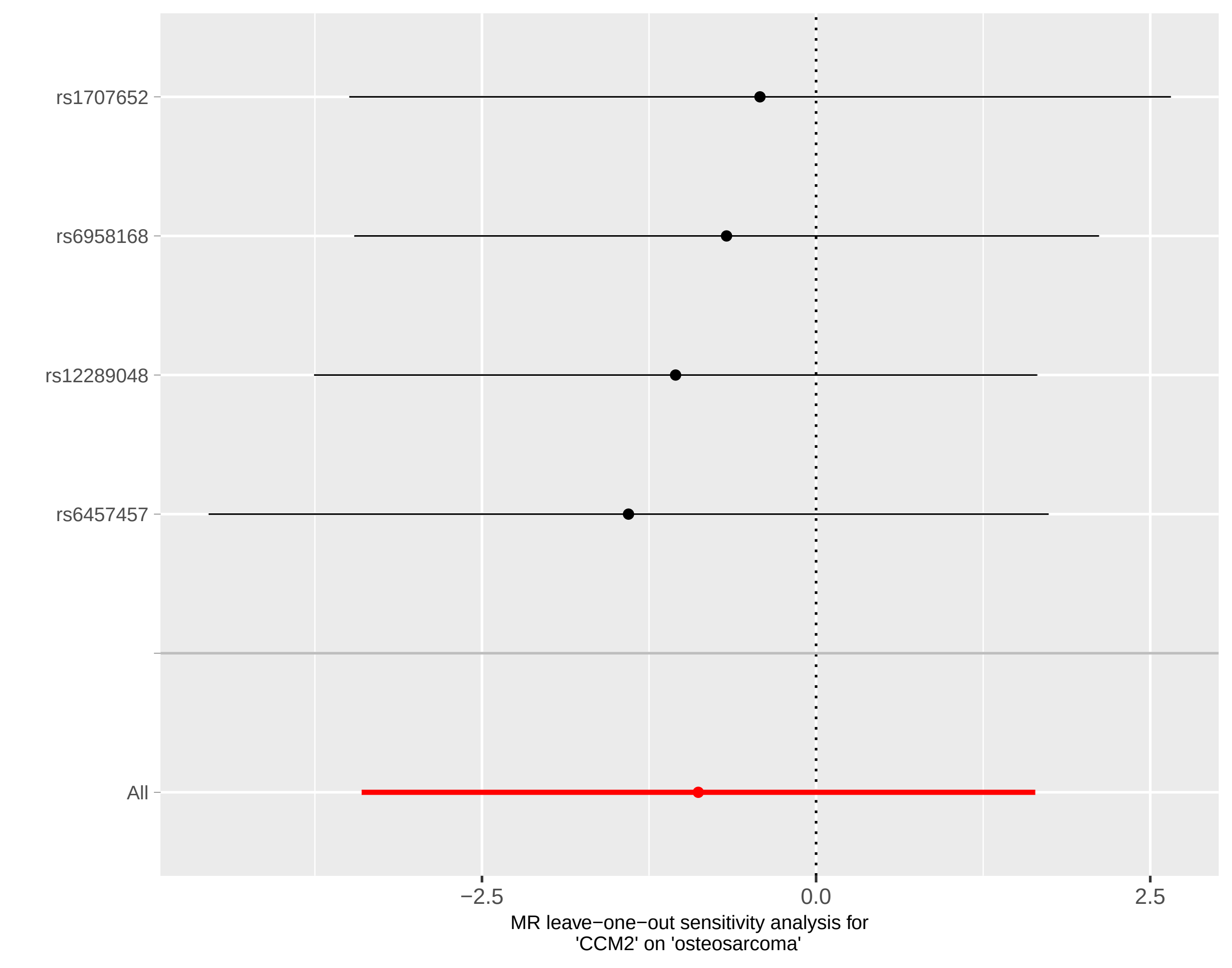

rs1707652
rs6958168
rs12289048
rs6457457
All
−2.5
0.0
2.5
MR leave−one−out sensitivity analysis for
'CCM2' on 'osteosarcoma'

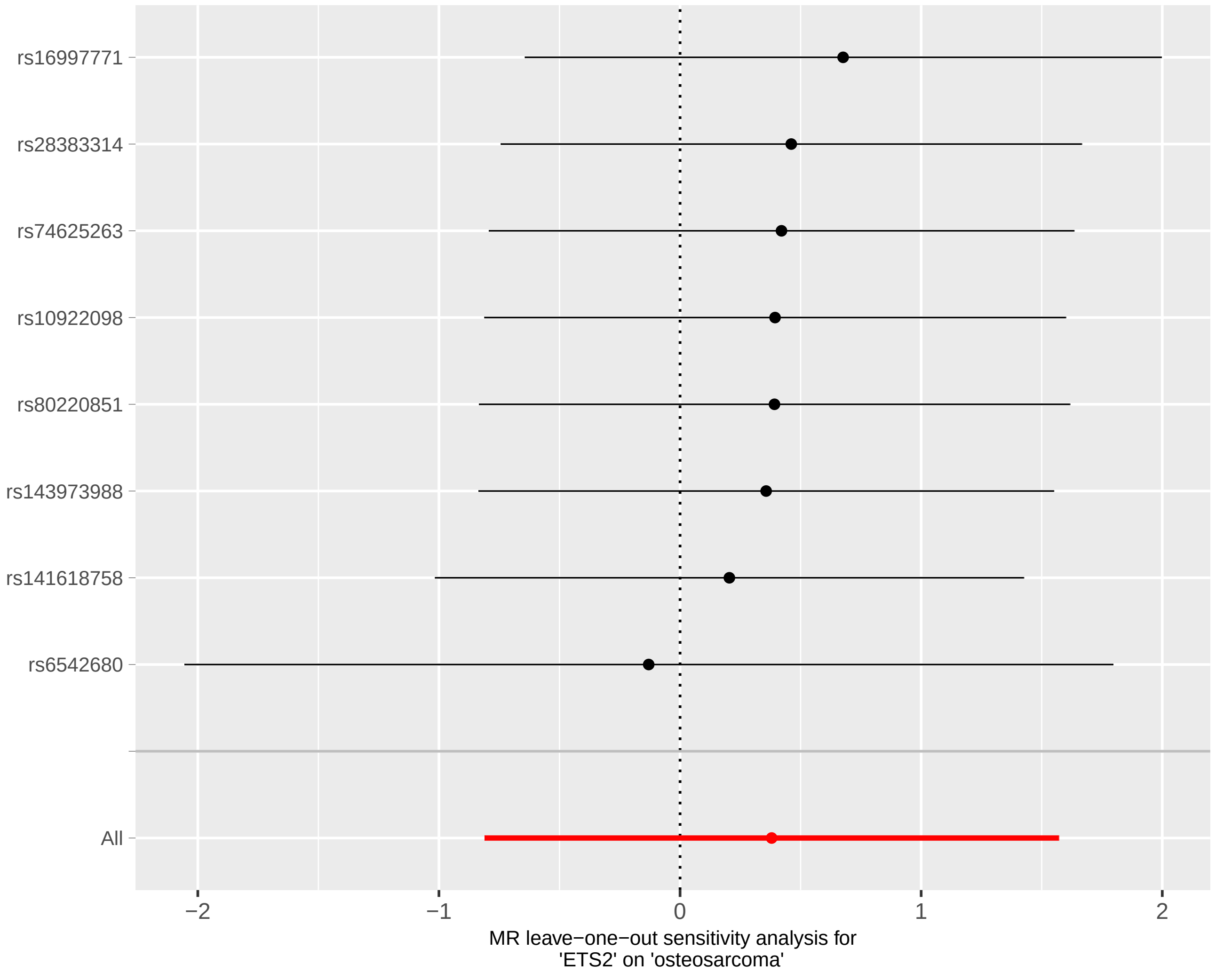

rs16997771
rs28383314
rs74625263
rs10922098
rs80220851
rs143973988
rs141618758
rs6542680
All
−2
−1
0
1
2
MR leave−one−out sensitivity analysis for
'ETS2' on 'osteosarcoma'

Insufficient number of SNPs

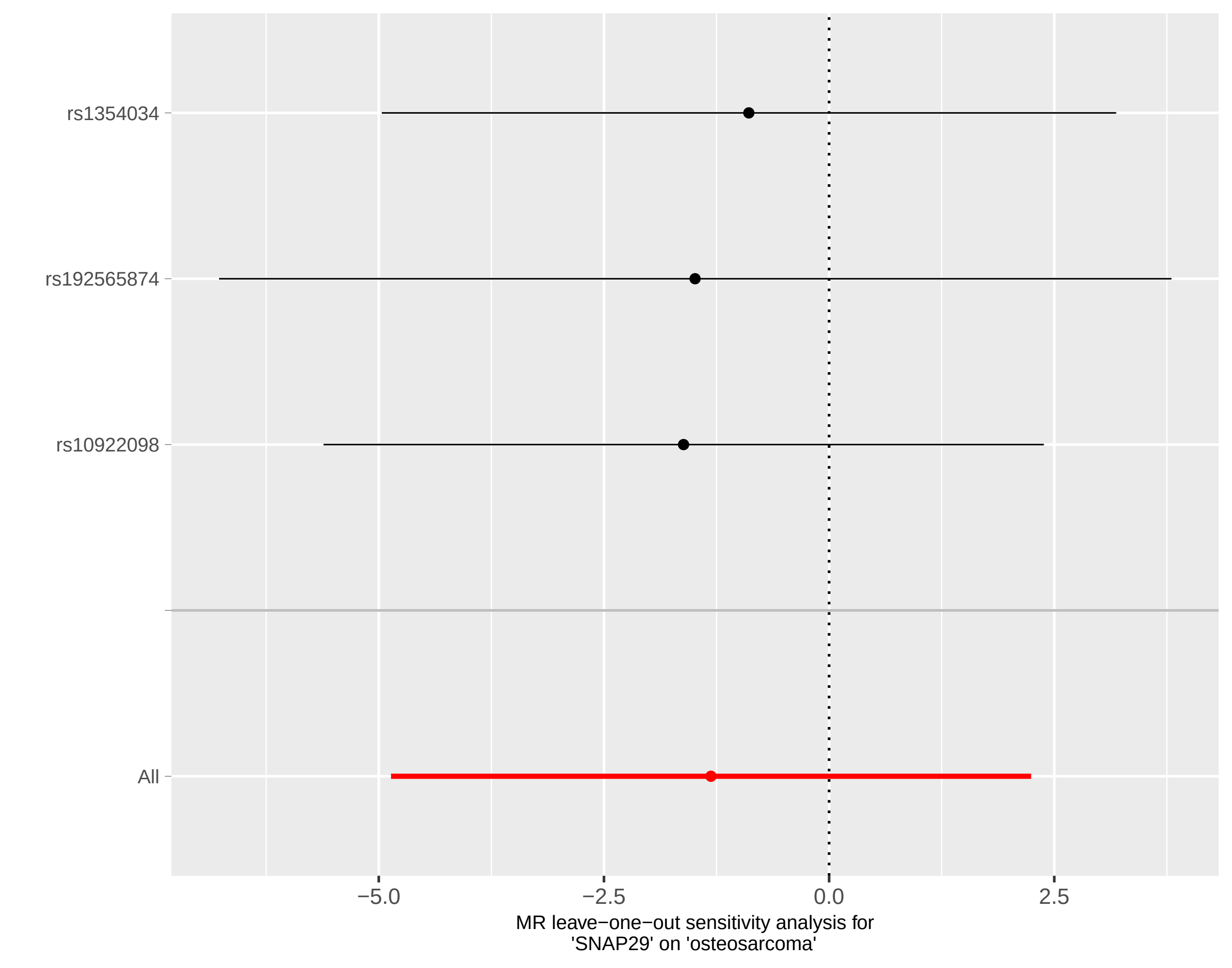
rs1354034
rs192565874
rs10922098
All
−5.0
−2.5
0.0
2.5
MR leave−one−out sensitivity analysis for
'SNAP29' on 'osteosarcoma'

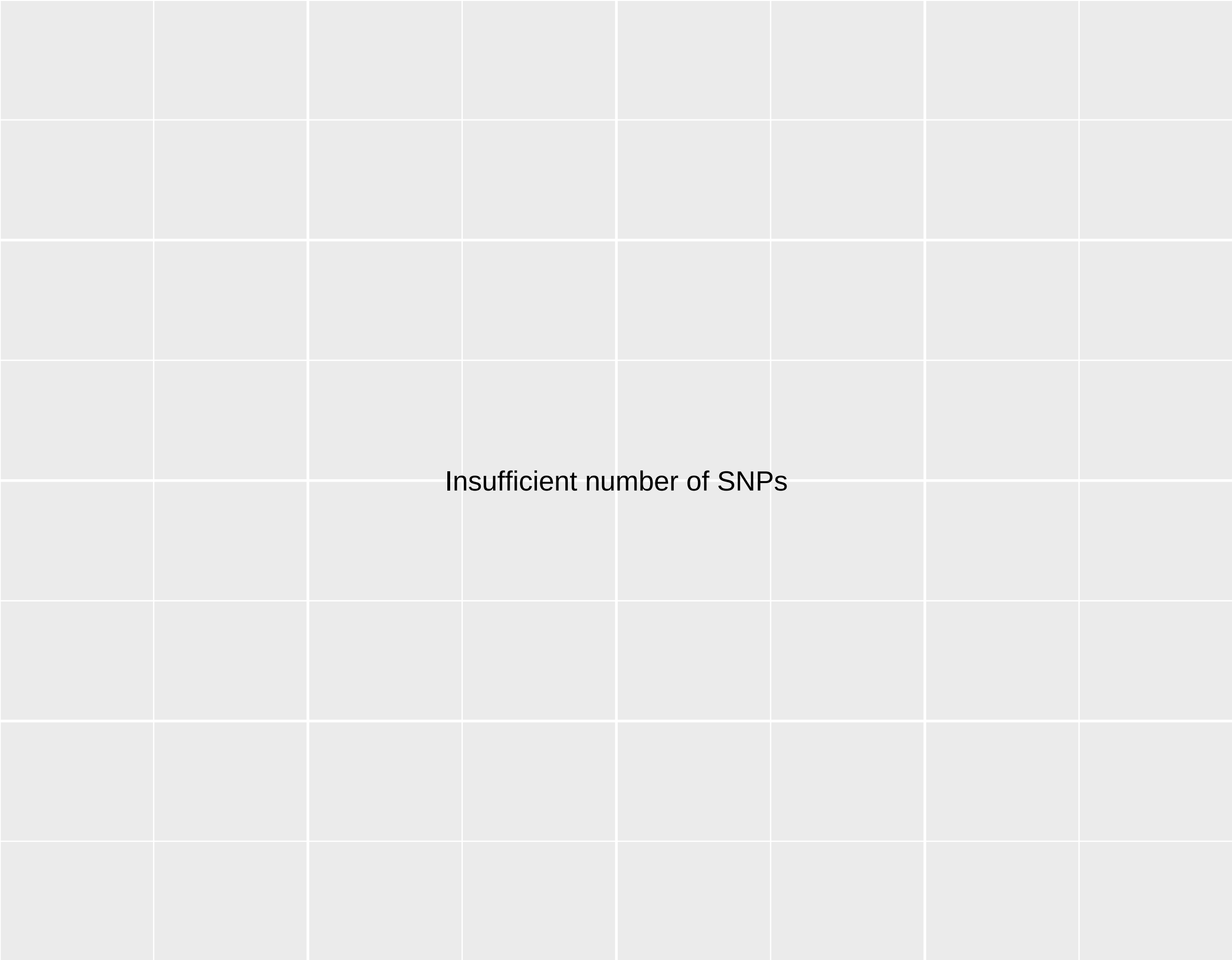
Insufficient number of SNPs

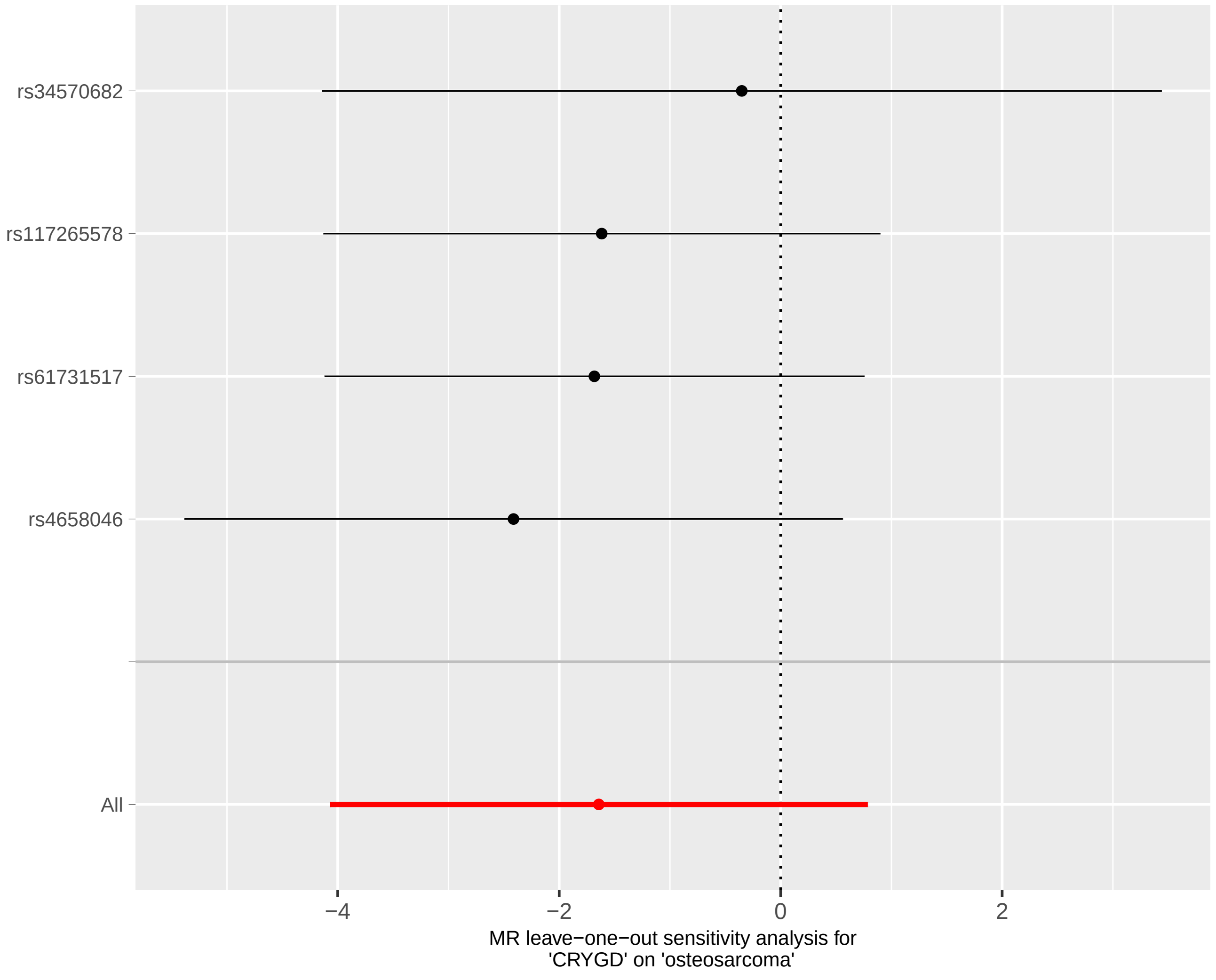

rs34570682
rs117265578
rs61731517
rs4658046
All
−4
−2
0
2
MR leave−one−out sensitivity analysis for
'CRYGD' on 'osteosarcoma'

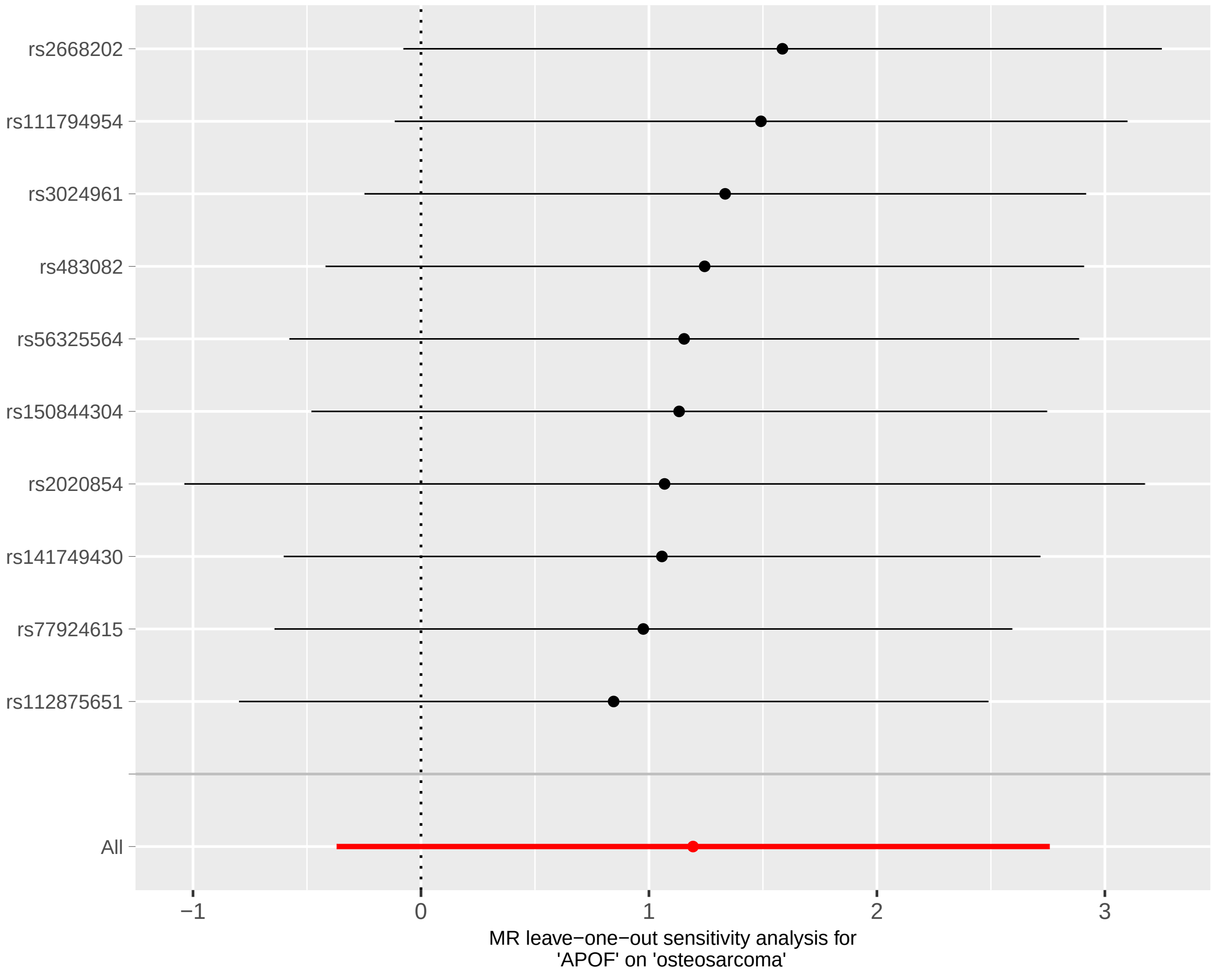
rs2668202
rs111794954
rs3024961
rs483082
rs56325564
rs150844304
rs2020854
rs141749430
rs77924615
rs112875651
All
−1
0
1
2
3
MR leave−one−out sensitivity analysis for
'APOF' on 'osteosarcoma'

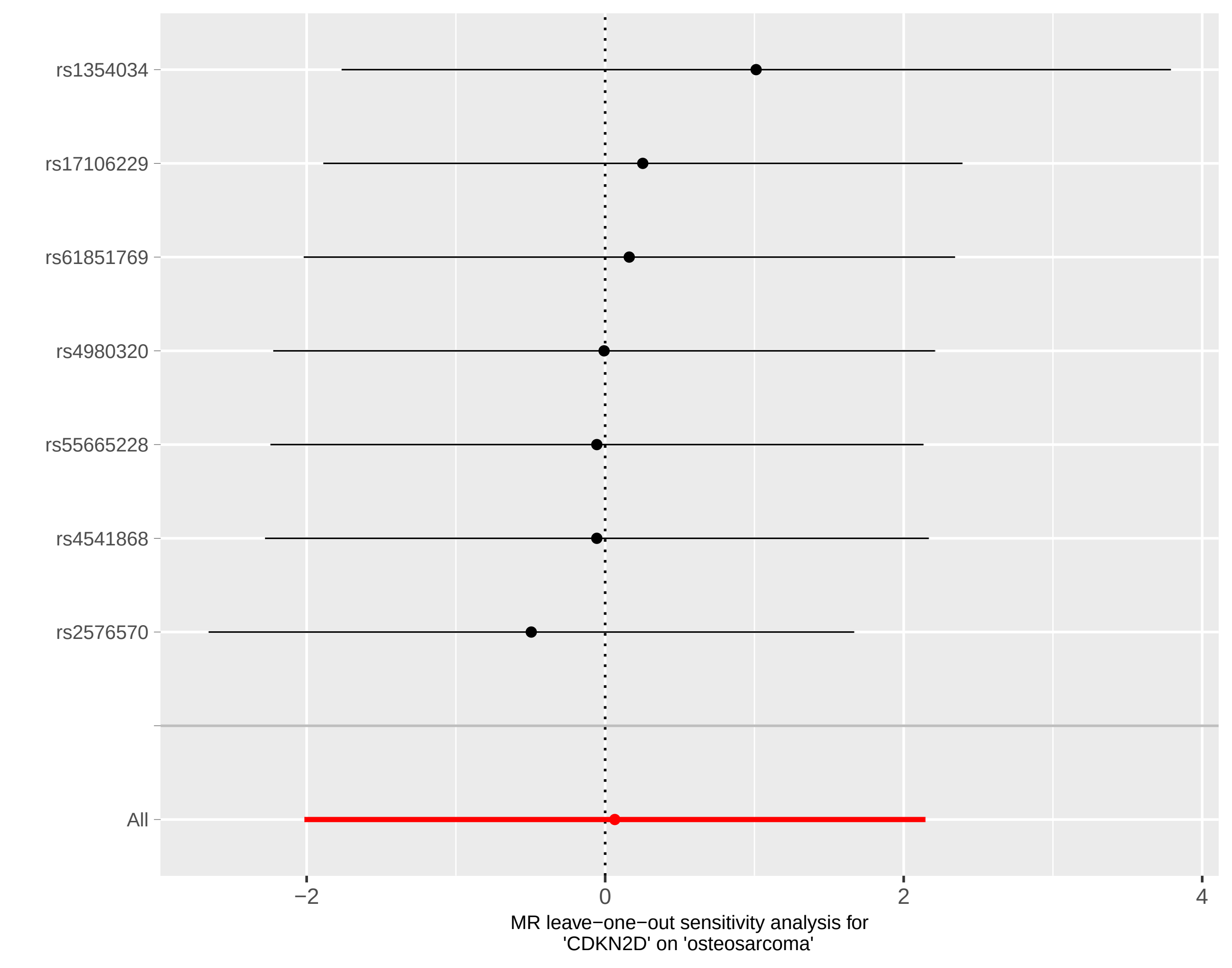
rs1354034
rs17106229
rs61851769
rs4980320
rs55665228
rs4541868
rs2576570
All
−2
0
2
4
MR leave−one−out sensitivity analysis for
'CDKN2D' on 'osteosarcoma'

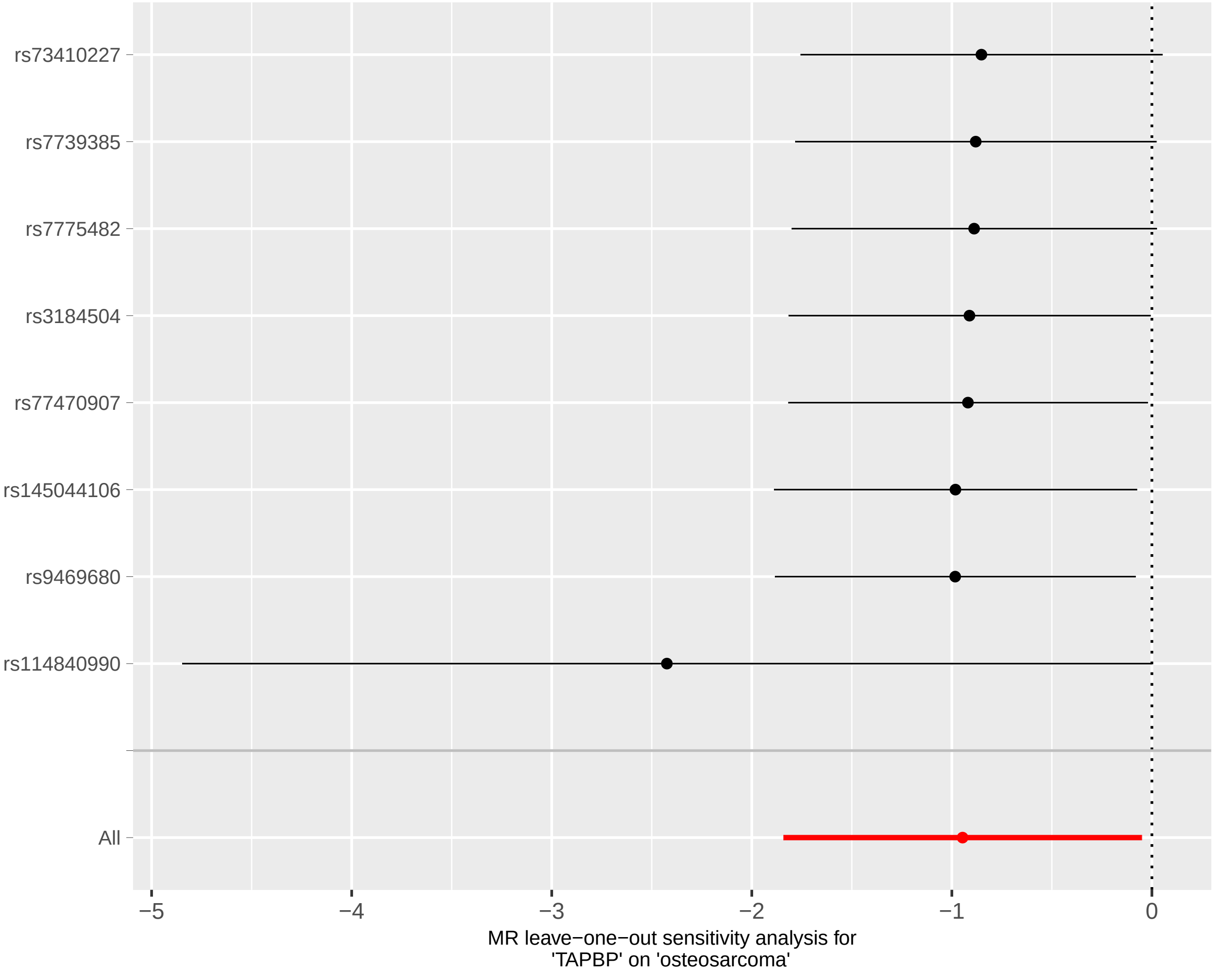
rs73410227
rs7739385
rs7775482
rs3184504
rs77470907
rs145044106
rs9469680
rs114840990
All
−5
−4
−3
−2
−1
0
MR leave−one−out sensitivity analysis for
'TAPBP' on 'osteosarcoma'

Insufficient number of SNPs

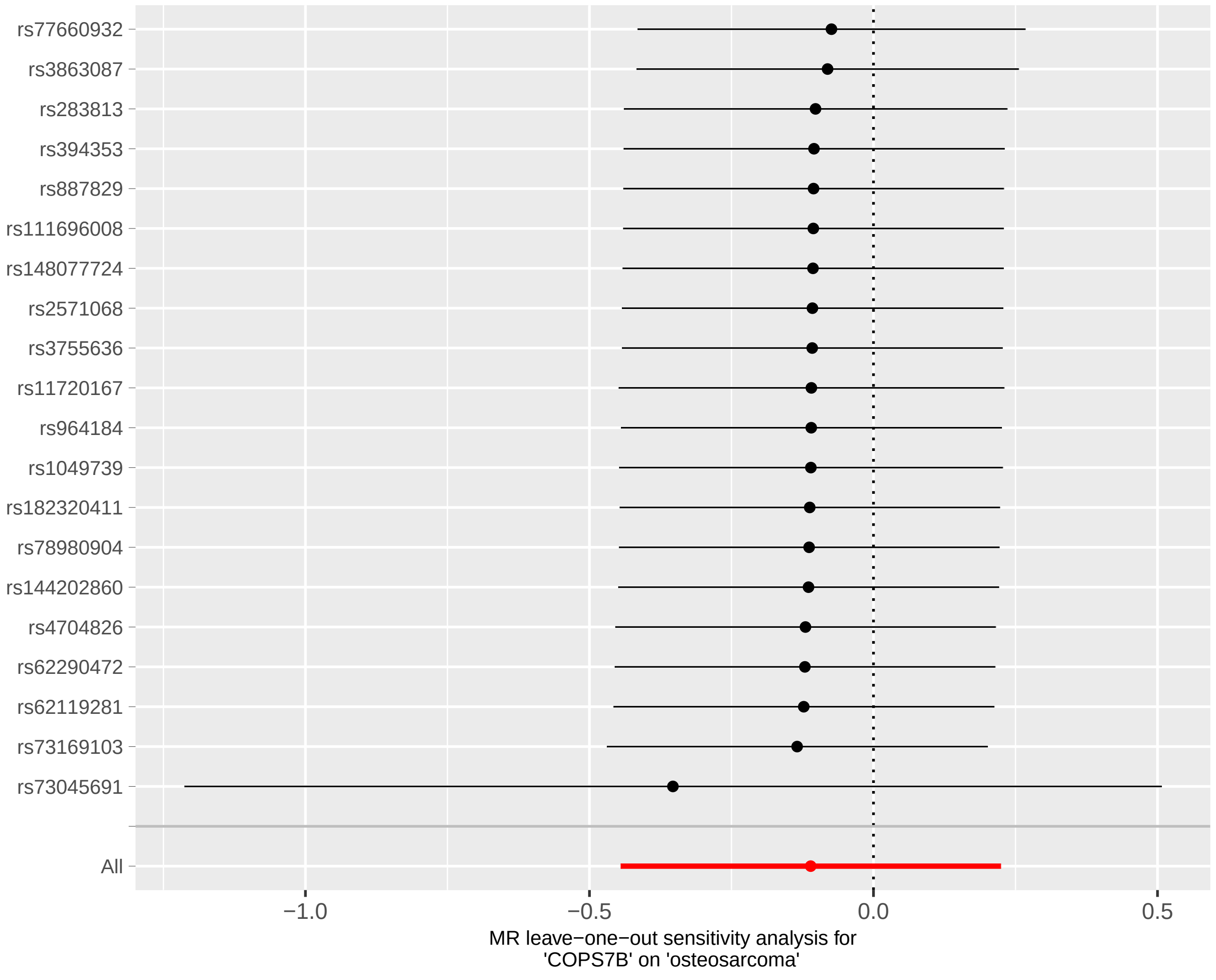
rs77660932
rs3863087
rs283813
rs394353
rs887829
rs111696008
rs148077724
rs2571068
rs3755636
rs11720167
rs964184
rs1049739
rs182320411
rs78980904
rs144202860
rs4704826
rs62290472
rs62119281
rs73169103
rs73045691
All
−1.0
−0.5
0.0
0.5
MR leave−one−out sensitivity analysis for
'COPS7B' on 'osteosarcoma'

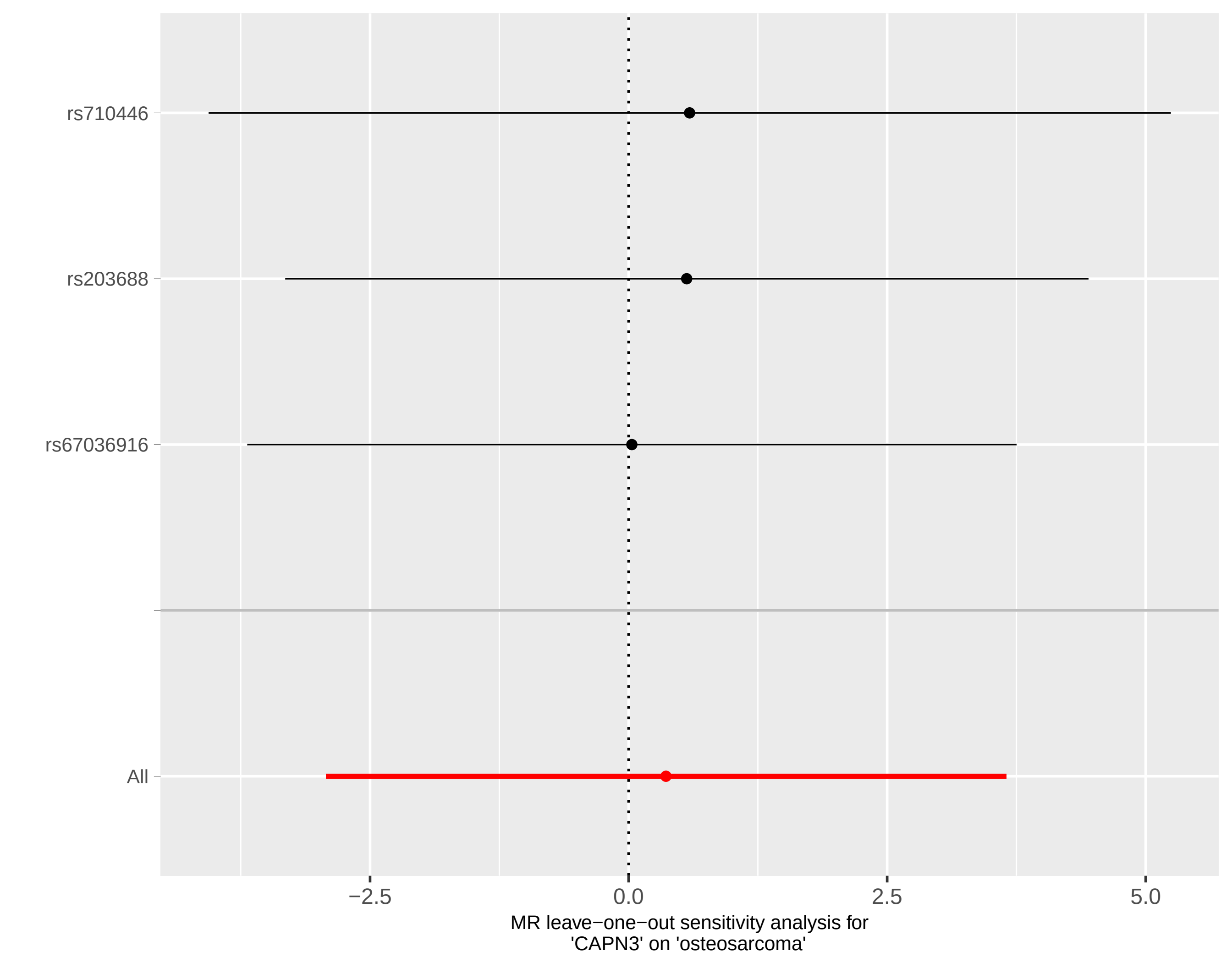
rs710446
rs203688
rs67036916
All
−2.5
0.0
2.5
5.0
MR leave−one−out sensitivity analysis for
'CAPN3' on 'osteosarcoma'

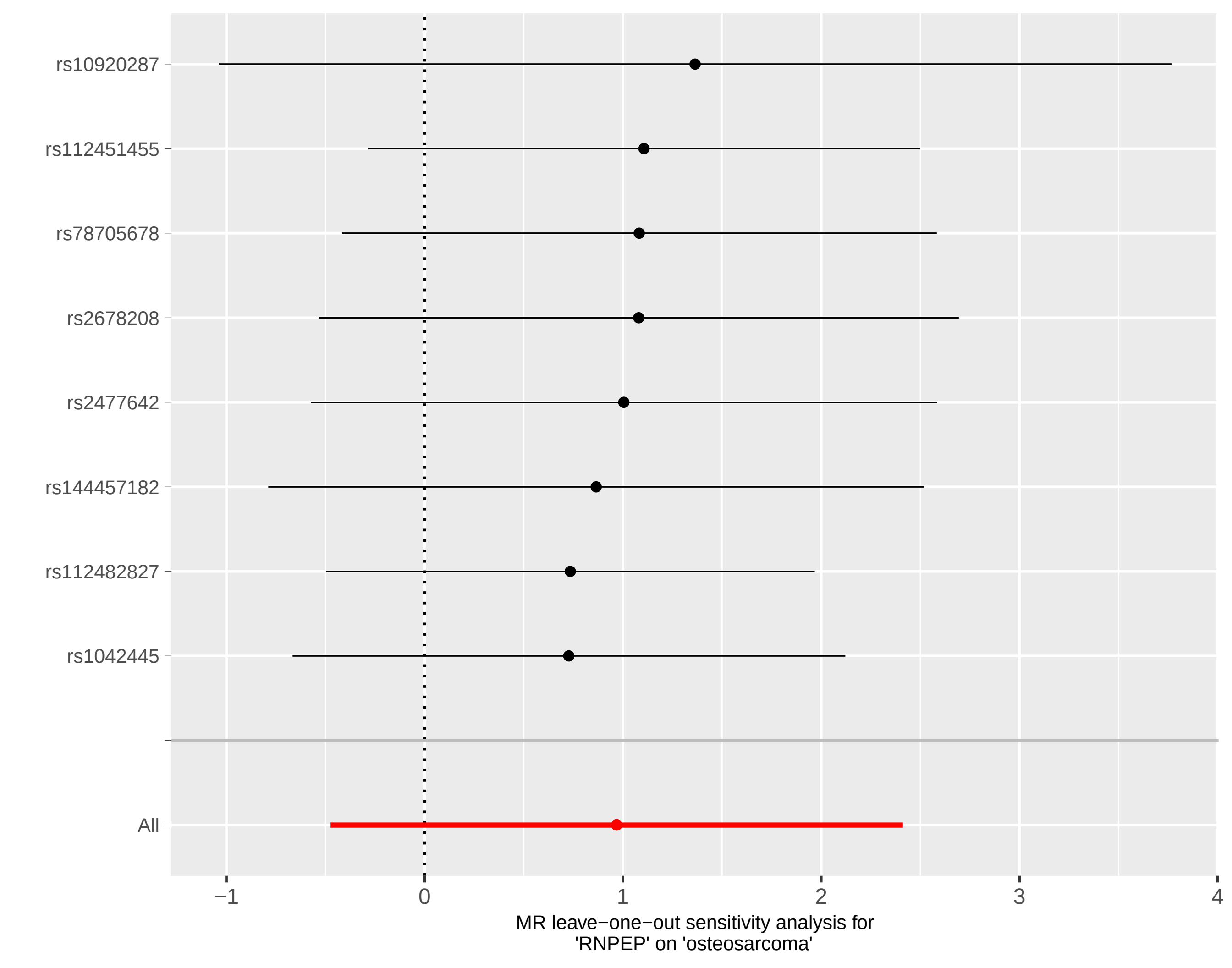
rs10920287
rs112451455
rs78705678
rs2678208
rs2477642
rs144457182
rs112482827
rs1042445
All
−1
0
1
2
3
4
MR leave−one−out sensitivity analysis for
'RNPEP' on 'osteosarcoma'

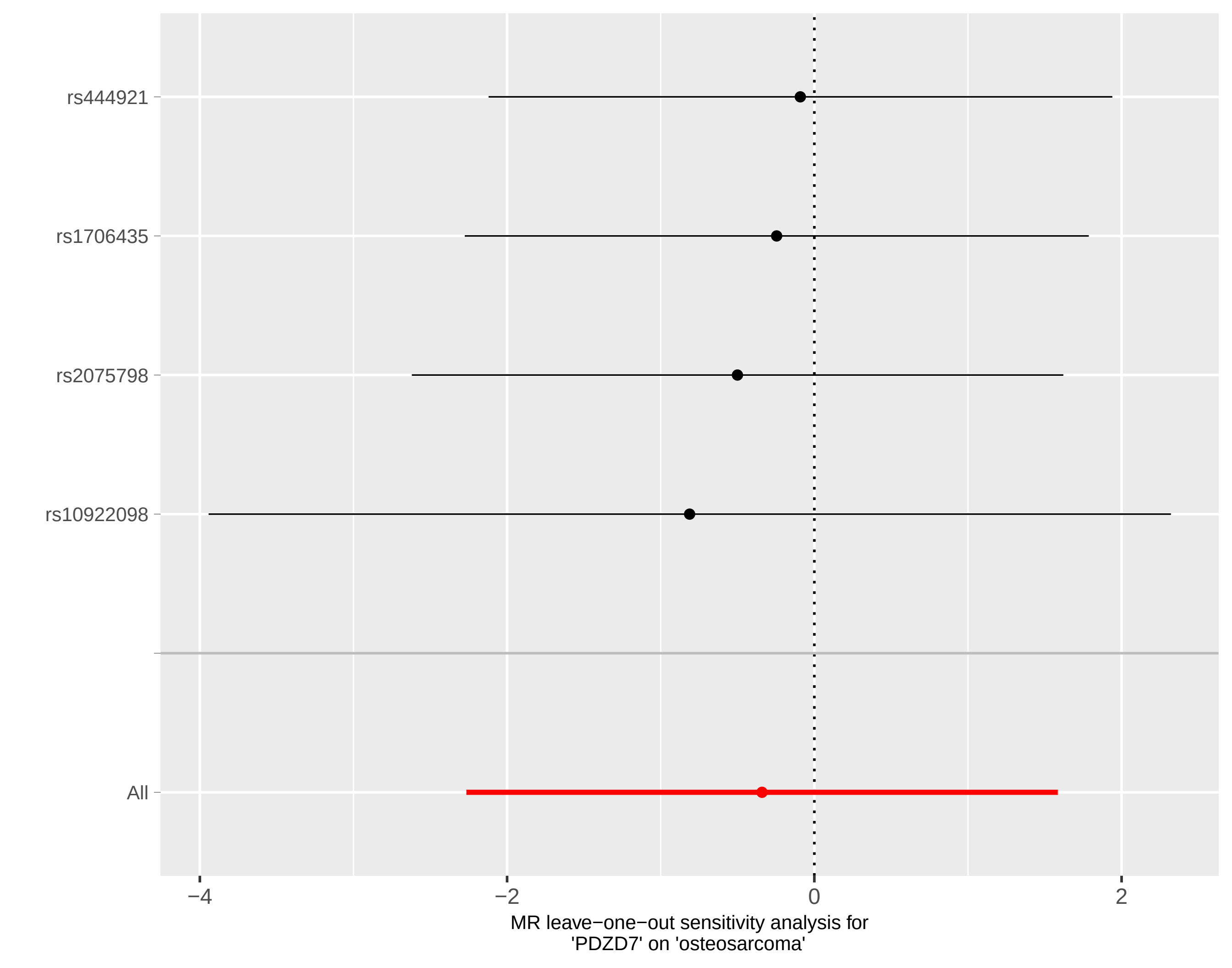

rs444921
rs1706435
rs2075798
rs10922098
All
−4
−2
0
2
MR leave−one−out sensitivity analysis for
'PDZD7' on 'osteosarcoma'

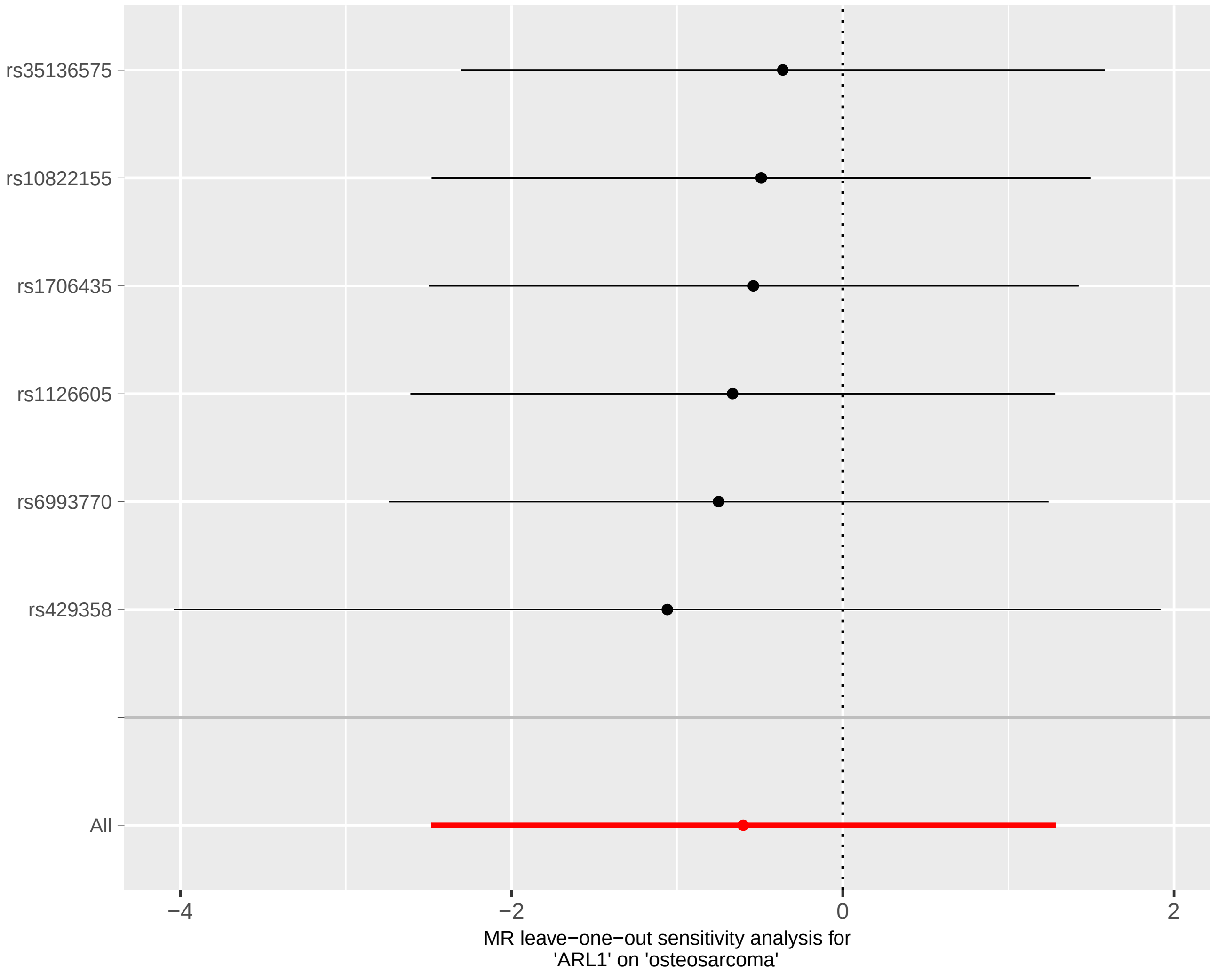
rs35136575
rs10822155
rs1706435
rs1126605
rs6993770
rs429358
All
−4
−2
0
2
MR leave−one−out sensitivity analysis for
'ARL1' on 'osteosarcoma'

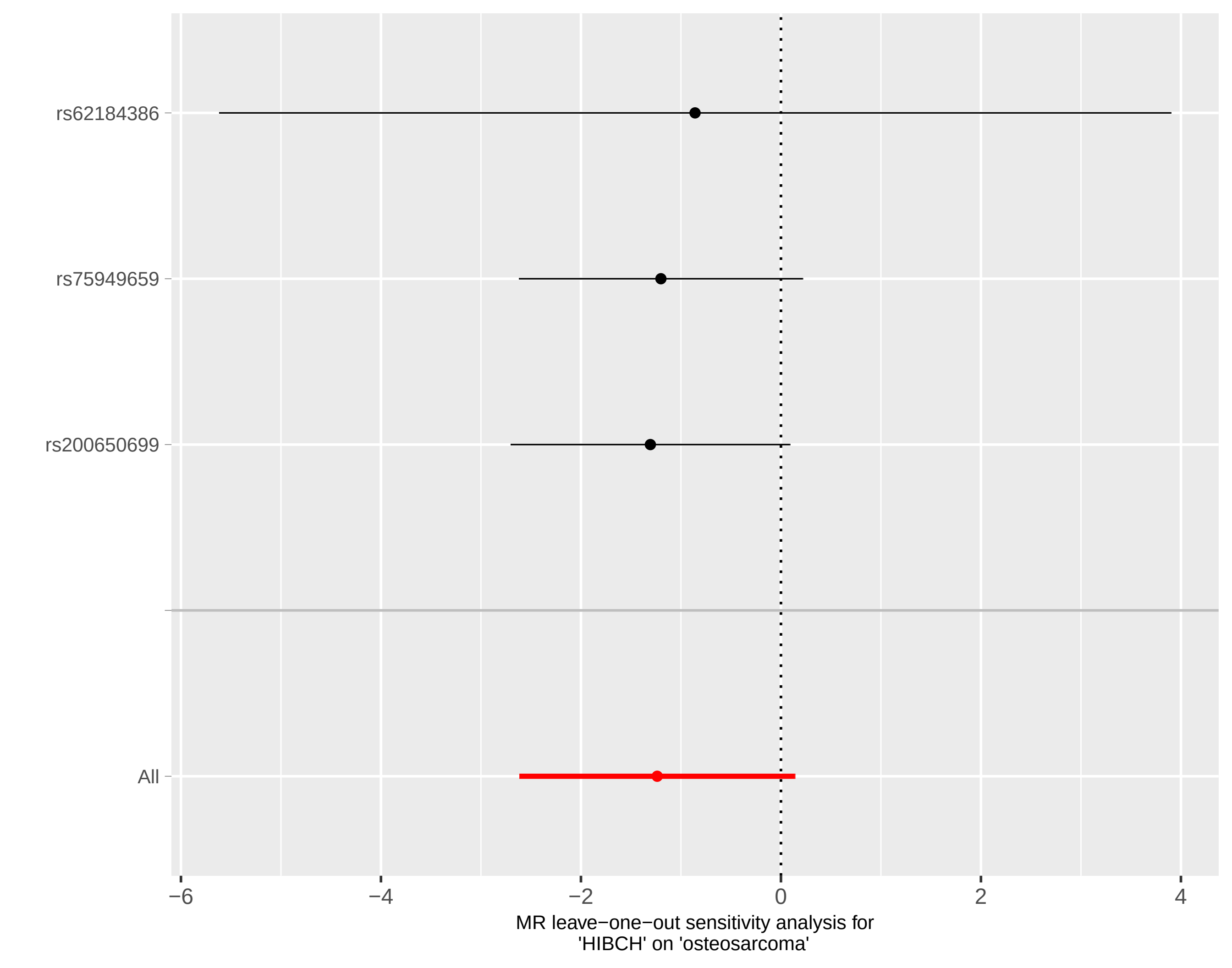
rs62184386
rs75949659
rs200650699
All
−6
−4
−2
0
2
4
MR leave−one−out sensitivity analysis for
'HIBCH' on 'osteosarcoma'

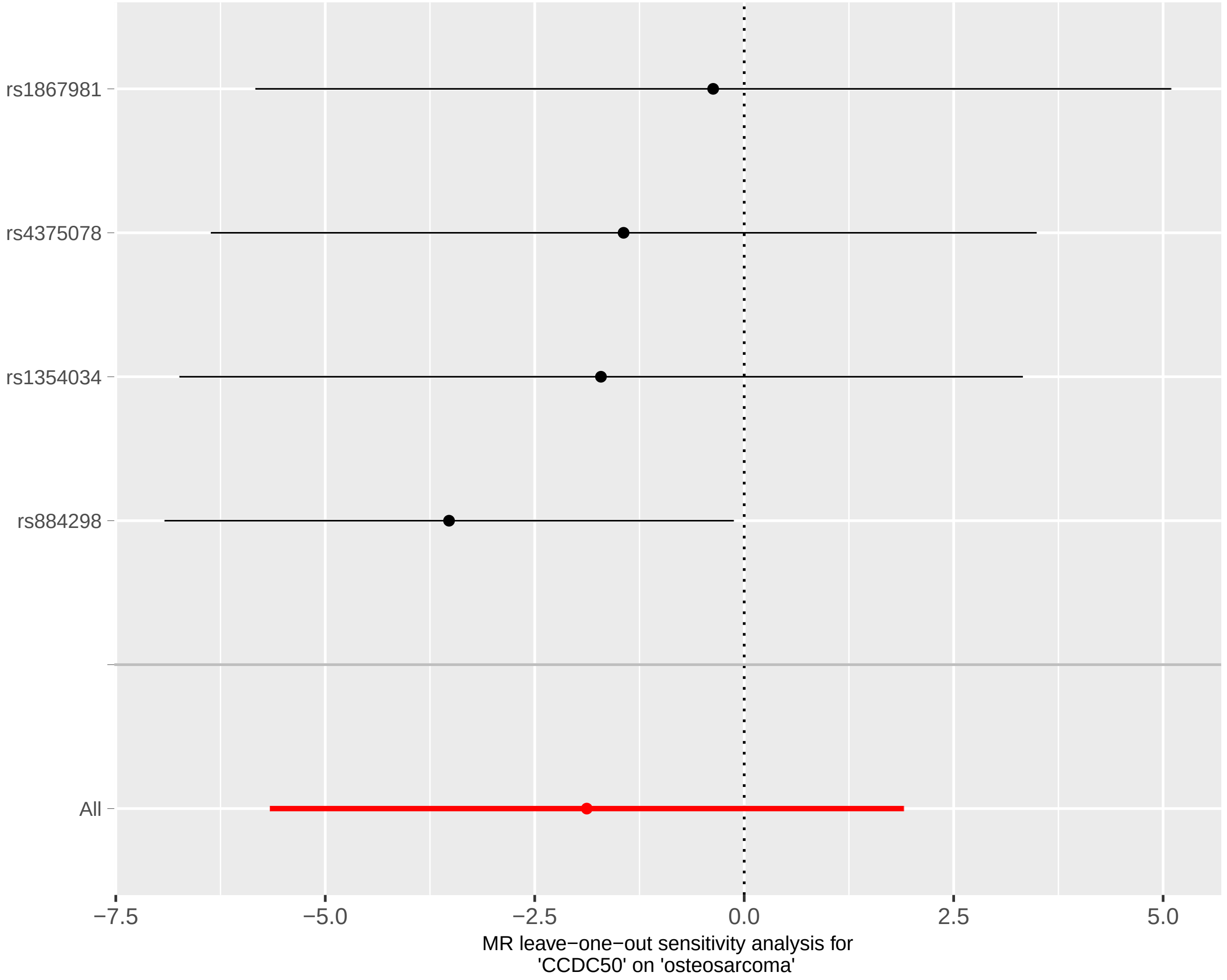

rs1867981
rs4375078
rs1354034
rs884298
All
−7.5
−5.0
−2.5
0.0
2.5
5.0
MR leave−one−out sensitivity analysis for
'CCDC50' on 'osteosarcoma'

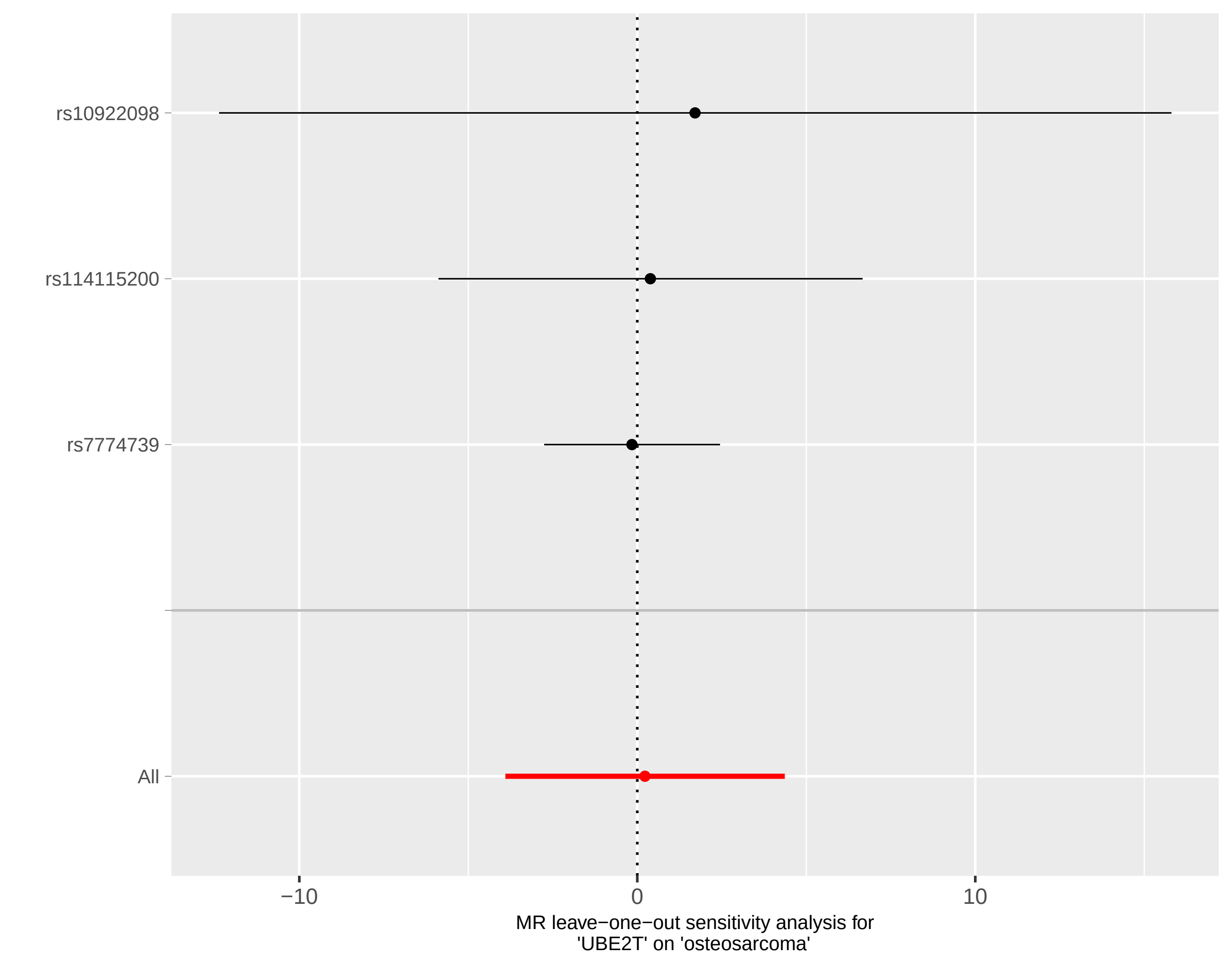
rs10922098
rs114115200
rs7774739
All
−10
0
10
MR leave−one−out sensitivity analysis for
'UBE2T' on 'osteosarcoma'

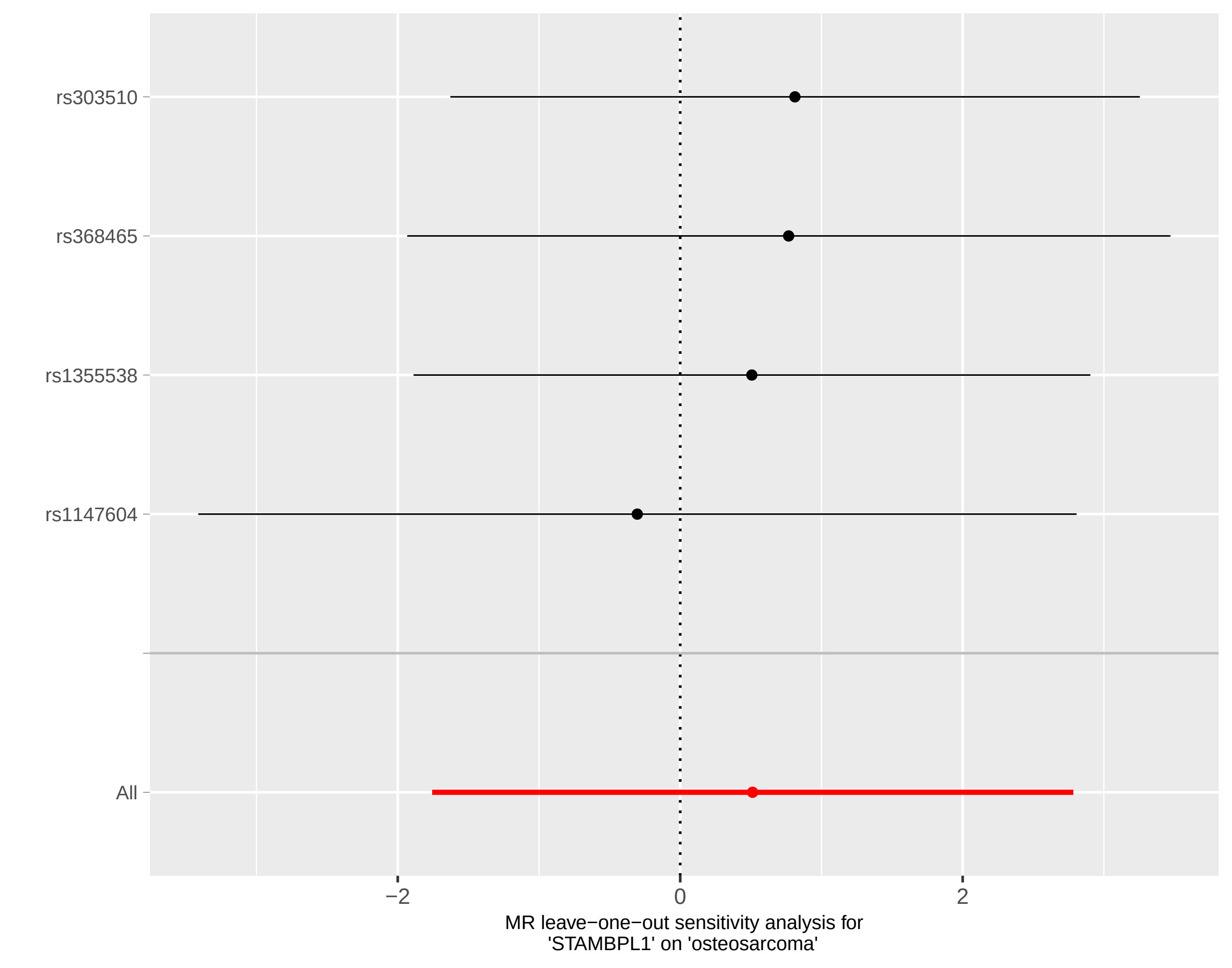
rs303510
rs368465
rs1355538
rs1147604
All
−2
0
2
MR leave−one−out sensitivity analysis for
'STAMBPL1' on 'osteosarcoma'

Insufficient number of SNPs

Insufficient number of SNPs

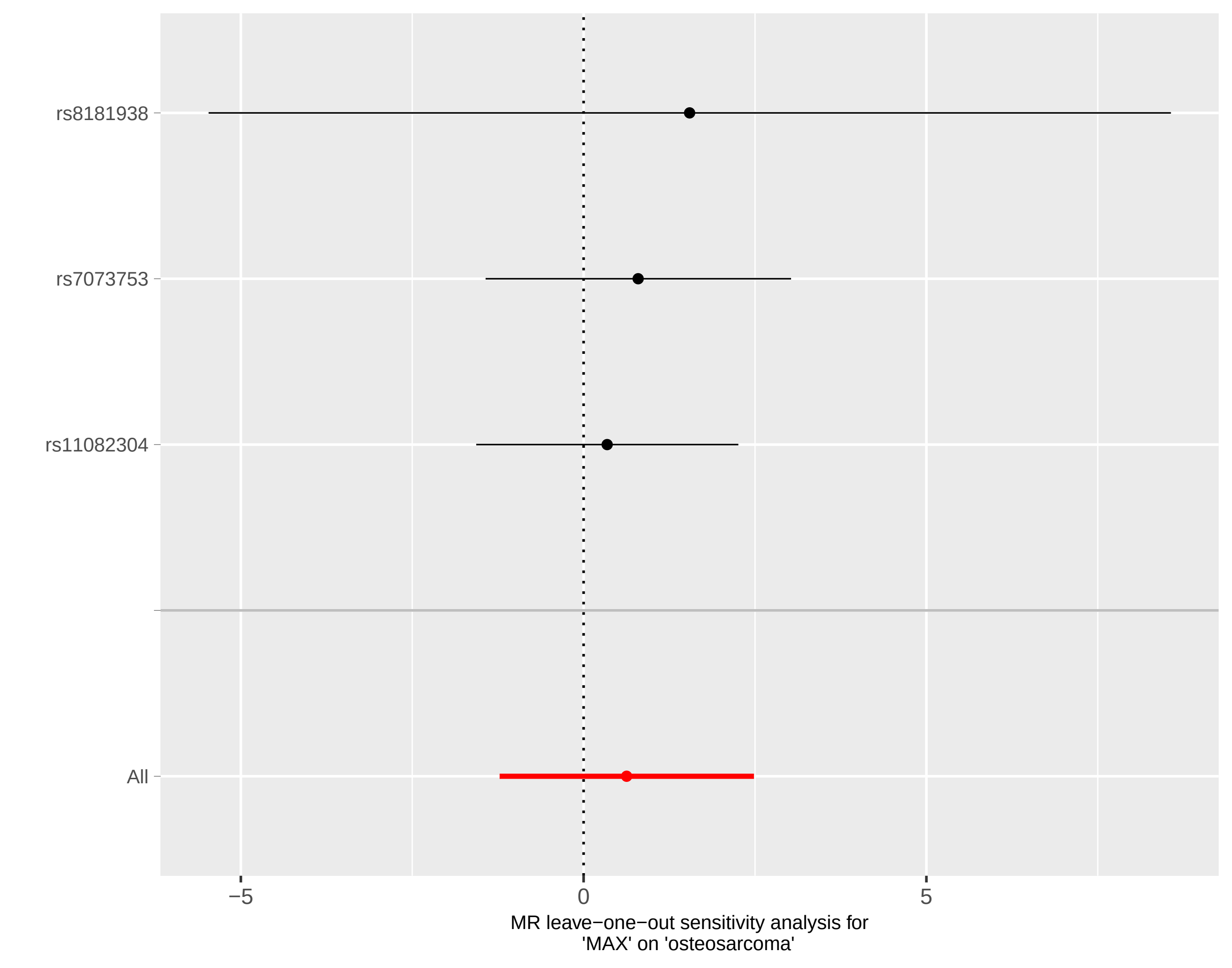

rs8181938
rs7073753
rs11082304
All
−5
0
5
MR leave−one−out sensitivity analysis for
'MAX' on 'osteosarcoma'

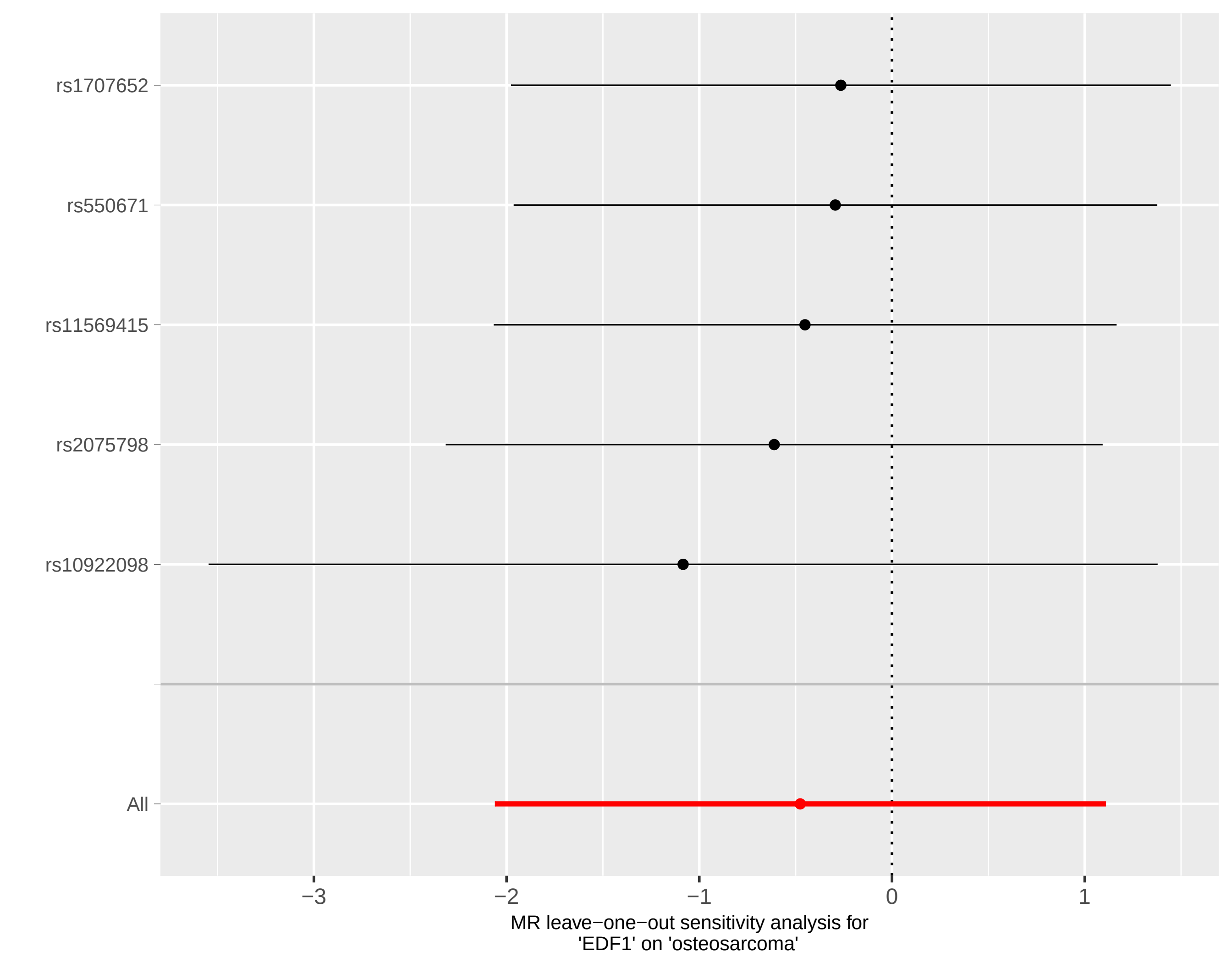

rs1707652
rs550671
rs11569415
rs2075798
rs10922098
All
−3
−2
−1
0
1
MR leave−one−out sensitivity analysis for
'EDF1' on 'osteosarcoma'

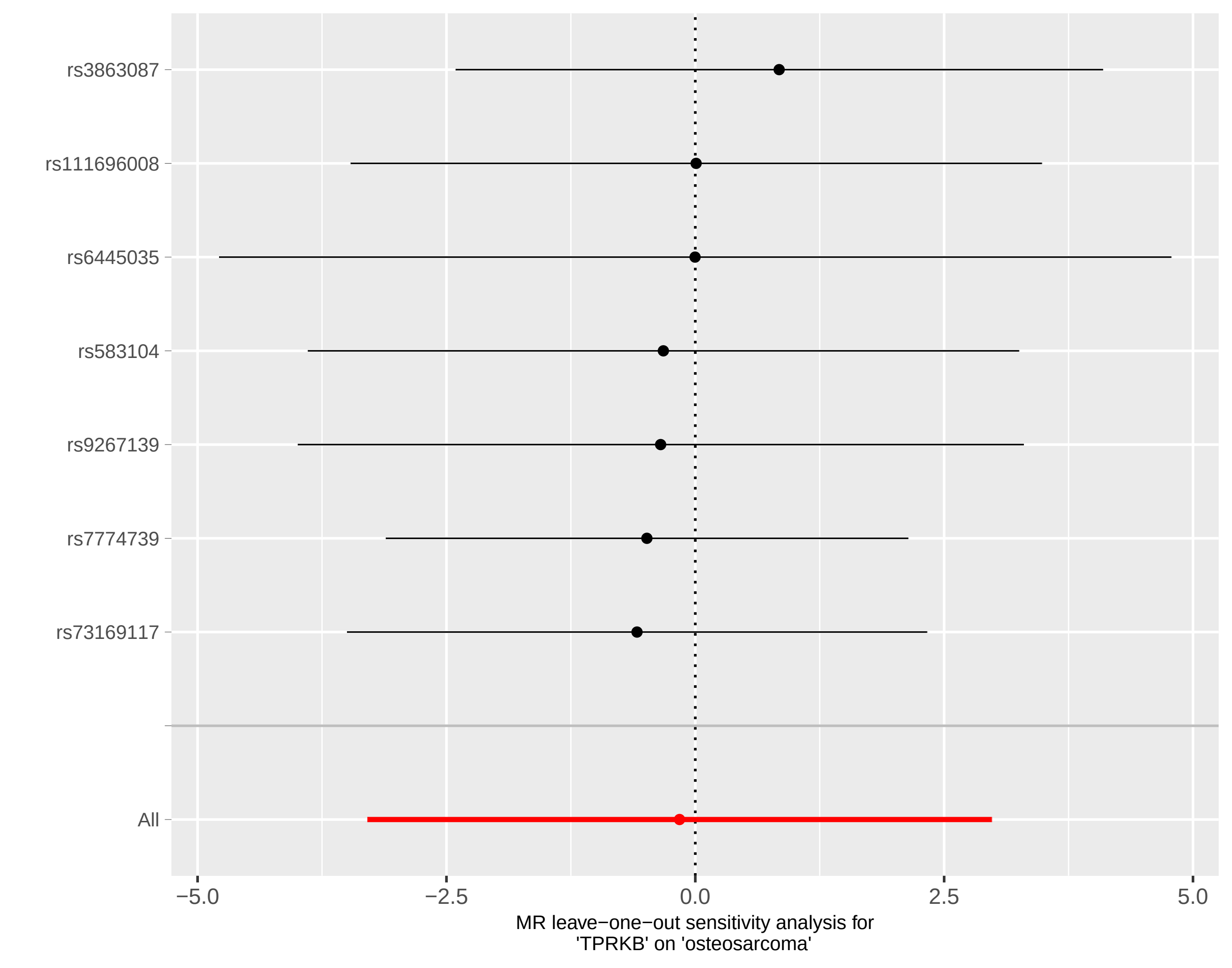
rs3863087
rs111696008
rs6445035
rs583104
rs9267139
rs7774739
rs73169117
All
−5.0
−2.5
0.0
2.5
5.0
MR leave−one−out sensitivity analysis for
'TPRKB' on 'osteosarcoma'

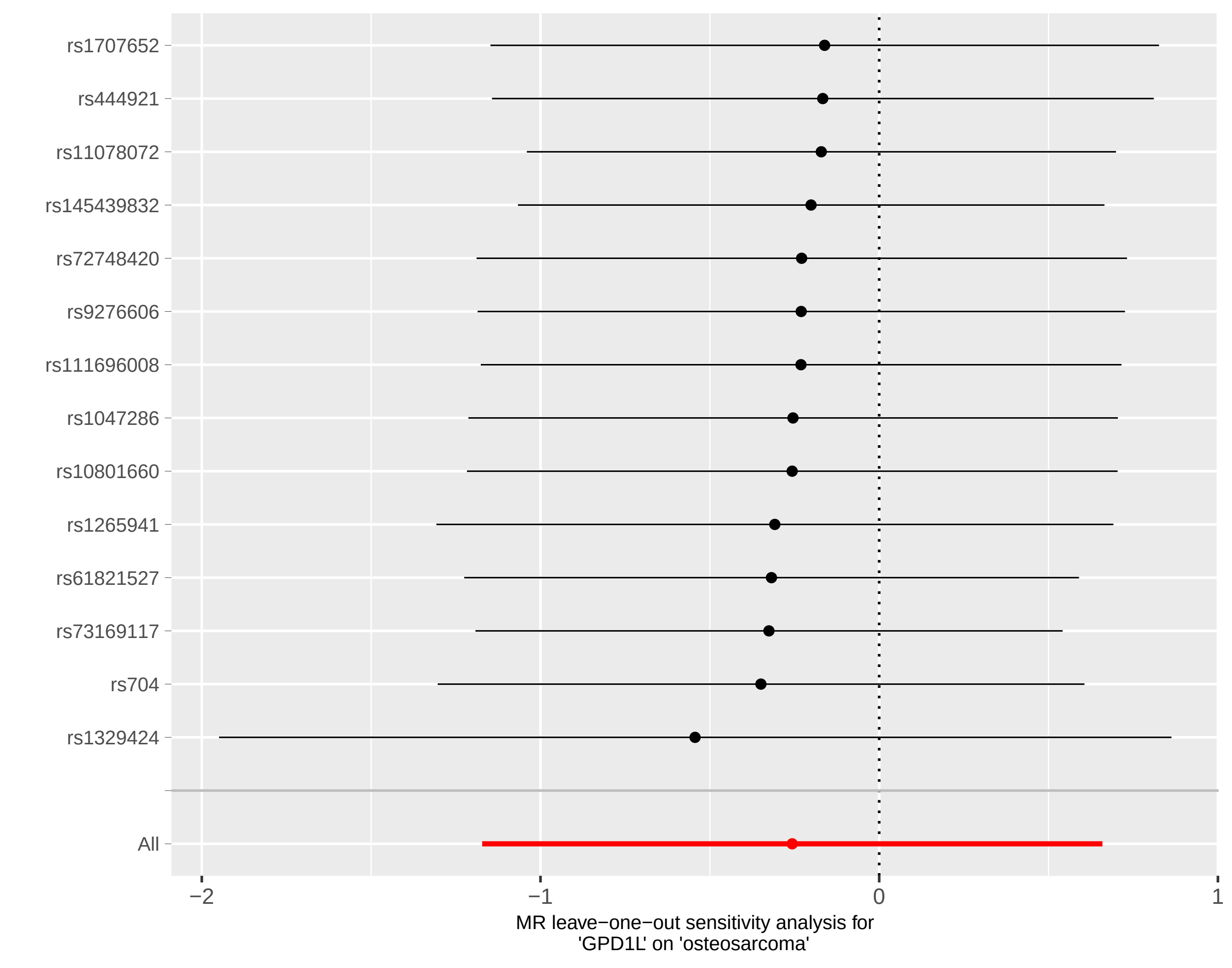
rs1707652
rs444921
rs11078072
rs145439832
rs72748420
rs9276606
rs111696008
rs1047286
rs10801660
rs1265941
rs61821527
rs73169117
rs704
rs1329424
All
−2
−1
0
1
MR leave−one−out sensitivity analysis for
'GPD1L' on 'osteosarcoma'

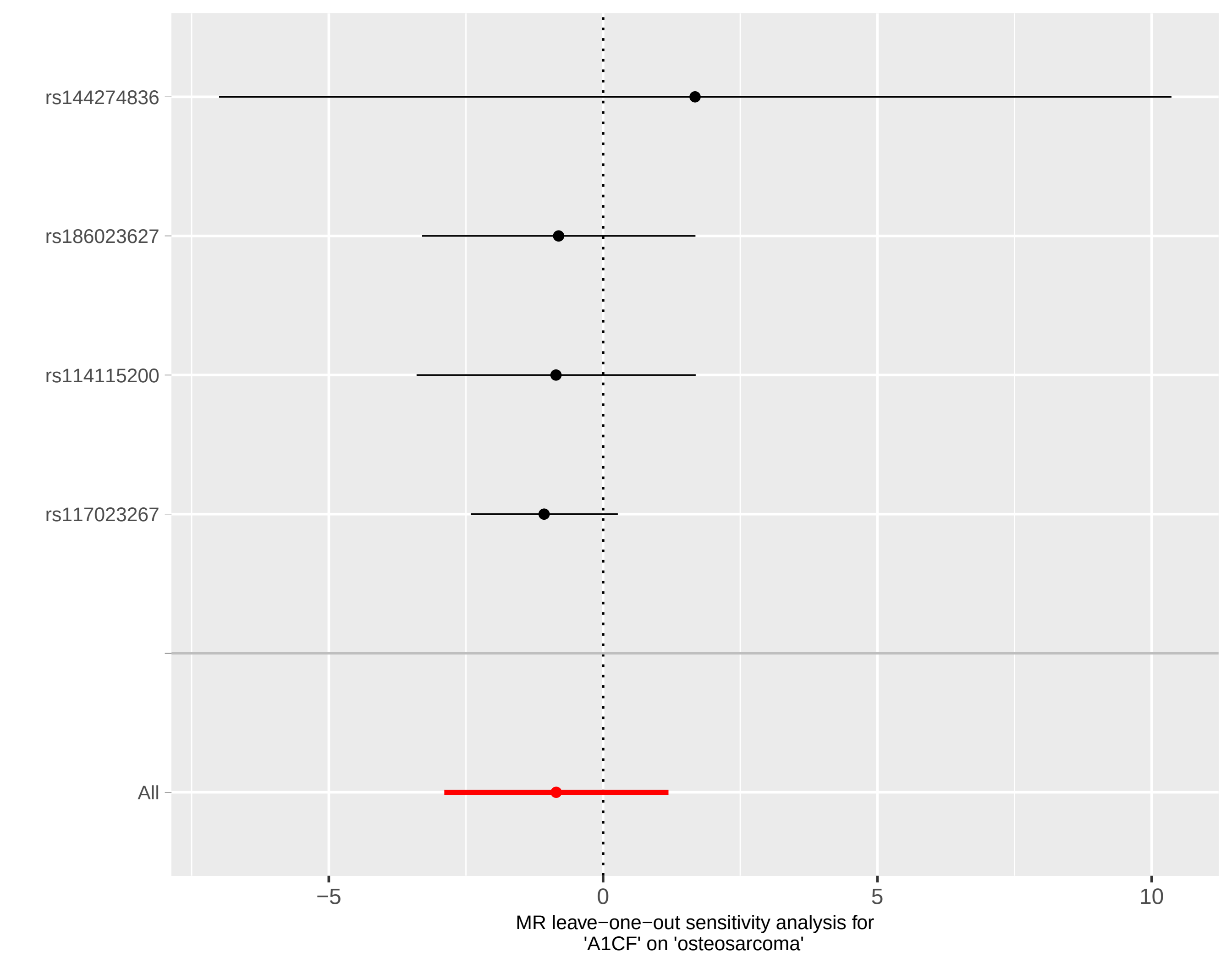
rs144274836
rs186023627
rs114115200
rs117023267
All
−5
0
5
10
MR leave−one−out sensitivity analysis for
'A1CF' on 'osteosarcoma'

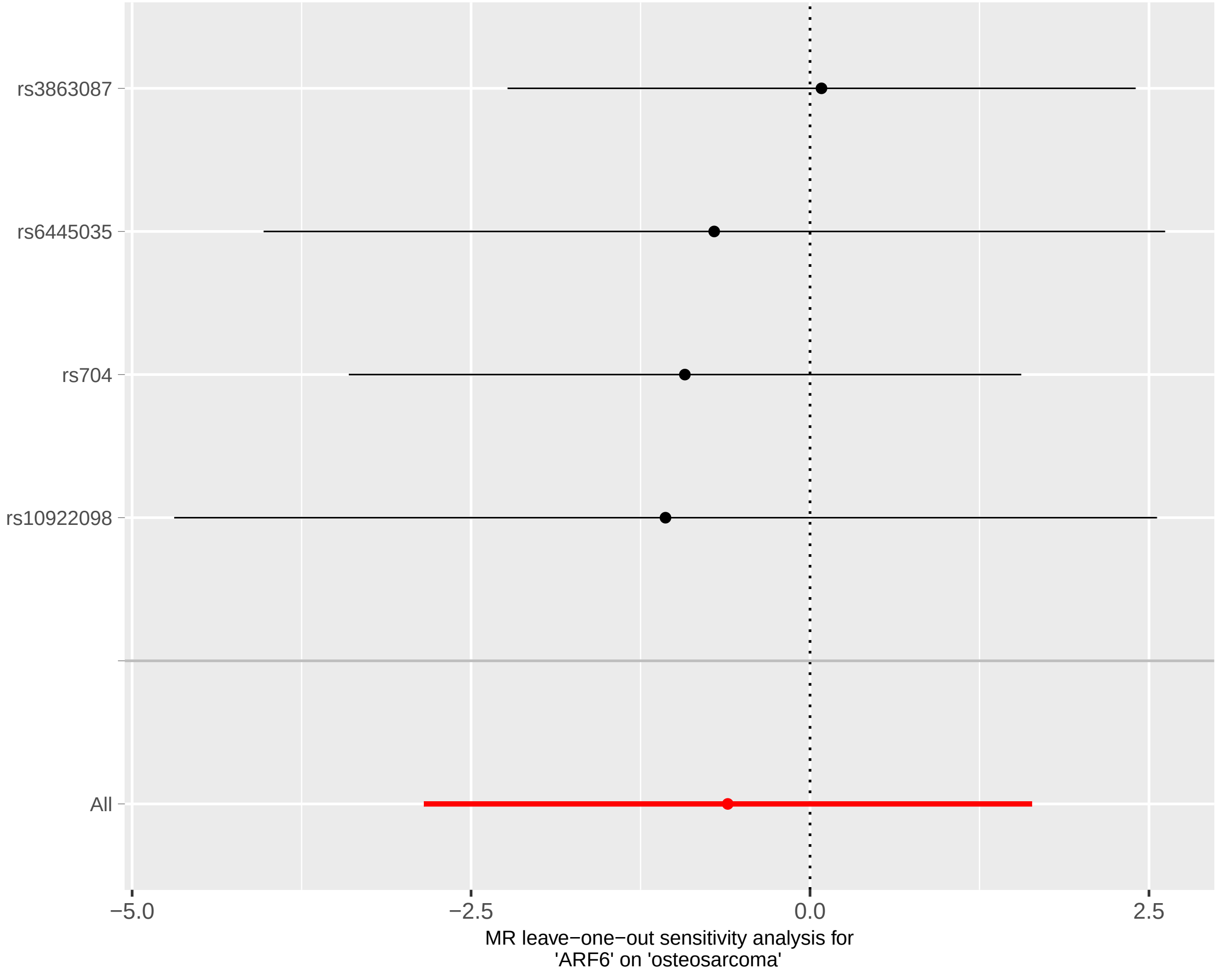
rs3863087
rs6445035
rs704
rs10922098
All
−5.0
−2.5
0.0
2.5
MR leave−one−out sensitivity analysis for
'ARF6' on 'osteosarcoma'

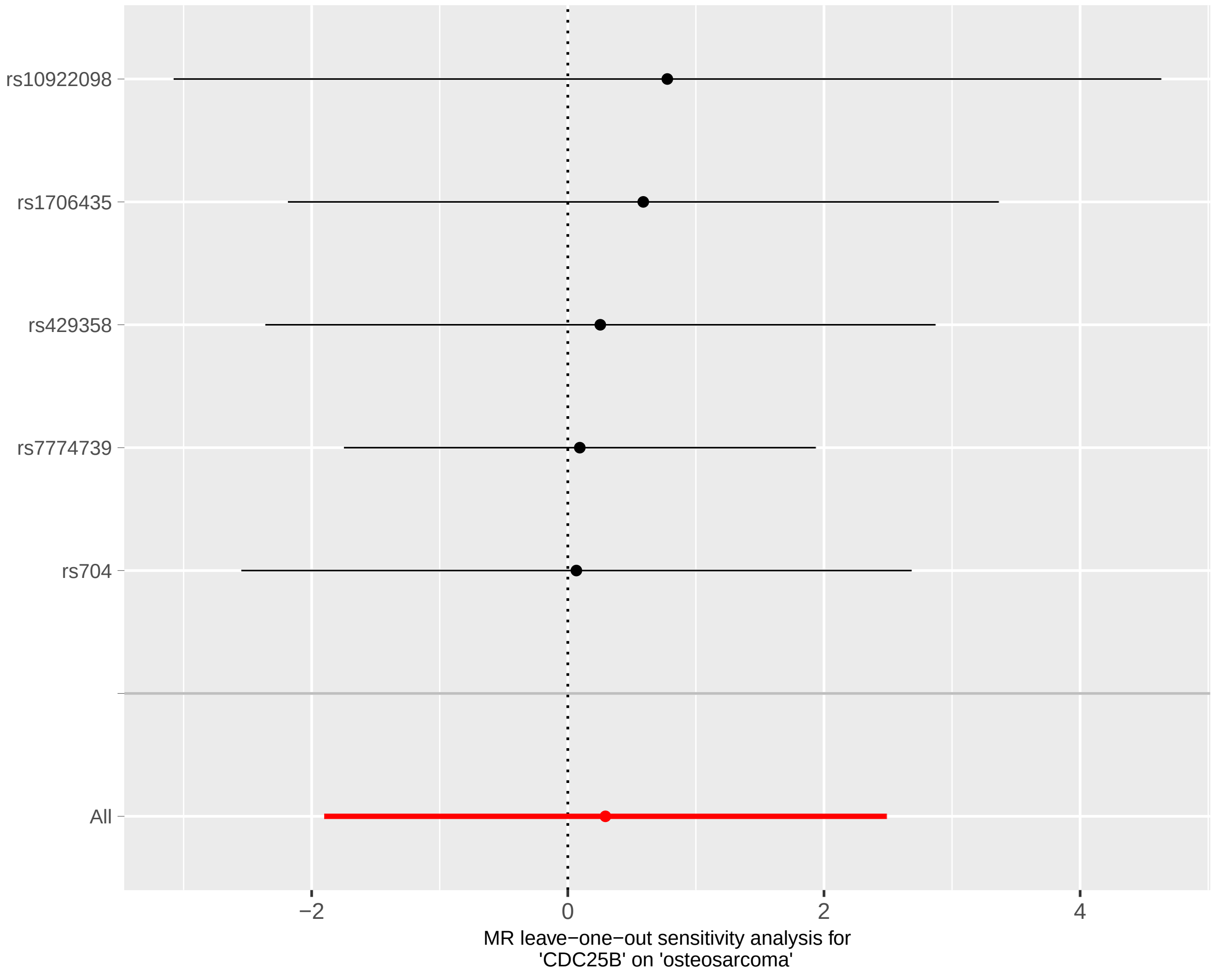
rs10922098
rs1706435
rs429358
rs7774739
rs704
All
−2
0
2
4
MR leave−one−out sensitivity analysis for
'CDC25B' on 'osteosarcoma'

Insufficient number of SNPs

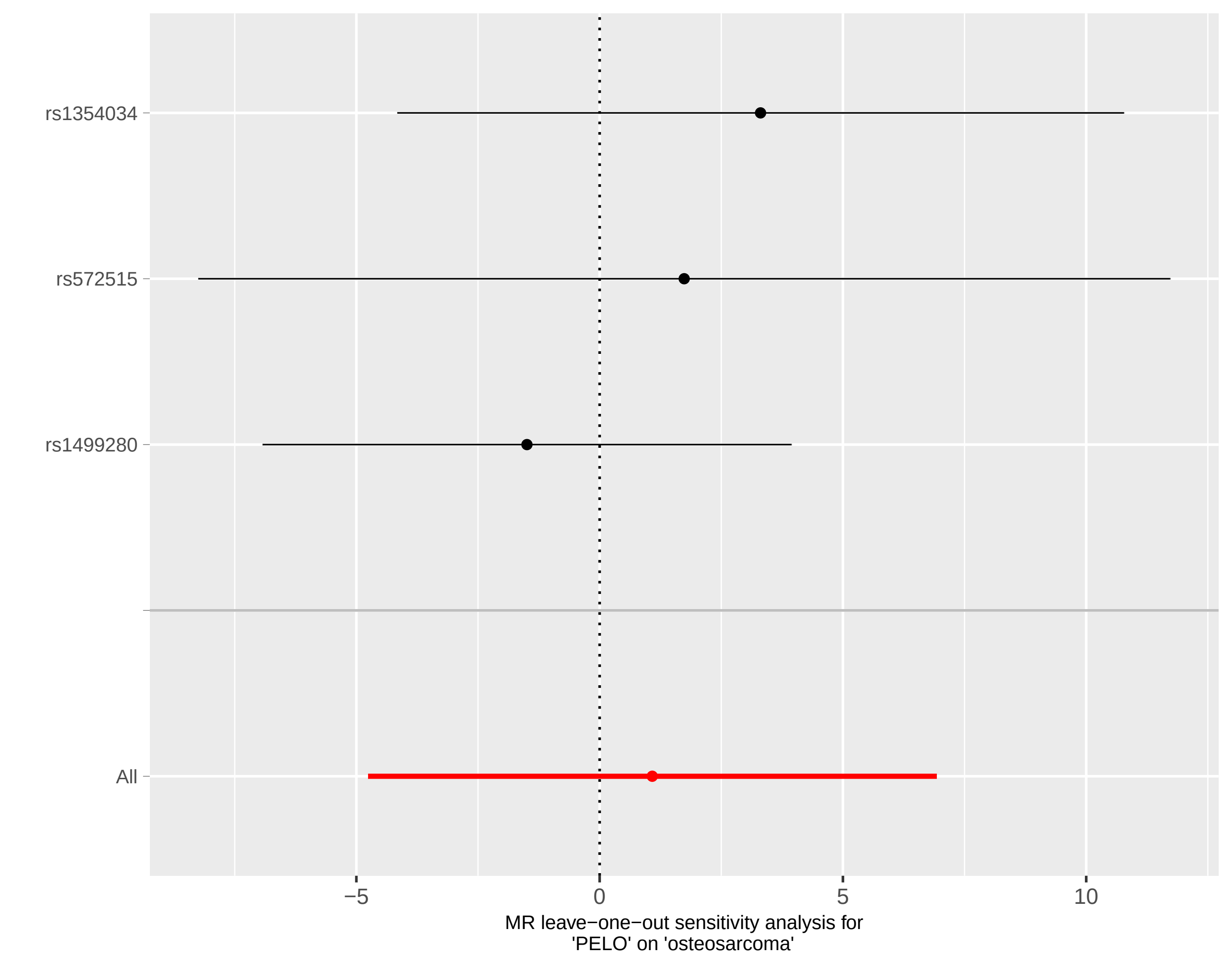
rs1354034
rs572515
rs1499280
All
−5
0
5
10
MR leave−one−out sensitivity analysis for
'PELO' on 'osteosarcoma'

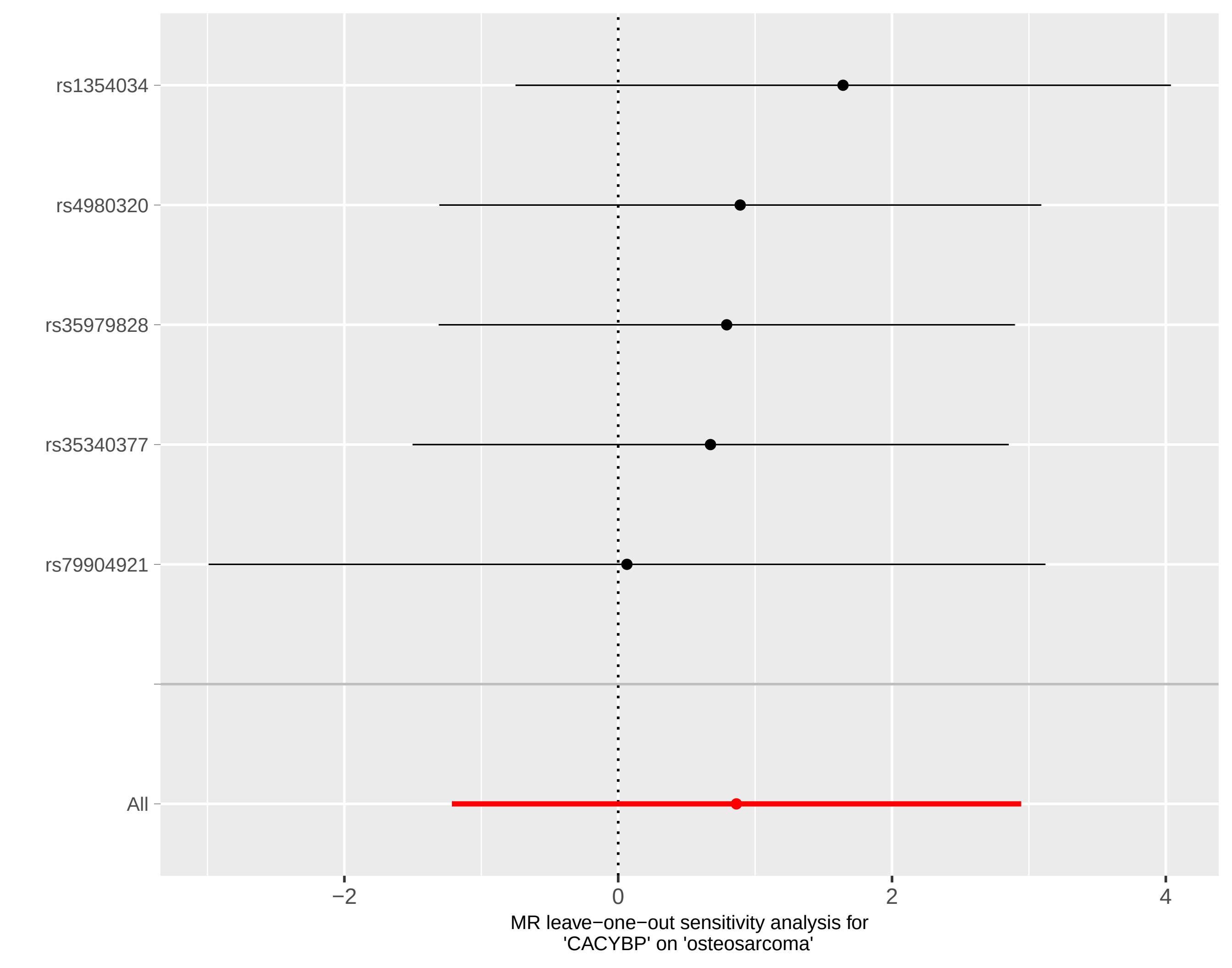
rs1354034
rs4980320
rs35979828
rs35340377
rs79904921
All
−2
0
2
4
MR leave−one−out sensitivity analysis for
'CACYBP' on 'osteosarcoma'

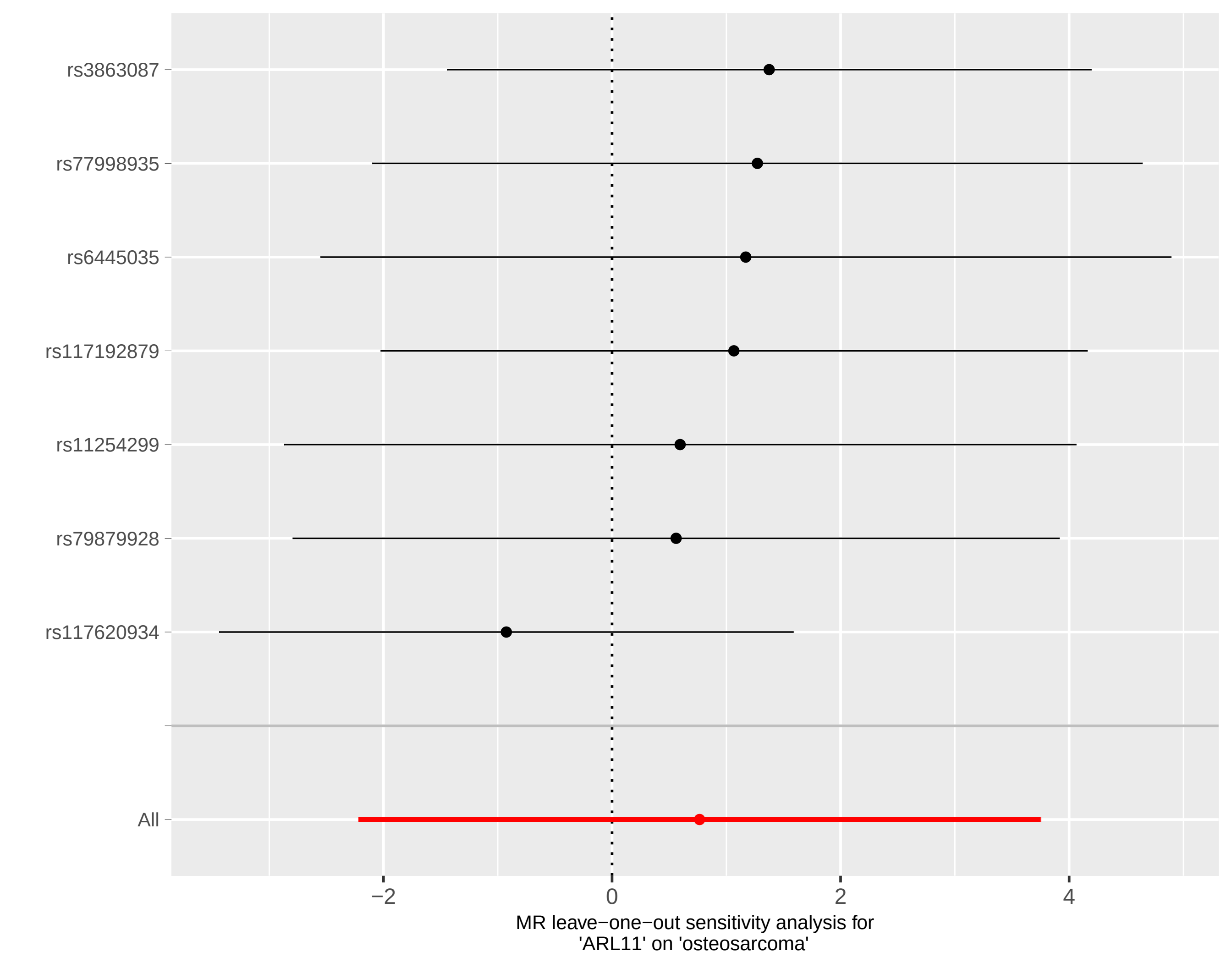
rs3863087
rs77998935
rs6445035
rs117192879
rs11254299
rs79879928
rs117620934
All
−2
0
2
4
MR leave−one−out sensitivity analysis for
'ARL11' on 'osteosarcoma'

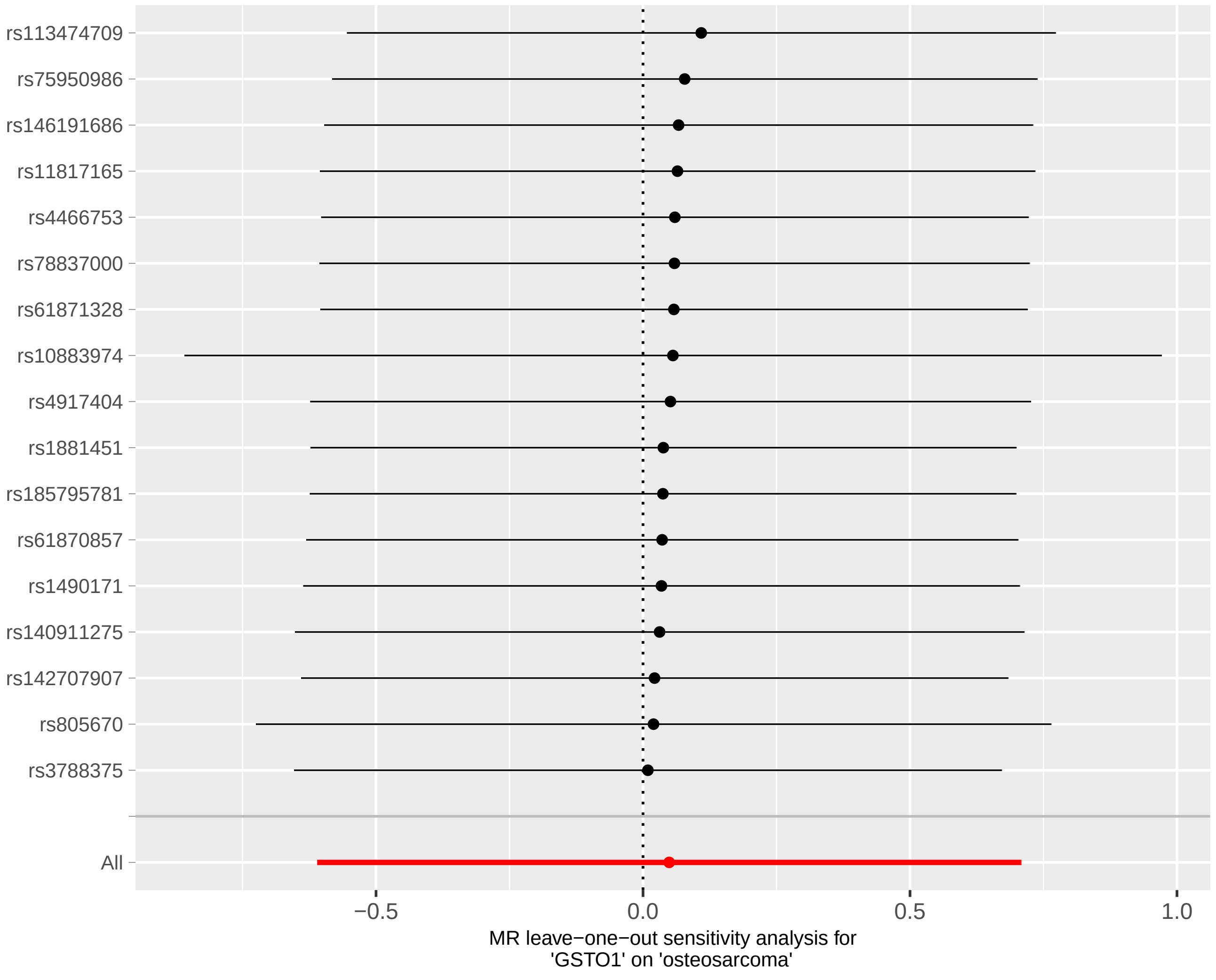

rs113474709
rs75950986
rs146191686
rs11817165
rs4466753
rs78837000
rs61871328
rs10883974
rs4917404
rs1881451
rs185795781
rs61870857
rs1490171
rs140911275
rs142707907
rs805670
rs3788375
All
−0.5
0.0
0.5
1.0
MR leave−one−out sensitivity analysis for
'GSTO1' on 'osteosarcoma'

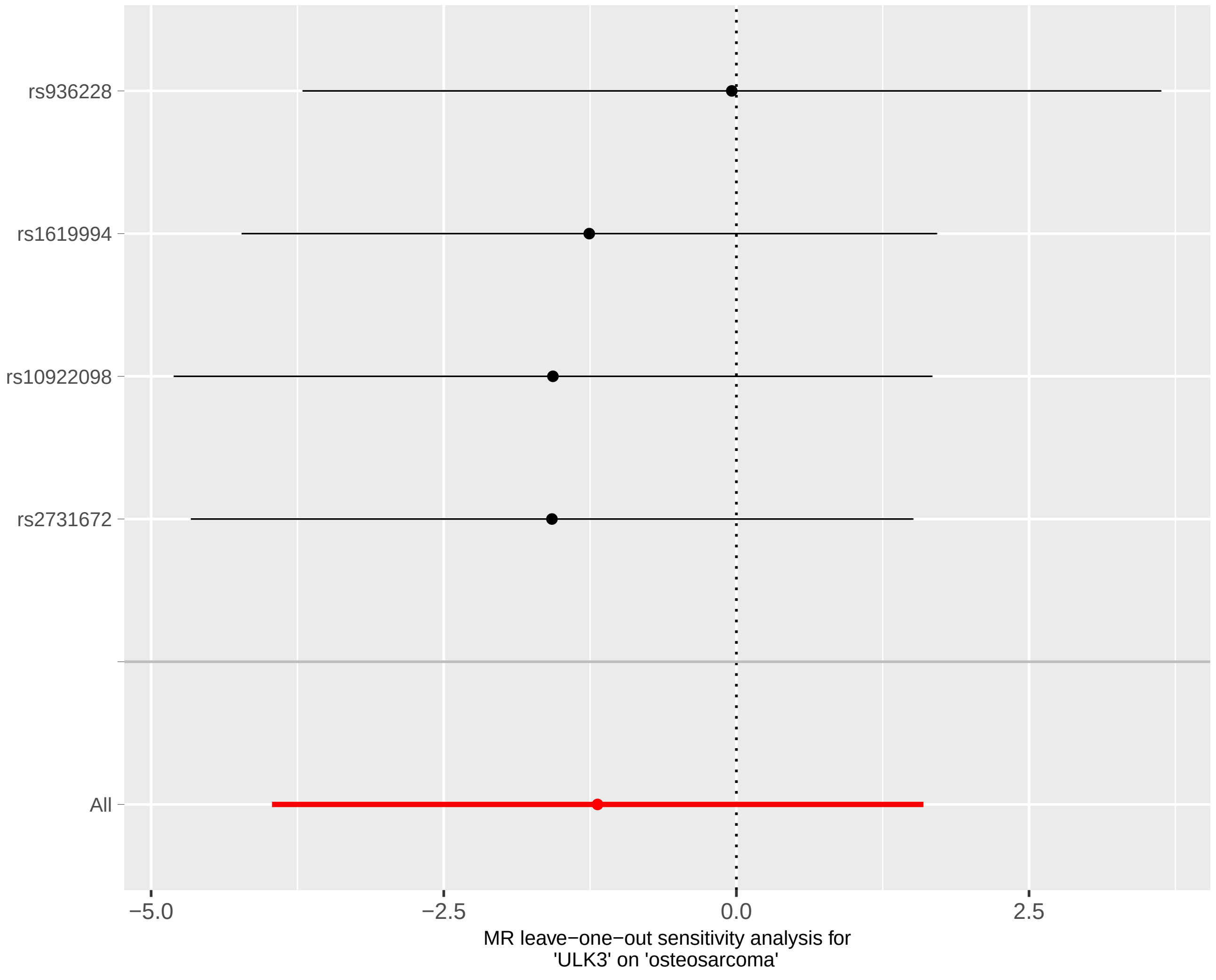
rs936228
rs1619994
rs10922098
rs2731672
All
−5.0
−2.5
0.0
2.5
MR leave−one−out sensitivity analysis for
'ULK3' on 'osteosarcoma'

Insufficient number of SNPs

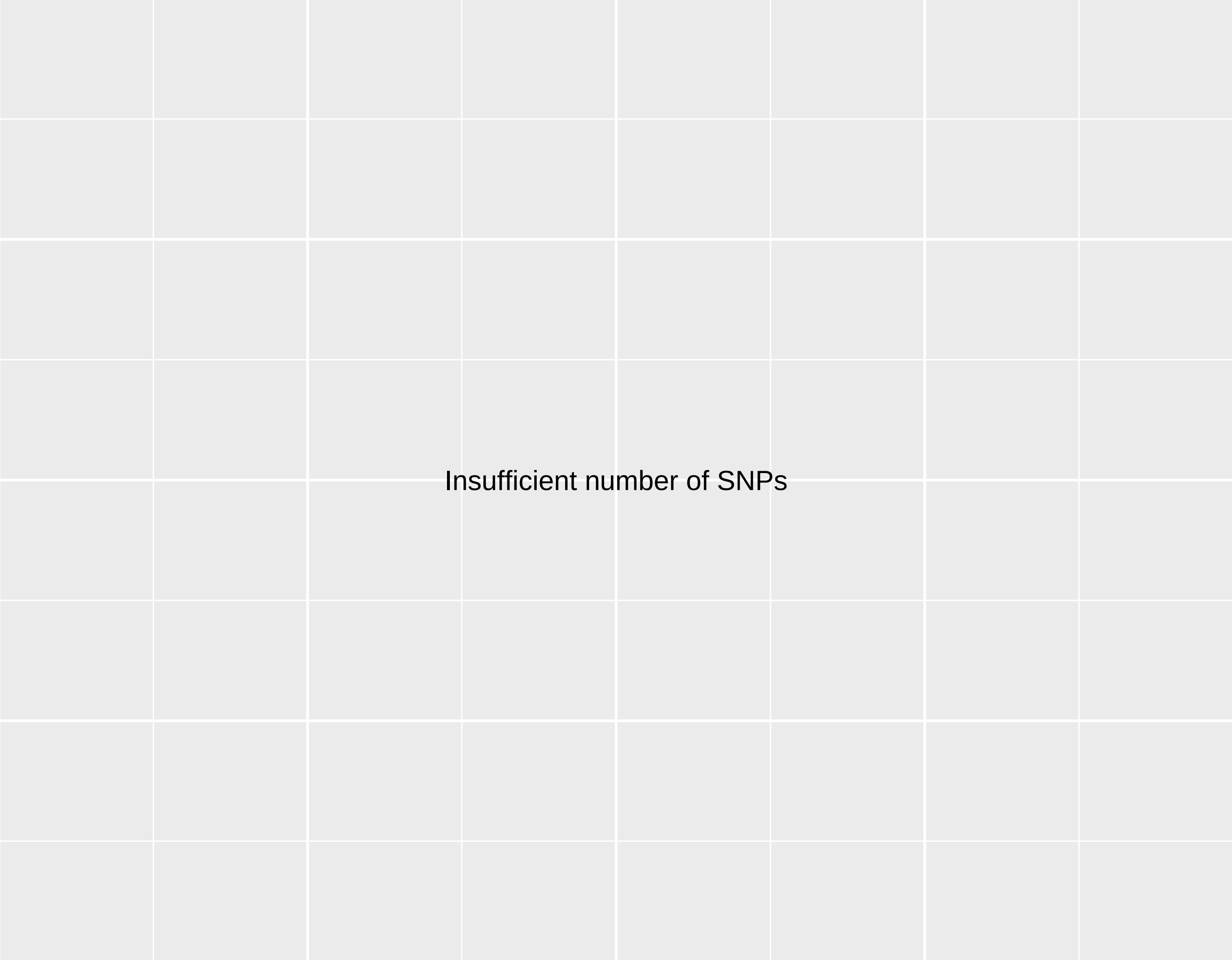
Insufficient number of SNPs

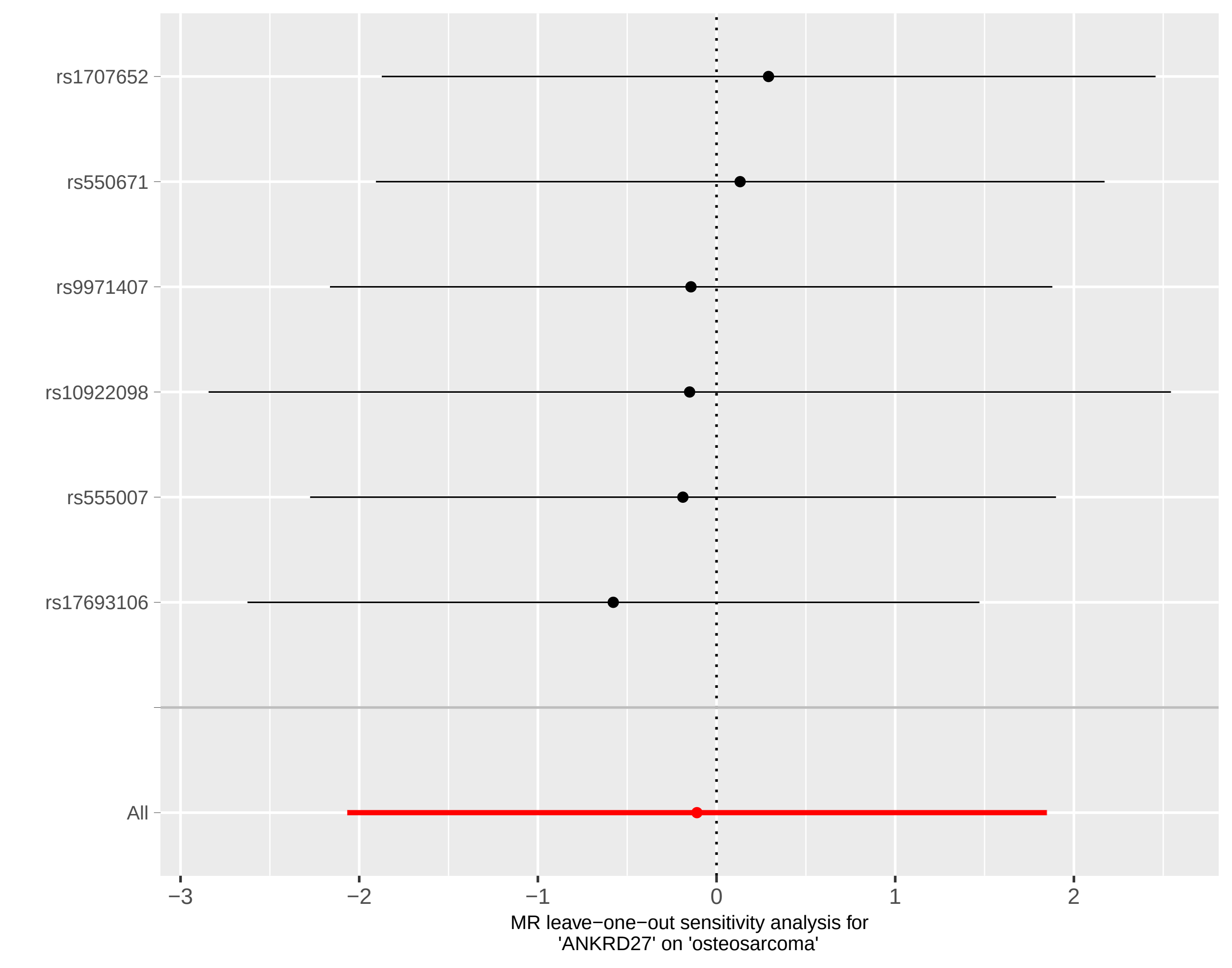
rs1707652
rs550671
rs9971407
rs10922098
rs555007
rs17693106
All
−3
−2
−1
0
1
2
MR leave−one−out sensitivity analysis for
'ANKRD27' on 'osteosarcoma'

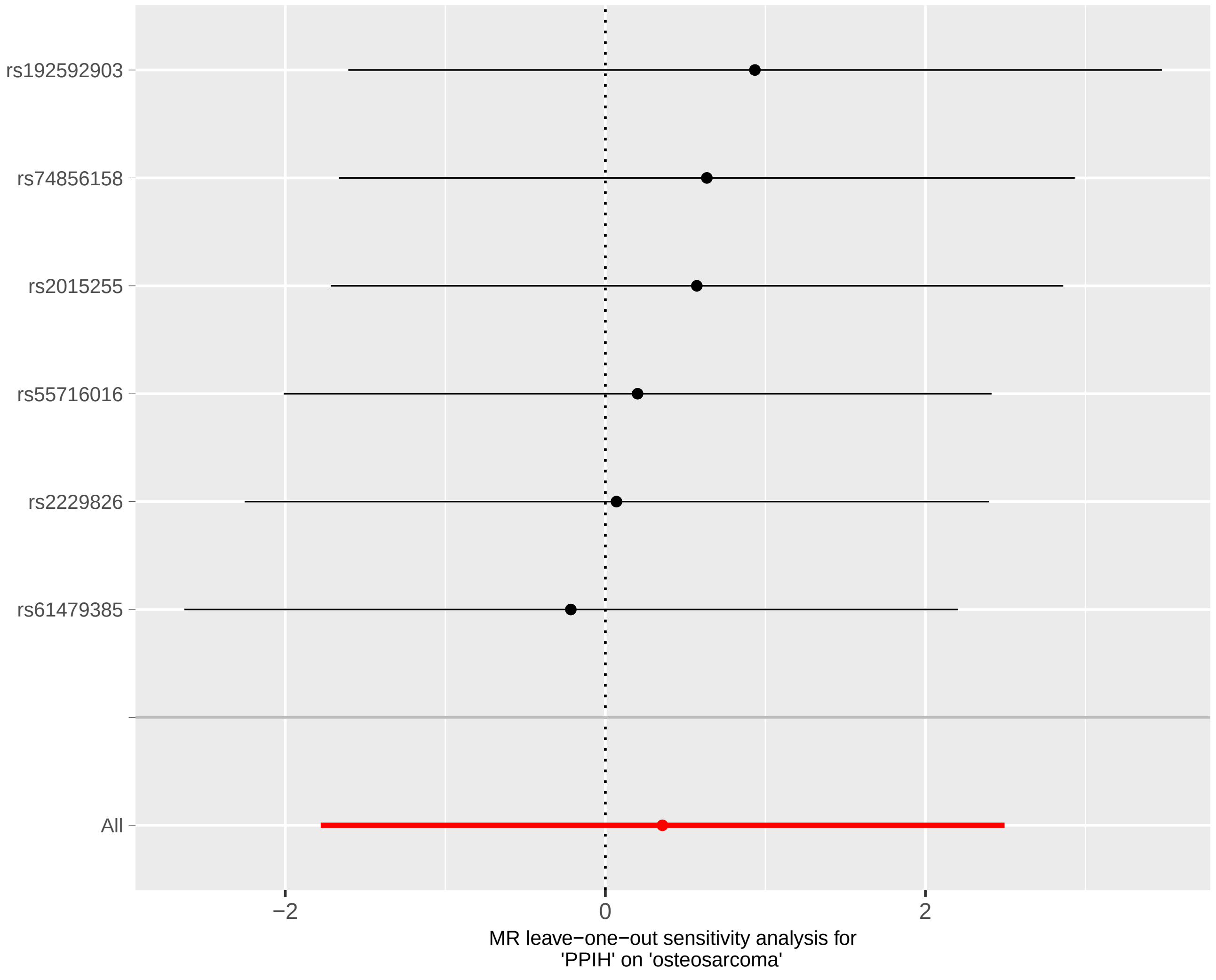
rs192592903
rs74856158
rs2015255
rs55716016
rs2229826
rs61479385
All
−2
0
2
MR leave−one−out sensitivity analysis for
'PPIH' on 'osteosarcoma'

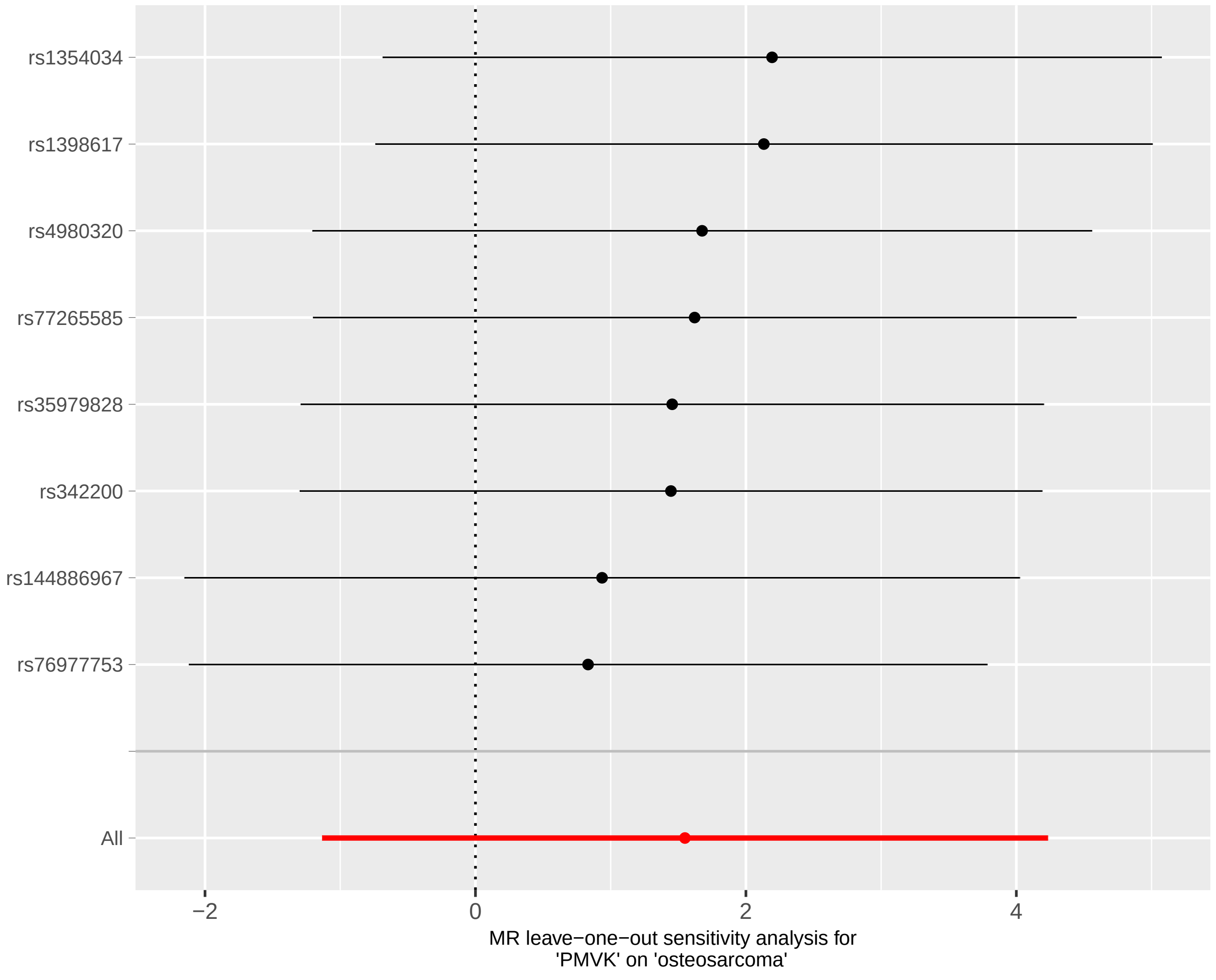
rs1354034
rs1398617
rs4980320
rs77265585
rs35979828
rs342200
rs144886967
rs76977753
All
−2
0
2
4
MR leave−one−out sensitivity analysis for
'PMVK' on 'osteosarcoma'

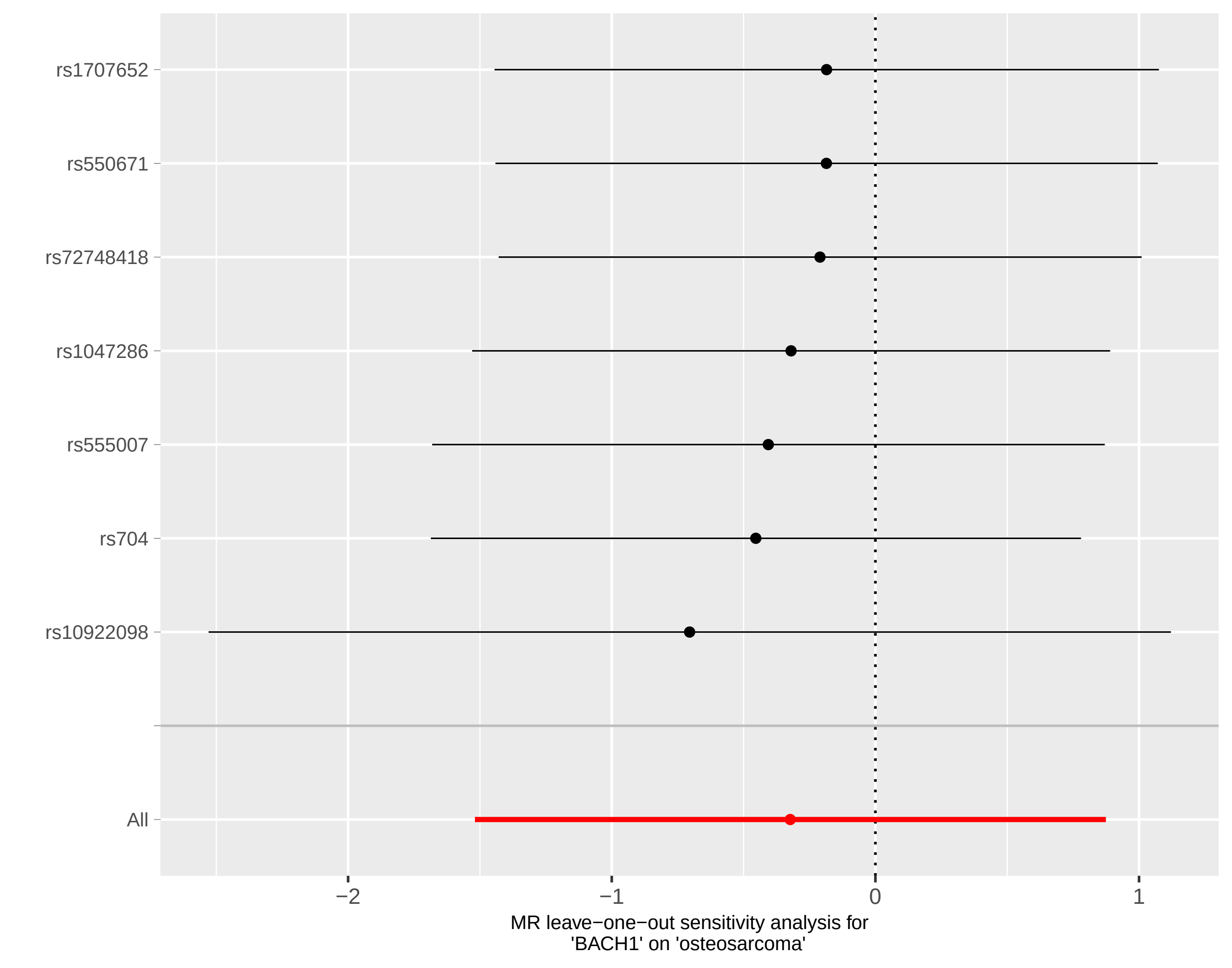

rs1707652
rs550671
rs72748418
rs1047286
rs555007
rs704
rs10922098
All
−2
−1
0
1
MR leave−one−out sensitivity analysis for
'BACH1' on 'osteosarcoma'

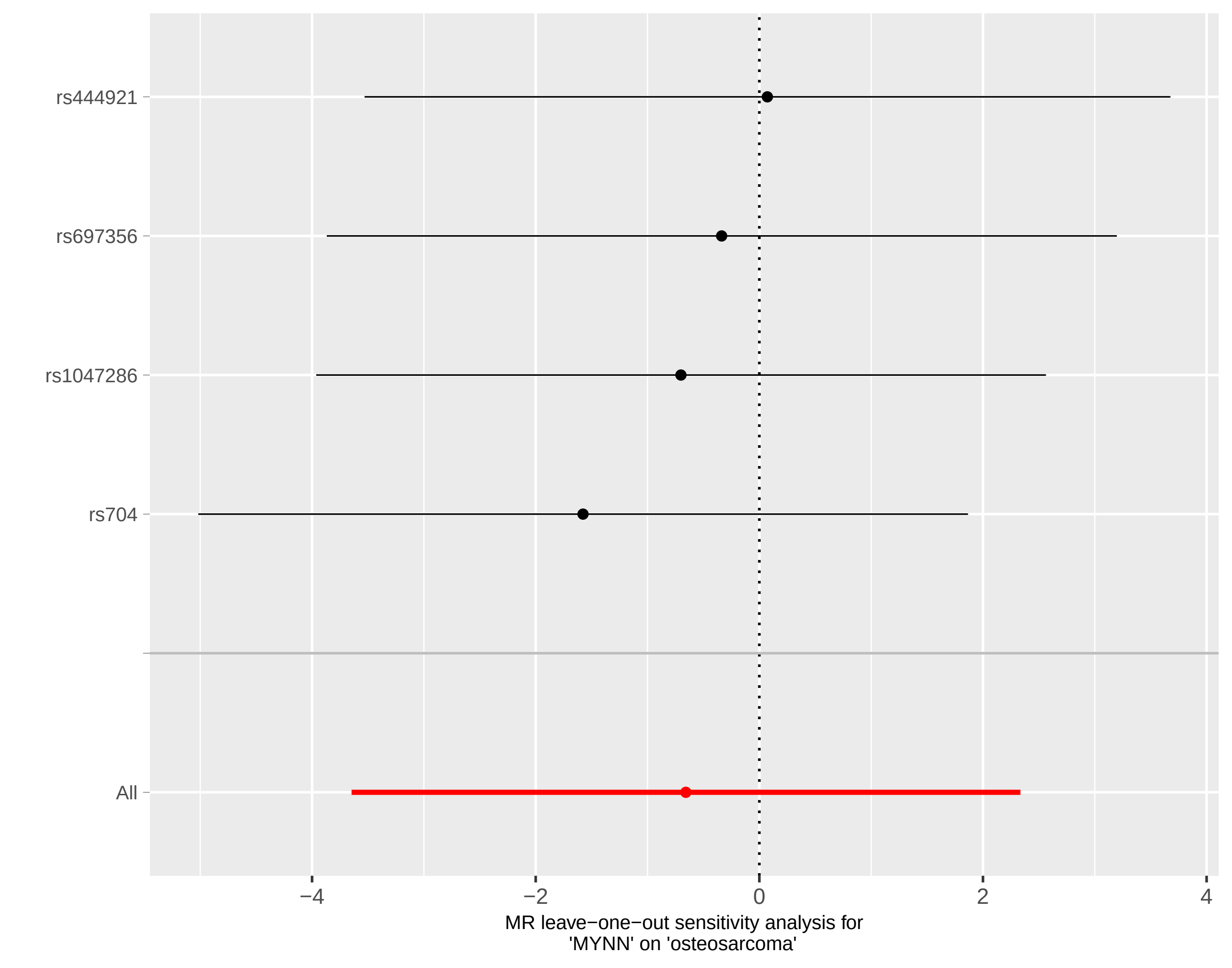
rs444921
rs697356
rs1047286
rs704
All
−4
−2
0
2
4
MR leave−one−out sensitivity analysis for
'MYNN' on 'osteosarcoma'

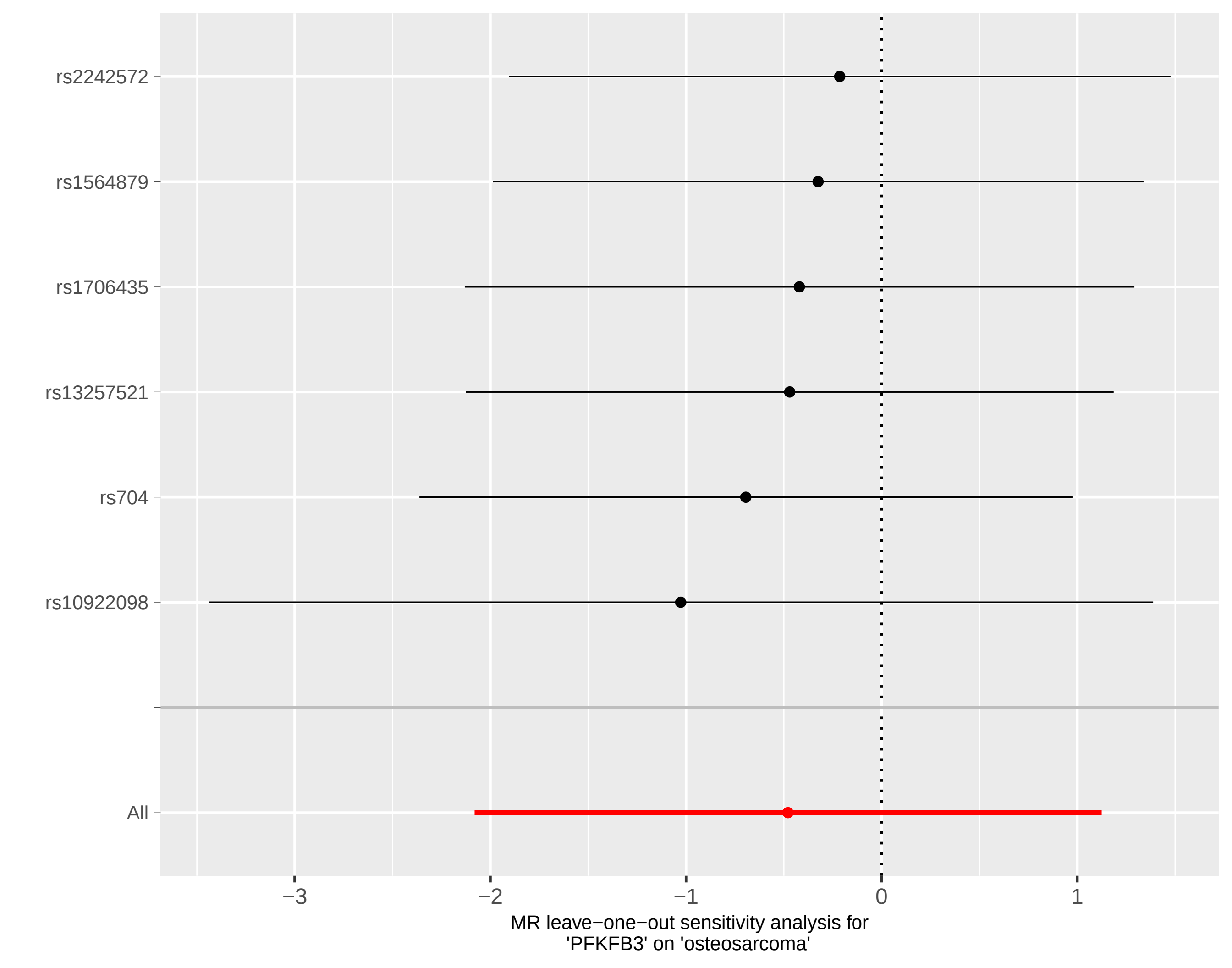
rs2242572
rs1564879
rs1706435
rs13257521
rs704
rs10922098
All
−3
−2
−1
0
1
MR leave−one−out sensitivity analysis for
'PFKFB3' on 'osteosarcoma'

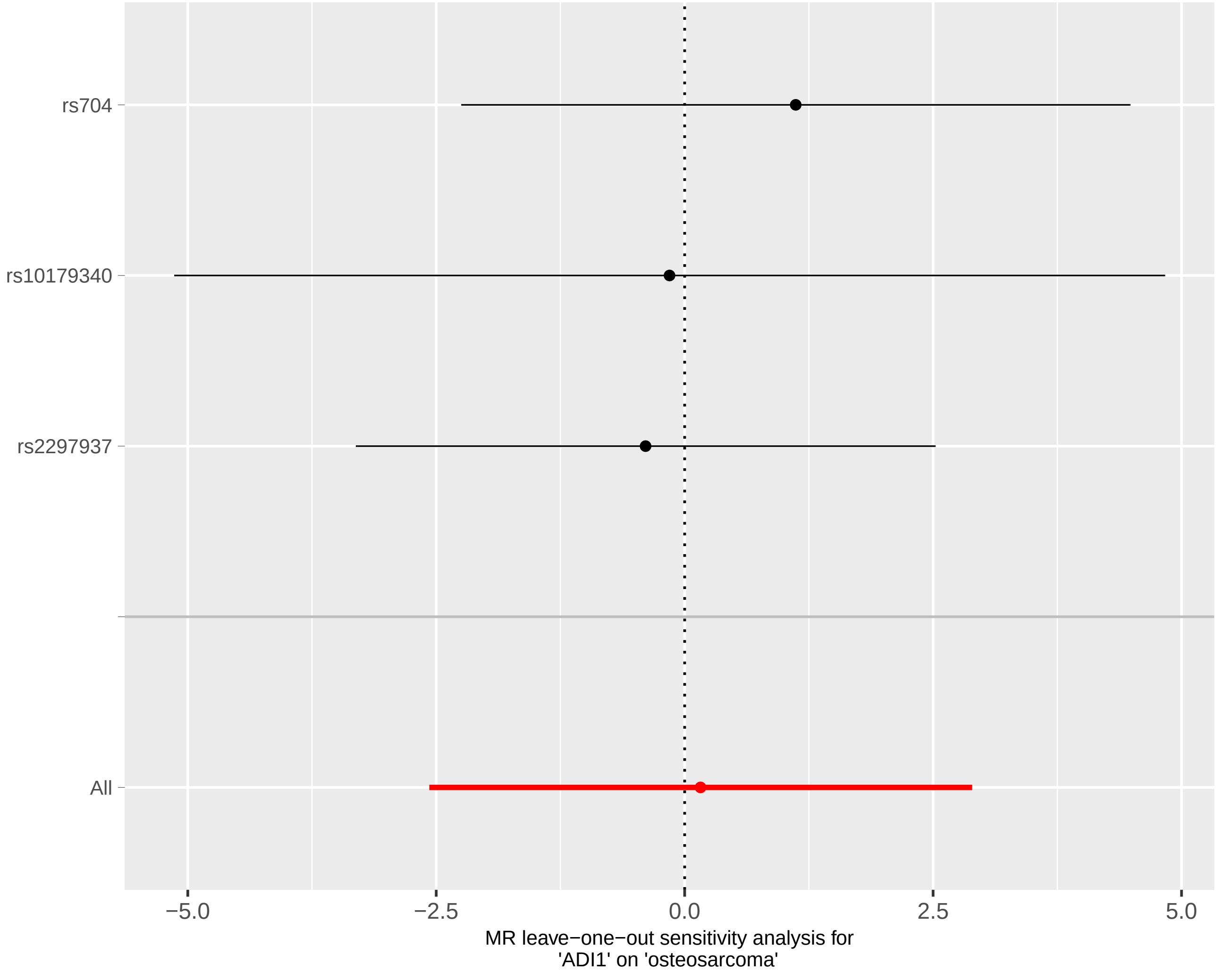
rs704
rs10179340
rs2297937
All
−5.0
−2.5
0.0
2.5
5.0
MR leave−one−out sensitivity analysis for
'ADI1' on 'osteosarcoma'

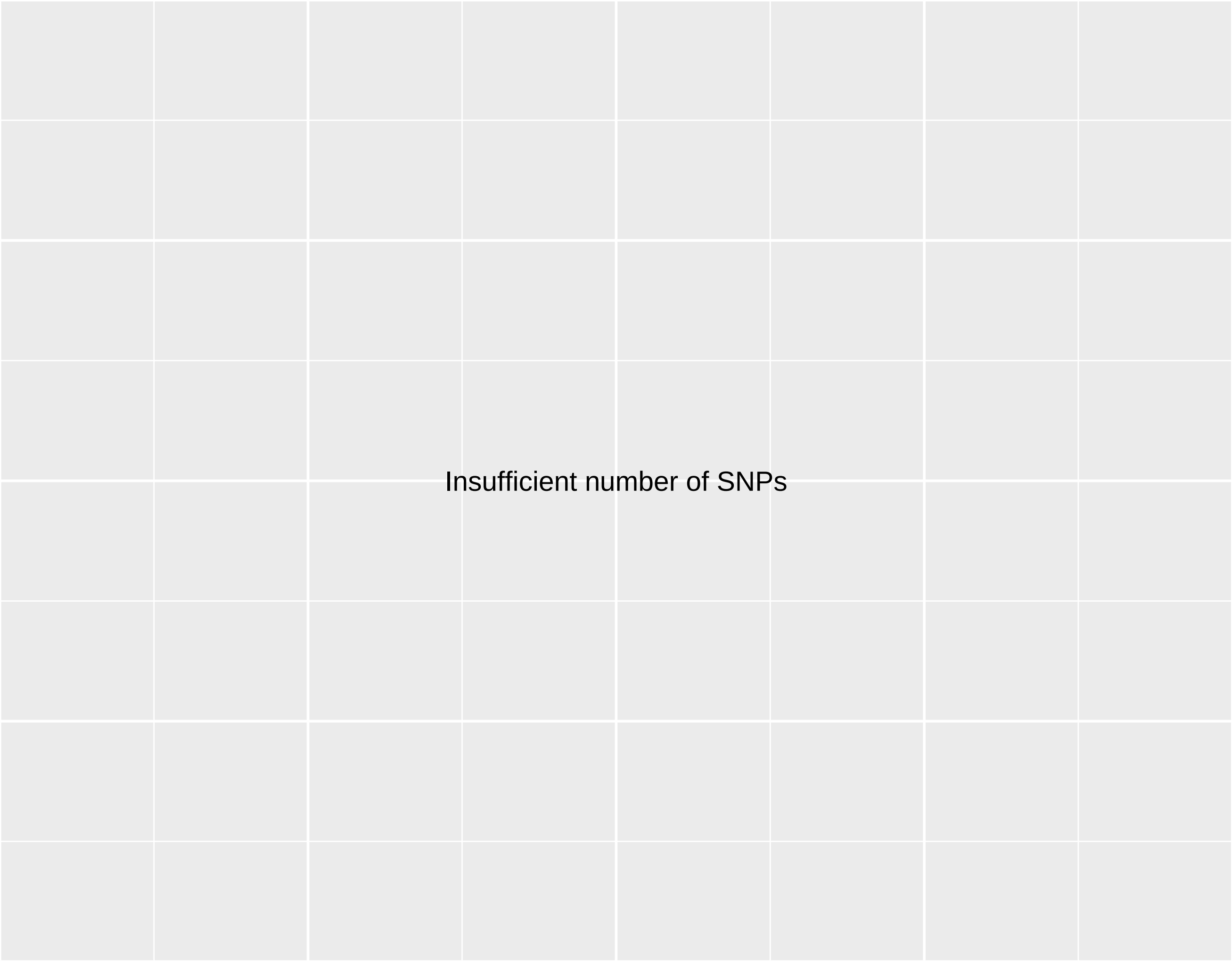
Insufficient number of SNPs

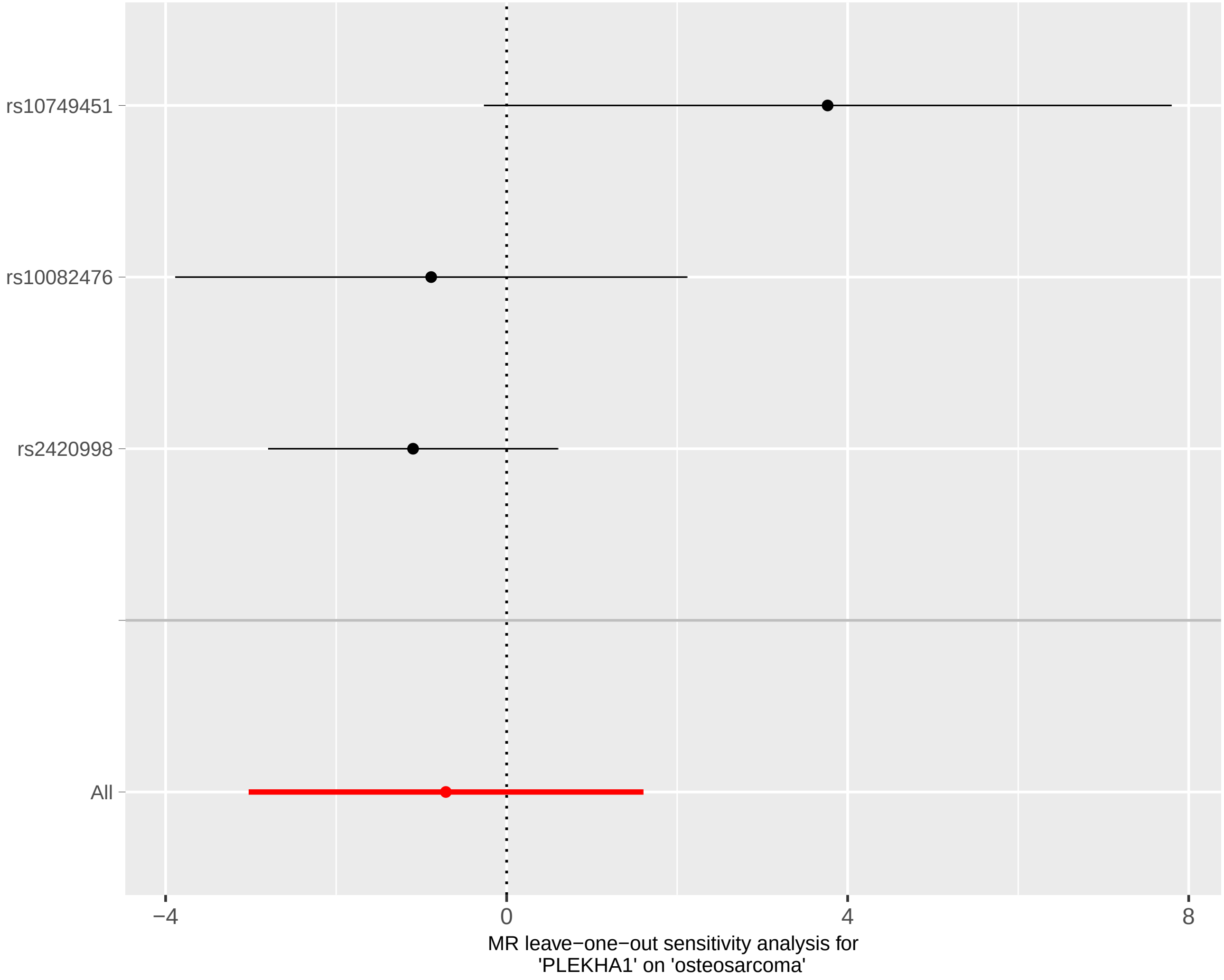
rs10749451
rs10082476
rs2420998
All
−4
0
4
8
MR leave−one−out sensitivity analysis for
'PLEKHA1' on 'osteosarcoma'

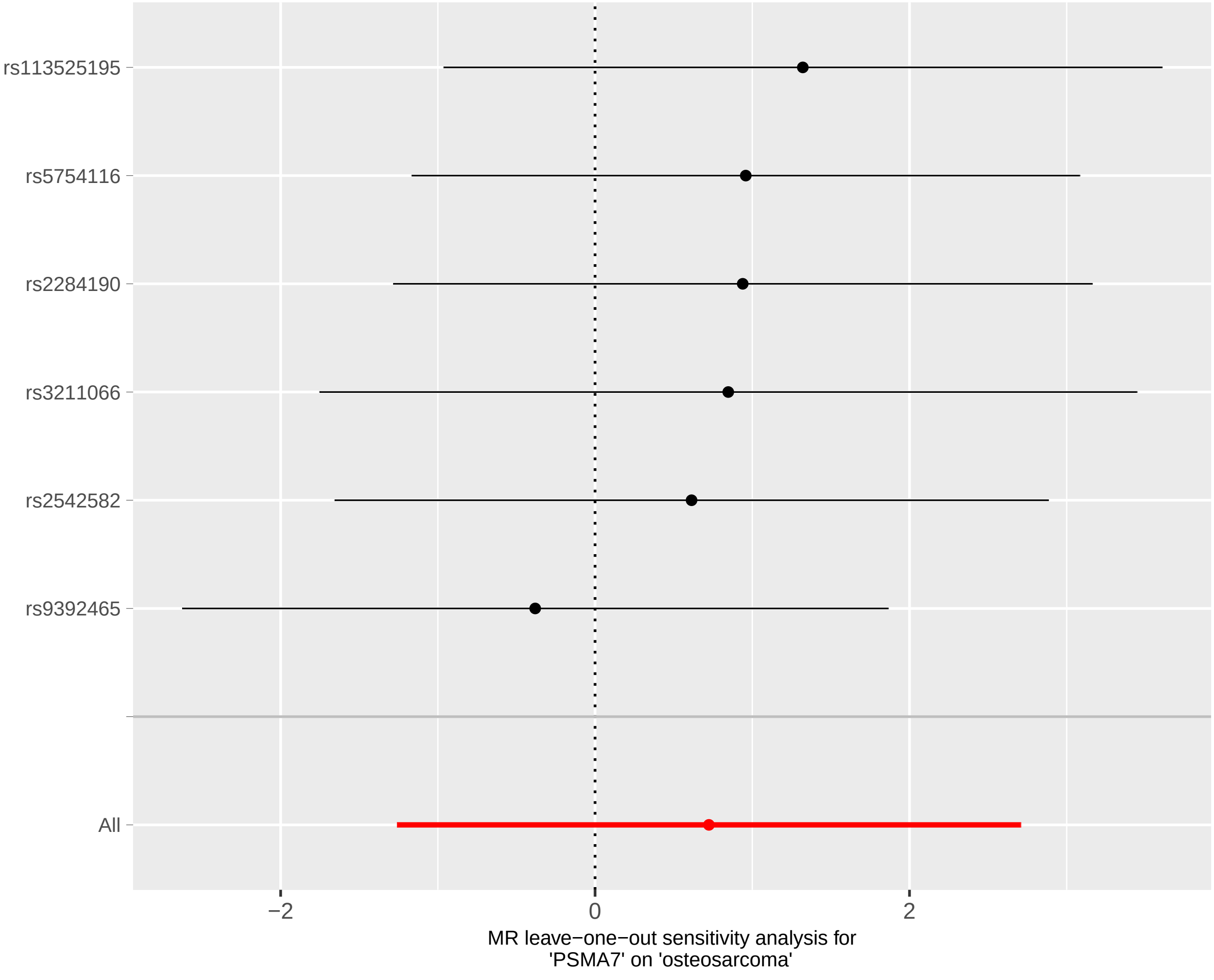

rs113525195
rs5754116
rs2284190
rs3211066
rs2542582
rs9392465
All
−2
0
2
MR leave−one−out sensitivity analysis for
'PSMA7' on 'osteosarcoma'

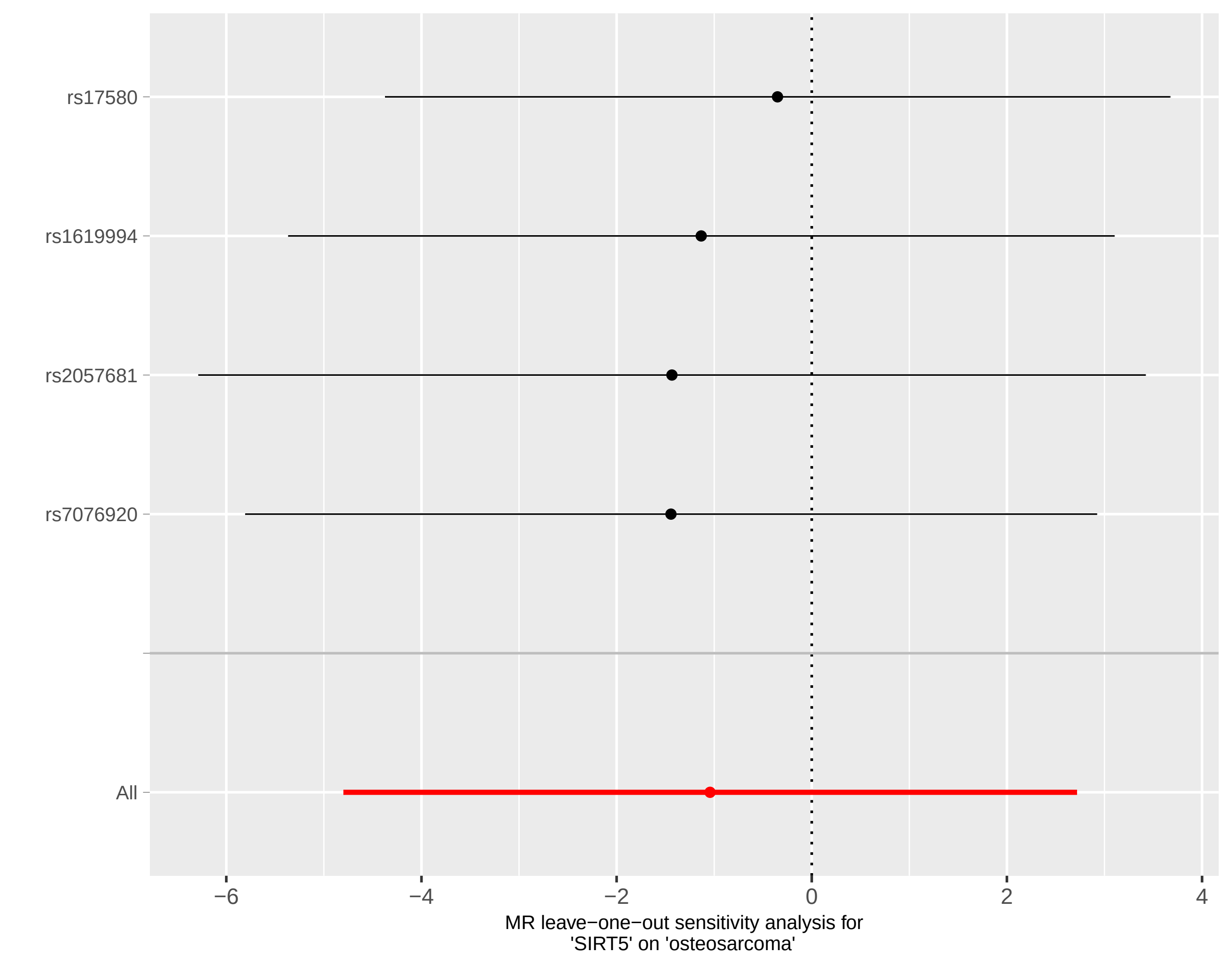

rs17580
rs1619994
rs2057681
rs7076920
All
−6
−4
−2
0
2
4
MR leave−one−out sensitivity analysis for
'SIRT5' on 'osteosarcoma'

Insufficient number of SNPs

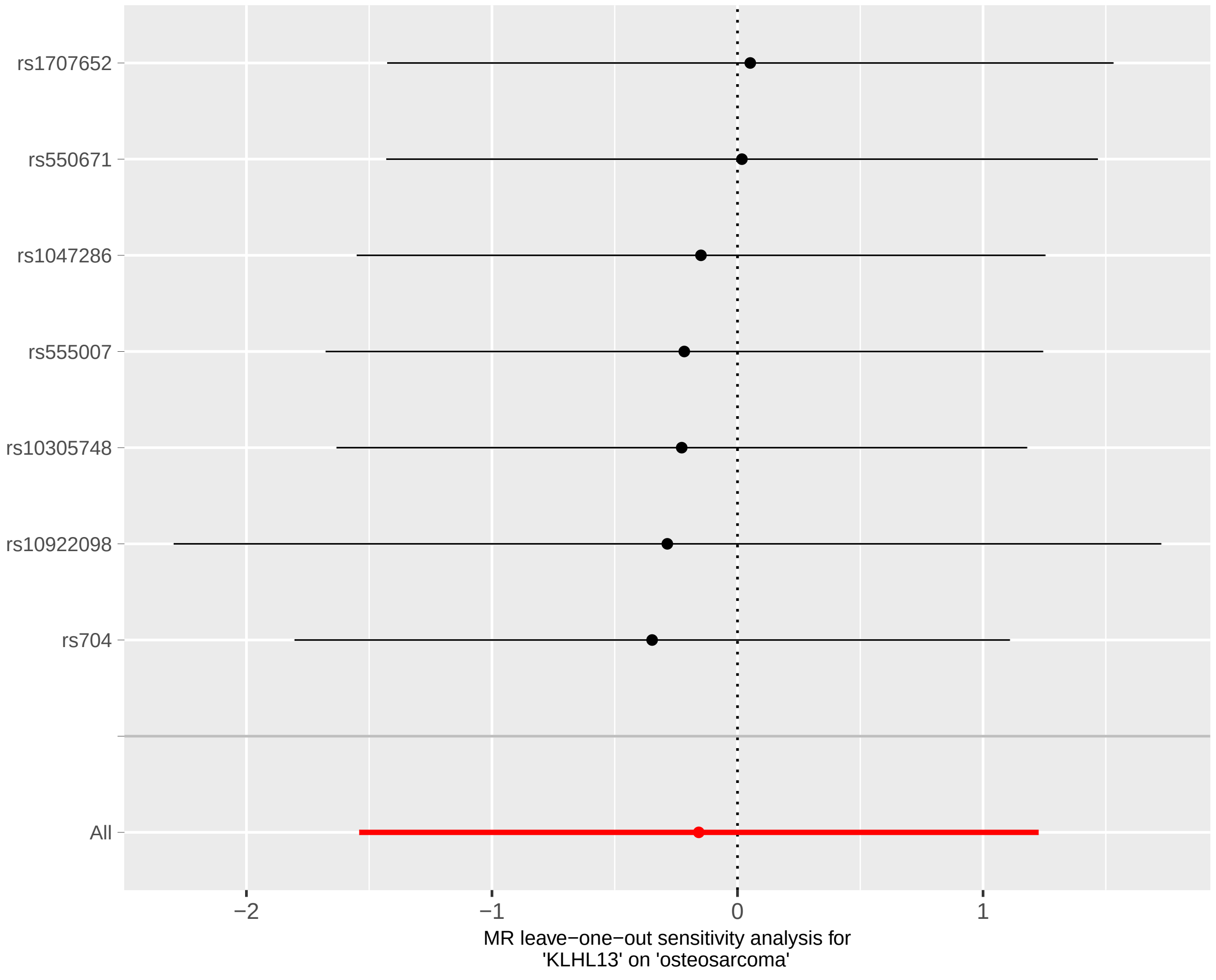

rs1707652
rs550671
rs1047286
rs555007
rs10305748
rs10922098
rs704
All
−2
−1
0
1
MR leave−one−out sensitivity analysis for
'KLHL13' on 'osteosarcoma'

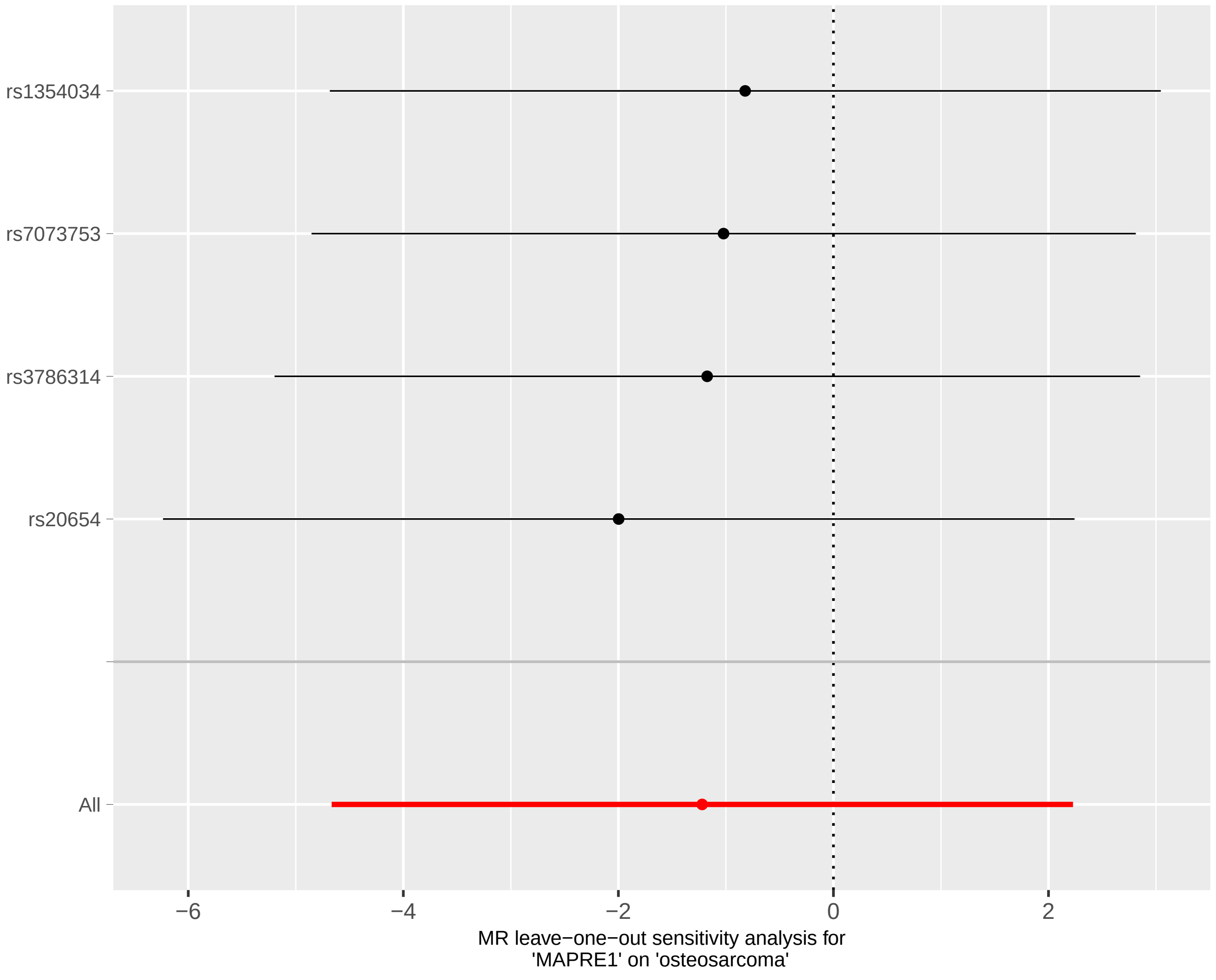

rs1354034
rs7073753
rs3786314
rs20654
All
−6
−4
−2
0
2
MR leave−one−out sensitivity analysis for
'MAPRE1' on 'osteosarcoma'

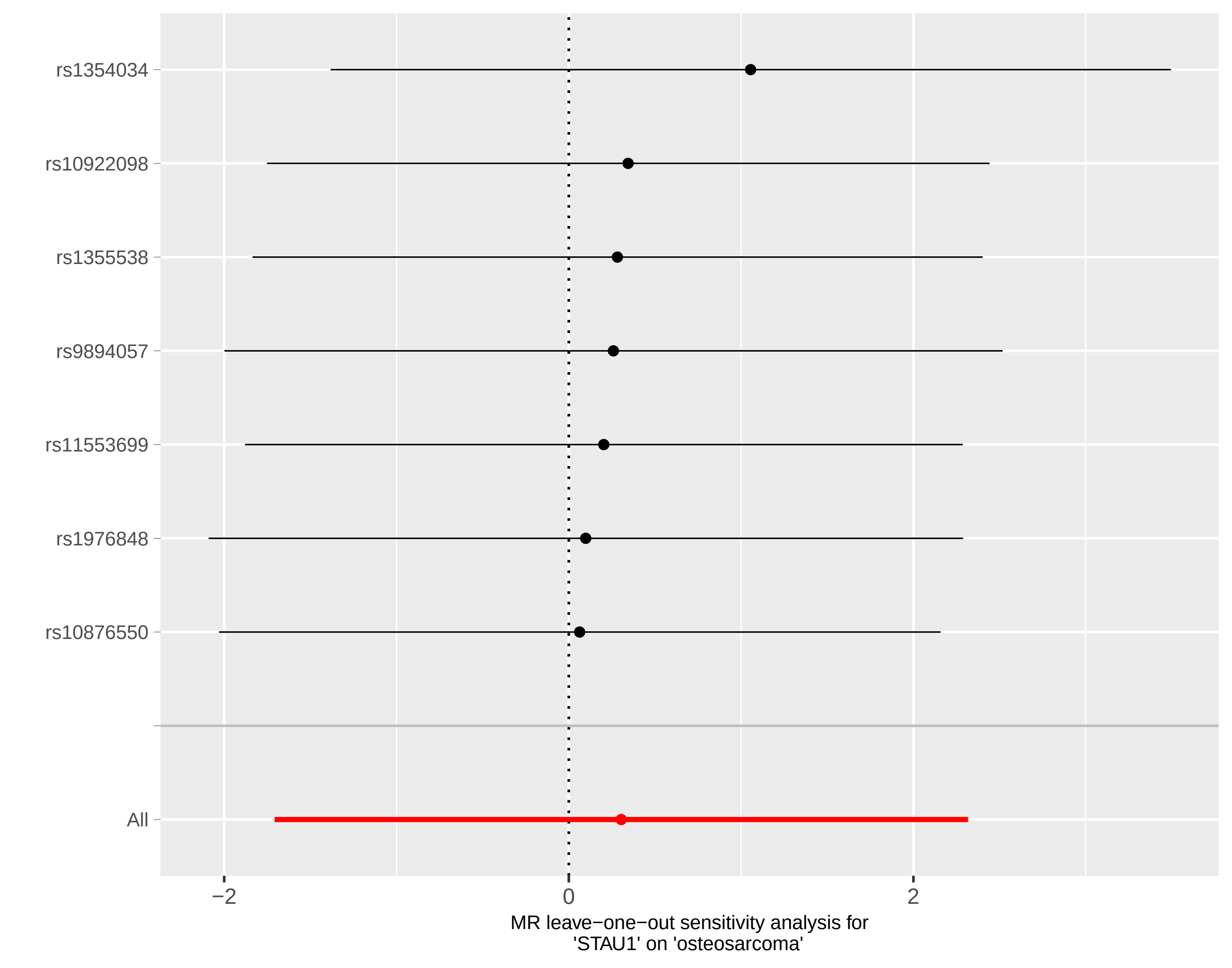

rs1354034
rs10922098
rs1355538
rs9894057
rs11553699
rs1976848
rs10876550
All
−2
0
2
MR leave−one−out sensitivity analysis for
'STAU1' on 'osteosarcoma'

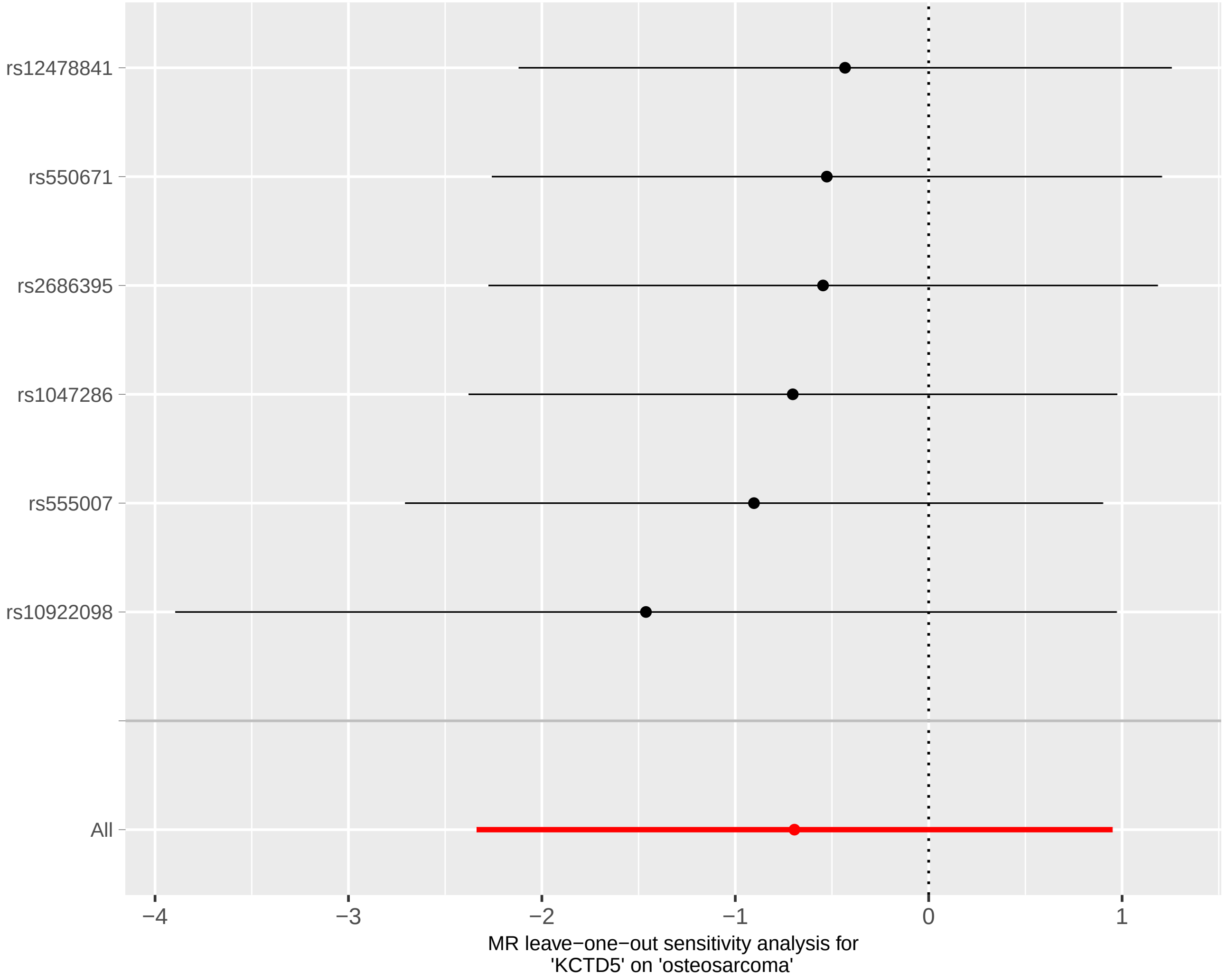
rs12478841
rs550671
rs2686395
rs1047286
rs555007
rs10922098
All
−4
−3
−2
−1
0
1
MR leave−one−out sensitivity analysis for
'KCTD5' on 'osteosarcoma'

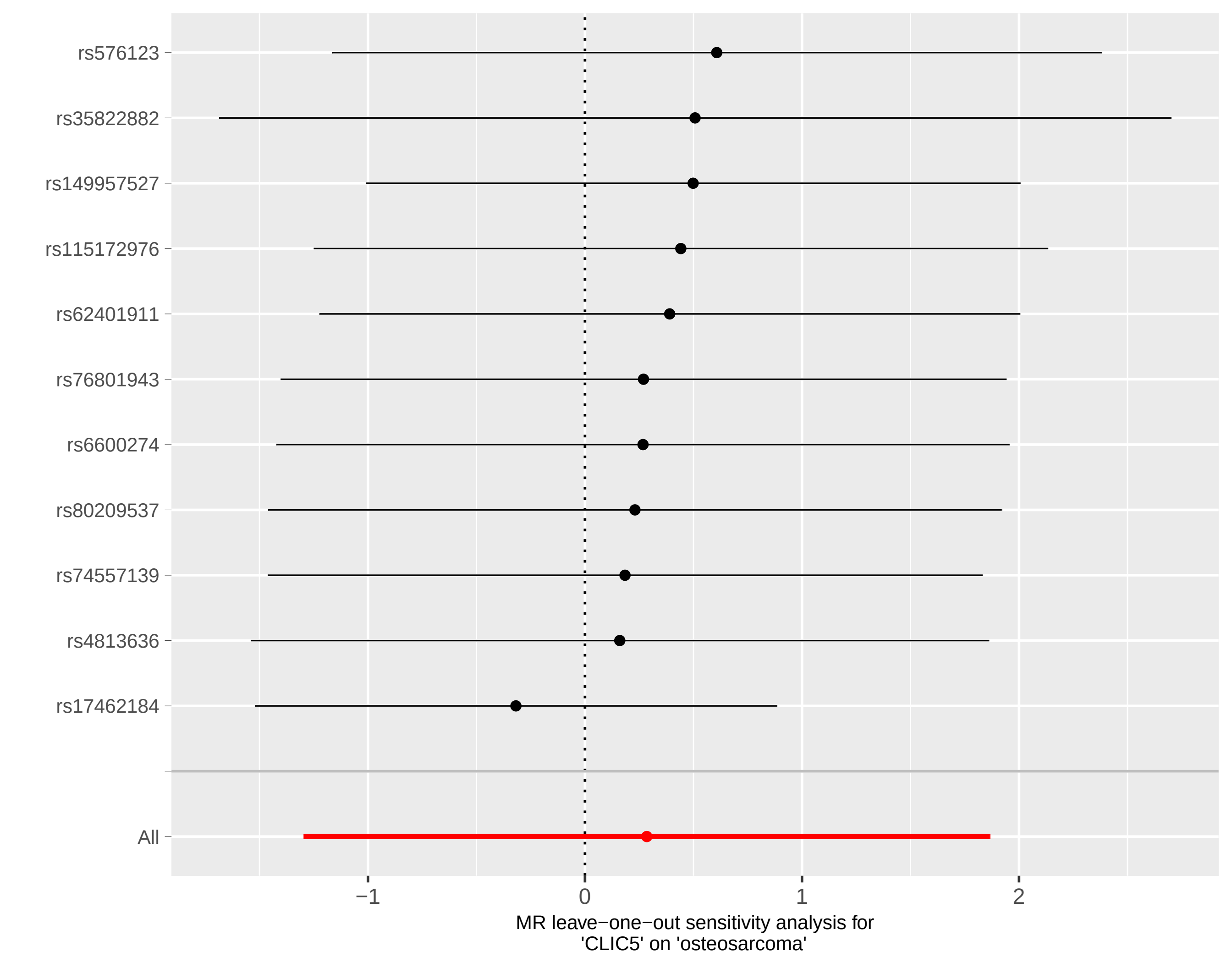

rs576123
rs35822882
rs149957527
rs115172976
rs62401911
rs76801943
rs6600274
rs80209537
rs74557139
rs4813636
rs17462184
All
−1
0
1
2
MR leave−one−out sensitivity analysis for
'CLIC5' on 'osteosarcoma'

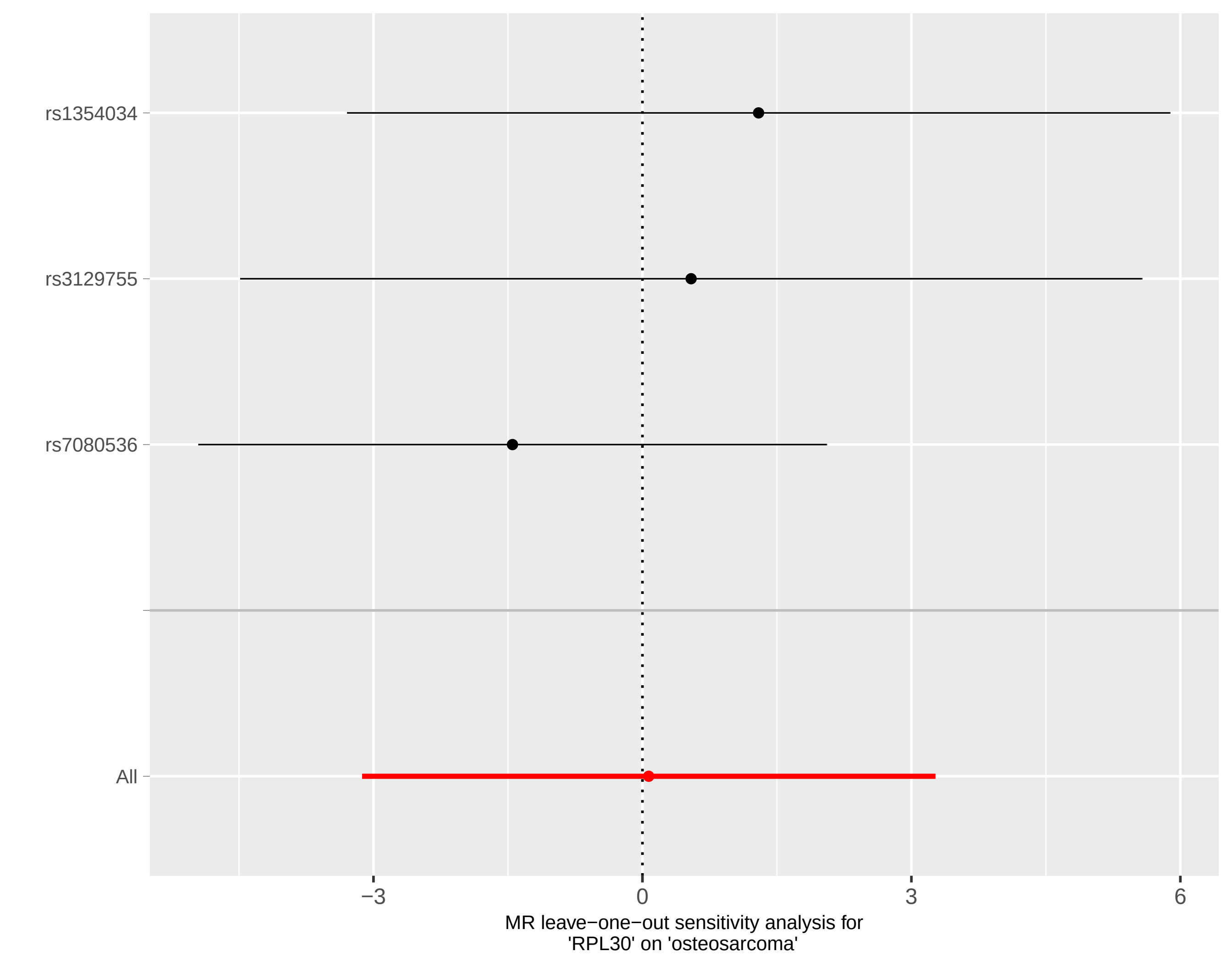
rs1354034
rs3129755
rs7080536
All
−3
0
3
6
MR leave−one−out sensitivity analysis for
'RPL30' on 'osteosarcoma'

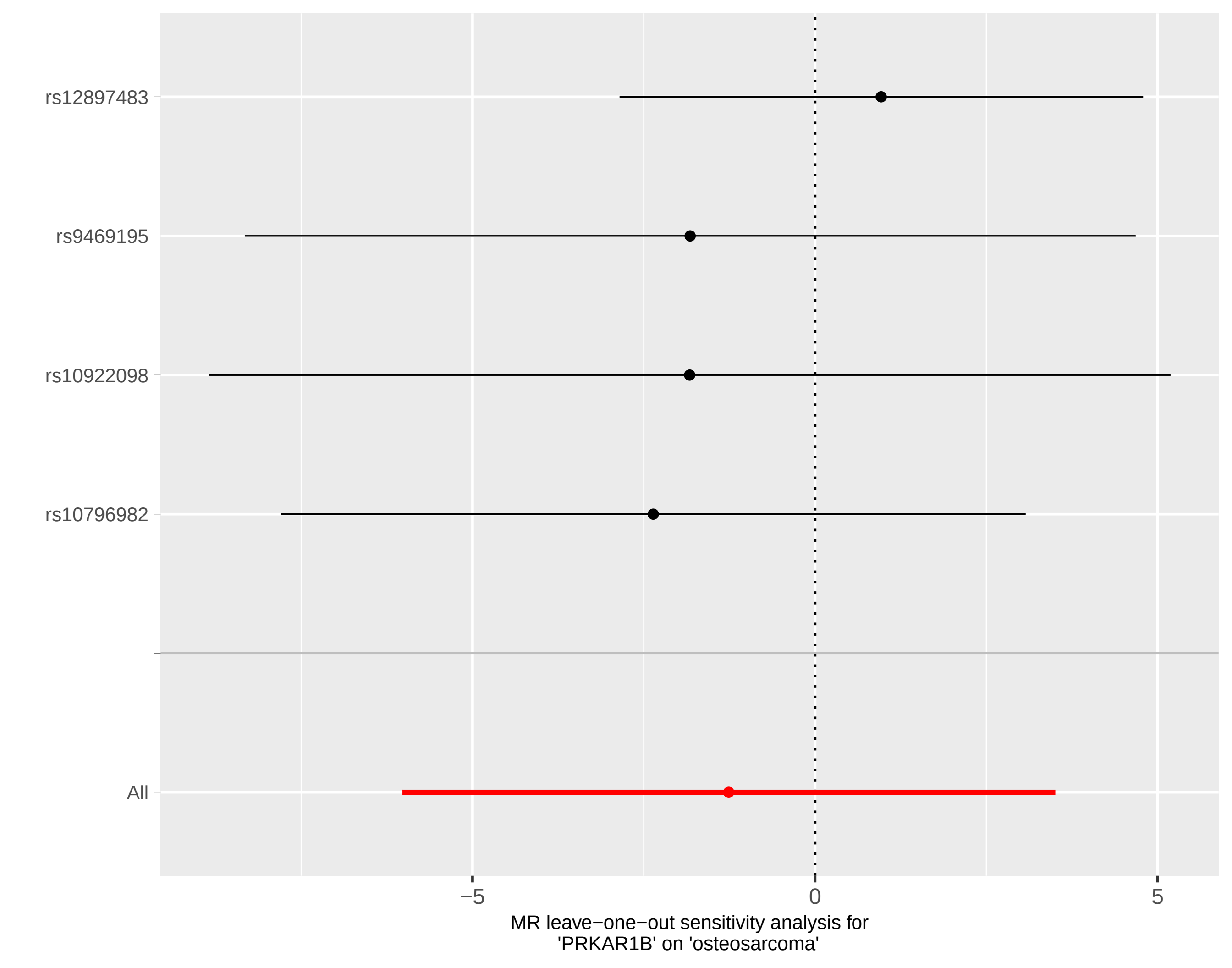
rs12897483
rs9469195
rs10922098
rs10796982
All
−5
0
5
MR leave−one−out sensitivity analysis for
'PRKAR1B' on 'osteosarcoma'

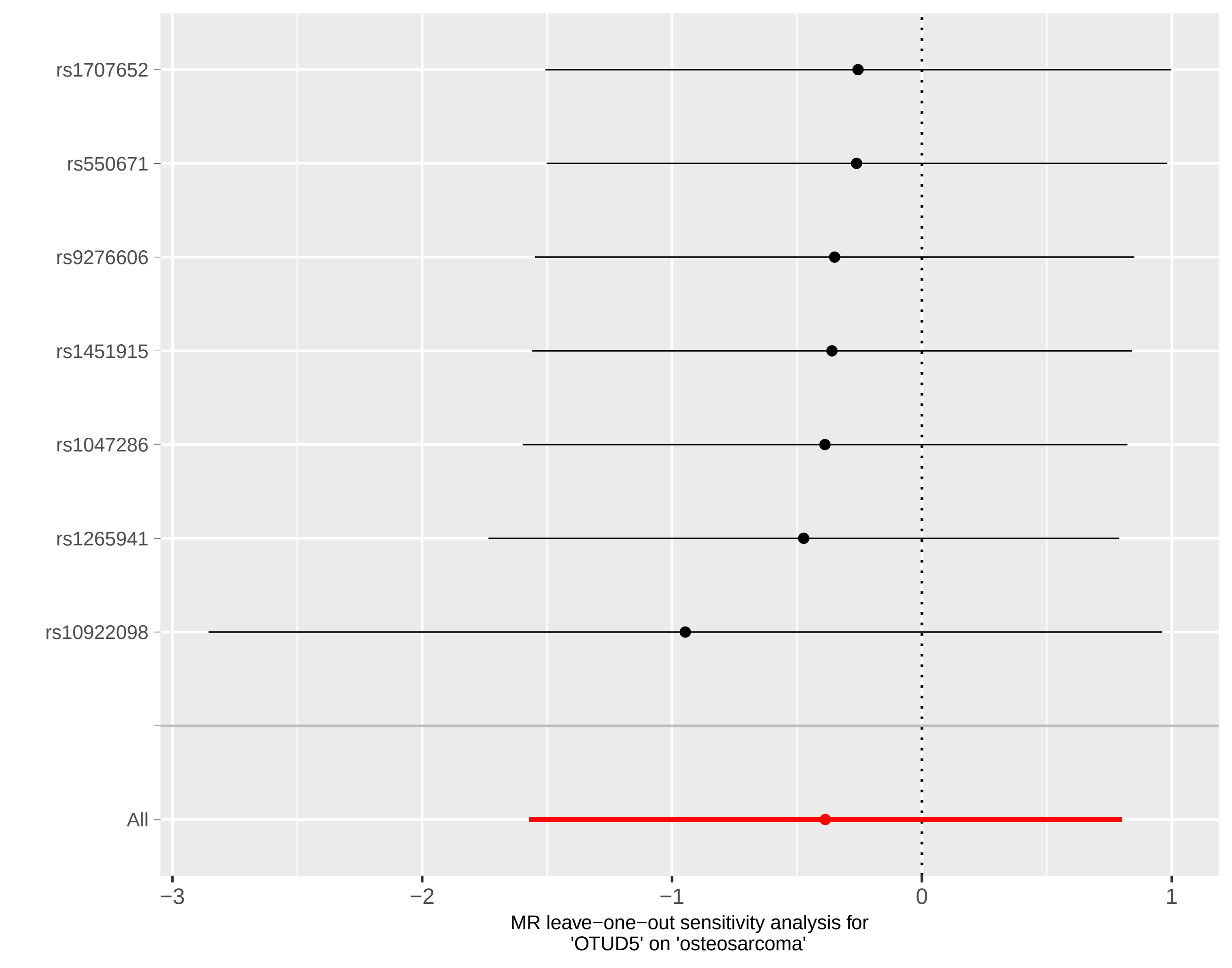
rs1707652
rs550671
rs9276606
rs1451915
rs1047286
rs1265941
rs10922098
All
−3
−2
−1
0
1
MR leave−one−out sensitivity analysis for
'OTUD5' on 'osteosarcoma'

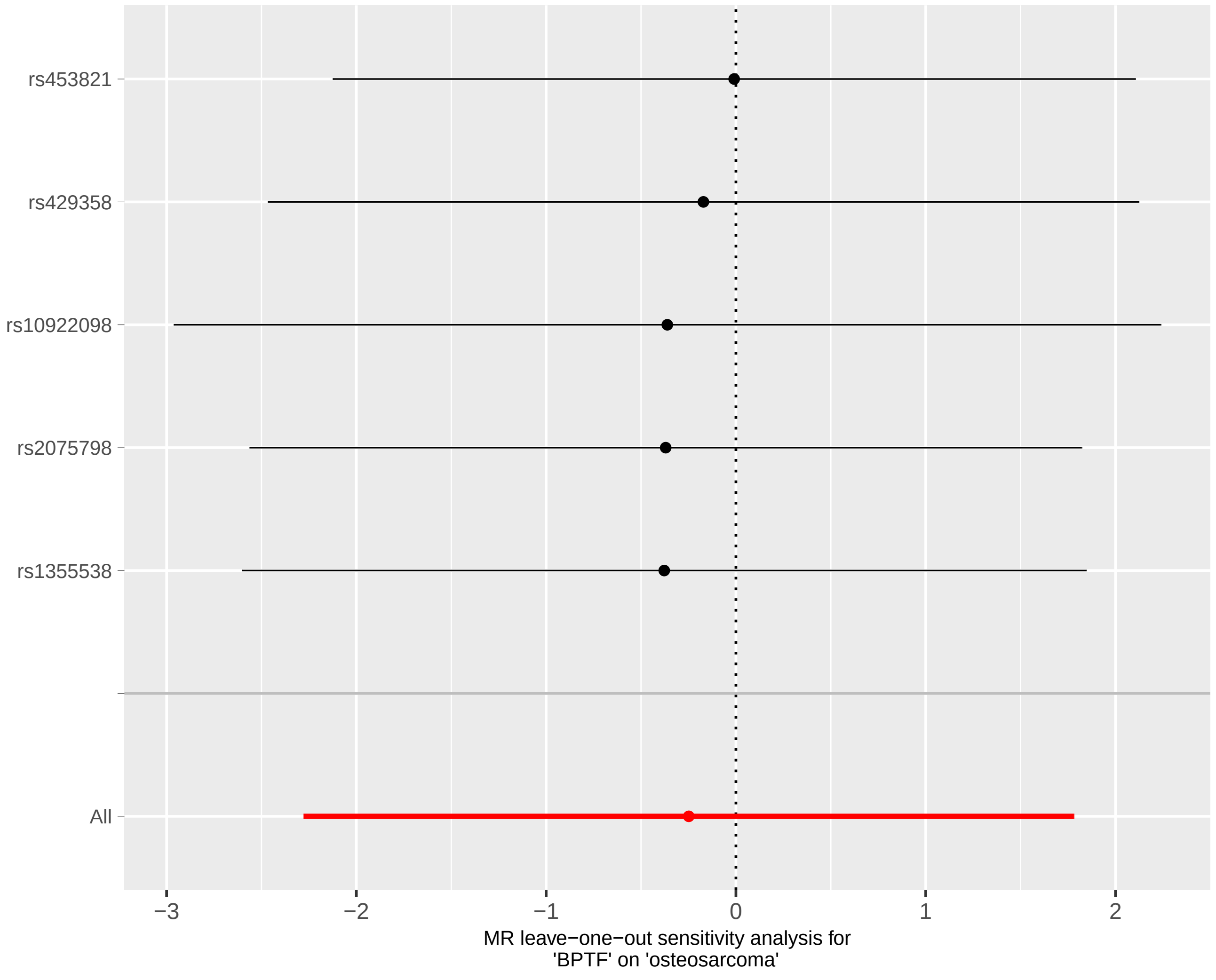
rs453821
rs429358
rs10922098
rs2075798
rs1355538
All
−3
−2
−1
0
1
2
MR leave−one−out sensitivity analysis for
'BPTF' on 'osteosarcoma'

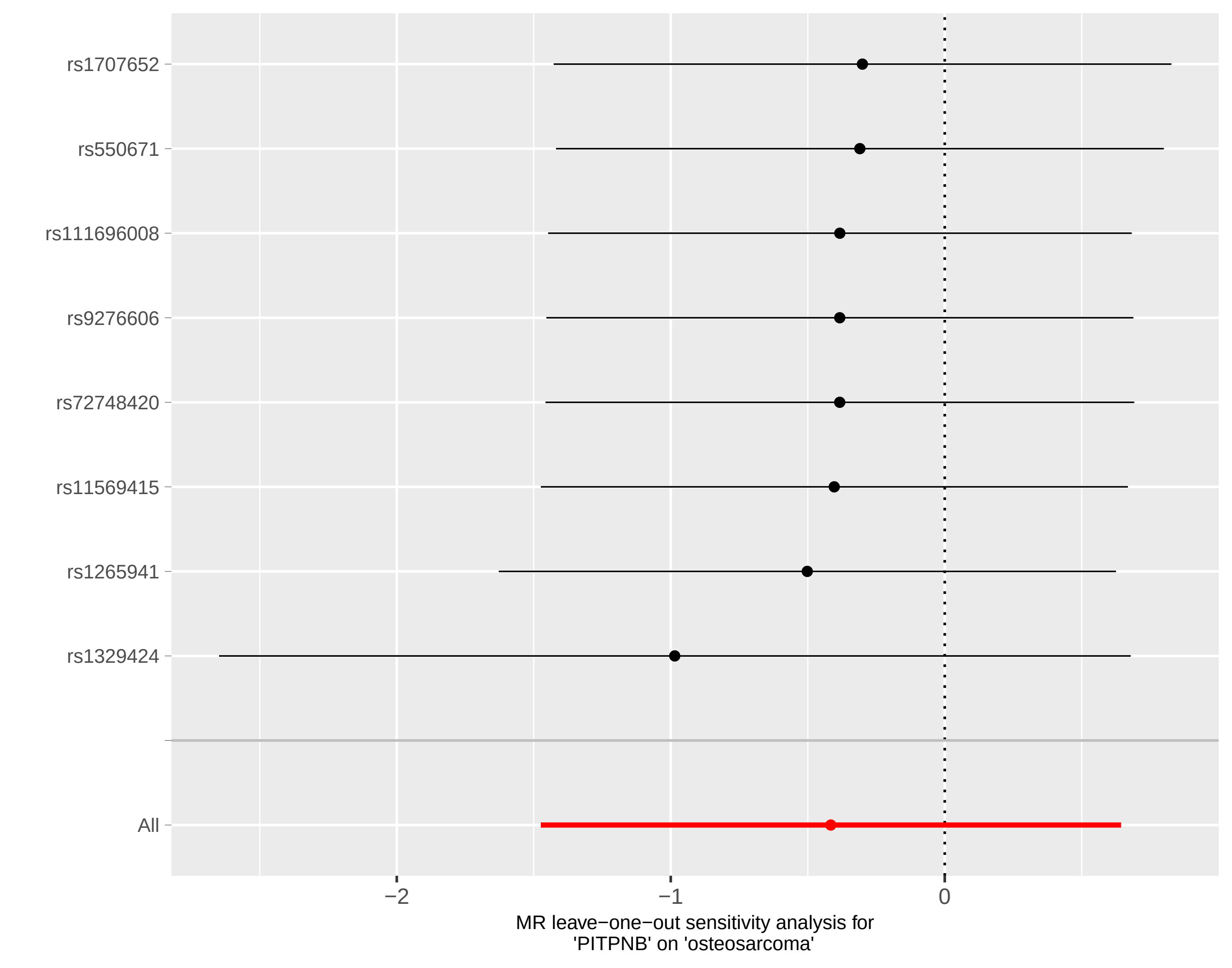
rs1707652
rs550671
rs111696008
rs9276606
rs72748420
rs11569415
rs1265941
rs1329424
All
−2
−1
0
MR leave−one−out sensitivity analysis for
'PITPNB' on 'osteosarcoma'

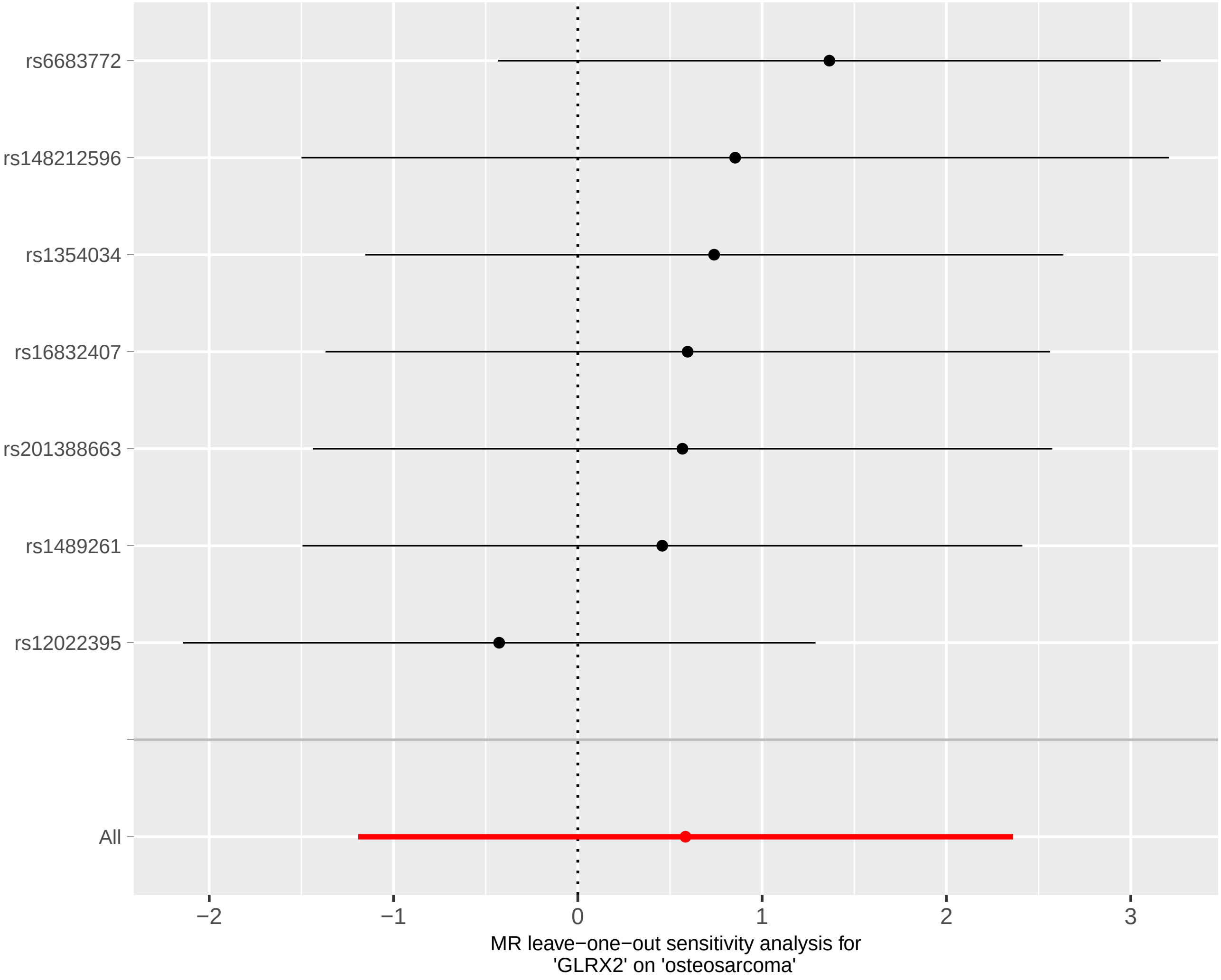
rs6683772
rs148212596
rs1354034
rs16832407
rs201388663
rs1489261
rs12022395
All
−2
−1
0
1
2
3
MR leave−one−out sensitivity analysis for
'GLRX2' on 'osteosarcoma'

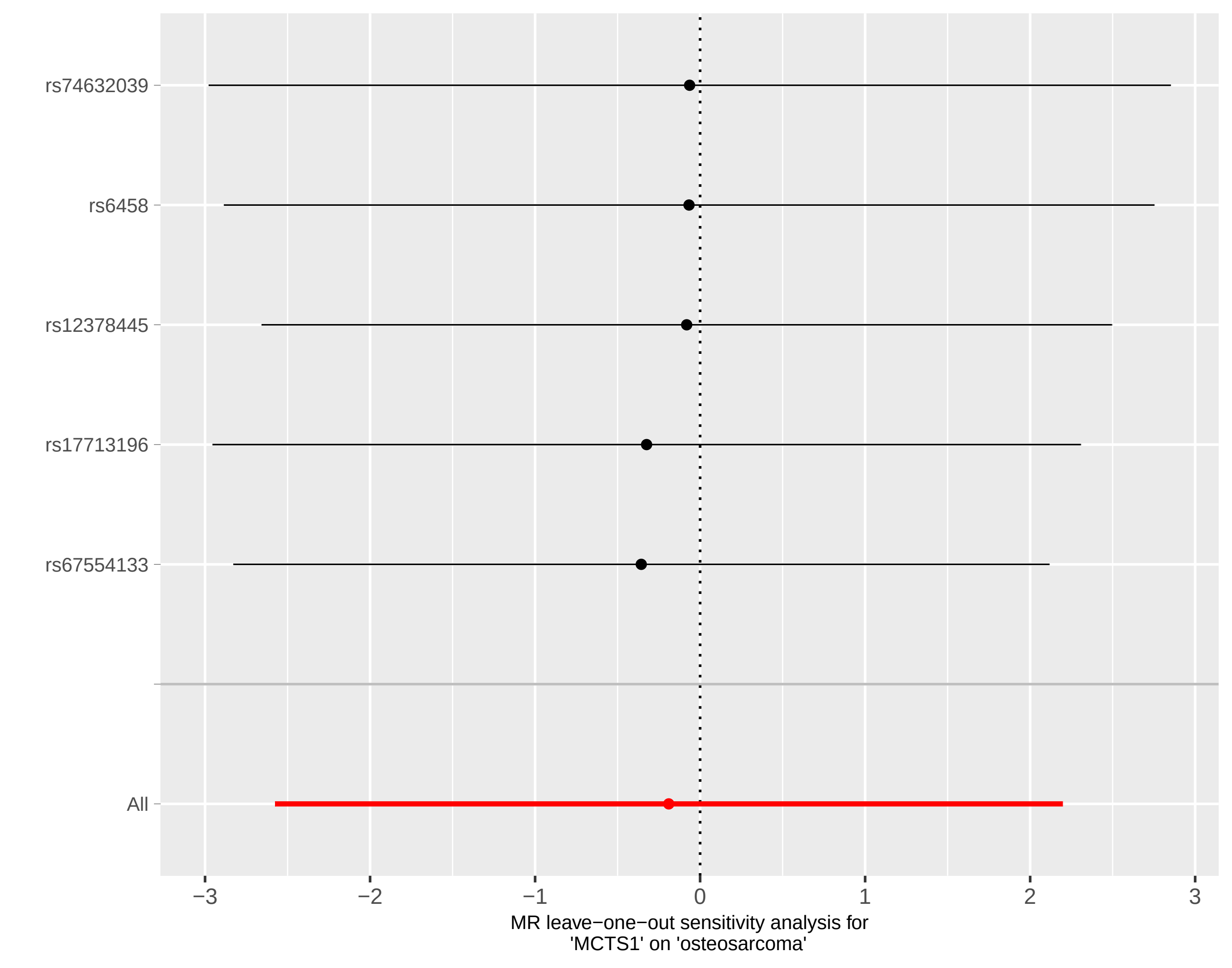

rs74632039
rs6458
rs12378445
rs17713196
rs67554133
All
−3
−2
−1
0
1
2
3
MR leave−one−out sensitivity analysis for
'MCTS1' on 'osteosarcoma'

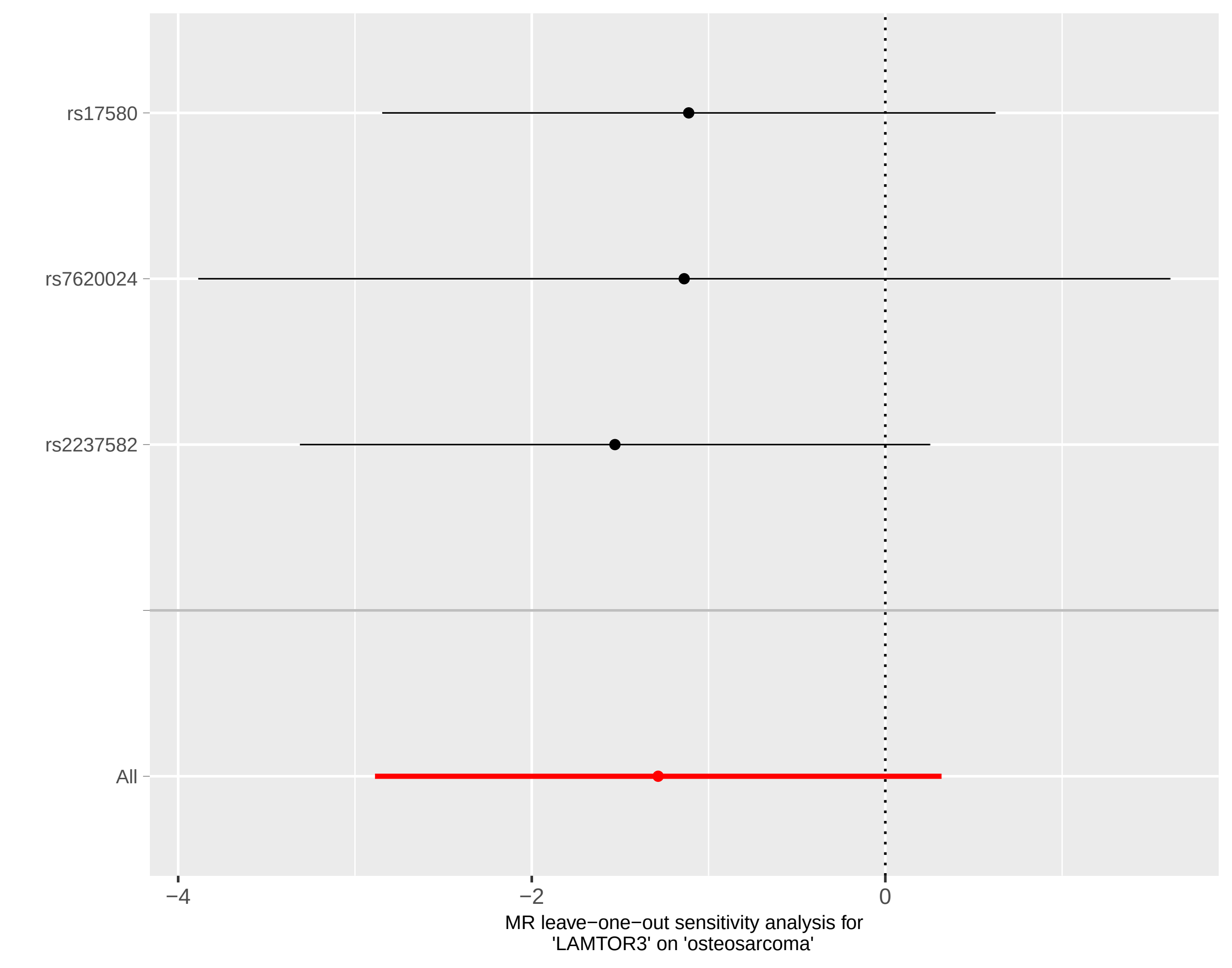
rs17580
rs7620024
rs2237582
All
−4
−2
0
MR leave−one−out sensitivity analysis for
'LAMTOR3' on 'osteosarcoma'

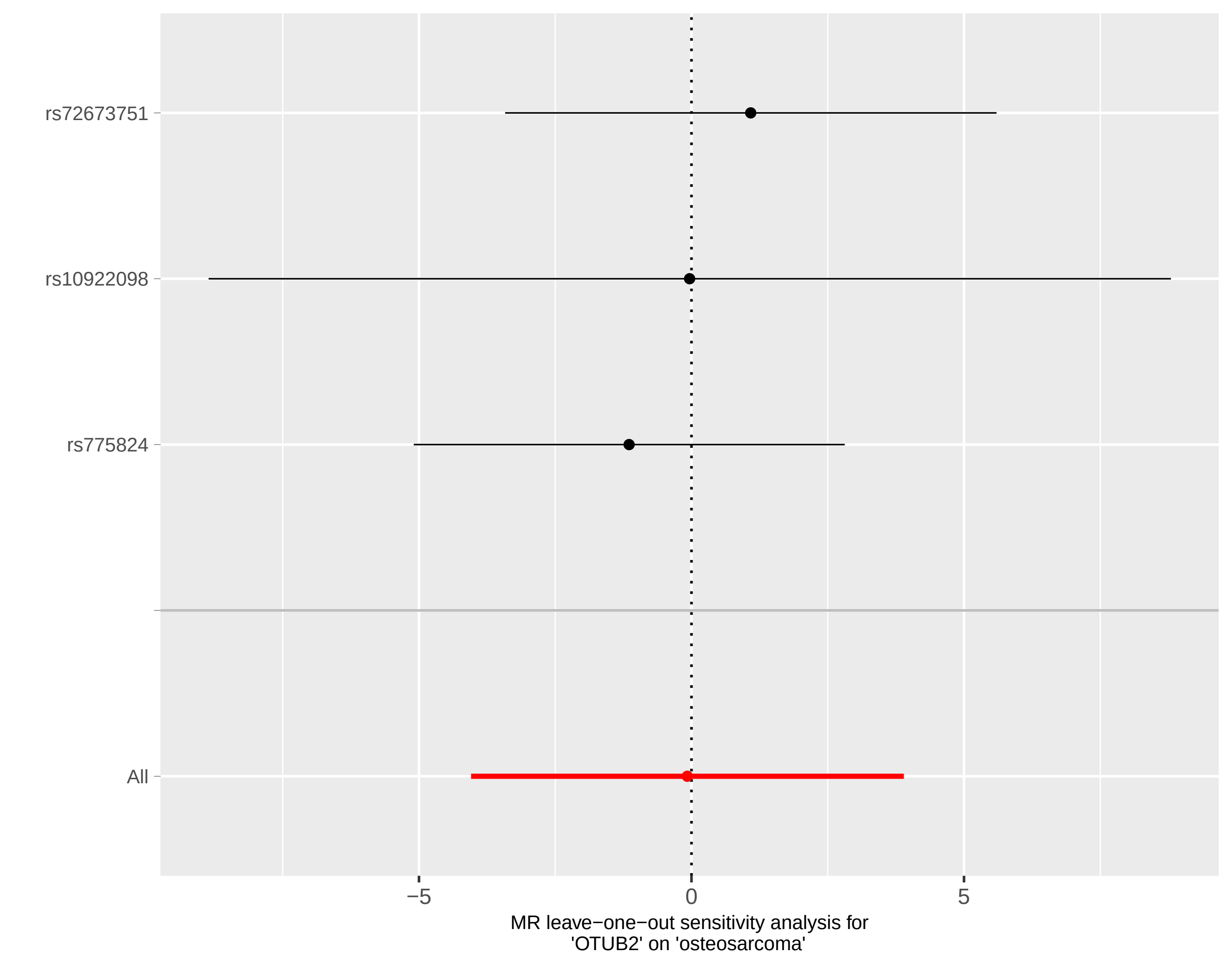

rs72673751
rs10922098
rs775824
All
−5
0
5
MR leave−one−out sensitivity analysis for
'OTUB2' on 'osteosarcoma'

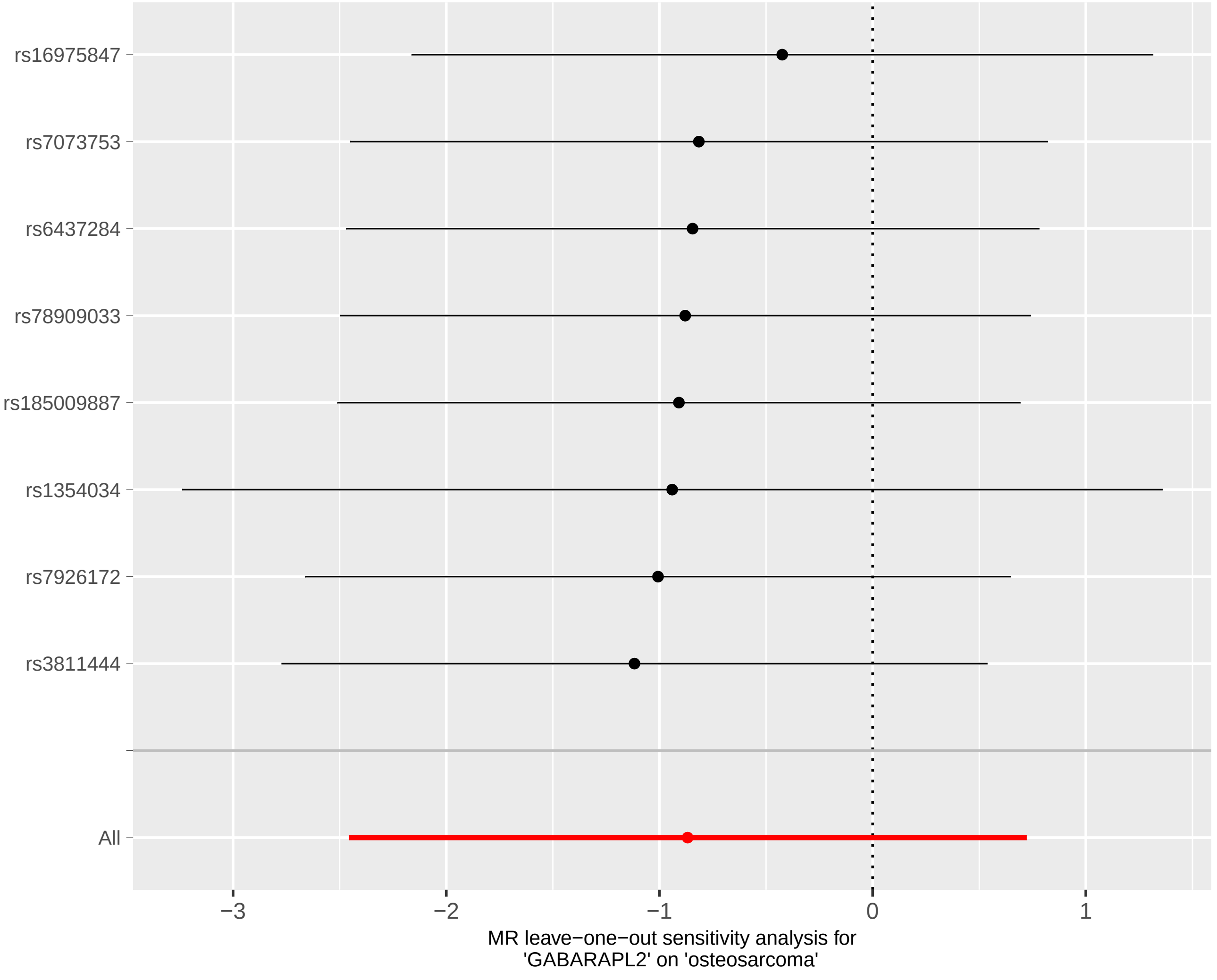

rs16975847
rs7073753
rs6437284
rs78909033
rs185009887
rs1354034
rs7926172
rs3811444
All
−3
−2
−1
0
1
MR leave−one−out sensitivity analysis for
'GABARAPL2' on 'osteosarcoma'

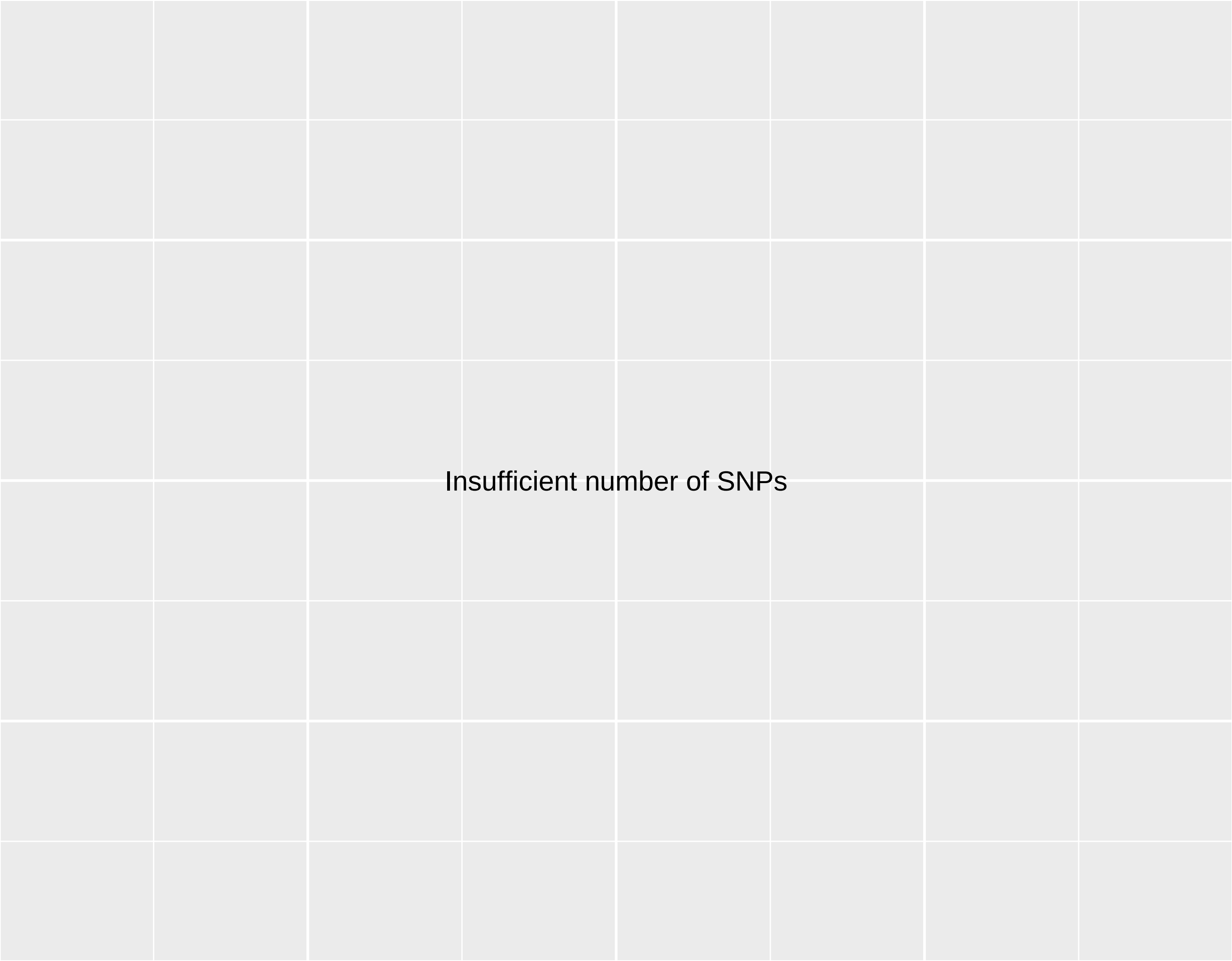
Insufficient number of SNPs

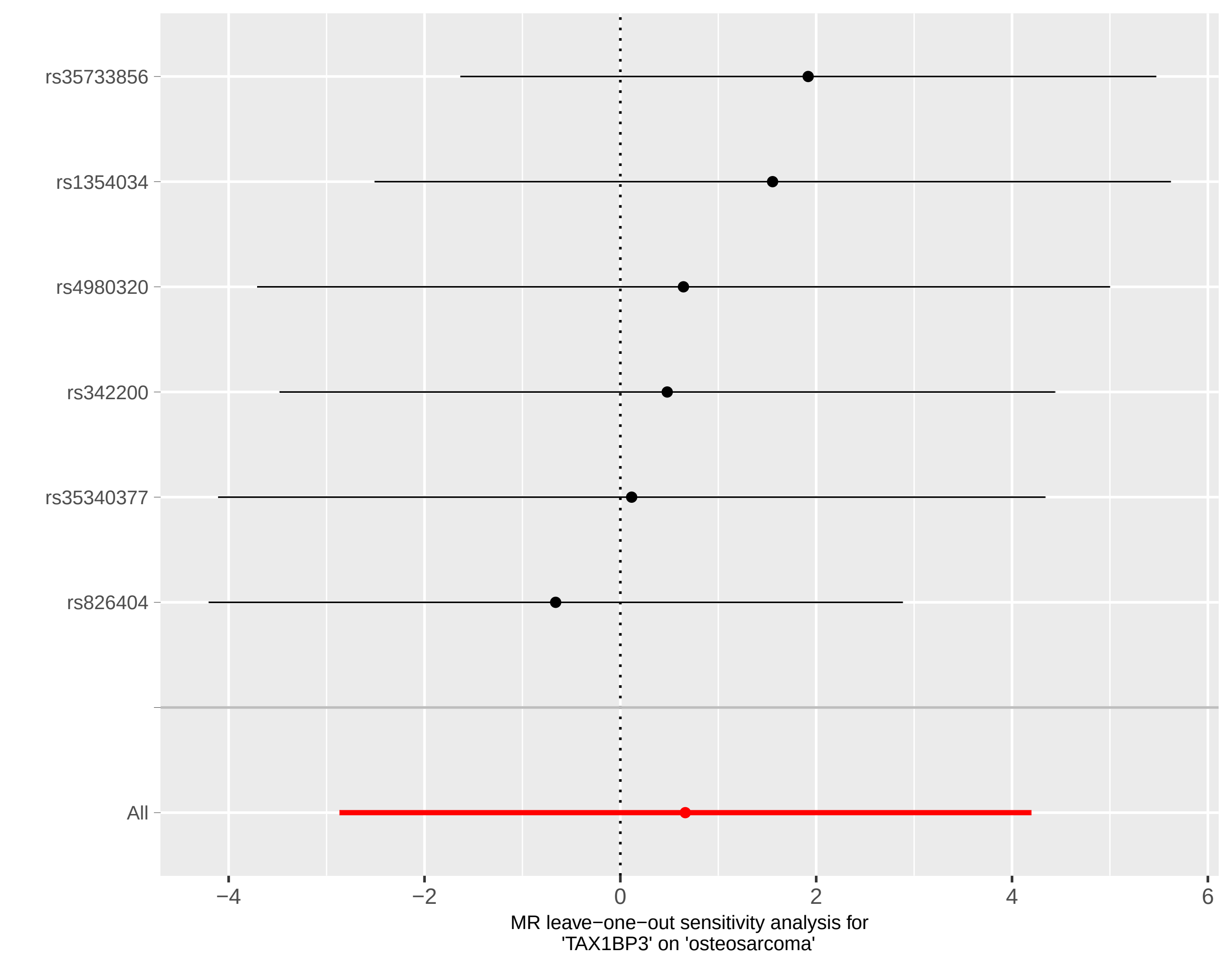

rs35733856
rs1354034
rs4980320
rs342200
rs35340377
rs826404
All
−4
−2
0
2
4
6
MR leave−one−out sensitivity analysis for
'TAX1BP3' on 'osteosarcoma'

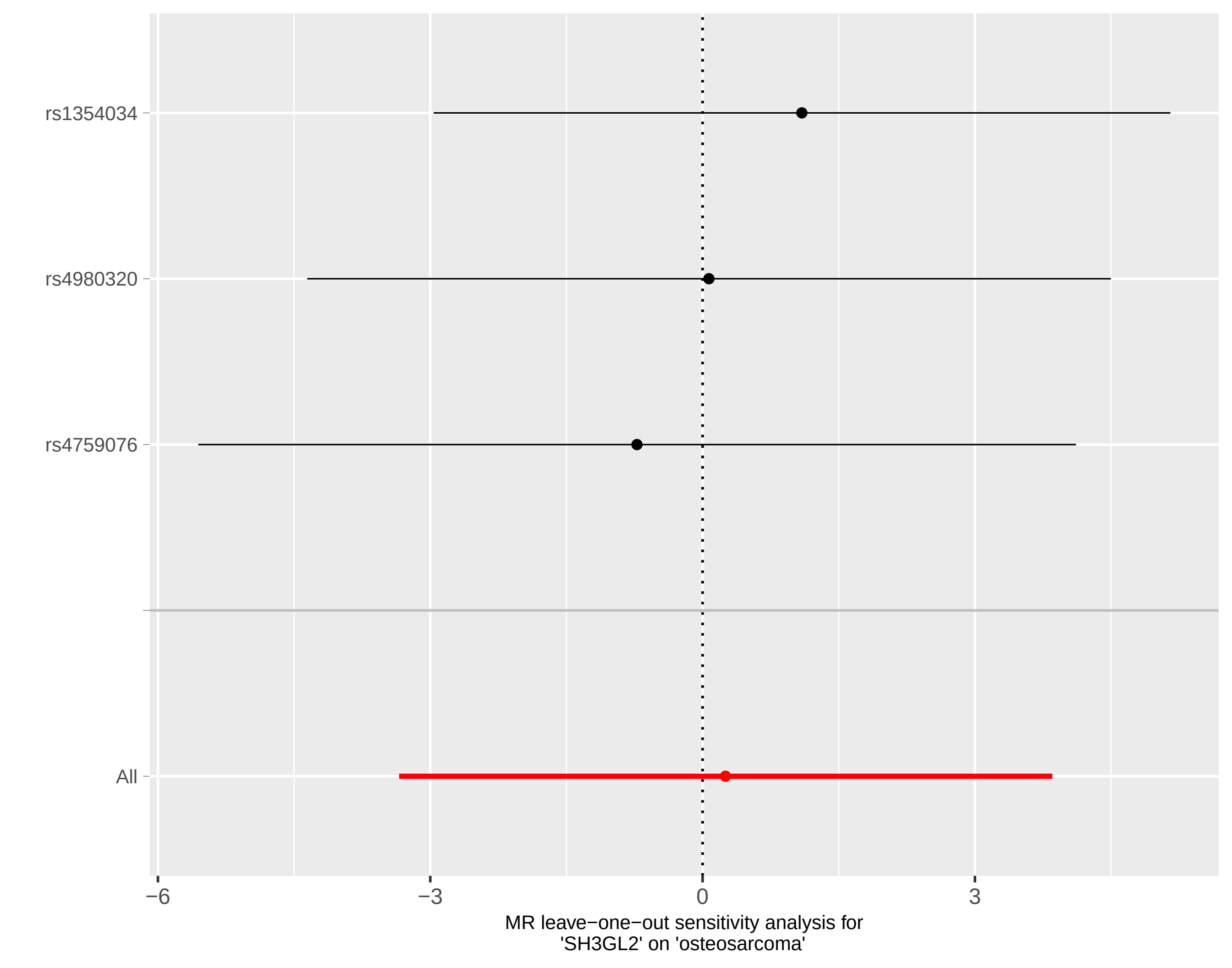

rs1354034
rs4980320
rs4759076
All
−6
−3
0
3
MR leave−one−out sensitivity analysis for
'SH3GL2' on 'osteosarcoma'

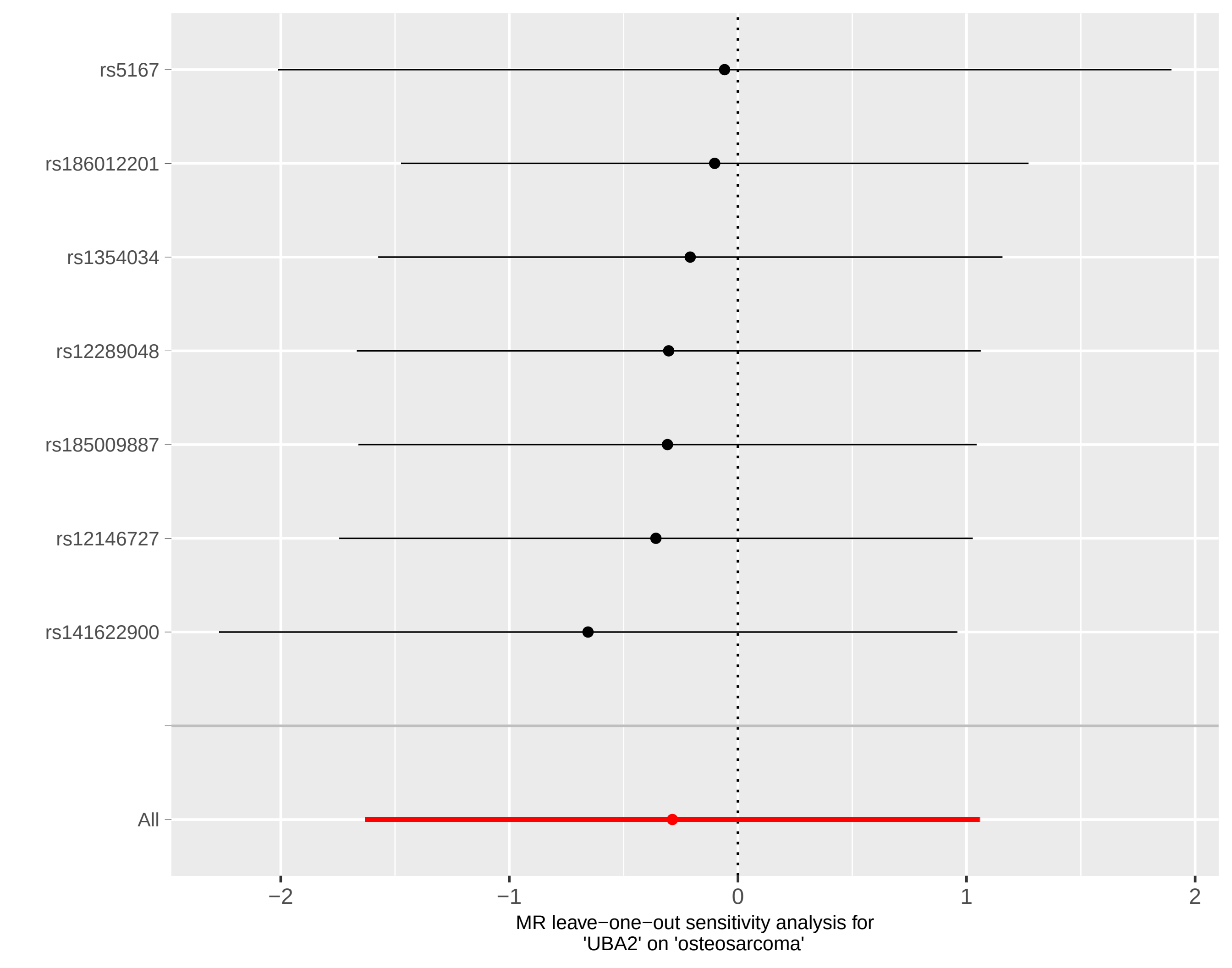

rs5167
rs186012201
rs1354034
rs12289048
rs185009887
rs12146727
rs141622900
All
−2
−1
0
1
2
MR leave−one−out sensitivity analysis for
'UBA2' on 'osteosarcoma'

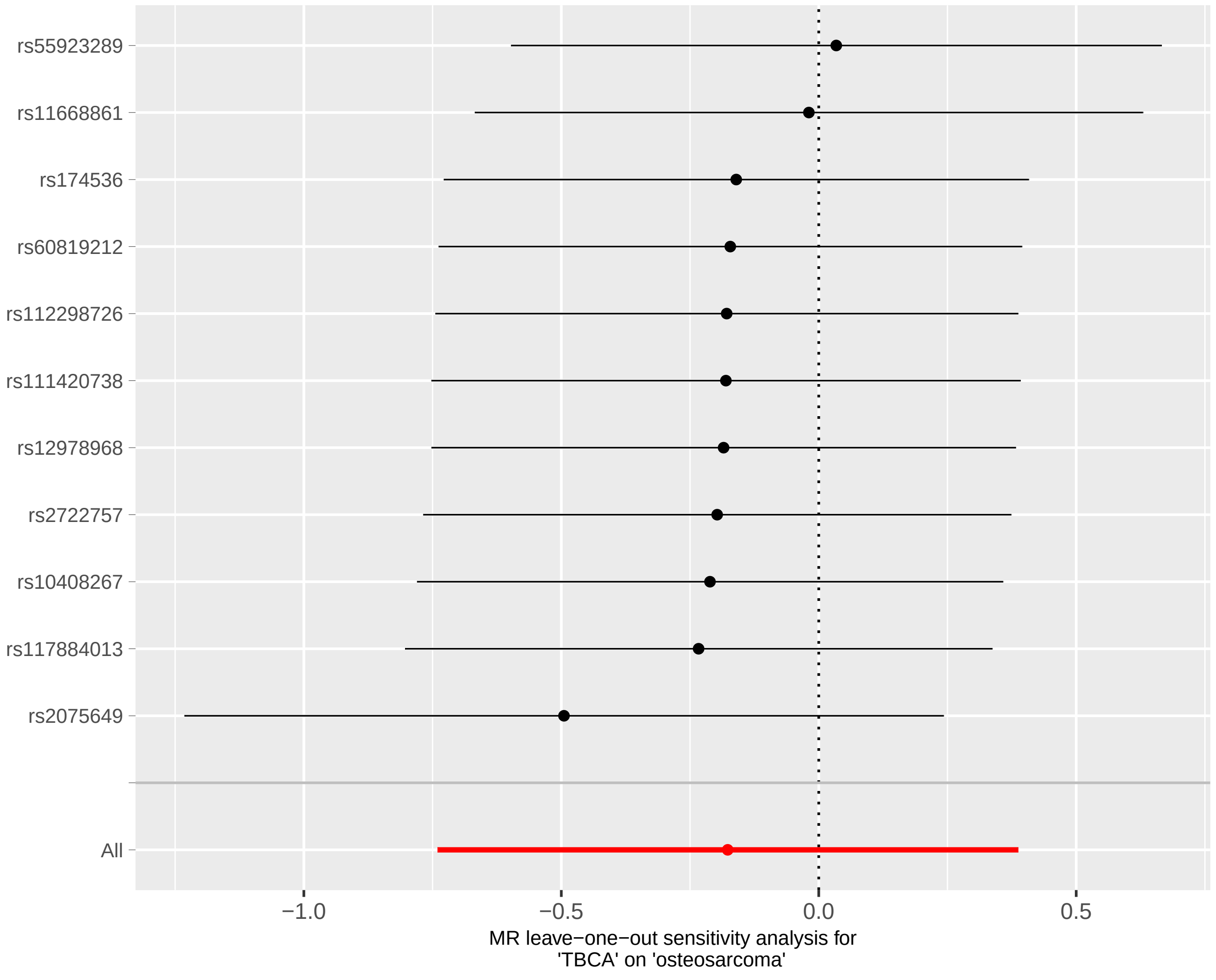
rs55923289
rs11668861
rs174536
rs60819212
rs112298726
rs111420738
rs12978968
rs2722757
rs10408267
rs117884013
rs2075649
All
−1.0
−0.5
0.0
0.5
MR leave−one−out sensitivity analysis for
'TBCA' on 'osteosarcoma'

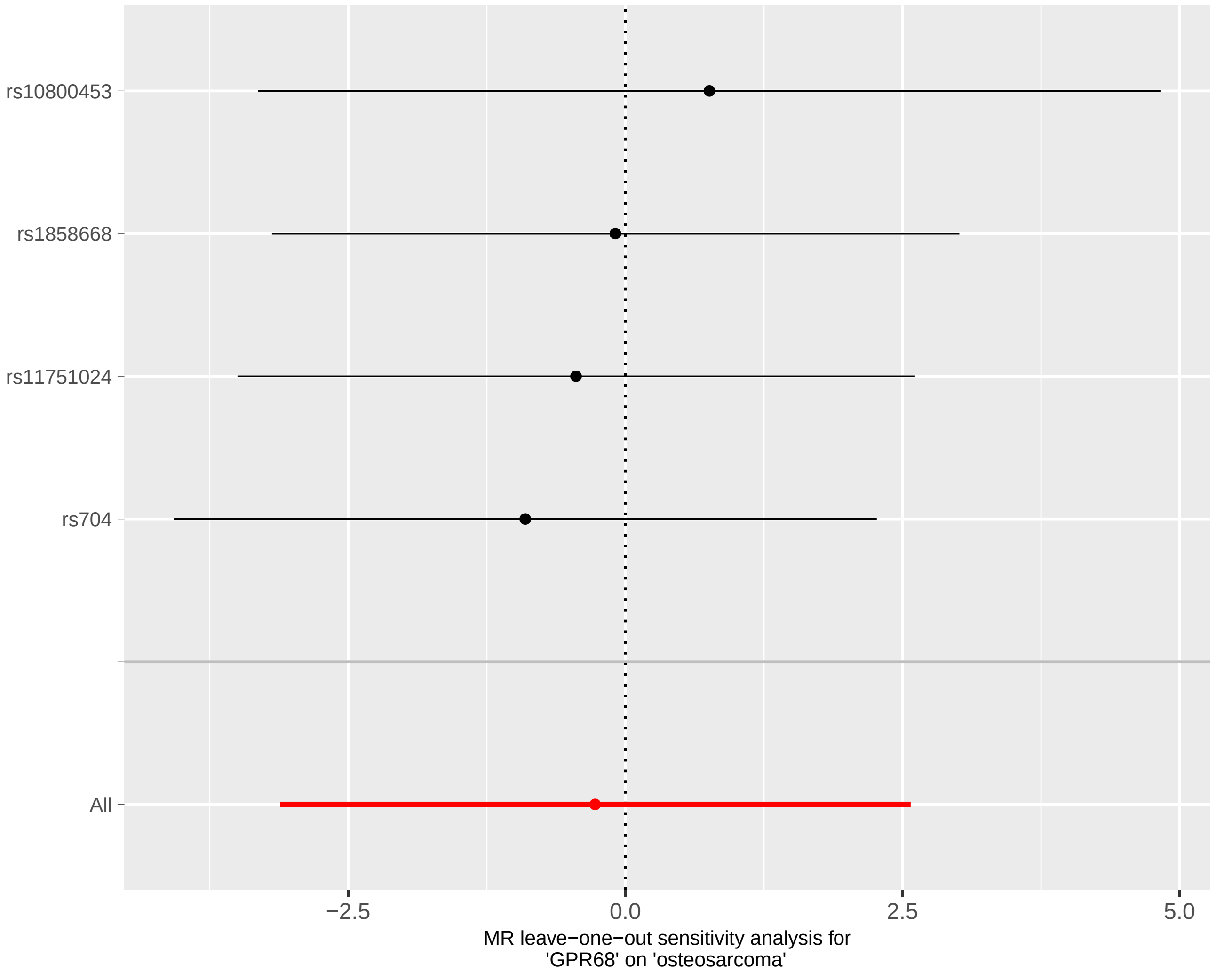
rs10800453
rs1858668
rs11751024
rs704
All
−2.5
0.0
2.5
5.0
MR leave−one−out sensitivity analysis for
'GPR68' on 'osteosarcoma'

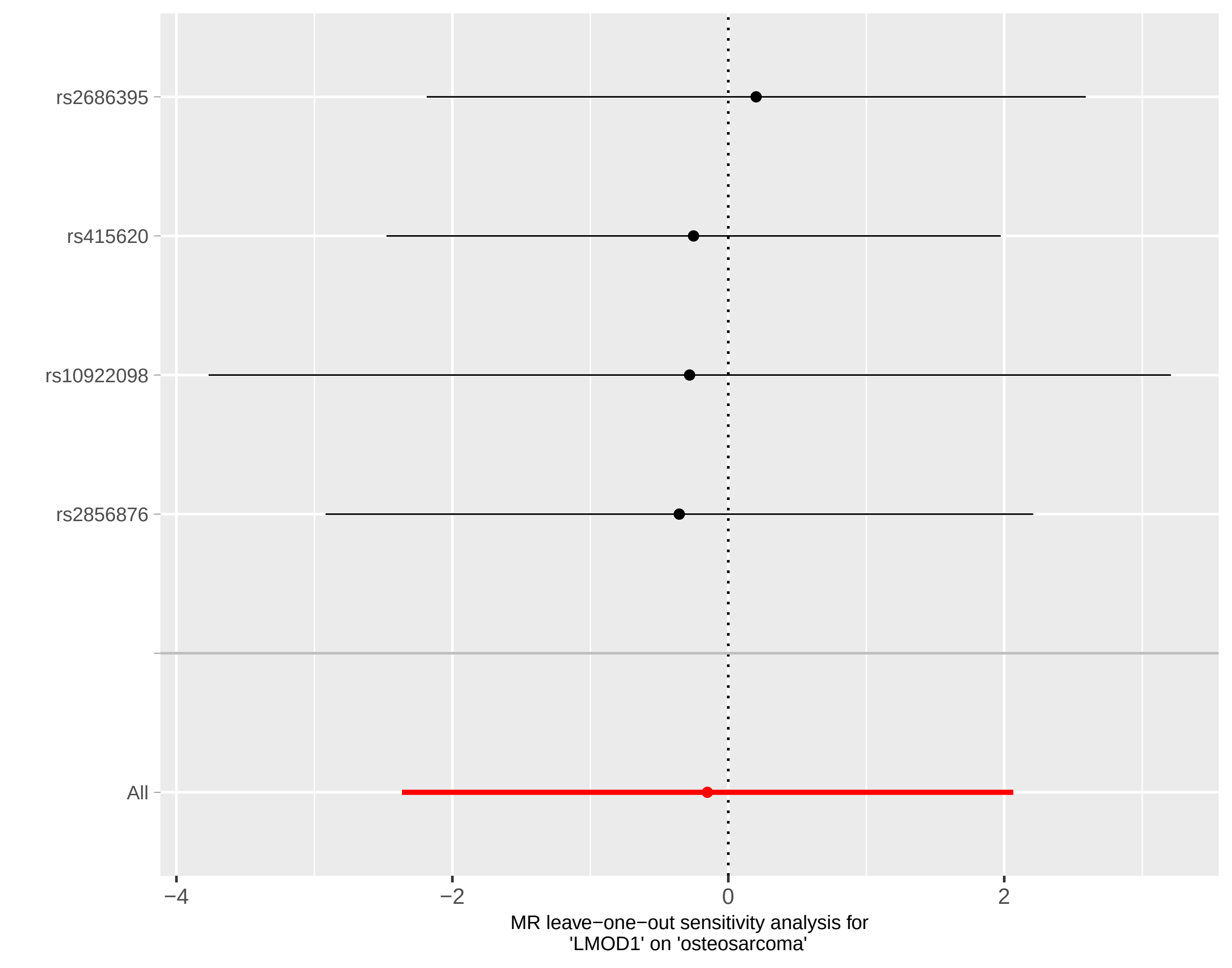
rs2686395
rs415620
rs10922098
rs2856876
All
−4
−2
0
2
MR leave−one−out sensitivity analysis for
'LMOD1' on 'osteosarcoma'

Insufficient number of SNPs

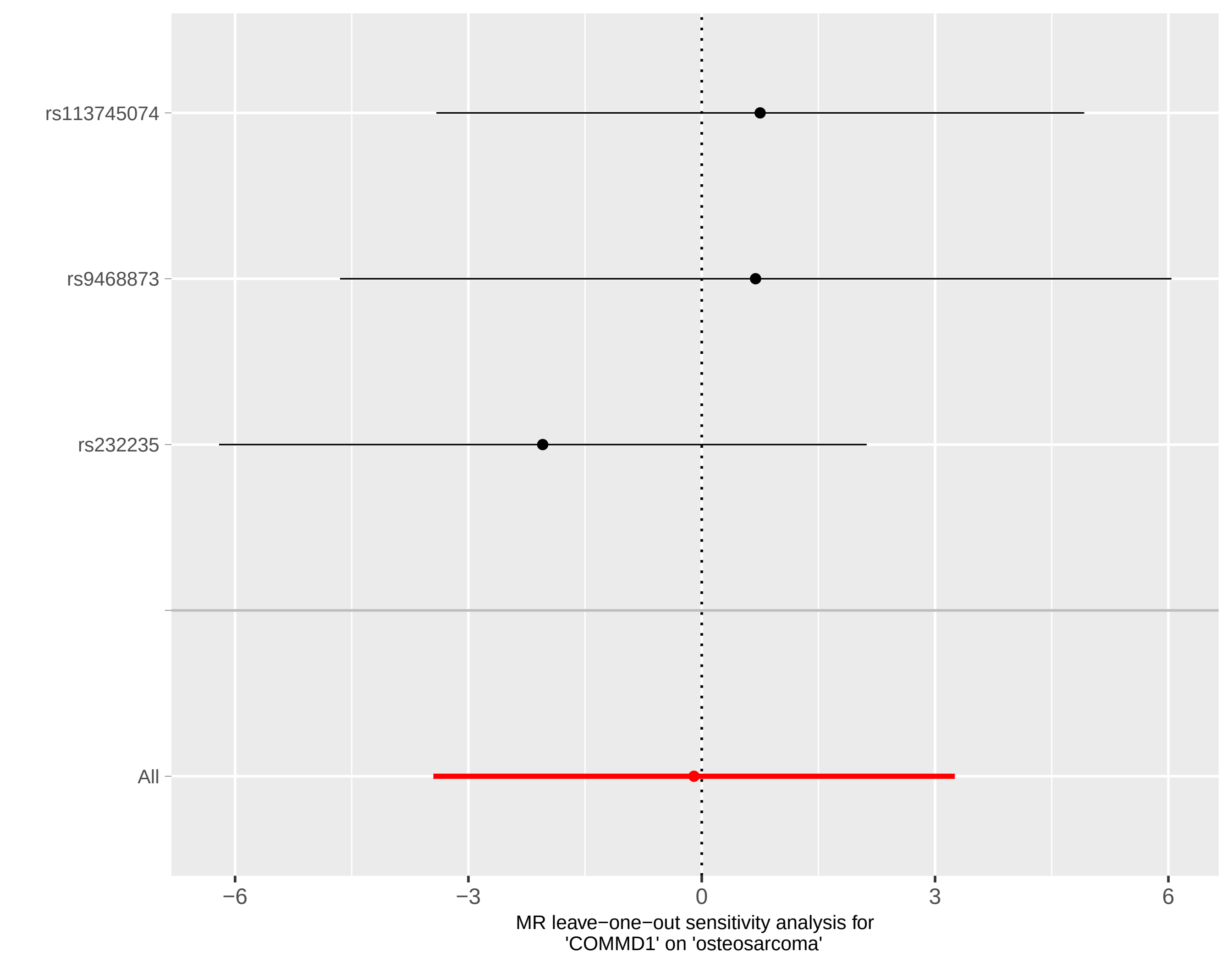

rs113745074
rs9468873
rs232235
All
−6
−3
0
3
6
MR leave−one−out sensitivity analysis for
'COMMD1' on 'osteosarcoma'

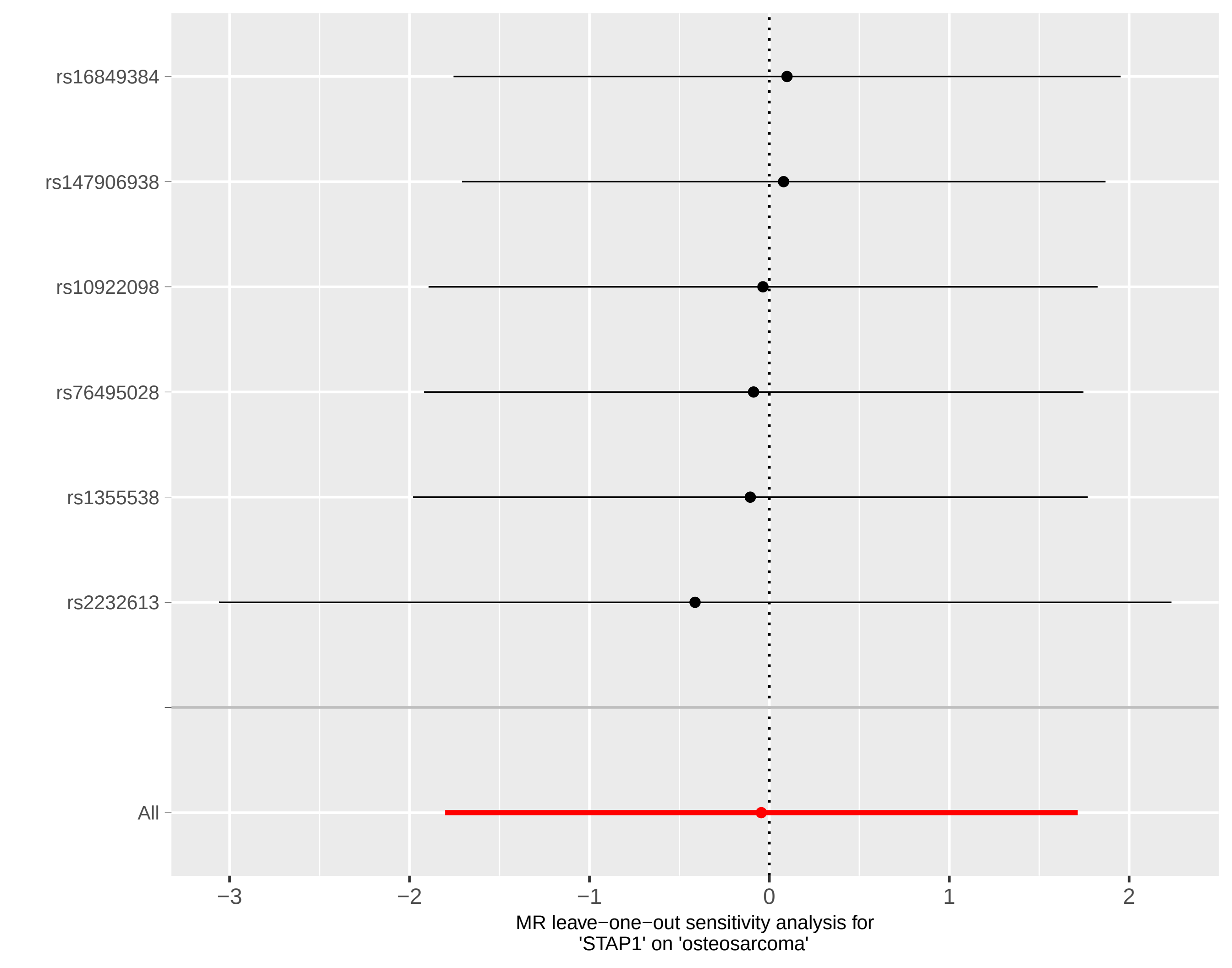

rs16849384
rs147906938
rs10922098
rs76495028
rs1355538
rs2232613
All
−3
−2
−1
0
1
2
MR leave−one−out sensitivity analysis for
'STAP1' on 'osteosarcoma'

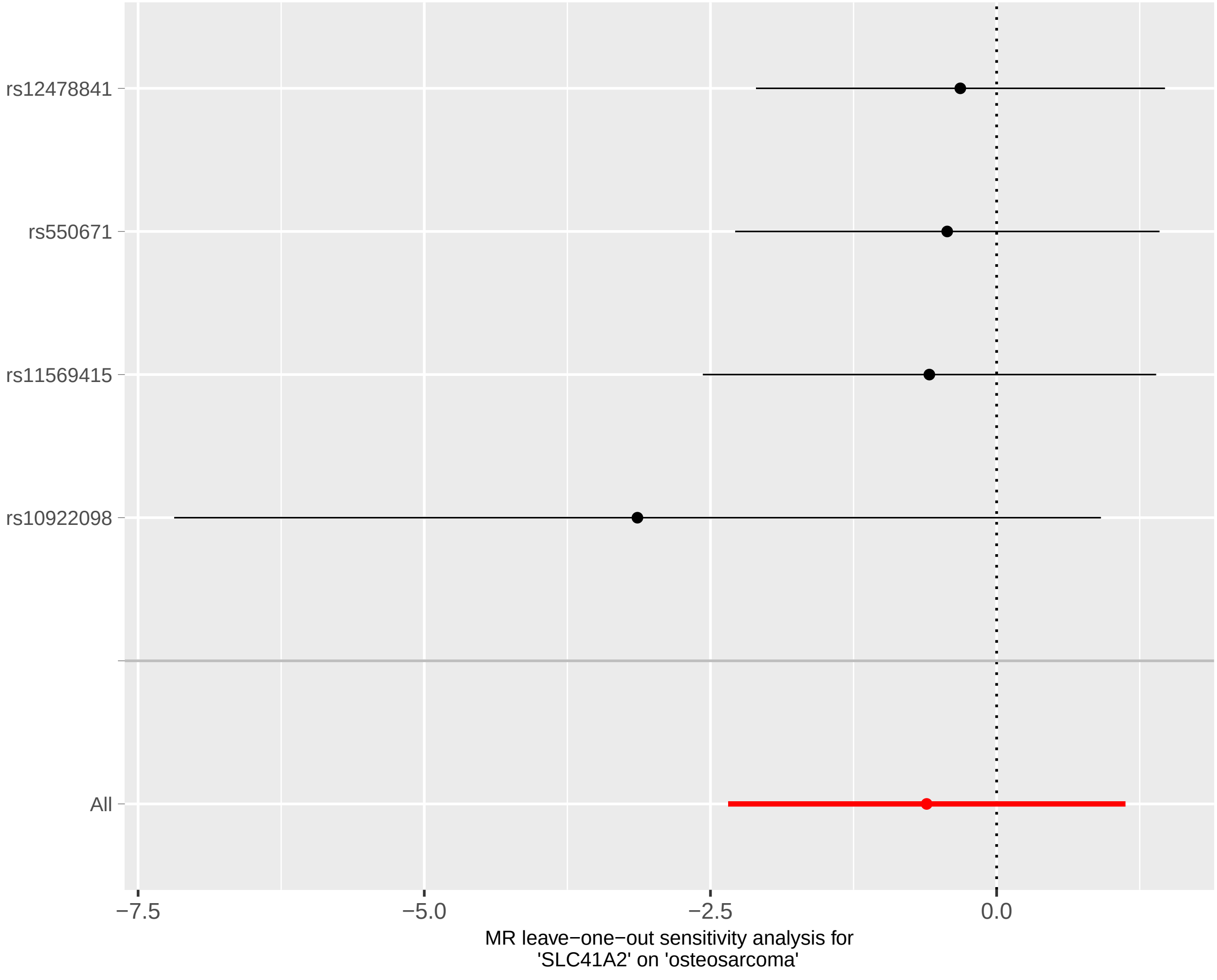

rs12478841
rs550671
rs11569415
rs10922098
All
−7.5
−5.0
−2.5
0.0
MR leave−one−out sensitivity analysis for
'SLC41A2' on 'osteosarcoma'

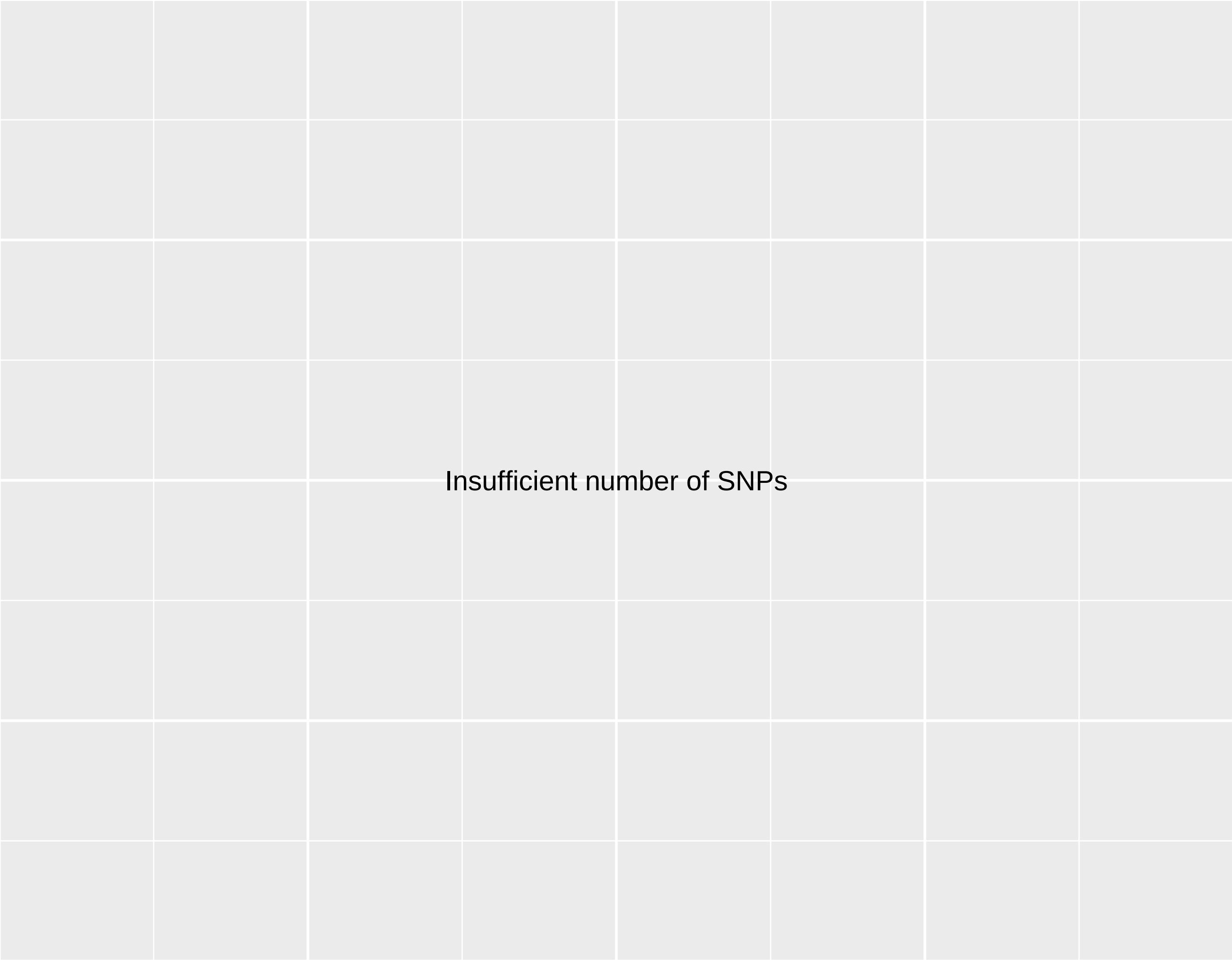
Insufficient number of SNPs

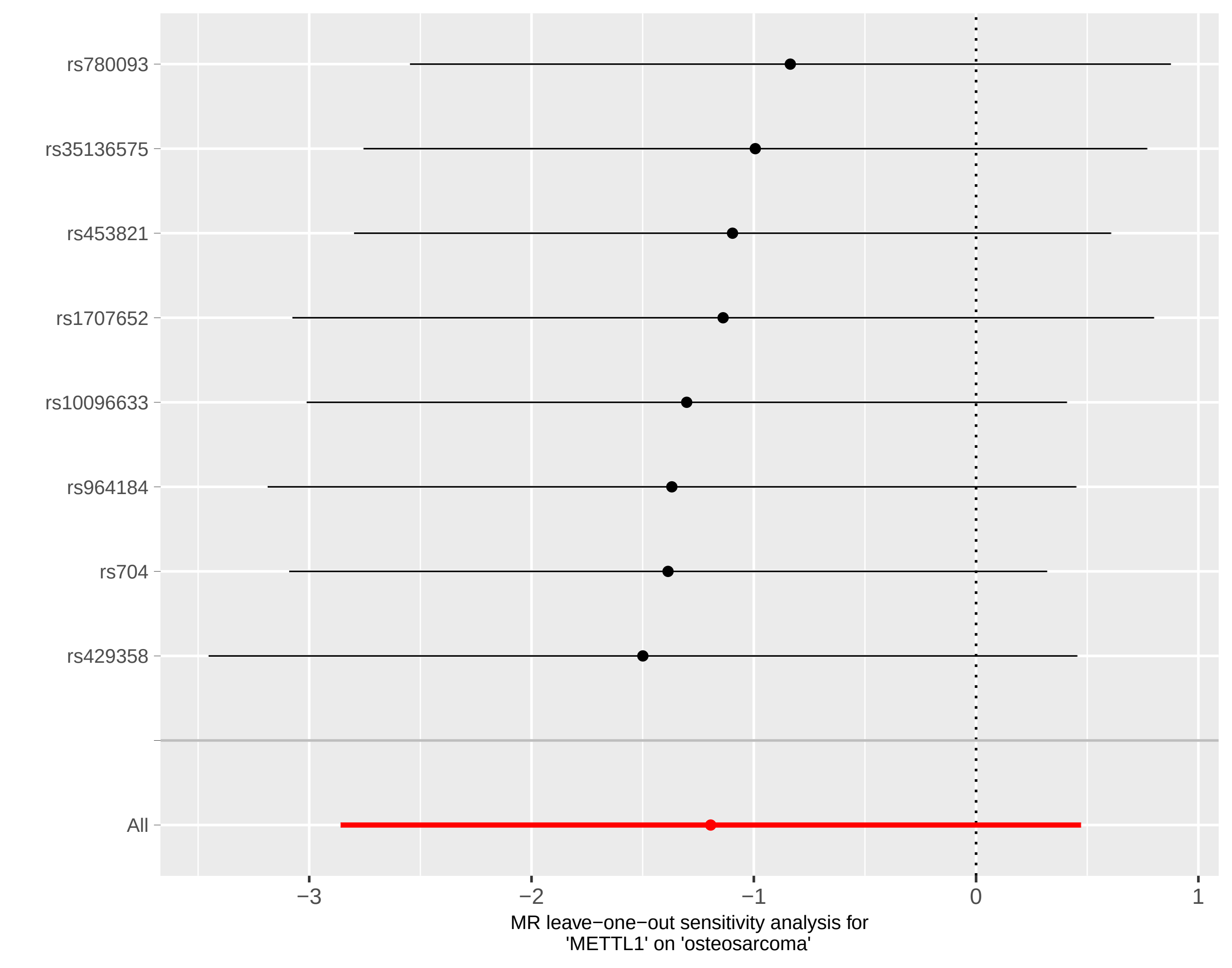
rs780093
rs35136575
rs453821
rs1707652
rs10096633
rs964184
rs704
rs429358
All
−3
−2
−1
0
1
MR leave−one−out sensitivity analysis for
'METTL1' on 'osteosarcoma'

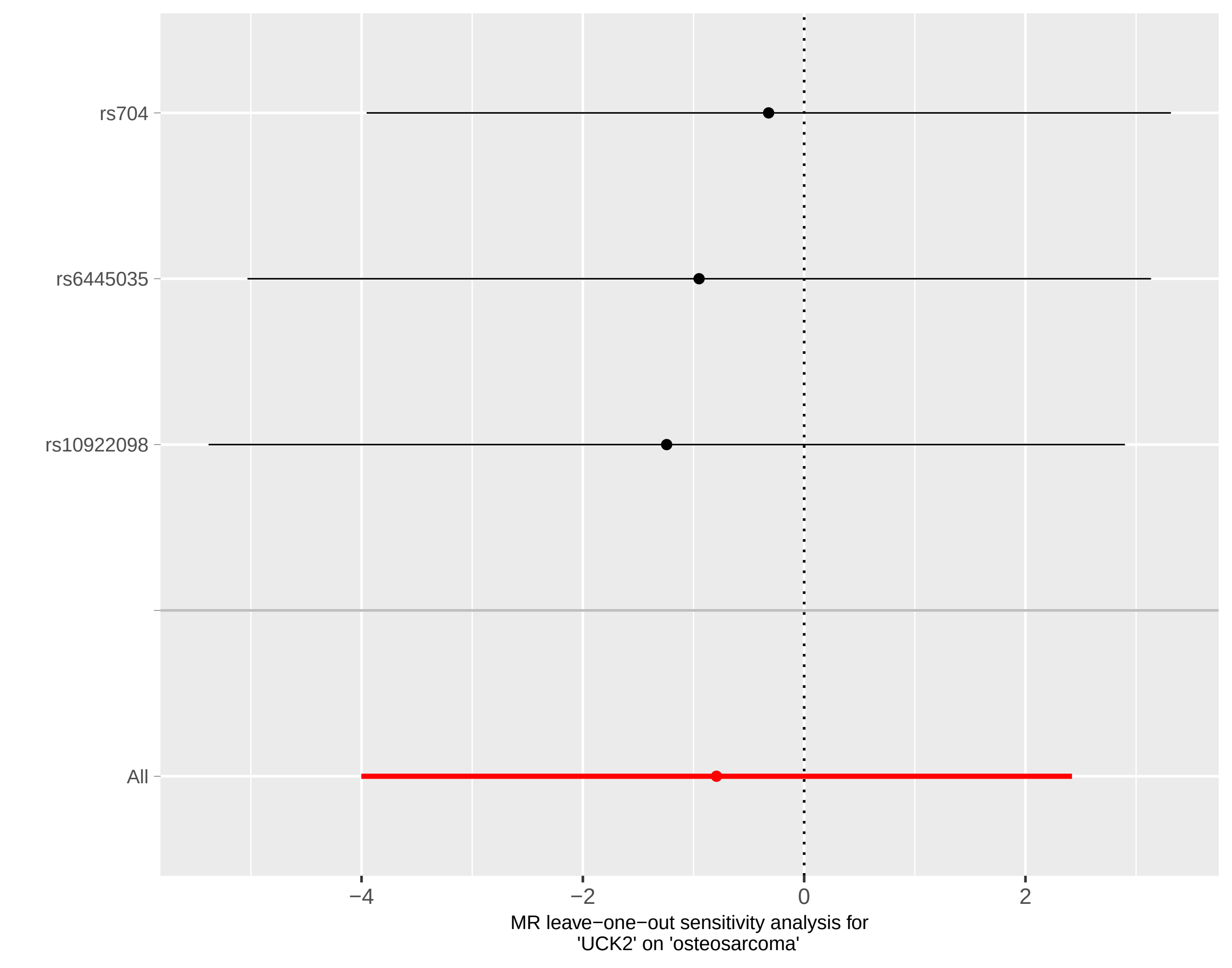
rs704
rs6445035
rs10922098
All
−4
−2
0
2
MR leave−one−out sensitivity analysis for
'UCK2' on 'osteosarcoma'

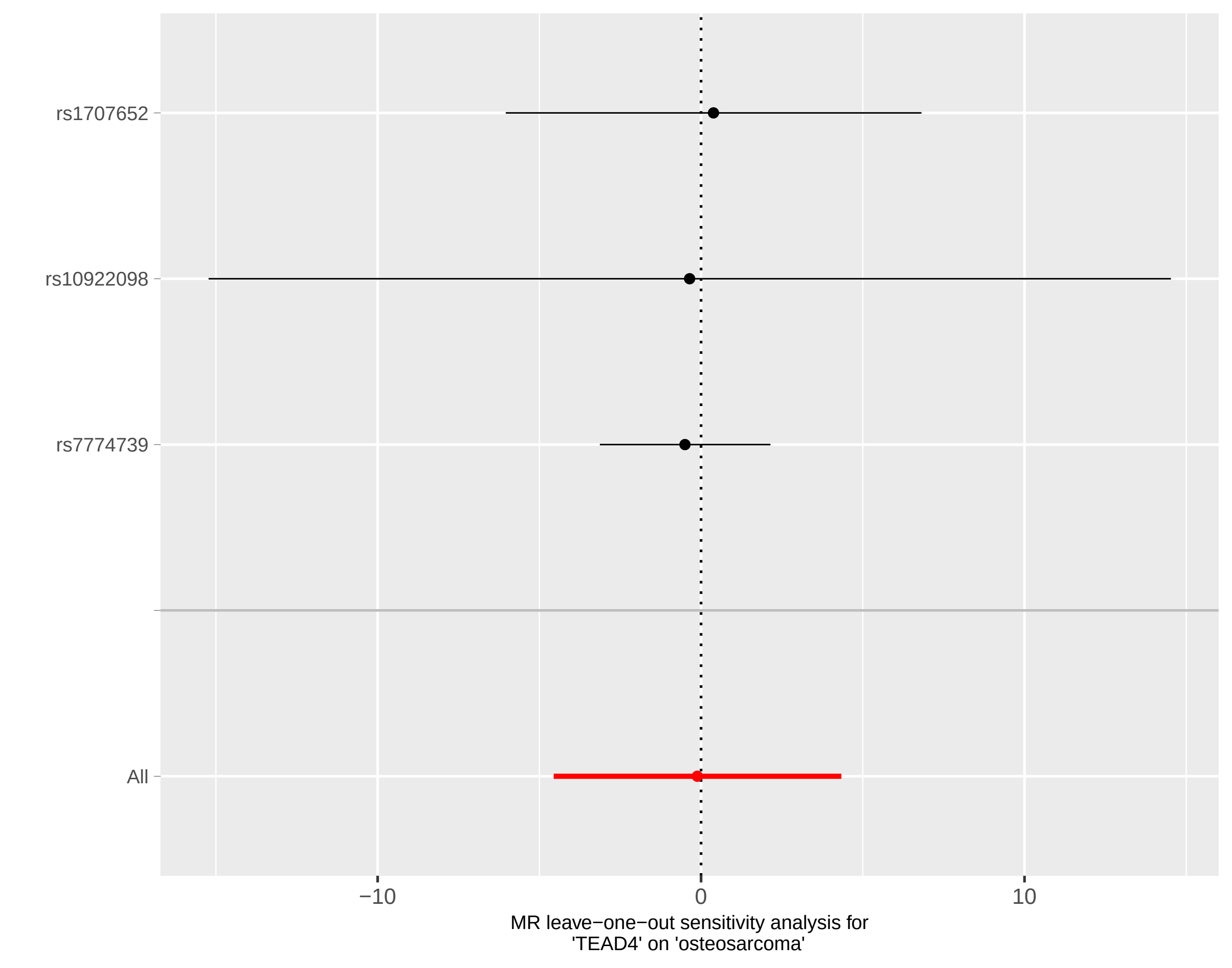
rs1707652
rs10922098
rs7774739
All
−10
0
10
MR leave−one−out sensitivity analysis for
'TEAD4' on 'osteosarcoma'

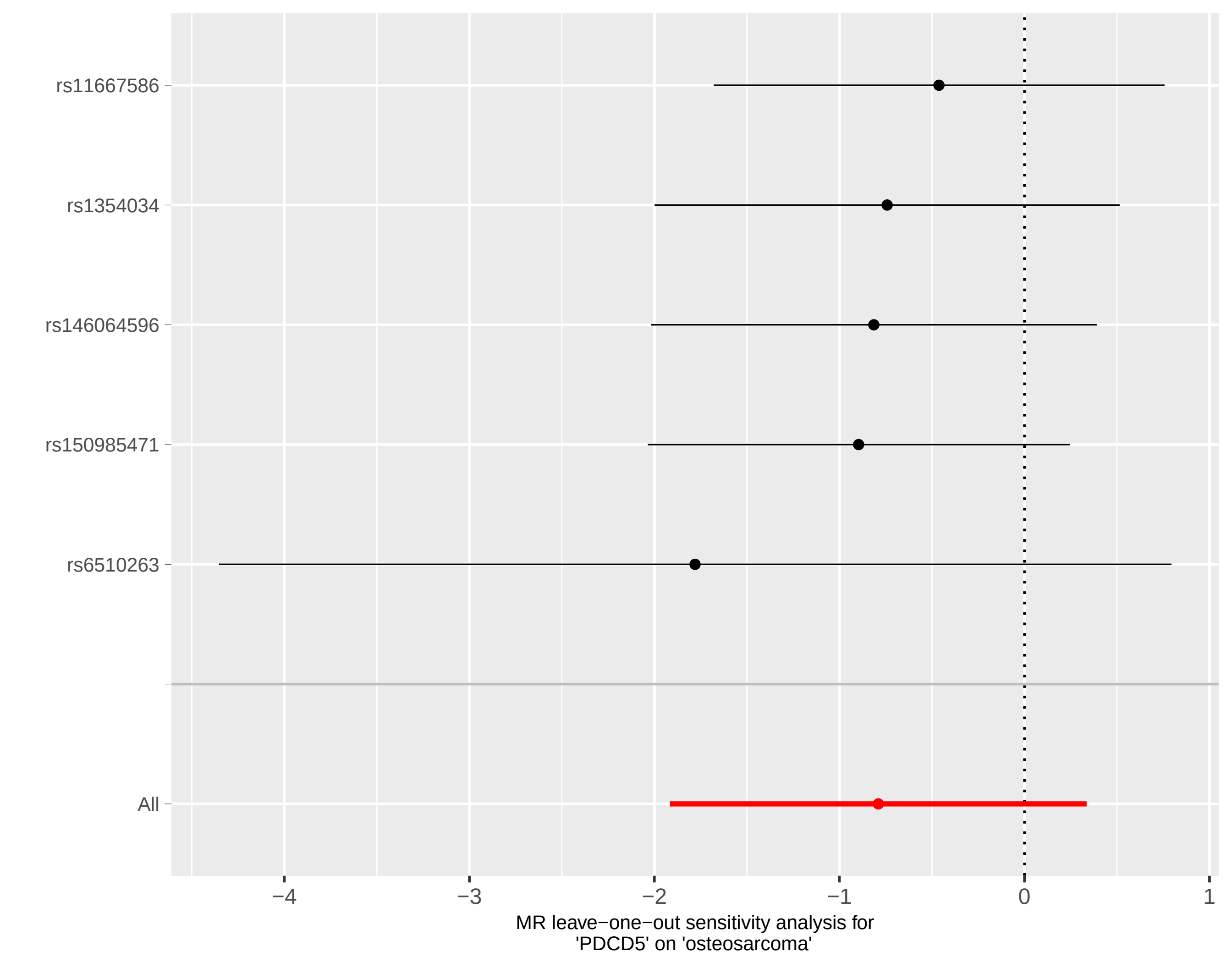
rs11667586
rs1354034
rs146064596
rs150985471
rs6510263
All
−4
−3
−2
−1
0
1
MR leave−one−out sensitivity analysis for
'PDCD5' on 'osteosarcoma'

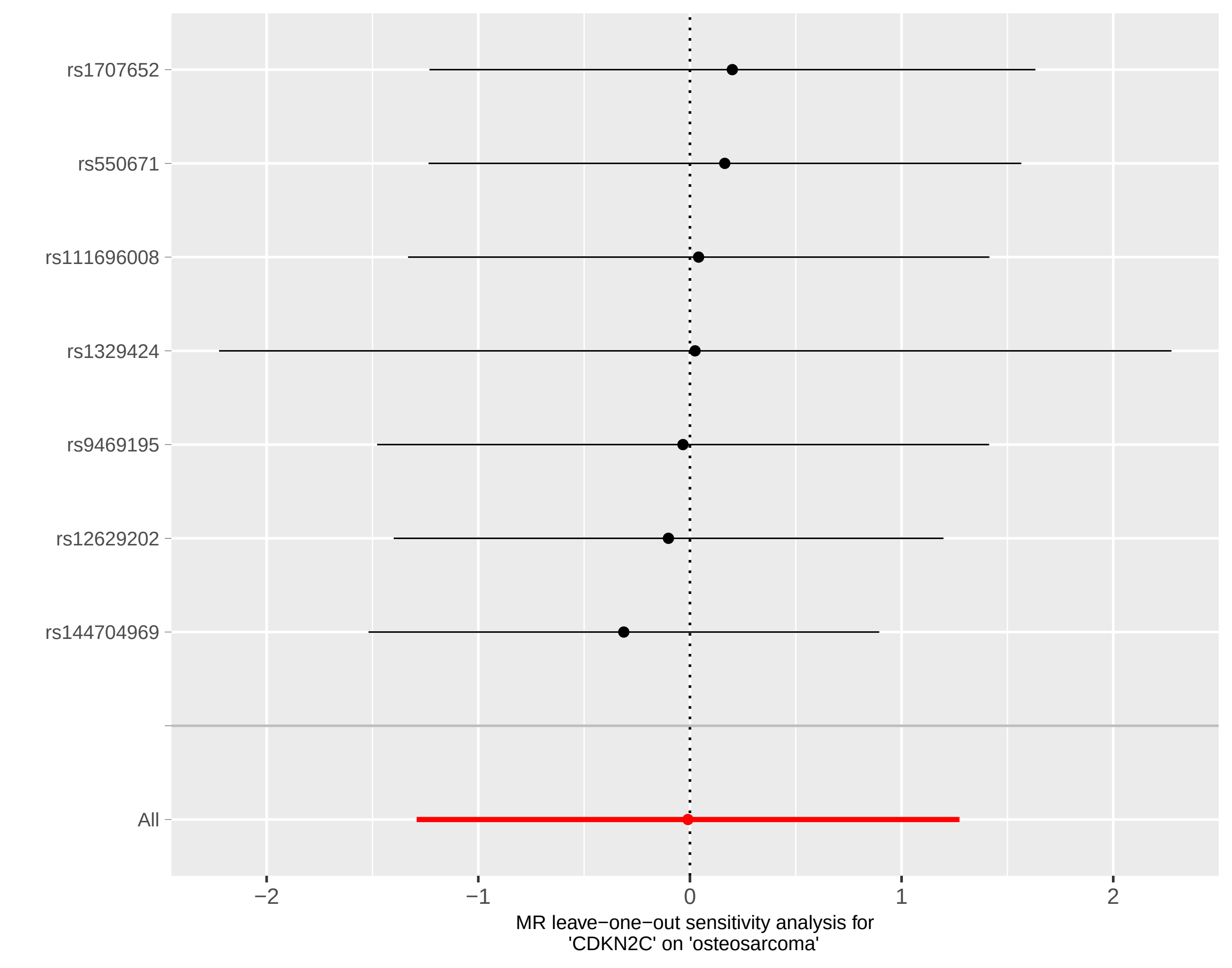
rs1707652
rs550671
rs111696008
rs1329424
rs9469195
rs12629202
rs144704969
All
−2
−1
0
1
2
MR leave−one−out sensitivity analysis for
'CDKN2C' on 'osteosarcoma'

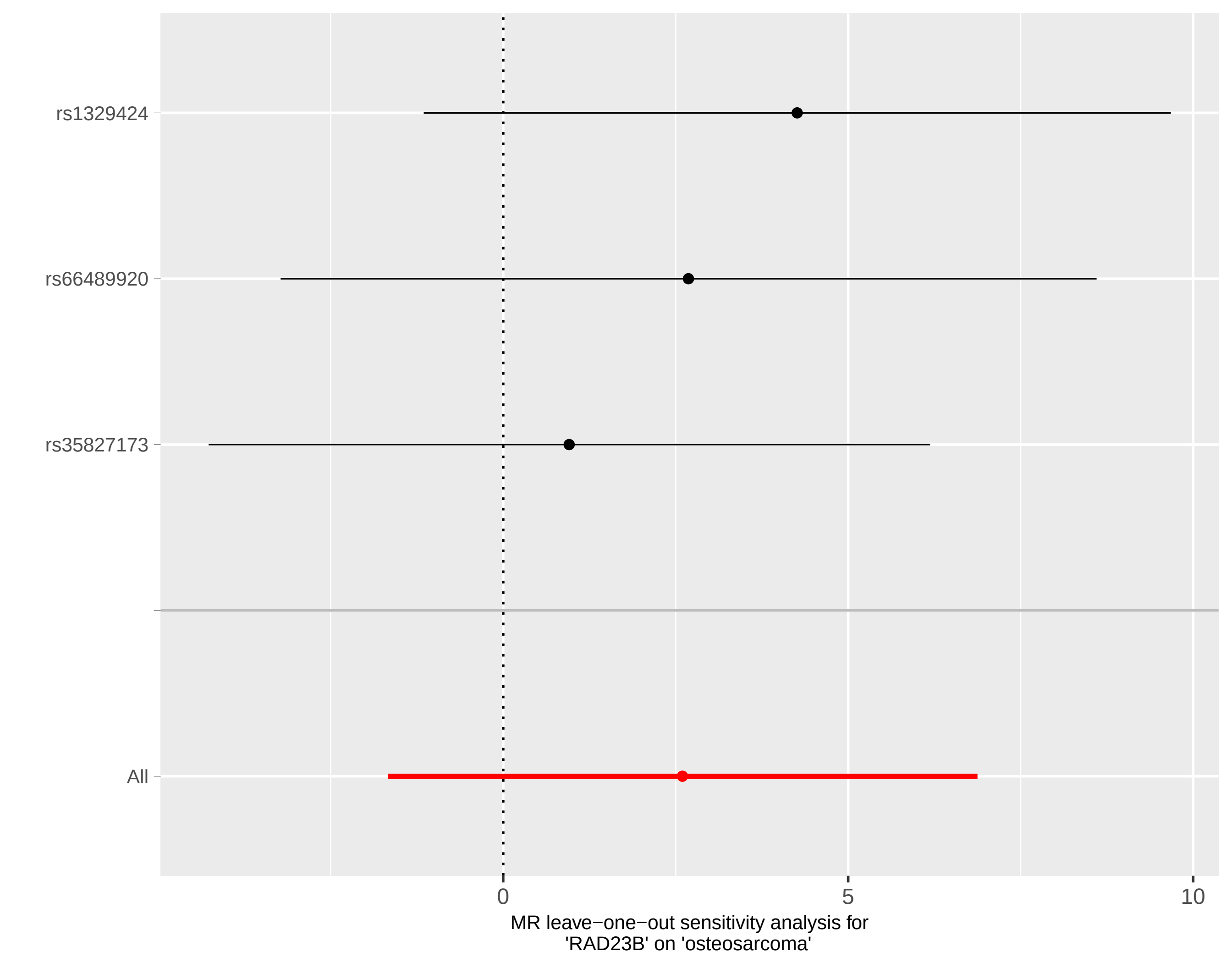

rs1329424
rs66489920
rs35827173
All
0
5
10
MR leave−one−out sensitivity analysis for
'RAD23B' on 'osteosarcoma'

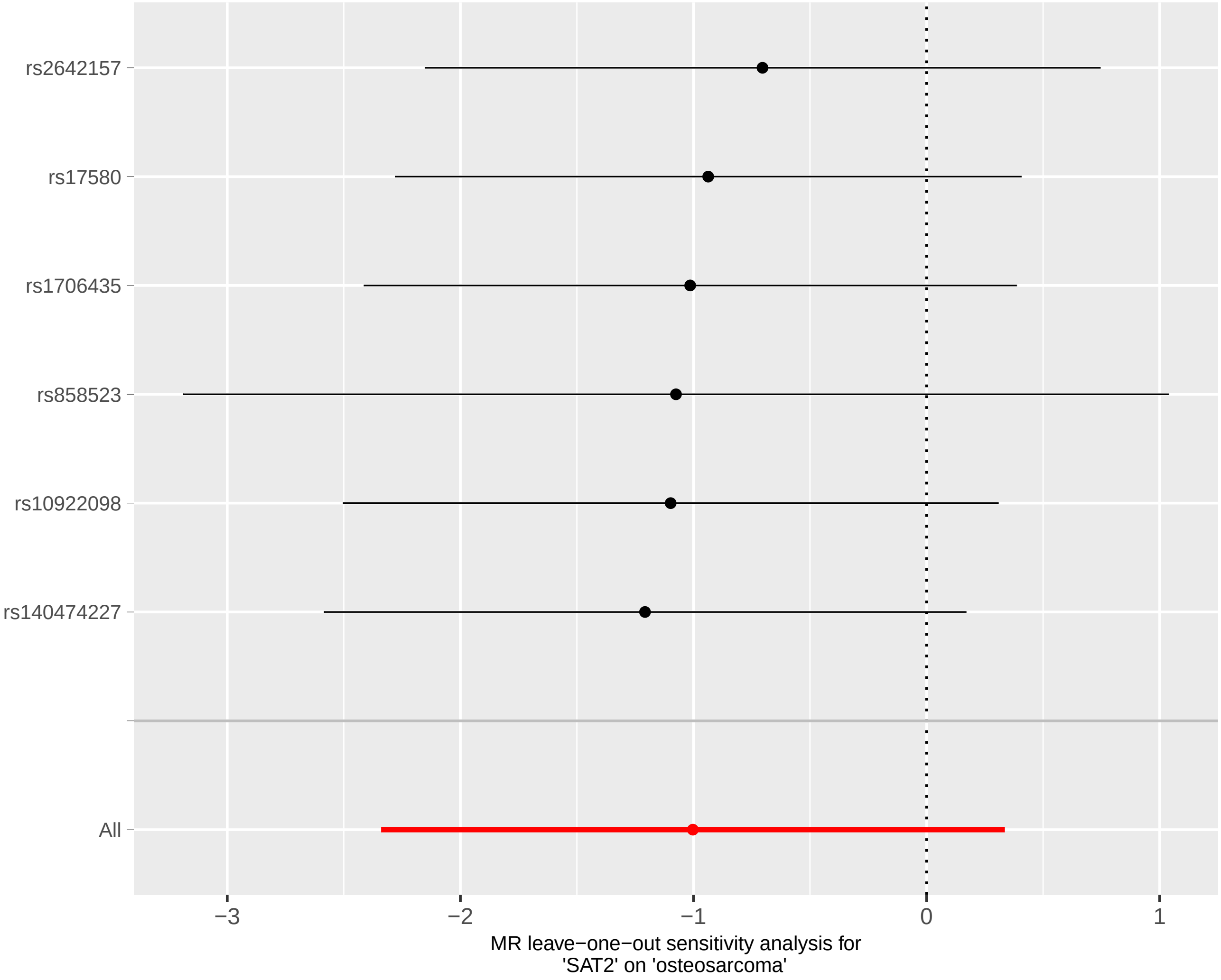
rs2642157
rs17580
rs1706435
rs858523
rs10922098
rs140474227
All
−3
−2
−1
0
1
MR leave−one−out sensitivity analysis for
'SAT2' on 'osteosarcoma'

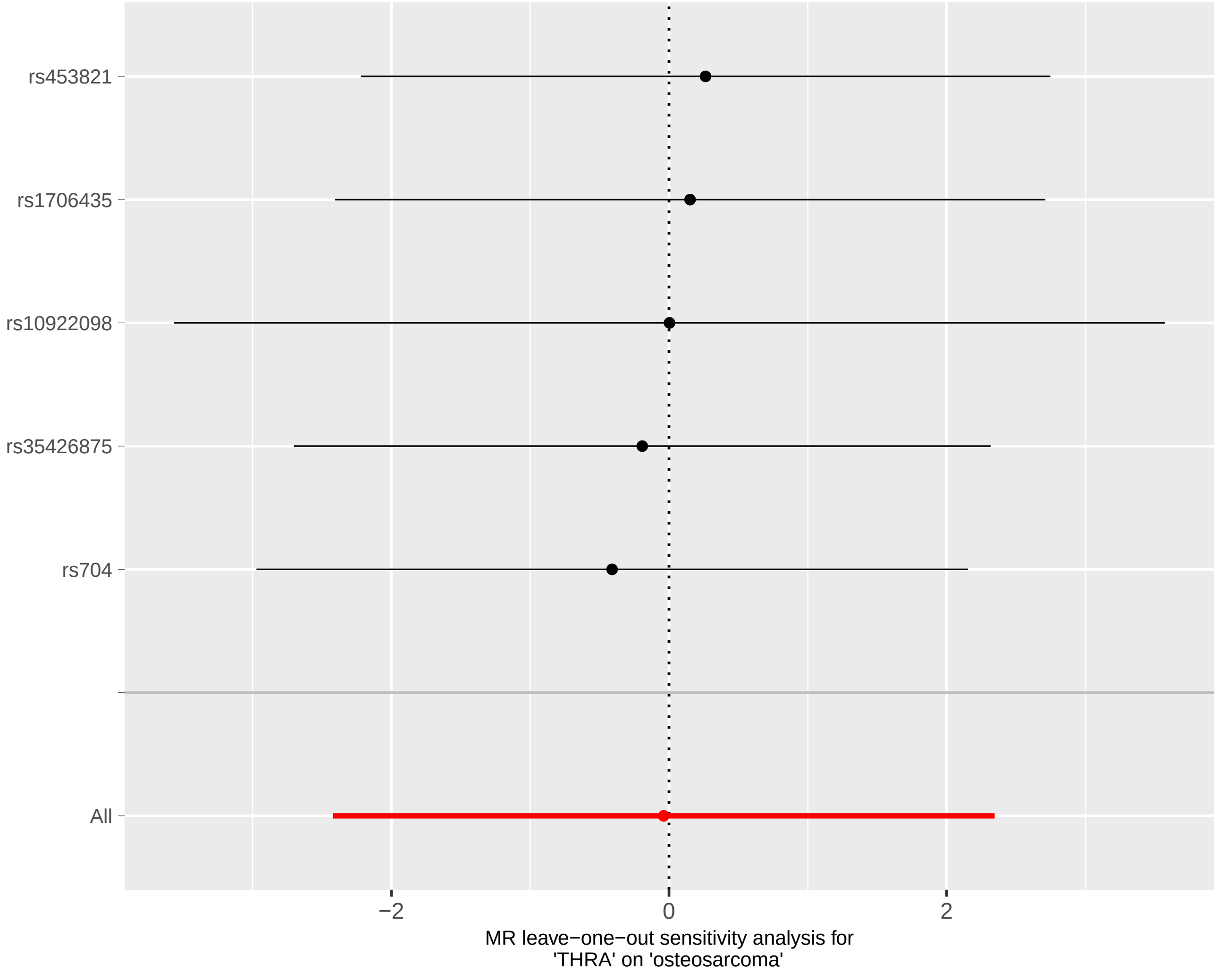
rs453821
rs1706435
rs10922098
rs35426875
rs704
All
−2
0
2
MR leave−one−out sensitivity analysis for
'THRA' on 'osteosarcoma'

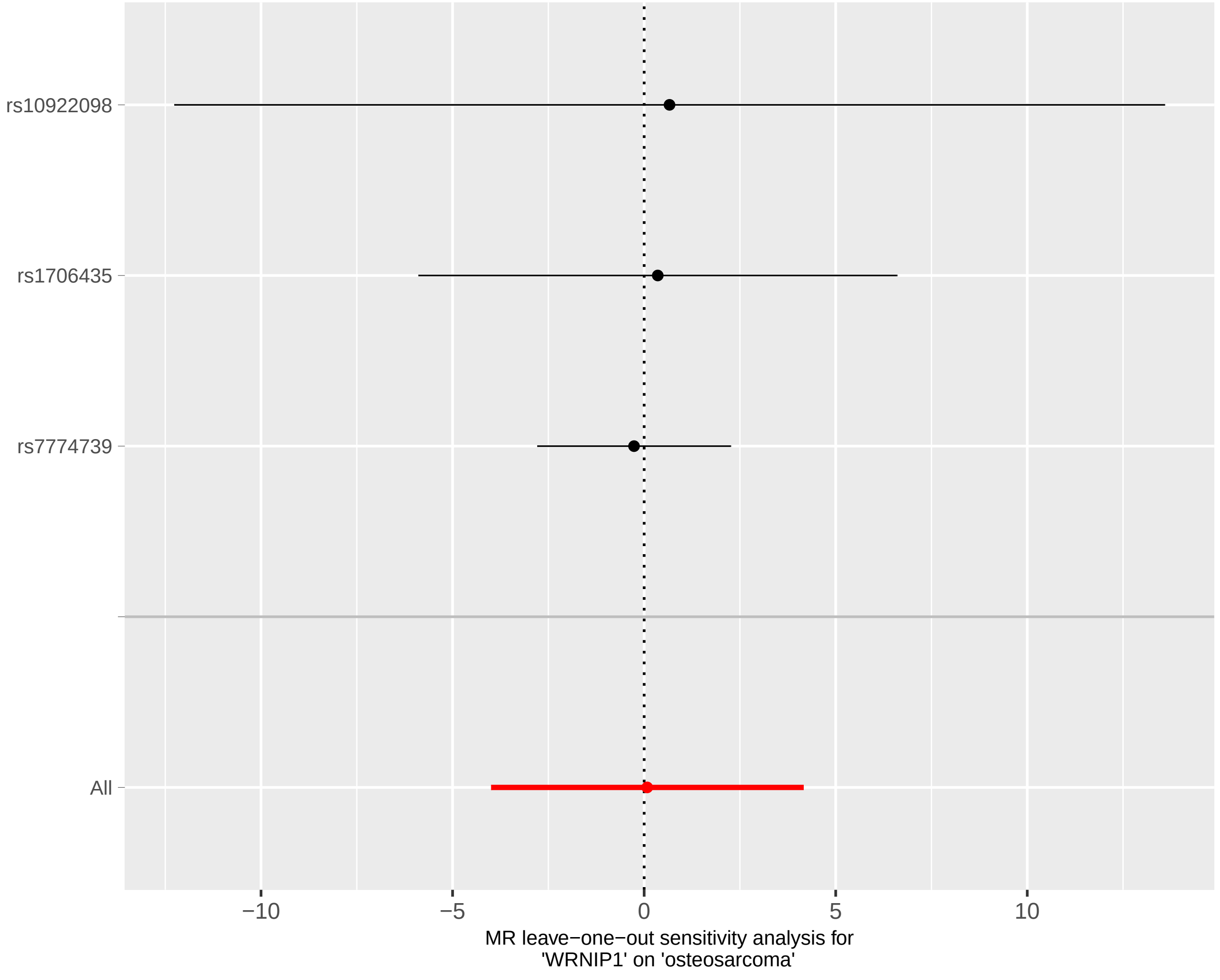
rs10922098
rs1706435
rs7774739
All
−10
−5
0
5
10
MR leave−one−out sensitivity analysis for
'WRNIP1' on 'osteosarcoma'

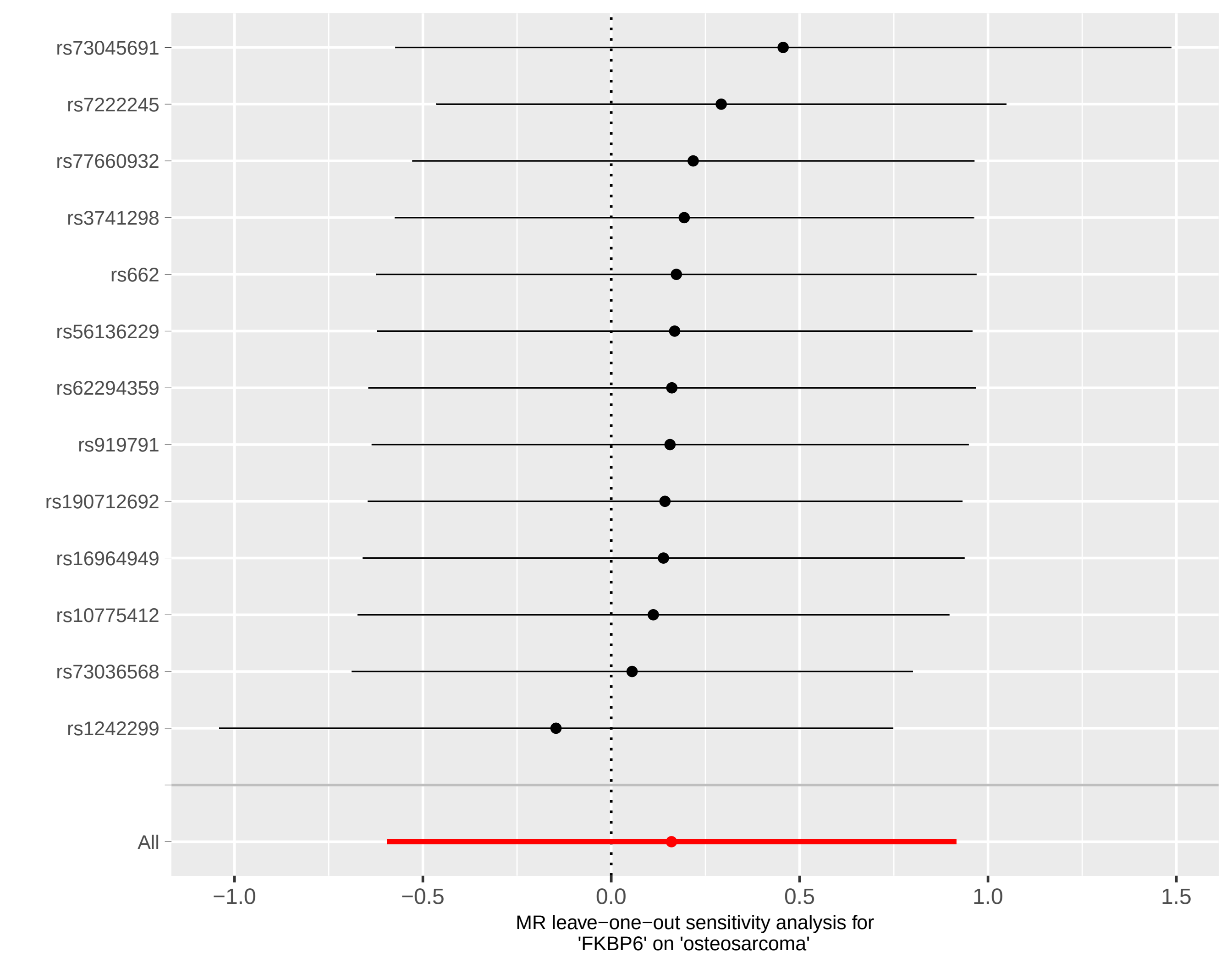

rs73045691
rs7222245
rs77660932
rs3741298
rs662
rs56136229
rs62294359
rs919791
rs190712692
rs16964949
rs10775412
rs73036568
rs1242299
All
−1.0
−0.5
0.0
0.5
1.0
1.5
MR leave−one−out sensitivity analysis for
'FKBP6' on 'osteosarcoma'

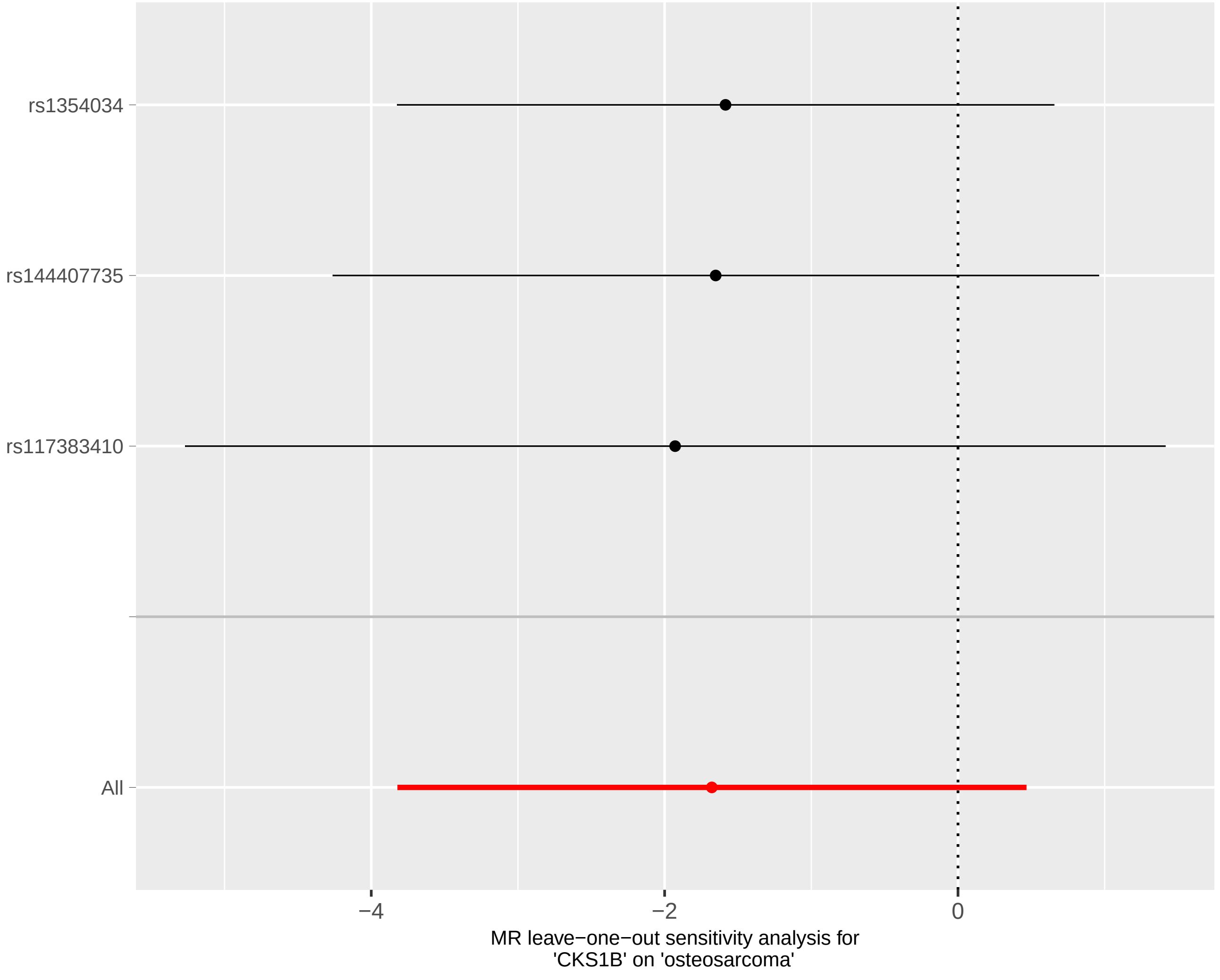
rs1354034
rs144407735
rs117383410
All
−4
−2
0
MR leave−one−out sensitivity analysis for
'CKS1B' on 'osteosarcoma'

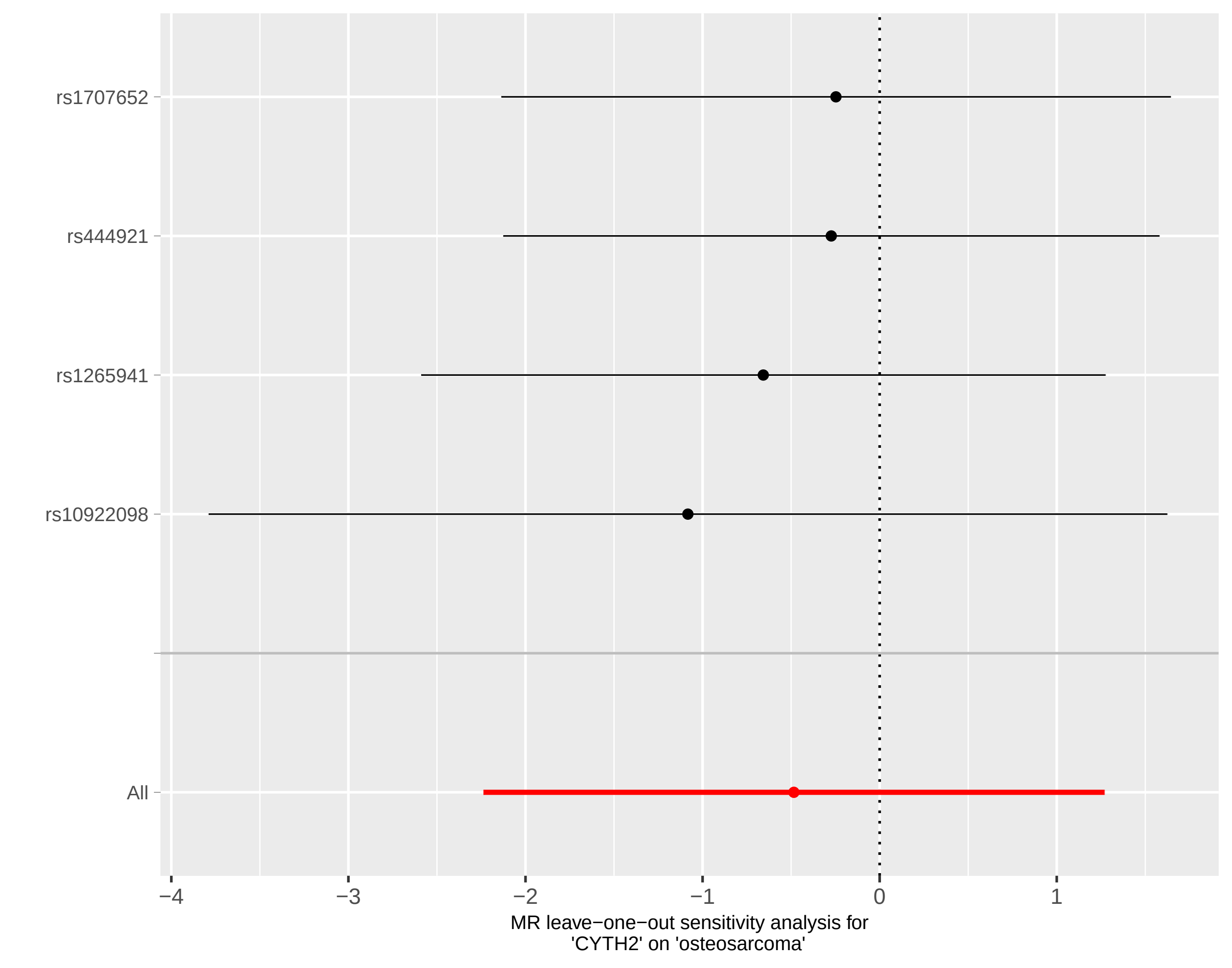
rs1707652
rs444921
rs1265941
rs10922098
All
−4
−3
−2
−1
0
1
MR leave−one−out sensitivity analysis for
'CYTH2' on 'osteosarcoma'

Insufficient number of SNPs

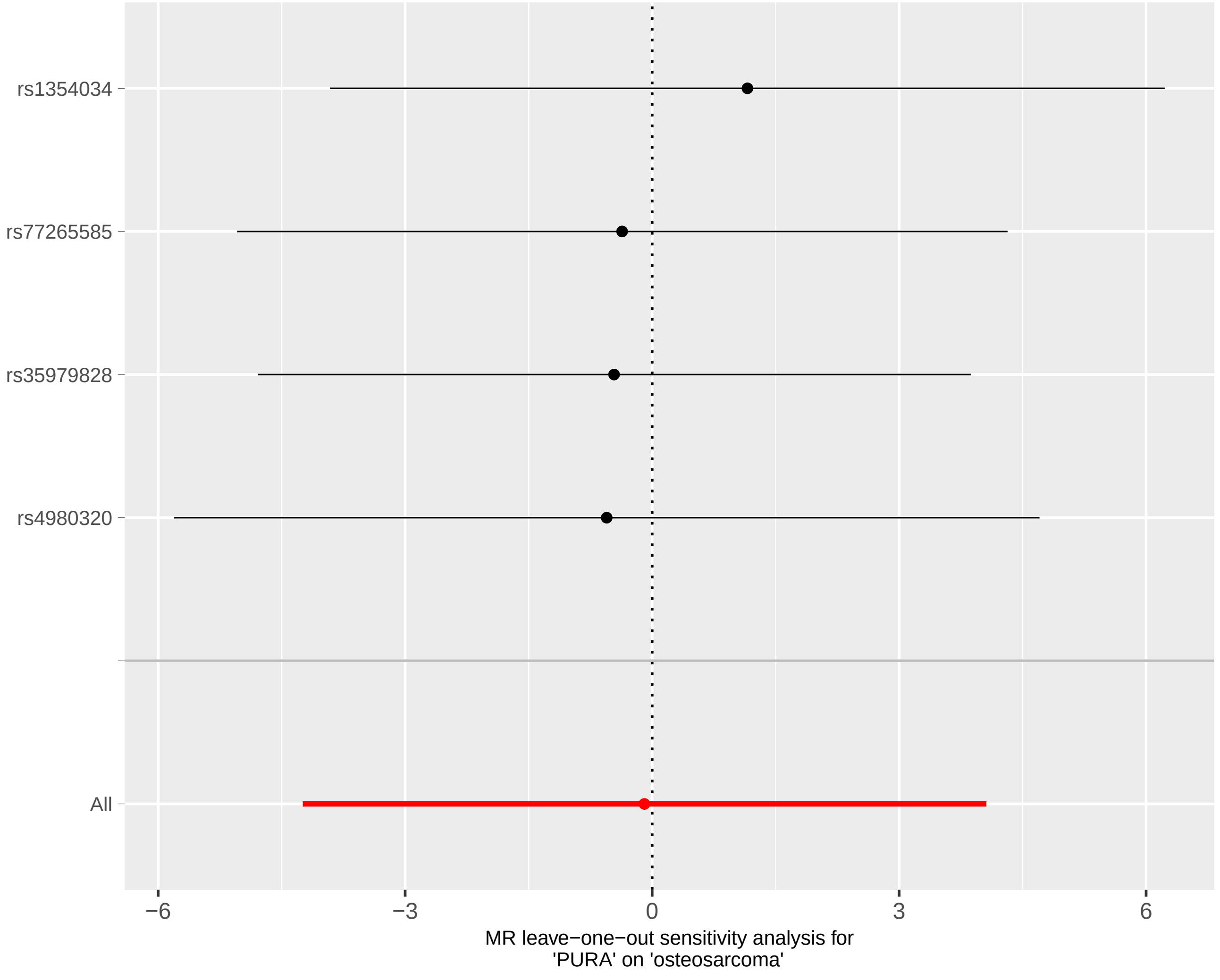
rs1354034
rs77265585
rs35979828
rs4980320
All
−6
−3
0
3
6
MR leave−one−out sensitivity analysis for
'PURA' on 'osteosarcoma'

Insufficient number of SNPs

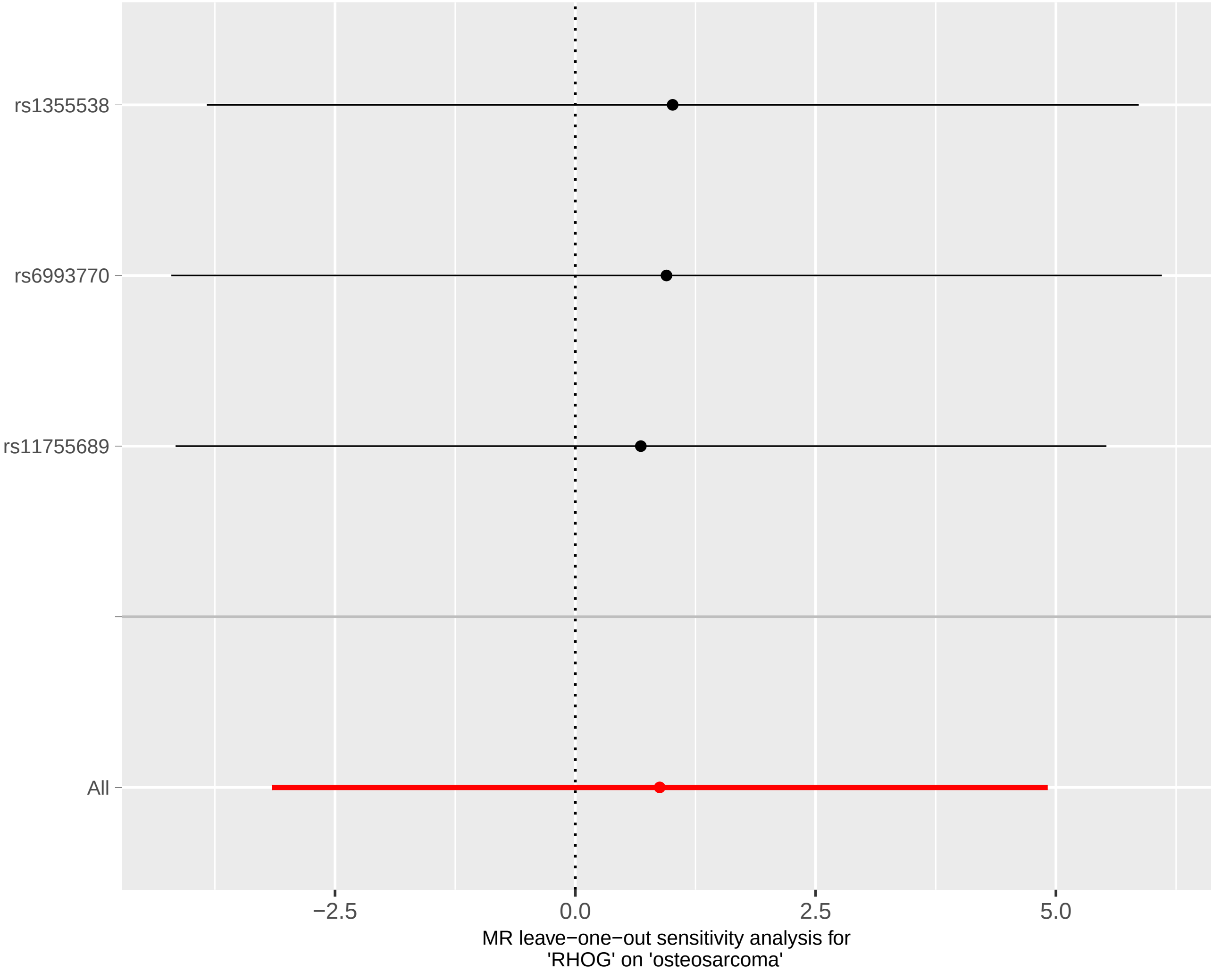

rs1355538
rs6993770
rs11755689
All
−2.5
0.0
2.5
5.0
MR leave−one−out sensitivity analysis for
'RHOG' on 'osteosarcoma'

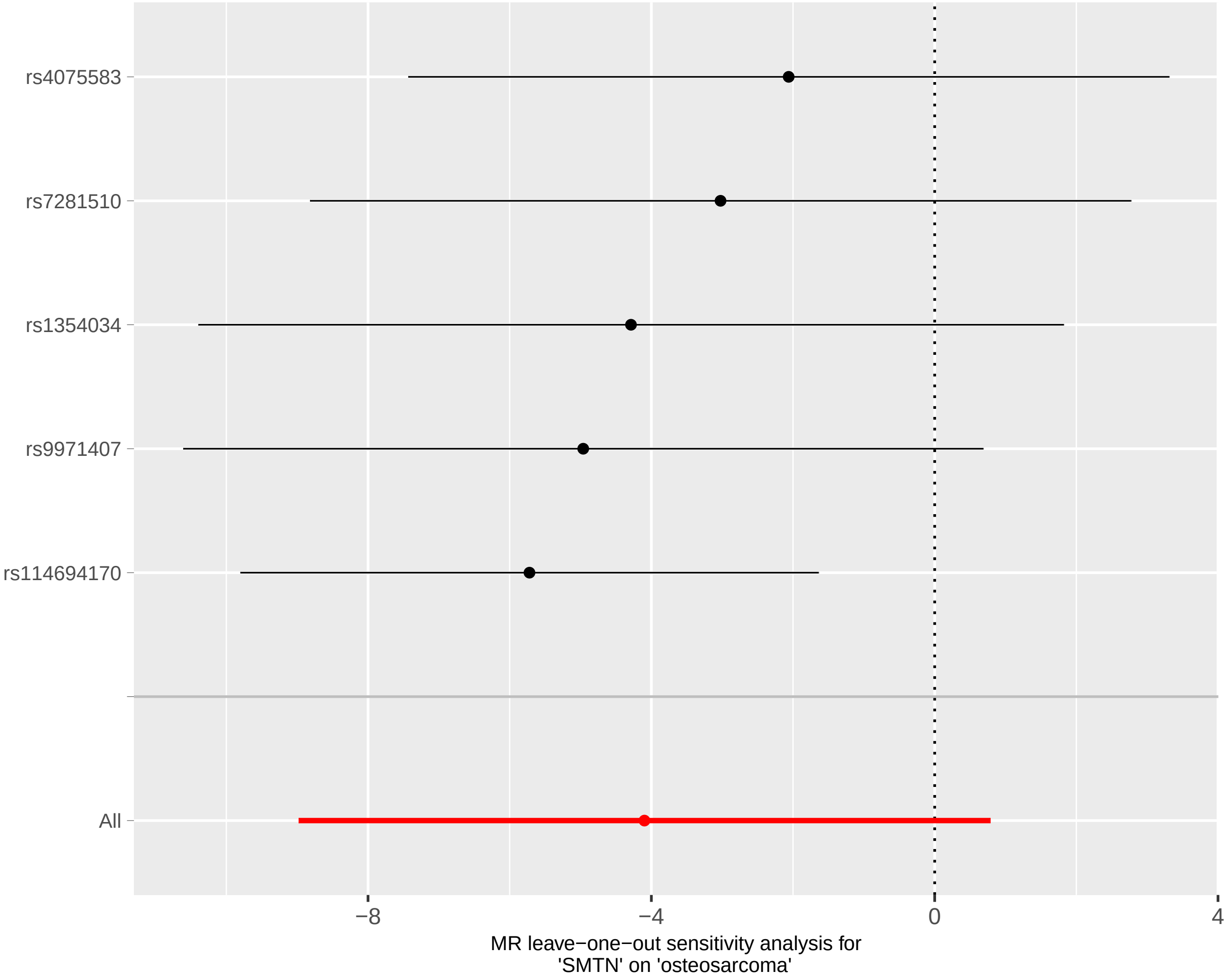
rs4075583
rs7281510
rs1354034
rs9971407
rs114694170
All
−8
−4
0
4
MR leave−one−out sensitivity analysis for
'SMTN' on 'osteosarcoma'

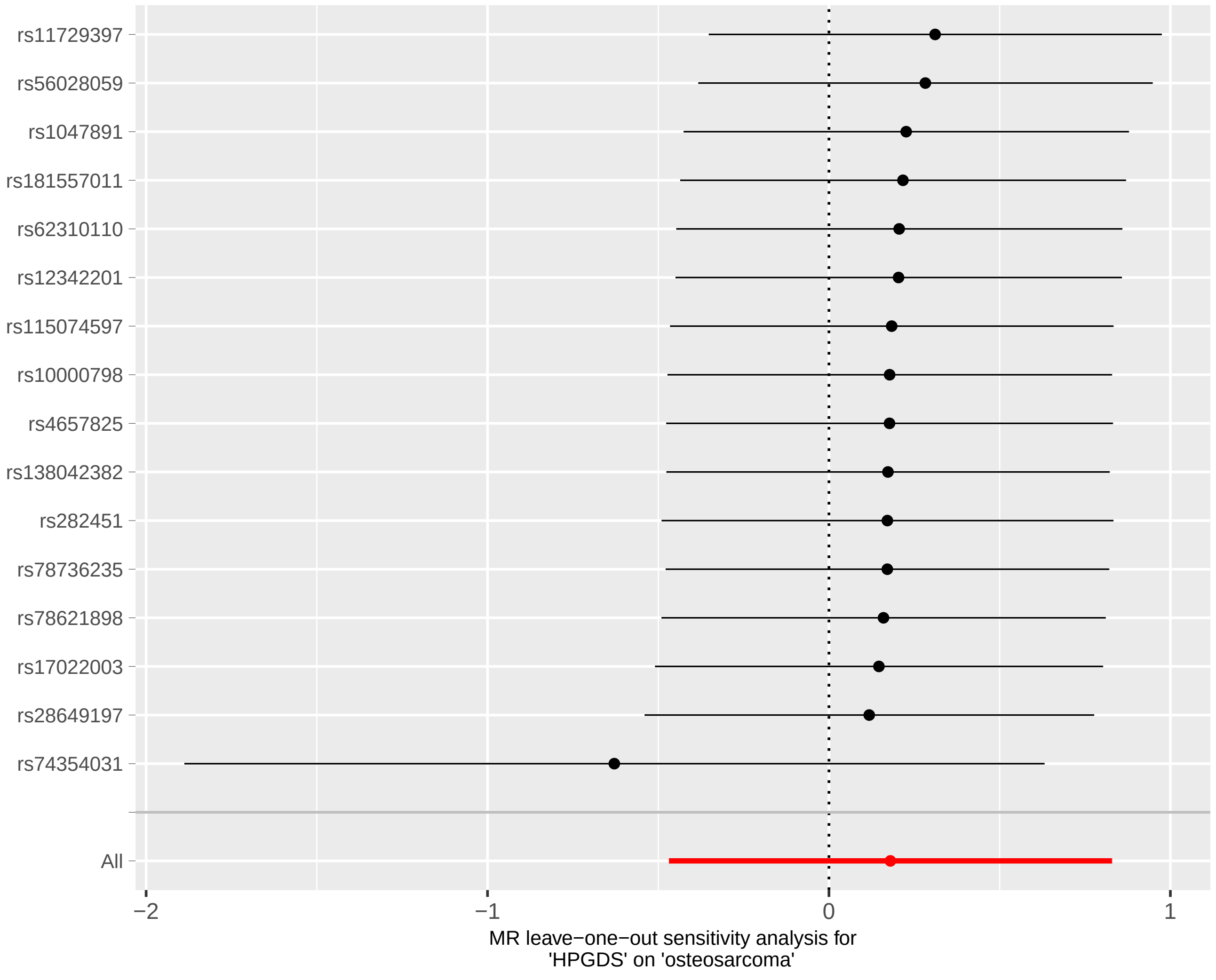
rs11729397
rs56028059
rs1047891
rs181557011
rs62310110
rs12342201
rs115074597
rs10000798
rs4657825
rs138042382
rs282451
rs78736235
rs78621898
rs17022003
rs28649197
rs74354031
All
−2
−1
0
1
MR leave−one−out sensitivity analysis for
'HPGDS' on 'osteosarcoma'

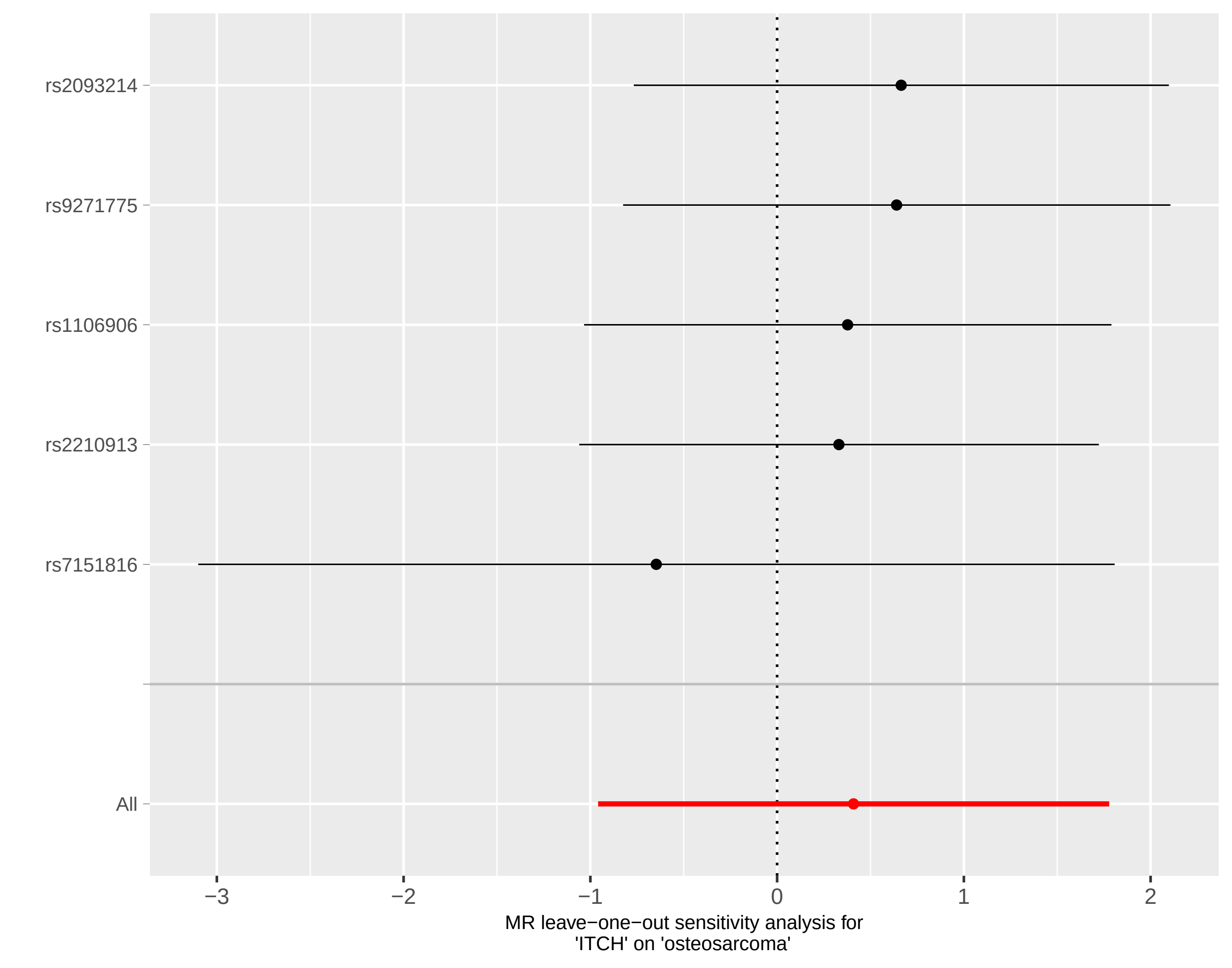
rs2093214
rs9271775
rs1106906
rs2210913
rs7151816
All
−3
−2
−1
0
1
2
MR leave−one−out sensitivity analysis for
'ITCH' on 'osteosarcoma'

Insufficient number of SNPs

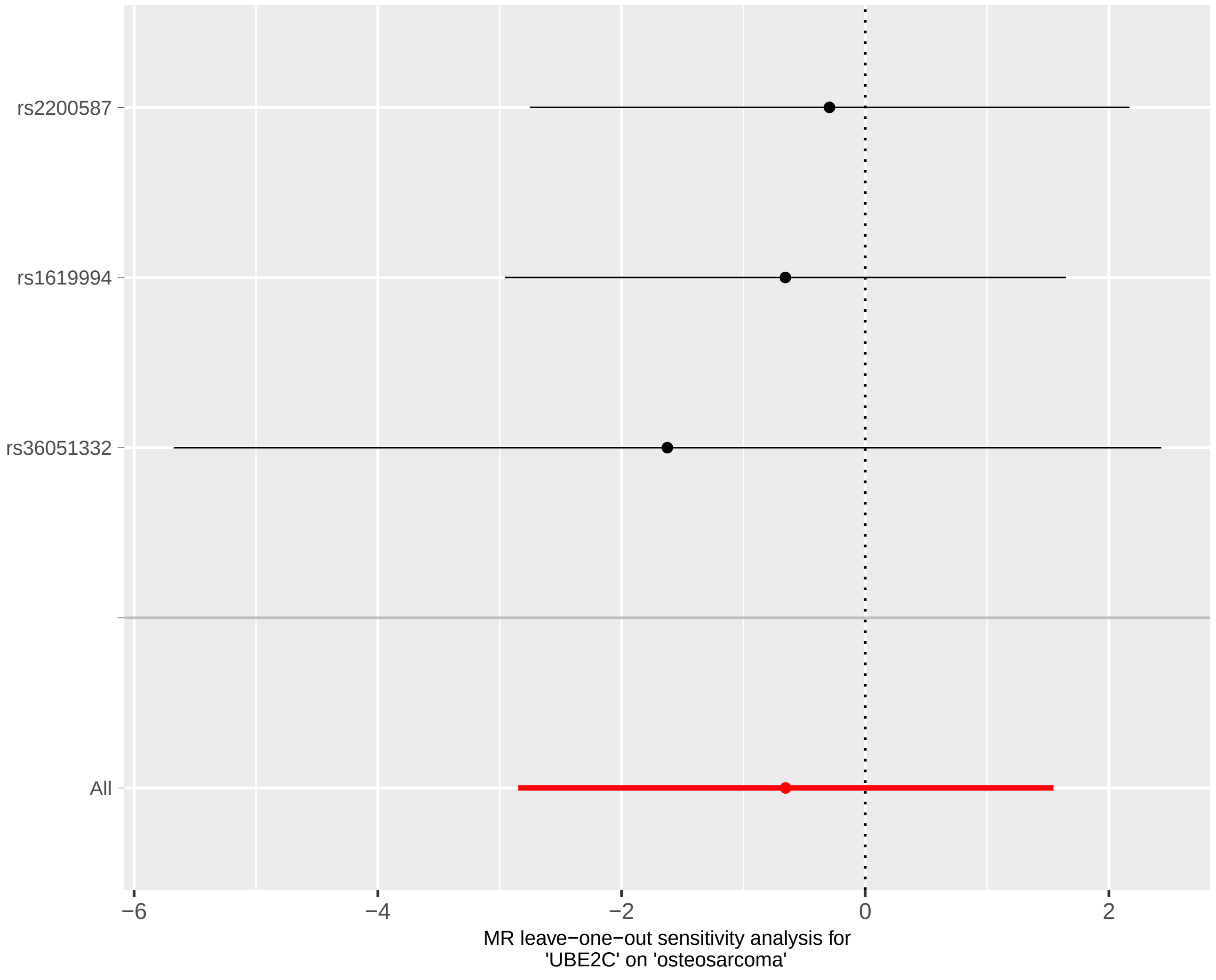

rs2200587
rs1619994
rs36051332
All
−6
−4
−2
0
2
MR leave−one−out sensitivity analysis for
'UBE2C' on 'osteosarcoma'

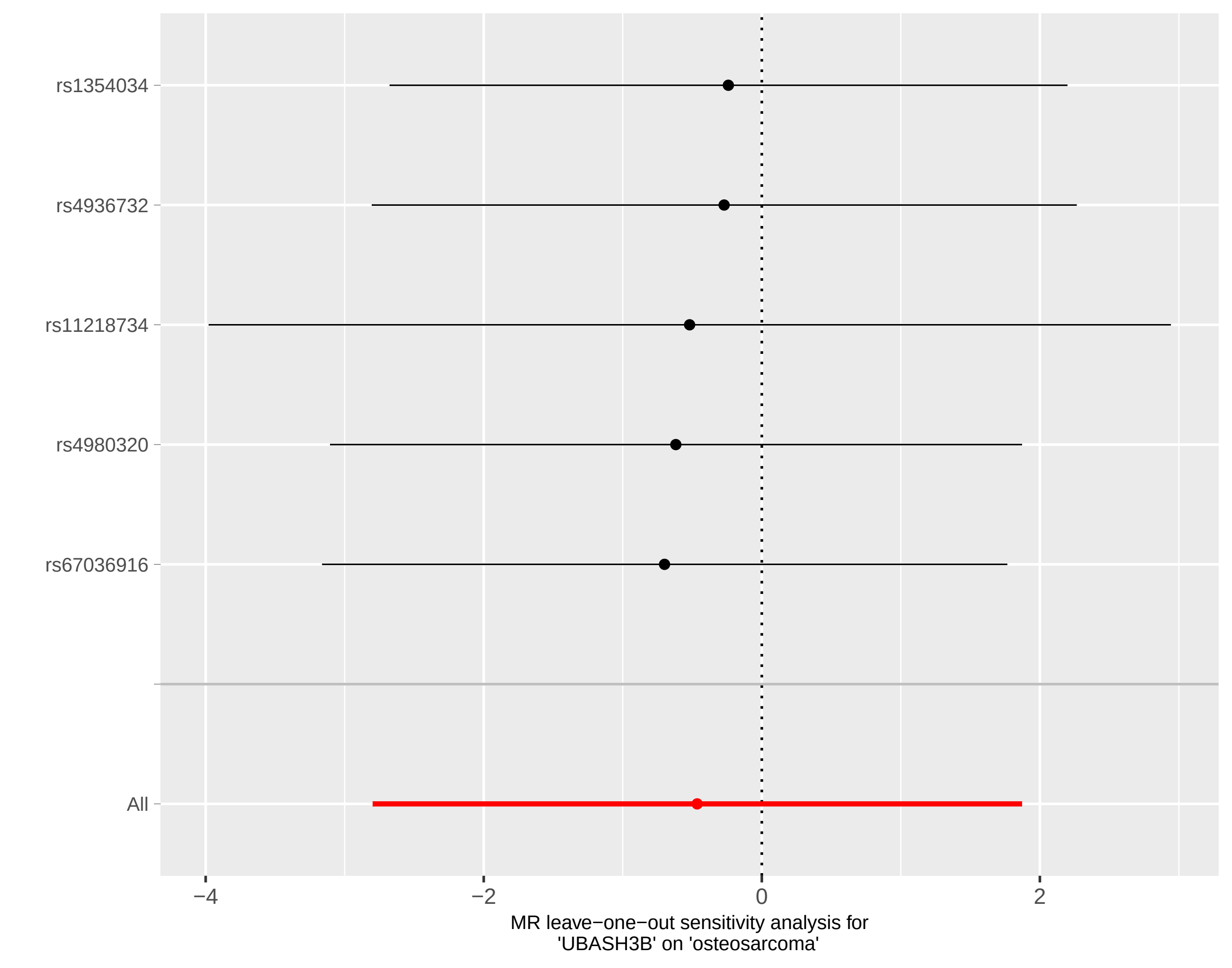
rs1354034
rs4936732
rs11218734
rs4980320
rs67036916
All
−4
−2
0
2
MR leave−one−out sensitivity analysis for
'UBASH3B' on 'osteosarcoma'

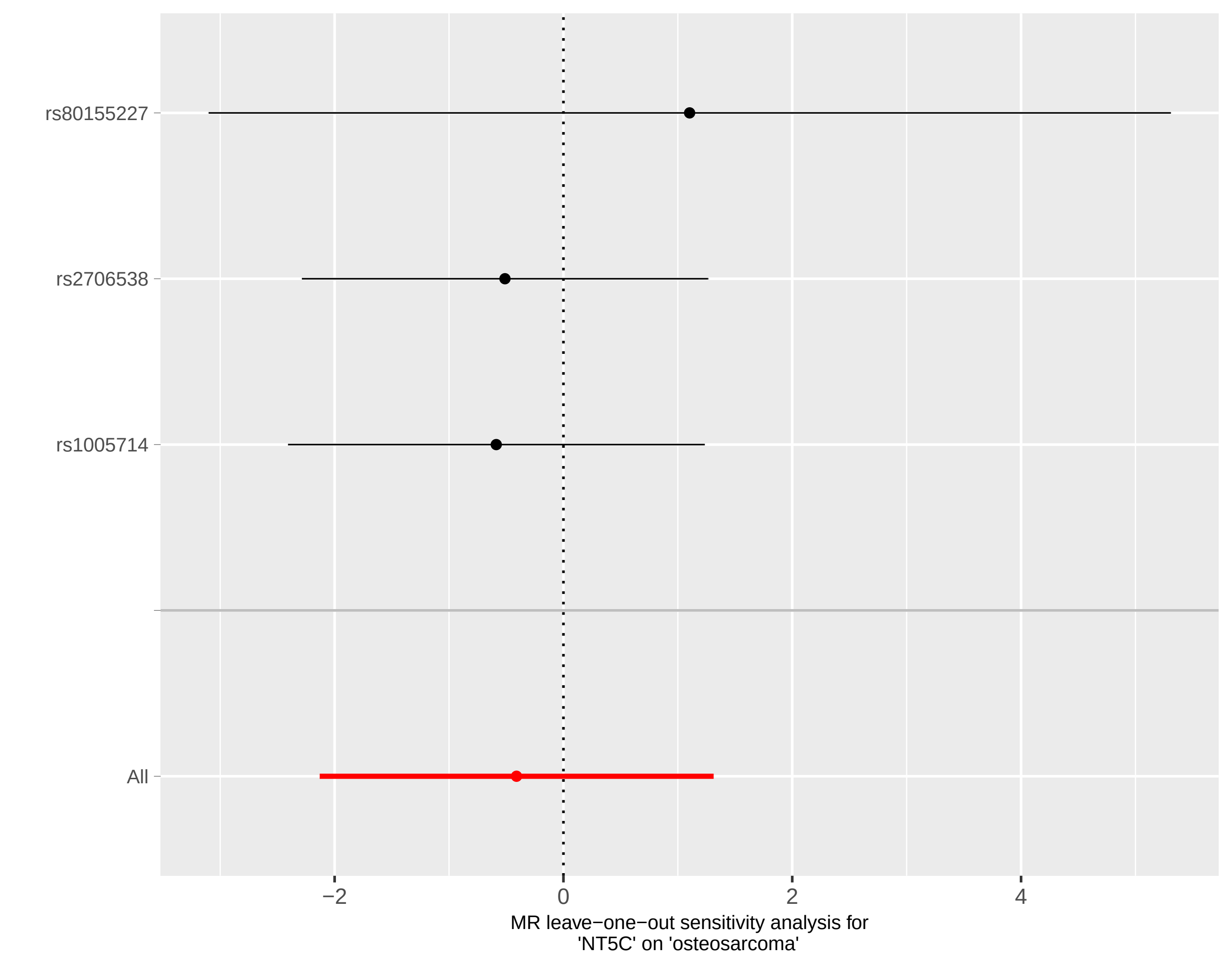
rs80155227
rs2706538
rs1005714
All
−2
0
2
4
MR leave−one−out sensitivity analysis for
'NT5C' on 'osteosarcoma'

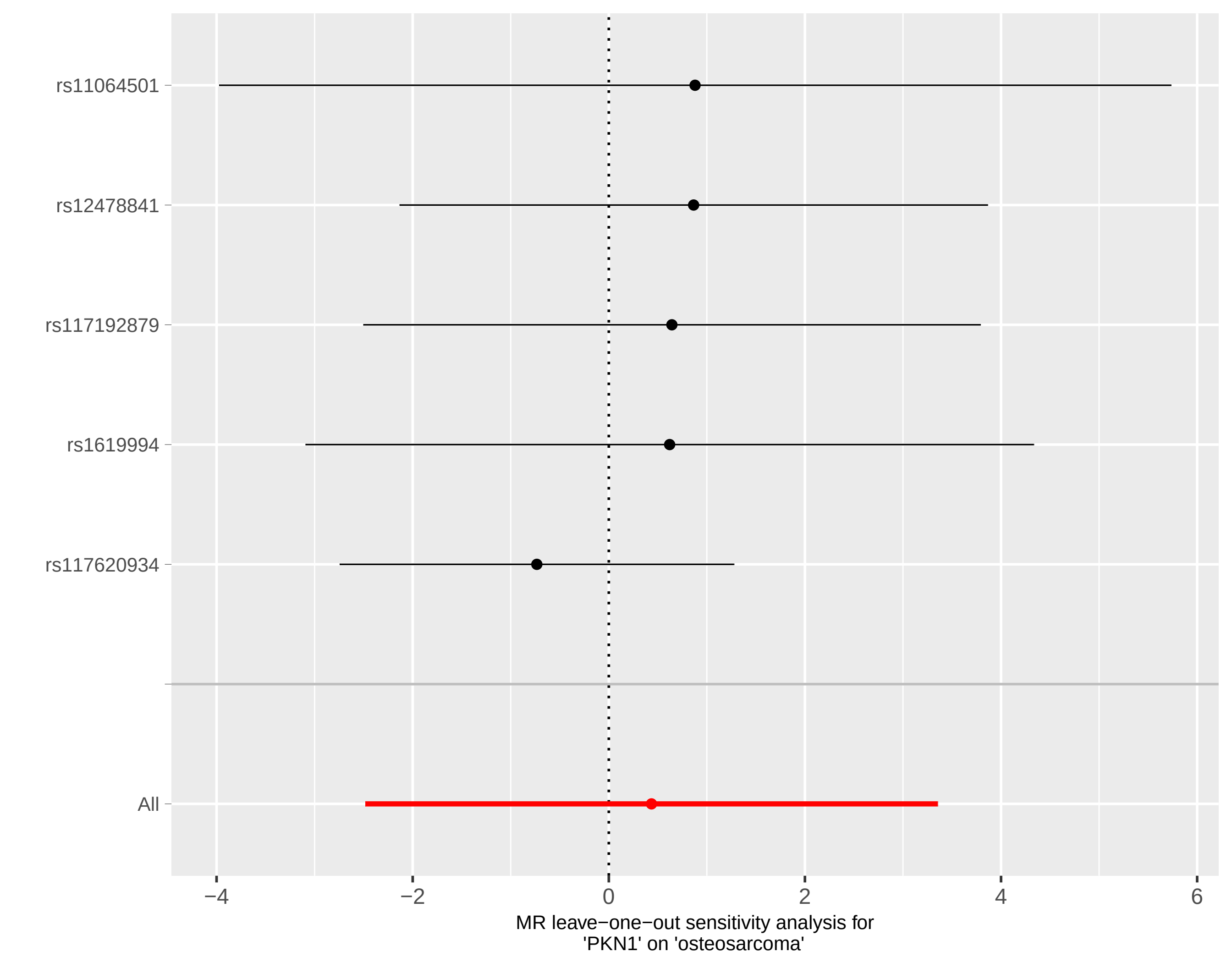

rs11064501
rs12478841
rs117192879
rs1619994
rs117620934
All
−4
−2
0
2
4
6
MR leave−one−out sensitivity analysis for
'PKN1' on 'osteosarcoma'

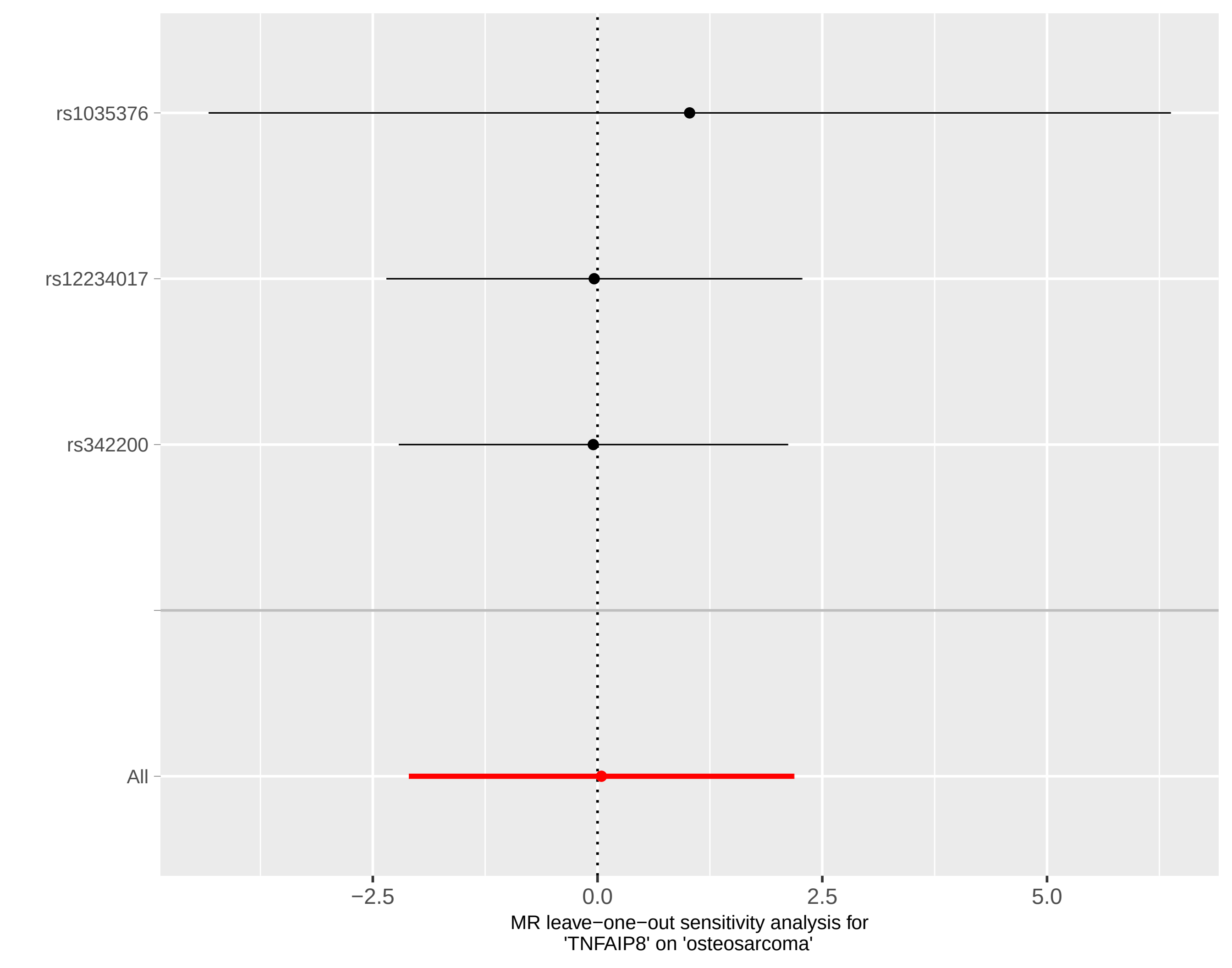
rs1035376
rs12234017
rs342200
All
−2.5
0.0
2.5
5.0
MR leave−one−out sensitivity analysis for
'TNFAIP8' on 'osteosarcoma'

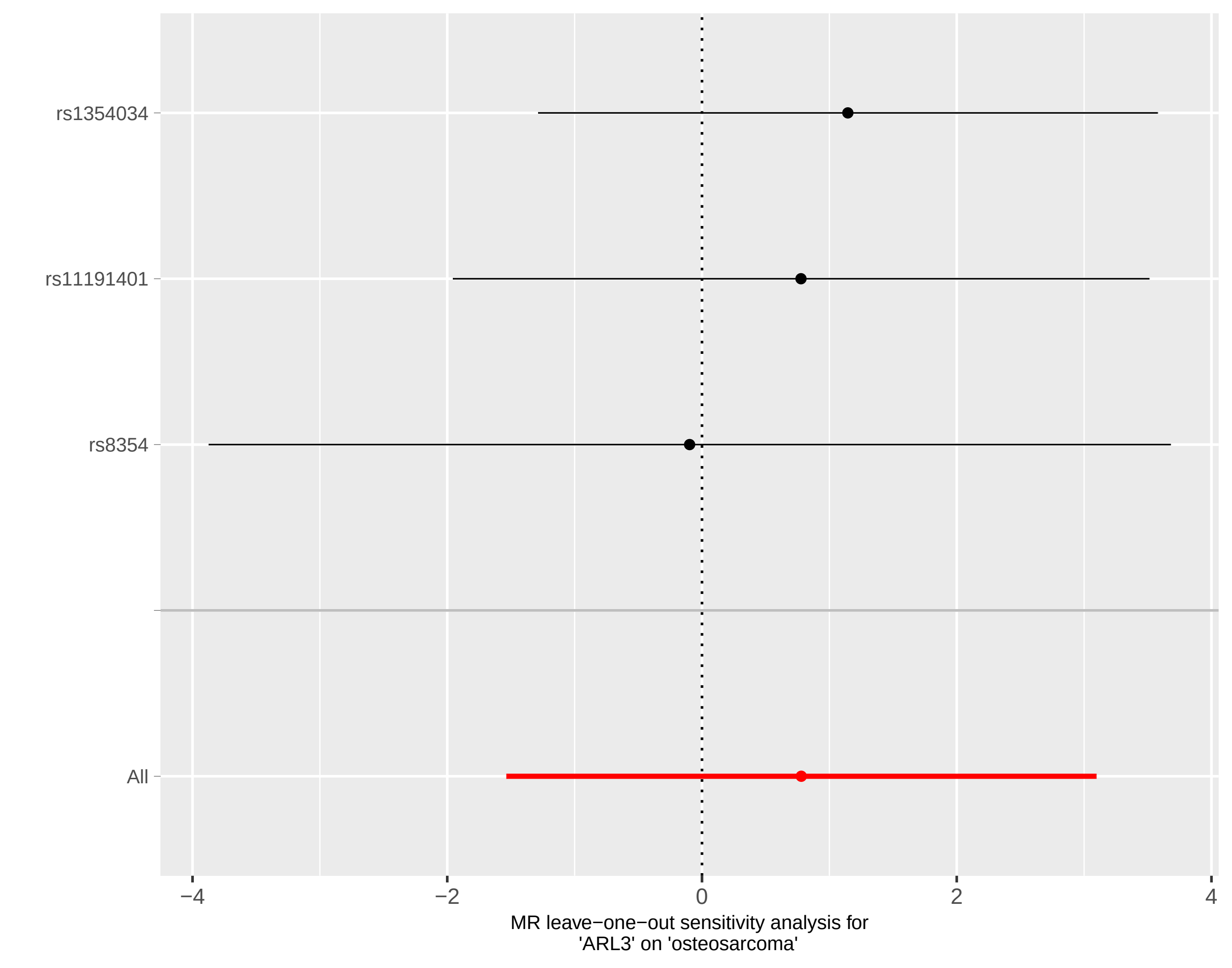

rs1354034
rs11191401
rs8354
All
−4
−2
0
2
4
MR leave−one−out sensitivity analysis for
'ARL3' on 'osteosarcoma'

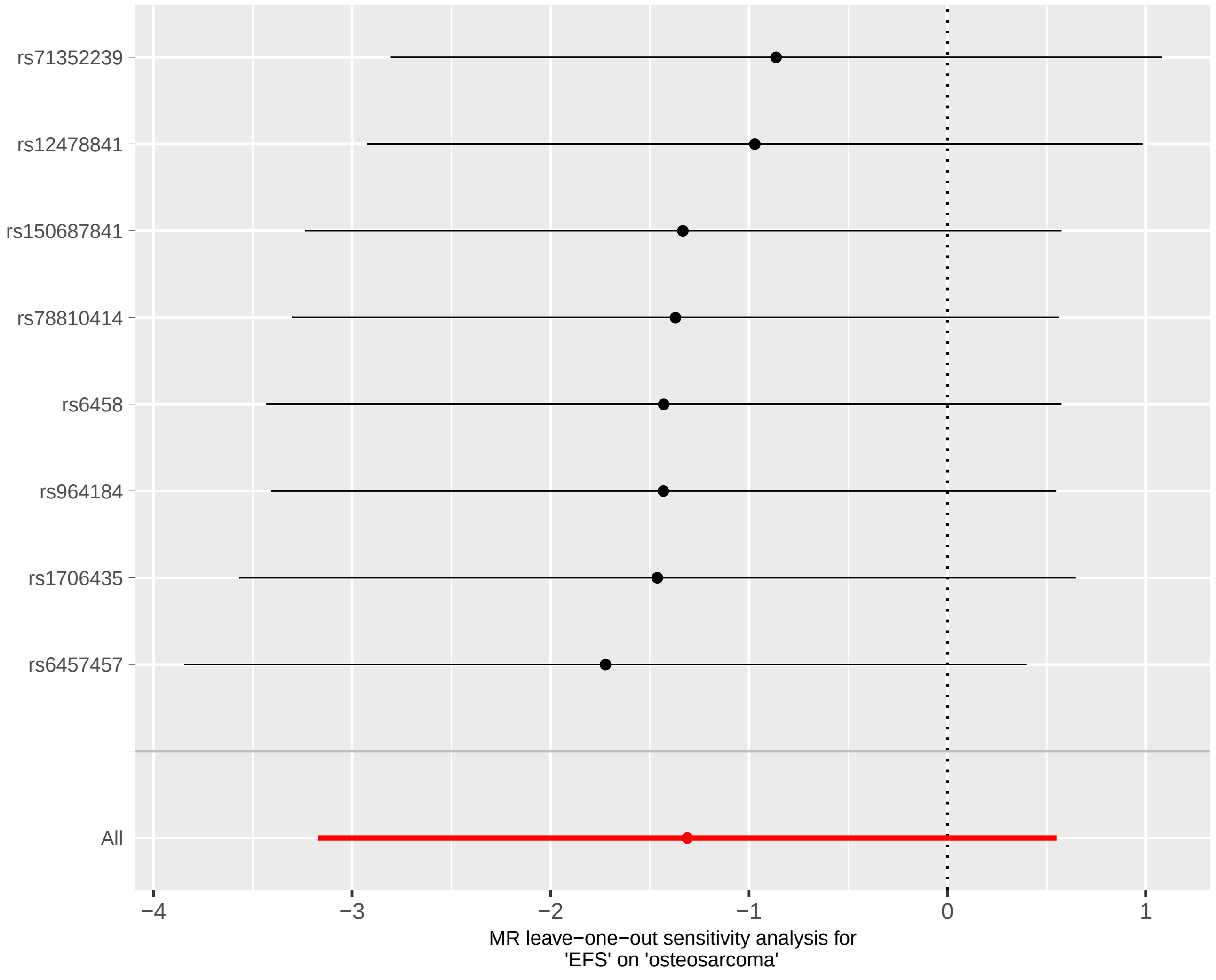

rs71352239
rs12478841
rs150687841
rs78810414
rs6458
rs964184
rs1706435
rs6457457
All
−4
−3
−2
−1
0
1
MR leave−one−out sensitivity analysis for
'EFS' on 'osteosarcoma'

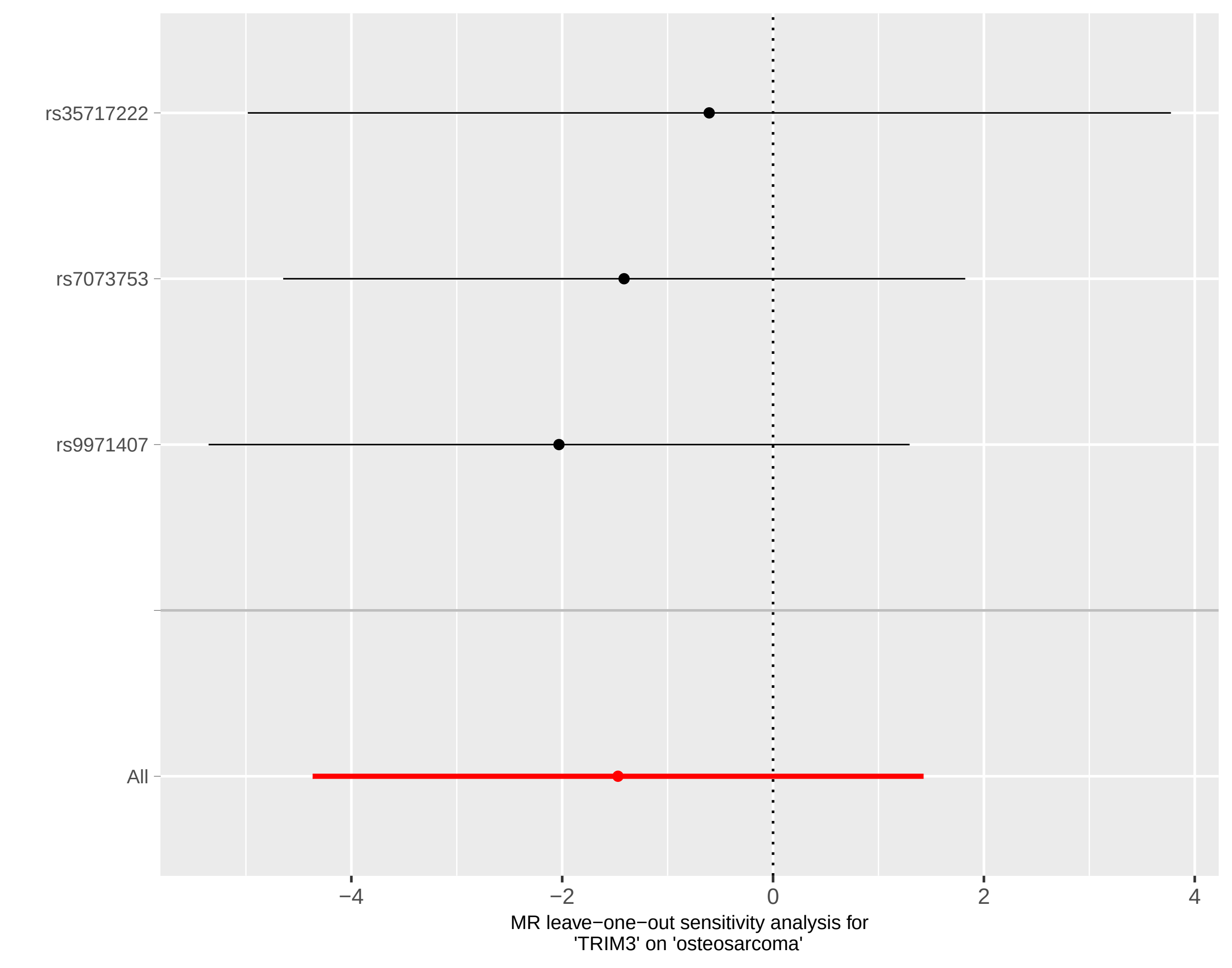

rs35717222
rs7073753
rs9971407
All
−4
−2
0
2
4
MR leave−one−out sensitivity analysis for
'TRIM3' on 'osteosarcoma'

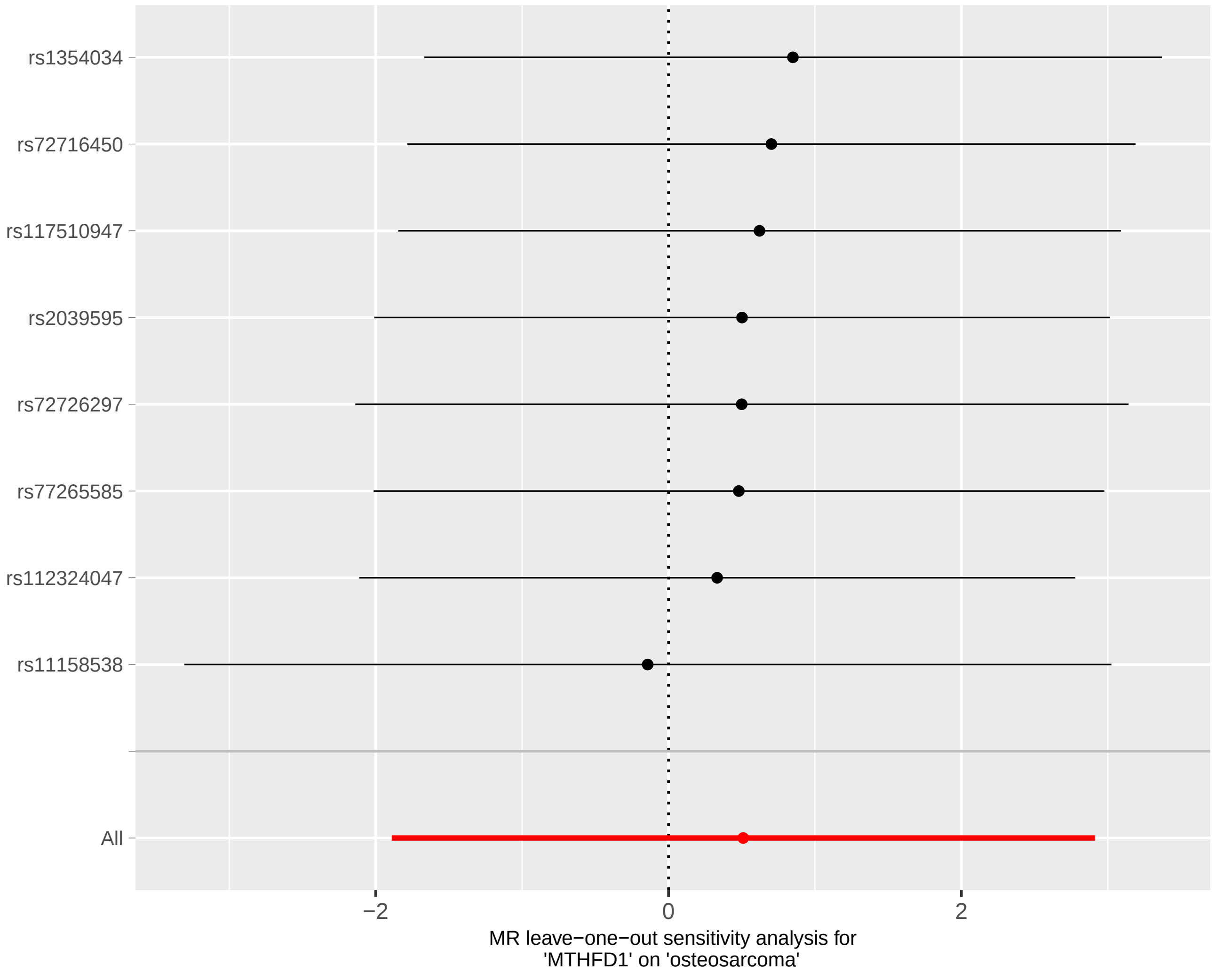
rs1354034
rs72716450
rs117510947
rs2039595
rs72726297
rs77265585
rs112324047
rs11158538
All
−2
0
2
MR leave−one−out sensitivity analysis for
'MTHFD1' on 'osteosarcoma'

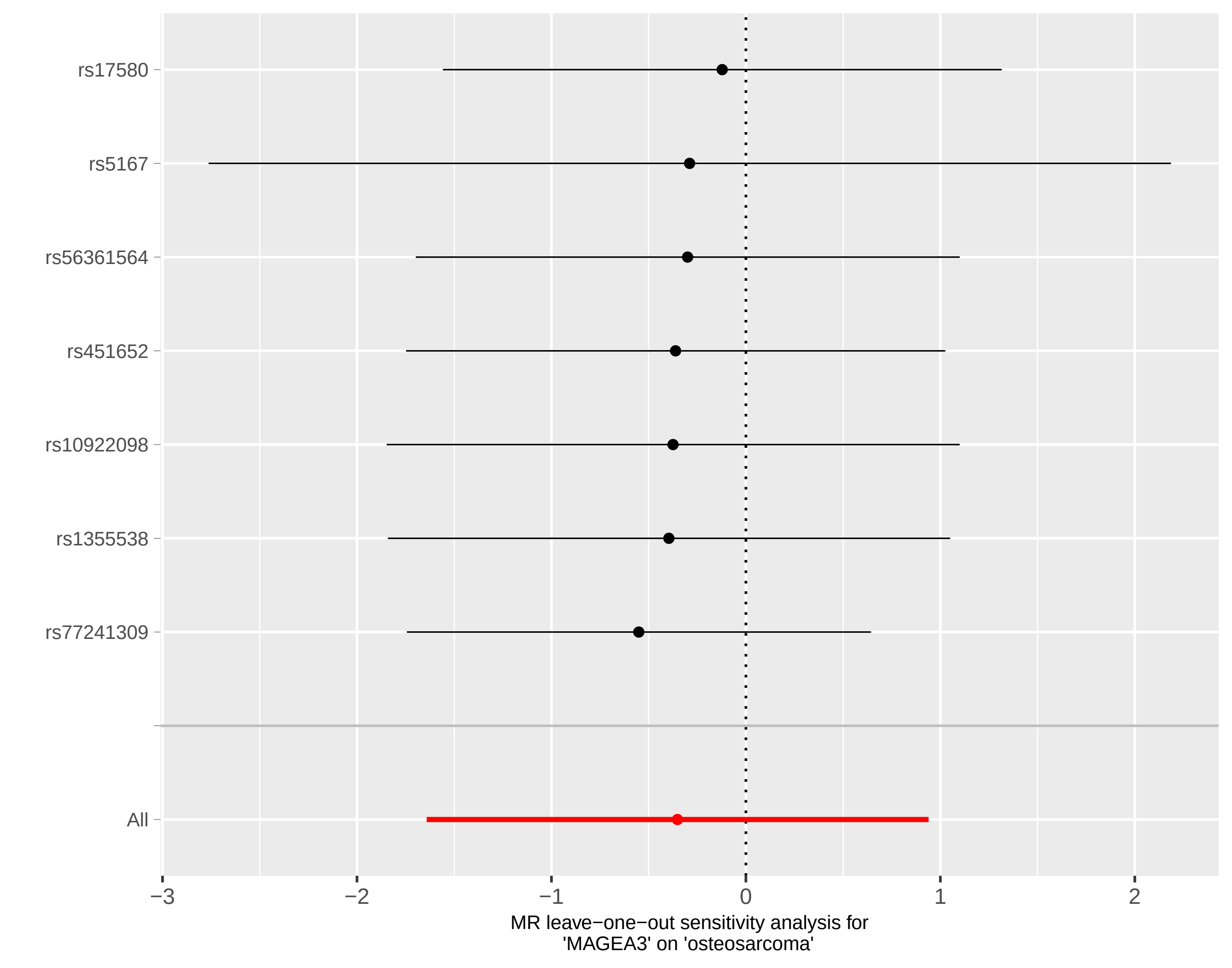

rs17580
rs5167
rs56361564
rs451652
rs10922098
rs1355538
rs77241309
All
−3
−2
−1
0
1
2
MR leave−one−out sensitivity analysis for
'MAGEA3' on 'osteosarcoma'

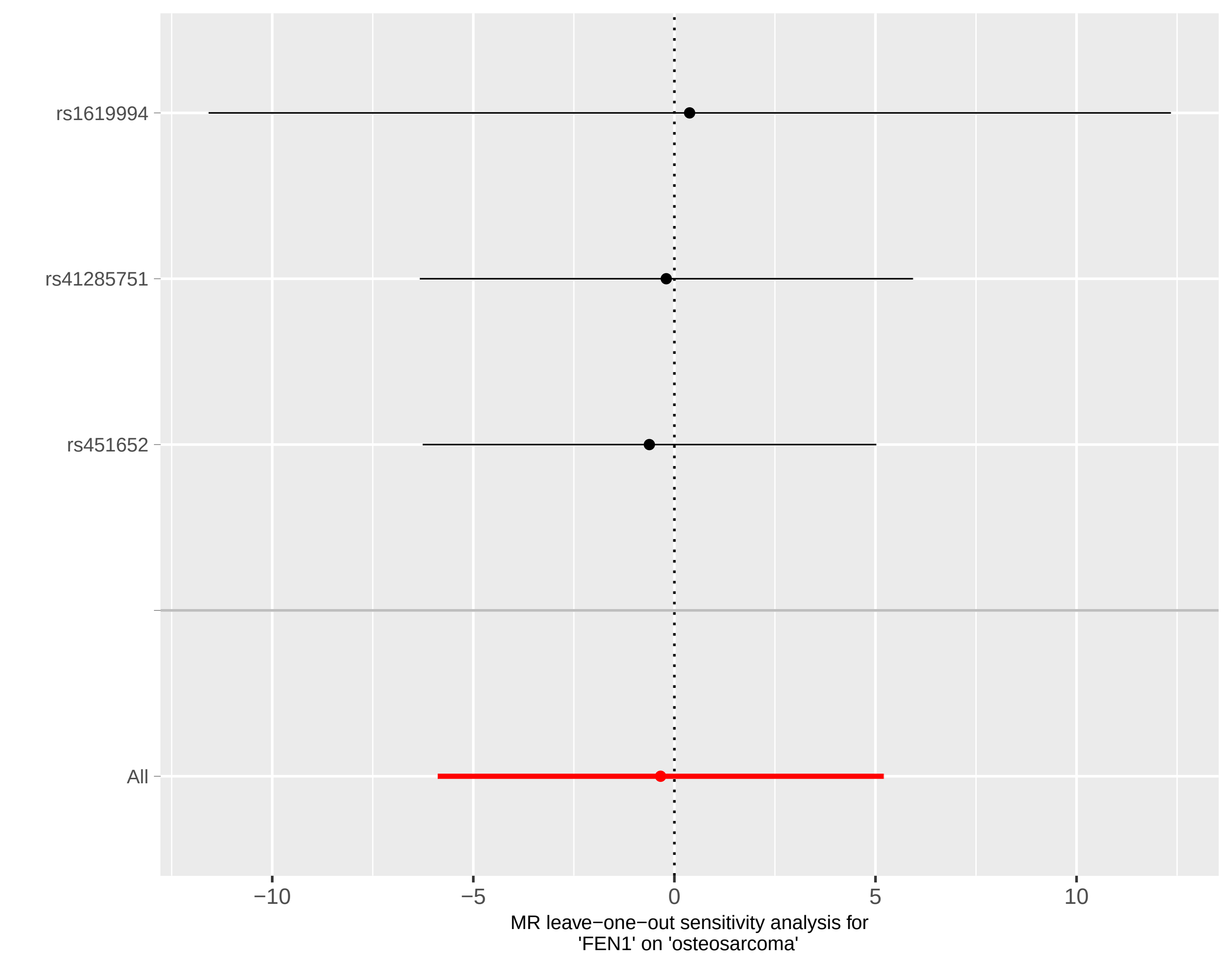
rs1619994
rs41285751
rs451652
All
−10
−5
0
5
10
MR leave−one−out sensitivity analysis for
'FEN1' on 'osteosarcoma'

Insufficient number of SNPs

Insufficient number of SNPs

Insufficient number of SNPs

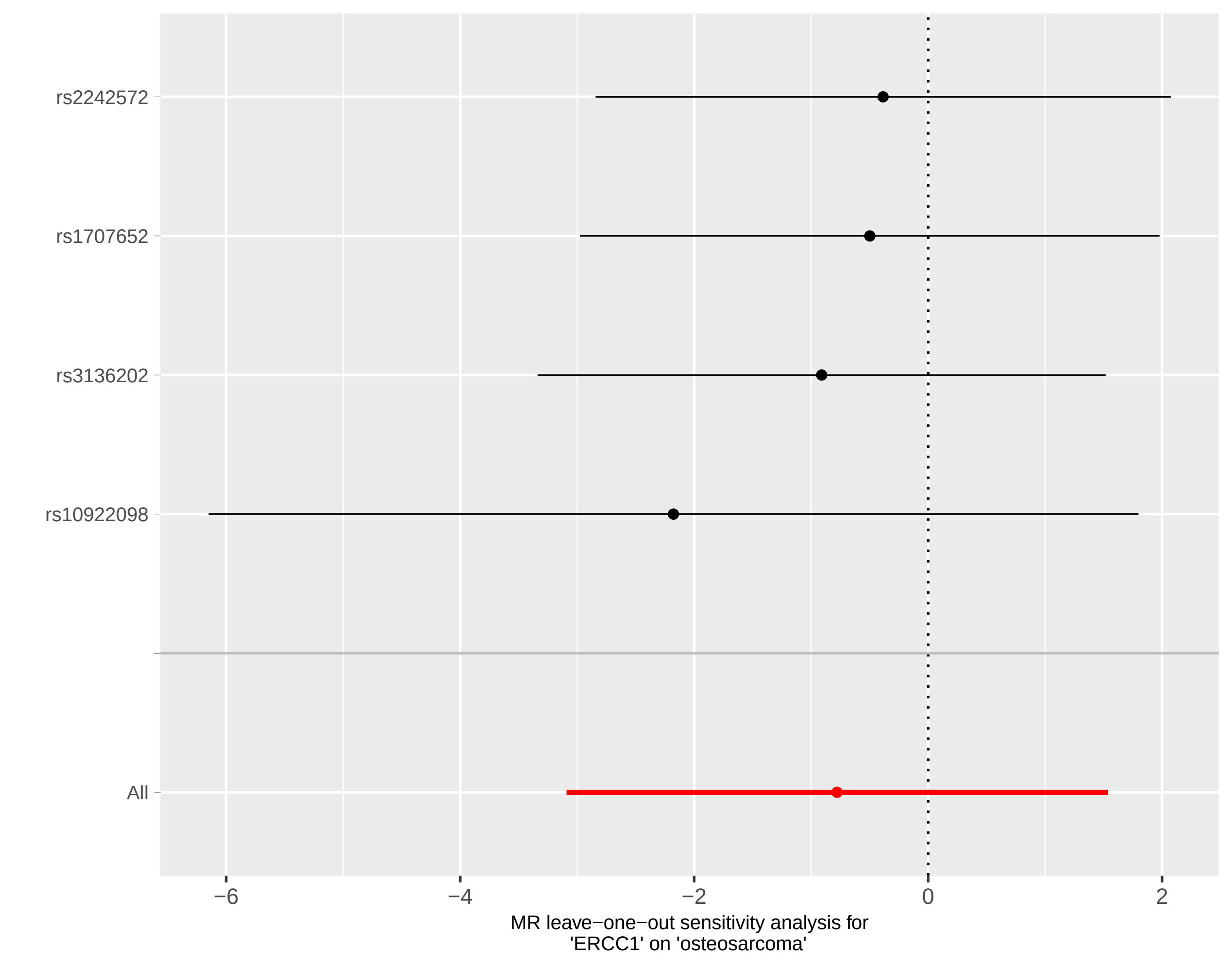

rs2242572
rs1707652
rs3136202
rs10922098
All
−6
−4
−2
0
2
MR leave−one−out sensitivity analysis for
'ERCC1' on 'osteosarcoma'

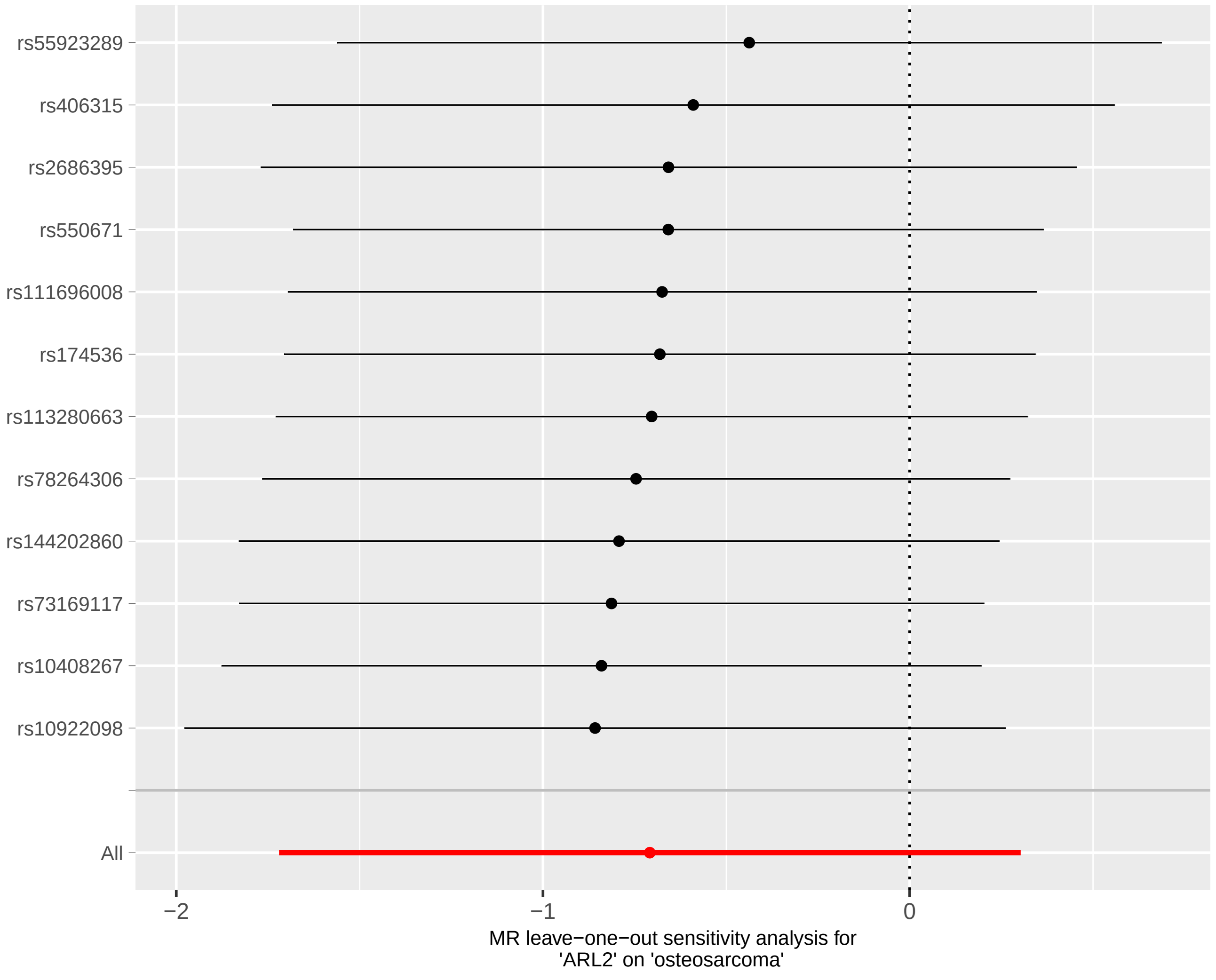

rs55923289
rs406315
rs2686395
rs550671
rs111696008
rs174536
rs113280663
rs78264306
rs144202860
rs73169117
rs10408267
rs10922098
All
−2
−1
0
MR leave−one−out sensitivity analysis for
'ARL2' on 'osteosarcoma'

Insufficient number of SNPs

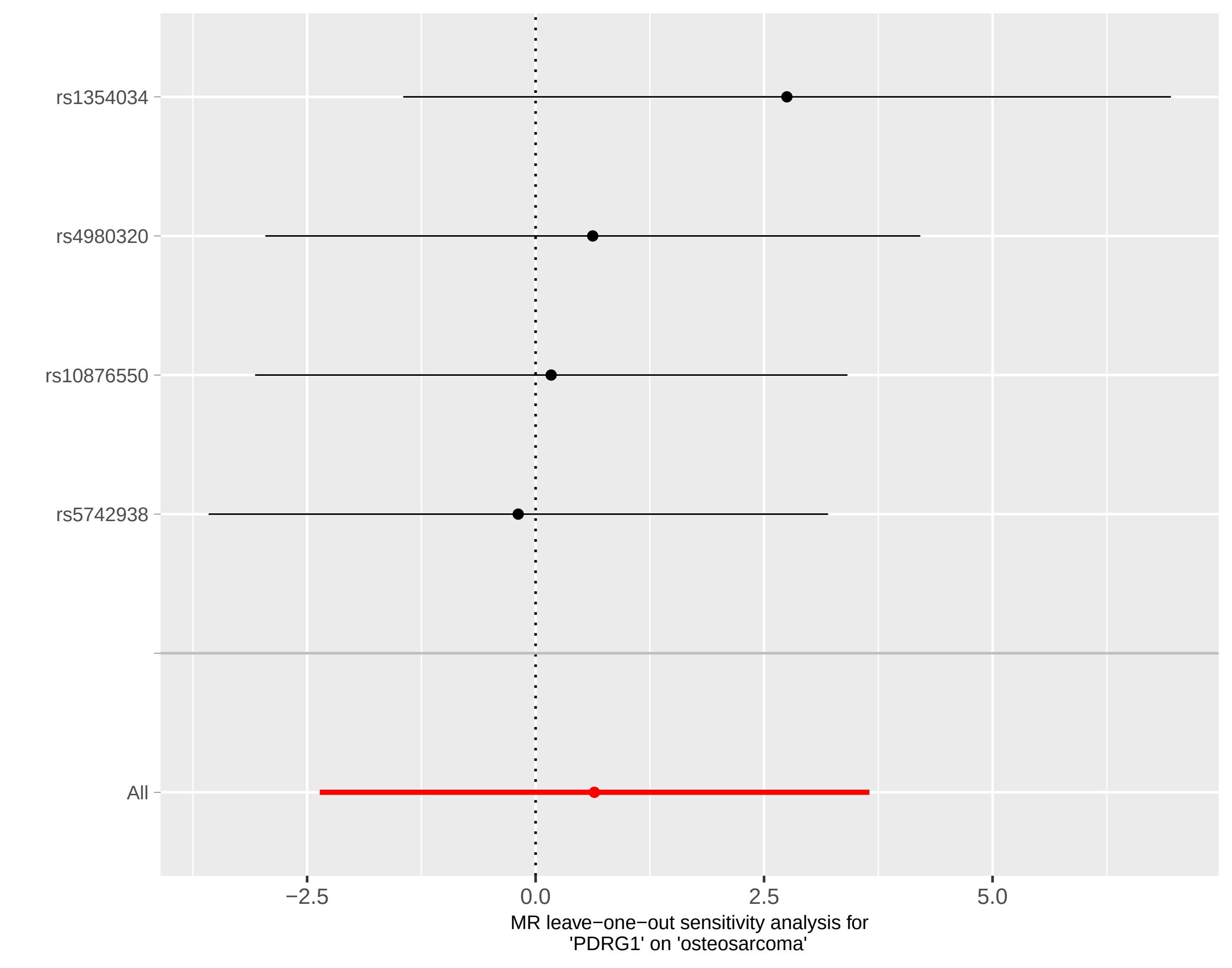

rs1354034
rs4980320
rs10876550
rs5742938
All
−2.5
0.0
2.5
5.0
MR leave−one−out sensitivity analysis for
'PDRG1' on 'osteosarcoma'

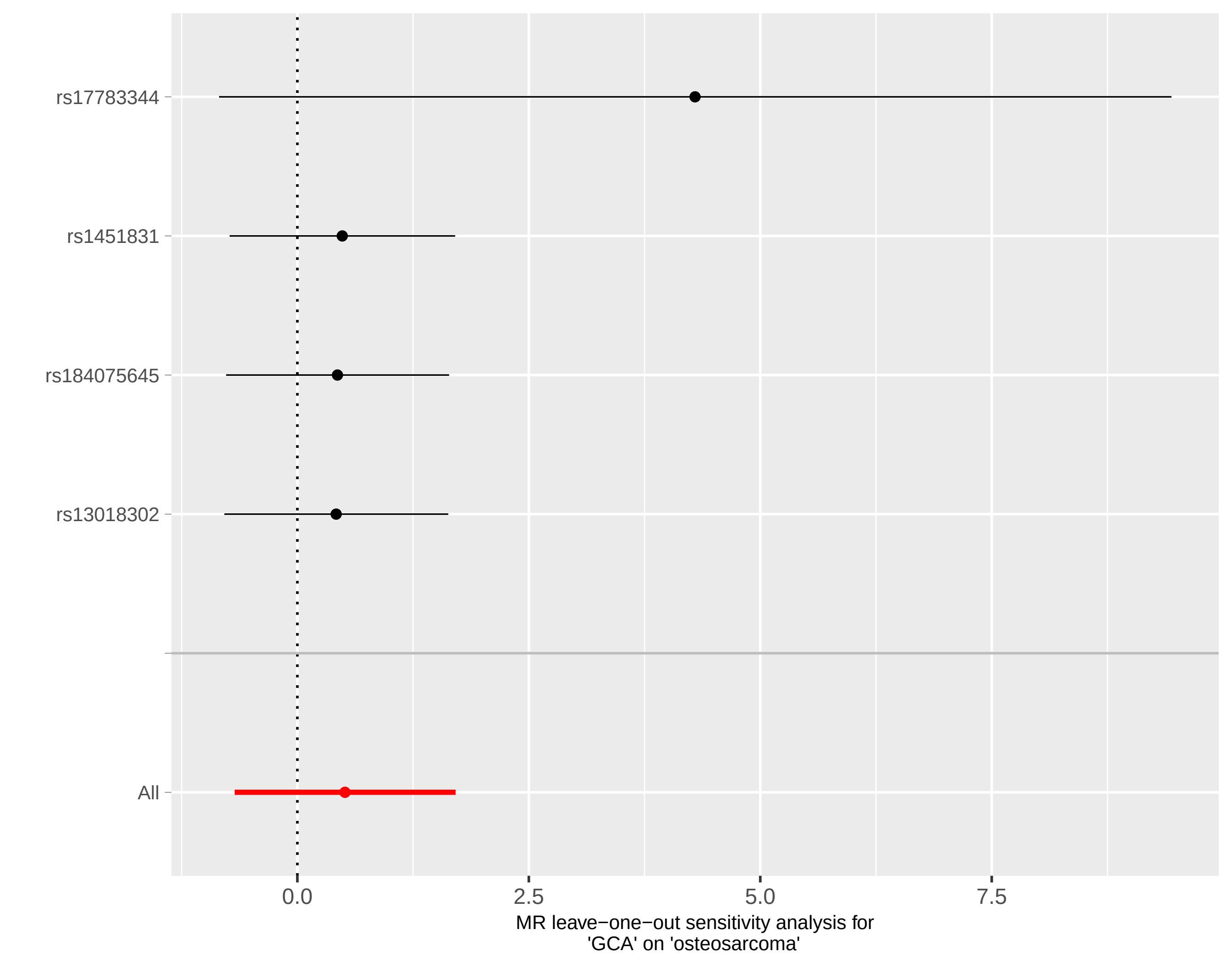
rs17783344
rs1451831
rs184075645
rs13018302
All
0.0
2.5
5.0
7.5
MR leave−one−out sensitivity analysis for
'GCA' on 'osteosarcoma'

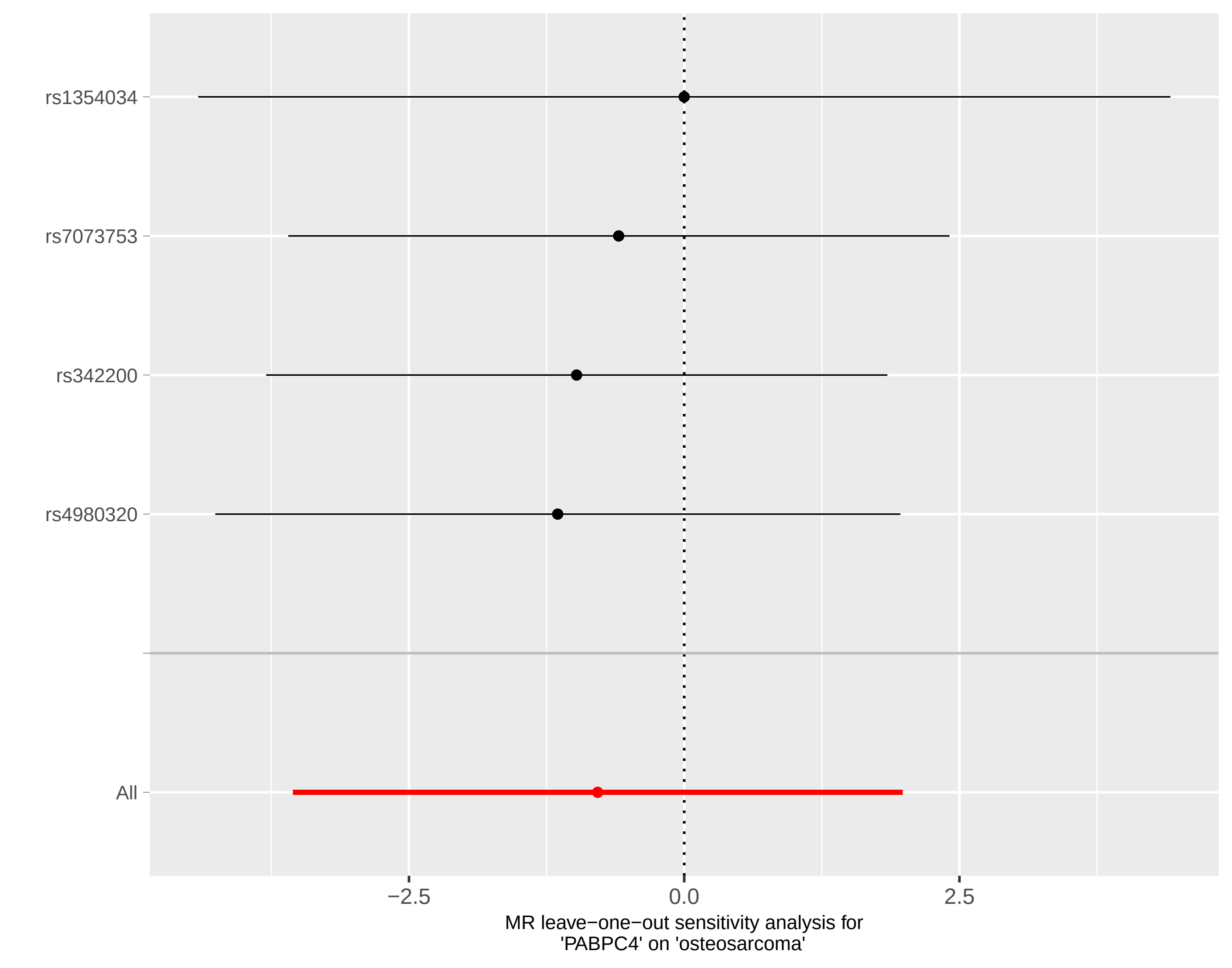

rs1354034
rs7073753
rs342200
rs4980320
All
−2.5
0.0
2.5
MR leave−one−out sensitivity analysis for
'PABPC4' on 'osteosarcoma'

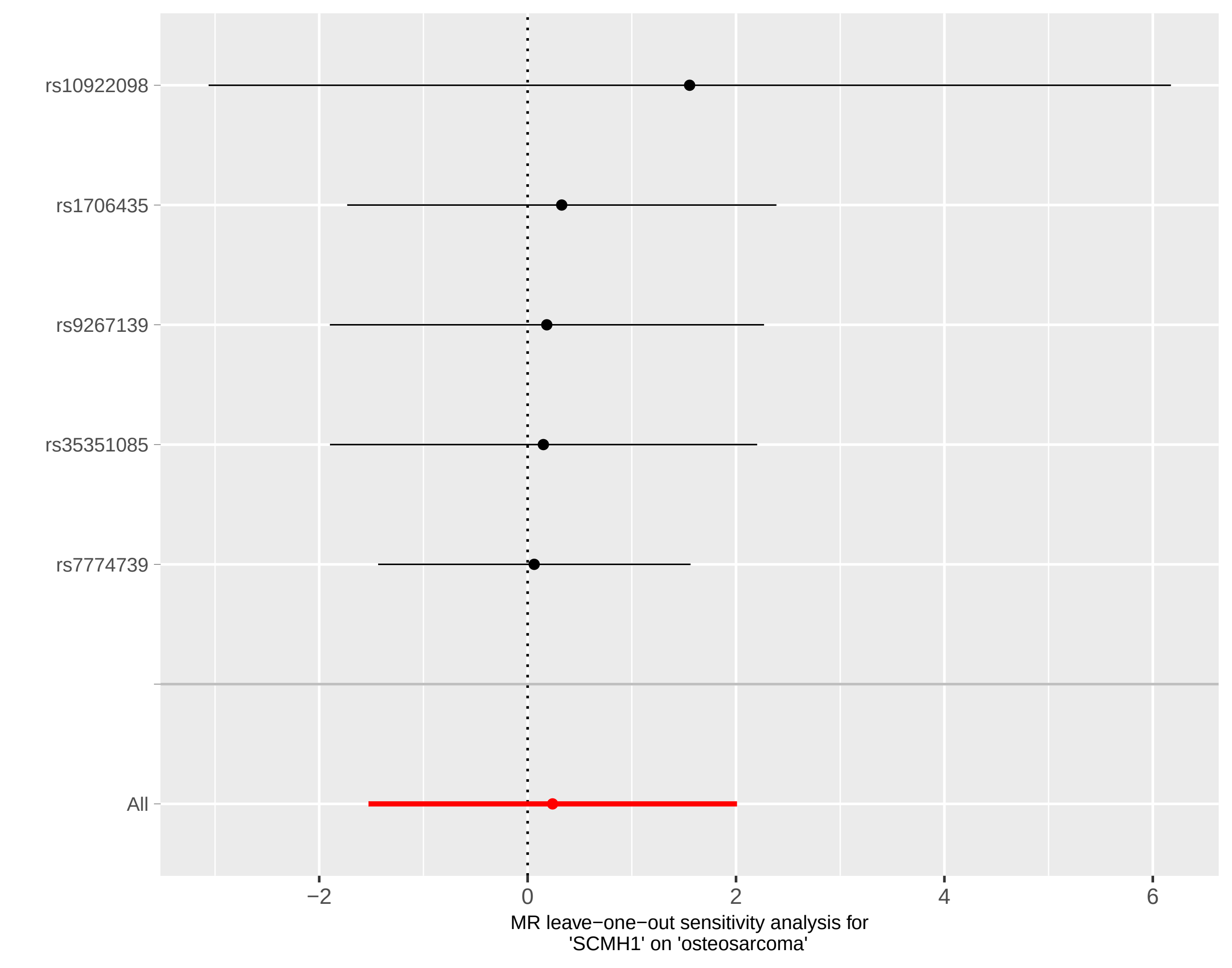
rs10922098
rs1706435
rs9267139
rs35351085
rs7774739
All
−2
0
2
4
6
MR leave−one−out sensitivity analysis for
'SCMH1' on 'osteosarcoma'

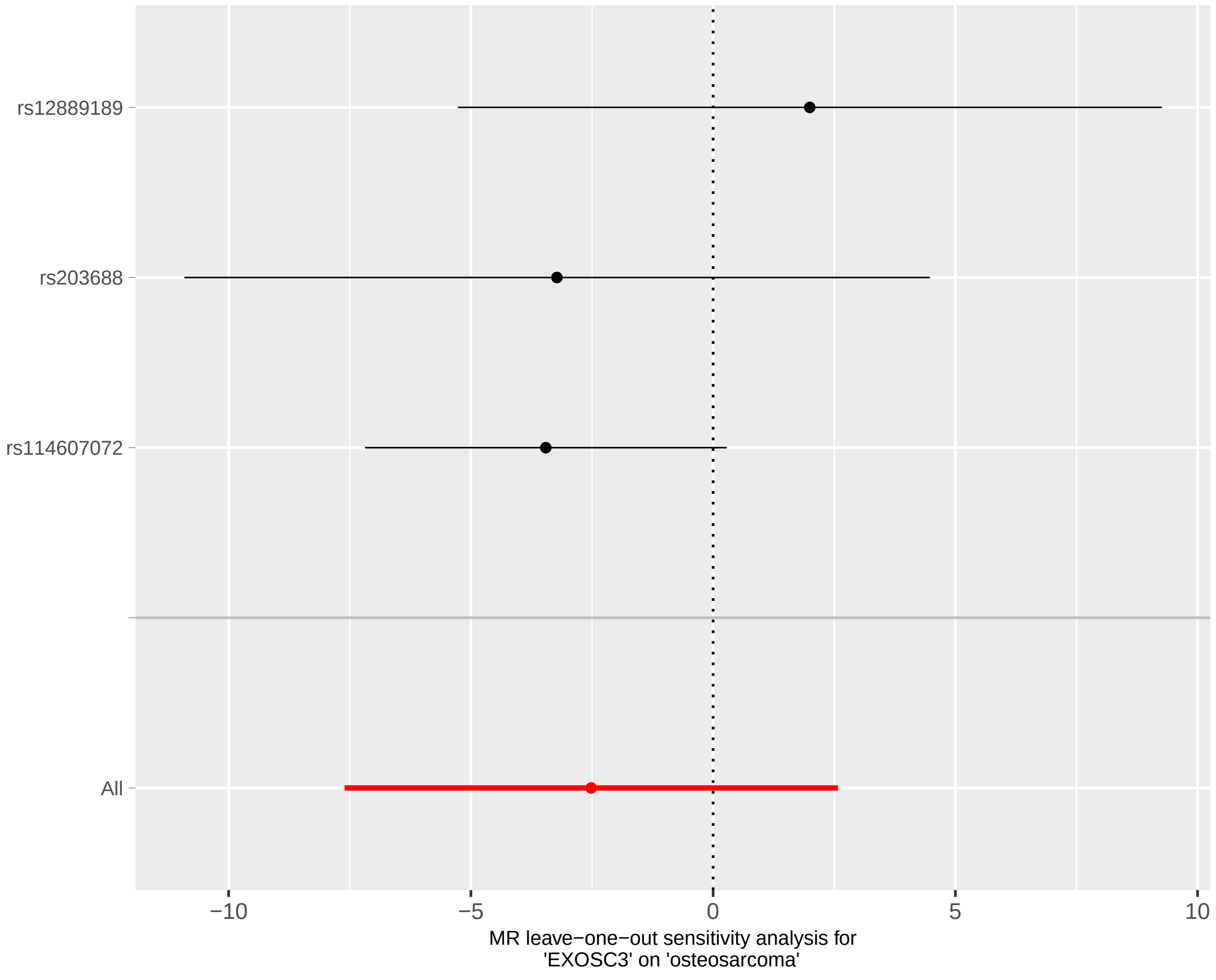
rs12889189
rs203688
rs114607072
All
−10
−5
0
5
10
MR leave−one−out sensitivity analysis for
'EXOSC3' on 'osteosarcoma'

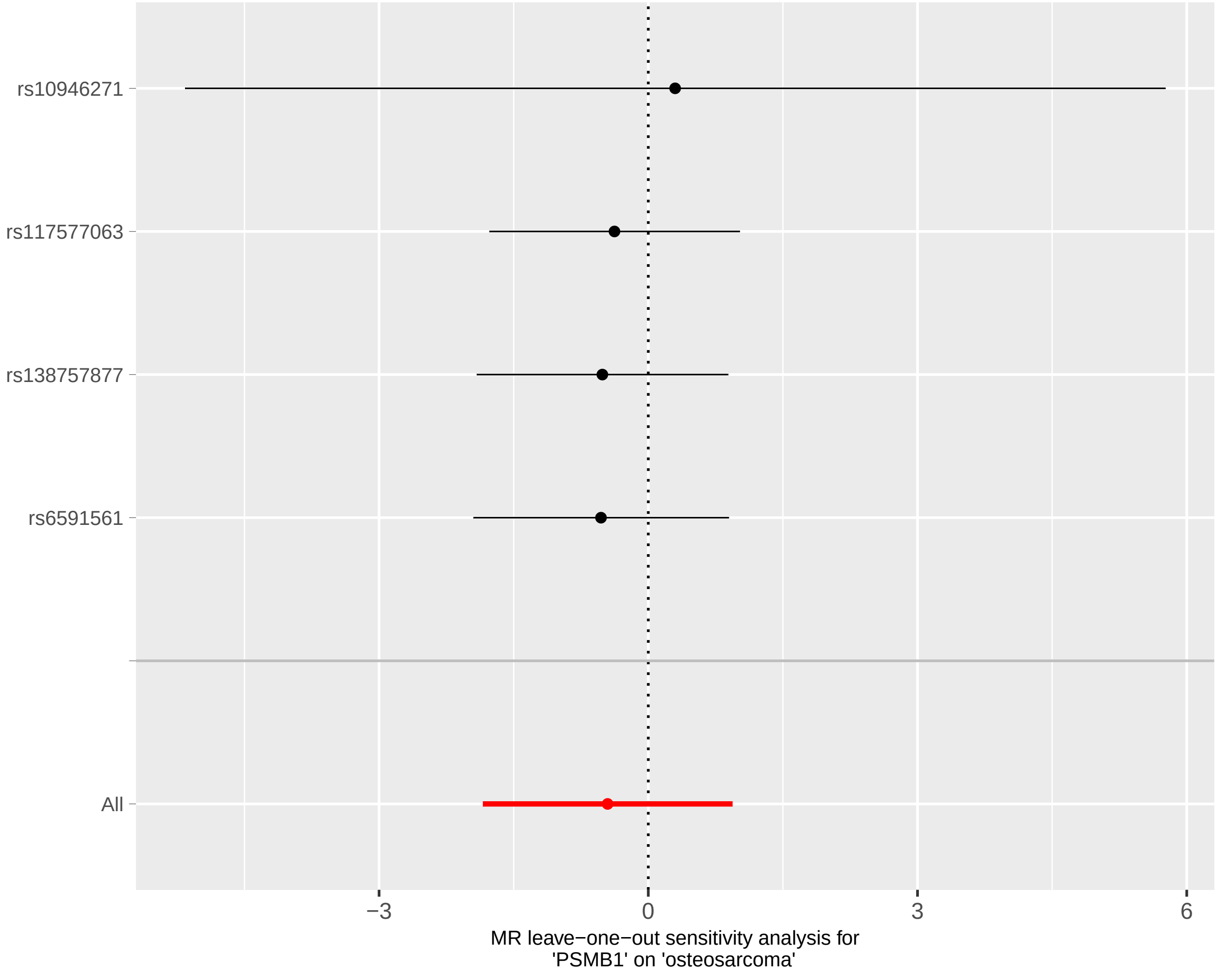

rs10946271
rs117577063
rs138757877
rs6591561
All
−3
0
3
6
MR leave−one−out sensitivity analysis for
'PSMB1' on 'osteosarcoma'

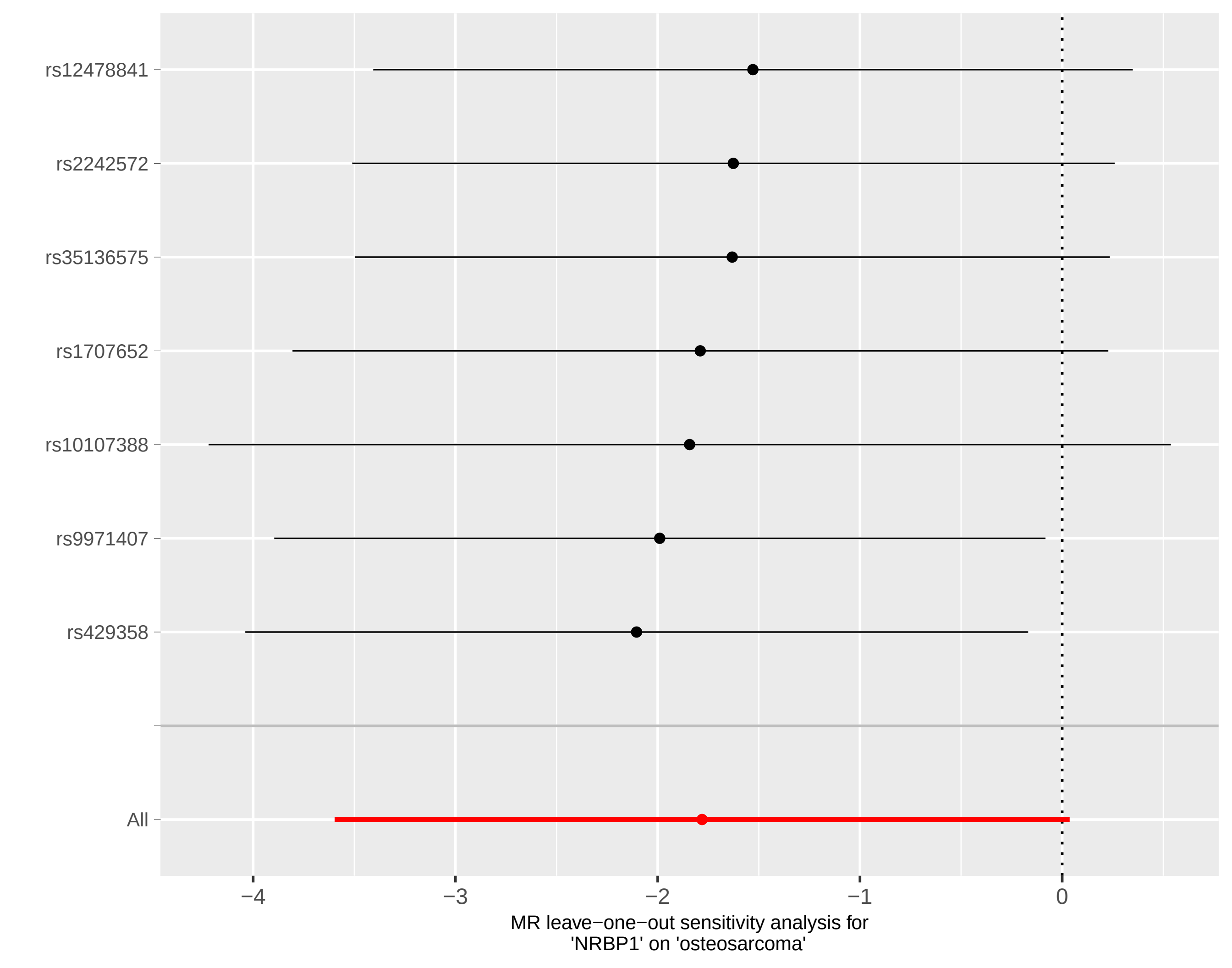
rs12478841
rs2242572
rs35136575
rs1707652
rs10107388
rs9971407
rs429358
All
−4
−3
−2
−1
0
MR leave−one−out sensitivity analysis for
'NRBP1' on 'osteosarcoma'

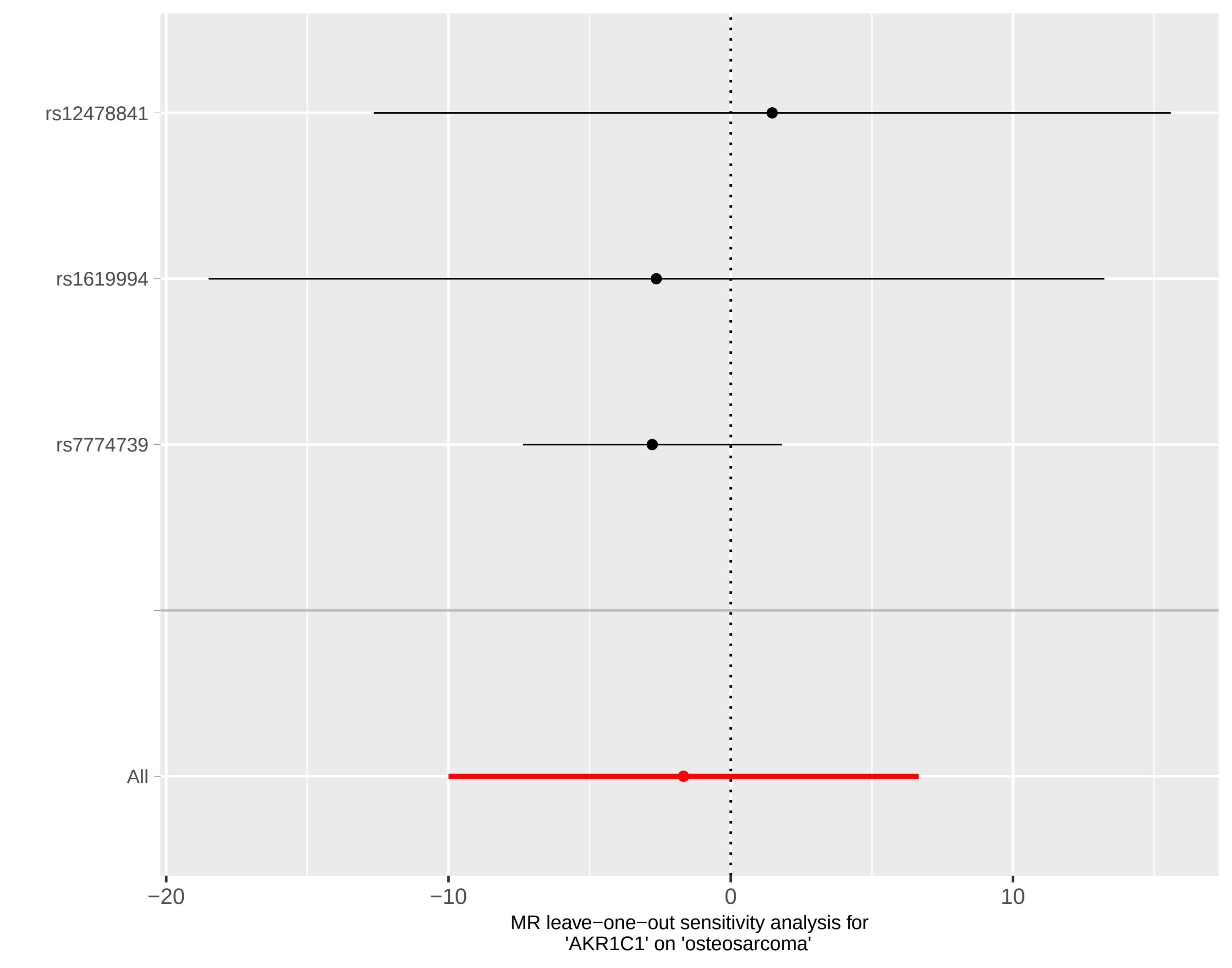

rs12478841
rs1619994
rs7774739
All
−20
−10
0
10
MR leave−one−out sensitivity analysis for
'AKR1C1' on 'osteosarcoma'

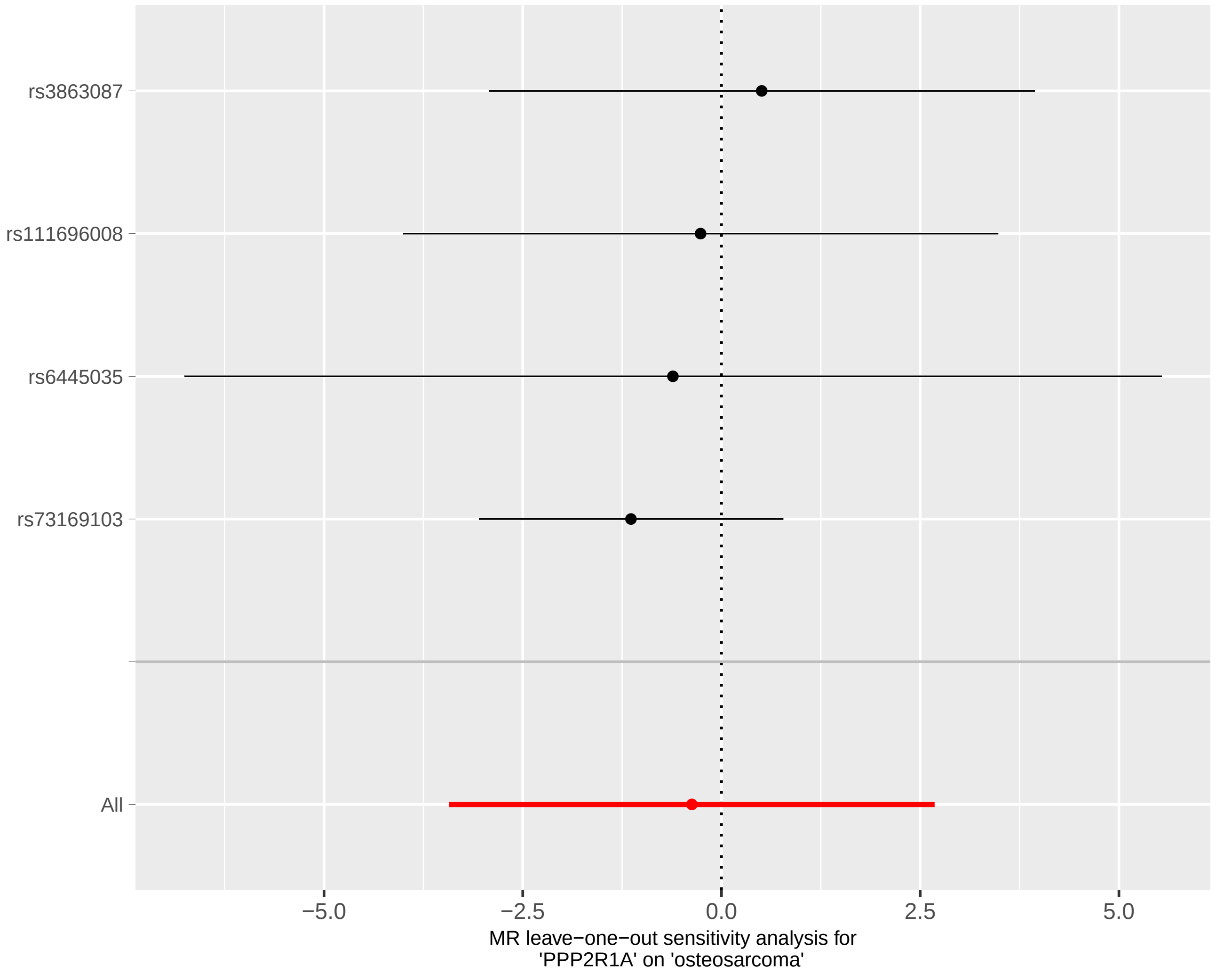
rs3863087
rs111696008
rs6445035
rs73169103
All
−5.0
−2.5
0.0
2.5
5.0
MR leave−one−out sensitivity analysis for
'PPP2R1A' on 'osteosarcoma'

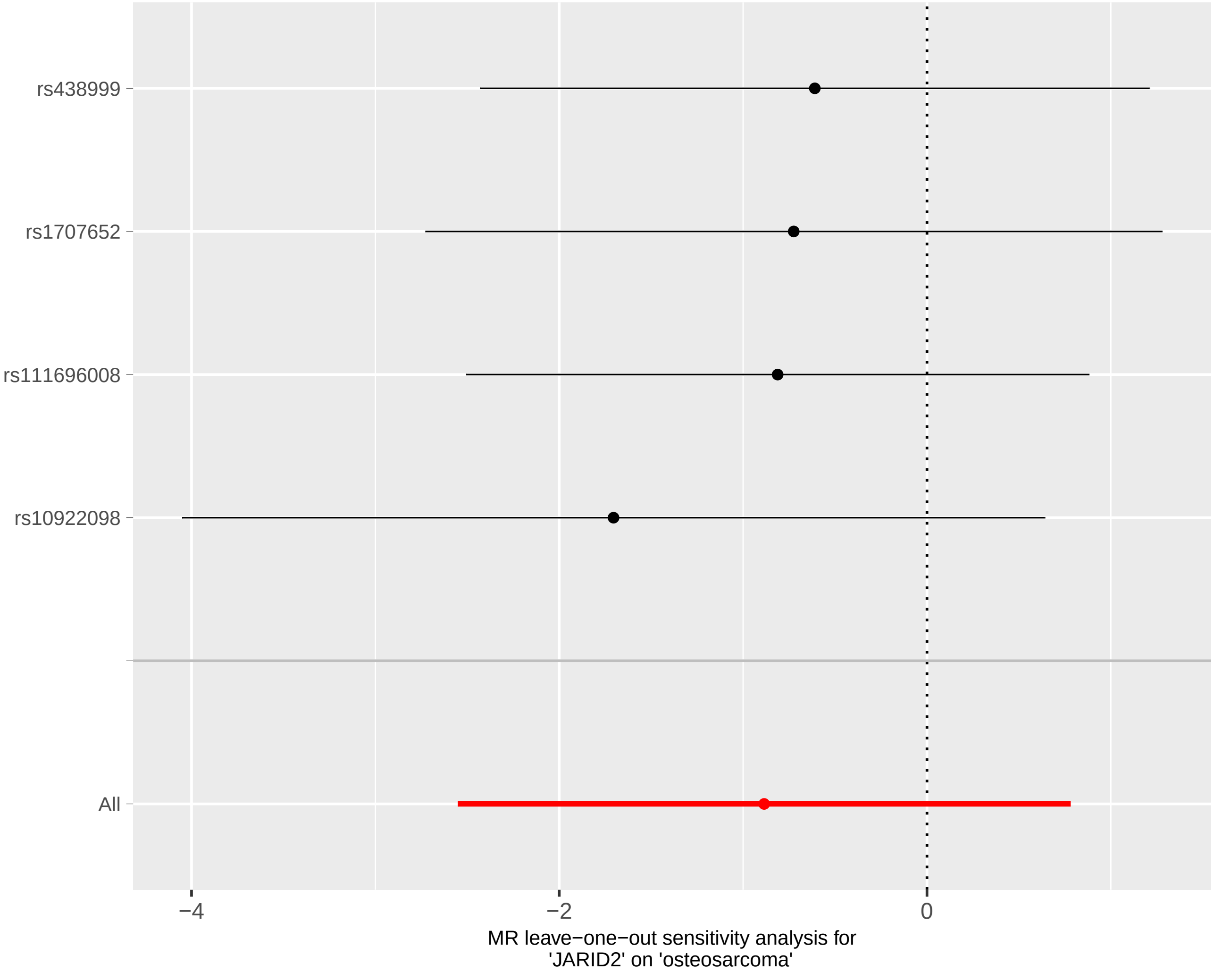

rs438999
rs1707652
rs111696008
rs10922098
All
−4
−2
0
MR leave−one−out sensitivity analysis for
'JARID2' on 'osteosarcoma'

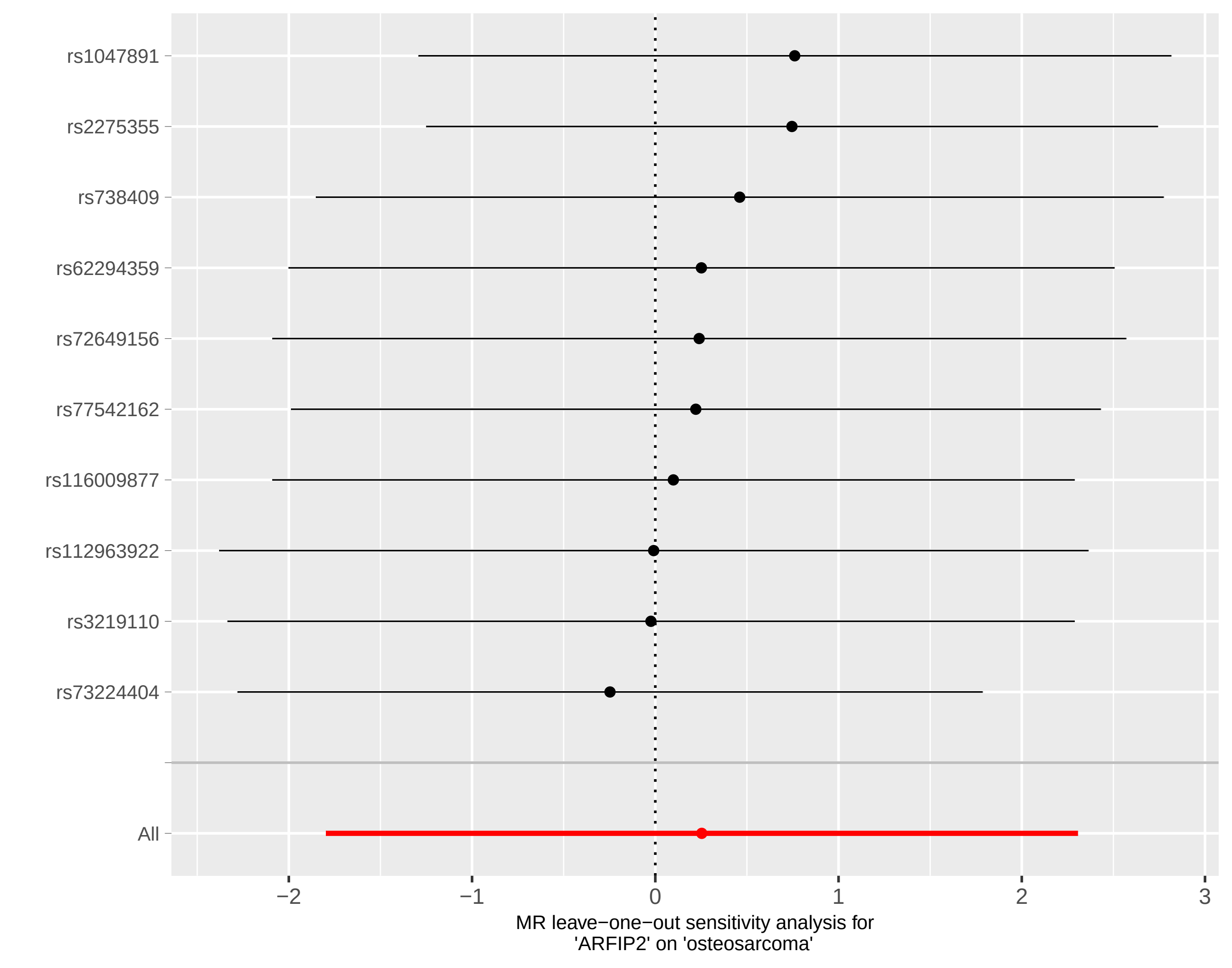

rs1047891
rs2275355
rs738409
rs62294359
rs72649156
rs77542162
rs116009877
rs112963922
rs3219110
rs73224404
All
−2
−1
0
1
2
3
MR leave−one−out sensitivity analysis for
'ARFIP2' on 'osteosarcoma'

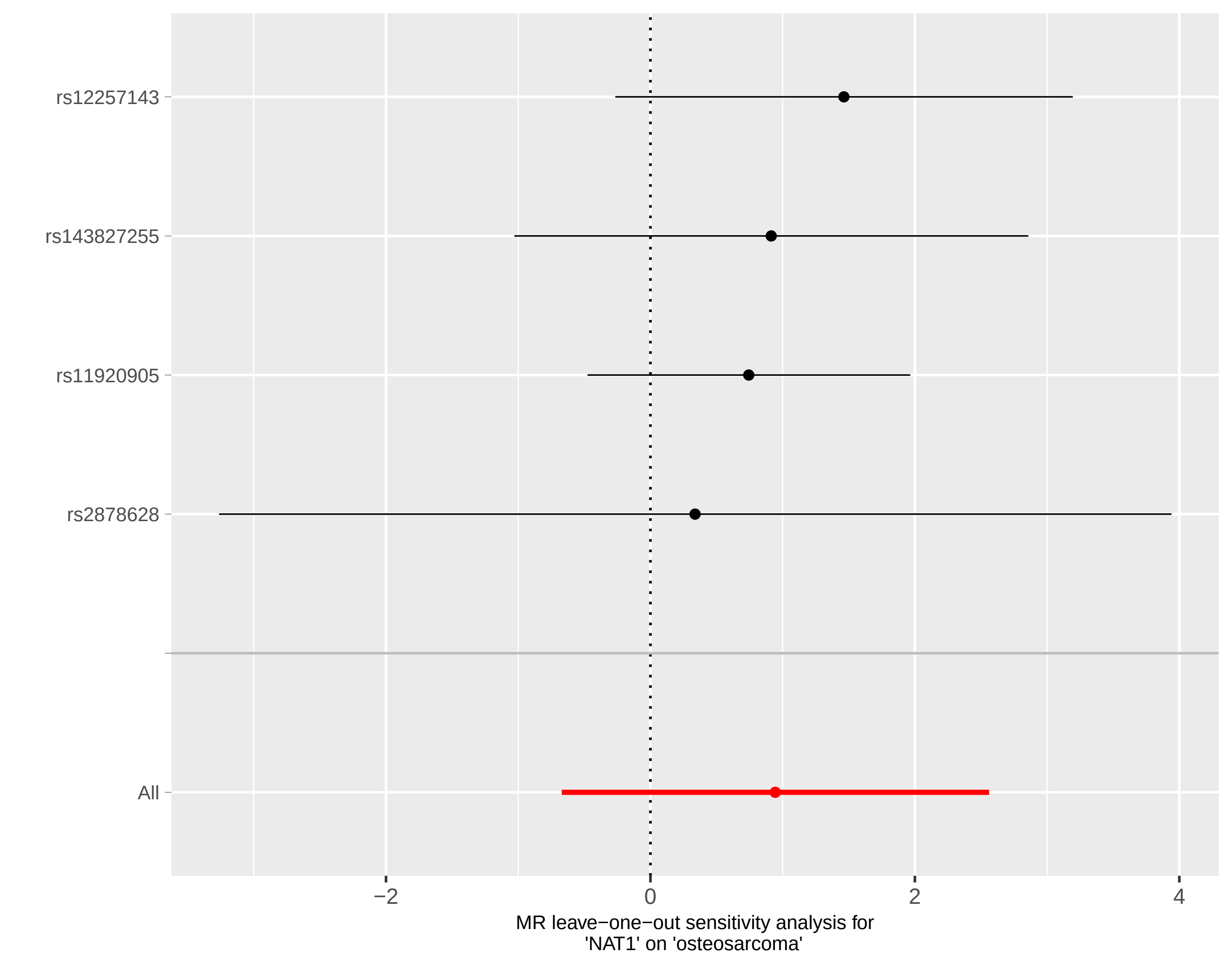
rs12257143
rs143827255
rs11920905
rs2878628
All
−2
0
2
4
MR leave−one−out sensitivity analysis for
'NAT1' on 'osteosarcoma'

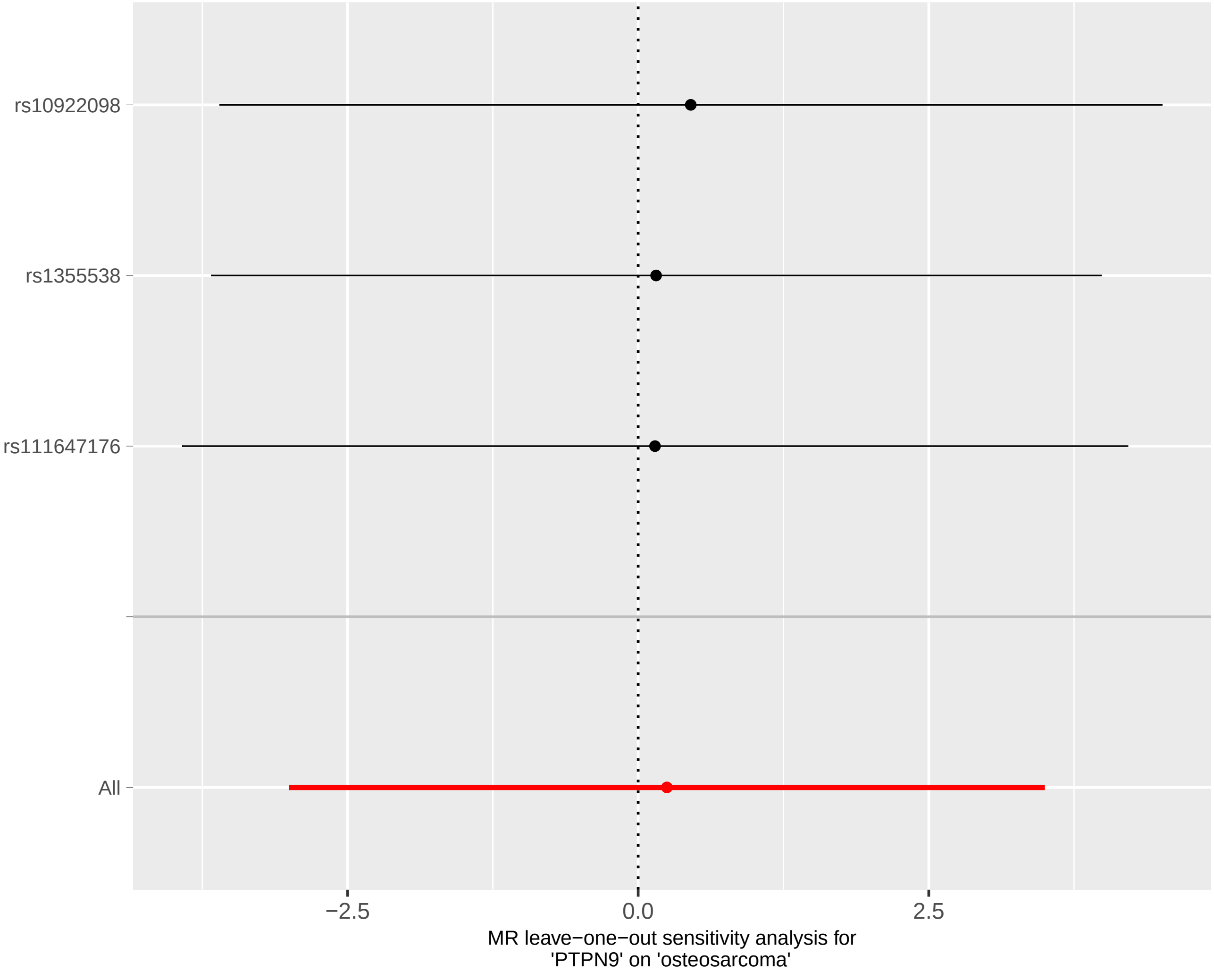

rs10922098
rs1355538
rs111647176
All
−2.5
0.0
2.5
MR leave−one−out sensitivity analysis for
'PTPN9' on 'osteosarcoma'

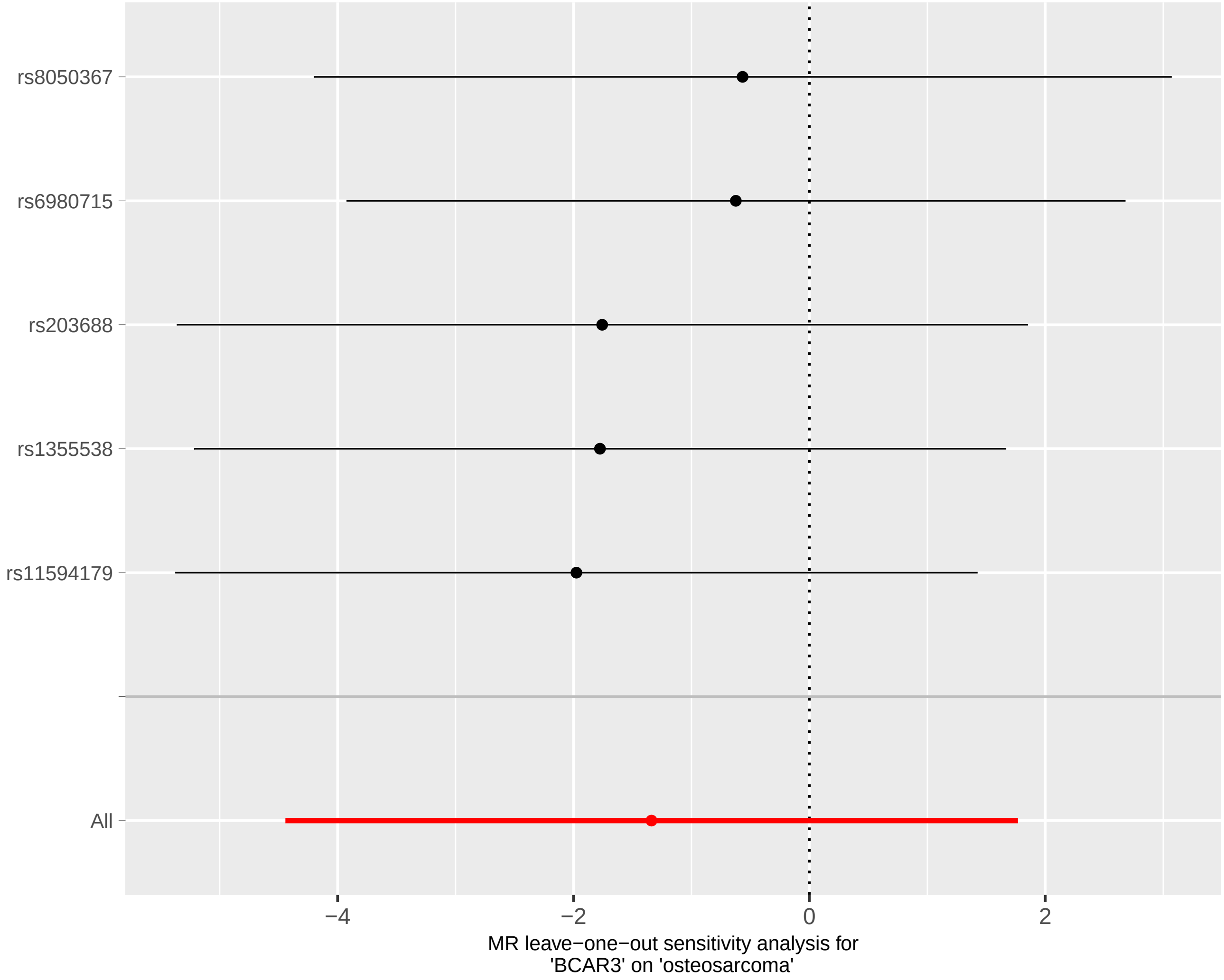

rs8050367
rs6980715
rs203688
rs1355538
rs11594179
All
−4
−2
0
2
MR leave−one−out sensitivity analysis for
'BCAR3' on 'osteosarcoma'

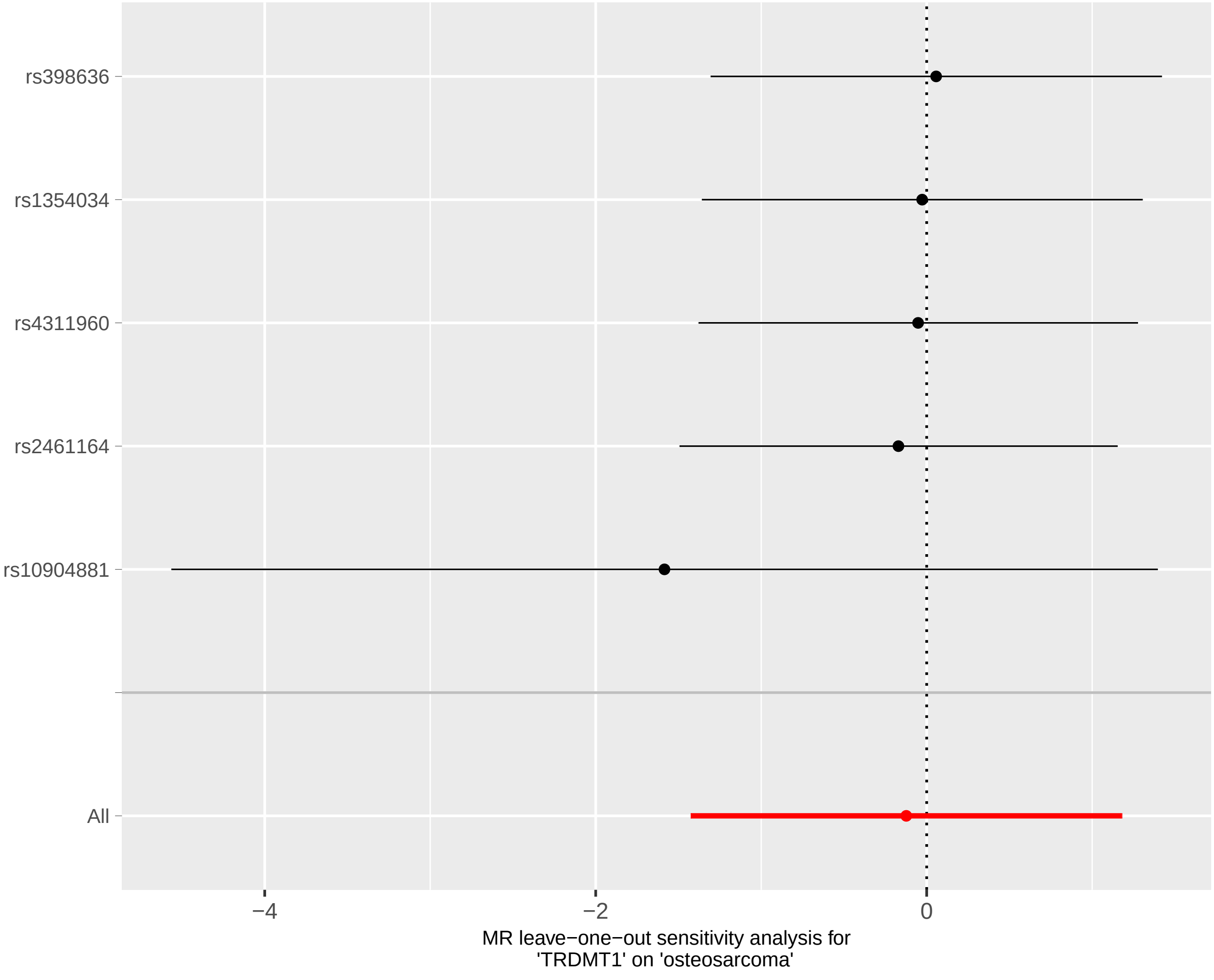

rs398636
rs1354034
rs4311960
rs2461164
rs10904881
All
−4
−2
0
MR leave−one−out sensitivity analysis for
'TRDMT1' on 'osteosarcoma'

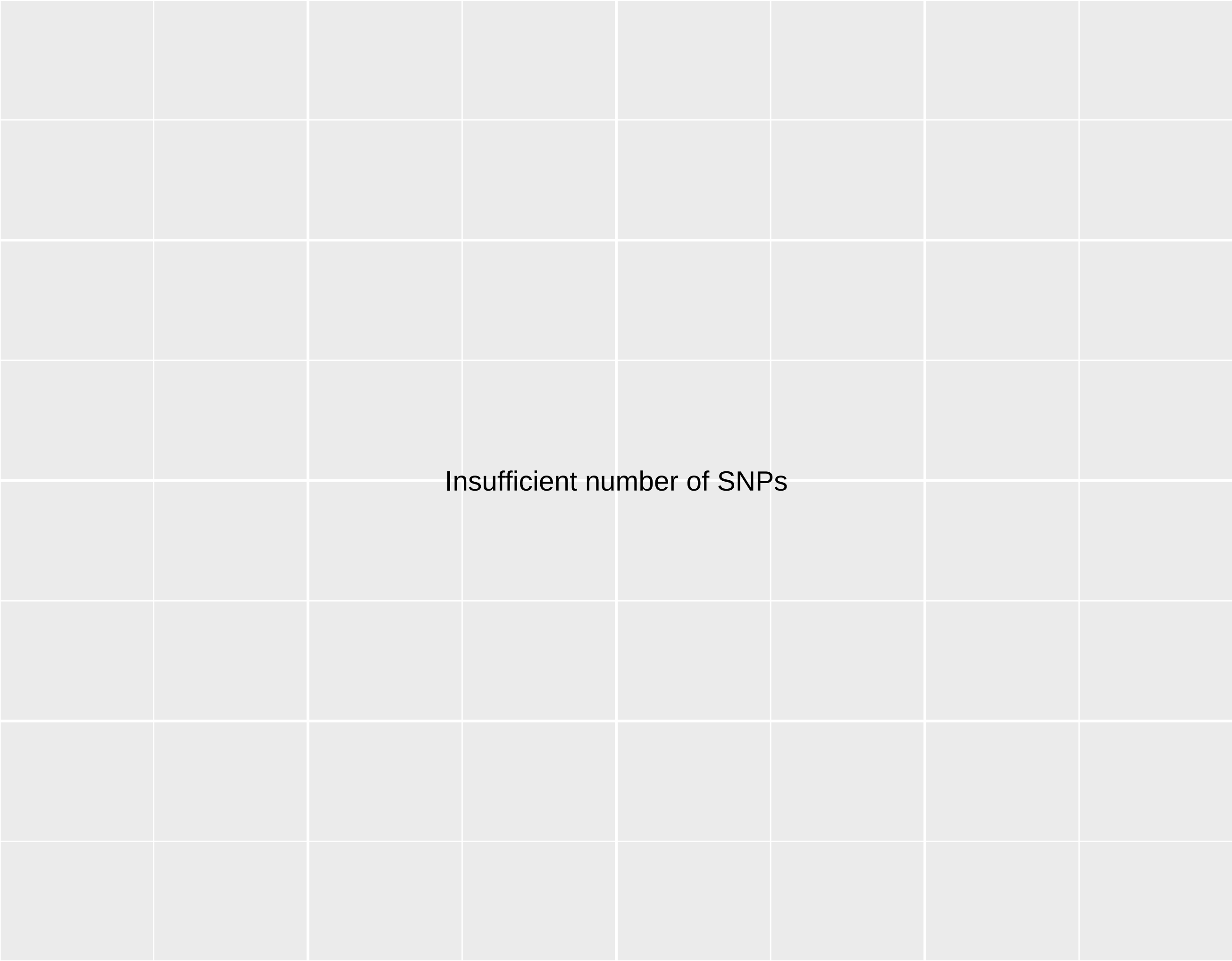
Insufficient number of SNPs

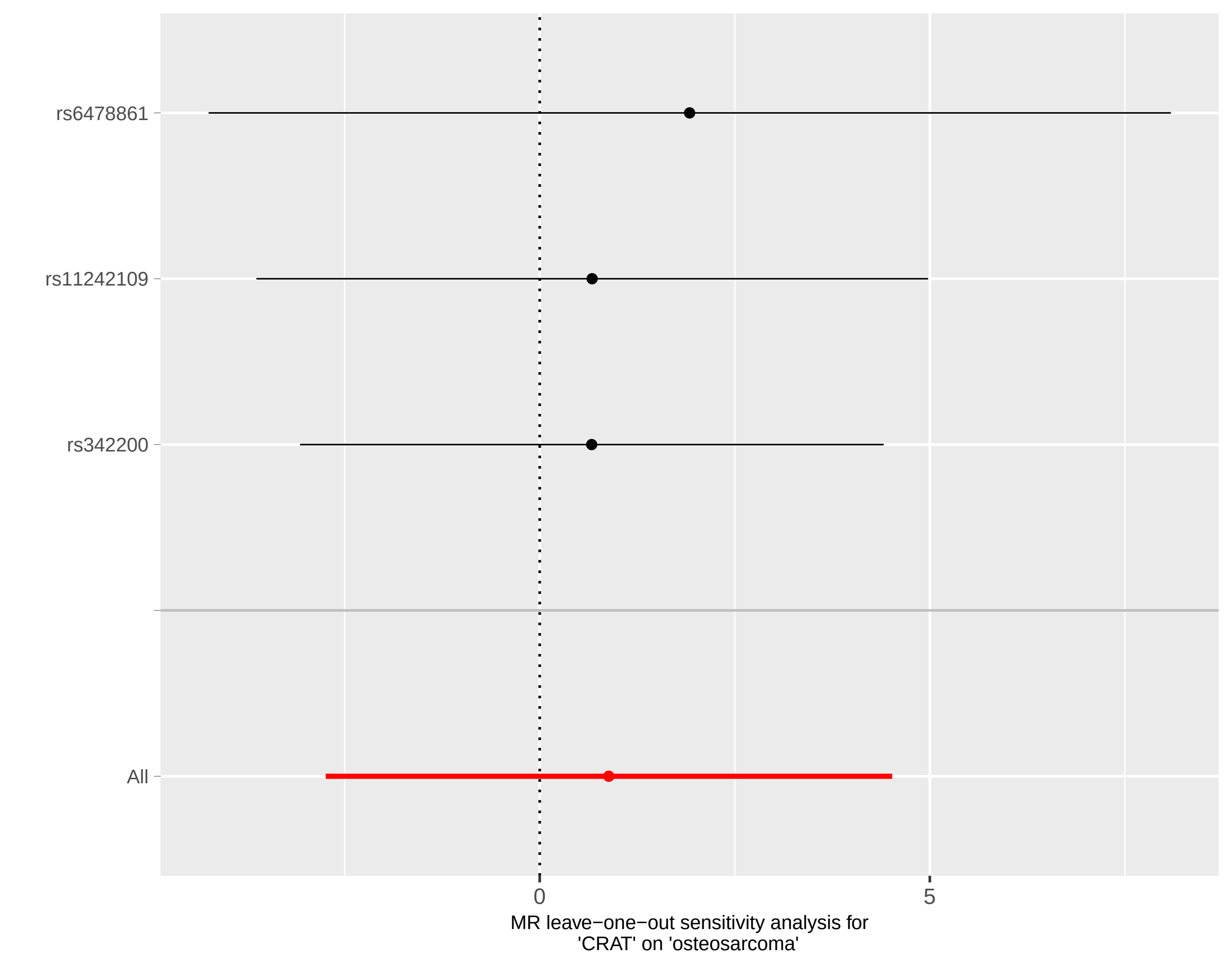
rs6478861
rs11242109
rs342200
All
0
5
MR leave−one−out sensitivity analysis for
'CRAT' on 'osteosarcoma'

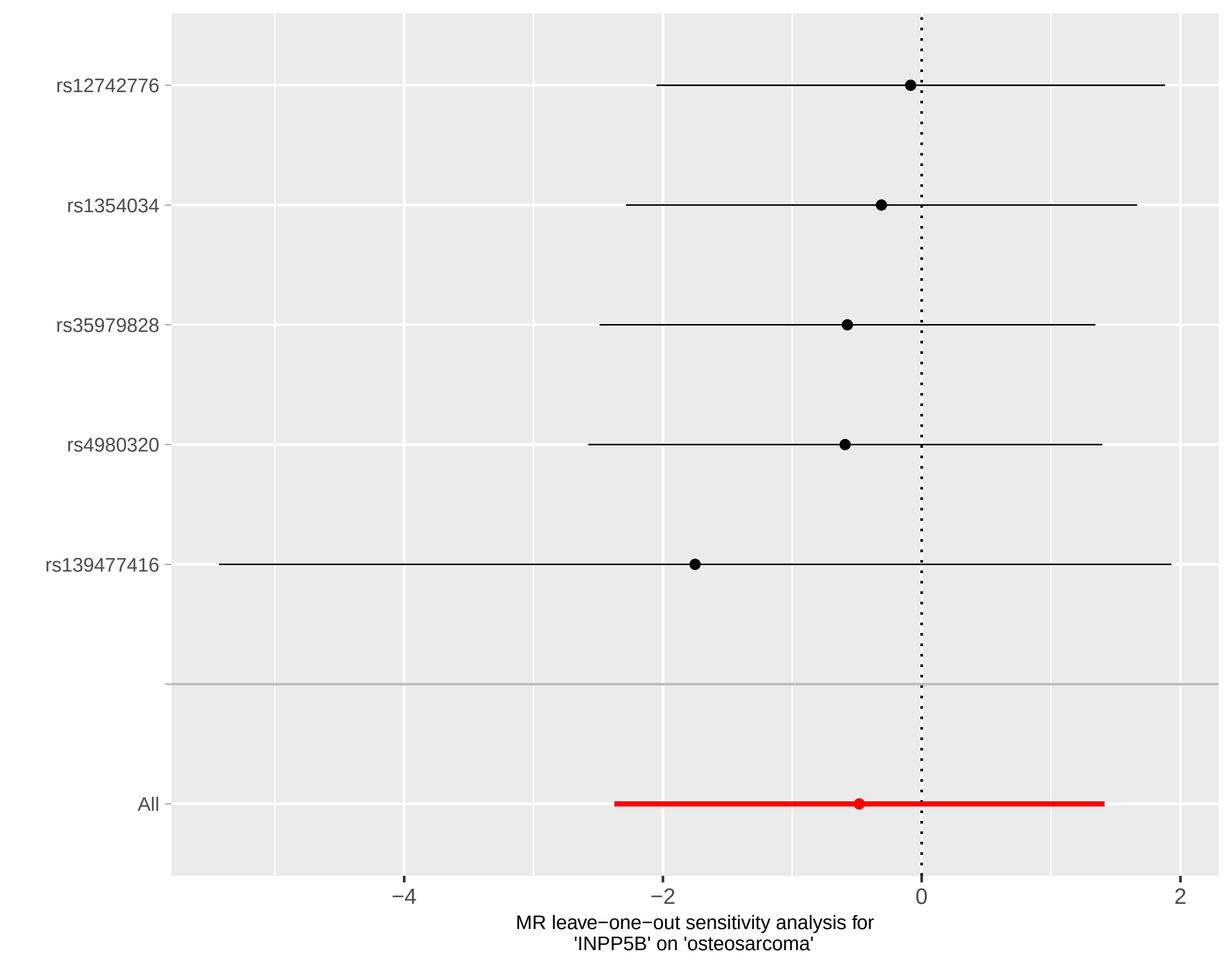
rs12742776
rs1354034
rs35979828
rs4980320
rs139477416
All
−4
−2
0
2
MR leave−one−out sensitivity analysis for
'INPP5B' on 'osteosarcoma'

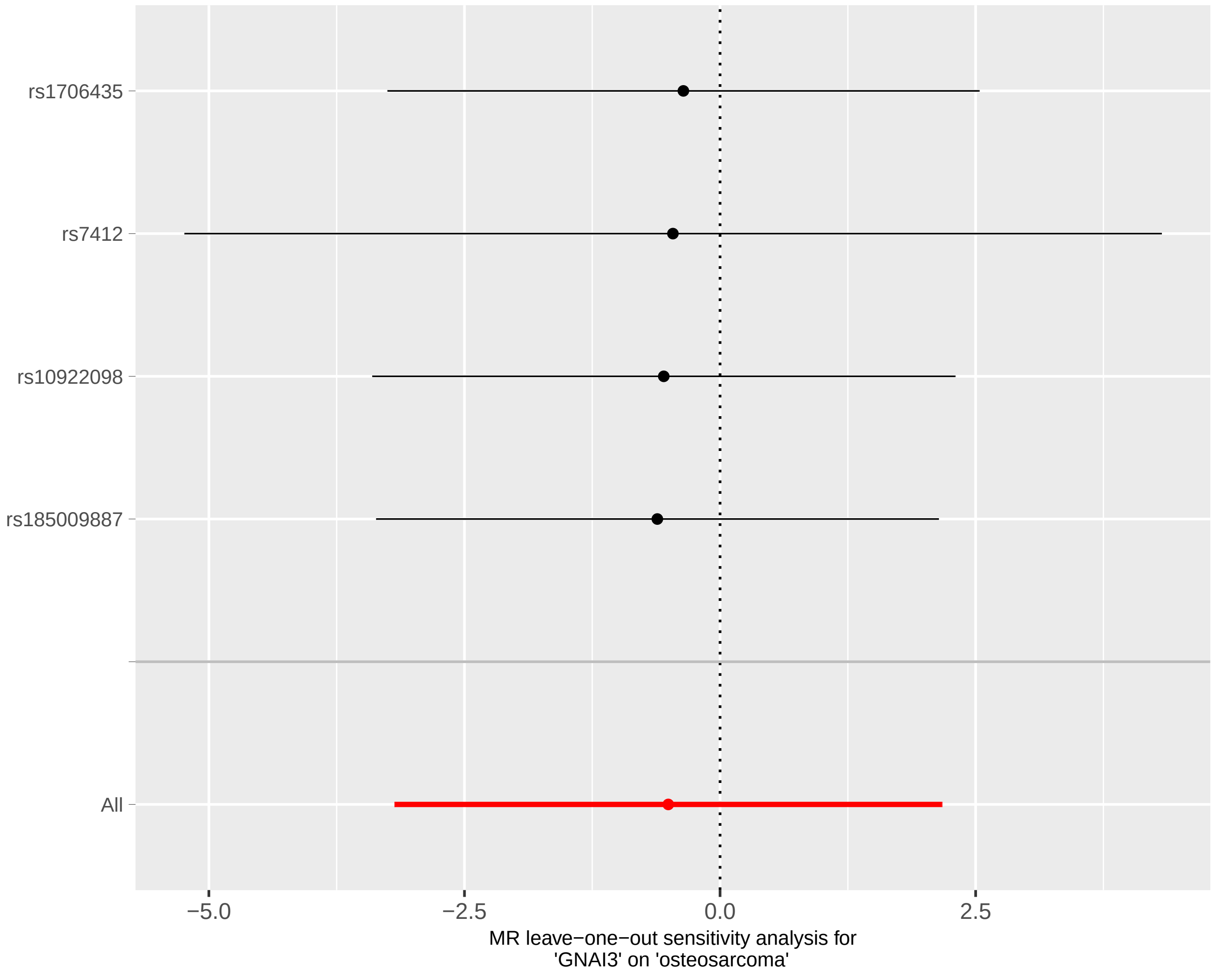

rs1706435
rs7412
rs10922098
rs185009887
All
−5.0
−2.5
0.0
2.5
MR leave−one−out sensitivity analysis for
'GNAI3' on 'osteosarcoma'

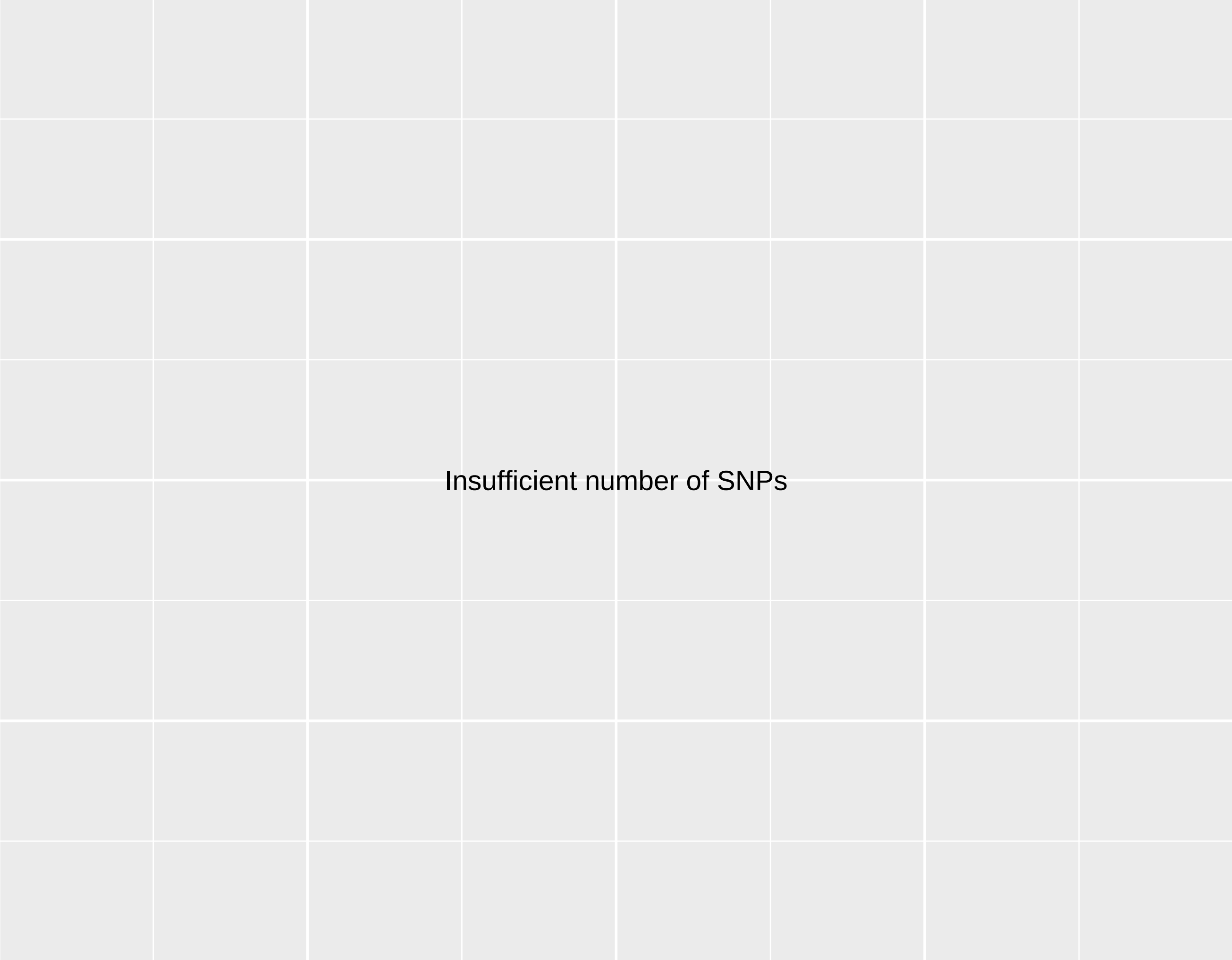
Insufficient number of SNPs

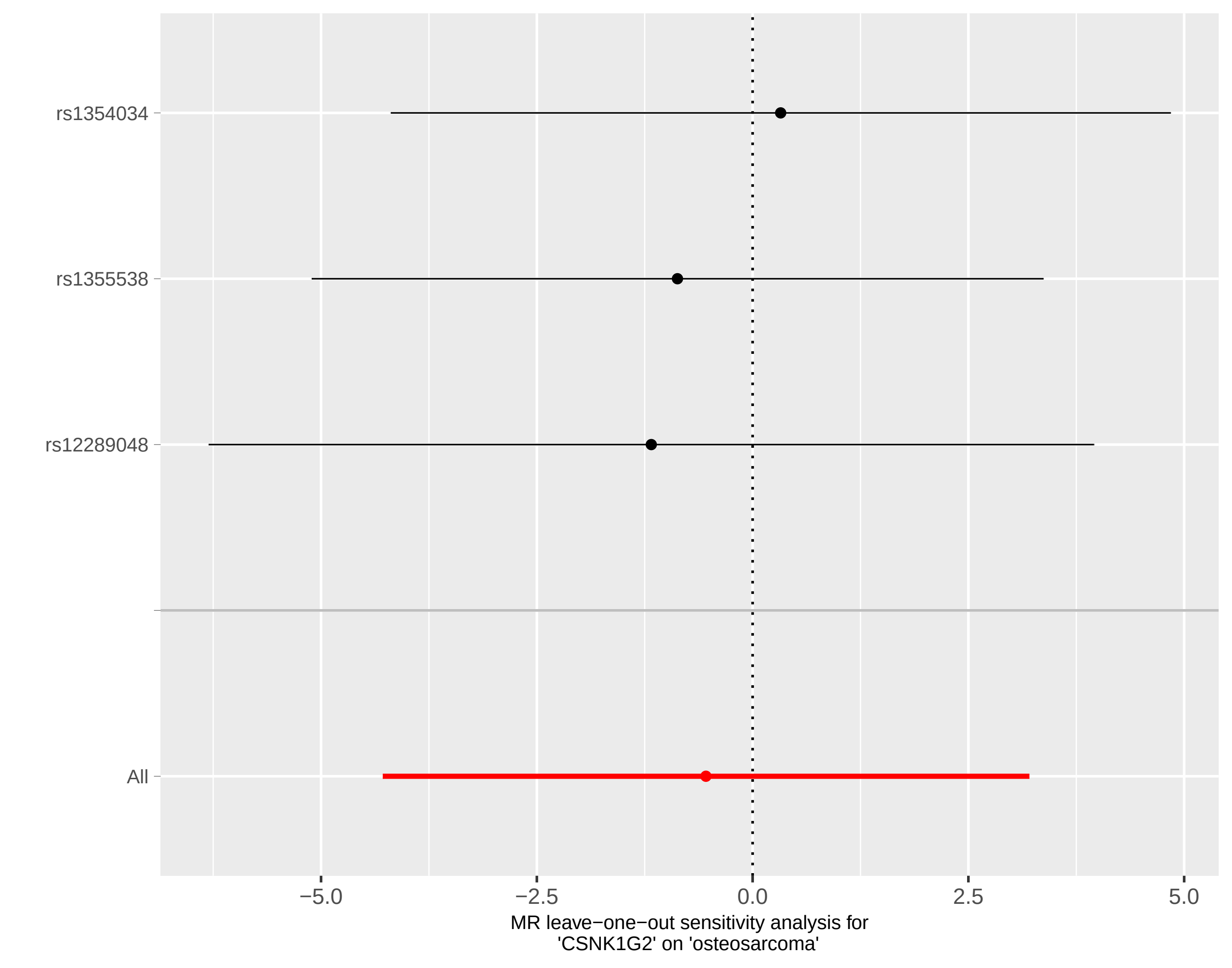

rs1354034
rs1355538
rs12289048
All
−5.0
−2.5
0.0
2.5
5.0
MR leave−one−out sensitivity analysis for
'CSNK1G2' on 'osteosarcoma'

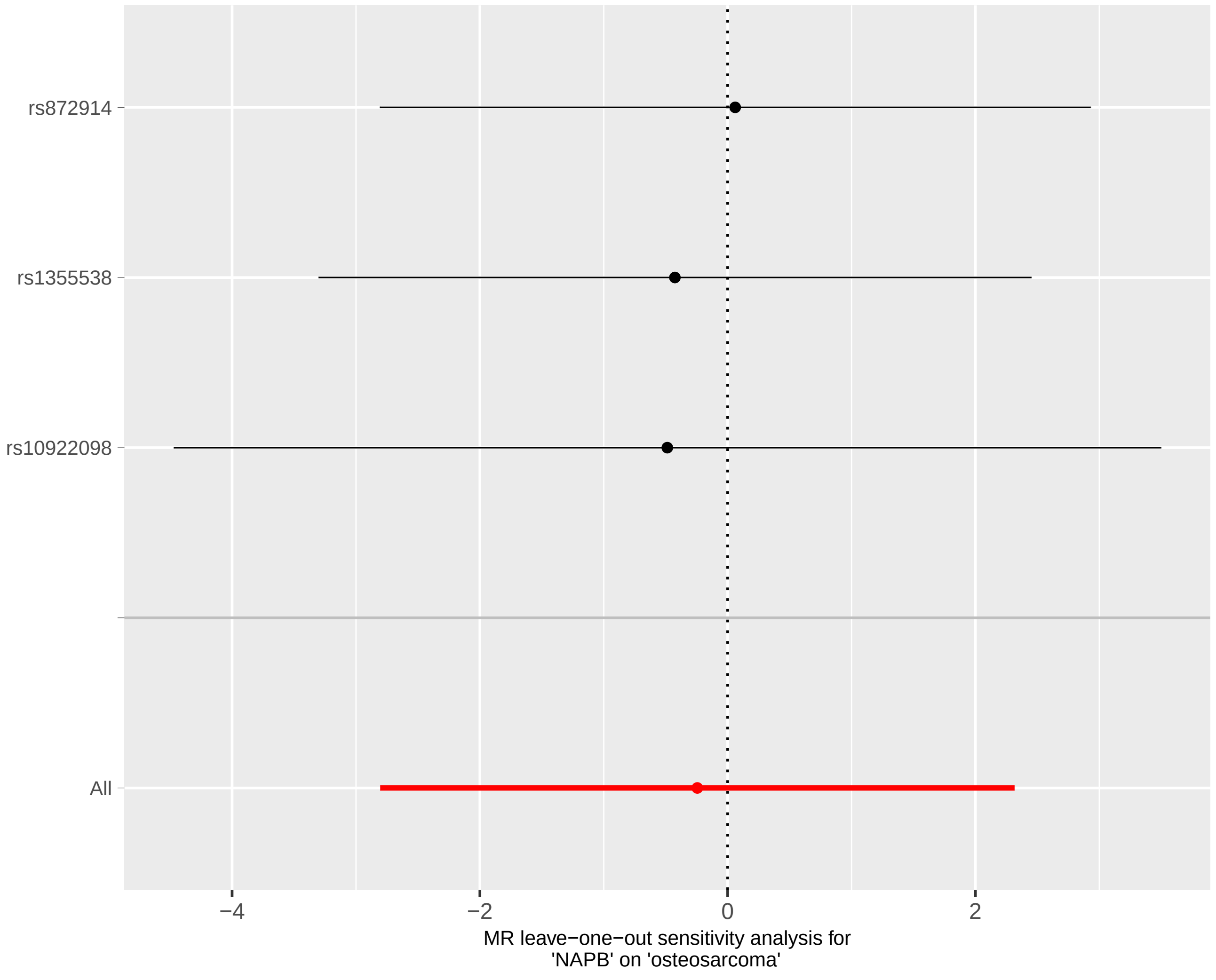

rs872914
rs1355538
rs10922098
All
−4
−2
0
2
MR leave−one−out sensitivity analysis for
'NAPB' on 'osteosarcoma'

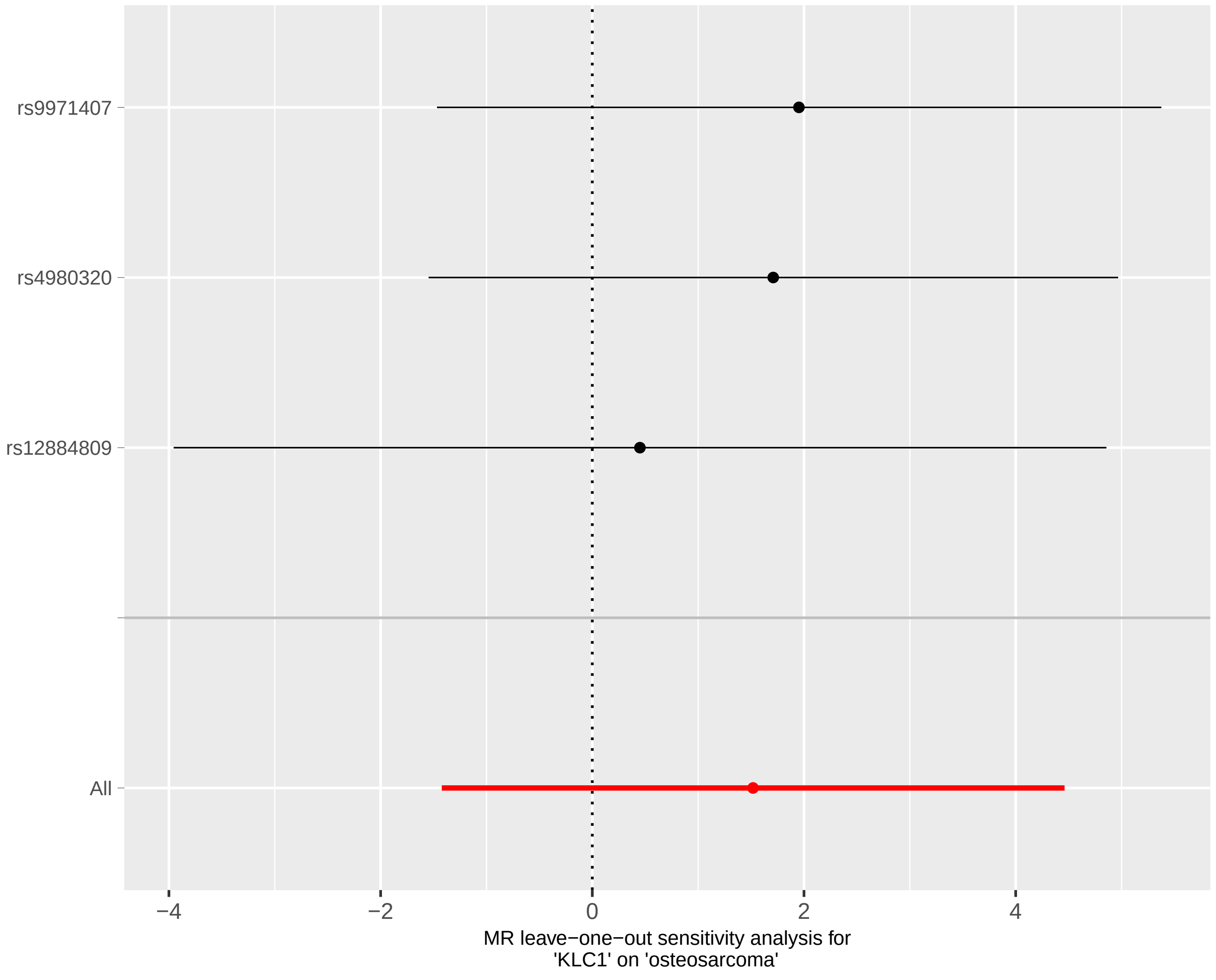

rs9971407
rs4980320
rs12884809
All
−4
−2
0
2
4
MR leave−one−out sensitivity analysis for
'KLC1' on 'osteosarcoma'

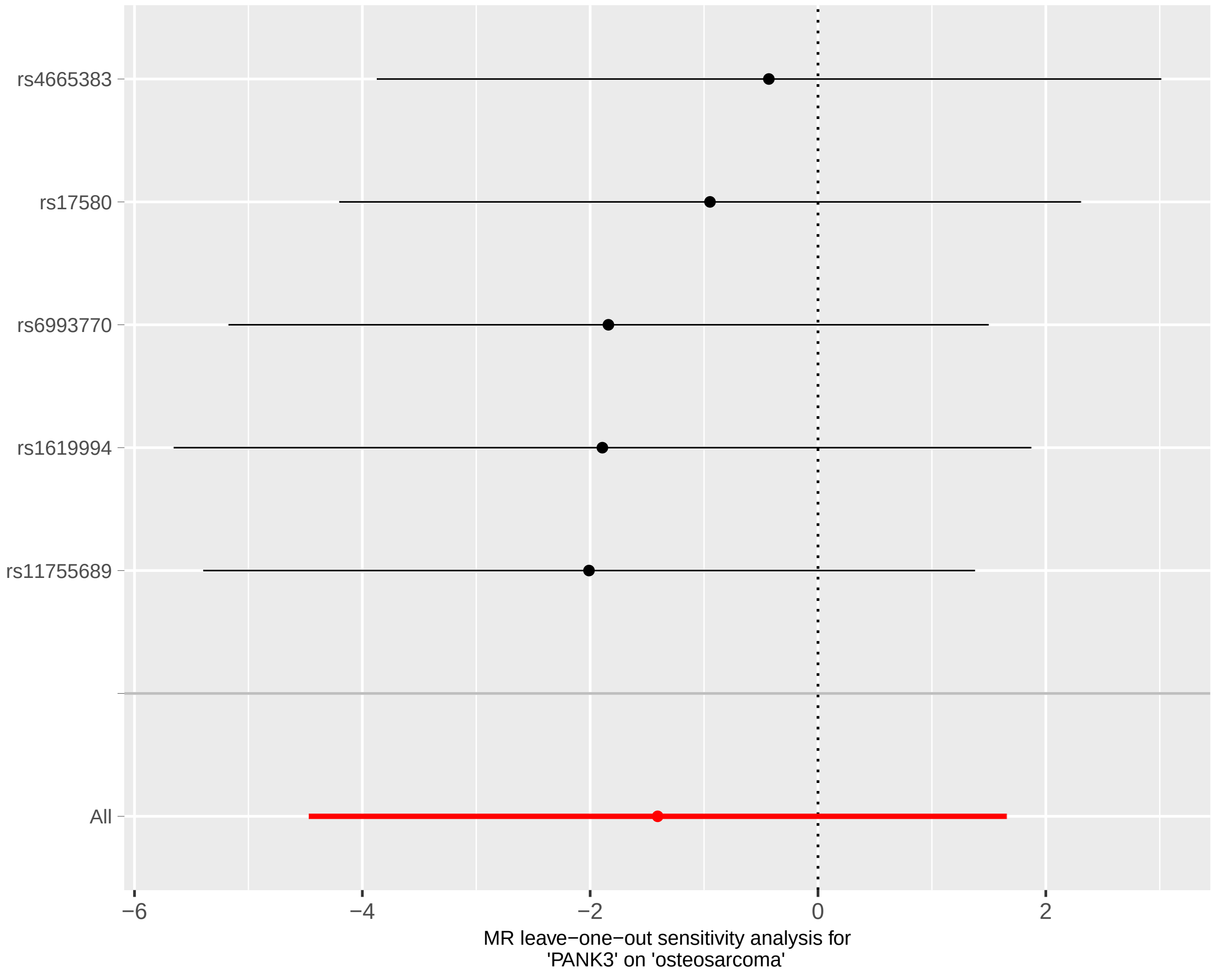
rs4665383
rs17580
rs6993770
rs1619994
rs11755689
All
−6
−4
−2
0
2
MR leave−one−out sensitivity analysis for
'PANK3' on 'osteosarcoma'

Insufficient number of SNPs

Insufficient number of SNPs

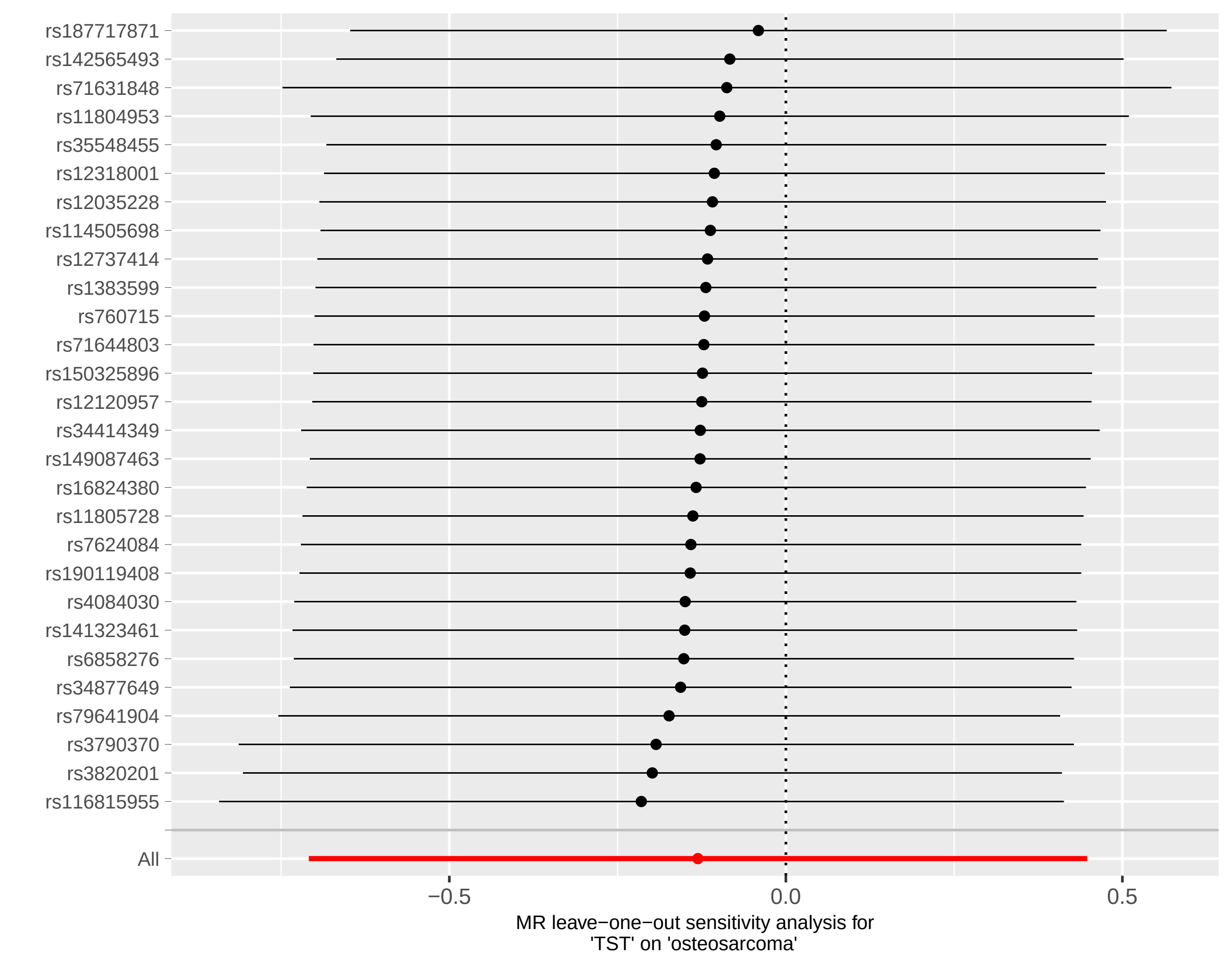

rs187717871
rs142565493
rs71631848
rs11804953
rs35548455
rs12318001
rs12035228
rs114505698
rs12737414
rs1383599
rs760715
rs71644803
rs150325896
rs12120957
rs34414349
rs149087463
rs16824380
rs11805728
rs7624084
rs190119408
rs4084030
rs141323461
rs6858276
rs34877649
rs79641904
rs3790370
rs3820201
rs116815955
All
−0.5
0.0
0.5
MR leave−one−out sensitivity analysis for
'TST' on 'osteosarcoma'

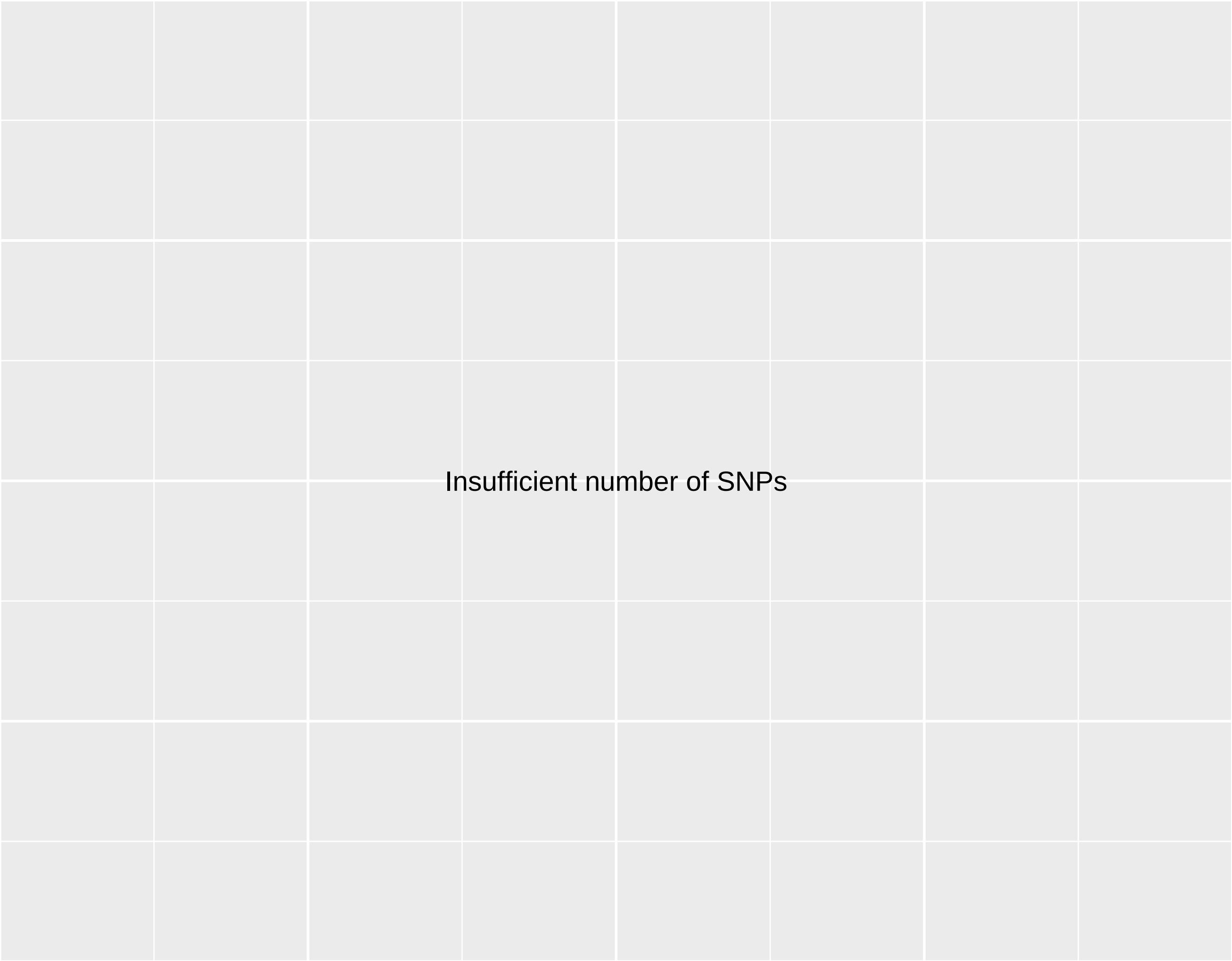
Insufficient number of SNPs

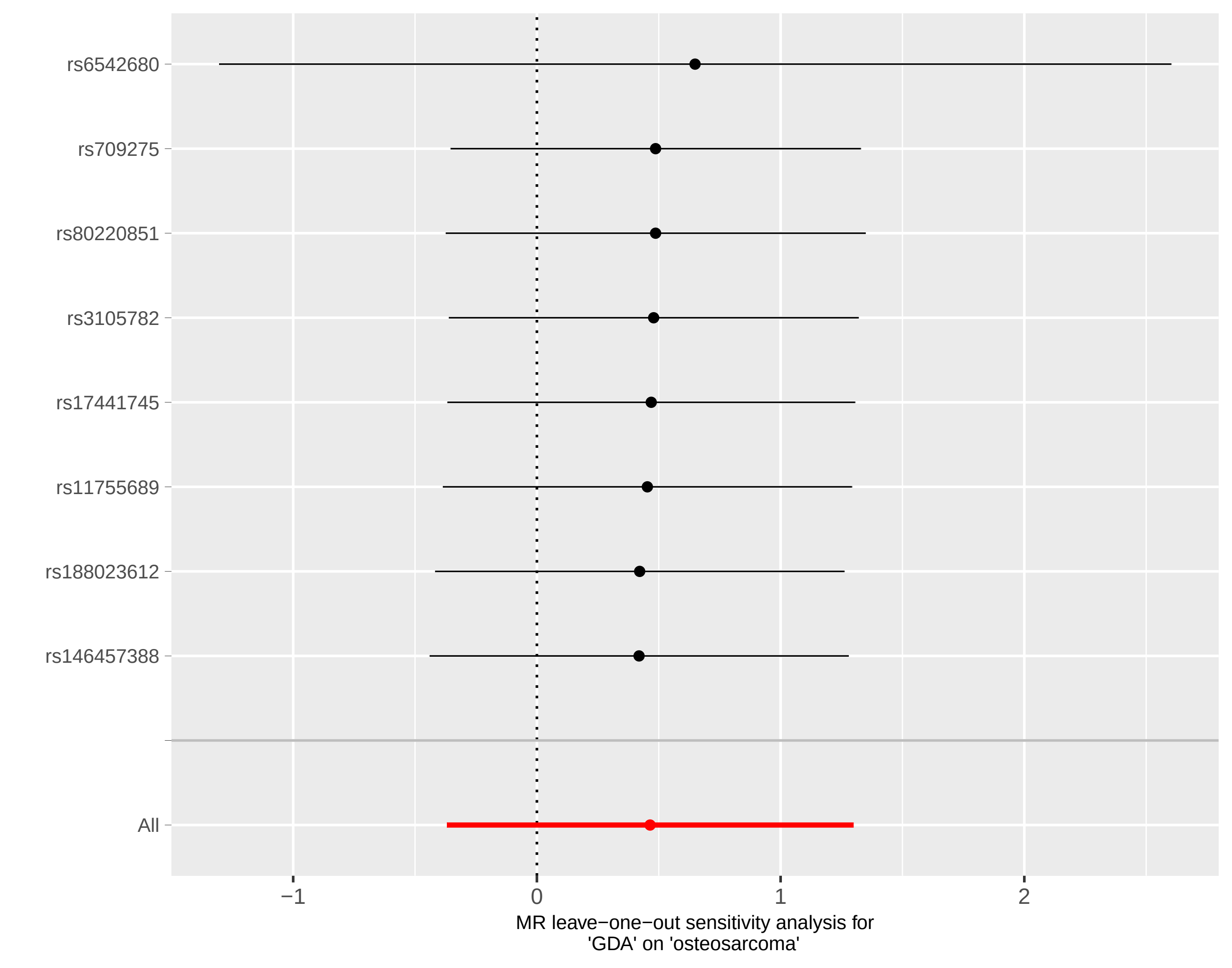

rs6542680
rs709275
rs80220851
rs3105782
rs17441745
rs11755689
rs188023612
rs146457388
All
−1
0
1
2
MR leave−one−out sensitivity analysis for
'GDA' on 'osteosarcoma'

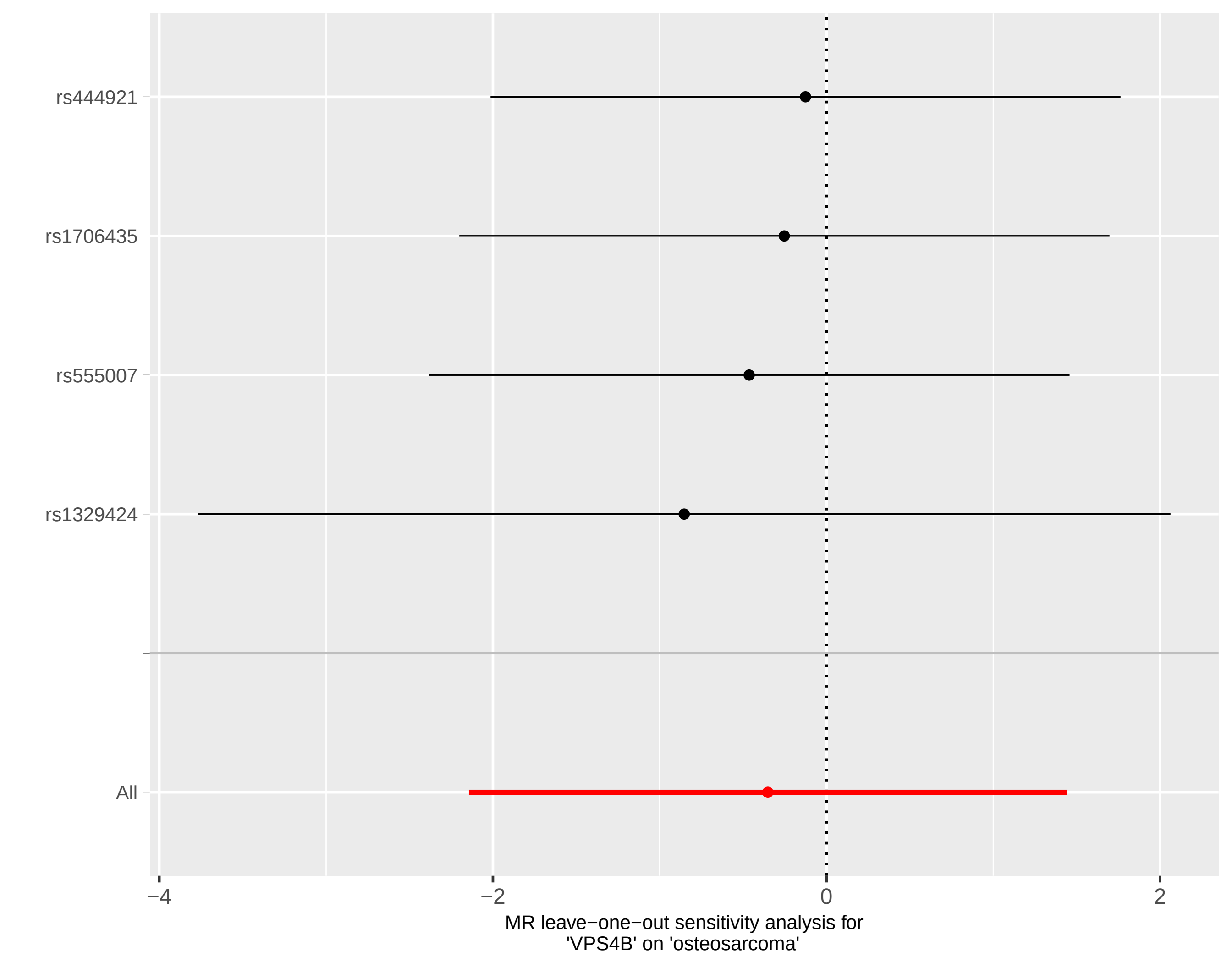

rs444921
rs1706435
rs555007
rs1329424
All
−4
−2
0
2
MR leave−one−out sensitivity analysis for
'VPS4B' on 'osteosarcoma'

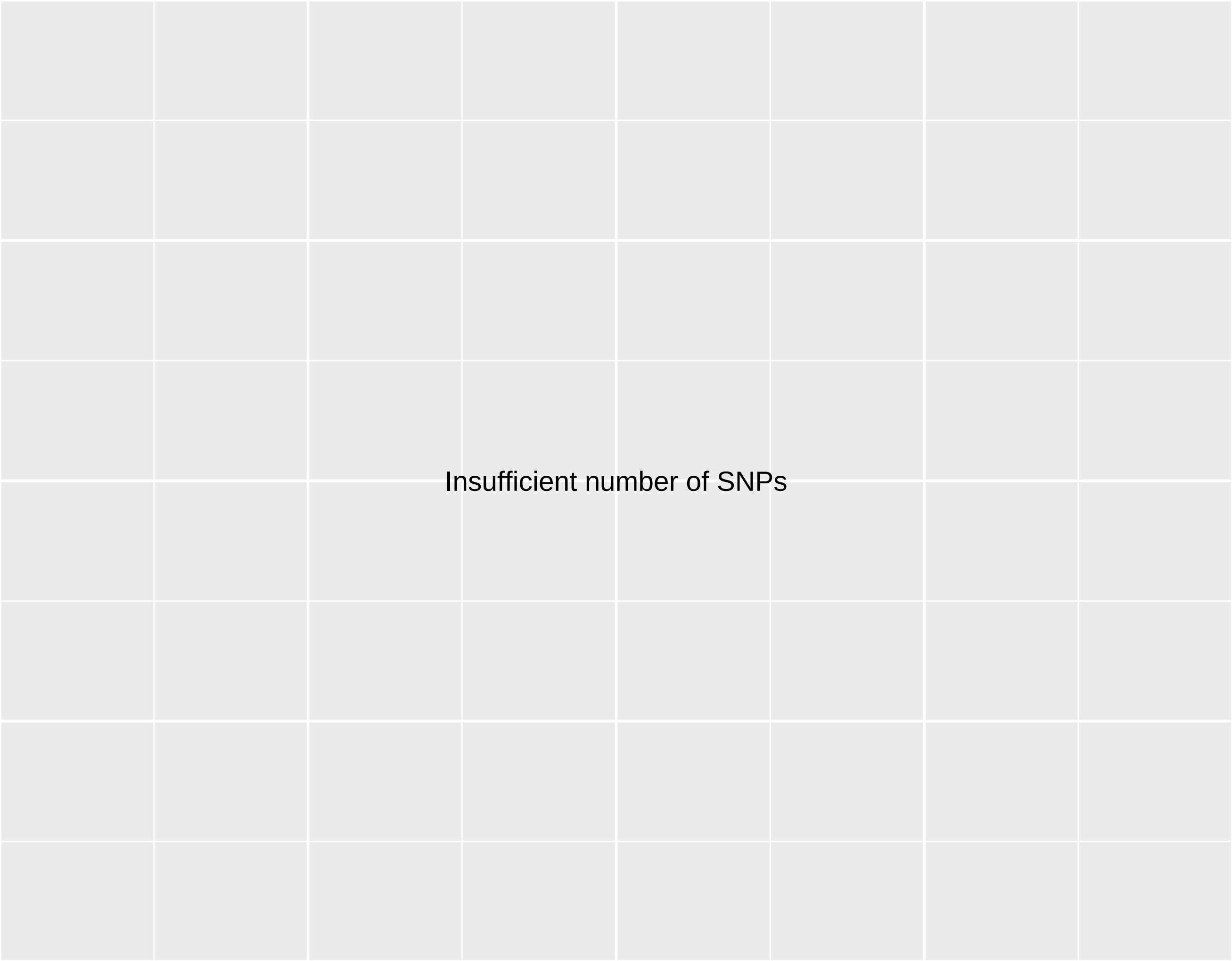
Insufficient number of SNPs

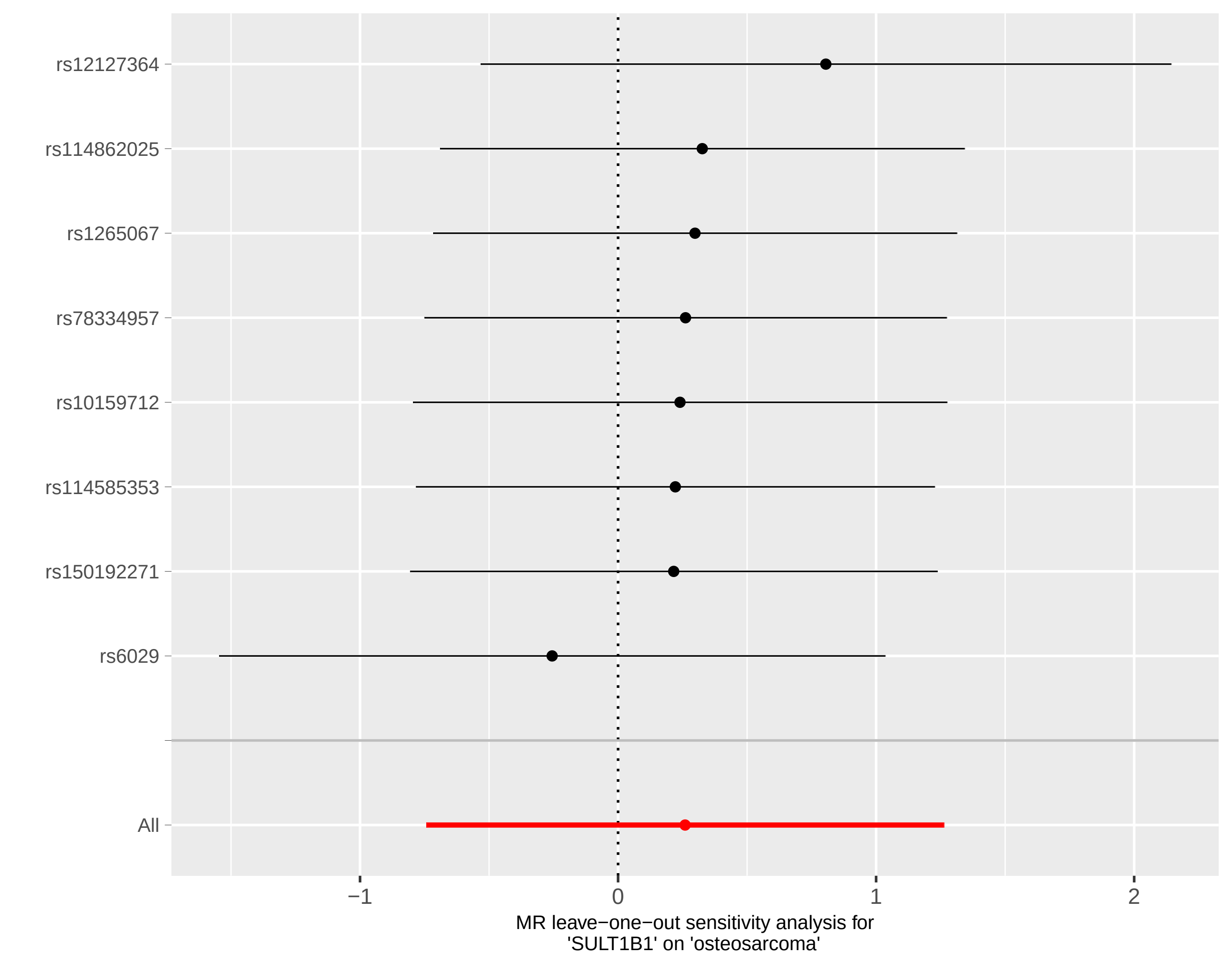
rs12127364
rs114862025
rs1265067
rs78334957
rs10159712
rs114585353
rs150192271
rs6029
All
−1
0
1
2
MR leave−one−out sensitivity analysis for
'SULT1B1' on 'osteosarcoma'

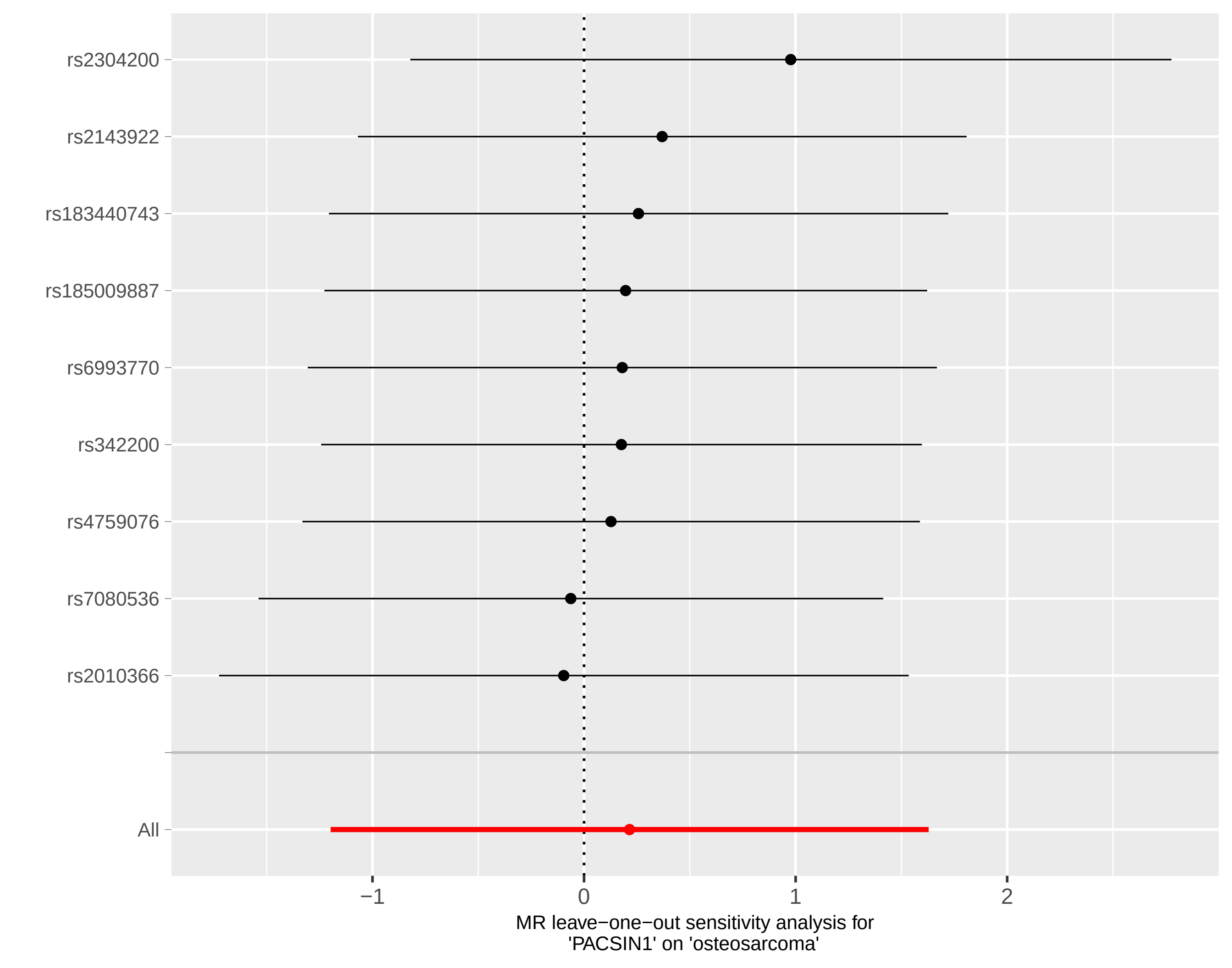
rs2304200
rs2143922
rs183440743
rs185009887
rs6993770
rs342200
rs4759076
rs7080536
rs2010366
All
−1
0
1
2
MR leave−one−out sensitivity analysis for
'PACSIN1' on 'osteosarcoma'

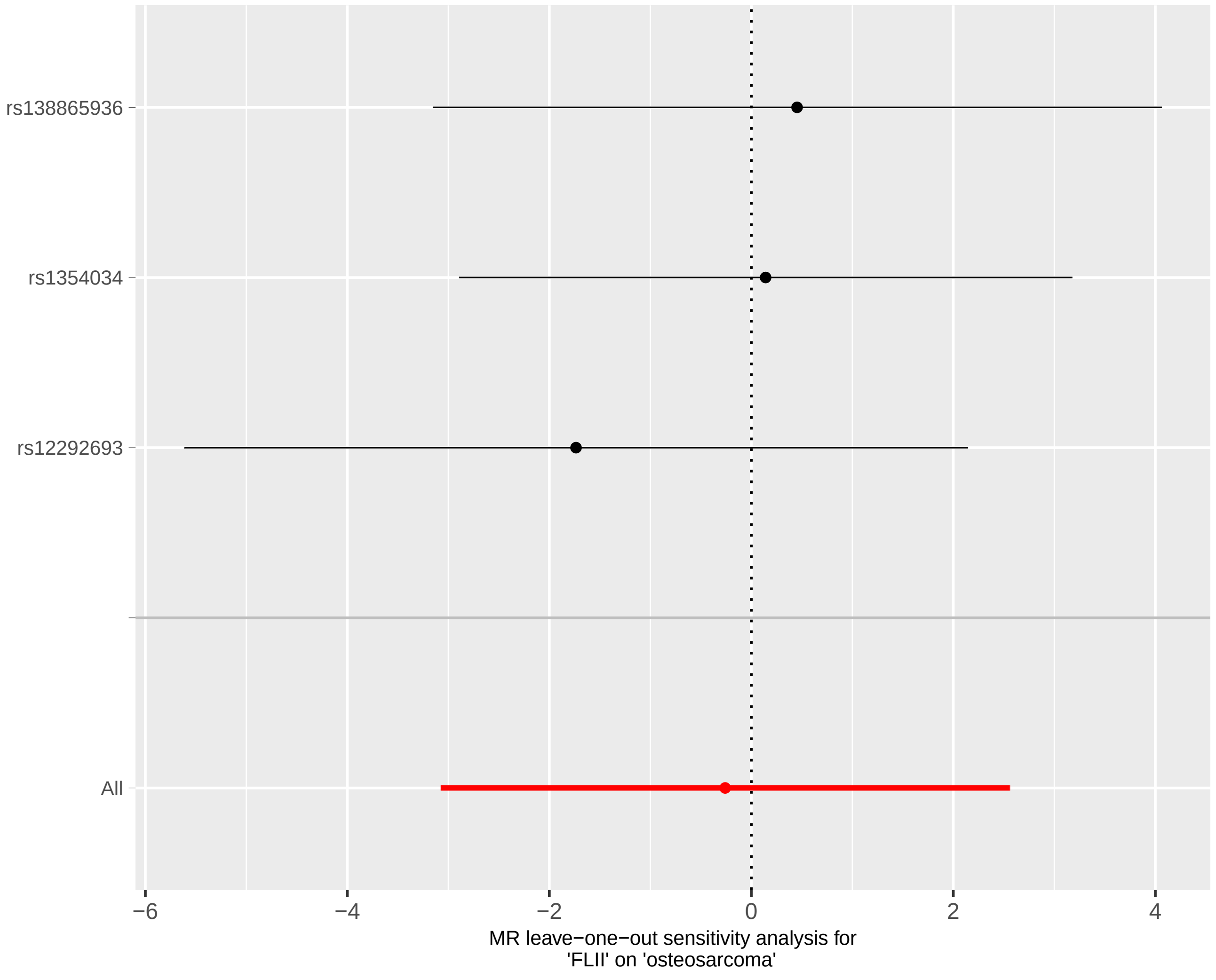
rs138865936
rs1354034
rs12292693
All
−6
−4
−2
0
2
4
MR leave−one−out sensitivity analysis for
'FLII' on 'osteosarcoma'

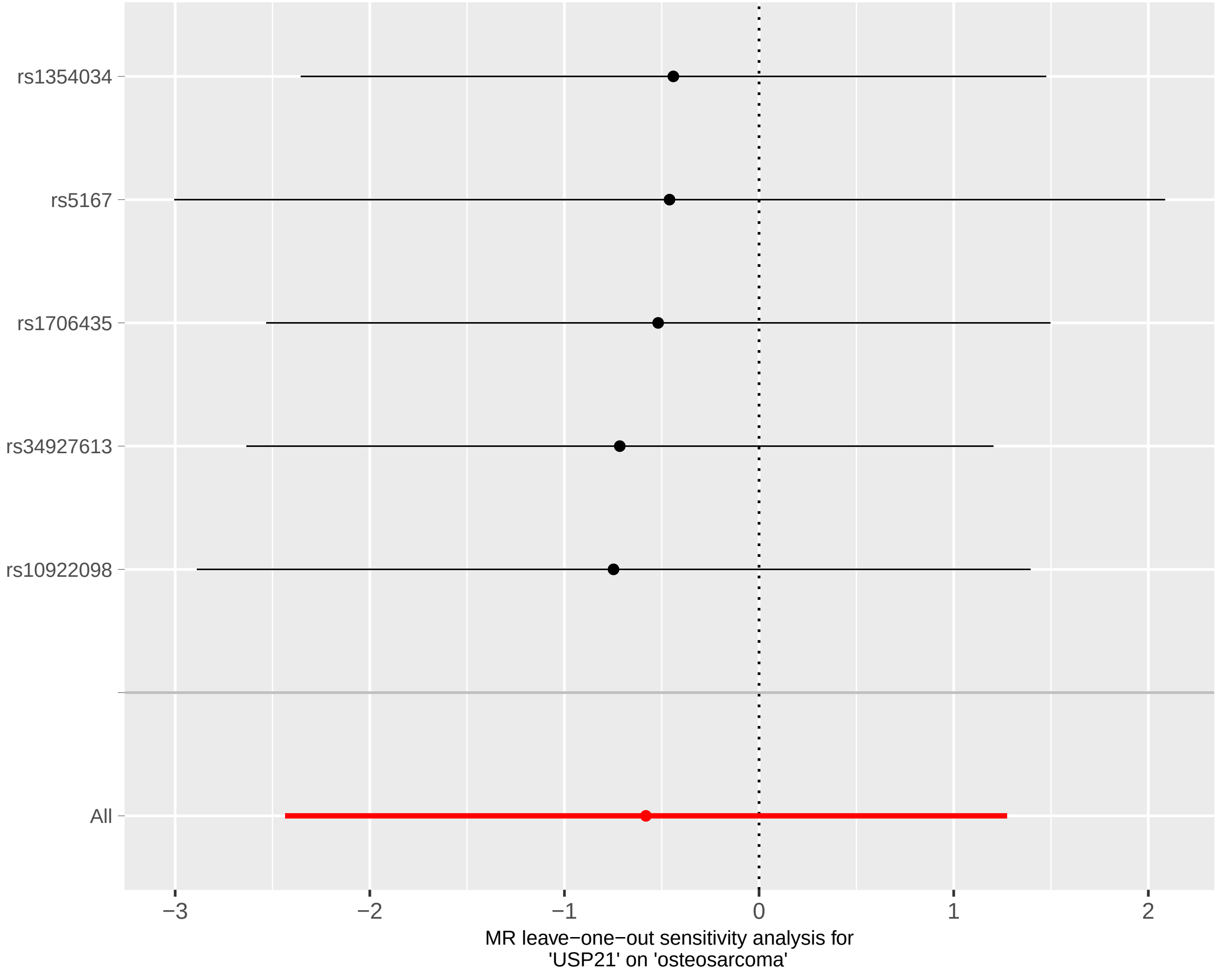
rs1354034
rs5167
rs1706435
rs34927613
rs10922098
All
−3
−2
−1
0
1
2
MR leave−one−out sensitivity analysis for
'USP21' on 'osteosarcoma'

Insufficient number of SNPs

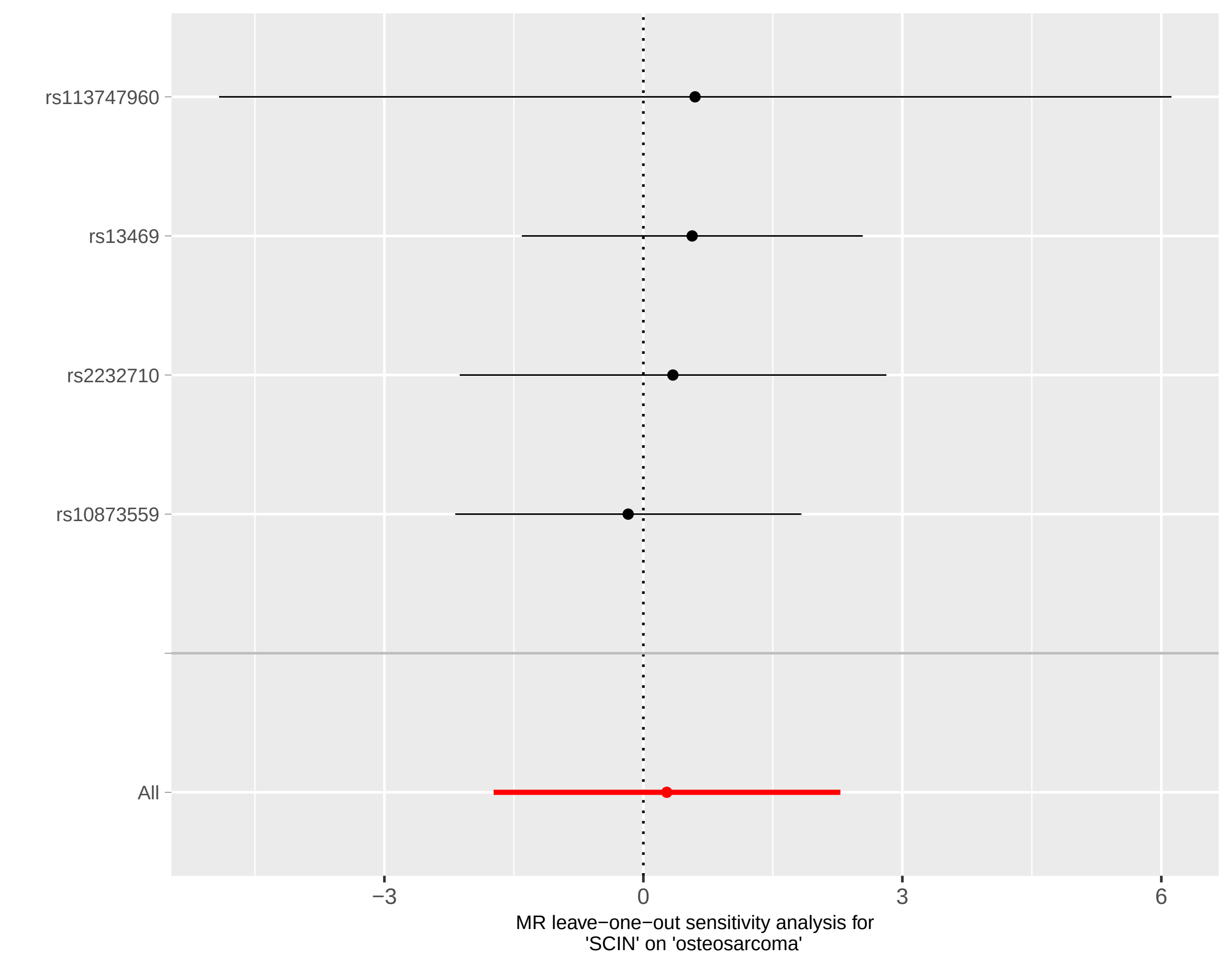

rs113747960
rs13469
rs2232710
rs10873559
All
−3
0
3
6
MR leave−one−out sensitivity analysis for
'SCIN' on 'osteosarcoma'

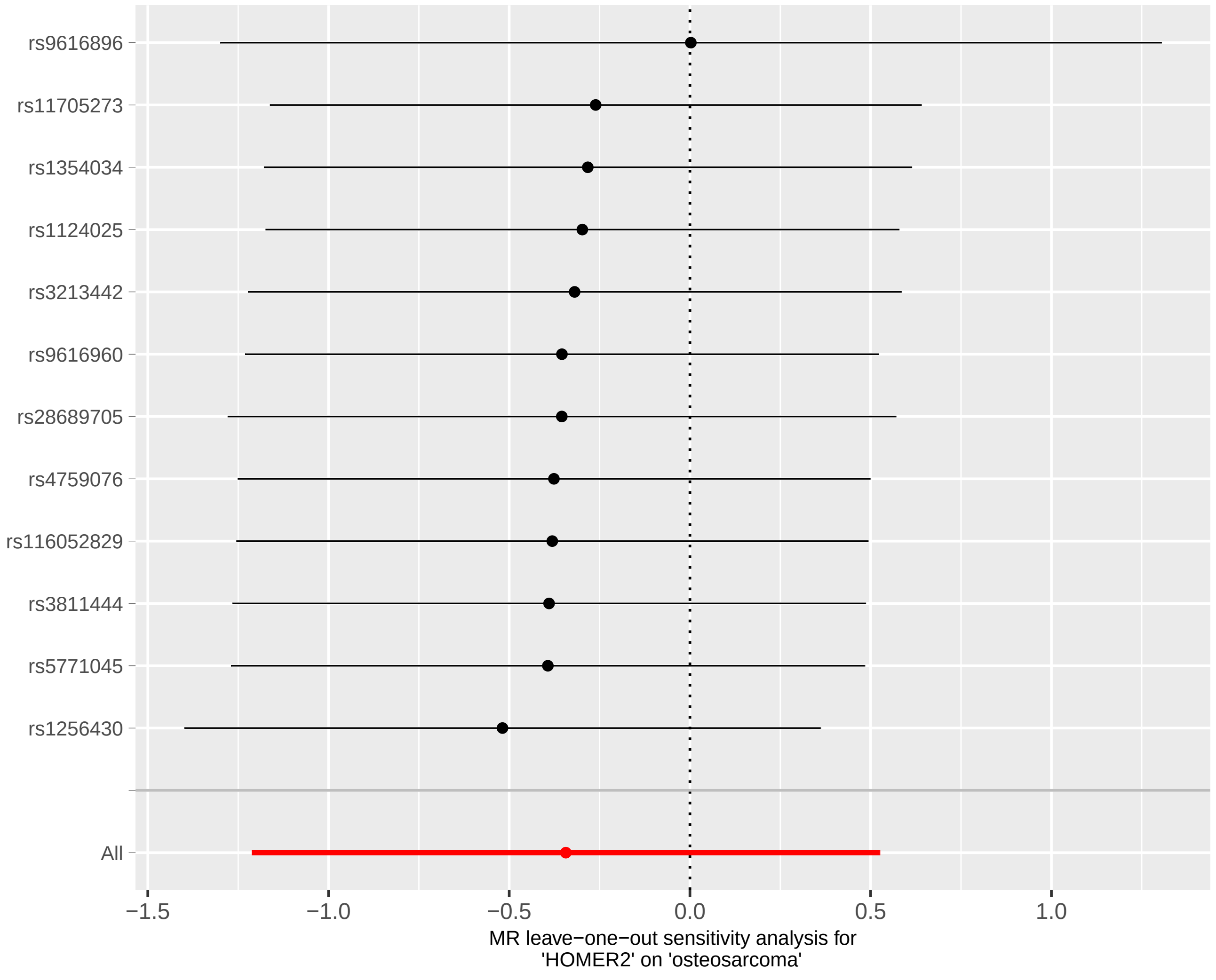
rs9616896
rs11705273
rs1354034
rs1124025
rs3213442
rs9616960
rs28689705
rs4759076
rs116052829
rs3811444
rs5771045
rs1256430
All
−1.5
−1.0
−0.5
0.0
0.5
1.0
MR leave−one−out sensitivity analysis for
'HOMER2' on 'osteosarcoma'

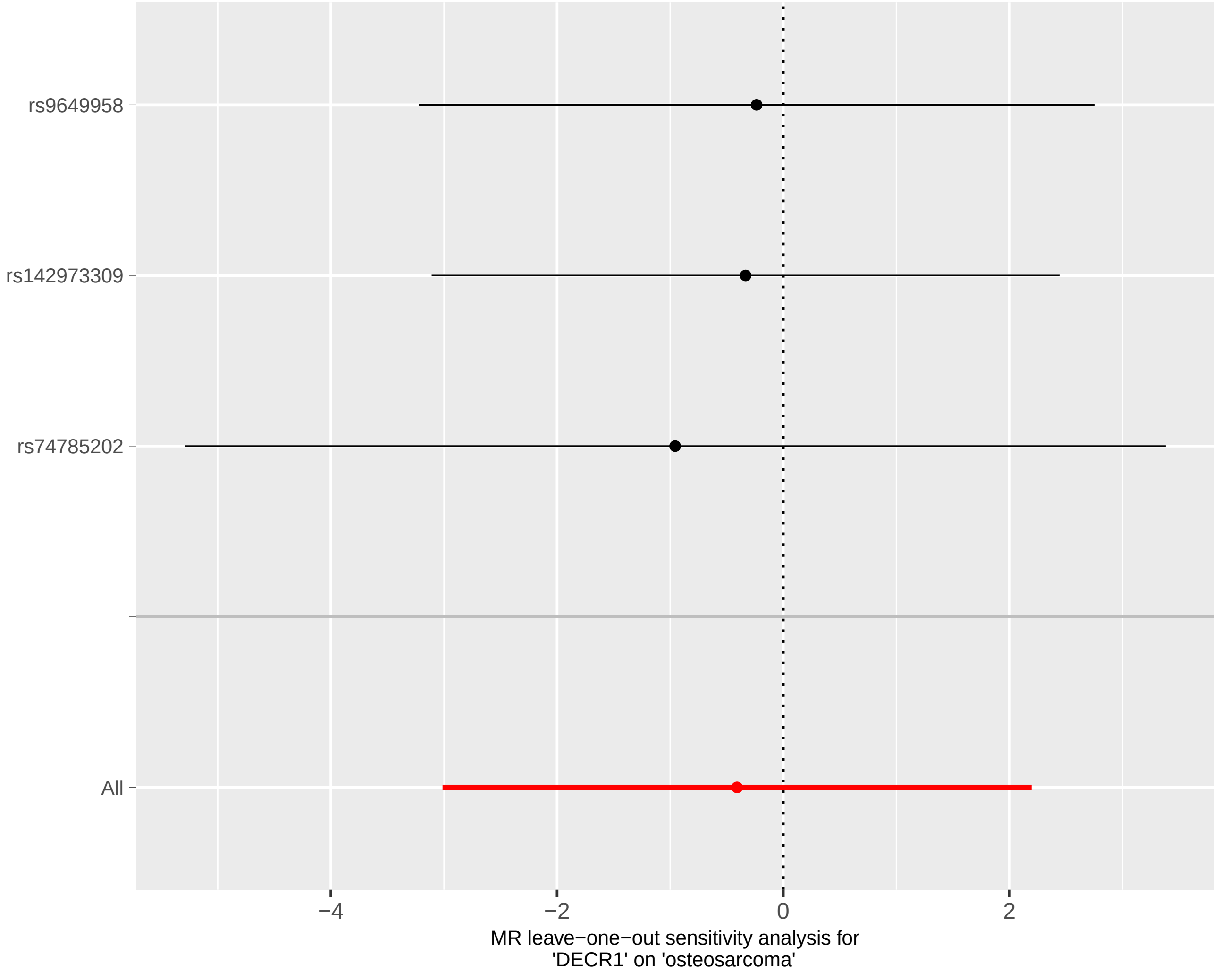

rs9649958
rs142973309
rs74785202
All
−4
−2
0
2
MR leave−one−out sensitivity analysis for
'DECR1' on 'osteosarcoma'

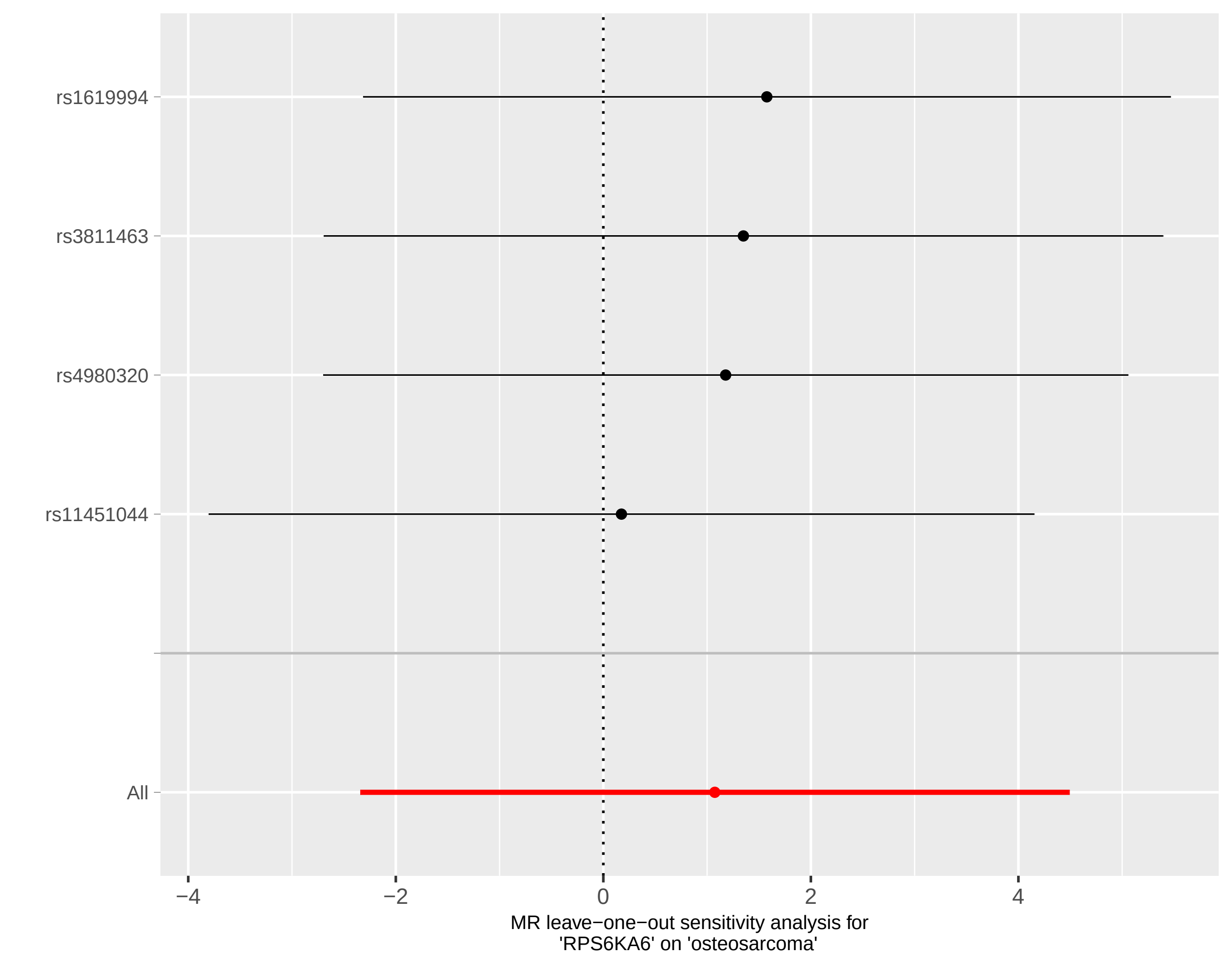
rs1619994
rs3811463
rs4980320
rs11451044
All
−4
−2
0
2
4
MR leave−one−out sensitivity analysis for
'RPS6KA6' on 'osteosarcoma'

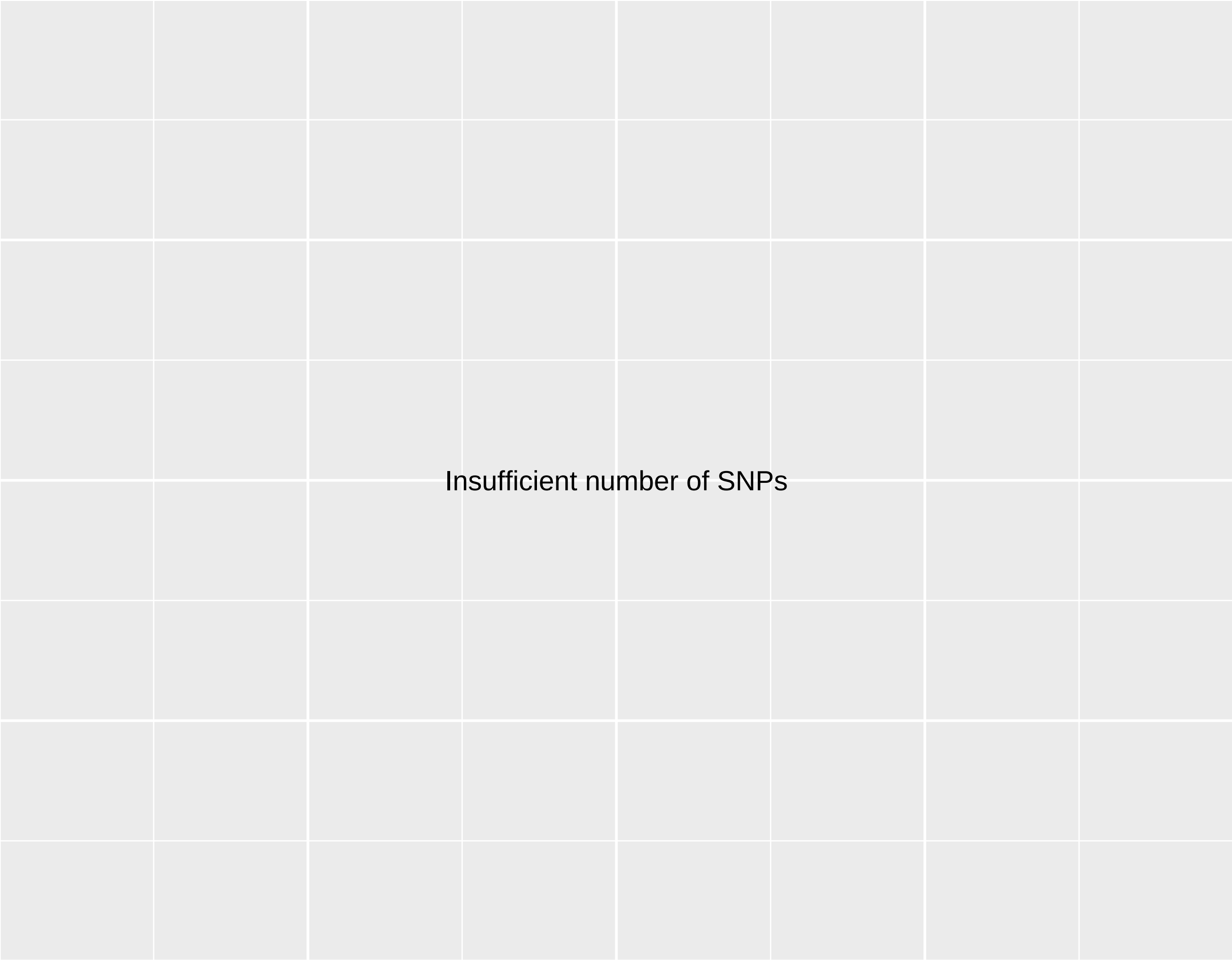
Insufficient number of SNPs

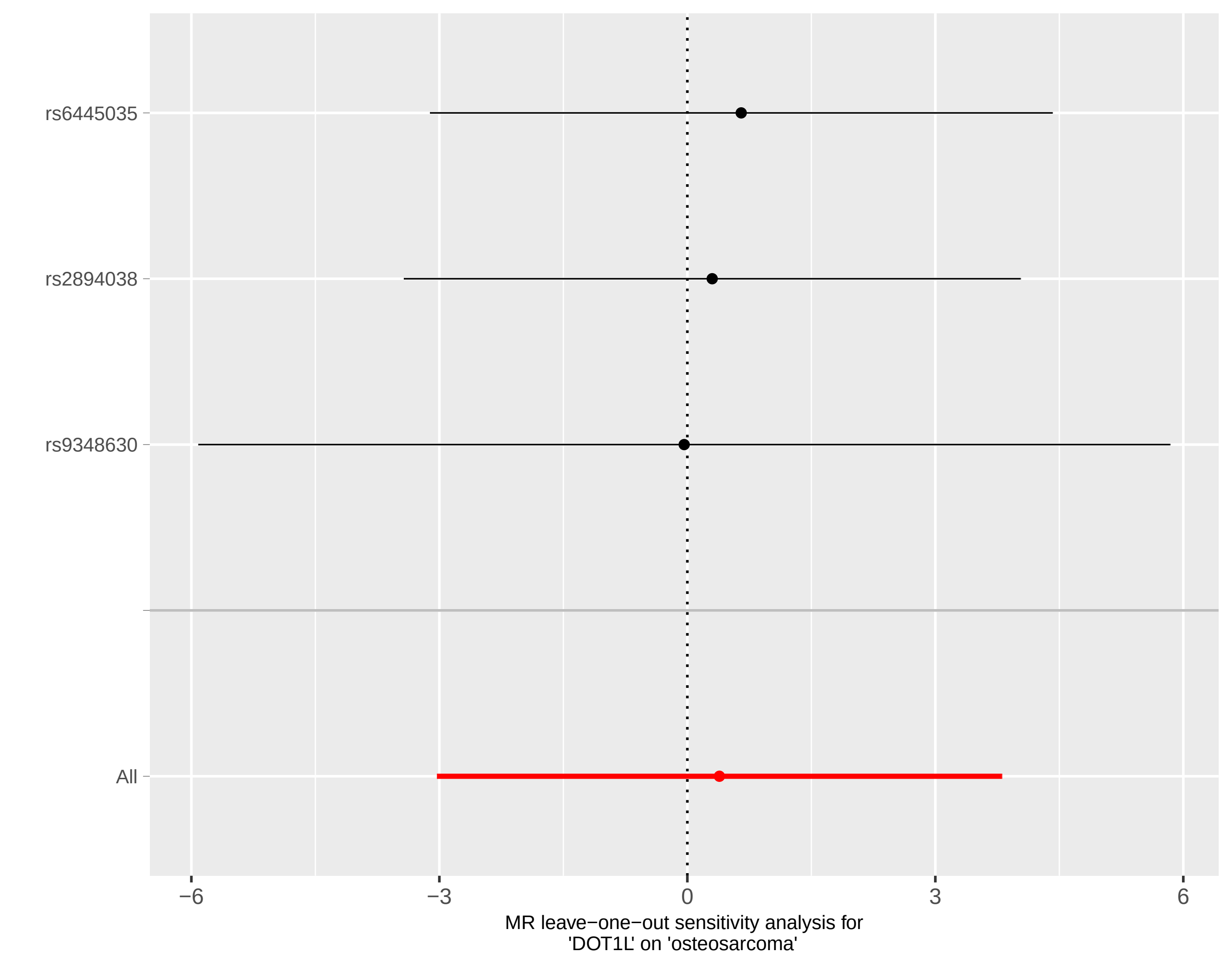
rs6445035
rs2894038
rs9348630
All
−6
−3
0
3
6
MR leave−one−out sensitivity analysis for
'DOT1L' on 'osteosarcoma'

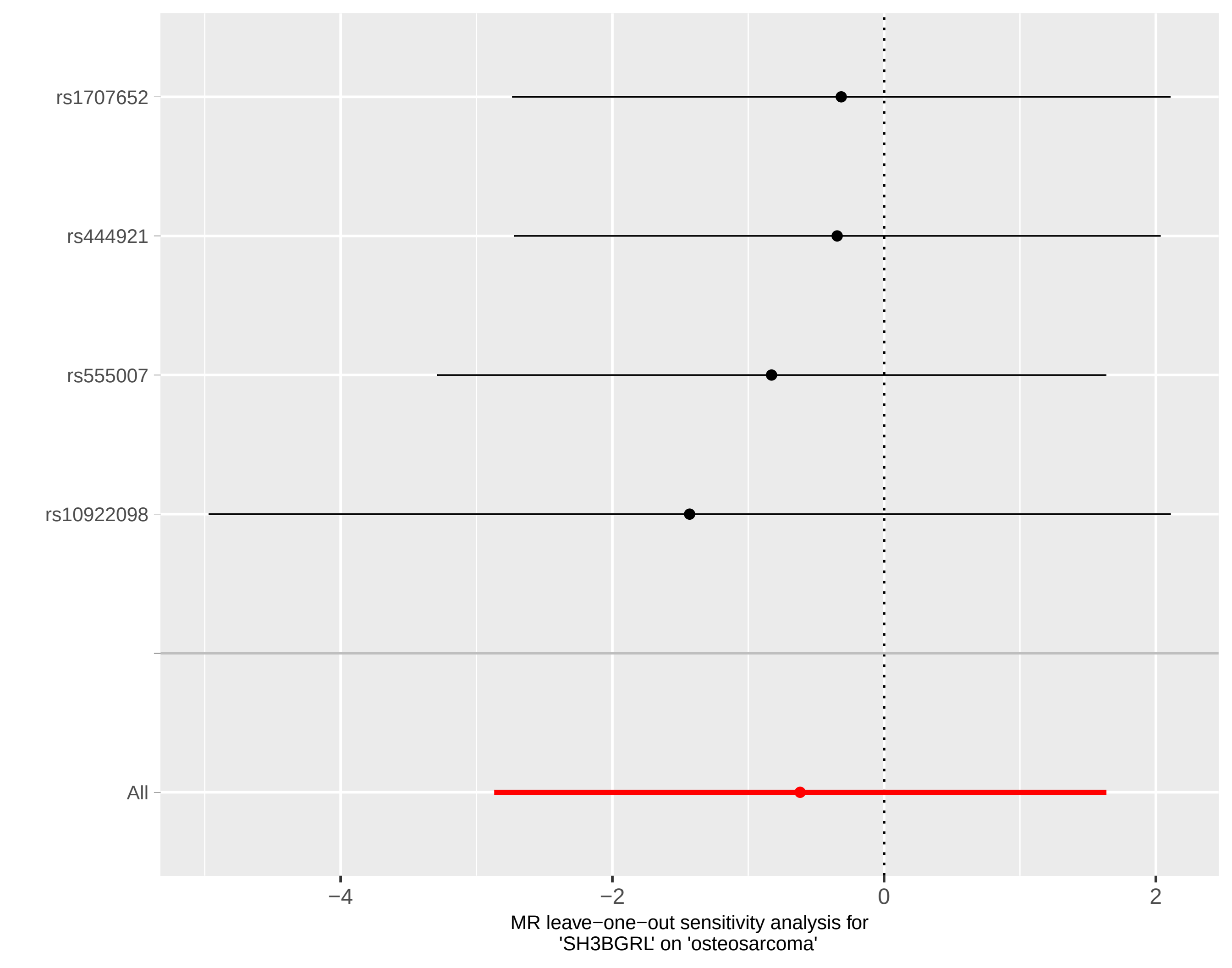
rs1707652
rs444921
rs555007
rs10922098
All
−4
−2
0
2
MR leave−one−out sensitivity analysis for
'SH3BGRL' on 'osteosarcoma'

Insufficient number of SNPs

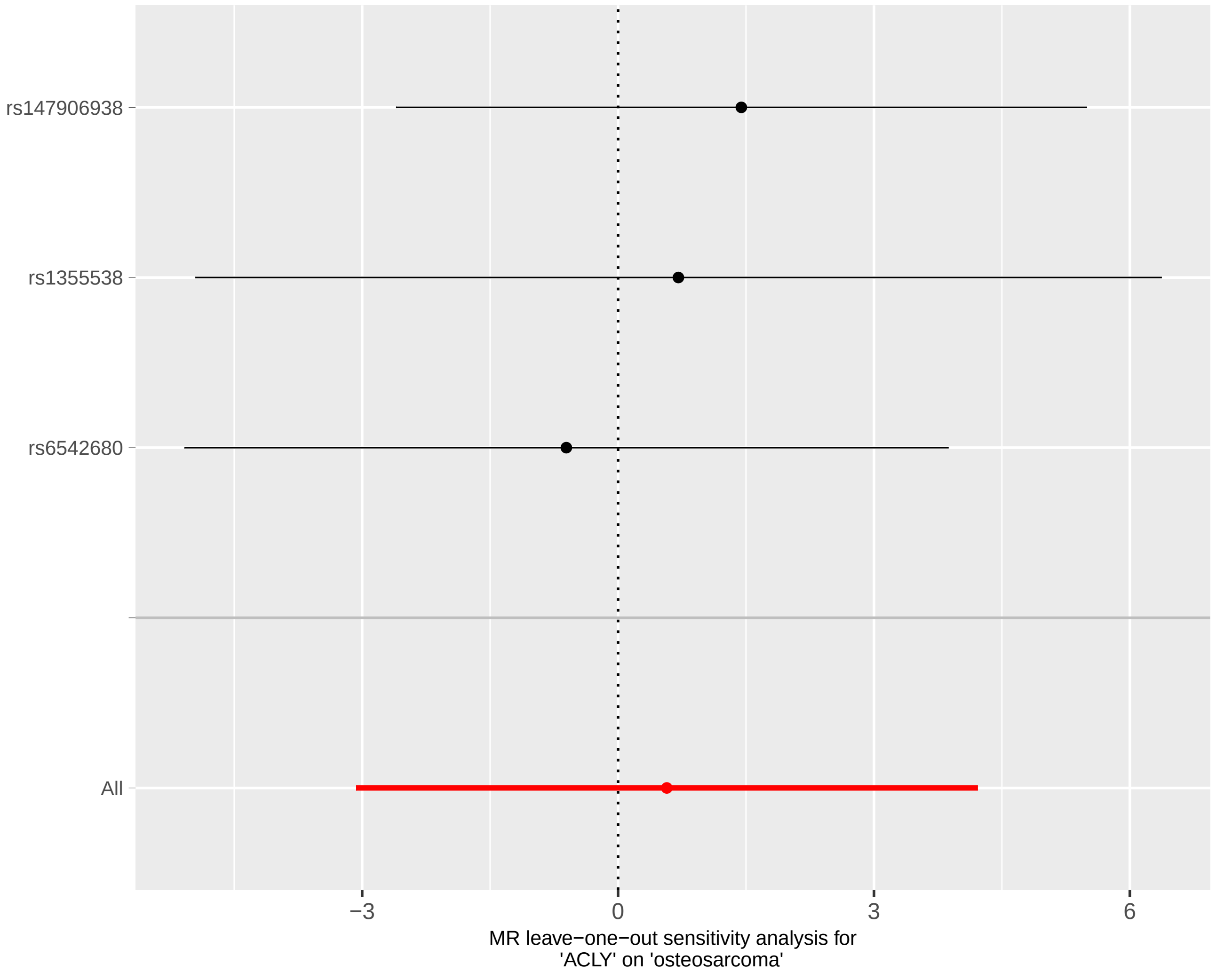

rs147906938
rs1355538
rs6542680
All
−3
0
3
6
MR leave−one−out sensitivity analysis for
'ACLY' on 'osteosarcoma'

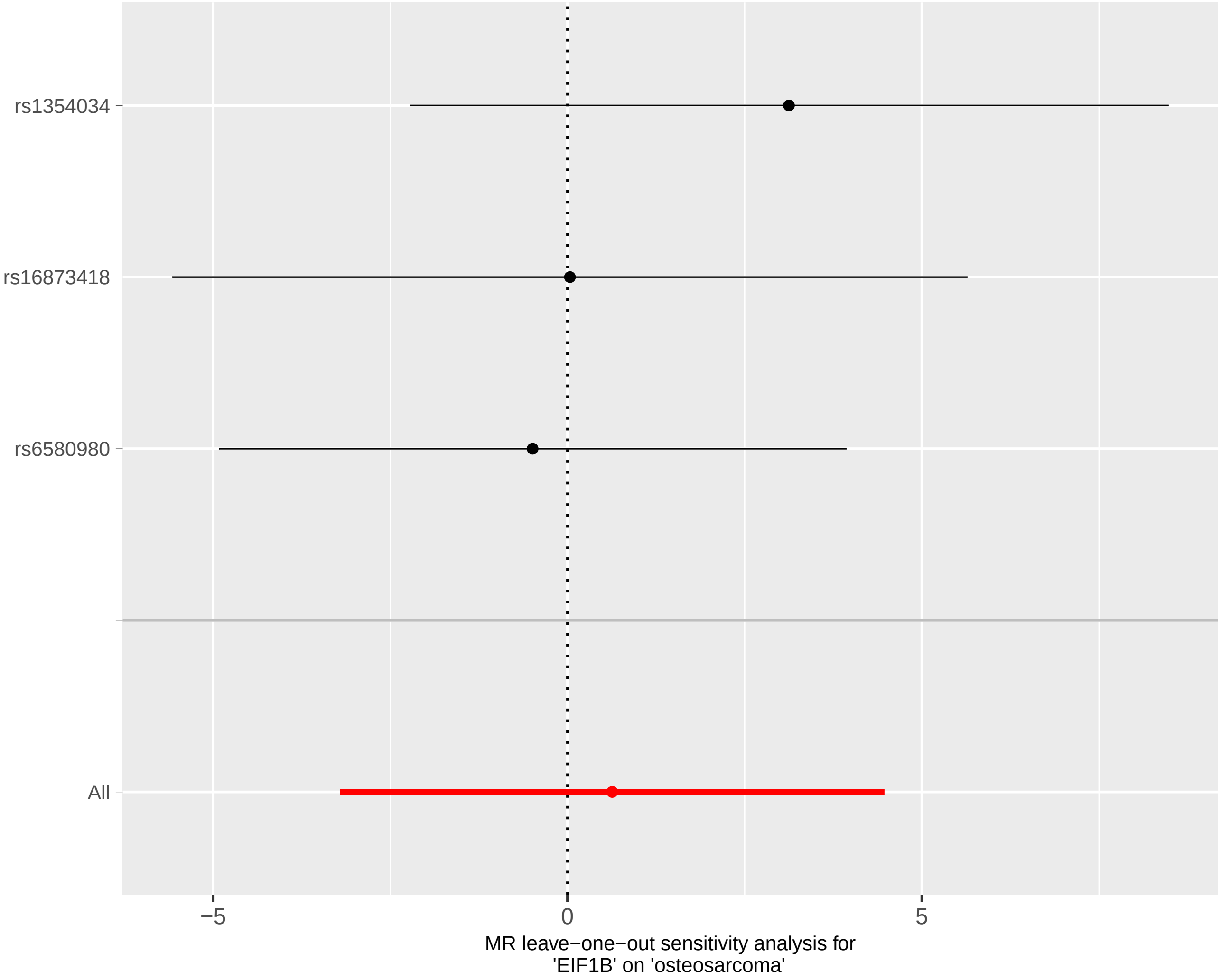
rs1354034
rs16873418
rs6580980
All
−5
0
5
MR leave−one−out sensitivity analysis for
'EIF1B' on 'osteosarcoma'

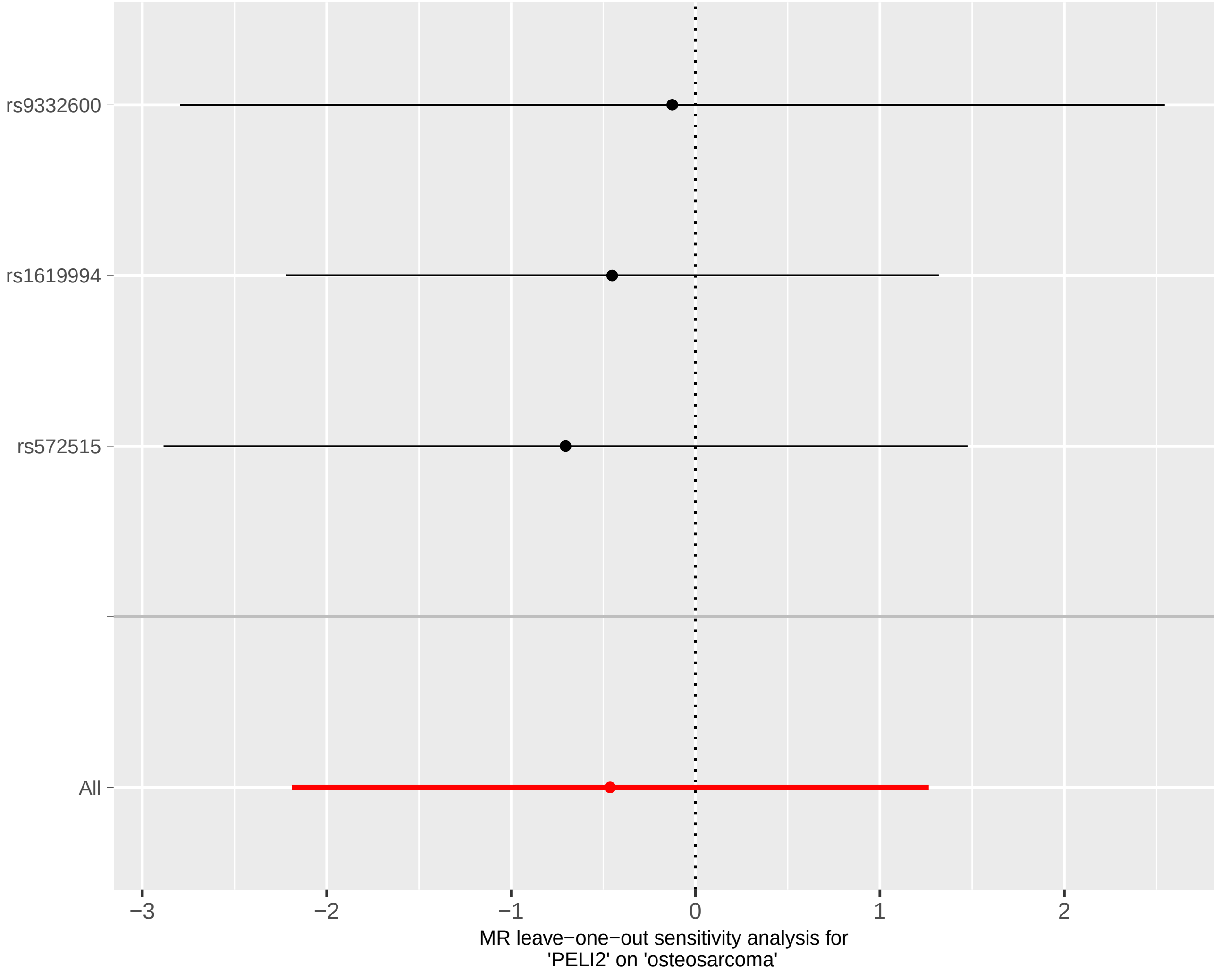
rs9332600
rs1619994
rs572515
All
−3
−2
−1
0
1
2
MR leave−one−out sensitivity analysis for
'PELI2' on 'osteosarcoma'

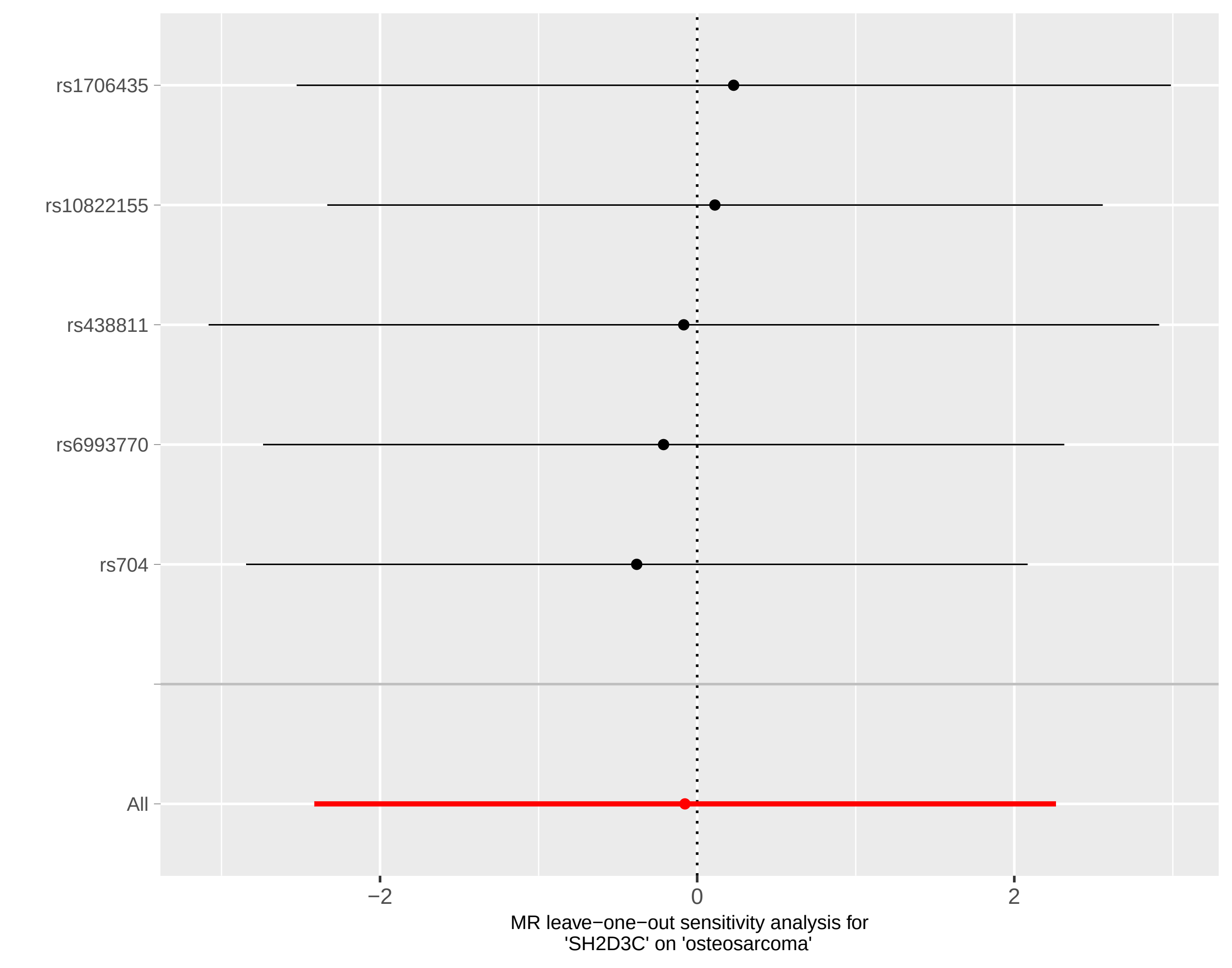
rs1706435
rs10822155
rs438811
rs6993770
rs704
All
−2
0
2
MR leave−one−out sensitivity analysis for
'SH2D3C' on 'osteosarcoma'

Insufficient number of SNPs

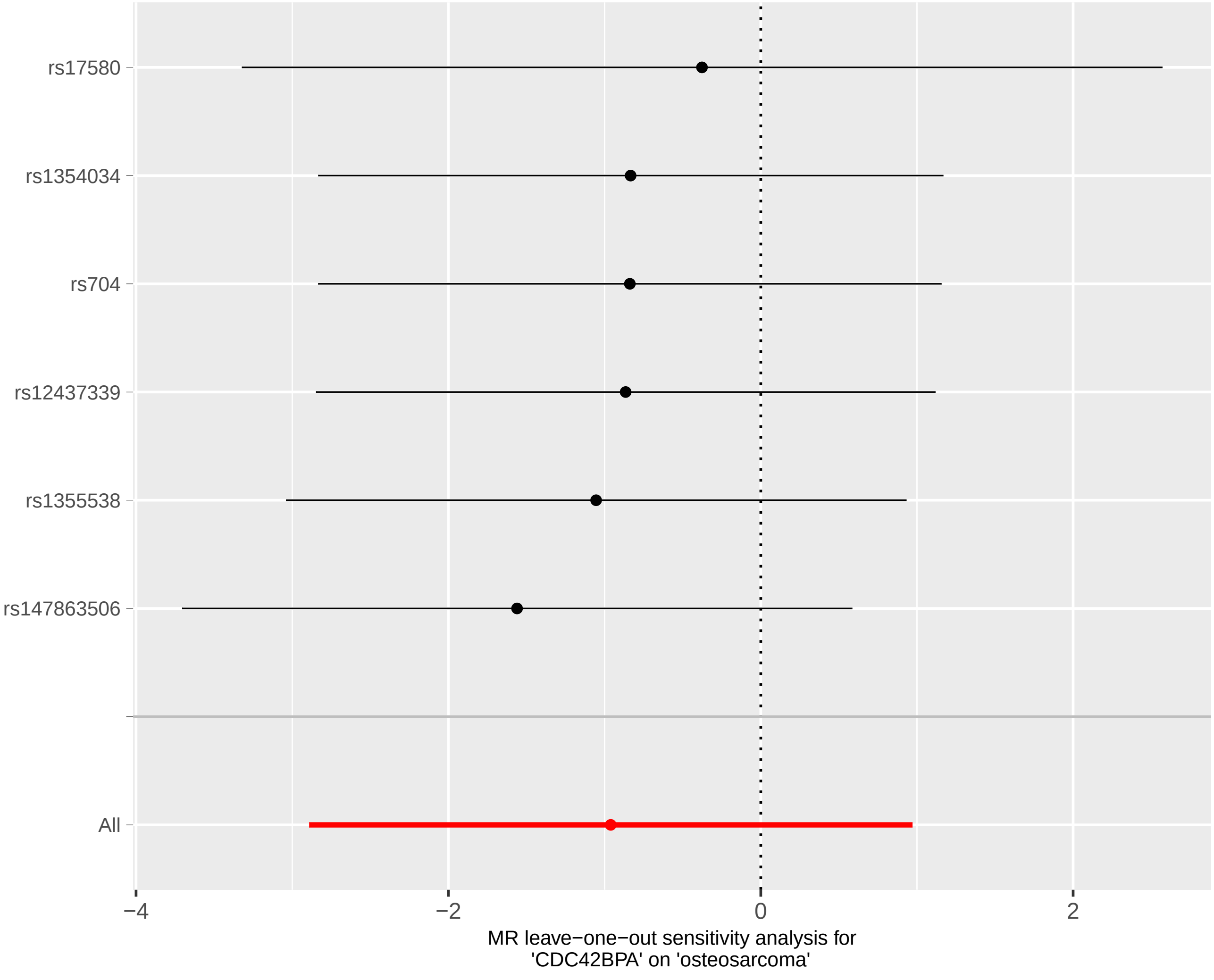

rs17580
rs1354034
rs704
rs12437339
rs1355538
rs147863506
All
−4
−2
0
2
MR leave−one−out sensitivity analysis for
'CDC42BPA' on 'osteosarcoma'

Insufficient number of SNPs

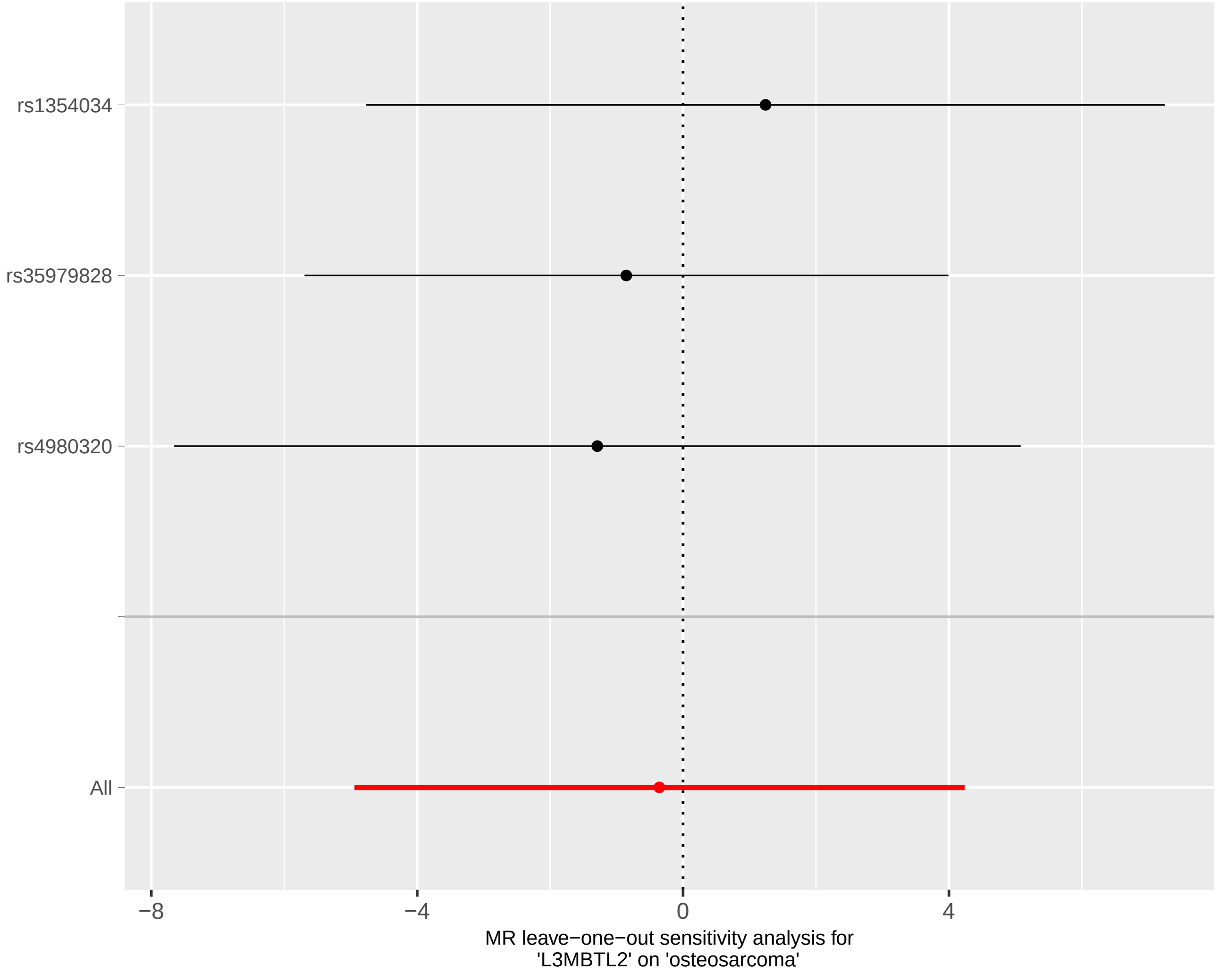

rs1354034
rs35979828
rs4980320
All
−8
−4
0
4
MR leave−one−out sensitivity analysis for
'L3MBTL2' on 'osteosarcoma'

Insufficient number of SNPs

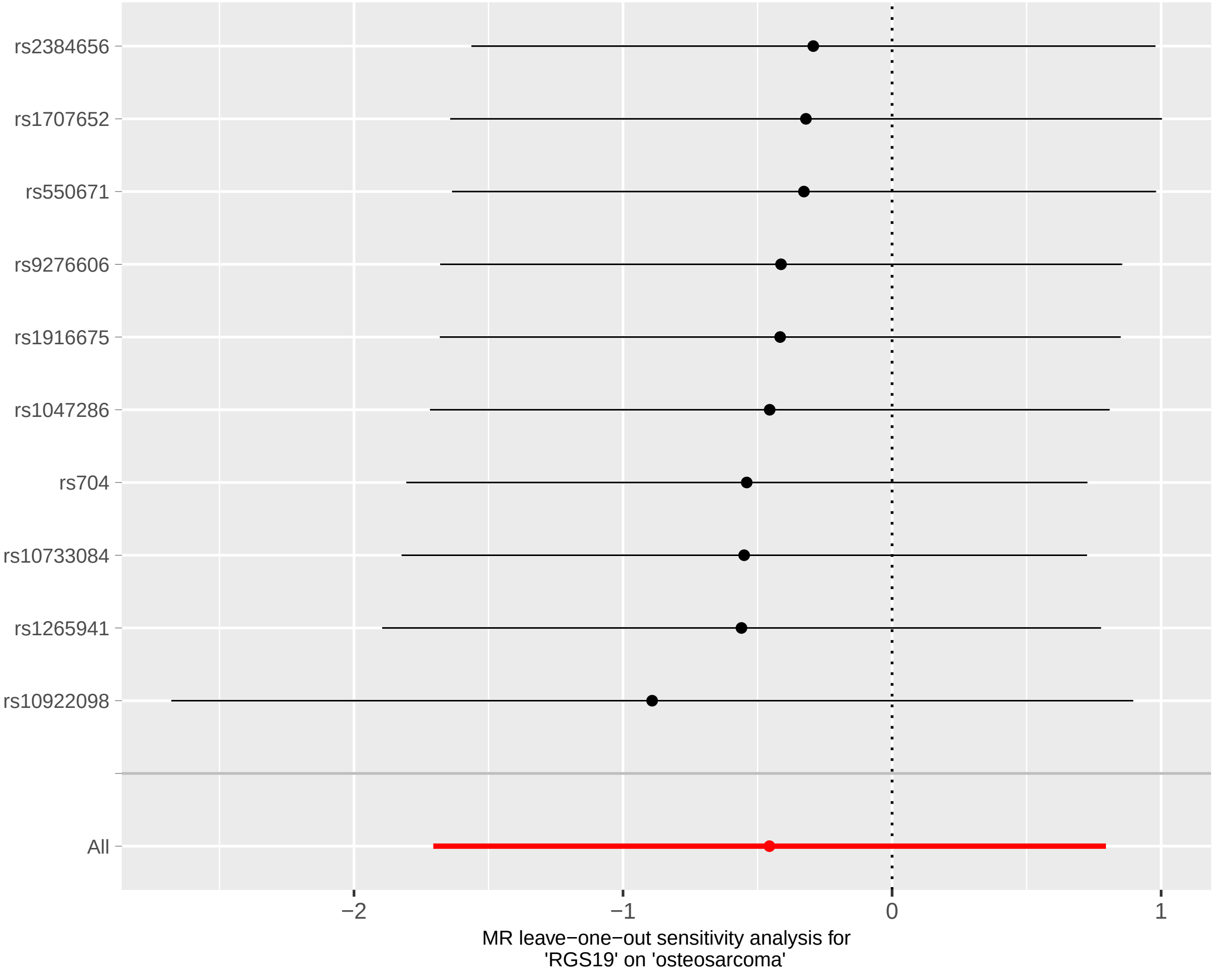

rs2384656
rs1707652
rs550671
rs9276606
rs1916675
rs1047286
rs704
rs10733084
rs1265941
rs10922098
All
−2
−1
0
1
MR leave−one−out sensitivity analysis for
'RGS19' on 'osteosarcoma'

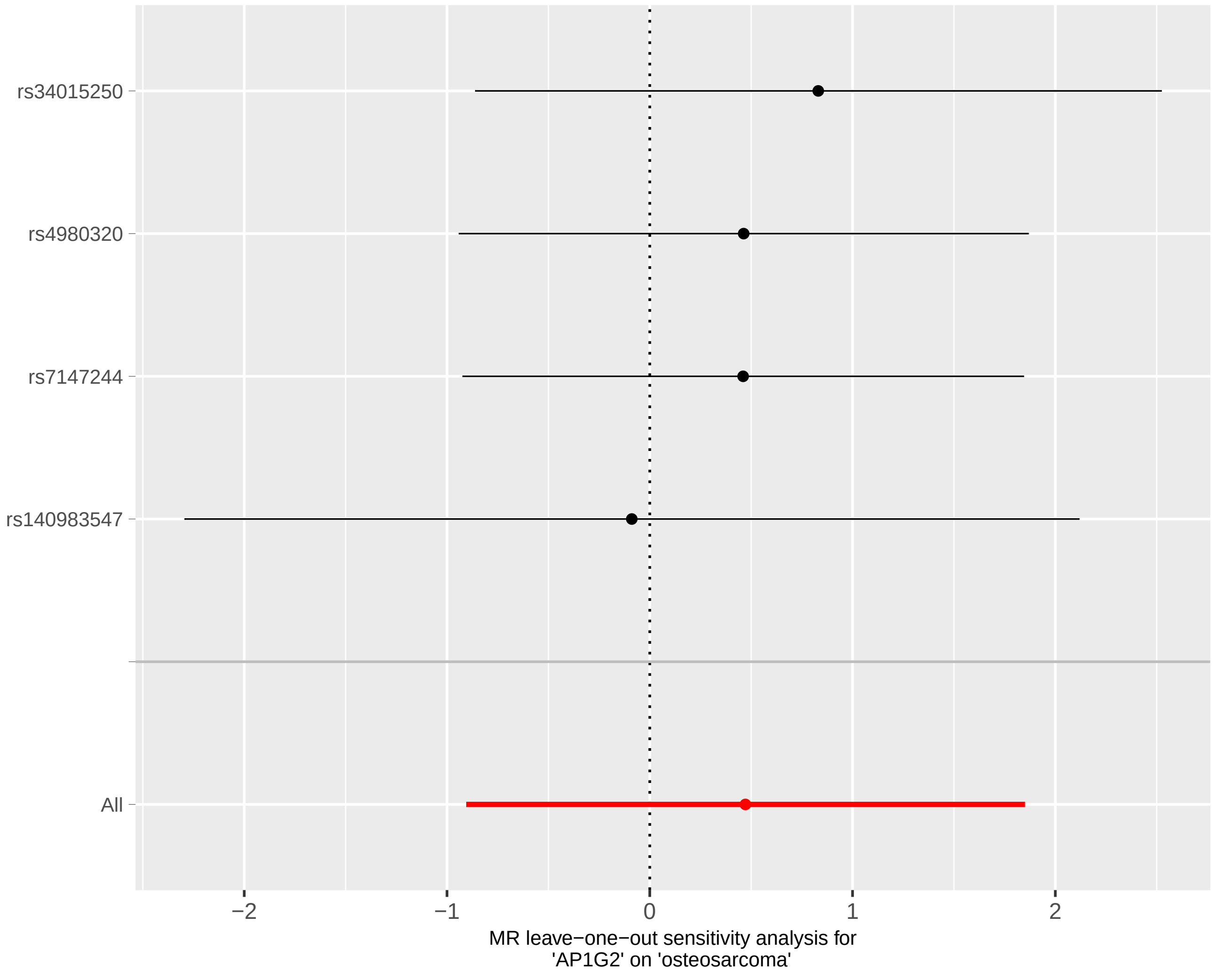
rs34015250
rs4980320
rs7147244
rs140983547
All
−2
−1
0
1
2
MR leave−one−out sensitivity analysis for
'AP1G2' on 'osteosarcoma'

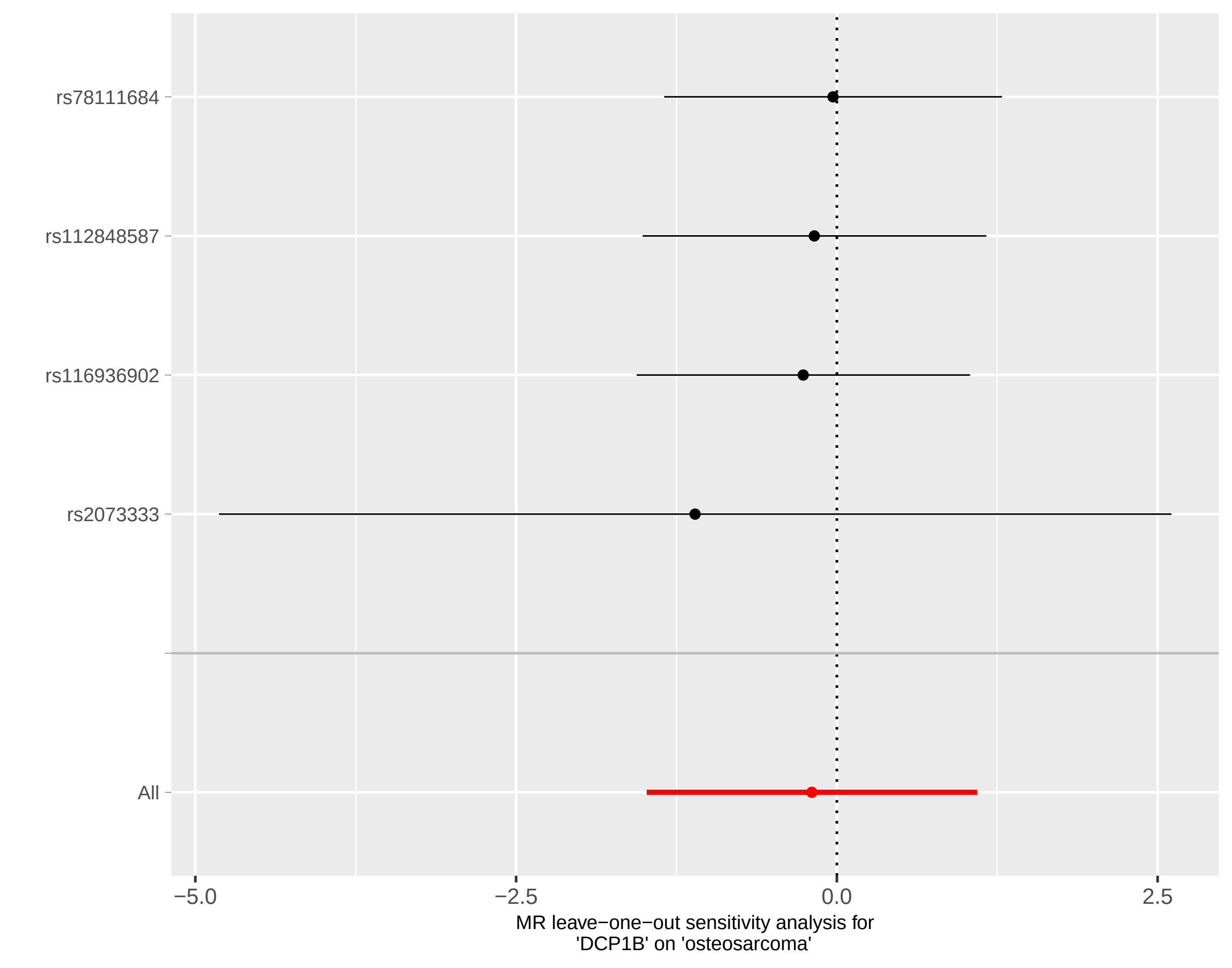
rs78111684
rs112848587
rs116936902
rs2073333
All
−5.0
−2.5
0.0
2.5
MR leave−one−out sensitivity analysis for
'DCP1B' on 'osteosarcoma'

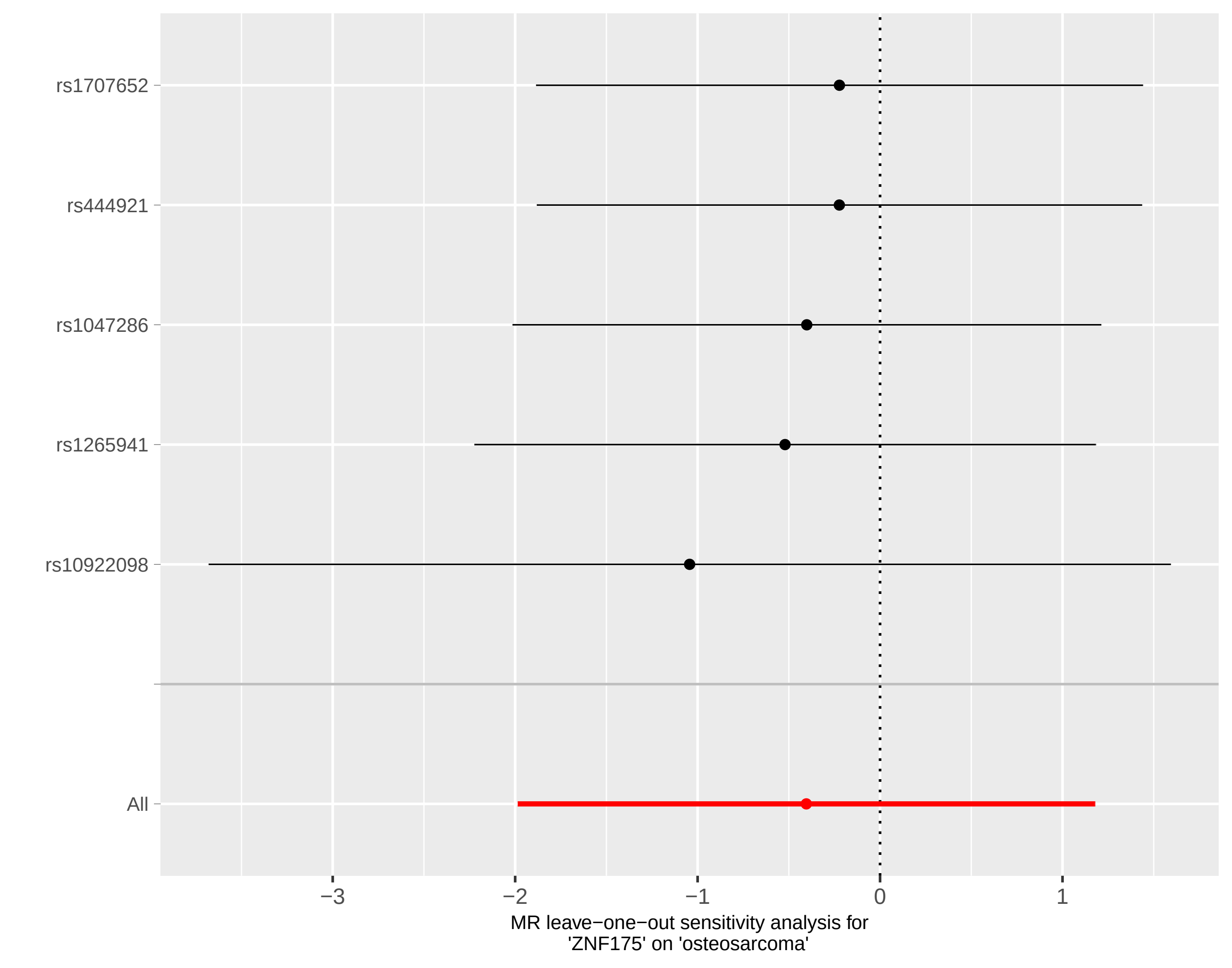

rs1707652
rs444921
rs1047286
rs1265941
rs10922098
All
−3
−2
−1
0
1
MR leave−one−out sensitivity analysis for
'ZNF175' on 'osteosarcoma'

Insufficient number of SNPs

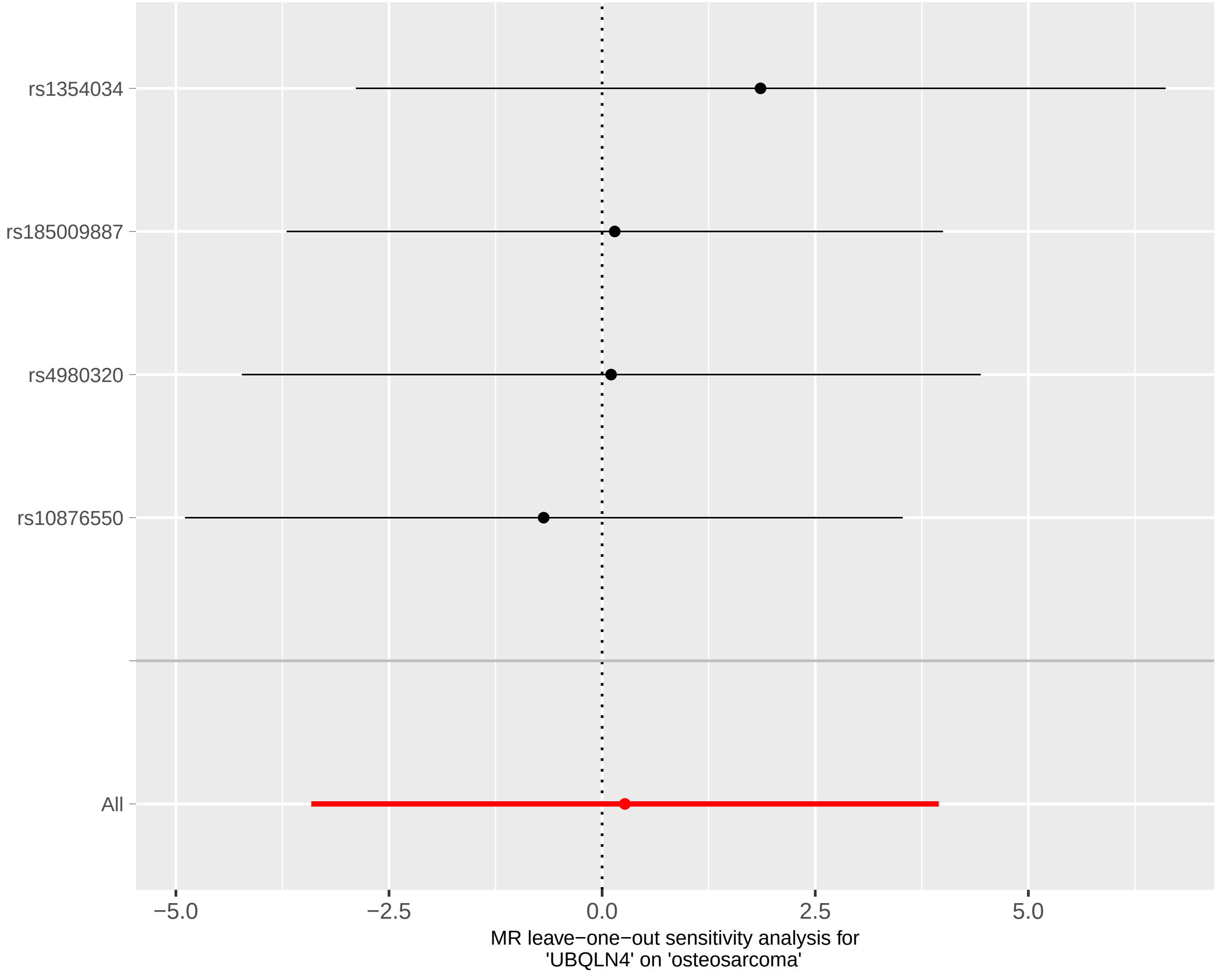
rs1354034
rs185009887
rs4980320
rs10876550
All
−5.0
−2.5
0.0
2.5
5.0
MR leave−one−out sensitivity analysis for
'UBQLN4' on 'osteosarcoma'

Insufficient number of SNPs

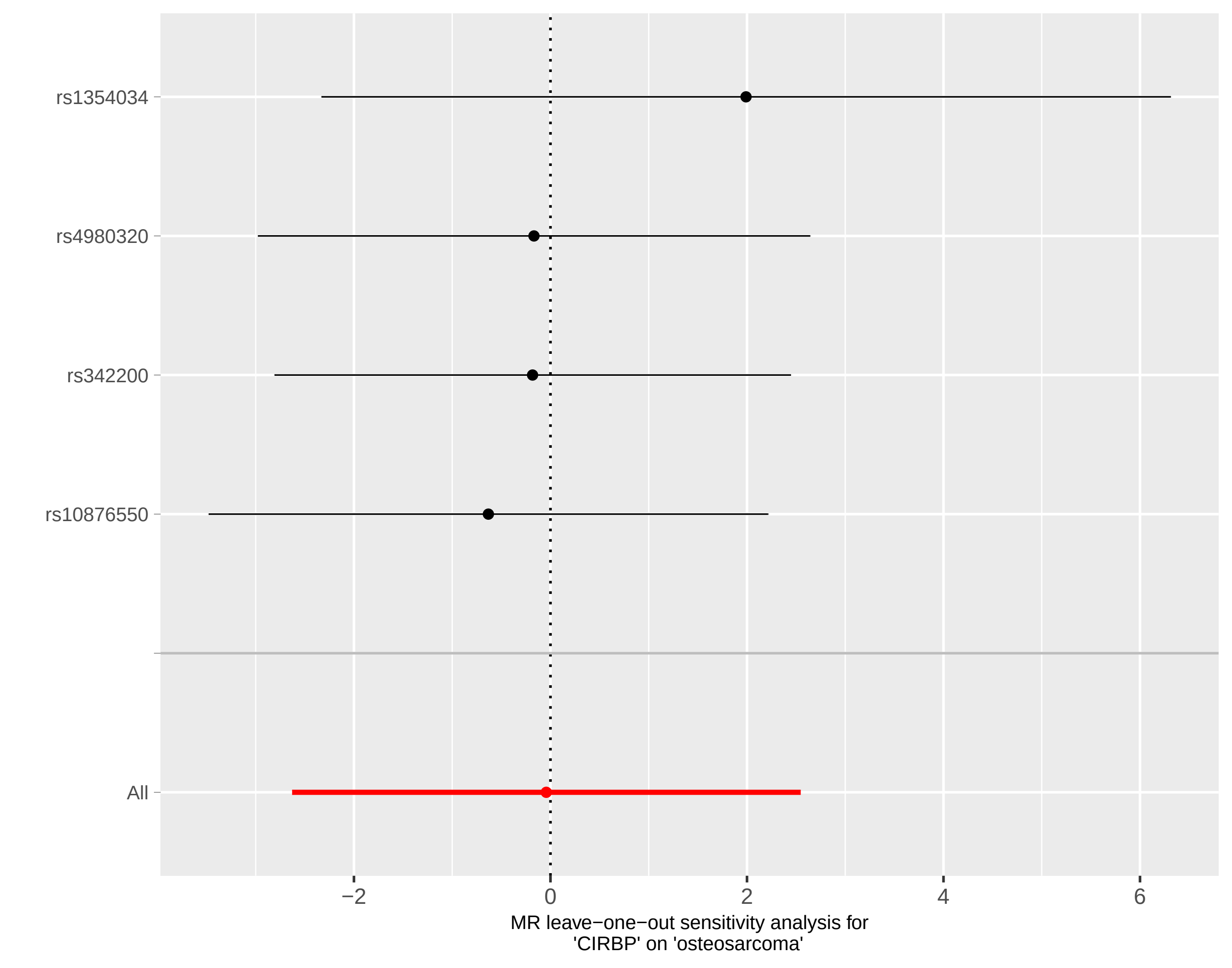
rs1354034
rs4980320
rs342200
rs10876550
All
−2
0
2
4
6
MR leave−one−out sensitivity analysis for
'CIRBP' on 'osteosarcoma'

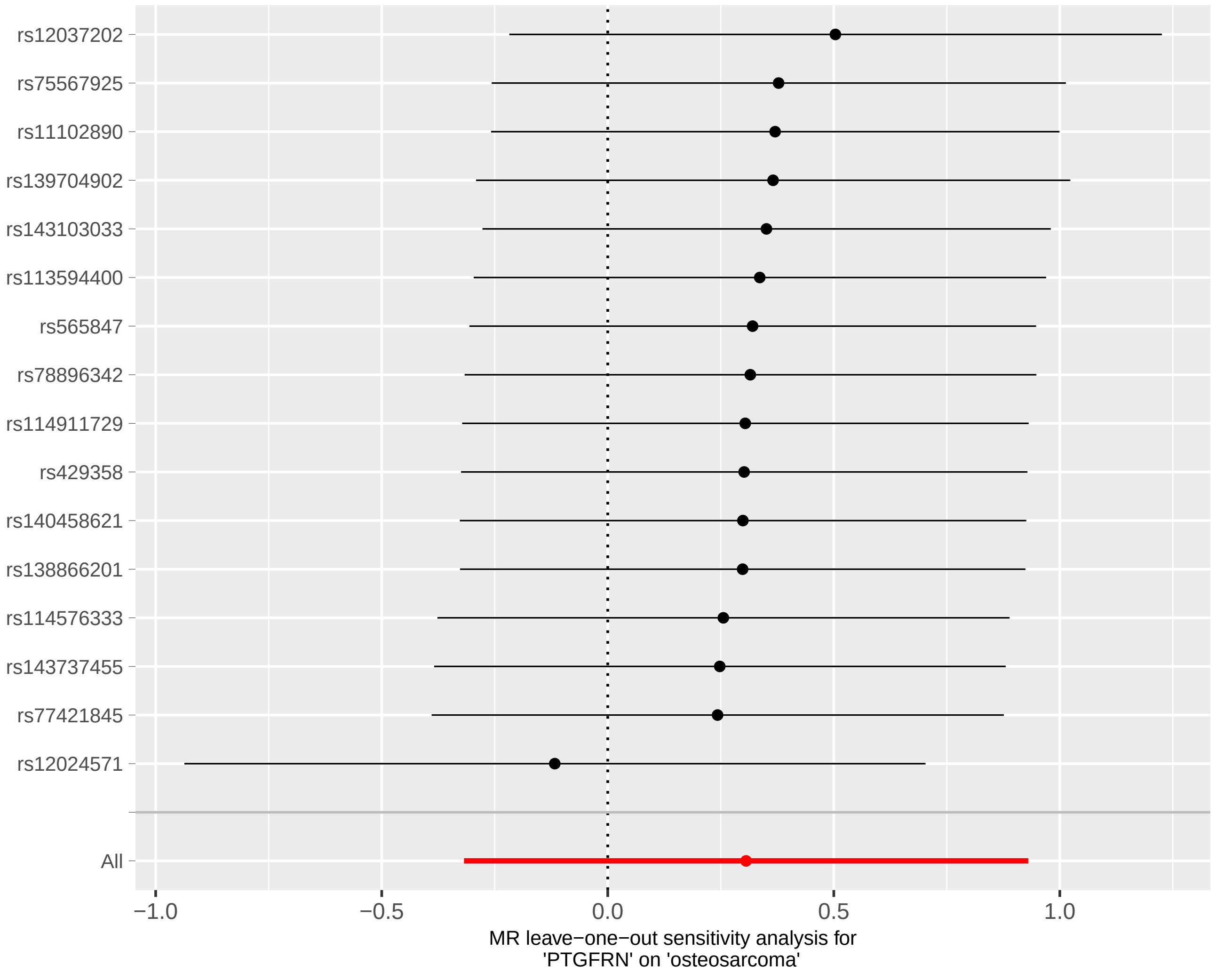
rs12037202
rs75567925
rs11102890
rs139704902
rs143103033
rs113594400
rs565847
rs78896342
rs114911729
rs429358
rs140458621
rs138866201
rs114576333
rs143737455
rs77421845
rs12024571
All
−1.0
−0.5
0.0
0.5
1.0
MR leave−one−out sensitivity analysis for
'PTGFRN' on 'osteosarcoma'

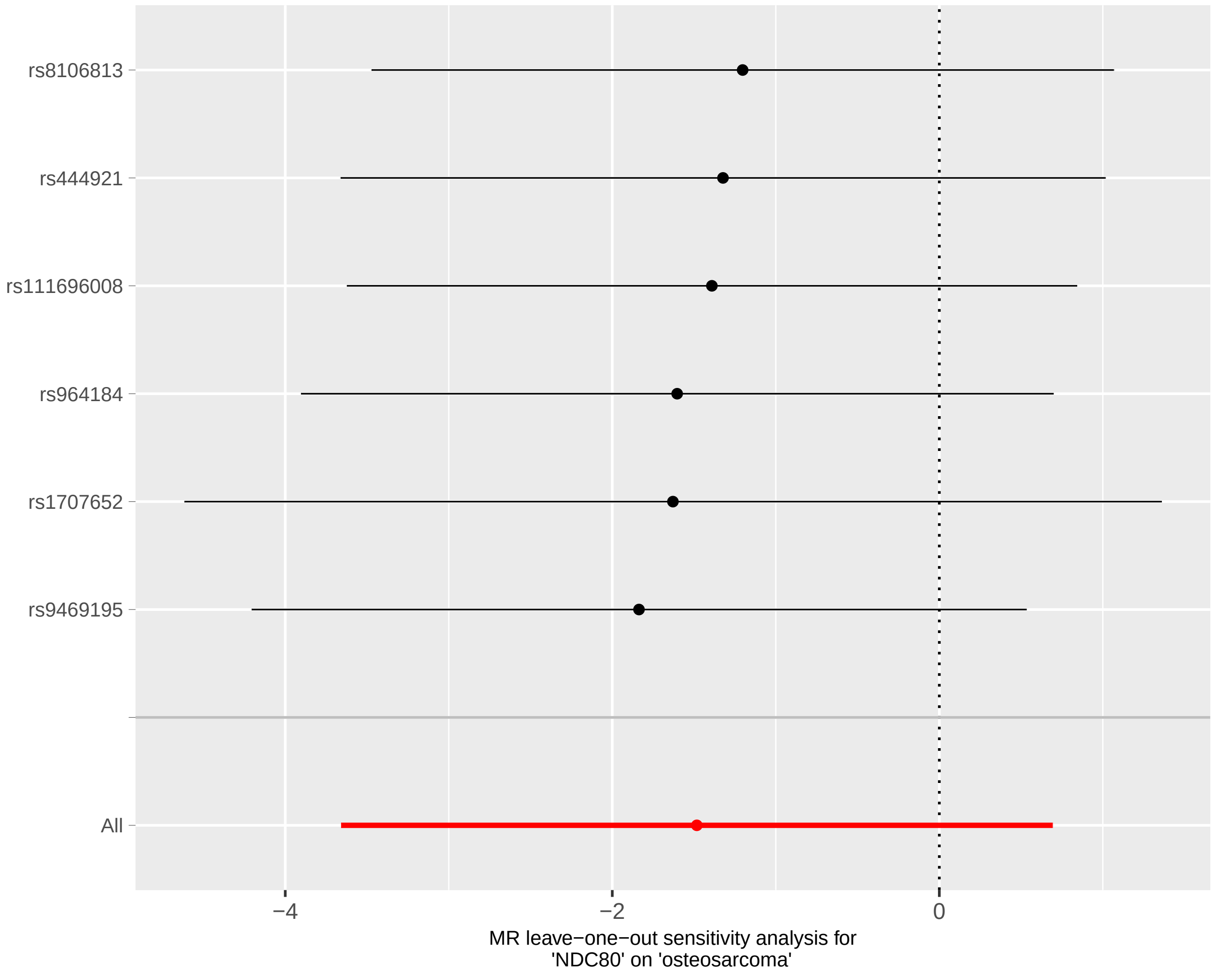
rs8106813
rs444921
rs111696008
rs964184
rs1707652
rs9469195
All
−4
−2
0
MR leave−one−out sensitivity analysis for
'NDC80' on 'osteosarcoma'

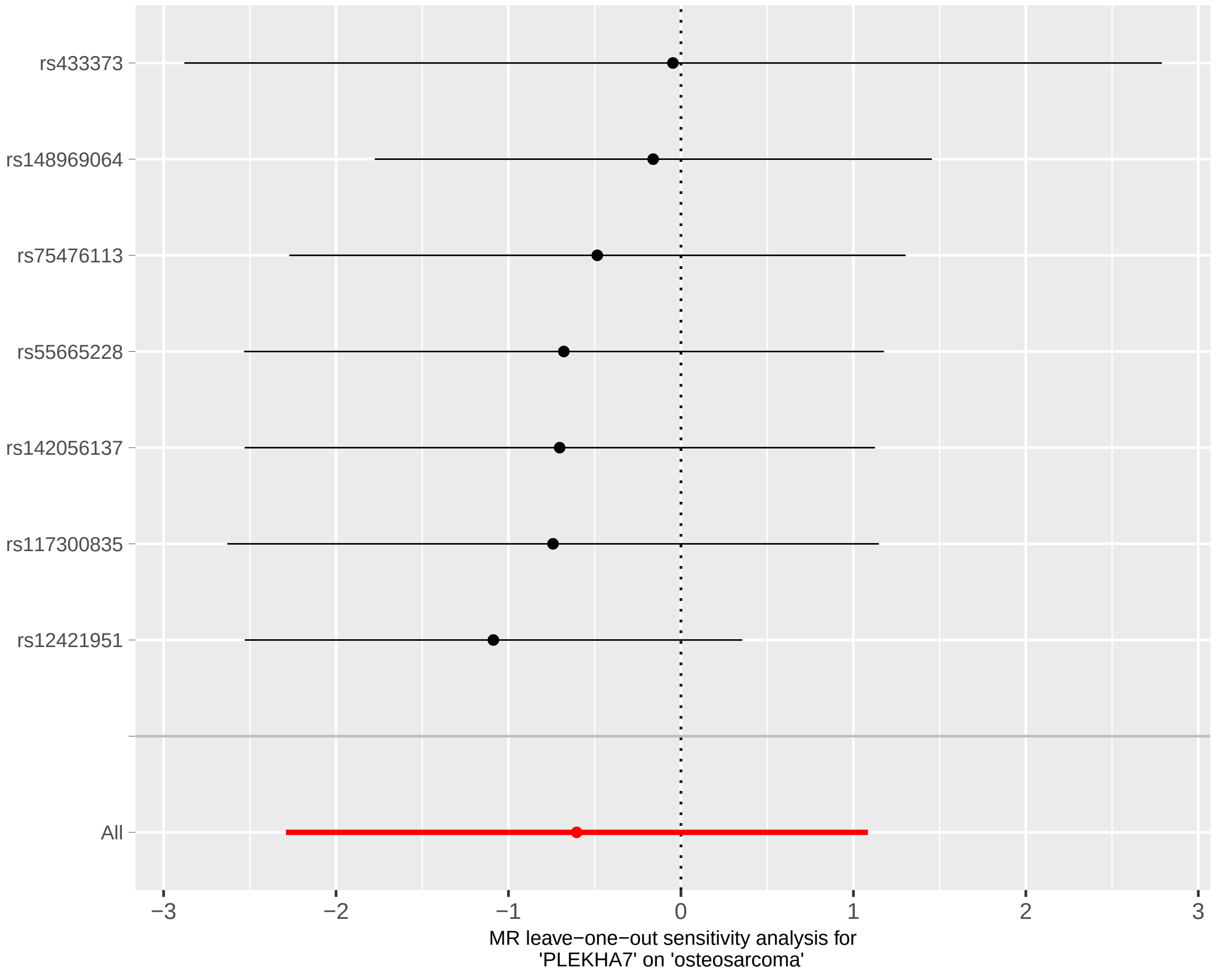

rs433373
rs148969064
rs75476113
rs55665228
rs142056137
rs117300835
rs12421951
All
−3
−2
−1
0
1
2
3
MR leave−one−out sensitivity analysis for
'PLEKHA7' on 'osteosarcoma'

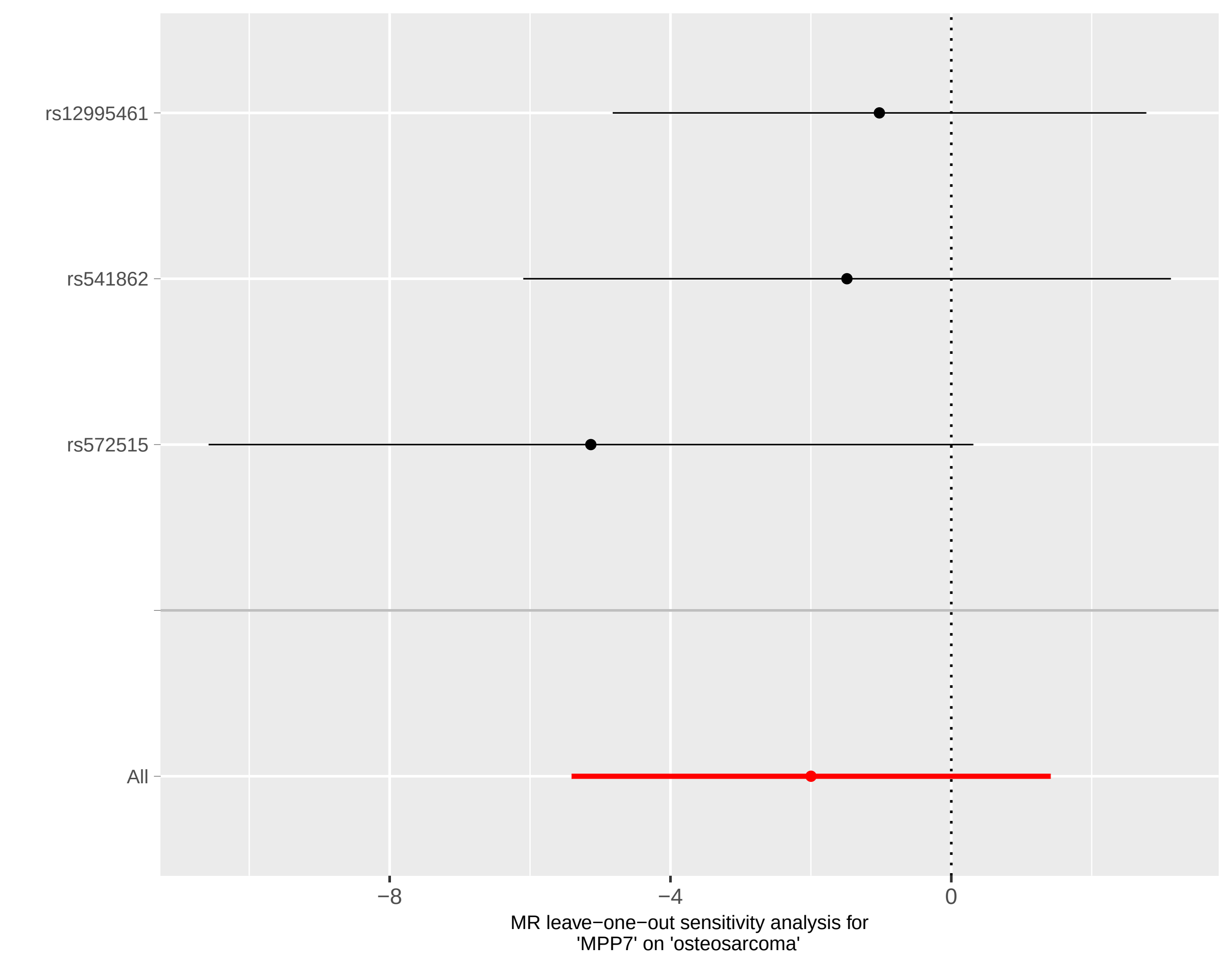
rs12995461
rs541862
rs572515
All
−8
−4
0
MR leave−one−out sensitivity analysis for
'MPP7' on 'osteosarcoma'

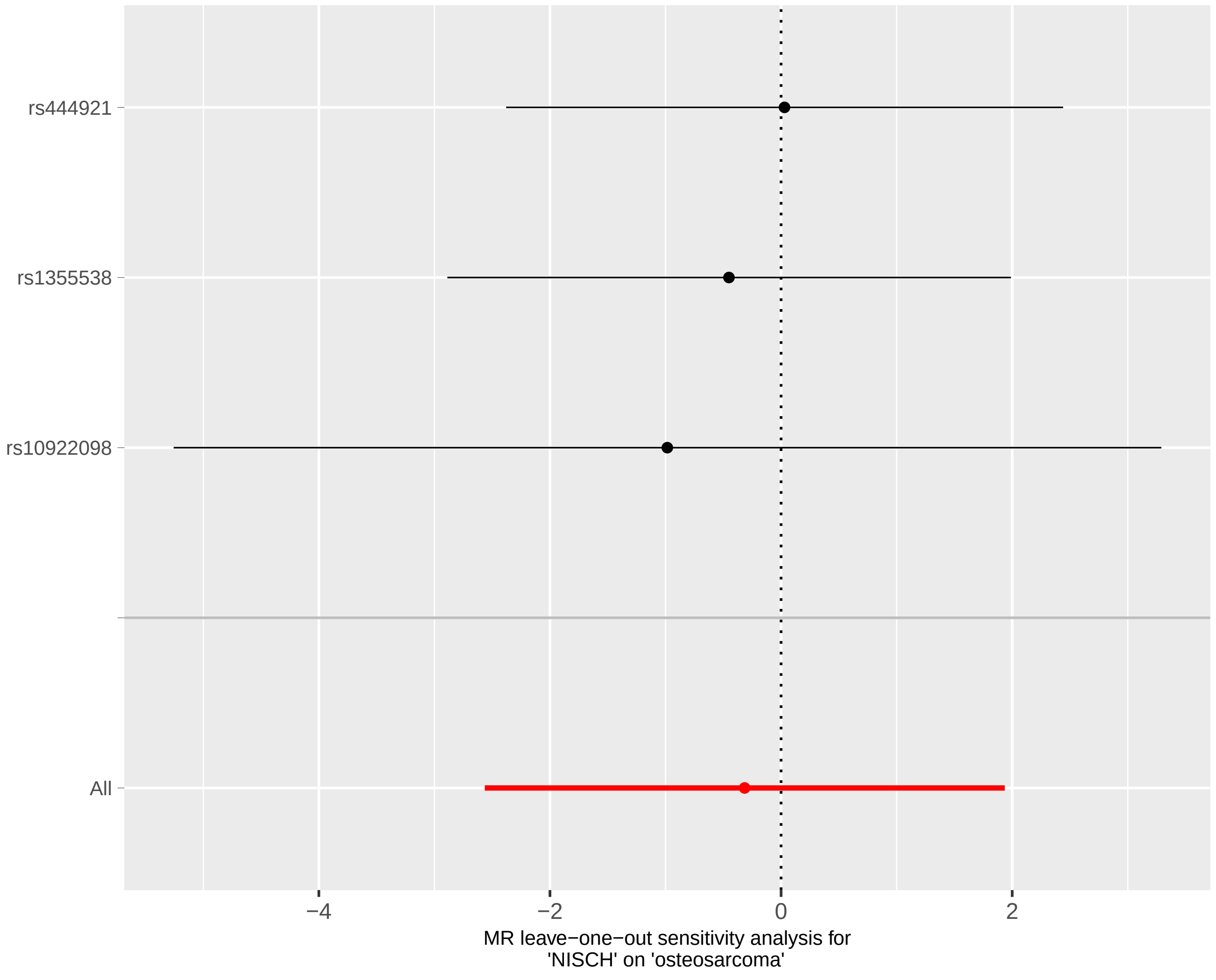
rs444921
rs1355538
rs10922098
All
−4
−2
0
2
MR leave−one−out sensitivity analysis for
'NISCH' on 'osteosarcoma'

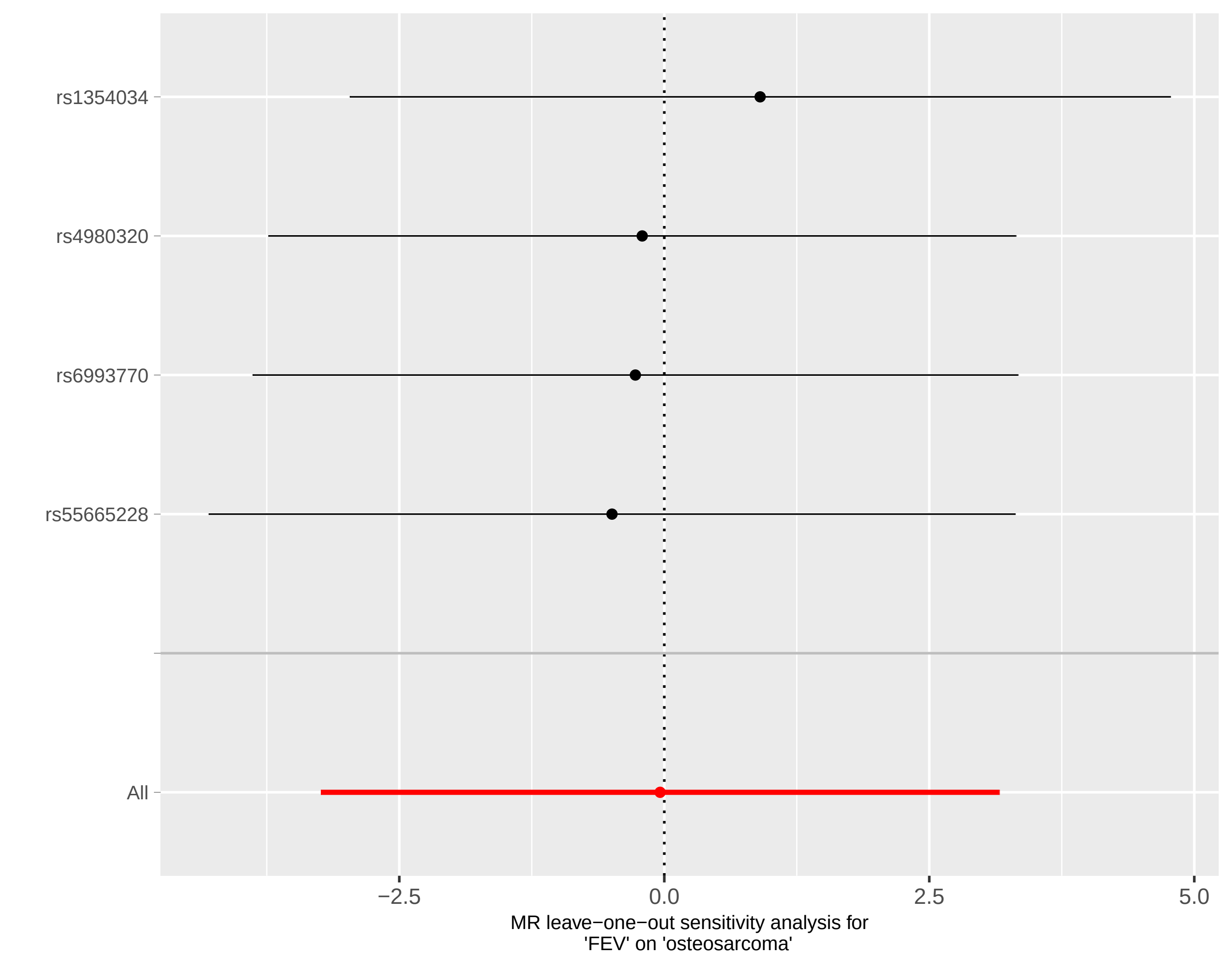
rs1354034
rs4980320
rs6993770
rs55665228
All
−2.5
0.0
2.5
5.0
MR leave−one−out sensitivity analysis for
'FEV' on 'osteosarcoma'

Insufficient number of SNPs

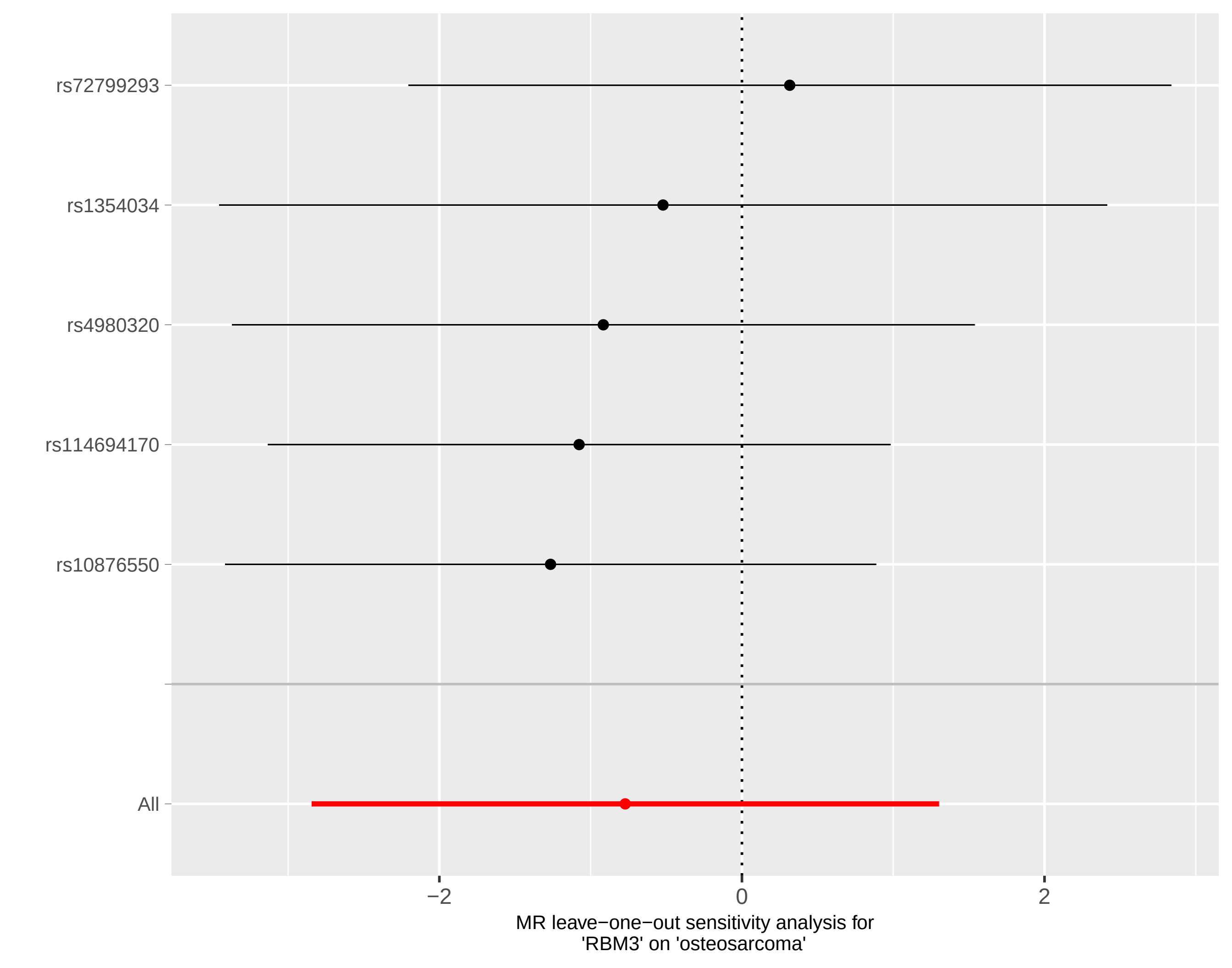
rs72799293
rs1354034
rs4980320
rs114694170
rs10876550
All
−2
0
2
MR leave−one−out sensitivity analysis for
'RBM3' on 'osteosarcoma'

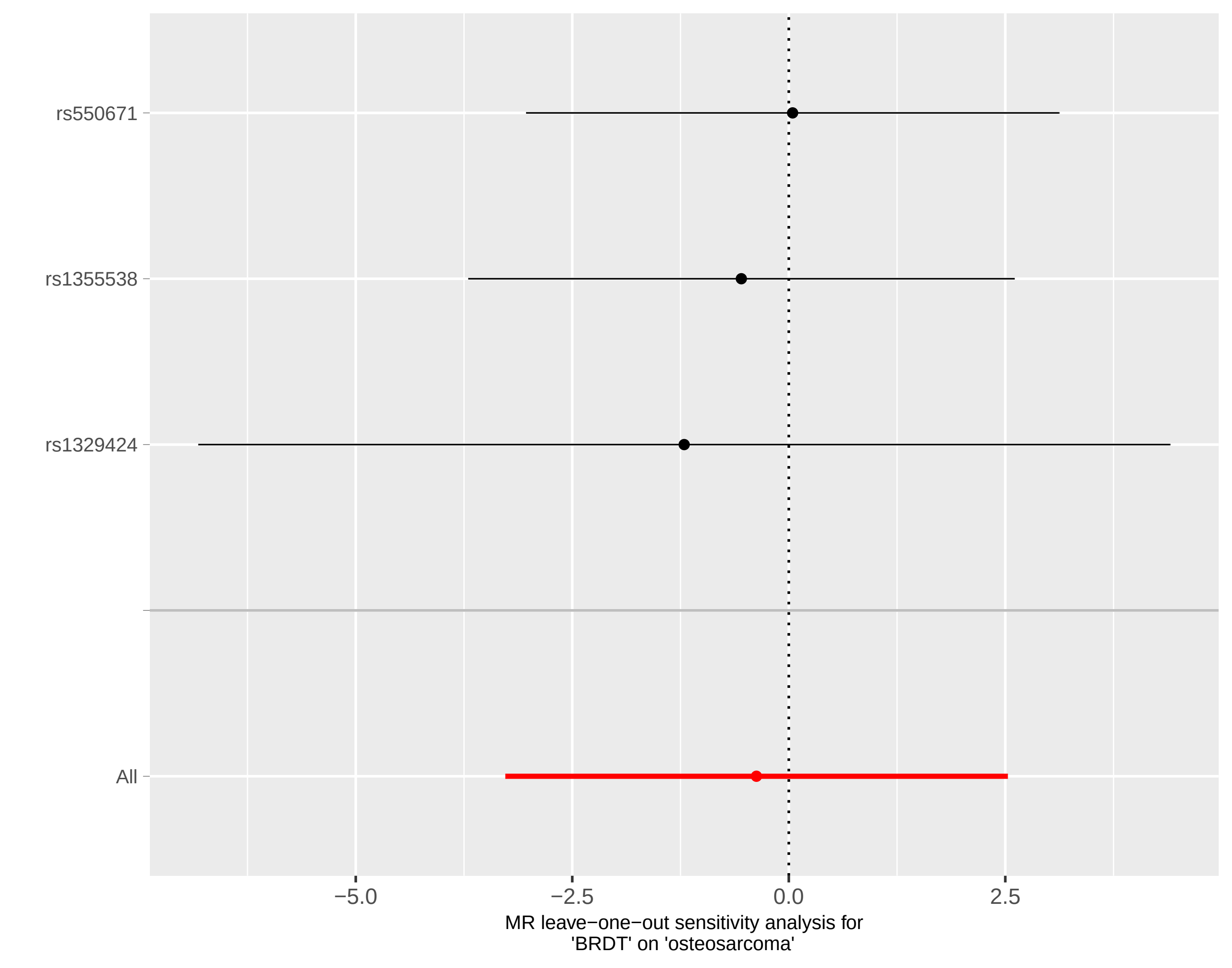
rs550671
rs1355538
rs1329424
All
−5.0
−2.5
0.0
2.5
MR leave−one−out sensitivity analysis for
'BRDT' on 'osteosarcoma'

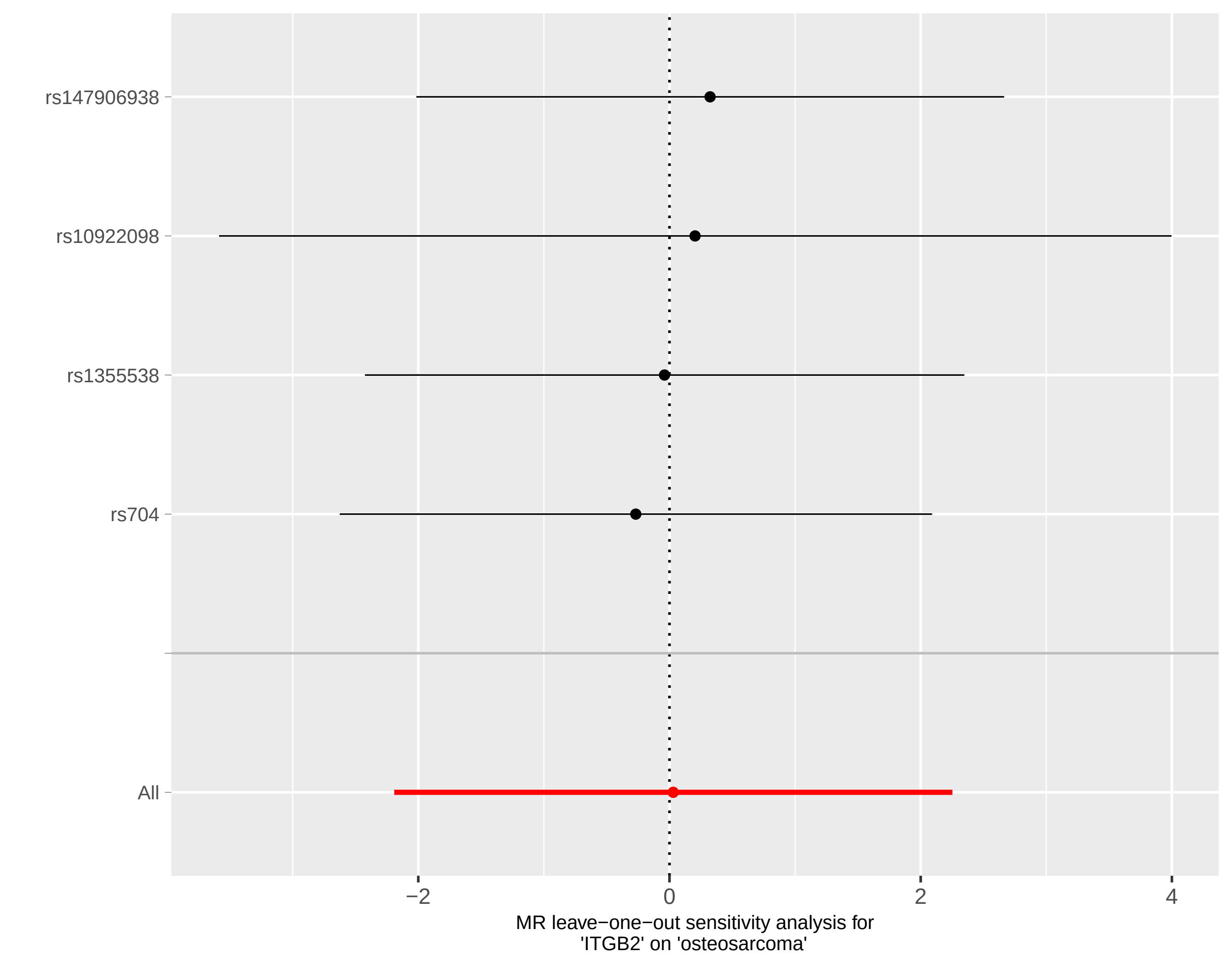
rs147906938
rs10922098
rs1355538
rs704
All
−2
0
2
4
MR leave−one−out sensitivity analysis for
'ITGB2' on 'osteosarcoma'

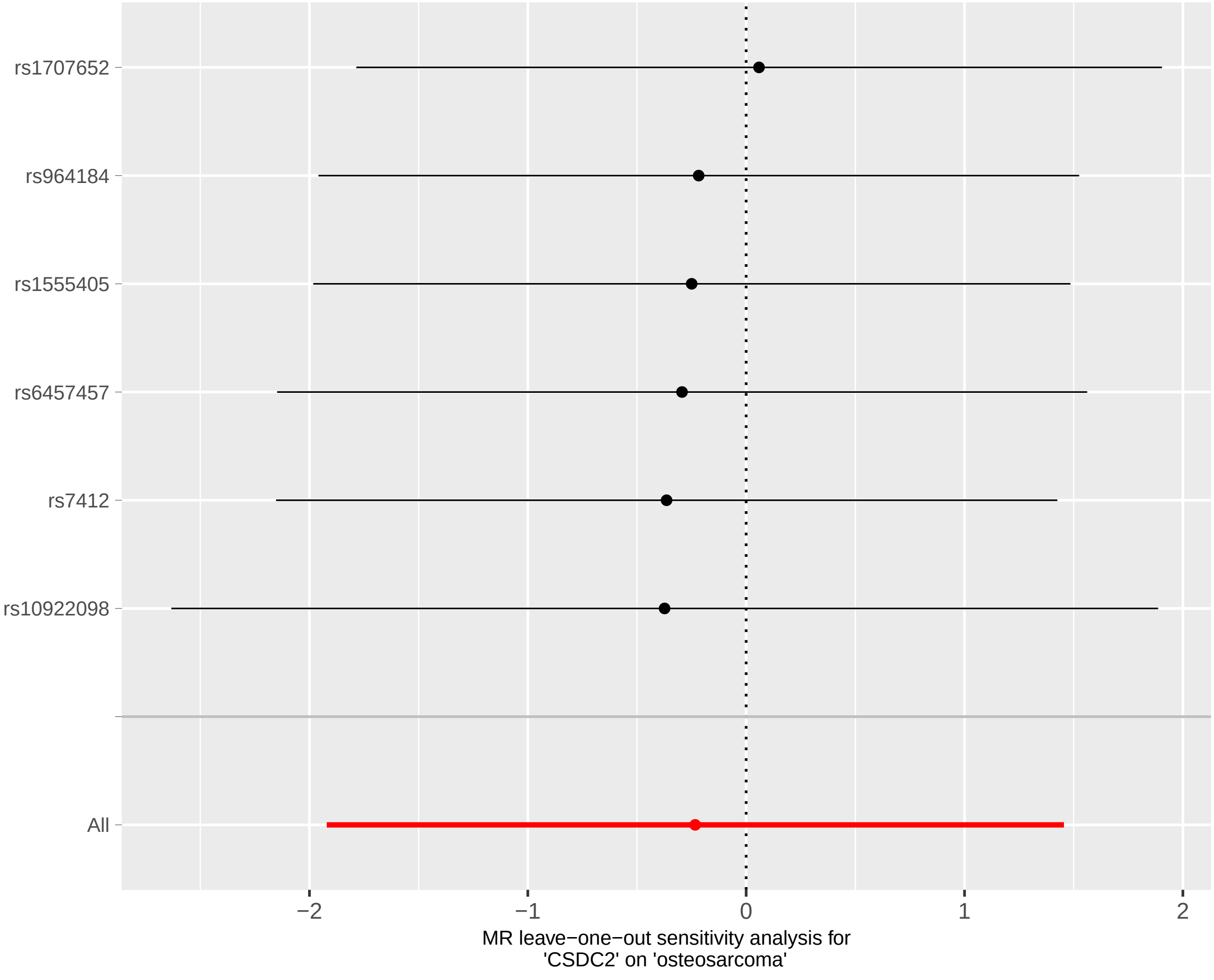
rs1707652
rs964184
rs1555405
rs6457457
rs7412
rs10922098
All
−2
−1
0
1
2
MR leave−one−out sensitivity analysis for
'CSDC2' on 'osteosarcoma'

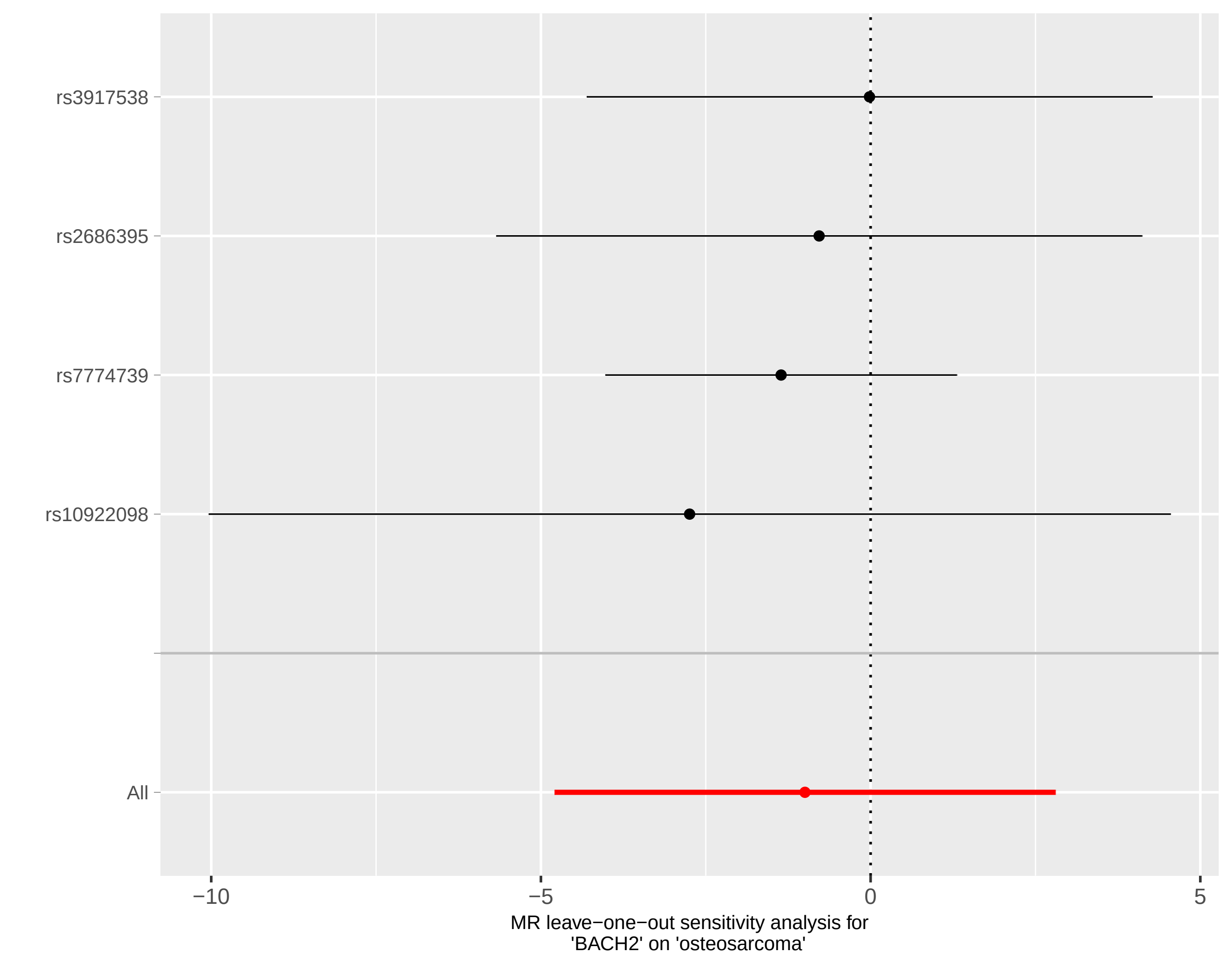
rs3917538
rs2686395
rs7774739
rs10922098
All
−10
−5
0
5
MR leave−one−out sensitivity analysis for
'BACH2' on 'osteosarcoma'

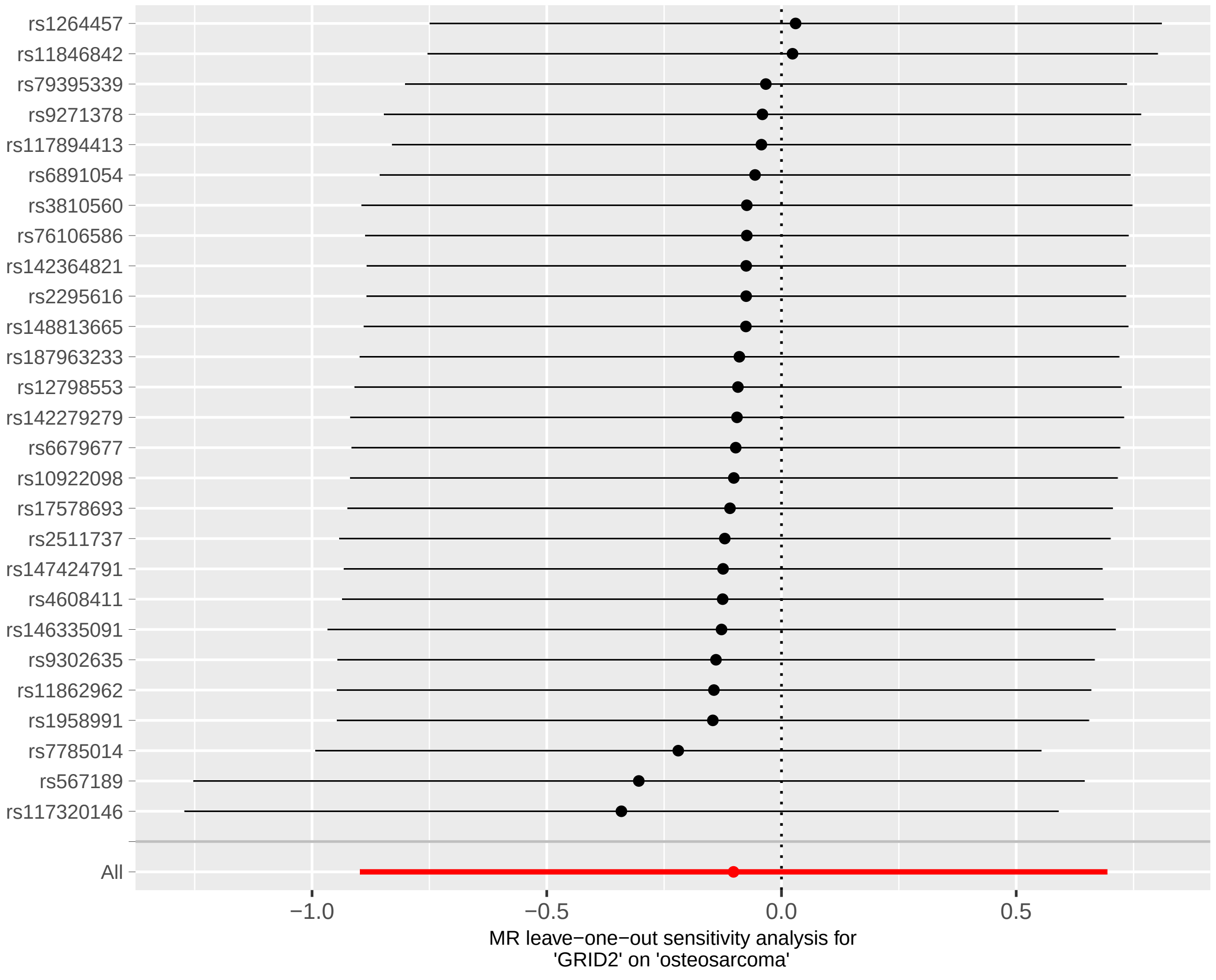

rs1264457
rs11846842
rs79395339
rs9271378
rs117894413
rs6891054
rs3810560
rs76106586
rs142364821
rs2295616
rs148813665
rs187963233
rs12798553
rs142279279
rs6679677
rs10922098
rs17578693
rs2511737
rs147424791
rs4608411
rs146335091
rs9302635
rs11862962
rs1958991
rs7785014
rs567189
rs117320146
All
−1.0
−0.5
0.0
0.5
MR leave−one−out sensitivity analysis for
'GRID2' on 'osteosarcoma'

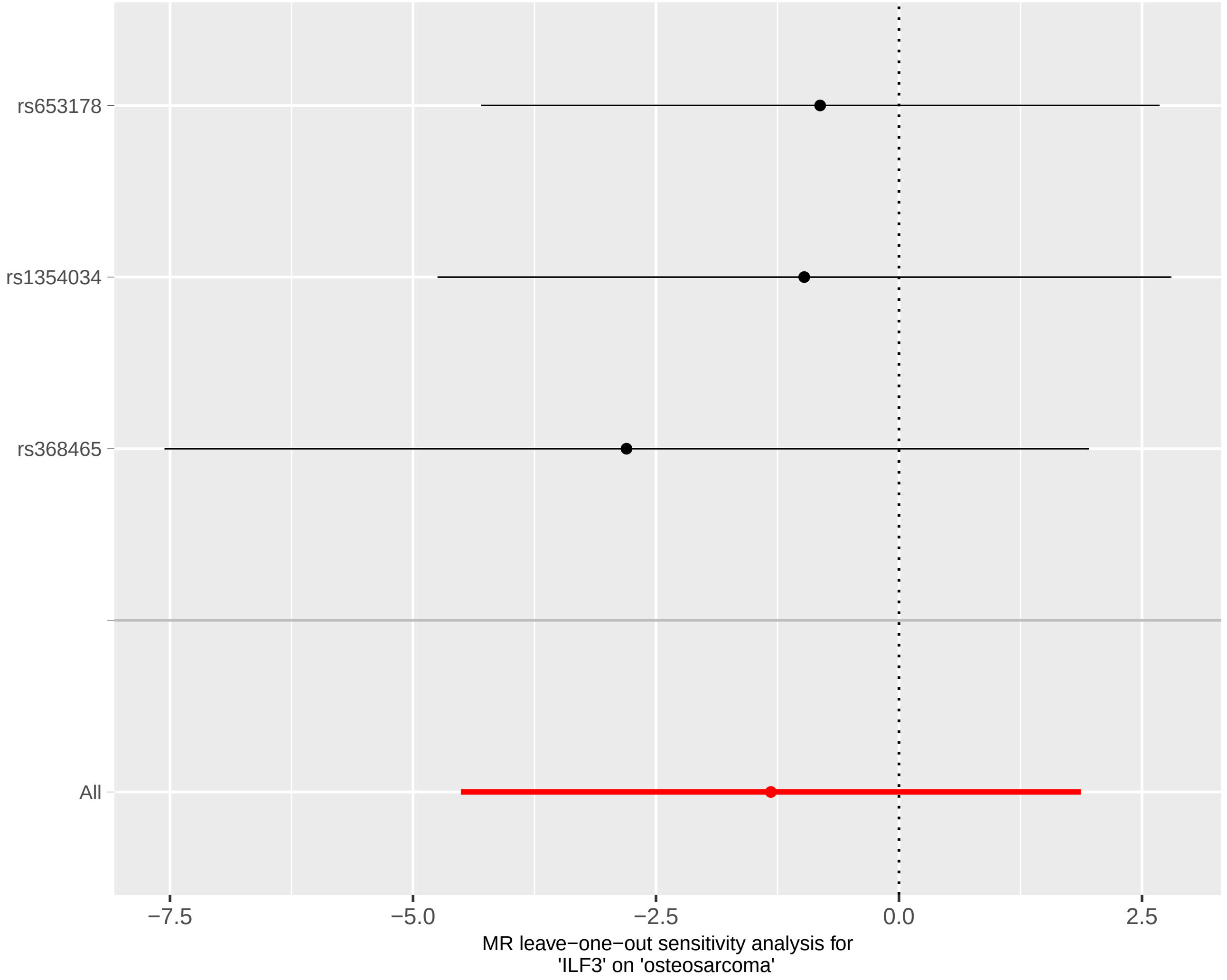
rs653178
rs1354034
rs368465
All
−7.5
−5.0
−2.5
0.0
2.5
MR leave−one−out sensitivity analysis for
'ILF3' on 'osteosarcoma'

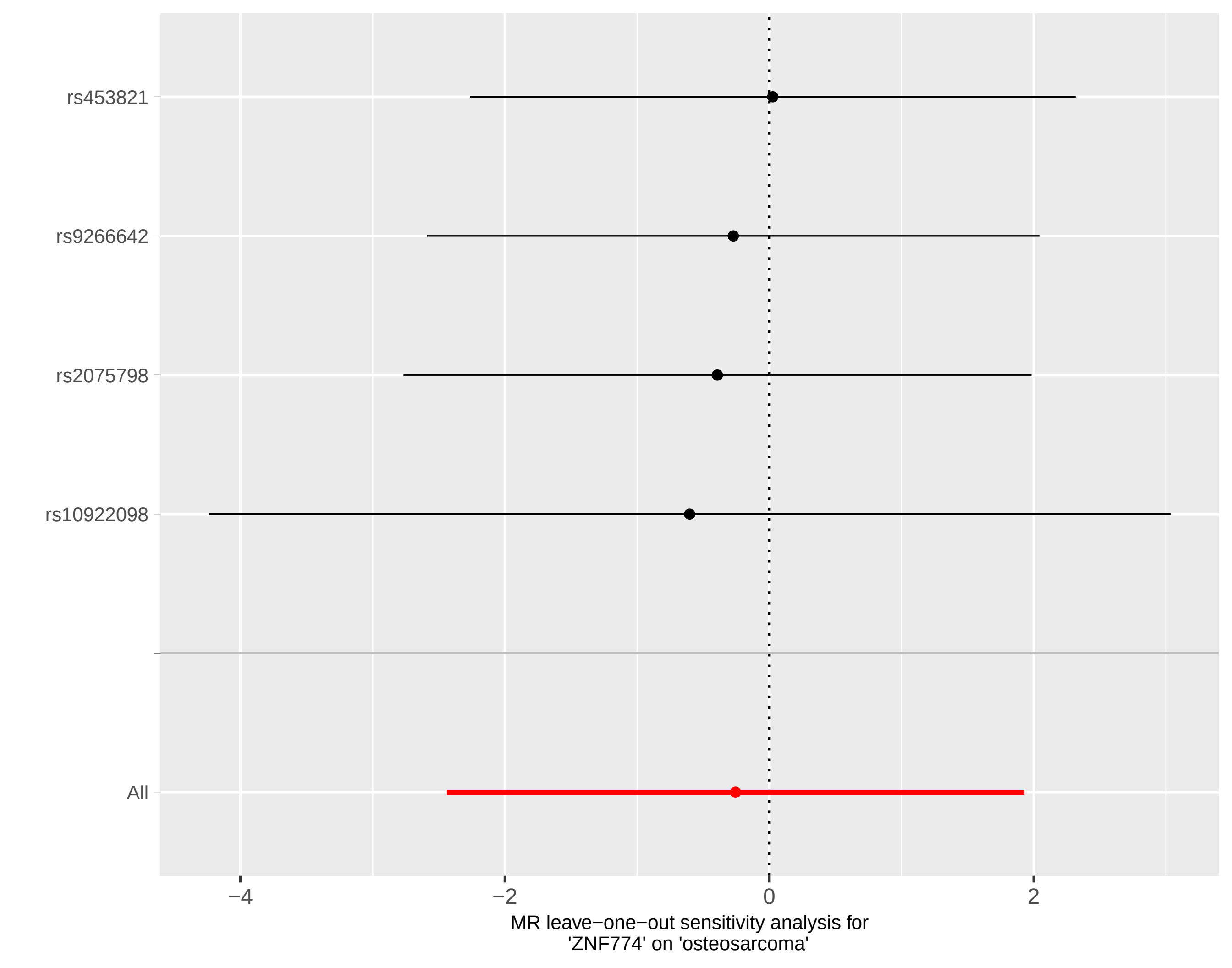
rs453821
rs9266642
rs2075798
rs10922098
All
−4
−2
0
2
MR leave−one−out sensitivity analysis for
'ZNF774' on 'osteosarcoma'

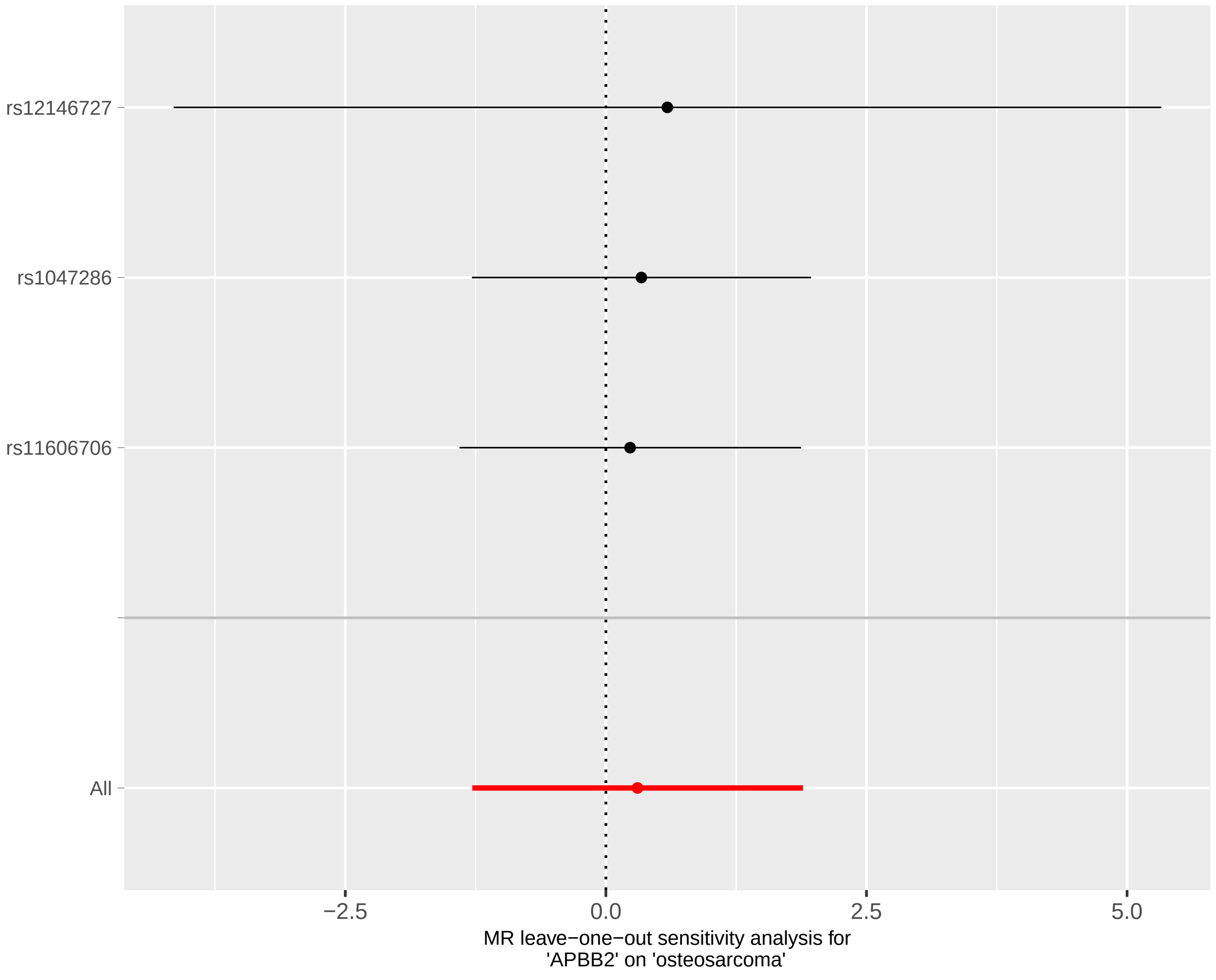
rs12146727
rs1047286
rs11606706
All
−2.5
0.0
2.5
5.0
MR leave−one−out sensitivity analysis for
'APBB2' on 'osteosarcoma'

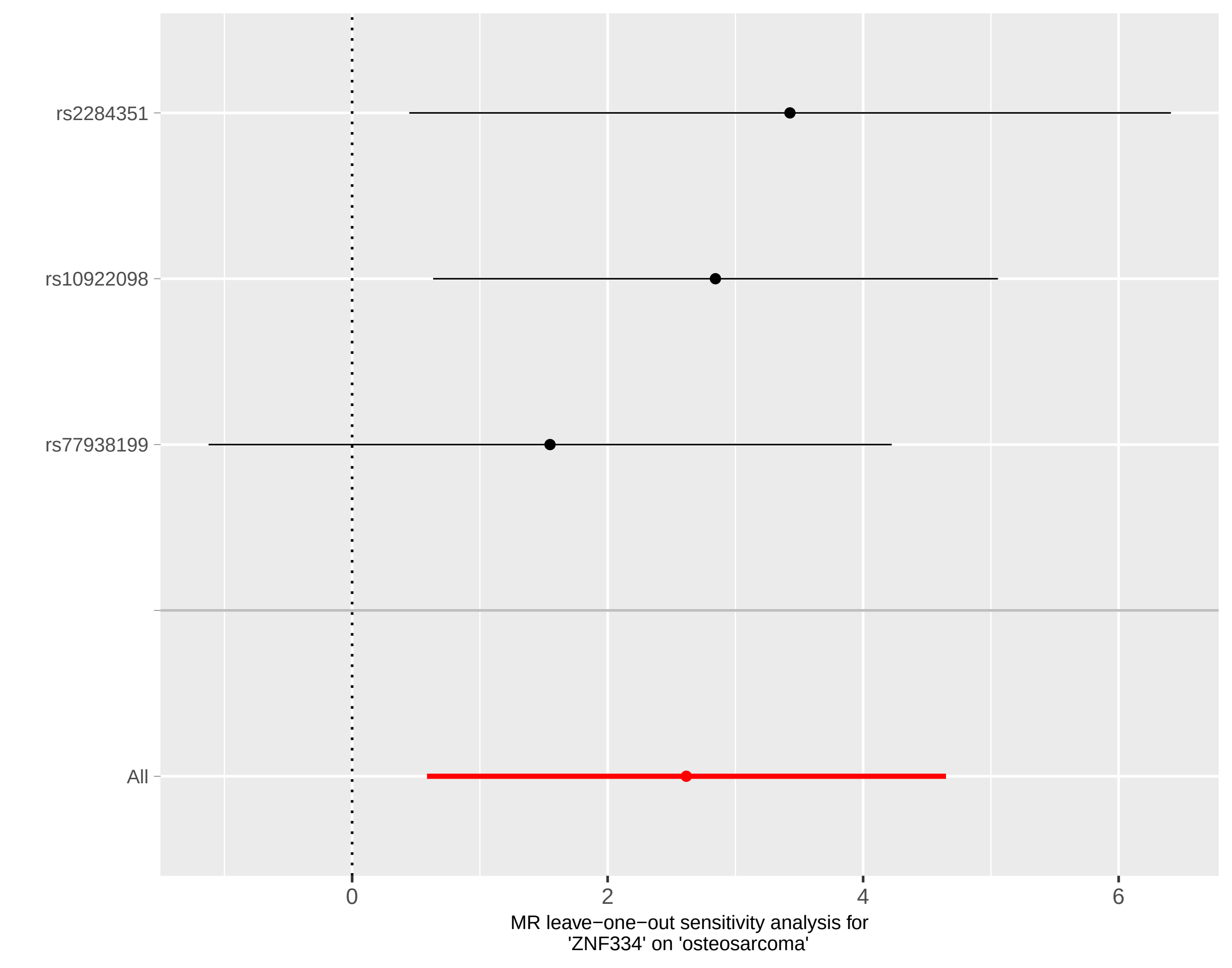

rs2284351
rs10922098
rs77938199
All
0
2
4
6
MR leave−one−out sensitivity analysis for
'ZNF334' on 'osteosarcoma'

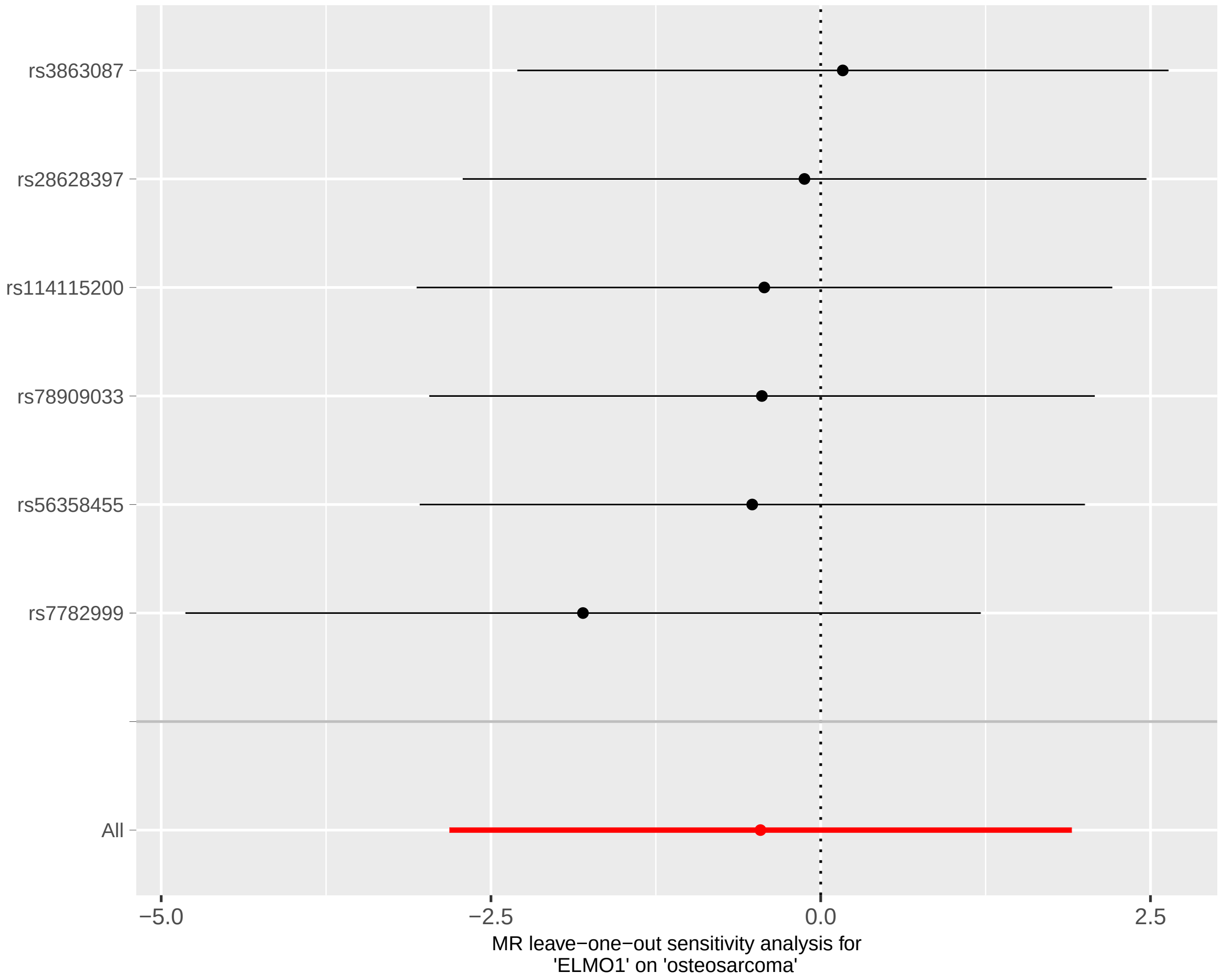
rs3863087
rs28628397
rs114115200
rs78909033
rs56358455
rs7782999
All
−5.0
−2.5
0.0
2.5
MR leave−one−out sensitivity analysis for
'ELMO1' on 'osteosarcoma'

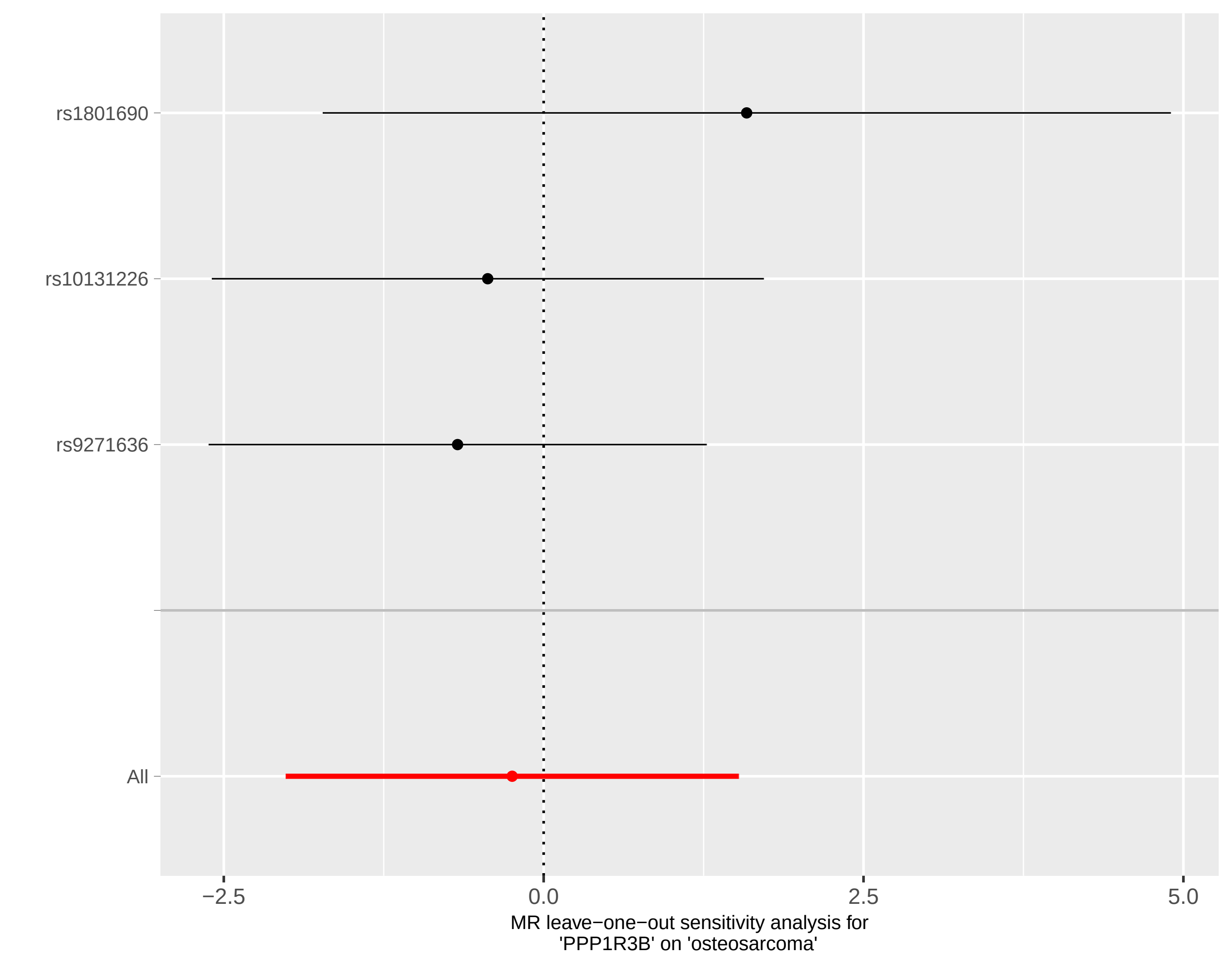

rs1801690
rs10131226
rs9271636
All
−2.5
0.0
2.5
5.0
MR leave−one−out sensitivity analysis for
'PPP1R3B' on 'osteosarcoma'

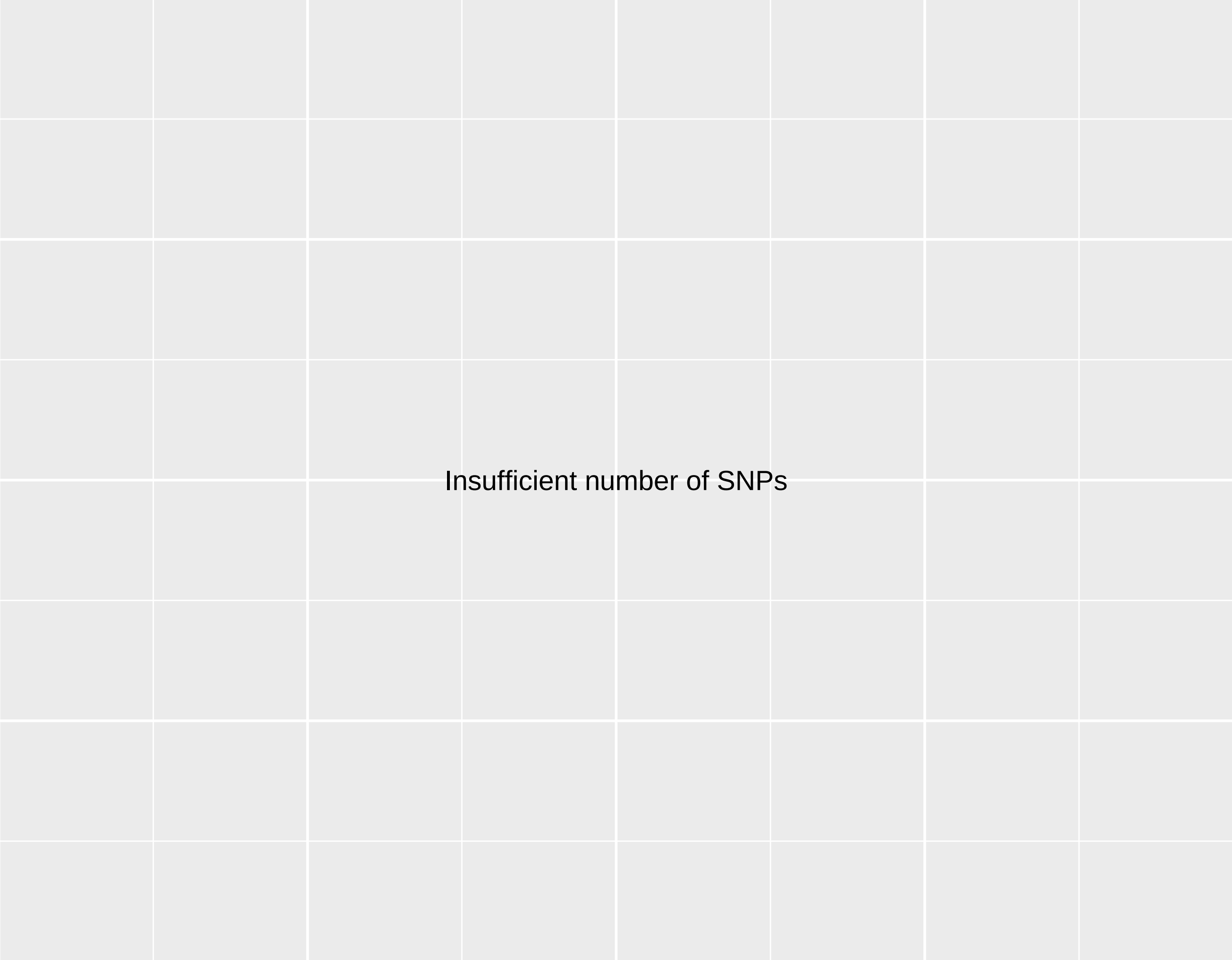
Insufficient number of SNPs

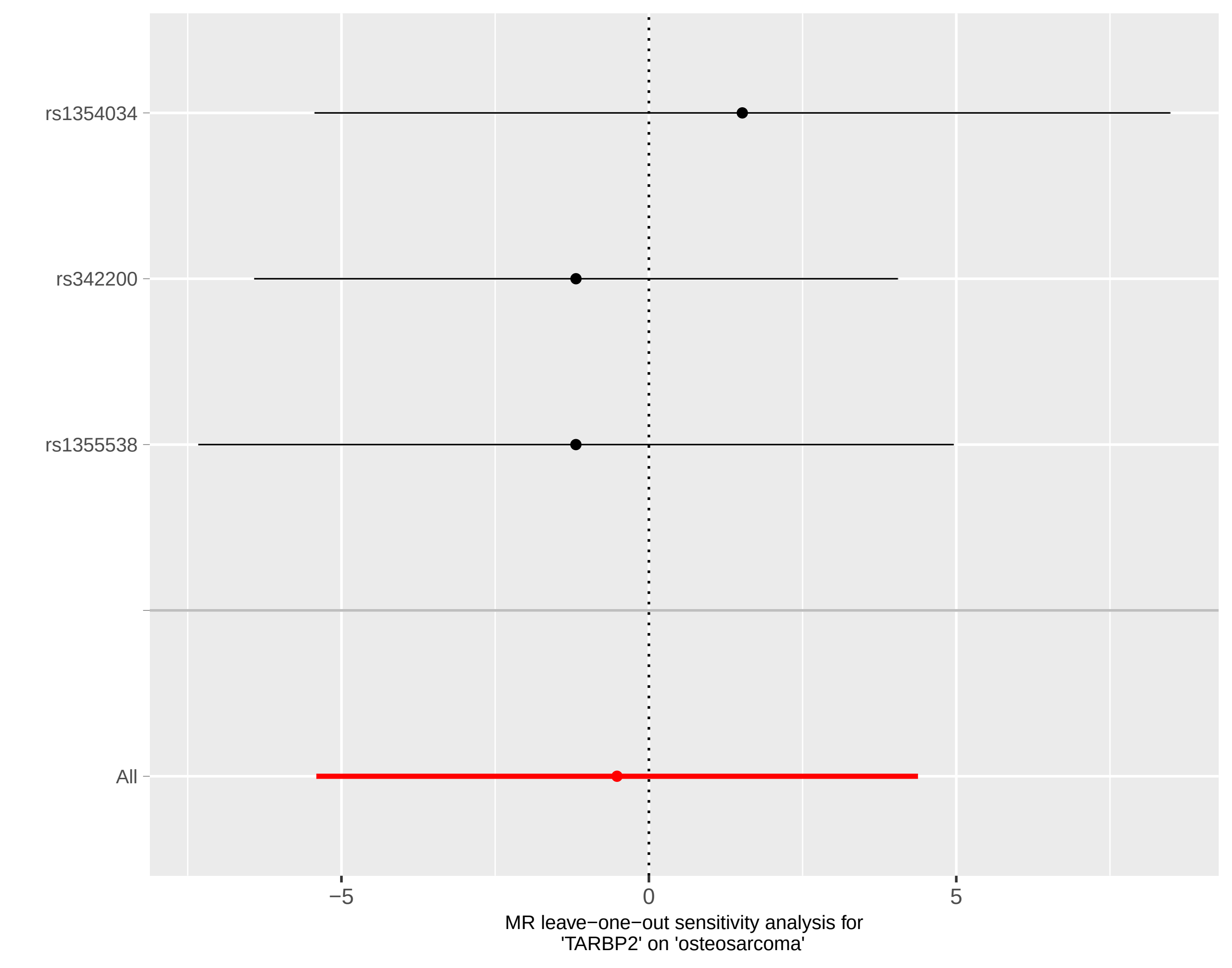
rs1354034
rs342200
rs1355538
All
−5
0
5
MR leave−one−out sensitivity analysis for
'TARBP2' on 'osteosarcoma'

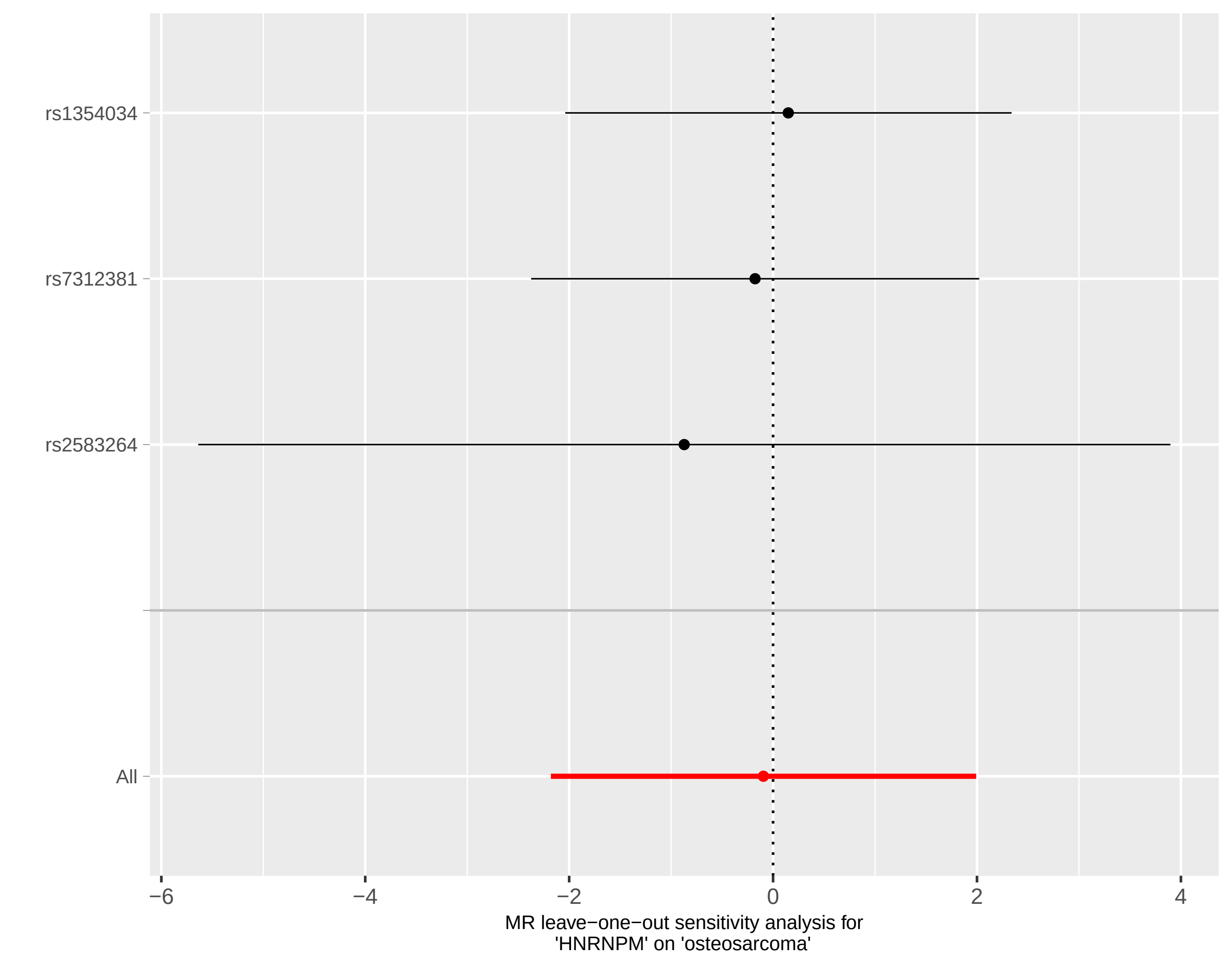
rs1354034
rs7312381
rs2583264
All
−6
−4
−2
0
2
4
MR leave−one−out sensitivity analysis for
'HNRNPM' on 'osteosarcoma'

Insufficient number of SNPs

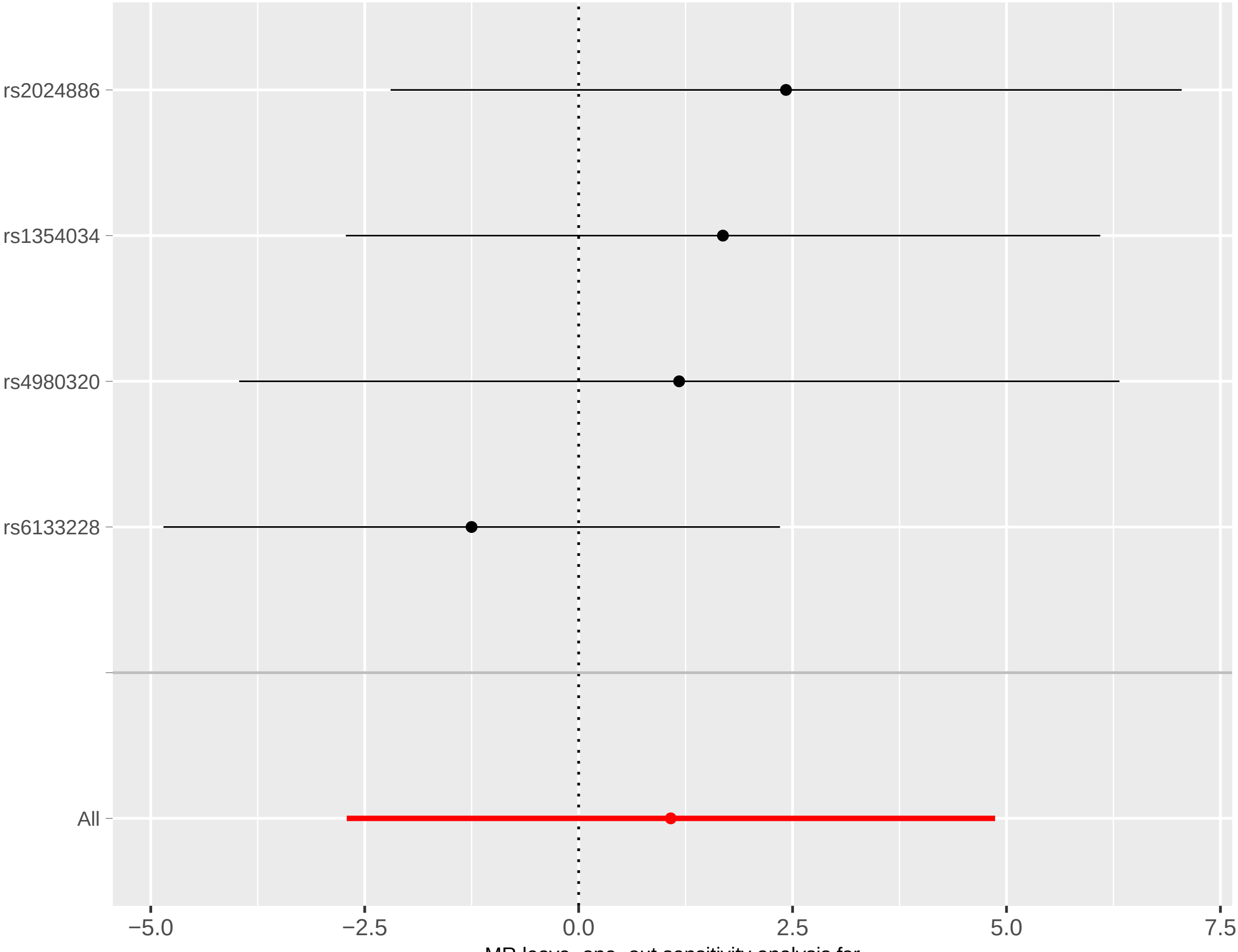

MR leave−one−out sensitivity analysis for
'GPCPD1' on 'osteosarcoma'

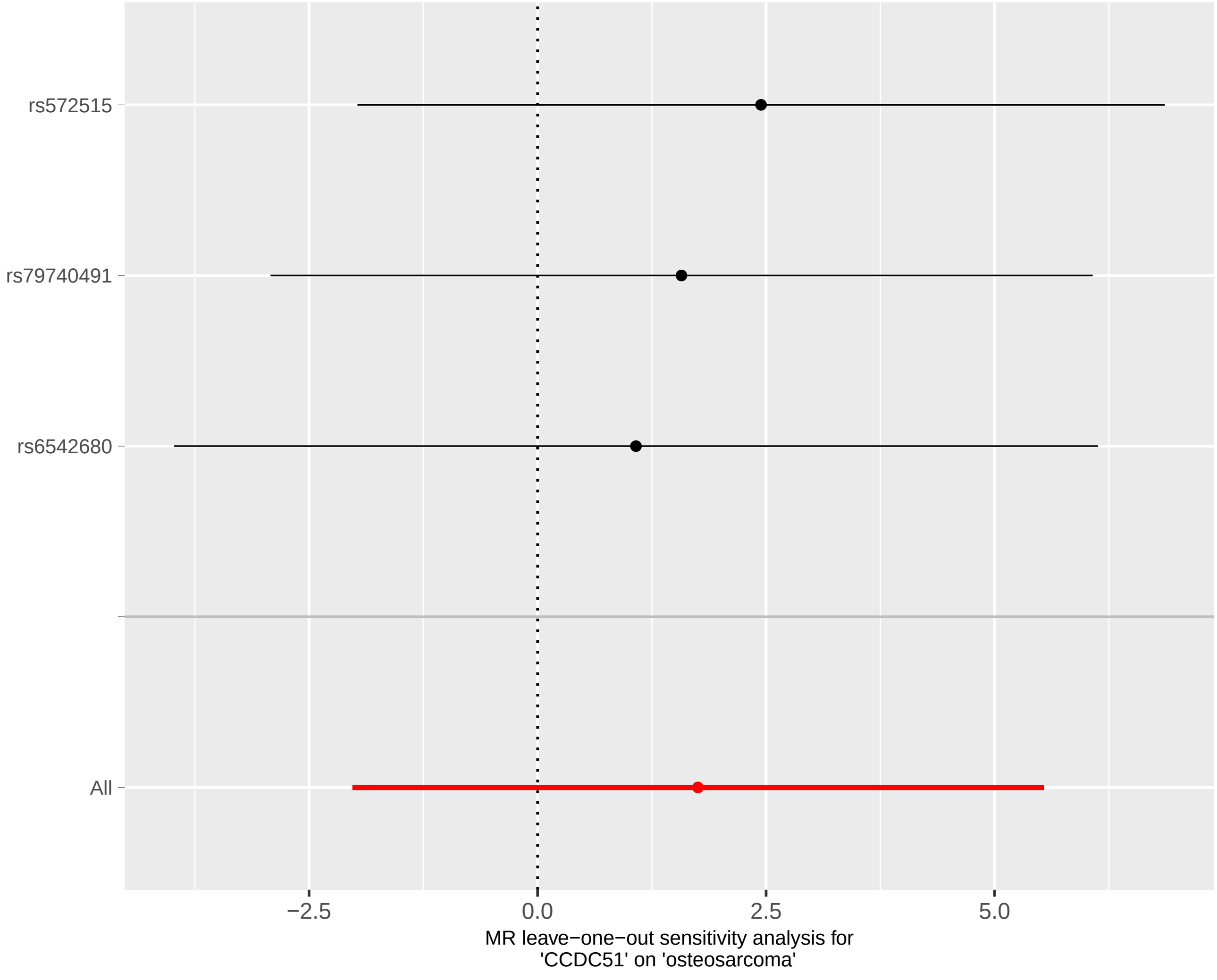
rs572515
rs79740491
rs6542680
All
−2.5
0.0
2.5
5.0
MR leave−one−out sensitivity analysis for
'CCDC51' on 'osteosarcoma'

Insufficient number of SNPs

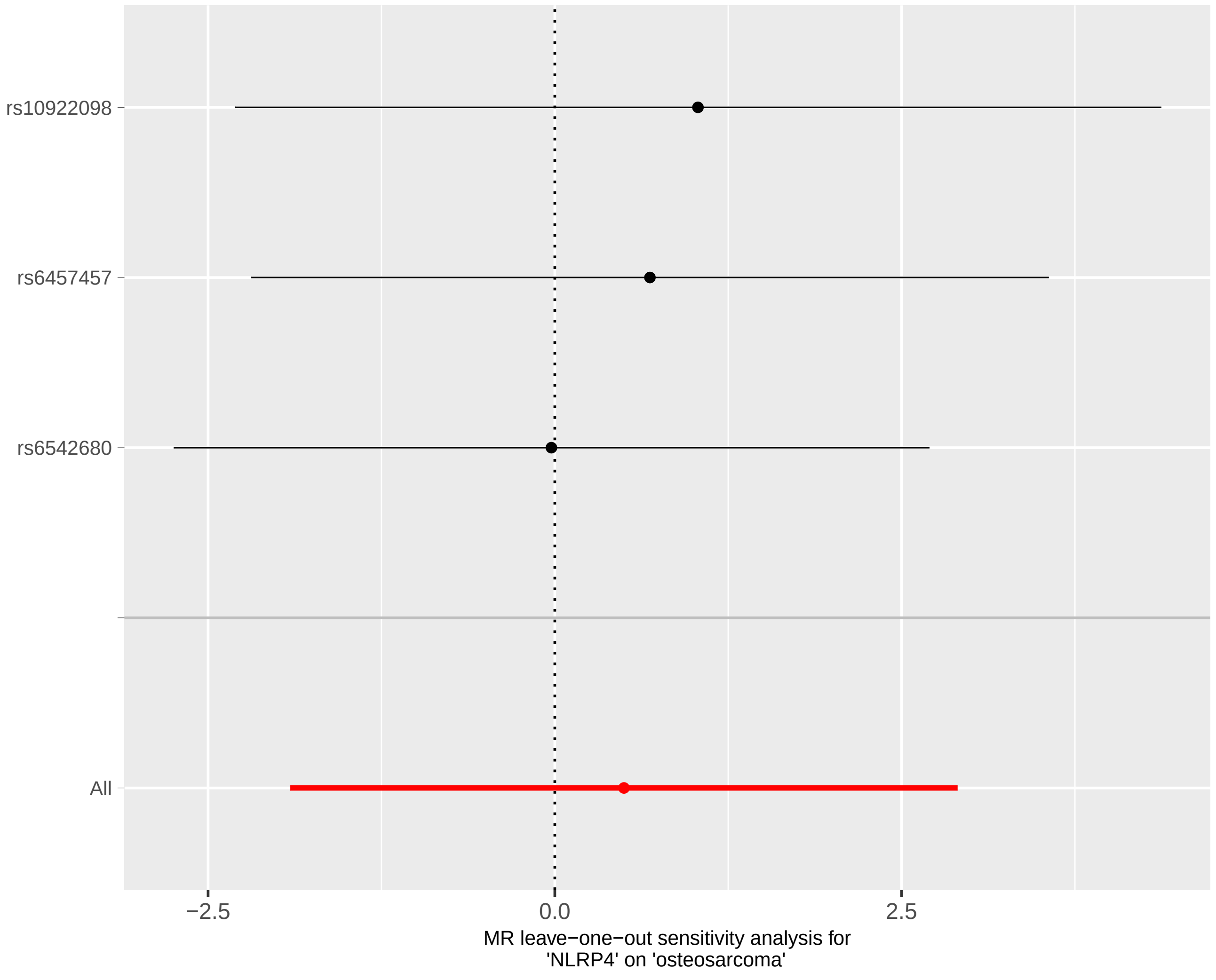

rs10922098
rs6457457
rs6542680
All
−2.5
0.0
2.5
MR leave−one−out sensitivity analysis for
'NLRP4' on 'osteosarcoma'

Insufficient number of SNPs

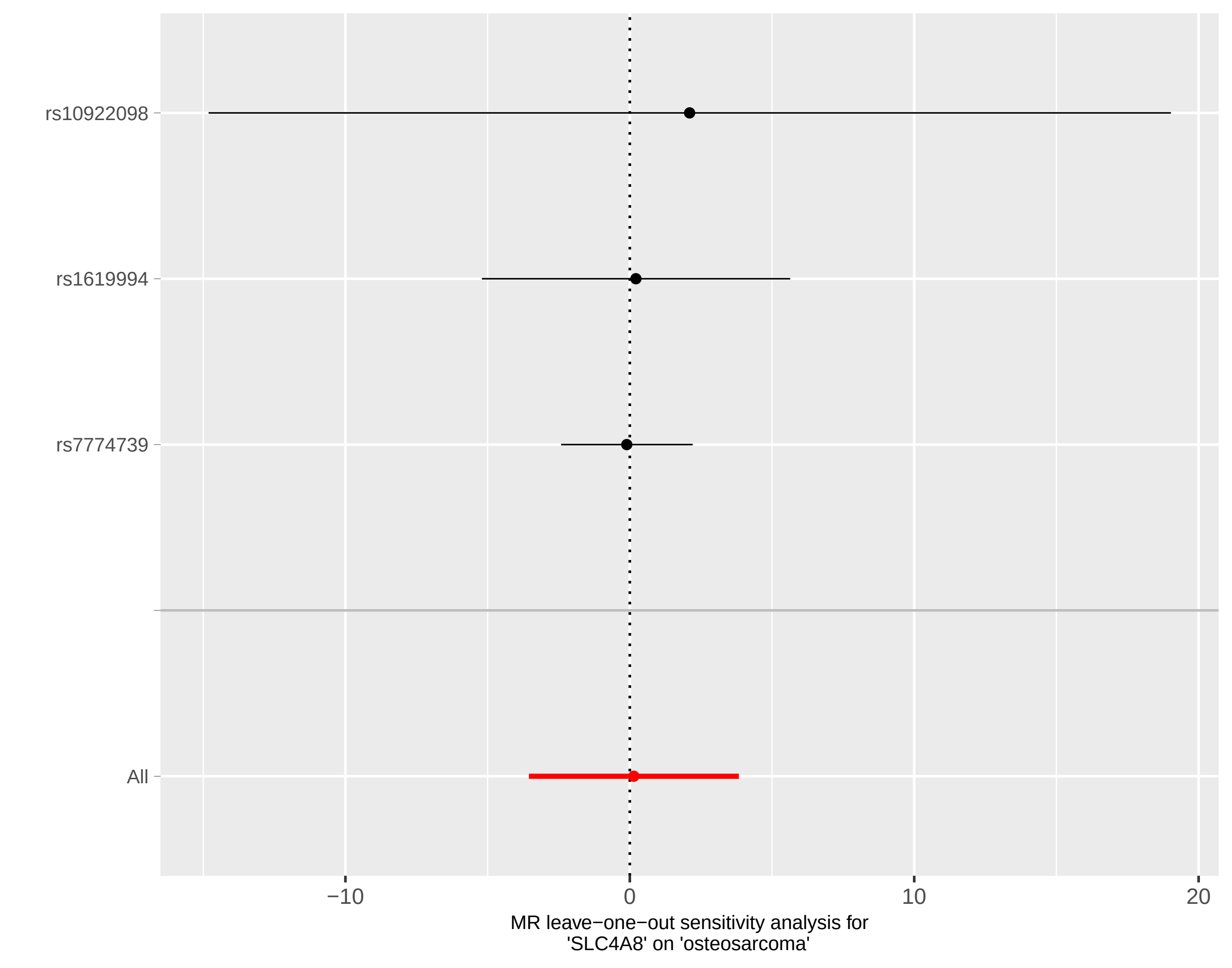

rs10922098
rs1619994
rs7774739
All
−10
0
10
20
MR leave−one−out sensitivity analysis for
'SLC4A8' on 'osteosarcoma'

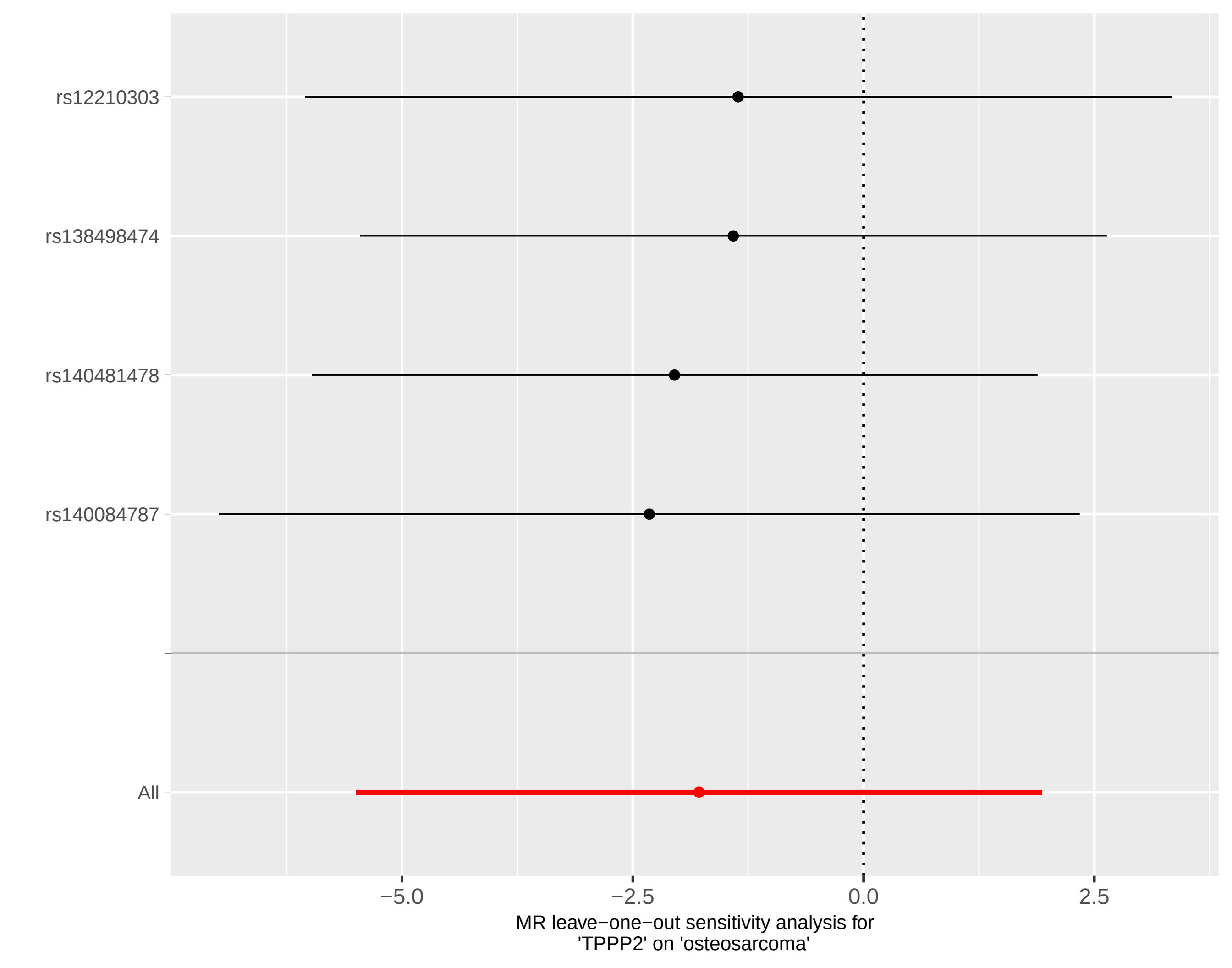
rs12210303
rs138498474
rs140481478
rs140084787
All
−5.0
−2.5
0.0
2.5
MR leave−one−out sensitivity analysis for
'TPPP2' on 'osteosarcoma'

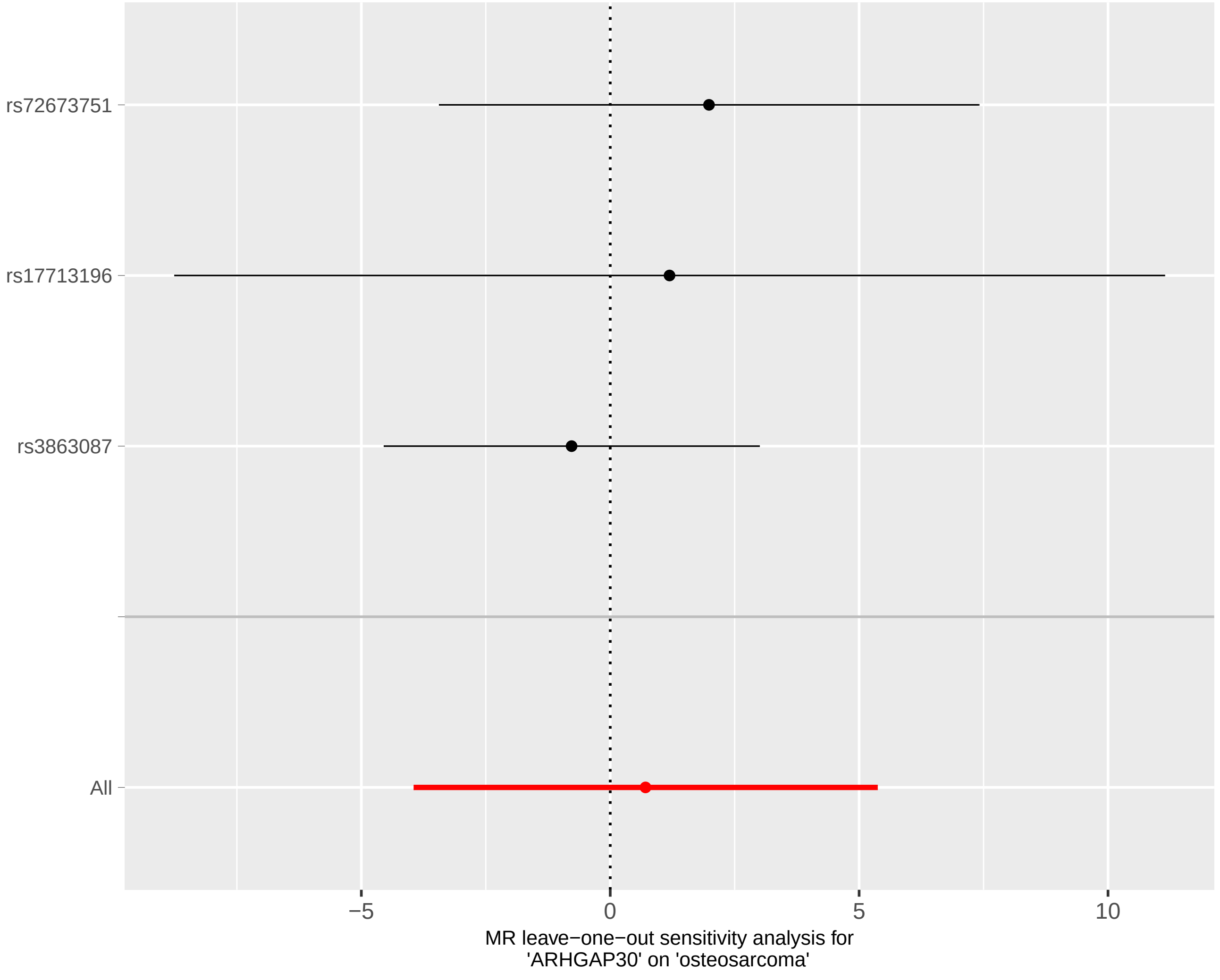

rs72673751
rs17713196
rs3863087
All
−5
0
5
10
MR leave−one−out sensitivity analysis for
'ARHGAP30' on 'osteosarcoma'

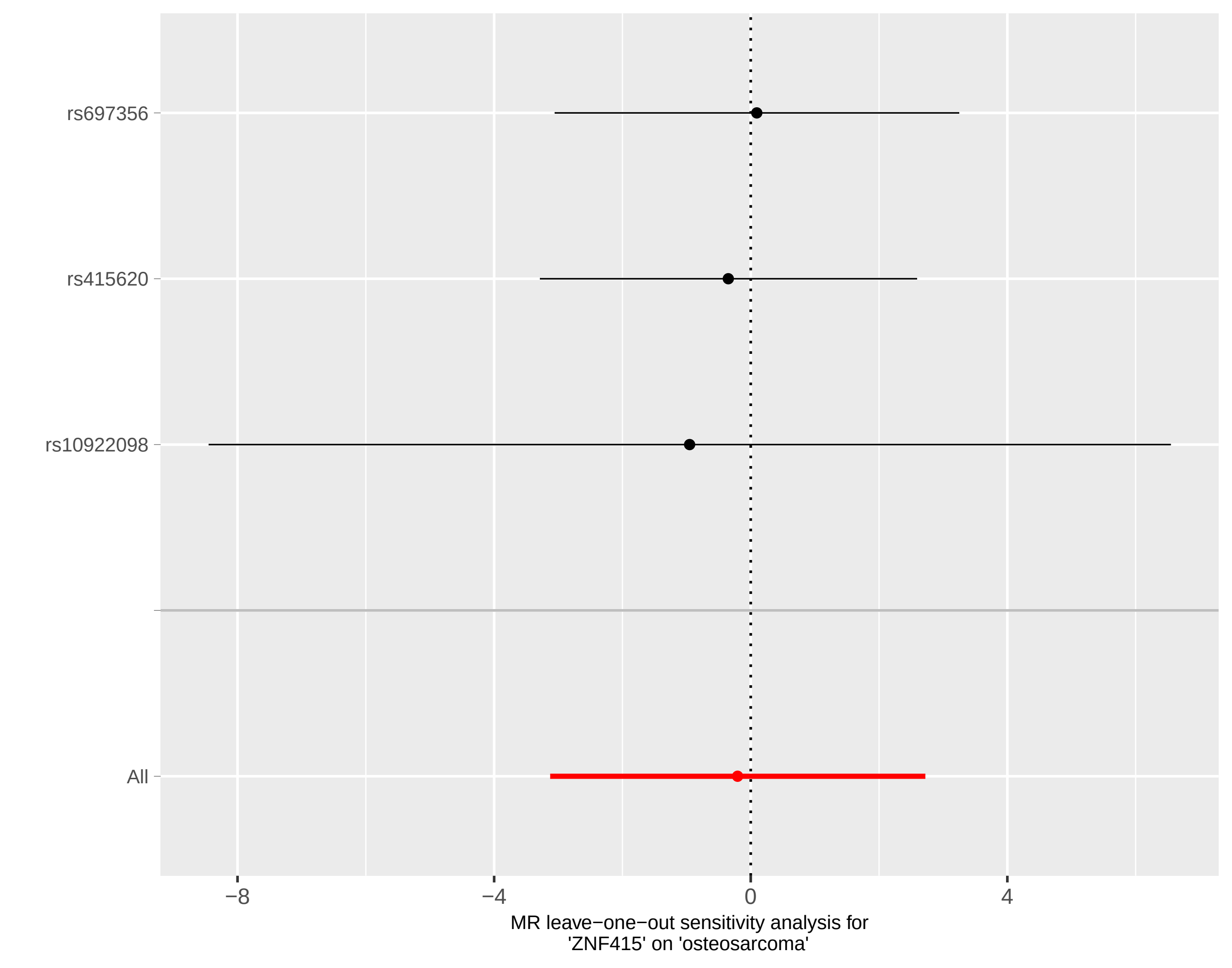
rs697356
rs415620
rs10922098
All
−8
−4
0
4
MR leave−one−out sensitivity analysis for
'ZNF415' on 'osteosarcoma'

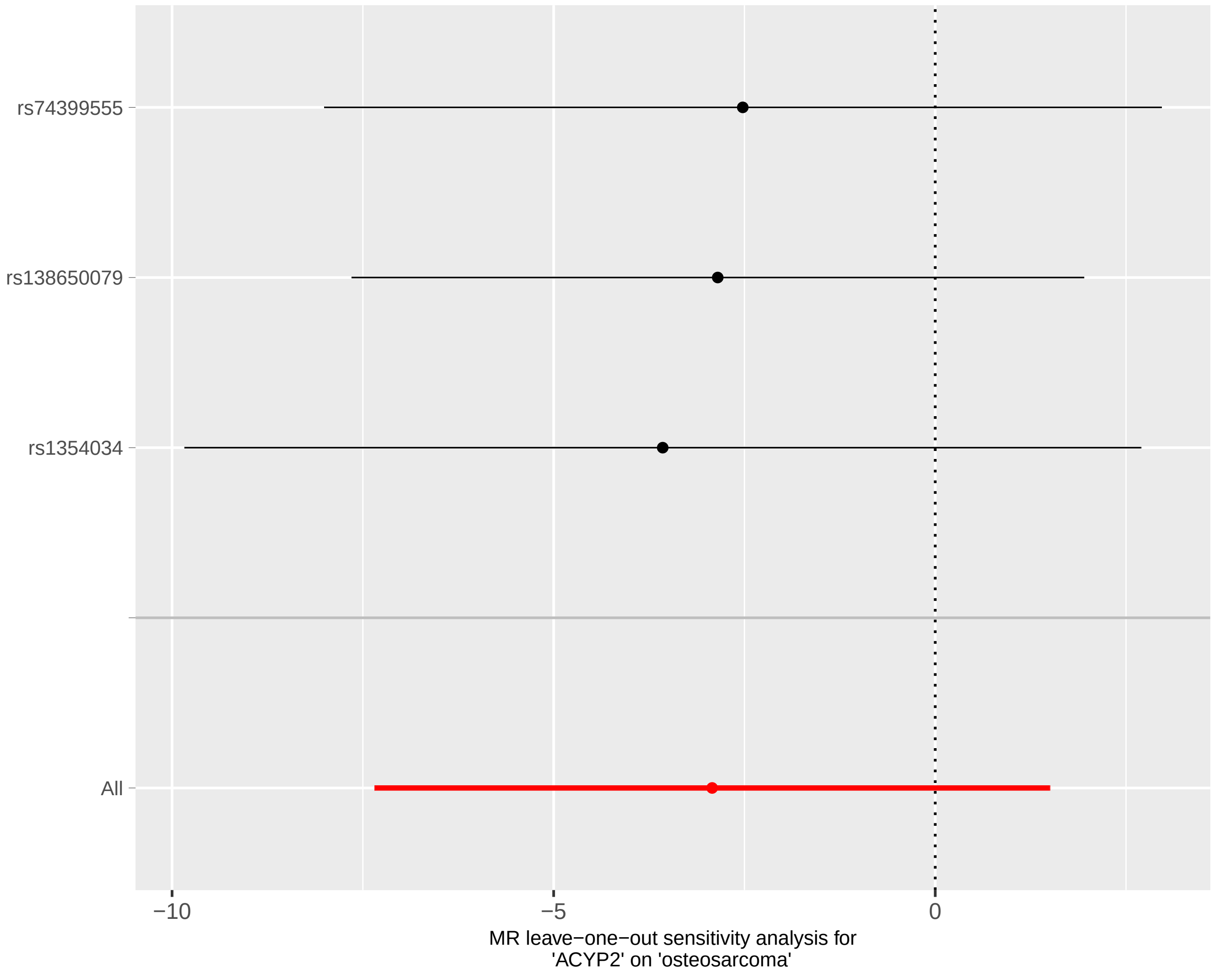
rs74399555
rs138650079
rs1354034
All
−10
−5
0
MR leave−one−out sensitivity analysis for
'ACYP2' on 'osteosarcoma'

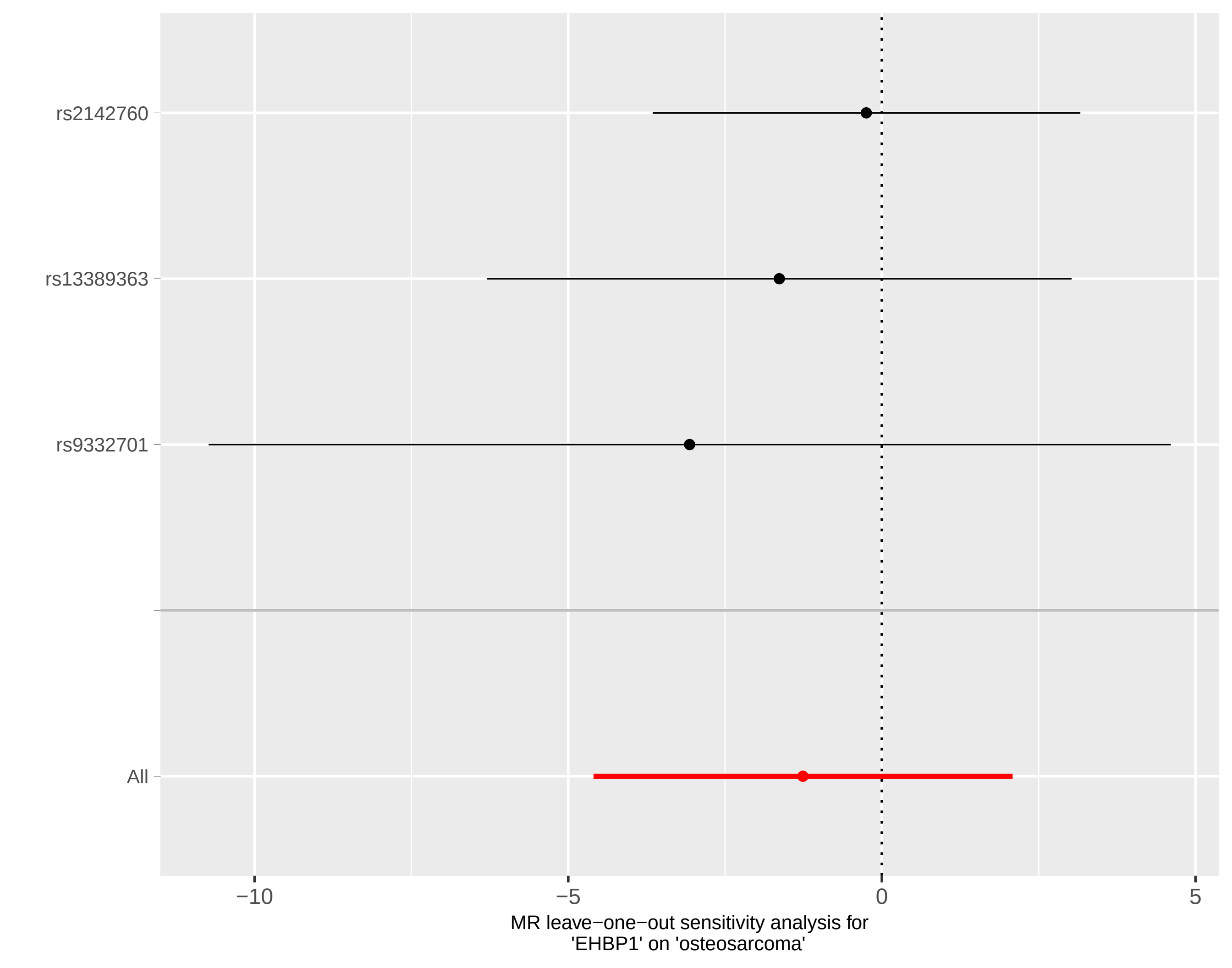
rs2142760
rs13389363
rs9332701
All
−10
−5
0
5
MR leave−one−out sensitivity analysis for
'EHBP1' on 'osteosarcoma'

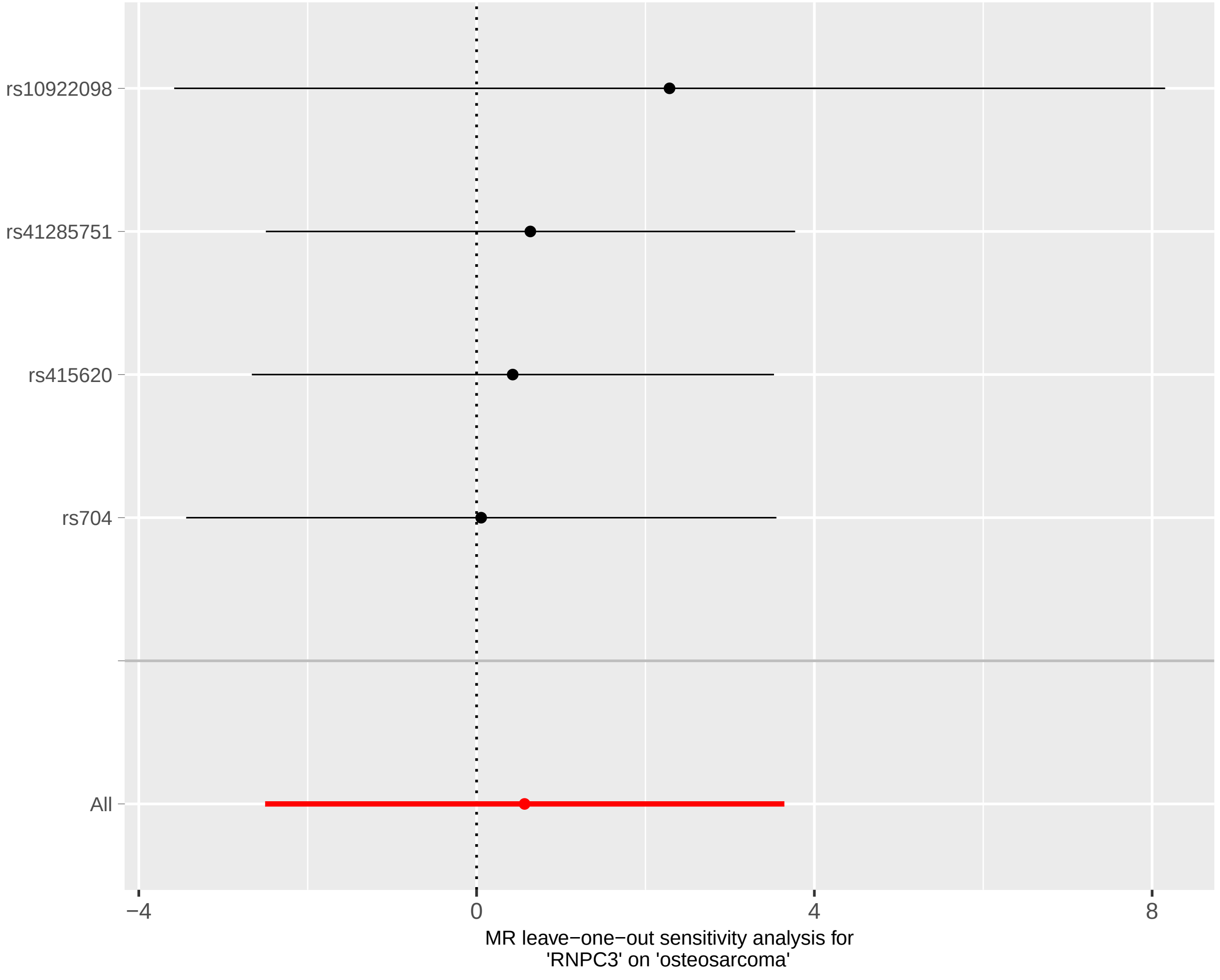

rs10922098
rs41285751
rs415620
rs704
All
−4
0
4
8
MR leave−one−out sensitivity analysis for
'RNPC3' on 'osteosarcoma'

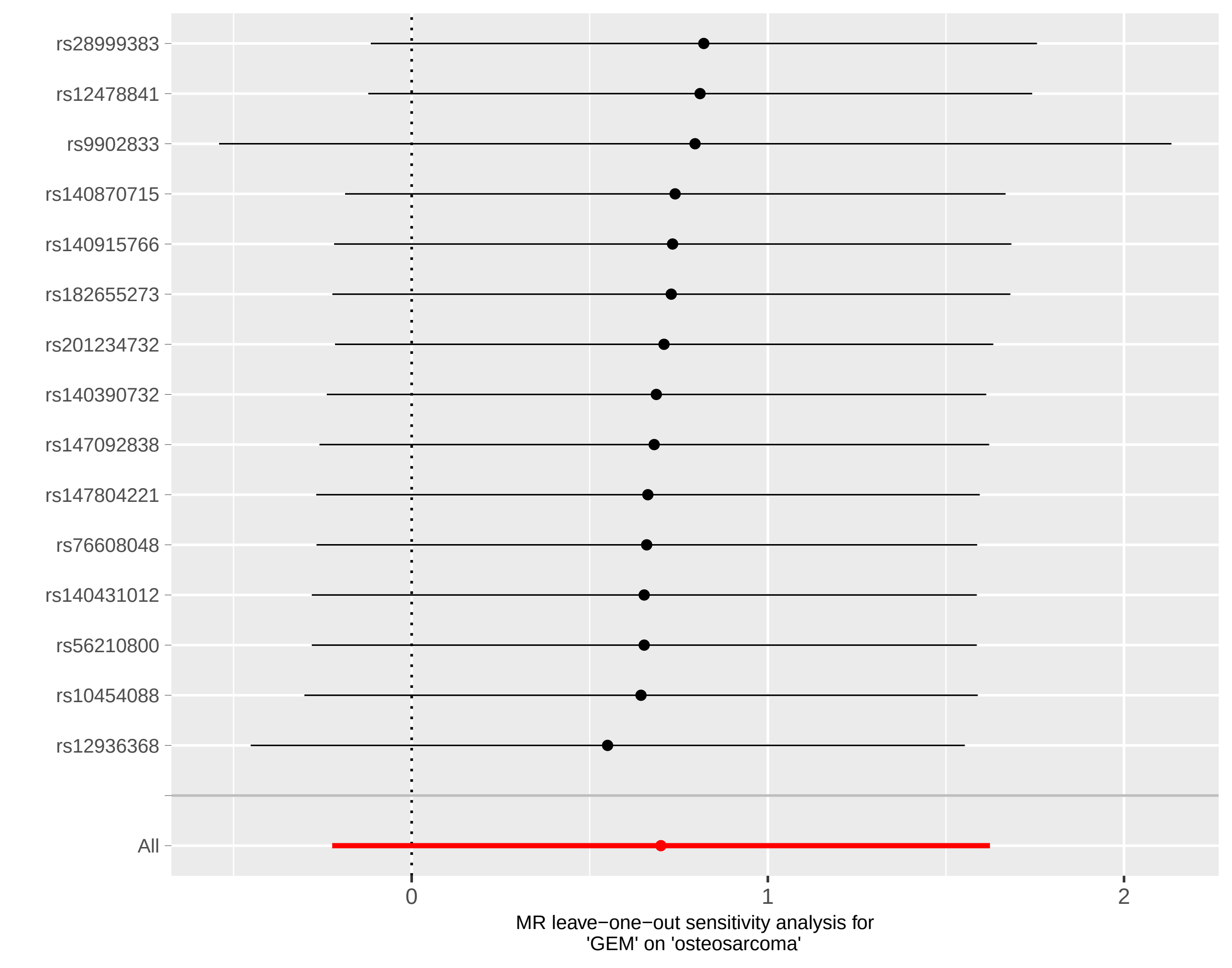

rs28999383
rs12478841
rs9902833
rs140870715
rs140915766
rs182655273
rs201234732
rs140390732
rs147092838
rs147804221
rs76608048
rs140431012
rs56210800
rs10454088
rs12936368
All
0
1
2
MR leave−one−out sensitivity analysis for
'GEM' on 'osteosarcoma'

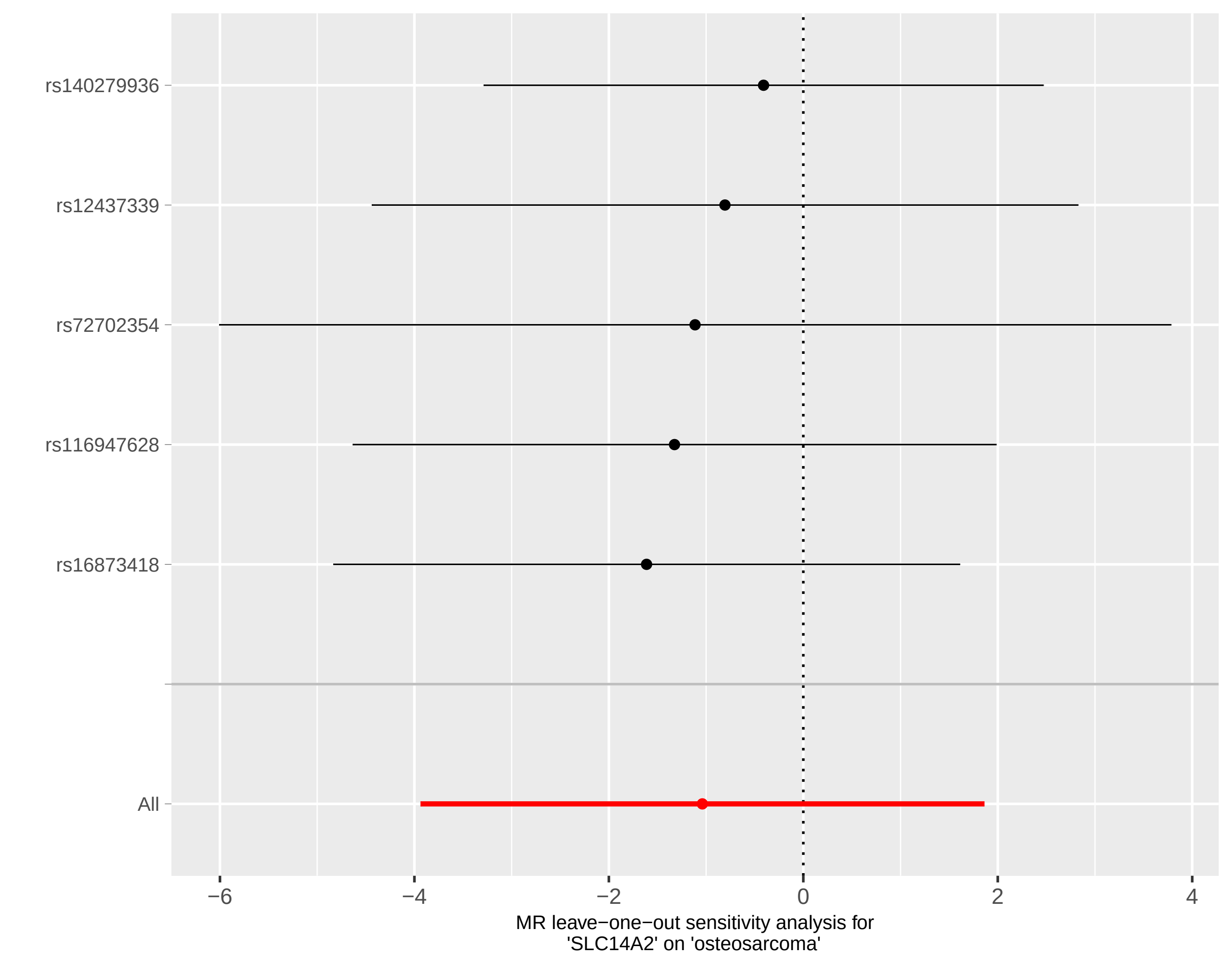
rs140279936
rs12437339
rs72702354
rs116947628
rs16873418
All
−6
−4
−2
0
2
4
MR leave−one−out sensitivity analysis for
'SLC14A2' on 'osteosarcoma'

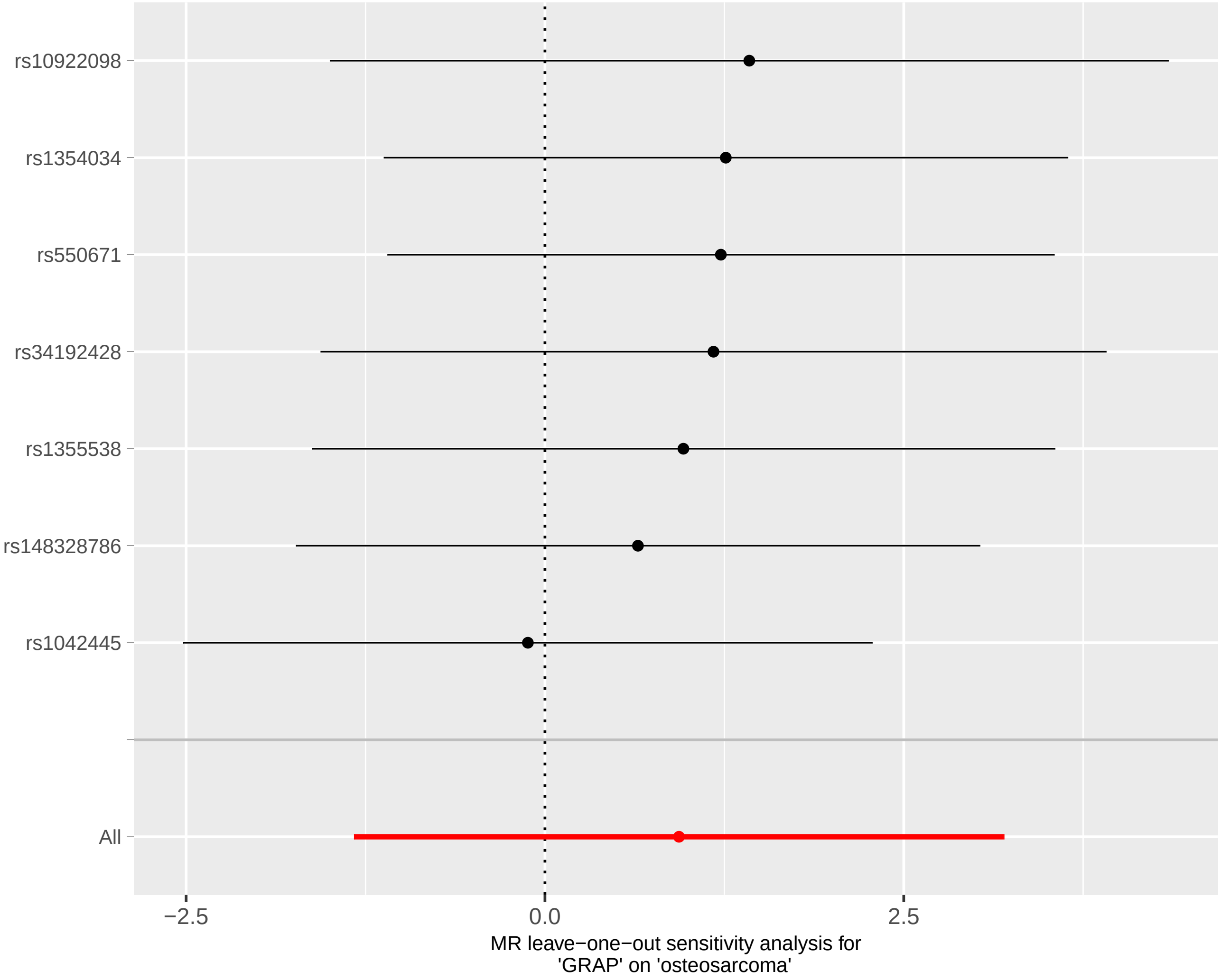
rs10922098
rs1354034
rs550671
rs34192428
rs1355538
rs148328786
rs1042445
All
−2.5
0.0
2.5
MR leave−one−out sensitivity analysis for
'GRAP' on 'osteosarcoma'

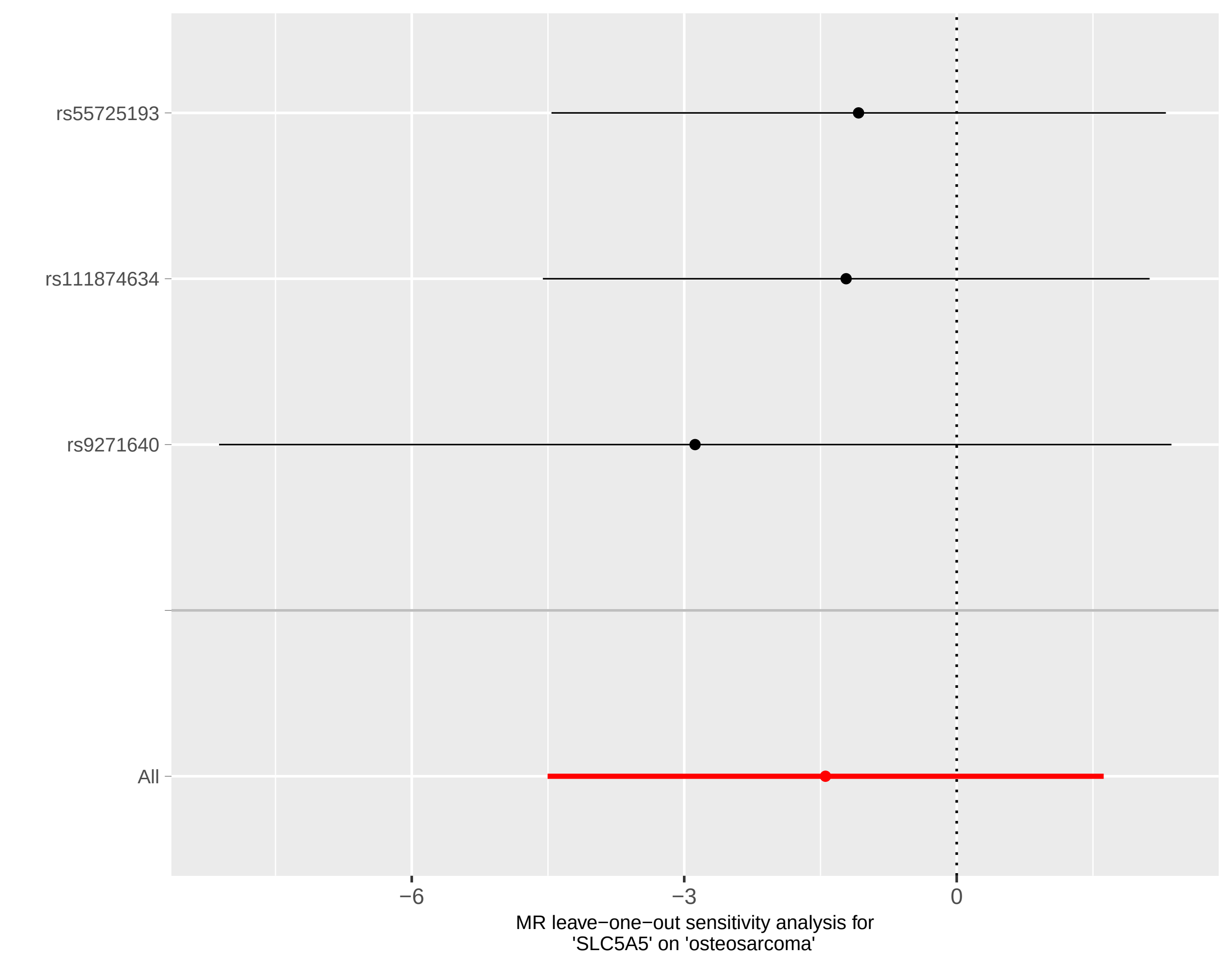

rs55725193
rs111874634
rs9271640
All
−6
−3
0
MR leave−one−out sensitivity analysis for
'SLC5A5' on 'osteosarcoma'

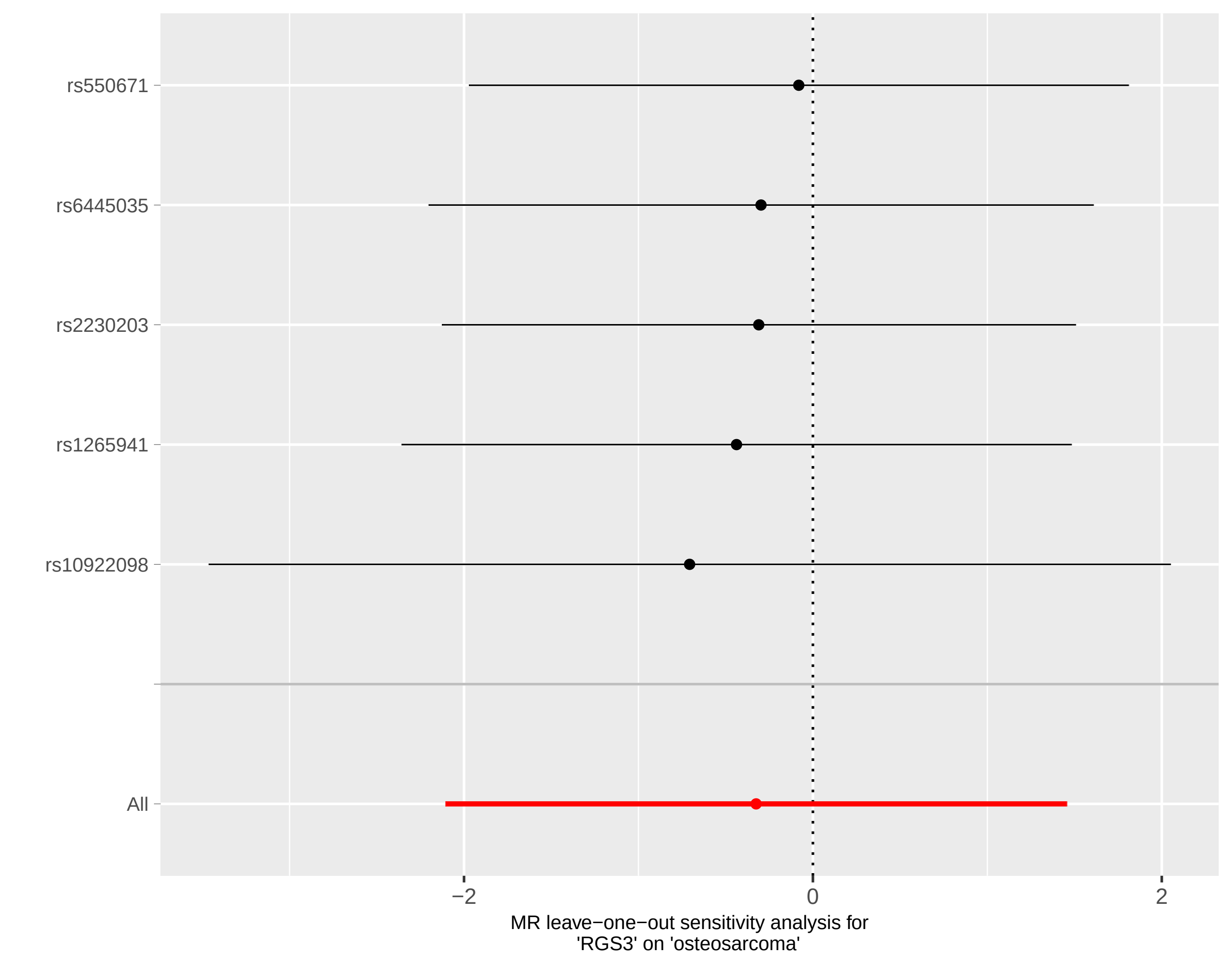

rs550671
rs6445035
rs2230203
rs1265941
rs10922098
All
−2
0
2
MR leave−one−out sensitivity analysis for
'RGS3' on 'osteosarcoma'

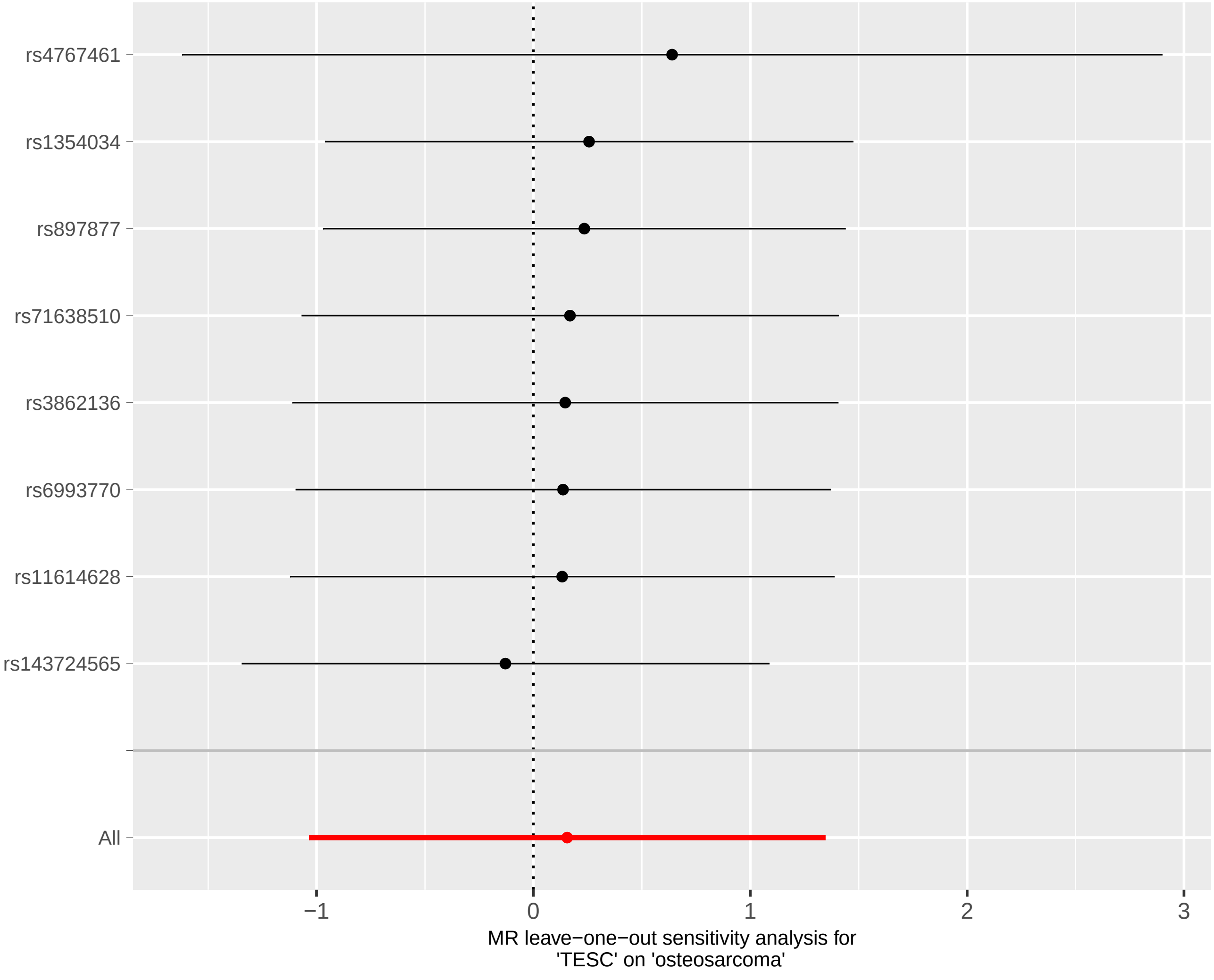

rs4767461
rs1354034
rs897877
rs71638510
rs3862136
rs6993770
rs11614628
rs143724565
All
−1
0
1
2
3
MR leave−one−out sensitivity analysis for
'TESC' on 'osteosarcoma'

Insufficient number of SNPs

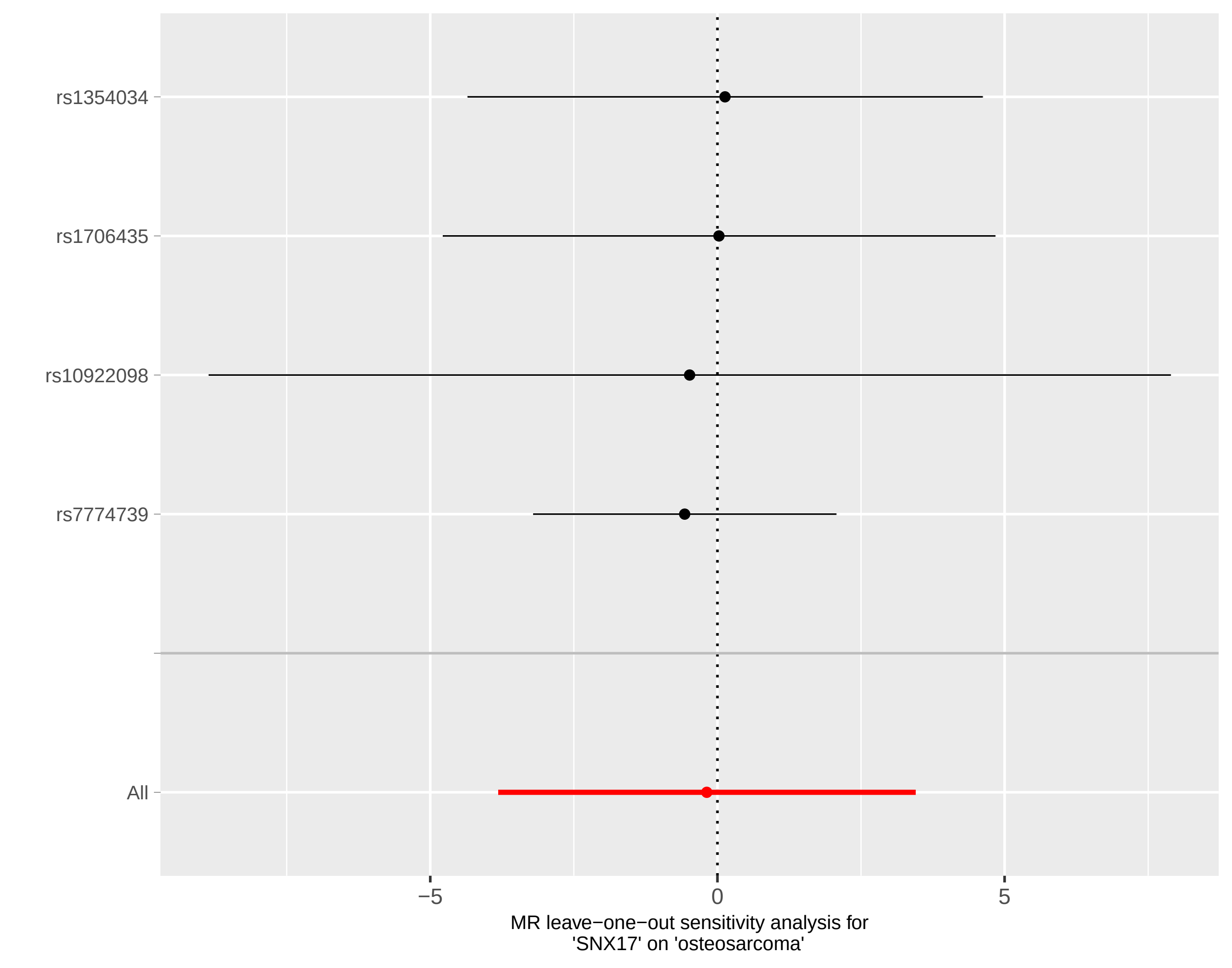
rs1354034
rs1706435
rs10922098
rs7774739
All
−5
0
5
MR leave−one−out sensitivity analysis for
'SNX17' on 'osteosarcoma'

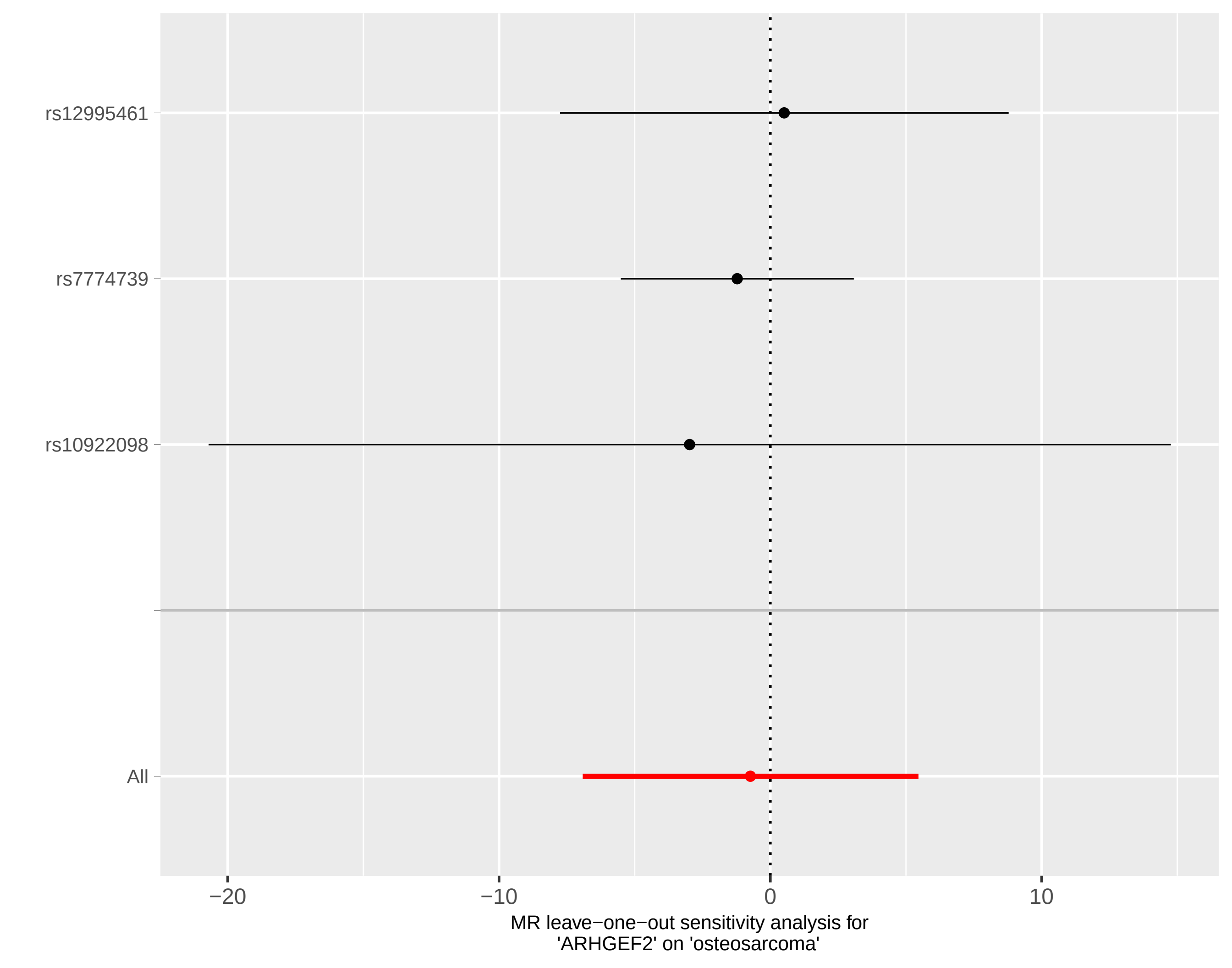
rs12995461
rs7774739
rs10922098
All
−20
−10
0
10
MR leave−one−out sensitivity analysis for
'ARHGEF2' on 'osteosarcoma'

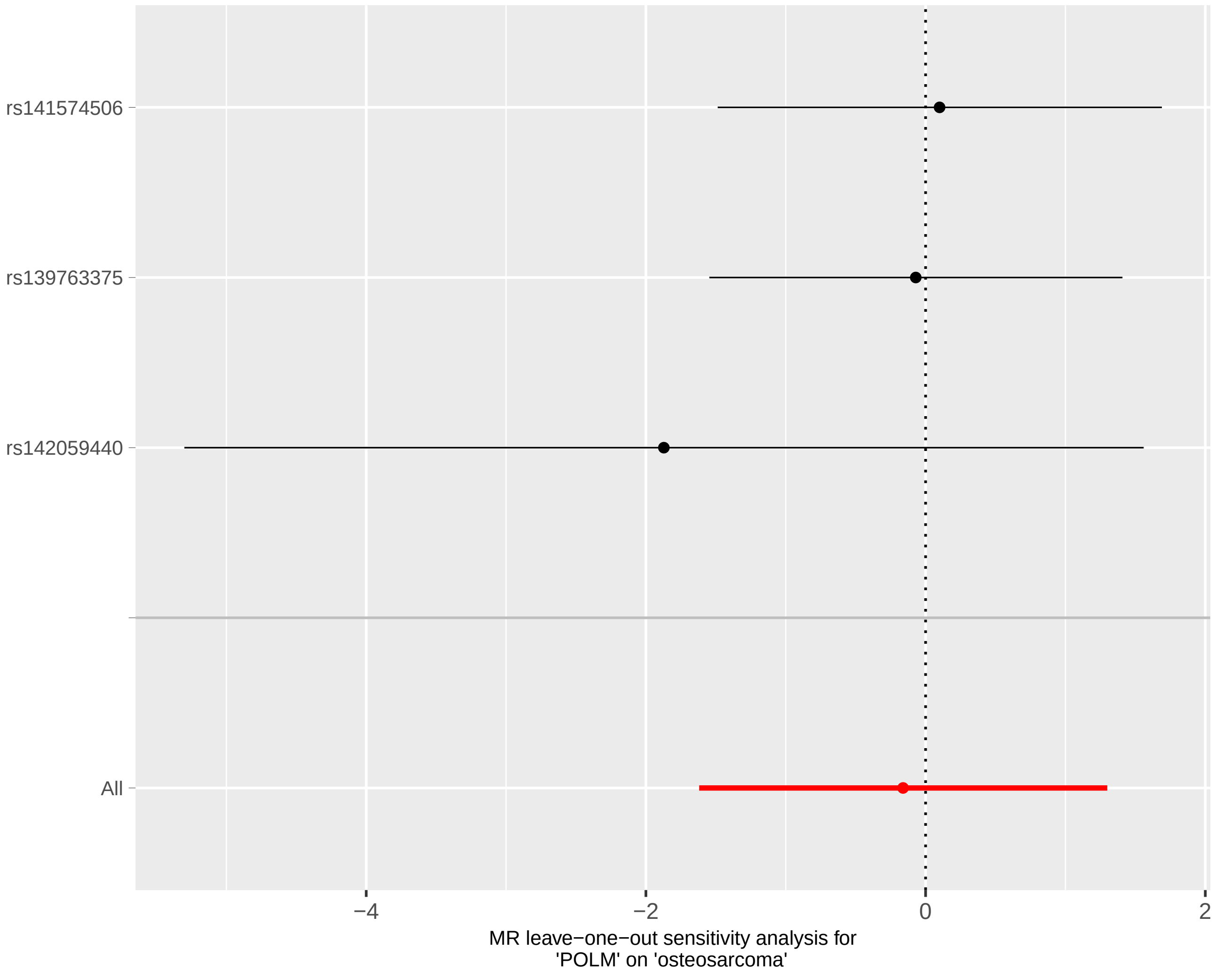
rs141574506
rs139763375
rs142059440
All
−4
−2
0
2
MR leave−one−out sensitivity analysis for
'POLM' on 'osteosarcoma'

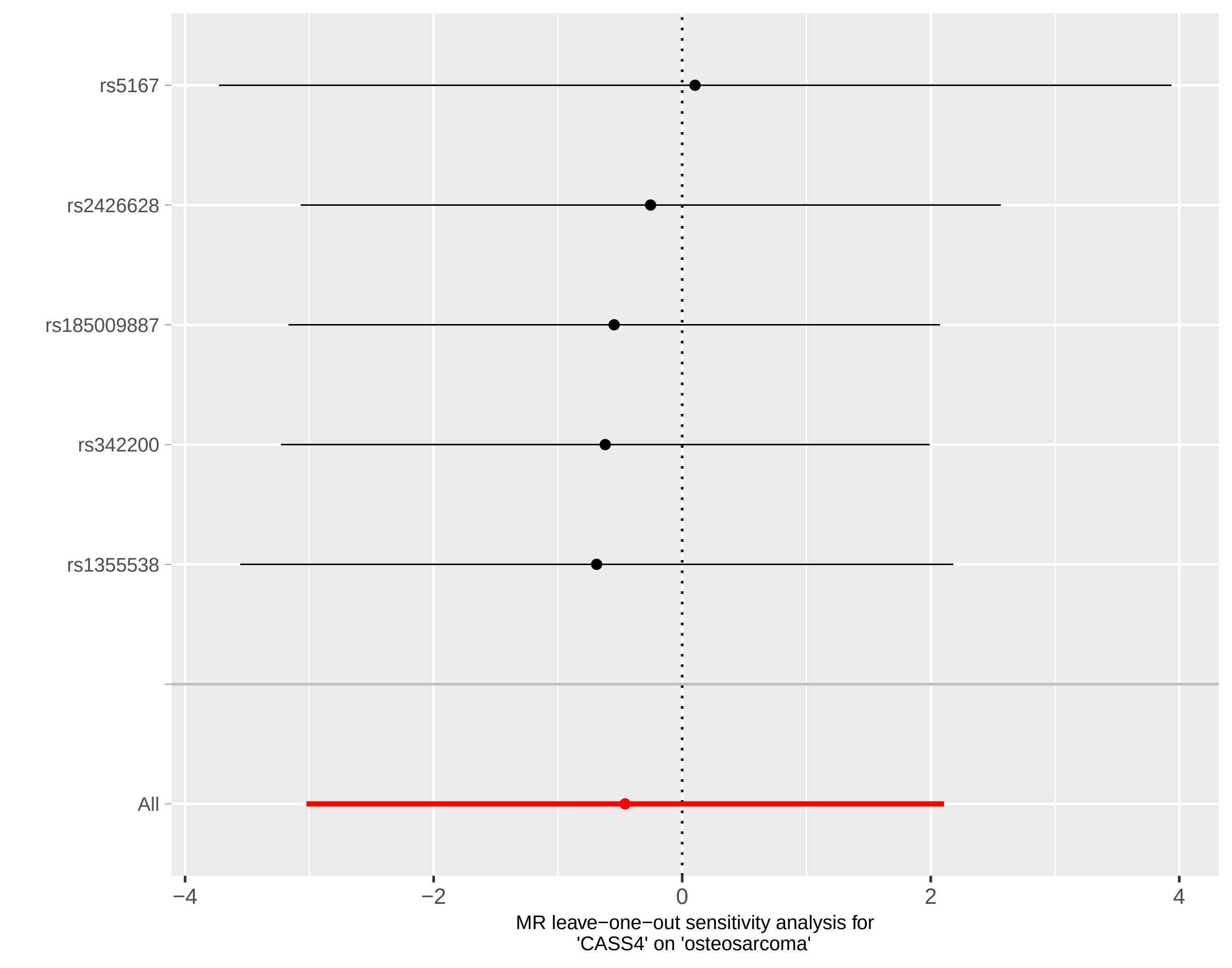
rs5167
rs2426628
rs185009887
rs342200
rs1355538
All
−4
−2
0
2
4
MR leave−one−out sensitivity analysis for
'CASS4' on 'osteosarcoma'

Insufficient number of SNPs

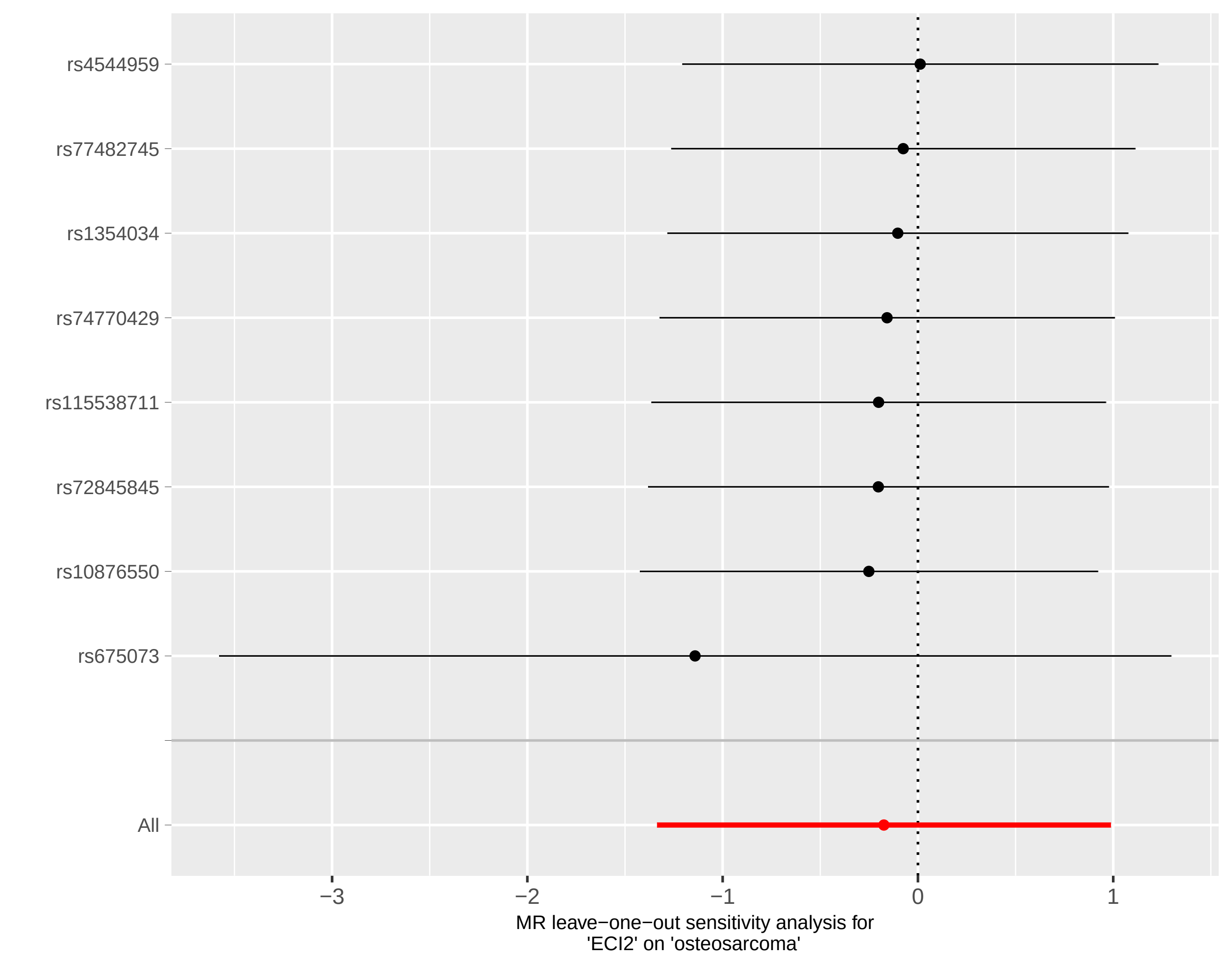

rs4544959
rs77482745
rs1354034
rs74770429
rs115538711
rs72845845
rs10876550
rs675073
All
−3
−2
−1
0
1
MR leave−one−out sensitivity analysis for
'ECI2' on 'osteosarcoma'

Insufficient number of SNPs

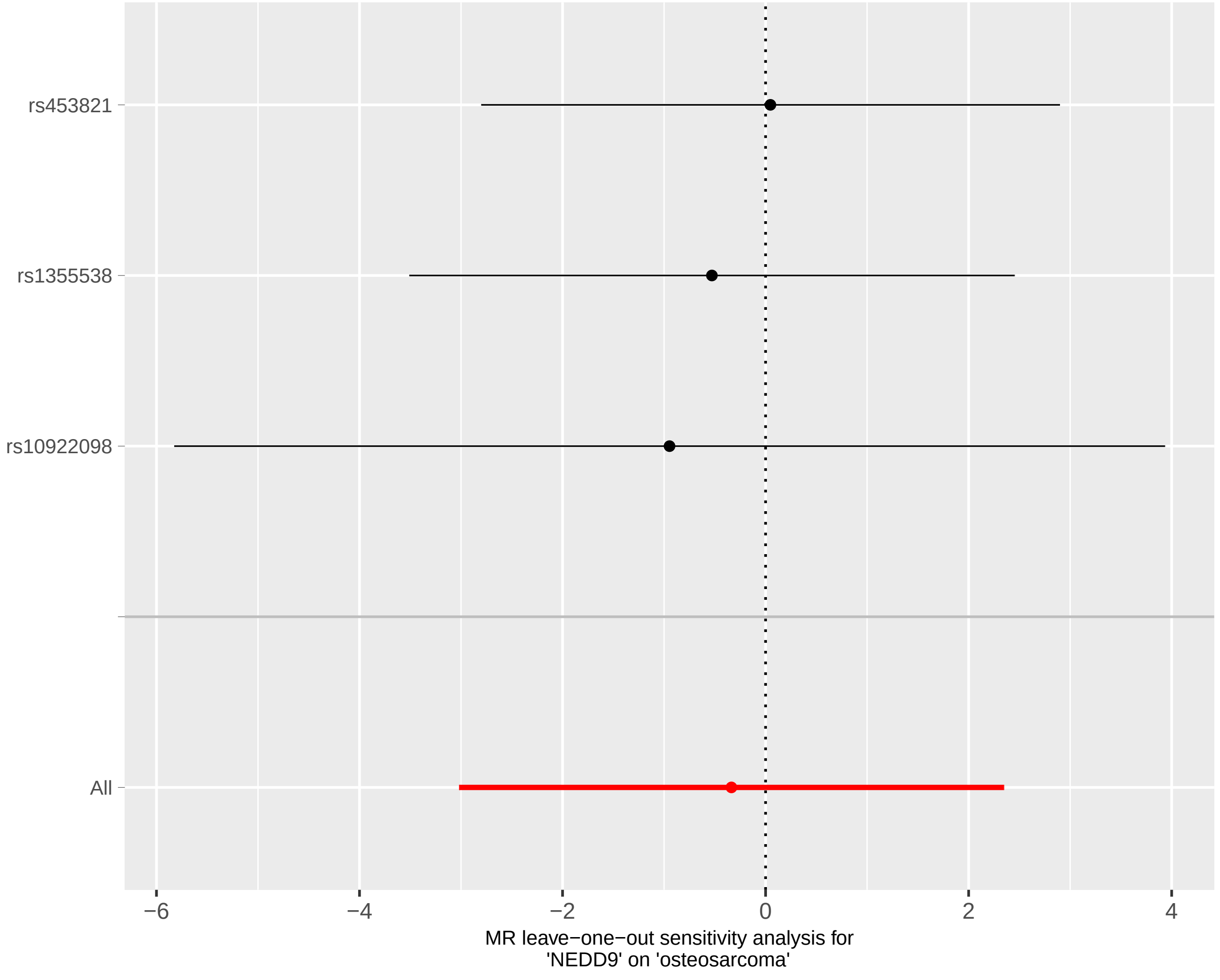
rs453821
rs1355538
rs10922098
All
−6
−4
−2
0
2
4
MR leave−one−out sensitivity analysis for
'NEDD9' on 'osteosarcoma'

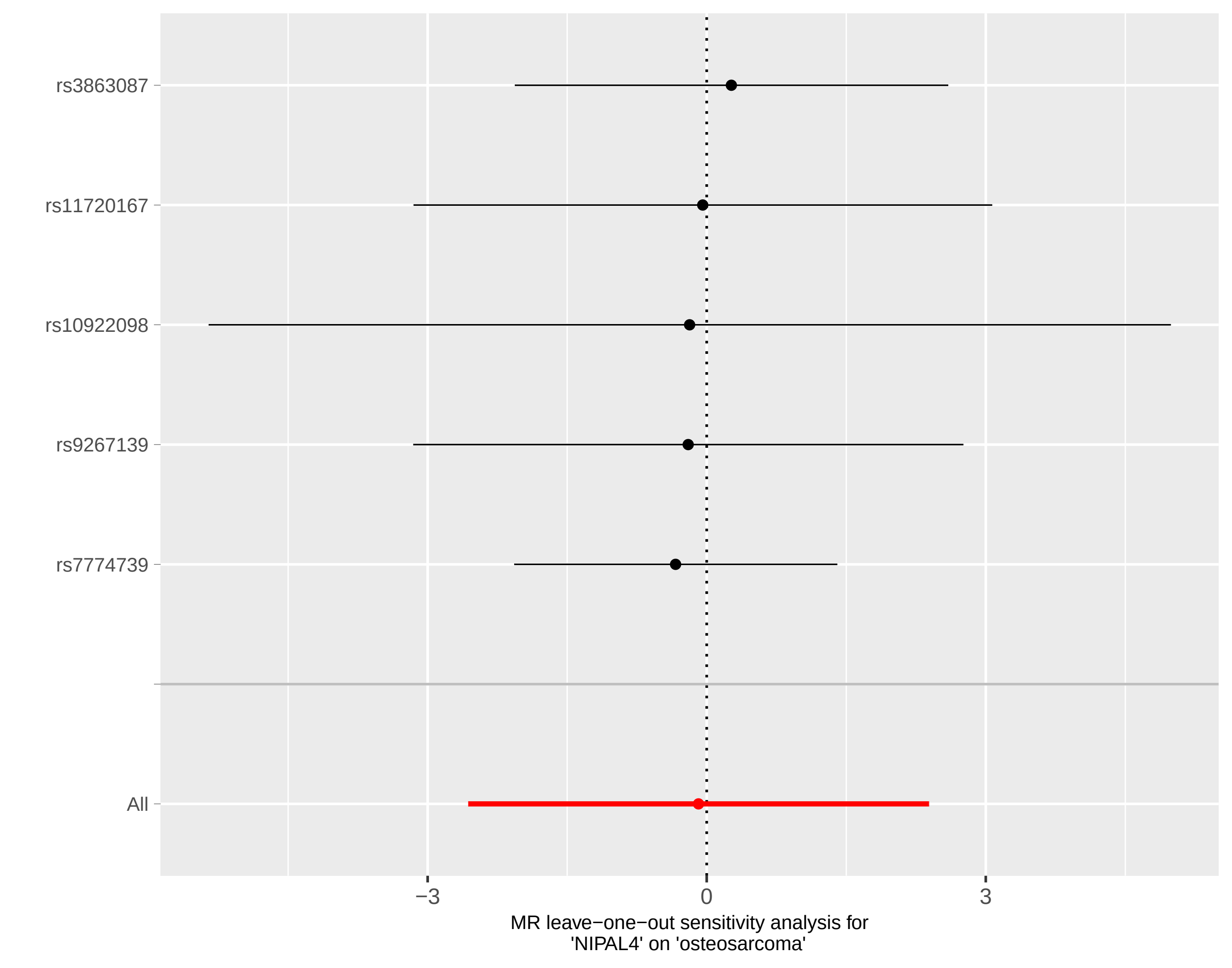
rs3863087
rs11720167
rs10922098
rs9267139
rs7774739
All
−3
0
3
MR leave−one−out sensitivity analysis for
'NIPAL4' on 'osteosarcoma'

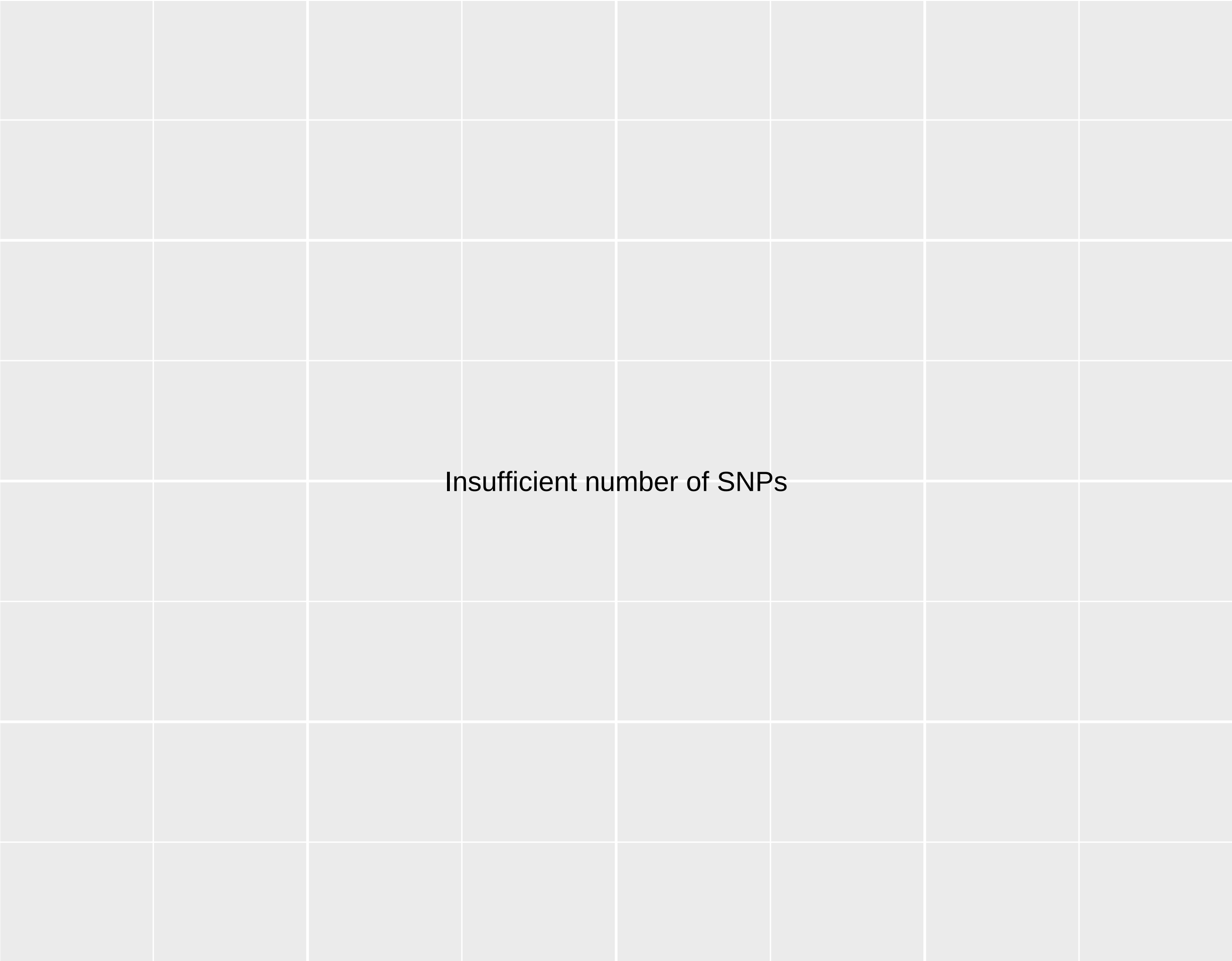
Insufficient number of SNPs

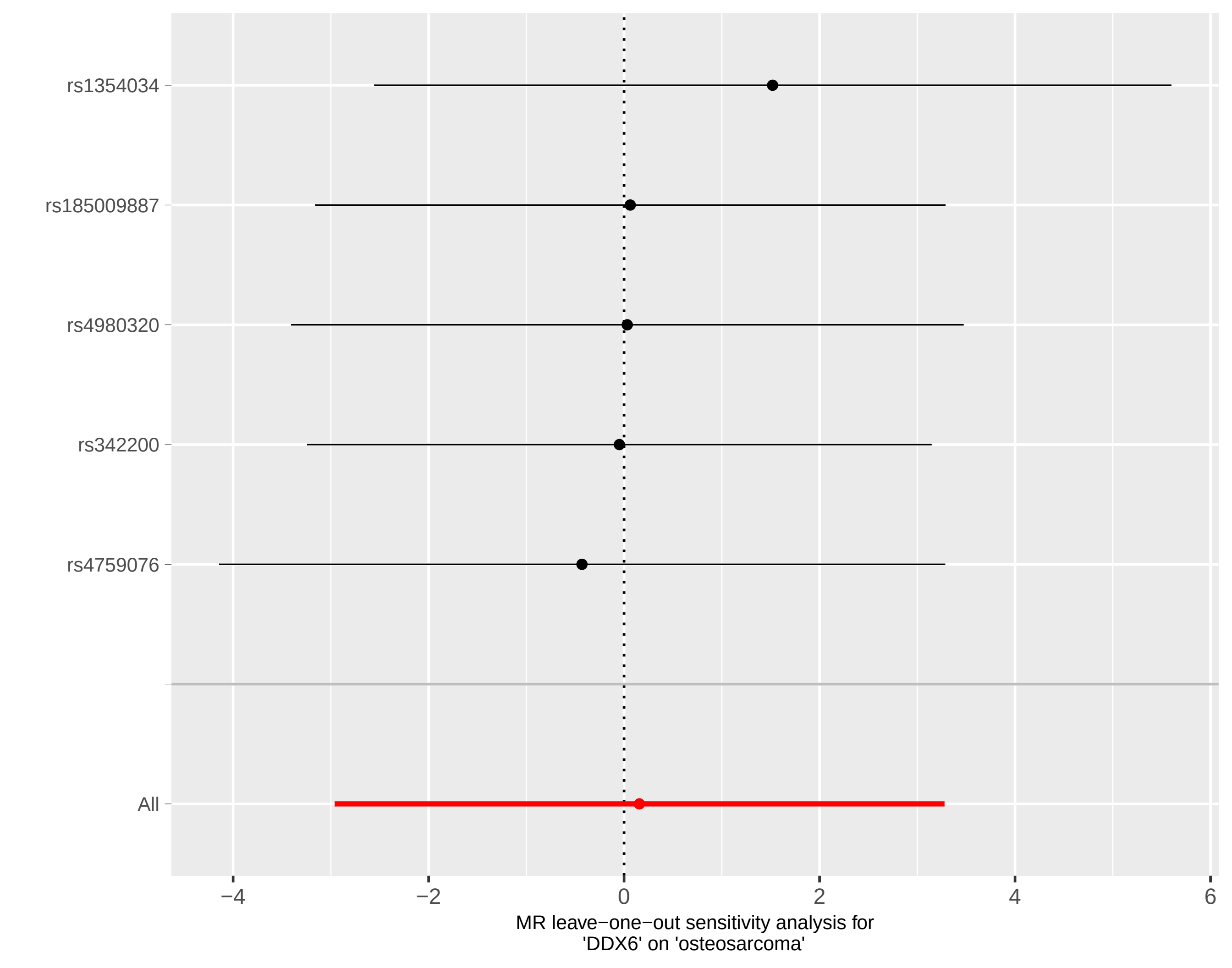
rs1354034
rs185009887
rs4980320
rs342200
rs4759076
All
−4
−2
0
2
4
6
MR leave−one−out sensitivity analysis for
'DDX6' on 'osteosarcoma'

Insufficient number of SNPs

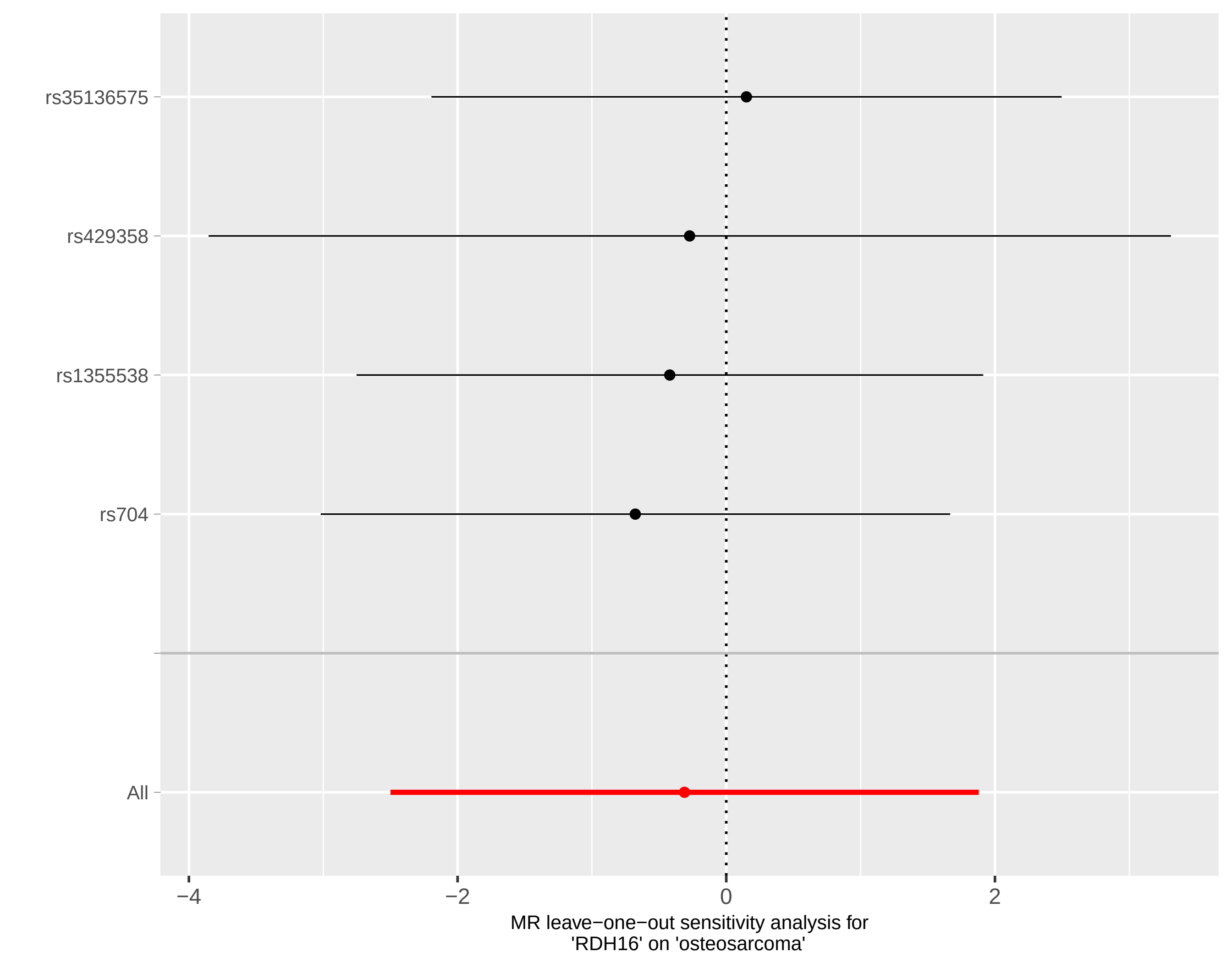
rs35136575
rs429358
rs1355538
rs704
All
−4
−2
0
2
MR leave−one−out sensitivity analysis for
'RDH16' on 'osteosarcoma'

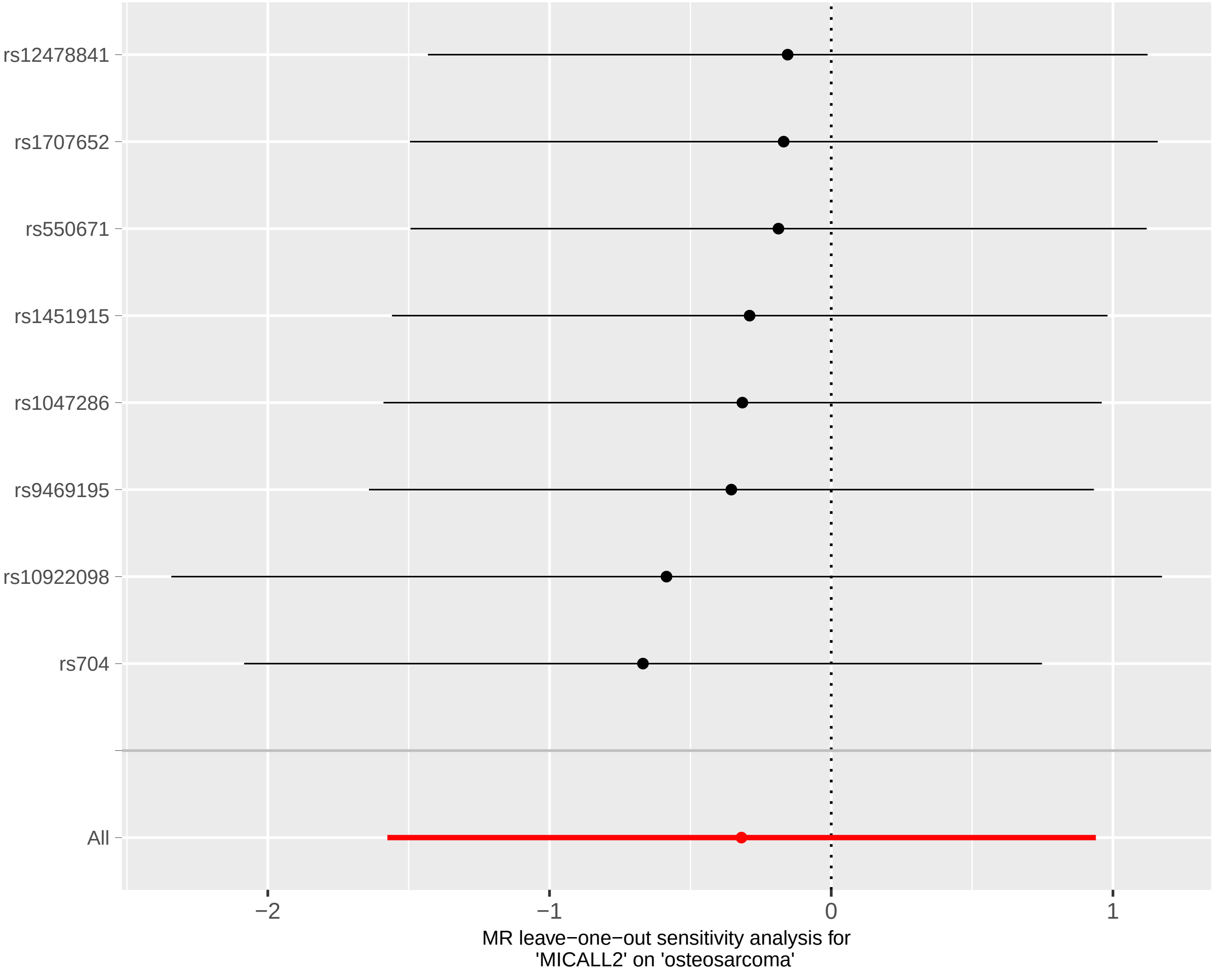
rs12478841
rs1707652
rs550671
rs1451915
rs1047286
rs9469195
rs10922098
rs704
All
−2
−1
0
1
MR leave−one−out sensitivity analysis for
'MICALL2' on 'osteosarcoma'

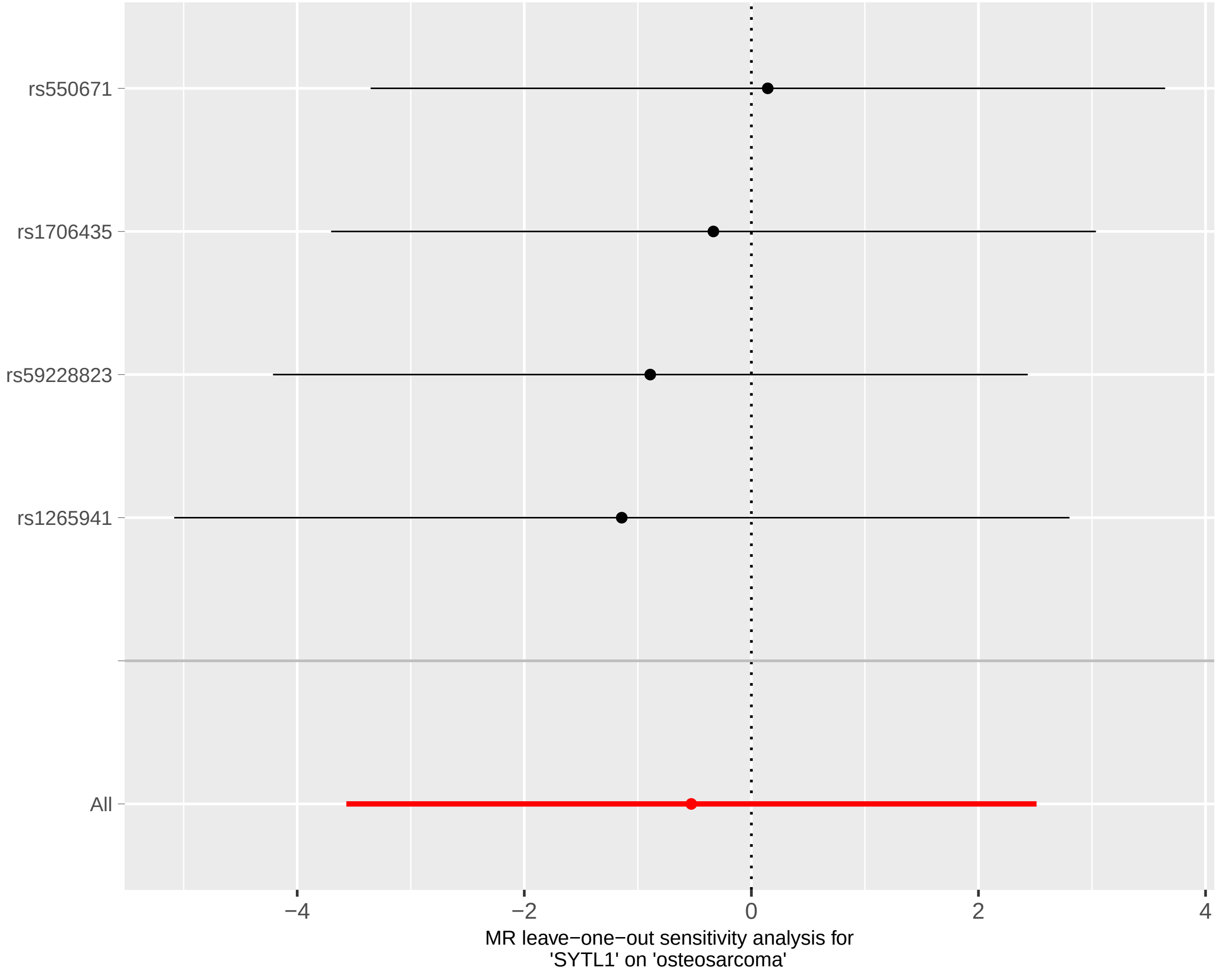
rs550671
rs1706435
rs59228823
rs1265941
All
−4
−2
0
2
4
MR leave−one−out sensitivity analysis for
'SYTL1' on 'osteosarcoma'

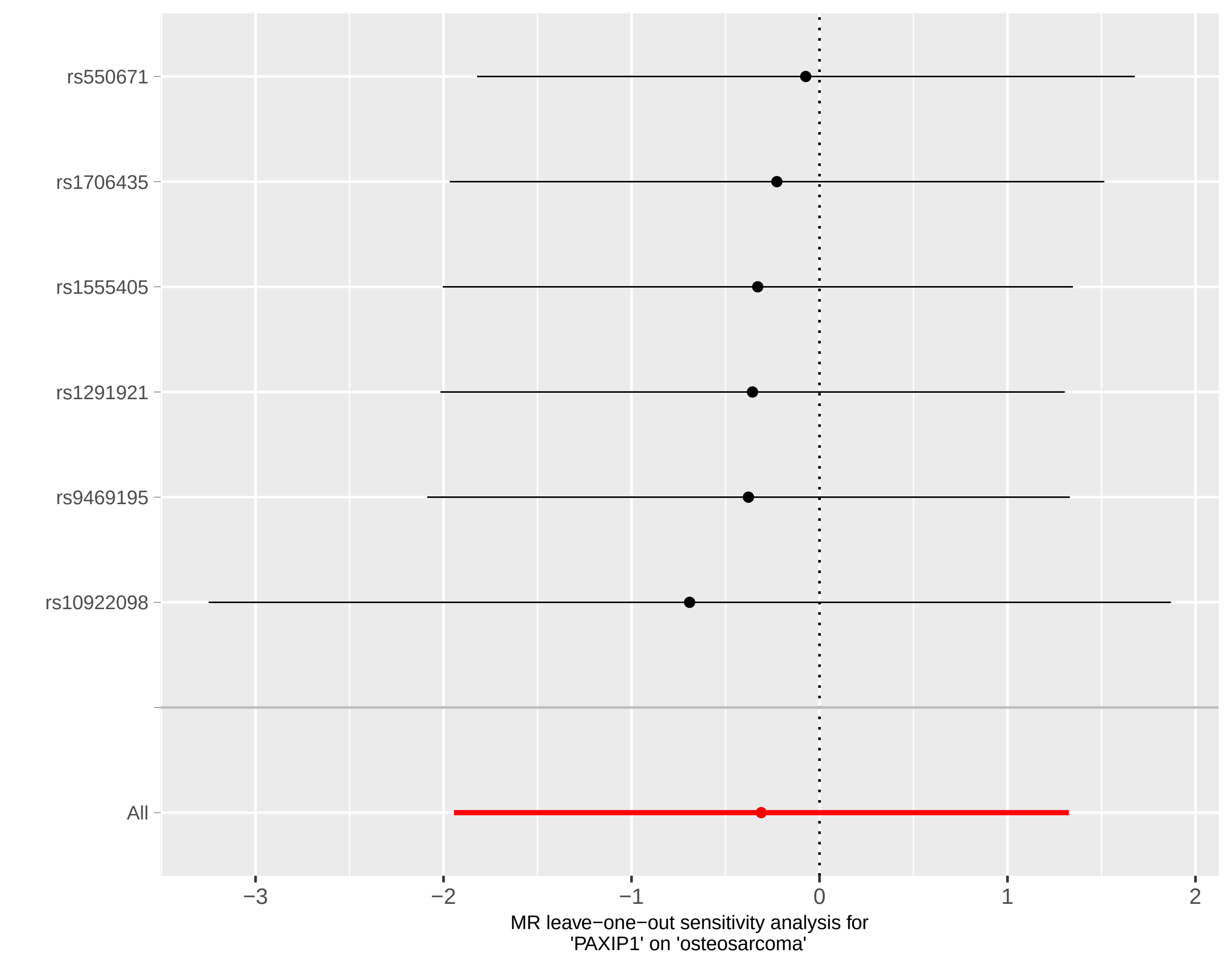

rs550671
rs1706435
rs1555405
rs1291921
rs9469195
rs10922098
All
−3
−2
−1
0
1
2
MR leave−one−out sensitivity analysis for
'PAXIP1' on 'osteosarcoma'

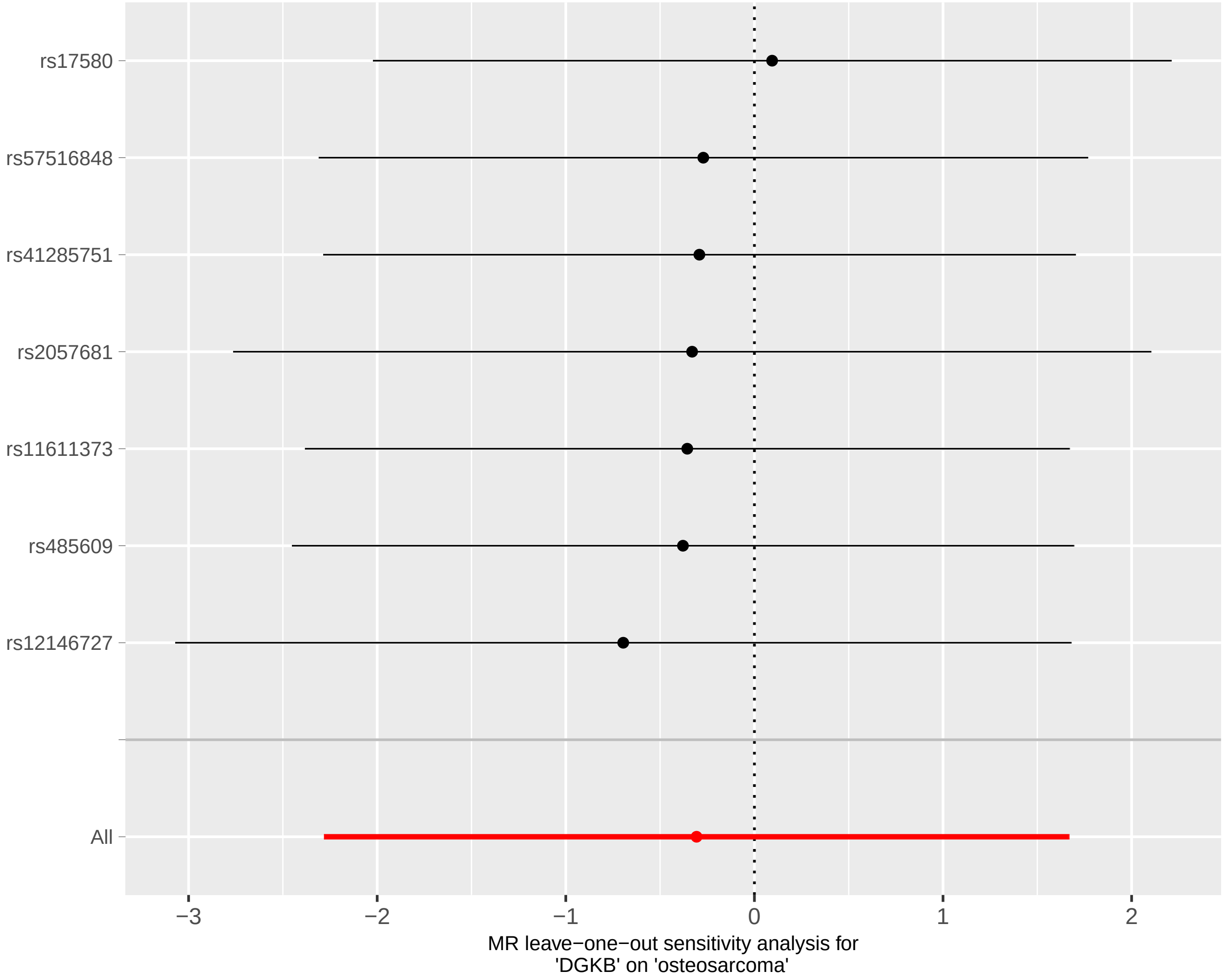
rs17580
rs57516848
rs41285751
rs2057681
rs11611373
rs485609
rs12146727
All
−3
−2
−1
0
1
2
MR leave−one−out sensitivity analysis for
'DGKB' on 'osteosarcoma'

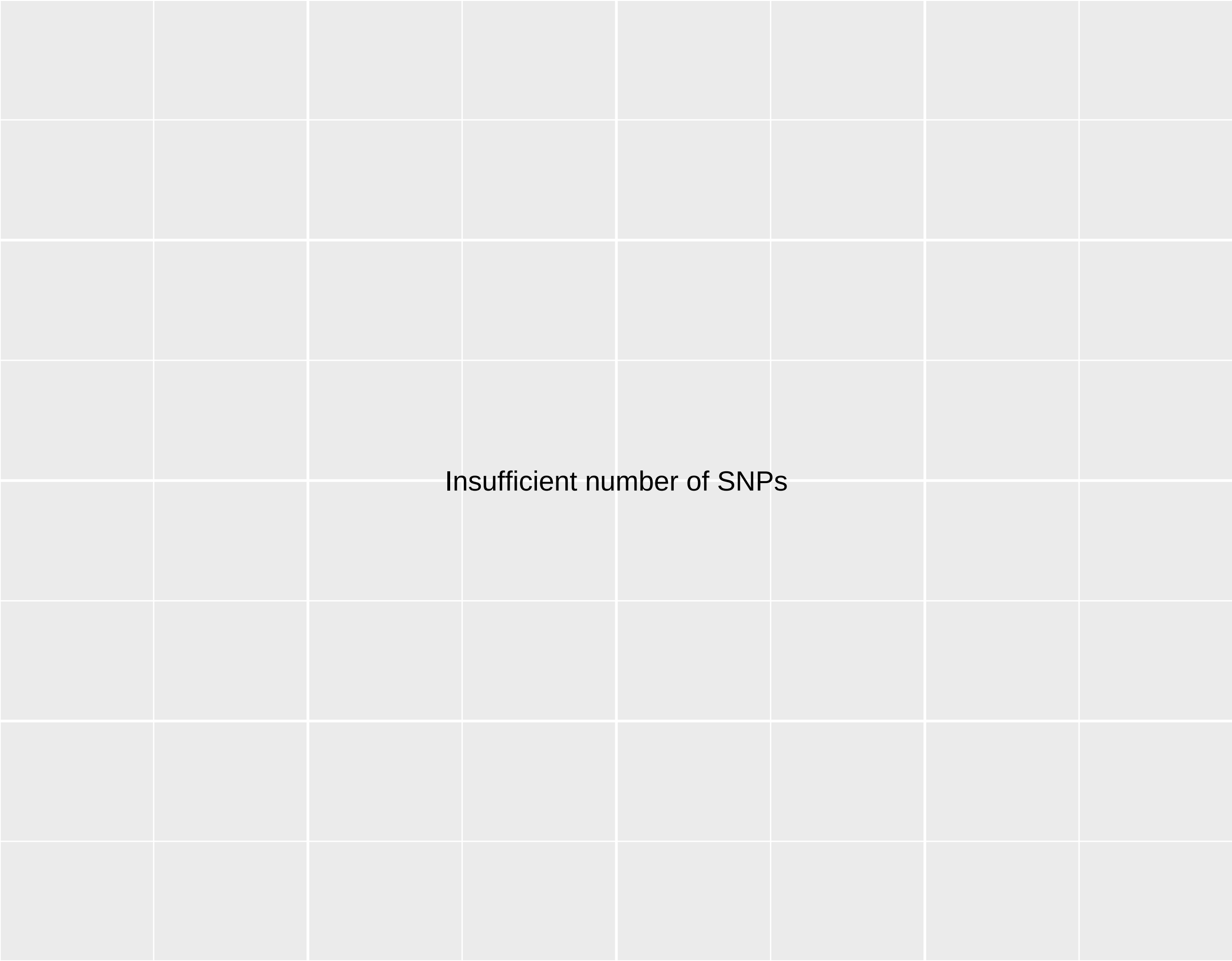
Insufficient number of SNPs

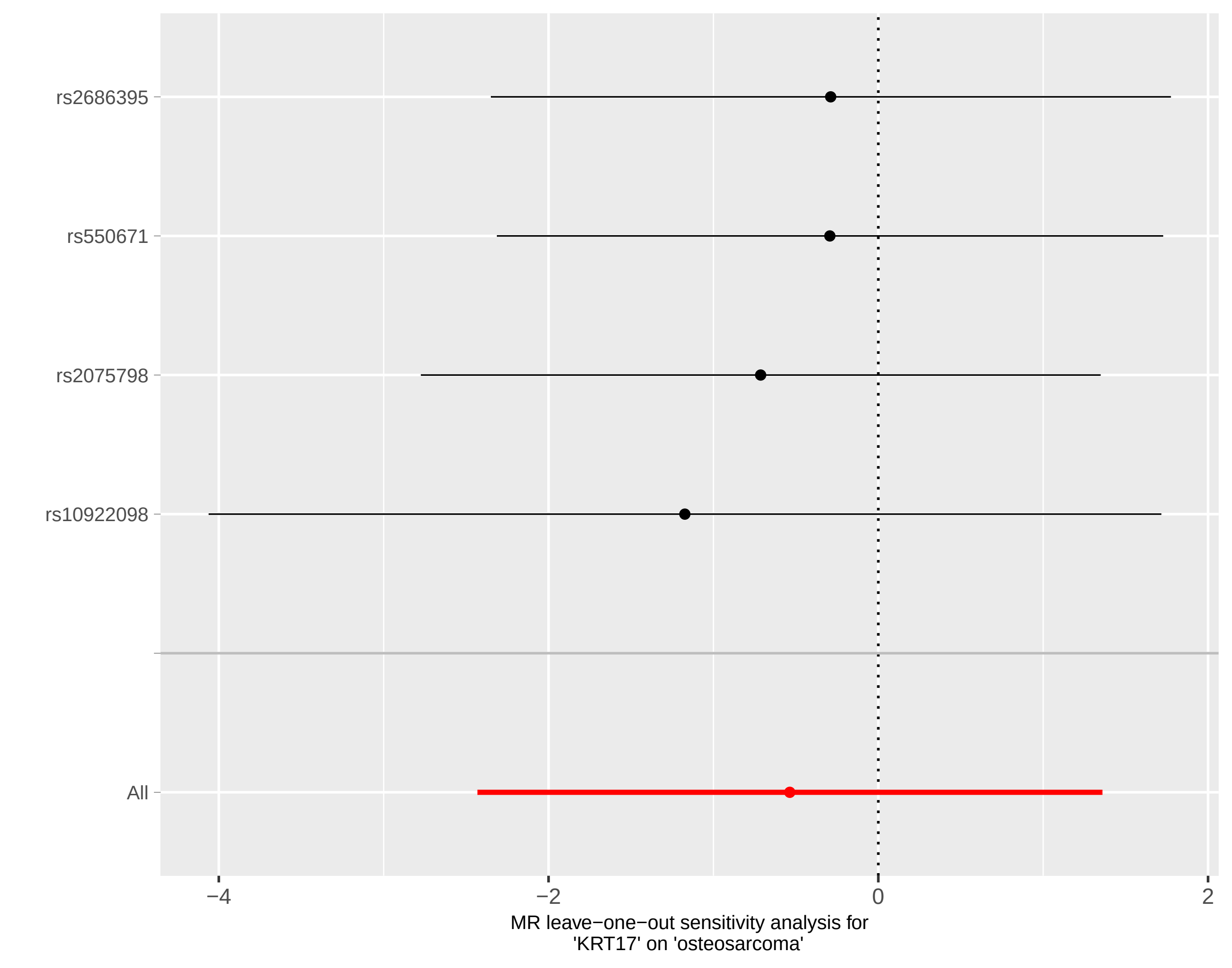
rs2686395
rs550671
rs2075798
rs10922098
All
−4
−2
0
2
MR leave−one−out sensitivity analysis for
'KRT17' on 'osteosarcoma'

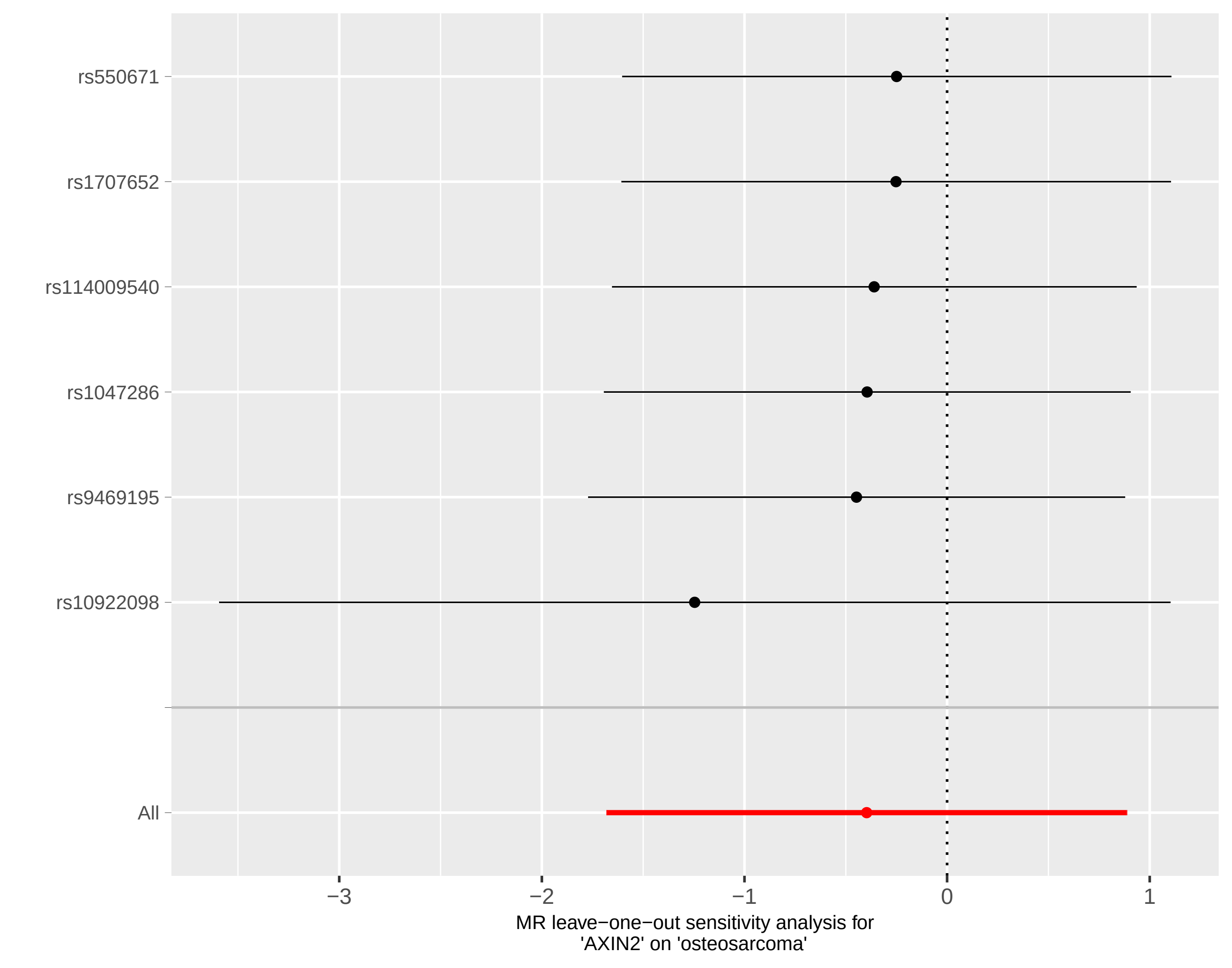
rs550671
rs1707652
rs114009540
rs1047286
rs9469195
rs10922098
All
−3
−2
−1
0
1
MR leave−one−out sensitivity analysis for
'AXIN2' on 'osteosarcoma'

Insufficient number of SNPs

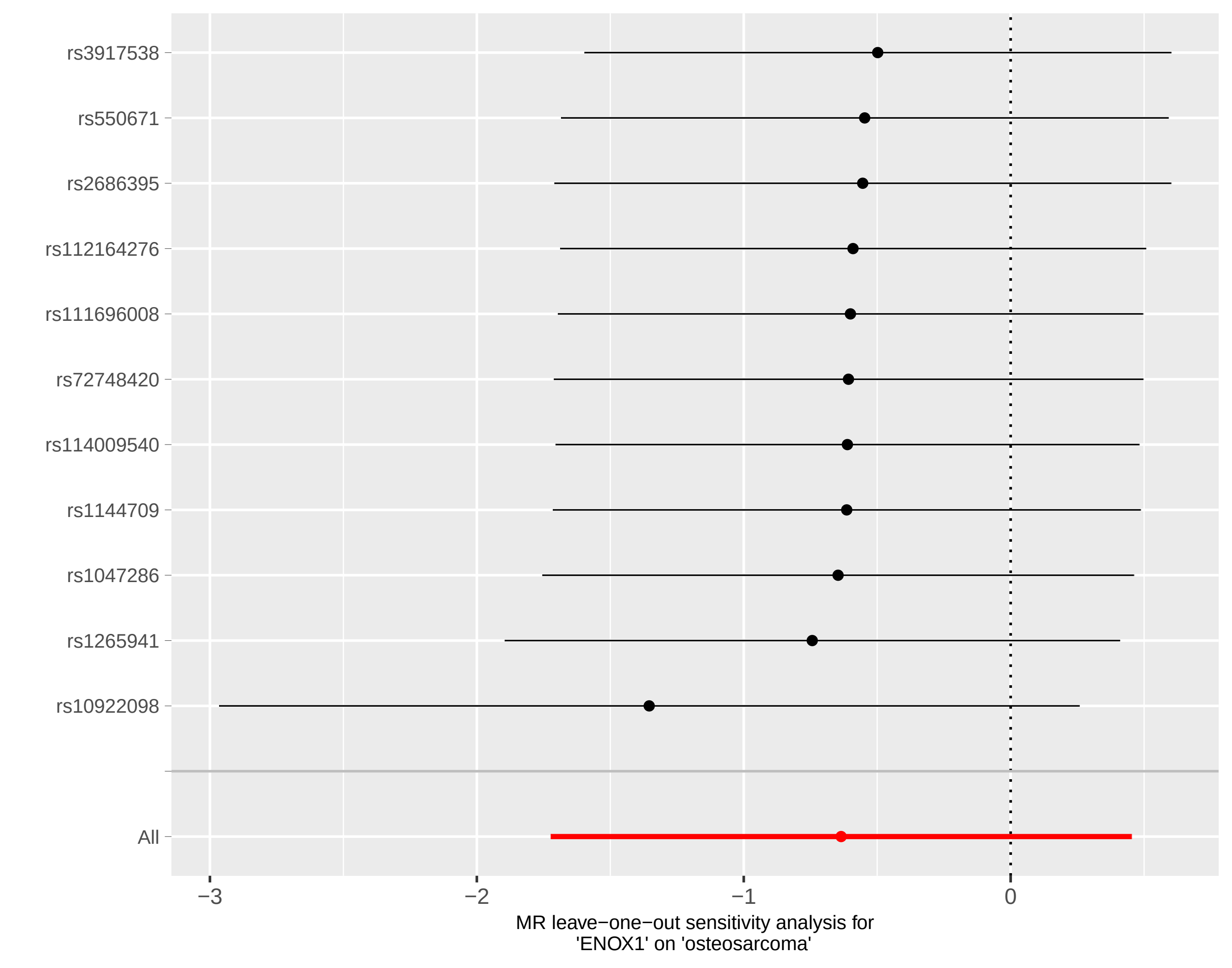
rs3917538
rs550671
rs2686395
rs112164276
rs111696008
rs72748420
rs114009540
rs1144709
rs1047286
rs1265941
rs10922098
All
−3
−2
−1
0
MR leave−one−out sensitivity analysis for
'ENOX1' on 'osteosarcoma'

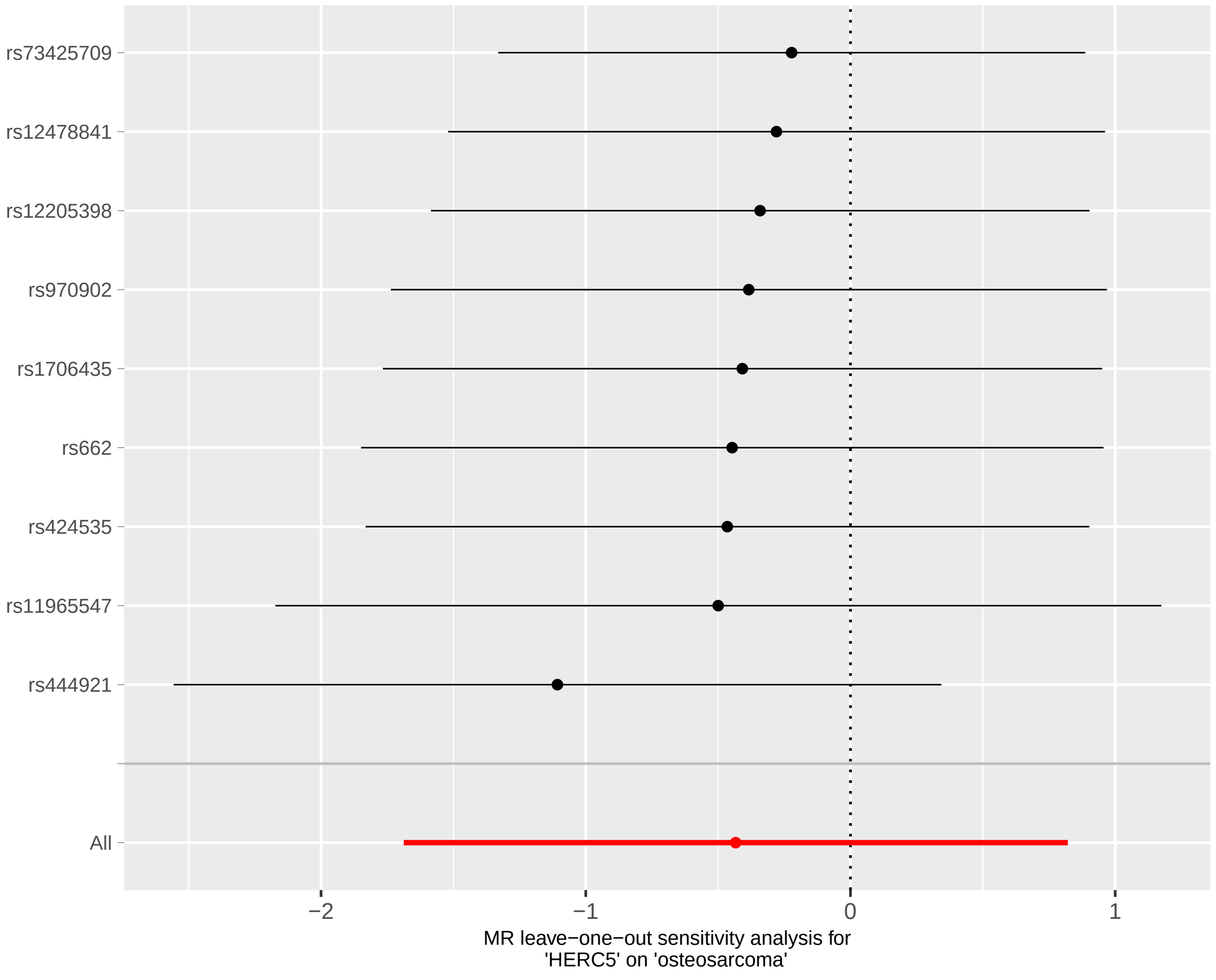
rs73425709
rs12478841
rs12205398
rs970902
rs1706435
rs662
rs424535
rs11965547
rs444921
All
−2
−1
0
1
MR leave−one−out sensitivity analysis for
'HERC5' on 'osteosarcoma'

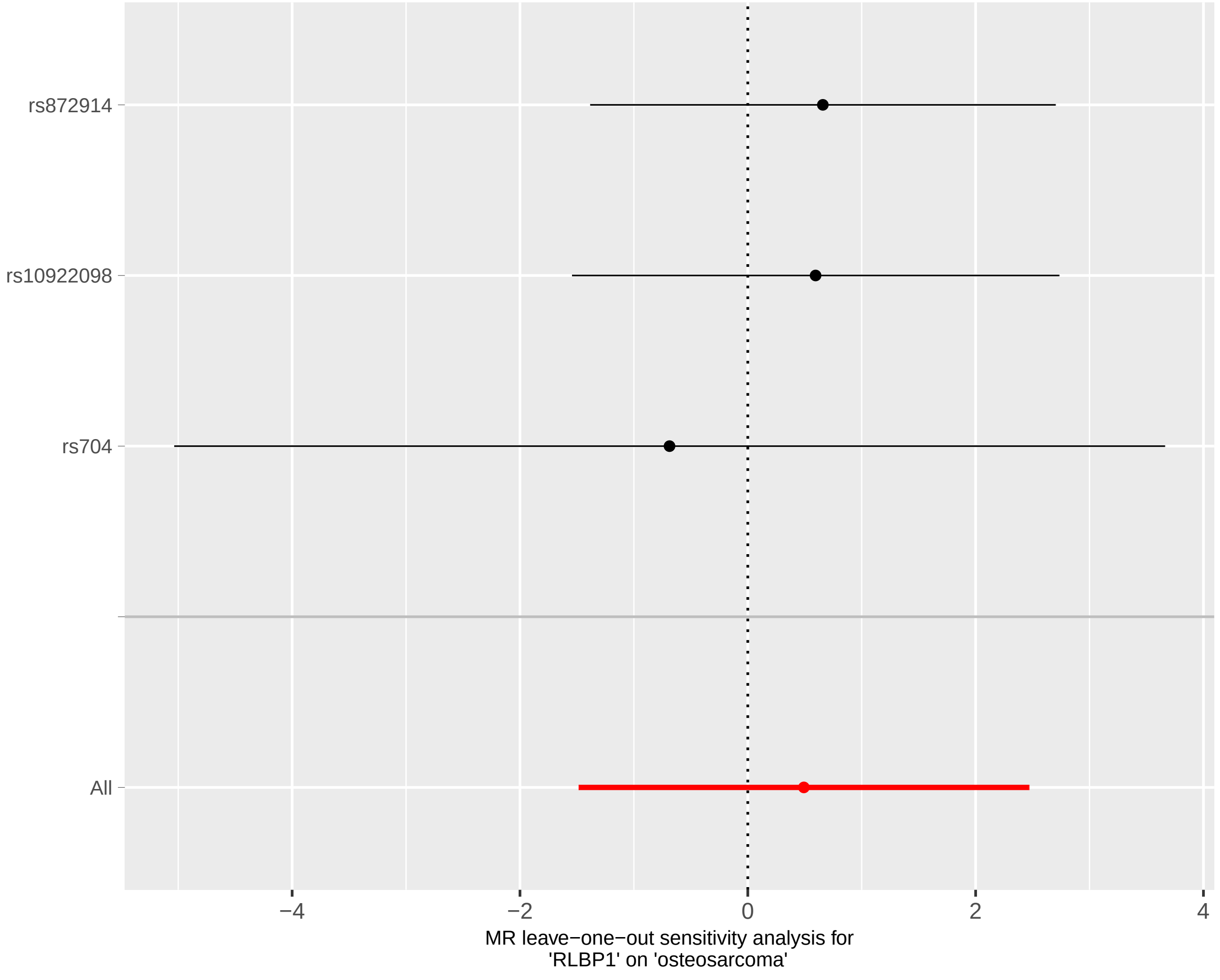
rs872914
rs10922098
rs704
All
−4
−2
0
2
4
MR leave−one−out sensitivity analysis for
'RLBP1' on 'osteosarcoma'

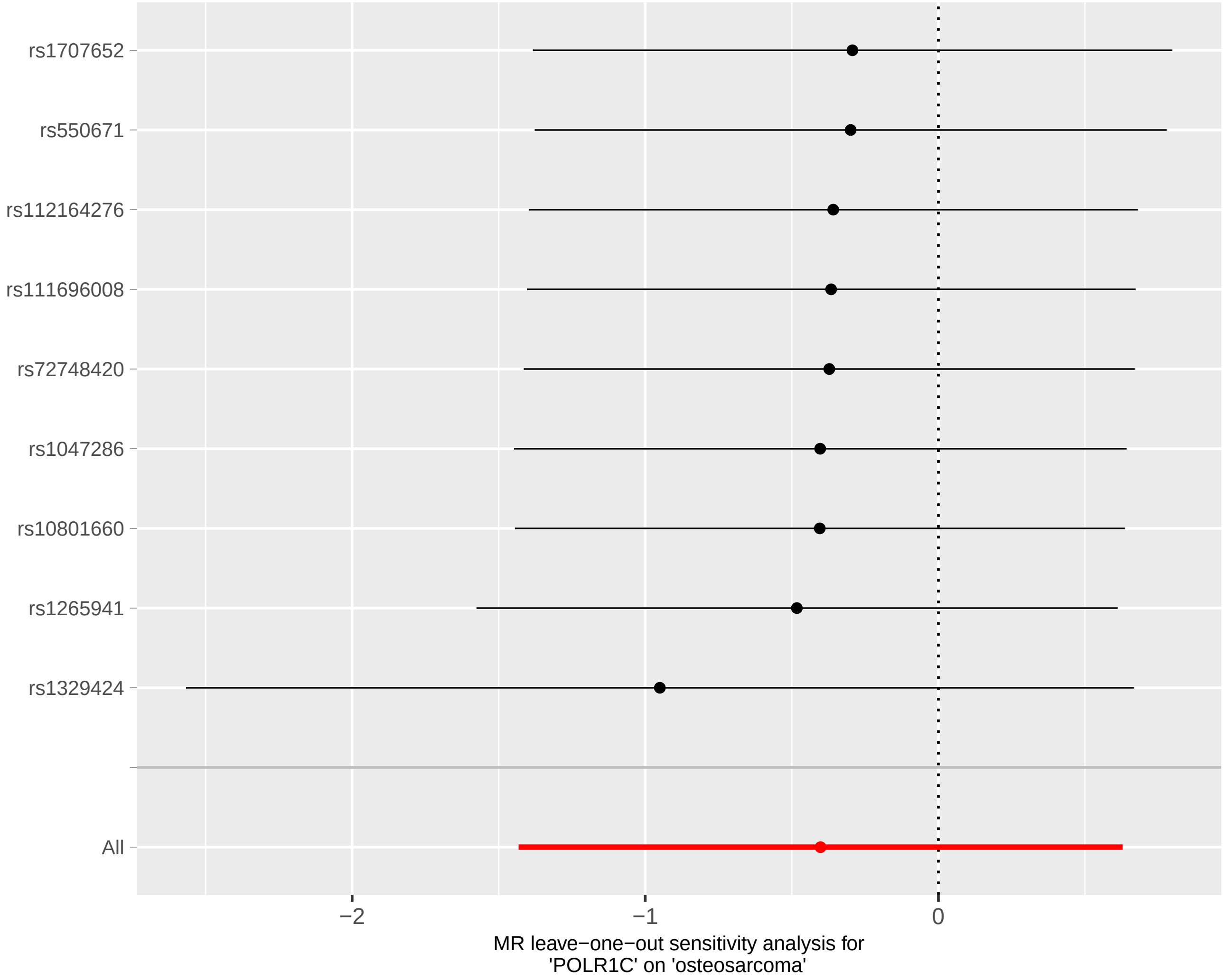

rs1707652
rs550671
rs112164276
rs111696008
rs72748420
rs1047286
rs10801660
rs1265941
rs1329424
All
−2
−1
0
MR leave−one−out sensitivity analysis for
'POLR1C' on 'osteosarcoma'

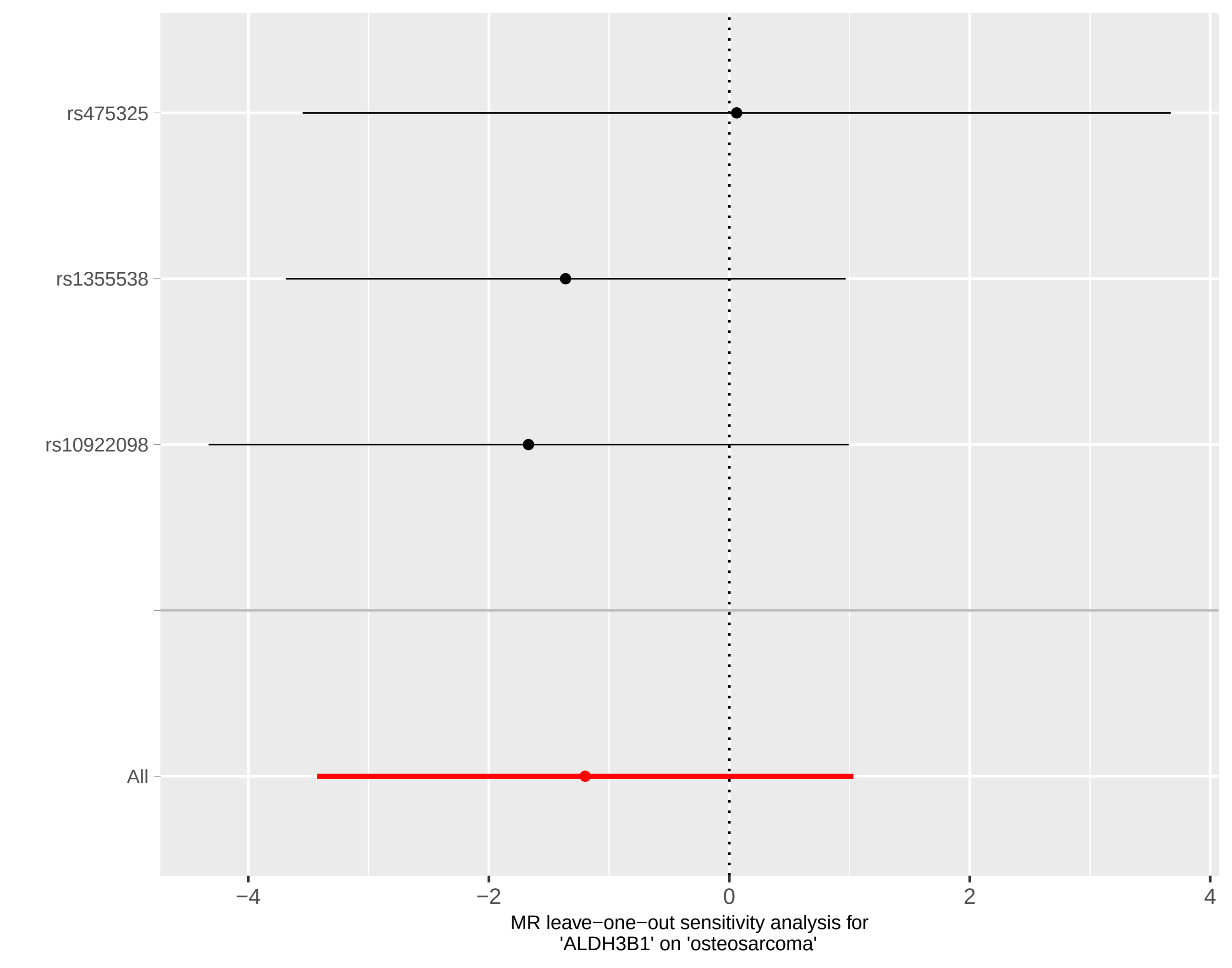

rs475325
rs1355538
rs10922098
All
−4
−2
0
2
4
MR leave−one−out sensitivity analysis for
'ALDH3B1' on 'osteosarcoma'

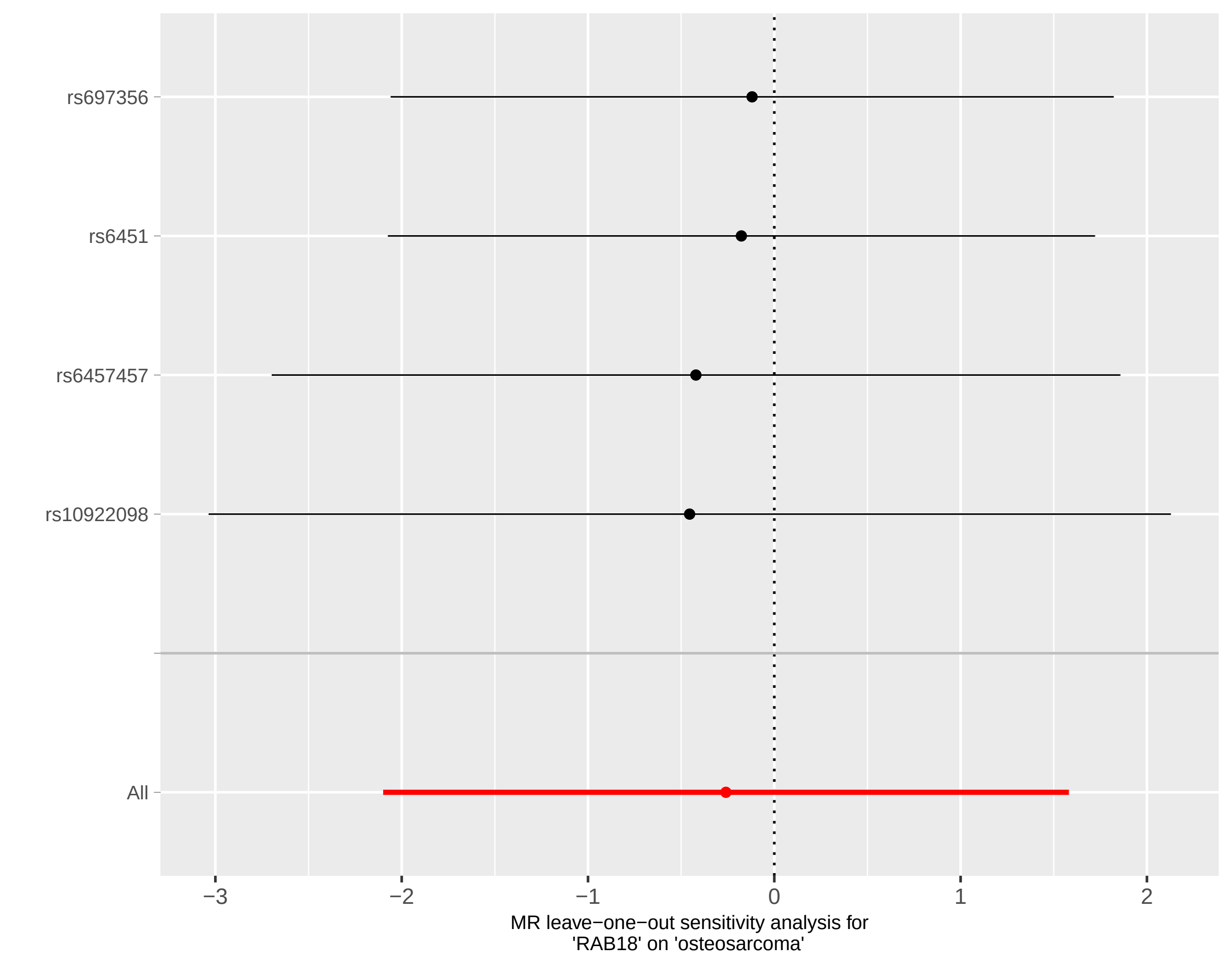
rs697356
rs6451
rs6457457
rs10922098
All
−3
−2
−1
0
1
2
MR leave−one−out sensitivity analysis for
'RAB18' on 'osteosarcoma'

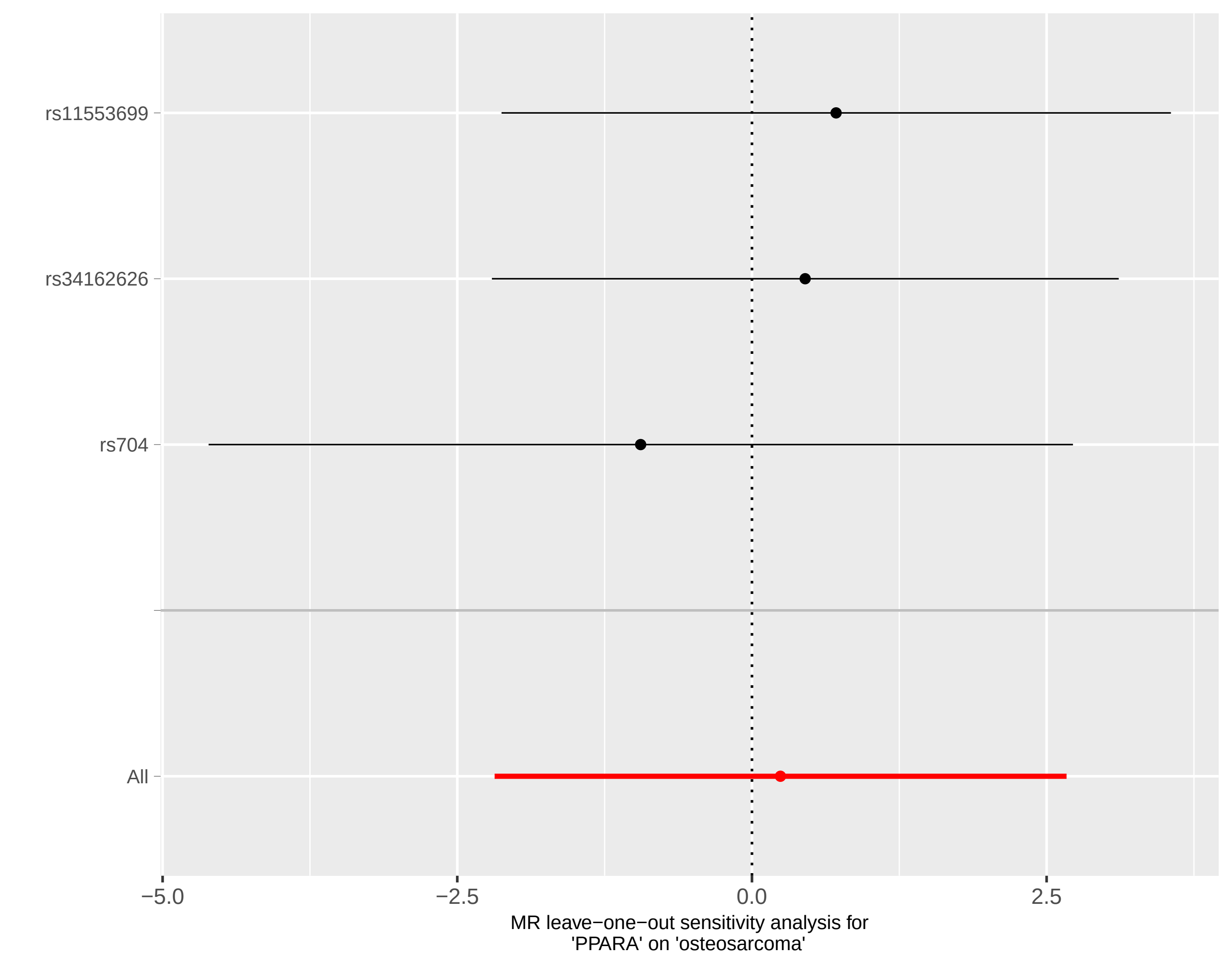
rs11553699
rs34162626
rs704
All
−5.0
−2.5
0.0
2.5
MR leave−one−out sensitivity analysis for
'PPARA' on 'osteosarcoma'

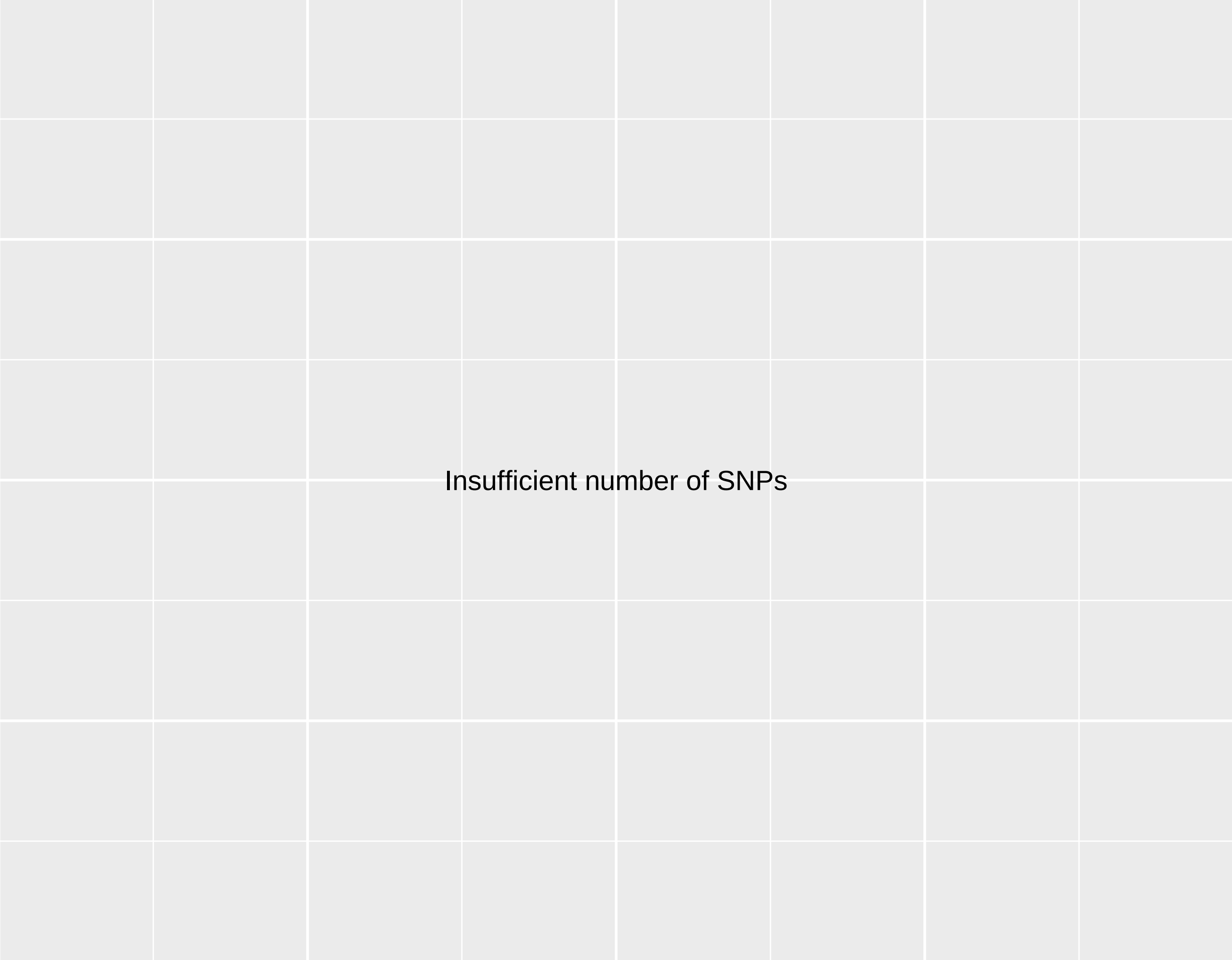
Insufficient number of SNPs

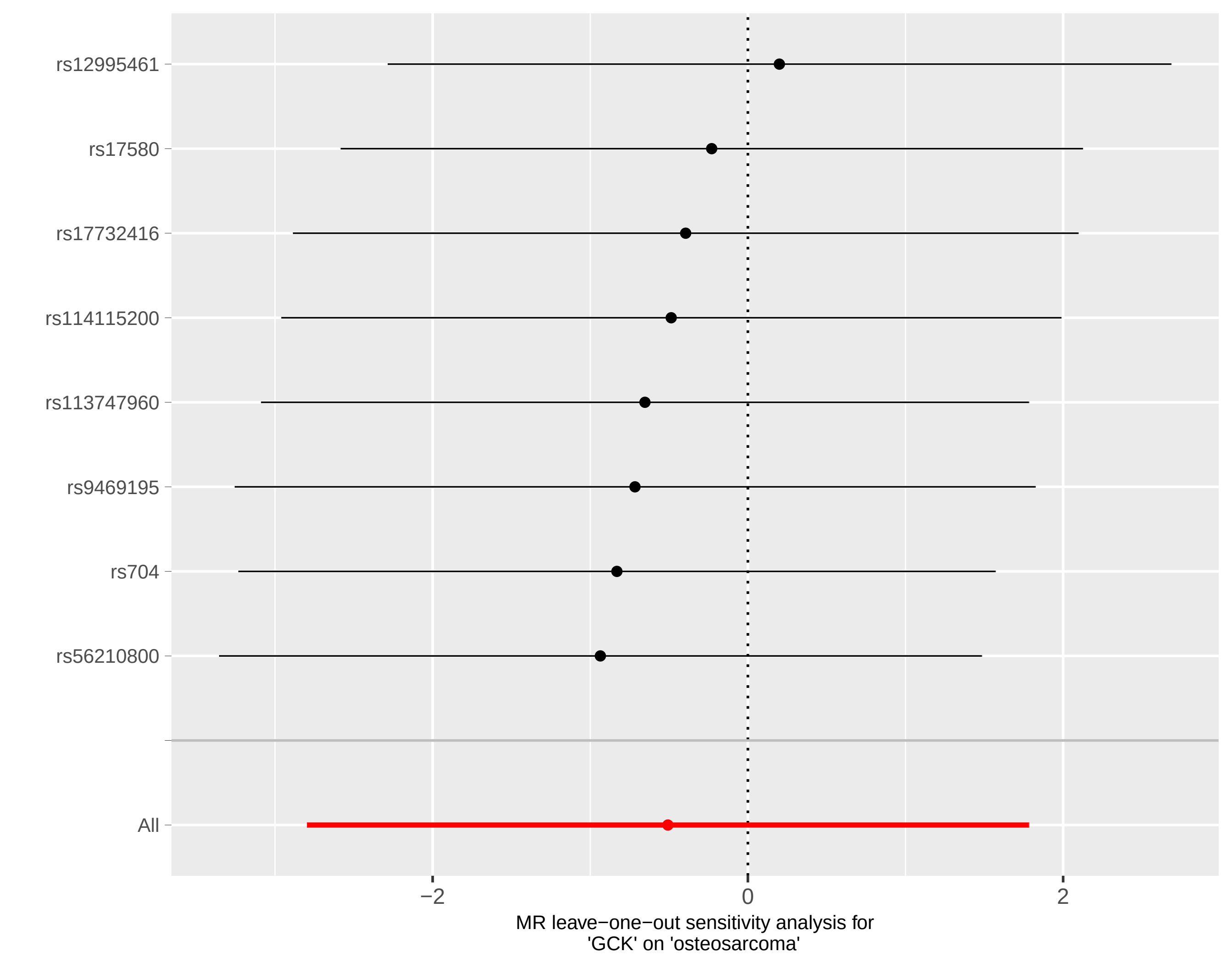

rs12995461
rs17580
rs17732416
rs114115200
rs113747960
rs9469195
rs704
rs56210800
All
−2
0
2
MR leave−one−out sensitivity analysis for
'GCK' on 'osteosarcoma'

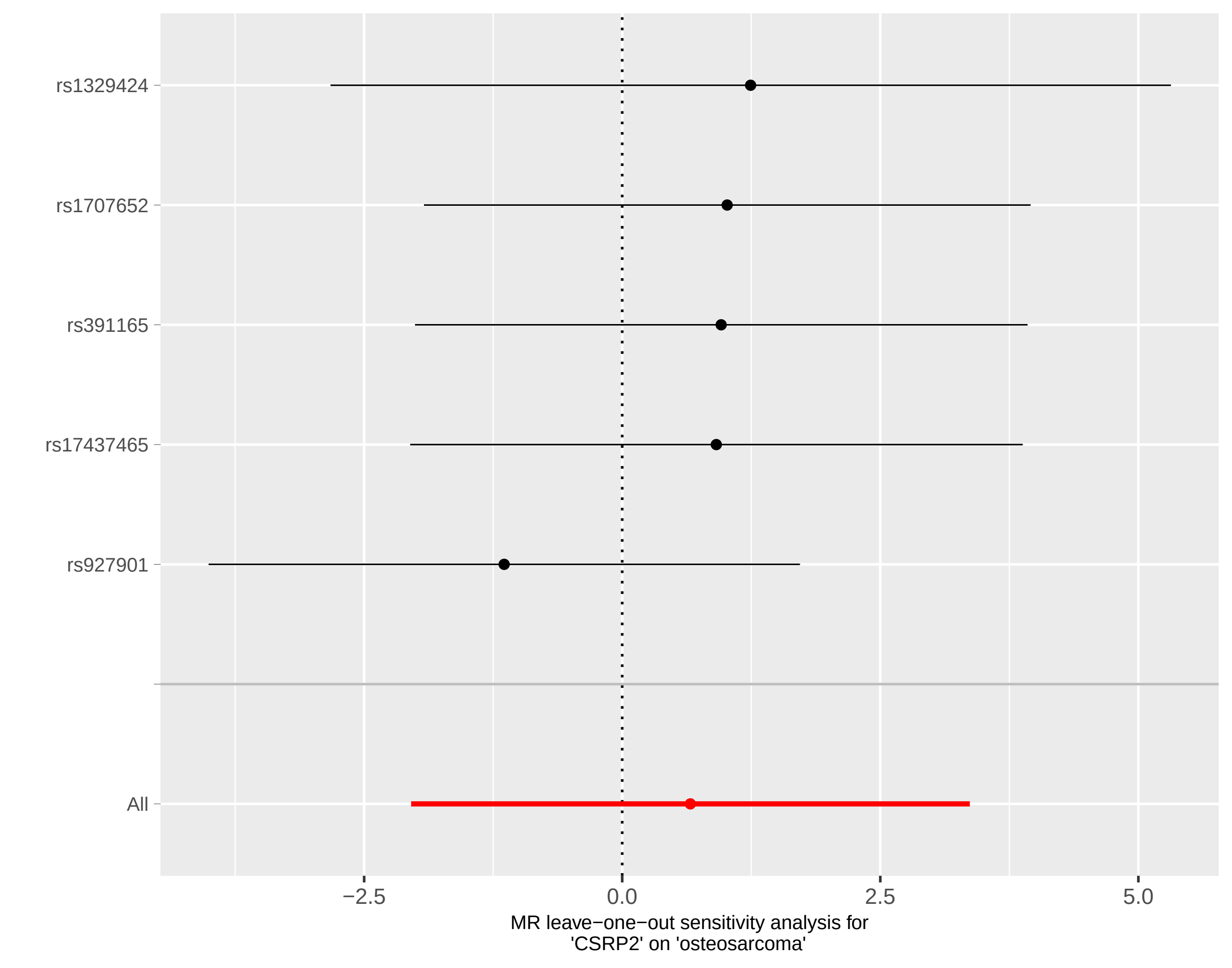
rs1329424
rs1707652
rs391165
rs17437465
rs927901
All
−2.5
0.0
2.5
5.0
MR leave−one−out sensitivity analysis for
'CSRP2' on 'osteosarcoma'

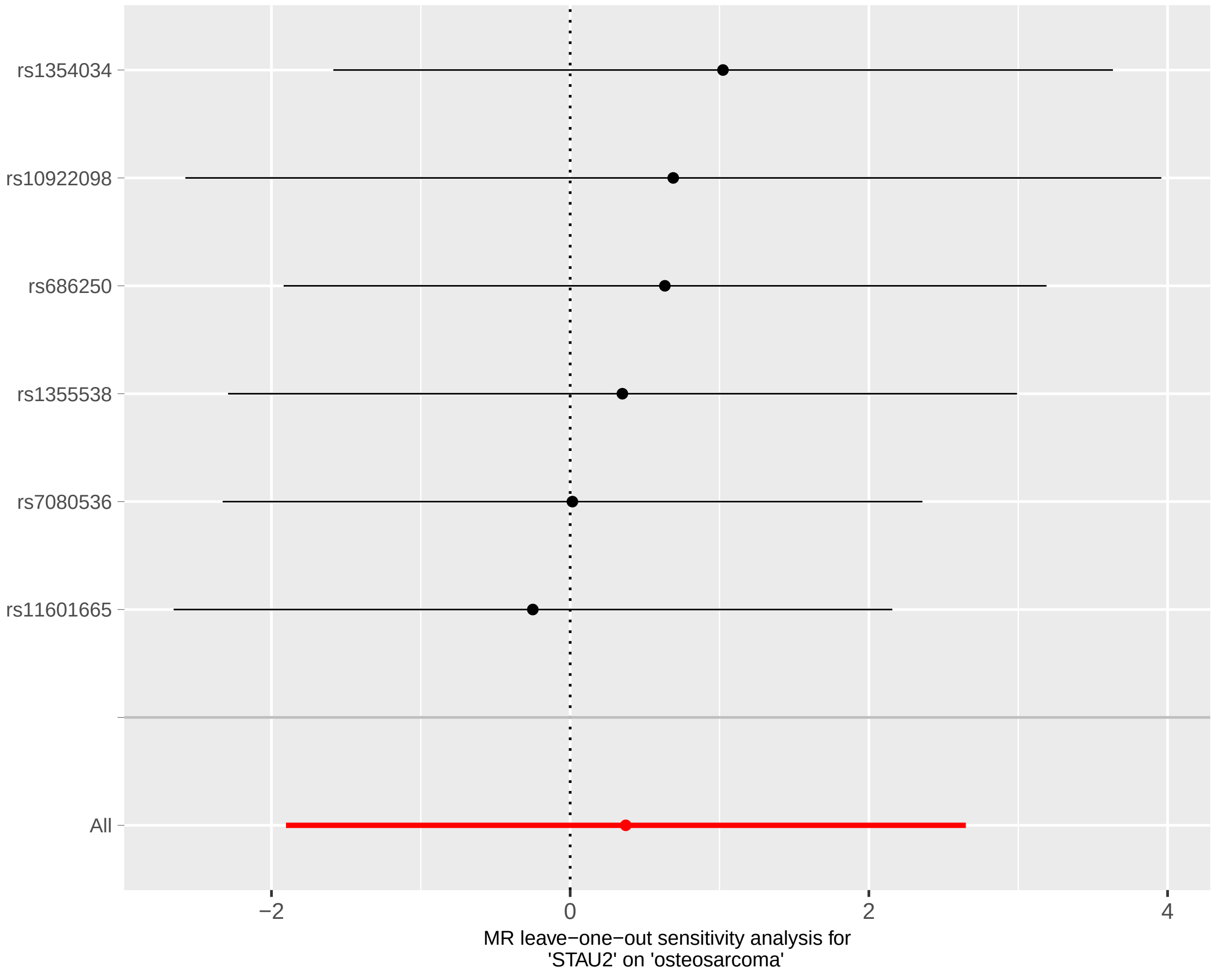
rs1354034
rs10922098
rs686250
rs1355538
rs7080536
rs11601665
All
−2
0
2
4
MR leave−one−out sensitivity analysis for
'STAU2' on 'osteosarcoma'

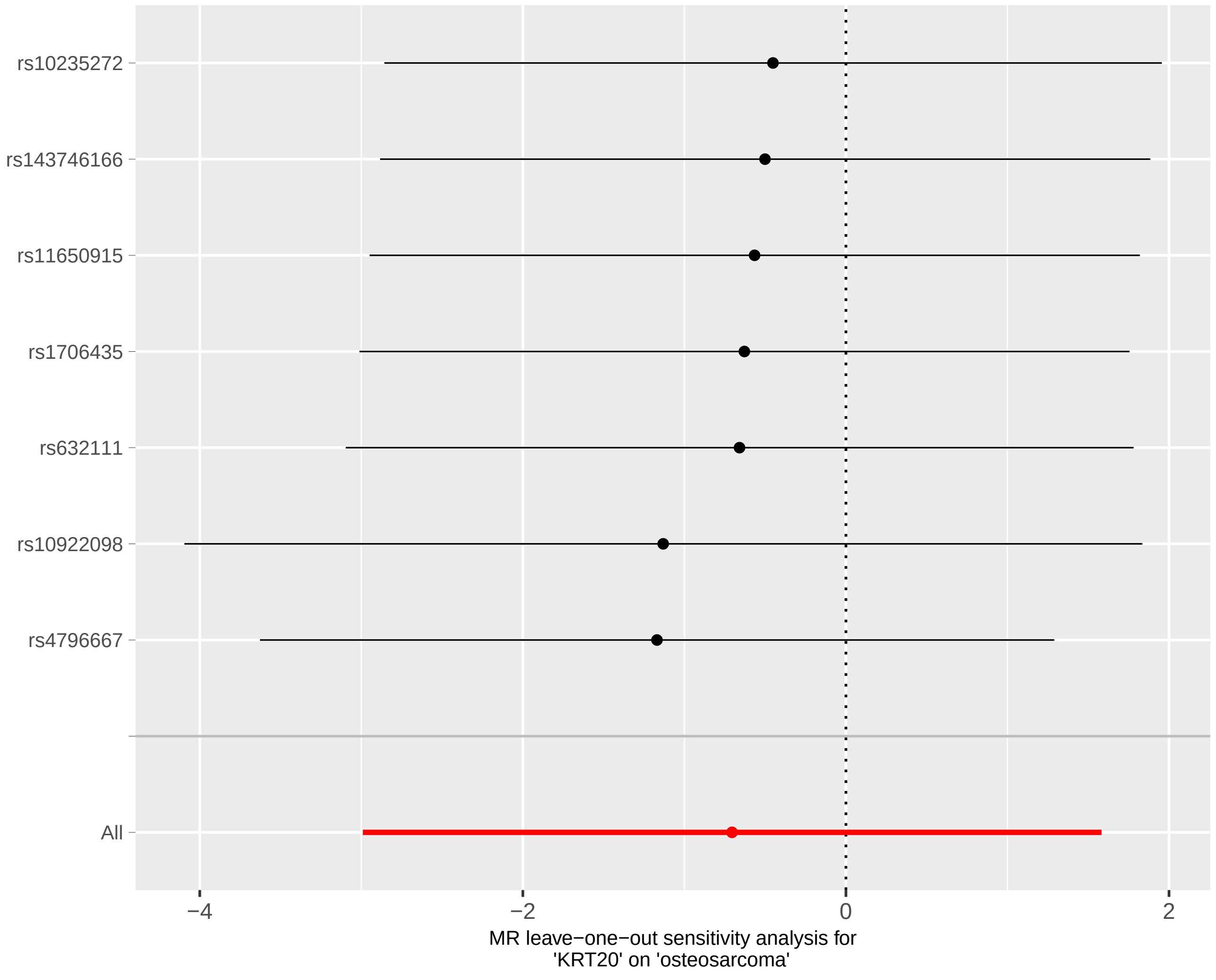
rs10235272
rs143746166
rs11650915
rs1706435
rs632111
rs10922098
rs4796667
All
−4
−2
0
2
MR leave−one−out sensitivity analysis for
'KRT20' on 'osteosarcoma'

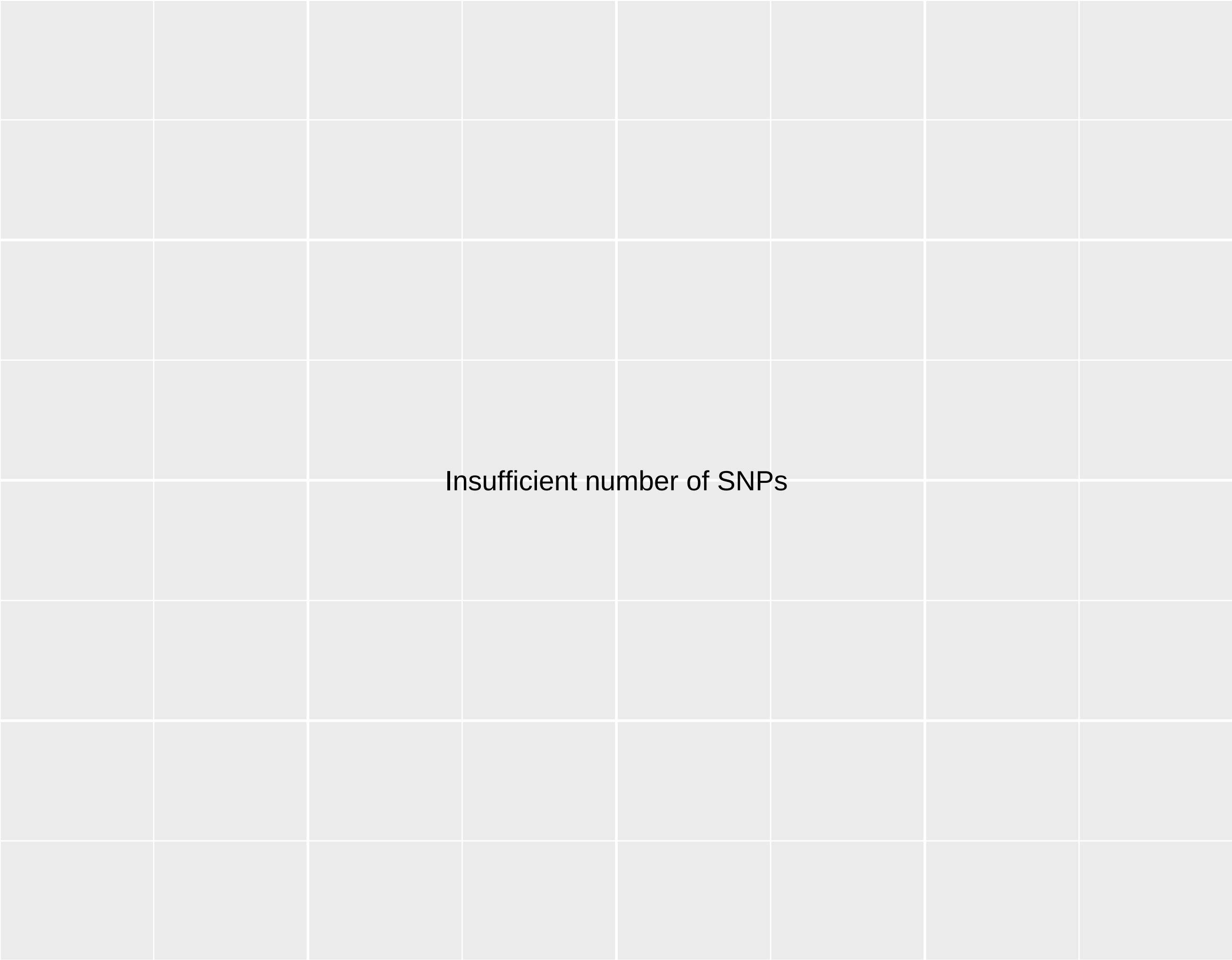
Insufficient number of SNPs

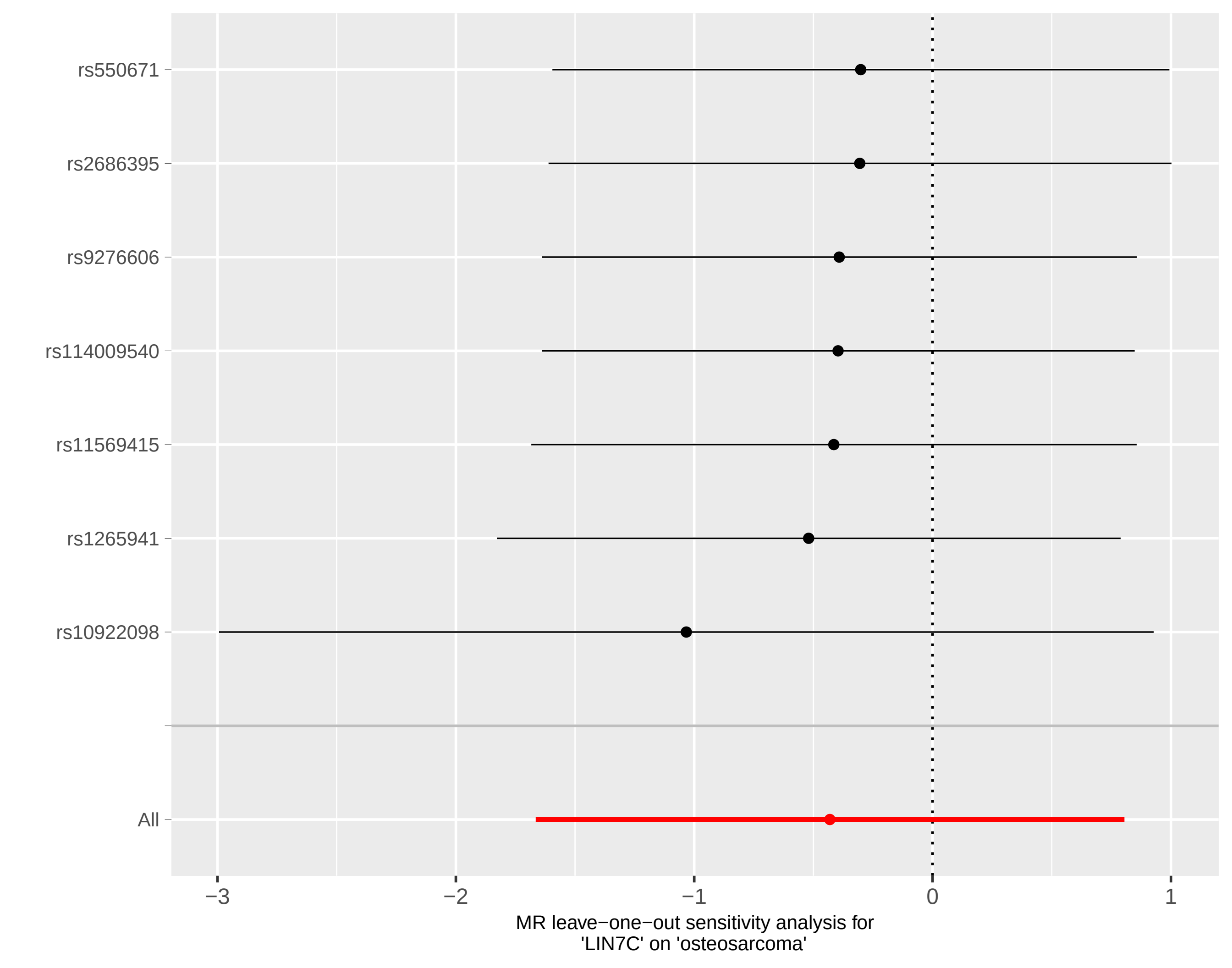

rs550671
rs2686395
rs9276606
rs114009540
rs11569415
rs1265941
rs10922098
All
−3
−2
−1
0
1
MR leave−one−out sensitivity analysis for
'LIN7C' on 'osteosarcoma'

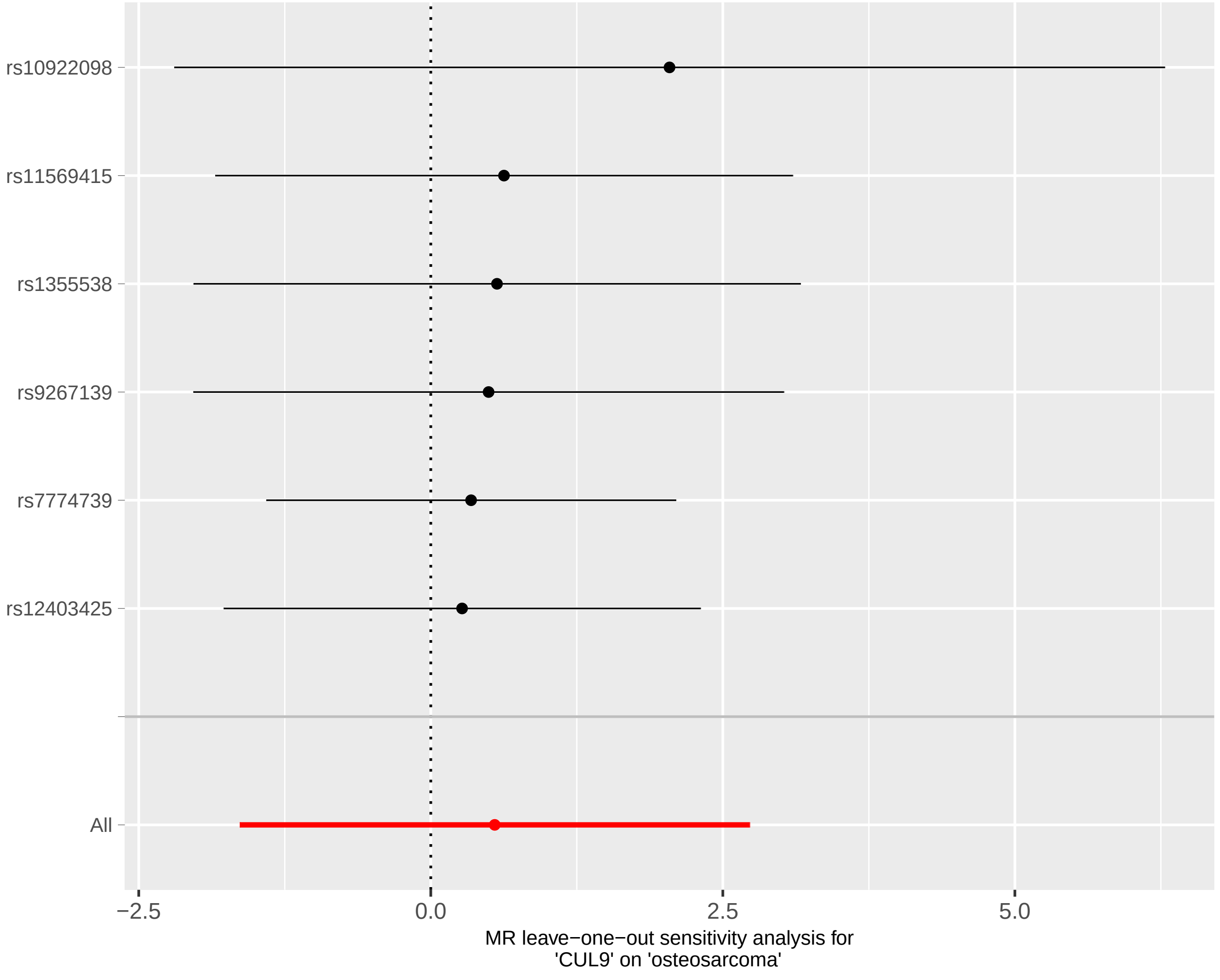
rs10922098
rs11569415
rs1355538
rs9267139
rs7774739
rs12403425
All
−2.5
0.0
2.5
5.0
MR leave−one−out sensitivity analysis for
'CUL9' on 'osteosarcoma'

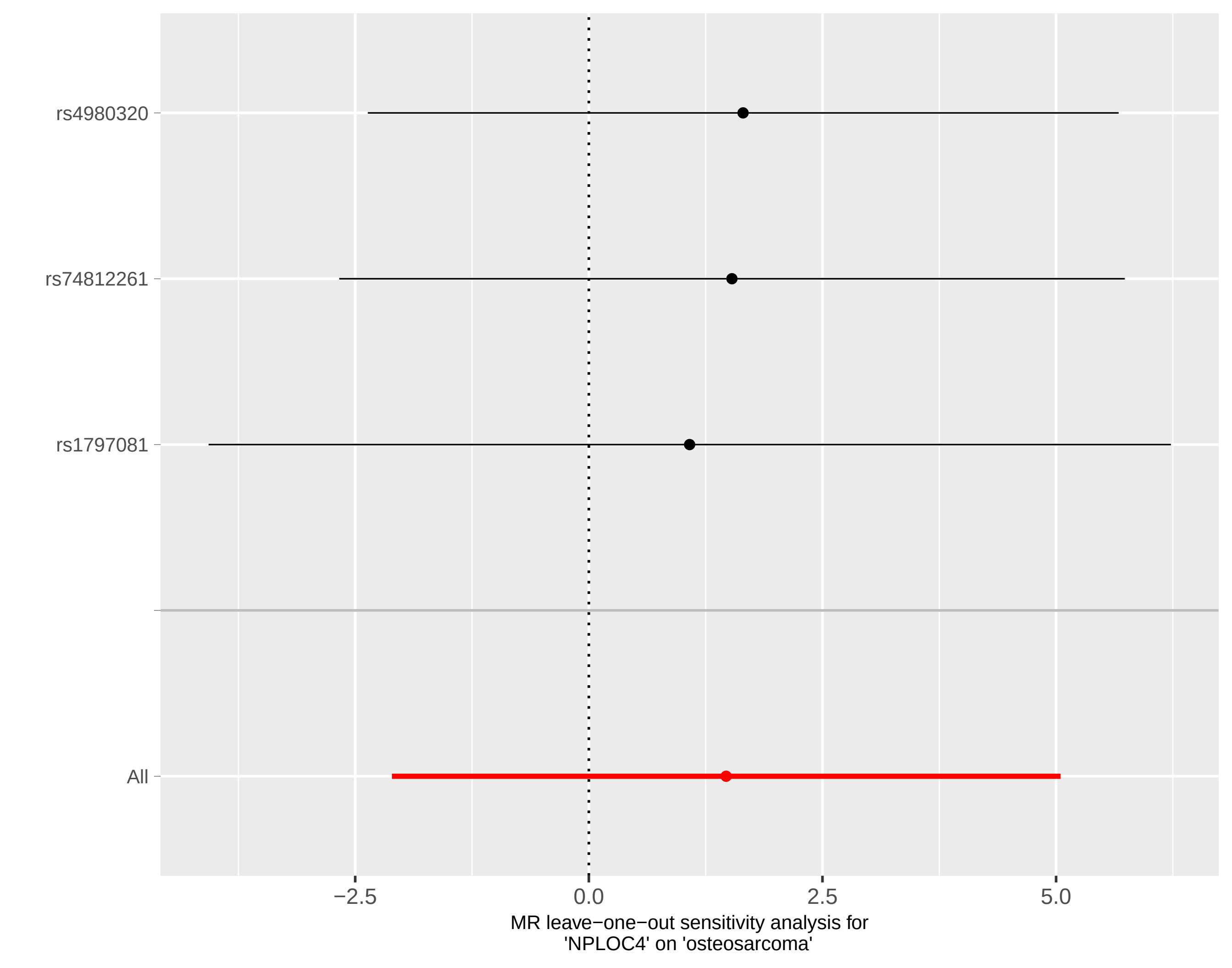
rs4980320
rs74812261
rs1797081
All
−2.5
0.0
2.5
5.0
MR leave−one−out sensitivity analysis for
'NPLOC4' on 'osteosarcoma'

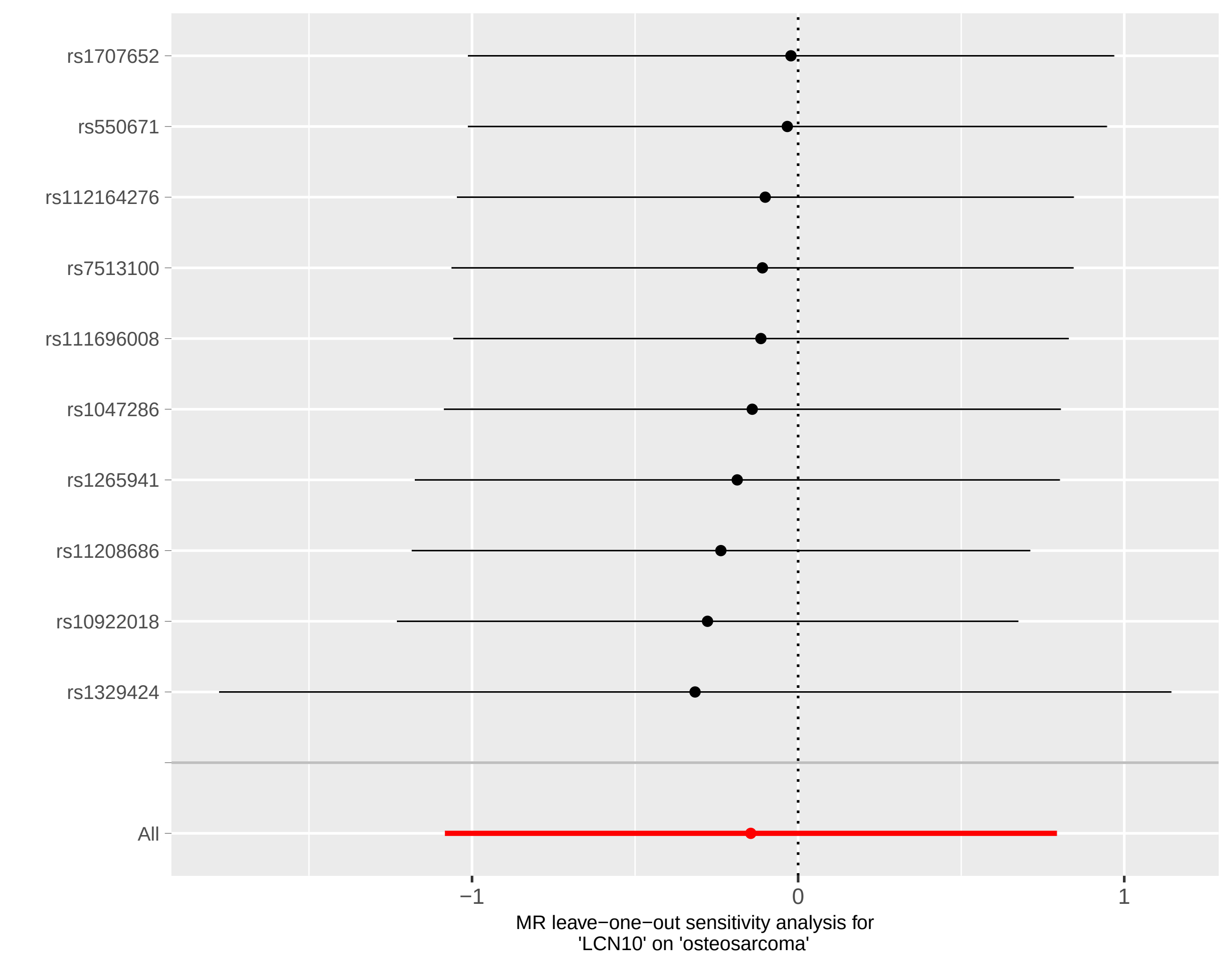

rs1707652
rs550671
rs112164276
rs7513100
rs111696008
rs1047286
rs1265941
rs11208686
rs10922018
rs1329424
All
−1
0
1
MR leave−one−out sensitivity analysis for
'LCN10' on 'osteosarcoma'

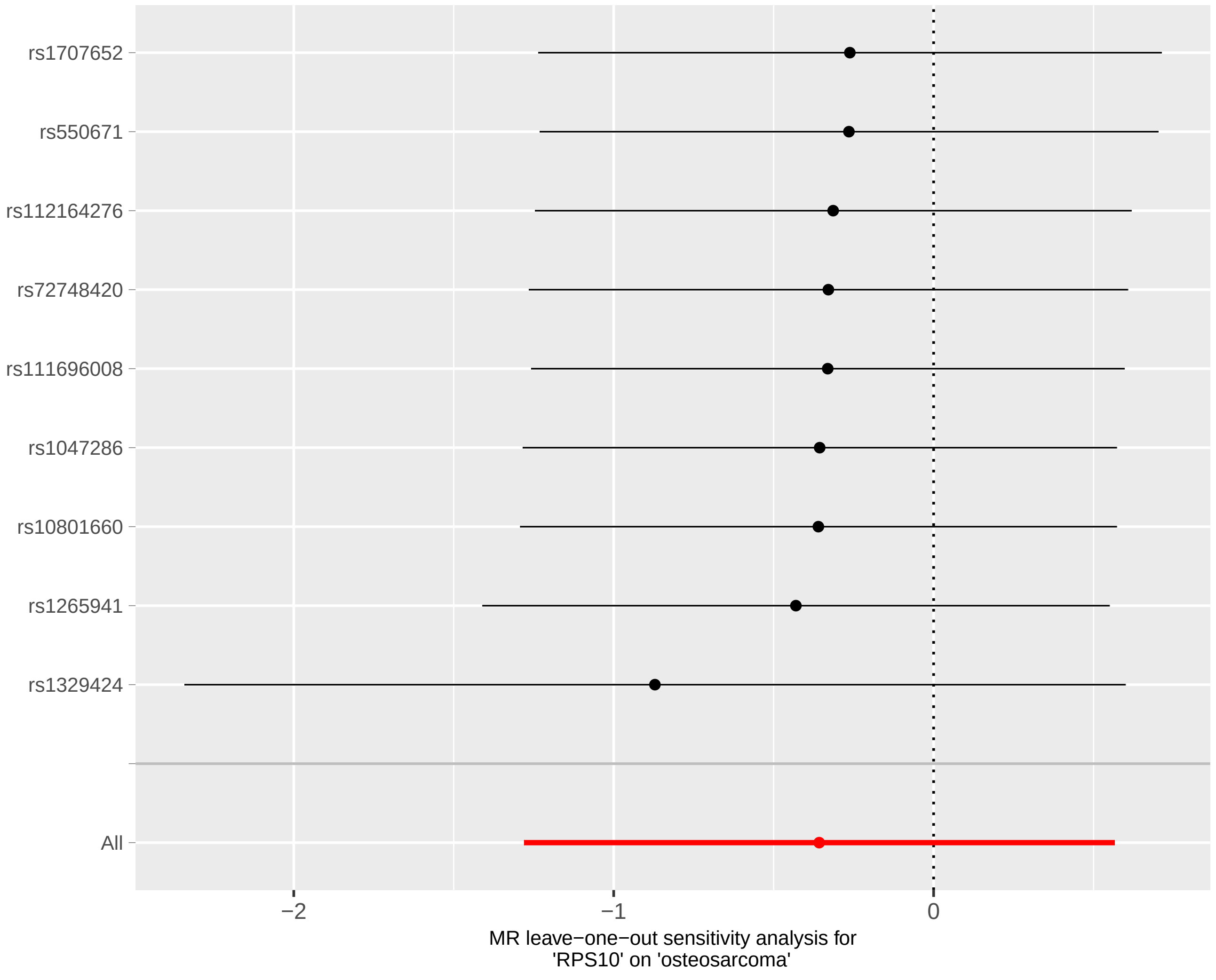
rs1707652
rs550671
rs112164276
rs72748420
rs111696008
rs1047286
rs10801660
rs1265941
rs1329424
All
−2
−1
0
MR leave−one−out sensitivity analysis for
'RPS10' on 'osteosarcoma'

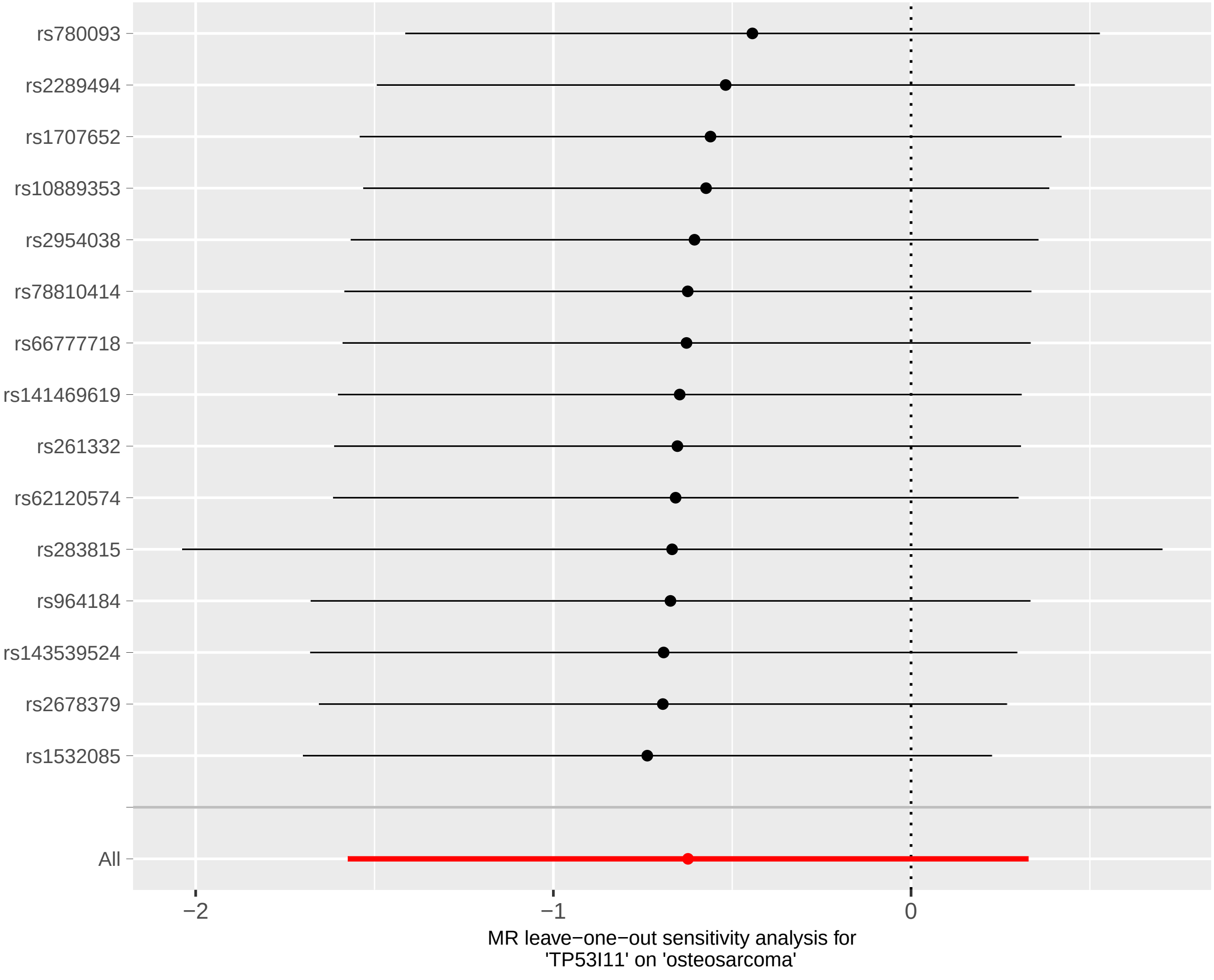
rs780093
rs2289494
rs1707652
rs10889353
rs2954038
rs78810414
rs66777718
rs141469619
rs261332
rs62120574
rs283815
rs964184
rs143539524
rs2678379
rs1532085
All
−2
−1
0
MR leave−one−out sensitivity analysis for
'TP53I11' on 'osteosarcoma'

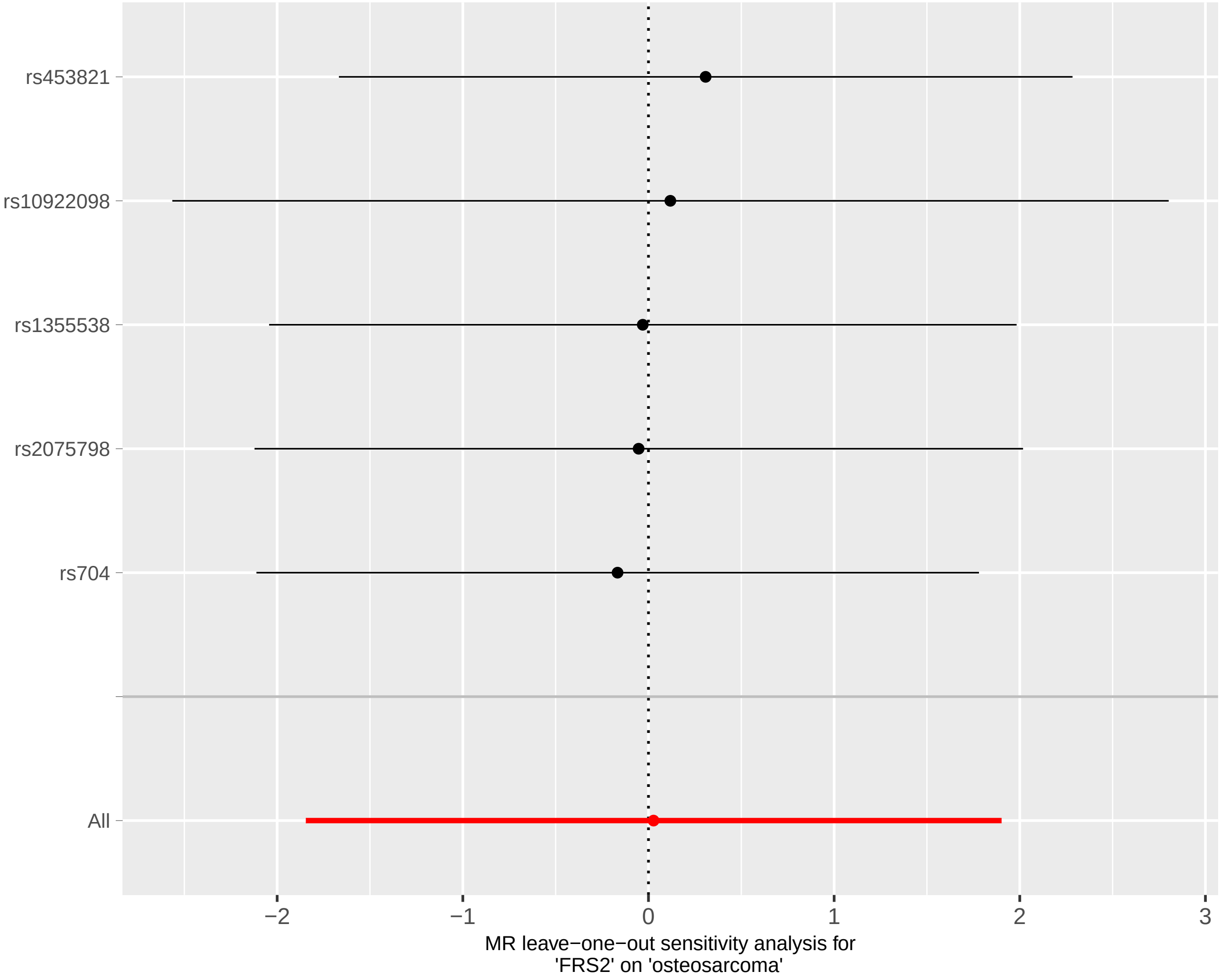

rs453821
rs10922098
rs1355538
rs2075798
rs704
All
−2
−1
0
1
2
3
MR leave−one−out sensitivity analysis for
'FRS2' on 'osteosarcoma'

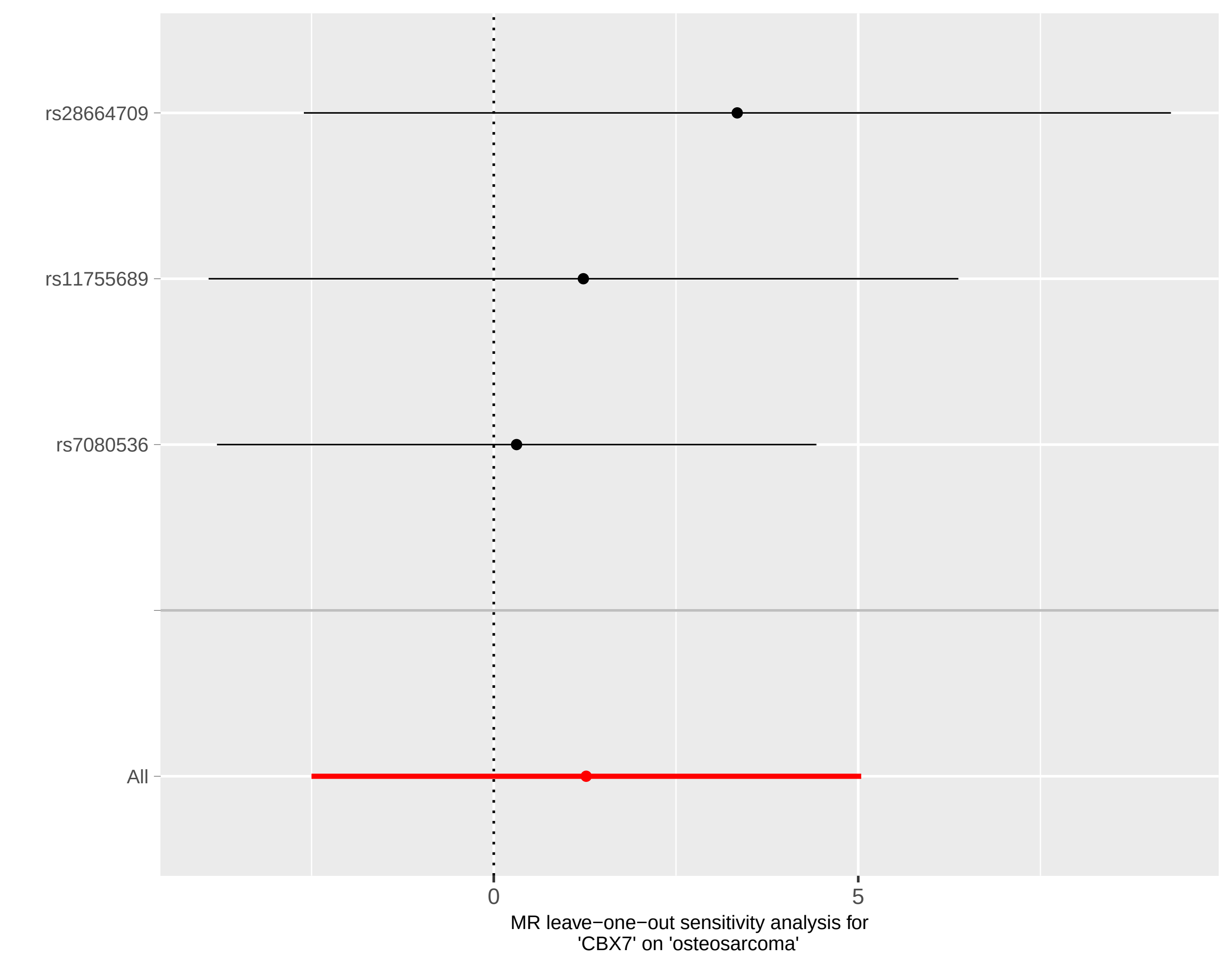

rs28664709
rs11755689
rs7080536
All
0
5
MR leave−one−out sensitivity analysis for
'CBX7' on 'osteosarcoma'

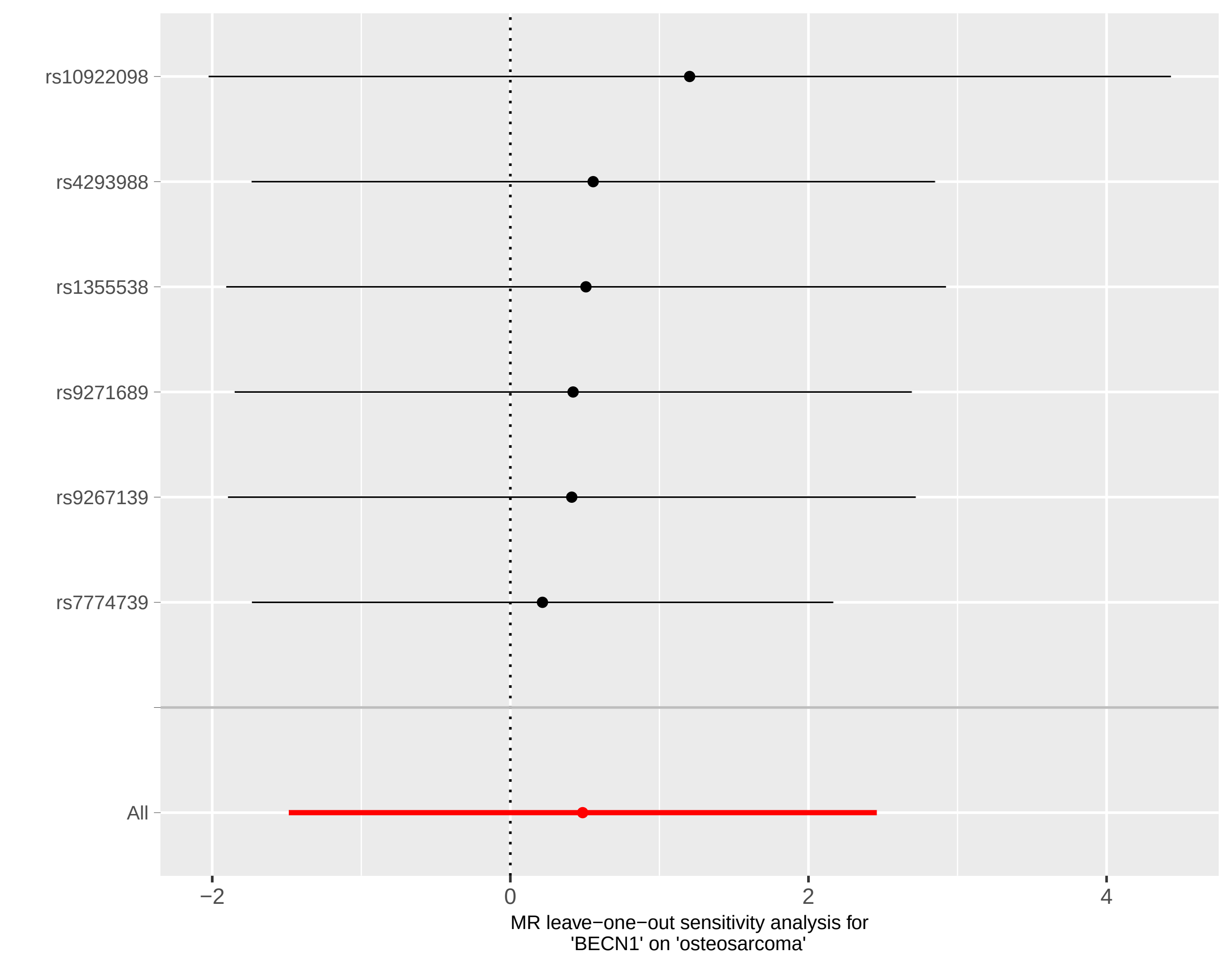
rs10922098
rs4293988
rs1355538
rs9271689
rs9267139
rs7774739
All
−2
0
2
4
MR leave−one−out sensitivity analysis for
'BECN1' on 'osteosarcoma'

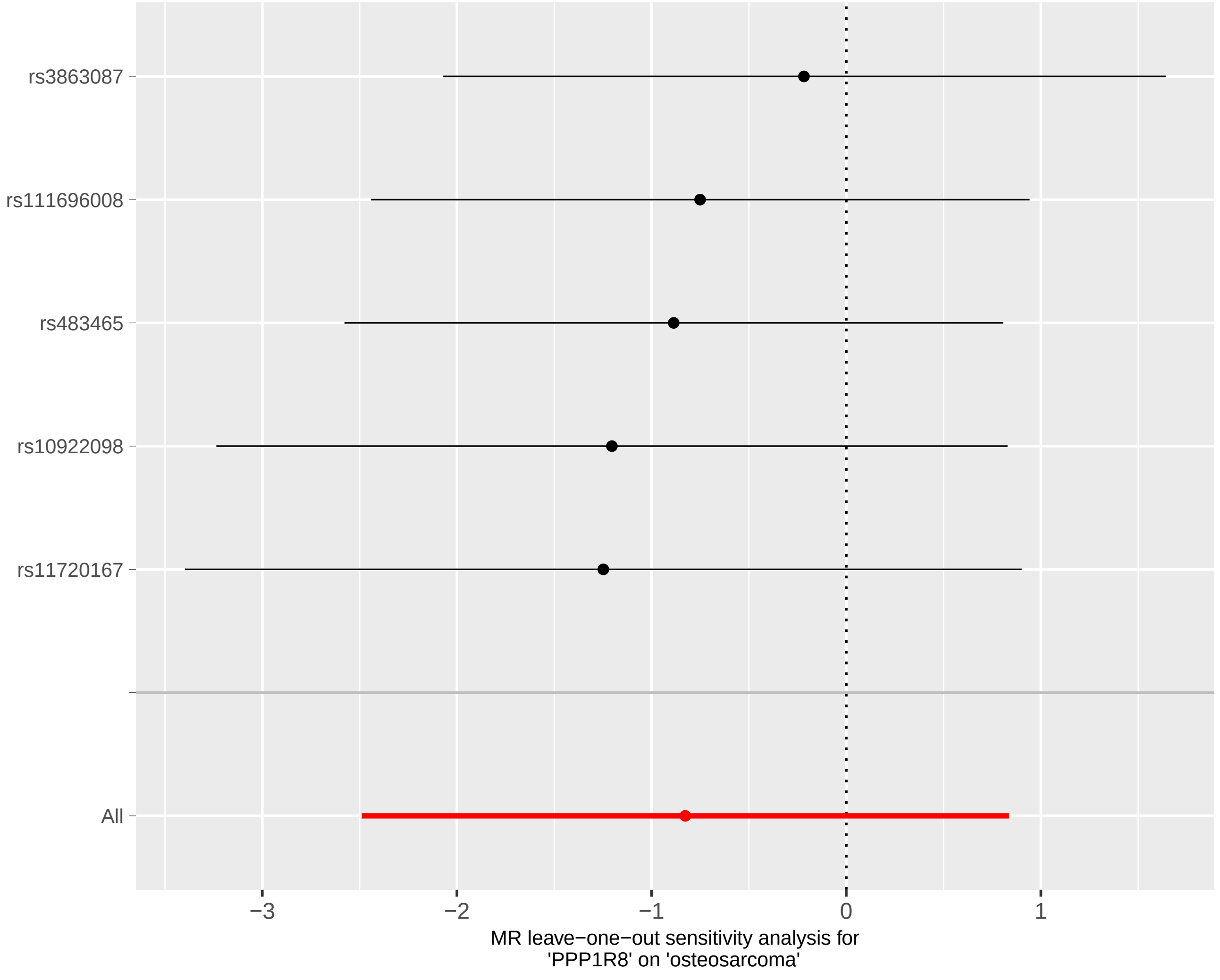

rs3863087
rs111696008
rs483465
rs10922098
rs11720167
All
−3
−2
−1
0
1
MR leave−one−out sensitivity analysis for
'PPP1R8' on 'osteosarcoma'

Insufficient number of SNPs

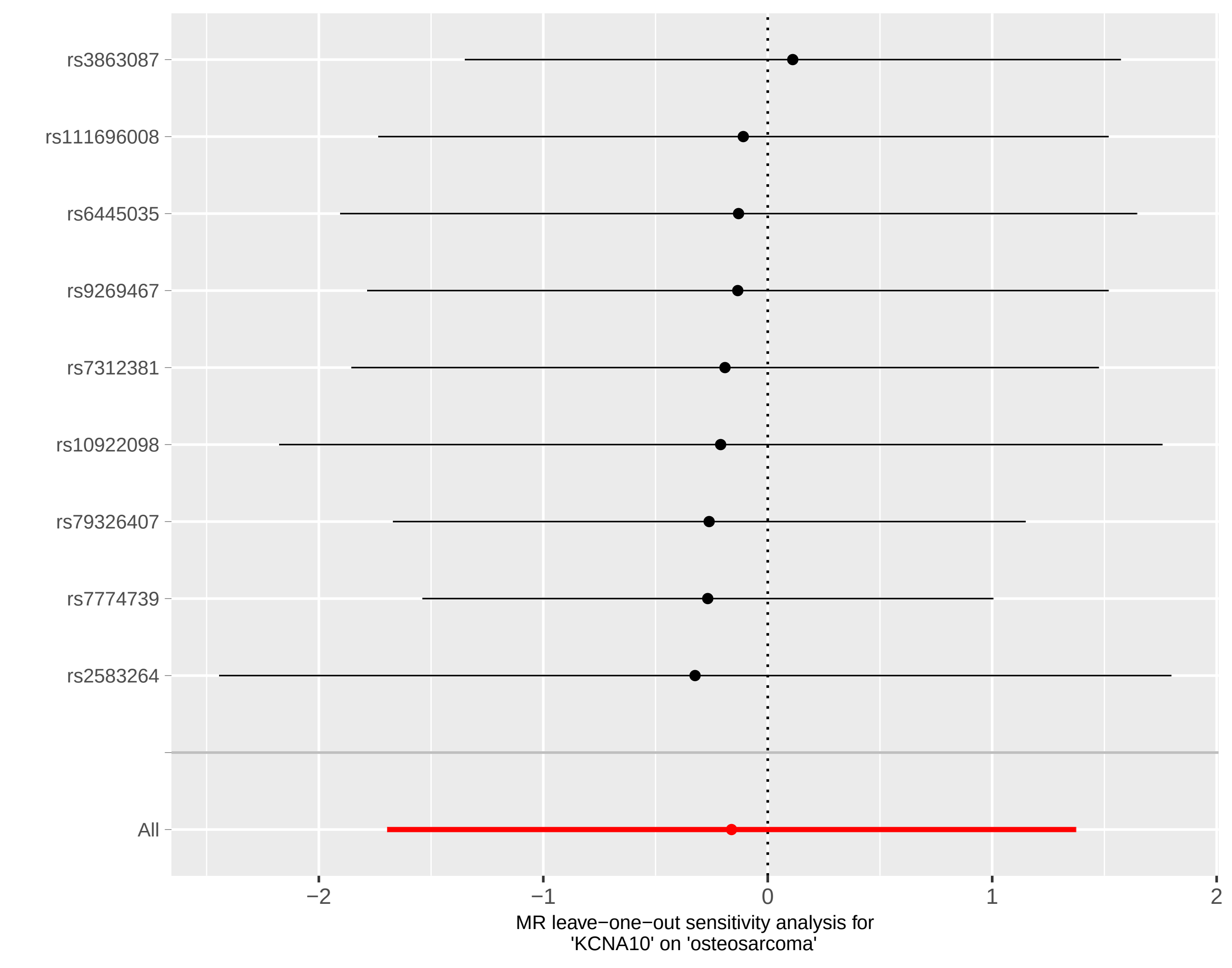
rs3863087
rs111696008
rs6445035
rs9269467
rs7312381
rs10922098
rs79326407
rs7774739
rs2583264
All
−2
−1
0
1
2
MR leave−one−out sensitivity analysis for
'KCNA10' on 'osteosarcoma'

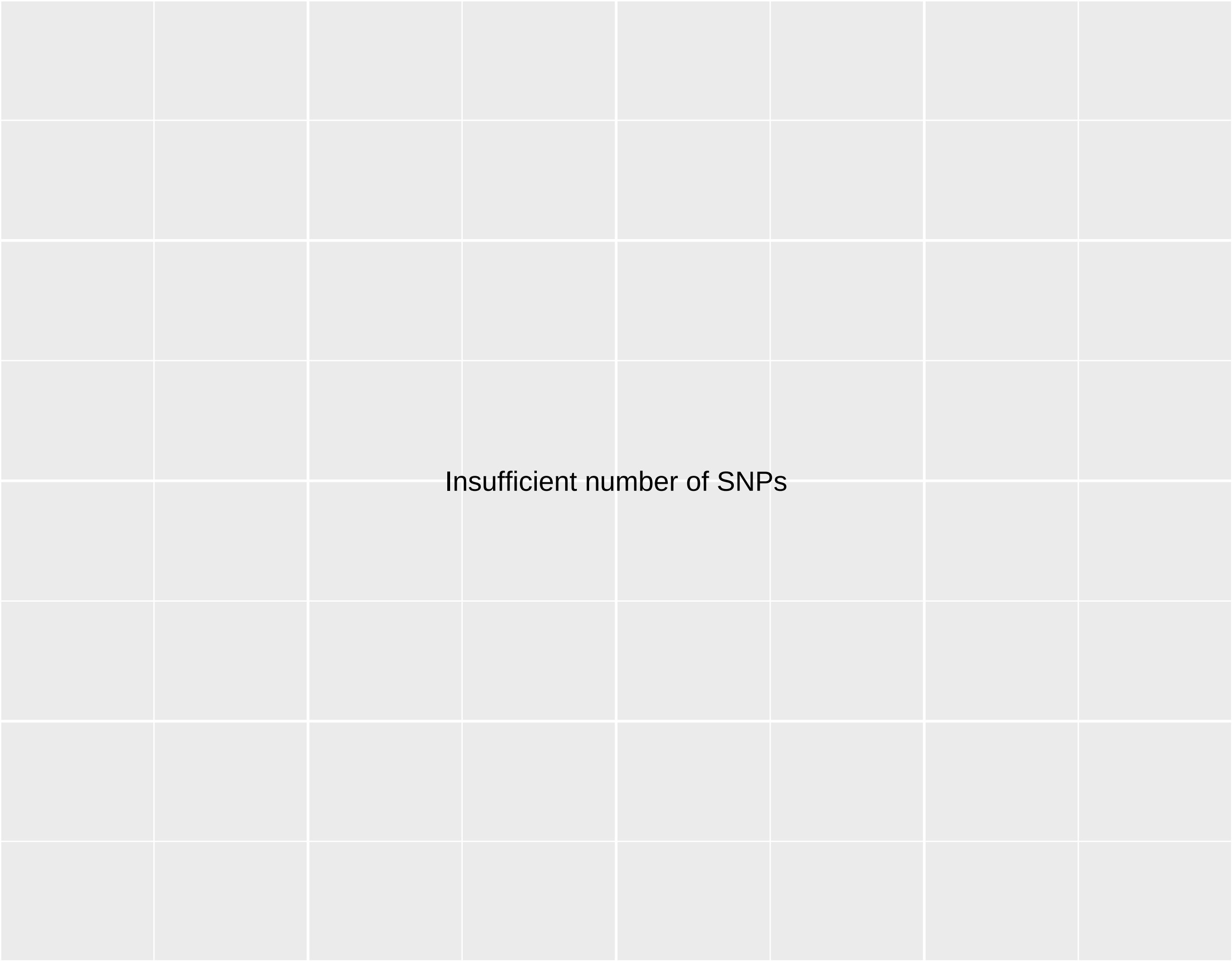
Insufficient number of SNPs

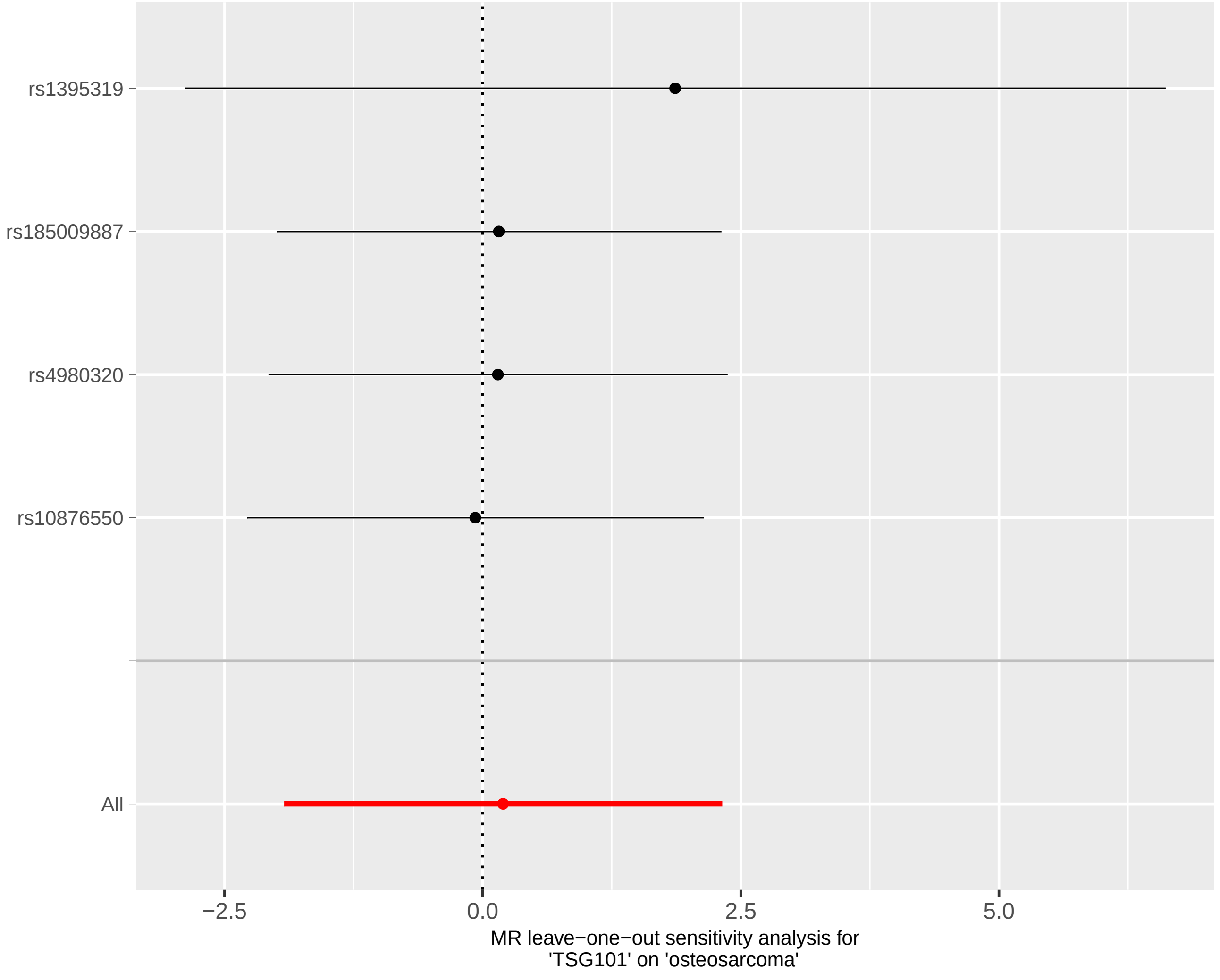
rs1395319
rs185009887
rs4980320
rs10876550
All
−2.5
0.0
2.5
5.0
MR leave−one−out sensitivity analysis for
'TSG101' on 'osteosarcoma'

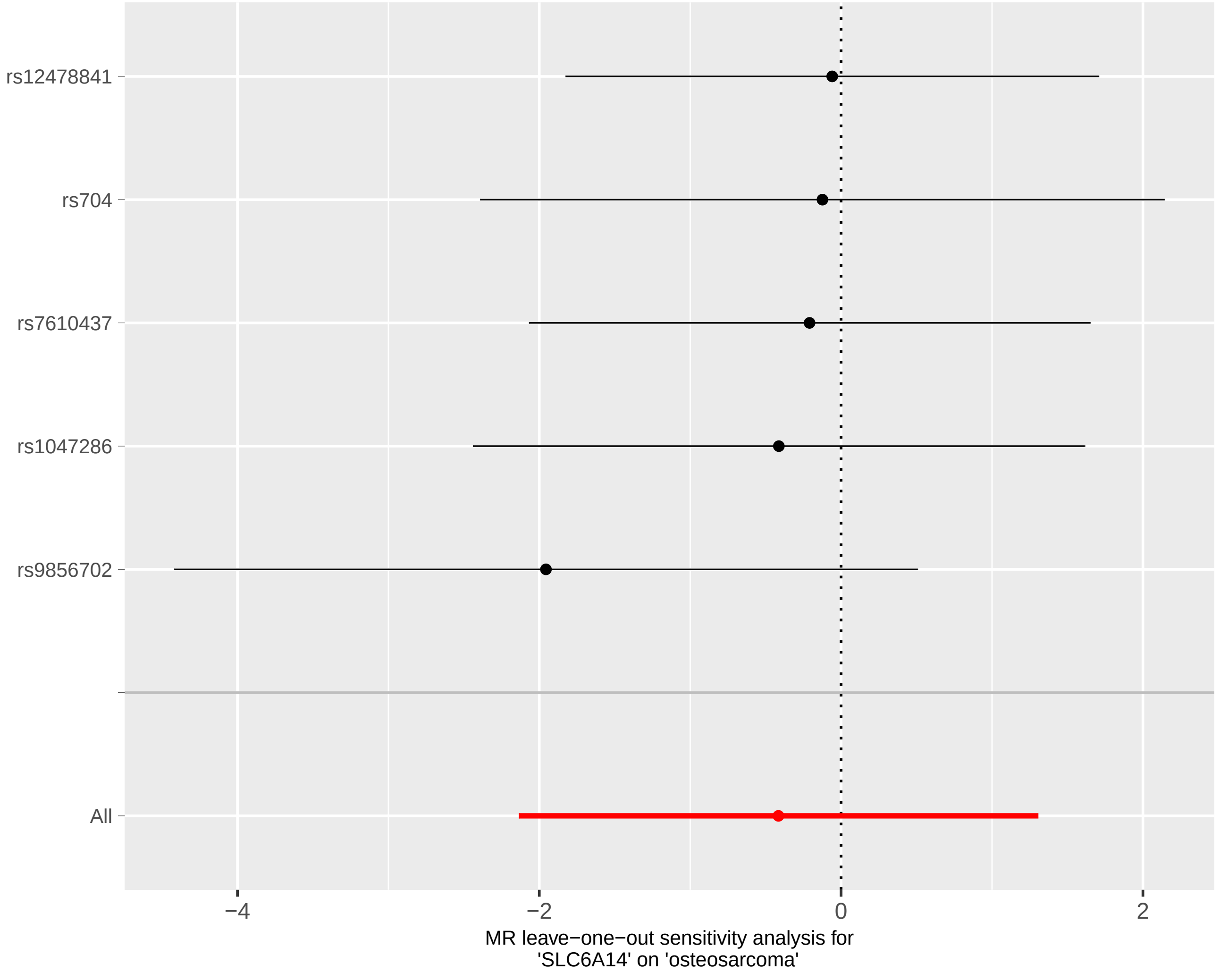
rs12478841
rs704
rs7610437
rs1047286
rs9856702
All
−4
−2
0
2
MR leave−one−out sensitivity analysis for
'SLC6A14' on 'osteosarcoma'

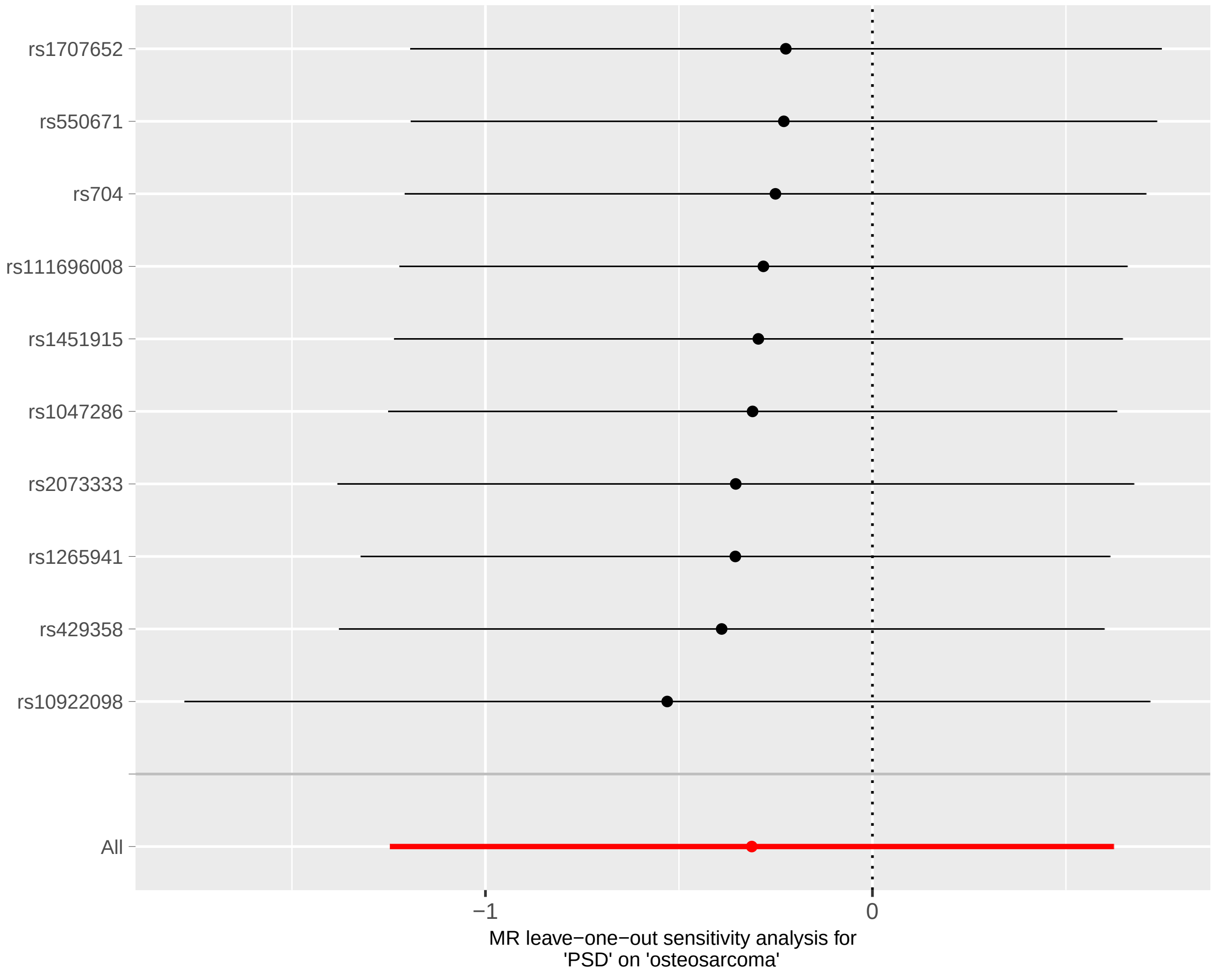
rs1707652
rs550671
rs704
rs111696008
rs1451915
rs1047286
rs2073333
rs1265941
rs429358
rs10922098
All
−1
0
MR leave−one−out sensitivity analysis for
'PSD' on 'osteosarcoma'

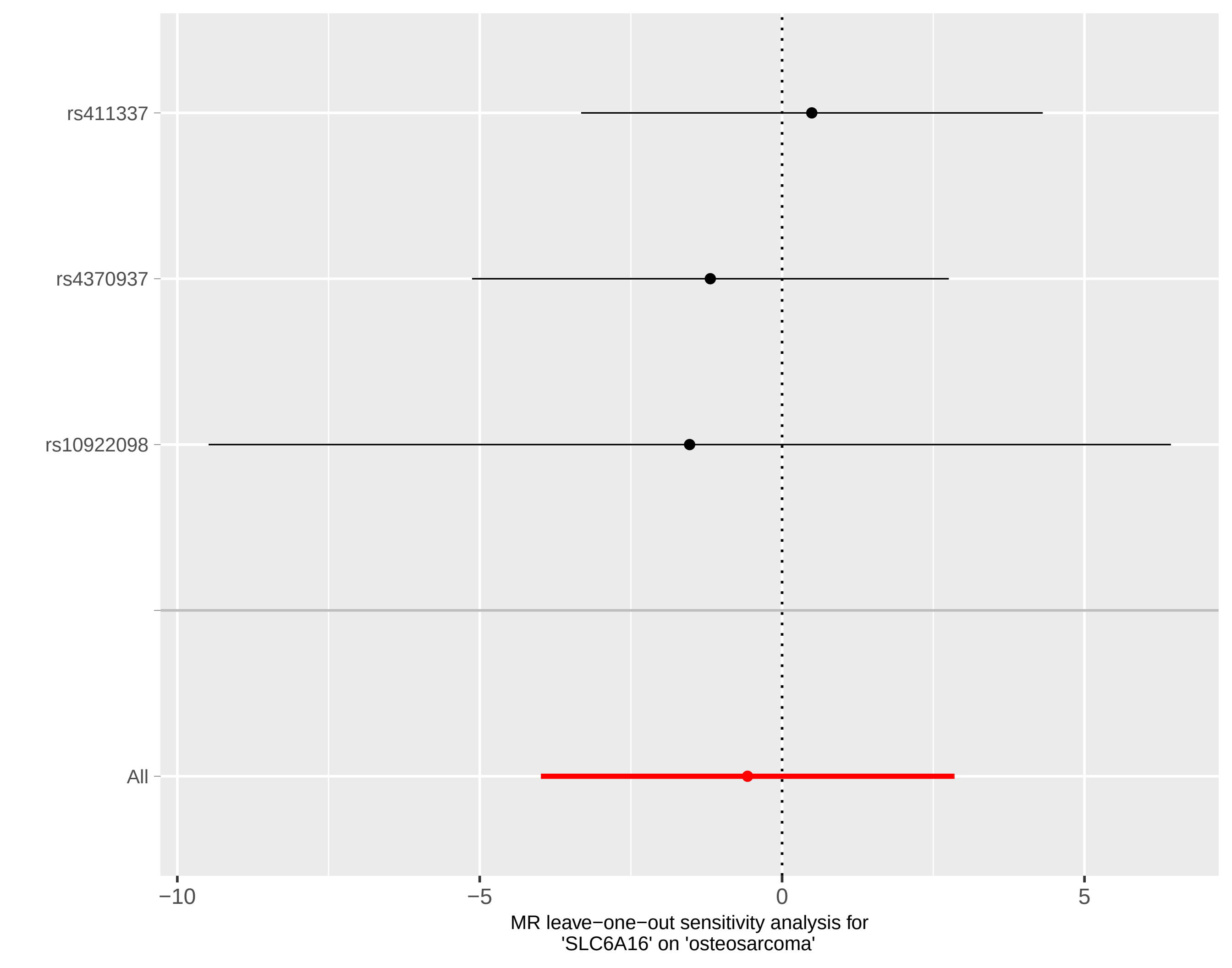

rs411337
rs4370937
rs10922098
All
−10
−5
0
5
MR leave−one−out sensitivity analysis for
'SLC6A16' on 'osteosarcoma'

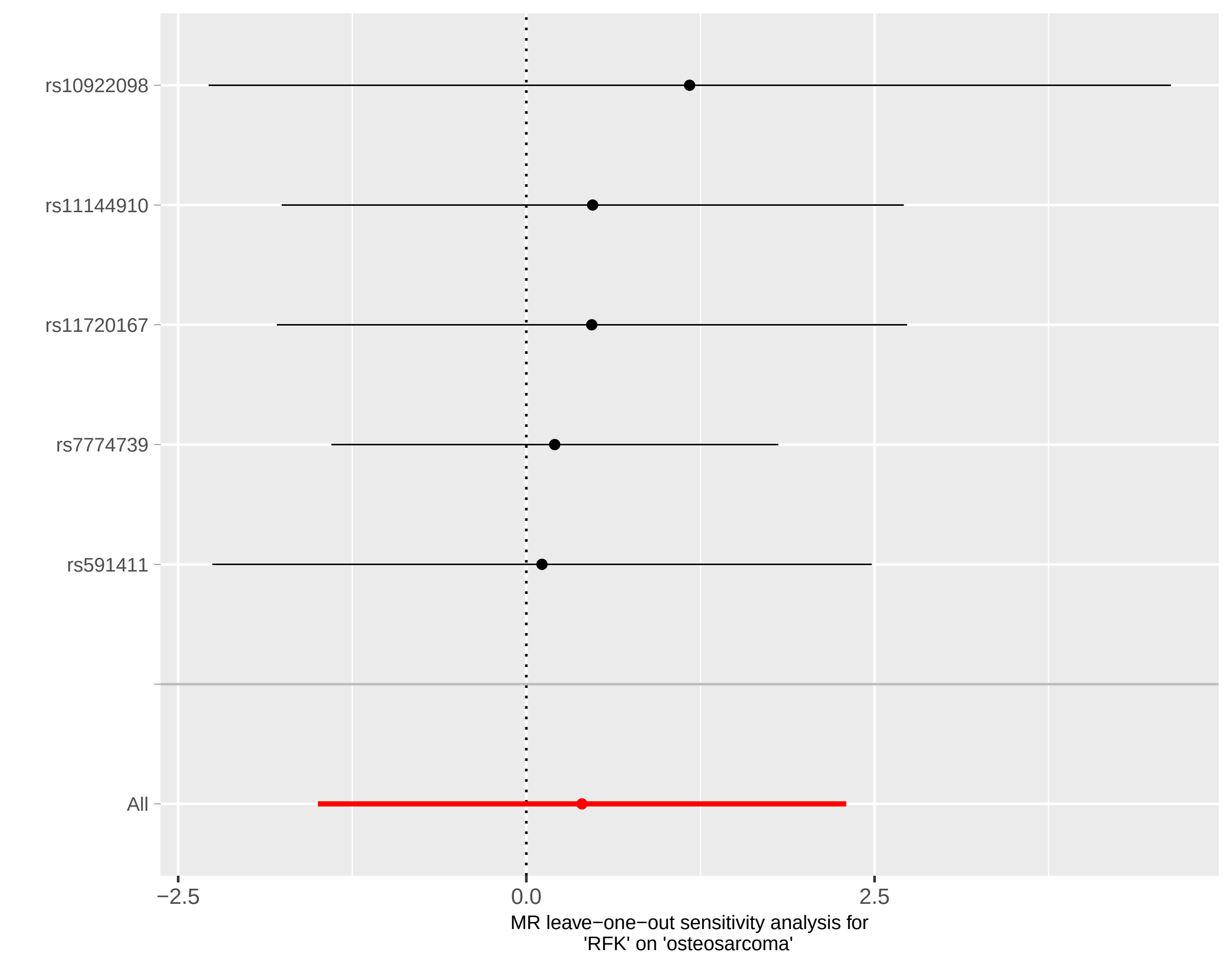
rs10922098
rs11144910
rs11720167
rs7774739
rs591411
All
−2.5
0.0
2.5
MR leave−one−out sensitivity analysis for
'RFK' on 'osteosarcoma'

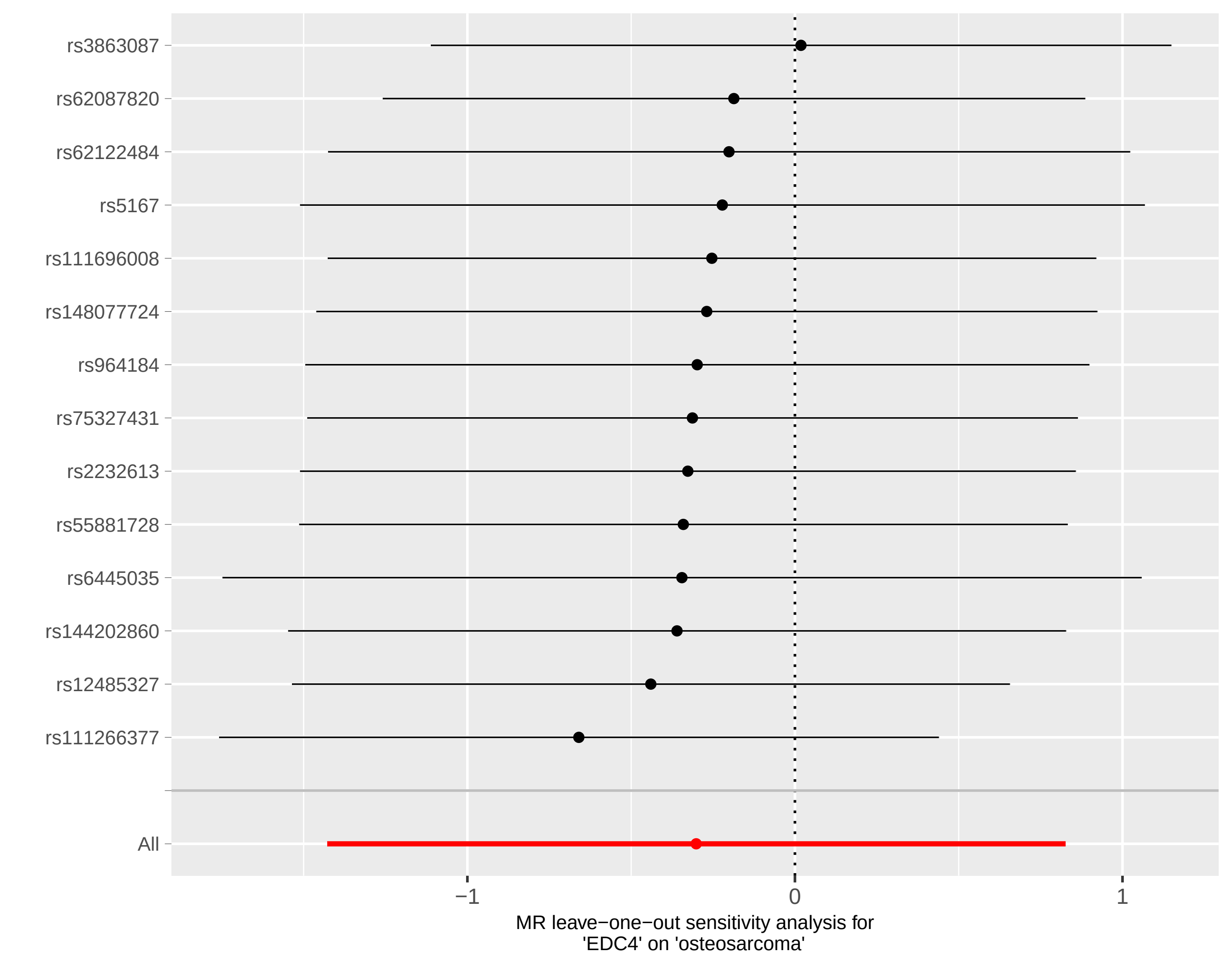
rs3863087
rs62087820
rs62122484
rs5167
rs111696008
rs148077724
rs964184
rs75327431
rs2232613
rs55881728
rs6445035
rs144202860
rs12485327
rs111266377
All
−1
0
1
MR leave−one−out sensitivity analysis for
'EDC4' on 'osteosarcoma'

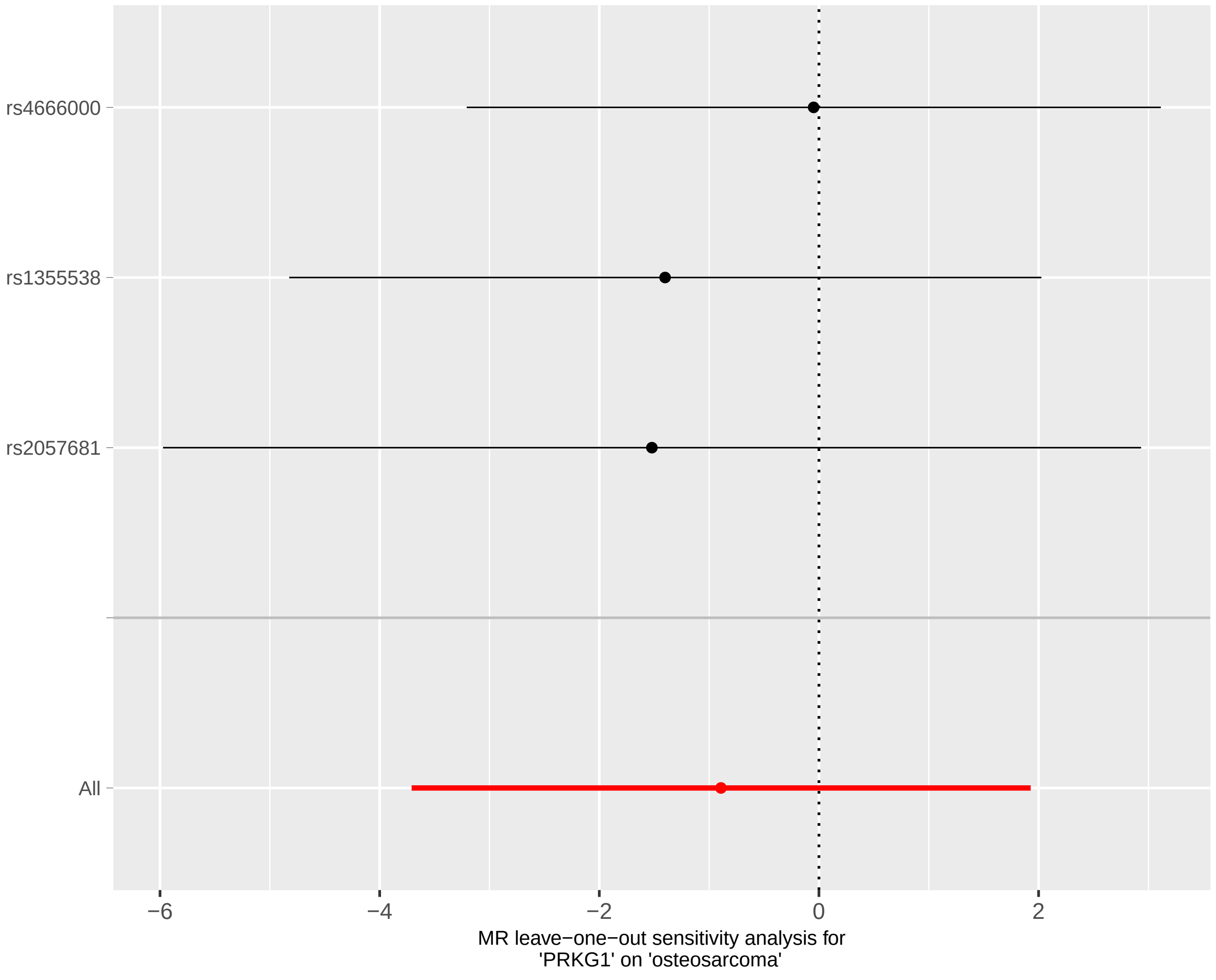

rs4666000
rs1355538
rs2057681
All
−6
−4
−2
0
2
MR leave−one−out sensitivity analysis for
'PRKG1' on 'osteosarcoma'

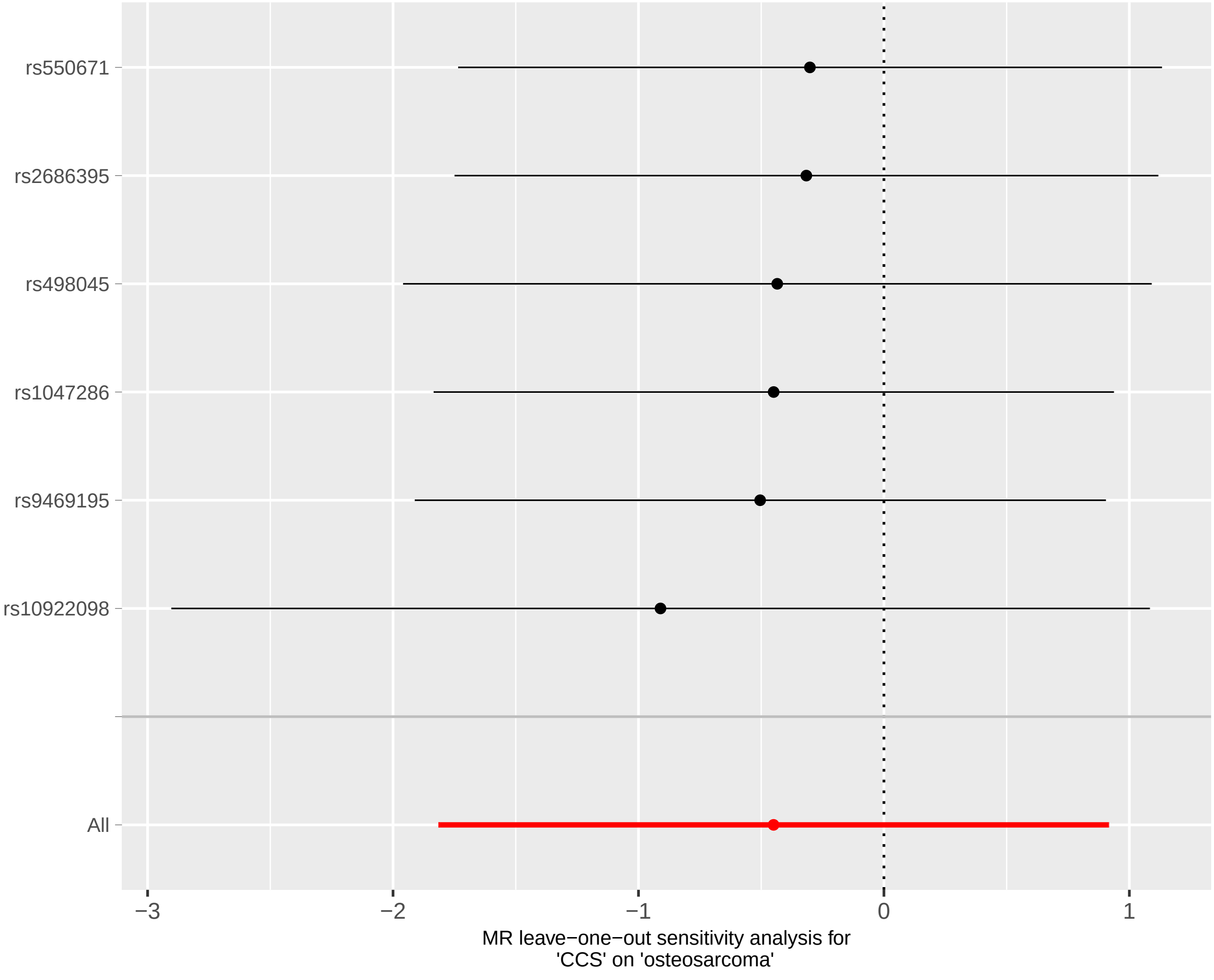
rs550671
rs2686395
rs498045
rs1047286
rs9469195
rs10922098
All
−3
−2
−1
0
1
MR leave−one−out sensitivity analysis for
'CCS' on 'osteosarcoma'

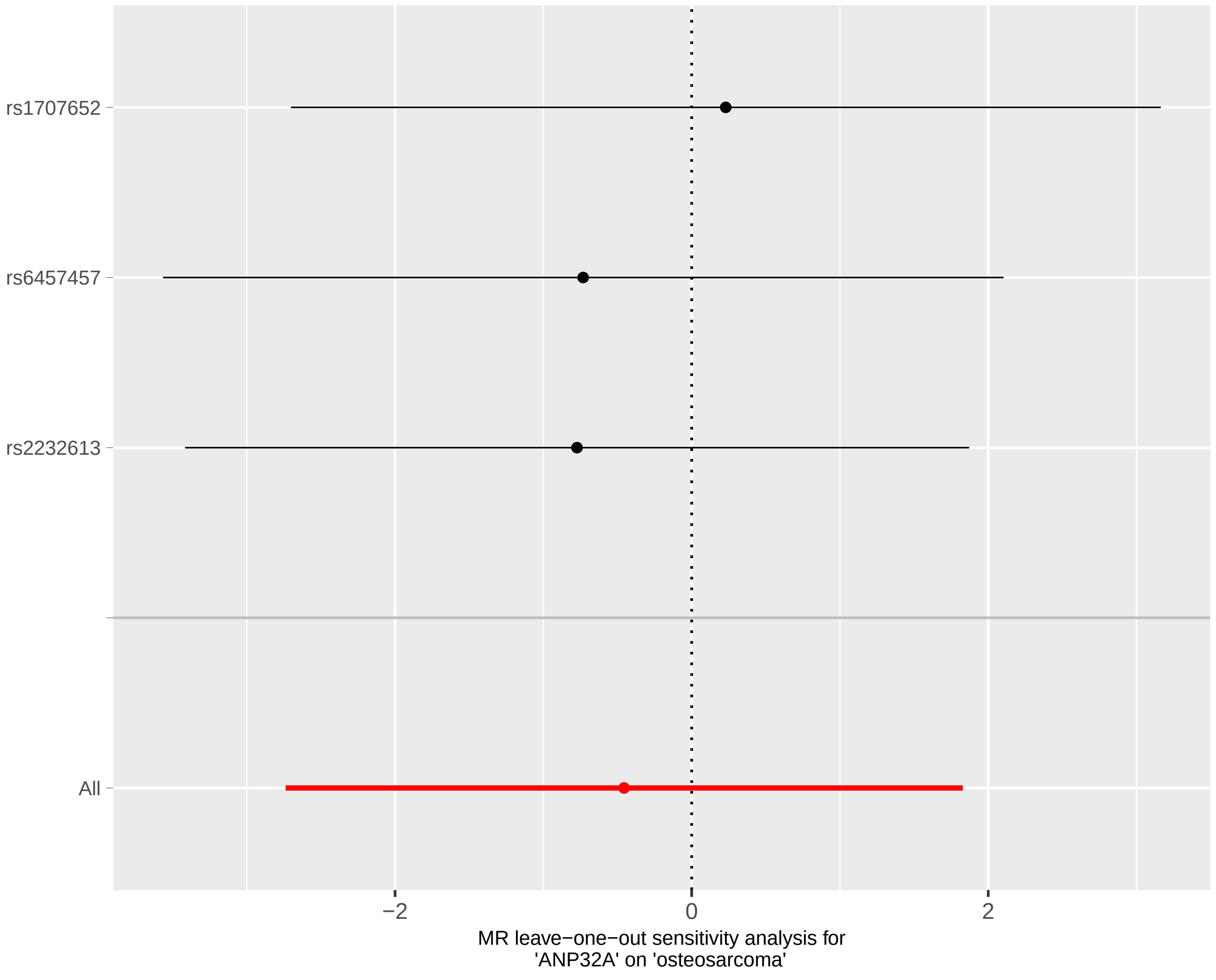

rs1707652
rs6457457
rs2232613
All
−2
0
2
MR leave−one−out sensitivity analysis for
'ANP32A' on 'osteosarcoma'

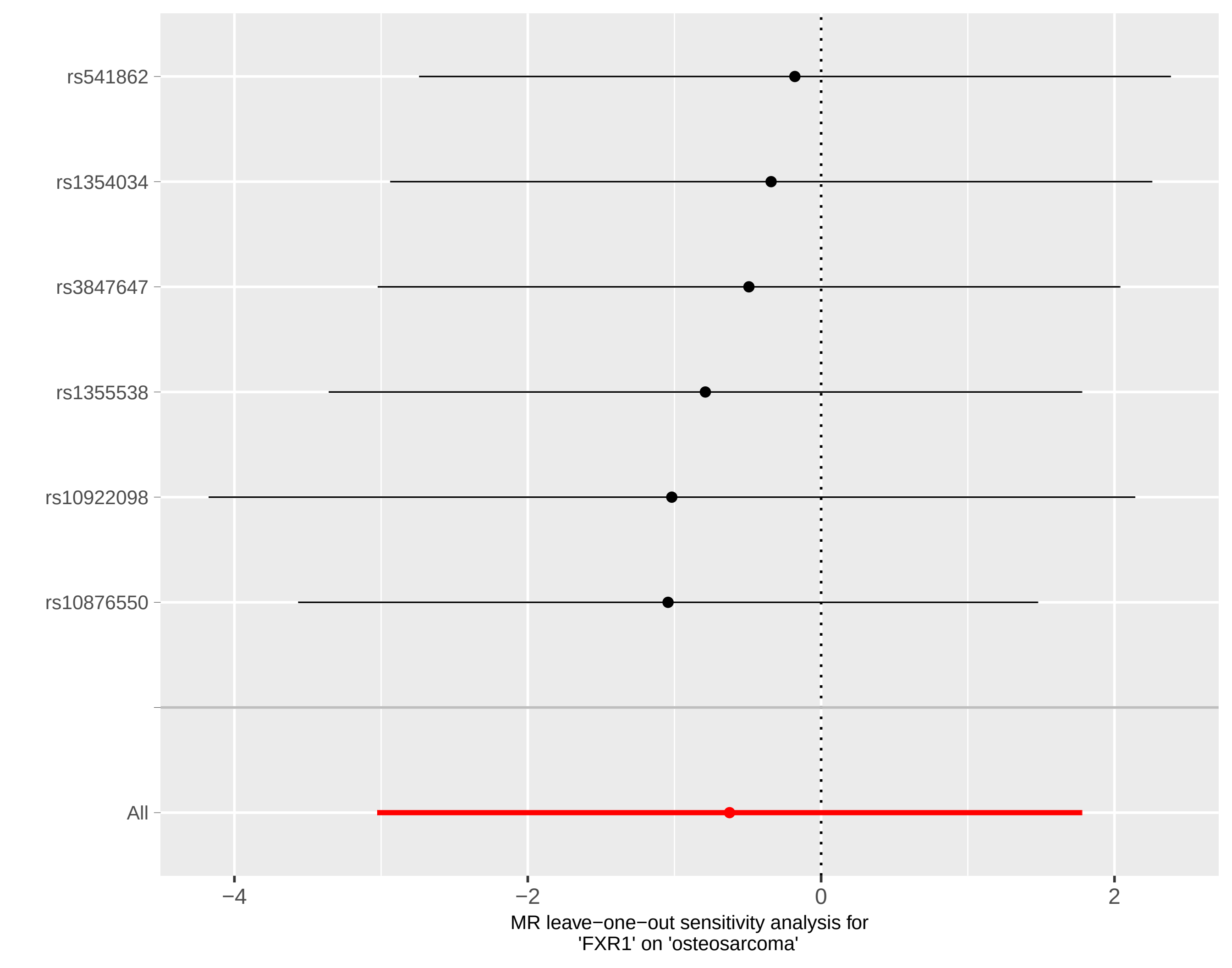

rs541862
rs1354034
rs3847647
rs1355538
rs10922098
rs10876550
All
−4
−2
0
2
MR leave−one−out sensitivity analysis for
'FXR1' on 'osteosarcoma'

Insufficient number of SNPs

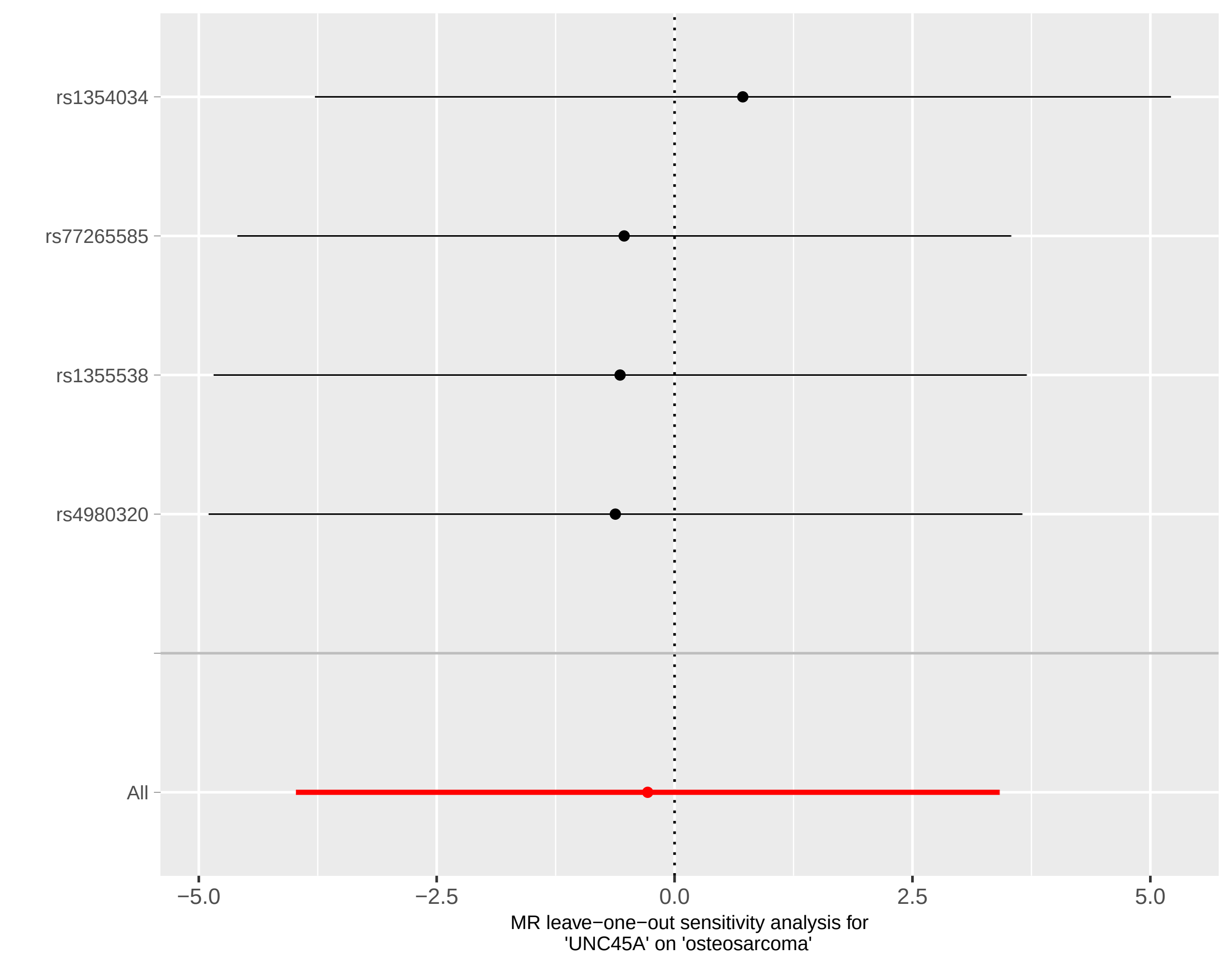

rs1354034
rs77265585
rs1355538
rs4980320
All
−5.0
−2.5
0.0
2.5
5.0
MR leave−one−out sensitivity analysis for
'UNC45A' on 'osteosarcoma'

Insufficient number of SNPs

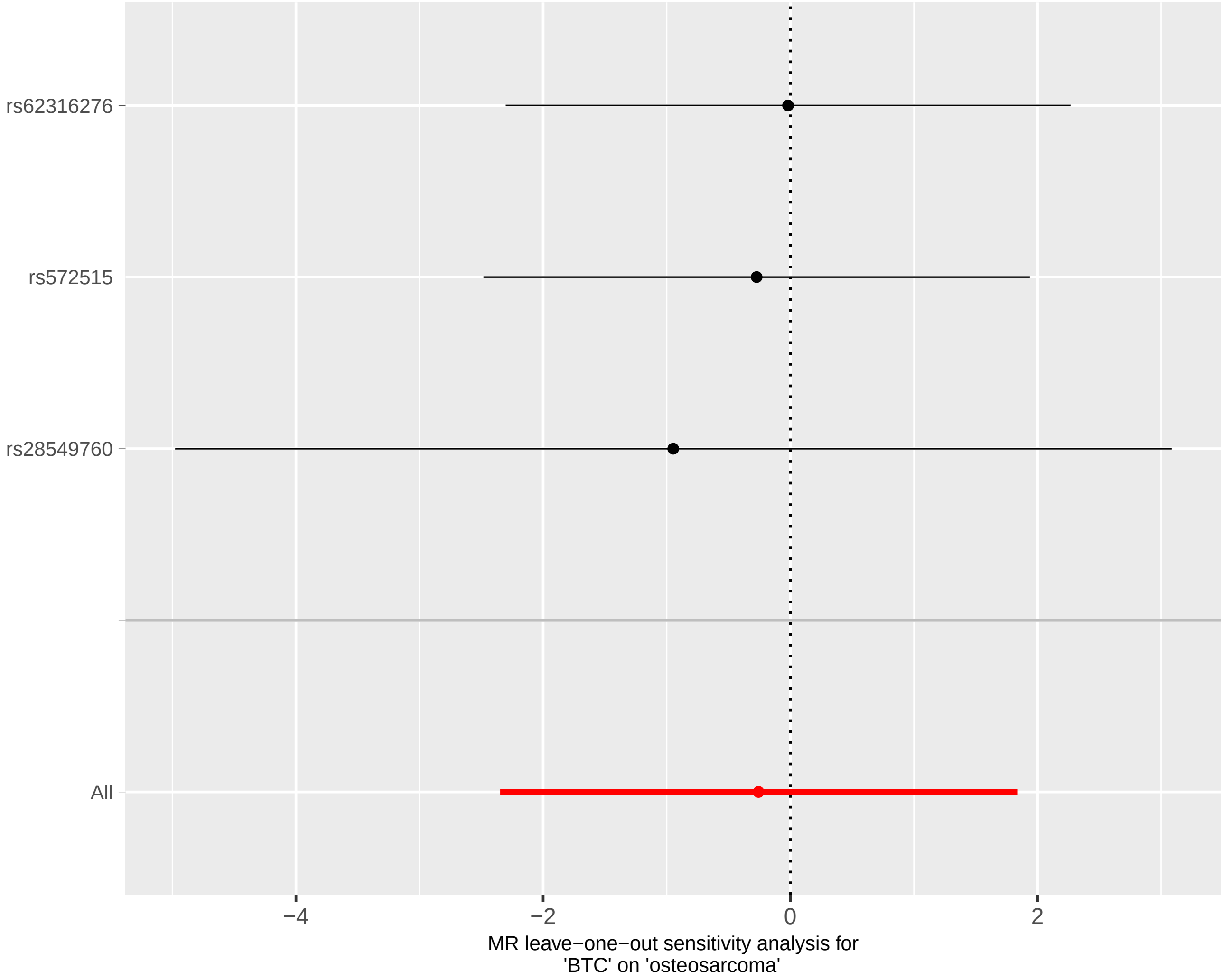

rs62316276
rs572515
rs28549760
All
−4
−2
0
2
MR leave−one−out sensitivity analysis for
'BTC' on 'osteosarcoma'

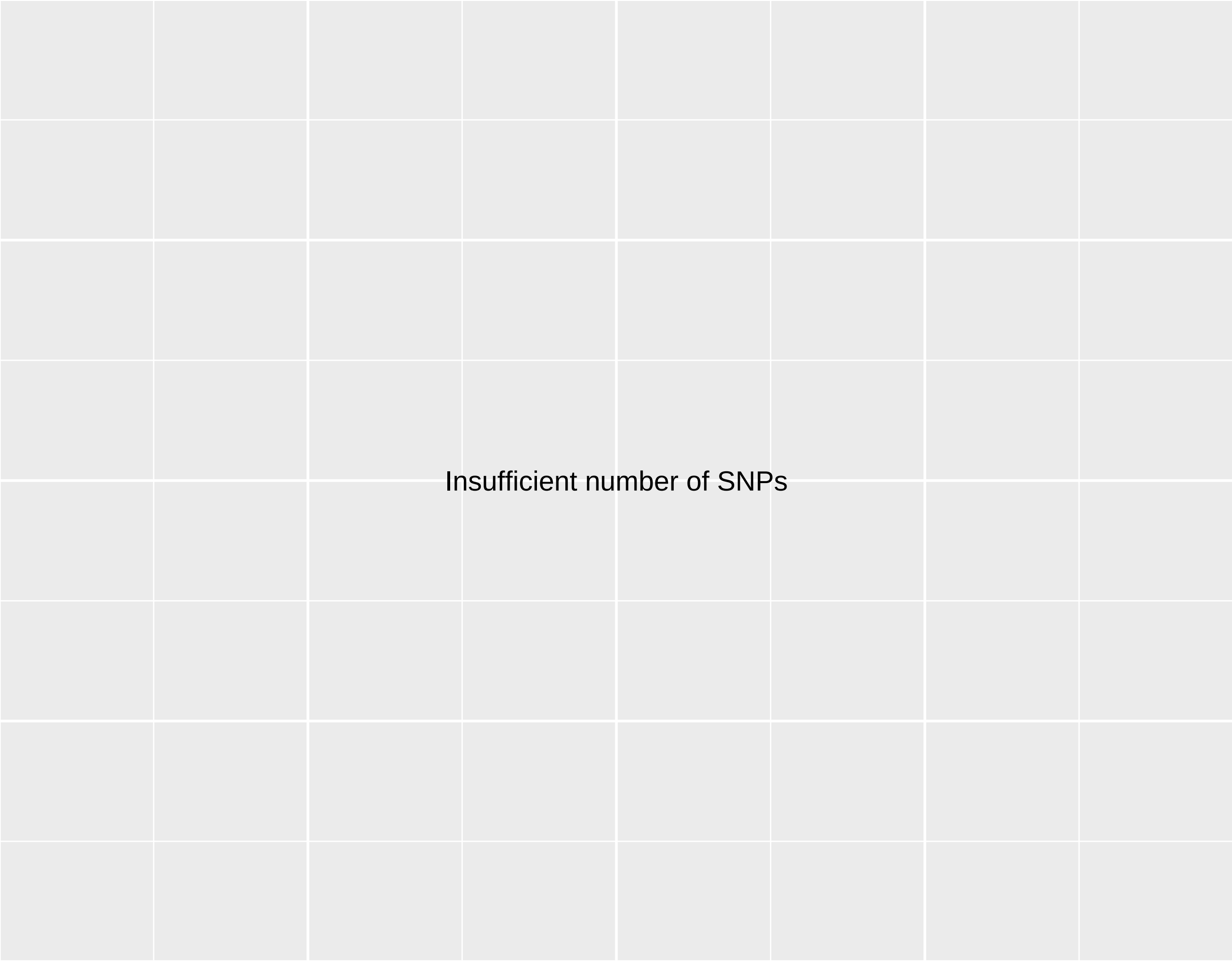
Insufficient number of SNPs

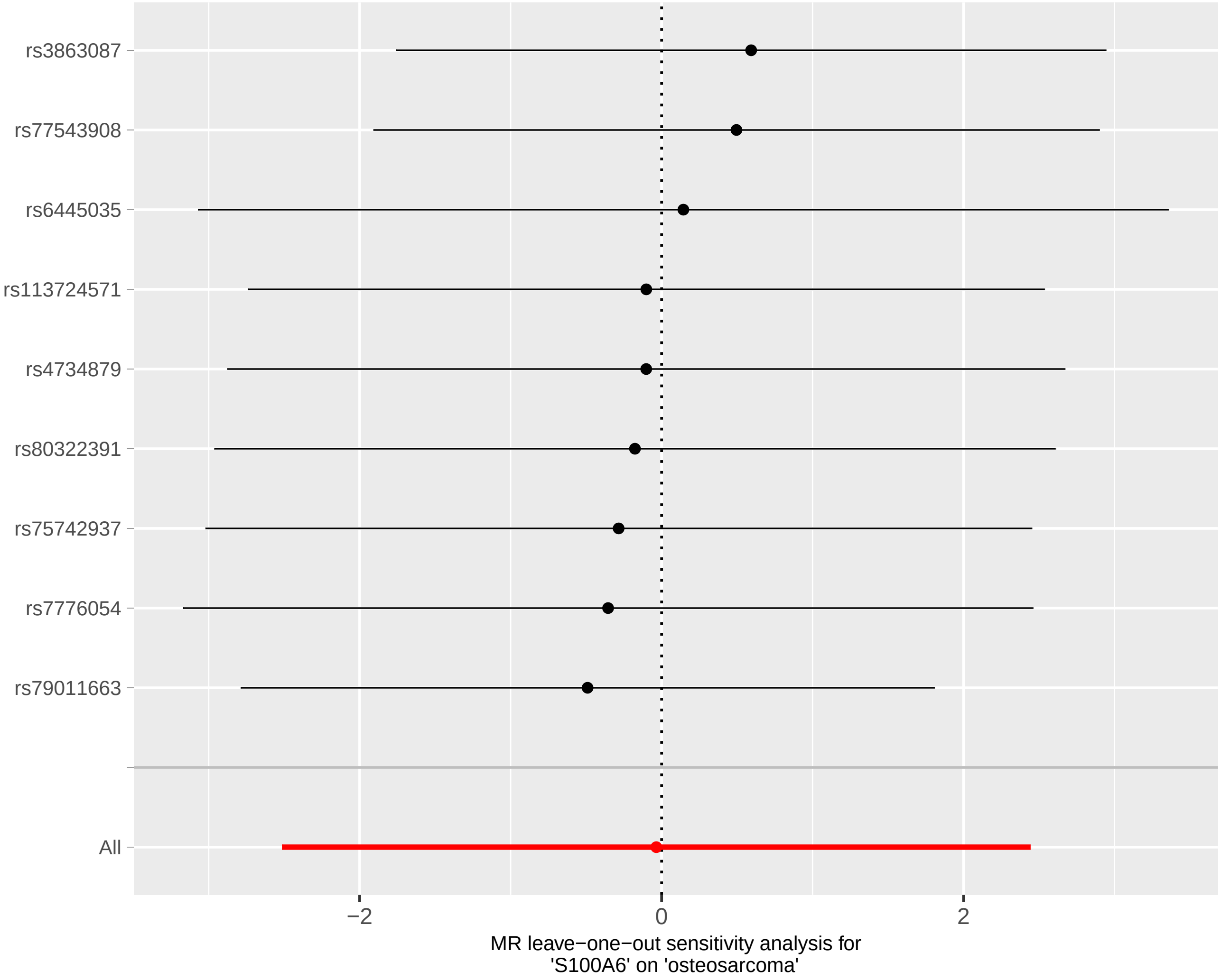

rs3863087
rs77543908
rs6445035
rs113724571
rs4734879
rs80322391
rs75742937
rs7776054
rs79011663
All
−2
0
2
MR leave−one−out sensitivity analysis for
'S100A6' on 'osteosarcoma'

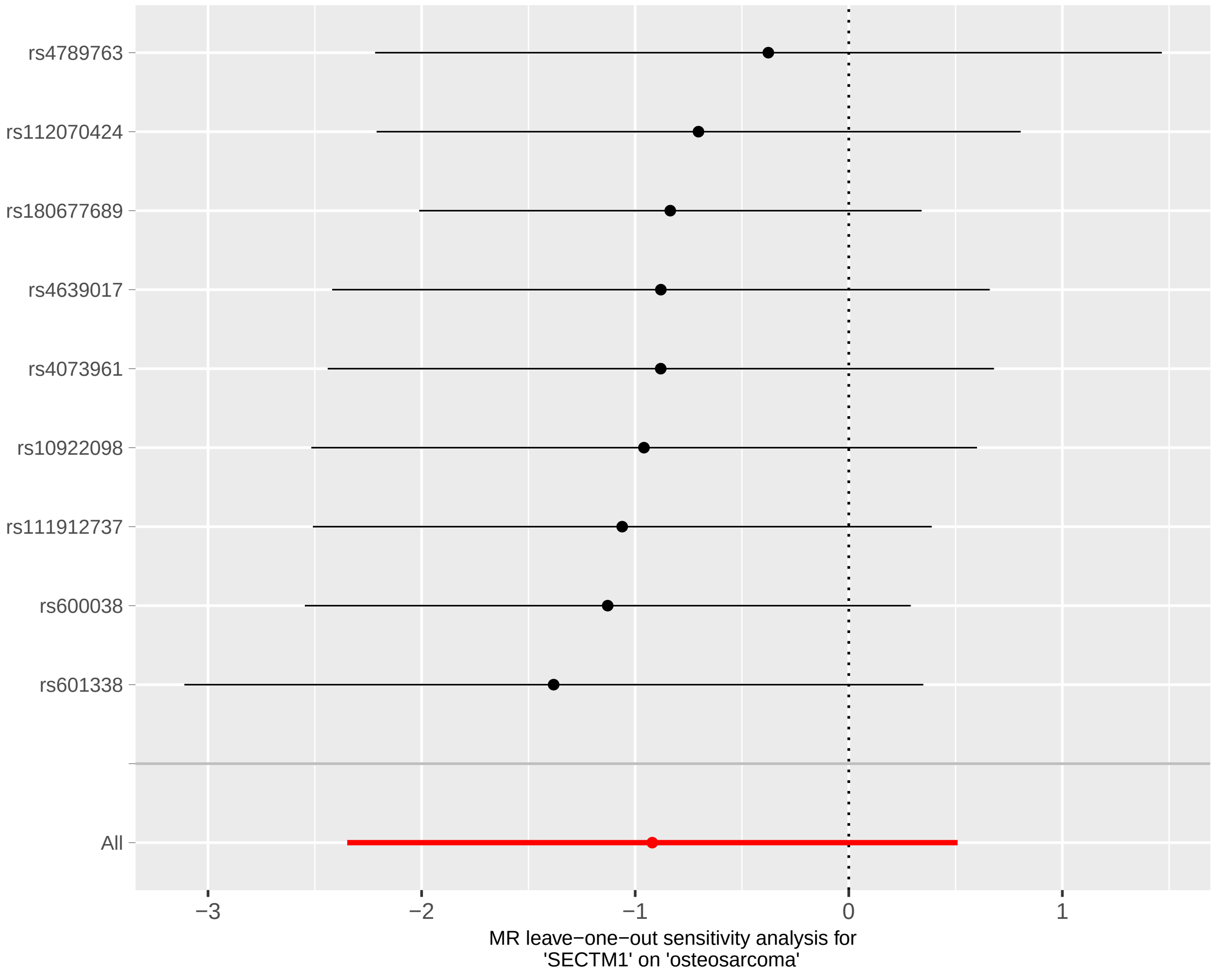
rs4789763
rs112070424
rs180677689
rs4639017
rs4073961
rs10922098
rs111912737
rs600038
rs601338
All
−3
−2
−1
0
1
MR leave−one−out sensitivity analysis for
'SECTM1' on 'osteosarcoma'

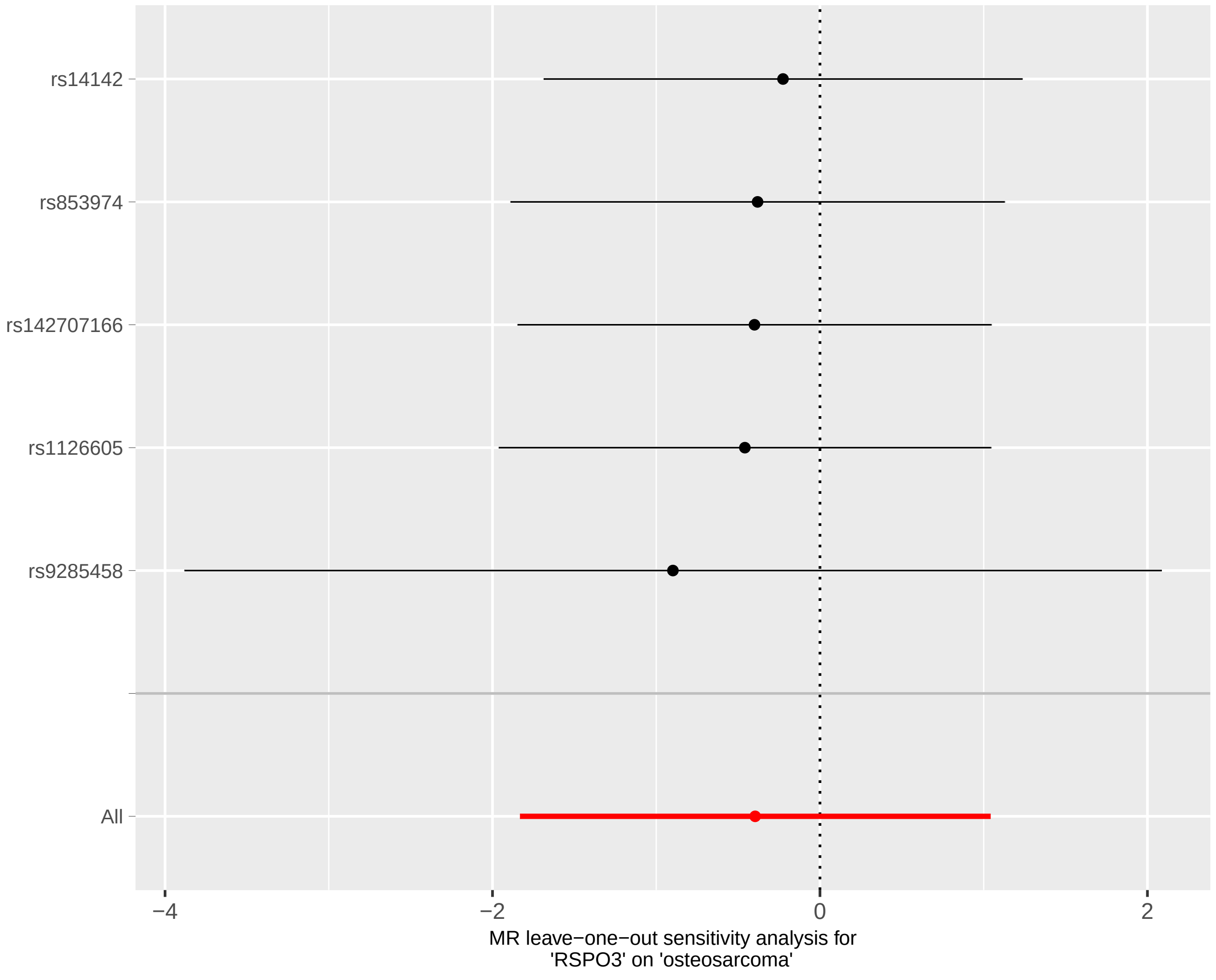

rs14142
rs853974
rs142707166
rs1126605
rs9285458
All
−4
−2
0
2
MR leave−one−out sensitivity analysis for
'RSPO3' on 'osteosarcoma'

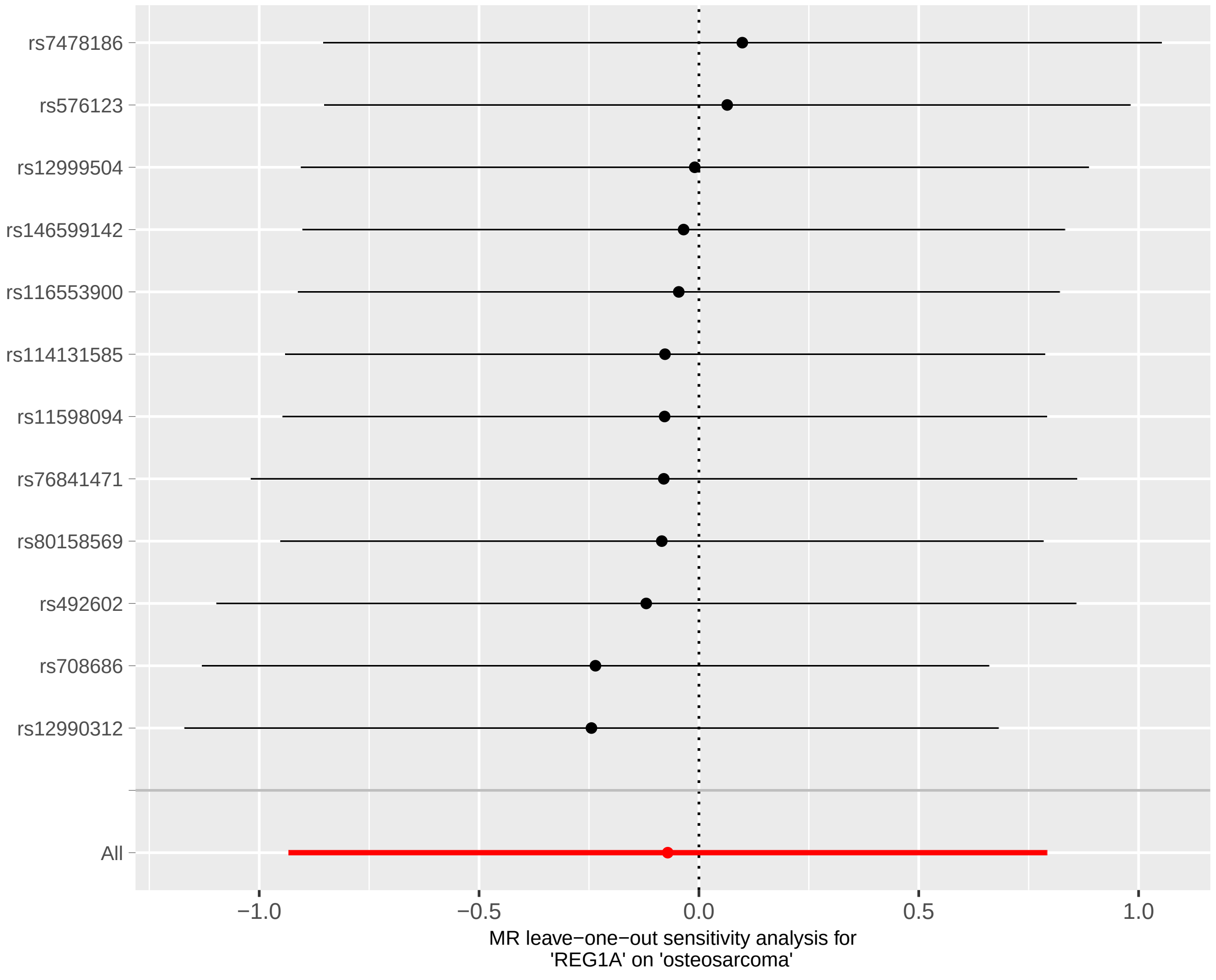

rs7478186
rs576123
rs12999504
rs146599142
rs116553900
rs114131585
rs11598094
rs76841471
rs80158569
rs492602
rs708686
rs12990312
All
−1.0
−0.5
0.0
0.5
1.0
MR leave−one−out sensitivity analysis for
'REG1A' on 'osteosarcoma'

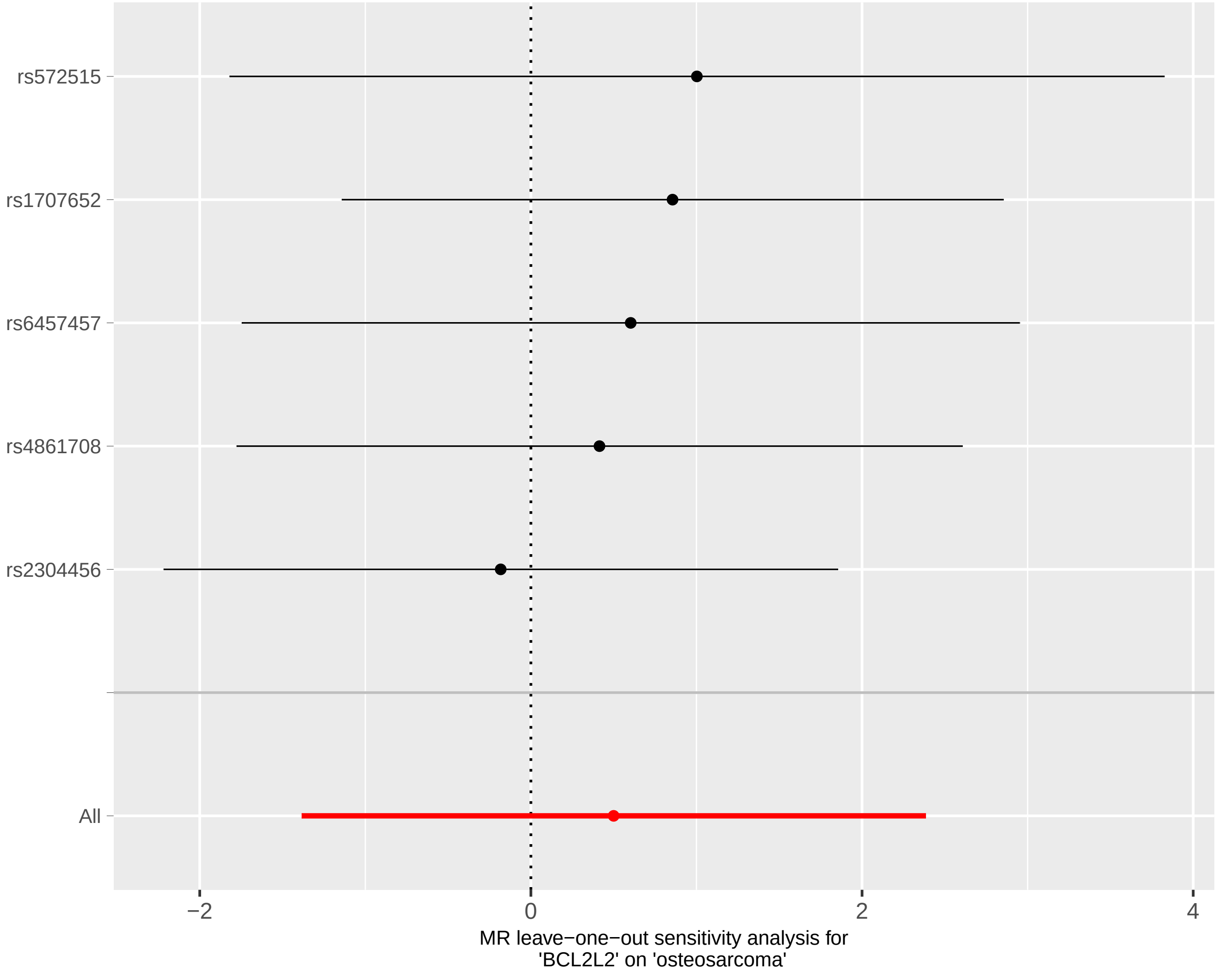

rs572515
rs1707652
rs6457457
rs4861708
rs2304456
All
−2
0
2
4
MR leave−one−out sensitivity analysis for
'BCL2L2' on 'osteosarcoma'

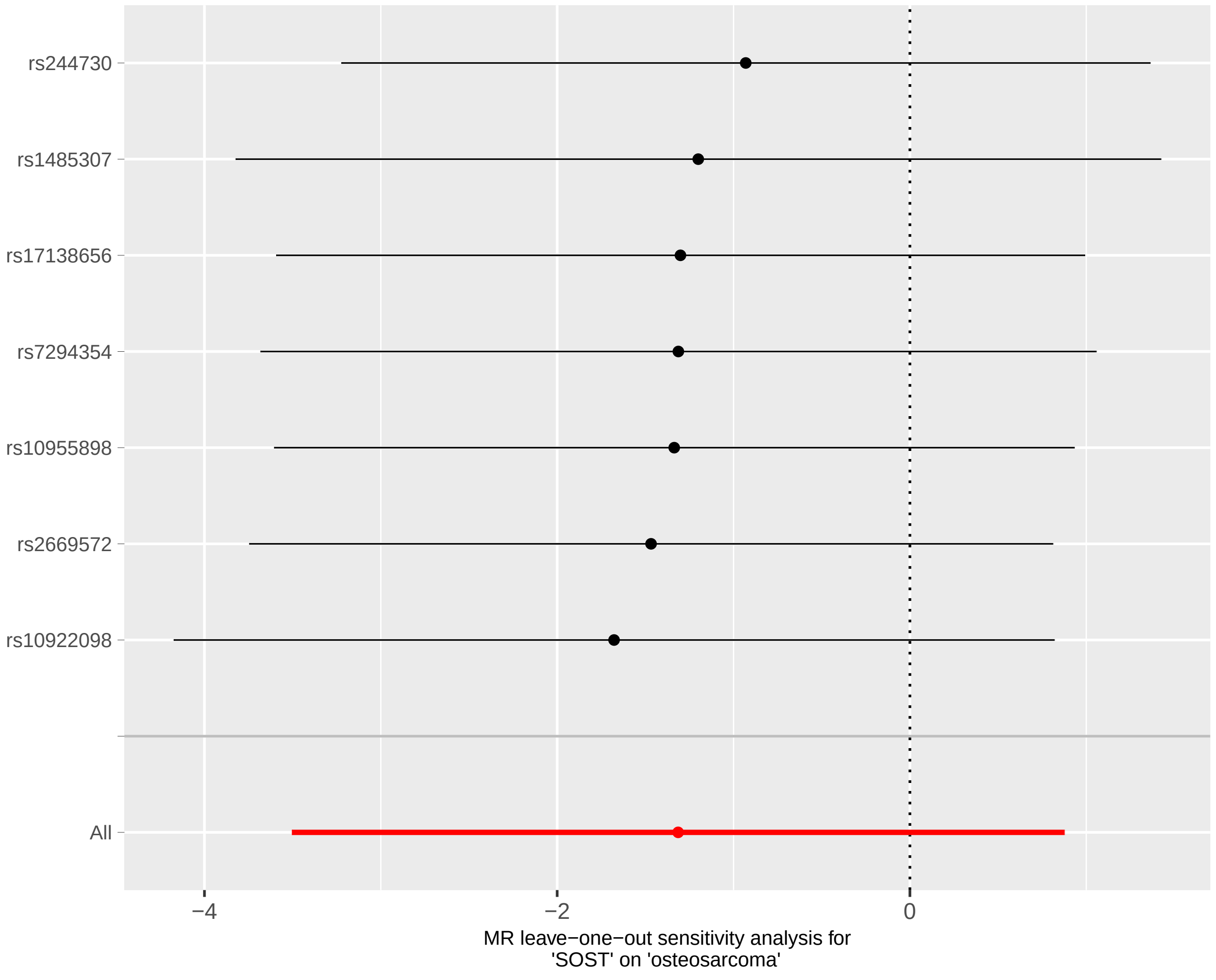
rs244730
rs1485307
rs17138656
rs7294354
rs10955898
rs2669572
rs10922098
All
−4
−2
0
MR leave−one−out sensitivity analysis for
'SOST' on 'osteosarcoma'

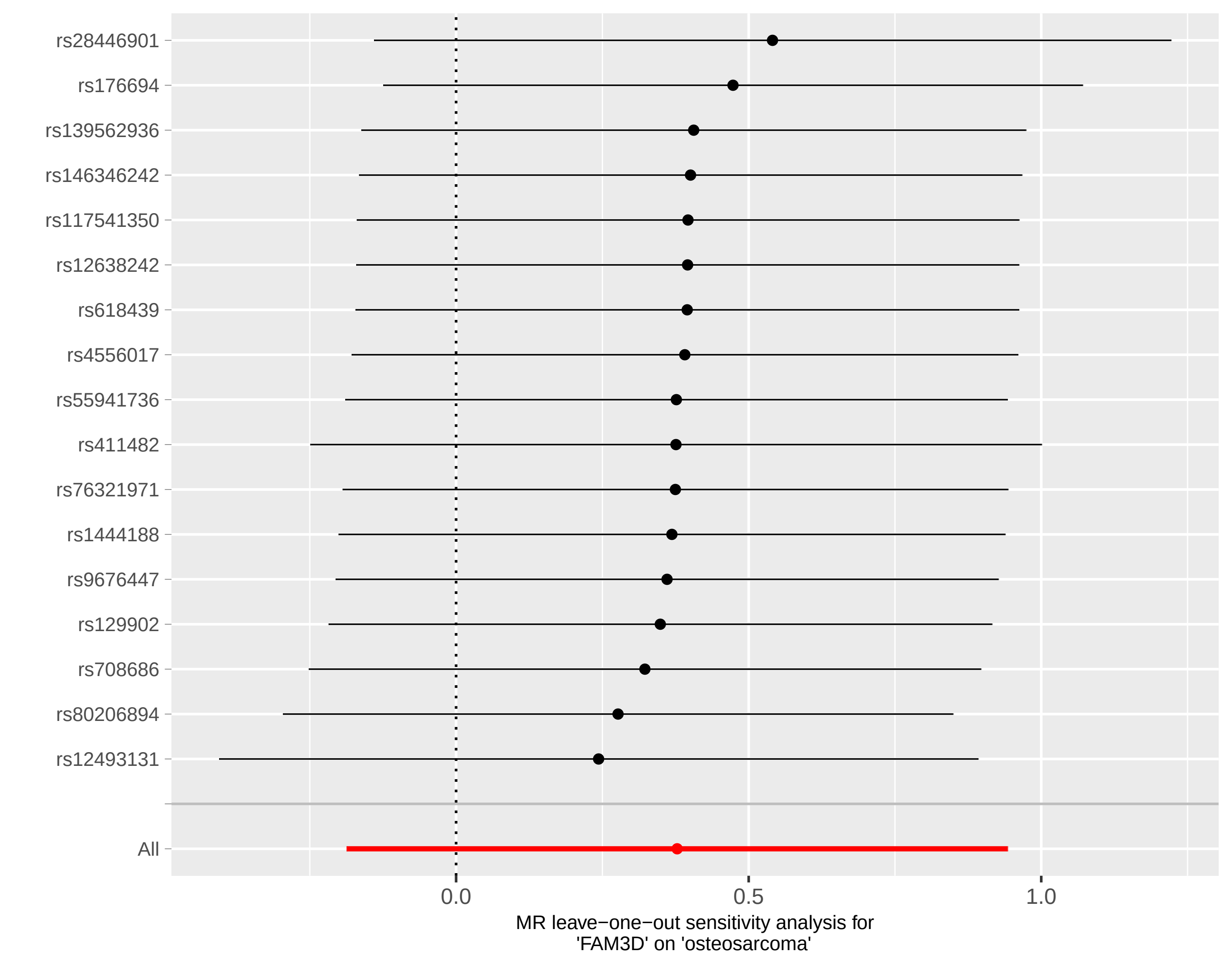

rs28446901
rs176694
rs139562936
rs146346242
rs117541350
rs12638242
rs618439
rs4556017
rs55941736
rs411482
rs76321971
rs1444188
rs9676447
rs129902
rs708686
rs80206894
rs12493131
All
0.0
0.5
1.0
MR leave−one−out sensitivity analysis for
'FAM3D' on 'osteosarcoma'

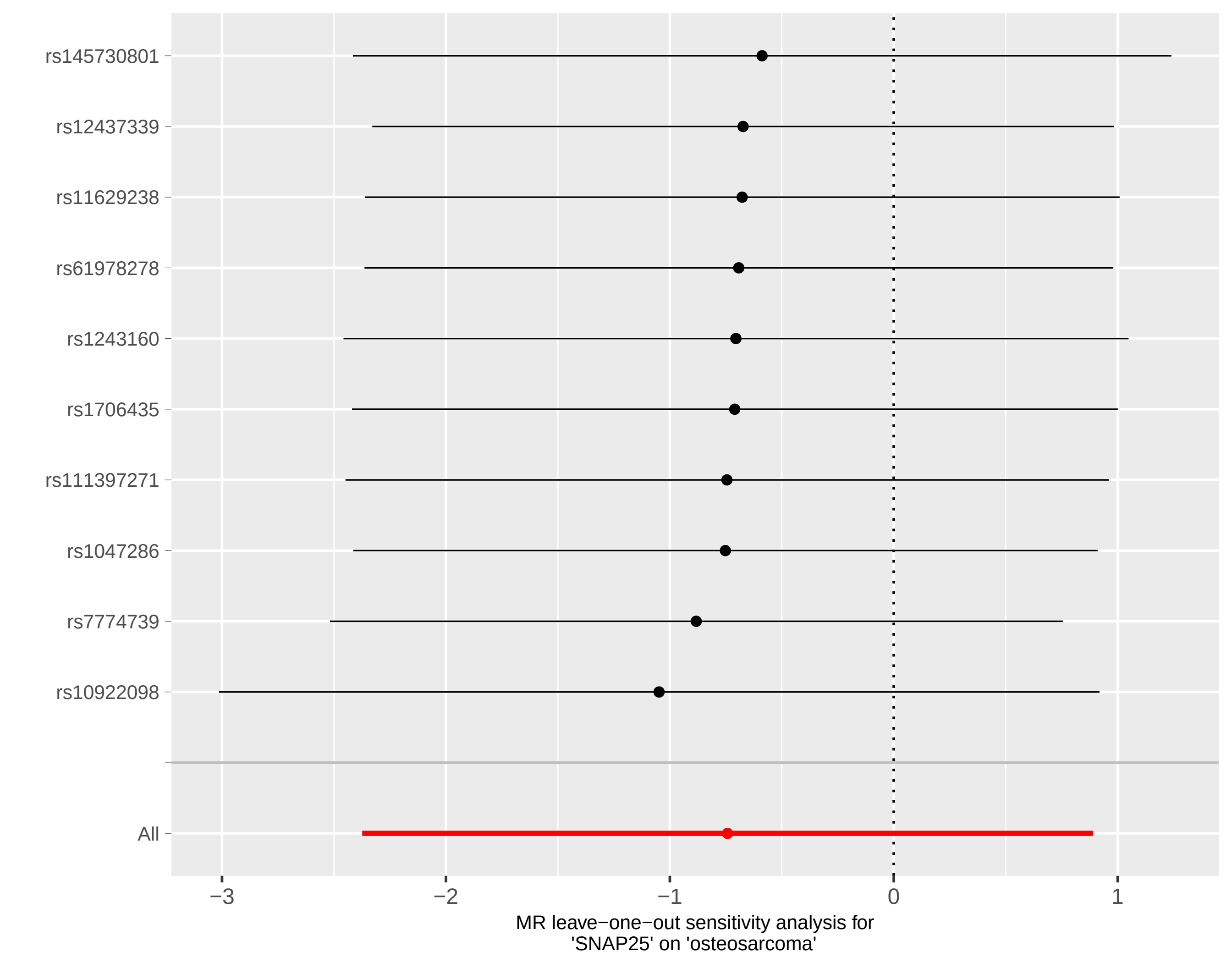
rs145730801
rs12437339
rs11629238
rs61978278
rs1243160
rs1706435
rs111397271
rs1047286
rs7774739
rs10922098
All
−3
−2
−1
0
1
MR leave−one−out sensitivity analysis for
'SNAP25' on 'osteosarcoma'

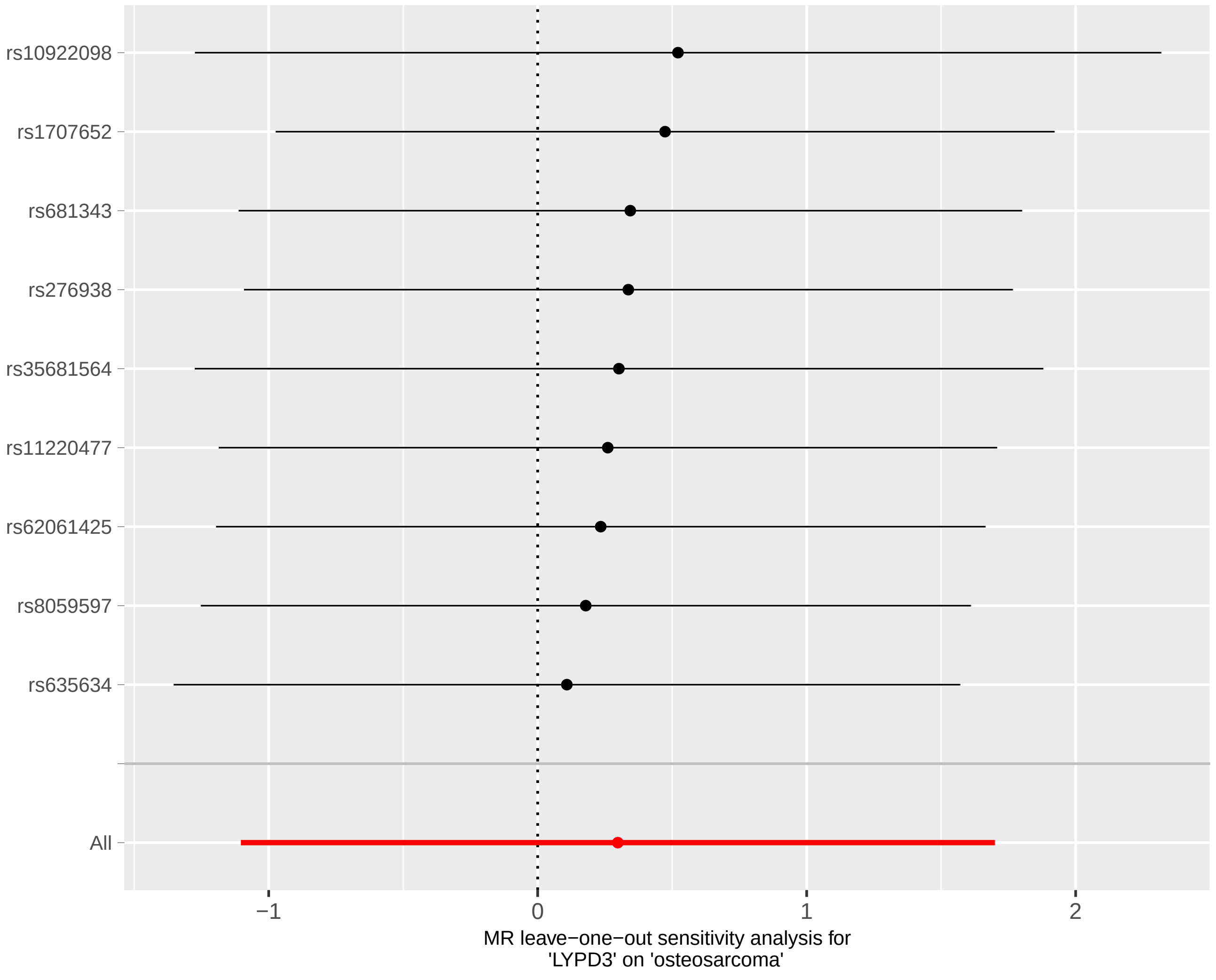

rs10922098
rs1707652
rs681343
rs276938
rs35681564
rs11220477
rs62061425
rs8059597
rs635634
All
−1
0
1
2
MR leave−one−out sensitivity analysis for
'LYPD3' on 'osteosarcoma'

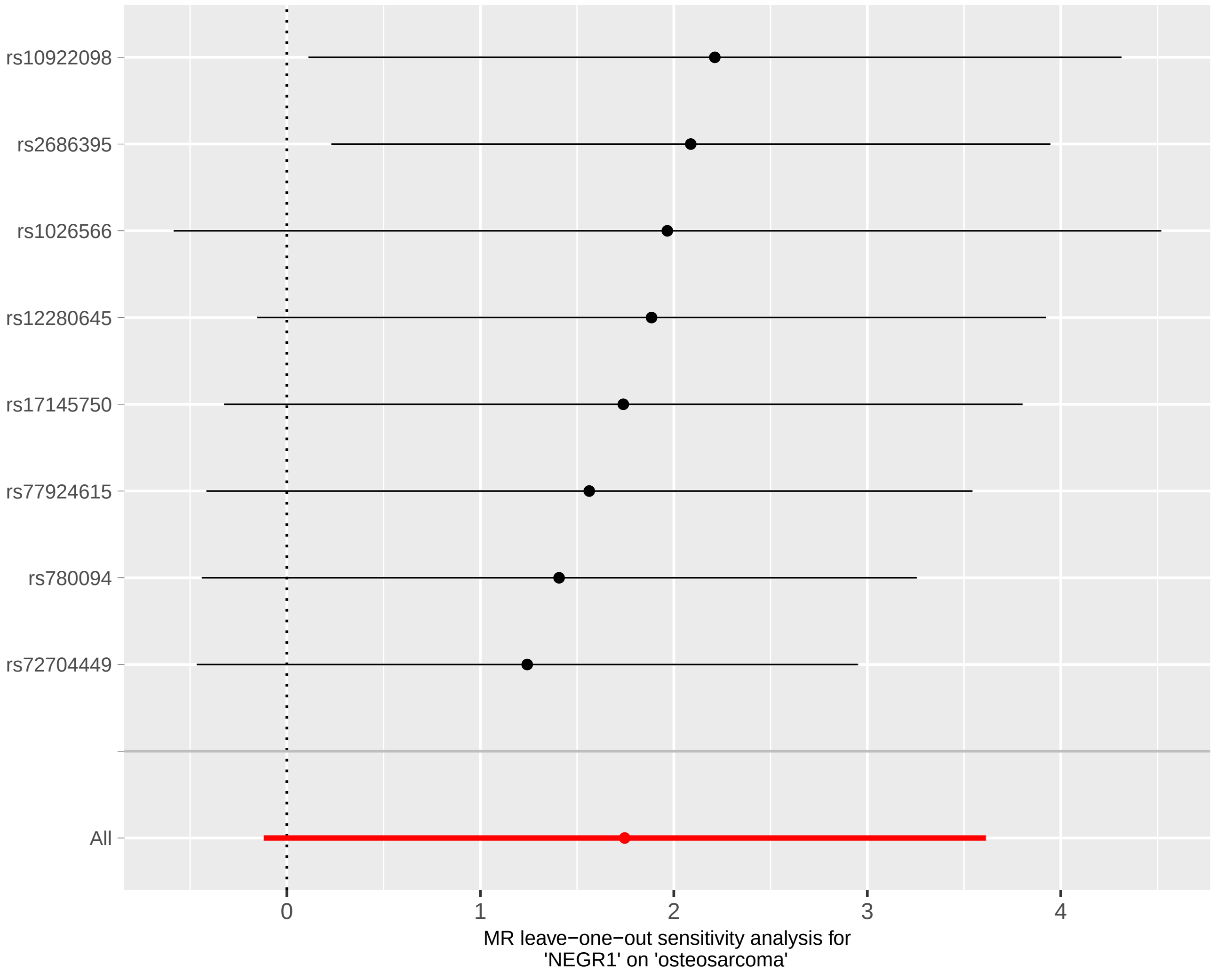
rs10922098
rs2686395
rs1026566
rs12280645
rs17145750
rs77924615
rs780094
rs72704449
All
0
1
2
3
4
MR leave−one−out sensitivity analysis for
'NEGR1' on 'osteosarcoma'

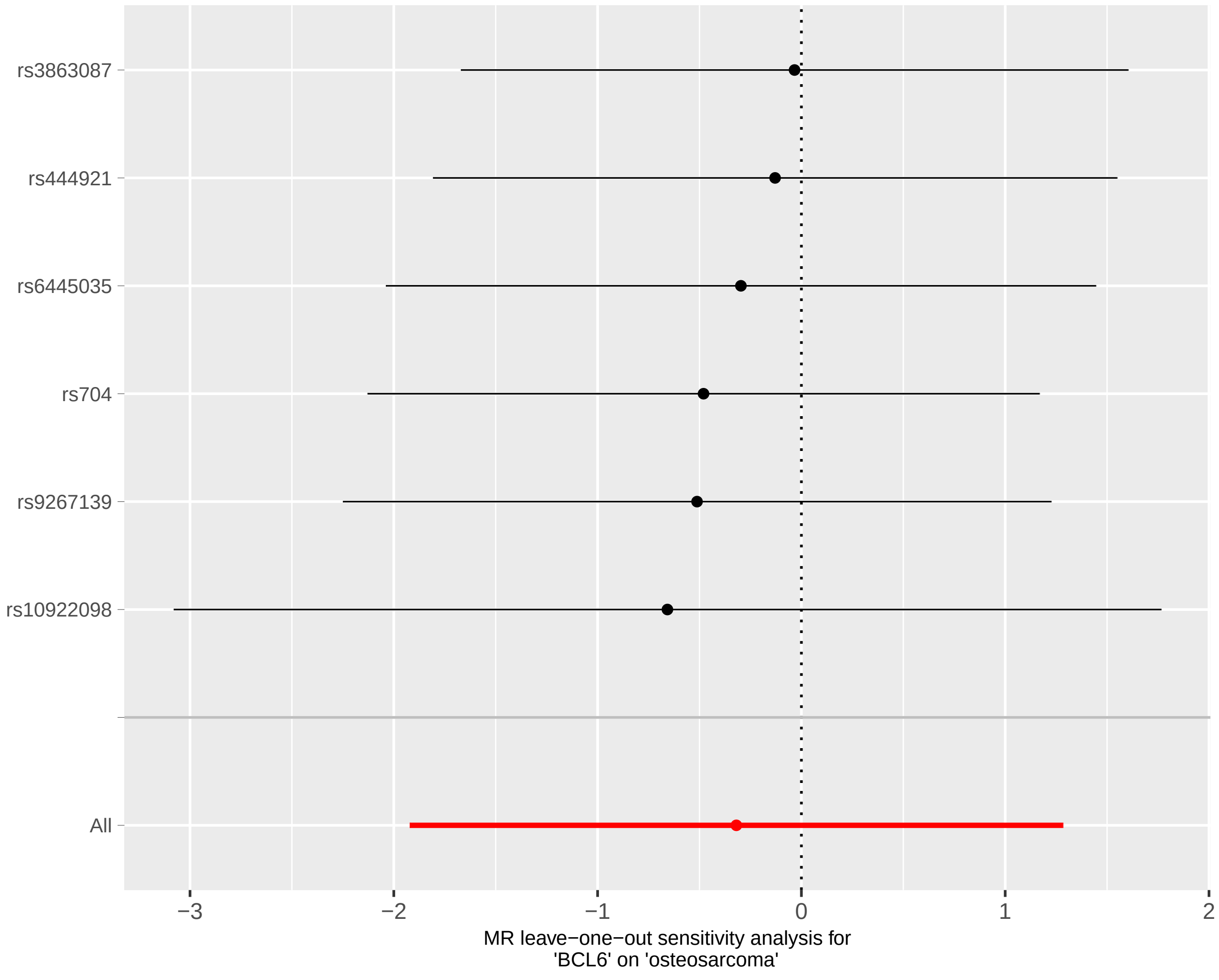
rs3863087
rs444921
rs6445035
rs704
rs9267139
rs10922098
All
−3
−2
−1
0
1
2
MR leave−one−out sensitivity analysis for
'BCL6' on 'osteosarcoma'

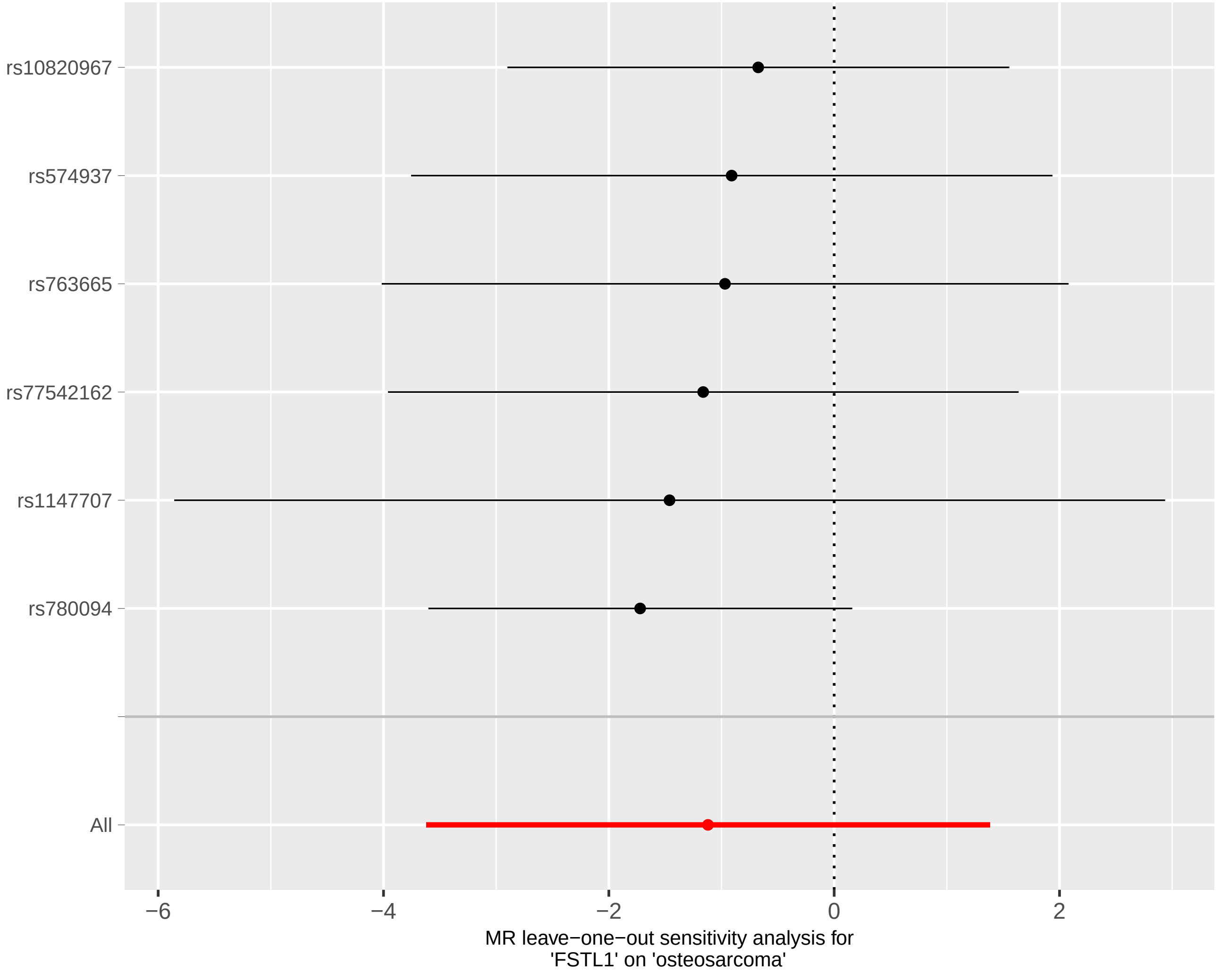
rs10820967
rs574937
rs763665
rs77542162
rs1147707
rs780094
All
−6
−4
−2
0
2
MR leave−one−out sensitivity analysis for
'FSTL1' on 'osteosarcoma'

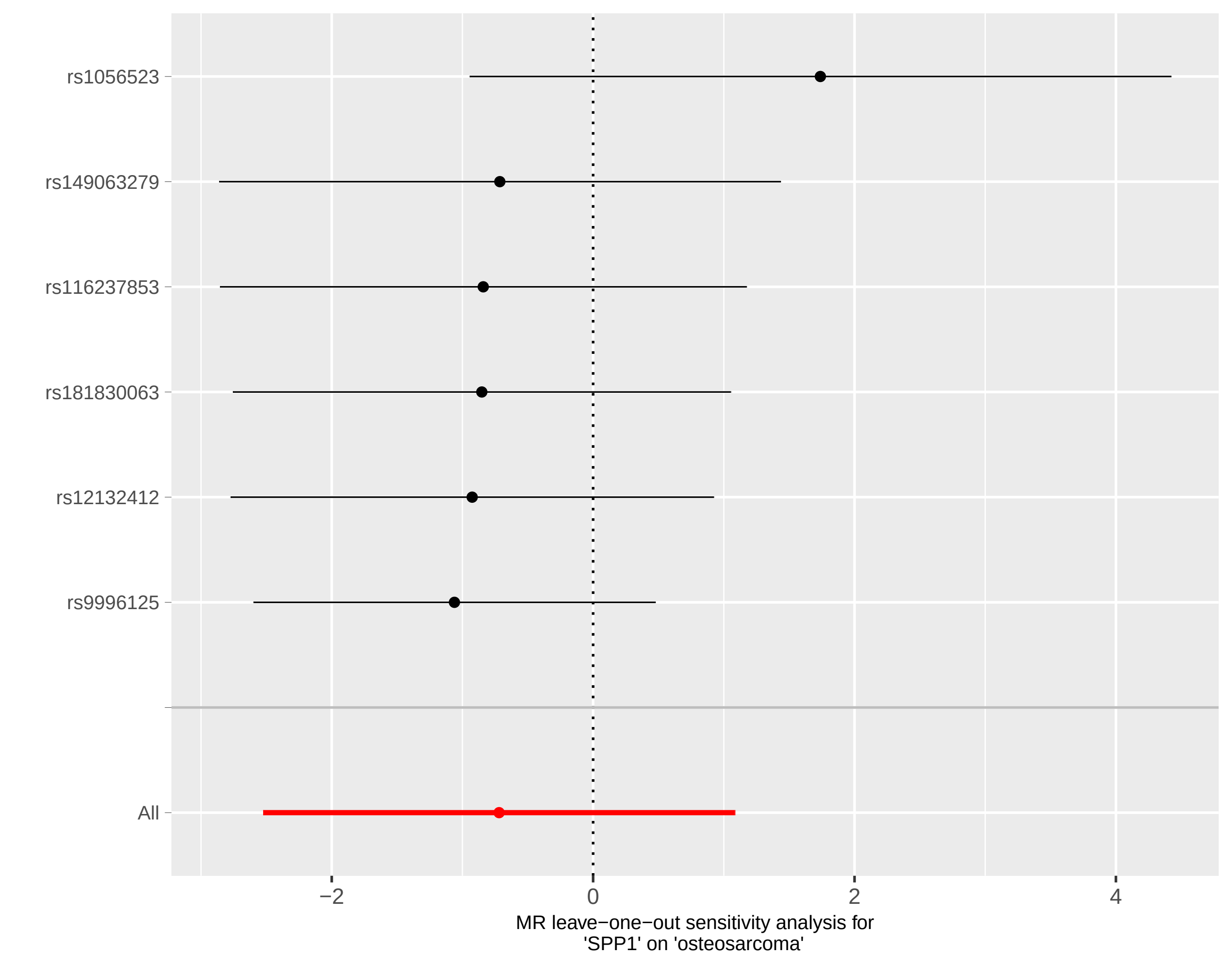

rs1056523
rs149063279
rs116237853
rs181830063
rs12132412
rs9996125
All
−2
0
2
4
MR leave−one−out sensitivity analysis for
'SPP1' on 'osteosarcoma'

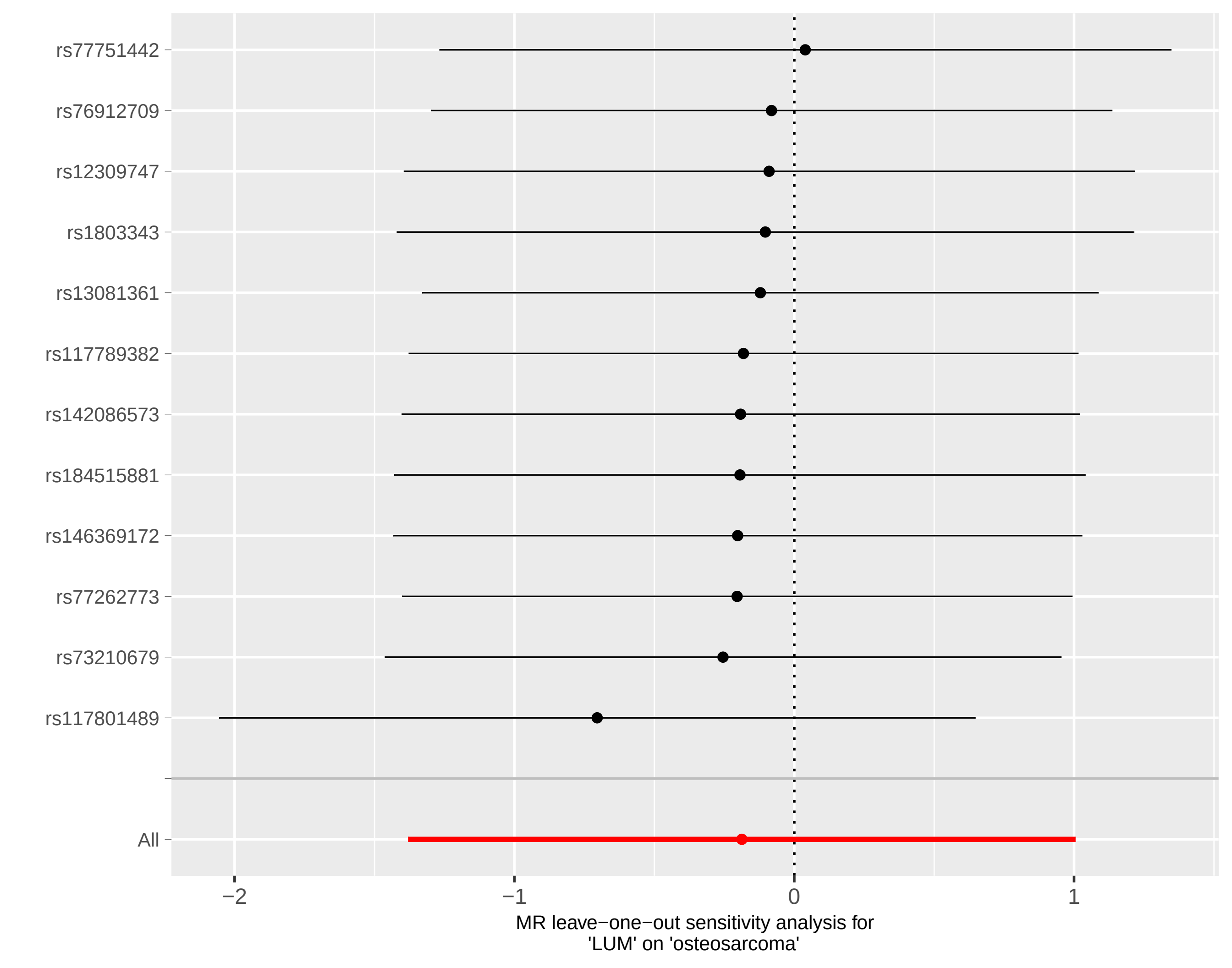
rs77751442
rs76912709
rs12309747
rs1803343
rs13081361
rs117789382
rs142086573
rs184515881
rs146369172
rs77262773
rs73210679
rs117801489
All
−2
−1
0
1
MR leave−one−out sensitivity analysis for
'LUM' on 'osteosarcoma'

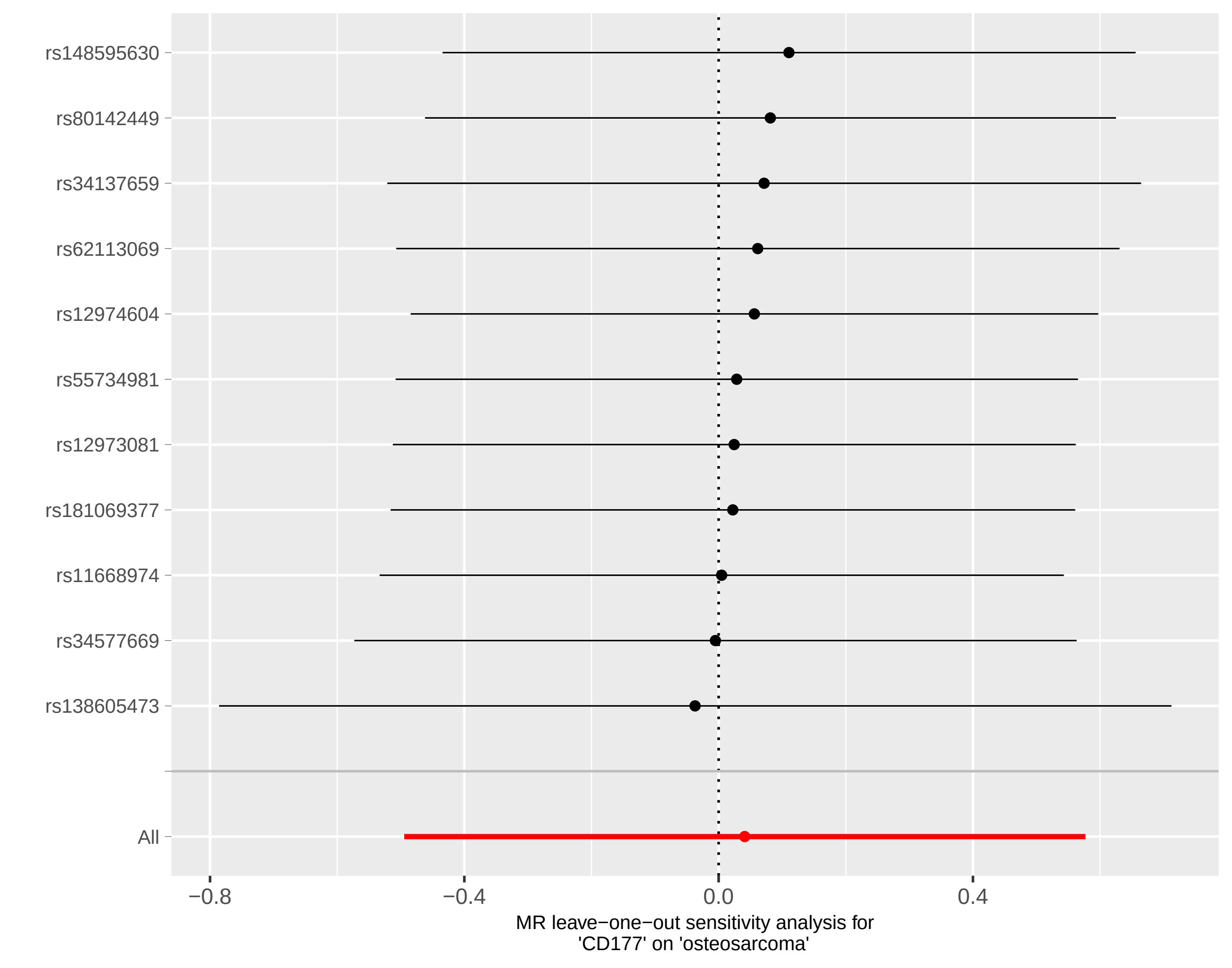
rs148595630
rs80142449
rs34137659
rs62113069
rs12974604
rs55734981
rs12973081
rs181069377
rs11668974
rs34577669
rs138605473
All
−0.8
−0.4
0.0
0.4
MR leave−one−out sensitivity analysis for
'CD177' on 'osteosarcoma'

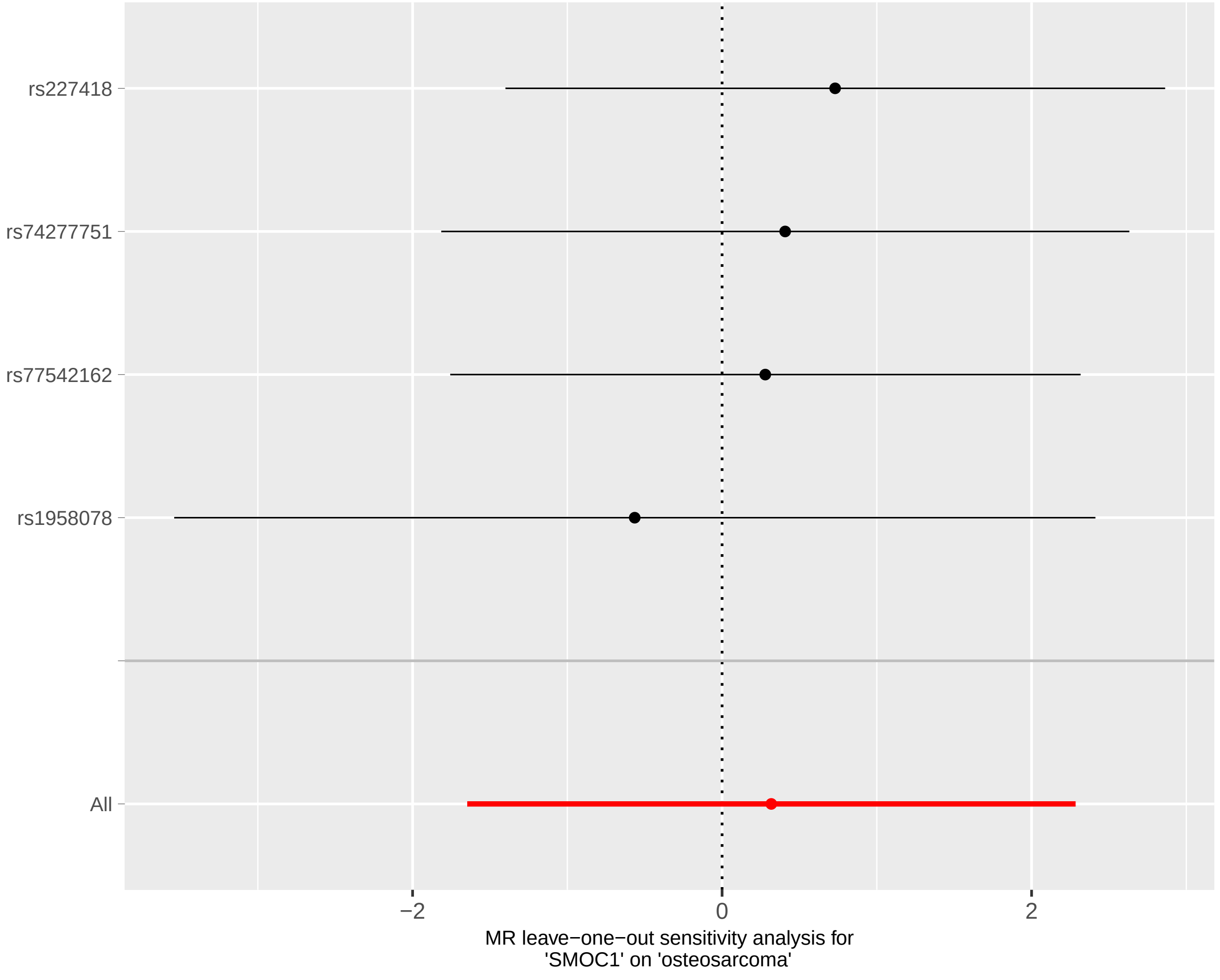
rs227418
rs74277751
rs77542162
rs1958078
All
−2
0
2
MR leave−one−out sensitivity analysis for
'SMOC1' on 'osteosarcoma'

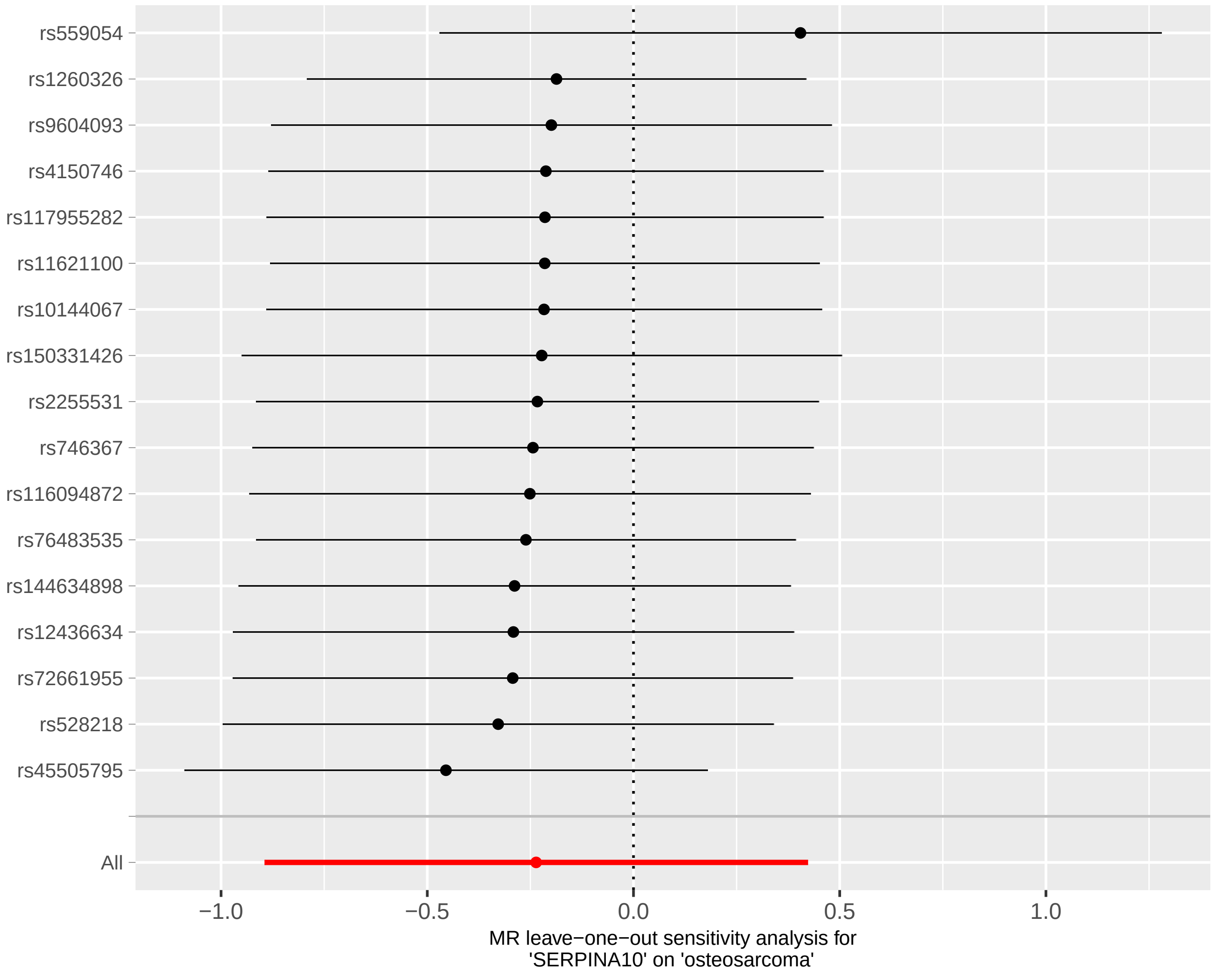
rs559054
rs1260326
rs9604093
rs4150746
rs117955282
rs11621100
rs10144067
rs150331426
rs2255531
rs746367
rs116094872
rs76483535
rs144634898
rs12436634
rs72661955
rs528218
rs45505795
All
−1.0
−0.5
0.0
0.5
1.0
MR leave−one−out sensitivity analysis for
'SERPINA10' on 'osteosarcoma'

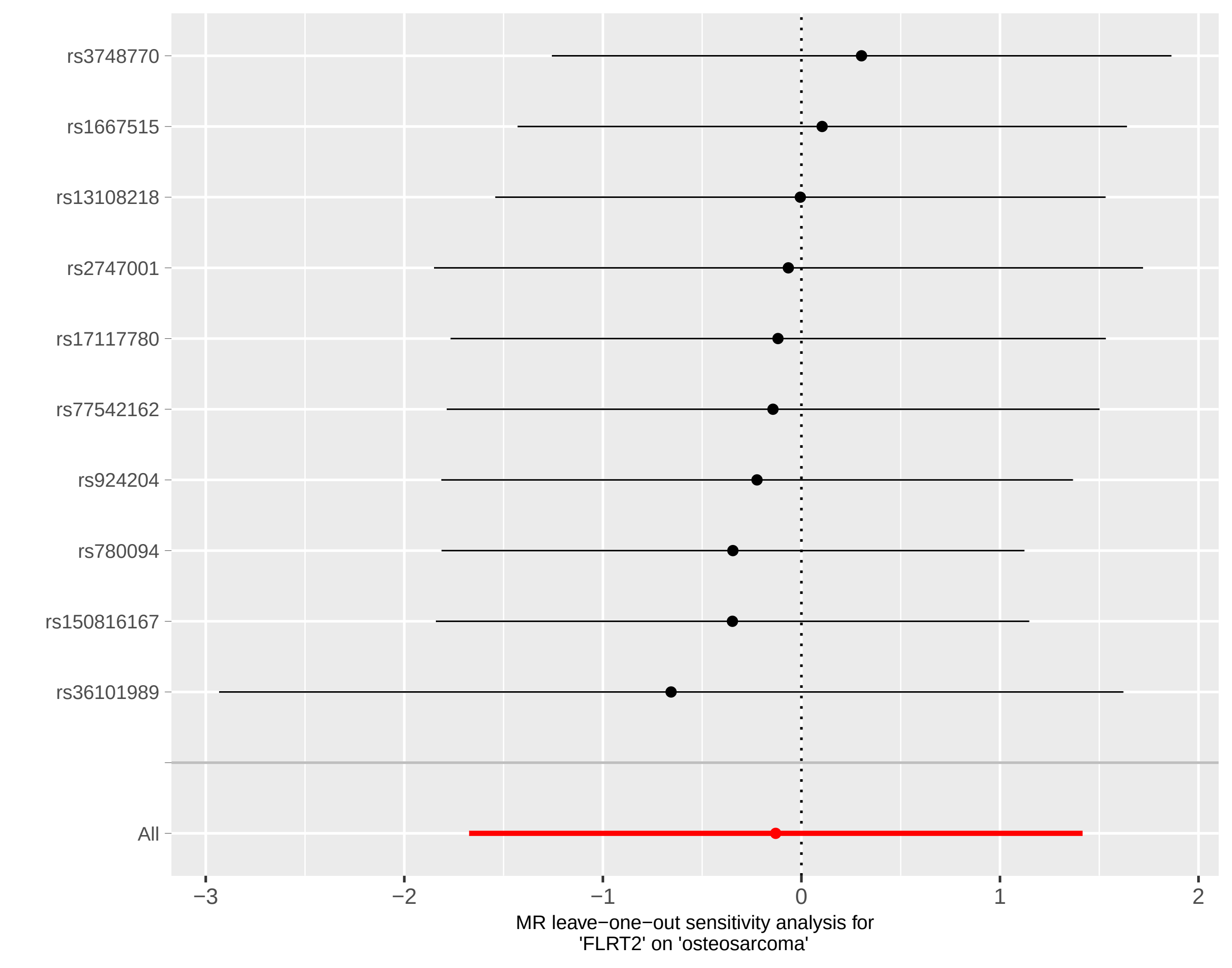

rs3748770
rs1667515
rs13108218
rs2747001
rs17117780
rs77542162
rs924204
rs780094
rs150816167
rs36101989
All
−3
−2
−1
0
1
2
MR leave−one−out sensitivity analysis for
'FLRT2' on 'osteosarcoma'

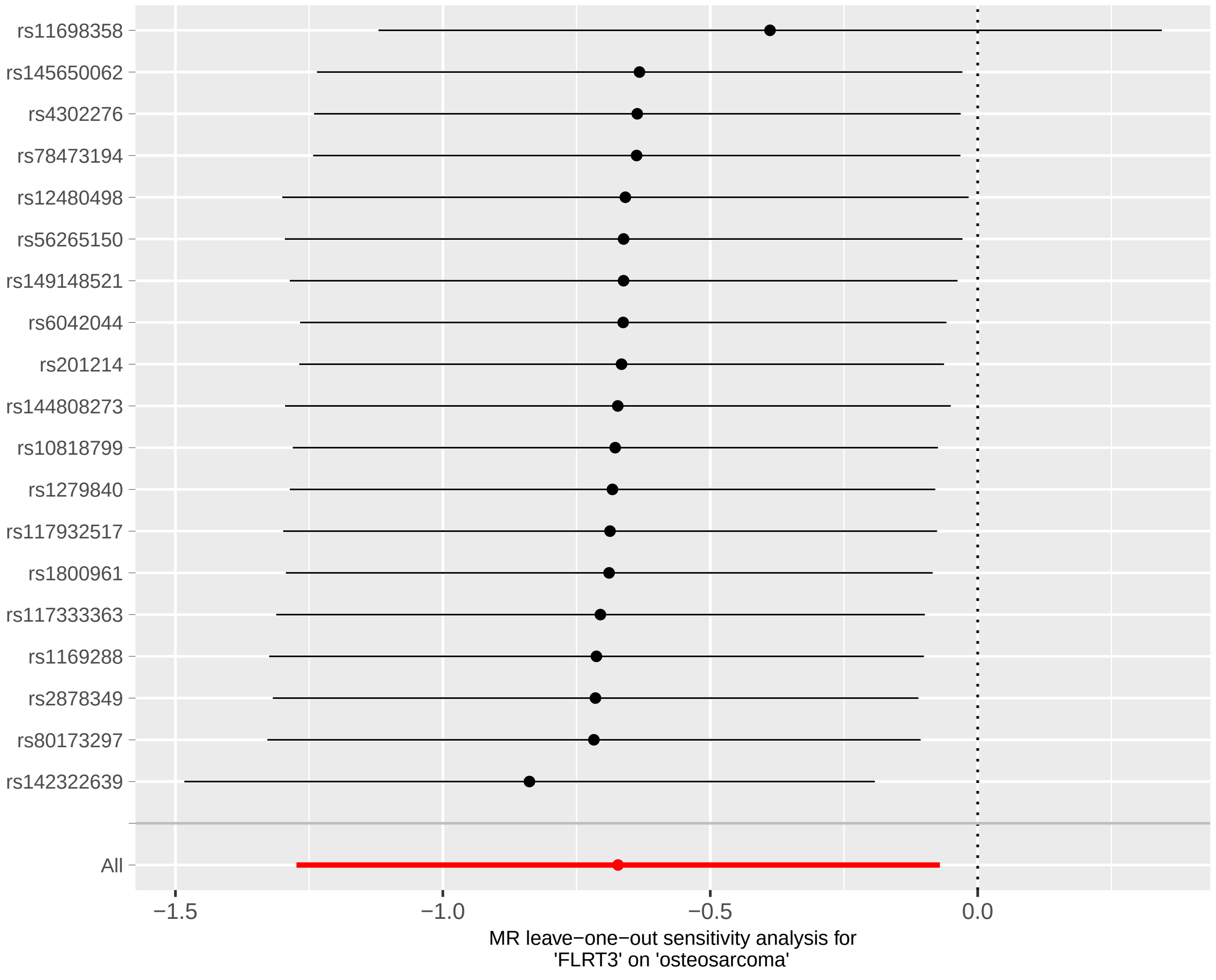
rs11698358
rs145650062
rs4302276
rs78473194
rs12480498
rs56265150
rs149148521
rs6042044
rs201214
rs144808273
rs10818799
rs1279840
rs117932517
rs1800961
rs117333363
rs1169288
rs2878349
rs80173297
rs142322639
All
−1.5
−1.0
−0.5
0.0
MR leave−one−out sensitivity analysis for
'FLRT3' on 'osteosarcoma'

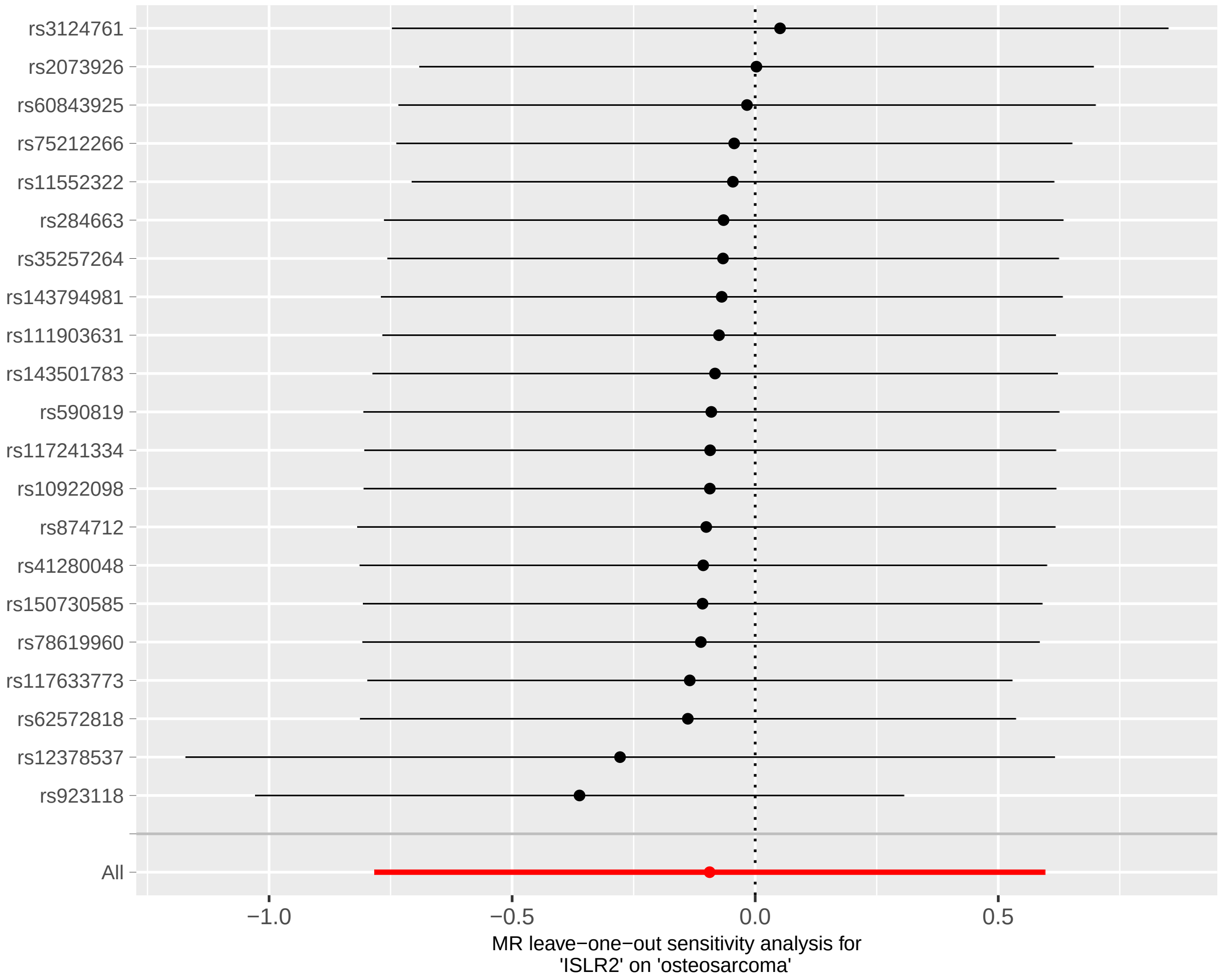
rs3124761
rs2073926
rs60843925
rs75212266
rs11552322
rs284663
rs35257264
rs143794981
rs111903631
rs143501783
rs590819
rs117241334
rs10922098
rs874712
rs41280048
rs150730585
rs78619960
rs117633773
rs62572818
rs12378537
rs923118
All
−1.0
−0.5
0.0
0.5
MR leave−one−out sensitivity analysis for
'ISLR2' on 'osteosarcoma'

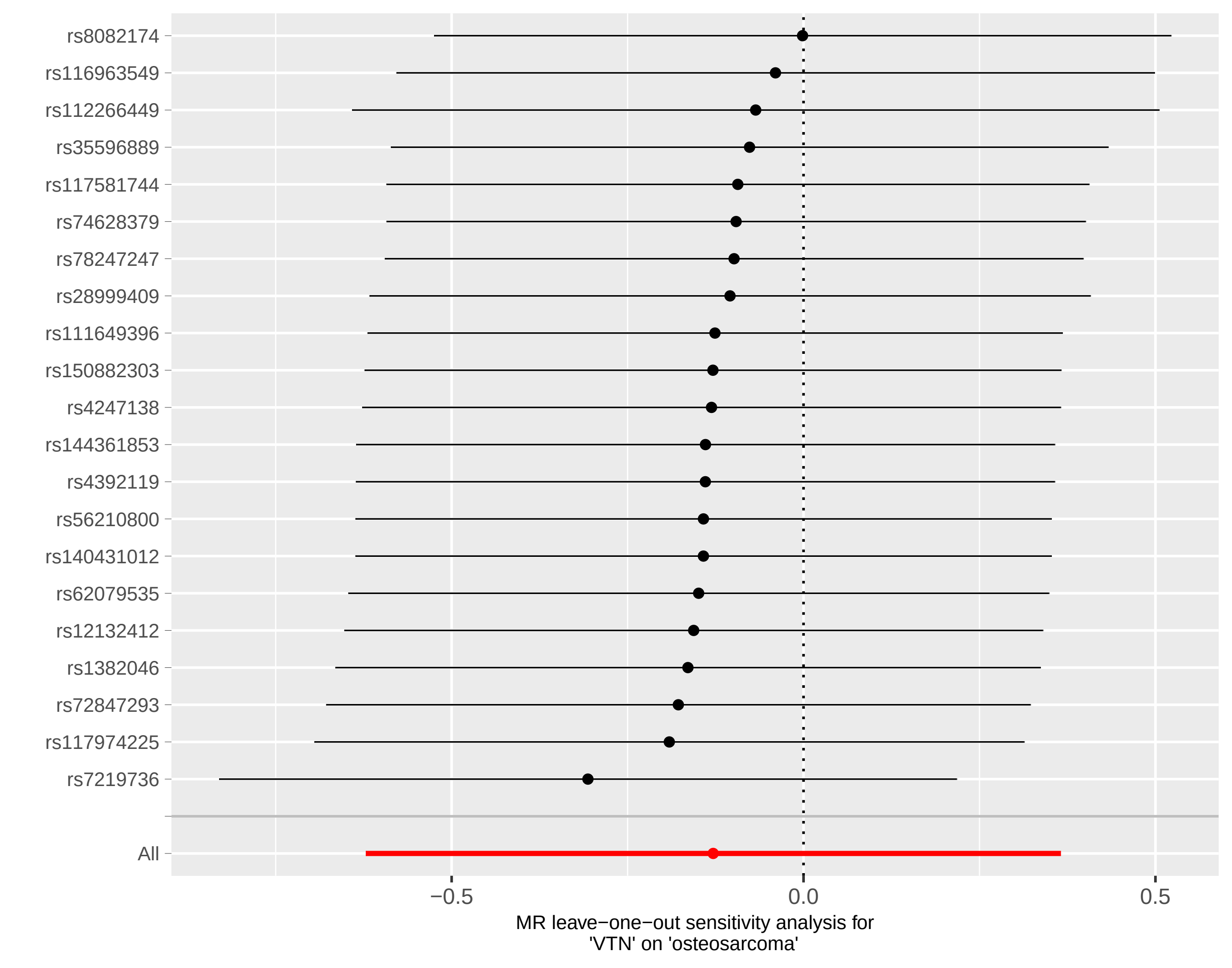

rs8082174
rs116963549
rs112266449
rs35596889
rs117581744
rs74628379
rs78247247
rs28999409
rs111649396
rs150882303
rs4247138
rs144361853
rs4392119
rs56210800
rs140431012
rs62079535
rs12132412
rs1382046
rs72847293
rs117974225
rs7219736
All
−0.5
0.0
0.5
MR leave−one−out sensitivity analysis for
'VTN' on 'osteosarcoma'

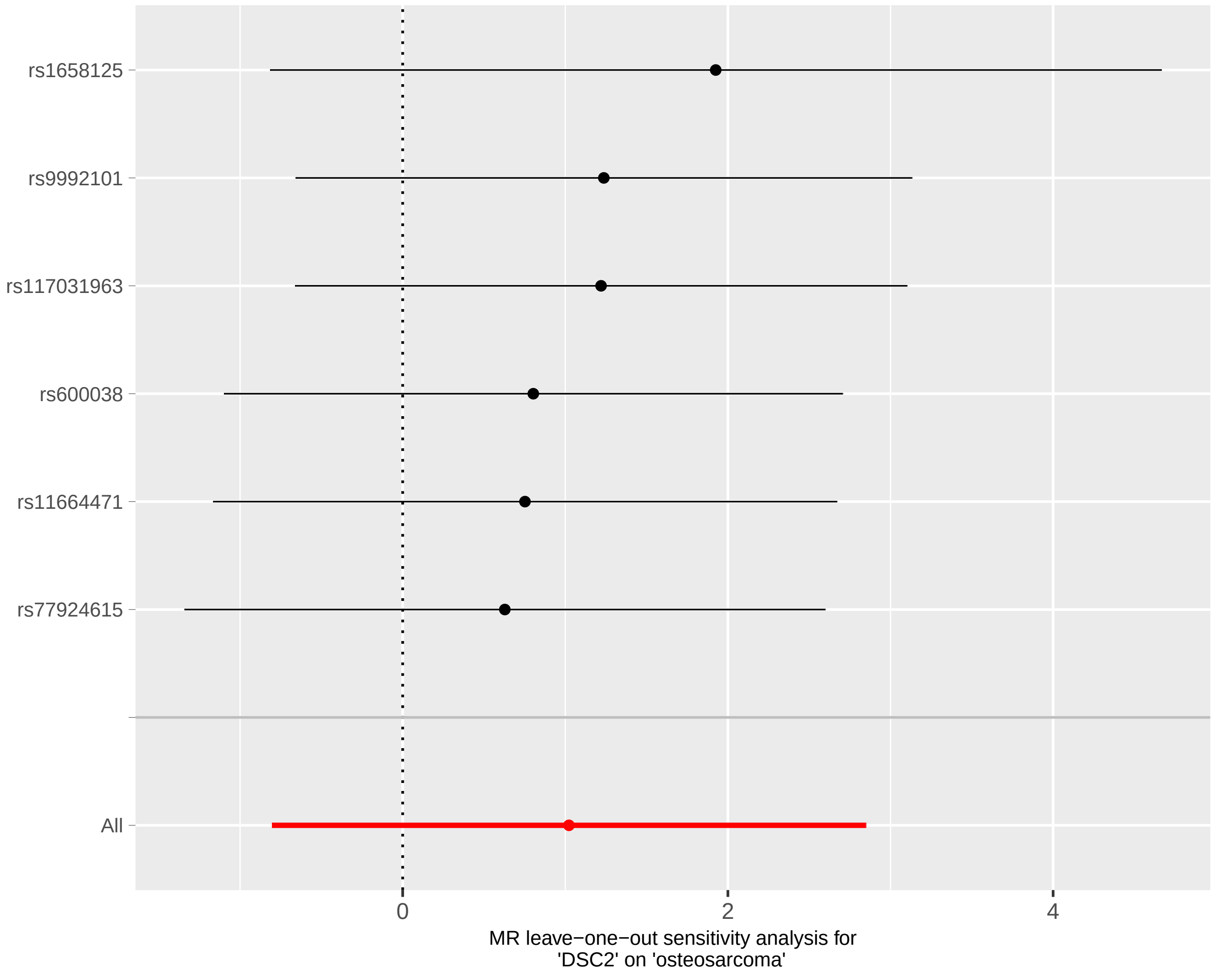

rs1658125
rs9992101
rs117031963
rs600038
rs11664471
rs77924615
All
0
2
4
MR leave−one−out sensitivity analysis for
'DSC2' on 'osteosarcoma'

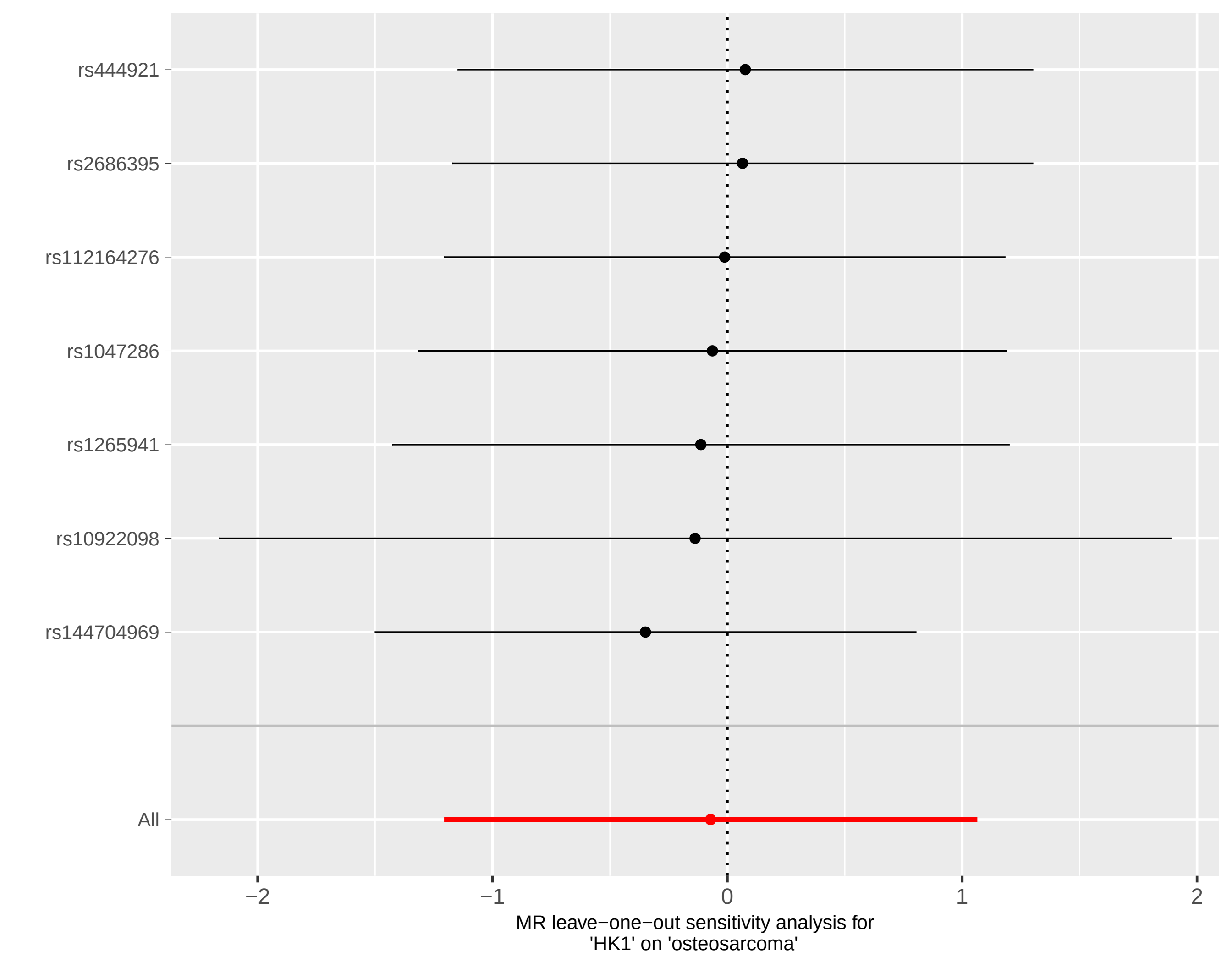
rs444921
rs2686395
rs112164276
rs1047286
rs1265941
rs10922098
rs144704969
All
−2
−1
0
1
2
MR leave−one−out sensitivity analysis for
'HK1' on 'osteosarcoma'

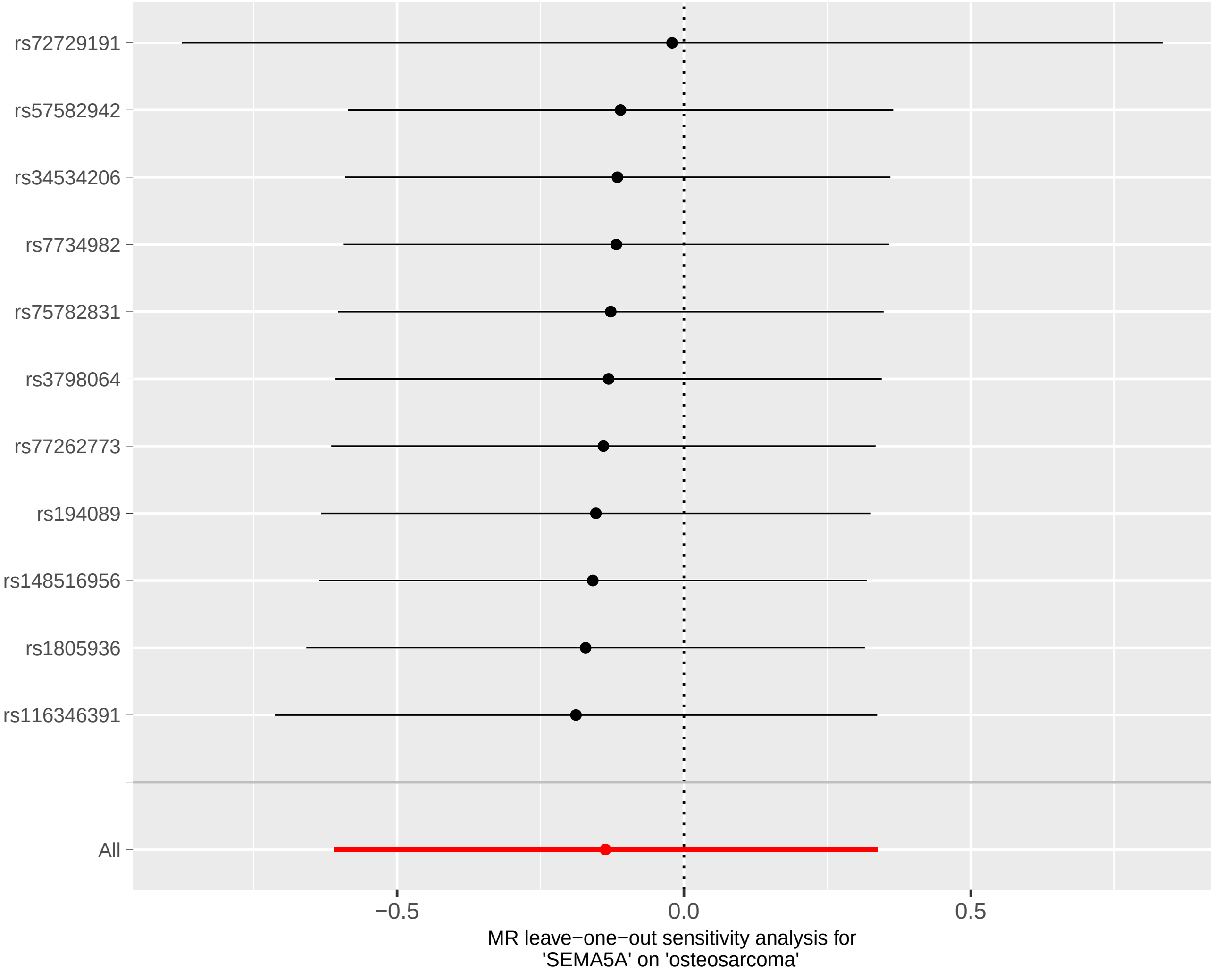
rs72729191
rs57582942
rs34534206
rs7734982
rs75782831
rs3798064
rs77262773
rs194089
rs148516956
rs1805936
rs116346391
All
−0.5
0.0
0.5
MR leave−one−out sensitivity analysis for
'SEMA5A' on 'osteosarcoma'

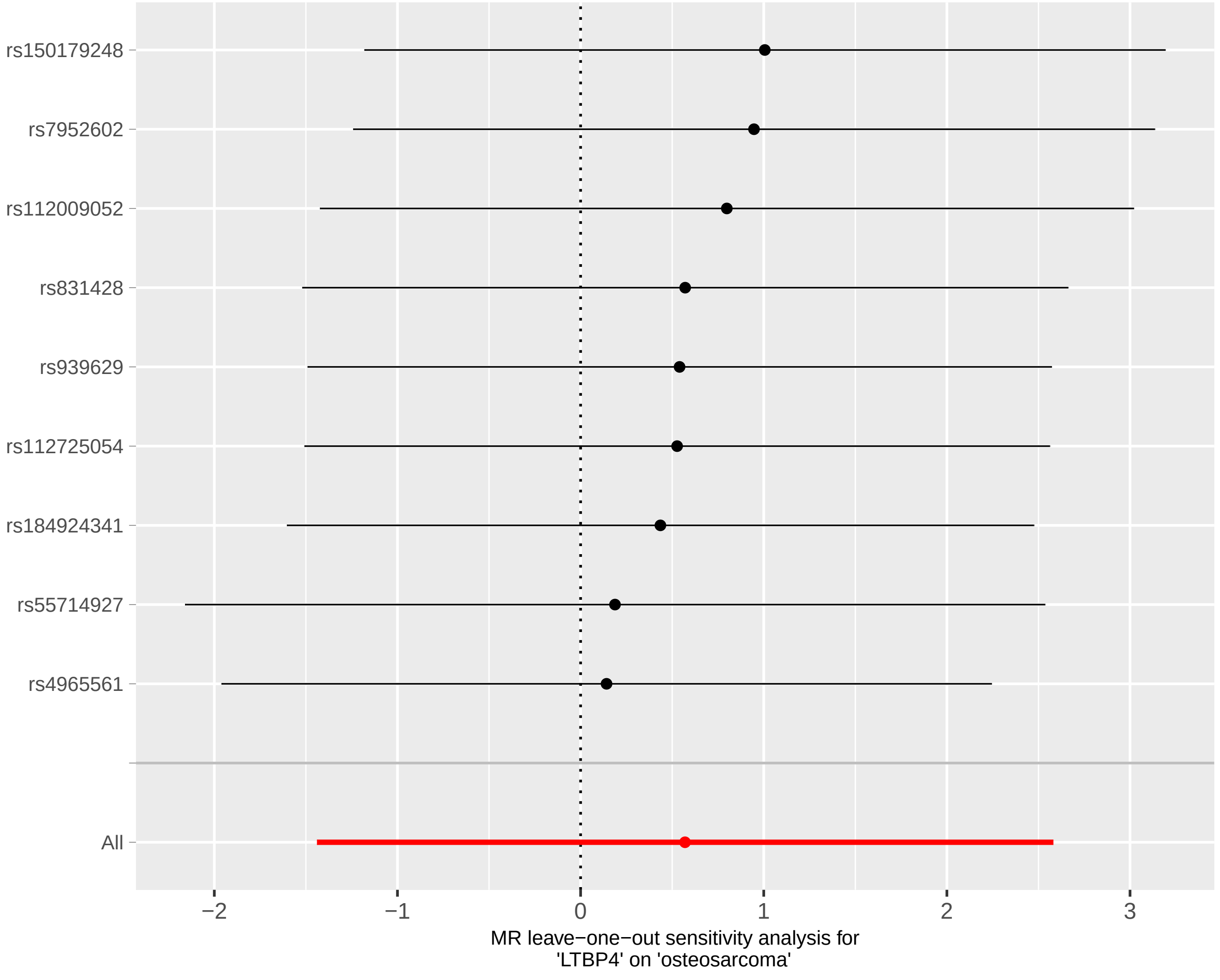
rs150179248
rs7952602
rs112009052
rs831428
rs939629
rs112725054
rs184924341
rs55714927
rs4965561
All
−2
−1
0
1
2
3
MR leave−one−out sensitivity analysis for
'LTBP4' on 'osteosarcoma'

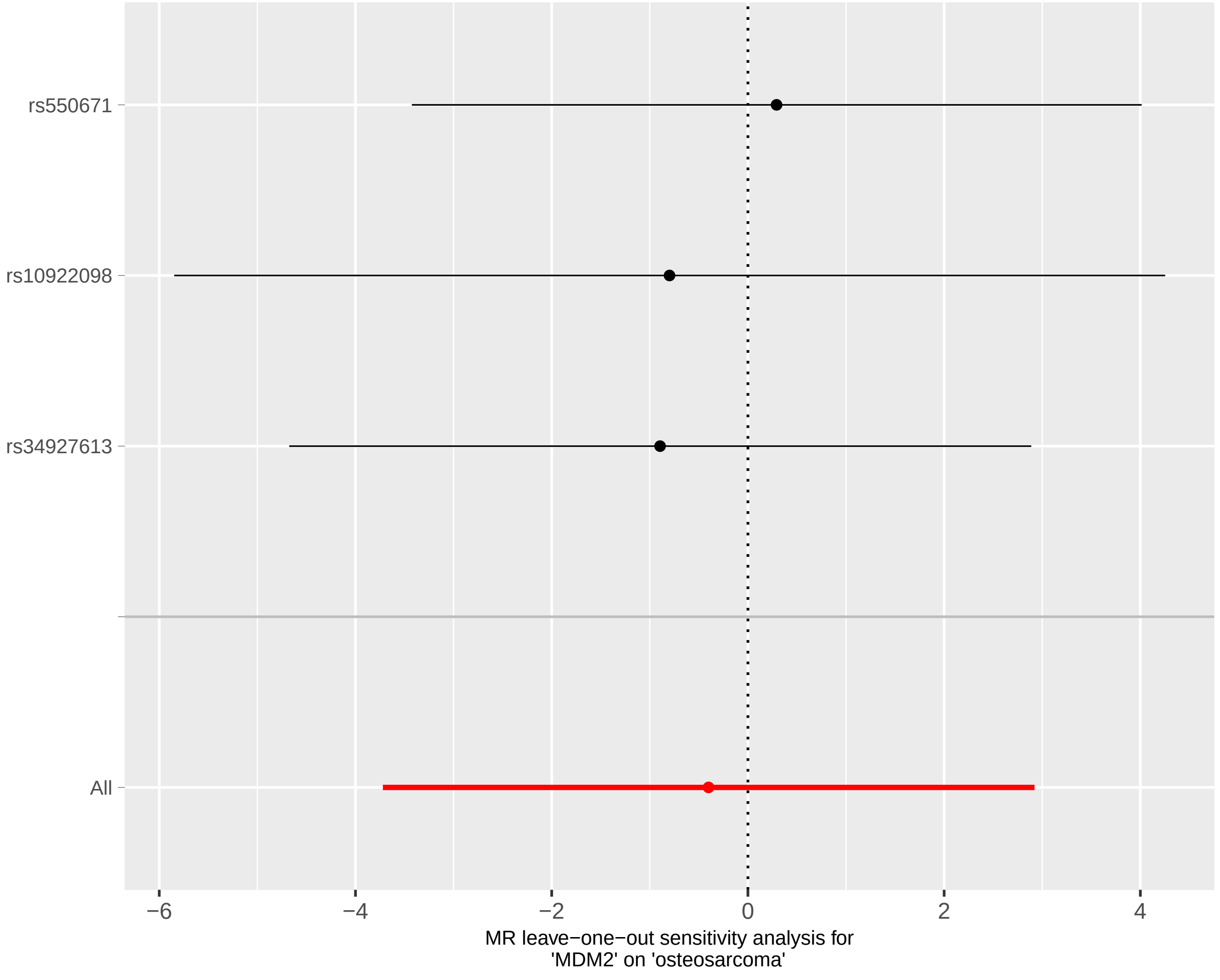
rs550671
rs10922098
rs34927613
All
−6
−4
−2
0
2
4
MR leave−one−out sensitivity analysis for
'MDM2' on 'osteosarcoma'

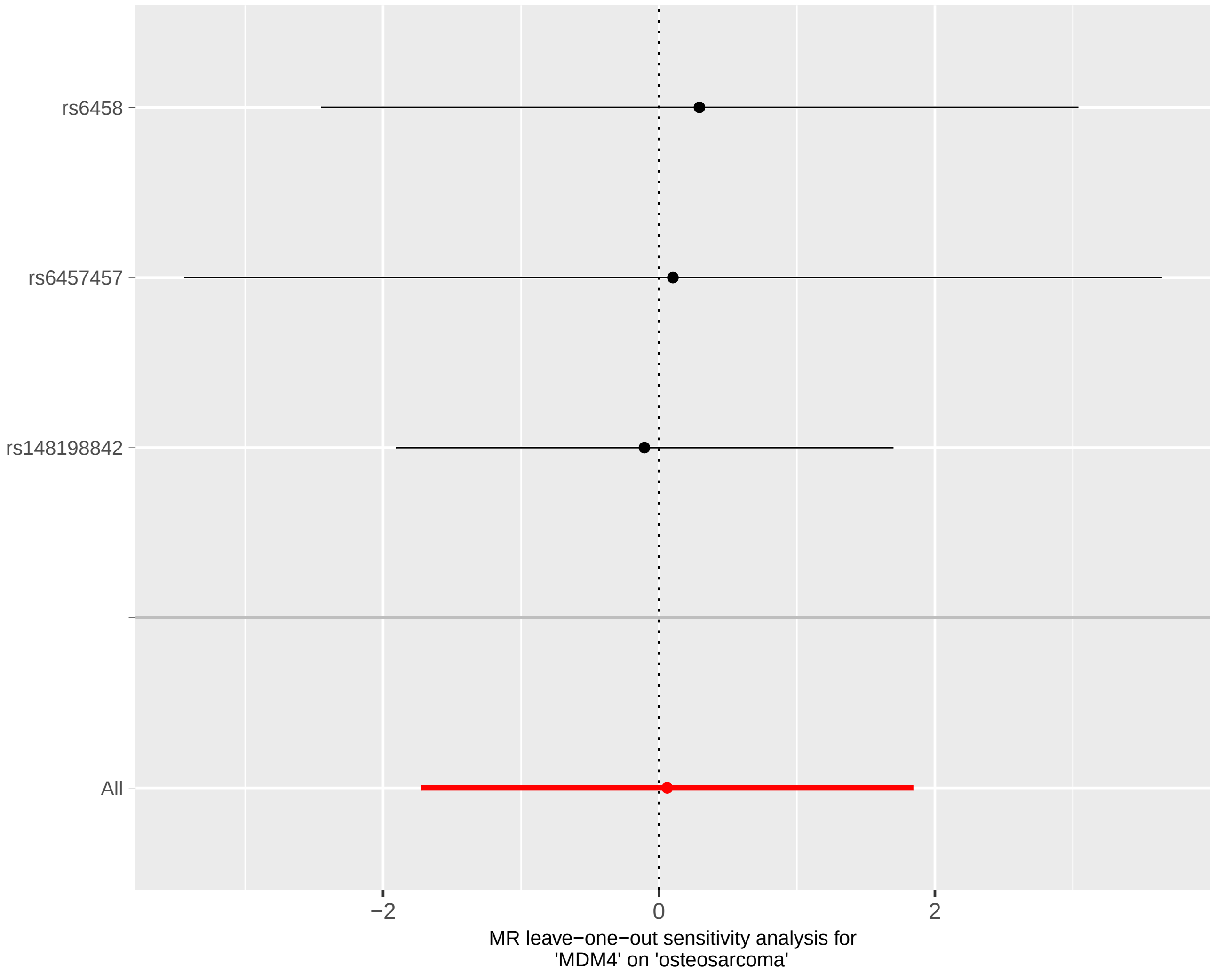
rs6458
rs6457457
rs148198842
All
−2
0
2
MR leave−one−out sensitivity analysis for
'MDM4' on 'osteosarcoma'

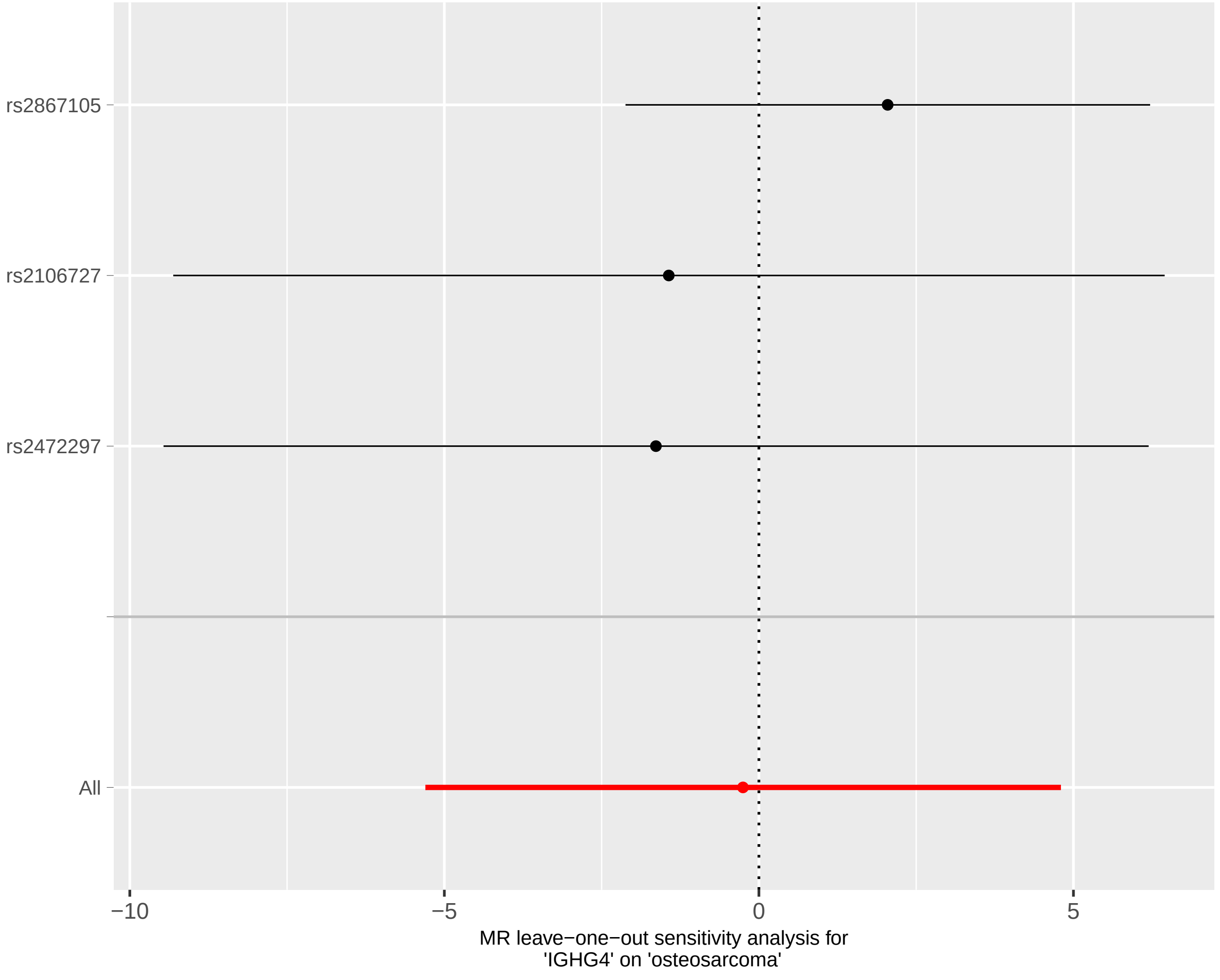
rs2867105
rs2106727
rs2472297
All
−10
−5
0
5
MR leave−one−out sensitivity analysis for
'IGHG4' on 'osteosarcoma'

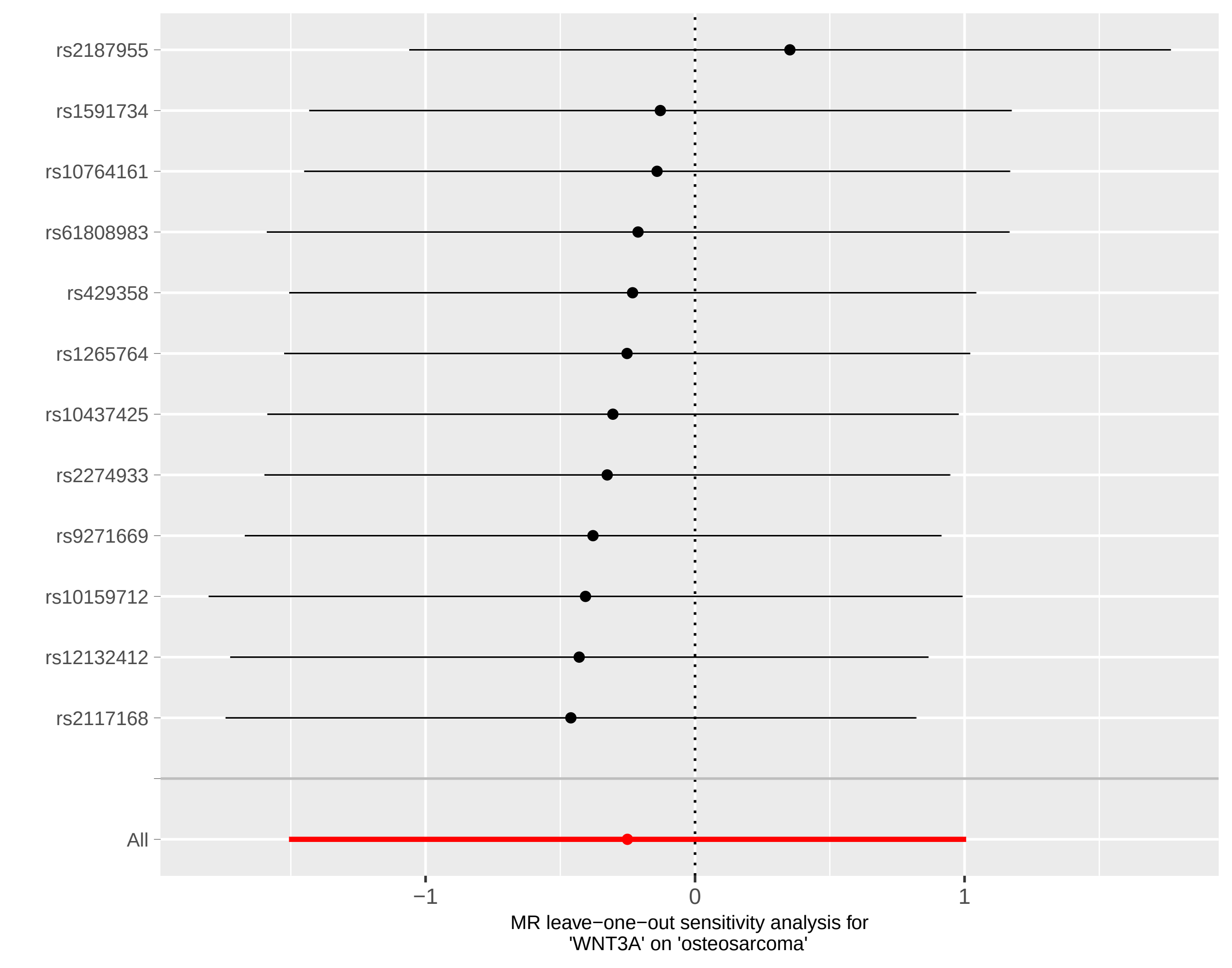

rs2187955
rs1591734
rs10764161
rs61808983
rs429358
rs1265764
rs10437425
rs2274933
rs9271669
rs10159712
rs12132412
rs2117168
All
−1
0
1
MR leave−one−out sensitivity analysis for
'WNT3A' on 'osteosarcoma'

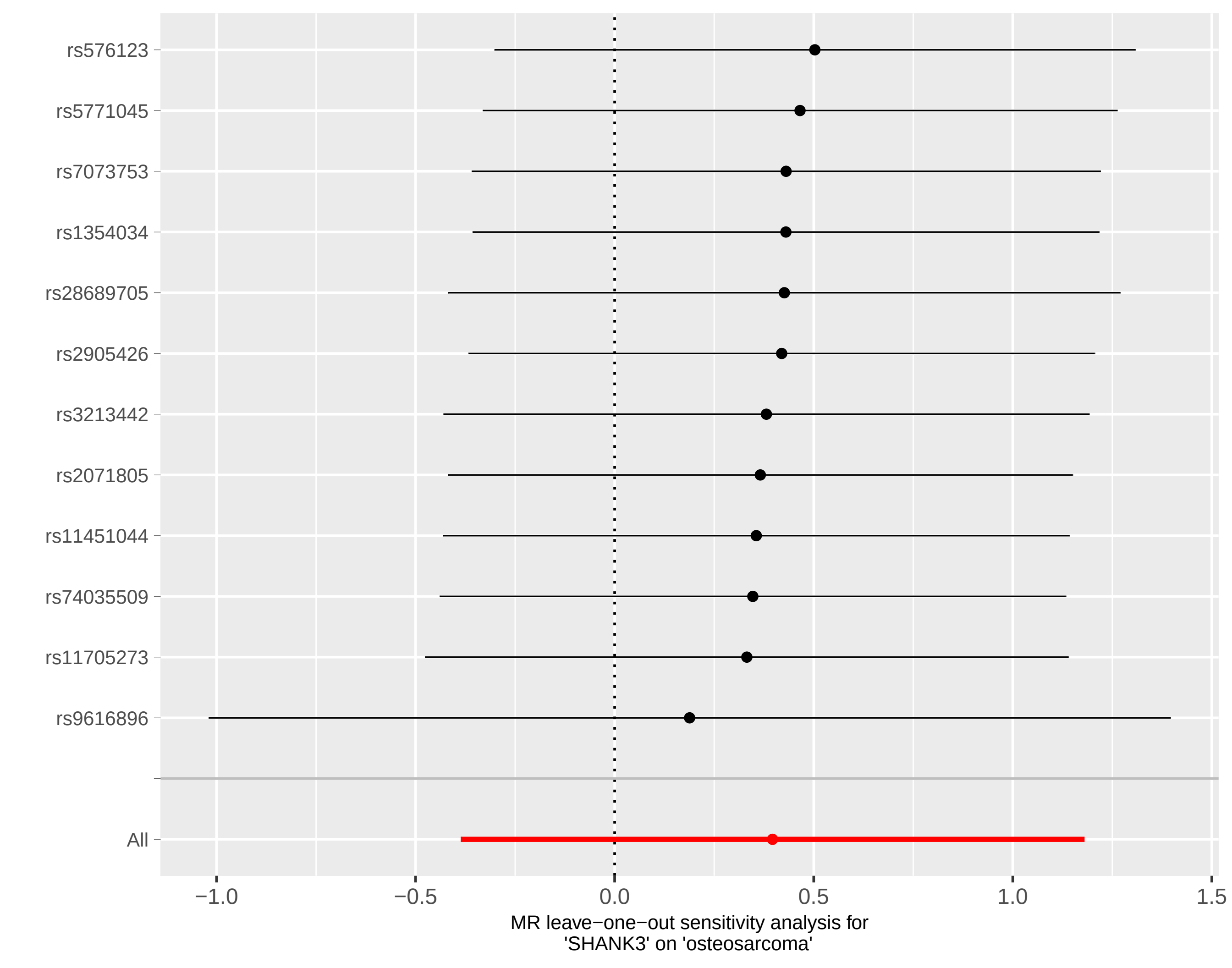

rs576123
rs5771045
rs7073753
rs1354034
rs28689705
rs2905426
rs3213442
rs2071805
rs11451044
rs74035509
rs11705273
rs9616896
All
−1.0
−0.5
0.0
0.5
1.0
1.5
MR leave−one−out sensitivity analysis for
'SHANK3' on 'osteosarcoma'

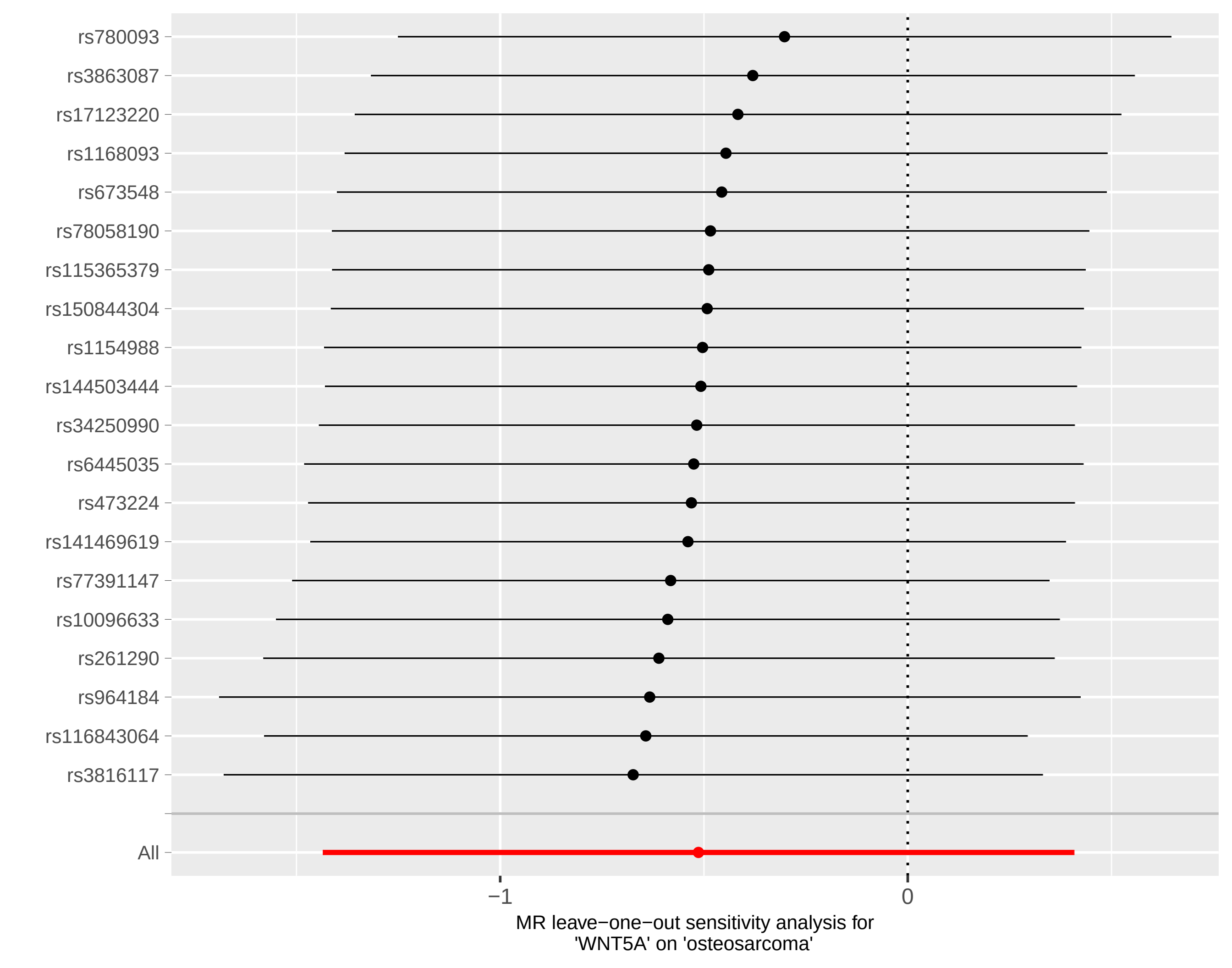

rs780093
rs3863087
rs17123220
rs1168093
rs673548
rs78058190
rs115365379
rs150844304
rs1154988
rs144503444
rs34250990
rs6445035
rs473224
rs141469619
rs77391147
rs10096633
rs261290
rs964184
rs116843064
rs3816117
All
−1
0
MR leave−one−out sensitivity analysis for
'WNT5A' on 'osteosarcoma'

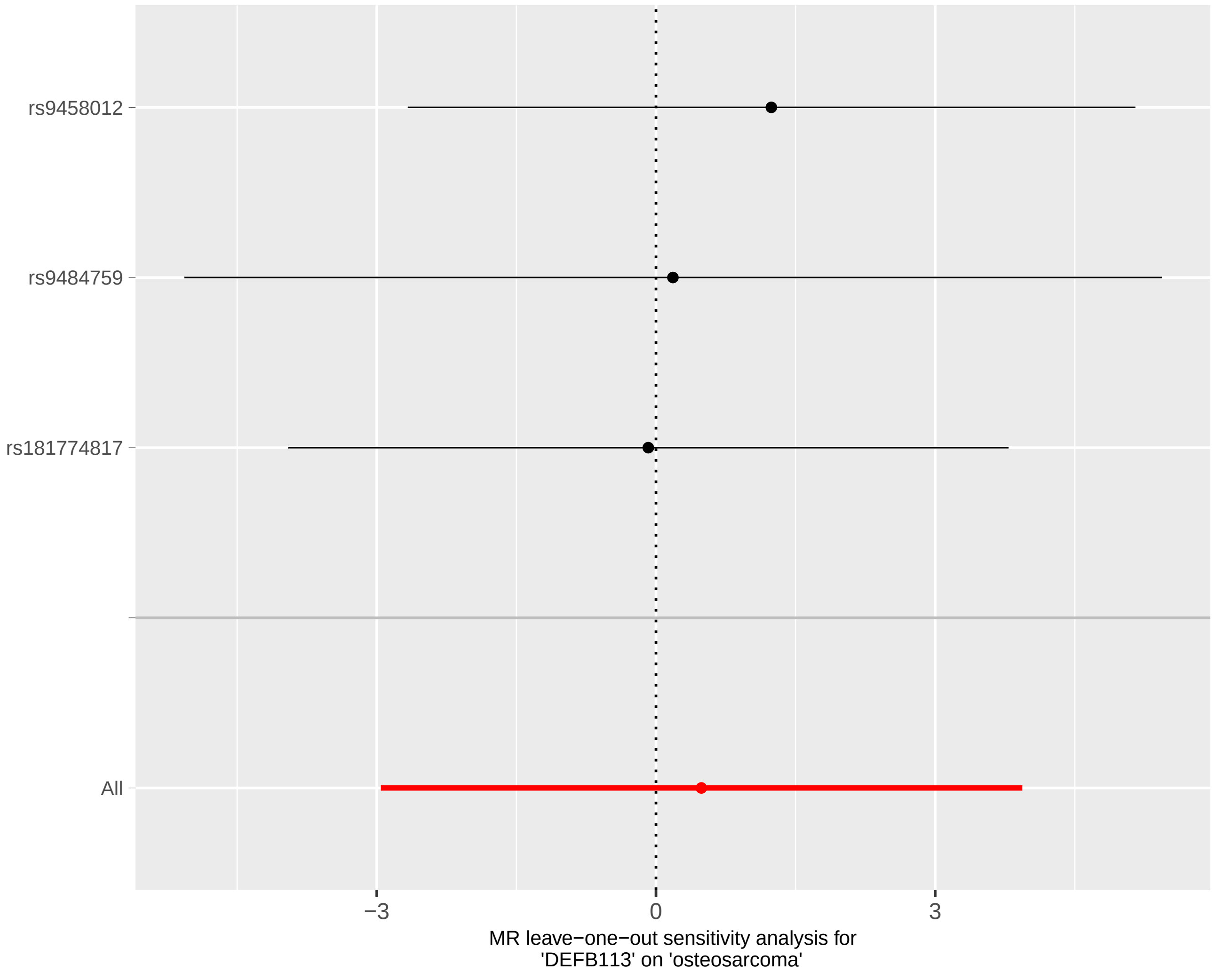
rs9458012
rs9484759
rs181774817
All
−3
0
3
MR leave−one−out sensitivity analysis for
'DEFB113' on 'osteosarcoma'

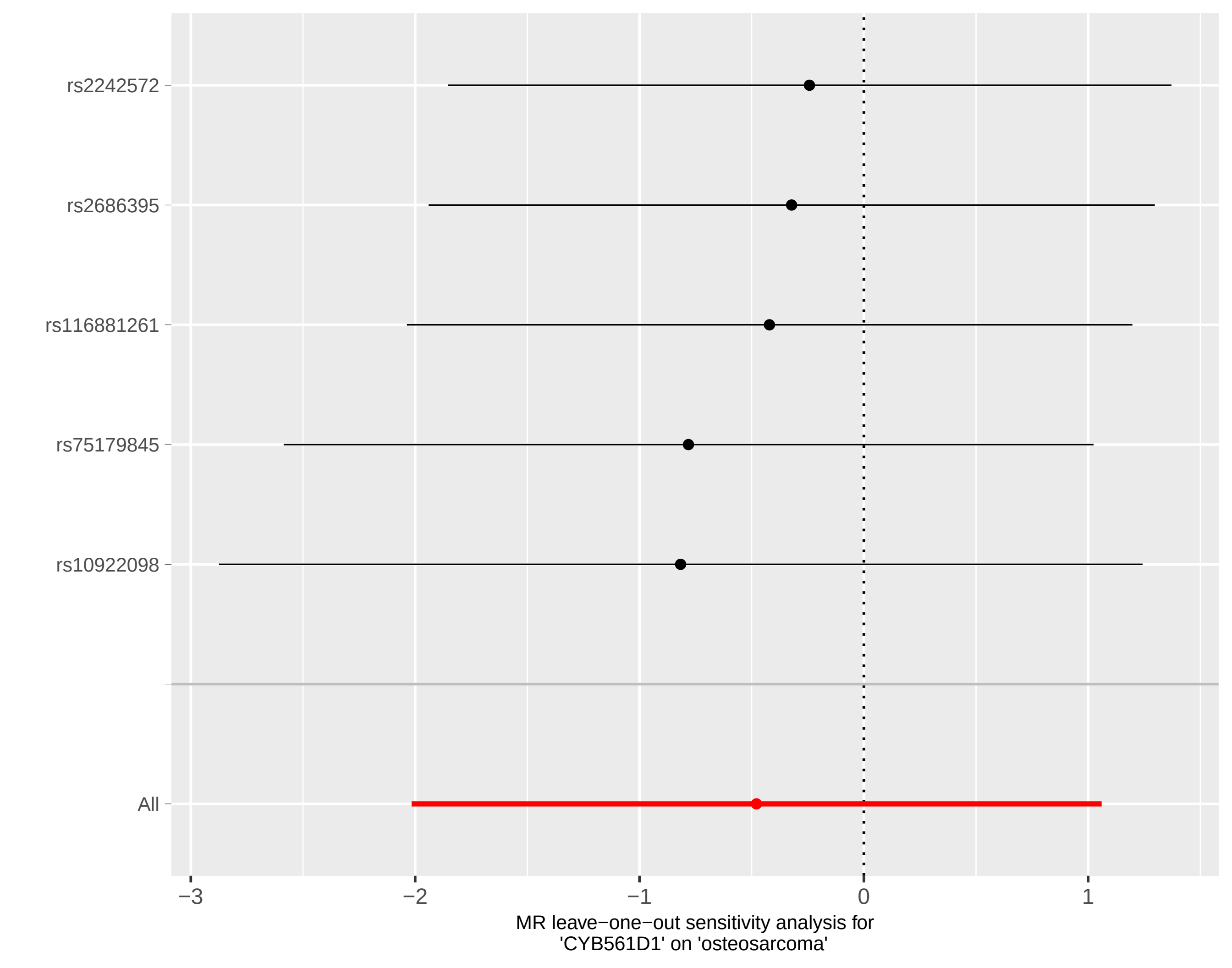
rs2242572
rs2686395
rs116881261
rs75179845
rs10922098
All
−3
−2
−1
0
1
MR leave−one−out sensitivity analysis for
'CYB561D1' on 'osteosarcoma'

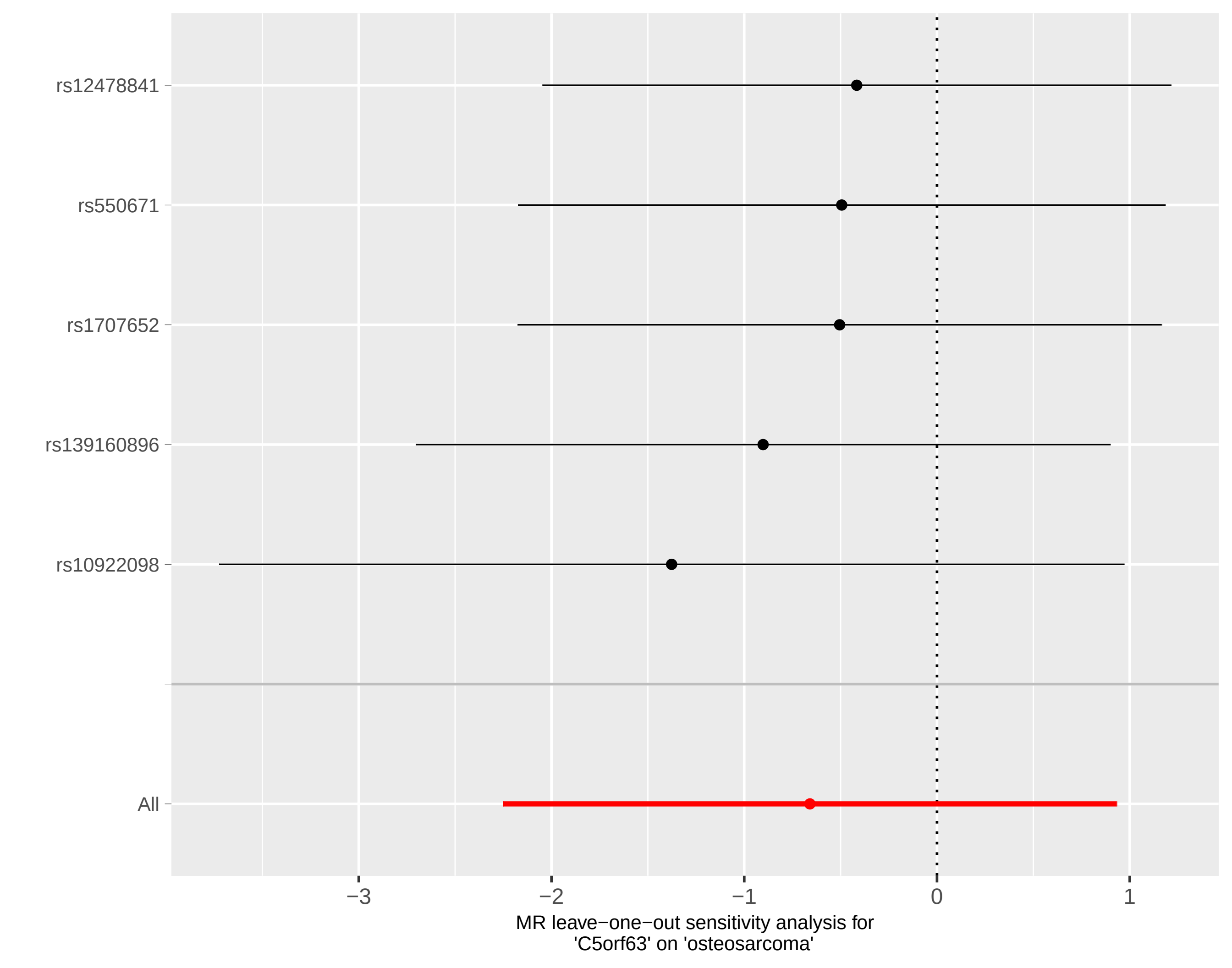

rs12478841
rs550671
rs1707652
rs139160896
rs10922098
All
−3
−2
−1
0
1
MR leave−one−out sensitivity analysis for
'C5orf63' on 'osteosarcoma'

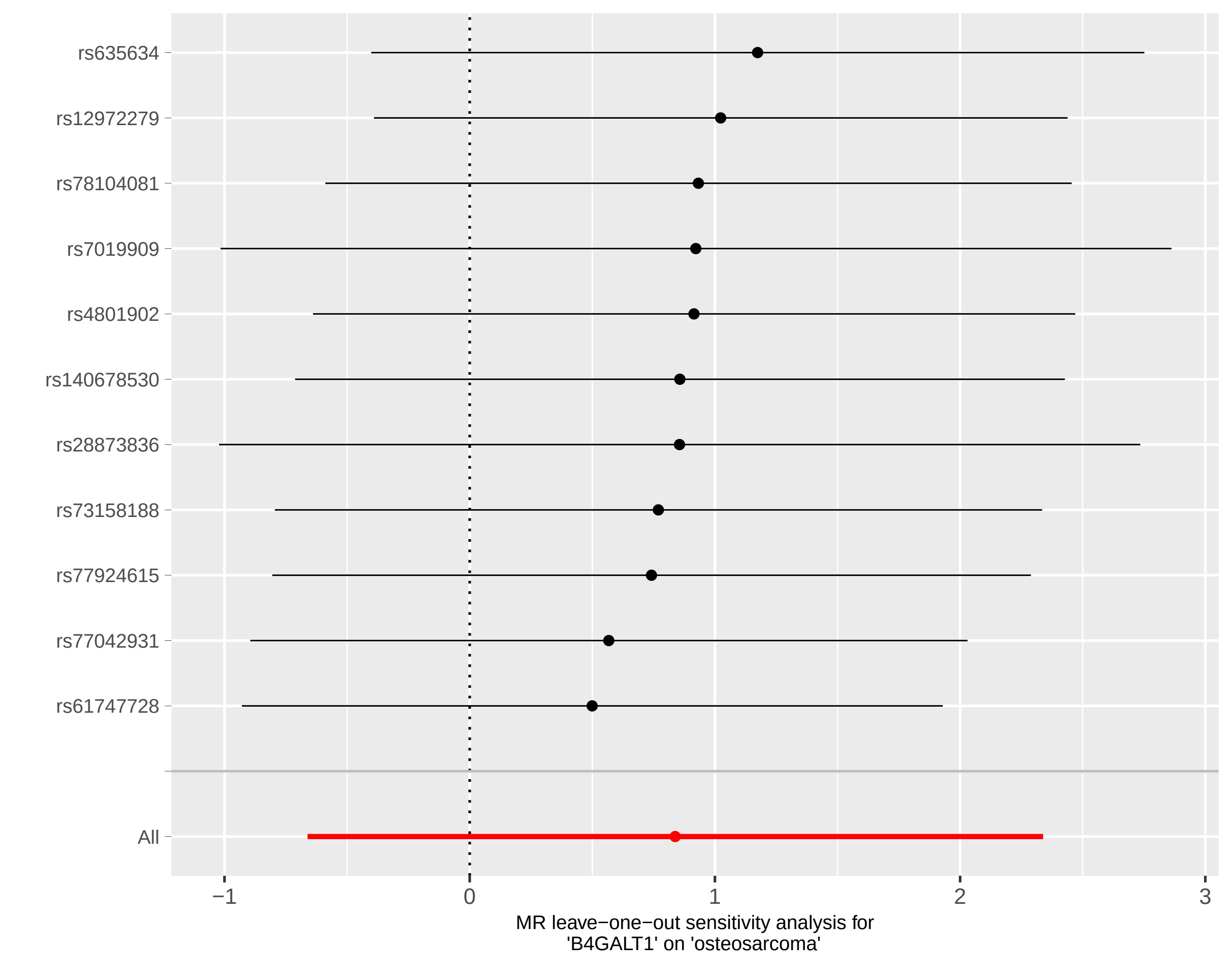
rs635634
rs12972279
rs78104081
rs7019909
rs4801902
rs140678530
rs28873836
rs73158188
rs77924615
rs77042931
rs61747728
All
−1
0
1
2
3
MR leave−one−out sensitivity analysis for
'B4GALT1' on 'osteosarcoma'

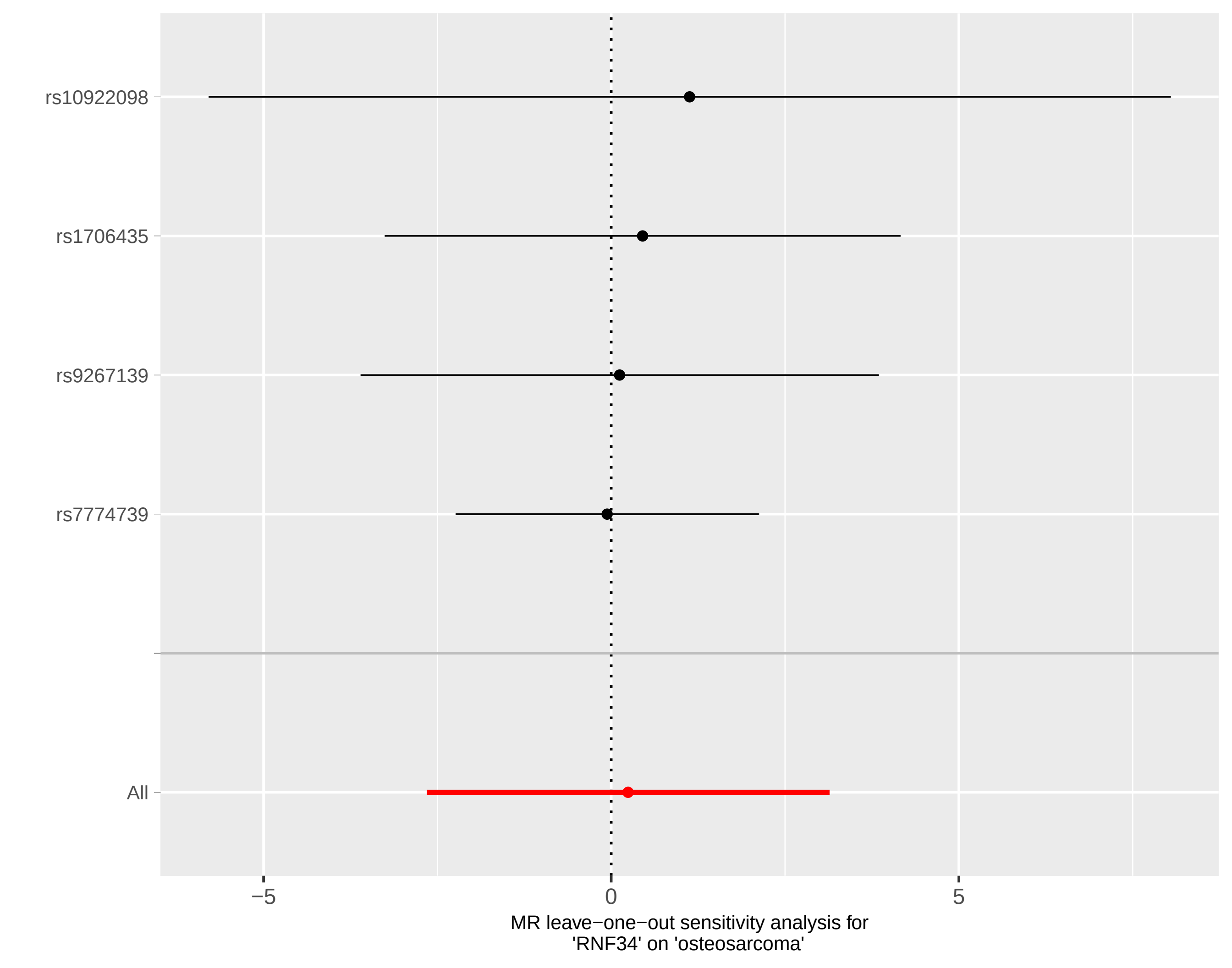

rs10922098
rs1706435
rs9267139
rs7774739
All
−5
0
5
MR leave−one−out sensitivity analysis for
'RNF34' on 'osteosarcoma'

Insufficient number of SNPs

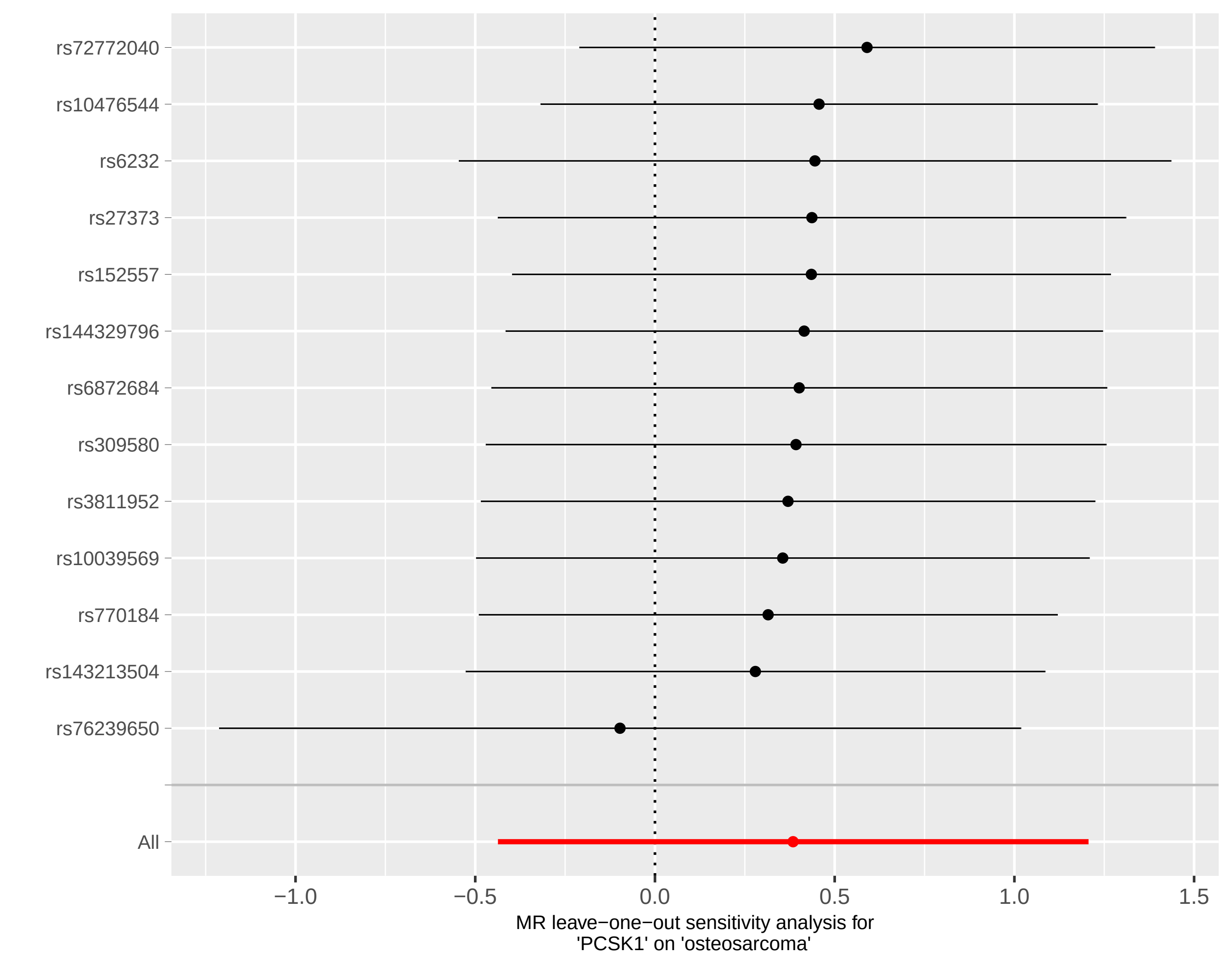

rs72772040
rs10476544
rs6232
rs27373
rs152557
rs144329796
rs6872684
rs309580
rs3811952
rs10039569
rs770184
rs143213504
rs76239650
All
−1.0
−0.5
0.0
0.5
1.0
1.5
MR leave−one−out sensitivity analysis for
'PCSK1' on 'osteosarcoma'

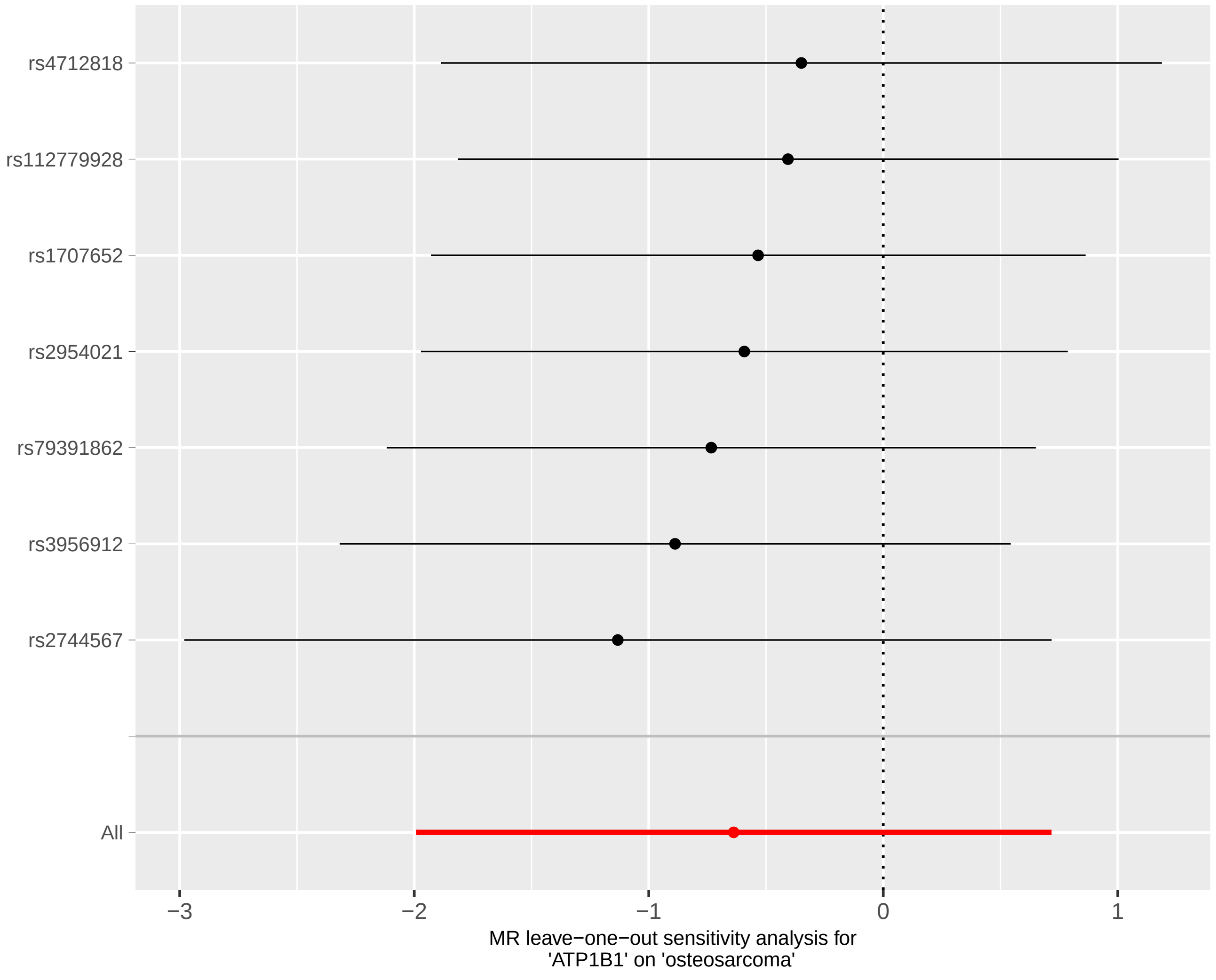

rs4712818
rs112779928
rs1707652
rs2954021
rs79391862
rs3956912
rs2744567
All
−3
−2
−1
0
1
MR leave−one−out sensitivity analysis for
'ATP1B1' on 'osteosarcoma'

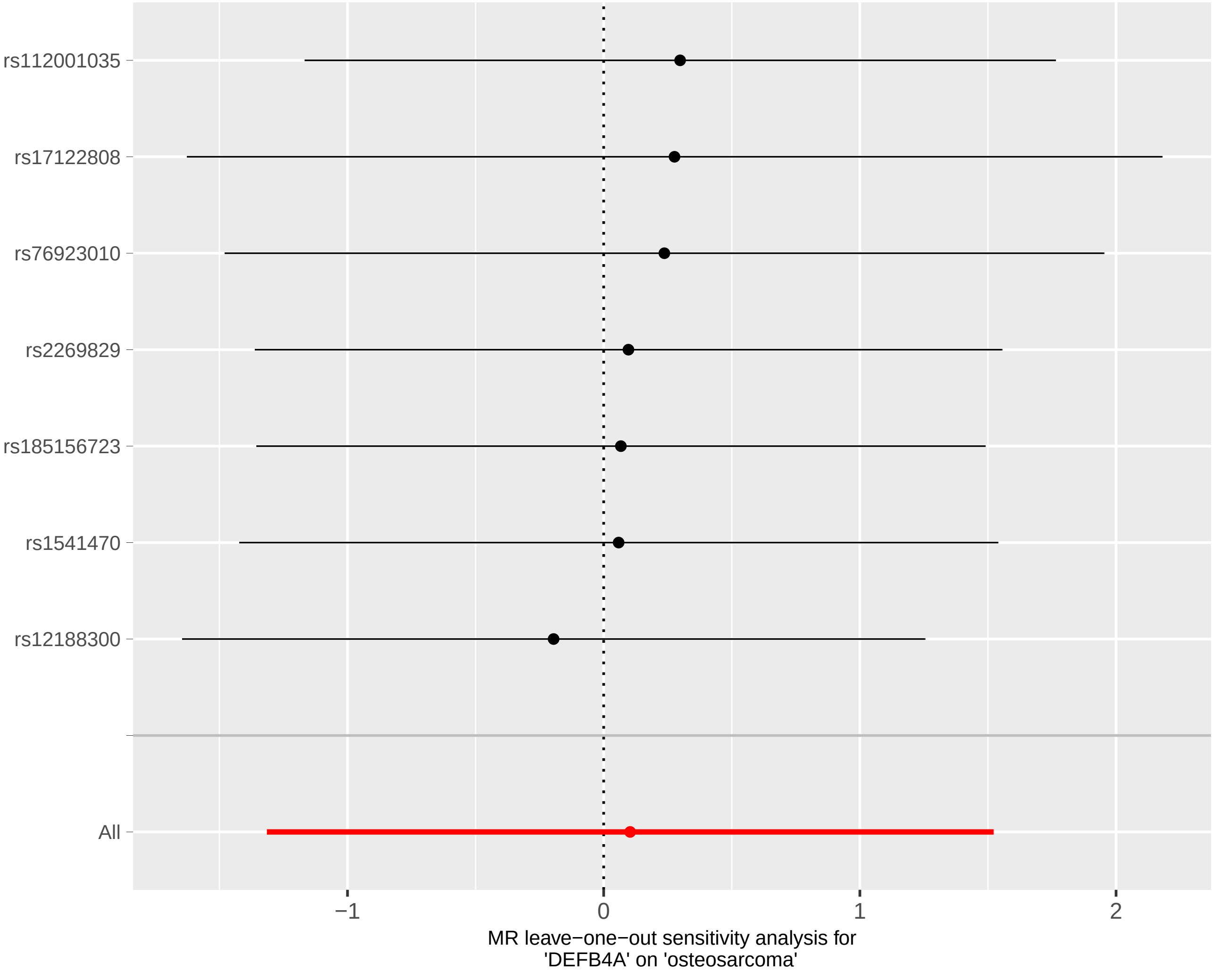
rs112001035
rs17122808
rs76923010
rs2269829
rs185156723
rs1541470
rs12188300
All
−1
0
1
2
MR leave−one−out sensitivity analysis for
'DEFB4A' on 'osteosarcoma'

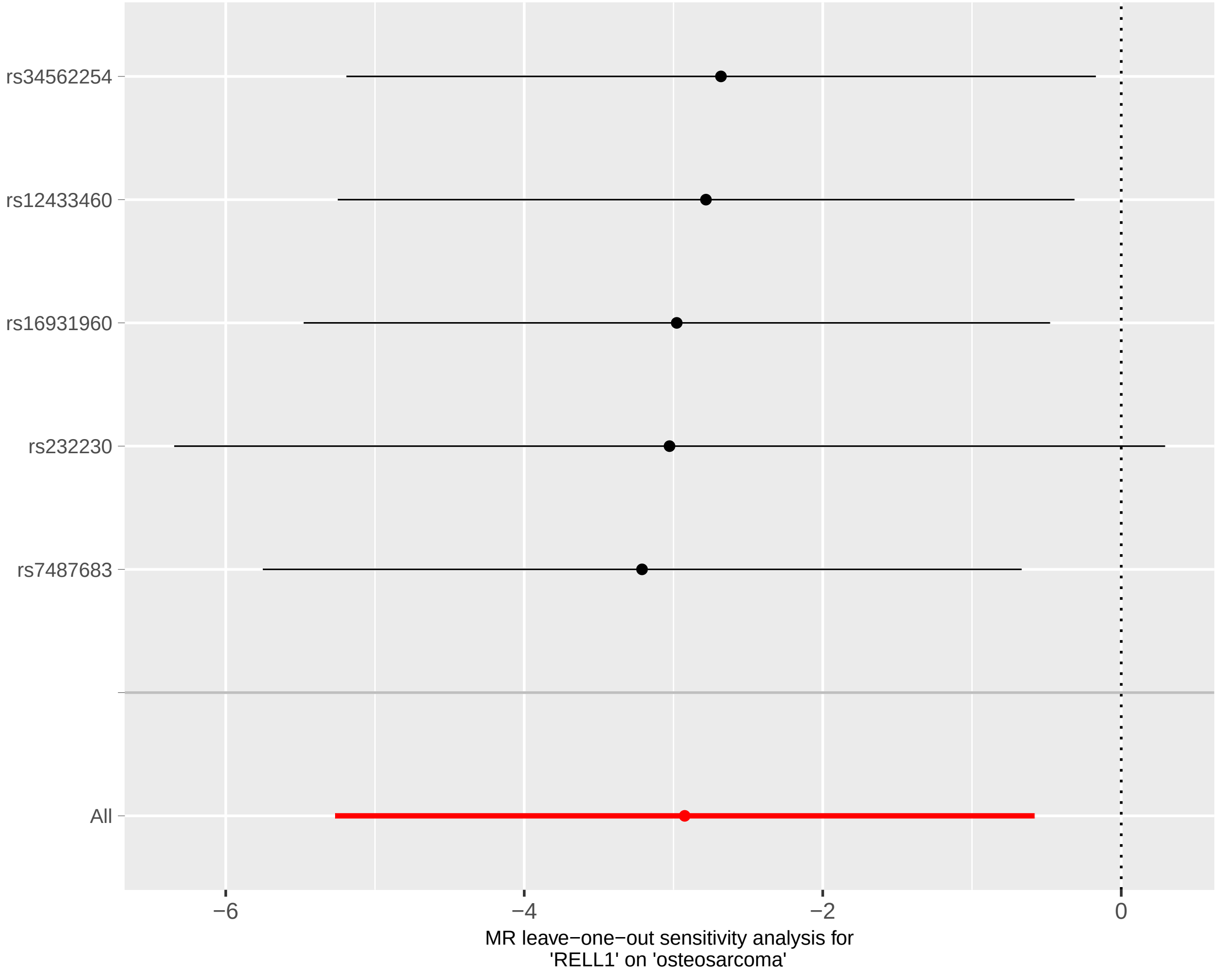
rs34562254
rs12433460
rs16931960
rs232230
rs7487683
All
−6
−4
−2
0
MR leave−one−out sensitivity analysis for
'RELL1' on 'osteosarcoma'

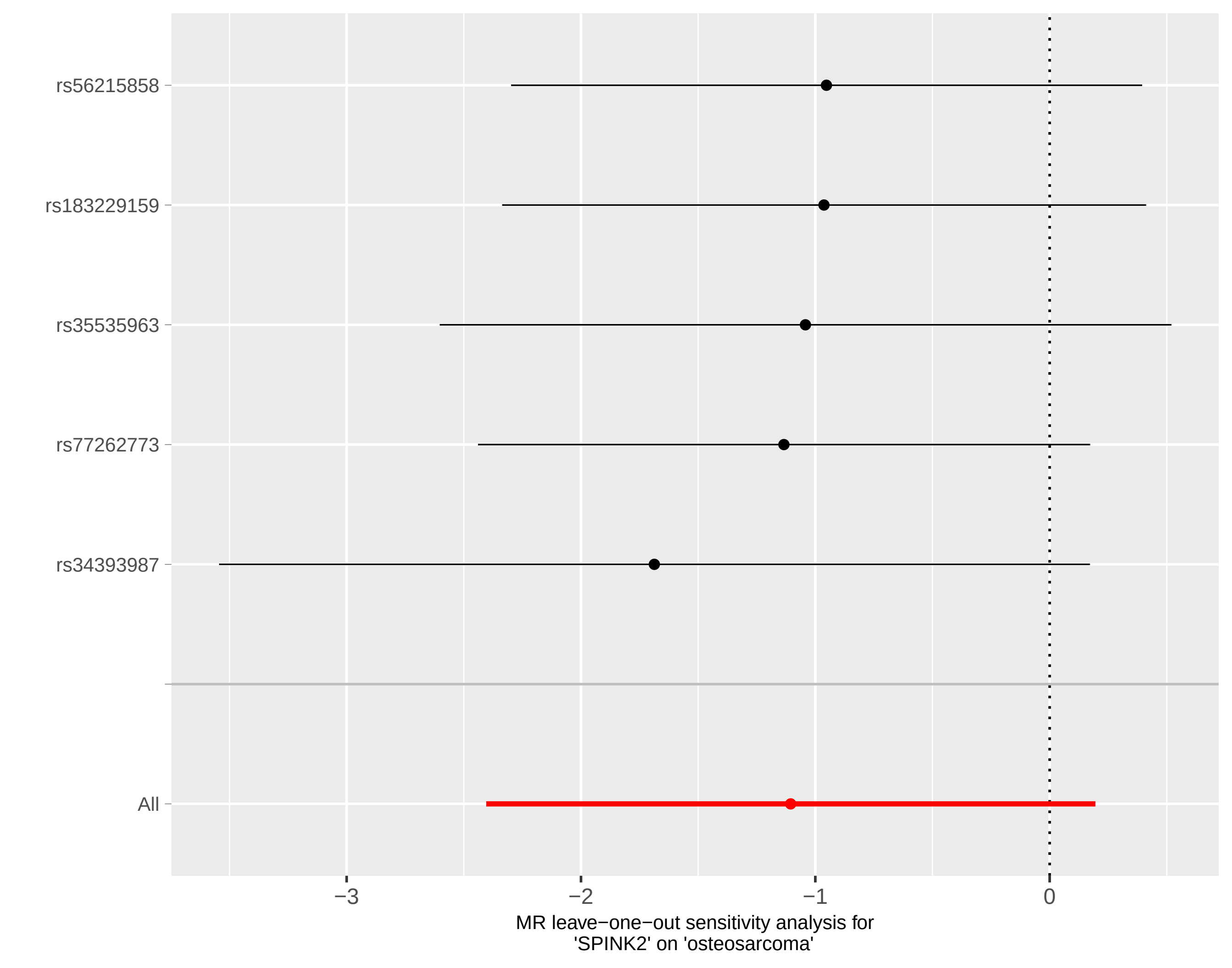
rs56215858
rs183229159
rs35535963
rs77262773
rs34393987
All
−3
−2
−1
0
MR leave−one−out sensitivity analysis for
'SPINK2' on 'osteosarcoma'

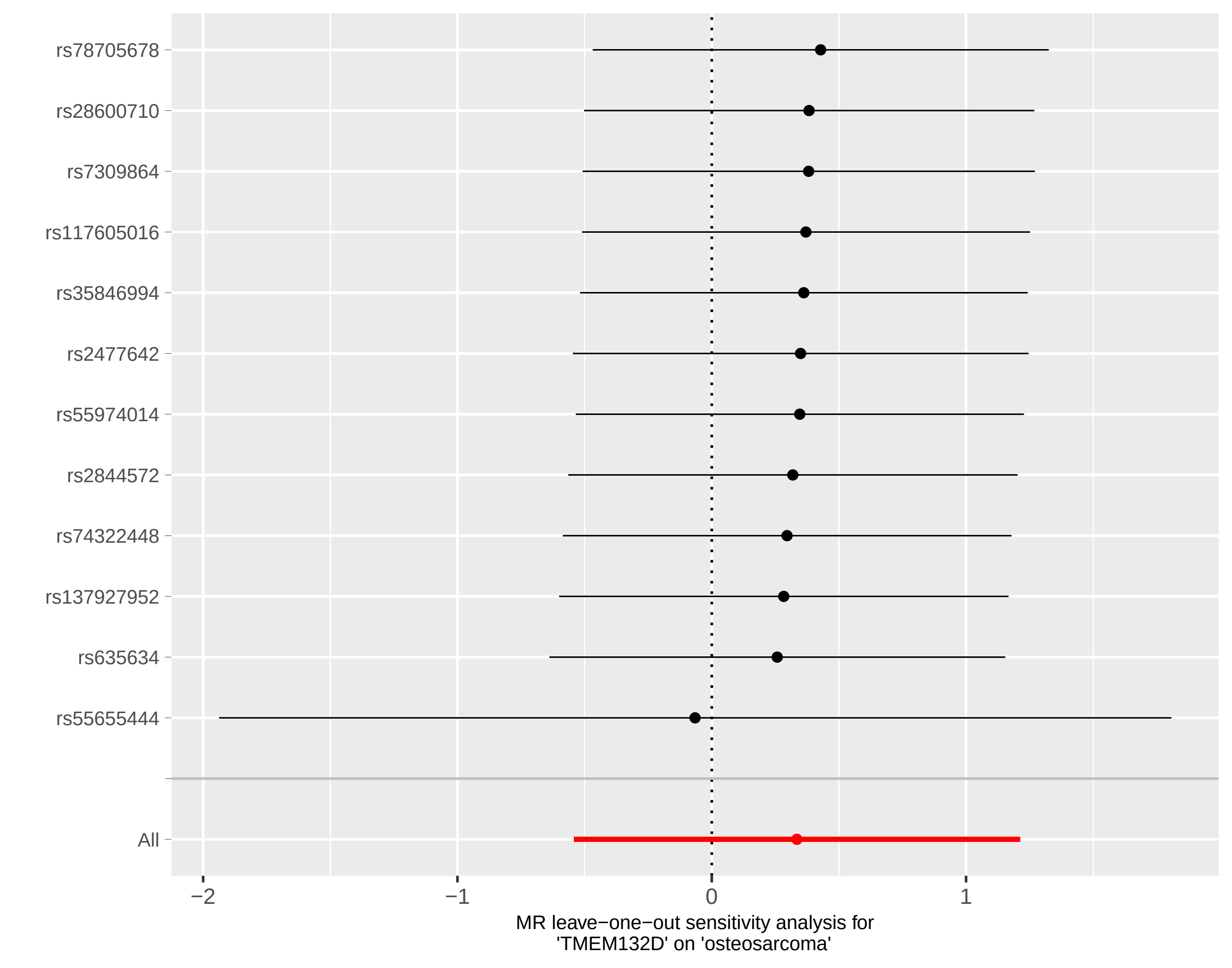
rs78705678
rs28600710
rs7309864
rs117605016
rs35846994
rs2477642
rs55974014
rs2844572
rs74322448
rs137927952
rs635634
rs55655444
All
−2
−1
0
1
MR leave−one−out sensitivity analysis for
'TMEM132D' on 'osteosarcoma'

Insufficient number of SNPs

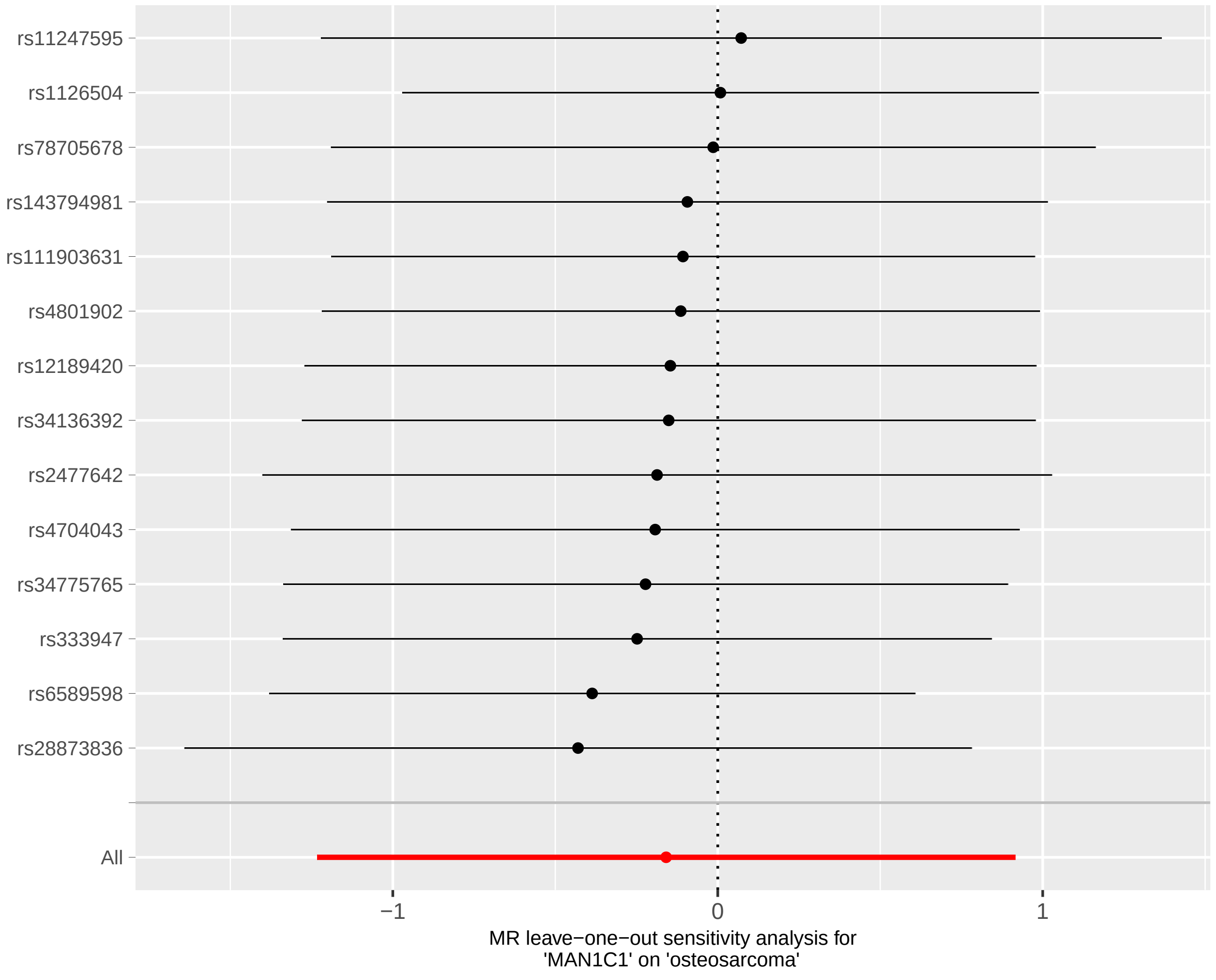
rs11247595
rs1126504
rs78705678
rs143794981
rs111903631
rs4801902
rs12189420
rs34136392
rs2477642
rs4704043
rs34775765
rs333947
rs6589598
rs28873836
All
−1
0
1
MR leave−one−out sensitivity analysis for
'MAN1C1' on 'osteosarcoma'

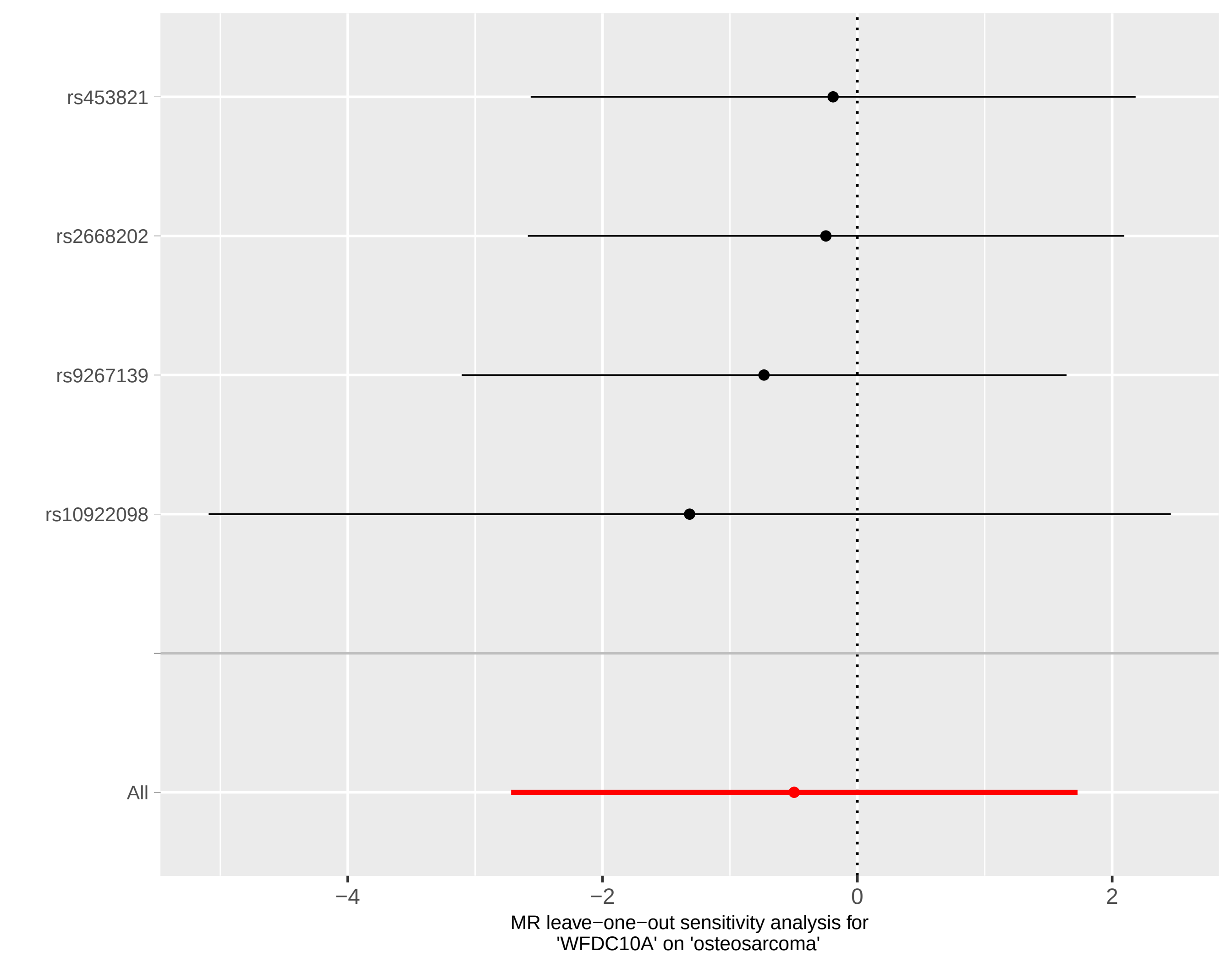

rs453821
rs2668202
rs9267139
rs10922098
All
−4
−2
0
2
MR leave−one−out sensitivity analysis for
'WFDC10A' on 'osteosarcoma'

Insufficient number of SNPs

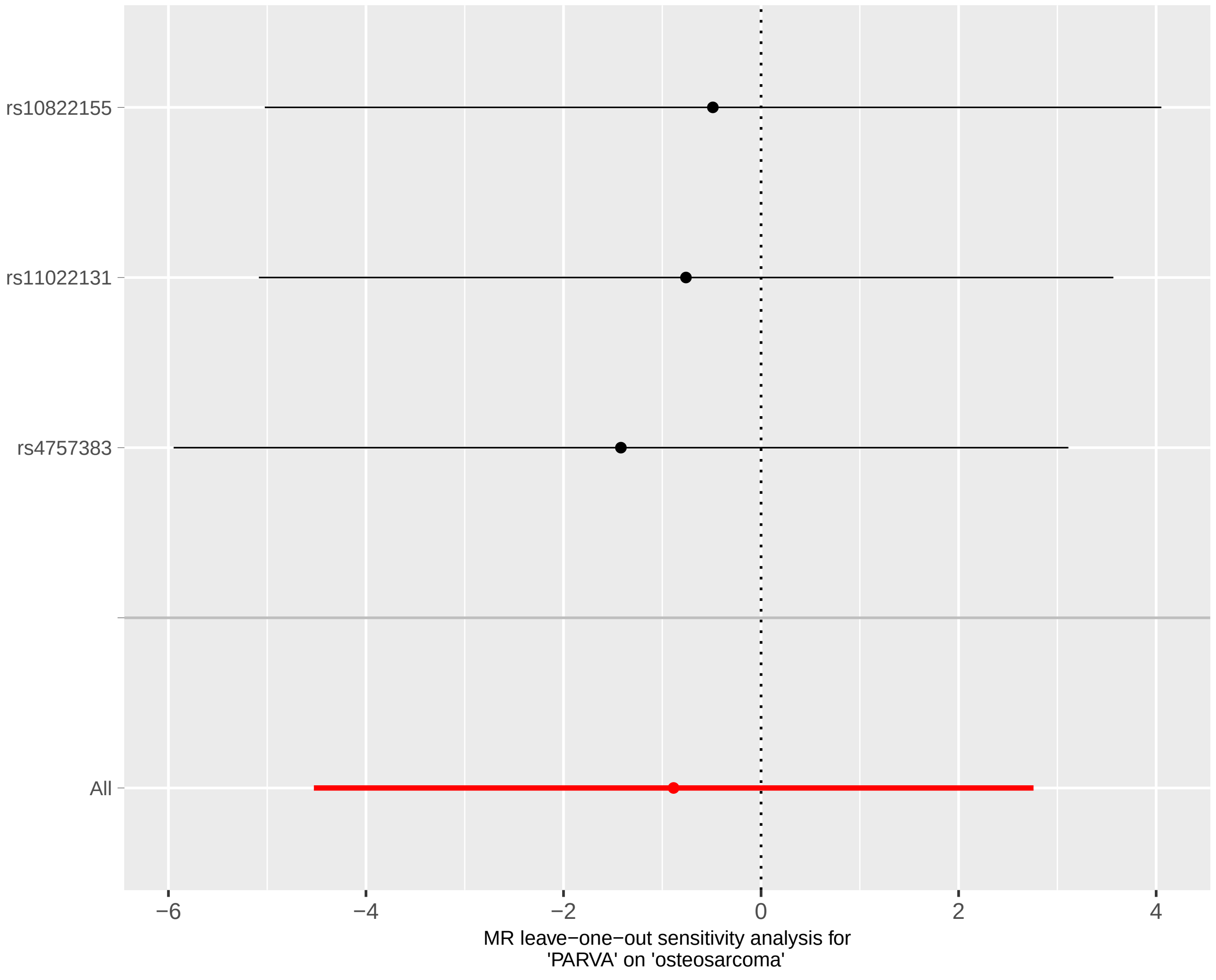

rs10822155
rs11022131
rs4757383
All
−6
−4
−2
0
2
4
MR leave−one−out sensitivity analysis for
'PARVA' on 'osteosarcoma'

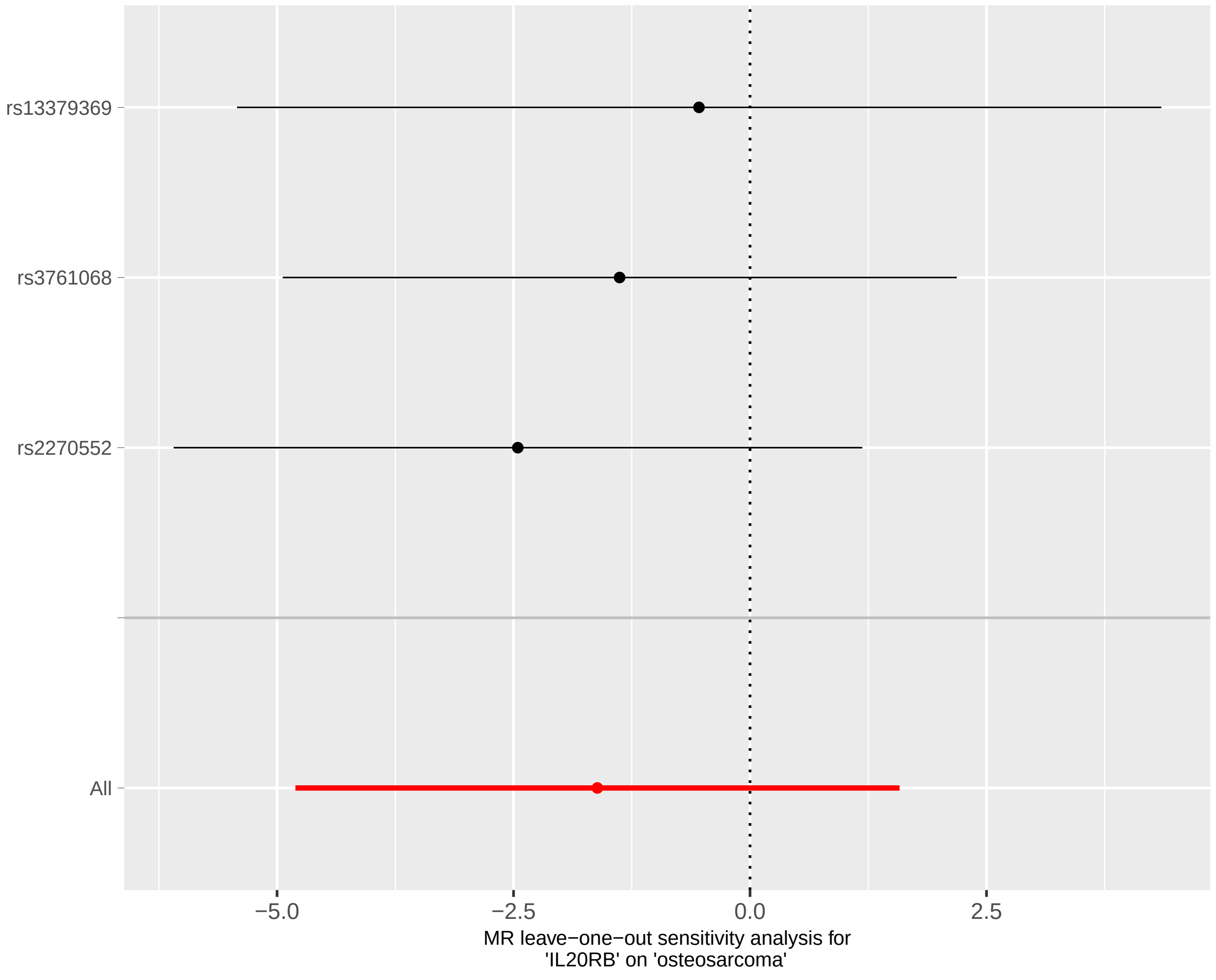

rs13379369
rs3761068
rs2270552
All
−5.0
−2.5
0.0
2.5
MR leave−one−out sensitivity analysis for
'IL20RB' on 'osteosarcoma'

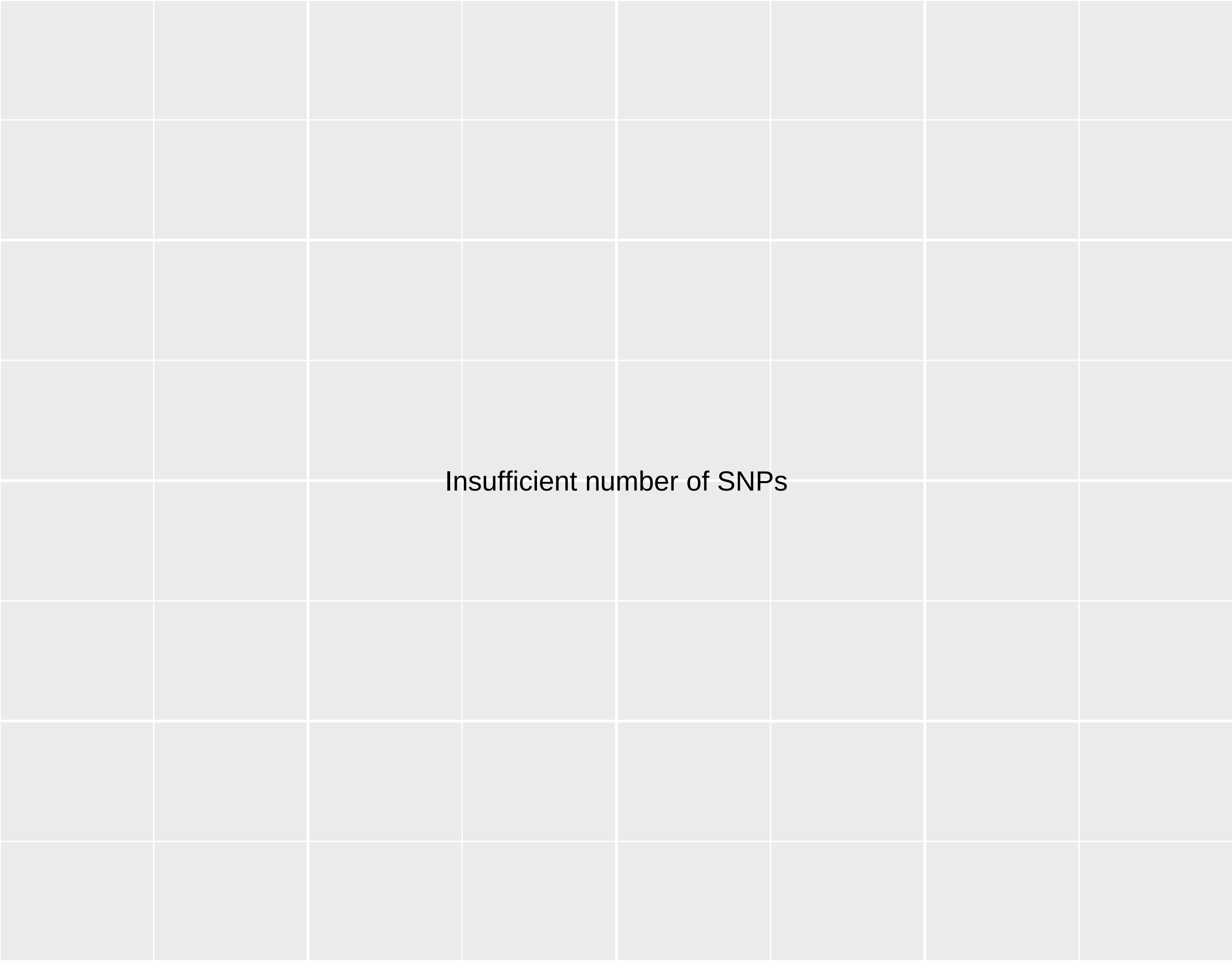
Insufficient number of SNPs

Insufficient number of SNPs

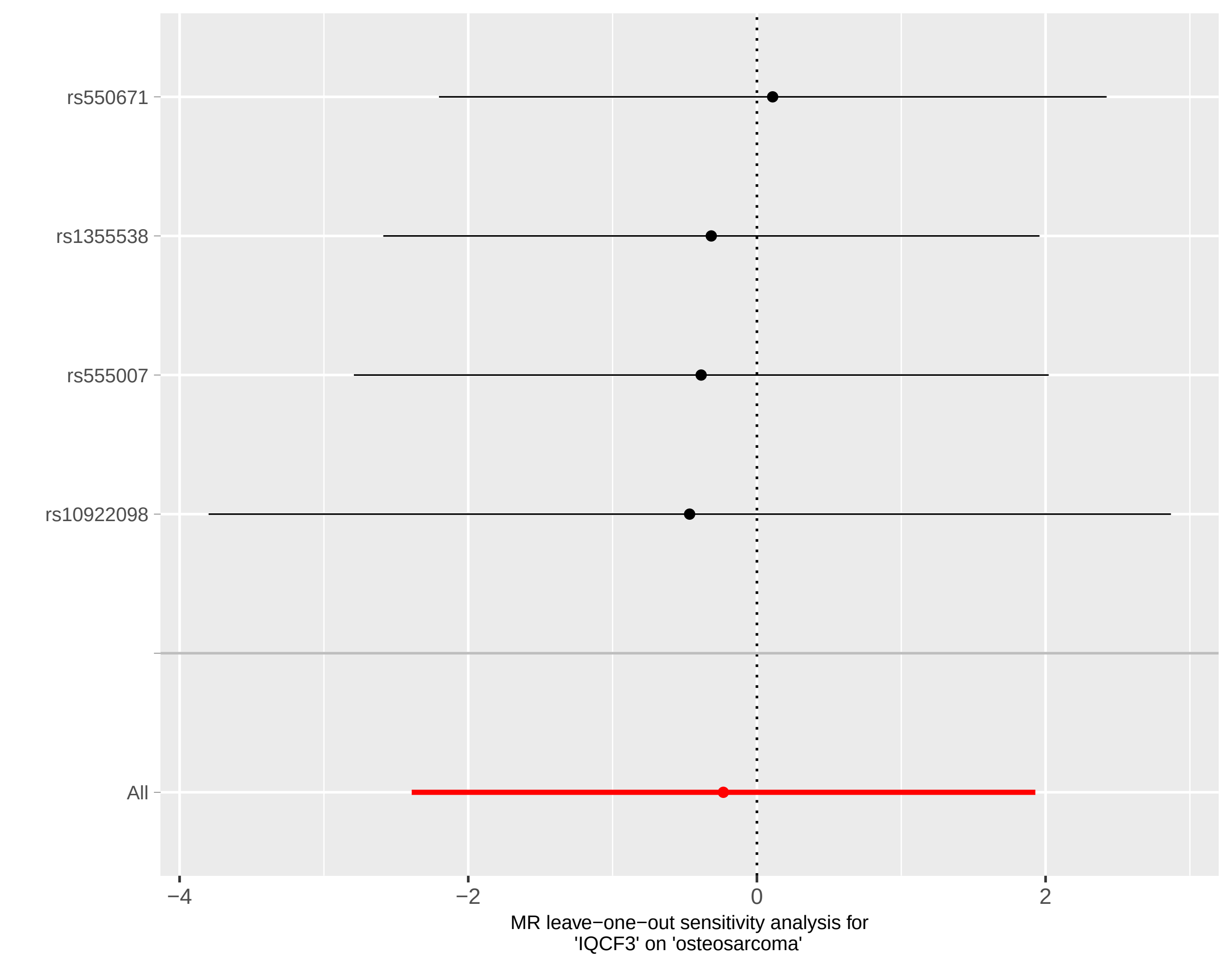

rs550671
rs1355538
rs555007
rs10922098
All
−4
−2
0
2
MR leave−one−out sensitivity analysis for
'IQCF3' on 'osteosarcoma'

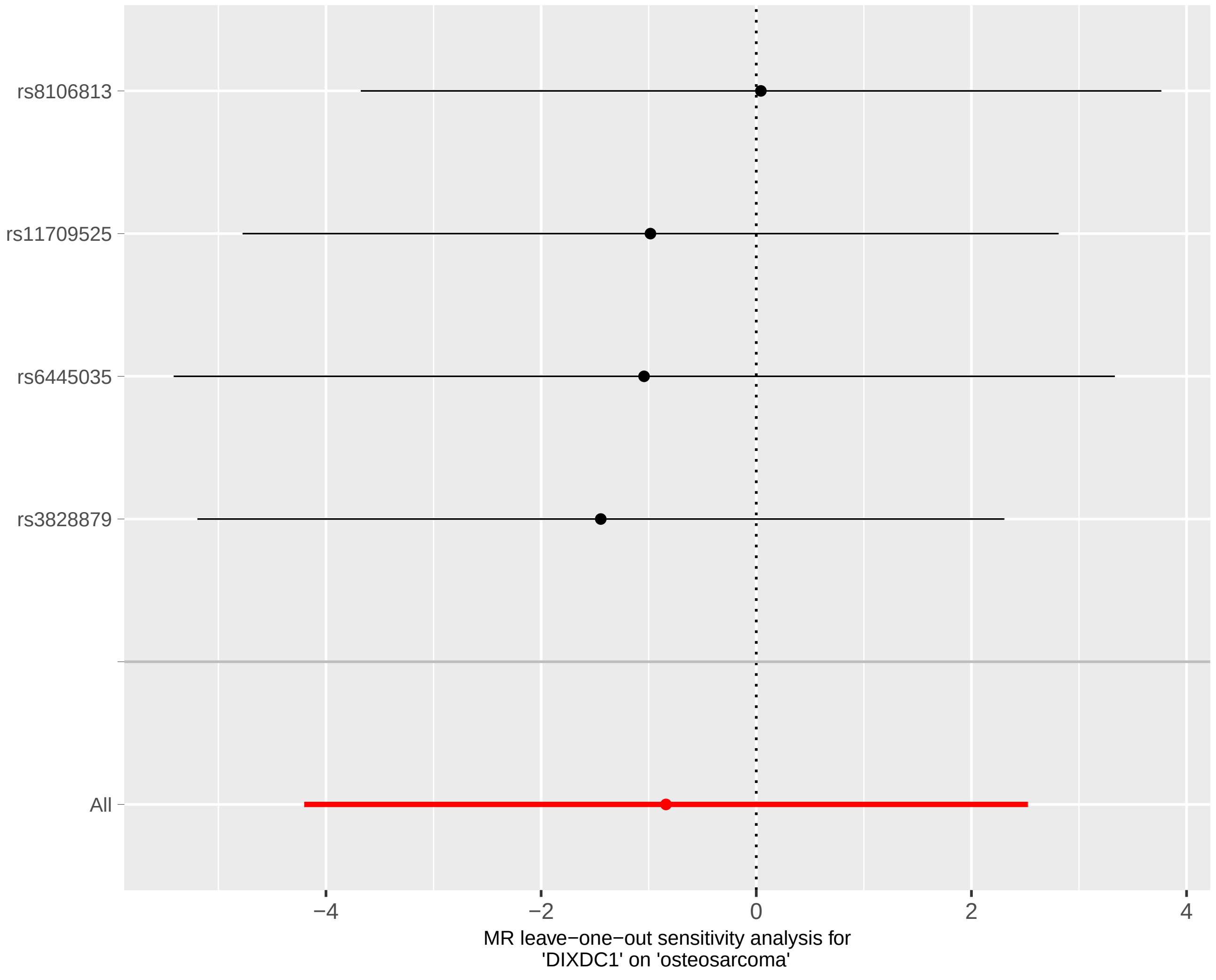
rs8106813
rs11709525
rs6445035
rs3828879
All
−4
−2
0
2
4
MR leave−one−out sensitivity analysis for
'DIXDC1' on 'osteosarcoma'

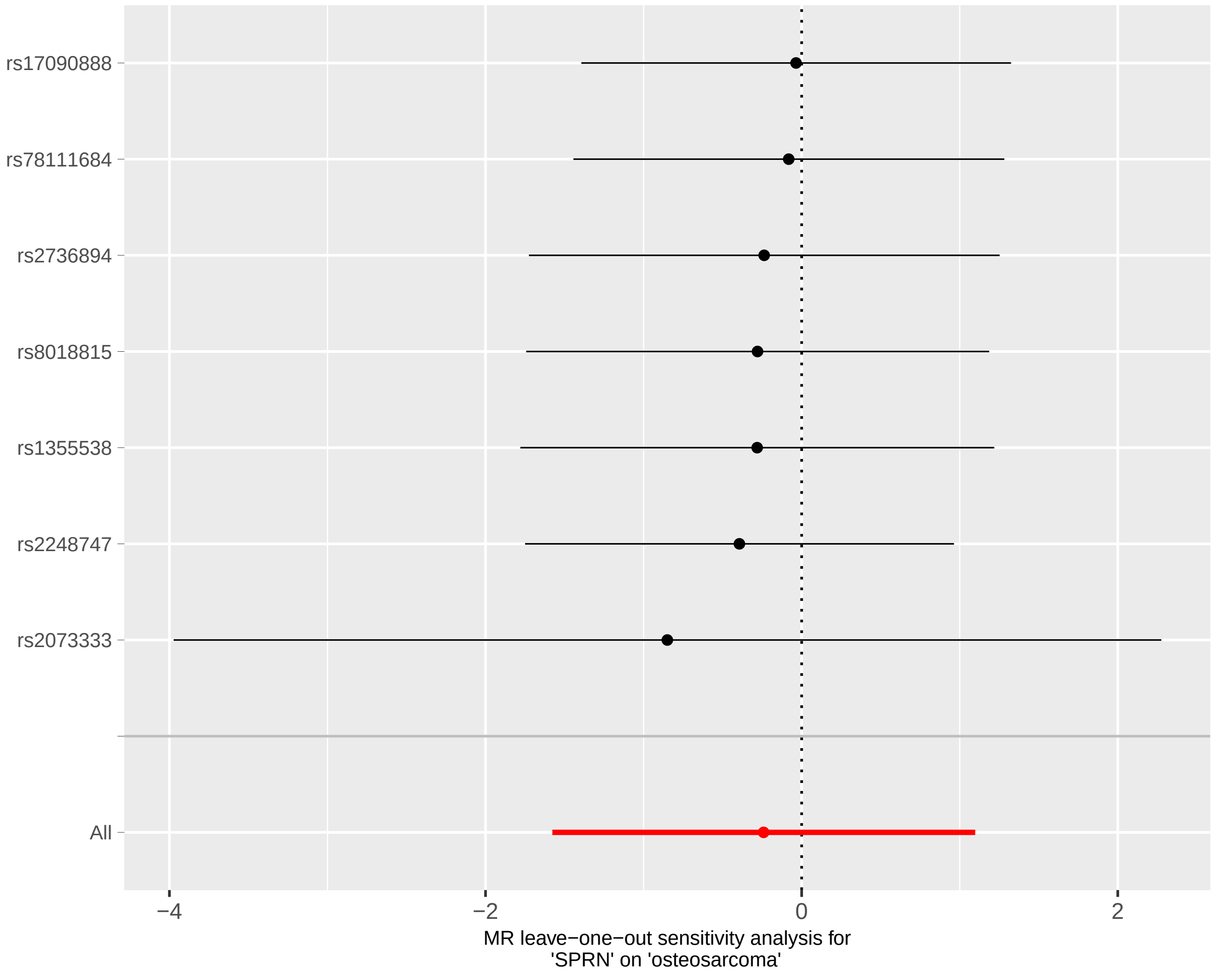
rs17090888
rs78111684
rs2736894
rs8018815
rs1355538
rs2248747
rs2073333
All
−4
−2
0
2
MR leave−one−out sensitivity analysis for
'SPRN' on 'osteosarcoma'

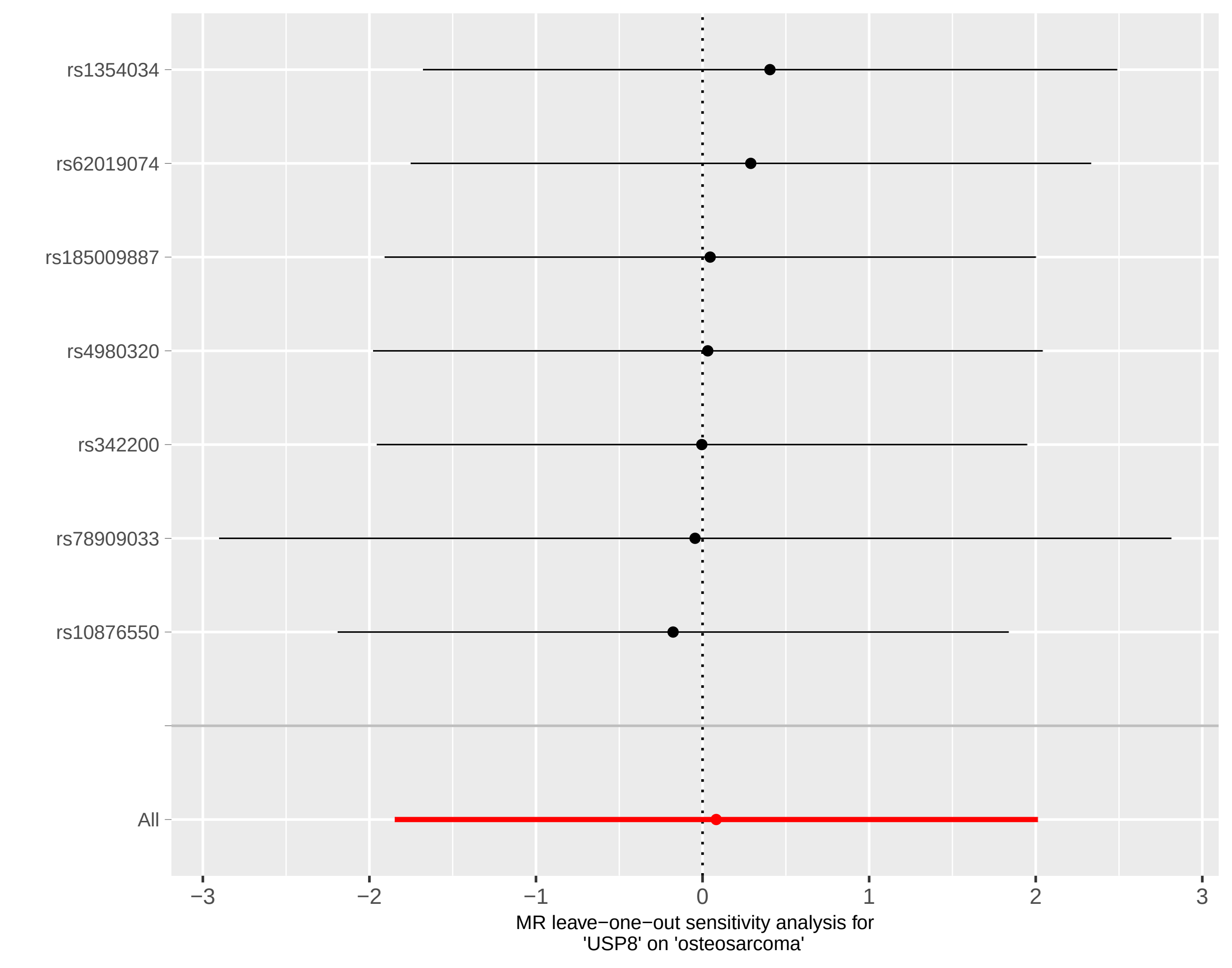

rs1354034
rs62019074
rs185009887
rs4980320
rs342200
rs78909033
rs10876550
All
−3
−2
−1
0
1
2
3
MR leave−one−out sensitivity analysis for
'USP8' on 'osteosarcoma'

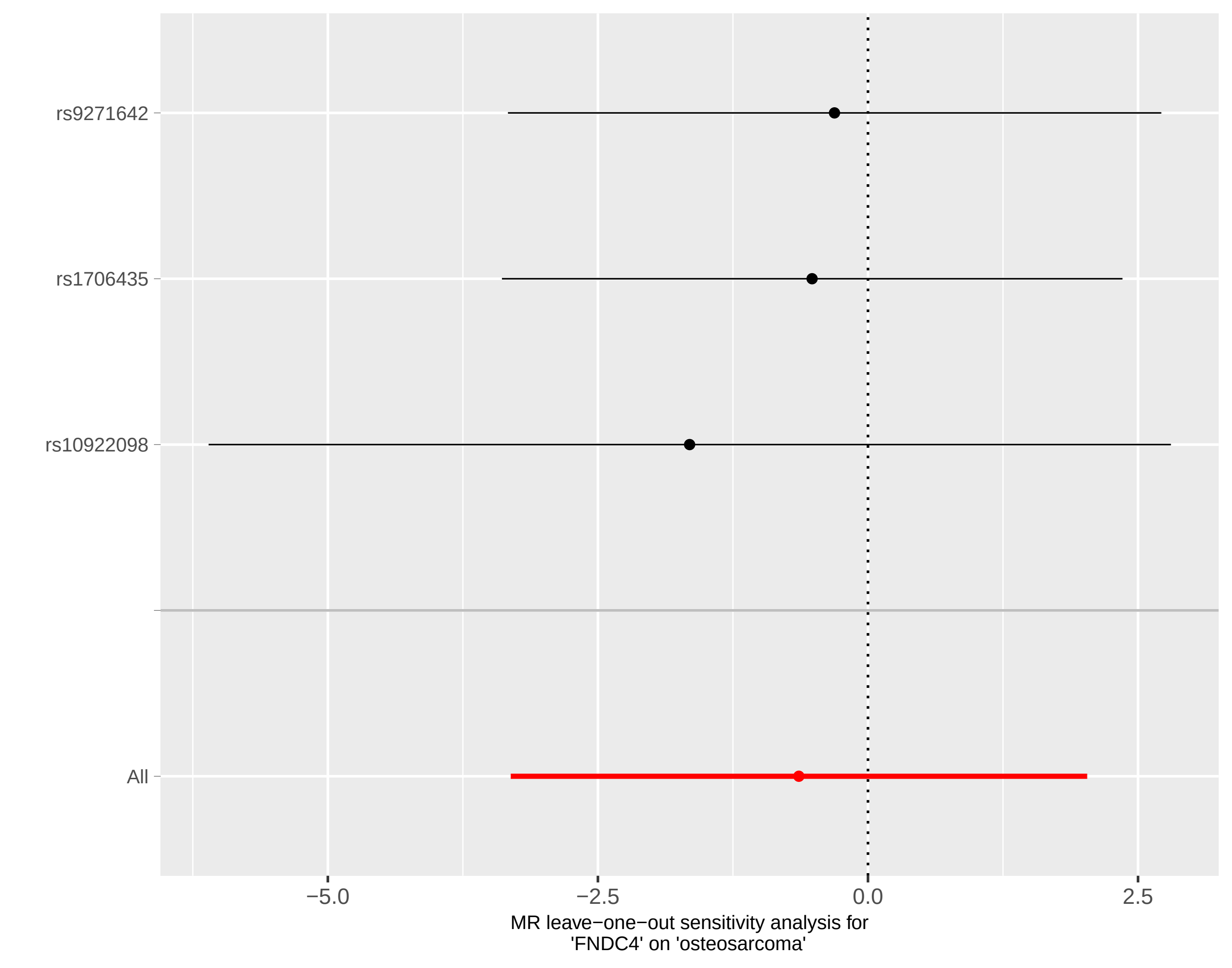

rs9271642
rs1706435
rs10922098
All
−5.0
−2.5
0.0
2.5
MR leave−one−out sensitivity analysis for
'FNDC4' on 'osteosarcoma'

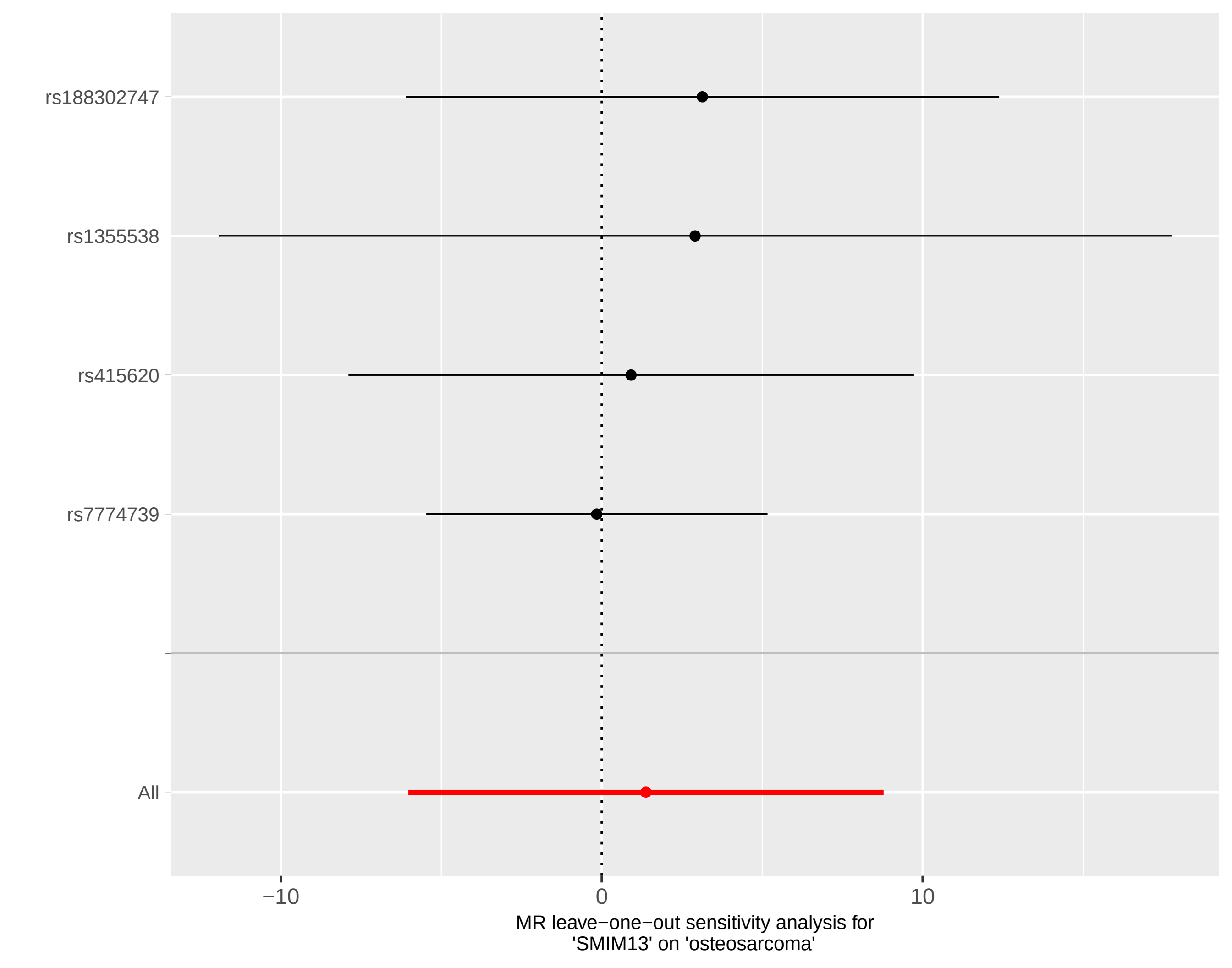
rs188302747
rs1355538
rs415620
rs7774739
All
−10
0
10
MR leave−one−out sensitivity analysis for
'SMIM13' on 'osteosarcoma'

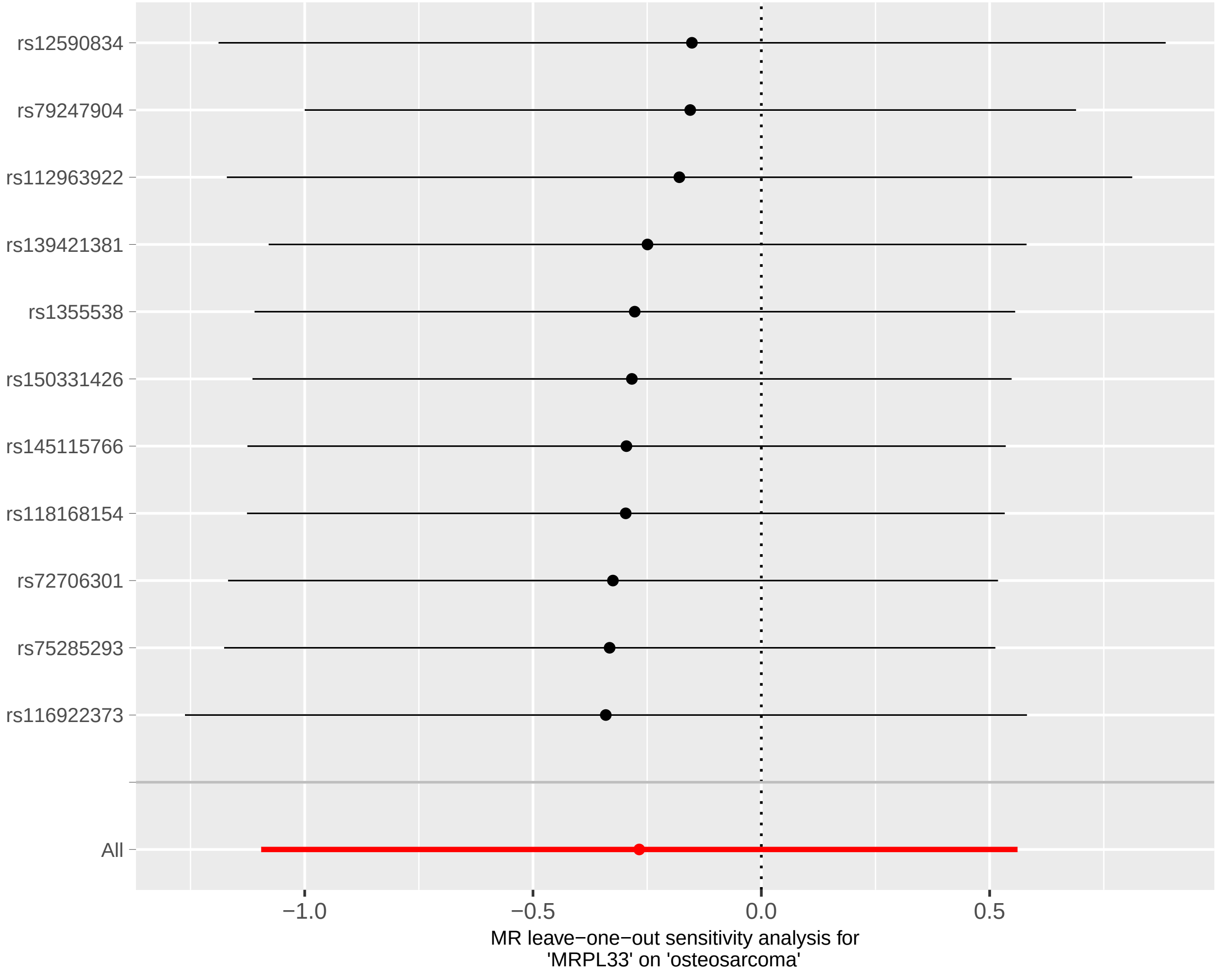

rs12590834
rs79247904
rs112963922
rs139421381
rs1355538
rs150331426
rs145115766
rs118168154
rs72706301
rs75285293
rs116922373
All
−1.0
−0.5
0.0
0.5
MR leave−one−out sensitivity analysis for
'MRPL33' on 'osteosarcoma'

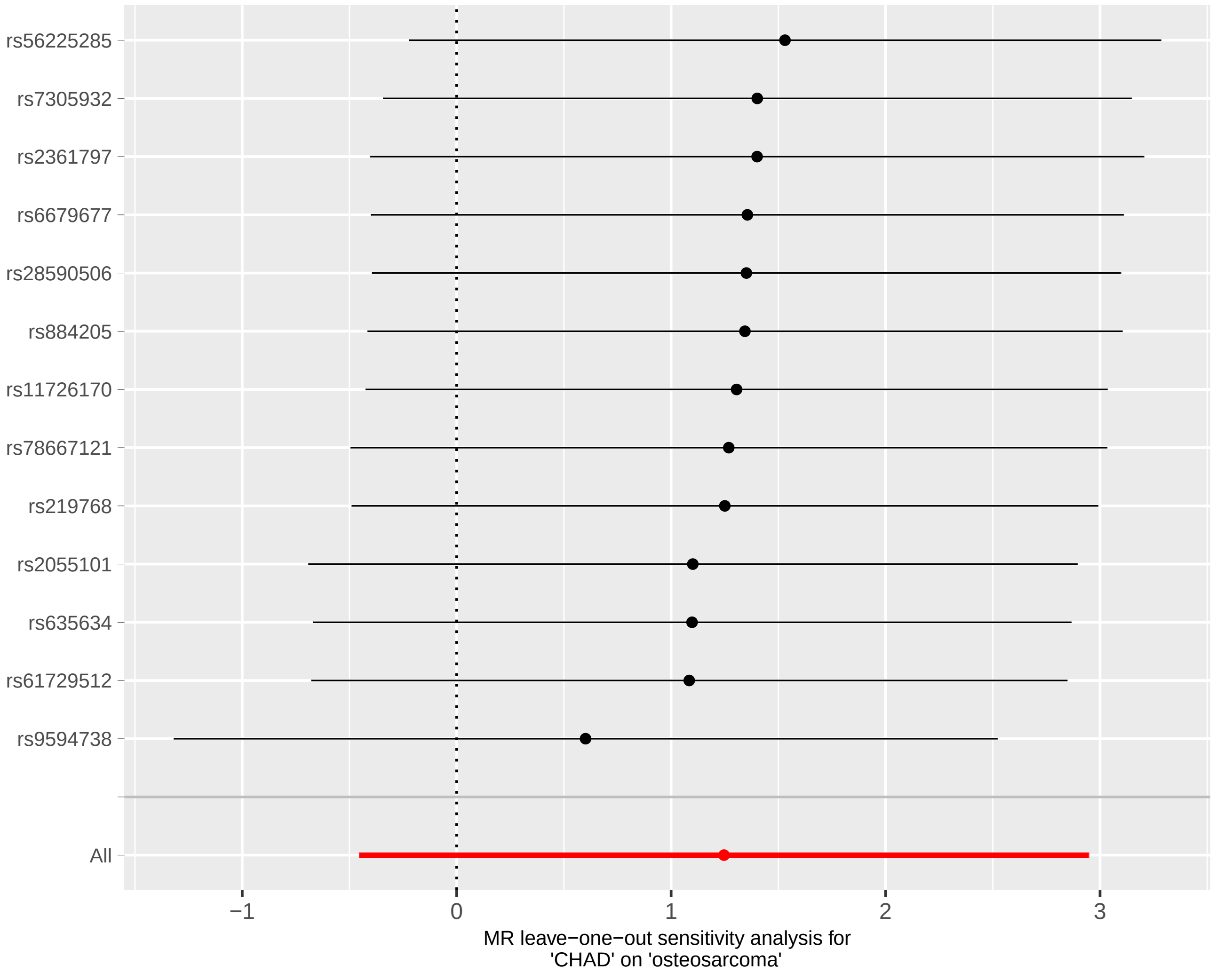
rs56225285
rs7305932
rs2361797
rs6679677
rs28590506
rs884205
rs11726170
rs78667121
rs219768
rs2055101
rs635634
rs61729512
rs9594738
All
−1
0
1
2
3
MR leave−one−out sensitivity analysis for
'CHAD' on 'osteosarcoma'

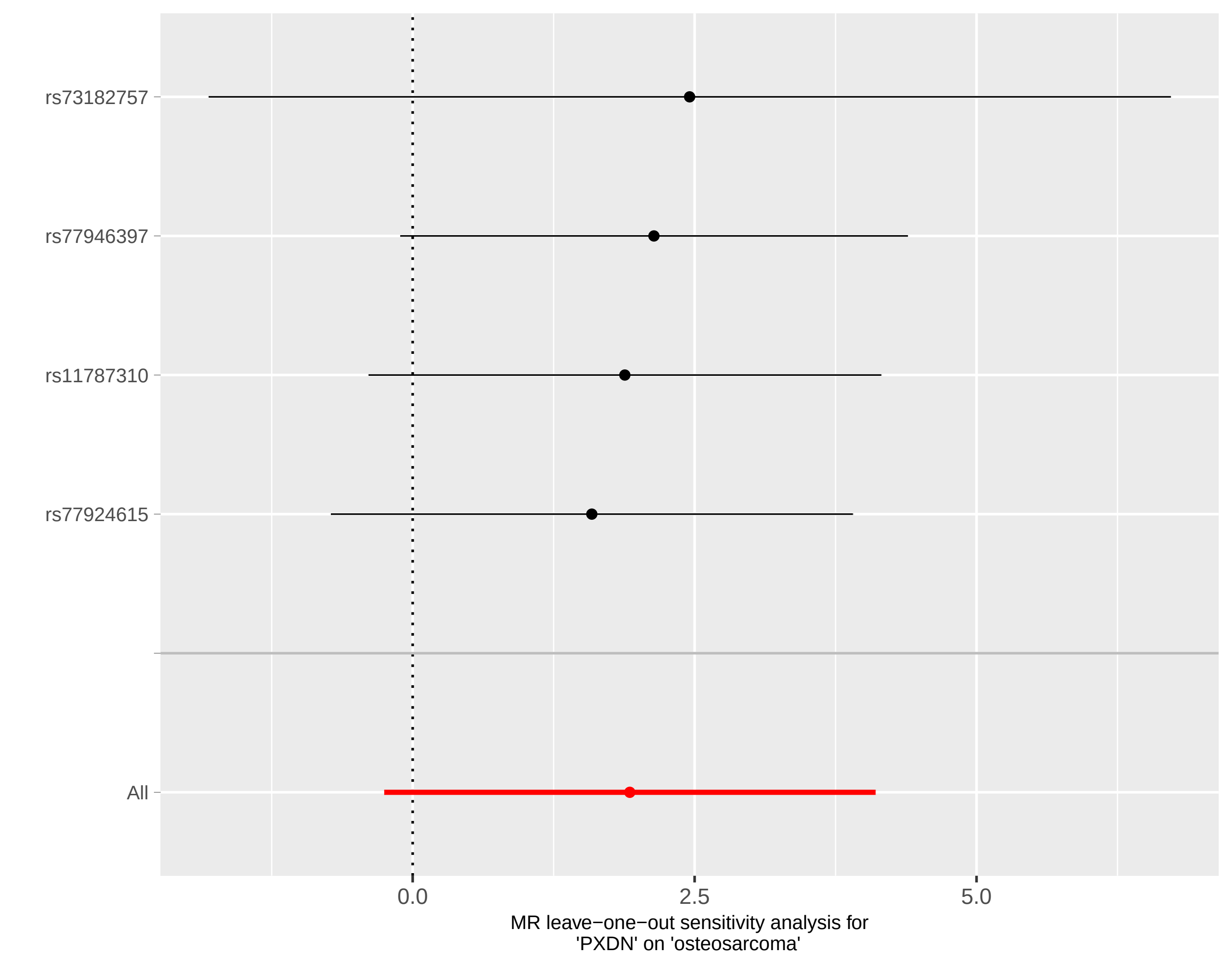
rs73182757
rs77946397
rs11787310
rs77924615
All
0.0
2.5
5.0
MR leave−one−out sensitivity analysis for
'PXDN' on 'osteosarcoma'

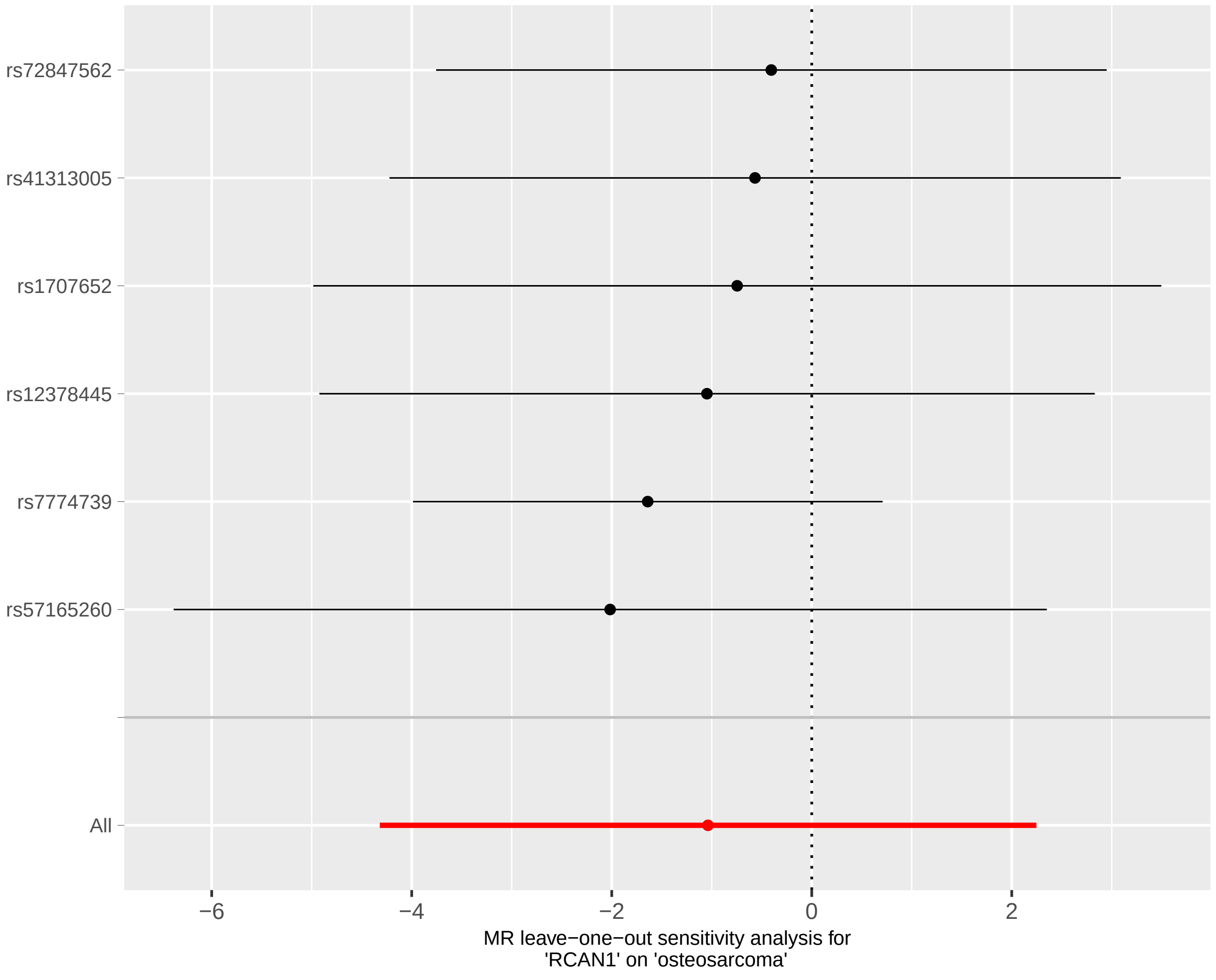
rs72847562
rs41313005
rs1707652
rs12378445
rs7774739
rs57165260
All
−6
−4
−2
0
2
MR leave−one−out sensitivity analysis for
'RCAN1' on 'osteosarcoma'

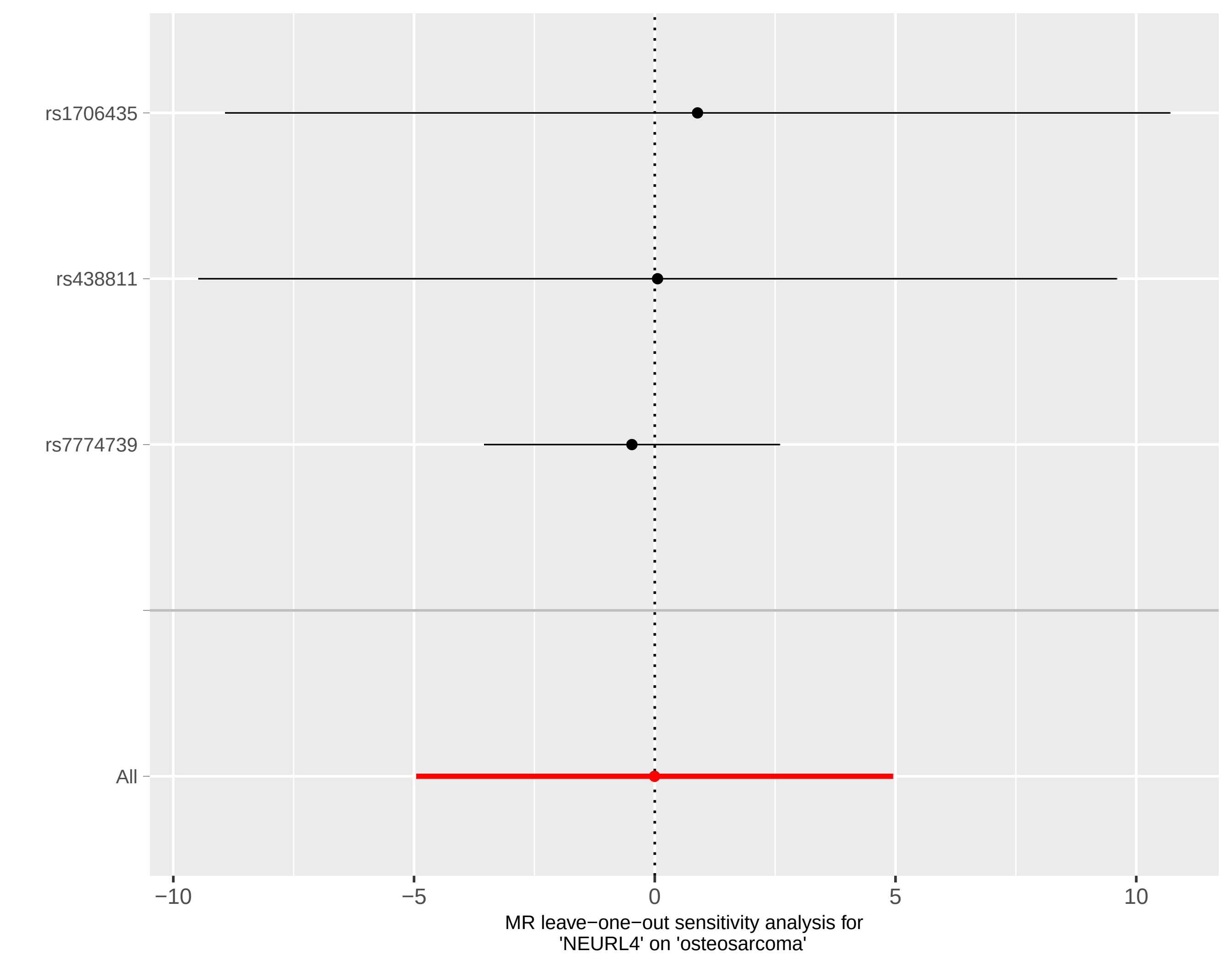

rs1706435
rs438811
rs7774739
All
−10
−5
0
5
10
MR leave−one−out sensitivity analysis for
'NEURL4' on 'osteosarcoma'

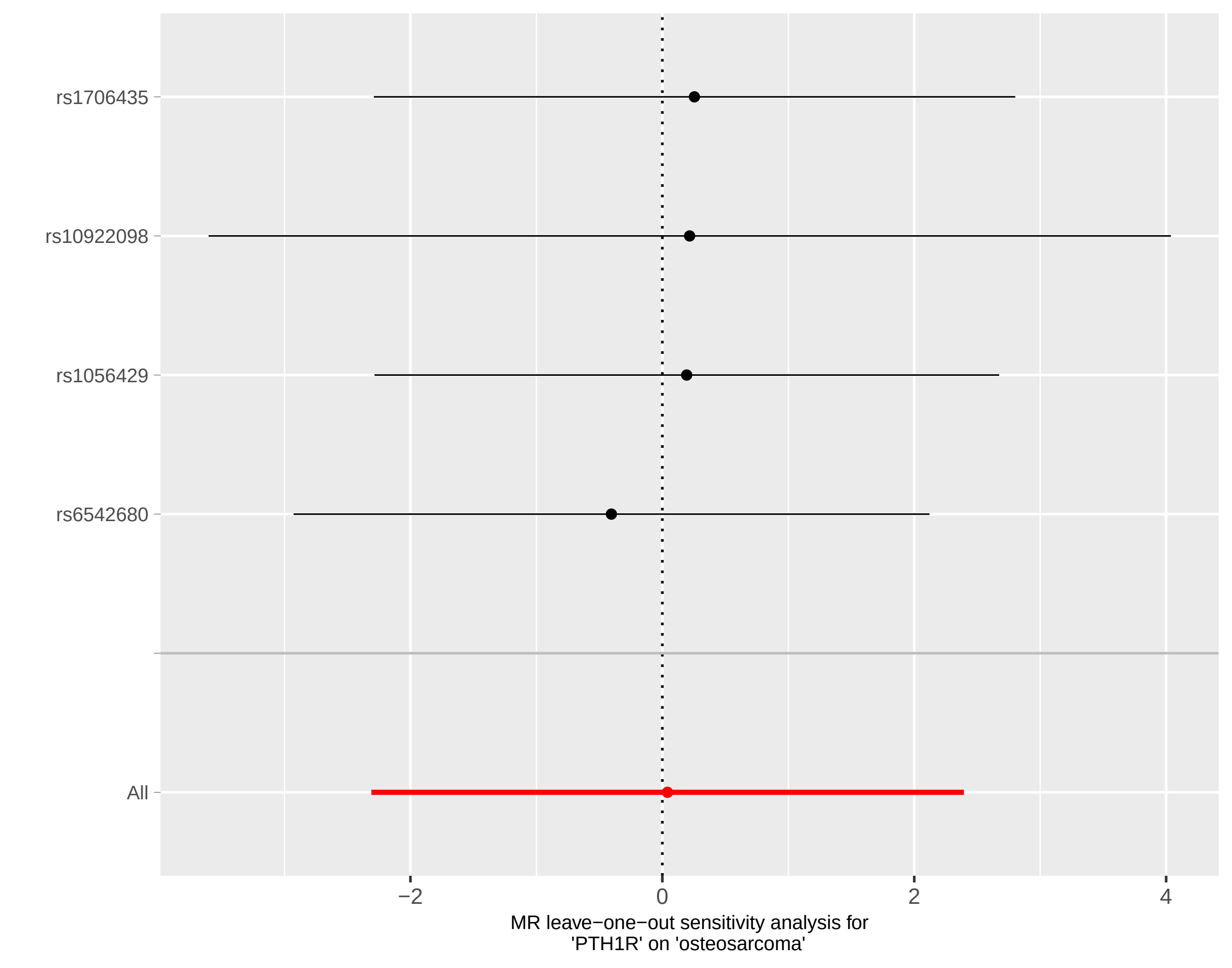
rs1706435
rs10922098
rs1056429
rs6542680
All
−2
0
2
4
MR leave−one−out sensitivity analysis for
'PTH1R' on 'osteosarcoma'

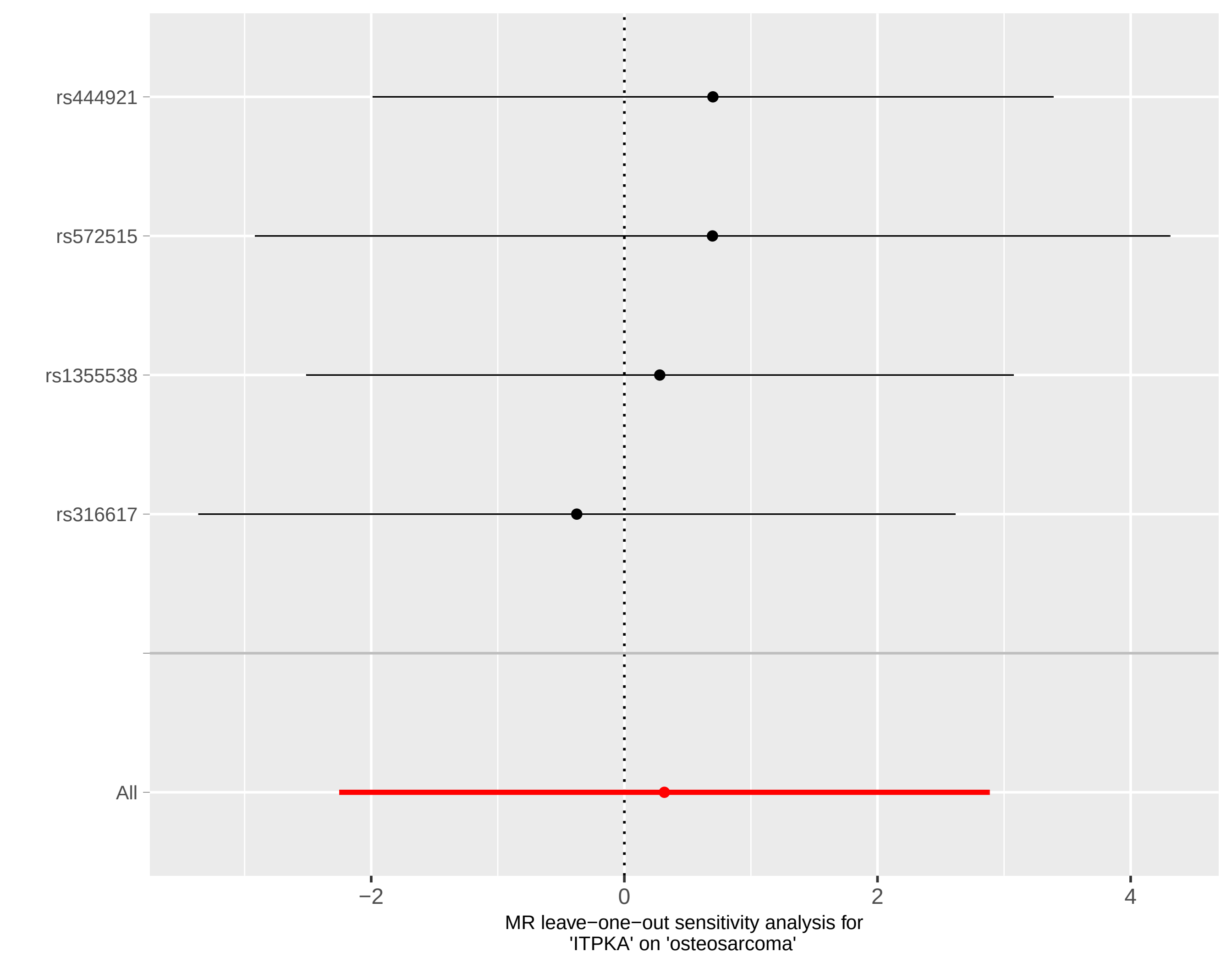
rs444921
rs572515
rs1355538
rs316617
All
−2
0
2
4
MR leave−one−out sensitivity analysis for
'ITPKA' on 'osteosarcoma'

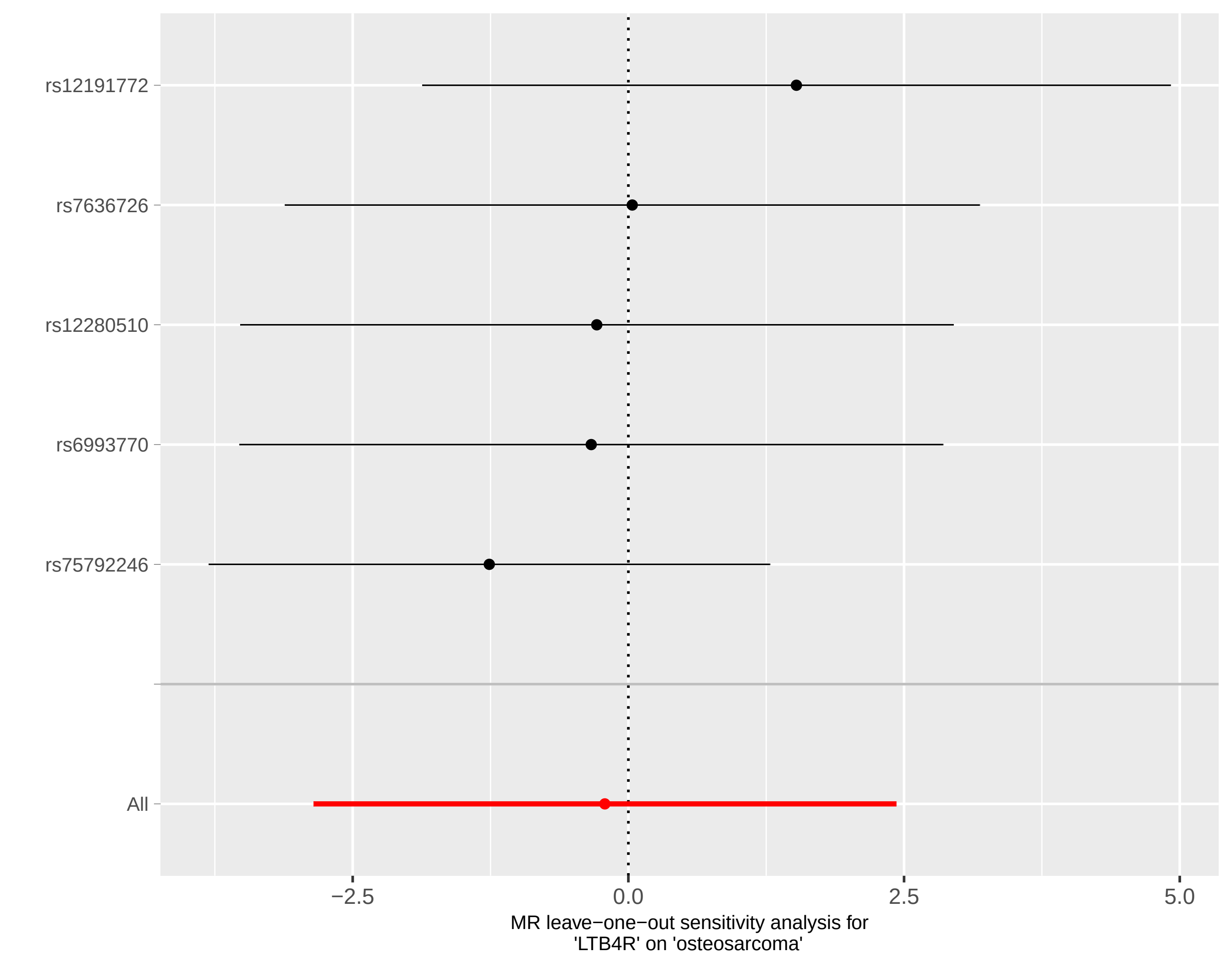
rs12191772
rs7636726
rs12280510
rs6993770
rs75792246
All
−2.5
0.0
2.5
5.0
MR leave−one−out sensitivity analysis for
'LTB4R' on 'osteosarcoma'

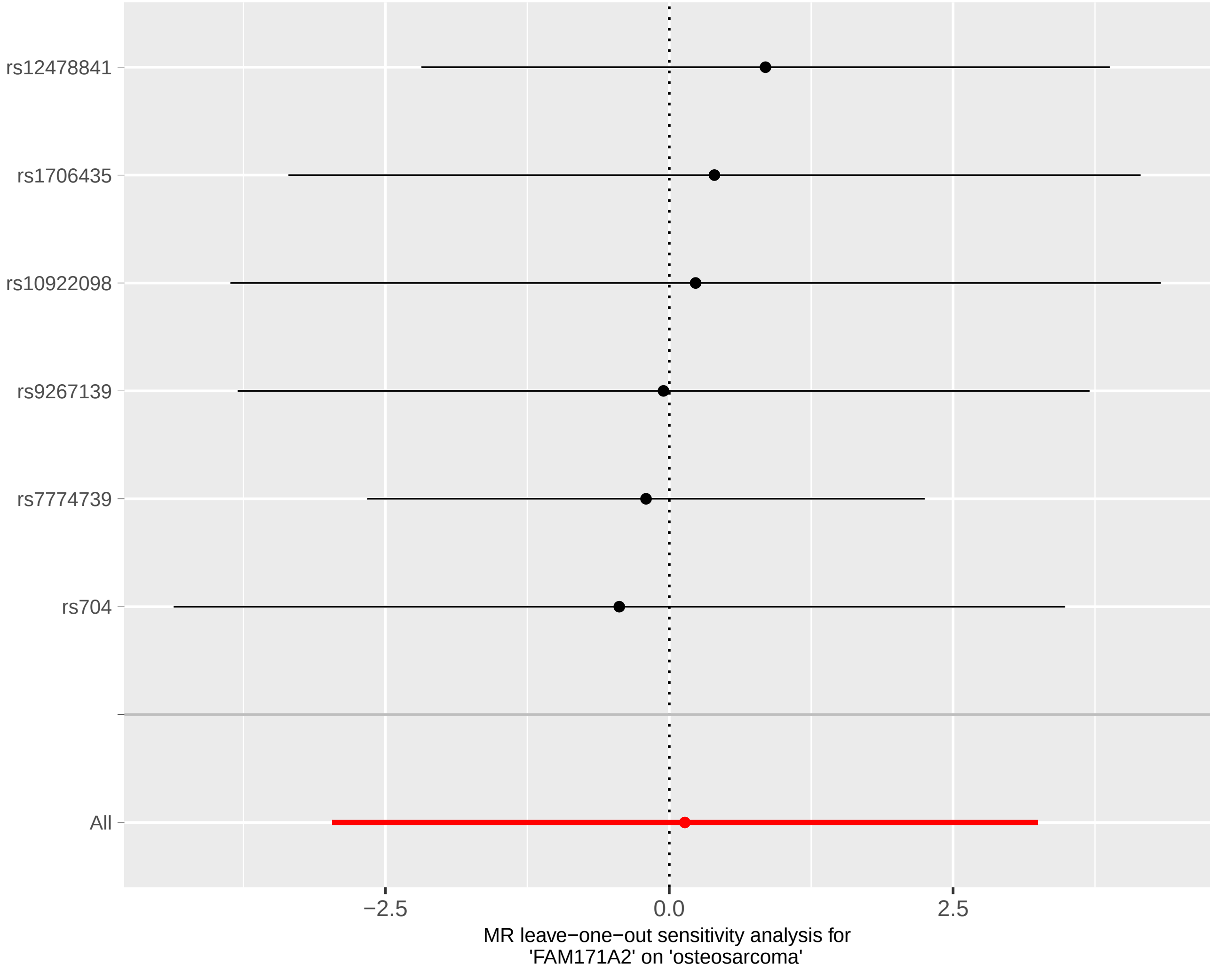

rs12478841
rs1706435
rs10922098
rs9267139
rs7774739
rs704
All
−2.5
0.0
2.5
MR leave−one−out sensitivity analysis for
'FAM171A2' on 'osteosarcoma'

Insufficient number of SNPs

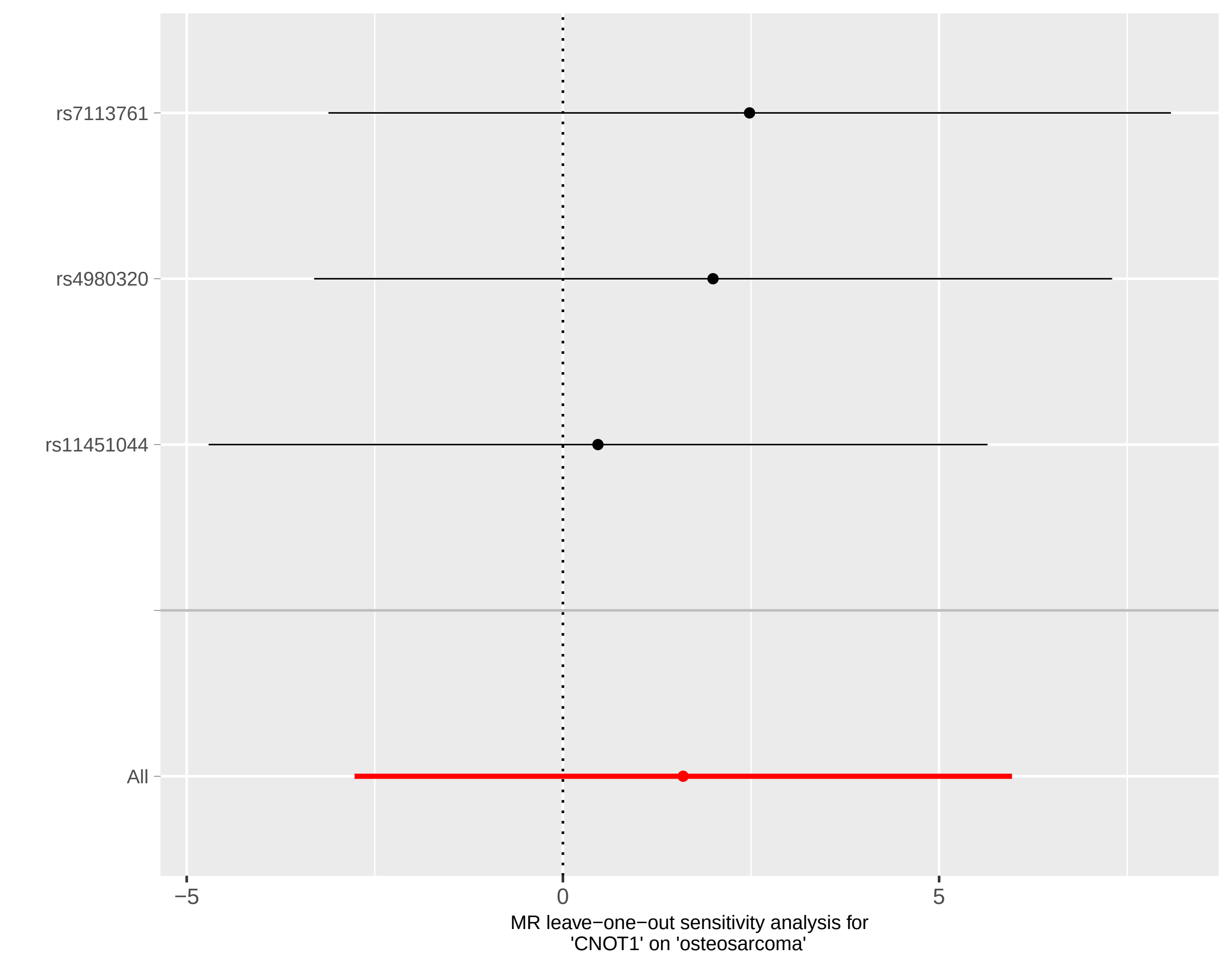
rs7113761
rs4980320
rs11451044
All
−5
0
5
MR leave−one−out sensitivity analysis for
'CNOT1' on 'osteosarcoma'

Insufficient number of SNPs

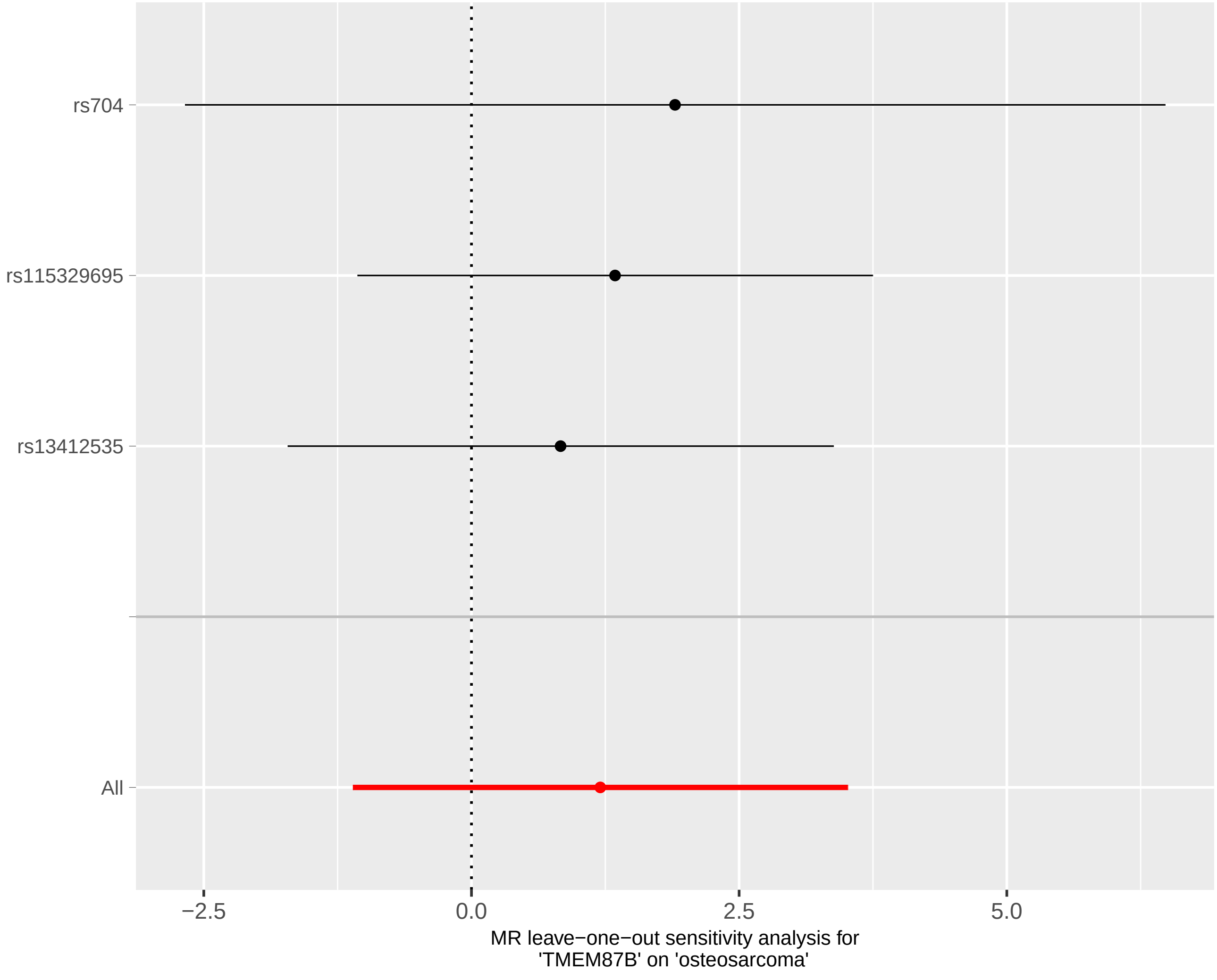

rs704
rs115329695
rs13412535
All
−2.5
0.0
2.5
5.0
MR leave−one−out sensitivity analysis for
'TMEM87B' on 'osteosarcoma'

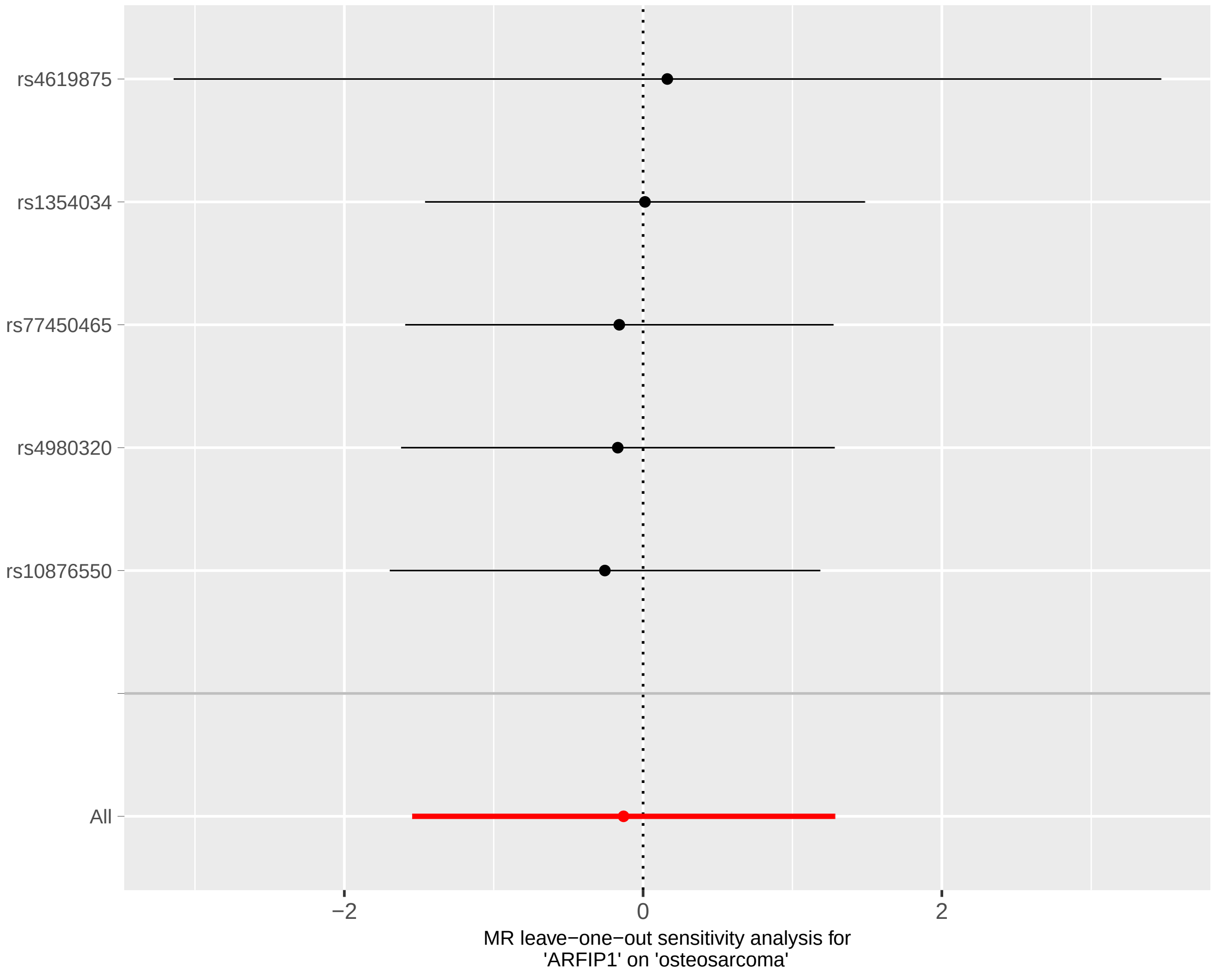

rs4619875
rs1354034
rs77450465
rs4980320
rs10876550
All
−2
0
2
MR leave−one−out sensitivity analysis for
'ARFIP1' on 'osteosarcoma'

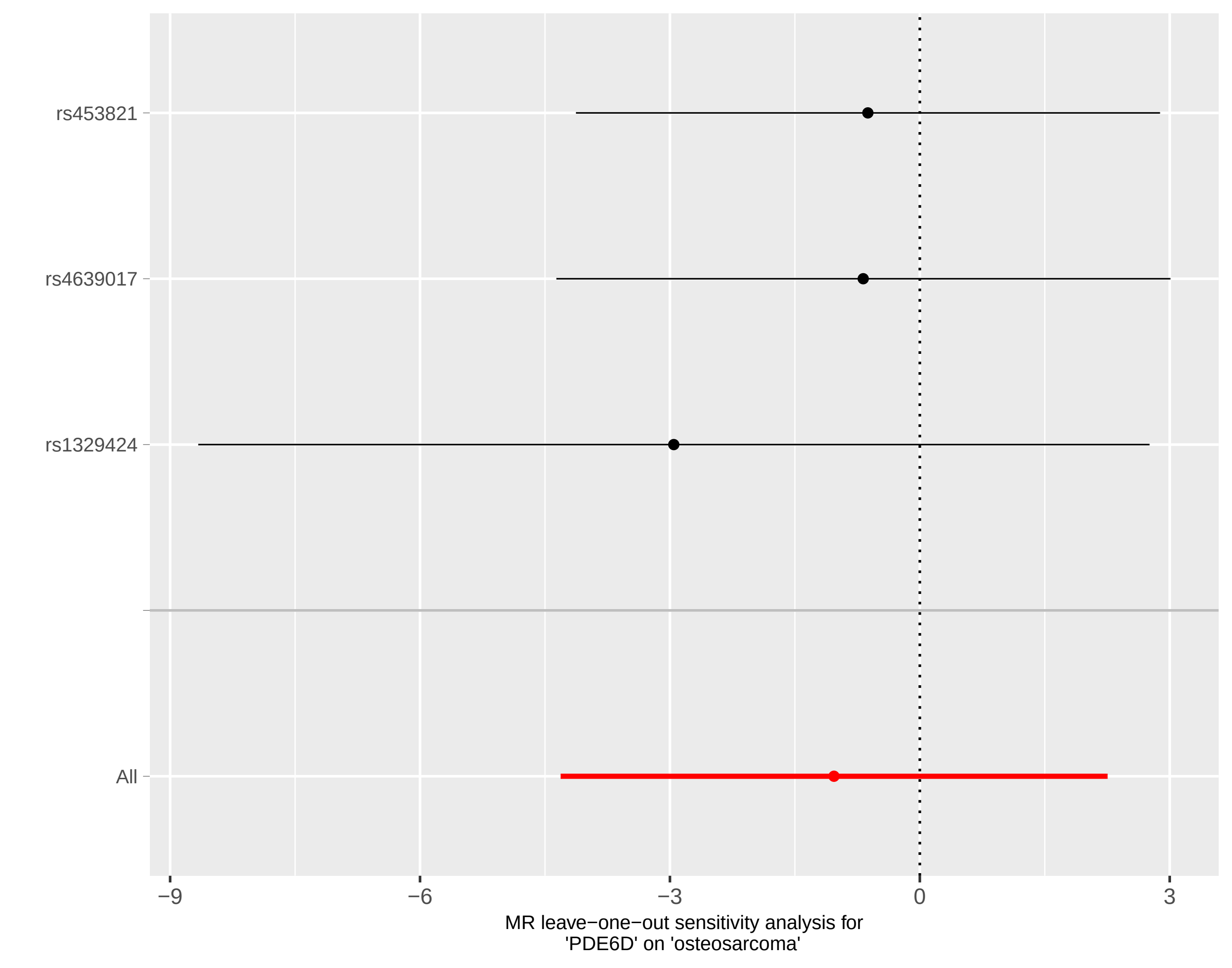
rs453821
rs4639017
rs1329424
All
−9
−6
−3
0
3
MR leave−one−out sensitivity analysis for
'PDE6D' on 'osteosarcoma'

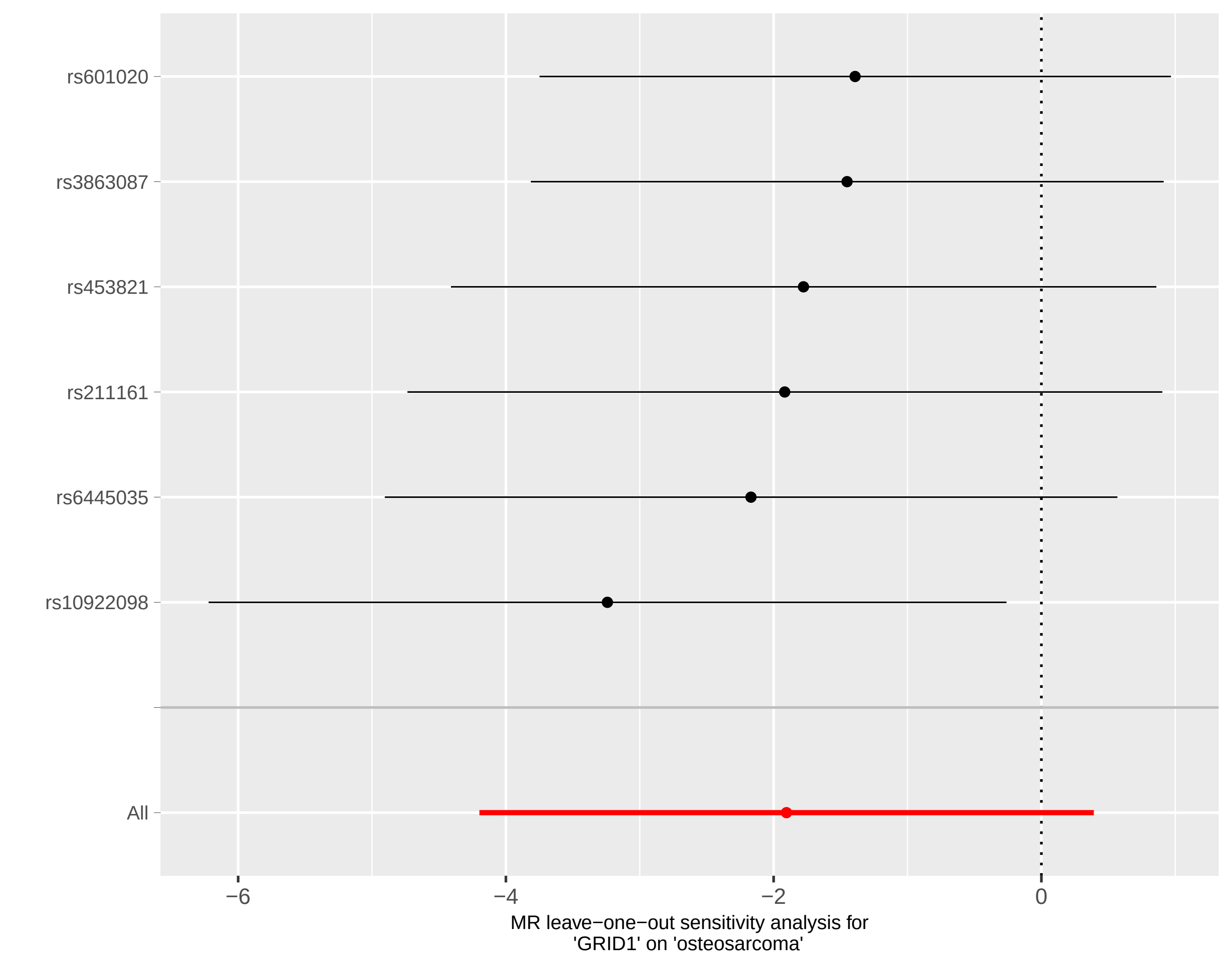

rs601020
rs3863087
rs453821
rs211161
rs6445035
rs10922098
All
−6
−4
−2
0
MR leave−one−out sensitivity analysis for
'GRID1' on 'osteosarcoma'

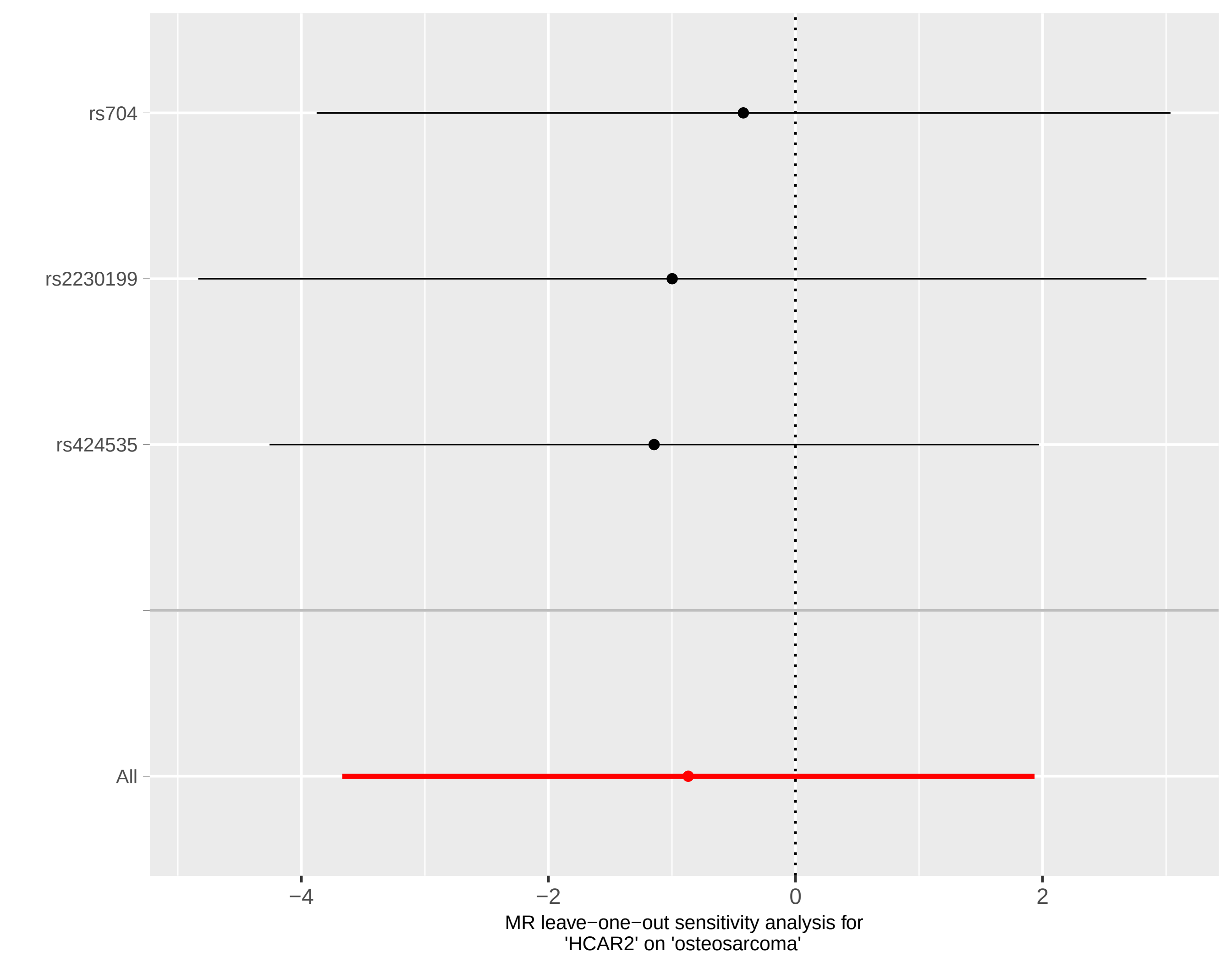

rs704
rs2230199
rs424535
All
−4
−2
0
2
MR leave−one−out sensitivity analysis for
'HCAR2' on 'osteosarcoma'

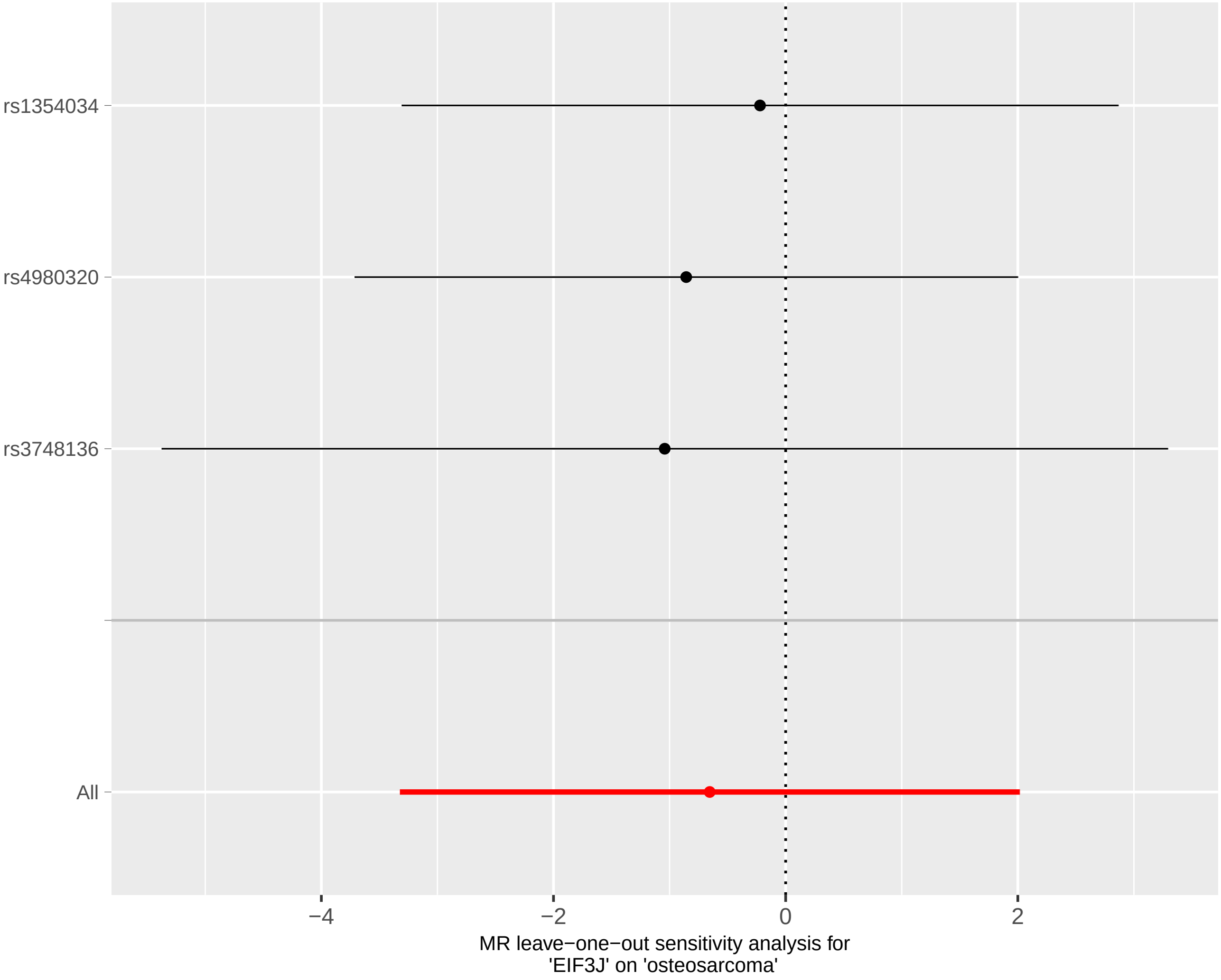
rs1354034
rs4980320
rs3748136
All
−4
−2
0
2
MR leave−one−out sensitivity analysis for
'EIF3J' on 'osteosarcoma'

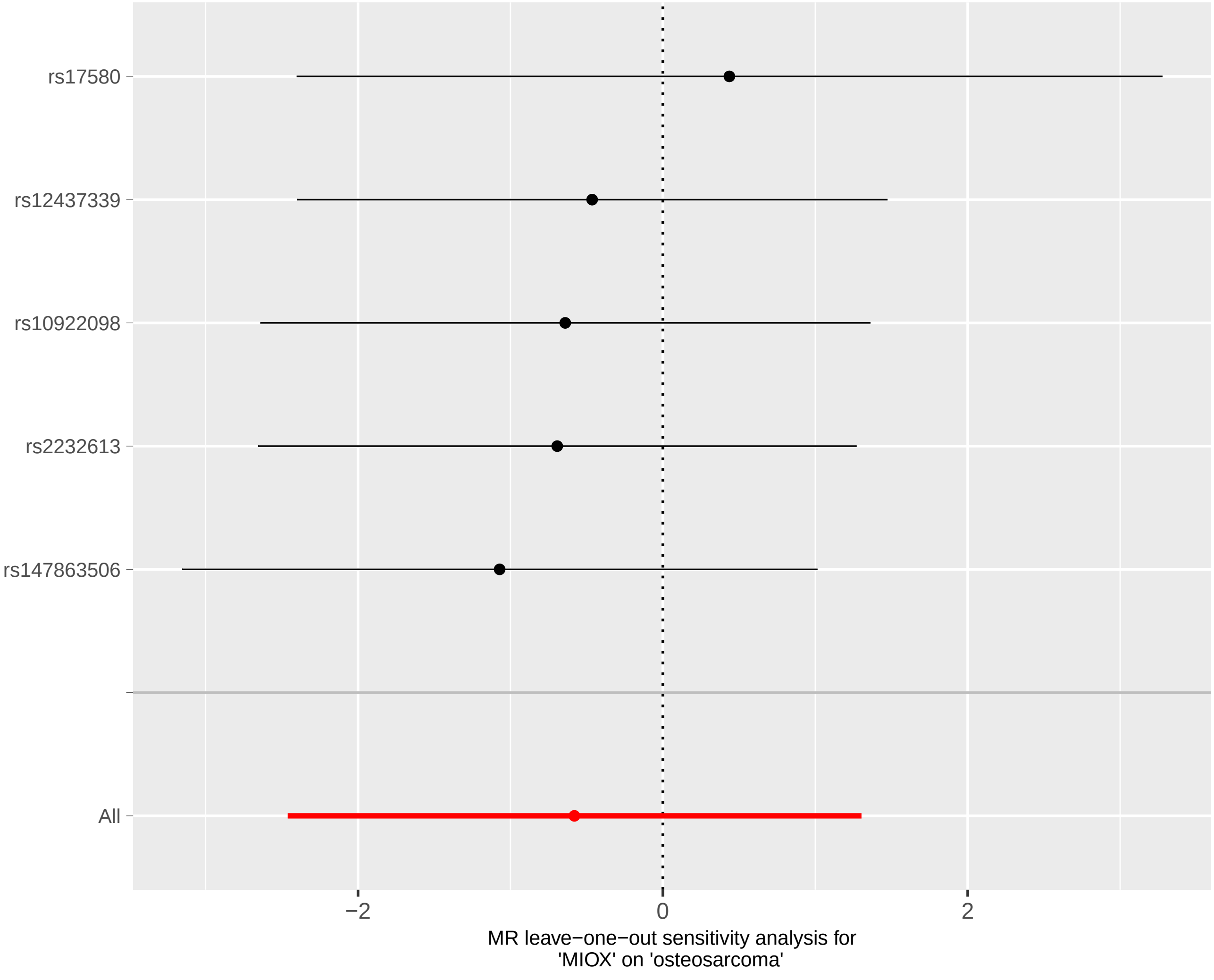
rs17580
rs12437339
rs10922098
rs2232613
rs147863506
All
−2
0
2
MR leave−one−out sensitivity analysis for
'MIOX' on 'osteosarcoma'

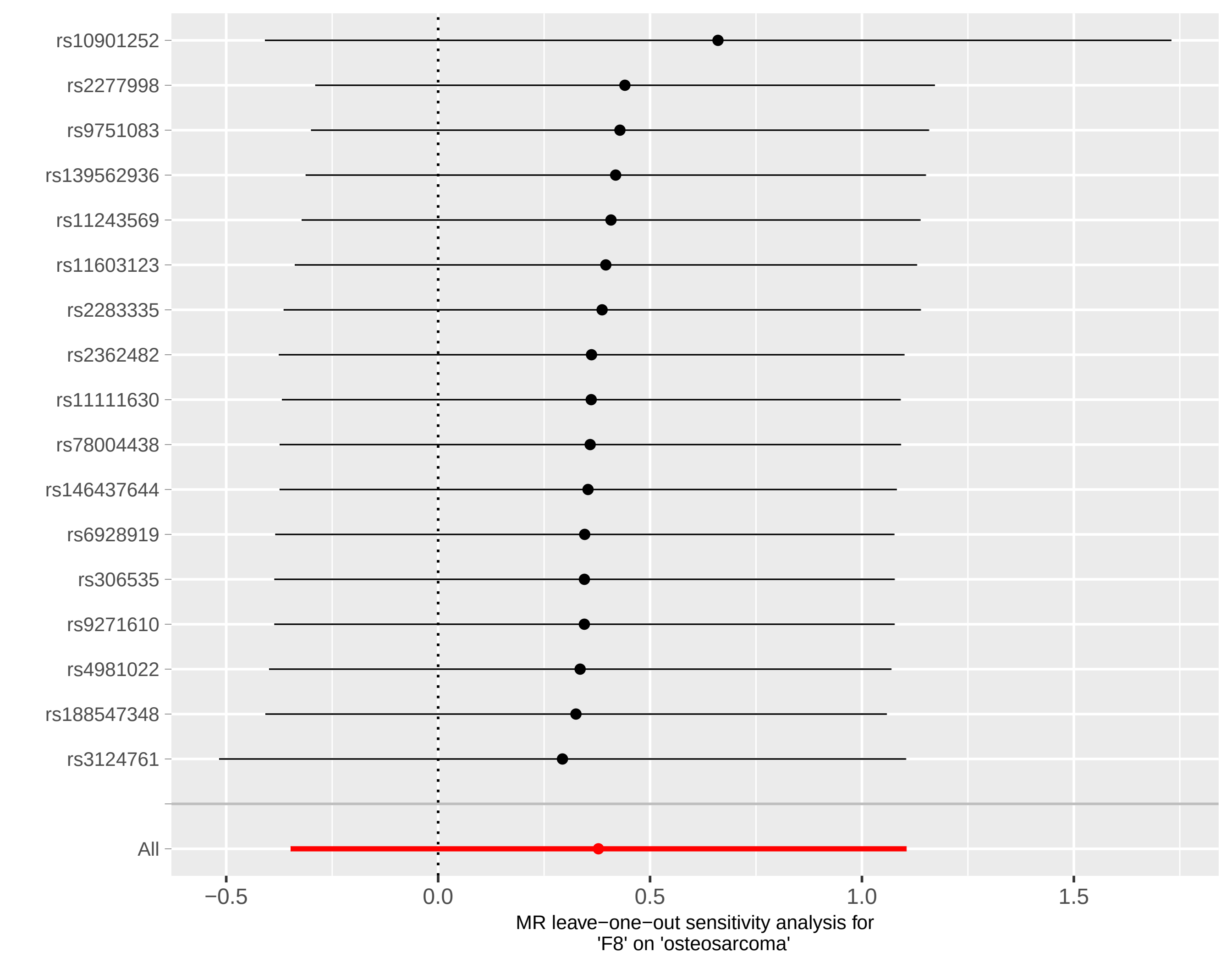
rs10901252
rs2277998
rs9751083
rs139562936
rs11243569
rs11603123
rs2283335
rs2362482
rs11111630
rs78004438
rs146437644
rs6928919
rs306535
rs9271610
rs4981022
rs188547348
rs3124761
All
−0.5
0.0
0.5
1.0
1.5
MR leave−one−out sensitivity analysis for
'F8' on 'osteosarcoma'

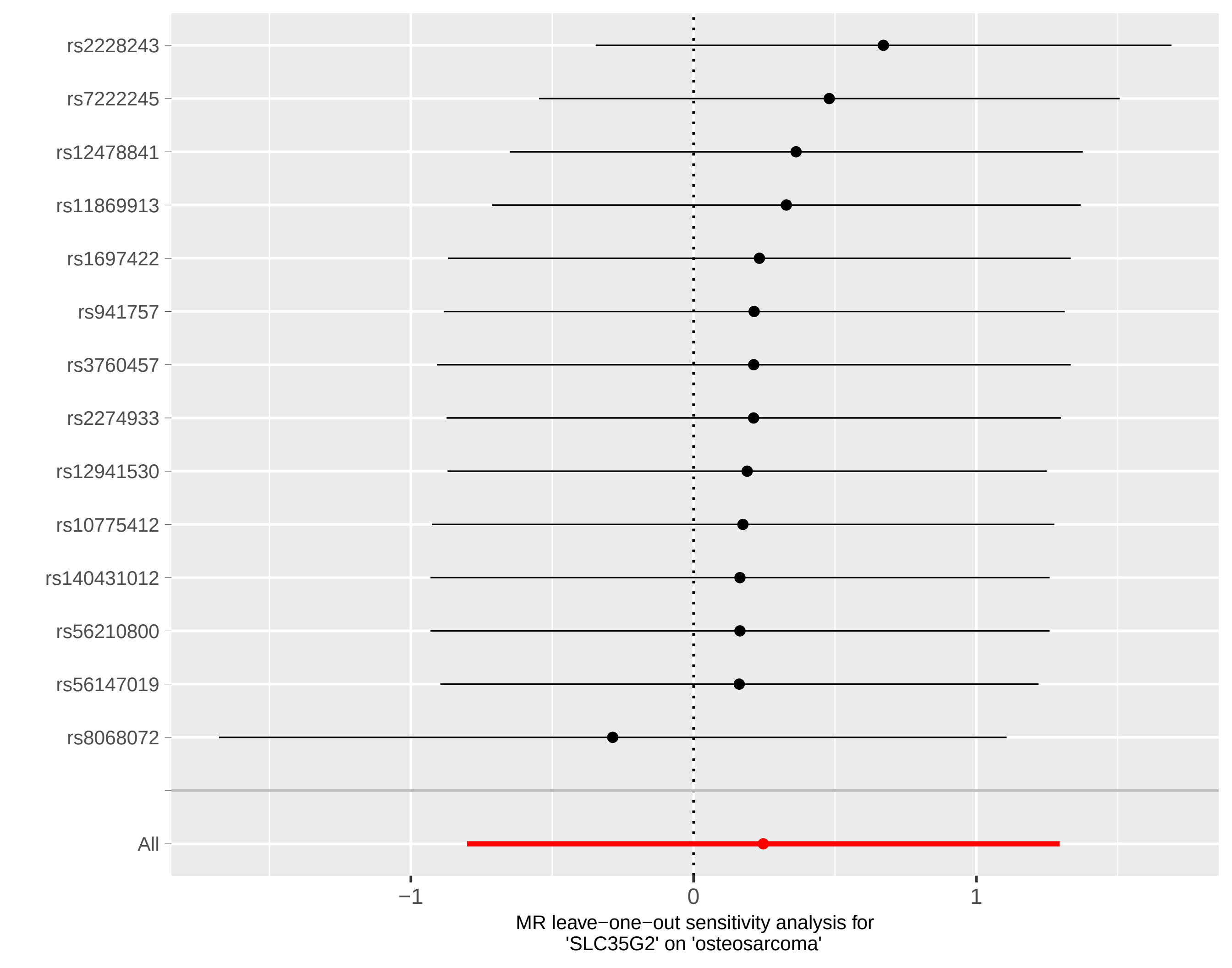
rs2228243
rs7222245
rs12478841
rs11869913
rs1697422
rs941757
rs3760457
rs2274933
rs12941530
rs10775412
rs140431012
rs56210800
rs56147019
rs8068072
All
−1
0
1
MR leave−one−out sensitivity analysis for
'SLC35G2' on 'osteosarcoma'

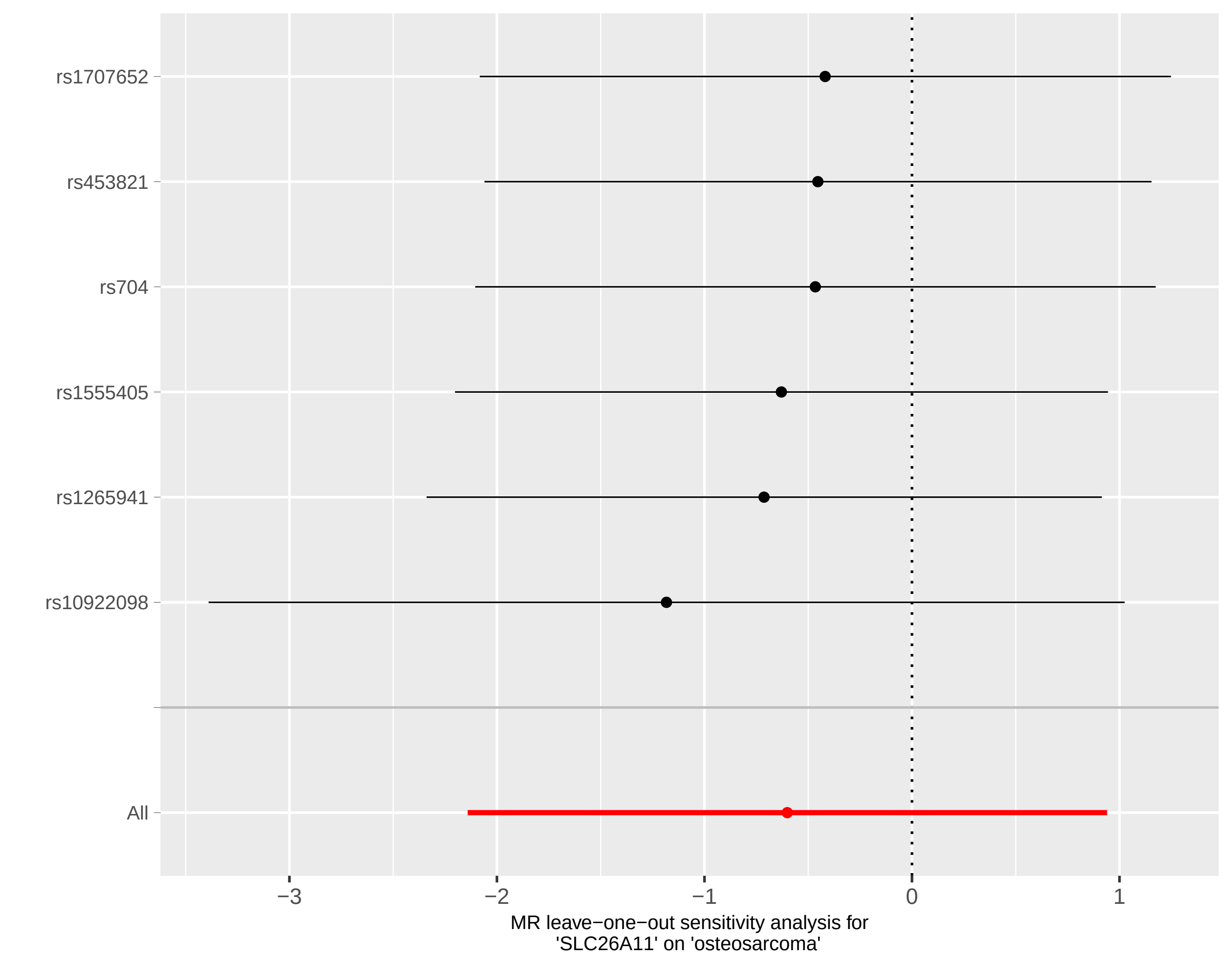

rs1707652
rs453821
rs704
rs1555405
rs1265941
rs10922098
All
−3
−2
−1
0
1
MR leave−one−out sensitivity analysis for
'SLC26A11' on 'osteosarcoma'

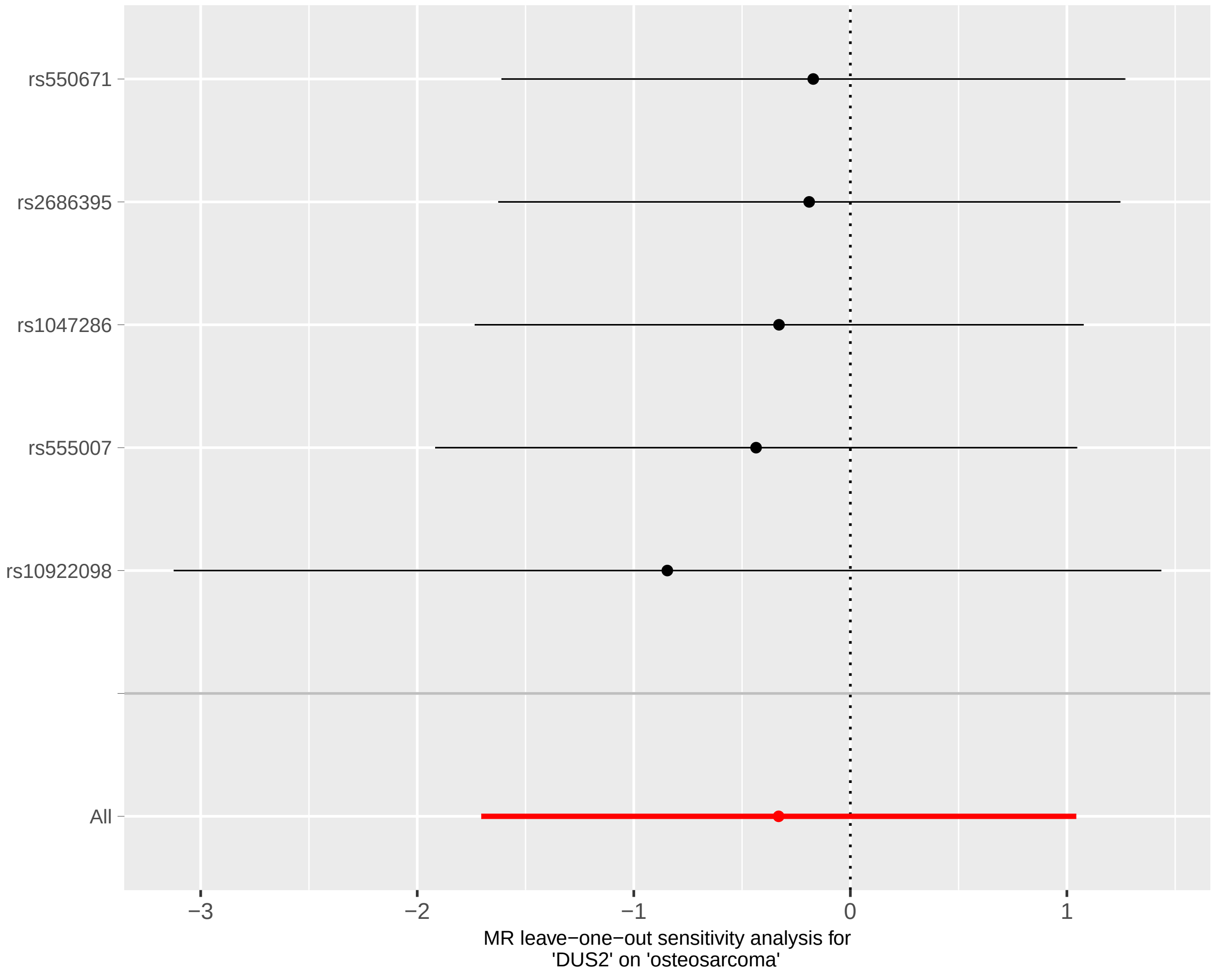

rs550671
rs2686395
rs1047286
rs555007
rs10922098
All
−3
−2
−1
0
1
MR leave−one−out sensitivity analysis for
'DUS2' on 'osteosarcoma'

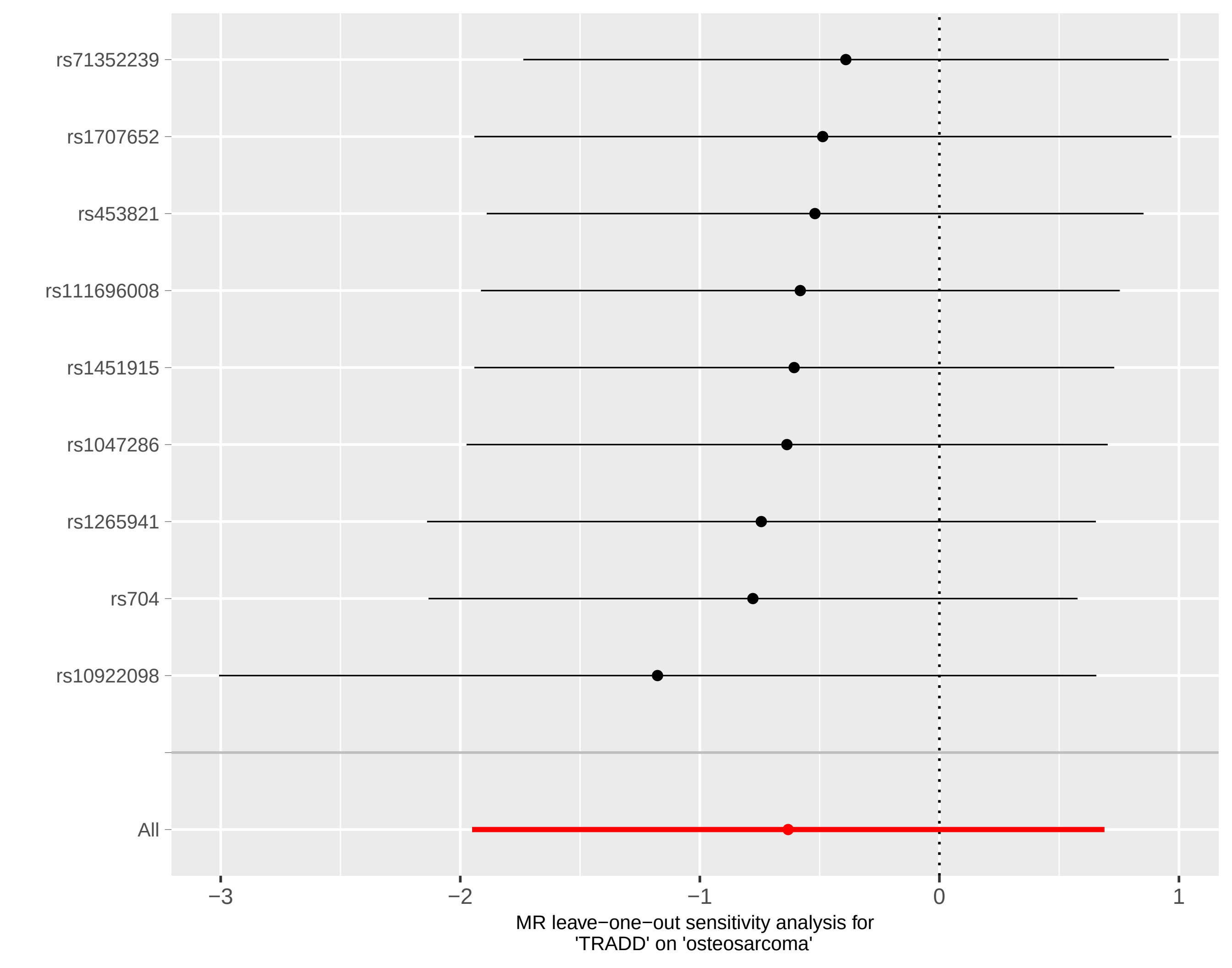

rs71352239
rs1707652
rs453821
rs111696008
rs1451915
rs1047286
rs1265941
rs704
rs10922098
All
−3
−2
−1
0
1
MR leave−one−out sensitivity analysis for
'TRADD' on 'osteosarcoma'

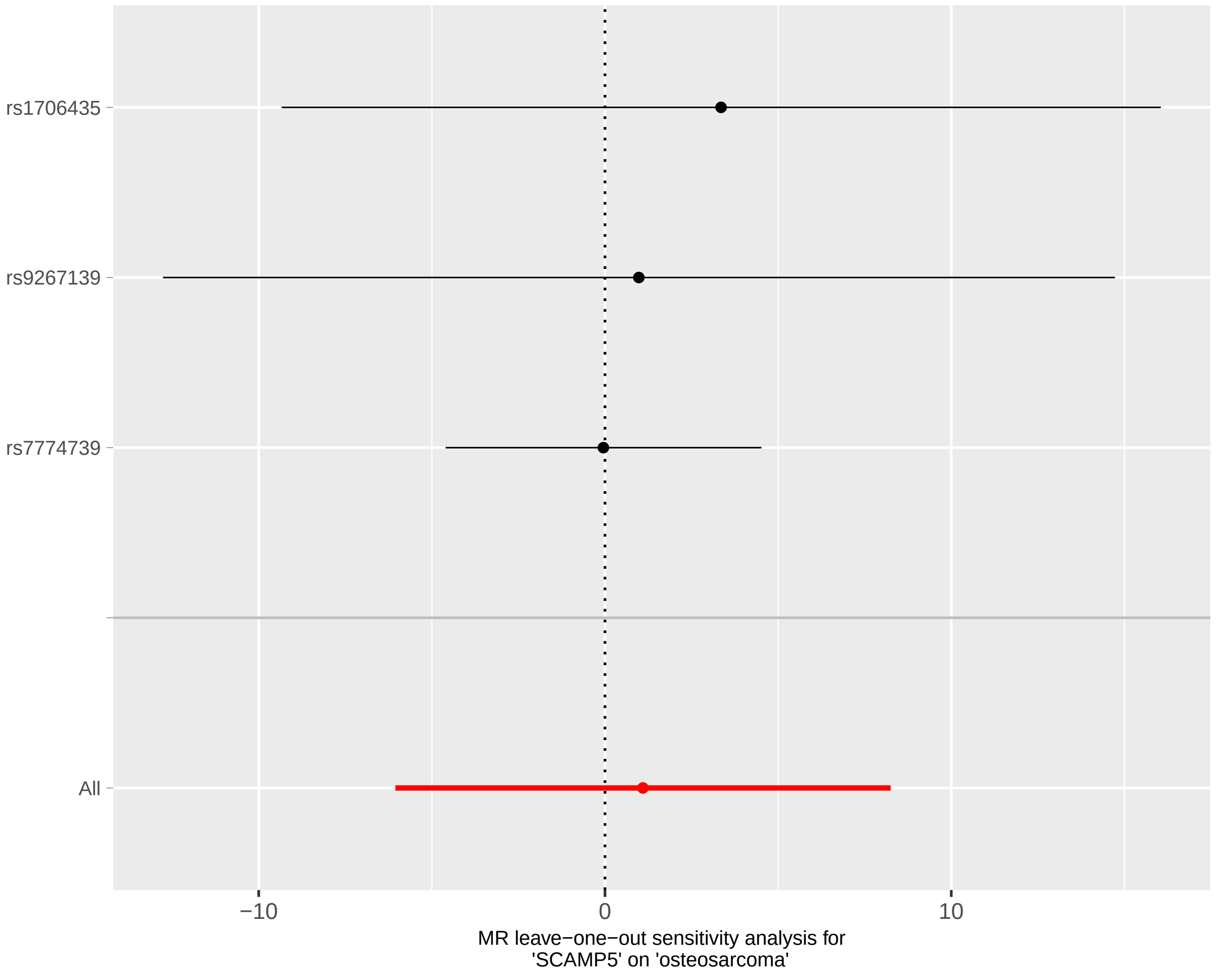
rs1706435
rs9267139
rs7774739
All
−10
0
10
MR leave−one−out sensitivity analysis for
'SCAMP5' on 'osteosarcoma'

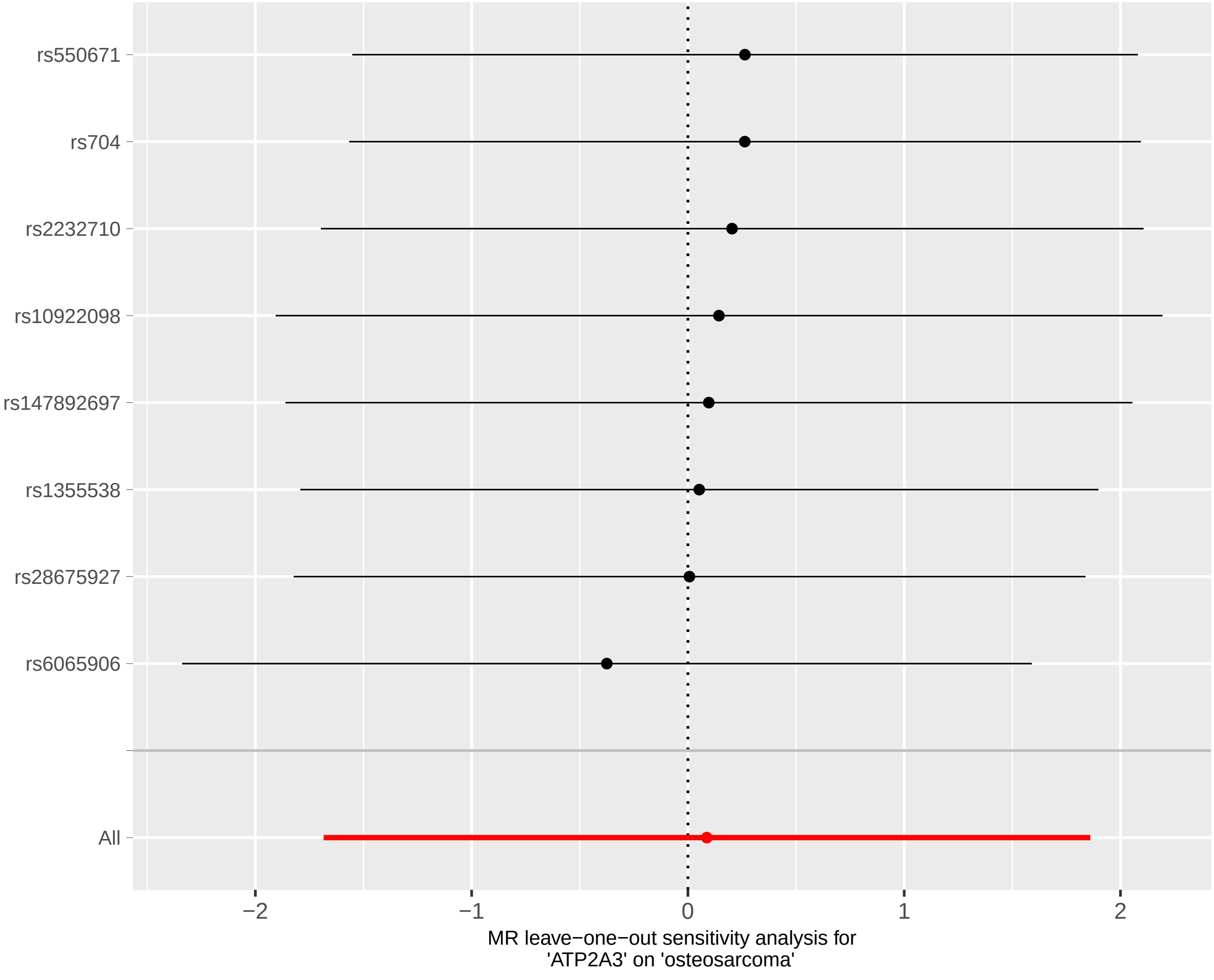
rs550671
rs704
rs2232710
rs10922098
rs147892697
rs1355538
rs28675927
rs6065906
All
−2
−1
0
1
2
MR leave−one−out sensitivity analysis for
'ATP2A3' on 'osteosarcoma'

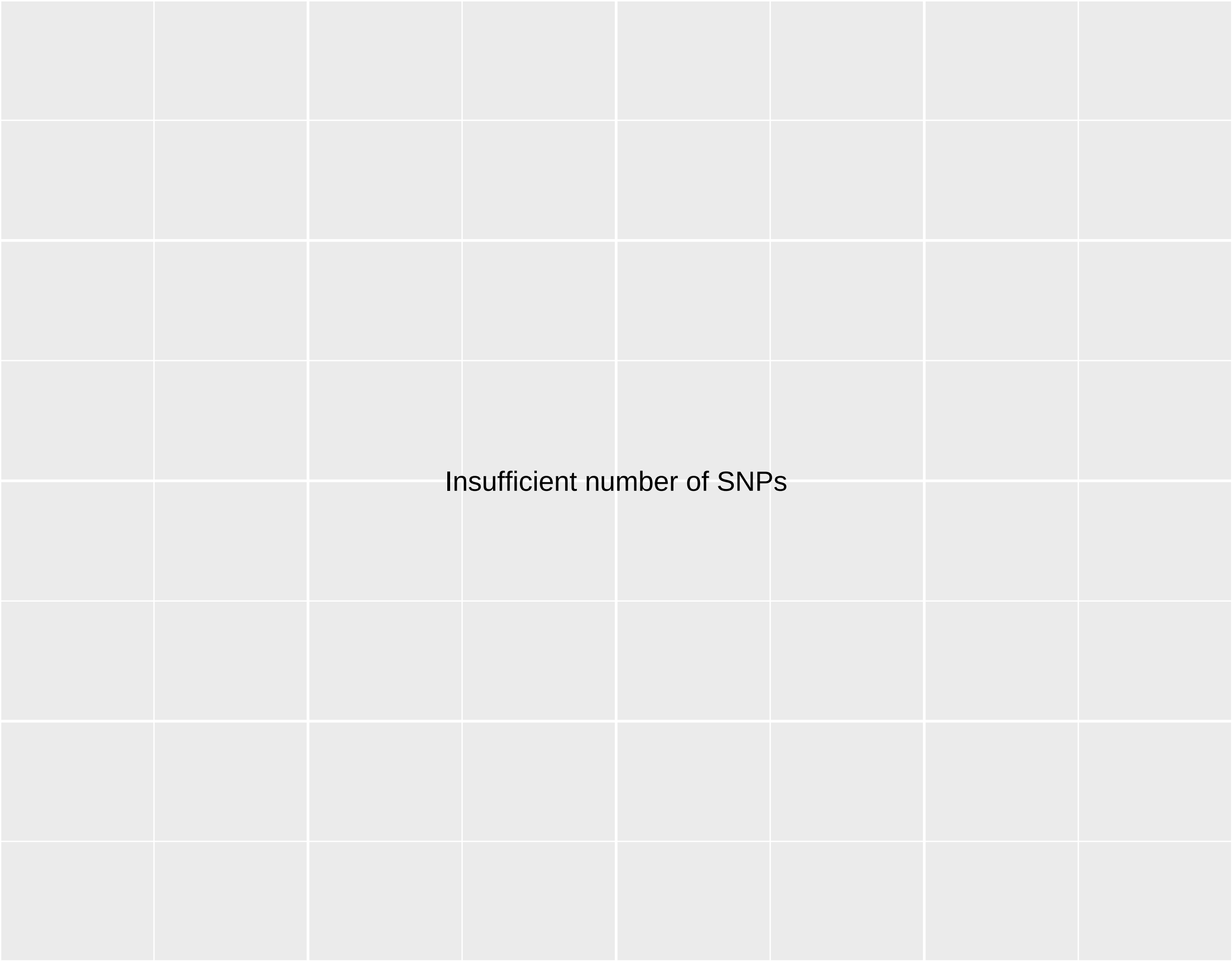
Insufficient number of SNPs

Insufficient number of SNPs

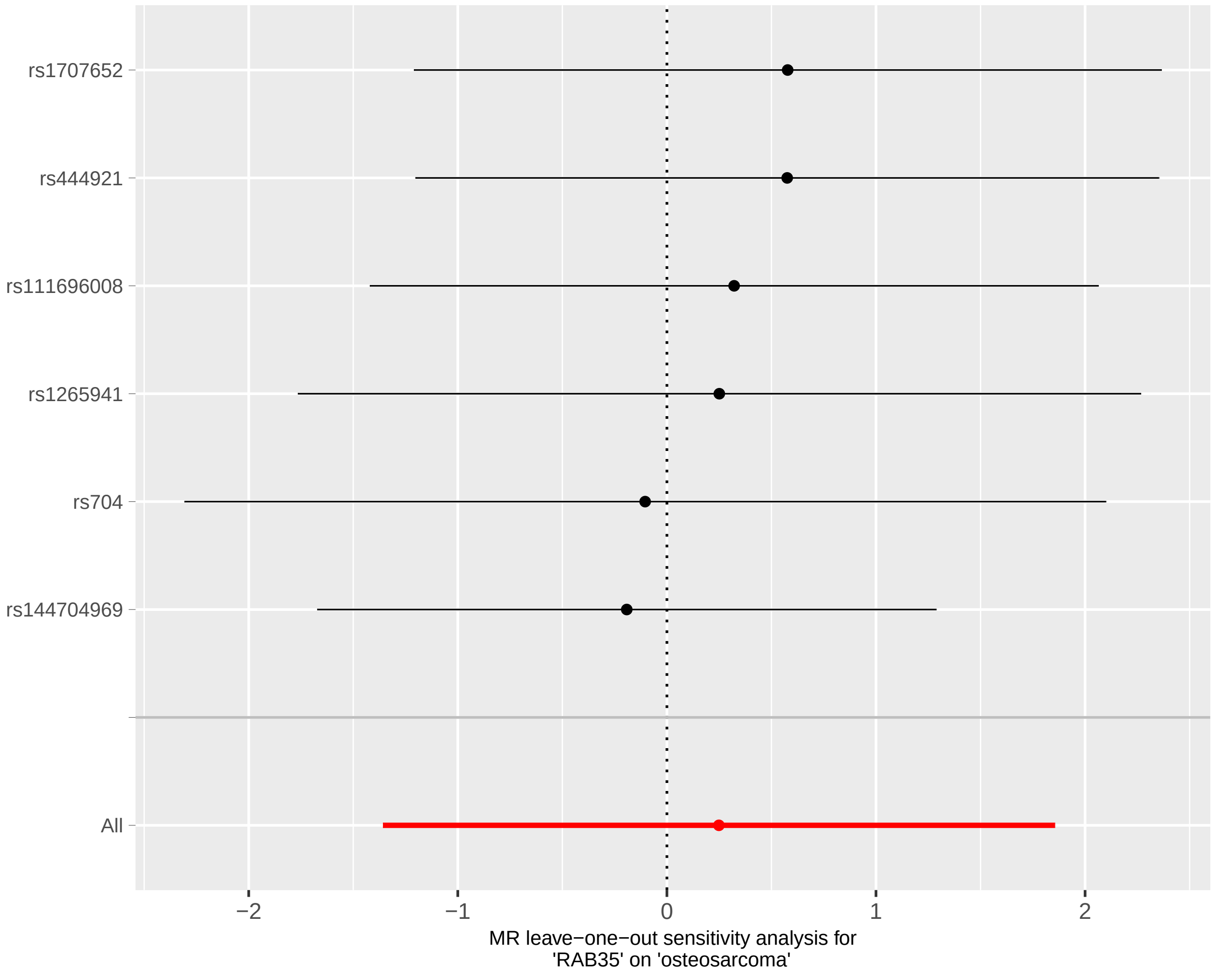
rs1707652
rs444921
rs111696008
rs1265941
rs704
rs144704969
All
−2
−1
0
1
2
MR leave−one−out sensitivity analysis for
'RAB35' on 'osteosarcoma'

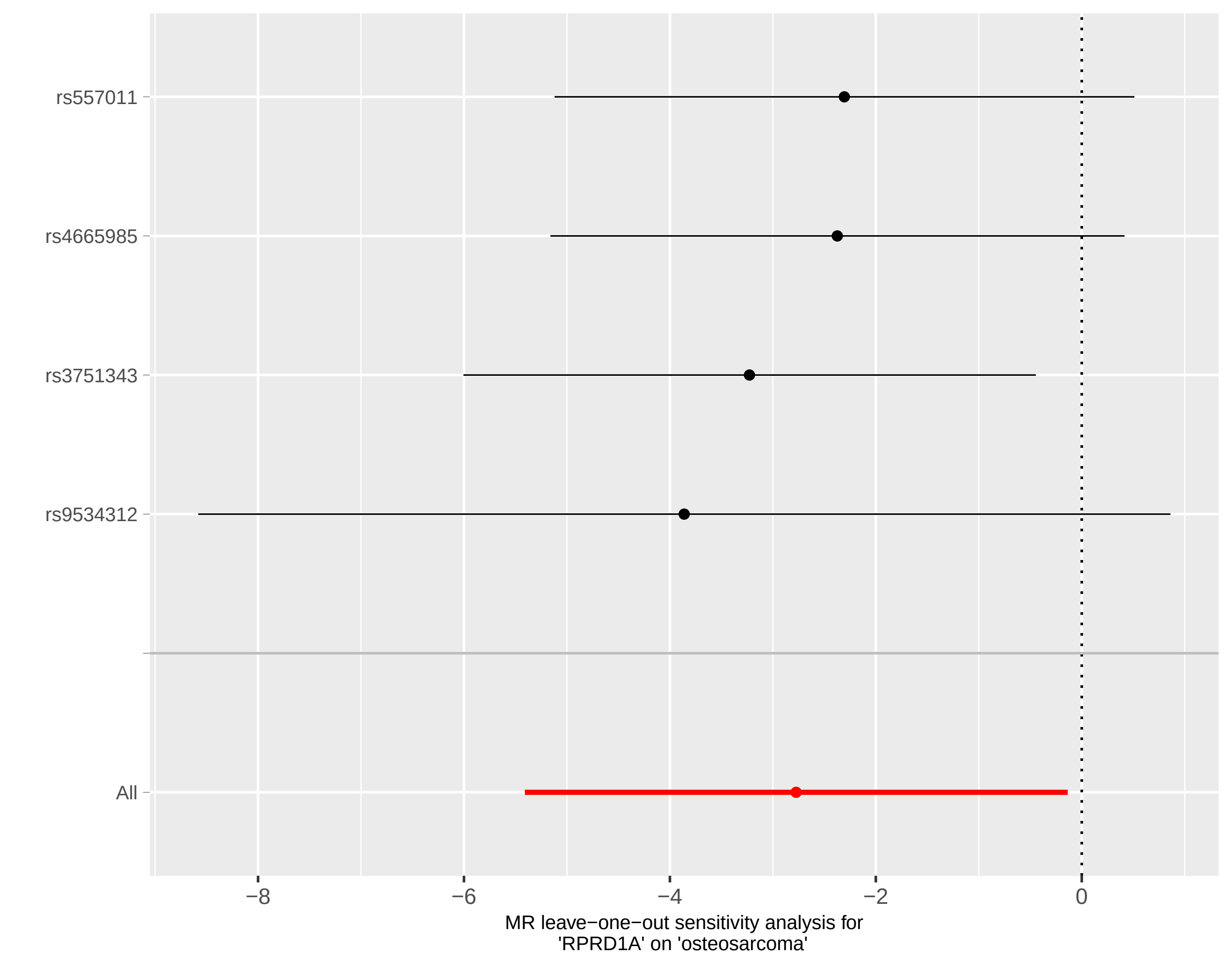
rs557011
rs4665985
rs3751343
rs9534312
All
−8
−6
−4
−2
0
MR leave−one−out sensitivity analysis for
'RPRD1A' on 'osteosarcoma'

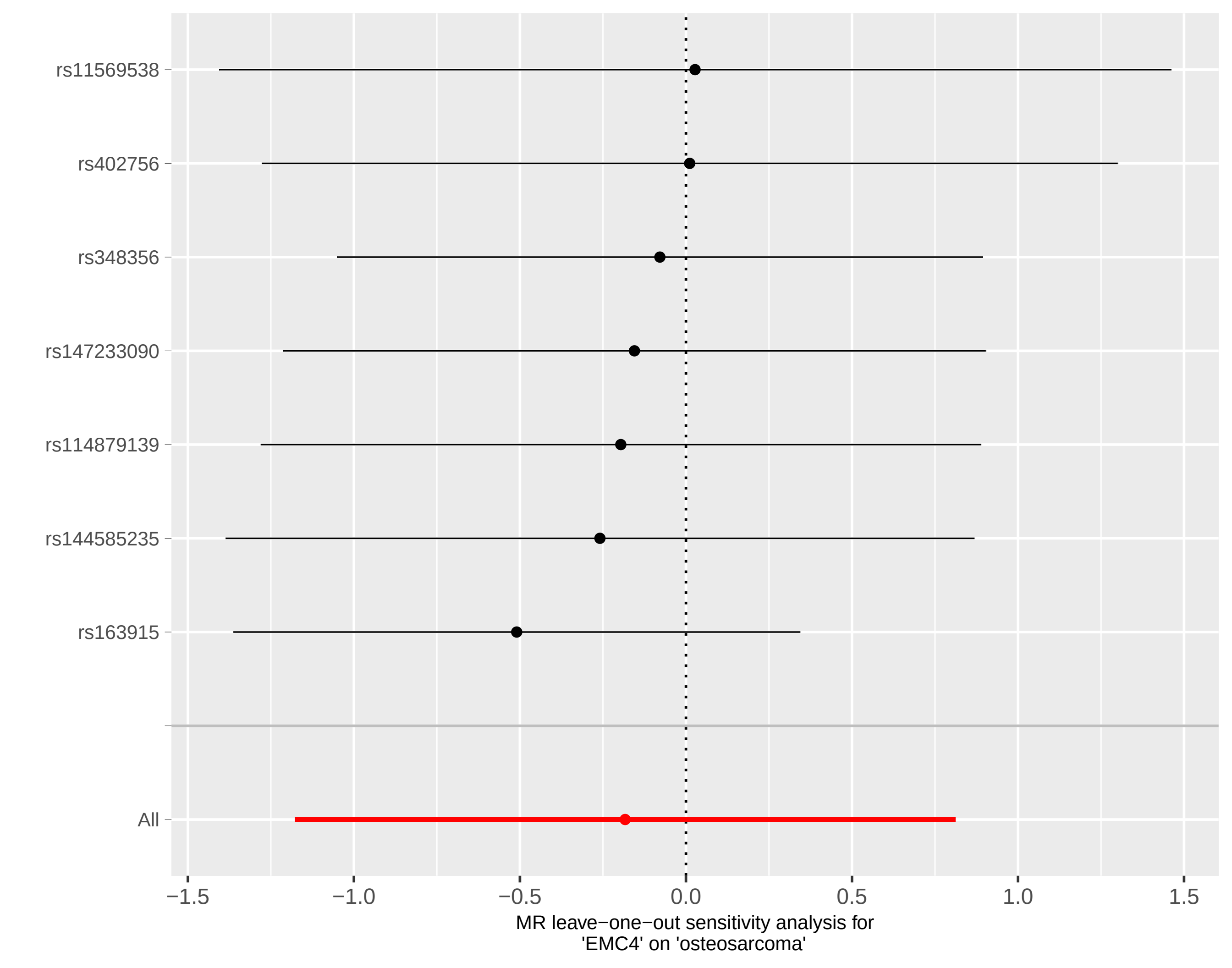

rs11569538
rs402756
rs348356
rs147233090
rs114879139
rs144585235
rs163915
All
−1.5
−1.0
−0.5
0.0
0.5
1.0
1.5
MR leave−one−out sensitivity analysis for
'EMC4' on 'osteosarcoma'

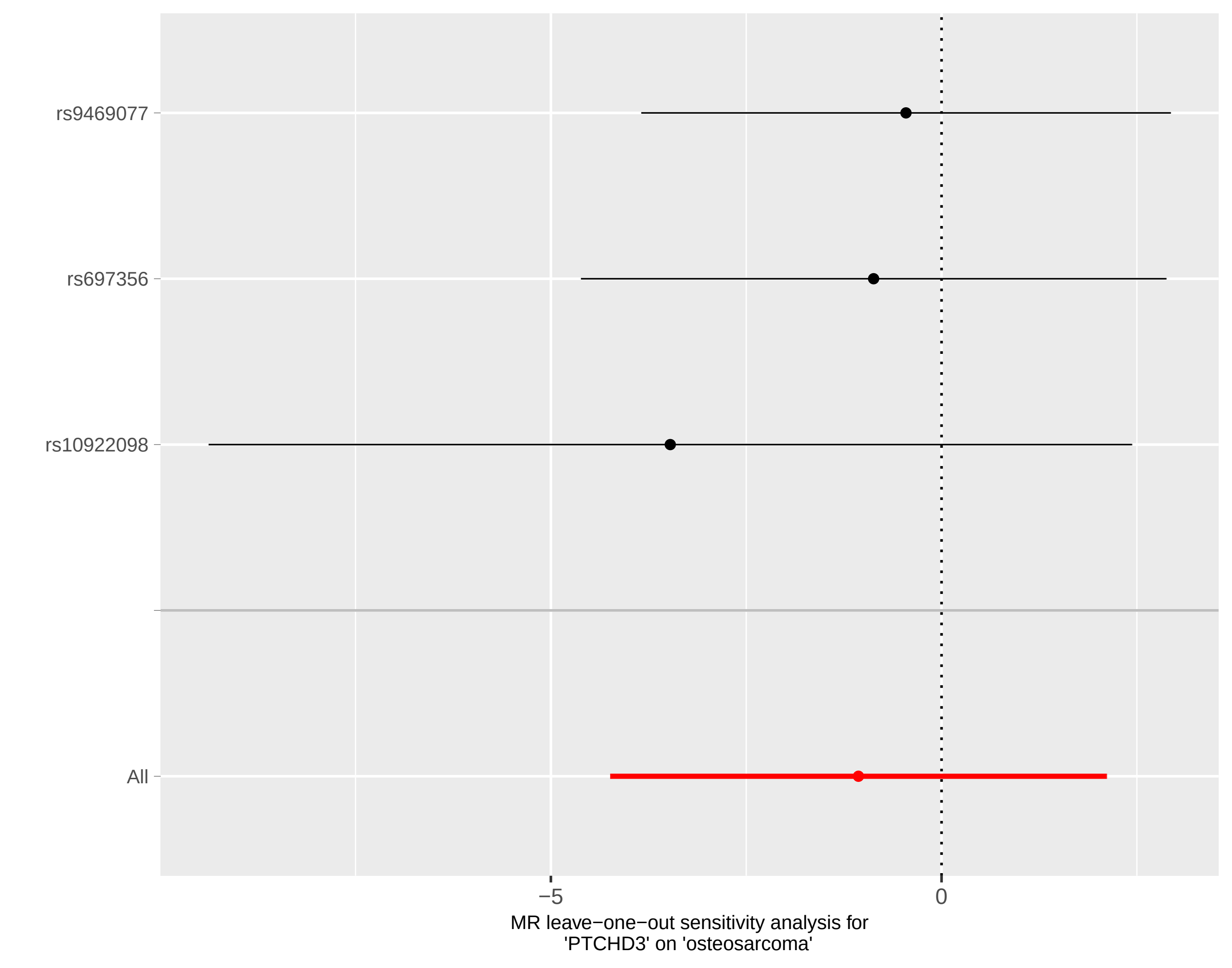

rs9469077
rs697356
rs10922098
All
−5
0
MR leave−one−out sensitivity analysis for
'PTCHD3' on 'osteosarcoma'

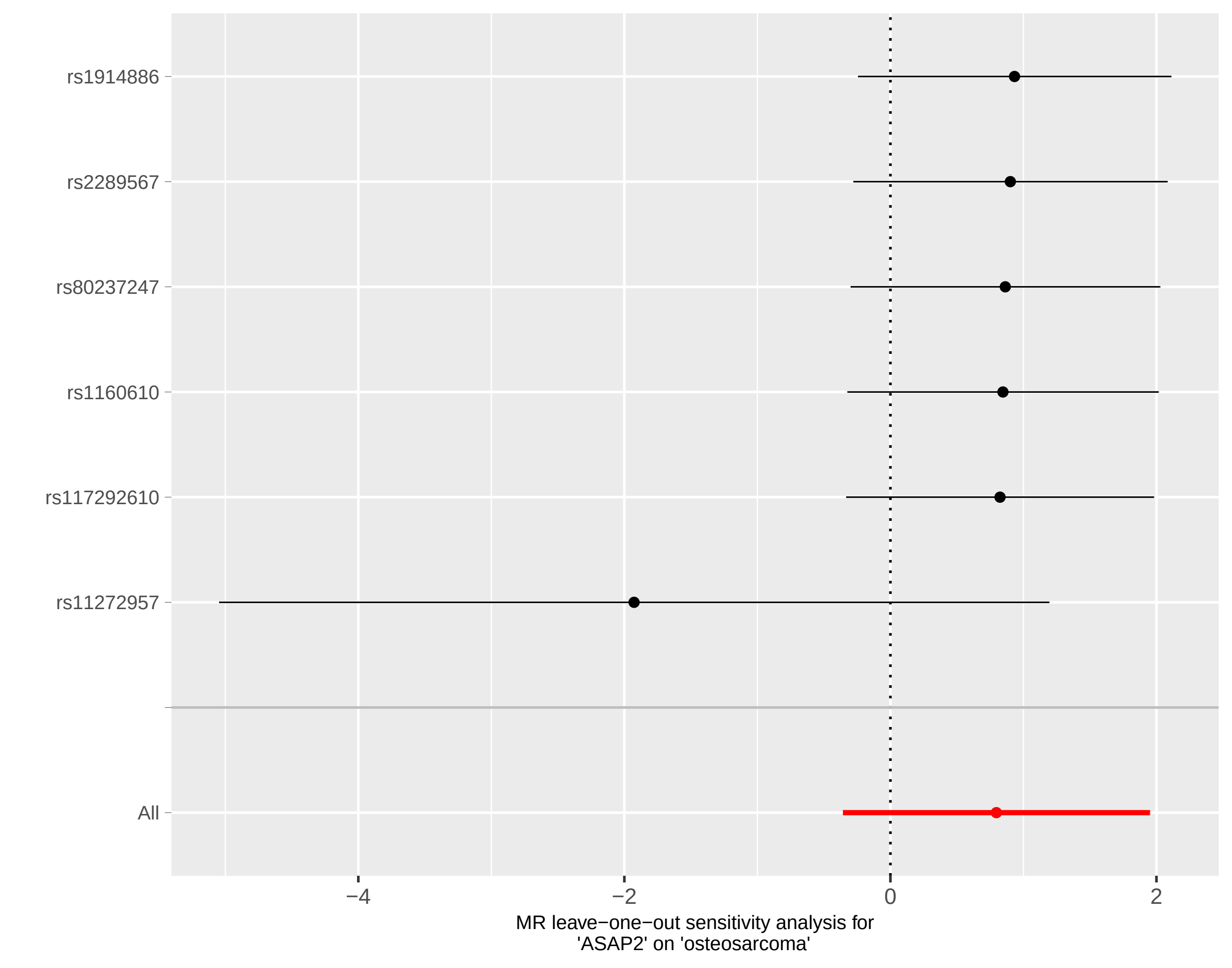

rs1914886
rs2289567
rs80237247
rs1160610
rs117292610
rs11272957
All
−4
−2
0
2
MR leave−one−out sensitivity analysis for
'ASAP2' on 'osteosarcoma'

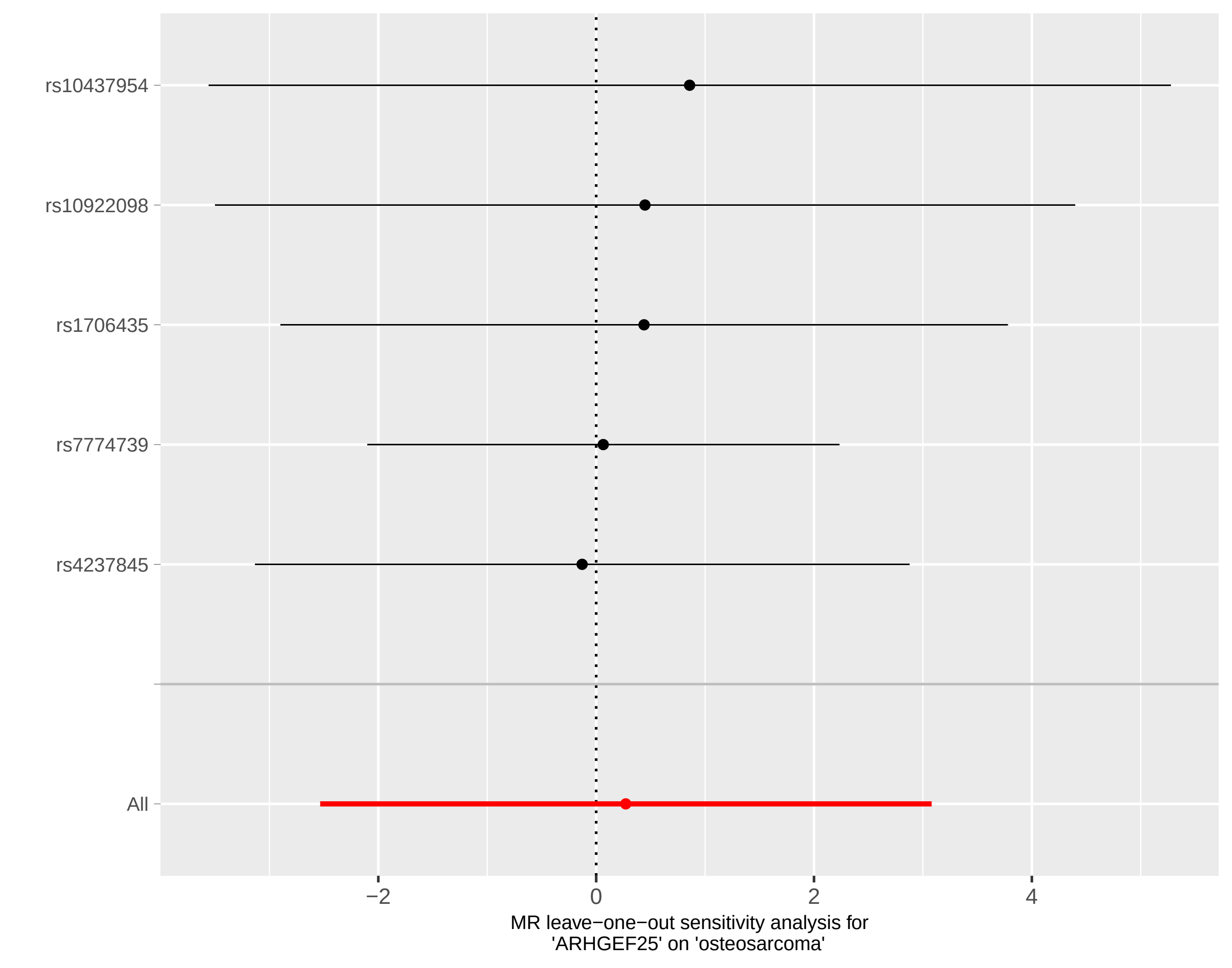

rs10437954
rs10922098
rs1706435
rs7774739
rs4237845
All
−2
0
2
4
MR leave−one−out sensitivity analysis for
'ARHGEF25' on 'osteosarcoma'

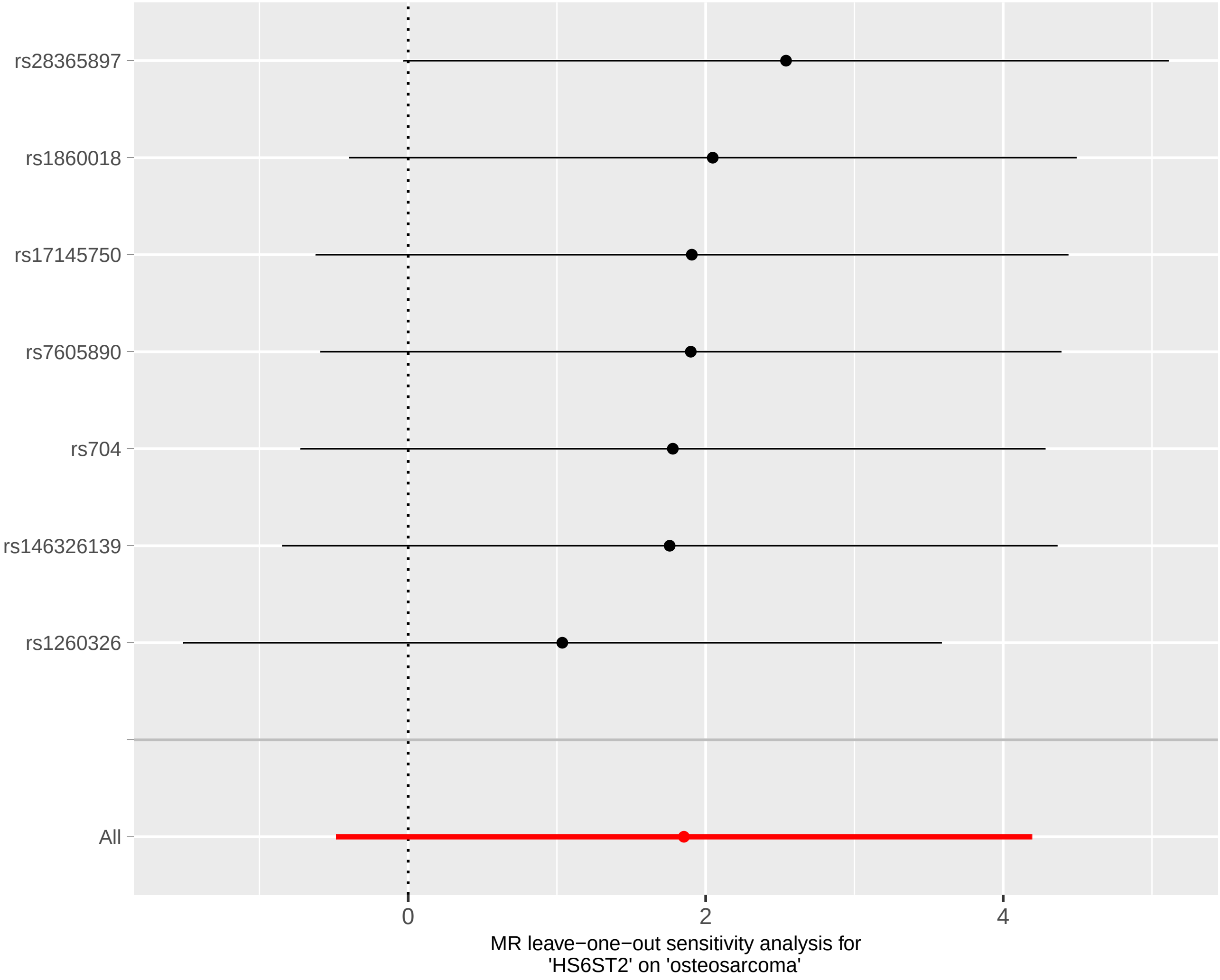

rs28365897
rs1860018
rs17145750
rs7605890
rs704
rs146326139
rs1260326
All
0
2
4
MR leave−one−out sensitivity analysis for
'HS6ST2' on 'osteosarcoma'

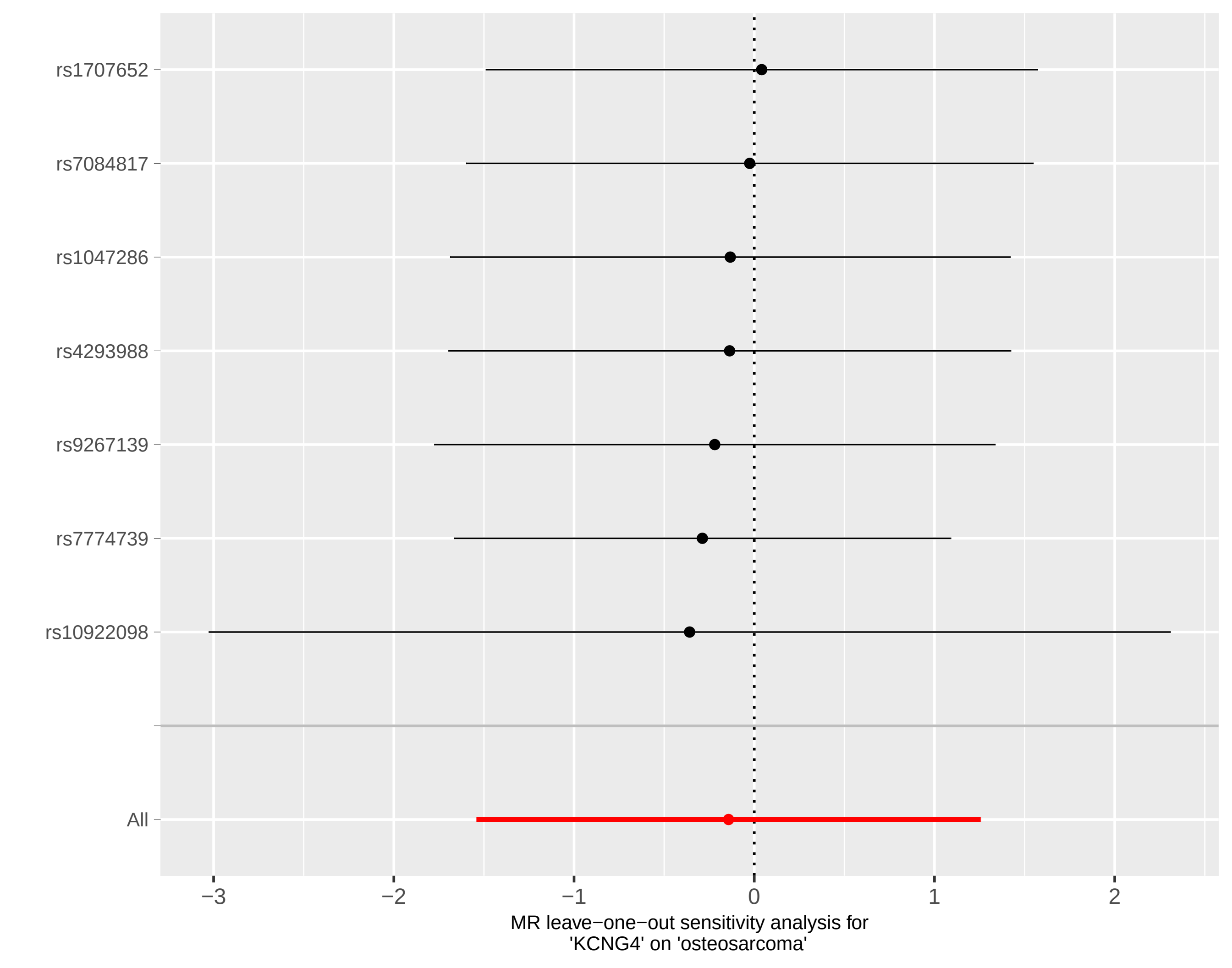

rs1707652
rs7084817
rs1047286
rs4293988
rs9267139
rs7774739
rs10922098
All
−3
−2
−1
0
1
2
MR leave−one−out sensitivity analysis for
'KCNG4' on 'osteosarcoma'

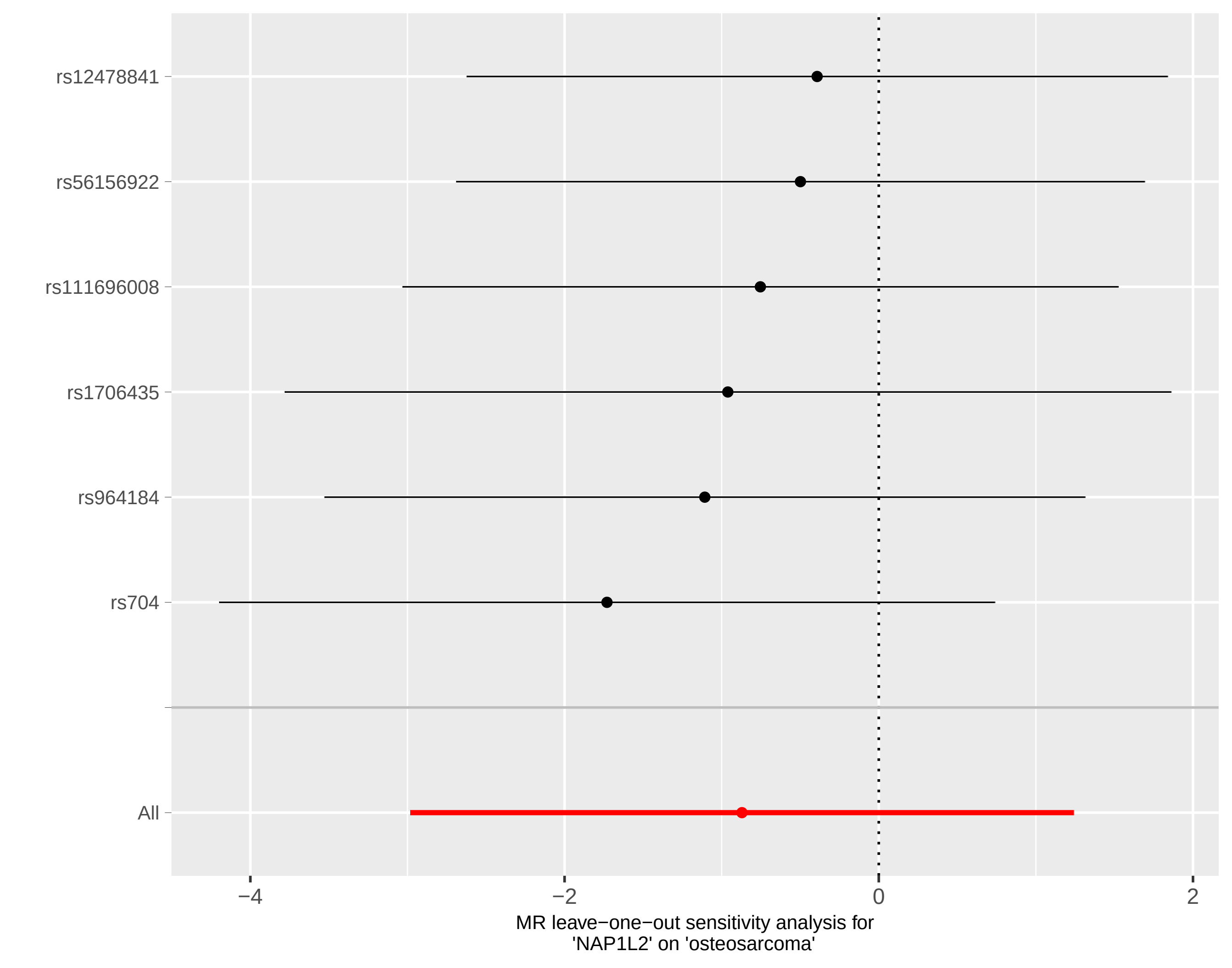
rs12478841
rs56156922
rs111696008
rs1706435
rs964184
rs704
All
−4
−2
0
2
MR leave−one−out sensitivity analysis for
'NAP1L2' on 'osteosarcoma'

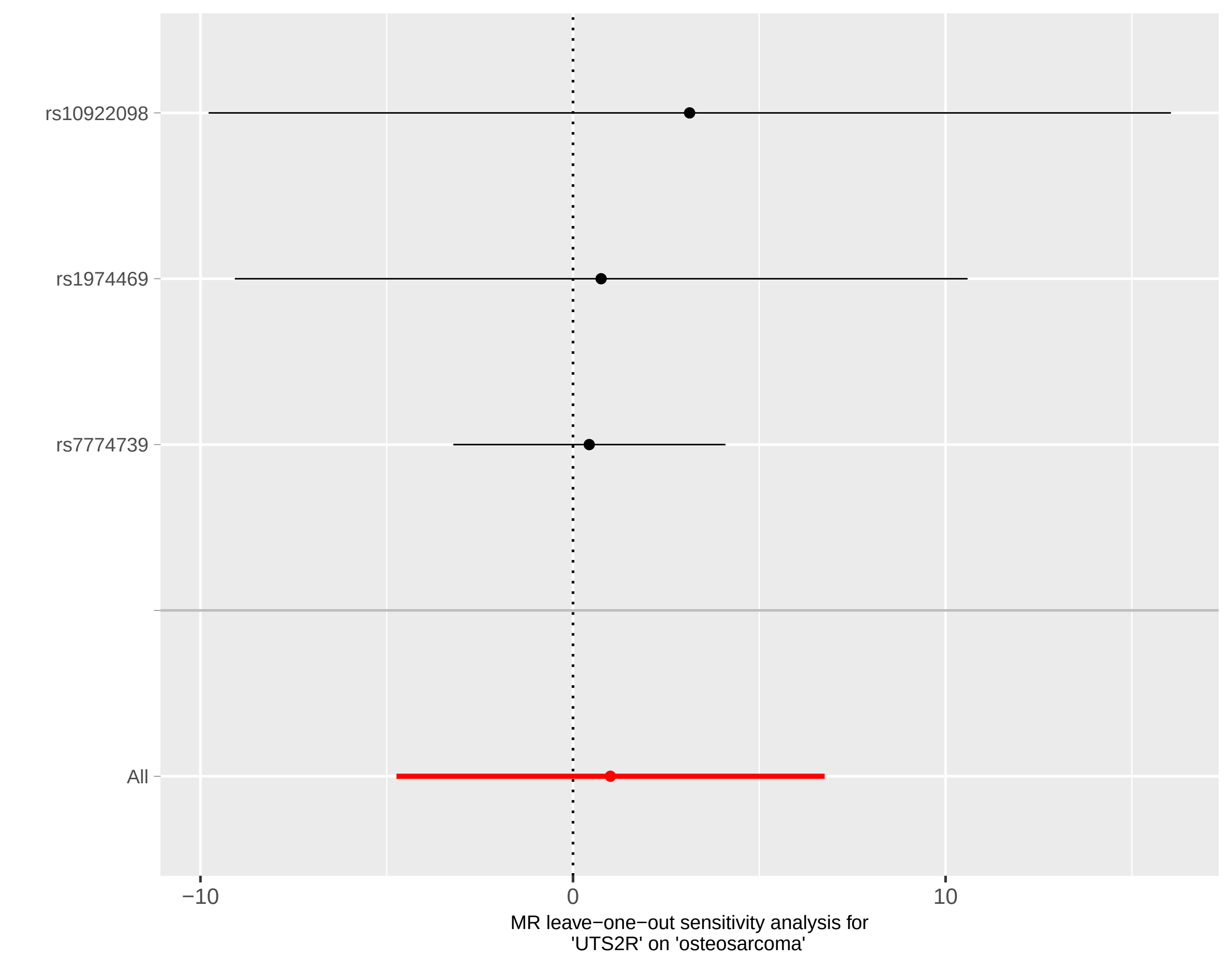
rs10922098
rs1974469
rs7774739
All
−10
0
10
MR leave−one−out sensitivity analysis for
'UTS2R' on 'osteosarcoma'

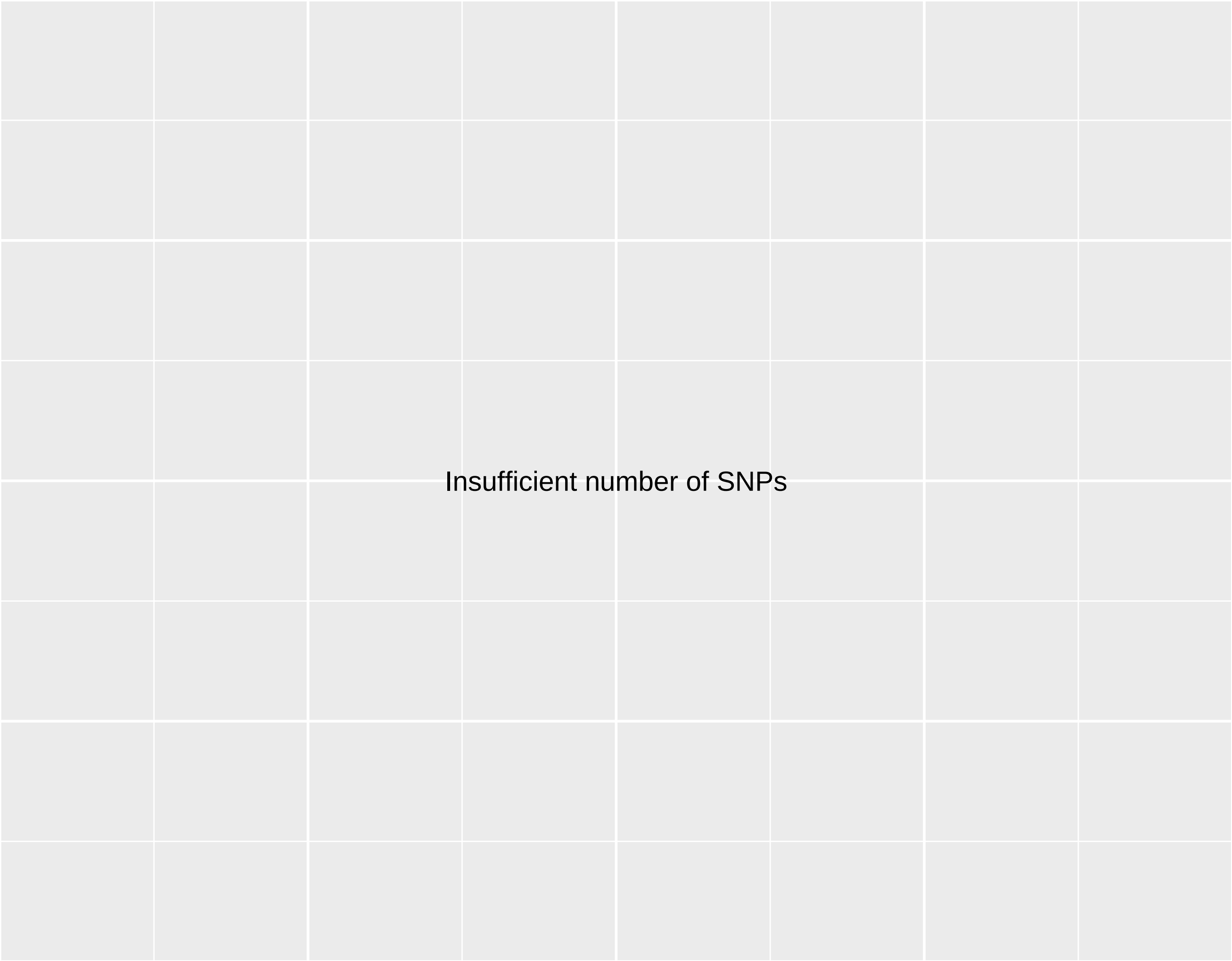
Insufficient number of SNPs

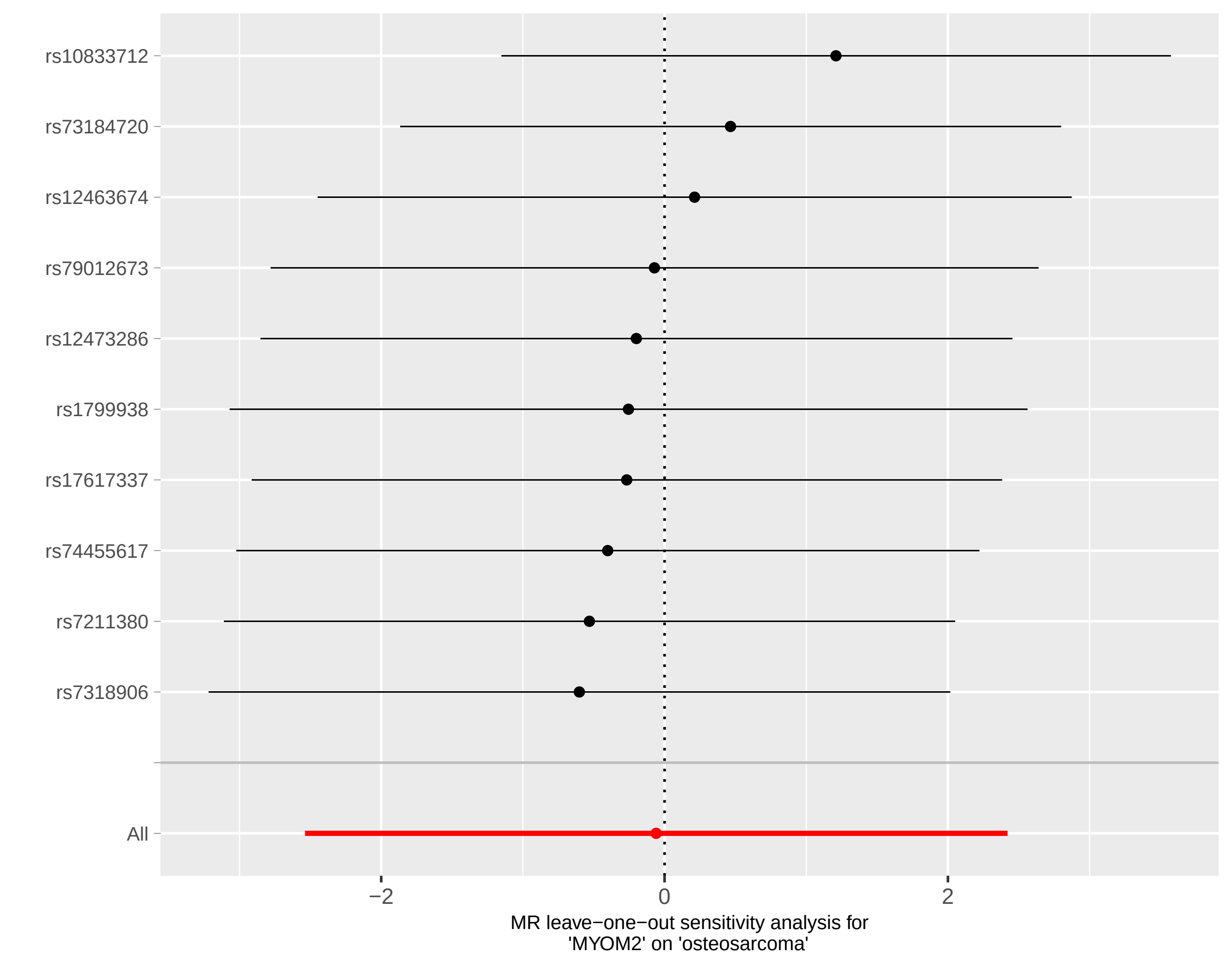

rs10833712
rs73184720
rs12463674
rs79012673
rs12473286
rs1799938
rs17617337
rs74455617
rs7211380
rs7318906
All
−2
0
2
MR leave−one−out sensitivity analysis for
'MYOM2' on 'osteosarcoma'

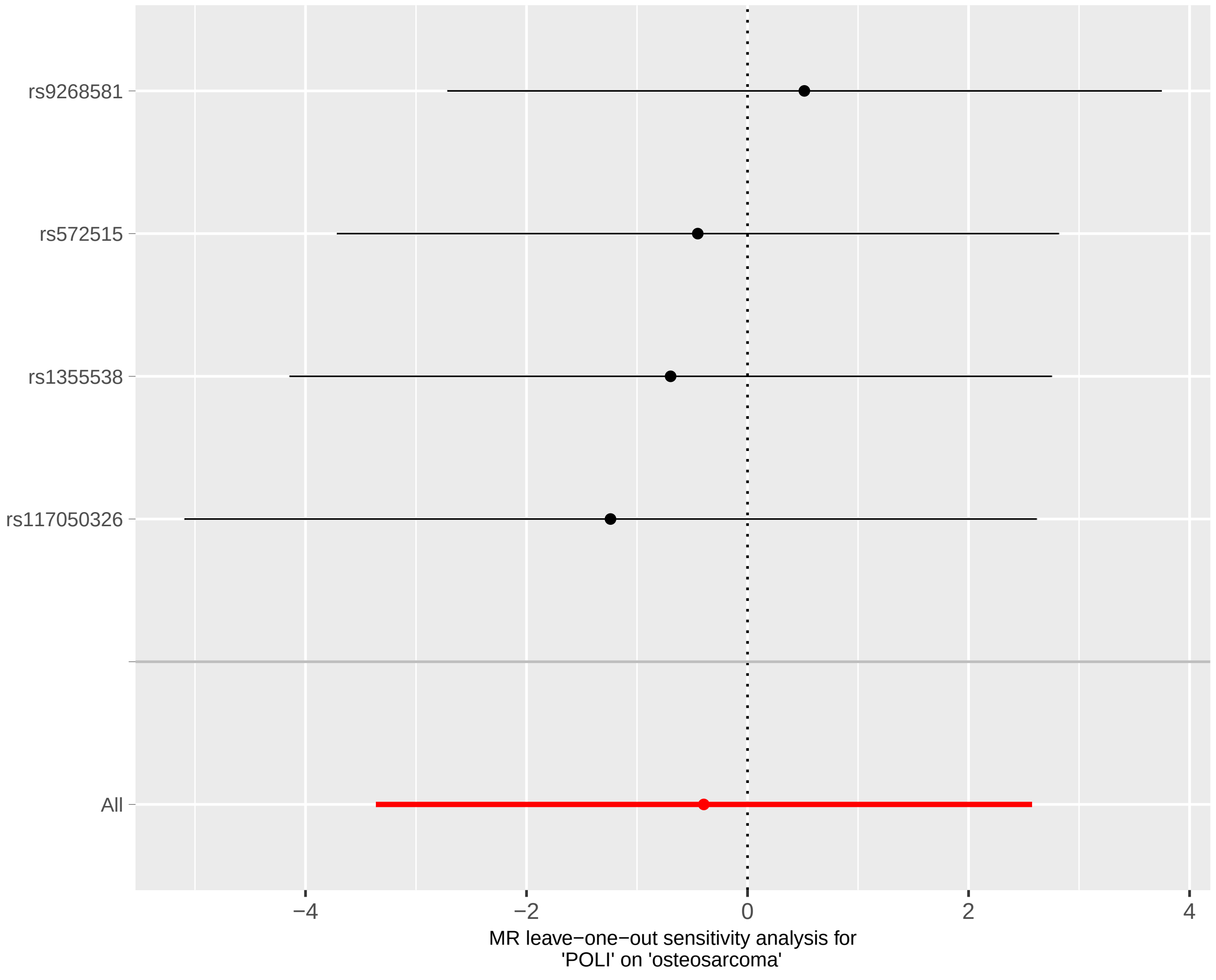

rs9268581
rs572515
rs1355538
rs117050326
All
−4
−2
0
2
4
MR leave−one−out sensitivity analysis for
'POLI' on 'osteosarcoma'

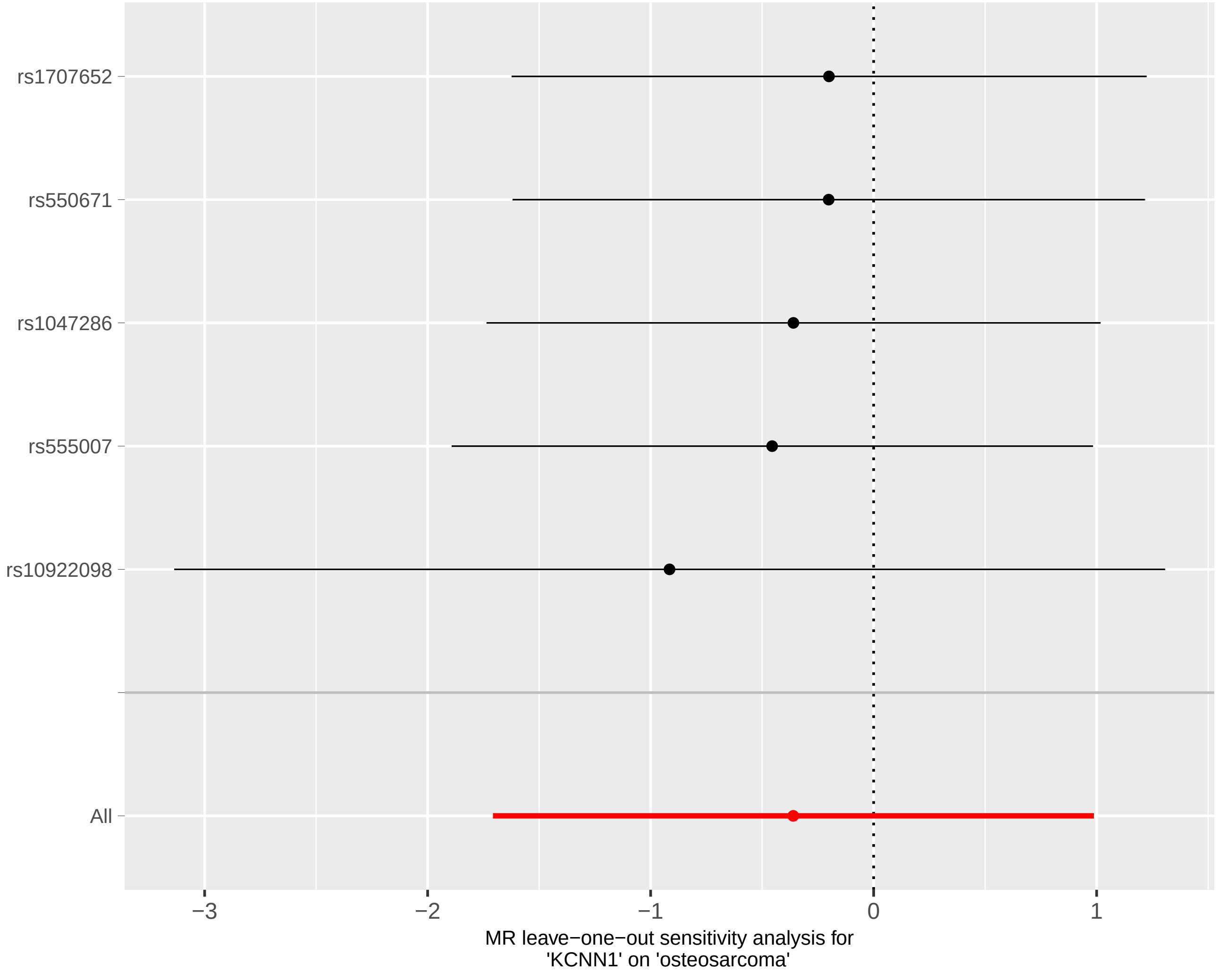
rs1707652
rs550671
rs1047286
rs555007
rs10922098
All
−3
−2
−1
0
1
MR leave−one−out sensitivity analysis for
'KCNN1' on 'osteosarcoma'

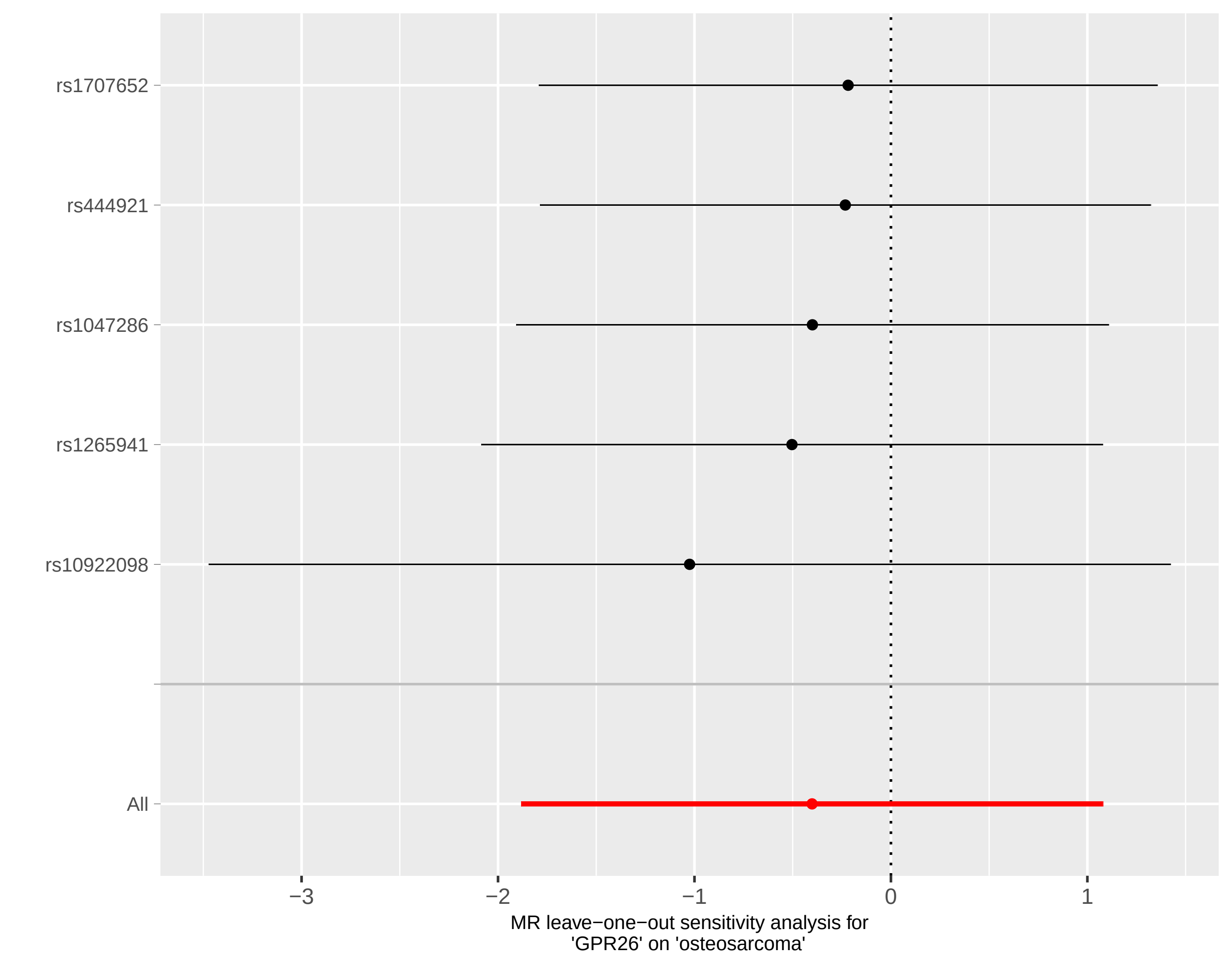

rs1707652
rs444921
rs1047286
rs1265941
rs10922098
All
−3
−2
−1
0
1
MR leave−one−out sensitivity analysis for
'GPR26' on 'osteosarcoma'

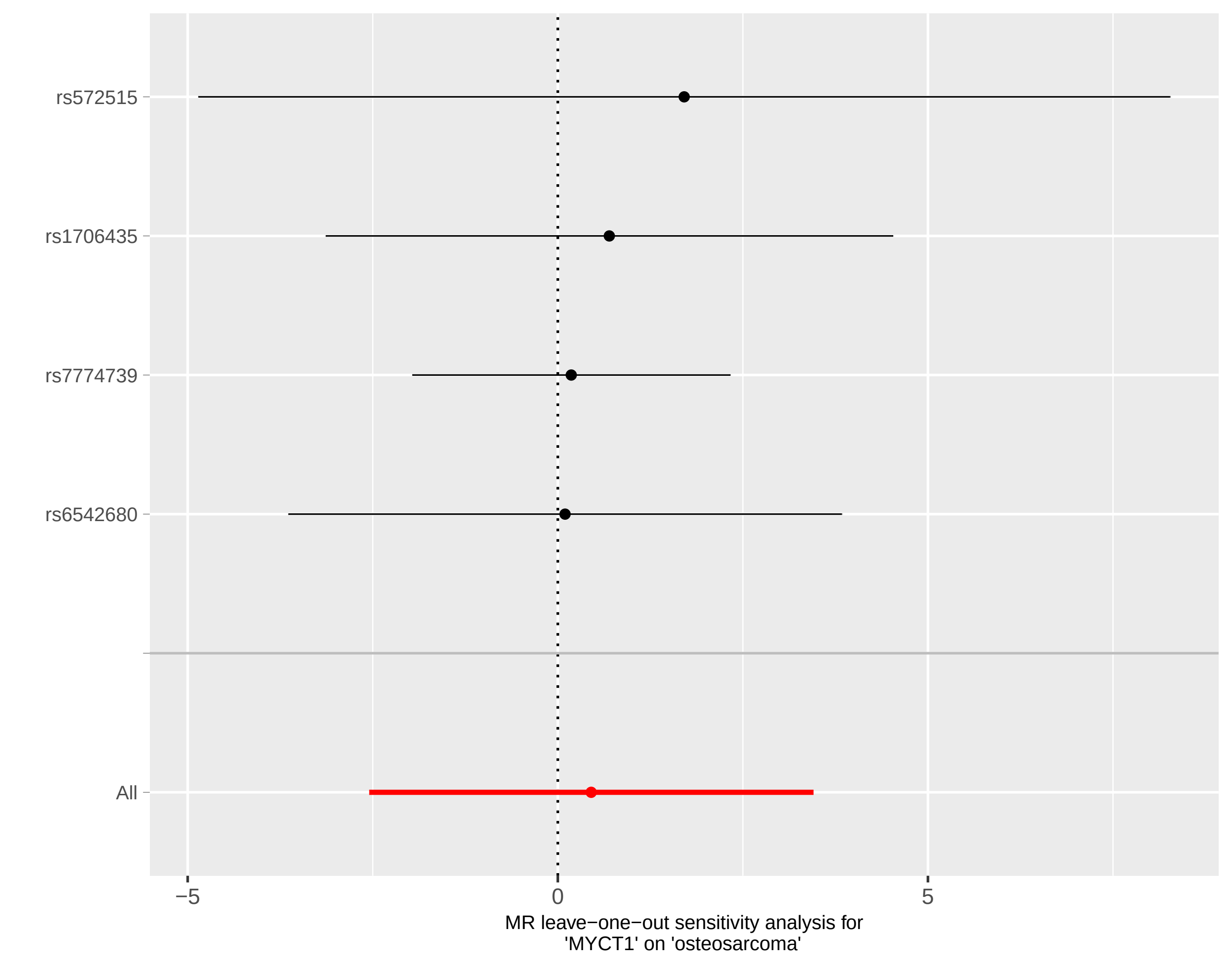
rs572515
rs1706435
rs7774739
rs6542680
All
−5
0
5
MR leave−one−out sensitivity analysis for
'MYCT1' on 'osteosarcoma'

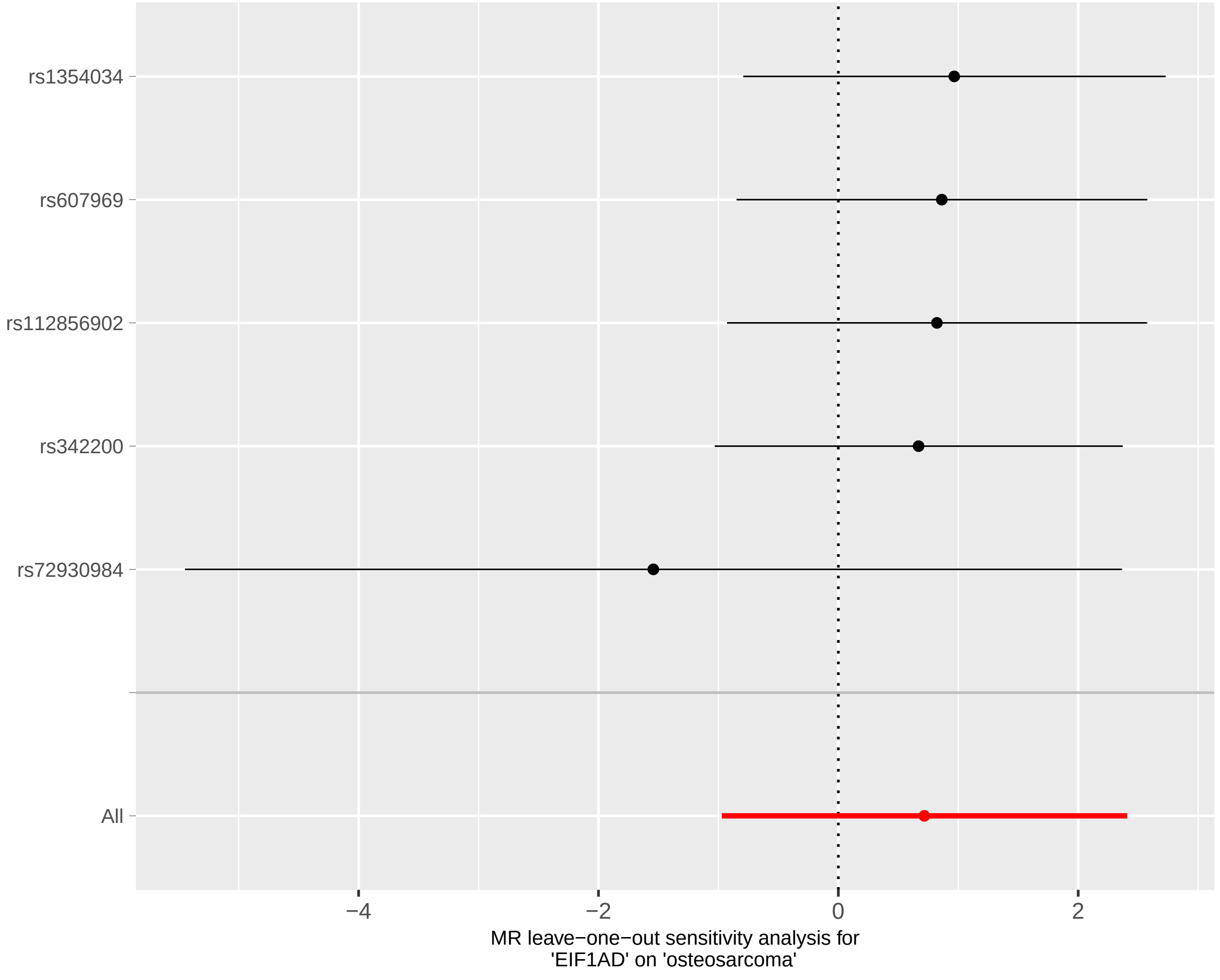

rs1354034
rs607969
rs112856902
rs342200
rs72930984
All
−4
−2
0
2
MR leave−one−out sensitivity analysis for
'EIF1AD' on 'osteosarcoma'

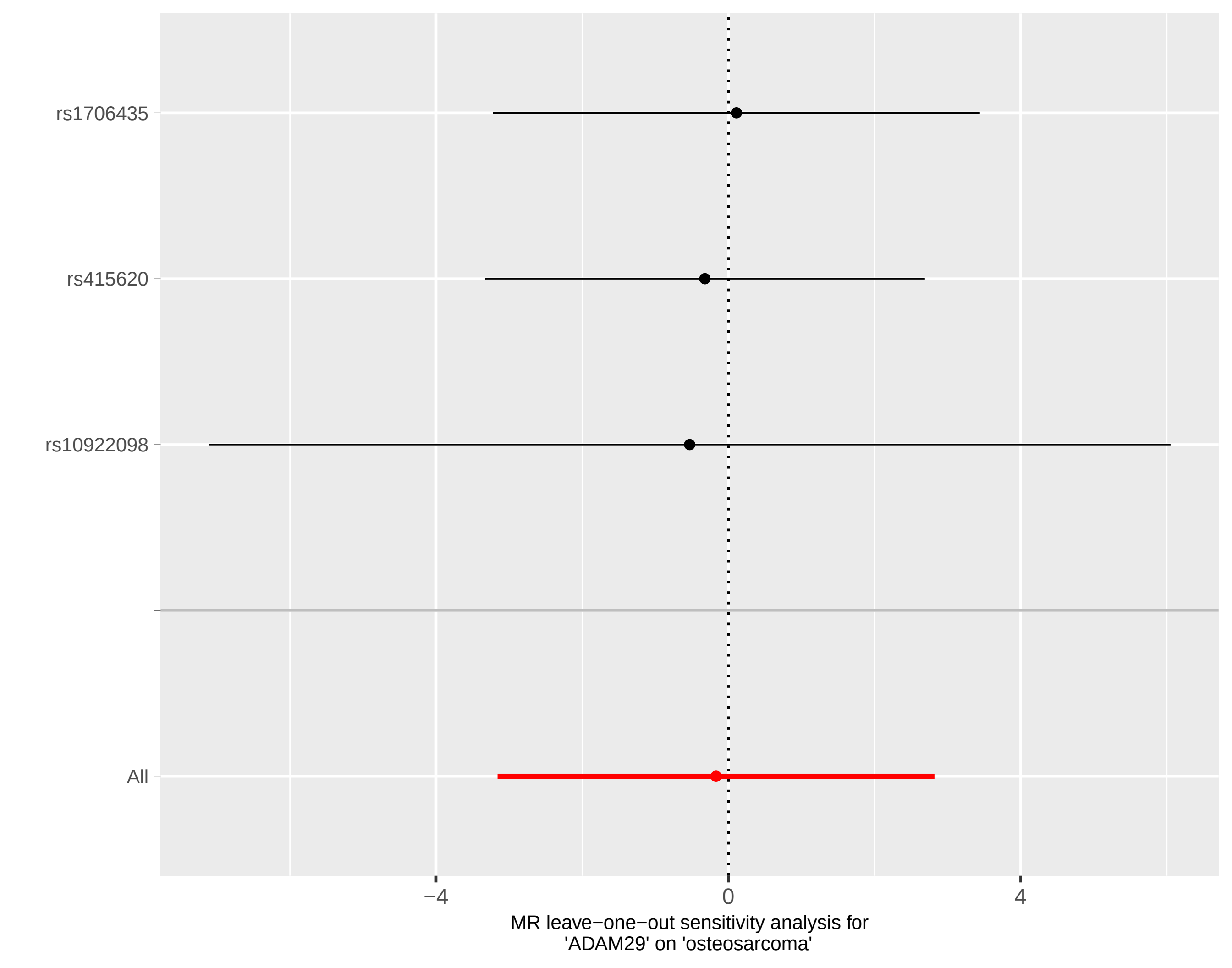

rs1706435
rs415620
rs10922098
All
−4
0
4
MR leave−one−out sensitivity analysis for
'ADAM29' on 'osteosarcoma'

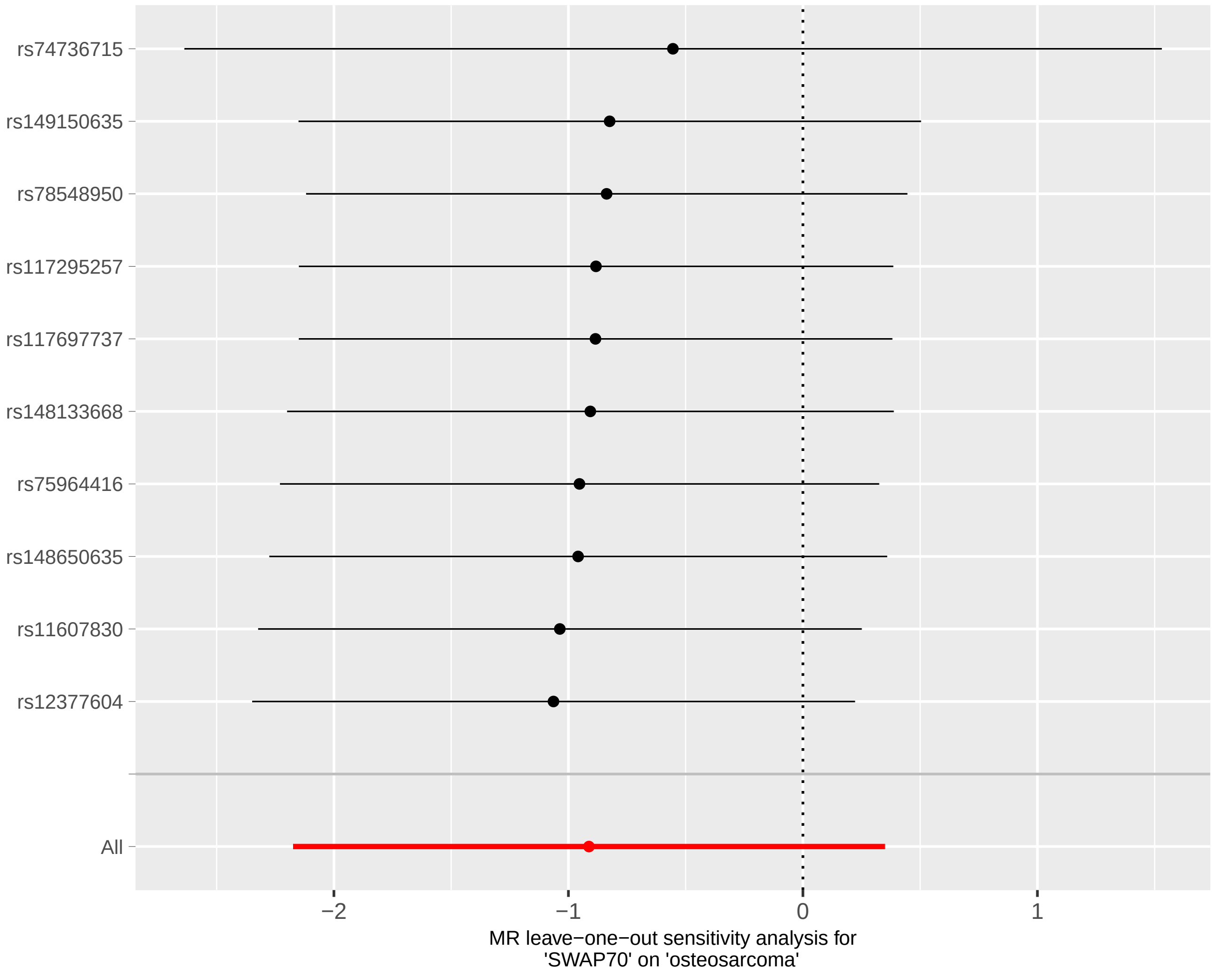

rs74736715
rs149150635
rs78548950
rs117295257
rs117697737
rs148133668
rs75964416
rs148650635
rs11607830
rs12377604
All
−2
−1
0
1
MR leave−one−out sensitivity analysis for
'SWAP70' on 'osteosarcoma'

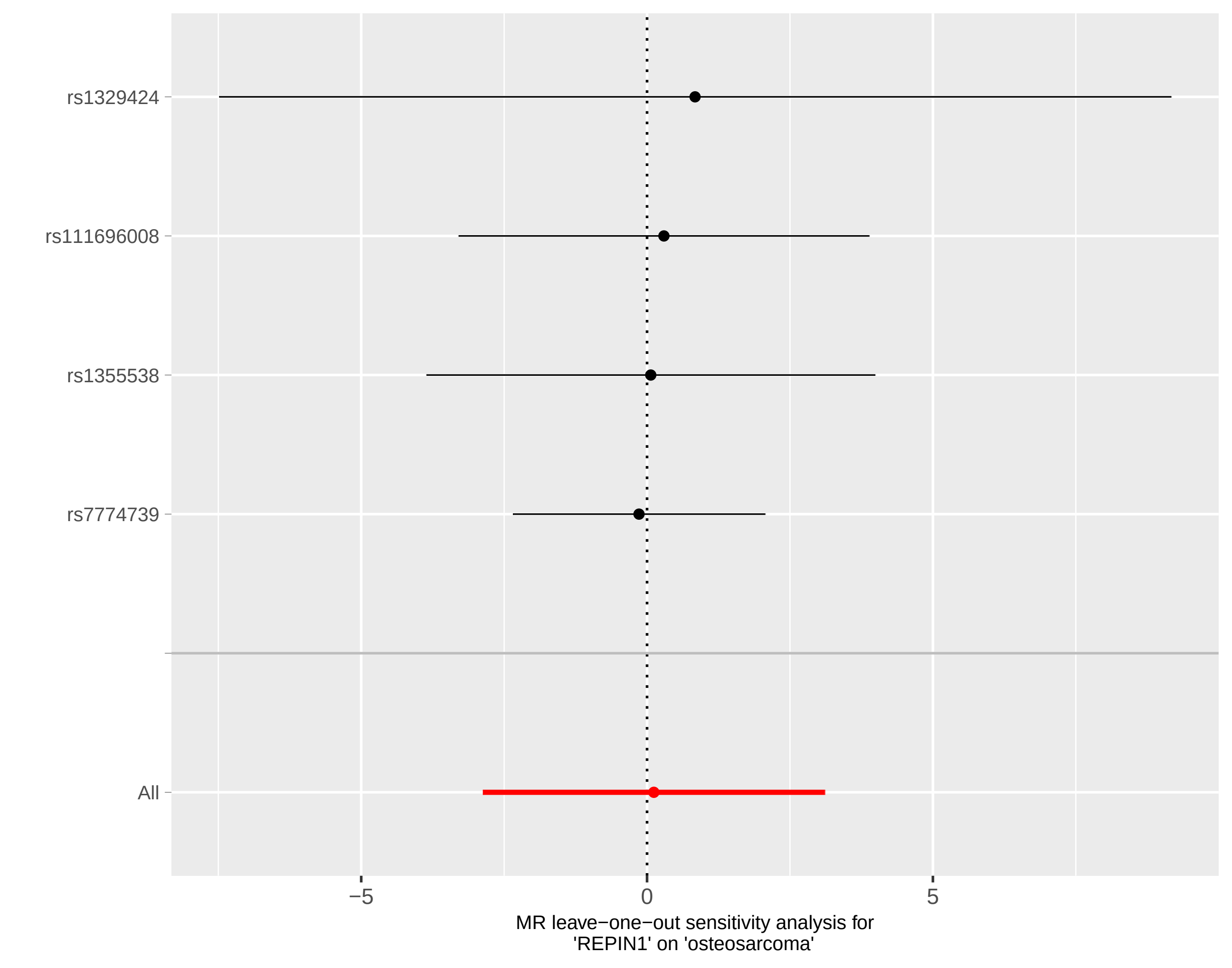

rs1329424
rs111696008
rs1355538
rs7774739
All
−5
0
5
MR leave−one−out sensitivity analysis for
'REPIN1' on 'osteosarcoma'

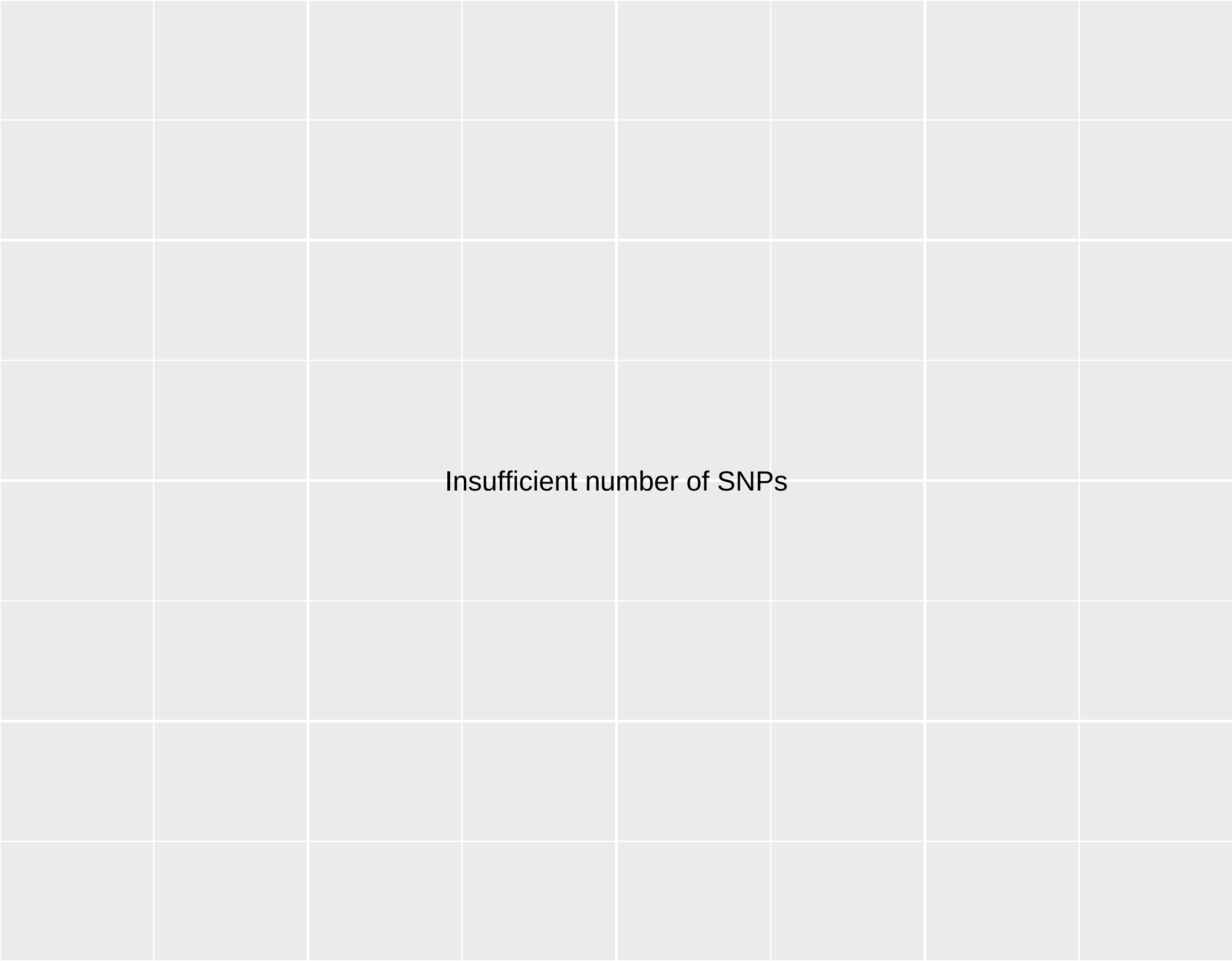
Insufficient number of SNPs

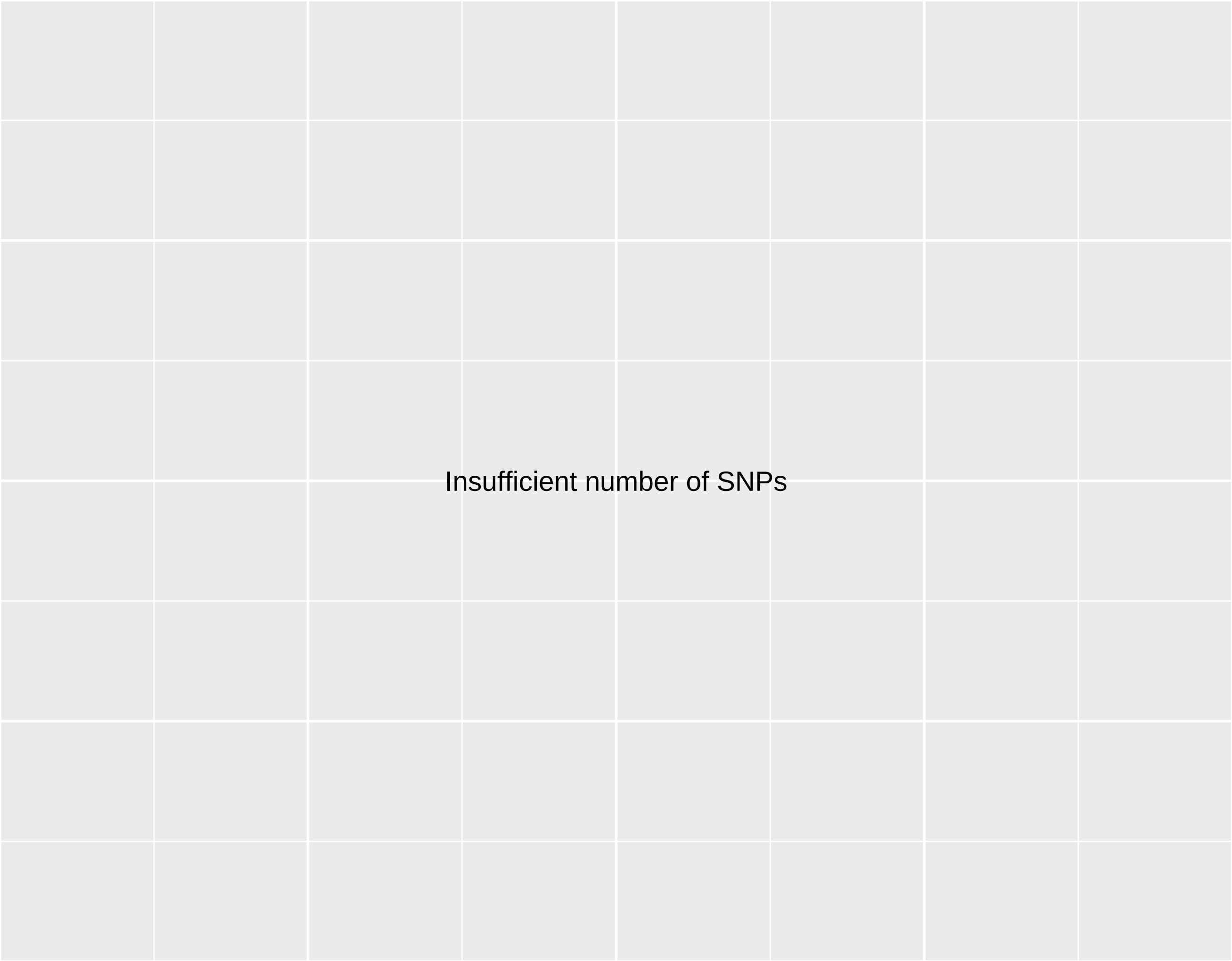
Insufficient number of SNPs

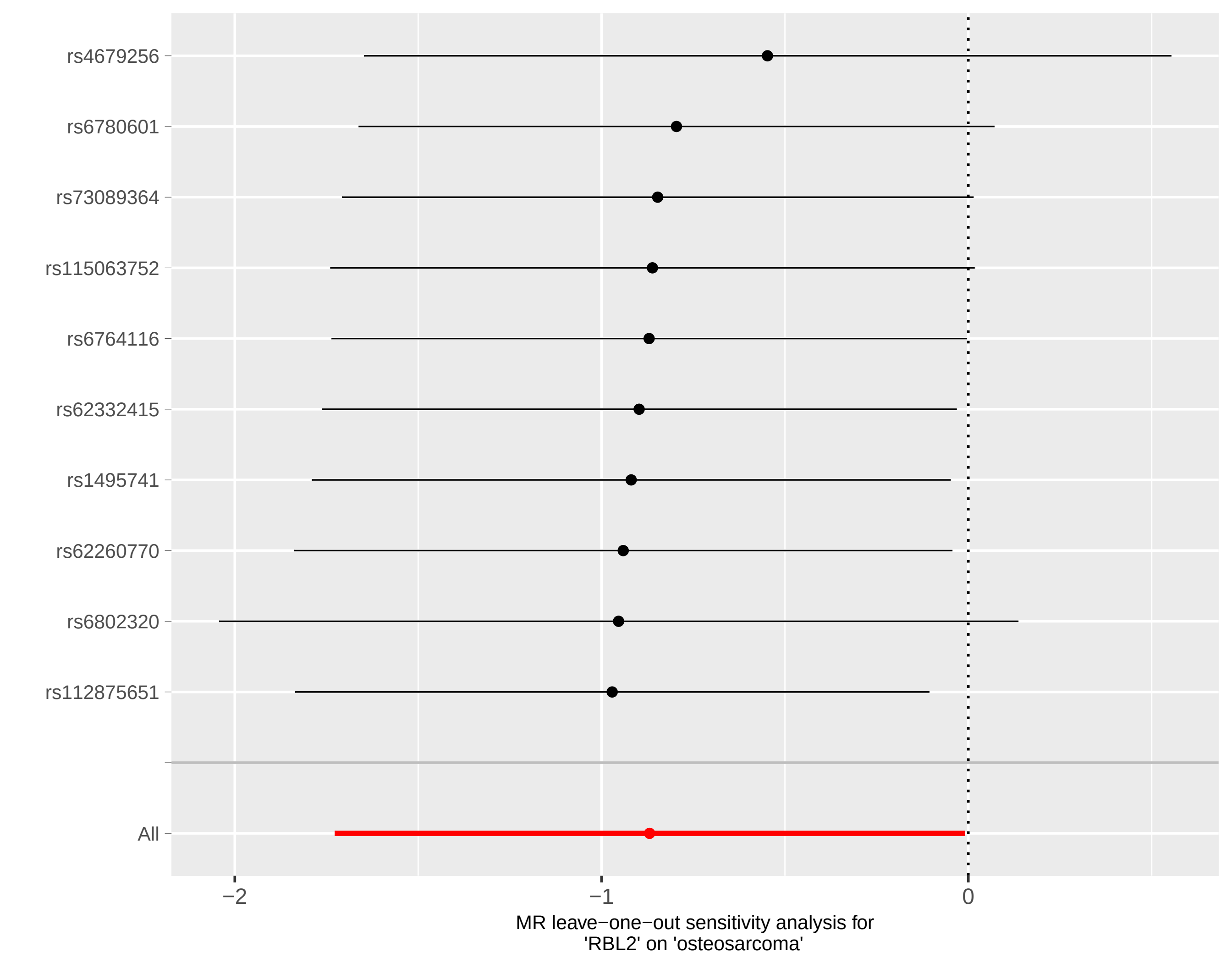

rs4679256
rs6780601
rs73089364
rs115063752
rs6764116
rs62332415
rs1495741
rs62260770
rs6802320
rs112875651
All
−2
−1
0
MR leave−one−out sensitivity analysis for
'RBL2' on 'osteosarcoma'

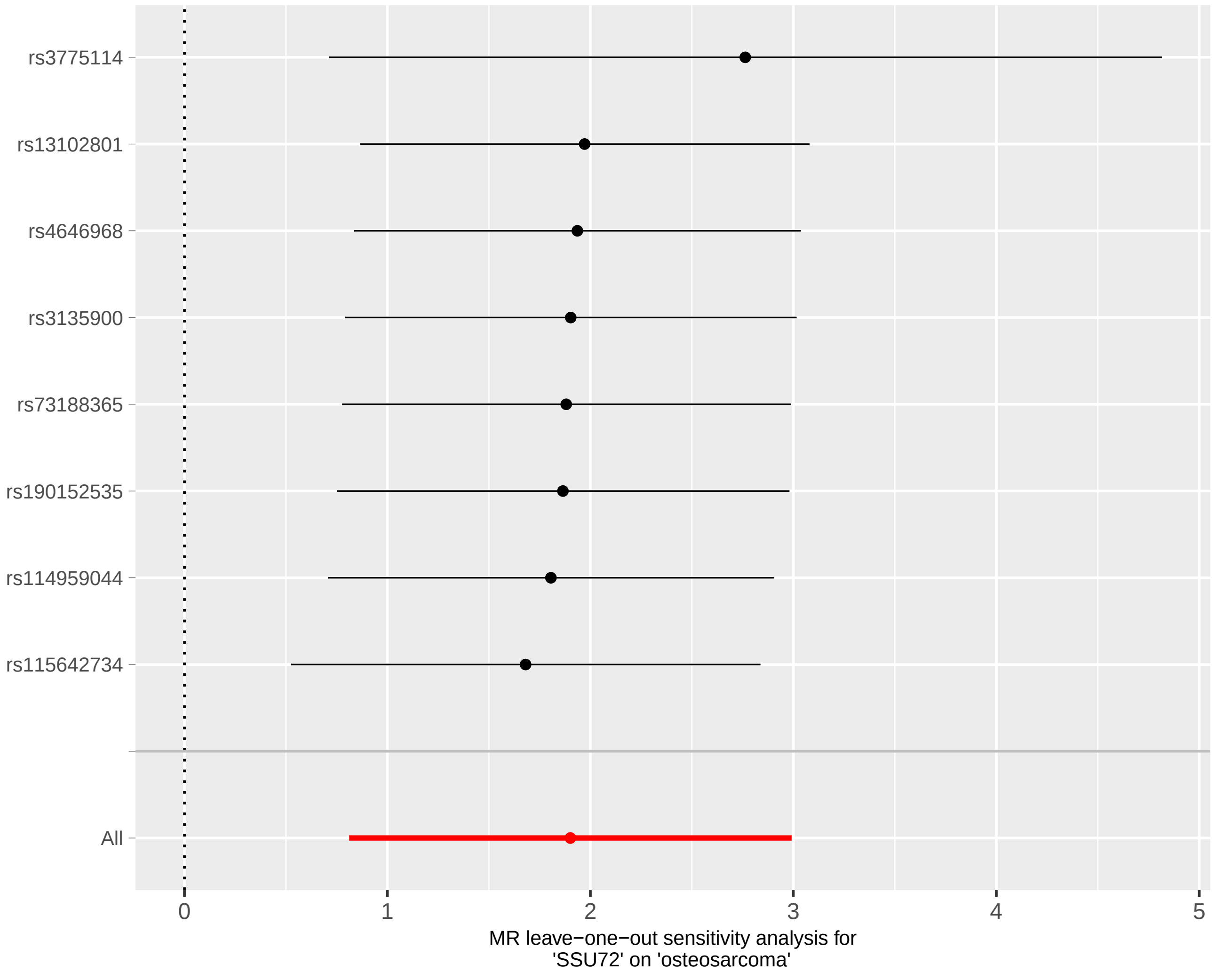
rs3775114
rs13102801
rs4646968
rs3135900
rs73188365
rs190152535
rs114959044
rs115642734
All
0
1
2
3
4
5
MR leave−one−out sensitivity analysis for
'SSU72' on 'osteosarcoma'

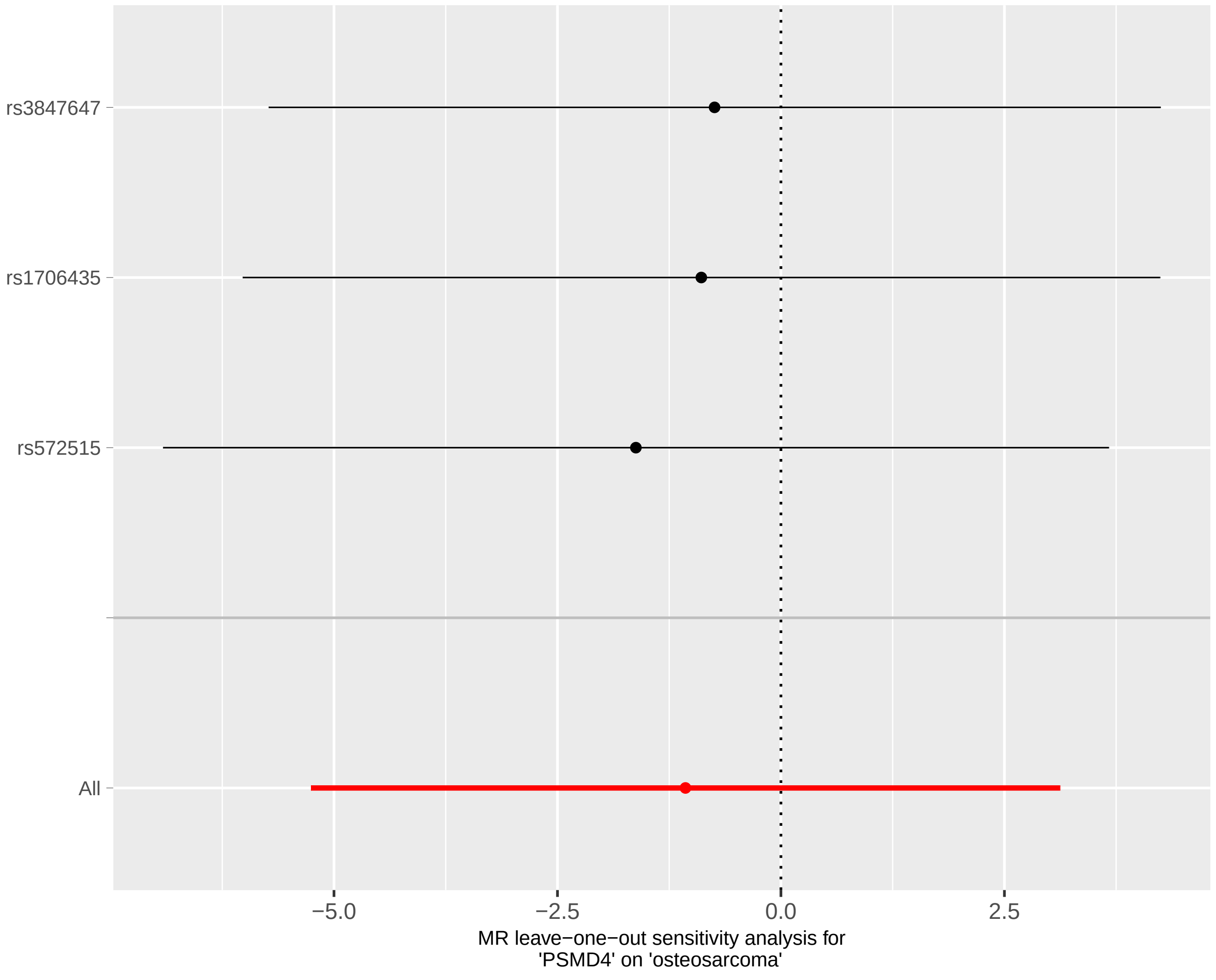

rs3847647
rs1706435
rs572515
All
−5.0
−2.5
0.0
2.5
MR leave−one−out sensitivity analysis for
'PSMD4' on 'osteosarcoma'

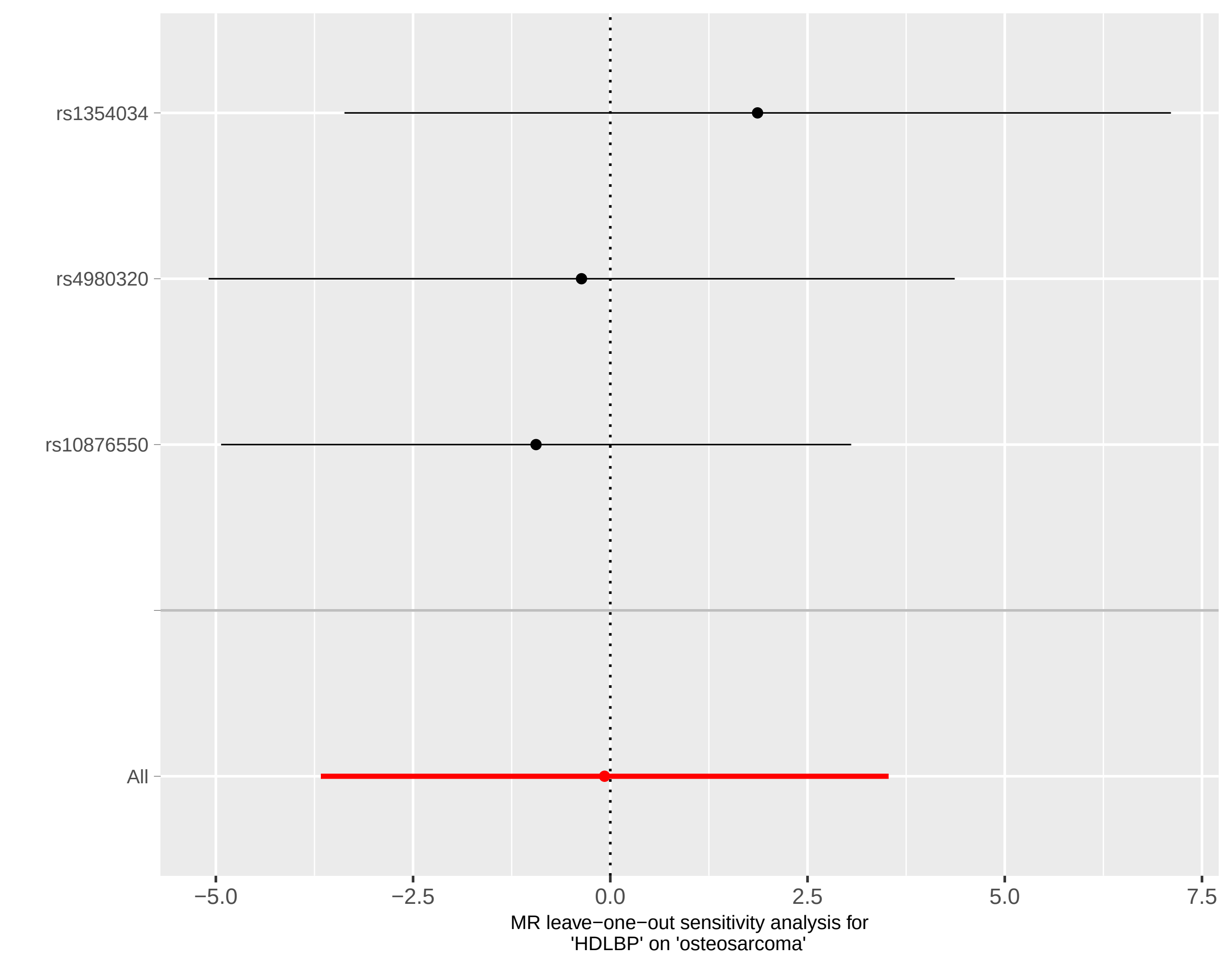

rs1354034
rs4980320
rs10876550
All
−5.0
−2.5
0.0
2.5
5.0
7.5
MR leave−one−out sensitivity analysis for
'HDLBP' on 'osteosarcoma'

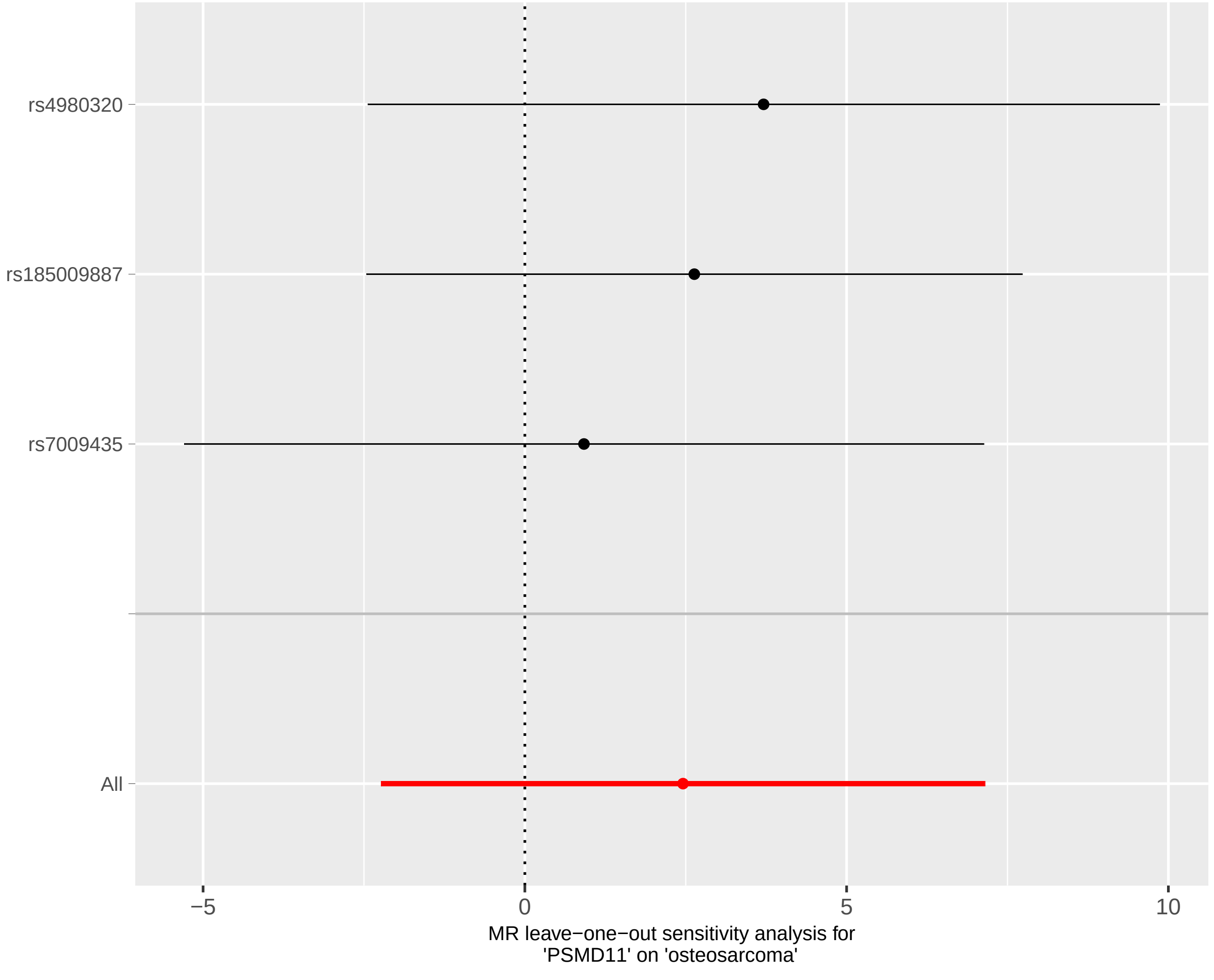

rs4980320
rs185009887
rs7009435
All
−5
0
5
10
MR leave−one−out sensitivity analysis for
'PSMD11' on 'osteosarcoma'

Insufficient number of SNPs

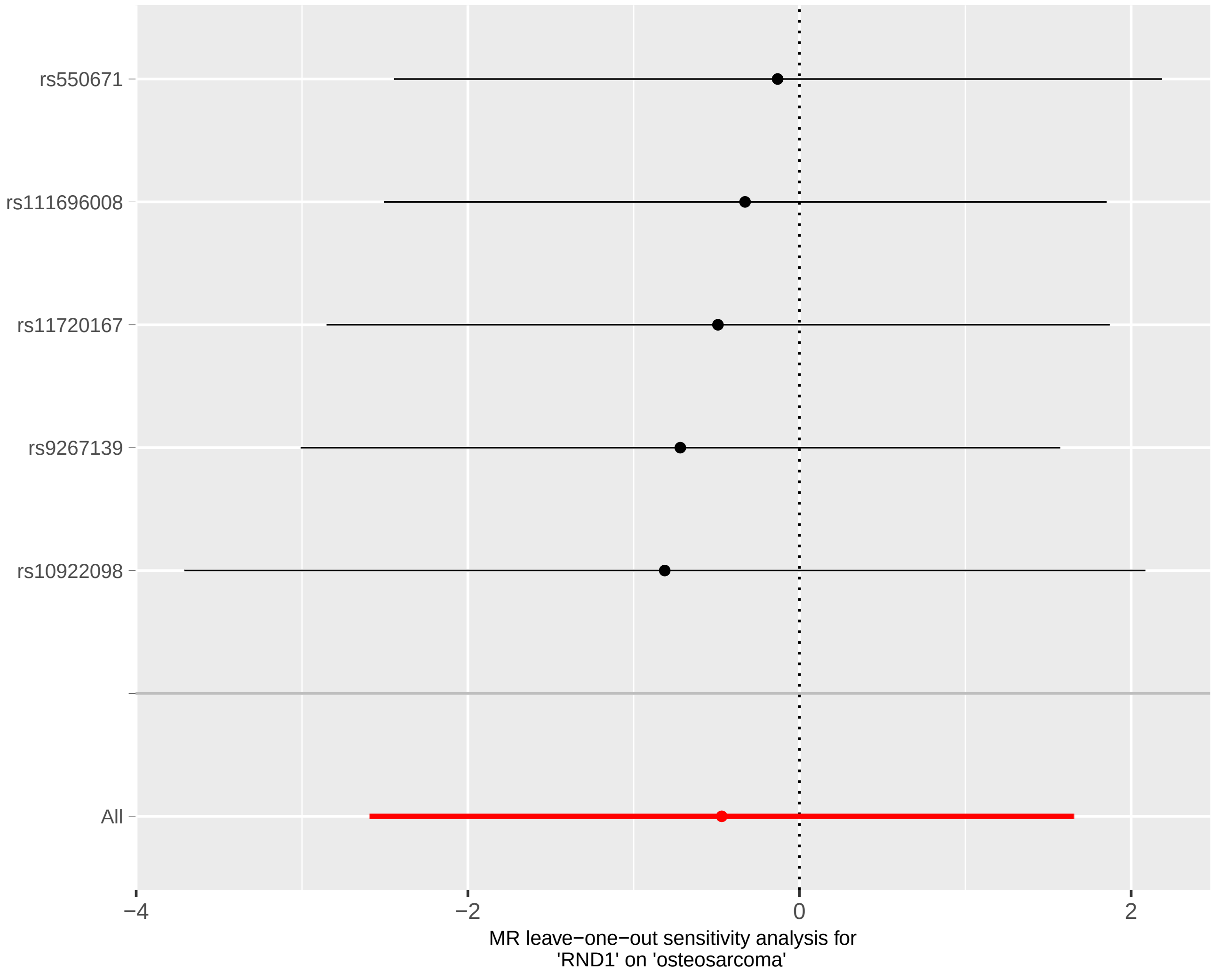
rs550671
rs111696008
rs11720167
rs9267139
rs10922098
All
−4
−2
0
2
MR leave−one−out sensitivity analysis for
'RND1' on 'osteosarcoma'

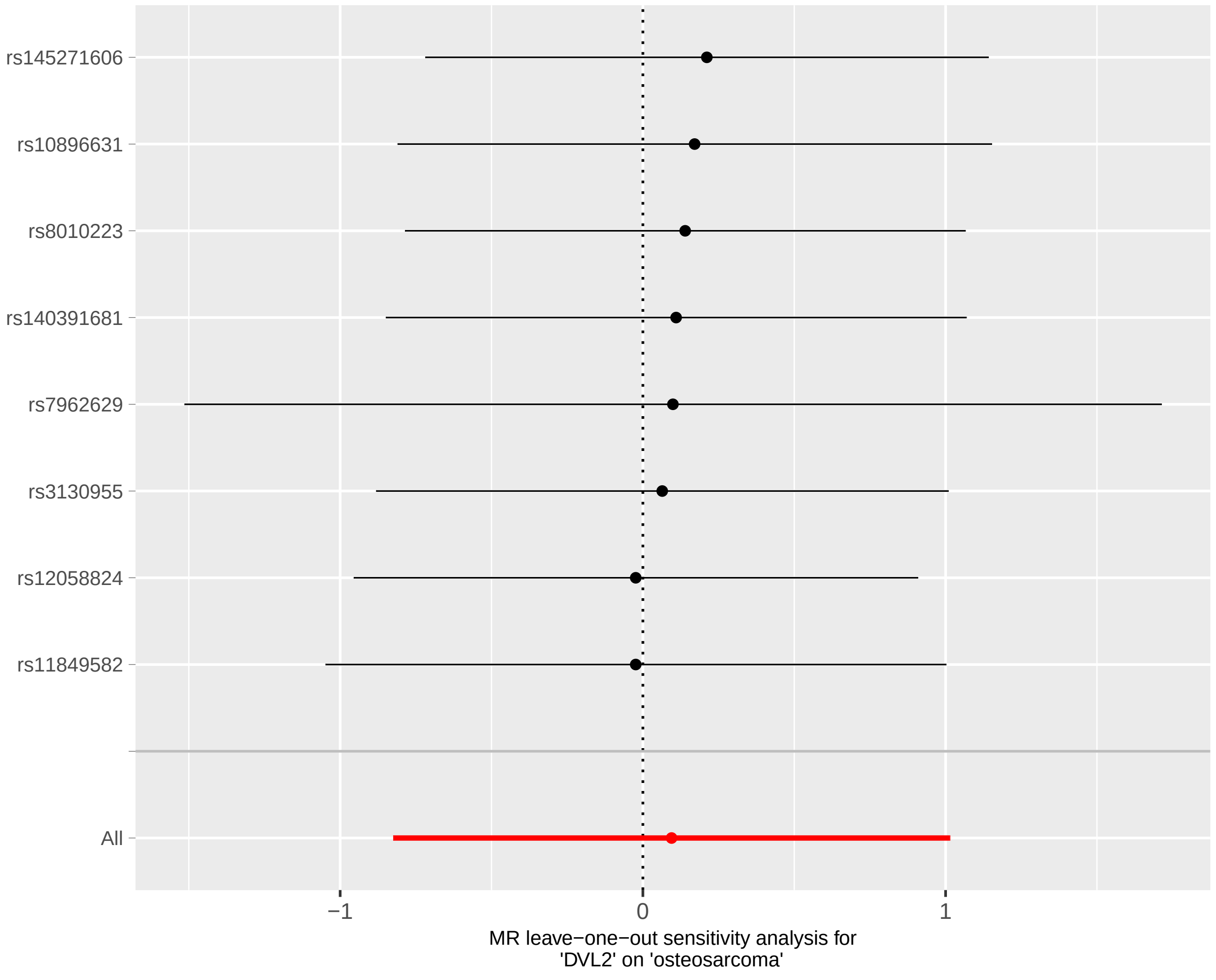
rs145271606
rs10896631
rs8010223
rs140391681
rs7962629
rs3130955
rs12058824
rs11849582
All
−1
0
1
MR leave−one−out sensitivity analysis for
'DVL2' on 'osteosarcoma'

Insufficient number of SNPs

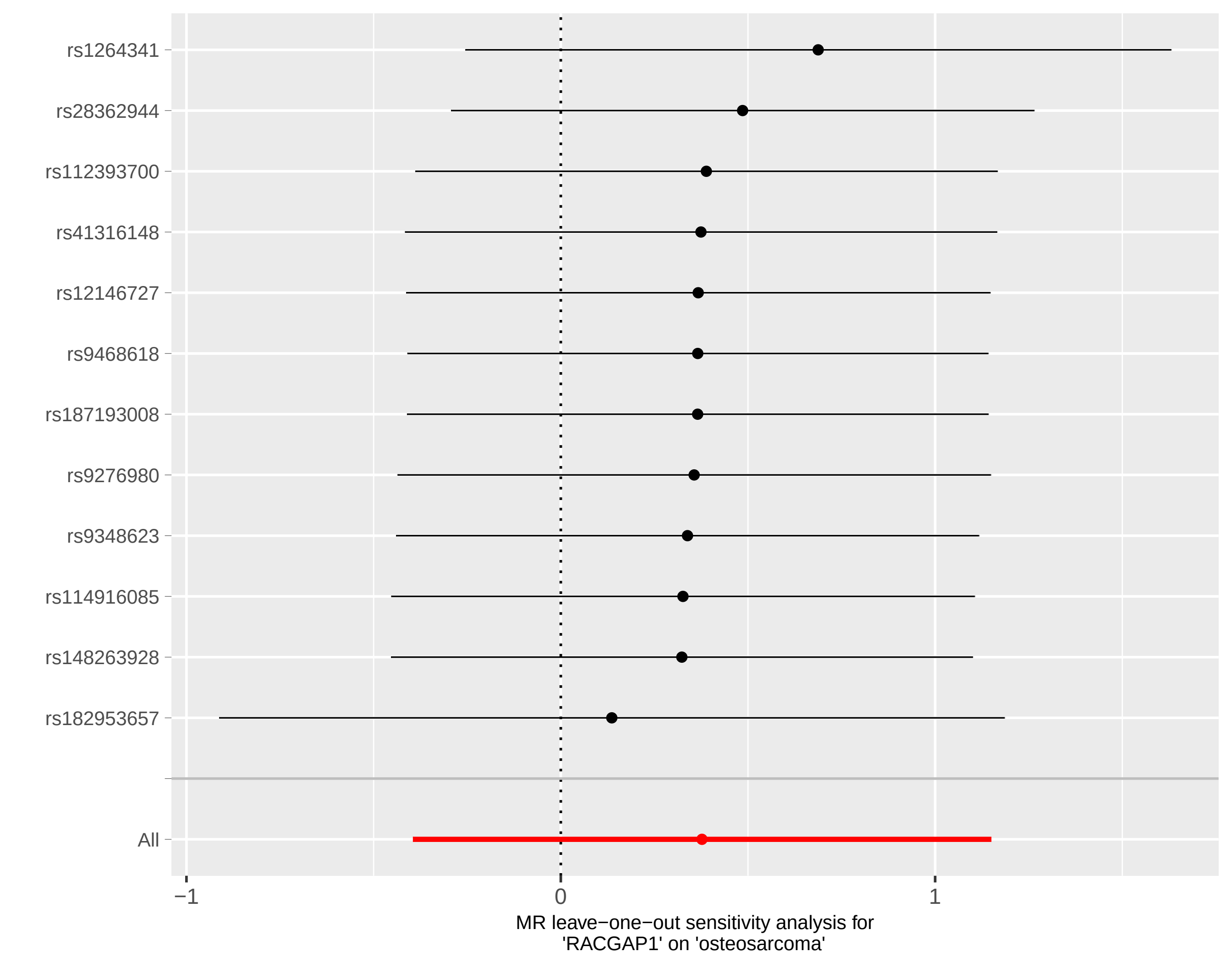
rs1264341
rs28362944
rs112393700
rs41316148
rs12146727
rs9468618
rs187193008
rs9276980
rs9348623
rs114916085
rs148263928
rs182953657
All
−1
0
1
MR leave−one−out sensitivity analysis for
'RACGAP1' on 'osteosarcoma'

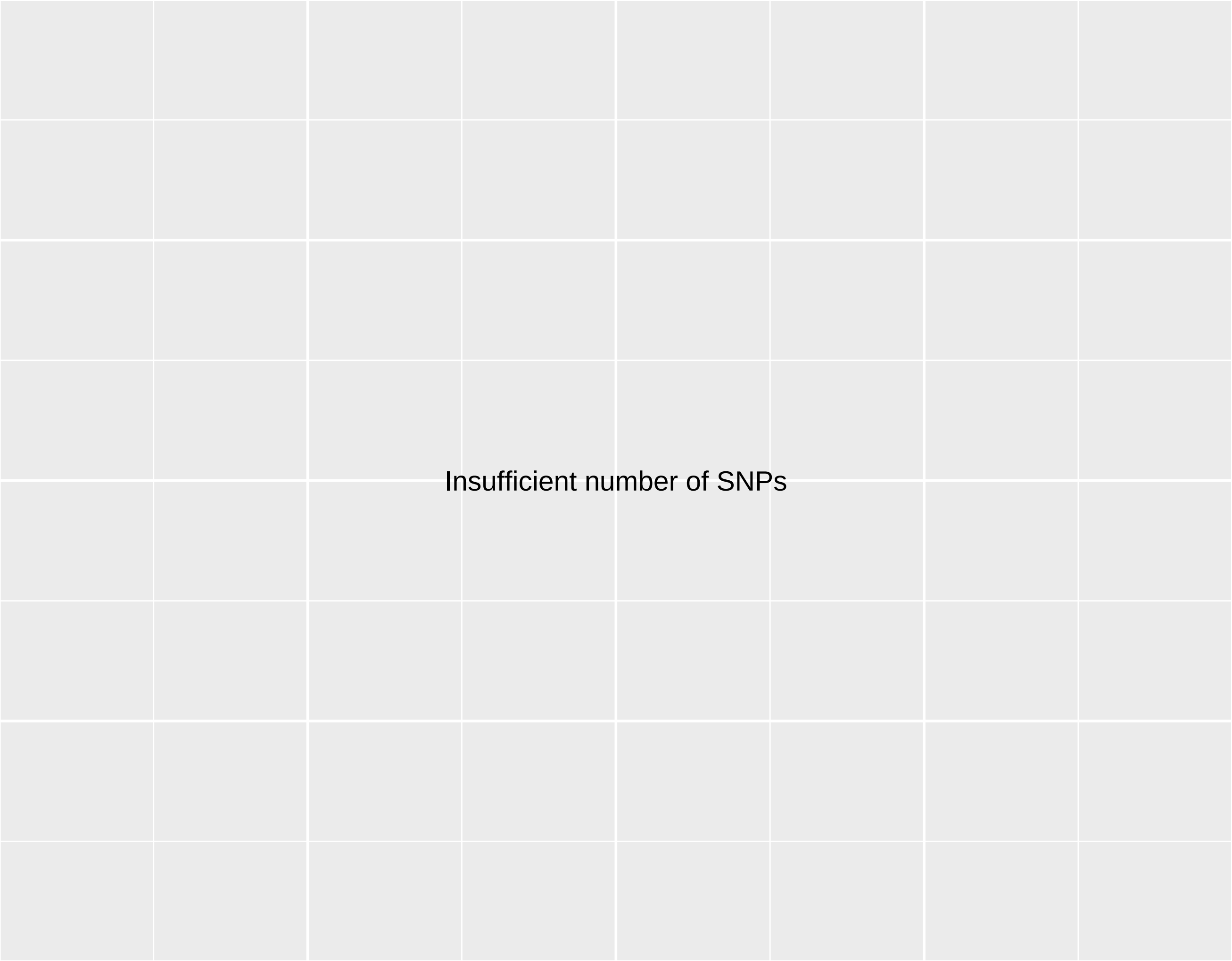
Insufficient number of SNPs

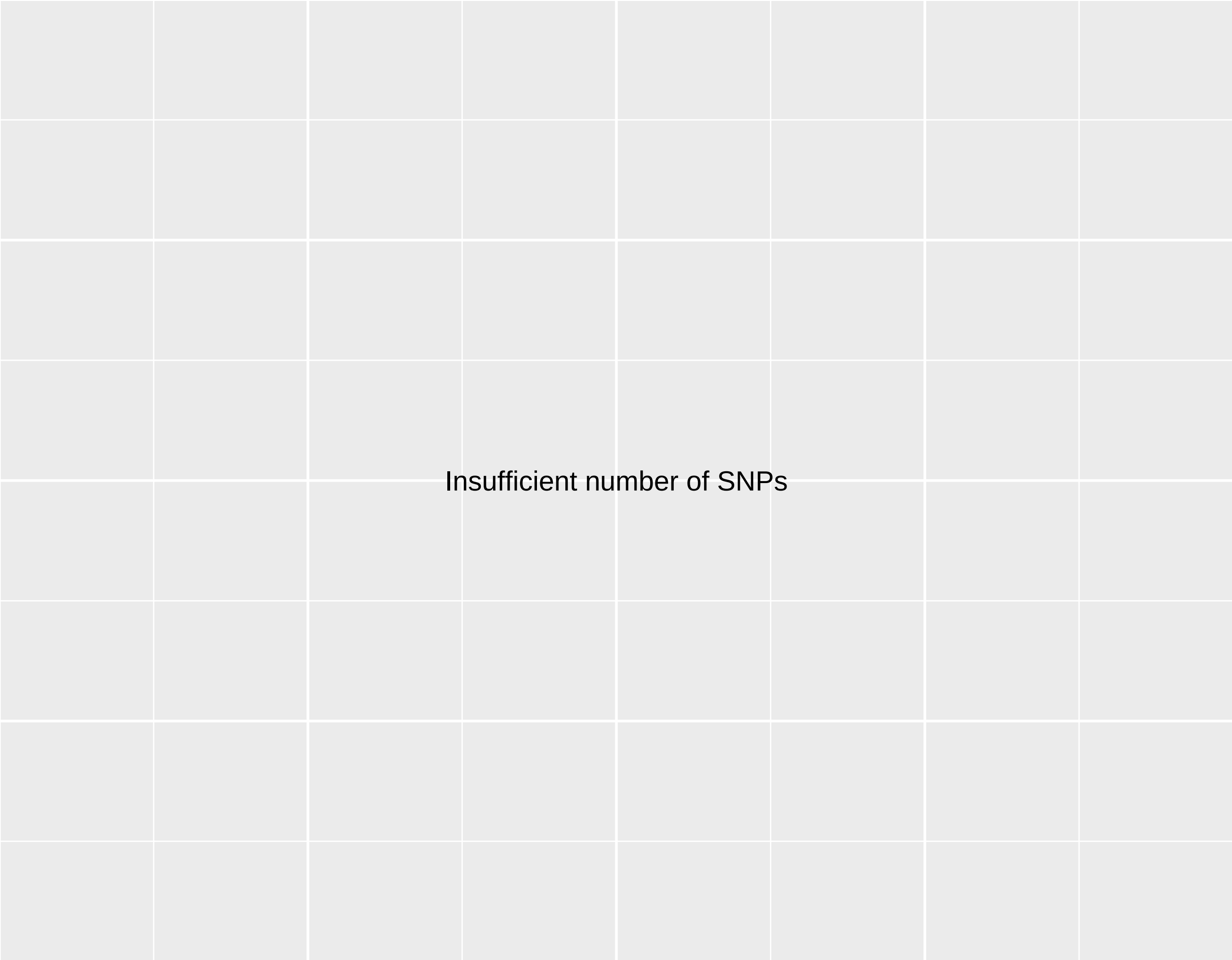
Insufficient number of SNPs

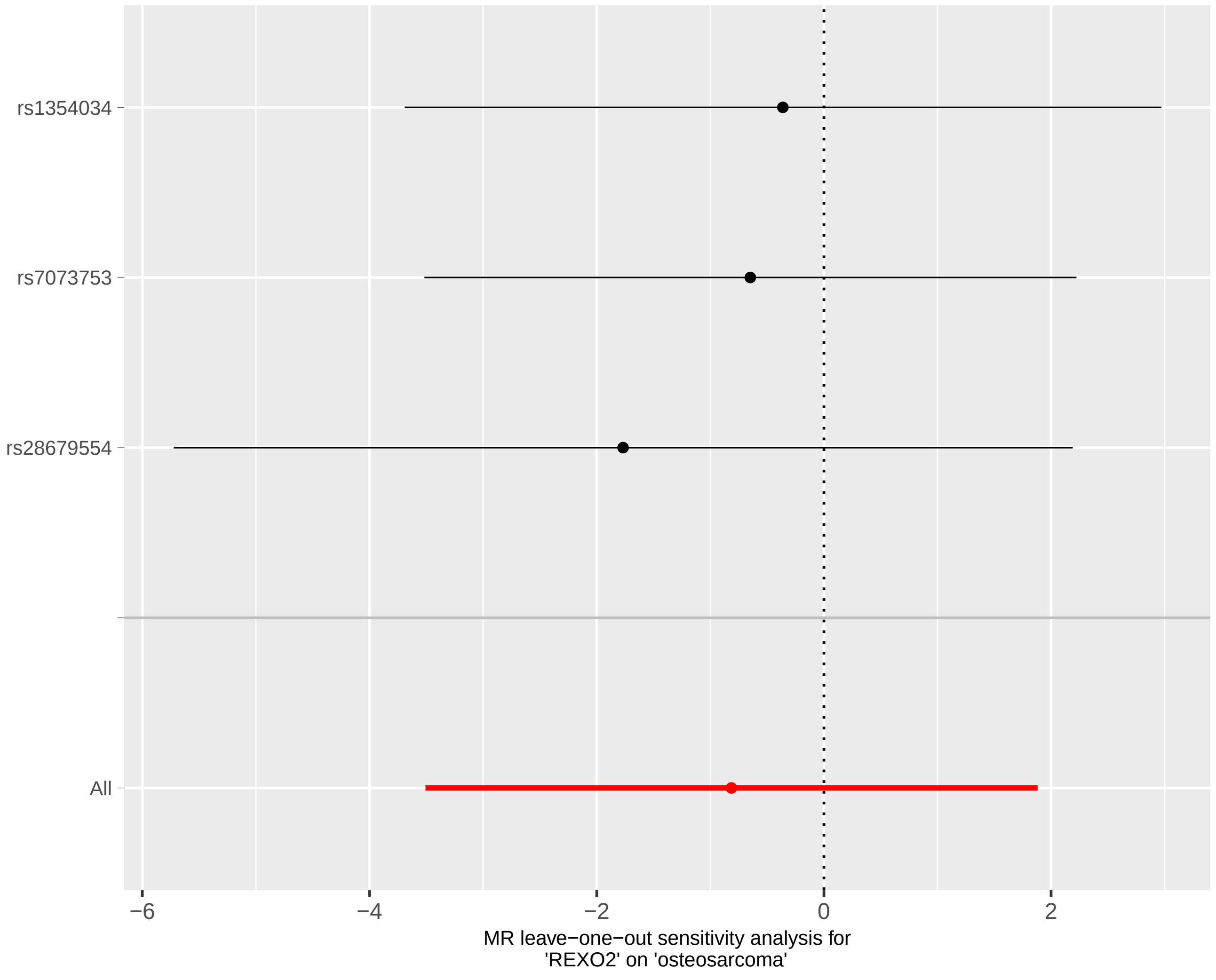
rs1354034
rs7073753
rs28679554
All
−6
−4
−2
0
2
MR leave−one−out sensitivity analysis for
'REXO2' on 'osteosarcoma'

Insufficient number of SNPs

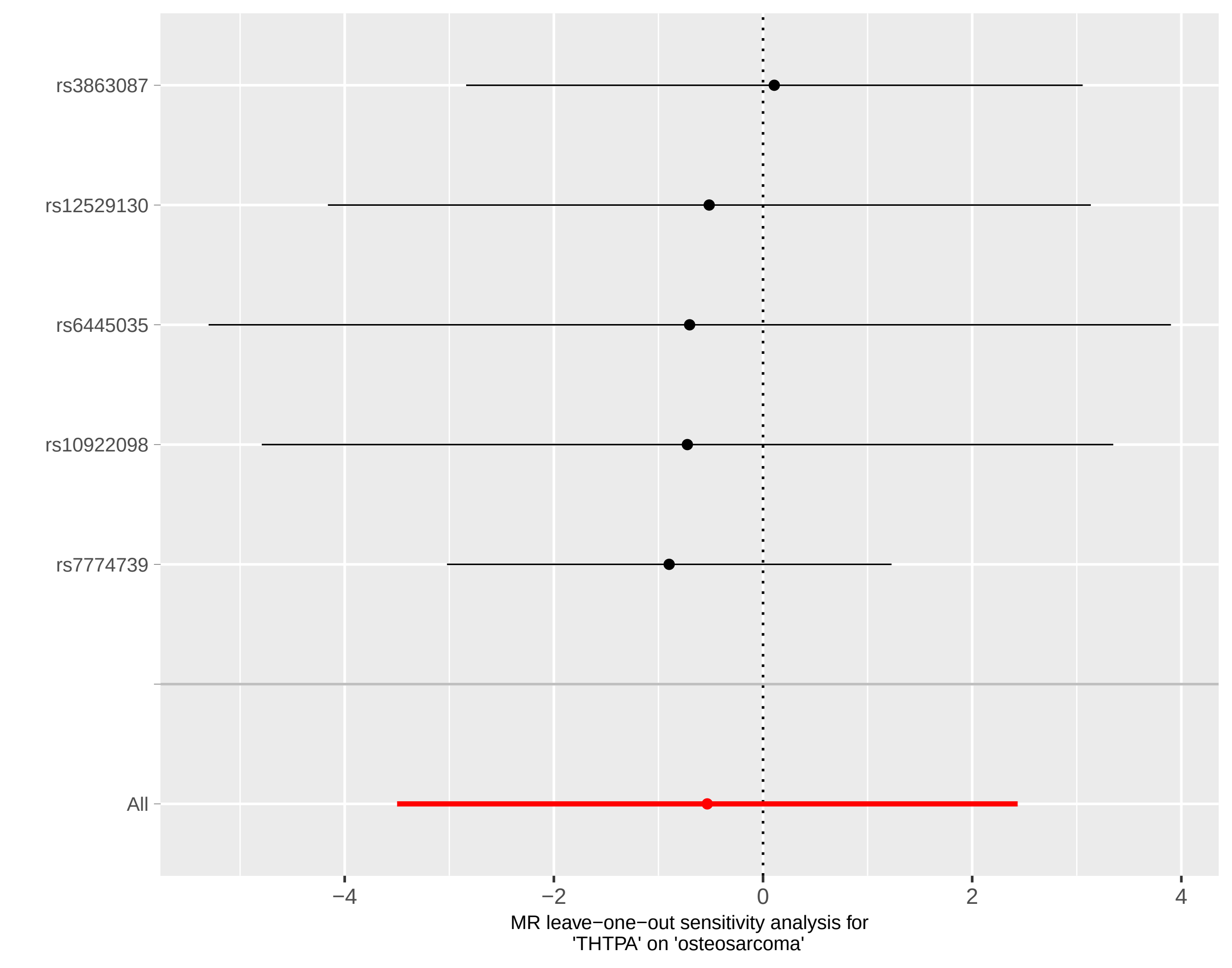
rs3863087
rs12529130
rs6445035
rs10922098
rs7774739
All
−4
−2
0
2
4
MR leave−one−out sensitivity analysis for
'THTPA' on 'osteosarcoma'

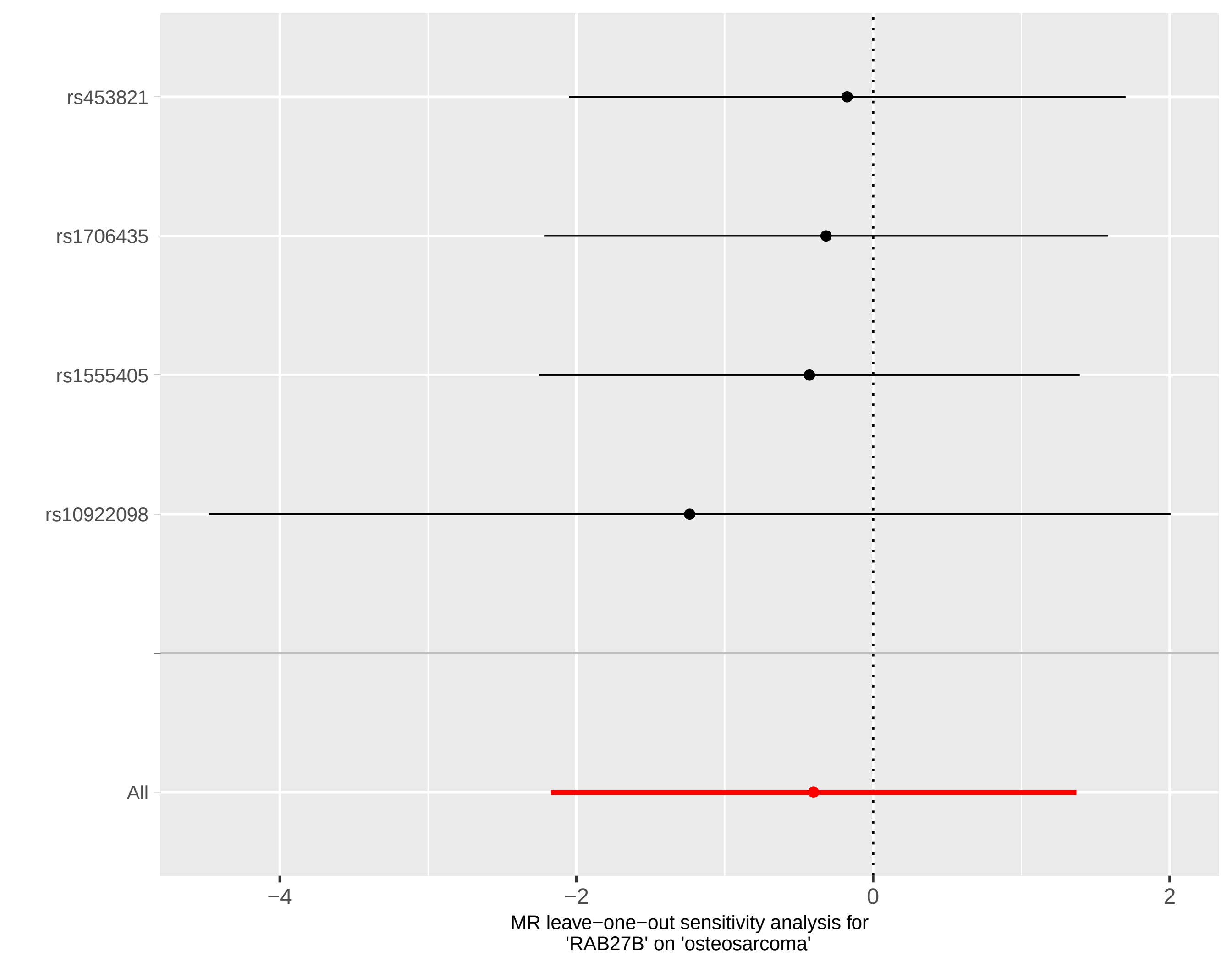
rs453821
rs1706435
rs1555405
rs10922098
All
−4
−2
0
2
MR leave−one−out sensitivity analysis for
'RAB27B' on 'osteosarcoma'

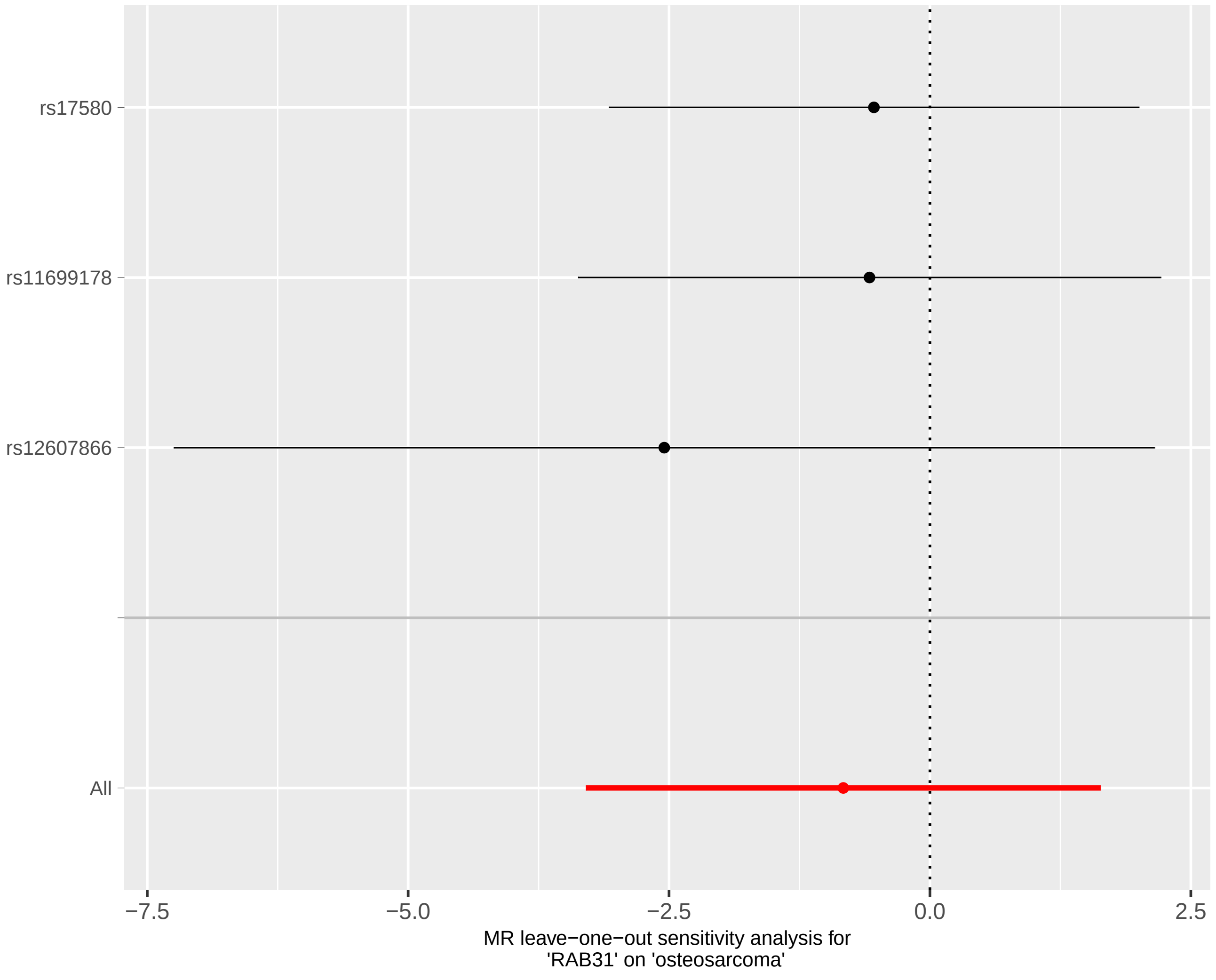
rs17580
rs11699178
rs12607866
All
−7.5
−5.0
−2.5
0.0
2.5
MR leave−one−out sensitivity analysis for
'RAB31' on 'osteosarcoma'

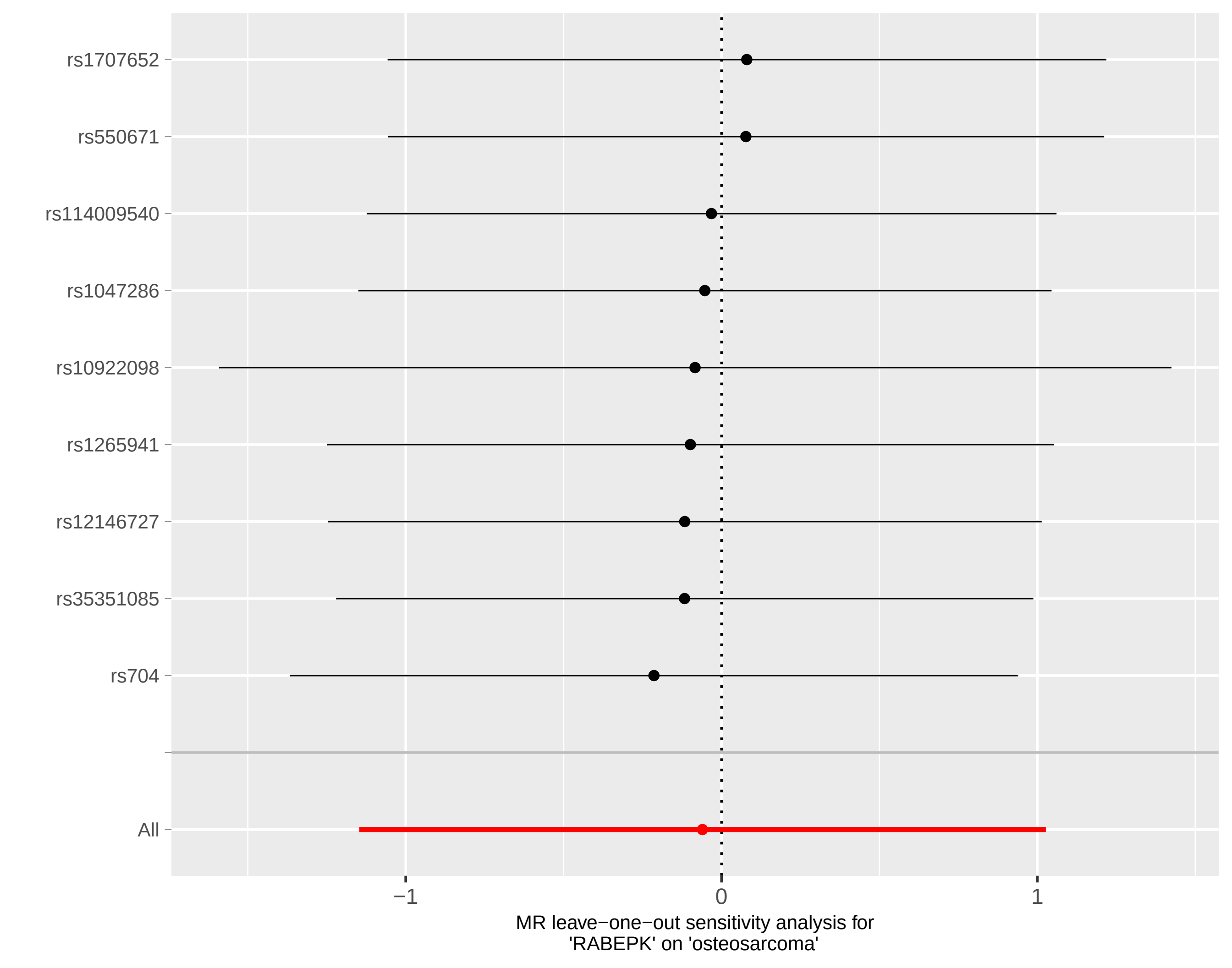
rs1707652
rs550671
rs114009540
rs1047286
rs10922098
rs1265941
rs12146727
rs35351085
rs704
All
−1
0
1
MR leave−one−out sensitivity analysis for
'RABEPK' on 'osteosarcoma'

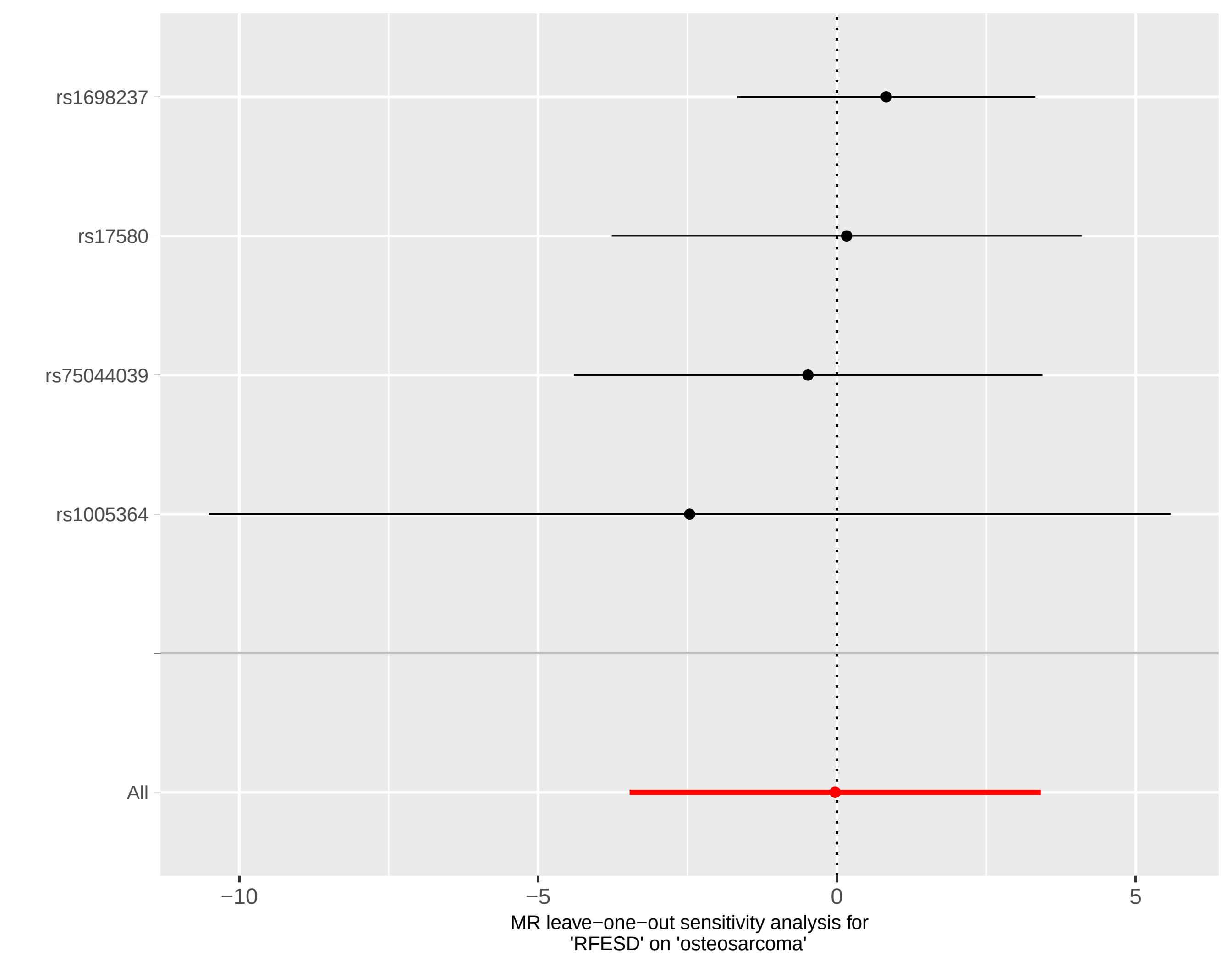

rs1698237
rs17580
rs75044039
rs1005364
All
−10
−5
0
5
MR leave−one−out sensitivity analysis for
'RFESD' on 'osteosarcoma'

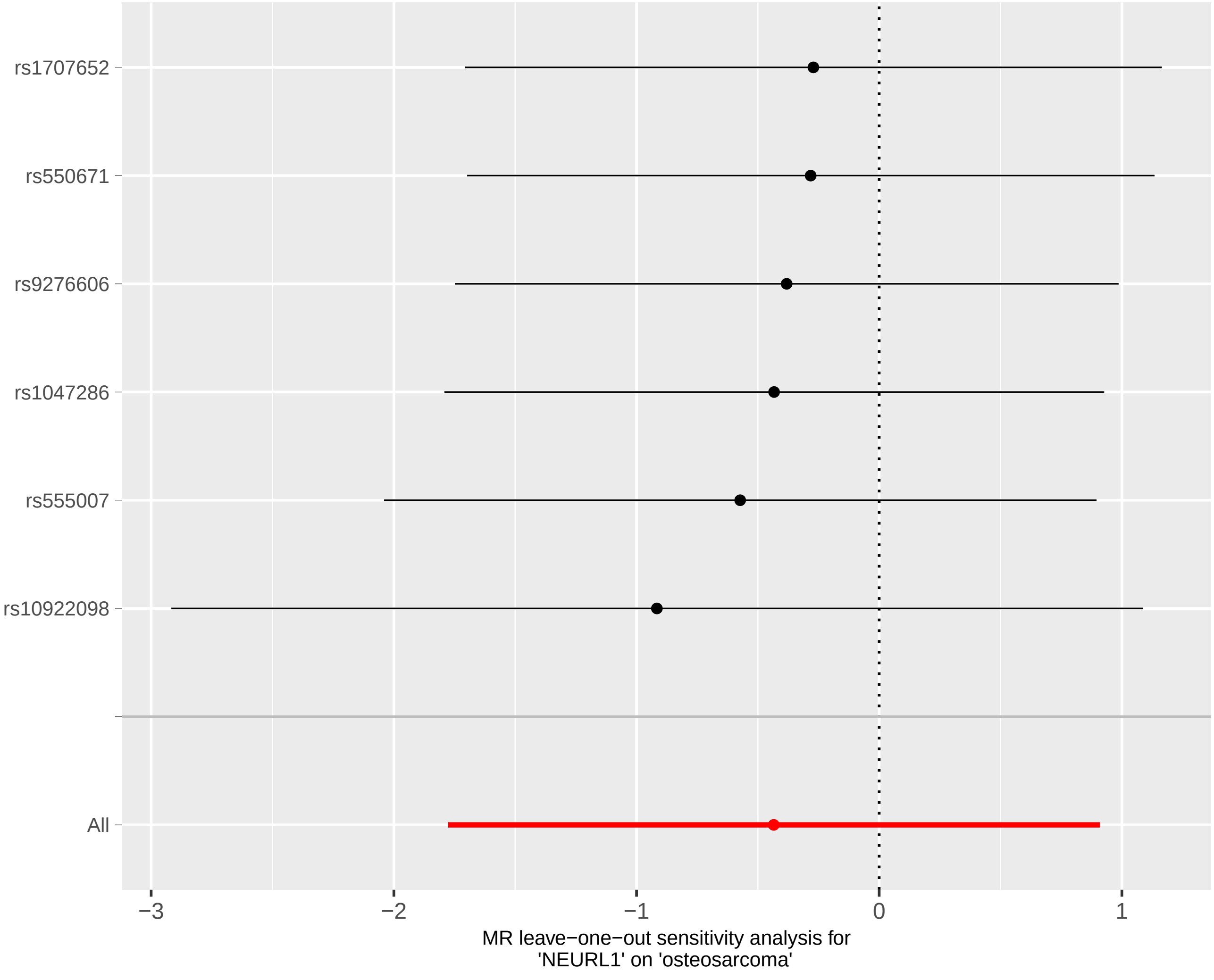
rs1707652
rs550671
rs9276606
rs1047286
rs555007
rs10922098
All
−3
−2
−1
0
1
MR leave−one−out sensitivity analysis for
'NEURL1' on 'osteosarcoma'

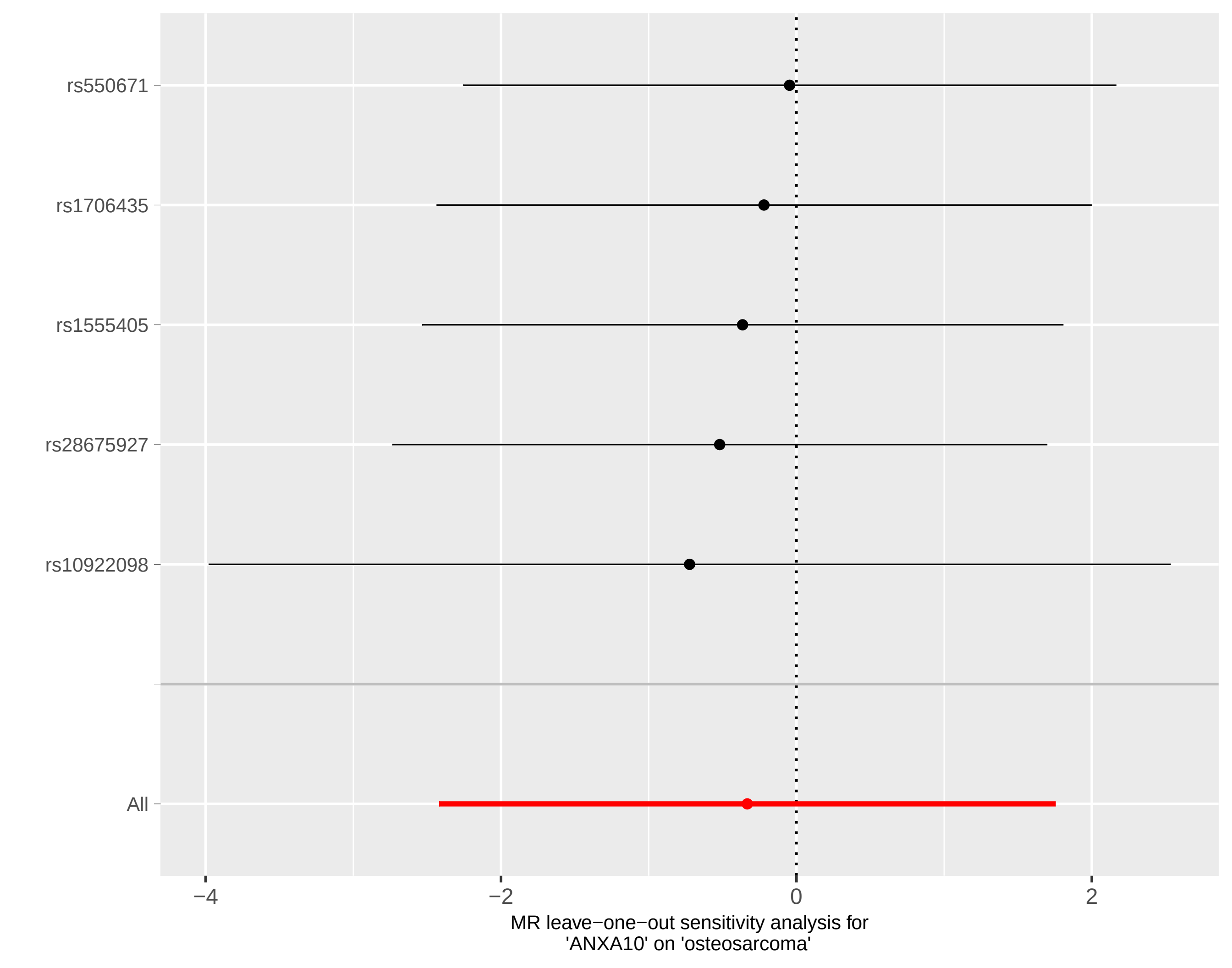

rs550671
rs1706435
rs1555405
rs28675927
rs10922098
All
−4
−2
0
2
MR leave−one−out sensitivity analysis for
'ANXA10' on 'osteosarcoma'

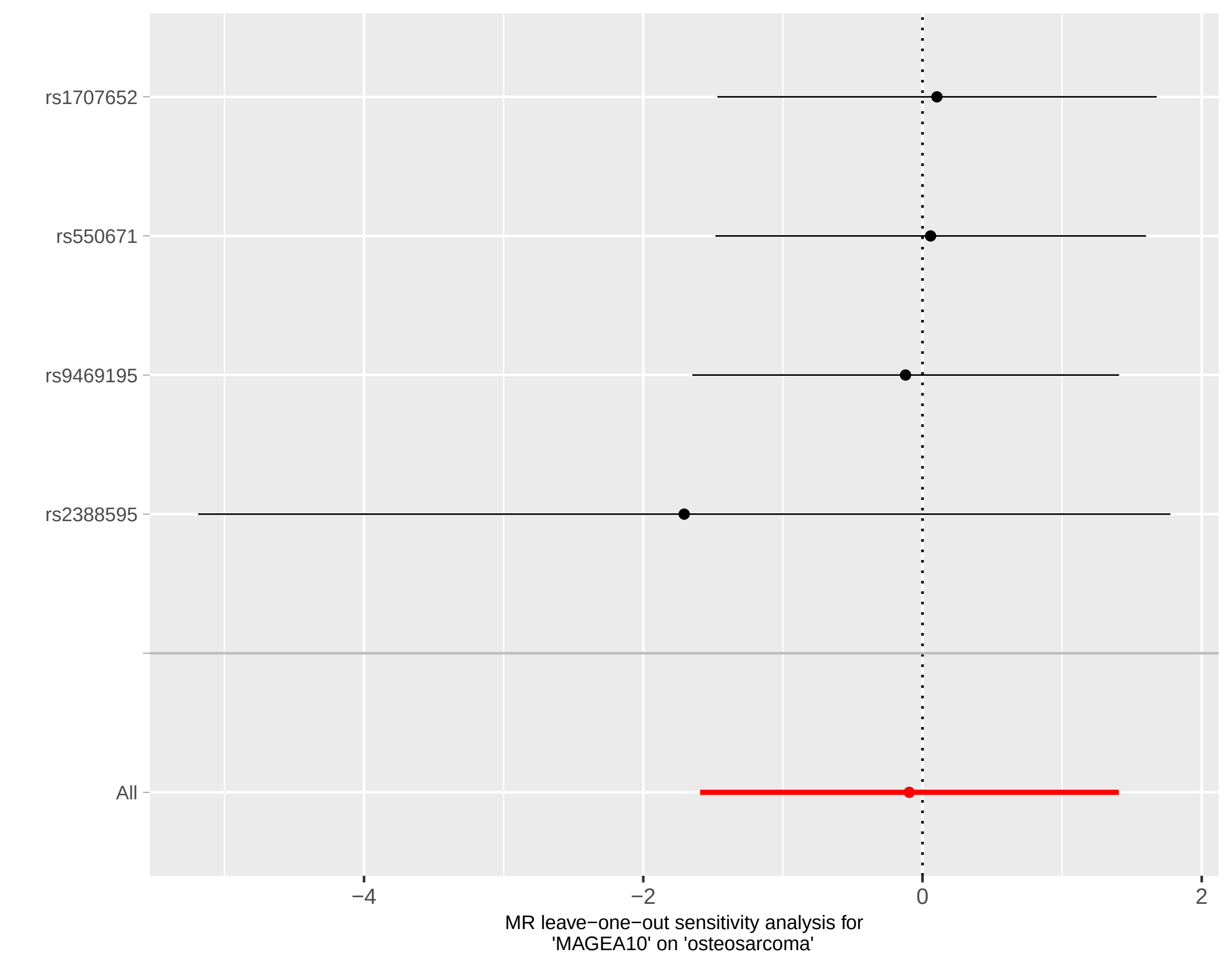
rs1707652
rs550671
rs9469195
rs2388595
All
−4
−2
0
2
MR leave−one−out sensitivity analysis for
'MAGEA10' on 'osteosarcoma'

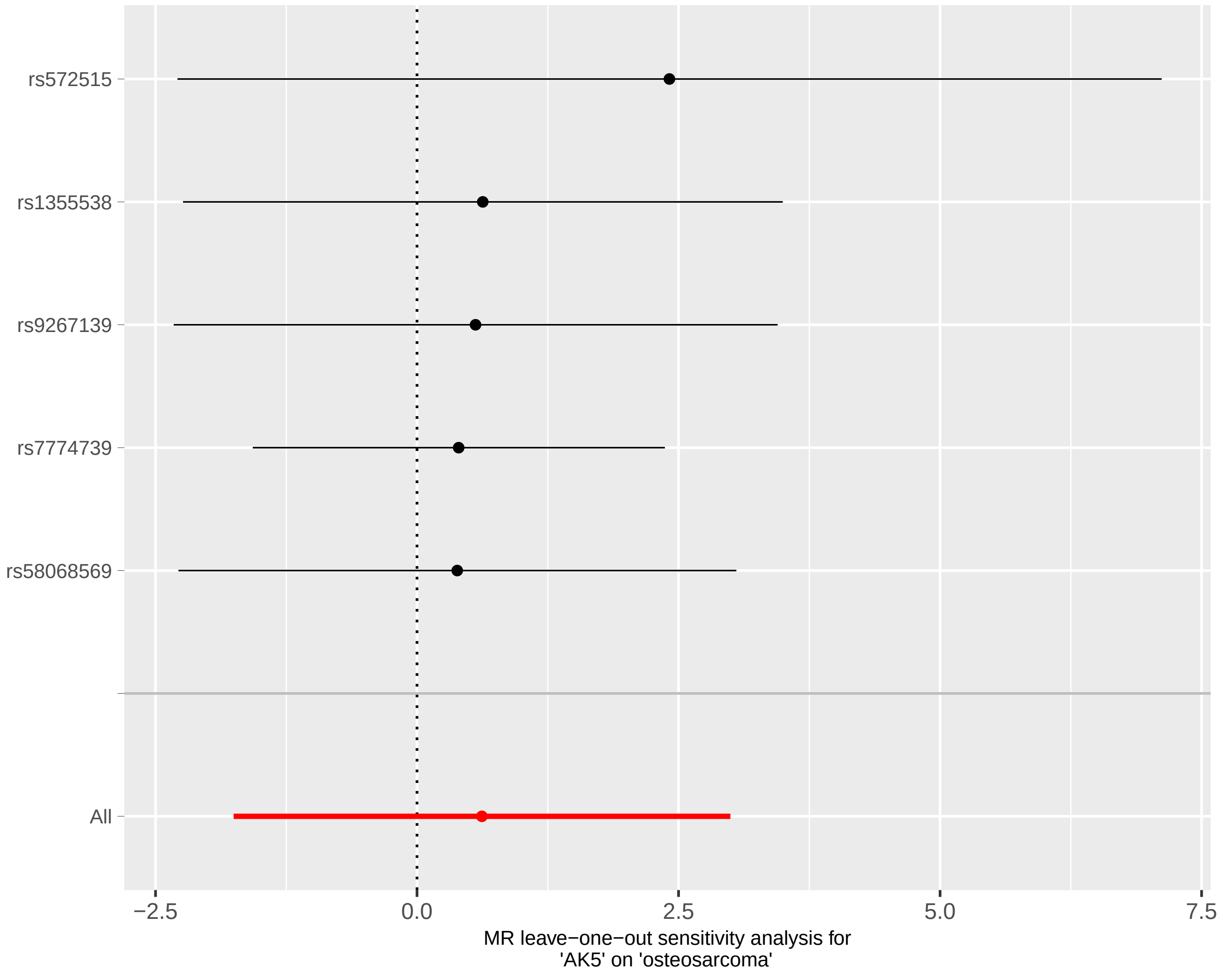

rs572515
rs1355538
rs9267139
rs7774739
rs58068569
All
−2.5
0.0
2.5
5.0
7.5
MR leave−one−out sensitivity analysis for
'AK5' on 'osteosarcoma'

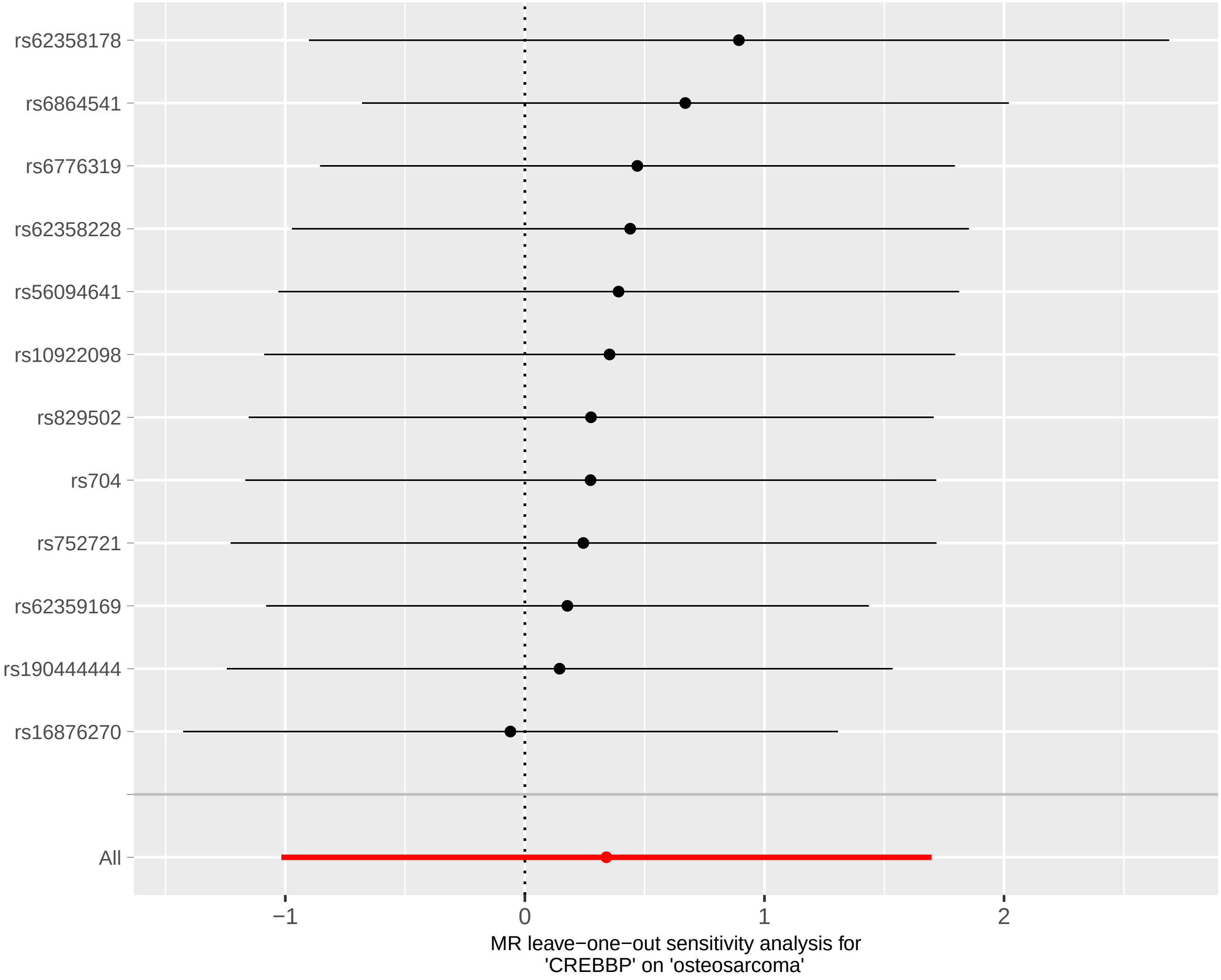

rs62358178
rs6864541
rs6776319
rs62358228
rs56094641
rs10922098
rs829502
rs704
rs752721
rs62359169
rs190444444
rs16876270
All
−1
0
1
2
MR leave−one−out sensitivity analysis for
'CREBBP' on 'osteosarcoma'

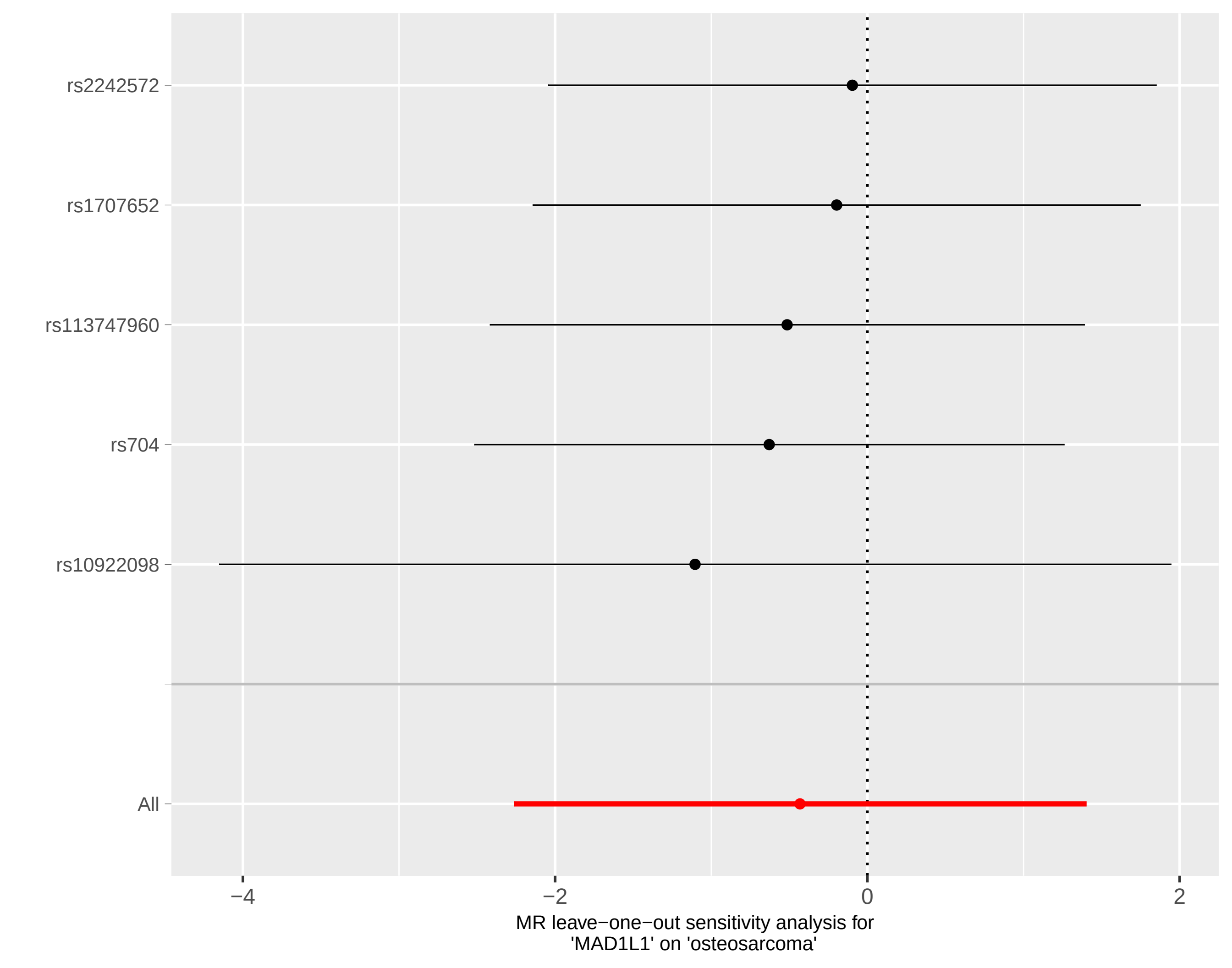
rs2242572
rs1707652
rs113747960
rs704
rs10922098
All
−4
−2
0
2
MR leave−one−out sensitivity analysis for
'MAD1L1' on 'osteosarcoma'

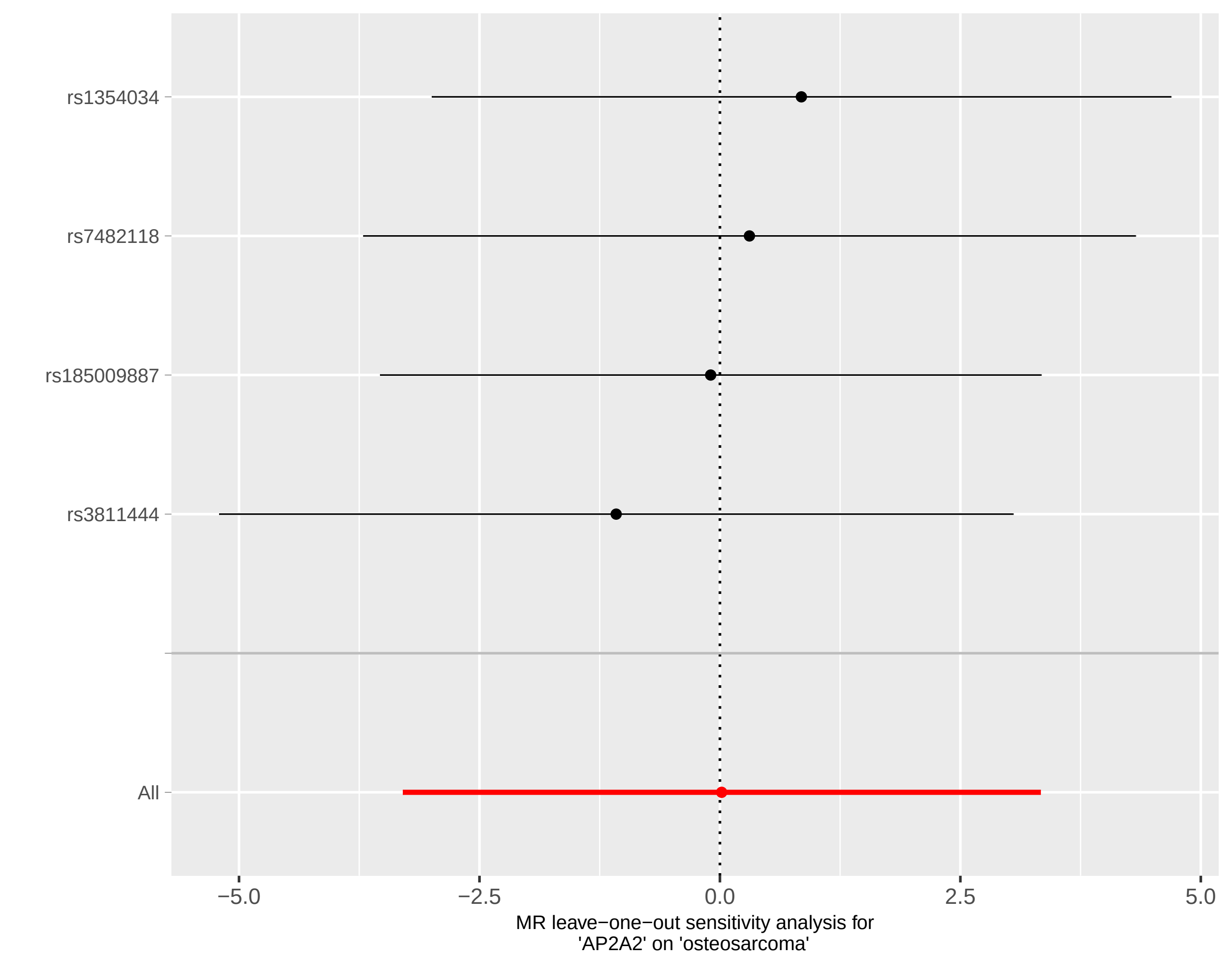
rs1354034
rs7482118
rs185009887
rs3811444
All
−5.0
−2.5
0.0
2.5
5.0
MR leave−one−out sensitivity analysis for
'AP2A2' on 'osteosarcoma'

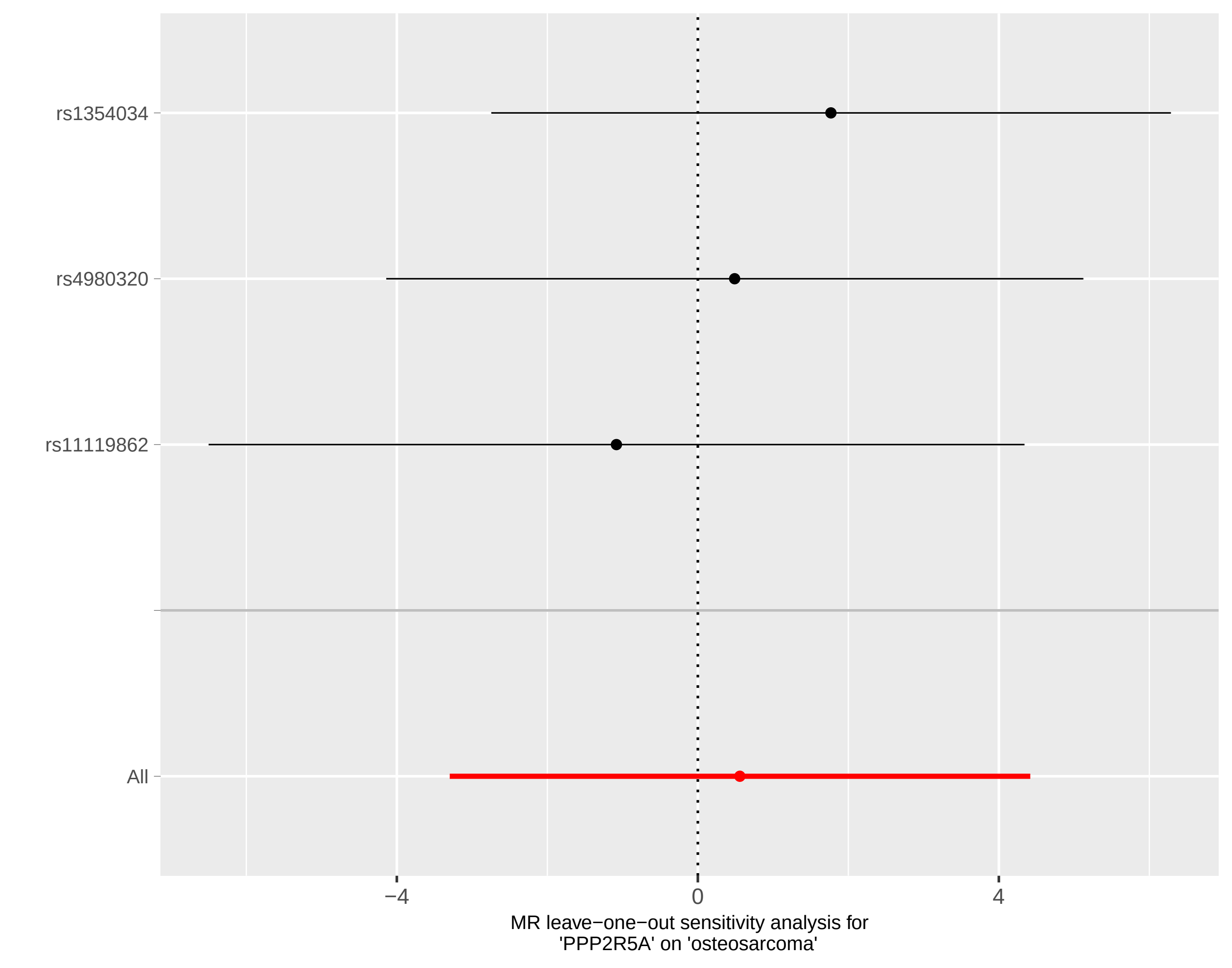
rs1354034
rs4980320
rs11119862
All
−4
0
4
MR leave−one−out sensitivity analysis for
'PPP2R5A' on 'osteosarcoma'

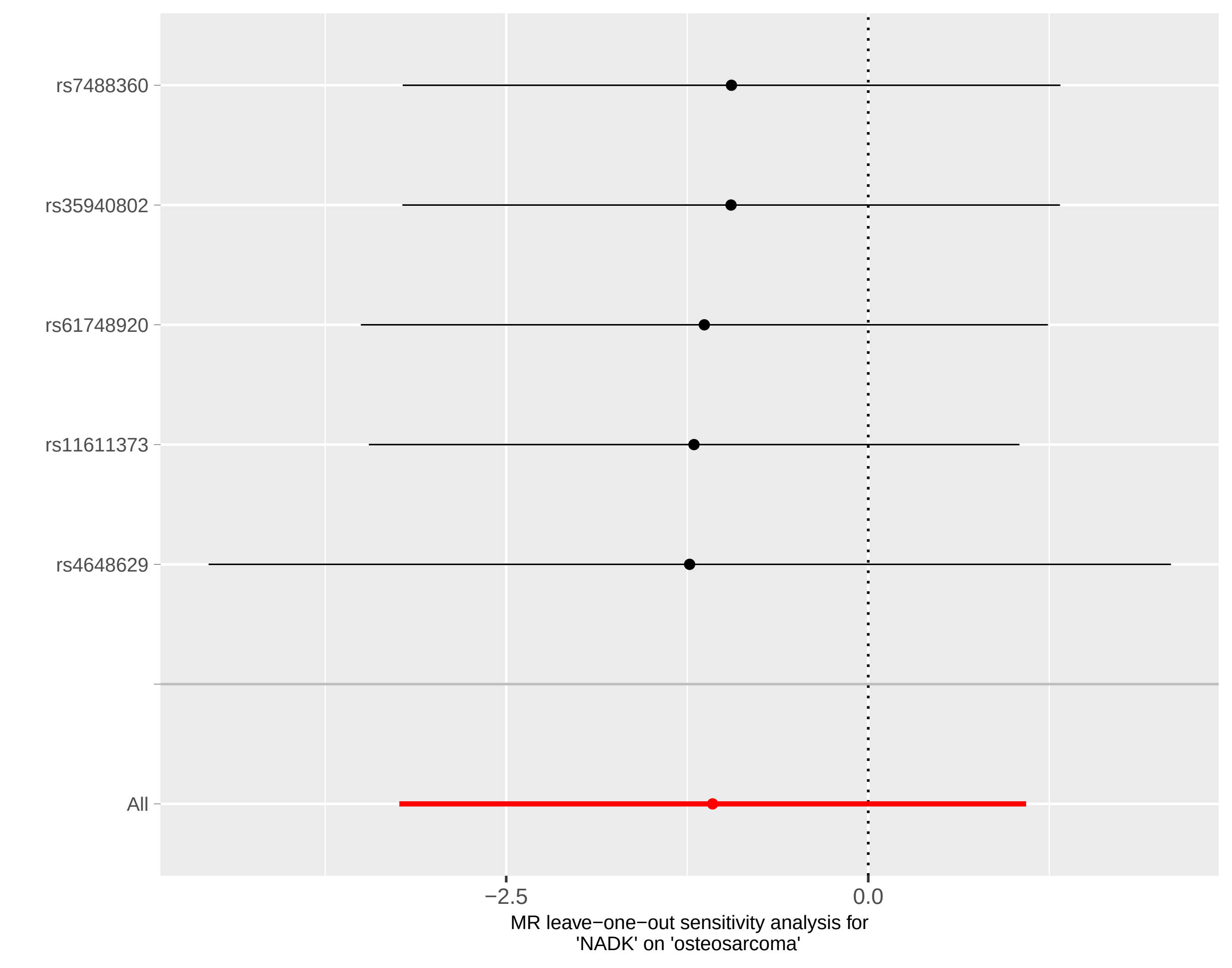
rs7488360
rs35940802
rs61748920
rs11611373
rs4648629
All
−2.5
0.0
MR leave−one−out sensitivity analysis for
'NADK' on 'osteosarcoma'

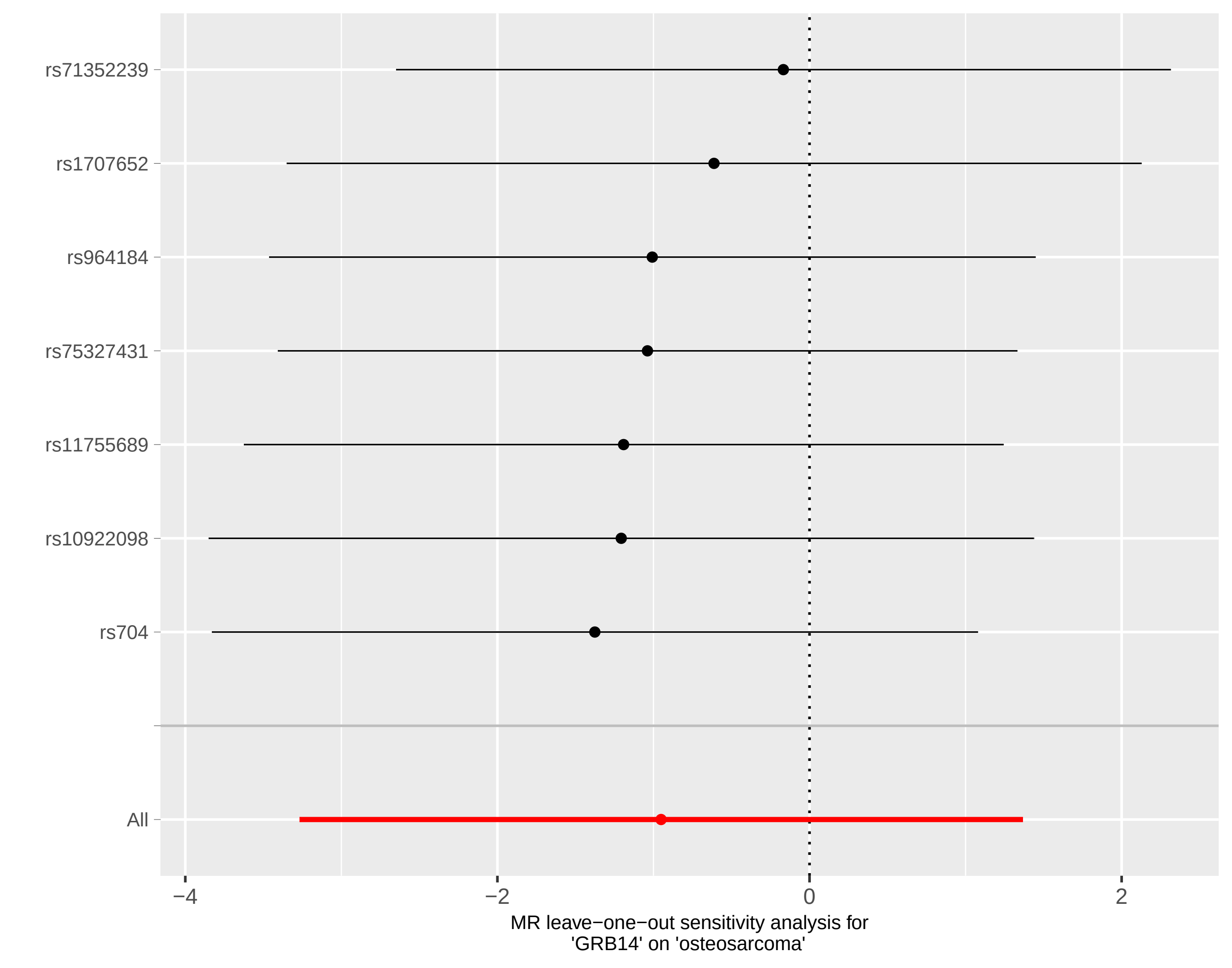

rs71352239
rs1707652
rs964184
rs75327431
rs11755689
rs10922098
rs704
All
−4
−2
0
2
MR leave−one−out sensitivity analysis for
'GRB14' on 'osteosarcoma'

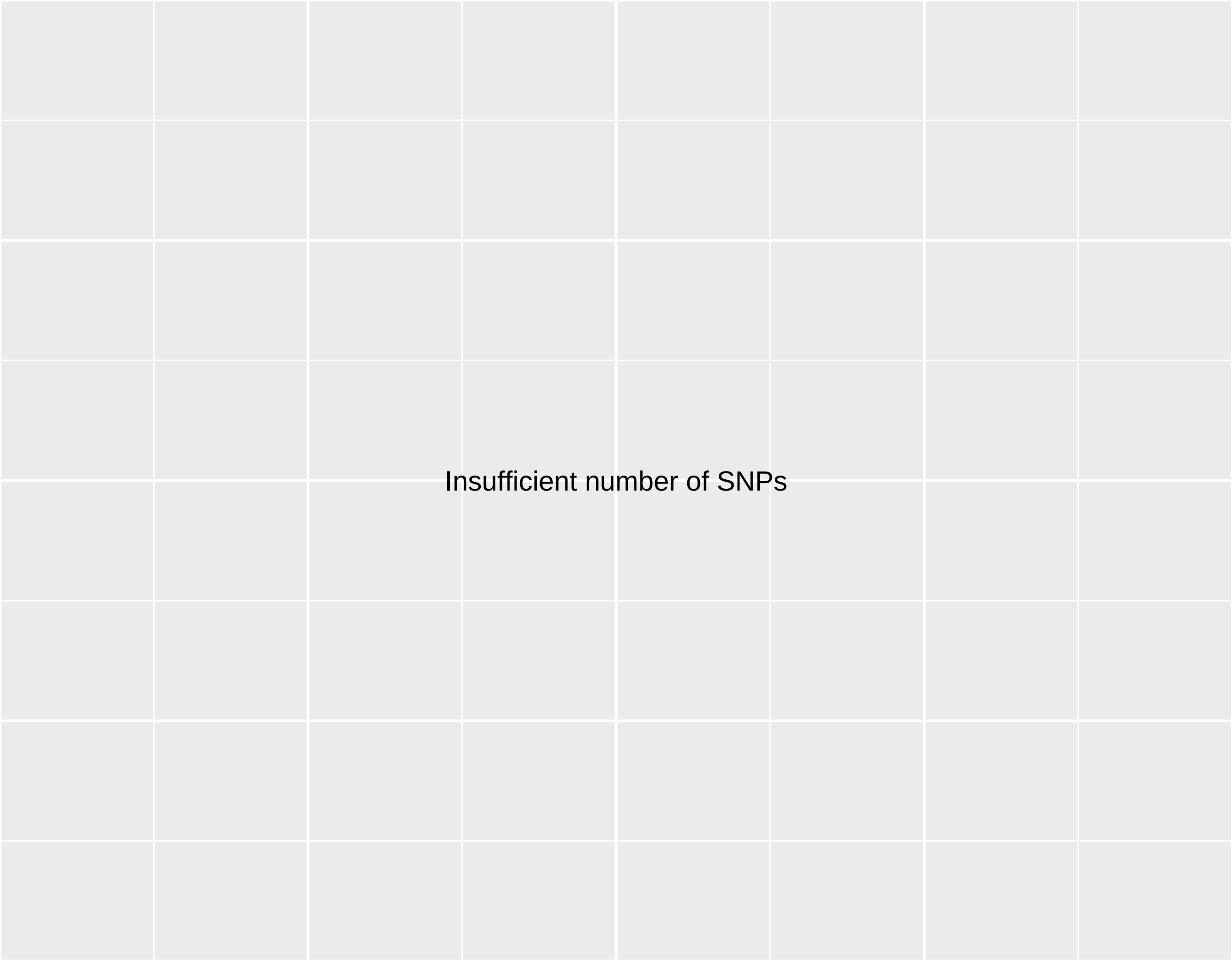
Insufficient number of SNPs

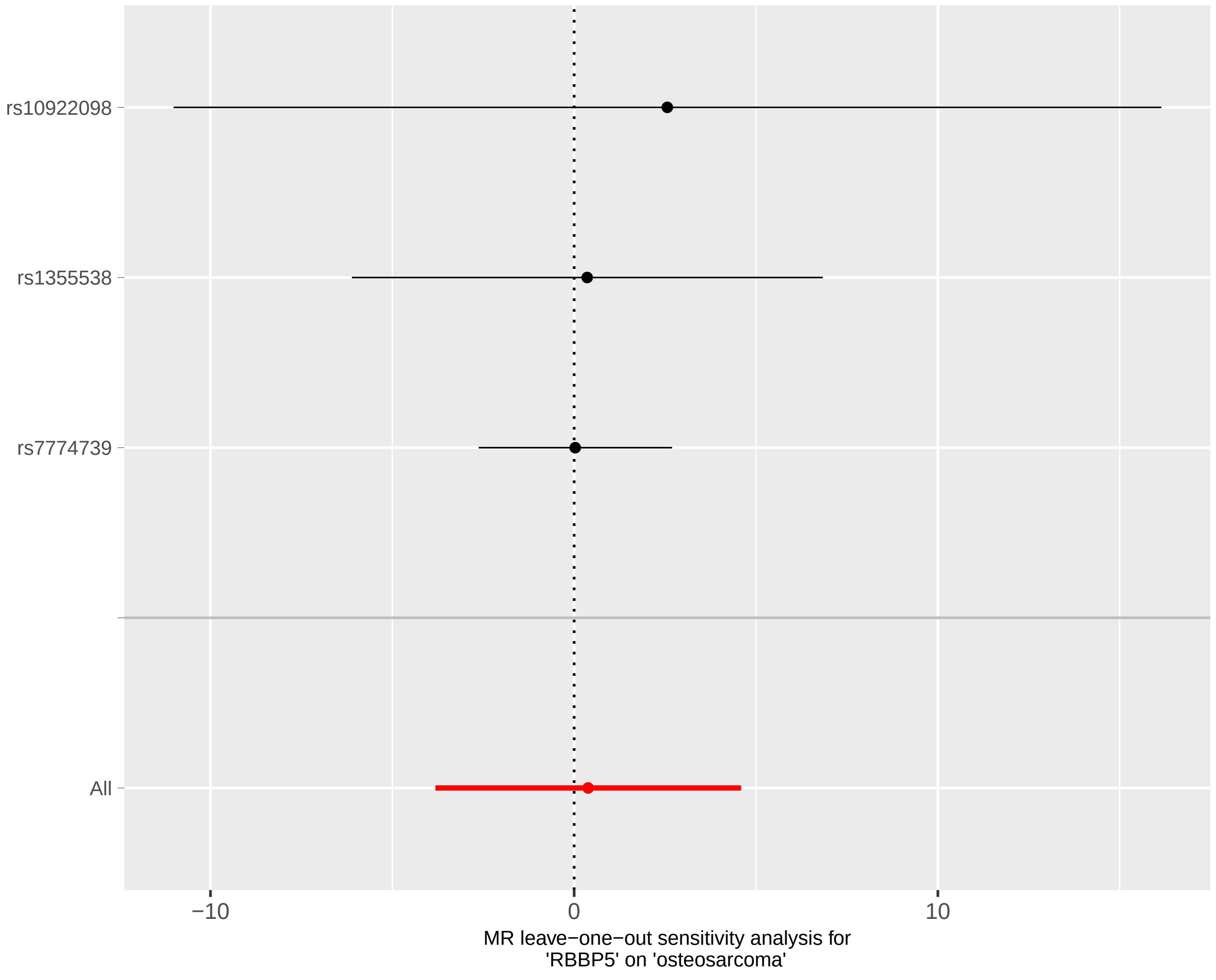

rs10922098
rs1355538
rs7774739
All
−10
0
10
MR leave−one−out sensitivity analysis for
'RBBP5' on 'osteosarcoma'

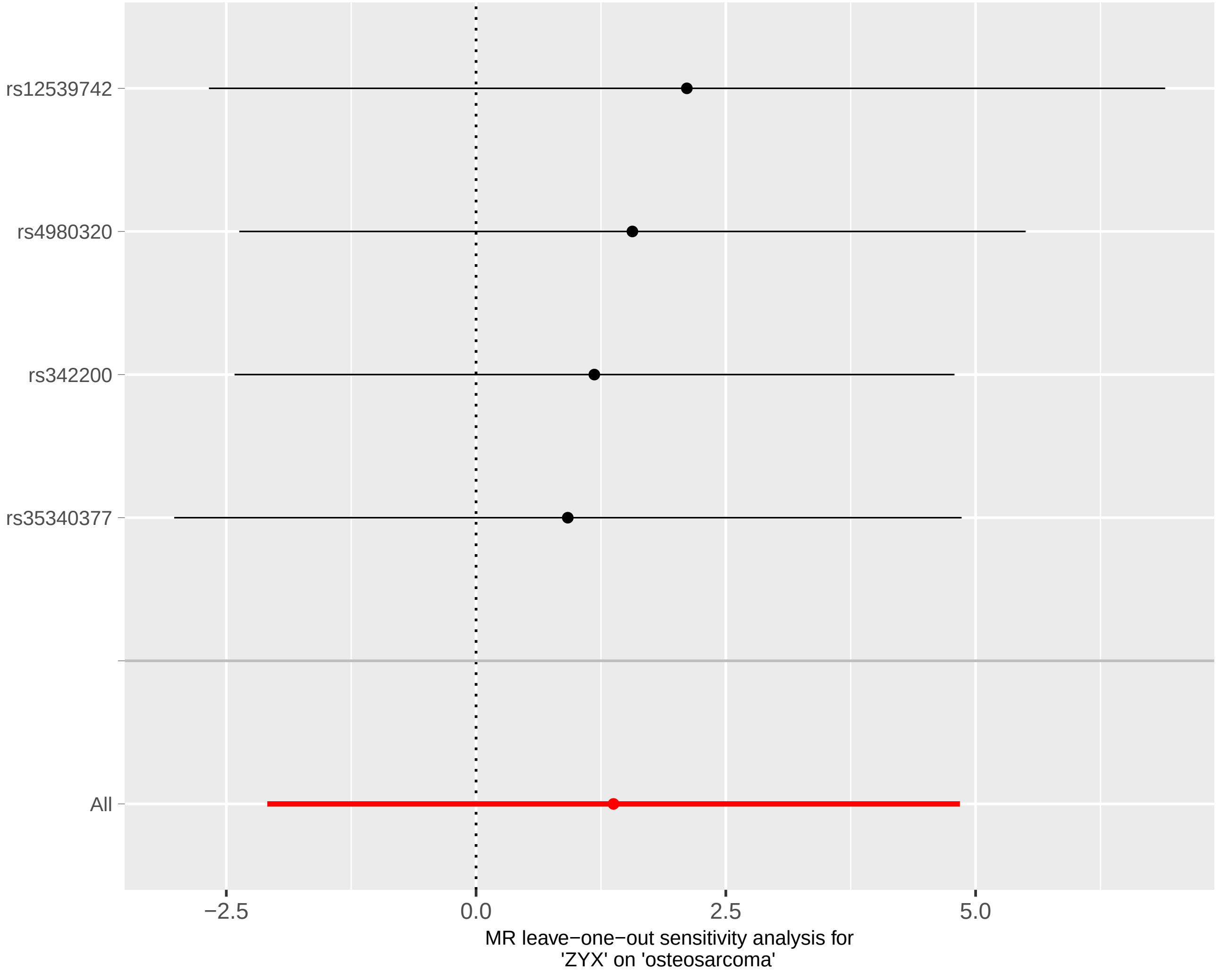
rs12539742
rs4980320
rs342200
rs35340377
All
−2.5
0.0
2.5
5.0
MR leave−one−out sensitivity analysis for
'ZYX' on 'osteosarcoma'

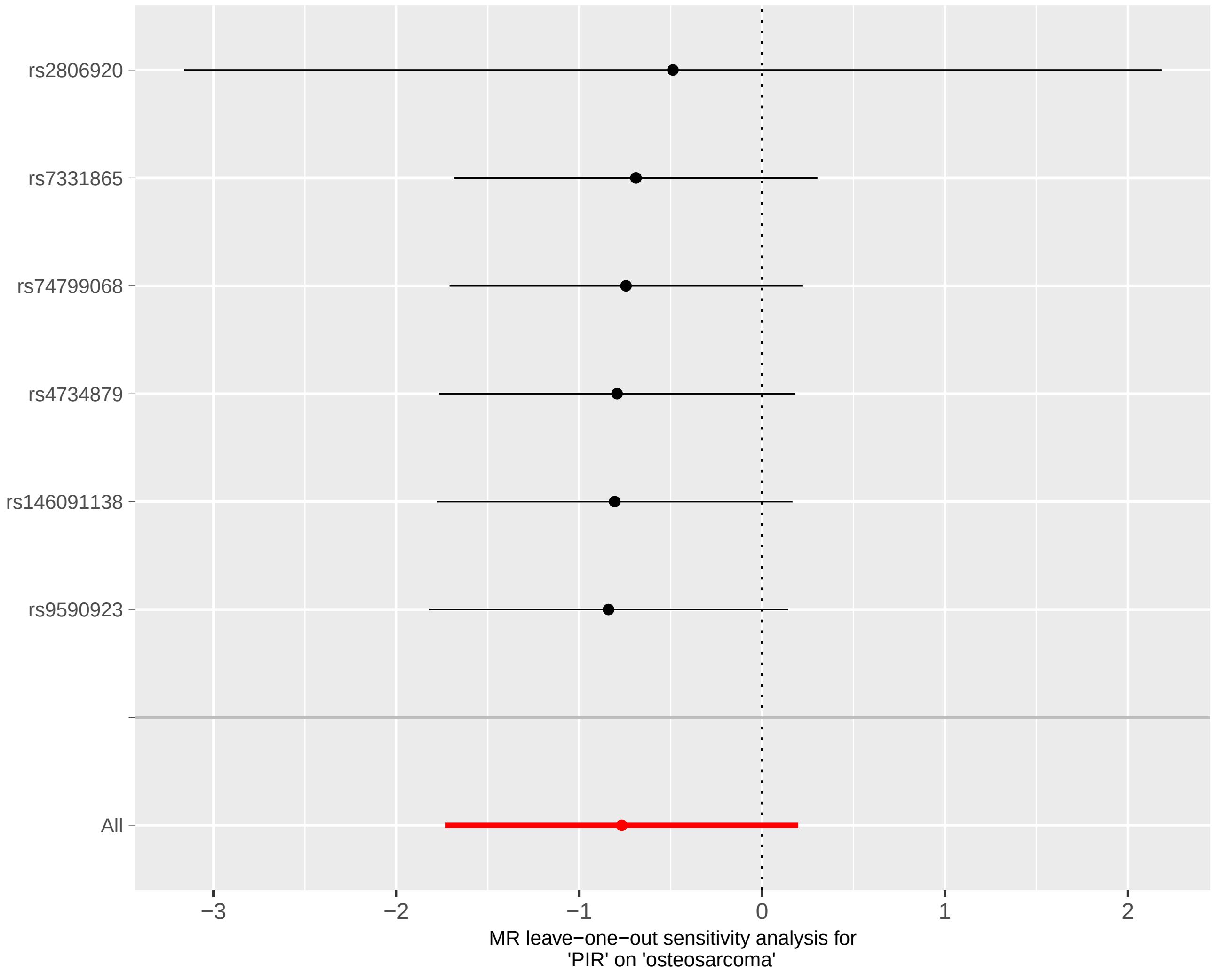
rs2806920
rs7331865
rs74799068
rs4734879
rs146091138
rs9590923
All
−3
−2
−1
0
1
2
MR leave−one−out sensitivity analysis for
'PIR' on 'osteosarcoma'

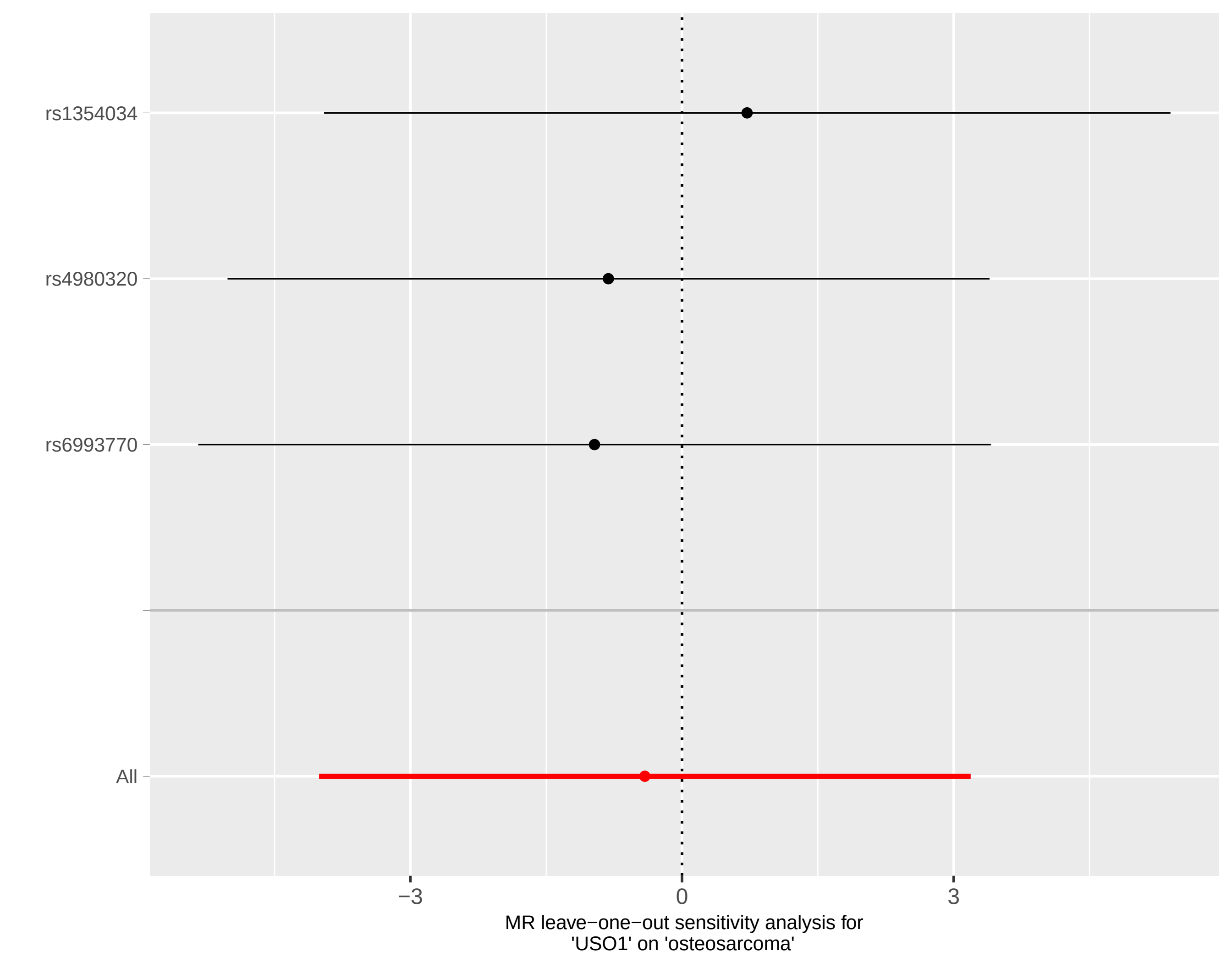
rs1354034
rs4980320
rs6993770
All
−3
0
3
MR leave−one−out sensitivity analysis for
'USO1' on 'osteosarcoma'

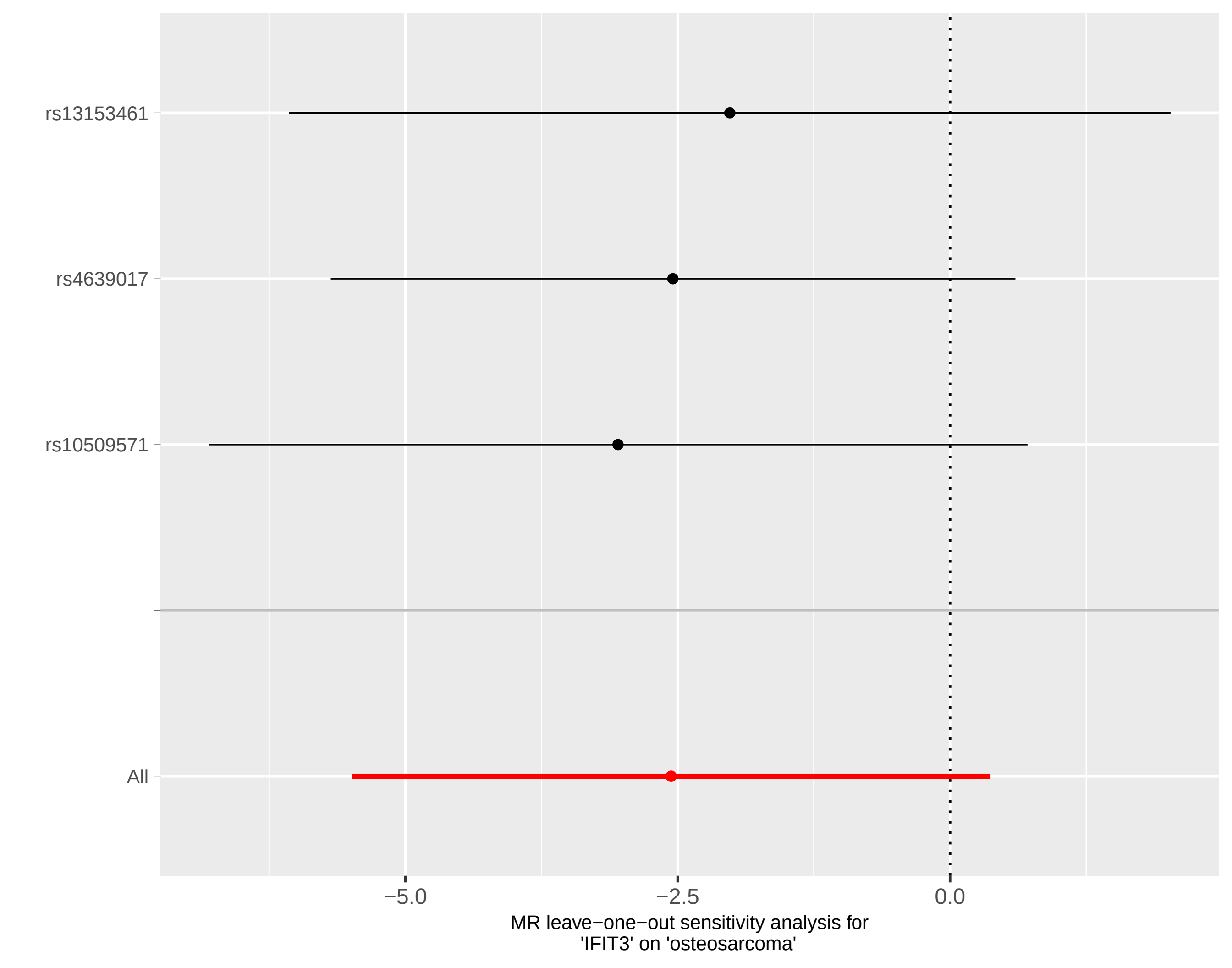
rs13153461
rs4639017
rs10509571
All
−5.0
−2.5
0.0
MR leave−one−out sensitivity analysis for
'IFIT3' on 'osteosarcoma'

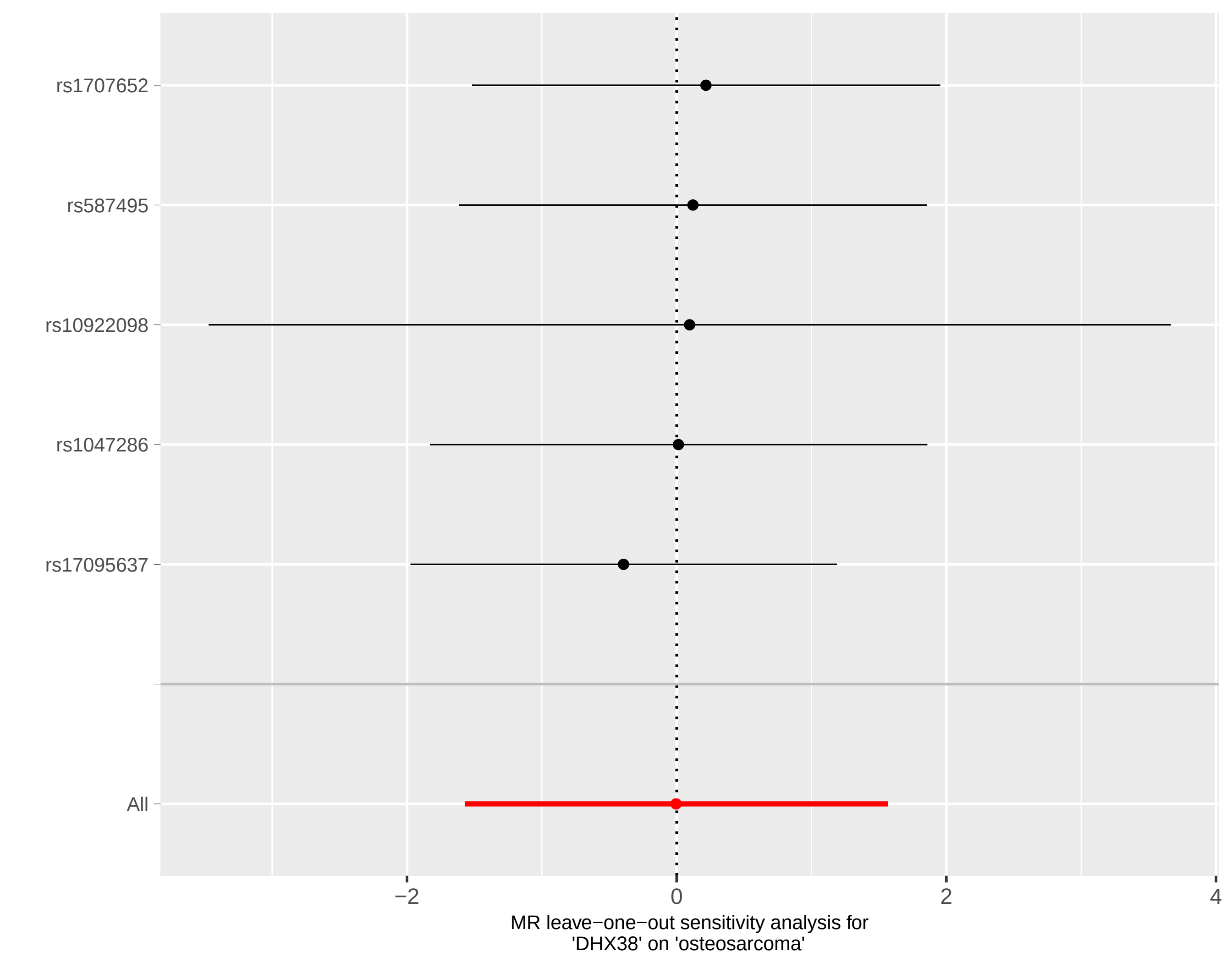
rs1707652
rs587495
rs10922098
rs1047286
rs17095637
All
−2
0
2
4
MR leave−one−out sensitivity analysis for
'DHX38' on 'osteosarcoma'

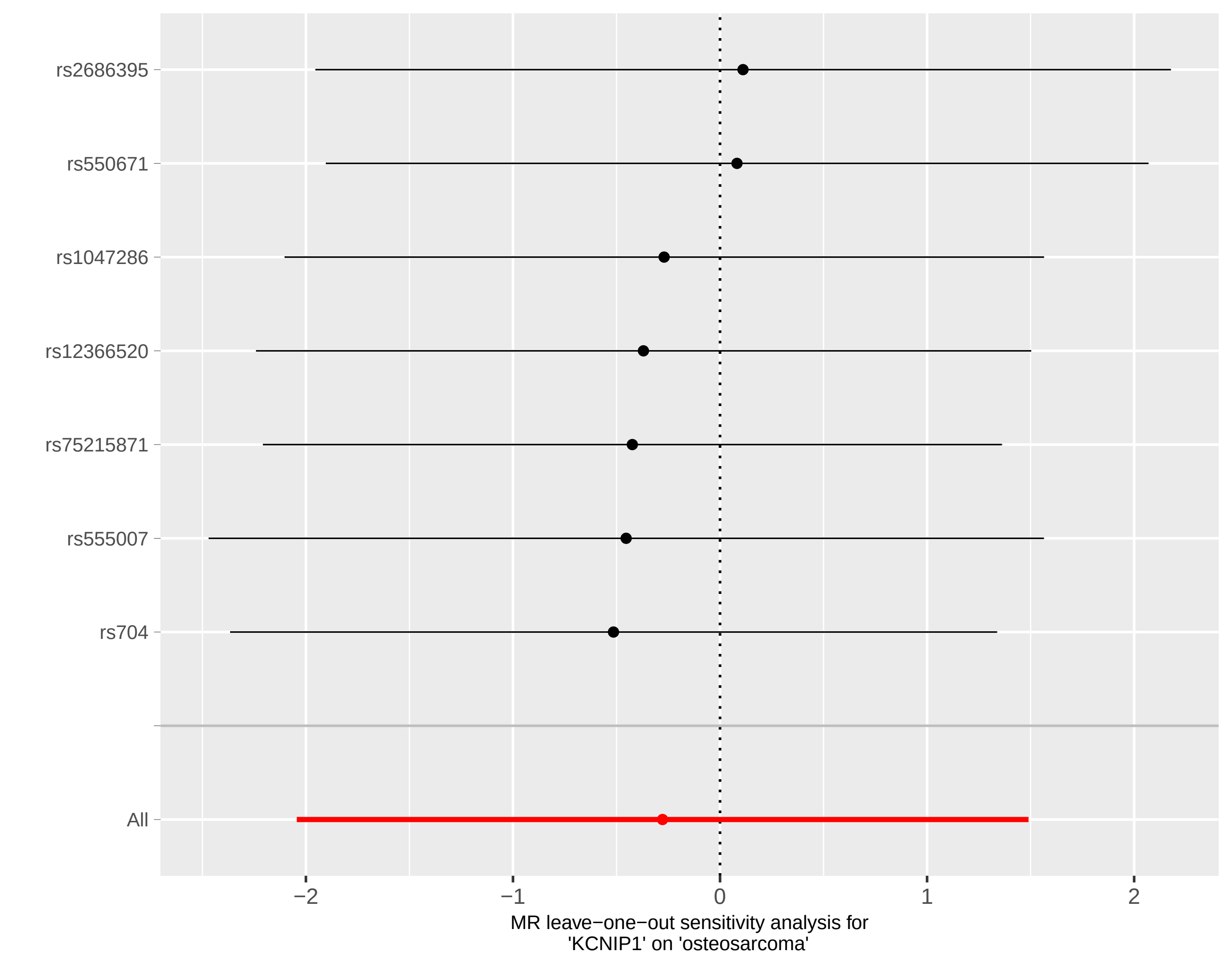

rs2686395
rs550671
rs1047286
rs12366520
rs75215871
rs555007
rs704
All
−2
−1
0
1
2
MR leave−one−out sensitivity analysis for
'KCNIP1' on 'osteosarcoma'

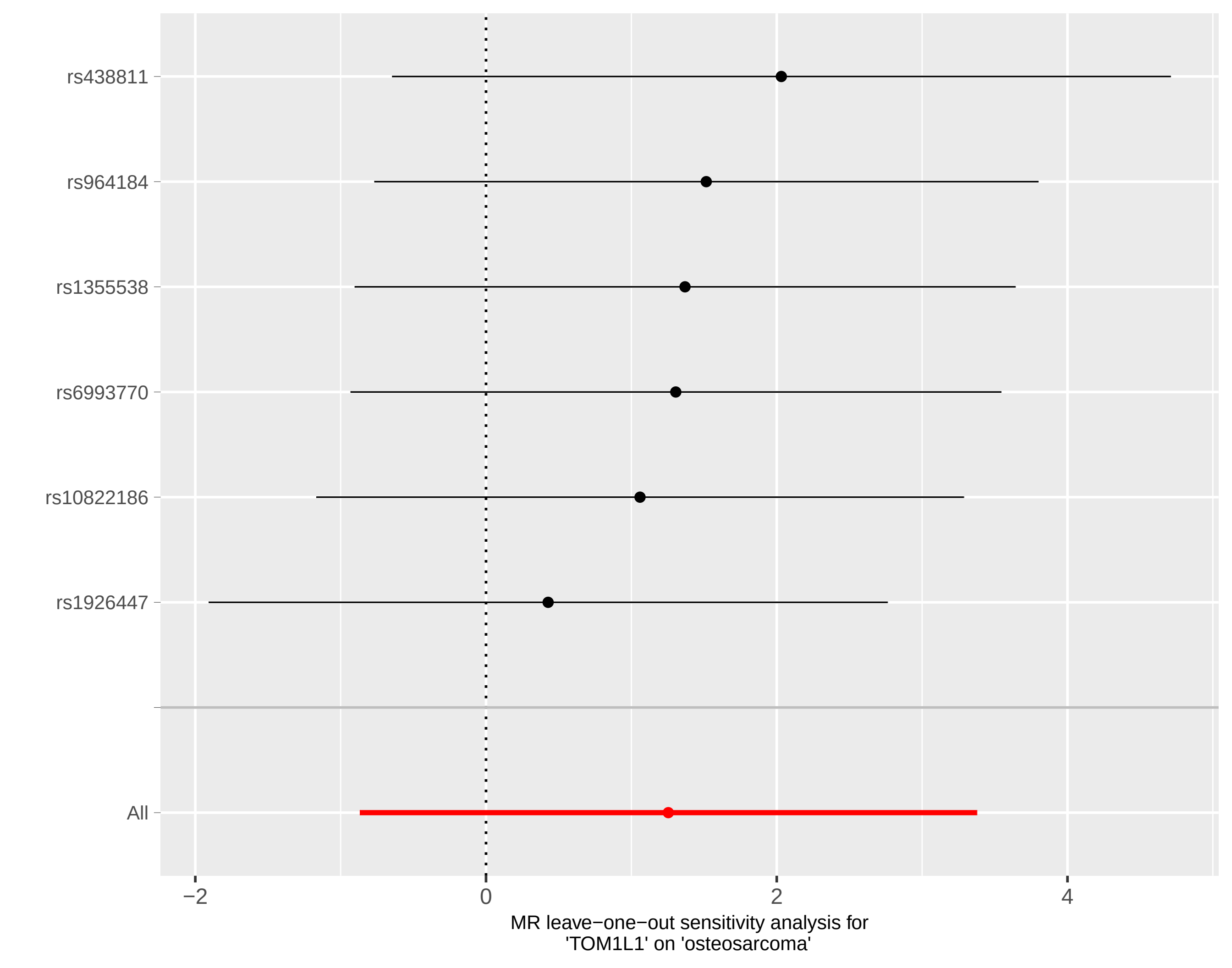

rs438811
rs964184
rs1355538
rs6993770
rs10822186
rs1926447
All
−2
0
2
4
MR leave−one−out sensitivity analysis for
'TOM1L1' on 'osteosarcoma'

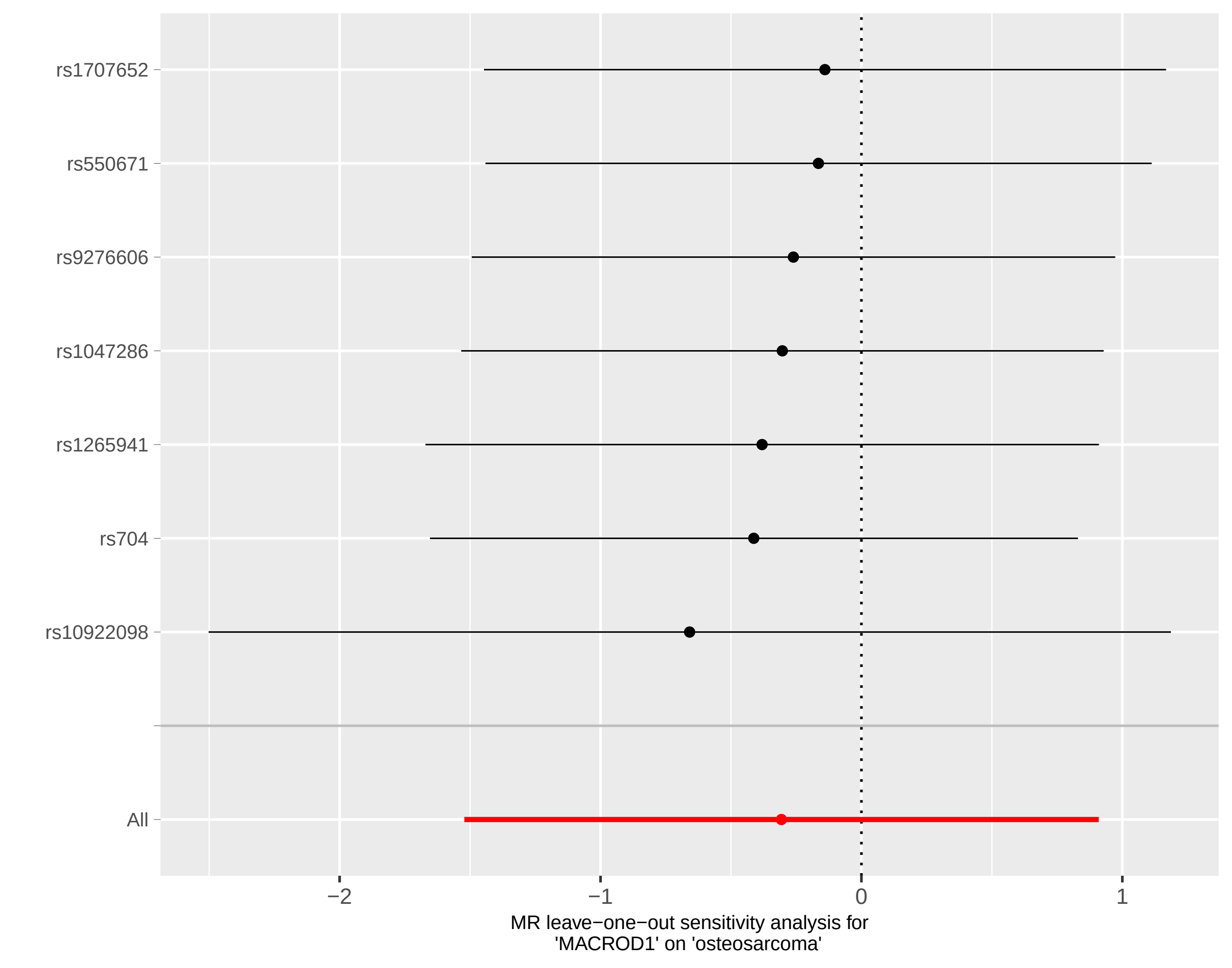

rs1707652
rs550671
rs9276606
rs1047286
rs1265941
rs704
rs10922098
All
−2
−1
0
1
MR leave−one−out sensitivity analysis for
'MACROD1' on 'osteosarcoma'

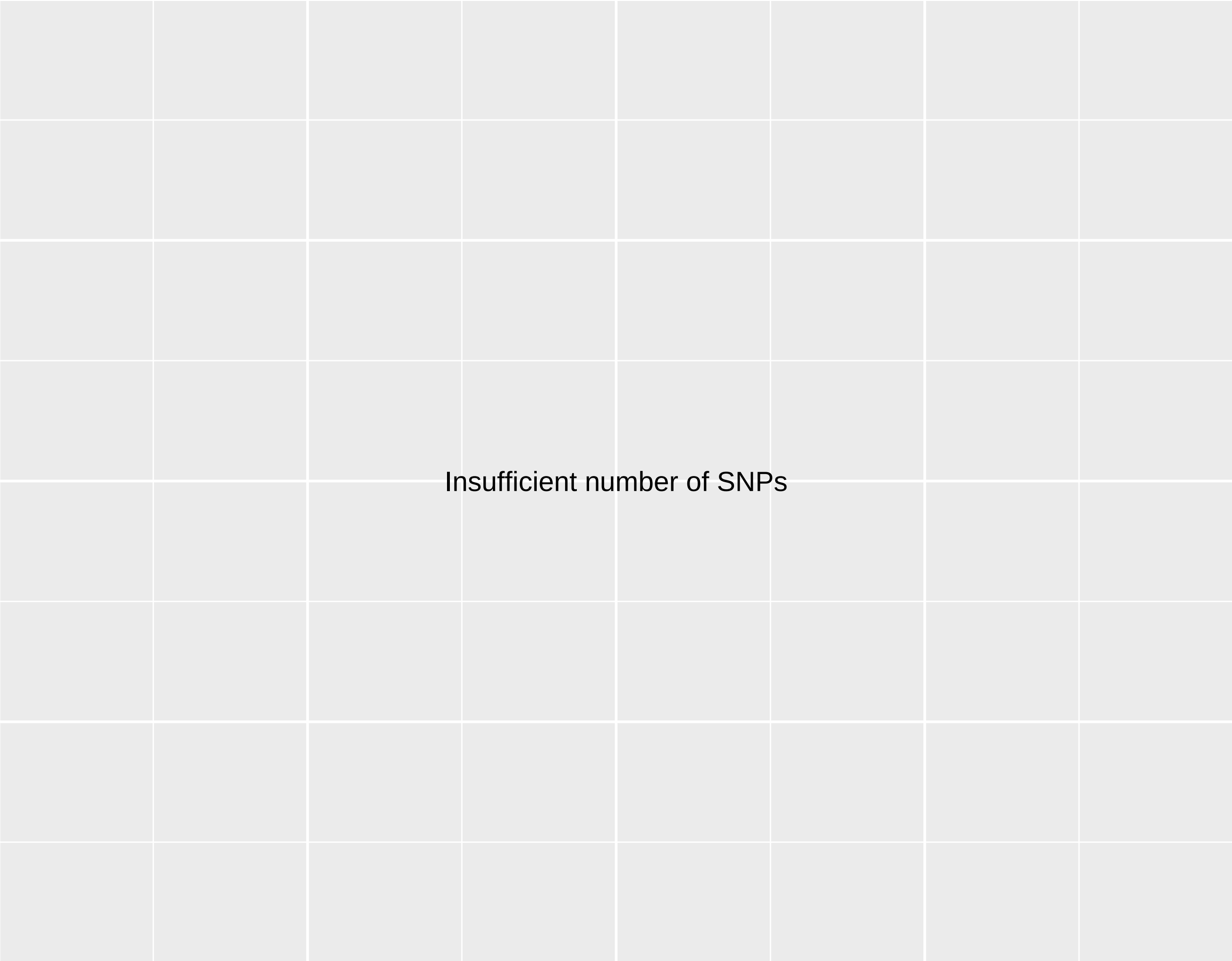
Insufficient number of SNPs

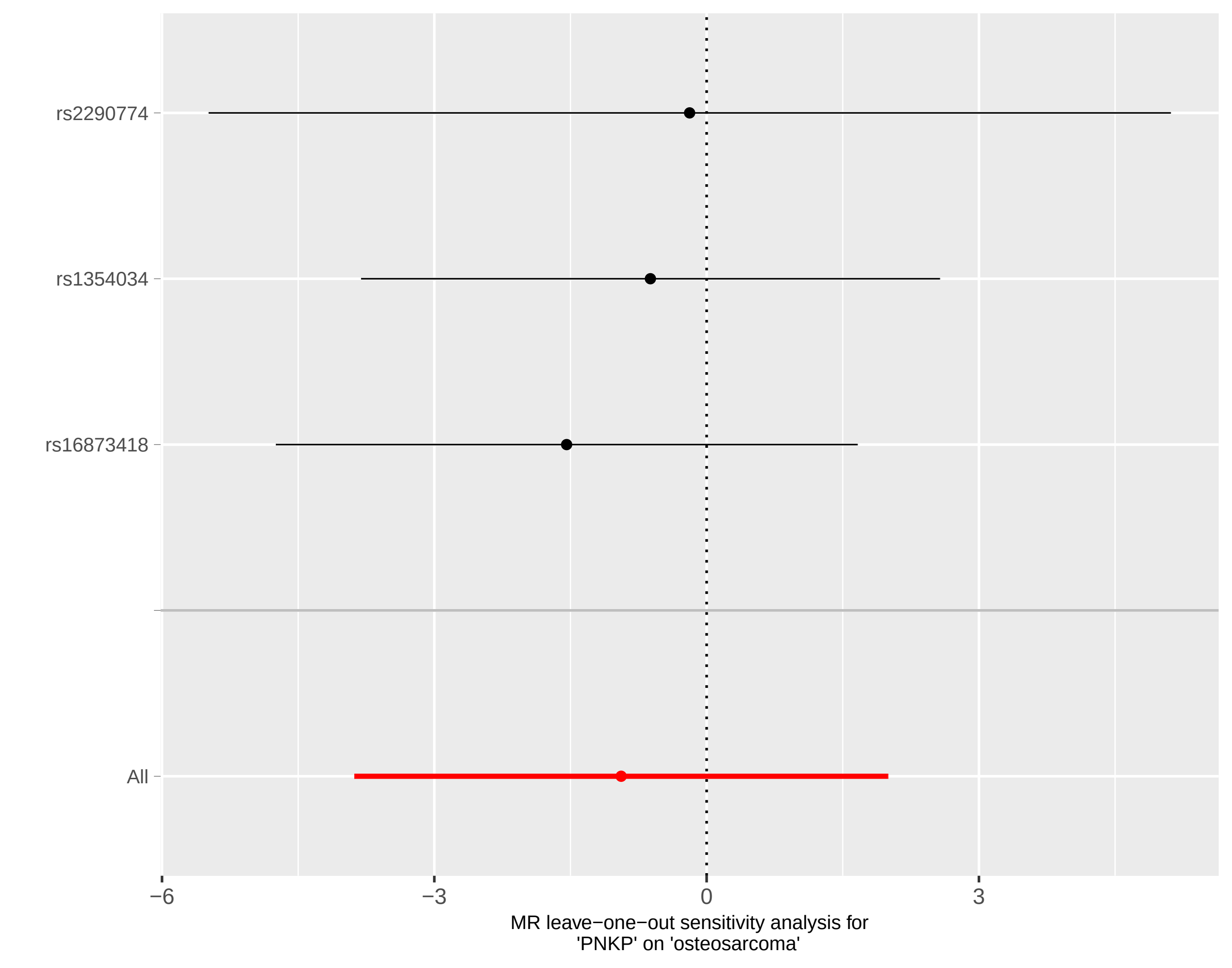
rs2290774
rs1354034
rs16873418
All
−6
−3
0
3
MR leave−one−out sensitivity analysis for
'PNKP' on 'osteosarcoma'

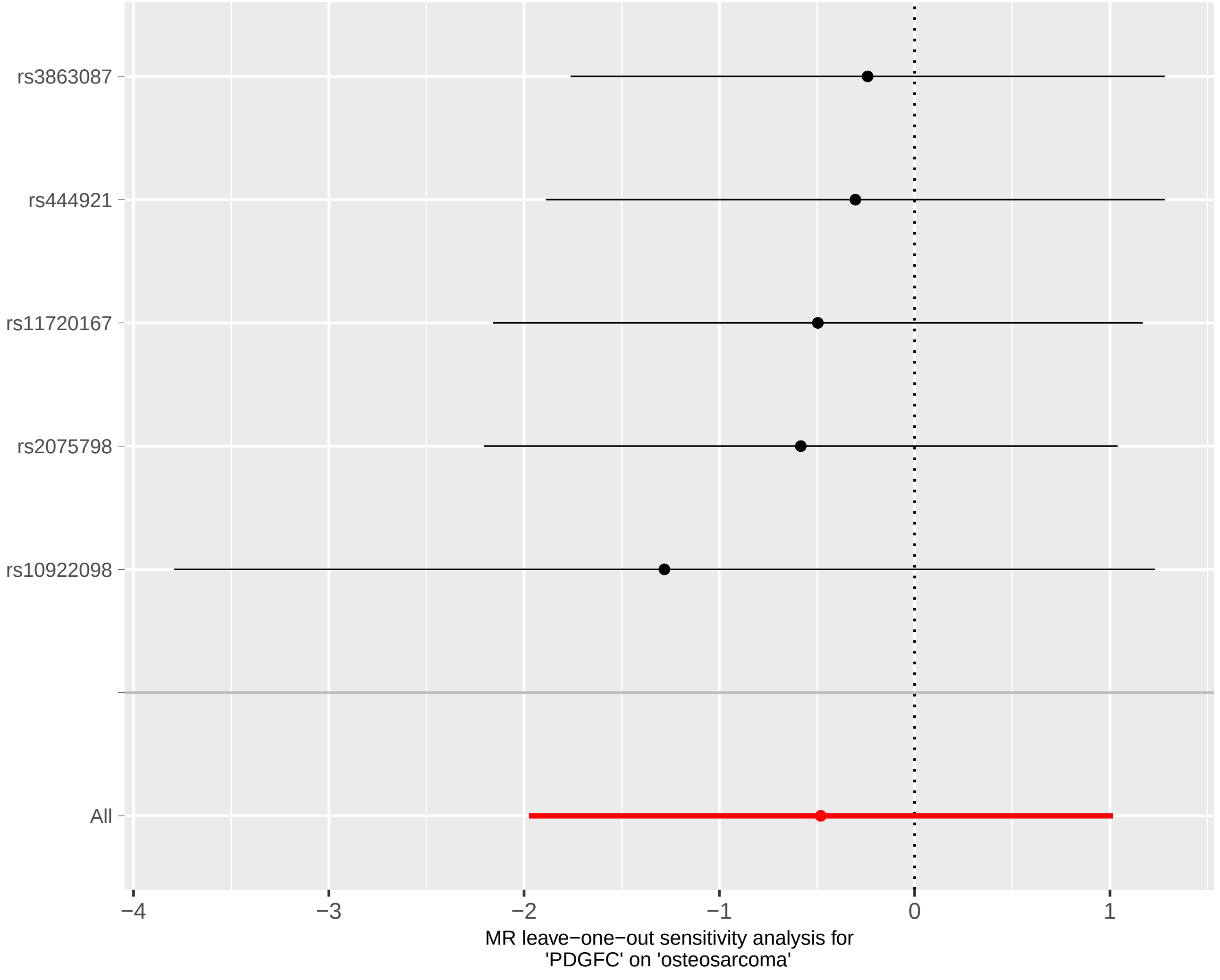

rs3863087
rs444921
rs11720167
rs2075798
rs10922098
All
−4
−3
−2
−1
0
1
MR leave−one−out sensitivity analysis for
'PDGFC' on 'osteosarcoma'

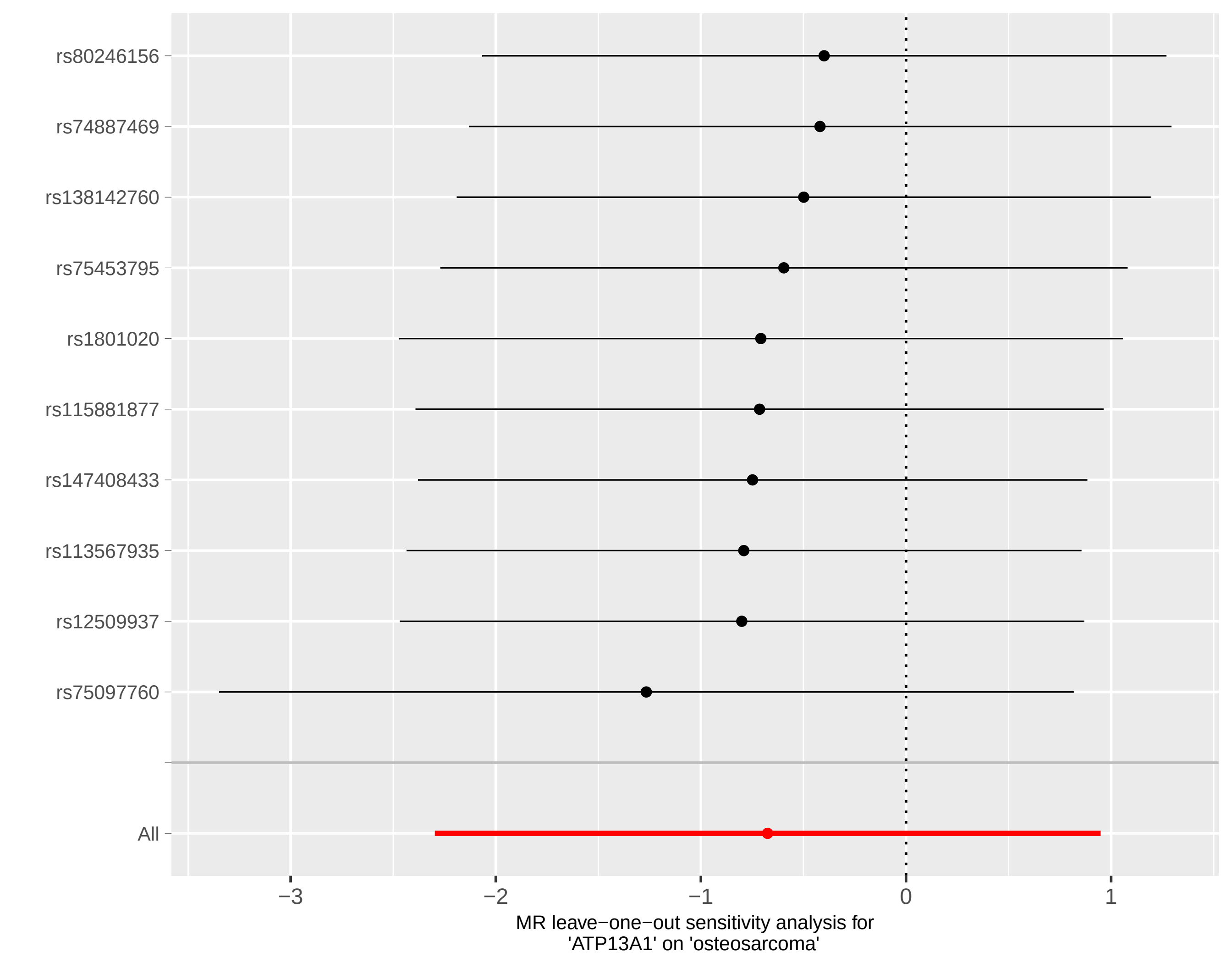

rs80246156
rs74887469
rs138142760
rs75453795
rs1801020
rs115881877
rs147408433
rs113567935
rs12509937
rs75097760
All
−3
−2
−1
0
1
MR leave−one−out sensitivity analysis for
'ATP13A1' on 'osteosarcoma'

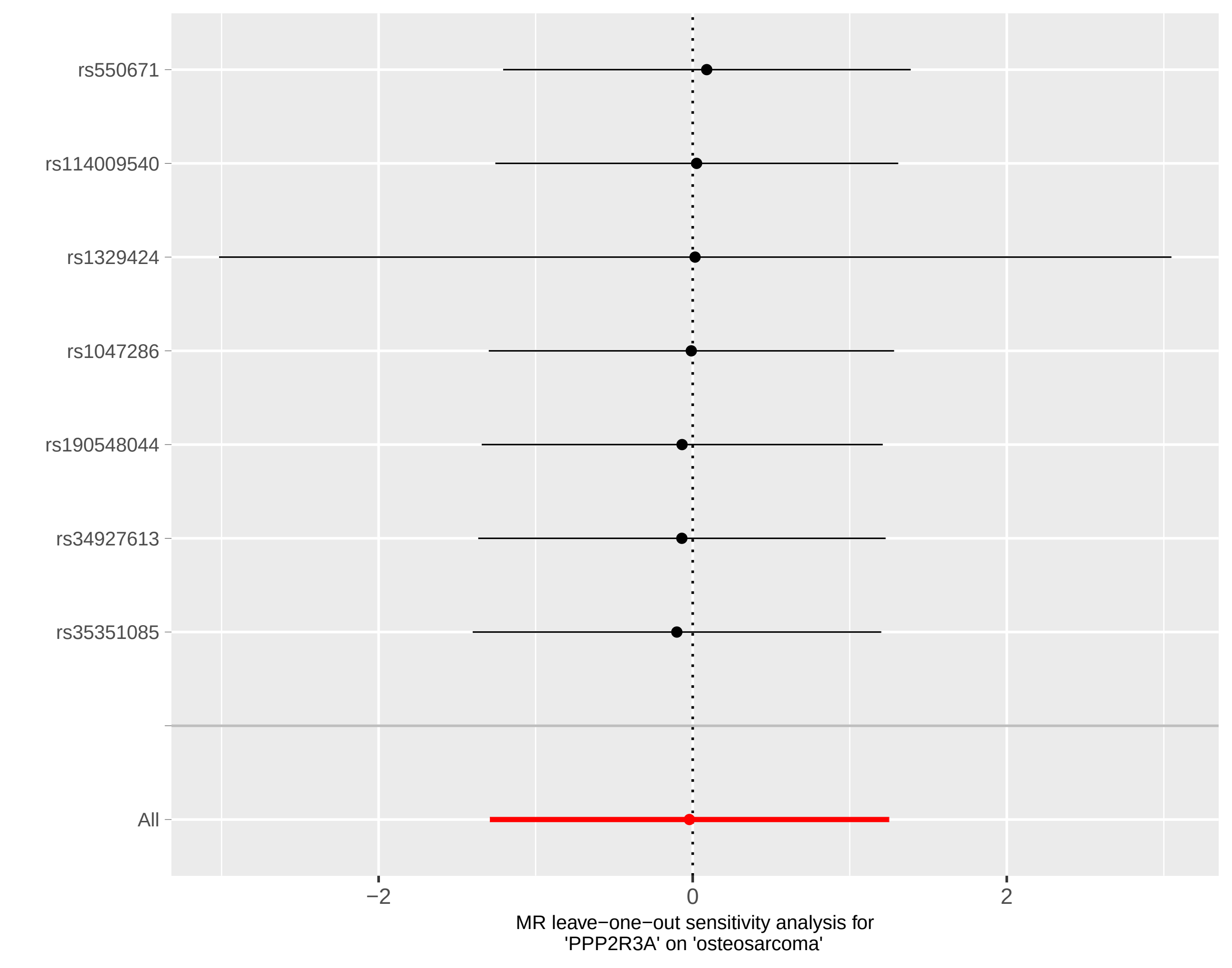
rs550671
rs114009540
rs1329424
rs1047286
rs190548044
rs34927613
rs35351085
All
−2
0
2
MR leave−one−out sensitivity analysis for
'PPP2R3A' on 'osteosarcoma'

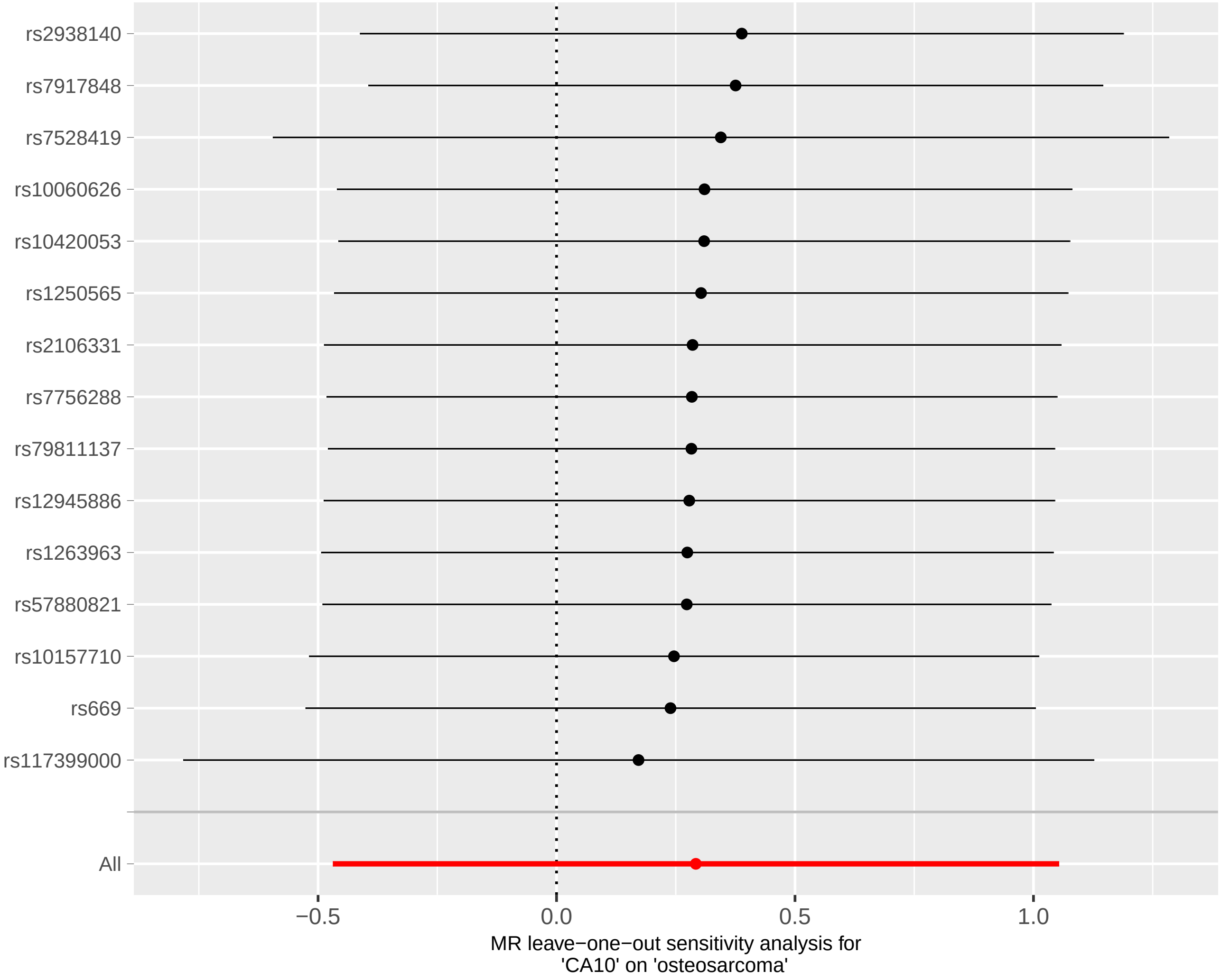
rs2938140
rs7917848
rs7528419
rs10060626
rs10420053
rs1250565
rs2106331
rs7756288
rs79811137
rs12945886
rs1263963
rs57880821
rs10157710
rs669
rs117399000
All
−0.5
0.0
0.5
1.0
MR leave−one−out sensitivity analysis for
'CA10' on 'osteosarcoma'

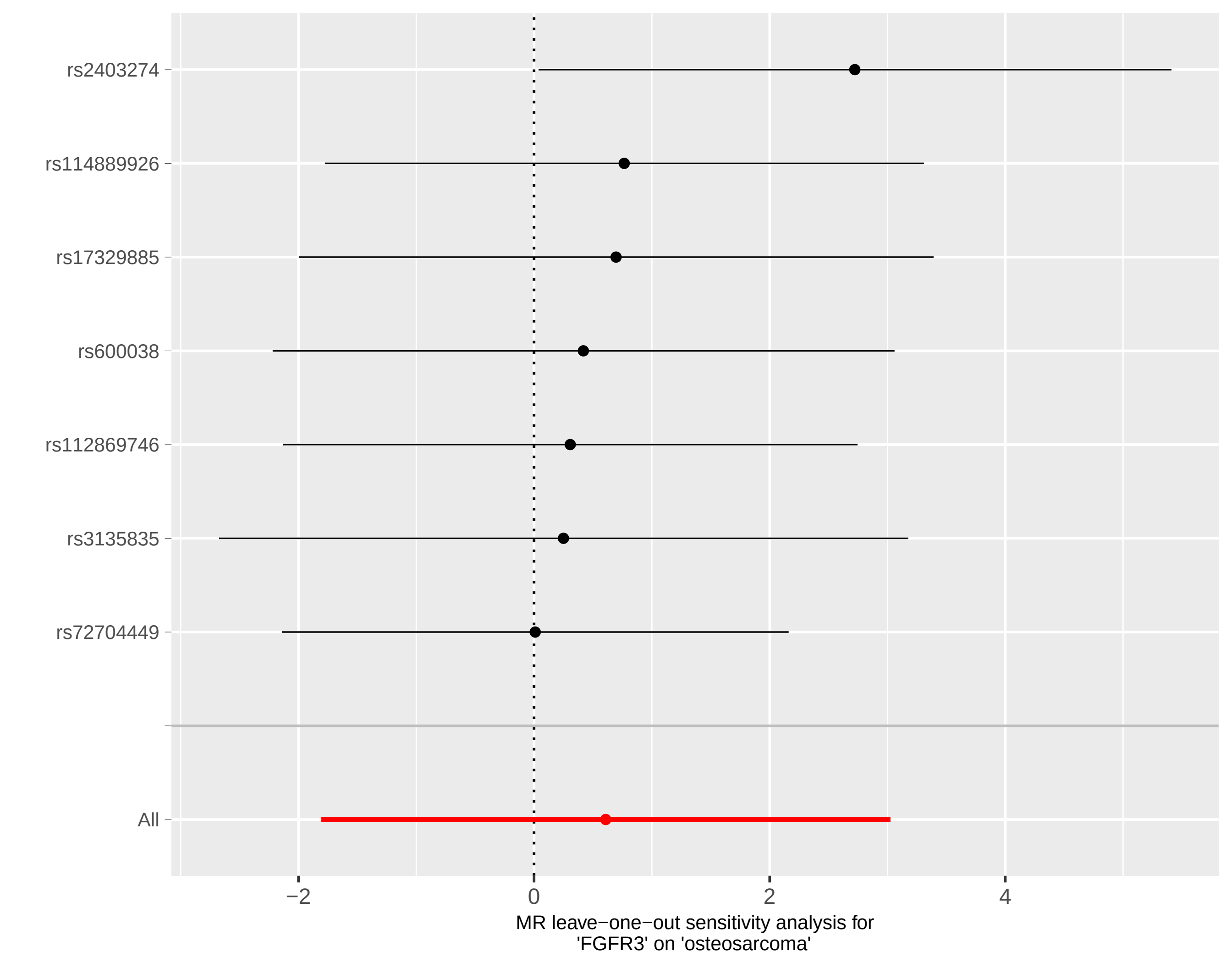

rs2403274
rs114889926
rs17329885
rs600038
rs112869746
rs3135835
rs72704449
All
−2
0
2
4
MR leave−one−out sensitivity analysis for
'FGFR3' on 'osteosarcoma'

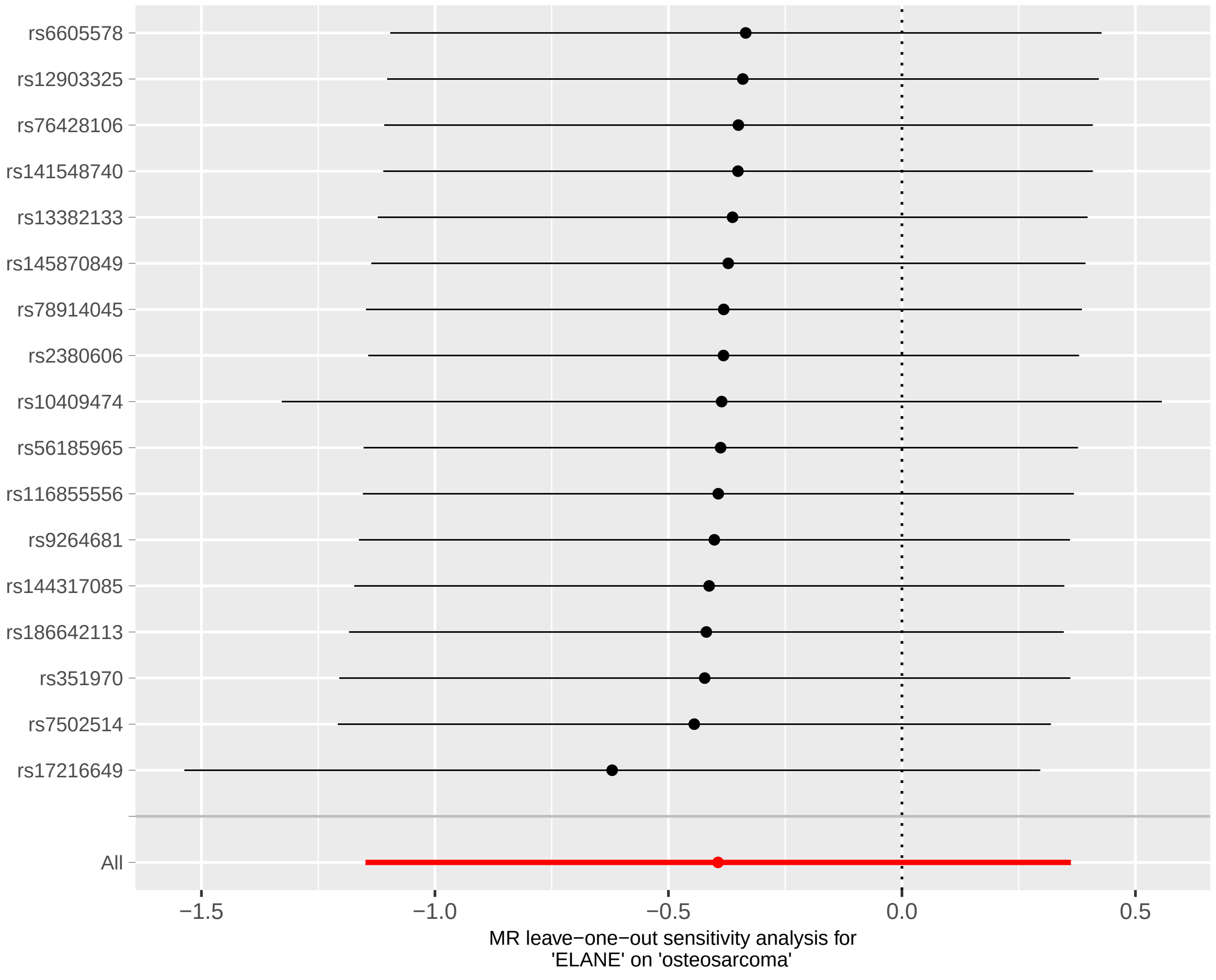
rs6605578
rs12903325
rs76428106
rs141548740
rs13382133
rs145870849
rs78914045
rs2380606
rs10409474
rs56185965
rs116855556
rs9264681
rs144317085
rs186642113
rs351970
rs7502514
rs17216649
All
−1.5
−1.0
−0.5
0.0
0.5
MR leave−one−out sensitivity analysis for
'ELANE' on 'osteosarcoma'

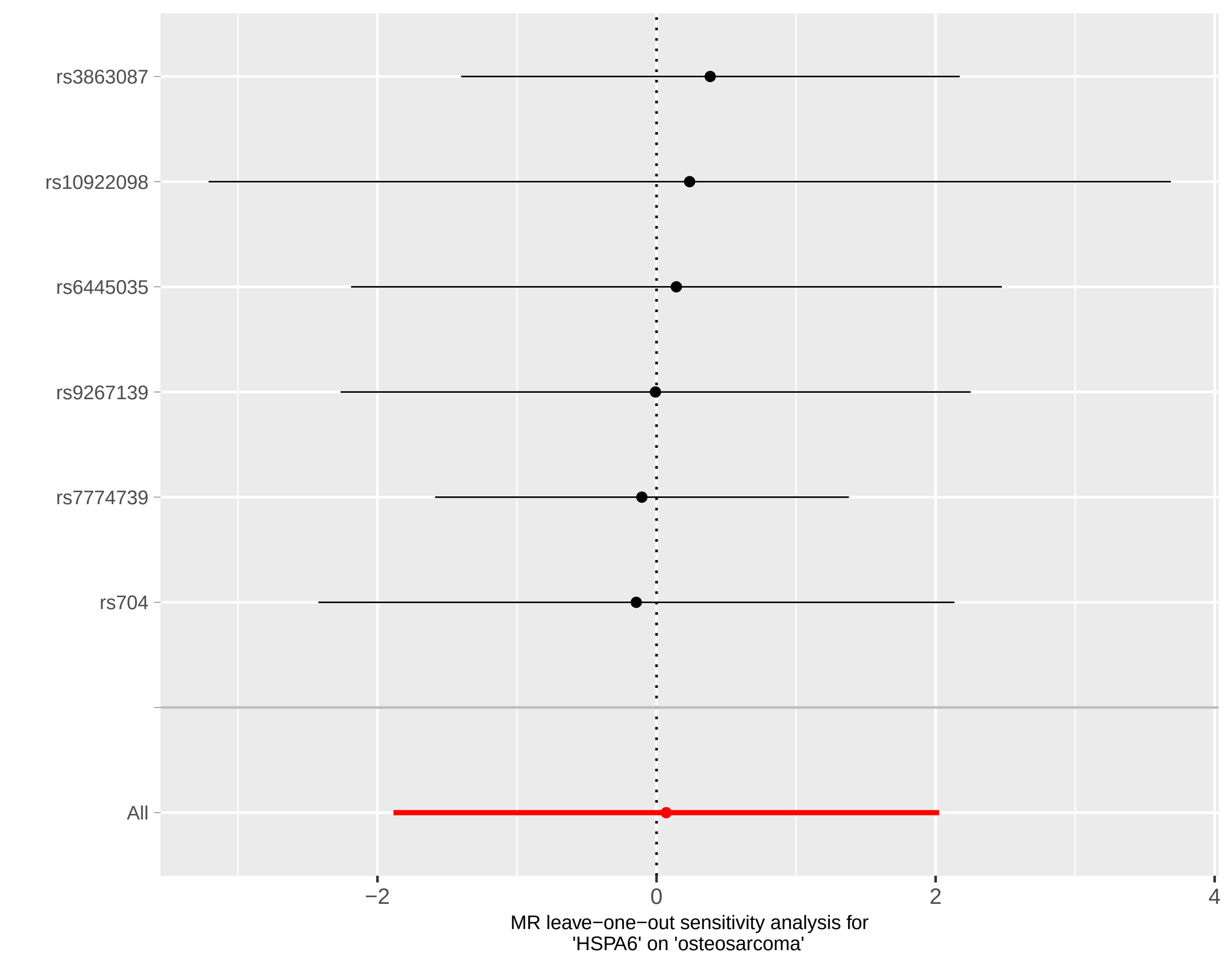
rs3863087
rs10922098
rs6445035
rs9267139
rs7774739
rs704
All
−2
0
2
4
MR leave−one−out sensitivity analysis for
'HSPA6' on 'osteosarcoma'

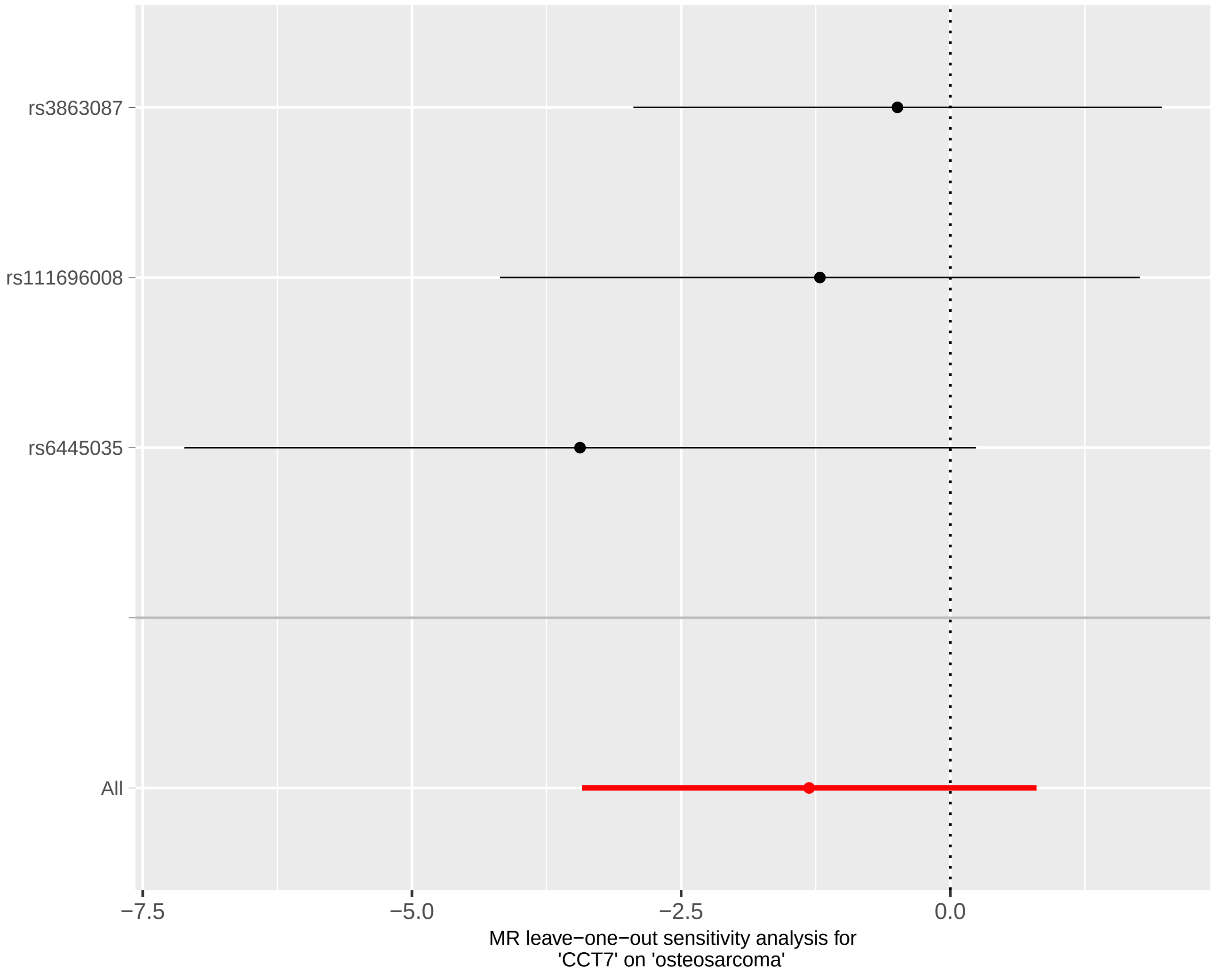
rs3863087
rs111696008
rs6445035
All
−7.5
−5.0
−2.5
0.0
MR leave−one−out sensitivity analysis for
'CCT7' on 'osteosarcoma'

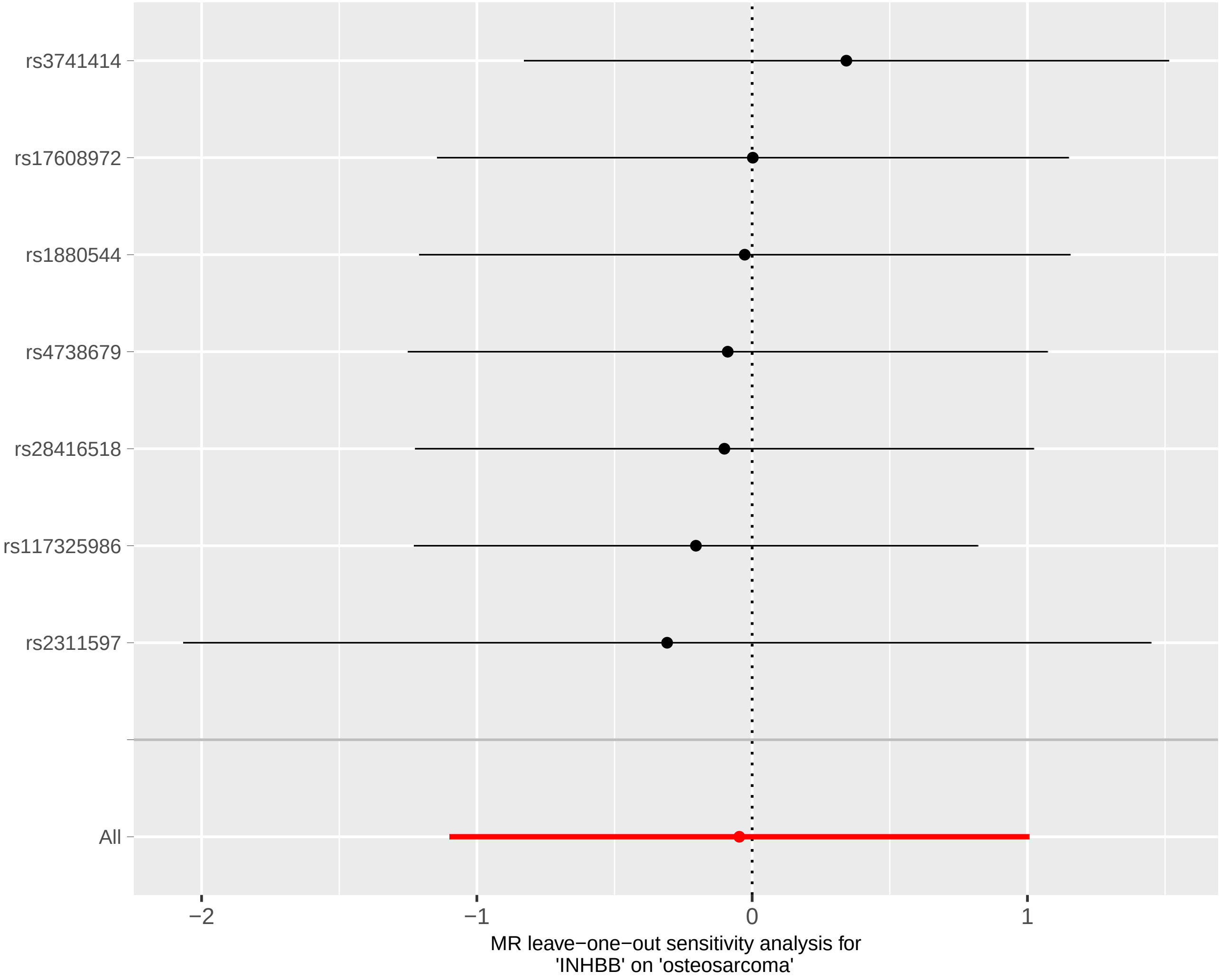

rs3741414
rs17608972
rs1880544
rs4738679
rs28416518
rs117325986
rs2311597
All
−2
−1
0
1
MR leave−one−out sensitivity analysis for
'INHBB' on 'osteosarcoma'

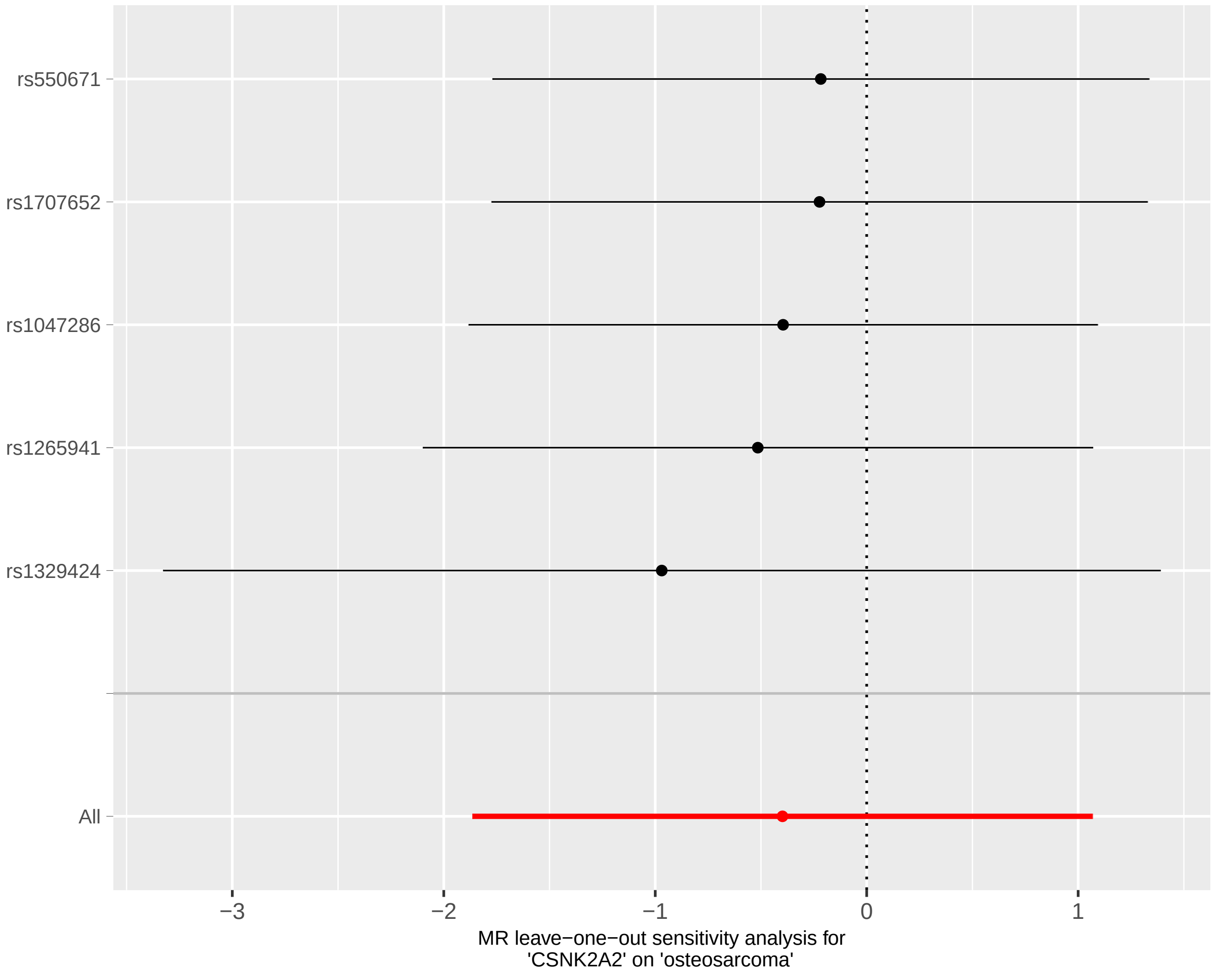
rs550671
rs1707652
rs1047286
rs1265941
rs1329424
All
−3
−2
−1
0
1
MR leave−one−out sensitivity analysis for
'CSNK2A2' on 'osteosarcoma'

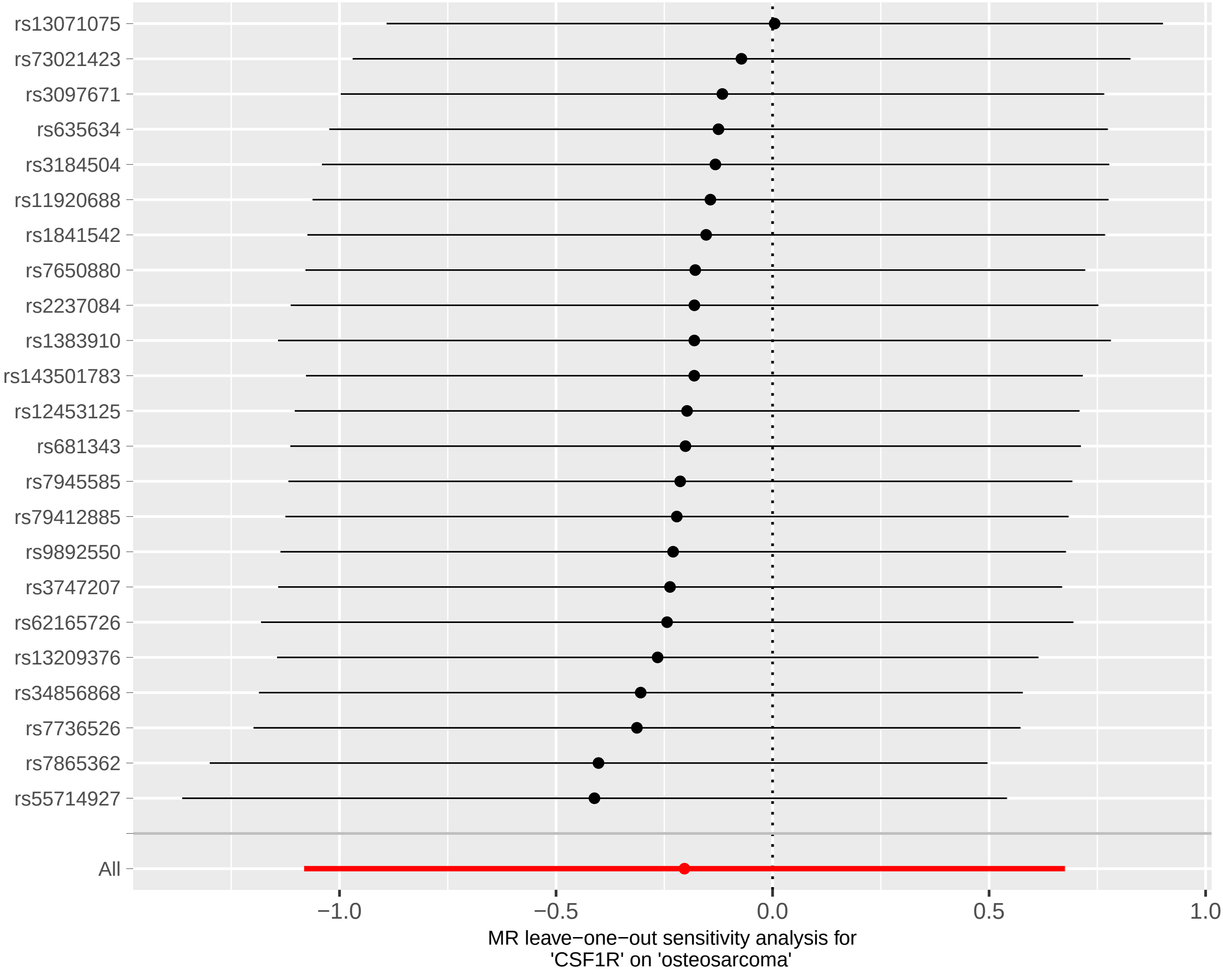
rs13071075
rs73021423
rs3097671
rs635634
rs3184504
rs11920688
rs1841542
rs7650880
rs2237084
rs1383910
rs143501783
rs12453125
rs681343
rs7945585
rs79412885
rs9892550
rs3747207
rs62165726
rs13209376
rs34856868
rs7736526
rs7865362
rs55714927
All
−1.0
−0.5
0.0
0.5
1.0
MR leave−one−out sensitivity analysis for
'CSF1R' on 'osteosarcoma'

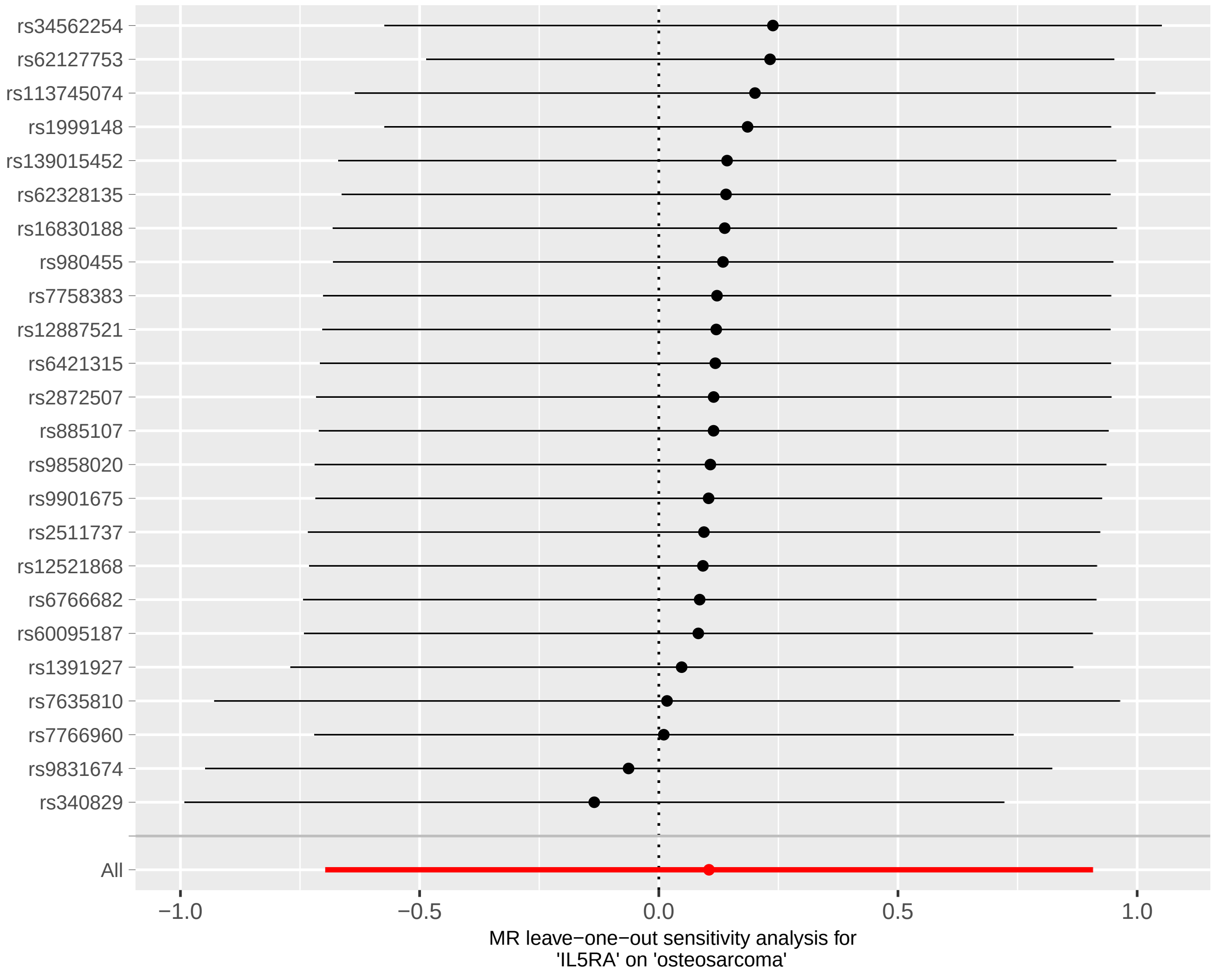

rs34562254
rs62127753
rs113745074
rs1999148
rs139015452
rs62328135
rs16830188
rs980455
rs7758383
rs12887521
rs6421315
rs2872507
rs885107
rs9858020
rs9901675
rs2511737
rs12521868
rs6766682
rs60095187
rs1391927
rs7635810
rs7766960
rs9831674
rs340829
All
−1.0
−0.5
0.0
0.5
1.0
MR leave−one−out sensitivity analysis for
'IL5RA' on 'osteosarcoma'

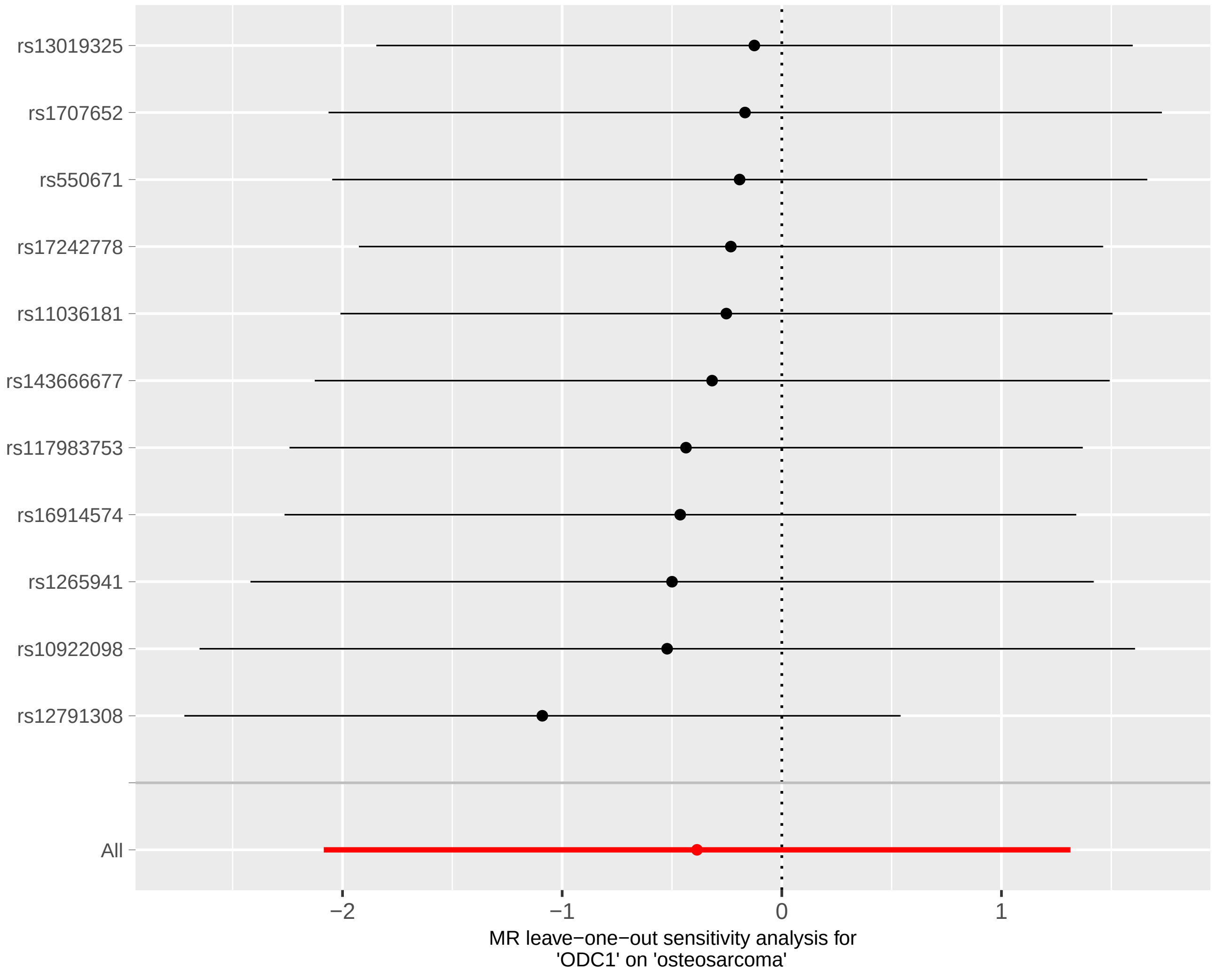
rs13019325
rs1707652
rs550671
rs17242778
rs11036181
rs143666677
rs117983753
rs16914574
rs1265941
rs10922098
rs12791308
All
−2
−1
0
1
MR leave−one−out sensitivity analysis for
'ODC1' on 'osteosarcoma'

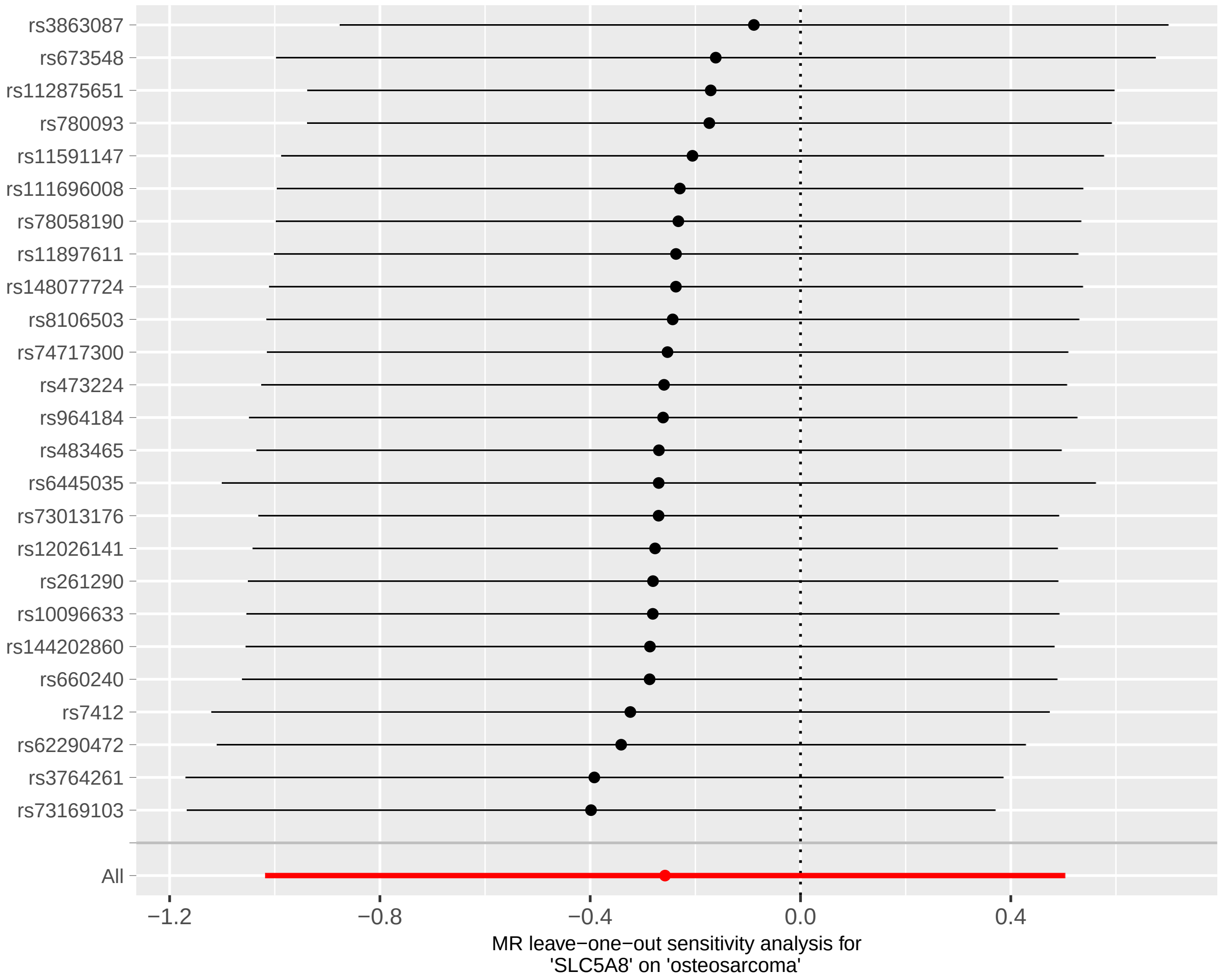

rs3863087
rs673548
rs112875651
rs780093
rs11591147
rs111696008
rs78058190
rs11897611
rs148077724
rs8106503
rs74717300
rs473224
rs964184
rs483465
rs6445035
rs73013176
rs12026141
rs261290
rs10096633
rs144202860
rs660240
rs7412
rs62290472
rs3764261
rs73169103
All
−1.2
−0.8
−0.4
0.0
0.4
MR leave−one−out sensitivity analysis for
'SLC5A8' on 'osteosarcoma'

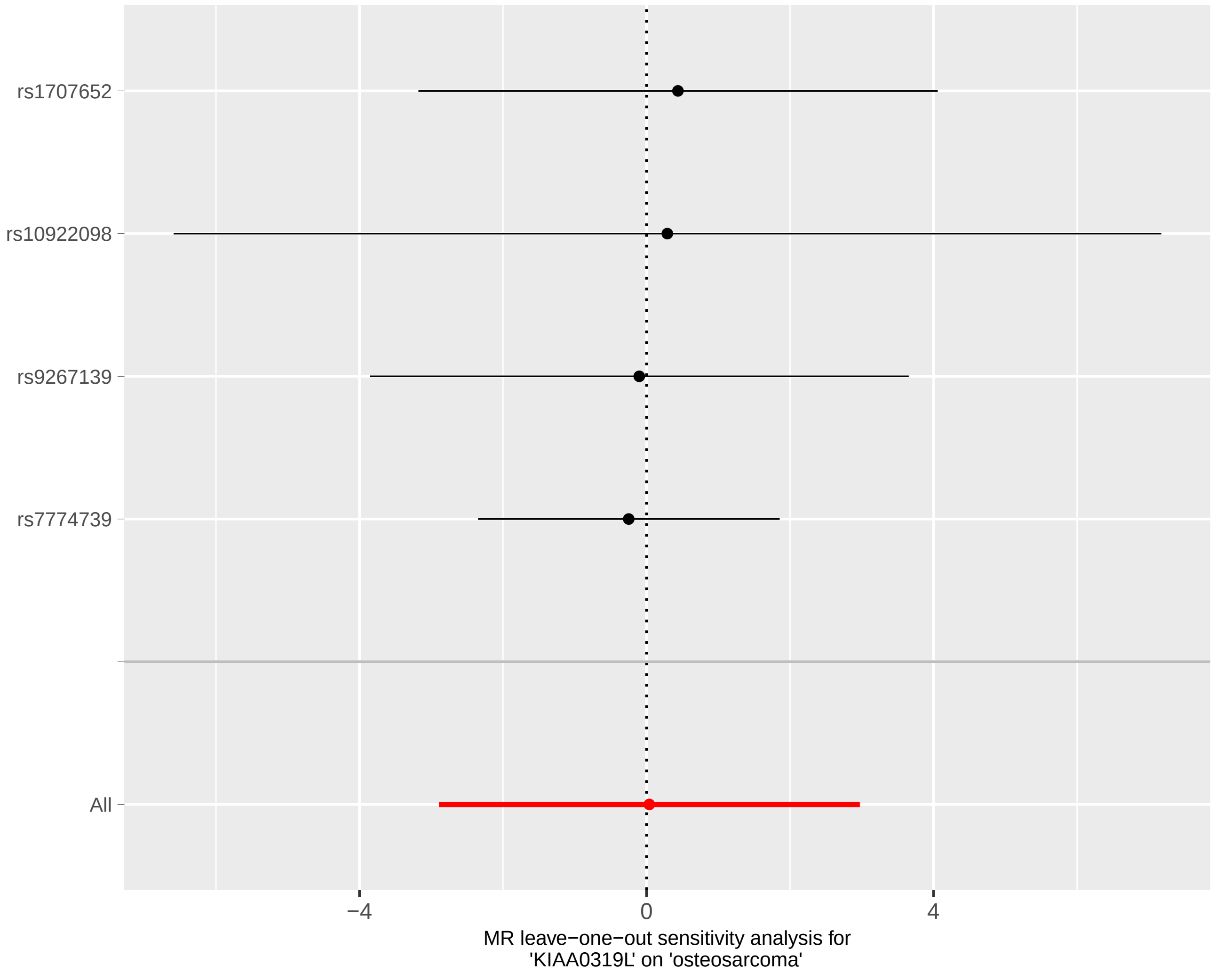
rs1707652
rs10922098
rs9267139
rs7774739
All
−4
0
4
MR leave−one−out sensitivity analysis for
'KIAA0319L' on 'osteosarcoma'

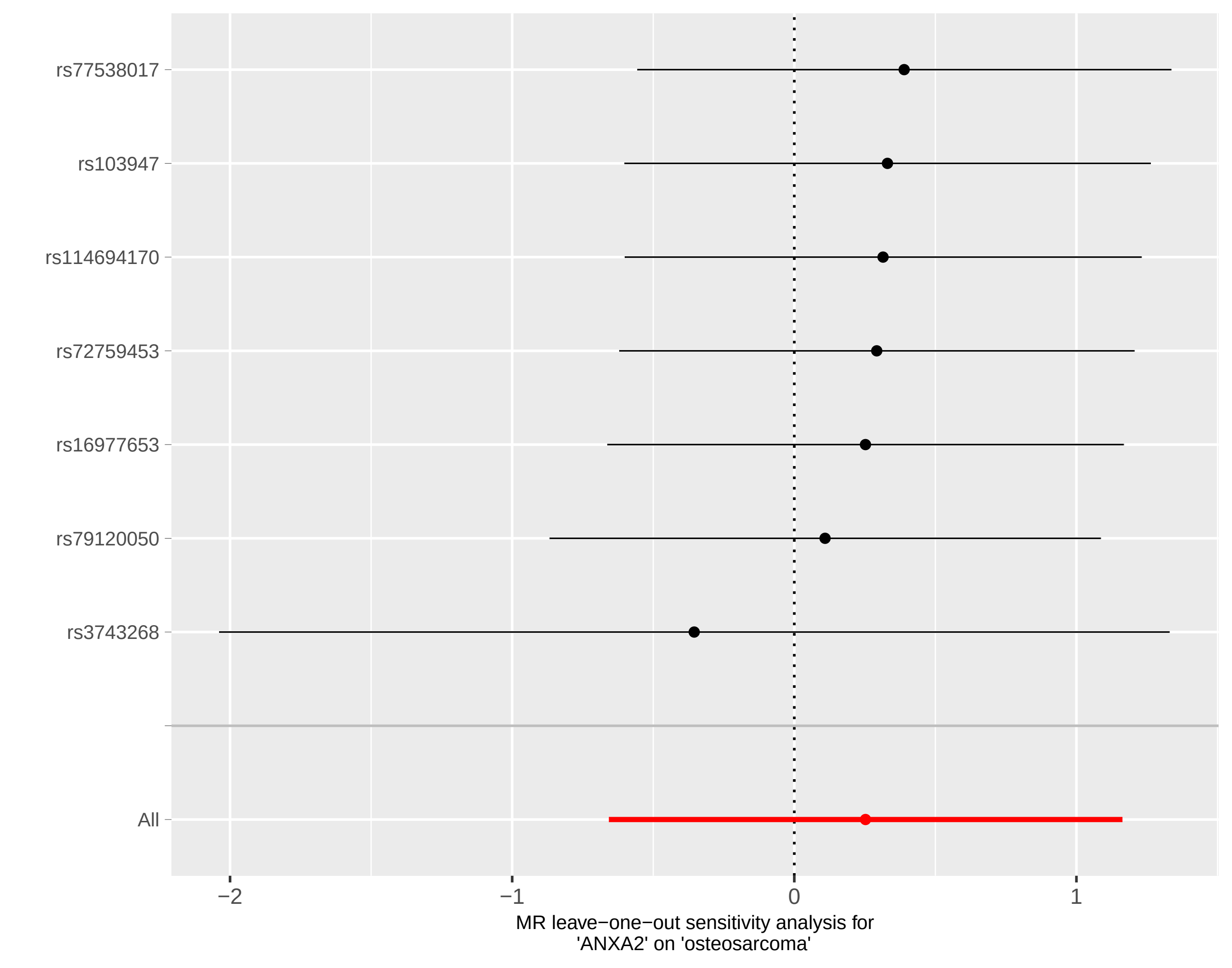
rs77538017
rs103947
rs114694170
rs72759453
rs16977653
rs79120050
rs3743268
All
−2
−1
0
1
MR leave−one−out sensitivity analysis for
'ANXA2' on 'osteosarcoma'

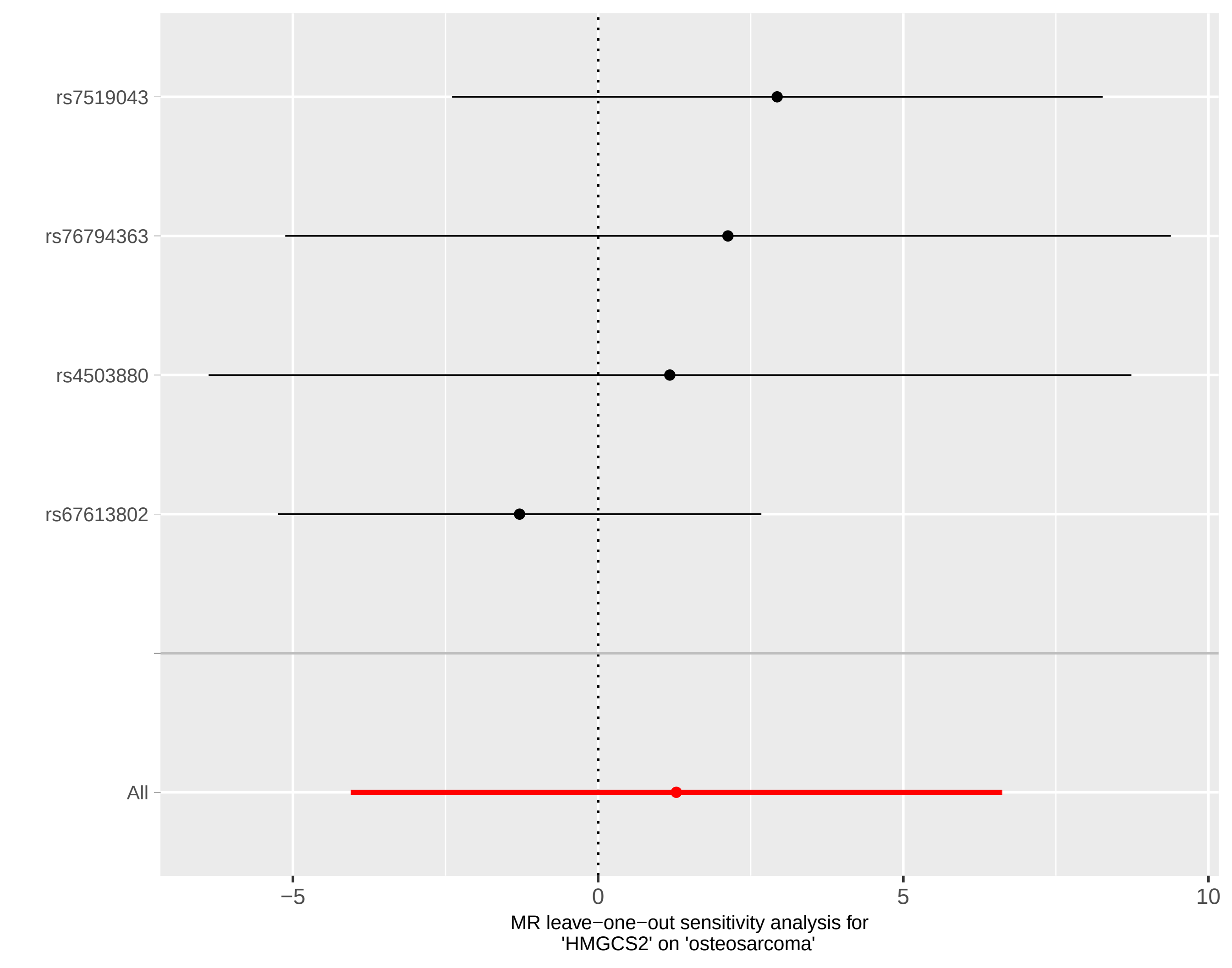

rs7519043
rs76794363
rs4503880
rs67613802
All
−5
0
5
10
MR leave−one−out sensitivity analysis for
'HMGCS2' on 'osteosarcoma'

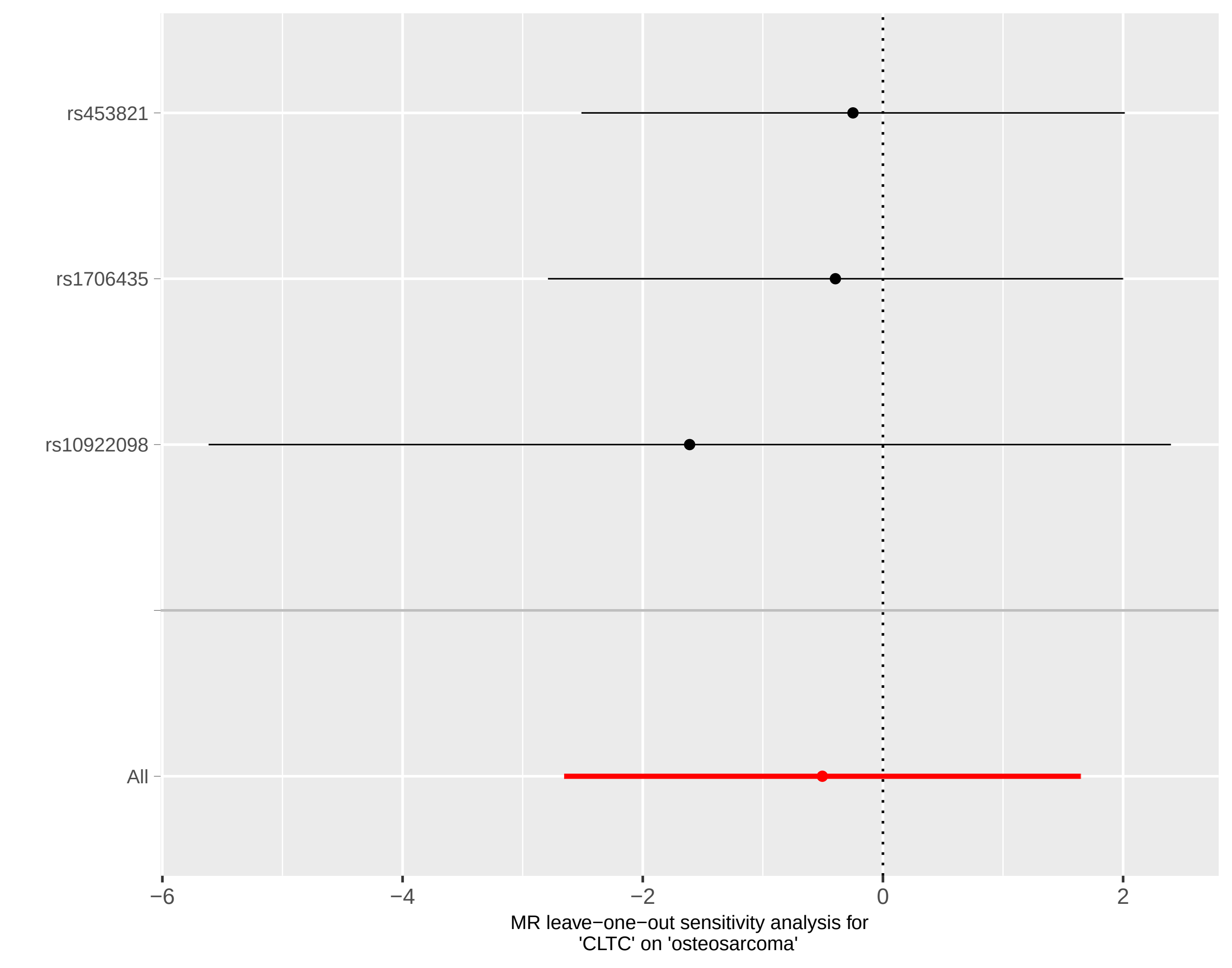

rs453821
rs1706435
rs10922098
All
−6
−4
−2
0
2
MR leave−one−out sensitivity analysis for
'CLTC' on 'osteosarcoma'

Insufficient number of SNPs

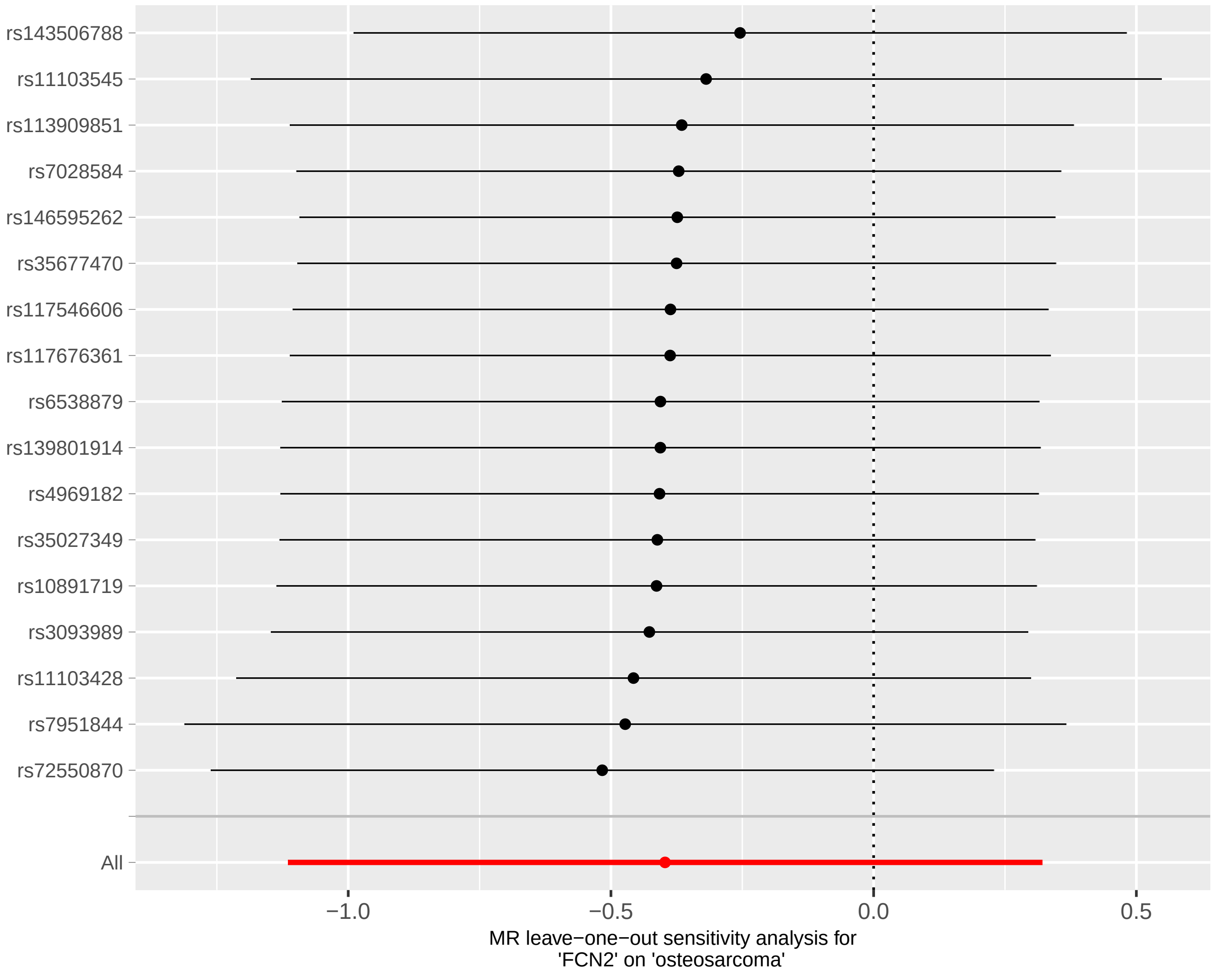
rs143506788
rs11103545
rs113909851
rs7028584
rs146595262
rs35677470
rs117546606
rs117676361
rs6538879
rs139801914
rs4969182
rs35027349
rs10891719
rs3093989
rs11103428
rs7951844
rs72550870
All
−1.0
−0.5
0.0
0.5
MR leave−one−out sensitivity analysis for
'FCN2' on 'osteosarcoma'

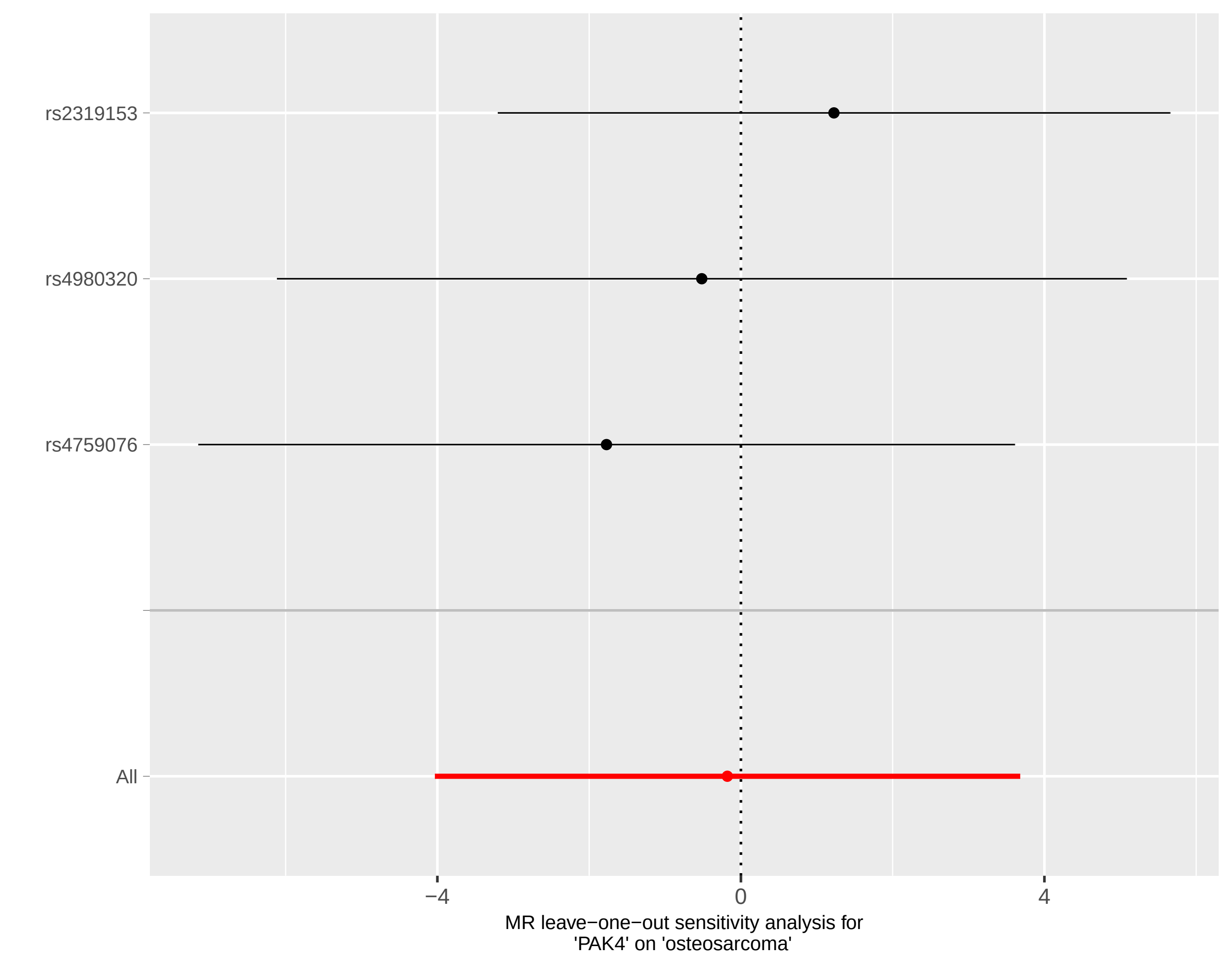

rs2319153
rs4980320
rs4759076
All
−4
0
4
MR leave−one−out sensitivity analysis for
'PAK4' on 'osteosarcoma'

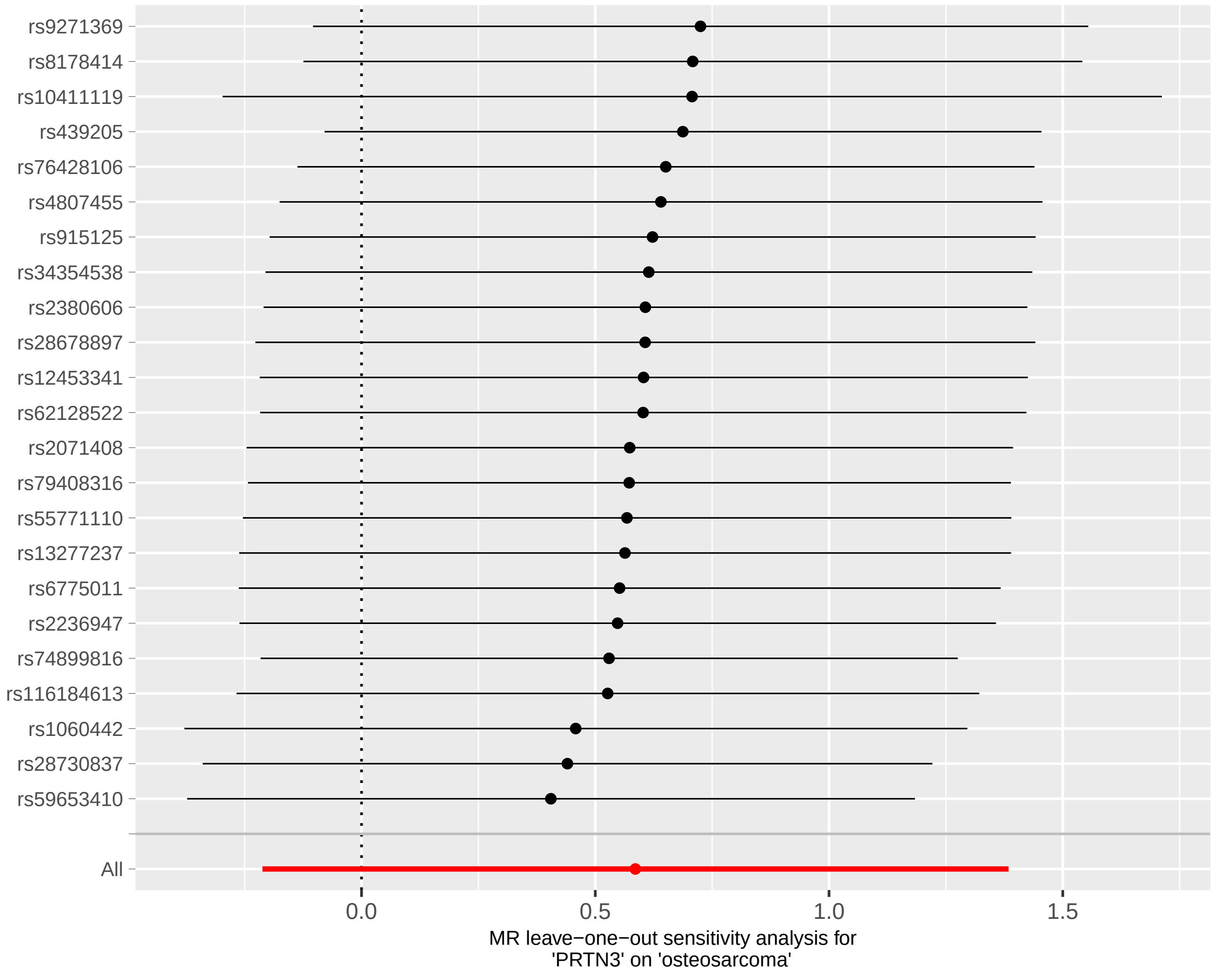
rs9271369
rs8178414
rs10411119
rs439205
rs76428106
rs4807455
rs915125
rs34354538
rs2380606
rs28678897
rs12453341
rs62128522
rs2071408
rs79408316
rs55771110
rs13277237
rs6775011
rs2236947
rs74899816
rs116184613
rs1060442
rs28730837
rs59653410
All
0.0
0.5
1.0
1.5
MR leave−one−out sensitivity analysis for
'PRTN3' on 'osteosarcoma'

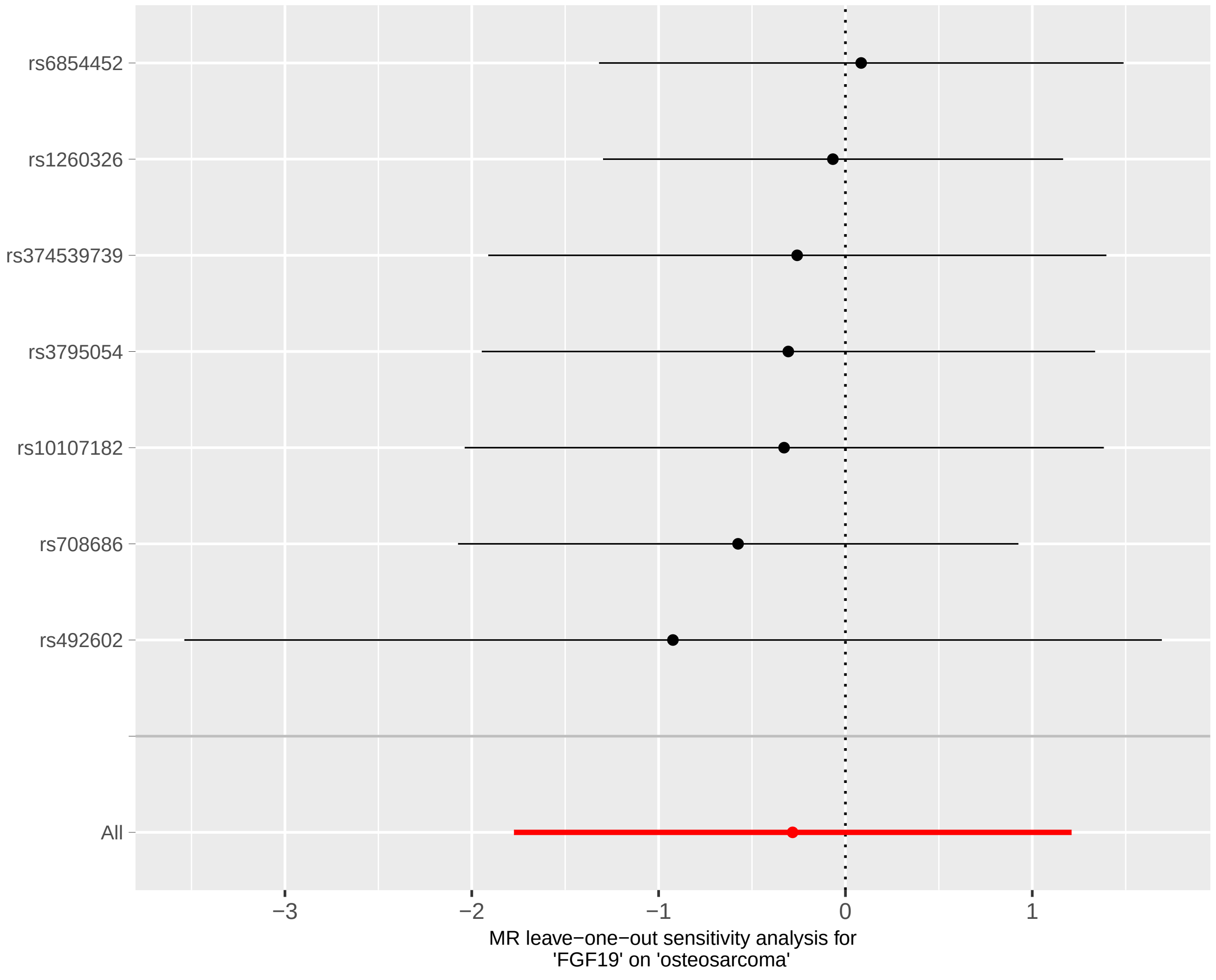
rs6854452
rs1260326
rs374539739
rs3795054
rs10107182
rs708686
rs492602
All
−3
−2
−1
0
1
MR leave−one−out sensitivity analysis for
'FGF19' on 'osteosarcoma'

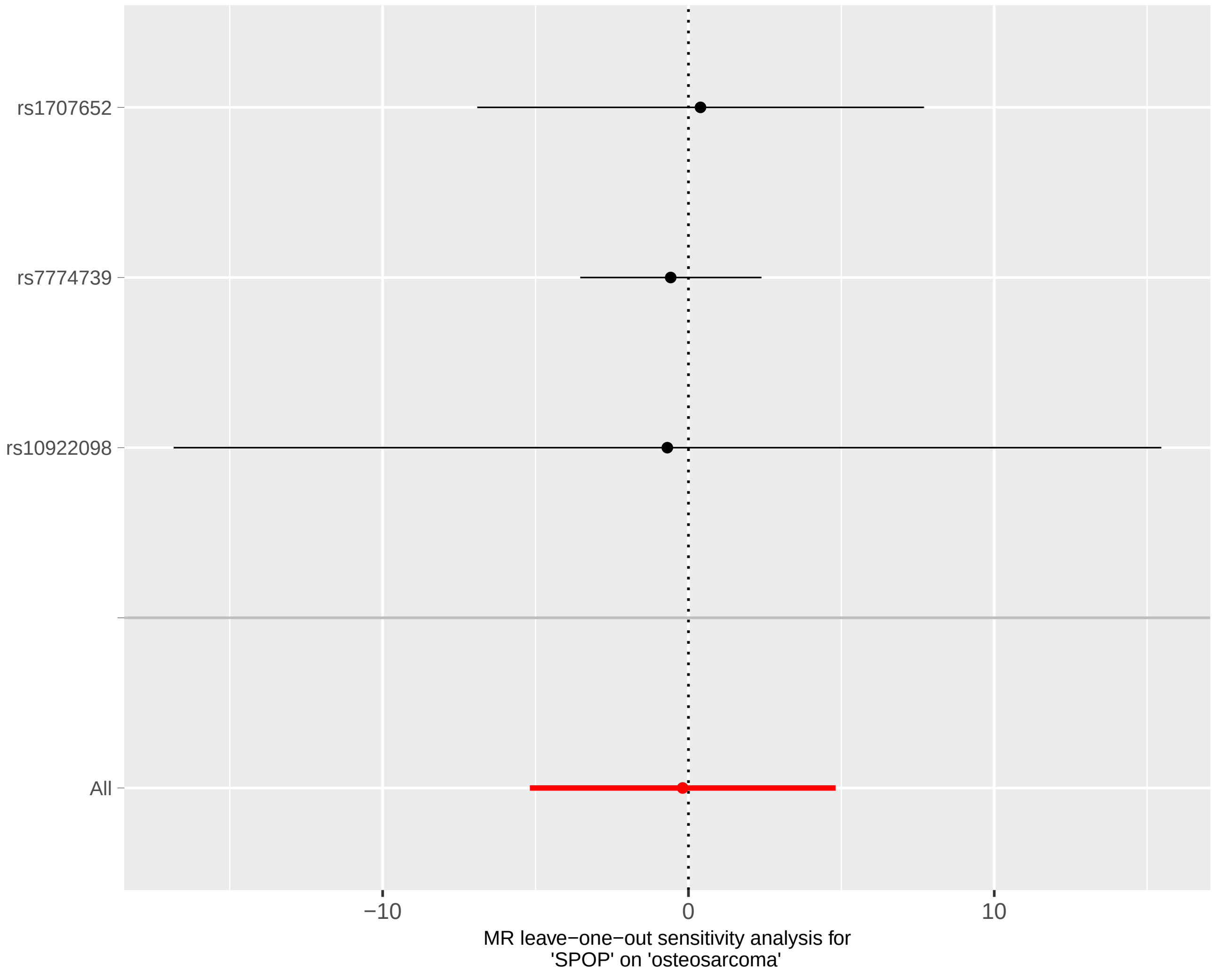

rs1707652
rs7774739
rs10922098
All
−10
0
10
MR leave−one−out sensitivity analysis for
'SPOP' on 'osteosarcoma'

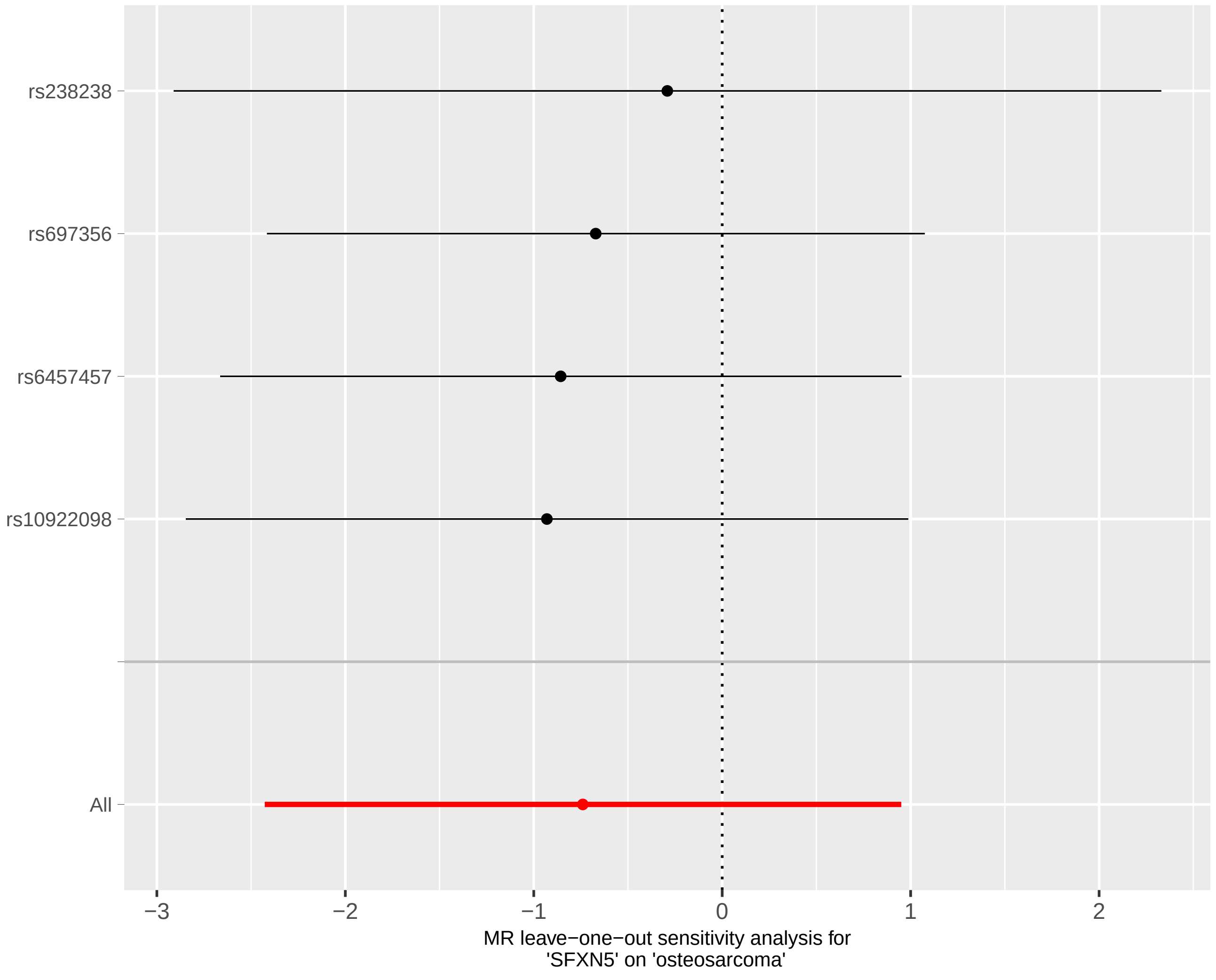
rs238238
rs697356
rs6457457
rs10922098
All
−3
−2
−1
0
1
2
MR leave−one−out sensitivity analysis for
'SFXN5' on 'osteosarcoma'

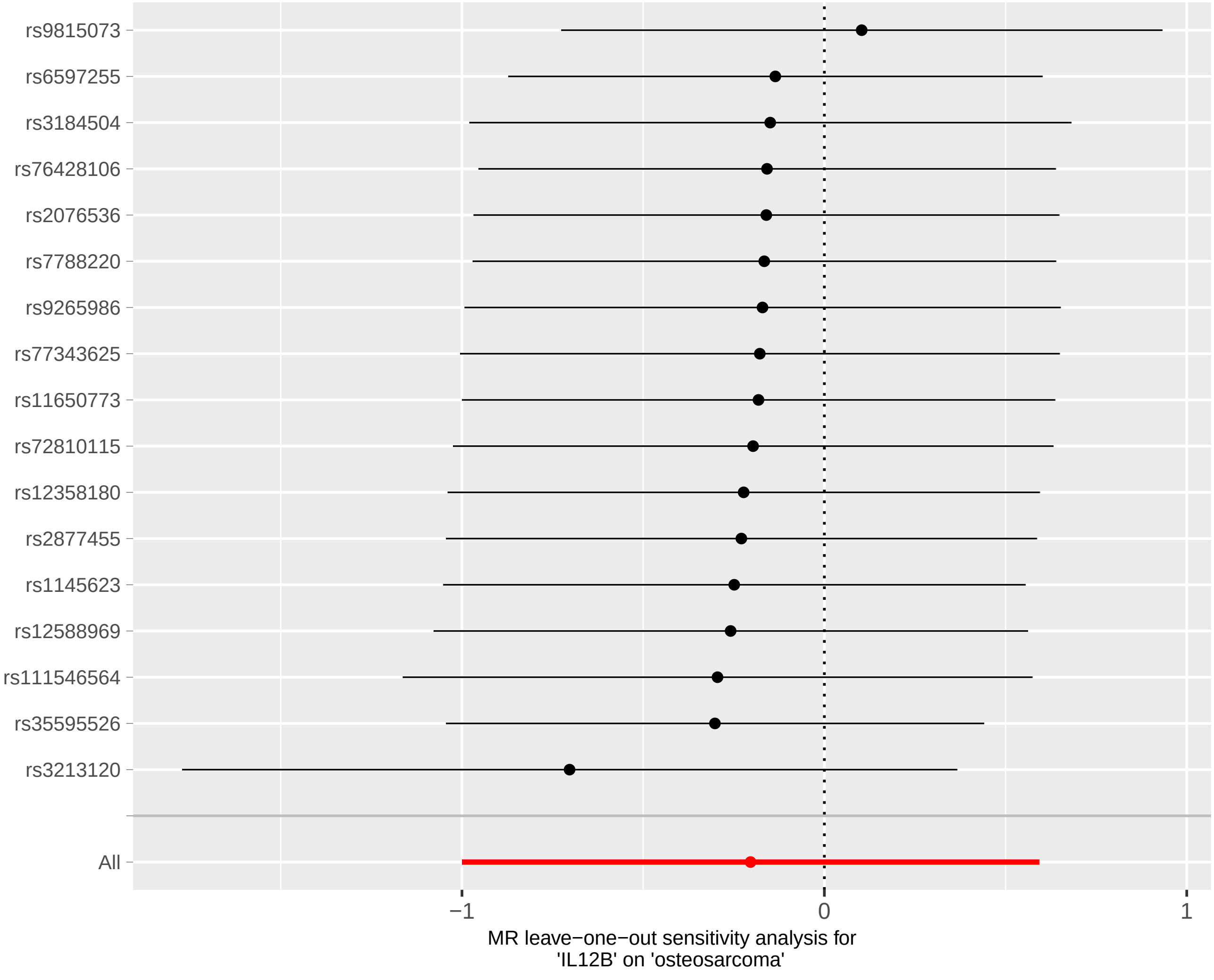
rs9815073
rs6597255
rs3184504
rs76428106
rs2076536
rs7788220
rs9265986
rs77343625
rs11650773
rs72810115
rs12358180
rs2877455
rs1145623
rs12588969
rs111546564
rs35595526
rs3213120
All
−1
0
1
MR leave−one−out sensitivity analysis for
'IL12B' on 'osteosarcoma'

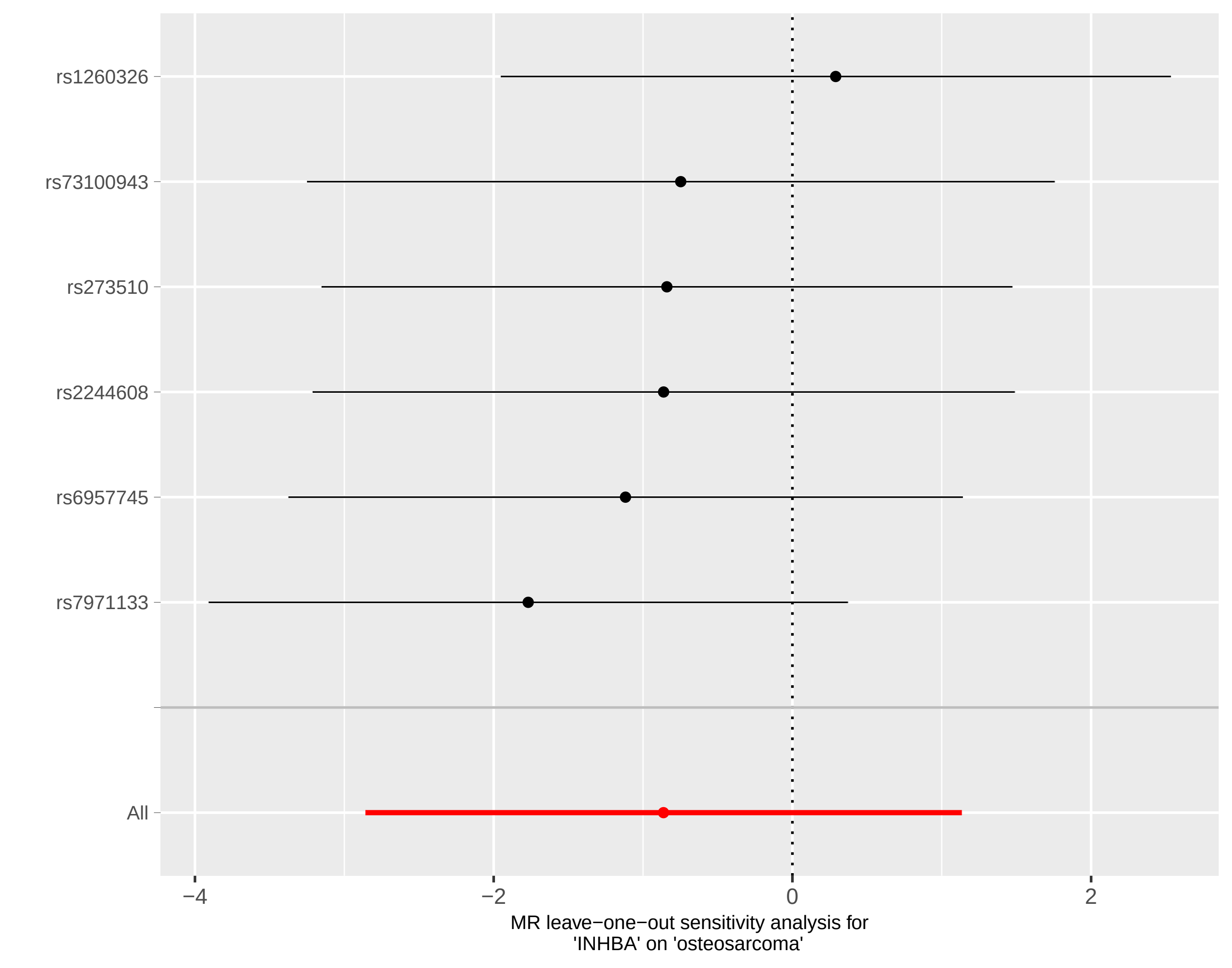
rs1260326
rs73100943
rs273510
rs2244608
rs6957745
rs7971133
All
−4
−2
0
2
MR leave−one−out sensitivity analysis for
'INHBA' on 'osteosarcoma'

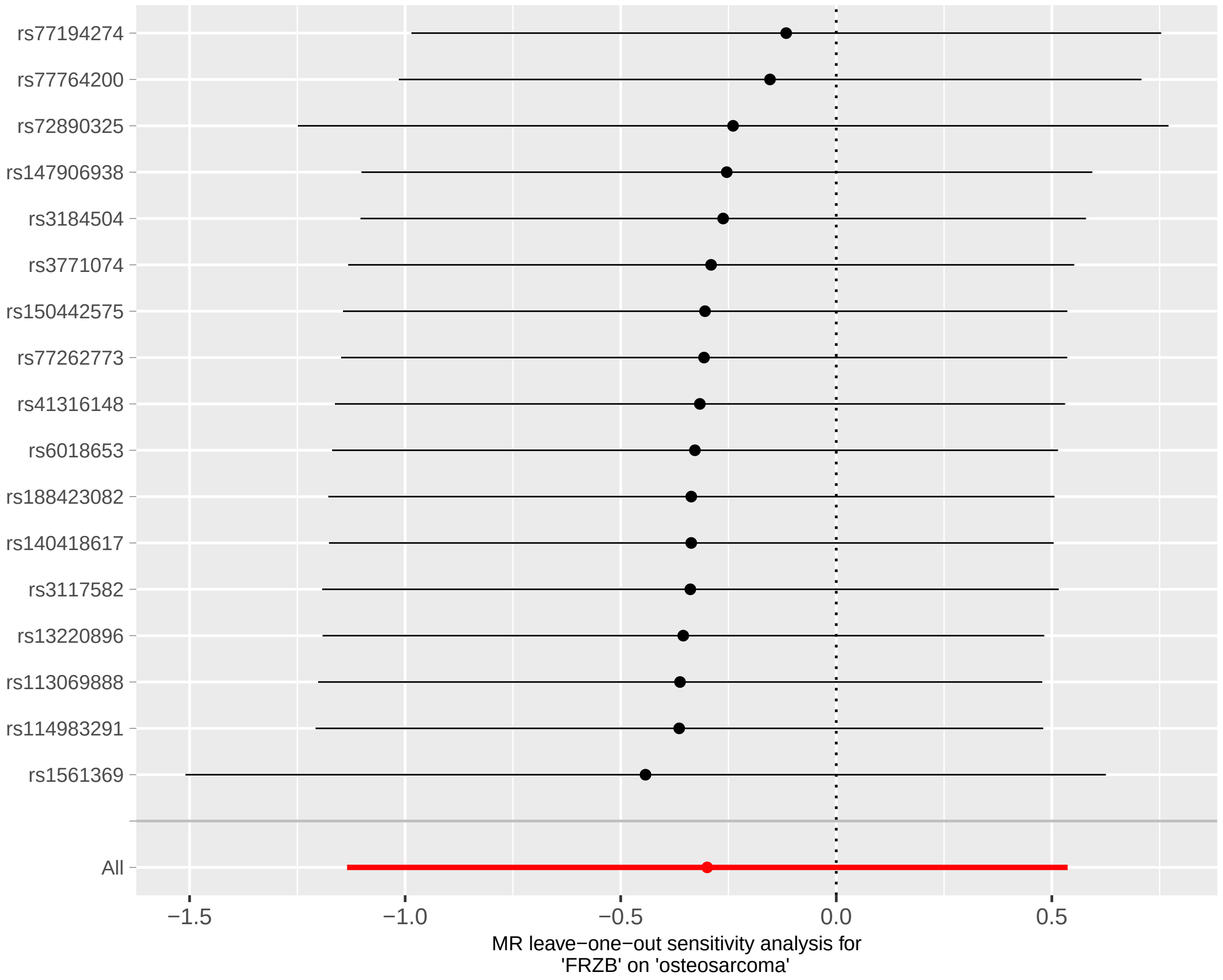
rs77194274
rs77764200
rs72890325
rs147906938
rs3184504
rs3771074
rs150442575
rs77262773
rs41316148
rs6018653
rs188423082
rs140418617
rs3117582
rs13220896
rs113069888
rs114983291
rs1561369
All
−1.5
−1.0
−0.5
0.0
0.5
MR leave−one−out sensitivity analysis for
'FRZB' on 'osteosarcoma'

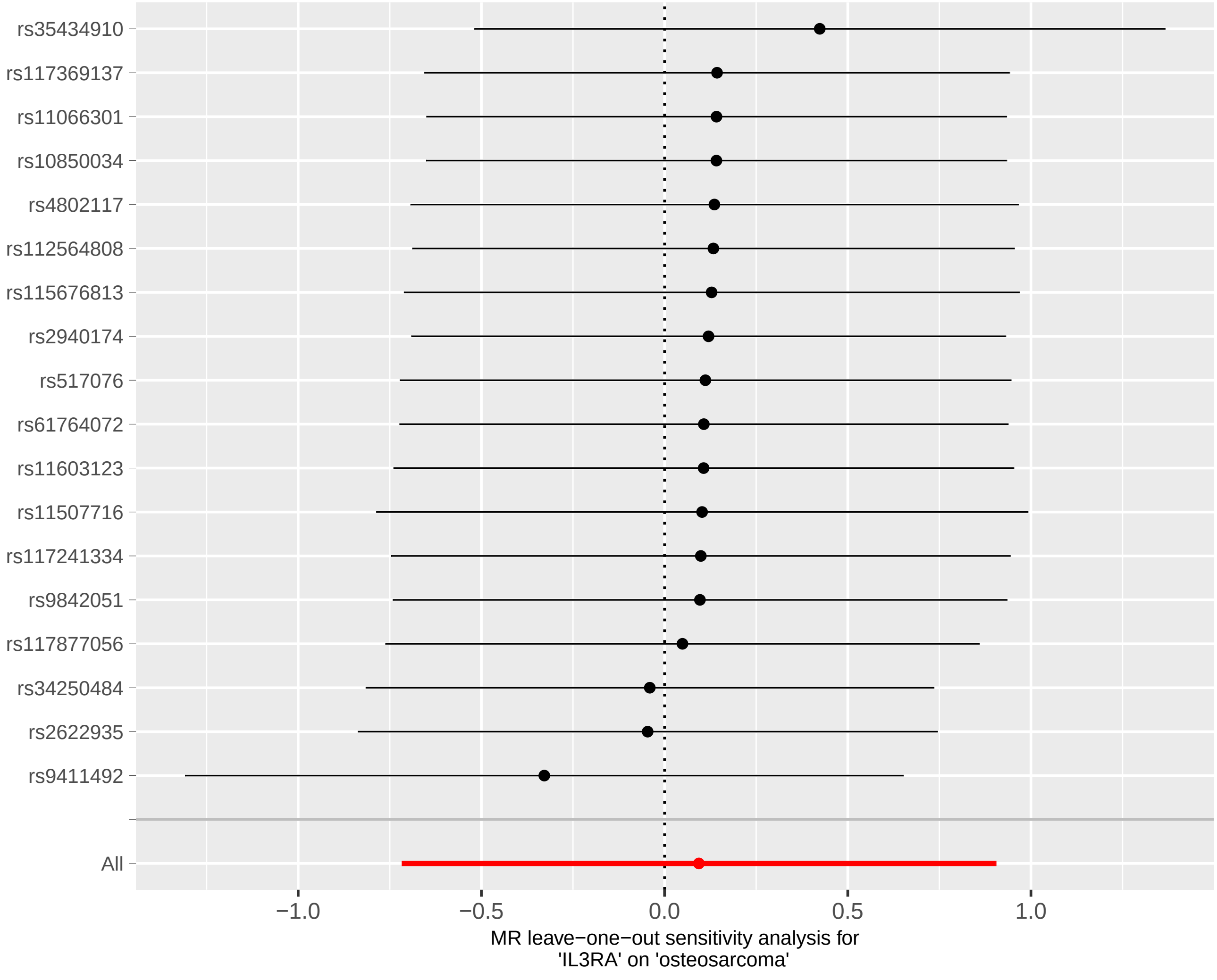

rs35434910
rs117369137
rs11066301
rs10850034
rs4802117
rs112564808
rs115676813
rs2940174
rs517076
rs61764072
rs11603123
rs11507716
rs117241334
rs9842051
rs117877056
rs34250484
rs2622935
rs9411492
All
−1.0
−0.5
0.0
0.5
1.0
MR leave−one−out sensitivity analysis for
'IL3RA' on 'osteosarcoma'

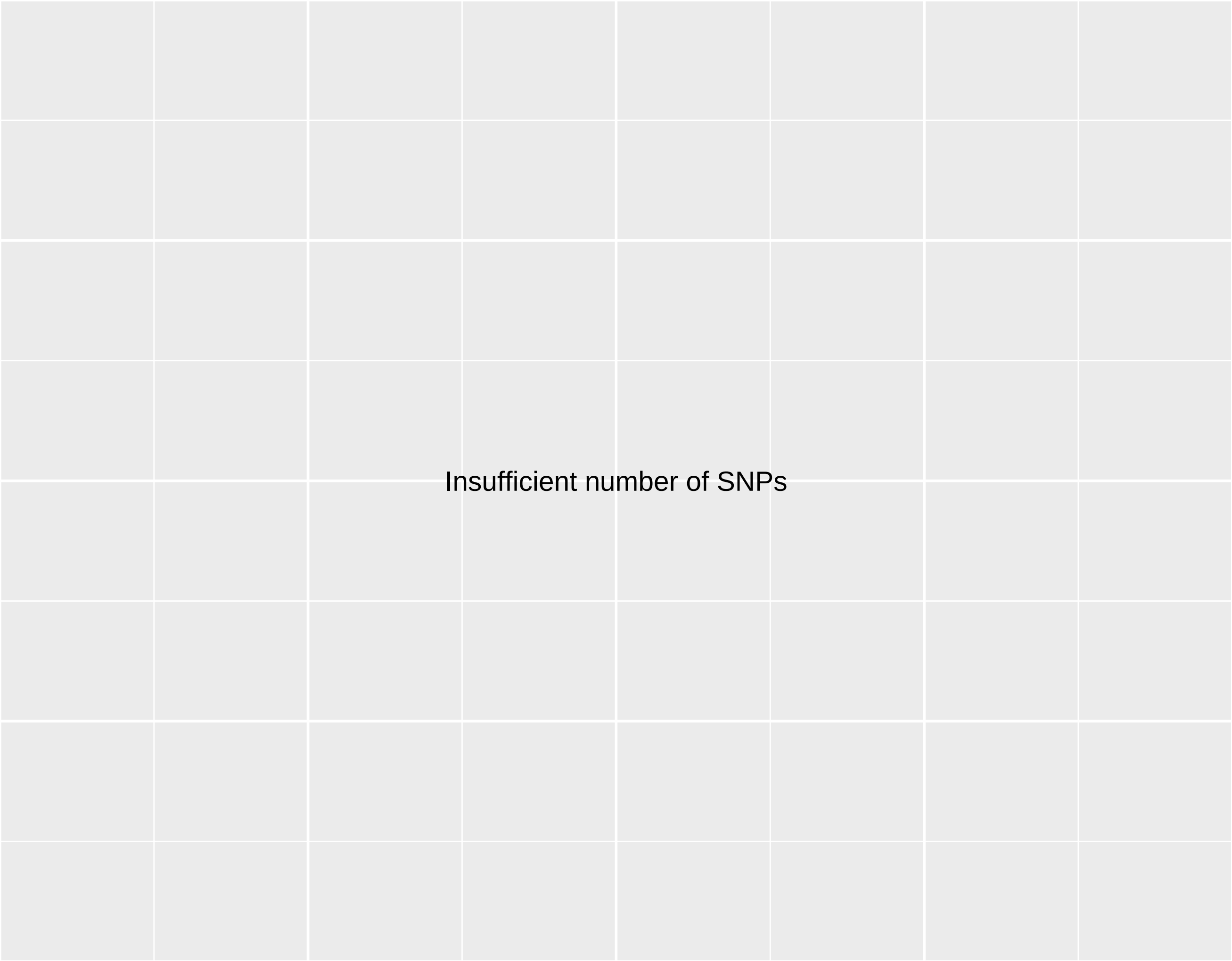
Insufficient number of SNPs

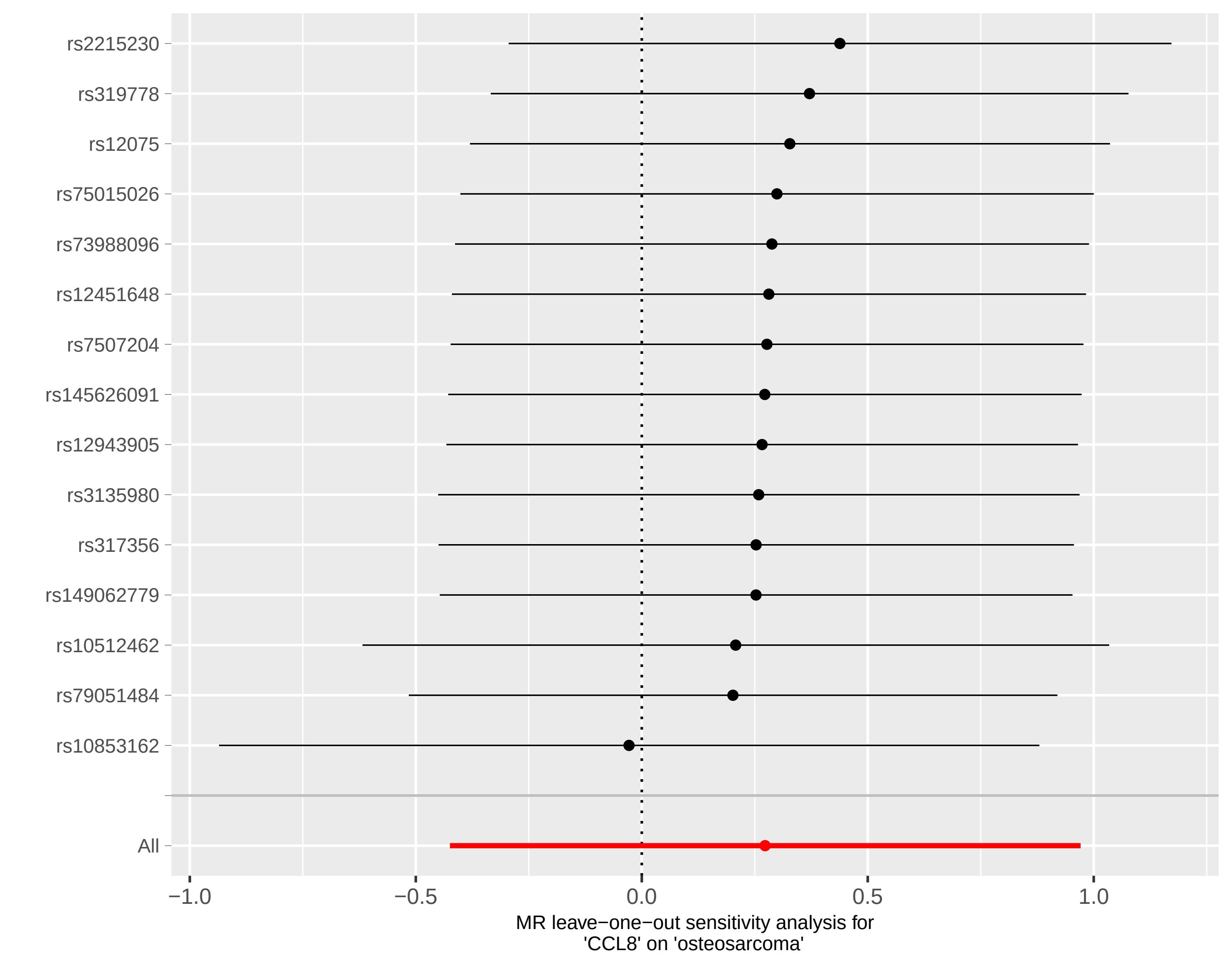
rs2215230
rs319778
rs12075
rs75015026
rs73988096
rs12451648
rs7507204
rs145626091
rs12943905
rs3135980
rs317356
rs149062779
rs10512462
rs79051484
rs10853162
All
−1.0
−0.5
0.0
0.5
1.0
MR leave−one−out sensitivity analysis for
'CCL8' on 'osteosarcoma'

Insufficient number of SNPs

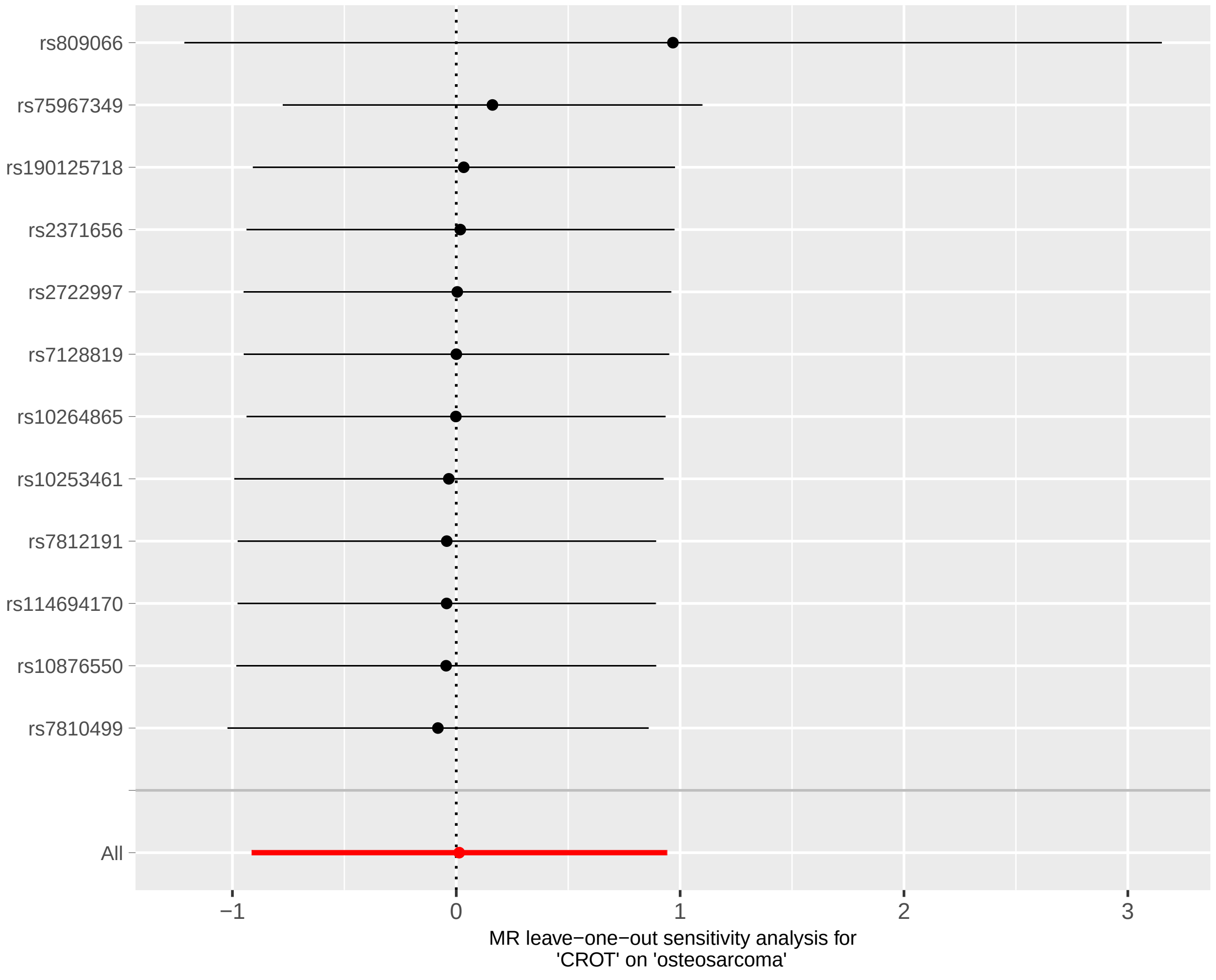
rs809066
rs75967349
rs190125718
rs2371656
rs2722997
rs7128819
rs10264865
rs10253461
rs7812191
rs114694170
rs10876550
rs7810499
All
−1
0
1
2
3
MR leave−one−out sensitivity analysis for
'CROT' on 'osteosarcoma'

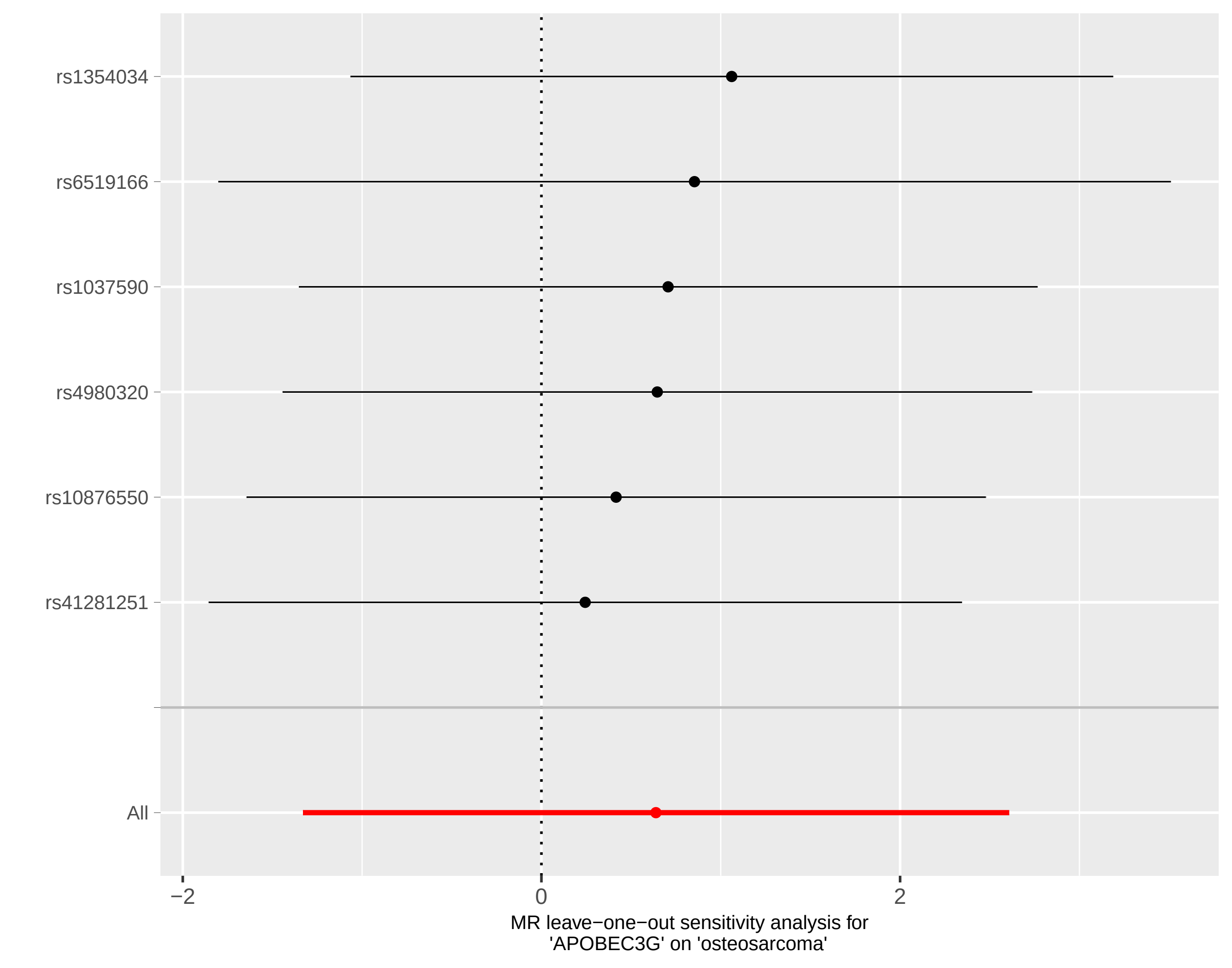
rs1354034
rs6519166
rs1037590
rs4980320
rs10876550
rs41281251
All
−2
0
2
MR leave−one−out sensitivity analysis for
'APOBEC3G' on 'osteosarcoma'

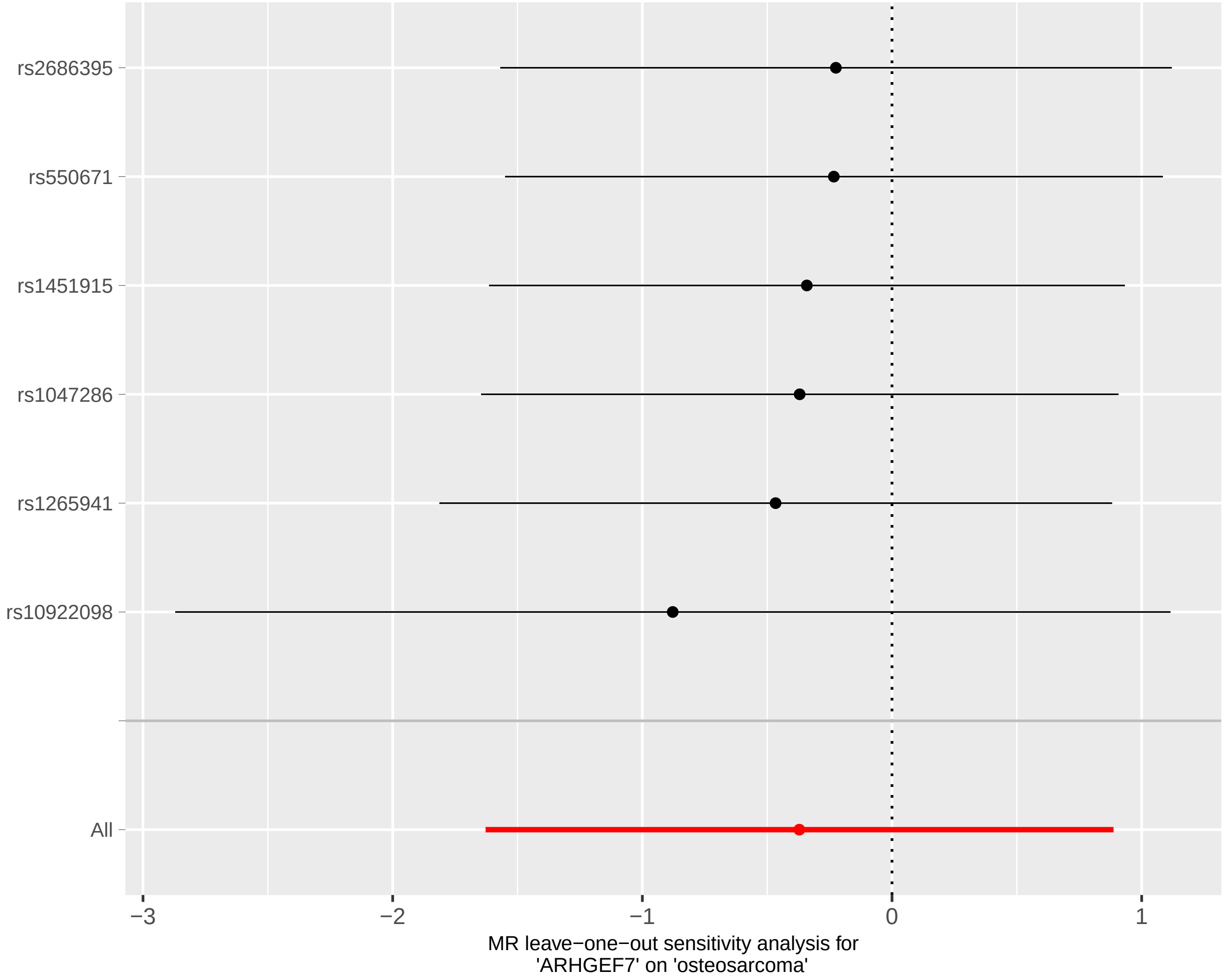
rs2686395
rs550671
rs1451915
rs1047286
rs1265941
rs10922098
All
−3
−2
−1
0
1
MR leave−one−out sensitivity analysis for
'ARHGEF7' on 'osteosarcoma'

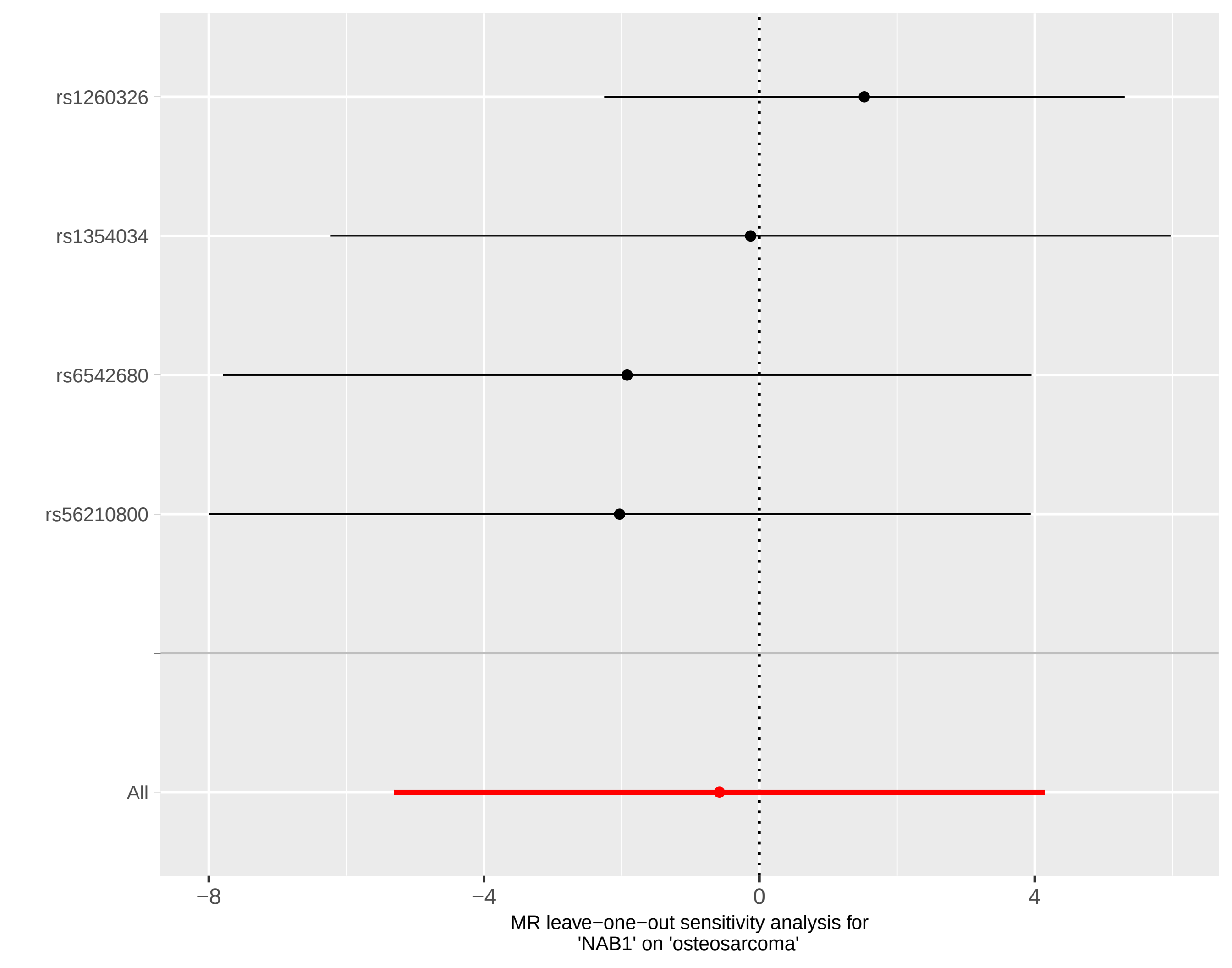

rs1260326
rs1354034
rs6542680
rs56210800
All
−8
−4
0
4
MR leave−one−out sensitivity analysis for
'NAB1' on 'osteosarcoma'

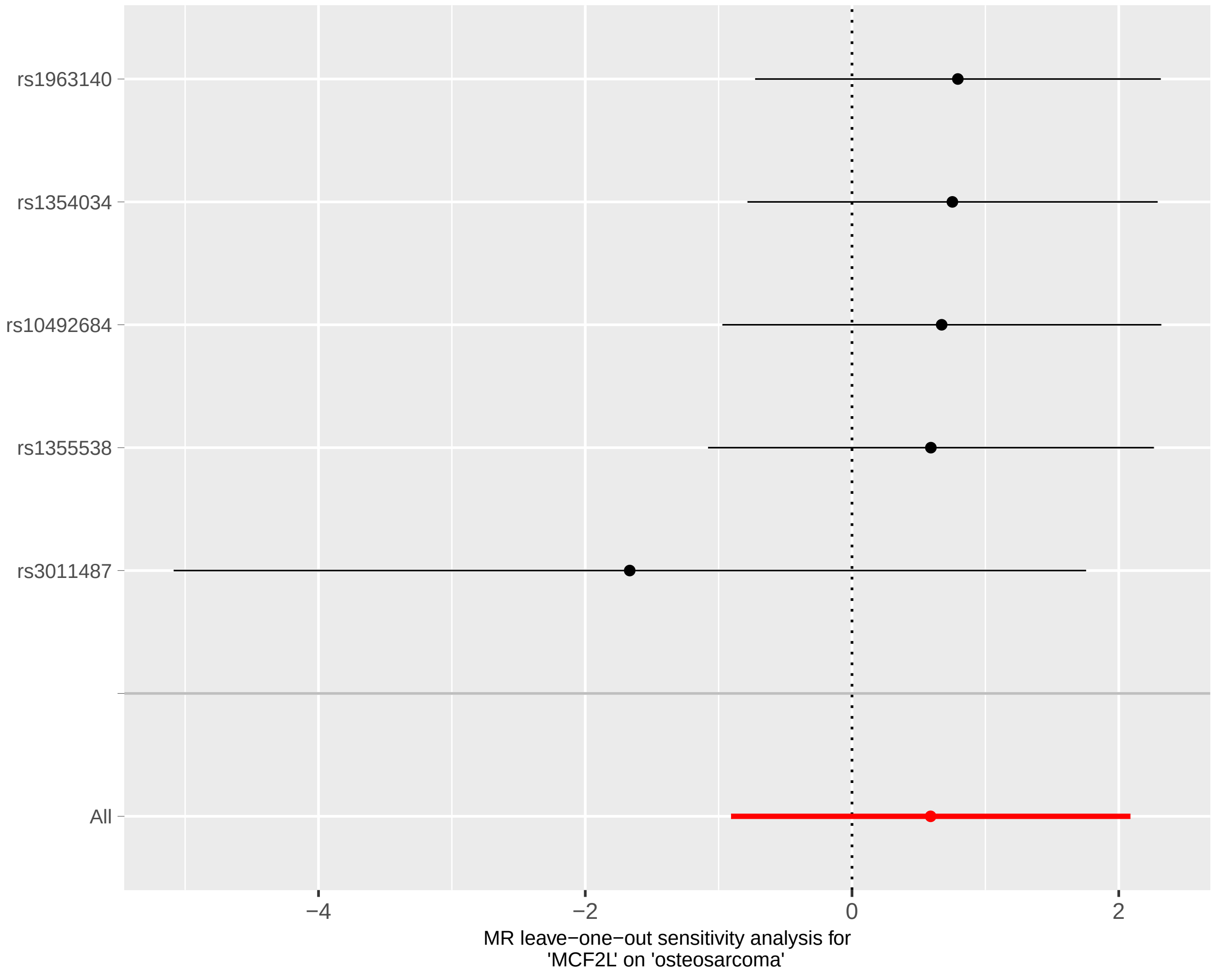
rs1963140
rs1354034
rs10492684
rs1355538
rs3011487
All
−4
−2
0
2
MR leave−one−out sensitivity analysis for
'MCF2L' on 'osteosarcoma'

Insufficient number of SNPs

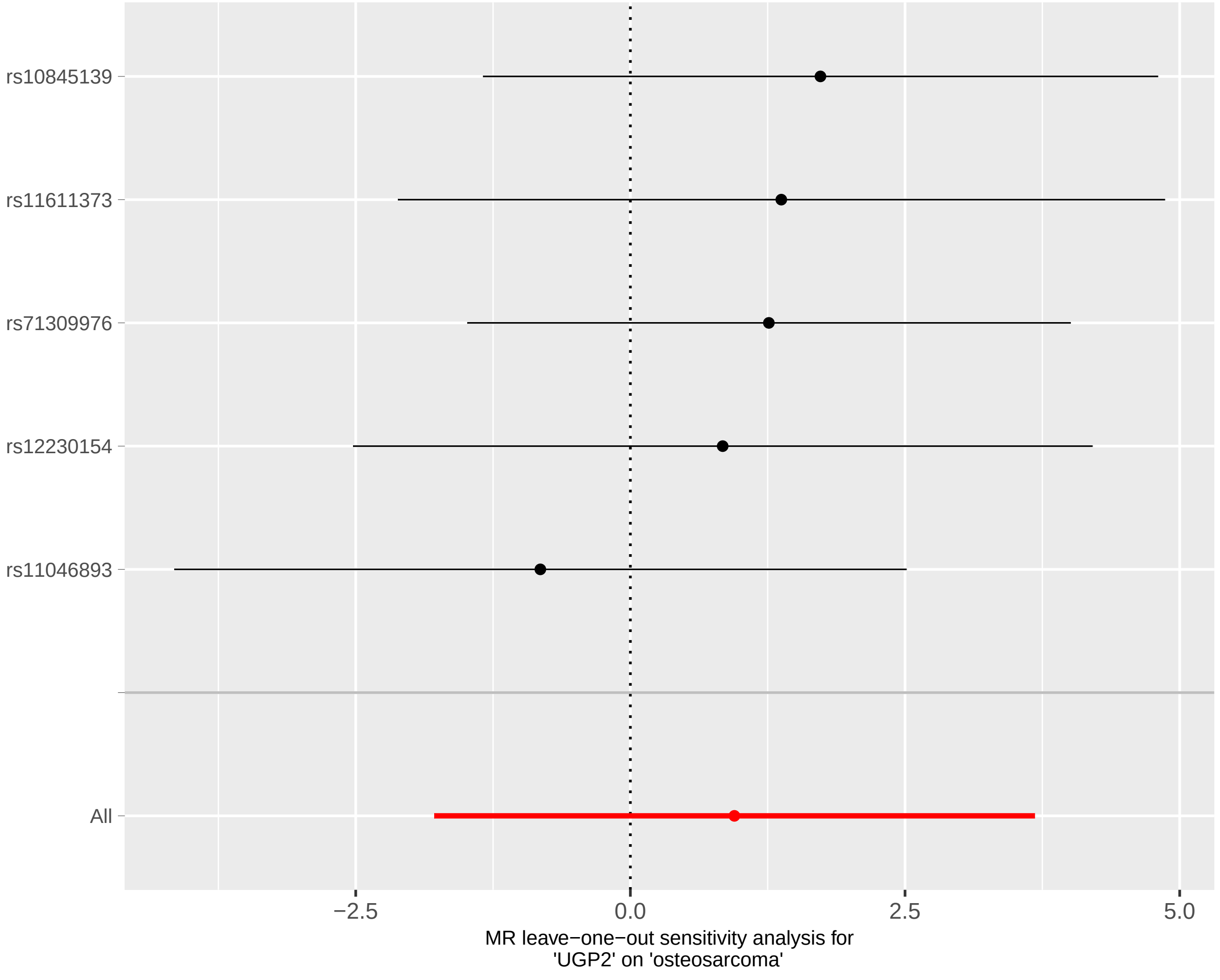
rs10845139
rs11611373
rs71309976
rs12230154
rs11046893
All
−2.5
0.0
2.5
5.0
MR leave−one−out sensitivity analysis for
'UGP2' on 'osteosarcoma'

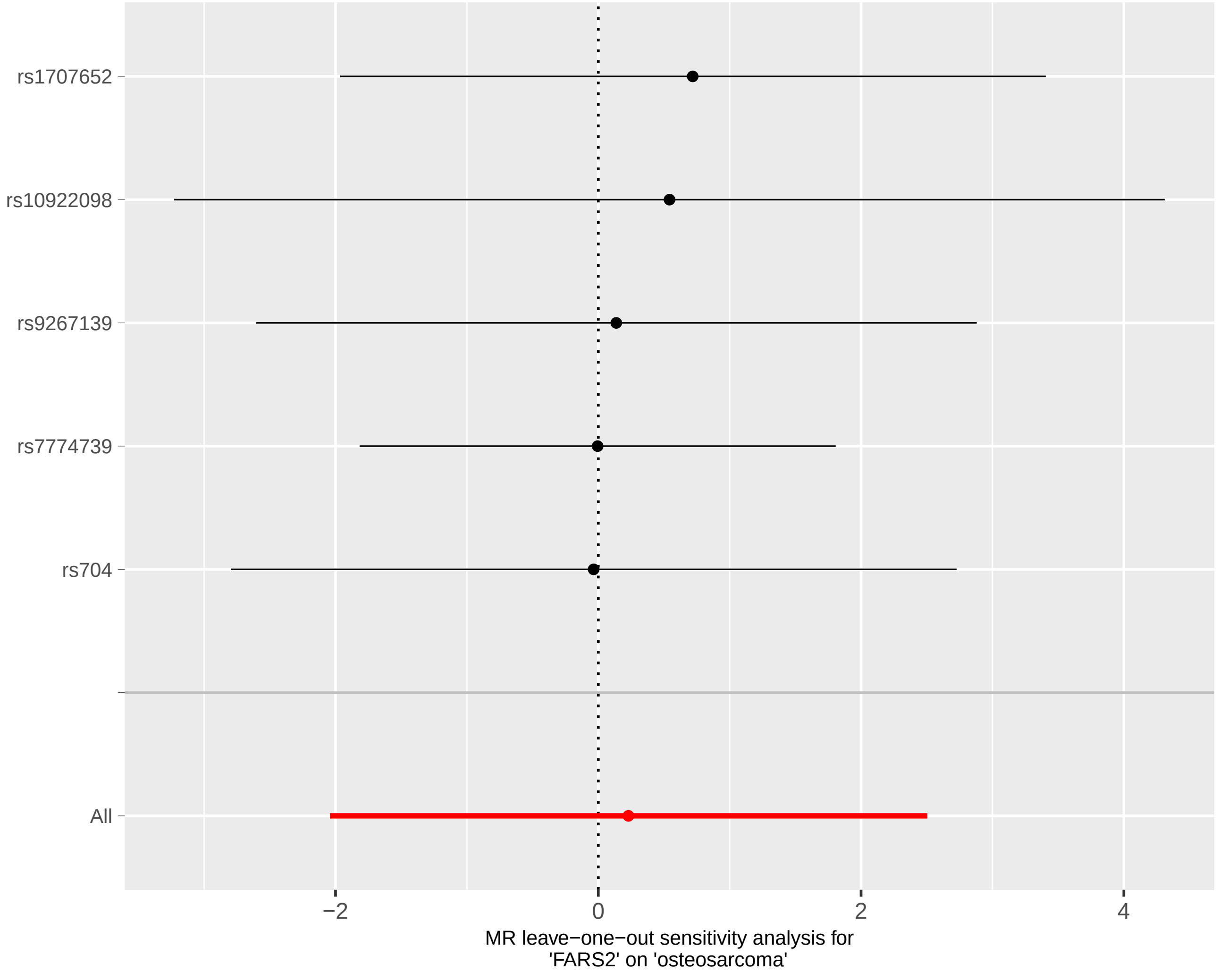

rs1707652
rs10922098
rs9267139
rs7774739
rs704
All
−2
0
2
4
MR leave−one−out sensitivity analysis for
'FARS2' on 'osteosarcoma'

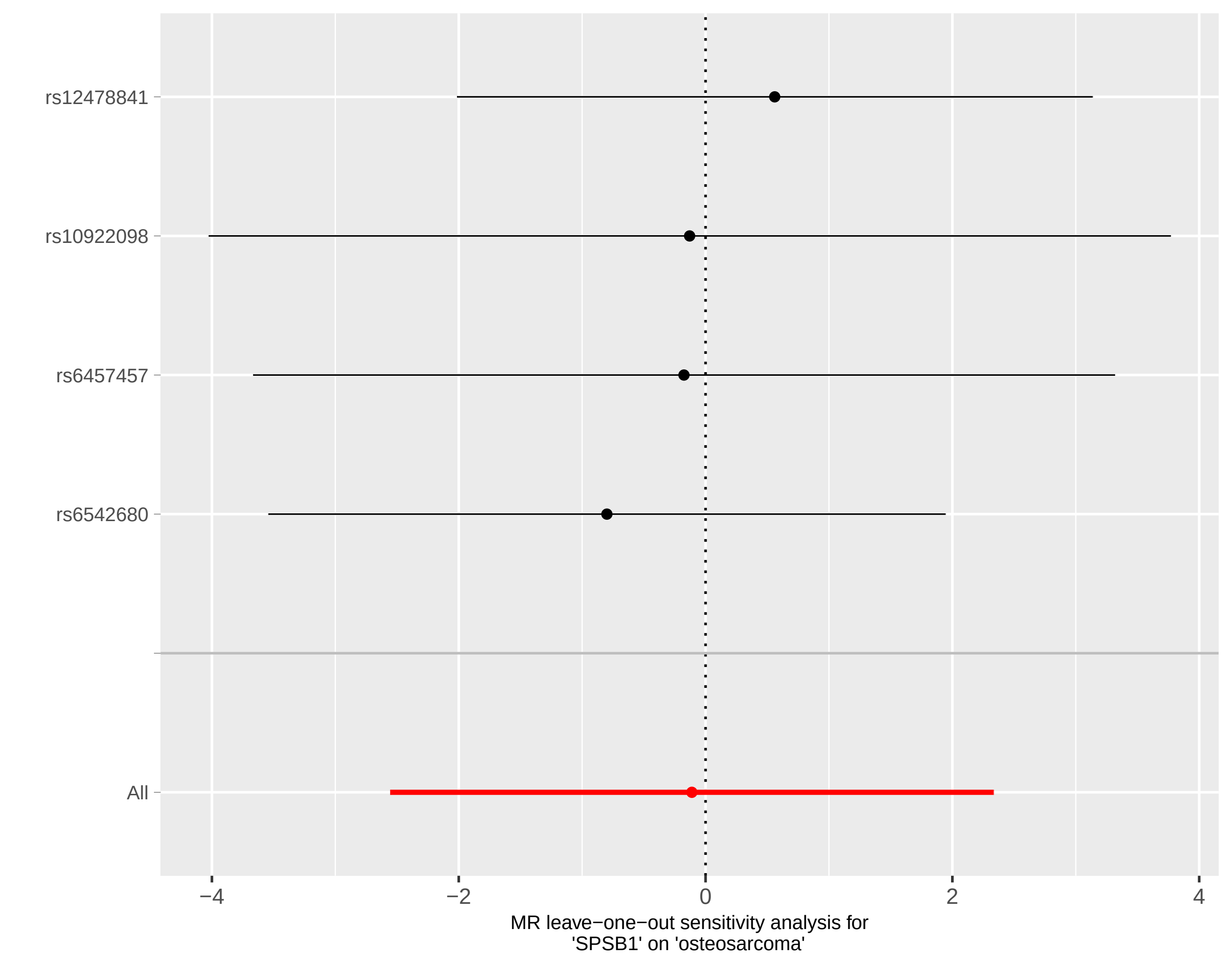
rs12478841
rs10922098
rs6457457
rs6542680
All
−4
−2
0
2
4
MR leave−one−out sensitivity analysis for
'SPSB1' on 'osteosarcoma'

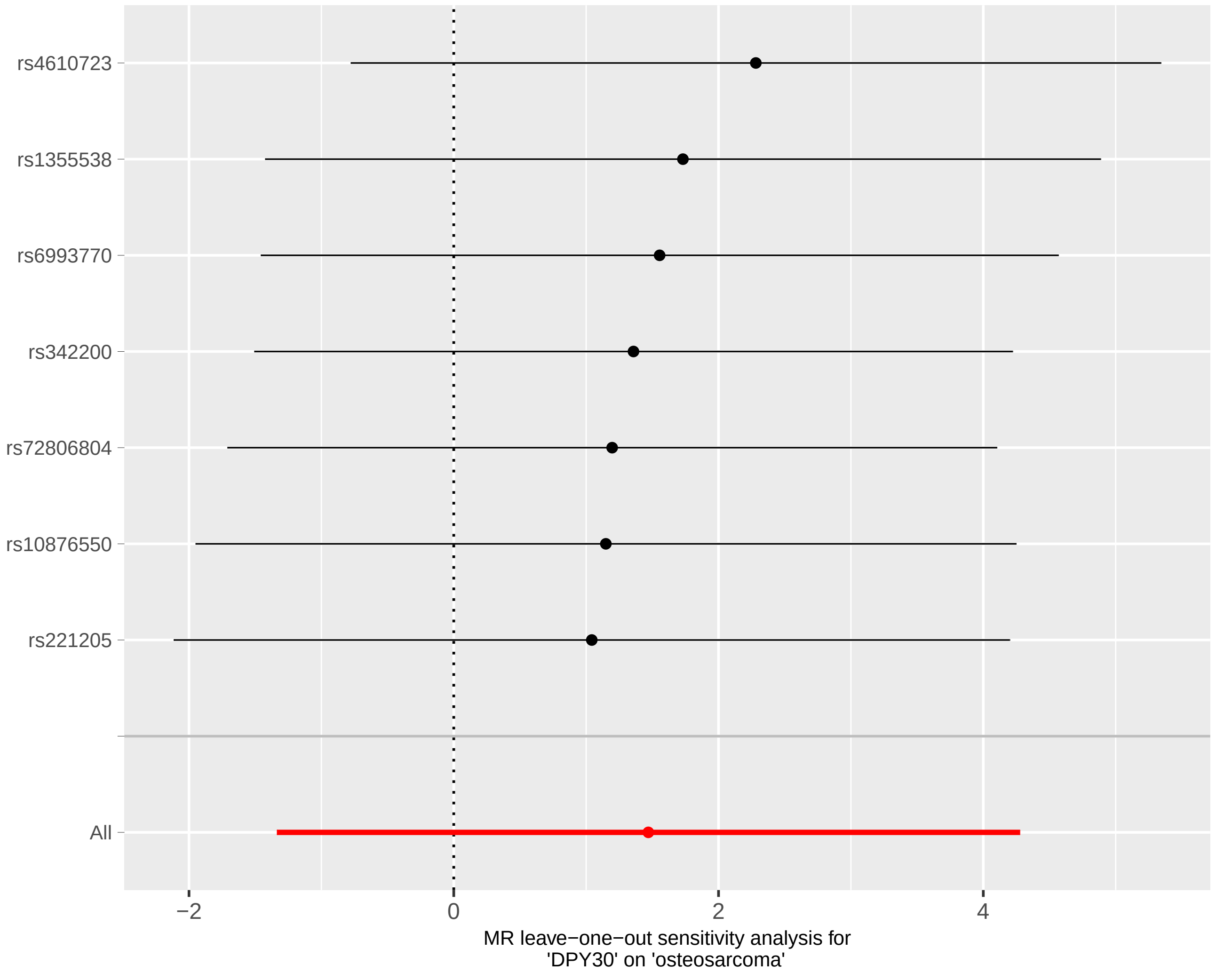

rs4610723
rs1355538
rs6993770
rs342200
rs72806804
rs10876550
rs221205
All
−2
0
2
4
MR leave−one−out sensitivity analysis for
'DPY30' on 'osteosarcoma'

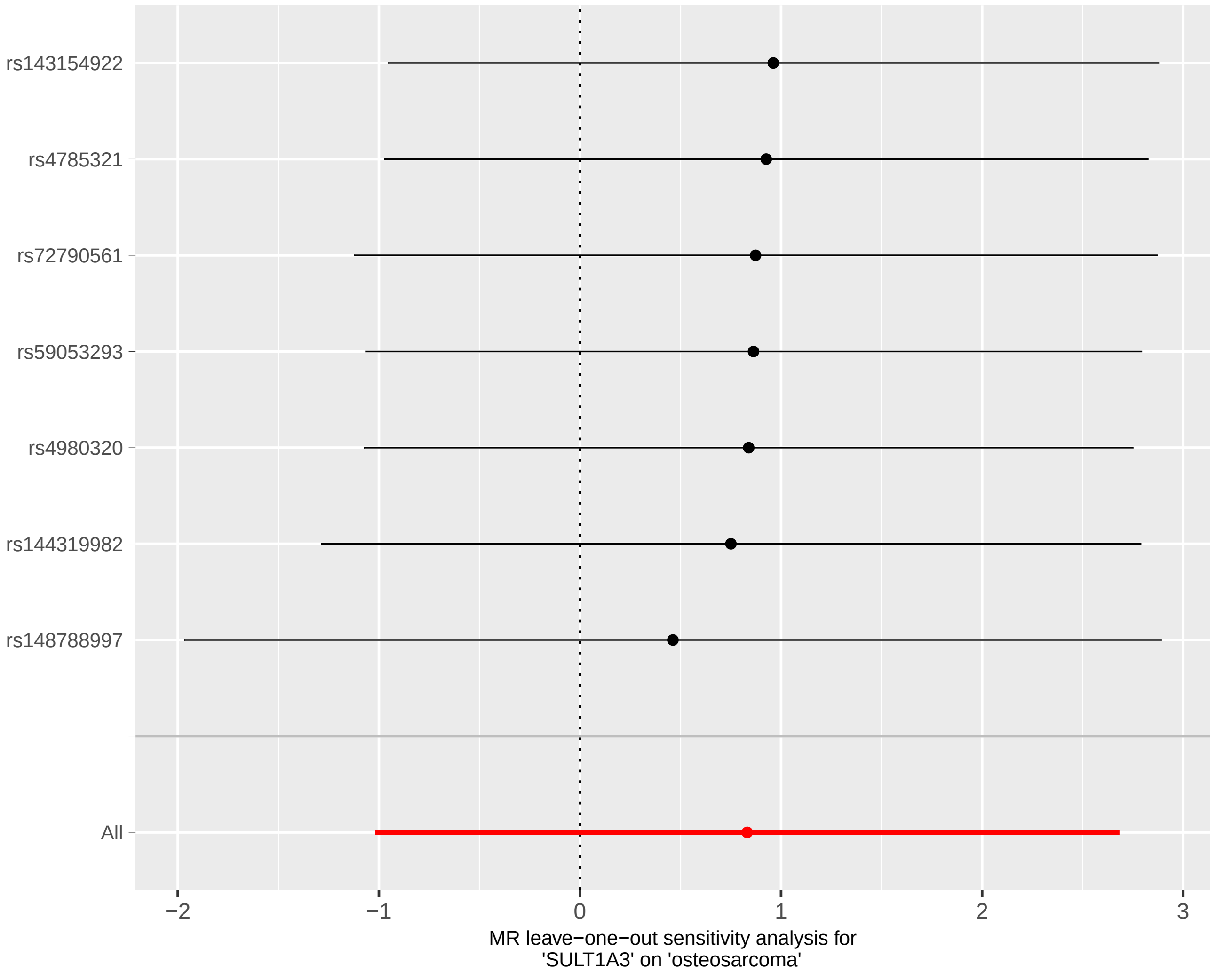
rs143154922
rs4785321
rs72790561
rs59053293
rs4980320
rs144319982
rs148788997
All
−2
−1
0
1
2
3
MR leave−one−out sensitivity analysis for
'SULT1A3' on 'osteosarcoma'

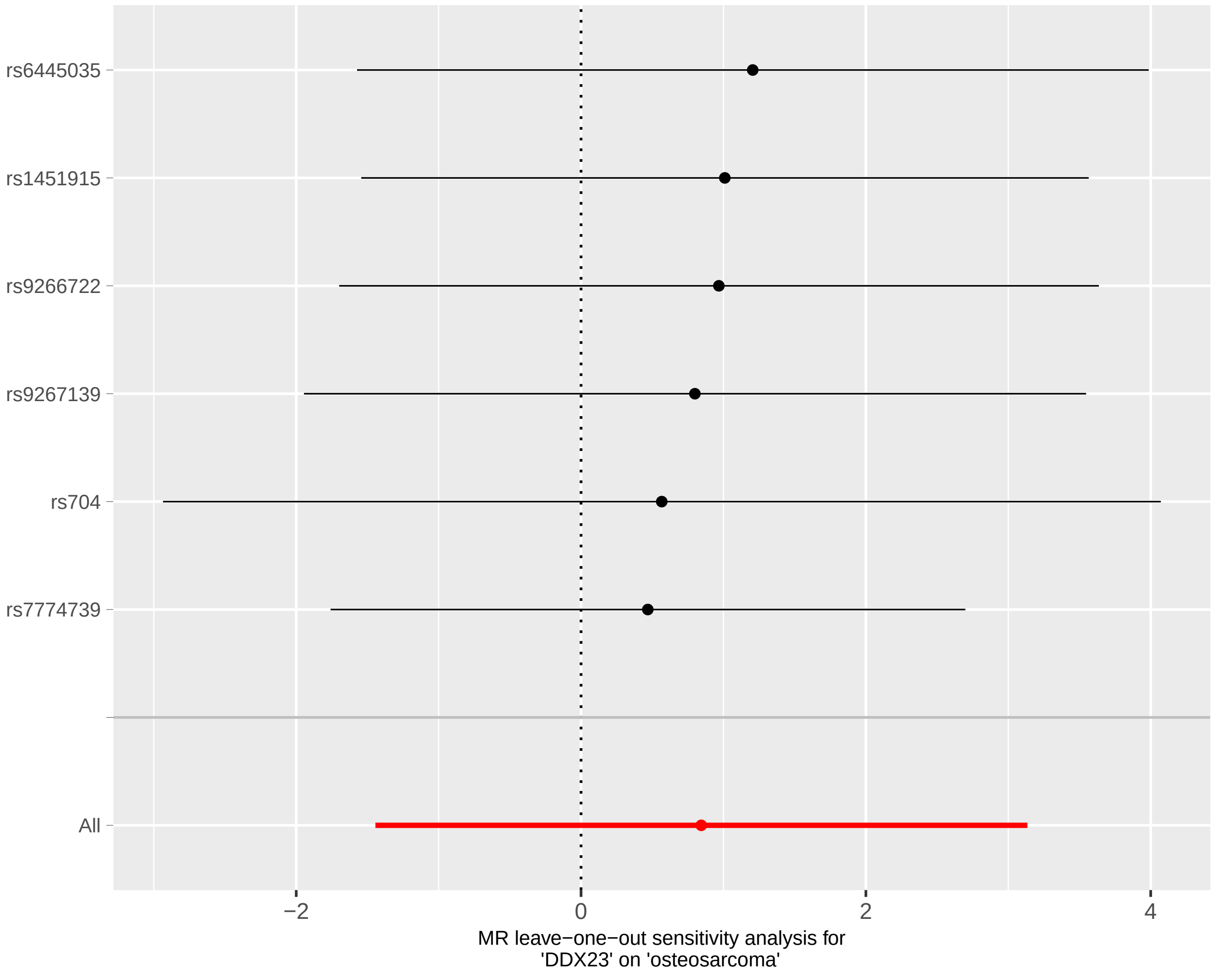

rs6445035
rs1451915
rs9266722
rs9267139
rs704
rs7774739
All
−2
0
2
4
MR leave−one−out sensitivity analysis for
'DDX23' on 'osteosarcoma'

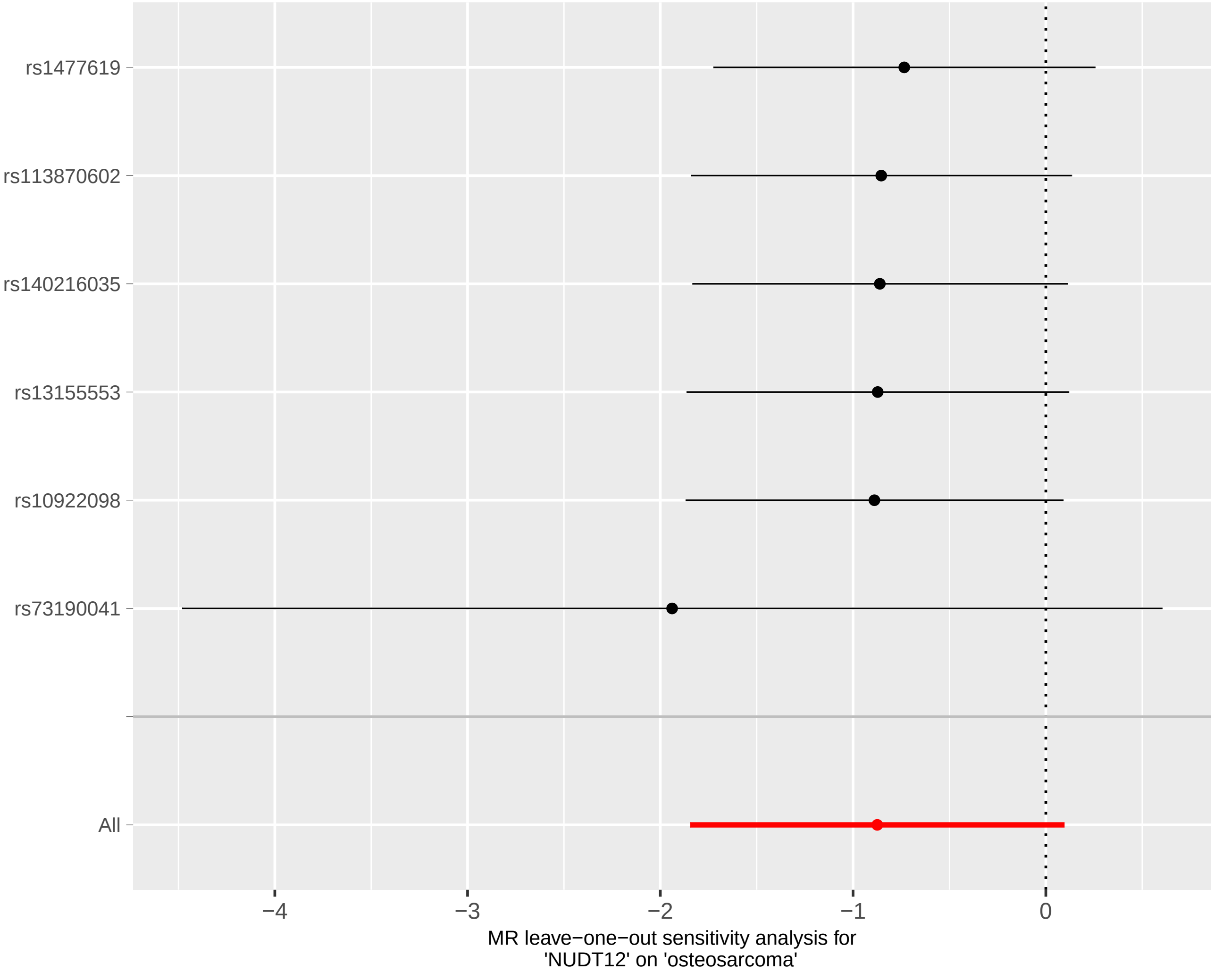
rs1477619
rs113870602
rs140216035
rs13155553
rs10922098
rs73190041
All
−4
−3
−2
−1
0
MR leave−one−out sensitivity analysis for
'NUDT12' on 'osteosarcoma'

Insufficient number of SNPs

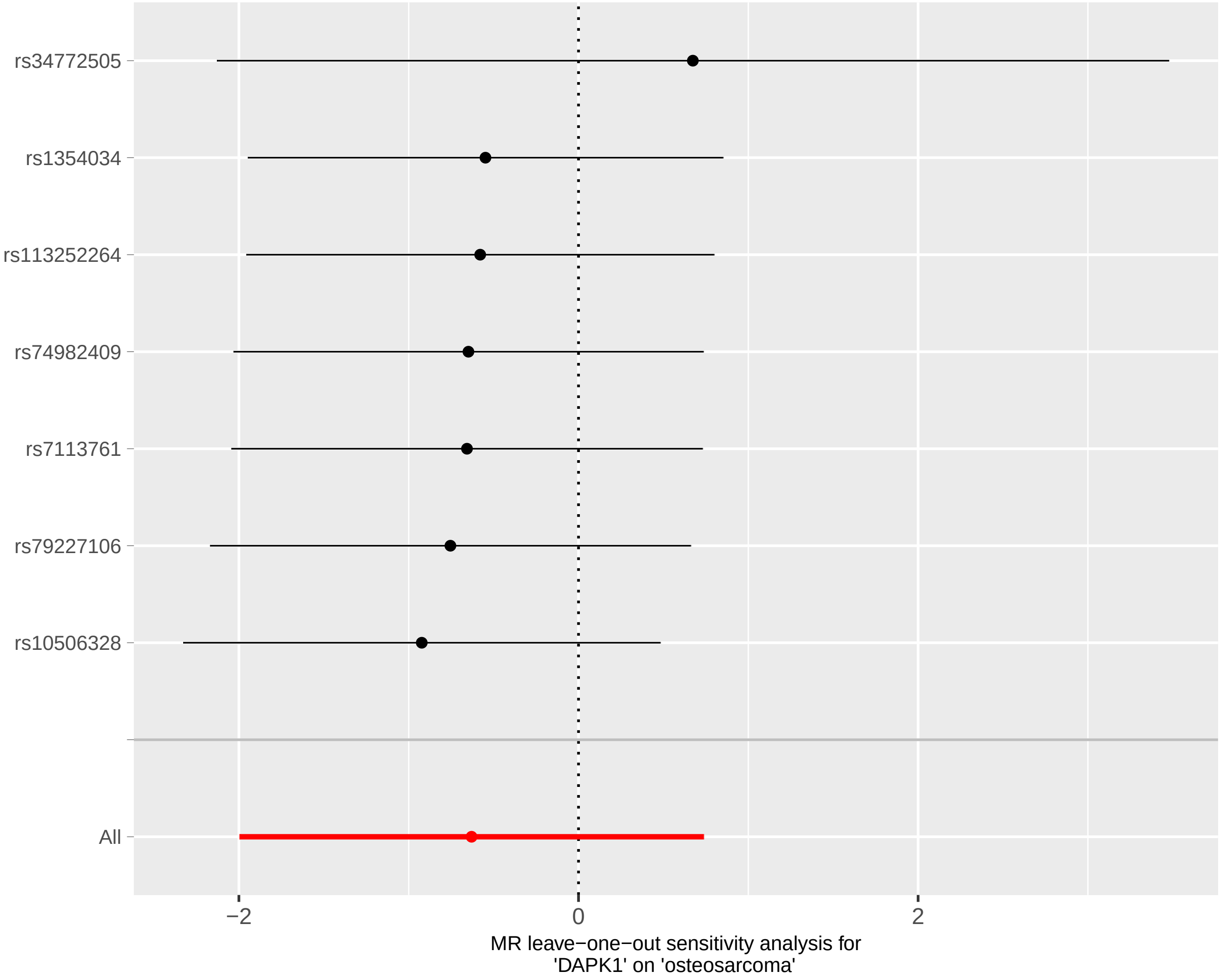
rs34772505
rs1354034
rs113252264
rs74982409
rs7113761
rs79227106
rs10506328
All
−2
0
2
MR leave−one−out sensitivity analysis for
'DAPK1' on 'osteosarcoma'

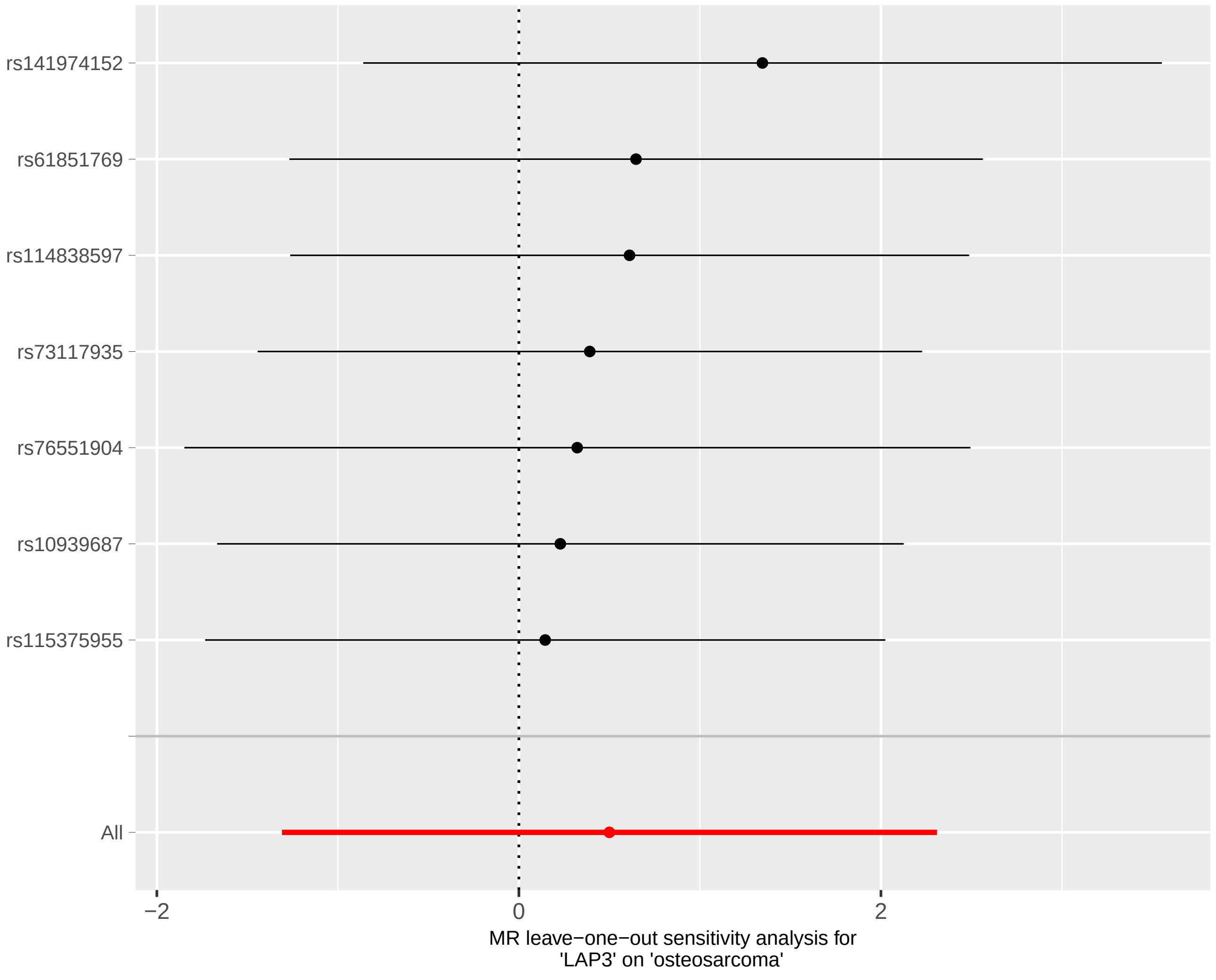

rs141974152
rs61851769
rs114838597
rs73117935
rs76551904
rs10939687
rs115375955
All
−2
0
2
MR leave−one−out sensitivity analysis for
'LAP3' on 'osteosarcoma'

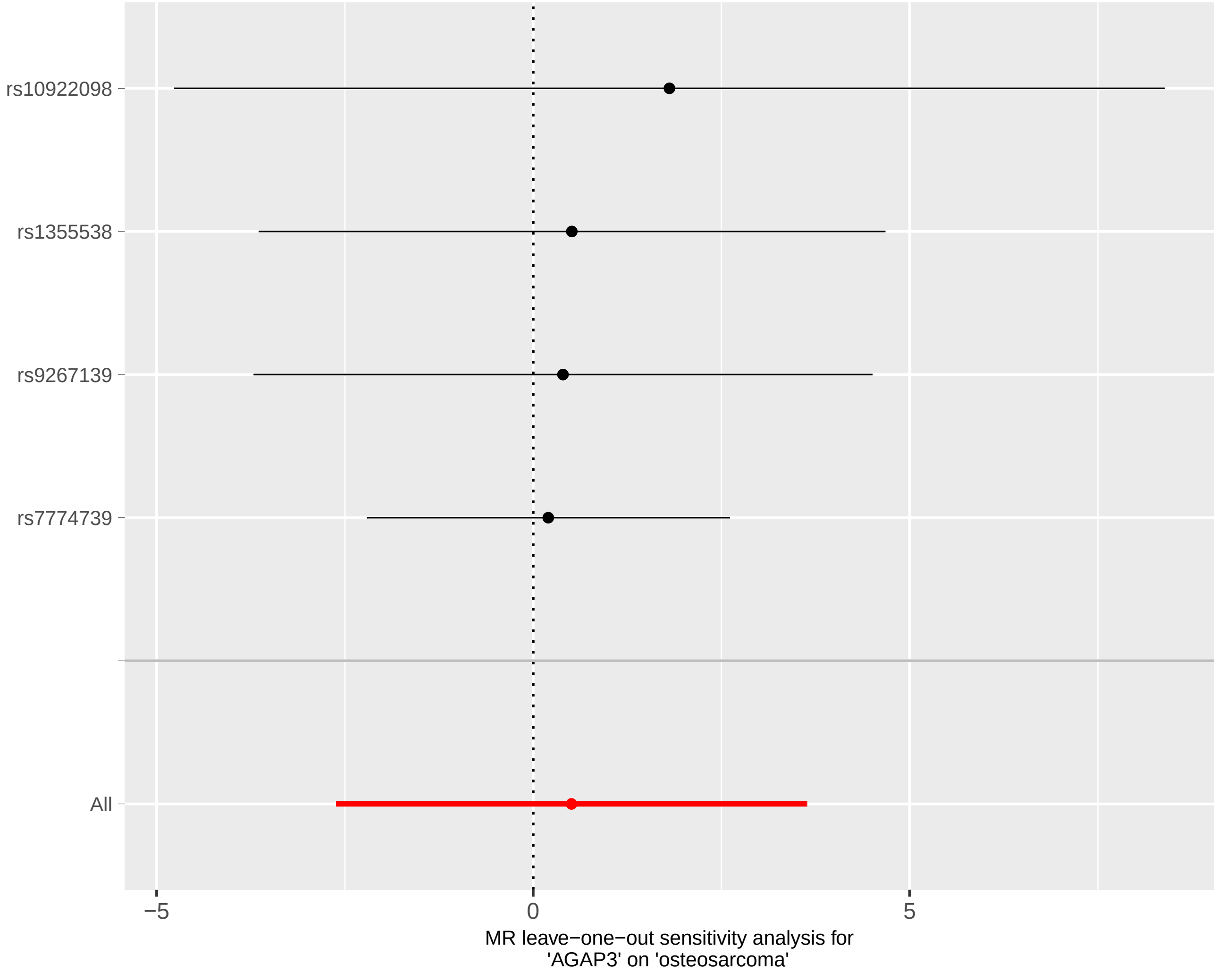
rs10922098
rs1355538
rs9267139
rs7774739
All
−5
0
5
MR leave−one−out sensitivity analysis for
'AGAP3' on 'osteosarcoma'

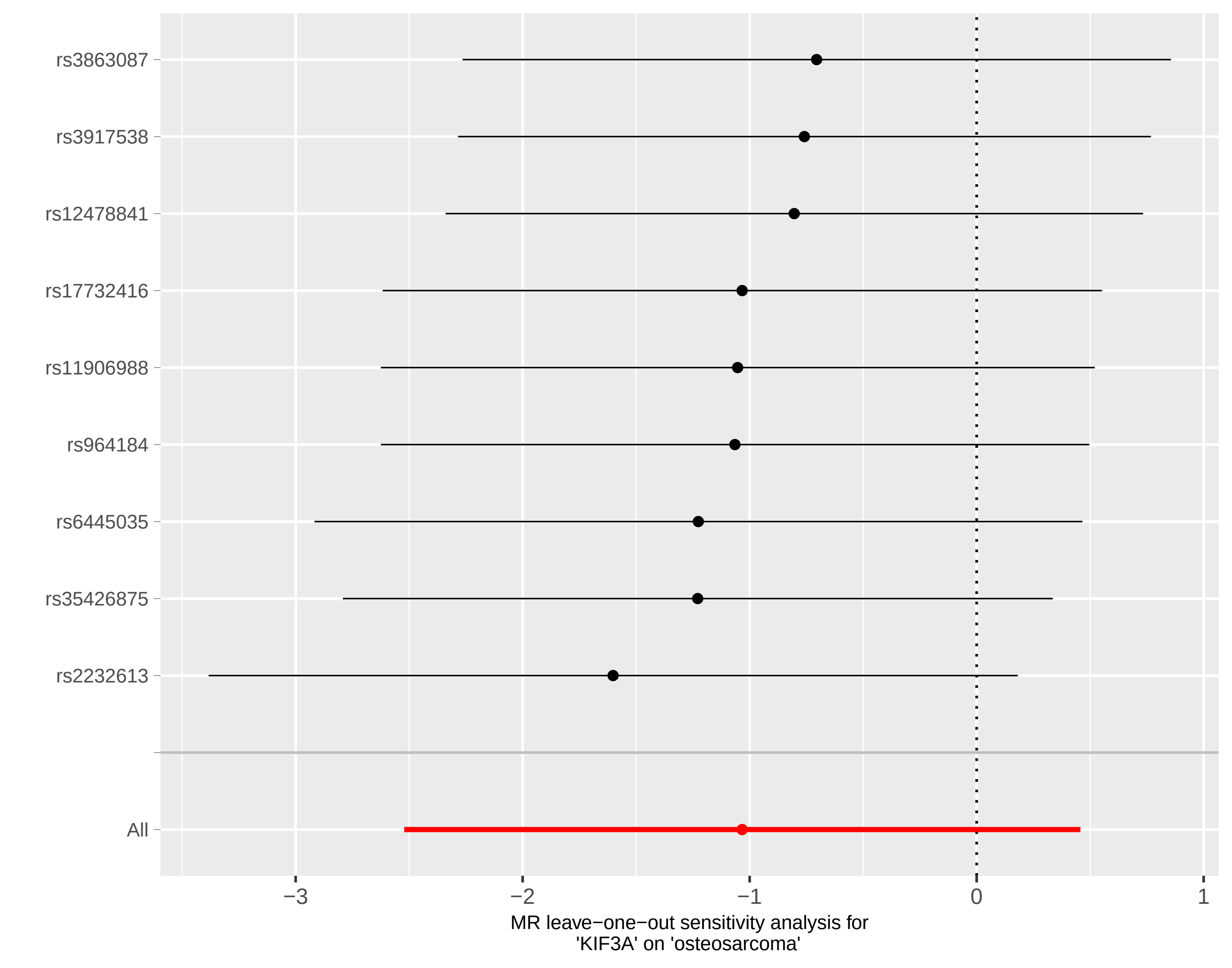
rs3863087
rs3917538
rs12478841
rs17732416
rs11906988
rs964184
rs6445035
rs35426875
rs2232613
All
−3
−2
−1
0
1
MR leave−one−out sensitivity analysis for
'KIF3A' on 'osteosarcoma'

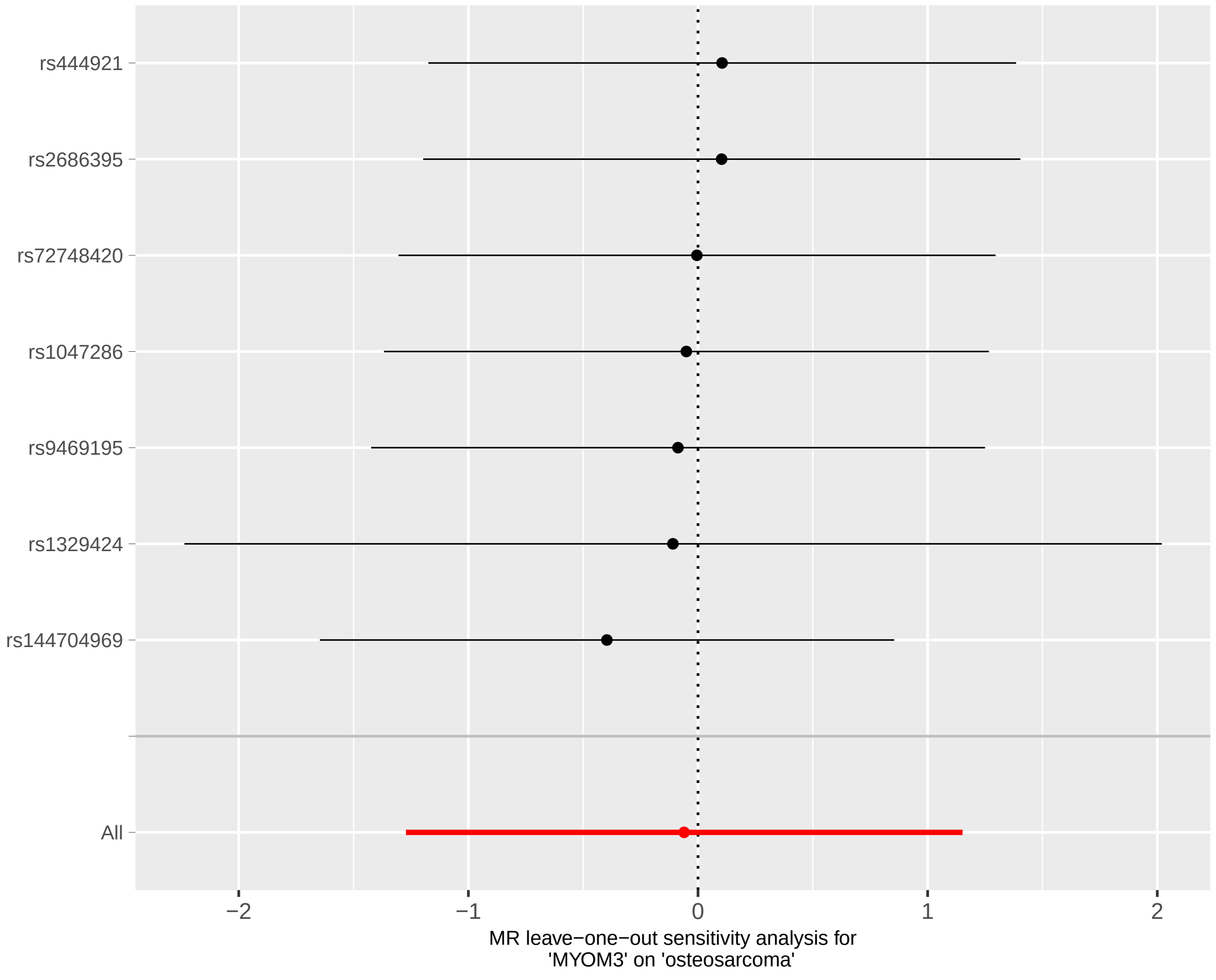

rs444921
rs2686395
rs72748420
rs1047286
rs9469195
rs1329424
rs144704969
All
−2
−1
0
1
2
MR leave−one−out sensitivity analysis for
'MYOM3' on 'osteosarcoma'

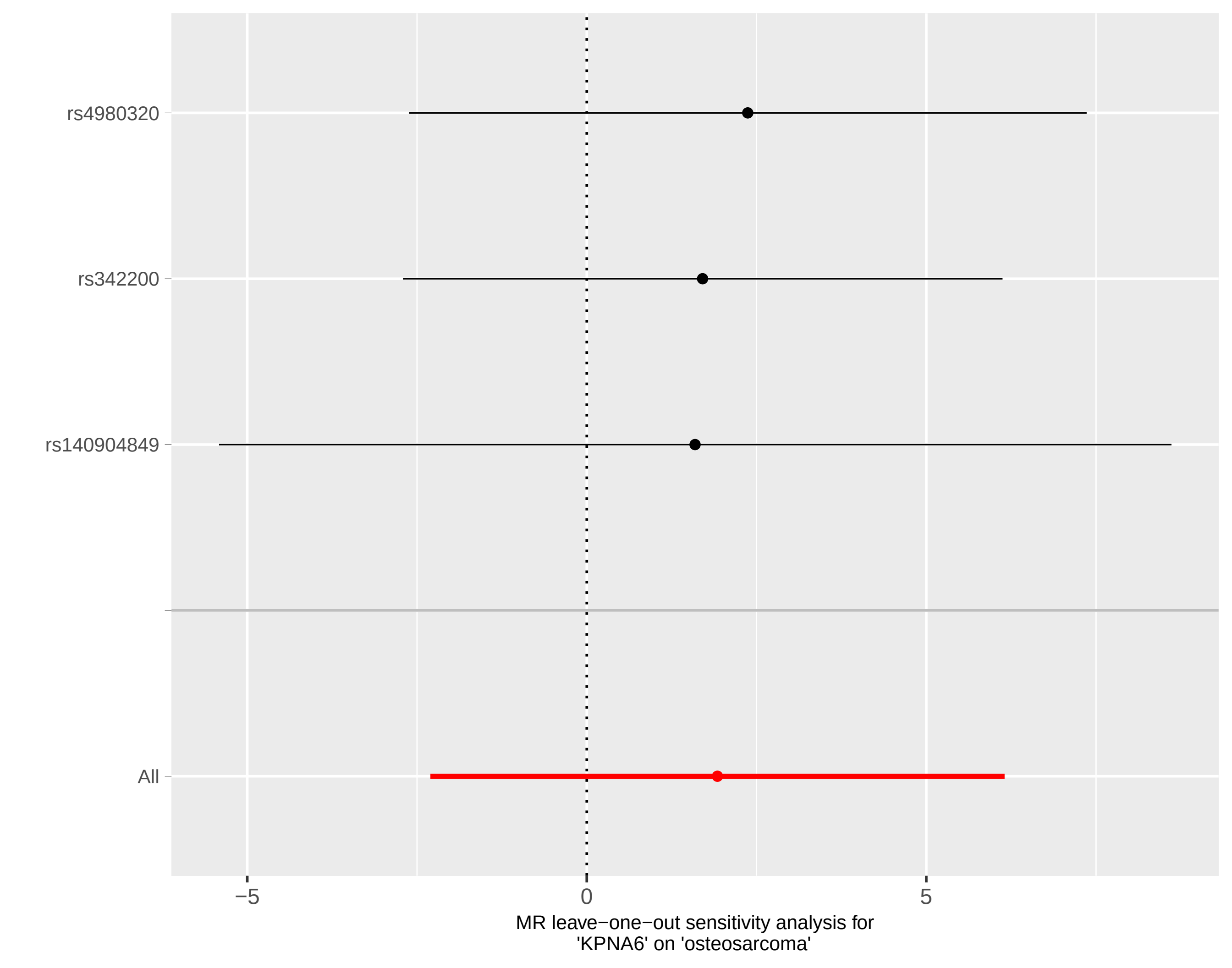
rs4980320
rs342200
rs140904849
All
−5
0
5
MR leave−one−out sensitivity analysis for
'KPNA6' on 'osteosarcoma'

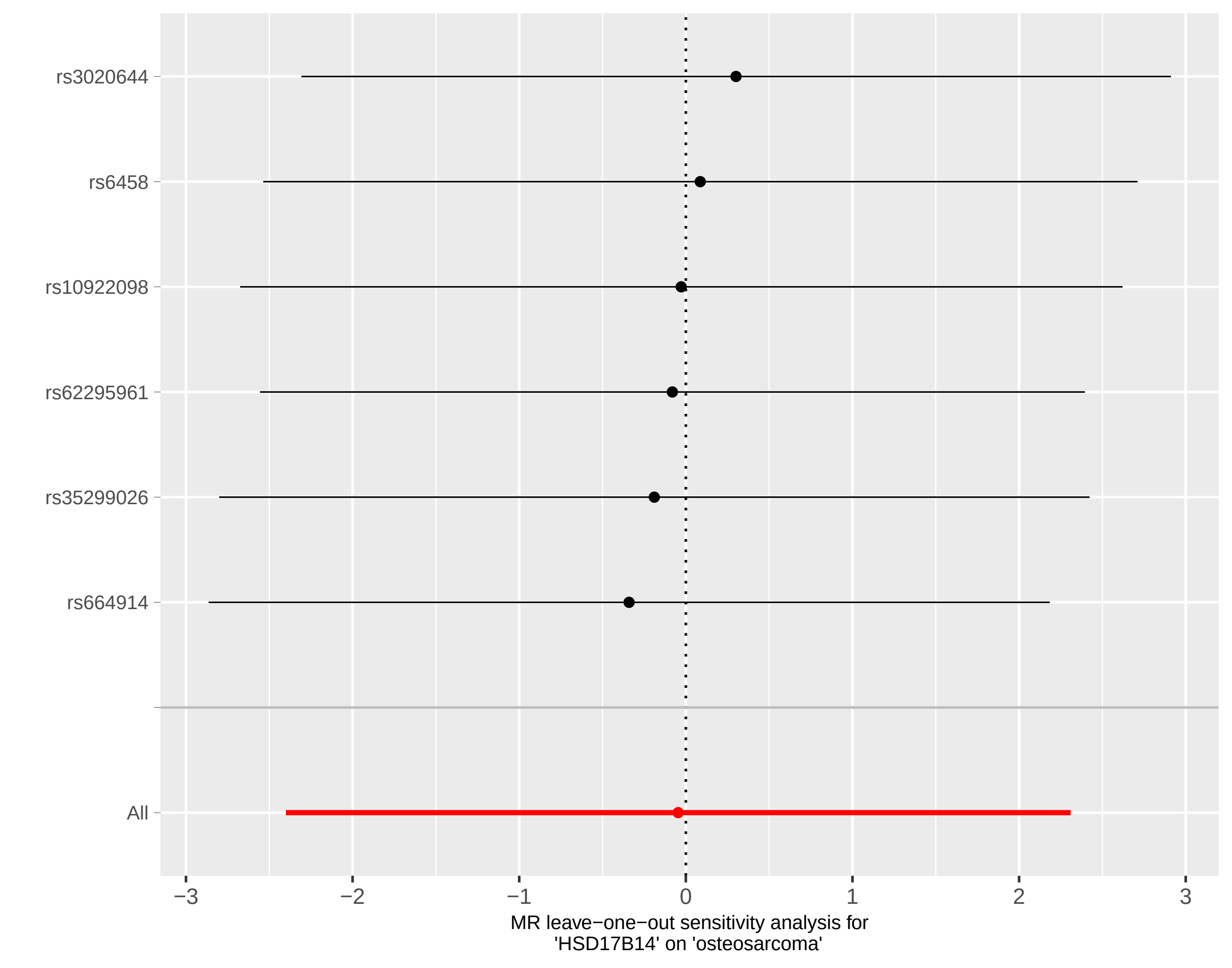

rs3020644
rs6458
rs10922098
rs62295961
rs35299026
rs664914
All
−3
−2
−1
0
1
2
3
MR leave−one−out sensitivity analysis for
'HSD17B14' on 'osteosarcoma'

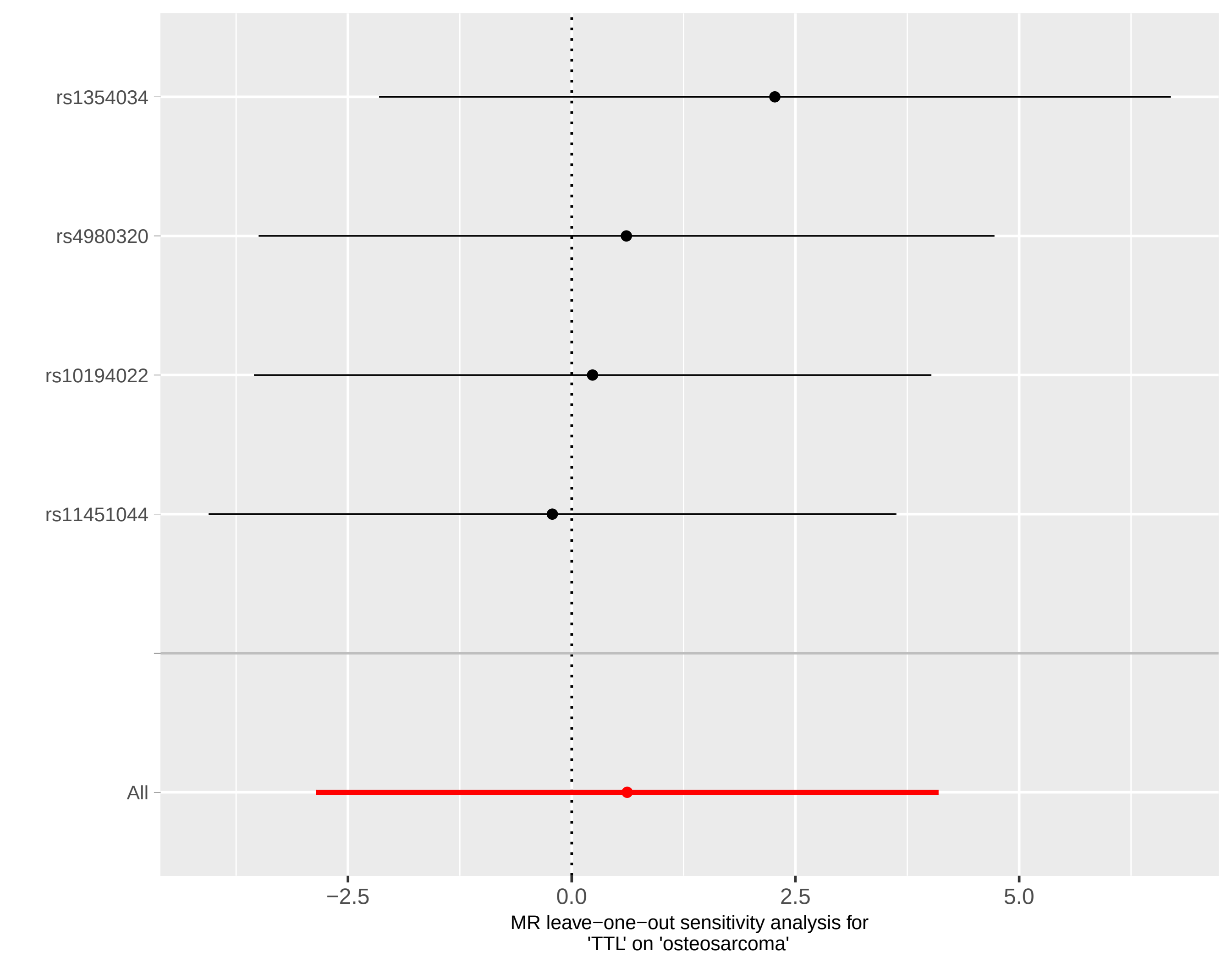

rs1354034
rs4980320
rs10194022
rs11451044
All
−2.5
0.0
2.5
5.0
MR leave−one−out sensitivity analysis for
'TTL' on 'osteosarcoma'

Insufficient number of SNPs

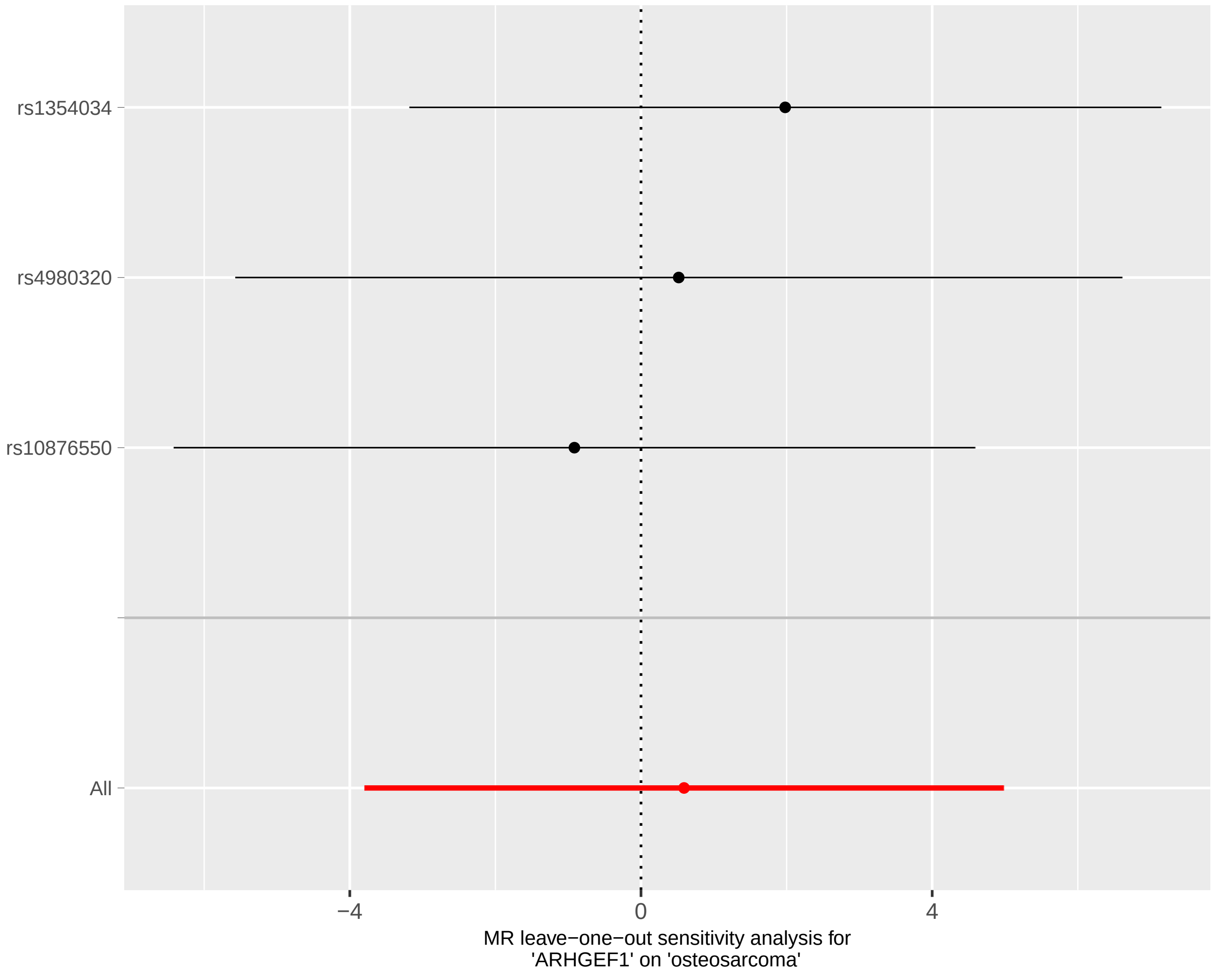
rs1354034
rs4980320
rs10876550
All
−4
0
4
MR leave−one−out sensitivity analysis for
'ARHGEF1' on 'osteosarcoma'

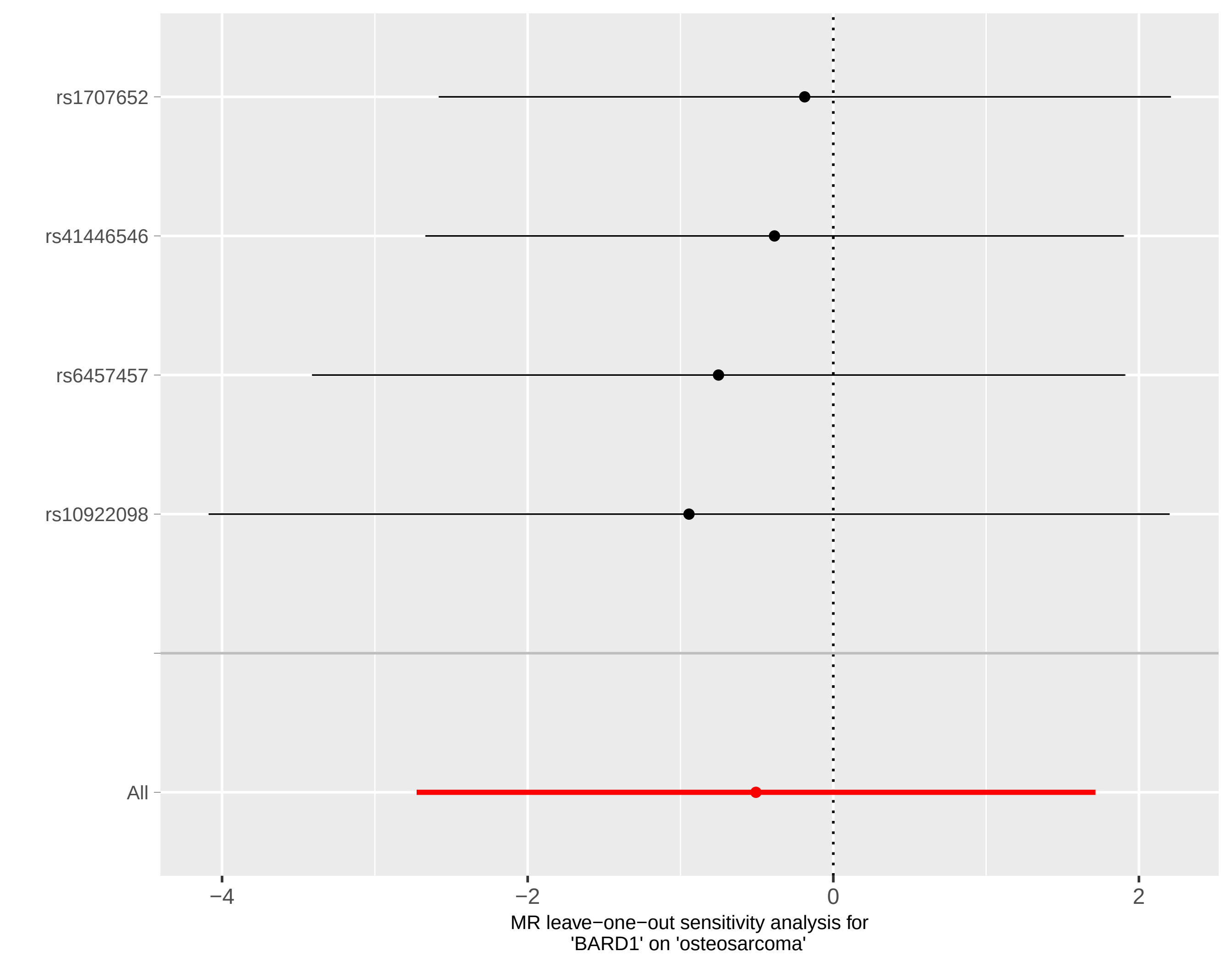
rs1707652
rs41446546
rs6457457
rs10922098
All
−4
−2
0
2
MR leave−one−out sensitivity analysis for
'BARD1' on 'osteosarcoma'

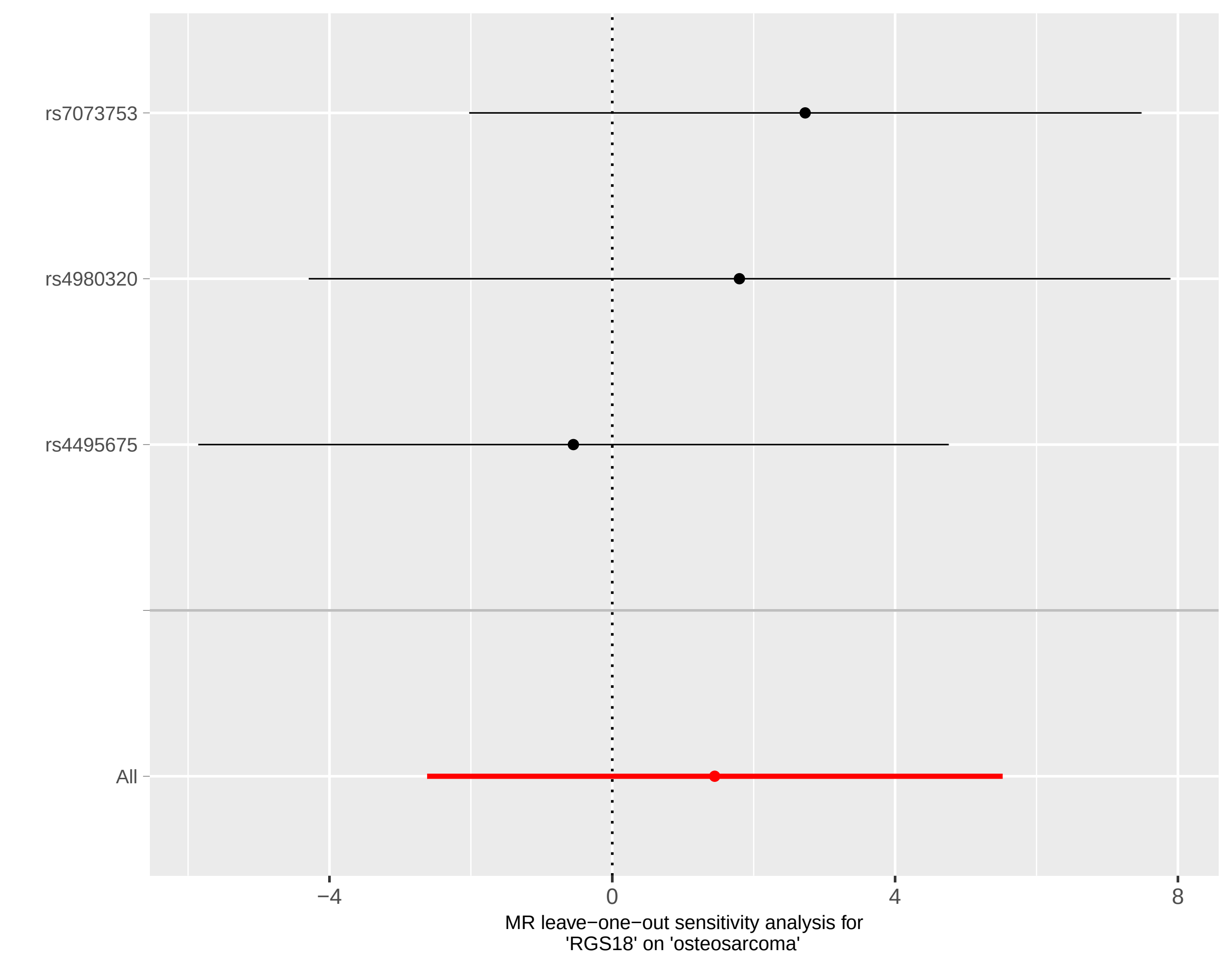

rs7073753
rs4980320
rs4495675
All
−4
0
4
8
MR leave−one−out sensitivity analysis for
'RGS18' on 'osteosarcoma'

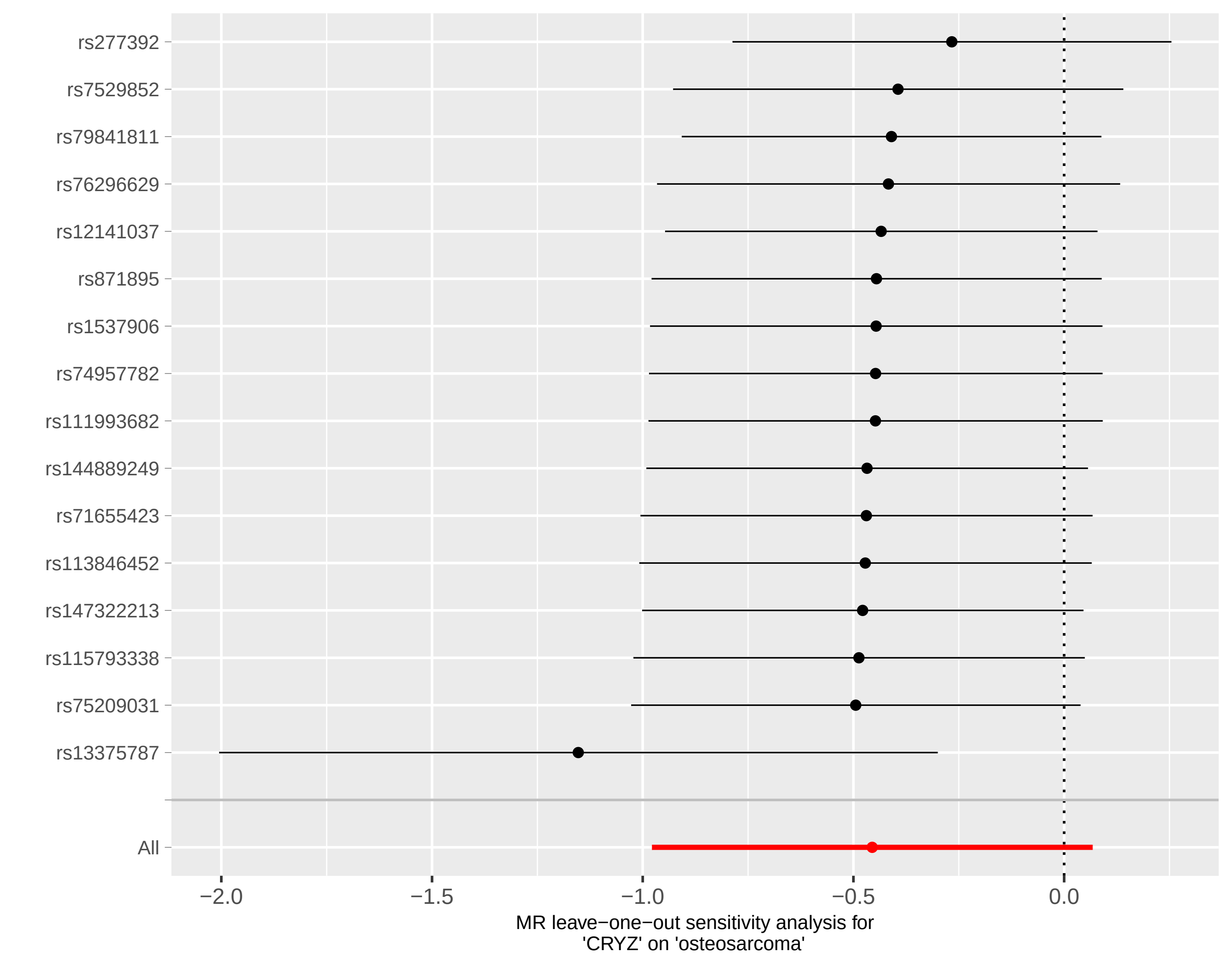
rs277392
rs7529852
rs79841811
rs76296629
rs12141037
rs871895
rs1537906
rs74957782
rs111993682
rs144889249
rs71655423
rs113846452
rs147322213
rs115793338
rs75209031
rs13375787
All
−2.0
−1.5
−1.0
−0.5
0.0
MR leave−one−out sensitivity analysis for
'CRYZ' on 'osteosarcoma'

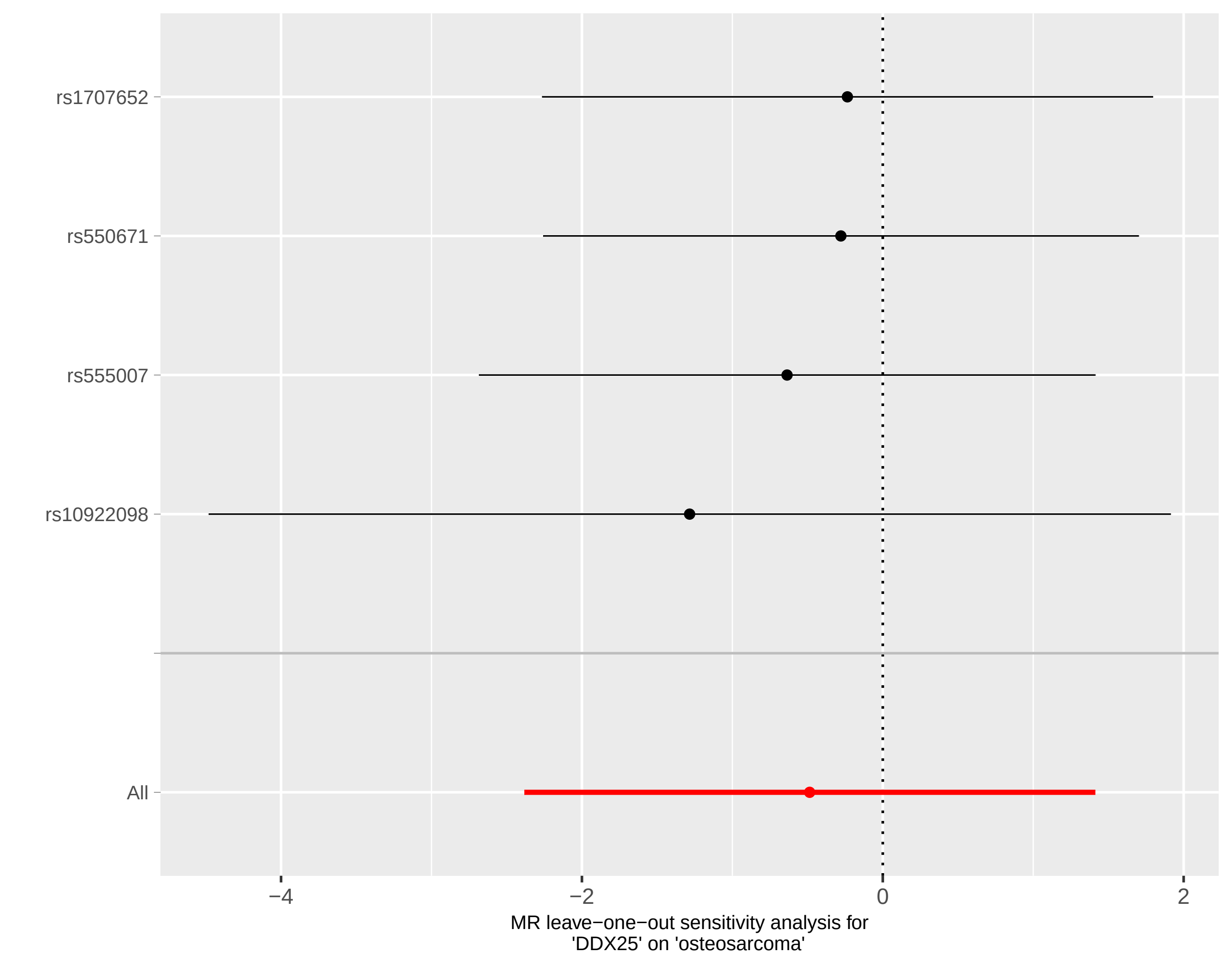

rs1707652
rs550671
rs555007
rs10922098
All
−4
−2
0
2
MR leave−one−out sensitivity analysis for
'DDX25' on 'osteosarcoma'

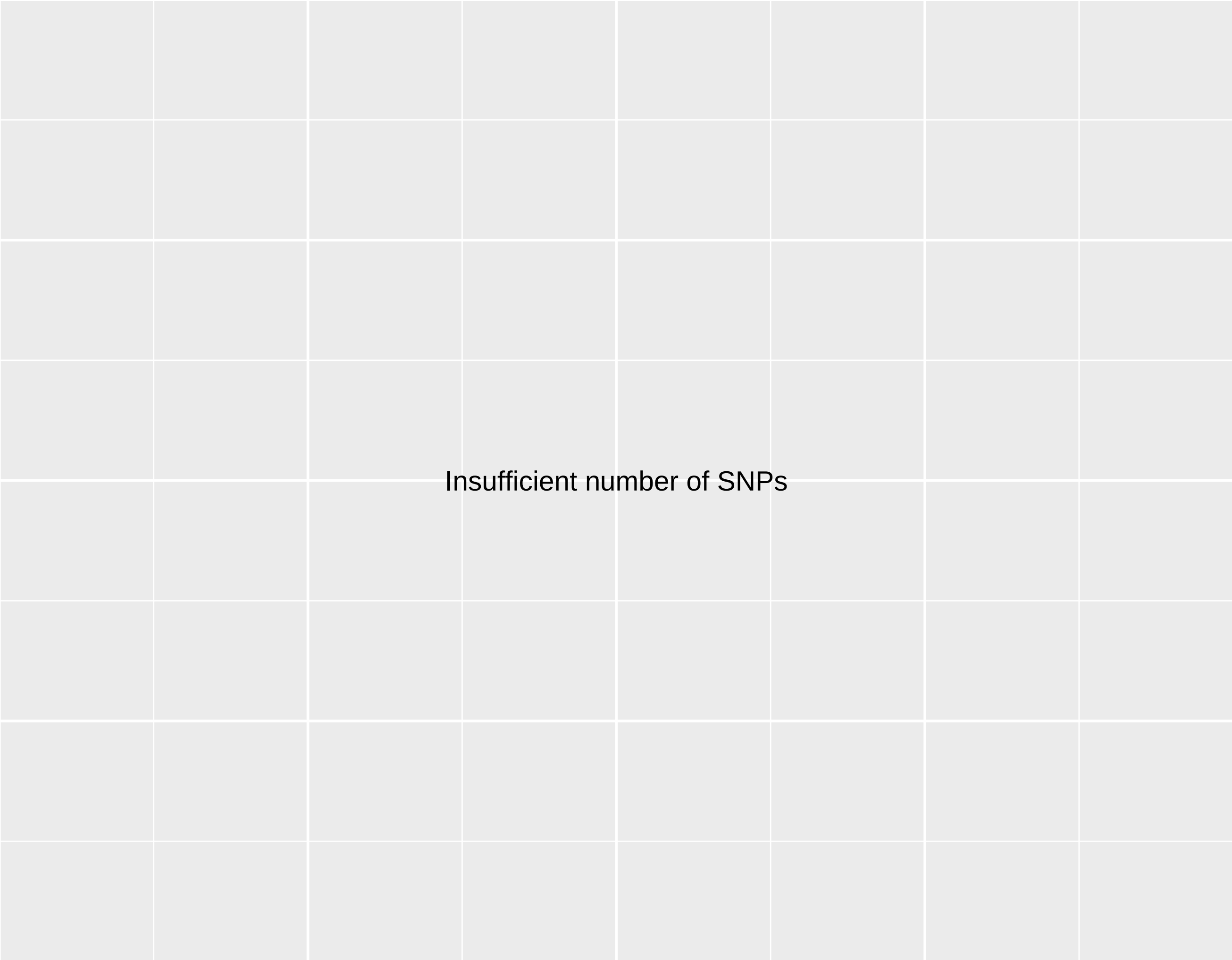
Insufficient number of SNPs

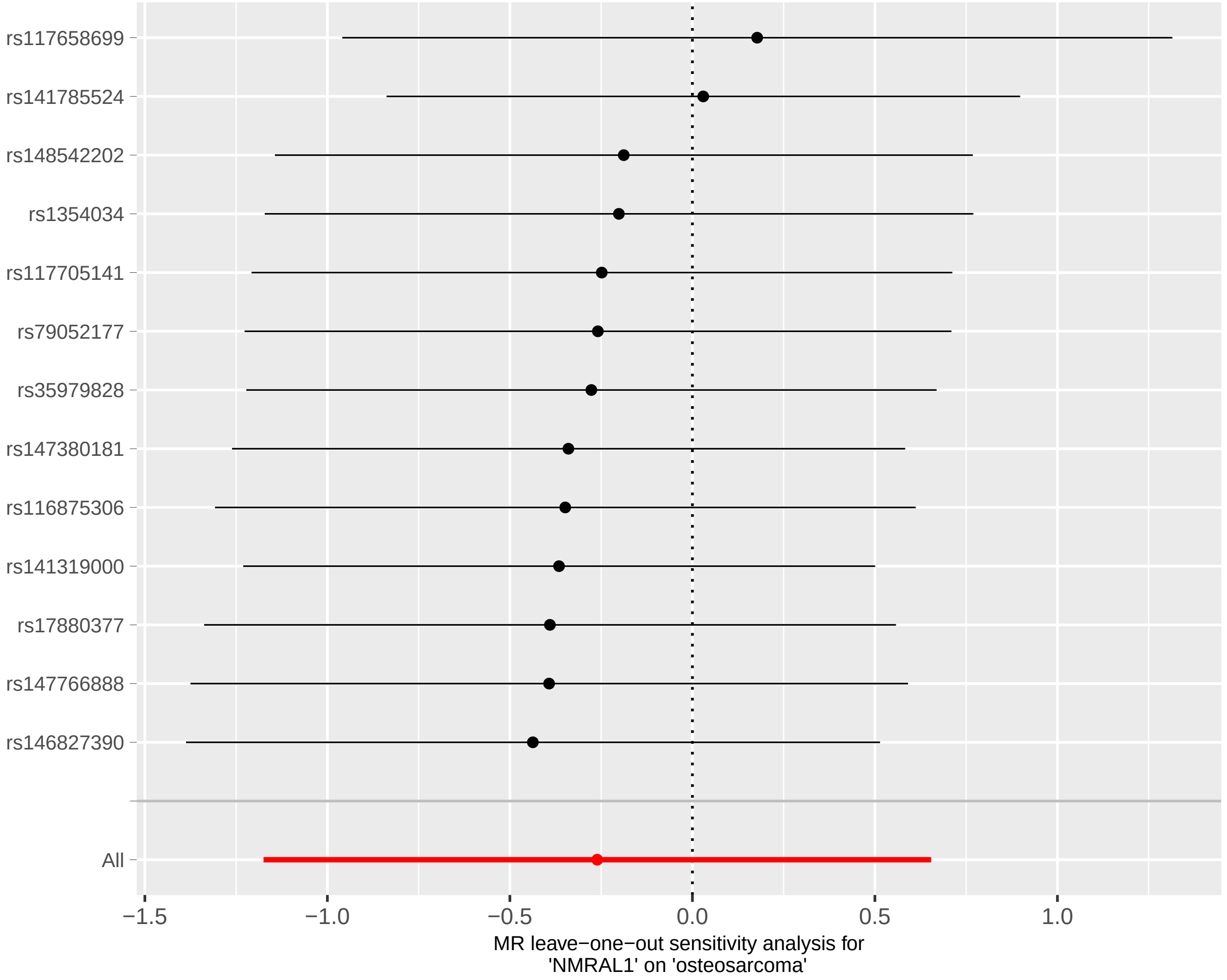
rs117658699
rs141785524
rs148542202
rs1354034
rs117705141
rs79052177
rs35979828
rs147380181
rs116875306
rs141319000
rs17880377
rs147766888
rs146827390
All
−1.5
−1.0
−0.5
0.0
0.5
1.0
MR leave−one−out sensitivity analysis for
'NMRAL1' on 'osteosarcoma'

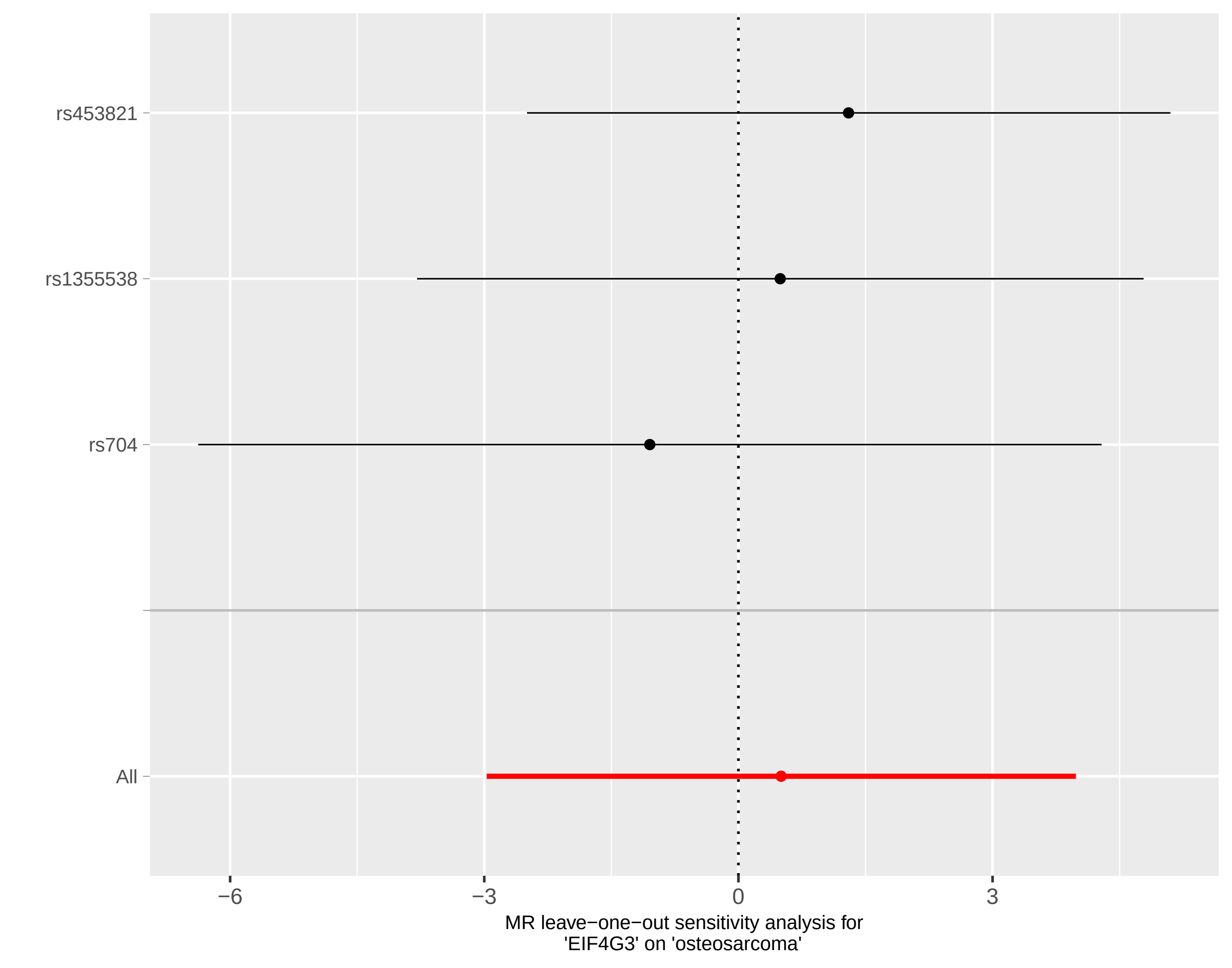
rs453821
rs1355538
rs704
All
−6
−3
0
3
MR leave−one−out sensitivity analysis for
'EIF4G3' on 'osteosarcoma'

Insufficient number of SNPs

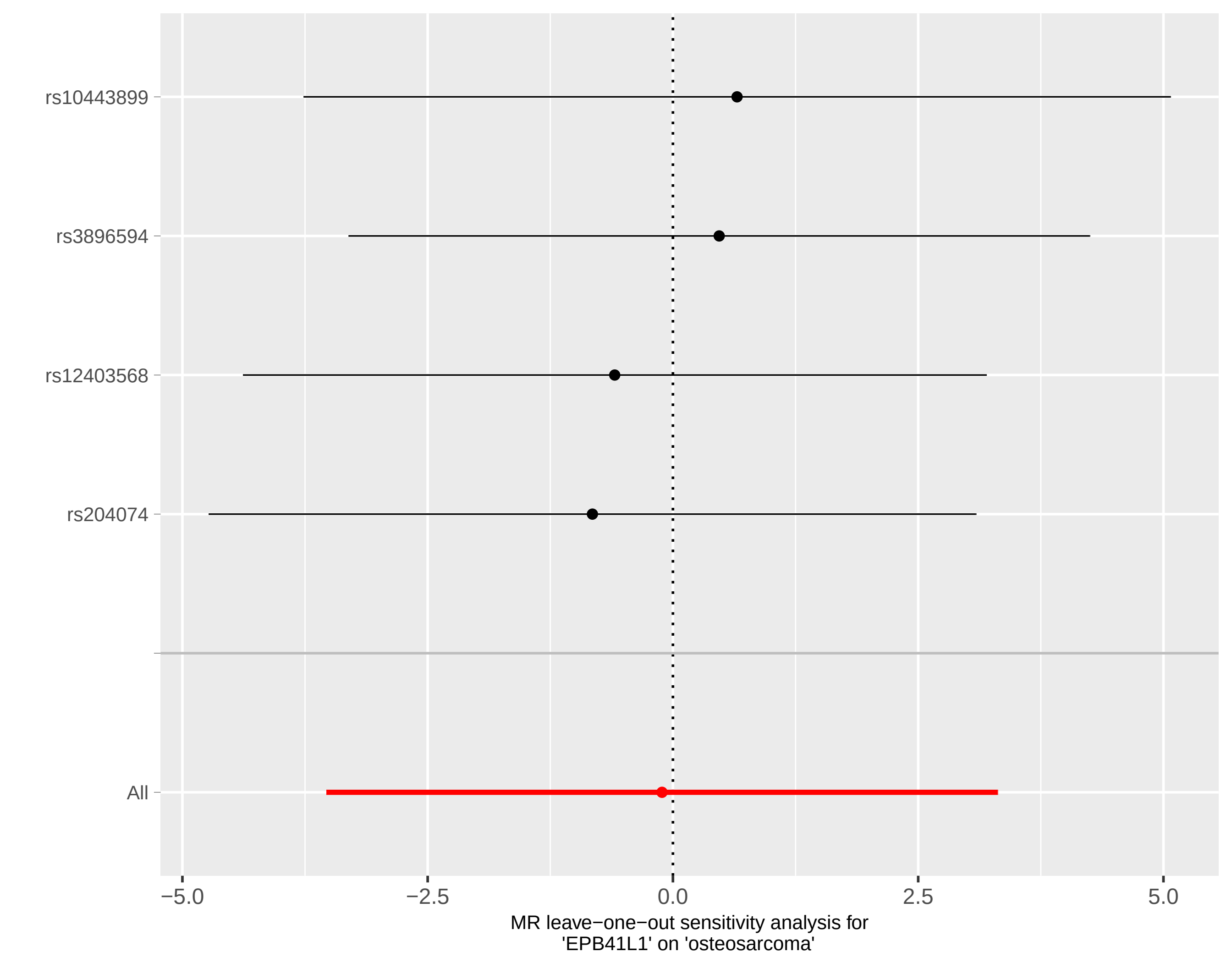
rs10443899
rs3896594
rs12403568
rs204074
All
−5.0
−2.5
0.0
2.5
5.0
MR leave−one−out sensitivity analysis for
'EPB41L1' on 'osteosarcoma'

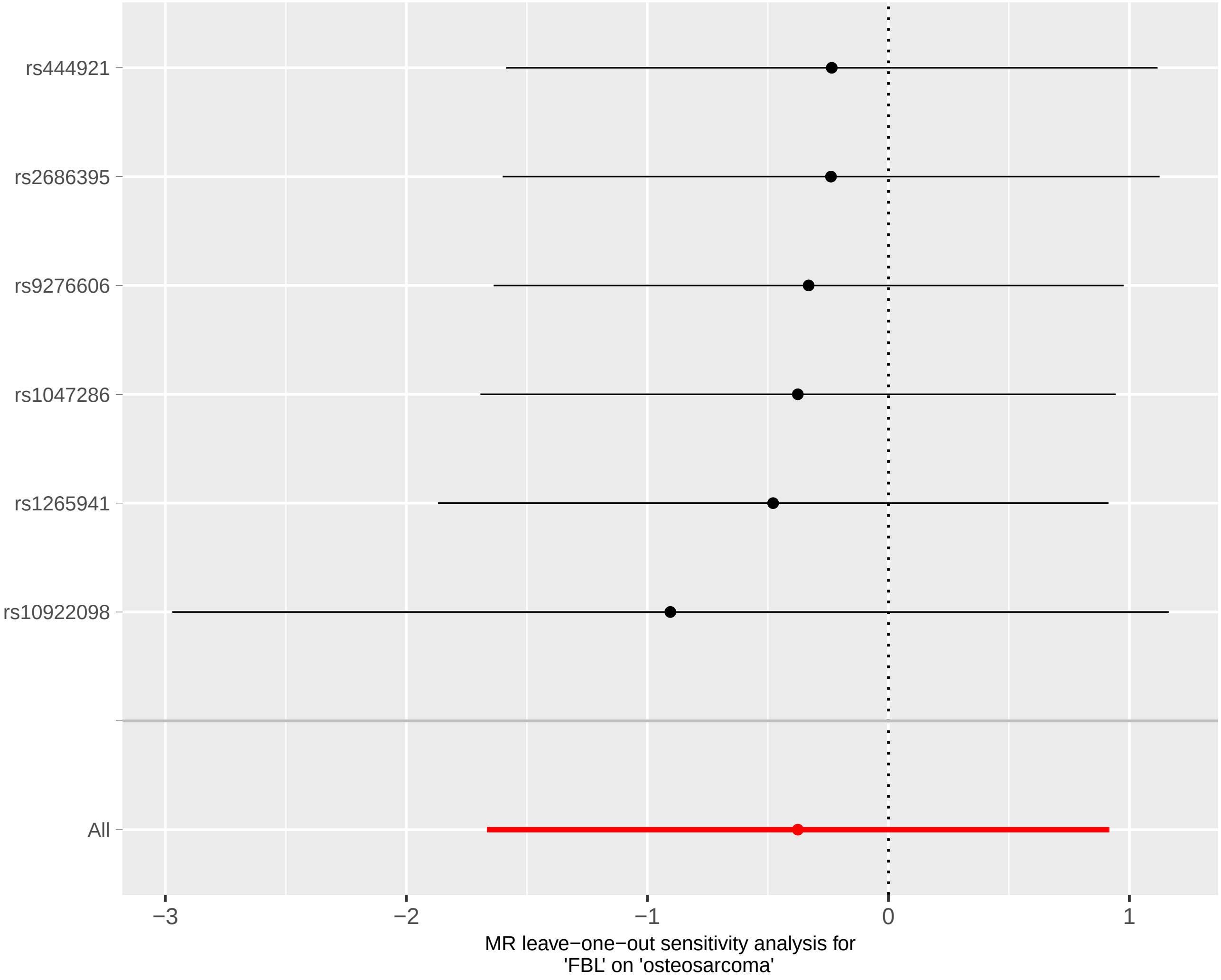
rs444921
rs2686395
rs9276606
rs1047286
rs1265941
rs10922098
All
−3
−2
−1
0
1
MR leave−one−out sensitivity analysis for
'FBL' on 'osteosarcoma'

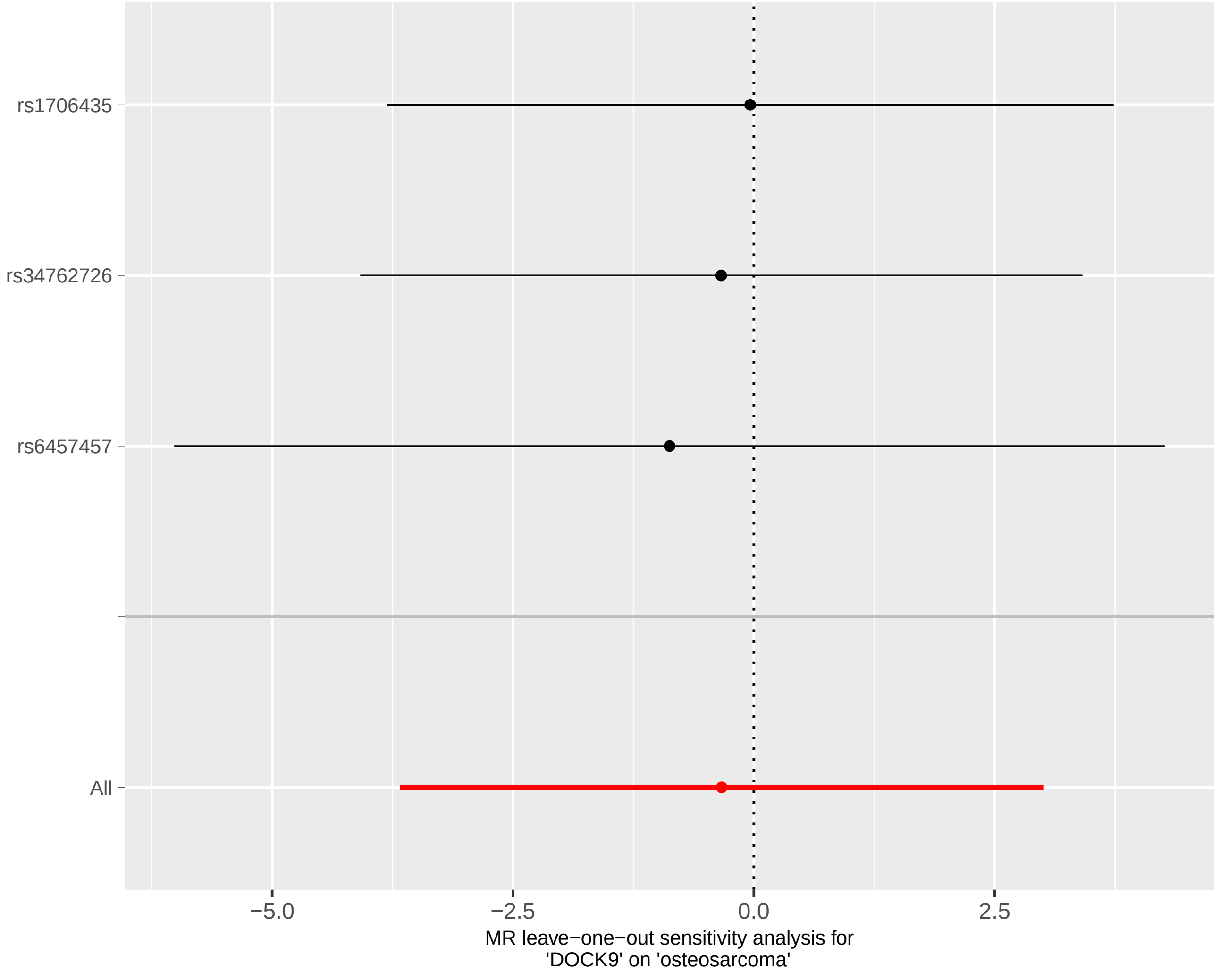
rs1706435
rs34762726
rs6457457
All
−5.0
−2.5
0.0
2.5
MR leave−one−out sensitivity analysis for
'DOCK9' on 'osteosarcoma'

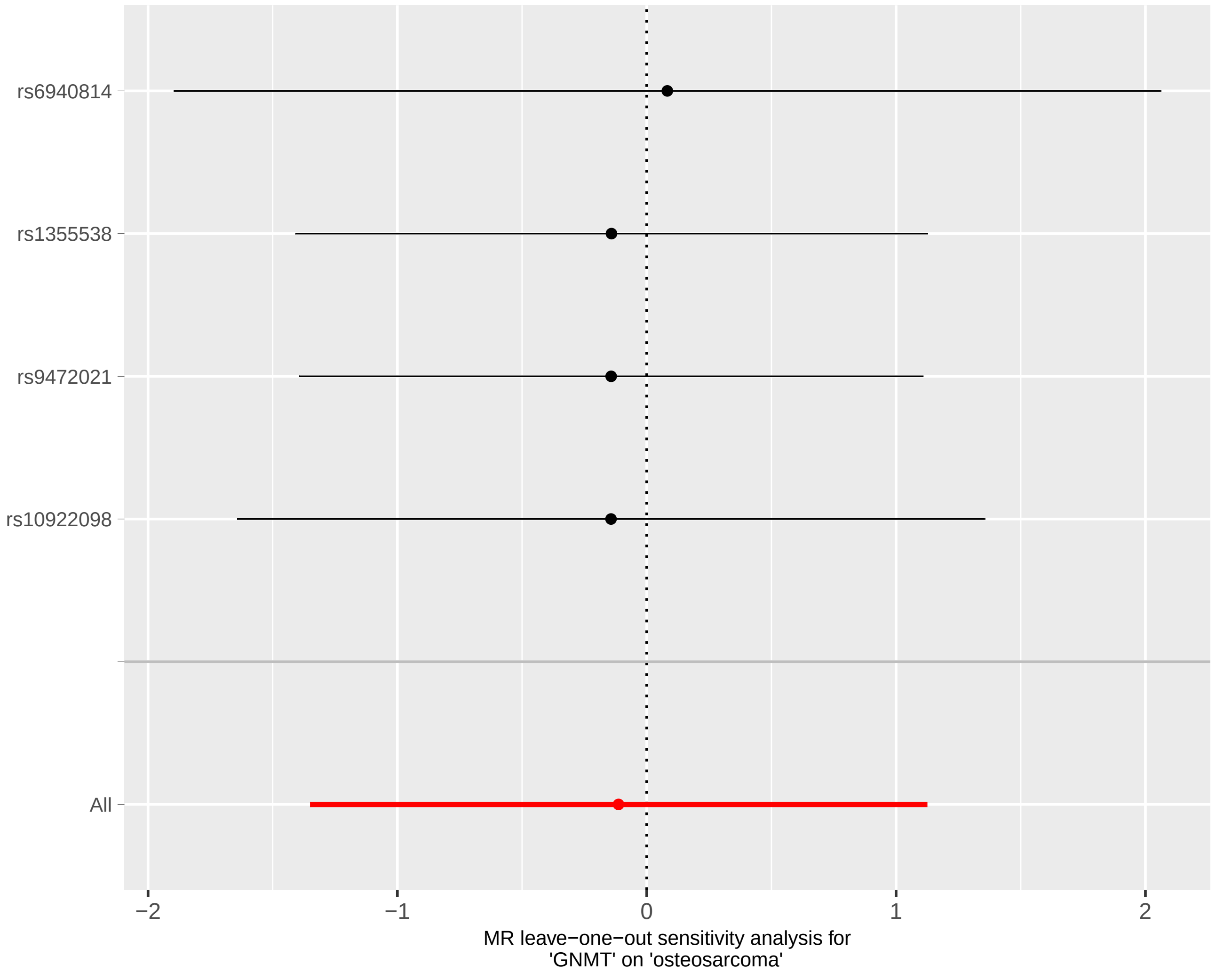

rs6940814
rs1355538
rs9472021
rs10922098
All
−2
−1
0
1
2
MR leave−one−out sensitivity analysis for
'GNMT' on 'osteosarcoma'

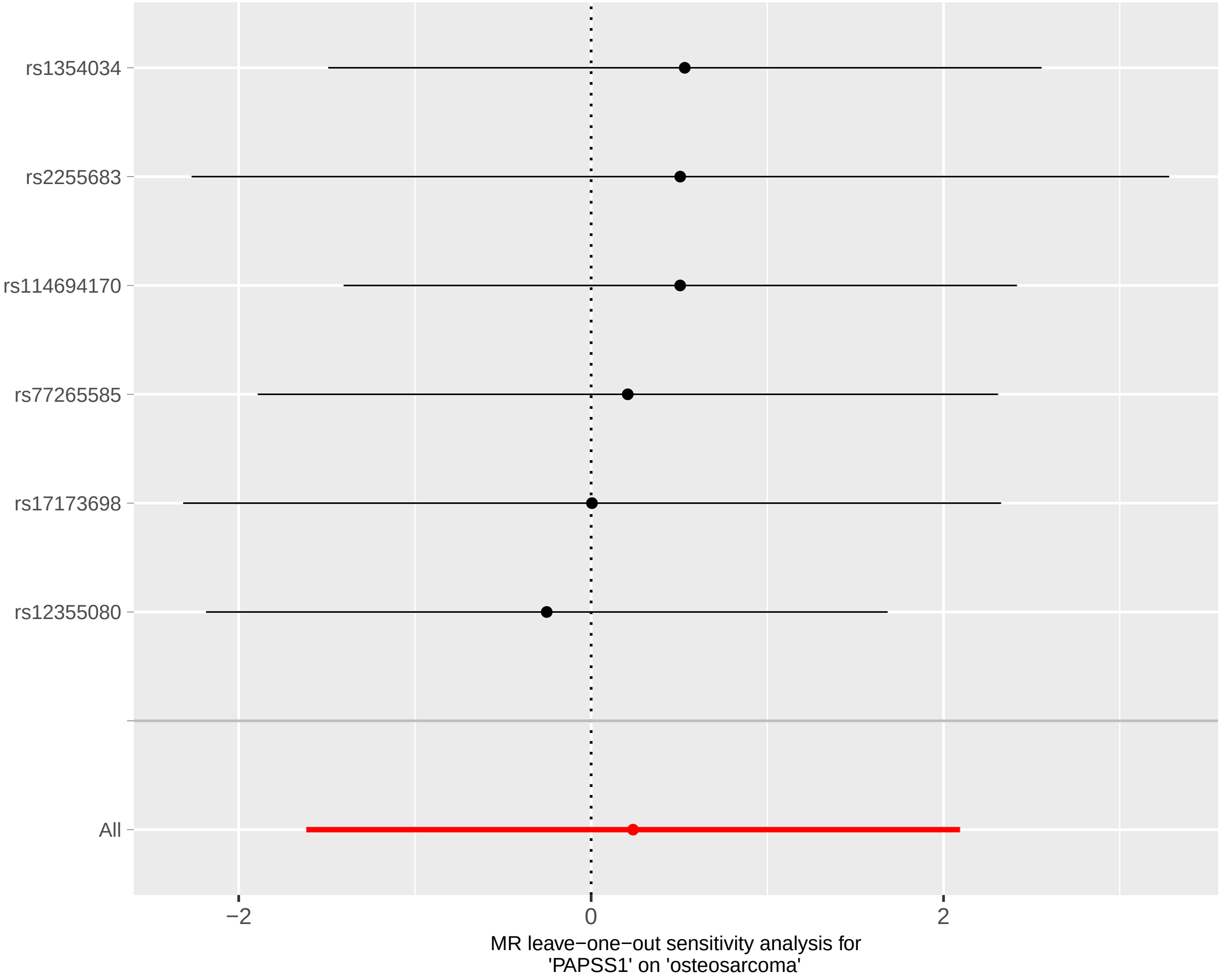
rs1354034
rs2255683
rs114694170
rs77265585
rs17173698
rs12355080
All
−2
0
2
MR leave−one−out sensitivity analysis for
'PAPSS1' on 'osteosarcoma'

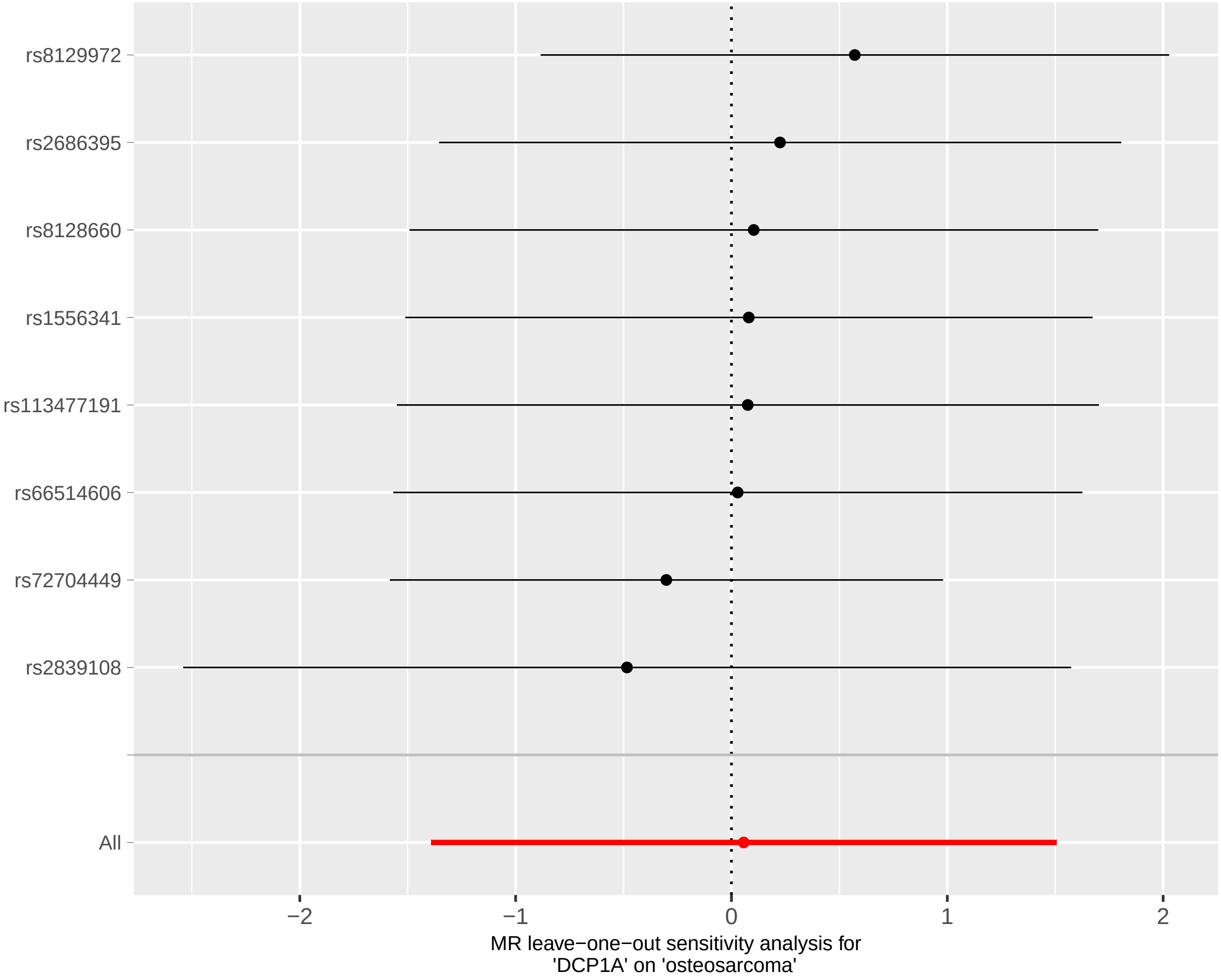
rs8129972
rs2686395
rs8128660
rs1556341
rs113477191
rs66514606
rs72704449
rs2839108
All
−2
−1
0
1
2
MR leave−one−out sensitivity analysis for
'DCP1A' on 'osteosarcoma'

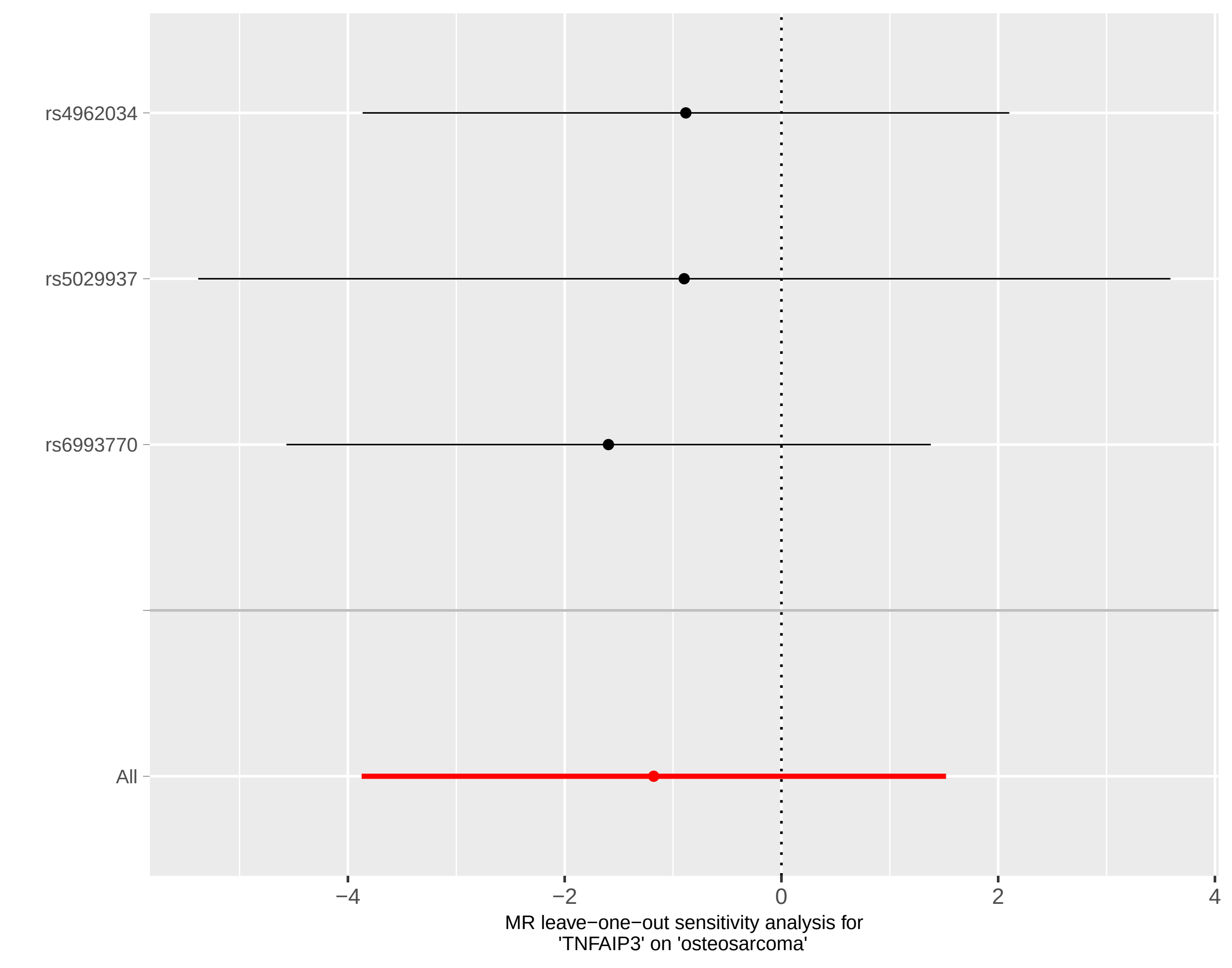

rs4962034
rs5029937
rs6993770
All
−4
−2
0
2
4
MR leave−one−out sensitivity analysis for
'TNFAIP3' on 'osteosarcoma'

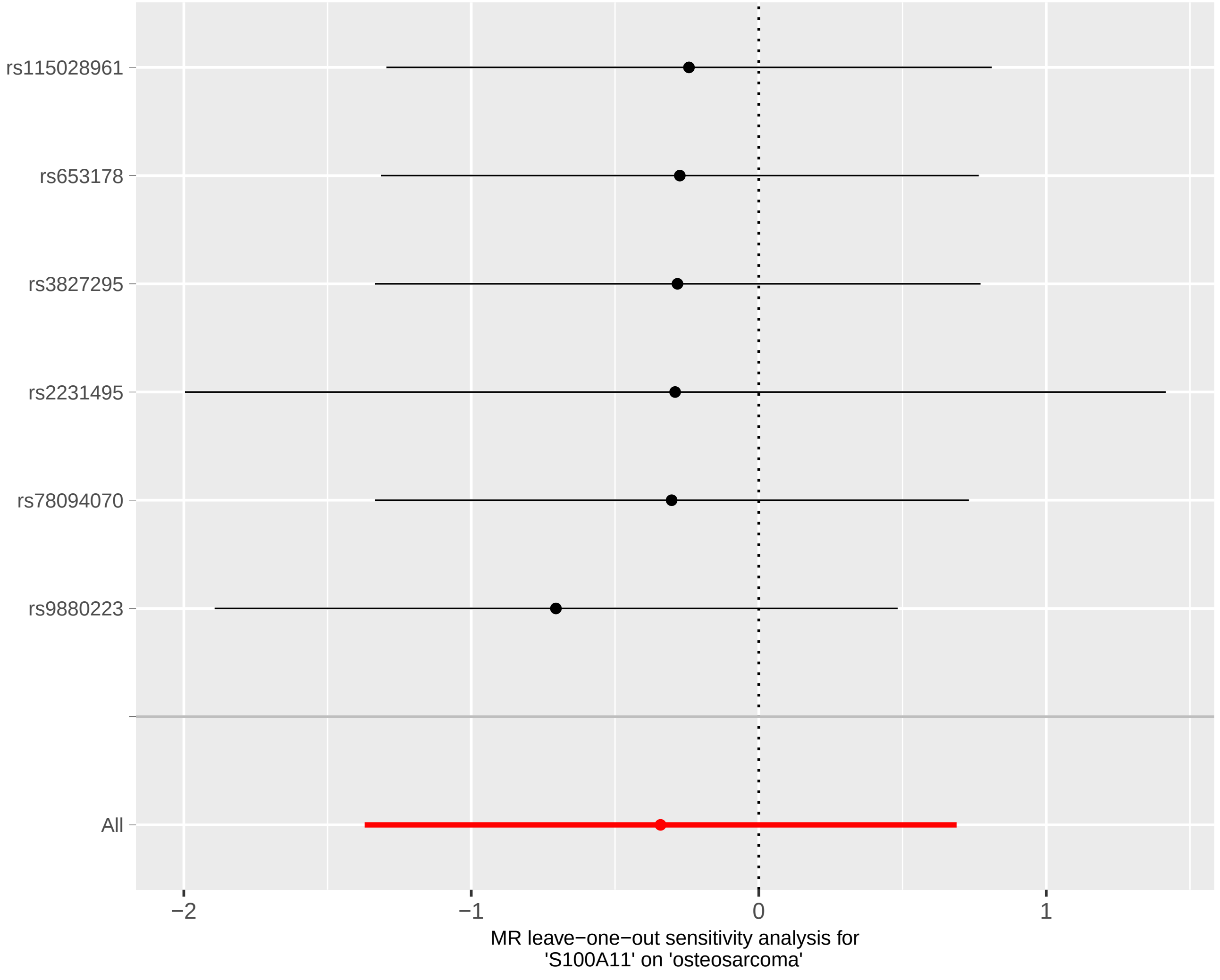
rs115028961
rs653178
rs3827295
rs2231495
rs78094070
rs9880223
All
−2
−1
0
1
MR leave−one−out sensitivity analysis for
'S100A11' on 'osteosarcoma'

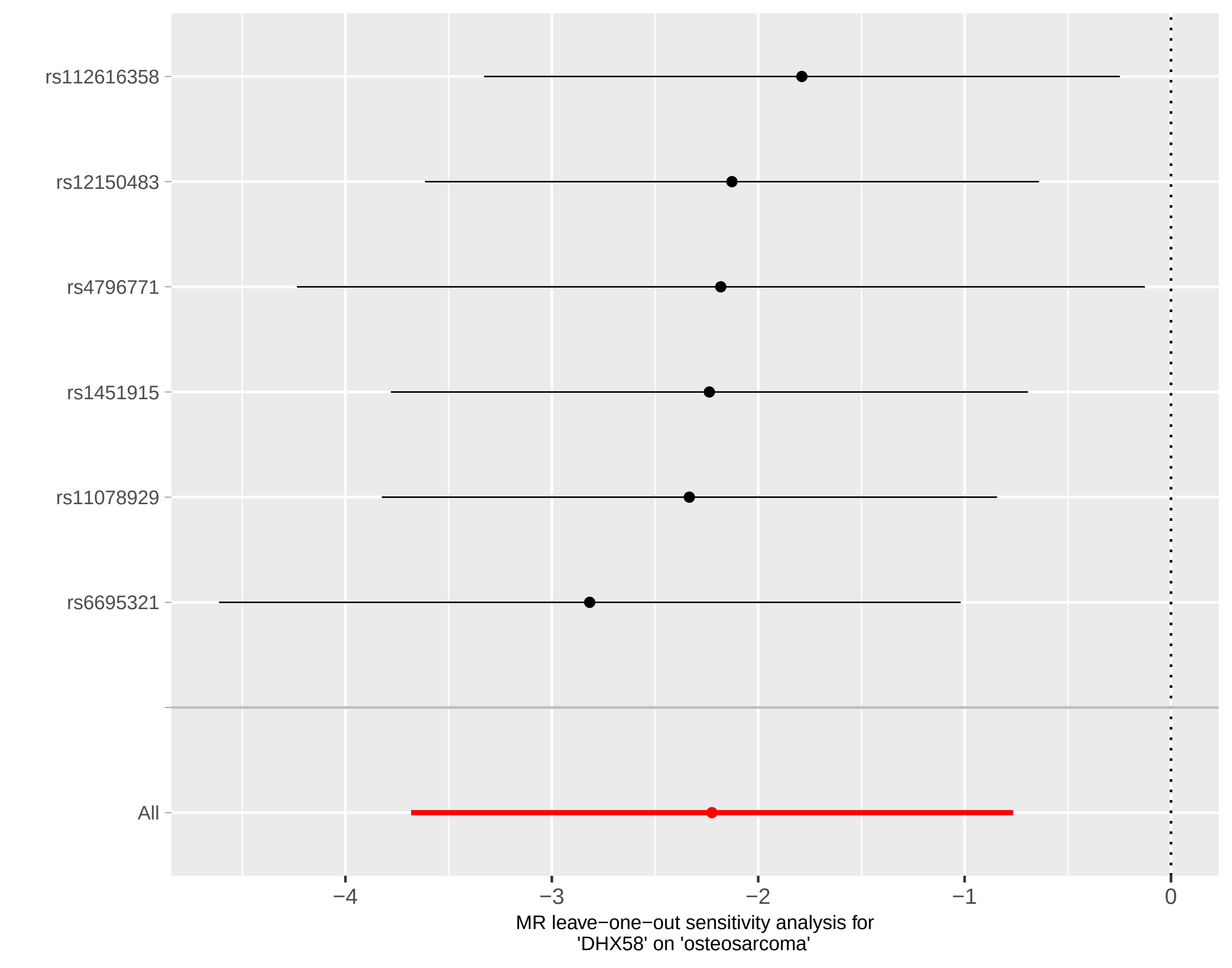

rs112616358
rs12150483
rs4796771
rs1451915
rs11078929
rs6695321
All
−4
−3
−2
−1
0
MR leave−one−out sensitivity analysis for
'DHX58' on 'osteosarcoma'

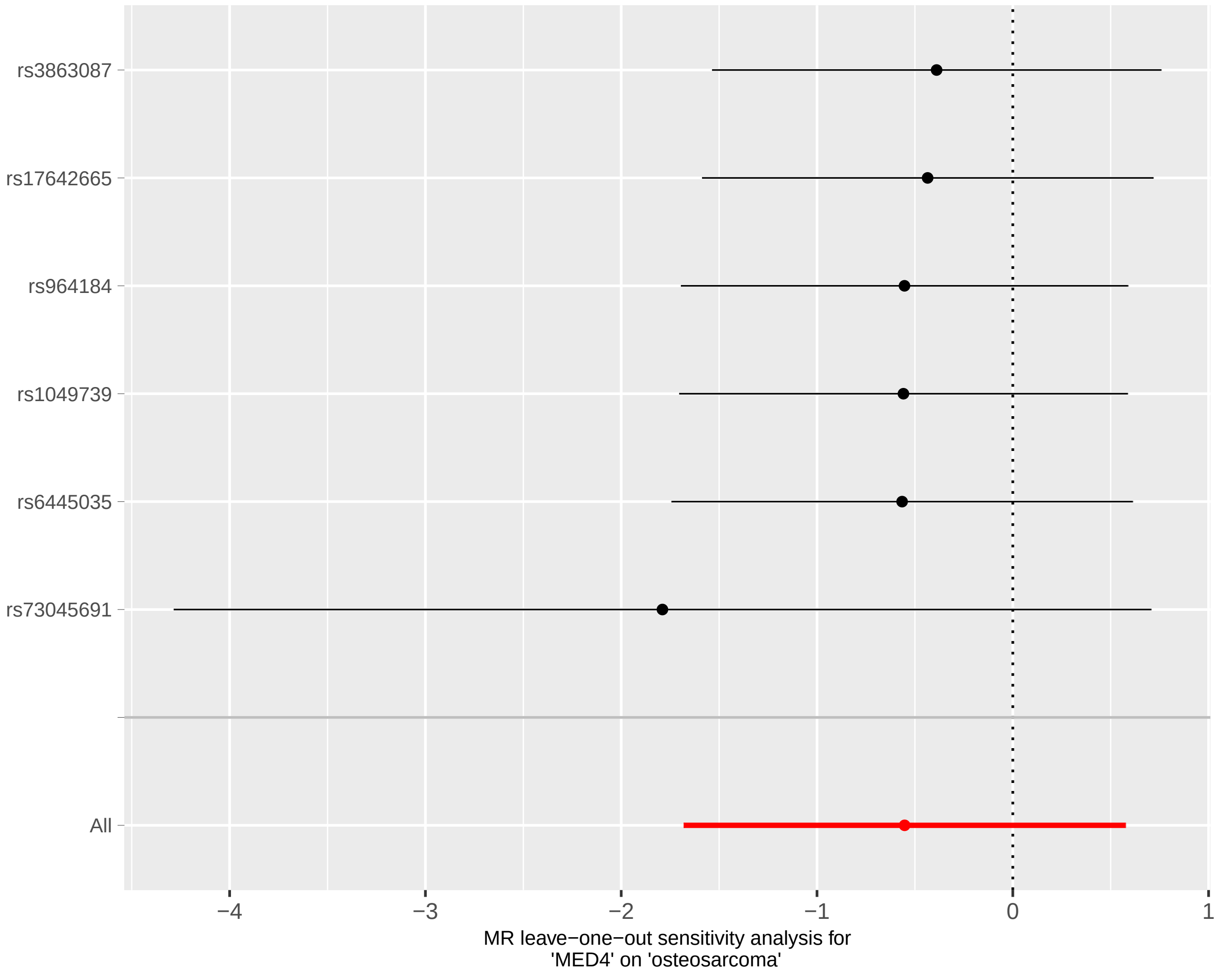
rs3863087
rs17642665
rs964184
rs1049739
rs6445035
rs73045691
All
−4
−3
−2
−1
0
1
MR leave−one−out sensitivity analysis for
'MED4' on 'osteosarcoma'

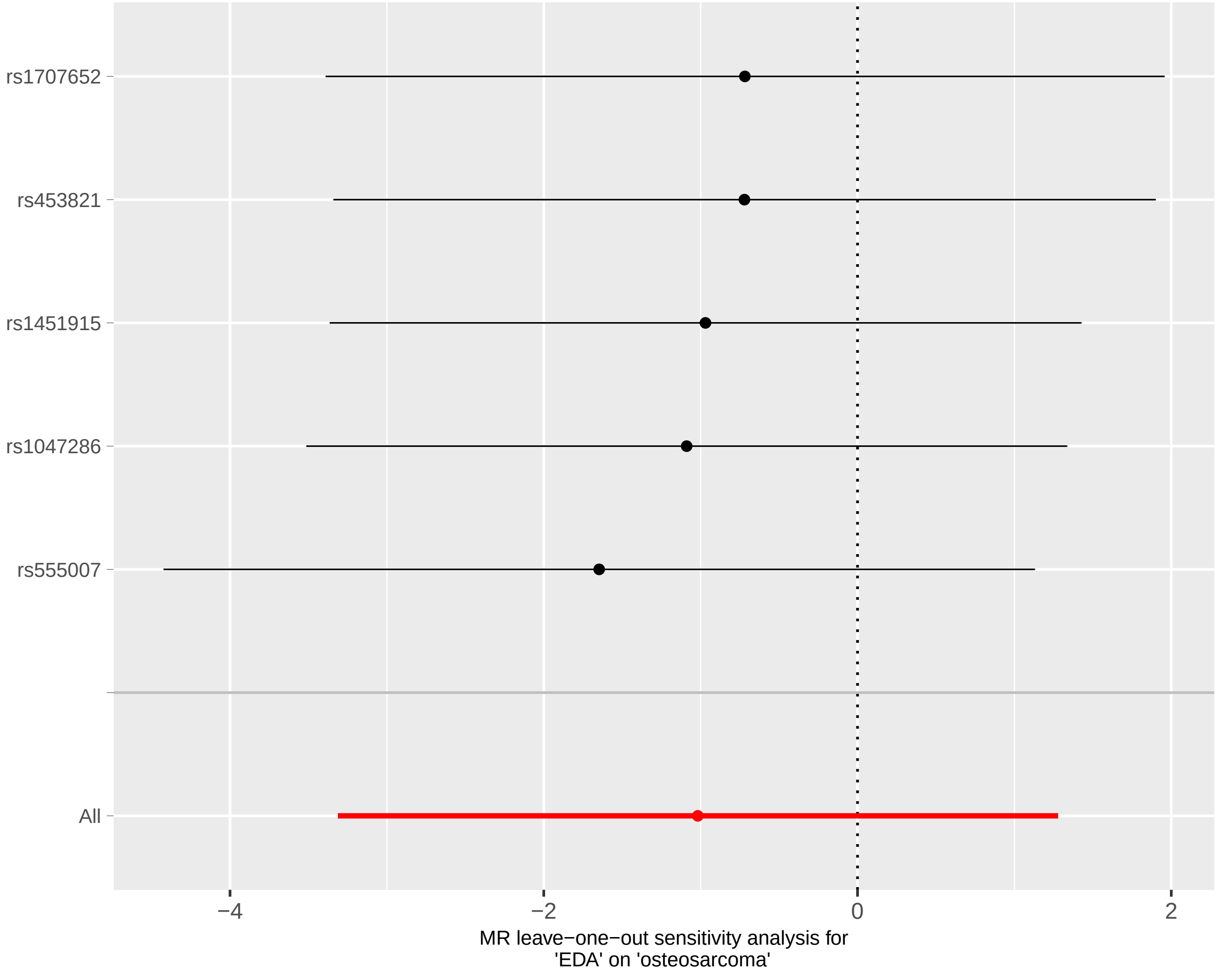

rs1707652
rs453821
rs1451915
rs1047286
rs555007
All
−4
−2
0
2
MR leave−one−out sensitivity analysis for
'EDA' on 'osteosarcoma'

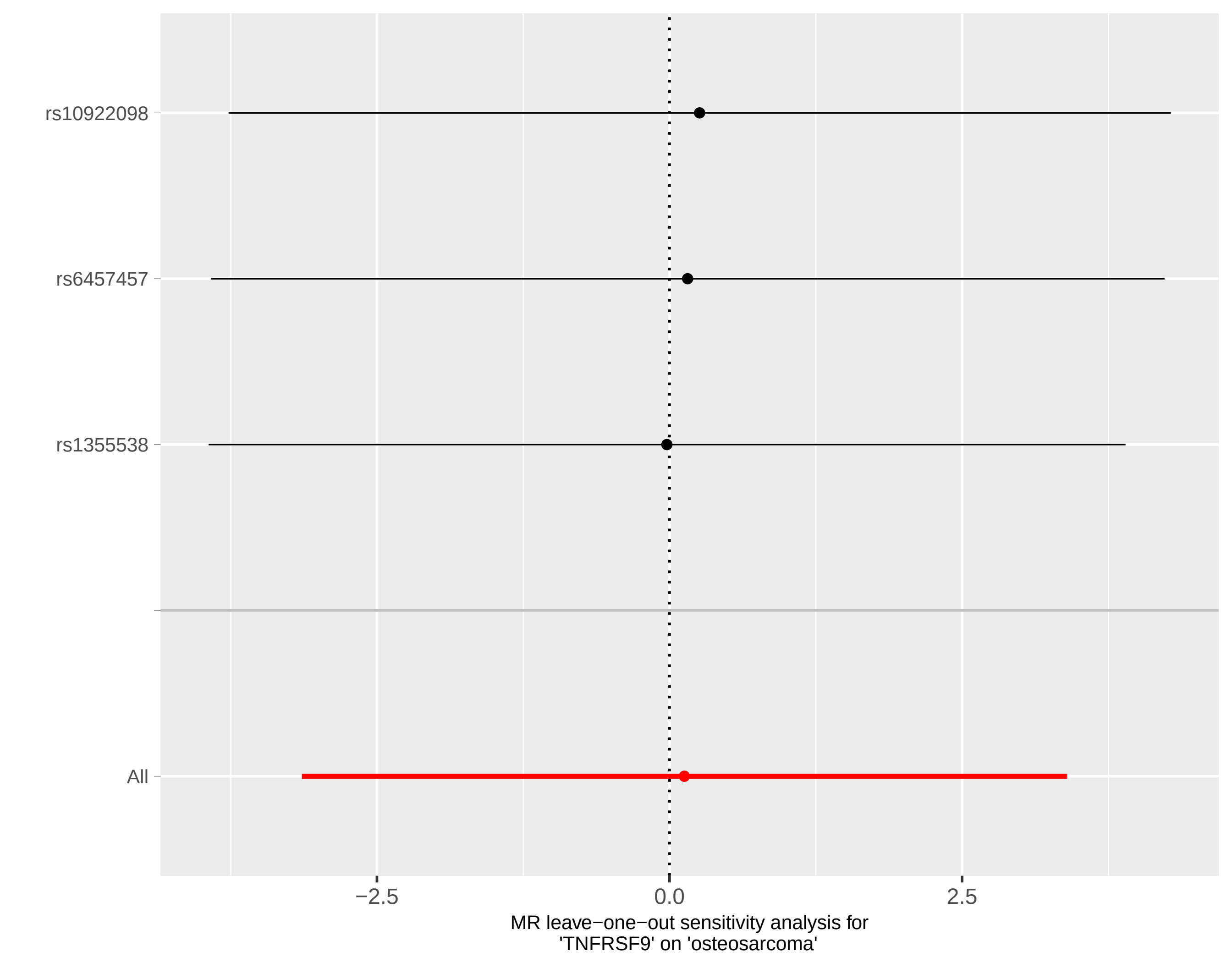
rs10922098
rs6457457
rs1355538
All
−2.5
0.0
2.5
MR leave−one−out sensitivity analysis for
'TNFRSF9' on 'osteosarcoma'

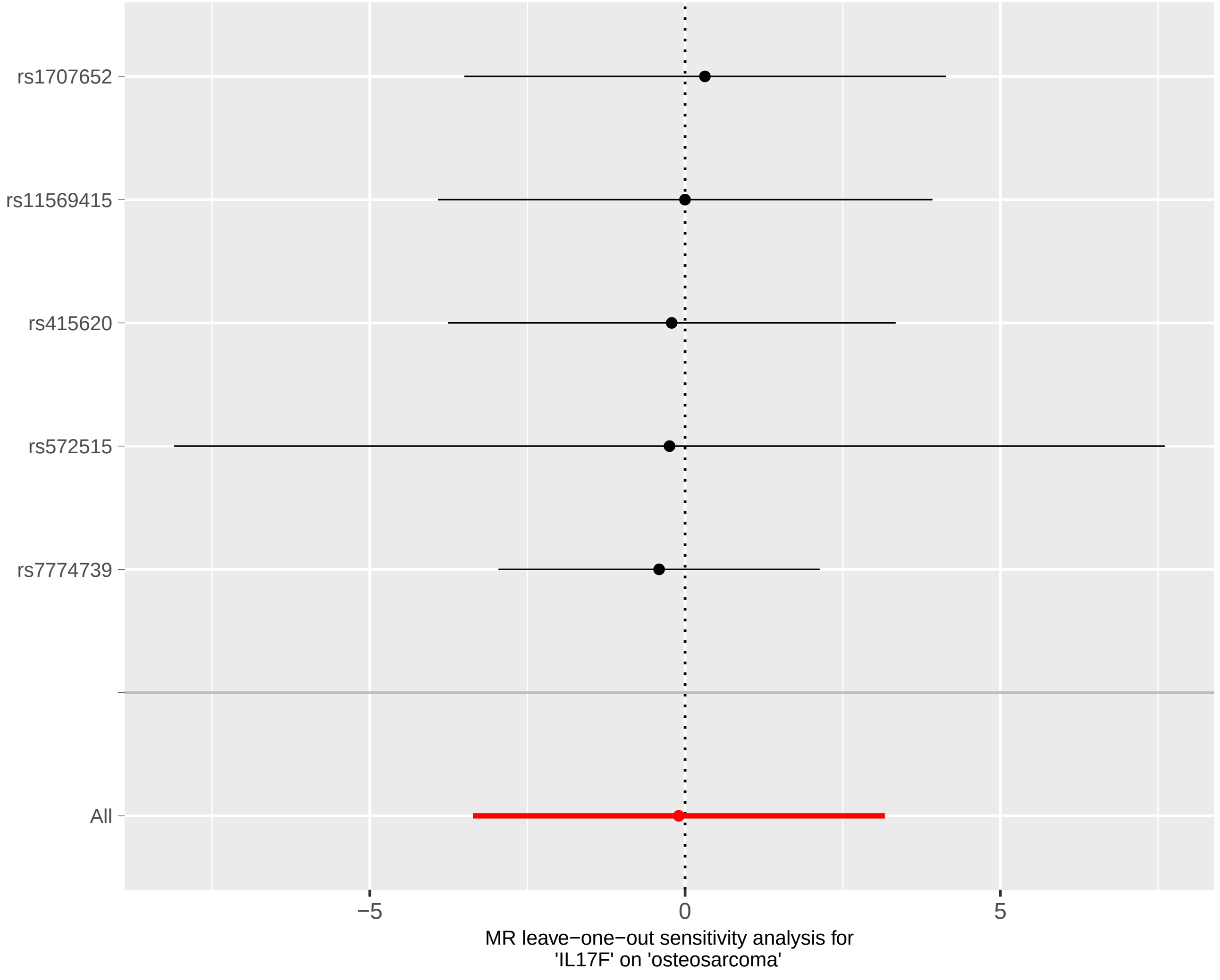

rs1707652
rs11569415
rs415620
rs572515
rs7774739
All
−5
0
5
MR leave−one−out sensitivity analysis for
'IL17F' on 'osteosarcoma'

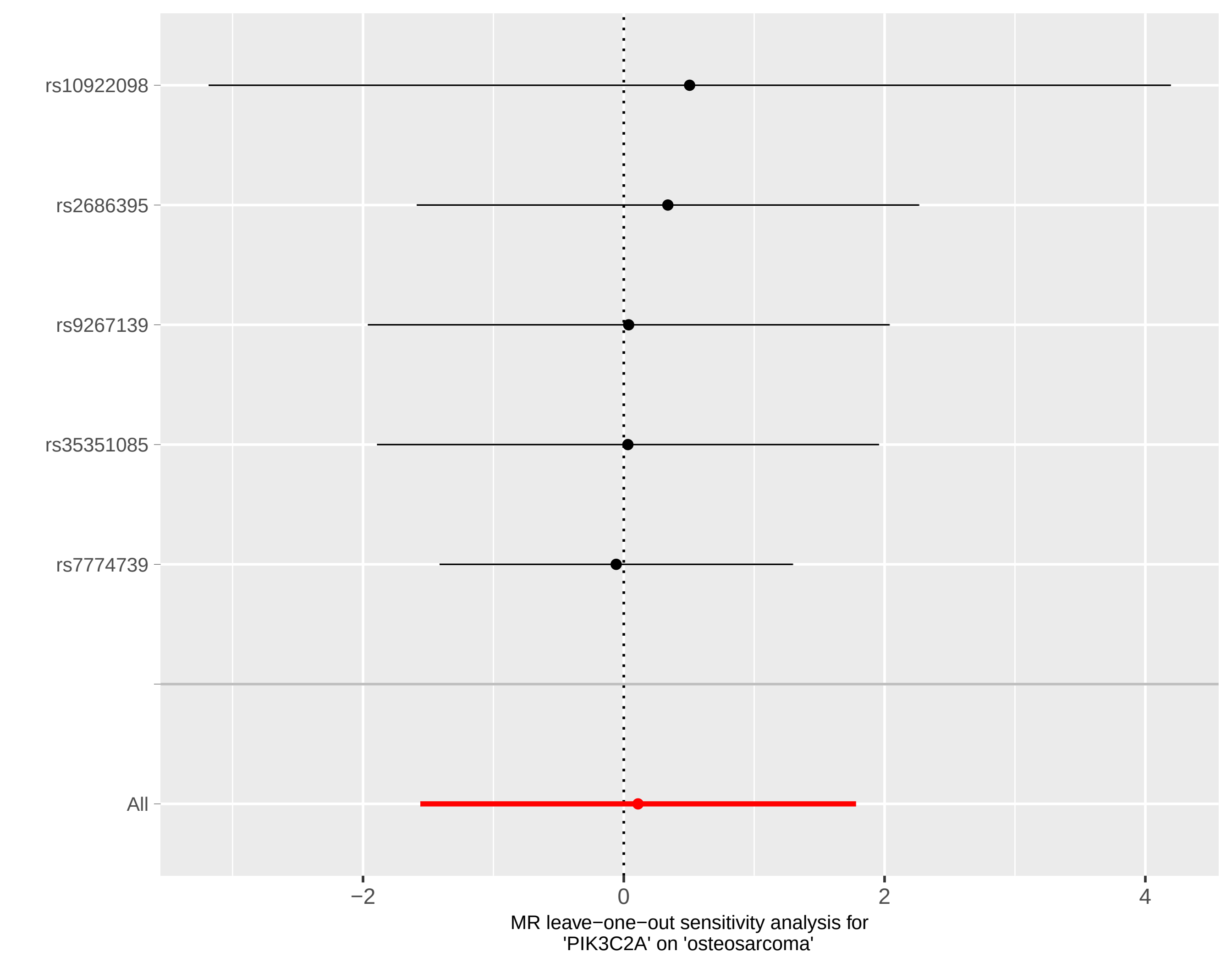

rs10922098
rs2686395
rs9267139
rs35351085
rs7774739
All
−2
0
2
4
MR leave−one−out sensitivity analysis for
'PIK3C2A' on 'osteosarcoma'

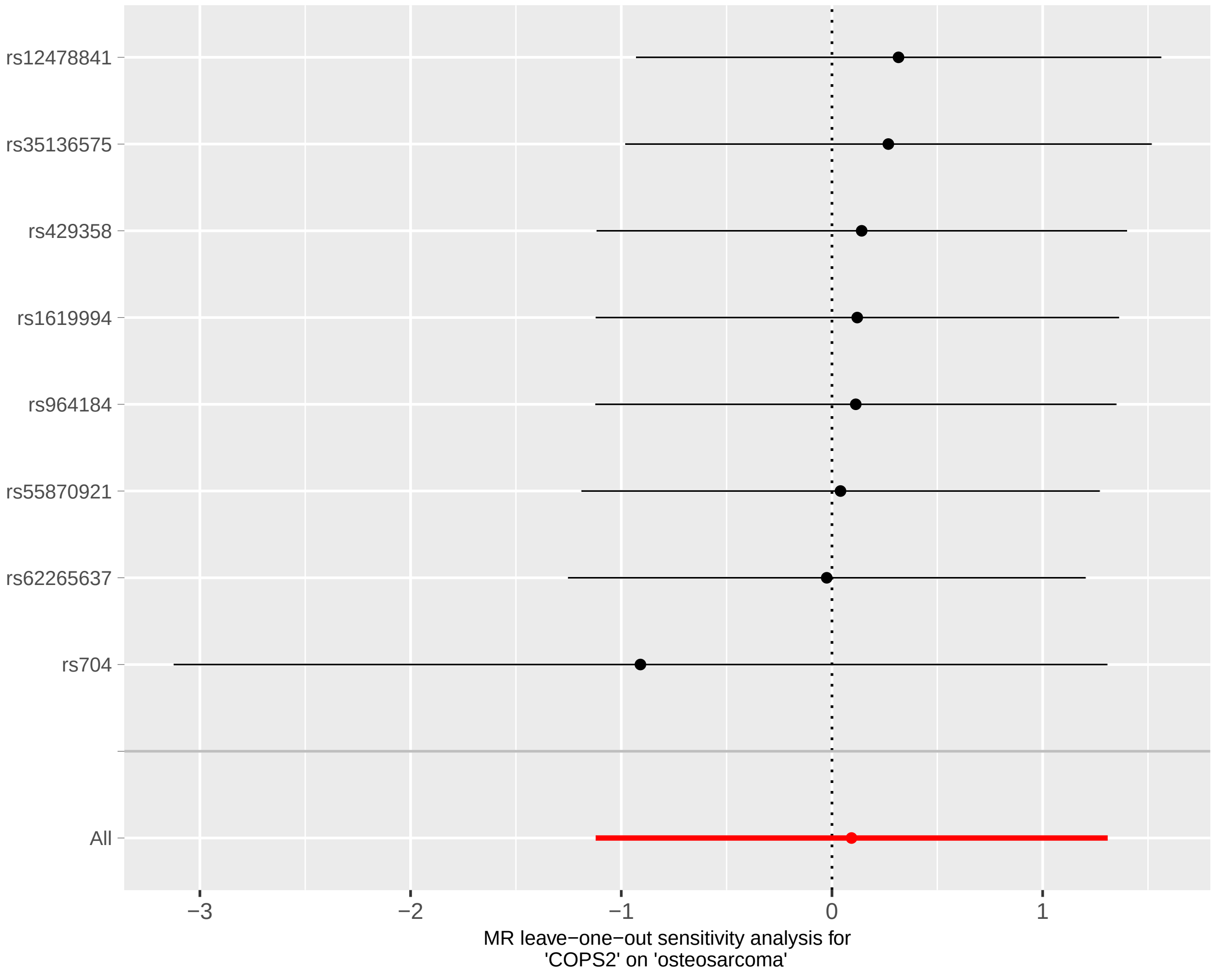

rs12478841
rs35136575
rs429358
rs1619994
rs964184
rs55870921
rs62265637
rs704
All
−3
−2
−1
0
1
MR leave−one−out sensitivity analysis for
'COPS2' on 'osteosarcoma'

Insufficient number of SNPs

Insufficient number of SNPs

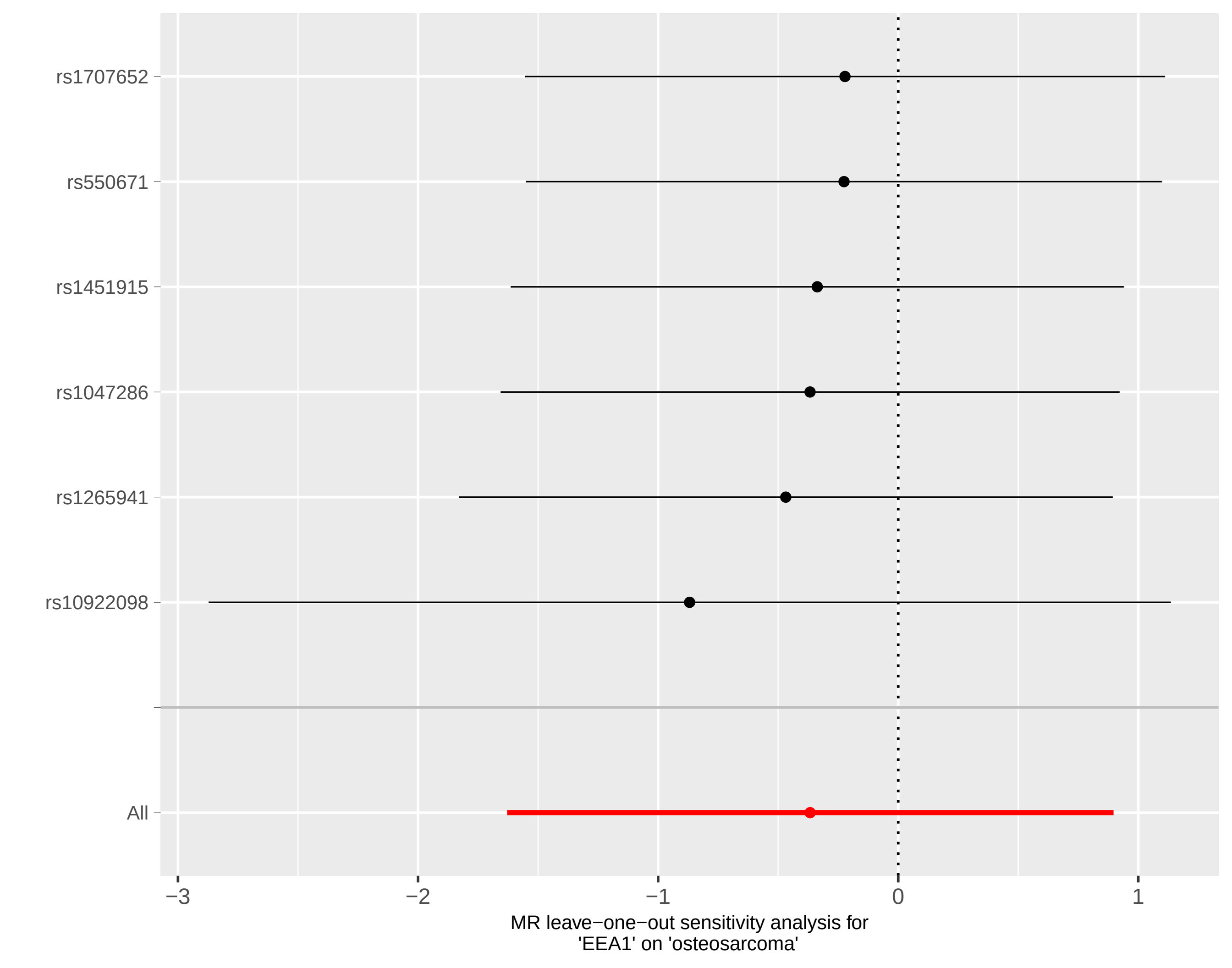

rs1707652
rs550671
rs1451915
rs1047286
rs1265941
rs10922098
All
−3
−2
−1
0
1
MR leave−one−out sensitivity analysis for
'EEA1' on 'osteosarcoma'

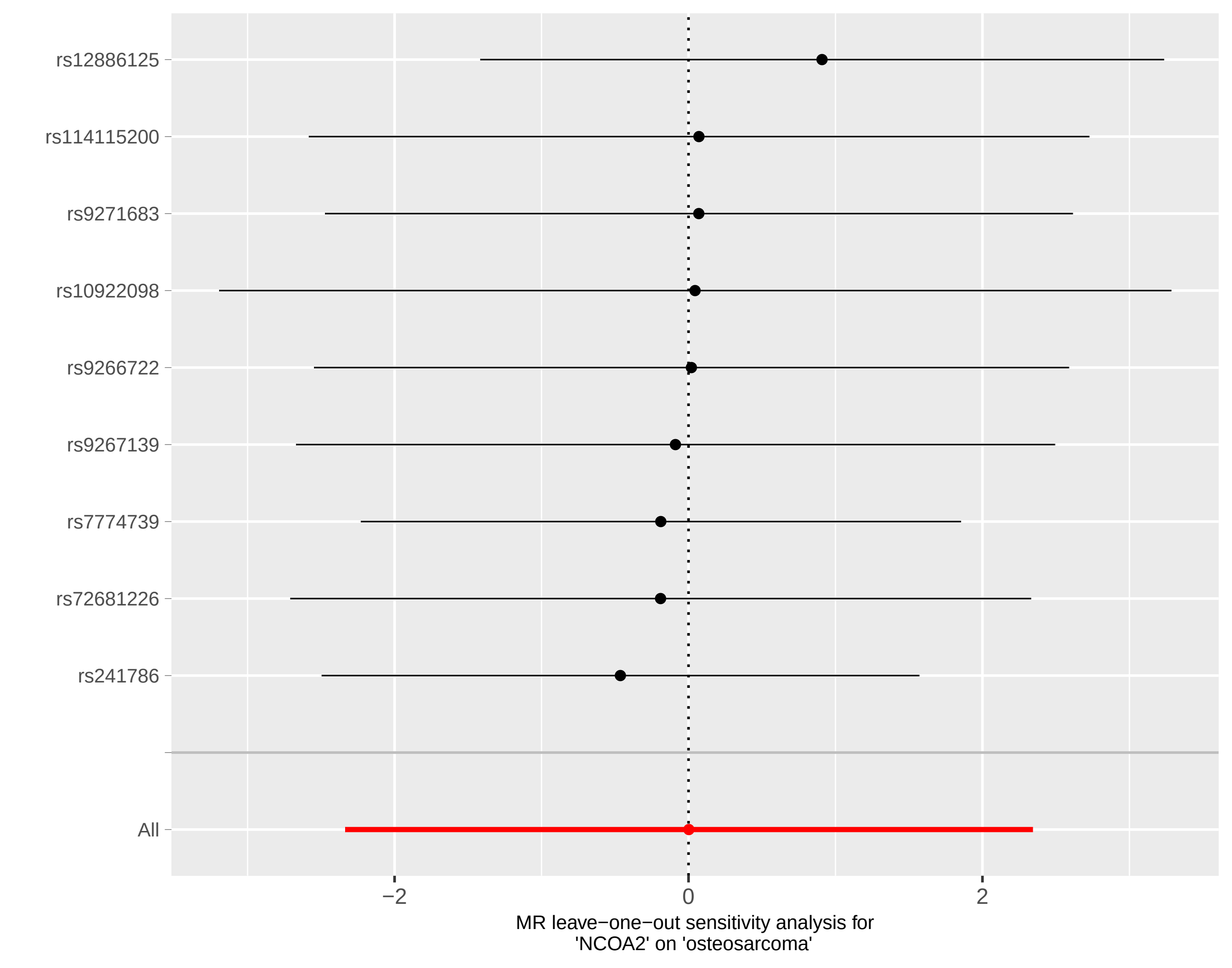
rs12886125
rs114115200
rs9271683
rs10922098
rs9266722
rs9267139
rs7774739
rs72681226
rs241786
All
−2
0
2
MR leave−one−out sensitivity analysis for
'NCOA2' on 'osteosarcoma'

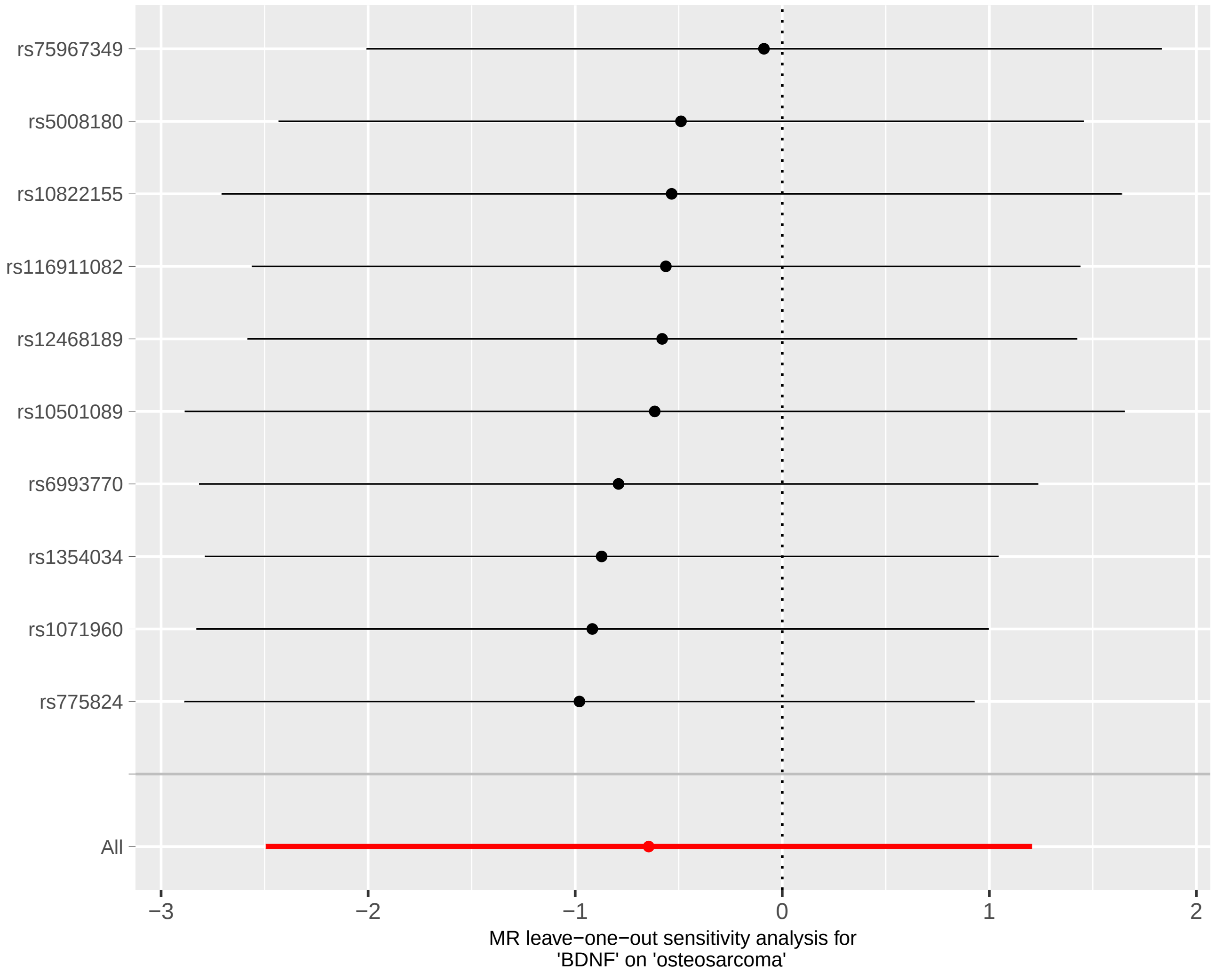
rs75967349
rs5008180
rs10822155
rs116911082
rs12468189
rs10501089
rs6993770
rs1354034
rs1071960
rs775824
All
−3
−2
−1
0
1
2
MR leave−one−out sensitivity analysis for
'BDNF' on 'osteosarcoma'

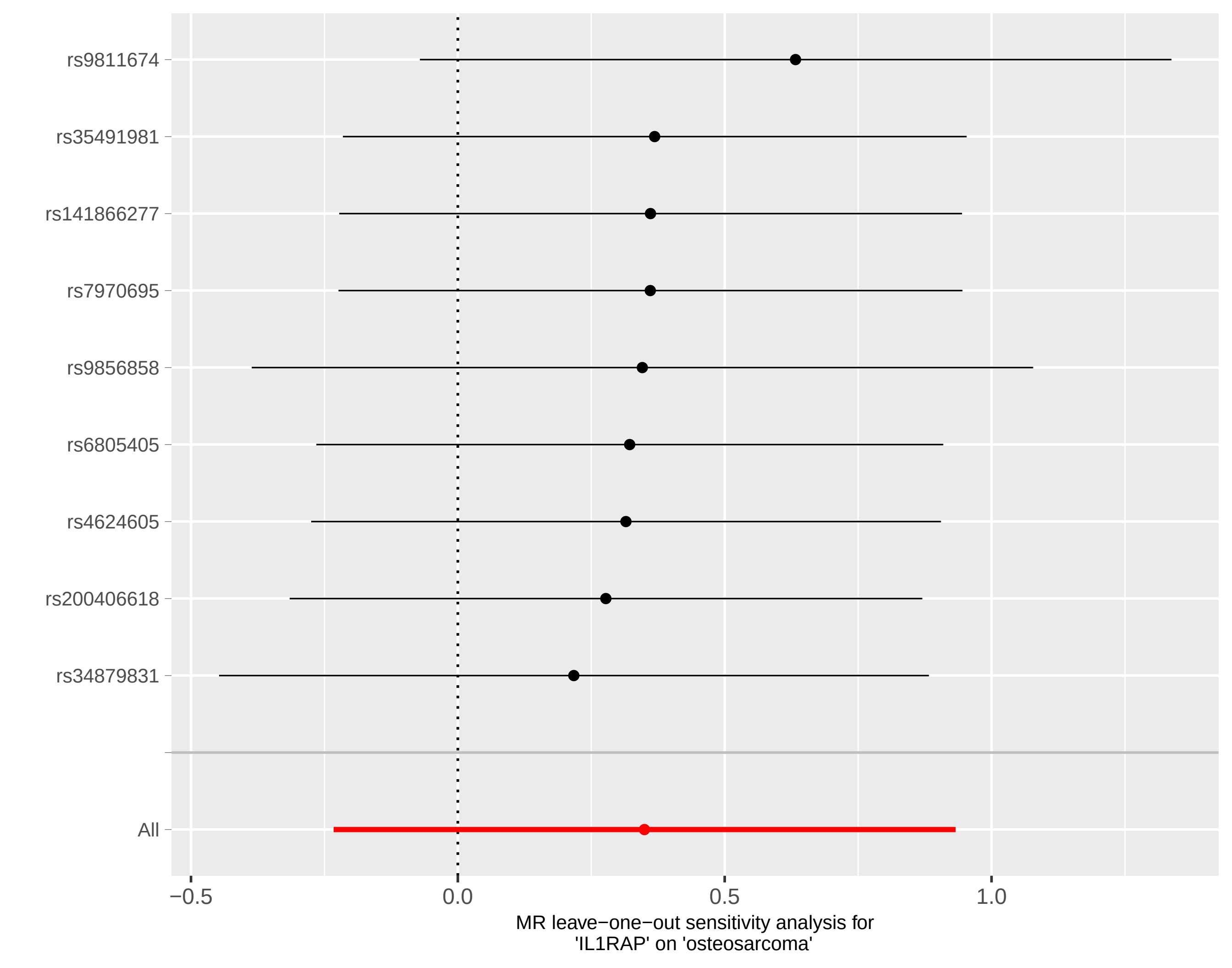

rs9811674
rs35491981
rs141866277
rs7970695
rs9856858
rs6805405
rs4624605
rs200406618
rs34879831
All
−0.5
0.0
0.5
1.0
MR leave−one−out sensitivity analysis for
'IL1RAP' on 'osteosarcoma'

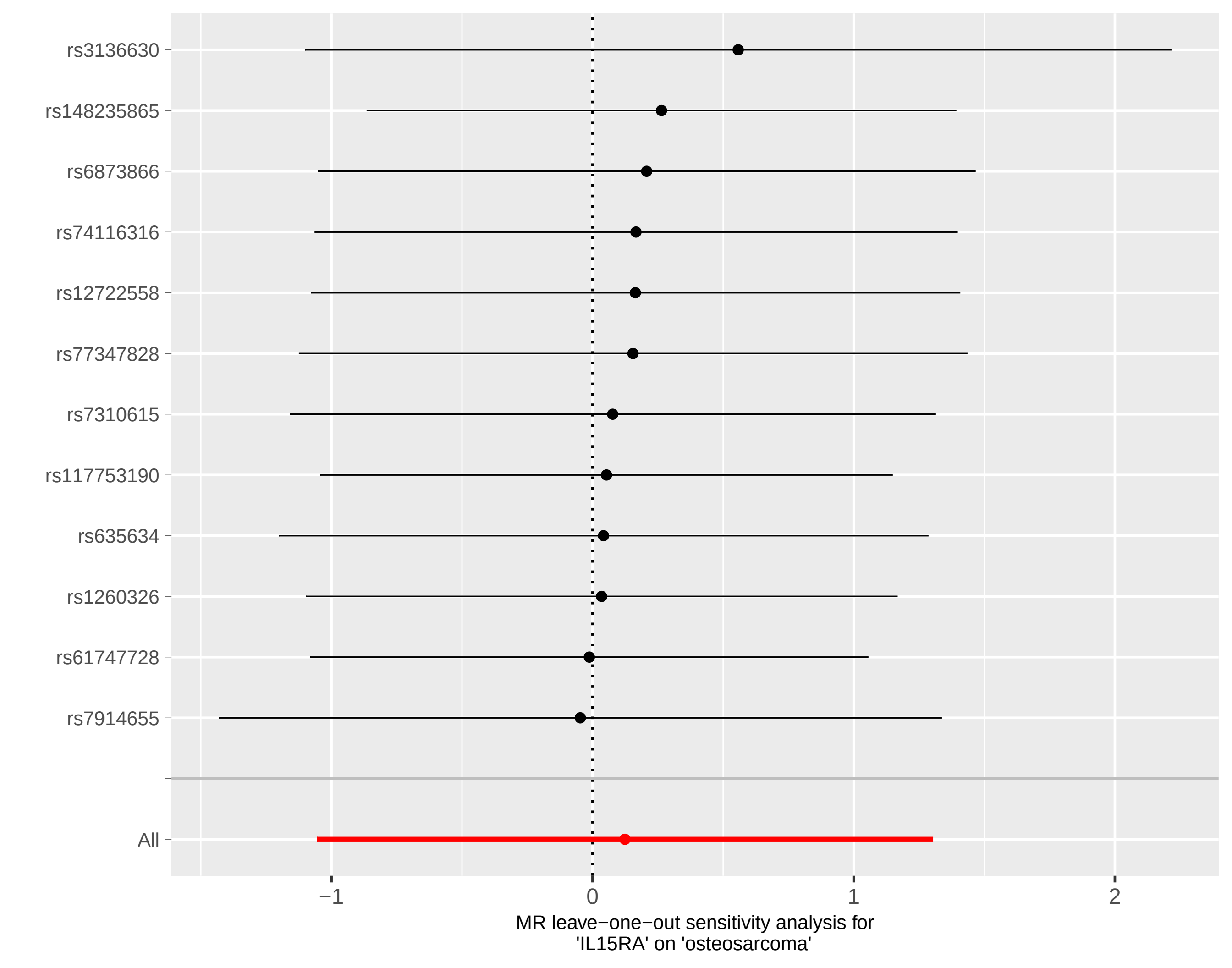
rs3136630
rs148235865
rs6873866
rs74116316
rs12722558
rs77347828
rs7310615
rs117753190
rs635634
rs1260326
rs61747728
rs7914655
All
−1
0
1
2
MR leave−one−out sensitivity analysis for
'IL15RA' on 'osteosarcoma'

Insufficient number of SNPs

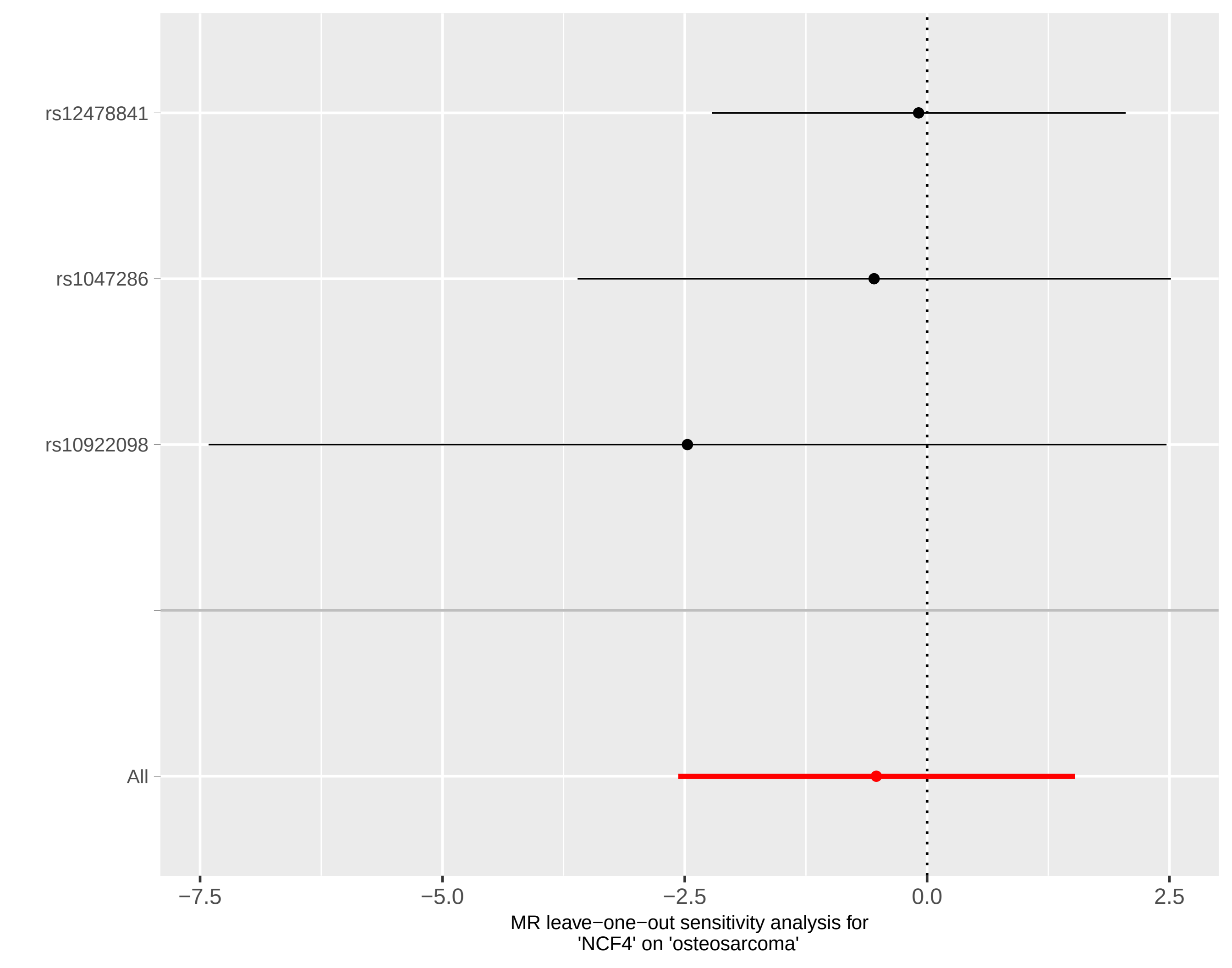
rs12478841
rs1047286
rs10922098
All
−7.5
−5.0
−2.5
0.0
2.5
MR leave−one−out sensitivity analysis for
'NCF4' on 'osteosarcoma'

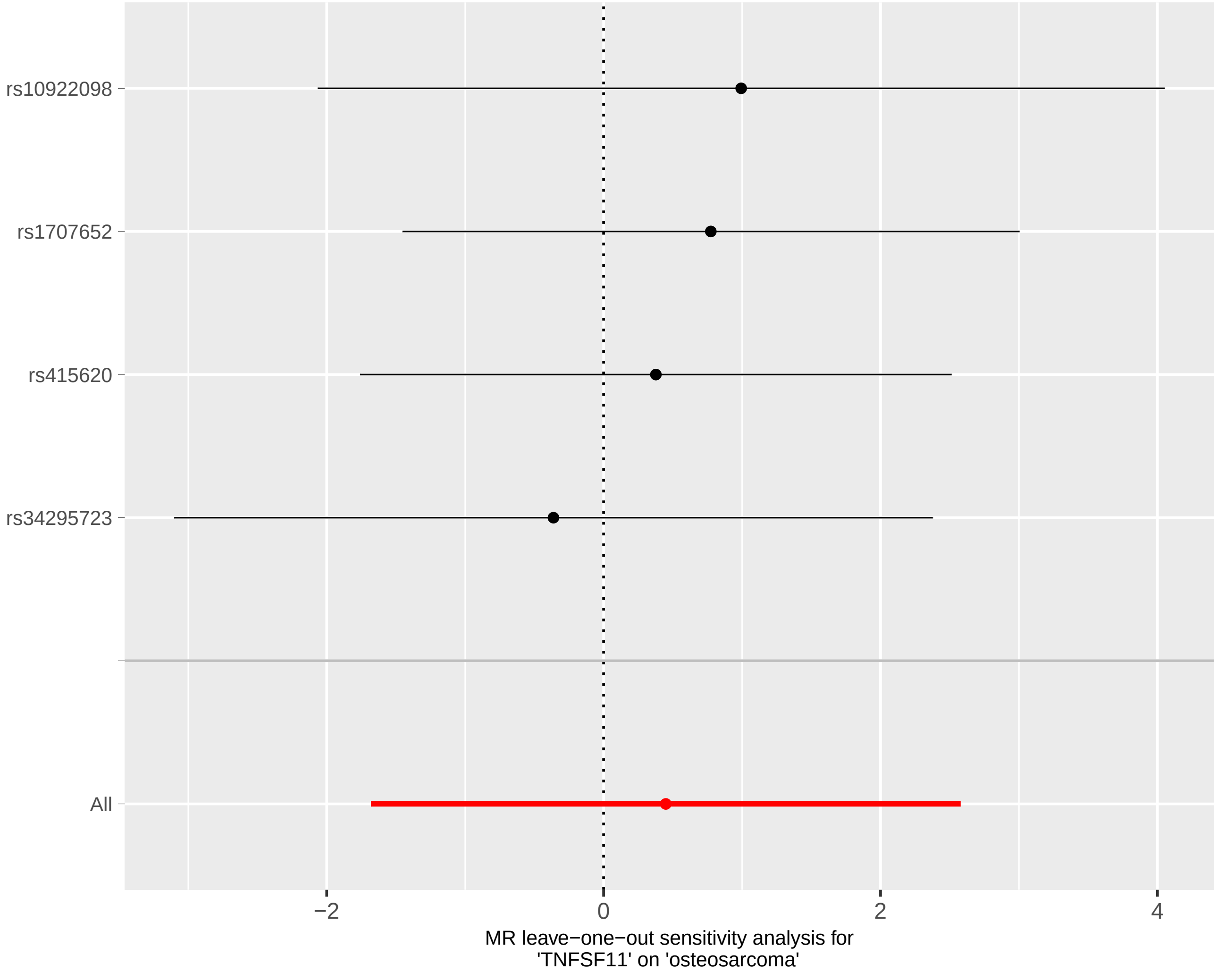
rs10922098
rs1707652
rs415620
rs34295723
All
−2
0
2
4
MR leave−one−out sensitivity analysis for
'TNFSF11' on 'osteosarcoma'

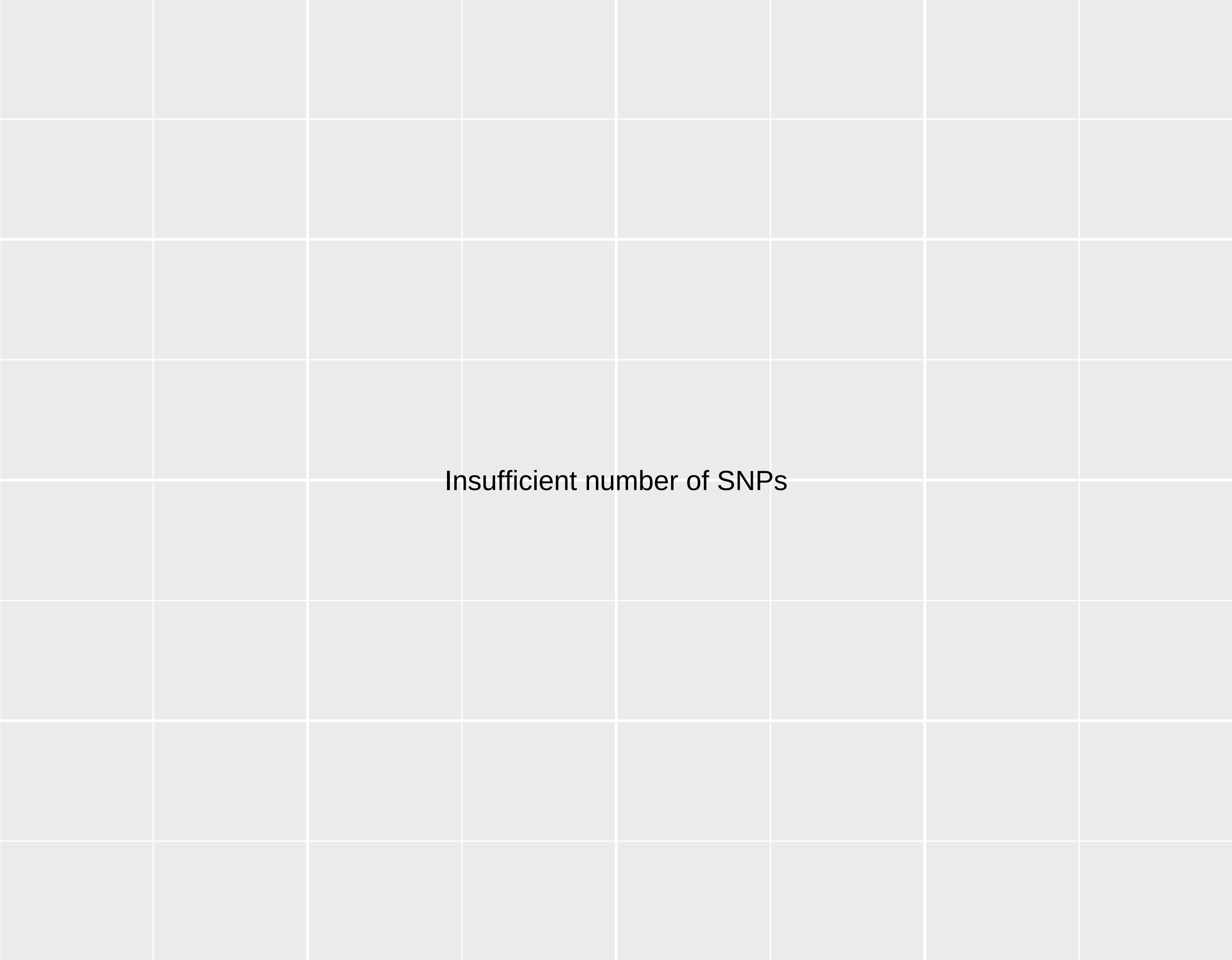
Insufficient number of SNPs

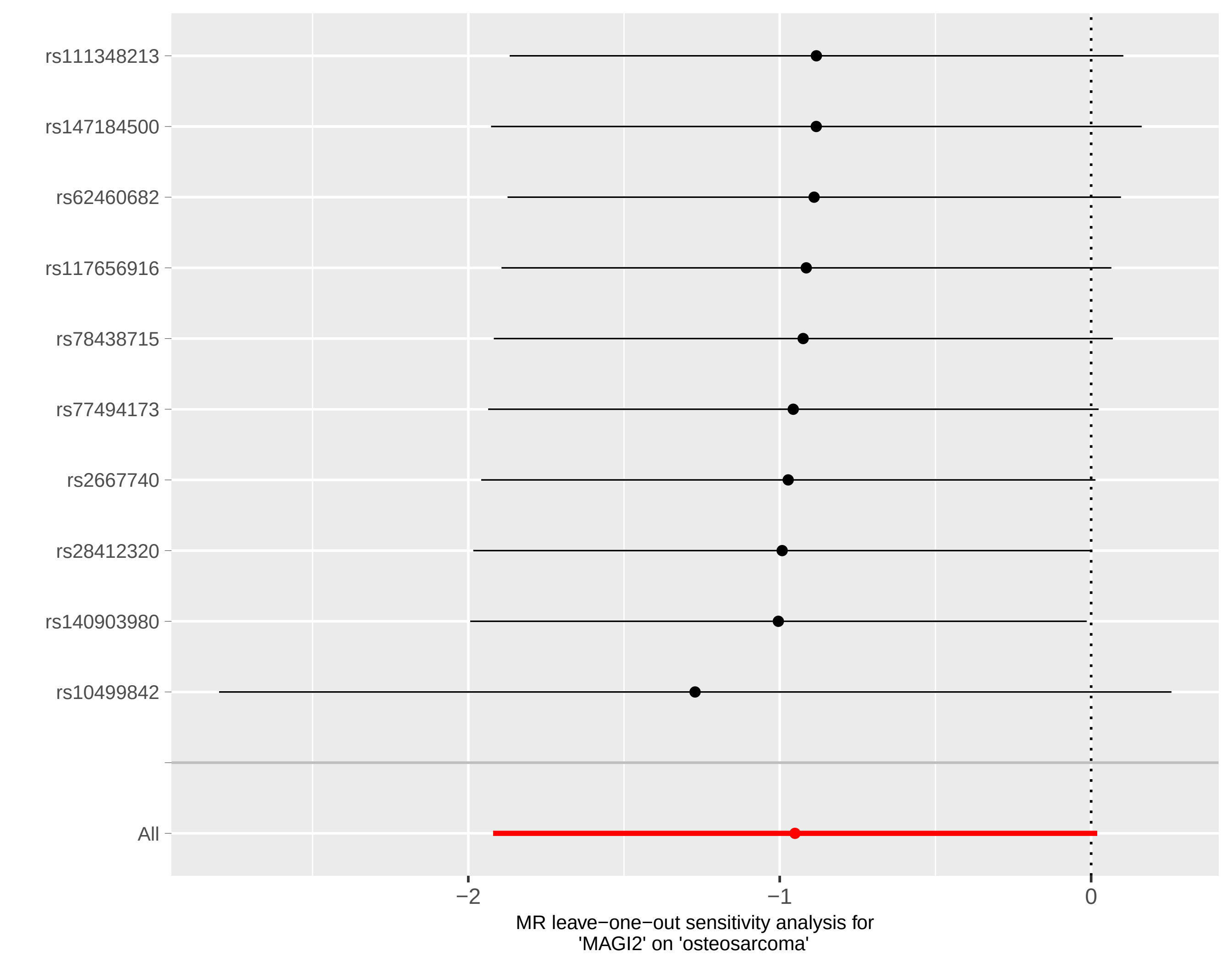
rs111348213
rs147184500
rs62460682
rs117656916
rs78438715
rs77494173
rs2667740
rs28412320
rs140903980
rs10499842
All
−2
−1
0
MR leave−one−out sensitivity analysis for
'MAGI2' on 'osteosarcoma'

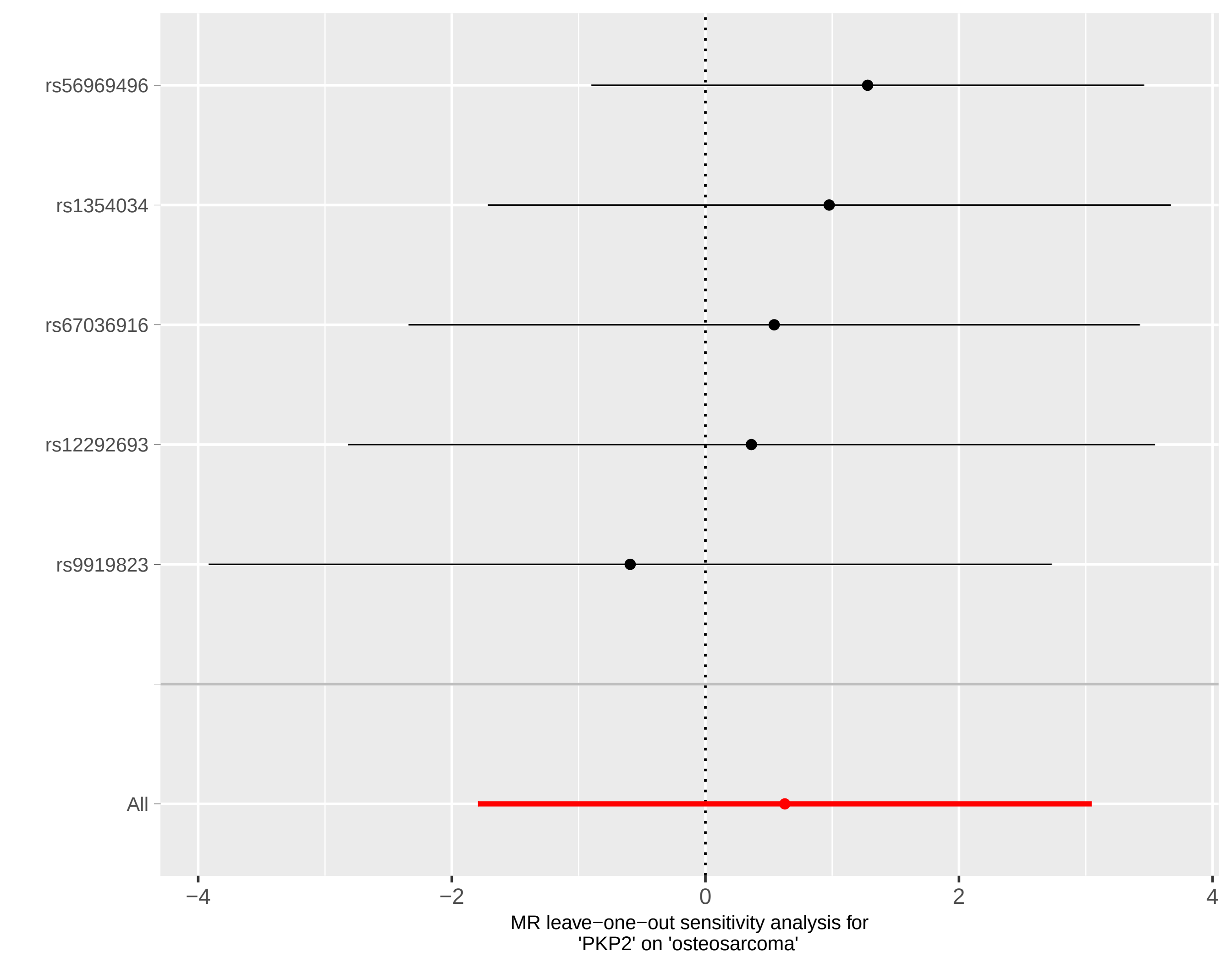

rs56969496
rs1354034
rs67036916
rs12292693
rs9919823
All
−4
−2
0
2
4
MR leave−one−out sensitivity analysis for
'PKP2' on 'osteosarcoma'

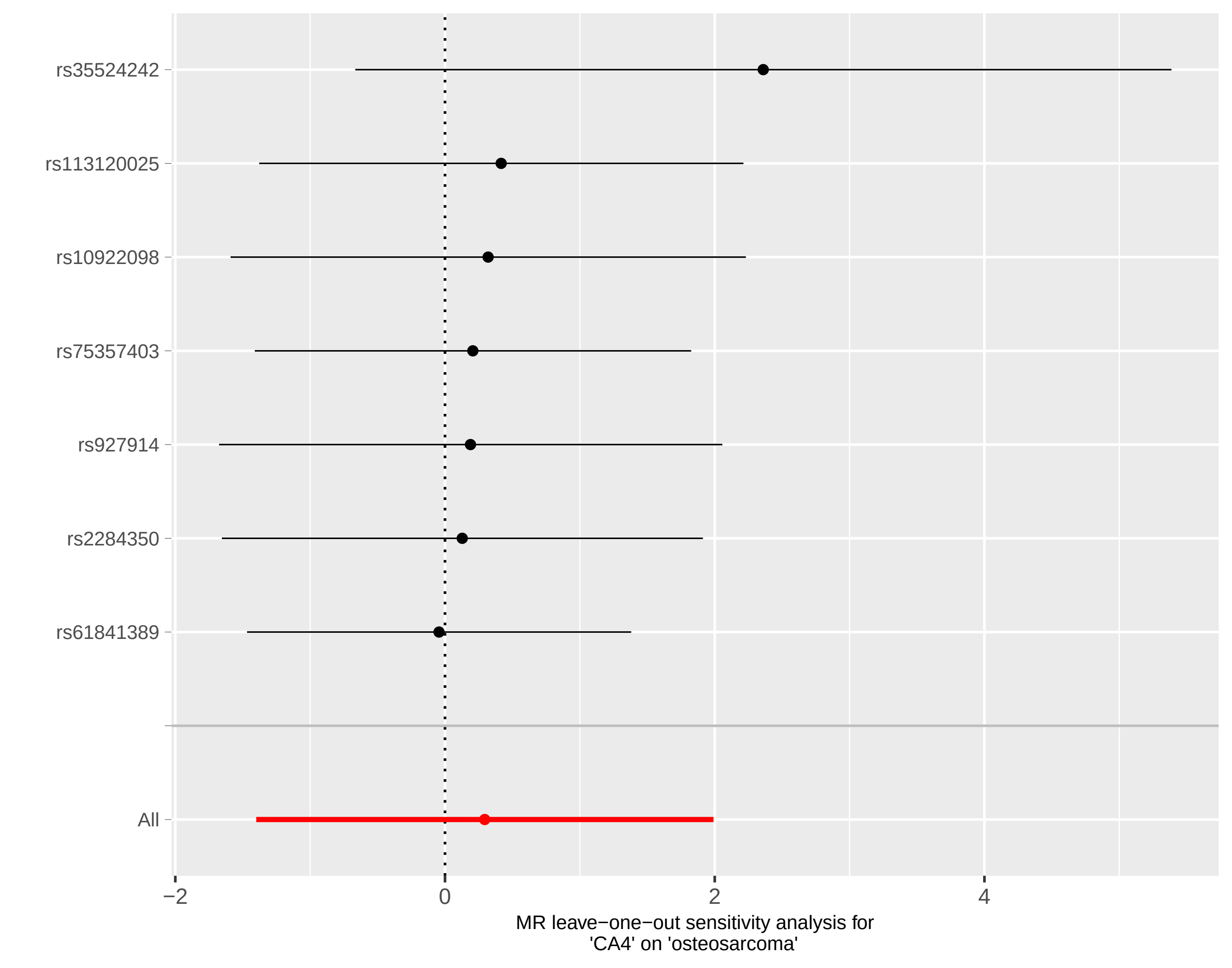

rs35524242
rs113120025
rs10922098
rs75357403
rs927914
rs2284350
rs61841389
All
−2
0
2
4
MR leave−one−out sensitivity analysis for
'CA4' on 'osteosarcoma'

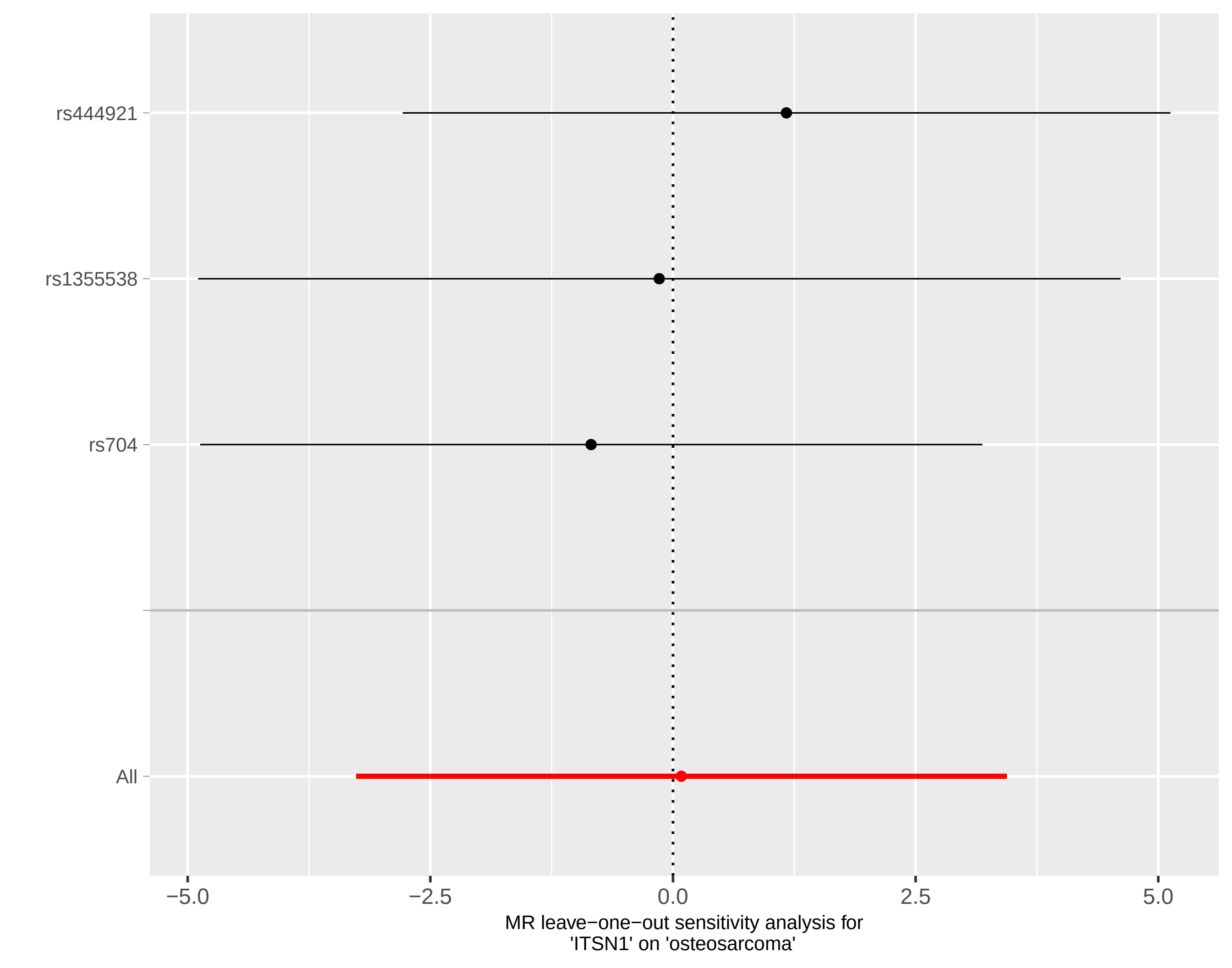
rs444921
rs1355538
rs704
All
−5.0
−2.5
0.0
2.5
5.0
MR leave−one−out sensitivity analysis for
'ITSN1' on 'osteosarcoma'

Insufficient number of SNPs

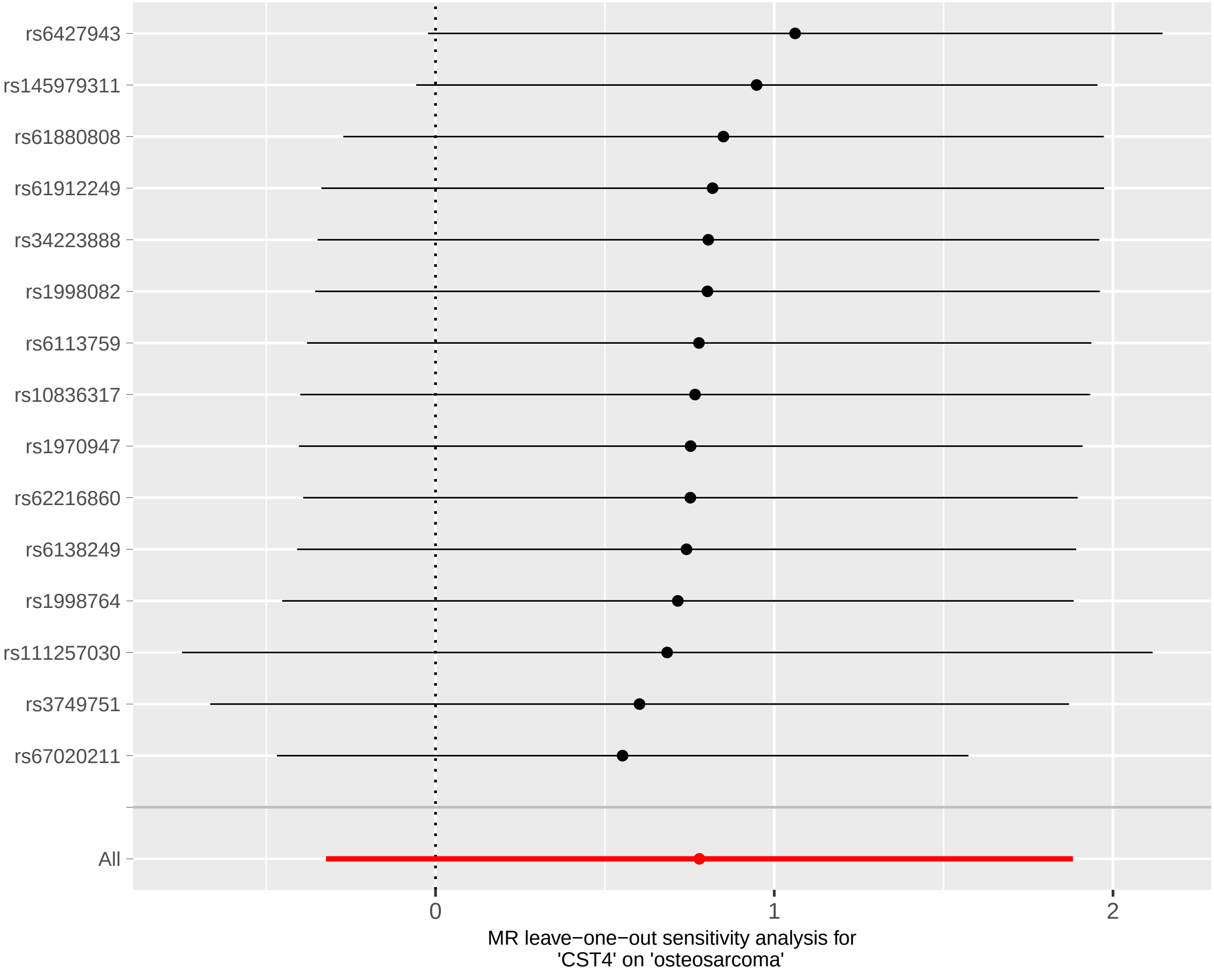
rs6427943
rs145979311
rs61880808
rs61912249
rs34223888
rs1998082
rs6113759
rs10836317
rs1970947
rs62216860
rs6138249
rs1998764
rs111257030
rs3749751
rs67020211
All
0
1
2
MR leave−one−out sensitivity analysis for
'CST4' on 'osteosarcoma'

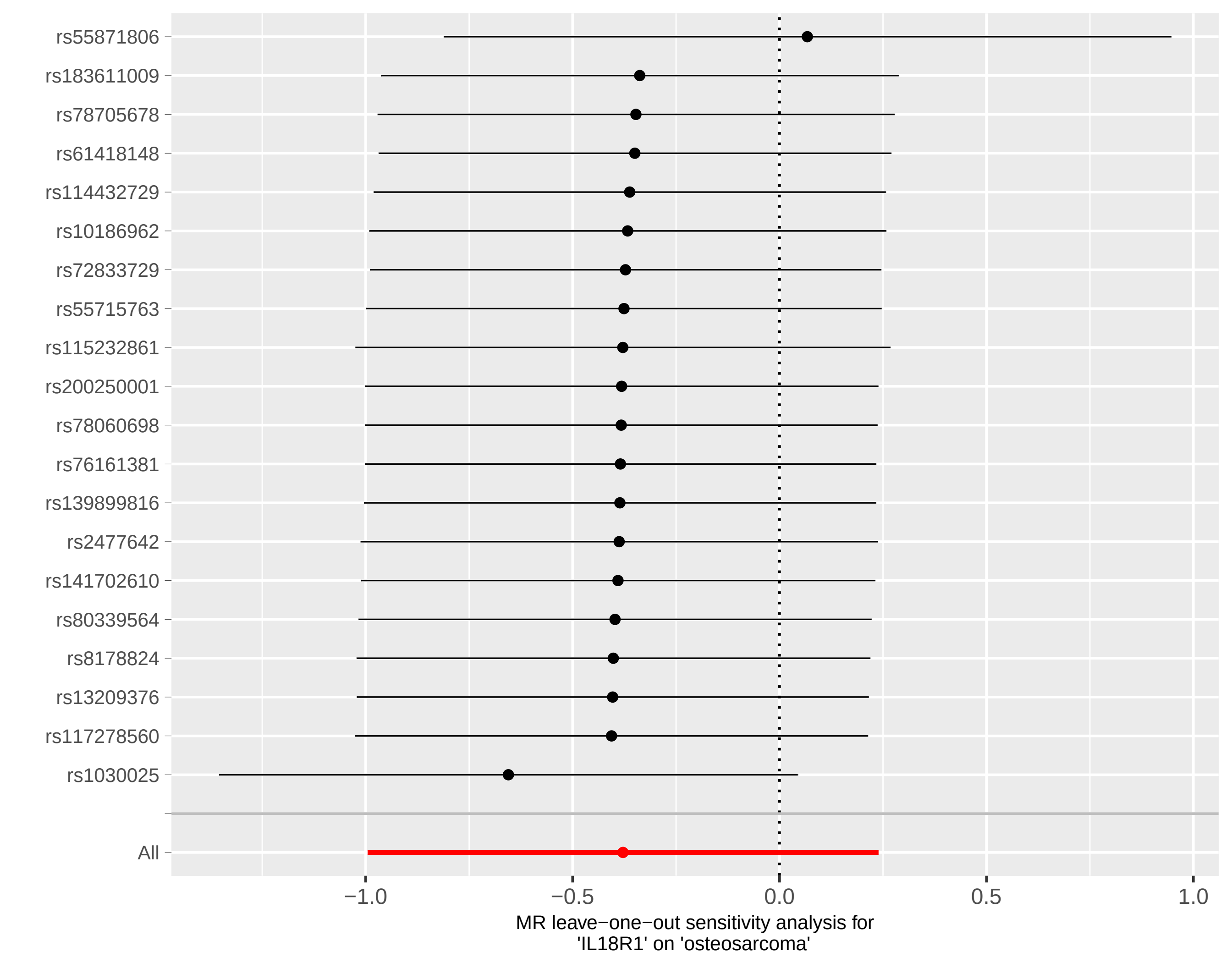
rs55871806
rs183611009
rs78705678
rs61418148
rs114432729
rs10186962
rs72833729
rs55715763
rs115232861
rs200250001
rs78060698
rs76161381
rs139899816
rs2477642
rs141702610
rs80339564
rs8178824
rs13209376
rs117278560
rs1030025
All
−1.0
−0.5
0.0
0.5
1.0
MR leave−one−out sensitivity analysis for
'IL18R1' on 'osteosarcoma'

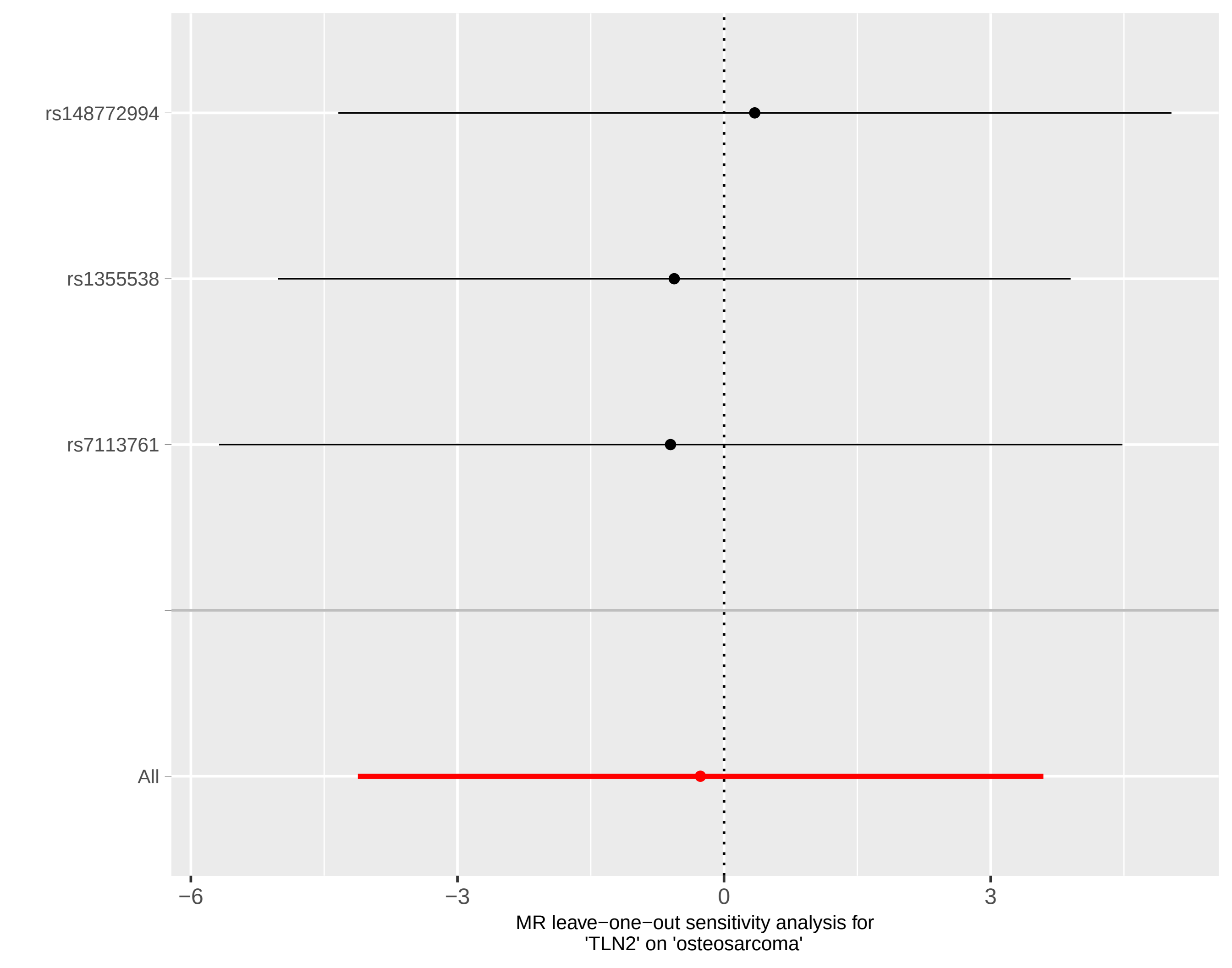

rs148772994
rs1355538
rs7113761
All
−6
−3
0
3
MR leave−one−out sensitivity analysis for
'TLN2' on 'osteosarcoma'

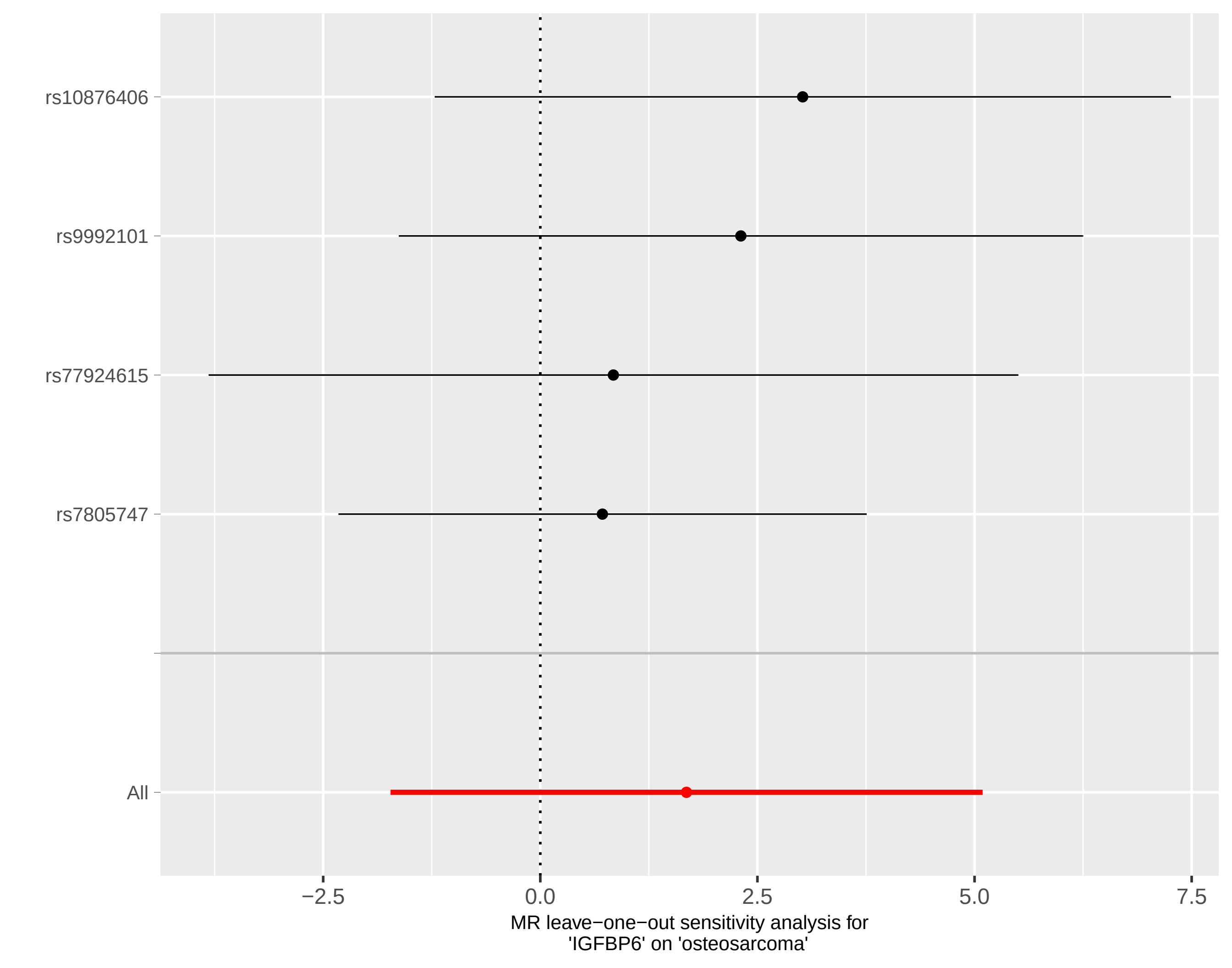

rs10876406
rs9992101
rs77924615
rs7805747
All
−2.5
0.0
2.5
5.0
7.5
MR leave−one−out sensitivity analysis for
'IGFBP6' on 'osteosarcoma'

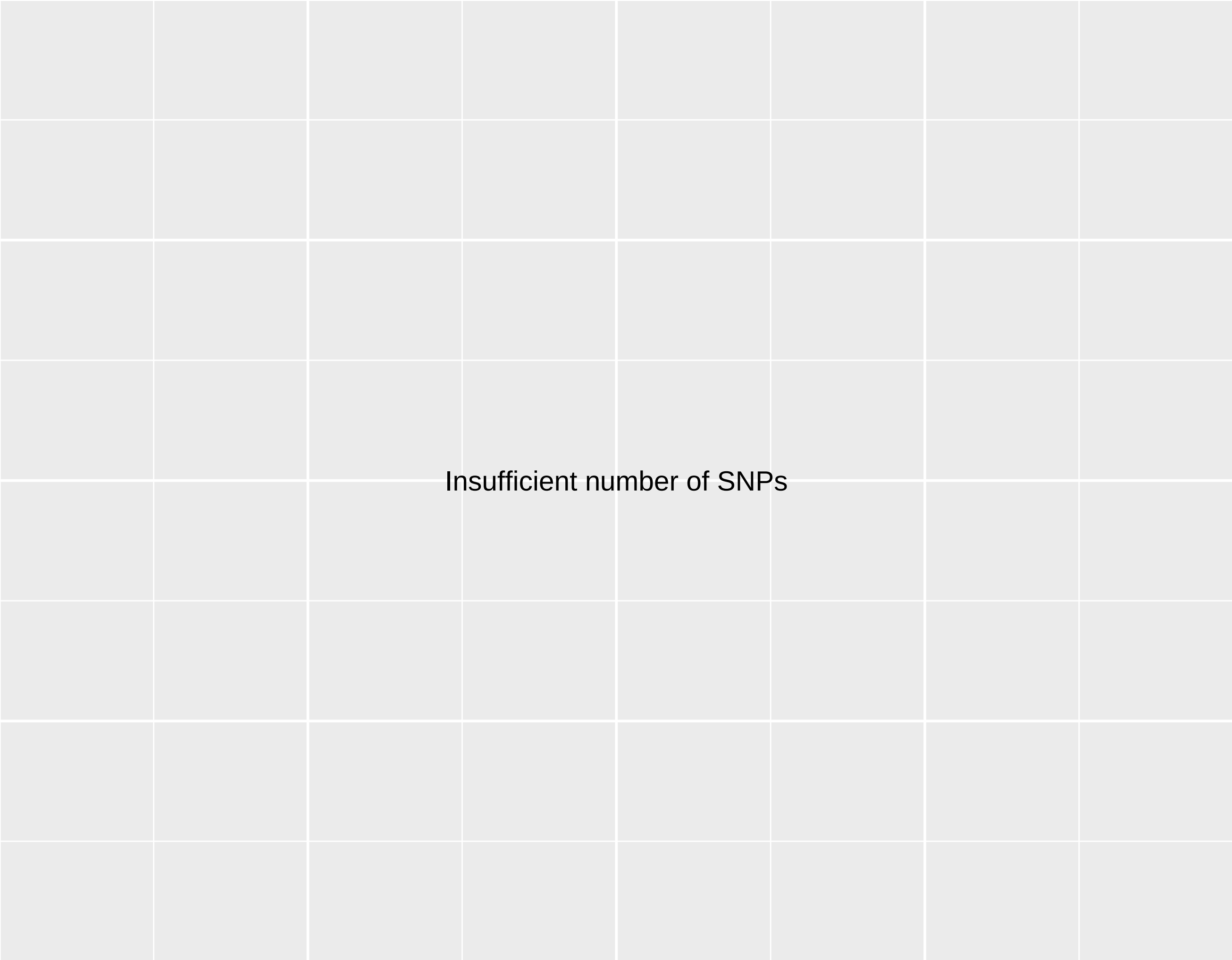
Insufficient number of SNPs

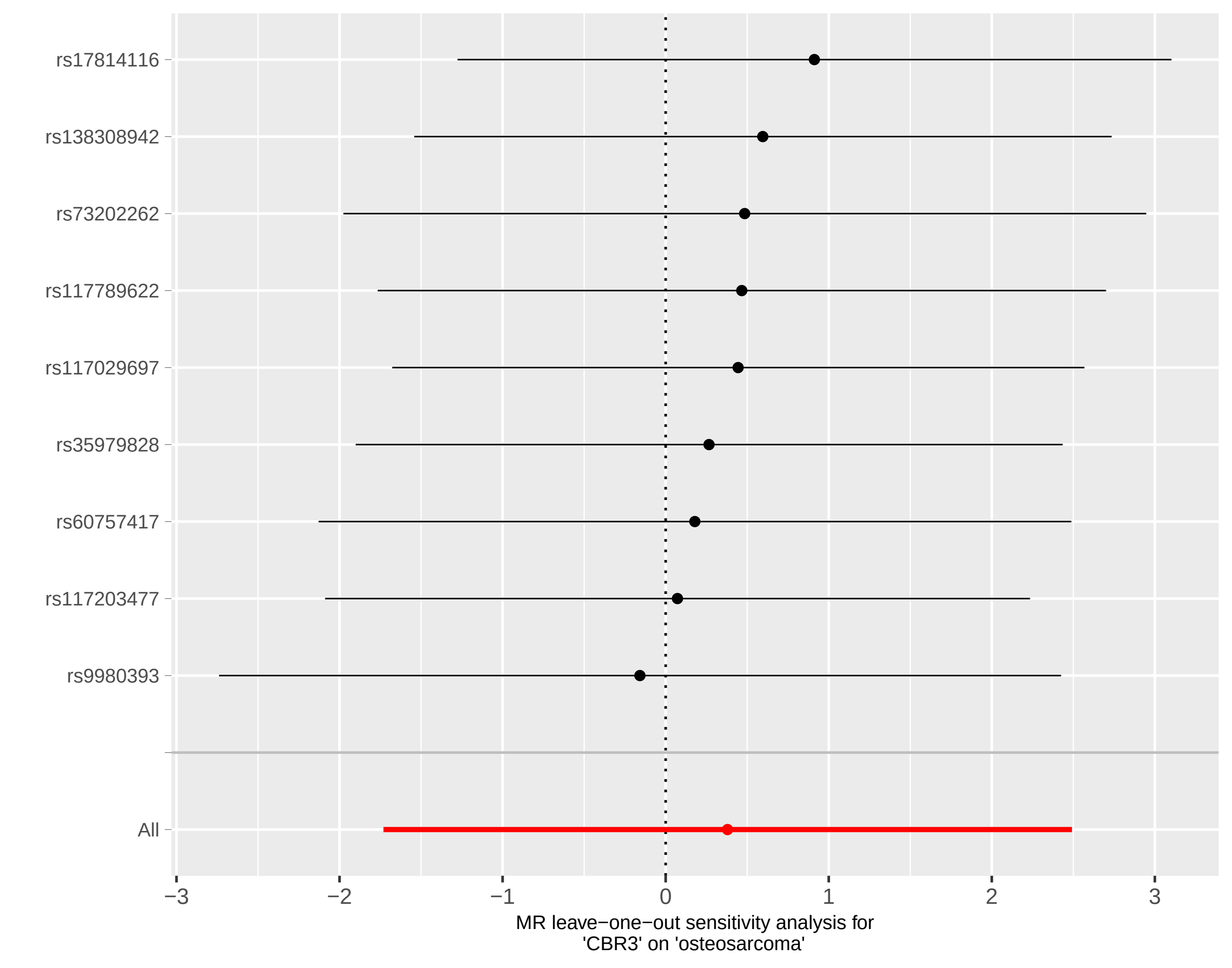

rs17814116
rs138308942
rs73202262
rs117789622
rs117029697
rs35979828
rs60757417
rs117203477
rs9980393
All
−3
−2
−1
0
1
2
3
MR leave−one−out sensitivity analysis for
'CBR3' on 'osteosarcoma'

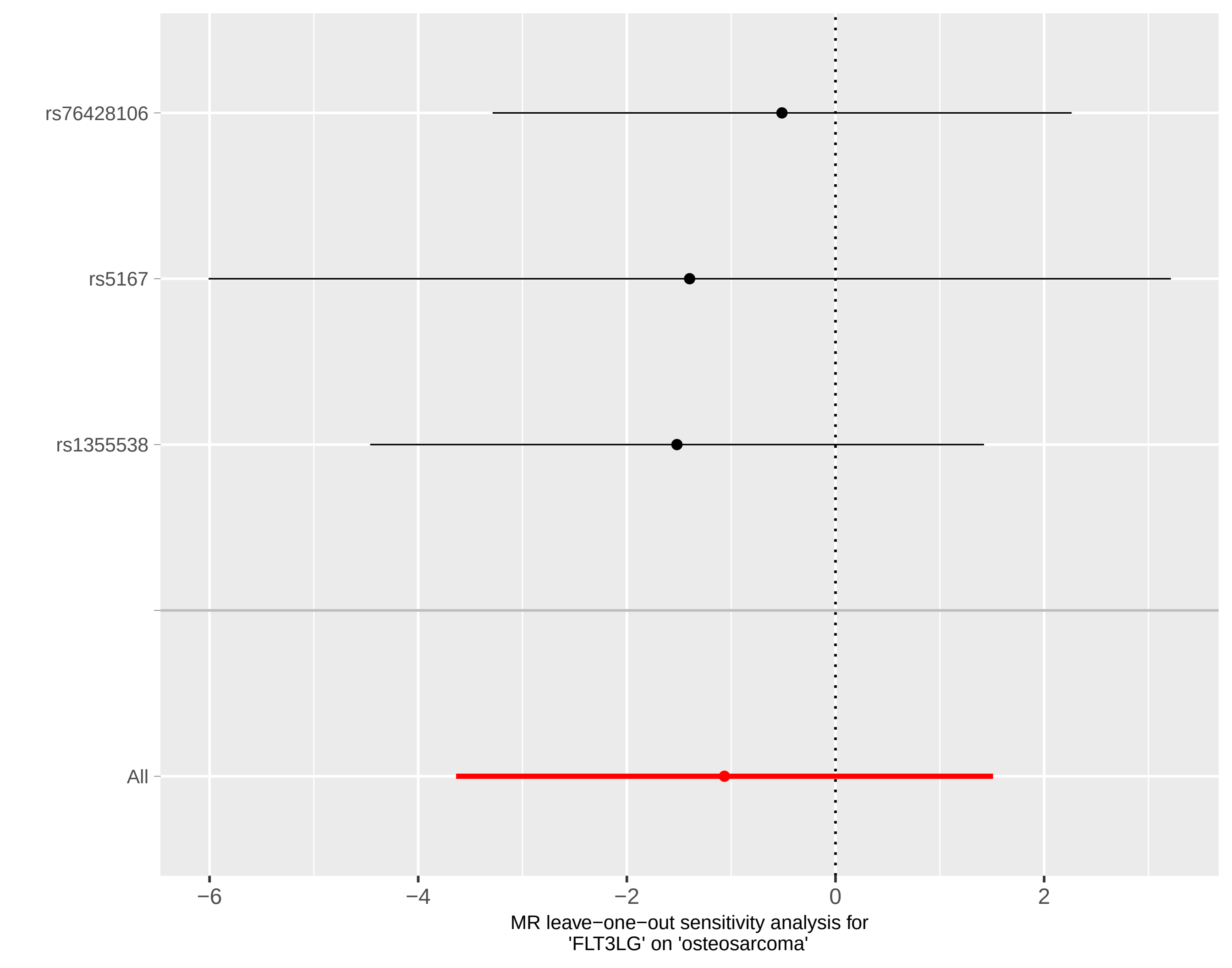
rs76428106
rs5167
rs1355538
All
−6
−4
−2
0
2
MR leave−one−out sensitivity analysis for
'FLT3LG' on 'osteosarcoma'

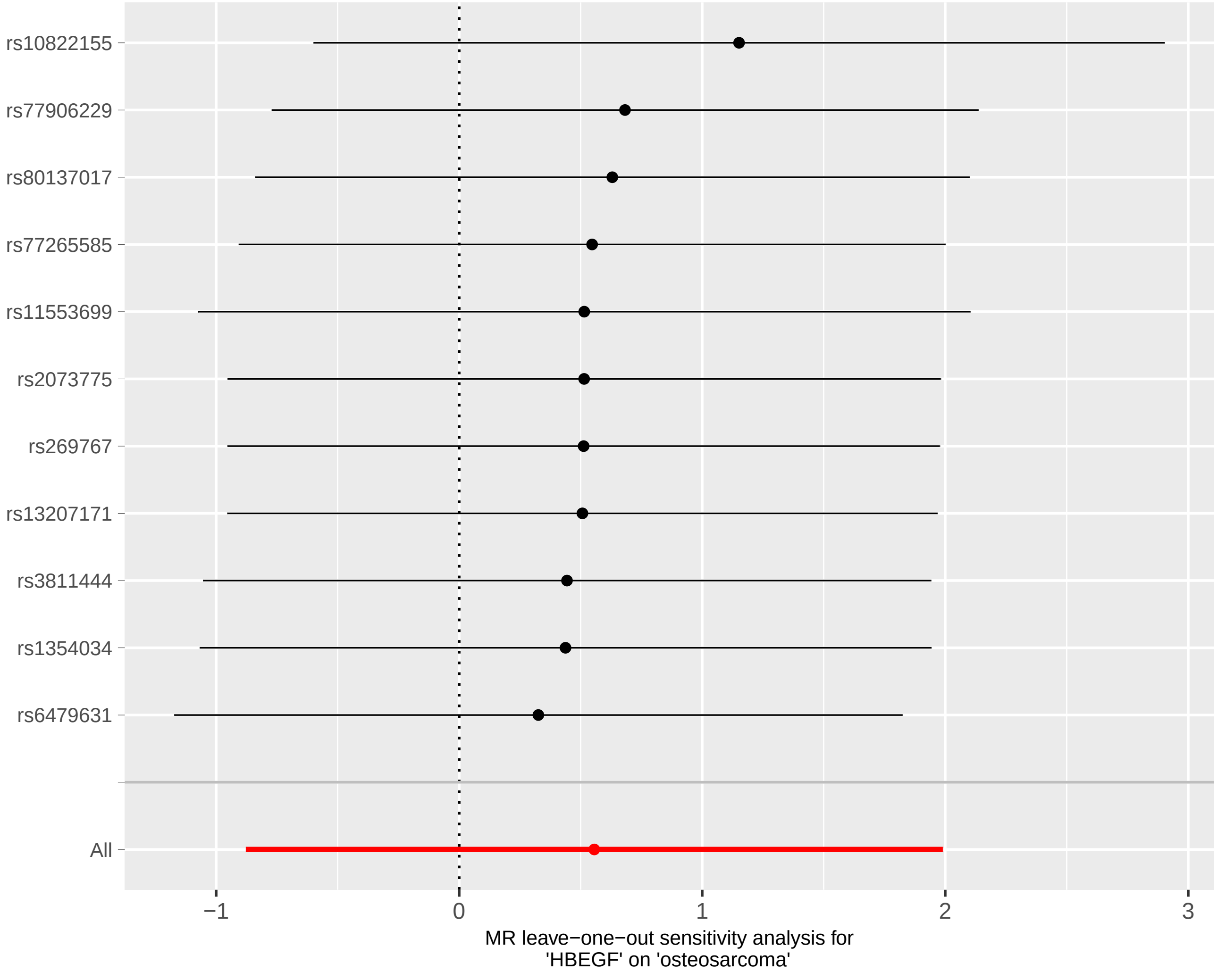

rs10822155
rs77906229
rs80137017
rs77265585
rs11553699
rs2073775
rs269767
rs13207171
rs3811444
rs1354034
rs6479631
All
−1
0
1
2
3
MR leave−one−out sensitivity analysis for
'HBEGF' on 'osteosarcoma'

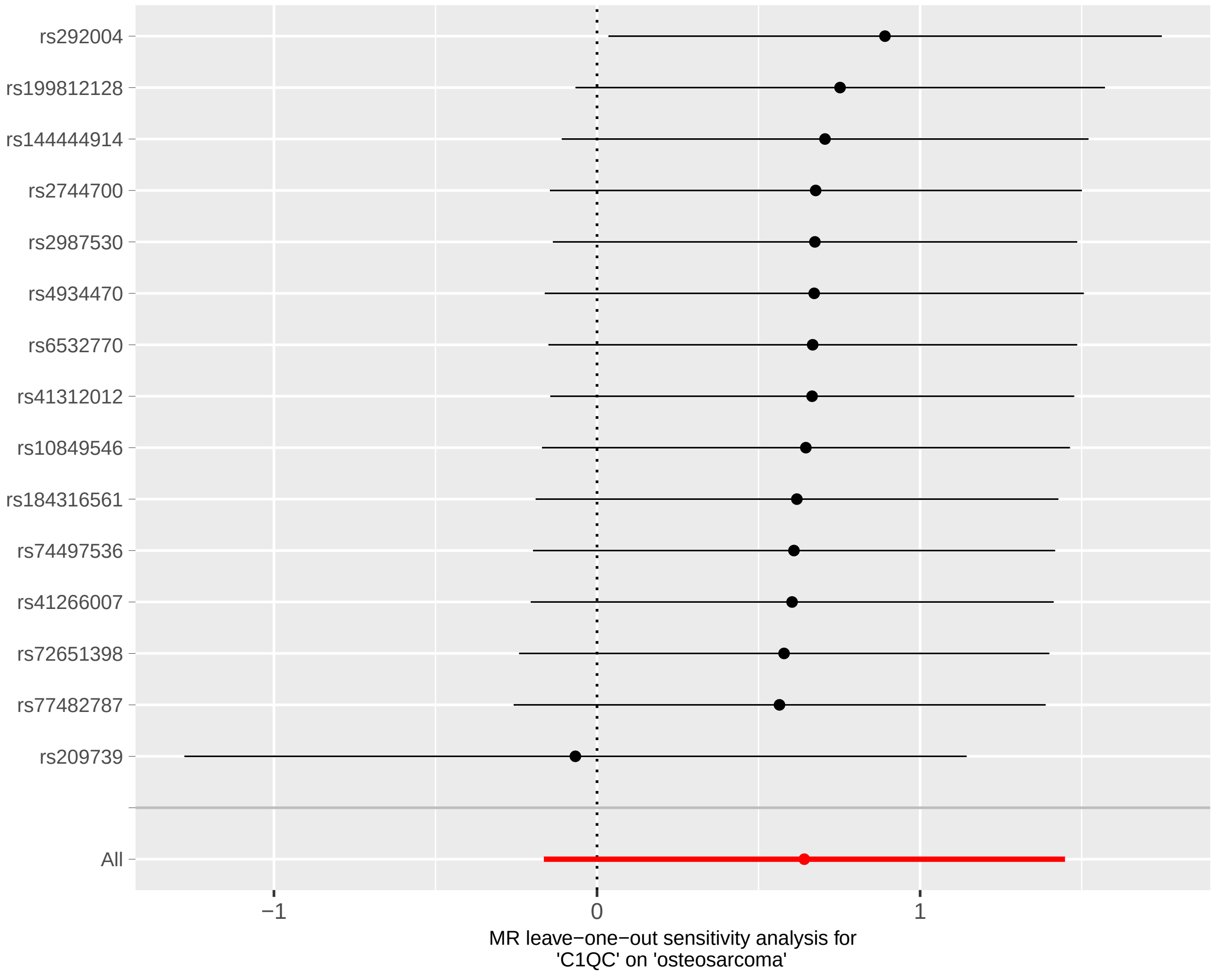
rs292004
rs199812128
rs144444914
rs2744700
rs2987530
rs4934470
rs6532770
rs41312012
rs10849546
rs184316561
rs74497536
rs41266007
rs72651398
rs77482787
rs209739
All
−1
0
1
MR leave−one−out sensitivity analysis for
'C1QC' on 'osteosarcoma'

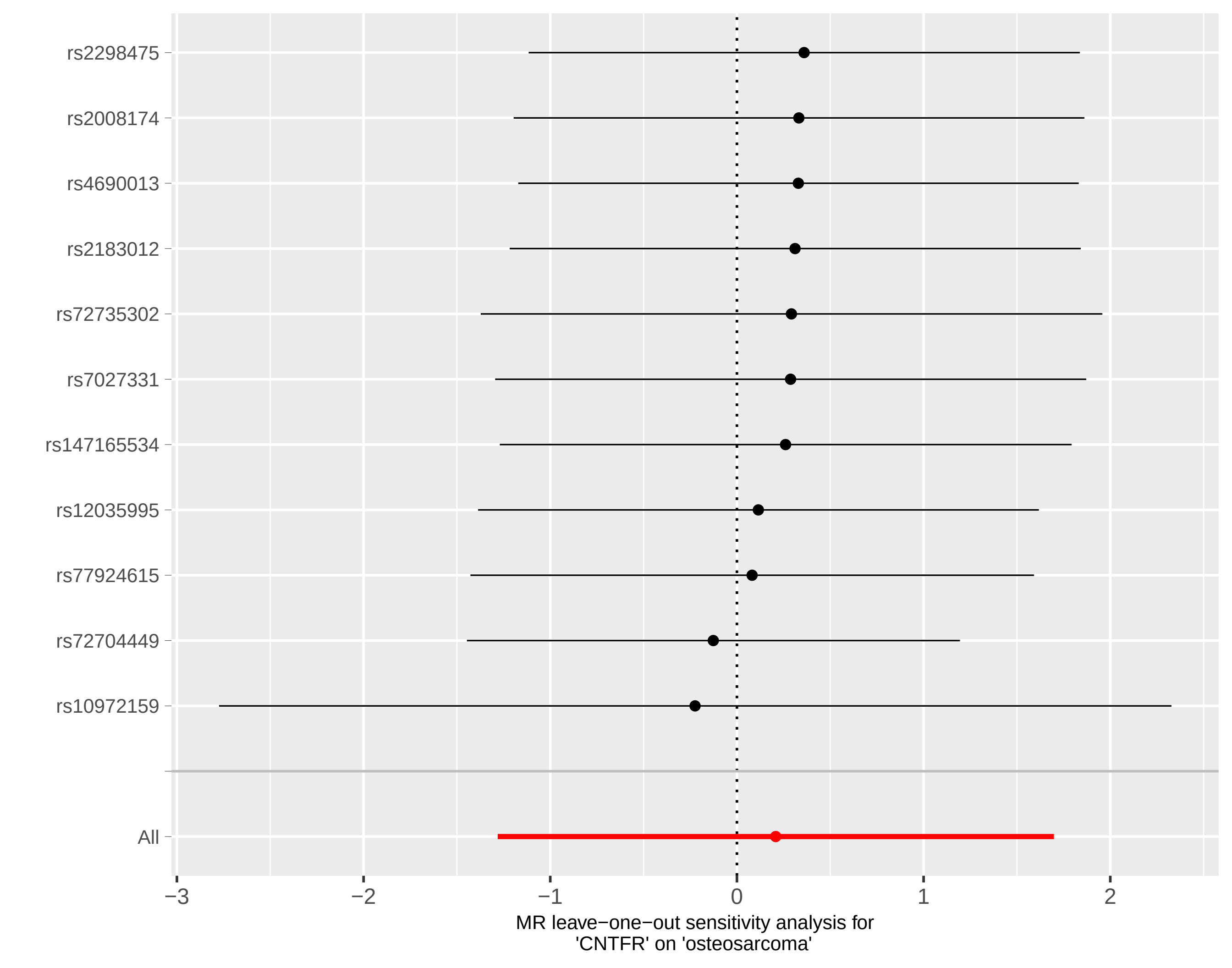
rs2298475
rs2008174
rs4690013
rs2183012
rs72735302
rs7027331
rs147165534
rs12035995
rs77924615
rs72704449
rs10972159
All
−3
−2
−1
0
1
2
MR leave−one−out sensitivity analysis for
'CNTFR' on 'osteosarcoma'

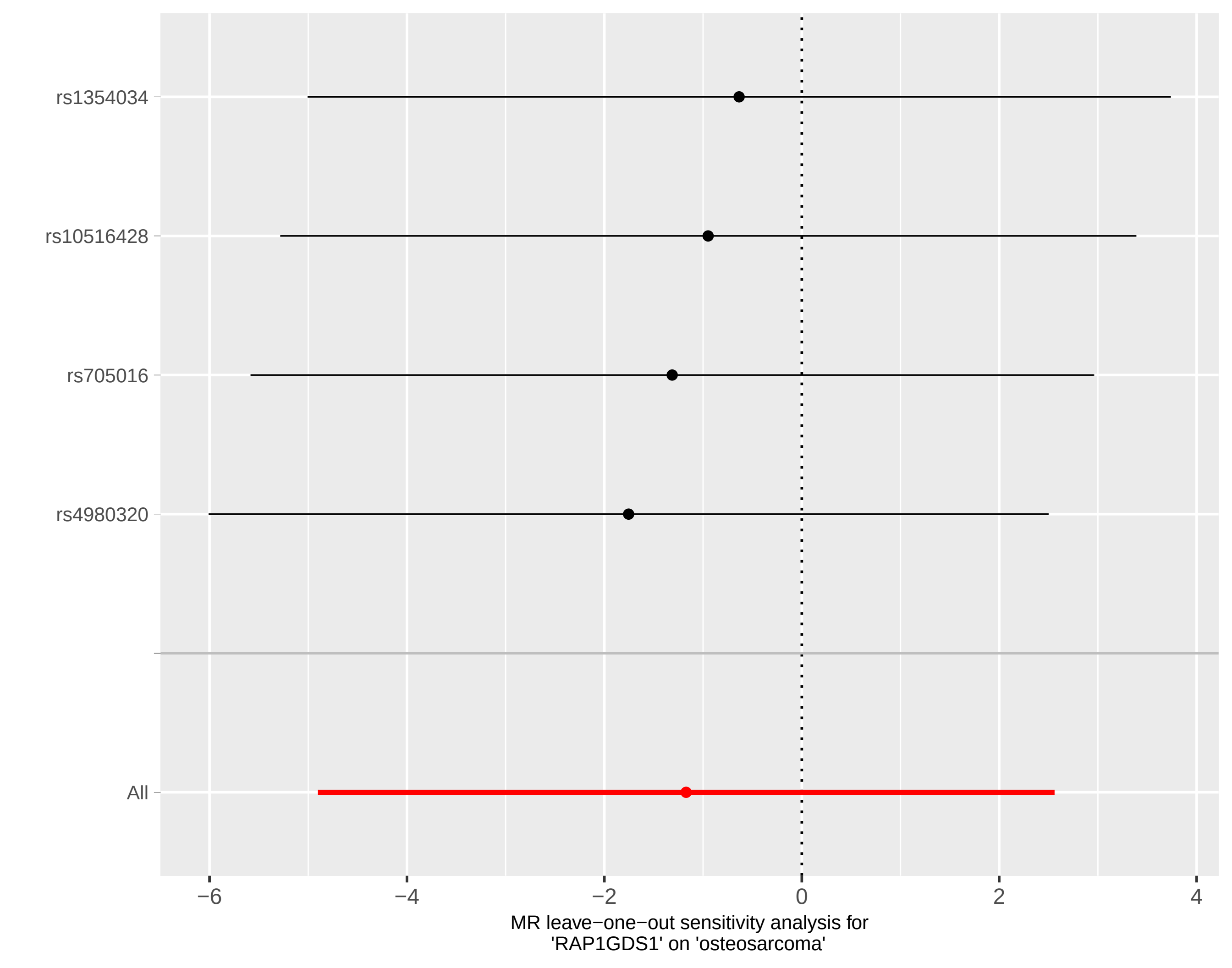
rs1354034
rs10516428
rs705016
rs4980320
All
−6
−4
−2
0
2
4
MR leave−one−out sensitivity analysis for
'RAP1GDS1' on 'osteosarcoma'

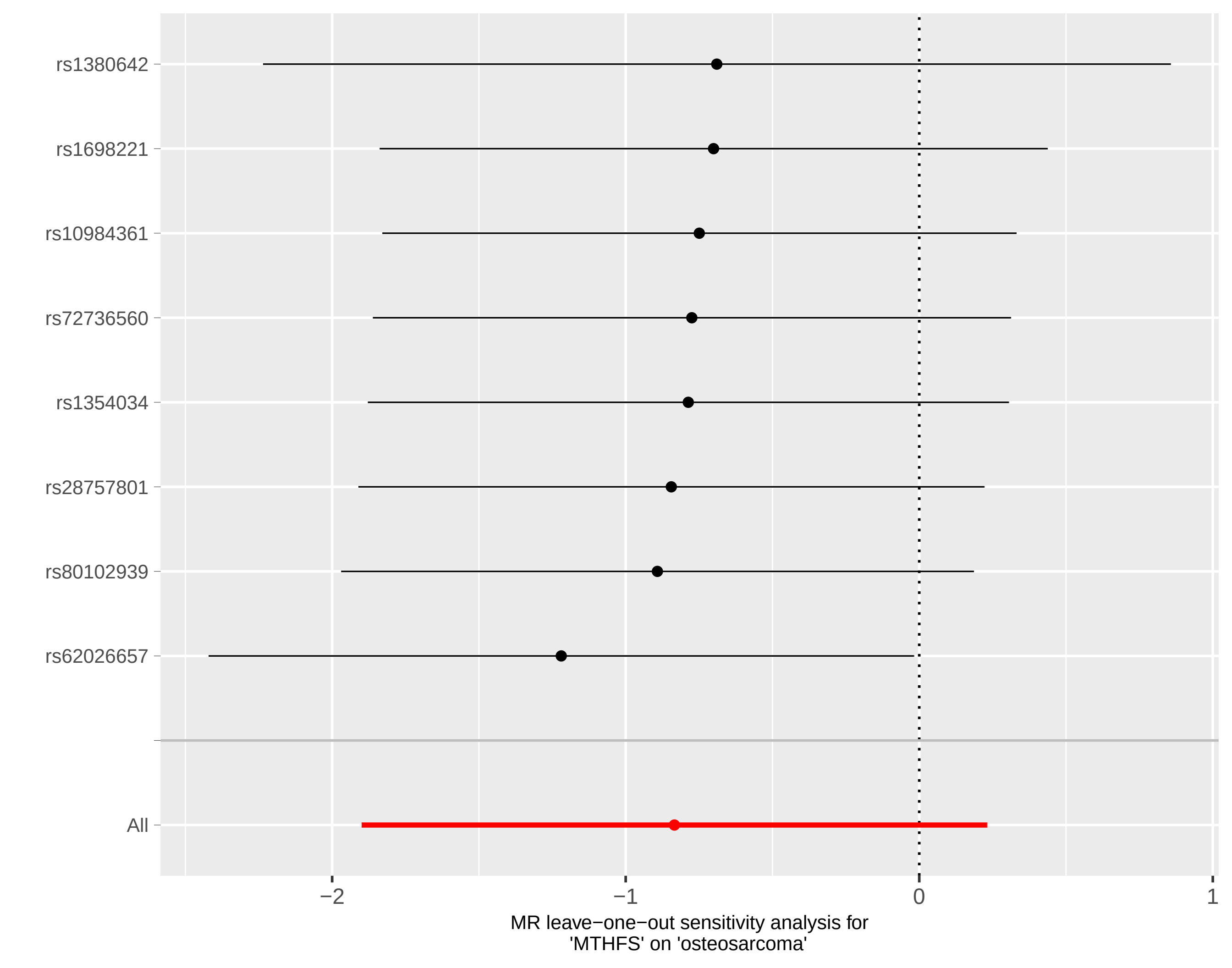
rs1380642
rs1698221
rs10984361
rs72736560
rs1354034
rs28757801
rs80102939
rs62026657
All
−2
−1
0
1
MR leave−one−out sensitivity analysis for
'MTHFS' on 'osteosarcoma'

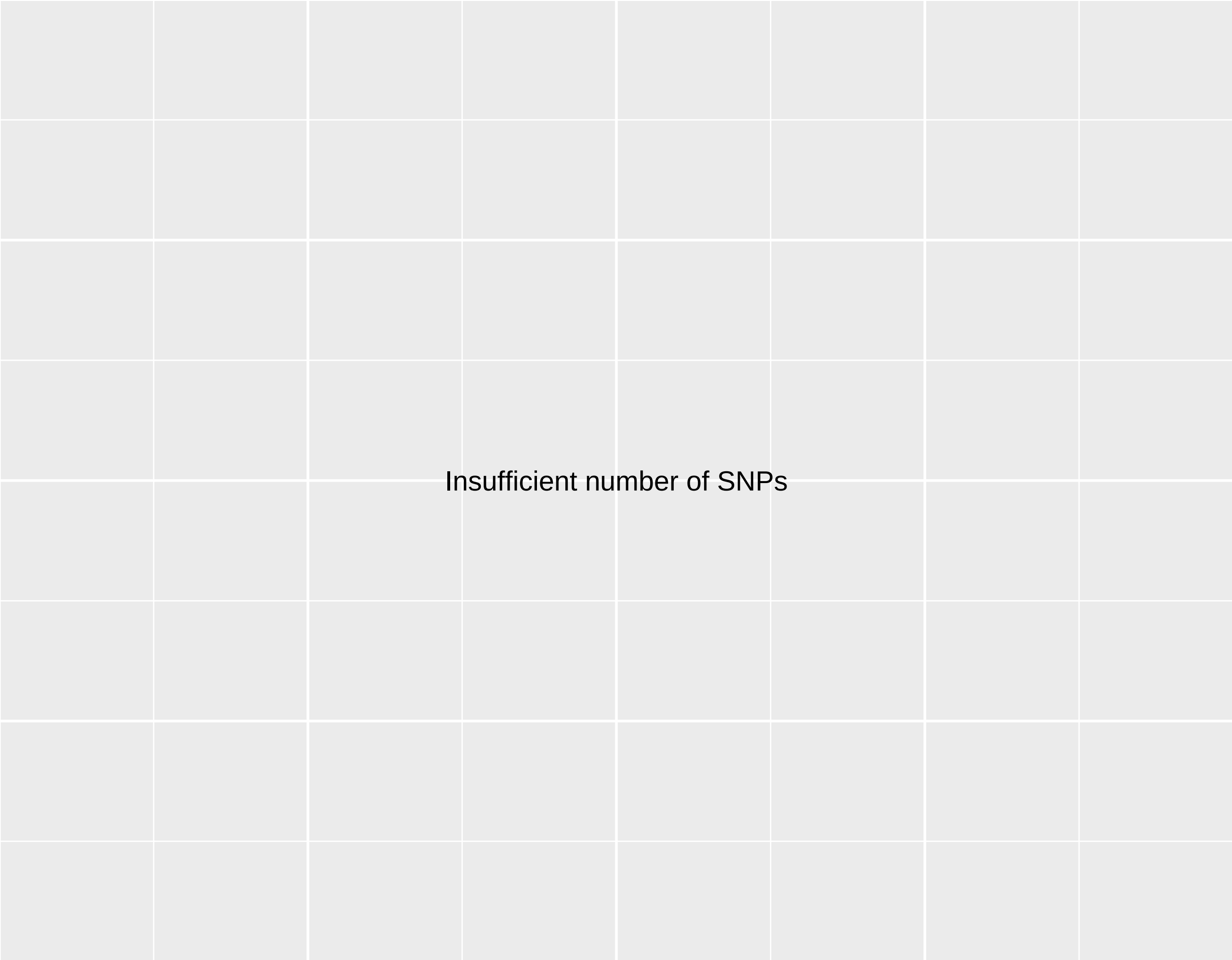
Insufficient number of SNPs

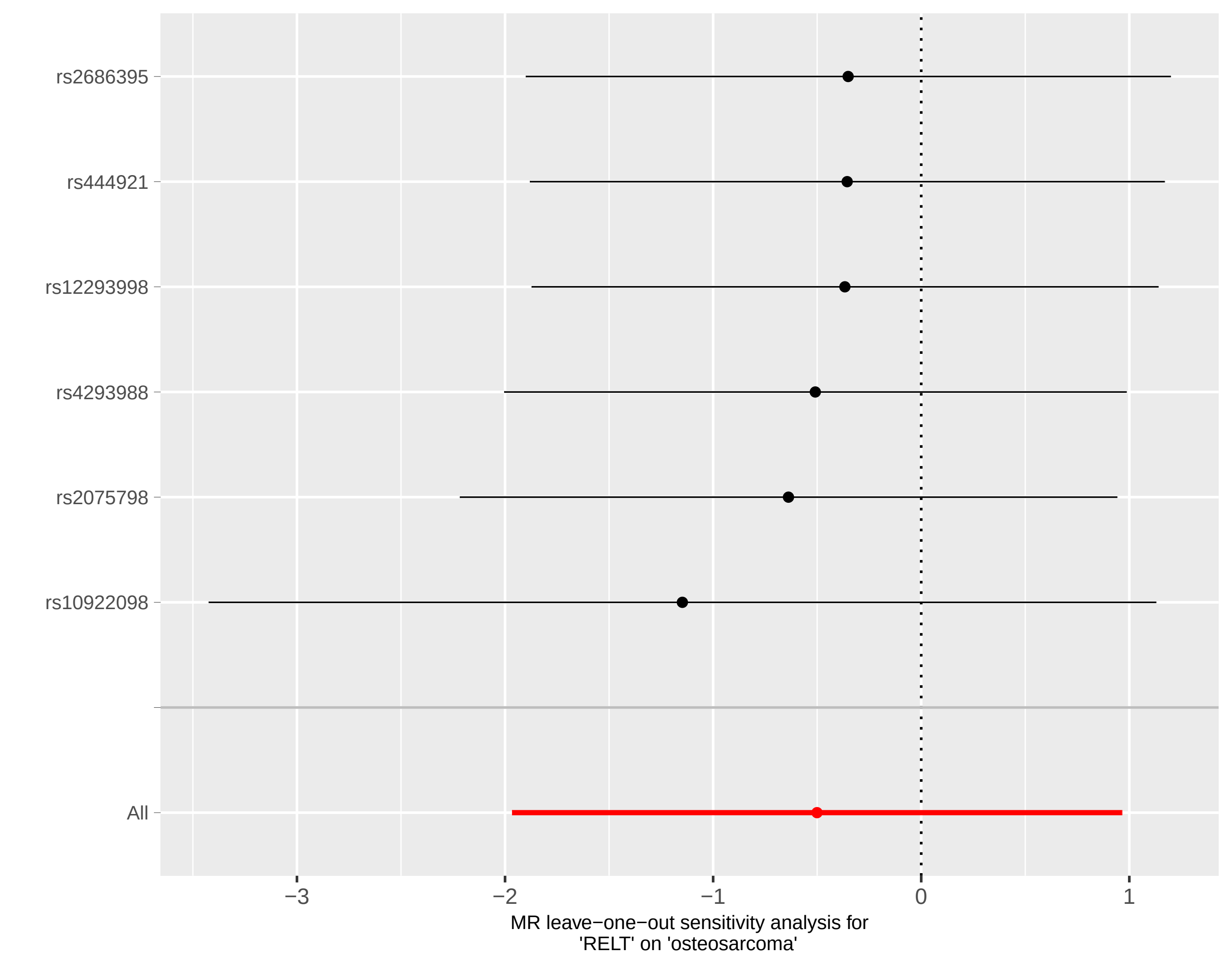
rs2686395
rs444921
rs12293998
rs4293988
rs2075798
rs10922098
All
−3
−2
−1
0
1
MR leave−one−out sensitivity analysis for
'RELT' on 'osteosarcoma'

Insufficient number of SNPs

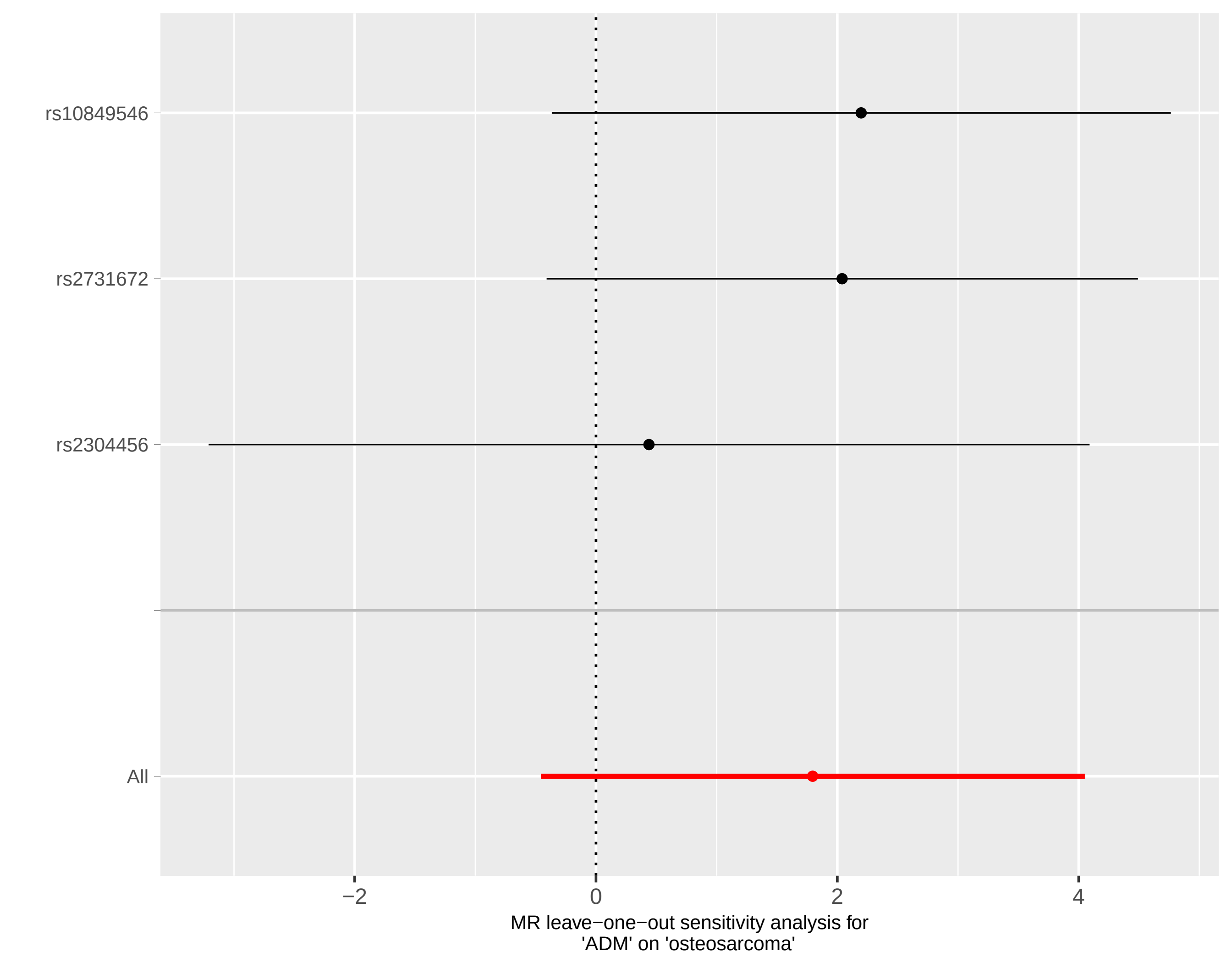
rs10849546
rs2731672
rs2304456
All
−2
0
2
4
MR leave−one−out sensitivity analysis for
'ADM' on 'osteosarcoma'

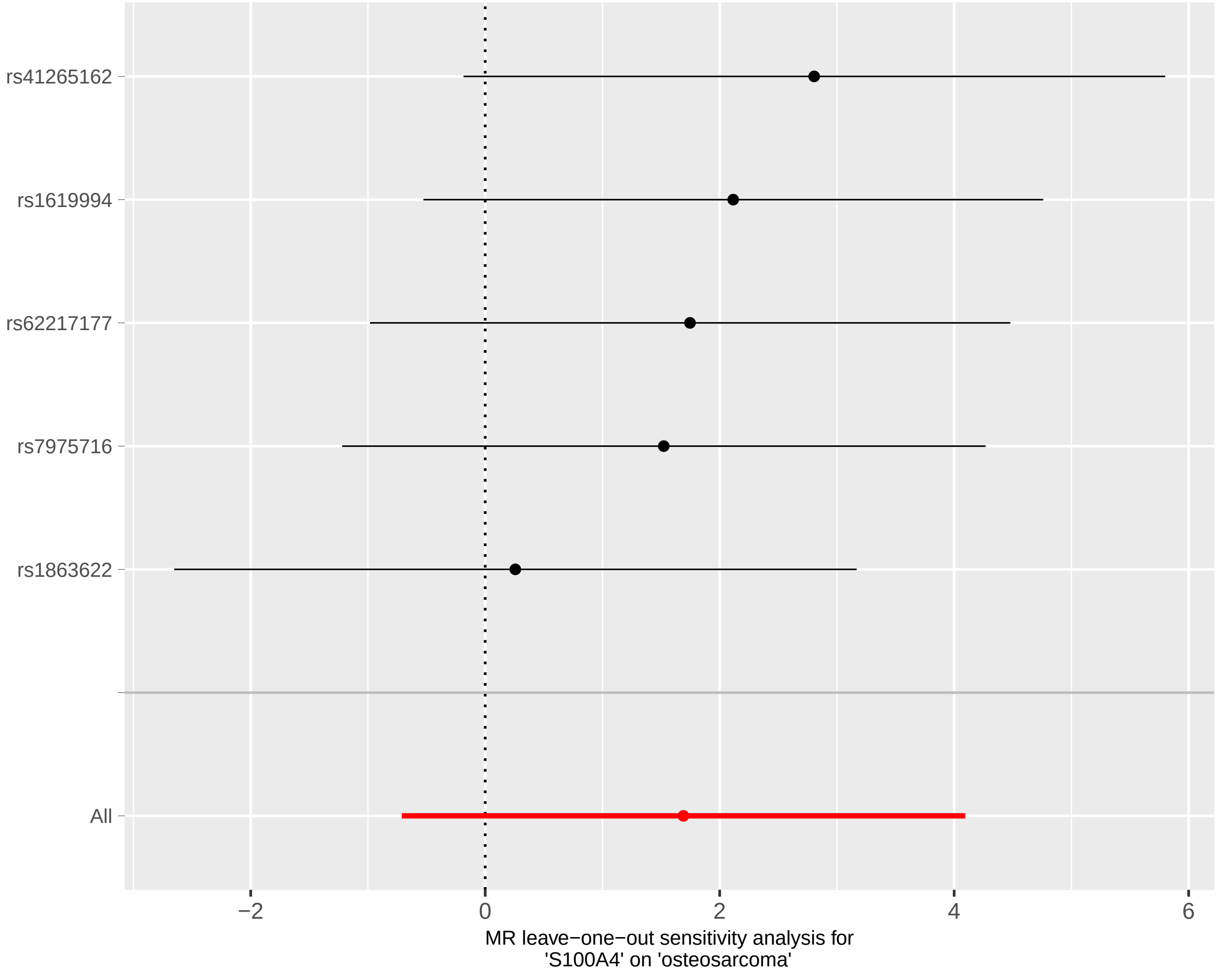
rs41265162
rs1619994
rs62217177
rs7975716
rs1863622
All
−2
0
2
4
6
MR leave−one−out sensitivity analysis for
'S100A4' on 'osteosarcoma'

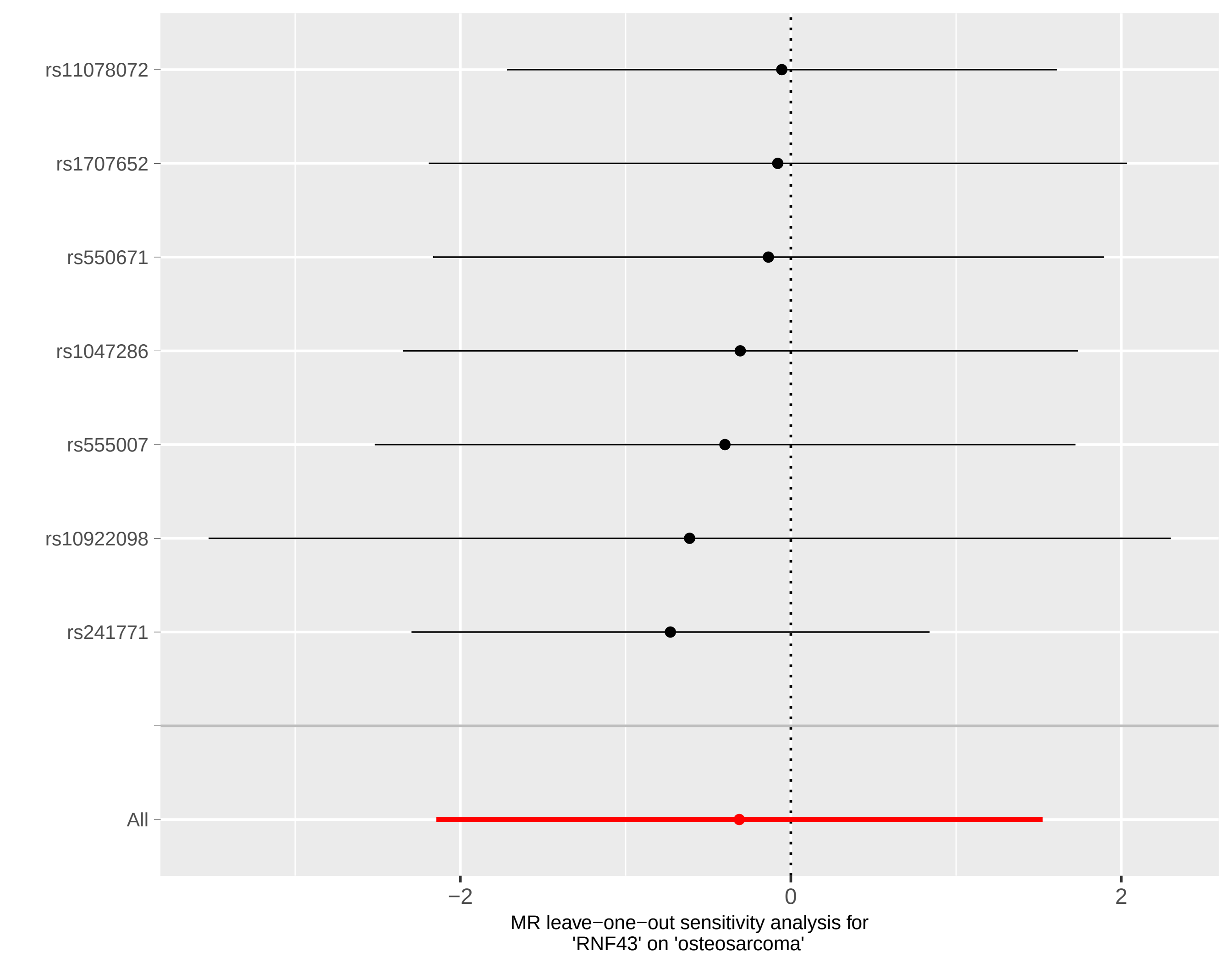

rs11078072
rs1707652
rs550671
rs1047286
rs555007
rs10922098
rs241771
All
−2
0
2
MR leave−one−out sensitivity analysis for
'RNF43' on 'osteosarcoma'

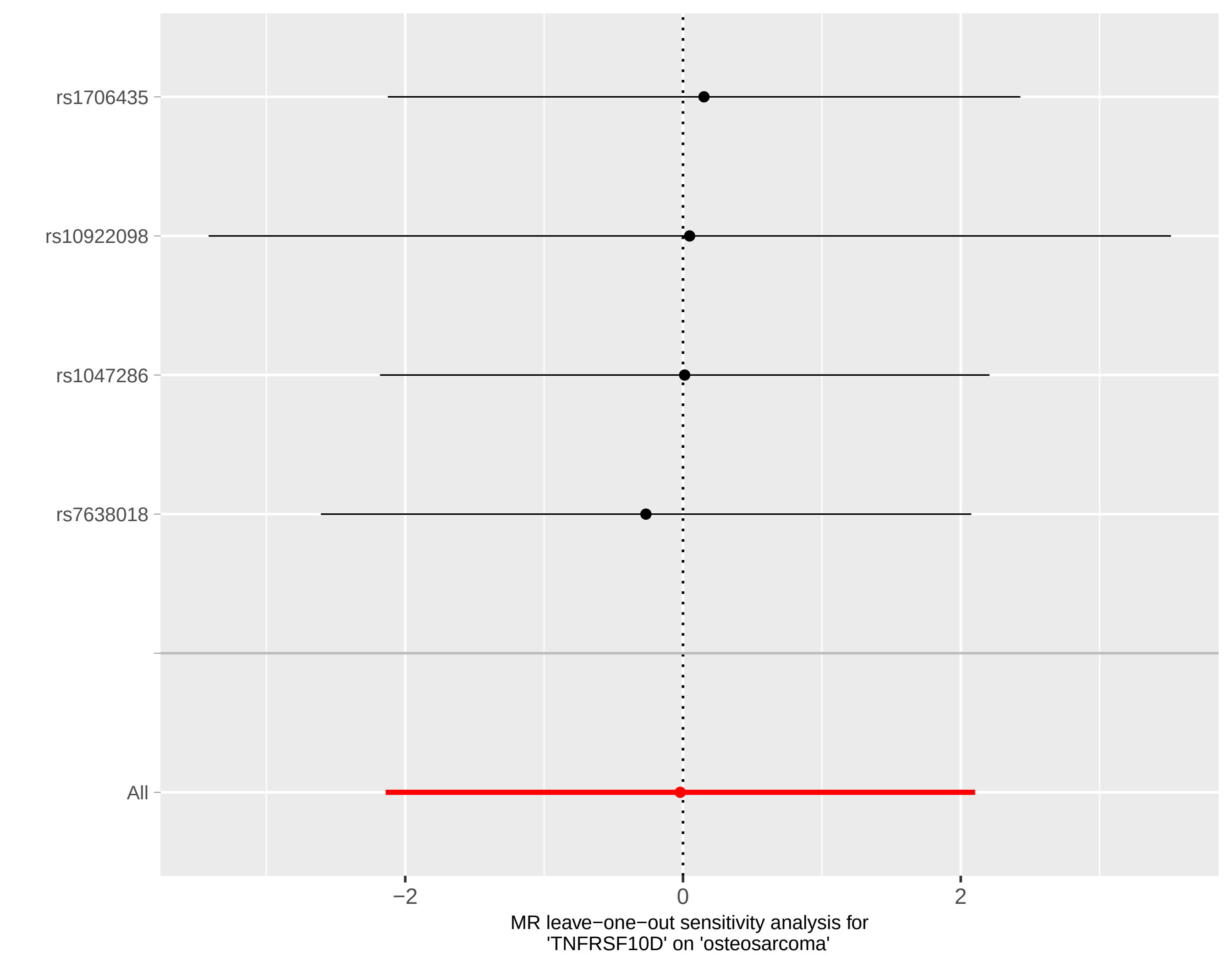
rs1706435
rs10922098
rs1047286
rs7638018
All
−2
0
2
MR leave−one−out sensitivity analysis for
'TNFRSF10D' on 'osteosarcoma'

Insufficient number of SNPs

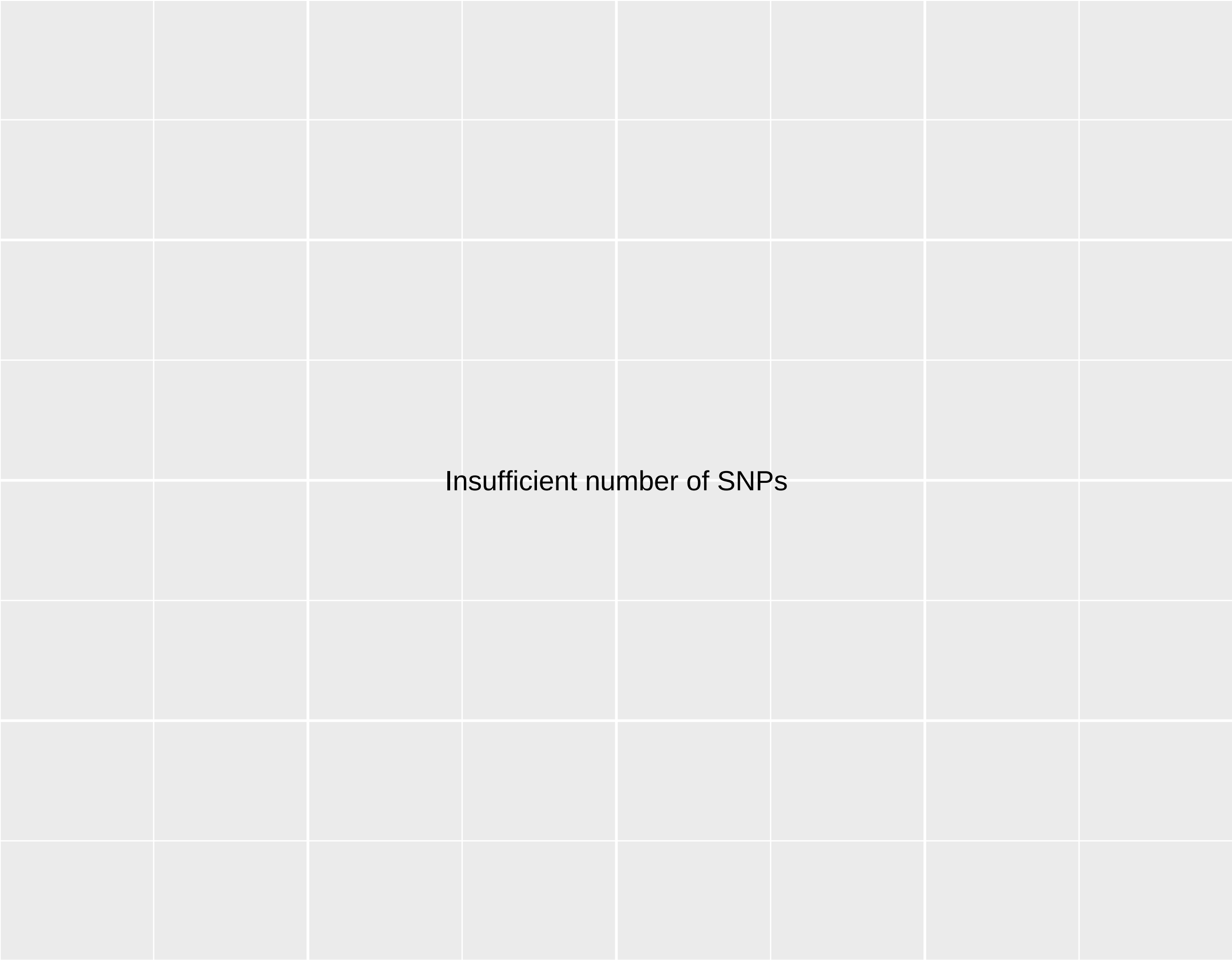
Insufficient number of SNPs

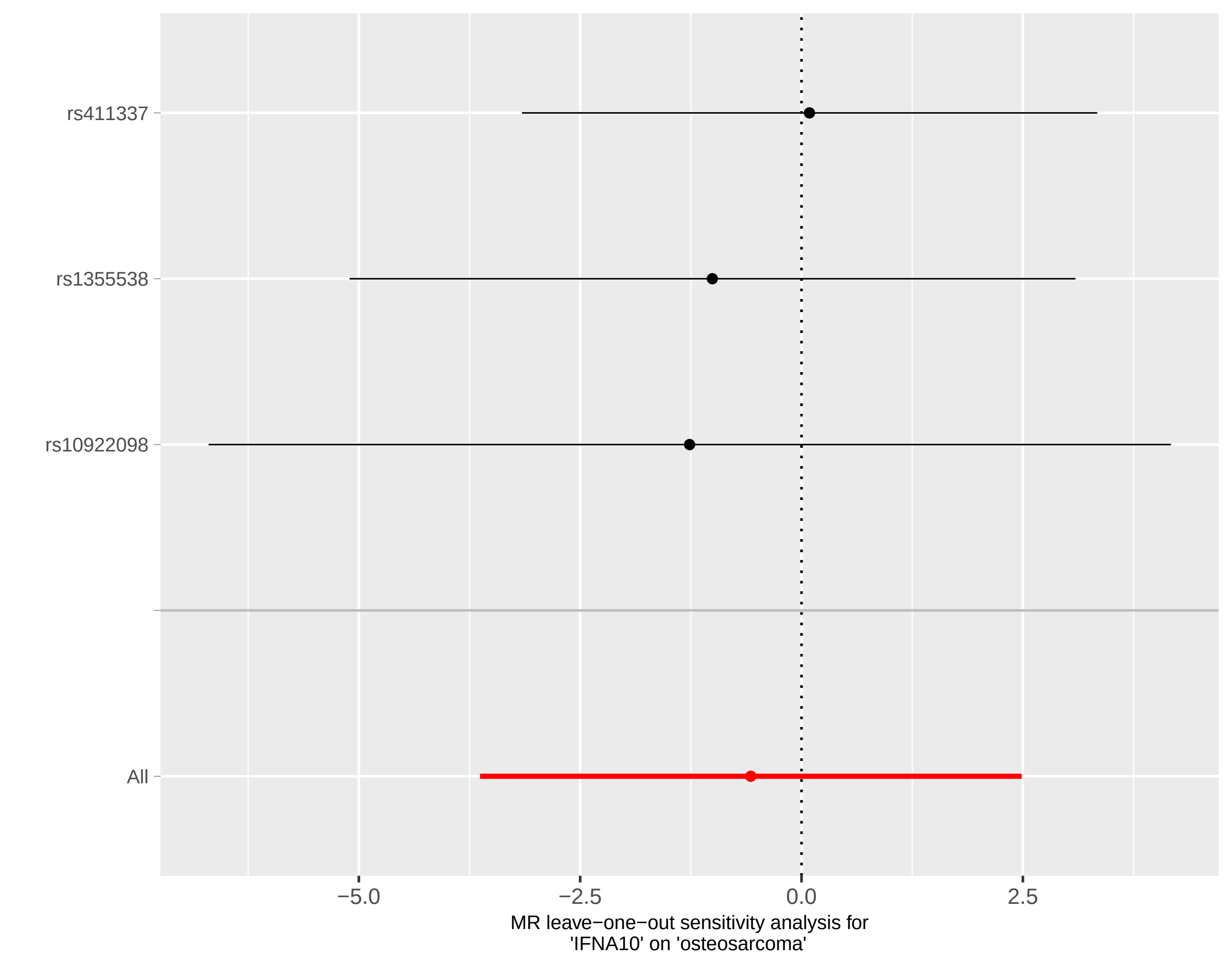
rs411337
rs1355538
rs10922098
All
−5.0
−2.5
0.0
2.5
MR leave−one−out sensitivity analysis for
'IFNA10' on 'osteosarcoma'

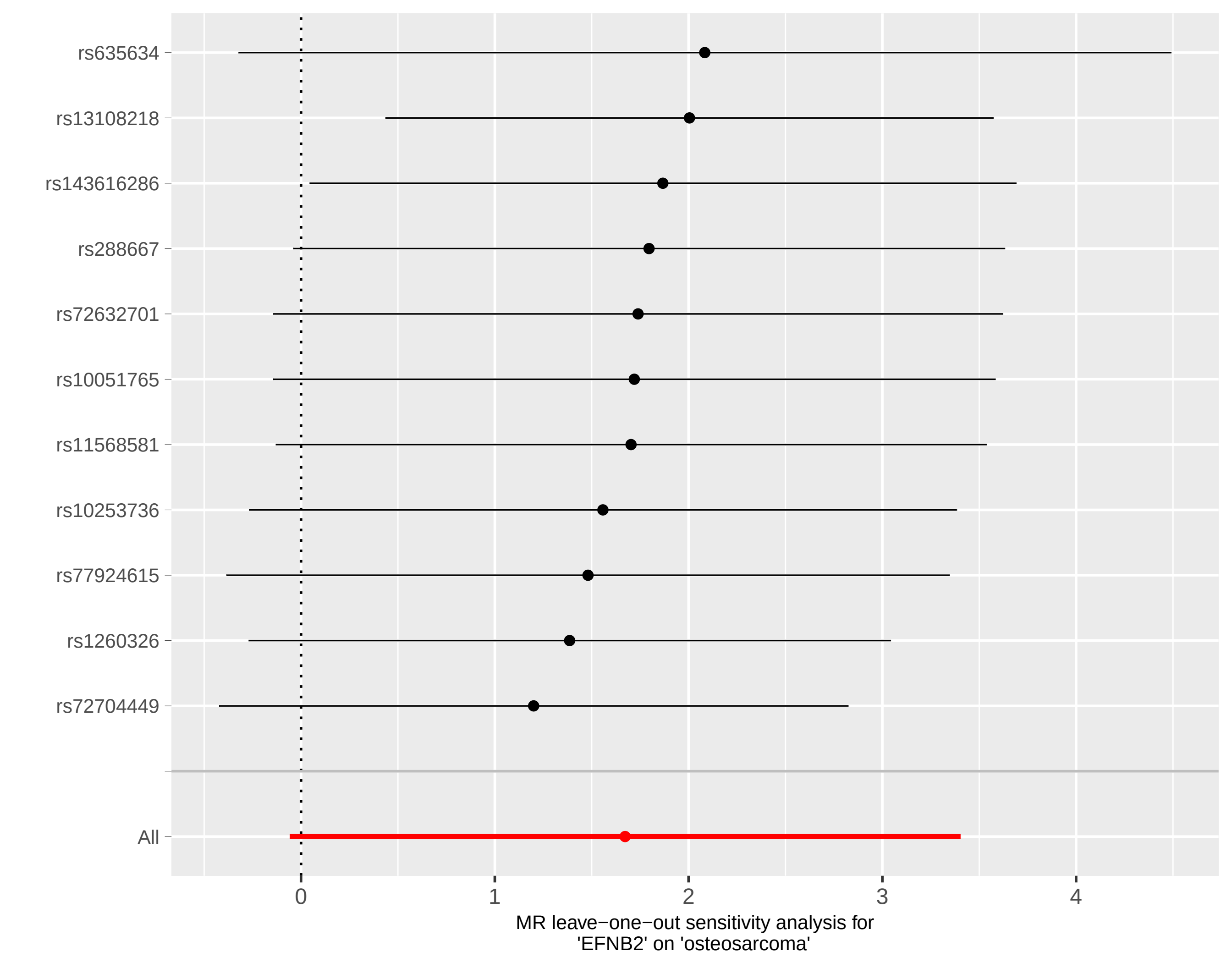
rs635634
rs13108218
rs143616286
rs288667
rs72632701
rs10051765
rs11568581
rs10253736
rs77924615
rs1260326
rs72704449
All
0
1
2
3
4
MR leave−one−out sensitivity analysis for
'EFNB2' on 'osteosarcoma'

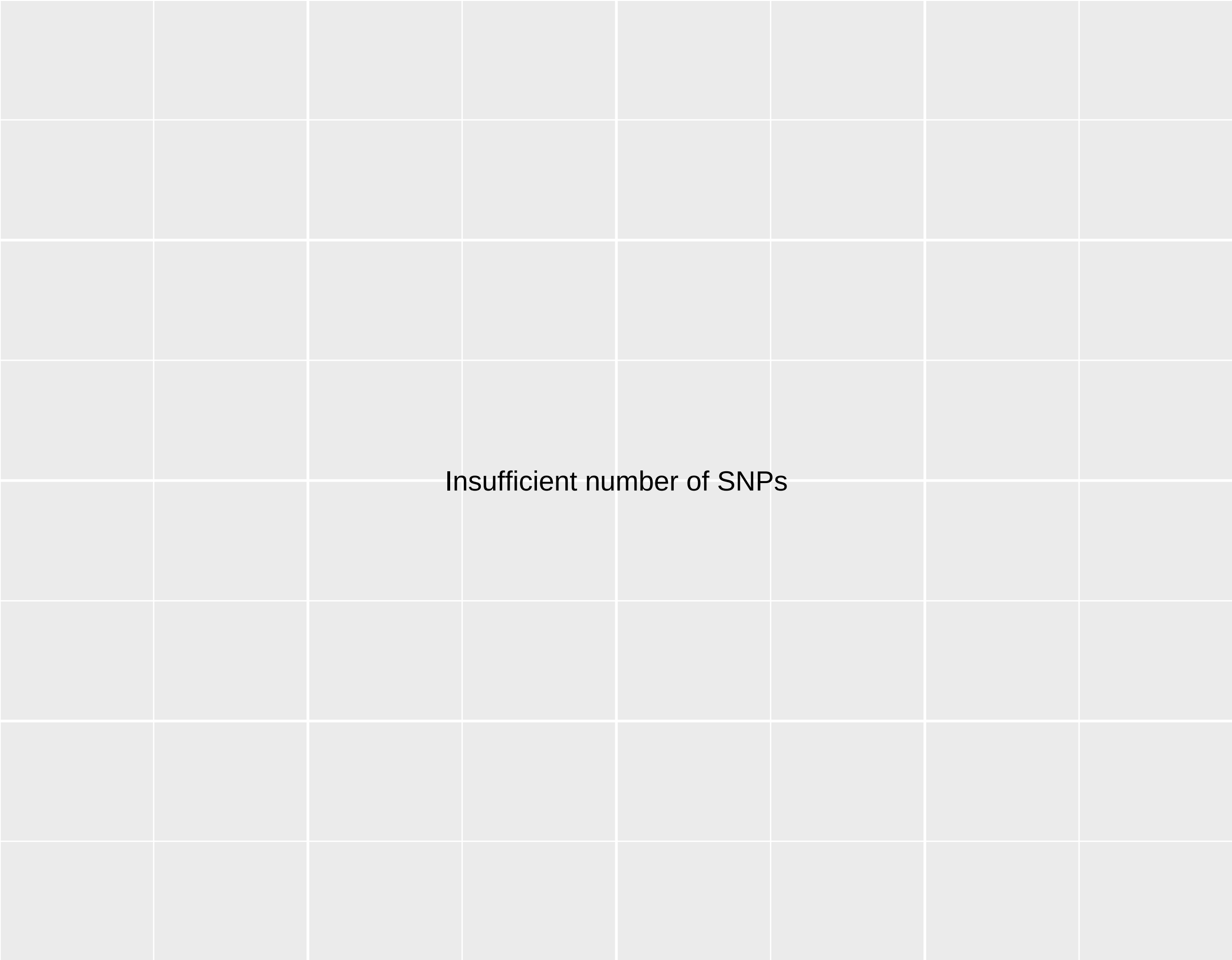
Insufficient number of SNPs

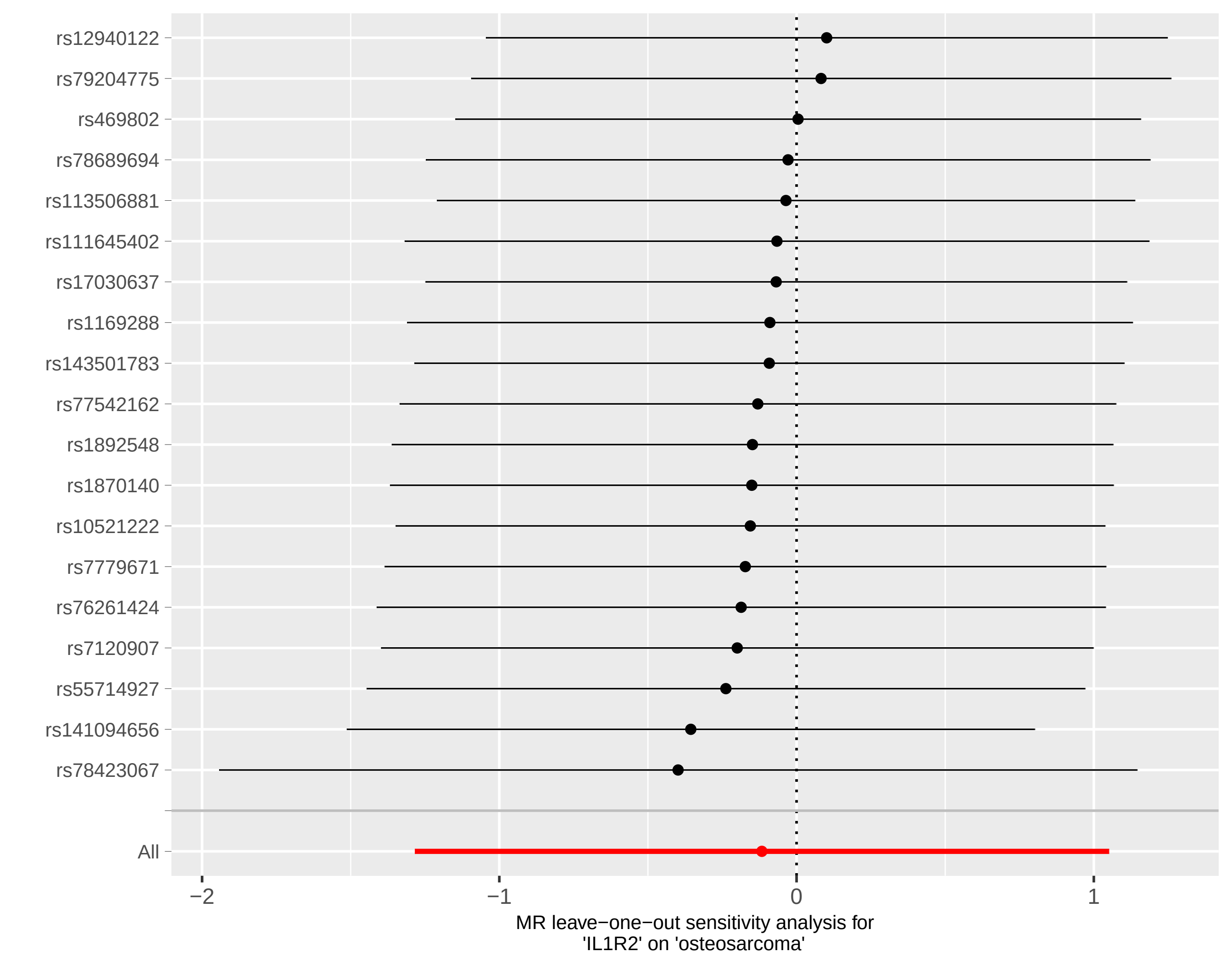
rs12940122
rs79204775
rs469802
rs78689694
rs113506881
rs111645402
rs17030637
rs1169288
rs143501783
rs77542162
rs1892548
rs1870140
rs10521222
rs7779671
rs76261424
rs7120907
rs55714927
rs141094656
rs78423067
All
−2
−1
0
1
MR leave−one−out sensitivity analysis for
'IL1R2' on 'osteosarcoma'

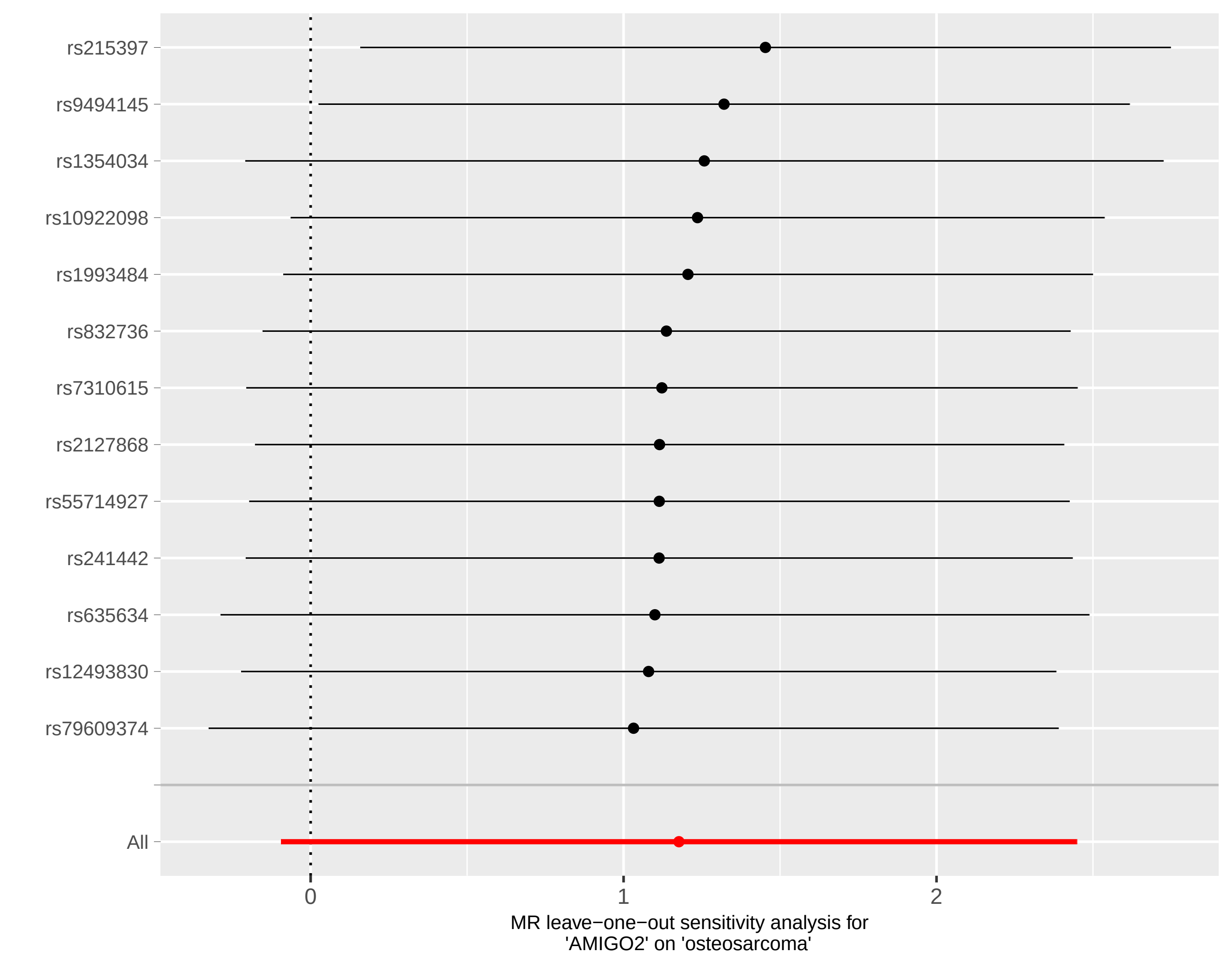

rs215397
rs9494145
rs1354034
rs10922098
rs1993484
rs832736
rs7310615
rs2127868
rs55714927
rs241442
rs635634
rs12493830
rs79609374
All
0
1
2
MR leave−one−out sensitivity analysis for
'AMIGO2' on 'osteosarcoma'

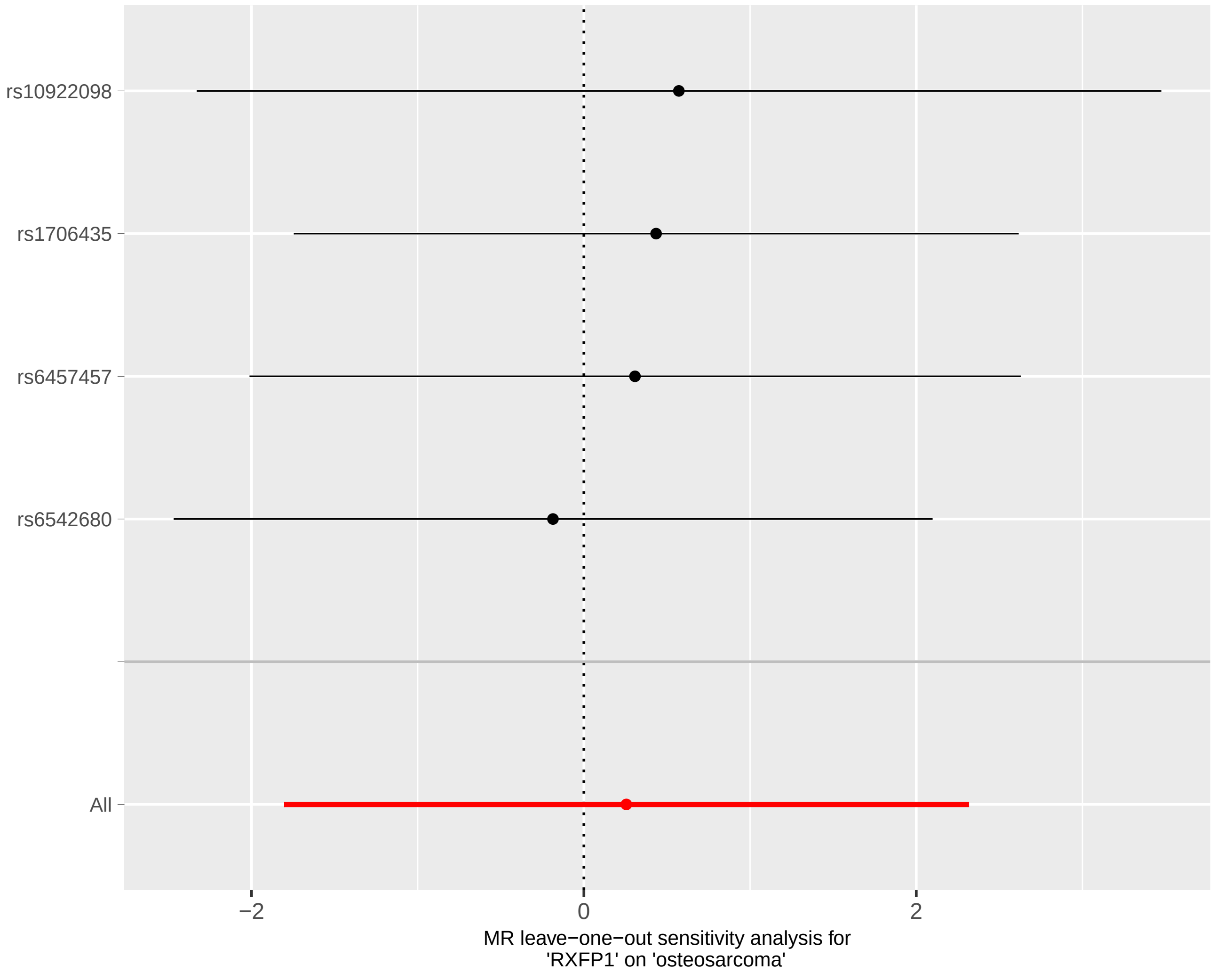

rs10922098
rs1706435
rs6457457
rs6542680
All
−2
0
2
MR leave−one−out sensitivity analysis for
'RXFP1' on 'osteosarcoma'

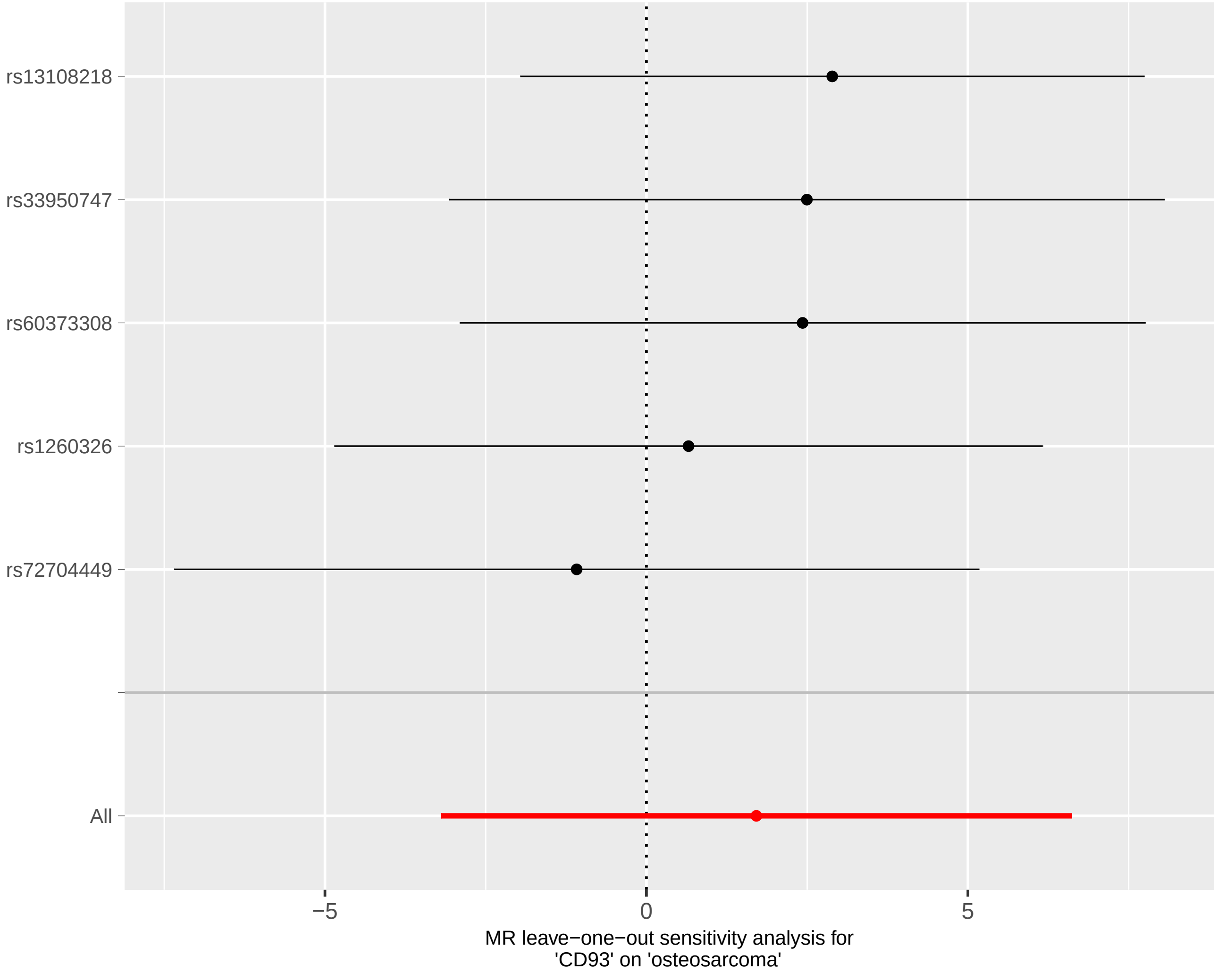
rs13108218
rs33950747
rs60373308
rs1260326
rs72704449
All
−5
0
5
MR leave−one−out sensitivity analysis for
'CD93' on 'osteosarcoma'

Insufficient number of SNPs

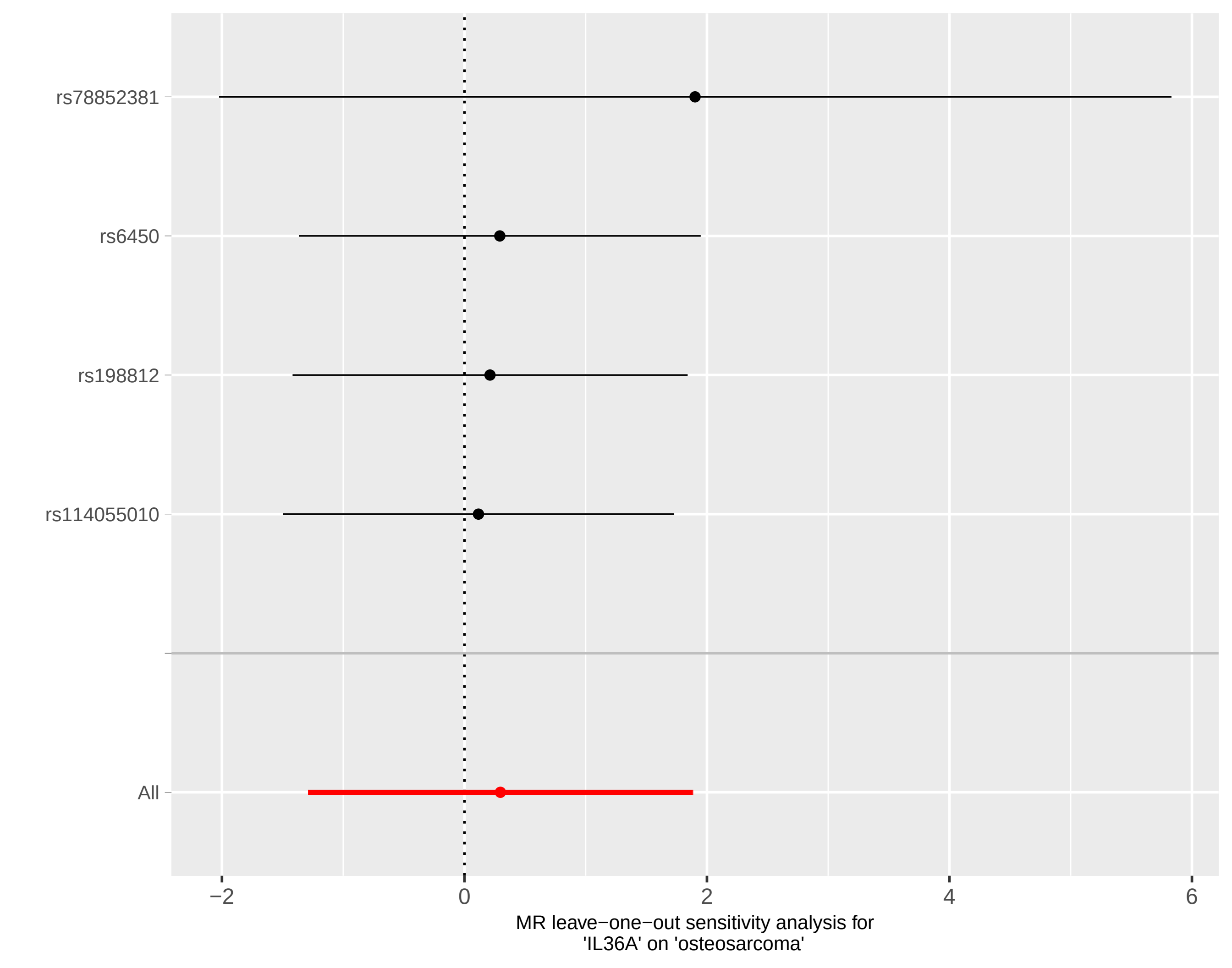

rs78852381
rs6450
rs198812
rs114055010
All
−2
0
2
4
6
MR leave−one−out sensitivity analysis for
'IL36A' on 'osteosarcoma'

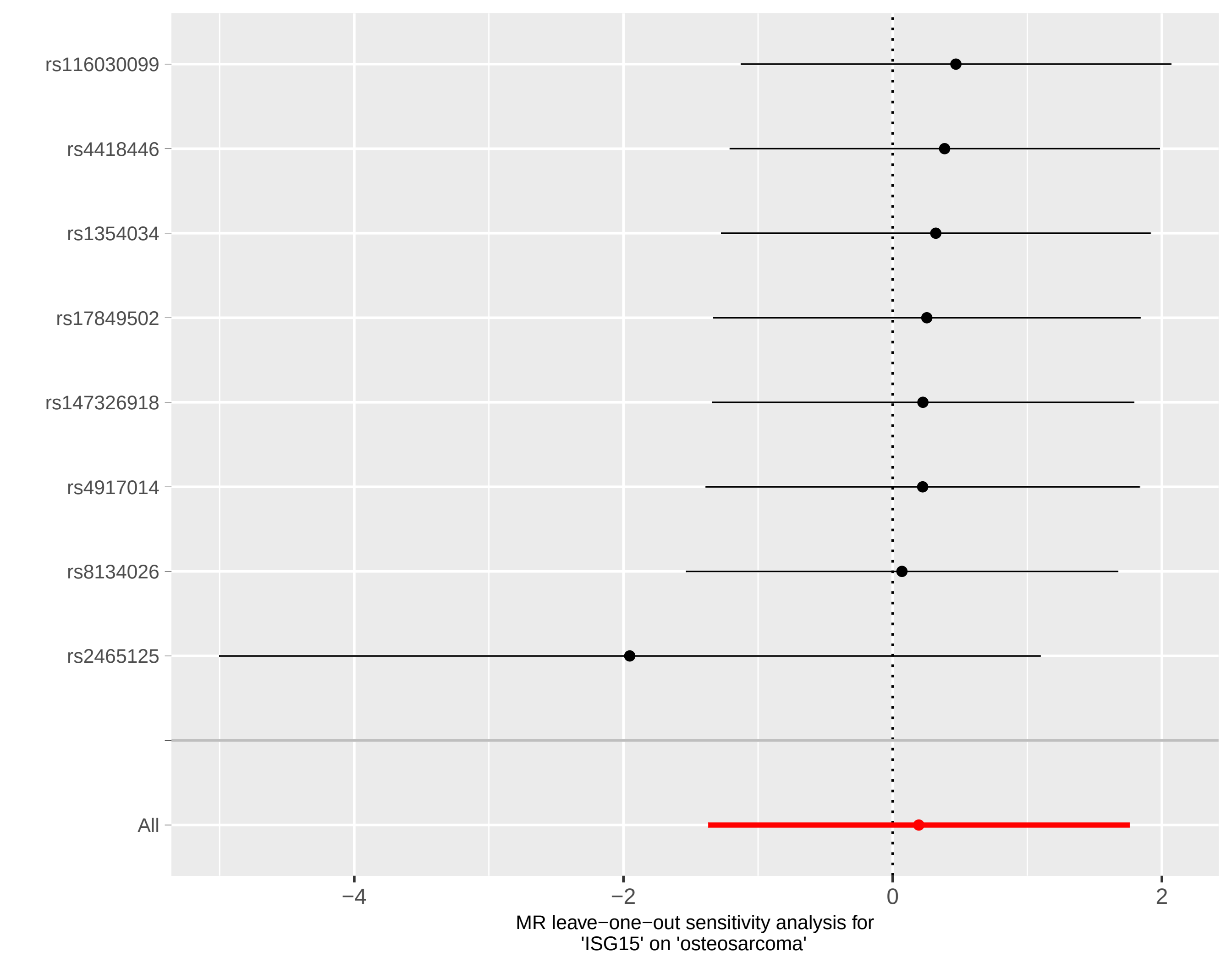
rs116030099
rs4418446
rs1354034
rs17849502
rs147326918
rs4917014
rs8134026
rs2465125
All
−4
−2
0
2
MR leave−one−out sensitivity analysis for
'ISG15' on 'osteosarcoma'

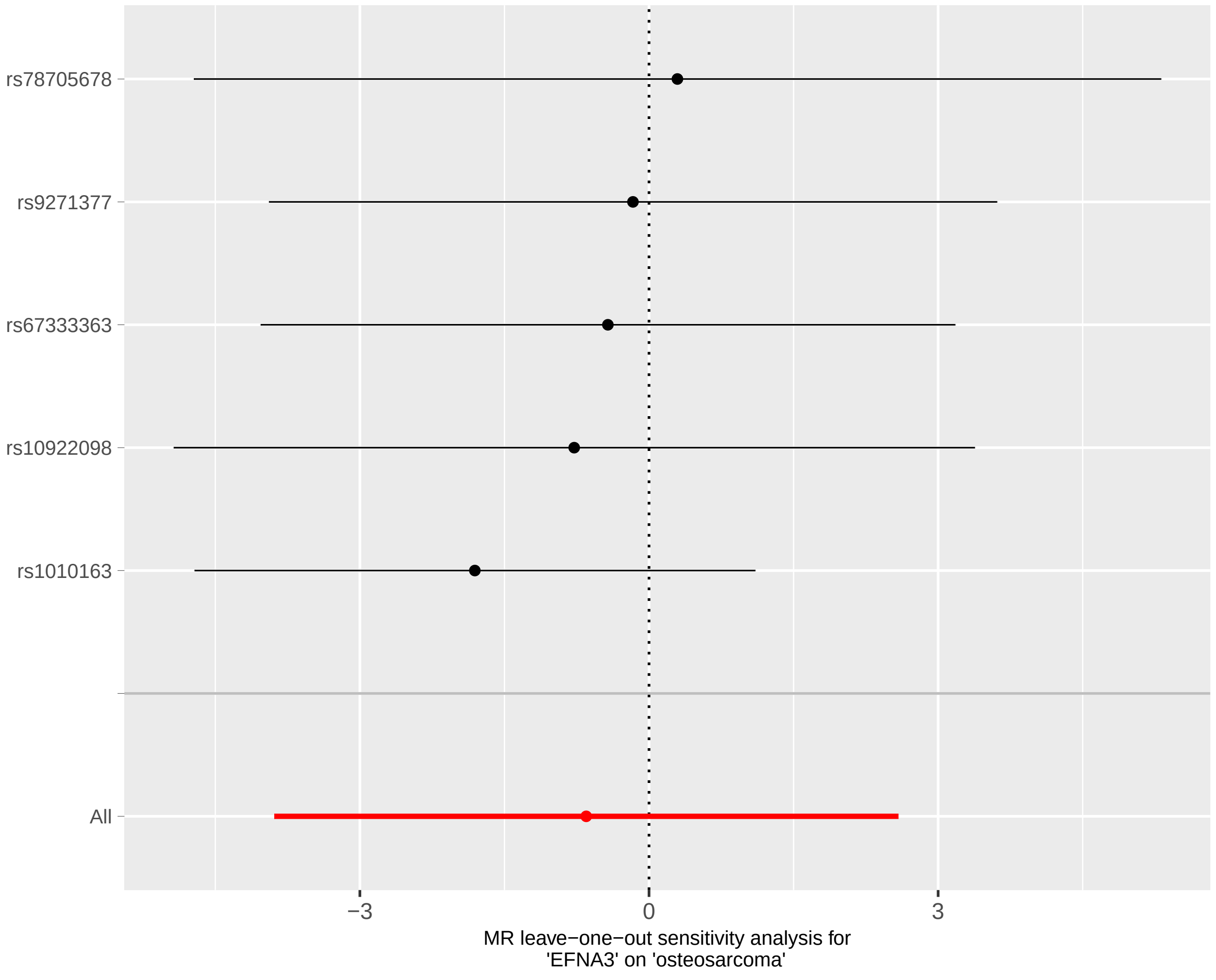
rs78705678
rs9271377
rs67333363
rs10922098
rs1010163
All
−3
0
3
MR leave−one−out sensitivity analysis for
'EFNA3' on 'osteosarcoma'

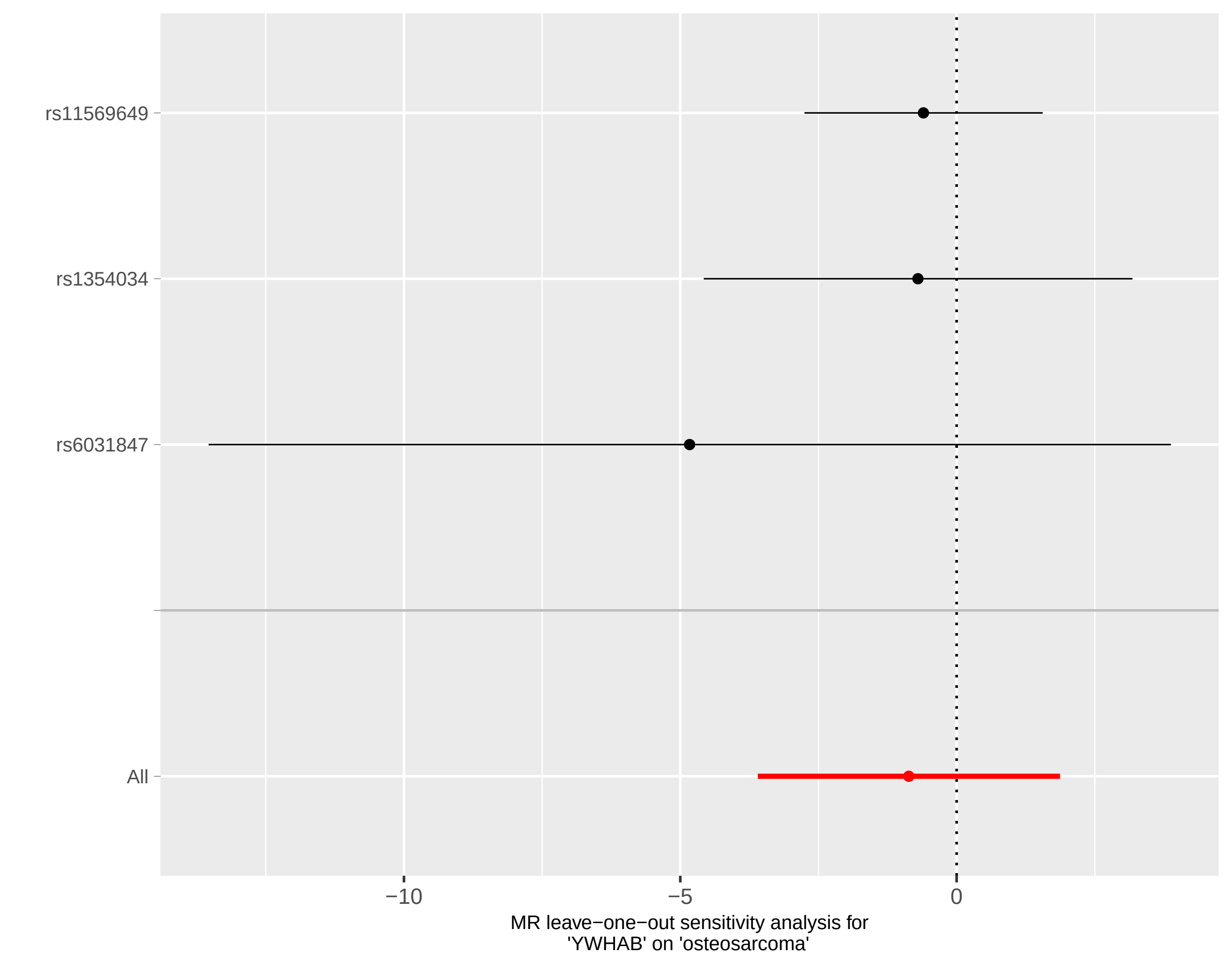
rs11569649
rs1354034
rs6031847
All
−10
−5
0
MR leave−one−out sensitivity analysis for
'YWHAB' on 'osteosarcoma'

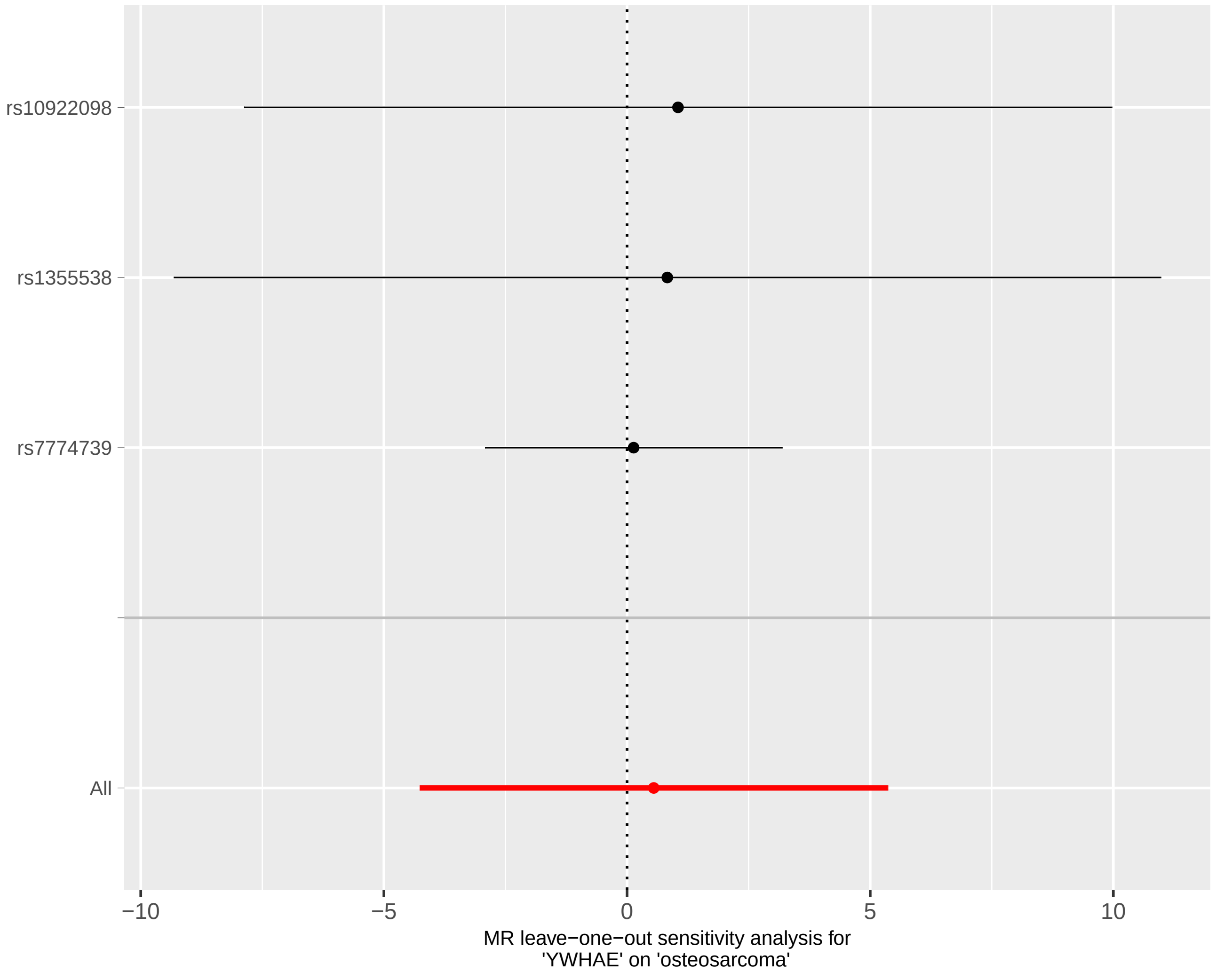
rs10922098
rs1355538
rs7774739
All
−10
−5
0
5
10
MR leave−one−out sensitivity analysis for
'YWHAE' on 'osteosarcoma'

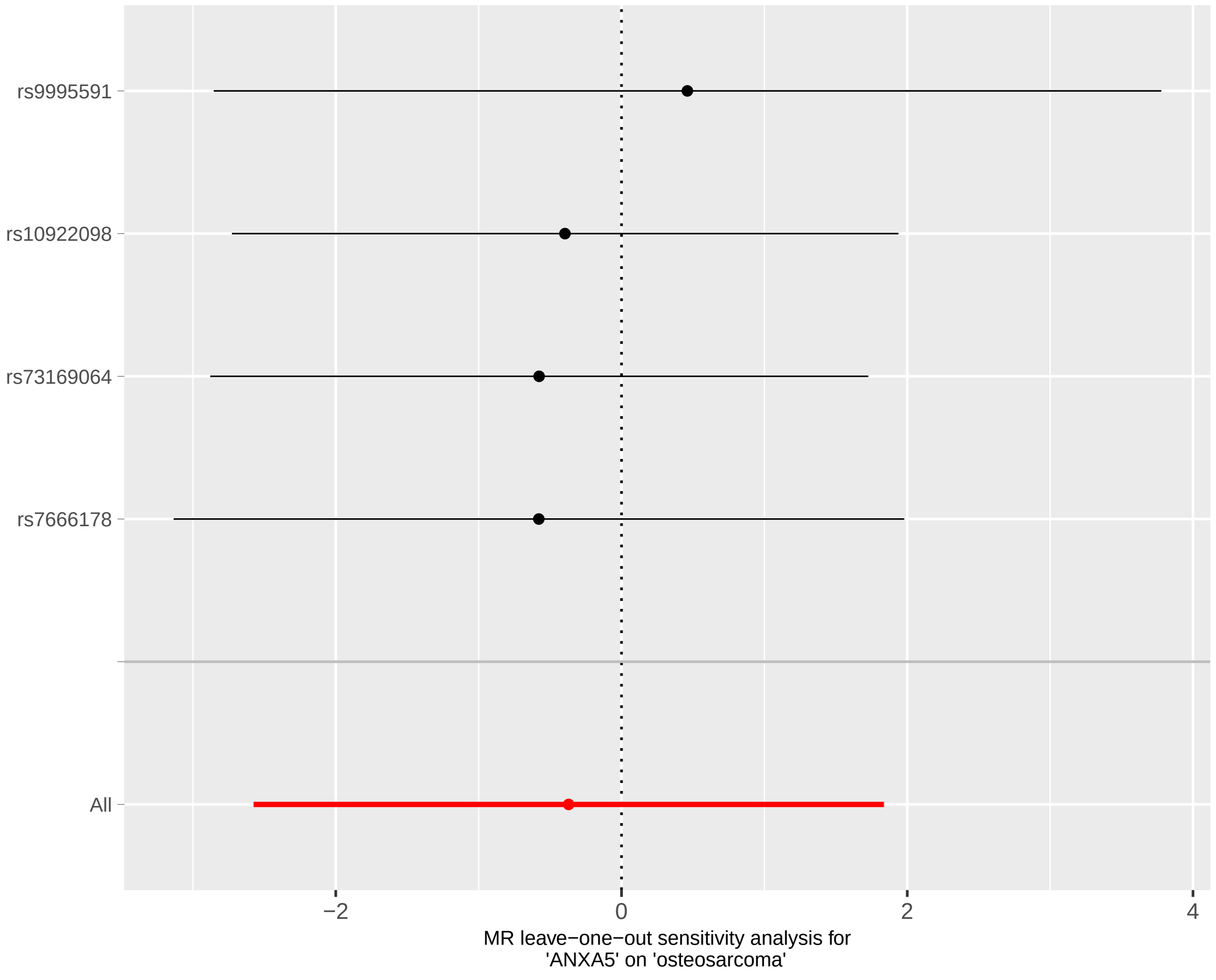

rs9995591
rs10922098
rs73169064
rs7666178
All
−2
0
2
4
MR leave−one−out sensitivity analysis for
'ANXA5' on 'osteosarcoma'

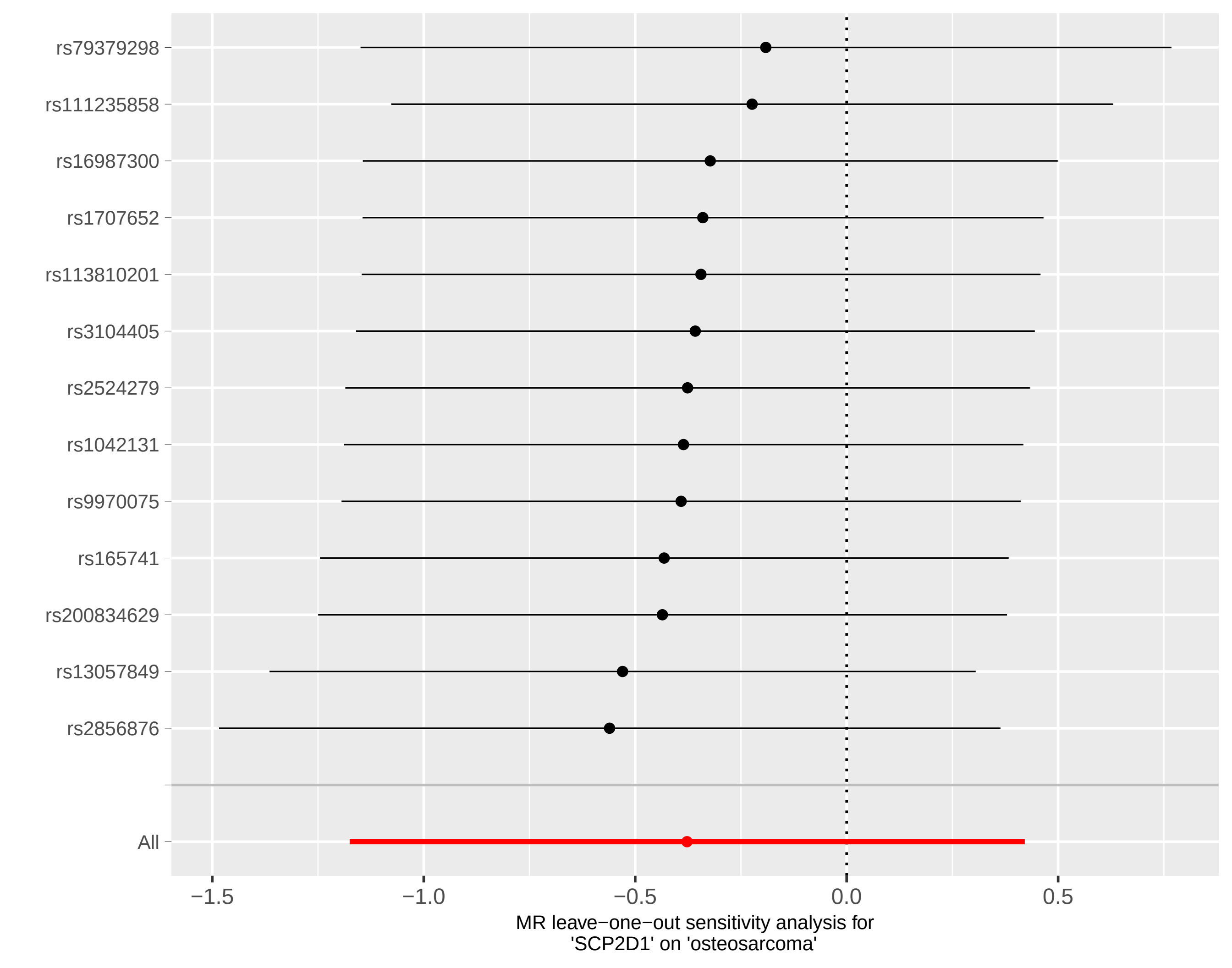
rs79379298
rs111235858
rs16987300
rs1707652
rs113810201
rs3104405
rs2524279
rs1042131
rs9970075
rs165741
rs200834629
rs13057849
rs2856876
All
−1.5
−1.0
−0.5
0.0
0.5
MR leave−one−out sensitivity analysis for
'SCP2D1' on 'osteosarcoma'

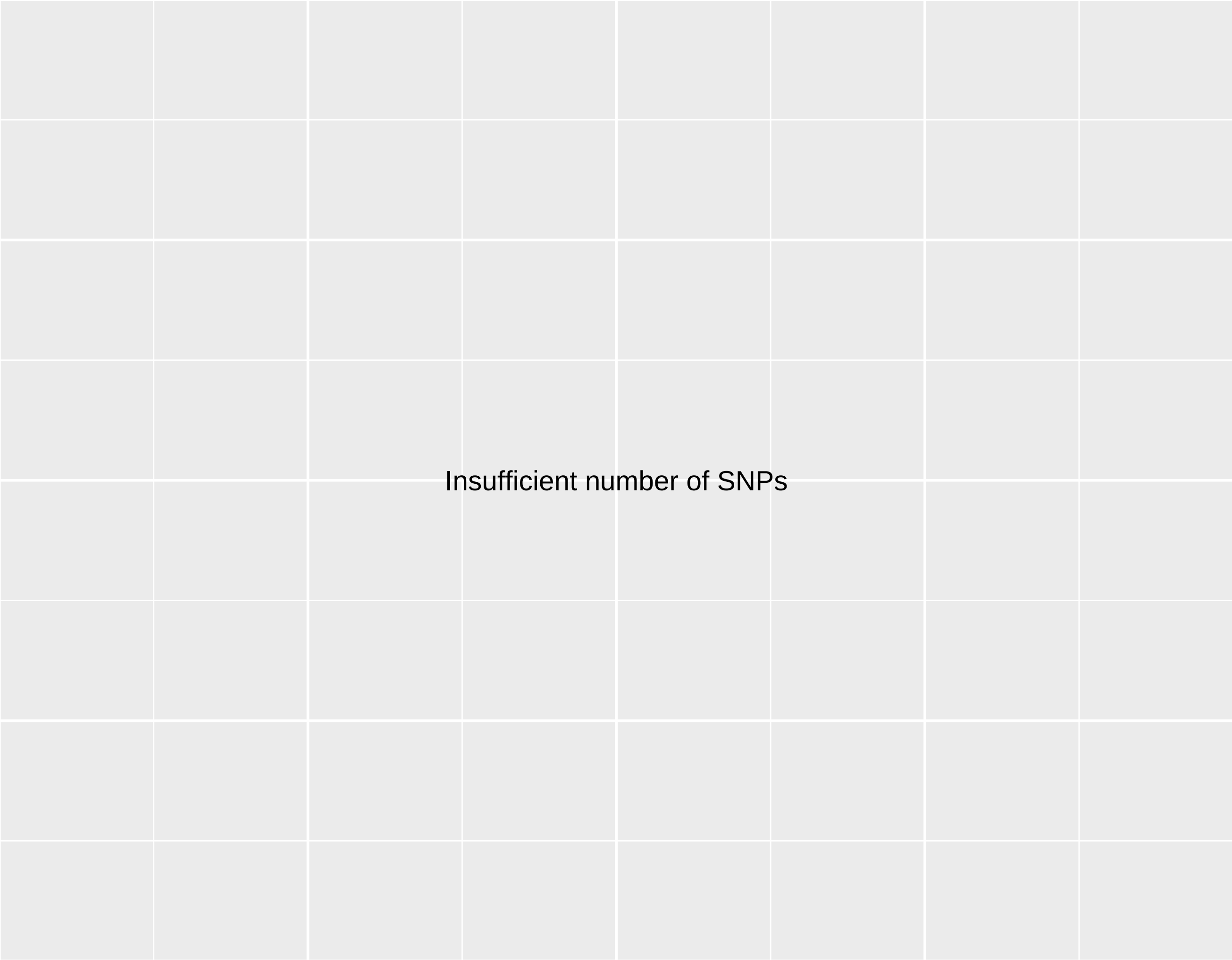
Insufficient number of SNPs

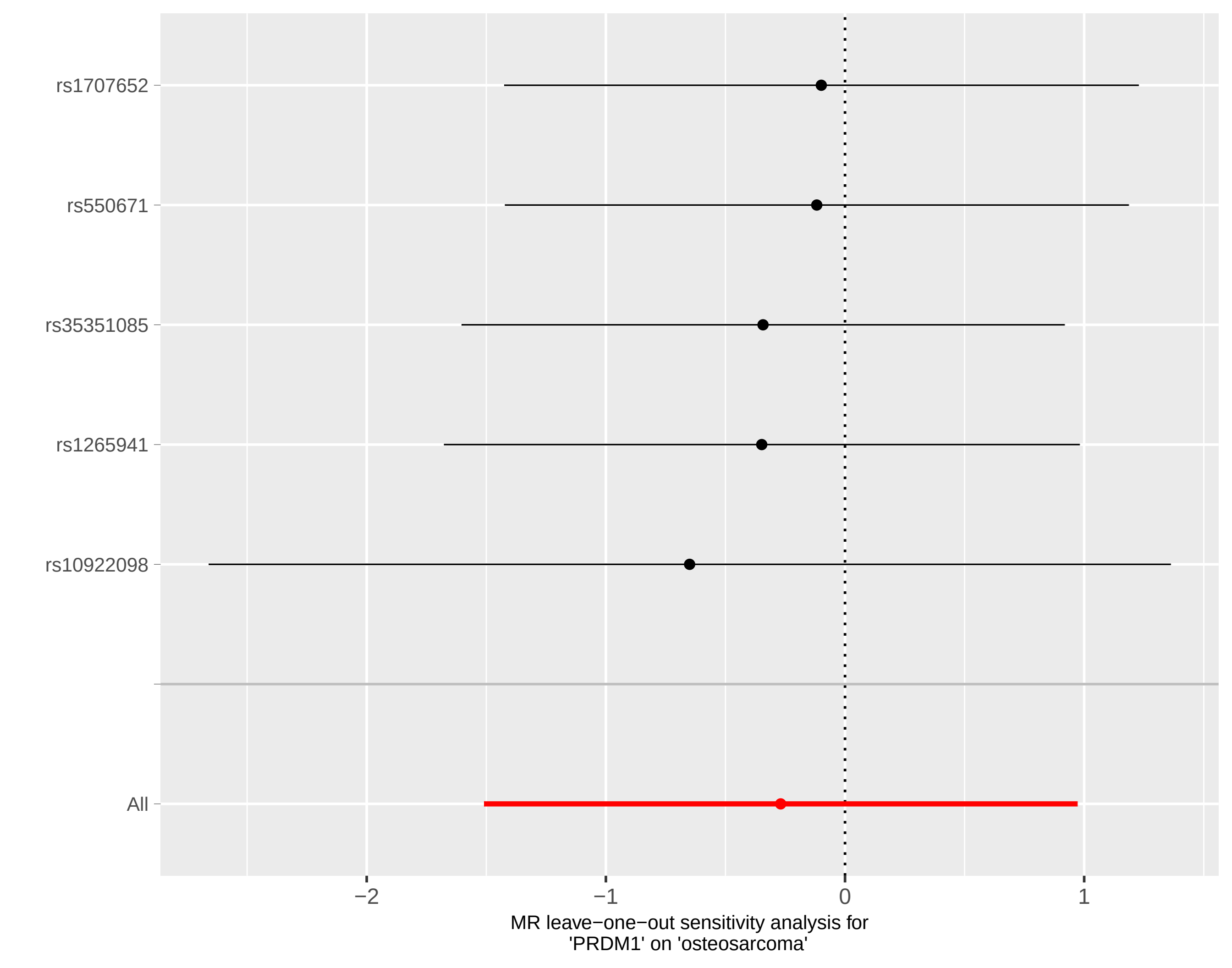
rs1707652
rs550671
rs35351085
rs1265941
rs10922098
All
−2
−1
0
1
MR leave−one−out sensitivity analysis for
'PRDM1' on 'osteosarcoma'

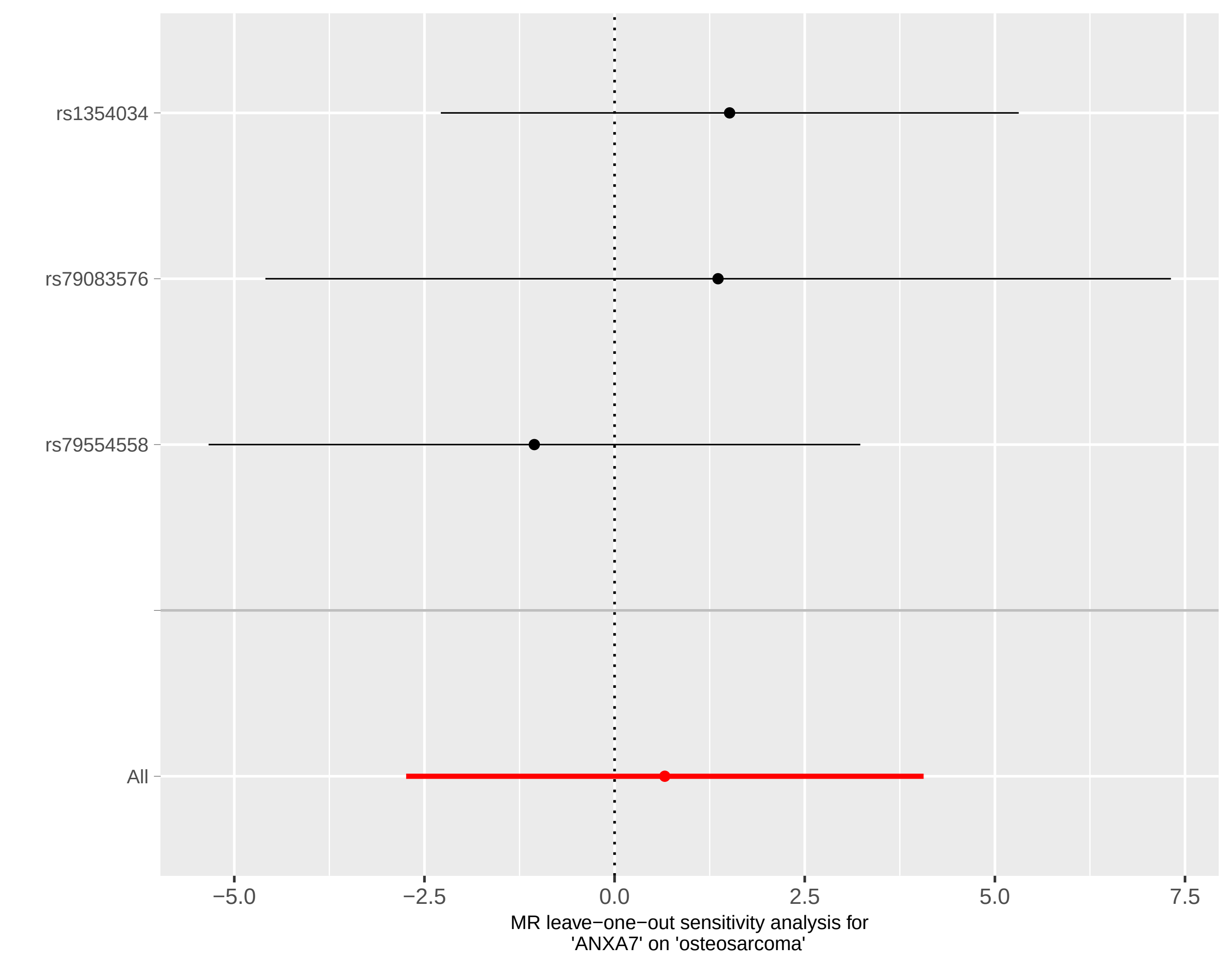

rs1354034
rs79083576
rs79554558
All
−5.0
−2.5
0.0
2.5
5.0
7.5
MR leave−one−out sensitivity analysis for
'ANXA7' on 'osteosarcoma'

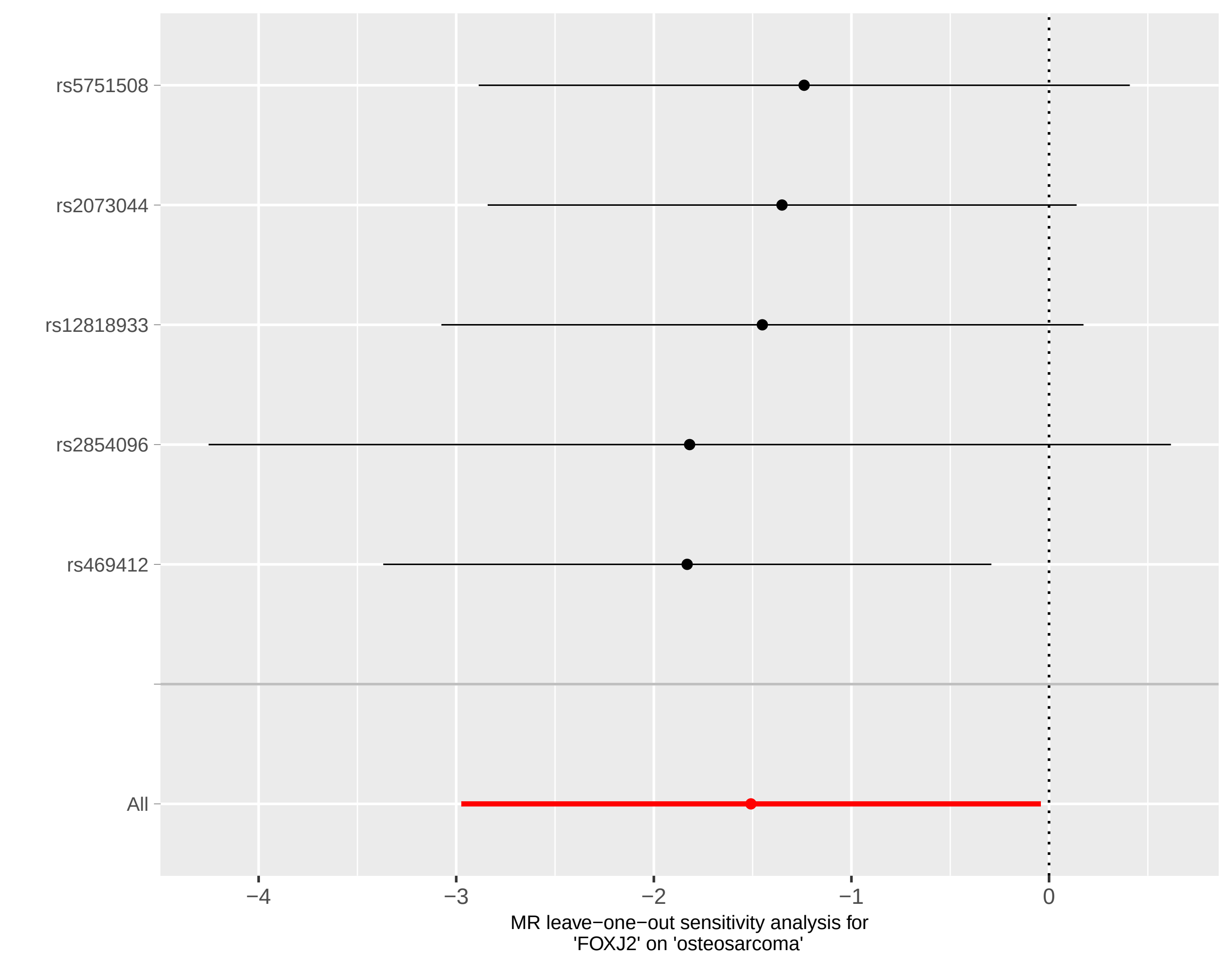
rs5751508
rs2073044
rs12818933
rs2854096
rs469412
All
−4
−3
−2
−1
0
MR leave−one−out sensitivity analysis for
'FOXJ2' on 'osteosarcoma'

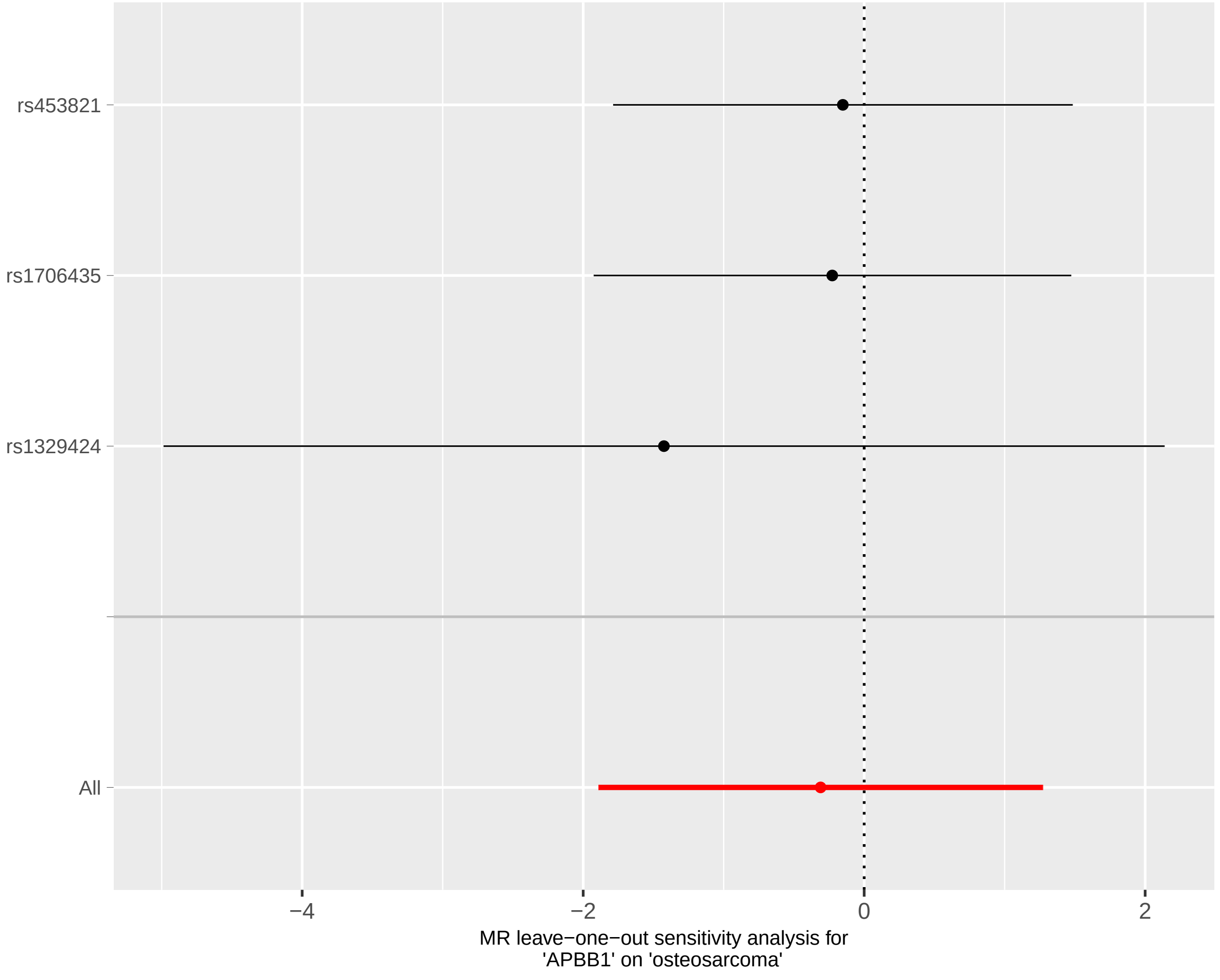

rs453821
rs1706435
rs1329424
All
−4
−2
0
2
MR leave−one−out sensitivity analysis for
'APBB1' on 'osteosarcoma'

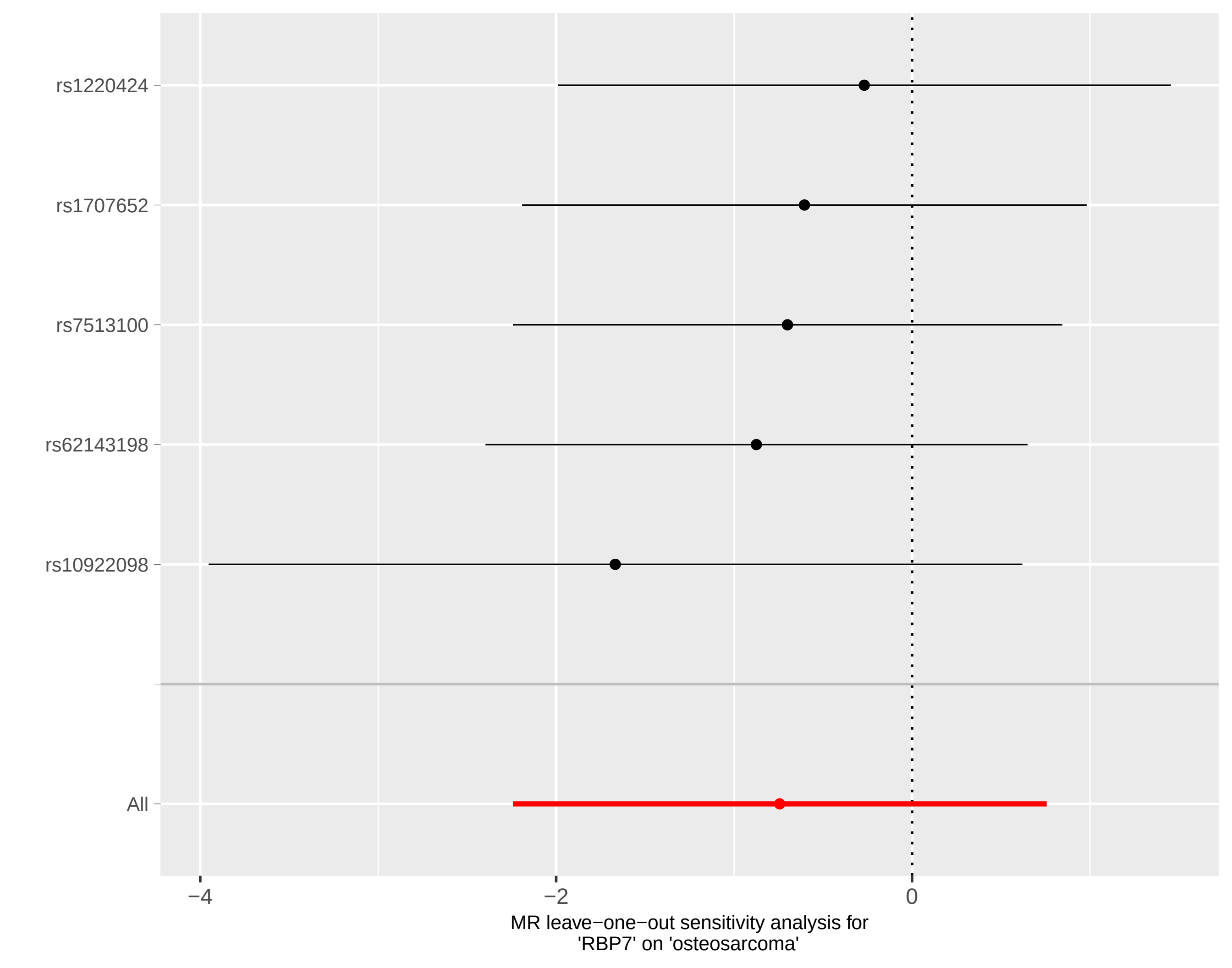
rs1220424
rs1707652
rs7513100
rs62143198
rs10922098
All
−4
−2
0
MR leave−one−out sensitivity analysis for
'RBP7' on 'osteosarcoma'

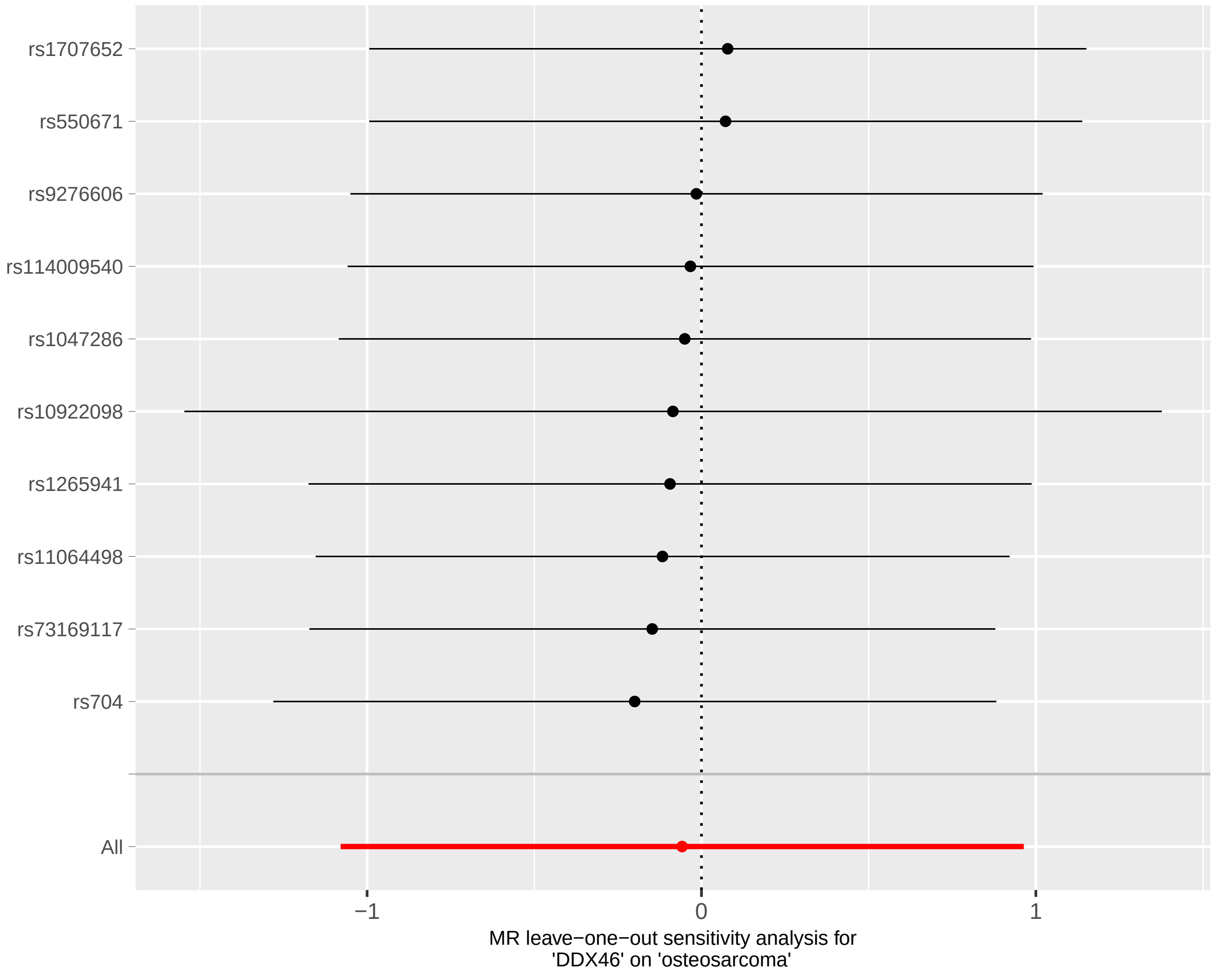
rs1707652
rs550671
rs9276606
rs114009540
rs1047286
rs10922098
rs1265941
rs11064498
rs73169117
rs704
All
−1
0
1
MR leave−one−out sensitivity analysis for
'DDX46' on 'osteosarcoma'

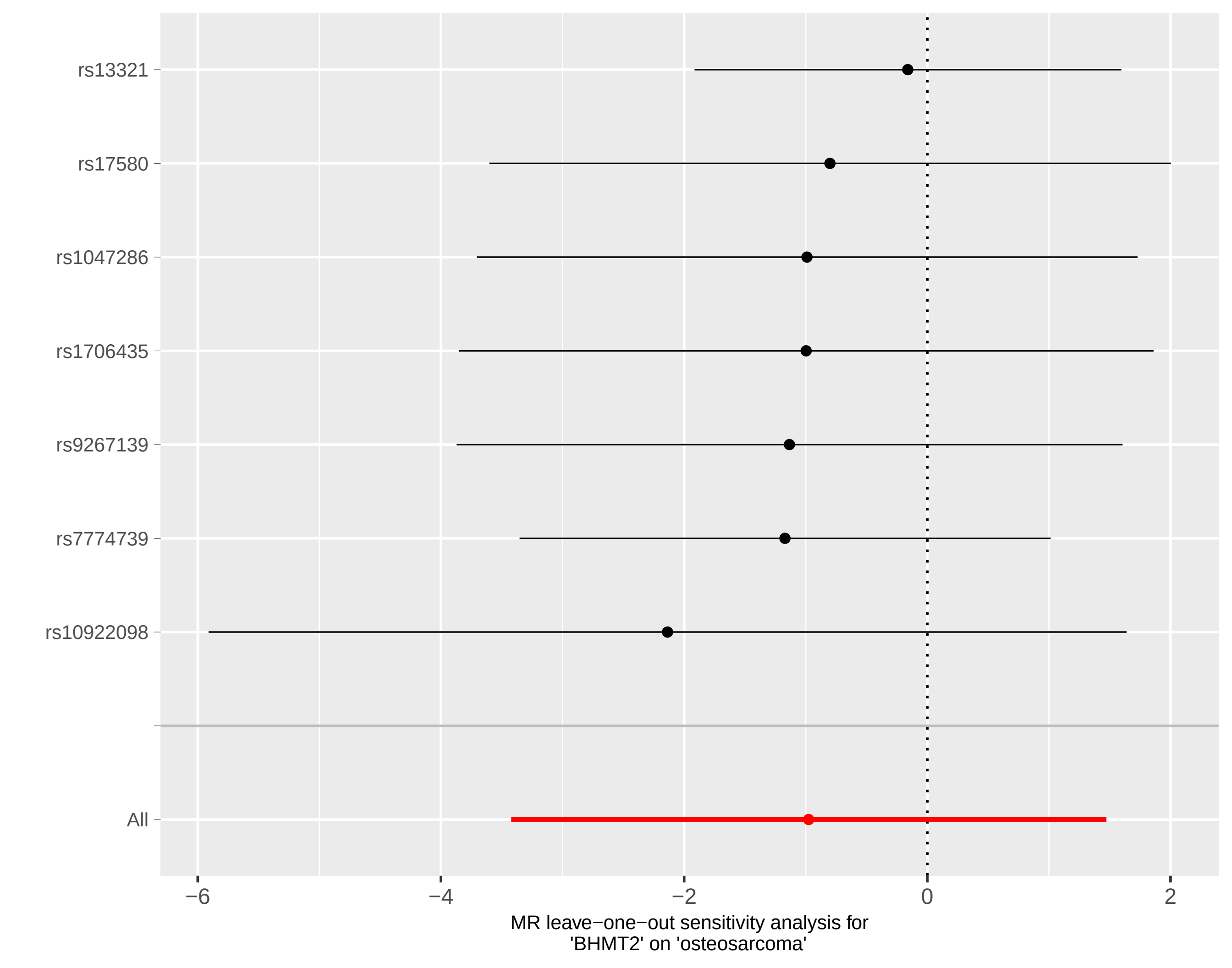

rs13321
rs17580
rs1047286
rs1706435
rs9267139
rs7774739
rs10922098
All
−6
−4
−2
0
2
MR leave−one−out sensitivity analysis for
'BHMT2' on 'osteosarcoma'

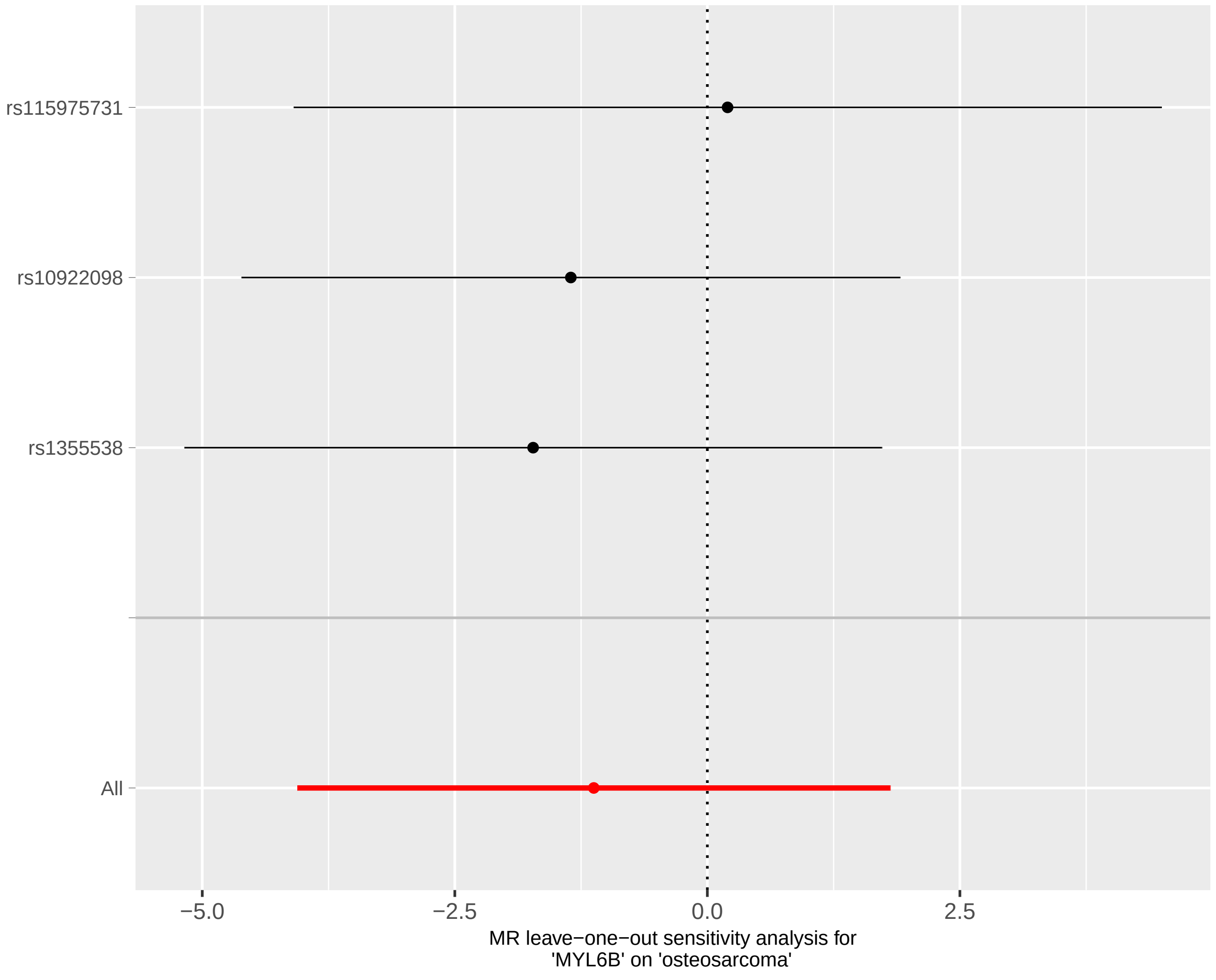

rs115975731
rs10922098
rs1355538
All
−5.0
−2.5
0.0
2.5
MR leave−one−out sensitivity analysis for
'MYL6B' on 'osteosarcoma'

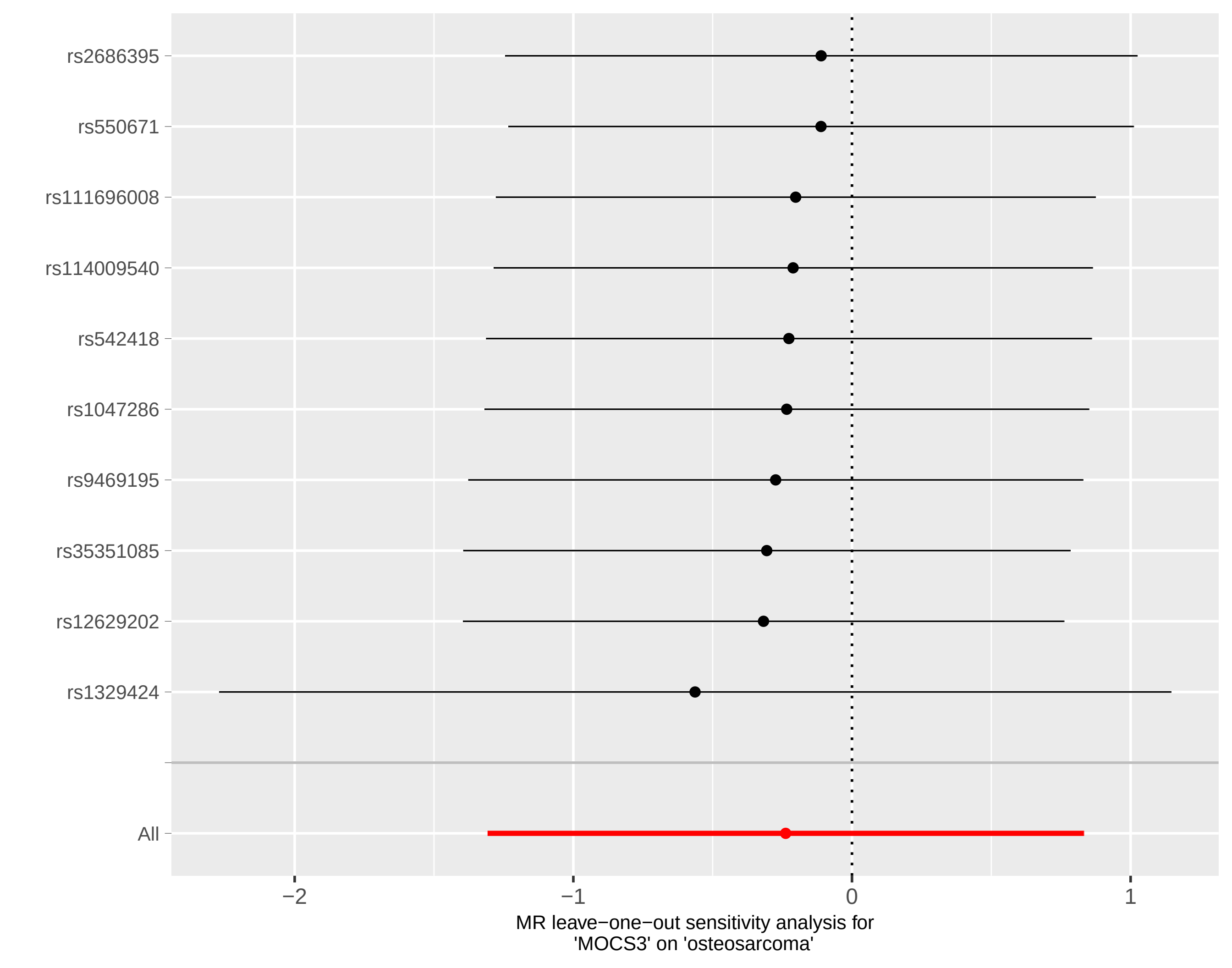
rs2686395
rs550671
rs111696008
rs114009540
rs542418
rs1047286
rs9469195
rs35351085
rs12629202
rs1329424
All
−2
−1
0
1
MR leave−one−out sensitivity analysis for
'MOCS3' on 'osteosarcoma'

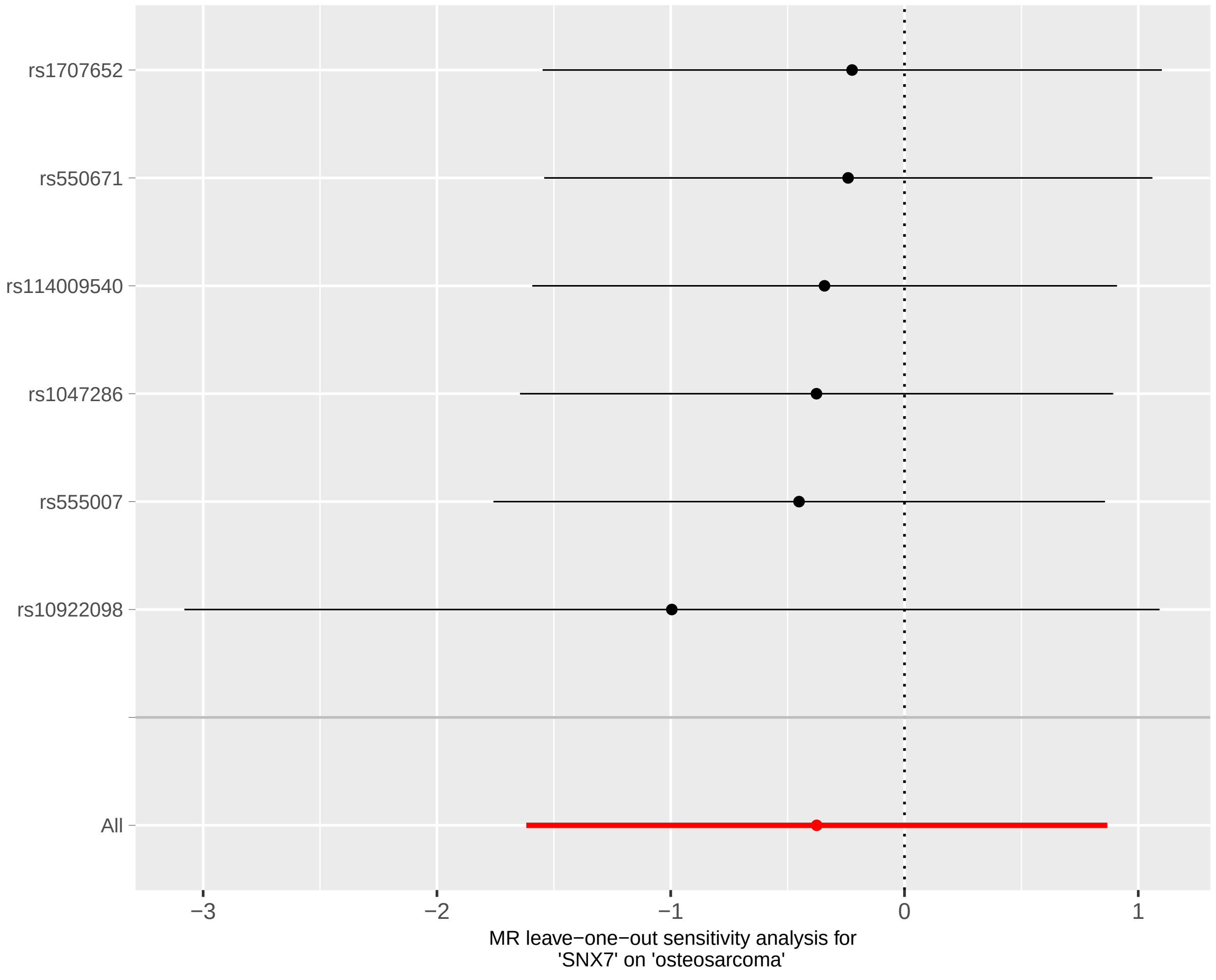
rs1707652
rs550671
rs114009540
rs1047286
rs555007
rs10922098
All
−3
−2
−1
0
1
MR leave−one−out sensitivity analysis for
'SNX7' on 'osteosarcoma'

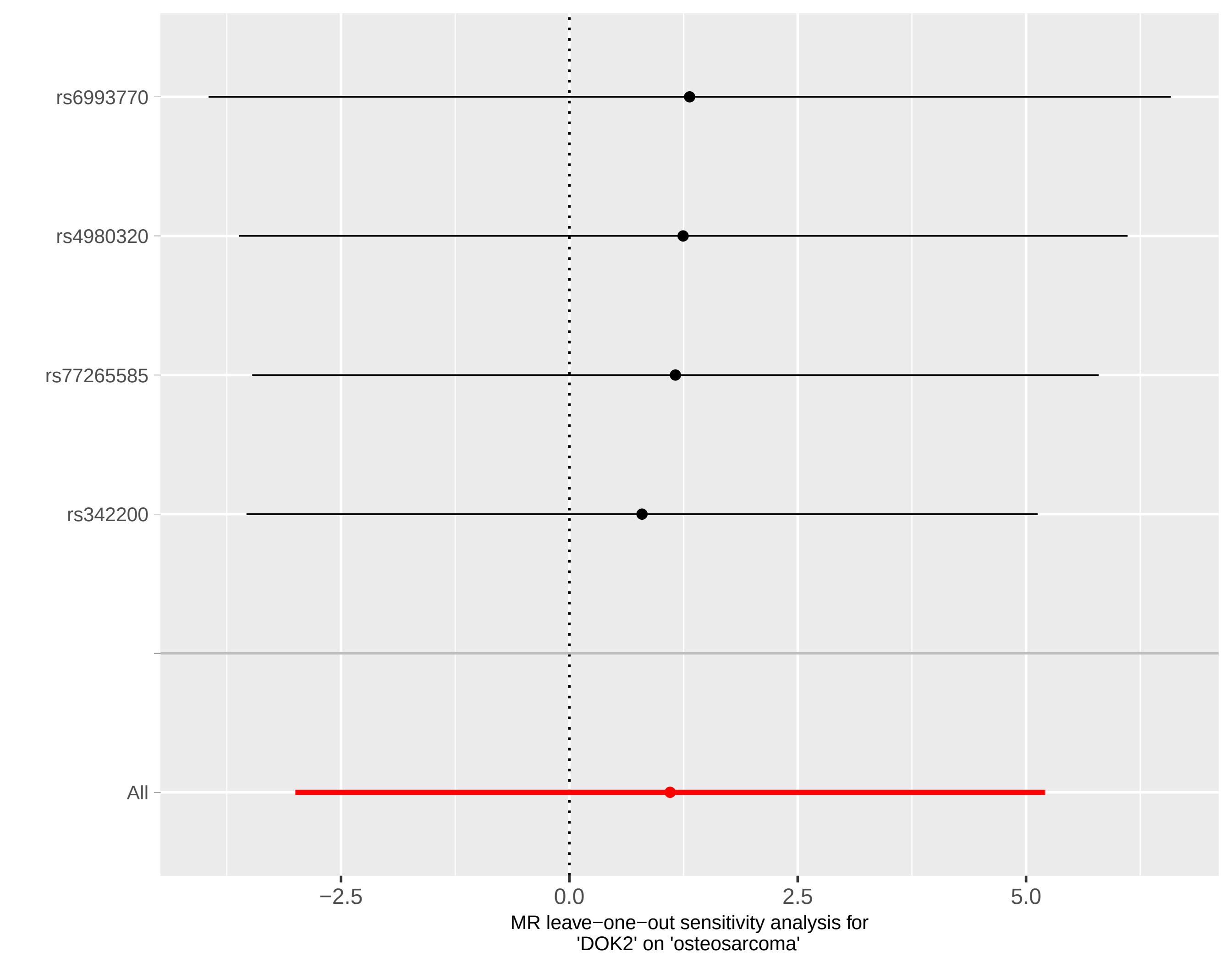

rs6993770
rs4980320
rs77265585
rs342200
All
−2.5
0.0
2.5
5.0
MR leave−one−out sensitivity analysis for
'DOK2' on 'osteosarcoma'

Insufficient number of SNPs

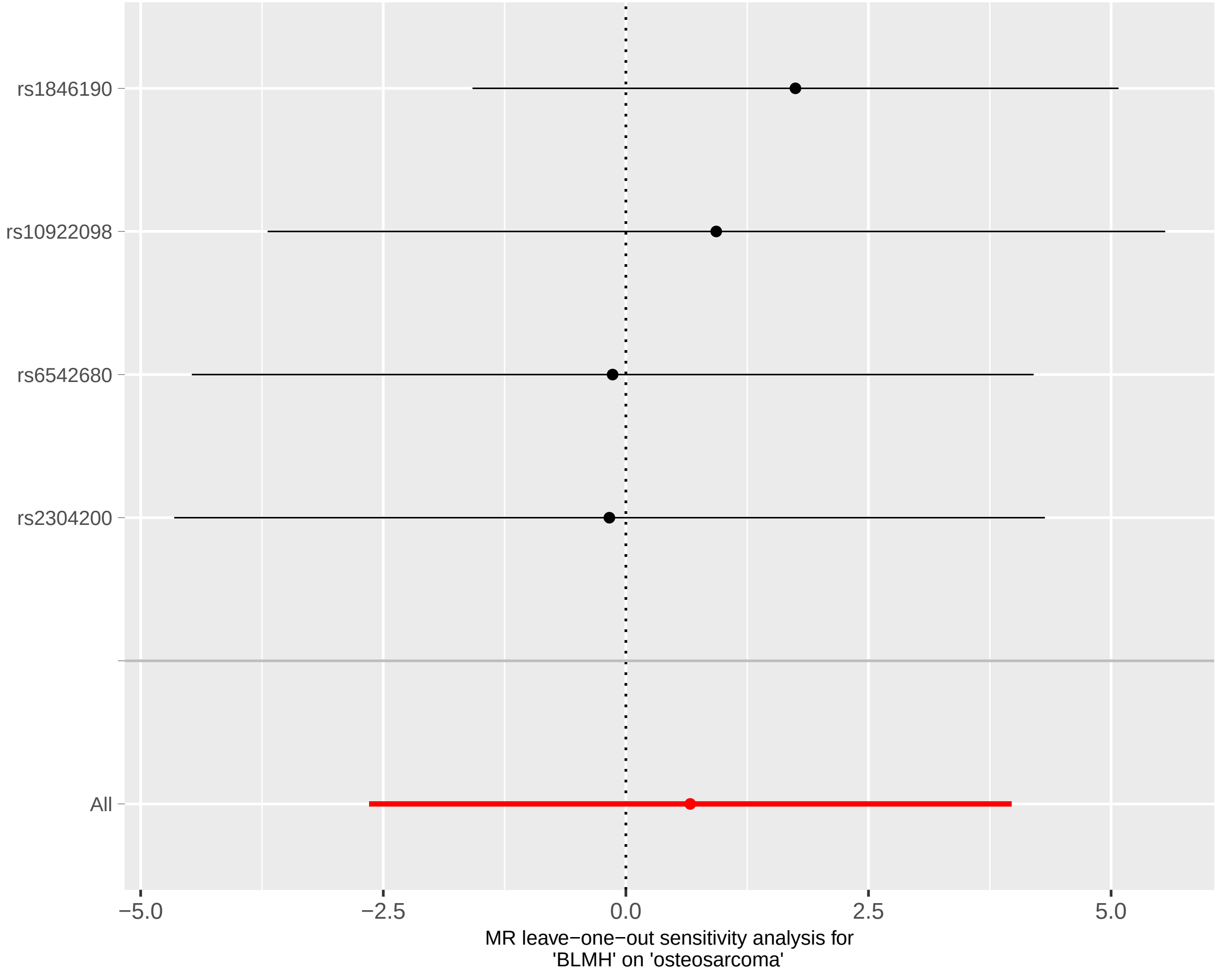

rs1846190
rs10922098
rs6542680
rs2304200
All
−5.0
−2.5
0.0
2.5
5.0
MR leave−one−out sensitivity analysis for
'BLMH' on 'osteosarcoma'

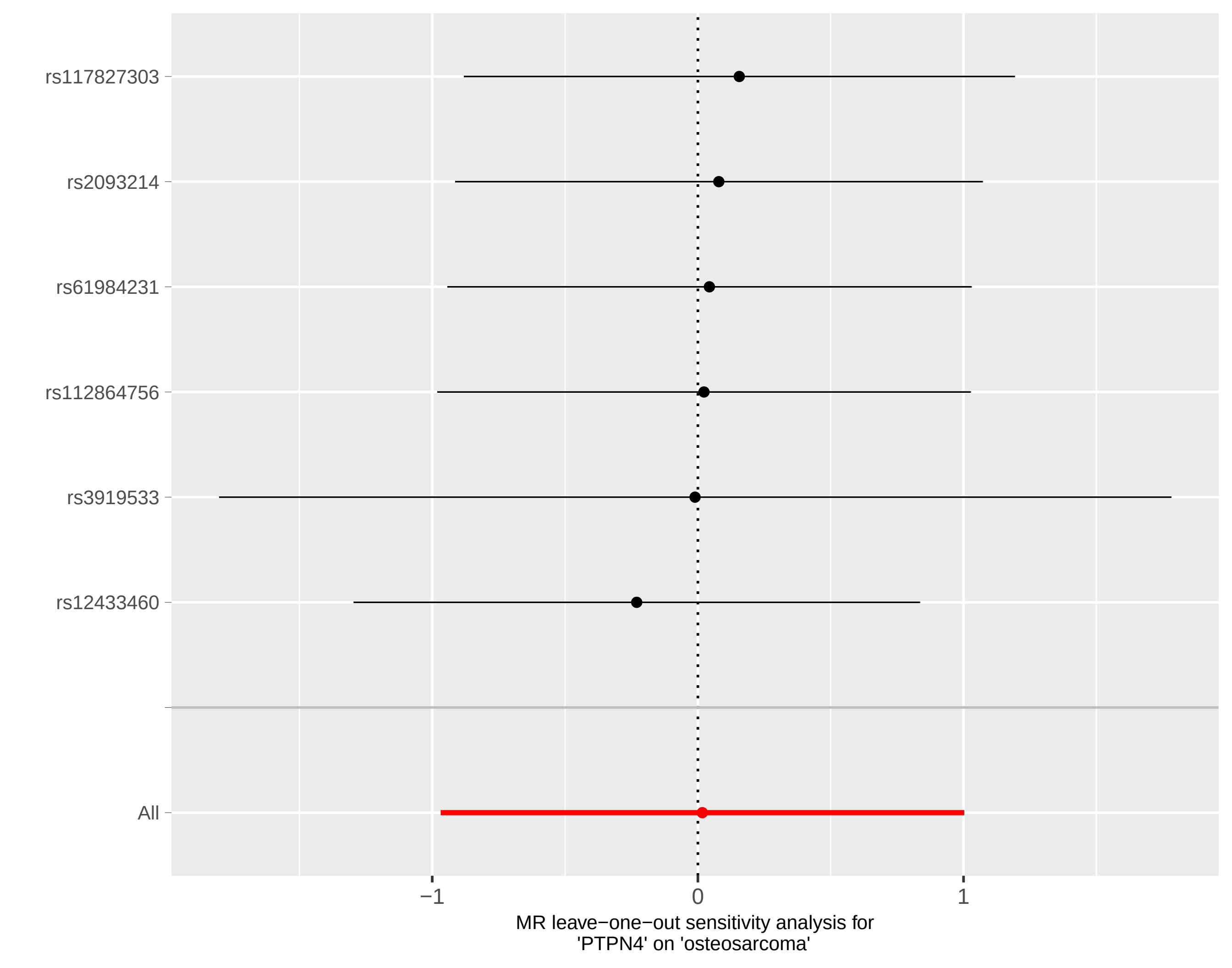
rs117827303
rs2093214
rs61984231
rs112864756
rs3919533
rs12433460
All
−1
0
1
MR leave−one−out sensitivity analysis for
'PTPN4' on 'osteosarcoma'

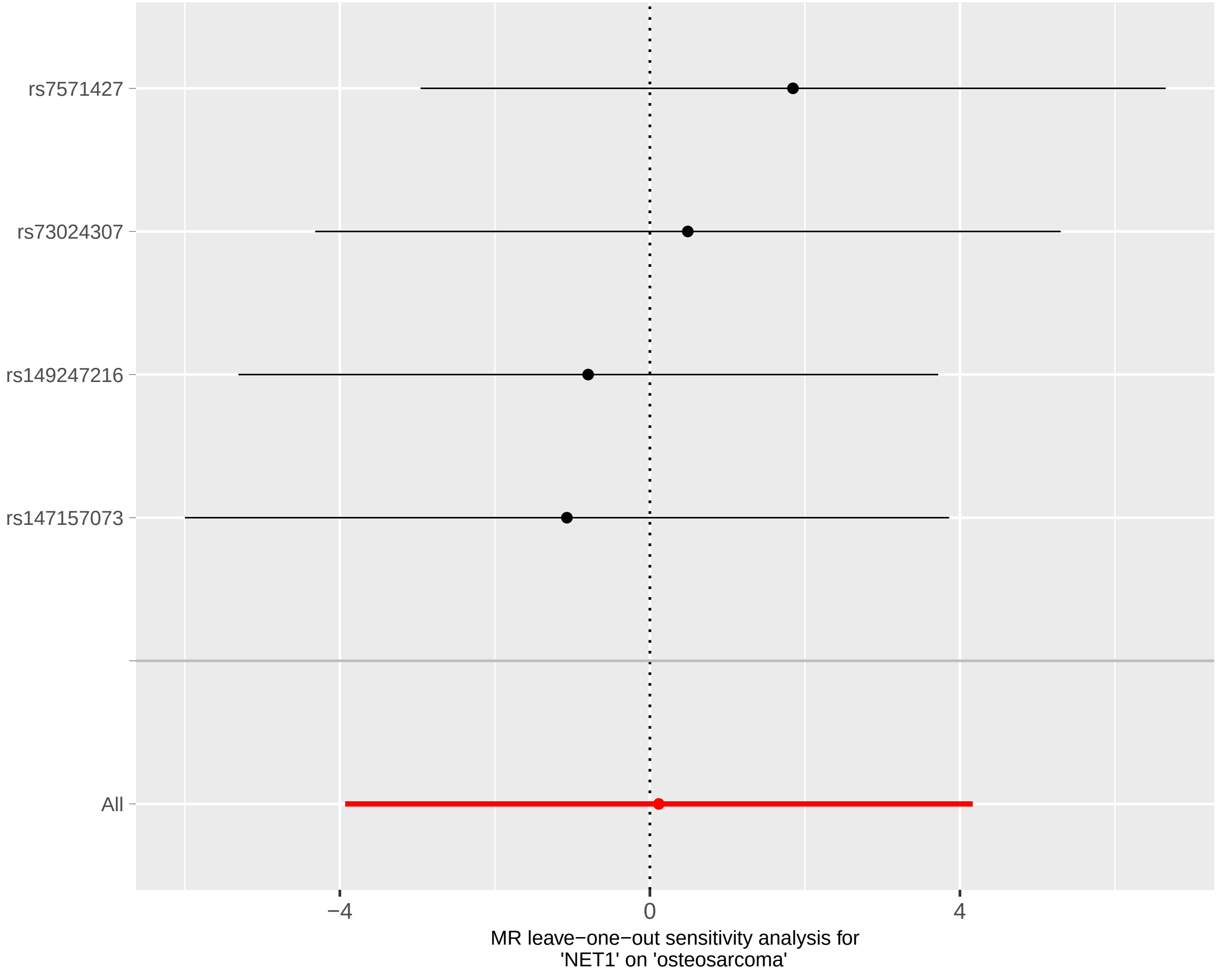

rs7571427
rs73024307
rs149247216
rs147157073
All
−4
0
4
MR leave−one−out sensitivity analysis for
'NET1' on 'osteosarcoma'

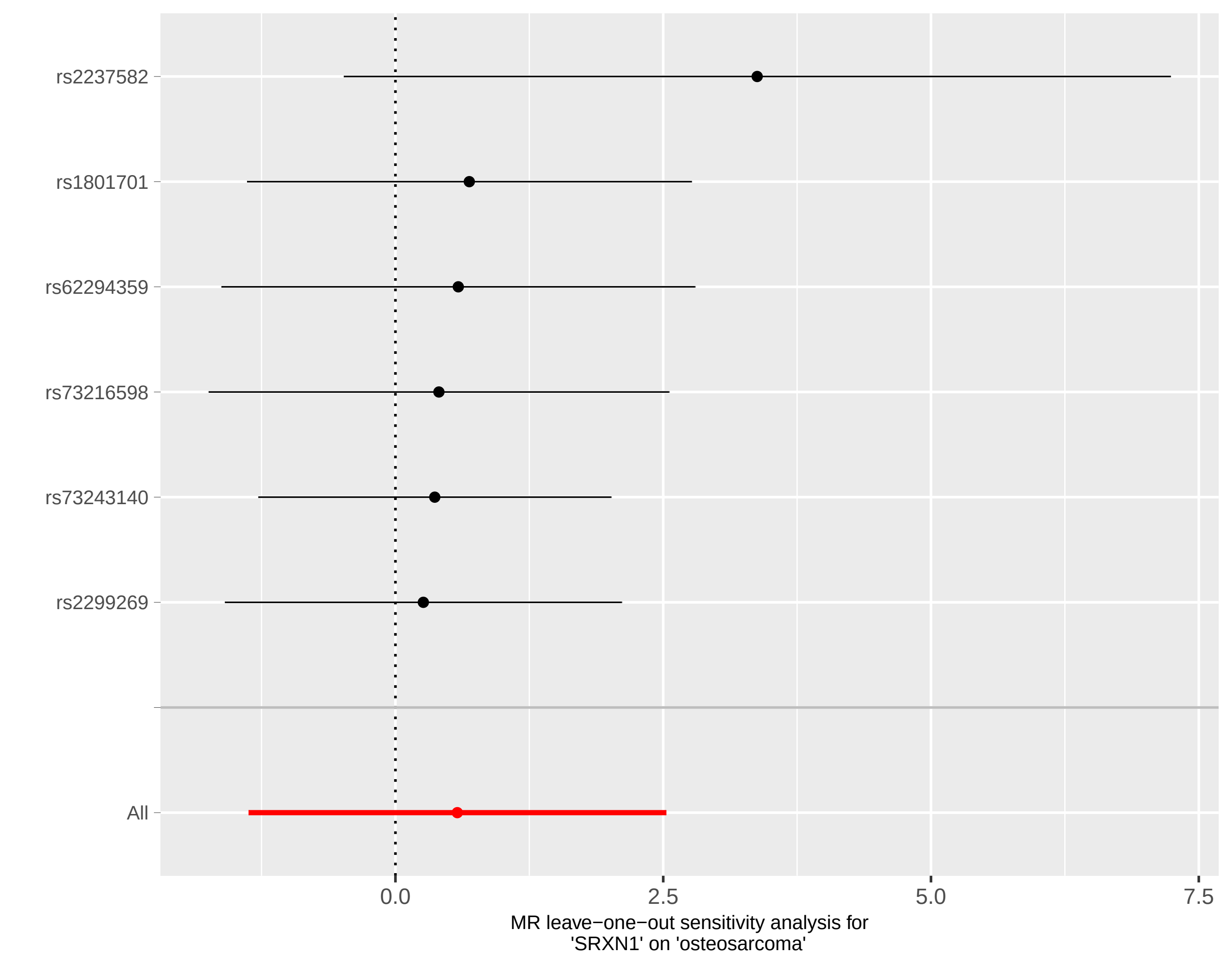

rs2237582
rs1801701
rs62294359
rs73216598
rs73243140
rs2299269
All
0.0
2.5
5.0
7.5
MR leave−one−out sensitivity analysis for
'SRXN1' on 'osteosarcoma'

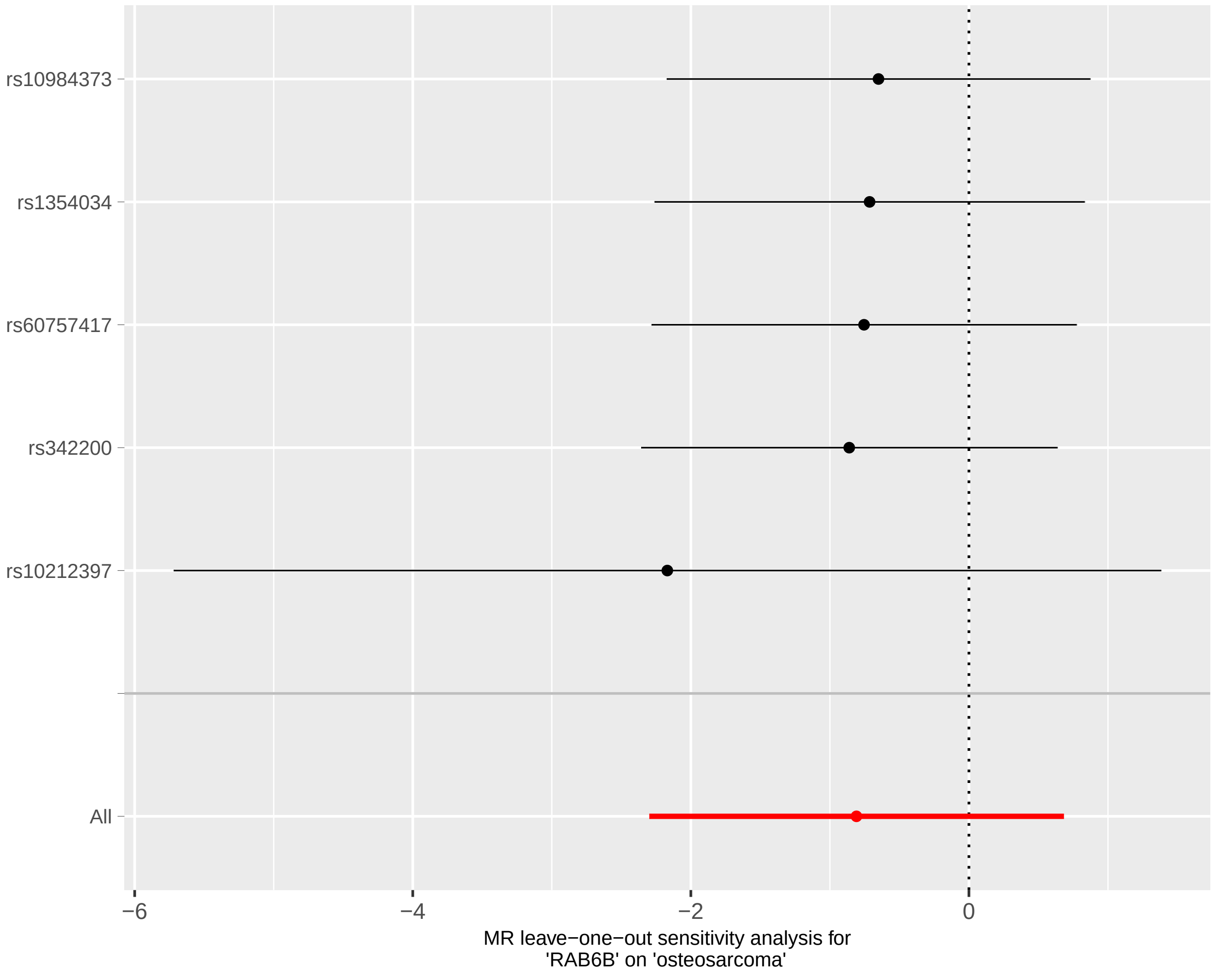
rs10984373
rs1354034
rs60757417
rs342200
rs10212397
All
−6
−4
−2
0
MR leave−one−out sensitivity analysis for
'RAB6B' on 'osteosarcoma'

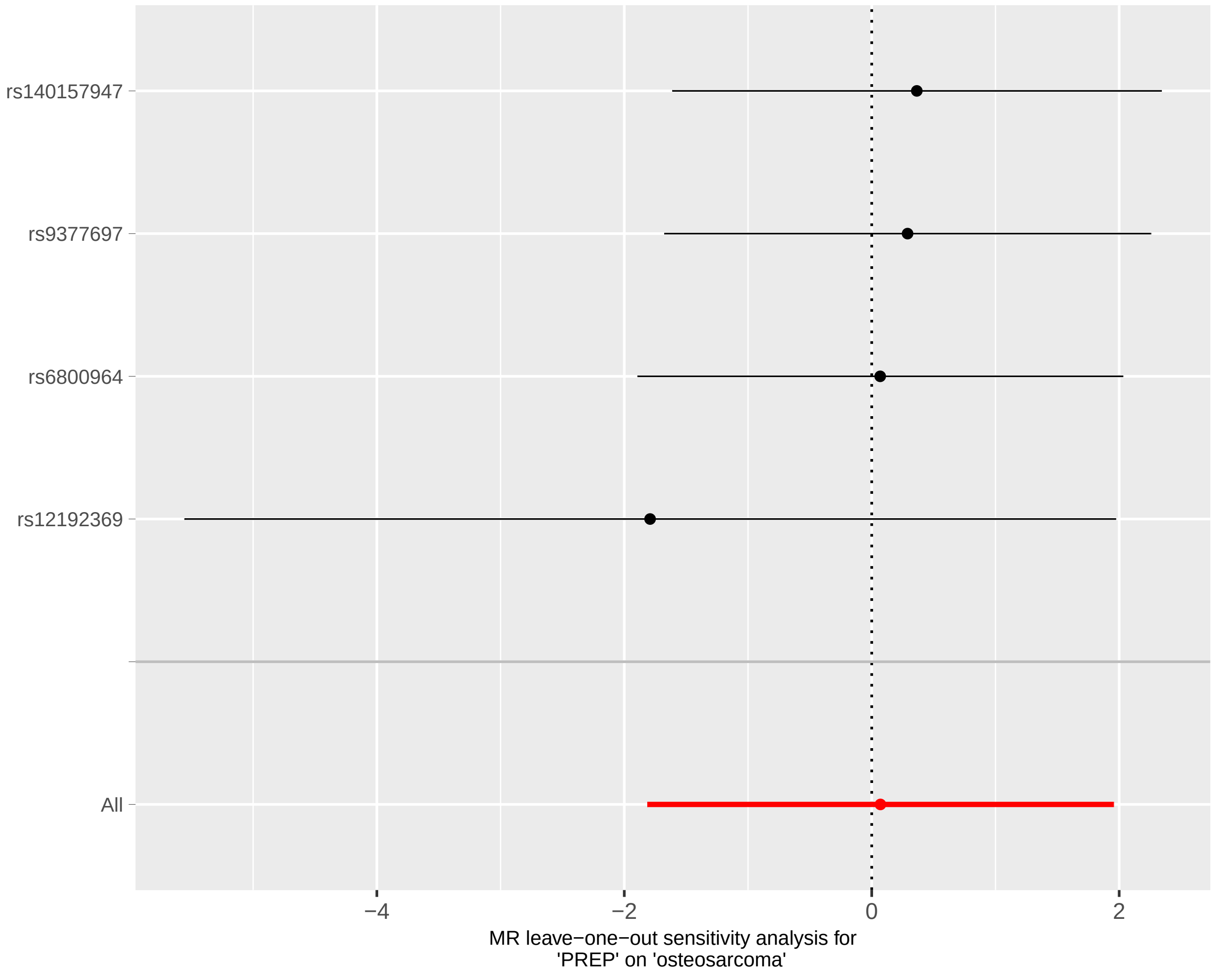

rs140157947
rs9377697
rs6800964
rs12192369
All
−4
−2
0
2
MR leave−one−out sensitivity analysis for
'PREP' on 'osteosarcoma'

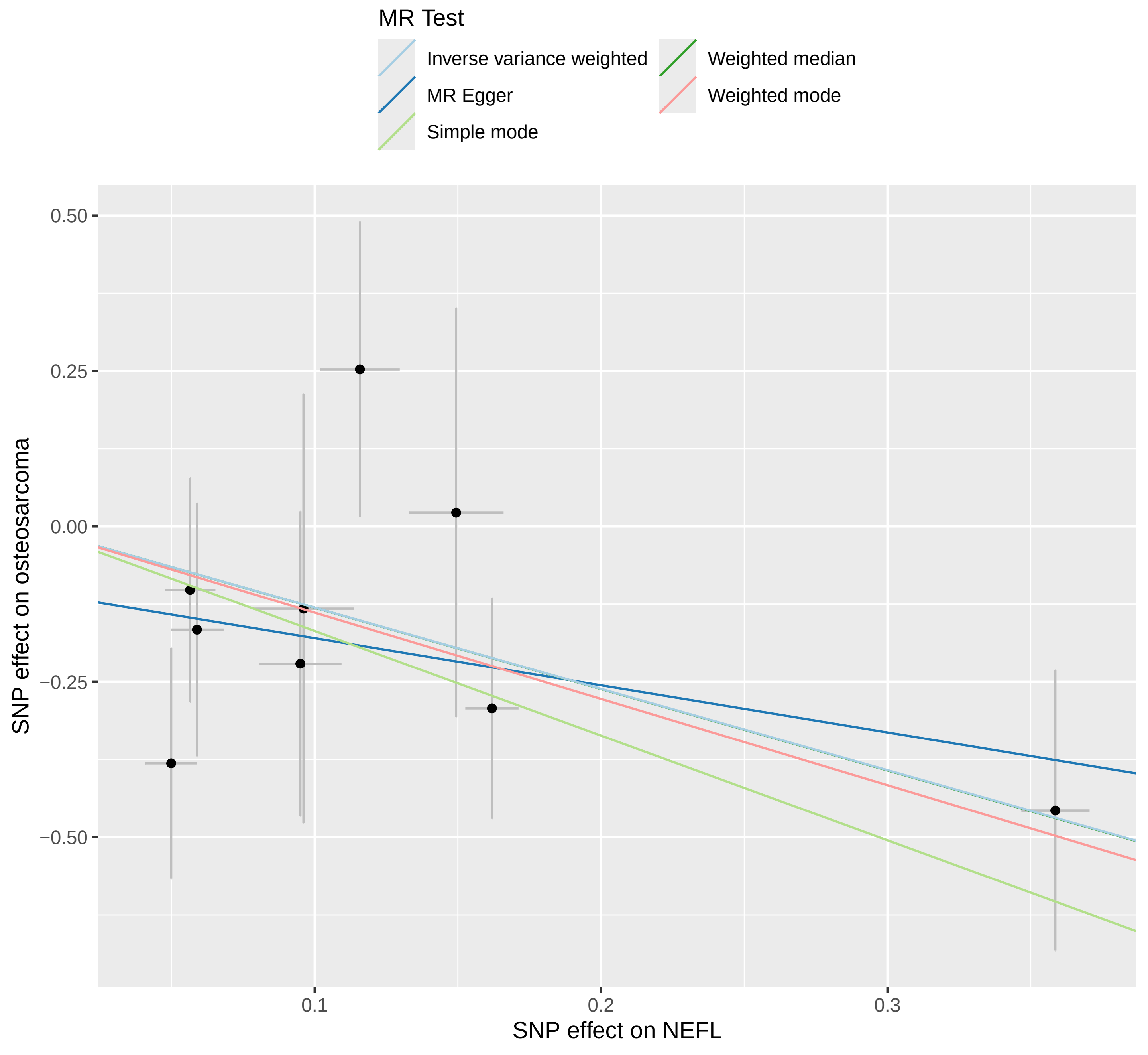
MR Test
Inverse variance weighted
MR Egger
Simple mode
Weighted median
Weighted mode
0.50
0.25
0.00
−0.25
−0.50
0.1
0.2
0.3
SNP effect on osteosarcoma
SNP effect on NEFL

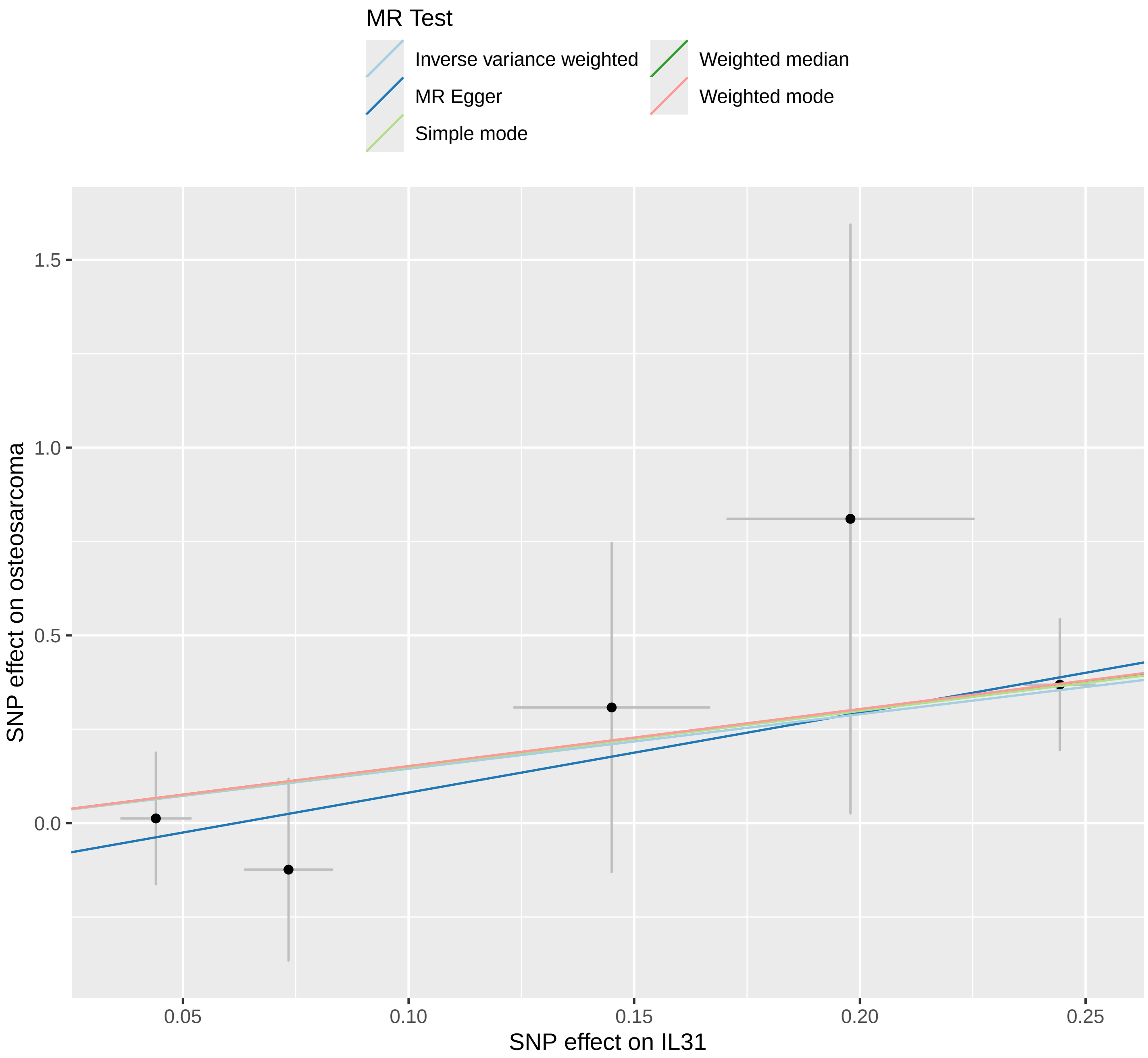
MR Test
Inverse variance weighted
MR Egger
Simple mode
Weighted median
Weighted mode
SNP effect on osteosarcoma
1.5
1.0
0.5
0.0
0.05
0.10
0.15
0.20
0.25
SNP effect on IL31

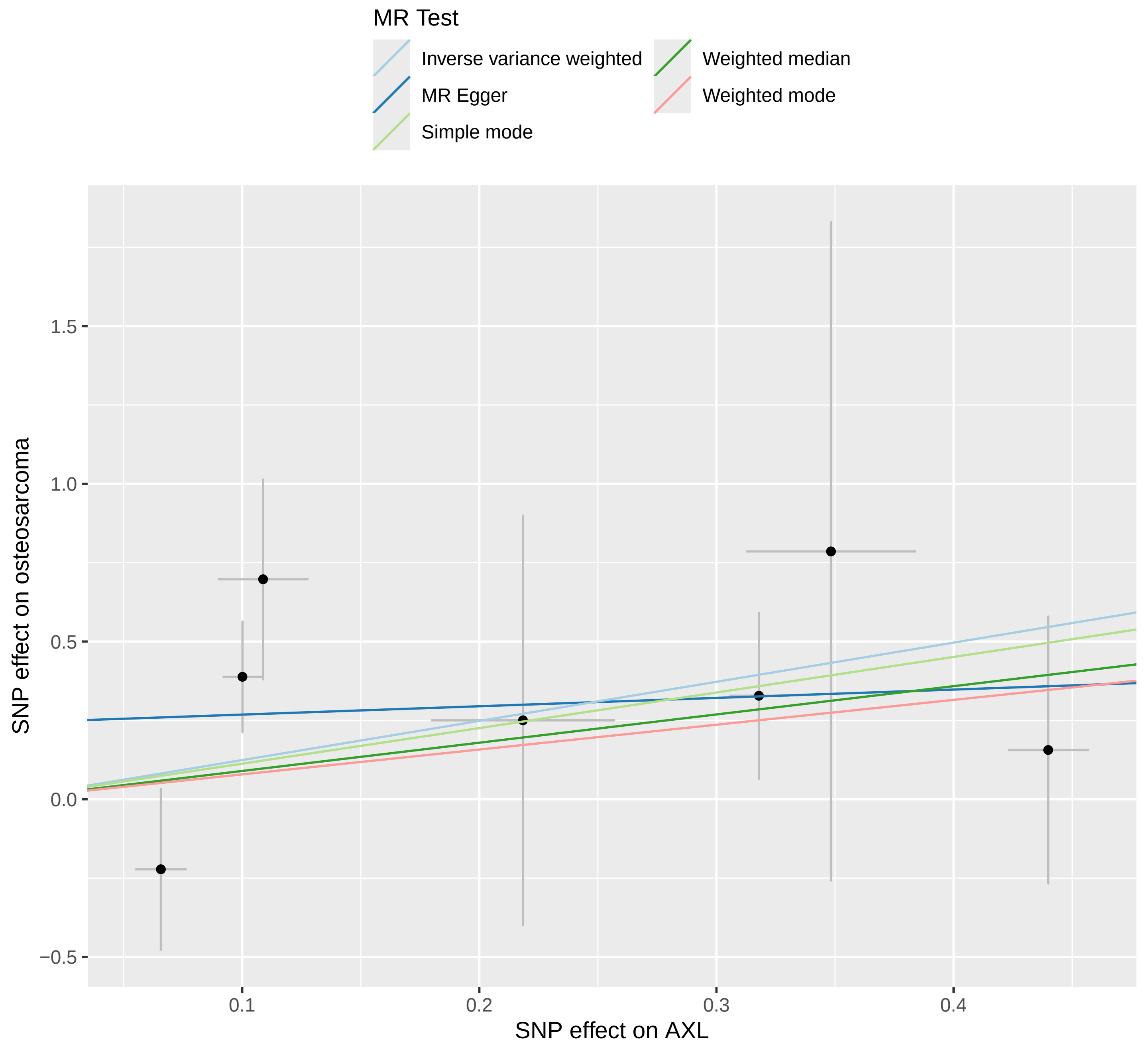
MR Test
Inverse variance weighted
MR Egger
Simple mode
Weighted median
Weighted mode
SNP effect on osteosarcoma
1.5
1.0
0.5
0.0
−0.5
0.1
0.2
0.3
0.4
SNP effect on AXL

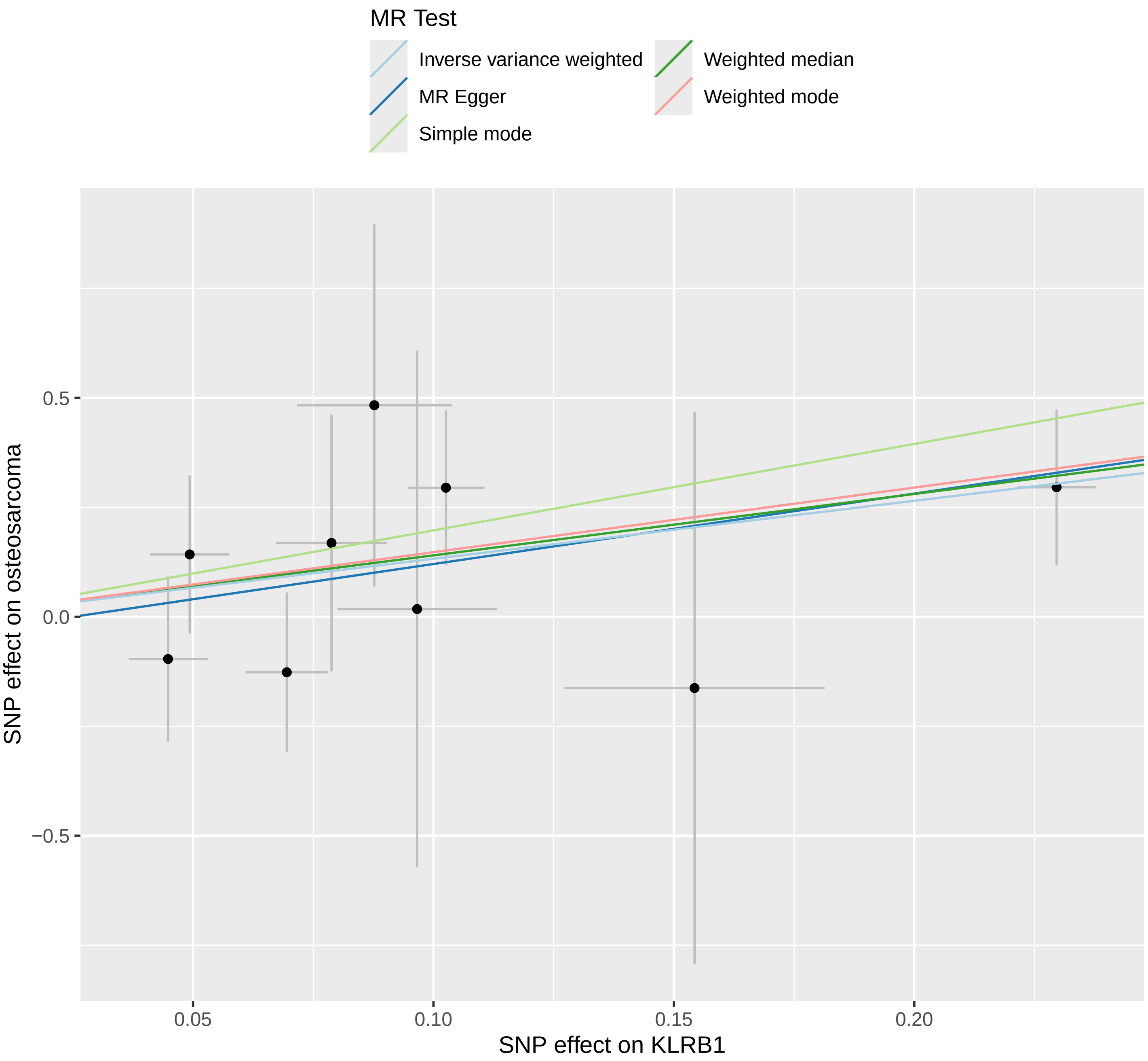

MR Test
Inverse variance weighted
MR Egger
Simple mode
Weighted median
Weighted mode
SNP effect on osteosarcoma
0.5
0.0
−0.5
0.05
0.10
0.15
0.20
SNP effect on KLRB1

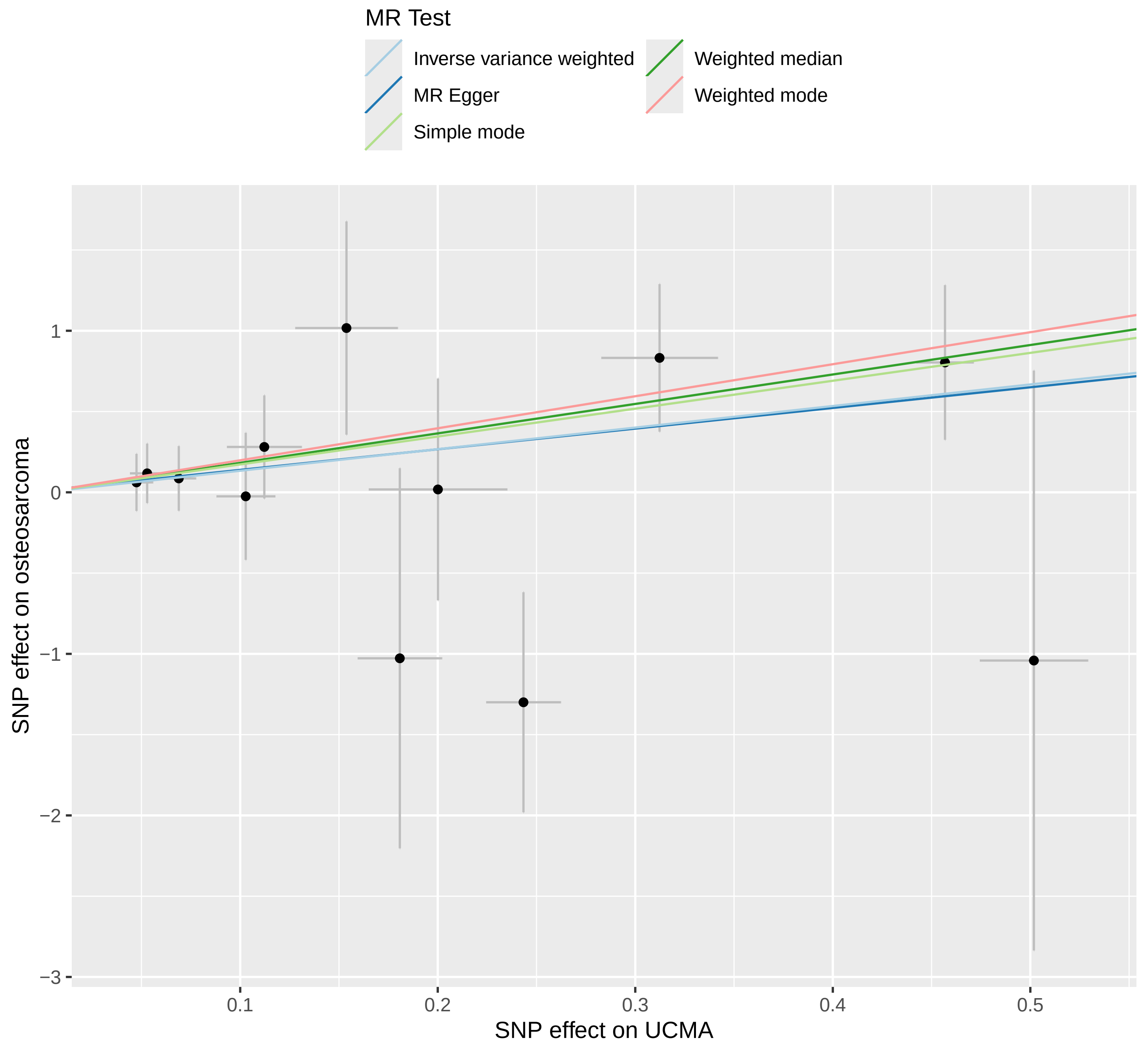
MR Test
Inverse variance weighted
MR Egger
Simple mode
Weighted median
Weighted mode
SNP effect on osteosarcoma
SNP effect on UCMA
1
0
−1
−2
−3
0.1
0.2
0.3
0.4
0.5

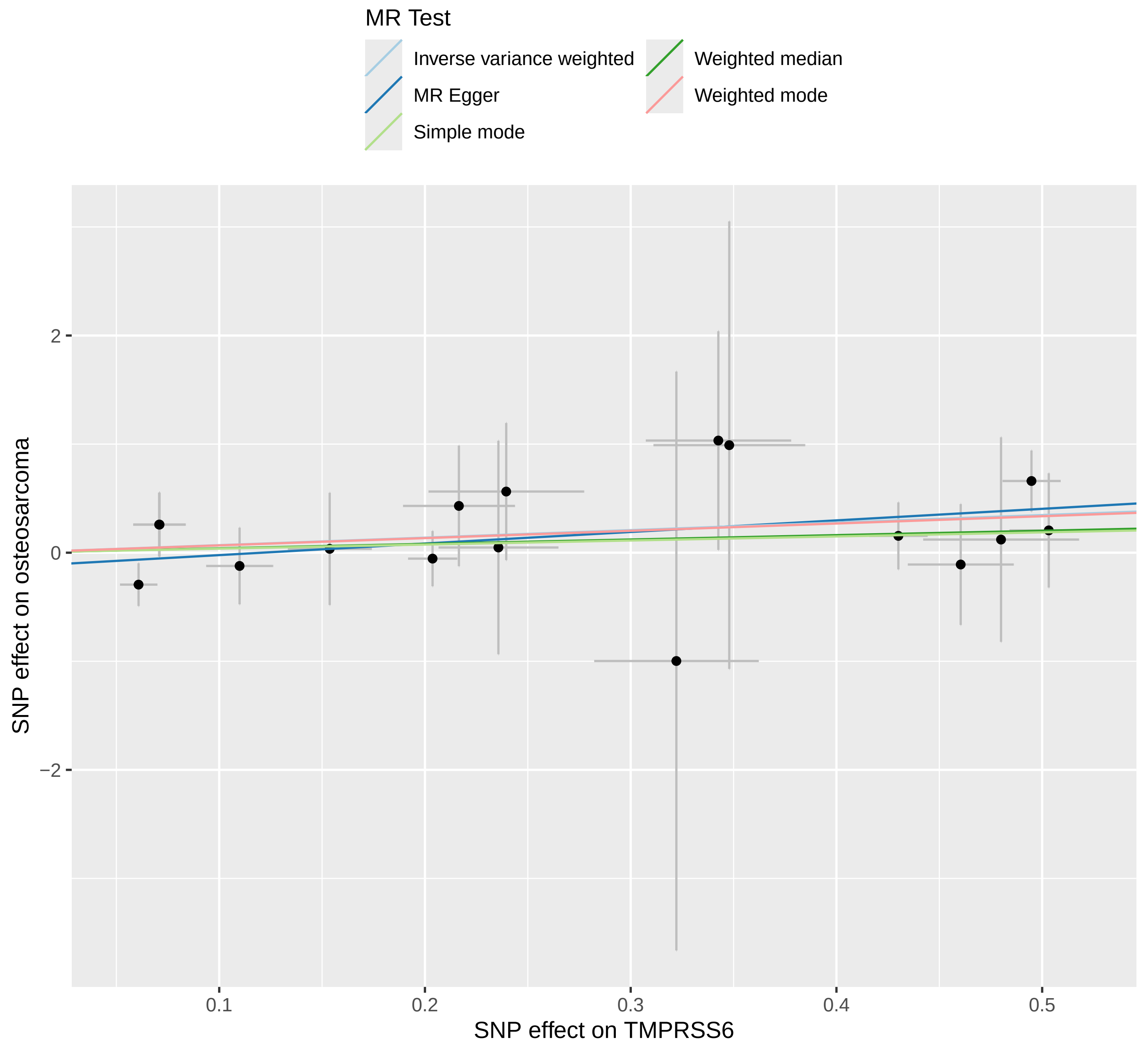

MR Test
Inverse variance weighted
MR Egger
Simple mode
Weighted median
Weighted mode
2
0
−2
0.1
0.2
0.3
0.4
0.5
SNP effect on TMPRSS6
SNP effect on osteosarcoma

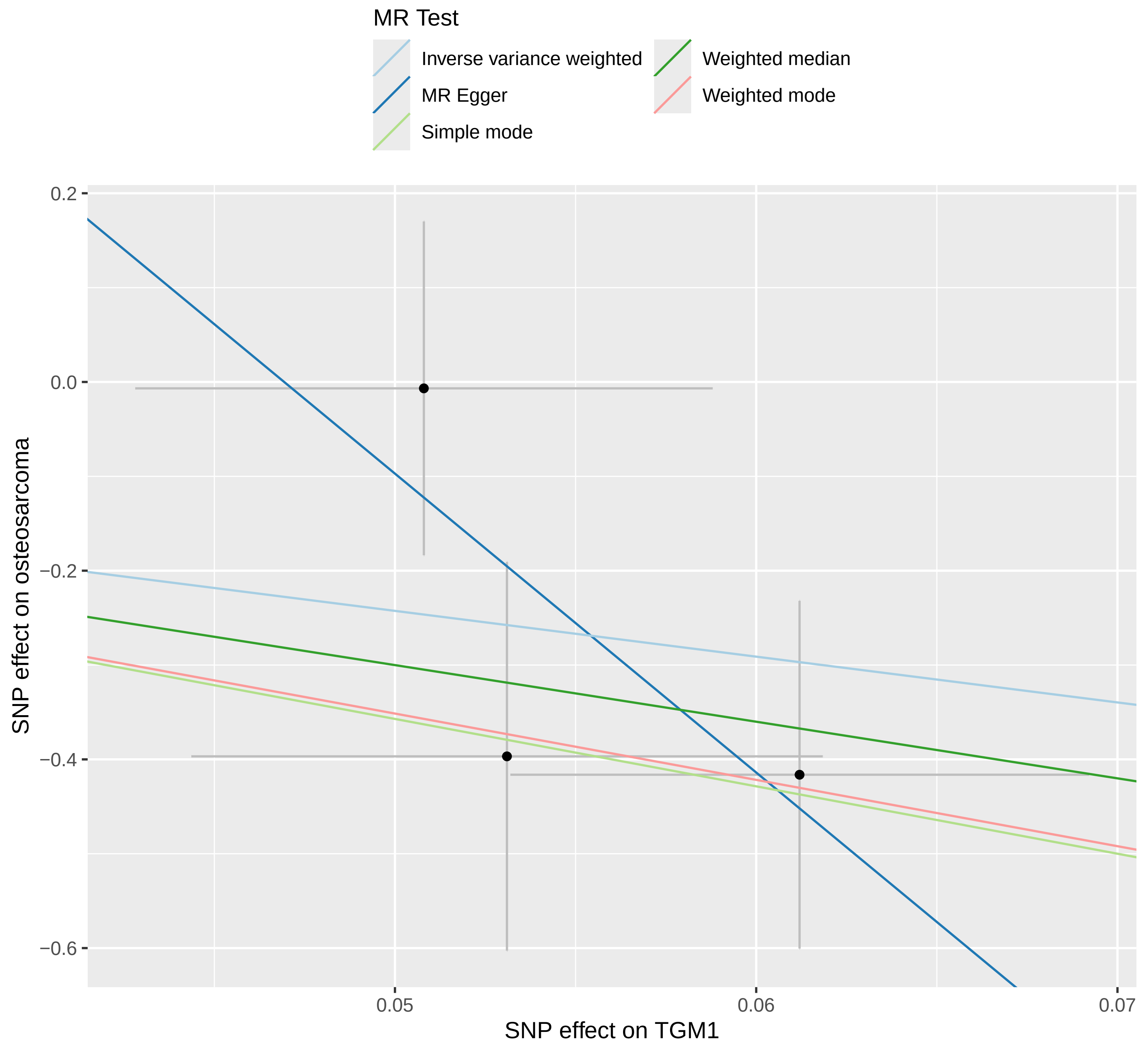
MR Test
Inverse variance weighted
MR Egger
Simple mode
Weighted median
Weighted mode
0.2
0.0
−0.2
−0.4
−0.6
SNP effect on osteosarcoma
0.05
0.06
0.07
SNP effect on TGM1

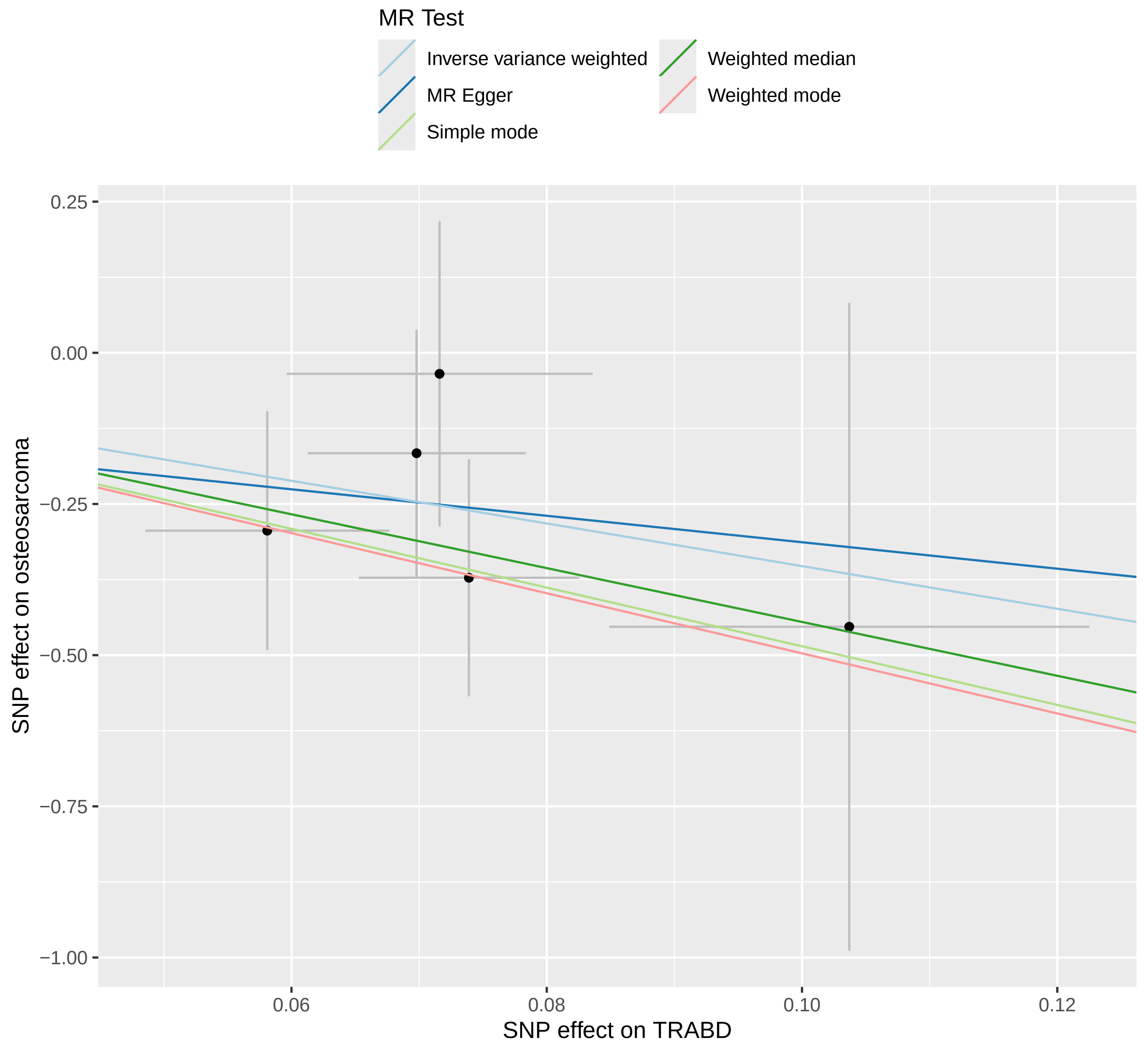

MR Test
Inverse variance weighted
MR Egger
Simple mode
Weighted median
Weighted mode
0.25
0.00
−0.25
−0.50
−0.75
−1.00
SNP effect on osteosarcoma
0.06
0.08
0.10
0.12
SNP effect on TRABD

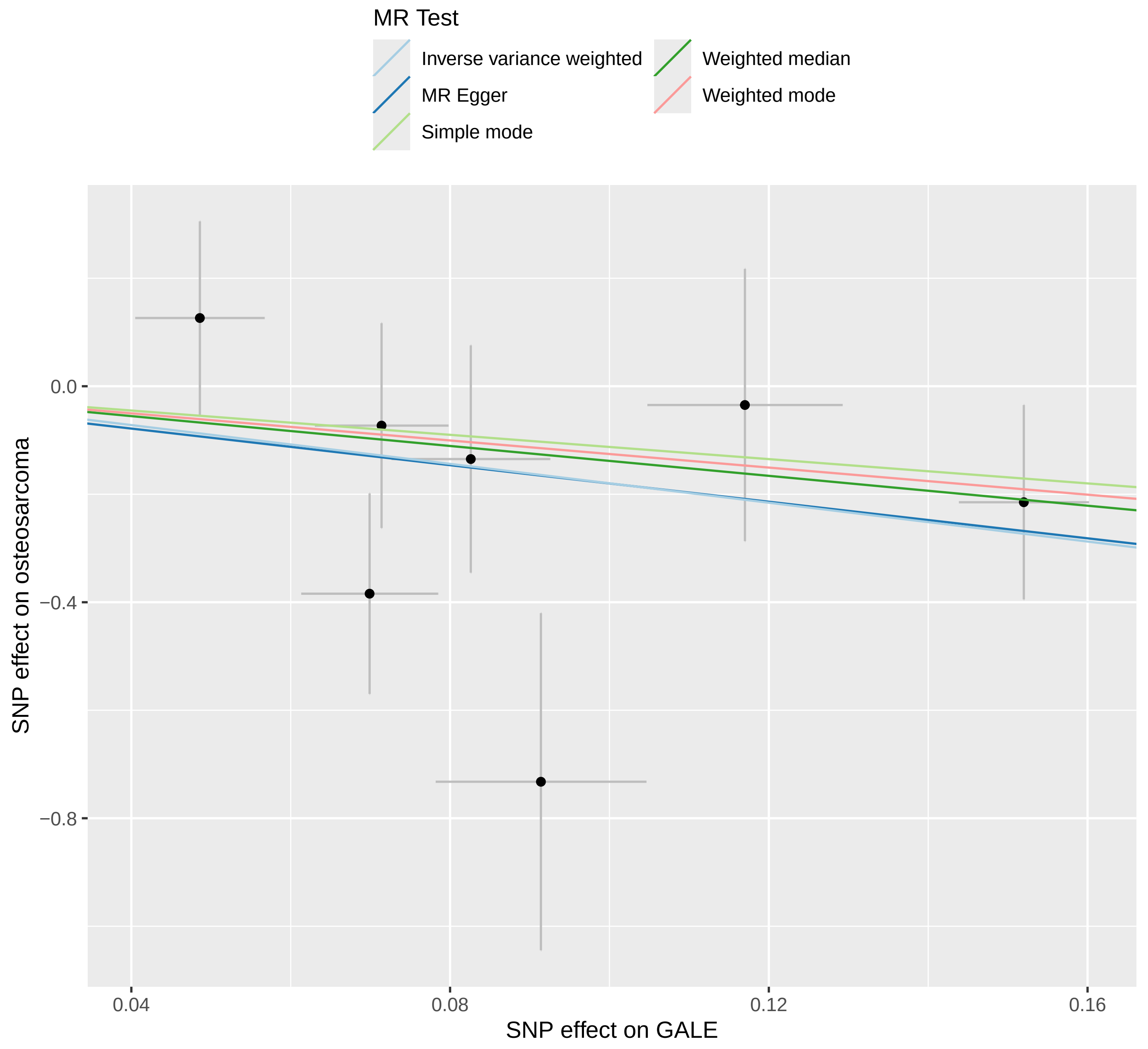

MR Test
Inverse variance weighted
MR Egger
Simple mode
Weighted median
Weighted mode
0.0
−0.4
−0.8
0.04
0.08
0.12
0.16
SNP effect on GALE
SNP effect on osteosarcoma

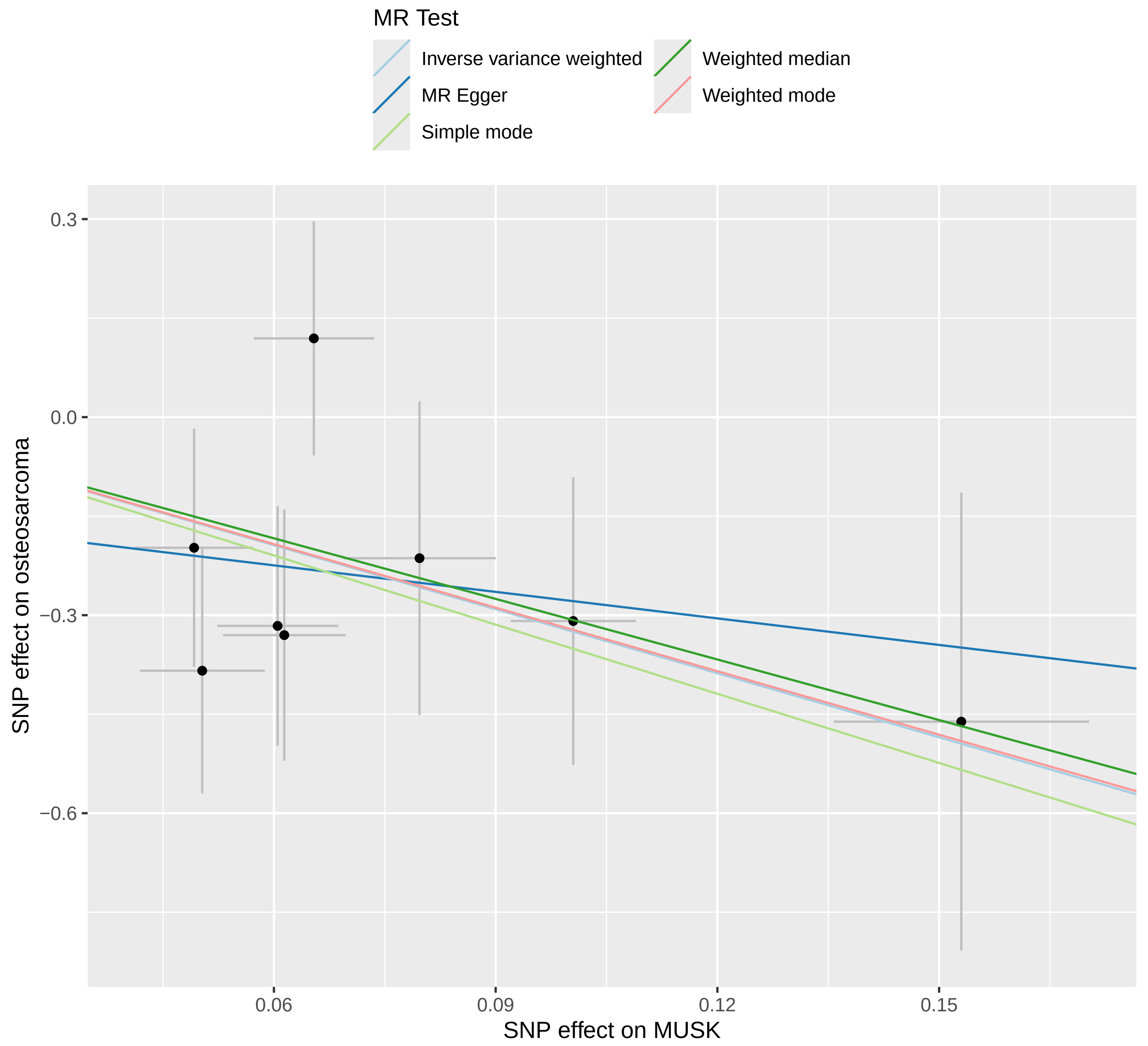
MR Test
Inverse variance weighted
MR Egger
Simple mode
Weighted median
Weighted mode
0.3
0.0
−0.3
−0.6
SNP effect on osteosarcoma
0.06
0.09
0.12
0.15
SNP effect on MUSK

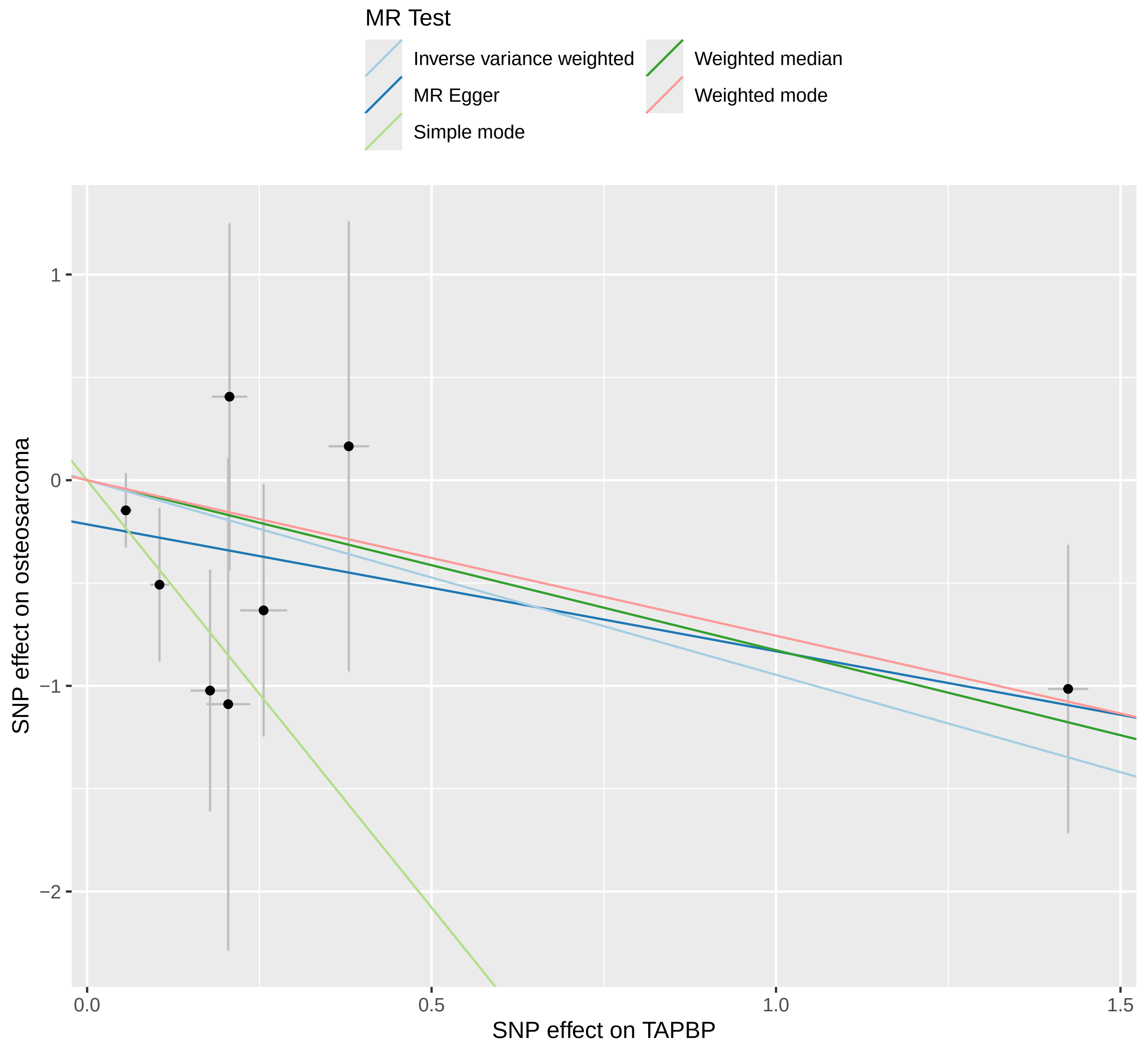
MR Test
Inverse variance weighted
MR Egger
Simple mode
Weighted median
Weighted mode
SNP effect on osteosarcoma
1
0
−1
−2
0.0
0.5
1.0
1.5
SNP effect on TAPBP

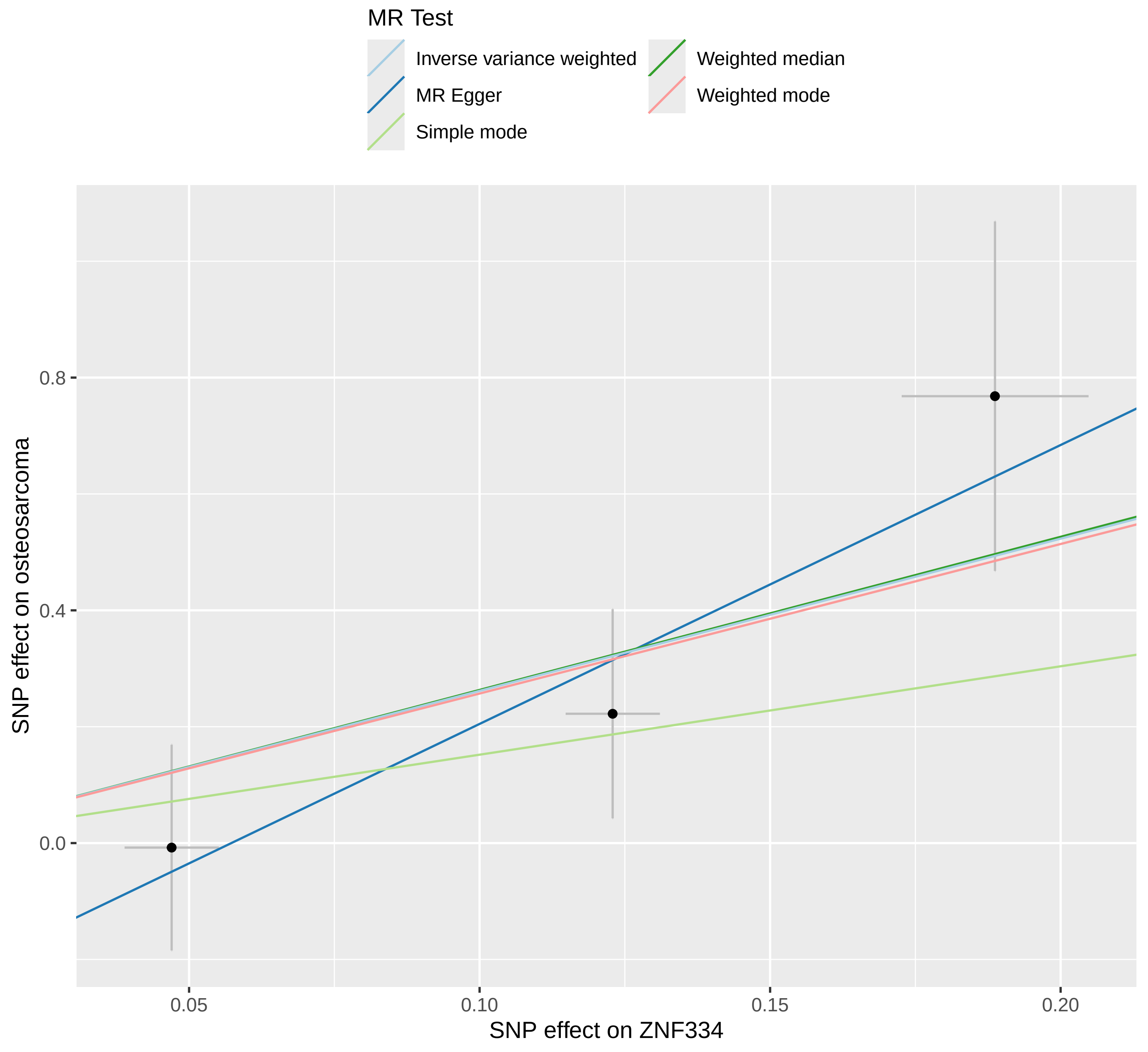

MR Test
Inverse variance weighted
MR Egger
Simple mode
Weighted median
Weighted mode
0.8
0.4
0.0
SNP effect on osteosarcoma
0.05
0.10
0.15
0.20
SNP effect on ZNF334

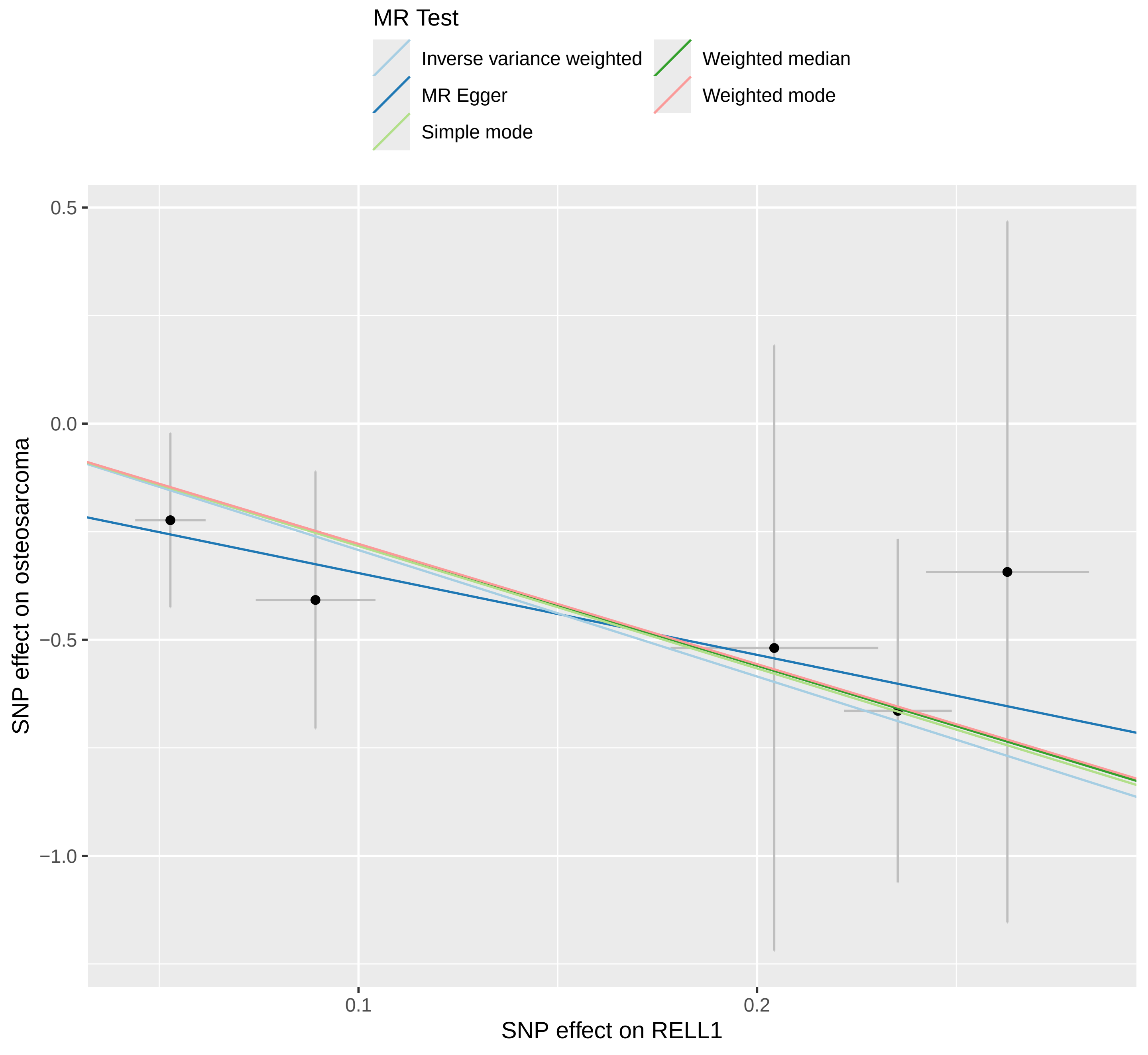
MR Test
Inverse variance weighted
MR Egger
Simple mode
Weighted median
Weighted mode
0.5
0.0
−0.5
−1.0
SNP effect on osteosarcoma
0.1
0.2
SNP effect on RELL1

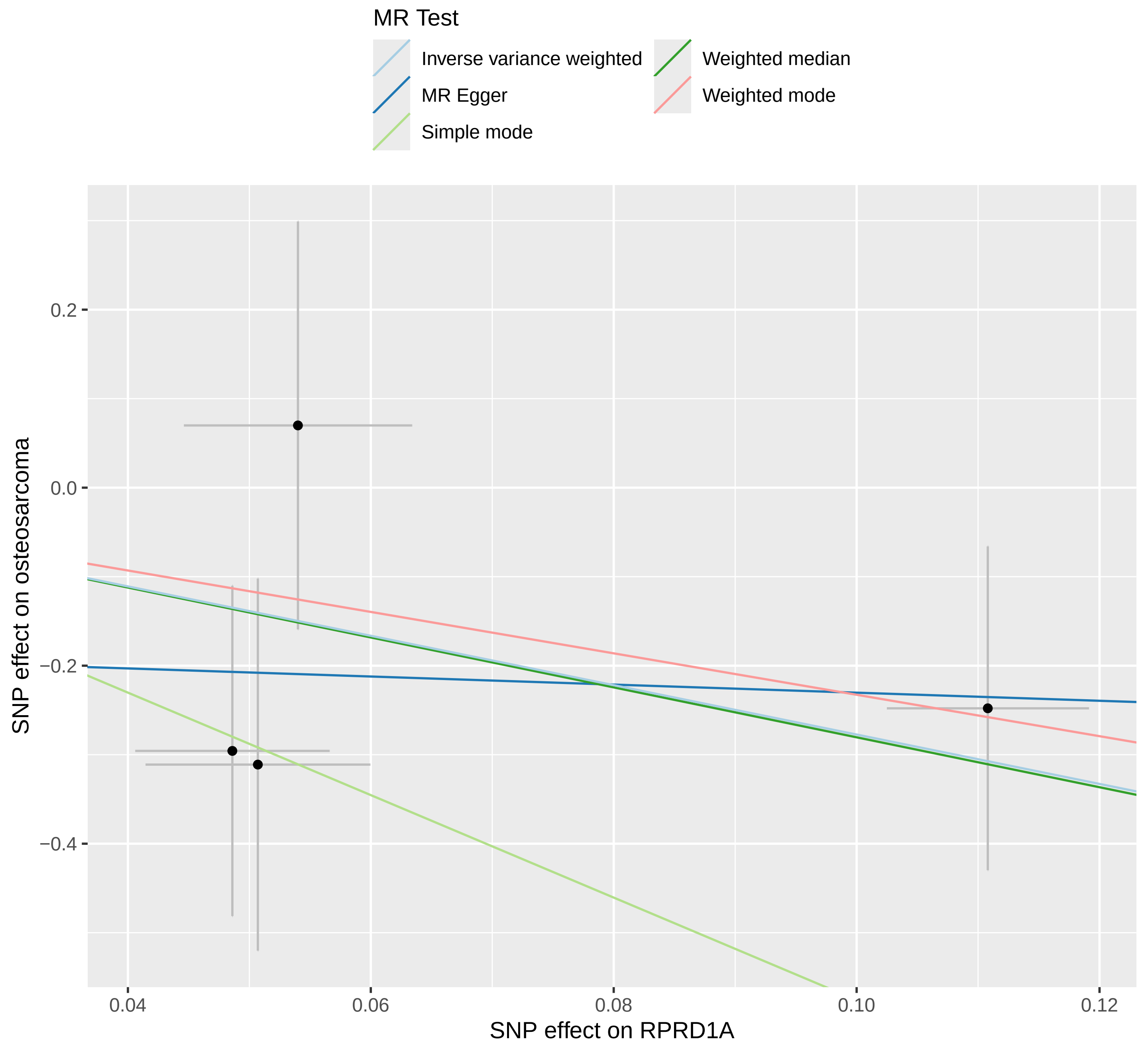
MR Test
Inverse variance weighted
MR Egger
Simple mode
Weighted median
Weighted mode
0.2
0.0
−0.2
−0.4
SNP effect on osteosarcoma
0.04
0.06
0.08
0.10
0.12
SNP effect on RPRD1A

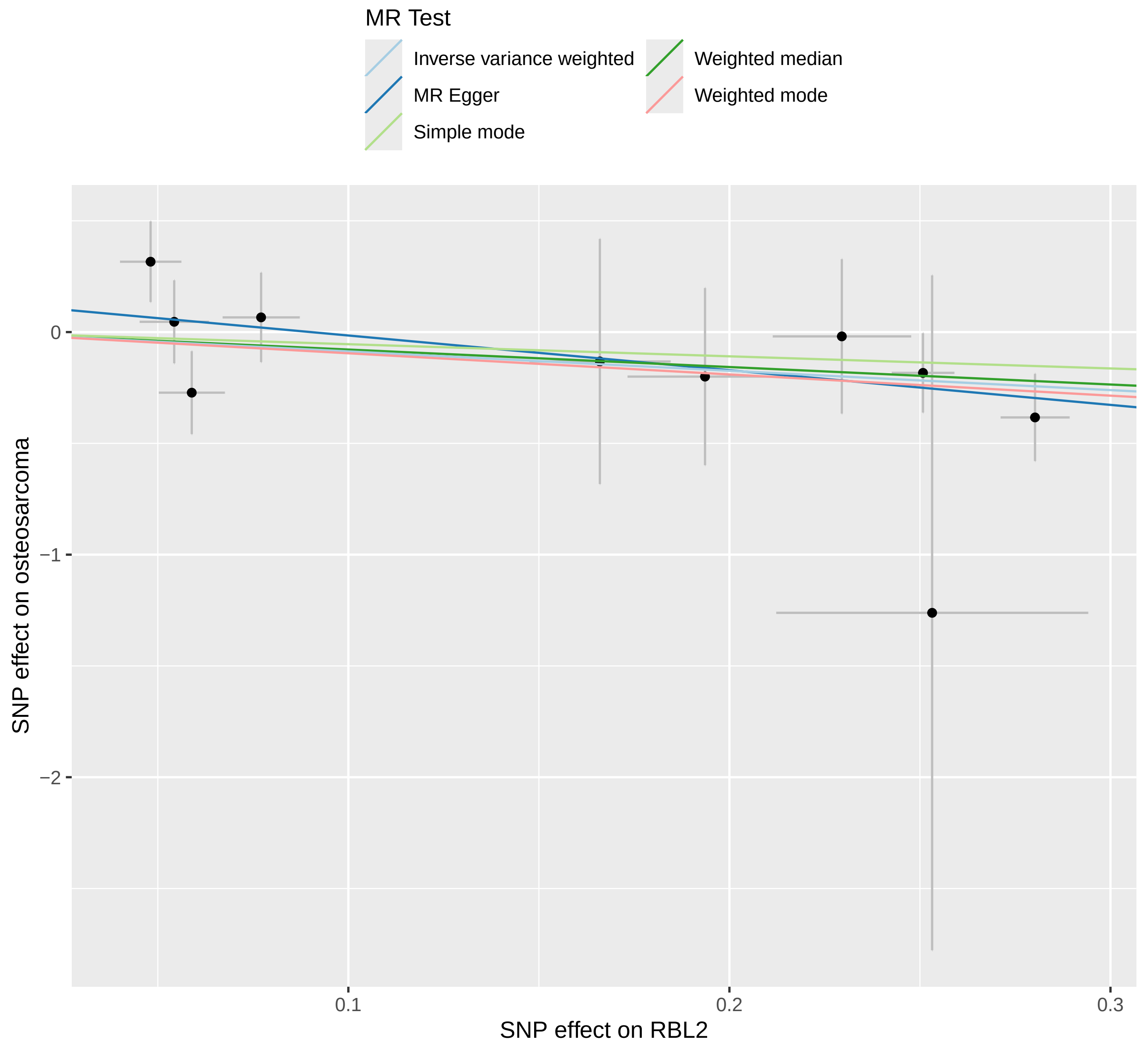

MR Test
Inverse variance weighted
MR Egger
Simple mode
Weighted median
Weighted mode
0
−1
−2
0.1
0.2
0.3
SNP effect on RBL2
SNP effect on osteosarcoma

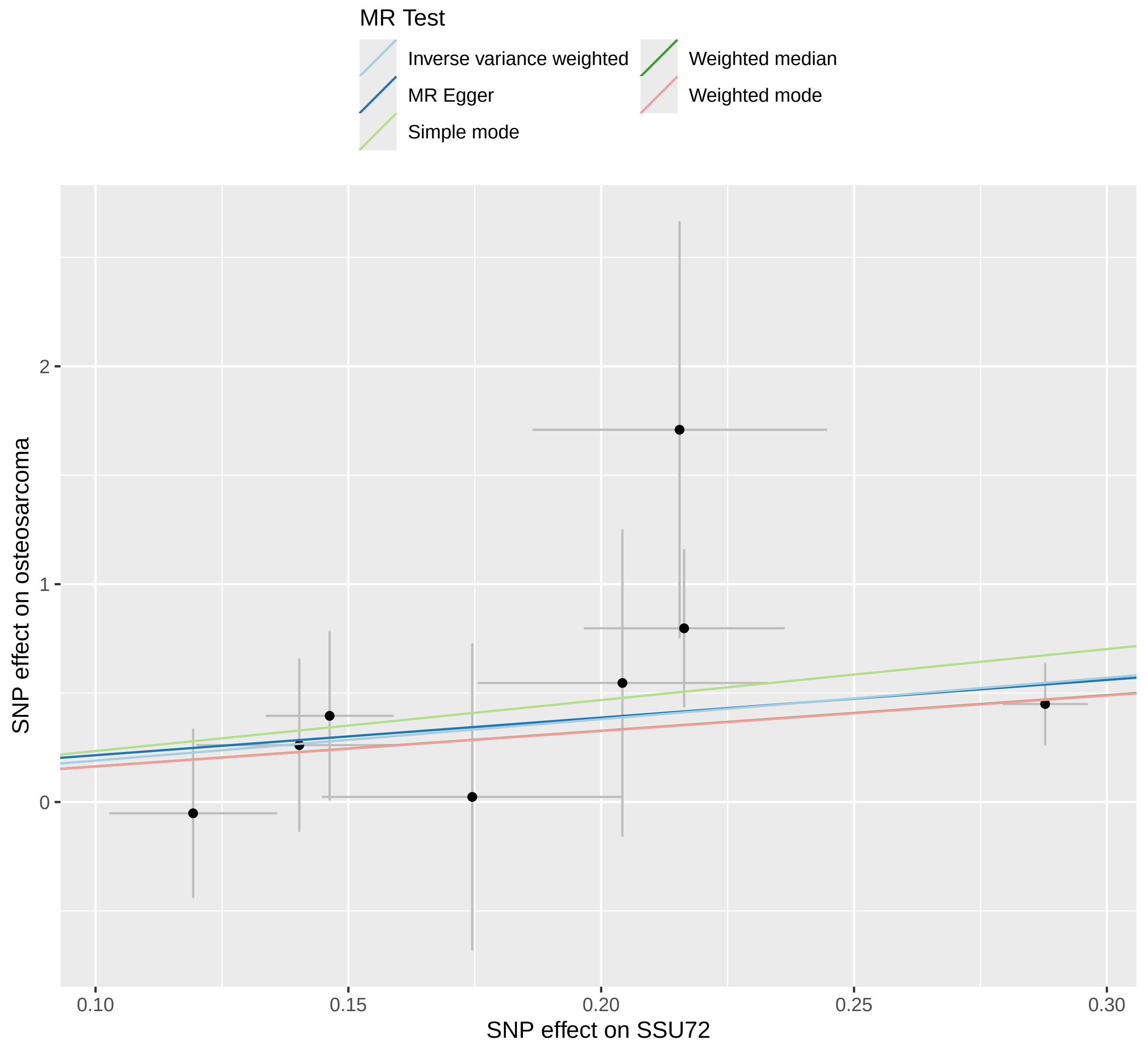
MR Test
Inverse variance weighted
MR Egger
Simple mode
Weighted median
Weighted mode
SNP effect on osteosarcoma
2
1
0
0.10
0.15
0.20
0.25
0.30
SNP effect on SSU72

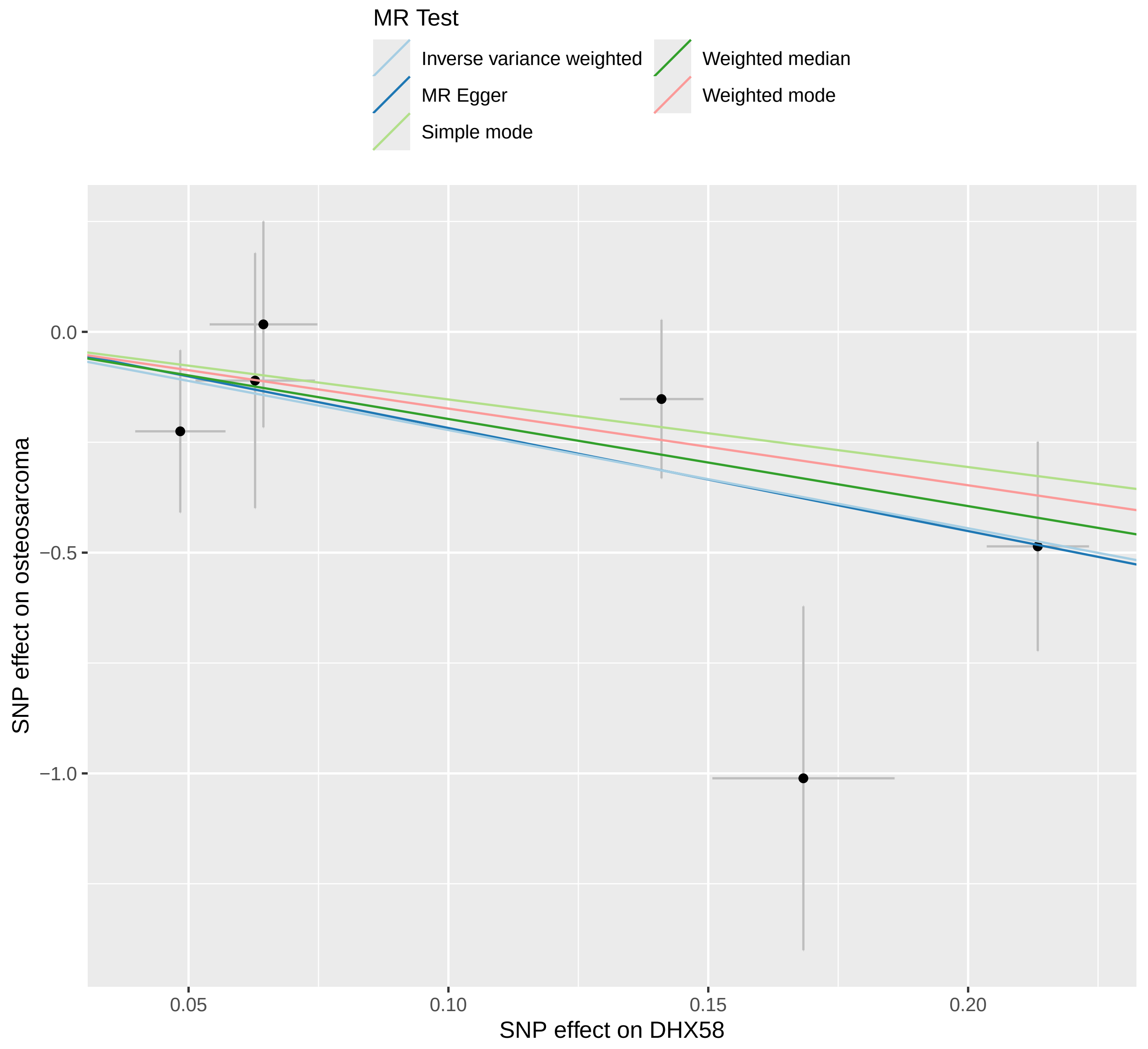
MR Test
Inverse variance weighted
MR Egger
Simple mode
Weighted median
Weighted mode
0.0
−0.5
−1.0
0.05
0.10
0.15
0.20
SNP effect on DHX58
SNP effect on osteosarcoma

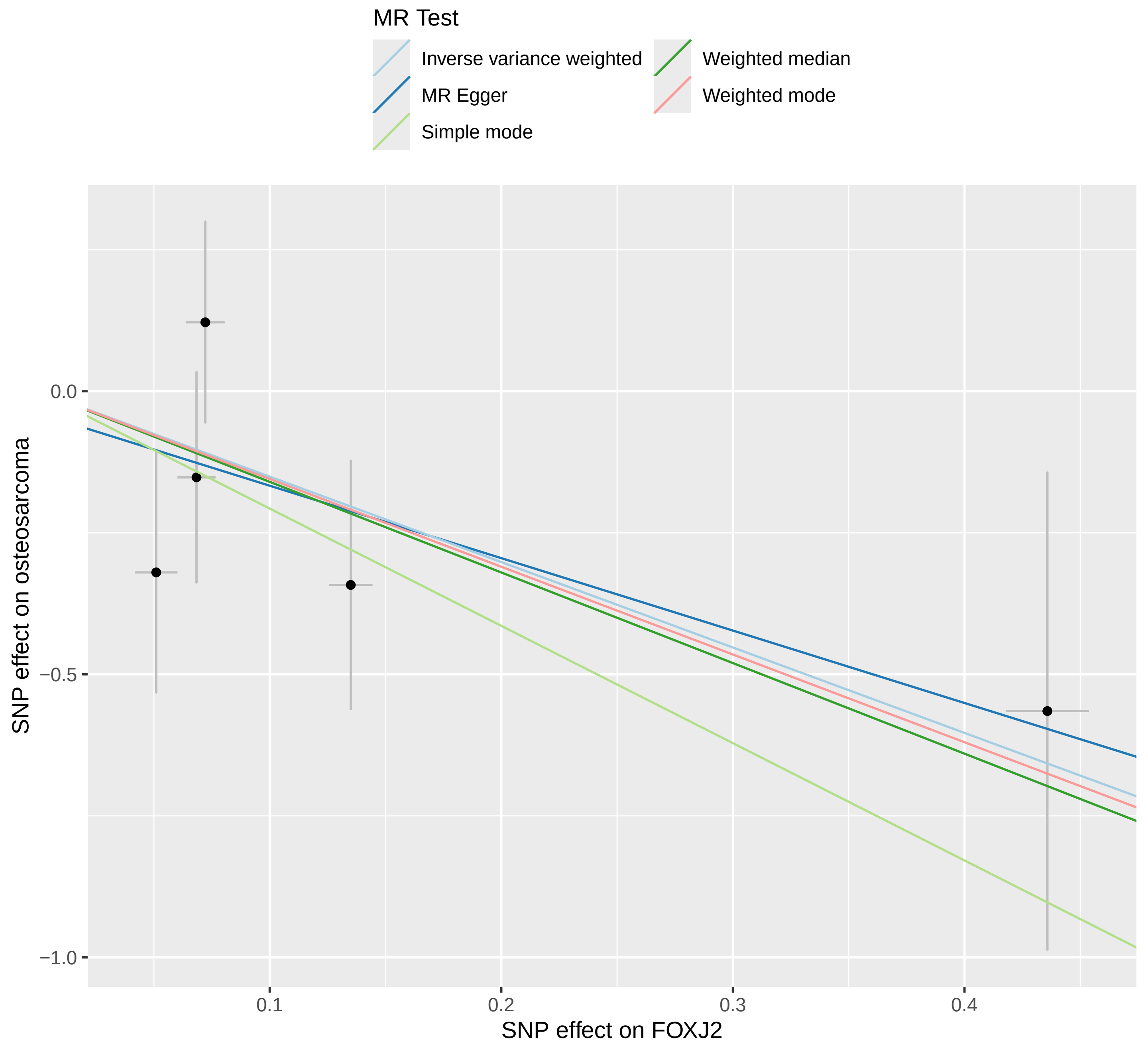
MR Test
Inverse variance weighted
MR Egger
Simple mode
Weighted median
Weighted mode
0.0
−0.5
−1.0
0.1
0.2
0.3
0.4
SNP effect on osteosarcoma
SNP effect on FOXJ2

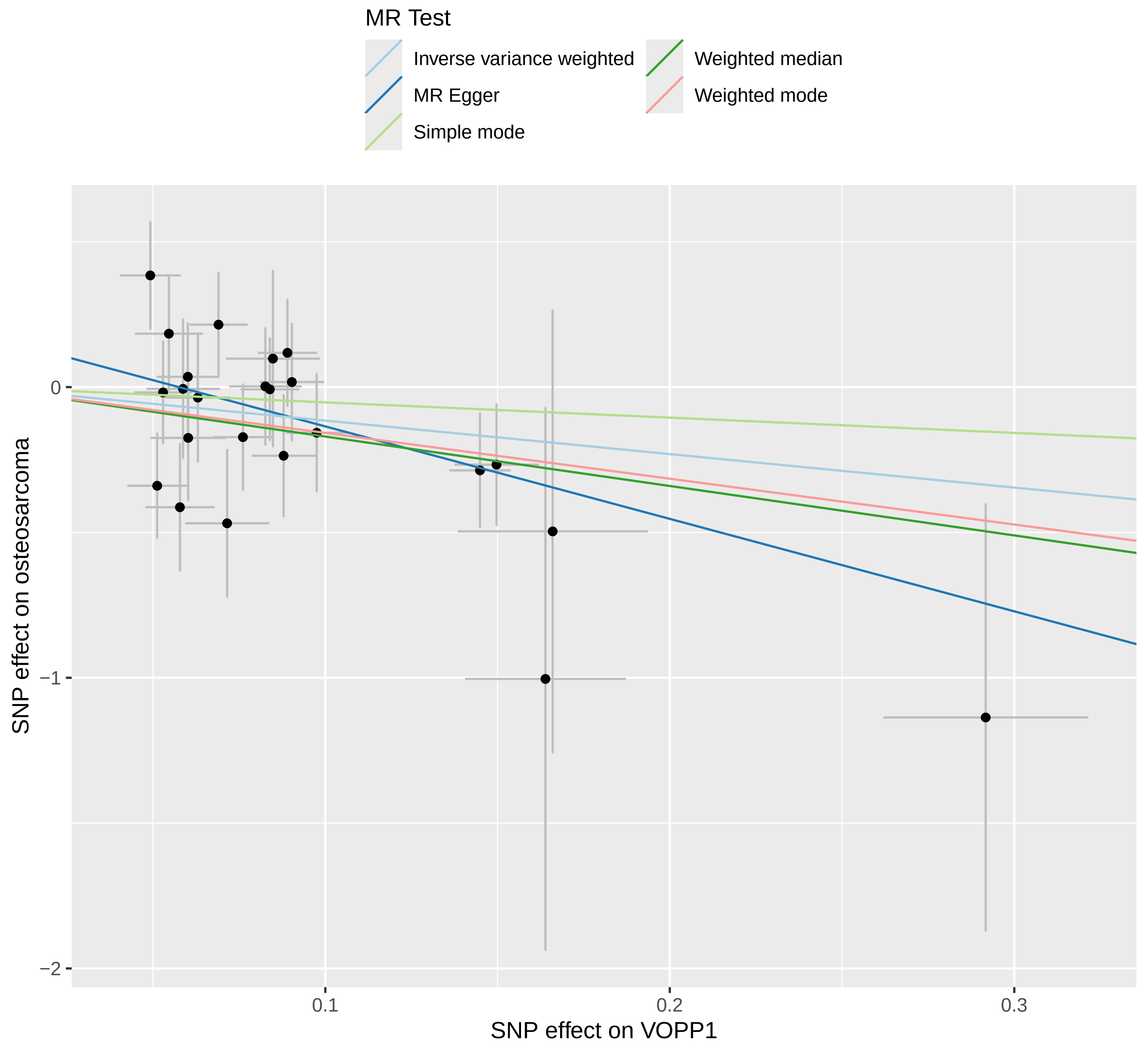

MR Test
Inverse variance weighted
MR Egger
Simple mode
Weighted median
Weighted mode
0
−1
−2
0.1
0.2
0.3
SNP effect on osteosarcoma
SNP effect on VOPP1

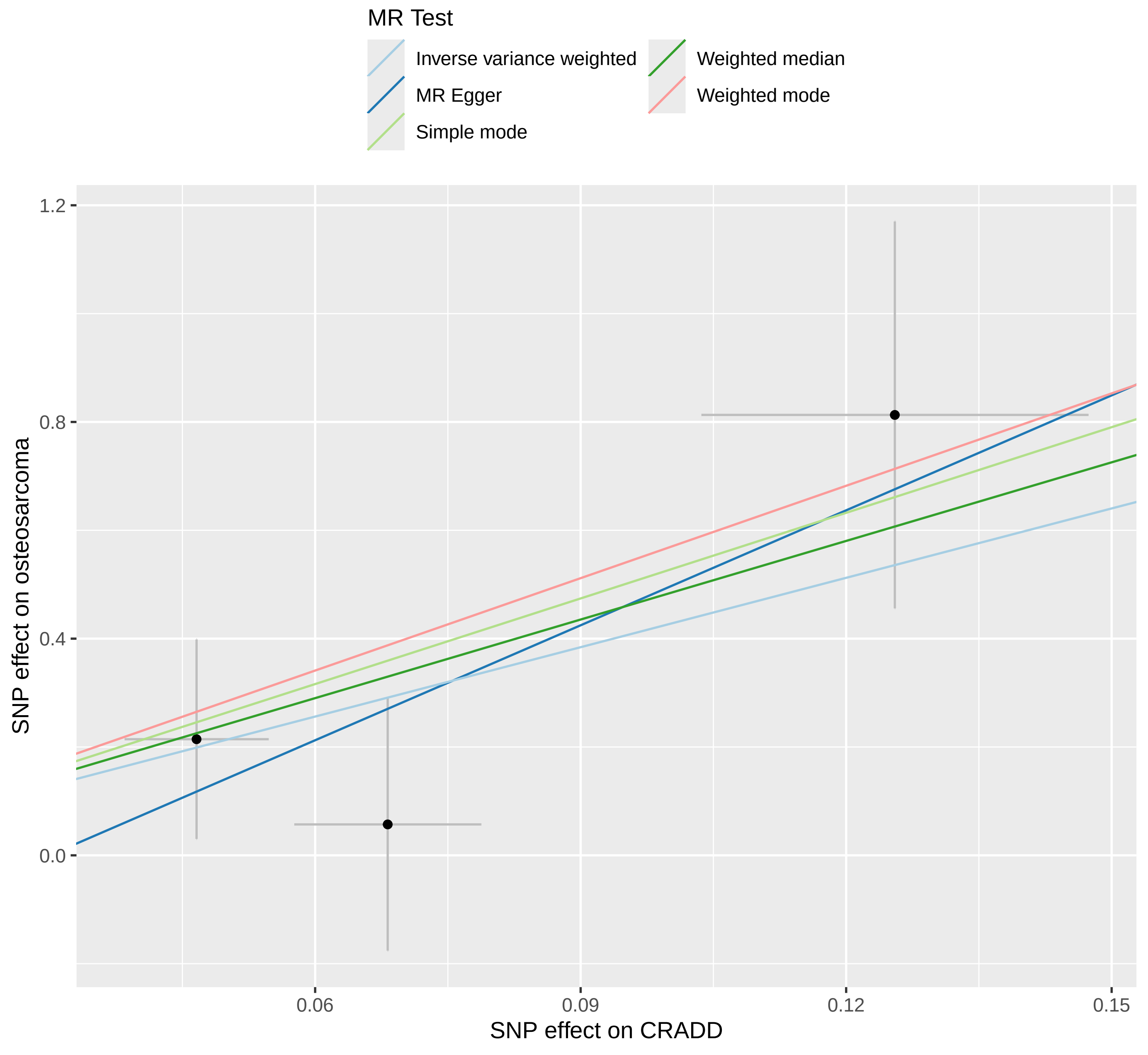
MR Test
Inverse variance weighted
MR Egger
Simple mode
Weighted median
Weighted mode
1.2
0.8
0.4
0.0
0.06
0.09
0.12
0.15
SNP effect on osteosarcoma
SNP effect on CRADD

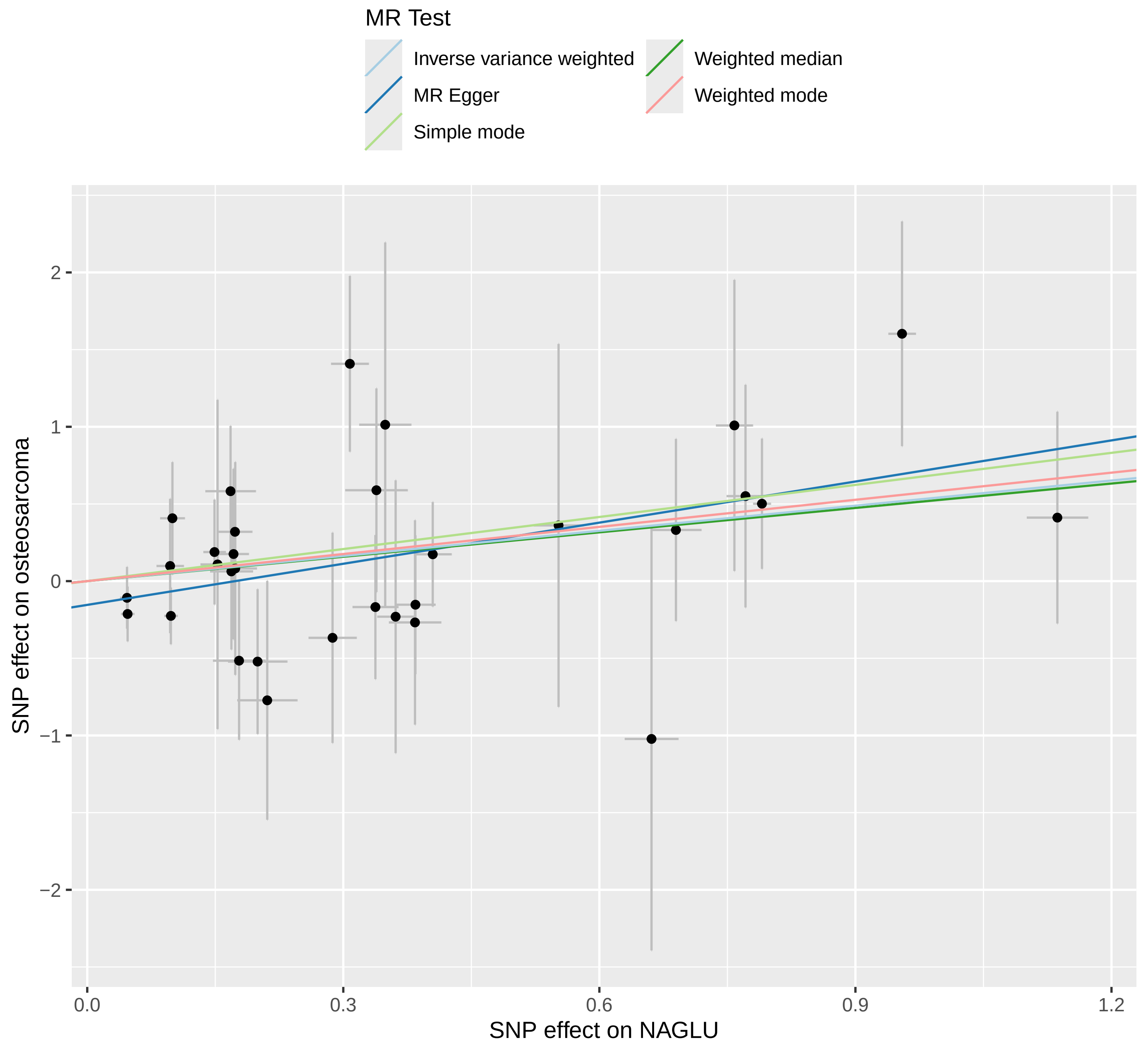

MR Test
Inverse variance weighted
MR Egger
Simple mode
Weighted median
Weighted mode
SNP effect on osteosarcoma
SNP effect on NAGLU
2
1
0
−1
−2
0.0
0.3
0.6
0.9
1.2

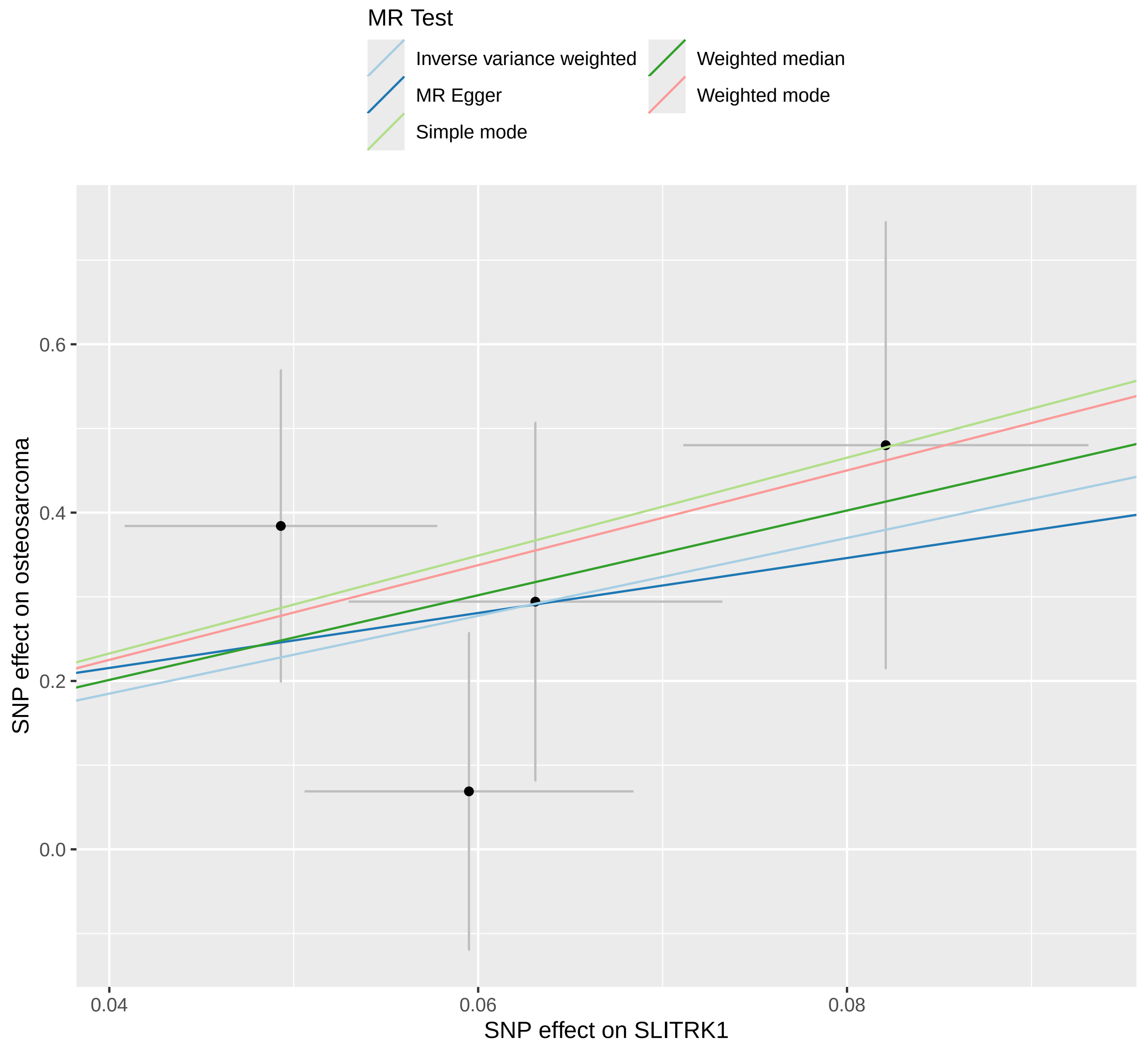
MR Test
Inverse variance weighted
MR Egger
Simple mode
Weighted median
Weighted mode
0.6
0.4
0.2
0.0
0.04
0.06
0.08
SNP effect on osteosarcoma
SNP effect on SLITRK1

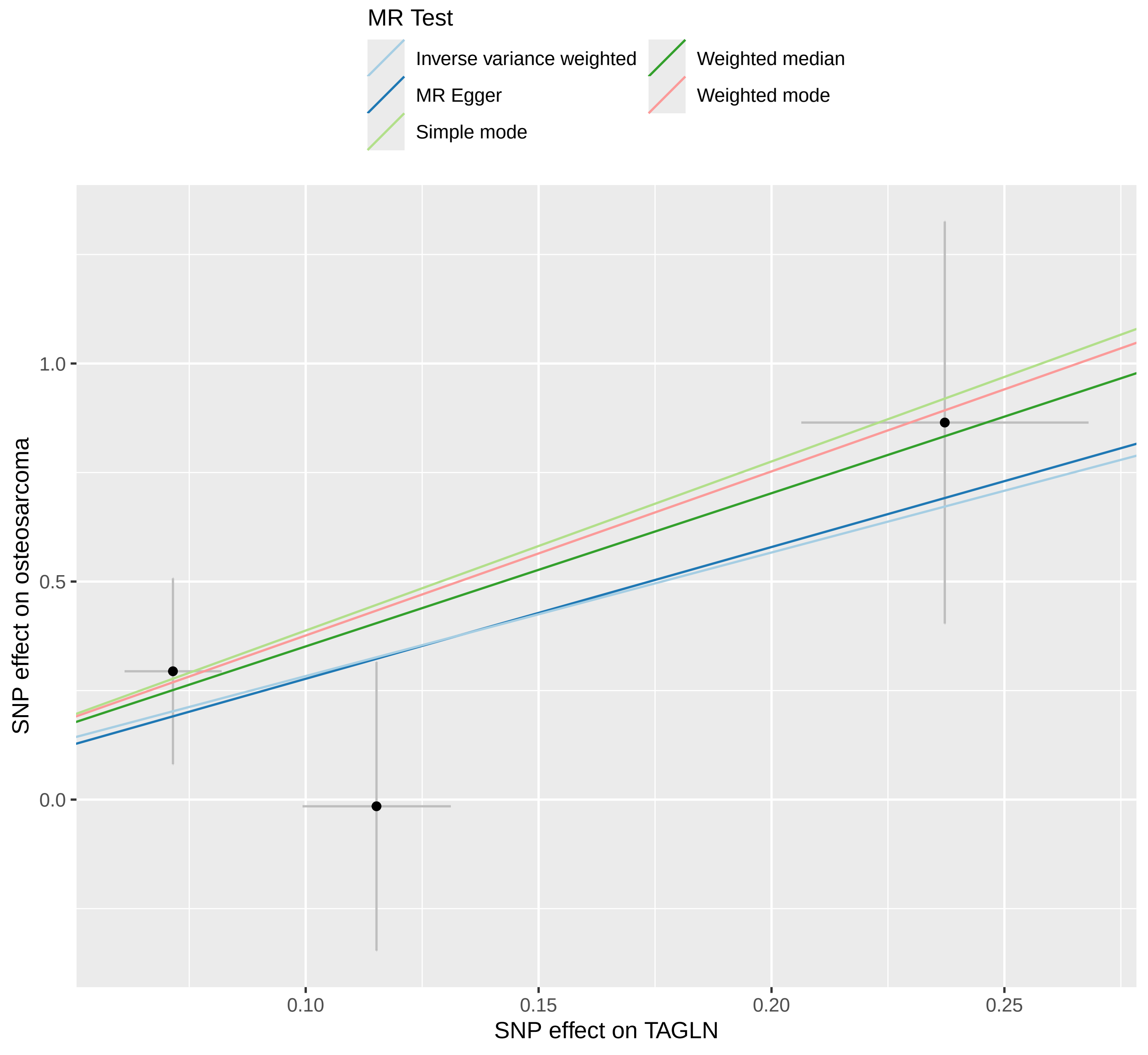

MR Test
Inverse variance weighted
MR Egger
Simple mode
Weighted median
Weighted mode
1.0
0.5
0.0
0.10
0.15
0.20
0.25
SNP effect on osteosarcoma
SNP effect on TAGLN

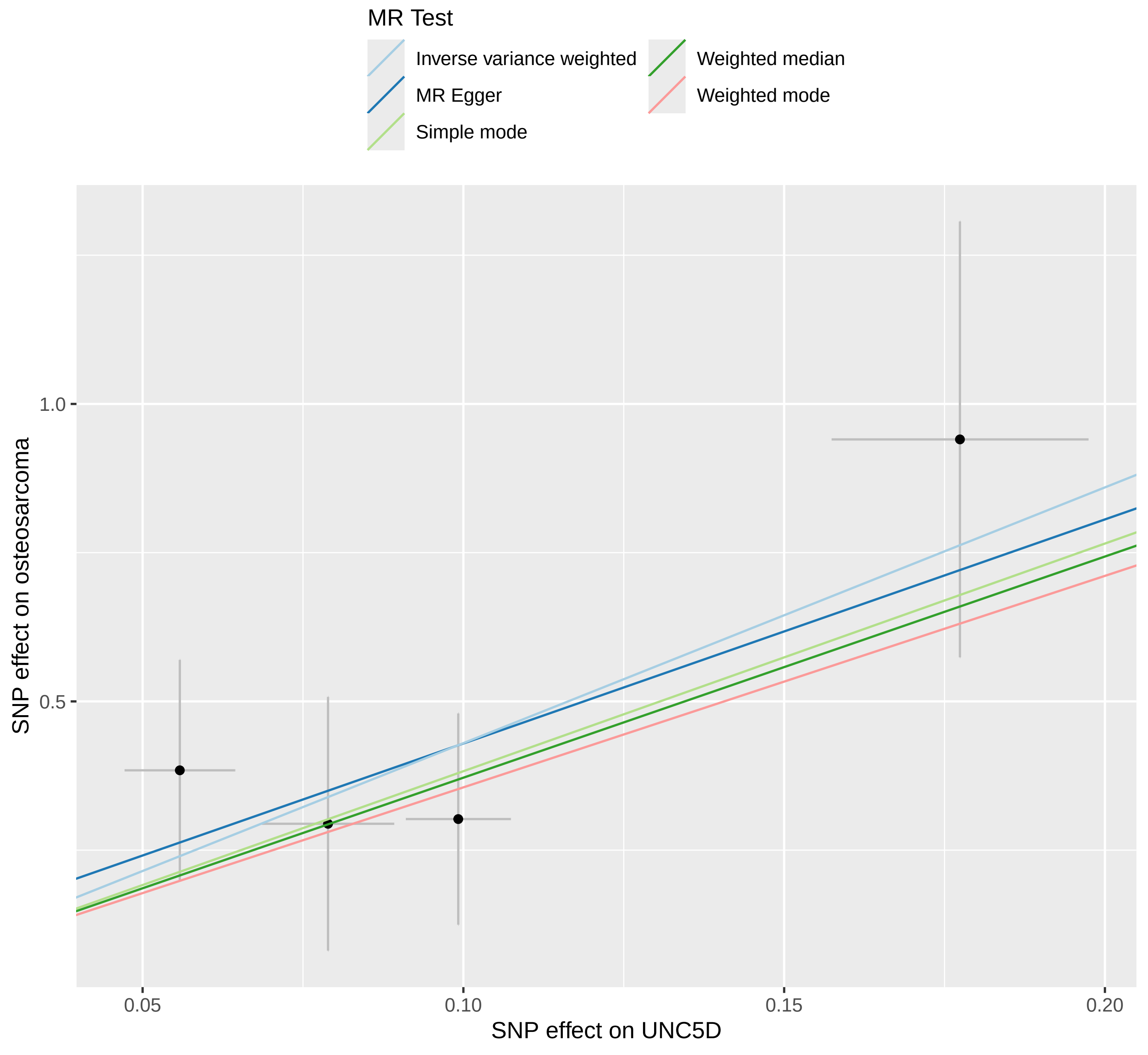
MR Test
Inverse variance weighted
MR Egger
Simple mode
Weighted median
Weighted mode
1.0
0.5
SNP effect on osteosarcoma
0.05
0.10
0.15
0.20
SNP effect on UNC5D

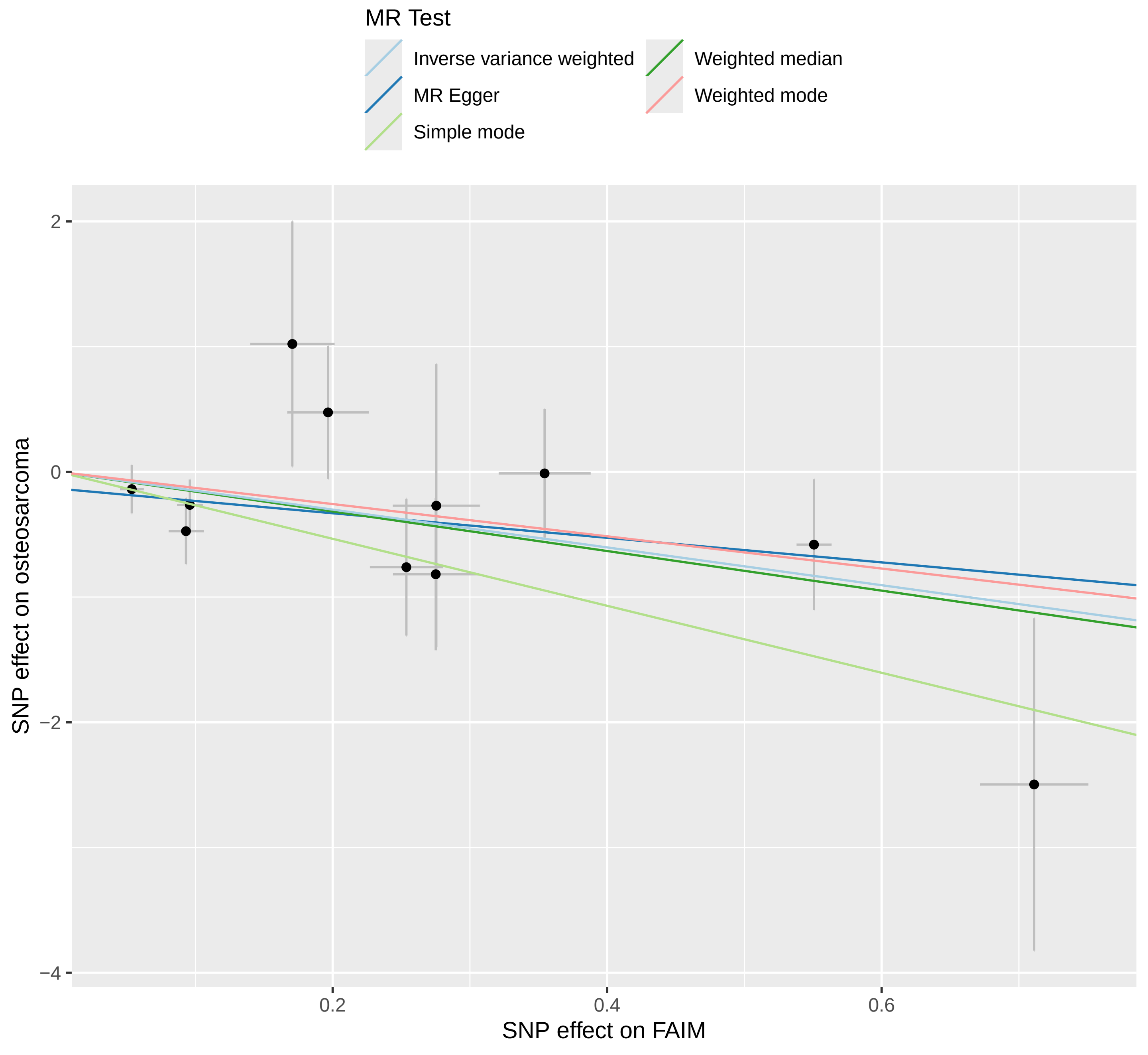
MR Test
Inverse variance weighted
MR Egger
Simple mode
Weighted median
Weighted mode
2
0
−2
−4
0.2
0.4
0.6
SNP effect on osteosarcoma
SNP effect on FAIM

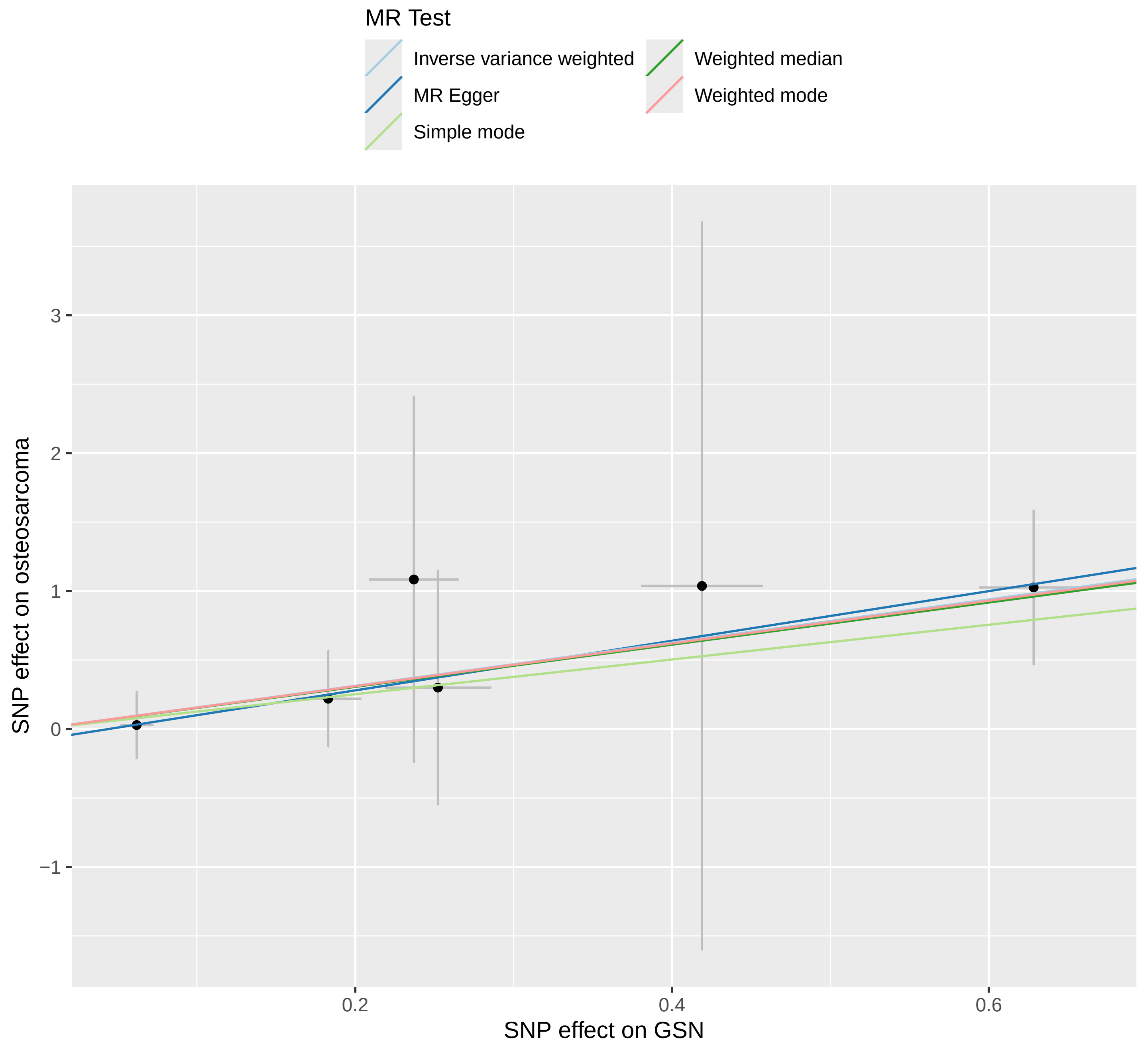
MR Test
Inverse variance weighted
MR Egger
Simple mode
Weighted median
Weighted mode
SNP effect on osteosarcoma
SNP effect on GSN
3
2
1
0
−1
0.2
0.4
0.6

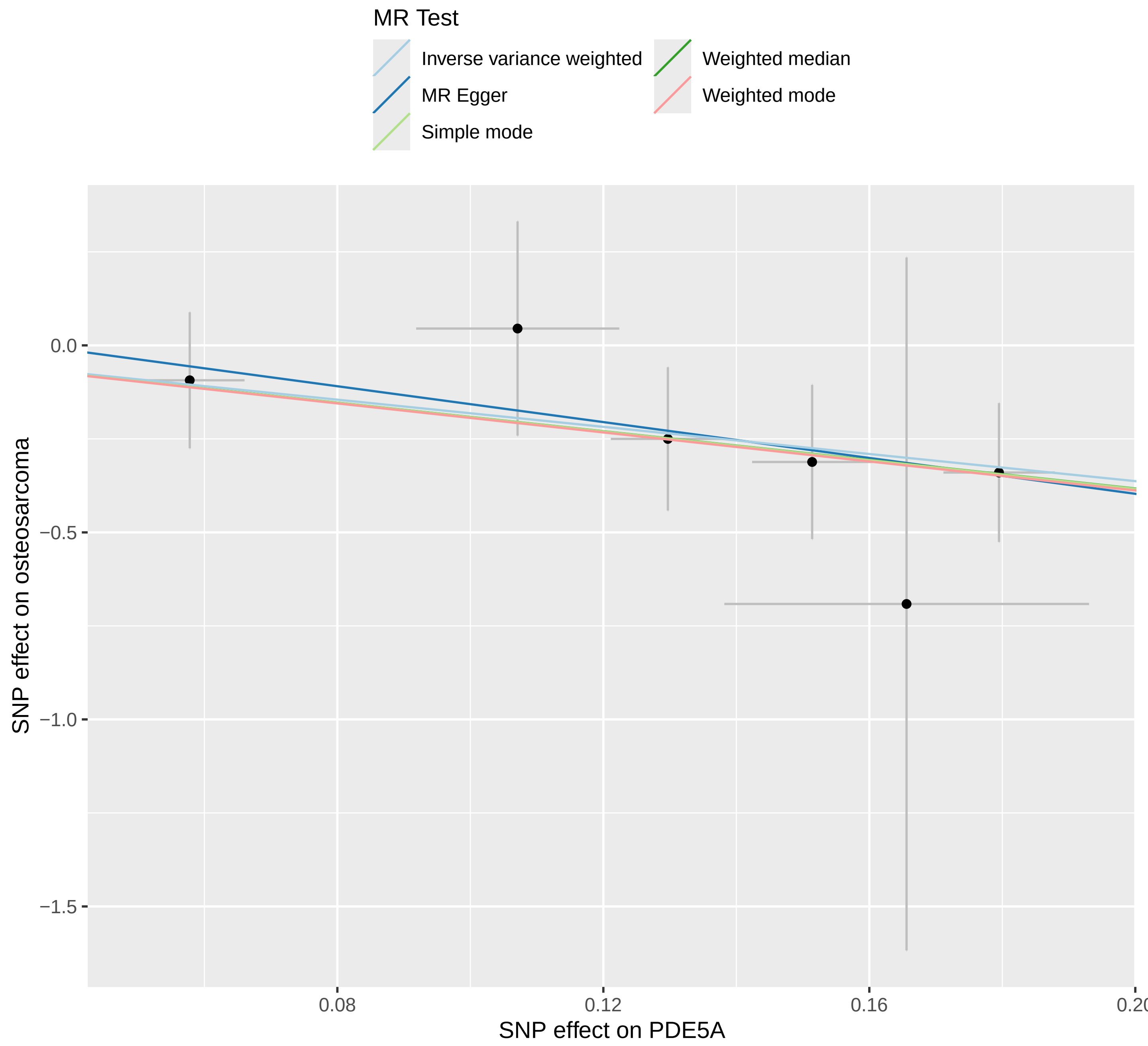
MR Test
Inverse variance weighted
MR Egger
Simple mode
Weighted median
Weighted mode
0.0
−0.5
−1.0
−1.5
SNP effect on osteosarcoma
0.08
0.12
0.16
0.20
SNP effect on PDE5A

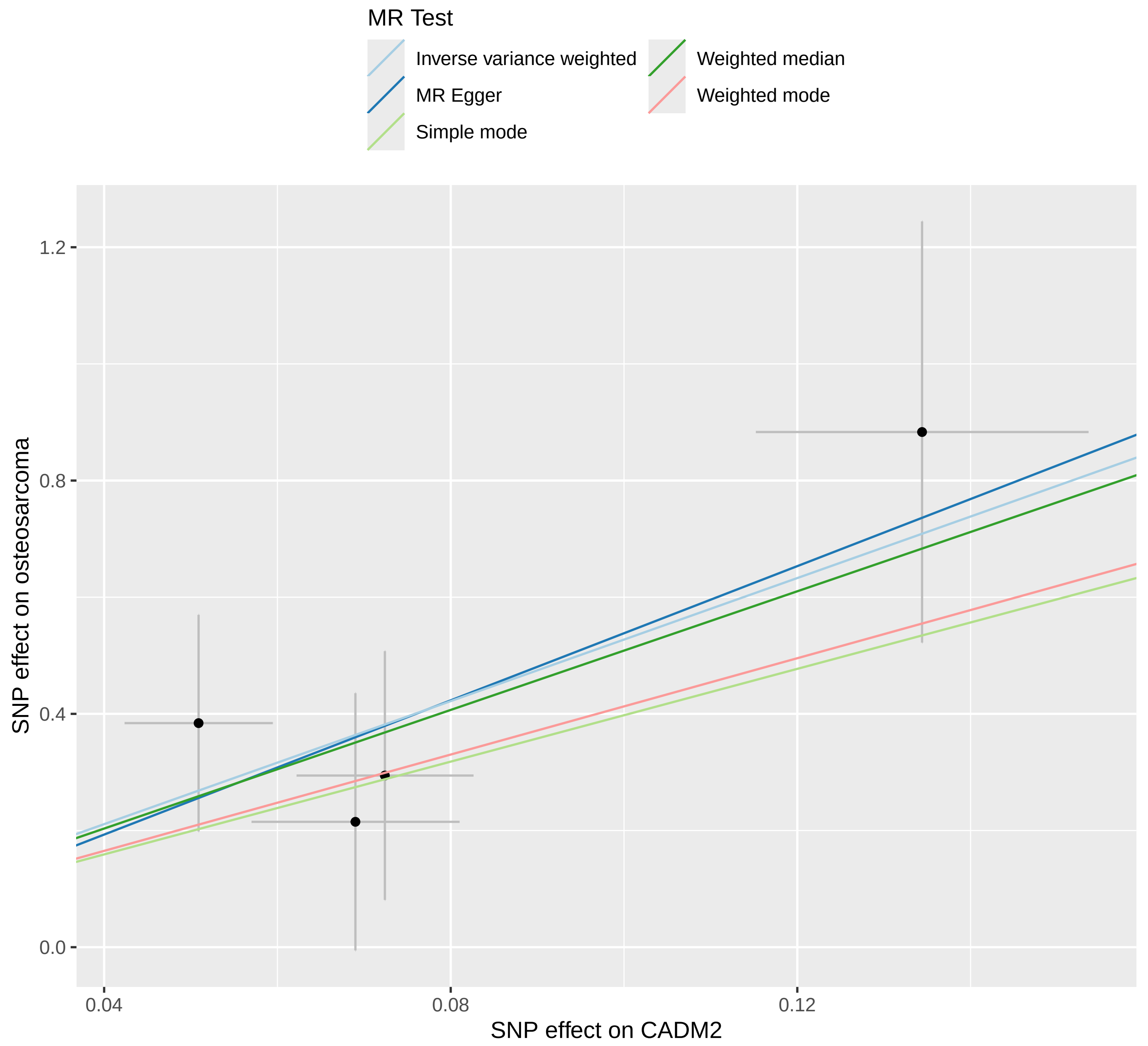
MR Test
Inverse variance weighted
MR Egger
Simple mode
Weighted median
Weighted mode
1.2
0.8
0.4
0.0
SNP effect on osteosarcoma
0.04
0.08
0.12
SNP effect on CADM2

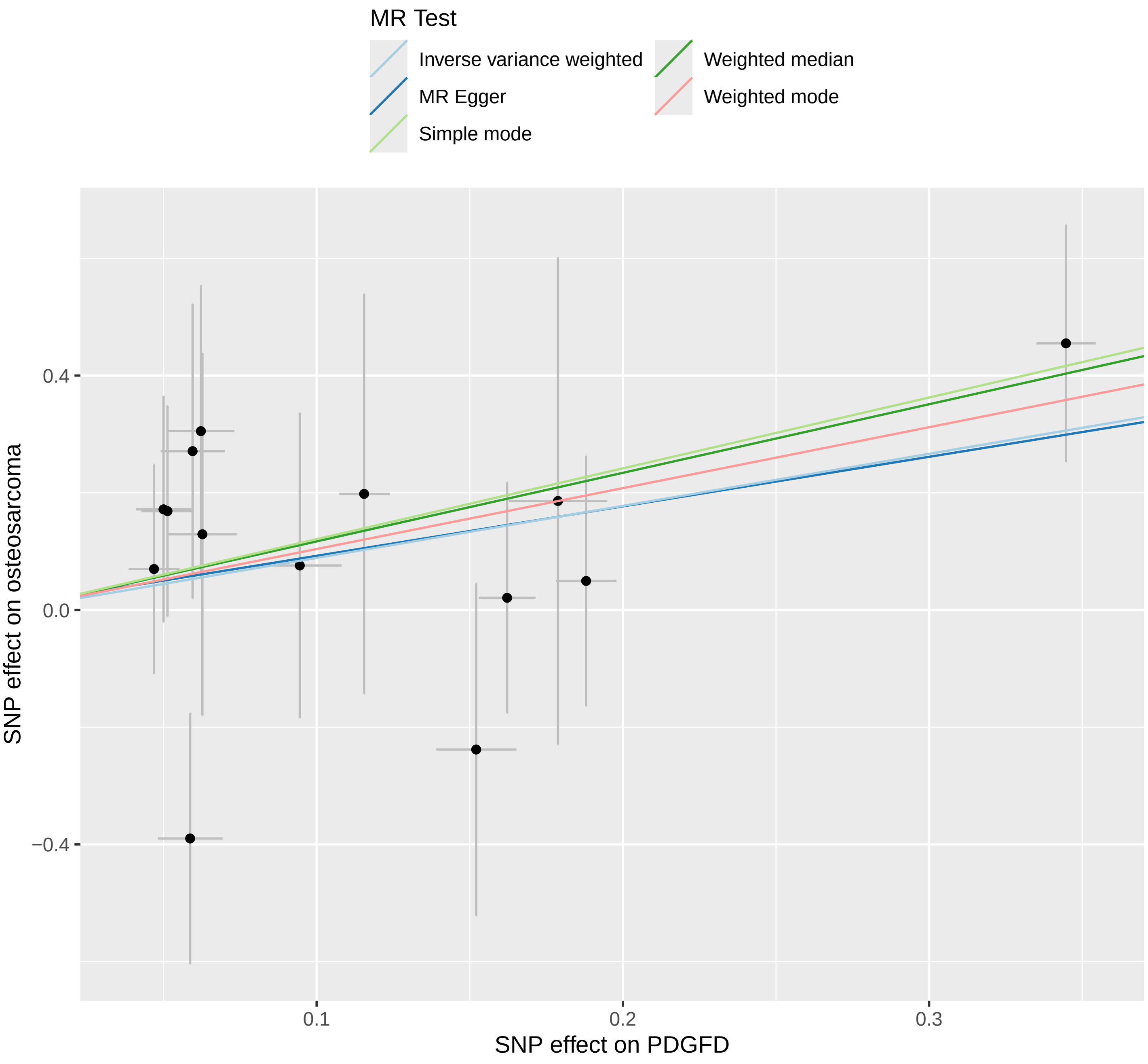
MR Test
Inverse variance weighted
MR Egger
Simple mode
Weighted median
Weighted mode
0.4
0.0
−0.4
SNP effect on osteosarcoma
0.1
0.2
0.3
SNP effect on PDGFD

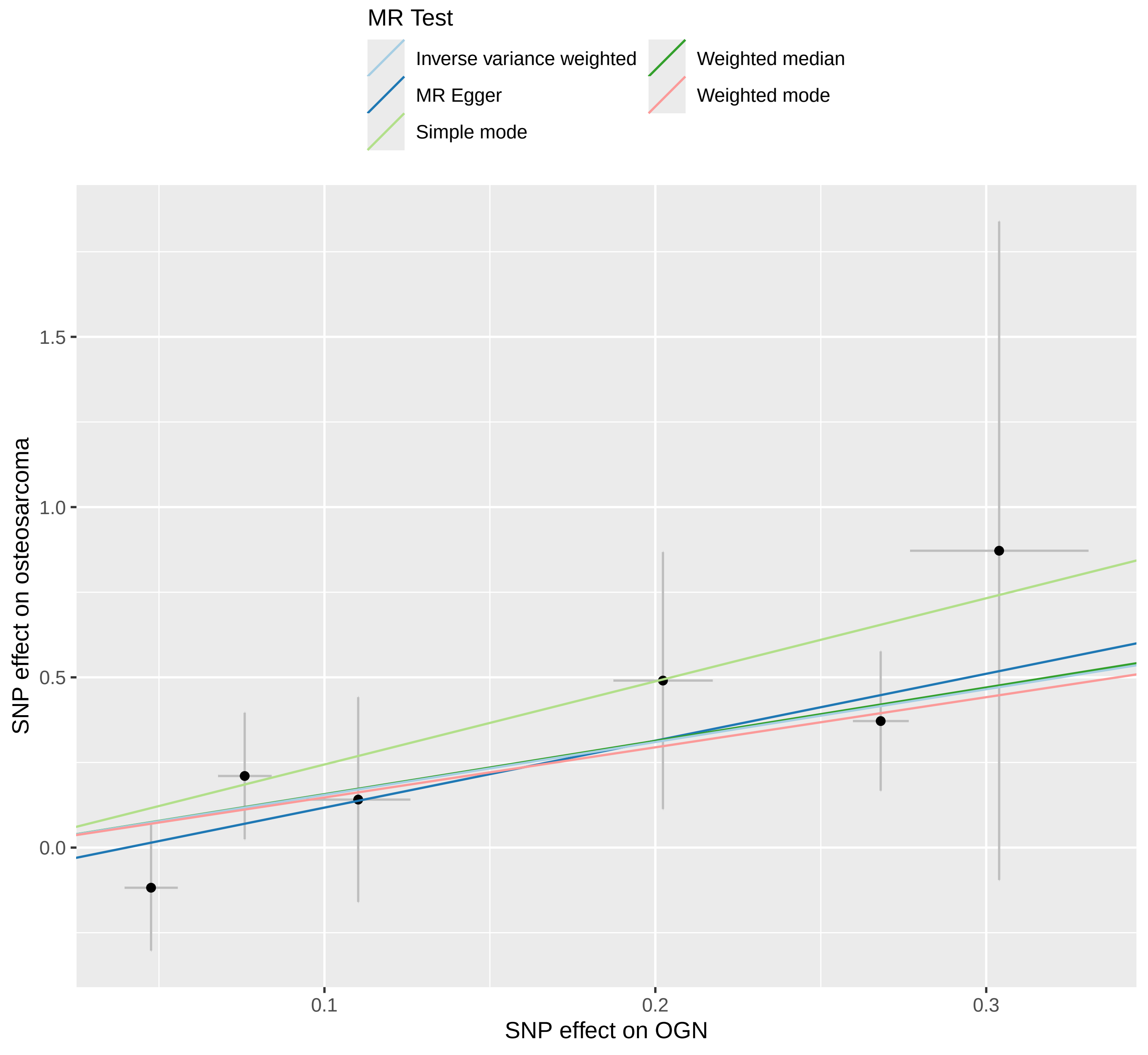
MR Test
Inverse variance weighted
MR Egger
Simple mode
Weighted median
Weighted mode
1.5
1.0
0.5
0.0
0.1
0.2
0.3
SNP effect on osteosarcoma
SNP effect on OGN

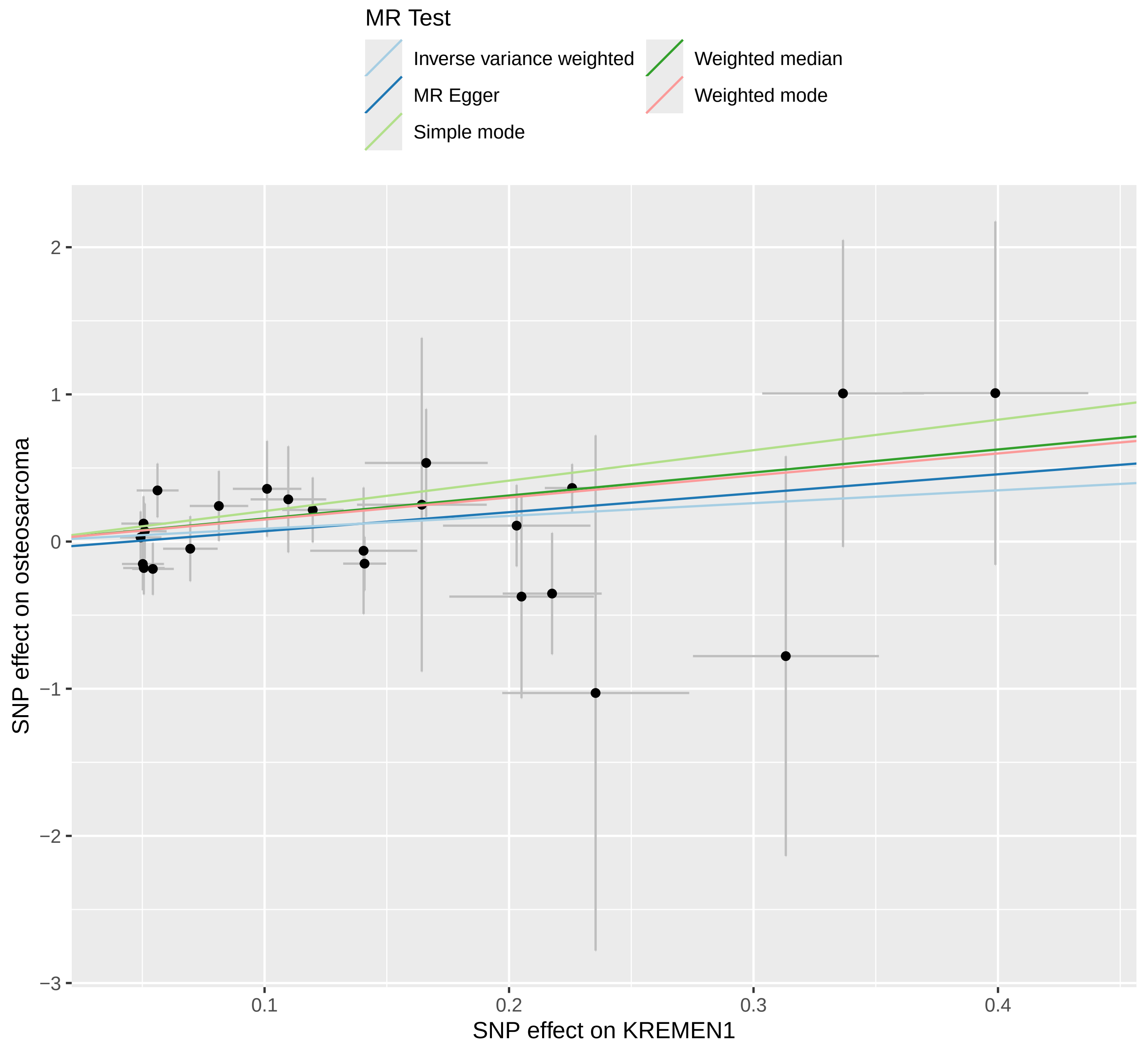
MR Test
Inverse variance weighted
MR Egger
Simple mode
Weighted median
Weighted mode
SNP effect on osteosarcoma
SNP effect on KREMEN1
2
1
0
−1
−2
−3
0.1
0.2
0.3
0.4

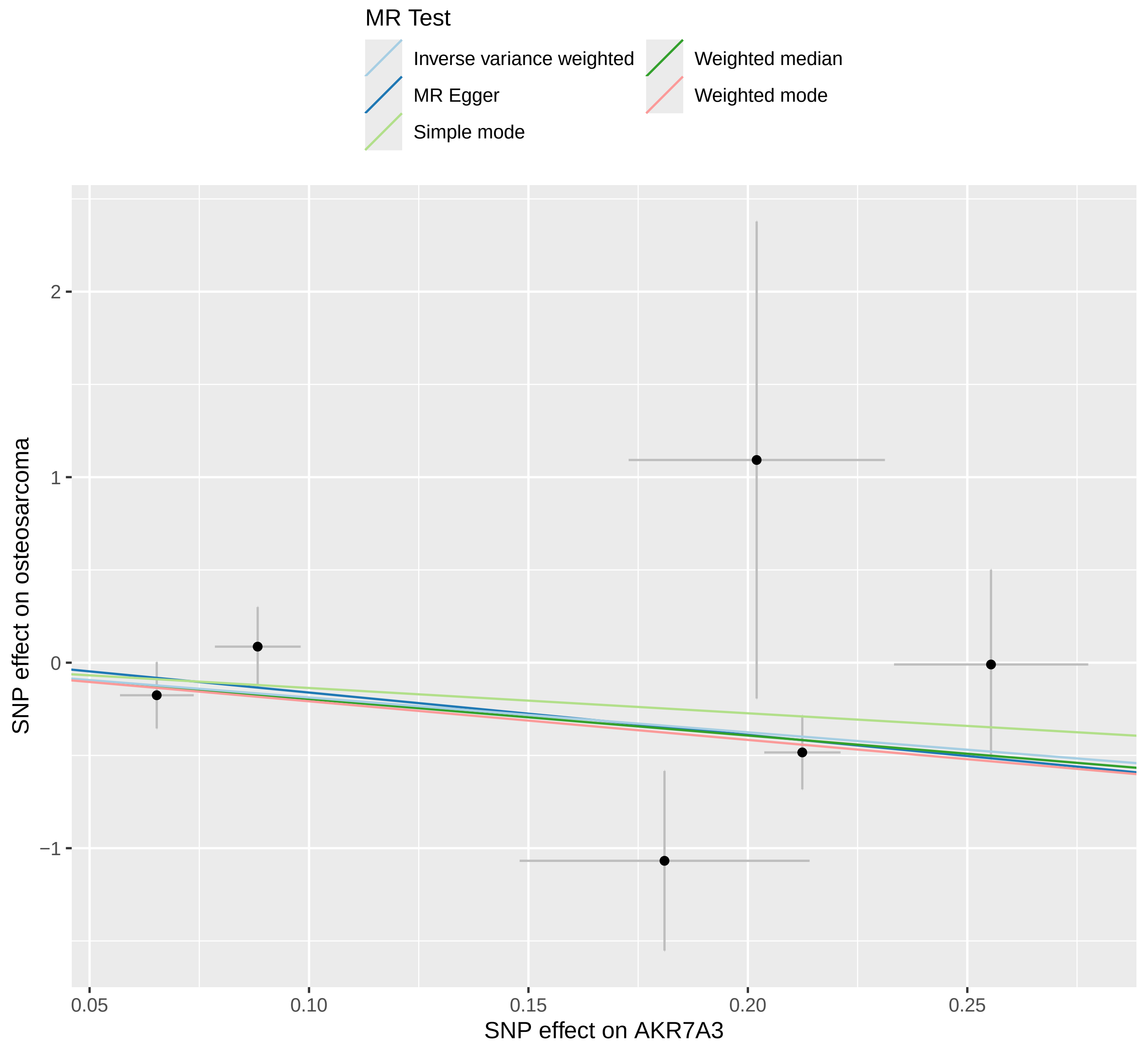
MR Test
Inverse variance weighted
MR Egger
Simple mode
Weighted median
Weighted mode
SNP effect on osteosarcoma
2
1
0
−1
0.05
0.10
0.15
0.20
0.25
SNP effect on AKR7A3

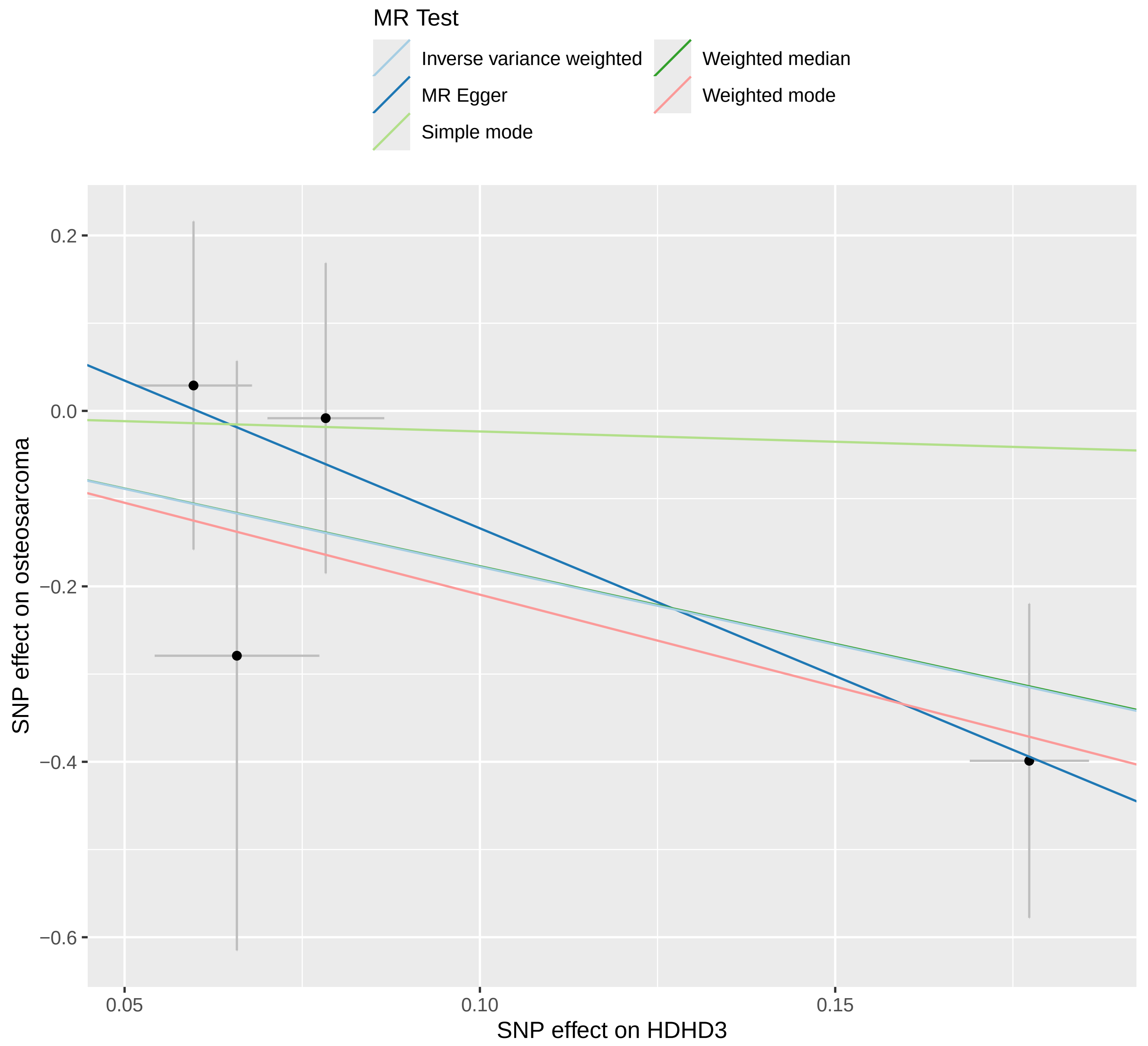
MR Test
Inverse variance weighted
MR Egger
Simple mode
Weighted median
Weighted mode
0.2
0.0
−0.2
−0.4
−0.6
0.05
0.10
0.15
SNP effect on HDHD3
SNP effect on osteosarcoma

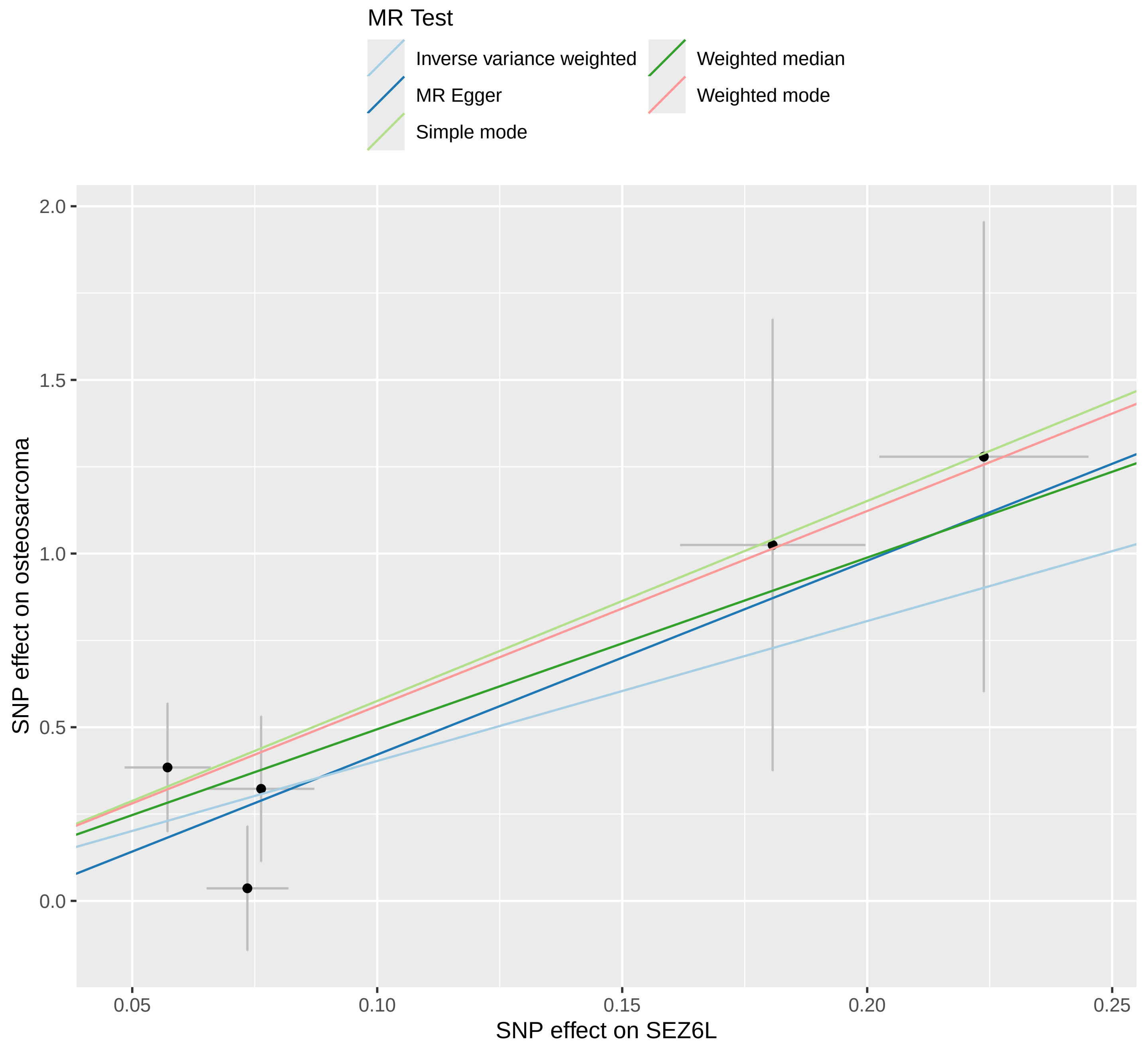
MR Test
Inverse variance weighted
MR Egger
Simple mode
Weighted median
Weighted mode
SNP effect on osteosarcoma
2.0
1.5
1.0
0.5
0.0
0.05
0.10
0.15
0.20
0.25
SNP effect on SEZ6L

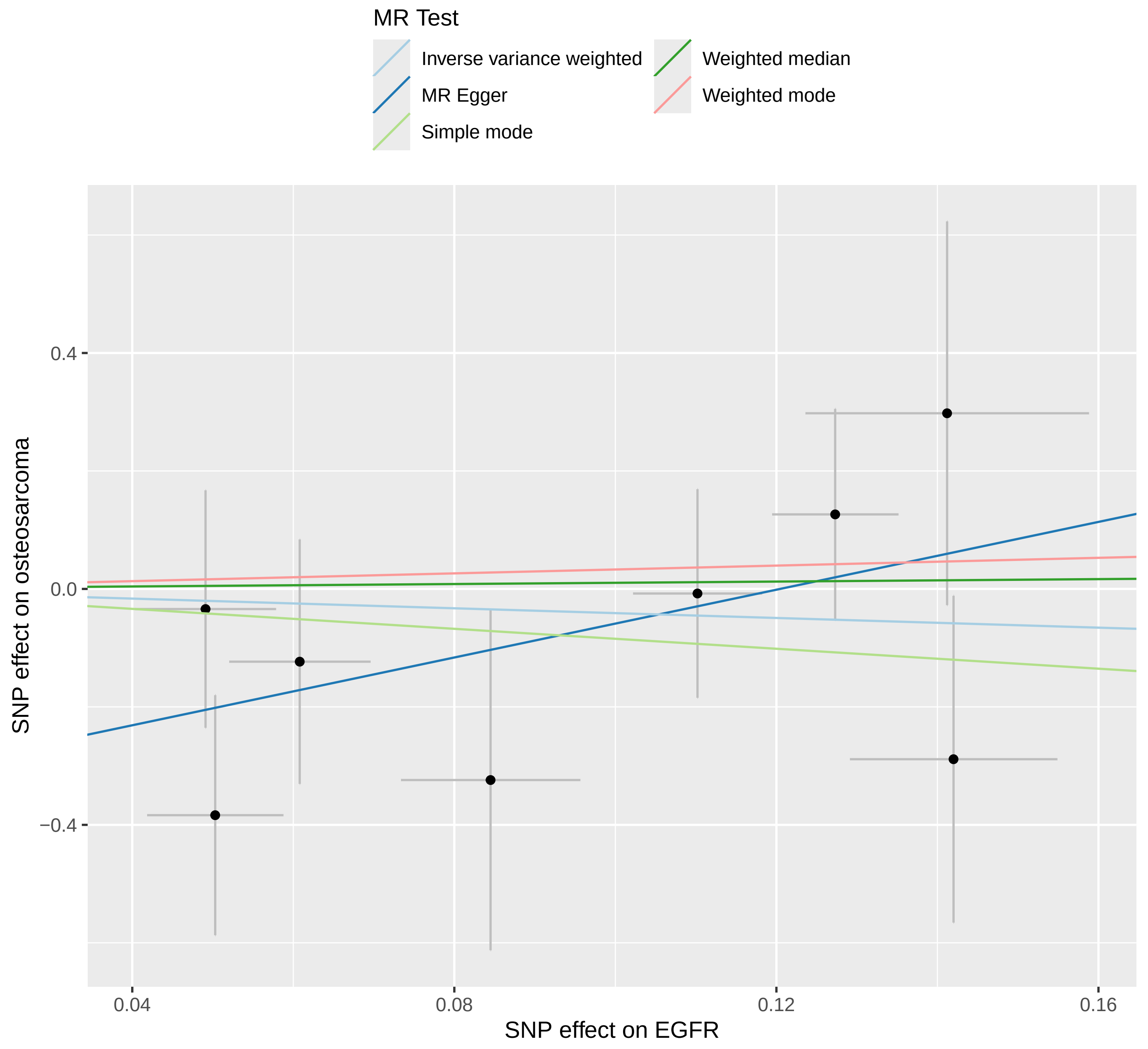
MR Test
Inverse variance weighted
MR Egger
Simple mode
Weighted median
Weighted mode
0.4
0.0
−0.4
SNP effect on osteosarcoma
0.04
0.08
0.12
0.16
SNP effect on EGFR

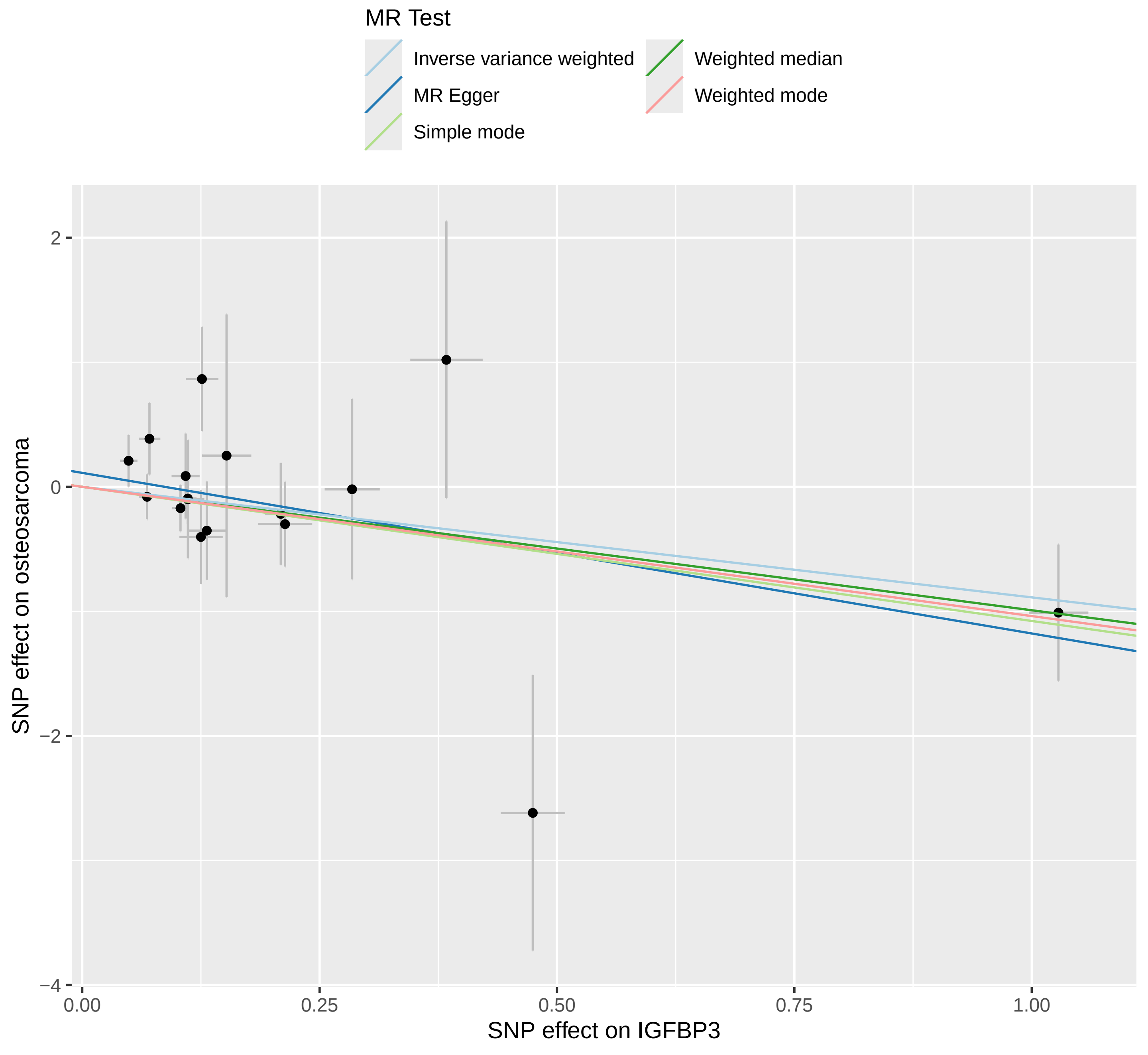

MR Test
Inverse variance weighted
MR Egger
Simple mode
Weighted median
Weighted mode
SNP effect on osteosarcoma
2
0
−2
−4
0.00
0.25
0.50
0.75
1.00
SNP effect on IGFBP3

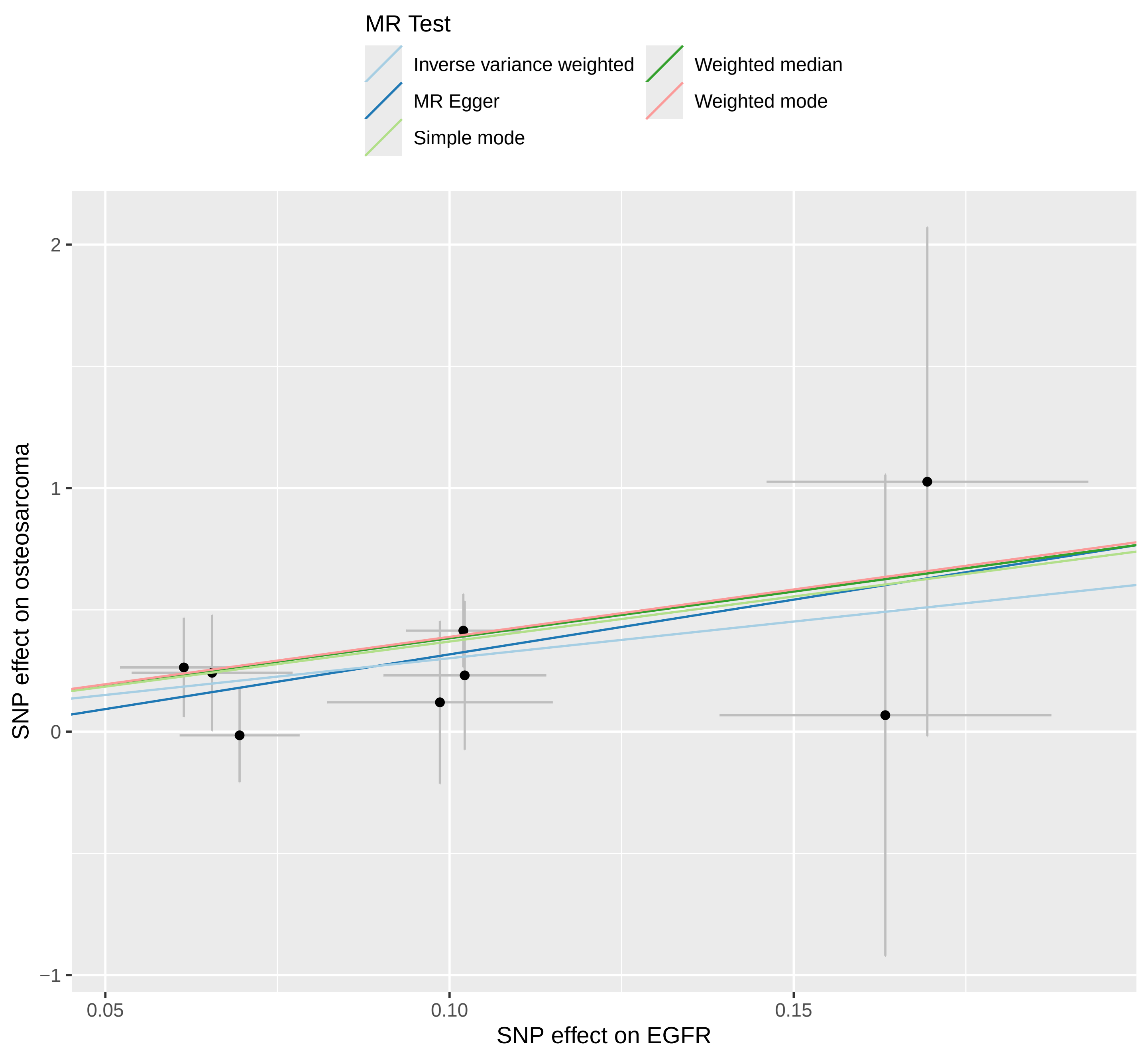

MR Test
Inverse variance weighted
MR Egger
Simple mode
Weighted median
Weighted mode
SNP effect on osteosarcoma
2
1
0
−1
0.05
0.10
0.15
SNP effect on EGFR

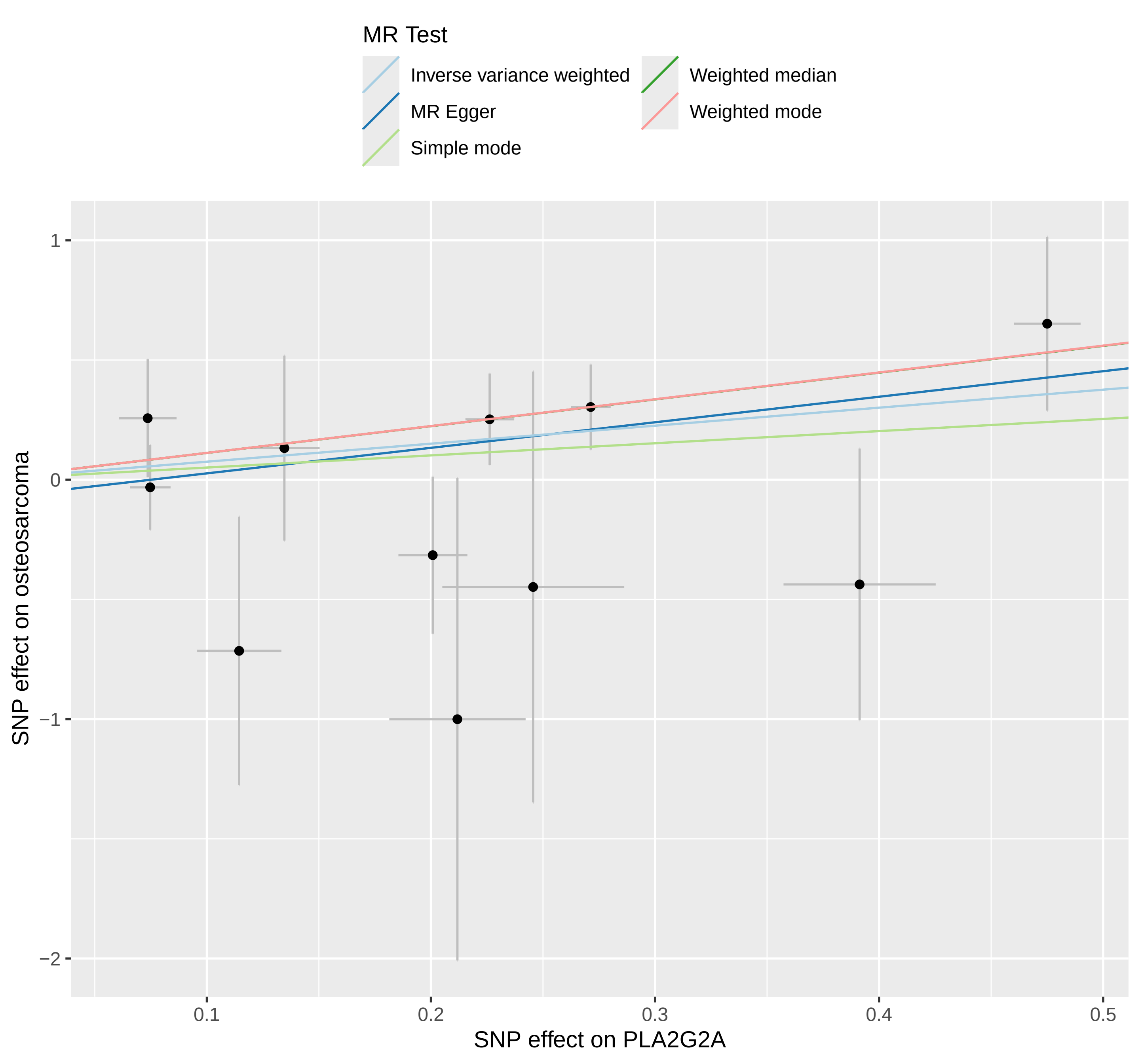
MR Test
Inverse variance weighted
MR Egger
Simple mode
Weighted median
Weighted mode
1
0
−1
−2
SNP effect on osteosarcoma
0.1
0.2
0.3
0.4
0.5
SNP effect on PLA2G2A

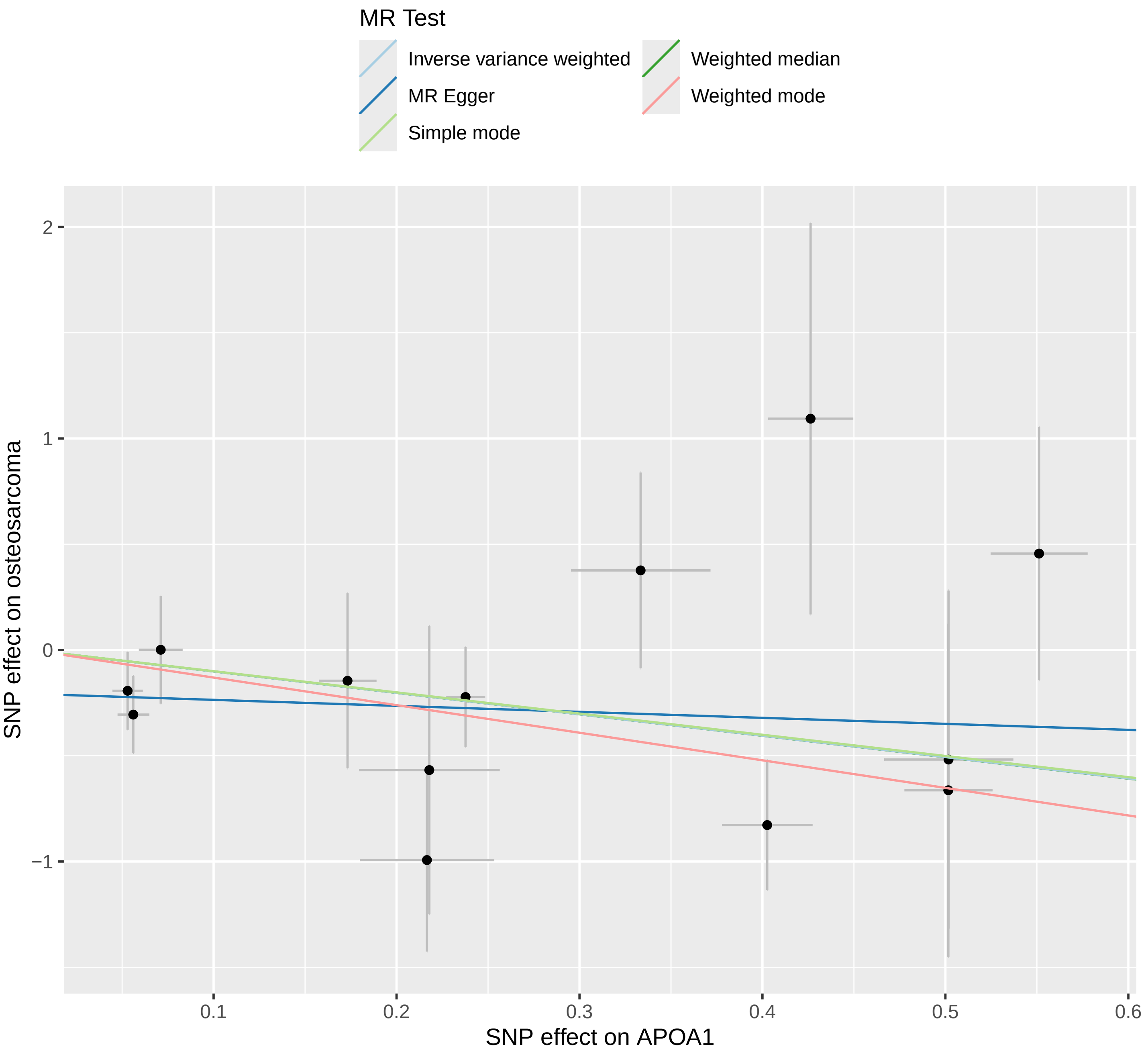
MR Test
Inverse variance weighted
MR Egger
Simple mode
Weighted median
Weighted mode
SNP effect on osteosarcoma
SNP effect on APOA1
2
1
0
−1
0.1
0.2
0.3
0.4
0.5
0.6

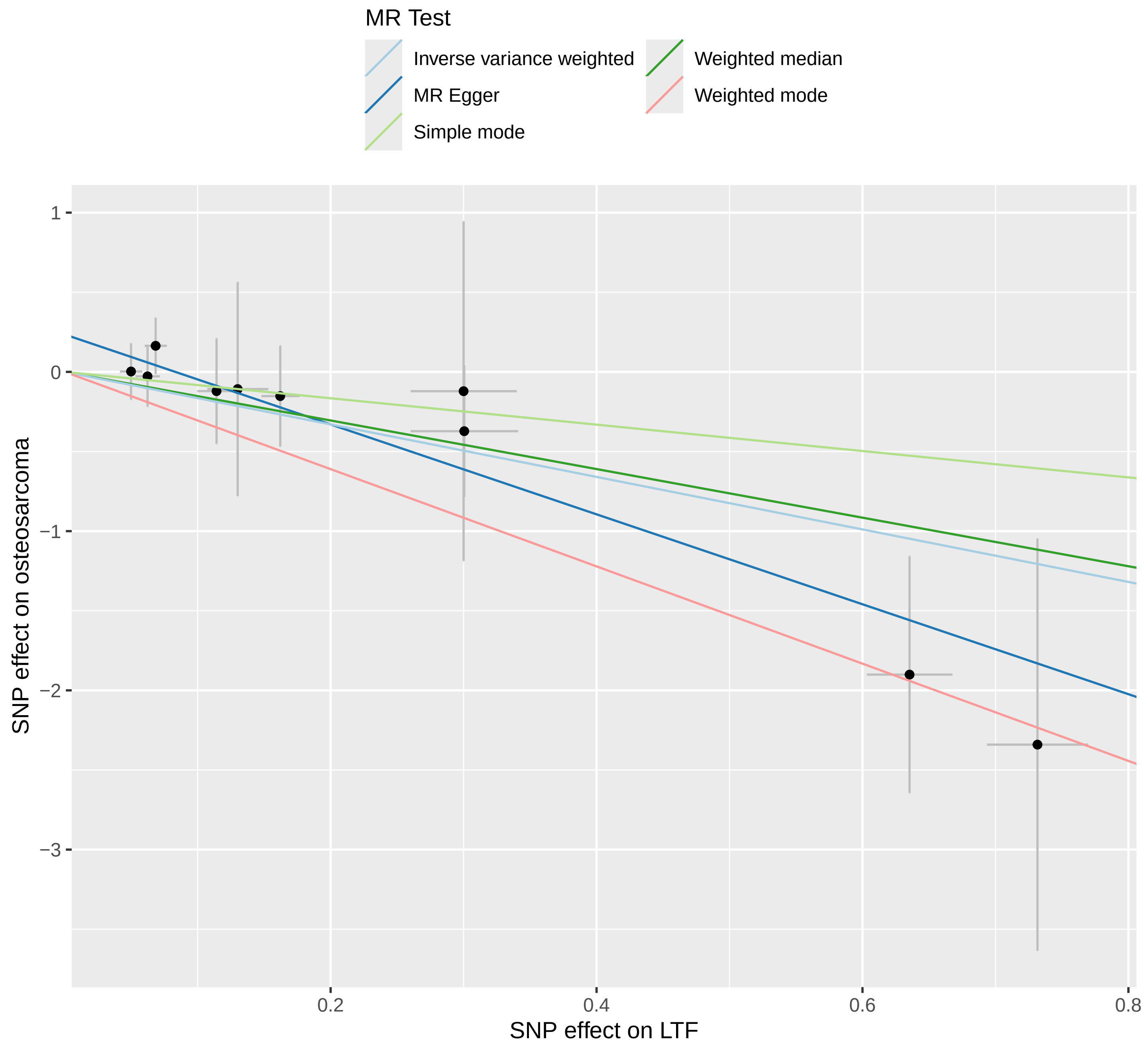

MR Test
Inverse variance weighted
MR Egger
Simple mode
Weighted median
Weighted mode
1
0
−1
−2
−3
0.2
0.4
0.6
0.8
SNP effect on osteosarcoma
SNP effect on LTF

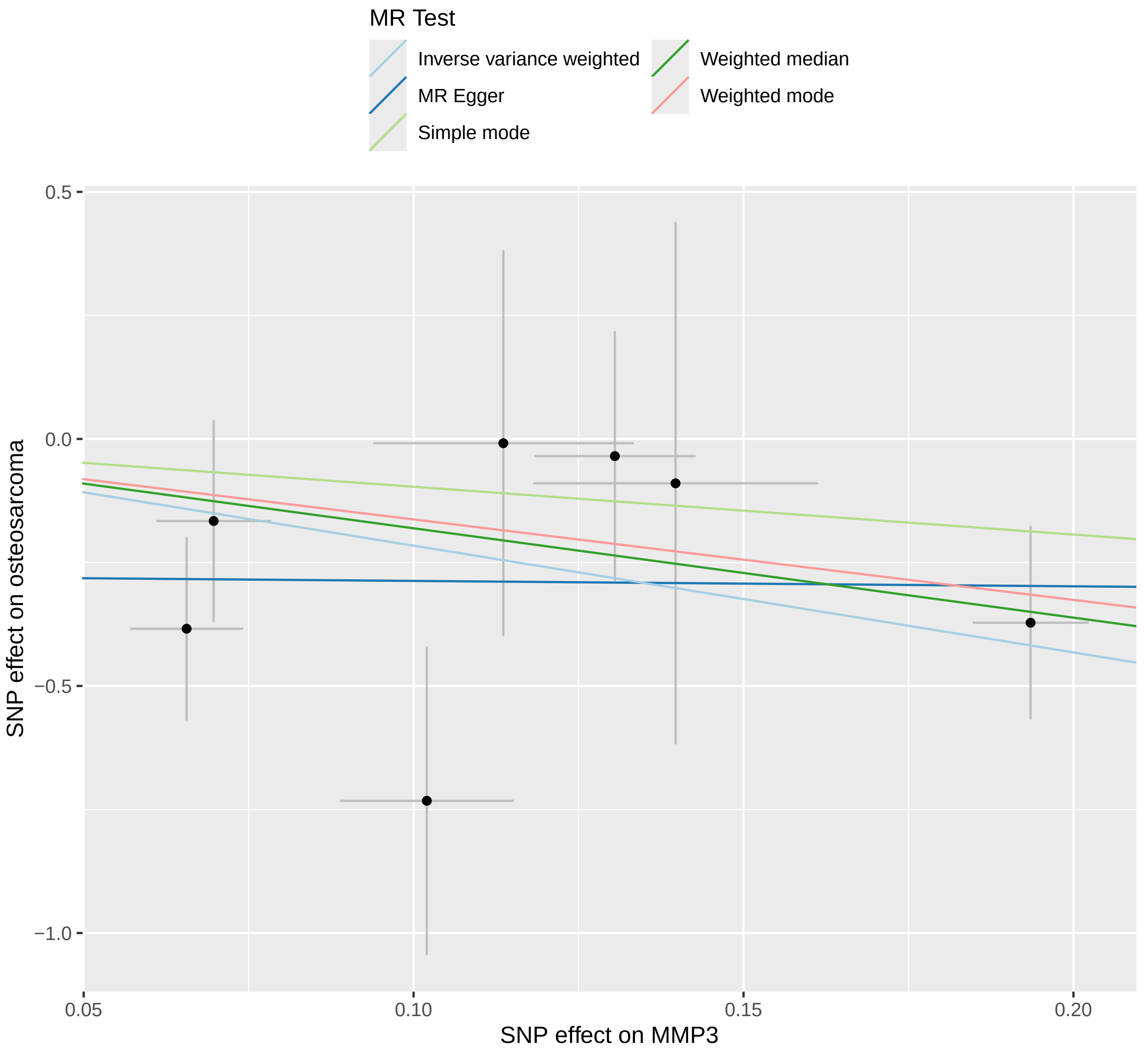
MR Test
Inverse variance weighted
MR Egger
Simple mode
Weighted median
Weighted mode
0.5
0.0
−0.5
−1.0
0.05
0.10
0.15
0.20
SNP effect on MMP3
SNP effect on osteosarcoma

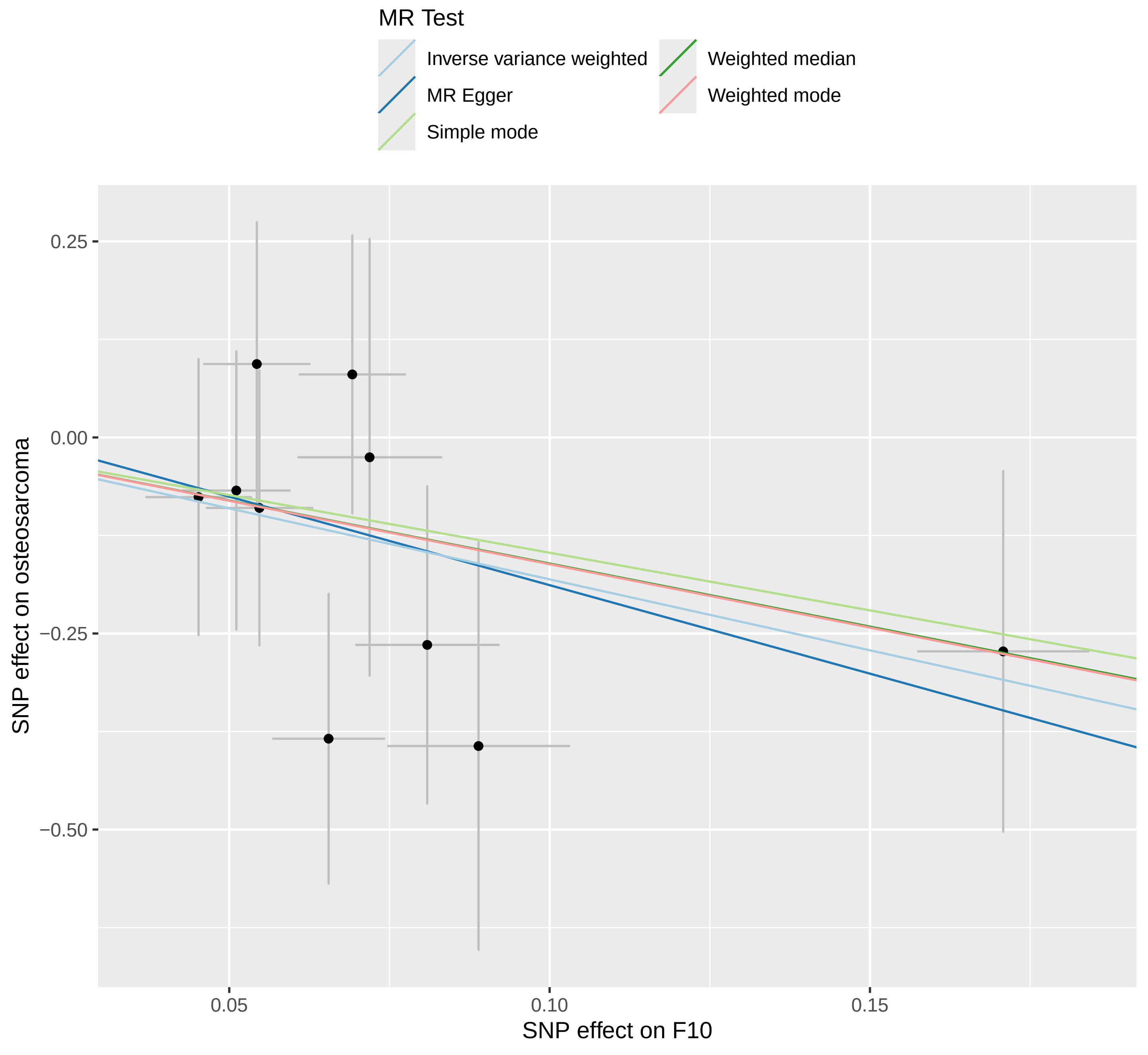

MR Test
Inverse variance weighted
MR Egger
Simple mode
Weighted median
Weighted mode
0.25
0.00
−0.25
−0.50
0.05
0.10
0.15
SNP effect on osteosarcoma
SNP effect on F10

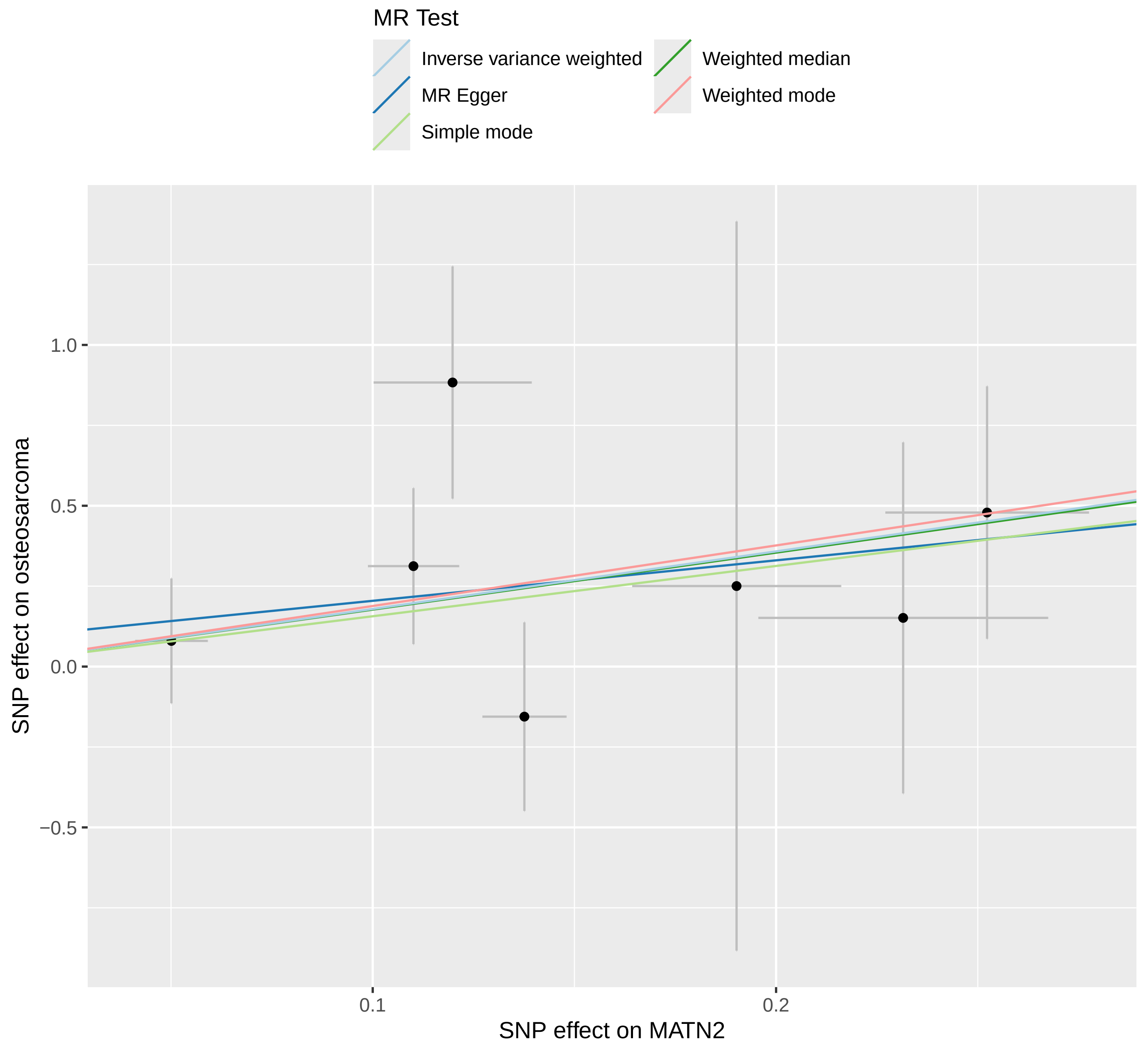
MR Test
Inverse variance weighted
MR Egger
Simple mode
Weighted median
Weighted mode
1.0
0.5
0.0
−0.5
SNP effect on osteosarcoma
0.1
0.2
SNP effect on MATN2

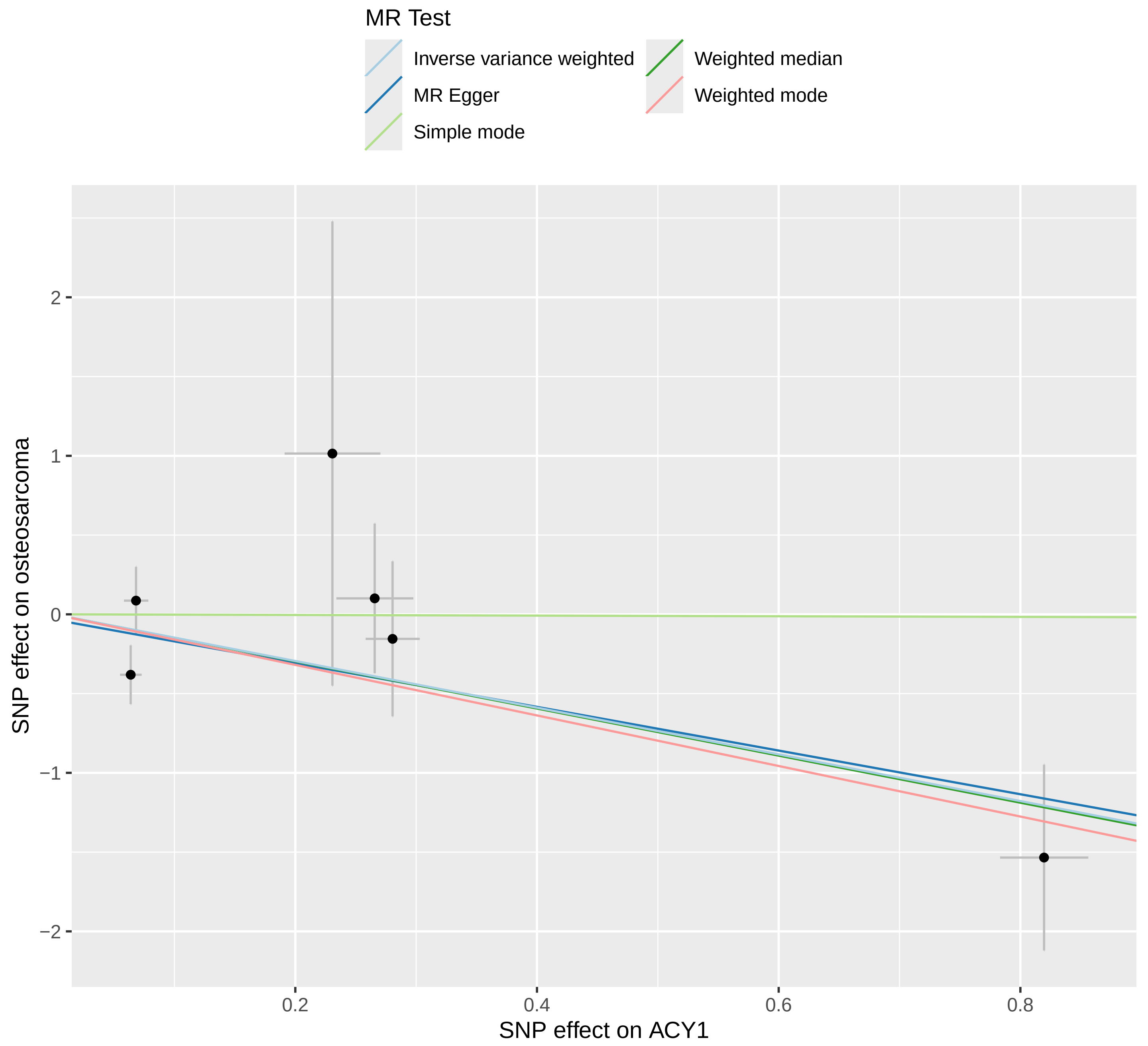
MR Test
Inverse variance weighted
MR Egger
Simple mode
Weighted median
Weighted mode
SNP effect on osteosarcoma
2
1
0
−1
−2
0.2
0.4
0.6
0.8
SNP effect on ACY1

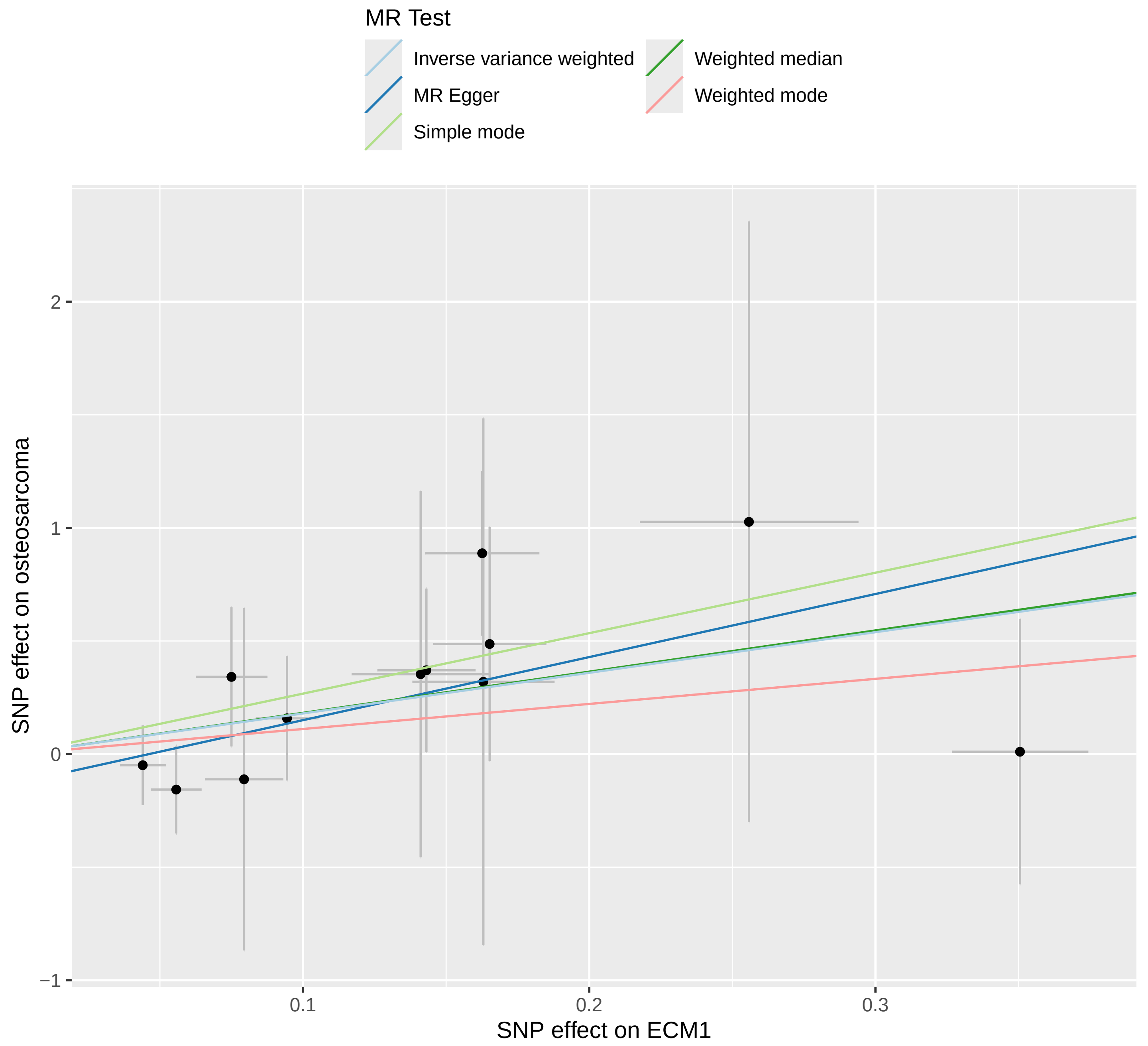
MR Test
Inverse variance weighted
MR Egger
Simple mode
Weighted median
Weighted mode
2
1
0
−1
0.1
0.2
0.3
SNP effect on osteosarcoma
SNP effect on ECM1

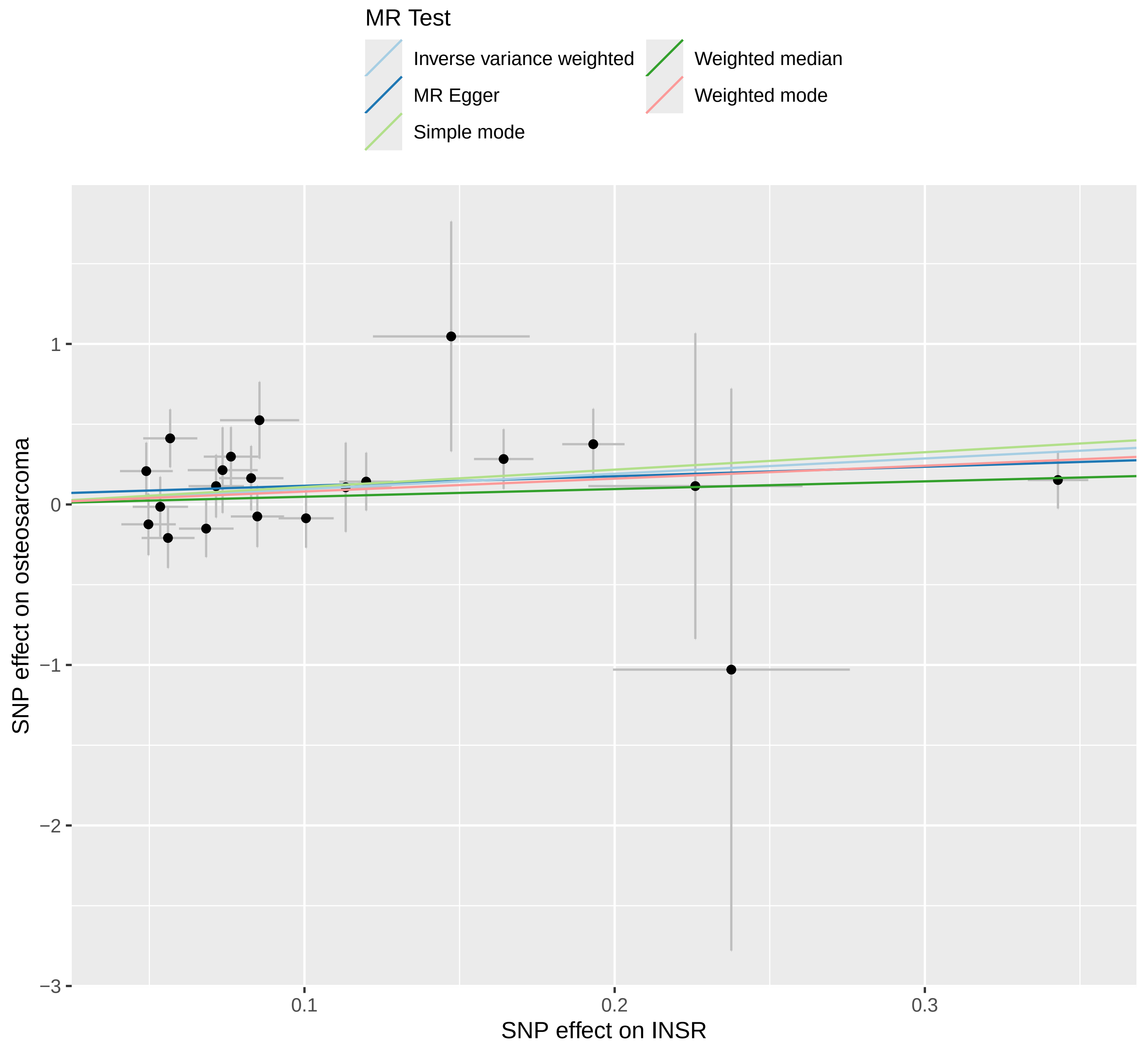

MR Test
Inverse variance weighted
MR Egger
Simple mode
Weighted median
Weighted mode
SNP effect on osteosarcoma
1
0
−1
−2
−3
0.1
0.2
0.3
SNP effect on INSR

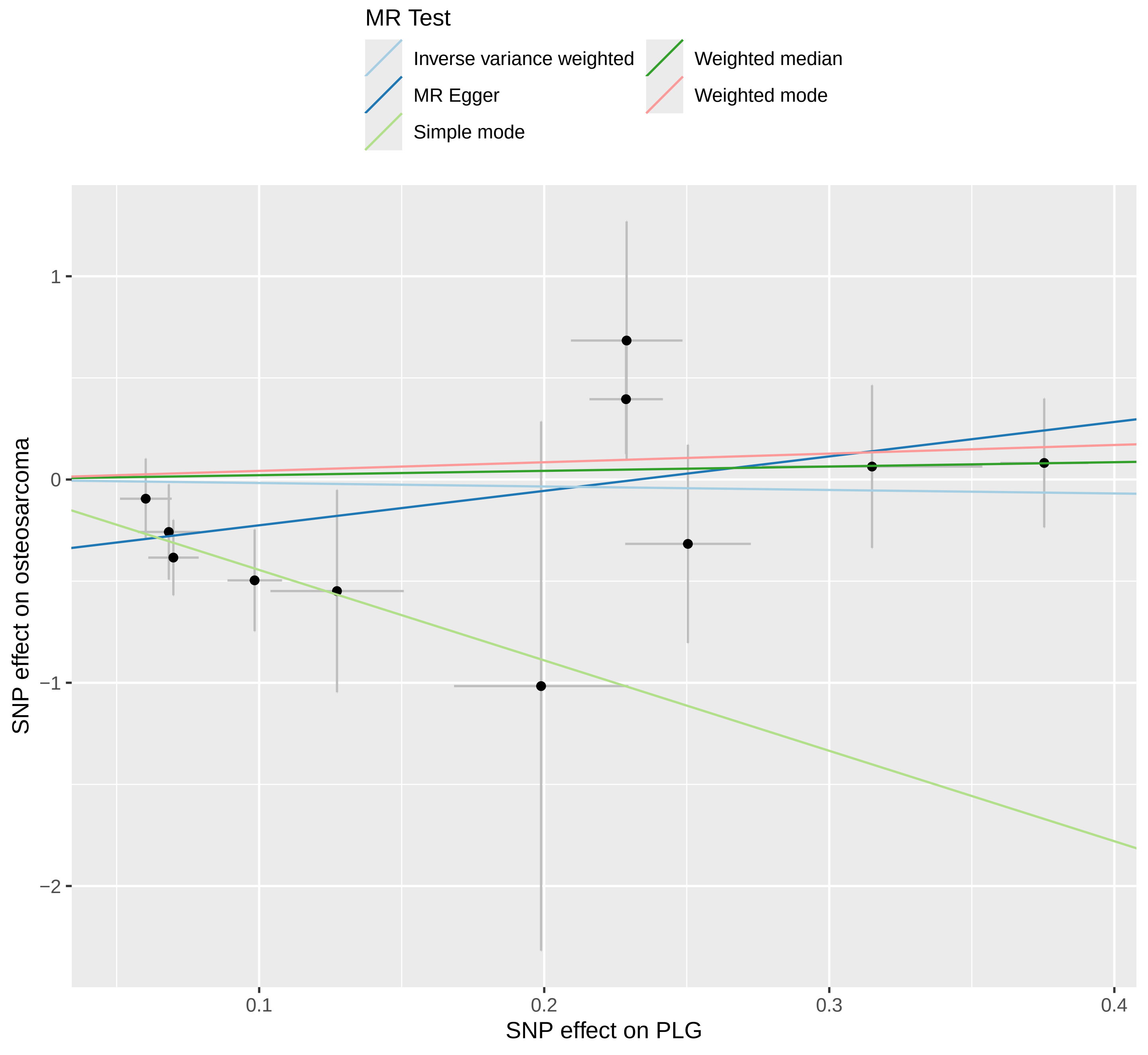
MR Test
Inverse variance weighted
MR Egger
Simple mode
Weighted median
Weighted mode
1
0
−1
−2
0.1
0.2
0.3
0.4
SNP effect on PLG
SNP effect on osteosarcoma

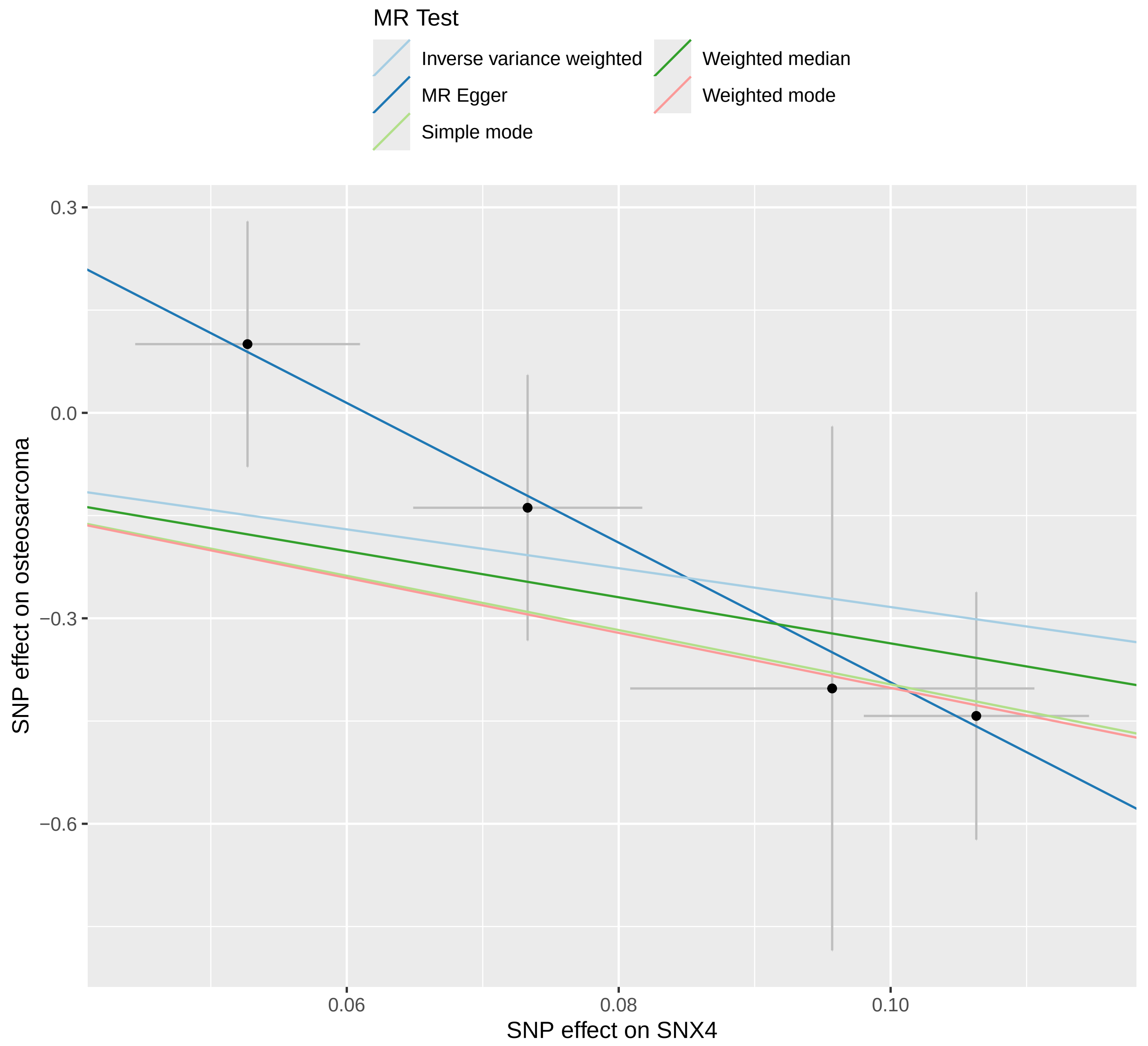
MR Test
Inverse variance weighted
MR Egger
Simple mode
Weighted median
Weighted mode
0.3
0.0
−0.3
−0.6
SNP effect on osteosarcoma
0.06
0.08
0.10
SNP effect on SNX4

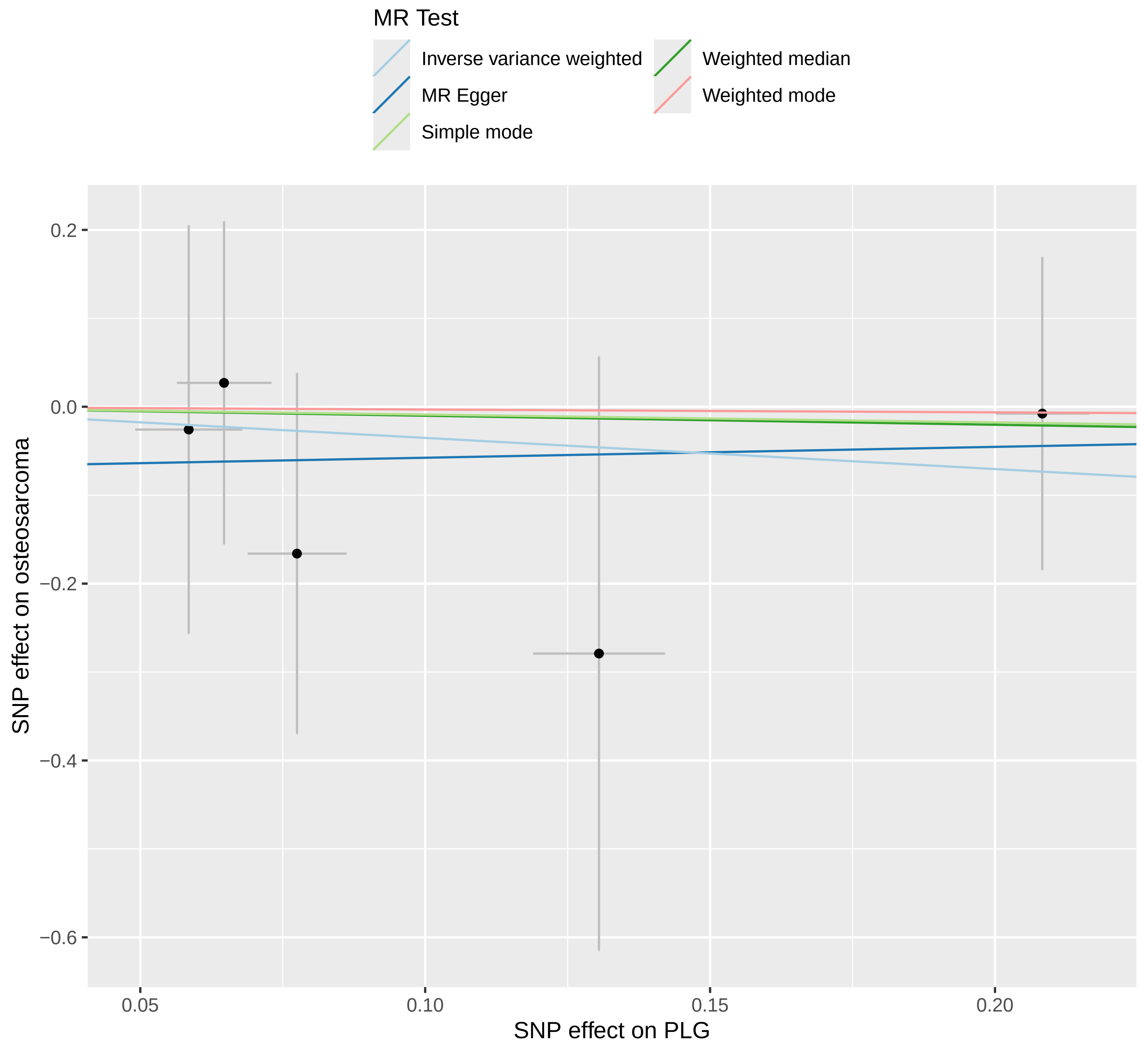
MR Test
Inverse variance weighted
MR Egger
Simple mode
Weighted median
Weighted mode
0.2
0.0
−0.2
−0.4
−0.6
SNP effect on osteosarcoma
0.05
0.10
0.15
0.20
SNP effect on PLG

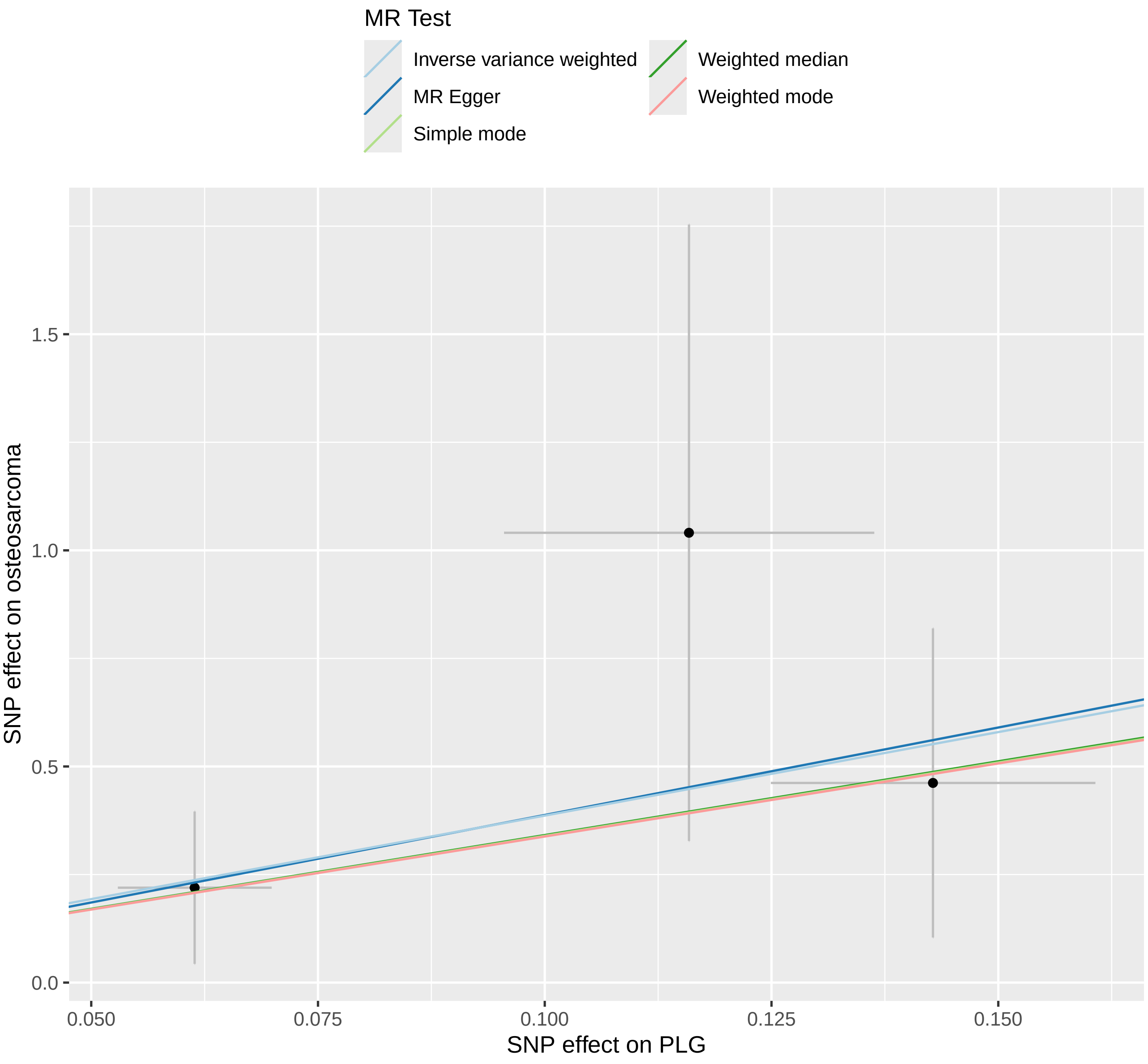

MR Test
Inverse variance weighted
MR Egger
Simple mode
Weighted median
Weighted mode
SNP effect on osteosarcoma
1.5
1.0
0.5
0.0
0.050
0.075
0.100
0.125
0.150
SNP effect on PLG

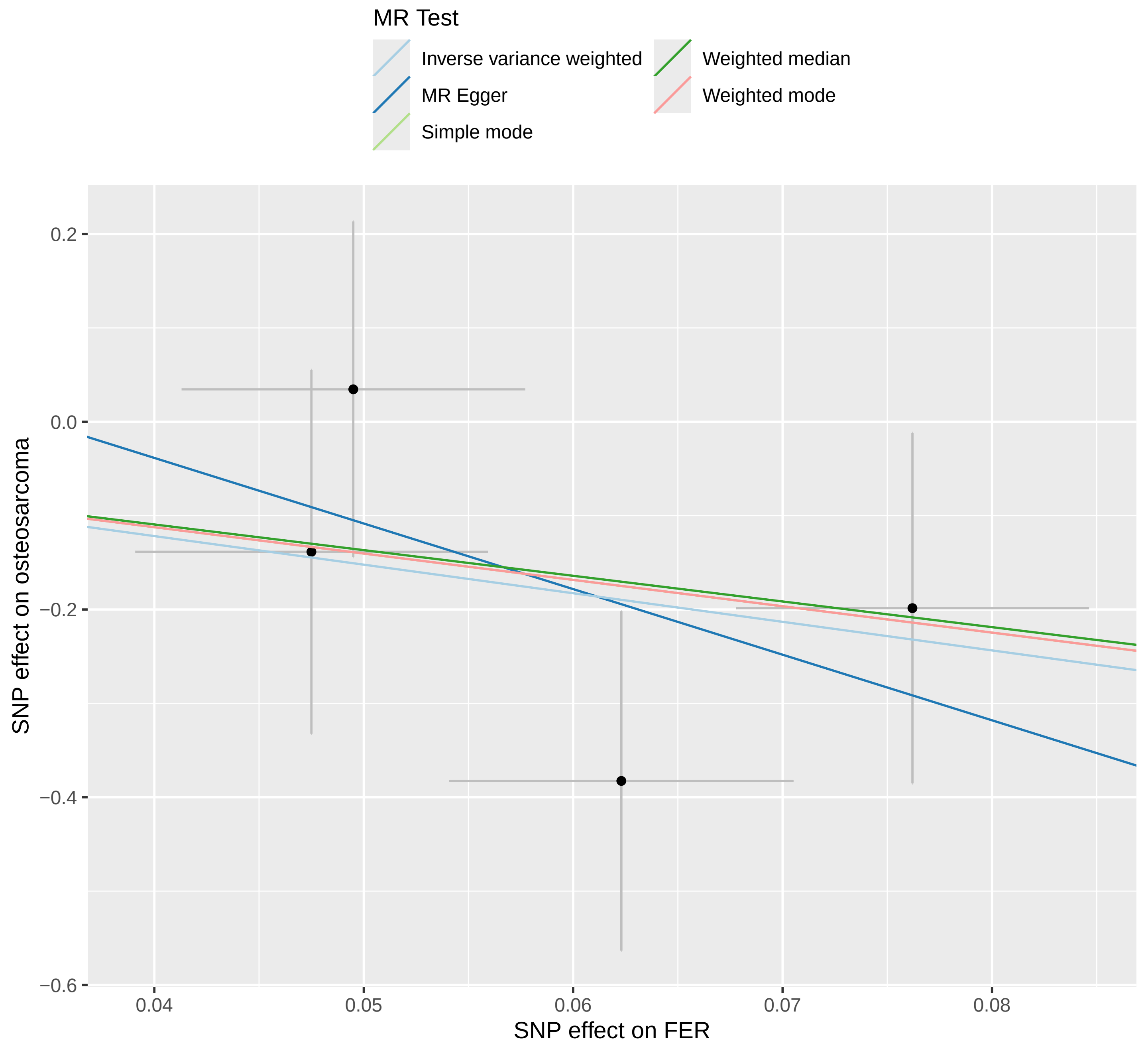
MR Test
Inverse variance weighted
MR Egger
Simple mode
Weighted median
Weighted mode
0.2
0.0
−0.2
−0.4
−0.6
0.04
0.05
0.06
0.07
0.08
SNP effect on FER
SNP effect on osteosarcoma

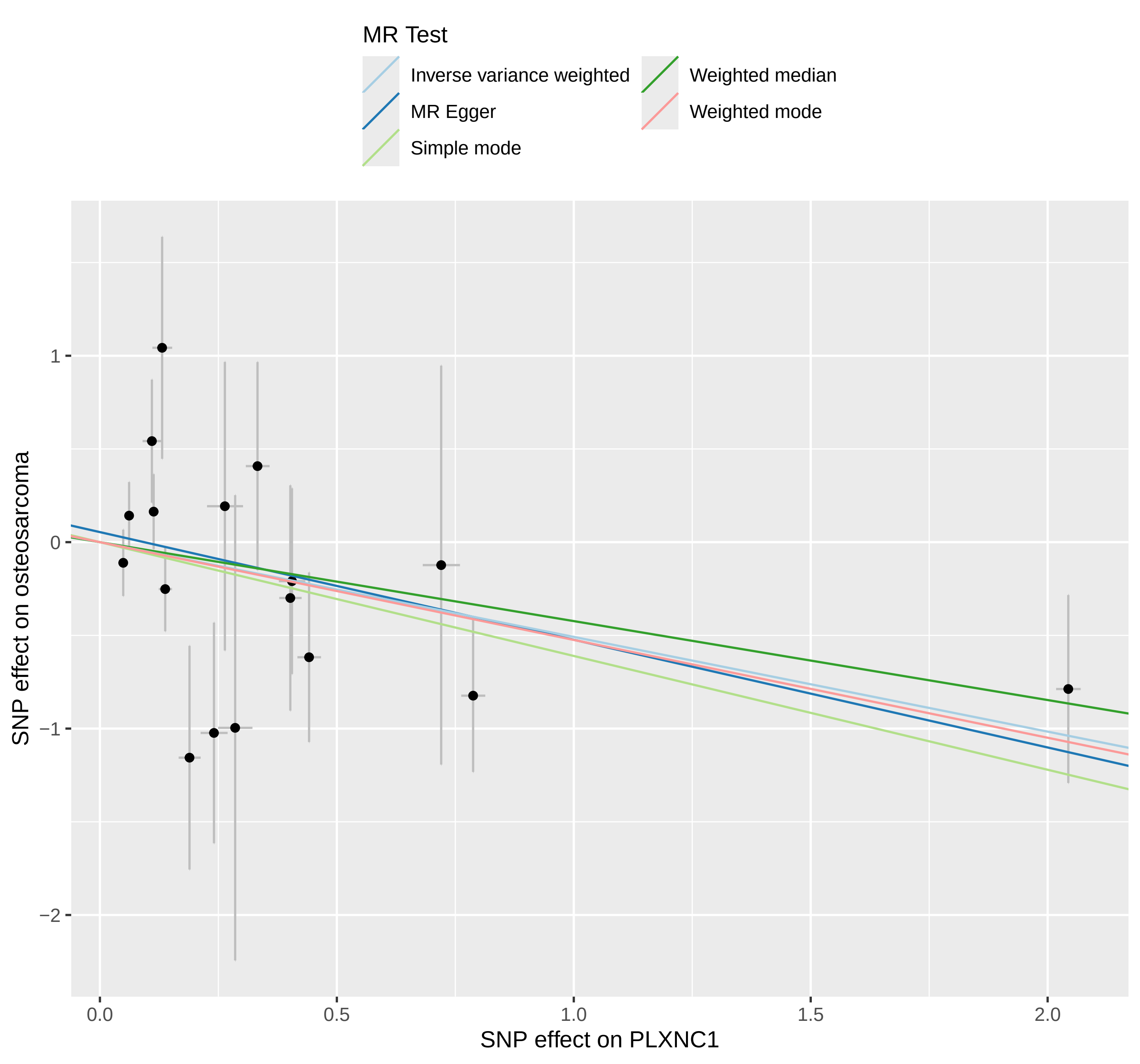

MR Test
Inverse variance weighted
MR Egger
Simple mode
Weighted median
Weighted mode
1
0
−1
−2
0.0
0.5
1.0
1.5
2.0
SNP effect on PLXNC1
SNP effect on osteosarcoma

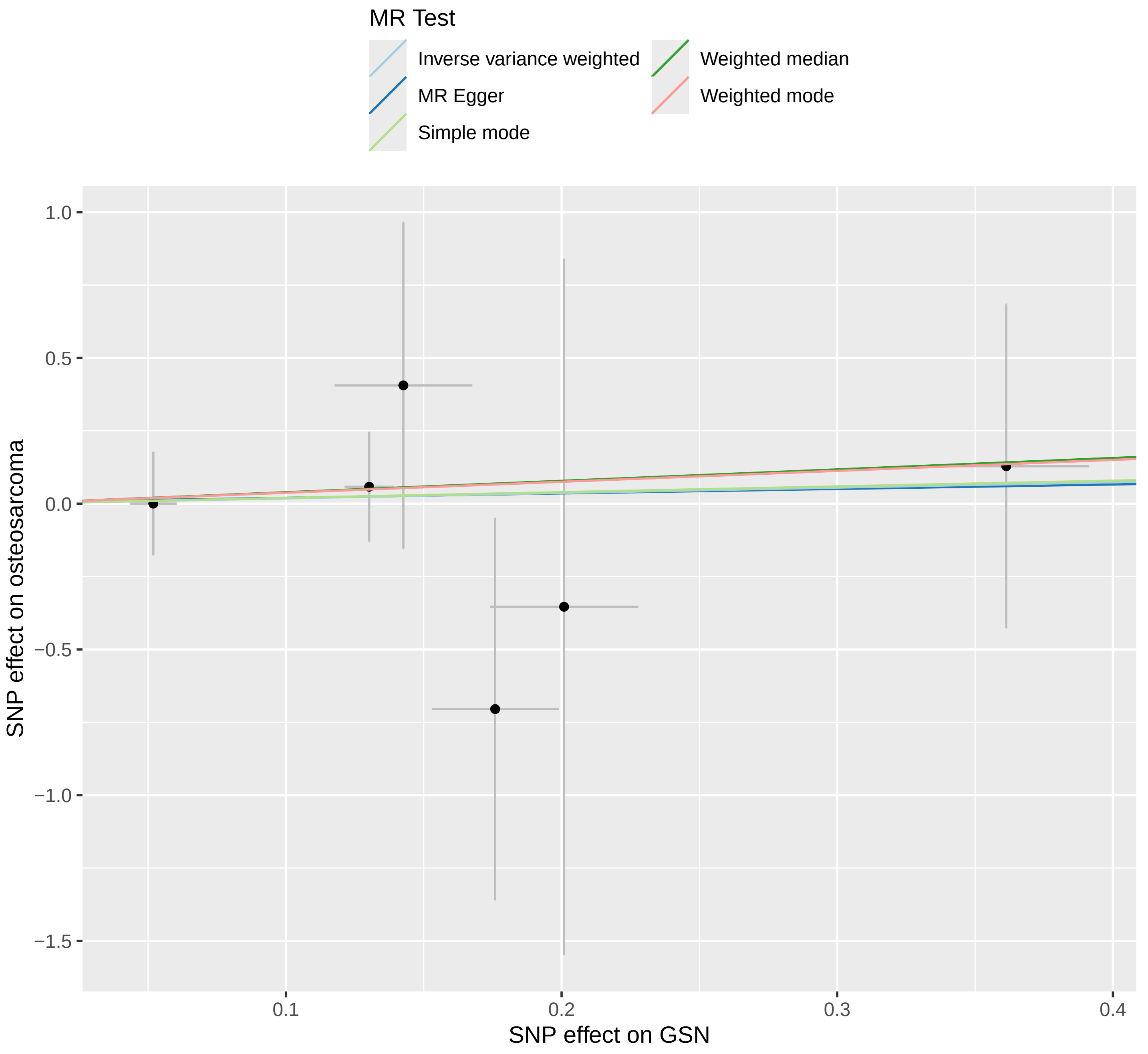
MR Test
Inverse variance weighted
MR Egger
Simple mode
Weighted median
Weighted mode
1.0
0.5
0.0
−0.5
−1.0
−1.5
0.1
0.2
0.3
0.4
SNP effect on GSN
SNP effect on osteosarcoma

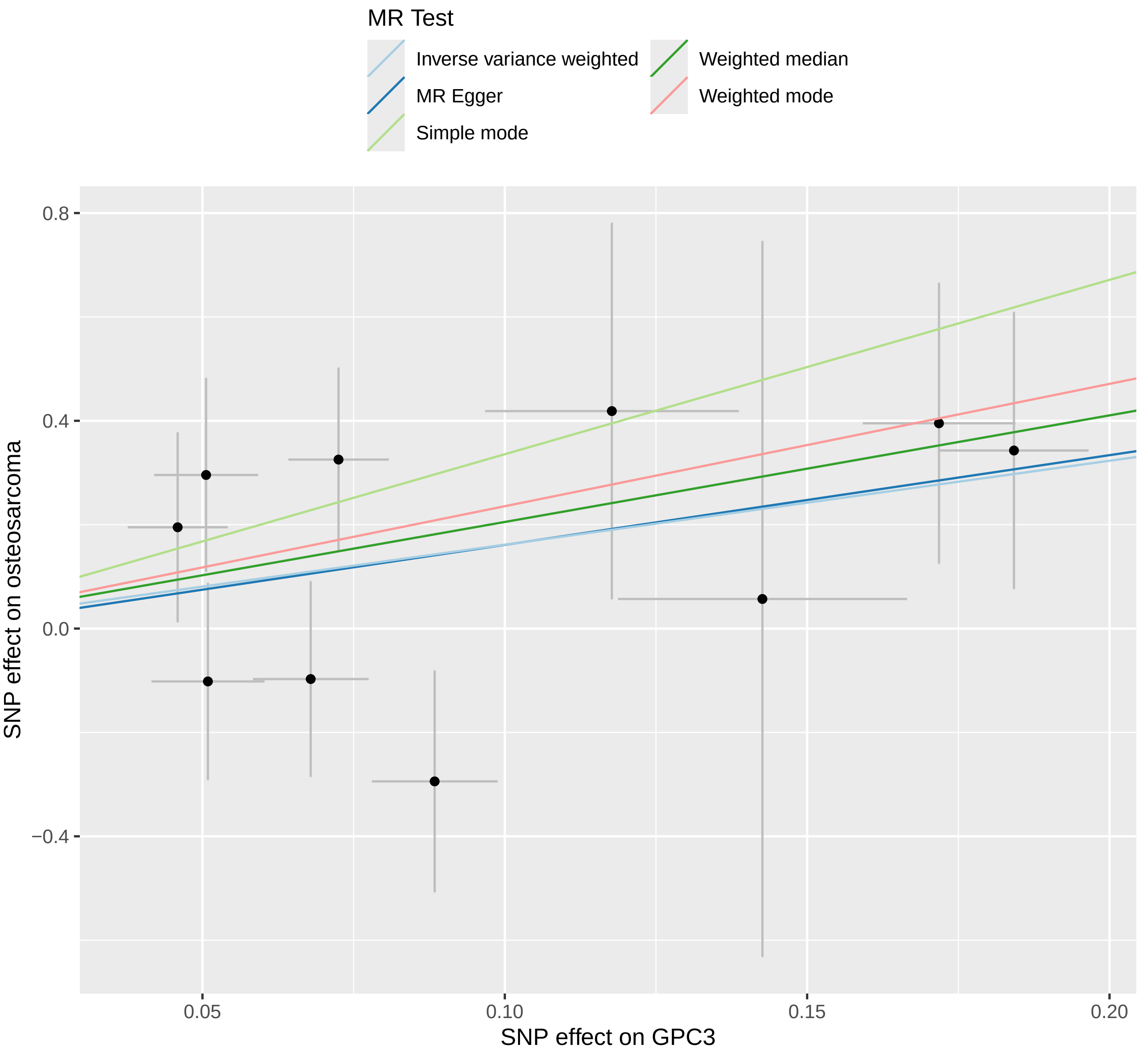
MR Test
Inverse variance weighted
MR Egger
Simple mode
Weighted median
Weighted mode
0.8
0.4
0.0
−0.4
SNP effect on osteosarcoma
0.05
0.10
0.15
0.20
SNP effect on GPC3

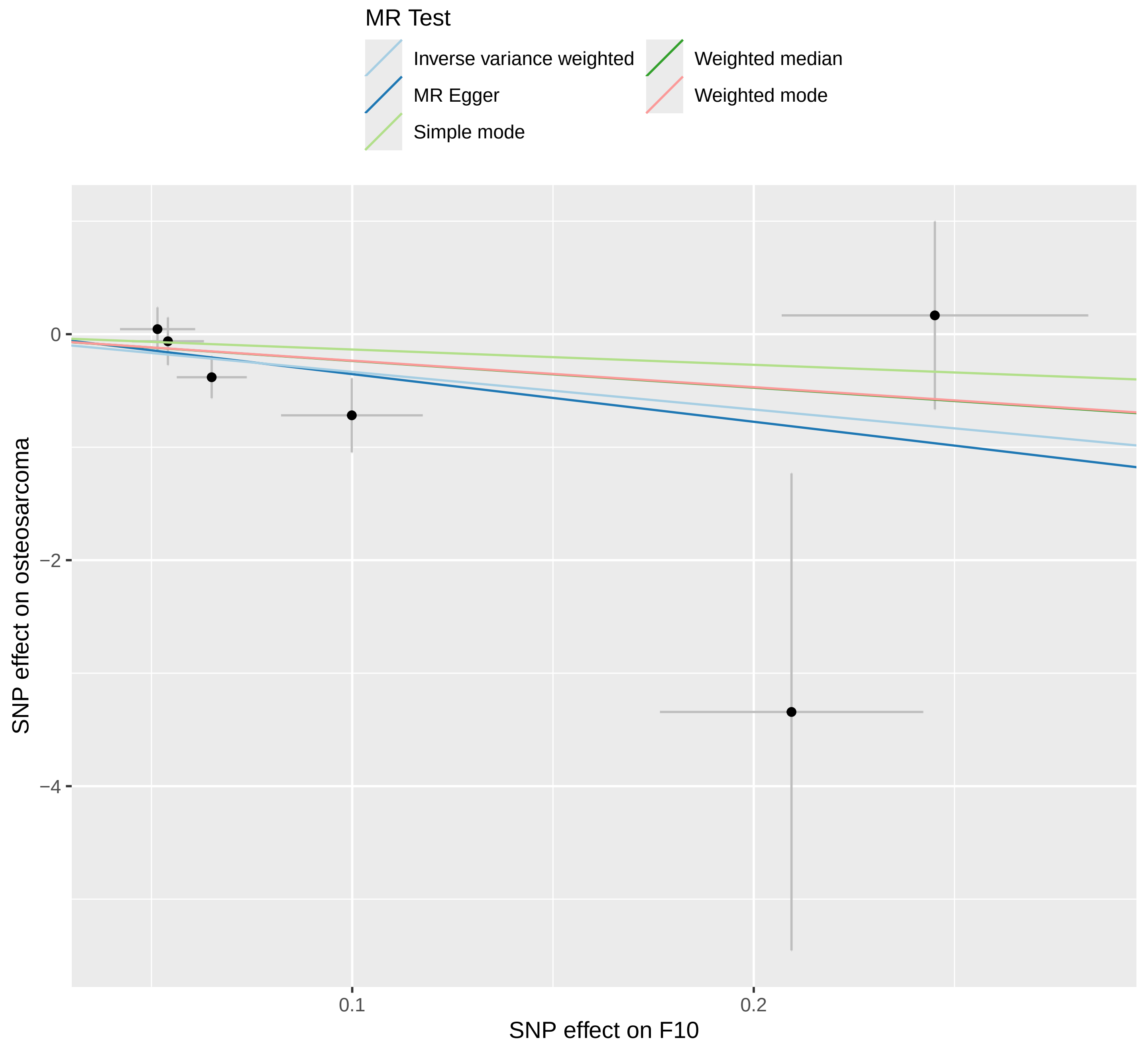

MR Test
Inverse variance weighted
MR Egger
Simple mode
Weighted median
Weighted mode
SNP effect on osteosarcoma
0
−2
−4
0.1
0.2
SNP effect on F10

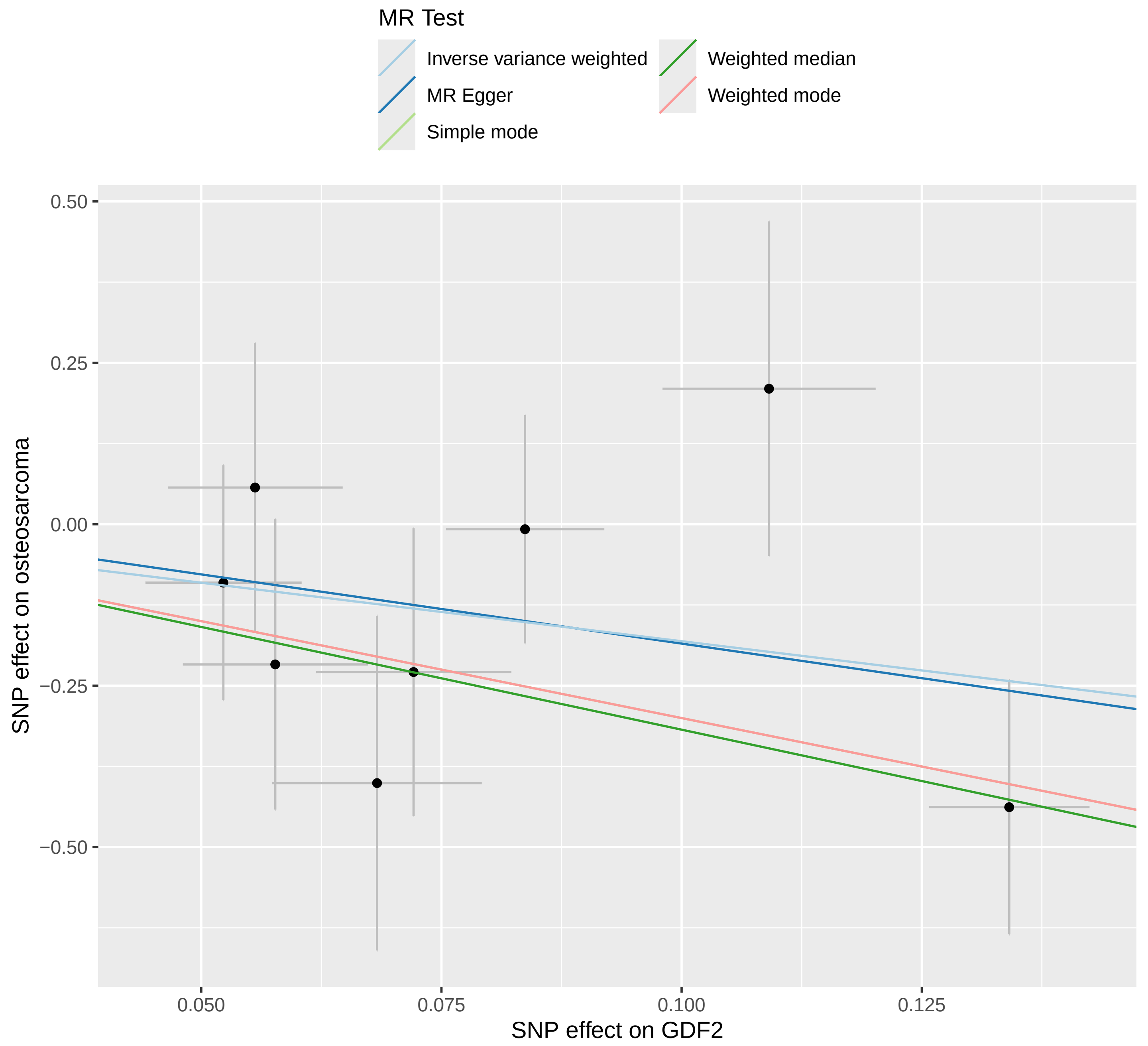
MR Test
Inverse variance weighted
MR Egger
Simple mode
Weighted median
Weighted mode
0.50
0.25
0.00
−0.25
−0.50
SNP effect on osteosarcoma
0.050
0.075
0.100
0.125
SNP effect on GDF2

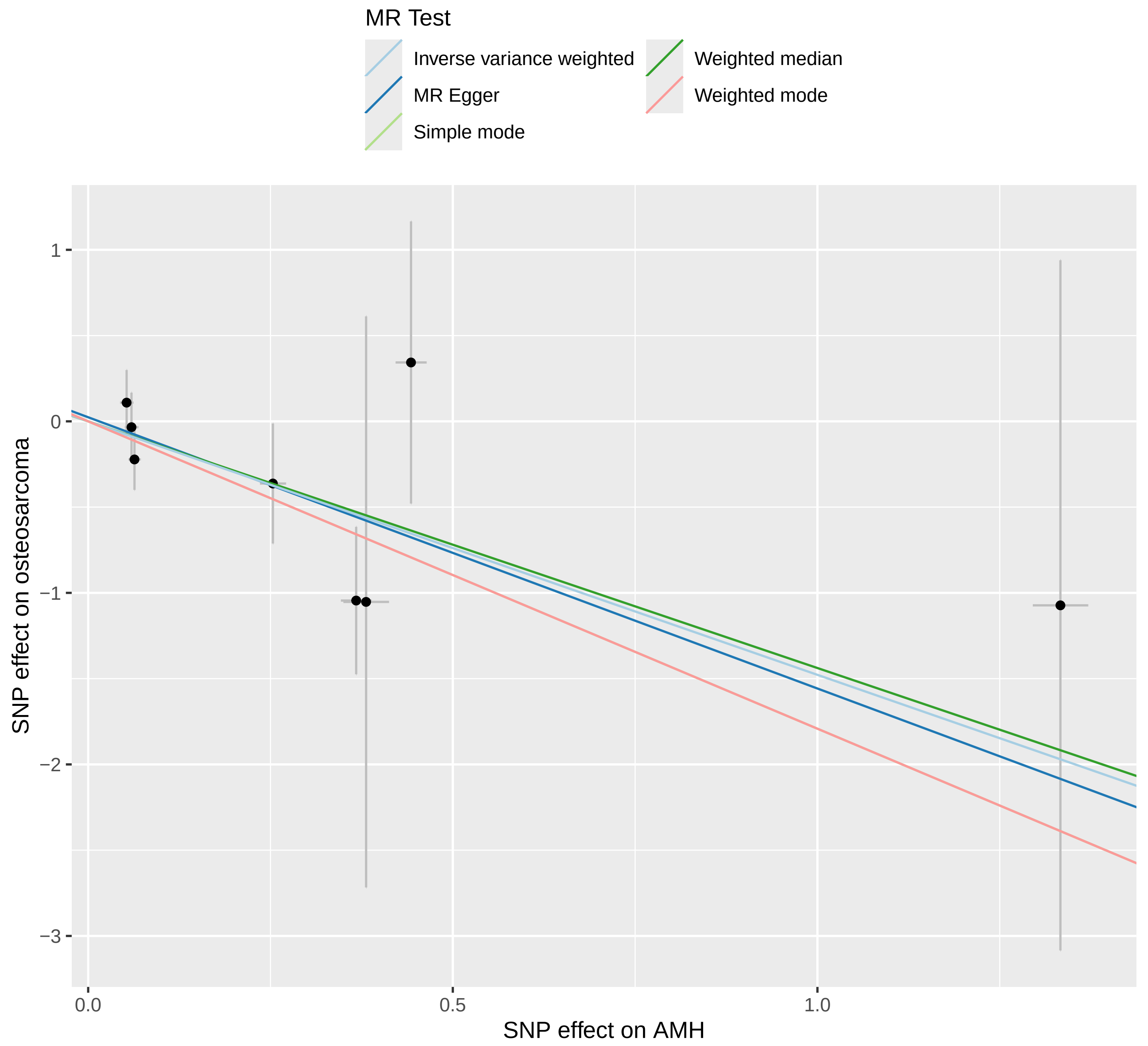

MR Test
Inverse variance weighted
MR Egger
Simple mode
Weighted median
Weighted mode
1
0
−1
−2
−3
SNP effect on osteosarcoma
0.0
0.5
1.0
SNP effect on AMH

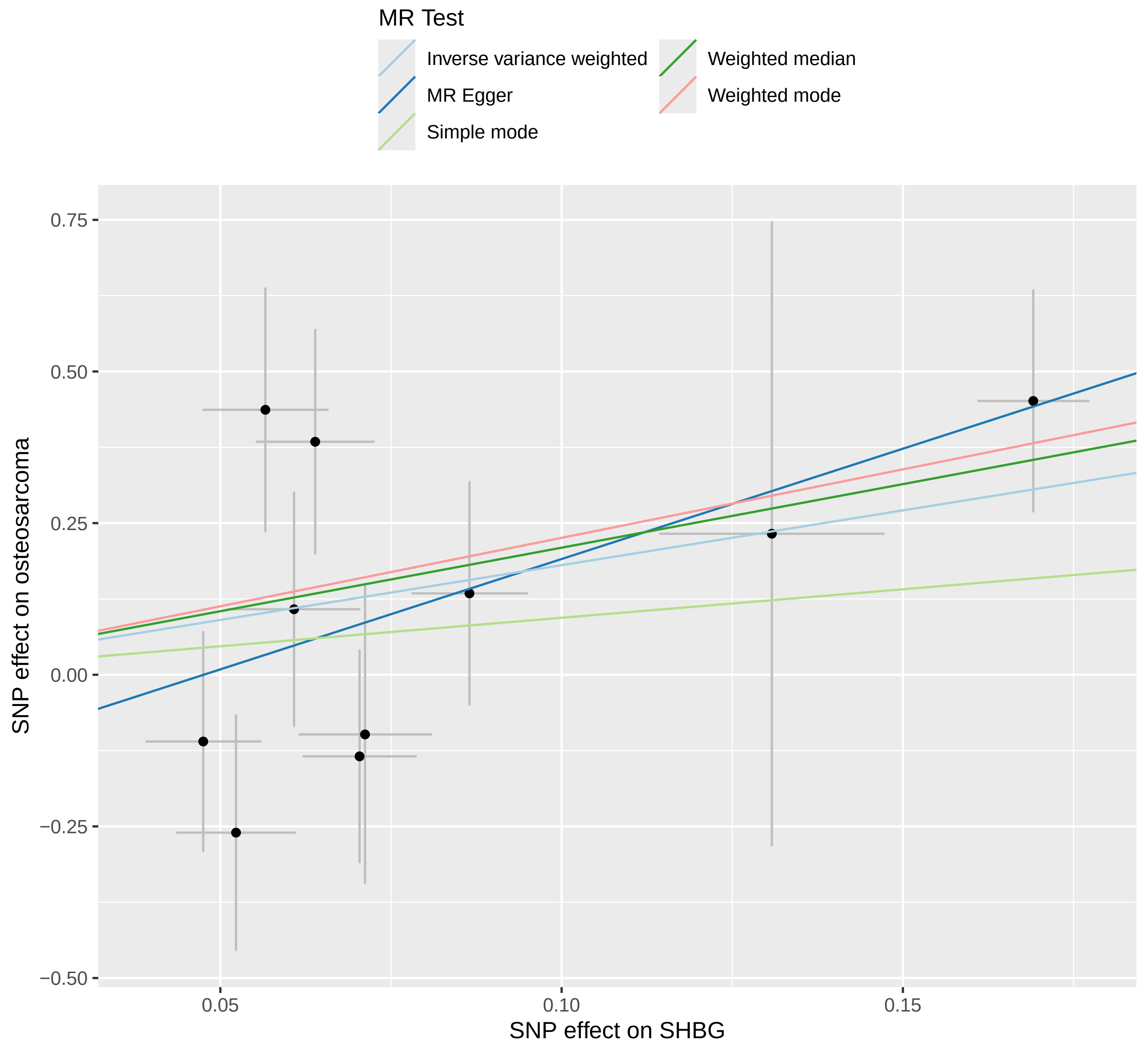

MR Test
Inverse variance weighted
MR Egger
Simple mode
Weighted median
Weighted mode
0.75
0.50
0.25
0.00
−0.25
−0.50
SNP effect on osteosarcoma
0.05
0.10
0.15
SNP effect on SHBG

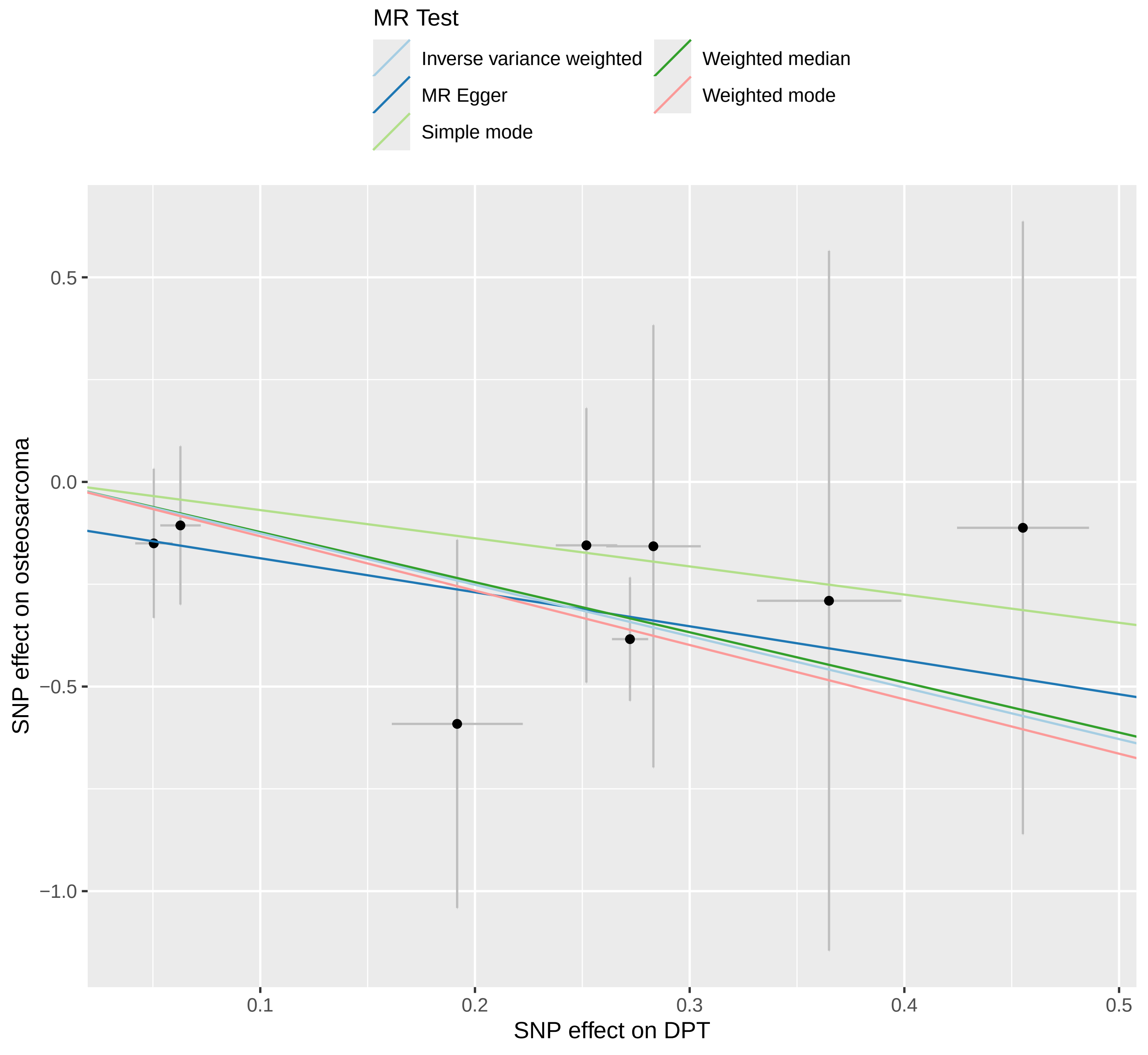

MR Test
Inverse variance weighted
MR Egger
Simple mode
Weighted median
Weighted mode
0.5
0.0
−0.5
−1.0
SNP effect on osteosarcoma
0.1
0.2
0.3
0.4
0.5
SNP effect on DPT

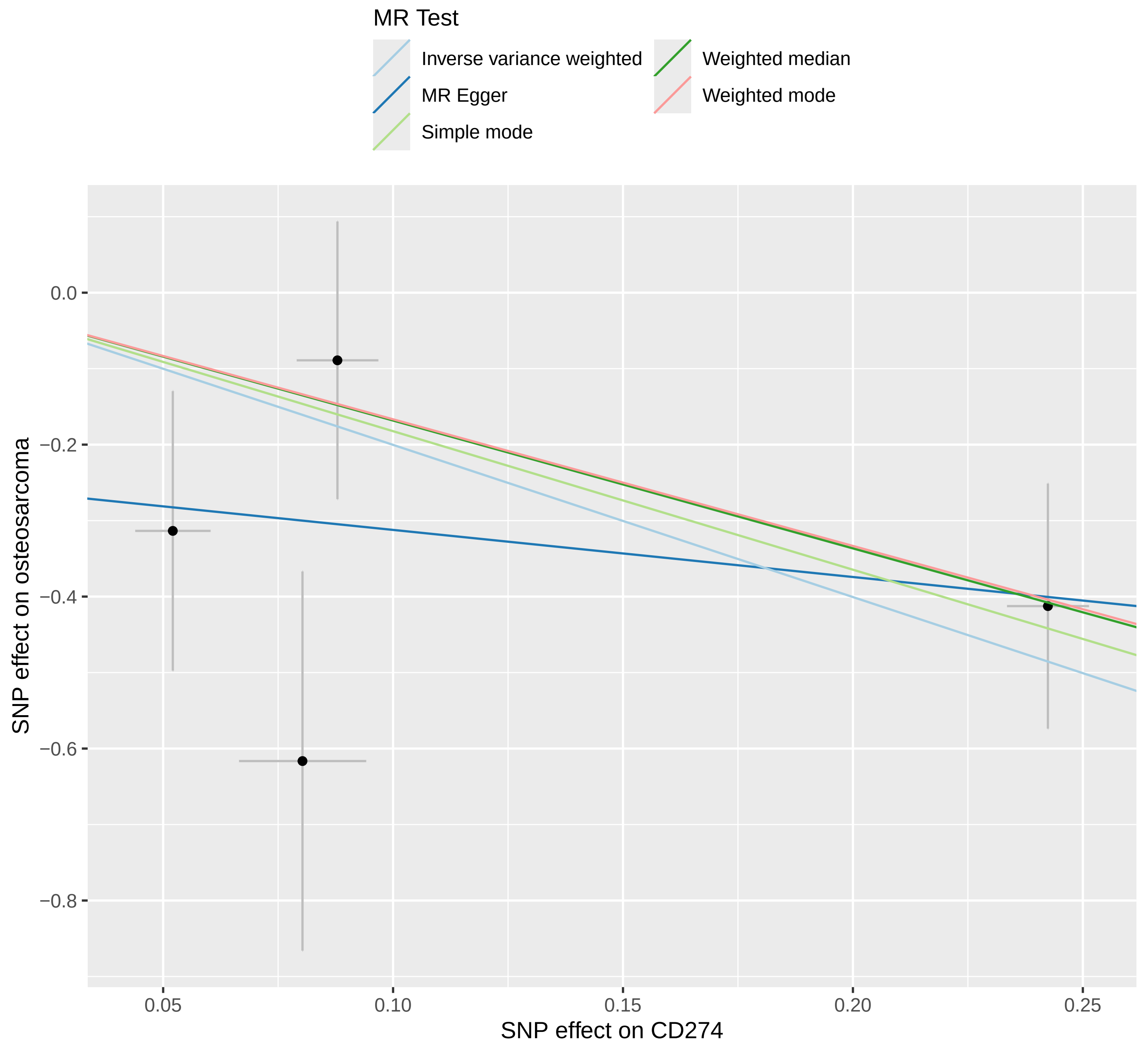
MR Test
Inverse variance weighted
MR Egger
Simple mode
Weighted median
Weighted mode
0.0
−0.2
−0.4
−0.6
−0.8
SNP effect on osteosarcoma
0.05
0.10
0.15
0.20
0.25
SNP effect on CD274

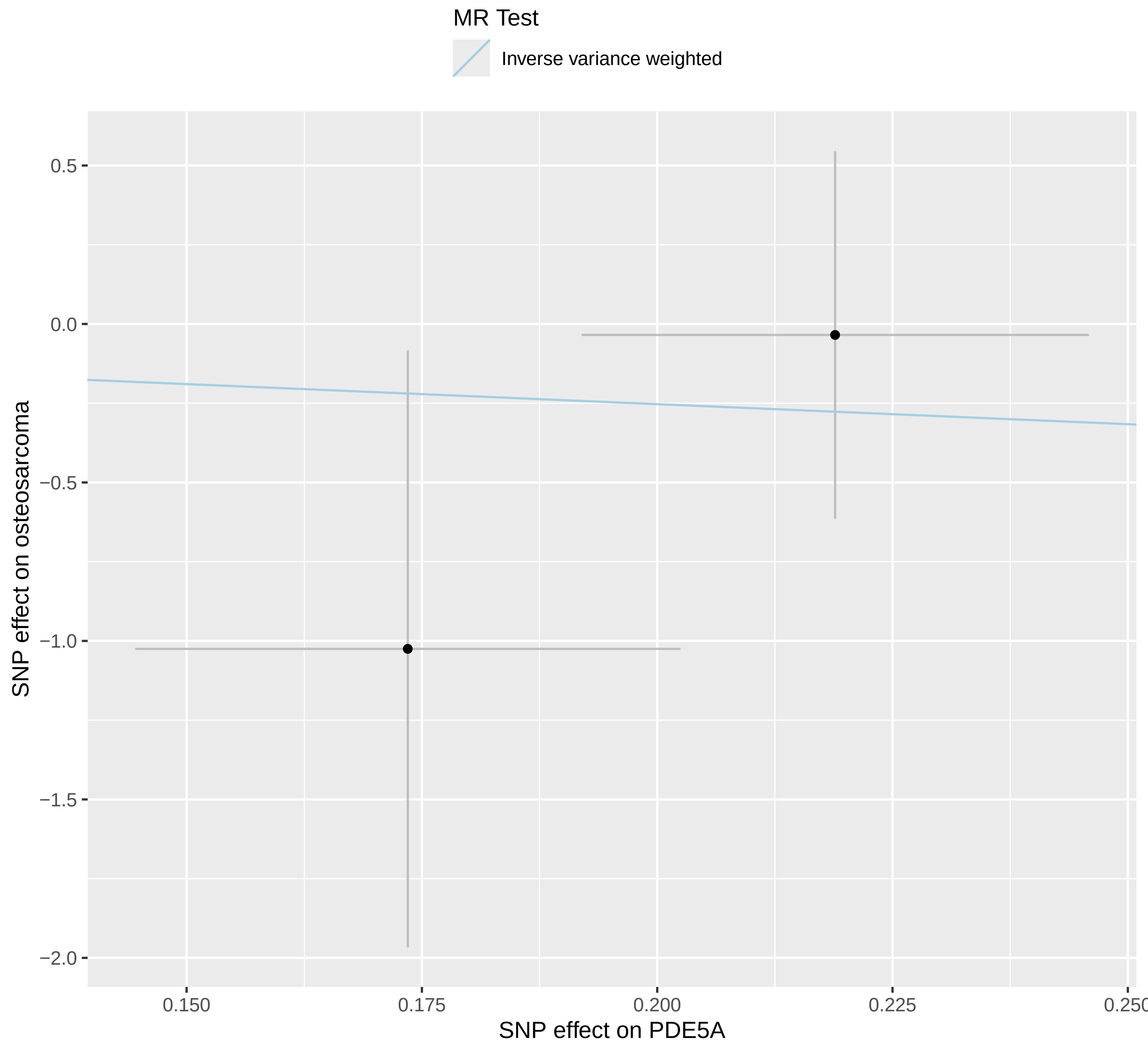
MR Test
Inverse variance weighted
0.5
0.0
−0.5
−1.0
−1.5
−2.0
0.150
0.175
0.200
0.225
0.250
SNP effect on PDE5A
SNP effect on osteosarcoma

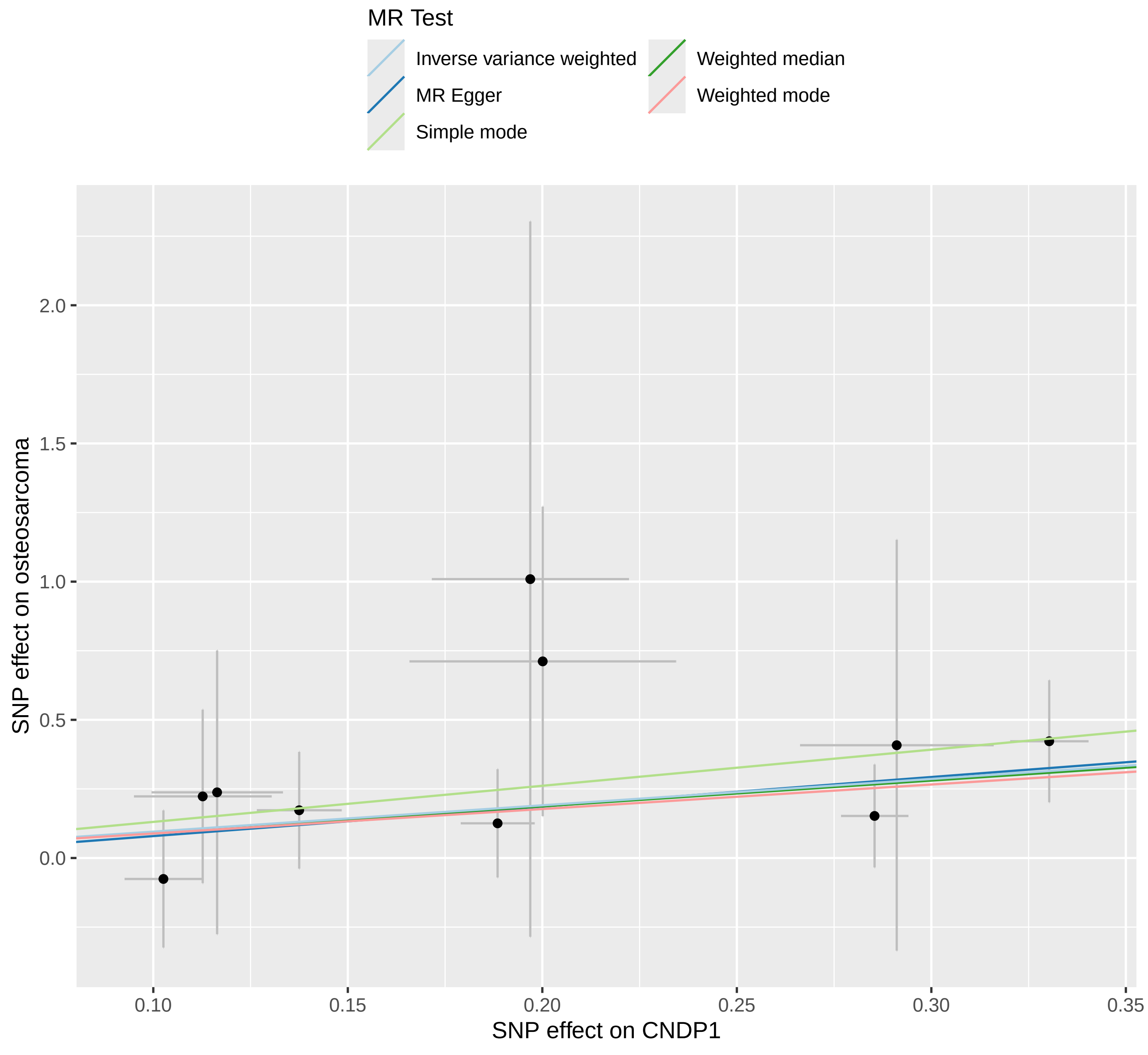
MR Test
Inverse variance weighted
MR Egger
Simple mode
Weighted median
Weighted mode
SNP effect on osteosarcoma
2.0
1.5
1.0
0.5
0.0
0.10
0.15
0.20
0.25
0.30
0.35
SNP effect on CNDP1

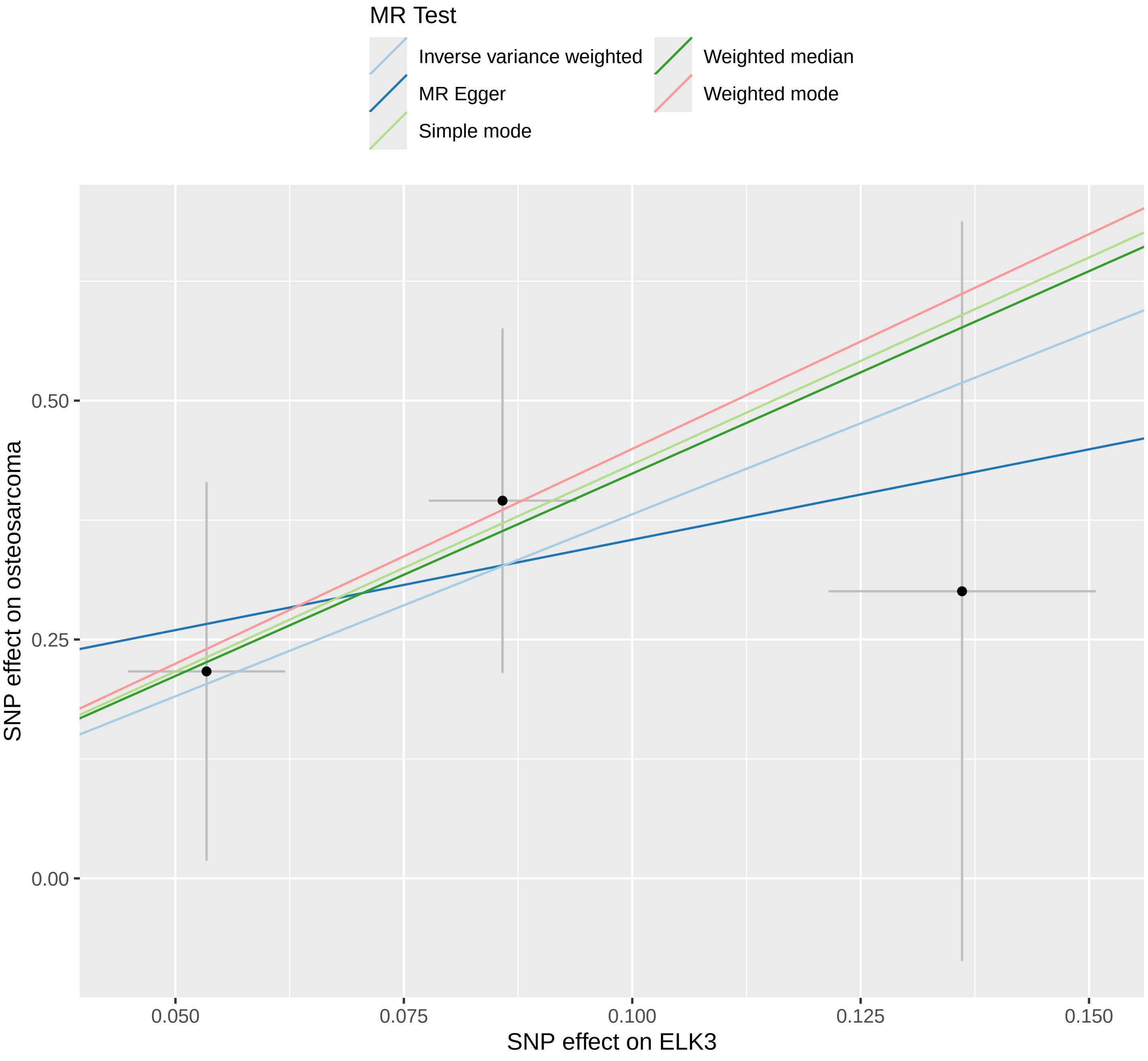
MR Test
Inverse variance weighted
MR Egger
Simple mode
Weighted median
Weighted mode
0.50
0.25
0.00
SNP effect on osteosarcoma
0.050
0.075
0.100
0.125
0.150
SNP effect on ELK3

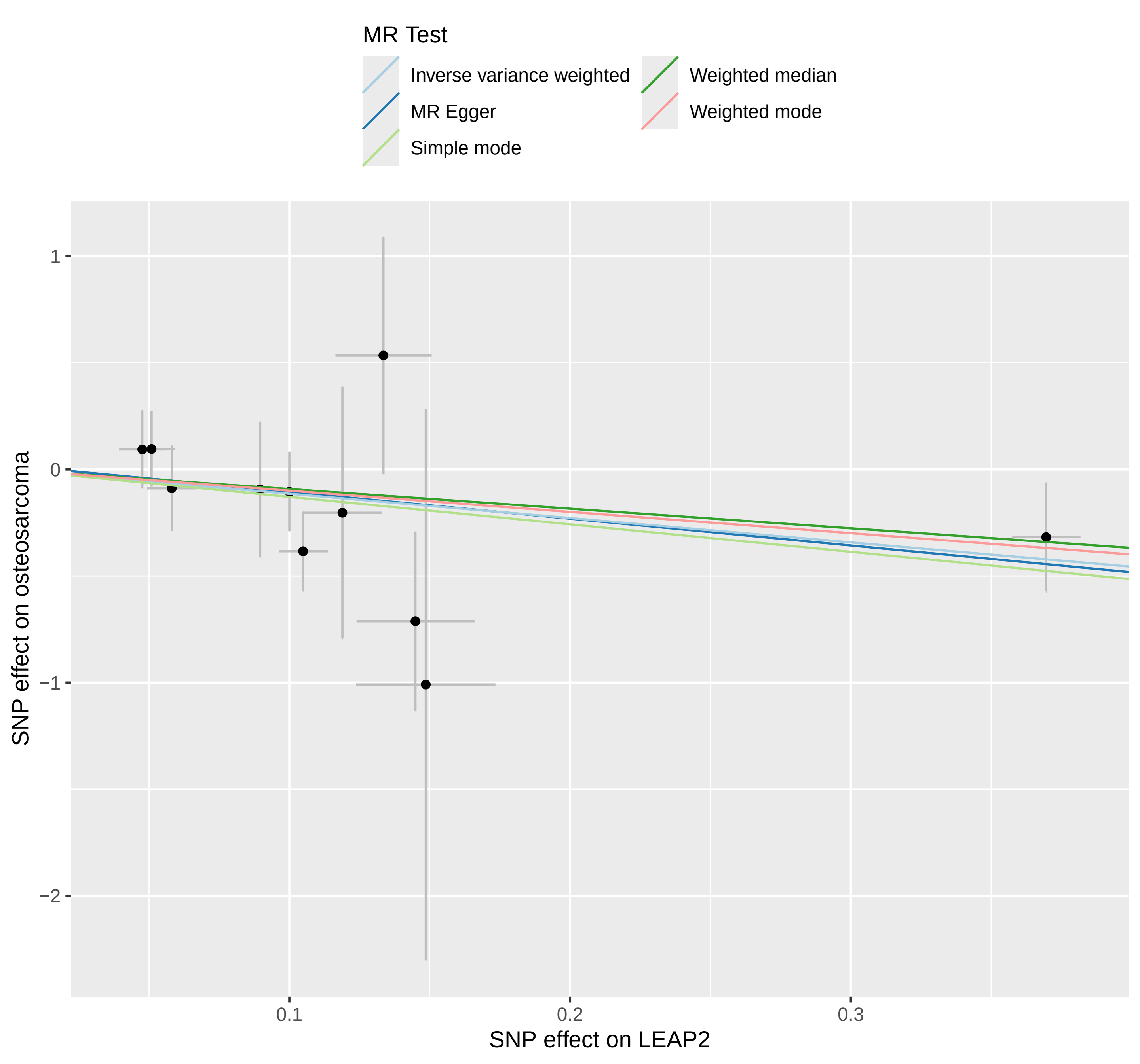
MR Test
Inverse variance weighted
MR Egger
Simple mode
Weighted median
Weighted mode
1
0
−1
−2
0.1
0.2
0.3
SNP effect on osteosarcoma
SNP effect on LEAP2

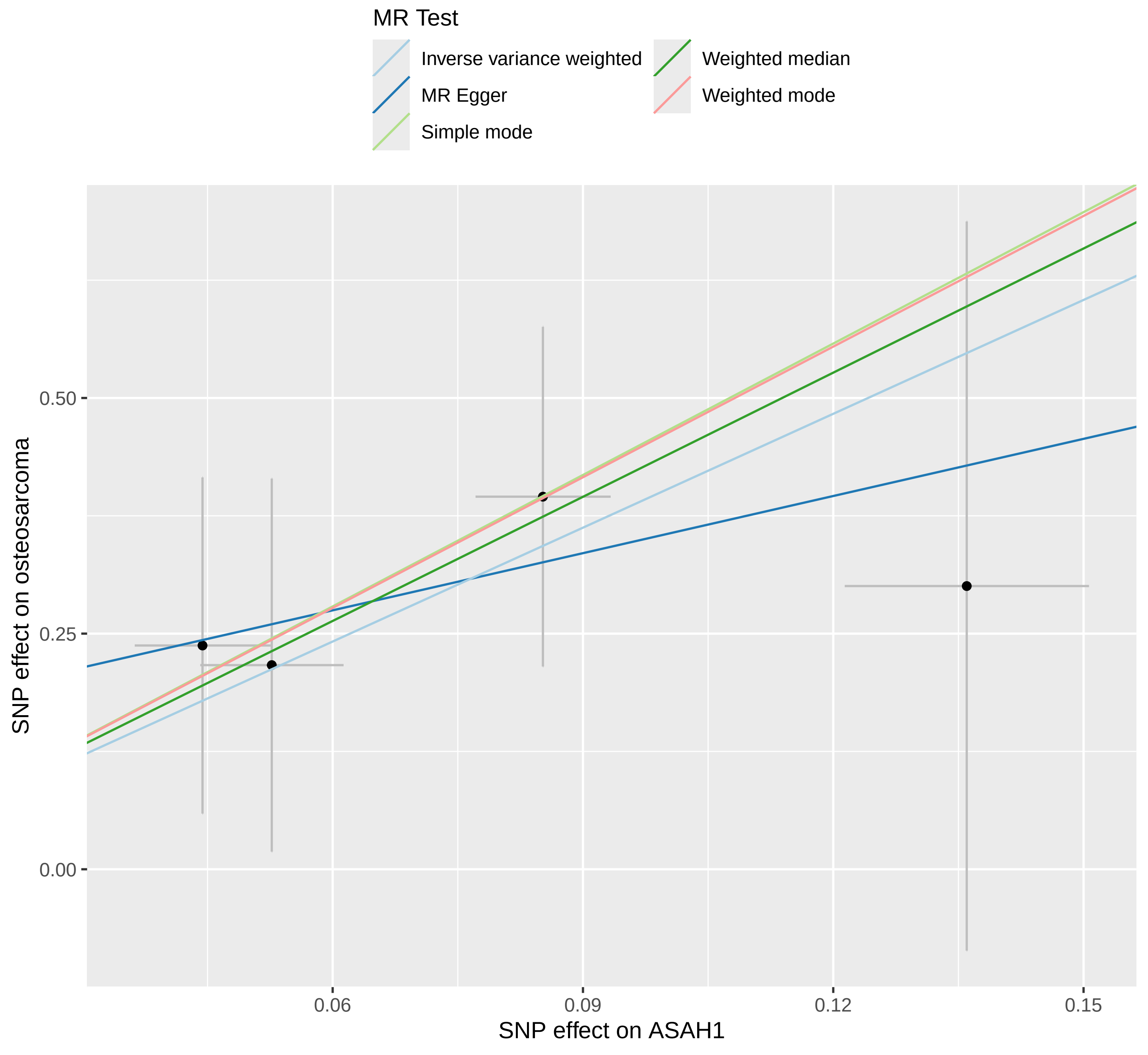
MR Test
Inverse variance weighted
MR Egger
Simple mode
Weighted median
Weighted mode
0.50
0.25
0.00
SNP effect on osteosarcoma
0.06
0.09
0.12
0.15
SNP effect on ASAH1

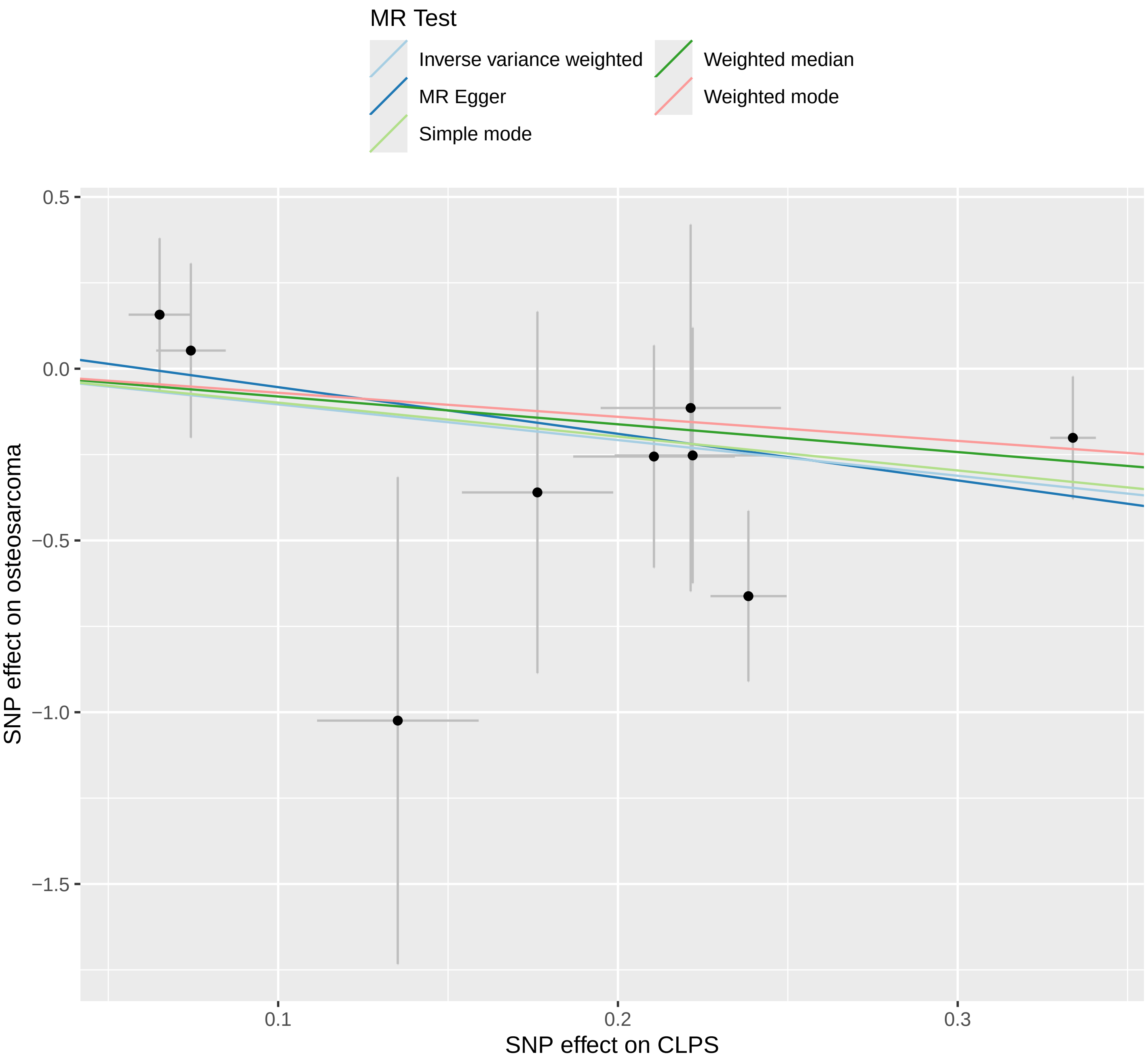
MR Test
Inverse variance weighted
MR Egger
Simple mode
Weighted median
Weighted mode
0.5
0.0
−0.5
−1.0
−1.5
SNP effect on osteosarcoma
0.1
0.2
0.3
SNP effect on CLPS

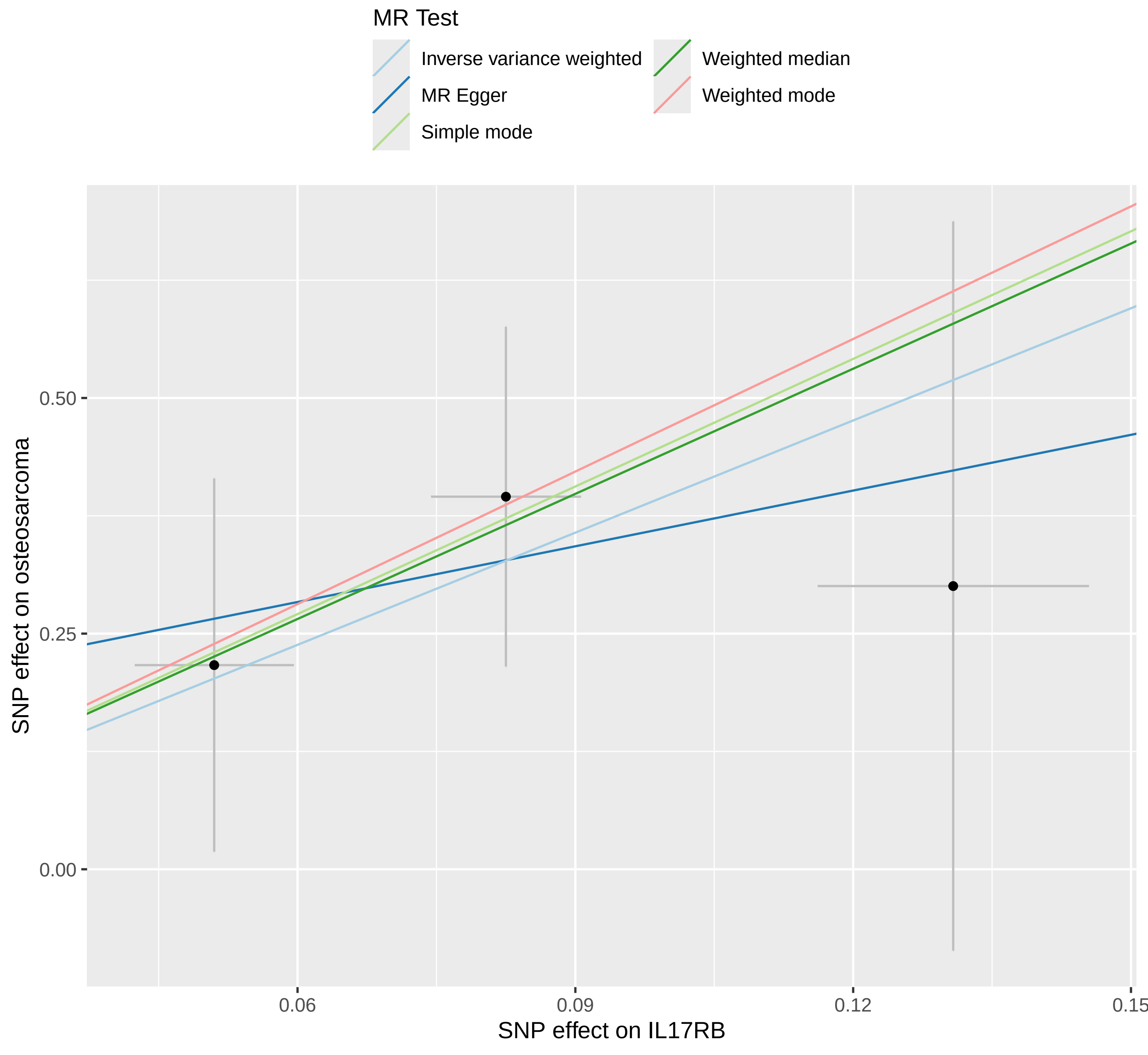

MR Test
Inverse variance weighted
MR Egger
Simple mode
Weighted median
Weighted mode
0.50
0.25
0.00
0.06
0.09
0.12
0.15
SNP effect on IL17RB
SNP effect on osteosarcoma

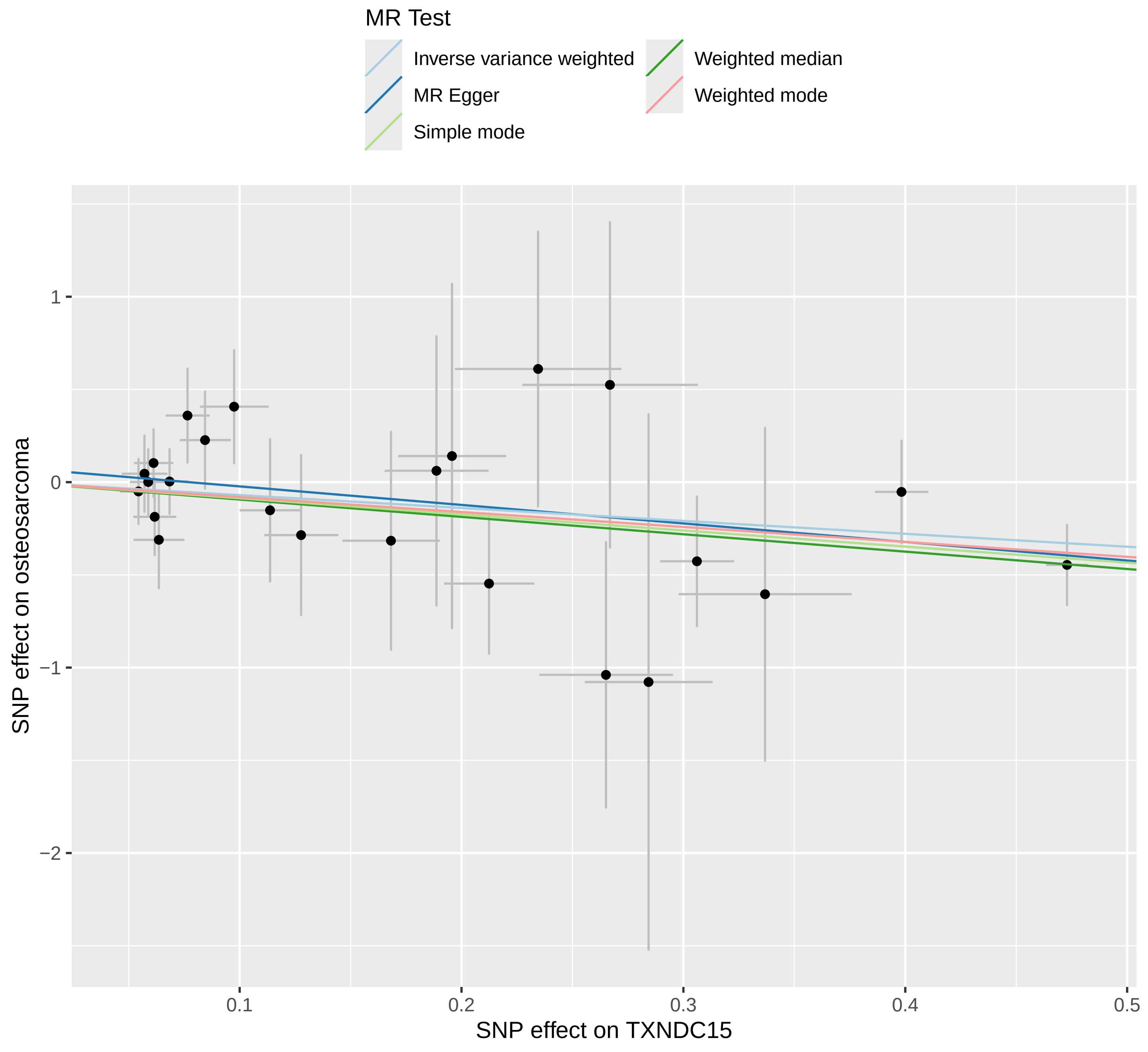
MR Test
Inverse variance weighted
MR Egger
Simple mode
Weighted median
Weighted mode
1
0
−1
−2
0.1
0.2
0.3
0.4
0.5
SNP effect on TXNDC15
SNP effect on osteosarcoma

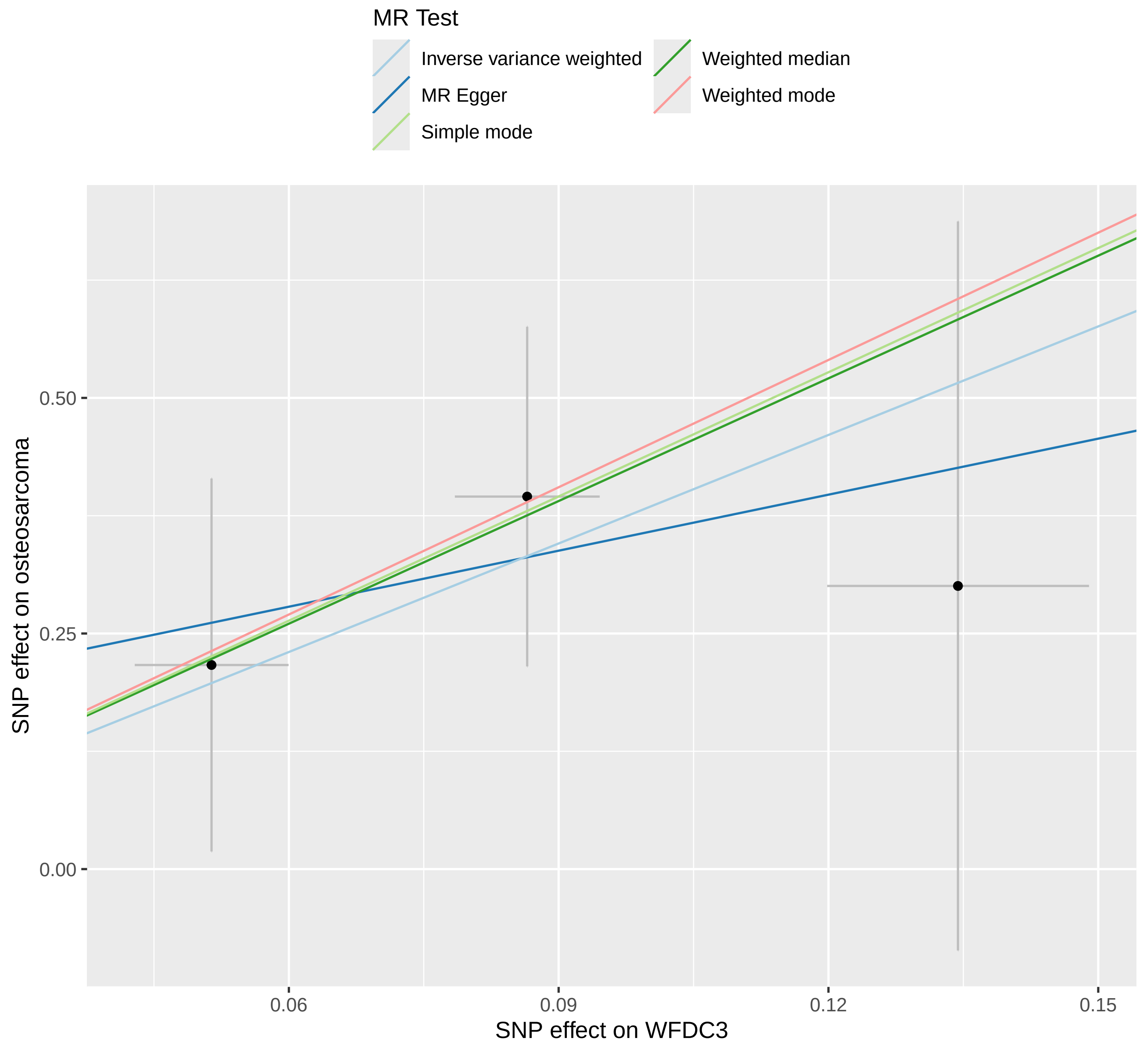

MR Test
Inverse variance weighted
MR Egger
Simple mode
Weighted median
Weighted mode
SNP effect on osteosarcoma
0.50
0.25
0.00
0.06
0.09
0.12
0.15
SNP effect on WFDC3

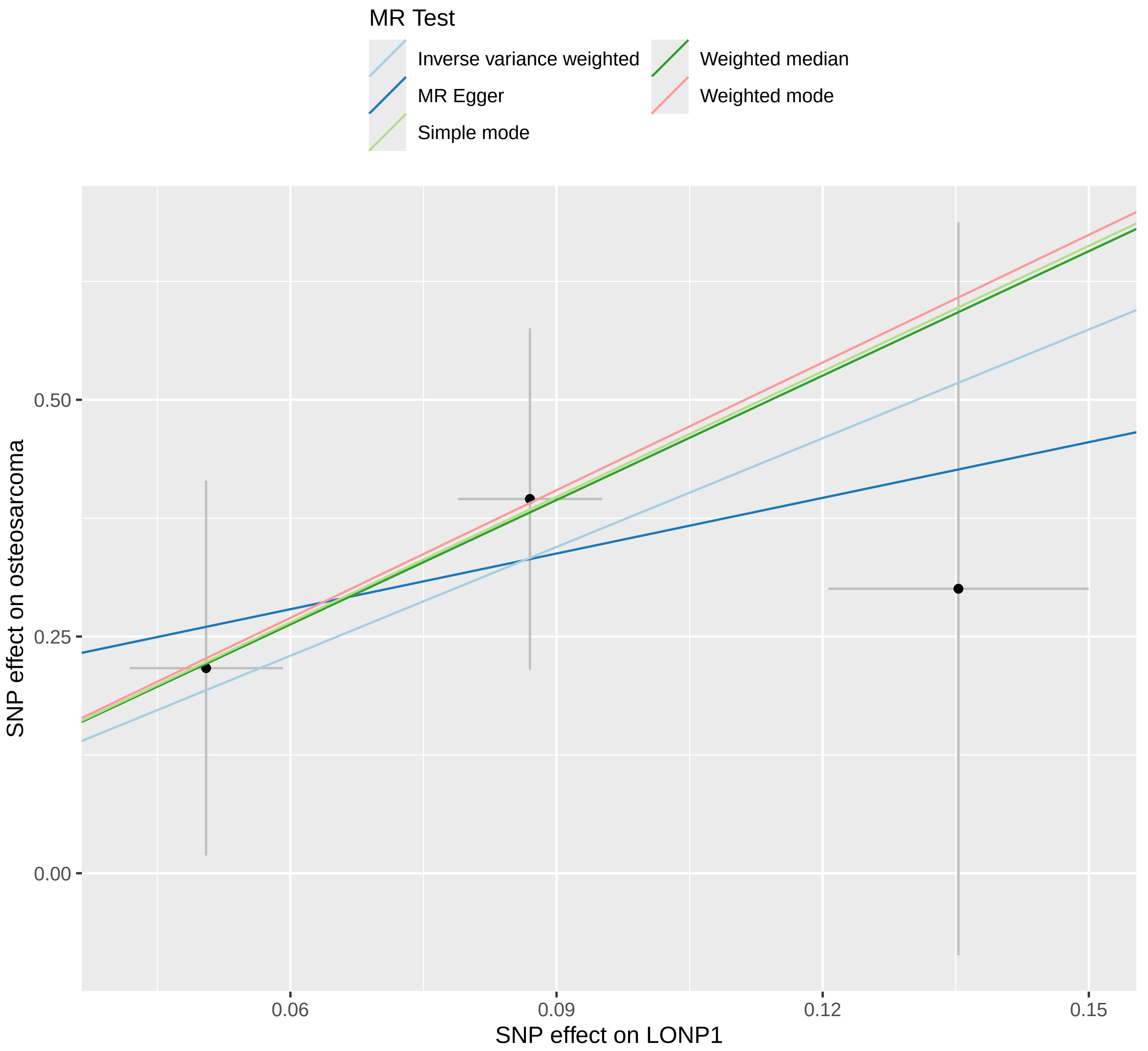
MR Test
Inverse variance weighted
MR Egger
Simple mode
Weighted median
Weighted mode
0.50
0.25
0.00
SNP effect on osteosarcoma
0.06
0.09
0.12
0.15
SNP effect on LONP1

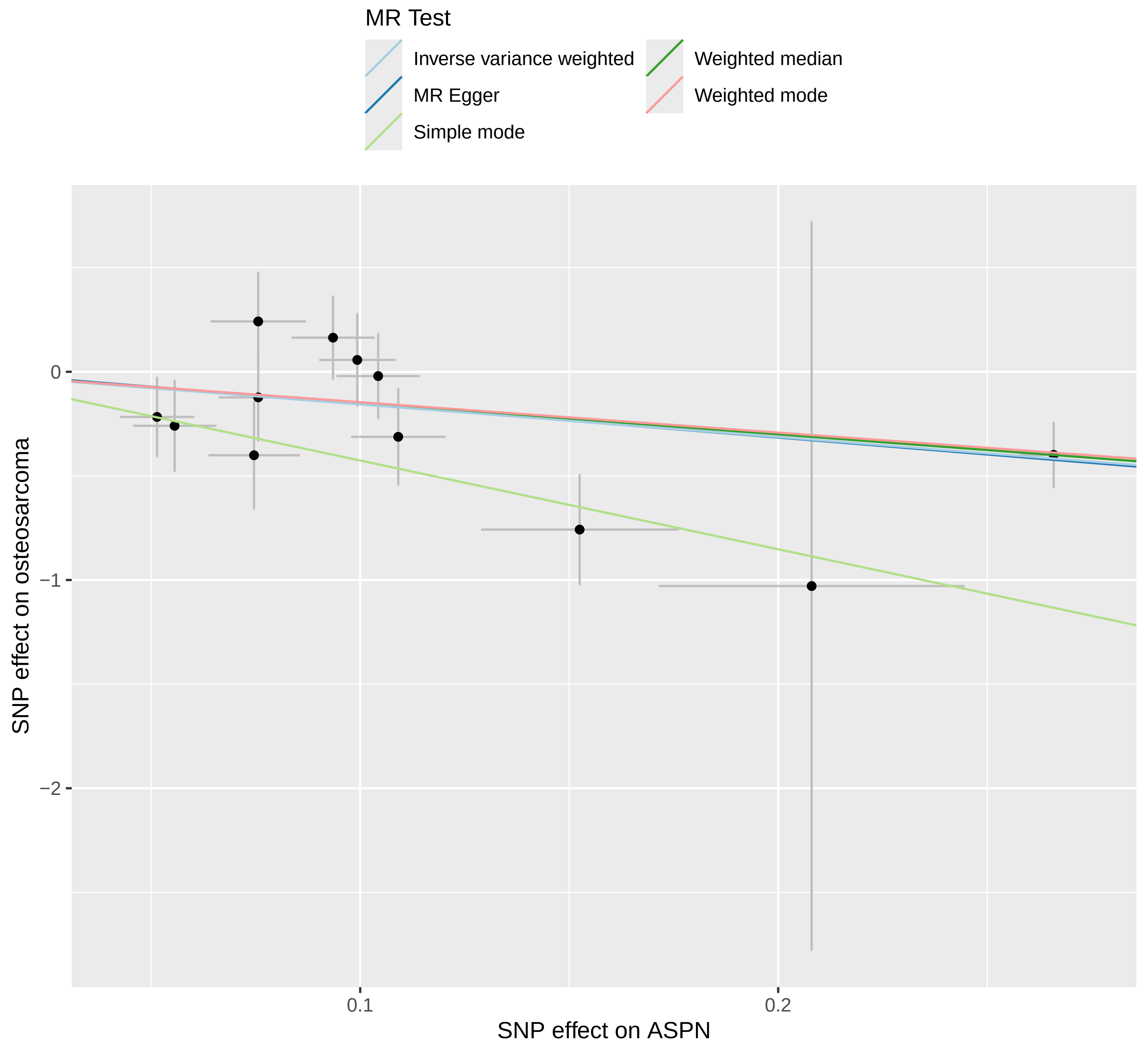

MR Test
Inverse variance weighted
MR Egger
Simple mode
Weighted median
Weighted mode
0
−1
−2
0.1
0.2
SNP effect on osteosarcoma
SNP effect on ASPN

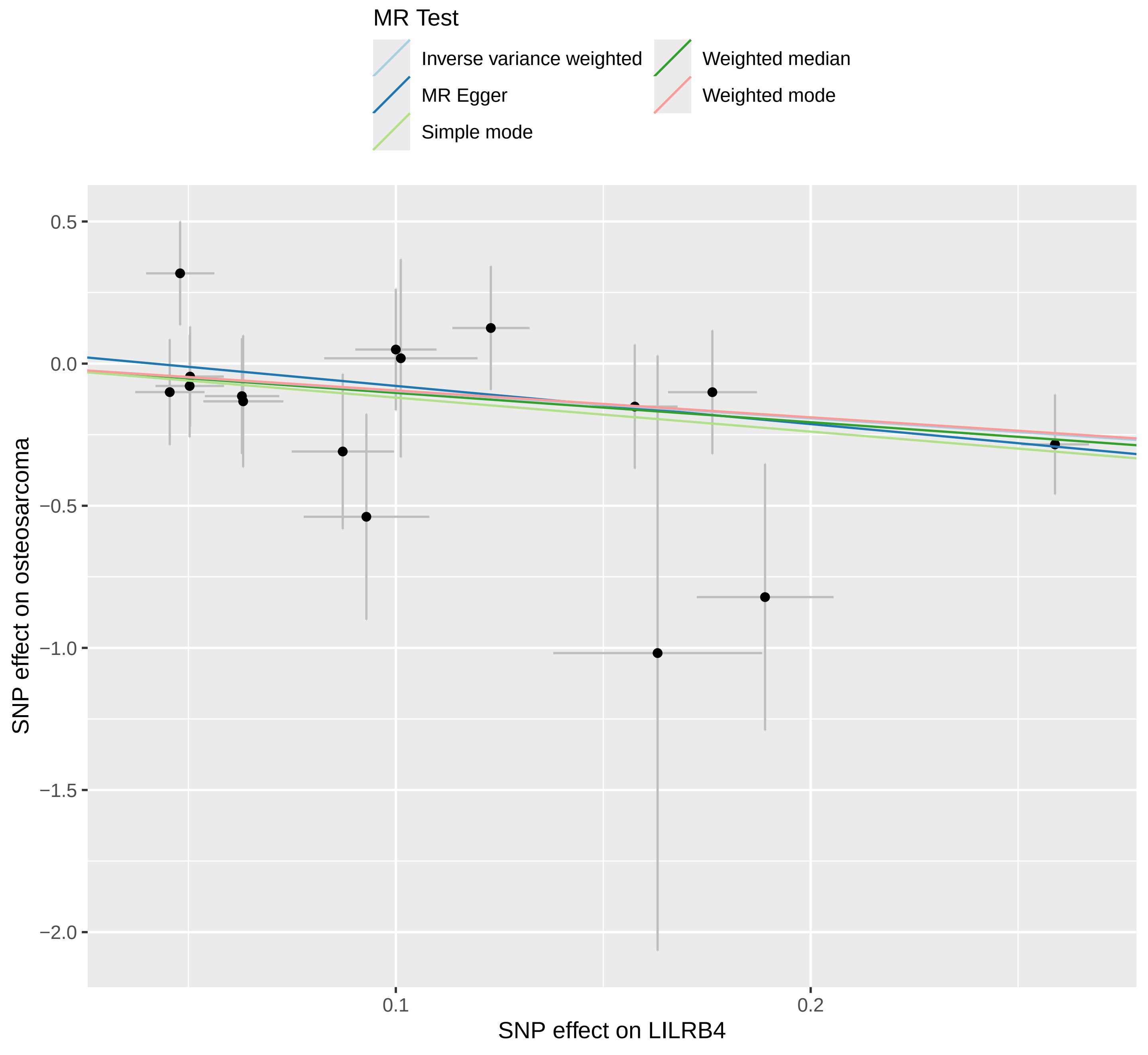
MR Test
Inverse variance weighted
MR Egger
Simple mode
Weighted median
Weighted mode
0.5
0.0
−0.5
−1.0
−1.5
−2.0
0.1
0.2
SNP effect on osteosarcoma
SNP effect on LILRB4

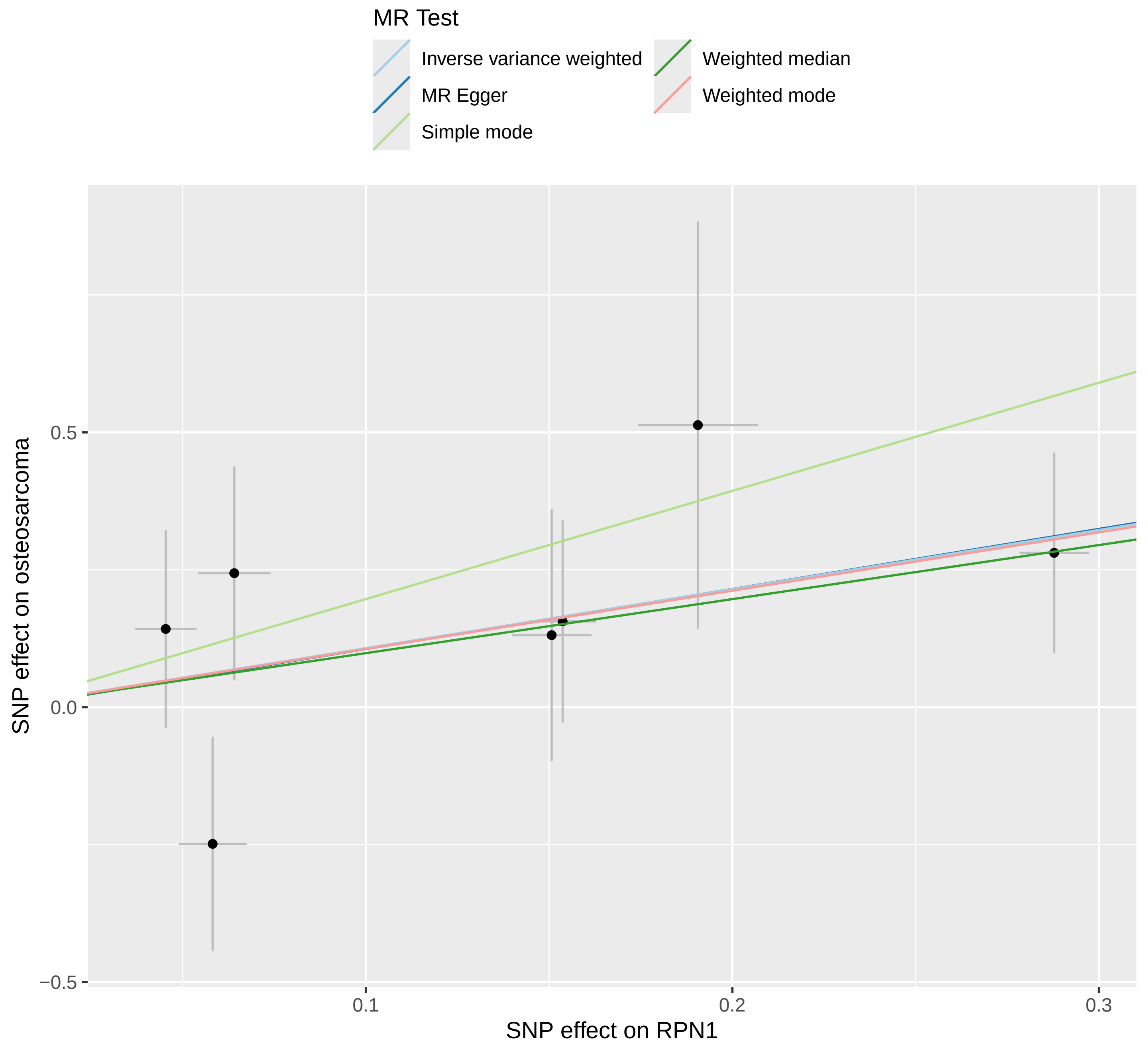

MR Test
Inverse variance weighted
MR Egger
Simple mode
Weighted median
Weighted mode
SNP effect on osteosarcoma
0.5
0.0
−0.5
0.1
0.2
0.3
SNP effect on RPN1

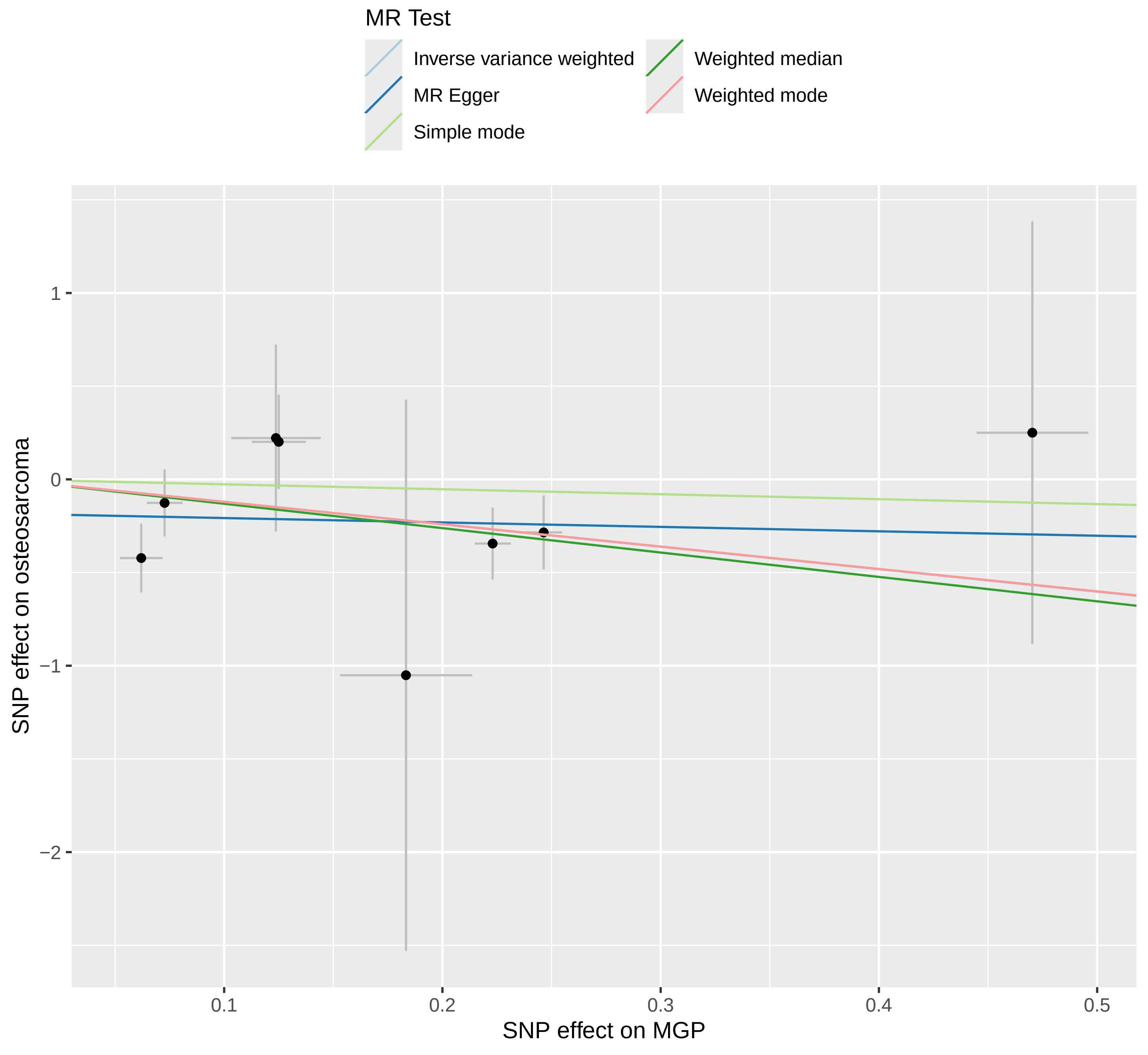
MR Test
Inverse variance weighted
MR Egger
Simple mode
Weighted median
Weighted mode
SNP effect on osteosarcoma
1
0
−1
−2
0.1
0.2
0.3
0.4
0.5
SNP effect on MGP

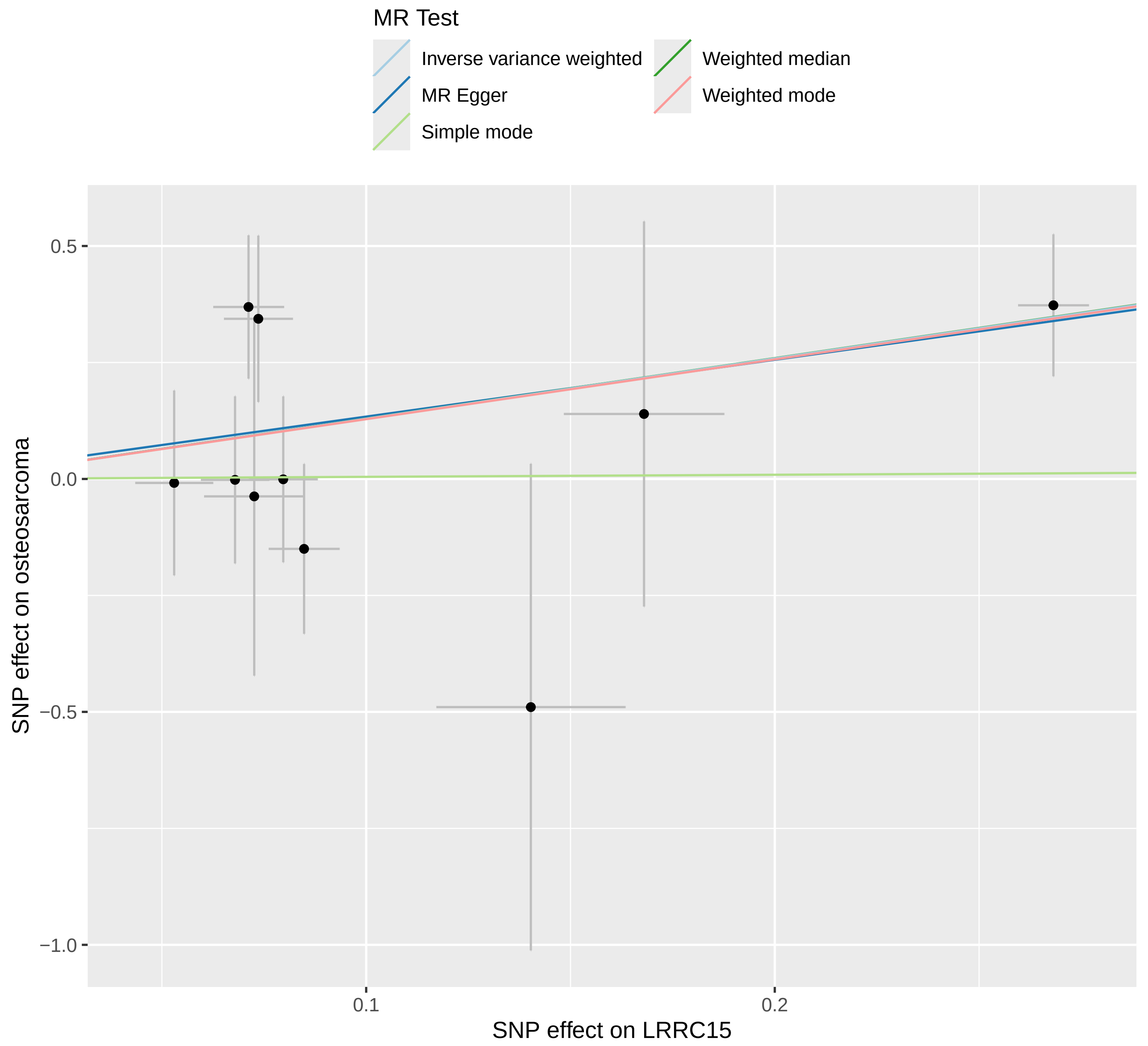
MR Test
Inverse variance weighted
MR Egger
Simple mode
Weighted median
Weighted mode
0.5
0.0
−0.5
−1.0
0.1
0.2
SNP effect on osteosarcoma
SNP effect on LRRC15

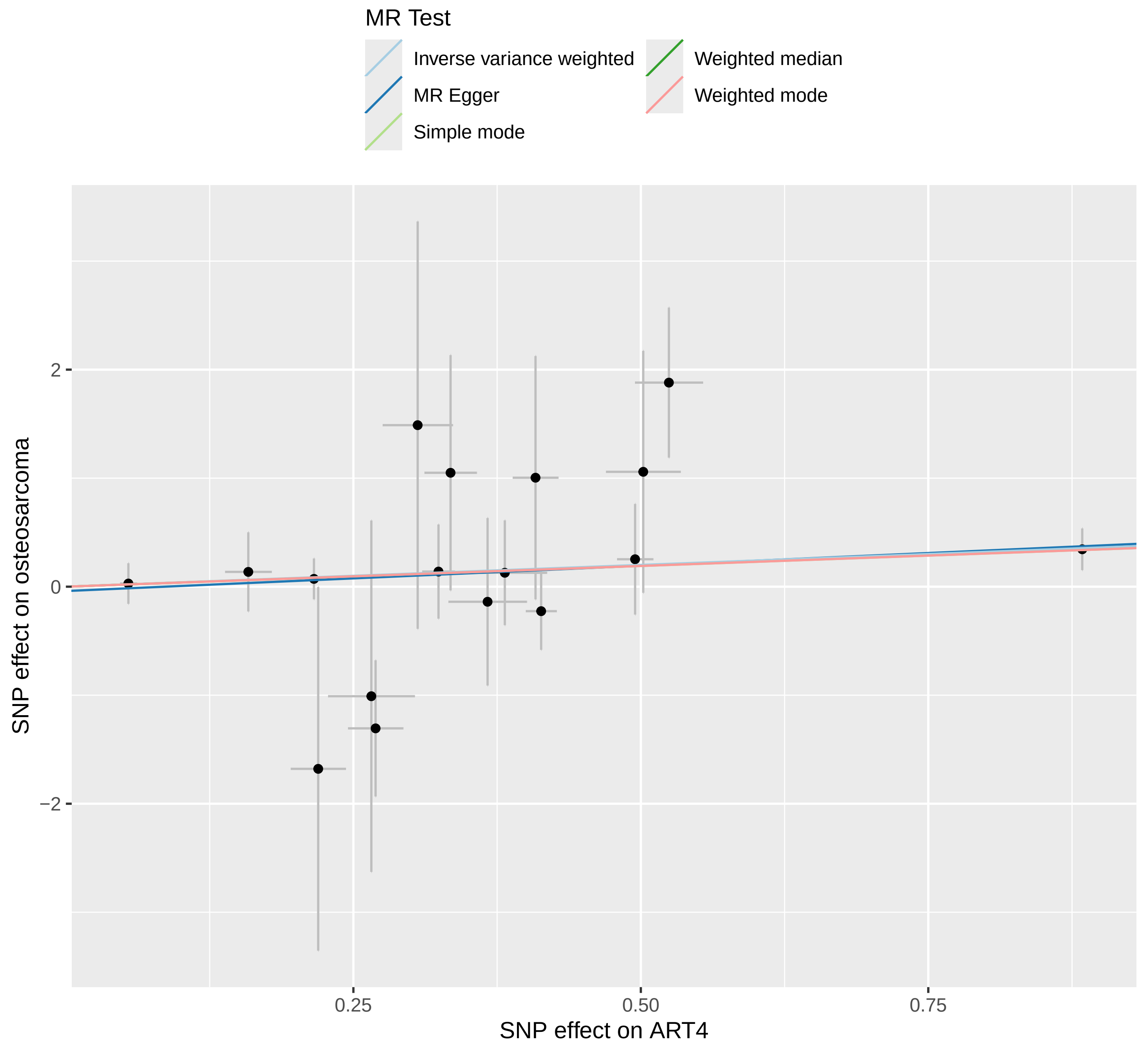
MR Test
Inverse variance weighted
MR Egger
Simple mode
Weighted median
Weighted mode
SNP effect on osteosarcoma
2
0
−2
0.25
0.50
0.75
SNP effect on ART4

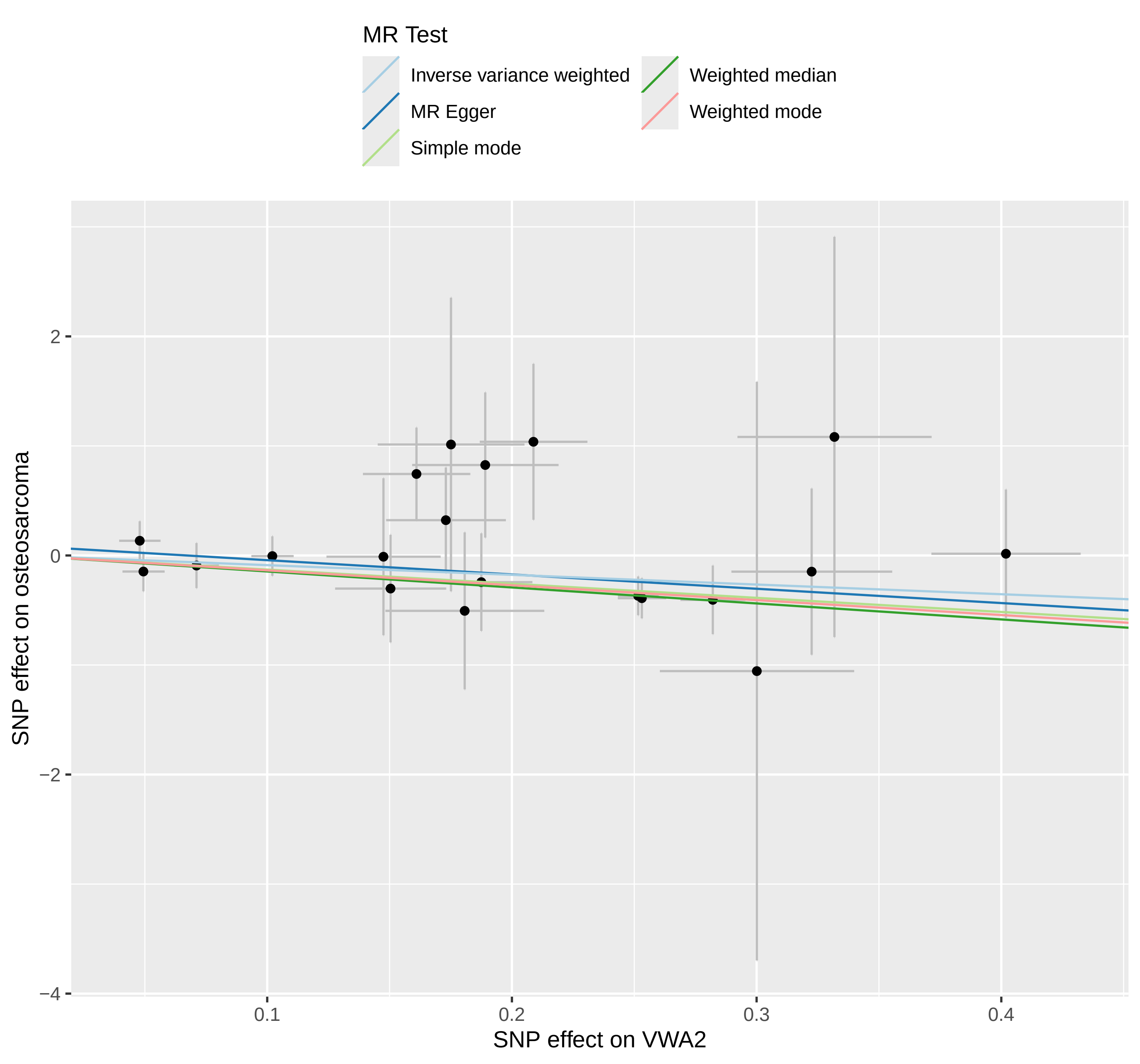
MR Test
Inverse variance weighted
MR Egger
Simple mode
Weighted median
Weighted mode
SNP effect on osteosarcoma
2
0
−2
−4
0.1
0.2
0.3
0.4
SNP effect on VWA2

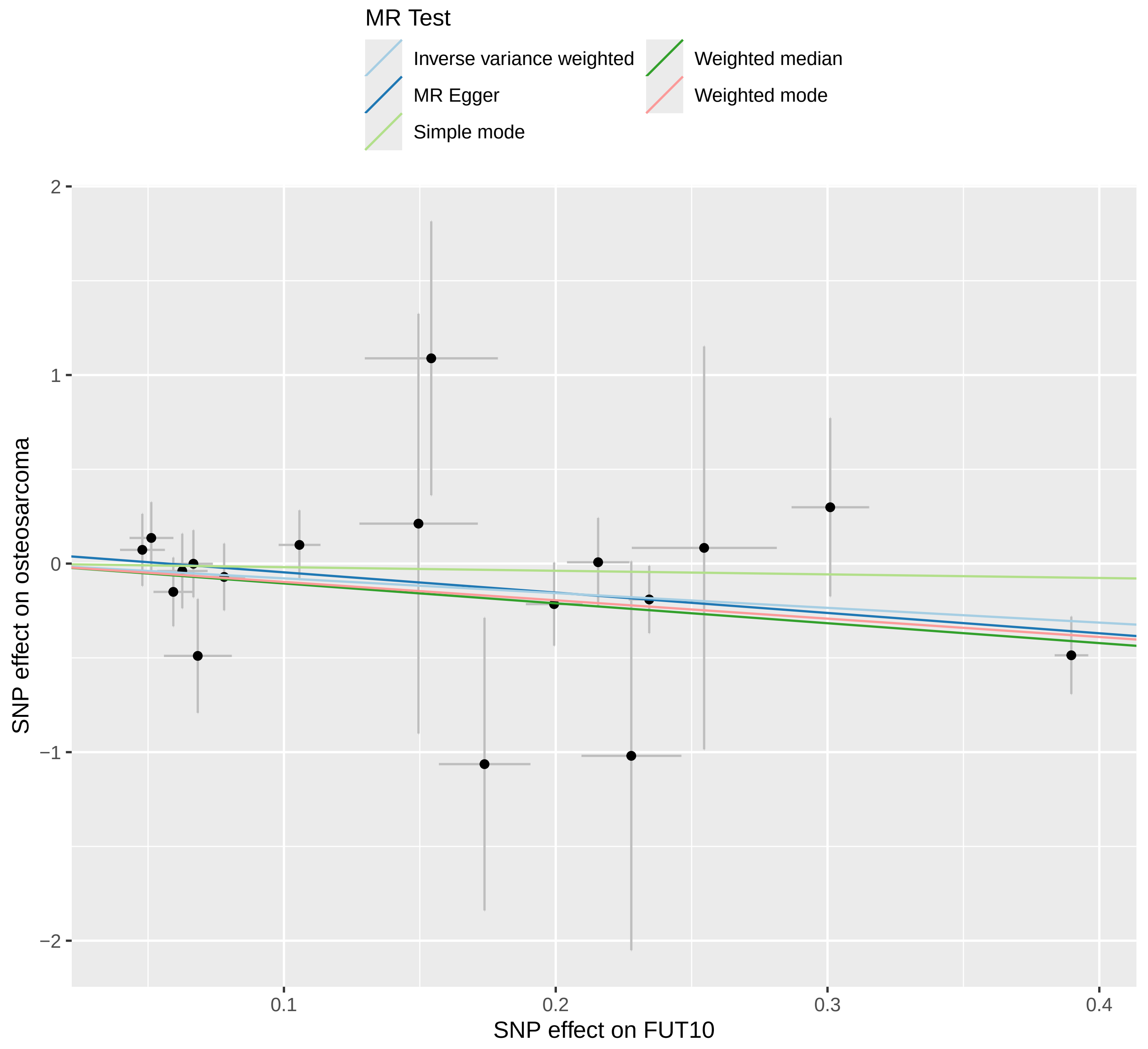
MR Test
Inverse variance weighted
MR Egger
Simple mode
Weighted median
Weighted mode
2
1
0
−1
−2
0.1
0.2
0.3
0.4
SNP effect on osteosarcoma
SNP effect on FUT10

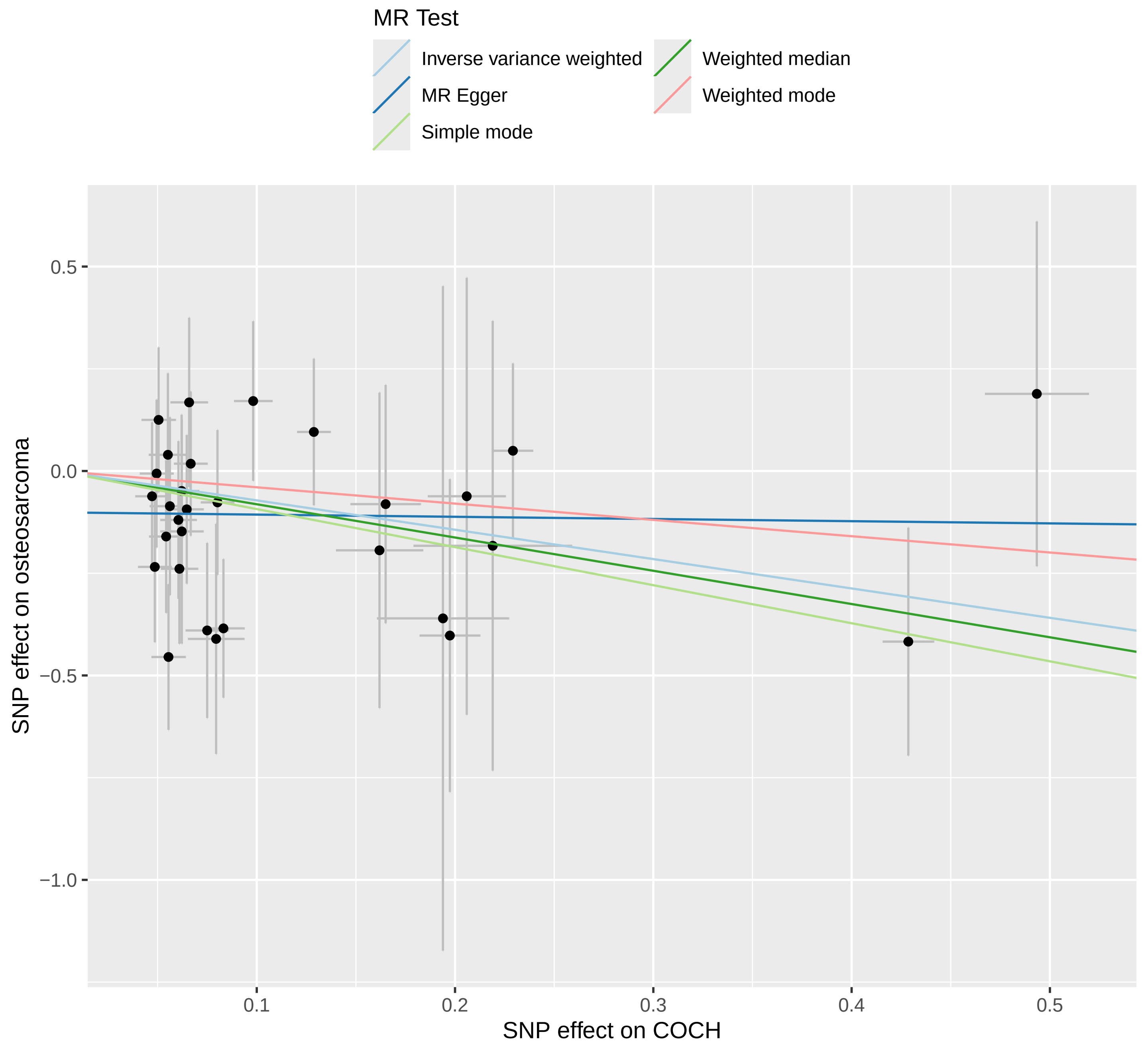

MR Test
Inverse variance weighted
MR Egger
Simple mode
Weighted median
Weighted mode
0.5
0.0
−0.5
−1.0
SNP effect on osteosarcoma
0.1
0.2
0.3
0.4
0.5
SNP effect on COCH

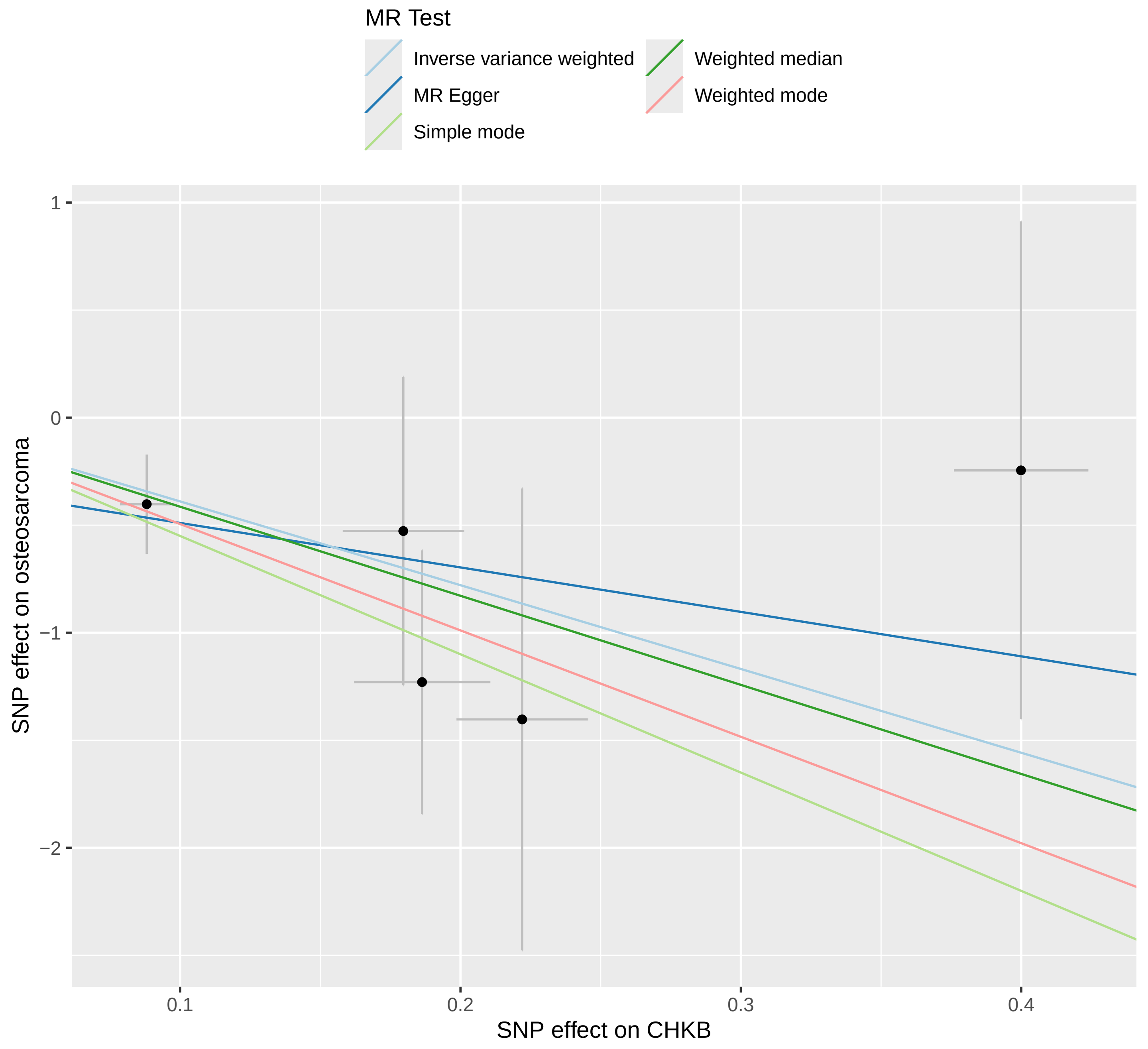
MR Test
Inverse variance weighted
MR Egger
Simple mode
Weighted median
Weighted mode
1
0
−1
−2
SNP effect on osteosarcoma
0.1
0.2
0.3
0.4
SNP effect on CHKB

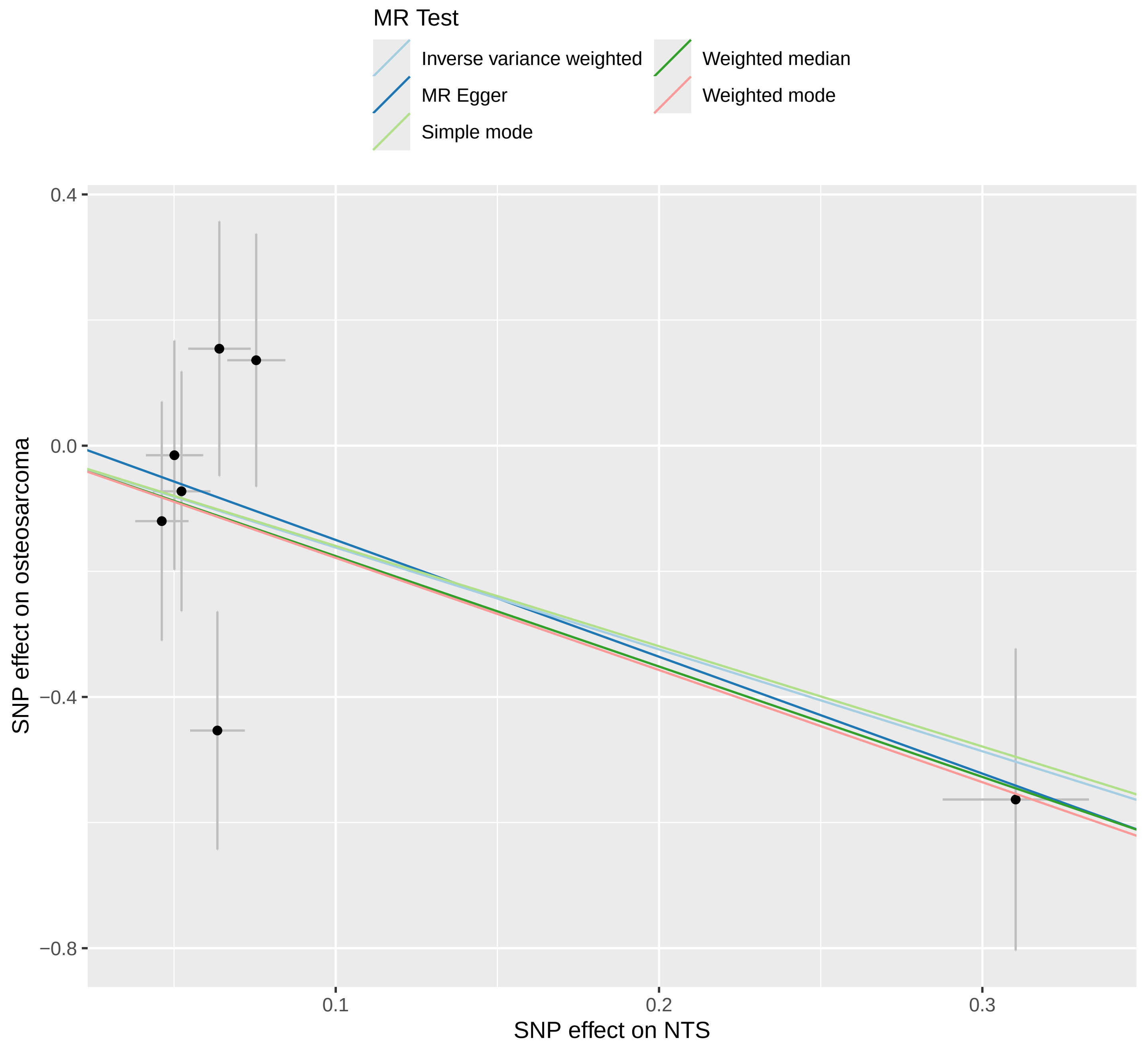

MR Test
Inverse variance weighted
MR Egger
Simple mode
Weighted median
Weighted mode
0.4
0.0
−0.4
−0.8
0.1
0.2
0.3
SNP effect on osteosarcoma
SNP effect on NTS

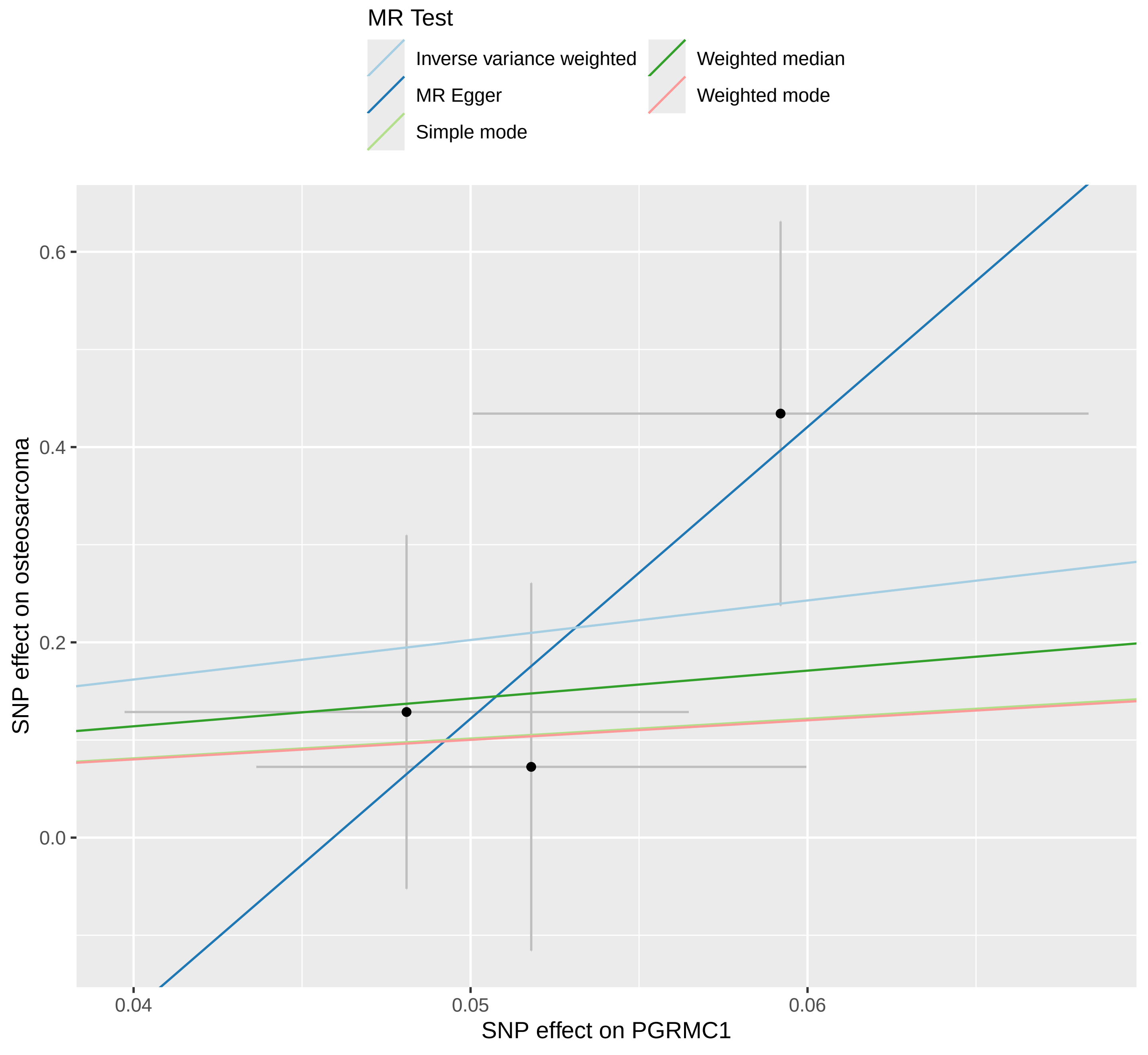

MR Test
Inverse variance weighted
MR Egger
Simple mode
Weighted median
Weighted mode
0.6
0.4
0.2
0.0
0.04
0.05
0.06
SNP effect on osteosarcoma
SNP effect on PGRMC1

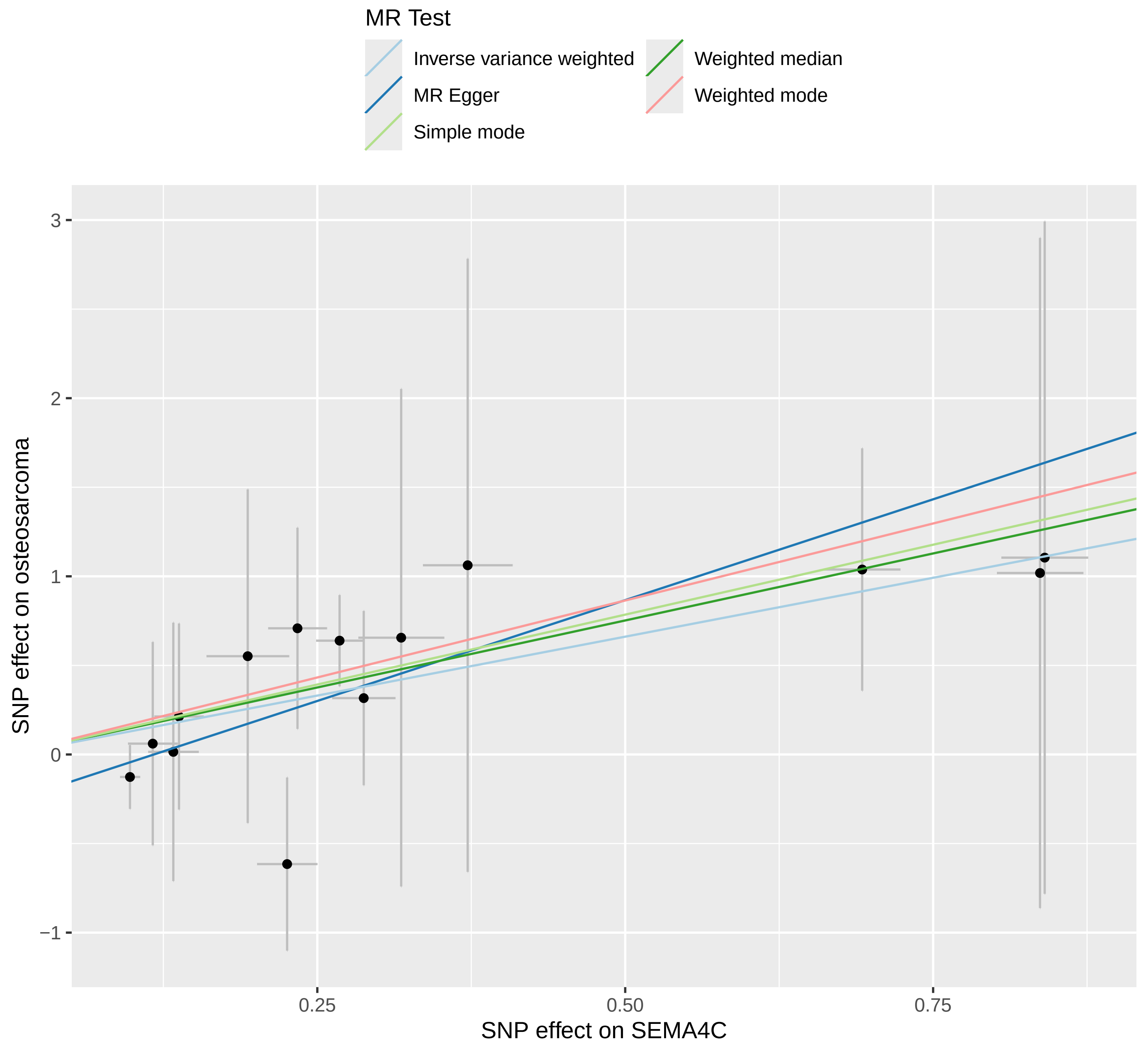
MR Test
Inverse variance weighted
MR Egger
Simple mode
Weighted median
Weighted mode
SNP effect on osteosarcoma
3
2
1
0
−1
0.25
0.50
0.75
SNP effect on SEMA4C

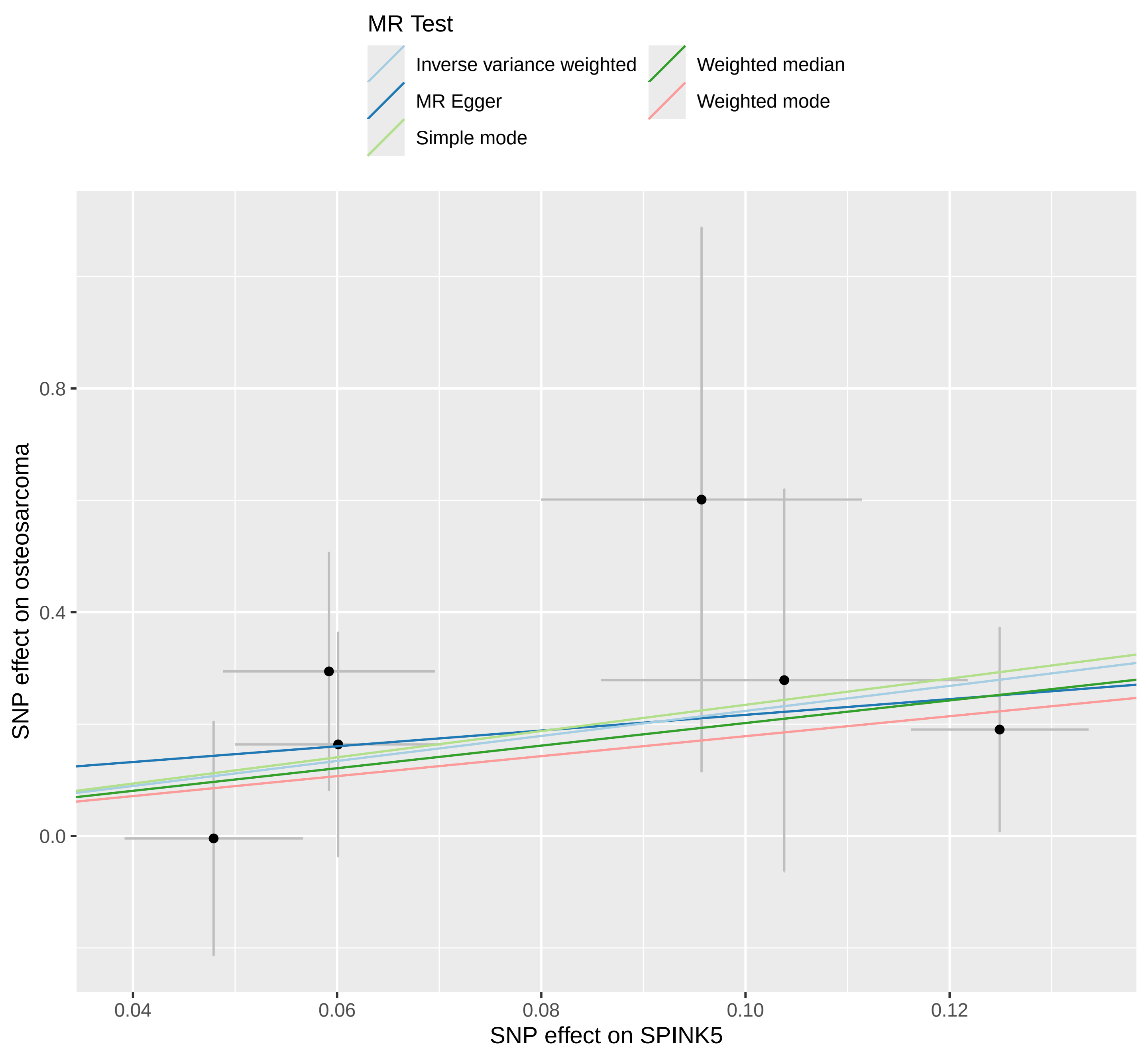
MR Test
Inverse variance weighted
MR Egger
Simple mode
Weighted median
Weighted mode
0.8
0.4
0.0
SNP effect on osteosarcoma
0.04
0.06
0.08
0.10
0.12
SNP effect on SPINK5

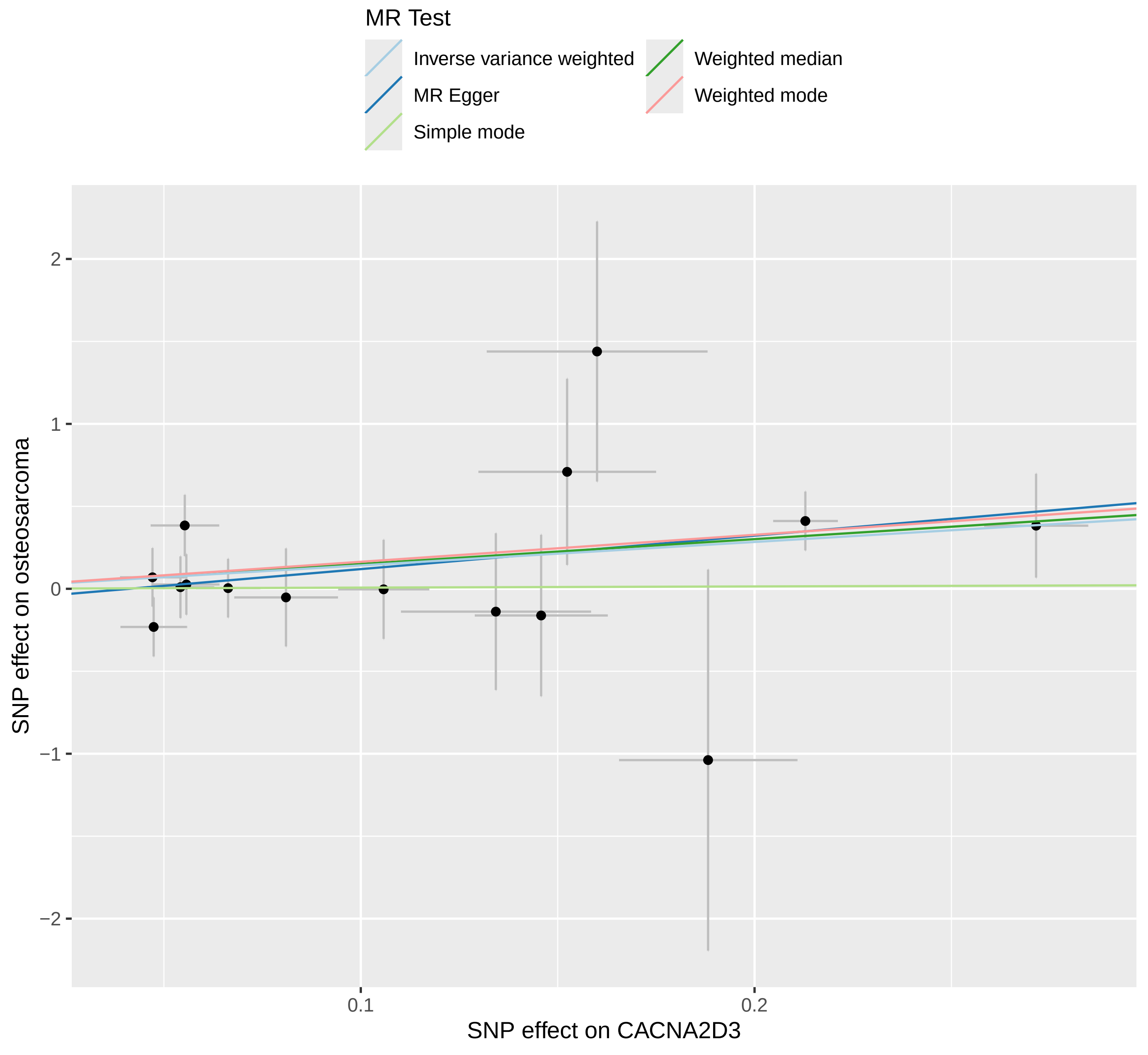
MR Test
Inverse variance weighted
MR Egger
Simple mode
Weighted median
Weighted mode
2
1
0
−1
−2
0.1
0.2
SNP effect on osteosarcoma
SNP effect on CACNA2D3

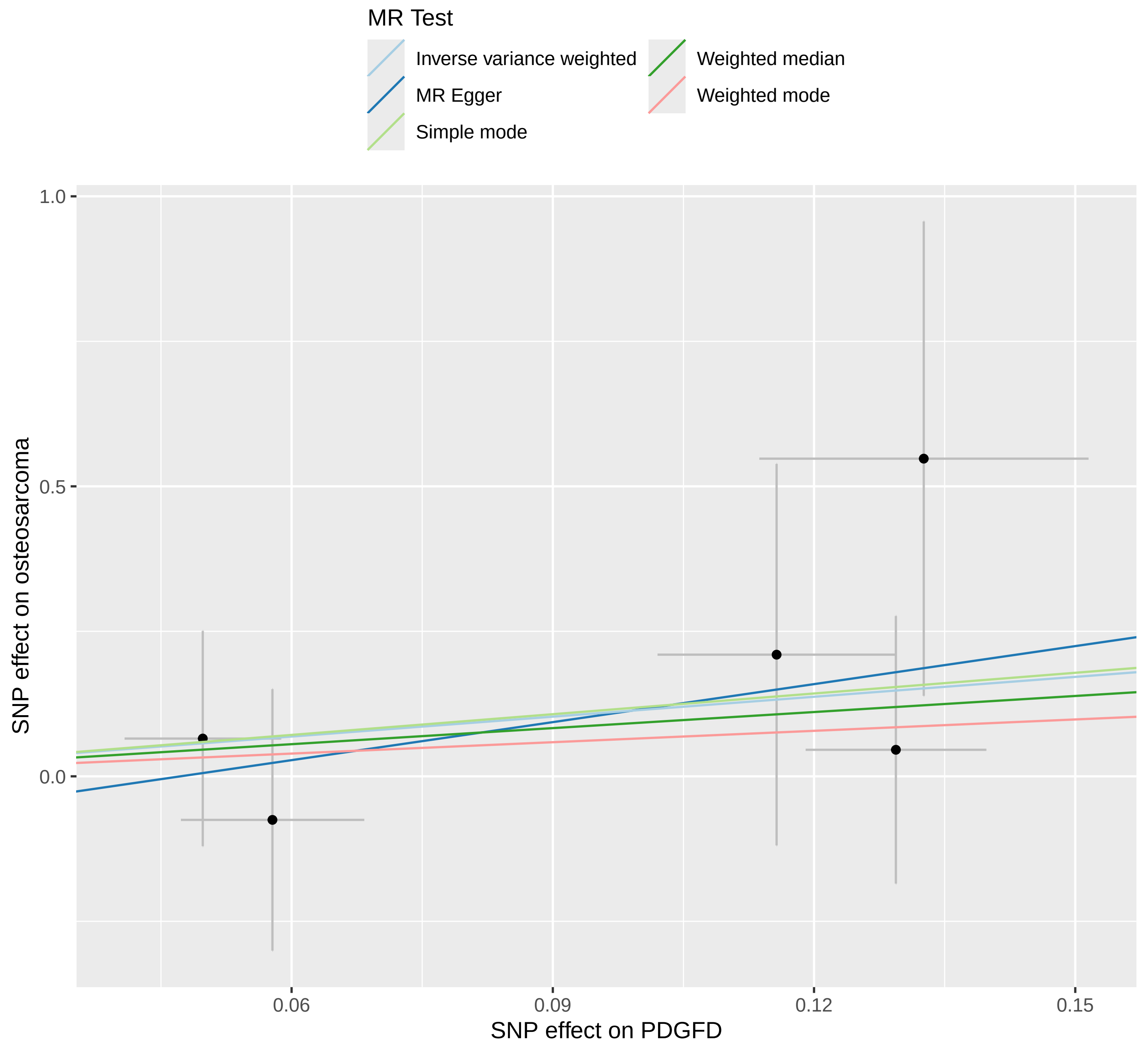
MR Test
Inverse variance weighted
MR Egger
Simple mode
Weighted median
Weighted mode
1.0
0.5
0.0
0.06
0.09
0.12
0.15
SNP effect on osteosarcoma
SNP effect on PDGFD

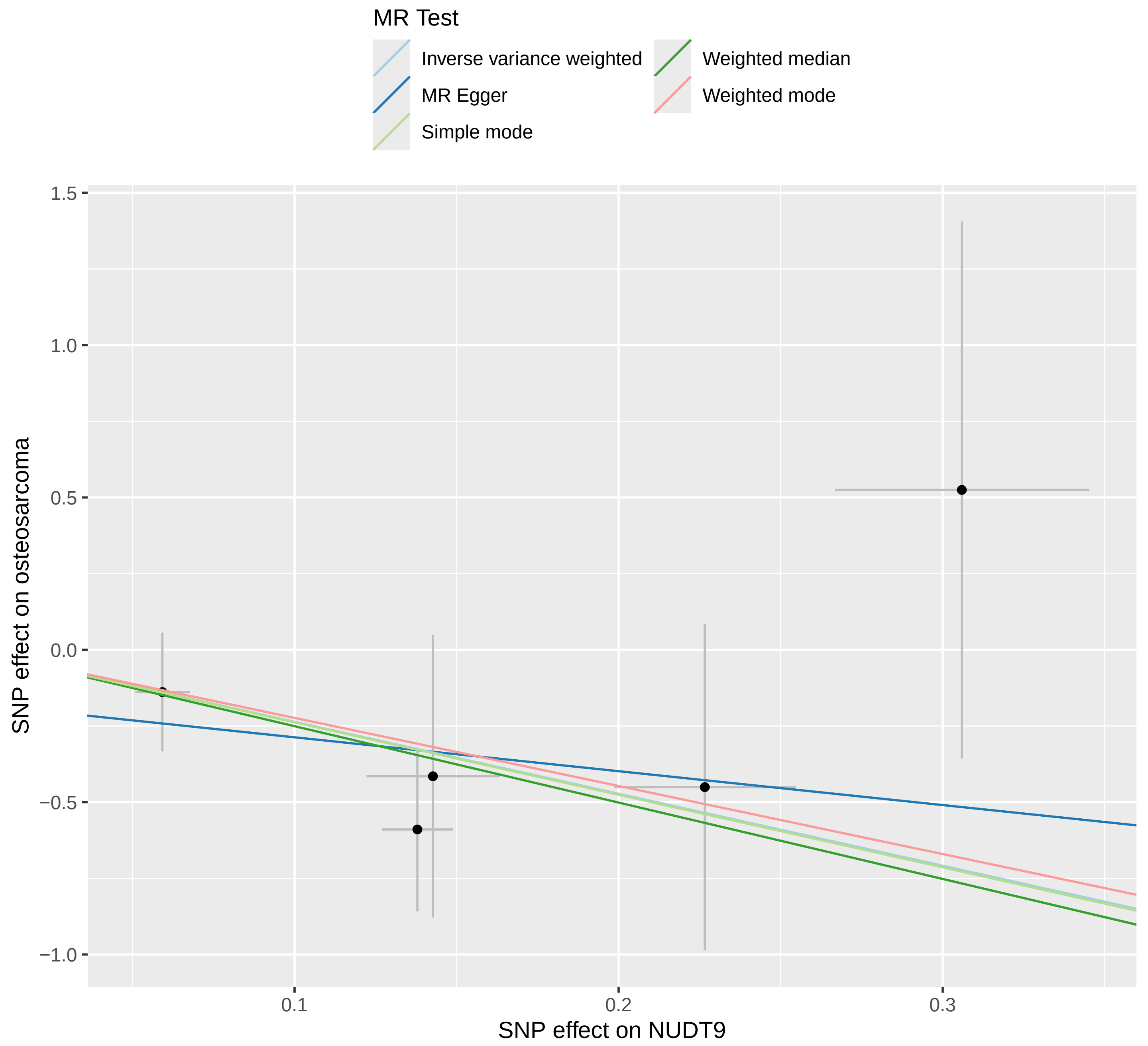
MR Test
Inverse variance weighted
MR Egger
Simple mode
Weighted median
Weighted mode
1.5
1.0
0.5
0.0
−0.5
−1.0
SNP effect on osteosarcoma
0.1
0.2
0.3
SNP effect on NUDT9

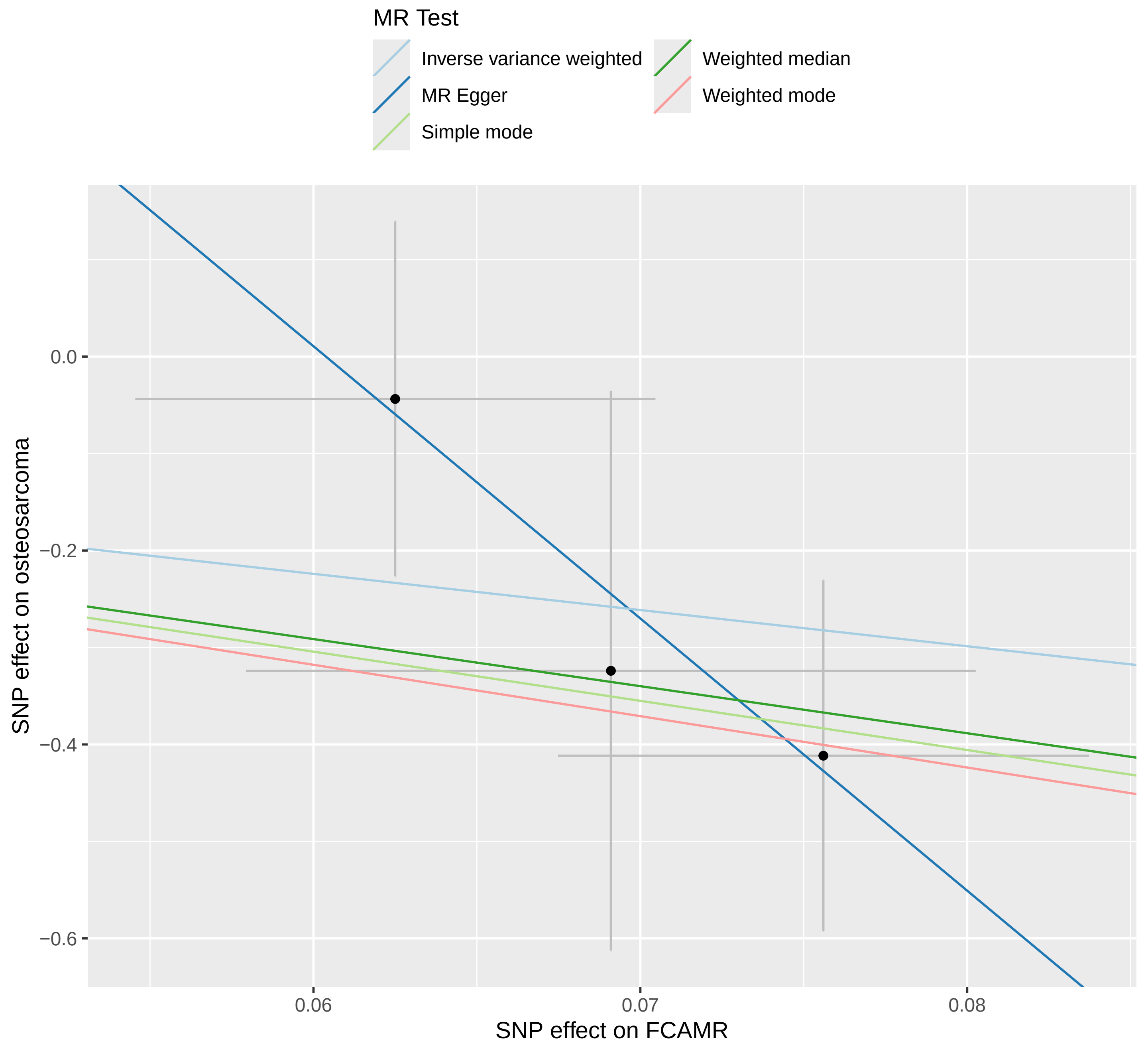
MR Test
Inverse variance weighted
MR Egger
Simple mode
Weighted median
Weighted mode
SNP effect on osteosarcoma
0.0
−0.2
−0.4
−0.6
0.06
0.07
0.08
SNP effect on FCAMR

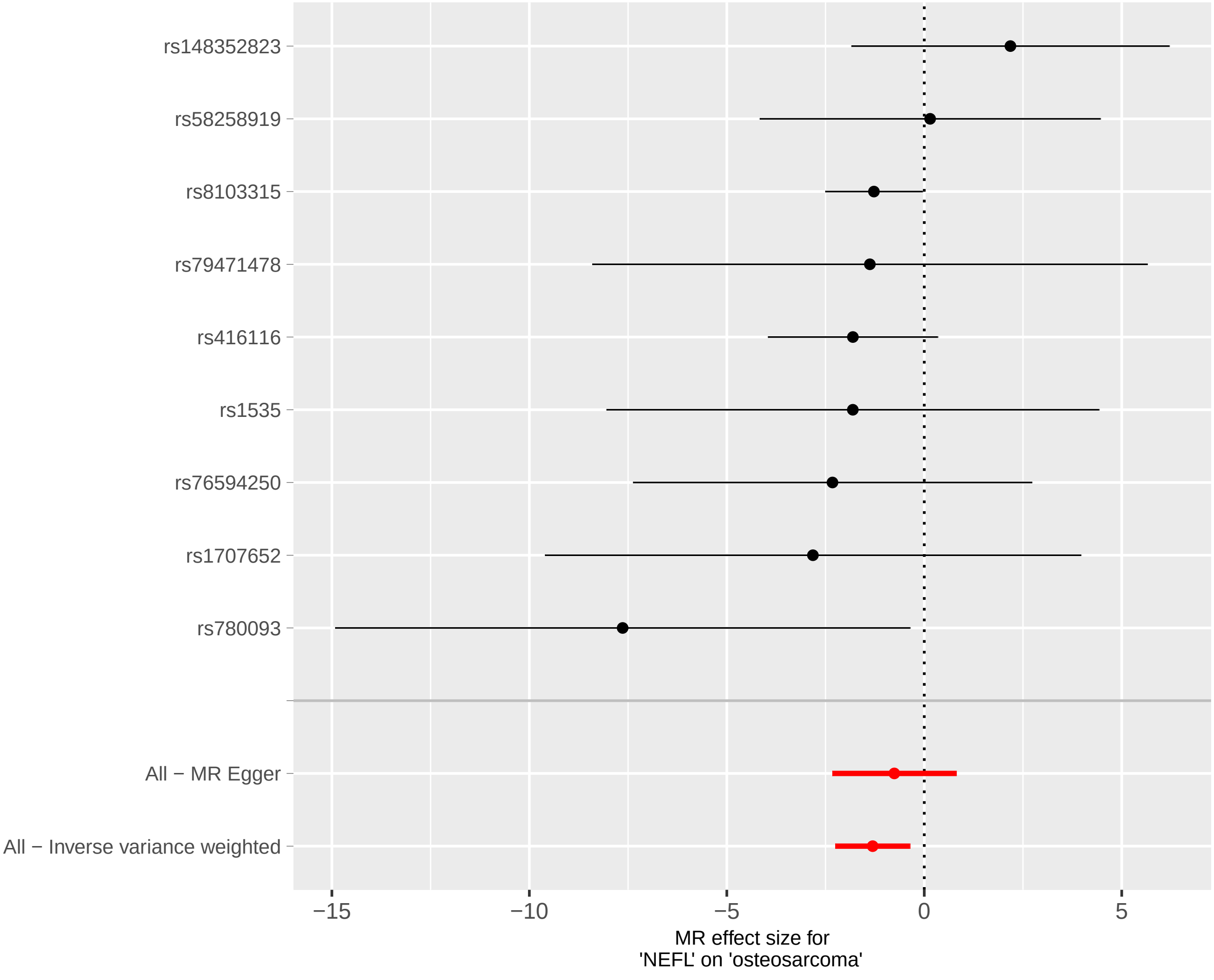

rs148352823
rs58258919
rs8103315
rs79471478
rs416116
rs1535
rs76594250
rs1707652
rs780093
All − MR Egger
All − Inverse variance weighted
−15
−10
−5
0
5
MR effect size for
'NEFL' on 'osteosarcoma'

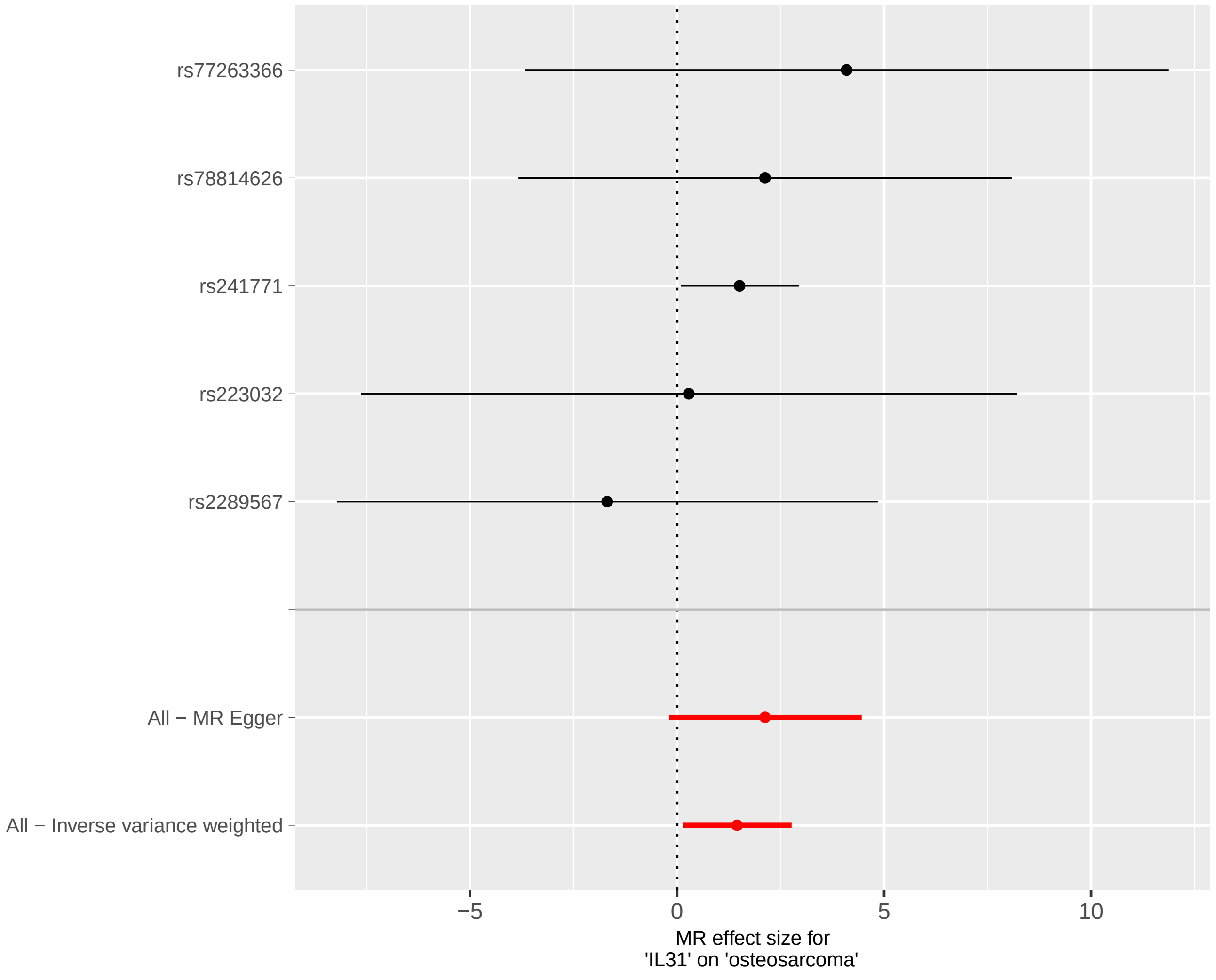
rs77263366
rs78814626
rs241771
rs223032
rs2289567
All − MR Egger
All − Inverse variance weighted
−5
0
5
10
MR effect size for
'IL31' on 'osteosarcoma'

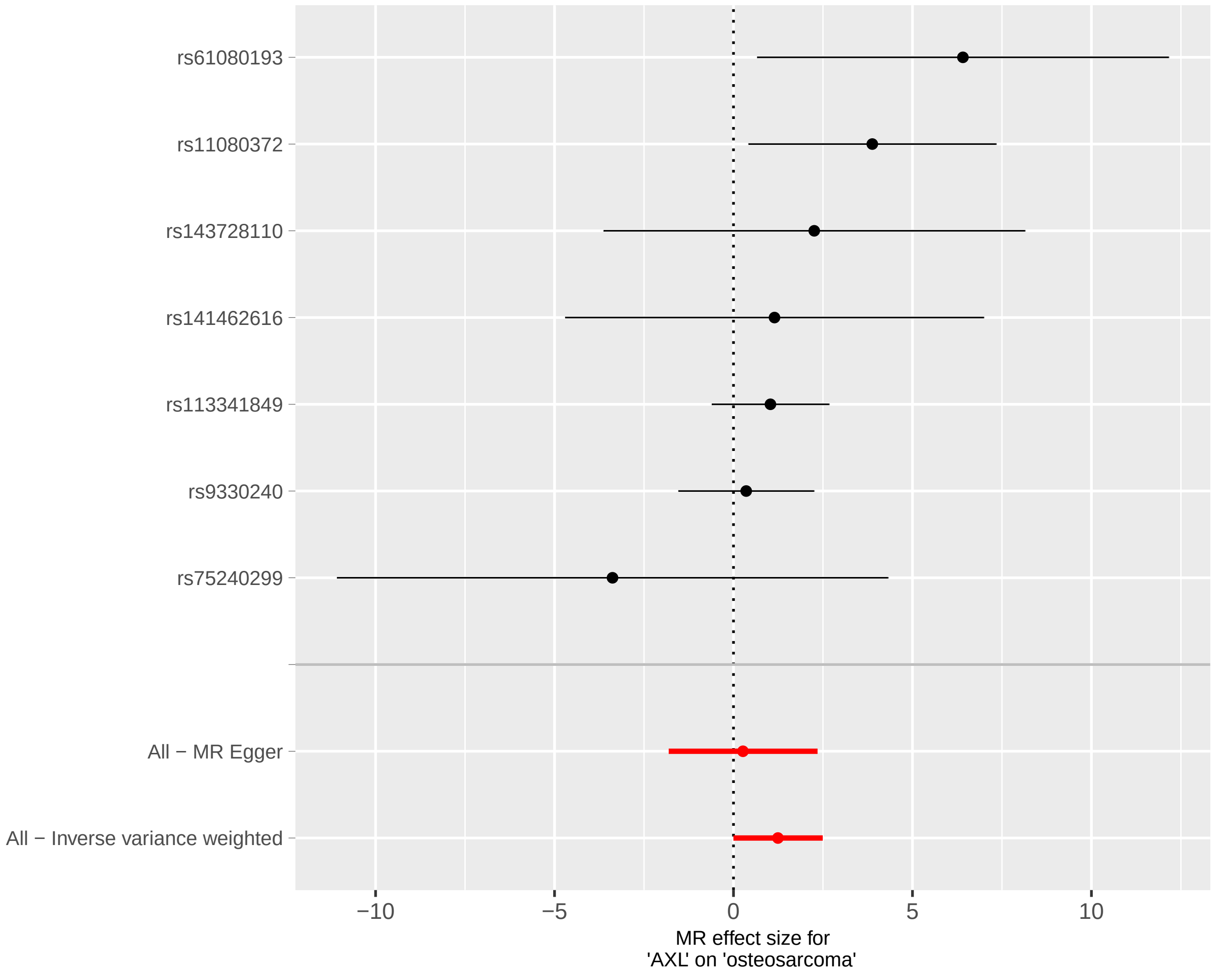

rs61080193
rs11080372
rs143728110
rs141462616
rs113341849
rs9330240
rs75240299
All − MR Egger
All − Inverse variance weighted
−10
−5
0
5
10
MR effect size for
'AXL' on 'osteosarcoma'

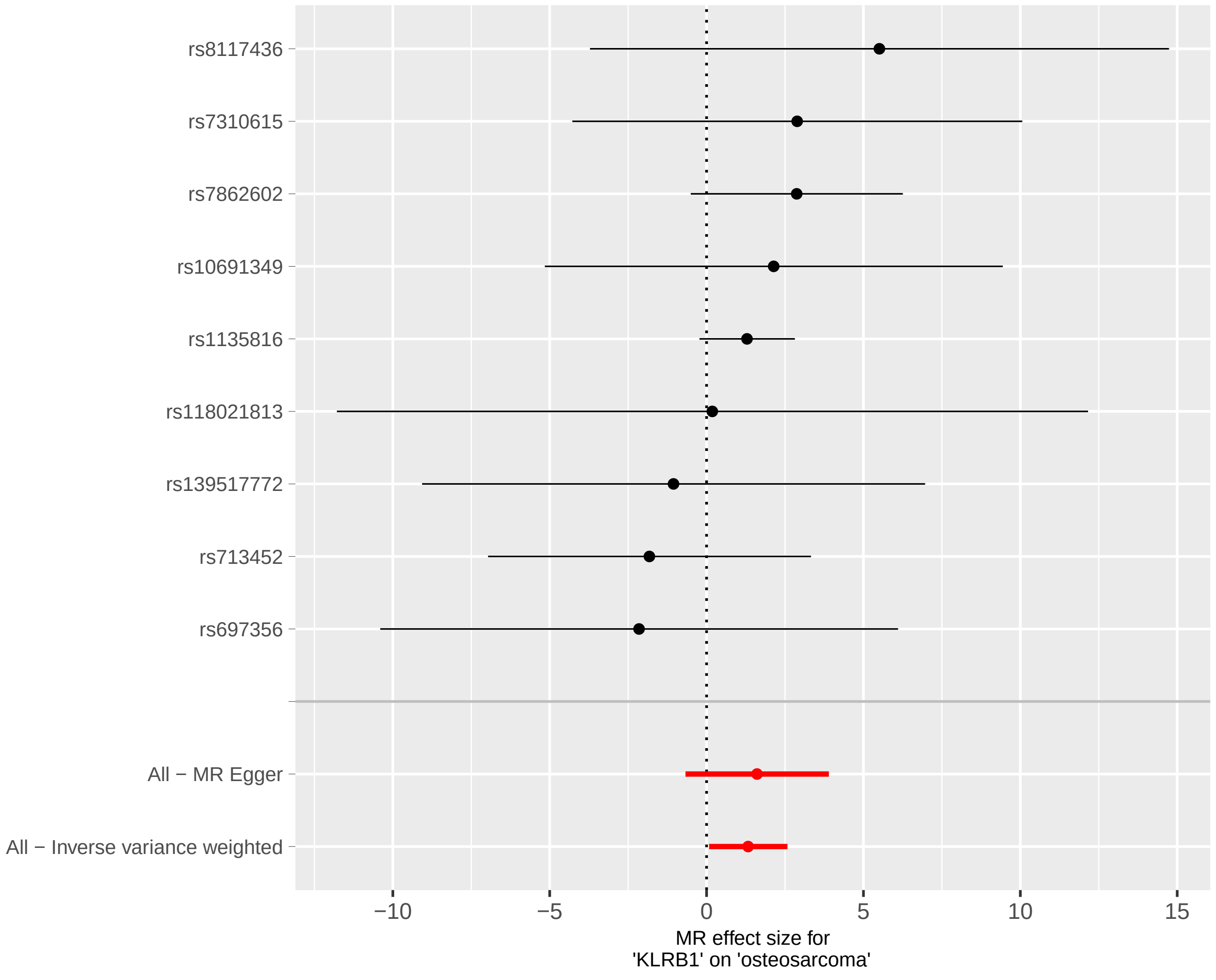
rs8117436
rs7310615
rs7862602
rs10691349
rs1135816
rs118021813
rs139517772
rs713452
rs697356
All − MR Egger
All − Inverse variance weighted
−10
−5
0
5
10
15
MR effect size for
'KLRB1' on 'osteosarcoma'

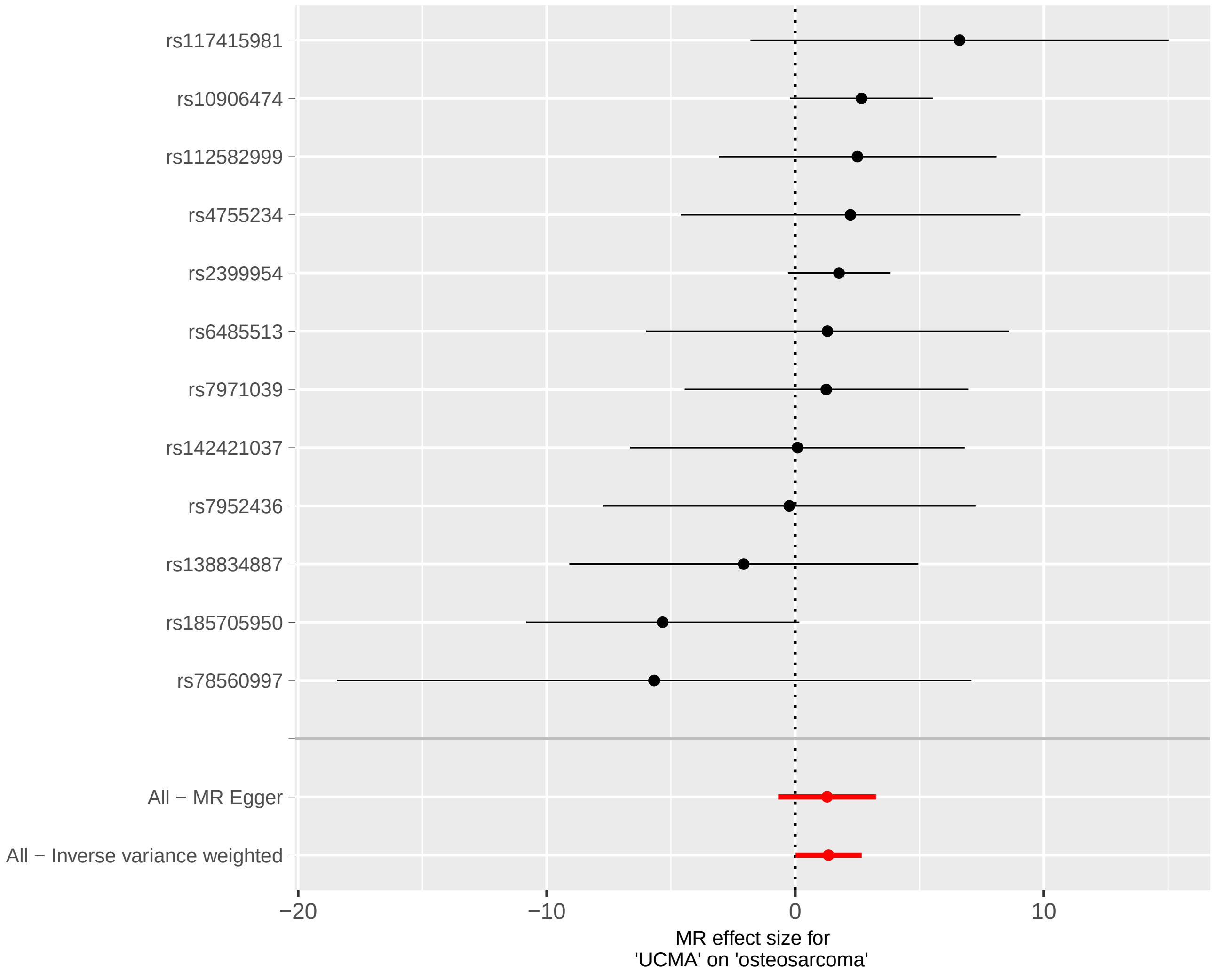
rs117415981
rs10906474
rs112582999
rs4755234
rs2399954
rs6485513
rs7971039
rs142421037
rs7952436
rs138834887
rs185705950
rs78560997
All − MR Egger
All − Inverse variance weighted
−20
−10
0
10
MR effect size for
'UCMA' on 'osteosarcoma'

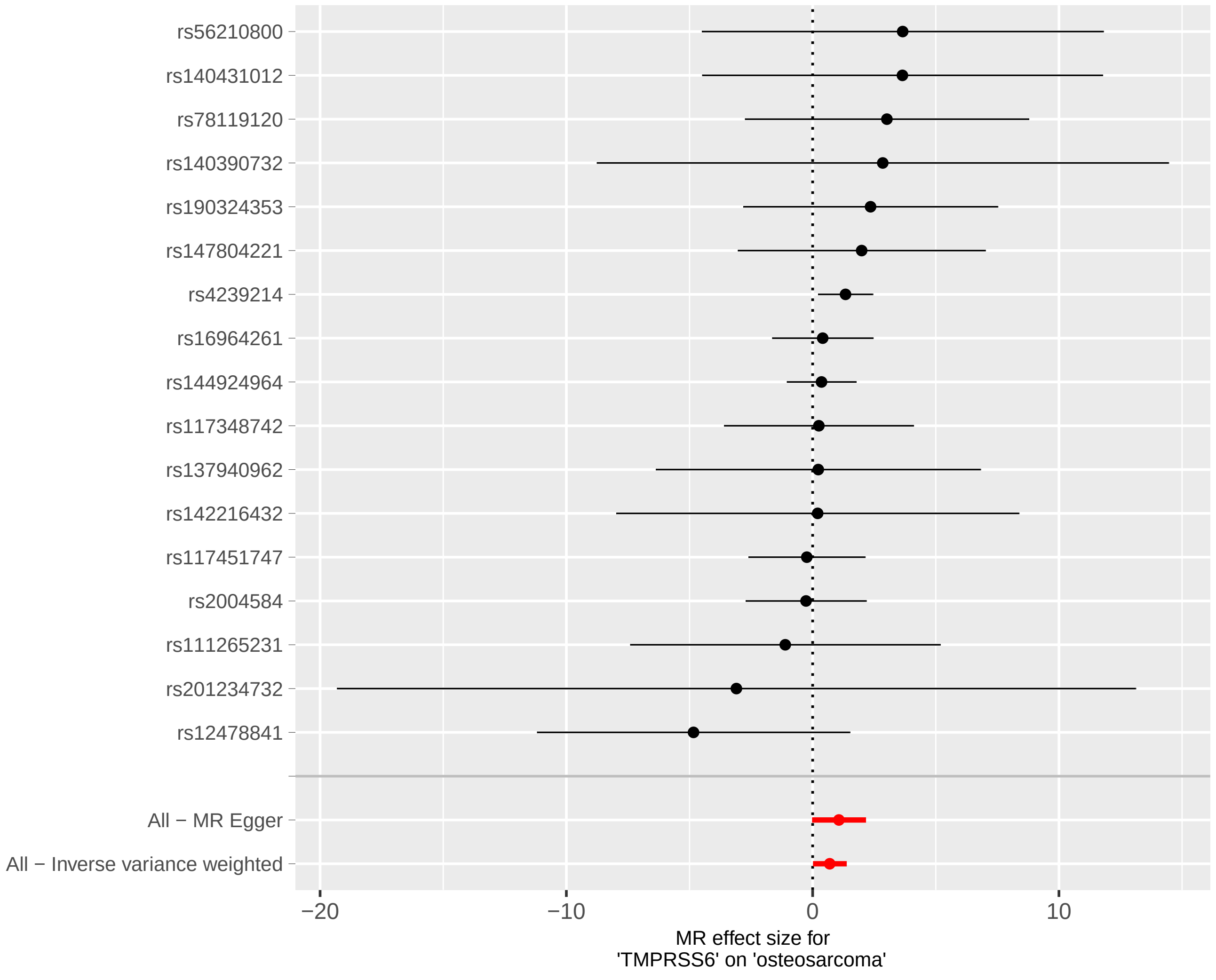
rs56210800
rs140431012
rs78119120
rs140390732
rs190324353
rs147804221
rs4239214
rs16964261
rs144924964
rs117348742
rs137940962
rs142216432
rs117451747
rs2004584
rs111265231
rs201234732
rs12478841
All − MR Egger
All − Inverse variance weighted
−20
−10
0
10
MR effect size for
'TMPRSS6' on 'osteosarcoma'

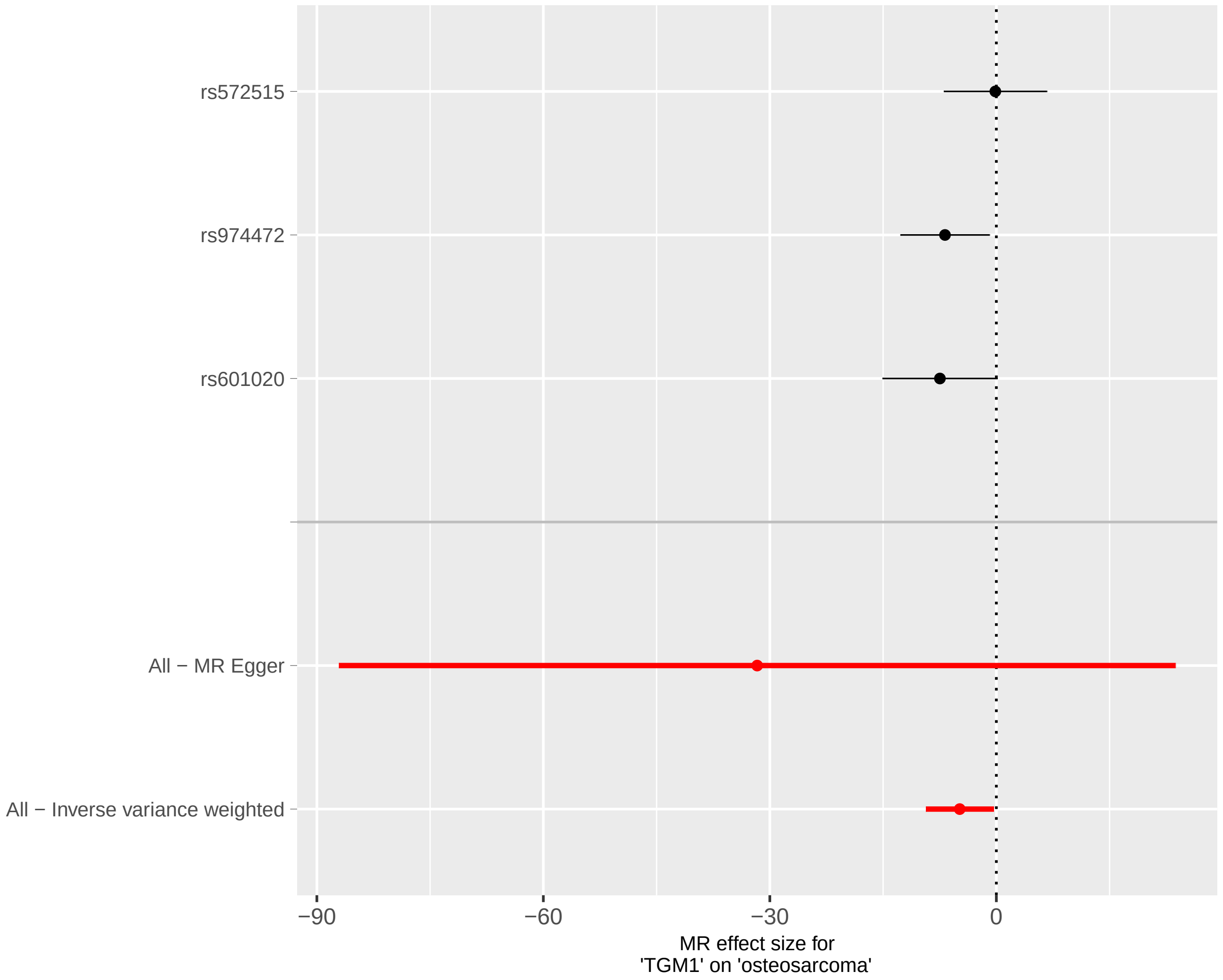

rs572515
rs974472
rs601020
All − MR Egger
All − Inverse variance weighted
−90
−60
−30
0
MR effect size for
'TGM1' on 'osteosarcoma'

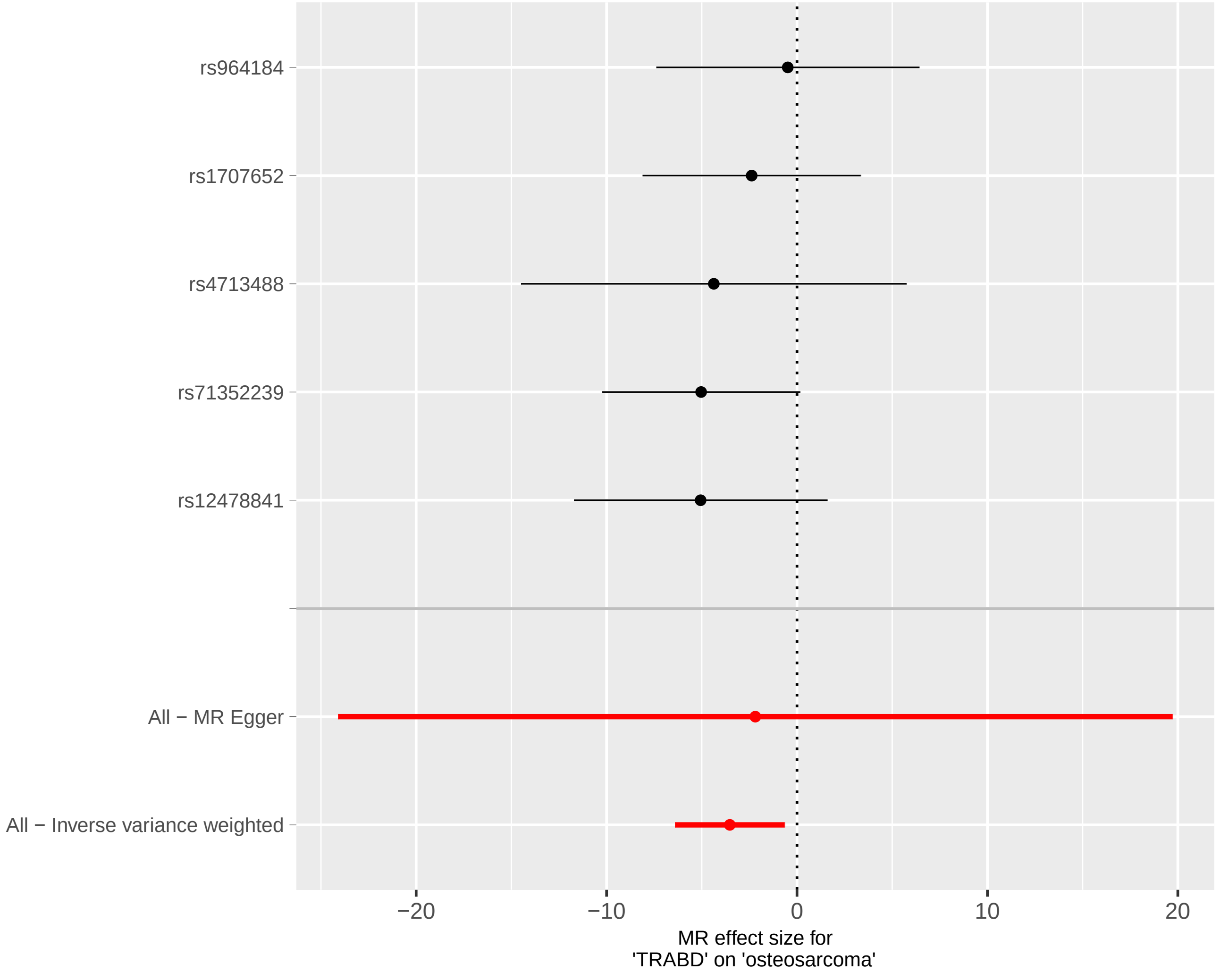

rs964184
rs1707652
rs4713488
rs71352239
rs12478841
All − MR Egger
All − Inverse variance weighted
−20
−10
0
10
20
MR effect size for
'TRABD' on 'osteosarcoma'

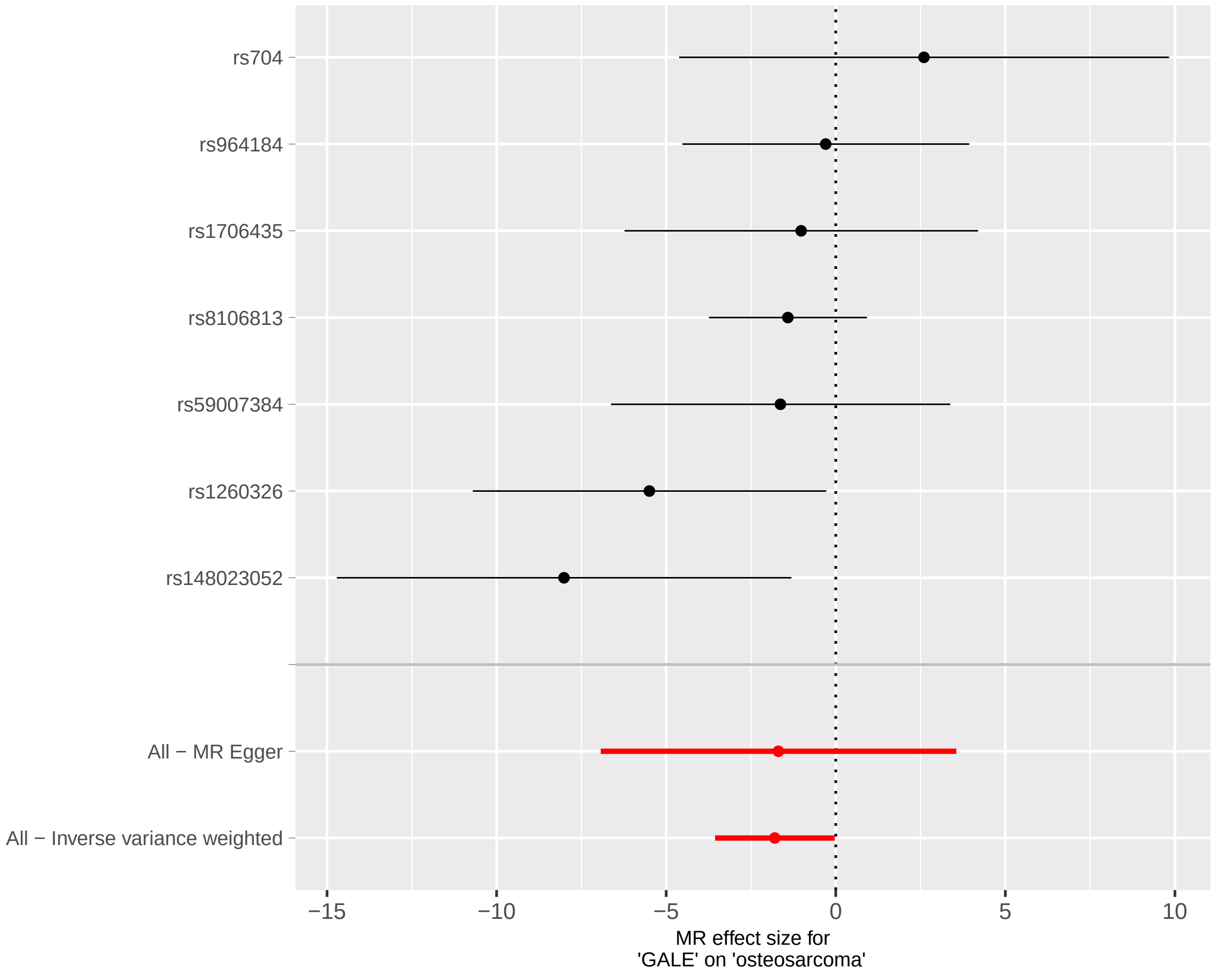
rs704
rs964184
rs1706435
rs8106813
rs59007384
rs1260326
rs148023052
All − MR Egger
All − Inverse variance weighted
−15
−10
−5
0
5
10
MR effect size for
'GALE' on 'osteosarcoma'

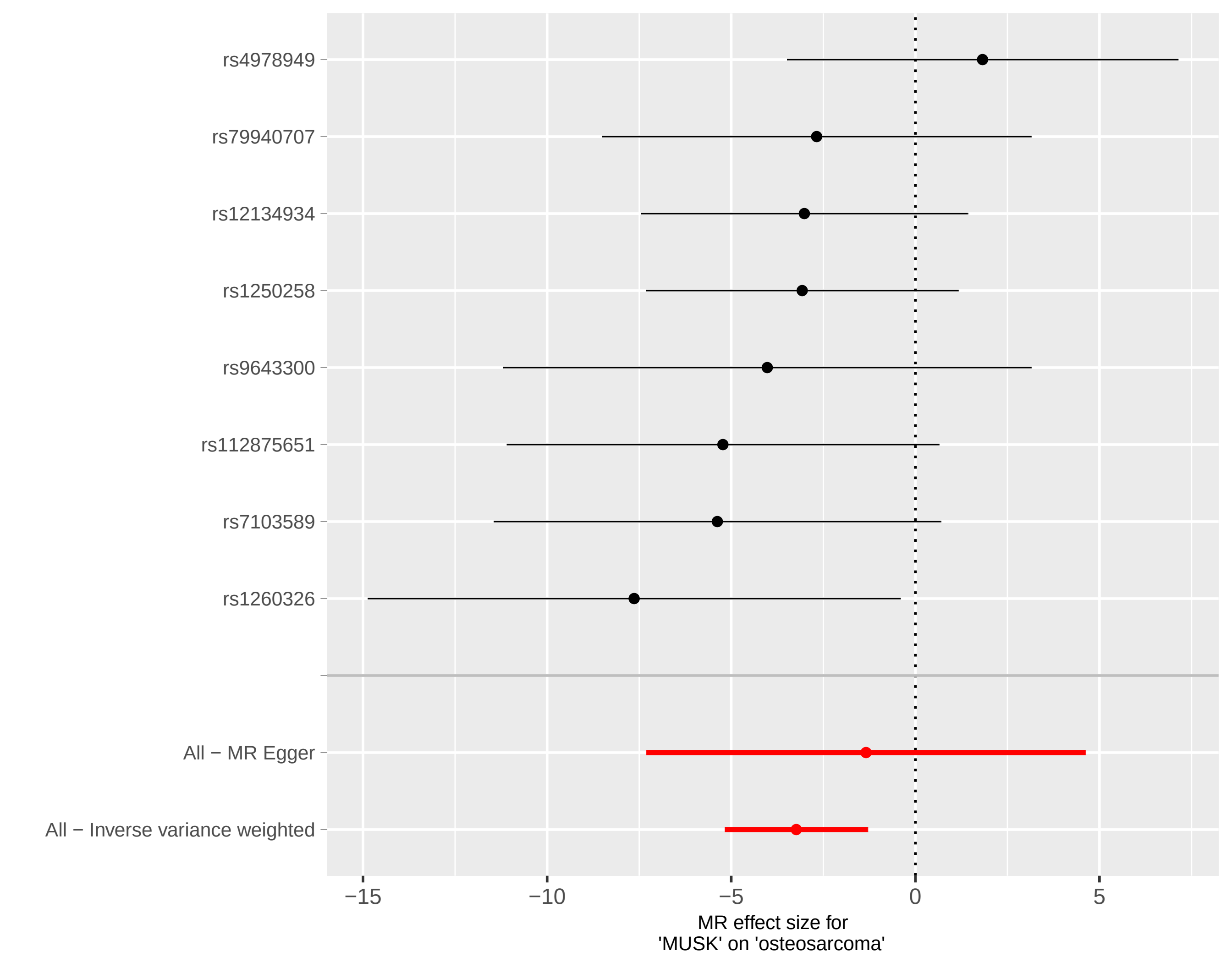
rs4978949
rs79940707
rs12134934
rs1250258
rs9643300
rs112875651
rs7103589
rs1260326
All − MR Egger
All − Inverse variance weighted
−15
−10
−5
0
5
MR effect size for
'MUSK' on 'osteosarcoma'

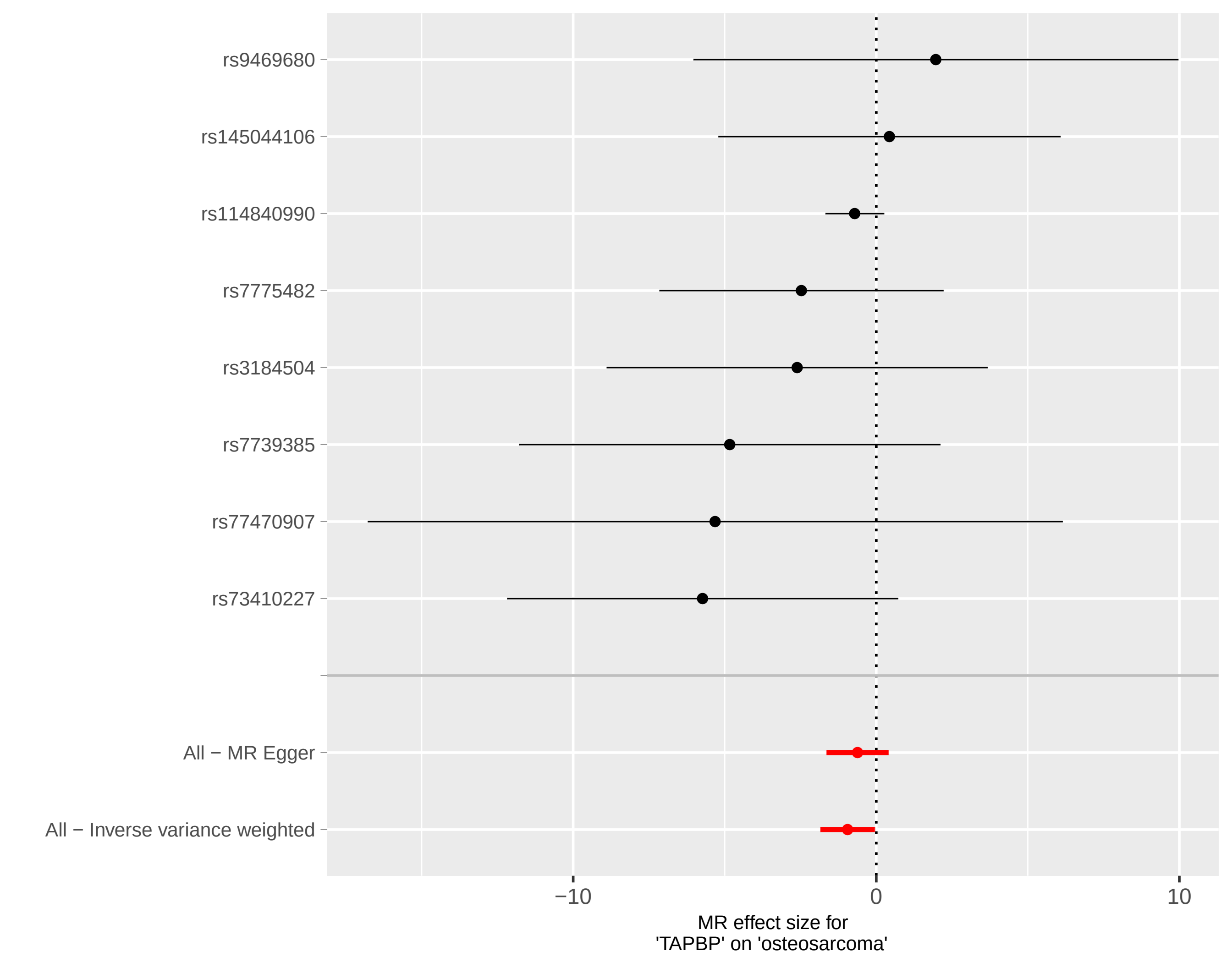
rs9469680
rs145044106
rs114840990
rs7775482
rs3184504
rs7739385
rs77470907
rs73410227
All − MR Egger
All − Inverse variance weighted
−10
0
10
MR effect size for
'TAPBP' on 'osteosarcoma'

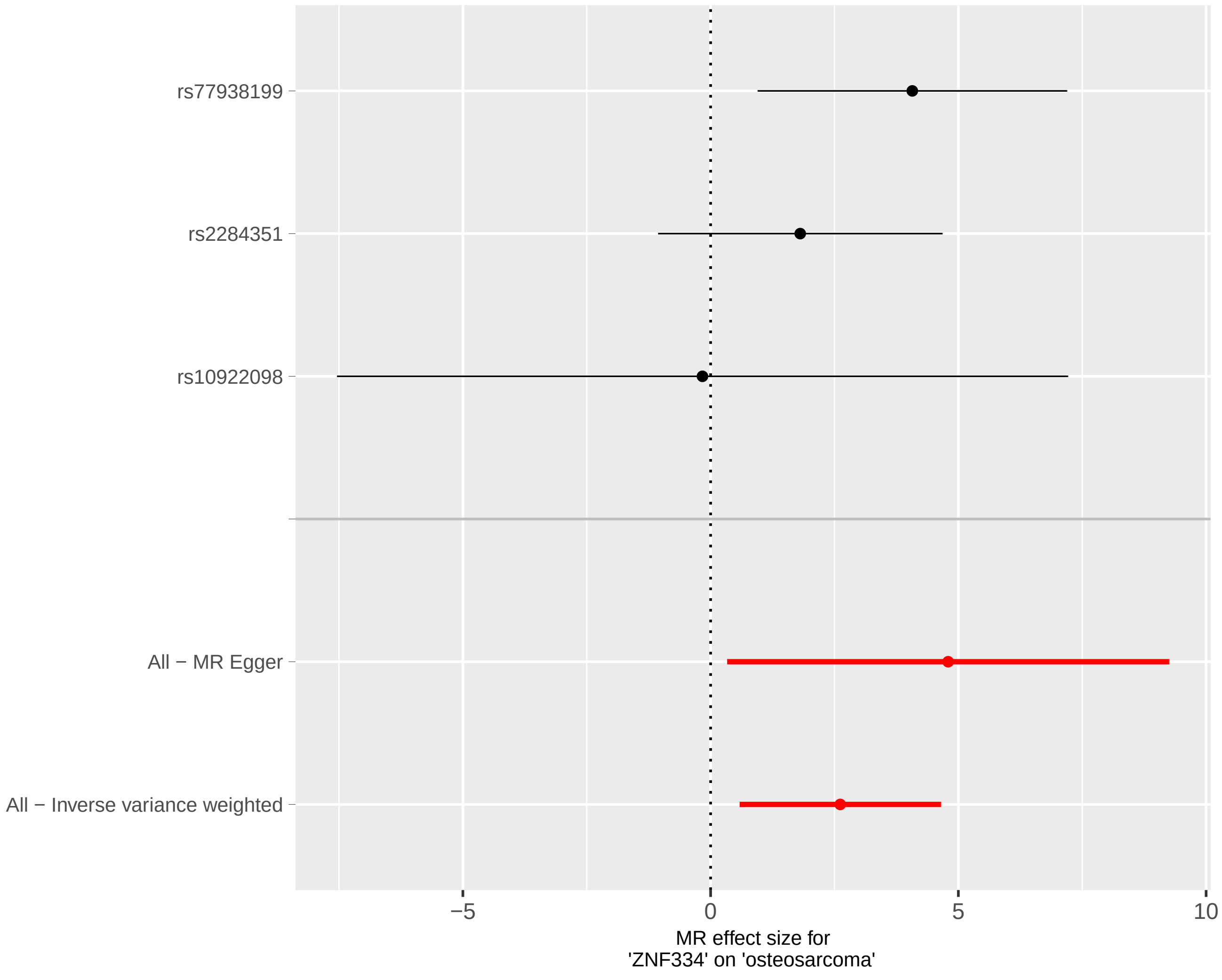
rs77938199
rs2284351
rs10922098
All − MR Egger
All − Inverse variance weighted
−5
0
5
10
MR effect size for
'ZNF334' on 'osteosarcoma'

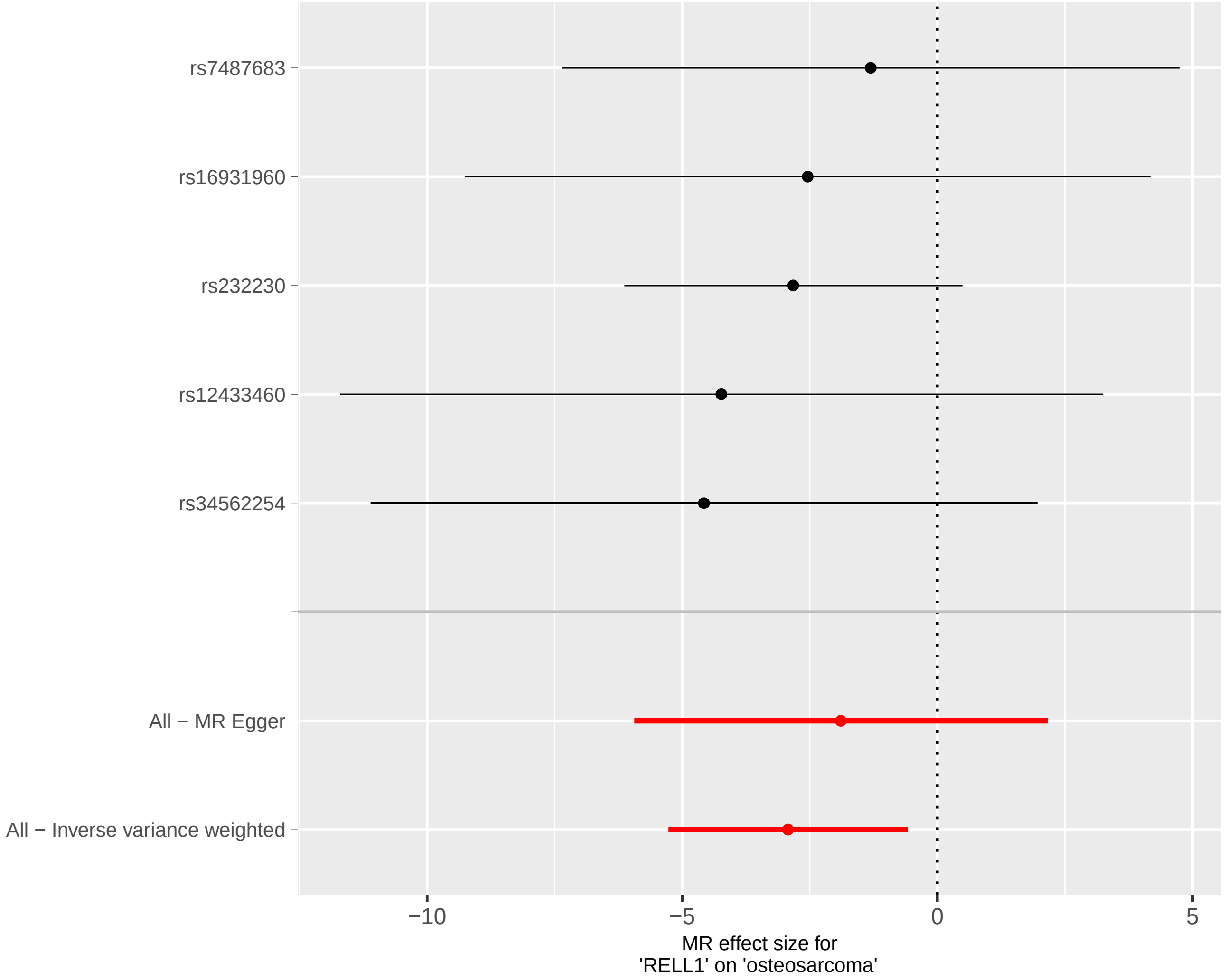

rs7487683
rs16931960
rs232230
rs12433460
rs34562254
All − MR Egger
All − Inverse variance weighted
−10
−5
0
5
MR effect size for
'RELL1' on 'osteosarcoma'

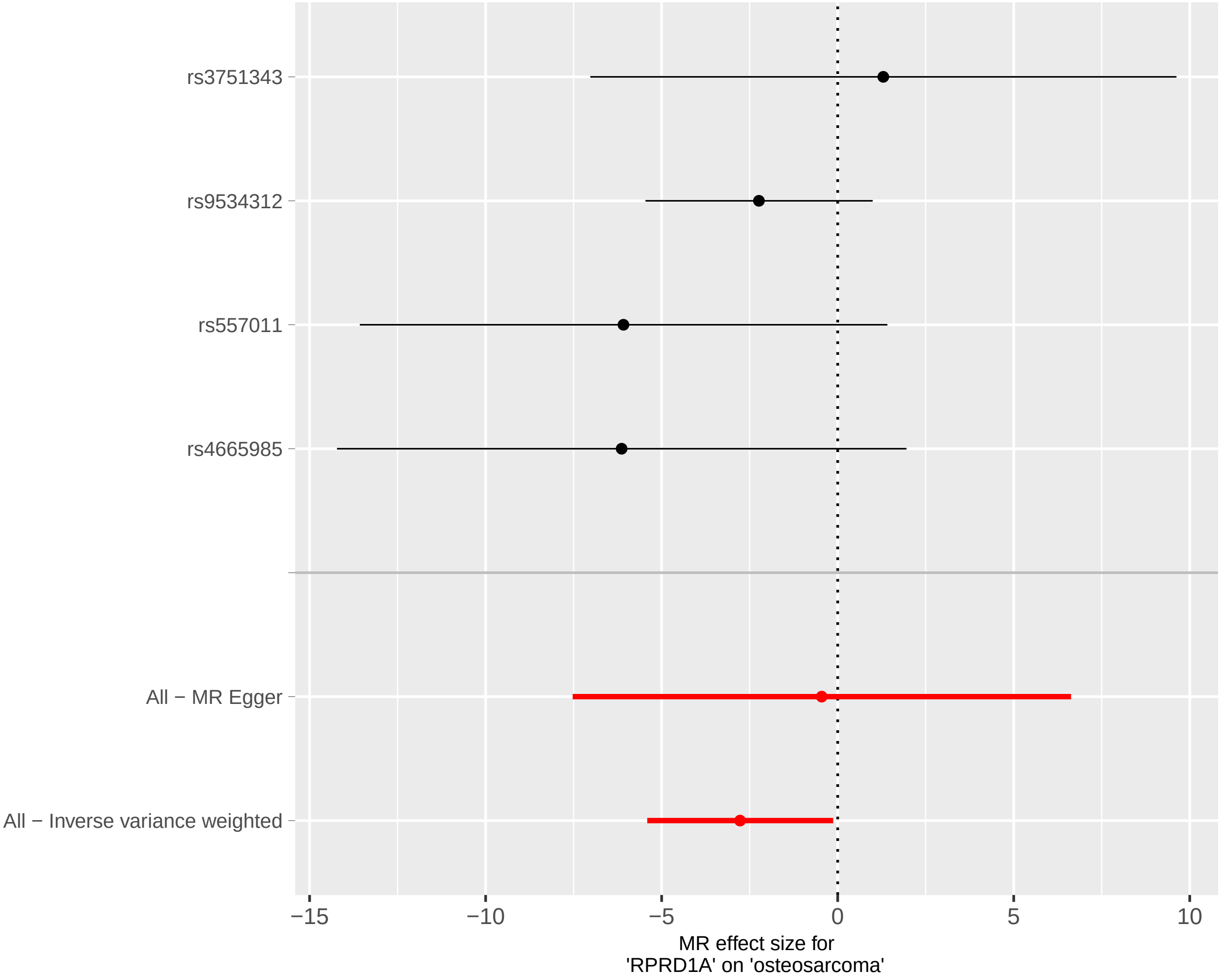
rs3751343
rs9534312
rs557011
rs4665985
All − MR Egger
All − Inverse variance weighted
−15
−10
−5
0
5
10
MR effect size for
'RPRD1A' on 'osteosarcoma'

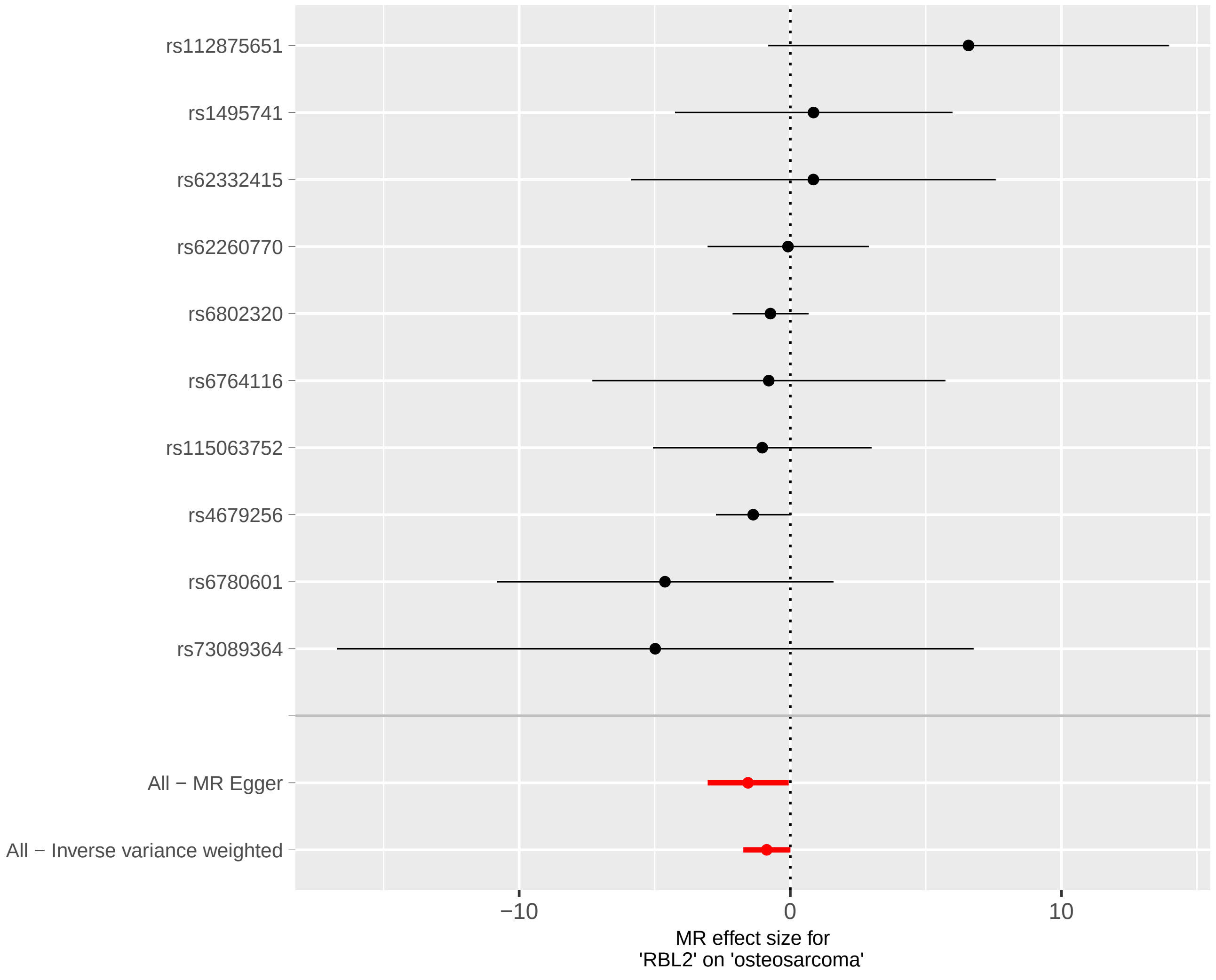

rs112875651
rs1495741
rs62332415
rs62260770
rs6802320
rs6764116
rs115063752
rs4679256
rs6780601
rs73089364
All − MR Egger
All − Inverse variance weighted
−10
0
10
MR effect size for
'RBL2' on 'osteosarcoma'

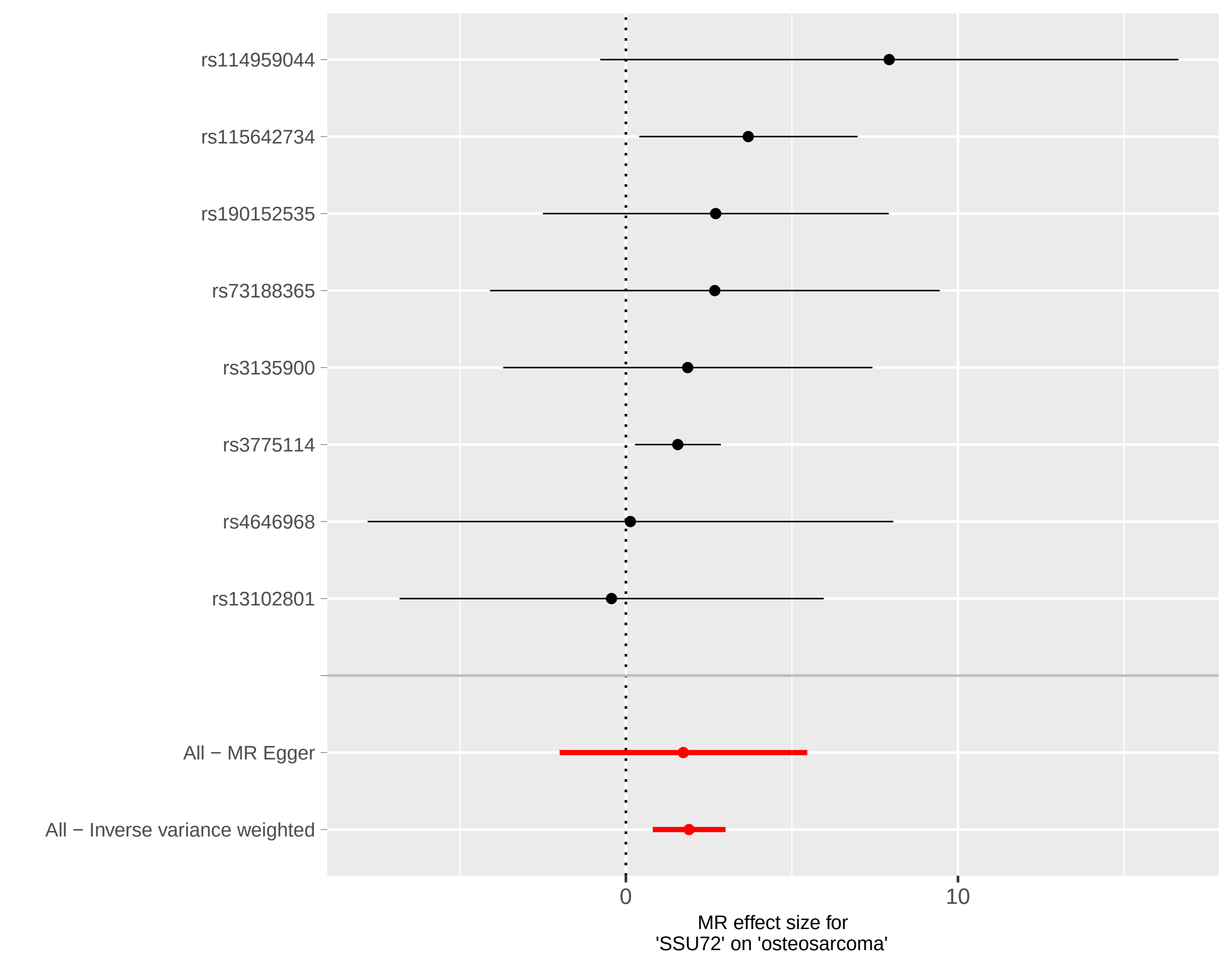
rs114959044
rs115642734
rs190152535
rs73188365
rs3135900
rs3775114
rs4646968
rs13102801
All − MR Egger
All − Inverse variance weighted
0
10
MR effect size for
'SSU72' on 'osteosarcoma'

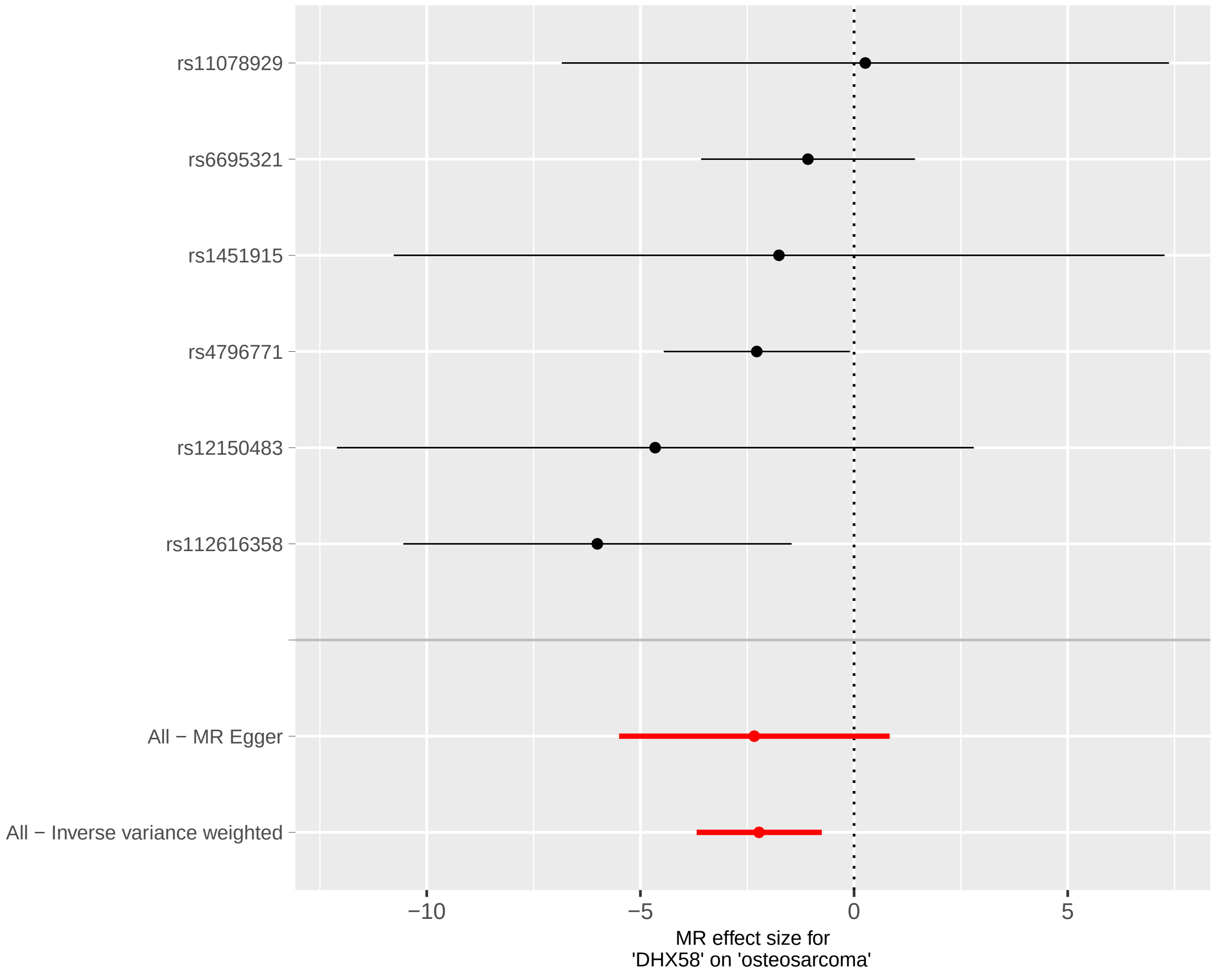
rs11078929
rs6695321
rs1451915
rs4796771
rs12150483
rs112616358
All − MR Egger
All − Inverse variance weighted
−10
−5
0
5
MR effect size for
'DHX58' on 'osteosarcoma'

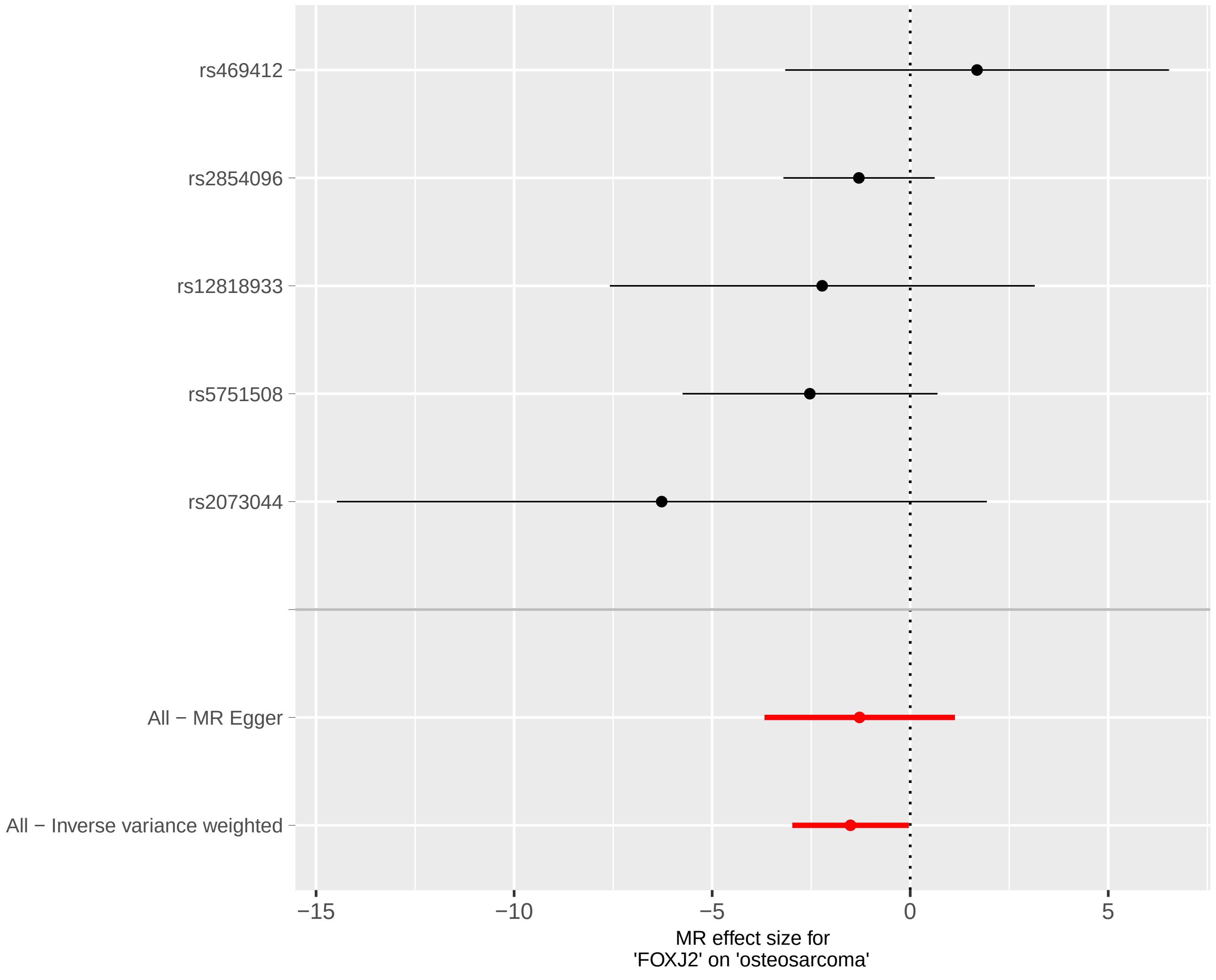
rs469412
rs2854096
rs12818933
rs5751508
rs2073044
All − MR Egger
All − Inverse variance weighted
−15
−10
−5
0
5
MR effect size for
'FOXJ2' on 'osteosarcoma'

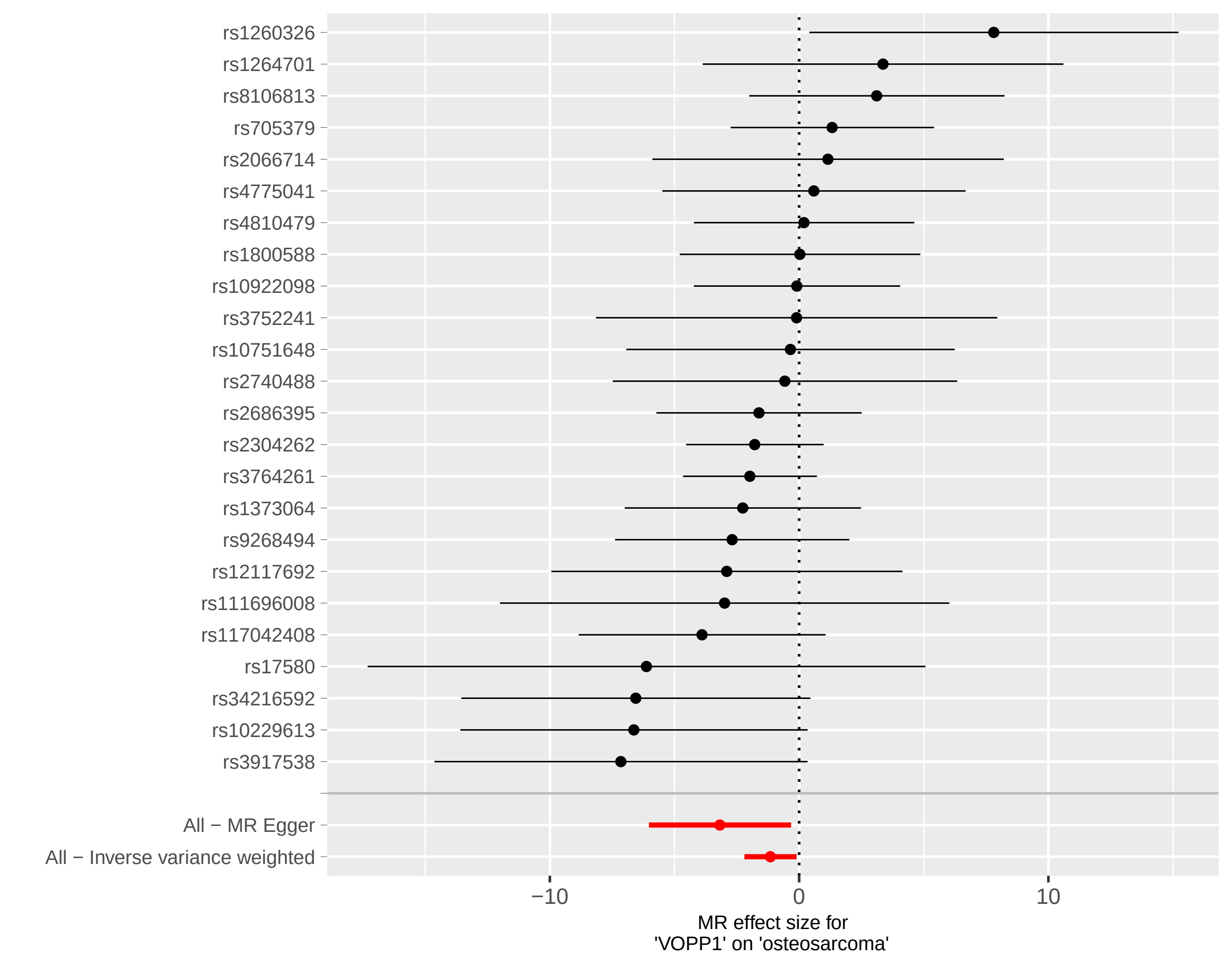
rs1260326
rs1264701
rs8106813
rs705379
rs2066714
rs4775041
rs4810479
rs1800588
rs10922098
rs3752241
rs10751648
rs2740488
rs2686395
rs2304262
rs3764261
rs1373064
rs9268494
rs12117692
rs111696008
rs117042408
rs17580
rs34216592
rs10229613
rs3917538
All − MR Egger
All − Inverse variance weighted
−10
0
10
MR effect size for
'VOPP1' on 'osteosarcoma'

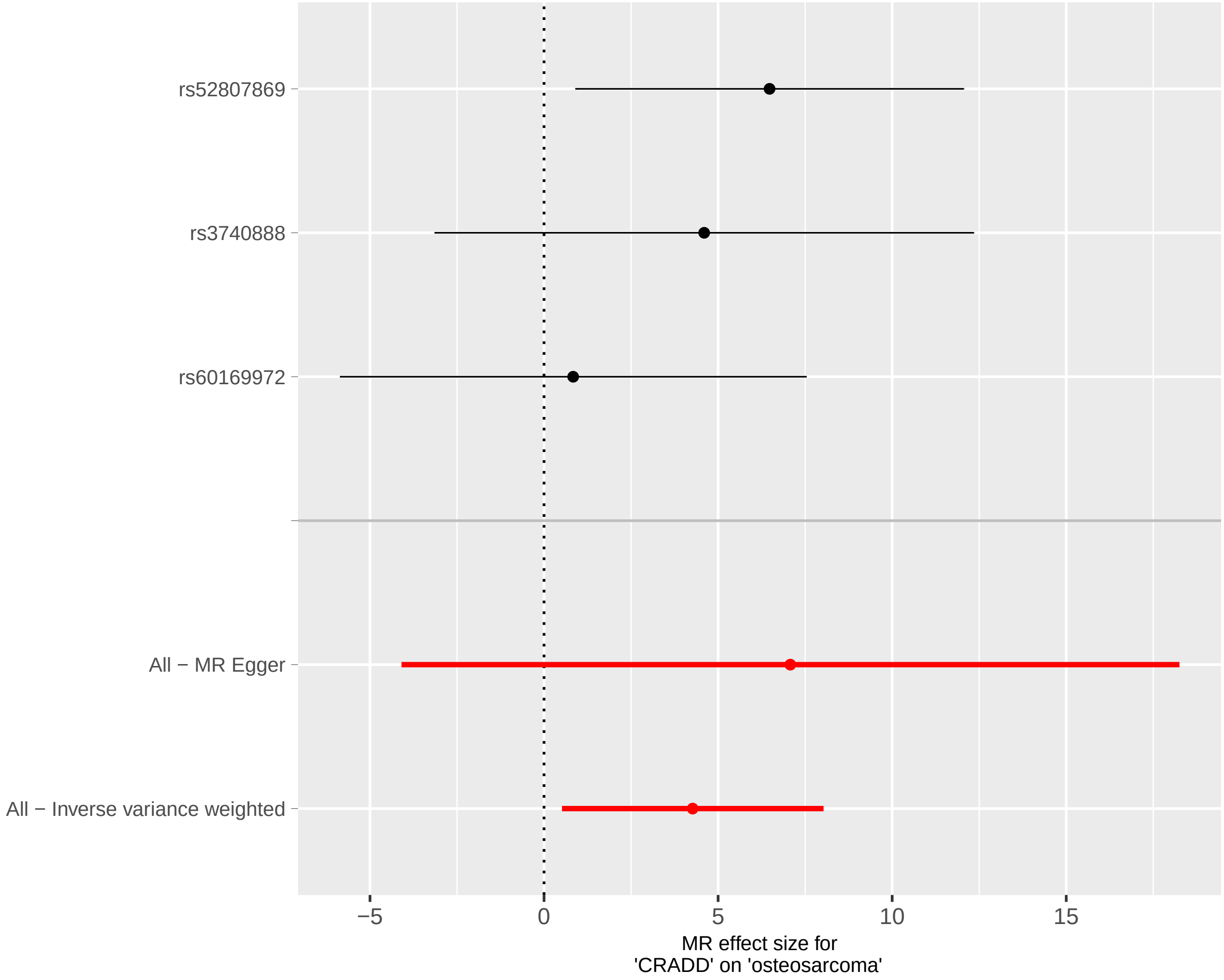
rs52807869
rs3740888
rs60169972
All − MR Egger
All − Inverse variance weighted
−5
0
5
10
15
MR effect size for
'CRADD' on 'osteosarcoma'

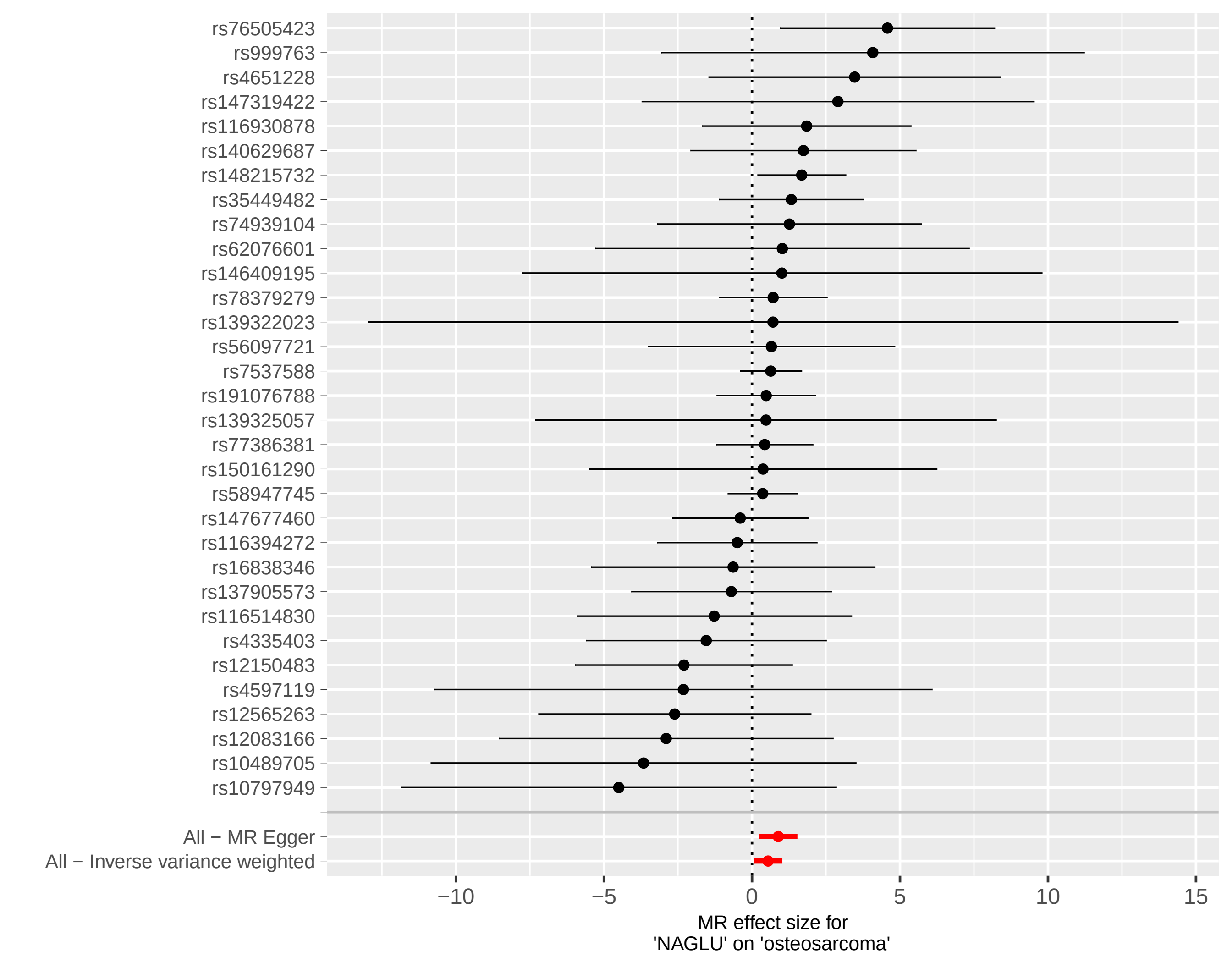

rs76505423
rs999763
rs4651228
rs147319422
rs116930878
rs140629687
rs148215732
rs35449482
rs74939104
rs62076601
rs146409195
rs78379279
rs139322023
rs56097721
rs7537588
rs191076788
rs139325057
rs77386381
rs150161290
rs58947745
rs147677460
rs116394272
rs16838346
rs137905573
rs116514830
rs4335403
rs12150483
rs4597119
rs12565263
rs12083166
rs10489705
rs10797949
All − MR Egger
All − Inverse variance weighted
−10
−5
0
5
10
15
MR effect size for
'NAGLU' on 'osteosarcoma'

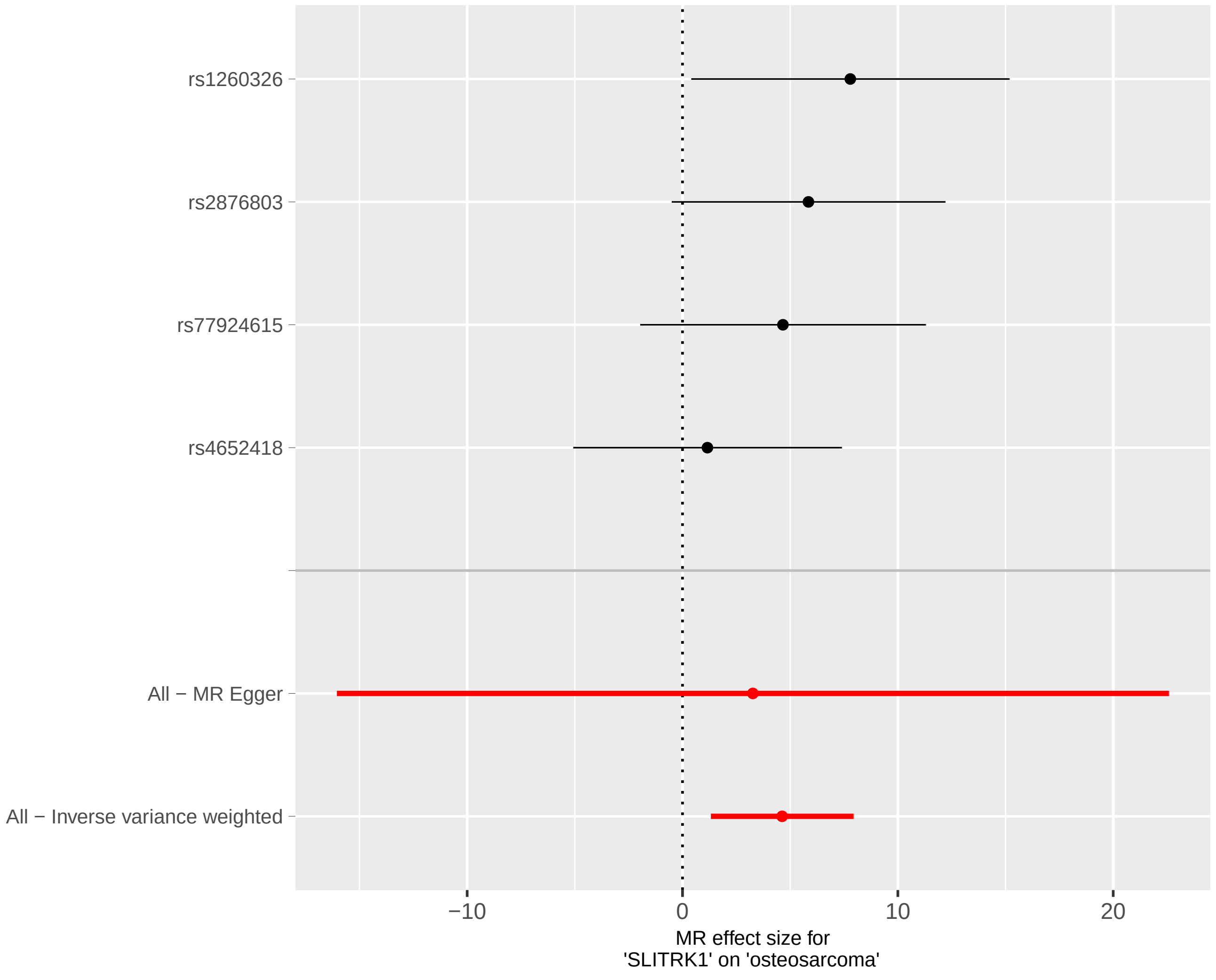

rs1260326
rs2876803
rs77924615
rs4652418
All − MR Egger
All − Inverse variance weighted
−10
0
10
20
MR effect size for
'SLITRK1' on 'osteosarcoma'

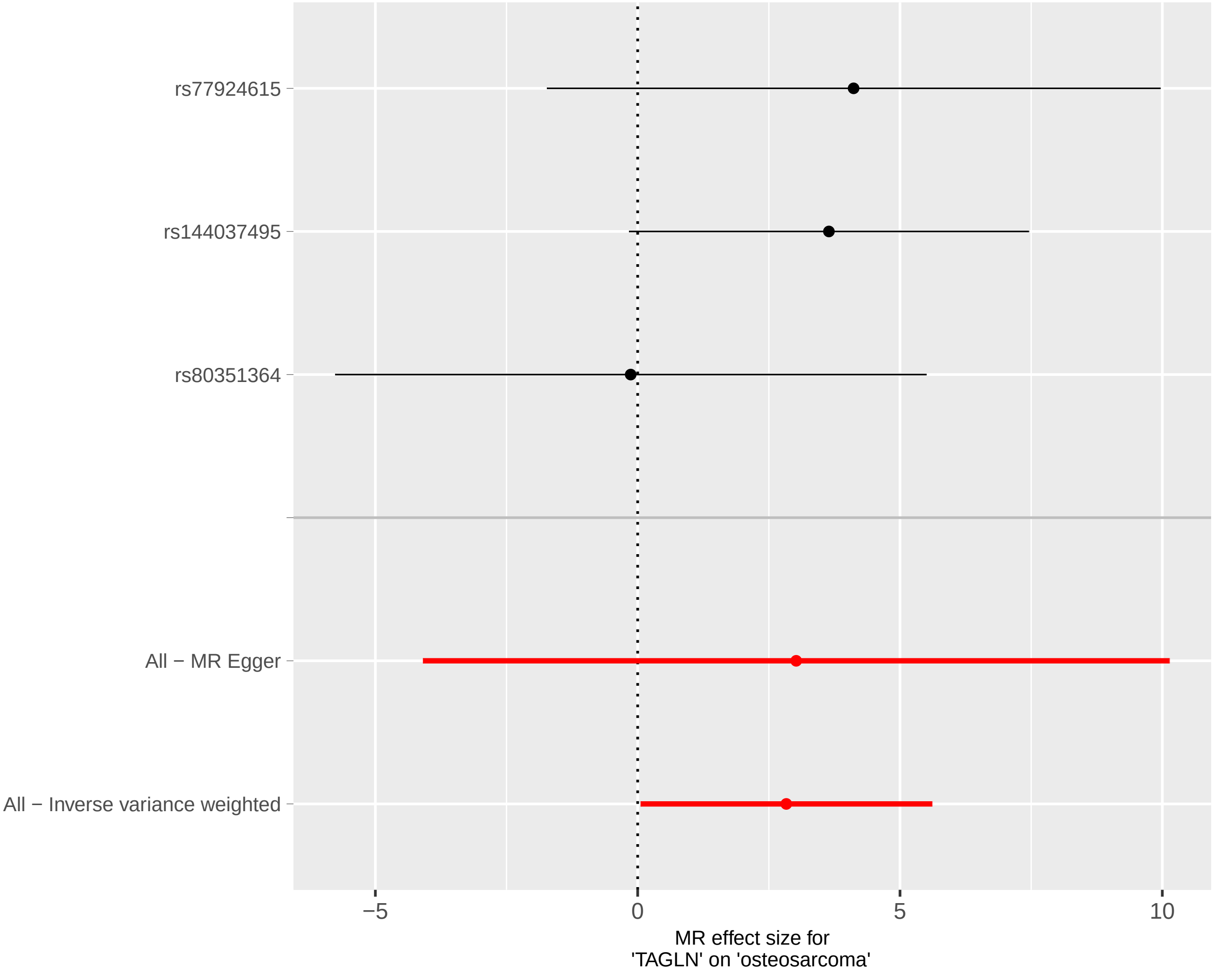

rs77924615
rs144037495
rs80351364
All − MR Egger
All − Inverse variance weighted
−5
0
5
10
MR effect size for
'TAGLN' on 'osteosarcoma'

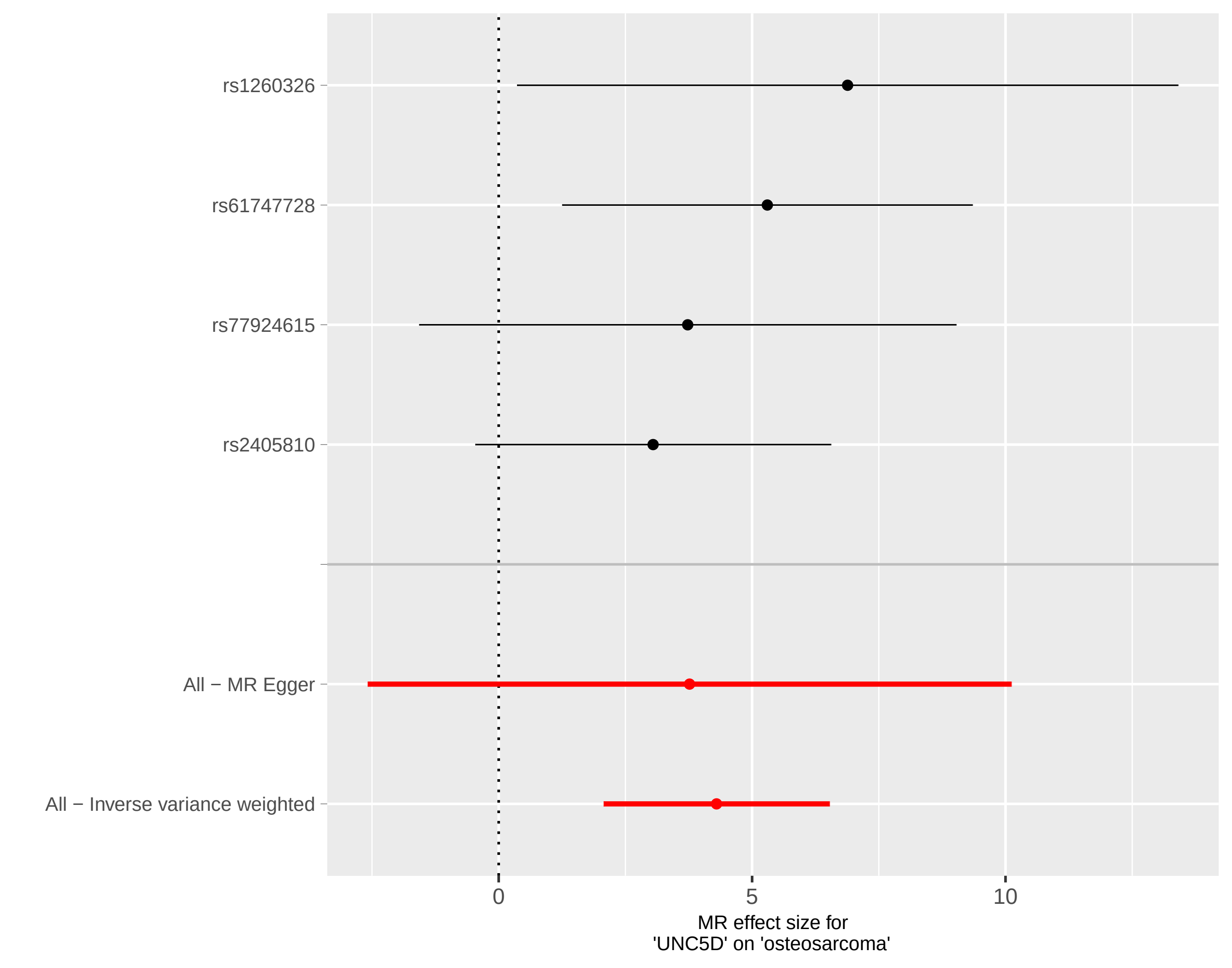

rs1260326
rs61747728
rs77924615
rs2405810
All − MR Egger
All − Inverse variance weighted
0
5
10
MR effect size for
'UNC5D' on 'osteosarcoma'

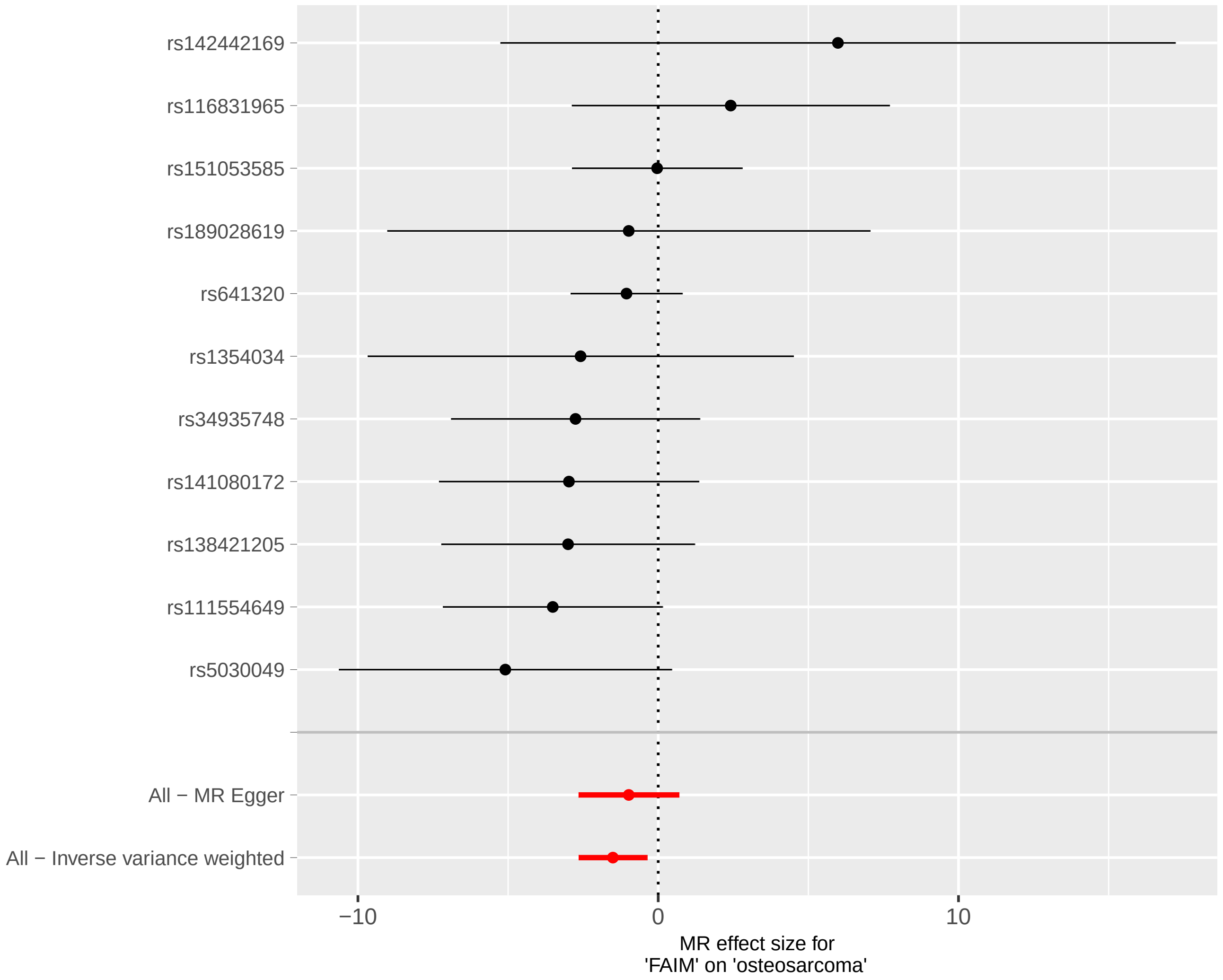
rs142442169
rs116831965
rs151053585
rs189028619
rs641320
rs1354034
rs34935748
rs141080172
rs138421205
rs111554649
rs5030049
All − MR Egger
All − Inverse variance weighted
−10
0
10
MR effect size for
'FAIM' on 'osteosarcoma'

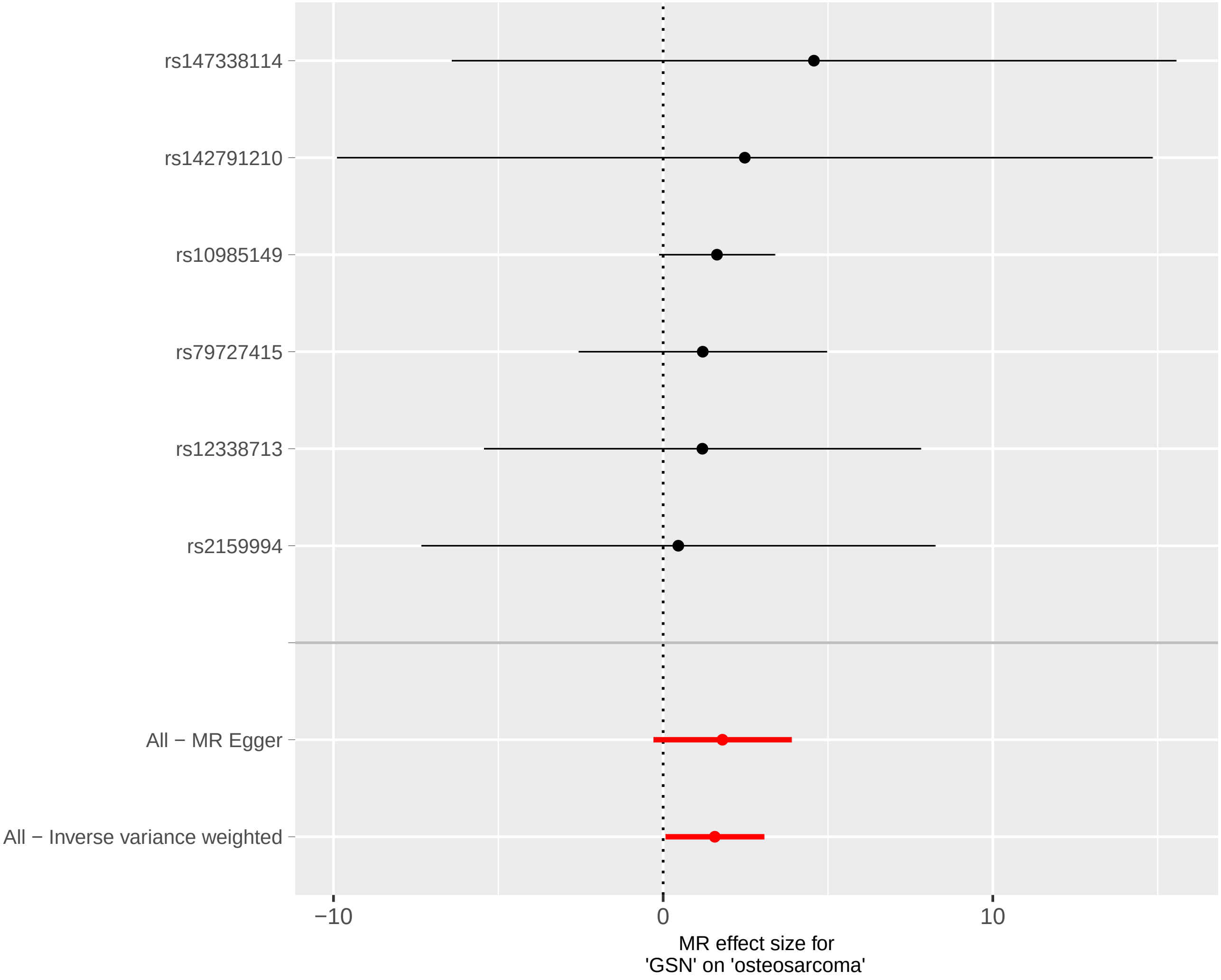
rs147338114
rs142791210
rs10985149
rs79727415
rs12338713
rs2159994
All − MR Egger
All − Inverse variance weighted
−10
0
10
MR effect size for
'GSN' on 'osteosarcoma'

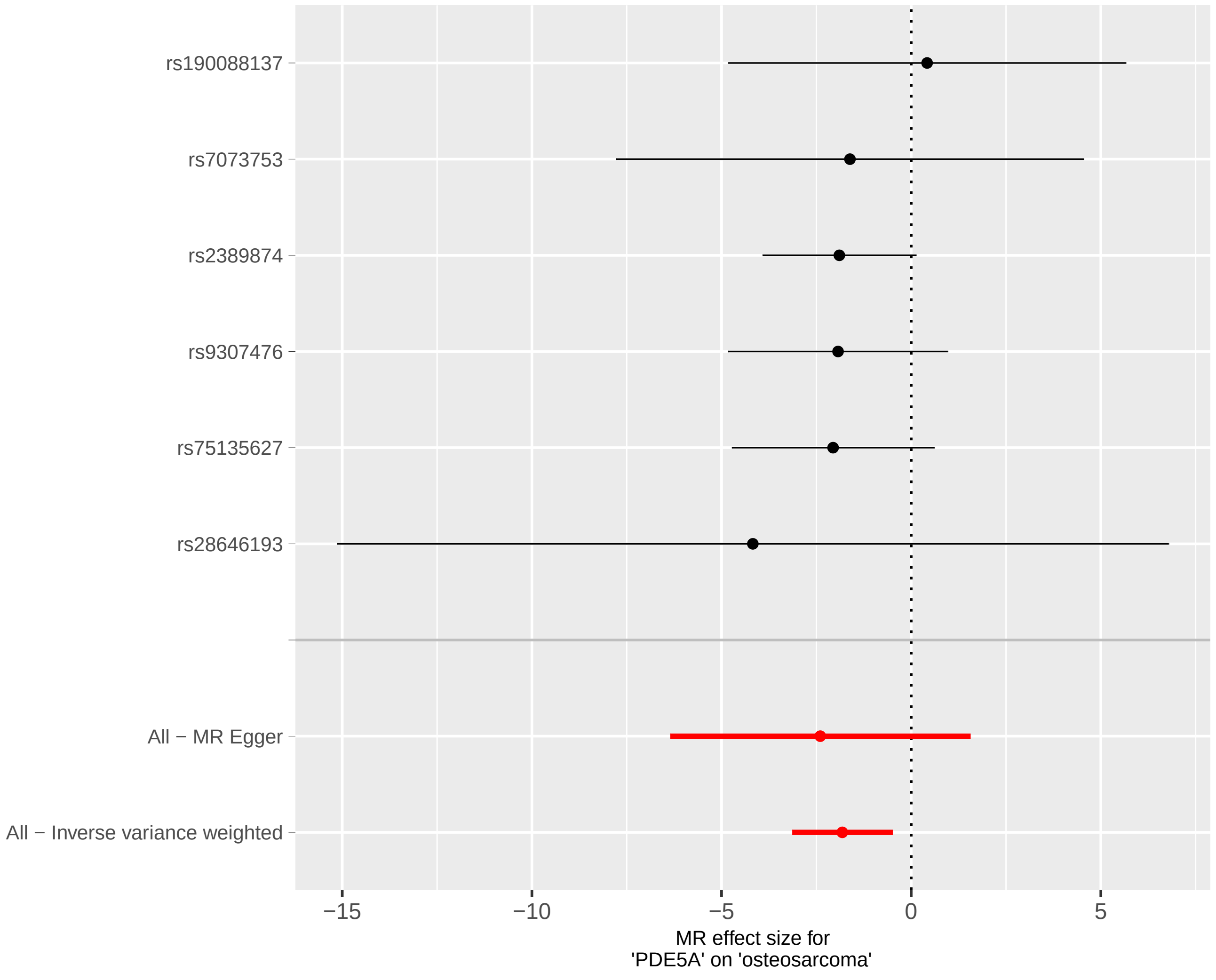
rs190088137
rs7073753
rs2389874
rs9307476
rs75135627
rs28646193
All − MR Egger
All − Inverse variance weighted
−15
−10
−5
0
5
MR effect size for
'PDE5A' on 'osteosarcoma'

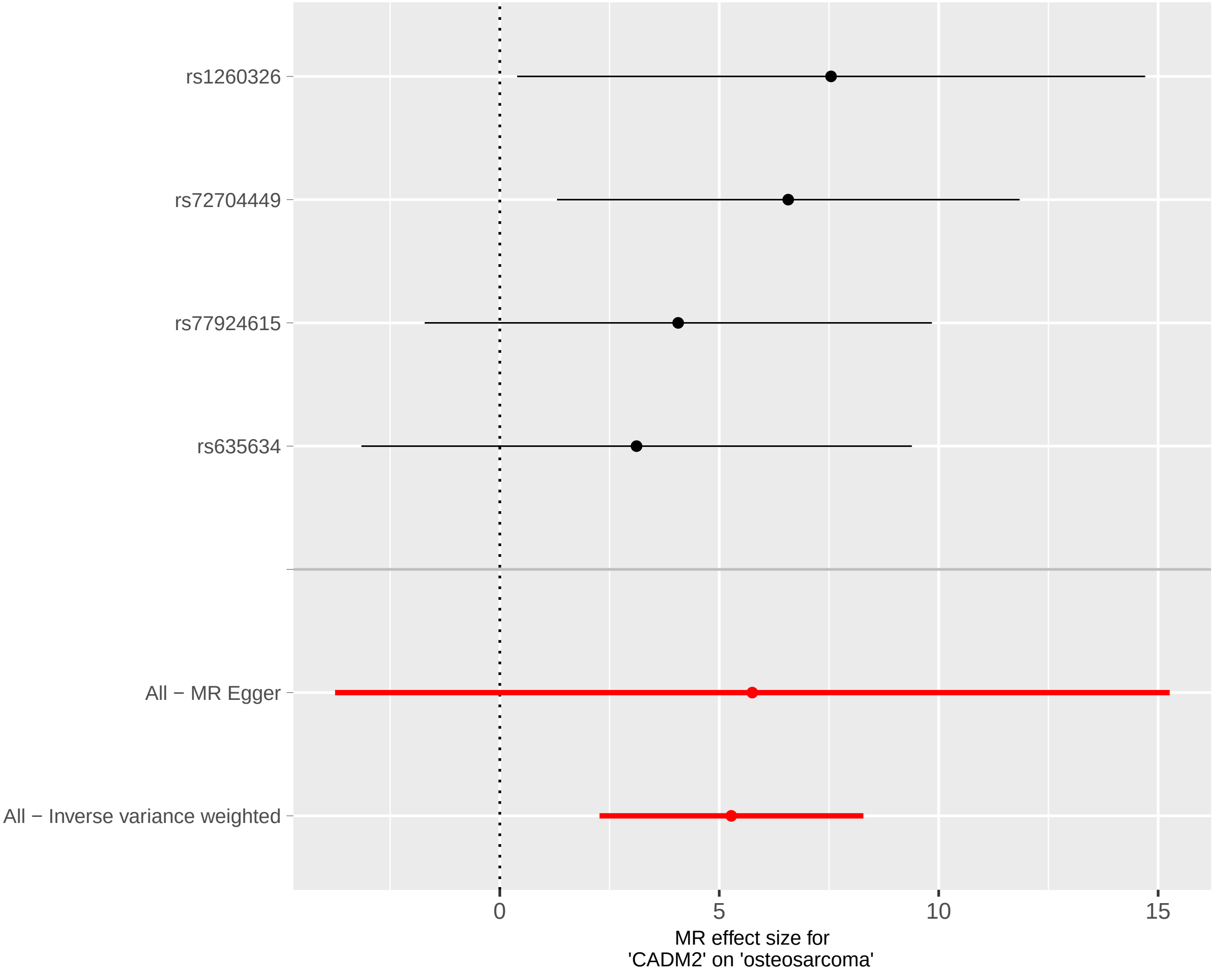

rs1260326
rs72704449
rs77924615
rs635634
All − MR Egger
All − Inverse variance weighted
0
5
10
15
MR effect size for
'CADM2' on 'osteosarcoma'

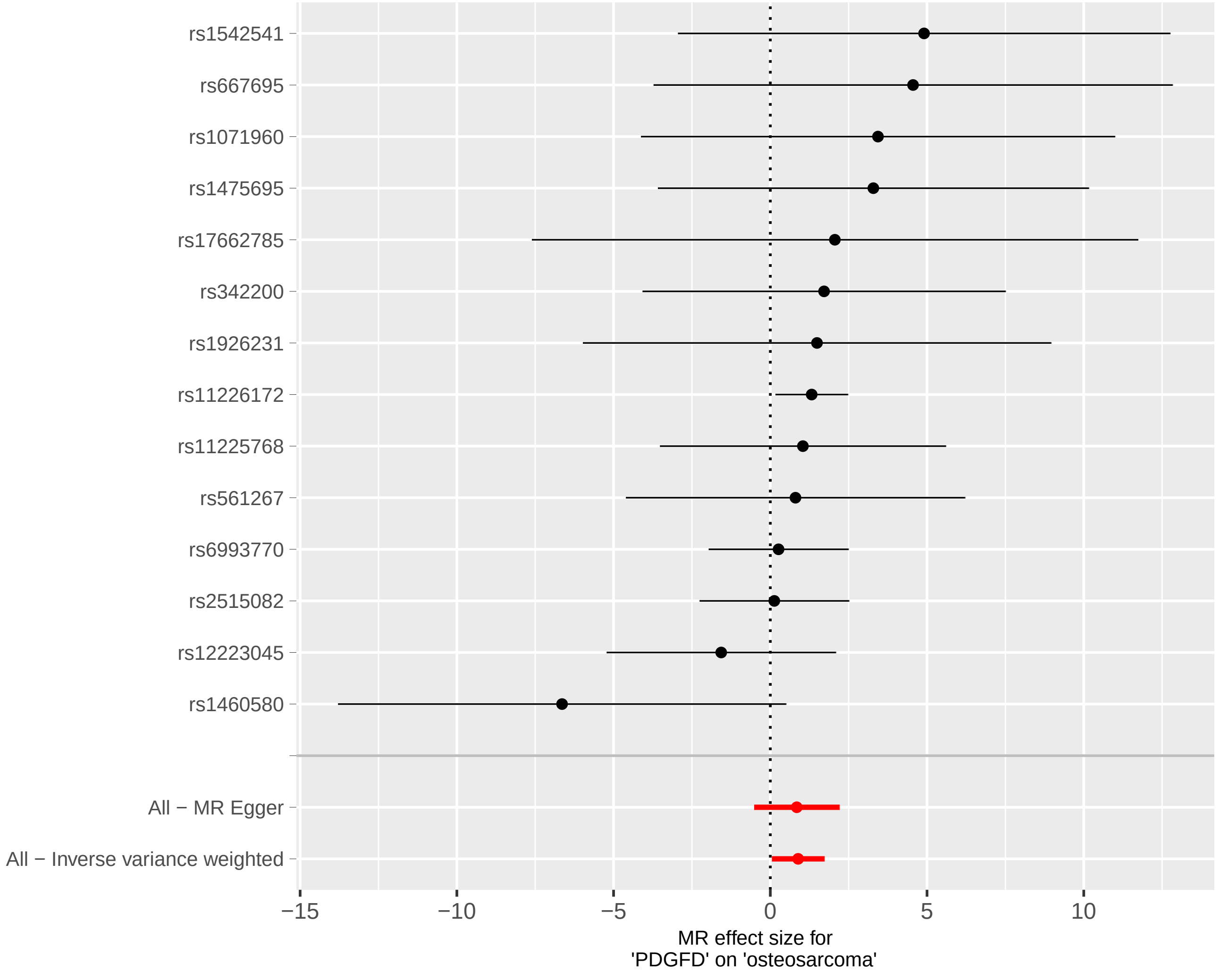

rs1542541
rs667695
rs1071960
rs1475695
rs17662785
rs342200
rs1926231
rs11226172
rs11225768
rs561267
rs6993770
rs2515082
rs12223045
rs1460580
All − MR Egger
All − Inverse variance weighted
−15
−10
−5
0
5
10
MR effect size for
'PDGFD' on 'osteosarcoma'

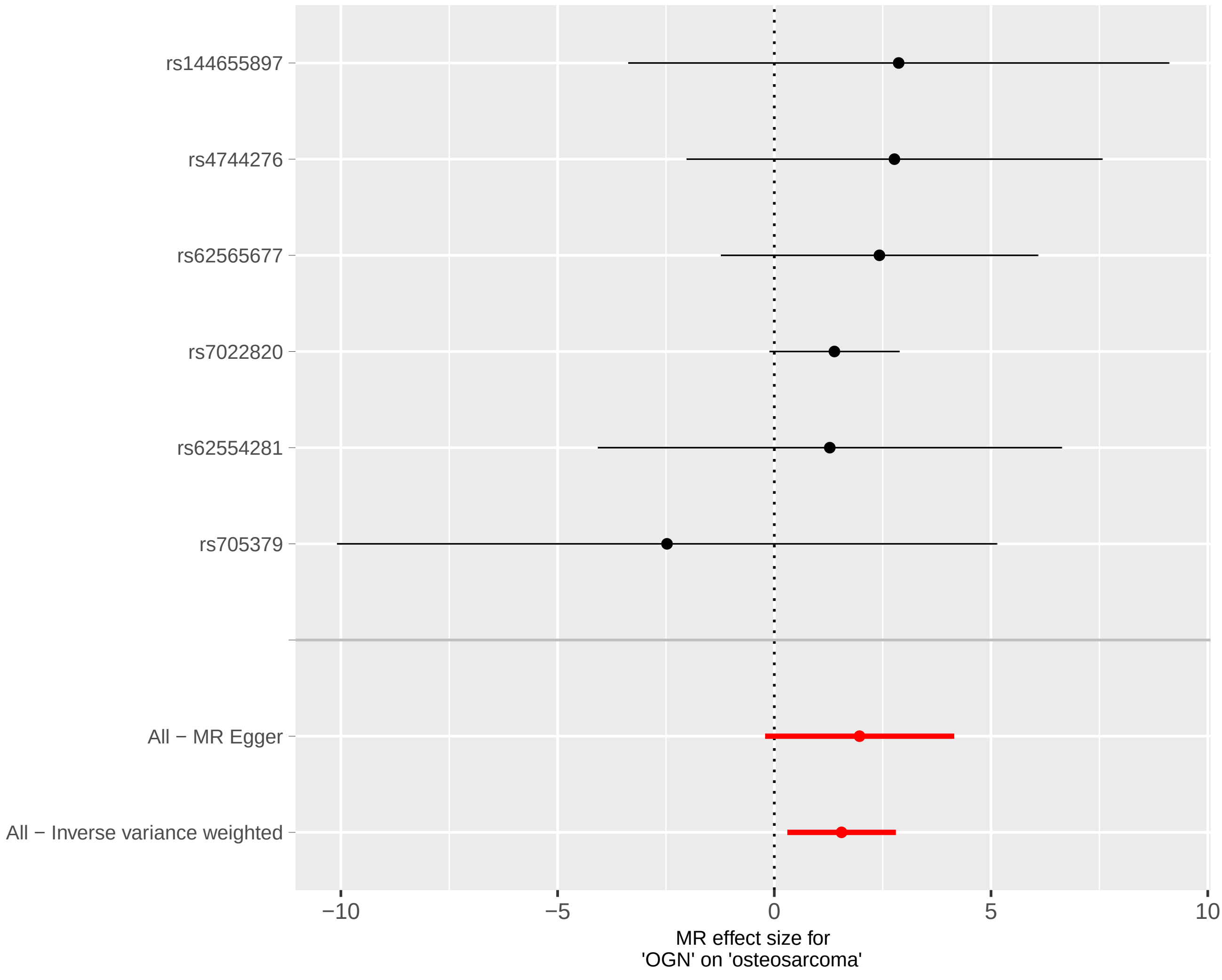
rs144655897
rs4744276
rs62565677
rs7022820
rs62554281
rs705379
All − MR Egger
All − Inverse variance weighted
−10
−5
0
5
10
MR effect size for
'OGN' on 'osteosarcoma'

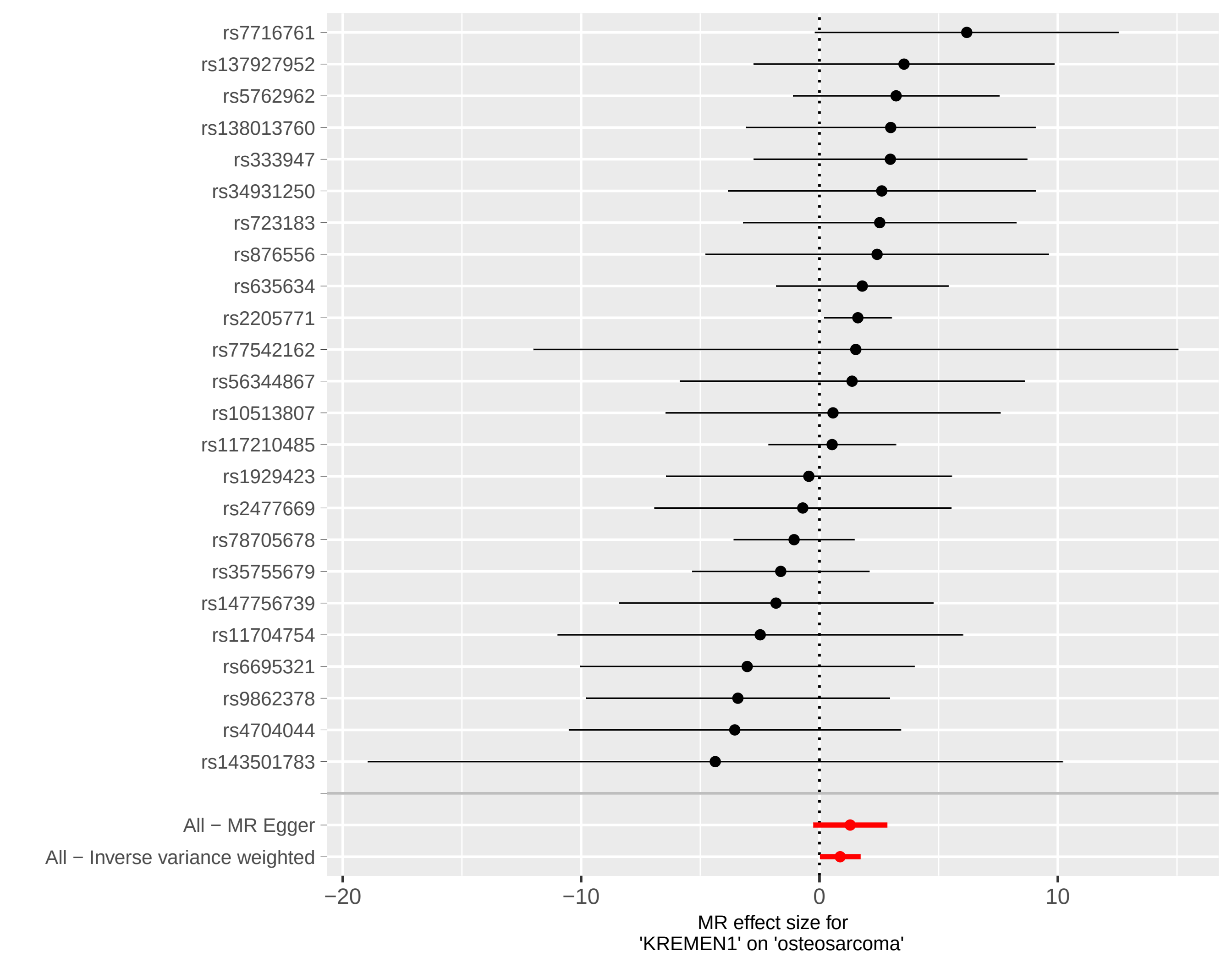

rs7716761
rs137927952
rs5762962
rs138013760
rs333947
rs34931250
rs723183
rs876556
rs635634
rs2205771
rs77542162
rs56344867
rs10513807
rs117210485
rs1929423
rs2477669
rs78705678
rs35755679
rs147756739
rs11704754
rs6695321
rs9862378
rs4704044
rs143501783
All − MR Egger
All − Inverse variance weighted
−20
−10
0
10
MR effect size for
'KREMEN1' on 'osteosarcoma'

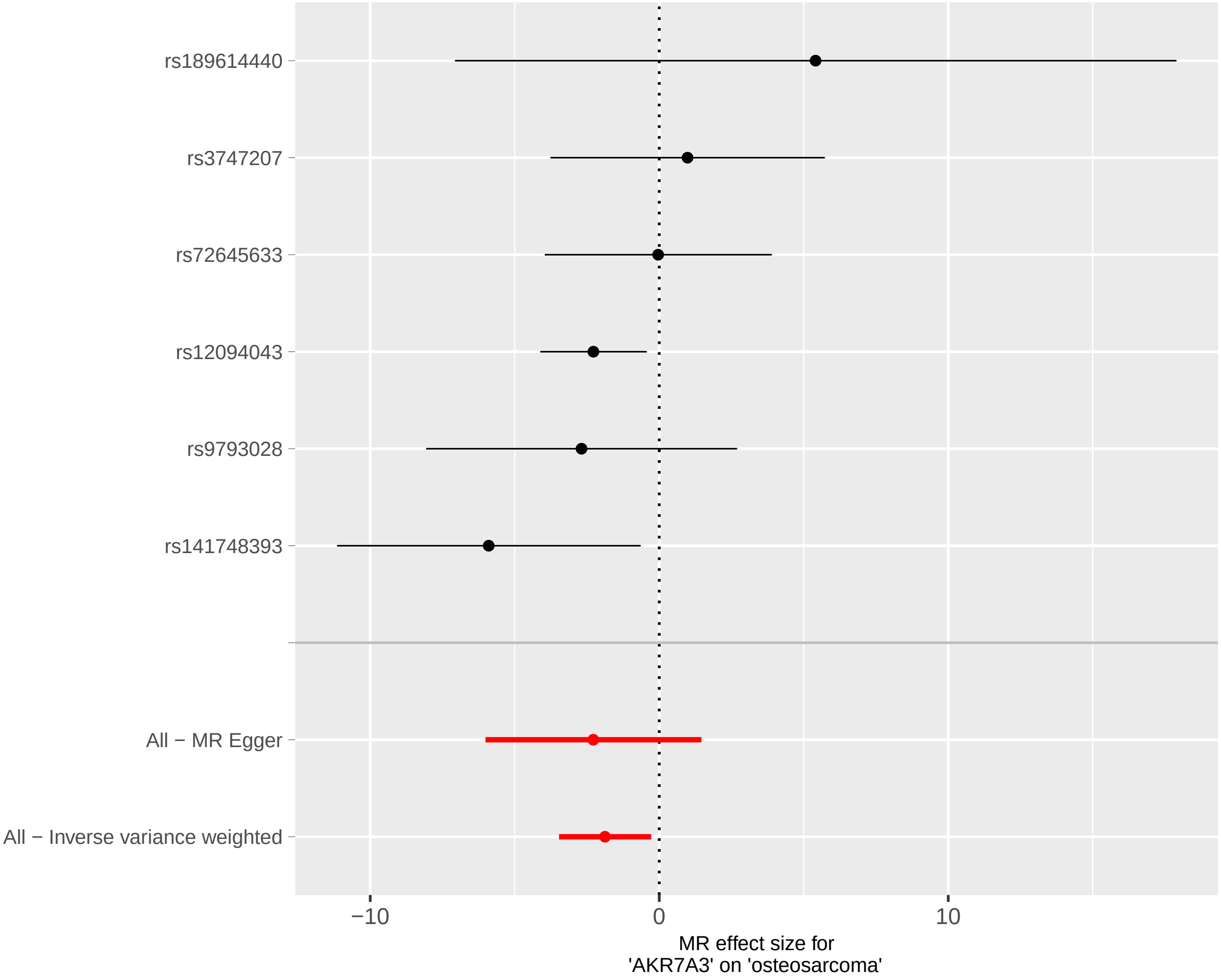
rs189614440
rs3747207
rs72645633
rs12094043
rs9793028
rs141748393
All − MR Egger
All − Inverse variance weighted
−10
0
10
MR effect size for
'AKR7A3' on 'osteosarcoma'

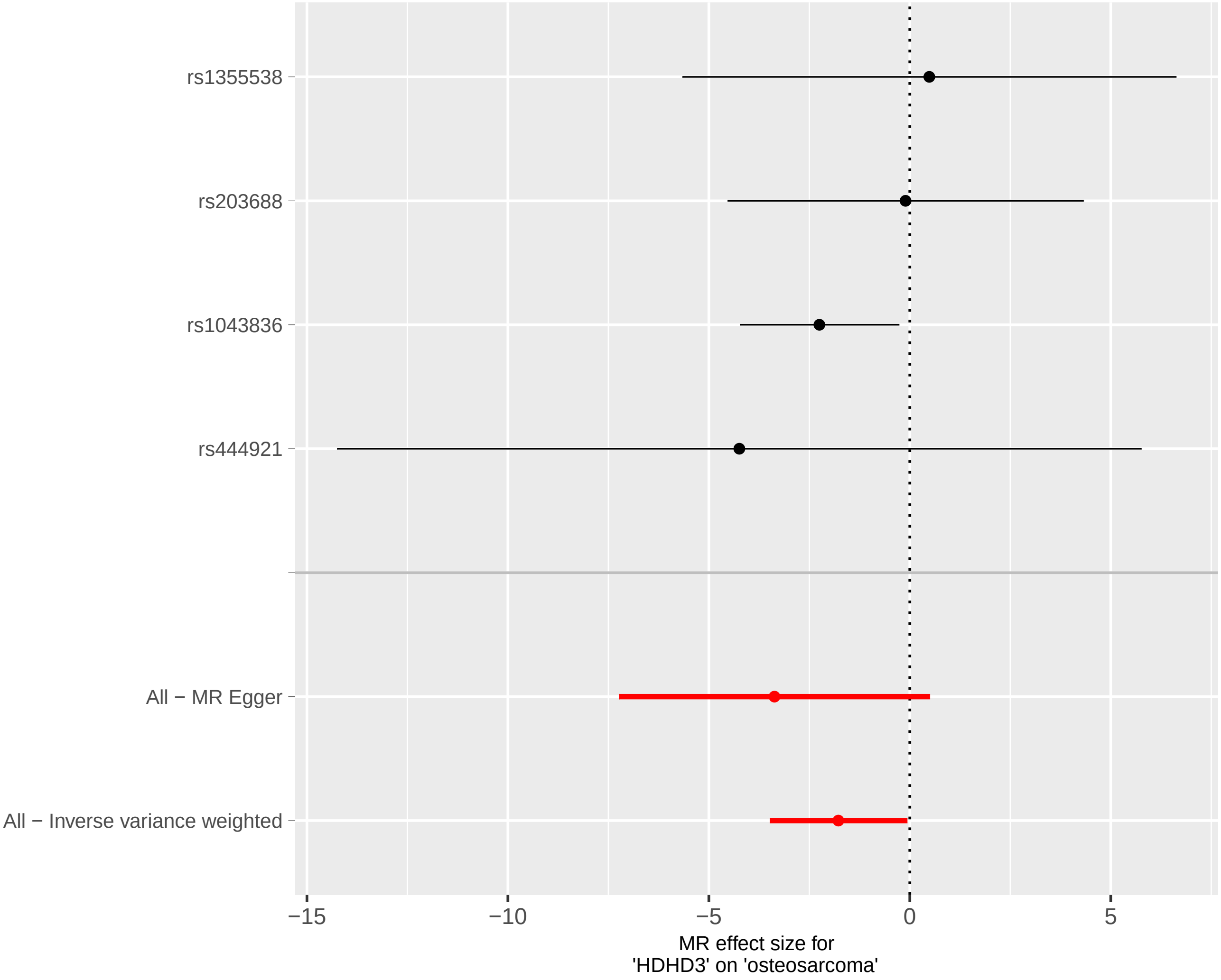
rs1355538
rs203688
rs1043836
rs444921
All − MR Egger
All − Inverse variance weighted
−15
−10
−5
0
5
MR effect size for
'HDHD3' on 'osteosarcoma'

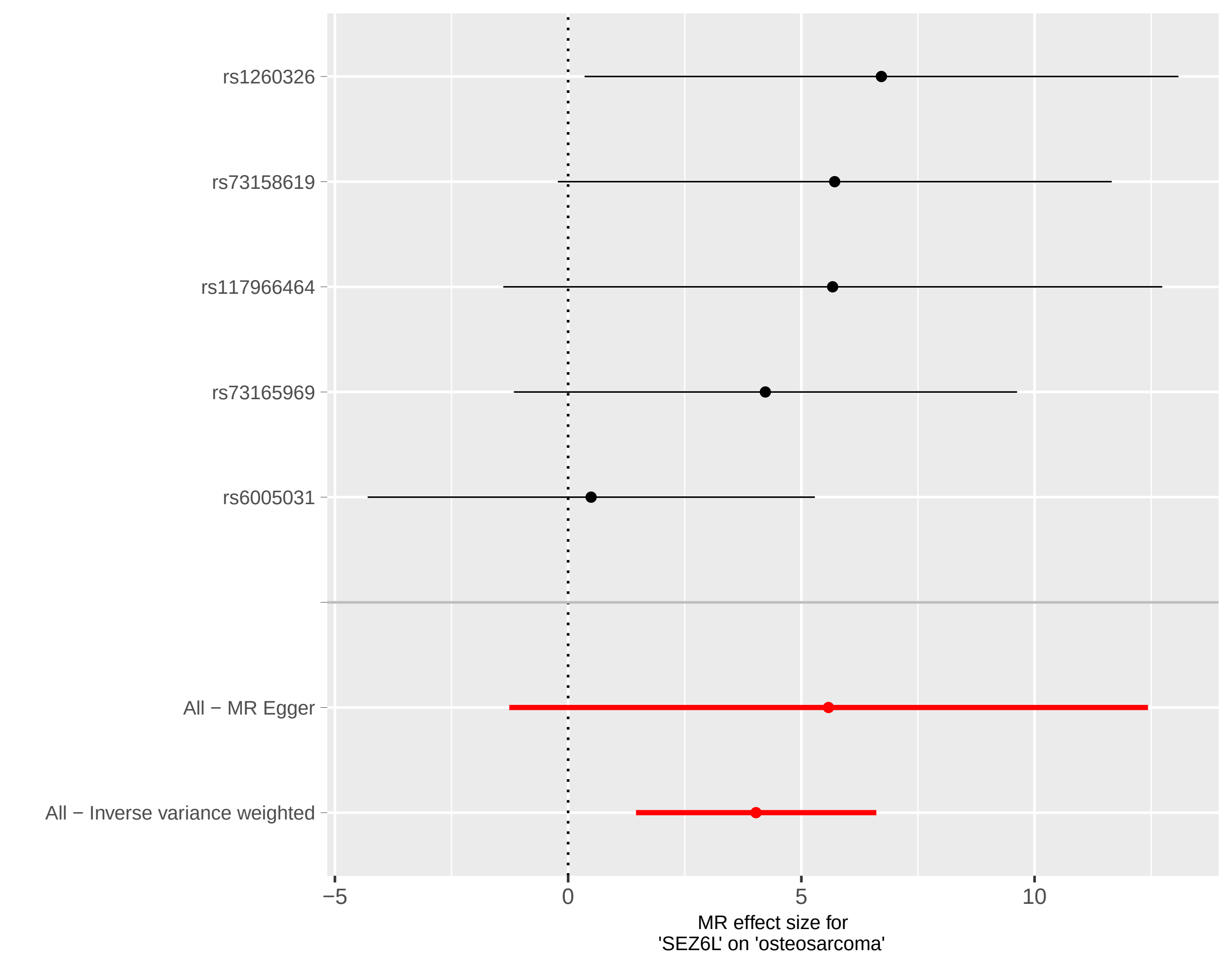

rs1260326
rs73158619
rs117966464
rs73165969
rs6005031
All − MR Egger
All − Inverse variance weighted
−5
0
5
10
MR effect size for
'SEZ6L' on 'osteosarcoma'

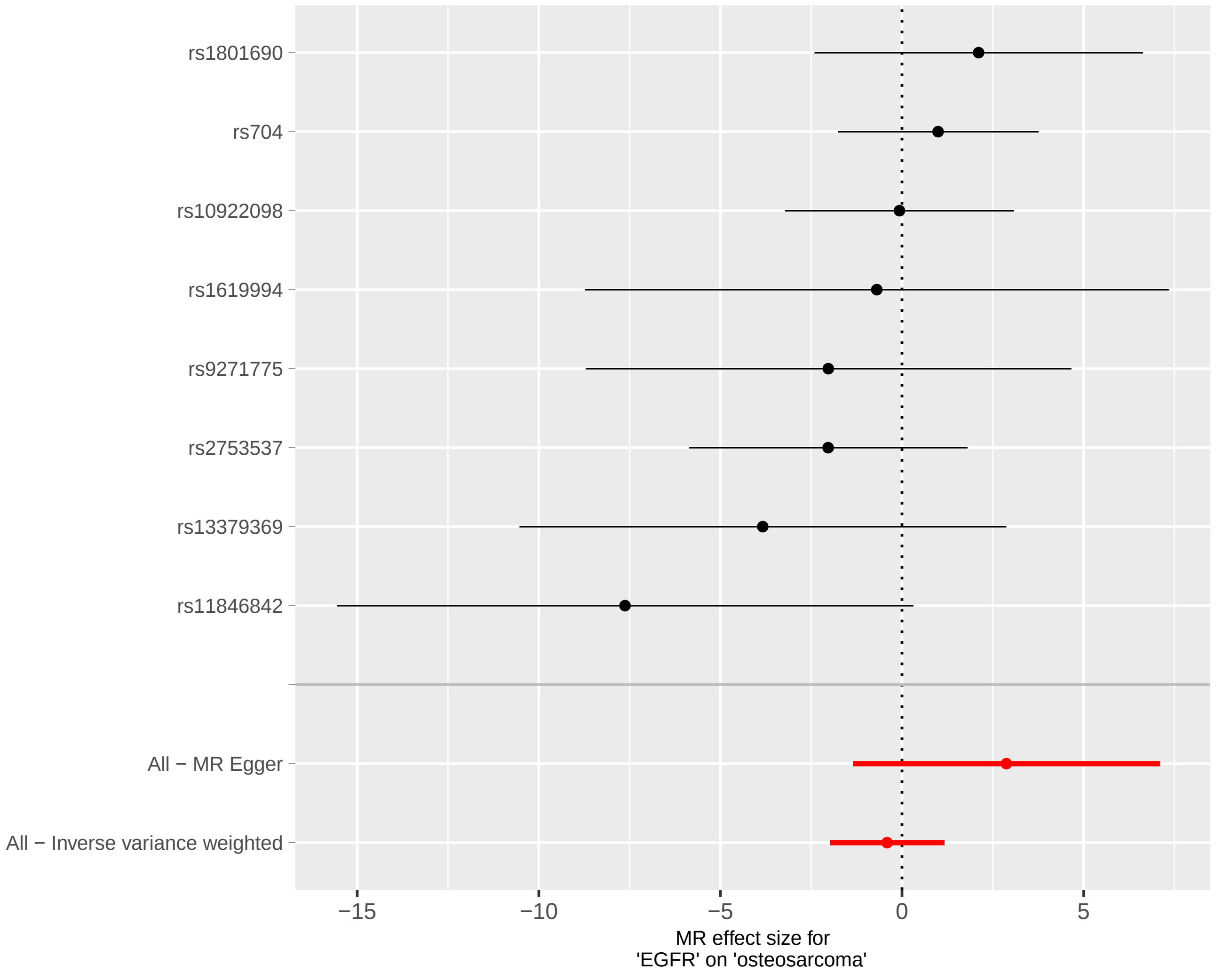
rs1801690
rs704
rs10922098
rs1619994
rs9271775
rs2753537
rs13379369
rs11846842
All − MR Egger
All − Inverse variance weighted
−15
−10
−5
0
5
MR effect size for
'EGFR' on 'osteosarcoma'

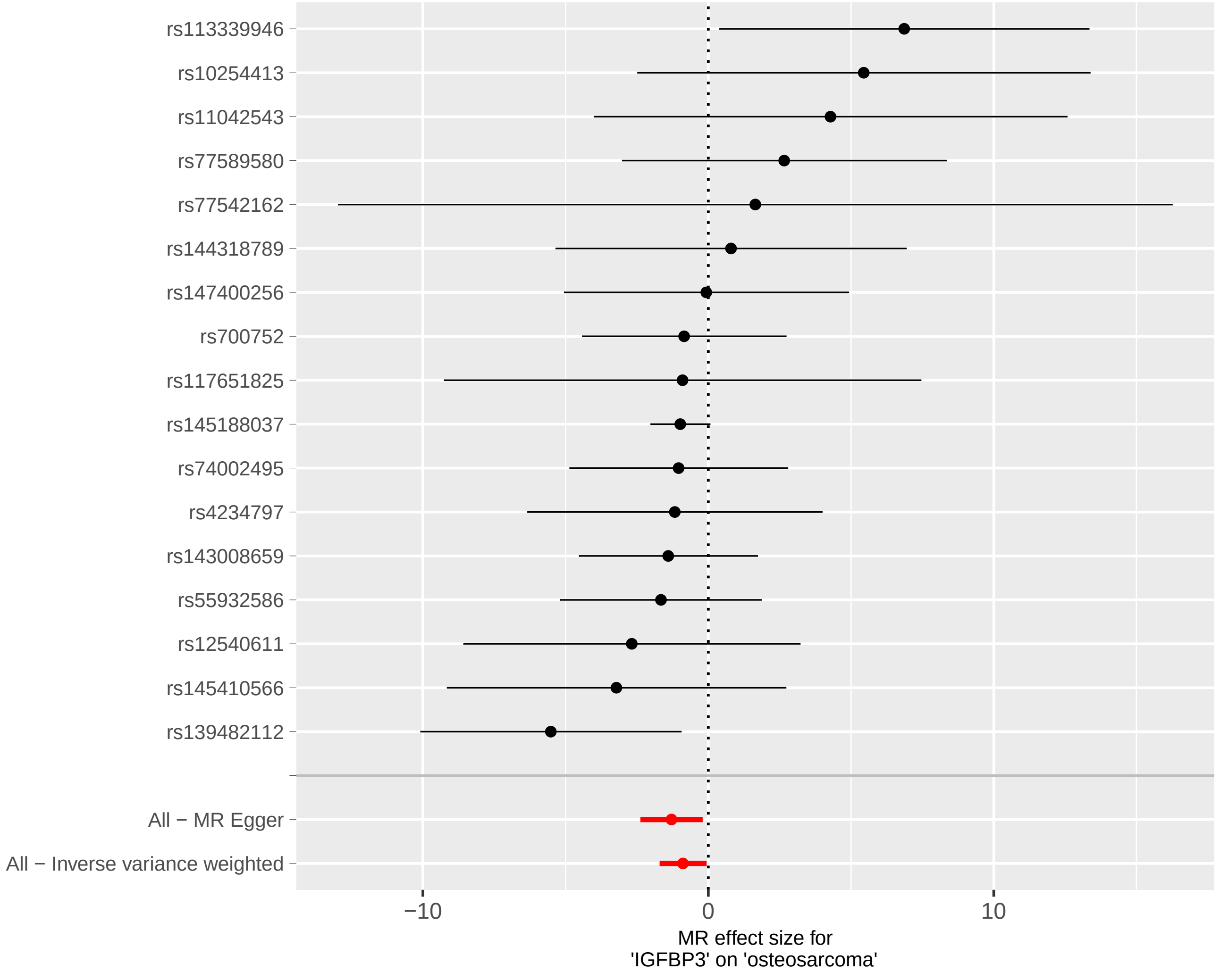
rs113339946
rs10254413
rs11042543
rs77589580
rs77542162
rs144318789
rs147400256
rs700752
rs117651825
rs145188037
rs74002495
rs4234797
rs143008659
rs55932586
rs12540611
rs145410566
rs139482112
All − MR Egger
All − Inverse variance weighted
−10
0
10
MR effect size for
'IGFBP3' on 'osteosarcoma'

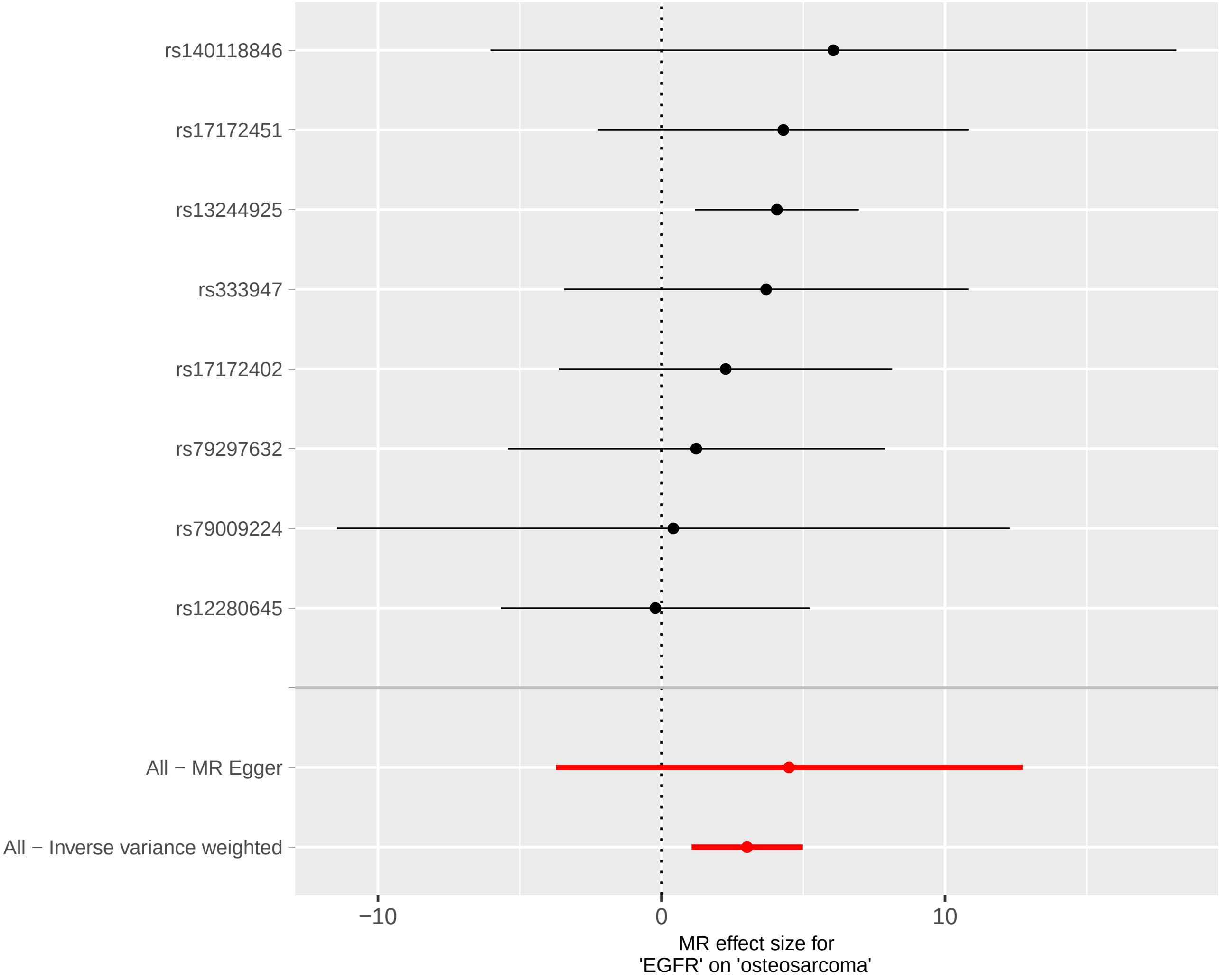
rs140118846
rs17172451
rs13244925
rs333947
rs17172402
rs79297632
rs79009224
rs12280645
All − MR Egger
All − Inverse variance weighted
−10
0
10
MR effect size for
'EGFR' on 'osteosarcoma'

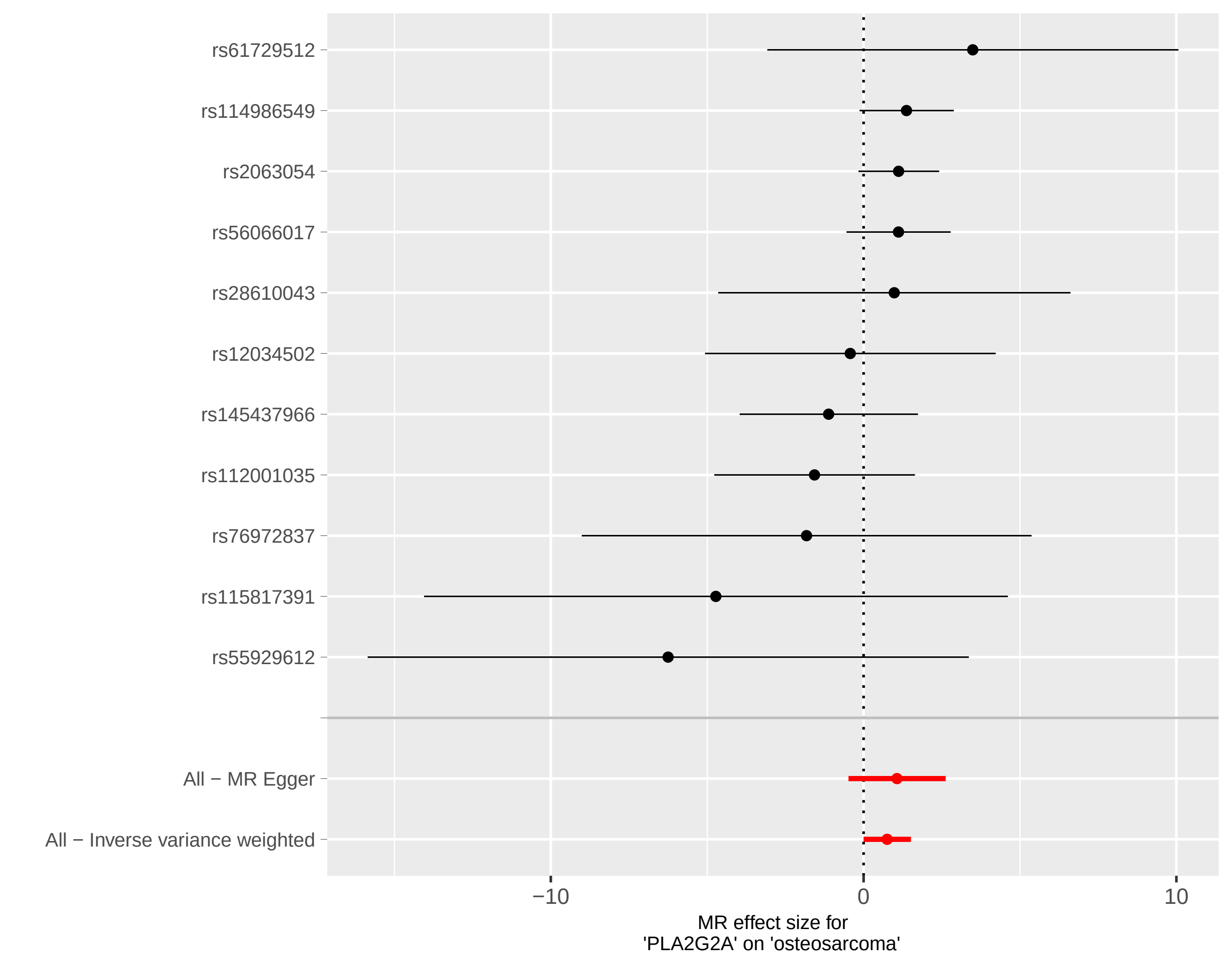
rs61729512
rs114986549
rs2063054
rs56066017
rs28610043
rs12034502
rs145437966
rs112001035
rs76972837
rs115817391
rs55929612
All − MR Egger
All − Inverse variance weighted
−10
0
10
MR effect size for
'PLA2G2A' on 'osteosarcoma'

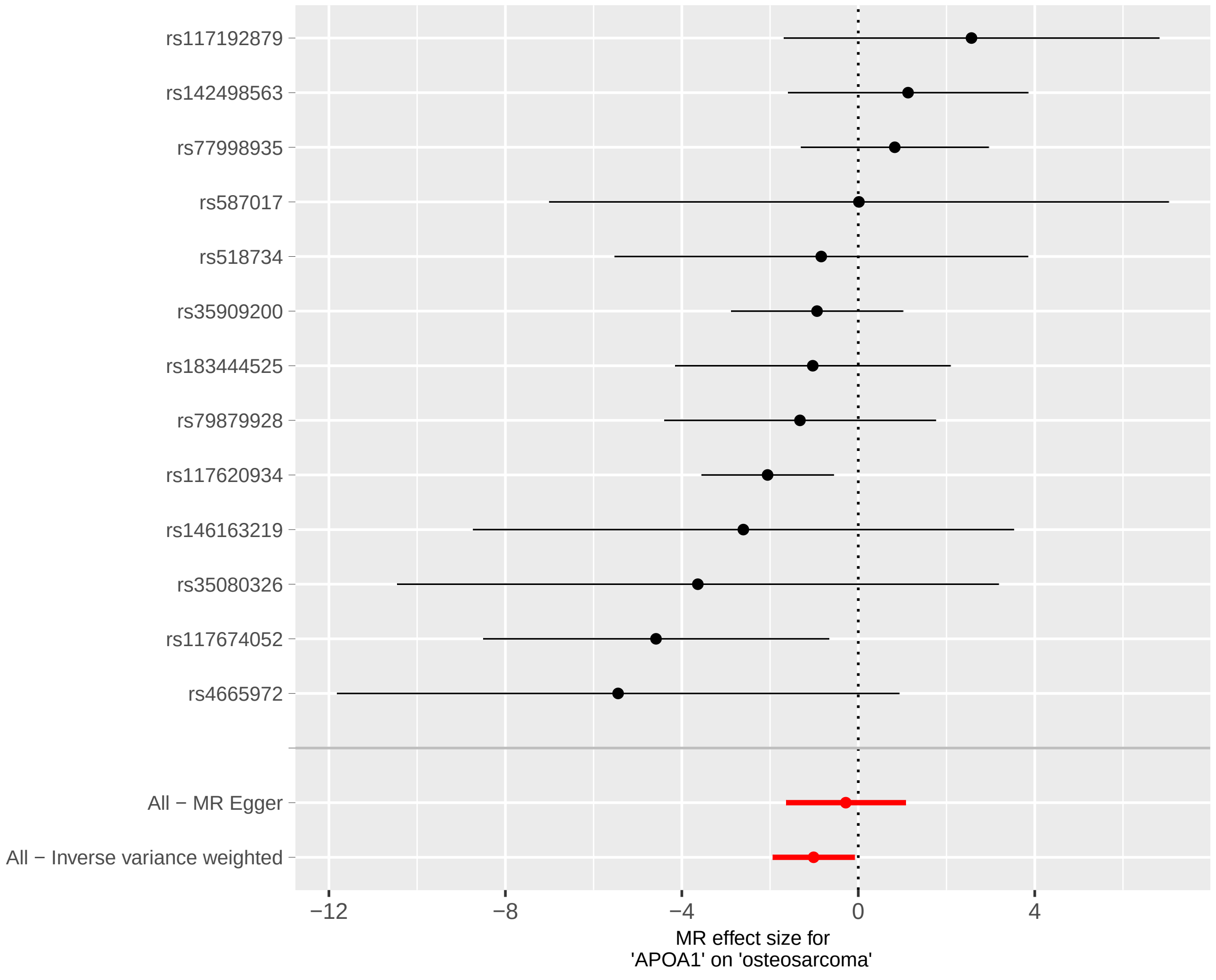
rs117192879
rs142498563
rs77998935
rs587017
rs518734
rs35909200
rs183444525
rs79879928
rs117620934
rs146163219
rs35080326
rs117674052
rs4665972
All − MR Egger
All − Inverse variance weighted
−12
−8
−4
0
4
MR effect size for
'APOA1' on 'osteosarcoma'

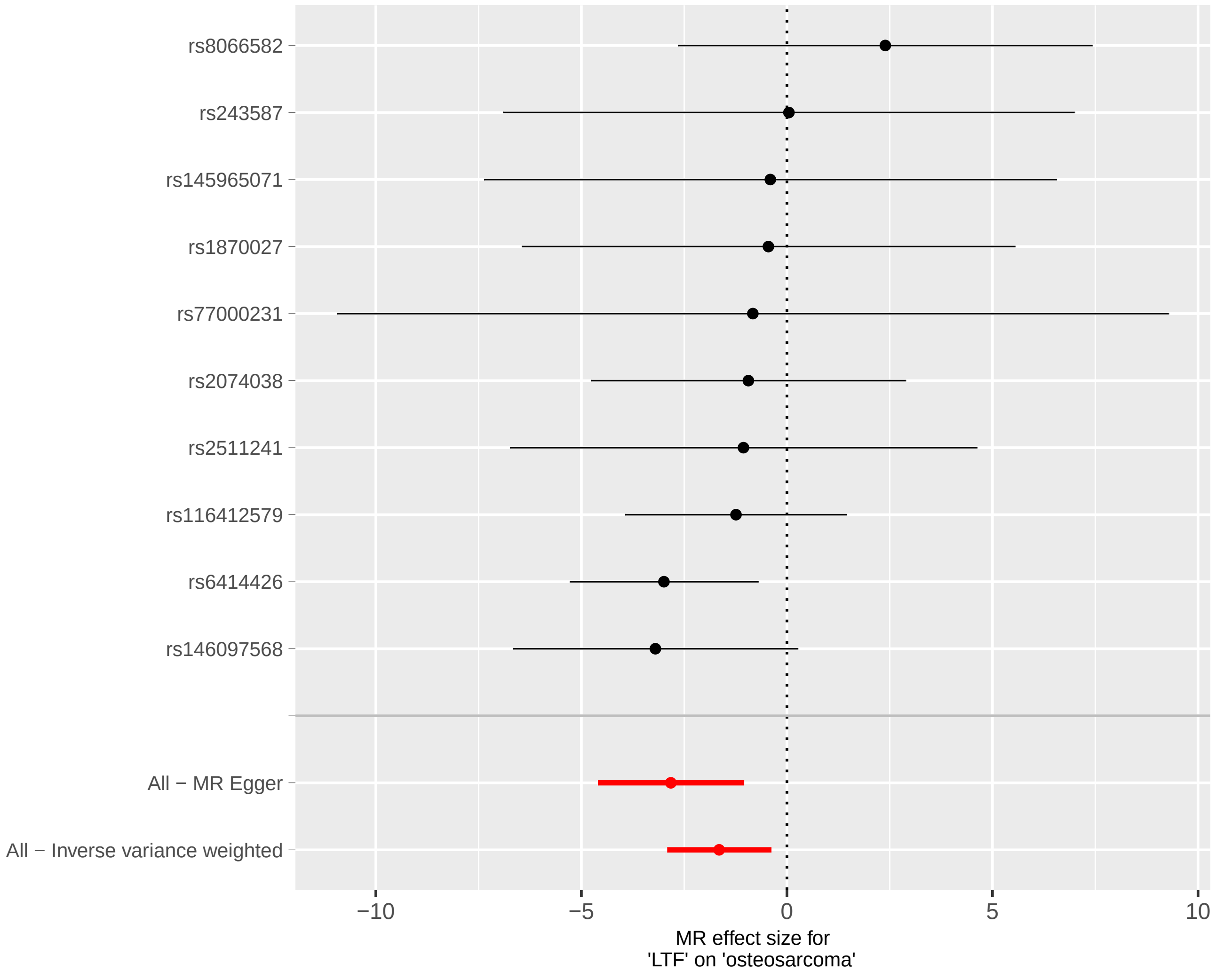
rs8066582
rs243587
rs145965071
rs1870027
rs77000231
rs2074038
rs2511241
rs116412579
rs6414426
rs146097568
All − MR Egger
All − Inverse variance weighted
−10
−5
0
5
10
MR effect size for
'LTF' on 'osteosarcoma'

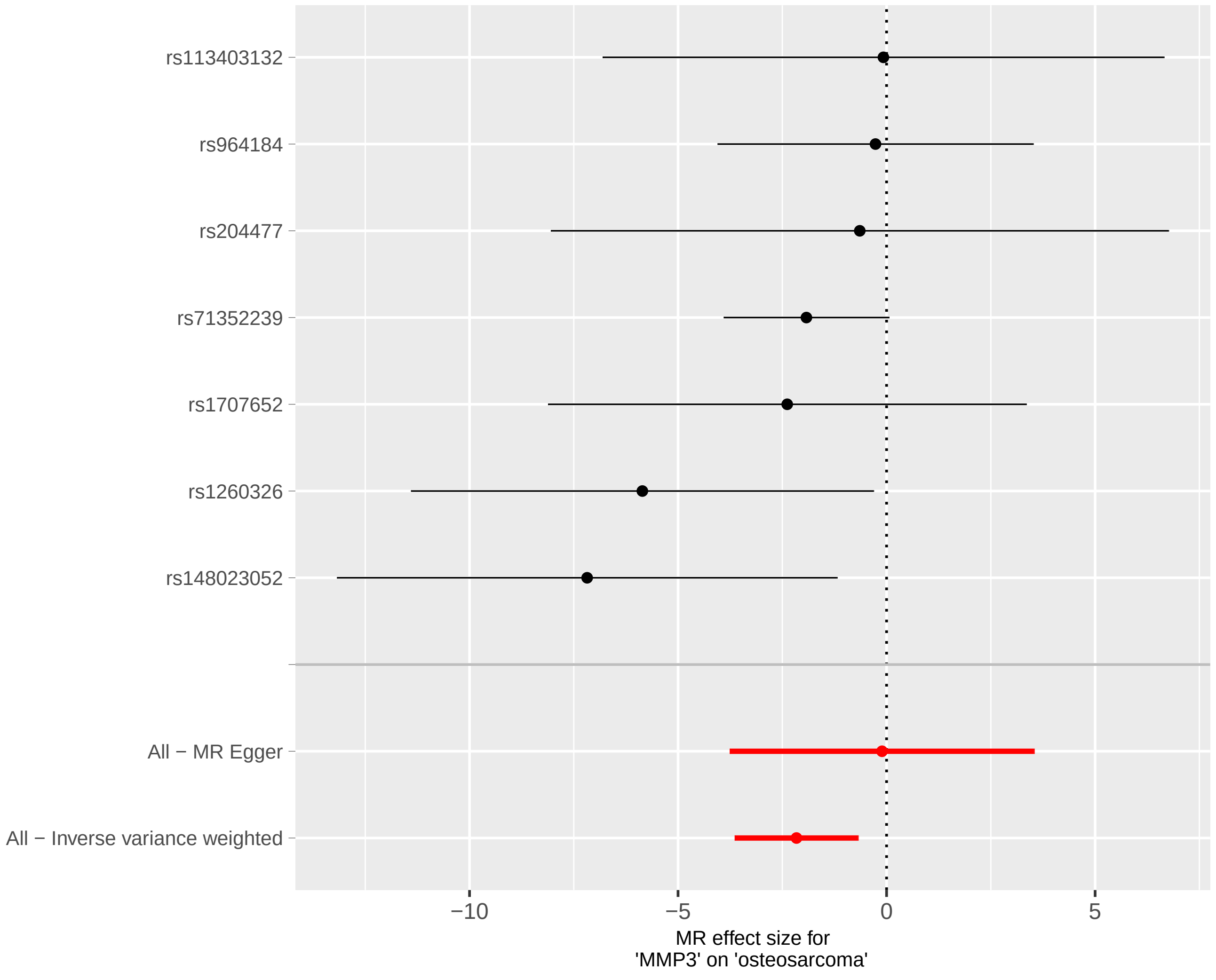
rs113403132
rs964184
rs204477
rs71352239
rs1707652
rs1260326
rs148023052
All − MR Egger
All − Inverse variance weighted
−10
−5
0
5
MR effect size for
'MMP3' on 'osteosarcoma'

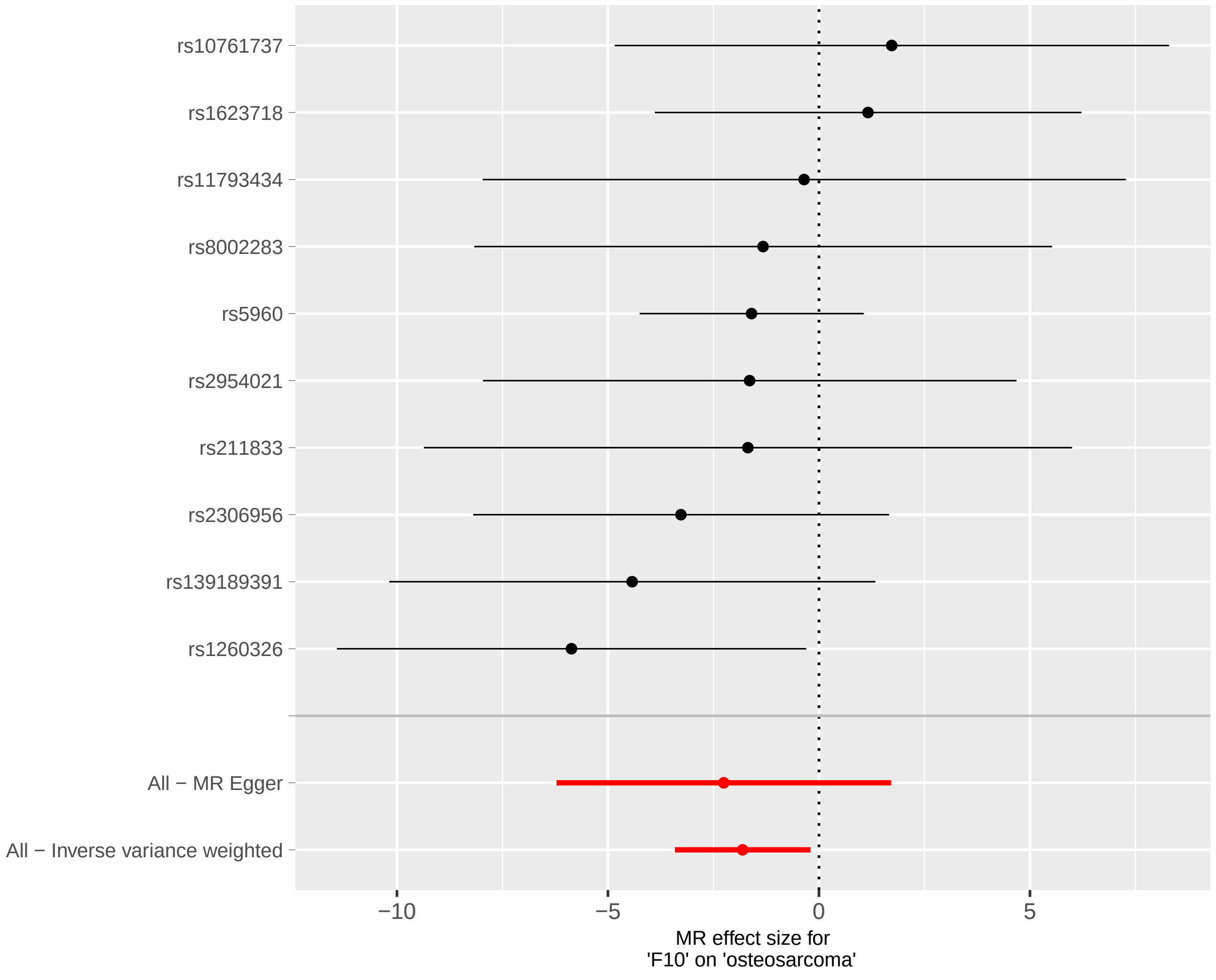
rs10761737
rs1623718
rs11793434
rs8002283
rs5960
rs2954021
rs211833
rs2306956
rs139189391
rs1260326
All − MR Egger
All − Inverse variance weighted
−10
−5
0
5
MR effect size for
'F10' on 'osteosarcoma'

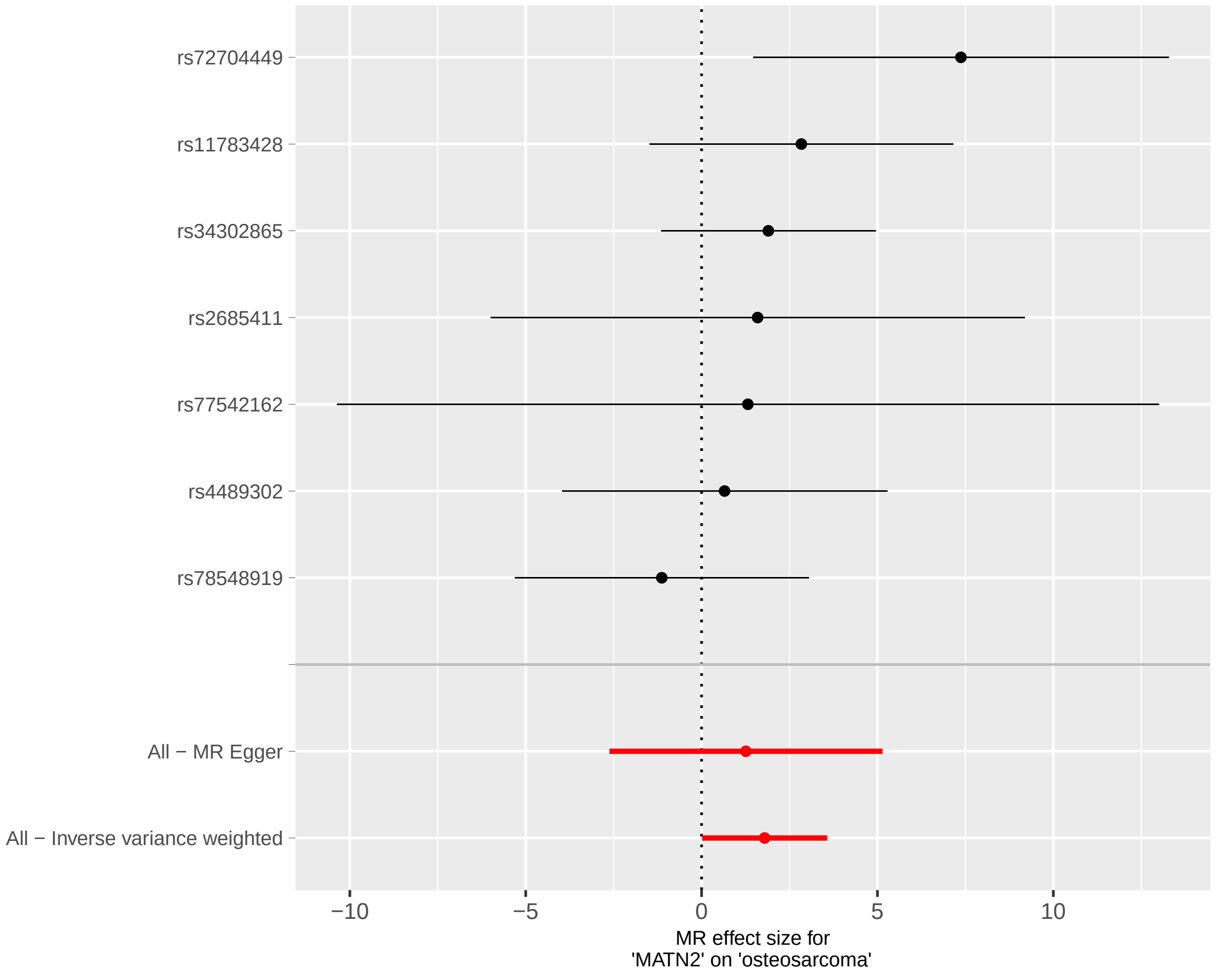

rs72704449
rs11783428
rs34302865
rs2685411
rs77542162
rs4489302
rs78548919
All − MR Egger
All − Inverse variance weighted
−10
−5
0
5
10
MR effect size for
'MATN2' on 'osteosarcoma'

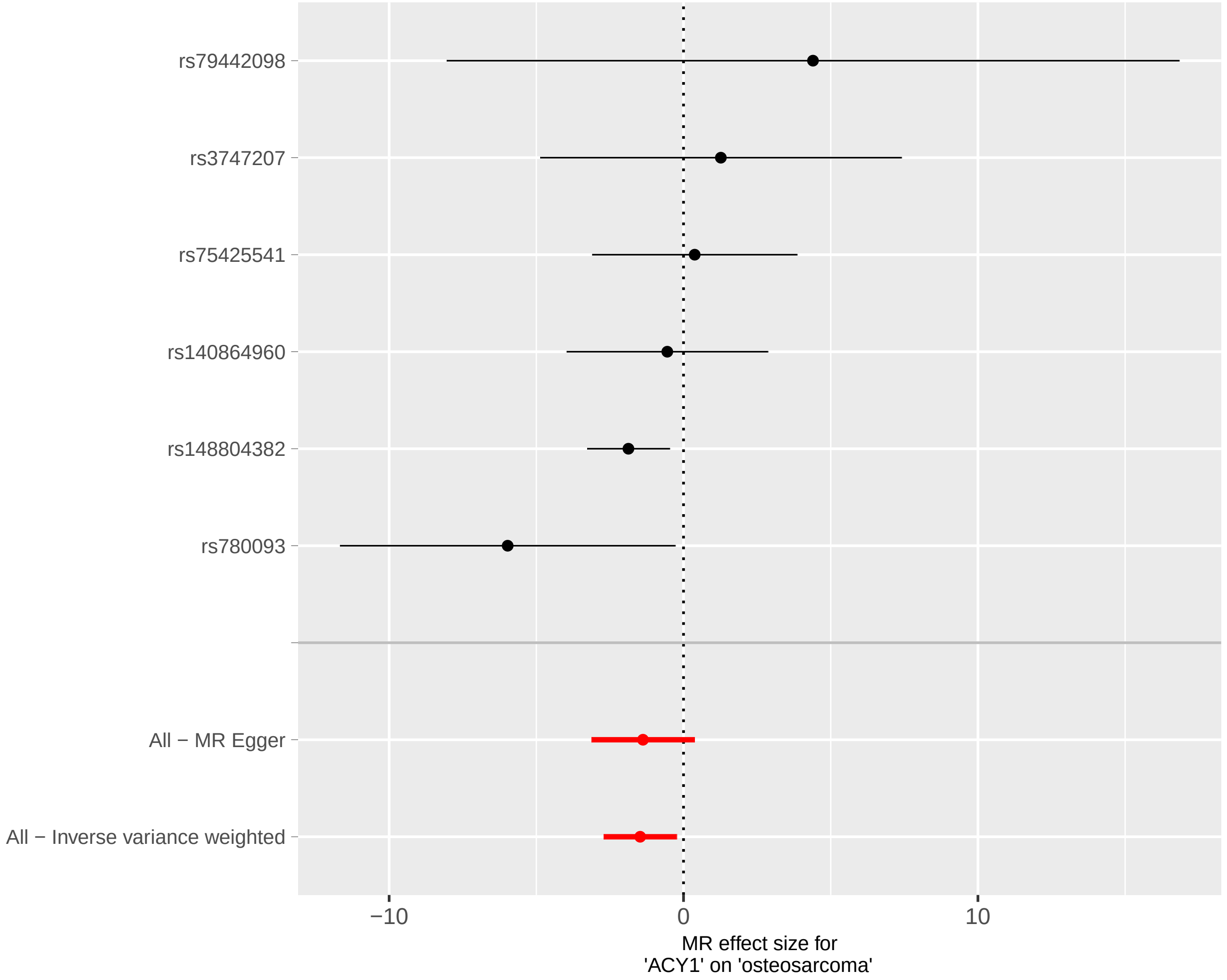
rs79442098
rs3747207
rs75425541
rs140864960
rs148804382
rs780093
All − MR Egger
All − Inverse variance weighted
−10
0
10
MR effect size for
'ACY1' on 'osteosarcoma'

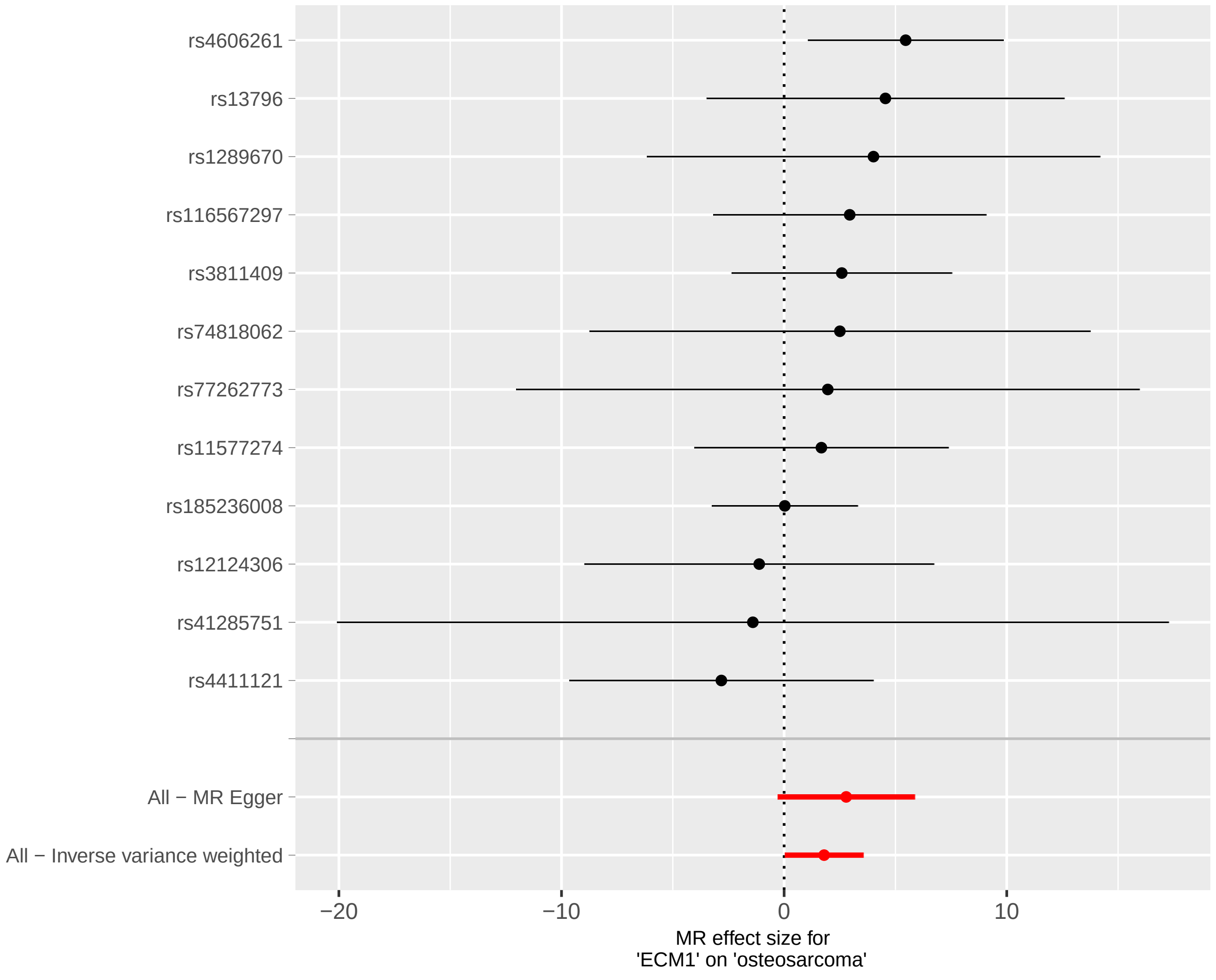

rs4606261
rs13796
rs1289670
rs116567297
rs3811409
rs74818062
rs77262773
rs11577274
rs185236008
rs12124306
rs41285751
rs4411121
All − MR Egger
All − Inverse variance weighted
−20
−10
0
10
MR effect size for
'ECM1' on 'osteosarcoma'

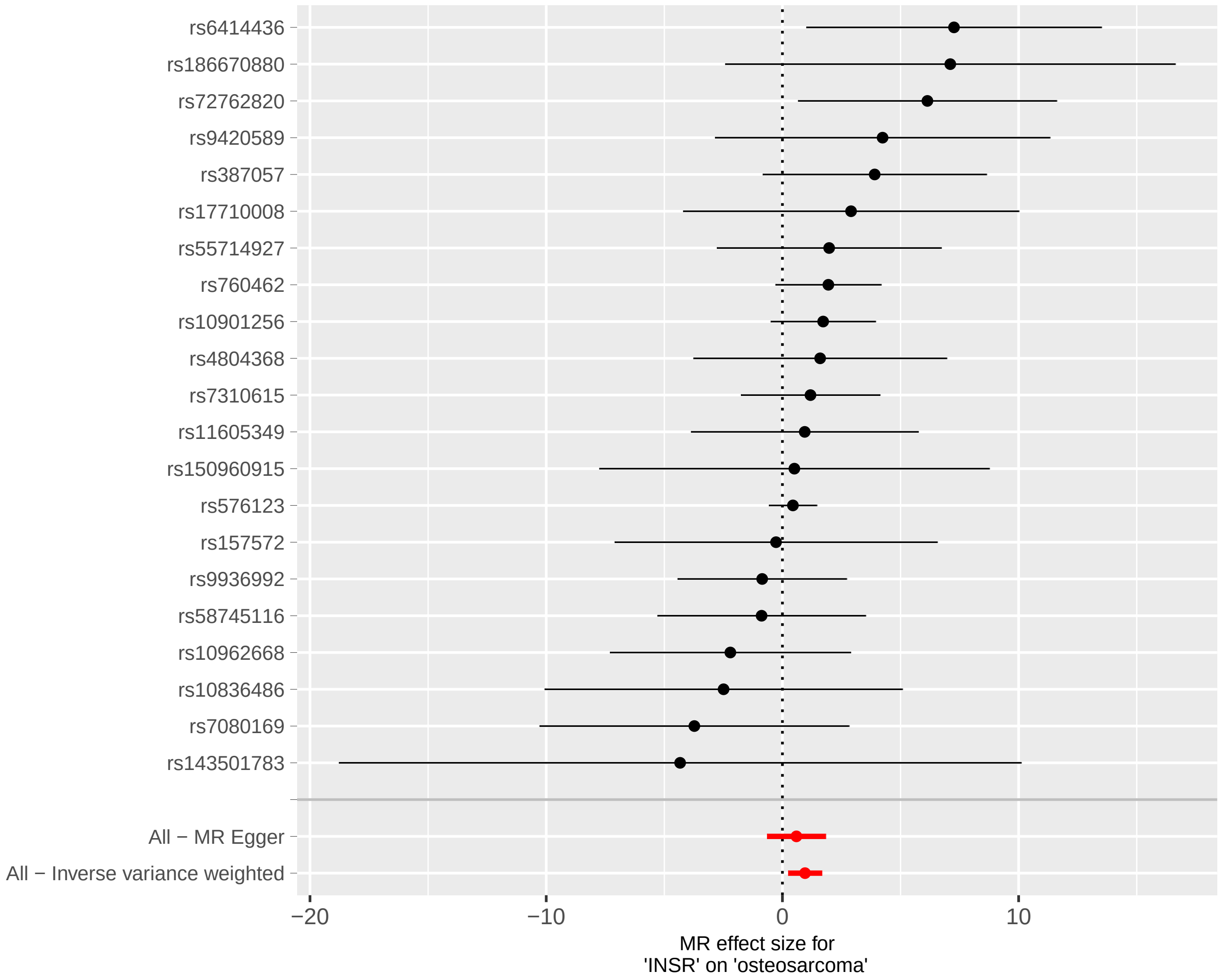

rs6414436
rs186670880
rs72762820
rs9420589
rs387057
rs17710008
rs55714927
rs760462
rs10901256
rs4804368
rs7310615
rs11605349
rs150960915
rs576123
rs157572
rs9936992
rs58745116
rs10962668
rs10836486
rs7080169
rs143501783
All − MR Egger
All − Inverse variance weighted
−20
−10
0
10
MR effect size for
'INSR' on 'osteosarcoma'

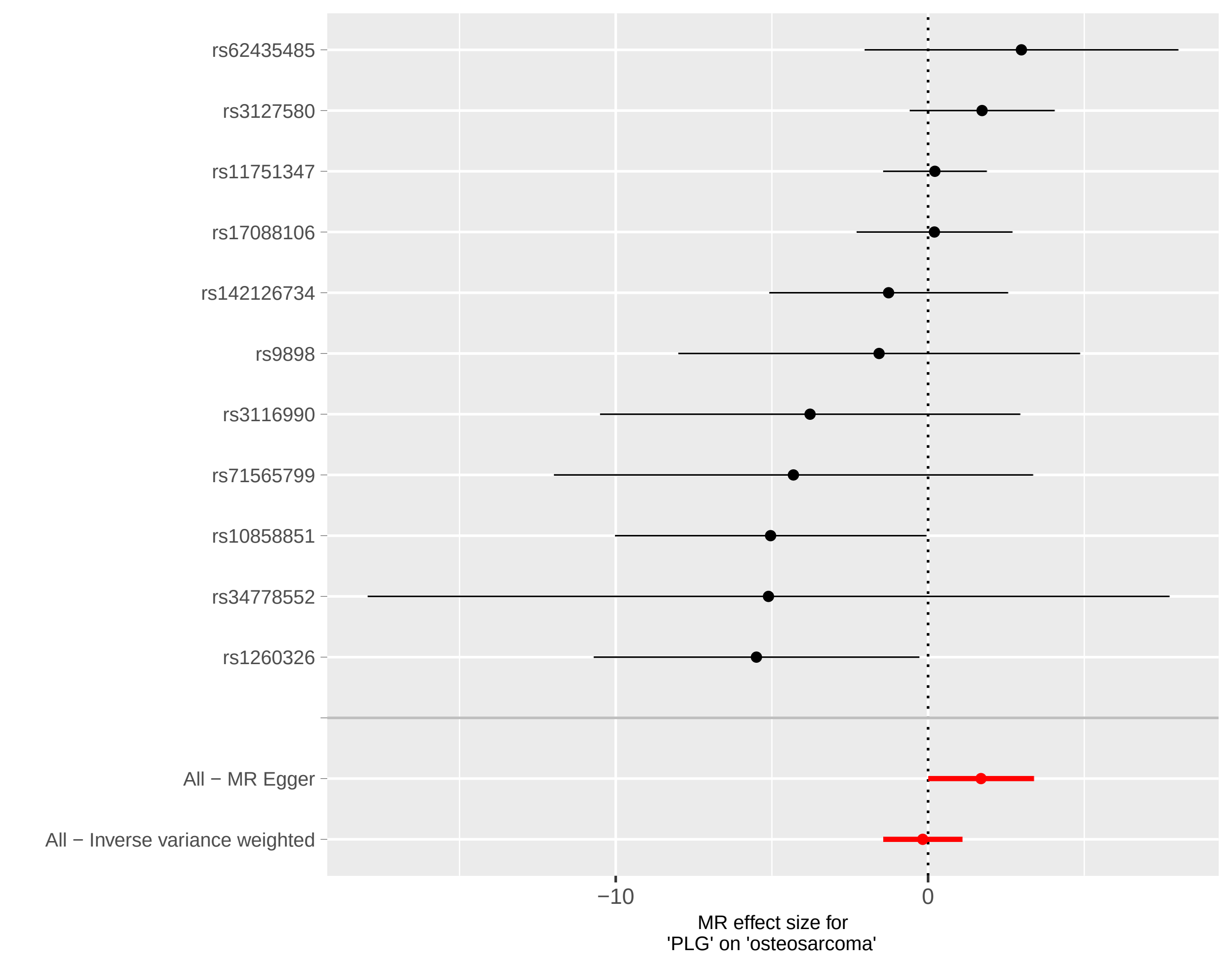
rs62435485
rs3127580
rs11751347
rs17088106
rs142126734
rs9898
rs3116990
rs71565799
rs10858851
rs34778552
rs1260326
All − MR Egger
All − Inverse variance weighted
−10
0
MR effect size for
'PLG' on 'osteosarcoma'

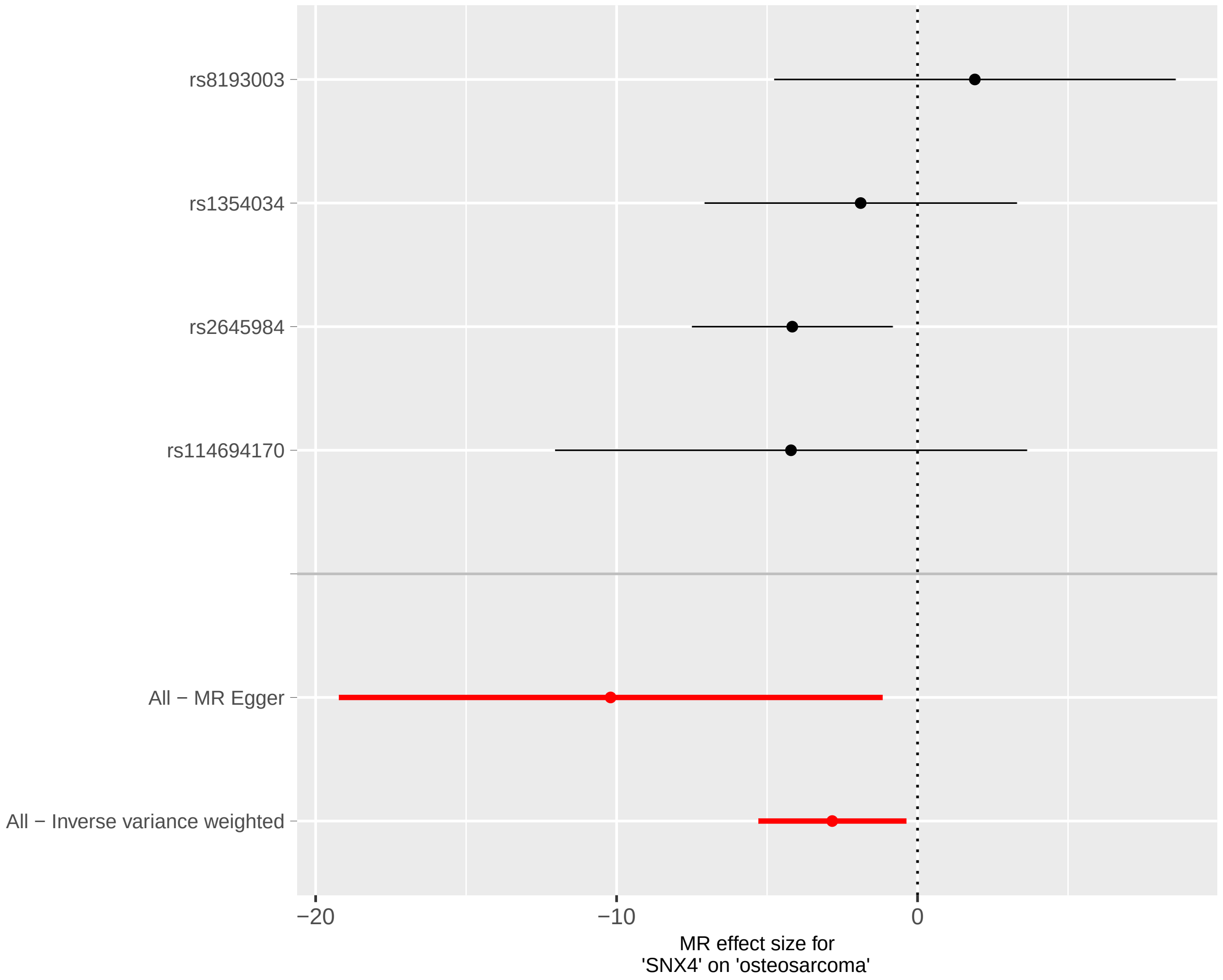
rs8193003
rs1354034
rs2645984
rs114694170
All − MR Egger
All − Inverse variance weighted
−20
−10
0
MR effect size for
'SNX4' on 'osteosarcoma'

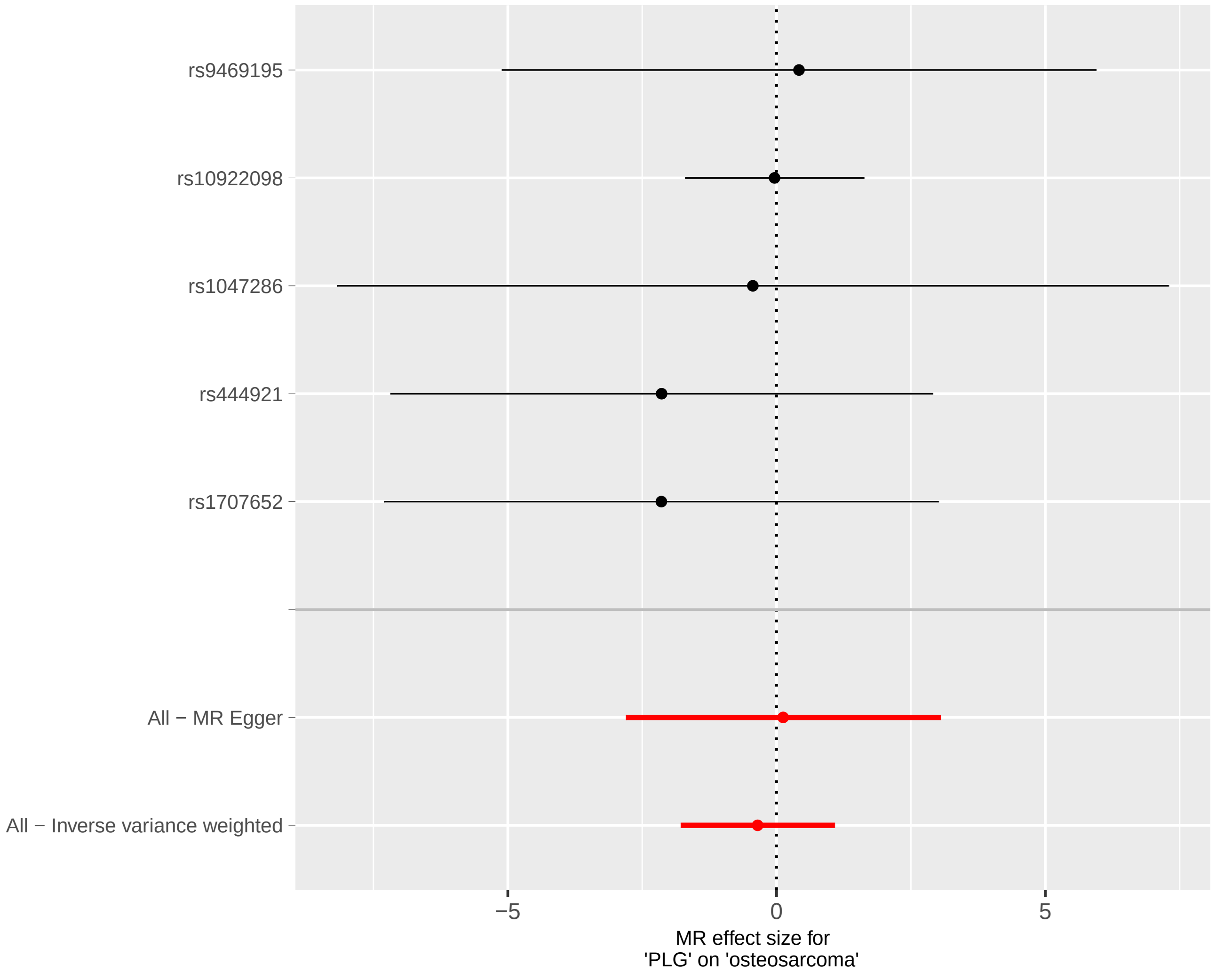
rs9469195
rs10922098
rs1047286
rs444921
rs1707652
All − MR Egger
All − Inverse variance weighted
−5
0
5
MR effect size for
'PLG' on 'osteosarcoma'

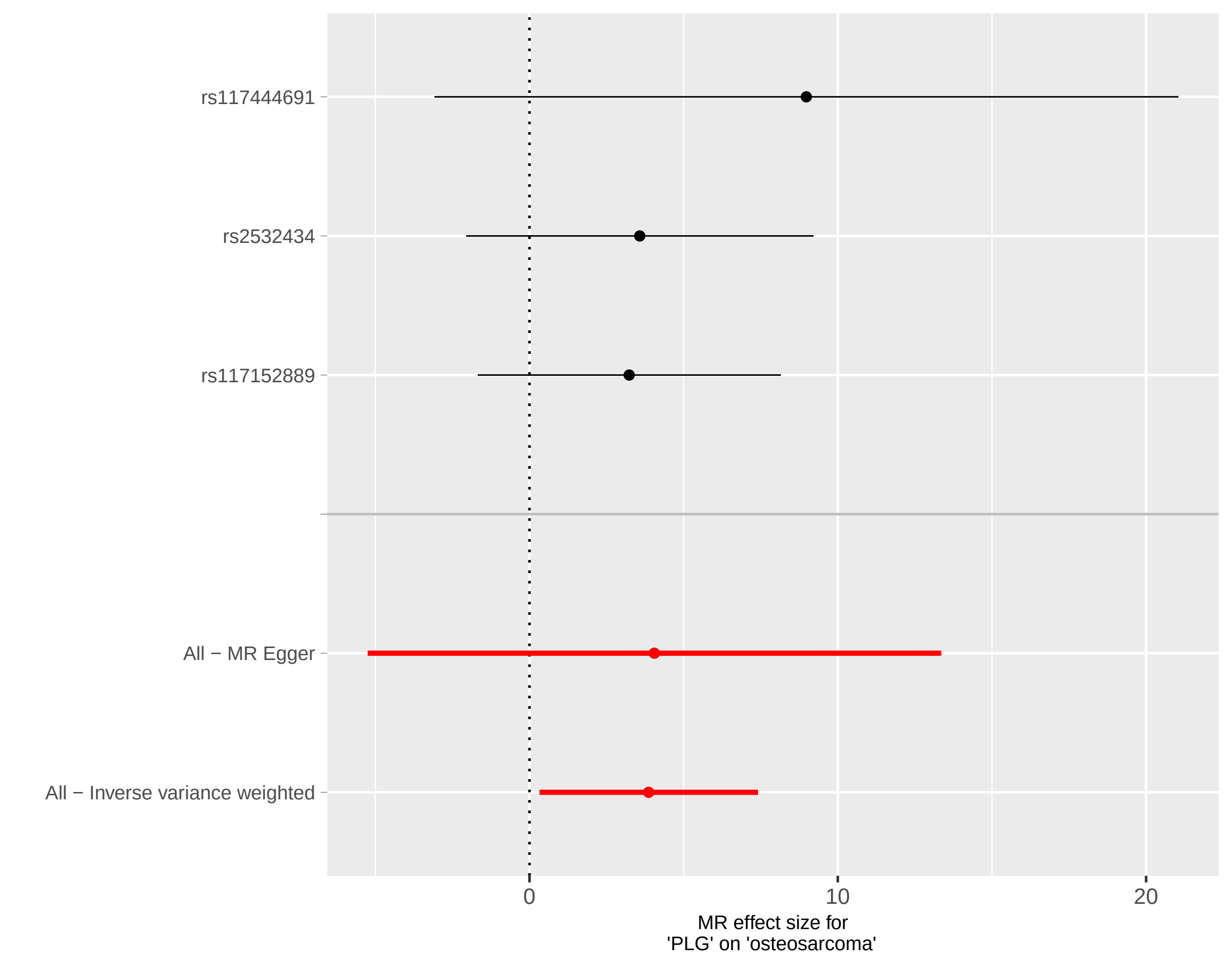
rs117444691
rs2532434
rs117152889
All − MR Egger
All − Inverse variance weighted
0
10
20
MR effect size for
'PLG' on 'osteosarcoma'

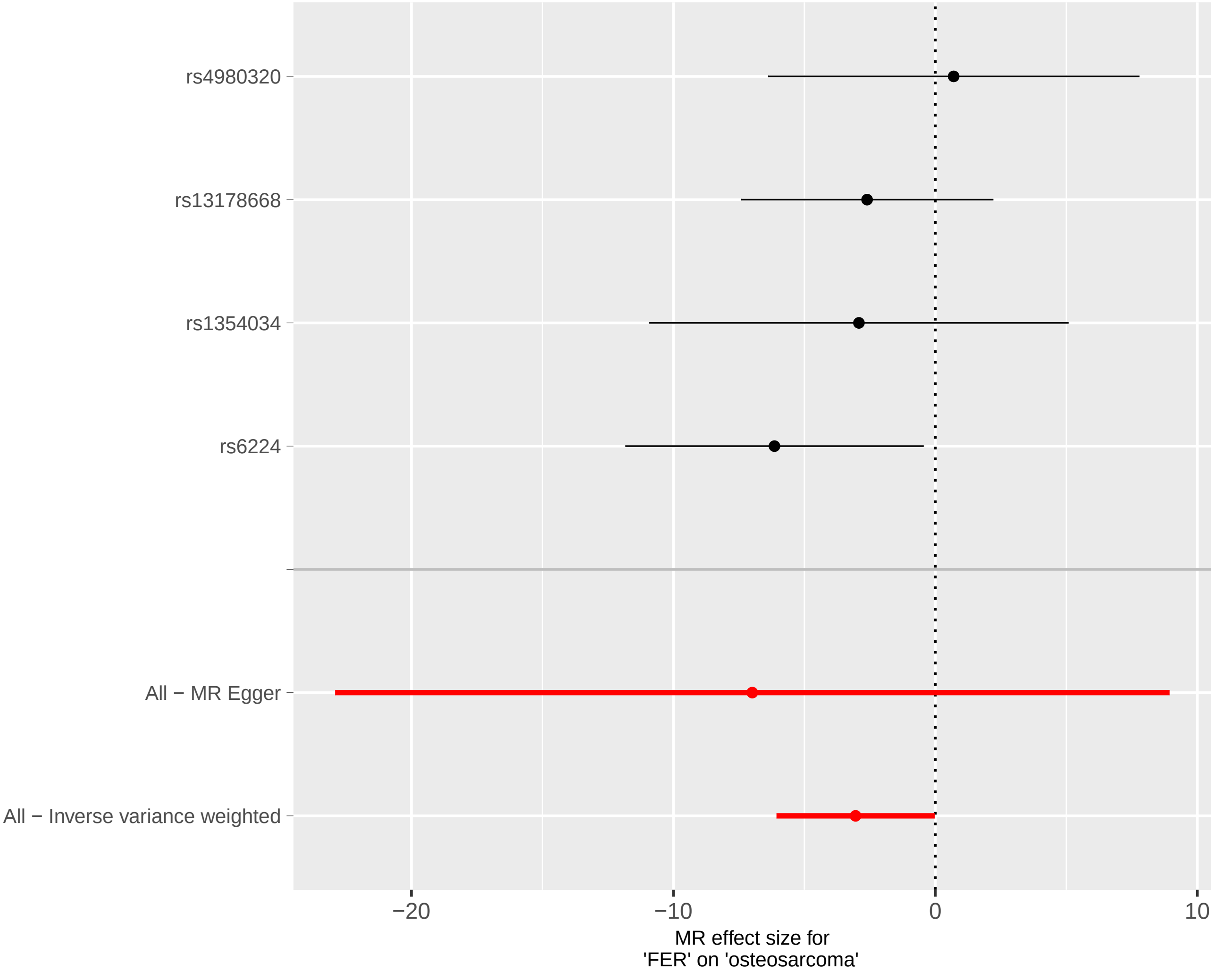
rs4980320
rs13178668
rs1354034
rs6224
All − MR Egger
All − Inverse variance weighted
−20
−10
0
10
MR effect size for
'FER' on 'osteosarcoma'

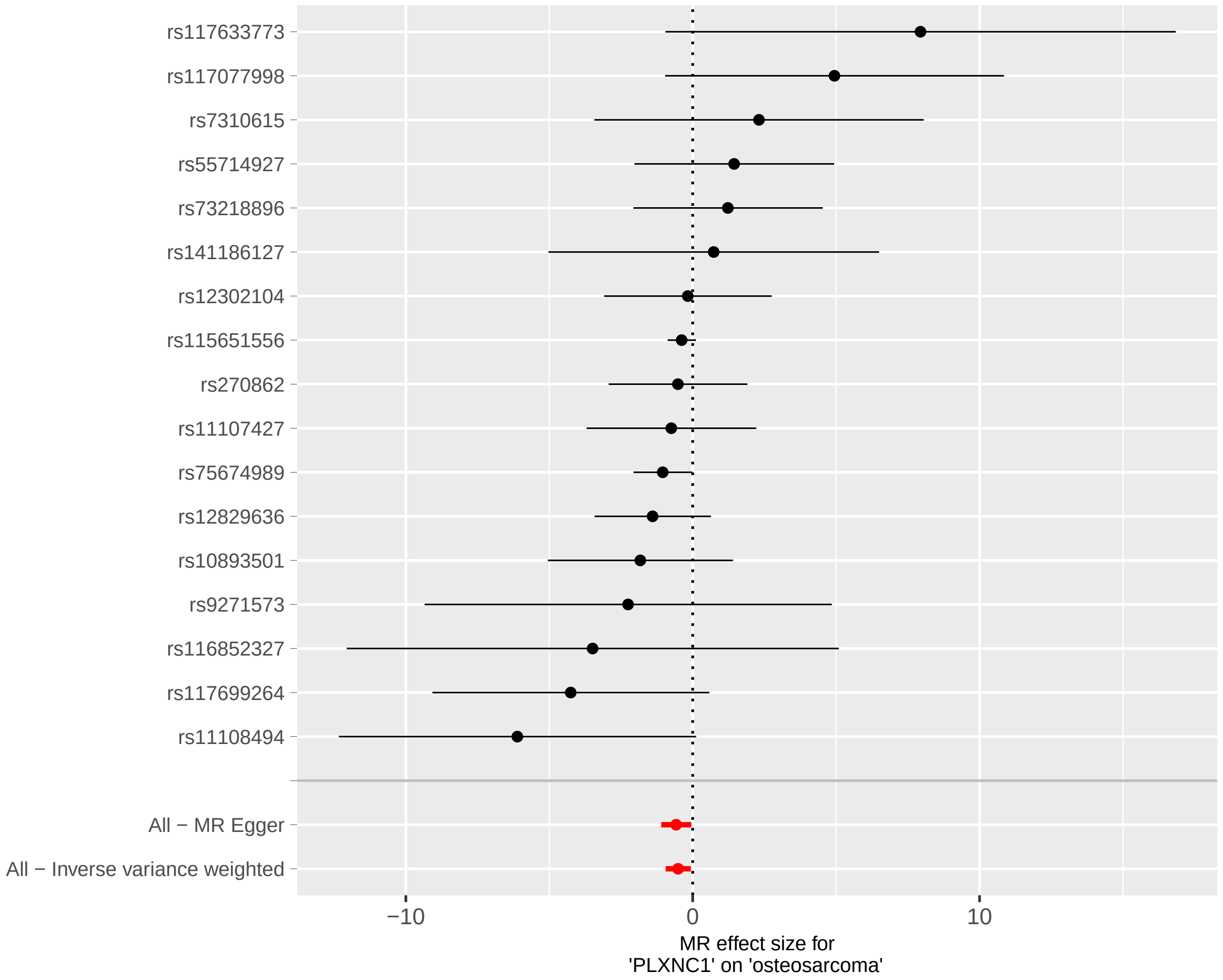
rs117633773
rs117077998
rs7310615
rs55714927
rs73218896
rs141186127
rs12302104
rs115651556
rs270862
rs11107427
rs75674989
rs12829636
rs10893501
rs9271573
rs116852327
rs117699264
rs11108494
All − MR Egger
All − Inverse variance weighted
−10
0
10
MR effect size for
'PLXNC1' on 'osteosarcoma'

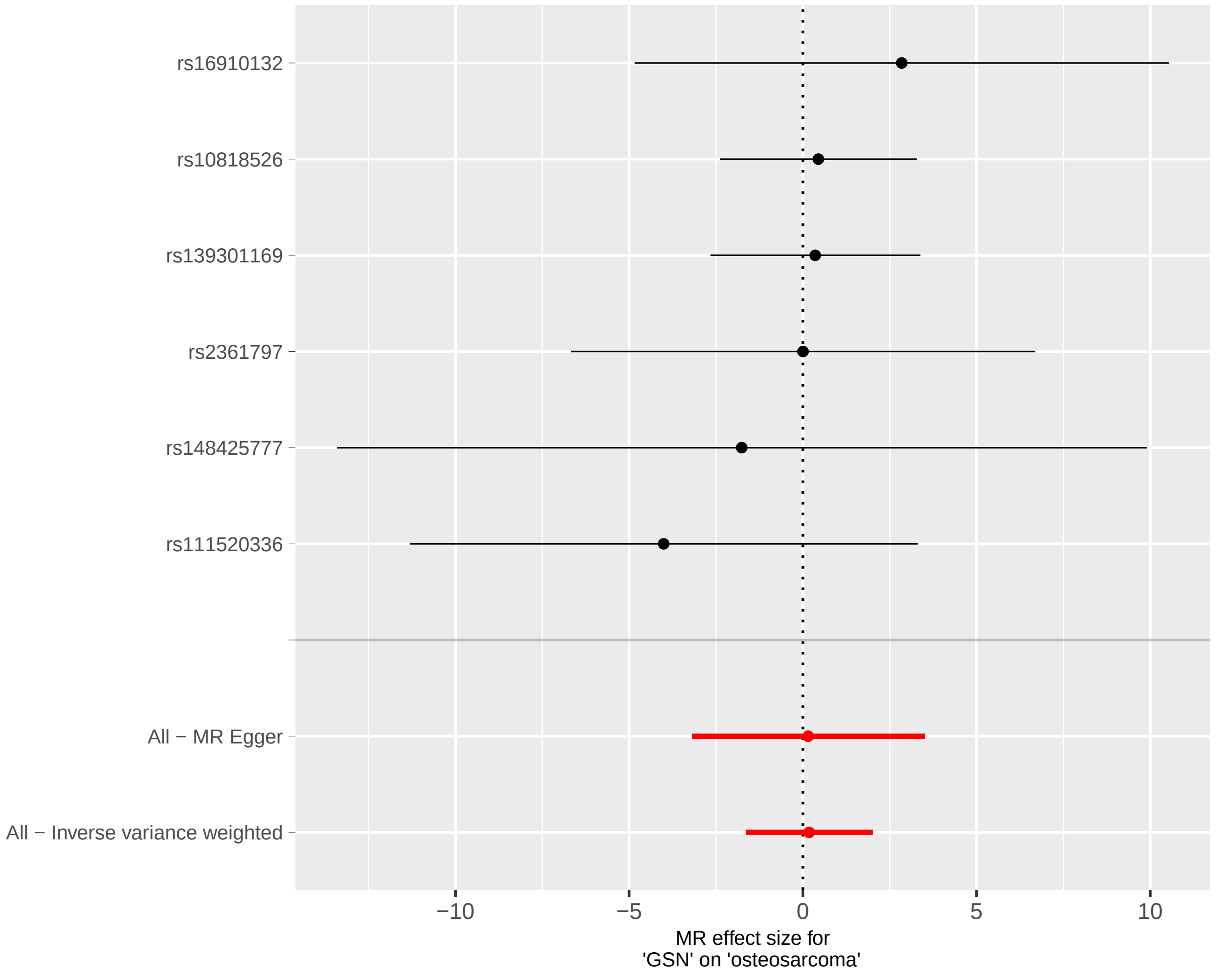
rs16910132
rs10818526
rs139301169
rs2361797
rs148425777
rs111520336
All − MR Egger
All − Inverse variance weighted
−10
−5
0
5
10
MR effect size for
'GSN' on 'osteosarcoma'

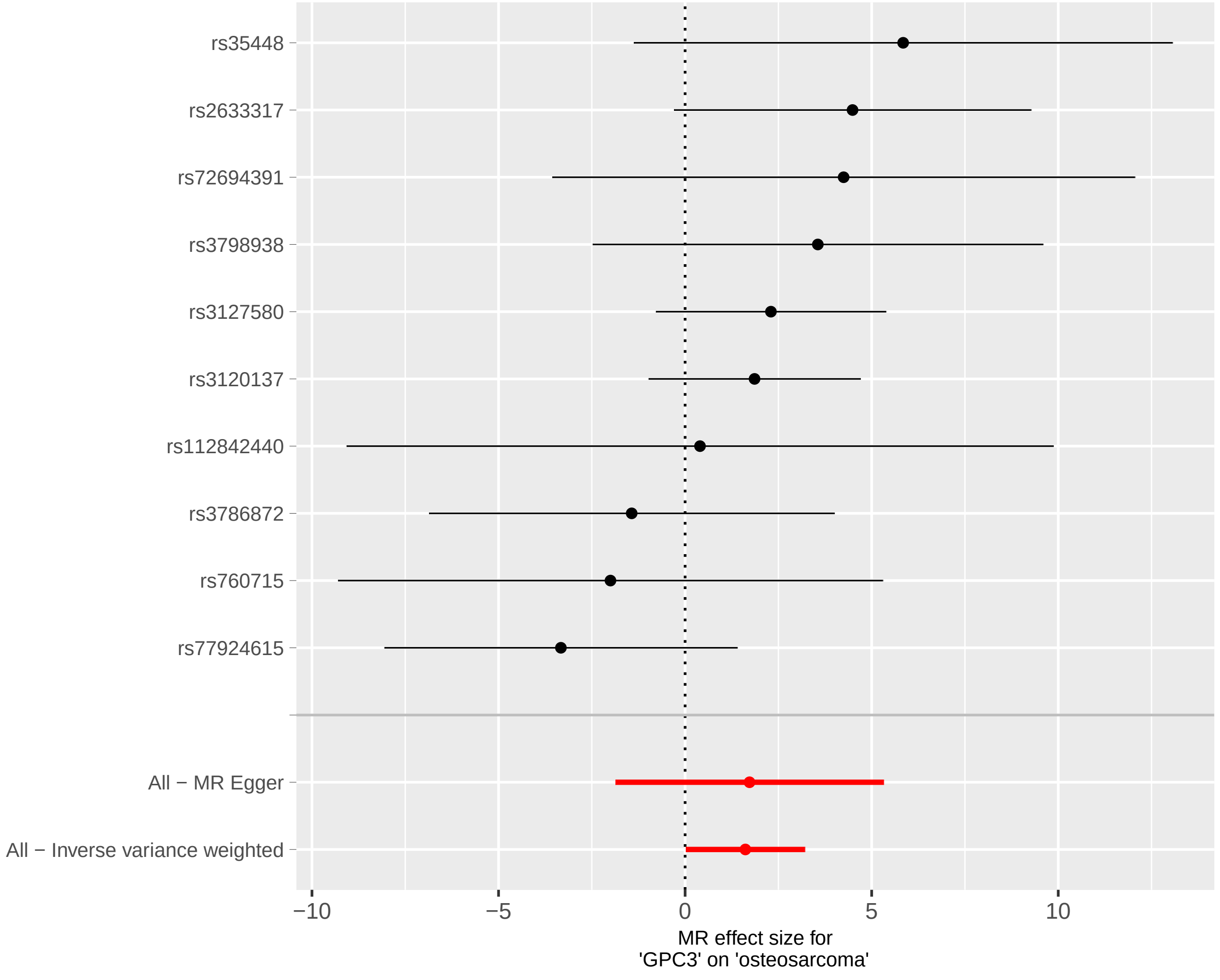

rs35448
rs2633317
rs72694391
rs3798938
rs3127580
rs3120137
rs112842440
rs3786872
rs760715
rs77924615
All − MR Egger
All − Inverse variance weighted
−10
−5
0
5
10
MR effect size for
'GPC3' on 'osteosarcoma'

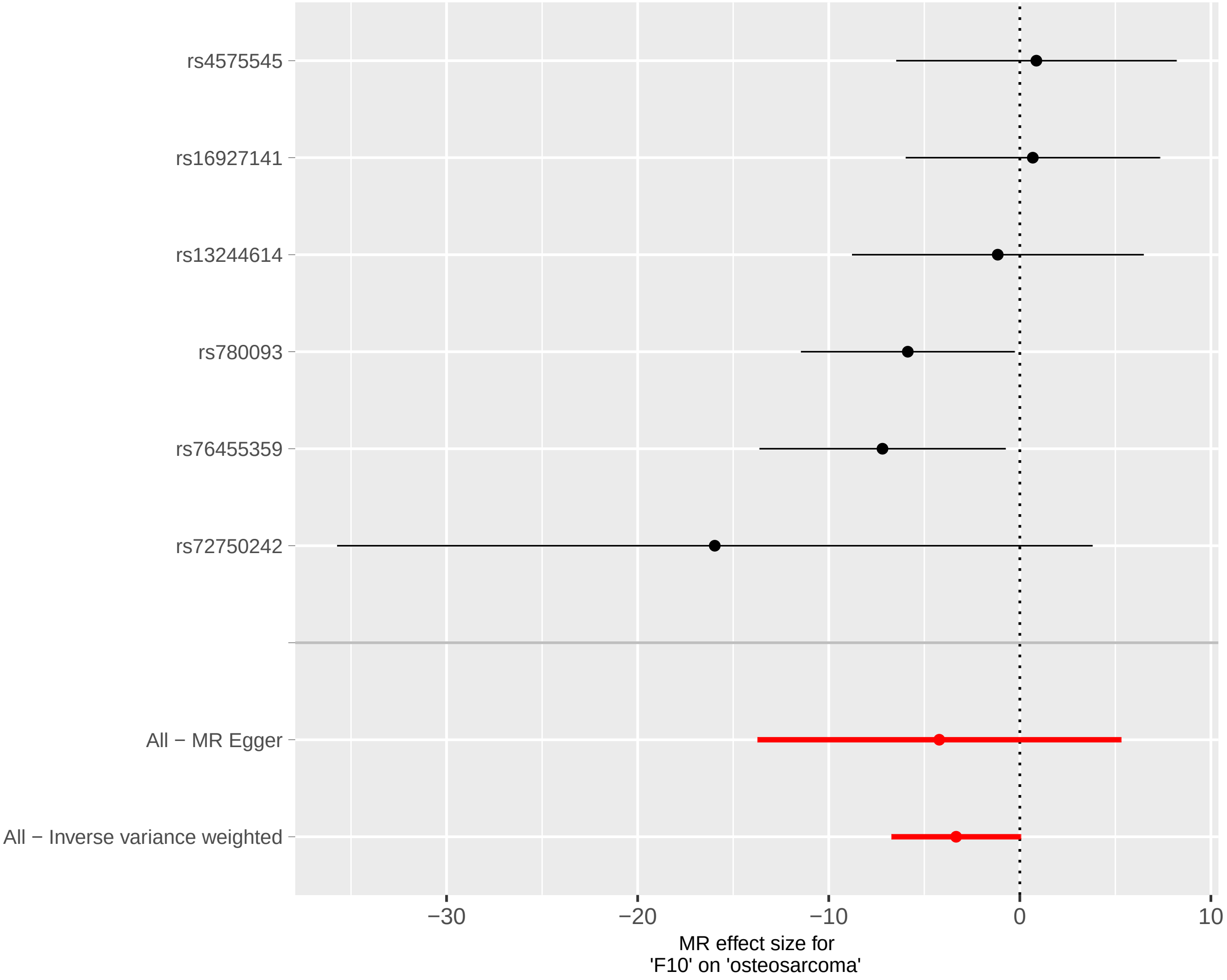
rs4575545
rs16927141
rs13244614
rs780093
rs76455359
rs72750242
All − MR Egger
All − Inverse variance weighted
−30
−20
−10
0
10
MR effect size for
'F10' on 'osteosarcoma'

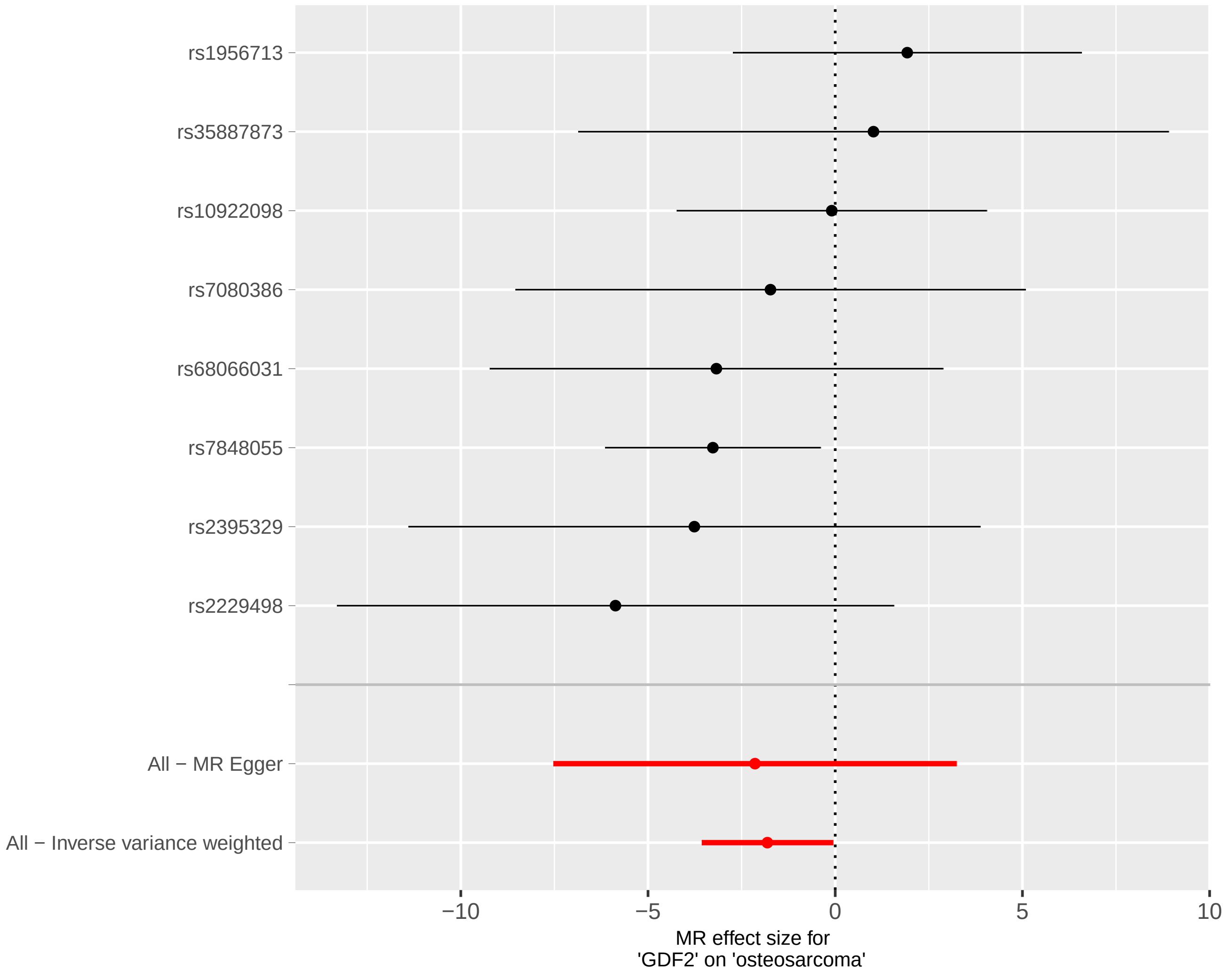

rs1956713
rs35887873
rs10922098
rs7080386
rs68066031
rs7848055
rs2395329
rs2229498
All − MR Egger
All − Inverse variance weighted
−10
−5
0
5
10
MR effect size for
'GDF2' on 'osteosarcoma'

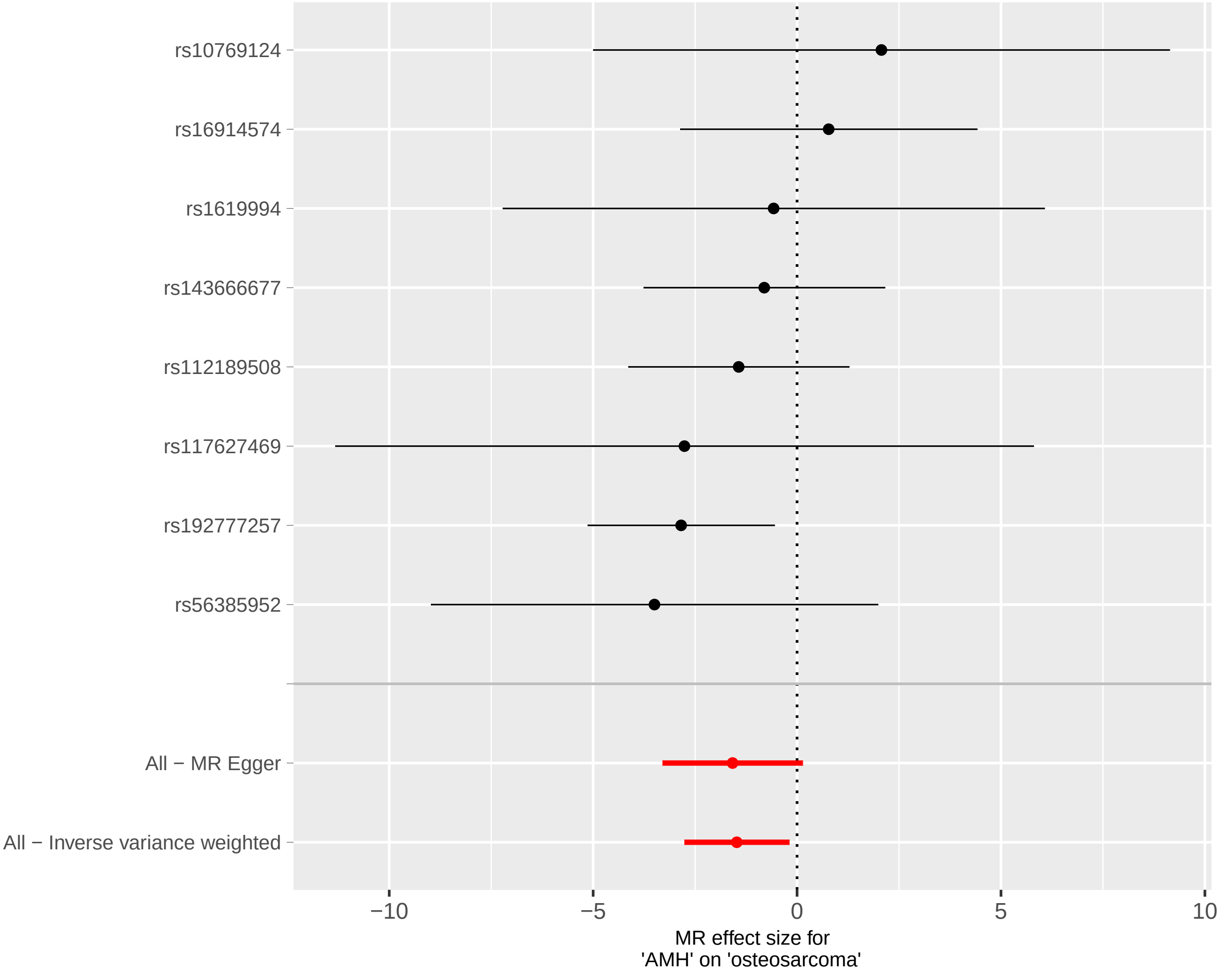

rs10769124
rs16914574
rs1619994
rs143666677
rs112189508
rs117627469
rs192777257
rs56385952
All − MR Egger
All − Inverse variance weighted
−10
−5
0
5
10
MR effect size for
'AMH' on 'osteosarcoma'

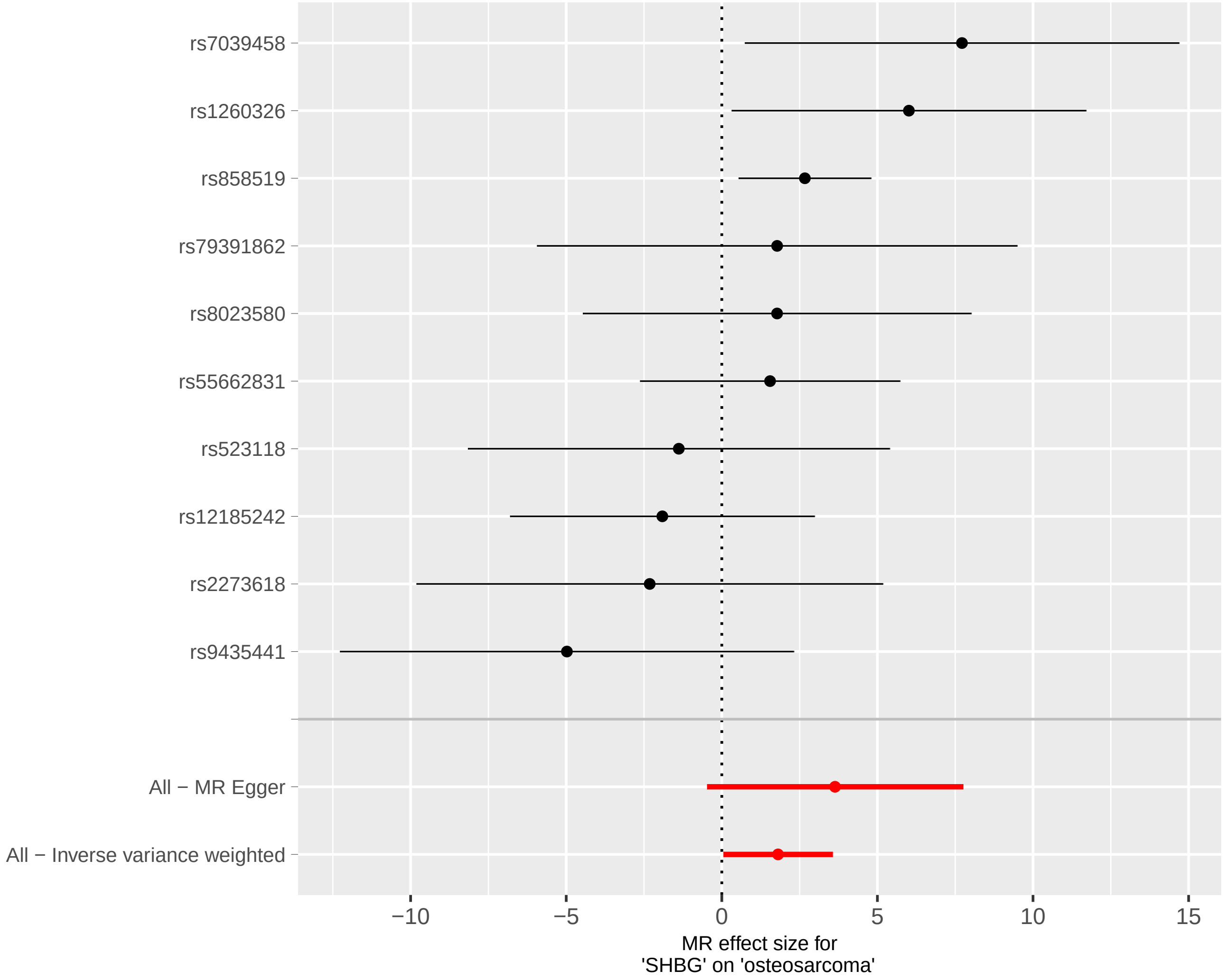

rs7039458
rs1260326
rs858519
rs79391862
rs8023580
rs55662831
rs523118
rs12185242
rs2273618
rs9435441
All − MR Egger
All − Inverse variance weighted
−10
−5
0
5
10
15
MR effect size for
'SHBG' on 'osteosarcoma'

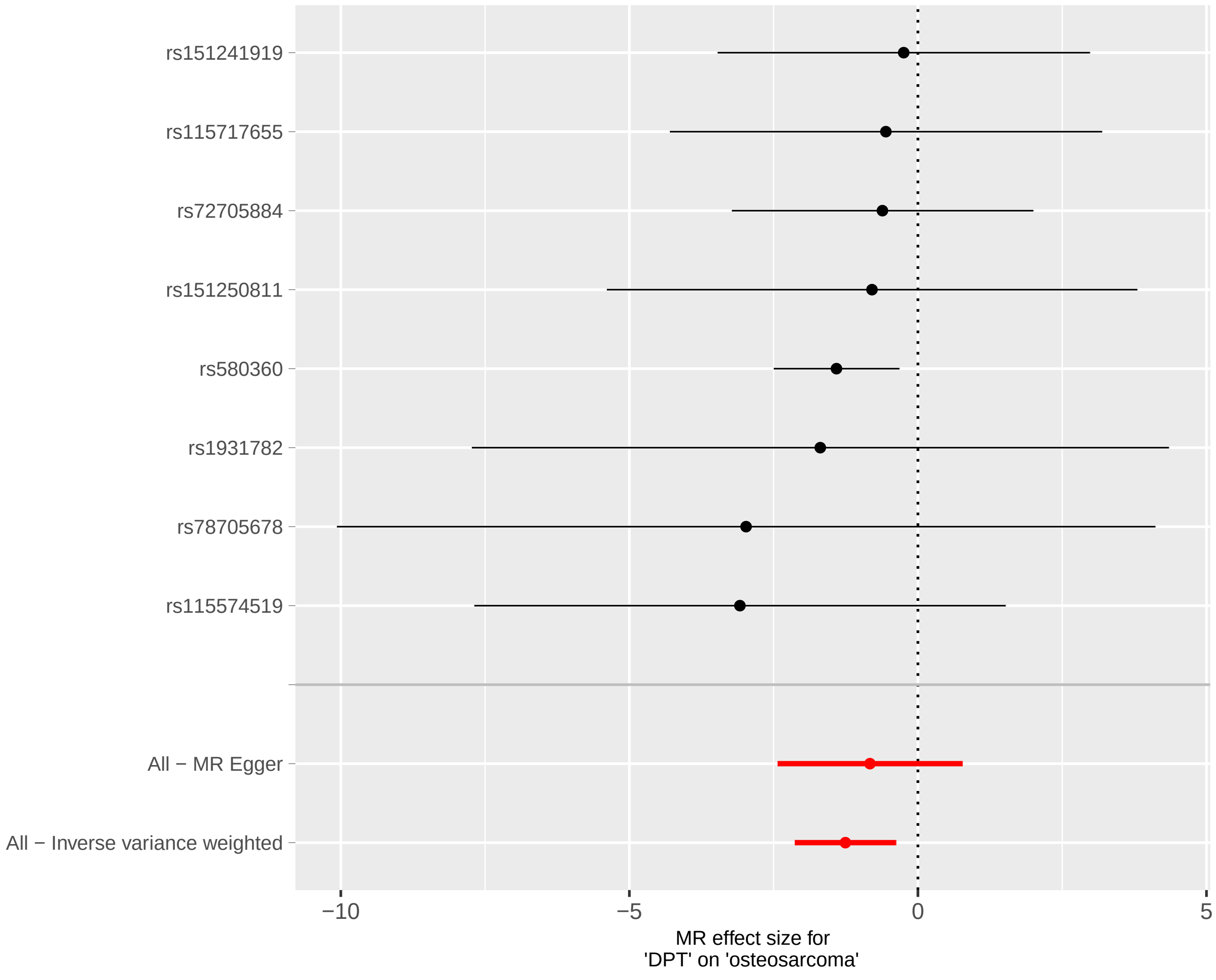

rs151241919
rs115717655
rs72705884
rs151250811
rs580360
rs1931782
rs78705678
rs115574519
All − MR Egger
All − Inverse variance weighted
−10
−5
0
5
MR effect size for
'DPT' on 'osteosarcoma'

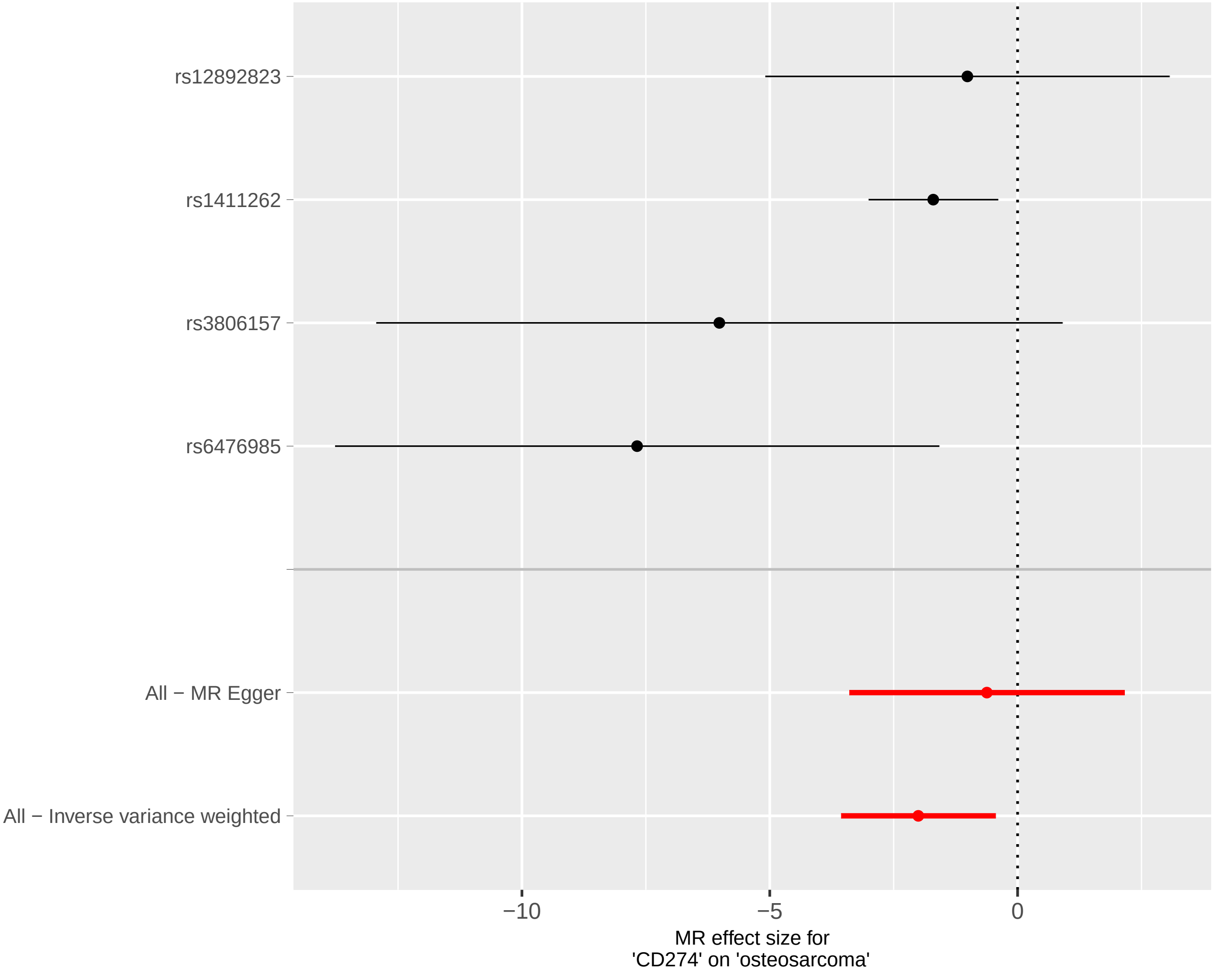
rs12892823
rs1411262
rs3806157
rs6476985
All − MR Egger
All − Inverse variance weighted
−10
−5
0
MR effect size for
'CD274' on 'osteosarcoma'

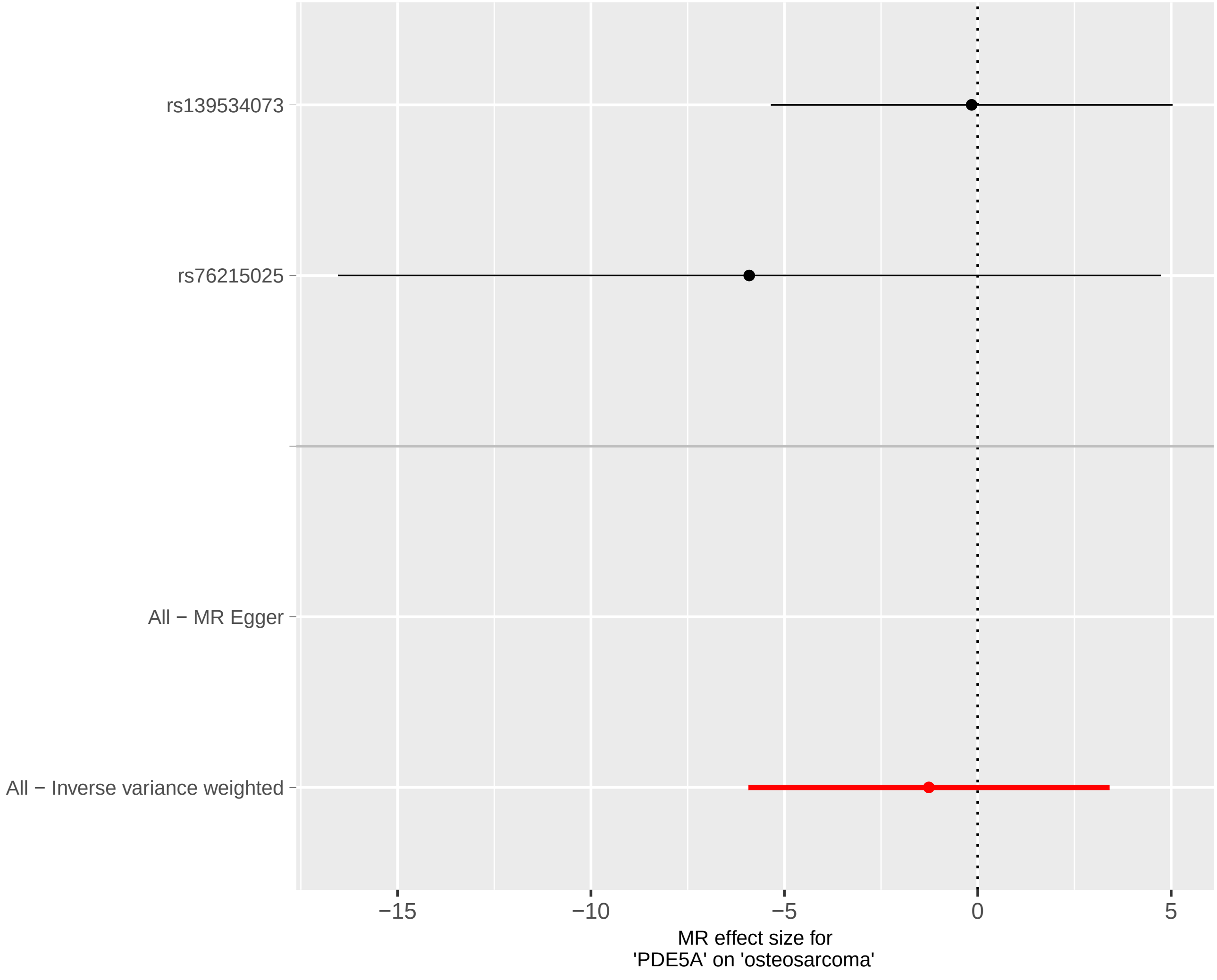

rs139534073
rs76215025
All − MR Egger
All − Inverse variance weighted
−15
−10
−5
0
5
MR effect size for
'PDE5A' on 'osteosarcoma'

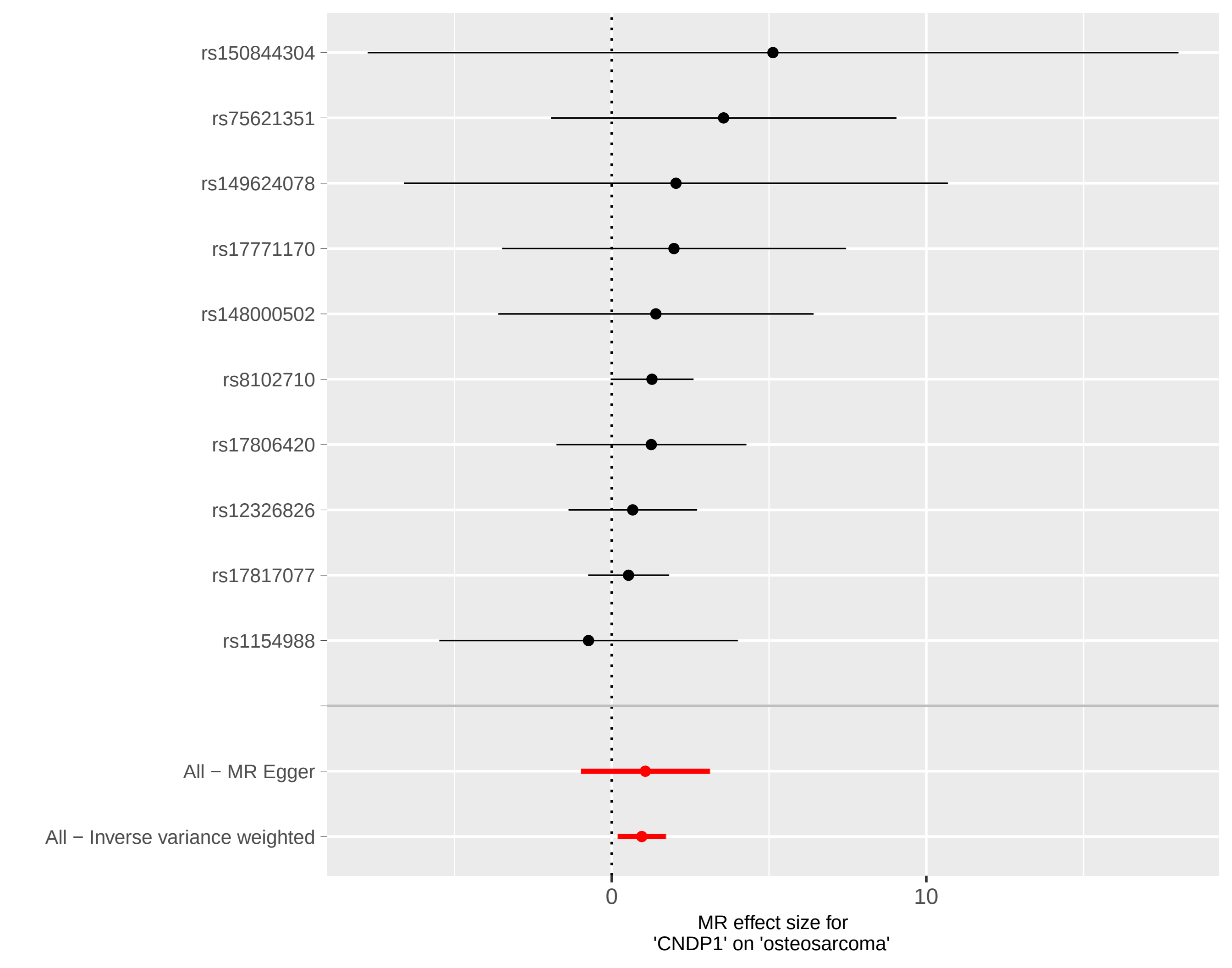
rs150844304
rs75621351
rs149624078
rs17771170
rs148000502
rs8102710
rs17806420
rs12326826
rs17817077
rs1154988
All − MR Egger
All − Inverse variance weighted
0
10
MR effect size for
'CNDP1' on 'osteosarcoma'

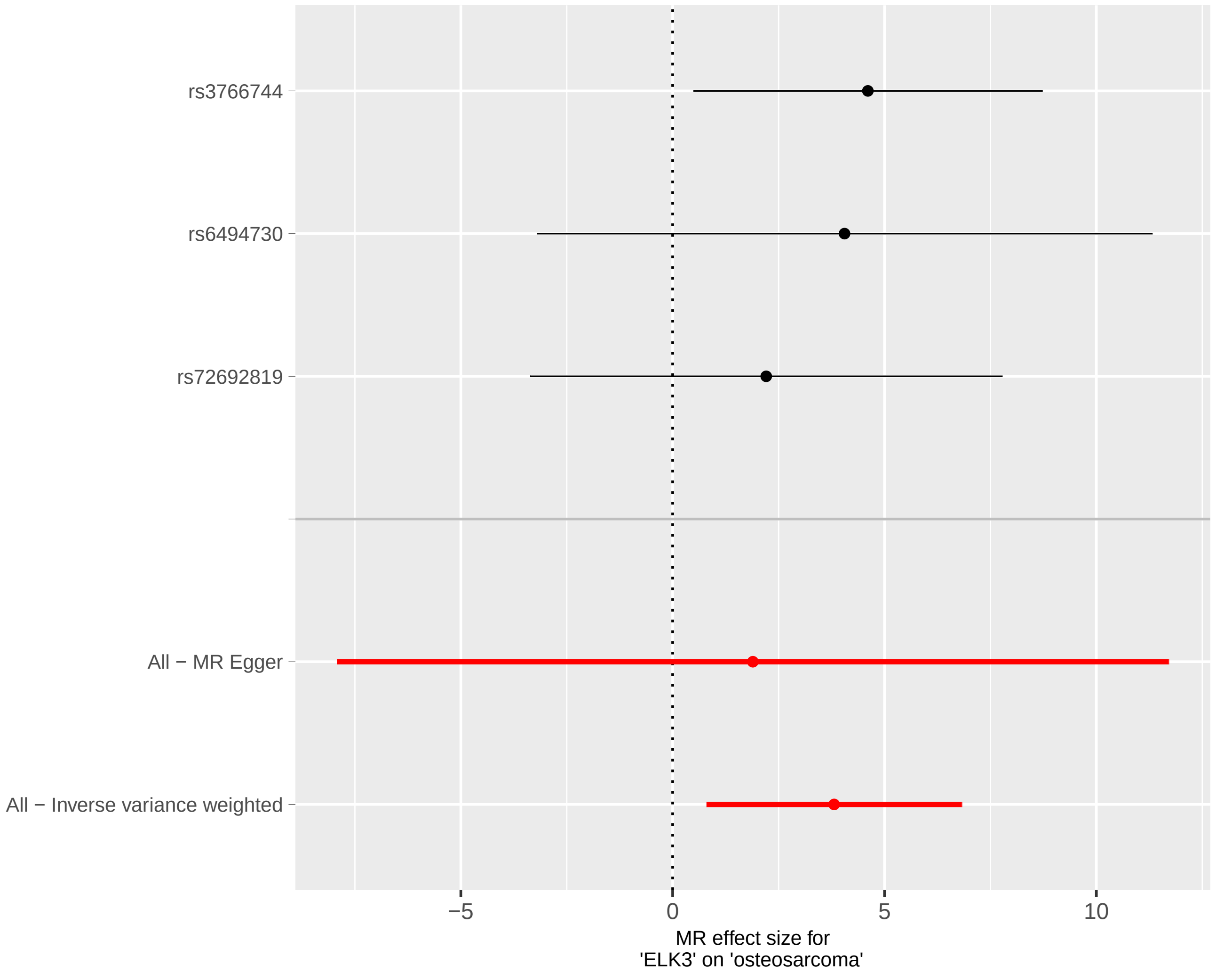
rs3766744
rs6494730
rs72692819
All − MR Egger
All − Inverse variance weighted
−5
0
5
10
MR effect size for
'ELK3' on 'osteosarcoma'

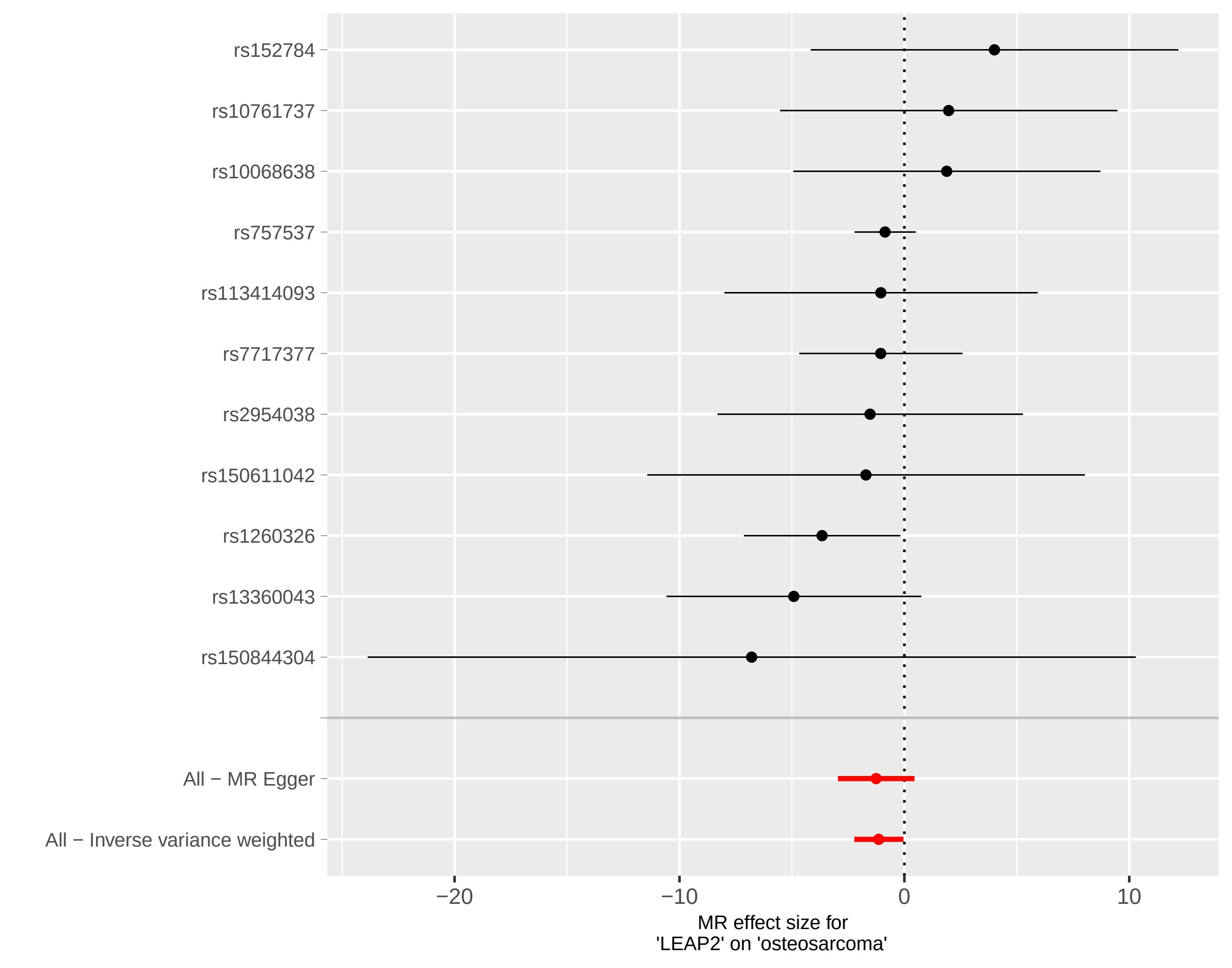

rs152784
rs10761737
rs10068638
rs757537
rs113414093
rs7717377
rs2954038
rs150611042
rs1260326
rs13360043
rs150844304
All − MR Egger
All − Inverse variance weighted
−20
−10
0
10
MR effect size for
'LEAP2' on 'osteosarcoma'

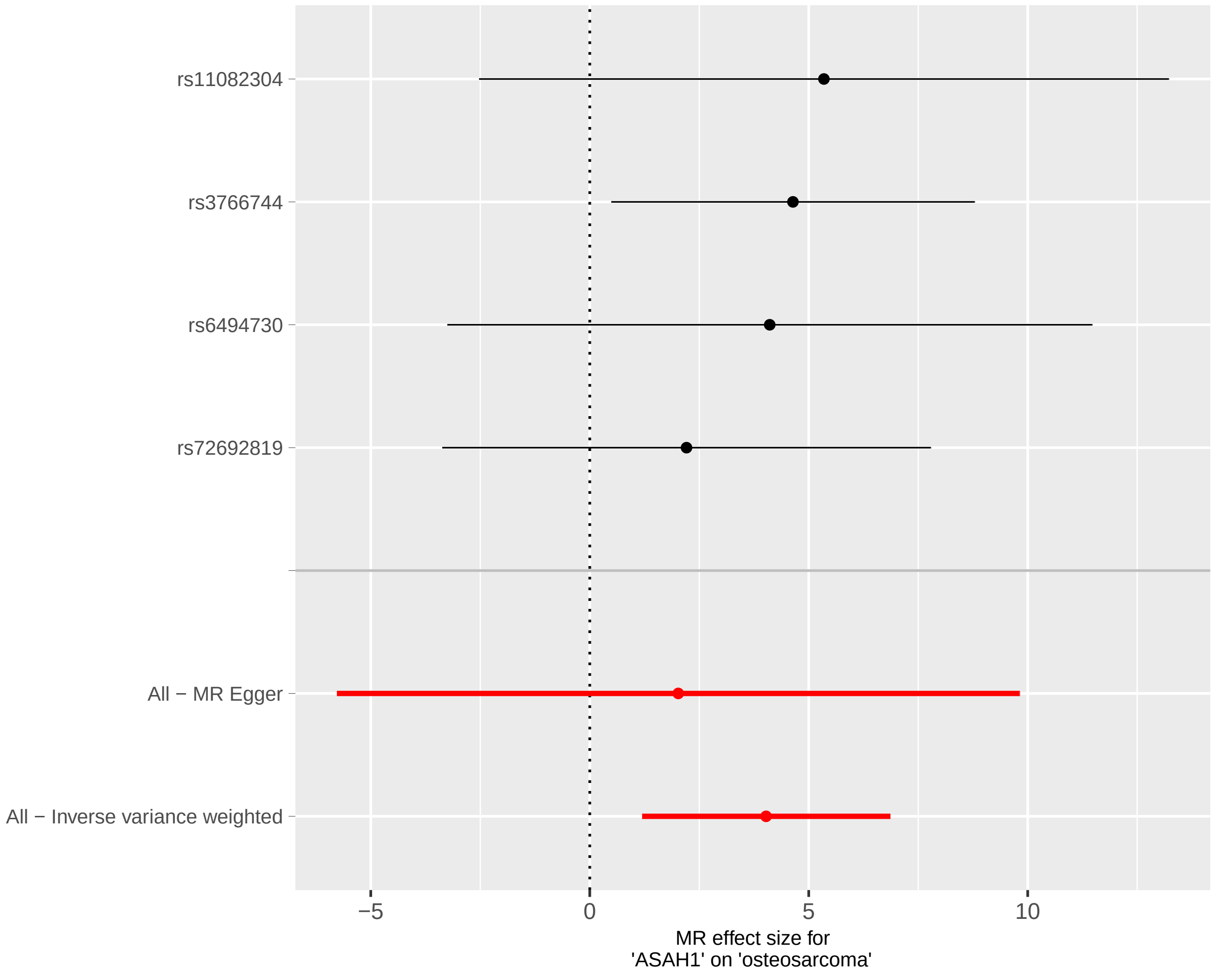
rs11082304
rs3766744
rs6494730
rs72692819
All − MR Egger
All − Inverse variance weighted
−5
0
5
10
MR effect size for
'ASAH1' on 'osteosarcoma'

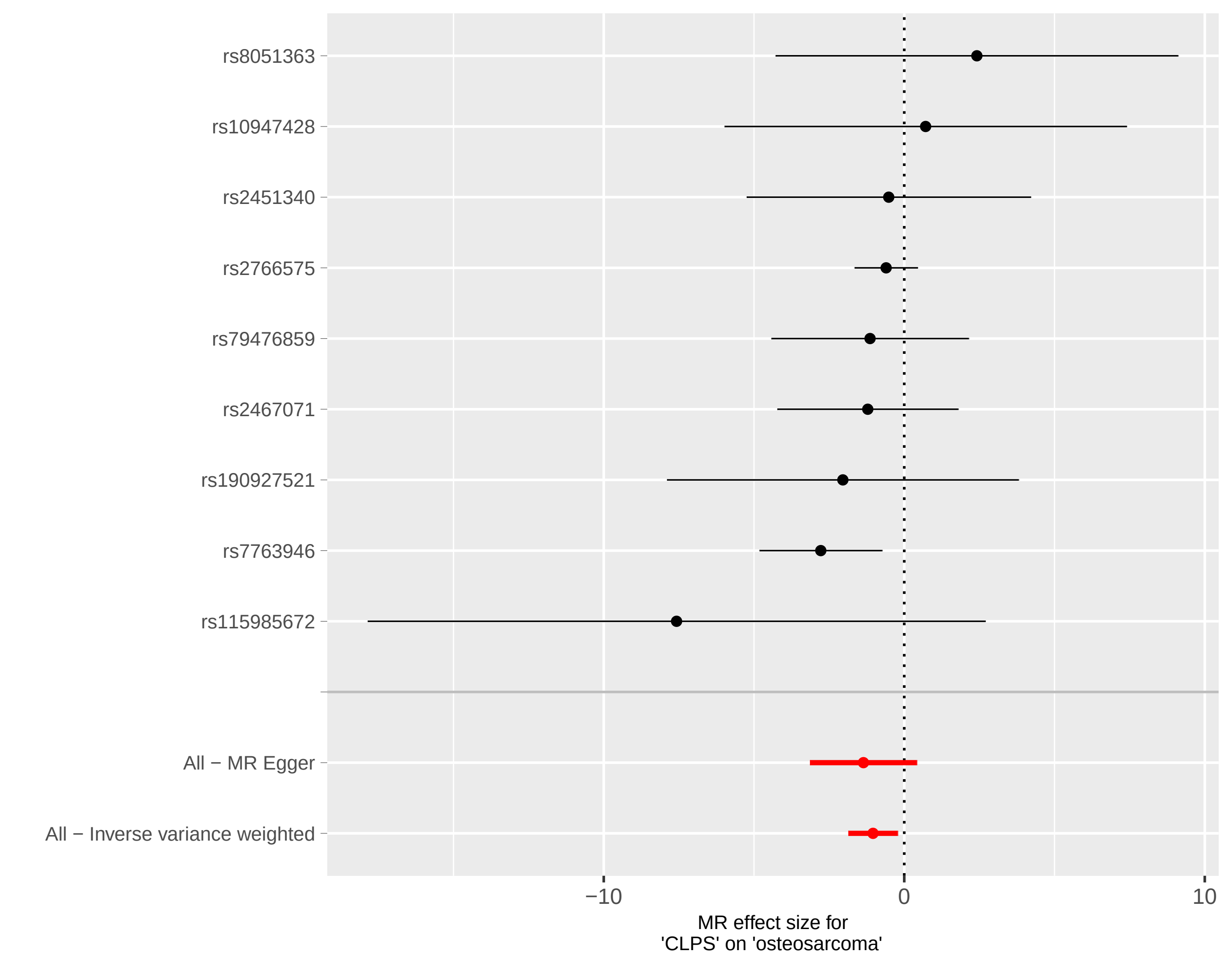
rs8051363
rs10947428
rs2451340
rs2766575
rs79476859
rs2467071
rs190927521
rs7763946
rs115985672
All − MR Egger
All − Inverse variance weighted
−10
0
10
MR effect size for
'CLPS' on 'osteosarcoma'

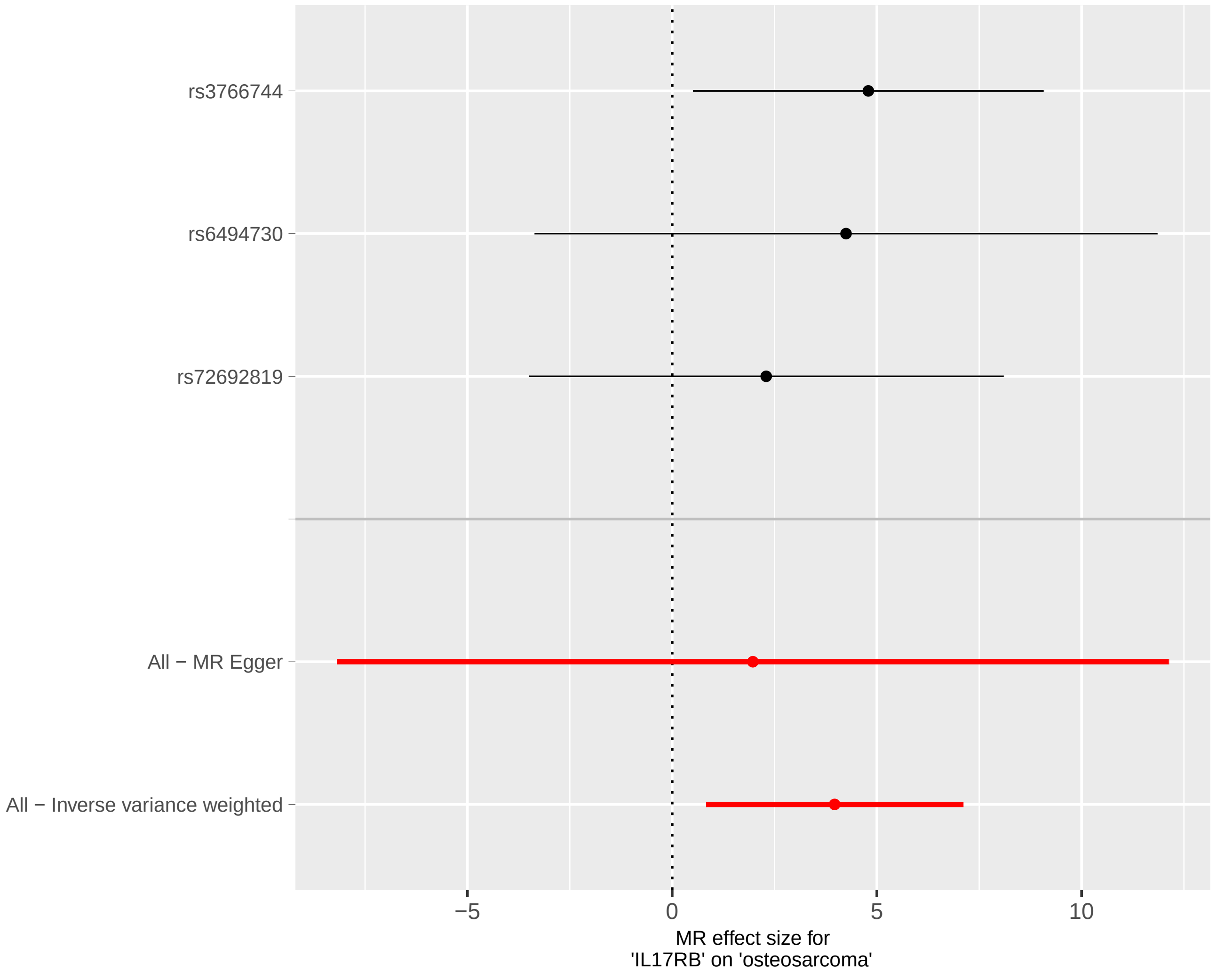
rs3766744
rs6494730
rs72692819
All − MR Egger
All − Inverse variance weighted
−5
0
5
10
MR effect size for
'IL17RB' on 'osteosarcoma'

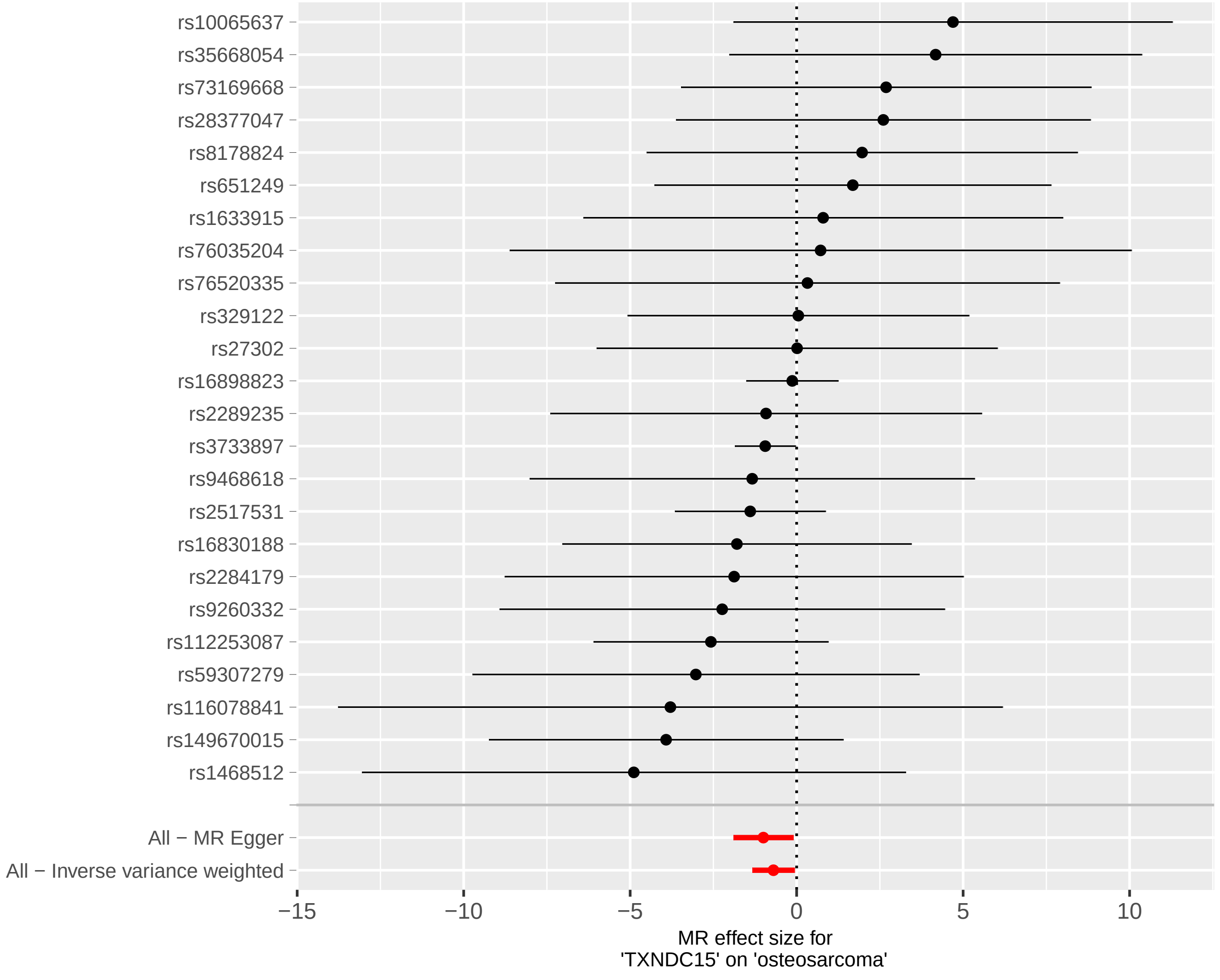

rs10065637
rs35668054
rs73169668
rs28377047
rs8178824
rs651249
rs1633915
rs76035204
rs76520335
rs329122
rs27302
rs16898823
rs2289235
rs3733897
rs9468618
rs2517531
rs16830188
rs2284179
rs9260332
rs112253087
rs59307279
rs116078841
rs149670015
rs1468512
All − MR Egger
All − Inverse variance weighted
−15
−10
−5
0
5
10
MR effect size for
'TXNDC15' on 'osteosarcoma'

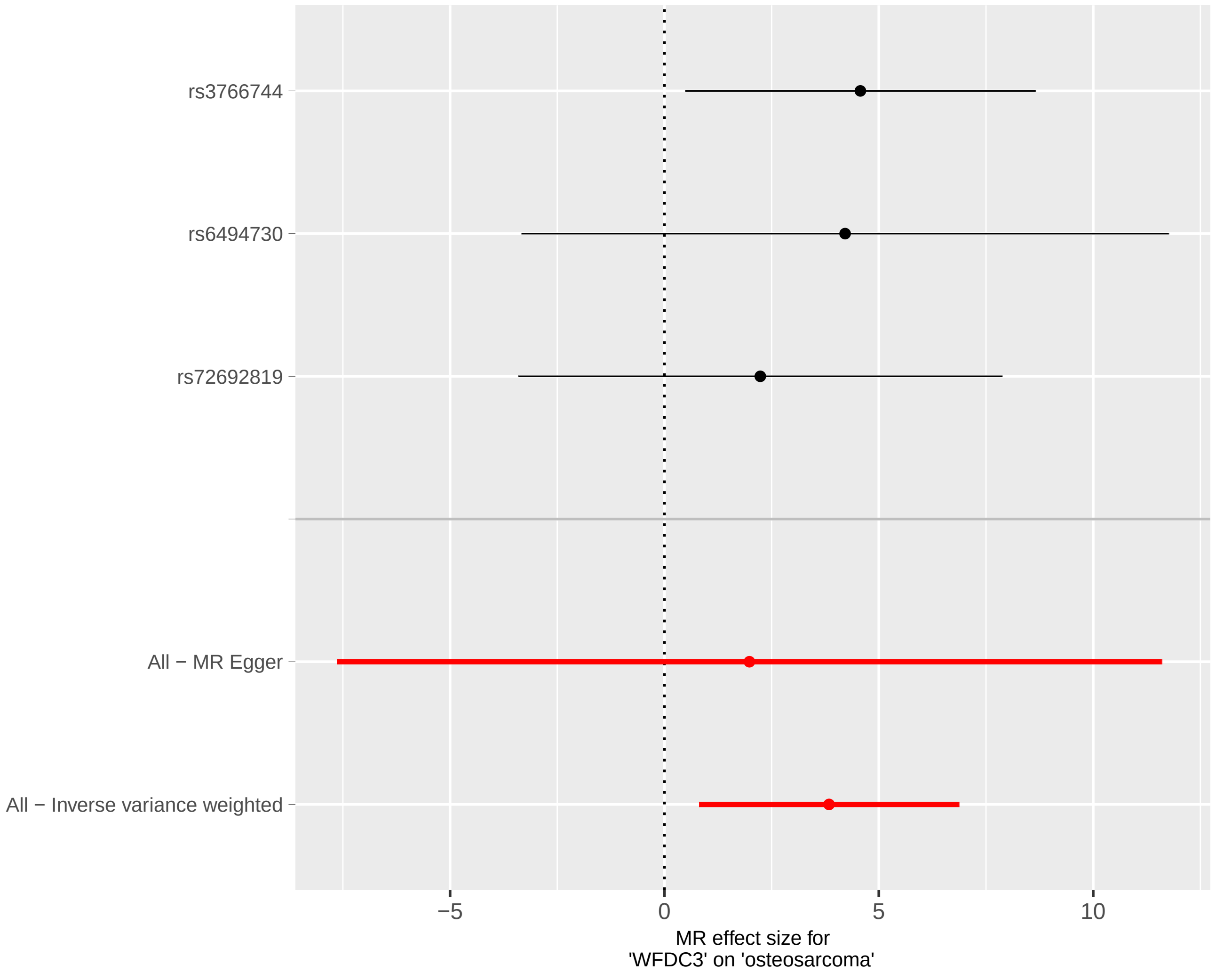

rs3766744
rs6494730
rs72692819
All − MR Egger
All − Inverse variance weighted
−5
0
5
10
MR effect size for
'WFDC3' on 'osteosarcoma'

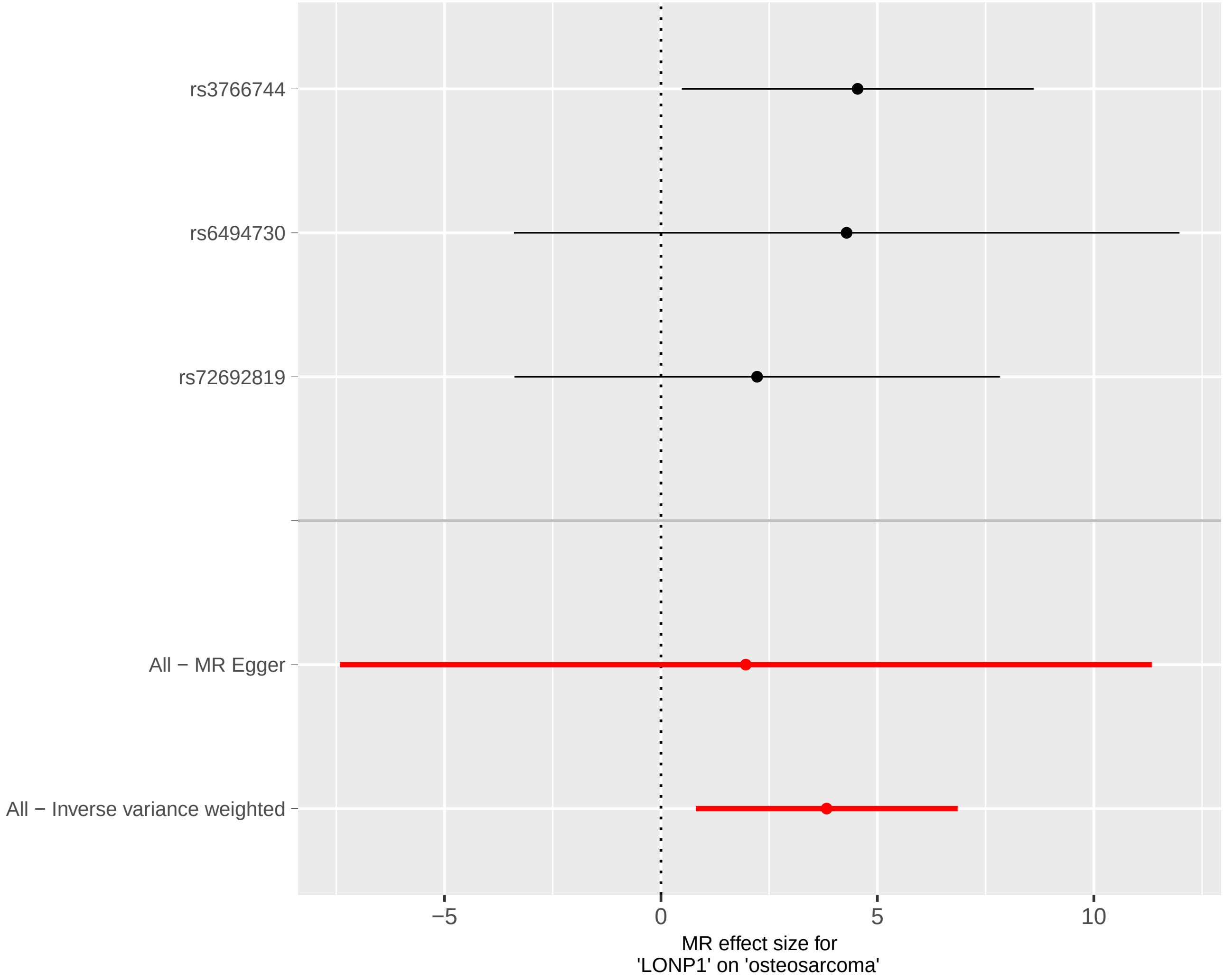
rs3766744
rs6494730
rs72692819
All − MR Egger
All − Inverse variance weighted
−5
0
5
10
MR effect size for
'LONP1' on 'osteosarcoma'

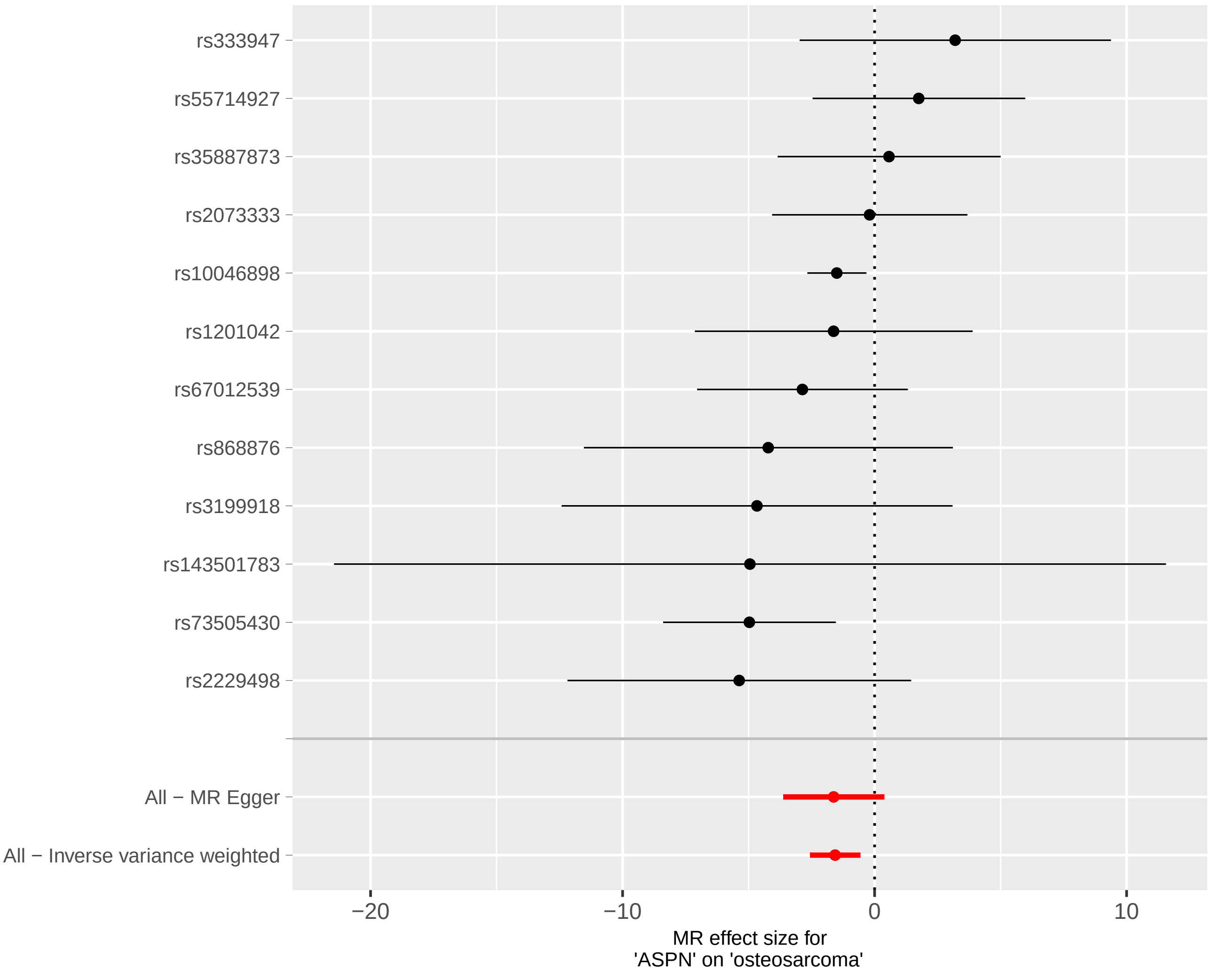

rs333947
rs55714927
rs35887873
rs2073333
rs10046898
rs1201042
rs67012539
rs868876
rs3199918
rs143501783
rs73505430
rs2229498
All − MR Egger
All − Inverse variance weighted
−20
−10
0
10
MR effect size for
'ASPN' on 'osteosarcoma'

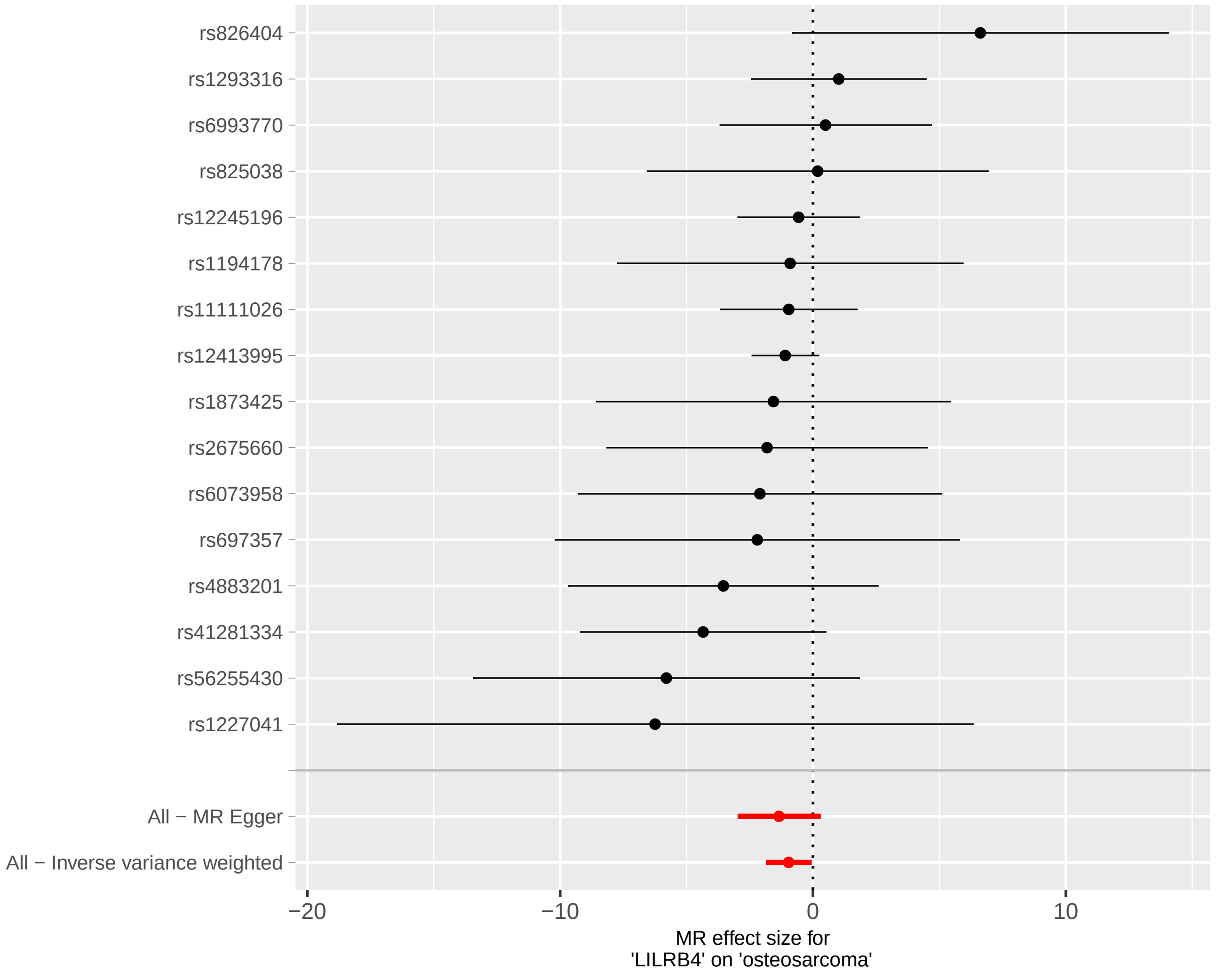
rs826404
rs1293316
rs6993770
rs825038
rs12245196
rs1194178
rs11111026
rs12413995
rs1873425
rs2675660
rs6073958
rs697357
rs4883201
rs41281334
rs56255430
rs1227041
All − MR Egger
All − Inverse variance weighted
−20
−10
0
10
MR effect size for
'LILRB4' on 'osteosarcoma'

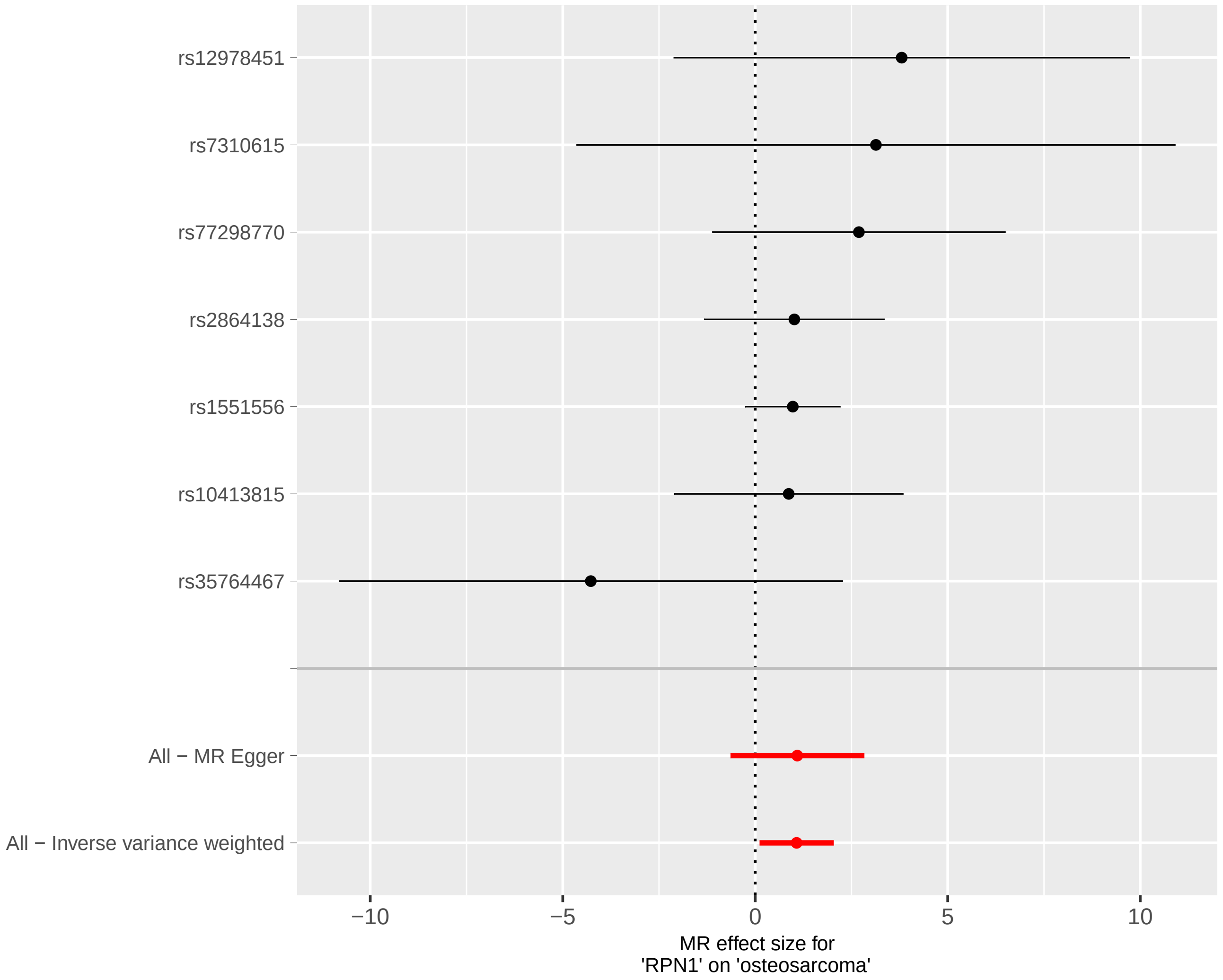

rs12978451
rs7310615
rs77298770
rs2864138
rs1551556
rs10413815
rs35764467
All − MR Egger
All − Inverse variance weighted
−10
−5
0
5
10
MR effect size for
'RPN1' on 'osteosarcoma'

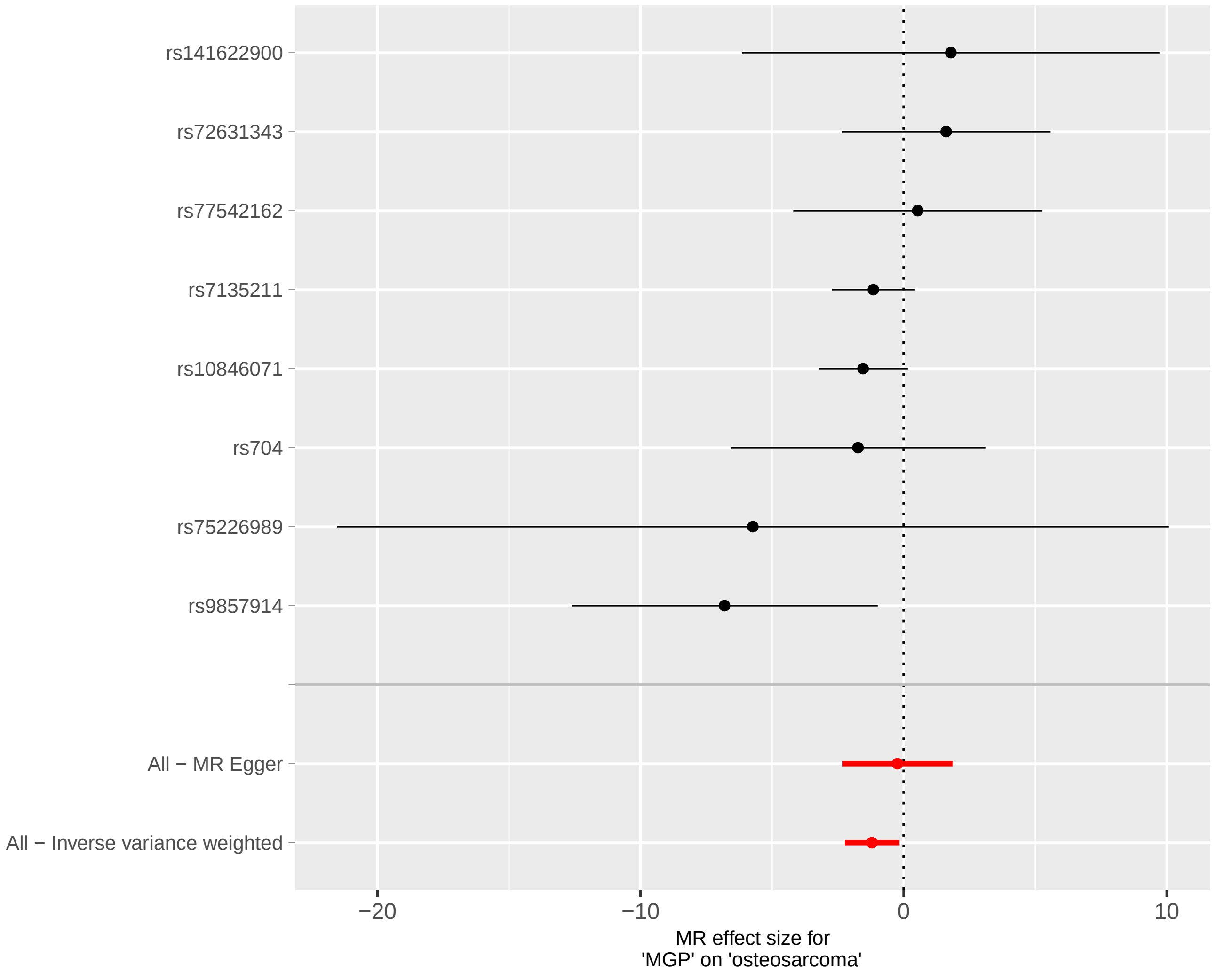
rs141622900
rs72631343
rs77542162
rs7135211
rs10846071
rs704
rs75226989
rs9857914
All − MR Egger
All − Inverse variance weighted
−20
−10
0
10
MR effect size for
'MGP' on 'osteosarcoma'

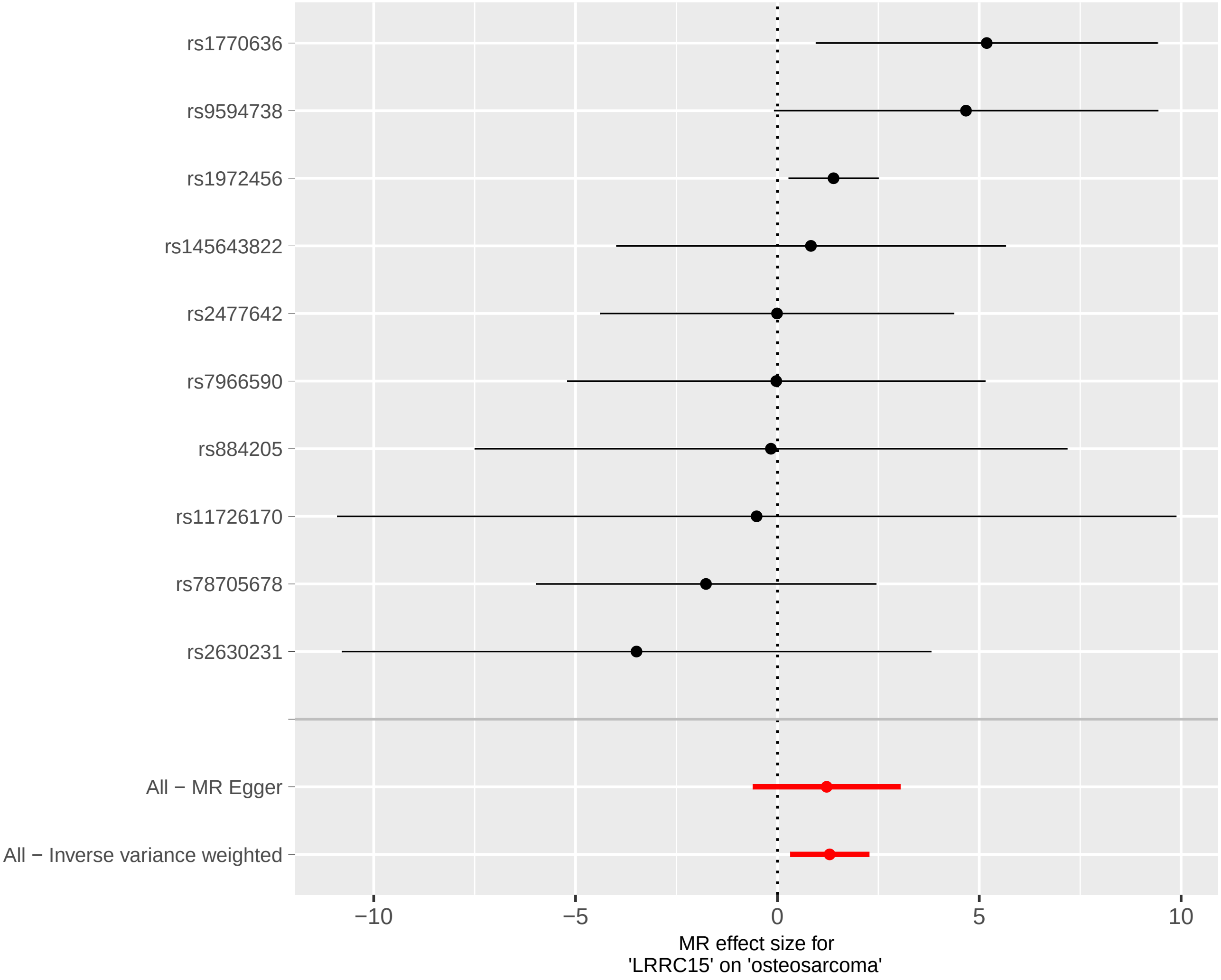
rs1770636
rs9594738
rs1972456
rs145643822
rs2477642
rs7966590
rs884205
rs11726170
rs78705678
rs2630231
All − MR Egger
All − Inverse variance weighted
−10
−5
0
5
10
MR effect size for
'LRRC15' on 'osteosarcoma'

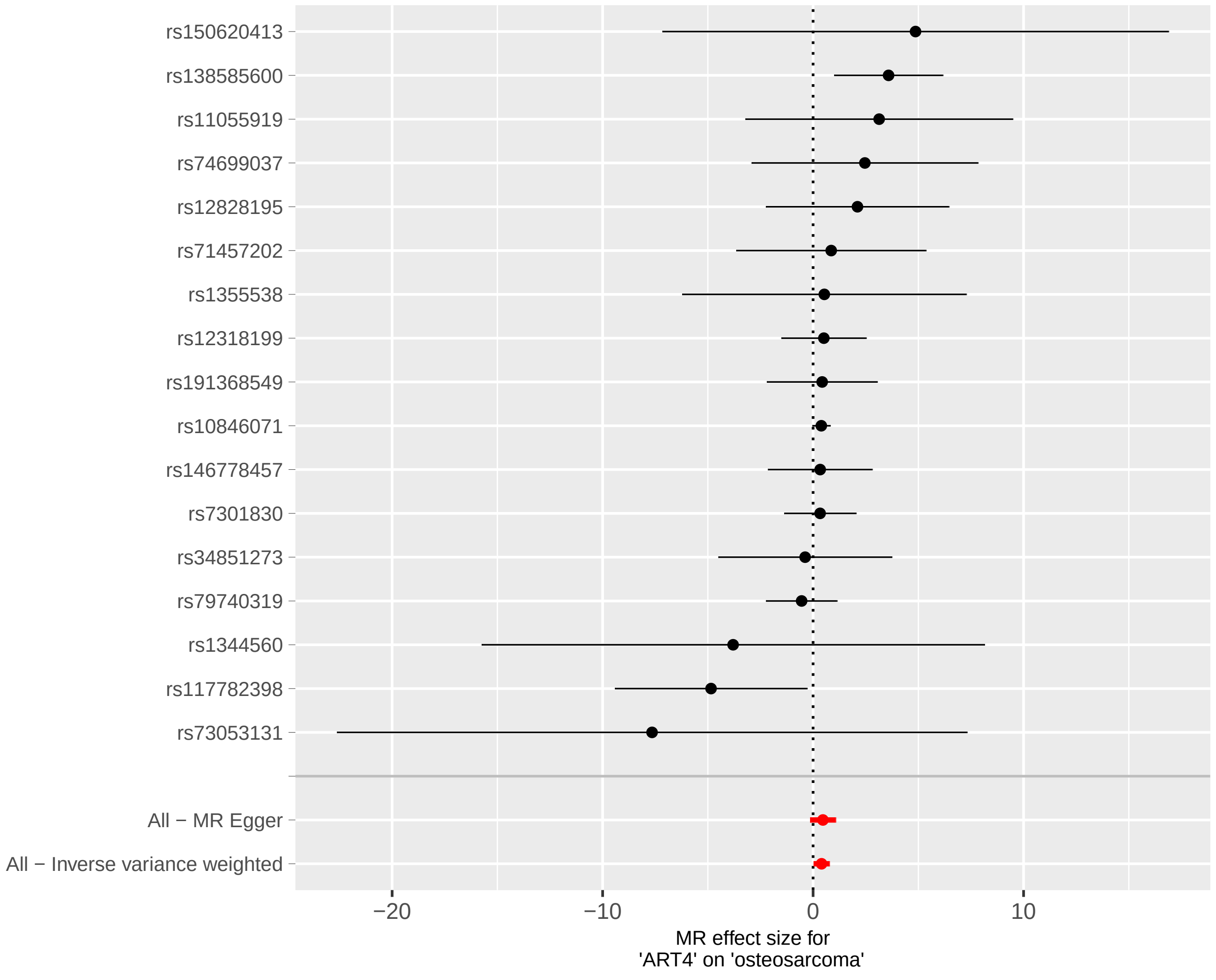
rs150620413
rs138585600
rs11055919
rs74699037
rs12828195
rs71457202
rs1355538
rs12318199
rs191368549
rs10846071
rs146778457
rs7301830
rs34851273
rs79740319
rs1344560
rs117782398
rs73053131
All − MR Egger
All − Inverse variance weighted
−20
−10
0
10
MR effect size for
'ART4' on 'osteosarcoma'

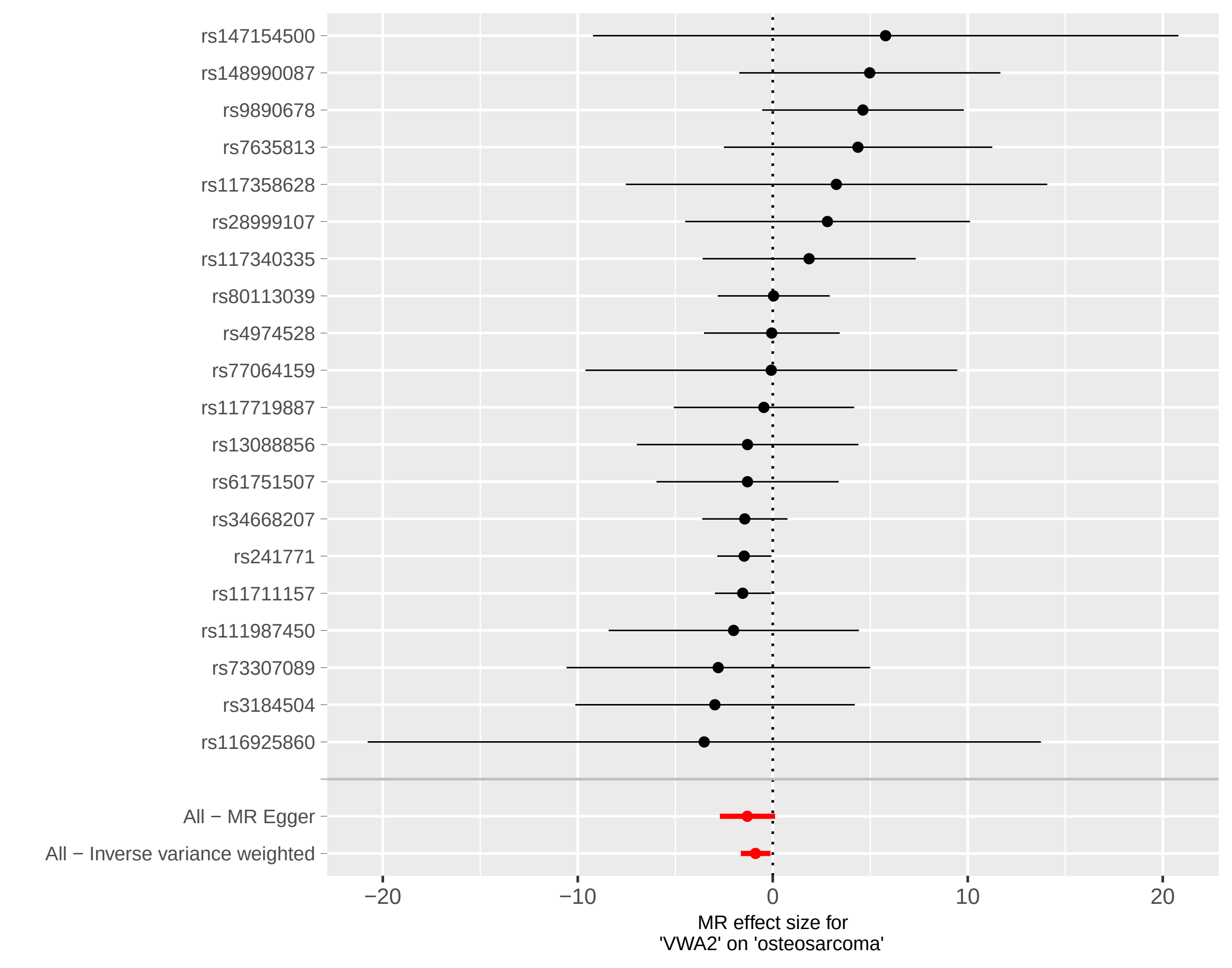
rs147154500
rs148990087
rs9890678
rs7635813
rs117358628
rs28999107
rs117340335
rs80113039
rs4974528
rs77064159
rs117719887
rs13088856
rs61751507
rs34668207
rs241771
rs11711157
rs111987450
rs73307089
rs3184504
rs116925860
All − MR Egger
All − Inverse variance weighted
−20
−10
0
10
20
MR effect size for
'VWA2' on 'osteosarcoma'

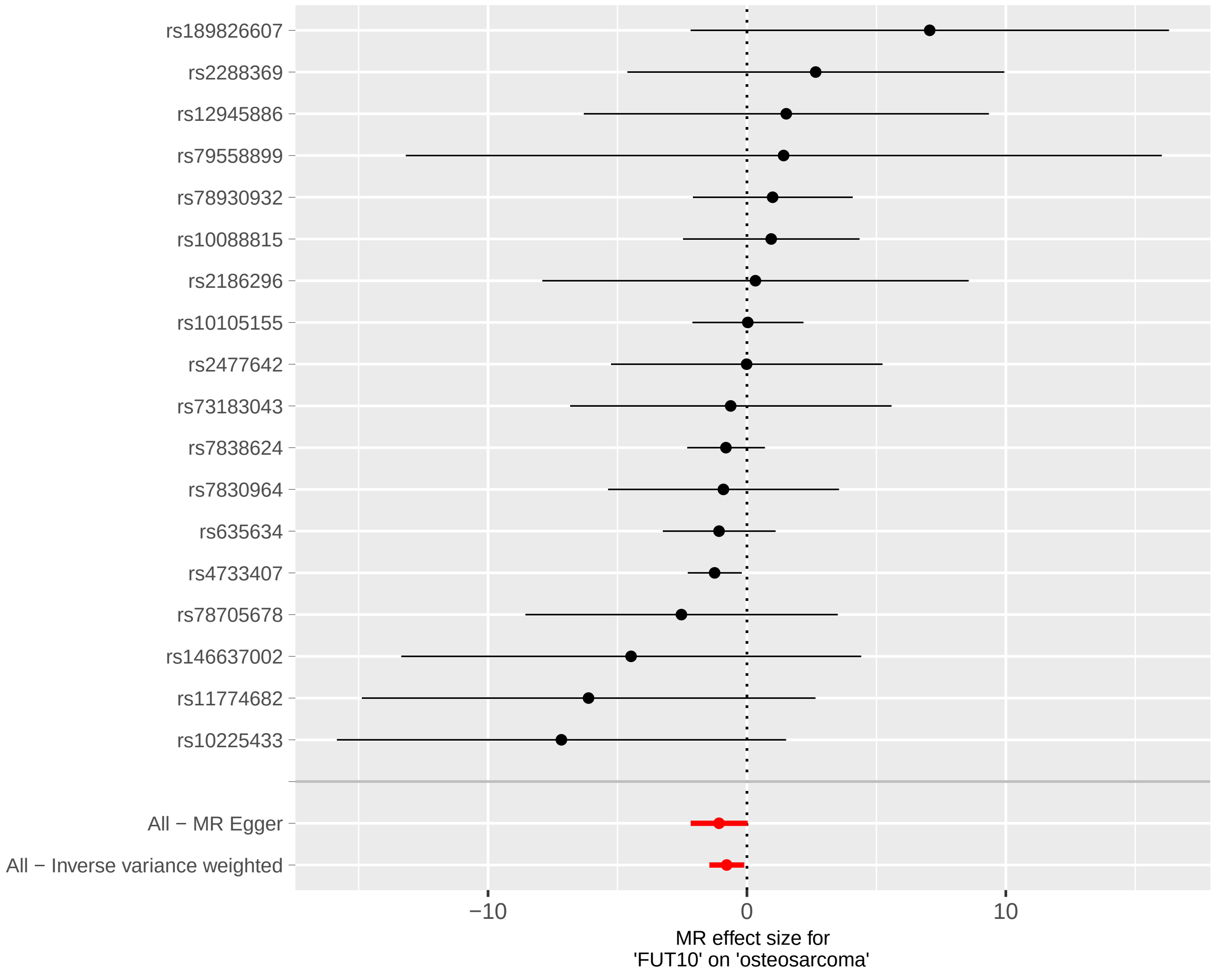

rs189826607
rs2288369
rs12945886
rs79558899
rs78930932
rs10088815
rs2186296
rs10105155
rs2477642
rs73183043
rs7838624
rs7830964
rs635634
rs4733407
rs78705678
rs146637002
rs11774682
rs10225433
All − MR Egger
All − Inverse variance weighted
−10
0
10
MR effect size for
'FUT10' on 'osteosarcoma'

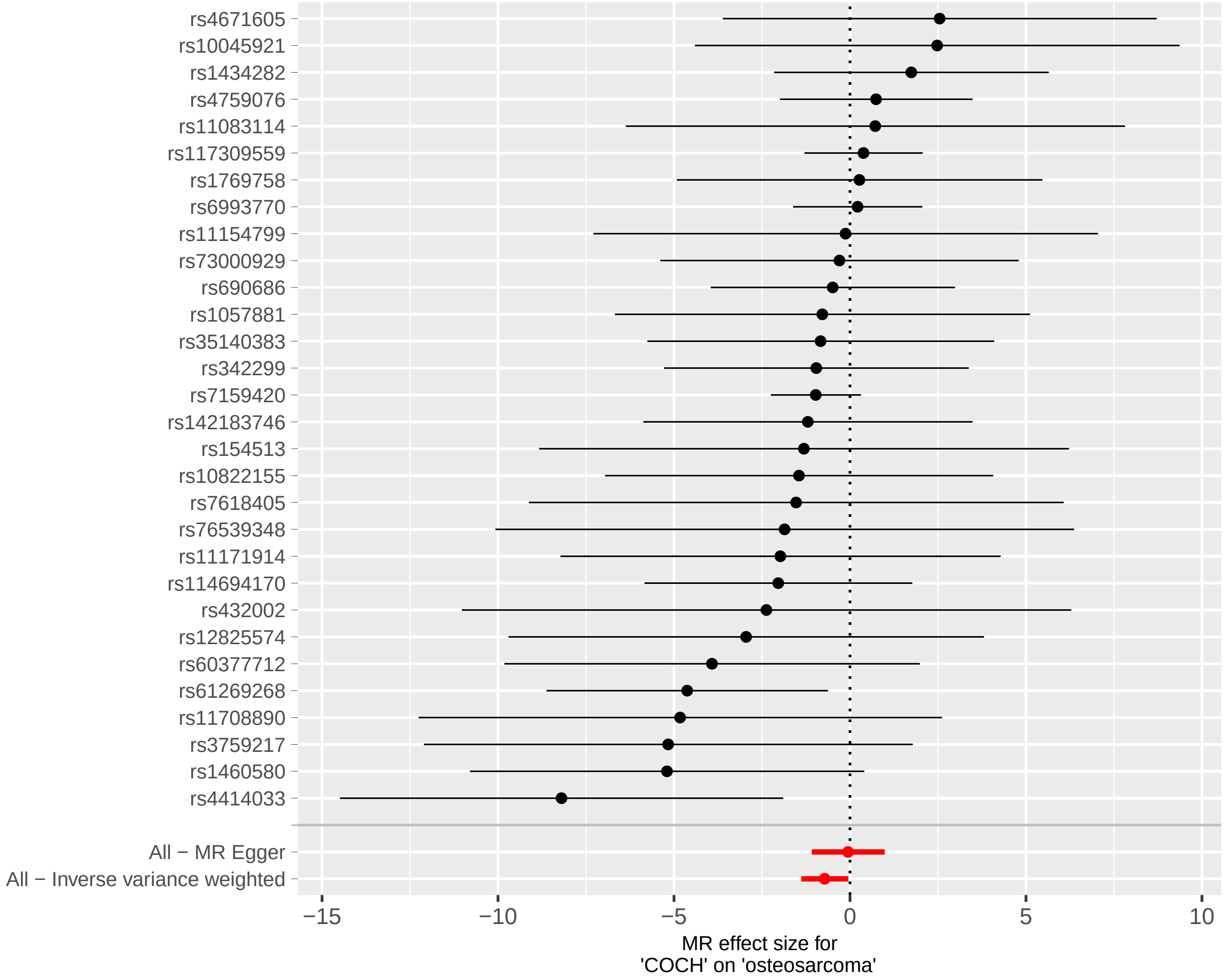
rs4671605
rs10045921
rs1434282
rs4759076
rs11083114
rs117309559
rs1769758
rs6993770
rs11154799
rs73000929
rs690686
rs1057881
rs35140383
rs342299
rs7159420
rs142183746
rs154513
rs10822155
rs7618405
rs76539348
rs11171914
rs114694170
rs432002
rs12825574
rs60377712
rs61269268
rs11708890
rs3759217
rs1460580
rs4414033
All − MR Egger
All − Inverse variance weighted
−15
−10
−5
0
5
10
MR effect size for
'COCH' on 'osteosarcoma'

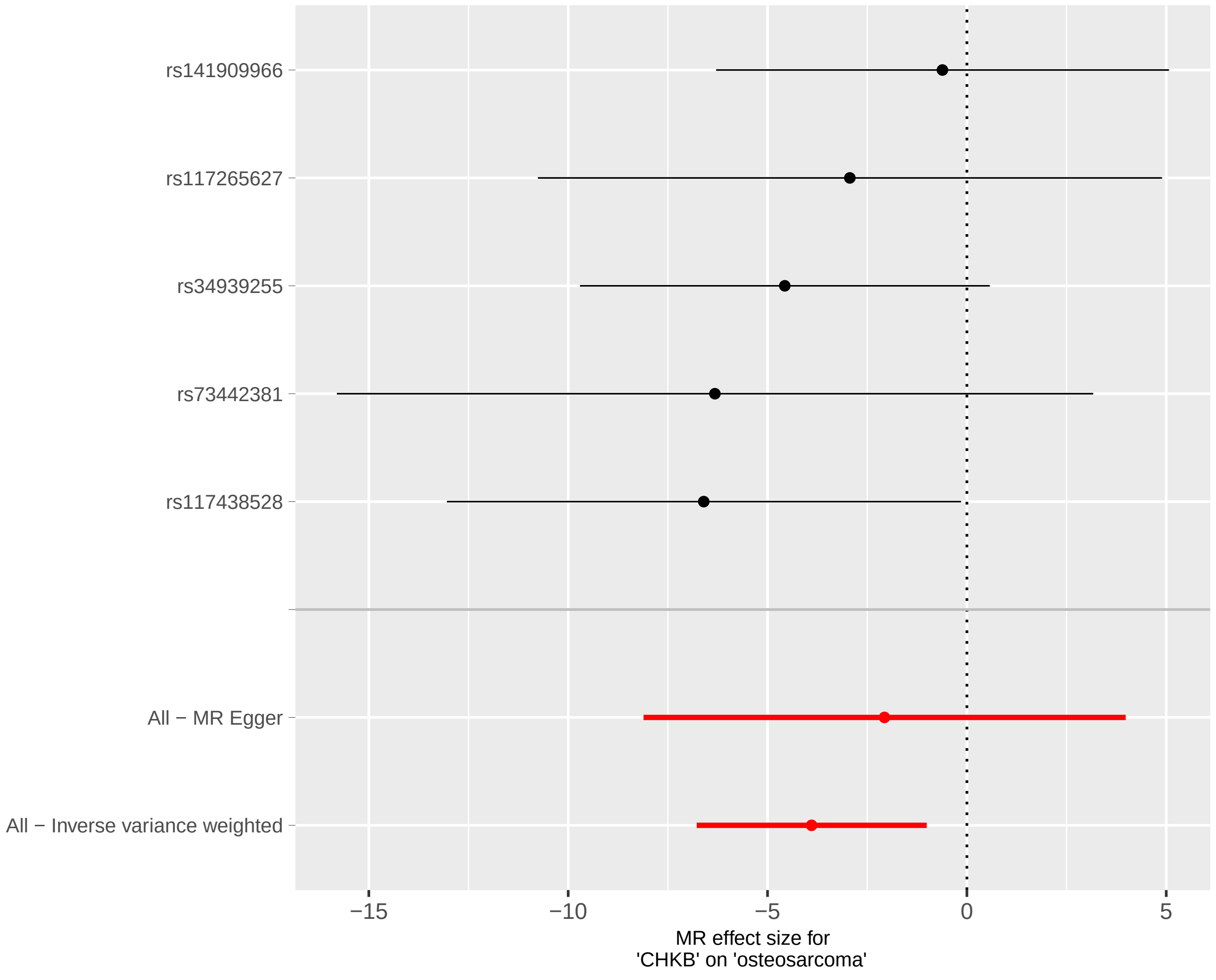

rs141909966
rs117265627
rs34939255
rs73442381
rs117438528
All − MR Egger
All − Inverse variance weighted
−15
−10
−5
0
5
MR effect size for
'CHKB' on 'osteosarcoma'

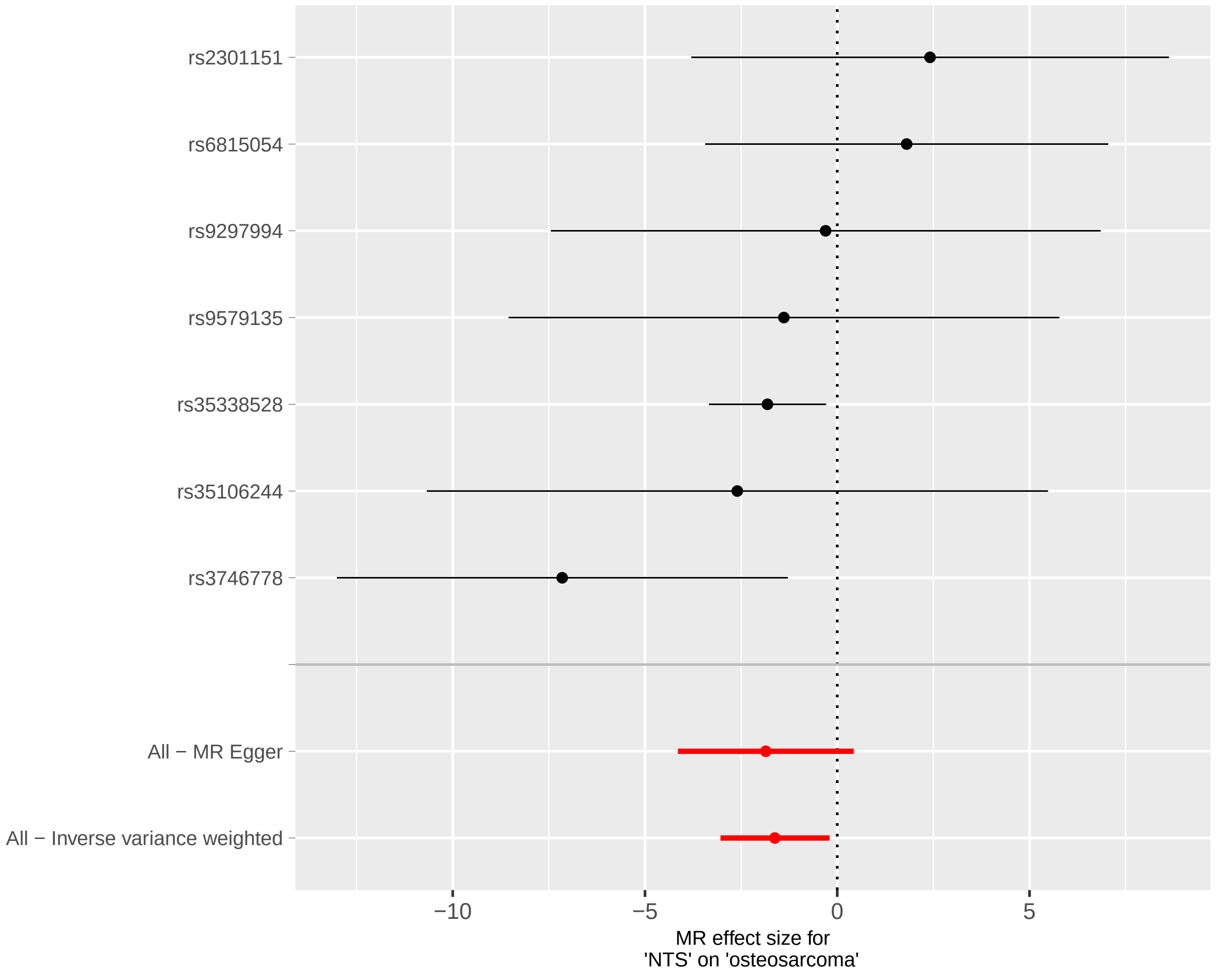
rs2301151
rs6815054
rs9297994
rs9579135
rs35338528
rs35106244
rs3746778
All − MR Egger
All − Inverse variance weighted
−10
−5
0
5
MR effect size for
'NTS' on 'osteosarcoma'

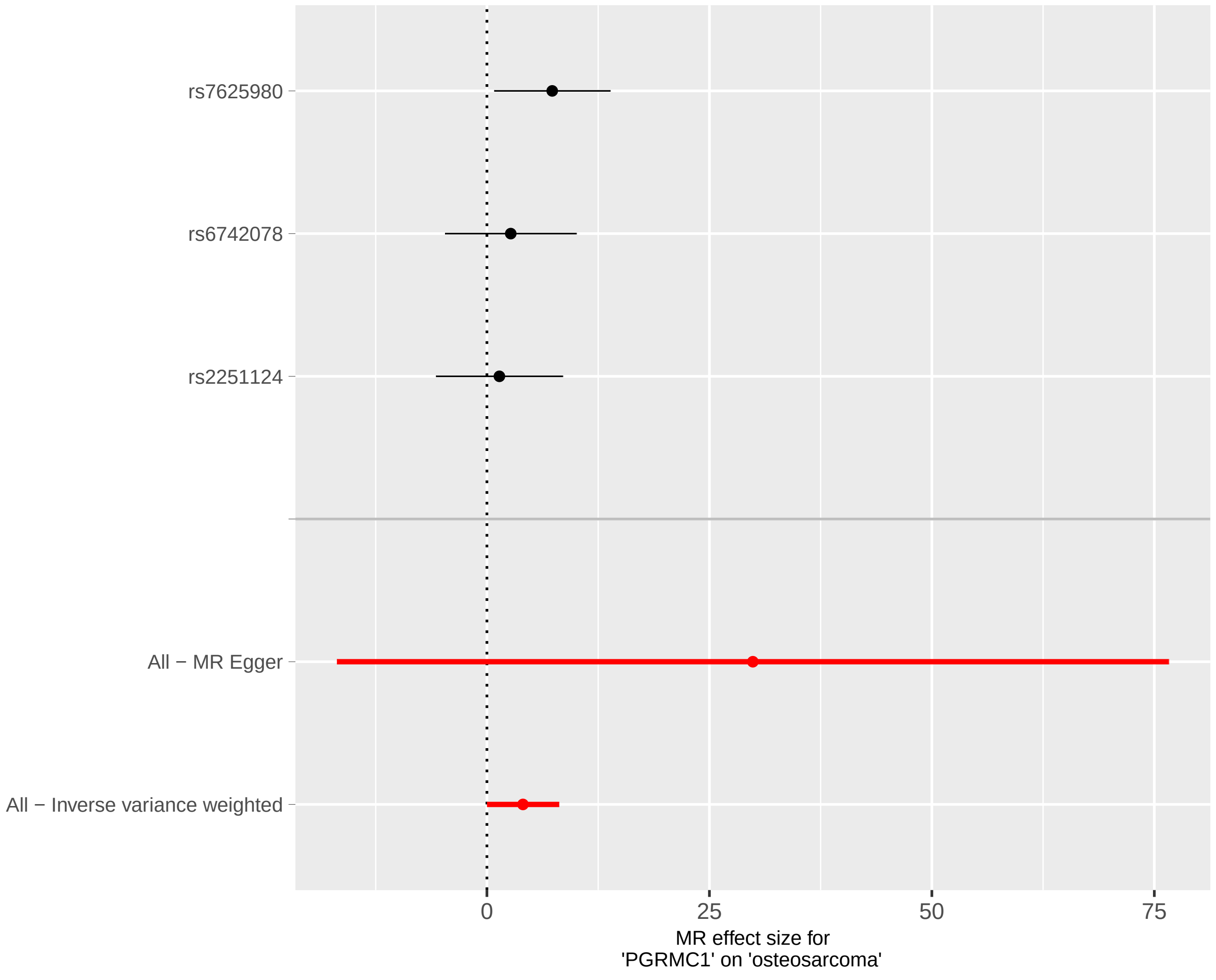
rs7625980
rs6742078
rs2251124
All − MR Egger
All − Inverse variance weighted
0
25
50
75
MR effect size for
'PGRMC1' on 'osteosarcoma'

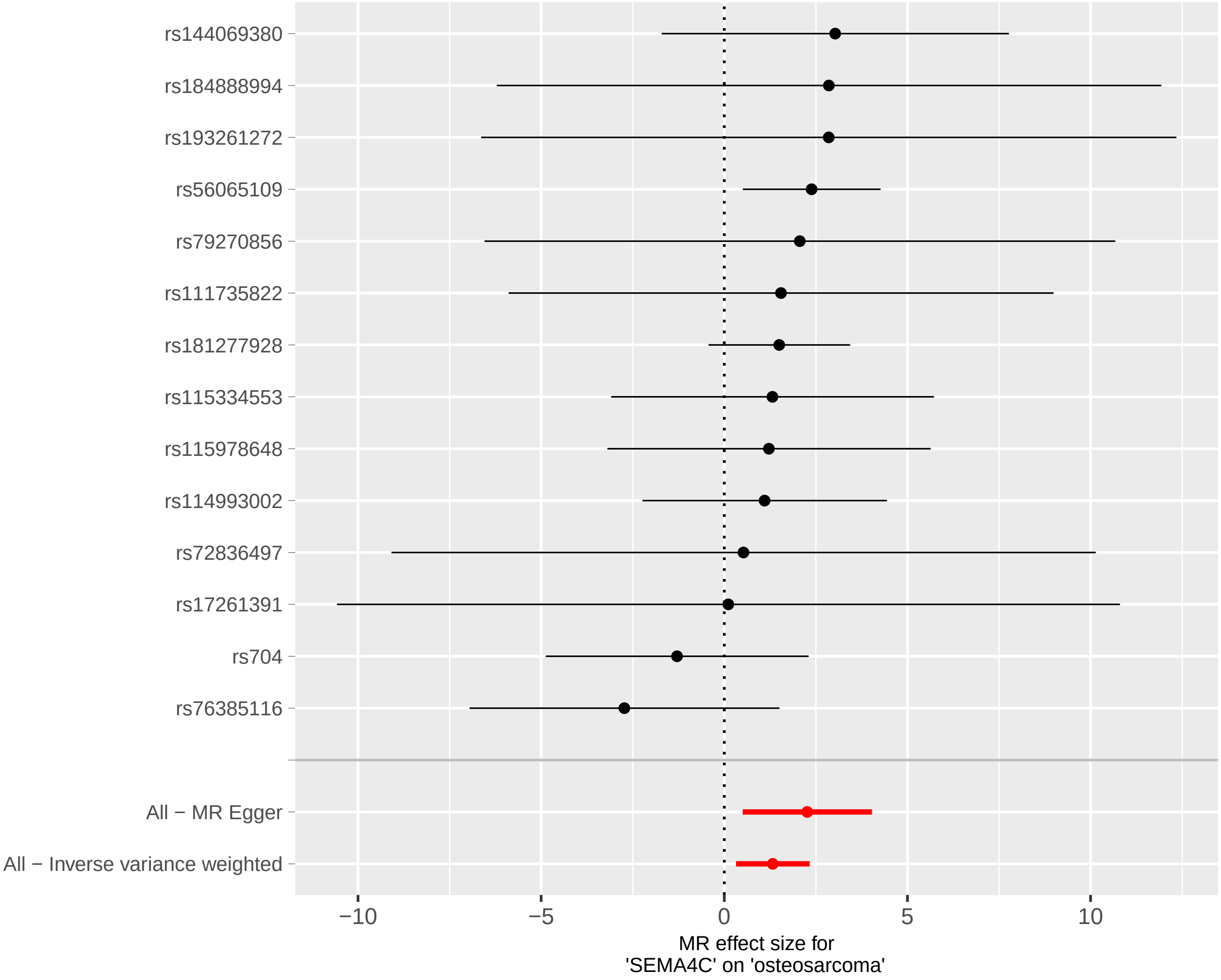

rs144069380
rs184888994
rs193261272
rs56065109
rs79270856
rs111735822
rs181277928
rs115334553
rs115978648
rs114993002
rs72836497
rs17261391
rs704
rs76385116
All − MR Egger
All − Inverse variance weighted
−10
−5
0
5
10
MR effect size for
'SEMA4C' on 'osteosarcoma'

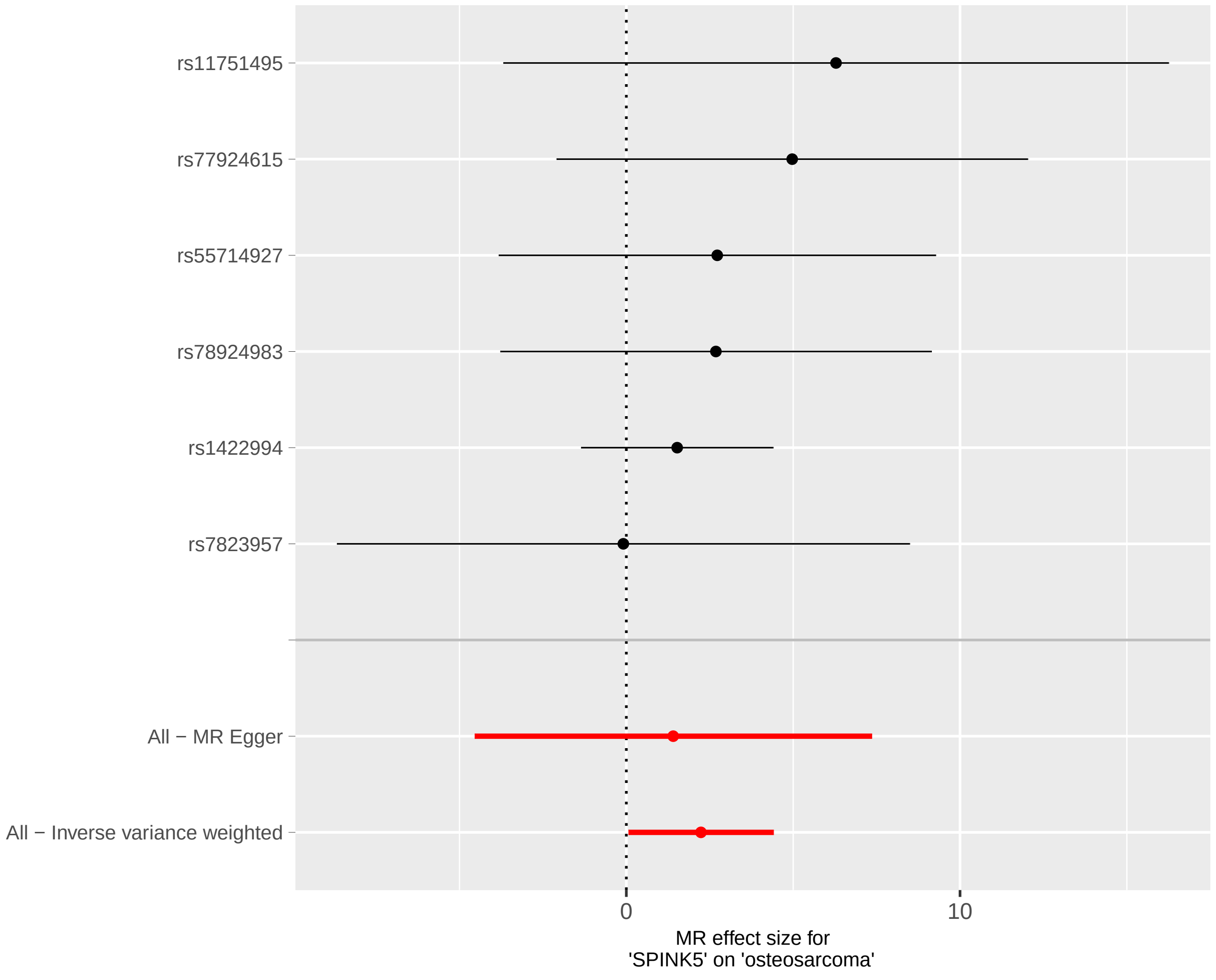
rs11751495
rs77924615
rs55714927
rs78924983
rs1422994
rs7823957
All − MR Egger
All − Inverse variance weighted
0
10
MR effect size for
'SPINK5' on 'osteosarcoma'

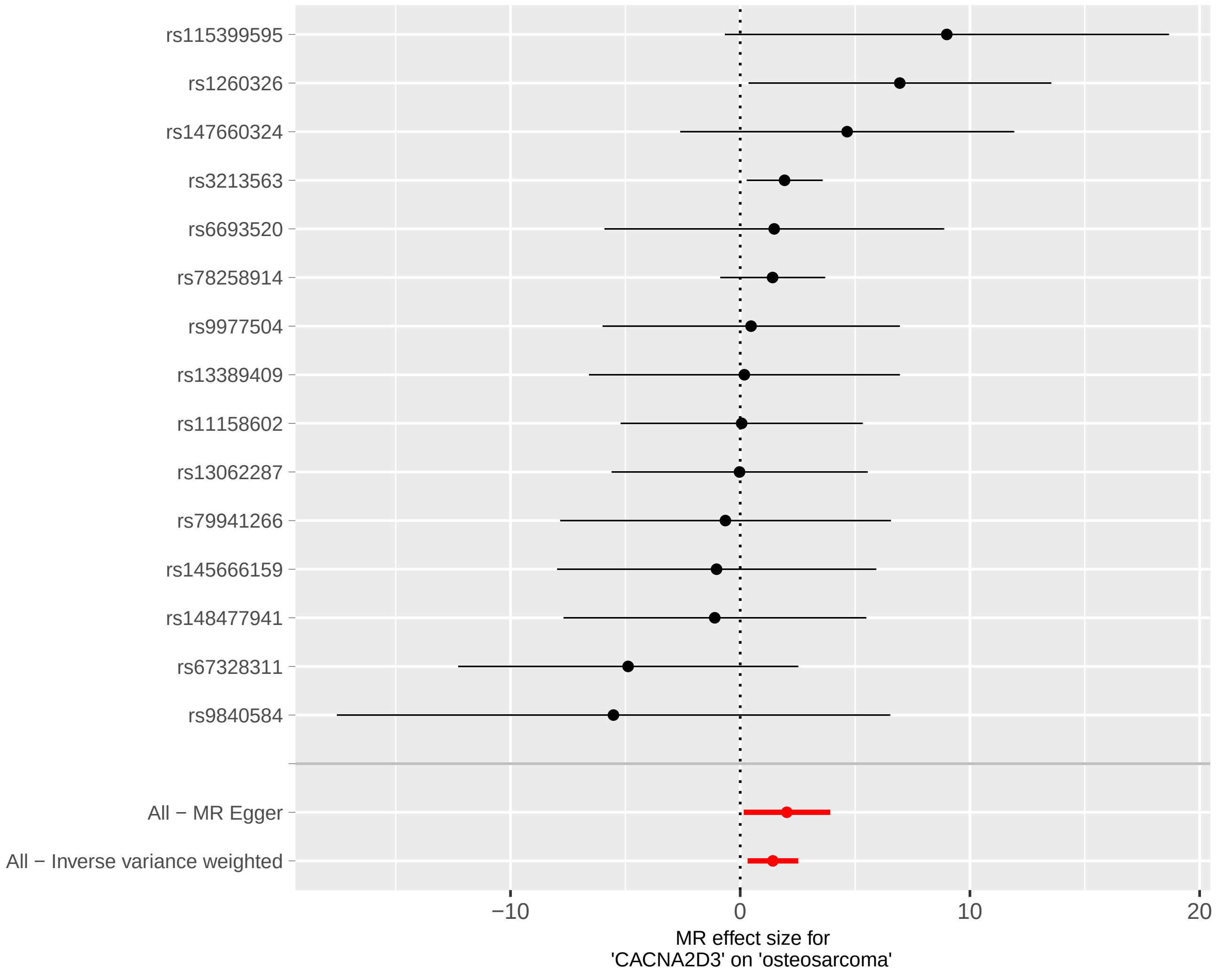
rs115399595
rs1260326
rs147660324
rs3213563
rs6693520
rs78258914
rs9977504
rs13389409
rs11158602
rs13062287
rs79941266
rs145666159
rs148477941
rs67328311
rs9840584
All − MR Egger
All − Inverse variance weighted
−10
0
10
20
MR effect size for
'CACNA2D3' on 'osteosarcoma'

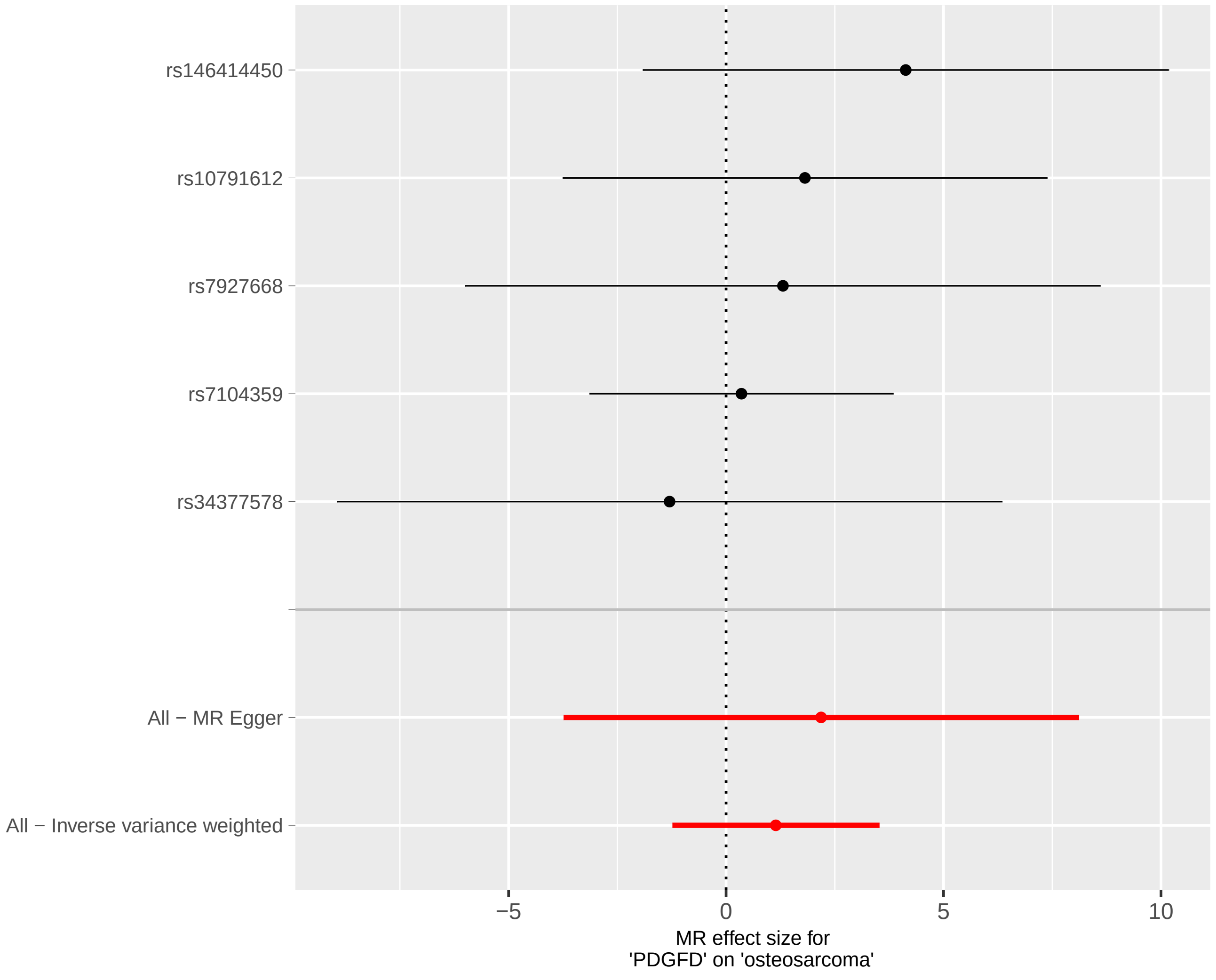

rs146414450
rs10791612
rs7927668
rs7104359
rs34377578
All − MR Egger
All − Inverse variance weighted
−5
0
5
10
MR effect size for
'PDGFD' on 'osteosarcoma'

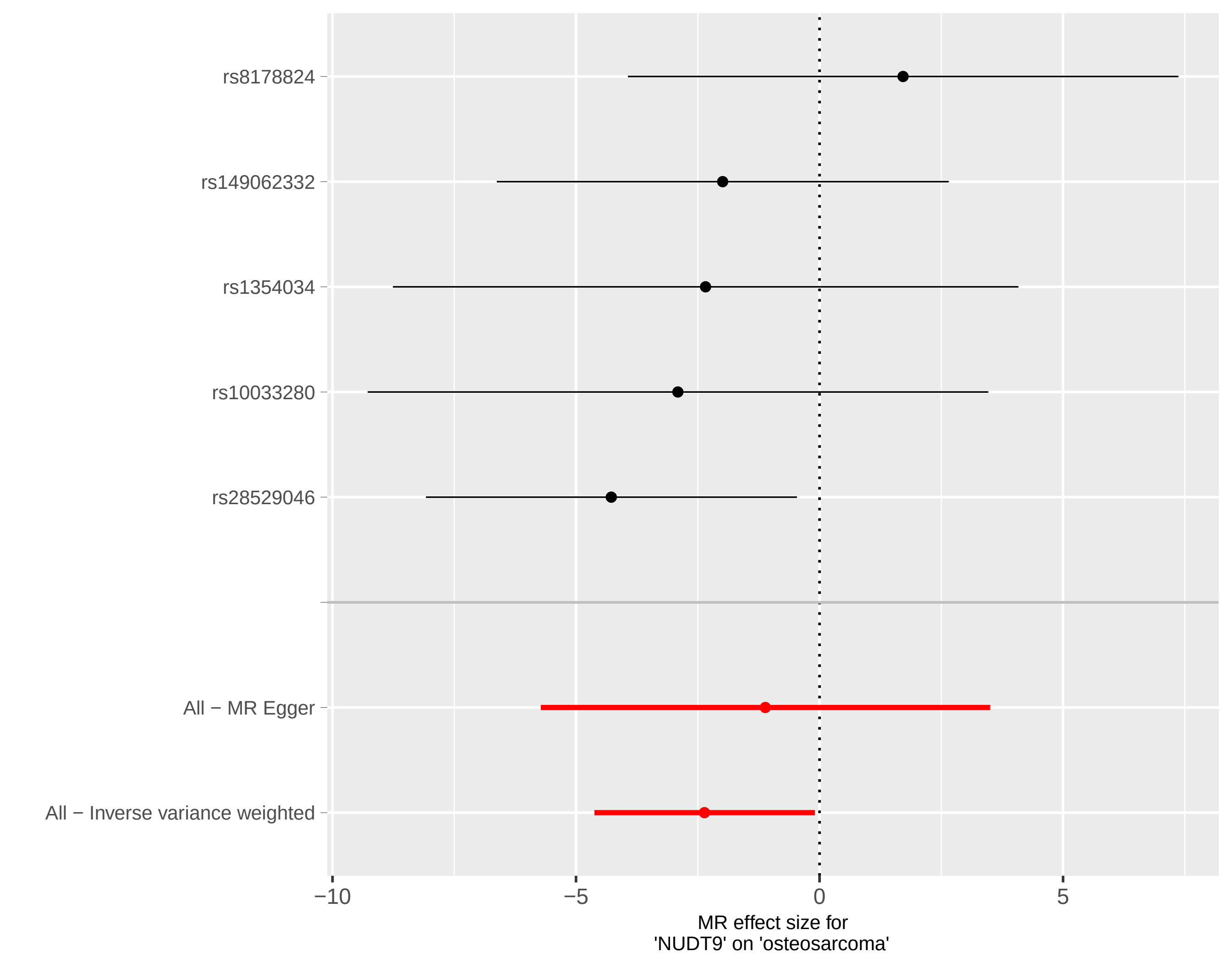
rs8178824
rs149062332
rs1354034
rs10033280
rs28529046
All − MR Egger
All − Inverse variance weighted
−10
−5
0
5
MR effect size for
'NUDT9' on 'osteosarcoma'

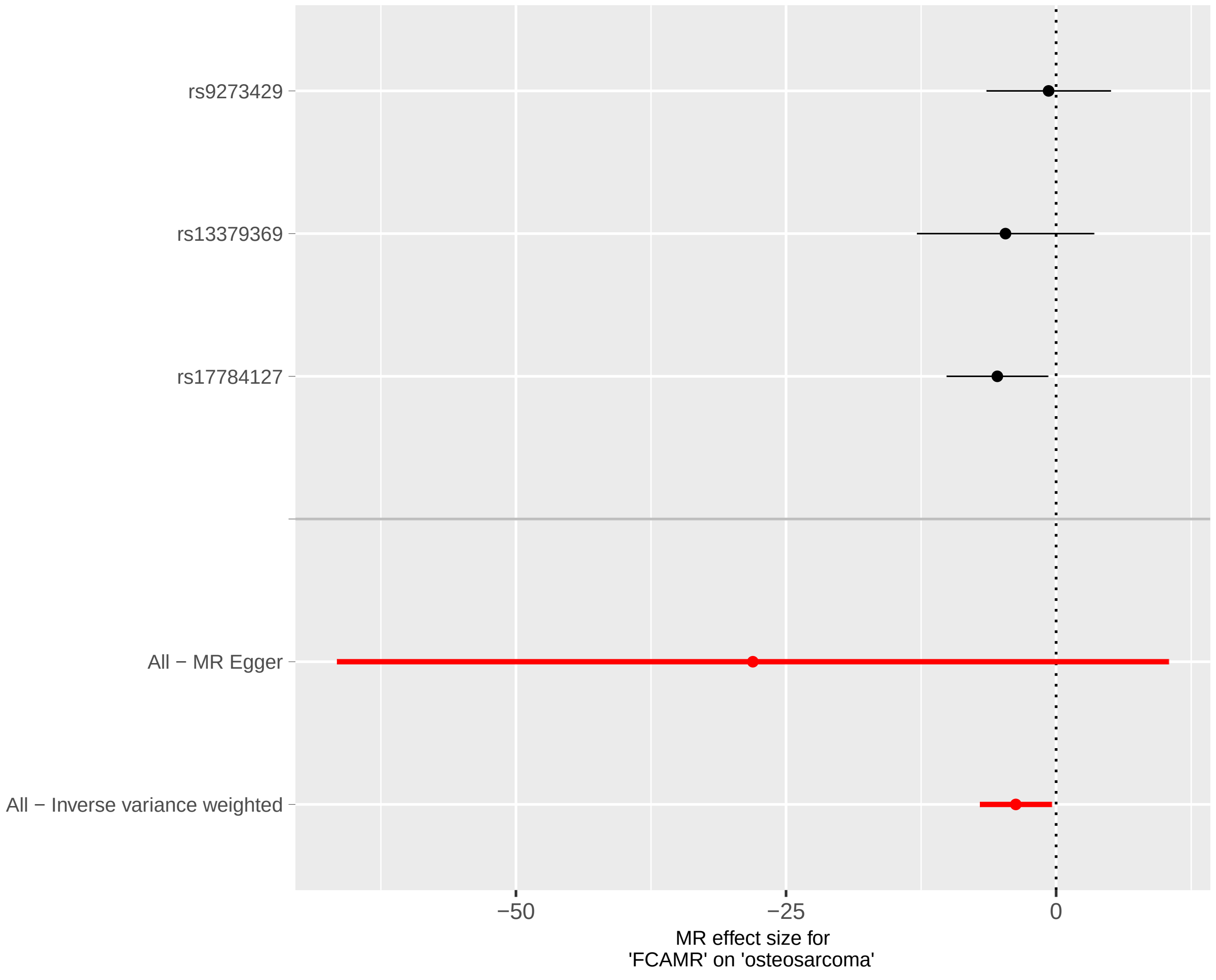
rs9273429
rs13379369
rs17784127
All − MR Egger
All − Inverse variance weighted
−50
−25
0
MR effect size for
'FCAMR' on 'osteosarcoma'

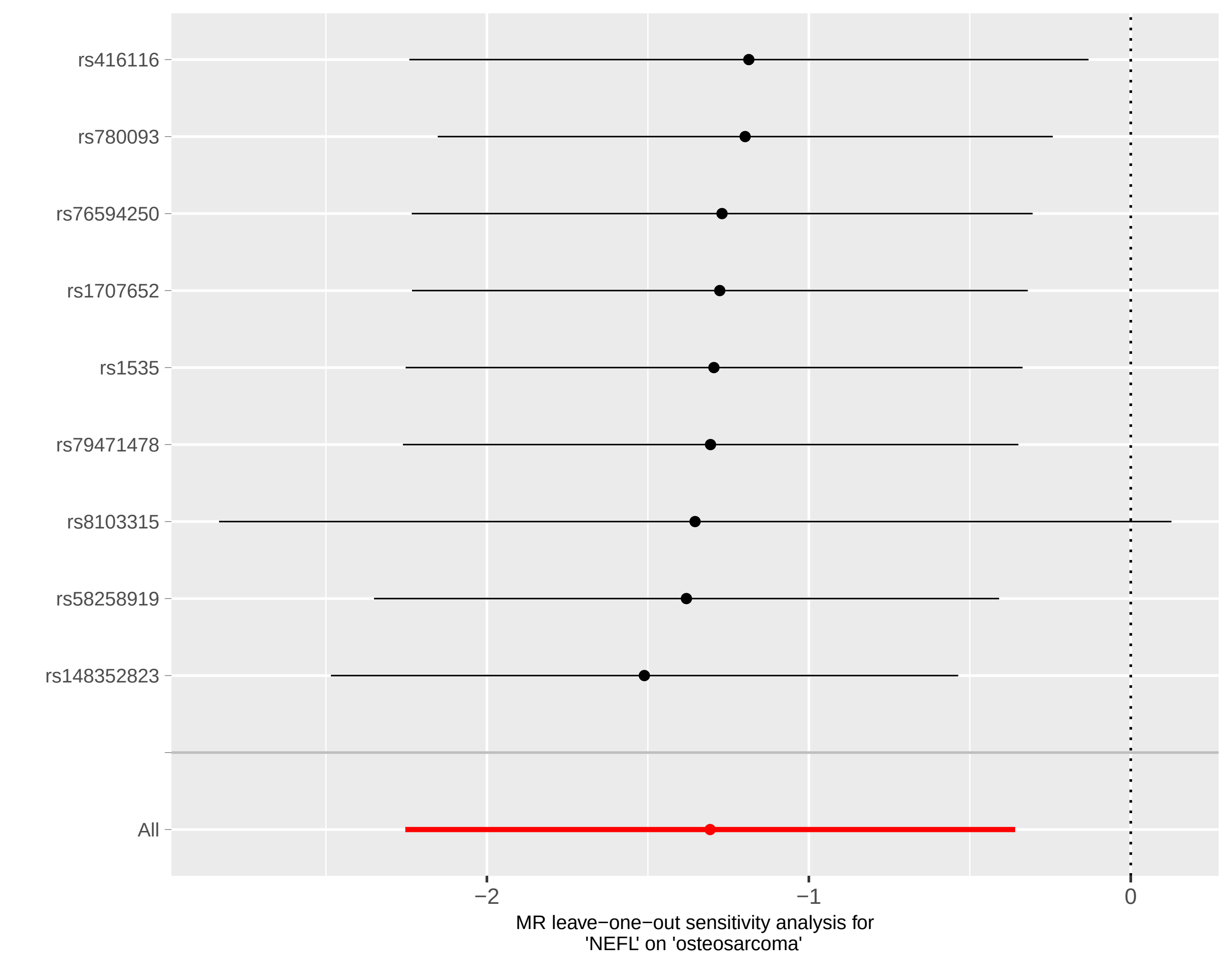

rs416116
rs780093
rs76594250
rs1707652
rs1535
rs79471478
rs8103315
rs58258919
rs148352823
All
−2
−1
0
MR leave−one−out sensitivity analysis for
'NEFL' on 'osteosarcoma'

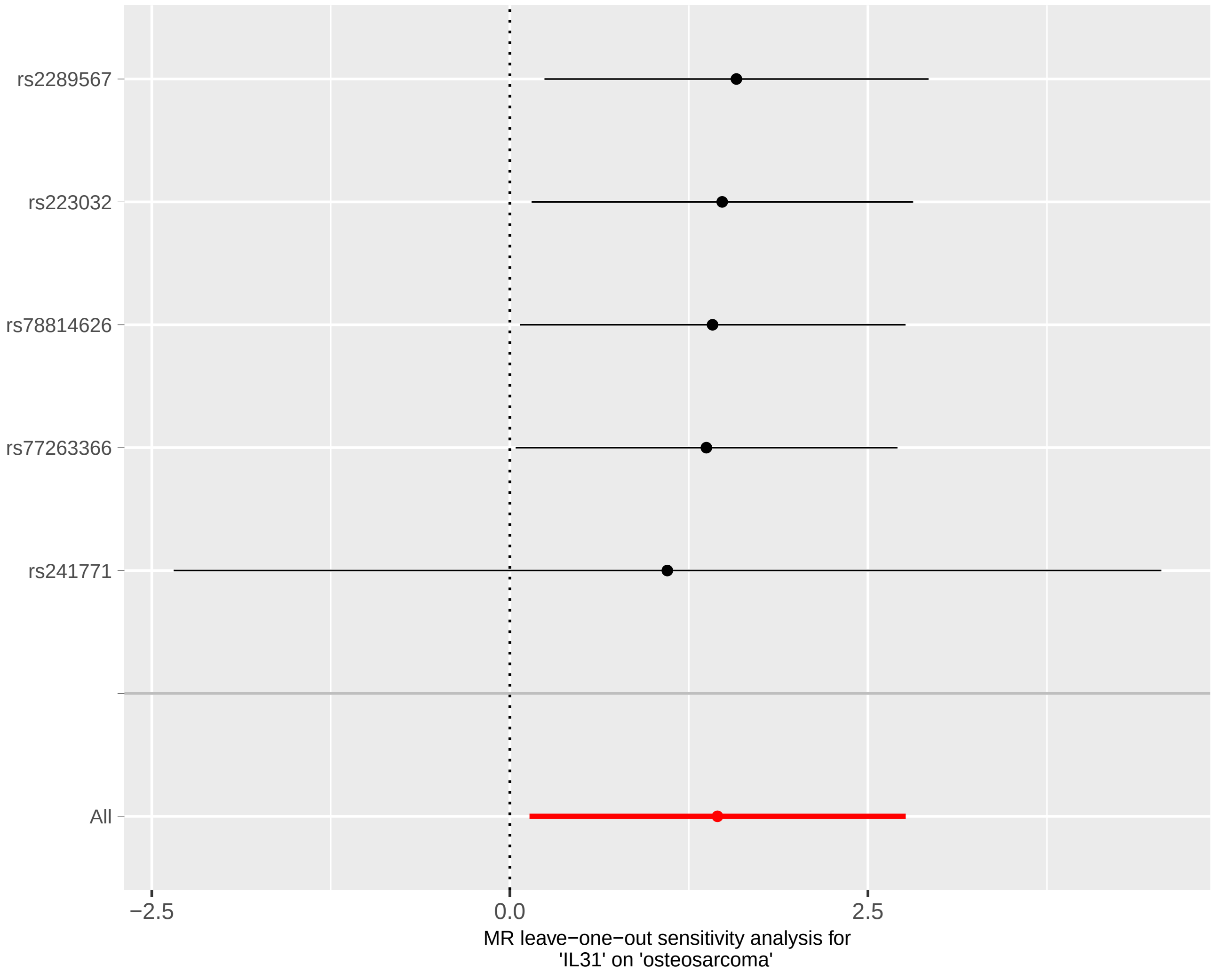

rs2289567
rs223032
rs78814626
rs77263366
rs241771
All
−2.5
0.0
2.5
MR leave−one−out sensitivity analysis for
'IL31' on 'osteosarcoma'

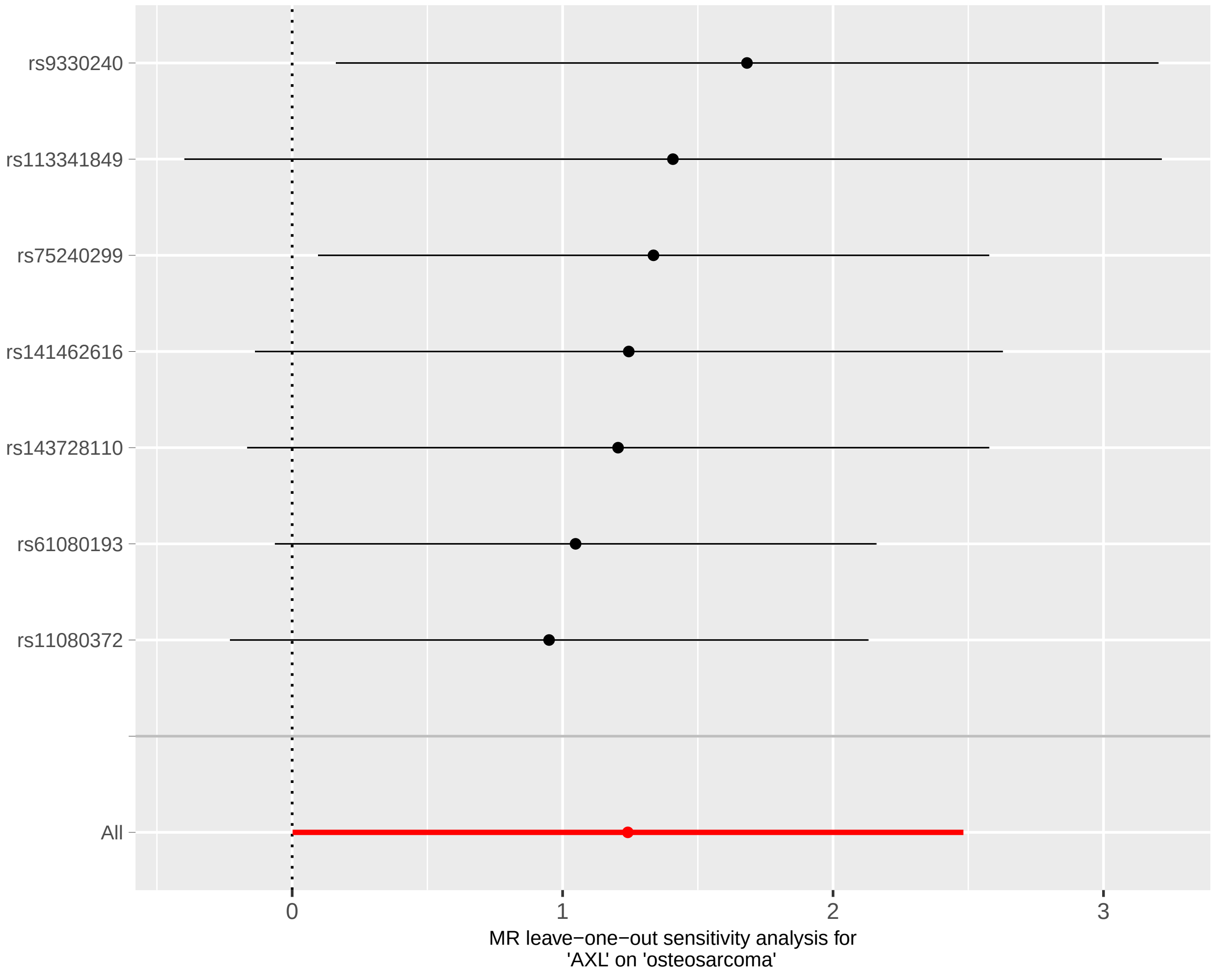
rs9330240
rs113341849
rs75240299
rs141462616
rs143728110
rs61080193
rs11080372
All
0
1
2
3
MR leave−one−out sensitivity analysis for
'AXL' on 'osteosarcoma'

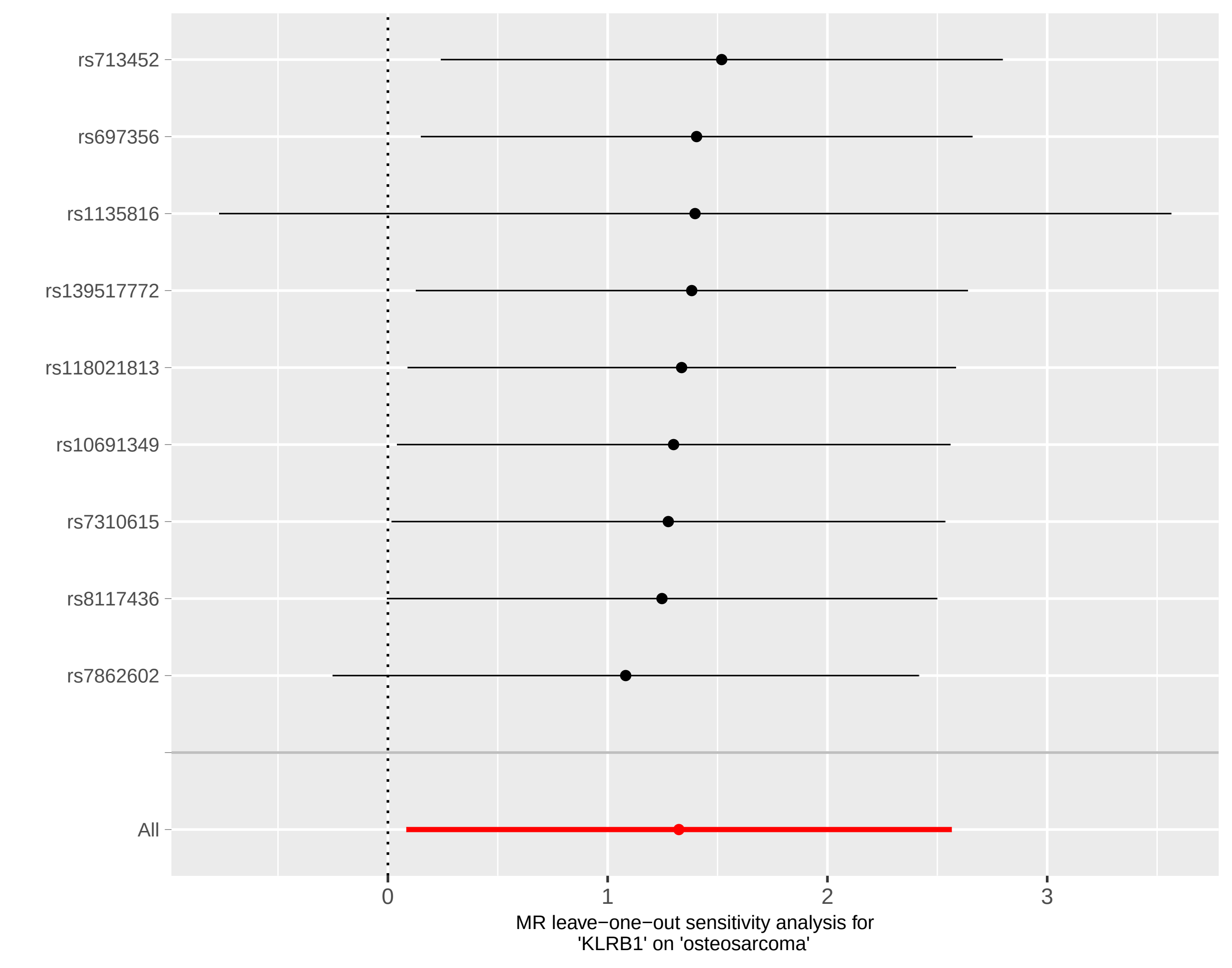
rs713452
rs697356
rs1135816
rs139517772
rs118021813
rs10691349
rs7310615
rs8117436
rs7862602
All
0
1
2
3
MR leave−one−out sensitivity analysis for
'KLRB1' on 'osteosarcoma'

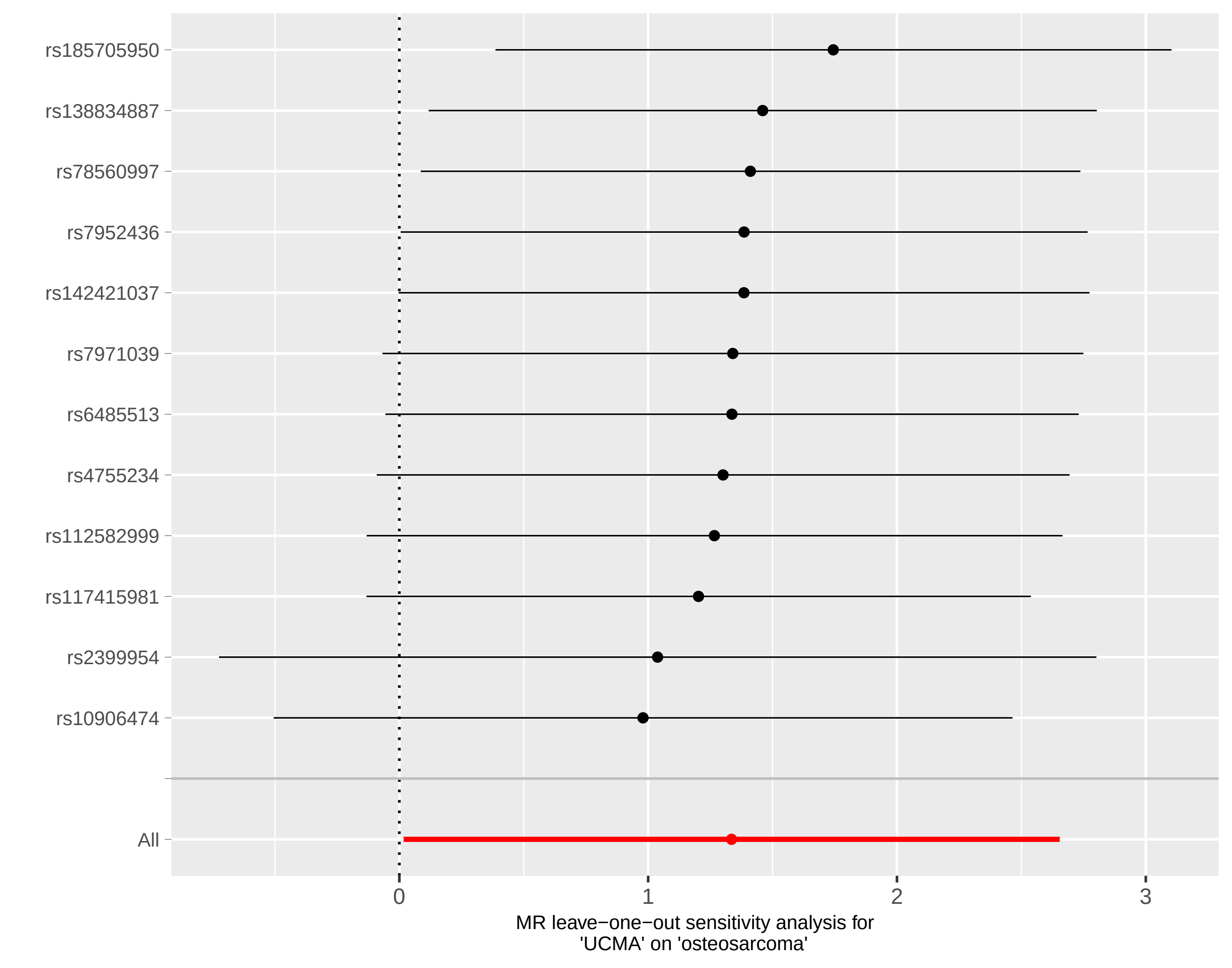

rs185705950
rs138834887
rs78560997
rs7952436
rs142421037
rs7971039
rs6485513
rs4755234
rs112582999
rs117415981
rs2399954
rs10906474
All
0
1
2
3
MR leave−one−out sensitivity analysis for
'UCMA' on 'osteosarcoma'

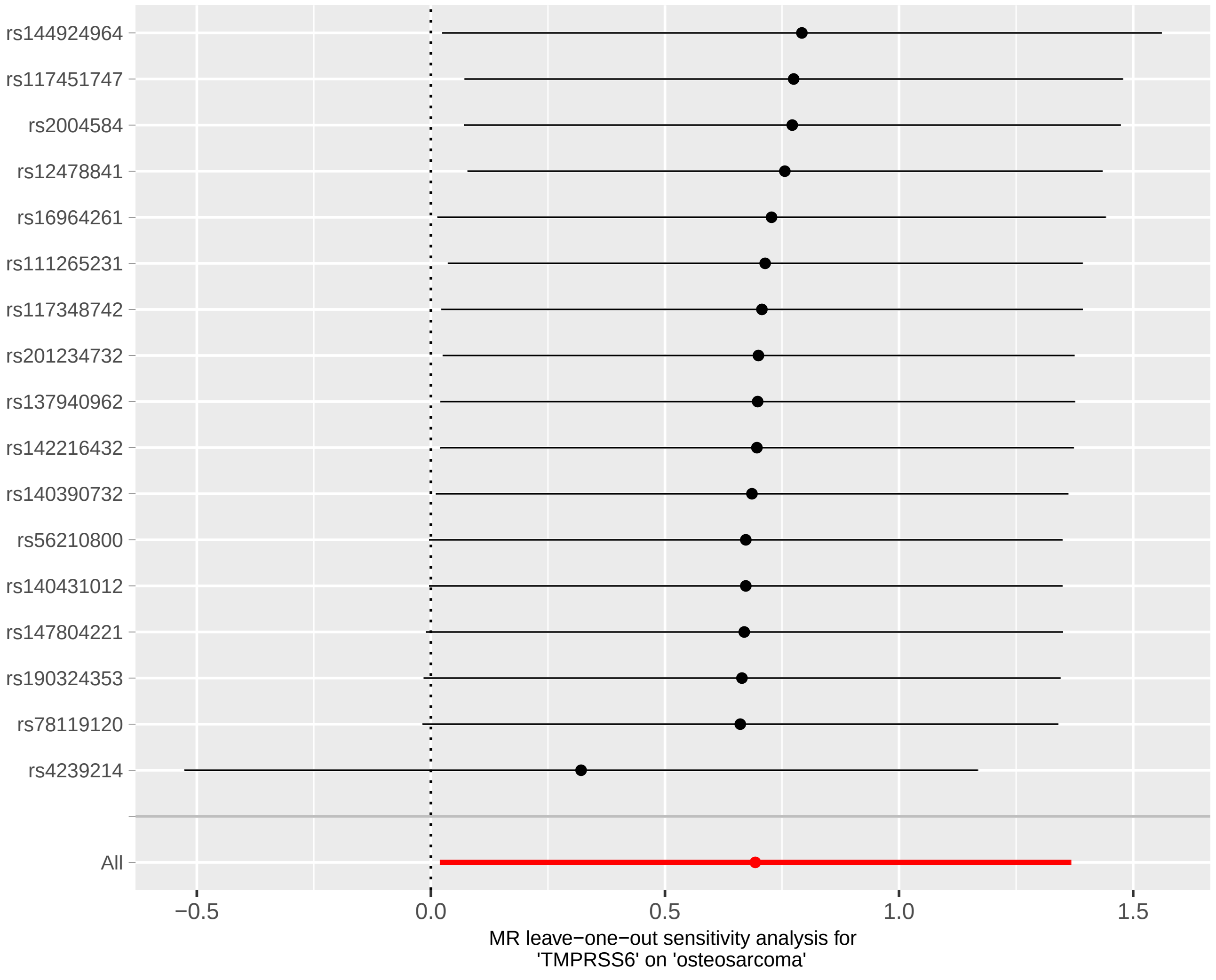
rs144924964
rs117451747
rs2004584
rs12478841
rs16964261
rs111265231
rs117348742
rs201234732
rs137940962
rs142216432
rs140390732
rs56210800
rs140431012
rs147804221
rs190324353
rs78119120
rs4239214
All
−0.5
0.0
0.5
1.0
1.5
MR leave−one−out sensitivity analysis for
'TMPRSS6' on 'osteosarcoma'

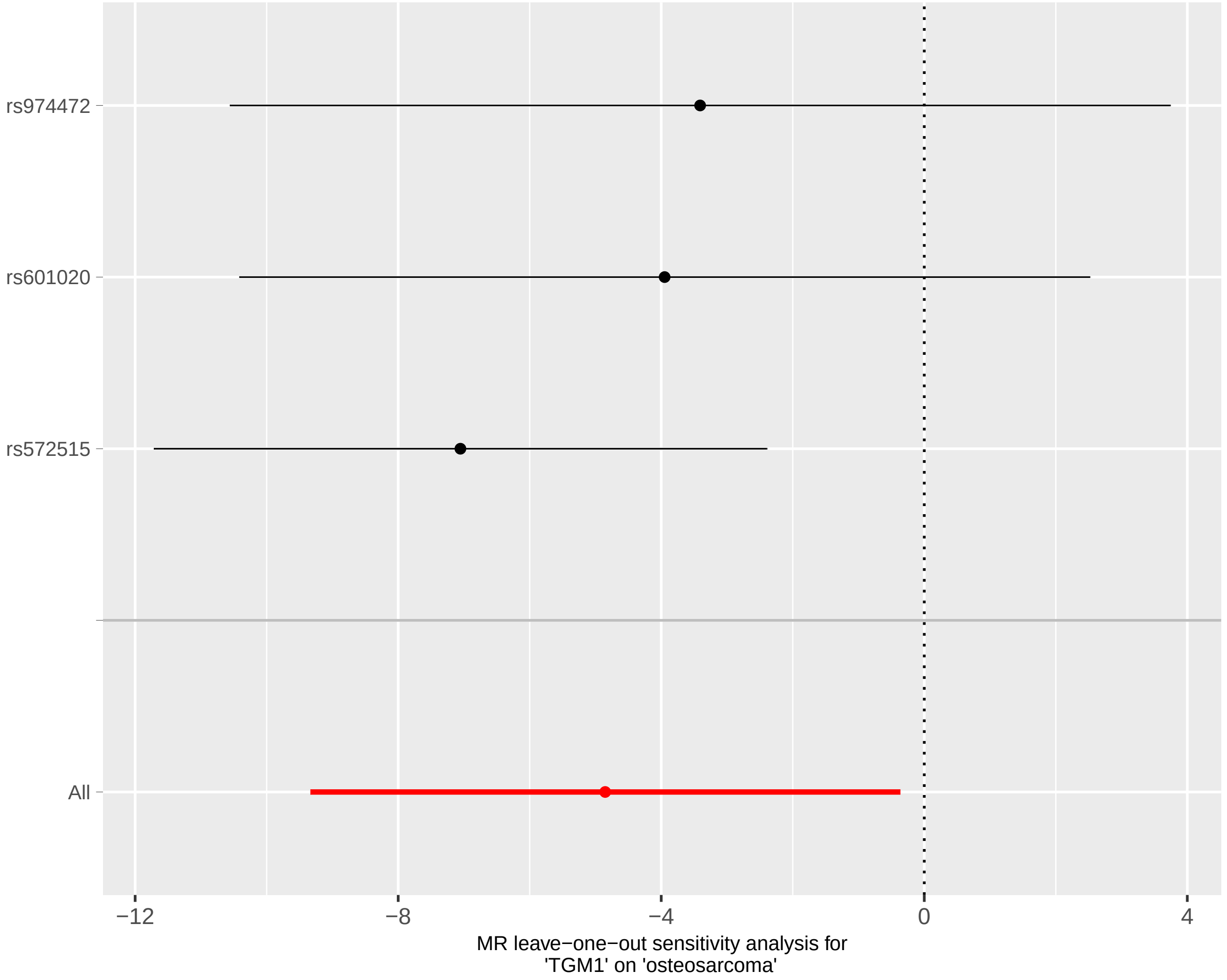

rs974472
rs601020
rs572515
All
−12
−8
−4
0
4
MR leave−one−out sensitivity analysis for
'TGM1' on 'osteosarcoma'

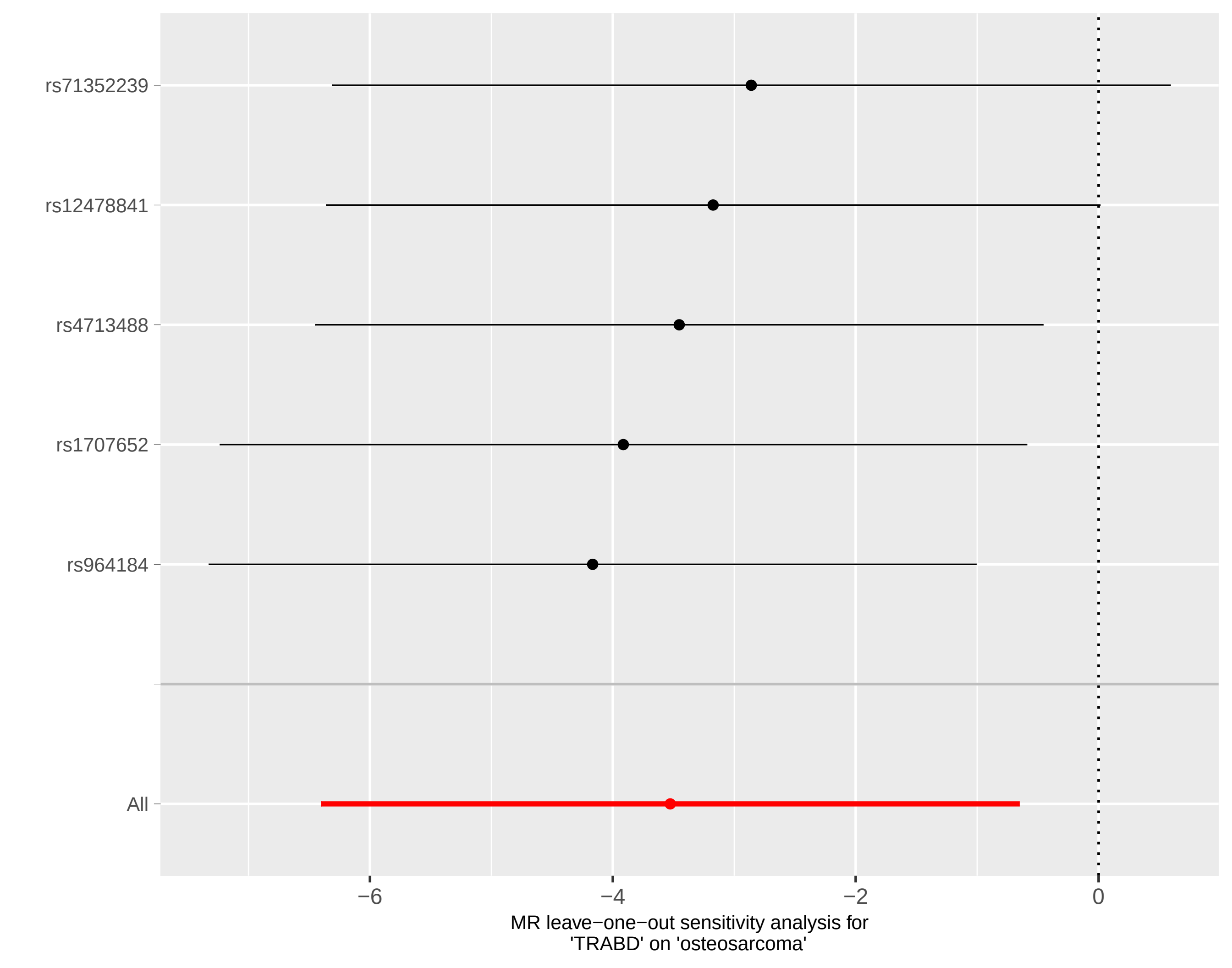
rs71352239
rs12478841
rs4713488
rs1707652
rs964184
All
−6
−4
−2
0
MR leave−one−out sensitivity analysis for
'TRABD' on 'osteosarcoma'

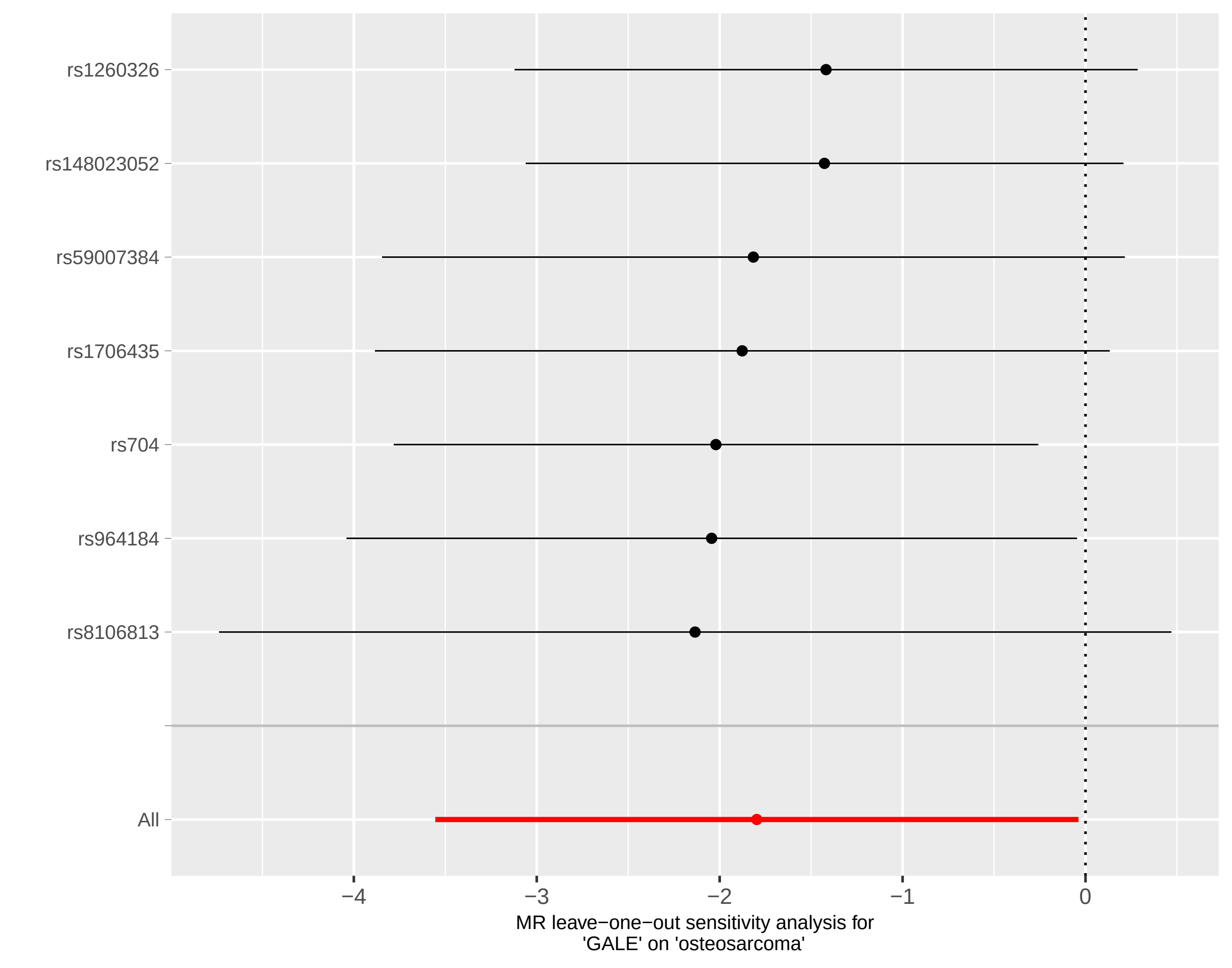
rs1260326
rs148023052
rs59007384
rs1706435
rs704
rs964184
rs8106813
All
−4
−3
−2
−1
0
MR leave−one−out sensitivity analysis for
'GALE' on 'osteosarcoma'

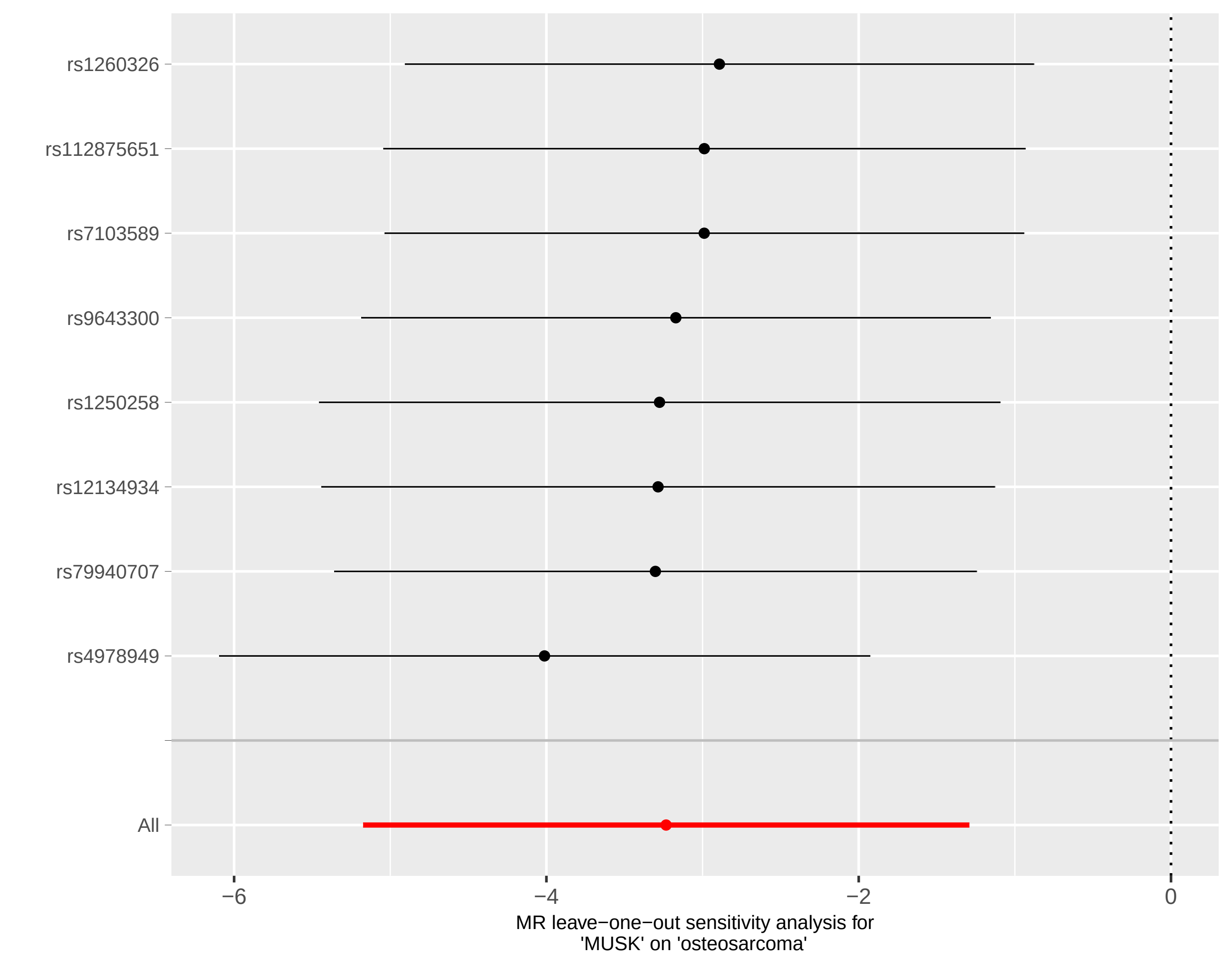

rs1260326
rs112875651
rs7103589
rs9643300
rs1250258
rs12134934
rs79940707
rs4978949
All
−6
−4
−2
0
MR leave−one−out sensitivity analysis for
'MUSK' on 'osteosarcoma'

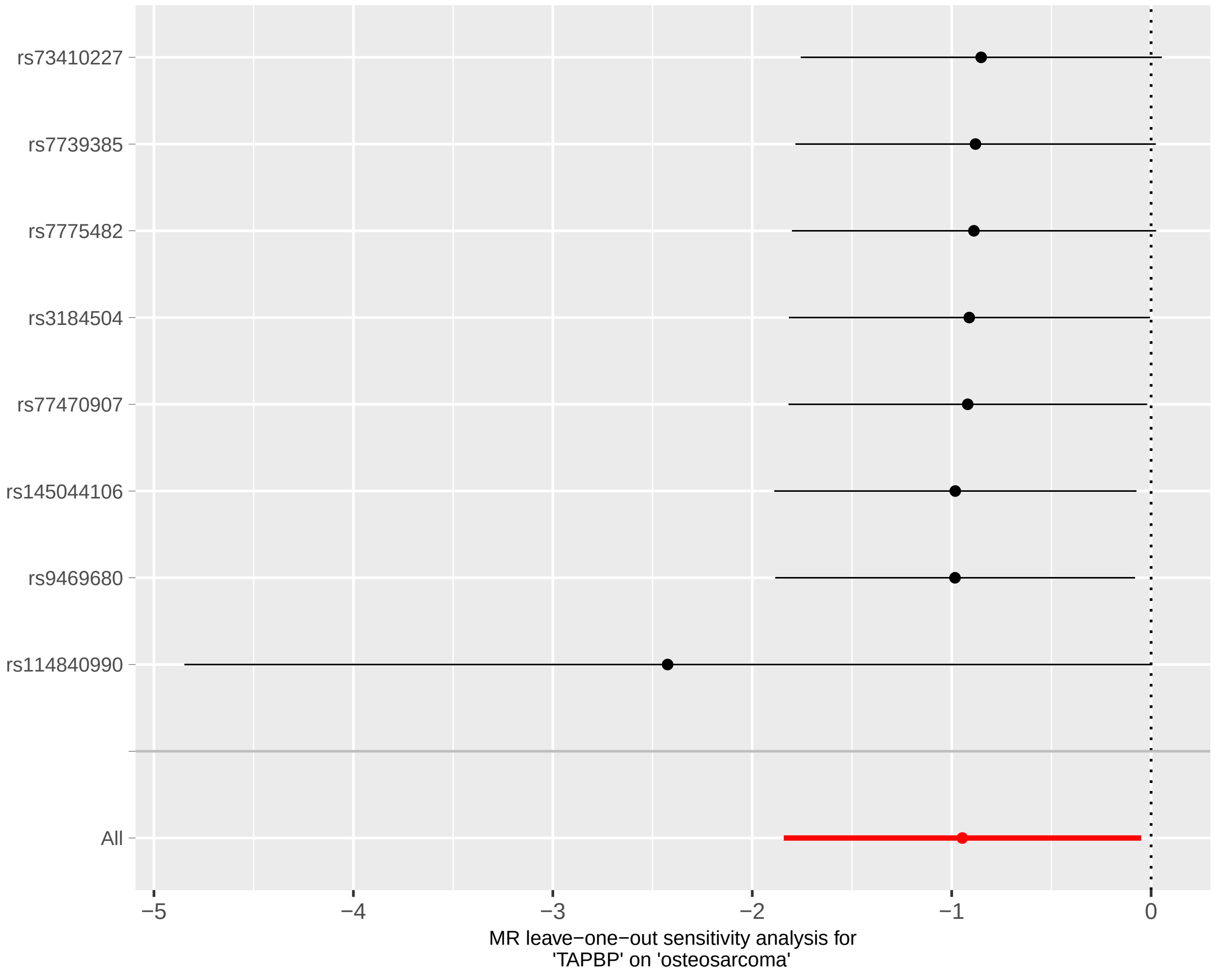

rs73410227
rs7739385
rs7775482
rs3184504
rs77470907
rs145044106
rs9469680
rs114840990
All
−5
−4
−3
−2
−1
0
MR leave−one−out sensitivity analysis for
'TAPBP' on 'osteosarcoma'

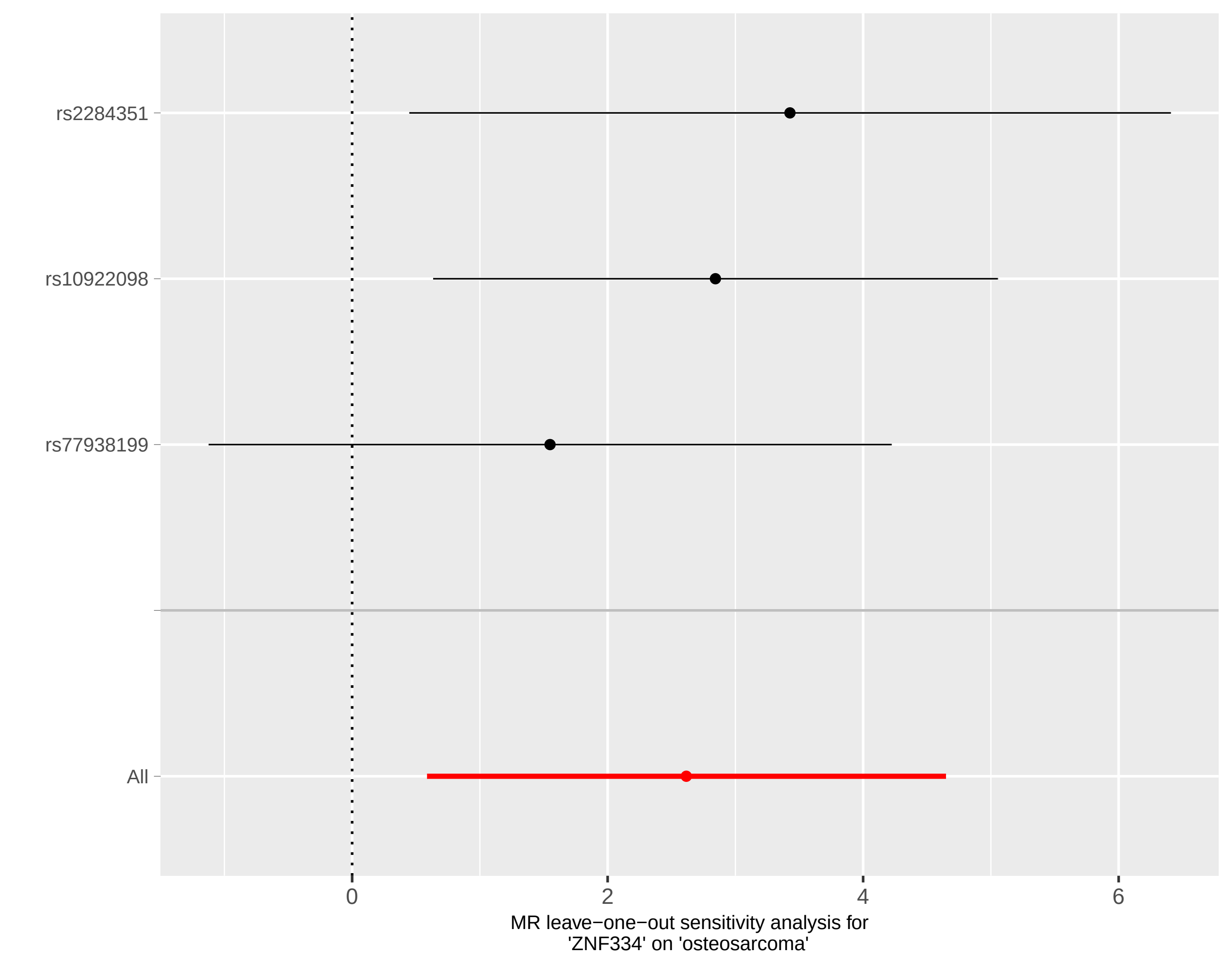

rs2284351
rs10922098
rs77938199
All
0
2
4
6
MR leave−one−out sensitivity analysis for
'ZNF334' on 'osteosarcoma'

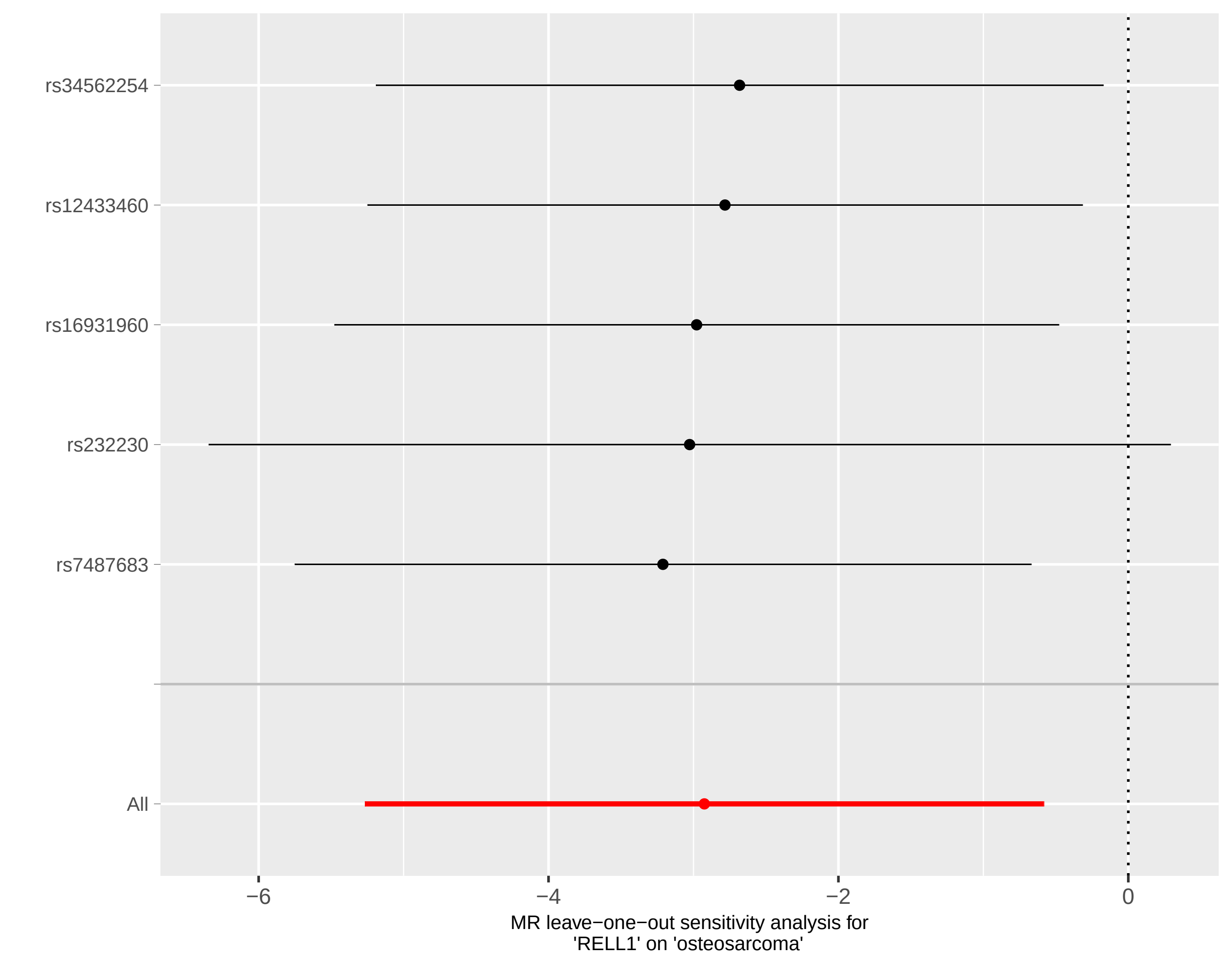

rs34562254
rs12433460
rs16931960
rs232230
rs7487683
All
−6
−4
−2
0
MR leave−one−out sensitivity analysis for
'RELL1' on 'osteosarcoma'

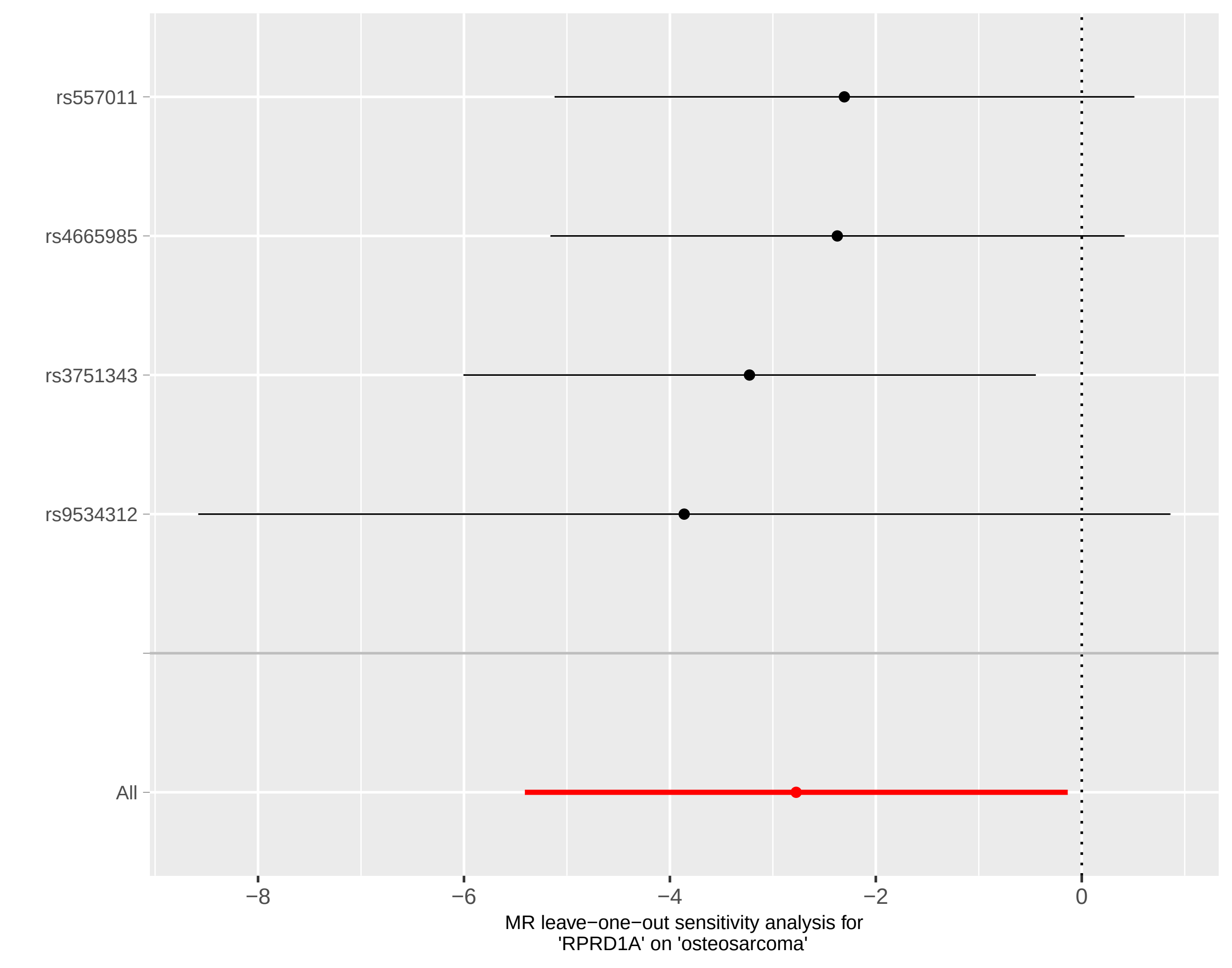
rs557011
rs4665985
rs3751343
rs9534312
All
−8
−6
−4
−2
0
MR leave−one−out sensitivity analysis for
'RPRD1A' on 'osteosarcoma'

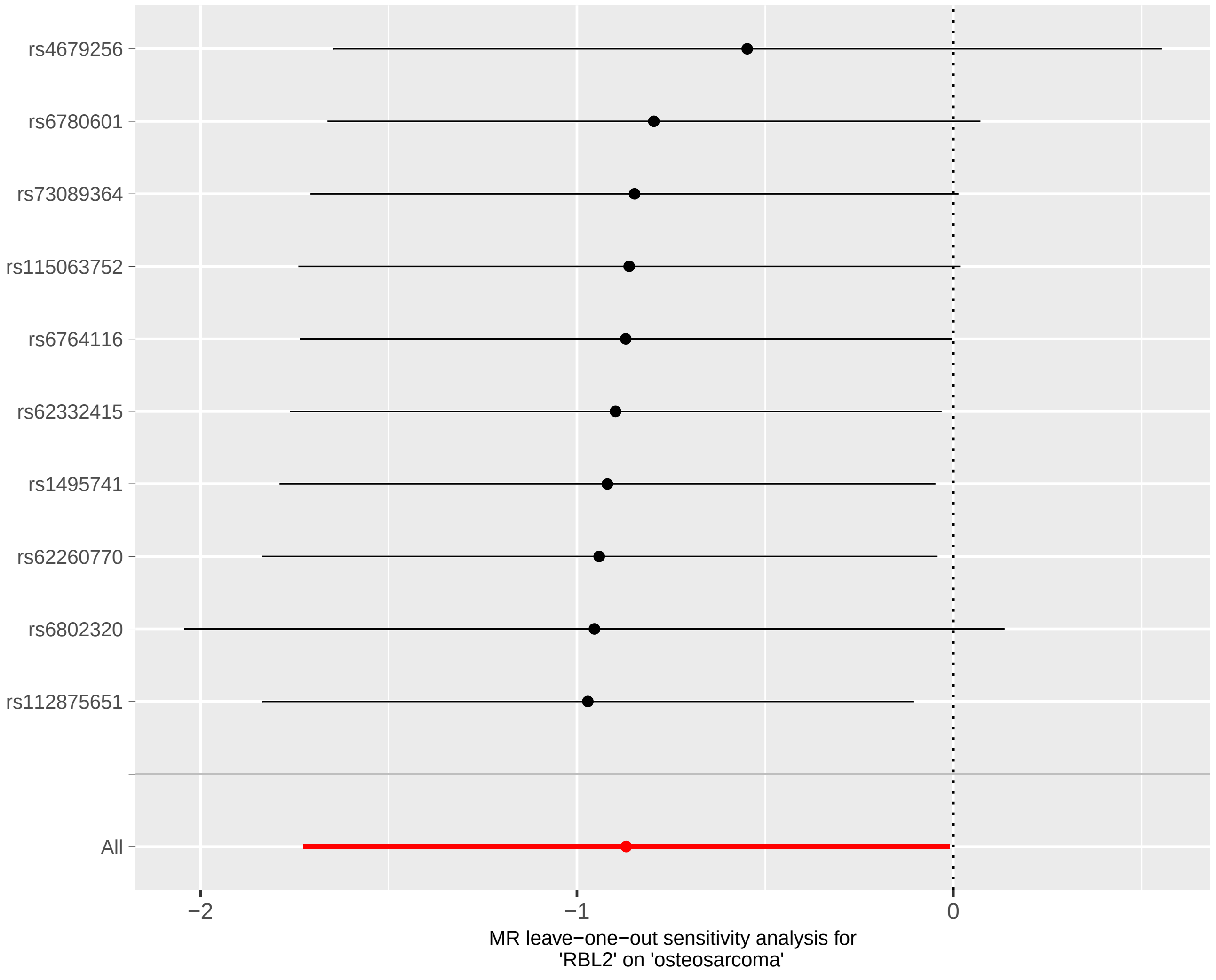
rs4679256
rs6780601
rs73089364
rs115063752
rs6764116
rs62332415
rs1495741
rs62260770
rs6802320
rs112875651
All
−2
−1
0
MR leave−one−out sensitivity analysis for
'RBL2' on 'osteosarcoma'

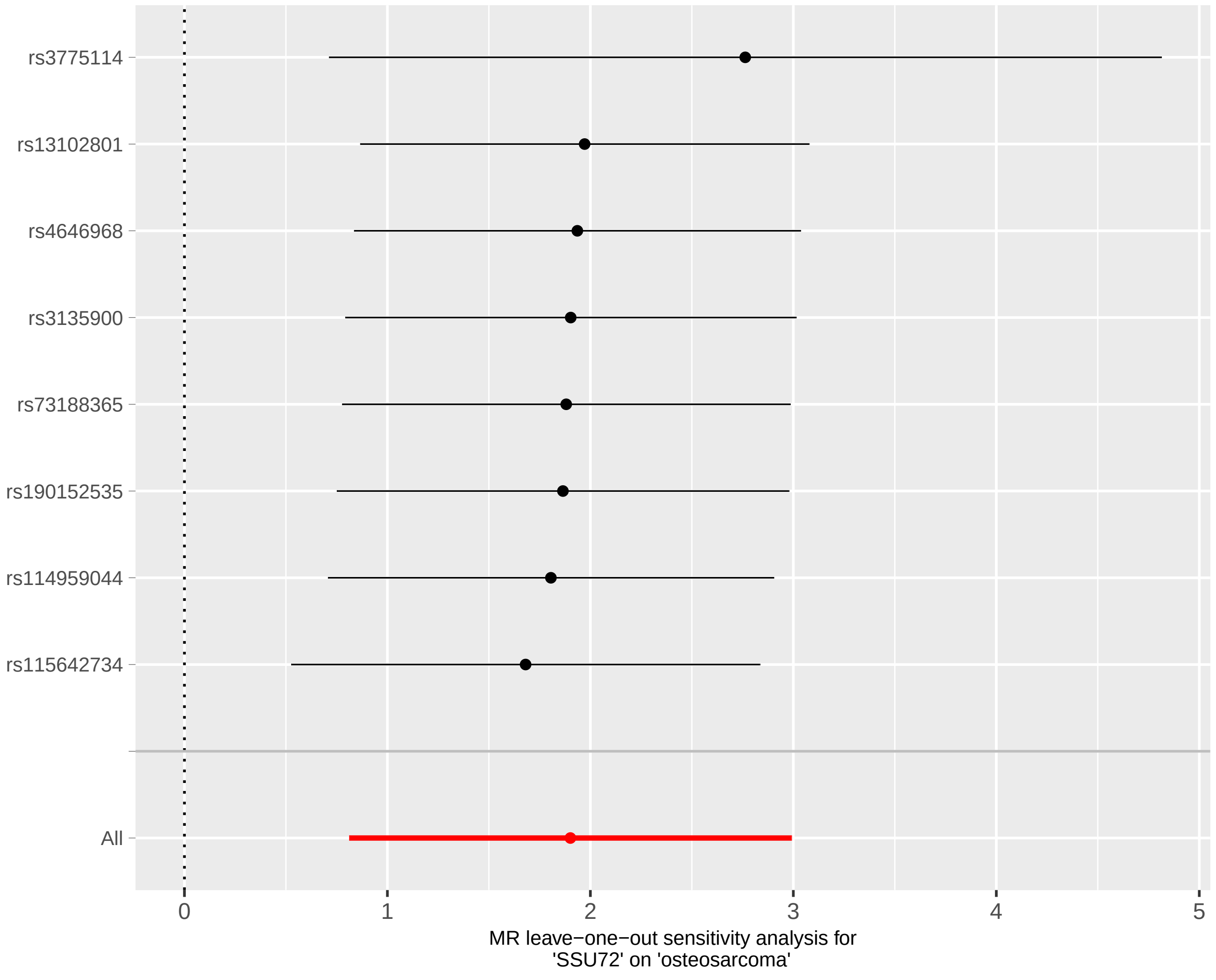
rs3775114
rs13102801
rs4646968
rs3135900
rs73188365
rs190152535
rs114959044
rs115642734
All
0
1
2
3
4
5
MR leave−one−out sensitivity analysis for
'SSU72' on 'osteosarcoma'

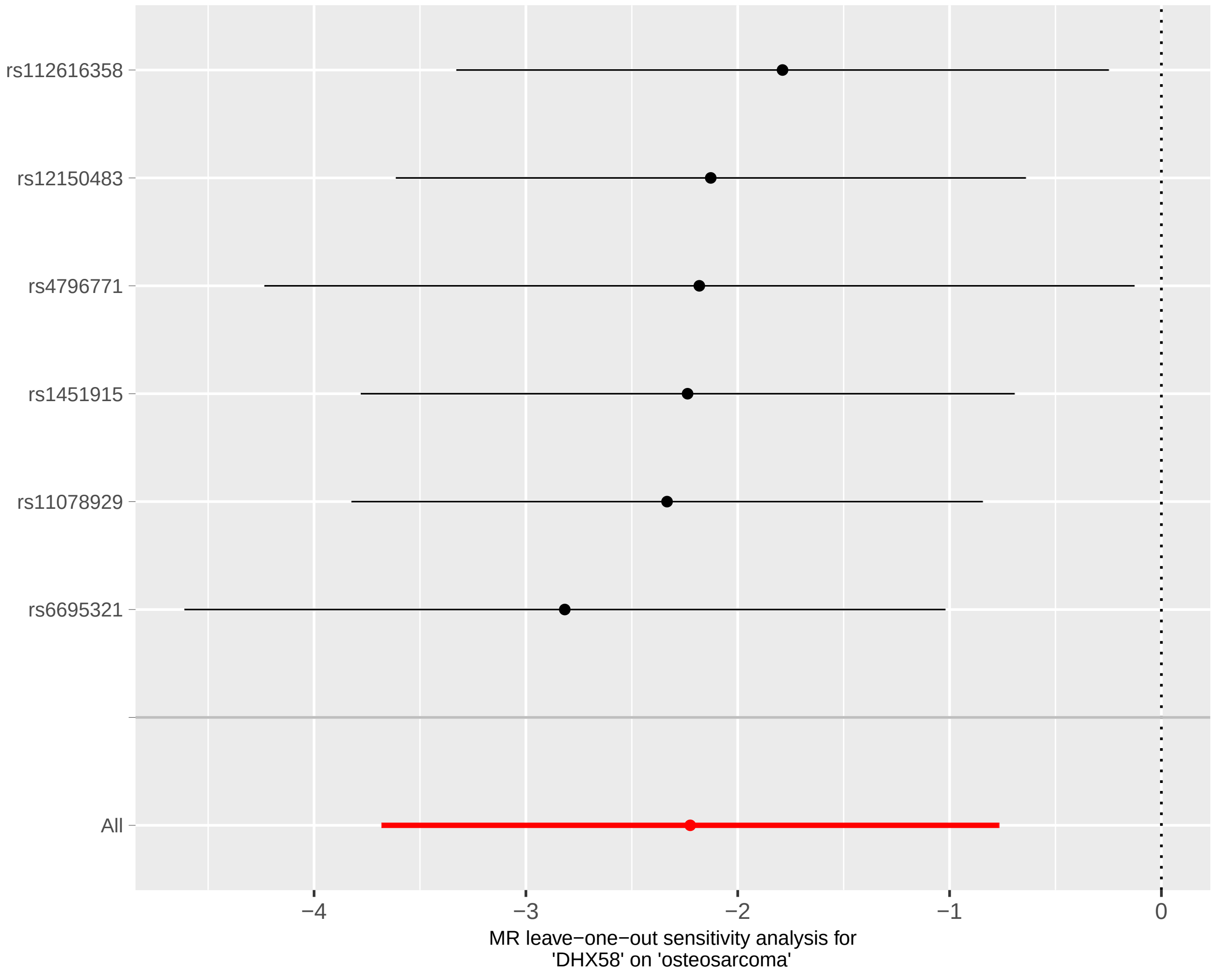

rs112616358
rs12150483
rs4796771
rs1451915
rs11078929
rs6695321
All
−4
−3
−2
−1
0
MR leave−one−out sensitivity analysis for
'DHX58' on 'osteosarcoma'

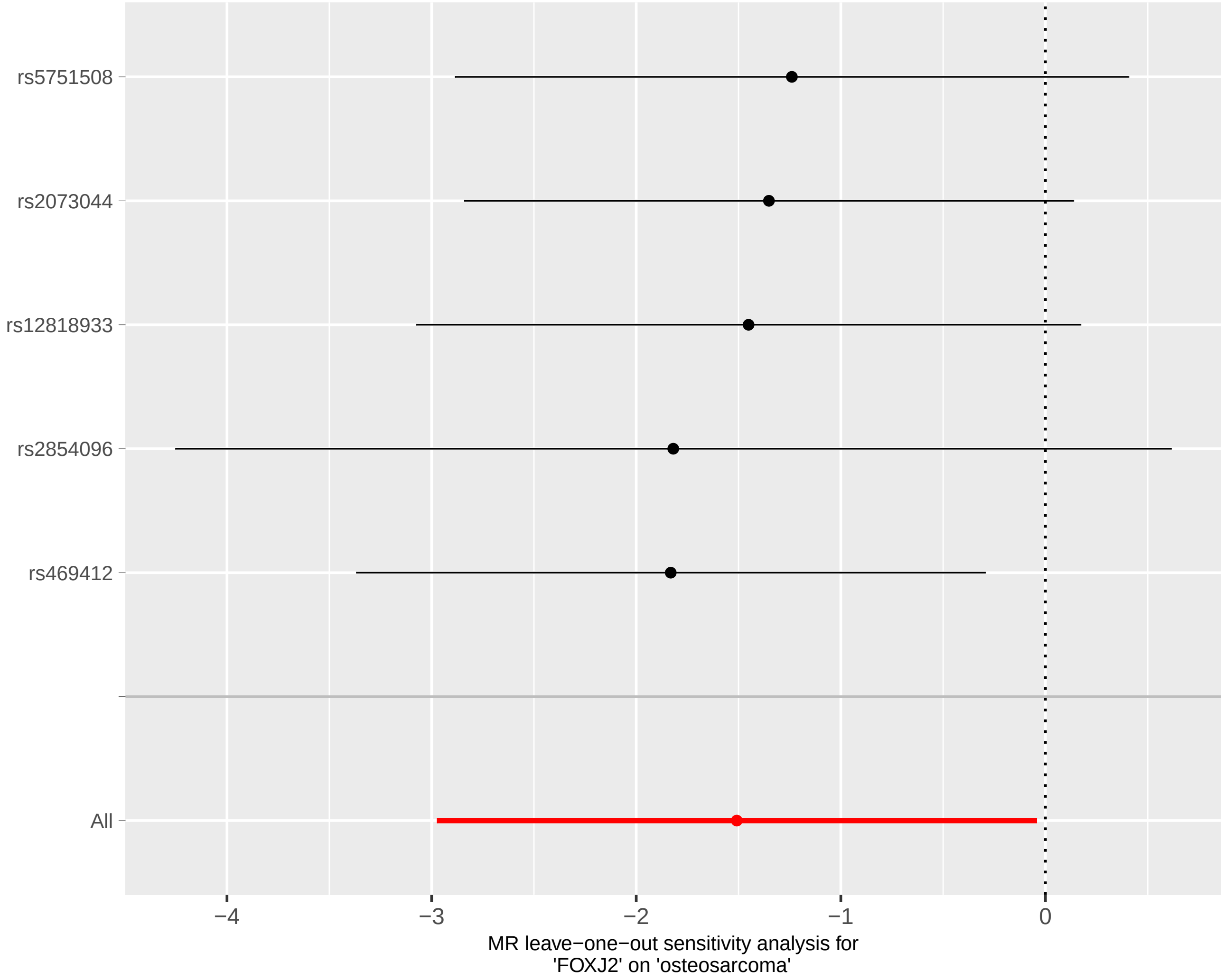

rs5751508
rs2073044
rs12818933
rs2854096
rs469412
All
−4
−3
−2
−1
0
MR leave−one−out sensitivity analysis for
'FOXJ2' on 'osteosarcoma'

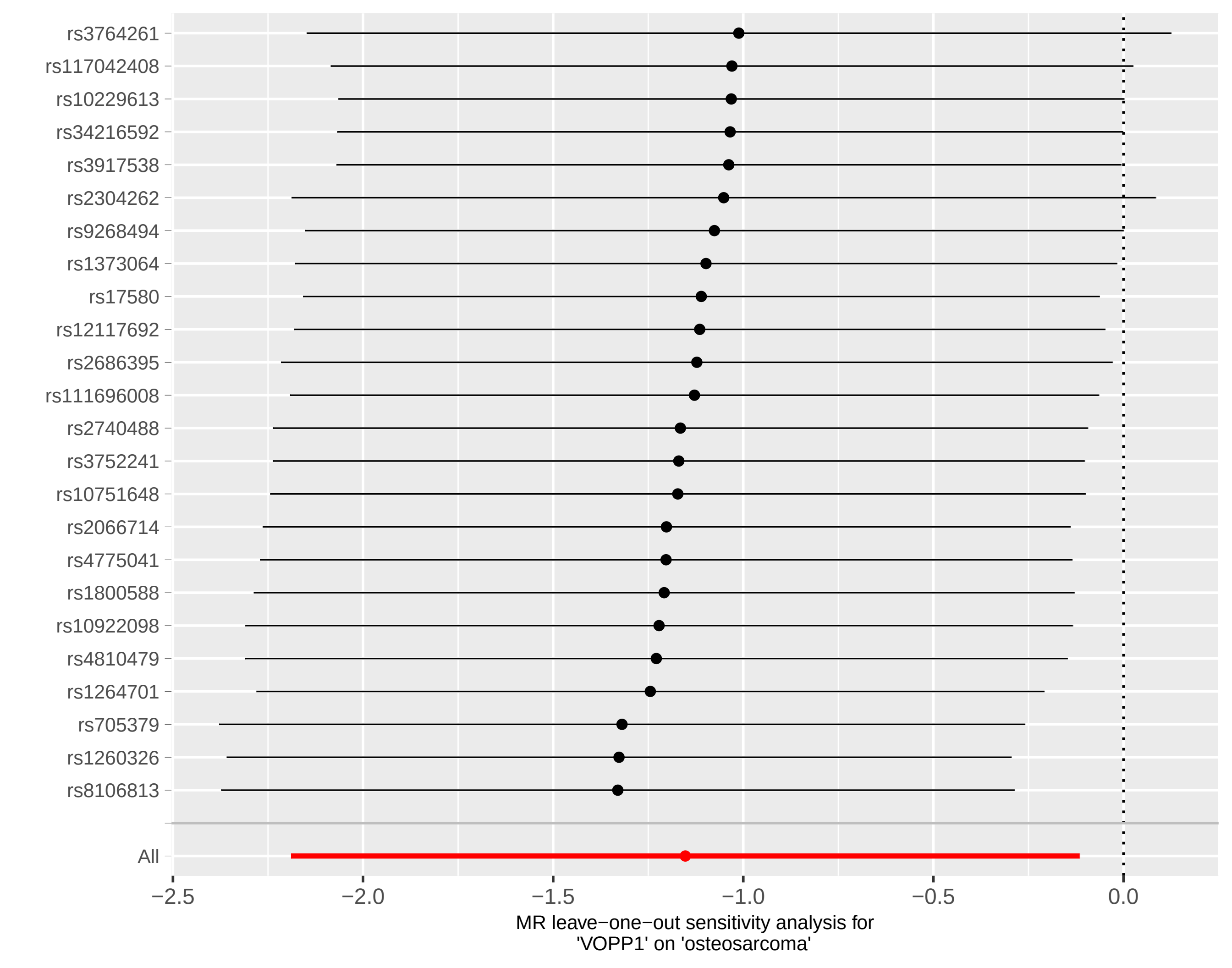

rs3764261
rs117042408
rs10229613
rs34216592
rs3917538
rs2304262
rs9268494
rs1373064
rs17580
rs12117692
rs2686395
rs111696008
rs2740488
rs3752241
rs10751648
rs2066714
rs4775041
rs1800588
rs10922098
rs4810479
rs1264701
rs705379
rs1260326
rs8106813
All
−2.5
−2.0
−1.5
−1.0
−0.5
0.0
MR leave−one−out sensitivity analysis for
'VOPP1' on 'osteosarcoma'

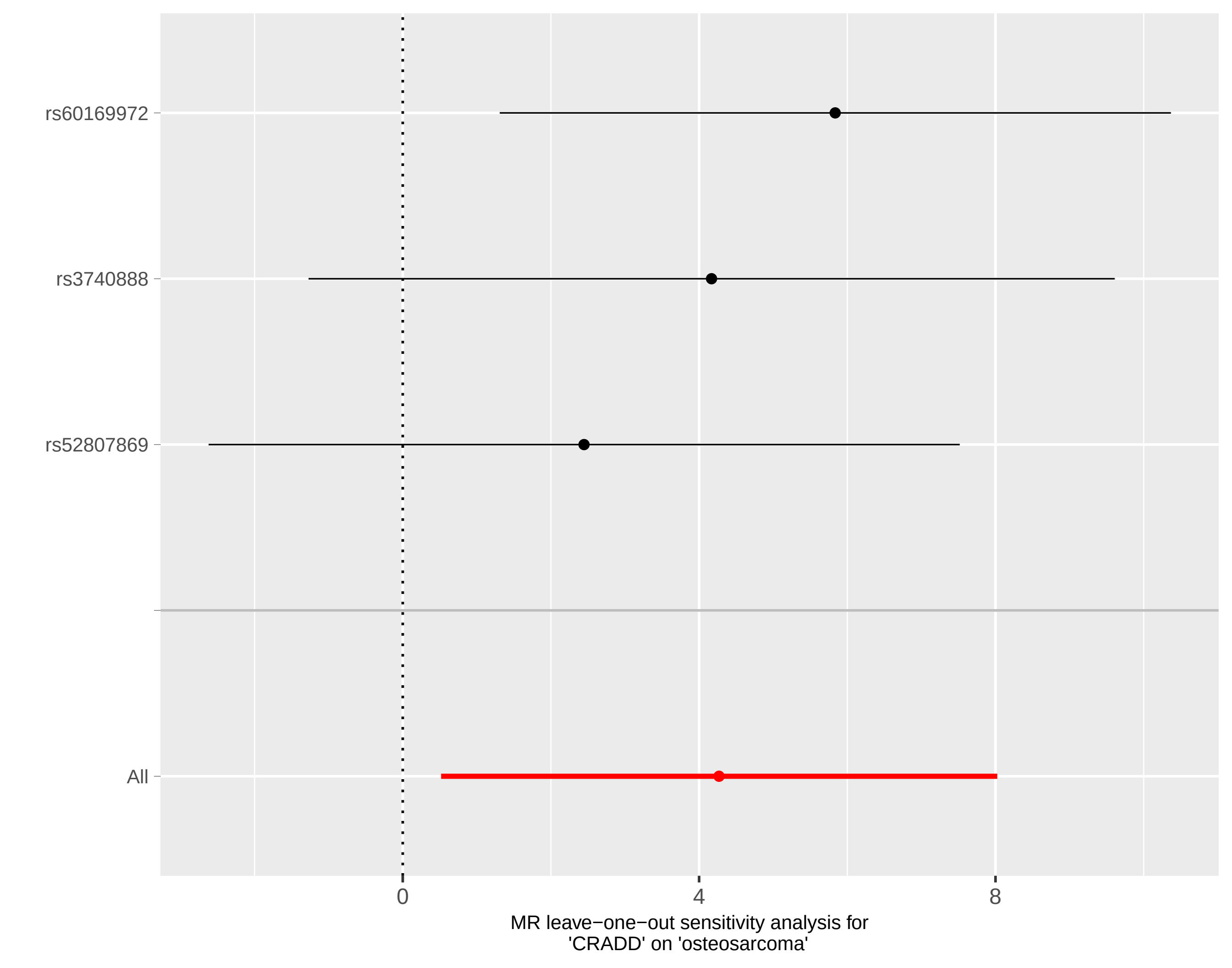
rs60169972
rs3740888
rs52807869
All
0
4
8
MR leave−one−out sensitivity analysis for
'CRADD' on 'osteosarcoma'

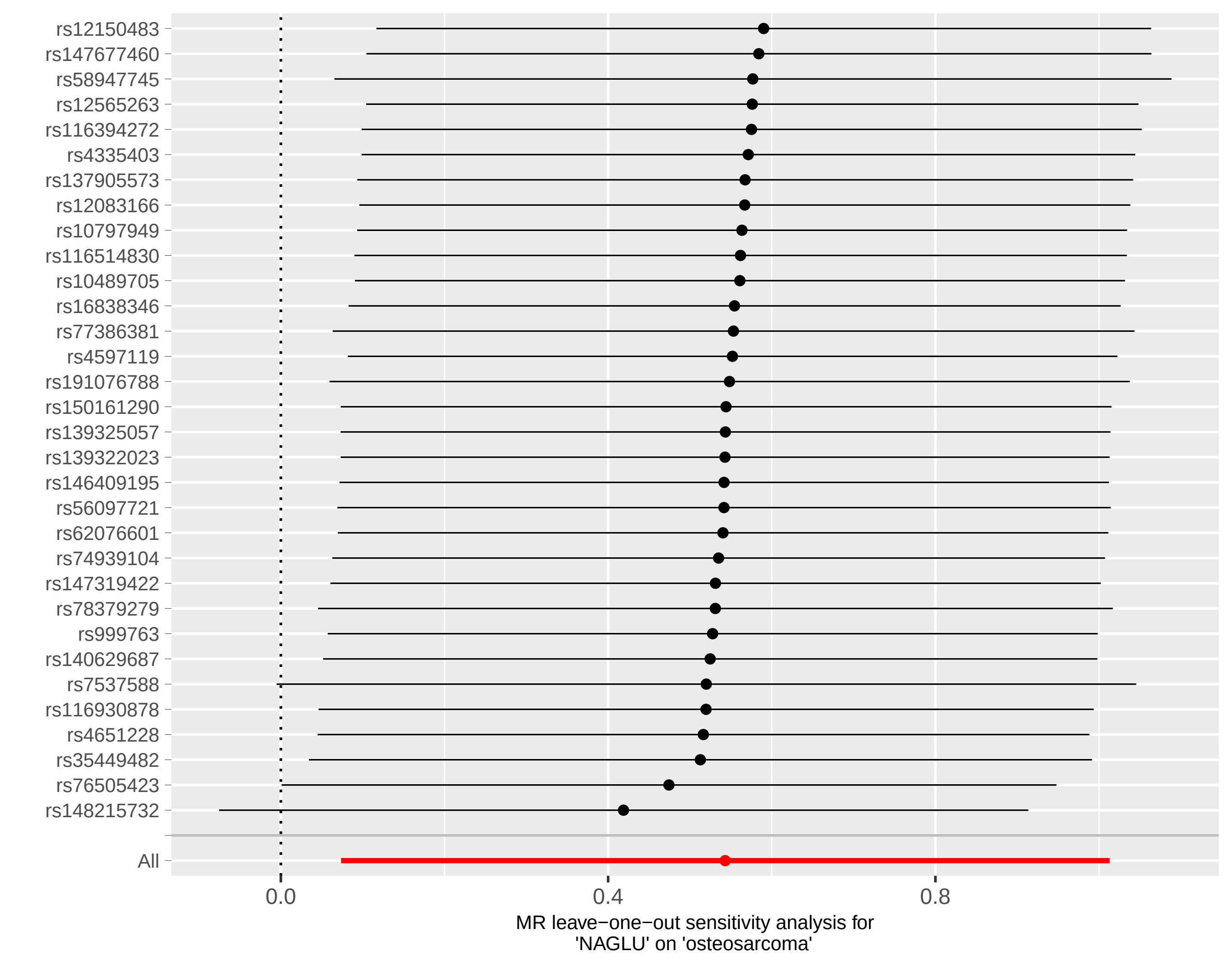
rs12150483
rs147677460
rs58947745
rs12565263
rs116394272
rs4335403
rs137905573
rs12083166
rs10797949
rs116514830
rs10489705
rs16838346
rs77386381
rs4597119
rs191076788
rs150161290
rs139325057
rs139322023
rs146409195
rs56097721
rs62076601
rs74939104
rs147319422
rs78379279
rs999763
rs140629687
rs7537588
rs116930878
rs4651228
rs35449482
rs76505423
rs148215732
All
0.0
0.4
0.8
MR leave−one−out sensitivity analysis for
'NAGLU' on 'osteosarcoma'

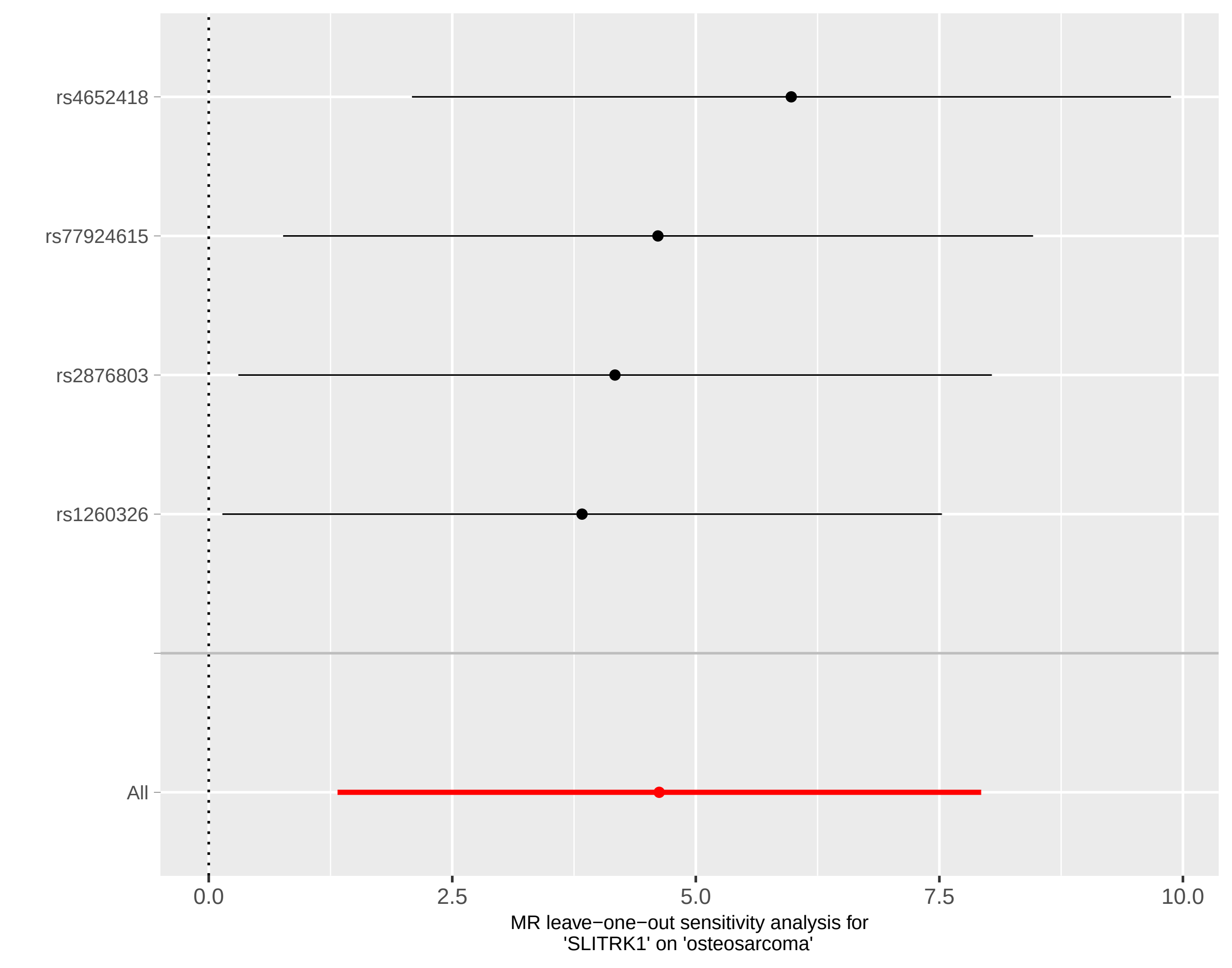
rs4652418
rs77924615
rs2876803
rs1260326
All
0.0
2.5
5.0
7.5
10.0
MR leave−one−out sensitivity analysis for
'SLITRK1' on 'osteosarcoma'

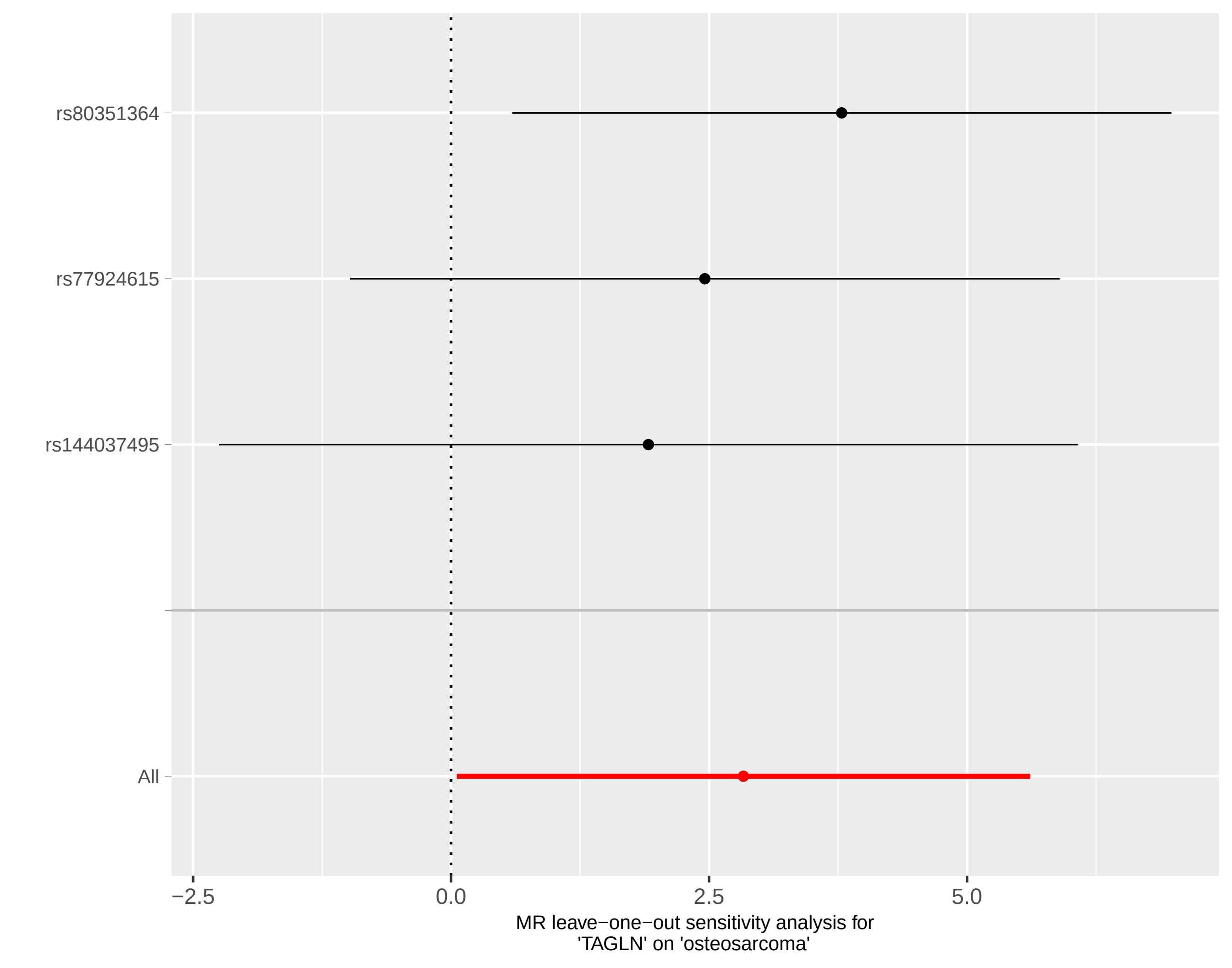
rs80351364
rs77924615
rs144037495
All
−2.5
0.0
2.5
5.0
MR leave−one−out sensitivity analysis for
'TAGLN' on 'osteosarcoma'

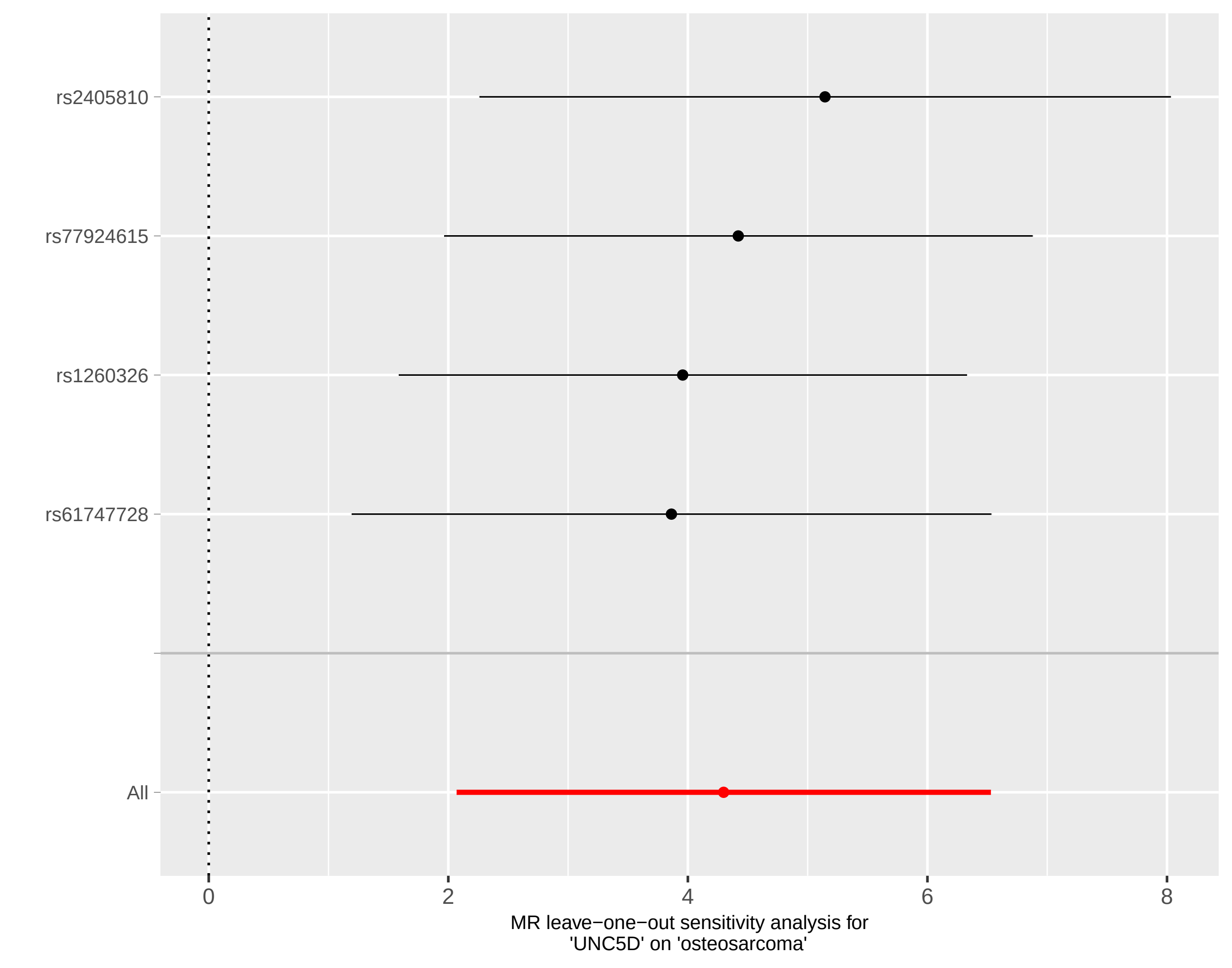
rs2405810
rs77924615
rs1260326
rs61747728
All
0
2
4
6
8
MR leave−one−out sensitivity analysis for
'UNC5D' on 'osteosarcoma'

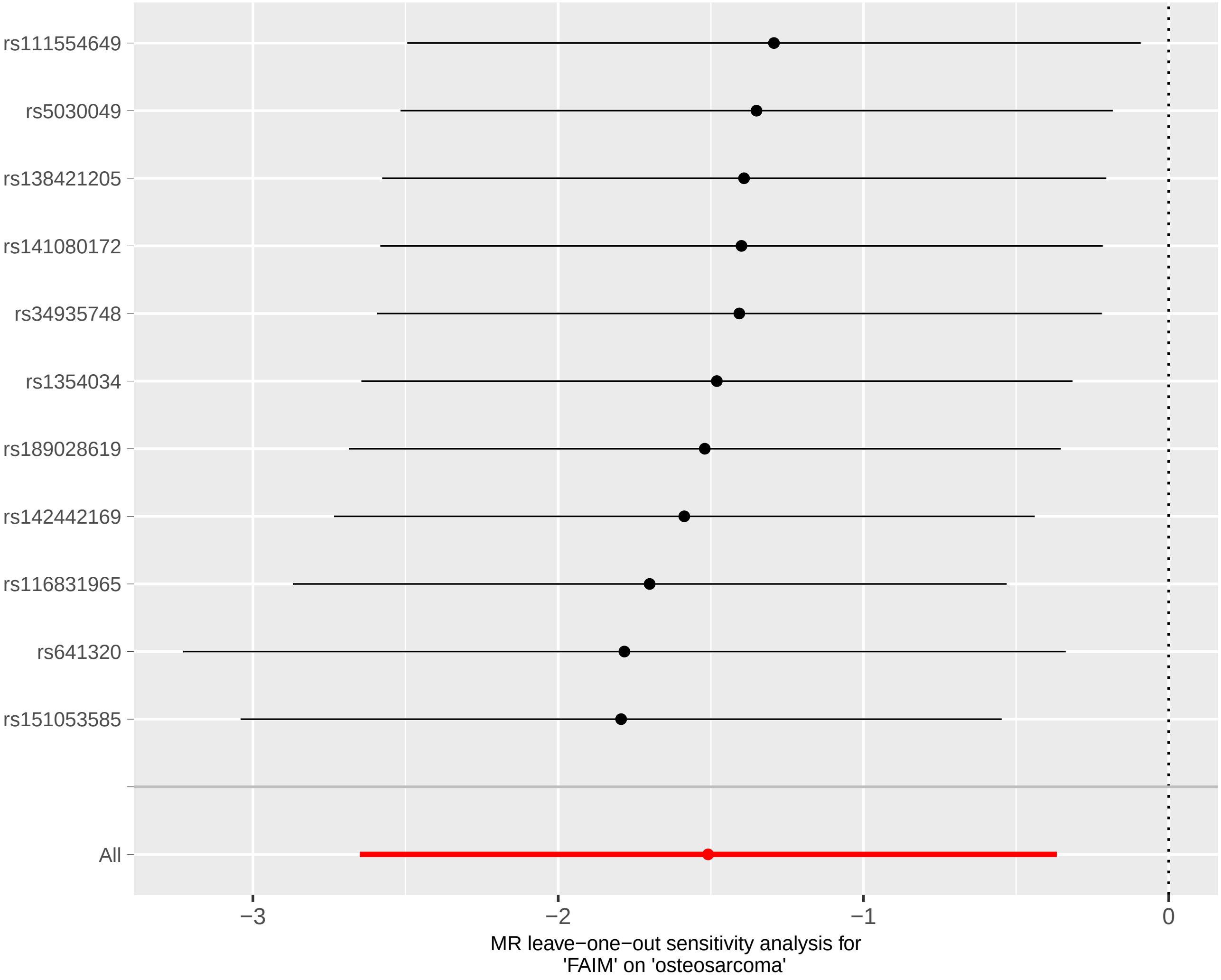
rs111554649
rs5030049
rs138421205
rs141080172
rs34935748
rs1354034
rs189028619
rs142442169
rs116831965
rs641320
rs151053585
All
−3
−2
−1
0
MR leave−one−out sensitivity analysis for
'FAIM' on 'osteosarcoma'

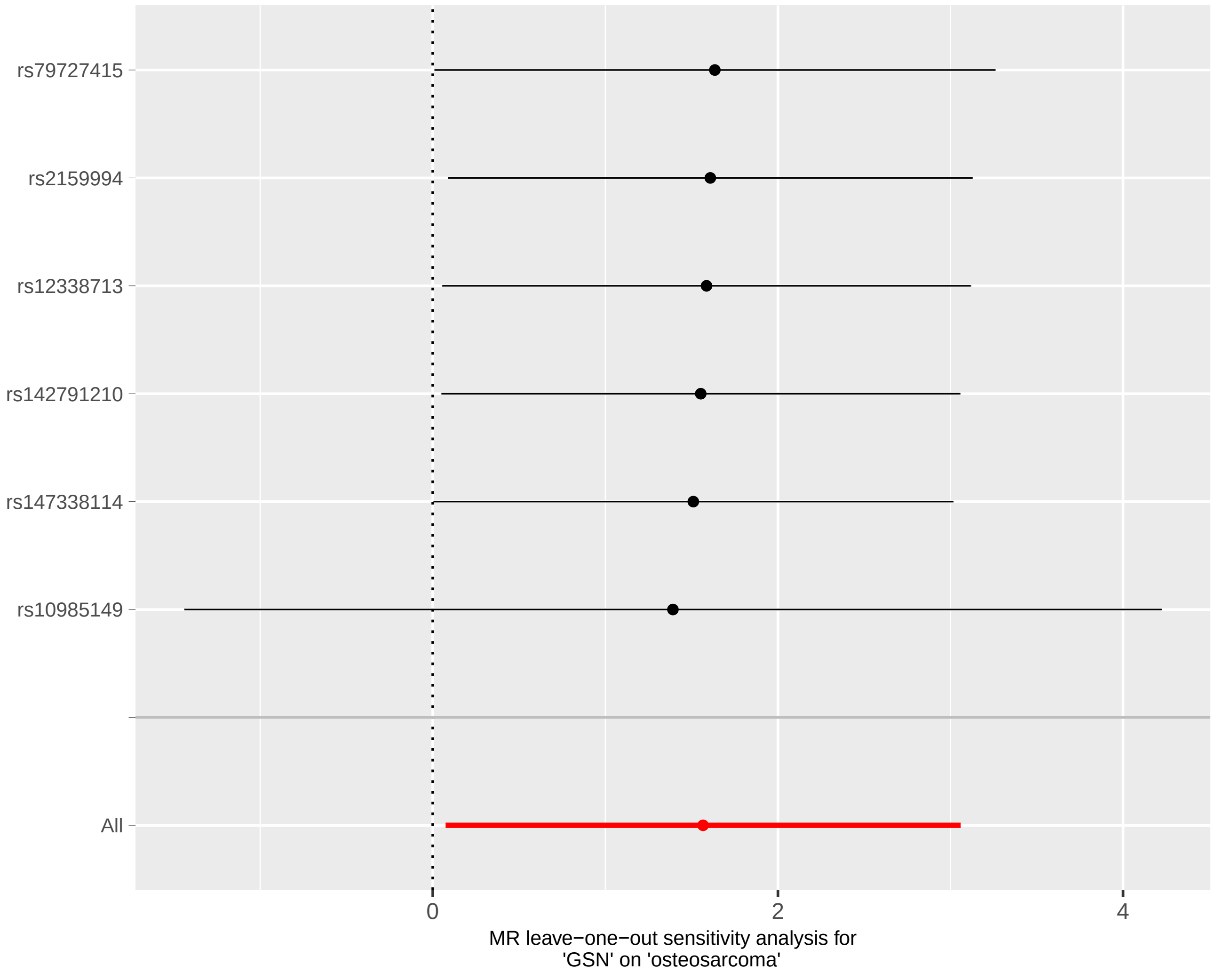
rs79727415
rs2159994
rs12338713
rs142791210
rs147338114
rs10985149
All
0
2
4
MR leave−one−out sensitivity analysis for
'GSN' on 'osteosarcoma'

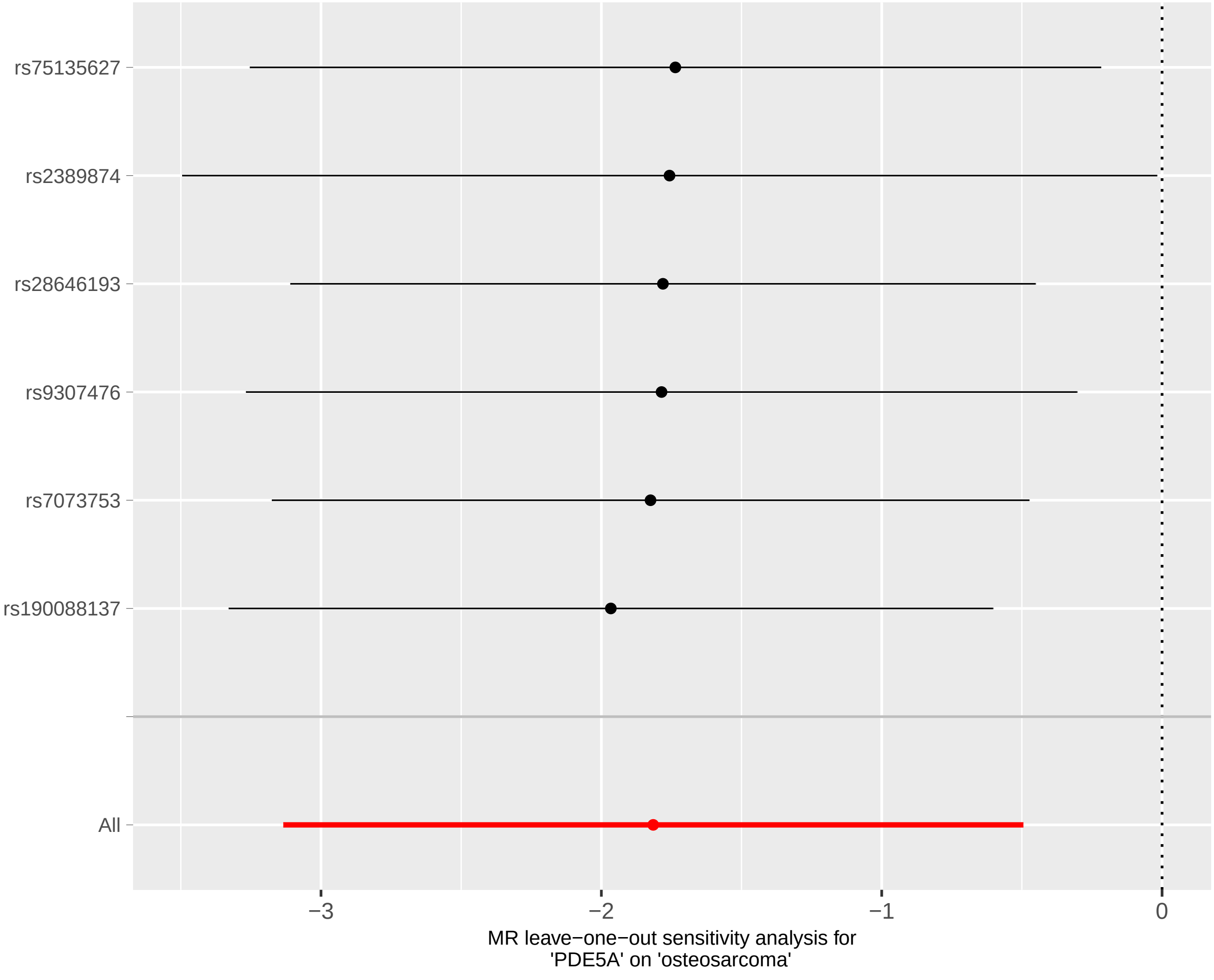
rs75135627
rs2389874
rs28646193
rs9307476
rs7073753
rs190088137
All
−3
−2
−1
0
MR leave−one−out sensitivity analysis for
'PDE5A' on 'osteosarcoma'

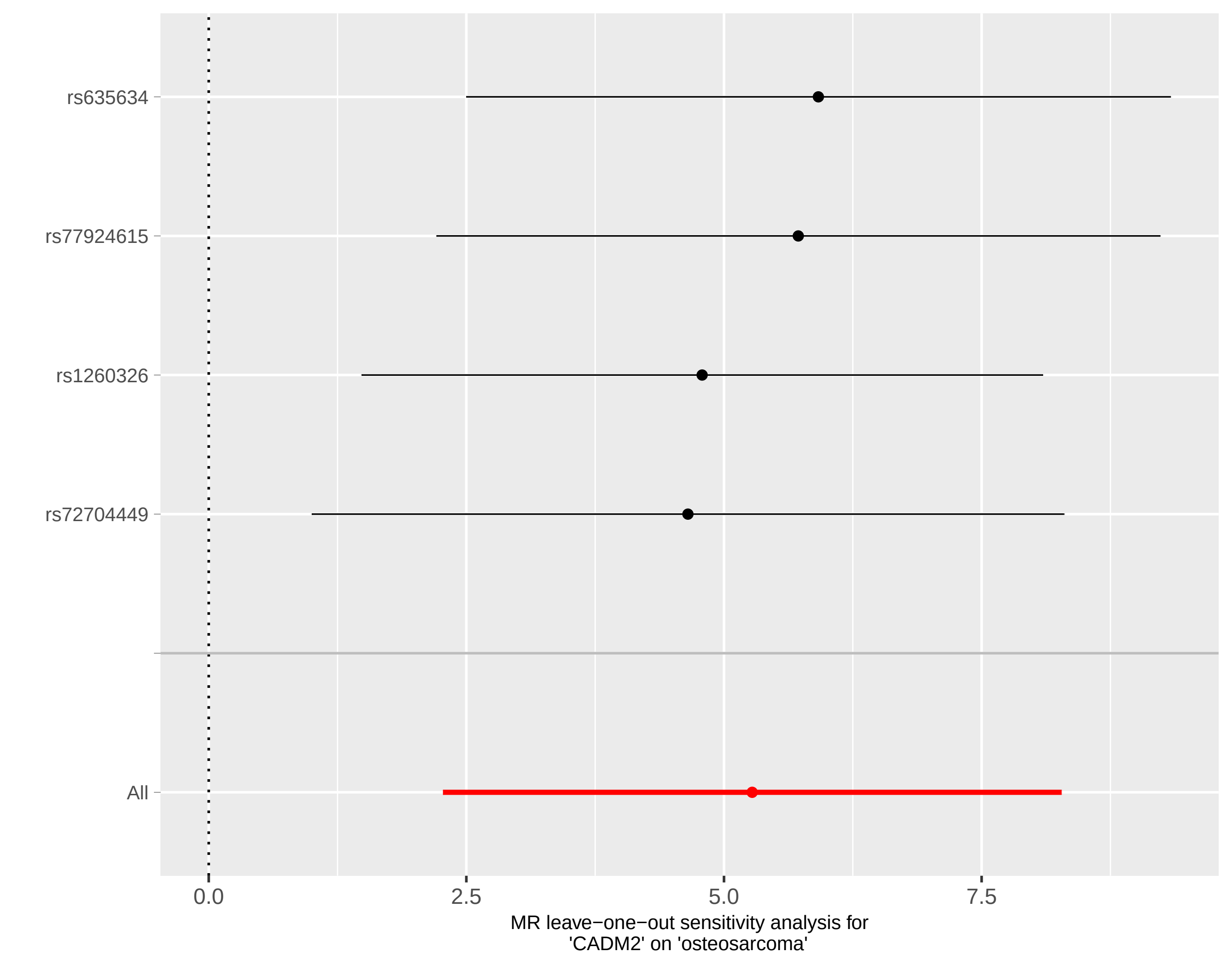
rs635634
rs77924615
rs1260326
rs72704449
All
0.0
2.5
5.0
7.5
MR leave−one−out sensitivity analysis for
'CADM2' on 'osteosarcoma'

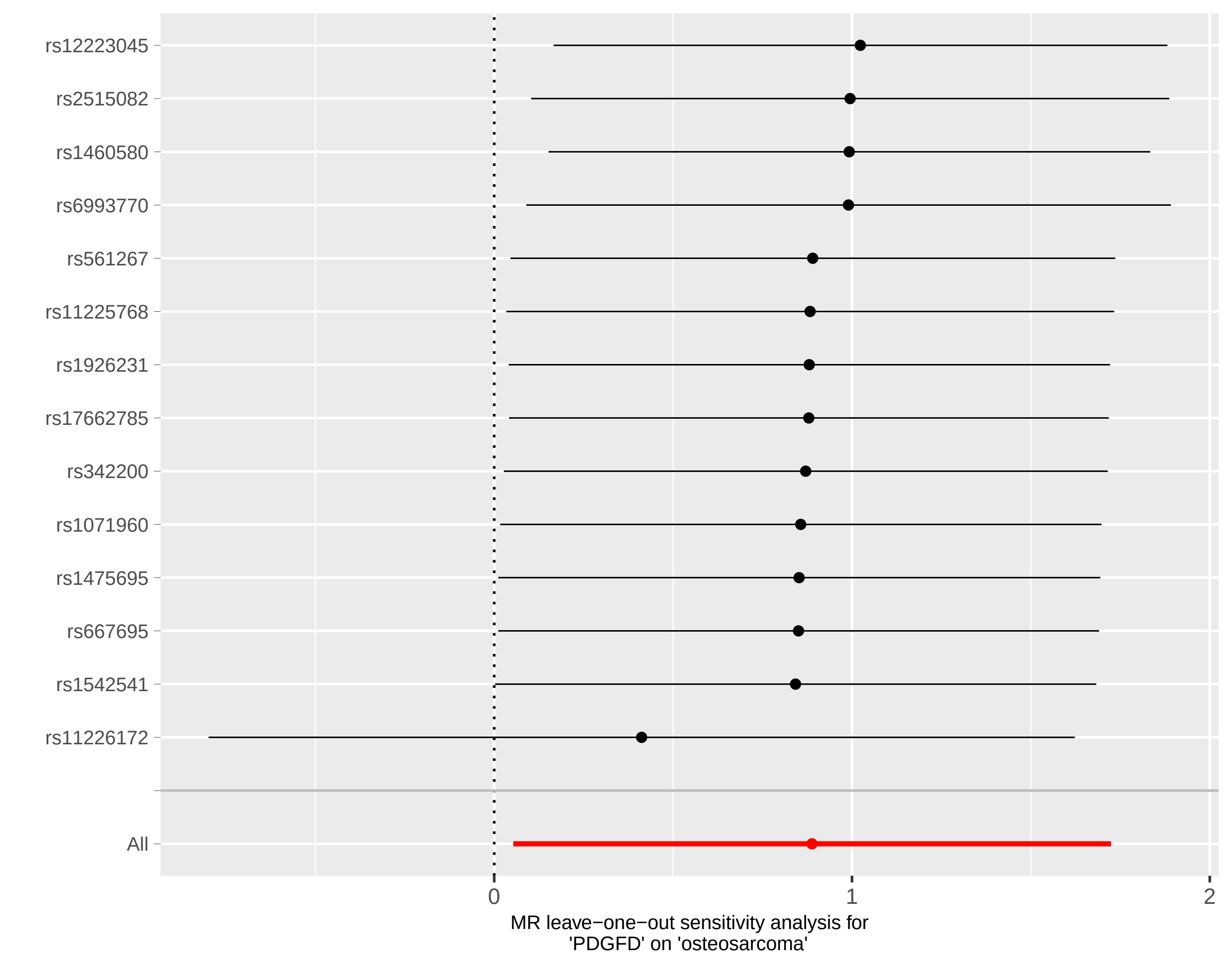

rs12223045
rs2515082
rs1460580
rs6993770
rs561267
rs11225768
rs1926231
rs17662785
rs342200
rs1071960
rs1475695
rs667695
rs1542541
rs11226172
All
0
1
2
MR leave−one−out sensitivity analysis for
'PDGFD' on 'osteosarcoma'

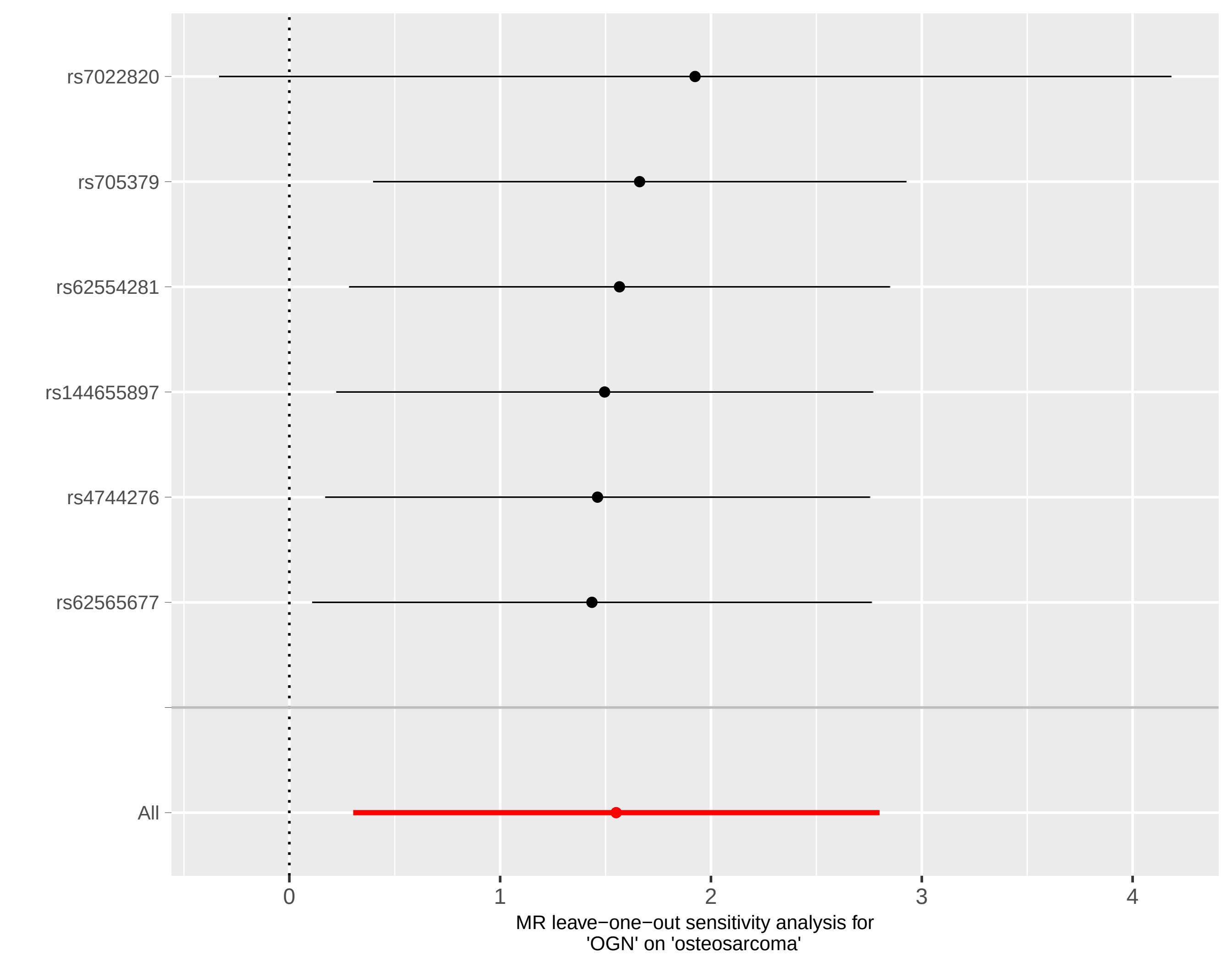

rs7022820
rs705379
rs62554281
rs144655897
rs4744276
rs62565677
All
0
1
2
3
4
MR leave−one−out sensitivity analysis for
'OGN' on 'osteosarcoma'

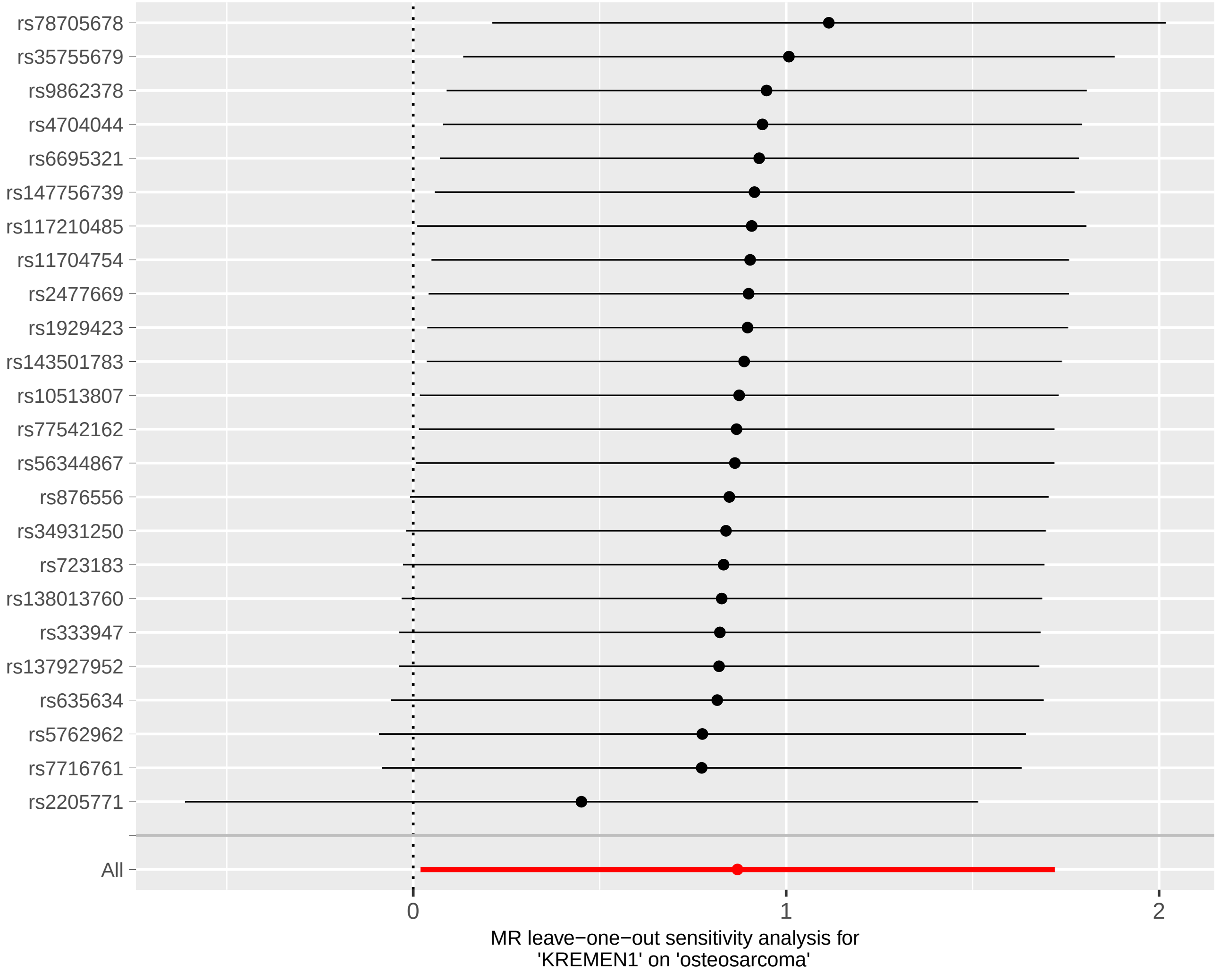

rs78705678
rs35755679
rs9862378
rs4704044
rs6695321
rs147756739
rs117210485
rs11704754
rs2477669
rs1929423
rs143501783
rs10513807
rs77542162
rs56344867
rs876556
rs34931250
rs723183
rs138013760
rs333947
rs137927952
rs635634
rs5762962
rs7716761
rs2205771
All
0
1
2
MR leave−one−out sensitivity analysis for
'KREMEN1' on 'osteosarcoma'

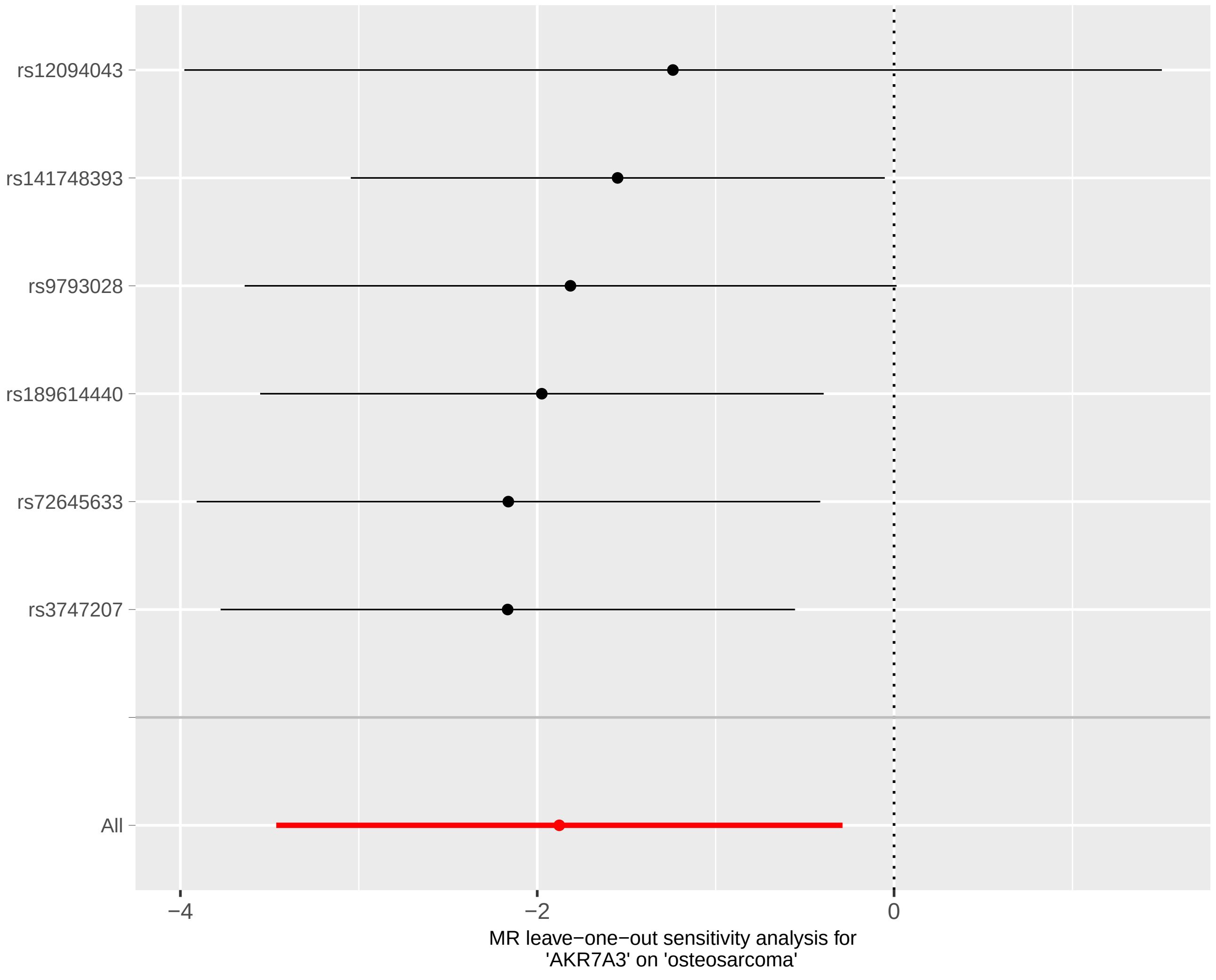
rs12094043
rs141748393
rs9793028
rs189614440
rs72645633
rs3747207
All
−4
−2
0
MR leave−one−out sensitivity analysis for
'AKR7A3' on 'osteosarcoma'

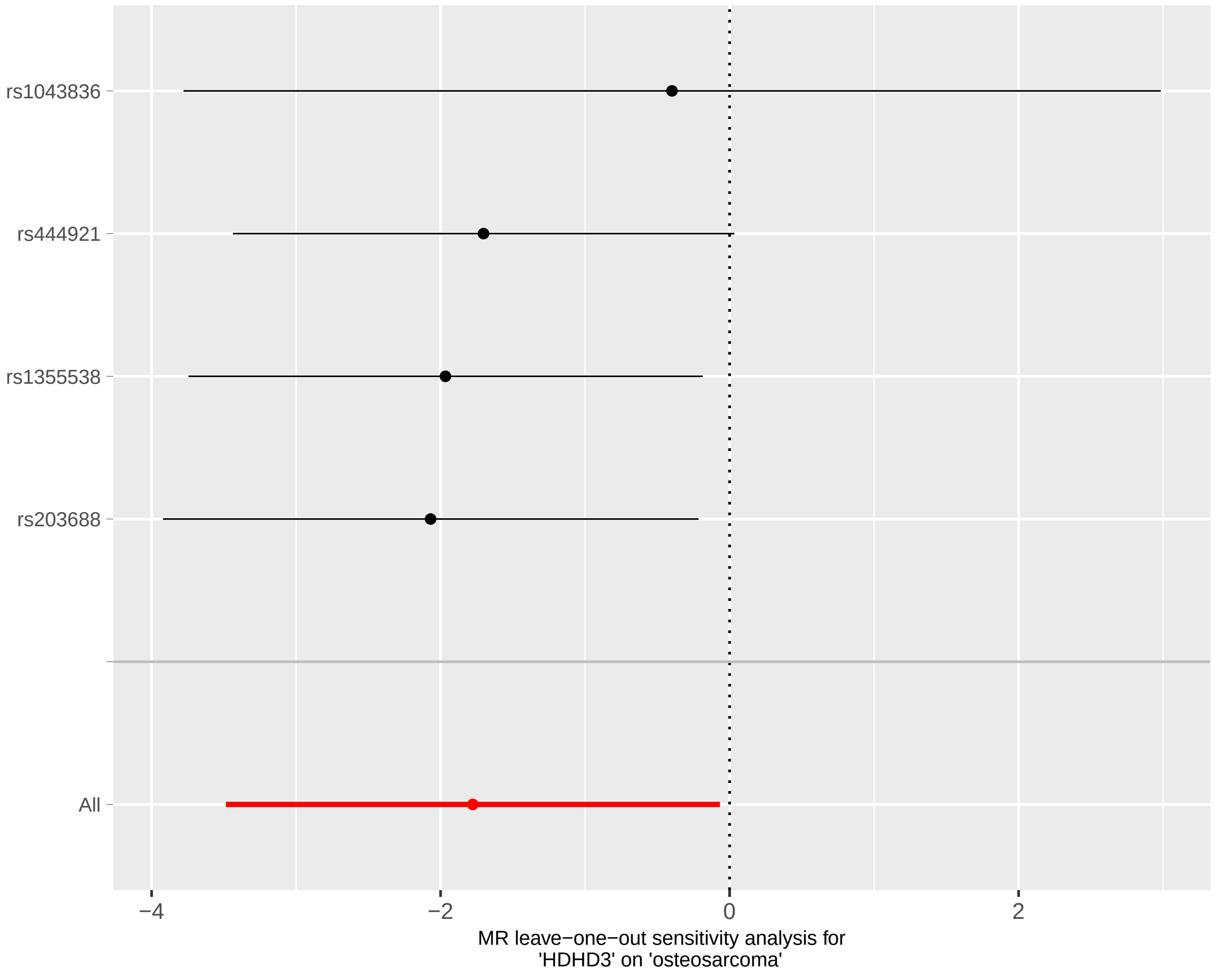
rs1043836
rs444921
rs1355538
rs203688
All
−4
−2
0
2
MR leave−one−out sensitivity analysis for
'HDHD3' on 'osteosarcoma'

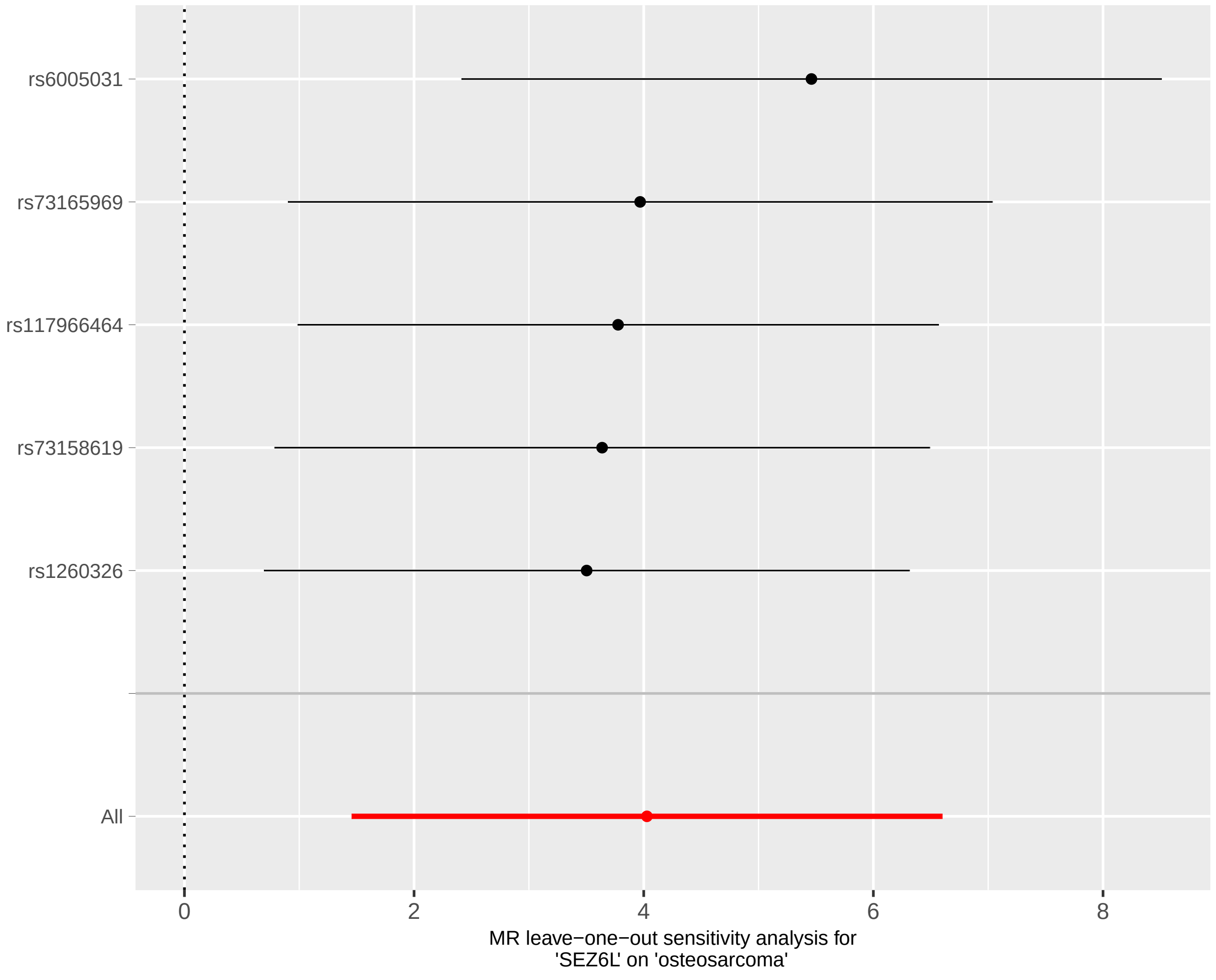
rs6005031
rs73165969
rs117966464
rs73158619
rs1260326
All
0
2
4
6
8
MR leave−one−out sensitivity analysis for
'SEZ6L' on 'osteosarcoma'

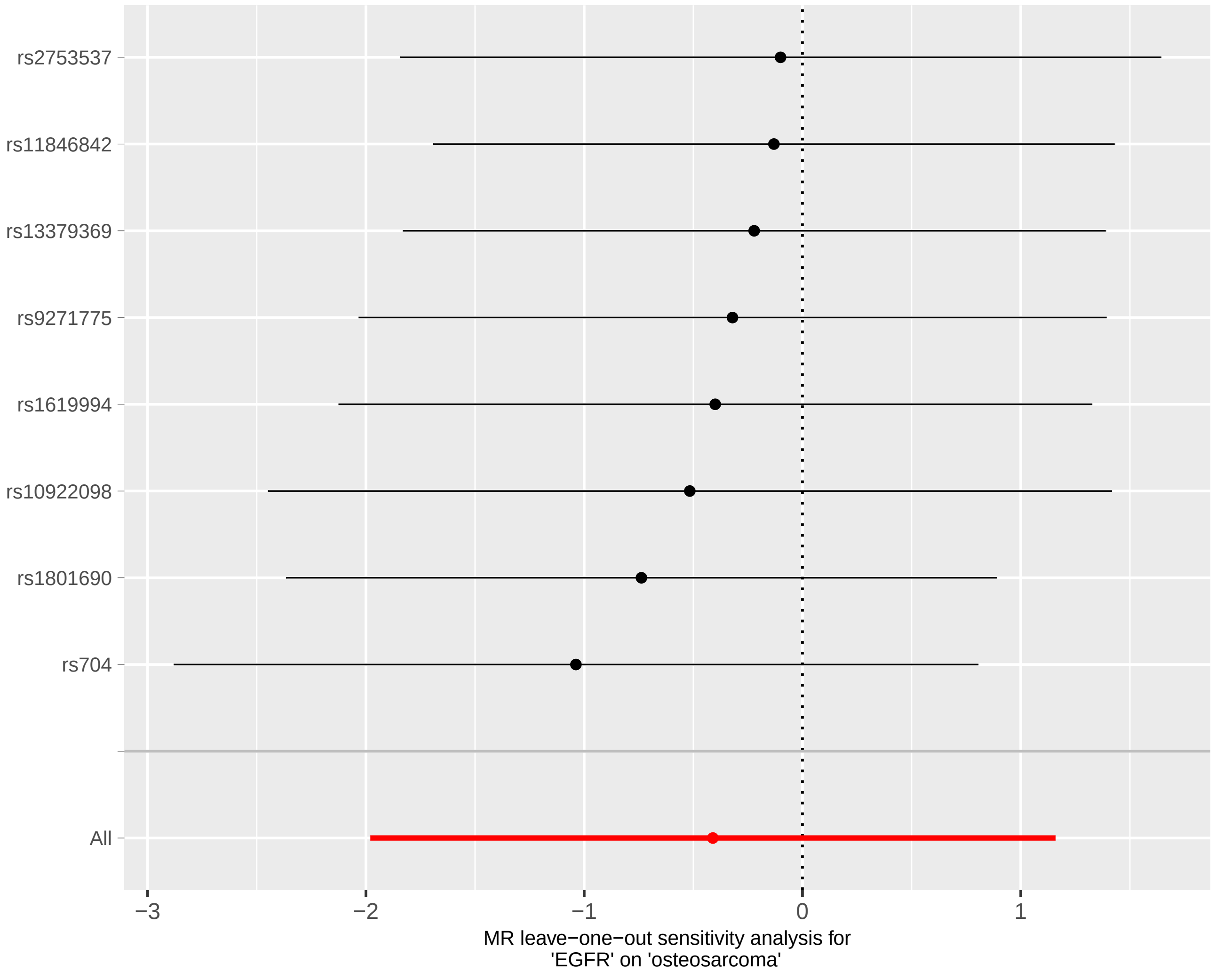

rs2753537
rs11846842
rs13379369
rs9271775
rs1619994
rs10922098
rs1801690
rs704
All
−3
−2
−1
0
1
MR leave−one−out sensitivity analysis for
'EGFR' on 'osteosarcoma'

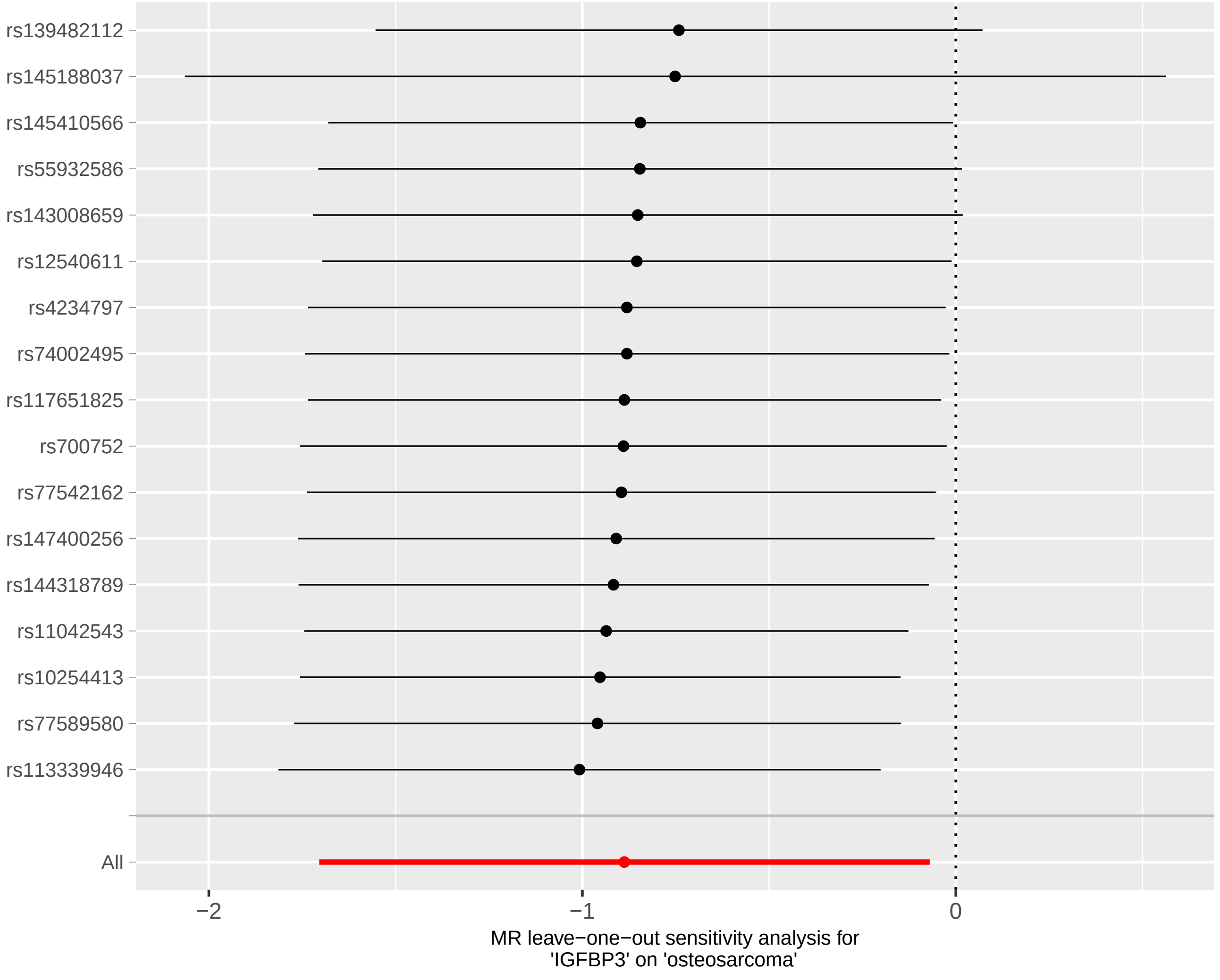
rs139482112
rs145188037
rs145410566
rs55932586
rs143008659
rs12540611
rs4234797
rs74002495
rs117651825
rs700752
rs77542162
rs147400256
rs144318789
rs11042543
rs10254413
rs77589580
rs113339946
All
−2
−1
0
MR leave−one−out sensitivity analysis for
'IGFBP3' on 'osteosarcoma'

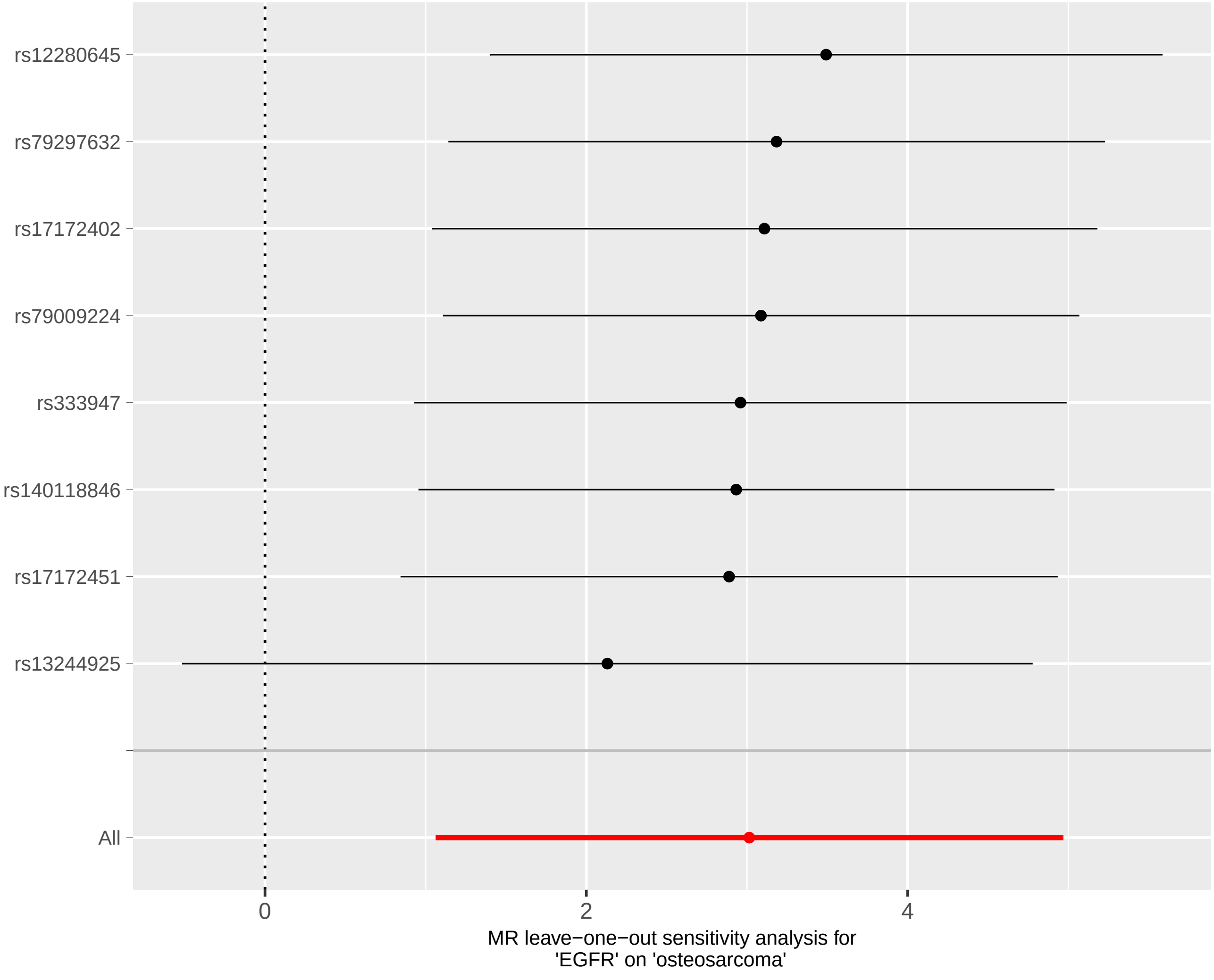

rs12280645
rs79297632
rs17172402
rs79009224
rs333947
rs140118846
rs17172451
rs13244925
All
0
2
4
MR leave−one−out sensitivity analysis for
'EGFR' on 'osteosarcoma'

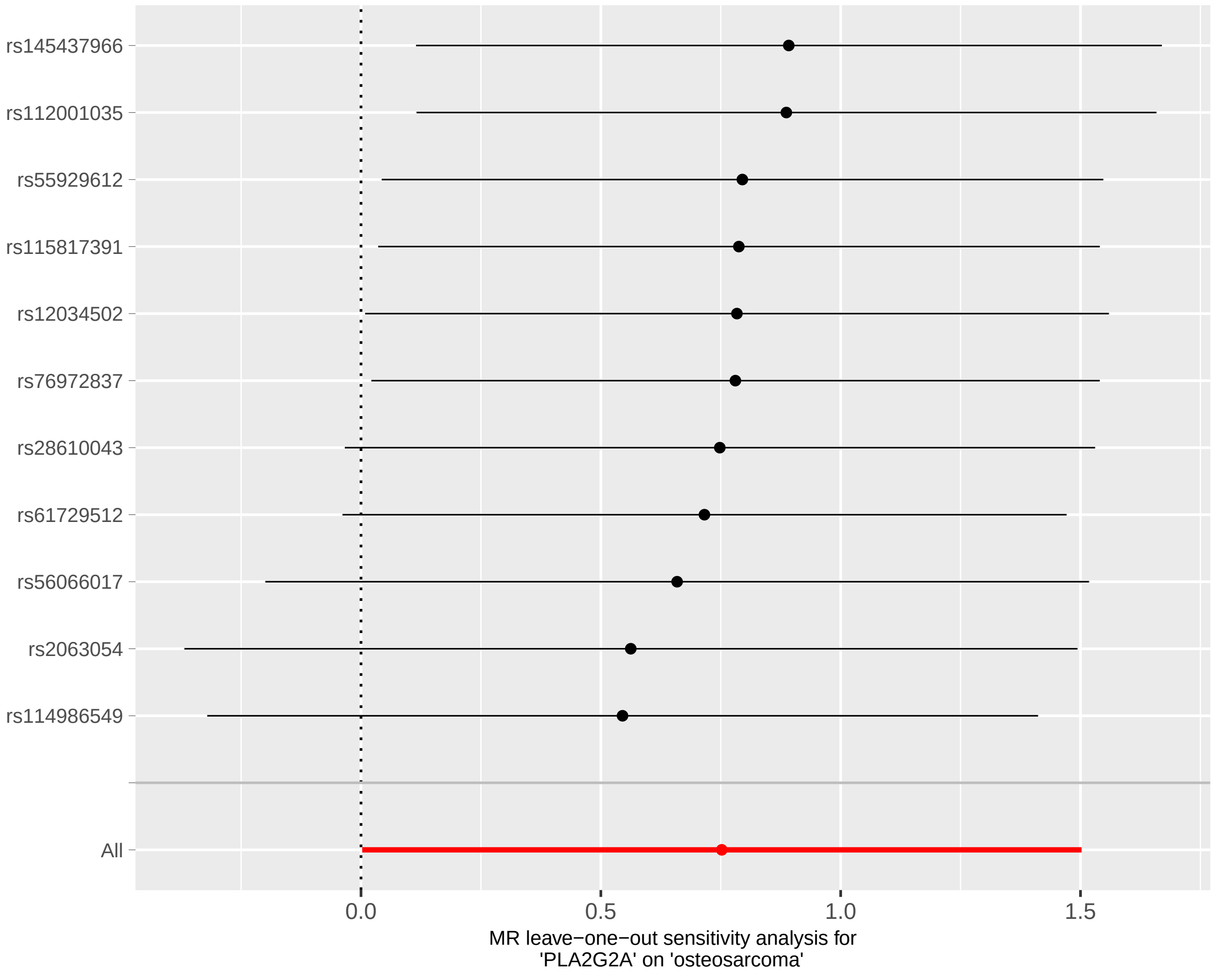
rs145437966
rs112001035
rs55929612
rs115817391
rs12034502
rs76972837
rs28610043
rs61729512
rs56066017
rs2063054
rs114986549
All
0.0
0.5
1.0
1.5
MR leave−one−out sensitivity analysis for
'PLA2G2A' on 'osteosarcoma'

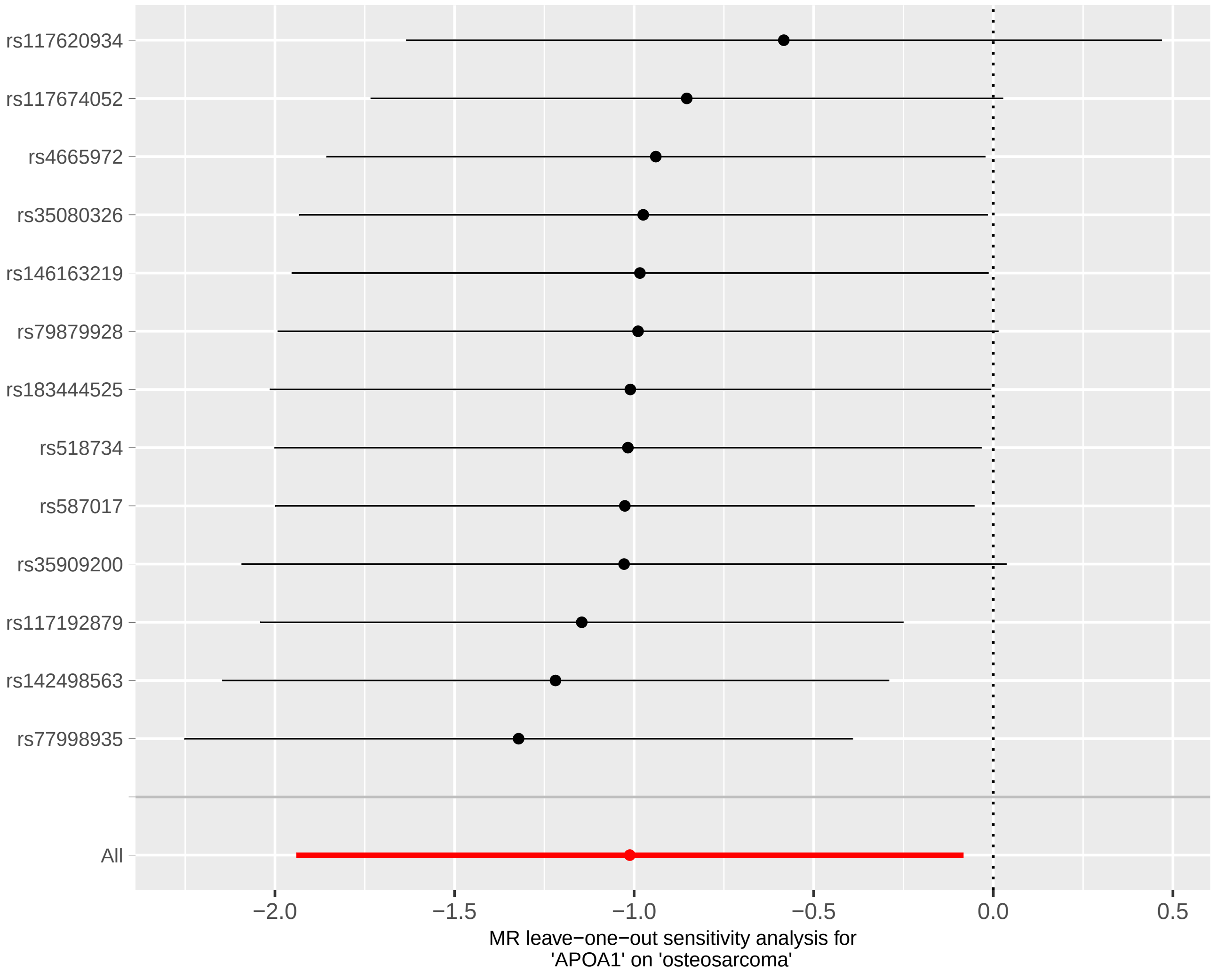
rs117620934
rs117674052
rs4665972
rs35080326
rs146163219
rs79879928
rs183444525
rs518734
rs587017
rs35909200
rs117192879
rs142498563
rs77998935
All
−2.0
−1.5
−1.0
−0.5
0.0
0.5
MR leave−one−out sensitivity analysis for
'APOA1' on 'osteosarcoma'

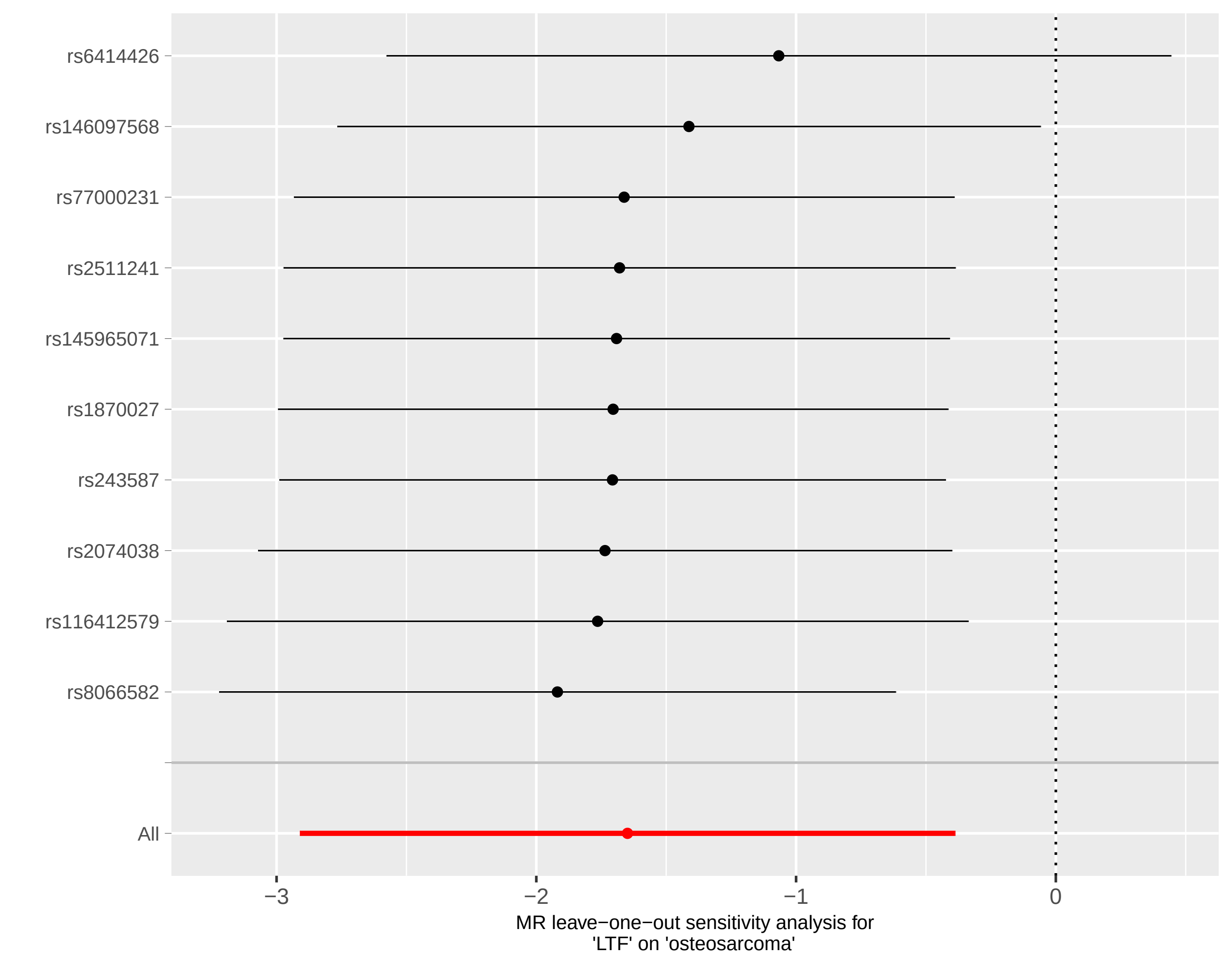
rs6414426
rs146097568
rs77000231
rs2511241
rs145965071
rs1870027
rs243587
rs2074038
rs116412579
rs8066582
All
−3
−2
−1
0
MR leave−one−out sensitivity analysis for
'LTF' on 'osteosarcoma'

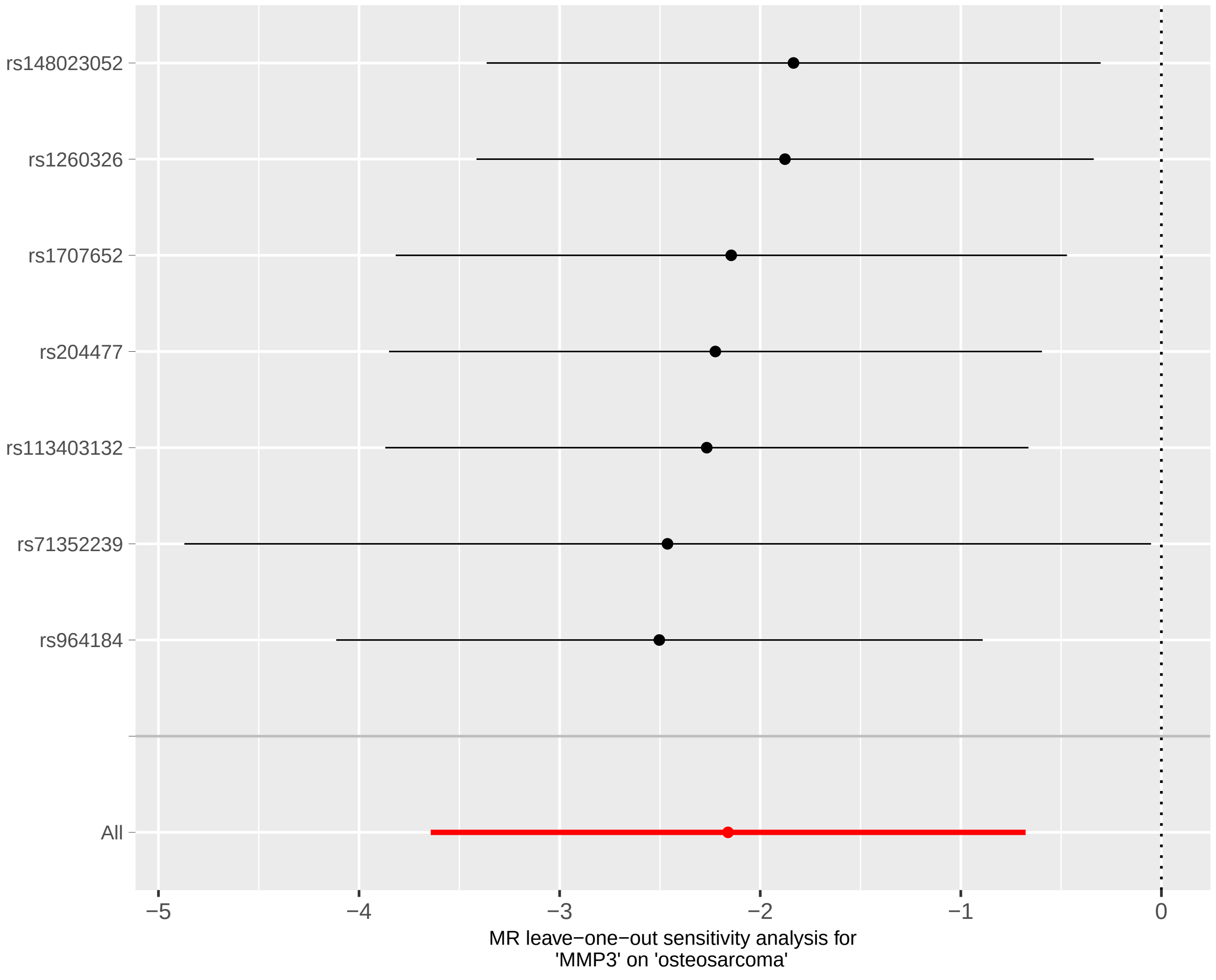
rs148023052
rs1260326
rs1707652
rs204477
rs113403132
rs71352239
rs964184
All
−5
−4
−3
−2
−1
0
MR leave−one−out sensitivity analysis for
'MMP3' on 'osteosarcoma'

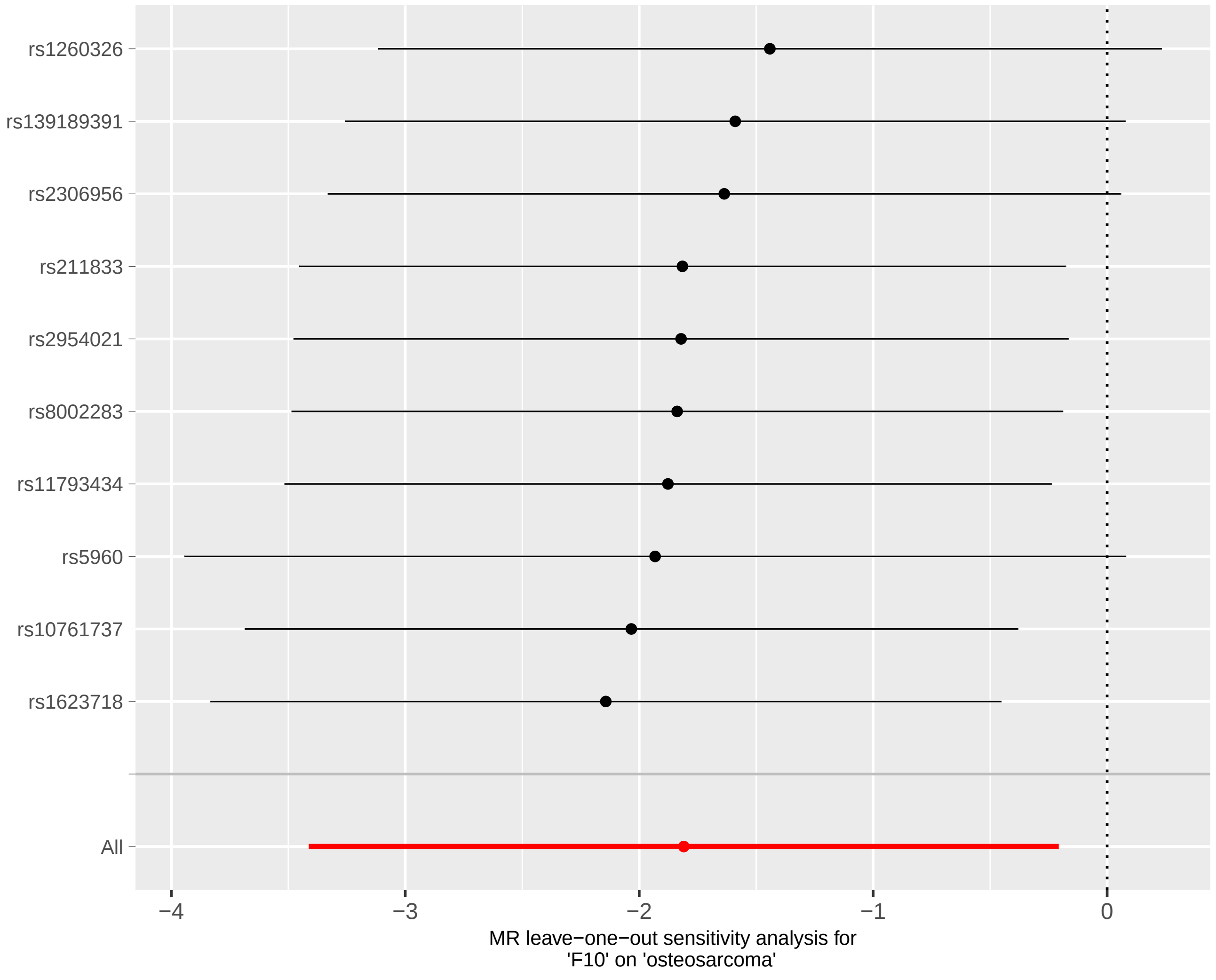
rs1260326
rs139189391
rs2306956
rs211833
rs2954021
rs8002283
rs11793434
rs5960
rs10761737
rs1623718
All
−4
−3
−2
−1
0
MR leave−one−out sensitivity analysis for
'F10' on 'osteosarcoma'

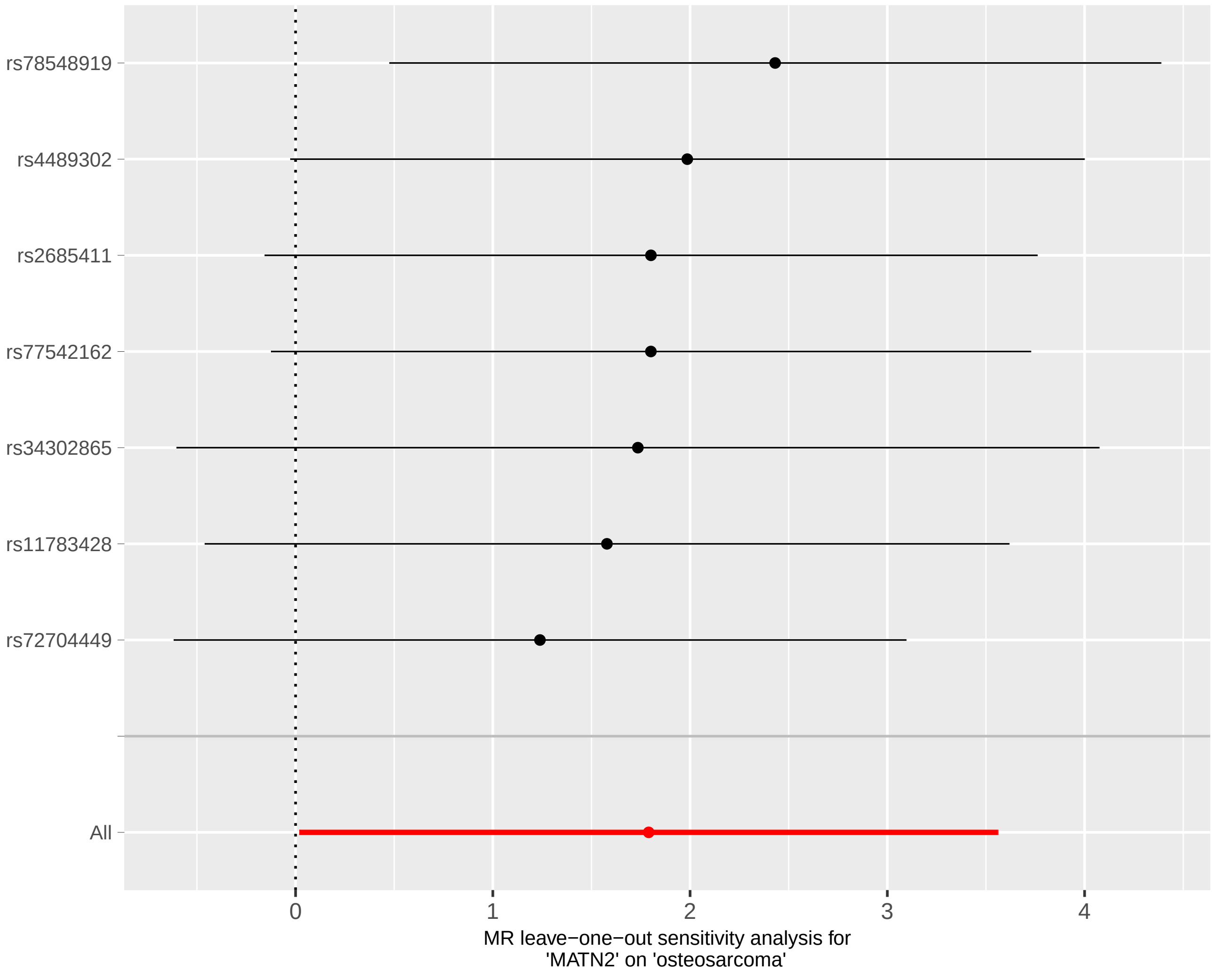

rs78548919
rs4489302
rs2685411
rs77542162
rs34302865
rs11783428
rs72704449
All
0
1
2
3
4
MR leave−one−out sensitivity analysis for
'MATN2' on 'osteosarcoma'

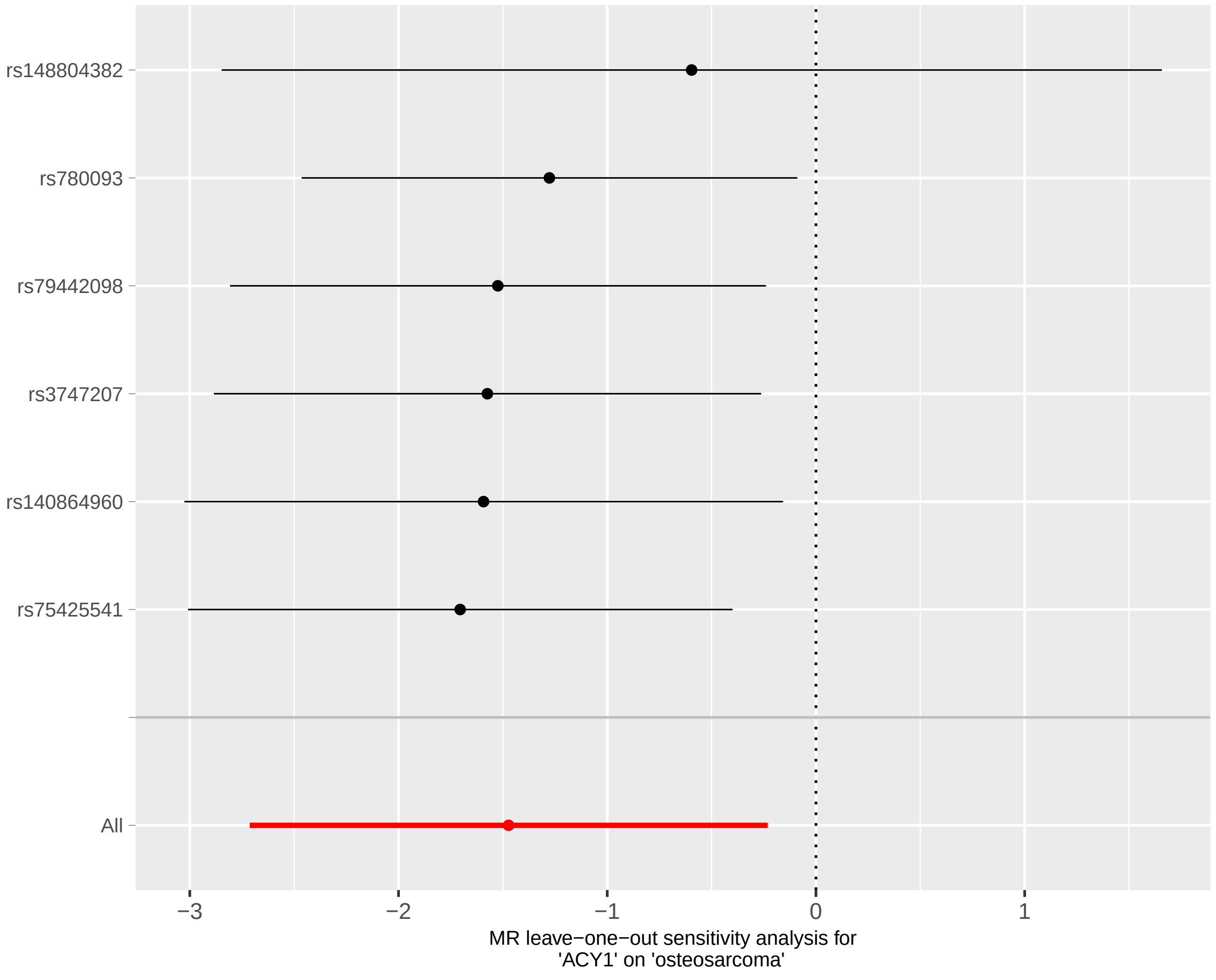

rs148804382
rs780093
rs79442098
rs3747207
rs140864960
rs75425541
All
−3
−2
−1
0
1
MR leave−one−out sensitivity analysis for
'ACY1' on 'osteosarcoma'

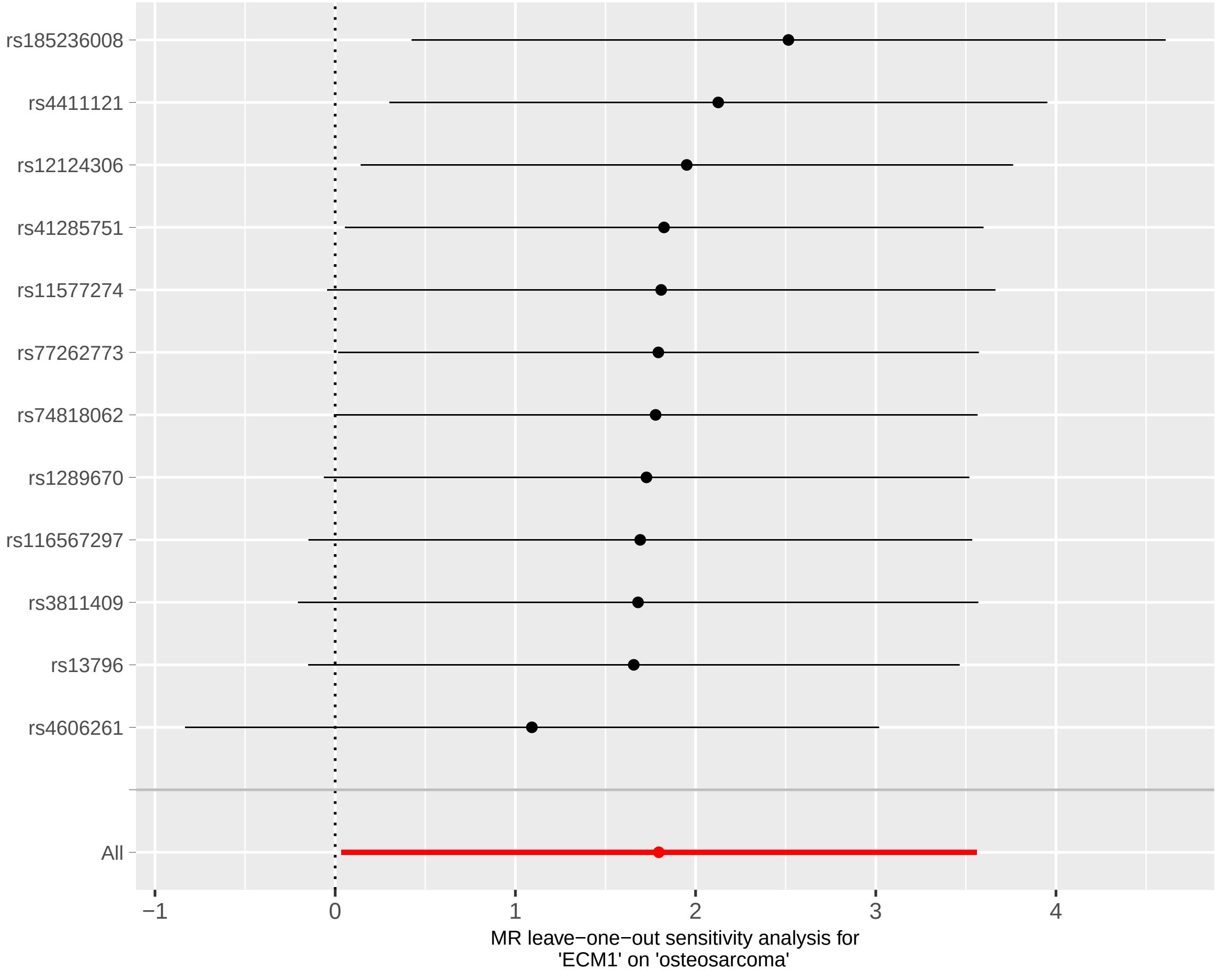
rs185236008
rs4411121
rs12124306
rs41285751
rs11577274
rs77262773
rs74818062
rs1289670
rs116567297
rs3811409
rs13796
rs4606261
All
−1
0
1
2
3
4
MR leave−one−out sensitivity analysis for
'ECM1' on 'osteosarcoma'

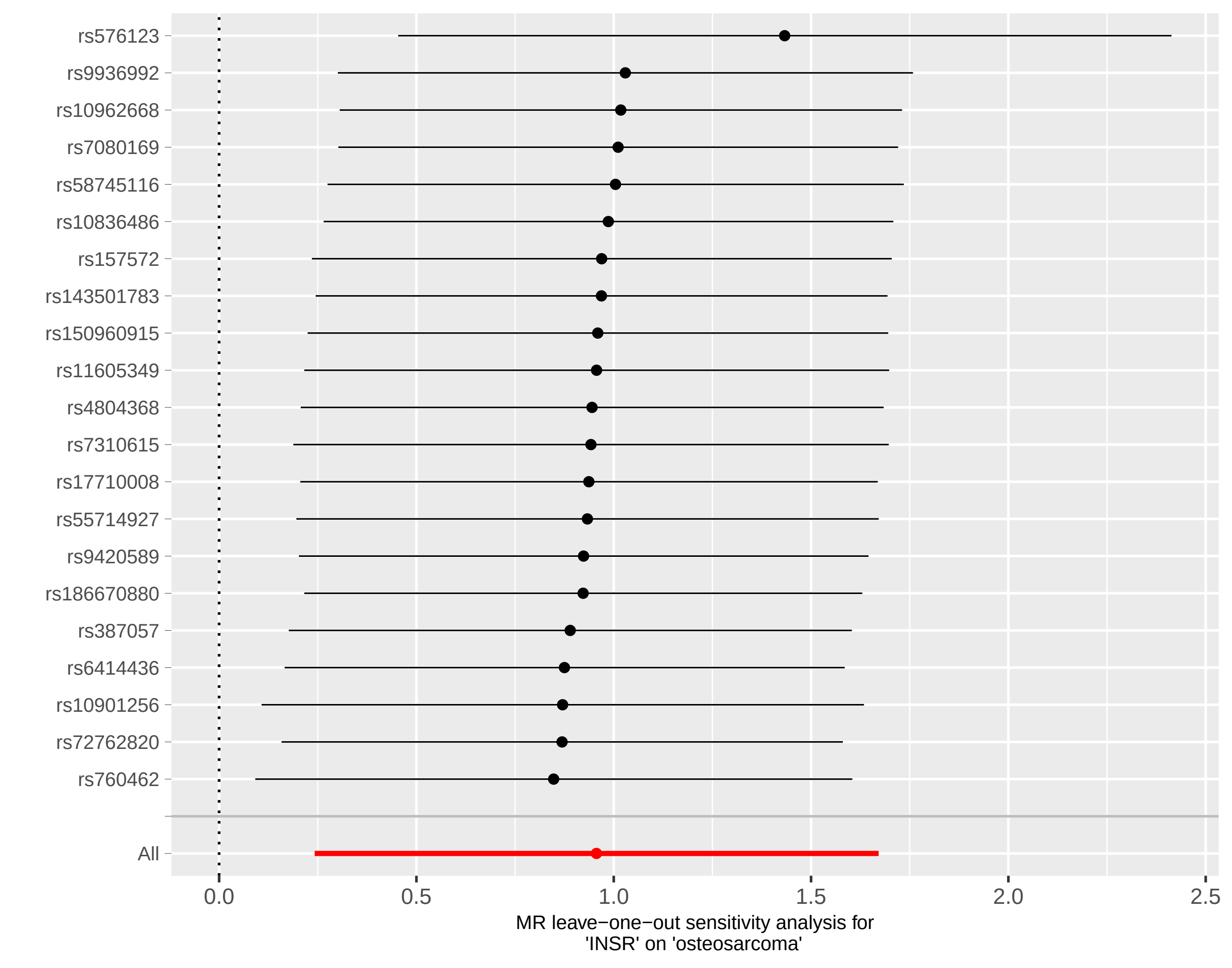
rs576123
rs9936992
rs10962668
rs7080169
rs58745116
rs10836486
rs157572
rs143501783
rs150960915
rs11605349
rs4804368
rs7310615
rs17710008
rs55714927
rs9420589
rs186670880
rs387057
rs6414436
rs10901256
rs72762820
rs760462
All
0.0
0.5
1.0
1.5
2.0
2.5
MR leave−one−out sensitivity analysis for
'INSR' on 'osteosarcoma'

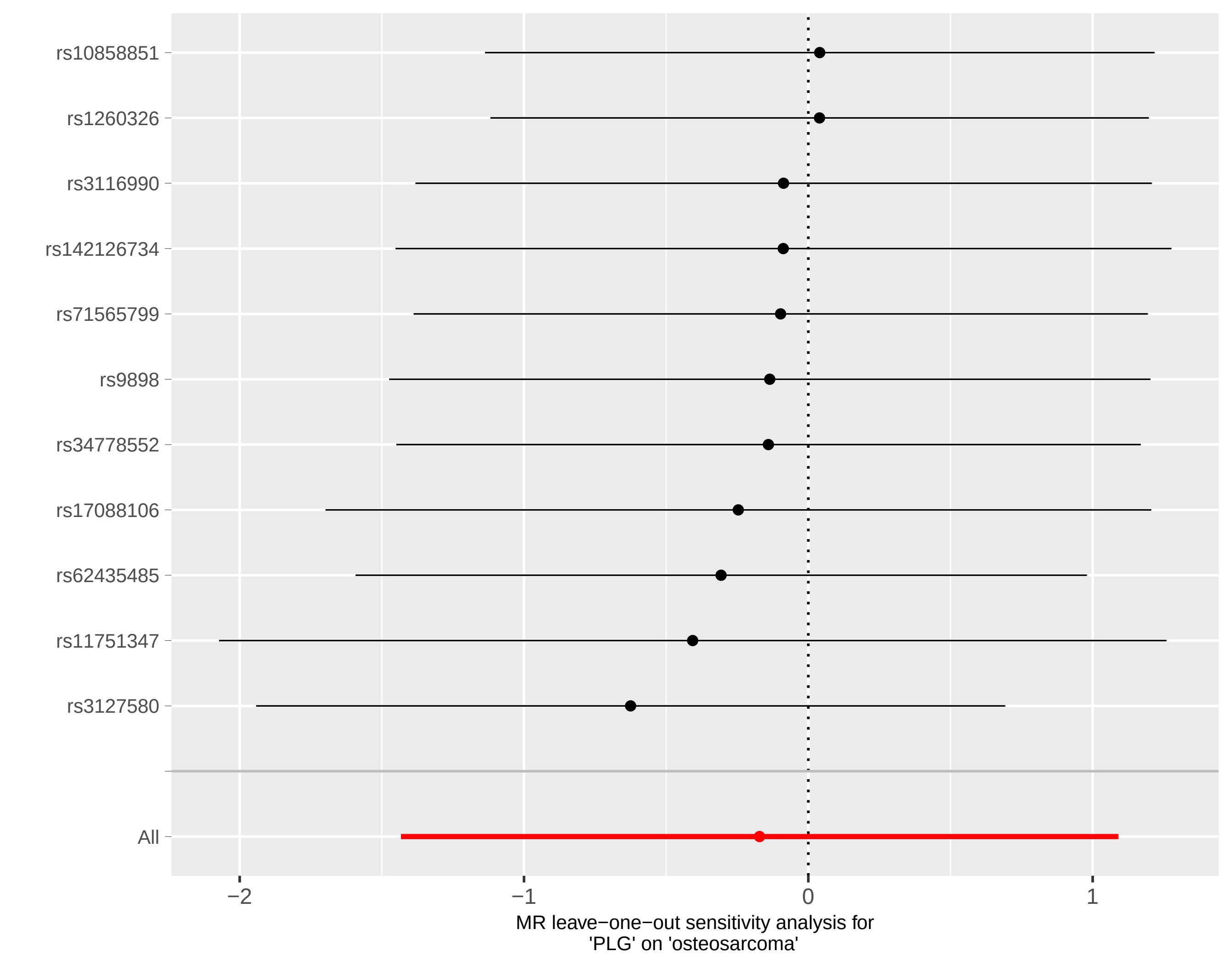
rs10858851
rs1260326
rs3116990
rs142126734
rs71565799
rs9898
rs34778552
rs17088106
rs62435485
rs11751347
rs3127580
All
−2
−1
0
1
MR leave−one−out sensitivity analysis for
'PLG' on 'osteosarcoma'

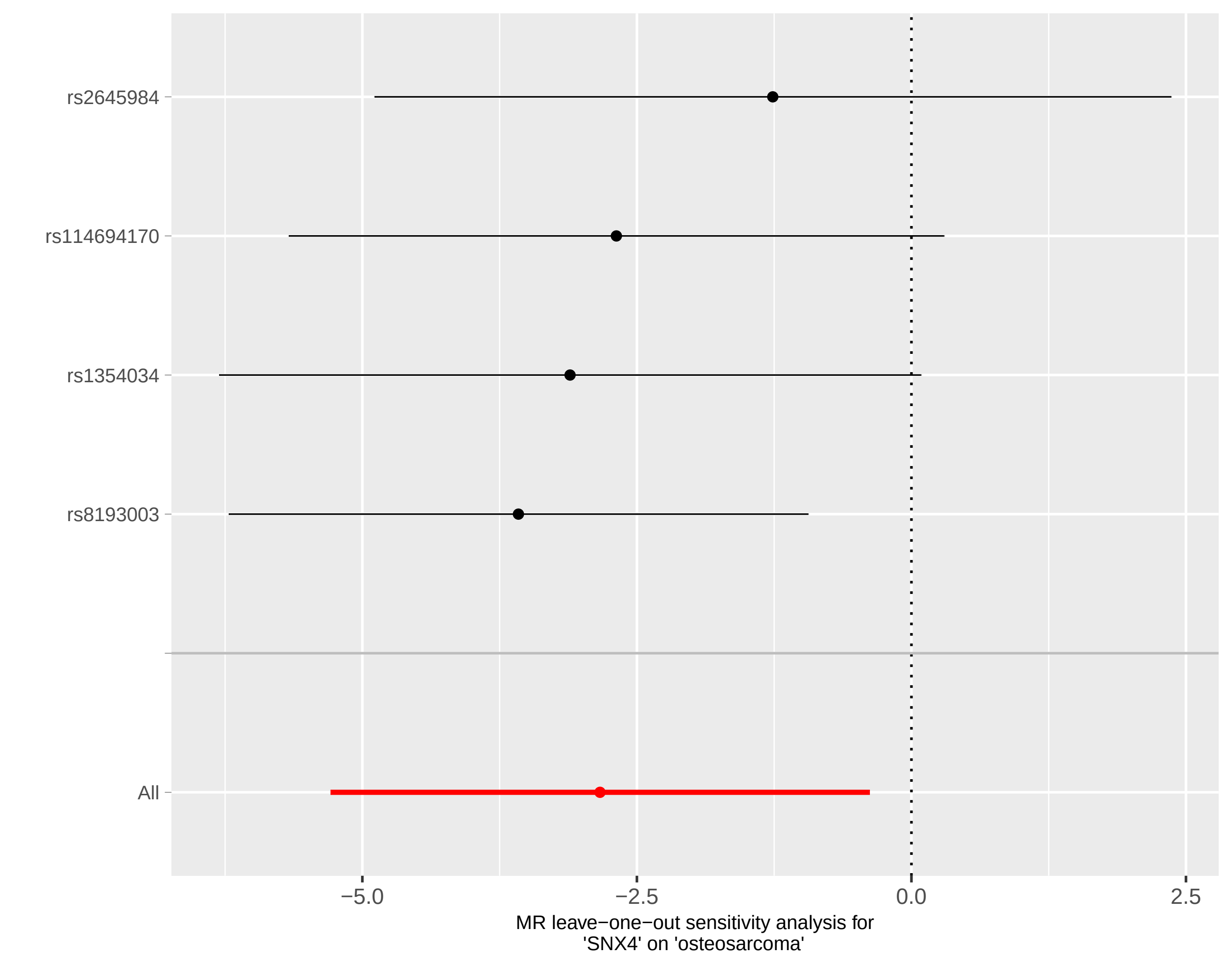

rs2645984
rs114694170
rs1354034
rs8193003
All
−5.0
−2.5
0.0
2.5
MR leave−one−out sensitivity analysis for
'SNX4' on 'osteosarcoma'

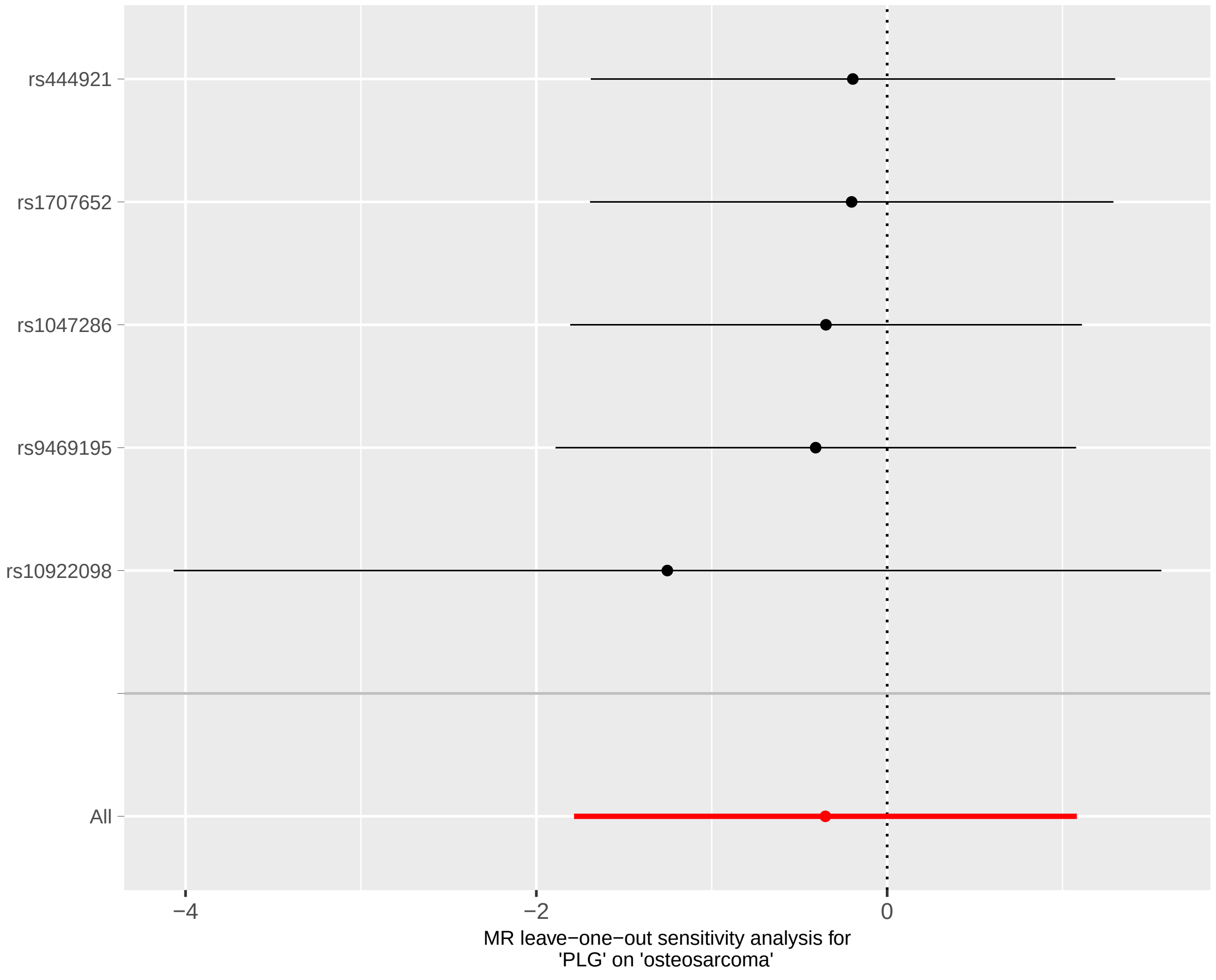
rs444921
rs1707652
rs1047286
rs9469195
rs10922098
All
−4
−2
0
MR leave−one−out sensitivity analysis for
'PLG' on 'osteosarcoma'

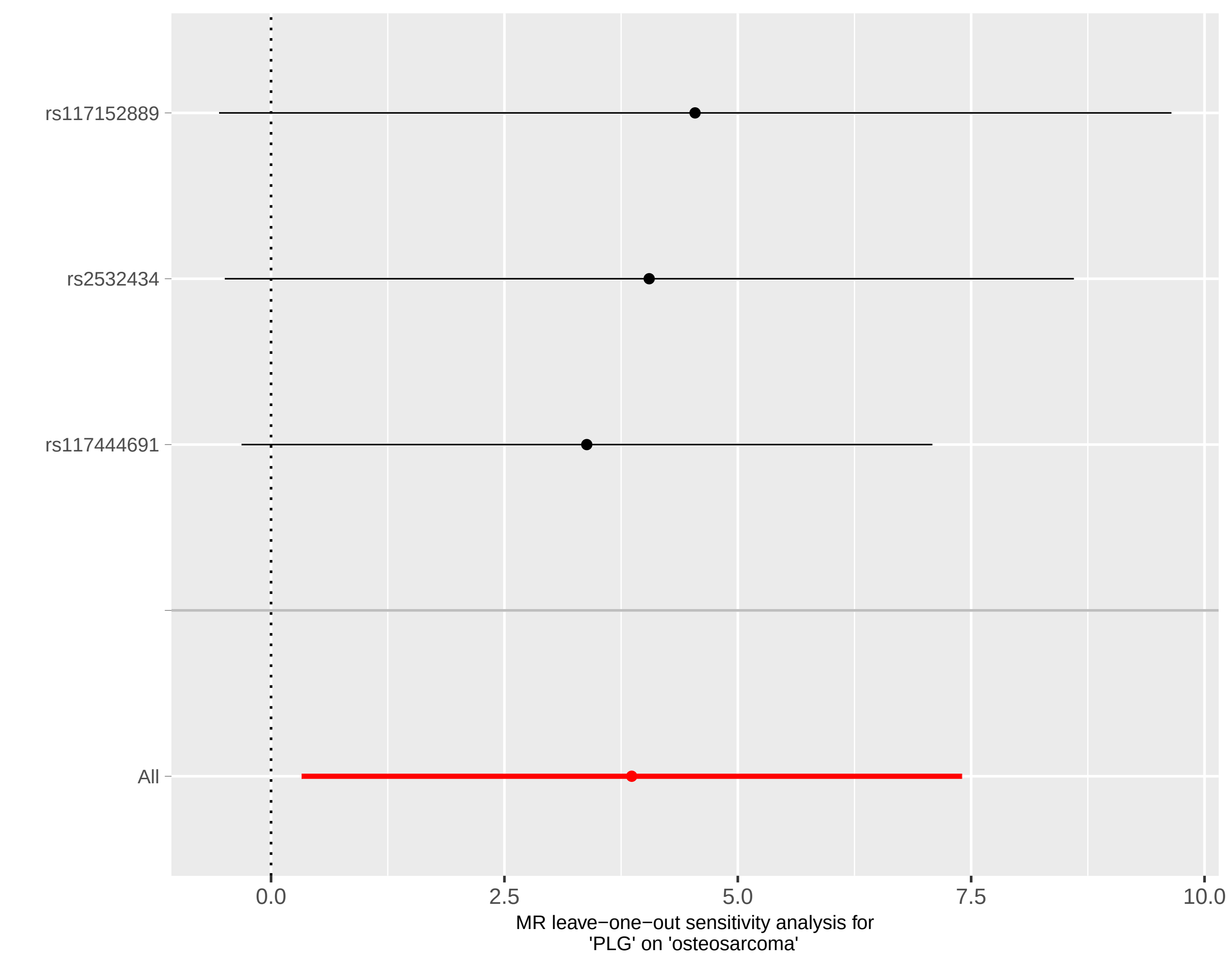

rs117152889
rs2532434
rs117444691
All
0.0
2.5
5.0
7.5
10.0
MR leave−one−out sensitivity analysis for
'PLG' on 'osteosarcoma'

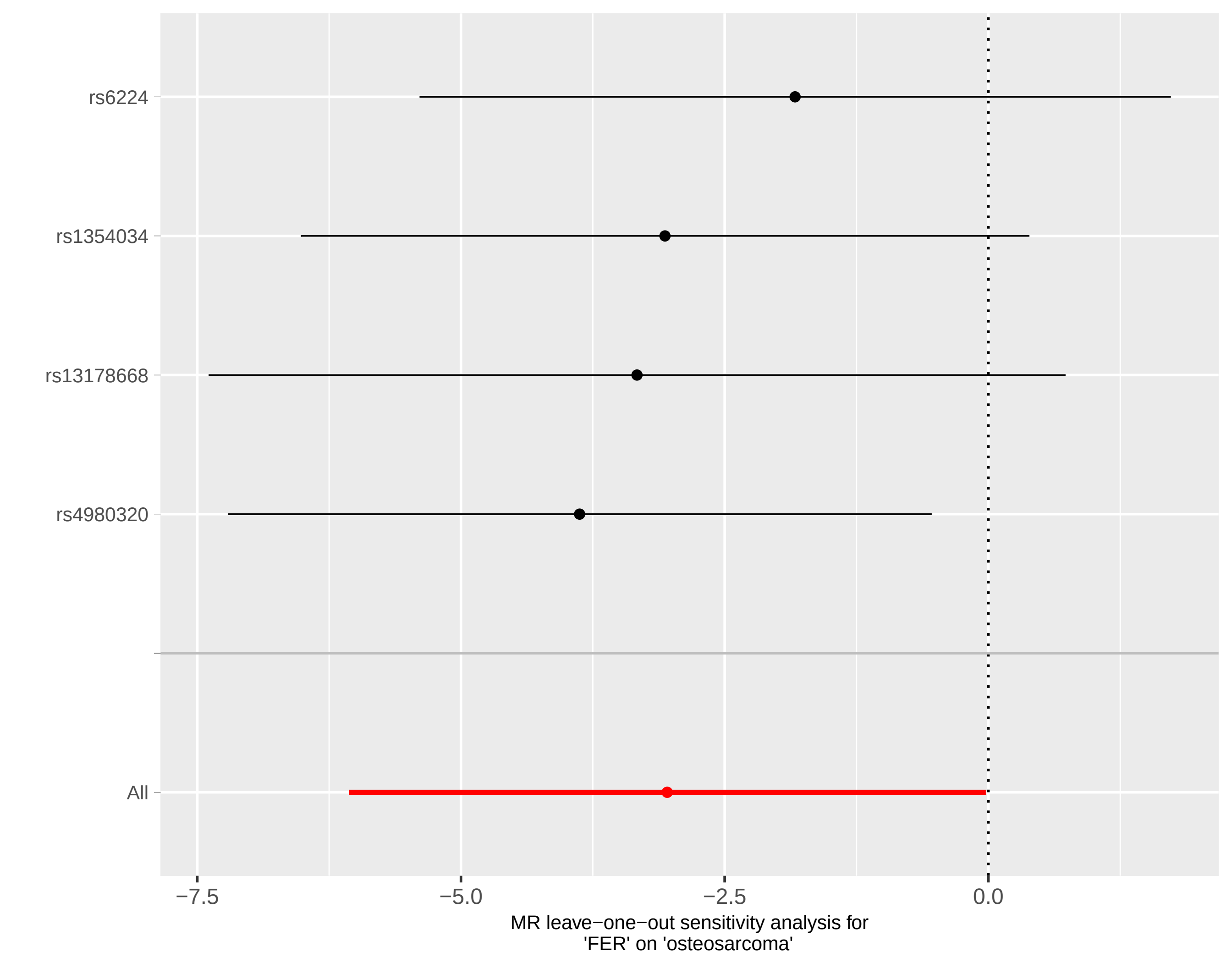

rs6224
rs1354034
rs13178668
rs4980320
All
−7.5
−5.0
−2.5
0.0
MR leave−one−out sensitivity analysis for
'FER' on 'osteosarcoma'

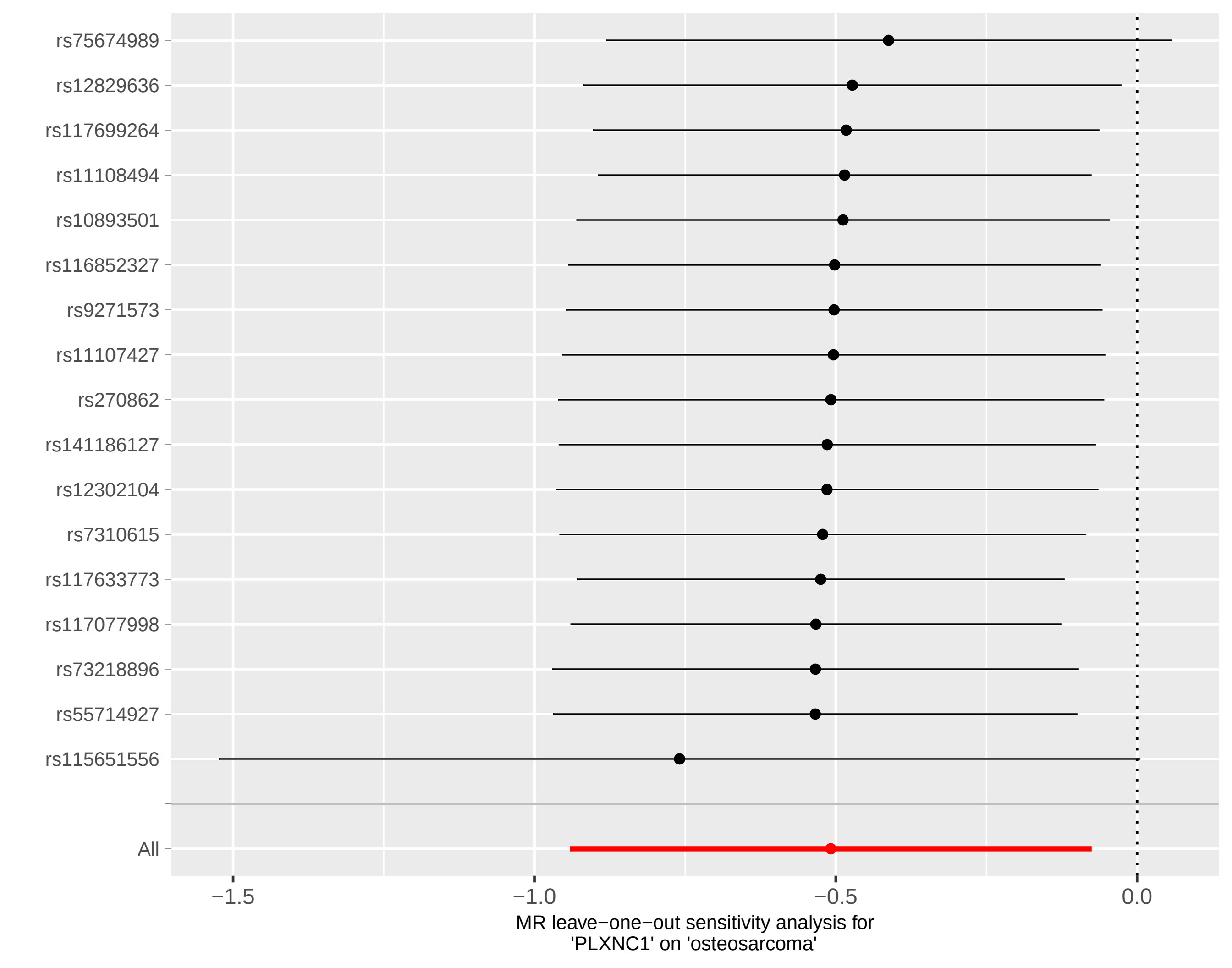
rs75674989
rs12829636
rs117699264
rs11108494
rs10893501
rs116852327
rs9271573
rs11107427
rs270862
rs141186127
rs12302104
rs7310615
rs117633773
rs117077998
rs73218896
rs55714927
rs115651556
All
−1.5
−1.0
−0.5
0.0
MR leave−one−out sensitivity analysis for
'PLXNC1' on 'osteosarcoma'

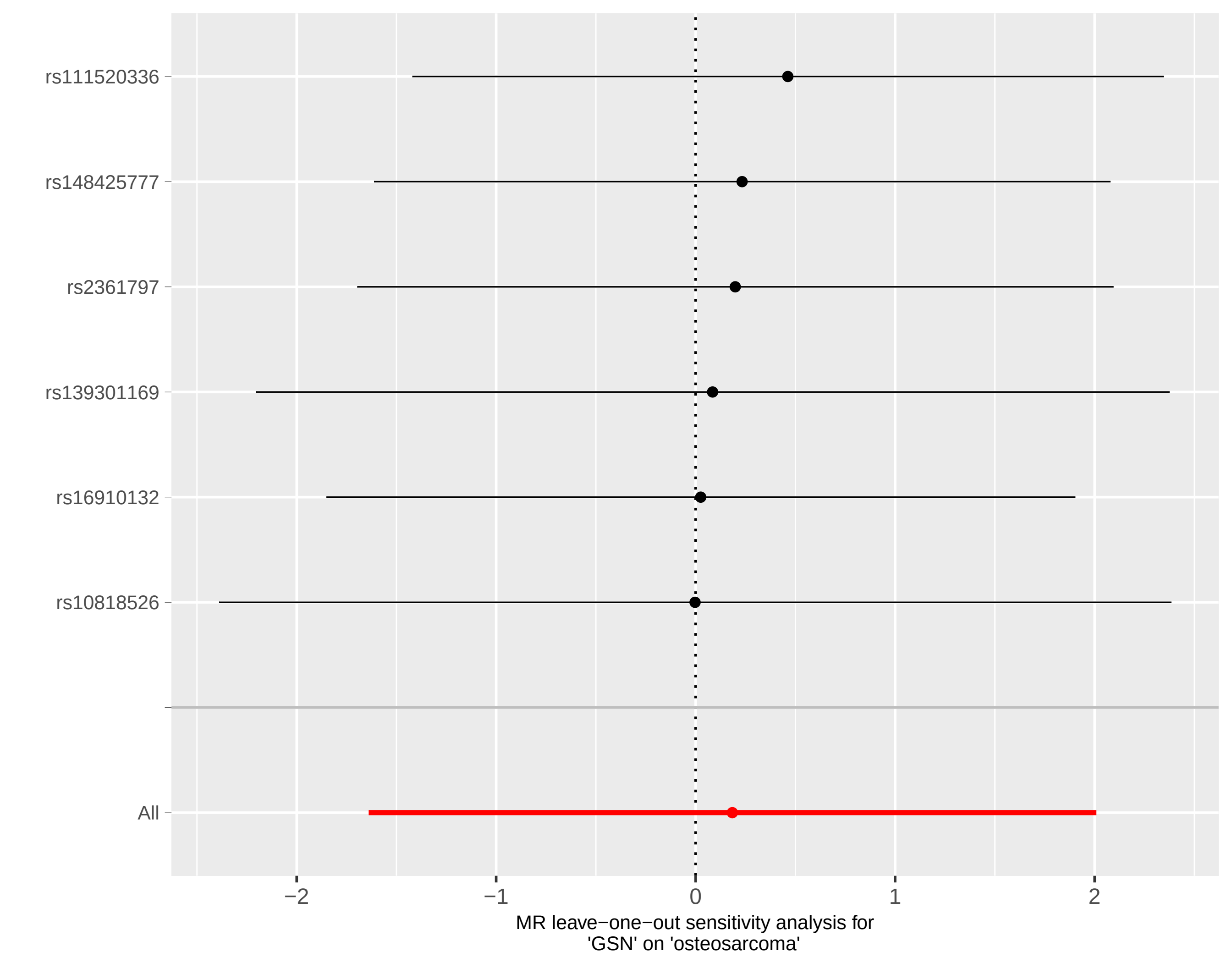
rs111520336
rs148425777
rs2361797
rs139301169
rs16910132
rs10818526
All
−2
−1
0
1
2
MR leave−one−out sensitivity analysis for
'GSN' on 'osteosarcoma'

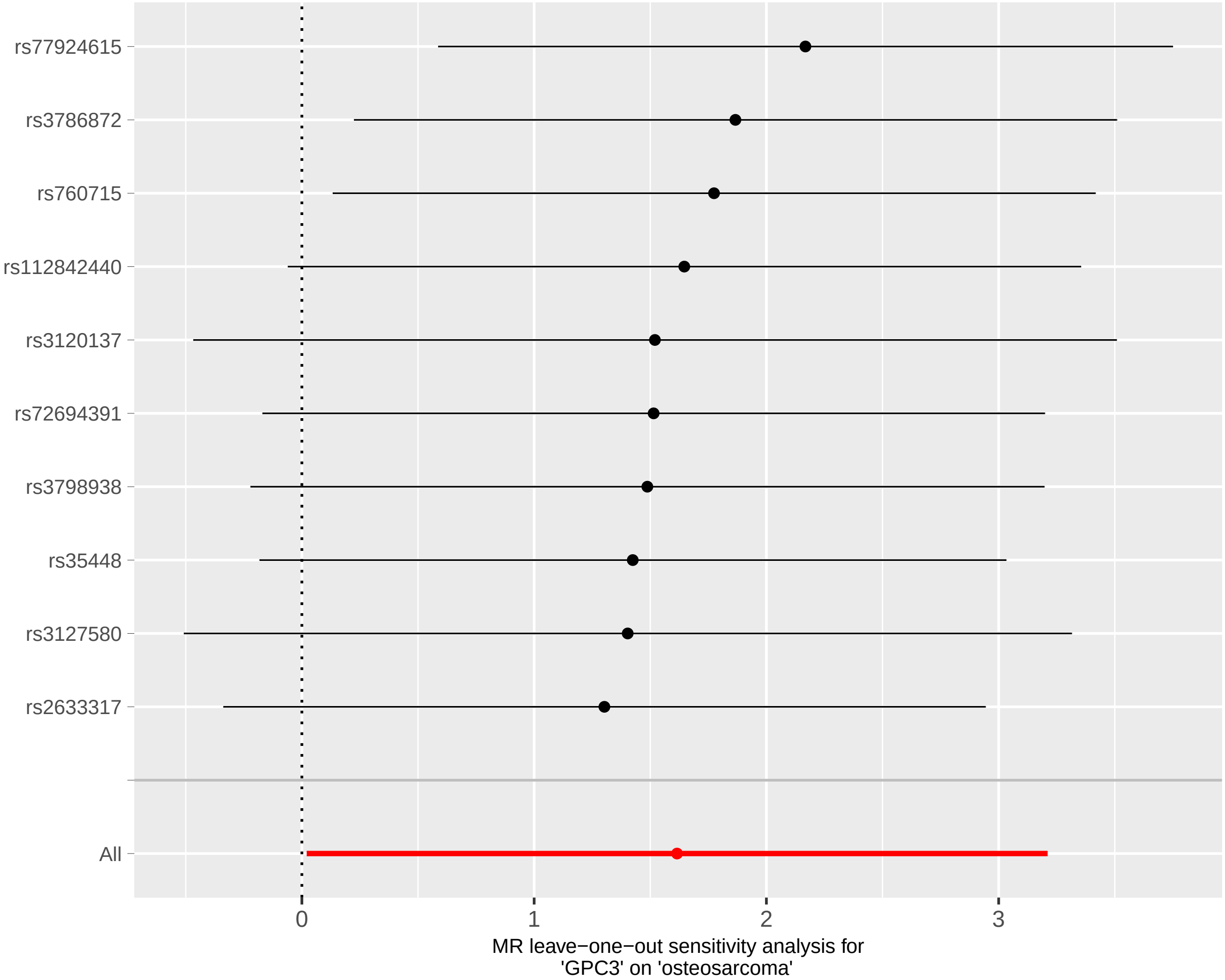
rs77924615
rs3786872
rs760715
rs112842440
rs3120137
rs72694391
rs3798938
rs35448
rs3127580
rs2633317
All
0
1
2
3
MR leave−one−out sensitivity analysis for
'GPC3' on 'osteosarcoma'

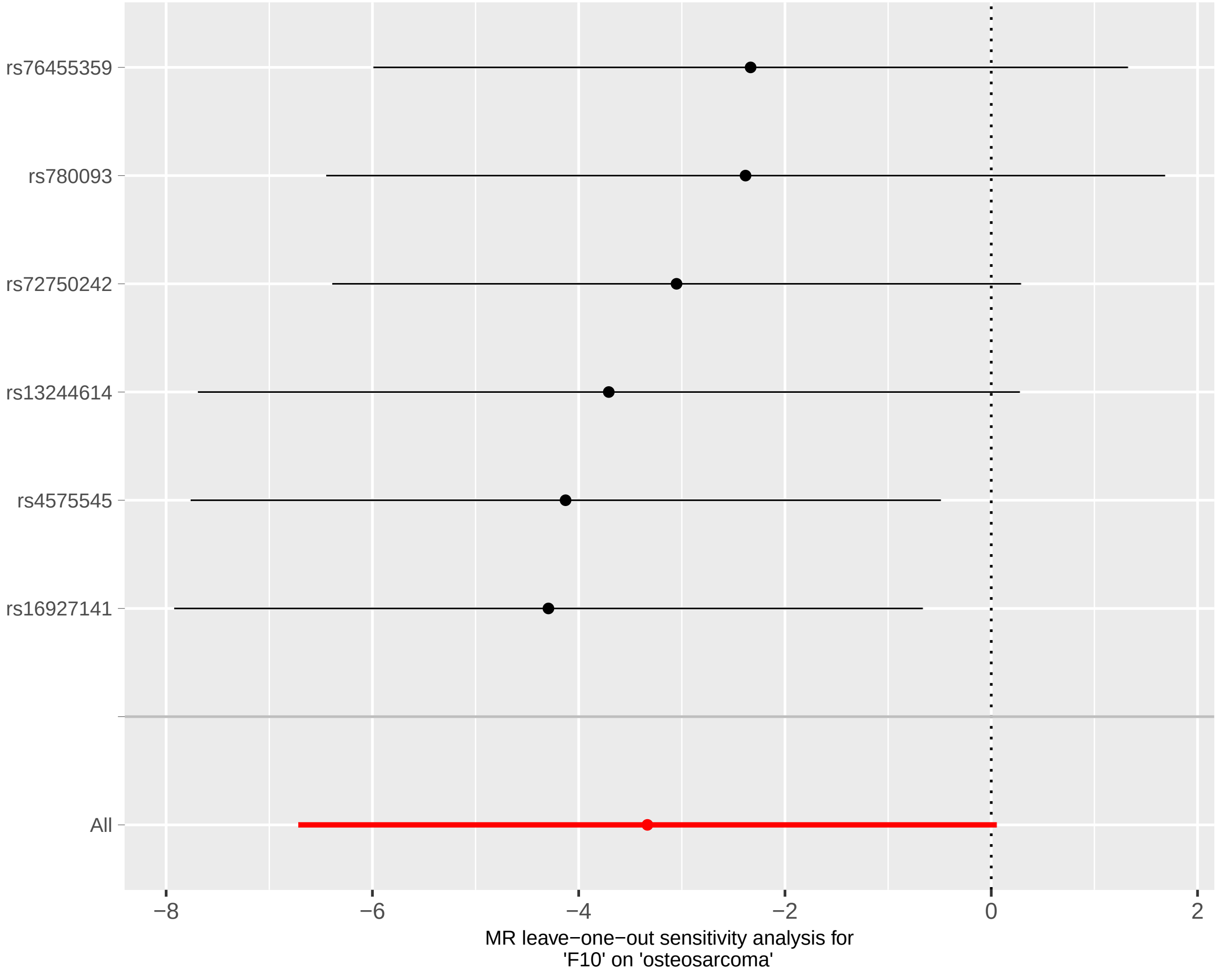

rs76455359
rs780093
rs72750242
rs13244614
rs4575545
rs16927141
All
−8
−6
−4
−2
0
2
MR leave−one−out sensitivity analysis for
'F10' on 'osteosarcoma'

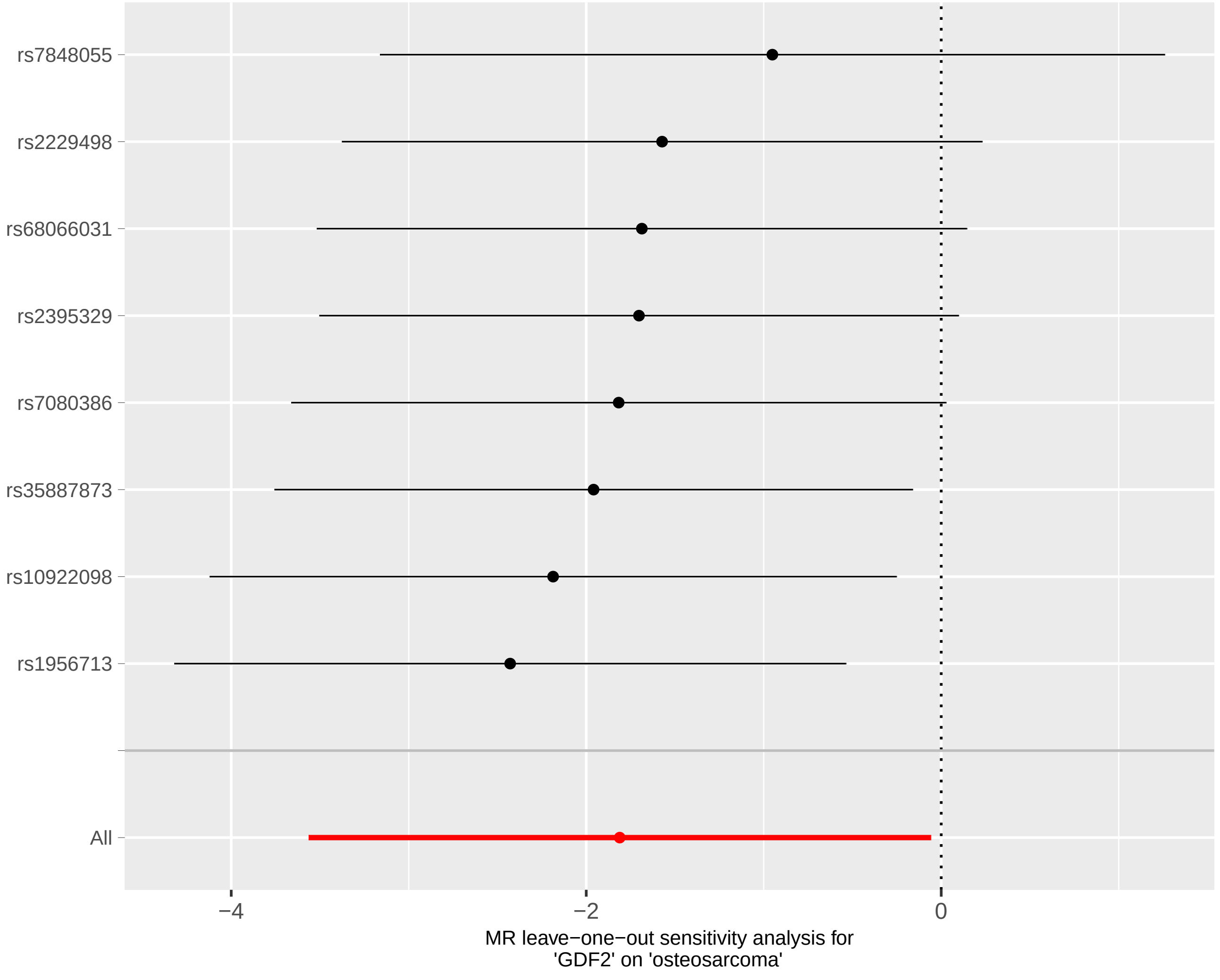
rs7848055
rs2229498
rs68066031
rs2395329
rs7080386
rs35887873
rs10922098
rs1956713
All
−4
−2
0
MR leave−one−out sensitivity analysis for
'GDF2' on 'osteosarcoma'

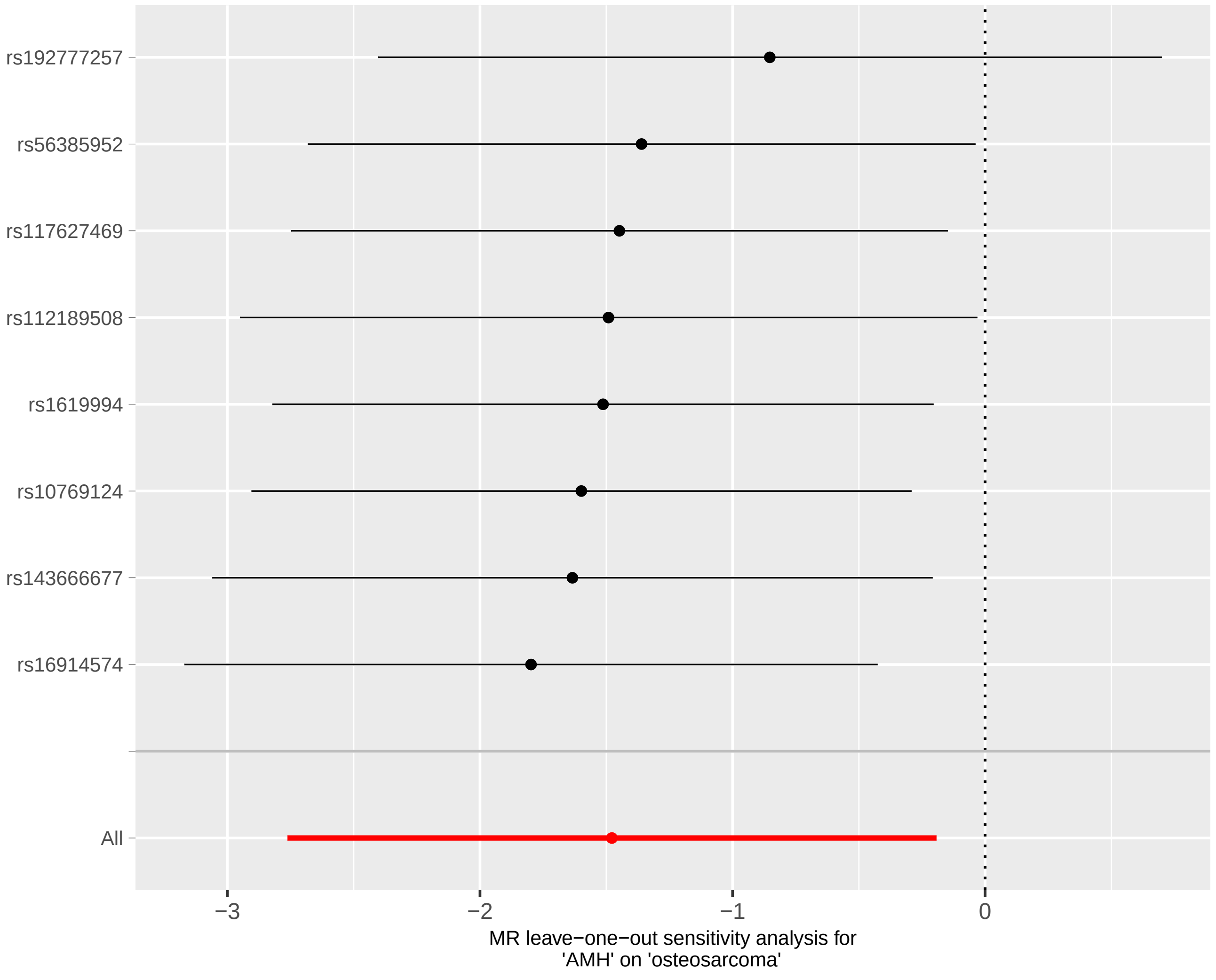
rs192777257
rs56385952
rs117627469
rs112189508
rs1619994
rs10769124
rs143666677
rs16914574
All
−3
−2
−1
0
MR leave−one−out sensitivity analysis for
'AMH' on 'osteosarcoma'

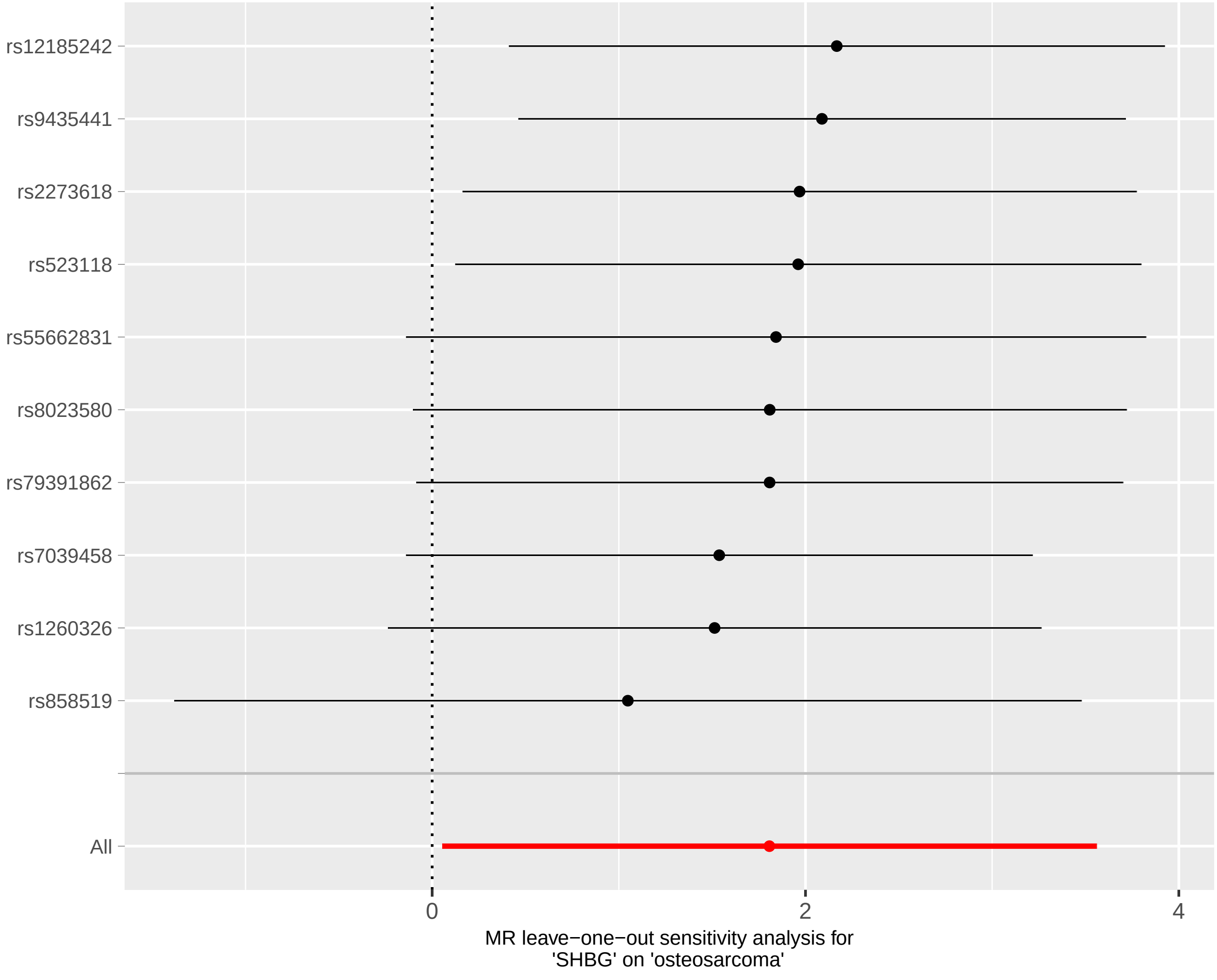
rs12185242
rs9435441
rs2273618
rs523118
rs55662831
rs8023580
rs79391862
rs7039458
rs1260326
rs858519
All
0
2
4
MR leave−one−out sensitivity analysis for
'SHBG' on 'osteosarcoma'

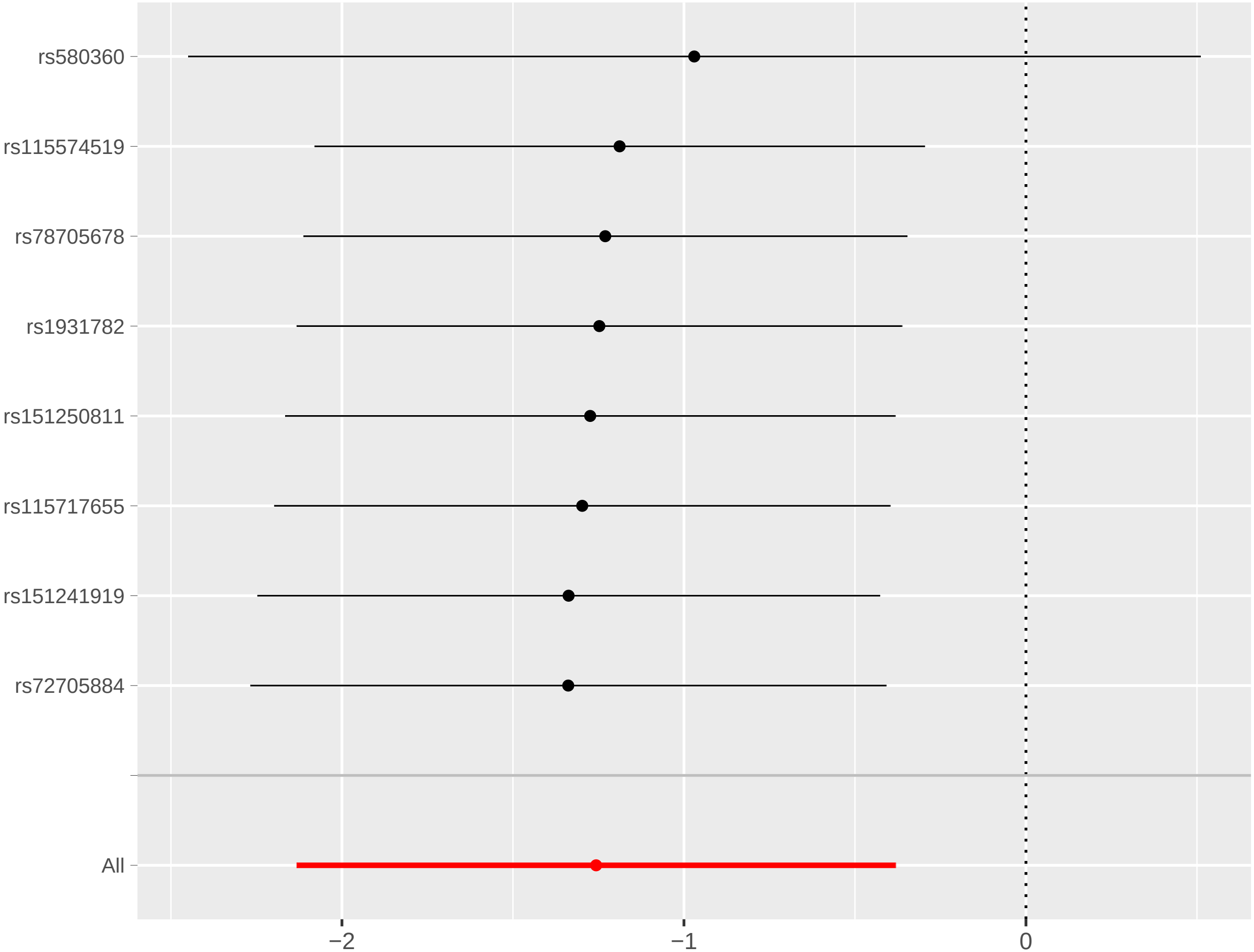

MR leave−one−out sensitivity analysis for
'DPT' on 'osteosarcoma'

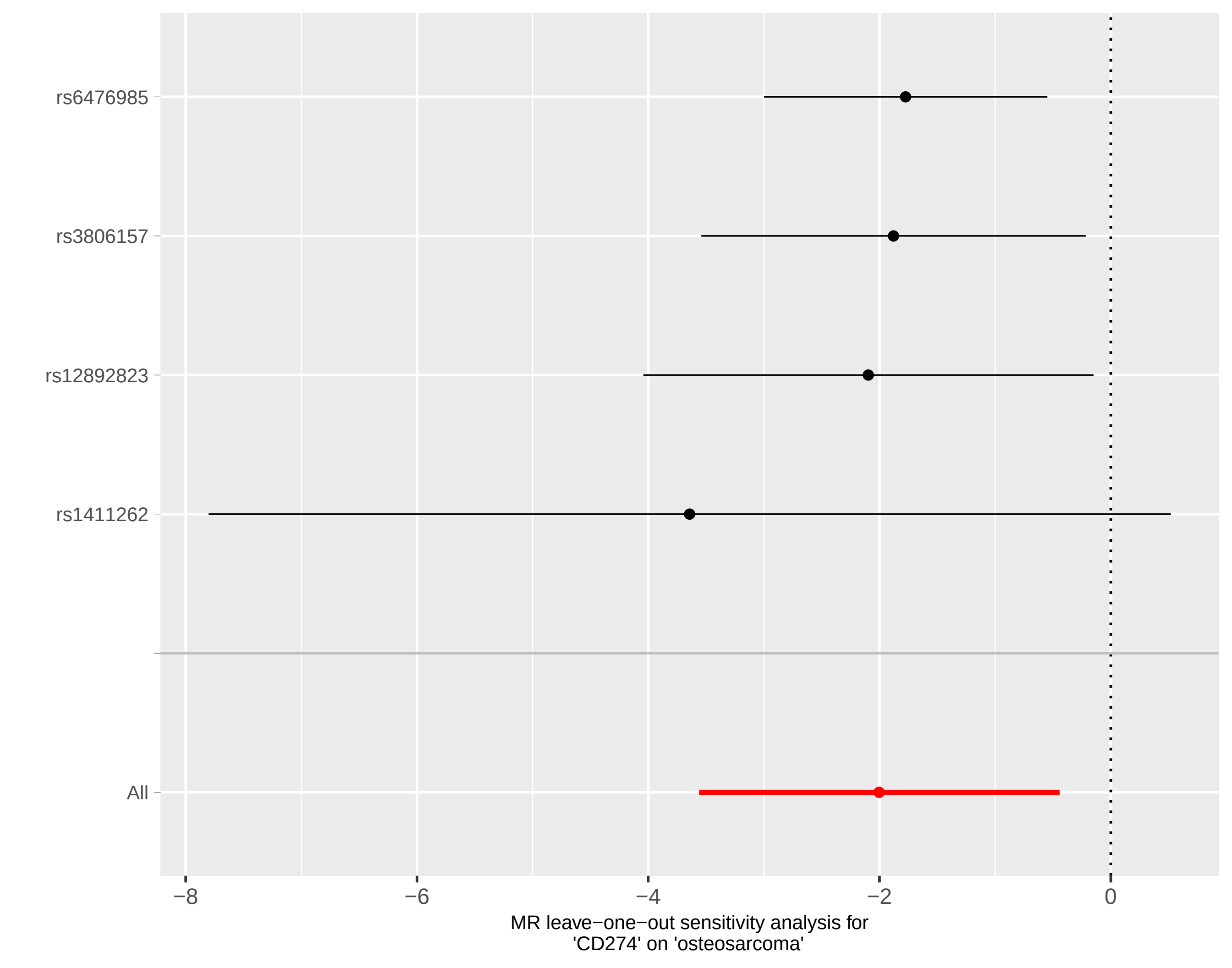

rs6476985
rs3806157
rs12892823
rs1411262
All
−8
−6
−4
−2
0
MR leave−one−out sensitivity analysis for
'CD274' on 'osteosarcoma'

Insufficient number of SNPs

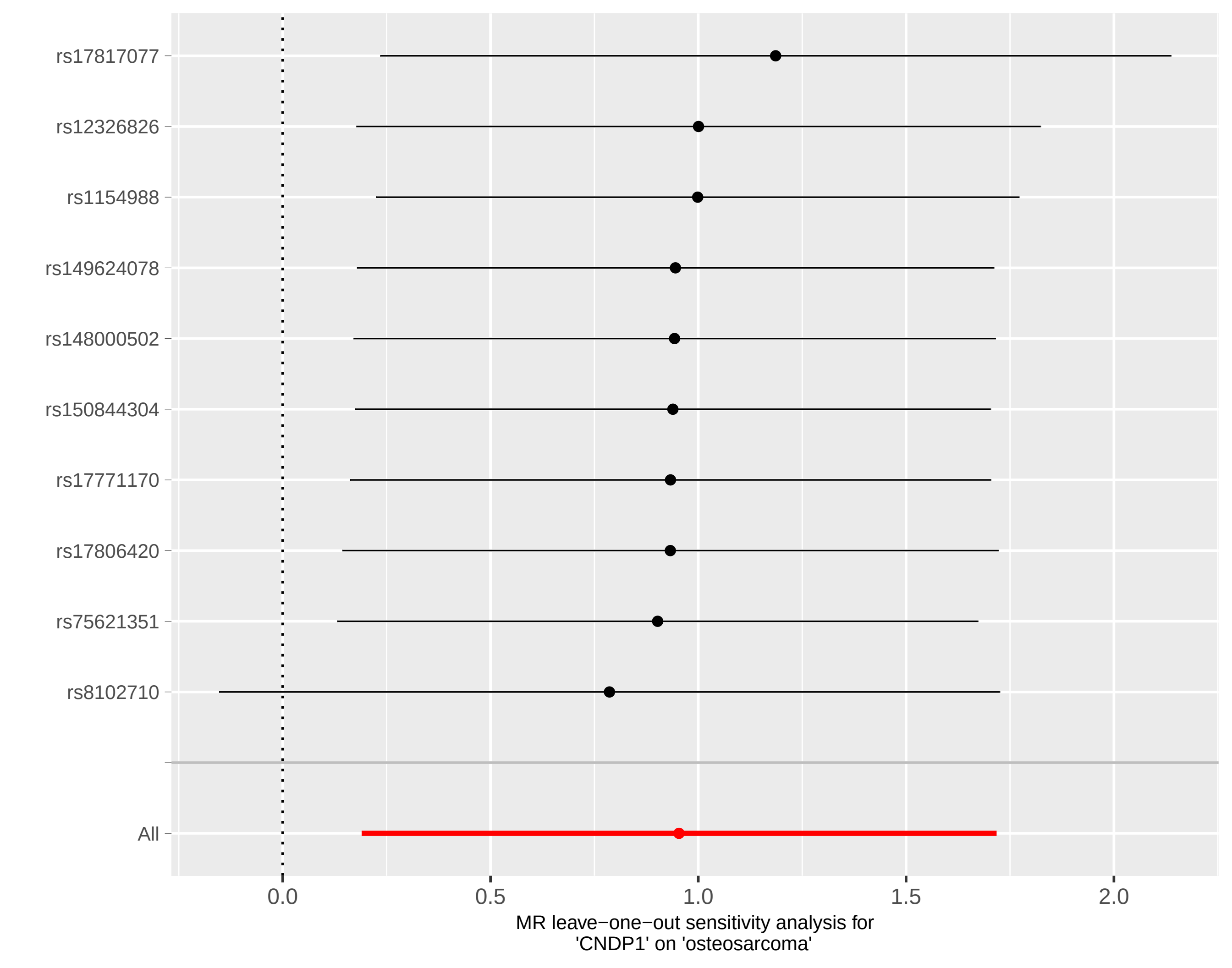
rs17817077
rs12326826
rs1154988
rs149624078
rs148000502
rs150844304
rs17771170
rs17806420
rs75621351
rs8102710
All
0.0
0.5
1.0
1.5
2.0
MR leave−one−out sensitivity analysis for
'CNDP1' on 'osteosarcoma'

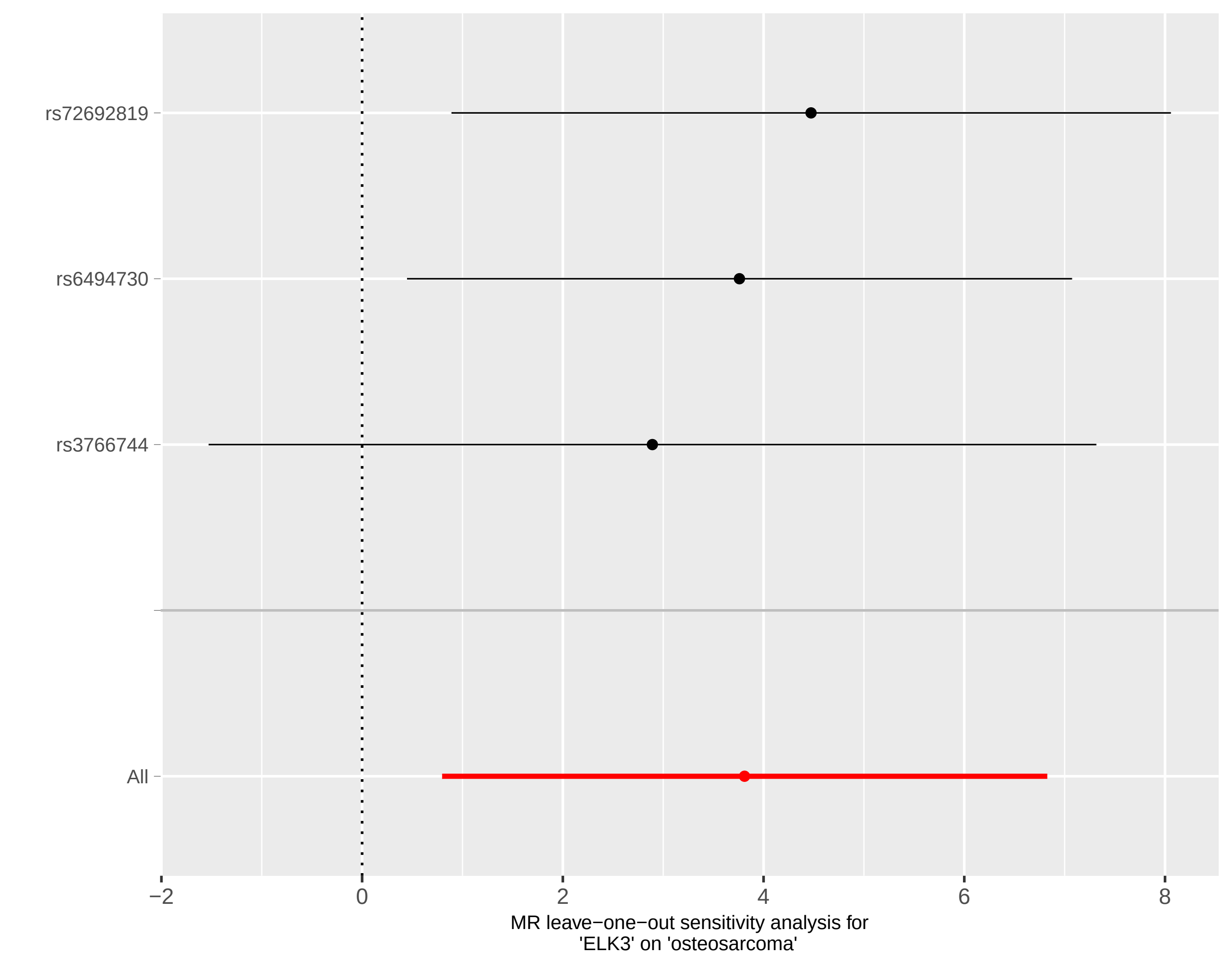

rs72692819
rs6494730
rs3766744
All
−2
0
2
4
6
8
MR leave−one−out sensitivity analysis for
'ELK3' on 'osteosarcoma'

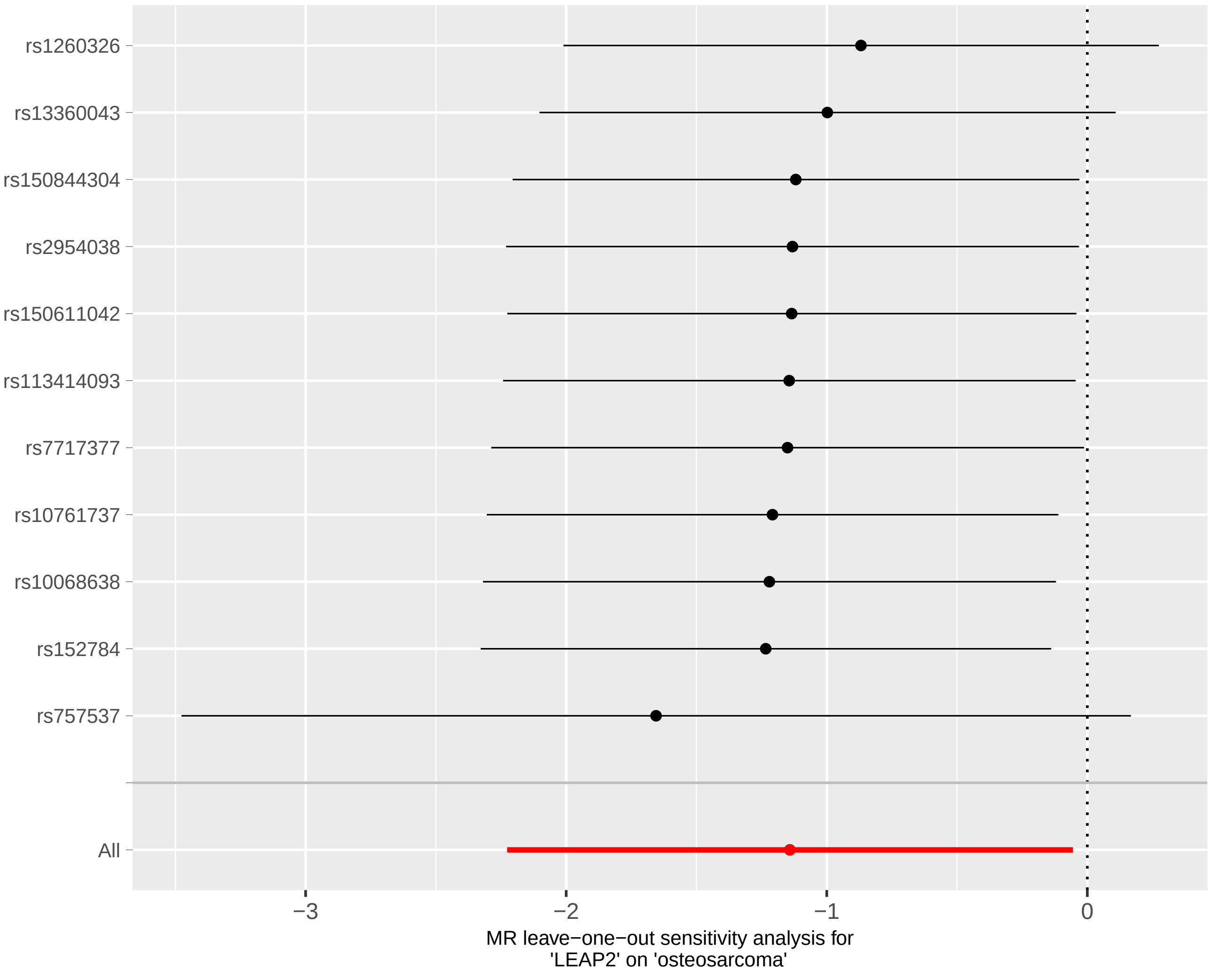
rs1260326
rs13360043
rs150844304
rs2954038
rs150611042
rs113414093
rs7717377
rs10761737
rs10068638
rs152784
rs757537
All
−3
−2
−1
0
MR leave−one−out sensitivity analysis for
'LEAP2' on 'osteosarcoma'

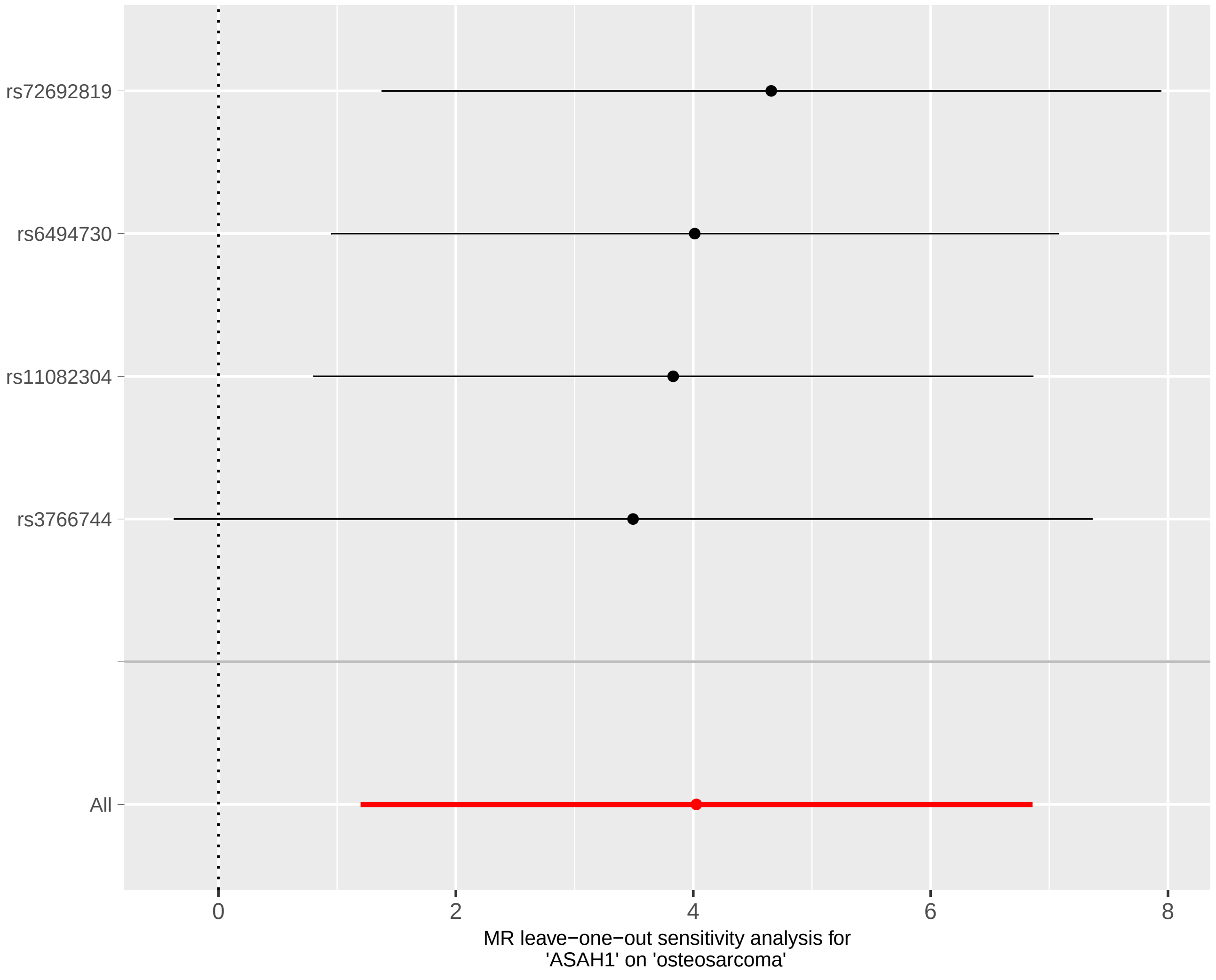
rs72692819
rs6494730
rs11082304
rs3766744
All
0
2
4
6
8
MR leave−one−out sensitivity analysis for
'ASAH1' on 'osteosarcoma'

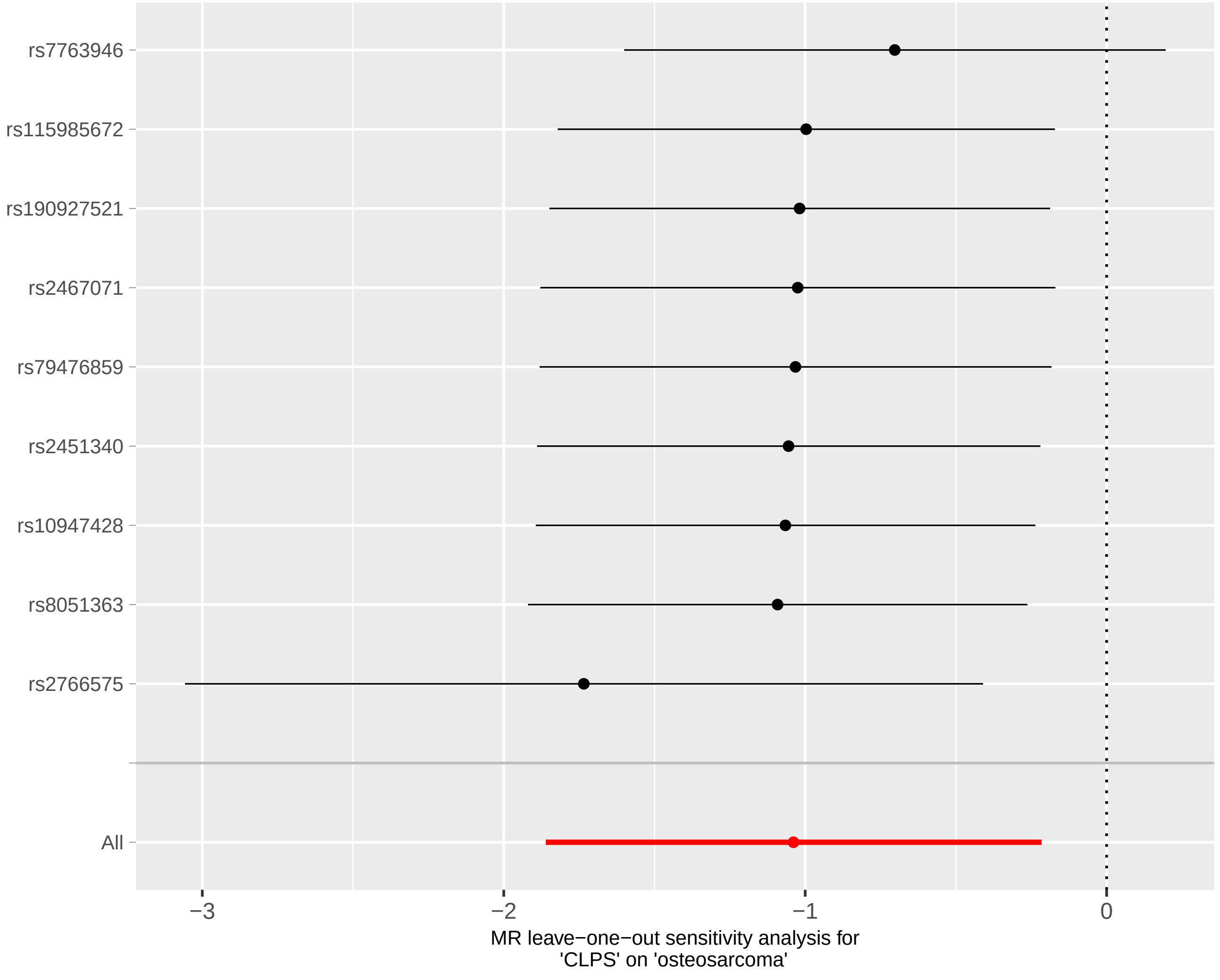
rs7763946
rs115985672
rs190927521
rs2467071
rs79476859
rs2451340
rs10947428
rs8051363
rs2766575
All
−3
−2
−1
0
MR leave−one−out sensitivity analysis for
'CLPS' on 'osteosarcoma'

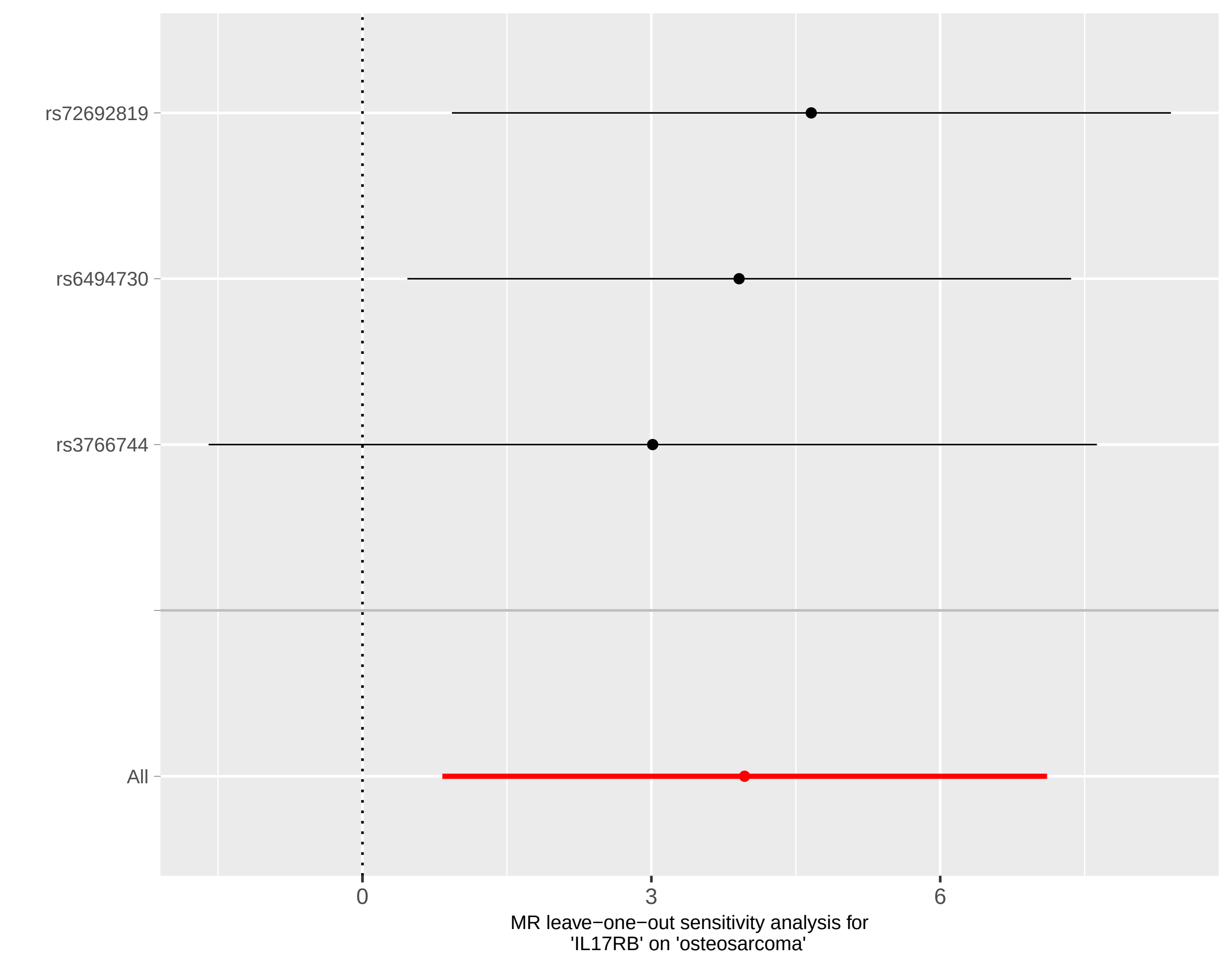
rs72692819
rs6494730
rs3766744
All
0
3
6
MR leave−one−out sensitivity analysis for
'IL17RB' on 'osteosarcoma'

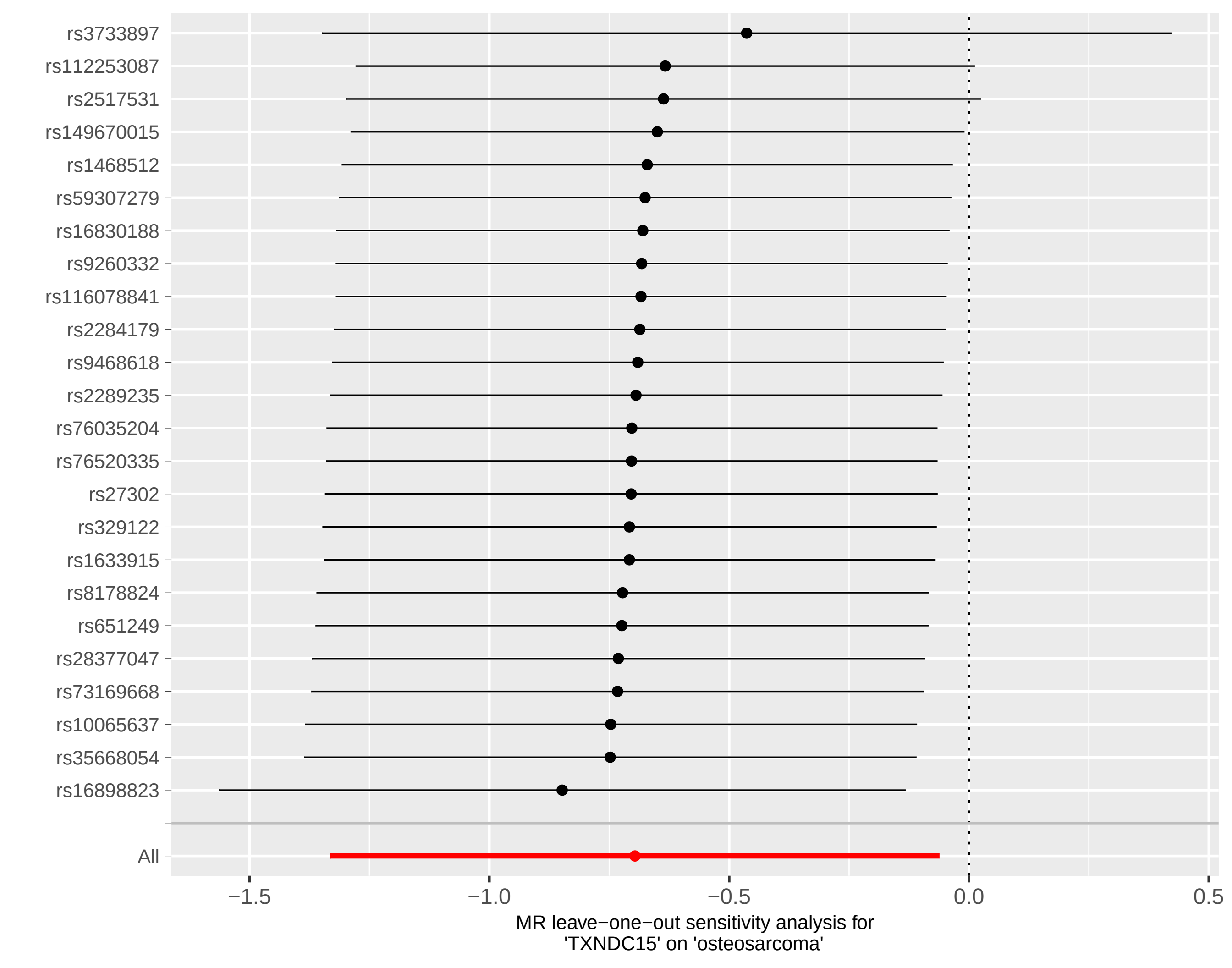
rs3733897
rs112253087
rs2517531
rs149670015
rs1468512
rs59307279
rs16830188
rs9260332
rs116078841
rs2284179
rs9468618
rs2289235
rs76035204
rs76520335
rs27302
rs329122
rs1633915
rs8178824
rs651249
rs28377047
rs73169668
rs10065637
rs35668054
rs16898823
All
−1.5
−1.0
−0.5
0.0
0.5
MR leave−one−out sensitivity analysis for
'TXNDC15' on 'osteosarcoma'

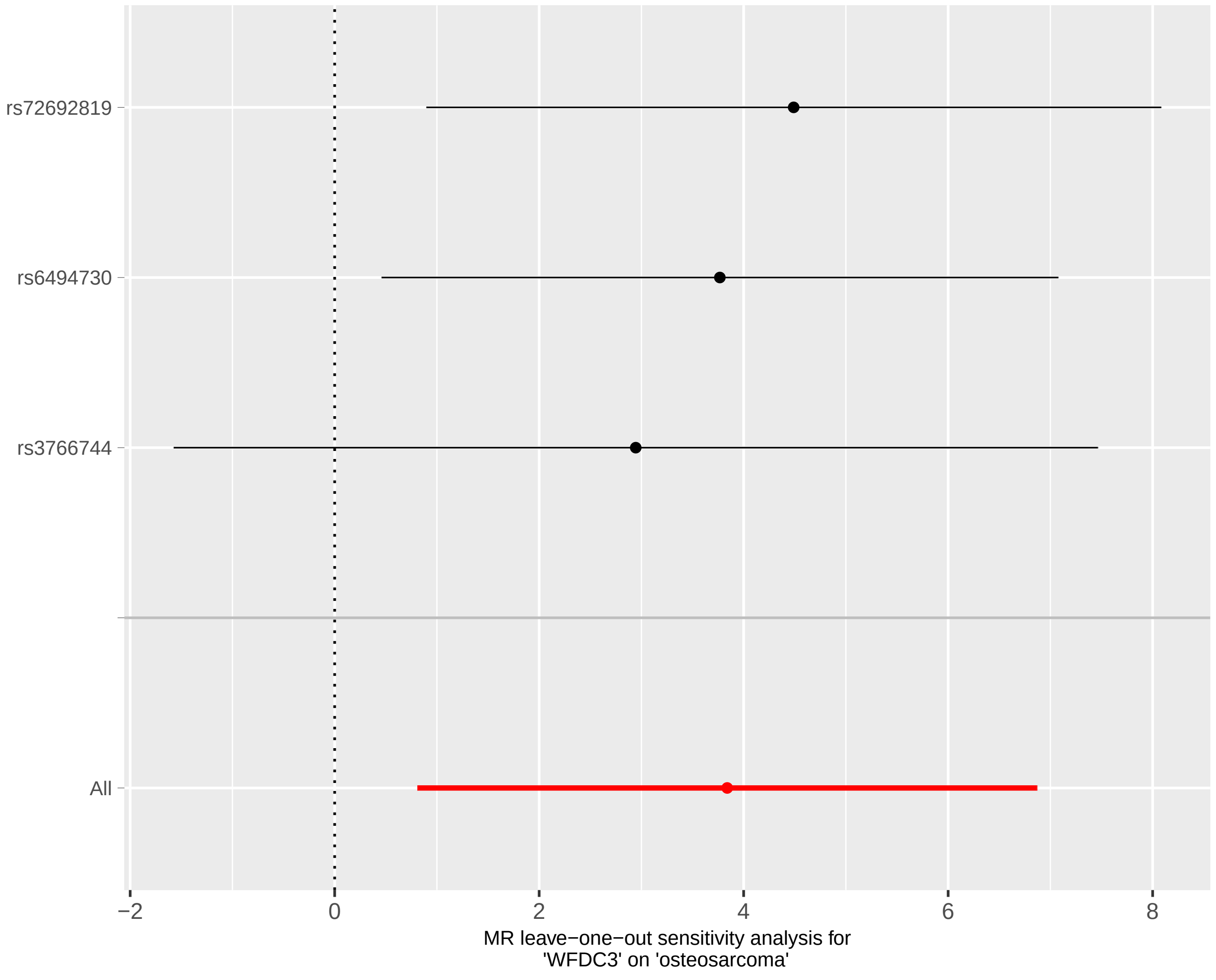

rs72692819
rs6494730
rs3766744
All
−2
0
2
4
6
8
MR leave−one−out sensitivity analysis for
'WFDC3' on 'osteosarcoma'

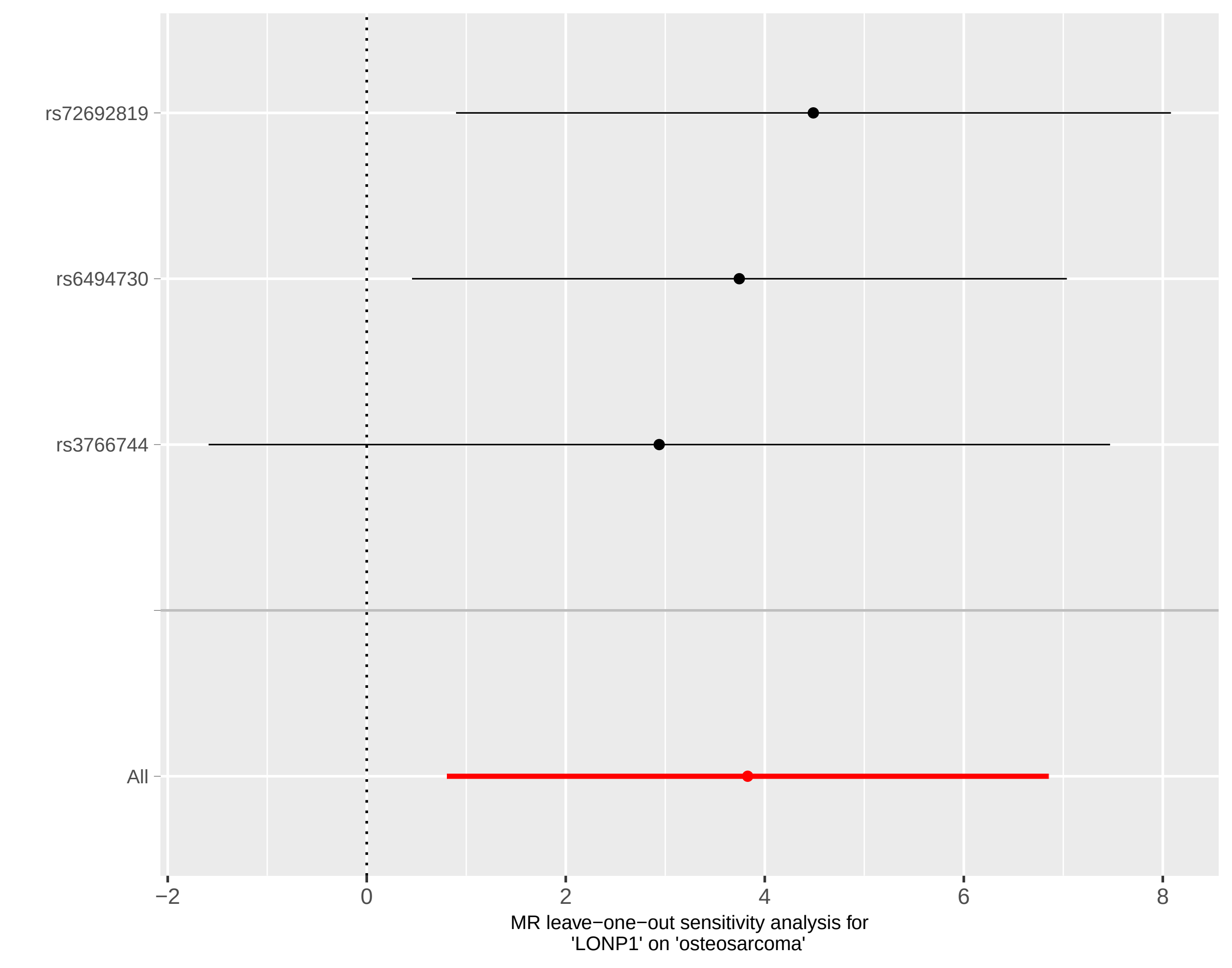
rs72692819
rs6494730
rs3766744
All
−2
0
2
4
6
8
MR leave−one−out sensitivity analysis for
'LONP1' on 'osteosarcoma'

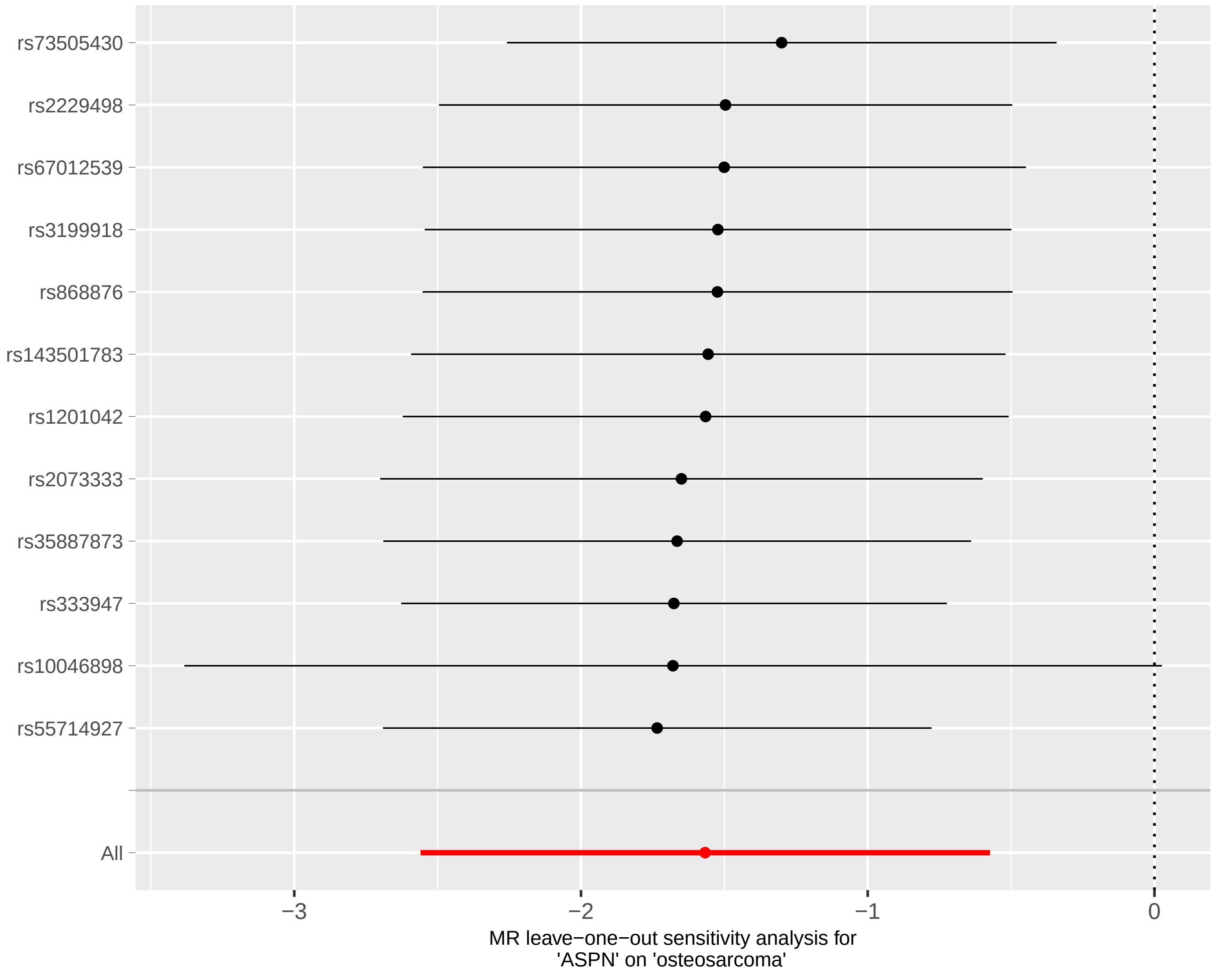
rs73505430
rs2229498
rs67012539
rs3199918
rs868876
rs143501783
rs1201042
rs2073333
rs35887873
rs333947
rs10046898
rs55714927
All
−3
−2
−1
0
MR leave−one−out sensitivity analysis for
'ASPN' on 'osteosarcoma'

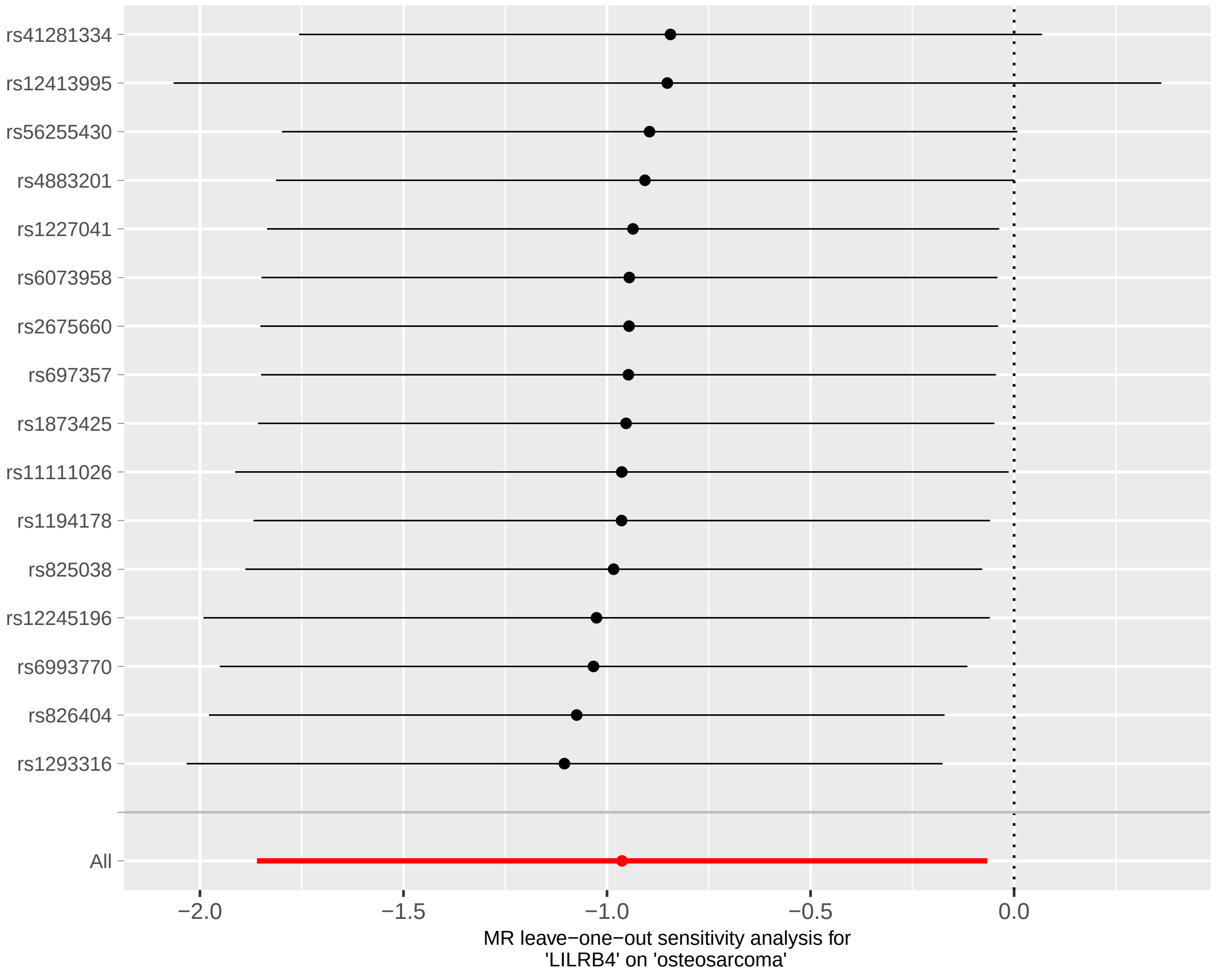
rs41281334
rs12413995
rs56255430
rs4883201
rs1227041
rs6073958
rs2675660
rs697357
rs1873425
rs11111026
rs1194178
rs825038
rs12245196
rs6993770
rs826404
rs1293316
All
−2.0
−1.5
−1.0
−0.5
0.0
MR leave−one−out sensitivity analysis for
'LILRB4' on 'osteosarcoma'

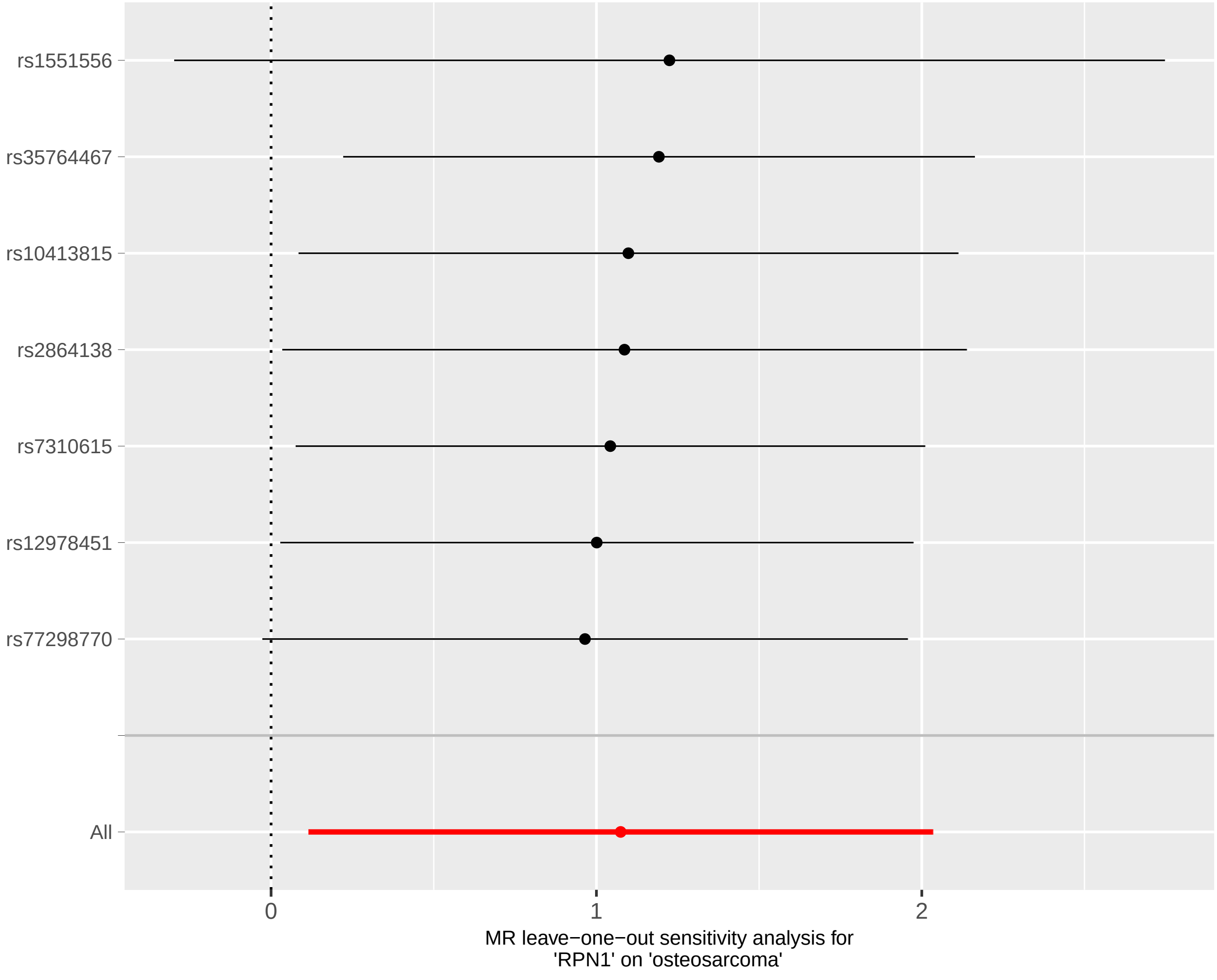
rs1551556
rs35764467
rs10413815
rs2864138
rs7310615
rs12978451
rs77298770
All
0
1
2
MR leave−one−out sensitivity analysis for
'RPN1' on 'osteosarcoma'

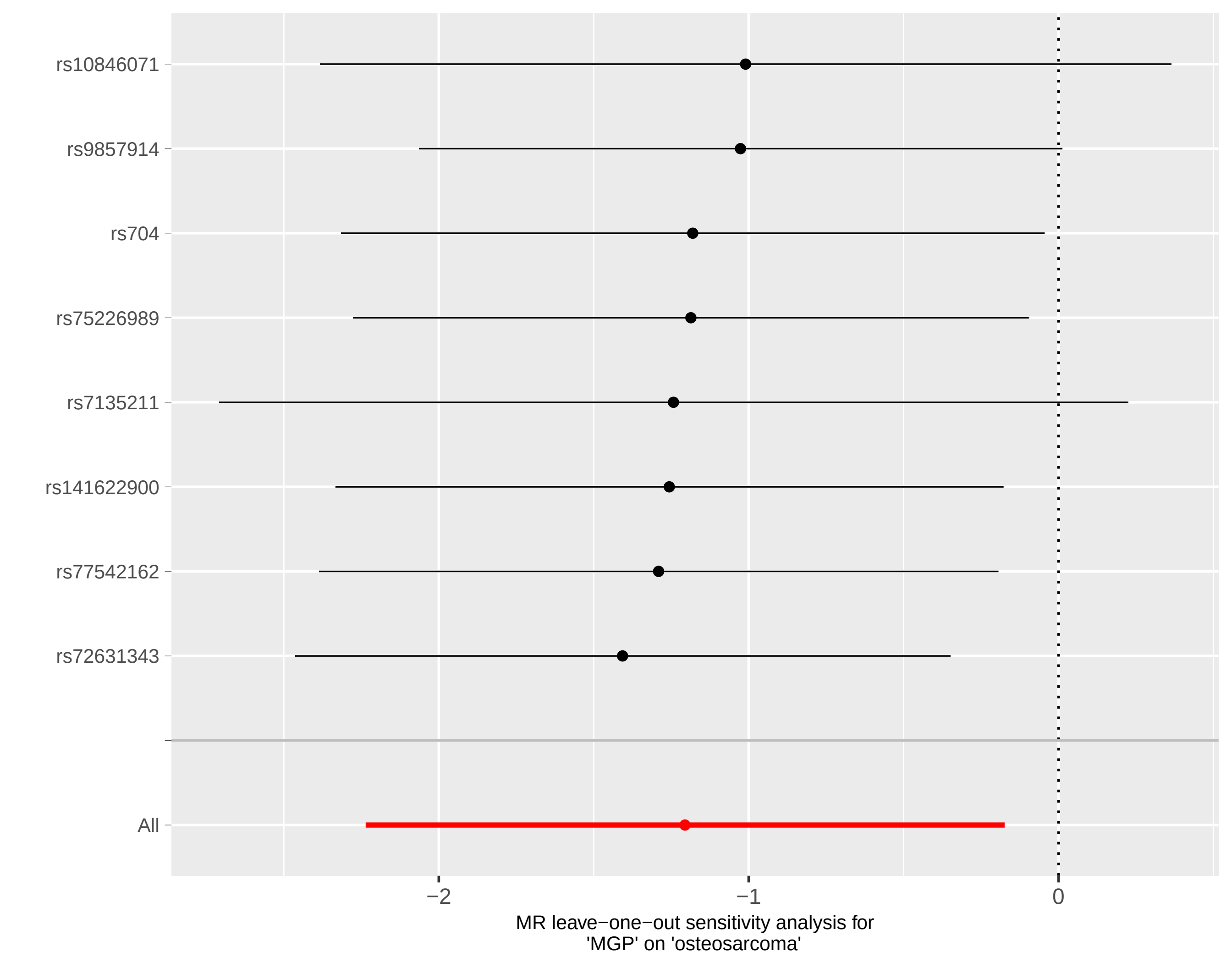
rs10846071
rs9857914
rs704
rs75226989
rs7135211
rs141622900
rs77542162
rs72631343
All
−2
−1
0
MR leave−one−out sensitivity analysis for
'MGP' on 'osteosarcoma'

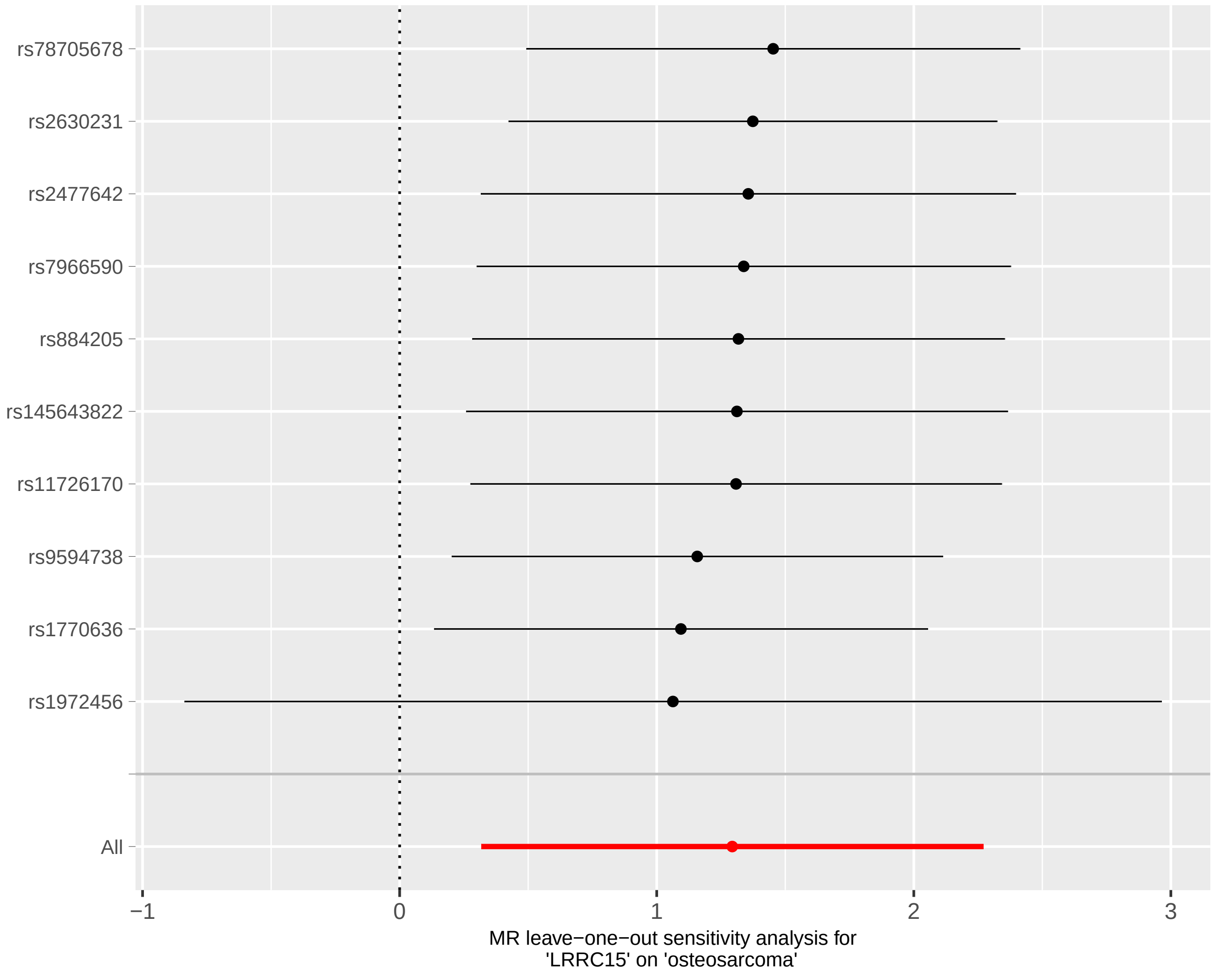

rs78705678
rs2630231
rs2477642
rs7966590
rs884205
rs145643822
rs11726170
rs9594738
rs1770636
rs1972456
All
−1
0
1
2
3
MR leave−one−out sensitivity analysis for
'LRRC15' on 'osteosarcoma'

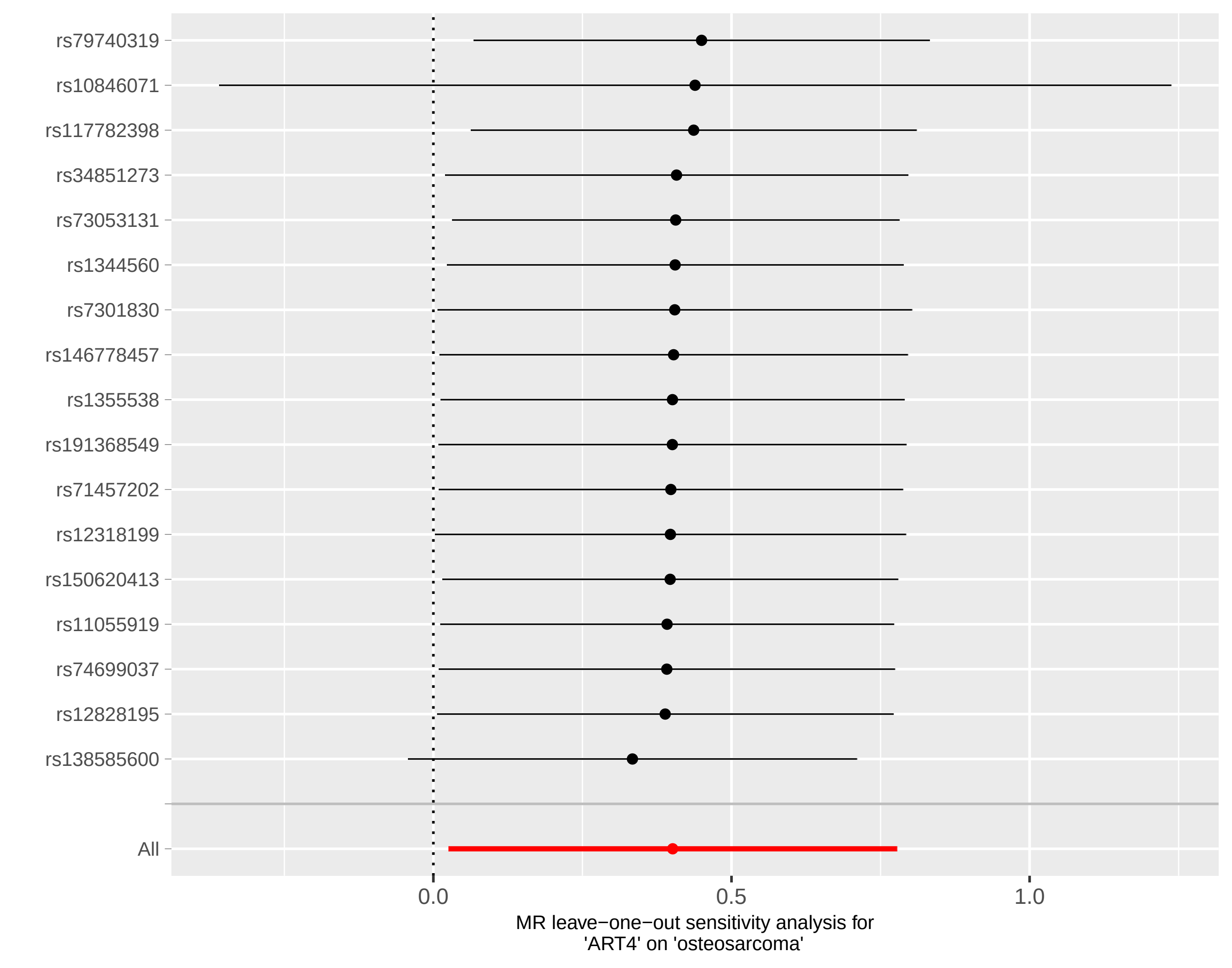
rs79740319
rs10846071
rs117782398
rs34851273
rs73053131
rs1344560
rs7301830
rs146778457
rs1355538
rs191368549
rs71457202
rs12318199
rs150620413
rs11055919
rs74699037
rs12828195
rs138585600
All
0.0
0.5
1.0
MR leave−one−out sensitivity analysis for
'ART4' on 'osteosarcoma'

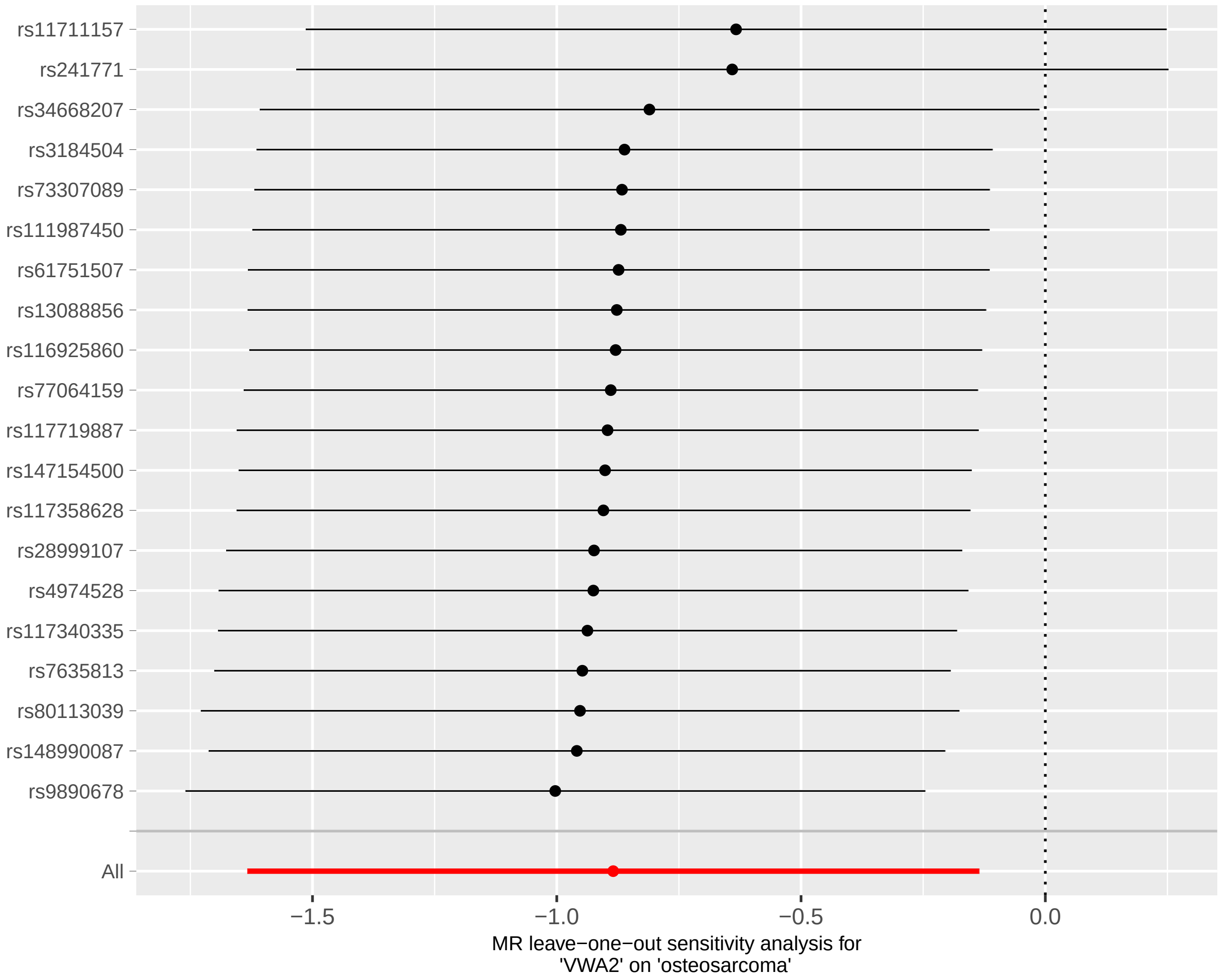
rs11711157
rs241771
rs34668207
rs3184504
rs73307089
rs111987450
rs61751507
rs13088856
rs116925860
rs77064159
rs117719887
rs147154500
rs117358628
rs28999107
rs4974528
rs117340335
rs7635813
rs80113039
rs148990087
rs9890678
All
−1.5
−1.0
−0.5
0.0
MR leave−one−out sensitivity analysis for
'VWA2' on 'osteosarcoma'

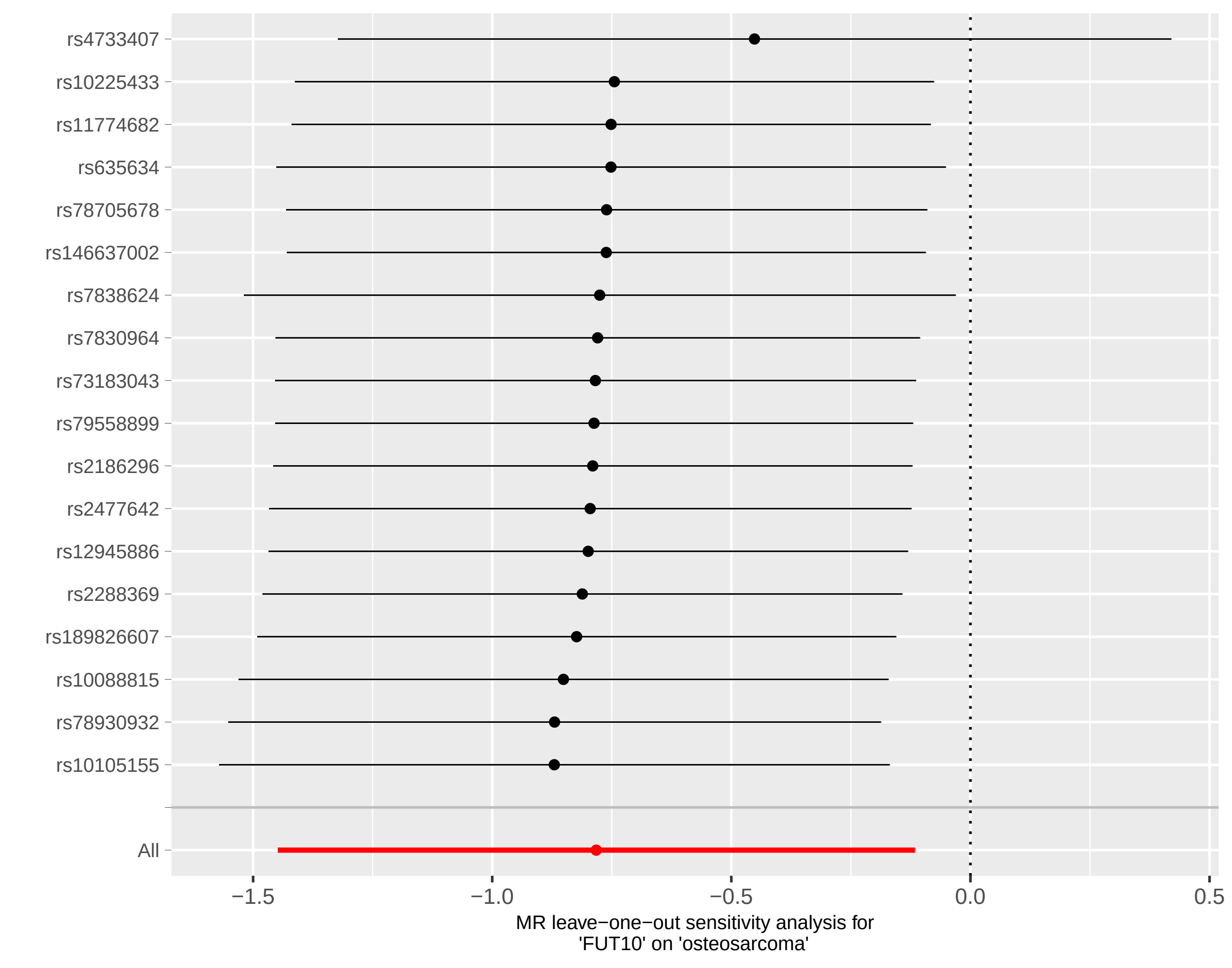
rs4733407
rs10225433
rs11774682
rs635634
rs78705678
rs146637002
rs7838624
rs7830964
rs73183043
rs79558899
rs2186296
rs2477642
rs12945886
rs2288369
rs189826607
rs10088815
rs78930932
rs10105155
All
−1.5
−1.0
−0.5
0.0
0.5
MR leave−one−out sensitivity analysis for
'FUT10' on 'osteosarcoma'

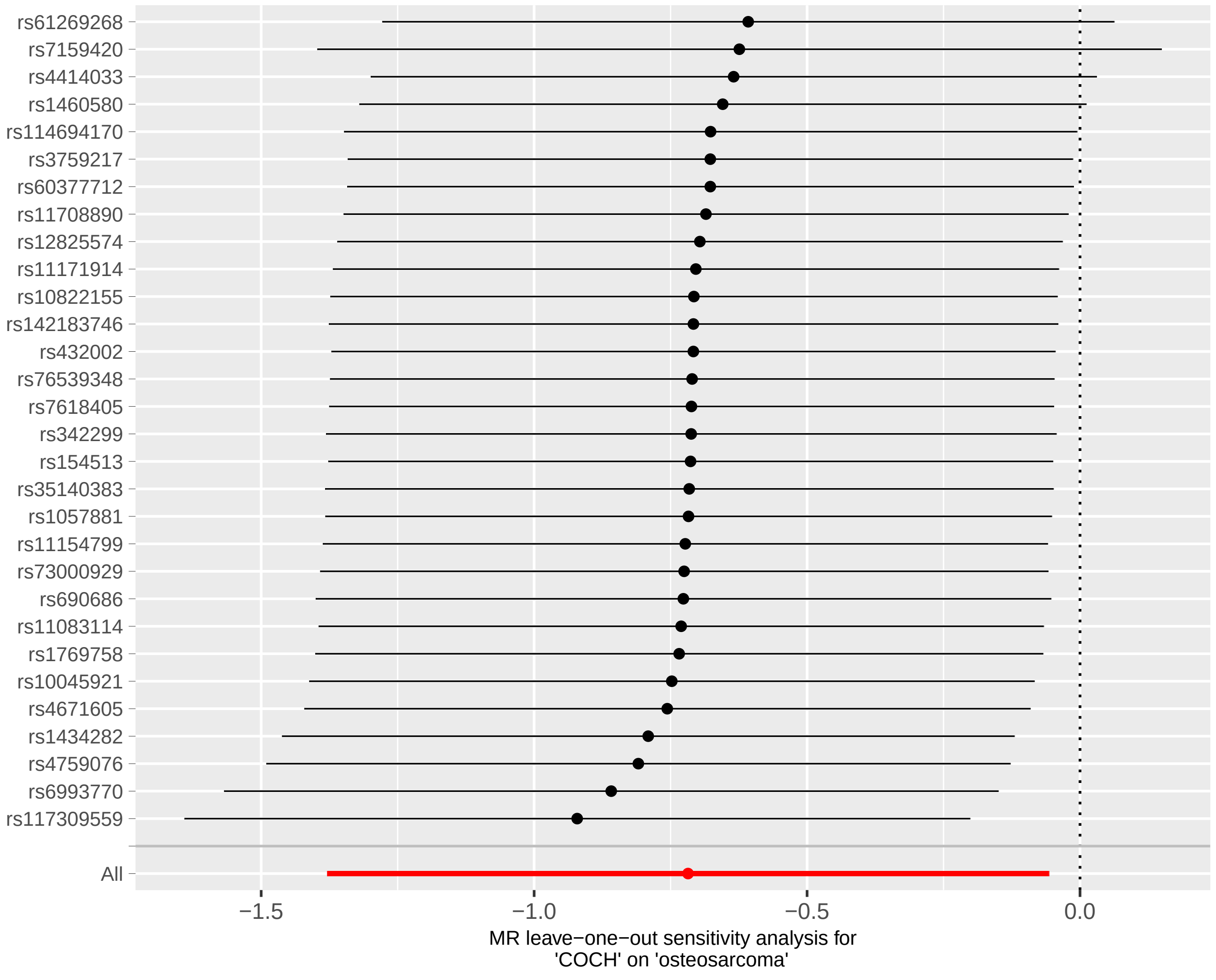

rs61269268
rs7159420
rs4414033
rs1460580
rs114694170
rs3759217
rs60377712
rs11708890
rs12825574
rs11171914
rs10822155
rs142183746
rs432002
rs76539348
rs7618405
rs342299
rs154513
rs35140383
rs1057881
rs11154799
rs73000929
rs690686
rs11083114
rs1769758
rs10045921
rs4671605
rs1434282
rs4759076
rs6993770
rs117309559
All
−1.5
−1.0
−0.5
0.0
MR leave−one−out sensitivity analysis for
'COCH' on 'osteosarcoma'

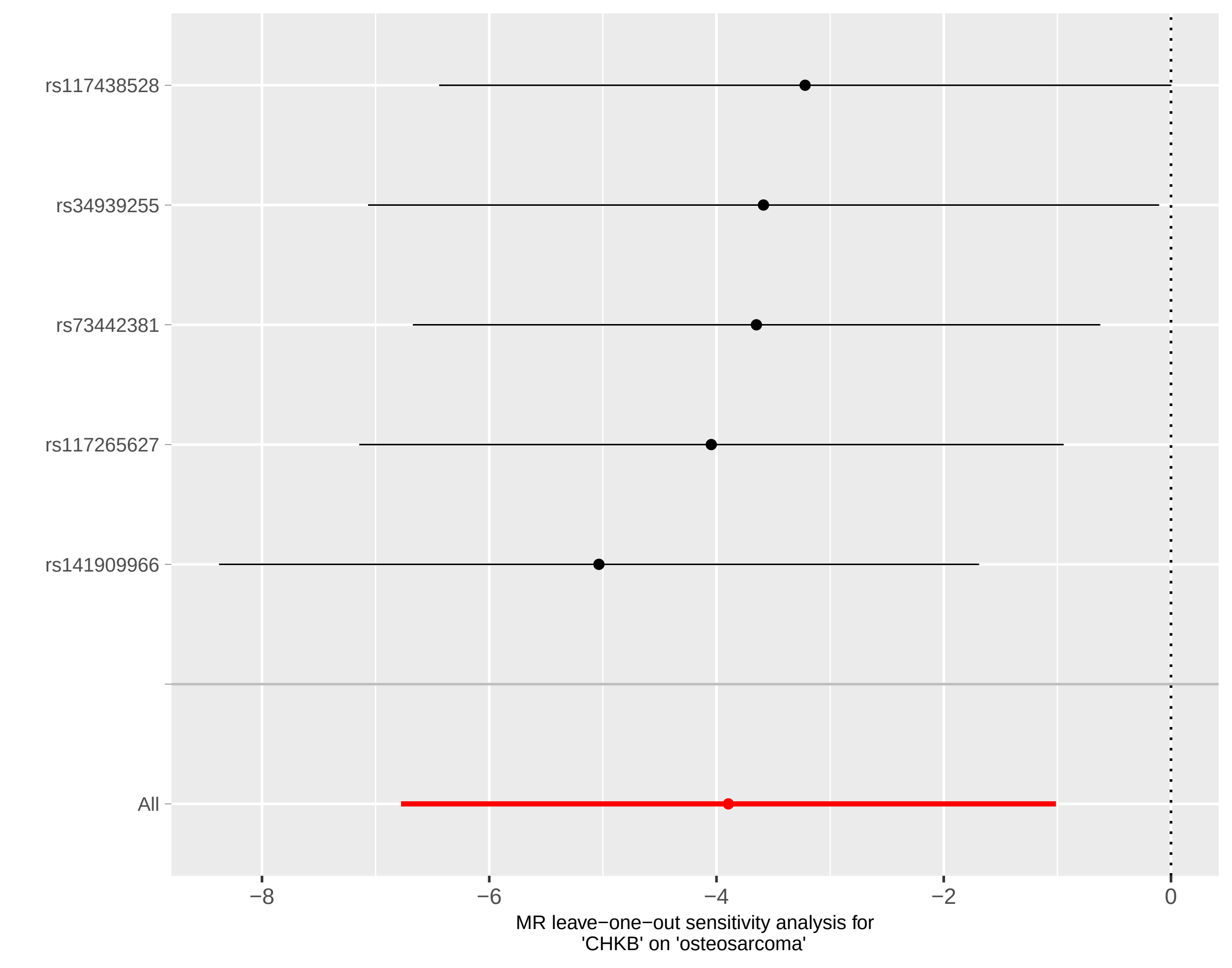
rs117438528
rs34939255
rs73442381
rs117265627
rs141909966
All
−8
−6
−4
−2
0
MR leave−one−out sensitivity analysis for
'CHKB' on 'osteosarcoma'

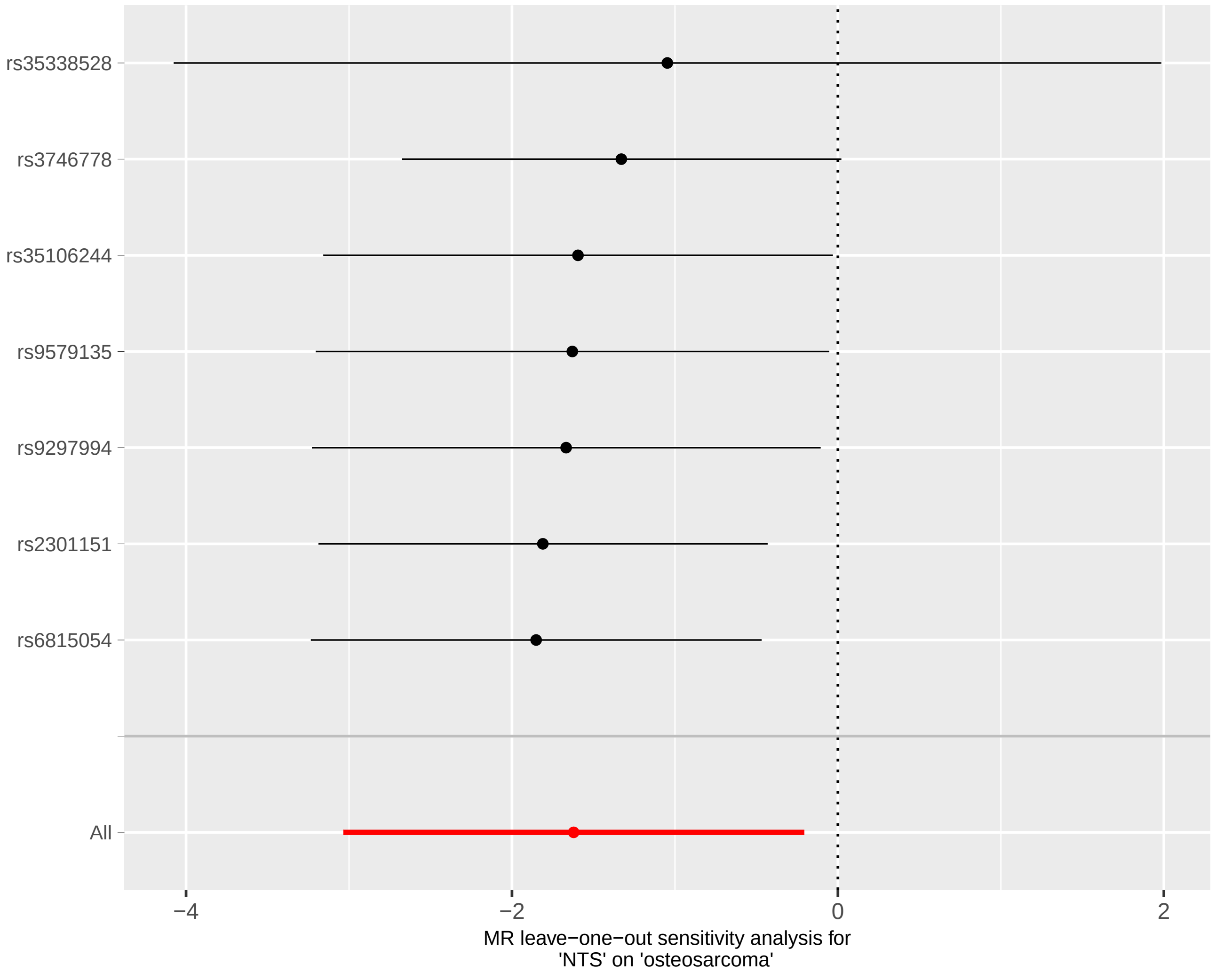

rs35338528
rs3746778
rs35106244
rs9579135
rs9297994
rs2301151
rs6815054
All
−4
−2
0
2
MR leave−one−out sensitivity analysis for
'NTS' on 'osteosarcoma'

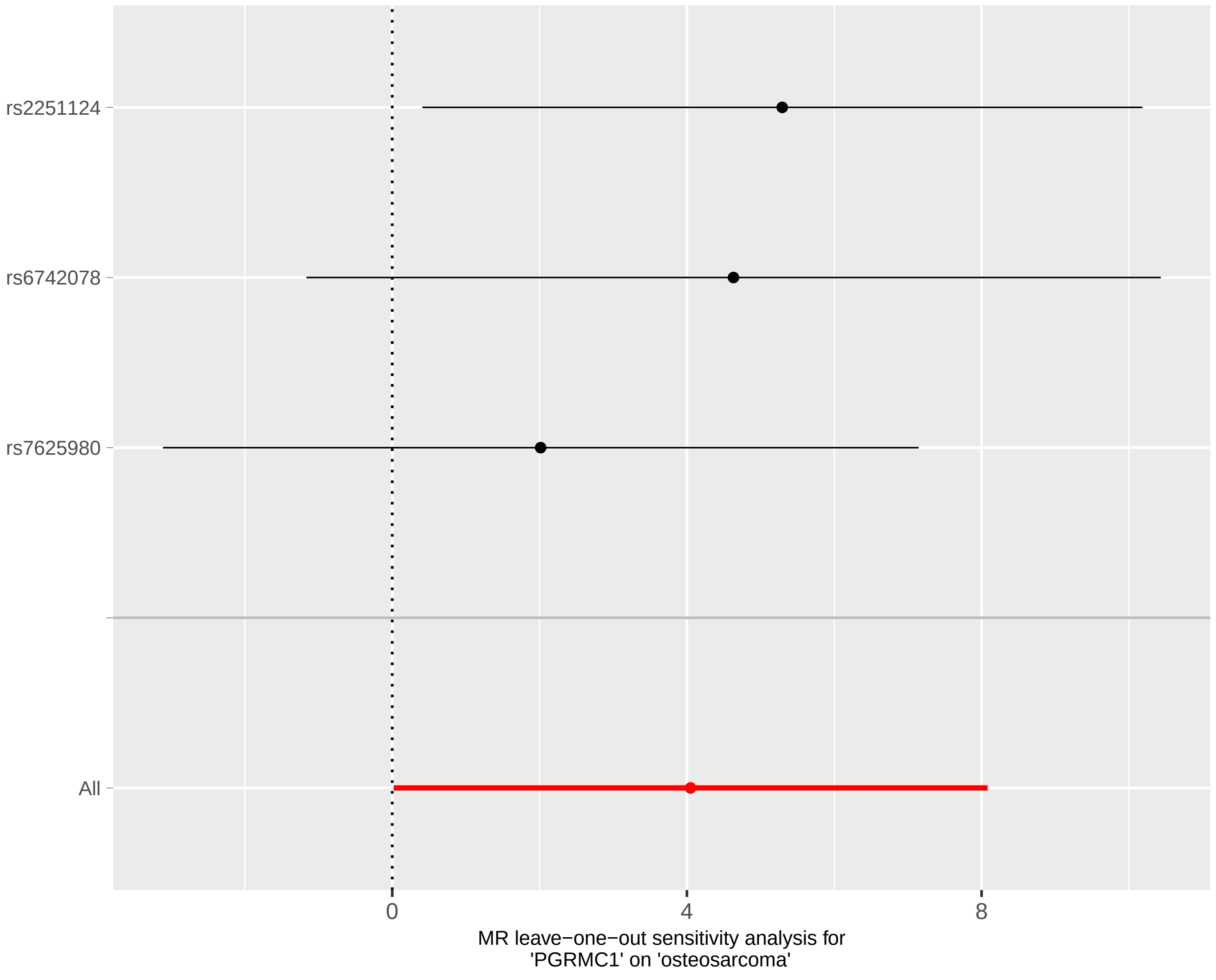
rs2251124
rs6742078
rs7625980
All
0
4
8
MR leave−one−out sensitivity analysis for
'PGRMC1' on 'osteosarcoma'

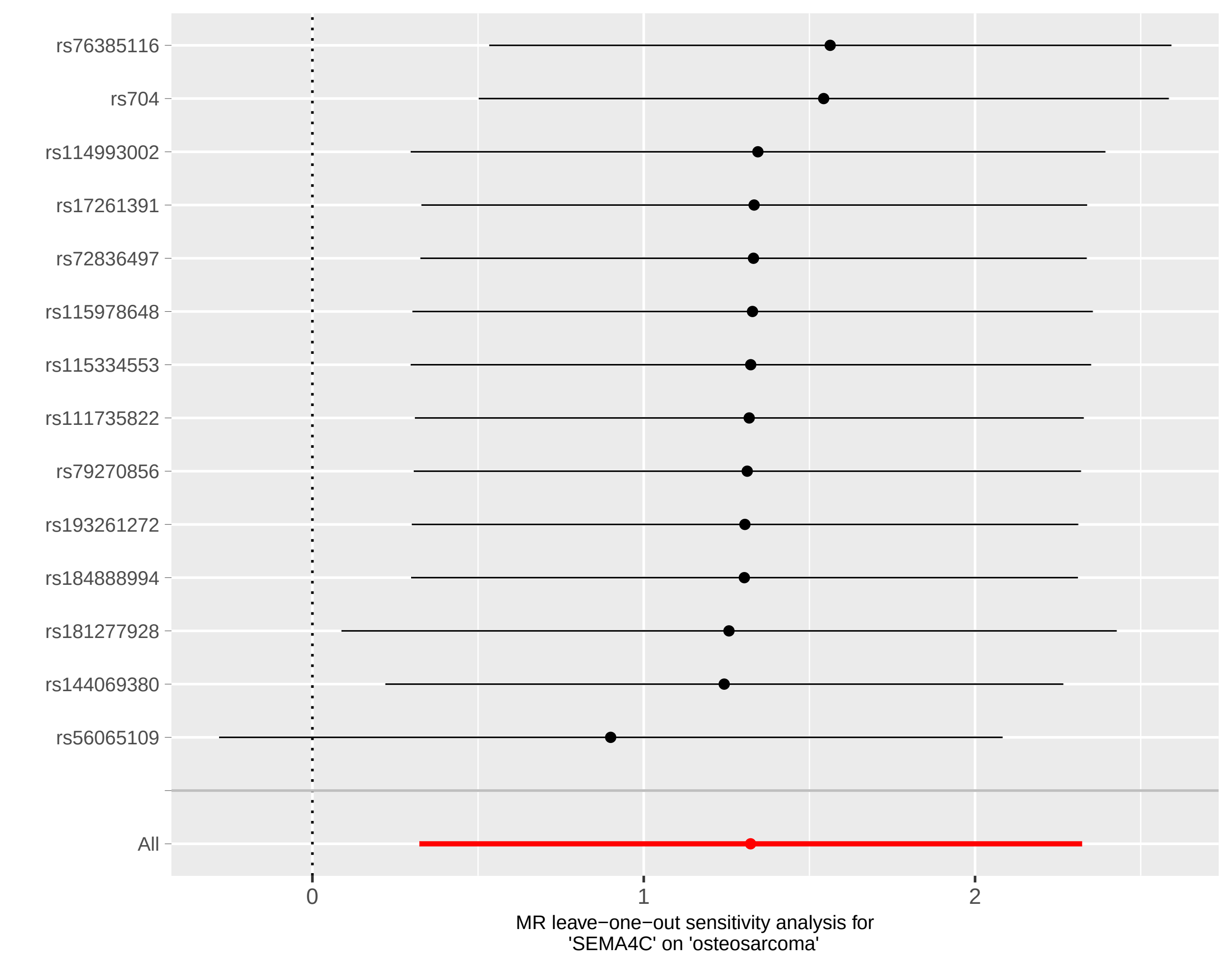

rs76385116
rs704
rs114993002
rs17261391
rs72836497
rs115978648
rs115334553
rs111735822
rs79270856
rs193261272
rs184888994
rs181277928
rs144069380
rs56065109
All
0
1
2
MR leave−one−out sensitivity analysis for
'SEMA4C' on 'osteosarcoma'

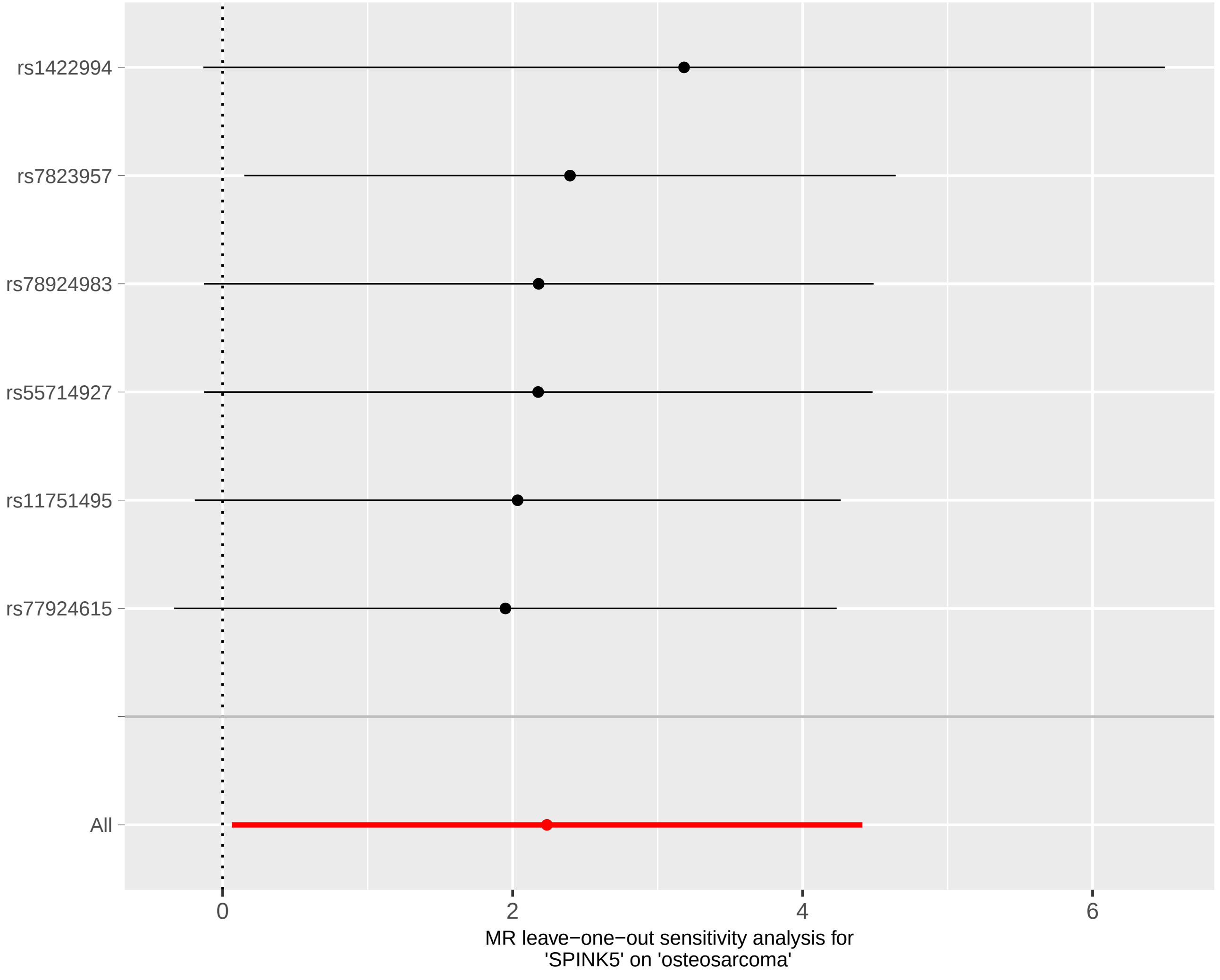

rs1422994
rs7823957
rs78924983
rs55714927
rs11751495
rs77924615
All
0
2
4
6
MR leave−one−out sensitivity analysis for
'SPINK5' on 'osteosarcoma'

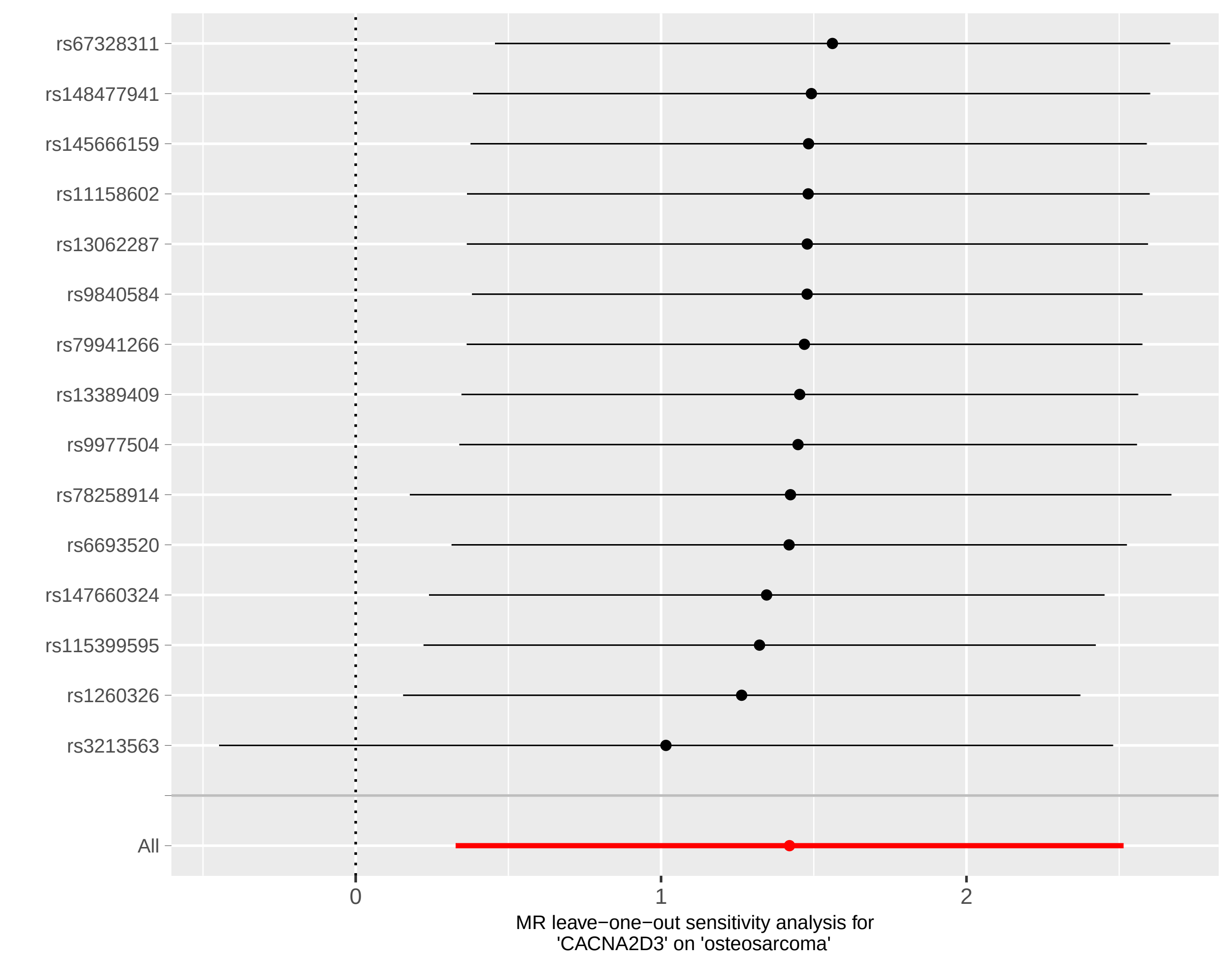
rs67328311
rs148477941
rs145666159
rs11158602
rs13062287
rs9840584
rs79941266
rs13389409
rs9977504
rs78258914
rs6693520
rs147660324
rs115399595
rs1260326
rs3213563
All
0
1
2
MR leave−one−out sensitivity analysis for
'CACNA2D3' on 'osteosarcoma'

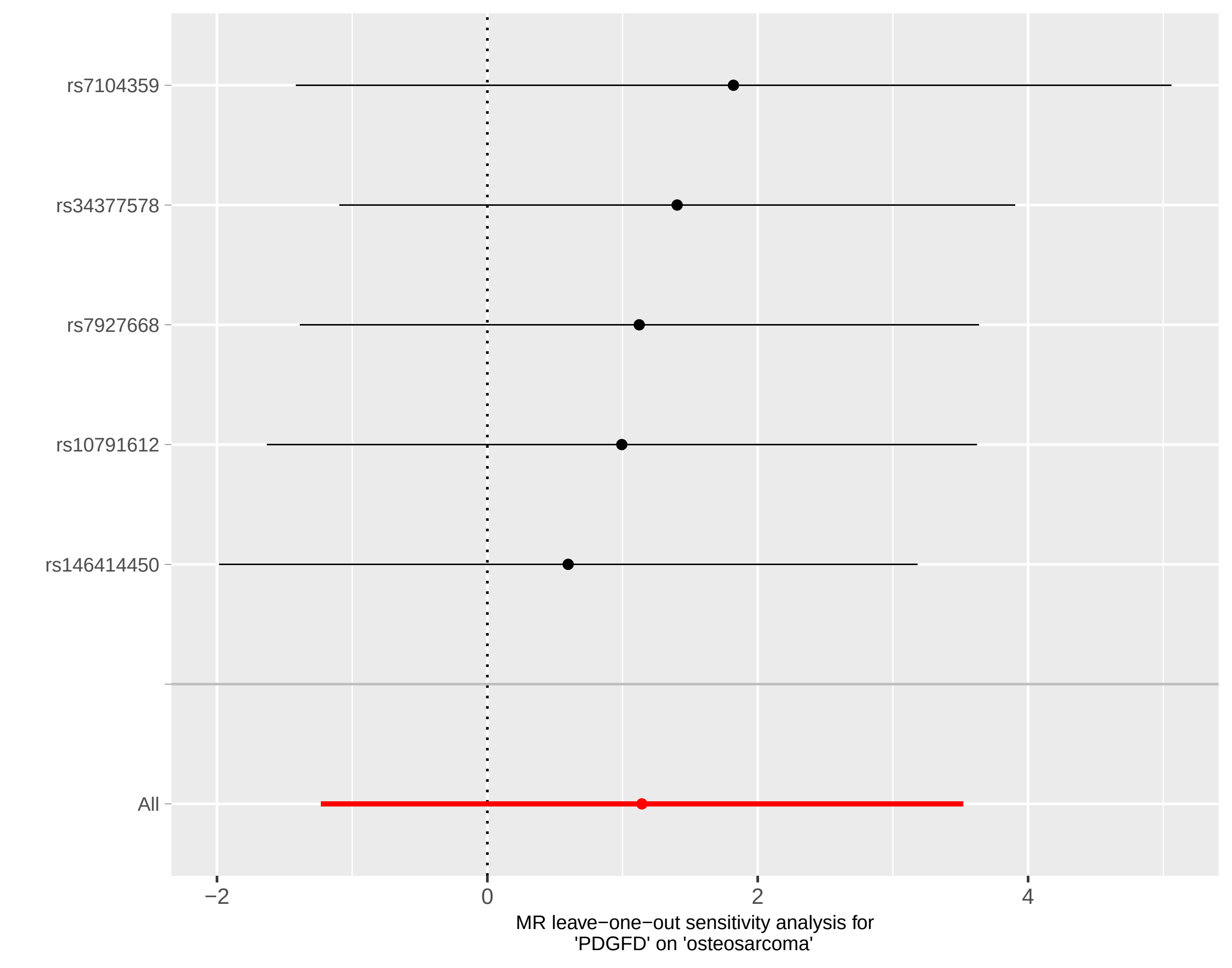
rs7104359
rs34377578
rs7927668
rs10791612
rs146414450
All
−2
0
2
4
MR leave−one−out sensitivity analysis for
'PDGFD' on 'osteosarcoma'

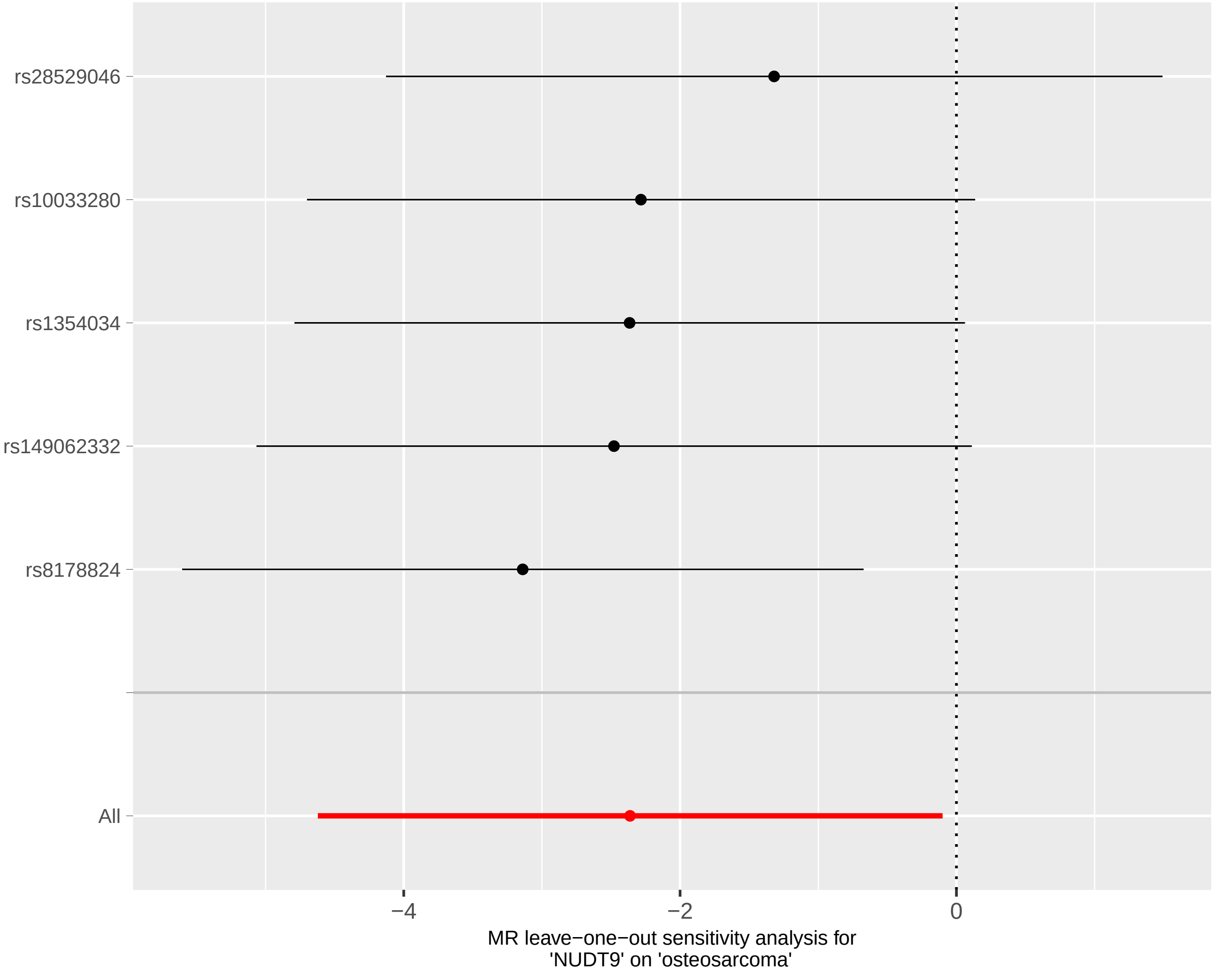
rs28529046
rs10033280
rs1354034
rs149062332
rs8178824
All
−4
−2
0
MR leave−one−out sensitivity analysis for
'NUDT9' on 'osteosarcoma'

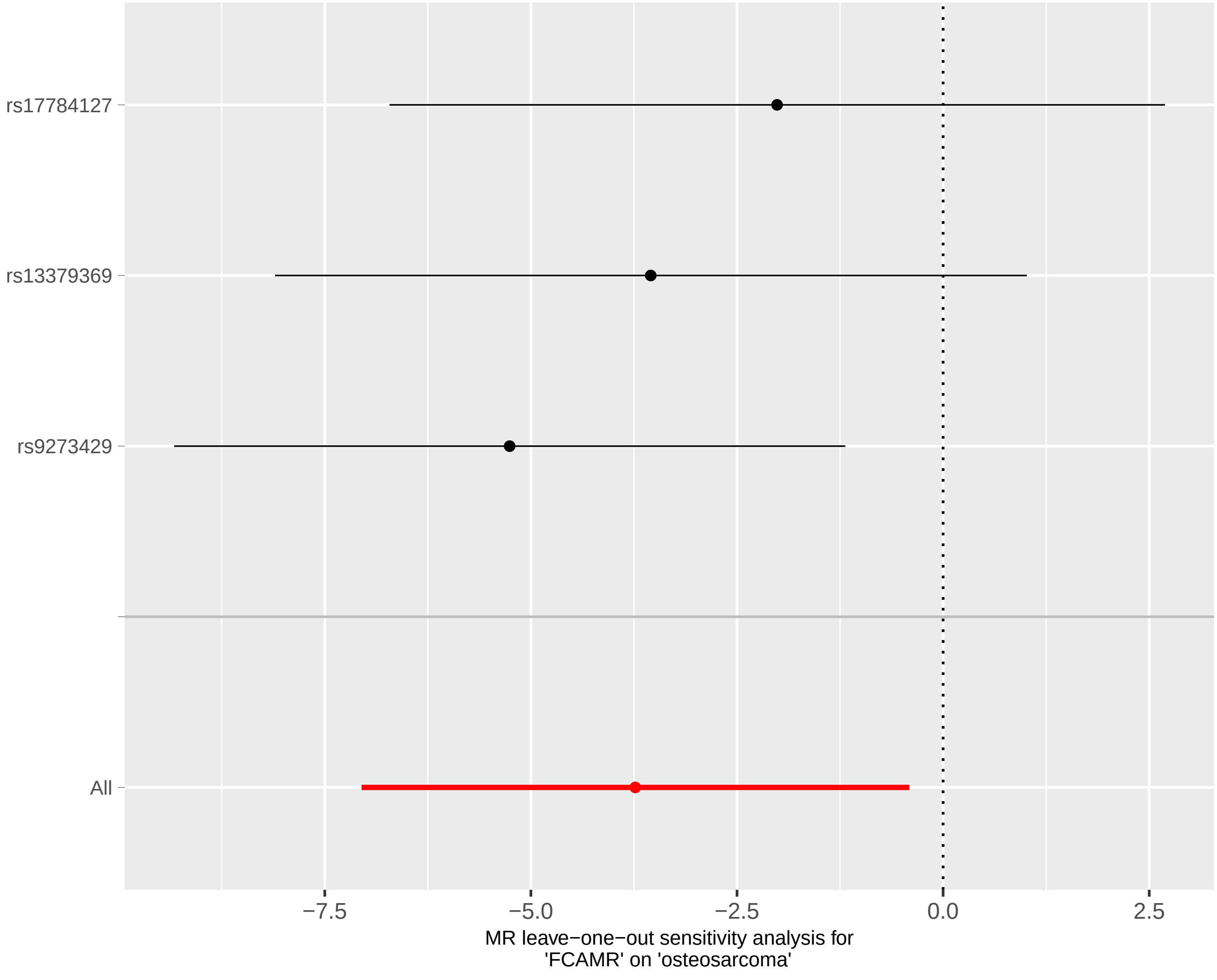
rs17784127
rs13379369
rs9273429
All
−7.5
−5.0
−2.5
0.0
2.5
MR leave−one−out sensitivity analysis for
'FCAMR' on 'osteosarcoma'
